# Uncovering deeply conserved motif combinations in rapidly evolving noncoding sequences

**DOI:** 10.1186/s13059-020-02247-1

**Published:** 2021-01-11

**Authors:** Caroline Jane Ross, Aviv Rom, Amit Spinrad, Dikla Gelbard-Solodkin, Neta Degani, Igor Ulitsky

**Affiliations:** Department of Biological Regulation, Weizmann Institute of Science, 76100 Rehovot, Israel

**Keywords:** Long noncoding RNA, Molecular evolution, Homology, Integer linear programming, OIP5-AS1, CHD2, CHASERR, MicroRNA, 3′UTR

## Abstract

**Background:**

Animal genomes contain thousands of long noncoding RNA (lncRNA) genes, a growing subset of which are thought to be functionally important. This functionality is often mediated by short sequence elements scattered throughout the RNA sequence that correspond to binding sites for small RNAs and RNA binding proteins. Throughout vertebrate evolution, the sequences of lncRNA genes changed extensively, so that it is often impossible to obtain significant alignments between sequences of lncRNAs from evolutionary distant species, even when synteny is evident. This often prohibits identifying conserved lncRNAs that are likely to be functional or prioritizing constrained regions for experimental interrogation.

**Results:**

We introduce here LncLOOM, a novel algorithmic framework for the discovery and evaluation of syntenic combinations of short motifs. LncLOOM is based on a graph representation of the input sequences and uses integer linear programming to efficiently compare dozens of sequences that have thousands of bases each and to evaluate the significance of the recovered motifs. We show that LncLOOM is capable of identifying specific, biologically relevant motifs which are conserved throughout vertebrates and beyond in lncRNAs and 3′UTRs, including novel functional RNA elements in the CHASERR lncRNA that are required for regulation of CHD2 expression.

**Conclusions:**

We expect that LncLOOM will become a broadly used approach for the discovery of functionally relevant elements in the noncoding genome.

**Supplementary Information:**

The online version contains supplementary material available at 10.1186/s13059-020-02247-1.

## Background

Tens of thousands of loci in the human genome encode long noncoding RNA (lncRNA) transcripts, which do not appear to code for functional proteins [1, 2]. These genes evolve much faster than most mRNAs [3]: there are no known homologs of vertebrate lncRNAs outside of vertebrates, and only ~ 100 lncRNAs have detectable conservation between mammals and fish [4]. On top of these, there are thousands of lncRNAs that are transcribed from syntenic regions in mammals and fish, suggesting that homologous lncRNAs may exist in species separated by large evolutionary distances, yet their sequence similarity evades detection by existing tools [4]. Furthermore, even lncRNAs with detectable similarity across long evolutionary distances frequently exhibit drastic changes in their exon-intron structure and overall length, often through species-specific acquisition of transposable elements [4]. These make it difficult to predict functionally important sequence elements by comparing lncRNAs from multiple species.

The use of comparative sequence analysis for identifying homologous sequences is an essential step in the functional inference and classification of genomic sequences. The conventional tools currently available have largely been developed for mRNAs and use alignment-based methods that search for significantly long stretches of high sequence conservation. These tools perform poorly when applied to rapidly evolving sequences such as lncRNAs, which lack long continuous stretches that are highly constrained at the sequence level. Pairs of sequences will often share short stretches of similarity, but these typically will not reach statistical significance (e.g., every 6mer would appear by chance once in every 4-kb sequence, and so two typical lncRNA sequences share many 6mers purely by chance). Multiple sequence alignment (MSA) can be a more sensitive method to identify shorter regions of homology, but MSAs are often inaccurate for ncRNA genes [5]. MSAs of dozens of sequences are typically difficult to parse visually, and they are unable to handle local sequence duplications and have difficulties with large independent insertions such as those resulting from species-specific transposable elements. MSA-based methods are thus difficult to use for homing in on specific motifs that are deeply conserved and may have biological relevance. One alternative to conventional sequence analysis is the SEEKR algorithm [6]. Developed primarily for the analysis of lncRNAs, SEEKR evaluates the functional similarity of sequences based on the abundance profiles of short *k*-mers. Such *k*-mers may correspond to specific protein binding sites, and SEEKR was shown to be effective for grouping different lncRNAs with putatively related functions within the same species [6]. However, more sensitive approaches are needed to confidently detect specific functional elements that have been evolutionarily conserved in orthologous lncRNAs in distantly related species.

With these limitations in mind, we developed the LncLOOM (Lncrna Linear Order cOnserved Motifs) framework, which is based on the identification of combinations of short motifs found in the same order in putatively homologous sequences from different species. There are two key assumptions in LncLOOM. First, that functional elements often require sequence conservation of short (6–12 nt) motifs, which may correspond to binding sites of RNA binding proteins (RBPs) or microRNAs (miRNAs), which recognize elements within this length range [7, 8]. Second, that the order of these elements is conserved across long evolutionary distances. This may occur either because the order is important for function, in particular if functionality of the specific elements is aided by a sequence or structural context in the longer RNA molecule, or because gain of these elements in evolution is sufficiently rare. These assumptions do not necessarily hold for many functional elements within biological sequences, but as we show, this model potentiates a powerful tool for identifying multiple functionally relevant and deeply conserved elements in various lncRNAs and is also directly applicable to other noncoding sequences, such as 3′UTRs.

## Results

### The LncLOOM framework

**Fig. 1:**
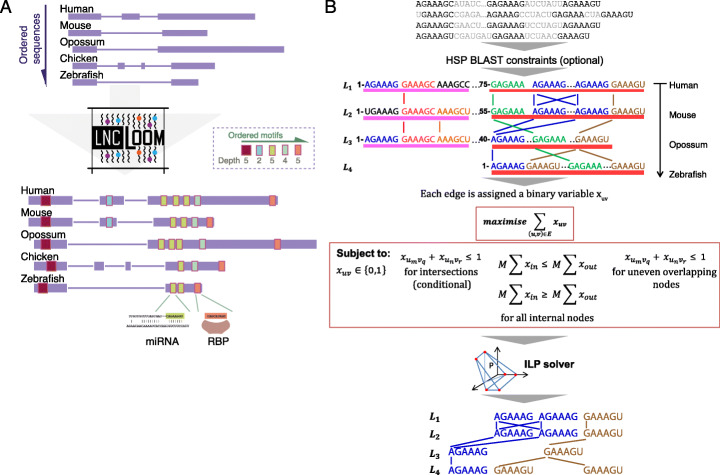
Overview of the LncLOOM framework. **a** Overview of the LncLOOM methodology. LncLOOM processes ordered lists of sequences and recovers a set of ordered motifs conserved to various depths that can be further annotated as miRNA or RBP binding sites. **b** Schematic diagram of graph construction and motif discovery using integer linear programming (ILP) to find long non-intersecting paths. Sequences are ordered with monotonically increasing evolutionary distance from the top layer (human). BLAST high-scoring segment pairs (HSPs) that can be used to constrain the placement of edges (see the “Methods” section) are depicted as pink and red blocks beneath each sequence. The graph is used for the construction of an ILP problem, and its solution is used for the construction of a set of long paths that correspond to conserved syntenic motifs

LncLOOM receives a collection of putatively homologous sequences of a genomic sequence of interest. Here, we focus on lncRNAs and 3′UTRs, but other elements, such as enhancers, can be readily used as well (see the “Discussion” section). For lncRNAs, we use only the exonic sequences for motif identification, but also visualize the positions of the exon-exon junctions. The input sequences are provided in a certain order (Fig. 1a), which ideally concurs with the evolutionary distances between the species, and which can be set automatically based on sequence similarity. The precise definitions of the data structures and algorithms used in LncLOOM appear in the “Methods” section, and an overview of the framework is presented in Fig. 1. LncLOOM represents each RNA sequence as a “layer” of nodes in a network graph (Fig. 1b), where each node represents a short *k*-mer. For the analysis presented here, we used *k* between 6 and 15, as *k*-mers shorter than 5 will typically result in graphs that are too large for efficient computation, and conserved *k*-mers longer than 15 are rare enough to be mostly found only once in each sequence (longer conserved *k*-mers will be recovered by merging of shorter *k*-mers (see below)). Although 6 is the minimum *k*-mer length allowed by LncLOOM, it is possible to initiate motif discovery with *k*-mers of any length to reduce graph complexity for the analysis of longer and/or more similar sequences. The order of the layers reflects the evolutionary distance of input sequences from a query sequence, which is placed in the first layer of the graph (human in the analyses described here), and sequences from the other species are placed in additional sequential layers of the graph. Edges in the graph connect between nodes with identical *k*-mers in consecutive layers. We note that it is possible to also connect “similar” *k*-mers, but this currently results in graphs that are too dense for practical applications of our approach (see the “Discussion” section), and so here we only connect nodes that are strictly identical. Under these definitions, our objective is to identify combinations of long “paths” in the graph that do not intersect each other and therefore connect short motifs that maintain the same order in different sequences. As we are usually interested in motifs that are present in the query sequence, we require that the paths begin in the top layer. The problem of identifying the maximal set of such paths is computationally hard, since for *k* = 1 it is the same as the longest common subsequence problem [9], but we show that it can be translated into a problem of solving an integer linear program (ILP), for which it is computationally hard to find an optimal solution, but efficient solvers are available [10] (Fig. 1b and the “Methods” section).

Once the graph is constructed, we begin by identifying paths for the largest *k* value, and then use these paths (if found) to constrain the possible locations of paths for smaller *k.* This approach allows us to favor longer conserved elements but also to identify significantly conserved short *k*-mers. Once all *k* values are tested, we merge the resulting graphs and obtain a combination of the motifs and the depths to which they are conserved. In order to compute the statistical significance of the motif conservation, we generate an MSA of the input sequences, shuffle the alignment columns, and so derive random sequences with an internal similarity structure similar to that of the input sequences. The full LncLOOM pipeline is then applied to these sequences, and for each motif found in the original input sequences to be conserved to layer *D*, we compute the empirical probability of identifying either precisely the same motif, or a combination of the same number of any motifs of that length, conserved to layer *D*. Additional *P* values are computed for a less stringent control, where random sequences with the same dinucleotide composition are generated and the inter-sequence similarity structure is not preserved.

A rich HTML-based suite is used to visualize these motifs in different ways, e.g., color coding them based on the depth of conservation and highlighting motifs in both the query sequence and in the other sequences (see Figs. 3 and 4 below for examples of LncLOOM output). The LncLOOM output also includes a color-coded custom track of motifs identified in the query sequence, which can be viewed in the UCSC genome browser. We annotate motifs using a set of seed sites of conserved microRNAs (from TargetScan [11]) and RBP binding sites found in eCLIP data from the ENCODE project [12].

### LncLOOM identifies deeply conserved elements in the *Cyrano* lncRNA

**Fig. 2:**
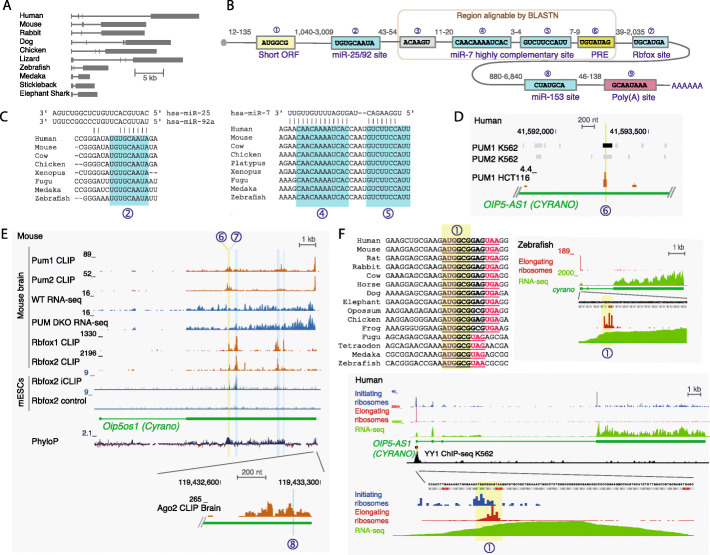
Conserved elements in the *Cyrano* lncRNA. **a** Outline of the genomic organization of *Cyrano* exons in select species. **b** Sequence elements identified by LncLOOM to be conserved in *Cyrano* in at least 17 species. The region containing elements found in the region alignable by BLAST between human and zebrafish Cyrano sequences is circled. Numbers between elements indicate the range distances between the elements in the 18 species. The circled number above each element indicates the element number used in the text and in the other panels. **c** Pairing between the predicted binding elements in *Cyrano* and the miR-25/92 and miR-7 miRNAs. **d** Evidence for binding of PUM1 and PUM2 to the UGUAUAG motif (shaded region) in the human genome. ENCODE project CLIP data (top, K562 cells) and [15] (bottom, HCT116 cells). Shading is based on strength of binding evidence, as defined by the ENCODE project. **e** Binding and regulation of the mouse *Cyrano* sequence by Pum1/2 and Rbfox1/2. Top: Pum1/2 CLIP and RNA-seq data from [16]. Middle: Rbfox1 CLIP from mouse brain [17] and from mESCs [18]. Binding motifs for Pumilio and Rbfox are highlighted in yellow and blue, respectively. PhyloP sequence conservation scores are from the UCSC genome browser. Bottom: Binding of Ago2 in the mouse brain to the region of the miR-153 binding site near the 3′ end of *Cyrano*. CLIP data from [19]. **f** Top left: Alignment of the region surrounding the conserved AUGGCG motif near the 5′ end of *Cyrano*. Top right and bottom: Composite Ribo-seq and RNA-seq data from multiple datasets curated in [20]. ChIP-seq data for YY1 in the K562 cell line from the ENCODE project. Shown is the read coverage and the IDR peaks

The *Cyrano* lncRNA (*OIP5-AS1* in human) is a broadly and highly expressed lncRNA [13, 14]. Despite being conserved throughout vertebrates, *Cyrano* exhibits ~ 5-fold variation in overall exonic sequence length (2340 nt in medaka to 10,155 nt in opossum, Fig. 2a). The previously identified 67 nt highly constrained element in *Cyrano* is the only region that BLAST reports with significant similarity when zebrafish and human sequences are compared [13]. Furthermore, the entire *Cyrano* locus is not alignable between mammals and fish in the 100-way whole genome alignment (UCSC genome browser). The highly conserved element contains an unusually extensively complementary miR-7 binding site, which is required for degradation of miR-7 by *Cyrano* [13, 14].

In order to identify additional conserved elements, we curated *Cyrano* sequences from 18 species where we could locate usable RNA-seq data, including eight mammals, chicken, *X. tropicalis*, seven vertebrate fish species, and the elephant shark (Additional file 2: Table S1). LncLOOM identified seven elements conserved in all species, nine conserved in all species except shark (Fig. 2b), and 37 motifs conserved throughout mammals. We focus here on the nine elements conserved in all species except shark (numbered 1–9 in Fig. 2b), seven of which were found to be statistically significant by both LncLOOM tests (*P* < 0.01). Only elements 3–6 fall within the 67-nt conserved region identifiable by BLAST, including two that correspond to pairing with the 5′ and 3′ of miR-7 (Fig. 2c), and another, UGUAUAG, that resembles a Pumilio Recognition Element (PRE, element #6). This element indeed binds PUM1 and PUM2 in CLIP data from human and mouse (Fig. 2d, e), and in the mouse neonatal brain, where *Cyrano* levels are relatively high, depletion of *Pum1* and *Pum2* leads to an increase in *Cyrano* expression (adjusted *P* value 3.49 × 10^−3^, data from [16], Fig. 2e), consistently with the functions of these proteins in RNA decay [21]. This repression is likely due to the combined effect of this highly conserved PRE and others—the 18 *Cyrano* sequences from different species had 3.2 consensus PREs on average (including two in the mouse sequence, compared to 1.3 on average in 1000 random shuffled sequences, *P* < 0.001, see the “Methods” section).

A putative biological function can be assigned to several additional conserved elements identified by LncLOOM within the *Cyrano* sequence. A 9mer conserved in all 18 input species, UGUGCAAUA (element #2 in Fig. 2b), is found ~ 60 nt upstream of the miR-7 binding site, outside of the region alignable by BLAST. This element corresponds to a miR-25/92 family seed match (Fig. 2c) and was recently shown to be bound and regulated by members of the miR-25/92 family in mouse embryonic heart [22]. At the 3′ end of *Cyrano*, one conserved element (GCAAUAAA) corresponds to the *Cyrano* polyadenylation signal (PAS) as well as a miR-137 site. Another sequence found ~ 100 nt upstream of the PAS, CUAUGCA, corresponds to a seed match of miR-153, and this region is bound by Ago2 in the mouse brain (Fig. 2e). Interestingly, *Cyrano* levels in HeLa cells are reduced by 41% and 11% following transfection of miR-137 and miR-153, respectively [23]. *Cyrano* is thus under highly conserved regulation by additional microRNAs beyond the reported interactions with miR-7 and miR-25/92.

Approximately 55 nt downstream of the conserved Pumilio binding site, there is a conserved WGCAUGA motif (W=A/U) that matches the consensus binding motif of the Rbfox RBPs. This motif is bound by Rbfox1/2 in mouse, as are additional regions containing instances of WGCAUGA in the 3′ half of *Cyrano* (Fig. 2e). In fact, analysis of the 18 *Cyrano* species showed significant enrichment of WGCAUGA (9.8 instances vs. 4.5 expected by chance, *P* < 0.001, see the “Methods” section). In contrast to the miRNA and the Pumilio binding sites, inspection of various RNA-seq datasets of Rbfox1/2 loss-of-function identified no effect on *Cyrano* levels (not shown), suggesting that the extensive and conserved binding by Rbfox1/2 might affect *Cyrano*’s functionality, rather than expression.

Another highly conserved 6mer, AUGGCG, is found at the very 5′ of *Cyrano*. Inspection of *Cyrano* sequences and Ribo-seq data from human, mouse, and zebrafish revealed that this 6mer corresponds to the first two codons of a conserved short 2–3 aa ORF (Fig. 2f). A clear ribosome association is found at the 5′ end of *Cyrano* at this ORF, with very limited numbers of ribosome protected fragments observed downstream to this element in both human and zebrafish (Fig. 2f), suggesting efficient translation and ribosome release at this short ORF. The context of the AUG start codon in the ORF perfectly matches the 12 bases of the TISU motif, a regulatory element influencing both transcription and translation. TISU is located at the 5′ end of transcripts and acts as a YY1 binding site that may dictate transcription initiation site and as a highly efficient and accurate cap-dependent translation initiator element, for translation that operates without scanning [24, 25]. The genomic region of this motif shows strong YY1 binding to the DNA (Fig. 2f). We speculate that this motif can have a dual function as a YY1 element regulating *Cyrano* expression, and as the beginning of the short ORF that may contribute to *Cyrano* function, as suggested for other lncRNAs [26]. Overall, putative biological functions could be postulated to eight of the nine conserved elements in *Cyrano*—four as miRNA binding sites, two as RBP binding sites, one as a conserved short ORF, and one as a PAS. These elements are separated by long stretches of non-conserved sequences (Fig. 2b), which underscores the power of combining LncLOOM with annotations and orthogonal data to uncover lncRNA biology.

As another example of the ability of LncLOOM to find conserved elements in transcripts known to be associated with the miRNA biology, we applied it to eight homologs of the *libra* lncRNA in zebrafish and *Nrep* protein in mammals. This is one of the few examples of a gene that morphed from a likely ancestral lncRNA to a protein-coding gene, while retaining substantial sequence homology in its 3′ region [13, 27]. *libra* causes degradation of miR-29b in zebrafish and mouse through a highly conserved and highly complementary site [27]. Comparing zebrafish *libra* with human and mouse sequences using BLASTN recovers an alignment of ~ 250 nt from the ~ 2.2-kb human sequence, and for spotted gar, there are additional short significant alignments (*E* value < 0.001). LncLOOM found 17 elements conserved between all species, and > 25 conserved in all species except zebrafish (Additional file 1: Fig. S1). These included the miR-29 site, as well as conserved binding sites for eight additional miRNAs, with three found outside of the region of alignment between mammalian and fish species by BLAST (Additional file 1: Fig. S1). It thus appears that *Cyrano* and *libra*, the two lncRNAs that were shown to effectively elicit target-directed miRNA degradation (TDMD), harbor several additional highly conserved miRNA binding sites, yet in contrast to the TDMD-mediated sites, these are canonical seed sites that likely affect lncRNA, rather than miRNA, levels.

### Conserved motifs in the sequence of the *CHASERR* lncRNA

In order to test the ability of LncLOOM to identify conserved modules in sequences that are not amenable for BLAST comparison, we focused on *CHASERR*, a lncRNA that we recently characterized as being essential for mouse viability [28]. *CHASERR* homologs are readily identifiable in different species based on the close proximity (< 2 kb) to the transcription start site of CHD2, as well as their characteristic 5-exon gene architecture [28]. We manually curated *CHASERR* sequences from 16 vertebrates, which were 579–1313 nt in length, and four of which were likely 5′-incomplete due to gaps in some of the genome assemblies around the extremely G/C-rich promoter and first exon of *CHASERR* [28] (Additional file 1: Fig. S2). BLASTN found significant (*E* value < 0.01) alignments between the human *CHASERR* and the nine sequences coming from amniotes, but not with any of the six other vertebrates. Conversely, when the zebrafish sequence was used as a query, BLAST only found homology in other fish species and in opossum. When the *CHASERR* sequences are fed into the ClustalO MSA [29], only three identical positions are found. The limited conservation of *CHASERR* is thus a challenge for analysis using commonly used tools for comparative genomics.

**Fig. 3:**
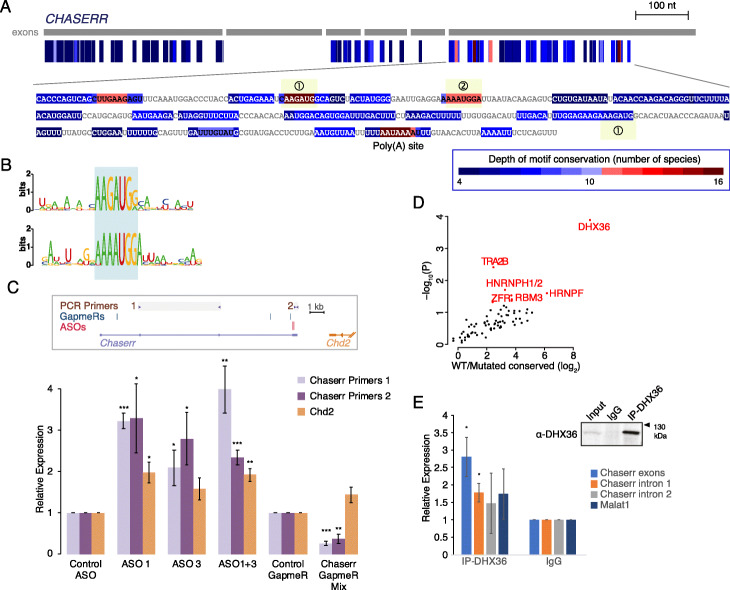
Conserved elements in the *CHASERR* lncRNA. **a** Human *CHASERR* gene structure is shown with motifs conserved in at least four species color-coded by their depth of conservation. The region of the last exon is magnified, and the motifs discussed in the text are highlighted. **b** Sequence logos of the sequences flanking the two most conserved motifs, with the shared AARAUGR motif shaded. **c** Top: mouse *Chaserr* locus with the positions of the primer pairs used for qRT-PCR, and the regions targeted by the GapmeRs (the same ones as used in [28]) and ASOs highlighted. Bottom: qRT-PCR with primers targeting *Chaserr* (shown on top) or *Chd2* exons in N2a cells treated with the indicated reagents, *n* = 4 for ASO treatments and *n* = 5 for GapmeRs. **d** Volcano plot for comparison of MS intensities between pulldown with the WT sequence of the Chaserr last exon and the last exon where the conserved elements were mutated (Additional File 1: Fig. S3A). **e** qRT-PCR using primers targeting the indicated regions following IP with the indicated antibody, *n* = 4. Top right: Western blot using anti-DHX36 antibody on the indicated sample

LncLOOM identified two *k*-mers as conserved in all the layers: AAUAAA at the 3′ end, which corresponds to the PAS, and AAGAUG, found once or twice in the last exon of all *CHASERR* sequences (motif 1 in Fig. 3a). Inspection of the *CHASERR* sequences found that the AAGAUG motif is substantially overrepresented—CHASERR homologs had 2.1 instances of it on average, compared to merely 0.45 expected by chance (*P* < 0.01). The context of the motif was also typically similar across these 34 instances, with the motif typically followed by a purine (Fig. 3b). An apparently related motif, AAAUGGA (motif 2 in Fig. 3a), was conserved in 11 of the sequences. Including flanking sequences, motif 2 shares an ARAUGR core with motif 1 (Fig. 3b). To the best of our knowledge, these sequences do not match the known binding preference of any RBP, and inspection of eCLIP data did not reveal an obvious candidate for a binder. We therefore further explored the functionality of these sequences experimentally.

To test the functional importance of the conserved elements, we designed antisense oligonucleotides (ASOs) complementary to the three instances of the conserved motifs in the mouse *Chaserr* (Additional file 1: Fig. S3A), and transfected them into Neuro2a (N2a) cells, where we had previously shown that depletion of *Chaserr* leads to an increase in *Chd2* RNA and protein levels [28]. Transfection of ASO1 and ASO3 individually or mixed led to a significant increase in *Chd2* levels, comparable to that caused by knockdown of *Chaserr* (Fig. 3c). Interestingly, ASO treatment led to an increase in *Chaserr* levels, as assessed by RT-PCR primer pairs found either upstream or downstream of the ASO-targeted region (Fig. 3c).

In order to identify proteins potentially binding the conserved regions, we used in vitro transcription to generate biotinylated RNAs containing the first 322 nt of the WT sequence of the last exon of mouse *Chaserr* (bases 559–880 of the RefSeq NR_037569.1 isoform), the same sequence with AUGG→UACC mutations in four conserved motifs, and a second mutant in which all seven of the AUGG sites in the last exon were mutated to UACC (Additional file 1: Fig. S3A). These sequences, alongside their antisense controls, were incubated with lysates from N2a cells and proteins that associated with the different RNA variants were isolated and identified using mass spectrometry. As typical in these experiments, a large number of proteins, 938, was identified as associating with the WT sequence (Additional file 2: Table S2), and 74 of these were enriched ≥ 3-fold compared to the antisense sequence; however, only 9 of these had ≥ 2-fold higher recovery when using the WT sequence compared to both mutants (Fig. 3d). We then examined public RNA-seq datasets and sought evidence for changes in *Chd2* and/or *Chaserr* levels when these proteins are perturbed. Such evidence was available for DHX36 and ZFR (Additional file 1: Fig. S3B-C). We validated the significant association of *Chaserr* with DHX36—the protein that showed the highest enrichment compared to the mutated sequences—using RNA immunoprecipitation (RIP) and a specific antibody (Fig. 3e). Interestingly, DHX36 is known to bind G-quadruplex sequences [30, 31], and the conserved elements indeed contain GG pairs, though those are quite far from each other, and typical G-quadruplexes contain runs of at least 3 Gs. QGRS mapper [32] predicts one G-quadruplex in the last exon of *Chaserr* (Additional file 1: Fig. S3A), but other tools, including G4RNA scanner [33], that integrate different scoring systems did not find any high-scoring G-quadruplexes in the last exon of *Chaserr*. It is also possible that a non-canonical G-quadruplex forming is formed in this sequence or that it has a different mode of recognition by DHX36.

LncLOOM is therefore capable of identifying functionally relevant elements within lncRNAs that can serve as a basis for the design of targeted reagents for perturbing their function, and enabling the use of proteomic methods for identifying specific, functionally relevant, lncRNA interaction partners.

### Deeply conserved elements within 3′UTRs of *DICER1* and Pumilio mRNAs

We next wanted to evaluate the applicability of LncLOOM beyond lncRNAs, and for comparing sequences across longer evolutionary distances. 3′UTRs can dictate RNA stability and translation efficiency of mRNAs, and they typically evolve much more rapidly than other mRNA regions [34]. Orthology between 3′UTRs is rather easy to define, based on their adjacent coding sequences, which are often readily comparable across very long evolutionary distances. However, there are very few known cases of long-range conservation of functional elements within 3′UTRs between vertebrates and invertebrates. In order to study 3′UTR conservation using LncLOOM, we first focused on genes that act in post-transcriptional regulation, as these typically undergo particularly complex post-transcriptional regulation. Using available RNA-seq and expressed sequence tag (EST) data, we compiled a collection of 3′UTR sequences of *DICER1*, which encodes a key component of the miRNA pathway, from 12 species, including eight vertebrates, lancelet, lamprey, sea urchin, *C. intestinalis*, and two DICERs in the fruit fly. Human *DICER1* 3'UTR could be aligned by BLASTN to the 3′UTRs from vertebrate species, but not beyond. LncLOOM identified 15 elements conserved in all the vertebrate sequences, six with lengths that were not found in random sequences (*P* < 0.01, Additional file 1: Fig. S4). Eight of the motifs were conserved beyond vertebrates (and could not be assessed by MSAs or BLAST), and one, corresponding to a binding site for the conserved miR-219, was found in all species, including the fly Dicer2 3′UTR.

**Fig. 4:**
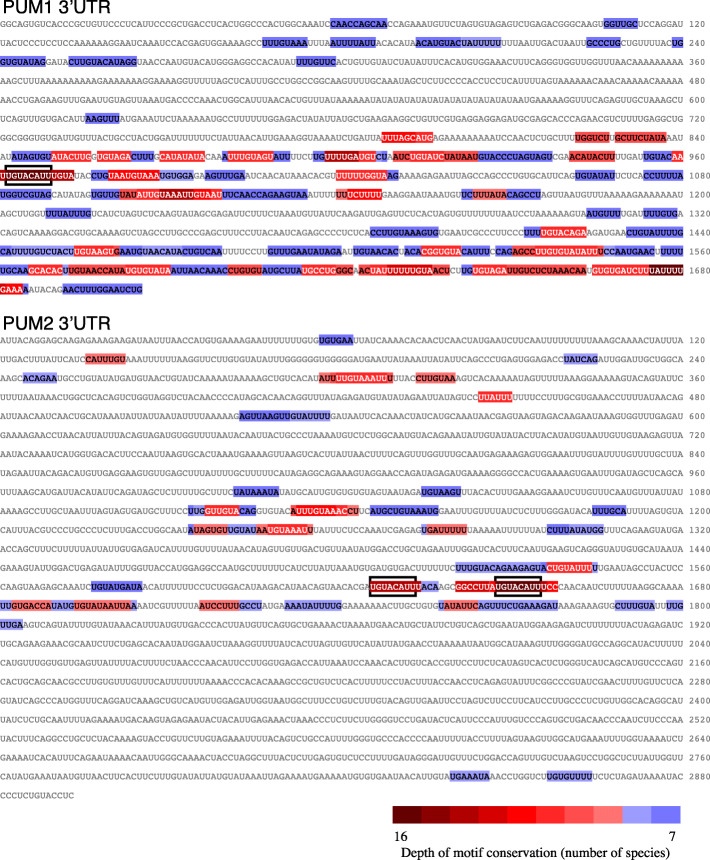
Conserved elements in the PUM1 and PUM2 3′UTRs. The human sequence is shown and the motifs conserved in at least seven species are color-coded based on their conservation. The occurrences of the ultra-conserved UGUACAUU motif are in a box

We then focused on 3′UTRs of the *PUM1* and *PUM2* mRNAs, which encode Pumilio proteins that post-transcriptionally repress gene expression. Pumilio proteins are deeply conserved, and there are two Pumilio proteins in vertebrates, PUM1 and PUM2, with a single ortholog in other chordates and in flies. We curated 3′UTR sequences from 12 vertebrates and four invertebrates (lamprey, lancelet, *C. intestinalis*, and fruit fly). Human and zebrafish 3′UTRs are readily alignable by BLASTN, and there is even significant homology between the 3′UTR of human PUM1 and those of the Pumilio mRNAs in lamprey and lancelet, but not of those in fly and *C. intestinalis*. LncLOOM identified eight elements conserved throughout vertebrate PUM1 3′UTRs, one of which, UGUACAUU, was conserved in all 16 analyzed 3′UTRs all the way to the fly *pum* 3′UTR (Fig. 4, top). In PUM2, there were three elements conserved throughout vertebrates, also including UGUACAUU, which was found in all the sequences (Fig. 4, bottom). Interestingly, this UGUACAUU motif partially matches the PRE consensus, UGUANAUA, and it is bound by both PUM1 and PUM2 in human ENCODE data, suggesting that this ancient element is part of the auto-regulatory program that is known to exist in Pumilio mRNAs [21]. LncLOOM is thus able to identify deeply conserved elements in 3′UTR sequences, including those separated by > 500 million years, where available tools do not detect significant sequence conservation.

### Systematic analysis of conserved motifs in 3′UTRs uncovers deeply conserved elements

**Fig. 5:**
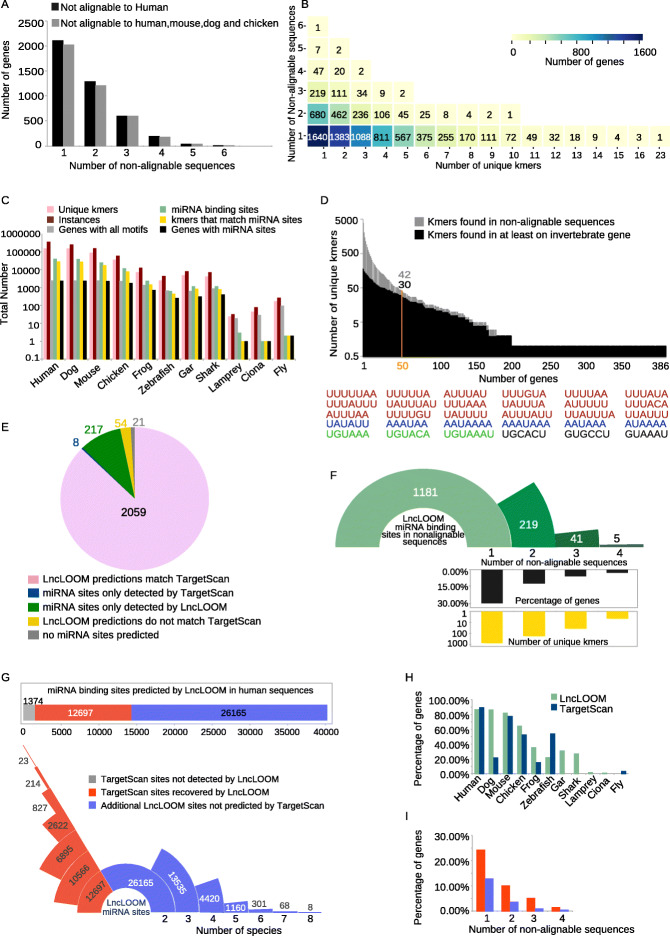
Global analysis of conserved motifs in 3′UTRs with LncLOOM. **a** Number of genes with various numbers of ortholog sequences that had no significant alignment to their human sequence (black) or to their mouse, dog, and chicken sequences (gray). **b** Distribution of combinations of unique *k*-mers conserved in the indicated number of sequences that did not align to the human 3′UTR sequence. **c** Quantification of the total number of unique *k*-mers (pink) and their total instances (dark red) that LncLOOM identified per species. The total number of broadly conserved miRNA binding sites is shown in green, and the number of unique *k*-mers that correspond to these sites in yellow. The number of genes that contained any *k*-mer is shown in gray, and the number of genes that contained at least one *k*-mer that corresponds to a miRNA site is shown in black. **d** Top: Distribution of unique *k*-mers that were identified in the first sequence non-alignable to human in multiple genes (gray). The number of *k*-mers detected in an invertebrate species in at least one gene is shown in black. Bottom: Unique *k*-mers common to at least 50 genes and detected in an invertebrate sequence. *k*-mers that resemble an ARE are colored red, those resembling a PAS are blue, and those resembling a PRE are green. **e** Comparison of genes that contained broadly conserved miRNA binding sites detected by LncLOOM and TargetScan in the human sequences of genes analyzed. **f** Number of broadly conserved miRNA bindings detected by LncLOOM per number of non-alignable sequences; the percentage of genes with a miRNA site detected per number of non-alignable layers (black) and the number of unique *k*-mers corresponding to the miRNA binding sites (yellow). **g** Top: Broadly conserved miRNA binding sites predicted by LncLOOM in human sequences. Sites predicted by TargetScan and recovered by LncLOOM are shown in red, and new sites (not previously predicted by TargetScan) in blue. Bottom: The conservation of these sites per number of species. **h** Comparison of the fractions of genes with at least one miRNA site detected in the indicated species by TargetScan and LncLOOM. Only sites found in TargetScanHuman were used. **i** Percentage of genes that contain a miRNA site detected by LncLOOM per number of non-alignable sequences: (red) miRNA sites that were previously predicted by TargetScan in the human sequence and recovered by LncLOOM in additional sequences, that were not part of the MSA used by TargetScan; (blue) new miRNA sites predicted by LncLOOM but not previously predicted by TargetScan in the human sequences

In order to broadly evaluate the predictive power of LncLOOM, we performed a comprehensive analysis of 3′UTR sequences. We focused on 3′UTRs that are well-defined based on the highly conserved coding sequence flanking them, allowing us to build a high-confidence input dataset spanning hundreds of millions of years of evolution, with which we could systematically study thousands of elements using LncLOOM. Our dataset was based on 2439 genes that had 3′UTR MSAs generated as part of the TargetScan7.2 miRNA target site prediction suite [11]. For each gene, we built a dataset of 3′UTR sequences for LncLOOM analysis that contained the aligned sequence from the TargetScan MSA in each of four species (human, mouse, dog, and chicken), only if those were 300–3000 nt long. For genes with several 3′UTR isoforms, we selected the longest 3′UTR. We then added to the dataset, where available, sequences of the 3′UTRs annotated in Ensembl in additional species, if those were longer than 200 bases. These included sequences from five non-amniote vertebrate species (frog, shark, zebrafish, gar, and lamprey) and two invertebrates (ciona and fly). As our main objective was to evaluate the ability of LncLOOM to identify deeply conserved elements, we focused only on genes that had a suitable sequence from at least one non-amniote. The numbers of sequences that could be analyzed at different depths are presented in Additional file 1: Fig. S5A. Of the 2439 3′UTR datasets, 2117 contained at least one sequence for which BLASTN did not report any significant alignment (*E* value < 0.05) to the human sequence, while 2031 datasets contained at least one sequence that did not have significant alignment to any of the four species (Fig. 5a). We could therefore analyze a large number of sequences where an MSA-based approach was potentially unable to interrogate the full depth of conservation.

We used LncLOOM to search for conserved motifs with a minimum length of 6 bases and with *P* < 0.05 in all LncLOOM tests. LncLOOM detected over 150,000 significant motifs in the human sequences, of which 27,826 (18.3%) corresponded to a seed site of a broadly conserved miRNA family (as defined by TargetScan). Eleven thousand seven hundred twenty-five *k*-mers were conserved beyond amniotes, of which 3897 were detected in at least one non-alignable sequence (Fig. 5 and Additional file 1: S5). LncLOOM detected at least one unique *k*-mer in the first non-alignable layer of 1640 of the 2117 genes that contained sequences that did not align to their respective human orthologs, while combinations of at least three unique *k*-mers were found in 1088 genes (Fig. 5b). When considering just sequences that did not align to either of the four amniote species, at least one unique *k-*mer was detected in the first non-alignable sequence in 1529 datasets (Additional file 1: Fig. S5B). In 114 genes, we found conservation beyond vertebrates and in 97 conservation all the way from human to the fruit fly. A total of 170 unique *k*-mers (265 instances) were found in fly genes, of which only two matched a broadly conserved miRNA binding site (Fig. 5c).

We next considered specific conserved *k*-mers shared between 3′UTRs of multiple genes. Within the *k*-mers detected in non-alignable sequences, 42 were common to at least 50 genes of which only two corresponded to a broadly conserved miRNA binding site and 30 were conserved in invertebrate sequences (Fig. 5d). Among these 30, 18 *k*-mers contained a UUU sequence in an A/U-rich context, resembling AU-rich elements (AREs), and 5 contained AUAA, resembling PASs. Other *k*-mers contained an UGUA core that resembles a PRE. These three groups of miRNA-unrelated elements are thus also often very deeply conserved in 3′UTRs, and these conserved occurrences can be detected by LncLOOM.

To assess the sensitivity of LncLOOM, we compared the binding sites of broadly conserved miRNAs that were identified by LncLOOM to TargetScan predictions for each of the 2439 genes, in 2121 of which TargetScan predicted binding sites in the human sequences. LncLOOM predicted binding sites in 2330 genes, including 217 for which the TargetScan alignments did not identify any broadly conserved sites (Fig. 5e). A summary of all miRNA sites predicted by LncLOOM can be found at https://github.com/LncLOOM/LncLOOM. In a substantial number of cases (29% of the 2117 genes), LncLOOM found a miRNA binding site significantly conserved in species where the 3′UTR was not alignable to the human sequence in the MSA (Fig. 5f). To compare LncLOOM and TargetScan predictions more precisely, we focused on the 2359 genes for which TargetScan predicted binding sites in the identical human transcript used for LncLOOM analysis (Fig. 5e), among which LncLOOM recovered 90.24% of all broadly conserved sites predicted by TargetScan in the human sequences (Fig. 5g). Within the 217 genes, 42 had sites conserved beyond mammals and in several genes conservation was found in fish and fruit fly species (Additional file 1: Fig. S5D). In addition to the miRNA sites recovered, LncLOOM identified a further 26,165 broadly conserved sites that had not been previously predicted (Fig. 5g). When comparing the depth of conservation, LncLOOM often detected the sites recovered by TargetScan in more distal species (Fig. 5h and Additional file 1: S5E). Importantly, 831 recovered and 331 new predictions were detected in non-alignable sequences in 24% and 13% of genes respectively (Fig. 5i and Additional file 1: S5F).

We conclude that LncLOOM is a powerful tool also for the analysis of 3′UTR sequences, revealing a greater depth of conservation of miRNA or other functional binding sites than what is possible by MSA-based approaches while having only a limited compromise on sensitivity.

## Discussion

We presented here a new framework for the detection of conserved elements in putatively orthologous biological sequences. This framework complements the SEEKR algorithm that uses *k*-mer profile similarity to cluster lncRNAs from the same species [6], and tailored approaches that combine sequence, structure, and synteny for sensitive search of homologs of specific lncRNAs with known functional elements in distant species [35, 36]. Contrary to SEEKR, LncLOOM requires that the linear order of conserved *k*-mers is maintained. Although the order of *k*-mers may not always be important for function, this constraint increases the precision of motif discovery as an ordered set of conserved *k*-mers is less likely to appear by chance and allows us to consider sets of conserved elements within the context of rapidly evolving sequences. As we show here, in many cases, those *k*-mers correspond to functionally relevant elements.

The main strength of our approach is the ability to detect conserved elements in sequences that have diverged beyond alignability and/or have accumulated substantial lineage-specific sequences such as transposable elements. Such integrations can dramatically alter the distances between conserved elements and are particularly common in lncRNAs [3]. As we show, LncLOOM is sensitive for detection of known functional elements, such as miRNA binding sites within 3′UTRs or lncRNAs, as well as for detection of completely new elements that we validated to play functional roles using existing data or targeted experiments. LncLOOM can be particularly useful when studying lncRNAs with partially known functions, but unknown protein binding partners. MS-based approaches for identifying such proteins typically yield hundreds to thousands of hits. As we show with *Chaserr*, targeted mutation of conserved regions allows homing in on specific, functionally relevant interactors.

With these strengths in mind, LncLOOM clearly also has its limitations. The requirement for perfect conservation of the motif sequence and the motif order might be too strict. Binding sites of RBPs can typically tolerate sequence variation, and motifs, especially those that appear multiple times in the sequence, can change their relative position across large evolutionary distances. Still, these constraints are currently required in order to keep the computational complexity at bay and, more crucially, discover motif combinations that are statistically significant. Another limitation is in the linear order of sequences that LncLOOM works with. In the future, we plan to extend LncLOOM to define relationships between sequences by a phylogenetic tree (with motifs conserved within monophyletic groups). Another extension that we believe would be particularly useful is to use the LncLOOM framework for first identifying a set of motifs in a set of known homologs of a lncRNA, and then use those for querying sequences of putative homologs (e.g., in transcriptomes of species where full genome sequence is unknown).

For the study of lncRNAs, input to LncLOOM requires sequences of homologs from multiple species. Despite the availability of a large number of RNA-seq datasets in many different species, and the increasing inclusion of lncRNAs in annotation databases such as ENSEMBL, this remains a major challenge that here we met through careful manual annotation of sequences from available annotations, ESTs, and RNA-seq data. Importantly, LncLOOM is capable of handling cases where parts of sequences are missing, e.g., due to gaps in genomic sequences, which tend to occur in particularly G/C-rich or repetitive regions, such as the first exon of *Chaserr*. Another challenge is the identification of homologs in species where there is no sequence similarity detectable by existing tools. Synteny can be useful in such cases (like in the case of *Chaserr*), though in many cases, lncRNAs would be syntenic to multiple other lncRNAs in a given species. lncRNAs are also often alternatively spliced [37]. We can envision an extension of LncLOOM to handle multiple possible alternative splicing isoforms, but for now, a simple solution is to use the union of all possible exons (in their linear order) as the “lncRNA sequence,” as we have done in the past in other contexts [4].

Once a set of putatively homologous sequences is available, in our experience so far, LncLOOM is most effective in cases where the sequences span a wide phylogenetic range, ideally one where for the most distant species there is limited or no homology detectable by BLAST (as we show for *Cyrano* and *CHASERR*). In these cases, LncLOOM can detect a few highly specific elements that can serve as a basis for functional experiments. When applied to more shallowly conserved functional lncRNAs, the large number of detected elements makes LncLOOM output more challenging to use. For example, when applied to NORAD [38, 39] sequences from nine mammals (4917–5458 nt in length), LncLOOM detects 46 significantly conserved elements shared in all species, including some as long as 34 nt (Additional file 3). Reassuringly, these elements included five binding sites for the Pumilio proteins, known functional binders of NORAD [38, 39]. Similarly, when applied to the longer XIST sequences from six mammals (10,466–25,215 nt in length), LncLOOM identifies 23 significantly conserved elements (Additional file 4).

Analysis of 19 sequences of MALAT1 (4992–12,676 nt in length, Additional file 5), a deeply conserved and extremely abundant lncRNA [13, 40, 41], illustrates a more complex scenario of high conservation only up to a certain evolutionary depth. Only six motifs are conserved in all sequences, including three that form part of the triple-helical motif at the 3′ end of MALAT1 [42, 43], and another motif in that region is conserved in 18 of the species. Nine additional short motifs are significantly conserved outside of amniotes (i.e., in at least 11 of the input species). In contrast to this relative scarcity of very deeply conserved motifs (given the length of the RNA), 122 *k*-mers are significantly conserved in at least one amniote species (turtle, alligator, or lizard).

We have invested a substantial effort in building a simple yet richly annotated HTML-based visualization engine for LncLOOM that allows the user to easily execute it on variable inputs and to obtain information on motifs conserved at different levels. We believe that this capability will make the tool widely useful for biologists studying lncRNAs as well as other biological sequences, enabling potent generation of hypotheses on functional elements that can be further dissected experimentally. We also believe that the framework that we present here for a graph-based discovery of conserved motif combinations is merely a starting point that will be extended further and serve as a basis for even more potent tools for biological discovery.

We show here that LncLOOM is capable of working with lncRNA and 3′UTR sequences, but importantly, it is also directly applicable to other types of biological sequences for which our assumptions of motif order conservation are reasonable, such as protein sequences and sequences of DNA enhancer elements (in these, motifs may correspond to transcription factor binding sites). The additional challenge in using LncLOOM for studying enhancer elements is that, in contrast to exonic sequences of lncRNAs, enhancer boundaries are more difficult to define. For instance, one may use boundaries of H3K27ac chromatin mark enrichment, but these are typically fuzzy, and ChIP-seq data are rarely available in matching tissues for multiple species, making it difficult to construct a set of putative orthologs.

## Conclusions

LncLOOM is a powerful new framework for the identification of conserved combinations of short sequence motifs in noncoding elements, capable of recovering both known and novel functional elements in lncRNAs and 3′UTRs, that can also be applied to other biological sequences. The assumption of linear order conservation is restrictive, but it is shared with alignment-based methods, and it is the basis for the statistical power to identify particularly deeply conserved motifs, shared between all vertebrates in several lncRNAs and conserved in 3′UTRs between vertebrates and insects. As we further demonstrate, once short functional motifs are identified, they can serve as a starting point for experimental determination of specific RNA binding partners and for design of effective antisense reagents for perturbation of lncRNA function.

## Methods

### Input to LncLOOM

LncLOOM works on a set of sequences from different species. Typically, each sequence corresponds to a putative homolog from a different species. Currently, we work with only one sequence isoform per species, though adaptations to cases where multiple sequences exist per species, e.g., alternative splicing products, are possible. The input sequences are typically constructed through manual inspection of RNA-seq and EST data and existing annotations. Sequences used as LncLOOM inputs are available within the LncLOOM implementation: https://github.com/LncLOOM/LncLOOM. We note that some of the input sequences might be incomplete, and our framework contains specific steps to accommodate such scenarios. Prior to graph building, the set is filtered to remove identical sequences. This can be further adjusted by the user to remove sequences with percentage identity above a threshold—in which case LncLOOM uses a MAFFT MSA [44] to compute percentage identity between each pair of sequences, and retain, among the similar sequences, the one that appears first in the input dataset.

### Sequence ordering

The LncLOOM framework is built around an ordered set of sequences that ideally should be from species with a monotonically increasing evolutionary distance with respect to the anchor (query) sequence (which is human in all the examples in this manuscript). The order of the sequences can be provided by the user or determined using BLAST [45]. If BLAST is used, the anchor sequence is defined to be the first sequence in the dataset. The second sequence is the one with the highest alignment score to the anchor sequence. Each subsequent sequence is then the one with the best alignment score to the preceding sequence among the sequences that have not been ordered yet. If no significant alignment is found, the next available sequence in the original input is selected.

### Overview of the LncLOOM method

Once the ordering of the sequences is established, LncLOOM identifies a set of combinations of short conserved *k*-mers for different values of *k*, by reducing each sequence of nucleotides to a sequence of *k*-mers, each represented by a node in a graph. Identical *k*-mers in adjacent sequences are connected in the graph, with additional constraints (Additional file 1: Fig. S6) and the use of ILP to find sets of long non-intersecting paths in these graphs. The set of paths identified in each graph is used to define constraints on graphs in subsequent iterations and to partition the graph (an example of graph partitioning is shown in Additional file 1: Fig. S7). Starting with the largest *k* and iteratively decreasing it, LncLOOM constructs an initial main graph for every *k*-mer length in the specified range. The main graph is constructed for all ordered sequences in the dataset and is then pruned layer-by-layer (until only the top two sequences remain) into a series of subgraphs for which the ILP problem of each is solved independently. At any given depth, a subgraph may be partitioned into an additional set of smaller subgraphs based on the paths found in previous iterations. In practice, this approach allows us to favor the identification of deeply conserved and longer motifs over shorter and less conserved ones and to also keep the size of the ILP program to below 1000 edges, which can be rapidly solved, keeping the overall runtime of LncLOOM to minutes even when applied to dozens of long sequences.

### Graph building

Given a dataset of lncRNA sequences from *D* species and *k*-mer length *k* (minimum 6 nt), LncLOOM constructs a directed graph *G* = (*V*, *E*), where *V* is the set of all nodes in the graph and *E* is the set of edges. The graph is composed of *D* layers, where *D* is the number of sequences in the dataset. Each sequence is modeled as a layer (*L*_1_, *L*_2_ ... *L*_*D*_), and layer *L*_*i*_, which corresponds to a sequence of length *N*(*i*), is composed of nodes (*v*_1_, *v*_2_ ...*v*_*N*(*i*)-*k* + 1_) where each node *v*_*n*_ represents the *k*-mer at position *n* in the *i*th sequence (Fig. 1b). All pairs of nodes that represent the same *k*-mer and are found in consecutive layers (*L*_*i*_ and *L*_*j*_ if *j* = *i* + 1) are connected by an edge *x*_*uv*_ = (u,v) where *u* ∈ *L*_*i*_ and *v* ∈ *L*_*j*_. Since each substring typically appears multiple times in a sequence, the number of edges may greatly exceed the number of nodes in the graph. Ordered combinations of *k*-mers that are deeply conserved correspond to long paths in *G* that do not intersect (i.e., for each ${x}_{u_m{v}_q},{x}_{u_n{v}_r}\in E,m<n\iff q<r,m\ne n\  and\ q\ne r\ \Big)$ and have a node in *L*_*1*_. Our goal is thus to find a set *S* in such that each edge is reachable from *L*_1_ via edges that are in *S* and no two edges in *S* intersect. Ideally, we would like to find the largest *S*, subject to potential additional constraints. For example, we may not be interested in short paths and so require that edges in *S* are all found on paths that reach to a certain layer.

### Identification of long non-intersecting paths using ILP

In the ILP problem, each edge in *G* is represented by a variable *x*_*uv*_ which is assigned a value of 1 if (*u,v)* is in *S*. The objective function is defined to maximize ∣*S*∣:

$$ \operatorname{maximize}{\sum}_{\left(u,v\right)\in E}{x}_{uv} $$

subject to: *x*_*uv*_ ∈ {0, 1}

The additional constraints imposed on this model are derived from several considerations. Firstly, LncLOOM aims to identify short conserved *k*-mers that appear in the same order in lncRNA sequences. However, it is unlikely that *k*-mers will appear only once in each sequence. Therefore, the constraints applied to the ILP model should allow for complex paths that contain multiple repeats of a single *k*-mer in one or more layers, provided it is not intersected by a path of a non-matching *k*-mer that has equal or greater depth (Fig. 1b and Additional file 1: S6A). To ensure selection of non-intersecting paths, the following constraint is imposed on any pair of edges that intersect between two consecutive layers:

$$ {x}_{u_m{v}_q}+{x}_{u_n{v}_r}\le 1, $$

If:

$$ m<n\ \mathrm{and}\ q>r\  OR\ m>n\ \mathrm{and}\ q<r $$

$$ {u}_m,{u}_n\in {L}_i $$

$$ {v}_q,{v}_r\in {L}_j $$

$$ j=i+1 $$

As the above constraint only considers the starting position of each node, it also excludes intersecting edges that connect identical *k*-mers that are repeated in two consecutive layers. In the case where a *k*-mer is repeated in both consecutive layers, a network of edges is constructed from each repeat-repeat connection (Additional file 1: Fig. S6B). This network of edges may override the selection of other paths that are equally conserved but connect fewer *k*-mers. Therefore, it is important to impose this constraint on edges that connect the identical *k*-mers, as it promotes the splitting of the complex path into multiple non-intersecting paths that are interspersed by paths of uniquely occurring *k*-mers. However, if the network of edges connecting the identical repeats is constrained only against each other in the absence of any other path, the ILP solver can select any possible solution of edges from the multiple repeat-repeat connections. This can lead to the suboptimal exclusion of repeated *k*-mers during subsequent iterations of graph refinement (scenario illustrated in Additional file 1: Fig. S6B). To avoid this scenario, the intersection constraint is only imposed on edges that connect identical *k*-mers if there is at least one other path, with equal depth, that intersects the network of repeated *k*-mers (Additional file 1: Fig. S6C-D).

To favor the selection of deeply conserved *k*-mers over repetitive shallower *k*-mers, the following two constraints are imposed on the successors and predecessors of each node *v* ∈ *L*_*i*_ *if* 1 < *i* < *y*:

$$ M{\sum}_{n\in Z}{x}_{vn}\le M{\sum}_{n\in P}{x}_{nv} $$

$$ M{\sum}_{n\in P}{x}_{nv}\le M{\sum}_{n\in Z}{x}_{vn} $$

where *Z* and *P* denote the respective subsets of all immediate successors and predecessors of node *v*, *y* is a minimum depth requirement, and *M* is a sufficiently large constant (in practice we used 100). Under this constraint, only paths that have continued connection from *L*_1_ to at least *L*_*y*_ are selected. At the same time, this constraint allows for the selection of connected complex paths that contain tandemly repeated *k*-mers in one or more layers (Fig. 1b).

In graph G, each layer *L*_*i*_ consists of nodes (*v*_1_, *v*_2_ ...*v*_*N*(*i*)-*k* + 1_) that start at every consecutive position in the sequence and have a length of *k* bases. It follows that from the set *S*, the set *S*_union_ can be formed by merging edges that connect adjacent nodes that overlap with each other. Once the ILP has been solved, these overlapping nodes will be combined into a single longer *k*-mer. This step may encounter a scenario where a set of adjacent *k*-mers represent a region of a sequence that contains a string of a single repeated base (see Additional file 1: Fig. S6A for an example). It is then possible that layer-specific insertions will be included in the resulting merged *k*-mer. To overcome this, the following constraint is imposed on any pair of edges that connect adjacent *k*-mers which overlap in either *L*_*i*_ or *L*_*j*_ such that the start and length of the overlapping region is equal between the two adjacent nodes in each layer:

$$ {x}_{u_m{v}_q}+{x}_{u_n{v}_r}\le 1 $$

If:

$$ n\le m+k-1\ \mathrm{and}\ m<n\ \mathrm{and}\ \left(m+k-1\right)-n\ne \left(q+k-1\right)-r $$

OR

$$ r\le q+k-1\ \mathrm{and}\ q<r\ \mathrm{and}\ \left(m+k-1\right)-n\ne \left(q+k-1\right)-r $$

$$ {u}_m,{u}_n\in {L}_i $$

$$ {v}_q,{v}_r\in {L}_j $$

$$ j=i+1 $$

ILP is a well-known NP-hard problem [46], which poses a major challenge in the scalability of LncLOOM to very long sequences or large datasets. To overcome this limitation, several steps have been included in the framework which reduce the complexity of the ILP of each graph and also favor the selection of deeply conserved *k*-mers. These include graph pruning, the partitioning of the graph based on simple paths, additional constraints on edge construction, and the iterative refinement of non-intersecting complex paths.

### Graph pruning

Two pruning steps are used in the LncLOOM framework. The first step involves the exclusion of nodes that correspond to *k*-mers which are excessively repeated in one or more layers. The number of allowed repeats per layer can be adjusted by the user and can greatly reduce the density of edges in longer sequences when a small *k* (e.g., 6) is used. For a given *k*-mer length, this step is performed during the construction of the initial graph on all sequences in the dataset and any excluded nodes are then excluded from all resulting subgraphs. The second pruning step is performed for each iteration of subgraph construction at a given level and excludes all nodes that do not have a connected path from *L*_1_ to the current depth.

### Partitioning the graph to reduce computational complexity

The constraints imposed on the ILP problem allow for the selection of *simple* or *complex* paths, where simple paths are defined as paths that contain only one node per layer. Simple paths consist of definitively selected edges that should not intersect shallower paths and therefore present boundaries at which the graph can be partitioned into smaller subgraphs that can be independently solved (Additional file 1: Fig. S7). Currently, these graphs are solved consecutively but in the future there is room for the use of parallel computing to handle larger datasets, provided that at least one simple path is found. The partition is based on simple paths of the current *k*-mer length that are found at each level in the layer-by-layer iterations. Each subgraph is constructed by selecting a subset of nodes that is located between two simple paths *τ*_*a*_ and *τ*_*b*_ with depth = *y*, where the boundaries are defined as the ending and starting positions of the nodes within each path: *W* = {*v*_*n*_| *q* + *k* − 1 < *n*, *n* + *k* − 1 < *r*, *v*_*q*_ ∈ *τ*_*a*_, *v*_*r*_ ∈ *τ*_*b*_} for each layer *L*_1_ to *L*_*y* − 1_ (the last layer is removed for the next iteration). In the case that *k*-mers of adjacent simple paths overlap, the *k*-mers are first combined and the boundaries are defined on the starting and ending position of the longer combined *k*-mer.

### Refinement of non-intersecting complex paths

In contrast to simple paths, complex paths can contain branches that connect repeated *k*-mers, particularly in paths that are selected in early iterations when the graph is not constrained. In an unconstrained graph, it is impossible to decipher which of the repeats appear by chance in each layer. Therefore, complex paths are not used to constrain edge selection in graphs in subsequent iterations. Instead, the set *S* that is found in each iteration is divided into (1) a subset of simple paths that are used for partitioning and edge constraint definition and (2) a subset of complex paths that are stored separately and continuously refined in the subsequent iterations. During refinement, the complex paths are optimized to remove branches that intersect with newly discovered paths (Additional file 1: Fig. S7). The refinement of complex paths is performed at two stages during the layer-by-layer eliminations. Firstly, before solving a subgraph that spans *y* layers, an individual graph of only complex paths is constructed from the subset *LC*_*d* = *y*_ of longer *k*-mers with depth = *y* and the subset *C*_*d* > *y*_ from paths of the current *k*-mer length that have a minimum depth of *y* + 1 (complex paths selected in previous iterations at the current *k*-mer length). A subset of refined complex paths, *C*_refined_, is then found according to the ILP problem described above. However, the following additional constraint is imposed to ensure the selection of all complex paths in *C*_*d* > *y*_ over any shallower path in *LC*_*d* = *y*_:

For every path, *τ* in *C*_*d* > *y*_

$$ \sum {x}_{uv}\ge 1\forall \left(u,v\right)\in \tau \mid u\in {L}_1\ \mathrm{and}\ v\in {L}_2 $$

Under this constraint, at least one repeated *k*-mer is selected from *L*_1_ for each path *τ* in *C*_*d* > *y*_. When this constraint is imposed together with the constraints described above, a refined path that spans at least *y* layers will be included in the solution. Once the set *C*_refined_ has been found, the subgraph of all *k*-mers of the current length and depth is constructed. All paths in *C*_refined_ are then added to the current subgraph and the ILP problem is solved with the additional constraint imposed to favor the selection of each path *τ* in *C*_refined_. This solution is then divided into a set of simple and complex paths for the next iteration. LncLOOM also includes an option to store and refine simple paths, such that simple paths of shorter *k*-mers with greater depth are favored over longer and shallower *k-*mers. However, if this option is applied, the graph is not partitioned and no constraints are imposed on edge construction in subsequent iterations. Therefore, this option is computationally expensive and can only be used to analyze a small dataset of short sequences.

### Using BLAST high-scoring pairs (HSPs) to reduce graph complexity

BLAST can also be used as an optional step in the process of LncLOOM graph construction. BLAST HSPs are local ungapped alignments between segments, with significant similarity, of sequences found in consecutive layers. We use these HSPs to constrain edge construction, such that any pair of nodes that are not contained within the same HSP between two consecutive layers are not connected. The HSPs that are found by BLAST are redundant in that HSPs may overlap one another and any segment may be matched to multiple segments in the target sequence. In regard to any set of HSPs that overlap each other, only the most significant pair is included in the HSPs used for graph construction. Similarly, in cases where one segment aligns with multiple segments in the target sequence, only the highest scoring alignment is included. The constraints that are derived from BLAST analysis can effectively decrease the number of possible paths in graphs and promote the correct placement of edges between layers where some of the sequences are incomplete (Fig. 1a).

### Graph size restriction

Although steps have been included to reduce the complexity of the ILP problem, in some scenarios, the graph is too large to be solved within a reasonable time. To address this bottleneck, the total number of edges in a graph is restricted. By default, the maximum number of edges allowed in the ILP problem is 1200, but it can be set to any number above 50. During any iteration, if the number of edges in a graph *G* exceeds the maximum limit, then the graph is divided into a series of subclusters in which the ILP problem is individually solved. Starting with the path that has the fewest edges (fewest repeated *k*-mers), an individual graph is constructed from each path *τ* in *G*, and only those paths in *C*_refined_ that intersect it. ILP is then used to optimize the allowed edges in this subcluster of *G*, *C*_refined_ is then updated to contain these edges and the path *τ* is removed from *G*. This process is repeated for each path that remains in *G* until all paths have been individually optimized against *C*_refined_ or the number of edges in *G* is the maximum limit, at which point all remaining paths in *G* are optimized against each other in a single ILP problem. If the number of edges in a graph constructed from an individual subcluster of intersecting paths exceeds the maximum limit, then ILP does not proceed and only the paths from *C*_refined_ are retained in the solution.

### Discovery of motifs in extended 5′ and 3′ regions of sequences

Input to LncLOOM may occasionally contain sequences that are 5′- or 3′-incomplete. As the data set is ordered by homology and not completeness, these sequences may be found in any layer in the graph and obstruct the layer-by-layer connection of nodes in these regions. To reduce the chance that conserved motifs are lost in this scenario, motif discovery is performed in three stages. In the first stage, LncLOOM identifies motifs from a primary graph that is constructed on all sequences in the dataset (a total of *D* sequences). LncLOOM then determines which sequences have a potentially extended 5′ or 3′ end by considering the position of the first and last motifs in each sequence relative to their median position across all sequences (Additional file 1: Fig. S8A). Based on this, LncLOOM builds and solves individual graphs of the extended 5′ and 3′ regions of the more complete sequences in the data set. To build the 5′ extended graph, LncLOOM first calculates the median position, *M*_*q*_, of the starting position of the first node ${v}_{q_i}\mid {v}_{q_i}\in S$ in each layer *L*_1_ to *L*_*D*_. A subset of nodes *W* = {*v*_*n*_| *n* + *k* − 1 < *q*_*i*_} is then extracted from each layer *L*_*i *_if q_*i*_ > *t* · *M*_*q*_, where *t* is some tolerance defined by the user. The nodes of the extended 3′ graph are extracted based on the ending positions of the last motifs relative to the length of each sequence. Specifically, LncLOOM calculates the median relative position, *M*_*Re*_, of the ending position of the last node ${v}_{r_i}\mid {v}_{r_i}\in S$ in each layer *L*_1_ to *L*_*D*_, where ${\mathit{\operatorname{Re}}}_i=\frac{r_i+k-1}{N(i)}$. A subset of nodes *W* = {*v*_*n*_| *n* > *r*_*i*_ + *k* − 1} is then extracted from each layer *L*_*i *_if Re_*i*_ < *M*_*Re*_ · (1 + *t*). By default, *t* = 0.5 for the extraction of both the 5′ and 3′ graph but a tolerance can be independently defined for each graph. This step of motif discovery only proceeds if nodes from an extended region of the anchor sequence have been included in the graph. To avoid a scenario where shallowly conserved motifs prevent identification of 5′ or 3′ truncations in deeper layers, for example because of motifs found close to the 5′ end are only conserved in the first two layers, a “minimum depth” parameter can be applied to select the positions of the first and last motifs in each sequence from a subset of motifs that are conserved to a specified depth. If the minimum depth parameter is applied, then all motifs that do not meet the specified depth requirement are also removed from the solution (scenario illustrated in Additional file 1: Fig. S8A).

### Calculation of motif modules and neighborhoods

Once the ILP problem has been solved for all subgraphs in the framework, each set of non-intersecting paths that was selected from the primary, 5′ extended, and 3′ extended graphs is processed into motifs modules and neighborhoods. A motif module is defined as an ordered combination of at least two unique motifs that is conserved in a set of sequences, where each motif is allowed to have any number of tandem repeats. By default, modules are calculated at every layer, *L*_*i*_ ∣ 2 ≤ *i* ≤ *D* of the graph by extracting paths that span all layers from *L*_1_ to *L*_*i*_. If a minimum depth *d* is specified in the parameters, then modules are calculated at every layer *L*_*i*_ ∣ *d* ≤ *i* ≤ *D*. As described above, motif discovery is performed through an iterative process of layer-by-layer elimination. This leads to the selection of longer regions of identity as the set of sequences continuously decreases to contain sequences that are more closely related. Consequently, shorter motifs that are more deeply conserved are often embedded in the longer motifs that are only conserved between the top layers (Additional file 1: Fig. S8B). We define these regions within the graph as motif neighborhoods, where each neighborhood comprises all nodes in the graph that are connected to a single region of overlapping nodes in *L*_1_, together with the flanking regions of each node in each layer. To calculate motif neighborhoods, LncLOOM first combines all overlapping nodes in *L*_1_ to form a set of reference *k*-mers that represent each neighborhood. For each reference *k*-mer, all paths that are connected to each shorter *k*-mer which is embedded within the reference *k*-mer are then included into that neighborhood. For each motif in each layer, the length of flanking regions is calculated relative to the position of the motif in the reference *k*-mer (Additional file 1: Fig. S8B). The motif modules and neighborhoods from each of the primary, 5′ extended, and 3′ extended graphs are presented in HTML and plain text file formats.

### Calculation of motif significance

Motif significance is inferred by calculating empirical *P* values of each motif in two genres of random datasets. Firstly, for a motif of length *k* that is conserved to *L*_*i*_, we determine the empirical probability of finding the exact motif found in the real dataset and any combination of the same number of any motifs of the same length or greater at least once in *L*_*i*_ of a set of random sequences that has the same percentage identity between consecutive layers as observed in the input sequences. This is achieved by using MAFFT [44] to generate an MSA of the input sequences, and then running multiple iterations of LncLOOM (100 for the analyses described in this manuscript) iterations in which the columns of the MSA are randomly shuffled. Secondly, we determine the empirical probability of finding the exact motif and any combination of the same number of any motifs of the same length at least once in *L*_*i*_ of a set of random sequences generated such that each layer has the same length and the same dinucleotide composition of its corresponding layer in the input sequences (but without preserving % identity between layers). Only the former *P* values were used in the analyses described in this manuscript. Multiprocessing has been implemented to execute the iterations in parallel.

### Functional annotation of motifs

LncLOOM has two optional annotation features. Firstly, the discovered motifs can be mapped to binding sites of miRNAs by identifying perfect base pairing with the seed regions of conserved (conserved throughout mammals) and broadly conserved (typically found throughout vertebrates) miRNAs from TargetScan [11]. For each motif, the type of pairing (6mer, 7mer, 7mer-A1, 7mer-M8, or 8mer) is determined in each sequence by considering the motif together with the immediate flanking base from both sides of the motif. A match is only found if the complete seed region (6mer) directly matches the motif. Secondly, motifs that are found in genes that are expressed in HepG2 or K562 cell lines can also be mapped to binding sites of RBPs identified by eCLIP in the ENCODE project [12]. To determine the chromosome coordinates of each motif in a selected query sequence, LncLOOM uses BLAT [47] to align the sequence to the genome and then calculates overlaps with the coordinates of binding sites of RBPs which are extracted from ENCODE bigBed files using the pyBigWig package. Alternatively, the user can also upload a BED file that specifies the chromosome coordinates and length of each exon in the query sequence. The extracted eCLIP data is filtered to exclude all peaks with enrichment < 2 over the mock input. RBPs that bind a large portion of the anchor sequence are marked, as the overlap of their binding peaks with any conserved motif is less likely to be functionally relevant for that specific motif.

### LncLOOM implementation and availability

LncLOOM has been open sourced and is available for download from https://github.com/lncLOOM/lncLOOM and is licensed under the Mozilla Public License 2.0. It is implemented in Python and is supported on Linux/Unix-based systems. Graph building is performed using the networkx package [48]. The integer programming problems are modeled using PuLP [49] and are solved by either the open source COIN-OR Branch-and-Cut solver (CBC) (https://www.coin-or.org/) or the commercial Gurobi solver (https://www.gurobi.com/). LncLOOM utilizes the following alignment programs during graph construction, motif annotation, and the empirical evaluation of motif significance: BLAST [45], BLAT [47], and MAFFT [44]. The multiprocessing python package is used to compute statistical iterations in parallel.

### Calculation of motif enrichment

For evaluating the enrichment of specific motifs in sequences, we generated 1000 sets of random sequences matching the dinucleotide composition of the input sequences and counted the occurrences of the motifs to compute the expected number of motifs and the empirical *P* values.

### Analysis of publicly available sequencing datasets

The following sequencing datasets found in GEO or SRA were used in this manuscript: GSE115429 (PUM1 CLIP in HCT116 cells), GSE95197 (PUM1/PUM2 CLIP and RNA-seq in mouse brain), SRP030031 (Rbfox1/Rbfox2 CLIP in mouse brain), GSE54794 (Rbfox2 CLIP in mESCs), GSE16338 (Ago2 CLIP mouse brain), and GSE140217 (miRNA overexpression in HeLa cells). ENCODE eCLIP datasets were taken from https://www.encodeproject.org/. Unless indicated otherwise, sequencing reads were mapped to the hg19 human genome assembly using STAR aligner [50] and gene expression levels were quantified using RSEM [51] and RefSeq annotations.

### LncLOOM analysis of lncRNAs and 3′UTRs

LncLOOM was used to analyze *Cyrano* sequences from 18 species, *libra* (*Nrep* in mammals) from 8 species, *CHASERR * sequences from 16 species, *DICER1* sequences from 12 species, and a *PUM1* and *PUM2* sequences from 16 species. For all genes, LncLOOM parameters were set to search for *k*-mers from 15 to 6 bases in length and the sequences were reordered by BLAST with the human sequence defined as the anchor sequence in each case. HSP constraints were not imposed. Motif significance was calculated over 100 iterations. The order of sequences for each gene as represented in the LncLOOM framework is shown in Additional file 6: Table S1.

LncLOOM was also used to analyze 2439 3′UTR genes. The datasets were constructed from 3′UTR MSAs generated by TargetScan7.2 miRNA target site prediction suite [11] and included the sequences of human, mouse, dog, and chicken that were between 300 and 3000 nt. Depending on availability and length (> 200 bases), sequences from frog, shark, zebrafish, gar and lamprey, cioan, and fly were obtained from Ensembl and added to their respective gene datasets. For each dataset, we used BLASTN, with a cutoff *E* value of 0.05, to classify which sequences in each of the respective species had no detectable alignment to their human ortholog, as well as those sequences that also did not align to mouse, dog, and chicken. *k*-mers identified by LncLOOM were matched to seeds of broadly conserved miRNA families, for which TargetScanHuman reported a human miRNA. To evaluate the sensitivity of LncLOOM, we compared the broadly conserved miRNA binding sites identified by LncLOOM to predictions reported by TargetScan (http://www.targetscan.org/cgi-bin/targetscan/data_download.vert72.cgi). Specifically, we only compared the miRNA sites from genes in which TargetScan reported sites in the identical representative human transcript as used in our LncLOOM datasets. In total, this corresponded to 2359 of the 2439 genes.

### Tissue culture

Neuro2a cells (ATCC) were routinely cultured in DMEM containing 10% fetal bovine serum and 100 U penicillin/0.1 mg ml^− 1^ streptomycin at 37 °C in a humidified incubator with 5% CO_2_. Cells were routinely tested for mycoplasma contamination and were not authenticated.

### Mass spectrometry sample preparation

Samples were subjected to in-solution tryptic digestion using suspension trapping (S-trap) as previously described [52]. Briefly, after pulldown proteins were eluted from the beads using 5% SDS in 50 mM Tris-HCl. Eluted proteins were reduced with 5 mM dithiothreitol and alkylated with 10 mM iodoacetamide in the dark. Each sample was loaded onto S-Trap microcolumns (Protifi, USA) according to the manufacturer’s instructions. After loading, samples were washed with 90:10% methanol/50 mM ammonium bicarbonate. Samples were then digested with trypsin for 1.5 h at 47 °C. The digested peptides were eluted using 50 mM ammonium bicarbonate. Trypsin was added to this fraction and incubated overnight at 37 °C. Two more elutions were made using 0.2% formic acid and 0.2% formic acid in 50% acetonitrile. The three elutions were pooled together and vacuum-centrifuged to dryness. Samples were kept at− 80 °C until further analysis.

### Liquid chromatography

ULC/MS grade solvents were used for all chromatographic steps. Dry digested samples were dissolved in 97:3% H_2_O/acetonitrile + 0.1% formic acid. Each sample was loaded using split-less nano-Ultra Performance Liquid Chromatography (10 kpsi nanoAcquity; Waters, Milford, MA, USA). The mobile phase was (A) H_2_O + 0.1% formic acid and (B) acetonitrile + 0.1% formic acid. Desalting of the samples was performed online using a reversed-phase Symmetry C18 trapping column (180 μm internal diameter, 20 mm length, 5 μm particle size; Waters). The peptides were then separated using a T3 HSS nano-column (75 μm internal diameter, 250 mm length, 1.8 μm particle size; Waters) at 0.35 μL/min. Peptides were eluted from the column into the mass spectrometer using the following gradient: 4 to 30%B in 55 min, 30 to 90%B in 5 min, maintained at 90% for 5 min, and then back to initial conditions.

### Mass spectrometry

The nanoUPLC was coupled online through a nanoESI emitter (10 μm tip; New Objective; Woburn, MA, USA) to a quadrupole orbitrap mass spectrometer (Q Exactive HF, Thermo Scientific) using a FlexIon nanospray apparatus (Proxeon).

Data was acquired in data-dependent acquisition (DDA) mode, using a Top10 method. MS1 resolution was set to 120,000 (at 200 *m*/*z*), mass range of 375–1650 *m*/*z*, AGC of 3e6, and maximum injection time was set to 60 ms. MS2 resolution was set to 15,000, quadrupole isolation 1.7 *m*/*z*, AGC of 1e5, dynamic exclusion of 20 s, and maximum injection time of 60 ms.

### Mass spectrometry data processing and analysis

Raw data was processed with MaxQuant v1.6.6.0 [53]. The data was searched with the Andromeda search engine against the mouse (*Mus musculus*) protein database as downloaded from Uniprot (www.uniprot.com), and appended with common lab protein contaminants. Enzyme specificity was set to trypsin and up to two missed cleavages were allowed. Fixed modification was set to carbamidomethylation of cysteines and variable modifications were set to oxidation of methionines, and protein N-terminal acetylation. Peptide precursor ions were searched with a maximum mass deviation of 4.5 ppm and fragment ions with a maximum mass deviation of 20 ppm. Peptide and protein identifications were filtered at an FDR of 1% using the decoy database strategy (MaxQuant’s “Revert” module). The minimal peptide length was 7 amino-acids and the minimum Andromeda score for modified peptides was 40. Peptide identifications were propagated across samples using the match-between-runs option checked. Searches were performed with the label-free quantification option selected. The quantitative comparisons were calculated using Perseus v1.6.0.7. Decoy hits were filtered out. A Student’s *t* test, after logarithmic transformation, was used to identify significant differences between the experimental groups, across the biological replica. Fold changes were calculated based on the ratio of geometric means of the different experimental groups.

### RNA-pulldown assay

Templates for in vitro transcription were generated by amplifying synthetic oligos (Twist Bioscience) and adding the T7 promoter to the 5′ end for sense sequences and to the 3′ end for antisense control sequences (see Additional file 6: Table S3 for full sequences). Biotinylated transcripts were produced using the MEGAscript T7 in vitro transcription reaction kit (Ambion) and Biotin RNA labeling mix (Roche). Template DNA was removed by treatment with DNaseI (Quanta). Neuro2a cells (ATCC) were lysed with RIPA buffer (10 mM Tris-HCl, pH 8.0, 1 mM EDTA, pH 8.0, 140 mM NaCl, 1% Triton X-100, 0.1% SDS, and 0.1% Na-DOC) supplemented with protease inhibitor cocktail (Sigma-Aldrich, #P8340) + 100 U/ml RNase inhibitor (#E4210-01), and 1 mM DTT for 15 min on ice. The lysate was cleared by centrifugation at 21130×*g* for 20 min at 4 °C. Streptavidin Magnetic Beads (NEB #S1420S) were washed twice in buffer A (NaOH 0.1 M and NaCl 0.05 M), once in buffer B (NaCl 0.05 M) and then resuspended in two tubes of binding/washing (NaCl 1 M, 5 mM Tris-HCl pH 7.5 and 0.5 mM EDTA supplement with PI + 100 U/ml RNase inhibitor, and 1 mM DTT). One tube of beads was washed three times in RIPA supplemented with PI and DTT 1 mM, after which cell lysate was added and pre-cleared with overhead rotation at 4 °C for 30 min. The second tube was equally divided into individual tubes for each RNA probe. Two to 10 pmol of the biotinylated transcripts were then added to the respective tubes and rotated overhead at 4 °C for 30 min. The beads were then washed three times in binding/washing buffer, after which equal amounts of the pre-cleared cell lysate were added to each sample of beads and RNA probe. The samples were then rotated overhead at 4 °C for 30 min. Following rotation, the beads were washed three times with high salt CEB (10 mM HEPES pH 7.5, 3 mM MgCl_2_, 250 mM NaCl, 1 mM DTT, and 10% glycerol). Proteins were then eluted from the beads in 5% SDS in 50 mM Tris pH 7.4 for 10 min in room temperature.

### Antisense oligonucleotide and LNA GapmeR transfections

ASOs (Integrated DNA Technologies) were designed to target the conserved ATGG sites that were identified by LncLOOM in the last exon of mouse *Chaserr* (Additional file 1: Fig. S3A). All ASOs were modified with 2′-O-methoxy-ethyl bases. LNA gapmers (Qiagen), targeted to *Chaserr* introns, were used for *Chaserr* knockdown (see Additional file 6: Table S4 for full oligo sequences). Transfection: 2 × 10^5^ Neuro2A cells were seeded in a six-well plate and transfected by using Lipofectamine 3000 (Life Technologies, L3000-008) following the manufacturer’s protocol with a mix of LNA1–4 or with ASO1, ASO2, ASO3, or a mix of either ASO1 and ASO3 or ASO1–3 to a final concentration of 25 nM. Endpoints for all experiments were at 48 h post transfection, after which the cells were collected with TRIZOL for RNA extraction and assessment by RT-qPCR analysis.

### RNA immunoprecipitation (RIP)

Neuro2a cells (ATCC) were collected, centrifuged at 94×*g* for 5 min at 4 °C, and washed twice with ice-cold phosphate-buffered saline (PBS) supplemented with ribonuclease inhibitor (100 U/mL, #E4210-01) and protease inhibitor cocktail (Sigma-Aldrich, #P8340). Next, cells were lysed in 1 mL of lysis buffer (5 mM PIPES, 200 mM KCl, 1 mM CaCl_2_, 1.5 mM MgCl_2_, 5% sucrose, 0.5% NP-40, supplemented with protease inhibitor cocktail + 100 U/ml RNase inhibitor, and 1 mM DTT) for 10 min on ice. Lysates were sonicated (Vibra-cell VCX-130) three times for 1 s ON, 30 s OFF at 30% amplitude, followed by centrifugation at 21130×*g* for 10 min at 4 °C. Supernatants were then transferred to new 2-mL tubes and supplemented with 1 mL of IP binding/washing buffer (150 mM KCl, 25 mM Tris (pH 7.5), 5 mM EDTA, 0.5% NP-40, supplemented with protease inhibitor cocktail + 100 U/ml RNase inhibitor, and 0.25 mM DTT). The samples were then rotated for 2–4 h at 4 °C with 5 μg of antibody per reaction. Fifty microliters of beads GenScript A/G beads (#L00277) per reaction was washed three times with IP binding/washing buffer, followed by addition to lysates for an overnight rotating incubation. After incubation, the beads were washed three times inIP binding/washing buffer. Ten percent of each sample was collected and boiled for 5 min at 95 °C for further analysis by western blot. The remaining beads were resuspended in 0.5 mL of TRIZOL for RNA extraction and assessment by RT-qPCR analysis where immunoprecipitation material was normalized to total cell lysate.

### Western blot

Protein samples collected from RIP were resolved on 8–10% SDS-PAGE gels and transferred to a polyvinylidene difluoride (PVDF) membrane. After blocking with 5% nonfat milk in PBS with 0.1% Tween-20 (PBST), the membranes were incubated with the primary antibody followed by the secondary antibody conjugated with horseradish peroxidase. Blots were quantified with Image Lab software. The primary antibody anti-Dhx36 (Bethyl, #A300-525A, 1:1000 dilution) and secondary antibody anti-rabbit (JIR #111-035, 1:10,000 dilution) were used.

### qRT-PCR

Total RNA was extracted from transfected N2a cells using TRIREAGENT (MRC) according to the manufacturer’s protocol. cDNA was synthesized using qScript Flex cDNA synthesis kit (95049, Quanta) with random primers. Fast SYBR Green master mix (4385614) was used for qPCR. Gene expression levels were normalized to the housekeeping genes Actin and *Gapdh.*

## Supplementary Information

**Additional file 1: Figure S1.** Conserved elements in the libra lncRNA. **Figure S2.** Gaps in the genomic assembly around the first exon in the *Chaserr* lncRNA locus. **Figure S3.** Functional characterization of the conserved elements in *Chaserr* lncRNA. **Figure S4.** Conserved elements. **Figure S5.** Additional analysis of LncLOOM motifs identified in 3’UTRs. **Figure S6.** Constraints imposed on the **Figure S7.** Partitioning of the LncLOOM graph and iterative refinement of selected repeated k-mers. LncLOOM graph.in the DICER 3’UTRs. **Figure S8.** Processing steps in the LncLOOM framework.

**Additional file 2: Supplementary Table S2.** Mass spectrometry results performed with fragments of mouse Chaserr Exon 5.

**Additional file 3.** LncLOOM output results for NORAD sequences from nine mammals.

**Additional file 4.** LncLOOM output results for XIST sequences from six mammals.

**Additional file 5.** LncLOOM output results for MALAT1 sequences from 19 vertebrates.

**Additional file 6: Table S1.** Order of sequences analyzed by LncLOOM. **Table S3.** Oligonucleotide sequences used for RNA pulldown. Mutated bases are underlined. **Table S4.** Oligonucleotide sequences of ASOs and LNA GapmeRs. **Table S5.** Primer sequences.

**Additional file 7:** Review history.

## Data Availability

LncLOOM code and the datasets used in this study are available in the GitHub repository https://github.com/lncLOOM/lncLOOM [54]. LncLOOM is licensed under the Mozilla Public License 2.0. A version of the source code used in this manuscript, along with the output generated for CHASERR, NORAD, XIST, and MALAT1 has been released on Zenodo (10.5281/zenodo.4320625, [55]). “The mass spectrometry proteomics data have been deposited to the ProteomeXchange Consortium via the PRIDE [56] partner repository with the dataset identifier PXD023093.”
