# Supplementary material for: Uncovering deeply conserved motif combinations in rapidly evolving noncoding sequences
Source: Genome Biol. 2021 Jan 11;22:29. doi: 10.1186/s13059-020-02247-1 (PMC7798263; doi:10.1186/s13059-020-02247-1)
Supplement: Supplementary file 1 — Additional file 1: Figure S1. Conserved elements in the libra lncRNA. Figure S2. Gaps in the genomic assembly around the first exon in the Chaserr lncRNA locus. Figure S3. Functional characterization of the conserved elements in Chaserr lncRNA. Figure S4. Conserved elements. Figure S5. Additional analysis of LncLOOM motifs identified in 3’UTRs. Figure S6. Constraints imposed on the Figure S7. Partitioning of the LncLOOM graph and iterative refinement of selected repeated k-mers. LncLOOM graph.in the DICER 3’UTRs. Figure S8. Processing steps in the LncLOOM framework. [file 13059_2020_2247_MOESM1_ESM.pdf]

Supplementary information for “Uncovering deeply conserved motif combinations in rapidly evolving noncoding sequences”

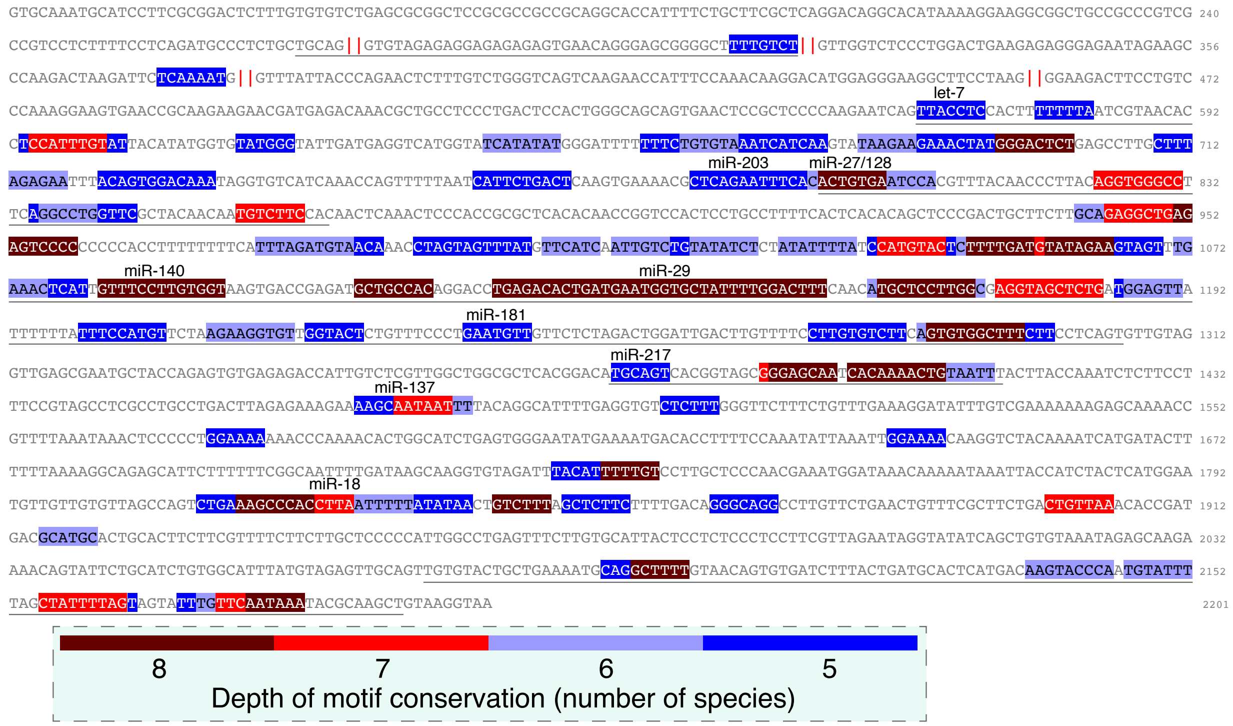

Figure S1. Conserved elements in the libra lncRNA. The human sequence is shown and the motifs conserved in at least five species are color-coded based on their conservation. Pairs of vertical lines represent intron positions. Motifs that match miRNA seed sites are indicated with the miRNA family name above the motif. Regions that are part of BLASTN alignments (E<0.001) between the human and spotted gar sequences are underlined.

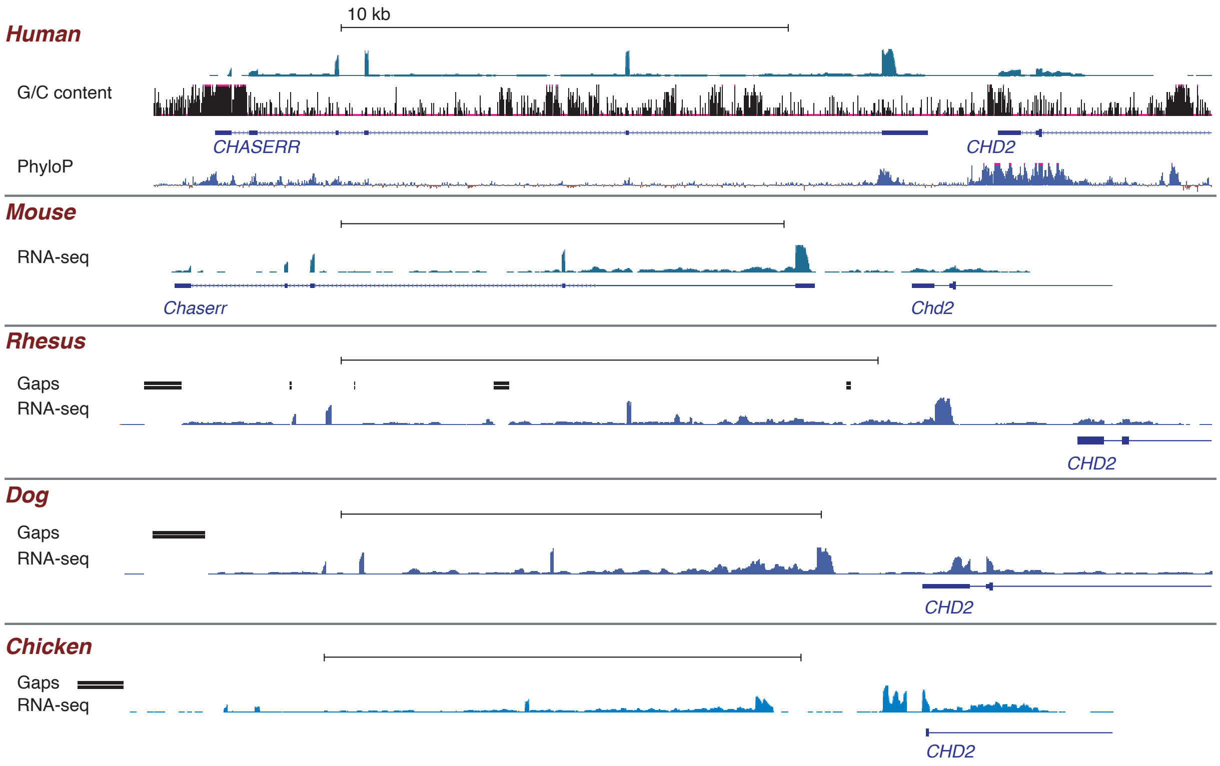

Figure S2. Gaps in the genomic assembly around the first exon in the *Chaserr* lncRNA locus. (A) Motifs conserved in at least four species, barcoded by their depth of conservation. **(B)** For each species, RNA-seq read coverage is shown, alongside gaps in the genome assembly (from the UCSC browser).

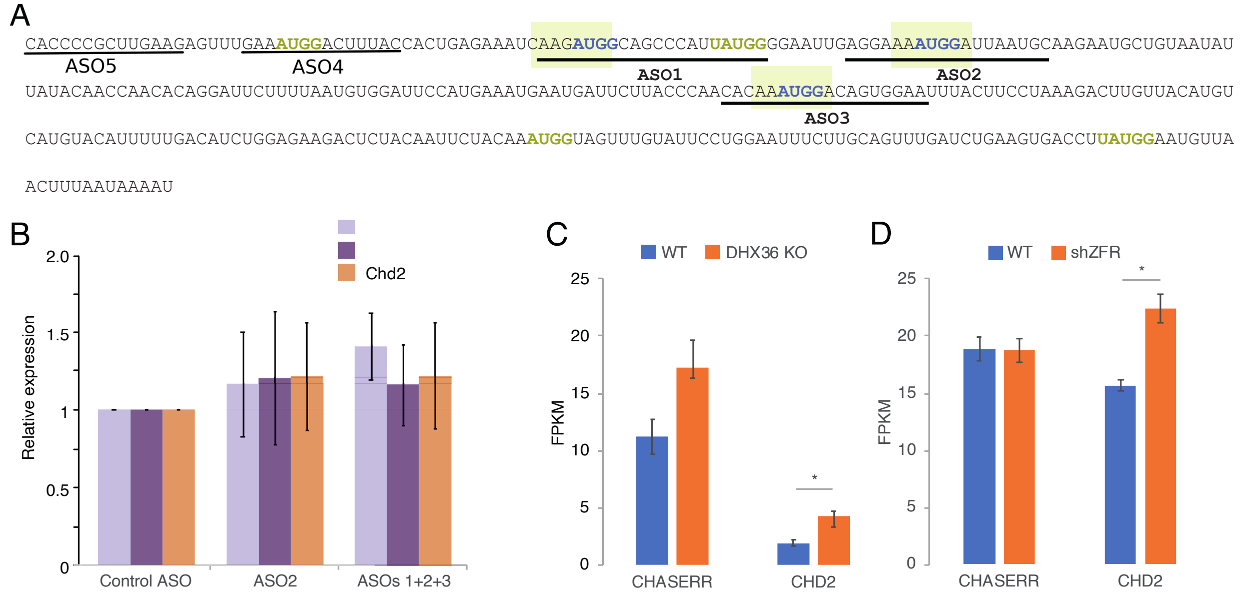

Figure S3. Functional characterization of the conserved elements in *Chaserr* lncRNA. (A) Sequence of the last exon of mouse *Chaserr*. The deeply conserved elements are shared. The conserved AUGG instances that were mutated in the MS baits are in blue and all the other AUGG instances are in green. Regions targeted by the ASOs are marked. **(B)** As in **Fig. 3C**, for the indicated ASO treatments. **(C)** RNA-seq quantification of the expression of the indicated gene in HEK293 cells with the indicated genotype, data from (Sauer et al. 2019). **(D)** RNA-seq quantification of the expression of the indicated genes in THP1 cells treated with a non-targeting shRNA (shNT) or an shRNA targeting ZFR. Data from (Haque et al. 2018).

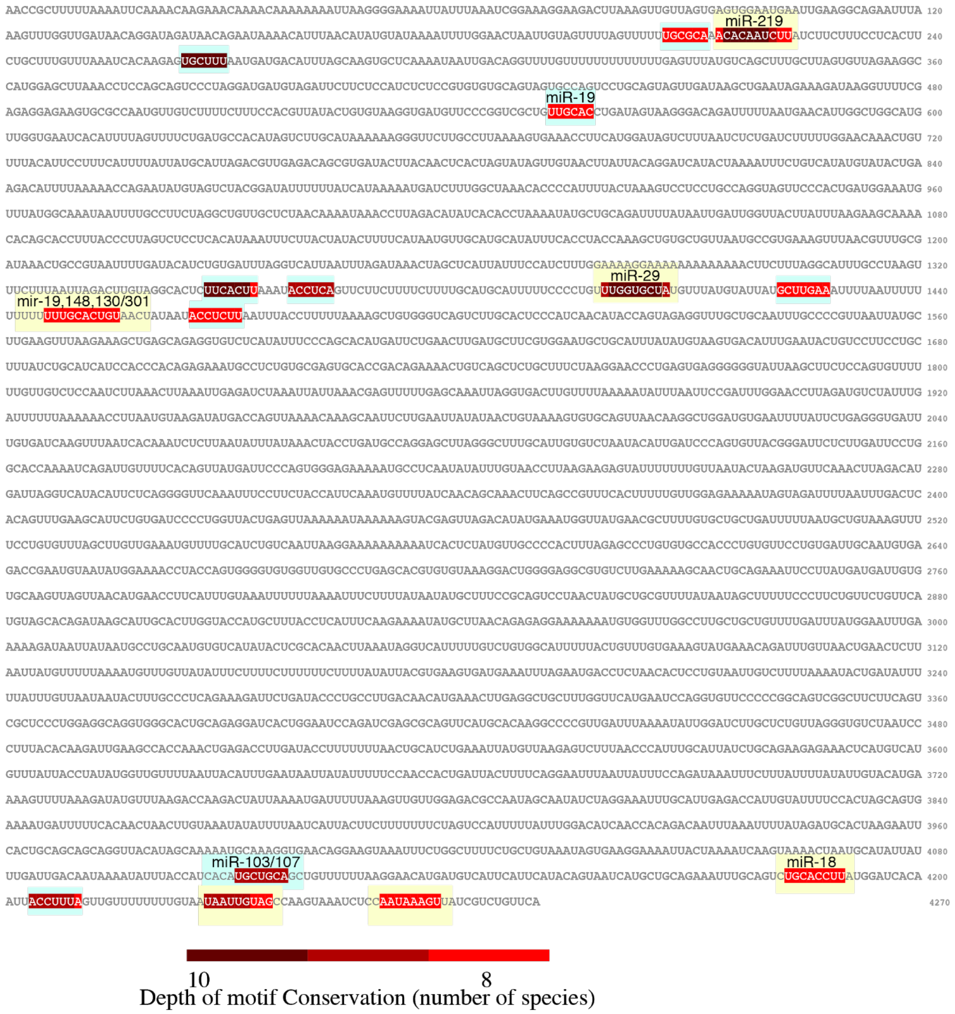

Figure S4. Conserved elements in the DICER 3'UTRs. The human sequence is shown and the motifs conserved in at least eight vertebrate species are color-coded based on their conservation (9 species - conserved in lancelet; 10 species - conserved in lancelet and sea urchin). Regions of motifs for which 100 random sequences preserving sequence identity do not contain any motif of this length are shaded in light yellow. Regions of motifs for which in random sequences the exact motif is not found are shaded in light cyan.

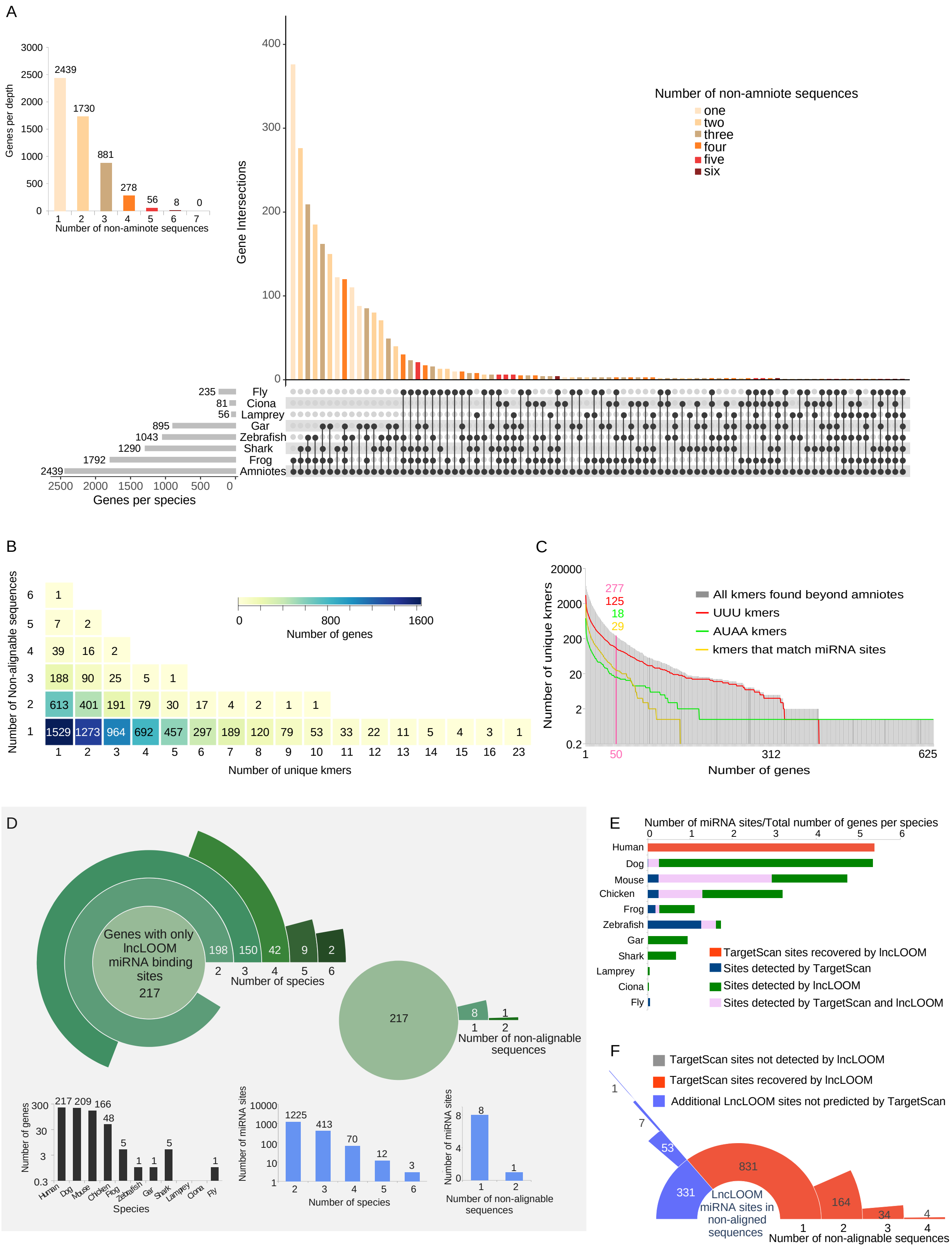
A
Genes per depth
2439
1730
881
278
56
8
0
Number of non-aminote sequences
Gene Intersections
Number of non-amniote sequences
one
two
three
four
five
six
Fly
Ciona
Lamprey
Gar
Zebrafish
Shark
Frog
Amniotes
235
81
56
895
1043
1290
1792
2439
Genes per species
B
Number of Non-alignable sequences
Number of genes
Number of unique kmers
C
Number of unique kmers
277
125
18
29
All kmers found beyond amniotes
UUU kmers
AUAA kmers
kmers that match miRNA sites
Number of genes
D
Genes with only lncLOOM miRNA binding sites 217
198
150
42
9
2
Number of species
217
8
1
Number of non-alignable sequences
Number of genes
Human
Dog
Mouse
Chicken
Frog
Zebrafish
Gar
Shark
Lamprey
Ciona
Fly
Species
Number of miRNA sites
1225
413
70
12
3
Number of species
Number of non-alignable sequences
E
Number of miRNA sites/Total number of genes per species
TargetScan sites recovered by lncLOOM
Sites detected by TargetScan
Sites detected by lncLOOM
Sites detected by TargetScan and lncLOOM
F
TargetScan sites not detected by lncLOOM
TargetScan sites recovered by lncLOOM
Additional LncLOOM sites not predicted by TargetScan
LncLOOM miRNA sites in non-aligned sequences
331
831
164
34
4
53
7
1
Number of non-alignable sequences

Figure S5. Additional analysis of LncLOOM motifs identified in 3'UTRs. (A) Distribution of orthologous 3'UTR sequences. Top left: Frequency of genes that were analysed at various depths. Top right: Distribution of various combinations of non-amniote sequences that were included in the 3'UTR sequence datasets. Bottom right: Overall number of genes analyzed in the indicated species. **(B)** Distribution of combinations of unique k-mers conserved per number of non-alignable sequences in 3'UTR datasets. Alignments to human, mouse, dog and chicken were considered. **(C)** Distribution of unique k-mers that were identified beyond amniotes and shared between multiple genes. Number of k-mers containing UUU (red line), AUAA (green line) or that matched a broadly conserved miRNA site (yellow line) are indicated. **(D)** Conservation of broadly conserved miRNA sites that were detected by LncLOOM in genes for which TargetScan did not report any predictions. (Top) Number of genes with a miRNA site detected per number of species (left) and number of non-alignable sequences (right). (Bottom left) Number of genes with a miRNA site detected per species. (Middle) Number of new miRNA sites detected per species. (Right) Number of new miRNA sites detected per number of non-alignable sequences. **(E)** Comparison of miRNA sites that have conservation detected per species by TargetScan and LncLOOM. Only sites that were previously identified by TargetScanHuman have been compared. **(F)** Conservation of miRNA sites detected by LncLOOM in sequences that had no alignment to the human sequence. Sites that were previously predicted by TargetScan in the human sequence are coloured red and new LncLOOM predictions are coloured blue.

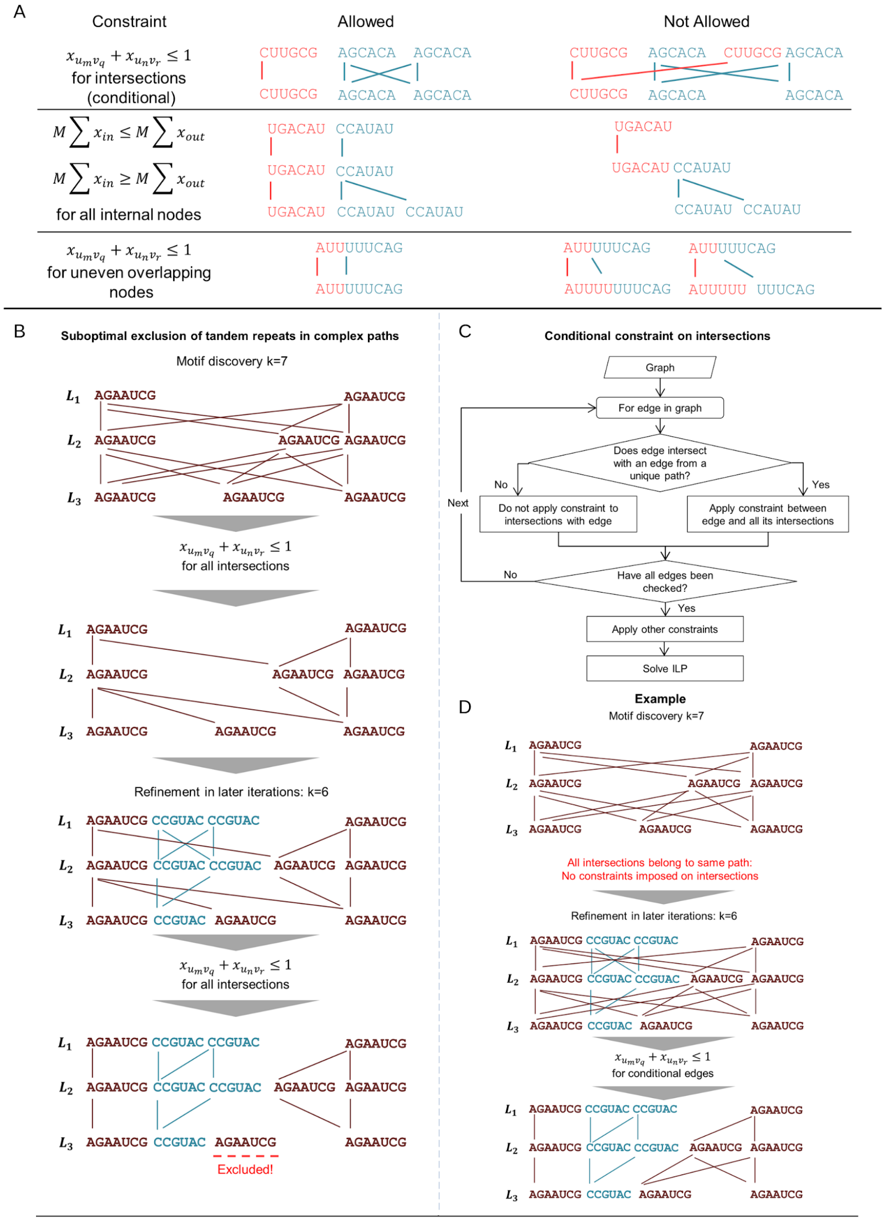

Figure S6. Constraints imposed on the LncLOOM graph. (A) Examples of scenarios in the LncLOOM graph and how those are represented in the ILP. **(B)** Conditional constraint on intersecting edges. An example of the suboptimal exclusion of repeated k-mers in complex paths during refinement in subsequent iterations that can occur if all intersections are constrained. **(C)** Flow diagram for defining conditional constraints on intersecting edges: a pair of intersecting edges is only constrained if there is at least one other edge, from a unique path, that intersects either of the edges. **(D)** Example demonstrating how the conditional constraint on intersections can mitigate the suboptimal exclusion of tandemly repeated k-mers.

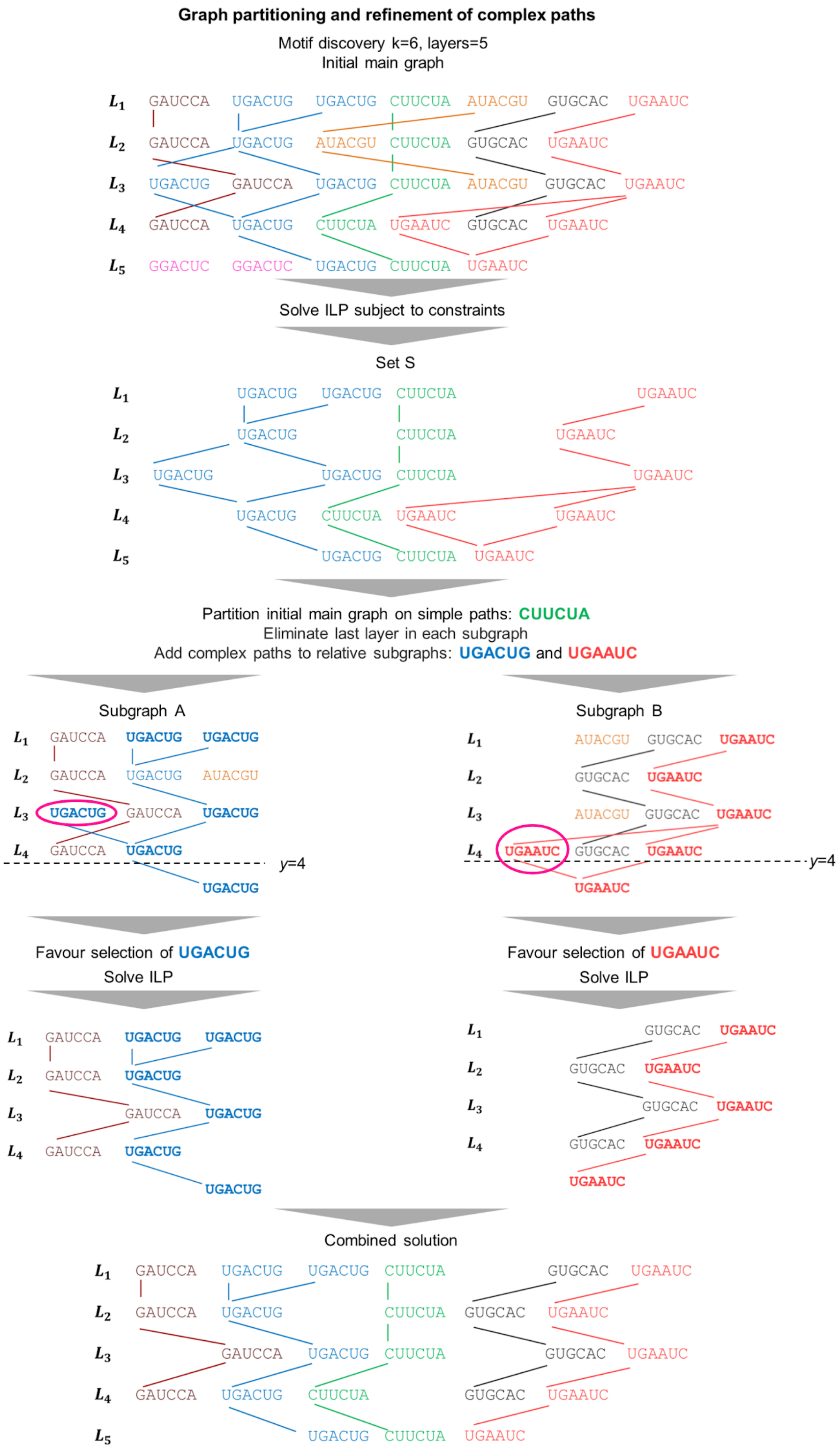

Figure S7. Partitioning of the LncLOOM graph and iterative refinement of selected repeated k-mers. Starting with the deepest layer in the graph, motif discovery is performed through an iterative process in which each step searches for motifs that are conserved at an increasingly shallower depth. Shown here is an example of motif discovery that begins in a graph of 5 layers. The graph is solved and the simple paths obtained in the solution (shown in green) are then used to partition the graph into subgraphs that are solved individually in the next iteration, which is performed on the top 4 layers of the graph. Each simple path is immediately added to the final solution, while complex paths (shown in blue and red) are refined during the subsequent iterations of motif discovery. In this case, the repeated k-mers that are removed during optimization are circled in pink.

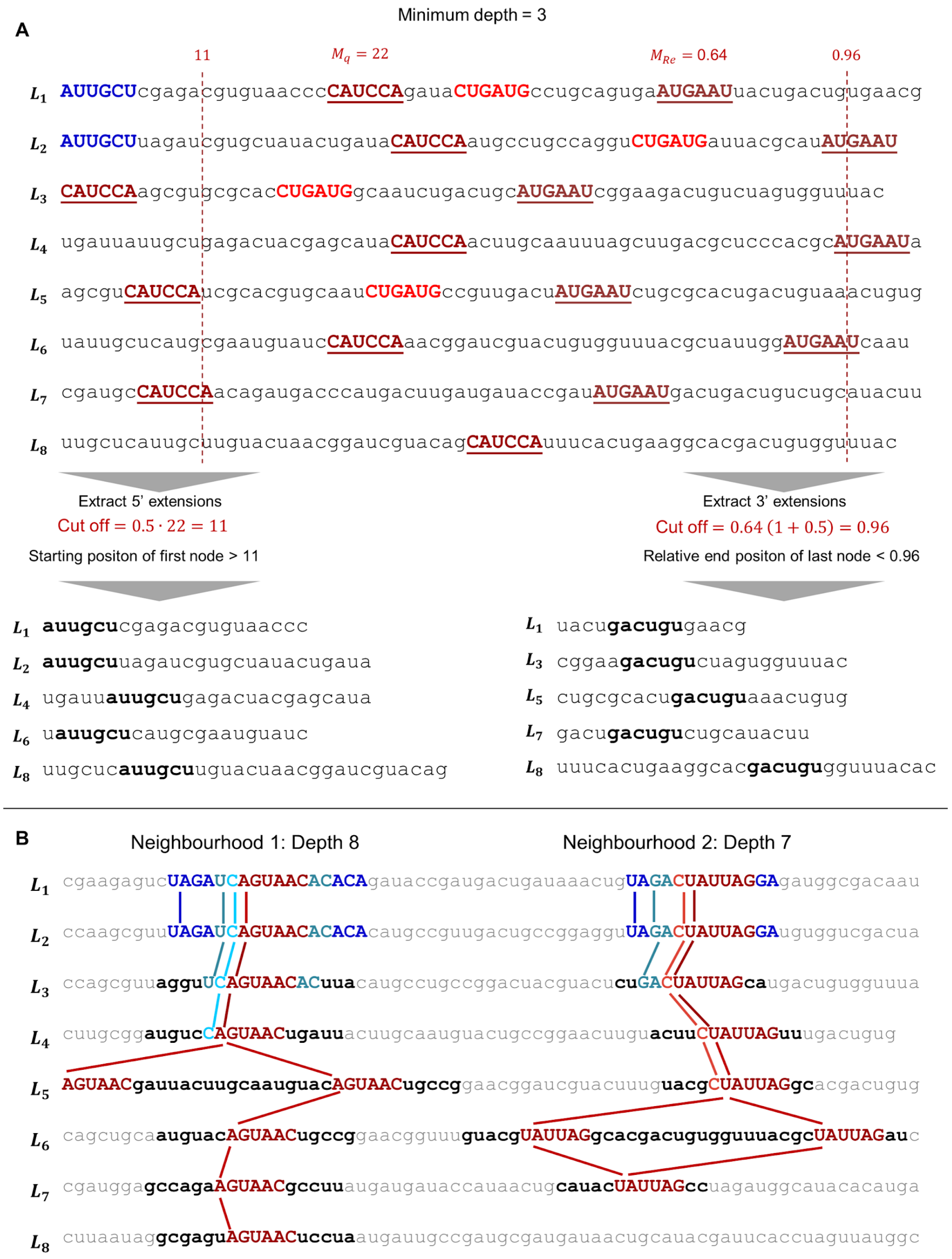

Figure S8. Processing steps in the LncLOOM framework. (A) Construction of the 5' and 3' graphs. LncLOOM uses the median positions of the first and last motifs identified in the primary ILP (in which the full-length of each sequence is considered) to predict and extract the 5' and 3' ends of individual sequences that are extended relative to other sequences in the graph. LncLOOM motif discovery is then performed on the subset of extracted 5' and 3' regions. In this example a minimum depth of 3 has been imposed, thus the AUUGCU (blue) motif that is only conserved in the top 2 sequences is ignored, and the CAUCCA (dark red and underlined) is considered as the first node instead. **(B)** Illustration of motif neighbourhoods. The reference sequence of each neighbourhood is determined by combining all overlapping k-mers in the anchor sequence. All k-mers that are conserved to respective depths in the graph and which are connected to one of the overlapping k-mers within the reference sequence, are then included within the neighbourhood.

References

Haque, Nazmul, Ryota Ouda, Chao Chen, Keiko Ozato, and J. Robert Hogg. 2018. “ZFR Coordinates Crosstalk between RNA Decay and Transcription in Innate Immunity.” *Nature Communications* 9 (1): 1145.

Sauer, Markus, Stefan A. Juranek, James Marks, Alessio De Magis, Hinke G. Kazemier, Daniel Hilbig, Daniel Benhalevy, Xiantao Wang, Markus Hafner, and Katrin Paeschke. 2019. “DHX36 Prevents the Accumulation of Translationally Inactive mRNAs with G4-Structures in Untranslated Regions.” *Nature Communications* 10 (1): 2421.
