# Supplementary material for: Uncovering deeply conserved motif combinations in rapidly evolving noncoding sequences
Source: Genome Biol. 2021 Jan 11;22:29. doi: 10.1186/s13059-020-02247-1 (PMC7798263; doi:10.1186/s13059-020-02247-1)
Supplement: Supplementary file 3 — Additional file 3. LncLOOM output results for NORAD sequences from nine mammals. [file 13059_2020_2247_MOESM3_ESM.gz › AdditionalFile3/NORAD_RESULTS.html]

LncLOOM Results


# NORAD Results

## Ulitsky Lab Weizmann Institute of Science

  

| All K-mers | Conservation by Species | Selected Species |
| --- | --- | --- |
| ▶ KMERS IN SEQUENCES | ▶ KMERS MAPPED TO ANCHOR SEQ. | ▶ DEEPEST. LEVEL SPECIFIC KMERS |
| ▶ BLOCK DIAGRAMS | ▶ BLOCK DIAGRAMS MAPPED TO ANCHOR SEQ. | ▶ DEEPEST. KMERS IN BLOCKS |
|  | ▶ MODULES |
