# Supplementary material for: Uncovering deeply conserved motif combinations in rapidly evolving noncoding sequences
Source: Genome Biol. 2021 Jan 11;22:29. doi: 10.1186/s13059-020-02247-1 (PMC7798263; doi:10.1186/s13059-020-02247-1)
Supplement: Supplementary file 3 — Additional file 3. LncLOOM output results for NORAD sequences from nine mammals. [file 13059_2020_2247_MOESM3_ESM.gz › AdditionalFile3/Html_Files/kmers_in_seqs_level.html]

 MOTIFS\_IN\_SEQS\_OVERLAP

# MOTIFS IN SEQUENCES

## Motifs conserved to (and beyond) MOUSE (depth:9)

  

NAVIGATE ▼

▶HUMAN (depth:1)▶BABOON (depth:2)▶DOG (depth:3)▶COW (depth:4)▶SHEEP (depth:5)▶PIG (depth:6)▶ARMADILLO (depth:7)▶GUINEAPIG (depth:8)▶MOUSE (depth:9)

  
  
  
  
Coloured by Conservation >>  

## >HUMAN (5443 bases)

```
 GGCACTTCCGGTACCGCTCCTCTCTTGCCAGCGCAGAGAAC

TGCCAA

TGCCAA  
Depth:9 (MOUSE)  
Ei-value:0.000, Pi-value:0.000  
Er-value:0.000, Pr-value:0.000  
No matches to eCLIP DataMATCHES To TargetScan▶ miR-182-5p:UUGGCAA▶ miR-96-5p/1271-5p:UUGGCAC

G

TCAGTTCCGG

TCAGTTCCGG  
Depth:9 (MOUSE)  
Ei-value:0.000, Pi-value:0.000  
Er-value:0.000, Pr-value:0.000  
eCLIP MATCHES▶EIF3G (bg=2.39%)No matches to TargetScan

TCCGGCAGAGATCGCGGAGAGACGCAGAACGCAGCCCGCTCCTCCAGGGCCCTCCAG

GCCCT

GCCCTC  
Depth:9 (MOUSE)  
Ei-value:0.000, Pi-value:0.000  
Er-value:0.000, Pr-value:0.000  
eCLIP MATCHES▶ddx3x (bg=13.89%)▶ddx6 (bg=23.92%)▶drosha (bg=11.37%)▶EIF3G (bg=2.39%)▶EIF3H (bg=7.83%)▶FMR1 (bg=6.3%)▶METAP2 (bg=6.14%)▶PUS1 (bg=2.87%)▶RPS3 (bg=4.15%)▶SDAD1 (bg=5.99%)▶SERBP1 (bg=0.81%)▶SF3B1 (bg=6.74%)▶WRN (bg=3.31%)▶ybx3 (bg=22.82%)No matches to TargetScan

 120  


C

GCCCTC  
Depth:9 (MOUSE)  
Ei-value:0.000, Pi-value:0.000  
Er-value:0.000, Pr-value:0.000  
eCLIP MATCHES▶ddx3x (bg=13.89%)▶ddx6 (bg=23.92%)▶drosha (bg=11.37%)▶EIF3G (bg=2.39%)▶EIF3H (bg=7.83%)▶FMR1 (bg=6.3%)▶METAP2 (bg=6.14%)▶PUS1 (bg=2.87%)▶RPS3 (bg=4.15%)▶SDAD1 (bg=5.99%)▶SERBP1 (bg=0.81%)▶SF3B1 (bg=6.74%)▶WRN (bg=3.31%)▶ybx3 (bg=22.82%)No matches to TargetScan

CGGCCCCGGGCCGGCGGGTGAACTGGGGGGCCCCGGGACAGGCCGAGCCCTCTGCCCTGCAGATAACGGAGGCCTCTGCTGTGGCTGCCCACTGGCTGTGCCCGCCCACTGGCTGTGCC 240  
 CAGACCTTGAAGCCGCAGCGAACCTCTCTTTCCCACCCCACCTCGGTGACTA

ATGGCGGC

ATGGCGGC  
Depth:9 (MOUSE)  
Ei-value:0.000, Pi-value:0.000  
Er-value:0.000, Pr-value:0.000  
eCLIP MATCHES▶BCCIP (bg=4.61%)▶ddx3x (bg=13.89%)▶ddx6 (bg=23.92%)▶dgcr8 (bg=19.31%)▶DHX30 (bg=5.05%)▶drosha (bg=11.37%)▶EIF3H (bg=7.83%)▶FMR1 (bg=6.3%)▶FTO (bg=7.53%)▶fubp3 (bg=23.31%)▶FXR2 (bg=7.51%)▶GEMIN5 (bg=2.46%)▶IGF2BP3 (bg=4.26%)▶METAP2 (bg=6.14%)▶NIP7 (bg=2.54%)▶PCBP1 (bg=2.3%)▶pum1 (bg=29.85%)▶pum2 (bg=21.55%)▶rbm15 (bg=11.81%)▶SDAD1 (bg=5.99%)▶WRN (bg=3.31%)No matches to TargetScan

CGTGGCGTCTCCCAGCCCGGACCCCGCCGGCACCCGGGTCTCCCGACCCAAGCCTCGACG 360  
 AAACCCCCGCAGAGCCGCCGGGACGCAGCGCCTTTGGGCGGCGCTGGGCGTGGTGGGCCGGGAAGTATGGCGGCAGCTCGAACGCCGCGCGGCGGAGGCCATTAAGGCGTGGACGGCCCG 480  
 GGAAGGCGGCCTAGGGACGCAAGCAGGCTCGGCCGCCTCTTTAGGCCACGGAGCCGCGCAGATCCGGTTCCCGGGTGACCACTCTGTCGCCATTGGGCGAGACCTACCTAGTCCTGACGA 600  
 CAACGGACAAAGGCCTTAAGGGGCCTGGAAGGTGAGCGAAGTCCCGAACGACGACGGGTGGAACGGTTAGCGGCCATCGGGCGGTTGGTCTTCATTCTACCAGACTTTGCTGTCGGAAGA 720  
 GAGAAATGGTAGAATGACAGGCCACGTTTGGCCCGTTGGAAATGCCCACCACCCTCT

GGGAAGA

GGGAAGA  
Depth:9 (MOUSE)  
Ei-value:0.000, Pi-value:0.000  
Er-value:0.000, Pr-value:0.000  
No matches to eCLIP DataNo matches to TargetScan

TTTACTGGCCGTTTATGGAAGG

CCTGTGTATATAATATGAAAAAGCTGCT

CCTGTGTATATAATATGAAAAAGCTGCT  
Depth:9 (MOUSE)  
Ei-value:0.000, Pi-value:0.000  
Er-value:0.000, Pr-value:0.000  
eCLIP MATCHES▶pum1 (bg=29.85%)MATCHES To TargetScan▶ miR-15-5p/16-5p/195-5p/424-5p/497-5p:AGCAGCA▶ miR-503-5p:AGCAGCG

CTCAAC 840  
 TCCACCC

CAACCTTT

CAACCTTT  
Depth:9 (MOUSE)  
Ei-value:0.000, Pi-value:0.000  
Er-value:0.000, Pr-value:0.000  
eCLIP MATCHES▶pum1 (bg=29.85%)No matches to TargetScan

TAATAGAAAACATTTGTCACATCTAGCCCTTCTAGATGGAA

AGAGGT

AGAGGT  
Depth:9 (MOUSE)  
Ei-value:0.000, Pi-value:0.000  
Er-value:0.000, Pr-value:0.000  
eCLIP MATCHES▶dgcr8 (bg=19.31%)▶IGF2BP2 (bg=3.44%)▶KHDRBS1 (bg=1.16%)▶lin28b (bg=16.96%)▶pum1 (bg=29.85%)▶pum2 (bg=21.55%)▶rbm15 (bg=11.81%)▶tia1 (bg=16.04%)No matches to TargetScan

TGCCGACGTATGATAAAATAGAGTTAGAAAGTTACACATCTTGTAAATTCTCATTTGT 960  
 TTAAAAGAAATCATAGAAAATACATGTCTTCTGGAGATGACTTTTG

GAAATG

GAAATG  
Depth:9 (MOUSE)  
Ei-value:0.000, Pi-value:0.000  
Er-value:0.000, Pr-value:0.000  
eCLIP MATCHES▶fubp3 (bg=23.31%)▶GRWD1 (bg=4.85%)▶PPIG (bg=0.88%)▶pum1 (bg=29.85%)▶pum2 (bg=21.55%)▶rbm15 (bg=11.81%)▶SND1 (bg=1.27%)▶ZNF622 (bg=1.54%)No matches to TargetScan

GAGTTGTTAAGACGGCCTCTGGAAGCGATACGTCCACGTTTGTTAAGTGGGTTAGATGACATGGAGCT 1080  
 GGAAGACCTGAGAAGGAAGAGAAGAAGGTTCTATGCTAGACTGGTCATATTT

AGAAGA

AGAAGA  
Depth:9 (MOUSE)  
Ei-value:0.000, Pi-value:0.000  
Er-value:0.000, Pr-value:0.000  
No matches to eCLIP DataNo matches to TargetScan

CATTTTCATATTCTATCCATTGTTTTGTGTGCATTTTATTCCTCACTACTGTGTATATAGTT 1200  
 GACAATGCTAAGCTTTTTTGAAATGTCTCTTCTTTTTAGATGTTCTG

AAGTGCCTG

AAGTGCCTG  
Depth:9 (MOUSE)  
Ei-value:0.000, Pi-value:0.000  
Er-value:0.000, Pr-value:0.000  
eCLIP MATCHES▶pum1 (bg=29.85%)▶PUS1 (bg=2.87%)▶tial1 (bg=14.09%)No matches to TargetScan

ATATGTTAAAATTAGAGGTAGCAAAATCACATTTTGTAAATACCTTTTTGTTACAATTCATAGG 1320  
 AAATATTTTTGGGGGGGAATGGCCAAATCACCTGTTGAGTAATACTCATTGTGT

TTGTGC

TTGTGC  
Depth:9 (MOUSE)  
Ei-value:0.000, Pi-value:0.000  
Er-value:0.000, Pr-value:0.000  
eCLIP MATCHES▶pum1 (bg=29.85%)▶tia1 (bg=16.04%)No matches to TargetScan

AGTGGTTCAGGGGAGGAGAGAGGAGGGGGAGGTGCAGAGAGCTCTATGCCATCCTGTTTA 1440  
 CAGCGAGGCAAGATGAATCATTATGTCTGTGCATTTTGTTTTACTTATCTGTGTATATAGTGTACATAAAGGACAGACGAGTCCTAATTGACAACATCTAGTCTTTCTG

GATGTT

GATGTT  
Depth:9 (MOUSE)  
Ei-value:0.000, Pi-value:0.000  
Er-value:0.000, Pr-value:0.000  
No matches to eCLIP DataNo matches to TargetScan


AAAGA

AAAGAGGTTGCCA  
Depth:9 (MOUSE)  
Ei-value:0.000, Pi-value:0.000  
Er-value:0.000, Pr-value:0.000  
No matches to eCLIP DataNo matches to TargetScan

 1560  


GGTTGCCA

AAAGAGGTTGCCA  
Depth:9 (MOUSE)  
Ei-value:0.000, Pi-value:0.000  
Er-value:0.000, Pr-value:0.000  
No matches to eCLIP DataNo matches to TargetScan

GTGTATGACAAAAGTAGAGTTAGTAAACTAATATATTTTGTAC

ATTTTGT

ATTTTGT  
Depth:9 (MOUSE)  
Ei-value:0.000, Pi-value:0.000  
Er-value:0.000, Pr-value:0.000  
No matches to eCLIP DataNo matches to TargetScan

TTTACAAGTCCTAGGAAAGATTGTCTTCTGAAAATTTGATGTCTTCTGGGTTGATGGAGATG 1680  
 GGAAGGGTTCTAGGCCAGAATGTTCACATTTGGAAGACTCTTTCAAATTATAACTGTTGTTACATGTTTGCAGTTTATTCAAGACTGCTGTATACATAGTAGACAAATTAAC

TCCTTA

TCCTTA  
Depth:9 (MOUSE)  
Ei-value:0.000, Pi-value:0.000  
Er-value:0.000, Pr-value:0.000  
No matches to eCLIP DataNo matches to TargetScan

CT 1800  
 TG

AAACATCT

AAACATCT  
Depth:9 (MOUSE)  
Ei-value:0.000, Pi-value:0.000  
Er-value:0.000, Pr-value:0.000  
No matches to eCLIP DataNo matches to TargetScan

AGTCTAT

CTAGATGTTTAGAAGTGCCC

CTAGATGTTTAGAAGTGCCC  
Depth:9 (MOUSE)  
Ei-value:0.000, Pi-value:0.000  
Er-value:0.000, Pr-value:0.000  
No matches to eCLIP DataNo matches to TargetScan

GAT

GTATGTTAAA

GTATGTTAAA  
Depth:9 (MOUSE)  
Ei-value:0.000, Pi-value:0.000  
Er-value:0.000, Pr-value:0.000  
No matches to eCLIP DataNo matches to TargetScan

TGTATAGGTAGTAAAATACCACTT

TGTAAATA

TGTAAATA  
Depth:9 (MOUSE)  
Ei-value:0.000, Pi-value:0.000  
Er-value:0.000, Pr-value:0.000  
No matches to eCLIP DataNo matches to TargetScan

TCTTTTTGCTAAAATTCATAGGAAATGCTTTTGGAAAT 1920  
 TGAATTGTGAAGCCACCTTTGTGAACAGTATAGTAATGTCTATACTTGTTCAATAGTTTAGAGGAGGTAGGAGGGAAGAAATTGCAAAAGGTAATATTACTAGTGTGTTCATACTTGGAC 2040  
 ATTTTCAGACACCATTTTTCTATATGTTTTGTGCATTTTGTTTTGCTCTGTATATAGTATATATAATGGACAAATAGTCCTAATTTTTCAACATCTAGTCTCTAGATGTTAAAGAGGTTG 2160  
 CCAGTGTATGACAAAGGAGTAAAATTAGCATATTTTGTACACTTTGTGTTGAAATTCGTAGGAAAACTTGTCTTCTGTAAAGACTTTTGCATAGGAATTTGTTTGACCATCTCTAAGCAT 2280  
 TACACGTGCCTGTACTTGTCCACTGGATTGAAGGCAGAGAAGGAAGGGAGGAGGGAATGATTCAAGGCCAAAATGGCCACATTTAGAAGATACCTCAGATGATAACCATTGTTATGTGTG 2400  
 TGCAATTTTATTTAACAGTGCTGTGTATGTGGTGGACAAGTTATATGAAATATCTAGTCTTTCTAGATATTTGGAAGTGCTTGATGTATTTAAAAGTGGTAGTAGAATAACACTTTGTAA 2520  
 ATAGCTTTTAAAAACTGATGGGAAATGCTGTTTGGAAGTGGAATTGTTGAACCACCTGGGAG

GTGGGAGGGAA

GTGGGAGGGAA  
Depth:9 (MOUSE)  
Ei-value:0.000, Pi-value:0.000  
Er-value:0.000, Pr-value:0.000  
eCLIP MATCHES▶ddx55 (bg=12.35%)▶FTO (bg=7.53%)▶GRSF1 (bg=3.8%)▶KHSRP (bg=8.1%)▶pum1 (bg=29.85%)▶SUB1 (bg=9.24%)MATCHES To TargetScan▶ miR-150-5p:CUCCCAA▶ miR-532-3p:CUCCCAC

GAAAT

TGCAAA

TGCAAA  
Depth:9 (MOUSE)  
Ei-value:0.000, Pi-value:0.000  
Er-value:0.000, Pr-value:0.000  
eCLIP MATCHES▶ddx55 (bg=12.35%)▶FTO (bg=7.53%)▶fubp3 (bg=23.31%)▶GRSF1 (bg=3.8%)▶KHSRP (bg=8.1%)▶pum1 (bg=29.85%)▶SUB1 (bg=9.24%)No matches to TargetScan

TGGTGTTTTGCCATTGTTTATTAGAAAATTTCAGCT 2640  
 TAATCCATTGTGTATATGTTACATGCATTTCATTTAACTTTGCTATAC

TGTATATA

TGTATATA  
Depth:9 (MOUSE)  
Ei-value:0.000, Pi-value:0.000  
Er-value:0.000, Pr-value:0.000  
eCLIP MATCHES▶dgcr8 (bg=19.31%)▶EIF4G2 (bg=0.7%)▶fam120a (bg=18.43%)▶FASTKD2 (bg=7.73%)▶fubp3 (bg=23.31%)▶HNRNPL (bg=3.4%)▶igf2bp1 (bg=11.67%)▶KHSRP (bg=8.1%)▶NCBP2 (bg=2.92%)▶NOLC1 (bg=6.58%)▶pum1 (bg=29.85%)▶pum2 (bg=21.55%)▶sf3a3 (bg=12.13%)▶SF3B1 (bg=6.74%)▶TBRG4 (bg=7.0%)▶tia1 (bg=16.04%)▶tial1 (bg=14.09%)▶ZC3H11A (bg=6.25%)No matches to TargetScan

TTGTATATATAACGGACAAATTAGTCCCGATTTTATA

ATATCTAG

ATATCTAG  
Depth:9 (MOUSE)  
Ei-value:0.000, Pi-value:0.000  
Er-value:0.000, Pr-value:0.000  
eCLIP MATCHES▶AKAP1 (bg=3.4%)▶ddx55 (bg=12.35%)▶dgcr8 (bg=19.31%)▶fam120a (bg=18.43%)▶fubp3 (bg=23.31%)▶GRSF1 (bg=3.8%)▶igf2bp1 (bg=11.67%)▶KHSRP (bg=8.1%)▶NKRF (bg=3.64%)▶PHF6 (bg=1.71%)▶pum1 (bg=29.85%)▶pum2 (bg=21.55%)▶sf3a3 (bg=12.13%)▶SF3B1 (bg=6.74%)▶tia1 (bg=16.04%)▶WRN (bg=3.31%)▶ZC3H11A (bg=6.25%)No matches to TargetScan

T

CTCTAG

CTCTAG  
Depth:9 (MOUSE)  
Ei-value:0.000, Pi-value:0.000  
Er-value:0.000, Pr-value:0.000  
eCLIP MATCHES▶ddx55 (bg=12.35%)▶dgcr8 (bg=19.31%)▶FASTKD2 (bg=7.73%)▶fubp3 (bg=23.31%)▶GRSF1 (bg=3.8%)▶KHSRP (bg=8.1%)▶pum1 (bg=29.85%)▶pum2 (bg=21.55%)▶tia1 (bg=16.04%)▶ZC3H11A (bg=6.25%)No matches to TargetScan

ATATT

AAAGAGG

AAAGAGGTTGCCAATGTATGACA  
Depth:9 (MOUSE)  
Ei-value:0.000, Pi-value:0.000  
Er-value:0.000, Pr-value:0.000  
eCLIP MATCHES▶ddx55 (bg=12.35%)▶dgcr8 (bg=19.31%)▶FASTKD2 (bg=7.73%)▶fubp3 (bg=23.31%)▶GRSF1 (bg=3.8%)▶NOLC1 (bg=6.58%)▶pum1 (bg=29.85%)▶pum2 (bg=21.55%)▶tial1 (bg=14.09%)MATCHES To TargetScan▶ miR-182-5p:UUGGCAA▶ miR-96-5p/1271-5p:UUGGCAC▶ miR-539-3p:UCAUACA

 2760  


TTGCCAATGTATGACA

AAAGAGGTTGCCAATGTATGACA  
Depth:9 (MOUSE)  
Ei-value:0.000, Pi-value:0.000  
Er-value:0.000, Pr-value:0.000  
eCLIP MATCHES▶ddx55 (bg=12.35%)▶dgcr8 (bg=19.31%)▶FASTKD2 (bg=7.73%)▶fubp3 (bg=23.31%)▶GRSF1 (bg=3.8%)▶NOLC1 (bg=6.58%)▶pum1 (bg=29.85%)▶pum2 (bg=21.55%)▶tial1 (bg=14.09%)MATCHES To TargetScan▶ miR-182-5p:UUGGCAA▶ miR-96-5p/1271-5p:UUGGCAC▶ miR-539-3p:UCAUACA

GAAGTAGA

GTTAGTAAACT

GTTAGTAAACT  
Depth:9 (MOUSE)  
Ei-value:0.000, Pi-value:0.000  
Er-value:0.000, Pr-value:0.000  
eCLIP MATCHES▶ddx55 (bg=12.35%)▶dgcr8 (bg=19.31%)▶FASTKD2 (bg=7.73%)▶NOLC1 (bg=6.58%)▶pum1 (bg=29.85%)▶pum2 (bg=21.55%)No matches to TargetScan

A

ACACATT

ACACATT  
Depth:9 (MOUSE)  
Ei-value:0.000, Pi-value:0.000  
Er-value:0.000, Pr-value:0.000  
eCLIP MATCHES▶NOLC1 (bg=6.58%)No matches to TargetScan

TTGTACACTTTGTTAAAATTTGTAGAAAGGC

TGTCTTCTGAA

TGTCTTCTGAA  
Depth:9 (MOUSE)  
Ei-value:0.000, Pi-value:0.000  
Er-value:0.000, Pr-value:0.000  
eCLIP MATCHES▶dgcr8 (bg=19.31%)▶pum2 (bg=21.55%)▶ybx3 (bg=22.82%)No matches to TargetScan

AAGGACTTTTGGAAGTGAGATAACATCAGCTCTAA 2880  
 GTGACAC

GTGCCT

GTGCCT  
Depth:9 (MOUSE)  
Ei-value:0.000, Pi-value:0.000  
Er-value:0.000, Pr-value:0.000  
eCLIP MATCHES▶lin28b (bg=16.96%)▶ybx3 (bg=22.82%)No matches to TargetScan

ATATCCATCAGGTTGGTGGTGGAGAGGAGTTGGAAGGAATGAAGGGTTCTAGACC

AGAATG

AGAATG  
Depth:9 (MOUSE)  
Ei-value:0.000, Pi-value:0.000  
Er-value:0.000, Pr-value:0.000  
eCLIP MATCHES▶AARS (bg=2.33%)▶FUS (bg=2.59%)▶lin28b (bg=16.96%)▶UTP3 (bg=2.81%)▶ybx3 (bg=22.82%)No matches to TargetScan

TTCGTATTT

AGAAGA

AGAAGA  
Depth:9 (MOUSE)  
Ei-value:0.000, Pi-value:0.000  
Er-value:0.000, Pr-value:0.000  
eCLIP MATCHES▶AARS (bg=2.33%)▶FUS (bg=2.59%)▶lin28b (bg=16.96%)No matches to TargetScan

CACTATCAGATATAACCATTGTTACATGTGT 3000  
 GTAGTTTATTCAACCCTACTG

TGTATATA

TGTATATA  
Depth:9 (MOUSE)  
Ei-value:0.000, Pi-value:0.000  
Er-value:0.000, Pr-value:0.000  
eCLIP MATCHES▶DDX21 (bg=1.64%)▶dgcr8 (bg=19.31%)▶fam120a (bg=18.43%)▶HNRNPL (bg=3.4%)▶igf2bp1 (bg=11.67%)▶pum2 (bg=21.55%)▶ZC3H11A (bg=6.25%)No matches to TargetScan

GCGGACAAACTTAAGTCCTTATT

TGAAACATCTAG

TGAAACATCTAG  
Depth:9 (MOUSE)  
Ei-value:0.000, Pi-value:0.000  
Er-value:0.000, Pr-value:0.000  
No matches to eCLIP DataNo matches to TargetScan

TCT

TTCTAG

TTCTAG  
Depth:9 (MOUSE)  
Ei-value:0.000, Pi-value:0.000  
Er-value:0.000, Pr-value:0.000  
No matches to eCLIP DataNo matches to TargetScan

A

TGTTTAGAAGTGCACAAAGTATGTTAAAAGTAGA

TGTTTAGAAGTGCACAAAGTATGTTAAAAGTAGA  
Depth:9 (MOUSE)  
Ei-value:0.000, Pi-value:0.000  
Er-value:0.000, Pr-value:0.000  
No matches to eCLIP DataNo matches to TargetScan

GGTAGTAAATAA 3120  
 CACATTTTGTAGCTATCCTTTTGATATGAAATATTGTCTTGGAAATTGATCAATTCTCTGAGCAGTACCCATTTTGATATTTGTGCTGGTTCAGGGGGAAGGAGGAGCACAAAGTGCAAA 3240  
 GGGCTTTCTACCAGTGTCCAGTGTGTTTATGAGGAGGCACATTGACCATTGTCCCTTATGTCTGCATTTTCATTTACTGTGCTG

TGTATA

TGTATA  
Depth:9 (MOUSE)  
Ei-value:0.000, Pi-value:0.000  
Er-value:0.000, Pr-value:0.000  
eCLIP MATCHES▶APOBEC3C (bg=3.95%)▶pum1 (bg=29.85%)▶pum2 (bg=21.55%)No matches to TargetScan

TAGTGTATATAAGCGGACATAGGAGTCCTA 3360  
 ATTTACGTCTAGTCGATGTTAAAAAGGTTGCCAGTATATGACAAAAGTAGAATTAGTAAACTACTACATTGAGTACACTTTGTGTTAAAATTCATAGGGAAGACTTCTTAAAAACAAGTG 3480  
 AAATTGTTAAAACCCCCCCTAAGCATTACAGATGGCTTATAGCTGTCCACGGGGTTGGTAGAGGTGGGAAAGGGAAGGGTTCTAGGCCAGAATGTTCCTATTTAGAAGACACTCAAATTA 3600  
 CAGTCTGTGTTATGTATGTATACCATTTATTCAATGCTACTGTGTATATAATGGAAAACTTAAGTCCAGTTTGAAACATCT

AGTCTT

AGTCTT  
Depth:9 (MOUSE)  
Ei-value:0.000, Pi-value:0.000  
Er-value:0.000, Pr-value:0.000  
eCLIP MATCHES▶fam120a (bg=18.43%)▶pum1 (bg=29.85%)▶pum2 (bg=21.55%)No matches to TargetScan

TCTAGGTGTTTAAAAGTGTACAACGGCCTGTCG 3720  
 CAGTGGCGCATGCCTGTAATCCCAGCACTTTGGGAGGCCGAGGCAGGCGGATCACGAGGTCAAGAGATCAGGACCATCTTGGCCAACATGGTGAAACCCCATCTTTACTAAAAATACAAA 3840  
 AATTAGCTGGTCGTGGTGGTGCCCACCTGTAGCCCCAGTTACTCGAGAGGCTGAGGCAGGAGAATCGCTTGAACTTGGGAGGCGGAAGTTGCAGTGAGCCAAGATCGCACCACTGCACTC 3960  
 CAGCCTGGCGACAGAGCGAGGCTCCGTTTCAAAAAAAAAAGTGCACAATGTAGGTTAACAGTAGAGGGCTTAAGTAACACCCCTCTAAGCATTTGTTTTCAGTACTTCCTAGGAGTGGTT 4080  
 GCATTTGGGAATGG

AATTGTTA

AATTGTTA  
Depth:9 (MOUSE)  
Ei-value:0.000, Pi-value:0.000  
Er-value:0.000, Pr-value:0.000  
No matches to eCLIP DataNo matches to TargetScan

AAACTTGATGCTTAGGAGCGAATGCAGACTATTCATTGGGTGTTTGGGGTGGGGGAAGGGGGGGTGGGCAGAGGAGGTATGCAGGGAGAGGGGTTCTG 4200  
 TGCTCCTGAGATTAGTTCAGATGGTCTAACCATTGTTCTATATGTGCATTTTAGTTAATATTGTGTATTAAAGGATAAGTCTTAATGCTCAAAGTATGTTAAAAATAGATGTAGTAAATC 4320  
 AGTCCCTTTGTGAATGTCCTTTTGTTAGTTTTTAGGAAGGCCTGTCCTCTGGGAGTGACCTTTATTAGTCCACCCCTTGGAGCTAGACATCCTGTACTTAGTCACGGGGATGGTGGAAGA 4440  
 GGGAGAAGAGGAAGGGTGAAGGGAAGGGCTCTTTGCTAGTATCTCCATATCTAGACGATGGTTTTAGATGATAACCACAGGTCTACAAGAGCGTTTTTAGTAAAGTGCCTGTGTTCATTG 4560  
 TGGACAAAGTTATTATTTTGCAACATCTAAGCTTTACGAATGGGGTGACAACTTATGATAAAAACTAGAGCTAGTGAATTAGCCTATTTGTAAATACCTTTGTTATAATTGATAGGATAC 4680  
 ATCTTGGACATGGAATTGTTAAGCCACCTCTGAGCAGTGTATGTCAGGACTTGTTCATTAGGTTGGCAGCAGAGGGGCAGAAGGAATTATACAGGTAGAGATGTATGCAGATGTGTCCAT 4800  
 ATATGTCCATATTTACATTTTGATAGCCATTGATGTATGCATCTCTTGGCTGTACTATAAGAACACATTAATTCAATGGAAATACACTTTGCTAATATTTTAATGGTATAGATCTGCTAA 4920  
 TGAATTCTCTTAAAAACATACTGTATTCTGTTGCTGTGTGTTTCATTTTAAATTGAGCATTAAGGGAATGCAGCATTTAAATCAGAACTCTGCCAATGCTTTTATCTAGAGGCGTGTTGC 5040  
 C

ATTTTTGT

ATTTTTGT  
Depth:9 (MOUSE)  
Ei-value:0.000, Pi-value:0.000  
Er-value:0.000, Pr-value:0.000  
eCLIP MATCHES▶EIF3H (bg=7.83%)▶HNRNPC (bg=1.49%)No matches to TargetScan

CTTATATGAAATTTCTGTCCCAAGAAAGGCAGGATTACATCTTTTTTTTTTTTTTTAGCAGTTTGAGTTGGTGTAGTGTA

TTCTTGGT

TTCTTGGT  
Depth:9 (MOUSE)  
Ei-value:0.000, Pi-value:0.000  
Er-value:0.000, Pr-value:0.000  
eCLIP MATCHES▶APOBEC3C (bg=3.95%)▶ddx55 (bg=12.35%)▶ddx6 (bg=23.92%)▶EIF3H (bg=7.83%)▶fam120a (bg=18.43%)▶fubp3 (bg=23.31%)▶FUS (bg=2.59%)▶tia1 (bg=16.04%)▶tial1 (bg=14.09%)▶ZC3H11A (bg=6.25%)No matches to TargetScan

TATCAGAATACTCATATAGCTTT 5160  
 GGGATTTTGAATTGGTA

AATATTCA

AATATTCA  
Depth:9 (MOUSE)  
Ei-value:0.000, Pi-value:0.000  
Er-value:0.000, Pr-value:0.000  
No matches to eCLIP DataNo matches to TargetScan

TGATGTGTGAAAAATCATGATACATACTGTACAGTCTCAGTCCCATAAAATTGGATGTTGTGCCTACACACAGGATCTAGAAGAATATGTCAAAC 5280  
 TATAAA

CTGCTT

CTGCTT  
Depth:9 (MOUSE)  
Ei-value:0.000, Pi-value:0.000  
Er-value:0.000, Pr-value:0.000  
eCLIP MATCHES▶fubp3 (bg=23.31%)▶KHSRP (bg=8.1%)▶NOLC1 (bg=6.58%)▶TARDBP (bg=1.75%)No matches to TargetScan

GTGATTGTGAA

TGACTT

TGACTT  
Depth:9 (MOUSE)  
Ei-value:0.000, Pi-value:0.000  
Er-value:0.000, Pr-value:0.000  
eCLIP MATCHES▶fubp3 (bg=23.31%)▶KHSRP (bg=8.1%)▶NOLC1 (bg=6.58%)▶SUB1 (bg=9.24%)▶TARDBP (bg=1.75%)▶ZC3H11A (bg=6.25%)MATCHES To TargetScan▶ miR-224-5p:AAGUCAC

TGTTCT

TTGCTT

TTGCTT  
Depth:9 (MOUSE)  
Ei-value:0.000, Pi-value:0.000  
Er-value:0.000, Pr-value:0.010  
eCLIP MATCHES▶ddx55 (bg=12.35%)▶ddx6 (bg=23.92%)▶fubp3 (bg=23.31%)▶KHSRP (bg=8.1%)▶NOLC1 (bg=6.58%)▶SUB1 (bg=9.24%)▶TARDBP (bg=1.75%)▶tia1 (bg=16.04%)▶tial1 (bg=14.09%)▶ZC3H11A (bg=6.25%)No matches to TargetScan

GTGTTTTTCAATTTCCTATAATGCACATACTAACTTTTAAA

AAATAAA

AAATAAA  
Depth:9 (MOUSE)  
Ei-value:0.000, Pi-value:0.000  
Er-value:0.000, Pr-value:0.000  
eCLIP MATCHES▶ZC3H11A (bg=6.25%)No matches to TargetScan

GGTTATTTTAAAAGCCTGTATTAAGCCCTCG 5400  
 TTGCTTGTAGAATAGAGTTAGTATACGCTACAGAAGCACAGGT                                                                              5443
```

---

## >BABOON (5458 bases)

```
 TCCCAGCGCAAGGGCACTTCCGGTACTCCTCCTCTCTCTCGCCAGCTCAGAGAAC

TGCCAA

TGCCAA  
Depth:9 (MOUSE)  
Ei-value:0.000, Pi-value:0.000  
Er-value:0.000, Pr-value:0.000  
MATCHES To TargetScan▶ miR-182-5p:UUGGCAA▶ miR-96-5p/1271-5p:UUGGCAC

G

TCAGTTCCGG

TCAGTTCCGG  
Depth:9 (MOUSE)  
Ei-value:0.000, Pi-value:0.000  
Er-value:0.000, Pr-value:0.000  
No matches to TargetScan

TCGGCAGAGAACGCGGAGAGACGCAGAACGCGGCCGGCTCCTTCAGGG 120  
 CCCTCCAG

GCCCTC

GCCCTC  
Depth:9 (MOUSE)  
Ei-value:0.000, Pi-value:0.000  
Er-value:0.000, Pr-value:0.000  
No matches to TargetScan

CGGCCCTGGGCCGGCGGGTGAACTGGGGGGCCCCGGGACAGGCCGAGCCCTGTGCCATGCAGATACCGGAGGCCTCTGCTGCGGCTGCCCACTGGCTGTGCCCAGG 240  
 CCTTGAAGCCGCAGCGAACCTCTCTTCCCCACCCCACCTCGGTGACTG

ATGGCGGC

ATGGCGGC  
Depth:9 (MOUSE)  
Ei-value:0.000, Pi-value:0.000  
Er-value:0.000, Pr-value:0.000  
No matches to TargetScan

GGCGGCCTCTCCCAGCCCGGACCCGGCCGGCCACCGGGTCTCCCGGCCCAAGCCTGCCGGGCCT 360  
 CAACGAAACCCCCGCAGAGCCGCCGGGACGCAGCGCTTTTGGGCTGCGGCGGGCGTGGCGGGCCGGGAAGCATGGCGGCCGCTCGAACGCCGCGCGGCGGAGGCCATTAGGGCGTGTAGG 480  
 GCCCAGGAAGGCGGCCTAGGGACGCAGGCAGGCTCGGCTGCCTCTTTAGCCCACGGAGCCGCGCAGATCCGGTTCCCGGGTGACCACTCTGTCGCCATTGGGCGAAACCTACCTAGTCCT 600  
 GACGACAACGGACAAAGGCCTTAACGGGCCTGGGAGGTGAGCGAAGCCCCGAACGACGACGGGTGGAACGATTAGCGGCCATCGGGCAGTTGGTCTTCGTTCTACCAGACTTTACTGTCG 720  
 GAAGAGAGAAATGGTAGAATGACAGGCCACGTTTGGCCCGTTGGAAATGCCCGCCACCCTCT

GGGAAGA

GGGAAGA  
Depth:9 (MOUSE)  
Ei-value:0.000, Pi-value:0.000  
Er-value:0.000, Pr-value:0.000  
No matches to TargetScan

TTTACTGGCCAATTTTGGAAGG

CCTGTGTATATAATATGAAAAAGCTGCT

CCTGTGTATATAATATGAAAAAGCTGCT  
Depth:9 (MOUSE)  
Ei-value:0.000, Pi-value:0.000  
Er-value:0.000, Pr-value:0.000  
MATCHES To TargetScan▶ miR-15-5p/16-5p/195-5p/424-5p/497-5p:AGCAGCA▶ miR-503-5p:AGCAGCG

C 840  
 TCAACTCCACCC

CAACCTTT

CAACCTTT  
Depth:9 (MOUSE)  
Ei-value:0.000, Pi-value:0.000  
Er-value:0.000, Pr-value:0.000  
No matches to TargetScan

TAATAGAAAACATTTGTCACATCTAGCCCTTTTAGATGGAA

AGAGGT

AGAGGT  
Depth:9 (MOUSE)  
Ei-value:0.000, Pi-value:0.000  
Er-value:0.000, Pr-value:0.000  
No matches to TargetScan

TGCCGACGTATGATAAAGTAGAGTTAGAAAGTCACACATCTTGTAAATTCTCA 960  
 TTTGTTTAAAAGAAATCATAGAAAATACGTGTCTTCTGGAGATGACTTTTG

GAAATG

GAAATG  
Depth:9 (MOUSE)  
Ei-value:0.000, Pi-value:0.000  
Er-value:0.000, Pr-value:0.000  
No matches to TargetScan

AAGTTGTTAGACGGCCTCTGGAAGCGATACGTCCACGTTAAGTGGGTTAGATGACATGGAGCT 1080  
 GGAAGACGTGAGAAGGAAGAGAAGGTTCTATGCTAGACTGGTCATATTT

AGAAGA

AGAAGA  
Depth:9 (MOUSE)  
Ei-value:0.000, Pi-value:0.000  
Er-value:0.000, Pr-value:0.000  
No matches to TargetScan

CATTTTCATATTCTATCCATTGTTTTGTGTGCATTTTATTCCTCACTACTGTGTATATAATTGAC 1200  
 AATGCTAAGCTTTTTTGAGATGTCTATTCTTTTTAGATGTTCTG

AAGTGCCTG

AAGTGCCTG  
Depth:9 (MOUSE)  
Ei-value:0.000, Pi-value:0.000  
Er-value:0.000, Pr-value:0.000  
No matches to TargetScan

ATATATGTTAAAATTAGAGGTAGCAAAATAACATTTTGTAAATATCTTTTTGTTACAATTCATAGGA 1320  
 AATGTTTTTTGGGGGGAATGGCCAAATCACCTGTTGAGTAATACTCATTGTGT

TTGTGC

TTGTGC  
Depth:9 (MOUSE)  
Ei-value:0.000, Pi-value:0.000  
Er-value:0.000, Pr-value:0.000  
No matches to TargetScan

AGTGGTTCGGGAAGGAGAGAGGAGGGGGAGGTGCAGAGAGCTATATGCCATCTTGCATGCA 1440  
 GCGAGGCAAGATGAATCATTATCTCTGTGCATTTTGTTTTACTTATCTGTGTATATAGTGTACATAAAGGACAGACGAGTCCTAATTGACAACATCTAGTCTTTCTG

GATGTT

GATGTT  
Depth:9 (MOUSE)  
Ei-value:0.000, Pi-value:0.000  
Er-value:0.000, Pr-value:0.000  
No matches to TargetScan


AAAGAGG

AAAGAGGTTGCCA  
Depth:9 (MOUSE)  
Ei-value:0.000, Pi-value:0.000  
Er-value:0.000, Pr-value:0.000  
No matches to TargetScan

 1560  


TTGCCA

AAAGAGGTTGCCA  
Depth:9 (MOUSE)  
Ei-value:0.000, Pi-value:0.000  
Er-value:0.000, Pr-value:0.000  
No matches to TargetScan

GTGTATGACAAAAGTAGAGTTTAAACTAATATATTTTGTAC

ATTTTGT

ATTTTGT  
Depth:9 (MOUSE)  
Ei-value:0.000, Pi-value:0.000  
Er-value:0.000, Pr-value:0.000  
No matches to TargetScan

TTTACAAGTCCTAGGAAAGATTGTCTTCTGAAAATTTGATGTCTTCTGGGTTGATGGAGATGGGAA 1680  
 GGGTTCTAGGCCAGAATGTTCACATTTGGAAGACTTAAATTATAAGTATTGTTACATGTTTGCAGTTTATTCAAGACTGCTATGTATATAGTGGACAAATTAAC

TCCTTA

TCCTTA  
Depth:9 (MOUSE)  
Ei-value:0.000, Pi-value:0.000  
Er-value:0.000, Pr-value:0.000  
No matches to TargetScan

CTTG

AAACAT

AAACATCT  
Depth:9 (MOUSE)  
Ei-value:0.000, Pi-value:0.000  
Er-value:0.000, Pr-value:0.000  
No matches to TargetScan

 1800  


CT

AAACATCT  
Depth:9 (MOUSE)  
Ei-value:0.000, Pi-value:0.000  
Er-value:0.000, Pr-value:0.000  
No matches to TargetScan

AGTCTAT

CTAGATGTTTAGAAGTGCCC

CTAGATGTTTAGAAGTGCCC  
Depth:9 (MOUSE)  
Ei-value:0.000, Pi-value:0.000  
Er-value:0.000, Pr-value:0.000  
No matches to TargetScan

AAT

GTATGTTAAA

GTATGTTAAA  
Depth:9 (MOUSE)  
Ei-value:0.000, Pi-value:0.000  
Er-value:0.000, Pr-value:0.000  
No matches to TargetScan

TGTAGAGGTAGTAAAATACCACTT

TGTAAATA

TGTAAATA  
Depth:9 (MOUSE)  
Ei-value:0.000, Pi-value:0.000  
Er-value:0.000, Pr-value:0.000  
No matches to TargetScan

TCTTTTTGCTAAAATTCATAGGAAATACTTTTGGAAATTGAATTGT 1920  
 GAAGCCACCTTTGAGCAGTATAGTAATGTCTATACTTGTTCAATGGTTTAGAGGAGGTGGGAGGGAAGAAATTGCAAAAGGTAATATACTAGTGTGTTCATACTTGGACATTTTCAGACA 2040  
 TTTTTCTGTATGTTTTGTGCATTTTGTTTTGCTCTGTATATAGTGTATATAATGGACAAATAGTCCTAATTTTTCAACATCTAGTCTCTAGATGTTAAAGAGGTTGCCAGTGTATGACAA 2160  
 AGTAGTAAAATTAGCATATTTTGTACGCTTTGTGTTGAAATTCATAGGAAAACTTGTCTTCTGTAAATGACTTTGCATAGGAATTTGTTCAACCATCTCTGAGCATTATACTTTCCTGTA 2280  
 CTTGTCCACTGGATTGAAGACAAAGAAGGAAGGAAGAGAGGAGGGAATGATTCAAGGCCAAAATGGCCACATTTAGAAGATACCTCAGATGATAACCATTGTTATATGTGTGCAATTTTA 2400  
 TTTAACAGTGCTCTGTACGTGGTGAACAAGTTATATGAAATATCTAGTCTTTCTAGATATTTGGAAGGAAGTGCTTGATGTATTTAAAAGTGGTAGTAGAATAACACTTTTTGTAAATAG 2520  
 CTTTTAAAAACTGATGGGAAATGCTGTTTGGAAGTGGAATTGTTGAACCATCTGGGAG

GTGGGAGGGAA

GTGGGAGGGAA  
Depth:9 (MOUSE)  
Ei-value:0.000, Pi-value:0.000  
Er-value:0.000, Pr-value:0.000  
MATCHES To TargetScan▶ miR-150-5p:CUCCCAA▶ miR-532-3p:CUCCCAC

AAAAC

TGCAAA

TGCAAA  
Depth:9 (MOUSE)  
Ei-value:0.000, Pi-value:0.000  
Er-value:0.000, Pr-value:0.000  
No matches to TargetScan

AGGTGTTTTGCCATTGTTTATTAGAAAATTTCAGCTTAAT 2640  
 CCATTGCCTGTATGTTACATGCATTTCATTTAACTTTGCTATAC

TGTATATA

TGTATATA  
Depth:9 (MOUSE)  
Ei-value:0.000, Pi-value:0.000  
Er-value:0.000, Pr-value:0.000  
No matches to TargetScan

TTGTGTATATACTGGACGAATGAGTCCTGATTTTGTA

ATATCTAG

ATATCTAG  
Depth:9 (MOUSE)  
Ei-value:0.000, Pi-value:0.000  
Er-value:0.000, Pr-value:0.000  
No matches to TargetScan

T

CTCTAG

CTCTAG  
Depth:9 (MOUSE)  
Ei-value:0.000, Pi-value:0.000  
Er-value:0.000, Pr-value:0.000  
No matches to TargetScan

ATATT

AAAGAGGTTGC

AAAGAGGTTGCCAATGTATGACA  
Depth:9 (MOUSE)  
Ei-value:0.000, Pi-value:0.000  
Er-value:0.000, Pr-value:0.000  
MATCHES To TargetScan▶ miR-182-5p:UUGGCAA▶ miR-96-5p/1271-5p:UUGGCAC▶ miR-539-3p:UCAUACA

 2760  


CAATGTATGACA

AAAGAGGTTGCCAATGTATGACA  
Depth:9 (MOUSE)  
Ei-value:0.000, Pi-value:0.000  
Er-value:0.000, Pr-value:0.000  
MATCHES To TargetScan▶ miR-182-5p:UUGGCAA▶ miR-96-5p/1271-5p:UUGGCAC▶ miR-539-3p:UCAUACA

GAAGTAGA

GTTAGTAAACT

GTTAGTAAACT  
Depth:9 (MOUSE)  
Ei-value:0.000, Pi-value:0.000  
Er-value:0.000, Pr-value:0.000  
No matches to TargetScan

A

ACACATT

ACACATT  
Depth:9 (MOUSE)  
Ei-value:0.000, Pi-value:0.000  
Er-value:0.000, Pr-value:0.000  
No matches to TargetScan

TTGTACACTTTGTTAAAATTTGTAGAAAGGC

TGTCTTCTGAA

TGTCTTCTGAA  
Depth:9 (MOUSE)  
Ei-value:0.000, Pi-value:0.000  
Er-value:0.000, Pr-value:0.000  
No matches to TargetScan

AAGGACTTTTGGAAGTGAAATGATAACATCAGCTCTAAG 2880  
 TGACAC

GTGCCT

GTGCCT  
Depth:9 (MOUSE)  
Ei-value:0.000, Pi-value:0.000  
Er-value:0.000, Pr-value:0.000  
No matches to TargetScan

ATATTCACCAGGTTGGTGGTGGAGAGGAGTTGGAAGGAATGAAGGGTTCTAGACC

AGAATG

AGAATG  
Depth:9 (MOUSE)  
Ei-value:0.000, Pi-value:0.000  
Er-value:0.000, Pr-value:0.000  
No matches to TargetScan

TTCCTATTT

AGAAGA

AGAAGA  
Depth:9 (MOUSE)  
Ei-value:0.000, Pi-value:0.000  
Er-value:0.000, Pr-value:0.000  
No matches to TargetScan

CACTTTGAGATATAACCATTGTTACCTGTGTG 3000  
 TAGTTTATTCAACACTACTG

TGTATATA

TGTATATA  
Depth:9 (MOUSE)  
Ei-value:0.000, Pi-value:0.000  
Er-value:0.000, Pr-value:0.000  
No matches to TargetScan

GCAGACAAACTTAAGTCCTTATT

TGAAACATCTAG

TGAAACATCTAG  
Depth:9 (MOUSE)  
Ei-value:0.000, Pi-value:0.000  
Er-value:0.000, Pr-value:0.000  
No matches to TargetScan

TCT

TTCTAG

TTCTAG  
Depth:9 (MOUSE)  
Ei-value:0.000, Pi-value:0.000  
Er-value:0.000, Pr-value:0.000  
No matches to TargetScan

A

TGTTTAGAAGTGCACAAAGTATGTTAAAAGTAGA

TGTTTAGAAGTGCACAAAGTATGTTAAAAGTAGA  
Depth:9 (MOUSE)  
Ei-value:0.000, Pi-value:0.000  
Er-value:0.000, Pr-value:0.000  
No matches to TargetScan

GGTAGTAAATAAC 3120  
 ACATTTTGTAGCTATCCTTTTGATATGAAATATTGTCTTGGAAACTGATCAATTCTCTGAGCAGTACCCATTTTGAGATATTTGTGCTGGTTCAGGGGGAAGGAGGAGCACAAAGTGCAA 3240  
 AGGGCTTTCTACCAGTGTCCAGTGTGTTTAAGAGGAGGCACATTGACCATTGTCCCTTGTGTCTGCATTTTCATTTACTGTGCTG

TGTATA

TGTATA  
Depth:9 (MOUSE)  
Ei-value:0.000, Pi-value:0.000  
Er-value:0.000, Pr-value:0.000  
No matches to TargetScan

TAGTGTATATAAGTGGACACAGGAGTCCT 3360  
 AATTTACATCTAGTCGATGTTAAAGAGGTTGCCAGTGTATGACAAAAGTAGAATTAGTAAACTGATACATCGAGTACTTTGTGTTAAAATTCACAGGGAAGACTTCTTAAAAACAGAAGG 3480  
 GAAATTGTTAAAATCCCCCCCCTAAGCATTACAGATGGCTTATAGCTGTCCACCAGGTTGGTAGAGGTGGGAAAGGGAAGGGTTCTAGGCCAGAATGTTCCTATTTAGAAGACACTCAAA 3600  
 TTATAGTCTGTGTTATGTATGTATACCATTTATTCAATGCTACTGTGTATATAATGGAAAACTTCAGTCCAGTTTGAAACATCT

AGTCTT

AGTCTT  
Depth:9 (MOUSE)  
Ei-value:0.000, Pi-value:0.000  
Er-value:0.000, Pr-value:0.000  
No matches to TargetScan

TCTAGGTGTTTAAAAGTGCACAACGGCCGG 3720  
 GCACAGTGGTTTAATGCCTGTAATCCCAGCACTTTGGGAGGCCGAGGCAGGCAGATCACGAGGTCGAGAGATCGAGACCATCTTGGCCAGCATGGTGAAACCACGTCTCTACTGAAAATA 3840  
 CAAAAATTAGCTGGTCGTTGGTGGTGTGCACCTGTAGTCCCAGCTACTTGGGAAGCTGAGGCAGGAGAATTGCTTGAACTTGGGAGGCGGAGCCGAGATCACACCACTGCACTCCAACCT 3960  
 GGCGACAGAGCAAGACTCCCTTTCAAAAAGAGGTGCACAATATAGGTTAAGAGTAGAGGGCTTAAGTAACACCCCTCTAAGCATTTGTTTTCAATACTTACTAGGAGTGGTTGCATTTGG 4080  
 GAATGG

AATTGTTA

AATTGTTA  
Depth:9 (MOUSE)  
Ei-value:0.000, Pi-value:0.000  
Er-value:0.000, Pr-value:0.000  
No matches to TargetScan

AAACTTGATGTTTAGGAGCGAATGCAGACTTCATTGGATGGTTGGGGTGGGGGAGGGGGGAGGAGGTATGCAGGGTTCTGTGCTCCTGAGATTAGTTCAGATGGTC 4200  
 TAACCATTGTTCTATATGTGCATTTTAGTTAATATTGTGTATTAAAGGATAAGTCTTAATGCTCAAAGTATGTTAAAAATAGATGTAGTAAACCAGTCCCTTTGTGAATGTCCTTTTGTT 4320  
 ATATTTTAGGAAGGCCTGTGTTCTGGGAGTGACCTTCATGAGTTCACCTCTTGGAGCTAGACATCCTATACTTAGTCACTGGGATGGTGGAAGAGGGAGACGAGGAAGGGTGAAGGGAAG 4440  
 GGCTCTTTGCTAGTATCTTCATATCTAGACGATGGTTTTAGATGATAACCACAGGTCTATAAGCATTTTTAGTAAAGTGCCTGTGTTCATTGTGGACAAAGTTCATTATTTTGTAACATC 4560  
 TAAGCTTTATGAATGGGGTGACAACTTATGATAAAAACTAGAGCTAGTGAATTAGCCAATTTGTAAATACCTTTGTTATAATTGATAGAAAAGATGCATCTTGGACATGGAATTGTTAAG 4680  
 CCACCTCTGAGCAGTGTATGTCAGGACTTACTTGTTCATTAGGTTGGCAGCAGAGAGGCAGAAGGAAGTATACAGGAAGAGGTGTATGCAGATGTGTCAATGTATGTCCATATTTACATT 4800  
 TTGATAGCCATTGATGTATGCATCTCTTTTAGCTGTACTATAGAAATACATTAAGTAATTCAATGGAAATATACTTTGCTAATATTTTAATGGTATAGATCTGCTAATGAATTCTCTTAA 4920  
 AAACGTTATACTTAGTATATTCTGTTGCTGTGTGTTTCATTTTAAATTGAGCATTAAGGGAATGCAGCATTTAAATCGGAACTCTGCCAATGCTTTTATCTAGAGGCGTGTTGCC

ATTTT

ATTTTTGT  
Depth:9 (MOUSE)  
Ei-value:0.000, Pi-value:0.000  
Er-value:0.000, Pr-value:0.000  
No matches to TargetScan

 5040  


TGT

ATTTTTGT  
Depth:9 (MOUSE)  
Ei-value:0.000, Pi-value:0.000  
Er-value:0.000, Pr-value:0.000  
No matches to TargetScan

CTTCTATGAAATTTTTGTCCCGAGAAAGGCAGGATTACATTTTTTTTTTTTTTTTTTTTTTTTAGCAGTTTGAGTTGGTGTAGTGTA

TTCTTGGT

TTCTTGGT  
Depth:9 (MOUSE)  
Ei-value:0.000, Pi-value:0.000  
Er-value:0.000, Pr-value:0.000  
No matches to TargetScan

TATCAAAATACTCATATAGCTT 5160  
 TGGGATTTTGAATTGGTA

AATATTCA

AATATTCA  
Depth:9 (MOUSE)  
Ei-value:0.000, Pi-value:0.000  
Er-value:0.000, Pr-value:0.000  
No matches to TargetScan

TGATGTGTGAAAAATCATGATACATACTGTACAATCTCAGTCCCATAAAATTGGATGTTTTGCCTACACACACACACGATCTAGAAGAACATGT 5280  
 CAAACTATAAA

CTGCTT

CTGCTT  
Depth:9 (MOUSE)  
Ei-value:0.000, Pi-value:0.000  
Er-value:0.000, Pr-value:0.000  
No matches to TargetScan

GTGATTTTTAA

TGACTT

TGACTT  
Depth:9 (MOUSE)  
Ei-value:0.000, Pi-value:0.000  
Er-value:0.000, Pr-value:0.000  
MATCHES To TargetScan▶ miR-224-5p:AAGUCAC

TGTTCT

TTGCTT

TTGCTT  
Depth:9 (MOUSE)  
Ei-value:0.000, Pi-value:0.000  
Er-value:0.000, Pr-value:0.010  
No matches to TargetScan

CTTGTGTTTTTCAGTTTCCTGTAATGCACATATTAACTTTTAAA

AAATAAA

AAATAAA  
Depth:9 (MOUSE)  
Ei-value:0.000, Pi-value:0.000  
Er-value:0.000, Pr-value:0.000  
No matches to TargetScan

GGTTATTTTAAAAGCCTGTATTA 5400  
 AGCCCTCGTTGCTTGTAGAATAGAGTCACTACACTCTACAGAAGCACAGGTTCATGCC                                                               5458
```

---

## >DOG (5115 bases)

```
 TTCGCGGTCCCGGCGCGAGGGCACTTCCGGTGCCGCACTCCTCTCGCCAGCAAAGAGAAC

TGCCAA

TGCCAA  
Depth:9 (MOUSE)  
Ei-value:0.000, Pi-value:0.000  
Er-value:0.000, Pr-value:0.000  
MATCHES To TargetScan▶ miR-182-5p:UUGGCAA▶ miR-96-5p/1271-5p:UUGGCAC

G

TCAGTTCCGG

TCAGTTCCGG  
Depth:9 (MOUSE)  
Ei-value:0.000, Pi-value:0.000  
Er-value:0.000, Pr-value:0.000  
No matches to TargetScan

CAGAAAGCGAGGTGAGAAGCGCAGGGCGGCGGTCTGTCCCGGG 120  
 CCCTCCAG

GCCCTC

GCCCTC  
Depth:9 (MOUSE)  
Ei-value:0.000, Pi-value:0.000  
Er-value:0.000, Pr-value:0.000  
No matches to TargetScan

CGGCCTGAGGCCGGGGGTGAACTGGGGGGCCCAAGGAGAGGTCAGGAGCTCGGACCTGCAGACACCCAGCGCCCTCGCCGCAGCTGCCCACCGGCCAGGCCCACAC 240  
 TTTGCAGCAGTTGTGAGCCTCTCCCCACCCCCACCCCCACCCCCACCCCCACGCCGGCGCCCATGGCTGCGGCCTCTCGGAGCCGGGCCTCGCCGGCTGCCCACTCTCCTGGCCCAGCTC 360  
 TGCGGAGCCTACGCGAAGCCCCCGCCCAGCCAGCCGGGCCGCGGAGCCTTTGGACGTCGGGTGTGGCGGGGTGAGAAGA

ATGGCGGC

ATGGCGGC  
Depth:9 (MOUSE)  
Ei-value:0.000, Pi-value:0.000  
Er-value:0.000, Pr-value:0.000  
No matches to TargetScan

CGCTCAAACGCCGGCCCGGCCCTTGGGGCCTCC 480  
 GAGGGCCTGAGAAGGCGGGTCTCCGCGGACGTAAGCTCGGCCGCCCTCGGGCTTTGGCGCCCCACAGACCCGGGCCCCGGGATGACCACTCCGCCCCAGGTGGACGAAACCTCACCTACG 600  
 GCGAACAGTGGACAGAGGCCCTAACGGCCGGAAGGTGAGCGAAGCTCCCCCCCGCCCCCCGCCCCGGTCGGCGACGGGTGGAACAGTTAATGGGCCAAAGGCGGTTGGTCTTCGTTCTAC 720  
 AGACATTGCTGTCGGAAGATCGAAATGGGAGAATTACGAACCGCGTTTGGCCAATAGGAAGACCACCCTTT

GGGAAGA

GGGAAGA  
Depth:9 (MOUSE)  
Ei-value:0.000, Pi-value:0.000  
Er-value:0.000, Pr-value:0.000  
No matches to TargetScan

TTTACTGGCCGTTTATAGAAGG

CCTGTGTATATAATATGAAA

CCTGTGTATATAATATGAAAAAGCTGCT  
Depth:9 (MOUSE)  
Ei-value:0.000, Pi-value:0.000  
Er-value:0.000, Pr-value:0.000  
MATCHES To TargetScan▶ miR-15-5p/16-5p/195-5p/424-5p/497-5p:AGCAGCA▶ miR-503-5p:AGCAGCG

 840  


AAGCTGCT

CCTGTGTATATAATATGAAAAAGCTGCT  
Depth:9 (MOUSE)  
Ei-value:0.000, Pi-value:0.000  
Er-value:0.000, Pr-value:0.000  
MATCHES To TargetScan▶ miR-15-5p/16-5p/195-5p/424-5p/497-5p:AGCAGCA▶ miR-503-5p:AGCAGCG

CTCAACTTACCCCC

CAACCTTT

CAACCTTT  
Depth:9 (MOUSE)  
Ei-value:0.000, Pi-value:0.000  
Er-value:0.000, Pr-value:0.000  
No matches to TargetScan

TCAAAAGAAAACTTTTGCCACATCTAGGCCTTCTAGATGTAA

AGAGGT

AGAGGT  
Depth:9 (MOUSE)  
Ei-value:0.000, Pi-value:0.000  
Er-value:0.000, Pr-value:0.000  
No matches to TargetScan

TGCCGACATATGATAAAGTAGAGTTAGAAAATCATACGTCTT 960  
 GTAAATGCCTGTTTGGTTTTTTGTGGGTTTTTTTTGTTTGTTTTTGTTTTTAAATCATAGAAATGTCTTTTG

GAAATG

GAAATG  
Depth:9 (MOUSE)  
Ei-value:0.000, Pi-value:0.000  
Er-value:0.000, Pr-value:0.000  
No matches to TargetScan

ACTTTTTGAAATGGAATTGTTAGATCACTTCTAGAAGCGACA 1080  
 TCAGCGACACGTTCACGTTTGTGCAGTGGGTAAGAGGACATGAAGTAGACGACCTTGAGGAGGAAGAAAAGAAGGTTCTGTGCCAGACTGGTCATACTT

AGAAGA

AGAAGA  
Depth:9 (MOUSE)  
Ei-value:0.000, Pi-value:0.000  
Er-value:0.000, Pr-value:0.000  
No matches to TargetScan

CATTTTCACATTATA 1200  
 ACCATTGTTTTGTGTGTGCATTTTATTCCTCAACTACTGTGTATATAGTTGACAATGCTAAGTACCTTTGTCAAATATCTAGTCTTTCTAGATGTTCTG

AAGTGCCTG

AAGTGCCTG  
Depth:9 (MOUSE)  
Ei-value:0.000, Pi-value:0.000  
Er-value:0.000, Pr-value:0.000  
No matches to TargetScan

ATACATGTTAAA 1320  
 AATAGAGGTAGTAAAAATACATTTTGTAAATATCTTCGTTGACATTCATAGGAAATGCTGTTTAATTTGCGGGGAATGGCCAAACCACCTCTTTGAATAGTATGCATTGTGT

TTGTGC

TTGTGC  
Depth:9 (MOUSE)  
Ei-value:0.000, Pi-value:0.000  
Er-value:0.000, Pr-value:0.000  
No matches to TargetScan

AC 1440  
 TGGTTCAAAGGAAGAGGGAGGAGAAGGAAGTGCAAAGAGCTTTGCCGGTGTGTTTATGGAGAGGGAAGATTAACCATTGTCCTTTATGTTCCTGCATTTTGTTTTACTTAGCTGTGTATA 1560  
 TAGTGTATATACTGGACAAATGAGTCCTAATTTTCAACATCTAGTCTTTTCTA

GATGTT

GATGTT  
Depth:9 (MOUSE)  
Ei-value:0.000, Pi-value:0.000  
Er-value:0.000, Pr-value:0.000  
No matches to TargetScan


AAAGAGGTTGCCA

AAAGAGGTTGCCA  
Depth:9 (MOUSE)  
Ei-value:0.000, Pi-value:0.000  
Er-value:0.000, Pr-value:0.000  
No matches to TargetScan

GTGTATGACAAAAATAGTAAACTAATATGTTTTGTAC

ATTTTGT

ATTTTGT  
Depth:9 (MOUSE)  
Ei-value:0.000, Pi-value:0.000  
Er-value:0.000, Pr-value:0.000  
No matches to TargetScan

GTTA 1680  
 AAATTCTTAGGAAGATTGTCTTCTGAAAATTGGAGCATTATAGCCCACTGGGCTGGTGGAGAAAGAAGCGAAGAGTTAGTCTTAGGCTAGAATGTTCTTATTTTGAAGACACTTTCAGAT 1800  
 TATAACTGTTACACGTGTGCAGTTTATTCAAGACTGCTATGTATATAGTGGACACATTAAG

TCCTTA

TCCTTA  
Depth:9 (MOUSE)  
Ei-value:0.000, Pi-value:0.000  
Er-value:0.000, Pr-value:0.000  
No matches to TargetScan

TTTA

AAACATCT

AAACATCT  
Depth:9 (MOUSE)  
Ei-value:0.000, Pi-value:0.000  
Er-value:0.000, Pr-value:0.000  
No matches to TargetScan

AGTCTGT

CTAGATGTTTAGAAGTGCCC

CTAGATGTTTAGAAGTGCCC  
Depth:9 (MOUSE)  
Ei-value:0.000, Pi-value:0.000  
Er-value:0.000, Pr-value:0.000  
No matches to TargetScan

AAC

GTATGTTAAA

GTATGTTAAA  
Depth:9 (MOUSE)  
Ei-value:0.000, Pi-value:0.000  
Er-value:0.000, Pr-value:0.000  
No matches to TargetScan

T 1920  
 GTAGAGGTAGTAAAATATCACTC

TGTAAATA

TGTAAATA  
Depth:9 (MOUSE)  
Ei-value:0.000, Pi-value:0.000  
Er-value:0.000, Pr-value:0.000  
No matches to TargetScan

TCTTTTTGCTAAAATTCATAGGAAATAACTCTTGGGAGTGGAATTGTTAAACCACCTCTGAGCAATATAGGCTATCATGTATATTCAGT 2040  
 GGTTTGGAGGAGGTGGAAGGGAAAGAATTGGAAAAGGTAATATGCTTGTGTGTTCATACTTGGACATTTTCAGATAAAACCATTTTTTTGTATGGTCTGTGCATTTTATTTTGCTGTGTA 2160  
 TATAGTGTATATAATGGACAAATGAGTCCTAGTTTTGCAACATCTAGTCTCTAGATGTTAAAGAGGTTGCCAATGTATGACAAAGTAGTTAAATTAGCACATTCTGTACACATTGTGTTG 2280  
 AAATTCAAAGGAAAGCTTTTTTTCTGTAAATAACTTTTGGATATGAATTTGTTCAACCACCTTTAAGCATTACACATGTCTGTACCTGTCTACTGGATTAGTGGGGGAGAGAAGGAAGTG 2400  
 AGGGAGGGAATAGTTTAGGCCAAAATGGTGGTCATATTTAGAAAATACCTCAGATTATACCTGTTGTTAGGTGTGTGCAATTCTATTTAACAGTGCTATGTACATAGTGGACAAGTTAGT 2520  
 TCTTATGTGAAATATCTAGTCTTTCTAGATACTTAGAAATGCTTGATGTATTTAGAAGTAGAGTAACGCTTCTTGTAAATAGCTTTTAAAGACTGATGGGAAATATTATCTATGGAAATA 2640  
 GAATTGCTAAAACCACCTCTCTGAACAGTATACTTGTTCAGTGGTTTGAGGGAG

GTGGGAGGGAA

GTGGGAGGGAA  
Depth:9 (MOUSE)  
Ei-value:0.000, Pi-value:0.000  
Er-value:0.000, Pr-value:0.000  
MATCHES To TargetScan▶ miR-150-5p:CUCCCAA▶ miR-532-3p:CUCCCAC

GAAAT

TGCAAA

TGCAAA  
Depth:9 (MOUSE)  
Ei-value:0.000, Pi-value:0.000  
Er-value:0.000, Pr-value:0.000  
No matches to TargetScan

AGATTTTTTGCTAGTGTGTGCTAGGAAATTTCAGCTTATCCATT 2760  
 GCGATATATGTTATGTGCATTCAACTCGAC

TGTATATA

TGTATATA  
Depth:9 (MOUSE)  
Ei-value:0.000, Pi-value:0.000  
Er-value:0.000, Pr-value:0.000  
No matches to TargetScan

TTGTATATATACTGGACAAATAGGTCCTAATTTTATA

ATATCTAG

ATATCTAG  
Depth:9 (MOUSE)  
Ei-value:0.000, Pi-value:0.000  
Er-value:0.000, Pr-value:0.000  
No matches to TargetScan

T

CTCTAG

CTCTAG  
Depth:9 (MOUSE)  
Ei-value:0.000, Pi-value:0.000  
Er-value:0.000, Pr-value:0.000  
No matches to TargetScan

ATATT

AAAGAGGTTGCCAATGTATGACA

AAAGAGGTTGCCAATGTATGACA  
Depth:9 (MOUSE)  
Ei-value:0.000, Pi-value:0.000  
Er-value:0.000, Pr-value:0.000  
MATCHES To TargetScan▶ miR-182-5p:UUGGCAA▶ miR-96-5p/1271-5p:UUGGCAC▶ miR-539-3p:UCAUACA

AA 2880  
 AGTAGA

GTTAGTAAACT

GTTAGTAAACT  
Depth:9 (MOUSE)  
Ei-value:0.000, Pi-value:0.000  
Er-value:0.000, Pr-value:0.000  
No matches to TargetScan

A

ACACATT

ACACATT  
Depth:9 (MOUSE)  
Ei-value:0.000, Pi-value:0.000  
Er-value:0.000, Pr-value:0.000  
No matches to TargetScan

TTGTACACTTTGTGTTAAAATTCATTGAAAGGC

TGTCTTCTGAA

TGTCTTCTGAA  
Depth:9 (MOUSE)  
Ei-value:0.000, Pi-value:0.000  
Er-value:0.000, Pr-value:0.000  
No matches to TargetScan

AAGGACTTTGAGAGAGAACATTGTAGTCACATCTCAGTGACAT

GTGCCT

GTGCCT  
Depth:9 (MOUSE)  
Ei-value:0.000, Pi-value:0.000  
Er-value:0.000, Pr-value:0.000  
No matches to TargetScan

GT 3000  
 CTACTCACCCTCTGGGTTGGCAGTGGAGAGAAGGGAGAAATGAAGGGTTCTAGACT

AGAATG

AGAATG  
Depth:9 (MOUSE)  
Ei-value:0.000, Pi-value:0.000  
Er-value:0.000, Pr-value:0.000  
No matches to TargetScan

TTCCTATGC

AGAAGA

AGAAGA  
Depth:9 (MOUSE)  
Ei-value:0.000, Pi-value:0.000  
Er-value:0.000, Pr-value:0.000  
No matches to TargetScan

CACTTTCAGATATAACCCATTGTTACATGTGTGTAGTTTATTC 3120  
 AACACTACTG

TGTATATA

TGTATATA  
Depth:9 (MOUSE)  
Ei-value:0.000, Pi-value:0.000  
Er-value:0.000, Pr-value:0.000  
No matches to TargetScan

GTGGACAAACTTAAGTCCTTATT

TGAAACATCTAG

TGAAACATCTAG  
Depth:9 (MOUSE)  
Ei-value:0.000, Pi-value:0.000  
Er-value:0.000, Pr-value:0.000  
No matches to TargetScan

CCT

TTCTAG

TTCTAG  
Depth:9 (MOUSE)  
Ei-value:0.000, Pi-value:0.000  
Er-value:0.000, Pr-value:0.000  
No matches to TargetScan

A

TGTTTAGAAGTGCACAAAGTATGTTAAAAGTAGA

TGTTTAGAAGTGCACAAAGTATGTTAAAAGTAGA  
Depth:9 (MOUSE)  
Ei-value:0.000, Pi-value:0.000  
Er-value:0.000, Pr-value:0.000  
No matches to TargetScan

GGTAGTAAAATAACACATCTTGT 3240  
 AGATAATCCTTTTGTTAAAATTCATATGAAATACTATCTTTTGAAAATGGTATGATCAAAGTGATCAAACCACCTCTCAGAGCAGCACACATTATATATCTGTGCTGGTTCAGGGAGAAA 3360  
 GGAGAAGAAAGTGCAAAGGGCTTTACACCAGTATGTTTATAGTTAGGTGGGATTAACCATTGTCCTGTAGGTCTGCATTTTGTTGTACTTAGCTA

TGTATA

TGTATA  
Depth:9 (MOUSE)  
Ei-value:0.000, Pi-value:0.000  
Er-value:0.000, Pr-value:0.000  
No matches to TargetScan

TAGTGTATATAAAGGACAA 3480  
 ATGAGTCCTAATTTACAACATCT

AGTCTT

AGTCTT  
Depth:9 (MOUSE)  
Ei-value:0.000, Pi-value:0.000  
Er-value:0.000, Pr-value:0.000  
No matches to TargetScan

TCTAGATGTTAAAGAGGTTGCCAGTGTATGACAAAAGTAGTAAACAAATACATAGTGTACACTTTGTGTTTAAATTCATAGGAAAGACTAT 3600  
 TCTGAAAACTAGAAGTGAAATTGTTAAATCCCCTCTAAGTATTACAGATGCTTATACTTTTCACTAGGTTGATATAGATGAGAAGAGGAAGGGTTAGGCCAGAATGTTCTTTCAGTGCTA 3720  
 CTATGTATGTAGTGGGCAAATTTTAAATACTTAACTTGAAACATCTAGTATTCTGGATGTTTGAAAGTGAACAACATGTTAGGAGTAGAGAGTTAAAGTAACAACACCCTTTAAGTATTA 3840  
 ATTTACAGGAATGGTTGTTACTTTGGGAATGG

AATTGTTA

AATTGTTA  
Depth:9 (MOUSE)  
Ei-value:0.000, Pi-value:0.000  
Er-value:0.000, Pr-value:0.000  
No matches to TargetScan

AACCTGATGTCTTGGAGAATATGCTGACTGTTCACTGGGGGACAGGGGCAGGGAAGAAGTGAGAAGAGAAGGGTTCTGTG 3960  
 CCCTTGAGTCTTTAGACTAAAATTCTGTTTAAAAGTAGAGGTAGTAAAATAATCCCTTTATAAATGTCCTTTTGTTAGTTTTTAGGAAGGACTGTTCTGGGAGTGTCTGTTAATCCACTT 4080  
 CTTAGAGCTAGATGTAGTCCTATACTTAGTCGTTAATATGGTGGAGGGAGCAGAGGAGGGGTGAAGGGAAGGGCTTTTTGCTAGTATCTCCACATCTAGAAGACAGTTTTAGGTTATAAC 4200  
 CATAGGTTTATATGTATGTTTTTATGAAGTACCTGTGTTTGTTGTGGAGAAGTTTCGTTATTTTGCAACATCTAAGCTTTTTGGATGTCCTGAGGTGACAATGTATGATAAAATGTACAG 4320  
 CTAGTGAATTAACCAATTTATAAGTTTCTCTTTGTTATCATTGATAGGAAAGAAGCATCTTGGACATGAGATTGTTAAGCTGTCTCTGAGAAATGTCAGAACTTACTAGGTTGGCAGCAG 4440  
 AGGGGCAGAAGGAAGTATAGAGGGAGAATTGTATGCGGATGTTTTCATATTTACATTTTAATGATAACGTGTATTAGTCTCTTTGGCTGTACTATAGGAATACATTAAGTGATTCAATGG 4560  
 AAACATACCTCTTTGCTAATTGAATATTATAGATTGGATGATGAATTCTTTTAGAAGCATTATATGTTGTGTACTCTGTTACTTTATGTCTCAGTTTTAATTGAACATTAAGGGAATGCA 4680  
 GTATTTTAATTGTAACTCTGTCAATATCTTTATCCAGAGTTCCTGTTGCC

ATTTTTGT

ATTTTTGT  
Depth:9 (MOUSE)  
Ei-value:0.000, Pi-value:0.000  
Er-value:0.000, Pr-value:0.000  
No matches to TargetScan

CGCCAAGAAAGGCAGGATTACATTTTTTTTTTTTTTTTCAGATGGAGTTGGTGTA

TTCTTGG

TTCTTGGT  
Depth:9 (MOUSE)  
Ei-value:0.000, Pi-value:0.000  
Er-value:0.000, Pr-value:0.000  
No matches to TargetScan

 4800  


T

TTCTTGGT  
Depth:9 (MOUSE)  
Ei-value:0.000, Pi-value:0.000  
Er-value:0.000, Pr-value:0.000  
No matches to TargetScan

TATCAAAATACTCATAGTTTGGGGACTTTGACATGATA

AATATTCA

AATATTCA  
Depth:9 (MOUSE)  
Ei-value:0.000, Pi-value:0.000  
Er-value:0.000, Pr-value:0.000  
No matches to TargetScan

TGGTGTGTAAAAGCATGATACATAACTGTACAATCTTACTTAAAATGCTTACTTGTGTAGATAGATACACACA 4920  
 CGGGACCTAGAAGTCAAACTGCAAA

CTGCTT

CTGCTT  
Depth:9 (MOUSE)  
Ei-value:0.000, Pi-value:0.000  
Er-value:0.000, Pr-value:0.000  
No matches to TargetScan

GTGATTATGGA

TGACTT

TGACTT  
Depth:9 (MOUSE)  
Ei-value:0.000, Pi-value:0.000  
Er-value:0.000, Pr-value:0.000  
MATCHES To TargetScan▶ miR-224-5p:AAGUCAC

TGTTCT

TTGCTT

TTGCTT  
Depth:9 (MOUSE)  
Ei-value:0.000, Pi-value:0.000  
Er-value:0.000, Pr-value:0.010  
No matches to TargetScan

CTTGTGTTTTTCAGTTTCCTTTTATGCACATATTAACTTTTAAA

AAATAAA

AAATAAA  
Depth:9 (MOUSE)  
Ei-value:0.000, Pi-value:0.000  
Er-value:0.000, Pr-value:0.000  
No matches to TargetScan

TTTTTAAAA 5040  
 AACCTATATGGAGTCCCTCCACTGCTTGCAAAGCCAGCACTCTCCTGGCAGTACAGTGTCCGCACCCTTCAGGTT                                              5115
```

---

## >COW (5258 bases)

```
 CGGGTCCCAGCGCGAAGGTACTTCCGGCGCCCCTCCTCTCTAGCCAGCAAAGAGAAC

TGCCAA

TGCCAA  
Depth:9 (MOUSE)  
Ei-value:0.000, Pi-value:0.000  
Er-value:0.000, Pr-value:0.000  
MATCHES To TargetScan▶ miR-182-5p:UUGGCAA▶ miR-96-5p/1271-5p:UUGGCAC

G

TCAGTTCCGG

TCAGTTCCGG  
Depth:9 (MOUSE)  
Ei-value:0.000, Pi-value:0.000  
Er-value:0.000, Pr-value:0.000  
No matches to TargetScan

CAGAGAGCGCGGCTAGAAGCGGATCGTGGCCTTCTACCCTGGGGCC 120  
 CTCCAG

GCCCTC

GCCCTC  
Depth:9 (MOUSE)  
Ei-value:0.000, Pi-value:0.000  
Er-value:0.000, Pr-value:0.000  
No matches to TargetScan

CGGCCTTTGGTCGGTAGGTGAACTGGGGGGCCCCGGGACATTCTCGGGTGTGTTTTGCAGAGCGGTCGCCGAGGCTGGCAGTTGGCAGGGCCCAAGACTGGAAGCAGC 240  
 CGCGAACCTCTCCCCCCACCCCCACCCCCACCCCCACCGCCGCGACCTAATGGCGTCGGCCCCTCCGAGCCAGGATCCTGCCGACCGCTGGGTCTCTTCACCCAGAGCTGCAGGGCTTGG 360  
 GCTTAGGCGAAGCCTCCGCCTGGCCACCGAGAACGTGGCGCCTTTCGGCGTCGGGCGTGGCAGGACTAGAAGA

ATGGCGGC

ATGGCGGC  
Depth:9 (MOUSE)  
Ei-value:0.000, Pi-value:0.000  
Er-value:0.000, Pr-value:0.000  
No matches to TargetScan

CGCTCTAACTCCGCGGAGGGCCGTTAGAGCCTCGGAGGC 480  
 CCGGGAAGGATTGTGGACGCCAGTTCGTTCACCCTTAGGCCTTGGGGCCACGCGGATCCGCGACCTGAGGTGACCGCTCTGTCCCAGTTGGACGAGACCTCACCTCGTCCGGACAACGAC 600  
 GGACAAAGGCCTTAACGTGCCCGCAAGGTGAGCGGAGCCGCGGTCGACGACGGGTGGAACGTTAGCGGGCCAGTGGGCTTCGATCGTCGTTCTACCGAGACATTGATGTCGGAAGATAGA 720  
 AATGGTAGAATAACATACCCCTTATGGCCAACAGAAAGTGCCCGGGGCCCTCT

GGGAAGA

GGGAAGA  
Depth:9 (MOUSE)  
Ei-value:0.000, Pi-value:0.000  
Er-value:0.000, Pr-value:0.000  
No matches to TargetScan

TTTACTGGCCGCTGATAAAGG

CCTGTGTATATAATATGAAAAAGCTGCT

CCTGTGTATATAATATGAAAAAGCTGCT  
Depth:9 (MOUSE)  
Ei-value:0.000, Pi-value:0.000  
Er-value:0.000, Pr-value:0.000  
MATCHES To TargetScan▶ miR-15-5p/16-5p/195-5p/424-5p/497-5p:AGCAGCA▶ miR-503-5p:AGCAGCG

CTCAACTTTAC 840  
 CCC

CAACCTTT

CAACCTTT  
Depth:9 (MOUSE)  
Ei-value:0.000, Pi-value:0.000  
Er-value:0.000, Pr-value:0.000  
No matches to TargetScan

CAAAAGAAAACTTTGGCTACATCTAGGCCTCCTAGATGTAA

AGAGGT

AGAGGT  
Depth:9 (MOUSE)  
Ei-value:0.000, Pi-value:0.000  
Er-value:0.000, Pr-value:0.000  
No matches to TargetScan

TGCCGACGTATGATAAAATAGTTAGAAAAATCACGTCTTGTAAATGCCCATTTGTTTAAAAA 960  
 AAAAAAAACAACTATAGAAAAGAAATGTCTTCTG

GAAATG

GAAATG  
Depth:9 (MOUSE)  
Ei-value:0.000, Pi-value:0.000  
Er-value:0.000, Pr-value:0.000  
No matches to TargetScan

ACTTTTCGAAATGGAGTTGTTTGATTACGTTTAGAAGCAACTTCCGGAGCCACCGAAGTCCCCGTTTGTTCAGTGGGTTG 1080  
 GAGGACGTCGAATGGAAGACCTTGAGGAGGAAGAATAGAAAGTTCTGTGCCAGACTGGTCATACTT

AGAAGA

AGAAGA  
Depth:9 (MOUSE)  
Ei-value:0.000, Pi-value:0.000  
Er-value:0.000, Pr-value:0.000  
No matches to TargetScan

CATTTCTATATAACCCTTGTTGTGTGTGTGCATTTTGTTCCTCACTAC 1200  
 TGTATATATAGTTAACAATAAGTACTTTTTTGAAATACCTAGTCTTTCTAGATGTTCTG

AAGTGCCTG

AAGTGCCTG  
Depth:9 (MOUSE)  
Ei-value:0.000, Pi-value:0.000  
Er-value:0.000, Pr-value:0.000  
No matches to TargetScan

ATATATGTTAAAAGTCGAGGTAGTAAAAGGACATTTTGTAAATACCTTTTTG 1320  
 TTAAAATTCATACGAAATGATGGGGAGTGGGGGGTTGGCCAGACCCCTTCCTGGAGCAGTACACTCTGTT

TTGTGC

TTGTGC  
Depth:9 (MOUSE)  
Ei-value:0.000, Pi-value:0.000  
Er-value:0.000, Pr-value:0.000  
No matches to TargetScan

ACTGGTCGGGAGAGGGGAGGGAAGTGCAGATCTCTGCCAGTGTG 1440  
 TTTATAGTGAGGCAAGAGGAACCATTGTCTTTTCCGTTAGTGCATTTTGTTTCACTCTGCTGTACATATAGTGTACATAATGGACAAATGAGTCCTAATTTACAACATCTAGTCTTTCTA 1560  


GATGTT

GATGTT  
Depth:9 (MOUSE)  
Ei-value:0.000, Pi-value:0.000  
Er-value:0.000, Pr-value:0.000  
No matches to TargetScan


AAAGAGGTTGCCA

AAAGAGGTTGCCA  
Depth:9 (MOUSE)  
Ei-value:0.000, Pi-value:0.000  
Er-value:0.000, Pr-value:0.000  
No matches to TargetScan

GTGTATGACAAAAGTAGAGTTAGTAAATGATACATTTTGTAC

ATTTTGT

ATTTTGT  
Depth:9 (MOUSE)  
Ei-value:0.000, Pi-value:0.000  
Er-value:0.000, Pr-value:0.000  
No matches to TargetScan

GTTAAAAGTTTCTAGGAAAGATTGTCTTATCAAAATTTGAGCATCATAGCCC 1680  
 CCTGGGTATGGTGGAAAGAAGACAGAAGAGAAGGGAAGGTCTTAGGCTAGAATACTCATATTTTGAAGACAATTTCAAGATTCTAACTGTTACATGTGGCAGTTTATTCAAGACTGCTTT 1800  
 GTATATAGTGGACAAATTAAG

TCCTTA

TCCTTA  
Depth:9 (MOUSE)  
Ei-value:0.000, Pi-value:0.000  
Er-value:0.000, Pr-value:0.000  
No matches to TargetScan

TTTG

AAACATCT

AAACATCT  
Depth:9 (MOUSE)  
Ei-value:0.000, Pi-value:0.000  
Er-value:0.000, Pr-value:0.000  
No matches to TargetScan

AGCCCAC

CTAGATGTTTAGAAGTGCCC

CTAGATGTTTAGAAGTGCCC  
Depth:9 (MOUSE)  
Ei-value:0.000, Pi-value:0.000  
Er-value:0.000, Pr-value:0.000  
No matches to TargetScan

GAC

GTATGTTAAA

GTATGTTAAA  
Depth:9 (MOUSE)  
Ei-value:0.000, Pi-value:0.000  
Er-value:0.000, Pr-value:0.000  
No matches to TargetScan

TGTAGAGGTAGTGAAATACCACTT

TGTAAATA

TGTAAATA  
Depth:9 (MOUSE)  
Ei-value:0.000, Pi-value:0.000  
Er-value:0.000, Pr-value:0.000  
No matches to TargetScan

CTTTTTGCT 1920  
 CAAATTCATAGGAAGTATTGTCTTTGAGAATGTTAAATGTTAAACCACTCTGTGAGCAGTATACTGTTGTCTACACTTGTTCAGTGGTTTGGAGGAGGTGGGAGGGAAAGAATTAACAAA 2040  
 AGGTAATATGCTAGTTGTTTGCACCTGACATTTTCAGATGAAACATTTTTTGTGTGTTATGTGCATTTTGTTTTGCTGTGTATATAGTGTATATAATGGACAAATGAGTCCTAATTTTGT 2160  
 GACATCTAATCTCTAGATGTTGAAAGAGGTTGCCAGTGTATGACAAAGTAGTAAAATTAGCACATTTTGTACACTTTGTGTTGAAATTCATAGGAATGCTTGTTTTCTGTAAATGATTTT 2280  
 TTTATATGAATGTATTCAGACACCTCTAAGCGTTATACATGCCTGTACTTGTCCACTGGATTGGTGGTGGAGAAAAGGAAGTGAGGGTGGGAATGGTTCAGGCCAAAATCGTCATATTTA 2400  
 GGAGATACCTCAAACTATAGCCATTGTTACCTGTGTGCAATTTTCCTTAACAGTACTGTGTACATAGTGGACAAGTAAGTTCTTATATGAAATATCTAGTCTTTCTAGATATTTAGAAGT 2520  
 GCTTGATGCATTTTACAGTAGACGTAGTAATGCTTCTTGTAAATAGCTTTTTAAAACTGATGGGAAATAACCGTAATTGGAATTGTTAAACCTCCTCTCCAAACAGTATACTCTGCTTTT 2640  
 TCACTGGGTTGAGGGAG

GTGGGAGGGAA

GTGGGAGGGAA  
Depth:9 (MOUSE)  
Ei-value:0.000, Pi-value:0.000  
Er-value:0.000, Pr-value:0.000  
MATCHES To TargetScan▶ miR-150-5p:CUCCCAA▶ miR-532-3p:CUCCCAC

GTT

TGCAAA

TGCAAA  
Depth:9 (MOUSE)  
Ei-value:0.000, Pi-value:0.000  
Er-value:0.000, Pr-value:0.000  
No matches to TargetScan

AGATGTTTTGCTAGTGTGTACTAGAACATTTCAGCTTACCATTGCTTTATATGTTAAGTGCATTTCATTTAACTTTATATATT 2760  
 A

TGTATATA

TGTATATA  
Depth:9 (MOUSE)  
Ei-value:0.000, Pi-value:0.000  
Er-value:0.000, Pr-value:0.000  
No matches to TargetScan

TTGGACAAATGGGTCCCAATTTTATA

ATATCTAG

ATATCTAG  
Depth:9 (MOUSE)  
Ei-value:0.000, Pi-value:0.000  
Er-value:0.000, Pr-value:0.000  
No matches to TargetScan

T

CTCTAG

CTCTAG  
Depth:9 (MOUSE)  
Ei-value:0.000, Pi-value:0.000  
Er-value:0.000, Pr-value:0.000  
No matches to TargetScan

ATATT

AAAGAGGTTGCCAATGTATGACA

AAAGAGGTTGCCAATGTATGACA  
Depth:9 (MOUSE)  
Ei-value:0.000, Pi-value:0.000  
Er-value:0.000, Pr-value:0.000  
MATCHES To TargetScan▶ miR-182-5p:UUGGCAA▶ miR-96-5p/1271-5p:UUGGCAC▶ miR-539-3p:UCAUACA

AAAGTAGG

GTTAGTAAACT

GTTAGTAAACT  
Depth:9 (MOUSE)  
Ei-value:0.000, Pi-value:0.000  
Er-value:0.000, Pr-value:0.000  
No matches to TargetScan

A

ACACATT

ACACATT  
Depth:9 (MOUSE)  
Ei-value:0.000, Pi-value:0.000  
Er-value:0.000, Pr-value:0.000  
No matches to TargetScan

TTGTACACTTTGTGT 2880  
 TACAATTCATTGGAAAGGC

TGTCTTCTGAA

TGTCTTCTGAA  
Depth:9 (MOUSE)  
Ei-value:0.000, Pi-value:0.000  
Er-value:0.000, Pr-value:0.000  
No matches to TargetScan

AAGGACCTTTGAAAGTGAAACTATCATAAATCACATCTAAGTGATACAT

GTGCCT

GTGCCT  
Depth:9 (MOUSE)  
Ei-value:0.000, Pi-value:0.000  
Er-value:0.000, Pr-value:0.000  
No matches to TargetScan

GTACTTGCCACTCACTGTTGGATGGATGGCAAAAA 3000  
 GGAGGAATTGGAAGAAATGAAAAGTTCTAGACC

AGAATG

AGAATG  
Depth:9 (MOUSE)  
Ei-value:0.000, Pi-value:0.000  
Er-value:0.000, Pr-value:0.000  
No matches to TargetScan

TTCCTTCGT

AGAAGA

AGAAGA  
Depth:9 (MOUSE)  
Ei-value:0.000, Pi-value:0.000  
Er-value:0.000, Pr-value:0.000  
No matches to TargetScan

TACCTTCAGGTATAACCATTGTTACATGTGTGTAGTTTATTCAACACTACTG

TGTATATA

TGTATATA  
Depth:9 (MOUSE)  
Ei-value:0.000, Pi-value:0.000  
Er-value:0.000, Pr-value:0.000  
No matches to TargetScan

GCGGAC 3120  
 AAACTTAAGTCCTTATT

TGAAACATCTAG

TGAAACATCTAG  
Depth:9 (MOUSE)  
Ei-value:0.000, Pi-value:0.000  
Er-value:0.000, Pr-value:0.000  
No matches to TargetScan

TCT

TTCTAG

TTCTAG  
Depth:9 (MOUSE)  
Ei-value:0.000, Pi-value:0.000  
Er-value:0.000, Pr-value:0.000  
No matches to TargetScan

A

TGTTTAGAAGTGCACAAAGTATGTTAAAAGTAGA

TGTTTAGAAGTGCACAAAGTATGTTAAAAGTAGA  
Depth:9 (MOUSE)  
Ei-value:0.000, Pi-value:0.000  
Er-value:0.000, Pr-value:0.000  
No matches to TargetScan

GGTAGTGAAATAACATTTTGTAGATATATCCTTTCATTAAGATTCAT 3240  
 ACGAAATACTCATTAAGAAATGGAATAATCAGAGTGATCAAACCACCTCTCAGAGCAGTACGTATTACTTTATTTGTGCTGGTTCAGGGAGAAAGTACAAGGGGGGTTATACCAGTATGT 3360  
 TTATGTTAGGCAAGGTTAACCGTTGTCCTGTATGTGTCTGCGTTTTGGTTTAGTTG

TGTATA

TGTATA  
Depth:9 (MOUSE)  
Ei-value:0.000, Pi-value:0.000  
Er-value:0.000, Pr-value:0.000  
No matches to TargetScan

TAGTGTACATAAGGGACAAACGTCCAAATTTACAACATCT

AGTCTT

AGTCTT  
Depth:9 (MOUSE)  
Ei-value:0.000, Pi-value:0.000  
Er-value:0.000, Pr-value:0.000  
No matches to TargetScan

CCTAGATGTTAA 3480  
 AGAGGTTGCCAGTGTATGACAAAAATAACCAATAAATACATTGTGTACACTTGATGTTAAAATTCATAGGCAAGATTTCTGAAAACTGGCAGAAGTGA

AATTGTTA

AATTGTTA  
Depth:9 (MOUSE)  
Ei-value:0.000, Pi-value:0.000  
Er-value:0.000, Pr-value:0.000  
No matches to TargetScan

AAATCCCCTCTAAG 3600  
 CATTATAGATGCTTATTAGACTTGTCCACTGGGTAGATAAAGGGGAAGGGTCCTTGGCTAGAGTGTTCTGTTTAGATGATACTTTGAGACTGTAGCCATTGTACGCTGTGTTCAGTGCTA 3720  
 CTGTGTGTGTGTGTAGTGGACAAACTTAAATCCTTATTTGAAACATCTAGCCTTTTAGATGTGTAAAAGTACACAGACTGTTTTGAAAGTAGAGAGTTAAAACACCTTTTAAGTTTTTTT 3840  
 TTTTTTTTTTTCTGTTAATAGTAATGGTTTTATTTGGGAATTGGGGTTGTCTTGGAGAGTAAGCTGAAGTTTACTGGGTGGTGGTGGGGGCGGGGGGGGGGGGGGCAGGGAAGGAAGTGC 3960  
 AGAGAGAAGGGCTGTGTGCCCATCAGTCTTTATAGACAAGTTCAGATTGTCTACCCATTGTTCTGTTTGTGCATTTTAGTTAATATTGCTATGTATTAACAAGCAAGTCCTAATGCCCAA 4080  
 AGTATATCAAAAGTAGAGGTAGTAAAATAACCCCTTAATGAATGCCCTTTTGTTGGCTTTTAGGAAGGGCTGTGTCTTCTGGGAGTATCTATGTTAATCCACCTCTTGGAACTAGATGTA 4200  
 GTCTGGTACTTAGTCGCTGAAATGCTGGAGGAGTAAGAGGAAGGGTGAGGGGAAGGGCTCTTGGCTAATAGCTCCCATTATCTGGAAGACAGTTTTAGGTTTTAACTATAGGTCTGTATG 4320  
 TGCATTTTTGTGAAGTACCTATGTTTATTGTGGACAAGCTTCATTACTTTGCAACATCTAGGCTTTTTAGATGTCCTGAGGTGACAATGTATGATATAAAGTAGAGCTAGTGAATTAACC 4440  
 AATTTGTAAATATCTTTGTTAGAATTGATTTTTTTTAAAAGGCAGCATCTTGGACCTGGAATTATTATACCTTCTCTGAGAACTGTACATCAGGACTTGCTCATCTAGGTTGGCAGCTGA 4560  
 GGGGAGGAAGGAAGTCTAGAGGGAGGGCTGTGTGTGGATGTGCTCATGTTTATATTTTGATGATAACCATTGATGTGCGTGCATCTCCTGGCTGTACTATATATAGGAATGCATTGTTTA 4680  
 ATAATTCAGCAGAAGCATACCTCCTTGCTAACACCGTAATAGTTTAGATCAGATGATGAACCCTCCTAGAACTATTATGCTTTGTGTGCTCTGTTGCTTTATGTCTCATTTTAATTGAAC 4800  
 ATTAAGGGAATGTAGTATTTTAATCATGATCTAGAGGCCTGTTGCC

ATTTTTGT

ATTTTTGT  
Depth:9 (MOUSE)  
Ei-value:0.000, Pi-value:0.000  
Er-value:0.000, Pr-value:0.000  
No matches to TargetScan

CTTTGAAATTTTTGTTGCCAAGTAAGGCAAGATTACATTTTTTTCCTTCCAGATTGAGTTGGTGTA 4920  
 GTATA

TTCTTGGT

TTCTTGGT  
Depth:9 (MOUSE)  
Ei-value:0.000, Pi-value:0.000  
Er-value:0.000, Pr-value:0.000  
No matches to TargetScan

TATCAAAATACTCATAGCTTTGGGACTTTGAAATGGTA

AATATTCA

AATATTCA  
Depth:9 (MOUSE)  
Ei-value:0.000, Pi-value:0.000  
Er-value:0.000, Pr-value:0.000  
No matches to TargetScan

TGATGTGTGAAACAGCATGATACATAACTGTAGGTCTTCATTACATAAAAATGGATGCTTG 5040  
 GTTGTGAAGATACACAAACGAGACCTAGAAGGACATGTCACGGTAAA

CTGCTT

CTGCTT  
Depth:9 (MOUSE)  
Ei-value:0.000, Pi-value:0.000  
Er-value:0.000, Pr-value:0.000  
No matches to TargetScan

GTGATTCTGAA

TGACTT

TGACTT  
Depth:9 (MOUSE)  
Ei-value:0.000, Pi-value:0.000  
Er-value:0.000, Pr-value:0.000  
MATCHES To TargetScan▶ miR-224-5p:AAGUCAC

CGTTTT

TTGCTT

TTGCTT  
Depth:9 (MOUSE)  
Ei-value:0.000, Pi-value:0.000  
Er-value:0.000, Pr-value:0.010  
No matches to TargetScan

CTTGTGCTTTTTGGTTTCCTATTATGCACATGTTAACT 5160  
 TTTAAG

AAATAAA

AAATAAA  
Depth:9 (MOUSE)  
Ei-value:0.000, Pi-value:0.000  
Er-value:0.000, Pr-value:0.000  
No matches to TargetScan

TGTTATTTTAAAAACCTTAATGAAGTTTCCATTCTTGTAAAGTTAGCATACACACTCCATAGAACATAGCGTCCTCGTCCTTCAT                       5258
```

---

## >SHEEP (4921 bases)

```
 AGCGCGAAGGCACTTCCGGTGCCCCTCCTCTCTAGCCAGCGAAGAGAAC

TGCCAA

TGCCAA  
Depth:9 (MOUSE)  
Ei-value:0.000, Pi-value:0.000  
Er-value:0.000, Pr-value:0.000  
MATCHES To TargetScan▶ miR-182-5p:UUGGCAA▶ miR-96-5p/1271-5p:UUGGCAC

C

TCAGTTCCGG

TCAGTTCCGG  
Depth:9 (MOUSE)  
Ei-value:0.000, Pi-value:0.000  
Er-value:0.000, Pr-value:0.000  
No matches to TargetScan

CAGAGAGCGCGGACAGAAGCGGATCGTGGCCTTCTACCCTGGGGCCCTCCAG

GC

GCCCTC  
Depth:9 (MOUSE)  
Ei-value:0.000, Pi-value:0.000  
Er-value:0.000, Pr-value:0.000  
No matches to TargetScan

 120  


CCTC

GCCCTC  
Depth:9 (MOUSE)  
Ei-value:0.000, Pi-value:0.000  
Er-value:0.000, Pr-value:0.000  
No matches to TargetScan

CGGCCTCTGGTCGGTAGGTGAACTGGGGGGCCCCGGGACATTCTCGGGTGTGTTTTGTAGAGCGGTCGCCGAGGCCGGCAGTTGGCAGGGCCCAAGACTGGAAGCAGCCTCGAAAC 240  
 TCTCCCCCTGCCCCCACCCCCCACCTCCACGACCTA

ATGGCGGC

ATGGCGGC  
Depth:9 (MOUSE)  
Ei-value:0.000, Pi-value:0.000  
Er-value:0.000, Pr-value:0.000  
No matches to TargetScan

GGCCCCTCCGAACCAGGATCCTGCCGACCGCTGGGTCTCTTCACCCAGACCTGCAGGGCTTGGGCTTAGGCGAAGC 360  
 CTCCGCCTGGTCACCGAGAACGTGGCGCCTTTGGGCGTCGGGCGTGGCAGGACTAGAAGAATGTGACGCCAGCTCGTCCATCCTTAAACTTTGGGCCACGCGGATCCGCAGACCTGGAGC 480  
 CGCGGTTGACGGCGGGTGGAACGTTAGCAGGCCAGCGGGCAGTGATCGTCGTTCTACCGAGACATTGATGTCGGAAGATAGAAATGGTAGAATAACATACCCCTTGTGGCCAACAGAAAG 600  
 TGCCCGCCGTCCTCT

GGGAAGA

GGGAAGA  
Depth:9 (MOUSE)  
Ei-value:0.000, Pi-value:0.000  
Er-value:0.000, Pr-value:0.000  
No matches to TargetScan

TTTACTGGCCGCTGATAAAGG

CCTGTGTATATAATATGAAAAAGCTGCT

CCTGTGTATATAATATGAAAAAGCTGCT  
Depth:9 (MOUSE)  
Ei-value:0.000, Pi-value:0.000  
Er-value:0.000, Pr-value:0.000  
MATCHES To TargetScan▶ miR-15-5p/16-5p/195-5p/424-5p/497-5p:AGCAGCA▶ miR-503-5p:AGCAGCG

CTCAACTTTACCCC

CAACCTTT

CAACCTTT  
Depth:9 (MOUSE)  
Ei-value:0.000, Pi-value:0.000  
Er-value:0.000, Pr-value:0.000  
No matches to TargetScan

CAAAAGAAAACTTTCGCTACATCTAGG 720  
 CCTCCTAGATGTGA

AGAGGT

AGAGGT  
Depth:9 (MOUSE)  
Ei-value:0.000, Pi-value:0.000  
Er-value:0.000, Pr-value:0.000  
No matches to TargetScan

TGCCGACGTATGATAAAATAGTTAGAAAAATCGCGTCTTGTAAATGCCCATTTGTTTAAAAAAAGAAAACTATAGAAAAGAAATGTCTTCTG

GAAATG

GAAATG  
Depth:9 (MOUSE)  
Ei-value:0.000, Pi-value:0.000  
Er-value:0.000, Pr-value:0.000  
No matches to TargetScan

AC 840  
 TTTTCGAAATGGAGTTGTTTGATTACCTCTAGAAGCAACTTCCGGAGCCACTGAAGTCCCCGTTTGTTCAGTGGGTTGGACGACGTCGAATAGAAGACCTTGAGGAGGAAGAATAGAAAG 960  
 TGCTATGTCAGACTGGTCATACTT

AGAAGA

AGAAGA  
Depth:9 (MOUSE)  
Ei-value:0.000, Pi-value:0.000  
Er-value:0.000, Pr-value:0.000  
No matches to TargetScan

CATTTCTATATAACCATTTTTTAGTGTGTGCATTTTATTCCTCACTACTGTATATATAGTTGAGAATAAGTACTTTTTGGAAATACCTAG 1080  
 TCTTTCTAGATGTTCTG

AAGTGCCTG

AAGTGCCTG  
Depth:9 (MOUSE)  
Ei-value:0.000, Pi-value:0.000  
Er-value:0.000, Pr-value:0.000  
No matches to TargetScan

ATATATGTTAAAAGTCGAGGTAGTAAAAAGACATTTTGTAAATACCTTTTTGTTAAAATTCATATGAAATGATGTTGGGGAGCGGGGGCGGGGT 1200  
 GGGACGGGGGGGGAGGATTGGCCAGATCACTTTCTGGAGCAGTACACTTTGTGTT

TTGTGC

TTGTGC  
Depth:9 (MOUSE)  
Ei-value:0.000, Pi-value:0.000  
Er-value:0.000, Pr-value:0.000  
No matches to TargetScan

ACTGGTTCAGTGTCGGGAGAGGGGAGAGAAGTGCAGATCTCTGTGCCAGTGTGTTTATA 1320  
 GTGAGGCGAGAGTAACCATTGTCTTTTCCGTTAGTGCGTTTTGTTTCACTTTGCTGTGTATATAGTGTATATAATGGACAAATGAGTCCTAATTTACAACATCTAGTCTTTCTA

GATGTT

GATGTT  
Depth:9 (MOUSE)  
Ei-value:0.000, Pi-value:0.000  
Er-value:0.000, Pr-value:0.000  
No matches to TargetScan

 1440  


GATGTT  
Depth:9 (MOUSE)  
Ei-value:0.000, Pi-value:0.000  
Er-value:0.000, Pr-value:0.000  
No matches to TargetScan


AAAGAGGTTGCCA

AAAGAGGTTGCCA  
Depth:9 (MOUSE)  
Ei-value:0.000, Pi-value:0.000  
Er-value:0.000, Pr-value:0.000  
No matches to TargetScan

GTGTATGACAAAAATAGAGTTAGTAAATAATACATTTTGTAC

ATTTTGT

ATTTTGT  
Depth:9 (MOUSE)  
Ei-value:0.000, Pi-value:0.000  
Er-value:0.000, Pr-value:0.000  
No matches to TargetScan

GTTAAAATTTCTAGGGAAGATTGTCTTCTGAAAATTTGAGCATTATAGCCCCCTGGGT 1560  
 ATGATGGTGGAGAGAAGAGAGGAGAGAAGGGGAGGGTCTTAGGCTAGAATGTTCATATTTTGAAGACAATTTCAAGATTATAACTGTTACATGTGGCAGTTTATTCAAGACTGCTTTGTA 1680  
 TATAGTGGACAAATGAAG

TCCTTA

TCCTTA  
Depth:9 (MOUSE)  
Ei-value:0.000, Pi-value:0.000  
Er-value:0.000, Pr-value:0.000  
No matches to TargetScan

TTTG

AAACATCT

AAACATCT  
Depth:9 (MOUSE)  
Ei-value:0.000, Pi-value:0.000  
Er-value:0.000, Pr-value:0.000  
No matches to TargetScan

AGCCCAC

CTAGATGTTTAGAAGTGCCC

CTAGATGTTTAGAAGTGCCC  
Depth:9 (MOUSE)  
Ei-value:0.000, Pi-value:0.000  
Er-value:0.000, Pr-value:0.000  
No matches to TargetScan

GAC

GTATGTTAAA

GTATGTTAAA  
Depth:9 (MOUSE)  
Ei-value:0.000, Pi-value:0.000  
Er-value:0.000, Pr-value:0.000  
No matches to TargetScan

TGTAGAGGTAGTGAAATACCGCTT

TGTAAATA

TGTAAATA  
Depth:9 (MOUSE)  
Ei-value:0.000, Pi-value:0.000  
Er-value:0.000, Pr-value:0.000  
No matches to TargetScan

ACTTTTTGCTCA 1800  
 AATTTGTAGAAGTACTGTCTTTGAGAATGTTAAATGTTAAGCCACTCTGAGCAGTATACTGTTGCTTACATTTGTTCAGTGGTTTGGAGGAGGTGGGAGGGAAAGAATTACAAAAGGTAA 1920  
 TATGCTAGTTGTTTGCACCTGGACATTTTCAGATGAAACCATTTTTTGTATATTACGTGCATTTTGTTTTGCTGTGTATATAGTGTATATAATGGACAAATGAGTCCTAATTTTGTGACA 2040  
 TCTAGTCTCTAGATGTTAAAGAGGTTGCCAGTGTATGACAAAGTAGTAAAATTAGCACATTTTGTACACTTTGTGTTGAAATTCCTAGGAATGCTGGTTTTCTGTAAATGATTTTTTAAT 2160  
 ATAAATGTATTCAGACACCTCTAAGTGTTAGACATGCCTGTACTTGTCCACTGGATTGGTGGTGGAGAAAAGGAAGTGAGGGTGGGAATGGTTCAGGCCAAAATGGTCATATTTAGGAGA 2280  
 TACCTCAAATTATAACCATTGTTACCTGTGTGCAATTTTACTTAACAGTACTGTGTATATAGTGGACAAGTAAGTTCTTACATGAAATATCTAGTCTTTCTAGATATTTAGAAGTGCTTG 2400  
 ATGCATTTTACAGTAGACATAGTGTTATAATGCTTCTTGTAAATAGCTTTTTAAAACTGATGGGAAATACTGTAATTGGAAATGGAATTGTTAAACCTCCTCTCTGAACAGTATACTCTG 2520  
 CTTTTTCATTGGGTTGAGGGAG

GTGGGAGGGAA

GTGGGAGGGAA  
Depth:9 (MOUSE)  
Ei-value:0.000, Pi-value:0.000  
Er-value:0.000, Pr-value:0.000  
MATCHES To TargetScan▶ miR-150-5p:CUCCCAA▶ miR-532-3p:CUCCCAC

GTT

TGCAAA

TGCAAA  
Depth:9 (MOUSE)  
Ei-value:0.000, Pi-value:0.000  
Er-value:0.000, Pr-value:0.000  
No matches to TargetScan

AGATGTTTTGCTAGTGTGTACTAGAACATTTCAGCTTACCATTGCTTTATATGTTAAGTACATTTCATTTAACTTTAC 2640  
 TGTGTATATTA

TGTATATA

TGTATATA  
Depth:9 (MOUSE)  
Ei-value:0.000, Pi-value:0.000  
Er-value:0.000, Pr-value:0.000  
No matches to TargetScan

CTGGACAAATGGGTCCCAATTTTATA

ATATCTAG

ATATCTAG  
Depth:9 (MOUSE)  
Ei-value:0.000, Pi-value:0.000  
Er-value:0.000, Pr-value:0.000  
No matches to TargetScan

T

CTCTAG

CTCTAG  
Depth:9 (MOUSE)  
Ei-value:0.000, Pi-value:0.000  
Er-value:0.000, Pr-value:0.000  
No matches to TargetScan

ATATT

AAAGAGGTTGCCAATGTATGACA

AAAGAGGTTGCCAATGTATGACA  
Depth:9 (MOUSE)  
Ei-value:0.000, Pi-value:0.000  
Er-value:0.000, Pr-value:0.000  
MATCHES To TargetScan▶ miR-182-5p:UUGGCAA▶ miR-96-5p/1271-5p:UUGGCAC▶ miR-539-3p:UCAUACA

AAAGTAGG

GTTAGTAAACT

GTTAGTAAACT  
Depth:9 (MOUSE)  
Ei-value:0.000, Pi-value:0.000  
Er-value:0.000, Pr-value:0.000  
No matches to TargetScan

A

ACACATT

ACACATT  
Depth:9 (MOUSE)  
Ei-value:0.000, Pi-value:0.000  
Er-value:0.000, Pr-value:0.000  
No matches to TargetScan

TTGTA 2760  
 CACTTTGTGTTACAATTCATTGGAAAGGC

TGTCTTCTGAA

TGTCTTCTGAA  
Depth:9 (MOUSE)  
Ei-value:0.000, Pi-value:0.000  
Er-value:0.000, Pr-value:0.000  
No matches to TargetScan

AAGGACCTTTGAAAGTGAAATTATAATAAATCACACGTAAGTGATACAT

GTGCCT

GTGCCT  
Depth:9 (MOUSE)  
Ei-value:0.000, Pi-value:0.000  
Er-value:0.000, Pr-value:0.000  
No matches to TargetScan

GTACTTCCACCCACTGTTGGATGGA 2880  
 TGGCAAAAAGGAGGAATTGGAAGAAATGAAAGGTTCTAGACC

AGAATG

AGAATG  
Depth:9 (MOUSE)  
Ei-value:0.000, Pi-value:0.000  
Er-value:0.000, Pr-value:0.000  
No matches to TargetScan

TTCCTTTGT

AGAAGA

AGAAGA  
Depth:9 (MOUSE)  
Ei-value:0.000, Pi-value:0.000  
Er-value:0.000, Pr-value:0.000  
No matches to TargetScan

TACCTTCAGGTATAACCATTGTTACATGTGTGTAGTTTATTCAACACTACTG

TGTAT

TGTATATA  
Depth:9 (MOUSE)  
Ei-value:0.000, Pi-value:0.000  
Er-value:0.000, Pr-value:0.000  
No matches to TargetScan

 3000  


ATA

TGTATATA  
Depth:9 (MOUSE)  
Ei-value:0.000, Pi-value:0.000  
Er-value:0.000, Pr-value:0.000  
No matches to TargetScan

GCGGACAAACAAGTCCTTATT

TGAAACATCTAG

TGAAACATCTAG  
Depth:9 (MOUSE)  
Ei-value:0.000, Pi-value:0.000  
Er-value:0.000, Pr-value:0.000  
No matches to TargetScan

TCT

TTCTAG

TTCTAG  
Depth:9 (MOUSE)  
Ei-value:0.000, Pi-value:0.000  
Er-value:0.000, Pr-value:0.000  
No matches to TargetScan

A

TGTTTAGAAGTGCACAAAGTATGTTAAAAGTAGA

TGTTTAGAAGTGCACAAAGTATGTTAAAAGTAGA  
Depth:9 (MOUSE)  
Ei-value:0.000, Pi-value:0.000  
Er-value:0.000, Pr-value:0.000  
No matches to TargetScan

GGTAGTGAAATTTGTAGATATATCCTTTTGTTAAGATTCA 3120  
 TACGAAATACTCGTTTAGAAATGGAATGATCAGAATGATCAAACCACCTCTCAGAGCAGTACATATTAATTTACTTGTGCTGGTTCAGGGAGAAAGTACAAGGGGGGGTTATACCAGTAT 3240  
 GTTTATGTTAGGATGGTTAACCATTGTCCTGTATGTGTCTGCGTTTTGGTTTACTTAGTTG

TGTATA

TGTATA  
Depth:9 (MOUSE)  
Ei-value:0.000, Pi-value:0.000  
Er-value:0.000, Pr-value:0.000  
No matches to TargetScan

TAGTGTACATAAGGGACAAACGTCCAAATTTACAACATCT

AGTCTT

AGTCTT  
Depth:9 (MOUSE)  
Ei-value:0.000, Pi-value:0.000  
Er-value:0.000, Pr-value:0.000  
No matches to TargetScan

CCTAGAT 3360  
 GTTAAAGAGGTTGCCAGTGTATGACAAAAATAACCAATAAATACATTGTGTACGCTTGATGTTAAAATTCATAGGCAAGATTTCTGAAAACAACTGGCAGAAGTGA

AATTGTTA

AATTGTTA  
Depth:9 (MOUSE)  
Ei-value:0.000, Pi-value:0.000  
Er-value:0.000, Pr-value:0.000  
No matches to TargetScan

AAATCC 3480  
 CCTCTAAGCATTATAGATGCTTATTAGACTTGTCCACTGGGTTGATAGAGAAGGGGAAGGGTCCTTGGCTAGCATGTTCCTGTTGAGATGATACTTTGAGACTATAGCCATTGTACACAG 3600  
 TGTATTCAGTGCTGCTGTGTGTATGTATAGTGGACAAACTTAAATCCTTATTTGAAACATCTAGCCTTTTAGATGTGTAAAAGTACACAACCTATTTTGAAAGTAGAGAGTTAAACACCT 3720  
 TTTAAGTTTTGGGGGTTTTTTTTCTGTTAATTAATAGGAATGGTTTTATTTGGGTAGTGGGGTTGTCTTGGAGAGGAAGCTGACGCTTACTGGGTGGTGGCGGGGGAGGGGGGGAATACA 3840  
 ATTCTATGAATTTAGTGTGTGAATTTTTTTTGTTAGTCCCGCTTCATGCTATGGAACCAAAGTTTCAAAATGAACTTCCAAAAGGCATTAGTATATTACAGCATTAAACCTATATACTAA 3960  
 AAGGTTATCATTTTCAAAATACTGTTGGGAGTAGAAAATAATACTTGCATATGTTTTTATCATTAAGTATTTATTATGTACAGTGAGCTTTAGAAATTATTGCTGACAATGCAAAAAATC 4080  
 CTGAGAGCTCTAATAACAATACACATAAATTAATGTTTTCTTGCCCTCTGGGAGGATTTTTTTCTAAGAATAAGTGAAGTCACTCAGTCGTGTCCGACTCTTTGTGACCCCATAGACTGT 4200  
 AGCCTACCAGGCTCCTCTGTCCATGGGATTTTCCAAGCAATAGTACTGGAGTGGATTGCCATTTCCTTCTCCATCTCCTAAGAATAGATACACTTTATTTAACTTTGGAAGCAACAAGGC 4320  
 TTAATGGAAATAACATTCTTGTTGGATGCTAAGAGTCCTTGTTATTCTATTTCCTTGGTGATCTTAAACAAGACGTTCAGTCTATGTCTTTCTTTCTCAGCACTAAAATGAAGAATGGTG 4440  
 GCTTTCTAAGGTAAATTATGGGTAGTGGCTAAGGTTTTCTATAGCTTCAGTATTGAATAAAAATATTTTATTTAACAGACATTTTAATCATAGCTCTGCCAATATGTTTATCTAGAGGCC 4560  
 TGTTGCC

ATTTTTGT

ATTTTTGT  
Depth:9 (MOUSE)  
Ei-value:0.000, Pi-value:0.000  
Er-value:0.000, Pr-value:0.000  
No matches to TargetScan

CTTTAAAATTTTTGTTGCCAAGAAAGGCAAGATTACATTTTTTTCCTTCCAGATTGAGTTGGTGTAGTGTA

TTCTTGGT

TTCTTGGT  
Depth:9 (MOUSE)  
Ei-value:0.000, Pi-value:0.000  
Er-value:0.000, Pr-value:0.000  
No matches to TargetScan

TATCAAAATACTCATAGCTTTGGGAC 4680  
 TTTGAAATGGTA

AATATTCA

AATATTCA  
Depth:9 (MOUSE)  
Ei-value:0.000, Pi-value:0.000  
Er-value:0.000, Pr-value:0.000  
No matches to TargetScan

TGATGTGTGAAACAGCATGATACATAACTGTATGGTCTTAATTACATAAAAATGAATGCTTAGTTGTGAAAATACACAAATGAGACCTAGAAGGAGATGT 4800  
 CAAACGGTAAA

CTGCTT

CTGCTT  
Depth:9 (MOUSE)  
Ei-value:0.000, Pi-value:0.000  
Er-value:0.000, Pr-value:0.000  
No matches to TargetScan

GTGATTATGAA

TGACTT

TGACTT  
Depth:9 (MOUSE)  
Ei-value:0.000, Pi-value:0.000  
Er-value:0.000, Pr-value:0.000  
MATCHES To TargetScan▶ miR-224-5p:AAGUCAC

CGTTTT

TTGCTT

TTGCTT  
Depth:9 (MOUSE)  
Ei-value:0.000, Pi-value:0.000  
Er-value:0.000, Pr-value:0.010  
No matches to TargetScan

CTTGTGCTTTTTGGTTTCCTATTATGTATATGTTAACTTTTAAG

AAATAAA

AAATAAA  
Depth:9 (MOUSE)  
Ei-value:0.000, Pi-value:0.000  
Er-value:0.000, Pr-value:0.000  
No matches to TargetScan

TGTTATTTTAAAAACCTTAATGA 4920  
 A                                                                                                                        4921
```

---

## >PIG (5203 bases)

```
 GCGTGGAATTTACCTCAAGTGACGCCTCCCTCGTCCTTCCCCTCGGAACGTTTCCGTGCCGGAACCTTGGCTCGAGGGGCCCGGAGCGGTGCGAAGGCACTTCCGGTGCCCCTTCTCGCT 120  
 TGCCAGTGAAGAGAAC

TGCCAA

TGCCAA  
Depth:9 (MOUSE)  
Ei-value:0.000, Pi-value:0.000  
Er-value:0.000, Pr-value:0.000  
MATCHES To TargetScan▶ miR-182-5p:UUGGCAA▶ miR-96-5p/1271-5p:UUGGCAC

G

TCAGTTCCGG

TCAGTTCCGG  
Depth:9 (MOUSE)  
Ei-value:0.000, Pi-value:0.000  
Er-value:0.000, Pr-value:0.000  
No matches to TargetScan

CAGAGAGCGCGGTGAGAAGCGGATCGCGGCCTTCTGTCCCGGG

GCCCTC

GCCCTC  
Depth:9 (MOUSE)  
Ei-value:0.000, Pi-value:0.000  
Er-value:0.000, Pr-value:0.000  
No matches to TargetScan

CGGGCCCTCTGGCCTCGGCCAGCGGTGAACTGGGGGGC 240  
 CCTGGGACAGGCCTGAACTGTGTCCTGCAGAGCTCCAGAGTCCTCGCGGCCGTCCATGGCCCAGGCCTTGGAGTAGCAGCGAACCTCTCCCCCACCCCCACCTCCGCGACCAA

ATGGCGG

ATGGCGGC  
Depth:9 (MOUSE)  
Ei-value:0.000, Pi-value:0.000  
Er-value:0.000, Pr-value:0.000  
No matches to TargetScan

 360  


C

ATGGCGGC  
Depth:9 (MOUSE)  
Ei-value:0.000, Pi-value:0.000  
Er-value:0.000, Pr-value:0.000  
No matches to TargetScan

GGACCCCGCGAGCCCAGGCCCAGCCGATCGCCGGGTCTCTTGGCCCAGCGCTGCAGGGCTTAGGCGAAGCCTCTGCCAAATCGCCTTTGGGAGTCCAGCGTGGCAGGGCGAGAAGA

ATG

ATGGCGGC  
Depth:9 (MOUSE)  
Ei-value:0.000, Pi-value:0.000  
Er-value:0.000, Pr-value:0.000  
No matches to TargetScan

 480  


GCGGC

ATGGCGGC  
Depth:9 (MOUSE)  
Ei-value:0.000, Pi-value:0.000  
Er-value:0.000, Pr-value:0.000  
No matches to TargetScan

CACTCGAACGCCATGGAGAGCCGTTAGAGCCTCGAGGGCCCGAGAAGGCTGCCACCCTTGGGCCTTGGAGCCGTGCGGATTTGGGACCTGTGGTGGCCGCTCTGTCCCGGTTGGA 600  
 CGAGACCTCACCTCATCCGGTAAACGACGGACAAAGGCCTTTACGGGCCCGGAAGGTGAGCGGAGCCCCATCAACGACGGGTGGAACGTTAGTGGGCCATCGTTGACTCTTGTTCTACCG 720  
 AGACCTTGAAGTTAGAAATGGTAGAATAACGTACCACATGTGGCCAATAGGAAGTGCCCACCACCTTTT

GGGAAGA

GGGAAGA  
Depth:9 (MOUSE)  
Ei-value:0.000, Pi-value:0.000  
Er-value:0.000, Pr-value:0.000  
No matches to TargetScan

TTTATTGGCCGTTTAGAGAAGG

CCTGTGTATATAATATGAAAAA

CCTGTGTATATAATATGAAAAAGCTGCT  
Depth:9 (MOUSE)  
Ei-value:0.000, Pi-value:0.000  
Er-value:0.000, Pr-value:0.000  
MATCHES To TargetScan▶ miR-15-5p/16-5p/195-5p/424-5p/497-5p:AGCAGCA▶ miR-503-5p:AGCAGCG

 840  


GCTGCT

CCTGTGTATATAATATGAAAAAGCTGCT  
Depth:9 (MOUSE)  
Ei-value:0.000, Pi-value:0.000  
Er-value:0.000, Pr-value:0.000  
MATCHES To TargetScan▶ miR-15-5p/16-5p/195-5p/424-5p/497-5p:AGCAGCA▶ miR-503-5p:AGCAGCG

CTCAACTTTCCCCC

CAACCTTT

CAACCTTT  
Depth:9 (MOUSE)  
Ei-value:0.000, Pi-value:0.000  
Er-value:0.000, Pr-value:0.000  
No matches to TargetScan

CAGAAGAAAACTTTTTGCTACATCTAGGCCTCCTAGATGTAA

AGAGGT

AGAGGT  
Depth:9 (MOUSE)  
Ei-value:0.000, Pi-value:0.000  
Er-value:0.000, Pr-value:0.000  
No matches to TargetScan

TGCCGACGTATGATAAAGTAGAGTTAGAAAATCGCATGTCTTGT 960  
 AAATGCCCATTTTGGGTTTTTTTTTTTTTAAATCAAAAGAAAAGGAATGTCTTCTG

GAAATG

GAAATG  
Depth:9 (MOUSE)  
Ei-value:0.000, Pi-value:0.000  
Er-value:0.000, Pr-value:0.000  
No matches to TargetScan

ACTTTGAGATGGAATTGTTTGACCACCTCTGAAGTGACATCTGGAGAGACAGAGATCC 1080  
 ACGTTTGTTCAGTGGGTTAGAGGACATGGAGTGGAAGATTTCGAGGAAGAAGAAGGTTCTGTGCCAGACTCGTCATACTT

AGAAGA

AGAAGA  
Depth:9 (MOUSE)  
Ei-value:0.000, Pi-value:0.000  
Er-value:0.000, Pr-value:0.000  
No matches to TargetScan

CATTTGCATATTATAACCATTGTTTTGTGTGTGC 1200  
 ATTTTATTCCTCACTTCTATATATAGTTGACAATAAGTACTTTTTGGAAATATCTAGGCTTTCTAGATTTTCTG

AAGTGCCTG

AAGTGCCTG  
Depth:9 (MOUSE)  
Ei-value:0.000, Pi-value:0.000  
Er-value:0.000, Pr-value:0.000  
No matches to TargetScan

ACATATGTTAAAAGTAGAGGTAGTAAAAAGACATCTT 1320  
 TTTGTGTATGATAGAAATATCTTTTTGTTAAAACTCATATGAAATATTGTTTATTTGGGGGGAGGGGGGTTGGCCAAACCACCTCTTTGAATAGTGCATTGTGT

TTGTGC

TTGTGC  
Depth:9 (MOUSE)  
Ei-value:0.000, Pi-value:0.000  
Er-value:0.000, Pr-value:0.000  
No matches to TargetScan

ACTGGTTCAA 1440  
 GGTCGGGGGAGGAGAAGGAAGTGCAAAGAGCTCTTTGCTAGTGTTTATAGCAAGGCAAGGTTAACCATTGTTCTTTTTACGTGCATTTCGTTTCACTTTGTGTGTATATAGTGTACATAA 1560  
 CGGACAAATGAGTCCTAATTTACAACATCTGGTCCTTCTA

GATGTT

GATGTT  
Depth:9 (MOUSE)  
Ei-value:0.000, Pi-value:0.000  
Er-value:0.000, Pr-value:0.000  
No matches to TargetScan


AAAGAGGTTGCCA

AAAGAGGTTGCCA  
Depth:9 (MOUSE)  
Ei-value:0.000, Pi-value:0.000  
Er-value:0.000, Pr-value:0.000  
No matches to TargetScan

GTGTATGATAGAAATAGAGATAGTAAACTAATACCATCCTATAC

ATTTTGT

ATTTTGT  
Depth:9 (MOUSE)  
Ei-value:0.000, Pi-value:0.000  
Er-value:0.000, Pr-value:0.000  
No matches to TargetScan

GTCCTGAAAA 1680  
 TGTGAGCATTATAGCCCTCTGGGTTGGTAGAGGGGGGAGAGAAGAGAAGGGGGGTGGTCTTATGCTAGAATGCTCACATTTGGAAGACACTTTCAGATTATAACTGTTACATGTCGCAGT 1800  
 TTATTGAGACTGCTCTGTATATAGTGGACAAATTAAG

TCCTTA

TCCTTA  
Depth:9 (MOUSE)  
Ei-value:0.000, Pi-value:0.000  
Er-value:0.000, Pr-value:0.000  
No matches to TargetScan

TTTG

AAACATCT

AAACATCT  
Depth:9 (MOUSE)  
Ei-value:0.000, Pi-value:0.000  
Er-value:0.000, Pr-value:0.000  
No matches to TargetScan

AGTCCAC

CTAGATGTTTAGAAGTGCCC

CTAGATGTTTAGAAGTGCCC  
Depth:9 (MOUSE)  
Ei-value:0.000, Pi-value:0.000  
Er-value:0.000, Pr-value:0.000  
No matches to TargetScan

GAC

GTATGTTAAA

GTATGTTAAA  
Depth:9 (MOUSE)  
Ei-value:0.000, Pi-value:0.000  
Er-value:0.000, Pr-value:0.000  
No matches to TargetScan

TGTAGAGGTAGTAAAATATCACTT

T

TGTAAATA  
Depth:9 (MOUSE)  
Ei-value:0.000, Pi-value:0.000  
Er-value:0.000, Pr-value:0.000  
No matches to TargetScan

 1920  


GTAAATA

TGTAAATA  
Depth:9 (MOUSE)  
Ei-value:0.000, Pi-value:0.000  
Er-value:0.000, Pr-value:0.000  
No matches to TargetScan

GCTTTTAGCTAAAATTCATAGGAAATATTGTCTTTTGAAAATGGAATTGTTAAACCACCTCTGTGACCAGTAGTTTGGAGTAGGTGAGAAGGAAAGAATTGCAAAAGGTAATA 2040  
 TGCTAGTGTGTTTGGACGTTTTCAGACAAAACCATTTTTTGTATGTTAGGTGCATTTTGCTTTTCTGTGTATATAGTGTATATAATGGACAAATGAGTCCTAATTTTGCAACATCTAGTC 2160  
 TCTAGATGTTAAAGAGGTTGCCAGTGTATGACAAAATAGTAAGATTTACATATTTTTTACACTTTGTGTTGAAATTTATAGGAAAGCTTGTCTTCTGTAGATGACTTTTGGATATGAATT 2280  
 TGTTCATTCACCTCTAAGCATTACACATTGCCTGTACTTGTCCACTGAATTCATGGTGGAGAGAAGGAAGTGAGGGAGGGAATGCTTCAGGCCAAAATGGTCATATTTAGAAGATACCTC 2400  
 AGATTATAACCATTGTTACACGTGTGCAATTTTATTTAACAGTGCTGTGTACTTAGTGAACAAGTAAGTTCTTATATGAAATATCTAGTCTTGCTGGGTATTTAGAAGTGCTTGATGTAT 2520  
 TTAAAAGTAGTAGTAGTAAAATAATGCTTCTTGTAAATAGCTTTTAAAAACATGGGAAATGCTGTCTTTGGAAATGGAATTGTTAAACCTCCTCTGACCAGTATACTCTGCTCATTCATT 2640  
 GGGTTGATGGAA

GTGGGAGGGAA

GTGGGAGGGAA  
Depth:9 (MOUSE)  
Ei-value:0.000, Pi-value:0.000  
Er-value:0.000, Pr-value:0.000  
MATCHES To TargetScan▶ miR-150-5p:CUCCCAA▶ miR-532-3p:CUCCCAC

GAT

TGCAAA

TGCAAA  
Depth:9 (MOUSE)  
Ei-value:0.000, Pi-value:0.000  
Er-value:0.000, Pr-value:0.000  
No matches to TargetScan

AACATGTTTTGCTAGTGTGTACTAAAAAATTTCACCTTATTGGTTGCTCTATGTTACATGCATTTTGTTTAACTTTAC

TGTATATA

TGTATATA  
Depth:9 (MOUSE)  
Ei-value:0.000, Pi-value:0.000  
Er-value:0.000, Pr-value:0.000  
No matches to TargetScan

TT 2760  
 A

TGTATATA

TGTATATA  
Depth:9 (MOUSE)  
Ei-value:0.000, Pi-value:0.000  
Er-value:0.000, Pr-value:0.000  
No matches to TargetScan

CTGGACAAATGGGTTCCAATTTTATA

ATATCTAG

ATATCTAG  
Depth:9 (MOUSE)  
Ei-value:0.000, Pi-value:0.000  
Er-value:0.000, Pr-value:0.000  
No matches to TargetScan

T

CTCTAG

CTCTAG  
Depth:9 (MOUSE)  
Ei-value:0.000, Pi-value:0.000  
Er-value:0.000, Pr-value:0.000  
No matches to TargetScan

ATATTAA

AAAGAGGTTGCCAATGTATGACA

AAAGAGGTTGCCAATGTATGACA  
Depth:9 (MOUSE)  
Ei-value:0.000, Pi-value:0.000  
Er-value:0.000, Pr-value:0.000  
MATCHES To TargetScan▶ miR-182-5p:UUGGCAA▶ miR-96-5p/1271-5p:UUGGCAC▶ miR-539-3p:UCAUACA

AAAGTAGA

GTTAGTAAACT

GTTAGTAAACT  
Depth:9 (MOUSE)  
Ei-value:0.000, Pi-value:0.000  
Er-value:0.000, Pr-value:0.000  
No matches to TargetScan

A

ACACATT

ACACATT  
Depth:9 (MOUSE)  
Ei-value:0.000, Pi-value:0.000  
Er-value:0.000, Pr-value:0.000  
No matches to TargetScan

TTGTACACTTGTG 2880  
 TTAAATTCCATTGAAAGGC

TGTCTTCTGAA

TGTCTTCTGAA  
Depth:9 (MOUSE)  
Ei-value:0.000, Pi-value:0.000  
Er-value:0.000, Pr-value:0.000  
No matches to TargetScan

AAGGACCTTTGGAAATAAAATTGTAAATCACATCTAAGTGACACAT

GTGCCT

GTGCCT  
Depth:9 (MOUSE)  
Ei-value:0.000, Pi-value:0.000  
Er-value:0.000, Pr-value:0.000  
No matches to TargetScan

GAACTCAGCCACTGTTGGATGGACAGTGGGAAGGAAGA 3000  
 GTTAGGAGCAATGAAGCATTCTAATAAGCT

AGAATG

AGAATG  
Depth:9 (MOUSE)  
Ei-value:0.000, Pi-value:0.000  
Er-value:0.000, Pr-value:0.000  
No matches to TargetScan

TTTCTGTGT

AGAAGA

AGAAGA  
Depth:9 (MOUSE)  
Ei-value:0.000, Pi-value:0.000  
Er-value:0.000, Pr-value:0.000  
No matches to TargetScan

CACTTTCATATATAACCATTTTTACATGTGTGTGGTTTATTCAACACTACTG

TGTATATA

TGTATATA  
Depth:9 (MOUSE)  
Ei-value:0.000, Pi-value:0.000  
Er-value:0.000, Pr-value:0.000  
No matches to TargetScan

TAGTGGACA 3120  
 AACTTAAGTCCTTATT

TGAAACATCTAG

TGAAACATCTAG  
Depth:9 (MOUSE)  
Ei-value:0.000, Pi-value:0.000  
Er-value:0.000, Pr-value:0.000  
No matches to TargetScan

TCT

TTCTAG

TTCTAG  
Depth:9 (MOUSE)  
Ei-value:0.000, Pi-value:0.000  
Er-value:0.000, Pr-value:0.000  
No matches to TargetScan

A

TGTTTAGAAGTGCACAAAGTATGTTAAAAGTAGA

TGTTTAGAAGTGCACAAAGTATGTTAAAAGTAGA  
Depth:9 (MOUSE)  
Ei-value:0.000, Pi-value:0.000  
Er-value:0.000, Pr-value:0.000  
No matches to TargetScan

GGTAGTGAAATAACATTTTGTAGATATCTTTTTGTTAAAATTCATATG 3240  
 AAATATTGCCTTTTAGAAATGGAATAATCAAACCACCTCTTTGAGCAGTGCACATTATTTTATTTGTGCTGGTTCAAGGAGGAAGAAAAAAGTACAAAGGGCTTTATACCAATATGTTTA 3360  
 TAGTTAGGAGAGATTAACCATCGTCCTGTATGTCTGTGTTTTGCTTTACTTAGTTG

TGTATA

TGTATA  
Depth:9 (MOUSE)  
Ei-value:0.000, Pi-value:0.000  
Er-value:0.000, Pr-value:0.000  
No matches to TargetScan

TAGTGTACATAAAGGACAGATGTCCAAATTTACATCTAGTCTTCCTAGATGTTAAAGA 3480  
 GGTTGCCAATGTATGACAAAAGTAGTTAGAAAATAAATACATTGTGAATACTTTGTTAAAATTCATACGAAAAATTGTTCTGAAAGCAACTGGAGGAGATGAAATTGTTAGAATCCCCTC 3600  
 TAAGCATTTCAGATGCTTTATTACACTTGCTTACTAGGTTGATAGAGATGAGAAGGGGAAGGGTTCTAGGCCCGTGTTCCTGTTTAGAAGACACTTTGAGATTATAGCCTTTGTGTGCAG 3720  
 TTTATTCAATACTACATGTATGTATAGTAAACTAAAATCCTTATTTGAAACATCT

AGTCTT

AGTCTT  
Depth:9 (MOUSE)  
Ei-value:0.000, Pi-value:0.000  
Er-value:0.000, Pr-value:0.000  
No matches to TargetScan

TCTAGATGTTTAAAAGTGTACAACATATGCTAAAAGTAGATAATTAACACCTTTTAAGA 3840  
 TTTTTTTCTGTTAATTCATAGGAATGGTCATATTTGGGGAATGGGATTGTTAAACTTGAGCCTTGGAGAGTATGCTGACTGATGACTGGGGTGGTGGGGGTTGGGGGGGACAGGGAAGGA 3960  
 AGCTCAGAGAAGGGTTCTGTGCTCATGAGTCTTTAGAAAGCTTCAGATTTTCTAACCATTGTTCTGTTTGTGTGTTTGAGTTAATATTGCTGTGTATTAAAGAATAAACAAGTCCCAGTG 4080  
 TCCAAAGTATATTGAAAGTAGAAGTAGTAAAATAATTCCTTTATAAATGCCCTTTTGTGGGTTTTTAGGAAGGGCTATGACTTCTGGGAGTGTCTATGTTAATCCACCTTTTGTAGCTAG 4200  
 ATGTAGTCCTGTAGTTAGTCACTGAAATGCTGGAAGAGGGTTAAGAGGAAGGGTGAAGGGAAGGGCTCTTGGCTATTATCTCCATATCTAGACAGTTTTAGTTTATAACCATAGGTCTAT 4320  
 ATGTGCATTTTTGTAAAGTTTCTGTTTATTGTGGACAAGGTTCATTCTTTATCTTGCAACATCTATGTCCCAGGATAACATATGATAAAAAGTAGAGTTAGTGAGTTAACCAATTTGTAA 4440  
 ATAAATATCTTTGTTACAATGATATAAAACAGCATCTTGGACATG

AATTGTTA

AATTGTTA  
Depth:9 (MOUSE)  
Ei-value:0.000, Pi-value:0.000  
Er-value:0.000, Pr-value:0.000  
No matches to TargetScan

AAGTCTCAGAAATGTATGTCAGGACTTGTTCACTAGGTTGGCAGCAGAGGGGCAGAAGGAAAGTATA 4560  
 AAGGGAGGGTTGTATGCGAATGTGTTCATATTTATATTTTGGTGATAACCATTGATGTGGGTGCATCTTTTGGCTGTAATATAAGCAATTCAGTGGAAACATACCTCCTTGCTAATATTT 4680  
 TAATAGTTTGGATAATTAGTCCTCTTAGAGGCATTATACTTTATGTACTCTGTTGCTTTATGTGTCATTTTTAATTGAACATAAGGGAATGTAGTATTTTAATCATAACTCTACCAATAT 4800  
 CTTTATCTTGAAGCCTGTTGCT

ATTTTTGT

ATTTTTGT  
Depth:9 (MOUSE)  
Ei-value:0.000, Pi-value:0.000  
Er-value:0.000, Pr-value:0.000  
No matches to TargetScan

CTTTTATGTAATTTTATCGCTAAGAAAGGCAGGATTACATTTTTTTTCCTTCCAGATCGAGTTGGTGTAGTATA

TTCTTGGT

TTCTTGGT  
Depth:9 (MOUSE)  
Ei-value:0.000, Pi-value:0.000  
Er-value:0.000, Pr-value:0.000  
No matches to TargetScan

TATCAAAA 4920  
 TAAAATACTCATATAGCTTTGGGACTTTGAAATGGTA

AATATTCA

AATATTCA  
Depth:9 (MOUSE)  
Ei-value:0.000, Pi-value:0.000  
Er-value:0.000, Pr-value:0.000  
No matches to TargetScan

TGATGTGTGAAAAAGCATGATACATAACTATGATCTTAATTACATAAAAATGGATGCTTCATTGTGTAGATACAC 5040  
 AAACGAAATCTAGAAGGACATATCAACCTGTAAA

CTGCTT

CTGCTT  
Depth:9 (MOUSE)  
Ei-value:0.000, Pi-value:0.000  
Er-value:0.000, Pr-value:0.000  
No matches to TargetScan

GATTATGGA

TGACTT

TGACTT  
Depth:9 (MOUSE)  
Ei-value:0.000, Pi-value:0.000  
Er-value:0.000, Pr-value:0.000  
MATCHES To TargetScan▶ miR-224-5p:AAGUCAC

TGTTTTTTGCTTCCTAT

TTGCTT

TTGCTT  
Depth:9 (MOUSE)  
Ei-value:0.000, Pi-value:0.000  
Er-value:0.000, Pr-value:0.010  
No matches to TargetScan

GTGTTTTTCAGTTTCCTATTATGCACATGTTAACTTTTAAG

A

AAATAAA  
Depth:9 (MOUSE)  
Ei-value:0.000, Pi-value:0.000  
Er-value:0.000, Pr-value:0.000  
No matches to TargetScan

 5160  


AATAAA

AAATAAA  
Depth:9 (MOUSE)  
Ei-value:0.000, Pi-value:0.000  
Er-value:0.000, Pr-value:0.000  
No matches to TargetScan

TTTTACTTAAAAAAGCCTTCATGAAGTCGCCATTGCT                                                                              5203
```

---

## >ARMADILLO (5039 bases)

```
 GGTTCCGGAGCAGAGTGTACTTCCGGTTCTTCTTCTCGCTCGCCCGCGGAGAGAAC

TGCCAA

TGCCAA  
Depth:9 (MOUSE)  
Ei-value:0.000, Pi-value:0.000  
Er-value:0.000, Pr-value:0.000  
MATCHES To TargetScan▶ miR-182-5p:UUGGCAA▶ miR-96-5p/1271-5p:UUGGCAC

G

TCAGTTCCGG

TCAGTTCCGG  
Depth:9 (MOUSE)  
Ei-value:0.000, Pi-value:0.000  
Er-value:0.000, Pr-value:0.000  
No matches to TargetScan

CAGAGAGCGCGGTGAAGAGCAGAGCGCGAATCCCCTCCCCGG

GCCCT

GCCCTC  
Depth:9 (MOUSE)  
Ei-value:0.000, Pi-value:0.000  
Er-value:0.000, Pr-value:0.000  
No matches to TargetScan

 120  


C

GCCCTC  
Depth:9 (MOUSE)  
Ei-value:0.000, Pi-value:0.000  
Er-value:0.000, Pr-value:0.000  
No matches to TargetScan

CGGCCTCTCGGCCCTCGGGTGGGTGAGCCGGGGGGCCCGGGGGTGGTGTGTGGTCCTTGCCCTGCAGAGACCCAGAGCCCTCGCCGCGATTGCTCACTGGCCCGGACCAGGCCCTGAGG 240  
 CAGCGGCGGAACCCTGCTCTCCCTTCCCCCATGATGTTCTGAAGGCAGCGGTGGCGGCCTCGCCCAGCCTGGGCCCCACCCGCGGCCAGATTTCCTTGCCCAAAATTGTCGGGCGGCGGC 360  
 TTAAGCCCCCAGCGGGCCACCGAGGACGCGGCCCCTTTGGGCGGCGGGCGCGGCGGGGAGAGAAGA

ATGGCGGC

ATGGCGGC  
Depth:9 (MOUSE)  
Ei-value:0.000, Pi-value:0.000  
Er-value:0.000, Pr-value:0.000  
No matches to TargetScan

TACGAACGAACGCCACGGAGGGCCGTTAGGCACCTCCAGGGCCCAG 480  
 AAGGCGCGCATGAGTGGAATCAAGTTCGATCACCTTTATAGGCCGTGGAAGCGTGCAGATCCGGGATGTGAGGTGATCGTTCAGTCCCGGTTGGACAACATGCCGCTTGCCCCGACAACA 600  
 ACGGACAAAGGCCTTAACGGGCCTGGAAGGTGAGCGGAAGCCTGGACAATGGCGGGTGGAACGGTTAGCGGCCATAGGGCGGCCAGTGATCGTTTTACCCGAACGTGCTTGATATTTGAA 720  
 GAGAGAAATAGTAGAATAACGTGCAACATTTGGGCAACAAGAAGTGTCCACAACCCTTT

GGGAAGA

GGGAAGA  
Depth:9 (MOUSE)  
Ei-value:0.000, Pi-value:0.000  
Er-value:0.000, Pr-value:0.000  
No matches to TargetScan

TTTACTGGCCATTTACAGAAGG

CCTGTGTATATAATATGAAAAAGCTGCT

CCTGTGTATATAATATGAAAAAGCTGCT  
Depth:9 (MOUSE)  
Ei-value:0.000, Pi-value:0.000  
Er-value:0.000, Pr-value:0.000  
MATCHES To TargetScan▶ miR-15-5p/16-5p/195-5p/424-5p/497-5p:AGCAGCA▶ miR-503-5p:AGCAGCG

ATCA 840  
 ACTTTTCCCC

CAACCTTT

CAACCTTT  
Depth:9 (MOUSE)  
Ei-value:0.000, Pi-value:0.000  
Er-value:0.000, Pr-value:0.000  
No matches to TargetScan

TAAAAGAAAACTTATGCTACATCTAGGCTTTCTAGATGAAA

AGAGGT

AGAGGT  
Depth:9 (MOUSE)  
Ei-value:0.000, Pi-value:0.000  
Er-value:0.000, Pr-value:0.000  
No matches to TargetScan

GCCGACGTATGATAAAGTAGAGTTAGAAAATCACATGTTTTGTAATGAGCATCTA 960  
 TTTAAAAAAAAAAATAGAAAAATATCTTCTG

GAAATG

GAAATG  
Depth:9 (MOUSE)  
Ei-value:0.000, Pi-value:0.000  
Er-value:0.000, Pr-value:0.000  
No matches to TargetScan

ACTTGGAGATGGAATTTAGACTGCCTCTGGAAGCGACACTGGGAGCCACGTACATCCATGTTTTGTGGGTTAGAGGAGGTGAA 1080  
 GCAGAGAAGGCTGAGAAGAGGATGAGAAGATTCTTTGCCAGACTGGTCATATTT

AGAAGA

AGAAGA  
Depth:9 (MOUSE)  
Ei-value:0.000, Pi-value:0.000  
Er-value:0.000, Pr-value:0.000  
No matches to TargetScan

TATTTTCATATTGTAACCATTGTTCTGTATGTCACTTTATTCCTCACTACCCTATGTATA 1200  
 GTTGACAATACTAAGTACTTTTTAAAAATATCTCTTCCTAGATGTTCTG

AAGTGCCTG

AAGTGCCTG  
Depth:9 (MOUSE)  
Ei-value:0.000, Pi-value:0.000  
Er-value:0.000, Pr-value:0.000  
No matches to TargetScan

ACGTACGTAAAAAGTAGAGGTAGTAAAAGAACACATTTTGTAAATATCTTTTTGTTAAAATT 1320  
 CATATAAAATATTATGGGGGGTTTTGGAATAGCCAAACCACCTCTCTGAATAGTTCACATT

TTGTGC

TTGTGC  
Depth:9 (MOUSE)  
Ei-value:0.000, Pi-value:0.000  
Er-value:0.000, Pr-value:0.000  
No matches to TargetScan

ACTGGTTTGGGGGTGGGGGGATAAGGAAGTGCAAGGGCTCTATGCTAGTGTGT 1440  
 TTAGAATGAGGCAAGATTAACCATTGTCCCTGTGTTCCCGCCCGCTTTTGGTTTTGTACTTTGCTGTGTATATAATGGACAAACACGTCCTAATTTACATCTAGTCTTCCA

GATGTT

GATGTT  
Depth:9 (MOUSE)  
Ei-value:0.000, Pi-value:0.000  
Er-value:0.000, Pr-value:0.000  
No matches to TargetScan

A

AA

AAAGAGGTTGCCA  
Depth:9 (MOUSE)  
Ei-value:0.000, Pi-value:0.000  
Er-value:0.000, Pr-value:0.000  
No matches to TargetScan

 1560  


AGAGGTTGCCA

AAAGAGGTTGCCA  
Depth:9 (MOUSE)  
Ei-value:0.000, Pi-value:0.000  
Er-value:0.000, Pr-value:0.000  
No matches to TargetScan

GTGTATGAAAAAATAGTAAATAC

ATTTTGT

ATTTTGT  
Depth:9 (MOUSE)  
Ei-value:0.000, Pi-value:0.000  
Er-value:0.000, Pr-value:0.000  
No matches to TargetScan

GTTAAAATTCCTAGGAAAGATTGTCTTCTGAAAACTTGAGCATTATAGCCCACAAGGAGGGATGTGGAGGGAGGGATAA 1680  
 AAGGAAAGGGTTTTAGACCAGAATGTTCATATTTAGAAGACACTTTCAGATTATAACTTGTTAAATGTGTGTTTAGTCAAGACCGCTCTGTATATAGTGGACAAAATAAG

TCCTTA

TCCTTA  
Depth:9 (MOUSE)  
Ei-value:0.000, Pi-value:0.000  
Er-value:0.000, Pr-value:0.000  
No matches to TargetScan

TTTG 1800  


AAACATCT

AAACATCT  
Depth:9 (MOUSE)  
Ei-value:0.000, Pi-value:0.000  
Er-value:0.000, Pr-value:0.000  
No matches to TargetScan

AGTCTGT

CTAGATGTTTAGAAGTGCCC

CTAGATGTTTAGAAGTGCCC  
Depth:9 (MOUSE)  
Ei-value:0.000, Pi-value:0.000  
Er-value:0.000, Pr-value:0.000  
No matches to TargetScan

GAC

GTATGTTAAA

GTATGTTAAA  
Depth:9 (MOUSE)  
Ei-value:0.000, Pi-value:0.000  
Er-value:0.000, Pr-value:0.000  
No matches to TargetScan

AGTAGAAGTAGTAAAACATTATTT

TGTAAATA

TGTAAATA  
Depth:9 (MOUSE)  
Ei-value:0.000, Pi-value:0.000  
Er-value:0.000, Pr-value:0.000  
No matches to TargetScan

TCATTTTGCTCAGTTTCATAGGAAATACTGTCTTTTGGAA 1920  
 ATAGGTTGCTAAACCGTCTCTCTGGGTAGCATACTACTGCCTATATATTCATTCATTGATTTGGAGGAGGTGGGAGGGAAGCAATTGCAAAAGGTAATTTGCTAGTATGTCCATACTTGG 2040  
 ATAATTTCAGGCAAAACTTTTCTGTTATGTTCTTTGCATTTTGTTTGGTTTGTATATAGTGTATATAGTGGACAAATGAGTCCTAATTTTGCAACATCTAGGGGTTCACAGTATATGACA 2160  
 AAGTAGAGTTAGTAAAATTAGCATGTTTTGTATACTTTGTGTTGAAATTCATTGGAAAGTTTGTCTTCTGTTATTGACTTTCGGTATGAATTTGTTCACCACCTCAGAGCATTGCACTTG 2280  
 CCTGTACTTGTCCACTGAACTGGTGGTGAGAAAAGGGAATGAGGAAGGAGTGGTTCCAGGCCAAGATGGTCATGTTTAGGAGATACCTCAGATTATAACCTTGTTATGTGTGTTCAATTT 2400  
 TATTTAACATTATGTATGTAGTGGACAAGTAAGTTCTTATTTGAAATATCTAGTCTTTCTAAATATTTTAGAAGTGCTTGATGTGCTTAAAAATAGAGTTAGTAAAAGAACACTGTAAAT 2520  
 AGCTTTTAAAAACTGGTGGGATATTGTCTTTGGAAATGGAACTGTACTCTTGTTCTTTGGTTTGAGGGAG

GTGGGAGGGAA

GTGGGAGGGAA  
Depth:9 (MOUSE)  
Ei-value:0.000, Pi-value:0.000  
Er-value:0.000, Pr-value:0.000  
MATCHES To TargetScan▶ miR-150-5p:CUCCCAA▶ miR-532-3p:CUCCCAC

GAAAG

TGCAAA

TGCAAA  
Depth:9 (MOUSE)  
Ei-value:0.000, Pi-value:0.000  
Er-value:0.000, Pr-value:0.000  
No matches to TargetScan

AGGTGTTTGCTAGTGTGTACTAGAAAAT 2640  
 TTCTGATCATCTTTTGCCCTGTATGTGCATTCATTTAAATTTGCTGTACTGTATATAT

TGTATATA

TGTATATA  
Depth:9 (MOUSE)  
Ei-value:0.000, Pi-value:0.000  
Er-value:0.000, Pr-value:0.000  
No matches to TargetScan

TACTGGACAAATGAGTCCTAATTTTACA

ATATCTAG

ATATCTAG  
Depth:9 (MOUSE)  
Ei-value:0.000, Pi-value:0.000  
Er-value:0.000, Pr-value:0.000  
No matches to TargetScan

T

CTCTAG

CTCTAG  
Depth:9 (MOUSE)  
Ei-value:0.000, Pi-value:0.000  
Er-value:0.000, Pr-value:0.000  
No matches to TargetScan

ATATT

AAAGAG

AAAGAGGTTGCCAATGTATGACA  
Depth:9 (MOUSE)  
Ei-value:0.000, Pi-value:0.000  
Er-value:0.000, Pr-value:0.000  
MATCHES To TargetScan▶ miR-182-5p:UUGGCAA▶ miR-96-5p/1271-5p:UUGGCAC▶ miR-539-3p:UCAUACA

 2760  


GTTGCCAATGTATGACA

AAAGAGGTTGCCAATGTATGACA  
Depth:9 (MOUSE)  
Ei-value:0.000, Pi-value:0.000  
Er-value:0.000, Pr-value:0.000  
MATCHES To TargetScan▶ miR-182-5p:UUGGCAA▶ miR-96-5p/1271-5p:UUGGCAC▶ miR-539-3p:UCAUACA

AAAGTAGA

GTTAGTAAACT

GTTAGTAAACT  
Depth:9 (MOUSE)  
Ei-value:0.000, Pi-value:0.000  
Er-value:0.000, Pr-value:0.000  
No matches to TargetScan

A

ACACATT

ACACATT  
Depth:9 (MOUSE)  
Ei-value:0.000, Pi-value:0.000  
Er-value:0.000, Pr-value:0.000  
No matches to TargetScan

TTGTACACTTTGTGTTAAAATTCATAGAAATGC

TGTCTTCTGAA

TGTCTTCTGAA  
Depth:9 (MOUSE)  
Ei-value:0.000, Pi-value:0.000  
Er-value:0.000, Pr-value:0.000  
No matches to TargetScan

AATGGCTTTTGGAAATGAAATTGTAAAACCAC 2880  
 ATCTAAGTGACAT

GTGCCT

GTGCCT  
Depth:9 (MOUSE)  
Ei-value:0.000, Pi-value:0.000  
Er-value:0.000, Pr-value:0.000  
No matches to TargetScan

GTAAATCAGCCACTGGGTTTGTAGTAAAGAGAAGGGATTGGGAGAAATGAAGGGCTCTAGACC

AGAATG

AGAATG  
Depth:9 (MOUSE)  
Ei-value:0.000, Pi-value:0.000  
Er-value:0.000, Pr-value:0.000  
No matches to TargetScan

TTTCTATTT

AGAAGA

AGAAGA  
Depth:9 (MOUSE)  
Ei-value:0.000, Pi-value:0.000  
Er-value:0.000, Pr-value:0.000  
No matches to TargetScan

CACTTTCAAATATAACC 3000  
 ATTGTTACATGTGCATAGTTTATTTAACACTACTG

TGTATATA

TGTATATA  
Depth:9 (MOUSE)  
Ei-value:0.000, Pi-value:0.000  
Er-value:0.000, Pr-value:0.000  
No matches to TargetScan

GTGGACGAATTAAGTCCTTATT

TGAAACATCTAG

TGAAACATCTAG  
Depth:9 (MOUSE)  
Ei-value:0.000, Pi-value:0.000  
Er-value:0.000, Pr-value:0.000  
No matches to TargetScan

ACT

TTCTAG

TTCTAG  
Depth:9 (MOUSE)  
Ei-value:0.000, Pi-value:0.000  
Er-value:0.000, Pr-value:0.000  
No matches to TargetScan

A

TGTTTAGAAGTGCACAAAGTATGTTAAAAGTAG

TGTTTAGAAGTGCACAAAGTATGTTAAAAGTAGA  
Depth:9 (MOUSE)  
Ei-value:0.000, Pi-value:0.000  
Er-value:0.000, Pr-value:0.000  
No matches to TargetScan

 3120  


A

TGTTTAGAAGTGCACAAAGTATGTTAAAAGTAGA  
Depth:9 (MOUSE)  
Ei-value:0.000, Pi-value:0.000  
Er-value:0.000, Pr-value:0.000  
No matches to TargetScan

GGTAGTGAAATAACATTGTAGATATTGTTTTGTTAAATTTCATATGAAAGATCTTTTGGAAATGGAGTGGTTATACTATGTGCTGTTTCAGGGAGGAGGGGGAAGGAAGTGCCAAGTGC 3240  
 TCTAAGGCAGTAAGTCTGTAGTTAGATAAGATAAACCATCATCTGTGTTCTGTGCATTTTCTTTTACTTTTCTGTGTAAATAG

TGTATA

TGTATA  
Depth:9 (MOUSE)  
Ei-value:0.000, Pi-value:0.000  
Er-value:0.000, Pr-value:0.000  
No matches to TargetScan

TAATGGACAAACTAGTCCTAATGTTATATCA 3360  
 TCT

AGTCTT

AGTCTT  
Depth:9 (MOUSE)  
Ei-value:0.000, Pi-value:0.000  
Er-value:0.000, Pr-value:0.000  
No matches to TargetScan

TCTAGATGTTAAAGAGGTTGCCAGTGTATTACAGAAGTAGAGTTAGTAAACTAATGCATTTTGTACTTTTTTTTTTGTTAAAATTCATAGGGAAGACTGTTGAAAACGACA 3480  
 GAATTAAATTTGTGAAAGCAAAATTGTTCAAAAAAAAAAATCCCCTCTAAGCATTGCAAATGCAAACCACTAAGTTGATAGAGGTCAGAAGGGGAAAGATTATAGGCCGGAATGTTCTTT 3600  
 TAAGAAGACATTTTTGGGTTATATCCTTTATTATGTGTGCGGTTTATTCAGTGCTACTGTGTGTATTGTGGACAAGCTTAAGTCCTTGCTTGAAACAGCTAGTCTTTCTAGATGTTTAGA 3720  
 AGTAAACAACATGTTAAAGTAGAAAGTGTAAAACAATACTTCAGATATGTTTTCTGTTAATACGTAGGAATGACTATTTGGGGAATGGGATTGTTAAACTAGATGTCTTGGAGCAGGATG 3840  
 CTGATGGGGTGGTGGTGGGGGCAGGGAAGGAAGTGCCCAGGGAAGGTTCTTTGCCTGTGAGTTGTGAGACAAGTTCAGATTGTCTGACCATTGTTCTCTAGGCATTTTAGTTATTACTGT 3960  
 GTATTACAGGACAAGACCTAATGTCCAAAGTATGTAAAAAATAGAGGTAGTAAAATAATCCCTATATAAAGGTTCTTGTGTTAGTTTTAGGATGTACTGTCTTCTGGGAGTGACCTCTGT 4080  
 CCATCCACCTTGAAATGGTGGGTGAGGAAGATGAGGAAGGGCAAAGGGAAGGGCTCCTTGGTAGTACTCTATATCTAGAAGACAGTTGTAGATTATAACCATAGTTCTATAAGTACATTT 4200  
 TTTGGTAAAAATACCTGTGTTAATTGCAGATATGGTTCATACTTTACTTCACATCTAAGCTTTTTAGATATCTTGAGGTGACAATGTATGATAAAATGTAGAGCTAGTGAATTAATGAAT 4320  
 TTATAAATATCTTTTTTTTTTTTTTTTTAGTTAATAGAAGCATTTTTGACGTGG

AATTGTTA

AATTGTTA  
Depth:9 (MOUSE)  
Ei-value:0.000, Pi-value:0.000  
Er-value:0.000, Pr-value:0.000  
No matches to TargetScan

AACCACCTCTGAGCAGTATGCACCAGGACTTGTTCATTAAATTGGCTGCAGAAGGGCA 4440  
 GGGGGAGGGGTATATGCAGATGTGTTCATATTTCCATTTGAAGCTAACAGTGTGTGAGCATCTCTTGGCTATACCATAGGAAAACATTGTTAATTCAATAGAAACATATGTTCTTGCTAA 4560  
 TAATTCAATATAGATCTGATAATGAATTTTCTTAGAAGCATTATACTTATTGTACTCTTGCTTTTGTCTCACTTTAACTTGAGCATTATTAAGGGAGCACAGTATTTGTAATTCTGCCAG 4680  
 TATCTATAGAGGCCTGTTGAT

ATTTTTGT

ATTTTTGT  
Depth:9 (MOUSE)  
Ei-value:0.000, Pi-value:0.000  
Er-value:0.000, Pr-value:0.000  
No matches to TargetScan

CCCCAAGAAAGGAATACATTTTTTTCCCGCTGAGATGGTGTCATATA

TTCTTGGT

TTCTTGGT  
Depth:9 (MOUSE)  
Ei-value:0.000, Pi-value:0.000  
Er-value:0.000, Pr-value:0.000  
No matches to TargetScan

CATCACAATATCTCATGGCTTTGGGGCTGTGAGTTG 4800  
 GTA

AATATTCA

AATATTCA  
Depth:9 (MOUSE)  
Ei-value:0.000, Pi-value:0.000  
Er-value:0.000, Pr-value:0.000  
No matches to TargetScan

TGATGTGTGATAAAGCATGATACATTCTGTGAGATCTTGACTAGATAAAAATGTATGCTCCGTTGTGTCTATACACACACAGGACCTAAAGGTTATGTCAAACTAAA

CT

CTGCTT  
Depth:9 (MOUSE)  
Ei-value:0.000, Pi-value:0.000  
Er-value:0.000, Pr-value:0.000  
No matches to TargetScan

 4920  


GCTT

CTGCTT  
Depth:9 (MOUSE)  
Ei-value:0.000, Pi-value:0.000  
Er-value:0.000, Pr-value:0.000  
No matches to TargetScan

GTGATTCTGGA

TGACTT

TGACTT  
Depth:9 (MOUSE)  
Ei-value:0.000, Pi-value:0.000  
Er-value:0.000, Pr-value:0.000  
MATCHES To TargetScan▶ miR-224-5p:AAGUCAC

TTGTGTTCT

TTGCTT

TTGCTT  
Depth:9 (MOUSE)  
Ei-value:0.000, Pi-value:0.000  
Er-value:0.000, Pr-value:0.010  
No matches to TargetScan

CTTGTGCTTTTCAGTTTTCTATATTGCACAATTAAAAGTTTGAAAAA

AAATAAA

AAATAAA  
Depth:9 (MOUSE)  
Ei-value:0.000, Pi-value:0.000  
Er-value:0.000, Pr-value:0.000  
No matches to TargetScan

TGTTACTTTAAAAACCTTCATGAAGCCTC  5039
```

---

## >GUINEAPIG (5080 bases)

```
 ATAGTTCTCTGGCTCTGGAGCTAAAGCACTTCCGGTACCGCTTCTCTATCACCAGCAAAGAGAAG

TGCCAA

TGCCAA  
Depth:9 (MOUSE)  
Ei-value:0.000, Pi-value:0.000  
Er-value:0.000, Pr-value:0.000  
MATCHES To TargetScan▶ miR-182-5p:UUGGCAA▶ miR-96-5p/1271-5p:UUGGCAC

G

TCAGTTCCGG

TCAGTTCCGG  
Depth:9 (MOUSE)  
Ei-value:0.000, Pi-value:0.000  
Er-value:0.000, Pr-value:0.000  
No matches to TargetScan

CGAAGAATACAGTGAAAAACGGAGCGCGGCCTGCTTCC 120  
 CCAGGGTCCTCCAG

GCCCTC

GCCCTC  
Depth:9 (MOUSE)  
Ei-value:0.000, Pi-value:0.000  
Er-value:0.000, Pr-value:0.000  
No matches to TargetScan

CCGCCGATGGCGGCGGGTGGCCTGGAGGGCCCAGGGATGGGCCGCGCTTCTGCCCTGTAGGCCTTCGGACCTCTCGCTGTGATTACCCGCTGGCAGGACC 240  
 TAGGCTTTGAAGCAGGGGAACATCTCTCTCACACCACCTCGATGGCGTG

ATGGCGGC

ATGGCGGC  
Depth:9 (MOUSE)  
Ei-value:0.000, Pi-value:0.000  
Er-value:0.000, Pr-value:0.000  
No matches to TargetScan

AGTGGCCTTGGTGACGGAATCCCTCGGGCTTCACAGTCTCCCGGCCCAACCCTGCAACCCTAG 360  
 GCGAAGACTCCGCCAGCCGTGGAGGACGTGGCACCTTTTGGCGGCTTGCAGCGCGGCATGGTAAGAAGAAGGGCGACCTCCAAGCCGCTGCAGAGGGCCGTTACATCTGGGGCCCGAGAA 480  
 GGCGGTATTGAAAGCTCGGCCACGTCGGGCCACAGAACTGCGCAGATCAGGGTCCGGGGGTGATTGCTGTGTCCTAGTTGGAGGAGACCACACCTTCTGGAGACCAAAACGAGTAAAGGC 600  
 CCCACCCGCCCTGGAAGGTGAGAGAGCCCAGGCCGAGGCCGAGTGGAAGGACTGGCGGGTCCTAGGCTGTGGCCAGAGAGAGAAACGGAAAGTAACATTCCAGGTTTGGCTGGTTGGAAA 720  
 TGCCCGTCGCCCTTT

GGGAAGA

GGGAAGA  
Depth:9 (MOUSE)  
Ei-value:0.000, Pi-value:0.000  
Er-value:0.000, Pr-value:0.000  
No matches to TargetScan

TTTACTGGCCGATTGTATAAGG

CCTGTGTATATAATATGAAAAAGCTGCT

CCTGTGTATATAATATGAAAAAGCTGCT  
Depth:9 (MOUSE)  
Ei-value:0.000, Pi-value:0.000  
Er-value:0.000, Pr-value:0.000  
MATCHES To TargetScan▶ miR-15-5p/16-5p/195-5p/424-5p/497-5p:AGCAGCA▶ miR-503-5p:AGCAGCG

CTCAACATTCCCCT

CAACCTTT

CAACCTTT  
Depth:9 (MOUSE)  
Ei-value:0.000, Pi-value:0.000  
Er-value:0.000, Pr-value:0.000  
No matches to TargetScan

TAAAAGAAAACTGCATGTGGACAAAT 840  
 ATAGATGTAA

AGAGGT

AGAGGT  
Depth:9 (MOUSE)  
Ei-value:0.000, Pi-value:0.000  
Er-value:0.000, Pr-value:0.000  
No matches to TargetScan

TGCTGACATATGATAAAGTAGTTAGGAAAACCACATGTCTTG

GAAATG

GAAATG  
Depth:9 (MOUSE)  
Ei-value:0.000, Pi-value:0.000  
Er-value:0.000, Pr-value:0.000  
No matches to TargetScan

CCCATTTGTTTAAAAAAAGGACAGAAAAGAAACCTTCTAGAAGCGACATATTGAAT 960  
 GGAGTTCGTTAGACCATCTGGAGAAGAGACACTAGGTGATTACGTCTACATTTGTTCTGTGAGTTGGAGACACGGAGCAGAAAACAAGAGAAGAGGTCCAGTACCAGACTGCTTACCTGT 1080  


AGAAGA

AGAAGA  
Depth:9 (MOUSE)  
Ei-value:0.000, Pi-value:0.000  
Er-value:0.000, Pr-value:0.000  
No matches to TargetScan

TGTTCCTATTGTAGCCATTGTTTTGTGTGTGCATTTTAAAATACATACATACATACATACATATATATATATGAATGACAGTGTTAAGTACTTTATTGAAATATCTGGTCTTTC 1200  
 GAGATTTTCTC

AAGTGCCTG

AAGTGCCTG  
Depth:9 (MOUSE)  
Ei-value:0.000, Pi-value:0.000  
Er-value:0.000, Pr-value:0.000  
No matches to TargetScan

AAACACGTTAAAAAATAGTAGTGAAGTAACGCATTTTGTAAATTTCTTATTAGAACTCACATAAAGTGTGATGGGGGAGTGCGGGGGATGGGAAGATCTA 1320  
 GGAAAACTTCCCTCTTGAGTATACATA

TTGTGC

TTGTGC  
Depth:9 (MOUSE)  
Ei-value:0.000, Pi-value:0.000  
Er-value:0.000, Pr-value:0.000  
No matches to TargetScan

ATGCAGTGGTTCTTGGAAGAAAGGAAATGGAGAGCGCTGTACAGCAAGGCAAGTTCCAGTGTCCCCTAGGTCTGTGGACTTTATCTT 1440  
 TGCTGTGTAAGTAGGGTATGTAAAGGACAAATGAAGCCTTTTCAACAAGTTGTCTTCCTA

GATGTT

GATGTT  
Depth:9 (MOUSE)  
Ei-value:0.000, Pi-value:0.000  
Er-value:0.000, Pr-value:0.000  
No matches to TargetScan


AAAGAGGTTGCCA

AAAGAGGTTGCCA  
Depth:9 (MOUSE)  
Ei-value:0.000, Pi-value:0.000  
Er-value:0.000, Pr-value:0.000  
No matches to TargetScan

ATTTTAATAGAGTTAGTAAACTGACAG

ATTTTGT

ATTTTGT  
Depth:9 (MOUSE)  
Ei-value:0.000, Pi-value:0.000  
Er-value:0.000, Pr-value:0.000  
No matches to TargetScan

ATTAAAA 1560  
 TTCACGGGGAAGATTGTCTTTTGAAGGTTTGATCATTTTAGCCTACTGGGTTAGTGGAGAAAAAGATTTTAGACTCATATATTCAAGACACTTTCAACTATTGTTACATGTGTGCAGTTT 1680  
 AATCAAGACTGCTGTGTATATATAATGGACAAATTGAG

TCCTTA

TCCTTA  
Depth:9 (MOUSE)  
Ei-value:0.000, Pi-value:0.000  
Er-value:0.000, Pr-value:0.000  
No matches to TargetScan

CTTG

AAACATCT

AAACATCT  
Depth:9 (MOUSE)  
Ei-value:0.000, Pi-value:0.000  
Er-value:0.000, Pr-value:0.000  
No matches to TargetScan

AGTCTTAT

CTAGATGTTTAGAAGTGCCC

CTAGATGTTTAGAAGTGCCC  
Depth:9 (MOUSE)  
Ei-value:0.000, Pi-value:0.000  
Er-value:0.000, Pr-value:0.000  
No matches to TargetScan

AAC

GTATGTTAAA

GTATGTTAAA  
Depth:9 (MOUSE)  
Ei-value:0.000, Pi-value:0.000  
Er-value:0.000, Pr-value:0.000  
No matches to TargetScan

TGTAGAAGTAGTAAAAATACCAC 1800  
 TT

TGTAAATA

TGTAAATA  
Depth:9 (MOUSE)  
Ei-value:0.000, Pi-value:0.000  
Er-value:0.000, Pr-value:0.000  
No matches to TargetScan

TCTTTTTGCTAAAGTTCATATGAAATACTGTCTTTTGGGAATGGAATGGTTAAACCACCTCTTGGGCAATATGATACTGCCTGTACTTGTTCAGTGGTGTGGAGGAG

GTG

GTGGGAGGGAA  
Depth:9 (MOUSE)  
Ei-value:0.000, Pi-value:0.000  
Er-value:0.000, Pr-value:0.000  
MATCHES To TargetScan▶ miR-150-5p:CUCCCAA▶ miR-532-3p:CUCCCAC

 1920  


GGAGGGAA

GTGGGAGGGAA  
Depth:9 (MOUSE)  
Ei-value:0.000, Pi-value:0.000  
Er-value:0.000, Pr-value:0.000  
MATCHES To TargetScan▶ miR-150-5p:CUCCCAA▶ miR-532-3p:CUCCCAC

GCAAT

TGCAAA

TGCAAA  
Depth:9 (MOUSE)  
Ei-value:0.000, Pi-value:0.000  
Er-value:0.000, Pr-value:0.000  
No matches to TargetScan

AGGTATGTGAGTGCATTTGTACTTGGACATTGTGAGACATCTTTTTTGTGTGTTTTGTTGTGTATATAGTATATGTAATGGACAACTCCAAATTTTGCAAC 2040  
 ATCTAGTTCCTAAATGTAGGAGTTTGCCAGTGTATGACACAAAGTAGTAAAGTTAGCACATTTTGTACACTGTGTAGAAATACACAGAAAGCTTGTCCTCTGTAAATGAATTTTGGATTC 2160  
 AGCCATCTCTAACATTACATATGCCTGTACTTGTCCATTGGGTTGAAGCAGAGAGAAGCAAATGGGGCCAAAATGGTCATACTTAGAAGATAACCTCAGATTACAACCATTACTATATGT 2280  
 GCCATATTGTCAGTGTTGTATGATAGCAGACAAGTTCTTATATGAAAAATCTATTCTAGATATGTAGAAGTGCTTGATACACTAAAGGCAGAAGTAGTAGAATATCACTTTGTAAATAGA 2400  
 TTTTATAAACTAATAGAAAATGCTATGTTTGCAAATAGAATGTTTAAACCACCTCTGAACAATAATACTGTGTGTACTTGCTCACTTGGTTGAGGGACGGGGAGGAAAGAAATTGTTGAA 2520  
 AGTGTTTTGTTAAATGTATGATAGAAAATTTCAGCTTATCCGTTGTTTTTATGTTTCATGAGTTTCATTTAACTTTGCTATGC

TGTATATA

TGTATATA  
Depth:9 (MOUSE)  
Ei-value:0.000, Pi-value:0.000  
Er-value:0.000, Pr-value:0.000  
No matches to TargetScan

TAGTGTACATACTGGACATATGAGTCCTA 2640  
 ATTTTATA

ATATCTAG

ATATCTAG  
Depth:9 (MOUSE)  
Ei-value:0.000, Pi-value:0.000  
Er-value:0.000, Pr-value:0.000  
No matches to TargetScan

G

CTCTAG

CTCTAG  
Depth:9 (MOUSE)  
Ei-value:0.000, Pi-value:0.000  
Er-value:0.000, Pr-value:0.000  
No matches to TargetScan

ATATT

AAAGAGGTTGCCAATGTATGACA

AAAGAGGTTGCCAATGTATGACA  
Depth:9 (MOUSE)  
Ei-value:0.000, Pi-value:0.000  
Er-value:0.000, Pr-value:0.000  
MATCHES To TargetScan▶ miR-182-5p:UUGGCAA▶ miR-96-5p/1271-5p:UUGGCAC▶ miR-539-3p:UCAUACA

AAAGTAGA

GTTAGTAAACT

GTTAGTAAACT  
Depth:9 (MOUSE)  
Ei-value:0.000, Pi-value:0.000  
Er-value:0.000, Pr-value:0.000  
No matches to TargetScan

A

ACACATT

ACACATT  
Depth:9 (MOUSE)  
Ei-value:0.000, Pi-value:0.000  
Er-value:0.000, Pr-value:0.000  
No matches to TargetScan

TTGTACACTTTGTGTTAAAATTCATAGGAAGGT

TGTCTTCTG

TGTCTTCTGAA  
Depth:9 (MOUSE)  
Ei-value:0.000, Pi-value:0.000  
Er-value:0.000, Pr-value:0.000  
No matches to TargetScan

 2760  


AA

TGTCTTCTGAA  
Depth:9 (MOUSE)  
Ei-value:0.000, Pi-value:0.000  
Er-value:0.000, Pr-value:0.000  
No matches to TargetScan

AAGGATTTTGGAAGTATAATTGTAGAGTCACATTTGAGTGGCACAT

GTGCCT

GTGCCT  
Depth:9 (MOUSE)  
Ei-value:0.000, Pi-value:0.000  
Er-value:0.000, Pr-value:0.000  
No matches to TargetScan

GTATTACAAGGAAAGAAGAAATTGGATGAAATGAAGGATTCTAGACC

AGAATG

AGAATG  
Depth:9 (MOUSE)  
Ei-value:0.000, Pi-value:0.000  
Er-value:0.000, Pr-value:0.000  
No matches to TargetScan

CTCCTACT

AGAAG

AGAAGA  
Depth:9 (MOUSE)  
Ei-value:0.000, Pi-value:0.000  
Er-value:0.000, Pr-value:0.000  
No matches to TargetScan

 2880  


A

AGAAGA  
Depth:9 (MOUSE)  
Ei-value:0.000, Pi-value:0.000  
Er-value:0.000, Pr-value:0.000  
No matches to TargetScan

CACTTTCAGATATAACCATTGTTACATGTGTATAGTTTATTCAACGCTACTG

TGTATATA

TGTATATA  
Depth:9 (MOUSE)  
Ei-value:0.000, Pi-value:0.000  
Er-value:0.000, Pr-value:0.000  
No matches to TargetScan

GTGGACAAACTCGAGTCCATATT

TGAAACATCTAG

TGAAACATCTAG  
Depth:9 (MOUSE)  
Ei-value:0.000, Pi-value:0.000  
Er-value:0.000, Pr-value:0.000  
No matches to TargetScan

TCT

TTCTAG

TTCTAG  
Depth:9 (MOUSE)  
Ei-value:0.000, Pi-value:0.000  
Er-value:0.000, Pr-value:0.000  
No matches to TargetScan

A

TGTTTAGAAGTGCA

TGTTTAGAAGTGCACAAAGTATGTTAAAAGTAGA  
Depth:9 (MOUSE)  
Ei-value:0.000, Pi-value:0.000  
Er-value:0.000, Pr-value:0.000  
No matches to TargetScan

 3000  


CAAAGTATGTTAAAAGTAGA

TGTTTAGAAGTGCACAAAGTATGTTAAAAGTAGA  
Depth:9 (MOUSE)  
Ei-value:0.000, Pi-value:0.000  
Er-value:0.000, Pr-value:0.000  
No matches to TargetScan

GGTAGTAAATAATACATTTTTTAGATATCCCTTGTTAAAATTGATGAAATACTGTCTTTAGGAGATAGAATGATCAAACCACTGATCTGAGCAGTAGACA 3120  
 TACTTATACTGGTTCAGAGAGGGAGGAGGAAAAGGGCCGTAAGTCAGTGTGGTTTAGGTGAAGTGAGATTGACTGCTGTCCCTTACGTCTGTTCTACTTTCCTG

TGTATA

TGTATA  
Depth:9 (MOUSE)  
Ei-value:0.000, Pi-value:0.000  
Er-value:0.000, Pr-value:0.000  
No matches to TargetScan

TAGT

TGTATA

TGTATA  
Depth:9 (MOUSE)  
Ei-value:0.000, Pi-value:0.000  
Er-value:0.000, Pr-value:0.000  
No matches to TargetScan

 3240  


TGTATA  
Depth:9 (MOUSE)  
Ei-value:0.000, Pi-value:0.000  
Er-value:0.000, Pr-value:0.000  
No matches to TargetScan

TAAAGGACAAAGTCCTAATTCACATCT

AGTCTT

AGTCTT  
Depth:9 (MOUSE)  
Ei-value:0.000, Pi-value:0.000  
Er-value:0.000, Pr-value:0.000  
No matches to TargetScan

TCTAGATGTTTAAGAGGTTGCTAGTGTATGACAGTAGAATAGTAAACTAACATGTTGAGTACACTTTATATTAAAATTCATTAGAAA 3360  
 CAGTGTTCTTAAAACCACTTAGTGAAATTTTGAAAGTCCCCTCTAAGTATTACATATGCATATATCTGTCCCCTGGGTTTAAAAAAAAGTGGGAAAGGGTAAGAGCTGGAAACTGGTGTG 3480  
 TTCCTATTTAGATGACACTTTAACATTACAGCCTTTGTTAAGTGTATGTGTGCAATTTACTCAGTCCTACTATATACATAGGACTTTTTAAGTTCTTACTGGAAACATCCATTATTTCTA 3600  
 GATGTTTTAAAGTTCACACATATAGCAGTTAAGGGCTAATATAATGTGCTTTAAGTATTTTTTGTGACTTCACAGAAATGGTTGTATGTTGGGAATGGAATTGTCAAATGGTATCTTGGA 3720  
 GAGTATGCCGGCTATTCATGGGACGTGGGAGGGAAAATAAGCAGAGAGGGACACTGAGTTCAGATTGTCTTGGCATTGTCCTATATGTATATTGTAGGTAGTGTTGCTGGGTATTAAAGG 3840  
 ATAAGTGTGTCTACCCAAATTATGTTAGTAGTAGCAAAATAATCCTTATATAAATGTCTTGTTAGTTTTTAGGAAAAATTATCTTCTGGAAGTGGCTTTGTTAAAGCCACCTCTTGGAGC 3960  
 TAGATGTGGTCCTGAACTTAGTCACTGAAGTGTTGAAGAGGGAAAAGGGCAGGTGAGGGTGTTAAGGGCTCTTTGTTAATATCTCCATATCTGGAAGATGATGTTAGATTGTACCTATAG 4080  
 GCCTGTATGTGCATTTTTGGTAAAGTACTTTGGCTTATTGTATACAAAATTCATACTCTATTGCACAGCATCTAAGCTTTATAGATACCCTAAAGTGACAATGATAAAAAGTAGAGGTAG 4200  
 TGAGTTAGATAACATTTAAGTATCTTTGTTCTTTAAGTATCTTAGTTTTTTTTTTTGGGGGGGGGGTAGGTGTGGTTTTTTTTTTTCTTCTTTTGGTACCGGGACTTGAACTCAACTTAG 4320  
 TGCTTGCCAGGCTGAGCTACATCCCCAGCCCTCTTTTTGTTATAATTGATAGAAAAGATGCATCTTGAACTTGG

AATTGTTA

AATTGTTA  
Depth:9 (MOUSE)  
Ei-value:0.000, Pi-value:0.000  
Er-value:0.000, Pr-value:0.000  
No matches to TargetScan

CGACACCTCTGGGCAGTGCCTTTCAGGACTTGTCATTA 4440  
 GGCTAGCAGCAGAGGGATAGAAATATGCATACAAGGAGAGGTGTGTGCAGAGGGGTCAGATTTCCATCTTCGTGAGAGGAGTCAGTGTCTTGTAGCTGTGCTGTGGAAATACATTATTCA 4560  
 GTGGGAACATGCTTTCTTGCTAATATCTTGTAGAGATTTGATAACTCAGATACATGGCTTGGTGTACTGTTTGTGTCTCATTTTAAATTGAGCATTAAGAAAGTGCCGTATTGTAATTGT 4680  
 AACTTTGCCAATACTTCTATCTAGAGGCCTGTTGCCATTTTTATCTTCAGTGAA

ATTTTTGT

ATTTTTGT  
Depth:9 (MOUSE)  
Ei-value:0.000, Pi-value:0.000  
Er-value:0.000, Pr-value:0.000  
No matches to TargetScan

TCCCAAGAAAGGCAAGATTACAATTTTTTTTCTAACAGATTGAGTTGATGTAGTGTG

T

TTCTTGGT  
Depth:9 (MOUSE)  
Ei-value:0.000, Pi-value:0.000  
Er-value:0.000, Pr-value:0.000  
No matches to TargetScan

 4800  


TCTTGGT

TTCTTGGT  
Depth:9 (MOUSE)  
Ei-value:0.000, Pi-value:0.000  
Er-value:0.000, Pr-value:0.000  
No matches to TargetScan

TATCAAAAATACTCTTGTAGCCTTGAGATTTTGAGTTGGCG

AATATTCA

AATATTCA  
Depth:9 (MOUSE)  
Ei-value:0.000, Pi-value:0.000  
Er-value:0.000, Pr-value:0.000  
No matches to TargetScan

TGATGTGTGGAAAAGCATGACACACACTGTGCAGACTGAATCACACAAAATGGCATGTCGTGTA 4920  
 TGTACACAGCAGGACCTAGAAGAACATGTCCAGCTAAG

CTGCTT

CTGCTT  
Depth:9 (MOUSE)  
Ei-value:0.000, Pi-value:0.000  
Er-value:0.000, Pr-value:0.000  
No matches to TargetScan

ATGACTGCGAA

TGACTT

TGACTT  
Depth:9 (MOUSE)  
Ei-value:0.000, Pi-value:0.000  
Er-value:0.000, Pr-value:0.000  
MATCHES To TargetScan▶ miR-224-5p:AAGUCAC

TGTTTT

TTGCTT

TTGCTT  
Depth:9 (MOUSE)  
Ei-value:0.000, Pi-value:0.000  
Er-value:0.000, Pr-value:0.010  
No matches to TargetScan

CCTGTGTTTTTGTTTCCTGTAATGCATATATTAACTTTTAAAA

AAAT

AAATAAA  
Depth:9 (MOUSE)  
Ei-value:0.000, Pi-value:0.000  
Er-value:0.000, Pr-value:0.000  
No matches to TargetScan

 5040  


AAA

AAATAAA  
Depth:9 (MOUSE)  
Ei-value:0.000, Pi-value:0.000  
Er-value:0.000, Pr-value:0.000  
No matches to TargetScan

GATTATTTTAAAACTTGTAAGAAGTCCCCATTGCTTG                                                                                 5080
```

---

## >MOUSE (4917 bases)

```
 GAGAAGAAT

TGCCAA

TGCCAA  
Depth:9 (MOUSE)  
Ei-value:0.000, Pi-value:0.000  
Er-value:0.000, Pr-value:0.000  
MATCHES To TargetScan▶ miR-182-5p:UUGGCAA▶ miR-96-5p/1271-5p:UUGGCAC

G

TCAGTTCCGG

TCAGTTCCGG  
Depth:9 (MOUSE)  
Ei-value:0.000, Pi-value:0.000  
Er-value:0.000, Pr-value:0.000  
No matches to TargetScan

CAGAGAGCGCAGCGAGCTCGGGAGTGTGGCCTTCTTCGCCCCGGGCCCCTCACGGCCTCGGCCCGCGCCGGGTGAGCCCGAGGGGCCCGGGAGC 120  
 AGCCTCGGTCCCTACTCTGCGGACGCCAGGA

GCCCTC

GCCCTC  
Depth:9 (MOUSE)  
Ei-value:0.000, Pi-value:0.000  
Er-value:0.000, Pr-value:0.000  
No matches to TargetScan


GCCCTC

GCCCTC  
Depth:9 (MOUSE)  
Ei-value:0.000, Pi-value:0.000  
Er-value:0.000, Pr-value:0.000  
No matches to TargetScan

GAGGCAGCCACCGGCTAGGCCACGCGGCCATTCCGCAGCGACTCTTGCCCTGCCAACGATGCGCCTGC

ATGGCGGC

ATGGCGGC  
Depth:9 (MOUSE)  
Ei-value:0.000, Pi-value:0.000  
Er-value:0.000, Pr-value:0.000  
No matches to TargetScan

G 240  
 GCCTCCCCGACTCCACCGGCGCCCTCTCTCCAGGGCCGGGCCCACCGAGGAGTCCCAGCCAGGCCTTCGAGGACGCCTCGAAAGTGGCGGCGAGCGTGGCATGAC

GGGAAGA

GGGAAGA  
Depth:9 (MOUSE)  
Ei-value:0.000, Pi-value:0.000  
Er-value:0.000, Pr-value:0.000  
No matches to TargetScan

ATGGCATC 360  
 CACCTGAAGGCCAAGGCTGGGCCTTCGGGGCTTCTGCGGCCTAAGGCGGCGGGTCTGAGCAAGAACACTCTGCCATCGCCGGCTCGGGACGGGAGGCCCACCCGTGGCCGAGCACGCAGA 480  
 CCTGGATGAACCGAGGCCTCGATGGGCCTGGACGAAGGTGAGCGGGGCCCCGAATCACGGCGAGAGGAACGATCGATTCTTGGGCCGCGGGTCTTCCCTCCACCCGAGAGAAGCCGAGAC 600  
 GCGACAACCGCAGGCGGCCATTCGGAAACGCTGTCGCCGTAGAAGT

CCTGTGTATATAATATGAAAAAGCTGCT

CCTGTGTATATAATATGAAAAAGCTGCT  
Depth:9 (MOUSE)  
Ei-value:0.000, Pi-value:0.000  
Er-value:0.000, Pr-value:0.000  
MATCHES To TargetScan▶ miR-15-5p/16-5p/195-5p/424-5p/497-5p:AGCAGCA▶ miR-503-5p:AGCAGCG

CT

CAACCTTT

CAACCTTT  
Depth:9 (MOUSE)  
Ei-value:0.000, Pi-value:0.000  
Er-value:0.000, Pr-value:0.000  
No matches to TargetScan

CCCCCGACCCTTTTTAGACTTCTGGCTAAATCTGGA 720  
 CGTCTAACTGTAGAGATTGCCGACGCAGGGTAAAGG

AGAGGT

AGAGGT  
Depth:9 (MOUSE)  
Ei-value:0.000, Pi-value:0.000  
Er-value:0.000, Pr-value:0.000  
No matches to TargetScan

AGGAGATGACGTGAATACCCAGTCGCCATAAAAAGAAGATCGTGGACACGTCTTCTGCAAGTGATTTT

GAAATG

GAAATG  
Depth:9 (MOUSE)  
Ei-value:0.000, Pi-value:0.000  
Er-value:0.000, Pr-value:0.000  
No matches to TargetScan

GAAT 840  
 TGTTTAGGCCGCCTCTAGAAAGACCCAGGAGCGACACTCGTCCCTGTTTGTTCAGTACCTTAGAGGATCCAGCAGGAGGA

AGAAGA

AGAAGA  
Depth:9 (MOUSE)  
Ei-value:0.000, Pi-value:0.000  
Er-value:0.000, Pr-value:0.000  
No matches to TargetScan

GGCCCGGAGCCAGATTGGTCGTGTGA

AGAAGA

AGAAGA  
Depth:9 (MOUSE)  
Ei-value:0.000, Pi-value:0.000  
Er-value:0.000, Pr-value:0.000  
No matches to TargetScan

CG 960  
 TGTCTCTCTTTTTTATCCTATTGTATTGTGTATGCATTTTAGTCCTCATTTCTTACCCCCCACACCATGTTTGCGTGTGTGCCTGTGTGTGTGTATTTGAGTGTGTTTGAAAATGCCAGG 1080  
 TTTTGGGGGTAATGTCTTTCTAGCTGCTCTG

AAGTGCCTG

AAGTGCCTG  
Depth:9 (MOUSE)  
Ei-value:0.000, Pi-value:0.000  
Er-value:0.000, Pr-value:0.000  
No matches to TargetScan

CCATATTTTATAAGTAGTAAACATGCTGTAAATACCTTTTTGTTAGGATTCATTTGAATTATTGTTTGGAGAAGAAAAGC 1200  
 CAAACCTCTCCTGAGTTGACCGCATTGTGT

TTGTGC

TTGTGC  
Depth:9 (MOUSE)  
Ei-value:0.000, Pi-value:0.000  
Er-value:0.000, Pr-value:0.000  
No matches to TargetScan

CCTGGTTCCCAGAAGGAAATCCAGAGCTCTAAGCTACTGTGGTCCGTGAGTGGAAAGGAACCACTATCTCCCACTATCCCATTT 1320  
 TGTTTGGCTTTTCTTTGTACATAGTGTATGTAAAGGACAAAGGGGTCCTAATTTACAACATCTAGTCACTCTA

GATGTT

GATGTT  
Depth:9 (MOUSE)  
Ei-value:0.000, Pi-value:0.000  
Er-value:0.000, Pr-value:0.000  
No matches to TargetScan


AAAGAGGTTGCCA

AAAGAGGTTGCCA  
Depth:9 (MOUSE)  
Ei-value:0.000, Pi-value:0.000  
Er-value:0.000, Pr-value:0.000  
No matches to TargetScan

GTGTATGACAAAGTAGAGTTAGTAGAAC 1440  
 AACACTTTTTTTTTTTTTTTTTTTTAC

ATTTTGT

ATTTTGT  
Depth:9 (MOUSE)  
Ei-value:0.000, Pi-value:0.000  
Er-value:0.000, Pr-value:0.000  
No matches to TargetScan

GTTAAAGTTGTAATTACTCTTGTTACGTGTGCCATTTACTGACTGCTGTGTATATAGTGGACAACAG

TCCTTA

TCCTTA  
Depth:9 (MOUSE)  
Ei-value:0.000, Pi-value:0.000  
Er-value:0.000, Pr-value:0.000  
No matches to TargetScan

CTTG

AAACATCT

AAACATCT  
Depth:9 (MOUSE)  
Ei-value:0.000, Pi-value:0.000  
Er-value:0.000, Pr-value:0.000  
No matches to TargetScan

G 1560  
 GTCTAT

CTAGATGTTTAGAAGTGCCC

CTAGATGTTTAGAAGTGCCC  
Depth:9 (MOUSE)  
Ei-value:0.000, Pi-value:0.000  
Er-value:0.000, Pr-value:0.000  
No matches to TargetScan

AAC

GTATGTTAAA

GTATGTTAAA  
Depth:9 (MOUSE)  
Ei-value:0.000, Pi-value:0.000  
Er-value:0.000, Pr-value:0.000  
No matches to TargetScan

TGTAGAGGTAGTGAAATATCACTT

TGTAAATA

TGTAAATA  
Depth:9 (MOUSE)  
Ei-value:0.000, Pi-value:0.000  
Er-value:0.000, Pr-value:0.000  
No matches to TargetScan

TCTTTTTGCAAACACTCATGAGTCGTTGTCTTTGGGGAGTGGGGTGATG 1680  
 AGCCCTCTGAGCAGTACTGTCTTTACTTGCTCCGGGGTTTGGAGGAGGTGTTGGGAGGCAGTGACAACGGCCATGTAGTGTGTGTGTATATGGGACCTTTTCAGATGCCATTTTTATATT 1800  
 CTTGCATTTTGTTTTGCTGTGTGTATACTGTATATAATGGACAAATATGTCTAAATTTTCAACATCTAGACTCTAGATGTTAAAGAGGTTGCCAATGTATGACAAAGTAGTAAATTTAGC 1920  
 ACATAGTGTACACCGTTAAAATTCATAGAAAATTTTGTTCTGTAAATGGCTTGGGGATAGGATTTGCTCAGCTGTATCTAAGCATTACACATACCTATATTTGTCTATTGGTTTGAAGGG 2040  
 AGAAGAAAGTGGGTCGAAGCCCAAATGGTTATGTTTAGAAGATACCTCAGATAATAACCATTGCTTTGCATACAGTTTTATTTACCAGTGCCATGTCAATAGCAGATAAGTTCTTACATG 2160  
 AAACATCTAGTCTTTTAGATATCTGAAAGTGCTTGATACATTTAAAGGTACAGGTAATAGAATAATATTTTGTGTATATAGTTTTTTTAAACTGGAGAAATGCTGTGTGTAGAAGTAGAA 2280  
 TTGTTAGCCCGCCTCTGAGAACAGTGTACCGGCTGTGCTTGTTCAGTGGATCAAGGAG

GTGGGAGGGAA

GTGGGAGGGAA  
Depth:9 (MOUSE)  
Ei-value:0.000, Pi-value:0.000  
Er-value:0.000, Pr-value:0.000  
MATCHES To TargetScan▶ miR-150-5p:CUCCCAA▶ miR-532-3p:CUCCCAC

GGAAC

TGCAAA

TGCAAA  
Depth:9 (MOUSE)  
Ei-value:0.000, Pi-value:0.000  
Er-value:0.000, Pr-value:0.000  
No matches to TargetScan

AAGATTCCATTTGTGTGCTAGACATTTCTAGCTTACCCAT 2400  
 CCCCCTGTGTGTTGTGTGCATCTCATGTAACTTTGCTATGC

TGTATATA

TGTATATA  
Depth:9 (MOUSE)  
Ei-value:0.000, Pi-value:0.000  
Er-value:0.000, Pr-value:0.000  
No matches to TargetScan

TTGTACATACTGGACAAACGAGTCCTATG

ATATCTAG

ATATCTAG  
Depth:9 (MOUSE)  
Ei-value:0.000, Pi-value:0.000  
Er-value:0.000, Pr-value:0.000  
No matches to TargetScan

T

CTCTAG

CTCTAG  
Depth:9 (MOUSE)  
Ei-value:0.000, Pi-value:0.000  
Er-value:0.000, Pr-value:0.000  
No matches to TargetScan

GTATT

AAAGAGGTTGCCAATGTATGAC

AAAGAGGTTGCCAATGTATGACA  
Depth:9 (MOUSE)  
Ei-value:0.000, Pi-value:0.000  
Er-value:0.000, Pr-value:0.000  
MATCHES To TargetScan▶ miR-182-5p:UUGGCAA▶ miR-96-5p/1271-5p:UUGGCAC▶ miR-539-3p:UCAUACA

 2520  


A

AAAGAGGTTGCCAATGTATGACA  
Depth:9 (MOUSE)  
Ei-value:0.000, Pi-value:0.000  
Er-value:0.000, Pr-value:0.000  
MATCHES To TargetScan▶ miR-182-5p:UUGGCAA▶ miR-96-5p/1271-5p:UUGGCAC▶ miR-539-3p:UCAUACA

AAAGTA

GTTAGTAAACT

GTTAGTAAACT  
Depth:9 (MOUSE)  
Ei-value:0.000, Pi-value:0.000  
Er-value:0.000, Pr-value:0.000  
No matches to TargetScan

C

ACACATT

ACACATT  
Depth:9 (MOUSE)  
Ei-value:0.000, Pi-value:0.000  
Er-value:0.000, Pr-value:0.000  
No matches to TargetScan

CTGTATACTCTGTTAGTTCATAGCAAGGT

TGTCTTCTGAA

TGTCTTCTGAA  
Depth:9 (MOUSE)  
Ei-value:0.000, Pi-value:0.000  
Er-value:0.000, Pr-value:0.000  
No matches to TargetScan

GAGTTGTGAAAGTGTATCATCCAAGTGACGAGTGACATAGGCACCC

GTGCCT

GTGCCT  
Depth:9 (MOUSE)  
Ei-value:0.000, Pi-value:0.000  
Er-value:0.000, Pr-value:0.000  
No matches to TargetScan

AT 2640  
 GTCACGGGACACAAGATGGGCTGAAGGGGAAGAAAGGTCGGAGACC

AGAATG

AGAATG  
Depth:9 (MOUSE)  
Ei-value:0.000, Pi-value:0.000  
Er-value:0.000, Pr-value:0.000  
No matches to TargetScan

TCCTCACC

AGAAGA

AGAAGA  
Depth:9 (MOUSE)  
Ei-value:0.000, Pi-value:0.000  
Er-value:0.000, Pr-value:0.000  
No matches to TargetScan

CACTTGGAGATCTAACTAGTTACATGTGTGTGGTTTCAGTACTACTA

TGTATAT

TGTATATA  
Depth:9 (MOUSE)  
Ei-value:0.000, Pi-value:0.000  
Er-value:0.000, Pr-value:0.000  
No matches to TargetScan

 2760  


A

TGTATATA  
Depth:9 (MOUSE)  
Ei-value:0.000, Pi-value:0.000  
Er-value:0.000, Pr-value:0.000  
No matches to TargetScan

GGGGACAAATTCATGTCCTCATC

TGAAACATCTAG

TGAAACATCTAG  
Depth:9 (MOUSE)  
Ei-value:0.000, Pi-value:0.000  
Er-value:0.000, Pr-value:0.000  
No matches to TargetScan

TCC

TTCTAG

TTCTAG  
Depth:9 (MOUSE)  
Ei-value:0.000, Pi-value:0.000  
Er-value:0.000, Pr-value:0.000  
No matches to TargetScan

G

TGTTTAGAAGTGCACAAAGTATGTTAAAAGTAGA

TGTTTAGAAGTGCACAAAGTATGTTAAAAGTAGA  
Depth:9 (MOUSE)  
Ei-value:0.000, Pi-value:0.000  
Er-value:0.000, Pr-value:0.000  
No matches to TargetScan

CATAGTAAATAATACGTGTTGTAGATTCTGTTTAAACTCA 2880  
 TGGTGAAGTGTTGTCTTTTGGAACTAGAAAGAGAAACTGCTTCTGAGCAATGCAGCCTTGCTTGTGCAGGTCTAGGAGGGAGGAAGAAAATGAATGAAGAGCCTAATGCTGATGCAAGTT 3000  
 TGGTAATACAAGGTTATCCATTGTTGCTTATATCTGTGCATCTTACTTTGCAA

TGTATA

TGTATA  
Depth:9 (MOUSE)  
Ei-value:0.000, Pi-value:0.000  
Er-value:0.000, Pr-value:0.000  
No matches to TargetScan

GAAAACATAGGTCCAAATTTGCAGCACCC

AGTCTT

AGTCTT  
Depth:9 (MOUSE)  
Ei-value:0.000, Pi-value:0.000  
Er-value:0.000, Pr-value:0.000  
No matches to TargetScan

CCTAGACTGTAAAGGTGCCAGTGTGT 3120  
 GACAAAGTAGAGTTAGTGAAAAGACATATTTATAGATTATGTTAAAATTTGTAGGAAAAAAACCTTAAAACCACTTAGGAAGTGA

AATTGTTA

AATTGTTA  
Depth:9 (MOUSE)  
Ei-value:0.000, Pi-value:0.000  
Er-value:0.000, Pr-value:0.000  
No matches to TargetScan

ATATTCCACAAAAACATGACAGATGCA 3240  
 TACACATGGCCCCTGGGGGGAGAGAACTGGAAGAGGGGCTGGACCAGAACCTTGCTGTTTAGAAGGCAGTCTGACCACAGCCTTTGTTAGGTGTGCAGTGTATTCACTGTGGTGTACACA 3360  
 TGCTGGGGAAACCAAAGTGCTTACTGGGAACATCTGGTCTCCAGCCTTTTAGGTGCATGATAGACCTGATGTGGTAATGCATGCCTTTAATTCTAGGCTGAGGCAGGTGGATCTCTGTTC 3480  
 TGGGTCAACCAGGGCTATAGACTTTGTCTCAAAACAGTGAGAAAGTGTACATAACGGATTGGTAGGGTGGCTCAGCATGTAAAGGTGCTTGCTACTAAGCTGGTGACCTGAGTTTGATTT 3600  
 CCGGGATCCACTTGATAAAAGCGATTCACAGCTTGTCTTTTAGCCTCCACACAAGTGCAGTGGCACATGTGCACACATGCATACAGTATTGCATACATGTAAAAATAGAGGGCTGACACC 3720  
 ACTTTGTTAACGATGATTTTGTTACAGGAATGGTCACACTGGGGGAATGGAGGTTAAGCTTGGCCTTTGAGCAGATGCTGTTACCAGGTGATGGAGGTGGGCTAGGGCAAAGCTTAGTGC 3840  
 TCAGAAAGAGGCACTGTAGCTGTTGGTTGGTTCGAGTTGTCTAGCCATTGGTTGGGTGTGCATTTCACTTAATGTTGCCAGGTAGTAGAGGTAAGTCCCAGTGCCTAAAGGATGTTAGAA 3960  
 GTAGCAAGAGTCACTAAAGGCCAGGCTCATCTTTGGGGAGGGCTGTTTTCTTGGTGTGGCCTTTGTTAATCCTCTCACTAGCATCATCTGCACTTAGGAGTTGTGCAGTGAAGAGGATGG 4080  
 CTGAGGGAAAGGCTGCTAGTGCCTCTGTCCAGGGGACAGCTCTACCCAGGGGTCCTATGTGCATCGTGGTAAAATCTTGTGTTCATTATGCACAGTTGTCATCTGAATGTTACATCCTAA 4200  
 AGGAAAGCACATGGTTAAGAGTAGAGCTAATGGCTATGGTGGAATTGACAAAAGGGTTCCTCTTGAACATGGAACTGCTAAGCCATGTCTGGCCAGTGTGGGGAAATTCATGTTGCTAGG 4320  
 CTGACTGGAGGGACACAGGTTCACAGGATGAGGTCTGCAGGTGTGTTGAGACTGACATCCTGAGAGCCCCTACCGTAGGTGTGTCTCCCAGCTGCTCCGTGTGGAATACATTTGTTGAGT 4440  
 GGGAAGATCCCTTCTACATATGCTAGTATATAGATCTGGTGATAAGCTTAGGAATGCTATACTTAGTGTGGTCTGTTGCTTTGTGTTTCCTTGTAAGCTGAGACTGAGCGAATGCAGAAT 4560  
 TTAATTGTAACTTTGCCAGTACATCTATCTAGAGGCCTGCTGCC

ATTTTTGT

ATTTTTGT  
Depth:9 (MOUSE)  
Ei-value:0.000, Pi-value:0.000  
Er-value:0.000, Pr-value:0.000  
No matches to TargetScan

CTTCTATGAAATTTCTGTTGCCAAGGAAGACAGGATTACAATTTTGTCTGACAGATTGAGATGGCATG 4680  
 GTGTA

TTCTTGGT

TTCTTGGT  
Depth:9 (MOUSE)  
Ei-value:0.000, Pi-value:0.000  
Er-value:0.000, Pr-value:0.000  
No matches to TargetScan

TATCAAAATCCCTGTGTAGTGTGGGGTTTTTAATTGGTG

AATATTCA

AATATTCA  
Depth:9 (MOUSE)  
Ei-value:0.000, Pi-value:0.000  
Er-value:0.000, Pr-value:0.000  
No matches to TargetScan

CAATGTGGGGAAAGCATGGTACATACTGTATAATCTCAATTACCCAAGTGTGTGTACGCT 4800  
 TGCAGGATCTAGAACAATACATCGAGCTGAG

CTGCTT

CTGCTT  
Depth:9 (MOUSE)  
Ei-value:0.000, Pi-value:0.000  
Er-value:0.000, Pr-value:0.000  
No matches to TargetScan

GTGACTGGG

TGACTT

TGACTT  
Depth:9 (MOUSE)  
Ei-value:0.000, Pi-value:0.000  
Er-value:0.000, Pr-value:0.000  
MATCHES To TargetScan▶ miR-224-5p:AAGUCAC

TGTTC

TTGCTT

TTGCTT  
Depth:9 (MOUSE)  
Ei-value:0.000, Pi-value:0.000  
Er-value:0.000, Pr-value:0.010  
No matches to TargetScan

TCTTGTTTCCTGTAATGCATATACTAAAA

AAATAAA

AAATAAA  
Depth:9 (MOUSE)  
Ei-value:0.000, Pi-value:0.000  
Er-value:0.000, Pr-value:0.000  
No matches to TargetScan

CAAAAGCTTCTTTAAAGA    4917
```

---
