# Supplementary material for: Uncovering deeply conserved motif combinations in rapidly evolving noncoding sequences
Source: Genome Biol. 2021 Jan 11;22:29. doi: 10.1186/s13059-020-02247-1 (PMC7798263; doi:10.1186/s13059-020-02247-1)
Supplement: Supplementary file 3 — Additional file 3. LncLOOM output results for NORAD sequences from nine mammals. [file 13059_2020_2247_MOESM3_ESM.gz › AdditionalFile3/Html_Files/kmers_in_blocks_level.html]

 MOTIFS IN BLOCKS

# MOTIFS IN BLOCK DIAGRAMS

## Motifs conserved to (and beyond) MOUSE (depth:9)

  

NAVIGATE ▼

▶HUMAN (depth:1)▶BABOON (depth:2)▶DOG (depth:3)▶COW (depth:4)▶SHEEP (depth:5)▶PIG (depth:6)▶ARMADILLO (depth:7)▶GUINEAPIG (depth:8)▶MOUSE (depth:9)

  
  
  

## >HUMAN (5443 bases)

```
 -----------------------------------------

TGCCAA

TGCCAA  
Depth:9 (MOUSE)  
Ei-value:0.000, Pi-value:0.000  
Er-value:0.000, Pr-value:0.000  
No matches to eCLIP DataMATCHES To TargetScan▶ miR-182-5p:UUGGCAA▶ miR-96-5p/1271-5p:UUGGCAC

-

TCAGTTCCGG

TCAGTTCCGG  
Depth:9 (MOUSE)  
Ei-value:0.000, Pi-value:0.000  
Er-value:0.000, Pr-value:0.000  
eCLIP MATCHES▶EIF3G (bg=2.39%)No matches to TargetScan

---------------------------------------------------------

GCCCT

GCCCTC  
Depth:9 (MOUSE)  
Ei-value:0.000, Pi-value:0.000  
Er-value:0.000, Pr-value:0.000  
eCLIP MATCHES▶ddx3x (bg=13.89%)▶ddx6 (bg=23.92%)▶drosha (bg=11.37%)▶EIF3G (bg=2.39%)▶EIF3H (bg=7.83%)▶FMR1 (bg=6.3%)▶METAP2 (bg=6.14%)▶PUS1 (bg=2.87%)▶RPS3 (bg=4.15%)▶SDAD1 (bg=5.99%)▶SERBP1 (bg=0.81%)▶SF3B1 (bg=6.74%)▶WRN (bg=3.31%)▶ybx3 (bg=22.82%)No matches to TargetScan

 120  


C

GCCCTC  
Depth:9 (MOUSE)  
Ei-value:0.000, Pi-value:0.000  
Er-value:0.000, Pr-value:0.000  
eCLIP MATCHES▶ddx3x (bg=13.89%)▶ddx6 (bg=23.92%)▶drosha (bg=11.37%)▶EIF3G (bg=2.39%)▶EIF3H (bg=7.83%)▶FMR1 (bg=6.3%)▶METAP2 (bg=6.14%)▶PUS1 (bg=2.87%)▶RPS3 (bg=4.15%)▶SDAD1 (bg=5.99%)▶SERBP1 (bg=0.81%)▶SF3B1 (bg=6.74%)▶WRN (bg=3.31%)▶ybx3 (bg=22.82%)No matches to TargetScan

----------------------------------------------------------------------------------------------------------------------- 240  
 ----------------------------------------------------

ATGGCGGC

ATGGCGGC  
Depth:9 (MOUSE)  
Ei-value:0.000, Pi-value:0.000  
Er-value:0.000, Pr-value:0.000  
eCLIP MATCHES▶BCCIP (bg=4.61%)▶ddx3x (bg=13.89%)▶ddx6 (bg=23.92%)▶dgcr8 (bg=19.31%)▶DHX30 (bg=5.05%)▶drosha (bg=11.37%)▶EIF3H (bg=7.83%)▶FMR1 (bg=6.3%)▶FTO (bg=7.53%)▶fubp3 (bg=23.31%)▶FXR2 (bg=7.51%)▶GEMIN5 (bg=2.46%)▶IGF2BP3 (bg=4.26%)▶METAP2 (bg=6.14%)▶NIP7 (bg=2.54%)▶PCBP1 (bg=2.3%)▶pum1 (bg=29.85%)▶pum2 (bg=21.55%)▶rbm15 (bg=11.81%)▶SDAD1 (bg=5.99%)▶WRN (bg=3.31%)No matches to TargetScan

------------------------------------------------------------ 360  
 ------------------------------------------------------------------------------------------------------------------------ 480  
 ------------------------------------------------------------------------------------------------------------------------ 600  
 ------------------------------------------------------------------------------------------------------------------------ 720  
 ---------------------------------------------------------

GGGAAGA

GGGAAGA  
Depth:9 (MOUSE)  
Ei-value:0.000, Pi-value:0.000  
Er-value:0.000, Pr-value:0.000  
No matches to eCLIP DataNo matches to TargetScan

----------------------

CCTGTGTATATAATATGAAAAAGCTGCT

CCTGTGTATATAATATGAAAAAGCTGCT  
Depth:9 (MOUSE)  
Ei-value:0.000, Pi-value:0.000  
Er-value:0.000, Pr-value:0.000  
eCLIP MATCHES▶pum1 (bg=29.85%)MATCHES To TargetScan▶ miR-15-5p/16-5p/195-5p/424-5p/497-5p:AGCAGCA▶ miR-503-5p:AGCAGCG

------ 840  
 -------

CAACCTTT

CAACCTTT  
Depth:9 (MOUSE)  
Ei-value:0.000, Pi-value:0.000  
Er-value:0.000, Pr-value:0.000  
eCLIP MATCHES▶pum1 (bg=29.85%)No matches to TargetScan

-----------------------------------------

AGAGGT

AGAGGT  
Depth:9 (MOUSE)  
Ei-value:0.000, Pi-value:0.000  
Er-value:0.000, Pr-value:0.000  
eCLIP MATCHES▶dgcr8 (bg=19.31%)▶IGF2BP2 (bg=3.44%)▶KHDRBS1 (bg=1.16%)▶lin28b (bg=16.96%)▶pum1 (bg=29.85%)▶pum2 (bg=21.55%)▶rbm15 (bg=11.81%)▶tia1 (bg=16.04%)No matches to TargetScan

---------------------------------------------------------- 960  
 ----------------------------------------------

GAAATG

GAAATG  
Depth:9 (MOUSE)  
Ei-value:0.000, Pi-value:0.000  
Er-value:0.000, Pr-value:0.000  
eCLIP MATCHES▶fubp3 (bg=23.31%)▶GRWD1 (bg=4.85%)▶PPIG (bg=0.88%)▶pum1 (bg=29.85%)▶pum2 (bg=21.55%)▶rbm15 (bg=11.81%)▶SND1 (bg=1.27%)▶ZNF622 (bg=1.54%)No matches to TargetScan

-------------------------------------------------------------------- 1080  
 ----------------------------------------------------

AGAAGA

AGAAGA  
Depth:9 (MOUSE)  
Ei-value:0.000, Pi-value:0.000  
Er-value:0.000, Pr-value:0.000  
No matches to eCLIP DataNo matches to TargetScan

-------------------------------------------------------------- 1200  
 -----------------------------------------------

AAGTGCCTG

AAGTGCCTG  
Depth:9 (MOUSE)  
Ei-value:0.000, Pi-value:0.000  
Er-value:0.000, Pr-value:0.000  
eCLIP MATCHES▶pum1 (bg=29.85%)▶PUS1 (bg=2.87%)▶tial1 (bg=14.09%)No matches to TargetScan

---------------------------------------------------------------- 1320  
 ------------------------------------------------------

TTGTGC

TTGTGC  
Depth:9 (MOUSE)  
Ei-value:0.000, Pi-value:0.000  
Er-value:0.000, Pr-value:0.000  
eCLIP MATCHES▶pum1 (bg=29.85%)▶tia1 (bg=16.04%)No matches to TargetScan

------------------------------------------------------------ 1440  
 -------------------------------------------------------------------------------------------------------------

GATGTT

GATGTT  
Depth:9 (MOUSE)  
Ei-value:0.000, Pi-value:0.000  
Er-value:0.000, Pr-value:0.000  
No matches to eCLIP DataNo matches to TargetScan


AAAGA

AAAGAGGTTGCCA  
Depth:9 (MOUSE)  
Ei-value:0.000, Pi-value:0.000  
Er-value:0.000, Pr-value:0.000  
No matches to eCLIP DataNo matches to TargetScan

 1560  


GGTTGCCA

AAAGAGGTTGCCA  
Depth:9 (MOUSE)  
Ei-value:0.000, Pi-value:0.000  
Er-value:0.000, Pr-value:0.000  
No matches to eCLIP DataNo matches to TargetScan

-------------------------------------------

ATTTTGT

ATTTTGT  
Depth:9 (MOUSE)  
Ei-value:0.000, Pi-value:0.000  
Er-value:0.000, Pr-value:0.000  
No matches to eCLIP DataNo matches to TargetScan

-------------------------------------------------------------- 1680  
 ----------------------------------------------------------------------------------------------------------------

TCCTTA

TCCTTA  
Depth:9 (MOUSE)  
Ei-value:0.000, Pi-value:0.000  
Er-value:0.000, Pr-value:0.000  
No matches to eCLIP DataNo matches to TargetScan

-- 1800  
 --

AAACATCT

AAACATCT  
Depth:9 (MOUSE)  
Ei-value:0.000, Pi-value:0.000  
Er-value:0.000, Pr-value:0.000  
No matches to eCLIP DataNo matches to TargetScan

-------

CTAGATGTTTAGAAGTGCCC

CTAGATGTTTAGAAGTGCCC  
Depth:9 (MOUSE)  
Ei-value:0.000, Pi-value:0.000  
Er-value:0.000, Pr-value:0.000  
No matches to eCLIP DataNo matches to TargetScan

---

GTATGTTAAA

GTATGTTAAA  
Depth:9 (MOUSE)  
Ei-value:0.000, Pi-value:0.000  
Er-value:0.000, Pr-value:0.000  
No matches to eCLIP DataNo matches to TargetScan

------------------------

TGTAAATA

TGTAAATA  
Depth:9 (MOUSE)  
Ei-value:0.000, Pi-value:0.000  
Er-value:0.000, Pr-value:0.000  
No matches to eCLIP DataNo matches to TargetScan

-------------------------------------- 1920  
 ------------------------------------------------------------------------------------------------------------------------ 2040  
 ------------------------------------------------------------------------------------------------------------------------ 2160  
 ------------------------------------------------------------------------------------------------------------------------ 2280  
 ------------------------------------------------------------------------------------------------------------------------ 2400  
 ------------------------------------------------------------------------------------------------------------------------ 2520  
 --------------------------------------------------------------

GTGGGAGGGAA

GTGGGAGGGAA  
Depth:9 (MOUSE)  
Ei-value:0.000, Pi-value:0.000  
Er-value:0.000, Pr-value:0.000  
eCLIP MATCHES▶ddx55 (bg=12.35%)▶FTO (bg=7.53%)▶GRSF1 (bg=3.8%)▶KHSRP (bg=8.1%)▶pum1 (bg=29.85%)▶SUB1 (bg=9.24%)MATCHES To TargetScan▶ miR-150-5p:CUCCCAA▶ miR-532-3p:CUCCCAC

-----

TGCAAA

TGCAAA  
Depth:9 (MOUSE)  
Ei-value:0.000, Pi-value:0.000  
Er-value:0.000, Pr-value:0.000  
eCLIP MATCHES▶ddx55 (bg=12.35%)▶FTO (bg=7.53%)▶fubp3 (bg=23.31%)▶GRSF1 (bg=3.8%)▶KHSRP (bg=8.1%)▶pum1 (bg=29.85%)▶SUB1 (bg=9.24%)No matches to TargetScan

------------------------------------ 2640  
 ------------------------------------------------

TGTATATA

TGTATATA  
Depth:9 (MOUSE)  
Ei-value:0.000, Pi-value:0.000  
Er-value:0.000, Pr-value:0.000  
eCLIP MATCHES▶dgcr8 (bg=19.31%)▶EIF4G2 (bg=0.7%)▶fam120a (bg=18.43%)▶FASTKD2 (bg=7.73%)▶fubp3 (bg=23.31%)▶HNRNPL (bg=3.4%)▶igf2bp1 (bg=11.67%)▶KHSRP (bg=8.1%)▶NCBP2 (bg=2.92%)▶NOLC1 (bg=6.58%)▶pum1 (bg=29.85%)▶pum2 (bg=21.55%)▶sf3a3 (bg=12.13%)▶SF3B1 (bg=6.74%)▶TBRG4 (bg=7.0%)▶tia1 (bg=16.04%)▶tial1 (bg=14.09%)▶ZC3H11A (bg=6.25%)No matches to TargetScan

-------------------------------------

ATATCTAG

ATATCTAG  
Depth:9 (MOUSE)  
Ei-value:0.000, Pi-value:0.000  
Er-value:0.000, Pr-value:0.000  
eCLIP MATCHES▶AKAP1 (bg=3.4%)▶ddx55 (bg=12.35%)▶dgcr8 (bg=19.31%)▶fam120a (bg=18.43%)▶fubp3 (bg=23.31%)▶GRSF1 (bg=3.8%)▶igf2bp1 (bg=11.67%)▶KHSRP (bg=8.1%)▶NKRF (bg=3.64%)▶PHF6 (bg=1.71%)▶pum1 (bg=29.85%)▶pum2 (bg=21.55%)▶sf3a3 (bg=12.13%)▶SF3B1 (bg=6.74%)▶tia1 (bg=16.04%)▶WRN (bg=3.31%)▶ZC3H11A (bg=6.25%)No matches to TargetScan

-

CTCTAG

CTCTAG  
Depth:9 (MOUSE)  
Ei-value:0.000, Pi-value:0.000  
Er-value:0.000, Pr-value:0.000  
eCLIP MATCHES▶ddx55 (bg=12.35%)▶dgcr8 (bg=19.31%)▶FASTKD2 (bg=7.73%)▶fubp3 (bg=23.31%)▶GRSF1 (bg=3.8%)▶KHSRP (bg=8.1%)▶pum1 (bg=29.85%)▶pum2 (bg=21.55%)▶tia1 (bg=16.04%)▶ZC3H11A (bg=6.25%)No matches to TargetScan

-----

AAAGAGG

AAAGAGGTTGCCAATGTATGACA  
Depth:9 (MOUSE)  
Ei-value:0.000, Pi-value:0.000  
Er-value:0.000, Pr-value:0.000  
eCLIP MATCHES▶ddx55 (bg=12.35%)▶dgcr8 (bg=19.31%)▶FASTKD2 (bg=7.73%)▶fubp3 (bg=23.31%)▶GRSF1 (bg=3.8%)▶NOLC1 (bg=6.58%)▶pum1 (bg=29.85%)▶pum2 (bg=21.55%)▶tial1 (bg=14.09%)MATCHES To TargetScan▶ miR-182-5p:UUGGCAA▶ miR-96-5p/1271-5p:UUGGCAC▶ miR-539-3p:UCAUACA

 2760  


TTGCCAATGTATGACA

AAAGAGGTTGCCAATGTATGACA  
Depth:9 (MOUSE)  
Ei-value:0.000, Pi-value:0.000  
Er-value:0.000, Pr-value:0.000  
eCLIP MATCHES▶ddx55 (bg=12.35%)▶dgcr8 (bg=19.31%)▶FASTKD2 (bg=7.73%)▶fubp3 (bg=23.31%)▶GRSF1 (bg=3.8%)▶NOLC1 (bg=6.58%)▶pum1 (bg=29.85%)▶pum2 (bg=21.55%)▶tial1 (bg=14.09%)MATCHES To TargetScan▶ miR-182-5p:UUGGCAA▶ miR-96-5p/1271-5p:UUGGCAC▶ miR-539-3p:UCAUACA

--------

GTTAGTAAACT

GTTAGTAAACT  
Depth:9 (MOUSE)  
Ei-value:0.000, Pi-value:0.000  
Er-value:0.000, Pr-value:0.000  
eCLIP MATCHES▶ddx55 (bg=12.35%)▶dgcr8 (bg=19.31%)▶FASTKD2 (bg=7.73%)▶NOLC1 (bg=6.58%)▶pum1 (bg=29.85%)▶pum2 (bg=21.55%)No matches to TargetScan

-

ACACATT

ACACATT  
Depth:9 (MOUSE)  
Ei-value:0.000, Pi-value:0.000  
Er-value:0.000, Pr-value:0.000  
eCLIP MATCHES▶NOLC1 (bg=6.58%)No matches to TargetScan

-------------------------------

TGTCTTCTGAA

TGTCTTCTGAA  
Depth:9 (MOUSE)  
Ei-value:0.000, Pi-value:0.000  
Er-value:0.000, Pr-value:0.000  
eCLIP MATCHES▶dgcr8 (bg=19.31%)▶pum2 (bg=21.55%)▶ybx3 (bg=22.82%)No matches to TargetScan

----------------------------------- 2880  
 -------

GTGCCT

GTGCCT  
Depth:9 (MOUSE)  
Ei-value:0.000, Pi-value:0.000  
Er-value:0.000, Pr-value:0.000  
eCLIP MATCHES▶lin28b (bg=16.96%)▶ybx3 (bg=22.82%)No matches to TargetScan

-------------------------------------------------------

AGAATG

AGAATG  
Depth:9 (MOUSE)  
Ei-value:0.000, Pi-value:0.000  
Er-value:0.000, Pr-value:0.000  
eCLIP MATCHES▶AARS (bg=2.33%)▶FUS (bg=2.59%)▶lin28b (bg=16.96%)▶UTP3 (bg=2.81%)▶ybx3 (bg=22.82%)No matches to TargetScan

---------

AGAAGA

AGAAGA  
Depth:9 (MOUSE)  
Ei-value:0.000, Pi-value:0.000  
Er-value:0.000, Pr-value:0.000  
eCLIP MATCHES▶AARS (bg=2.33%)▶FUS (bg=2.59%)▶lin28b (bg=16.96%)No matches to TargetScan

------------------------------- 3000  
 ---------------------

TGTATATA

TGTATATA  
Depth:9 (MOUSE)  
Ei-value:0.000, Pi-value:0.000  
Er-value:0.000, Pr-value:0.000  
eCLIP MATCHES▶DDX21 (bg=1.64%)▶dgcr8 (bg=19.31%)▶fam120a (bg=18.43%)▶HNRNPL (bg=3.4%)▶igf2bp1 (bg=11.67%)▶pum2 (bg=21.55%)▶ZC3H11A (bg=6.25%)No matches to TargetScan

-----------------------

TGAAACATCTAG

TGAAACATCTAG  
Depth:9 (MOUSE)  
Ei-value:0.000, Pi-value:0.000  
Er-value:0.000, Pr-value:0.000  
No matches to eCLIP DataNo matches to TargetScan

---

TTCTAG

TTCTAG  
Depth:9 (MOUSE)  
Ei-value:0.000, Pi-value:0.000  
Er-value:0.000, Pr-value:0.000  
No matches to eCLIP DataNo matches to TargetScan

-

TGTTTAGAAGTGCACAAAGTATGTTAAAAGTAGA

TGTTTAGAAGTGCACAAAGTATGTTAAAAGTAGA  
Depth:9 (MOUSE)  
Ei-value:0.000, Pi-value:0.000  
Er-value:0.000, Pr-value:0.000  
No matches to eCLIP DataNo matches to TargetScan

------------ 3120  
 ------------------------------------------------------------------------------------------------------------------------ 3240  
 ------------------------------------------------------------------------------------

TGTATA

TGTATA  
Depth:9 (MOUSE)  
Ei-value:0.000, Pi-value:0.000  
Er-value:0.000, Pr-value:0.000  
eCLIP MATCHES▶APOBEC3C (bg=3.95%)▶pum1 (bg=29.85%)▶pum2 (bg=21.55%)No matches to TargetScan

------------------------------ 3360  
 ------------------------------------------------------------------------------------------------------------------------ 3480  
 ------------------------------------------------------------------------------------------------------------------------ 3600  
 ---------------------------------------------------------------------------------

AGTCTT

AGTCTT  
Depth:9 (MOUSE)  
Ei-value:0.000, Pi-value:0.000  
Er-value:0.000, Pr-value:0.000  
eCLIP MATCHES▶fam120a (bg=18.43%)▶pum1 (bg=29.85%)▶pum2 (bg=21.55%)No matches to TargetScan

--------------------------------- 3720  
 ------------------------------------------------------------------------------------------------------------------------ 3840  
 ------------------------------------------------------------------------------------------------------------------------ 3960  
 ------------------------------------------------------------------------------------------------------------------------ 4080  
 --------------

AATTGTTA

AATTGTTA  
Depth:9 (MOUSE)  
Ei-value:0.000, Pi-value:0.000  
Er-value:0.000, Pr-value:0.000  
No matches to eCLIP DataNo matches to TargetScan

-------------------------------------------------------------------------------------------------- 4200  
 ------------------------------------------------------------------------------------------------------------------------ 4320  
 ------------------------------------------------------------------------------------------------------------------------ 4440  
 ------------------------------------------------------------------------------------------------------------------------ 4560  
 ------------------------------------------------------------------------------------------------------------------------ 4680  
 ------------------------------------------------------------------------------------------------------------------------ 4800  
 ------------------------------------------------------------------------------------------------------------------------ 4920  
 ------------------------------------------------------------------------------------------------------------------------ 5040  
 -

ATTTTTGT

ATTTTTGT  
Depth:9 (MOUSE)  
Ei-value:0.000, Pi-value:0.000  
Er-value:0.000, Pr-value:0.000  
eCLIP MATCHES▶EIF3H (bg=7.83%)▶HNRNPC (bg=1.49%)No matches to TargetScan

--------------------------------------------------------------------------------

TTCTTGGT

TTCTTGGT  
Depth:9 (MOUSE)  
Ei-value:0.000, Pi-value:0.000  
Er-value:0.000, Pr-value:0.000  
eCLIP MATCHES▶APOBEC3C (bg=3.95%)▶ddx55 (bg=12.35%)▶ddx6 (bg=23.92%)▶EIF3H (bg=7.83%)▶fam120a (bg=18.43%)▶fubp3 (bg=23.31%)▶FUS (bg=2.59%)▶tia1 (bg=16.04%)▶tial1 (bg=14.09%)▶ZC3H11A (bg=6.25%)No matches to TargetScan

----------------------- 5160  
 -----------------

AATATTCA

AATATTCA  
Depth:9 (MOUSE)  
Ei-value:0.000, Pi-value:0.000  
Er-value:0.000, Pr-value:0.000  
No matches to eCLIP DataNo matches to TargetScan

----------------------------------------------------------------------------------------------- 5280  
 ------

CTGCTT

CTGCTT  
Depth:9 (MOUSE)  
Ei-value:0.000, Pi-value:0.000  
Er-value:0.000, Pr-value:0.000  
eCLIP MATCHES▶fubp3 (bg=23.31%)▶KHSRP (bg=8.1%)▶NOLC1 (bg=6.58%)▶TARDBP (bg=1.75%)No matches to TargetScan

-----------

TGACTT

TGACTT  
Depth:9 (MOUSE)  
Ei-value:0.000, Pi-value:0.000  
Er-value:0.000, Pr-value:0.000  
eCLIP MATCHES▶fubp3 (bg=23.31%)▶KHSRP (bg=8.1%)▶NOLC1 (bg=6.58%)▶SUB1 (bg=9.24%)▶TARDBP (bg=1.75%)▶ZC3H11A (bg=6.25%)MATCHES To TargetScan▶ miR-224-5p:AAGUCAC

------

TTGCTT

TTGCTT  
Depth:9 (MOUSE)  
Ei-value:0.000, Pi-value:0.000  
Er-value:0.000, Pr-value:0.010  
eCLIP MATCHES▶ddx55 (bg=12.35%)▶ddx6 (bg=23.92%)▶fubp3 (bg=23.31%)▶KHSRP (bg=8.1%)▶NOLC1 (bg=6.58%)▶SUB1 (bg=9.24%)▶TARDBP (bg=1.75%)▶tia1 (bg=16.04%)▶tial1 (bg=14.09%)▶ZC3H11A (bg=6.25%)No matches to TargetScan

-----------------------------------------

AAATAAA

AAATAAA  
Depth:9 (MOUSE)  
Ei-value:0.000, Pi-value:0.000  
Er-value:0.000, Pr-value:0.000  
eCLIP MATCHES▶ZC3H11A (bg=6.25%)No matches to TargetScan

------------------------------- 5400  
 -------------------------------------------                                                                              5443
```

---

## >BABOON (5458 bases)

```
 -------------------------------------------------------

TGCCAA

TGCCAA  
Depth:9 (MOUSE)  
Ei-value:0.000, Pi-value:0.000  
Er-value:0.000, Pr-value:0.000  
MATCHES To TargetScan▶ miR-182-5p:UUGGCAA▶ miR-96-5p/1271-5p:UUGGCAC

-

TCAGTTCCGG

TCAGTTCCGG  
Depth:9 (MOUSE)  
Ei-value:0.000, Pi-value:0.000  
Er-value:0.000, Pr-value:0.000  
No matches to TargetScan

------------------------------------------------ 120  
 --------

GCCCTC

GCCCTC  
Depth:9 (MOUSE)  
Ei-value:0.000, Pi-value:0.000  
Er-value:0.000, Pr-value:0.000  
No matches to TargetScan

---------------------------------------------------------------------------------------------------------- 240  
 ------------------------------------------------

ATGGCGGC

ATGGCGGC  
Depth:9 (MOUSE)  
Ei-value:0.000, Pi-value:0.000  
Er-value:0.000, Pr-value:0.000  
No matches to TargetScan

---------------------------------------------------------------- 360  
 ------------------------------------------------------------------------------------------------------------------------ 480  
 ------------------------------------------------------------------------------------------------------------------------ 600  
 ------------------------------------------------------------------------------------------------------------------------ 720  
 --------------------------------------------------------------

GGGAAGA

GGGAAGA  
Depth:9 (MOUSE)  
Ei-value:0.000, Pi-value:0.000  
Er-value:0.000, Pr-value:0.000  
No matches to TargetScan

----------------------

CCTGTGTATATAATATGAAAAAGCTGCT

CCTGTGTATATAATATGAAAAAGCTGCT  
Depth:9 (MOUSE)  
Ei-value:0.000, Pi-value:0.000  
Er-value:0.000, Pr-value:0.000  
MATCHES To TargetScan▶ miR-15-5p/16-5p/195-5p/424-5p/497-5p:AGCAGCA▶ miR-503-5p:AGCAGCG

- 840  
 ------------

CAACCTTT

CAACCTTT  
Depth:9 (MOUSE)  
Ei-value:0.000, Pi-value:0.000  
Er-value:0.000, Pr-value:0.000  
No matches to TargetScan

-----------------------------------------

AGAGGT

AGAGGT  
Depth:9 (MOUSE)  
Ei-value:0.000, Pi-value:0.000  
Er-value:0.000, Pr-value:0.000  
No matches to TargetScan

----------------------------------------------------- 960  
 ---------------------------------------------------

GAAATG

GAAATG  
Depth:9 (MOUSE)  
Ei-value:0.000, Pi-value:0.000  
Er-value:0.000, Pr-value:0.000  
No matches to TargetScan

--------------------------------------------------------------- 1080  
 -------------------------------------------------

AGAAGA

AGAAGA  
Depth:9 (MOUSE)  
Ei-value:0.000, Pi-value:0.000  
Er-value:0.000, Pr-value:0.000  
No matches to TargetScan

----------------------------------------------------------------- 1200  
 --------------------------------------------

AAGTGCCTG

AAGTGCCTG  
Depth:9 (MOUSE)  
Ei-value:0.000, Pi-value:0.000  
Er-value:0.000, Pr-value:0.000  
No matches to TargetScan

------------------------------------------------------------------- 1320  
 -----------------------------------------------------

TTGTGC

TTGTGC  
Depth:9 (MOUSE)  
Ei-value:0.000, Pi-value:0.000  
Er-value:0.000, Pr-value:0.000  
No matches to TargetScan

------------------------------------------------------------- 1440  
 -----------------------------------------------------------------------------------------------------------

GATGTT

GATGTT  
Depth:9 (MOUSE)  
Ei-value:0.000, Pi-value:0.000  
Er-value:0.000, Pr-value:0.000  
No matches to TargetScan


AAAGAGG

AAAGAGGTTGCCA  
Depth:9 (MOUSE)  
Ei-value:0.000, Pi-value:0.000  
Er-value:0.000, Pr-value:0.000  
No matches to TargetScan

 1560  


TTGCCA

AAAGAGGTTGCCA  
Depth:9 (MOUSE)  
Ei-value:0.000, Pi-value:0.000  
Er-value:0.000, Pr-value:0.000  
No matches to TargetScan

-----------------------------------------

ATTTTGT

ATTTTGT  
Depth:9 (MOUSE)  
Ei-value:0.000, Pi-value:0.000  
Er-value:0.000, Pr-value:0.000  
No matches to TargetScan

------------------------------------------------------------------ 1680  
 --------------------------------------------------------------------------------------------------------

TCCTTA

TCCTTA  
Depth:9 (MOUSE)  
Ei-value:0.000, Pi-value:0.000  
Er-value:0.000, Pr-value:0.000  
No matches to TargetScan

----

AAACAT

AAACATCT  
Depth:9 (MOUSE)  
Ei-value:0.000, Pi-value:0.000  
Er-value:0.000, Pr-value:0.000  
No matches to TargetScan

 1800  


CT

AAACATCT  
Depth:9 (MOUSE)  
Ei-value:0.000, Pi-value:0.000  
Er-value:0.000, Pr-value:0.000  
No matches to TargetScan

-------

CTAGATGTTTAGAAGTGCCC

CTAGATGTTTAGAAGTGCCC  
Depth:9 (MOUSE)  
Ei-value:0.000, Pi-value:0.000  
Er-value:0.000, Pr-value:0.000  
No matches to TargetScan

---

GTATGTTAAA

GTATGTTAAA  
Depth:9 (MOUSE)  
Ei-value:0.000, Pi-value:0.000  
Er-value:0.000, Pr-value:0.000  
No matches to TargetScan

------------------------

TGTAAATA

TGTAAATA  
Depth:9 (MOUSE)  
Ei-value:0.000, Pi-value:0.000  
Er-value:0.000, Pr-value:0.000  
No matches to TargetScan

---------------------------------------------- 1920  
 ------------------------------------------------------------------------------------------------------------------------ 2040  
 ------------------------------------------------------------------------------------------------------------------------ 2160  
 ------------------------------------------------------------------------------------------------------------------------ 2280  
 ------------------------------------------------------------------------------------------------------------------------ 2400  
 ------------------------------------------------------------------------------------------------------------------------ 2520  
 ----------------------------------------------------------

GTGGGAGGGAA

GTGGGAGGGAA  
Depth:9 (MOUSE)  
Ei-value:0.000, Pi-value:0.000  
Er-value:0.000, Pr-value:0.000  
MATCHES To TargetScan▶ miR-150-5p:CUCCCAA▶ miR-532-3p:CUCCCAC

-----

TGCAAA

TGCAAA  
Depth:9 (MOUSE)  
Ei-value:0.000, Pi-value:0.000  
Er-value:0.000, Pr-value:0.000  
No matches to TargetScan

---------------------------------------- 2640  
 --------------------------------------------

TGTATATA

TGTATATA  
Depth:9 (MOUSE)  
Ei-value:0.000, Pi-value:0.000  
Er-value:0.000, Pr-value:0.000  
No matches to TargetScan

-------------------------------------

ATATCTAG

ATATCTAG  
Depth:9 (MOUSE)  
Ei-value:0.000, Pi-value:0.000  
Er-value:0.000, Pr-value:0.000  
No matches to TargetScan

-

CTCTAG

CTCTAG  
Depth:9 (MOUSE)  
Ei-value:0.000, Pi-value:0.000  
Er-value:0.000, Pr-value:0.000  
No matches to TargetScan

-----

AAAGAGGTTGC

AAAGAGGTTGCCAATGTATGACA  
Depth:9 (MOUSE)  
Ei-value:0.000, Pi-value:0.000  
Er-value:0.000, Pr-value:0.000  
MATCHES To TargetScan▶ miR-182-5p:UUGGCAA▶ miR-96-5p/1271-5p:UUGGCAC▶ miR-539-3p:UCAUACA

 2760  


CAATGTATGACA

AAAGAGGTTGCCAATGTATGACA  
Depth:9 (MOUSE)  
Ei-value:0.000, Pi-value:0.000  
Er-value:0.000, Pr-value:0.000  
MATCHES To TargetScan▶ miR-182-5p:UUGGCAA▶ miR-96-5p/1271-5p:UUGGCAC▶ miR-539-3p:UCAUACA

--------

GTTAGTAAACT

GTTAGTAAACT  
Depth:9 (MOUSE)  
Ei-value:0.000, Pi-value:0.000  
Er-value:0.000, Pr-value:0.000  
No matches to TargetScan

-

ACACATT

ACACATT  
Depth:9 (MOUSE)  
Ei-value:0.000, Pi-value:0.000  
Er-value:0.000, Pr-value:0.000  
No matches to TargetScan

-------------------------------

TGTCTTCTGAA

TGTCTTCTGAA  
Depth:9 (MOUSE)  
Ei-value:0.000, Pi-value:0.000  
Er-value:0.000, Pr-value:0.000  
No matches to TargetScan

--------------------------------------- 2880  
 ------

GTGCCT

GTGCCT  
Depth:9 (MOUSE)  
Ei-value:0.000, Pi-value:0.000  
Er-value:0.000, Pr-value:0.000  
No matches to TargetScan

-------------------------------------------------------

AGAATG

AGAATG  
Depth:9 (MOUSE)  
Ei-value:0.000, Pi-value:0.000  
Er-value:0.000, Pr-value:0.000  
No matches to TargetScan

---------

AGAAGA

AGAAGA  
Depth:9 (MOUSE)  
Ei-value:0.000, Pi-value:0.000  
Er-value:0.000, Pr-value:0.000  
No matches to TargetScan

-------------------------------- 3000  
 --------------------

TGTATATA

TGTATATA  
Depth:9 (MOUSE)  
Ei-value:0.000, Pi-value:0.000  
Er-value:0.000, Pr-value:0.000  
No matches to TargetScan

-----------------------

TGAAACATCTAG

TGAAACATCTAG  
Depth:9 (MOUSE)  
Ei-value:0.000, Pi-value:0.000  
Er-value:0.000, Pr-value:0.000  
No matches to TargetScan

---

TTCTAG

TTCTAG  
Depth:9 (MOUSE)  
Ei-value:0.000, Pi-value:0.000  
Er-value:0.000, Pr-value:0.000  
No matches to TargetScan

-

TGTTTAGAAGTGCACAAAGTATGTTAAAAGTAGA

TGTTTAGAAGTGCACAAAGTATGTTAAAAGTAGA  
Depth:9 (MOUSE)  
Ei-value:0.000, Pi-value:0.000  
Er-value:0.000, Pr-value:0.000  
No matches to TargetScan

------------- 3120  
 ------------------------------------------------------------------------------------------------------------------------ 3240  
 -------------------------------------------------------------------------------------

TGTATA

TGTATA  
Depth:9 (MOUSE)  
Ei-value:0.000, Pi-value:0.000  
Er-value:0.000, Pr-value:0.000  
No matches to TargetScan

----------------------------- 3360  
 ------------------------------------------------------------------------------------------------------------------------ 3480  
 ------------------------------------------------------------------------------------------------------------------------ 3600  
 ------------------------------------------------------------------------------------

AGTCTT

AGTCTT  
Depth:9 (MOUSE)  
Ei-value:0.000, Pi-value:0.000  
Er-value:0.000, Pr-value:0.000  
No matches to TargetScan

------------------------------ 3720  
 ------------------------------------------------------------------------------------------------------------------------ 3840  
 ------------------------------------------------------------------------------------------------------------------------ 3960  
 ------------------------------------------------------------------------------------------------------------------------ 4080  
 ------

AATTGTTA

AATTGTTA  
Depth:9 (MOUSE)  
Ei-value:0.000, Pi-value:0.000  
Er-value:0.000, Pr-value:0.000  
No matches to TargetScan

---------------------------------------------------------------------------------------------------------- 4200  
 ------------------------------------------------------------------------------------------------------------------------ 4320  
 ------------------------------------------------------------------------------------------------------------------------ 4440  
 ------------------------------------------------------------------------------------------------------------------------ 4560  
 ------------------------------------------------------------------------------------------------------------------------ 4680  
 ------------------------------------------------------------------------------------------------------------------------ 4800  
 ------------------------------------------------------------------------------------------------------------------------ 4920  
 -------------------------------------------------------------------------------------------------------------------

ATTTT

ATTTTTGT  
Depth:9 (MOUSE)  
Ei-value:0.000, Pi-value:0.000  
Er-value:0.000, Pr-value:0.000  
No matches to TargetScan

 5040  


TGT

ATTTTTGT  
Depth:9 (MOUSE)  
Ei-value:0.000, Pi-value:0.000  
Er-value:0.000, Pr-value:0.000  
No matches to TargetScan

---------------------------------------------------------------------------------------

TTCTTGGT

TTCTTGGT  
Depth:9 (MOUSE)  
Ei-value:0.000, Pi-value:0.000  
Er-value:0.000, Pr-value:0.000  
No matches to TargetScan

---------------------- 5160  
 ------------------

AATATTCA

AATATTCA  
Depth:9 (MOUSE)  
Ei-value:0.000, Pi-value:0.000  
Er-value:0.000, Pr-value:0.000  
No matches to TargetScan

---------------------------------------------------------------------------------------------- 5280  
 -----------

CTGCTT

CTGCTT  
Depth:9 (MOUSE)  
Ei-value:0.000, Pi-value:0.000  
Er-value:0.000, Pr-value:0.000  
No matches to TargetScan

-----------

TGACTT

TGACTT  
Depth:9 (MOUSE)  
Ei-value:0.000, Pi-value:0.000  
Er-value:0.000, Pr-value:0.000  
MATCHES To TargetScan▶ miR-224-5p:AAGUCAC

------

TTGCTT

TTGCTT  
Depth:9 (MOUSE)  
Ei-value:0.000, Pi-value:0.000  
Er-value:0.000, Pr-value:0.010  
No matches to TargetScan

--------------------------------------------

AAATAAA

AAATAAA  
Depth:9 (MOUSE)  
Ei-value:0.000, Pi-value:0.000  
Er-value:0.000, Pr-value:0.000  
No matches to TargetScan

----------------------- 5400  
 ----------------------------------------------------------                                                               5458
```

---

## >DOG (5115 bases)

```
 ------------------------------------------------------------

TGCCAA

TGCCAA  
Depth:9 (MOUSE)  
Ei-value:0.000, Pi-value:0.000  
Er-value:0.000, Pr-value:0.000  
MATCHES To TargetScan▶ miR-182-5p:UUGGCAA▶ miR-96-5p/1271-5p:UUGGCAC

-

TCAGTTCCGG

TCAGTTCCGG  
Depth:9 (MOUSE)  
Ei-value:0.000, Pi-value:0.000  
Er-value:0.000, Pr-value:0.000  
No matches to TargetScan

------------------------------------------- 120  
 --------

GCCCTC

GCCCTC  
Depth:9 (MOUSE)  
Ei-value:0.000, Pi-value:0.000  
Er-value:0.000, Pr-value:0.000  
No matches to TargetScan

---------------------------------------------------------------------------------------------------------- 240  
 ------------------------------------------------------------------------------------------------------------------------ 360  
 -------------------------------------------------------------------------------

ATGGCGGC

ATGGCGGC  
Depth:9 (MOUSE)  
Ei-value:0.000, Pi-value:0.000  
Er-value:0.000, Pr-value:0.000  
No matches to TargetScan

--------------------------------- 480  
 ------------------------------------------------------------------------------------------------------------------------ 600  
 ------------------------------------------------------------------------------------------------------------------------ 720  
 -----------------------------------------------------------------------

GGGAAGA

GGGAAGA  
Depth:9 (MOUSE)  
Ei-value:0.000, Pi-value:0.000  
Er-value:0.000, Pr-value:0.000  
No matches to TargetScan

----------------------

CCTGTGTATATAATATGAAA

CCTGTGTATATAATATGAAAAAGCTGCT  
Depth:9 (MOUSE)  
Ei-value:0.000, Pi-value:0.000  
Er-value:0.000, Pr-value:0.000  
MATCHES To TargetScan▶ miR-15-5p/16-5p/195-5p/424-5p/497-5p:AGCAGCA▶ miR-503-5p:AGCAGCG

 840  


AAGCTGCT

CCTGTGTATATAATATGAAAAAGCTGCT  
Depth:9 (MOUSE)  
Ei-value:0.000, Pi-value:0.000  
Er-value:0.000, Pr-value:0.000  
MATCHES To TargetScan▶ miR-15-5p/16-5p/195-5p/424-5p/497-5p:AGCAGCA▶ miR-503-5p:AGCAGCG

--------------

CAACCTTT

CAACCTTT  
Depth:9 (MOUSE)  
Ei-value:0.000, Pi-value:0.000  
Er-value:0.000, Pr-value:0.000  
No matches to TargetScan

------------------------------------------

AGAGGT

AGAGGT  
Depth:9 (MOUSE)  
Ei-value:0.000, Pi-value:0.000  
Er-value:0.000, Pr-value:0.000  
No matches to TargetScan

------------------------------------------ 960  
 ------------------------------------------------------------------------

GAAATG

GAAATG  
Depth:9 (MOUSE)  
Ei-value:0.000, Pi-value:0.000  
Er-value:0.000, Pr-value:0.000  
No matches to TargetScan

------------------------------------------ 1080  
 ---------------------------------------------------------------------------------------------------

AGAAGA

AGAAGA  
Depth:9 (MOUSE)  
Ei-value:0.000, Pi-value:0.000  
Er-value:0.000, Pr-value:0.000  
No matches to TargetScan

--------------- 1200  
 ---------------------------------------------------------------------------------------------------

AAGTGCCTG

AAGTGCCTG  
Depth:9 (MOUSE)  
Ei-value:0.000, Pi-value:0.000  
Er-value:0.000, Pr-value:0.000  
No matches to TargetScan

------------ 1320  
 ----------------------------------------------------------------------------------------------------------------

TTGTGC

TTGTGC  
Depth:9 (MOUSE)  
Ei-value:0.000, Pi-value:0.000  
Er-value:0.000, Pr-value:0.000  
No matches to TargetScan

-- 1440  
 ------------------------------------------------------------------------------------------------------------------------ 1560  
 -----------------------------------------------------

GATGTT

GATGTT  
Depth:9 (MOUSE)  
Ei-value:0.000, Pi-value:0.000  
Er-value:0.000, Pr-value:0.000  
No matches to TargetScan


AAAGAGGTTGCCA

AAAGAGGTTGCCA  
Depth:9 (MOUSE)  
Ei-value:0.000, Pi-value:0.000  
Er-value:0.000, Pr-value:0.000  
No matches to TargetScan

-------------------------------------

ATTTTGT

ATTTTGT  
Depth:9 (MOUSE)  
Ei-value:0.000, Pi-value:0.000  
Er-value:0.000, Pr-value:0.000  
No matches to TargetScan

---- 1680  
 ------------------------------------------------------------------------------------------------------------------------ 1800  
 -------------------------------------------------------------

TCCTTA

TCCTTA  
Depth:9 (MOUSE)  
Ei-value:0.000, Pi-value:0.000  
Er-value:0.000, Pr-value:0.000  
No matches to TargetScan

----

AAACATCT

AAACATCT  
Depth:9 (MOUSE)  
Ei-value:0.000, Pi-value:0.000  
Er-value:0.000, Pr-value:0.000  
No matches to TargetScan

-------

CTAGATGTTTAGAAGTGCCC

CTAGATGTTTAGAAGTGCCC  
Depth:9 (MOUSE)  
Ei-value:0.000, Pi-value:0.000  
Er-value:0.000, Pr-value:0.000  
No matches to TargetScan

---

GTATGTTAAA

GTATGTTAAA  
Depth:9 (MOUSE)  
Ei-value:0.000, Pi-value:0.000  
Er-value:0.000, Pr-value:0.000  
No matches to TargetScan

- 1920  
 -----------------------

TGTAAATA

TGTAAATA  
Depth:9 (MOUSE)  
Ei-value:0.000, Pi-value:0.000  
Er-value:0.000, Pr-value:0.000  
No matches to TargetScan

----------------------------------------------------------------------------------------- 2040  
 ------------------------------------------------------------------------------------------------------------------------ 2160  
 ------------------------------------------------------------------------------------------------------------------------ 2280  
 ------------------------------------------------------------------------------------------------------------------------ 2400  
 ------------------------------------------------------------------------------------------------------------------------ 2520  
 ------------------------------------------------------------------------------------------------------------------------ 2640  
 ------------------------------------------------------

GTGGGAGGGAA

GTGGGAGGGAA  
Depth:9 (MOUSE)  
Ei-value:0.000, Pi-value:0.000  
Er-value:0.000, Pr-value:0.000  
MATCHES To TargetScan▶ miR-150-5p:CUCCCAA▶ miR-532-3p:CUCCCAC

-----

TGCAAA

TGCAAA  
Depth:9 (MOUSE)  
Ei-value:0.000, Pi-value:0.000  
Er-value:0.000, Pr-value:0.000  
No matches to TargetScan

-------------------------------------------- 2760  
 ------------------------------

TGTATATA

TGTATATA  
Depth:9 (MOUSE)  
Ei-value:0.000, Pi-value:0.000  
Er-value:0.000, Pr-value:0.000  
No matches to TargetScan

-------------------------------------

ATATCTAG

ATATCTAG  
Depth:9 (MOUSE)  
Ei-value:0.000, Pi-value:0.000  
Er-value:0.000, Pr-value:0.000  
No matches to TargetScan

-

CTCTAG

CTCTAG  
Depth:9 (MOUSE)  
Ei-value:0.000, Pi-value:0.000  
Er-value:0.000, Pr-value:0.000  
No matches to TargetScan

-----

AAAGAGGTTGCCAATGTATGACA

AAAGAGGTTGCCAATGTATGACA  
Depth:9 (MOUSE)  
Ei-value:0.000, Pi-value:0.000  
Er-value:0.000, Pr-value:0.000  
MATCHES To TargetScan▶ miR-182-5p:UUGGCAA▶ miR-96-5p/1271-5p:UUGGCAC▶ miR-539-3p:UCAUACA

-- 2880  
 ------

GTTAGTAAACT

GTTAGTAAACT  
Depth:9 (MOUSE)  
Ei-value:0.000, Pi-value:0.000  
Er-value:0.000, Pr-value:0.000  
No matches to TargetScan

-

ACACATT

ACACATT  
Depth:9 (MOUSE)  
Ei-value:0.000, Pi-value:0.000  
Er-value:0.000, Pr-value:0.000  
No matches to TargetScan

---------------------------------

TGTCTTCTGAA

TGTCTTCTGAA  
Depth:9 (MOUSE)  
Ei-value:0.000, Pi-value:0.000  
Er-value:0.000, Pr-value:0.000  
No matches to TargetScan

-------------------------------------------

GTGCCT

GTGCCT  
Depth:9 (MOUSE)  
Ei-value:0.000, Pi-value:0.000  
Er-value:0.000, Pr-value:0.000  
No matches to TargetScan

-- 3000  
 --------------------------------------------------------

AGAATG

AGAATG  
Depth:9 (MOUSE)  
Ei-value:0.000, Pi-value:0.000  
Er-value:0.000, Pr-value:0.000  
No matches to TargetScan

---------

AGAAGA

AGAAGA  
Depth:9 (MOUSE)  
Ei-value:0.000, Pi-value:0.000  
Er-value:0.000, Pr-value:0.000  
No matches to TargetScan

------------------------------------------- 3120  
 ----------

TGTATATA

TGTATATA  
Depth:9 (MOUSE)  
Ei-value:0.000, Pi-value:0.000  
Er-value:0.000, Pr-value:0.000  
No matches to TargetScan

-----------------------

TGAAACATCTAG

TGAAACATCTAG  
Depth:9 (MOUSE)  
Ei-value:0.000, Pi-value:0.000  
Er-value:0.000, Pr-value:0.000  
No matches to TargetScan

---

TTCTAG

TTCTAG  
Depth:9 (MOUSE)  
Ei-value:0.000, Pi-value:0.000  
Er-value:0.000, Pr-value:0.000  
No matches to TargetScan

-

TGTTTAGAAGTGCACAAAGTATGTTAAAAGTAGA

TGTTTAGAAGTGCACAAAGTATGTTAAAAGTAGA  
Depth:9 (MOUSE)  
Ei-value:0.000, Pi-value:0.000  
Er-value:0.000, Pr-value:0.000  
No matches to TargetScan

----------------------- 3240  
 ------------------------------------------------------------------------------------------------------------------------ 3360  
 -----------------------------------------------------------------------------------------------

TGTATA

TGTATA  
Depth:9 (MOUSE)  
Ei-value:0.000, Pi-value:0.000  
Er-value:0.000, Pr-value:0.000  
No matches to TargetScan

------------------- 3480  
 -----------------------

AGTCTT

AGTCTT  
Depth:9 (MOUSE)  
Ei-value:0.000, Pi-value:0.000  
Er-value:0.000, Pr-value:0.000  
No matches to TargetScan

------------------------------------------------------------------------------------------- 3600  
 ------------------------------------------------------------------------------------------------------------------------ 3720  
 ------------------------------------------------------------------------------------------------------------------------ 3840  
 --------------------------------

AATTGTTA

AATTGTTA  
Depth:9 (MOUSE)  
Ei-value:0.000, Pi-value:0.000  
Er-value:0.000, Pr-value:0.000  
No matches to TargetScan

-------------------------------------------------------------------------------- 3960  
 ------------------------------------------------------------------------------------------------------------------------ 4080  
 ------------------------------------------------------------------------------------------------------------------------ 4200  
 ------------------------------------------------------------------------------------------------------------------------ 4320  
 ------------------------------------------------------------------------------------------------------------------------ 4440  
 ------------------------------------------------------------------------------------------------------------------------ 4560  
 ------------------------------------------------------------------------------------------------------------------------ 4680  
 --------------------------------------------------

ATTTTTGT

ATTTTTGT  
Depth:9 (MOUSE)  
Ei-value:0.000, Pi-value:0.000  
Er-value:0.000, Pr-value:0.000  
No matches to TargetScan

-------------------------------------------------------

TTCTTGG

TTCTTGGT  
Depth:9 (MOUSE)  
Ei-value:0.000, Pi-value:0.000  
Er-value:0.000, Pr-value:0.000  
No matches to TargetScan

 4800  


T

TTCTTGGT  
Depth:9 (MOUSE)  
Ei-value:0.000, Pi-value:0.000  
Er-value:0.000, Pr-value:0.000  
No matches to TargetScan

--------------------------------------

AATATTCA

AATATTCA  
Depth:9 (MOUSE)  
Ei-value:0.000, Pi-value:0.000  
Er-value:0.000, Pr-value:0.000  
No matches to TargetScan

------------------------------------------------------------------------- 4920  
 -------------------------

CTGCTT

CTGCTT  
Depth:9 (MOUSE)  
Ei-value:0.000, Pi-value:0.000  
Er-value:0.000, Pr-value:0.000  
No matches to TargetScan

-----------

TGACTT

TGACTT  
Depth:9 (MOUSE)  
Ei-value:0.000, Pi-value:0.000  
Er-value:0.000, Pr-value:0.000  
MATCHES To TargetScan▶ miR-224-5p:AAGUCAC

------

TTGCTT

TTGCTT  
Depth:9 (MOUSE)  
Ei-value:0.000, Pi-value:0.000  
Er-value:0.000, Pr-value:0.010  
No matches to TargetScan

--------------------------------------------

AAATAAA

AAATAAA  
Depth:9 (MOUSE)  
Ei-value:0.000, Pi-value:0.000  
Er-value:0.000, Pr-value:0.000  
No matches to TargetScan

--------- 5040  
 ---------------------------------------------------------------------------                                              5115
```

---

## >COW (5258 bases)

```
 ---------------------------------------------------------

TGCCAA

TGCCAA  
Depth:9 (MOUSE)  
Ei-value:0.000, Pi-value:0.000  
Er-value:0.000, Pr-value:0.000  
MATCHES To TargetScan▶ miR-182-5p:UUGGCAA▶ miR-96-5p/1271-5p:UUGGCAC

-

TCAGTTCCGG

TCAGTTCCGG  
Depth:9 (MOUSE)  
Ei-value:0.000, Pi-value:0.000  
Er-value:0.000, Pr-value:0.000  
No matches to TargetScan

---------------------------------------------- 120  
 ------

GCCCTC

GCCCTC  
Depth:9 (MOUSE)  
Ei-value:0.000, Pi-value:0.000  
Er-value:0.000, Pr-value:0.000  
No matches to TargetScan

------------------------------------------------------------------------------------------------------------ 240  
 ------------------------------------------------------------------------------------------------------------------------ 360  
 -------------------------------------------------------------------------

ATGGCGGC

ATGGCGGC  
Depth:9 (MOUSE)  
Ei-value:0.000, Pi-value:0.000  
Er-value:0.000, Pr-value:0.000  
No matches to TargetScan

--------------------------------------- 480  
 ------------------------------------------------------------------------------------------------------------------------ 600  
 ------------------------------------------------------------------------------------------------------------------------ 720  
 -----------------------------------------------------

GGGAAGA

GGGAAGA  
Depth:9 (MOUSE)  
Ei-value:0.000, Pi-value:0.000  
Er-value:0.000, Pr-value:0.000  
No matches to TargetScan

---------------------

CCTGTGTATATAATATGAAAAAGCTGCT

CCTGTGTATATAATATGAAAAAGCTGCT  
Depth:9 (MOUSE)  
Ei-value:0.000, Pi-value:0.000  
Er-value:0.000, Pr-value:0.000  
MATCHES To TargetScan▶ miR-15-5p/16-5p/195-5p/424-5p/497-5p:AGCAGCA▶ miR-503-5p:AGCAGCG

----------- 840  
 ---

CAACCTTT

CAACCTTT  
Depth:9 (MOUSE)  
Ei-value:0.000, Pi-value:0.000  
Er-value:0.000, Pr-value:0.000  
No matches to TargetScan

-----------------------------------------

AGAGGT

AGAGGT  
Depth:9 (MOUSE)  
Ei-value:0.000, Pi-value:0.000  
Er-value:0.000, Pr-value:0.000  
No matches to TargetScan

-------------------------------------------------------------- 960  
 ----------------------------------

GAAATG

GAAATG  
Depth:9 (MOUSE)  
Ei-value:0.000, Pi-value:0.000  
Er-value:0.000, Pr-value:0.000  
No matches to TargetScan

-------------------------------------------------------------------------------- 1080  
 ------------------------------------------------------------------

AGAAGA

AGAAGA  
Depth:9 (MOUSE)  
Ei-value:0.000, Pi-value:0.000  
Er-value:0.000, Pr-value:0.000  
No matches to TargetScan

------------------------------------------------ 1200  
 -----------------------------------------------------------

AAGTGCCTG

AAGTGCCTG  
Depth:9 (MOUSE)  
Ei-value:0.000, Pi-value:0.000  
Er-value:0.000, Pr-value:0.000  
No matches to TargetScan

---------------------------------------------------- 1320  
 ----------------------------------------------------------------------

TTGTGC

TTGTGC  
Depth:9 (MOUSE)  
Ei-value:0.000, Pi-value:0.000  
Er-value:0.000, Pr-value:0.000  
No matches to TargetScan

-------------------------------------------- 1440  
 ------------------------------------------------------------------------------------------------------------------------ 1560  


GATGTT

GATGTT  
Depth:9 (MOUSE)  
Ei-value:0.000, Pi-value:0.000  
Er-value:0.000, Pr-value:0.000  
No matches to TargetScan


AAAGAGGTTGCCA

AAAGAGGTTGCCA  
Depth:9 (MOUSE)  
Ei-value:0.000, Pi-value:0.000  
Er-value:0.000, Pr-value:0.000  
No matches to TargetScan

------------------------------------------

ATTTTGT

ATTTTGT  
Depth:9 (MOUSE)  
Ei-value:0.000, Pi-value:0.000  
Er-value:0.000, Pr-value:0.000  
No matches to TargetScan

---------------------------------------------------- 1680  
 ------------------------------------------------------------------------------------------------------------------------ 1800  
 ---------------------

TCCTTA

TCCTTA  
Depth:9 (MOUSE)  
Ei-value:0.000, Pi-value:0.000  
Er-value:0.000, Pr-value:0.000  
No matches to TargetScan

----

AAACATCT

AAACATCT  
Depth:9 (MOUSE)  
Ei-value:0.000, Pi-value:0.000  
Er-value:0.000, Pr-value:0.000  
No matches to TargetScan

-------

CTAGATGTTTAGAAGTGCCC

CTAGATGTTTAGAAGTGCCC  
Depth:9 (MOUSE)  
Ei-value:0.000, Pi-value:0.000  
Er-value:0.000, Pr-value:0.000  
No matches to TargetScan

---

GTATGTTAAA

GTATGTTAAA  
Depth:9 (MOUSE)  
Ei-value:0.000, Pi-value:0.000  
Er-value:0.000, Pr-value:0.000  
No matches to TargetScan

------------------------

TGTAAATA

TGTAAATA  
Depth:9 (MOUSE)  
Ei-value:0.000, Pi-value:0.000  
Er-value:0.000, Pr-value:0.000  
No matches to TargetScan

--------- 1920  
 ------------------------------------------------------------------------------------------------------------------------ 2040  
 ------------------------------------------------------------------------------------------------------------------------ 2160  
 ------------------------------------------------------------------------------------------------------------------------ 2280  
 ------------------------------------------------------------------------------------------------------------------------ 2400  
 ------------------------------------------------------------------------------------------------------------------------ 2520  
 ------------------------------------------------------------------------------------------------------------------------ 2640  
 -----------------

GTGGGAGGGAA

GTGGGAGGGAA  
Depth:9 (MOUSE)  
Ei-value:0.000, Pi-value:0.000  
Er-value:0.000, Pr-value:0.000  
MATCHES To TargetScan▶ miR-150-5p:CUCCCAA▶ miR-532-3p:CUCCCAC

---

TGCAAA

TGCAAA  
Depth:9 (MOUSE)  
Ei-value:0.000, Pi-value:0.000  
Er-value:0.000, Pr-value:0.000  
No matches to TargetScan

----------------------------------------------------------------------------------- 2760  
 -

TGTATATA

TGTATATA  
Depth:9 (MOUSE)  
Ei-value:0.000, Pi-value:0.000  
Er-value:0.000, Pr-value:0.000  
No matches to TargetScan

--------------------------

ATATCTAG

ATATCTAG  
Depth:9 (MOUSE)  
Ei-value:0.000, Pi-value:0.000  
Er-value:0.000, Pr-value:0.000  
No matches to TargetScan

-

CTCTAG

CTCTAG  
Depth:9 (MOUSE)  
Ei-value:0.000, Pi-value:0.000  
Er-value:0.000, Pr-value:0.000  
No matches to TargetScan

-----

AAAGAGGTTGCCAATGTATGACA

AAAGAGGTTGCCAATGTATGACA  
Depth:9 (MOUSE)  
Ei-value:0.000, Pi-value:0.000  
Er-value:0.000, Pr-value:0.000  
MATCHES To TargetScan▶ miR-182-5p:UUGGCAA▶ miR-96-5p/1271-5p:UUGGCAC▶ miR-539-3p:UCAUACA

--------

GTTAGTAAACT

GTTAGTAAACT  
Depth:9 (MOUSE)  
Ei-value:0.000, Pi-value:0.000  
Er-value:0.000, Pr-value:0.000  
No matches to TargetScan

-

ACACATT

ACACATT  
Depth:9 (MOUSE)  
Ei-value:0.000, Pi-value:0.000  
Er-value:0.000, Pr-value:0.000  
No matches to TargetScan

--------------- 2880  
 -------------------

TGTCTTCTGAA

TGTCTTCTGAA  
Depth:9 (MOUSE)  
Ei-value:0.000, Pi-value:0.000  
Er-value:0.000, Pr-value:0.000  
No matches to TargetScan

-------------------------------------------------

GTGCCT

GTGCCT  
Depth:9 (MOUSE)  
Ei-value:0.000, Pi-value:0.000  
Er-value:0.000, Pr-value:0.000  
No matches to TargetScan

----------------------------------- 3000  
 ---------------------------------

AGAATG

AGAATG  
Depth:9 (MOUSE)  
Ei-value:0.000, Pi-value:0.000  
Er-value:0.000, Pr-value:0.000  
No matches to TargetScan

---------

AGAAGA

AGAAGA  
Depth:9 (MOUSE)  
Ei-value:0.000, Pi-value:0.000  
Er-value:0.000, Pr-value:0.000  
No matches to TargetScan

----------------------------------------------------

TGTATATA

TGTATATA  
Depth:9 (MOUSE)  
Ei-value:0.000, Pi-value:0.000  
Er-value:0.000, Pr-value:0.000  
No matches to TargetScan

------ 3120  
 -----------------

TGAAACATCTAG

TGAAACATCTAG  
Depth:9 (MOUSE)  
Ei-value:0.000, Pi-value:0.000  
Er-value:0.000, Pr-value:0.000  
No matches to TargetScan

---

TTCTAG

TTCTAG  
Depth:9 (MOUSE)  
Ei-value:0.000, Pi-value:0.000  
Er-value:0.000, Pr-value:0.000  
No matches to TargetScan

-

TGTTTAGAAGTGCACAAAGTATGTTAAAAGTAGA

TGTTTAGAAGTGCACAAAGTATGTTAAAAGTAGA  
Depth:9 (MOUSE)  
Ei-value:0.000, Pi-value:0.000  
Er-value:0.000, Pr-value:0.000  
No matches to TargetScan

----------------------------------------------- 3240  
 ------------------------------------------------------------------------------------------------------------------------ 3360  
 --------------------------------------------------------

TGTATA

TGTATA  
Depth:9 (MOUSE)  
Ei-value:0.000, Pi-value:0.000  
Er-value:0.000, Pr-value:0.000  
No matches to TargetScan

----------------------------------------

AGTCTT

AGTCTT  
Depth:9 (MOUSE)  
Ei-value:0.000, Pi-value:0.000  
Er-value:0.000, Pr-value:0.000  
No matches to TargetScan

------------ 3480  
 --------------------------------------------------------------------------------------------------

AATTGTTA

AATTGTTA  
Depth:9 (MOUSE)  
Ei-value:0.000, Pi-value:0.000  
Er-value:0.000, Pr-value:0.000  
No matches to TargetScan

-------------- 3600  
 ------------------------------------------------------------------------------------------------------------------------ 3720  
 ------------------------------------------------------------------------------------------------------------------------ 3840  
 ------------------------------------------------------------------------------------------------------------------------ 3960  
 ------------------------------------------------------------------------------------------------------------------------ 4080  
 ------------------------------------------------------------------------------------------------------------------------ 4200  
 ------------------------------------------------------------------------------------------------------------------------ 4320  
 ------------------------------------------------------------------------------------------------------------------------ 4440  
 ------------------------------------------------------------------------------------------------------------------------ 4560  
 ------------------------------------------------------------------------------------------------------------------------ 4680  
 ------------------------------------------------------------------------------------------------------------------------ 4800  
 ----------------------------------------------

ATTTTTGT

ATTTTTGT  
Depth:9 (MOUSE)  
Ei-value:0.000, Pi-value:0.000  
Er-value:0.000, Pr-value:0.000  
No matches to TargetScan

------------------------------------------------------------------ 4920  
 -----

TTCTTGGT

TTCTTGGT  
Depth:9 (MOUSE)  
Ei-value:0.000, Pi-value:0.000  
Er-value:0.000, Pr-value:0.000  
No matches to TargetScan

--------------------------------------

AATATTCA

AATATTCA  
Depth:9 (MOUSE)  
Ei-value:0.000, Pi-value:0.000  
Er-value:0.000, Pr-value:0.000  
No matches to TargetScan

------------------------------------------------------------- 5040  
 -----------------------------------------------

CTGCTT

CTGCTT  
Depth:9 (MOUSE)  
Ei-value:0.000, Pi-value:0.000  
Er-value:0.000, Pr-value:0.000  
No matches to TargetScan

-----------

TGACTT

TGACTT  
Depth:9 (MOUSE)  
Ei-value:0.000, Pi-value:0.000  
Er-value:0.000, Pr-value:0.000  
MATCHES To TargetScan▶ miR-224-5p:AAGUCAC

------

TTGCTT

TTGCTT  
Depth:9 (MOUSE)  
Ei-value:0.000, Pi-value:0.000  
Er-value:0.000, Pr-value:0.010  
No matches to TargetScan

-------------------------------------- 5160  
 ------

AAATAAA

AAATAAA  
Depth:9 (MOUSE)  
Ei-value:0.000, Pi-value:0.000  
Er-value:0.000, Pr-value:0.000  
No matches to TargetScan

-------------------------------------------------------------------------------------                       5258
```

---

## >SHEEP (4921 bases)

```
 -------------------------------------------------

TGCCAA

TGCCAA  
Depth:9 (MOUSE)  
Ei-value:0.000, Pi-value:0.000  
Er-value:0.000, Pr-value:0.000  
MATCHES To TargetScan▶ miR-182-5p:UUGGCAA▶ miR-96-5p/1271-5p:UUGGCAC

-

TCAGTTCCGG

TCAGTTCCGG  
Depth:9 (MOUSE)  
Ei-value:0.000, Pi-value:0.000  
Er-value:0.000, Pr-value:0.000  
No matches to TargetScan

----------------------------------------------------

GC

GCCCTC  
Depth:9 (MOUSE)  
Ei-value:0.000, Pi-value:0.000  
Er-value:0.000, Pr-value:0.000  
No matches to TargetScan

 120  


CCTC

GCCCTC  
Depth:9 (MOUSE)  
Ei-value:0.000, Pi-value:0.000  
Er-value:0.000, Pr-value:0.000  
No matches to TargetScan

-------------------------------------------------------------------------------------------------------------------- 240  
 ------------------------------------

ATGGCGGC

ATGGCGGC  
Depth:9 (MOUSE)  
Ei-value:0.000, Pi-value:0.000  
Er-value:0.000, Pr-value:0.000  
No matches to TargetScan

---------------------------------------------------------------------------- 360  
 ------------------------------------------------------------------------------------------------------------------------ 480  
 ------------------------------------------------------------------------------------------------------------------------ 600  
 ---------------

GGGAAGA

GGGAAGA  
Depth:9 (MOUSE)  
Ei-value:0.000, Pi-value:0.000  
Er-value:0.000, Pr-value:0.000  
No matches to TargetScan

---------------------

CCTGTGTATATAATATGAAAAAGCTGCT

CCTGTGTATATAATATGAAAAAGCTGCT  
Depth:9 (MOUSE)  
Ei-value:0.000, Pi-value:0.000  
Er-value:0.000, Pr-value:0.000  
MATCHES To TargetScan▶ miR-15-5p/16-5p/195-5p/424-5p/497-5p:AGCAGCA▶ miR-503-5p:AGCAGCG

--------------

CAACCTTT

CAACCTTT  
Depth:9 (MOUSE)  
Ei-value:0.000, Pi-value:0.000  
Er-value:0.000, Pr-value:0.000  
No matches to TargetScan

--------------------------- 720  
 --------------

AGAGGT

AGAGGT  
Depth:9 (MOUSE)  
Ei-value:0.000, Pi-value:0.000  
Er-value:0.000, Pr-value:0.000  
No matches to TargetScan

--------------------------------------------------------------------------------------------

GAAATG

GAAATG  
Depth:9 (MOUSE)  
Ei-value:0.000, Pi-value:0.000  
Er-value:0.000, Pr-value:0.000  
No matches to TargetScan

-- 840  
 ------------------------------------------------------------------------------------------------------------------------ 960  
 ------------------------

AGAAGA

AGAAGA  
Depth:9 (MOUSE)  
Ei-value:0.000, Pi-value:0.000  
Er-value:0.000, Pr-value:0.000  
No matches to TargetScan

------------------------------------------------------------------------------------------ 1080  
 -----------------

AAGTGCCTG

AAGTGCCTG  
Depth:9 (MOUSE)  
Ei-value:0.000, Pi-value:0.000  
Er-value:0.000, Pr-value:0.000  
No matches to TargetScan

---------------------------------------------------------------------------------------------- 1200  
 -------------------------------------------------------

TTGTGC

TTGTGC  
Depth:9 (MOUSE)  
Ei-value:0.000, Pi-value:0.000  
Er-value:0.000, Pr-value:0.000  
No matches to TargetScan

----------------------------------------------------------- 1320  
 ------------------------------------------------------------------------------------------------------------------

GATGTT

GATGTT  
Depth:9 (MOUSE)  
Ei-value:0.000, Pi-value:0.000  
Er-value:0.000, Pr-value:0.000  
No matches to TargetScan

 1440  


GATGTT  
Depth:9 (MOUSE)  
Ei-value:0.000, Pi-value:0.000  
Er-value:0.000, Pr-value:0.000  
No matches to TargetScan


AAAGAGGTTGCCA

AAAGAGGTTGCCA  
Depth:9 (MOUSE)  
Ei-value:0.000, Pi-value:0.000  
Er-value:0.000, Pr-value:0.000  
No matches to TargetScan

------------------------------------------

ATTTTGT

ATTTTGT  
Depth:9 (MOUSE)  
Ei-value:0.000, Pi-value:0.000  
Er-value:0.000, Pr-value:0.000  
No matches to TargetScan

---------------------------------------------------------- 1560  
 ------------------------------------------------------------------------------------------------------------------------ 1680  
 ------------------

TCCTTA

TCCTTA  
Depth:9 (MOUSE)  
Ei-value:0.000, Pi-value:0.000  
Er-value:0.000, Pr-value:0.000  
No matches to TargetScan

----

AAACATCT

AAACATCT  
Depth:9 (MOUSE)  
Ei-value:0.000, Pi-value:0.000  
Er-value:0.000, Pr-value:0.000  
No matches to TargetScan

-------

CTAGATGTTTAGAAGTGCCC

CTAGATGTTTAGAAGTGCCC  
Depth:9 (MOUSE)  
Ei-value:0.000, Pi-value:0.000  
Er-value:0.000, Pr-value:0.000  
No matches to TargetScan

---

GTATGTTAAA

GTATGTTAAA  
Depth:9 (MOUSE)  
Ei-value:0.000, Pi-value:0.000  
Er-value:0.000, Pr-value:0.000  
No matches to TargetScan

------------------------

TGTAAATA

TGTAAATA  
Depth:9 (MOUSE)  
Ei-value:0.000, Pi-value:0.000  
Er-value:0.000, Pr-value:0.000  
No matches to TargetScan

------------ 1800  
 ------------------------------------------------------------------------------------------------------------------------ 1920  
 ------------------------------------------------------------------------------------------------------------------------ 2040  
 ------------------------------------------------------------------------------------------------------------------------ 2160  
 ------------------------------------------------------------------------------------------------------------------------ 2280  
 ------------------------------------------------------------------------------------------------------------------------ 2400  
 ------------------------------------------------------------------------------------------------------------------------ 2520  
 ----------------------

GTGGGAGGGAA

GTGGGAGGGAA  
Depth:9 (MOUSE)  
Ei-value:0.000, Pi-value:0.000  
Er-value:0.000, Pr-value:0.000  
MATCHES To TargetScan▶ miR-150-5p:CUCCCAA▶ miR-532-3p:CUCCCAC

---

TGCAAA

TGCAAA  
Depth:9 (MOUSE)  
Ei-value:0.000, Pi-value:0.000  
Er-value:0.000, Pr-value:0.000  
No matches to TargetScan

------------------------------------------------------------------------------ 2640  
 -----------

TGTATATA

TGTATATA  
Depth:9 (MOUSE)  
Ei-value:0.000, Pi-value:0.000  
Er-value:0.000, Pr-value:0.000  
No matches to TargetScan

--------------------------

ATATCTAG

ATATCTAG  
Depth:9 (MOUSE)  
Ei-value:0.000, Pi-value:0.000  
Er-value:0.000, Pr-value:0.000  
No matches to TargetScan

-

CTCTAG

CTCTAG  
Depth:9 (MOUSE)  
Ei-value:0.000, Pi-value:0.000  
Er-value:0.000, Pr-value:0.000  
No matches to TargetScan

-----

AAAGAGGTTGCCAATGTATGACA

AAAGAGGTTGCCAATGTATGACA  
Depth:9 (MOUSE)  
Ei-value:0.000, Pi-value:0.000  
Er-value:0.000, Pr-value:0.000  
MATCHES To TargetScan▶ miR-182-5p:UUGGCAA▶ miR-96-5p/1271-5p:UUGGCAC▶ miR-539-3p:UCAUACA

--------

GTTAGTAAACT

GTTAGTAAACT  
Depth:9 (MOUSE)  
Ei-value:0.000, Pi-value:0.000  
Er-value:0.000, Pr-value:0.000  
No matches to TargetScan

-

ACACATT

ACACATT  
Depth:9 (MOUSE)  
Ei-value:0.000, Pi-value:0.000  
Er-value:0.000, Pr-value:0.000  
No matches to TargetScan

----- 2760  
 -----------------------------

TGTCTTCTGAA

TGTCTTCTGAA  
Depth:9 (MOUSE)  
Ei-value:0.000, Pi-value:0.000  
Er-value:0.000, Pr-value:0.000  
No matches to TargetScan

-------------------------------------------------

GTGCCT

GTGCCT  
Depth:9 (MOUSE)  
Ei-value:0.000, Pi-value:0.000  
Er-value:0.000, Pr-value:0.000  
No matches to TargetScan

------------------------- 2880  
 ------------------------------------------

AGAATG

AGAATG  
Depth:9 (MOUSE)  
Ei-value:0.000, Pi-value:0.000  
Er-value:0.000, Pr-value:0.000  
No matches to TargetScan

---------

AGAAGA

AGAAGA  
Depth:9 (MOUSE)  
Ei-value:0.000, Pi-value:0.000  
Er-value:0.000, Pr-value:0.000  
No matches to TargetScan

----------------------------------------------------

TGTAT

TGTATATA  
Depth:9 (MOUSE)  
Ei-value:0.000, Pi-value:0.000  
Er-value:0.000, Pr-value:0.000  
No matches to TargetScan

 3000  


ATA

TGTATATA  
Depth:9 (MOUSE)  
Ei-value:0.000, Pi-value:0.000  
Er-value:0.000, Pr-value:0.000  
No matches to TargetScan

---------------------

TGAAACATCTAG

TGAAACATCTAG  
Depth:9 (MOUSE)  
Ei-value:0.000, Pi-value:0.000  
Er-value:0.000, Pr-value:0.000  
No matches to TargetScan

---

TTCTAG

TTCTAG  
Depth:9 (MOUSE)  
Ei-value:0.000, Pi-value:0.000  
Er-value:0.000, Pr-value:0.000  
No matches to TargetScan

-

TGTTTAGAAGTGCACAAAGTATGTTAAAAGTAGA

TGTTTAGAAGTGCACAAAGTATGTTAAAAGTAGA  
Depth:9 (MOUSE)  
Ei-value:0.000, Pi-value:0.000  
Er-value:0.000, Pr-value:0.000  
No matches to TargetScan

---------------------------------------- 3120  
 ------------------------------------------------------------------------------------------------------------------------ 3240  
 -------------------------------------------------------------

TGTATA

TGTATA  
Depth:9 (MOUSE)  
Ei-value:0.000, Pi-value:0.000  
Er-value:0.000, Pr-value:0.000  
No matches to TargetScan

----------------------------------------

AGTCTT

AGTCTT  
Depth:9 (MOUSE)  
Ei-value:0.000, Pi-value:0.000  
Er-value:0.000, Pr-value:0.000  
No matches to TargetScan

------- 3360  
 ----------------------------------------------------------------------------------------------------------

AATTGTTA

AATTGTTA  
Depth:9 (MOUSE)  
Ei-value:0.000, Pi-value:0.000  
Er-value:0.000, Pr-value:0.000  
No matches to TargetScan

------ 3480  
 ------------------------------------------------------------------------------------------------------------------------ 3600  
 ------------------------------------------------------------------------------------------------------------------------ 3720  
 ------------------------------------------------------------------------------------------------------------------------ 3840  
 ------------------------------------------------------------------------------------------------------------------------ 3960  
 ------------------------------------------------------------------------------------------------------------------------ 4080  
 ------------------------------------------------------------------------------------------------------------------------ 4200  
 ------------------------------------------------------------------------------------------------------------------------ 4320  
 ------------------------------------------------------------------------------------------------------------------------ 4440  
 ------------------------------------------------------------------------------------------------------------------------ 4560  
 -------

ATTTTTGT

ATTTTTGT  
Depth:9 (MOUSE)  
Ei-value:0.000, Pi-value:0.000  
Er-value:0.000, Pr-value:0.000  
No matches to TargetScan

-----------------------------------------------------------------------

TTCTTGGT

TTCTTGGT  
Depth:9 (MOUSE)  
Ei-value:0.000, Pi-value:0.000  
Er-value:0.000, Pr-value:0.000  
No matches to TargetScan

-------------------------- 4680  
 ------------

AATATTCA

AATATTCA  
Depth:9 (MOUSE)  
Ei-value:0.000, Pi-value:0.000  
Er-value:0.000, Pr-value:0.000  
No matches to TargetScan

---------------------------------------------------------------------------------------------------- 4800  
 -----------

CTGCTT

CTGCTT  
Depth:9 (MOUSE)  
Ei-value:0.000, Pi-value:0.000  
Er-value:0.000, Pr-value:0.000  
No matches to TargetScan

-----------

TGACTT

TGACTT  
Depth:9 (MOUSE)  
Ei-value:0.000, Pi-value:0.000  
Er-value:0.000, Pr-value:0.000  
MATCHES To TargetScan▶ miR-224-5p:AAGUCAC

------

TTGCTT

TTGCTT  
Depth:9 (MOUSE)  
Ei-value:0.000, Pi-value:0.000  
Er-value:0.000, Pr-value:0.010  
No matches to TargetScan

--------------------------------------------

AAATAAA

AAATAAA  
Depth:9 (MOUSE)  
Ei-value:0.000, Pi-value:0.000  
Er-value:0.000, Pr-value:0.000  
No matches to TargetScan

----------------------- 4920  
 -                                                                                                                        4921
```

---

## >PIG (5203 bases)

```
 ------------------------------------------------------------------------------------------------------------------------ 120  
 ----------------

TGCCAA

TGCCAA  
Depth:9 (MOUSE)  
Ei-value:0.000, Pi-value:0.000  
Er-value:0.000, Pr-value:0.000  
MATCHES To TargetScan▶ miR-182-5p:UUGGCAA▶ miR-96-5p/1271-5p:UUGGCAC

-

TCAGTTCCGG

TCAGTTCCGG  
Depth:9 (MOUSE)  
Ei-value:0.000, Pi-value:0.000  
Er-value:0.000, Pr-value:0.000  
No matches to TargetScan

-------------------------------------------

GCCCTC

GCCCTC  
Depth:9 (MOUSE)  
Ei-value:0.000, Pi-value:0.000  
Er-value:0.000, Pr-value:0.000  
No matches to TargetScan

-------------------------------------- 240  
 -----------------------------------------------------------------------------------------------------------------

ATGGCGG

ATGGCGGC  
Depth:9 (MOUSE)  
Ei-value:0.000, Pi-value:0.000  
Er-value:0.000, Pr-value:0.000  
No matches to TargetScan

 360  


C

ATGGCGGC  
Depth:9 (MOUSE)  
Ei-value:0.000, Pi-value:0.000  
Er-value:0.000, Pr-value:0.000  
No matches to TargetScan

--------------------------------------------------------------------------------------------------------------------

ATG

ATGGCGGC  
Depth:9 (MOUSE)  
Ei-value:0.000, Pi-value:0.000  
Er-value:0.000, Pr-value:0.000  
No matches to TargetScan

 480  


GCGGC

ATGGCGGC  
Depth:9 (MOUSE)  
Ei-value:0.000, Pi-value:0.000  
Er-value:0.000, Pr-value:0.000  
No matches to TargetScan

------------------------------------------------------------------------------------------------------------------- 600  
 ------------------------------------------------------------------------------------------------------------------------ 720  
 ---------------------------------------------------------------------

GGGAAGA

GGGAAGA  
Depth:9 (MOUSE)  
Ei-value:0.000, Pi-value:0.000  
Er-value:0.000, Pr-value:0.000  
No matches to TargetScan

----------------------

CCTGTGTATATAATATGAAAAA

CCTGTGTATATAATATGAAAAAGCTGCT  
Depth:9 (MOUSE)  
Ei-value:0.000, Pi-value:0.000  
Er-value:0.000, Pr-value:0.000  
MATCHES To TargetScan▶ miR-15-5p/16-5p/195-5p/424-5p/497-5p:AGCAGCA▶ miR-503-5p:AGCAGCG

 840  


GCTGCT

CCTGTGTATATAATATGAAAAAGCTGCT  
Depth:9 (MOUSE)  
Ei-value:0.000, Pi-value:0.000  
Er-value:0.000, Pr-value:0.000  
MATCHES To TargetScan▶ miR-15-5p/16-5p/195-5p/424-5p/497-5p:AGCAGCA▶ miR-503-5p:AGCAGCG

--------------

CAACCTTT

CAACCTTT  
Depth:9 (MOUSE)  
Ei-value:0.000, Pi-value:0.000  
Er-value:0.000, Pr-value:0.000  
No matches to TargetScan

------------------------------------------

AGAGGT

AGAGGT  
Depth:9 (MOUSE)  
Ei-value:0.000, Pi-value:0.000  
Er-value:0.000, Pr-value:0.000  
No matches to TargetScan

-------------------------------------------- 960  
 --------------------------------------------------------

GAAATG

GAAATG  
Depth:9 (MOUSE)  
Ei-value:0.000, Pi-value:0.000  
Er-value:0.000, Pr-value:0.000  
No matches to TargetScan

---------------------------------------------------------- 1080  
 --------------------------------------------------------------------------------

AGAAGA

AGAAGA  
Depth:9 (MOUSE)  
Ei-value:0.000, Pi-value:0.000  
Er-value:0.000, Pr-value:0.000  
No matches to TargetScan

---------------------------------- 1200  
 --------------------------------------------------------------------------

AAGTGCCTG

AAGTGCCTG  
Depth:9 (MOUSE)  
Ei-value:0.000, Pi-value:0.000  
Er-value:0.000, Pr-value:0.000  
No matches to TargetScan

------------------------------------- 1320  
 --------------------------------------------------------------------------------------------------------

TTGTGC

TTGTGC  
Depth:9 (MOUSE)  
Ei-value:0.000, Pi-value:0.000  
Er-value:0.000, Pr-value:0.000  
No matches to TargetScan

---------- 1440  
 ------------------------------------------------------------------------------------------------------------------------ 1560  
 ----------------------------------------

GATGTT

GATGTT  
Depth:9 (MOUSE)  
Ei-value:0.000, Pi-value:0.000  
Er-value:0.000, Pr-value:0.000  
No matches to TargetScan


AAAGAGGTTGCCA

AAAGAGGTTGCCA  
Depth:9 (MOUSE)  
Ei-value:0.000, Pi-value:0.000  
Er-value:0.000, Pr-value:0.000  
No matches to TargetScan

--------------------------------------------

ATTTTGT

ATTTTGT  
Depth:9 (MOUSE)  
Ei-value:0.000, Pi-value:0.000  
Er-value:0.000, Pr-value:0.000  
No matches to TargetScan

---------- 1680  
 ------------------------------------------------------------------------------------------------------------------------ 1800  
 -------------------------------------

TCCTTA

TCCTTA  
Depth:9 (MOUSE)  
Ei-value:0.000, Pi-value:0.000  
Er-value:0.000, Pr-value:0.000  
No matches to TargetScan

----

AAACATCT

AAACATCT  
Depth:9 (MOUSE)  
Ei-value:0.000, Pi-value:0.000  
Er-value:0.000, Pr-value:0.000  
No matches to TargetScan

-------

CTAGATGTTTAGAAGTGCCC

CTAGATGTTTAGAAGTGCCC  
Depth:9 (MOUSE)  
Ei-value:0.000, Pi-value:0.000  
Er-value:0.000, Pr-value:0.000  
No matches to TargetScan

---

GTATGTTAAA

GTATGTTAAA  
Depth:9 (MOUSE)  
Ei-value:0.000, Pi-value:0.000  
Er-value:0.000, Pr-value:0.000  
No matches to TargetScan

------------------------

T

TGTAAATA  
Depth:9 (MOUSE)  
Ei-value:0.000, Pi-value:0.000  
Er-value:0.000, Pr-value:0.000  
No matches to TargetScan

 1920  


GTAAATA

TGTAAATA  
Depth:9 (MOUSE)  
Ei-value:0.000, Pi-value:0.000  
Er-value:0.000, Pr-value:0.000  
No matches to TargetScan

----------------------------------------------------------------------------------------------------------------- 2040  
 ------------------------------------------------------------------------------------------------------------------------ 2160  
 ------------------------------------------------------------------------------------------------------------------------ 2280  
 ------------------------------------------------------------------------------------------------------------------------ 2400  
 ------------------------------------------------------------------------------------------------------------------------ 2520  
 ------------------------------------------------------------------------------------------------------------------------ 2640  
 ------------

GTGGGAGGGAA

GTGGGAGGGAA  
Depth:9 (MOUSE)  
Ei-value:0.000, Pi-value:0.000  
Er-value:0.000, Pr-value:0.000  
MATCHES To TargetScan▶ miR-150-5p:CUCCCAA▶ miR-532-3p:CUCCCAC

---

TGCAAA

TGCAAA  
Depth:9 (MOUSE)  
Ei-value:0.000, Pi-value:0.000  
Er-value:0.000, Pr-value:0.000  
No matches to TargetScan

------------------------------------------------------------------------------

TGTATATA

TGTATATA  
Depth:9 (MOUSE)  
Ei-value:0.000, Pi-value:0.000  
Er-value:0.000, Pr-value:0.000  
No matches to TargetScan

-- 2760  
 -

TGTATATA

TGTATATA  
Depth:9 (MOUSE)  
Ei-value:0.000, Pi-value:0.000  
Er-value:0.000, Pr-value:0.000  
No matches to TargetScan

--------------------------

ATATCTAG

ATATCTAG  
Depth:9 (MOUSE)  
Ei-value:0.000, Pi-value:0.000  
Er-value:0.000, Pr-value:0.000  
No matches to TargetScan

-

CTCTAG

CTCTAG  
Depth:9 (MOUSE)  
Ei-value:0.000, Pi-value:0.000  
Er-value:0.000, Pr-value:0.000  
No matches to TargetScan

-------

AAAGAGGTTGCCAATGTATGACA

AAAGAGGTTGCCAATGTATGACA  
Depth:9 (MOUSE)  
Ei-value:0.000, Pi-value:0.000  
Er-value:0.000, Pr-value:0.000  
MATCHES To TargetScan▶ miR-182-5p:UUGGCAA▶ miR-96-5p/1271-5p:UUGGCAC▶ miR-539-3p:UCAUACA

--------

GTTAGTAAACT

GTTAGTAAACT  
Depth:9 (MOUSE)  
Ei-value:0.000, Pi-value:0.000  
Er-value:0.000, Pr-value:0.000  
No matches to TargetScan

-

ACACATT

ACACATT  
Depth:9 (MOUSE)  
Ei-value:0.000, Pi-value:0.000  
Er-value:0.000, Pr-value:0.000  
No matches to TargetScan

------------- 2880  
 -------------------

TGTCTTCTGAA

TGTCTTCTGAA  
Depth:9 (MOUSE)  
Ei-value:0.000, Pi-value:0.000  
Er-value:0.000, Pr-value:0.000  
No matches to TargetScan

----------------------------------------------

GTGCCT

GTGCCT  
Depth:9 (MOUSE)  
Ei-value:0.000, Pi-value:0.000  
Er-value:0.000, Pr-value:0.000  
No matches to TargetScan

-------------------------------------- 3000  
 ------------------------------

AGAATG

AGAATG  
Depth:9 (MOUSE)  
Ei-value:0.000, Pi-value:0.000  
Er-value:0.000, Pr-value:0.000  
No matches to TargetScan

---------

AGAAGA

AGAAGA  
Depth:9 (MOUSE)  
Ei-value:0.000, Pi-value:0.000  
Er-value:0.000, Pr-value:0.000  
No matches to TargetScan

----------------------------------------------------

TGTATATA

TGTATATA  
Depth:9 (MOUSE)  
Ei-value:0.000, Pi-value:0.000  
Er-value:0.000, Pr-value:0.000  
No matches to TargetScan

--------- 3120  
 ----------------

TGAAACATCTAG

TGAAACATCTAG  
Depth:9 (MOUSE)  
Ei-value:0.000, Pi-value:0.000  
Er-value:0.000, Pr-value:0.000  
No matches to TargetScan

---

TTCTAG

TTCTAG  
Depth:9 (MOUSE)  
Ei-value:0.000, Pi-value:0.000  
Er-value:0.000, Pr-value:0.000  
No matches to TargetScan

-

TGTTTAGAAGTGCACAAAGTATGTTAAAAGTAGA

TGTTTAGAAGTGCACAAAGTATGTTAAAAGTAGA  
Depth:9 (MOUSE)  
Ei-value:0.000, Pi-value:0.000  
Er-value:0.000, Pr-value:0.000  
No matches to TargetScan

------------------------------------------------ 3240  
 ------------------------------------------------------------------------------------------------------------------------ 3360  
 --------------------------------------------------------

TGTATA

TGTATA  
Depth:9 (MOUSE)  
Ei-value:0.000, Pi-value:0.000  
Er-value:0.000, Pr-value:0.000  
No matches to TargetScan

---------------------------------------------------------- 3480  
 ------------------------------------------------------------------------------------------------------------------------ 3600  
 ------------------------------------------------------------------------------------------------------------------------ 3720  
 -------------------------------------------------------

AGTCTT

AGTCTT  
Depth:9 (MOUSE)  
Ei-value:0.000, Pi-value:0.000  
Er-value:0.000, Pr-value:0.000  
No matches to TargetScan

----------------------------------------------------------- 3840  
 ------------------------------------------------------------------------------------------------------------------------ 3960  
 ------------------------------------------------------------------------------------------------------------------------ 4080  
 ------------------------------------------------------------------------------------------------------------------------ 4200  
 ------------------------------------------------------------------------------------------------------------------------ 4320  
 ------------------------------------------------------------------------------------------------------------------------ 4440  
 ---------------------------------------------

AATTGTTA

AATTGTTA  
Depth:9 (MOUSE)  
Ei-value:0.000, Pi-value:0.000  
Er-value:0.000, Pr-value:0.000  
No matches to TargetScan

------------------------------------------------------------------- 4560  
 ------------------------------------------------------------------------------------------------------------------------ 4680  
 ------------------------------------------------------------------------------------------------------------------------ 4800  
 ----------------------

ATTTTTGT

ATTTTTGT  
Depth:9 (MOUSE)  
Ei-value:0.000, Pi-value:0.000  
Er-value:0.000, Pr-value:0.000  
No matches to TargetScan

--------------------------------------------------------------------------

TTCTTGGT

TTCTTGGT  
Depth:9 (MOUSE)  
Ei-value:0.000, Pi-value:0.000  
Er-value:0.000, Pr-value:0.000  
No matches to TargetScan

-------- 4920  
 -------------------------------------

AATATTCA

AATATTCA  
Depth:9 (MOUSE)  
Ei-value:0.000, Pi-value:0.000  
Er-value:0.000, Pr-value:0.000  
No matches to TargetScan

--------------------------------------------------------------------------- 5040  
 ----------------------------------

CTGCTT

CTGCTT  
Depth:9 (MOUSE)  
Ei-value:0.000, Pi-value:0.000  
Er-value:0.000, Pr-value:0.000  
No matches to TargetScan

---------

TGACTT

TGACTT  
Depth:9 (MOUSE)  
Ei-value:0.000, Pi-value:0.000  
Er-value:0.000, Pr-value:0.000  
MATCHES To TargetScan▶ miR-224-5p:AAGUCAC

-----------------

TTGCTT

TTGCTT  
Depth:9 (MOUSE)  
Ei-value:0.000, Pi-value:0.000  
Er-value:0.000, Pr-value:0.010  
No matches to TargetScan

-----------------------------------------

A

AAATAAA  
Depth:9 (MOUSE)  
Ei-value:0.000, Pi-value:0.000  
Er-value:0.000, Pr-value:0.000  
No matches to TargetScan

 5160  


AATAAA

AAATAAA  
Depth:9 (MOUSE)  
Ei-value:0.000, Pi-value:0.000  
Er-value:0.000, Pr-value:0.000  
No matches to TargetScan

-------------------------------------                                                                              5203
```

---

## >ARMADILLO (5039 bases)

```
 --------------------------------------------------------

TGCCAA

TGCCAA  
Depth:9 (MOUSE)  
Ei-value:0.000, Pi-value:0.000  
Er-value:0.000, Pr-value:0.000  
MATCHES To TargetScan▶ miR-182-5p:UUGGCAA▶ miR-96-5p/1271-5p:UUGGCAC

-

TCAGTTCCGG

TCAGTTCCGG  
Depth:9 (MOUSE)  
Ei-value:0.000, Pi-value:0.000  
Er-value:0.000, Pr-value:0.000  
No matches to TargetScan

------------------------------------------

GCCCT

GCCCTC  
Depth:9 (MOUSE)  
Ei-value:0.000, Pi-value:0.000  
Er-value:0.000, Pr-value:0.000  
No matches to TargetScan

 120  


C

GCCCTC  
Depth:9 (MOUSE)  
Ei-value:0.000, Pi-value:0.000  
Er-value:0.000, Pr-value:0.000  
No matches to TargetScan

----------------------------------------------------------------------------------------------------------------------- 240  
 ------------------------------------------------------------------------------------------------------------------------ 360  
 ------------------------------------------------------------------

ATGGCGGC

ATGGCGGC  
Depth:9 (MOUSE)  
Ei-value:0.000, Pi-value:0.000  
Er-value:0.000, Pr-value:0.000  
No matches to TargetScan

---------------------------------------------- 480  
 ------------------------------------------------------------------------------------------------------------------------ 600  
 ------------------------------------------------------------------------------------------------------------------------ 720  
 -----------------------------------------------------------

GGGAAGA

GGGAAGA  
Depth:9 (MOUSE)  
Ei-value:0.000, Pi-value:0.000  
Er-value:0.000, Pr-value:0.000  
No matches to TargetScan

----------------------

CCTGTGTATATAATATGAAAAAGCTGCT

CCTGTGTATATAATATGAAAAAGCTGCT  
Depth:9 (MOUSE)  
Ei-value:0.000, Pi-value:0.000  
Er-value:0.000, Pr-value:0.000  
MATCHES To TargetScan▶ miR-15-5p/16-5p/195-5p/424-5p/497-5p:AGCAGCA▶ miR-503-5p:AGCAGCG

---- 840  
 ----------

CAACCTTT

CAACCTTT  
Depth:9 (MOUSE)  
Ei-value:0.000, Pi-value:0.000  
Er-value:0.000, Pr-value:0.000  
No matches to TargetScan

-----------------------------------------

AGAGGT

AGAGGT  
Depth:9 (MOUSE)  
Ei-value:0.000, Pi-value:0.000  
Er-value:0.000, Pr-value:0.000  
No matches to TargetScan

------------------------------------------------------- 960  
 -------------------------------

GAAATG

GAAATG  
Depth:9 (MOUSE)  
Ei-value:0.000, Pi-value:0.000  
Er-value:0.000, Pr-value:0.000  
No matches to TargetScan

----------------------------------------------------------------------------------- 1080  
 ------------------------------------------------------

AGAAGA

AGAAGA  
Depth:9 (MOUSE)  
Ei-value:0.000, Pi-value:0.000  
Er-value:0.000, Pr-value:0.000  
No matches to TargetScan

------------------------------------------------------------ 1200  
 -------------------------------------------------

AAGTGCCTG

AAGTGCCTG  
Depth:9 (MOUSE)  
Ei-value:0.000, Pi-value:0.000  
Er-value:0.000, Pr-value:0.000  
No matches to TargetScan

-------------------------------------------------------------- 1320  
 -------------------------------------------------------------

TTGTGC

TTGTGC  
Depth:9 (MOUSE)  
Ei-value:0.000, Pi-value:0.000  
Er-value:0.000, Pr-value:0.000  
No matches to TargetScan

----------------------------------------------------- 1440  
 ---------------------------------------------------------------------------------------------------------------

GATGTT

GATGTT  
Depth:9 (MOUSE)  
Ei-value:0.000, Pi-value:0.000  
Er-value:0.000, Pr-value:0.000  
No matches to TargetScan

-

AA

AAAGAGGTTGCCA  
Depth:9 (MOUSE)  
Ei-value:0.000, Pi-value:0.000  
Er-value:0.000, Pr-value:0.000  
No matches to TargetScan

 1560  


AGAGGTTGCCA

AAAGAGGTTGCCA  
Depth:9 (MOUSE)  
Ei-value:0.000, Pi-value:0.000  
Er-value:0.000, Pr-value:0.000  
No matches to TargetScan

-----------------------

ATTTTGT

ATTTTGT  
Depth:9 (MOUSE)  
Ei-value:0.000, Pi-value:0.000  
Er-value:0.000, Pr-value:0.000  
No matches to TargetScan

------------------------------------------------------------------------------- 1680  
 --------------------------------------------------------------------------------------------------------------

TCCTTA

TCCTTA  
Depth:9 (MOUSE)  
Ei-value:0.000, Pi-value:0.000  
Er-value:0.000, Pr-value:0.000  
No matches to TargetScan

---- 1800  


AAACATCT

AAACATCT  
Depth:9 (MOUSE)  
Ei-value:0.000, Pi-value:0.000  
Er-value:0.000, Pr-value:0.000  
No matches to TargetScan

-------

CTAGATGTTTAGAAGTGCCC

CTAGATGTTTAGAAGTGCCC  
Depth:9 (MOUSE)  
Ei-value:0.000, Pi-value:0.000  
Er-value:0.000, Pr-value:0.000  
No matches to TargetScan

---

GTATGTTAAA

GTATGTTAAA  
Depth:9 (MOUSE)  
Ei-value:0.000, Pi-value:0.000  
Er-value:0.000, Pr-value:0.000  
No matches to TargetScan

------------------------

TGTAAATA

TGTAAATA  
Depth:9 (MOUSE)  
Ei-value:0.000, Pi-value:0.000  
Er-value:0.000, Pr-value:0.000  
No matches to TargetScan

---------------------------------------- 1920  
 ------------------------------------------------------------------------------------------------------------------------ 2040  
 ------------------------------------------------------------------------------------------------------------------------ 2160  
 ------------------------------------------------------------------------------------------------------------------------ 2280  
 ------------------------------------------------------------------------------------------------------------------------ 2400  
 ------------------------------------------------------------------------------------------------------------------------ 2520  
 ----------------------------------------------------------------------

GTGGGAGGGAA

GTGGGAGGGAA  
Depth:9 (MOUSE)  
Ei-value:0.000, Pi-value:0.000  
Er-value:0.000, Pr-value:0.000  
MATCHES To TargetScan▶ miR-150-5p:CUCCCAA▶ miR-532-3p:CUCCCAC

-----

TGCAAA

TGCAAA  
Depth:9 (MOUSE)  
Ei-value:0.000, Pi-value:0.000  
Er-value:0.000, Pr-value:0.000  
No matches to TargetScan

---------------------------- 2640  
 ----------------------------------------------------------

TGTATATA

TGTATATA  
Depth:9 (MOUSE)  
Ei-value:0.000, Pi-value:0.000  
Er-value:0.000, Pr-value:0.000  
No matches to TargetScan

----------------------------

ATATCTAG

ATATCTAG  
Depth:9 (MOUSE)  
Ei-value:0.000, Pi-value:0.000  
Er-value:0.000, Pr-value:0.000  
No matches to TargetScan

-

CTCTAG

CTCTAG  
Depth:9 (MOUSE)  
Ei-value:0.000, Pi-value:0.000  
Er-value:0.000, Pr-value:0.000  
No matches to TargetScan

-----

AAAGAG

AAAGAGGTTGCCAATGTATGACA  
Depth:9 (MOUSE)  
Ei-value:0.000, Pi-value:0.000  
Er-value:0.000, Pr-value:0.000  
MATCHES To TargetScan▶ miR-182-5p:UUGGCAA▶ miR-96-5p/1271-5p:UUGGCAC▶ miR-539-3p:UCAUACA

 2760  


GTTGCCAATGTATGACA

AAAGAGGTTGCCAATGTATGACA  
Depth:9 (MOUSE)  
Ei-value:0.000, Pi-value:0.000  
Er-value:0.000, Pr-value:0.000  
MATCHES To TargetScan▶ miR-182-5p:UUGGCAA▶ miR-96-5p/1271-5p:UUGGCAC▶ miR-539-3p:UCAUACA

--------

GTTAGTAAACT

GTTAGTAAACT  
Depth:9 (MOUSE)  
Ei-value:0.000, Pi-value:0.000  
Er-value:0.000, Pr-value:0.000  
No matches to TargetScan

-

ACACATT

ACACATT  
Depth:9 (MOUSE)  
Ei-value:0.000, Pi-value:0.000  
Er-value:0.000, Pr-value:0.000  
No matches to TargetScan

---------------------------------

TGTCTTCTGAA

TGTCTTCTGAA  
Depth:9 (MOUSE)  
Ei-value:0.000, Pi-value:0.000  
Er-value:0.000, Pr-value:0.000  
No matches to TargetScan

-------------------------------- 2880  
 -------------

GTGCCT

GTGCCT  
Depth:9 (MOUSE)  
Ei-value:0.000, Pi-value:0.000  
Er-value:0.000, Pr-value:0.000  
No matches to TargetScan

---------------------------------------------------------------

AGAATG

AGAATG  
Depth:9 (MOUSE)  
Ei-value:0.000, Pi-value:0.000  
Er-value:0.000, Pr-value:0.000  
No matches to TargetScan

---------

AGAAGA

AGAAGA  
Depth:9 (MOUSE)  
Ei-value:0.000, Pi-value:0.000  
Er-value:0.000, Pr-value:0.000  
No matches to TargetScan

----------------- 3000  
 -----------------------------------

TGTATATA

TGTATATA  
Depth:9 (MOUSE)  
Ei-value:0.000, Pi-value:0.000  
Er-value:0.000, Pr-value:0.000  
No matches to TargetScan

----------------------

TGAAACATCTAG

TGAAACATCTAG  
Depth:9 (MOUSE)  
Ei-value:0.000, Pi-value:0.000  
Er-value:0.000, Pr-value:0.000  
No matches to TargetScan

---

TTCTAG

TTCTAG  
Depth:9 (MOUSE)  
Ei-value:0.000, Pi-value:0.000  
Er-value:0.000, Pr-value:0.000  
No matches to TargetScan

-

TGTTTAGAAGTGCACAAAGTATGTTAAAAGTAG

TGTTTAGAAGTGCACAAAGTATGTTAAAAGTAGA  
Depth:9 (MOUSE)  
Ei-value:0.000, Pi-value:0.000  
Er-value:0.000, Pr-value:0.000  
No matches to TargetScan

 3120  


A

TGTTTAGAAGTGCACAAAGTATGTTAAAAGTAGA  
Depth:9 (MOUSE)  
Ei-value:0.000, Pi-value:0.000  
Er-value:0.000, Pr-value:0.000  
No matches to TargetScan

----------------------------------------------------------------------------------------------------------------------- 3240  
 -----------------------------------------------------------------------------------

TGTATA

TGTATA  
Depth:9 (MOUSE)  
Ei-value:0.000, Pi-value:0.000  
Er-value:0.000, Pr-value:0.000  
No matches to TargetScan

------------------------------- 3360  
 ---

AGTCTT

AGTCTT  
Depth:9 (MOUSE)  
Ei-value:0.000, Pi-value:0.000  
Er-value:0.000, Pr-value:0.000  
No matches to TargetScan

--------------------------------------------------------------------------------------------------------------- 3480  
 ------------------------------------------------------------------------------------------------------------------------ 3600  
 ------------------------------------------------------------------------------------------------------------------------ 3720  
 ------------------------------------------------------------------------------------------------------------------------ 3840  
 ------------------------------------------------------------------------------------------------------------------------ 3960  
 ------------------------------------------------------------------------------------------------------------------------ 4080  
 ------------------------------------------------------------------------------------------------------------------------ 4200  
 ------------------------------------------------------------------------------------------------------------------------ 4320  
 ------------------------------------------------------

AATTGTTA

AATTGTTA  
Depth:9 (MOUSE)  
Ei-value:0.000, Pi-value:0.000  
Er-value:0.000, Pr-value:0.000  
No matches to TargetScan

---------------------------------------------------------- 4440  
 ------------------------------------------------------------------------------------------------------------------------ 4560  
 ------------------------------------------------------------------------------------------------------------------------ 4680  
 ---------------------

ATTTTTGT

ATTTTTGT  
Depth:9 (MOUSE)  
Ei-value:0.000, Pi-value:0.000  
Er-value:0.000, Pr-value:0.000  
No matches to TargetScan

-----------------------------------------------

TTCTTGGT

TTCTTGGT  
Depth:9 (MOUSE)  
Ei-value:0.000, Pi-value:0.000  
Er-value:0.000, Pr-value:0.000  
No matches to TargetScan

------------------------------------ 4800  
 ---

AATATTCA

AATATTCA  
Depth:9 (MOUSE)  
Ei-value:0.000, Pi-value:0.000  
Er-value:0.000, Pr-value:0.000  
No matches to TargetScan

-----------------------------------------------------------------------------------------------------------

CT

CTGCTT  
Depth:9 (MOUSE)  
Ei-value:0.000, Pi-value:0.000  
Er-value:0.000, Pr-value:0.000  
No matches to TargetScan

 4920  


GCTT

CTGCTT  
Depth:9 (MOUSE)  
Ei-value:0.000, Pi-value:0.000  
Er-value:0.000, Pr-value:0.000  
No matches to TargetScan

-----------

TGACTT

TGACTT  
Depth:9 (MOUSE)  
Ei-value:0.000, Pi-value:0.000  
Er-value:0.000, Pr-value:0.000  
MATCHES To TargetScan▶ miR-224-5p:AAGUCAC

---------

TTGCTT

TTGCTT  
Depth:9 (MOUSE)  
Ei-value:0.000, Pi-value:0.000  
Er-value:0.000, Pr-value:0.010  
No matches to TargetScan

-----------------------------------------------

AAATAAA

AAATAAA  
Depth:9 (MOUSE)  
Ei-value:0.000, Pi-value:0.000  
Er-value:0.000, Pr-value:0.000  
No matches to TargetScan

-----------------------------  5039
```

---

## >GUINEAPIG (5080 bases)

```
 -----------------------------------------------------------------

TGCCAA

TGCCAA  
Depth:9 (MOUSE)  
Ei-value:0.000, Pi-value:0.000  
Er-value:0.000, Pr-value:0.000  
MATCHES To TargetScan▶ miR-182-5p:UUGGCAA▶ miR-96-5p/1271-5p:UUGGCAC

-

TCAGTTCCGG

TCAGTTCCGG  
Depth:9 (MOUSE)  
Ei-value:0.000, Pi-value:0.000  
Er-value:0.000, Pr-value:0.000  
No matches to TargetScan

-------------------------------------- 120  
 --------------

GCCCTC

GCCCTC  
Depth:9 (MOUSE)  
Ei-value:0.000, Pi-value:0.000  
Er-value:0.000, Pr-value:0.000  
No matches to TargetScan

---------------------------------------------------------------------------------------------------- 240  
 -------------------------------------------------

ATGGCGGC

ATGGCGGC  
Depth:9 (MOUSE)  
Ei-value:0.000, Pi-value:0.000  
Er-value:0.000, Pr-value:0.000  
No matches to TargetScan

--------------------------------------------------------------- 360  
 ------------------------------------------------------------------------------------------------------------------------ 480  
 ------------------------------------------------------------------------------------------------------------------------ 600  
 ------------------------------------------------------------------------------------------------------------------------ 720  
 ---------------

GGGAAGA

GGGAAGA  
Depth:9 (MOUSE)  
Ei-value:0.000, Pi-value:0.000  
Er-value:0.000, Pr-value:0.000  
No matches to TargetScan

----------------------

CCTGTGTATATAATATGAAAAAGCTGCT

CCTGTGTATATAATATGAAAAAGCTGCT  
Depth:9 (MOUSE)  
Ei-value:0.000, Pi-value:0.000  
Er-value:0.000, Pr-value:0.000  
MATCHES To TargetScan▶ miR-15-5p/16-5p/195-5p/424-5p/497-5p:AGCAGCA▶ miR-503-5p:AGCAGCG

--------------

CAACCTTT

CAACCTTT  
Depth:9 (MOUSE)  
Ei-value:0.000, Pi-value:0.000  
Er-value:0.000, Pr-value:0.000  
No matches to TargetScan

-------------------------- 840  
 ----------

AGAGGT

AGAGGT  
Depth:9 (MOUSE)  
Ei-value:0.000, Pi-value:0.000  
Er-value:0.000, Pr-value:0.000  
No matches to TargetScan

------------------------------------------

GAAATG

GAAATG  
Depth:9 (MOUSE)  
Ei-value:0.000, Pi-value:0.000  
Er-value:0.000, Pr-value:0.000  
No matches to TargetScan

-------------------------------------------------------- 960  
 ------------------------------------------------------------------------------------------------------------------------ 1080  


AGAAGA

AGAAGA  
Depth:9 (MOUSE)  
Ei-value:0.000, Pi-value:0.000  
Er-value:0.000, Pr-value:0.000  
No matches to TargetScan

------------------------------------------------------------------------------------------------------------------ 1200  
 -----------

AAGTGCCTG

AAGTGCCTG  
Depth:9 (MOUSE)  
Ei-value:0.000, Pi-value:0.000  
Er-value:0.000, Pr-value:0.000  
No matches to TargetScan

---------------------------------------------------------------------------------------------------- 1320  
 ---------------------------

TTGTGC

TTGTGC  
Depth:9 (MOUSE)  
Ei-value:0.000, Pi-value:0.000  
Er-value:0.000, Pr-value:0.000  
No matches to TargetScan

--------------------------------------------------------------------------------------- 1440  
 ------------------------------------------------------------

GATGTT

GATGTT  
Depth:9 (MOUSE)  
Ei-value:0.000, Pi-value:0.000  
Er-value:0.000, Pr-value:0.000  
No matches to TargetScan


AAAGAGGTTGCCA

AAAGAGGTTGCCA  
Depth:9 (MOUSE)  
Ei-value:0.000, Pi-value:0.000  
Er-value:0.000, Pr-value:0.000  
No matches to TargetScan

---------------------------

ATTTTGT

ATTTTGT  
Depth:9 (MOUSE)  
Ei-value:0.000, Pi-value:0.000  
Er-value:0.000, Pr-value:0.000  
No matches to TargetScan

------- 1560  
 ------------------------------------------------------------------------------------------------------------------------ 1680  
 --------------------------------------

TCCTTA

TCCTTA  
Depth:9 (MOUSE)  
Ei-value:0.000, Pi-value:0.000  
Er-value:0.000, Pr-value:0.000  
No matches to TargetScan

----

AAACATCT

AAACATCT  
Depth:9 (MOUSE)  
Ei-value:0.000, Pi-value:0.000  
Er-value:0.000, Pr-value:0.000  
No matches to TargetScan

--------

CTAGATGTTTAGAAGTGCCC

CTAGATGTTTAGAAGTGCCC  
Depth:9 (MOUSE)  
Ei-value:0.000, Pi-value:0.000  
Er-value:0.000, Pr-value:0.000  
No matches to TargetScan

---

GTATGTTAAA

GTATGTTAAA  
Depth:9 (MOUSE)  
Ei-value:0.000, Pi-value:0.000  
Er-value:0.000, Pr-value:0.000  
No matches to TargetScan

----------------------- 1800  
 --

TGTAAATA

TGTAAATA  
Depth:9 (MOUSE)  
Ei-value:0.000, Pi-value:0.000  
Er-value:0.000, Pr-value:0.000  
No matches to TargetScan

-----------------------------------------------------------------------------------------------------------

GTG

GTGGGAGGGAA  
Depth:9 (MOUSE)  
Ei-value:0.000, Pi-value:0.000  
Er-value:0.000, Pr-value:0.000  
MATCHES To TargetScan▶ miR-150-5p:CUCCCAA▶ miR-532-3p:CUCCCAC

 1920  


GGAGGGAA

GTGGGAGGGAA  
Depth:9 (MOUSE)  
Ei-value:0.000, Pi-value:0.000  
Er-value:0.000, Pr-value:0.000  
MATCHES To TargetScan▶ miR-150-5p:CUCCCAA▶ miR-532-3p:CUCCCAC

-----

TGCAAA

TGCAAA  
Depth:9 (MOUSE)  
Ei-value:0.000, Pi-value:0.000  
Er-value:0.000, Pr-value:0.000  
No matches to TargetScan

----------------------------------------------------------------------------------------------------- 2040  
 ------------------------------------------------------------------------------------------------------------------------ 2160  
 ------------------------------------------------------------------------------------------------------------------------ 2280  
 ------------------------------------------------------------------------------------------------------------------------ 2400  
 ------------------------------------------------------------------------------------------------------------------------ 2520  
 -----------------------------------------------------------------------------------

TGTATATA

TGTATATA  
Depth:9 (MOUSE)  
Ei-value:0.000, Pi-value:0.000  
Er-value:0.000, Pr-value:0.000  
No matches to TargetScan

----------------------------- 2640  
 --------

ATATCTAG

ATATCTAG  
Depth:9 (MOUSE)  
Ei-value:0.000, Pi-value:0.000  
Er-value:0.000, Pr-value:0.000  
No matches to TargetScan

-

CTCTAG

CTCTAG  
Depth:9 (MOUSE)  
Ei-value:0.000, Pi-value:0.000  
Er-value:0.000, Pr-value:0.000  
No matches to TargetScan

-----

AAAGAGGTTGCCAATGTATGACA

AAAGAGGTTGCCAATGTATGACA  
Depth:9 (MOUSE)  
Ei-value:0.000, Pi-value:0.000  
Er-value:0.000, Pr-value:0.000  
MATCHES To TargetScan▶ miR-182-5p:UUGGCAA▶ miR-96-5p/1271-5p:UUGGCAC▶ miR-539-3p:UCAUACA

--------

GTTAGTAAACT

GTTAGTAAACT  
Depth:9 (MOUSE)  
Ei-value:0.000, Pi-value:0.000  
Er-value:0.000, Pr-value:0.000  
No matches to TargetScan

-

ACACATT

ACACATT  
Depth:9 (MOUSE)  
Ei-value:0.000, Pi-value:0.000  
Er-value:0.000, Pr-value:0.000  
No matches to TargetScan

---------------------------------

TGTCTTCTG

TGTCTTCTGAA  
Depth:9 (MOUSE)  
Ei-value:0.000, Pi-value:0.000  
Er-value:0.000, Pr-value:0.000  
No matches to TargetScan

 2760  


AA

TGTCTTCTGAA  
Depth:9 (MOUSE)  
Ei-value:0.000, Pi-value:0.000  
Er-value:0.000, Pr-value:0.000  
No matches to TargetScan

----------------------------------------------

GTGCCT

GTGCCT  
Depth:9 (MOUSE)  
Ei-value:0.000, Pi-value:0.000  
Er-value:0.000, Pr-value:0.000  
No matches to TargetScan

-----------------------------------------------

AGAATG

AGAATG  
Depth:9 (MOUSE)  
Ei-value:0.000, Pi-value:0.000  
Er-value:0.000, Pr-value:0.000  
No matches to TargetScan

--------

AGAAG

AGAAGA  
Depth:9 (MOUSE)  
Ei-value:0.000, Pi-value:0.000  
Er-value:0.000, Pr-value:0.000  
No matches to TargetScan

 2880  


A

AGAAGA  
Depth:9 (MOUSE)  
Ei-value:0.000, Pi-value:0.000  
Er-value:0.000, Pr-value:0.000  
No matches to TargetScan

----------------------------------------------------

TGTATATA

TGTATATA  
Depth:9 (MOUSE)  
Ei-value:0.000, Pi-value:0.000  
Er-value:0.000, Pr-value:0.000  
No matches to TargetScan

-----------------------

TGAAACATCTAG

TGAAACATCTAG  
Depth:9 (MOUSE)  
Ei-value:0.000, Pi-value:0.000  
Er-value:0.000, Pr-value:0.000  
No matches to TargetScan

---

TTCTAG

TTCTAG  
Depth:9 (MOUSE)  
Ei-value:0.000, Pi-value:0.000  
Er-value:0.000, Pr-value:0.000  
No matches to TargetScan

-

TGTTTAGAAGTGCA

TGTTTAGAAGTGCACAAAGTATGTTAAAAGTAGA  
Depth:9 (MOUSE)  
Ei-value:0.000, Pi-value:0.000  
Er-value:0.000, Pr-value:0.000  
No matches to TargetScan

 3000  


CAAAGTATGTTAAAAGTAGA

TGTTTAGAAGTGCACAAAGTATGTTAAAAGTAGA  
Depth:9 (MOUSE)  
Ei-value:0.000, Pi-value:0.000  
Er-value:0.000, Pr-value:0.000  
No matches to TargetScan

---------------------------------------------------------------------------------------------------- 3120  
 --------------------------------------------------------------------------------------------------------

TGTATA

TGTATA  
Depth:9 (MOUSE)  
Ei-value:0.000, Pi-value:0.000  
Er-value:0.000, Pr-value:0.000  
No matches to TargetScan

----

TGTATA

TGTATA  
Depth:9 (MOUSE)  
Ei-value:0.000, Pi-value:0.000  
Er-value:0.000, Pr-value:0.000  
No matches to TargetScan

 3240  


TGTATA  
Depth:9 (MOUSE)  
Ei-value:0.000, Pi-value:0.000  
Er-value:0.000, Pr-value:0.000  
No matches to TargetScan

---------------------------

AGTCTT

AGTCTT  
Depth:9 (MOUSE)  
Ei-value:0.000, Pi-value:0.000  
Er-value:0.000, Pr-value:0.000  
No matches to TargetScan

--------------------------------------------------------------------------------------- 3360  
 ------------------------------------------------------------------------------------------------------------------------ 3480  
 ------------------------------------------------------------------------------------------------------------------------ 3600  
 ------------------------------------------------------------------------------------------------------------------------ 3720  
 ------------------------------------------------------------------------------------------------------------------------ 3840  
 ------------------------------------------------------------------------------------------------------------------------ 3960  
 ------------------------------------------------------------------------------------------------------------------------ 4080  
 ------------------------------------------------------------------------------------------------------------------------ 4200  
 ------------------------------------------------------------------------------------------------------------------------ 4320  
 --------------------------------------------------------------------------

AATTGTTA

AATTGTTA  
Depth:9 (MOUSE)  
Ei-value:0.000, Pi-value:0.000  
Er-value:0.000, Pr-value:0.000  
No matches to TargetScan

-------------------------------------- 4440  
 ------------------------------------------------------------------------------------------------------------------------ 4560  
 ------------------------------------------------------------------------------------------------------------------------ 4680  
 ------------------------------------------------------

ATTTTTGT

ATTTTTGT  
Depth:9 (MOUSE)  
Ei-value:0.000, Pi-value:0.000  
Er-value:0.000, Pr-value:0.000  
No matches to TargetScan

---------------------------------------------------------

T

TTCTTGGT  
Depth:9 (MOUSE)  
Ei-value:0.000, Pi-value:0.000  
Er-value:0.000, Pr-value:0.000  
No matches to TargetScan

 4800  


TCTTGGT

TTCTTGGT  
Depth:9 (MOUSE)  
Ei-value:0.000, Pi-value:0.000  
Er-value:0.000, Pr-value:0.000  
No matches to TargetScan

-----------------------------------------

AATATTCA

AATATTCA  
Depth:9 (MOUSE)  
Ei-value:0.000, Pi-value:0.000  
Er-value:0.000, Pr-value:0.000  
No matches to TargetScan

---------------------------------------------------------------- 4920  
 --------------------------------------

CTGCTT

CTGCTT  
Depth:9 (MOUSE)  
Ei-value:0.000, Pi-value:0.000  
Er-value:0.000, Pr-value:0.000  
No matches to TargetScan

-----------

TGACTT

TGACTT  
Depth:9 (MOUSE)  
Ei-value:0.000, Pi-value:0.000  
Er-value:0.000, Pr-value:0.000  
MATCHES To TargetScan▶ miR-224-5p:AAGUCAC

------

TTGCTT

TTGCTT  
Depth:9 (MOUSE)  
Ei-value:0.000, Pi-value:0.000  
Er-value:0.000, Pr-value:0.010  
No matches to TargetScan

-------------------------------------------

AAAT

AAATAAA  
Depth:9 (MOUSE)  
Ei-value:0.000, Pi-value:0.000  
Er-value:0.000, Pr-value:0.000  
No matches to TargetScan

 5040  


AAA

AAATAAA  
Depth:9 (MOUSE)  
Ei-value:0.000, Pi-value:0.000  
Er-value:0.000, Pr-value:0.000  
No matches to TargetScan

-------------------------------------                                                                                 5080
```

---

## >MOUSE (4917 bases)

```
 ---------

TGCCAA

TGCCAA  
Depth:9 (MOUSE)  
Ei-value:0.000, Pi-value:0.000  
Er-value:0.000, Pr-value:0.000  
MATCHES To TargetScan▶ miR-182-5p:UUGGCAA▶ miR-96-5p/1271-5p:UUGGCAC

-

TCAGTTCCGG

TCAGTTCCGG  
Depth:9 (MOUSE)  
Ei-value:0.000, Pi-value:0.000  
Er-value:0.000, Pr-value:0.000  
No matches to TargetScan

---------------------------------------------------------------------------------------------- 120  
 -------------------------------

GCCCTC

GCCCTC  
Depth:9 (MOUSE)  
Ei-value:0.000, Pi-value:0.000  
Er-value:0.000, Pr-value:0.000  
No matches to TargetScan


GCCCTC

GCCCTC  
Depth:9 (MOUSE)  
Ei-value:0.000, Pi-value:0.000  
Er-value:0.000, Pr-value:0.000  
No matches to TargetScan

--------------------------------------------------------------------

ATGGCGGC

ATGGCGGC  
Depth:9 (MOUSE)  
Ei-value:0.000, Pi-value:0.000  
Er-value:0.000, Pr-value:0.000  
No matches to TargetScan

- 240  
 ---------------------------------------------------------------------------------------------------------

GGGAAGA

GGGAAGA  
Depth:9 (MOUSE)  
Ei-value:0.000, Pi-value:0.000  
Er-value:0.000, Pr-value:0.000  
No matches to TargetScan

-------- 360  
 ------------------------------------------------------------------------------------------------------------------------ 480  
 ------------------------------------------------------------------------------------------------------------------------ 600  
 ----------------------------------------------

CCTGTGTATATAATATGAAAAAGCTGCT

CCTGTGTATATAATATGAAAAAGCTGCT  
Depth:9 (MOUSE)  
Ei-value:0.000, Pi-value:0.000  
Er-value:0.000, Pr-value:0.000  
MATCHES To TargetScan▶ miR-15-5p/16-5p/195-5p/424-5p/497-5p:AGCAGCA▶ miR-503-5p:AGCAGCG

--

CAACCTTT

CAACCTTT  
Depth:9 (MOUSE)  
Ei-value:0.000, Pi-value:0.000  
Er-value:0.000, Pr-value:0.000  
No matches to TargetScan

------------------------------------ 720  
 ------------------------------------

AGAGGT

AGAGGT  
Depth:9 (MOUSE)  
Ei-value:0.000, Pi-value:0.000  
Er-value:0.000, Pr-value:0.000  
No matches to TargetScan

--------------------------------------------------------------------

GAAATG

GAAATG  
Depth:9 (MOUSE)  
Ei-value:0.000, Pi-value:0.000  
Er-value:0.000, Pr-value:0.000  
No matches to TargetScan

---- 840  
 --------------------------------------------------------------------------------

AGAAGA

AGAAGA  
Depth:9 (MOUSE)  
Ei-value:0.000, Pi-value:0.000  
Er-value:0.000, Pr-value:0.000  
No matches to TargetScan

--------------------------

AGAAGA

AGAAGA  
Depth:9 (MOUSE)  
Ei-value:0.000, Pi-value:0.000  
Er-value:0.000, Pr-value:0.000  
No matches to TargetScan

-- 960  
 ------------------------------------------------------------------------------------------------------------------------ 1080  
 -------------------------------

AAGTGCCTG

AAGTGCCTG  
Depth:9 (MOUSE)  
Ei-value:0.000, Pi-value:0.000  
Er-value:0.000, Pr-value:0.000  
No matches to TargetScan

-------------------------------------------------------------------------------- 1200  
 ------------------------------

TTGTGC

TTGTGC  
Depth:9 (MOUSE)  
Ei-value:0.000, Pi-value:0.000  
Er-value:0.000, Pr-value:0.000  
No matches to TargetScan

------------------------------------------------------------------------------------ 1320  
 -------------------------------------------------------------------------

GATGTT

GATGTT  
Depth:9 (MOUSE)  
Ei-value:0.000, Pi-value:0.000  
Er-value:0.000, Pr-value:0.000  
No matches to TargetScan


AAAGAGGTTGCCA

AAAGAGGTTGCCA  
Depth:9 (MOUSE)  
Ei-value:0.000, Pi-value:0.000  
Er-value:0.000, Pr-value:0.000  
No matches to TargetScan

---------------------------- 1440  
 ---------------------------

ATTTTGT

ATTTTGT  
Depth:9 (MOUSE)  
Ei-value:0.000, Pi-value:0.000  
Er-value:0.000, Pr-value:0.000  
No matches to TargetScan

-------------------------------------------------------------------

TCCTTA

TCCTTA  
Depth:9 (MOUSE)  
Ei-value:0.000, Pi-value:0.000  
Er-value:0.000, Pr-value:0.000  
No matches to TargetScan

----

AAACATCT

AAACATCT  
Depth:9 (MOUSE)  
Ei-value:0.000, Pi-value:0.000  
Er-value:0.000, Pr-value:0.000  
No matches to TargetScan

- 1560  
 ------

CTAGATGTTTAGAAGTGCCC

CTAGATGTTTAGAAGTGCCC  
Depth:9 (MOUSE)  
Ei-value:0.000, Pi-value:0.000  
Er-value:0.000, Pr-value:0.000  
No matches to TargetScan

---

GTATGTTAAA

GTATGTTAAA  
Depth:9 (MOUSE)  
Ei-value:0.000, Pi-value:0.000  
Er-value:0.000, Pr-value:0.000  
No matches to TargetScan

------------------------

TGTAAATA

TGTAAATA  
Depth:9 (MOUSE)  
Ei-value:0.000, Pi-value:0.000  
Er-value:0.000, Pr-value:0.000  
No matches to TargetScan

------------------------------------------------- 1680  
 ------------------------------------------------------------------------------------------------------------------------ 1800  
 ------------------------------------------------------------------------------------------------------------------------ 1920  
 ------------------------------------------------------------------------------------------------------------------------ 2040  
 ------------------------------------------------------------------------------------------------------------------------ 2160  
 ------------------------------------------------------------------------------------------------------------------------ 2280  
 ----------------------------------------------------------

GTGGGAGGGAA

GTGGGAGGGAA  
Depth:9 (MOUSE)  
Ei-value:0.000, Pi-value:0.000  
Er-value:0.000, Pr-value:0.000  
MATCHES To TargetScan▶ miR-150-5p:CUCCCAA▶ miR-532-3p:CUCCCAC

-----

TGCAAA

TGCAAA  
Depth:9 (MOUSE)  
Ei-value:0.000, Pi-value:0.000  
Er-value:0.000, Pr-value:0.000  
No matches to TargetScan

---------------------------------------- 2400  
 -----------------------------------------

TGTATATA

TGTATATA  
Depth:9 (MOUSE)  
Ei-value:0.000, Pi-value:0.000  
Er-value:0.000, Pr-value:0.000  
No matches to TargetScan

-----------------------------

ATATCTAG

ATATCTAG  
Depth:9 (MOUSE)  
Ei-value:0.000, Pi-value:0.000  
Er-value:0.000, Pr-value:0.000  
No matches to TargetScan

-

CTCTAG

CTCTAG  
Depth:9 (MOUSE)  
Ei-value:0.000, Pi-value:0.000  
Er-value:0.000, Pr-value:0.000  
No matches to TargetScan

-----

AAAGAGGTTGCCAATGTATGAC

AAAGAGGTTGCCAATGTATGACA  
Depth:9 (MOUSE)  
Ei-value:0.000, Pi-value:0.000  
Er-value:0.000, Pr-value:0.000  
MATCHES To TargetScan▶ miR-182-5p:UUGGCAA▶ miR-96-5p/1271-5p:UUGGCAC▶ miR-539-3p:UCAUACA

 2520  


A

AAAGAGGTTGCCAATGTATGACA  
Depth:9 (MOUSE)  
Ei-value:0.000, Pi-value:0.000  
Er-value:0.000, Pr-value:0.000  
MATCHES To TargetScan▶ miR-182-5p:UUGGCAA▶ miR-96-5p/1271-5p:UUGGCAC▶ miR-539-3p:UCAUACA

------

GTTAGTAAACT

GTTAGTAAACT  
Depth:9 (MOUSE)  
Ei-value:0.000, Pi-value:0.000  
Er-value:0.000, Pr-value:0.000  
No matches to TargetScan

-

ACACATT

ACACATT  
Depth:9 (MOUSE)  
Ei-value:0.000, Pi-value:0.000  
Er-value:0.000, Pr-value:0.000  
No matches to TargetScan

-----------------------------

TGTCTTCTGAA

TGTCTTCTGAA  
Depth:9 (MOUSE)  
Ei-value:0.000, Pi-value:0.000  
Er-value:0.000, Pr-value:0.000  
No matches to TargetScan

----------------------------------------------

GTGCCT

GTGCCT  
Depth:9 (MOUSE)  
Ei-value:0.000, Pi-value:0.000  
Er-value:0.000, Pr-value:0.000  
No matches to TargetScan

-- 2640  
 ----------------------------------------------

AGAATG

AGAATG  
Depth:9 (MOUSE)  
Ei-value:0.000, Pi-value:0.000  
Er-value:0.000, Pr-value:0.000  
No matches to TargetScan

--------

AGAAGA

AGAAGA  
Depth:9 (MOUSE)  
Ei-value:0.000, Pi-value:0.000  
Er-value:0.000, Pr-value:0.000  
No matches to TargetScan

-----------------------------------------------

TGTATAT

TGTATATA  
Depth:9 (MOUSE)  
Ei-value:0.000, Pi-value:0.000  
Er-value:0.000, Pr-value:0.000  
No matches to TargetScan

 2760  


A

TGTATATA  
Depth:9 (MOUSE)  
Ei-value:0.000, Pi-value:0.000  
Er-value:0.000, Pr-value:0.000  
No matches to TargetScan

-----------------------

TGAAACATCTAG

TGAAACATCTAG  
Depth:9 (MOUSE)  
Ei-value:0.000, Pi-value:0.000  
Er-value:0.000, Pr-value:0.000  
No matches to TargetScan

---

TTCTAG

TTCTAG  
Depth:9 (MOUSE)  
Ei-value:0.000, Pi-value:0.000  
Er-value:0.000, Pr-value:0.000  
No matches to TargetScan

-

TGTTTAGAAGTGCACAAAGTATGTTAAAAGTAGA

TGTTTAGAAGTGCACAAAGTATGTTAAAAGTAGA  
Depth:9 (MOUSE)  
Ei-value:0.000, Pi-value:0.000  
Er-value:0.000, Pr-value:0.000  
No matches to TargetScan

---------------------------------------- 2880  
 ------------------------------------------------------------------------------------------------------------------------ 3000  
 -----------------------------------------------------

TGTATA

TGTATA  
Depth:9 (MOUSE)  
Ei-value:0.000, Pi-value:0.000  
Er-value:0.000, Pr-value:0.000  
No matches to TargetScan

-----------------------------

AGTCTT

AGTCTT  
Depth:9 (MOUSE)  
Ei-value:0.000, Pi-value:0.000  
Er-value:0.000, Pr-value:0.000  
No matches to TargetScan

-------------------------- 3120  
 -------------------------------------------------------------------------------------

AATTGTTA

AATTGTTA  
Depth:9 (MOUSE)  
Ei-value:0.000, Pi-value:0.000  
Er-value:0.000, Pr-value:0.000  
No matches to TargetScan

--------------------------- 3240  
 ------------------------------------------------------------------------------------------------------------------------ 3360  
 ------------------------------------------------------------------------------------------------------------------------ 3480  
 ------------------------------------------------------------------------------------------------------------------------ 3600  
 ------------------------------------------------------------------------------------------------------------------------ 3720  
 ------------------------------------------------------------------------------------------------------------------------ 3840  
 ------------------------------------------------------------------------------------------------------------------------ 3960  
 ------------------------------------------------------------------------------------------------------------------------ 4080  
 ------------------------------------------------------------------------------------------------------------------------ 4200  
 ------------------------------------------------------------------------------------------------------------------------ 4320  
 ------------------------------------------------------------------------------------------------------------------------ 4440  
 ------------------------------------------------------------------------------------------------------------------------ 4560  
 --------------------------------------------

ATTTTTGT

ATTTTTGT  
Depth:9 (MOUSE)  
Ei-value:0.000, Pi-value:0.000  
Er-value:0.000, Pr-value:0.000  
No matches to TargetScan

-------------------------------------------------------------------- 4680  
 -----

TTCTTGGT

TTCTTGGT  
Depth:9 (MOUSE)  
Ei-value:0.000, Pi-value:0.000  
Er-value:0.000, Pr-value:0.000  
No matches to TargetScan

---------------------------------------

AATATTCA

AATATTCA  
Depth:9 (MOUSE)  
Ei-value:0.000, Pi-value:0.000  
Er-value:0.000, Pr-value:0.000  
No matches to TargetScan

------------------------------------------------------------ 4800  
 -------------------------------

CTGCTT

CTGCTT  
Depth:9 (MOUSE)  
Ei-value:0.000, Pi-value:0.000  
Er-value:0.000, Pr-value:0.000  
No matches to TargetScan

---------

TGACTT

TGACTT  
Depth:9 (MOUSE)  
Ei-value:0.000, Pi-value:0.000  
Er-value:0.000, Pr-value:0.000  
MATCHES To TargetScan▶ miR-224-5p:AAGUCAC

-----

TTGCTT

TTGCTT  
Depth:9 (MOUSE)  
Ei-value:0.000, Pi-value:0.000  
Er-value:0.000, Pr-value:0.010  
No matches to TargetScan

-----------------------------

AAATAAA

AAATAAA  
Depth:9 (MOUSE)  
Ei-value:0.000, Pi-value:0.000  
Er-value:0.000, Pr-value:0.000  
No matches to TargetScan

------------------    4917
```

---
