# Supplementary material for: Uncovering deeply conserved motif combinations in rapidly evolving noncoding sequences
Source: Genome Biol. 2021 Jan 11;22:29. doi: 10.1186/s13059-020-02247-1 (PMC7798263; doi:10.1186/s13059-020-02247-1)
Supplement: Supplementary file 3 — Additional file 3. LncLOOM output results for NORAD sequences from nine mammals. [file 13059_2020_2247_MOESM3_ESM.gz › AdditionalFile3/Html_Files/miRNA_Matches.html]

 miRNA Matches

# Matches to miRNA Families Retrieved from TargetScan

  

\*\*SEED defined as positions 2-7 of mature miRNA sequence  
\*\*MOTIF MATCHES that correspond to the reverse complement of miRNA SEEDS are displayed from 5`-3` relative to lincRNA sequence

  

| Seed Matches to the miRNA miR-411-3p | | | | | |
| --- | --- | --- | --- | --- | --- |
| Seed | Conservation | Species | Matches | | |
|  |  |  | Sequence | Motif | Type |
| AUGUAAC | Conserved | Human (Homo sapiens)  Mouse (Mus musculus)  Rat (Rattus norvegicus) | HUMAN | TTGTTACATGTTTGCAGTTTATTCAAGACTGCT | 7mer-m8 |
|  |  |  | HUMAN | TATGTTACATGCATTTCATTTAACTTTGCTATACTGTATATATTGT | 7mer-m8 |
|  |  |  | HUMAN | TATGTCCATATTTACATTTTGATAGCCATTGATGTATGCATCTCTT | 6mer |
|  |  |  | HUMAN | CATATTTACATTTT | 6mer |
|  |  |  | HUMAN | AGAAAGGCAGGATTACAT | 6mer |
|  |  |  | HUMAN | GATTACAT | 6mer |
|  |  |  | BABOON | TTGTTACATGTTTGCAGTTTATTCAAGACTGCT | 7mer-m8 |
|  |  |  | BABOON | TATGTTACATGCATTTCATTTAACTTTGCTATACTGTATATATTGT | 7mer-m8 |
|  |  |  | BABOON | TATGTCCATATTTACATTTTGATAGCCATTGATGTATGCATCTCTT | 6mer |
|  |  |  | BABOON | CATATTTACATTTT | 6mer |
|  |  |  | BABOON | AGAAAGGCAGGATTACAT | 6mer |
|  |  |  | BABOON | GATTACAT | 6mer |
|  |  |  | DOG | CATATTTACATTTT | 6mer |
|  |  |  | DOG | AGAAAGGCAGGATTACAT | 6mer |
|  |  |  | DOG | GATTACAT | 6mer |
|  |  |  | COW | GATTACAT | 6mer |
|  |  |  | SHEEP | GATTACAT | 6mer |
|  |  |  | PIG | GATTACAT | 6mer |

  
  
  
  

| Seed Matches to the miRNA miR-501-3p/502-3p | | | | | |
| --- | --- | --- | --- | --- | --- |
| Seed | Conservation | Species | Matches | | |
|  |  |  | Sequence | Motif | Type |
| AUGCACC | Conserved | Human (Homo sapiens) | HUMAN | AGAAGGTTCTATGCTAGACTGGTCATATTTAGAAGACATTTTCATATTCTATCCATTGTTTTGTGTGCATTTTATTCCTCACTACTGTGTATATA | 6mer |
|  |  |  | HUMAN | TGTGTGCATTTTATTCCTC | 6mer |
|  |  |  | HUMAN | TGTGTGCATTTT | 6mer |
|  |  |  | HUMAN | TCTGTGCATTTTGTTTTACTTATCTGTGTATATAGTGTACATAAAGGACAGACGAGTCCTAATTGACAACATCTAGTCTTTCTGGATGTTAAAGAGGTTGCCAGTGTATGACAAAAGTAGAGTT | 6mer |
|  |  |  | HUMAN | TATGTTTTGTGCATTTTGTTTTGCTCTGTATATAGT | 6mer |
|  |  |  | HUMAN | TGTGCATTTT | 6mer |
|  |  |  | HUMAN | GTGCATTTT | 6mer |
|  |  |  | HUMAN | GGGTTCTGTGCTCCTGAGATTAGTTCAGATGGTCTAACCATTGTTCTATATGTGCATTTTAGTTAATATTGTGTATTAAAGGATAAGTCTTAATGCTCAAAGTATGTTAAAAATAGATGTAGTAAA | 6mer |
|  |  |  | BABOON | AGAAGGTTCTATGCTAGACTGGTCATATTTAGAAGACATTTTCATATTCTATCCATTGTTTTGTGTGCATTTTATTCCTCACTACTGTGTATATA | 6mer |
|  |  |  | BABOON | TGTGTGCATTTTATTCCTC | 6mer |
|  |  |  | BABOON | TGTGTGCATTTT | 6mer |
|  |  |  | BABOON | TCTGTGCATTTTGTTTTACTTATCTGTGTATATAGTGTACATAAAGGACAGACGAGTCCTAATTGACAACATCTAGTCTTTCTGGATGTTAAAGAGGTTGCCAGTGTATGACAAAAGTAGAGTT | 6mer |
|  |  |  | BABOON | TATGTTTTGTGCATTTTGTTTTGCTCTGTATATAGT | 6mer |
|  |  |  | BABOON | TGTGCATTTT | 6mer |
|  |  |  | BABOON | GTGCATTTT | 6mer |
|  |  |  | BABOON | GGGTTCTGTGCTCCTGAGATTAGTTCAGATGGTCTAACCATTGTTCTATATGTGCATTTTAGTTAATATTGTGTATTAAAGGATAAGTCTTAATGCTCAAAGTATGTTAAAAATAGATGTAGTAAA | 6mer |
|  |  |  | DOG | TGTGTGCATTTTATTCCTC | 6mer |
|  |  |  | DOG | TGTGTGCATTTT | 6mer |
|  |  |  | DOG | TGTGCATTTT | 6mer |
|  |  |  | DOG | GTGCATTTT | 6mer |
|  |  |  | COW | TGTGTGCATTTT | 6mer |
|  |  |  | COW | TGTGCATTTT | 6mer |
|  |  |  | COW | GTGCATTTT | 6mer |
|  |  |  | SHEEP | TGTGTGCATTTT | 6mer |
|  |  |  | SHEEP | GTGCATTTT | 6mer |
|  |  |  | PIG | TGTGTGCATTTT | 6mer |
|  |  |  | PIG | GTGCATTTT | 7mer-m8 |

  
  
  
  

| Seed Matches to the miRNA miR-875-5p | | | | | |
| --- | --- | --- | --- | --- | --- |
| Seed | Conservation | Species | Matches | | |
|  |  |  | Sequence | Motif | Type |
| AUACCUC | Conserved | Human (Homo sapiens)  Rhesus (Macaca mulatta)  Mouse (Mus musculus) | HUMAN | GAGGAGGTATGCA | 7mer-m8 |
|  |  |  | BABOON | GAGGAGGTATGCA | 7mer-m8 |

  
  
  
  

| Seed Matches to the miRNA miR-122-5p | | | | | |
| --- | --- | --- | --- | --- | --- |
| Seed | Conservation | Species | Matches | | |
|  |  |  | Sequence | Motif | Type |
| GGAGUGU | Broadly Conserved | Human (Homo sapiens)  Chicken (Gallus gallus)  Rhesus (Macaca mulatta)  Mouse (Mus musculus)  Rat (Rattus norvegicus)  Opossum (Monodelphis domestica) | HUMAN | CACCACTGCACTCCA | 7mer-A1 |
|  |  |  | BABOON | CACCACTGCACTCCA | 7mer-A1 |

  
  
  
  

| Seed Matches to the miRNA miR-382-5p | | | | | |
| --- | --- | --- | --- | --- | --- |
| Seed | Conservation | Species | Matches | | |
|  |  |  | Sequence | Motif | Type |
| AAGUUGU | Conserved | Human (Homo sapiens)  Rhesus (Macaca mulatta)  Mouse (Mus musculus)  Rat (Rattus norvegicus) | HUMAN | GAATGGGGTGACAACTTATGATAAAAACTAGAGCTAGTGAATTAGCC | 8mer |
|  |  |  | BABOON | GAATGGGGTGACAACTTATGATAAAAACTAGAGCTAGTGAATTAGCC | 8mer |

  
  
  
  

| Seed Matches to the miRNA miR-218-5p | | | | | |
| --- | --- | --- | --- | --- | --- |
| Seed | Conservation | Species | Matches | | |
|  |  |  | Sequence | Motif | Type |
| UGUGCUU | Broadly Conserved | Human (Homo sapiens)  Chicken (Gallus gallus)  Rhesus (Macaca mulatta)  Mouse (Mus musculus)  Rat (Rattus norvegicus)  Opossum (Monodelphis domestica) | HUMAN | TATTTGTGCTGGTTCAGGGGGAAGGAGGAGCACAAAGTGCAAAGGGCTTTCTACCAGTGTCCAGTGTGTTTA | 7mer-A1 |
|  |  |  | HUMAN | CTACAGAAGCACAGGT | 7mer-m8 |
|  |  |  | BABOON | TATTTGTGCTGGTTCAGGGGGAAGGAGGAGCACAAAGTGCAAAGGGCTTTCTACCAGTGTCCAGTGTGTTTA | 7mer-A1 |
|  |  |  | BABOON | CTACAGAAGCACAGGT | 7mer-m8 |

  
  
  
  

| Seed Matches to the miRNA miR-505-3p.2 | | | | | |
| --- | --- | --- | --- | --- | --- |
| Seed | Conservation | Species | Matches | | |
|  |  |  | Sequence | Motif | Type |
| UCAACAC | Conserved | Human (Homo sapiens)  Mouse (Mus musculus) | HUMAN | GGGGGGAATGGCCAAATCACCTGTTGAGTAATACTCATTGTGTTTGTGCAGTGGTTC | 6mer |
|  |  |  | HUMAN | CTTTGTGTTGAAATTC | 8mer |
|  |  |  | HUMAN | TTGTGTTGAAATTC | 8mer |
|  |  |  | HUMAN | TTGTGTTGAAATT | 8mer |
|  |  |  | HUMAN | TTGTAAATAGCTTTTAAAAACTGATGGGAAATGCTGTTTGGAAGTGGAATTGTTGAACCA | 7mer-A1 |
|  |  |  | BABOON | GGGGGGAATGGCCAAATCACCTGTTGAGTAATACTCATTGTGTTTGTGCAGTGGTTC | 6mer |
|  |  |  | BABOON | CTTTGTGTTGAAATTC | 8mer |
|  |  |  | BABOON | TTGTGTTGAAATTC | 8mer |
|  |  |  | BABOON | TTGTGTTGAAATT | 8mer |
|  |  |  | BABOON | TTGTAAATAGCTTTTAAAAACTGATGGGAAATGCTGTTTGGAAGTGGAATTGTTGAACCA | 7mer-A1 |
|  |  |  | DOG | TTGTGTTGAAATTC | 8mer |
|  |  |  | DOG | TTGTGTTGAAATT | 8mer |
|  |  |  | COW | TTGTGTTGAAATTC | 8mer |
|  |  |  | COW | TTGTGTTGAAATT | 8mer |
|  |  |  | SHEEP | TTGTGTTGAAATTC | 8mer |
|  |  |  | SHEEP | TTGTGTTGAAATT | 8mer |
|  |  |  | PIG | TTGTGTTGAAATT | 8mer |
|  |  |  | ARMADILLO | TTGTGTTGAAATT | 8mer |

  
  
  
  

| Seed Matches to the miRNA miR-421 | | | | | |
| --- | --- | --- | --- | --- | --- |
| Seed | Conservation | Species | Matches | | |
|  |  |  | Sequence | Motif | Type |
| UCAACAG | Conserved | Human (Homo sapiens)  Rhesus (Macaca mulatta)  Chimp (Pan troglodytes)  Dog (Canis lupus familiaris)  Cow (Bos taurus) | HUMAN | GGGGGGAATGGCCAAATCACCTGTTGAGTAATACTCATTGTGTTTGTGCAGTGGTTC | 7mer-m8 |
|  |  |  | HUMAN | CTTTGTGTTGAAATTC | 7mer-A1 |
|  |  |  | HUMAN | TTGTGTTGAAATTC | 7mer-A1 |
|  |  |  | HUMAN | TTGTGTTGAAATT | 7mer-A1 |
|  |  |  | HUMAN | TTGTAAATAGCTTTTAAAAACTGATGGGAAATGCTGTTTGGAAGTGGAATTGTTGAACCA | 7mer-A1 |
|  |  |  | BABOON | GGGGGGAATGGCCAAATCACCTGTTGAGTAATACTCATTGTGTTTGTGCAGTGGTTC | 7mer-m8 |
|  |  |  | BABOON | CTTTGTGTTGAAATTC | 7mer-A1 |
|  |  |  | BABOON | TTGTGTTGAAATTC | 7mer-A1 |
|  |  |  | BABOON | TTGTGTTGAAATT | 7mer-A1 |
|  |  |  | BABOON | TTGTAAATAGCTTTTAAAAACTGATGGGAAATGCTGTTTGGAAGTGGAATTGTTGAACCA | 7mer-A1 |
|  |  |  | DOG | TTGTGTTGAAATTC | 7mer-A1 |
|  |  |  | DOG | TTGTGTTGAAATT | 7mer-A1 |
|  |  |  | COW | TTGTGTTGAAATTC | 7mer-A1 |
|  |  |  | COW | TTGTGTTGAAATT | 7mer-A1 |
|  |  |  | SHEEP | TTGTGTTGAAATTC | 7mer-A1 |
|  |  |  | SHEEP | TTGTGTTGAAATT | 7mer-A1 |
|  |  |  | PIG | TTGTGTTGAAATT | 7mer-A1 |
|  |  |  | ARMADILLO | TTGTGTTGAAATT | 7mer-A1 |

  
  
  
  

| Seed Matches to the miRNA miR-140-5p | | | | | |
| --- | --- | --- | --- | --- | --- |
| Seed | Conservation | Species | Matches | | |
|  |  |  | Sequence | Motif | Type |
| AGUGGUU | Broadly Conserved | Human (Homo sapiens)  Rhesus (Macaca mulatta)  Mouse (Mus musculus)  Rat (Rattus norvegicus)  Opossum (Monodelphis domestica) | HUMAN | CCACGGAGCCGCGCAGATCCGGTTCCCGGGTGACCACTCTGTCGCCATTGGGCGA | 6mer |
|  |  |  | HUMAN | TGACCACTC | 6mer |
|  |  |  | HUMAN | AGGTAGTAAAATACCACTTTGTAAATATCTTTTTGCTAAAATTCATAGGAAAT | 6mer |
|  |  |  | HUMAN | CACCACTGCACTCCA | 6mer |
|  |  |  | BABOON | CCACGGAGCCGCGCAGATCCGGTTCCCGGGTGACCACTCTGTCGCCATTGGGCGA | 6mer |
|  |  |  | BABOON | TGACCACTC | 6mer |
|  |  |  | BABOON | AGGTAGTAAAATACCACTTTGTAAATATCTTTTTGCTAAAATTCATAGGAAAT | 6mer |
|  |  |  | BABOON | CACCACTGCACTCCA | 6mer |
|  |  |  | DOG | TGACCACTC | 6mer |

  
  
  
  

| Seed Matches to the miRNA miR-448 | | | | | |
| --- | --- | --- | --- | --- | --- |
| Seed | Conservation | Species | Matches | | |
|  |  |  | Sequence | Motif | Type |
| UGCAUAU | Conserved | Human (Homo sapiens)  Rhesus (Macaca mulatta)  Chimp (Pan troglodytes)  Dog (Canis lupus familiaris)  Cow (Bos taurus) | HUMAN | GAGGAGGTATGCA | 6mer |
|  |  |  | HUMAN | TGTATGCAGATGTGTC | 6mer |
|  |  |  | HUMAN | TATGTCCATATTTACATTTTGATAGCCATTGATGTATGCATCTCTT | 6mer |
|  |  |  | BABOON | GAGGAGGTATGCA | 6mer |
|  |  |  | BABOON | TGTATGCAGATGTGTC | 6mer |
|  |  |  | BABOON | TATGTCCATATTTACATTTTGATAGCCATTGATGTATGCATCTCTT | 6mer |

  
  
  
  

| Seed Matches to the miRNA miR-499a-5p | | | | | |
| --- | --- | --- | --- | --- | --- |
| Seed | Conservation | Species | Matches | | |
|  |  |  | Sequence | Motif | Type |
| UAAGACU | Broadly Conserved | Human (Homo sapiens) | HUMAN | GGGTTCTGTGCTCCTGAGATTAGTTCAGATGGTCTAACCATTGTTCTATATGTGCATTTTAGTTAATATTGTGTATTAAAGGATAAGTCTTAATGCTCAAAGTATGTTAAAAATAGATGTAGTAAA | 8mer |
|  |  |  | BABOON | GGGTTCTGTGCTCCTGAGATTAGTTCAGATGGTCTAACCATTGTTCTATATGTGCATTTTAGTTAATATTGTGTATTAAAGGATAAGTCTTAATGCTCAAAGTATGTTAAAAATAGATGTAGTAAA | 8mer |

  
  
  
  

| Seed Matches to the miRNA miR-28-3p | | | | | |
| --- | --- | --- | --- | --- | --- |
| Seed | Conservation | Species | Matches | | |
|  |  |  | Sequence | Motif | Type |
| ACUAGAU | Conserved | Human (Homo sapiens)  Rhesus (Macaca mulatta)  Mouse (Mus musculus)  Rat (Rattus norvegicus) | HUMAN | TCTGTGCATTTTGTTTTACTTATCTGTGTATATAGTGTACATAAAGGACAGACGAGTCCTAATTGACAACATCTAGTCTTTCTGGATGTTAAAGAGGTTGCCAGTGTATGACAAAAGTAGAGTT | 7mer-m8 |
|  |  |  | HUMAN | CAACATCTAGTCTTT | 7mer-m8 |
|  |  |  | HUMAN | GACAAATTAACTCCTTACTTGAAACATCTAGTCTATCTAGATGTTTAGAAGTGCCC | 7mer-m8 |
|  |  |  | HUMAN | AAACATCTAGTCT | 7mer-m8 |
|  |  |  | HUMAN | TATATAATGGACAAATAGTCCTAATTTTTCAACATCTAGTCTCTAGATGTTAAAGAGGTTGCCAGTGTATGACAAAG | 7mer-m8 |
|  |  |  | HUMAN | CAACATCTAGTCTCTAGATGTTAAAGAGGTTGCCA | 7mer-m8 |
|  |  |  | HUMAN | ACAAGTTATATGAAATATCTAGTCTTTCTAGATATTTGGAAG | 7mer-m8 |
|  |  |  | HUMAN | TGAAATATCTAGTCTTTCTAGATA | 7mer-m8 |
|  |  |  | HUMAN | TGAAATATCTAGTCTT | 7mer-m8 |
|  |  |  | HUMAN | TAATATCTAGTCTCTAGATATTAAAGAGGTTGCCAATGTATGACAGAAGTAGAGTTAGTAAACTAACACATTTTGTACACTTTGTTAAAATTTGTAGAAAGGCTGTCTTCTGAAAAGGACTTTTGGAAGTGA | 7mer-m8 |
|  |  |  | HUMAN | TAATATCTAGTCTCTAGATATTAAAGAGGTTGCCAATGTATGACA | 7mer-m8 |
|  |  |  | HUMAN | TAATATCTAGTCTCTAGATATT | 7mer-m8 |
|  |  |  | HUMAN | AATATCTAGTCTCTAGATATT | 7mer-m8 |
|  |  |  | HUMAN | GACAAACTTAAGTCCTTATTTGAAACATCTAGTCTTTCTAGATGTTTAGAAGTGCACAAAGTATGTTAAAAGTAGAGGTAGTAAATAACACATTTTGTAGCTATCCTTTTGATATGAAATATTGTCTTGGAAA | 7mer-m8 |
|  |  |  | HUMAN | TCTAGTCGATGTTAAA | 6mer |
|  |  |  | HUMAN | AGTCCAGTTTGAAACATCTAGTCTTTCTAGGTGTTTAAAAGTG | 7mer-m8 |
|  |  |  | HUMAN | AACATCTAGTCTTTCTAG | 7mer-m8 |
|  |  |  | HUMAN | AACATCTAGTCTT | 7mer-m8 |
|  |  |  | HUMAN | CATCTAGTCTT | 7mer-m8 |
|  |  |  | BABOON | TCTGTGCATTTTGTTTTACTTATCTGTGTATATAGTGTACATAAAGGACAGACGAGTCCTAATTGACAACATCTAGTCTTTCTGGATGTTAAAGAGGTTGCCAGTGTATGACAAAAGTAGAGTT | 7mer-m8 |
|  |  |  | BABOON | CAACATCTAGTCTTT | 7mer-m8 |
|  |  |  | BABOON | GACAAATTAACTCCTTACTTGAAACATCTAGTCTATCTAGATGTTTAGAAGTGCCC | 7mer-m8 |
|  |  |  | BABOON | AAACATCTAGTCT | 7mer-m8 |
|  |  |  | BABOON | TATATAATGGACAAATAGTCCTAATTTTTCAACATCTAGTCTCTAGATGTTAAAGAGGTTGCCAGTGTATGACAAAG | 7mer-m8 |
|  |  |  | BABOON | CAACATCTAGTCTCTAGATGTTAAAGAGGTTGCCA | 7mer-m8 |
|  |  |  | BABOON | ACAAGTTATATGAAATATCTAGTCTTTCTAGATATTTGGAAG | 7mer-m8 |
|  |  |  | BABOON | TGAAATATCTAGTCTTTCTAGATA | 7mer-m8 |
|  |  |  | BABOON | TGAAATATCTAGTCTT | 7mer-m8 |
|  |  |  | BABOON | TAATATCTAGTCTCTAGATATTAAAGAGGTTGCCAATGTATGACAGAAGTAGAGTTAGTAAACTAACACATTTTGTACACTTTGTTAAAATTTGTAGAAAGGCTGTCTTCTGAAAAGGACTTTTGGAAGTGA | 7mer-m8 |
|  |  |  | BABOON | TAATATCTAGTCTCTAGATATTAAAGAGGTTGCCAATGTATGACA | 7mer-m8 |
|  |  |  | BABOON | TAATATCTAGTCTCTAGATATT | 7mer-m8 |
|  |  |  | BABOON | AATATCTAGTCTCTAGATATT | 7mer-m8 |
|  |  |  | BABOON | GACAAACTTAAGTCCTTATTTGAAACATCTAGTCTTTCTAGATGTTTAGAAGTGCACAAAGTATGTTAAAAGTAGAGGTAGTAAATAACACATTTTGTAGCTATCCTTTTGATATGAAATATTGTCTTGGAAA | 7mer-m8 |
|  |  |  | BABOON | TCTAGTCGATGTTAAA | 7mer-m8 |
|  |  |  | BABOON | AGTCCAGTTTGAAACATCTAGTCTTTCTAGGTGTTTAAAAGTG | 7mer-m8 |
|  |  |  | BABOON | AACATCTAGTCTTTCTAG | 7mer-m8 |
|  |  |  | BABOON | AACATCTAGTCTT | 7mer-m8 |
|  |  |  | BABOON | CATCTAGTCTT | 7mer-m8 |
|  |  |  | DOG | CAACATCTAGTCTTT | 7mer-m8 |
|  |  |  | DOG | AAACATCTAGTCT | 7mer-m8 |
|  |  |  | DOG | CAACATCTAGTCTCTAGATGTTAAAGAGGTTGCCA | 7mer-m8 |
|  |  |  | DOG | TGAAATATCTAGTCTTTCTAGATA | 7mer-m8 |
|  |  |  | DOG | TGAAATATCTAGTCTT | 7mer-m8 |
|  |  |  | DOG | TAATATCTAGTCTCTAGATATTAAAGAGGTTGCCAATGTATGACA | 7mer-m8 |
|  |  |  | DOG | TAATATCTAGTCTCTAGATATT | 7mer-m8 |
|  |  |  | DOG | AATATCTAGTCTCTAGATATT | 7mer-m8 |
|  |  |  | DOG | AACATCTAGTCTTTCTAG | 7mer-m8 |
|  |  |  | DOG | AACATCTAGTCTT | 7mer-m8 |
|  |  |  | DOG | CATCTAGTCTT | 7mer-m8 |
|  |  |  | COW | CAACATCTAGTCTTT | 7mer-m8 |
|  |  |  | COW | TGAAATATCTAGTCTTTCTAGATA | 7mer-m8 |
|  |  |  | COW | TGAAATATCTAGTCTT | 7mer-m8 |
|  |  |  | COW | TAATATCTAGTCTCTAGATATTAAAGAGGTTGCCAATGTATGACA | 7mer-m8 |
|  |  |  | COW | TAATATCTAGTCTCTAGATATT | 7mer-m8 |
|  |  |  | COW | AATATCTAGTCTCTAGATATT | 7mer-m8 |
|  |  |  | COW | AACATCTAGTCTT | 7mer-m8 |
|  |  |  | COW | CATCTAGTCTT | 7mer-m8 |
|  |  |  | SHEEP | CAACATCTAGTCTTT | 7mer-m8 |
|  |  |  | SHEEP | TGAAATATCTAGTCTTTCTAGATA | 7mer-m8 |
|  |  |  | SHEEP | TGAAATATCTAGTCTT | 7mer-m8 |
|  |  |  | SHEEP | TAATATCTAGTCTCTAGATATTAAAGAGGTTGCCAATGTATGACA | 7mer-m8 |
|  |  |  | SHEEP | TAATATCTAGTCTCTAGATATT | 7mer-m8 |
|  |  |  | SHEEP | AATATCTAGTCTCTAGATATT | 7mer-m8 |
|  |  |  | SHEEP | AACATCTAGTCTT | 7mer-m8 |
|  |  |  | SHEEP | CATCTAGTCTT | 7mer-m8 |
|  |  |  | PIG | TGAAATATCTAGTCTT | 7mer-m8 |
|  |  |  | PIG | TAATATCTAGTCTCTAGATATT | 7mer-m8 |
|  |  |  | PIG | AATATCTAGTCTCTAGATATT | 7mer-m8 |
|  |  |  | PIG | AACATCTAGTCTT | 7mer-m8 |
|  |  |  | PIG | CATCTAGTCTT | 7mer-m8 |
|  |  |  | ARMADILLO | TGAAATATCTAGTCTT | 7mer-m8 |
|  |  |  | ARMADILLO | AATATCTAGTCTCTAGATATT | 7mer-m8 |
|  |  |  | ARMADILLO | CATCTAGTCTT | 7mer-m8 |
|  |  |  | GUINEAPIG | CATCTAGTCTT | 7mer-m8 |

  
  
  
  

| Seed Matches to the miRNA miR-323-3p | | | | | |
| --- | --- | --- | --- | --- | --- |
| Seed | Conservation | Species | Matches | | |
|  |  |  | Sequence | Motif | Type |
| ACAUUAC | Conserved | Human (Homo sapiens)  Mouse (Mus musculus)  Rat (Rattus norvegicus) | HUMAN | CAGTATAGTAATGTCTATACTTGTTCAAT | 7mer-m8 |
|  |  |  | BABOON | CAGTATAGTAATGTCTATACTTGTTCAAT | 7mer-m8 |

  
  
  
  

| Seed Matches to the miRNA miR-331-3p | | | | | |
| --- | --- | --- | --- | --- | --- |
| Seed | Conservation | Species | Matches | | |
|  |  |  | Sequence | Motif | Type |
| CCCCUGG | Conserved | Human (Homo sapiens)  Rhesus (Macaca mulatta)  Cow (Bos taurus)  Mouse (Mus musculus)  Rat (Rattus norvegicus) | HUMAN | TATTTGTGCTGGTTCAGGGGGAAGGAGGAGCACAAAGTGCAAAGGGCTTTCTACCAGTGTCCAGTGTGTTTA | 6mer |
|  |  |  | BABOON | TATTTGTGCTGGTTCAGGGGGAAGGAGGAGCACAAAGTGCAAAGGGCTTTCTACCAGTGTCCAGTGTGTTTA | 6mer |

  
  
  
  

| Seed Matches to the miRNA miR-153-3p | | | | | |
| --- | --- | --- | --- | --- | --- |
| Seed | Conservation | Species | Matches | | |
|  |  |  | Sequence | Motif | Type |
| UGCAUAG | Broadly Conserved | Human (Homo sapiens)  Chicken (Gallus gallus)  Rhesus (Macaca mulatta)  Mouse (Mus musculus)  Rat (Rattus norvegicus)  Opossum (Monodelphis domestica) | HUMAN | GAGGAGGTATGCA | 6mer |
|  |  |  | HUMAN | TGTATGCAGATGTGTC | 6mer |
|  |  |  | HUMAN | TATGTCCATATTTACATTTTGATAGCCATTGATGTATGCATCTCTT | 6mer |
|  |  |  | BABOON | GAGGAGGTATGCA | 6mer |
|  |  |  | BABOON | TGTATGCAGATGTGTC | 6mer |
|  |  |  | BABOON | TATGTCCATATTTACATTTTGATAGCCATTGATGTATGCATCTCTT | 6mer |

  
  
  
  

| Seed Matches to the miRNA miR-410-3p | | | | | |
| --- | --- | --- | --- | --- | --- |
| Seed | Conservation | Species | Matches | | |
|  |  |  | Sequence | Motif | Type |
| AUAUAAC | Conserved | Human (Homo sapiens)  Rhesus (Macaca mulatta) | HUMAN | ACAAGTTATATGAAATATCTAGTCTTTCTAGATATTTGGAAG | 7mer-m8 |
|  |  |  | BABOON | ACAAGTTATATGAAATATCTAGTCTTTCTAGATATTTGGAAG | 7mer-m8 |

  
  
  
  

| Seed Matches to the miRNA miR-382-3p | | | | | |
| --- | --- | --- | --- | --- | --- |
| Seed | Conservation | Species | Matches | | |
|  |  |  | Sequence | Motif | Type |
| AUCAUUC | Conserved | Human (Homo sapiens)  Rhesus (Macaca mulatta)  Mouse (Mus musculus)  Rat (Rattus norvegicus) | HUMAN | GAGGAGGGAATGATTCAAGGCCAAAATGGCCACATTTAGAAGATACCTCAGATGATAACCATTGTTAT | 7mer-m8 |
|  |  |  | BABOON | GAGGAGGGAATGATTCAAGGCCAAAATGGCCACATTTAGAAGATACCTCAGATGATAACCATTGTTAT | 7mer-m8 |

  
  
  
  

| Seed Matches to the miRNA miR-378-3p | | | | | |
| --- | --- | --- | --- | --- | --- |
| Seed | Conservation | Species | Matches | | |
|  |  |  | Sequence | Motif | Type |
| CUGGACU | Conserved | Human (Homo sapiens)  Mouse (Mus musculus)  Rat (Rattus norvegicus) | HUMAN | TATTTGTGCTGGTTCAGGGGGAAGGAGGAGCACAAAGTGCAAAGGGCTTTCTACCAGTGTCCAGTGTGTTTA | 6mer |
|  |  |  | HUMAN | AGTCCAGTTTGAAACATCTAGTCTTTCTAGGTGTTTAAAAGTG | 7mer-m8 |
|  |  |  | BABOON | TATTTGTGCTGGTTCAGGGGGAAGGAGGAGCACAAAGTGCAAAGGGCTTTCTACCAGTGTCCAGTGTGTTTA | 6mer |
|  |  |  | BABOON | AGTCCAGTTTGAAACATCTAGTCTTTCTAGGTGTTTAAAAGTG | 7mer-m8 |

  
  
  
  

| Seed Matches to the miRNA miR-125-5p | | | | | |
| --- | --- | --- | --- | --- | --- |
| Seed | Conservation | Species | Matches | | |
|  |  |  | Sequence | Motif | Type |
| CCCUGAG | Broadly Conserved | Human (Homo sapiens)  Chicken (Gallus gallus)  Opossum (Monodelphis domestica) | HUMAN | TATTTGTGCTGGTTCAGGGGGAAGGAGGAGCACAAAGTGCAAAGGGCTTTCTACCAGTGTCCAGTGTGTTTA | 6mer |
|  |  |  | HUMAN | TGTGCTGGTTCAGGG | 6mer |
|  |  |  | BABOON | TATTTGTGCTGGTTCAGGGGGAAGGAGGAGCACAAAGTGCAAAGGGCTTTCTACCAGTGTCCAGTGTGTTTA | 6mer |
|  |  |  | BABOON | TGTGCTGGTTCAGGG | 6mer |
|  |  |  | DOG | TGTGCTGGTTCAGGG | 7mer-A1 |
|  |  |  | COW | TGTGCTGGTTCAGGG | 7mer-A1 |
|  |  |  | SHEEP | TGTGCTGGTTCAGGG | 7mer-A1 |

  
  
  
  

| Seed Matches to the miRNA miR-431-5p | | | | | |
| --- | --- | --- | --- | --- | --- |
| Seed | Conservation | Species | Matches | | |
|  |  |  | Sequence | Motif | Type |
| GUCUUGC | Conserved | Human (Homo sapiens)  Mouse (Mus musculus) | HUMAN | TTGTTACATGTTTGCAGTTTATTCAAGACTGCT | 6mer |
|  |  |  | HUMAN | TGCAGTTTATTCAAGACTGCT | 6mer |
|  |  |  | HUMAN | GCAGTTTATTCAAGACTGCT | 6mer |
|  |  |  | BABOON | TTGTTACATGTTTGCAGTTTATTCAAGACTGCT | 6mer |
|  |  |  | BABOON | TGCAGTTTATTCAAGACTGCT | 6mer |
|  |  |  | BABOON | GCAGTTTATTCAAGACTGCT | 6mer |
|  |  |  | DOG | TGCAGTTTATTCAAGACTGCT | 6mer |
|  |  |  | DOG | GCAGTTTATTCAAGACTGCT | 6mer |
|  |  |  | COW | GCAGTTTATTCAAGACTGCT | 6mer |
|  |  |  | SHEEP | GCAGTTTATTCAAGACTGCT | 6mer |

  
  
  
  

| Seed Matches to the miRNA miR-296-5p | | | | | |
| --- | --- | --- | --- | --- | --- |
| Seed | Conservation | Species | Matches | | |
|  |  |  | Sequence | Motif | Type |
| GGGCCCC | Conserved | Human (Homo sapiens)  Rhesus (Macaca mulatta)  Mouse (Mus musculus)  Rat (Rattus norvegicus) | HUMAN | CAGGGCCCTCCAGGCCCTCCGGCCC | 6mer |
|  |  |  | HUMAN | GGGCCCTCCAGGCCCTCCGGCC | 6mer |
|  |  |  | HUMAN | GGGCCGGCGGGTGAACTGGGGGGCCCCGGGACAGGCCGAGCCCT | 7mer-m8 |
|  |  |  | HUMAN | GGGTGAACTGGGGGGCCC | 7mer-m8 |
|  |  |  | HUMAN | GGTGAACTGGGGGGCCC | 7mer-m8 |
|  |  |  | HUMAN | GGGGGGCCC | 7mer-m8 |
|  |  |  | HUMAN | GGGCCC | 7mer-m8 |
|  |  |  | BABOON | CAGGGCCCTCCAGGCCCTCCGGCCC | 6mer |
|  |  |  | BABOON | GGGCCCTCCAGGCCCTCCGGCC | 6mer |
|  |  |  | BABOON | GGGCCGGCGGGTGAACTGGGGGGCCCCGGGACAGGCCGAGCCCT | 7mer-m8 |
|  |  |  | BABOON | GGGTGAACTGGGGGGCCC | 7mer-m8 |
|  |  |  | BABOON | GGTGAACTGGGGGGCCC | 7mer-m8 |
|  |  |  | BABOON | GGGGGGCCC | 7mer-m8 |
|  |  |  | BABOON | GGGCCC | 7mer-m8 |
|  |  |  | DOG | GGGCCCTCCAGGCCCTCCGGCC | 6mer |
|  |  |  | DOG | GGGTGAACTGGGGGGCCC | 8mer |
|  |  |  | DOG | GGTGAACTGGGGGGCCC | 8mer |
|  |  |  | DOG | GGGGGGCCC | 8mer |
|  |  |  | DOG | GGGCCC | 8mer |
|  |  |  | COW | GGGCCCTCCAGGCCCTCCGGCC | 7mer-m8 |
|  |  |  | COW | GGTGAACTGGGGGGCCC | 7mer-m8 |
|  |  |  | COW | GGGGGGCCC | 7mer-m8 |
|  |  |  | COW | GGGCCC | 7mer-m8 |
|  |  |  | SHEEP | GGGCCCTCCAGGCCCTCCGGCC | 7mer-m8 |
|  |  |  | SHEEP | GGTGAACTGGGGGGCCC | 7mer-m8 |
|  |  |  | SHEEP | GGGGGGCCC | 7mer-m8 |
|  |  |  | SHEEP | GGGCCC | 7mer-m8 |
|  |  |  | PIG | GGTGAACTGGGGGGCCC | 7mer-m8 |
|  |  |  | PIG | GGGGGGCCC | 7mer-m8 |
|  |  |  | PIG | GGGCCC | 7mer-m8 |
|  |  |  | ARMADILLO | GGGGGGCCC | 7mer-m8 |
|  |  |  | ARMADILLO | GGGCCC | 7mer-m8 |
|  |  |  | GUINEAPIG | GGGCCC | 7mer-A1 |

  
  
  
  

| Seed Matches to the miRNA miR-433-3p | | | | | |
| --- | --- | --- | --- | --- | --- |
| Seed | Conservation | Species | Matches | | |
|  |  |  | Sequence | Motif | Type |
| UCAUGAU | Conserved | Human (Homo sapiens)  Rhesus (Macaca mulatta)  Mouse (Mus musculus)  Rat (Rattus norvegicus) | HUMAN | AATACTCATATAGCTTTGGGATTTTGAATTGGTAAATATTCATGATGTGTGAAAAATCATGATACATACTGTACA | 7mer-m8 |
|  |  |  | BABOON | AATACTCATATAGCTTTGGGATTTTGAATTGGTAAATATTCATGATGTGTGAAAAATCATGATACATACTGTACA | 7mer-m8 |

  
  
  
  

| Seed Matches to the miRNA miR-491-5p | | | | | |
| --- | --- | --- | --- | --- | --- |
| Seed | Conservation | Species | Matches | | |
|  |  |  | Sequence | Motif | Type |
| GUGGGGA | Conserved | Human (Homo sapiens)  Rhesus (Macaca mulatta)  Mouse (Mus musculus) | HUMAN | CCCACCCCACCTCGGTGACT | 6mer |
|  |  |  | BABOON | CCCACCCCACCTCGGTGACT | 6mer |

  
  
  
  

| Seed Matches to the miRNA miR-338-3p | | | | | |
| --- | --- | --- | --- | --- | --- |
| Seed | Conservation | Species | Matches | | |
|  |  |  | Sequence | Motif | Type |
| CCAGCAU | Broadly Conserved | Human (Homo sapiens)  Rhesus (Macaca mulatta)  Mouse (Mus musculus)  Rat (Rattus norvegicus) | HUMAN | TATTTGTGCTGGTTCAGGGGGAAGGAGGAGCACAAAGTGCAAAGGGCTTTCTACCAGTGTCCAGTGTGTTTA | 6mer |
|  |  |  | HUMAN | TGTGCTGGTTCAGGG | 6mer |
|  |  |  | HUMAN | TGTGCTGGTTCA | 6mer |
|  |  |  | BABOON | TATTTGTGCTGGTTCAGGGGGAAGGAGGAGCACAAAGTGCAAAGGGCTTTCTACCAGTGTCCAGTGTGTTTA | 6mer |
|  |  |  | BABOON | TGTGCTGGTTCAGGG | 6mer |
|  |  |  | BABOON | TGTGCTGGTTCA | 6mer |
|  |  |  | DOG | TGTGCTGGTTCAGGG | 6mer |
|  |  |  | DOG | TGTGCTGGTTCA | 6mer |
|  |  |  | COW | TGTGCTGGTTCAGGG | 6mer |
|  |  |  | COW | TGTGCTGGTTCA | 6mer |
|  |  |  | SHEEP | TGTGCTGGTTCAGGG | 6mer |
|  |  |  | SHEEP | TGTGCTGGTTCA | 6mer |
|  |  |  | PIG | TGTGCTGGTTCA | 6mer |

  
  
  
  

| Seed Matches to the miRNA miR-374-5p | | | | | |
| --- | --- | --- | --- | --- | --- |
| Seed | Conservation | Species | Matches | | |
|  |  |  | Sequence | Motif | Type |
| UAUAAUA | Conserved | Human (Homo sapiens)  Rhesus (Macaca mulatta)  Rat (Rattus norvegicus) | HUMAN | AAATTATAA | 7mer-A1 |
|  |  |  | HUMAN | ATTATAA | 7mer-A1 |
|  |  |  | BABOON | AAATTATAA | 7mer-A1 |
|  |  |  | BABOON | ATTATAA | 7mer-A1 |
|  |  |  | DOG | ATTATAA | 7mer-A1 |

  
  
  
  

| Seed Matches to the miRNA miR-302c-3p.2/520-3p | | | | | |
| --- | --- | --- | --- | --- | --- |
| Seed | Conservation | Species | Matches | | |
|  |  |  | Sequence | Motif | Type |
| AGUGCUU | Broadly Conserved | Human (Homo sapiens) | HUMAN | ATGCCTGTAATCCCAGCACTTTGGGAGGCCGAGGCAGGC | 6mer |
|  |  |  | BABOON | ATGCCTGTAATCCCAGCACTTTGGGAGGCCGAGGCAGGC | 6mer |

  
  
  
  

| Seed Matches to the miRNA miR-371-5p | | | | | |
| --- | --- | --- | --- | --- | --- |
| Seed | Conservation | Species | Matches | | |
|  |  |  | Sequence | Motif | Type |
| CUCAAAC | Conserved | Human (Homo sapiens)  Rhesus (Macaca mulatta) | HUMAN | AGCAGTTTGAGTTGGTGTAGTGTATTCTTGGTTATCA | 7mer-m8 |
|  |  |  | BABOON | AGCAGTTTGAGTTGGTGTAGTGTATTCTTGGTTATCA | 7mer-m8 |

  
  
  
  

| Seed Matches to the miRNA miR-134-5p | | | | | |
| --- | --- | --- | --- | --- | --- |
| Seed | Conservation | Species | Matches | | |
|  |  |  | Sequence | Motif | Type |
| GUGACUG | Conserved | Human (Homo sapiens)  Rhesus (Macaca mulatta)  Mouse (Mus musculus)  Rat (Rattus norvegicus) | HUMAN | TACTTAGTCAC | 6mer |
|  |  |  | BABOON | TACTTAGTCAC | 6mer |

  
  
  
  

| Seed Matches to the miRNA miR-1306-5p | | | | | |
| --- | --- | --- | --- | --- | --- |
| Seed | Conservation | Species | Matches | | |
|  |  |  | Sequence | Motif | Type |
| CACCUCC | Conserved | Human (Homo sapiens)  Rhesus (Macaca mulatta)  Mouse (Mus musculus)  Rat (Rattus norvegicus) | HUMAN | AGGAGAGAGGAGGGGGAGGTGCAGAGAGCT | 7mer-m8 |
|  |  |  | HUMAN | CTGGGAGGTGGGAGGGAA | 7mer-m8 |
|  |  |  | HUMAN | GGGAGGTGGGAGGGAA | 7mer-m8 |
|  |  |  | HUMAN | GGTTGGTAGAGGTGGGAAAGGGAAGGGTTCTAGGCCAGAATGTTCCTATTTAGAAGACACTCAAATTA | 6mer |
|  |  |  | BABOON | AGGAGAGAGGAGGGGGAGGTGCAGAGAGCT | 7mer-m8 |
|  |  |  | BABOON | CTGGGAGGTGGGAGGGAA | 7mer-m8 |
|  |  |  | BABOON | GGGAGGTGGGAGGGAA | 7mer-m8 |
|  |  |  | BABOON | GGTTGGTAGAGGTGGGAAAGGGAAGGGTTCTAGGCCAGAATGTTCCTATTTAGAAGACACTCAAATTA | 6mer |
|  |  |  | DOG | GGGAGGTGGGAGGGAA | 7mer-m8 |
|  |  |  | COW | GGGAGGTGGGAGGGAA | 7mer-m8 |
|  |  |  | SHEEP | GGGAGGTGGGAGGGAA | 7mer-m8 |

  
  
  
  

| Seed Matches to the miRNA miR-208-3p | | | | | |
| --- | --- | --- | --- | --- | --- |
| Seed | Conservation | Species | Matches | | |
|  |  |  | Sequence | Motif | Type |
| UAAGACG | Broadly Conserved | Human (Homo sapiens)  Rhesus (Macaca mulatta)  Mouse (Mus musculus)  Rat (Rattus norvegicus)  Opossum (Monodelphis domestica) | HUMAN | GGGTTCTGTGCTCCTGAGATTAGTTCAGATGGTCTAACCATTGTTCTATATGTGCATTTTAGTTAATATTGTGTATTAAAGGATAAGTCTTAATGCTCAAAGTATGTTAAAAATAGATGTAGTAAA | 7mer-A1 |
|  |  |  | BABOON | GGGTTCTGTGCTCCTGAGATTAGTTCAGATGGTCTAACCATTGTTCTATATGTGCATTTTAGTTAATATTGTGTATTAAAGGATAAGTCTTAATGCTCAAAGTATGTTAAAAATAGATGTAGTAAA | 7mer-A1 |

  
  
  
  

| Seed Matches to the miRNA miR-543 | | | | | |
| --- | --- | --- | --- | --- | --- |
| Seed | Conservation | Species | Matches | | |
|  |  |  | Sequence | Motif | Type |
| AACAUUC | Conserved | Human (Homo sapiens)  Chimp (Pan troglodytes)  Dog (Canis lupus familiaris)  Cow (Bos taurus) | HUMAN | TAAACTAATATATTTTGTACATTTTGTTTTACAAGTCCTAGGAAAGATTGTCTTCTGAAAATTTGATGTCTTCTGGGTTGATGGAGATGGGAAGGGTTCTAGGCCAGAATGTTCACATTTGGAAGACT | 7mer-m8 |
|  |  |  | HUMAN | AGAATGTTC | 7mer-m8 |
|  |  |  | HUMAN | CAGGTTGGTGGTGGAGAGGAGTTGGAAGGAATGAAGGGTTCTAGACCAGAATGTTC | 7mer-m8 |
|  |  |  | HUMAN | AGAATGTTC | 7mer-m8 |
|  |  |  | HUMAN | AGAATGTT | 7mer-m8 |
|  |  |  | HUMAN | GGTTGGTAGAGGTGGGAAAGGGAAGGGTTCTAGGCCAGAATGTTCCTATTTAGAAGACACTCAAATTA | 7mer-m8 |
|  |  |  | BABOON | TAAACTAATATATTTTGTACATTTTGTTTTACAAGTCCTAGGAAAGATTGTCTTCTGAAAATTTGATGTCTTCTGGGTTGATGGAGATGGGAAGGGTTCTAGGCCAGAATGTTCACATTTGGAAGACT | 7mer-m8 |
|  |  |  | BABOON | AGAATGTTC | 7mer-m8 |
|  |  |  | BABOON | CAGGTTGGTGGTGGAGAGGAGTTGGAAGGAATGAAGGGTTCTAGACCAGAATGTTC | 7mer-m8 |
|  |  |  | BABOON | AGAATGTTC | 7mer-m8 |
|  |  |  | BABOON | AGAATGTT | 7mer-m8 |
|  |  |  | BABOON | GGTTGGTAGAGGTGGGAAAGGGAAGGGTTCTAGGCCAGAATGTTCCTATTTAGAAGACACTCAAATTA | 7mer-m8 |
|  |  |  | DOG | AGAATGTTC | 7mer-m8 |
|  |  |  | DOG | AGAATGTTC | 7mer-m8 |
|  |  |  | DOG | AGAATGTT | 7mer-m8 |
|  |  |  | COW | AGAATGTTC | 7mer-m8 |
|  |  |  | COW | AGAATGTT | 7mer-m8 |
|  |  |  | SHEEP | AGAATGTTC | 7mer-m8 |
|  |  |  | SHEEP | AGAATGTT | 7mer-m8 |
|  |  |  | PIG | AGAATGTT | 7mer-m8 |
|  |  |  | ARMADILLO | AGAATGTT | 7mer-m8 |

  
  
  
  

| Seed Matches to the miRNA miR-495-3p | | | | | |
| --- | --- | --- | --- | --- | --- |
| Seed | Conservation | Species | Matches | | |
|  |  |  | Sequence | Motif | Type |
| AACAAAC | Conserved | Human (Homo sapiens)  Rhesus (Macaca mulatta)  Mouse (Mus musculus) | HUMAN | ACACATCTTGTAAATTCTCATTTGTTTAAAAGAAATCATAGAAAATAC | 6mer |
|  |  |  | HUMAN | TTTGTTT | 6mer |
|  |  |  | HUMAN | CTTTTTGTTACAATTCATAGGAAAT | 7mer-A1 |
|  |  |  | HUMAN | TCTGTGCATTTTGTTTTACTTATCTGTGTATATAGTGTACATAAAGGACAGACGAGTCCTAATTGACAACATCTAGTCTTTCTGGATGTTAAAGAGGTTGCCAGTGTATGACAAAAGTAGAGTT | 6mer |
|  |  |  | HUMAN | TGCATTTTGTTTTACTTA | 6mer |
|  |  |  | HUMAN | TGCATTTTGTTT | 6mer |
|  |  |  | HUMAN | TTTTGTTT | 6mer |
|  |  |  | HUMAN | TAAACTAATATATTTTGTACATTTTGTTTTACAAGTCCTAGGAAAGATTGTCTTCTGAAAATTTGATGTCTTCTGGGTTGATGGAGATGGGAAGGGTTCTAGGCCAGAATGTTCACATTTGGAAGACT | 6mer |
|  |  |  | HUMAN | TATGTTTTGTGCATTTTGTTTTGCTCTGTATATAGT | 6mer |
|  |  |  | HUMAN | TTTGCATAGGAATTTGTT | 6mer |
|  |  |  | HUMAN | GAATTTGTT | 6mer |
|  |  |  | HUMAN | TAATATCTAGTCTCTAGATATTAAAGAGGTTGCCAATGTATGACAGAAGTAGAGTTAGTAAACTAACACATTTTGTACACTTTGTTAAAATTTGTAGAAAGGCTGTCTTCTGAAAAGGACTTTTGGAAGTGA | 7mer-A1 |
|  |  |  | HUMAN | AGTAGAGGGCTTAAGTAACACCCCTCTAAGCATTTGTTTTCA | 6mer |
|  |  |  | HUMAN | CAGTCCCTTTGTGAATGTCCTTTTGTTA | 7mer-A1 |
|  |  |  | HUMAN | AATGTCCTTTTGTTA | 7mer-A1 |
|  |  |  | HUMAN | CCTTTTGTT | 7mer-A1 |
|  |  |  | HUMAN | ATTTGTAAATACCTTTGTTATAATTGATAG | 7mer-A1 |
|  |  |  | HUMAN | CTTTGTTAT | 7mer-A1 |
|  |  |  | HUMAN | CTTTGTTA | 7mer-A1 |
|  |  |  | HUMAN | TTTGTTA | 7mer-A1 |
|  |  |  | HUMAN | AATGACTTTGTTCTTTGCTT | 6mer |
|  |  |  | HUMAN | ATGACTTTGTTCTTTGCTT | 6mer |
|  |  |  | BABOON | ACACATCTTGTAAATTCTCATTTGTTTAAAAGAAATCATAGAAAATAC | 6mer |
|  |  |  | BABOON | TTTGTTT | 6mer |
|  |  |  | BABOON | CTTTTTGTTACAATTCATAGGAAAT | 7mer-A1 |
|  |  |  | BABOON | TCTGTGCATTTTGTTTTACTTATCTGTGTATATAGTGTACATAAAGGACAGACGAGTCCTAATTGACAACATCTAGTCTTTCTGGATGTTAAAGAGGTTGCCAGTGTATGACAAAAGTAGAGTT | 6mer |
|  |  |  | BABOON | TGCATTTTGTTTTACTTA | 6mer |
|  |  |  | BABOON | TGCATTTTGTTT | 6mer |
|  |  |  | BABOON | TTTTGTTT | 6mer |
|  |  |  | BABOON | TAAACTAATATATTTTGTACATTTTGTTTTACAAGTCCTAGGAAAGATTGTCTTCTGAAAATTTGATGTCTTCTGGGTTGATGGAGATGGGAAGGGTTCTAGGCCAGAATGTTCACATTTGGAAGACT | 6mer |
|  |  |  | BABOON | TATGTTTTGTGCATTTTGTTTTGCTCTGTATATAGT | 6mer |
|  |  |  | BABOON | TTTGCATAGGAATTTGTT | 6mer |
|  |  |  | BABOON | GAATTTGTT | 6mer |
|  |  |  | BABOON | TAATATCTAGTCTCTAGATATTAAAGAGGTTGCCAATGTATGACAGAAGTAGAGTTAGTAAACTAACACATTTTGTACACTTTGTTAAAATTTGTAGAAAGGCTGTCTTCTGAAAAGGACTTTTGGAAGTGA | 7mer-A1 |
|  |  |  | BABOON | AGTAGAGGGCTTAAGTAACACCCCTCTAAGCATTTGTTTTCA | 6mer |
|  |  |  | BABOON | CAGTCCCTTTGTGAATGTCCTTTTGTTA | 7mer-A1 |
|  |  |  | BABOON | AATGTCCTTTTGTTA | 7mer-A1 |
|  |  |  | BABOON | CCTTTTGTT | 7mer-A1 |
|  |  |  | BABOON | ATTTGTAAATACCTTTGTTATAATTGATAG | 7mer-A1 |
|  |  |  | BABOON | CTTTGTTAT | 7mer-A1 |
|  |  |  | BABOON | CTTTGTTA | 7mer-A1 |
|  |  |  | BABOON | TTTGTTA | 7mer-A1 |
|  |  |  | BABOON | AATGACTTTGTTCTTTGCTT | 6mer |
|  |  |  | BABOON | ATGACTTTGTTCTTTGCTT | 6mer |
|  |  |  | DOG | TTTGTTT | 6mer |
|  |  |  | DOG | TTTGTTT | 6mer |
|  |  |  | DOG | TGCATTTTGTTTTACTTA | 6mer |
|  |  |  | DOG | TGCATTTTGTTT | 6mer |
|  |  |  | DOG | TTTTGTTT | 6mer |
|  |  |  | DOG | GAATTTGTT | 6mer |
|  |  |  | DOG | AATGTCCTTTTGTTA | 7mer-A1 |
|  |  |  | DOG | CCTTTTGTT | 7mer-A1 |
|  |  |  | DOG | CTTTGTTAT | 7mer-A1 |
|  |  |  | DOG | CTTTGTTA | 7mer-A1 |
|  |  |  | DOG | TTTGTTA | 7mer-A1 |
|  |  |  | DOG | ATGACTTTGTTCTTTGCTT | 6mer |
|  |  |  | COW | TTTGTTT | 6mer |
|  |  |  | COW | TGCATTTTGTTT | 6mer |
|  |  |  | COW | TTTTGTTT | 6mer |
|  |  |  | COW | CCTTTTGTT | 6mer |
|  |  |  | COW | CTTTGTTA | 7mer-A1 |
|  |  |  | COW | TTTGTTA | 7mer-A1 |
|  |  |  | SHEEP | TTTGTTT | 6mer |
|  |  |  | SHEEP | TTTTGTTT | 6mer |
|  |  |  | SHEEP | TTTGTTA | 7mer-A1 |

  
  
  
  

| Seed Matches to the miRNA miR-34-5p/449-5p | | | | | |
| --- | --- | --- | --- | --- | --- |
| Seed | Conservation | Species | Matches | | |
|  |  |  | Sequence | Motif | Type |
| GGCAGUG | Broadly Conserved | Human (Homo sapiens)  Chicken (Gallus gallus)  Rhesus (Macaca mulatta)  Mouse (Mus musculus)  Rat (Rattus norvegicus)  Opossum (Monodelphis domestica) | HUMAN | CAGAGAACTGCCAAGTCAGTTCCGGTC | 7mer-A1 |
|  |  |  | HUMAN | AGAGAACTGCCAAGTCAGTTCCGG | 7mer-A1 |
|  |  |  | HUMAN | AGAGAACTGCCAA | 7mer-A1 |
|  |  |  | BABOON | CAGAGAACTGCCAAGTCAGTTCCGGTC | 7mer-A1 |
|  |  |  | BABOON | AGAGAACTGCCAAGTCAGTTCCGG | 7mer-A1 |
|  |  |  | BABOON | AGAGAACTGCCAA | 7mer-A1 |
|  |  |  | DOG | AGAGAACTGCCAAGTCAGTTCCGG | 7mer-A1 |
|  |  |  | DOG | AGAGAACTGCCAA | 7mer-A1 |
|  |  |  | COW | AGAGAACTGCCAAGTCAGTTCCGG | 7mer-A1 |
|  |  |  | COW | AGAGAACTGCCAA | 7mer-A1 |
|  |  |  | SHEEP | AGAGAACTGCCAA | 7mer-A1 |
|  |  |  | PIG | AGAGAACTGCCAA | 7mer-A1 |
|  |  |  | ARMADILLO | AGAGAACTGCCAA | 7mer-A1 |

  
  
  
  

| Seed Matches to the miRNA miR-154-3p/487-3p | | | | | |
| --- | --- | --- | --- | --- | --- |
| Seed | Conservation | Species | Matches | | |
|  |  |  | Sequence | Motif | Type |
| AUCAUAC | Conserved | Human (Homo sapiens) | HUMAN | TAGATGGAAAGAGGTTGCCGACGTATGATAAA | 8mer |
|  |  |  | HUMAN | TATGATAAA | 8mer |
|  |  |  | HUMAN | GAATGGGGTGACAACTTATGATAAAAACTAGAGCTAGTGAATTAGCC | 7mer-A1 |
|  |  |  | HUMAN | TATGATAAAA | 7mer-A1 |
|  |  |  | HUMAN | TATGATA | 7mer-A1 |
|  |  |  | BABOON | TAGATGGAAAGAGGTTGCCGACGTATGATAAA | 8mer |
|  |  |  | BABOON | TATGATAAA | 8mer |
|  |  |  | BABOON | GAATGGGGTGACAACTTATGATAAAAACTAGAGCTAGTGAATTAGCC | 7mer-A1 |
|  |  |  | BABOON | TATGATAAAA | 7mer-A1 |
|  |  |  | BABOON | TATGATA | 7mer-A1 |
|  |  |  | DOG | TATGATAAA | 7mer-A1 |
|  |  |  | DOG | TATGATAAAA | 8mer |
|  |  |  | DOG | TATGATA | 8mer |
|  |  |  | COW | TATGATAAA | 8mer |
|  |  |  | COW | TATGATA | 8mer |
|  |  |  | SHEEP | TATGATAAA | 8mer |
|  |  |  | PIG | TATGATAAA | 8mer |
|  |  |  | ARMADILLO | TATGATAAA | 8mer |
|  |  |  | GUINEAPIG | TATGATAAA | 7mer-A1 |

  
  
  
  

| Seed Matches to the miRNA miR-670-3p | | | | | |
| --- | --- | --- | --- | --- | --- |
| Seed | Conservation | Species | Matches | | |
|  |  |  | Sequence | Motif | Type |
| UUCCUCA | Conserved | Human (Homo sapiens)  Rhesus (Macaca mulatta)  Mouse (Mus musculus) | HUMAN | GAGGAAGGGTGAAGGGAAGGGCTCTTTGCTAGTATCT | 6mer |
|  |  |  | BABOON | GAGGAAGGGTGAAGGGAAGGGCTCTTTGCTAGTATCT | 6mer |

  
  
  
  

| Seed Matches to the miRNA miR-150-5p | | | | | |
| --- | --- | --- | --- | --- | --- |
| Seed | Conservation | Species | Matches | | |
|  |  |  | Sequence | Motif | Type |
| CUCCCAA | Broadly Conserved | Human (Homo sapiens)  Rhesus (Macaca mulatta)  Mouse (Mus musculus)  Rat (Rattus norvegicus)  Opossum (Monodelphis domestica) | HUMAN | CTGGGAGGTGGGAGGGAA | 6mer |
|  |  |  | HUMAN | GGGAGGTGGGAGGGAA | 6mer |
|  |  |  | HUMAN | GTGGGAGGGAA | 6mer |
|  |  |  | HUMAN | ATGCCTGTAATCCCAGCACTTTGGGAGGCCGAGGCAGGC | 7mer-m8 |
|  |  |  | HUMAN | GCTTGAACTTGGGAGGCGGA | 7mer-m8 |
|  |  |  | HUMAN | TCTGGGAGTGACCTT | 6mer |
|  |  |  | HUMAN | TCTGGGAGTG | 6mer |
|  |  |  | HUMAN | TCTGGGAGT | 6mer |
|  |  |  | BABOON | CTGGGAGGTGGGAGGGAA | 6mer |
|  |  |  | BABOON | GGGAGGTGGGAGGGAA | 6mer |
|  |  |  | BABOON | GTGGGAGGGAA | 6mer |
|  |  |  | BABOON | ATGCCTGTAATCCCAGCACTTTGGGAGGCCGAGGCAGGC | 7mer-m8 |
|  |  |  | BABOON | GCTTGAACTTGGGAGGCGGA | 7mer-m8 |
|  |  |  | BABOON | TCTGGGAGTGACCTT | 6mer |
|  |  |  | BABOON | TCTGGGAGTG | 6mer |
|  |  |  | BABOON | TCTGGGAGT | 6mer |
|  |  |  | DOG | GGGAGGTGGGAGGGAA | 6mer |
|  |  |  | DOG | GTGGGAGGGAA | 6mer |
|  |  |  | DOG | TCTGGGAGTG | 6mer |
|  |  |  | DOG | TCTGGGAGT | 6mer |
|  |  |  | COW | GGGAGGTGGGAGGGAA | 6mer |
|  |  |  | COW | GTGGGAGGGAA | 6mer |
|  |  |  | COW | TCTGGGAGT | 6mer |
|  |  |  | SHEEP | GGGAGGTGGGAGGGAA | 6mer |
|  |  |  | SHEEP | GTGGGAGGGAA | 6mer |
|  |  |  | PIG | GTGGGAGGGAA | 6mer |
|  |  |  | ARMADILLO | GTGGGAGGGAA | 6mer |
|  |  |  | GUINEAPIG | GTGGGAGGGAA | 6mer |
|  |  |  | MOUSE | GTGGGAGGGAA | 6mer |

  
  
  
  

| Seed Matches to the miRNA miR-532-3p | | | | | |
| --- | --- | --- | --- | --- | --- |
| Seed | Conservation | Species | Matches | | |
|  |  |  | Sequence | Motif | Type |
| CUCCCAC | Conserved | Human (Homo sapiens)  Rhesus (Macaca mulatta)  Mouse (Mus musculus)  Rat (Rattus norvegicus) | HUMAN | CTGGGAGGTGGGAGGGAA | 7mer-m8 |
|  |  |  | HUMAN | GGGAGGTGGGAGGGAA | 7mer-m8 |
|  |  |  | HUMAN | GTGGGAGGGAA | 7mer-m8 |
|  |  |  | HUMAN | ATGCCTGTAATCCCAGCACTTTGGGAGGCCGAGGCAGGC | 6mer |
|  |  |  | HUMAN | GCTTGAACTTGGGAGGCGGA | 6mer |
|  |  |  | HUMAN | TCTGGGAGTGACCTT | 6mer |
|  |  |  | HUMAN | TCTGGGAGTG | 6mer |
|  |  |  | HUMAN | TCTGGGAGT | 6mer |
|  |  |  | BABOON | CTGGGAGGTGGGAGGGAA | 7mer-m8 |
|  |  |  | BABOON | GGGAGGTGGGAGGGAA | 7mer-m8 |
|  |  |  | BABOON | GTGGGAGGGAA | 7mer-m8 |
|  |  |  | BABOON | ATGCCTGTAATCCCAGCACTTTGGGAGGCCGAGGCAGGC | 6mer |
|  |  |  | BABOON | GCTTGAACTTGGGAGGCGGA | 6mer |
|  |  |  | BABOON | TCTGGGAGTGACCTT | 6mer |
|  |  |  | BABOON | TCTGGGAGTG | 6mer |
|  |  |  | BABOON | TCTGGGAGT | 6mer |
|  |  |  | DOG | GGGAGGTGGGAGGGAA | 7mer-m8 |
|  |  |  | DOG | GTGGGAGGGAA | 7mer-m8 |
|  |  |  | DOG | TCTGGGAGTG | 6mer |
|  |  |  | DOG | TCTGGGAGT | 6mer |
|  |  |  | COW | GGGAGGTGGGAGGGAA | 7mer-m8 |
|  |  |  | COW | GTGGGAGGGAA | 7mer-m8 |
|  |  |  | COW | TCTGGGAGT | 6mer |
|  |  |  | SHEEP | GGGAGGTGGGAGGGAA | 7mer-m8 |
|  |  |  | SHEEP | GTGGGAGGGAA | 7mer-m8 |
|  |  |  | PIG | GTGGGAGGGAA | 7mer-m8 |
|  |  |  | ARMADILLO | GTGGGAGGGAA | 7mer-m8 |
|  |  |  | GUINEAPIG | GTGGGAGGGAA | 7mer-m8 |
|  |  |  | MOUSE | GTGGGAGGGAA | 7mer-m8 |

  
  
  
  

| Seed Matches to the miRNA miR-181-5p | | | | | |
| --- | --- | --- | --- | --- | --- |
| Seed | Conservation | Species | Matches | | |
|  |  |  | Sequence | Motif | Type |
| ACAUUCA | Broadly Conserved | Human (Homo sapiens)  X. tropicalis (Xenopus tropicalis)  Chicken (Gallus gallus)  Rhesus (Macaca mulatta)  Chimp (Pan troglodytes)  Mouse (Mus musculus)  Rat (Rattus norvegicus)  Opossum (Monodelphis domestica) | HUMAN | TAAACTAATATATTTTGTACATTTTGTTTTACAAGTCCTAGGAAAGATTGTCTTCTGAAAATTTGATGTCTTCTGGGTTGATGGAGATGGGAAGGGTTCTAGGCCAGAATGTTCACATTTGGAAGACT | 6mer |
|  |  |  | HUMAN | AGAATGTTC | 6mer |
|  |  |  | HUMAN | CAGGTTGGTGGTGGAGAGGAGTTGGAAGGAATGAAGGGTTCTAGACCAGAATGTTC | 6mer |
|  |  |  | HUMAN | AGAATGTTC | 6mer |
|  |  |  | HUMAN | AGAATGTT | 6mer |
|  |  |  | HUMAN | GGTTGGTAGAGGTGGGAAAGGGAAGGGTTCTAGGCCAGAATGTTCCTATTTAGAAGACACTCAAATTA | 6mer |
|  |  |  | HUMAN | CAGTCCCTTTGTGAATGTCCTTTTGTTA | 7mer-m8 |
|  |  |  | BABOON | TAAACTAATATATTTTGTACATTTTGTTTTACAAGTCCTAGGAAAGATTGTCTTCTGAAAATTTGATGTCTTCTGGGTTGATGGAGATGGGAAGGGTTCTAGGCCAGAATGTTCACATTTGGAAGACT | 6mer |
|  |  |  | BABOON | AGAATGTTC | 6mer |
|  |  |  | BABOON | CAGGTTGGTGGTGGAGAGGAGTTGGAAGGAATGAAGGGTTCTAGACCAGAATGTTC | 6mer |
|  |  |  | BABOON | AGAATGTTC | 6mer |
|  |  |  | BABOON | AGAATGTT | 6mer |
|  |  |  | BABOON | GGTTGGTAGAGGTGGGAAAGGGAAGGGTTCTAGGCCAGAATGTTCCTATTTAGAAGACACTCAAATTA | 6mer |
|  |  |  | BABOON | CAGTCCCTTTGTGAATGTCCTTTTGTTA | 7mer-m8 |
|  |  |  | DOG | AGAATGTTC | 6mer |
|  |  |  | DOG | AGAATGTTC | 6mer |
|  |  |  | DOG | AGAATGTT | 6mer |
|  |  |  | COW | AGAATGTTC | 6mer |
|  |  |  | COW | AGAATGTT | 6mer |
|  |  |  | SHEEP | AGAATGTTC | 6mer |
|  |  |  | SHEEP | AGAATGTT | 6mer |
|  |  |  | PIG | AGAATGTT | 6mer |
|  |  |  | ARMADILLO | AGAATGTT | 6mer |

  
  
  
  

| Seed Matches to the miRNA miR-136-5p | | | | | |
| --- | --- | --- | --- | --- | --- |
| Seed | Conservation | Species | Matches | | |
|  |  |  | Sequence | Motif | Type |
| CUCCAUU | Conserved | Human (Homo sapiens)  Mouse (Mus musculus)  Rat (Rattus norvegicus) | HUMAN | TTAAGTGGGTTAGATGACATGGAGCTGGAAGAC | 6mer |
|  |  |  | HUMAN | TAAACTAATATATTTTGTACATTTTGTTTTACAAGTCCTAGGAAAGATTGTCTTCTGAAAATTTGATGTCTTCTGGGTTGATGGAGATGGGAAGGGTTCTAGGCCAGAATGTTCACATTTGGAAGACT | 7mer-A1 |
|  |  |  | BABOON | TTAAGTGGGTTAGATGACATGGAGCTGGAAGAC | 6mer |
|  |  |  | BABOON | TAAACTAATATATTTTGTACATTTTGTTTTACAAGTCCTAGGAAAGATTGTCTTCTGAAAATTTGATGTCTTCTGGGTTGATGGAGATGGGAAGGGTTCTAGGCCAGAATGTTCACATTTGGAAGACT | 7mer-A1 |

  
  
  
  

| Seed Matches to the miRNA miR-802 | | | | | |
| --- | --- | --- | --- | --- | --- |
| Seed | Conservation | Species | Matches | | |
|  |  |  | Sequence | Motif | Type |
| CAGUAAC | Broadly Conserved | Human (Homo sapiens) | HUMAN | CCACCCTCTGGGAAGATTTACTGGCC | 6mer |
|  |  |  | HUMAN | TGGGAAGATTTACTGGCC | 6mer |
|  |  |  | HUMAN | TGTCTGCATTTTCATTTACTGTGCTGTGTATATAGTGTATATAAG | 6mer |
|  |  |  | BABOON | CCACCCTCTGGGAAGATTTACTGGCC | 6mer |
|  |  |  | BABOON | TGGGAAGATTTACTGGCC | 6mer |
|  |  |  | BABOON | TGTCTGCATTTTCATTTACTGTGCTGTGTATATAGTGTATATAAG | 6mer |
|  |  |  | DOG | TGGGAAGATTTACTGGCC | 6mer |
|  |  |  | COW | TGGGAAGATTTACTGGCC | 6mer |
|  |  |  | SHEEP | TGGGAAGATTTACTGGCC | 6mer |

  
  
  
  

| Seed Matches to the miRNA miR-615-3p | | | | | |
| --- | --- | --- | --- | --- | --- |
| Seed | Conservation | Species | Matches | | |
|  |  |  | Sequence | Motif | Type |
| CCGAGCC | Conserved | Human (Homo sapiens)  Rhesus (Macaca mulatta)  Mouse (Mus musculus) | HUMAN | GCAGGCTCGGC | 7mer-m8 |
|  |  |  | HUMAN | GCTCGGC | 7mer-m8 |
|  |  |  | BABOON | GCAGGCTCGGC | 7mer-m8 |
|  |  |  | BABOON | GCTCGGC | 7mer-m8 |
|  |  |  | DOG | GCTCGGC | 6mer |

  
  
  
  

| Seed Matches to the miRNA miR-204-5p/211-5p | | | | | |
| --- | --- | --- | --- | --- | --- |
| Seed | Conservation | Species | Matches | | |
|  |  |  | Sequence | Motif | Type |
| UCCCUUU | Broadly Conserved | Human (Homo sapiens)  Mouse (Mus musculus)  Rat (Rattus norvegicus) | HUMAN | GGTTGGTAGAGGTGGGAAAGGGAAGGGTTCTAGGCCAGAATGTTCCTATTTAGAAGACACTCAAATTA | 8mer |
|  |  |  | HUMAN | GAGGAAGGGTGAAGGGAAGGGCTCTTTGCTAGTATCT | 7mer-A1 |
|  |  |  | HUMAN | GGGTGAAGGGAAGGGCT | 7mer-A1 |
|  |  |  | HUMAN | TATTCTGTTGCTGTGTGTTTCATTTTAAATTGAGCATTAAGGGAATGCAGCATTTAAATC | 7mer-A1 |
|  |  |  | HUMAN | CATTAAGGGAATGCAG | 7mer-A1 |
|  |  |  | HUMAN | CATTAAGGGAATG | 7mer-A1 |
|  |  |  | BABOON | GGTTGGTAGAGGTGGGAAAGGGAAGGGTTCTAGGCCAGAATGTTCCTATTTAGAAGACACTCAAATTA | 8mer |
|  |  |  | BABOON | GAGGAAGGGTGAAGGGAAGGGCTCTTTGCTAGTATCT | 7mer-A1 |
|  |  |  | BABOON | GGGTGAAGGGAAGGGCT | 7mer-A1 |
|  |  |  | BABOON | TATTCTGTTGCTGTGTGTTTCATTTTAAATTGAGCATTAAGGGAATGCAGCATTTAAATC | 7mer-A1 |
|  |  |  | BABOON | CATTAAGGGAATGCAG | 7mer-A1 |
|  |  |  | BABOON | CATTAAGGGAATG | 7mer-A1 |
|  |  |  | DOG | GGGTGAAGGGAAGGGCT | 7mer-A1 |
|  |  |  | DOG | CATTAAGGGAATGCAG | 7mer-A1 |
|  |  |  | DOG | CATTAAGGGAATG | 7mer-A1 |
|  |  |  | COW | CATTAAGGGAATG | 7mer-A1 |

  
  
  
  

| Seed Matches to the miRNA miR-493-5p | | | | | |
| --- | --- | --- | --- | --- | --- |
| Seed | Conservation | Species | Matches | | |
|  |  |  | Sequence | Motif | Type |
| UGUACAU | Conserved | Human (Homo sapiens)  Rhesus (Macaca mulatta)  Mouse (Mus musculus)  Rat (Rattus norvegicus) | HUMAN | TCTGTGCATTTTGTTTTACTTATCTGTGTATATAGTGTACATAAAGGACAGACGAGTCCTAATTGACAACATCTAGTCTTTCTGGATGTTAAAGAGGTTGCCAGTGTATGACAAAAGTAGAGTT | 6mer |
|  |  |  | HUMAN | TAAACTAATATATTTTGTACATTTTGTTTTACAAGTCCTAGGAAAGATTGTCTTCTGAAAATTTGATGTCTTCTGGGTTGATGGAGATGGGAAGGGTTCTAGGCCAGAATGTTCACATTTGGAAGACT | 6mer |
|  |  |  | HUMAN | TTTTGTACATTTTGT | 6mer |
|  |  |  | HUMAN | TAATATCTAGTCTCTAGATATTAAAGAGGTTGCCAATGTATGACAGAAGTAGAGTTAGTAAACTAACACATTTTGTACACTTTGTTAAAATTTGTAGAAAGGCTGTCTTCTGAAAAGGACTTTTGGAAGTGA | 6mer |
|  |  |  | HUMAN | AAGTAGAGTTAGTAAACTAACACATTTTGTACACTTTGT | 6mer |
|  |  |  | HUMAN | GTTAGTAAACTAACACATTTTGTACACTTTGT | 6mer |
|  |  |  | HUMAN | GTTAGTAAACTAACACATTTTGTACACTT | 6mer |
|  |  |  | HUMAN | AATACTCATATAGCTTTGGGATTTTGAATTGGTAAATATTCATGATGTGTGAAAAATCATGATACATACTGTACA | 6mer |
|  |  |  | HUMAN | CTGTACA | 6mer |
|  |  |  | BABOON | TCTGTGCATTTTGTTTTACTTATCTGTGTATATAGTGTACATAAAGGACAGACGAGTCCTAATTGACAACATCTAGTCTTTCTGGATGTTAAAGAGGTTGCCAGTGTATGACAAAAGTAGAGTT | 6mer |
|  |  |  | BABOON | TAAACTAATATATTTTGTACATTTTGTTTTACAAGTCCTAGGAAAGATTGTCTTCTGAAAATTTGATGTCTTCTGGGTTGATGGAGATGGGAAGGGTTCTAGGCCAGAATGTTCACATTTGGAAGACT | 6mer |
|  |  |  | BABOON | TTTTGTACATTTTGT | 6mer |
|  |  |  | BABOON | TAATATCTAGTCTCTAGATATTAAAGAGGTTGCCAATGTATGACAGAAGTAGAGTTAGTAAACTAACACATTTTGTACACTTTGTTAAAATTTGTAGAAAGGCTGTCTTCTGAAAAGGACTTTTGGAAGTGA | 6mer |
|  |  |  | BABOON | AAGTAGAGTTAGTAAACTAACACATTTTGTACACTTTGT | 6mer |
|  |  |  | BABOON | GTTAGTAAACTAACACATTTTGTACACTTTGT | 6mer |
|  |  |  | BABOON | GTTAGTAAACTAACACATTTTGTACACTT | 6mer |
|  |  |  | BABOON | AATACTCATATAGCTTTGGGATTTTGAATTGGTAAATATTCATGATGTGTGAAAAATCATGATACATACTGTACA | 7mer-A1 |
|  |  |  | BABOON | CTGTACA | 7mer-A1 |
|  |  |  | DOG | TTTTGTACATTTTGT | 6mer |
|  |  |  | DOG | AAGTAGAGTTAGTAAACTAACACATTTTGTACACTTTGT | 6mer |
|  |  |  | DOG | GTTAGTAAACTAACACATTTTGTACACTTTGT | 6mer |
|  |  |  | DOG | GTTAGTAAACTAACACATTTTGTACACTT | 6mer |
|  |  |  | DOG | CTGTACA | 7mer-A1 |
|  |  |  | COW | TTTTGTACATTTTGT | 6mer |
|  |  |  | COW | GTTAGTAAACTAACACATTTTGTACACTTTGT | 6mer |
|  |  |  | COW | GTTAGTAAACTAACACATTTTGTACACTT | 6mer |
|  |  |  | SHEEP | TTTTGTACATTTTGT | 6mer |
|  |  |  | SHEEP | GTTAGTAAACTAACACATTTTGTACACTTTGT | 6mer |
|  |  |  | SHEEP | GTTAGTAAACTAACACATTTTGTACACTT | 6mer |
|  |  |  | PIG | GTTAGTAAACTAACACATTTTGTACACTT | 6mer |
|  |  |  | ARMADILLO | GTTAGTAAACTAACACATTTTGTACACTT | 6mer |
|  |  |  | GUINEAPIG | GTTAGTAAACTAACACATTTTGTACACTT | 6mer |

  
  
  
  

| Seed Matches to the miRNA miR-135-5p | | | | | |
| --- | --- | --- | --- | --- | --- |
| Seed | Conservation | Species | Matches | | |
|  |  |  | Sequence | Motif | Type |
| AUGGCUU | Broadly Conserved | Human (Homo sapiens)  Chicken (Gallus gallus)  Rhesus (Macaca mulatta)  Dog (Canis lupus familiaris)  Mouse (Mus musculus)  Rat (Rattus norvegicus)  Opossum (Monodelphis domestica) | HUMAN | TATGTCCATATTTACATTTTGATAGCCATTGATGTATGCATCTCTT | 6mer |
|  |  |  | BABOON | TATGTCCATATTTACATTTTGATAGCCATTGATGTATGCATCTCTT | 6mer |

  
  
  
  

| Seed Matches to the miRNA miR-1251-5p | | | | | |
| --- | --- | --- | --- | --- | --- |
| Seed | Conservation | Species | Matches | | |
|  |  |  | Sequence | Motif | Type |
| CUCUAGC | Conserved | Human (Homo sapiens)  Rhesus (Macaca mulatta)  Mouse (Mus musculus)  Opossum (Monodelphis domestica) | HUMAN | GAATGGGGTGACAACTTATGATAAAAACTAGAGCTAGTGAATTAGCC | 6mer |
|  |  |  | HUMAN | GAACTCTGCCAATGCTTTTATCTAGAGGCGTGTTGCCATTTTTGTCTT | 6mer |
|  |  |  | BABOON | GAATGGGGTGACAACTTATGATAAAAACTAGAGCTAGTGAATTAGCC | 6mer |
|  |  |  | BABOON | GAACTCTGCCAATGCTTTTATCTAGAGGCGTGTTGCCATTTTTGTCTT | 6mer |

  
  
  
  

| Seed Matches to the miRNA miR-423-3p | | | | | |
| --- | --- | --- | --- | --- | --- |
| Seed | Conservation | Species | Matches | | |
|  |  |  | Sequence | Motif | Type |
| GCUCGGU | Conserved | Human (Homo sapiens)  Rhesus (Macaca mulatta)  Mouse (Mus musculus)  Rat (Rattus norvegicus) | HUMAN | GGGCCGGCGGGTGAACTGGGGGGCCCCGGGACAGGCCGAGCCCT | 6mer |
|  |  |  | BABOON | GGGCCGGCGGGTGAACTGGGGGGCCCCGGGACAGGCCGAGCCCT | 6mer |

  
  
  
  

| Seed Matches to the miRNA miR-423-5p | | | | | |
| --- | --- | --- | --- | --- | --- |
| Seed | Conservation | Species | Matches | | |
|  |  |  | Sequence | Motif | Type |
| GAGGGGC | Conserved | Human (Homo sapiens)  Rhesus (Macaca mulatta)  Cow (Bos taurus)  Mouse (Mus musculus) | HUMAN | AGTAGAGGGCTTAAGTAACACCCCTCTAAGCATTTGTTTTCA | 6mer |
|  |  |  | HUMAN | CCCCTCTAAG | 6mer |
|  |  |  | BABOON | AGTAGAGGGCTTAAGTAACACCCCTCTAAGCATTTGTTTTCA | 6mer |
|  |  |  | BABOON | CCCCTCTAAG | 6mer |
|  |  |  | DOG | CCCCTCTAAG | 6mer |

  
  
  
  

| Seed Matches to the miRNA miR-214-5p | | | | | |
| --- | --- | --- | --- | --- | --- |
| Seed | Conservation | Species | Matches | | |
|  |  |  | Sequence | Motif | Type |
| GCCUGUC | Broadly Conserved | Human (Homo sapiens)  Rhesus (Macaca mulatta)  Mouse (Mus musculus)  Opossum (Monodelphis domestica) | HUMAN | GGGCCGGCGGGTGAACTGGGGGGCCCCGGGACAGGCCGAGCCCT | 7mer-m8 |
|  |  |  | HUMAN | CTGTCGGAAGAGAGAAATGGTAGAATGACAGGCCACGTTTGGCCCGTTGGAAATGCCC | 7mer-m8 |
|  |  |  | BABOON | GGGCCGGCGGGTGAACTGGGGGGCCCCGGGACAGGCCGAGCCCT | 7mer-m8 |
|  |  |  | BABOON | CTGTCGGAAGAGAGAAATGGTAGAATGACAGGCCACGTTTGGCCCGTTGGAAATGCCC | 7mer-m8 |

  
  
  
  

| Seed Matches to the miRNA miR-877-5p | | | | | |
| --- | --- | --- | --- | --- | --- |
| Seed | Conservation | Species | Matches | | |
|  |  |  | Sequence | Motif | Type |
| UAGAGGA | Conserved | Human (Homo sapiens)  Rhesus (Macaca mulatta)  Mouse (Mus musculus) | HUMAN | AGTAGAGGGCTTAAGTAACACCCCTCTAAGCATTTGTTTTCA | 7mer-A1 |
|  |  |  | HUMAN | CCCCTCTAAG | 7mer-A1 |
|  |  |  | BABOON | AGTAGAGGGCTTAAGTAACACCCCTCTAAGCATTTGTTTTCA | 7mer-A1 |
|  |  |  | BABOON | CCCCTCTAAG | 7mer-A1 |
|  |  |  | DOG | CCCCTCTAAG | 7mer-A1 |

  
  
  
  

| Seed Matches to the miRNA miR-124-3p.2/506-3p | | | | | |
| --- | --- | --- | --- | --- | --- |
| Seed | Conservation | Species | Matches | | |
|  |  |  | Sequence | Motif | Type |
| UAAGGCA | Broadly Conserved | Human (Homo sapiens) | HUMAN | ACCTACCTAGTCCTGACGACAACGGACAAAGGCCTTAA | 7mer-A1 |
|  |  |  | BABOON | ACCTACCTAGTCCTGACGACAACGGACAAAGGCCTTAA | 7mer-A1 |

  
  
  
  

| Seed Matches to the miRNA miR-760 | | | | | |
| --- | --- | --- | --- | --- | --- |
| Seed | Conservation | Species | Matches | | |
|  |  |  | Sequence | Motif | Type |
| GGCUCUG | Conserved | Human (Homo sapiens)  Rhesus (Macaca mulatta)  Chimp (Pan troglodytes) | HUMAN | ACGAAACCCCCGCAGAGCCGCCGGGACGCAGCGC | 7mer-m8 |
|  |  |  | BABOON | ACGAAACCCCCGCAGAGCCGCCGGGACGCAGCGC | 7mer-m8 |

  
  
  
  

| Seed Matches to the miRNA miR-744-5p | | | | | |
| --- | --- | --- | --- | --- | --- |
| Seed | Conservation | Species | Matches | | |
|  |  |  | Sequence | Motif | Type |
| GCGGGGC | Conserved | Human (Homo sapiens)  Mouse (Mus musculus) | HUMAN | ACGAAACCCCCGCAGAGCCGCCGGGACGCAGCGC | 7mer-A1 |
|  |  |  | BABOON | ACGAAACCCCCGCAGAGCCGCCGGGACGCAGCGC | 7mer-A1 |

  
  
  
  

| Seed Matches to the miRNA miR-455-5p | | | | | |
| --- | --- | --- | --- | --- | --- |
| Seed | Conservation | Species | Matches | | |
|  |  |  | Sequence | Motif | Type |
| AUGUGCC | Broadly Conserved | Human (Homo sapiens)  Chicken (Gallus gallus)  Rhesus (Macaca mulatta)  Cow (Bos taurus)  Mouse (Mus musculus)  Rat (Rattus norvegicus)  Opossum (Monodelphis domestica) | HUMAN | GAGGAGGCACATTGACCATTGTCCCTT | 7mer-m8 |
|  |  |  | HUMAN | TAATGCACATA | 7mer-A1 |
|  |  |  | HUMAN | ATGCACATA | 7mer-A1 |
|  |  |  | HUMAN | ATGCACAT | 7mer-A1 |
|  |  |  | BABOON | GAGGAGGCACATTGACCATTGTCCCTT | 7mer-m8 |
|  |  |  | BABOON | TAATGCACATA | 7mer-A1 |
|  |  |  | BABOON | ATGCACATA | 7mer-A1 |
|  |  |  | BABOON | ATGCACAT | 7mer-A1 |
|  |  |  | DOG | ATGCACATA | 7mer-A1 |
|  |  |  | DOG | ATGCACAT | 7mer-A1 |
|  |  |  | COW | ATGCACAT | 6mer |

  
  
  
  

| Seed Matches to the miRNA miR-483-3p.2 | | | | | |
| --- | --- | --- | --- | --- | --- |
| Seed | Conservation | Species | Matches | | |
|  |  |  | Sequence | Motif | Type |
| CACUCCU | Conserved | Human (Homo sapiens)  Mouse (Mus musculus) | HUMAN | CTAGGAGTGGTTGCATTTGGGAATGGAATTGTTAAAACTTGATG | 7mer-m8 |
|  |  |  | HUMAN | TCTGGGAGTGACCTT | 7mer-A1 |
|  |  |  | HUMAN | TCTGGGAGTG | 7mer-A1 |
|  |  |  | BABOON | CTAGGAGTGGTTGCATTTGGGAATGGAATTGTTAAAACTTGATG | 7mer-m8 |
|  |  |  | BABOON | TCTGGGAGTGACCTT | 7mer-A1 |
|  |  |  | BABOON | TCTGGGAGTG | 7mer-A1 |
|  |  |  | DOG | TCTGGGAGTG | 6mer |

  
  
  
  

| Seed Matches to the miRNA miR-330-3p.2 | | | | | |
| --- | --- | --- | --- | --- | --- |
| Seed | Conservation | Species | Matches | | |
|  |  |  | Sequence | Motif | Type |
| AAAGCAC | Conserved | Human (Homo sapiens)  Mouse (Mus musculus) | HUMAN | GAACTCTGCCAATGCTTTTATCTAGAGGCGTGTTGCCATTTTTGTCTT | 6mer |
|  |  |  | BABOON | GAACTCTGCCAATGCTTTTATCTAGAGGCGTGTTGCCATTTTTGTCTT | 6mer |

  
  
  
  

| Seed Matches to the miRNA miR-18-5p | | | | | |
| --- | --- | --- | --- | --- | --- |
| Seed | Conservation | Species | Matches | | |
|  |  |  | Sequence | Motif | Type |
| AAGGUGC | Broadly Conserved | Human (Homo sapiens)  X. tropicalis (Xenopus tropicalis)  Chicken (Gallus gallus)  Mouse (Mus musculus)  Rat (Rattus norvegicus)  Opossum (Monodelphis domestica) | HUMAN | CTTTTGGAAATTGAATTGTGAAGCCACCTTTG | 6mer |
|  |  |  | BABOON | CTTTTGGAAATTGAATTGTGAAGCCACCTTTG | 6mer |

  
  
  
  

| Seed Matches to the miRNA miR-496.1 | | | | | |
| --- | --- | --- | --- | --- | --- |
| Seed | Conservation | Species | Matches | | |
|  |  |  | Sequence | Motif | Type |
| GAGUAUU | Conserved | Human (Homo sapiens) | HUMAN | GGGGGGAATGGCCAAATCACCTGTTGAGTAATACTCATTGTGTTTGTGCAGTGGTTC | 8mer |
|  |  |  | HUMAN | AATACTCATATAGCTTTGGGATTTTGAATTGGTAAATATTCATGATGTGTGAAAAATCATGATACATACTGTACA | 8mer |
|  |  |  | HUMAN | AATACTCATA | 8mer |
|  |  |  | BABOON | GGGGGGAATGGCCAAATCACCTGTTGAGTAATACTCATTGTGTTTGTGCAGTGGTTC | 8mer |
|  |  |  | BABOON | AATACTCATATAGCTTTGGGATTTTGAATTGGTAAATATTCATGATGTGTGAAAAATCATGATACATACTGTACA | 8mer |
|  |  |  | BABOON | AATACTCATA | 8mer |
|  |  |  | DOG | AATACTCATA | 8mer |
|  |  |  | COW | AATACTCATA | 8mer |
|  |  |  | SHEEP | AATACTCATA | 8mer |
|  |  |  | PIG | AATACTCATA | 8mer |

  
  
  
  

| Seed Matches to the miRNA miR-1298-5p | | | | | |
| --- | --- | --- | --- | --- | --- |
| Seed | Conservation | Species | Matches | | |
|  |  |  | Sequence | Motif | Type |
| UCAUUCG | Conserved | Human (Homo sapiens)  Rhesus (Macaca mulatta)  Mouse (Mus musculus) | HUMAN | CTGTCGGAAGAGAGAAATGGTAGAATGACAGGCCACGTTTGGCCCGTTGGAAATGCCC | 6mer |
|  |  |  | HUMAN | GAGGAGGGAATGATTCAAGGCCAAAATGGCCACATTTAGAAGATACCTCAGATGATAACCATTGTTAT | 6mer |
|  |  |  | HUMAN | CAGGTTGGTGGTGGAGAGGAGTTGGAAGGAATGAAGGGTTCTAGACCAGAATGTTC | 7mer-A1 |
|  |  |  | BABOON | CTGTCGGAAGAGAGAAATGGTAGAATGACAGGCCACGTTTGGCCCGTTGGAAATGCCC | 6mer |
|  |  |  | BABOON | GAGGAGGGAATGATTCAAGGCCAAAATGGCCACATTTAGAAGATACCTCAGATGATAACCATTGTTAT | 6mer |
|  |  |  | BABOON | CAGGTTGGTGGTGGAGAGGAGTTGGAAGGAATGAAGGGTTCTAGACCAGAATGTTC | 7mer-A1 |

  
  
  
  

| Seed Matches to the miRNA miR-145-5p | | | | | |
| --- | --- | --- | --- | --- | --- |
| Seed | Conservation | Species | Matches | | |
|  |  |  | Sequence | Motif | Type |
| UCCAGUU | Broadly Conserved | Human (Homo sapiens)  Rhesus (Macaca mulatta)  Mouse (Mus musculus)  Rat (Rattus norvegicus)  Opossum (Monodelphis domestica) | HUMAN | CCTGTACTTGTCCACTGGATTGAAG | 6mer |
|  |  |  | HUMAN | ACTGGATT | 6mer |
|  |  |  | BABOON | CCTGTACTTGTCCACTGGATTGAAG | 6mer |
|  |  |  | BABOON | ACTGGATT | 6mer |
|  |  |  | DOG | ACTGGATT | 6mer |
|  |  |  | COW | ACTGGATT | 6mer |
|  |  |  | SHEEP | ACTGGATT | 6mer |

  
  
  
  

| Seed Matches to the miRNA miR-203a-3p.1 | | | | | |
| --- | --- | --- | --- | --- | --- |
| Seed | Conservation | Species | Matches | | |
|  |  |  | Sequence | Motif | Type |
| GAAAUGU | Broadly Conserved | Human (Homo sapiens) | HUMAN | TATGTTACATGCATTTCATTTAACTTTGCTATACTGTATATATTGT | 7mer-A1 |
|  |  |  | BABOON | TATGTTACATGCATTTCATTTAACTTTGCTATACTGTATATATTGT | 7mer-A1 |

  
  
  
  

| Seed Matches to the miRNA miR-155-5p | | | | | |
| --- | --- | --- | --- | --- | --- |
| Seed | Conservation | Species | Matches | | |
|  |  |  | Sequence | Motif | Type |
| UAAUGCU | Broadly Conserved | Human (Homo sapiens)  Mouse (Mus musculus)  Rat (Rattus norvegicus) | HUMAN | AGCATTA | 7mer-m8 |
|  |  |  | HUMAN | CCCCCCCTAAGCATTACAGATGGCTTATAGCTGTCCAC | 7mer-m8 |
|  |  |  | HUMAN | TATTCTGTTGCTGTGTGTTTCATTTTAAATTGAGCATTAAGGGAATGCAGCATTTAAATC | 8mer |
|  |  |  | BABOON | AGCATTA | 7mer-m8 |
|  |  |  | BABOON | CCCCCCCTAAGCATTACAGATGGCTTATAGCTGTCCAC | 7mer-m8 |
|  |  |  | BABOON | TATTCTGTTGCTGTGTGTTTCATTTTAAATTGAGCATTAAGGGAATGCAGCATTTAAATC | 8mer |
|  |  |  | DOG | AGCATTA | 7mer-m8 |

  
  
  
  

| Seed Matches to the miRNA miR-26-5p | | | | | |
| --- | --- | --- | --- | --- | --- |
| Seed | Conservation | Species | Matches | | |
|  |  |  | Sequence | Motif | Type |
| UCAAGUA | Broadly Conserved | Human (Homo sapiens)  Chicken (Gallus gallus)  Rhesus (Macaca mulatta)  Mouse (Mus musculus)  Rat (Rattus norvegicus)  Opossum (Monodelphis domestica) | HUMAN | GACAAATTAACTCCTTACTTGAAACATCTAGTCTATCTAGATGTTTAGAAGTGCCC | 8mer |
|  |  |  | HUMAN | CTAGGAGTGGTTGCATTTGGGAATGGAATTGTTAAAACTTGATG | 6mer |
|  |  |  | BABOON | GACAAATTAACTCCTTACTTGAAACATCTAGTCTATCTAGATGTTTAGAAGTGCCC | 8mer |
|  |  |  | BABOON | CTAGGAGTGGTTGCATTTGGGAATGGAATTGTTAAAACTTGATG | 6mer |

  
  
  
  

| Seed Matches to the miRNA miR-219a-2-3p | | | | | |
| --- | --- | --- | --- | --- | --- |
| Seed | Conservation | Species | Matches | | |
|  |  |  | Sequence | Motif | Type |
| GAAUUGU | Conserved | Human (Homo sapiens)  Mouse (Mus musculus)  Rat (Rattus norvegicus) | HUMAN | CTTTTTGTTACAATTCATAGGAAAT | 8mer |
|  |  |  | HUMAN | TGATCAATTCTCTGAGCAGTACCCATTTTGA | 6mer |
|  |  |  | BABOON | CTTTTTGTTACAATTCATAGGAAAT | 8mer |
|  |  |  | BABOON | TGATCAATTCTCTGAGCAGTACCCATTTTGA | 6mer |

  
  
  
  

| Seed Matches to the miRNA miR-1224-5p | | | | | |
| --- | --- | --- | --- | --- | --- |
| Seed | Conservation | Species | Matches | | |
|  |  |  | Sequence | Motif | Type |
| UGAGGAC | Conserved | Human (Homo sapiens)  Rhesus (Macaca mulatta)  Chimp (Pan troglodytes)  Mouse (Mus musculus) | HUMAN | AGAAGGTTCTATGCTAGACTGGTCATATTTAGAAGACATTTTCATATTCTATCCATTGTTTTGTGTGCATTTTATTCCTCACTACTGTGTATATA | 6mer |
|  |  |  | BABOON | AGAAGGTTCTATGCTAGACTGGTCATATTTAGAAGACATTTTCATATTCTATCCATTGTTTTGTGTGCATTTTATTCCTCACTACTGTGTATATA | 6mer |

  
  
  
  

| Seed Matches to the miRNA miR-489-3p | | | | | |
| --- | --- | --- | --- | --- | --- |
| Seed | Conservation | Species | Matches | | |
|  |  |  | Sequence | Motif | Type |
| UGACAUC | Broadly Conserved | Human (Homo sapiens)  Rhesus (Macaca mulatta)  Rat (Rattus norvegicus) | HUMAN | CATCTTGGACATGGAATTGTTAAGCCACCTCTGAGCAGTGTATGTCAGGACTT | 6mer |
|  |  |  | HUMAN | ATGTCAG | 6mer |
|  |  |  | HUMAN | ATGTCAAACTATAAACTGCTTGTGATT | 7mer-A1 |
|  |  |  | BABOON | CATCTTGGACATGGAATTGTTAAGCCACCTCTGAGCAGTGTATGTCAGGACTT | 6mer |
|  |  |  | BABOON | ATGTCAG | 6mer |
|  |  |  | BABOON | ATGTCAAACTATAAACTGCTTGTGATT | 7mer-A1 |
|  |  |  | DOG | ATGTCAG | 6mer |

  
  
  
  

| Seed Matches to the miRNA miR-217 | | | | | |
| --- | --- | --- | --- | --- | --- |
| Seed | Conservation | Species | Matches | | |
|  |  |  | Sequence | Motif | Type |
| ACUGCAU | Broadly Conserved | Human (Homo sapiens)  X. tropicalis (Xenopus tropicalis)  Rhesus (Macaca mulatta)  Chimp (Pan troglodytes)  Dog (Canis lupus familiaris)  Cow (Bos taurus) | HUMAN | GGGGGGAATGGCCAAATCACCTGTTGAGTAATACTCATTGTGTTTGTGCAGTGGTTC | 6mer |
|  |  |  | HUMAN | TTGTTACATGTTTGCAGTTTATTCAAGACTGCT | 6mer |
|  |  |  | HUMAN | TGCAGTTTATTCAAGACTGCT | 6mer |
|  |  |  | BABOON | GGGGGGAATGGCCAAATCACCTGTTGAGTAATACTCATTGTGTTTGTGCAGTGGTTC | 6mer |
|  |  |  | BABOON | TTGTTACATGTTTGCAGTTTATTCAAGACTGCT | 6mer |
|  |  |  | BABOON | TGCAGTTTATTCAAGACTGCT | 6mer |
|  |  |  | DOG | TGCAGTTTATTCAAGACTGCT | 6mer |

  
  
  
  

| Seed Matches to the miRNA miR-486-5p | | | | | |
| --- | --- | --- | --- | --- | --- |
| Seed | Conservation | Species | Matches | | |
|  |  |  | Sequence | Motif | Type |
| CCUGUAC | Conserved | Human (Homo sapiens)  Rhesus (Macaca mulatta)  Mouse (Mus musculus) | HUMAN | TATACAGG | 6mer |
|  |  |  | BABOON | TATACAGG | 7mer-A1 |

  
  
  
  

| Seed Matches to the miRNA miR-329-3p/362-3p | | | | | |
| --- | --- | --- | --- | --- | --- |
| Seed | Conservation | Species | Matches | | |
|  |  |  | Sequence | Motif | Type |
| ACACACC | Conserved | Human (Homo sapiens)  Rhesus (Macaca mulatta)  Mouse (Mus musculus)  Rat (Rattus norvegicus) | HUMAN | ACTAGTGTGTTCATACTTGGACATTTTCAGACA | 6mer |
|  |  |  | HUMAN | GTGTGTTCATACTTGGACATTTTCAGA | 6mer |
|  |  |  | HUMAN | TGTGTGTAGTTTATTCAAC | 7mer-A1 |
|  |  |  | HUMAN | TGTGTGT | 7mer-A1 |
|  |  |  | HUMAN | TATTTGTGCTGGTTCAGGGGGAAGGAGGAGCACAAAGTGCAAAGGGCTTTCTACCAGTGTCCAGTGTGTTTA | 6mer |
|  |  |  | HUMAN | TATTCTGTTGCTGTGTGTTTCATTTTAAATTGAGCATTAAGGGAATGCAGCATTTAAATC | 6mer |
|  |  |  | BABOON | ACTAGTGTGTTCATACTTGGACATTTTCAGACA | 6mer |
|  |  |  | BABOON | GTGTGTTCATACTTGGACATTTTCAGA | 6mer |
|  |  |  | BABOON | TGTGTGTAGTTTATTCAAC | 7mer-A1 |
|  |  |  | BABOON | TGTGTGT | 7mer-A1 |
|  |  |  | BABOON | TATTTGTGCTGGTTCAGGGGGAAGGAGGAGCACAAAGTGCAAAGGGCTTTCTACCAGTGTCCAGTGTGTTTA | 6mer |
|  |  |  | BABOON | TATTCTGTTGCTGTGTGTTTCATTTTAAATTGAGCATTAAGGGAATGCAGCATTTAAATC | 6mer |
|  |  |  | DOG | GTGTGTTCATACTTGGACATTTTCAGA | 6mer |
|  |  |  | DOG | TGTGTGTAGTTTATTCAAC | 7mer-A1 |
|  |  |  | DOG | TGTGTGT | 7mer-A1 |
|  |  |  | COW | TGTGTGTAGTTTATTCAAC | 7mer-A1 |
|  |  |  | COW | TGTGTGT | 7mer-A1 |
|  |  |  | SHEEP | TGTGTGTAGTTTATTCAAC | 7mer-A1 |
|  |  |  | SHEEP | TGTGTGT | 7mer-A1 |
|  |  |  | PIG | TGTGTGT | 6mer |

  
  
  
  

| Seed Matches to the miRNA miR-130-3p/301-3p/454-3p | | | | | |
| --- | --- | --- | --- | --- | --- |
| Seed | Conservation | Species | Matches | | |
|  |  |  | Sequence | Motif | Type |
| AGUGCAA | Broadly Conserved | Human (Homo sapiens)  Chicken (Gallus gallus)  Rhesus (Macaca mulatta) | HUMAN | CACCACTGCACTCCA | 6mer |
|  |  |  | BABOON | CACCACTGCACTCCA | 6mer |

  
  
  
  

| Seed Matches to the miRNA miR-101-3p.1 | | | | | |
| --- | --- | --- | --- | --- | --- |
| Seed | Conservation | Species | Matches | | |
|  |  |  | Sequence | Motif | Type |
| ACAGUAC | Broadly Conserved | Human (Homo sapiens)  Mouse (Mus musculus) | HUMAN | AGAAGGTTCTATGCTAGACTGGTCATATTTAGAAGACATTTTCATATTCTATCCATTGTTTTGTGTGCATTTTATTCCTCACTACTGTGTATATA | 6mer |
|  |  |  | HUMAN | ACTACTGTGTATATA | 6mer |
|  |  |  | HUMAN | TATGTTACATGCATTTCATTTAACTTTGCTATACTGTATATATTGT | 7mer-A1 |
|  |  |  | HUMAN | CTACTGTGTATATAGC | 6mer |
|  |  |  | HUMAN | CTACTGTGTATATAG | 6mer |
|  |  |  | HUMAN | CTACTGTGTATATA | 6mer |
|  |  |  | HUMAN | TGTCTGCATTTTCATTTACTGTGCTGTGTATATAGTGTATATAAG | 6mer |
|  |  |  | HUMAN | AGTCTGTGTTATGTATGTATACCATTTATTCAATGCTACTGTGTATATAATGGAAAACTT | 6mer |
|  |  |  | HUMAN | AATACTCATATAGCTTTGGGATTTTGAATTGGTAAATATTCATGATGTGTGAAAAATCATGATACATACTGTACA | 7mer-A1 |
|  |  |  | BABOON | AGAAGGTTCTATGCTAGACTGGTCATATTTAGAAGACATTTTCATATTCTATCCATTGTTTTGTGTGCATTTTATTCCTCACTACTGTGTATATA | 6mer |
|  |  |  | BABOON | ACTACTGTGTATATA | 6mer |
|  |  |  | BABOON | TATGTTACATGCATTTCATTTAACTTTGCTATACTGTATATATTGT | 7mer-A1 |
|  |  |  | BABOON | CTACTGTGTATATAGC | 6mer |
|  |  |  | BABOON | CTACTGTGTATATAG | 6mer |
|  |  |  | BABOON | CTACTGTGTATATA | 6mer |
|  |  |  | BABOON | TGTCTGCATTTTCATTTACTGTGCTGTGTATATAGTGTATATAAG | 6mer |
|  |  |  | BABOON | AGTCTGTGTTATGTATGTATACCATTTATTCAATGCTACTGTGTATATAATGGAAAACTT | 6mer |
|  |  |  | BABOON | AATACTCATATAGCTTTGGGATTTTGAATTGGTAAATATTCATGATGTGTGAAAAATCATGATACATACTGTACA | 7mer-A1 |
|  |  |  | DOG | ACTACTGTGTATATA | 6mer |
|  |  |  | DOG | CTACTGTGTATATAG | 6mer |
|  |  |  | DOG | CTACTGTGTATATA | 6mer |
|  |  |  | COW | CTACTGTGTATATAG | 6mer |
|  |  |  | COW | CTACTGTGTATATA | 6mer |
|  |  |  | SHEEP | CTACTGTGTATATAG | 6mer |
|  |  |  | SHEEP | CTACTGTGTATATA | 6mer |
|  |  |  | PIG | CTACTGTGTATATA | 6mer |
|  |  |  | ARMADILLO | CTACTGTGTATATA | 6mer |
|  |  |  | GUINEAPIG | CTACTGTGTATATA | 6mer |

  
  
  
  

| Seed Matches to the miRNA miR-320 | | | | | |
| --- | --- | --- | --- | --- | --- |
| Seed | Conservation | Species | Matches | | |
|  |  |  | Sequence | Motif | Type |
| AAAGCUG | Conserved | Human (Homo sapiens)  X. tropicalis (Xenopus tropicalis)  Rhesus (Macaca mulatta)  Chimp (Pan troglodytes)  Dog (Canis lupus familiaris) | HUMAN | TTGACAATGCTAAGCTTTTTTGA | 6mer |
|  |  |  | HUMAN | TTGTAAATAGCTTTTAAAAACTGATGGGAAATGCTGTTTGGAAGTGGAATTGTTGAACCA | 6mer |
|  |  |  | HUMAN | TTGTAAATAGCTTTTAAA | 6mer |
|  |  |  | HUMAN | TTGTAAATAGCTTTT | 6mer |
|  |  |  | HUMAN | TGTAAATAGCTTTT | 6mer |
|  |  |  | HUMAN | AACATCTAAGCTTTA | 7mer-A1 |
|  |  |  | HUMAN | AACATCTAAGCTTT | 7mer-A1 |
|  |  |  | HUMAN | AATACTCATATAGCTTTGGGATTTTGAATTGGTAAATATTCATGATGTGTGAAAAATCATGATACATACTGTACA | 6mer |
|  |  |  | BABOON | TTGACAATGCTAAGCTTTTTTGA | 6mer |
|  |  |  | BABOON | TTGTAAATAGCTTTTAAAAACTGATGGGAAATGCTGTTTGGAAGTGGAATTGTTGAACCA | 6mer |
|  |  |  | BABOON | TTGTAAATAGCTTTTAAA | 6mer |
|  |  |  | BABOON | TTGTAAATAGCTTTT | 6mer |
|  |  |  | BABOON | TGTAAATAGCTTTT | 6mer |
|  |  |  | BABOON | AACATCTAAGCTTTA | 7mer-A1 |
|  |  |  | BABOON | AACATCTAAGCTTT | 7mer-A1 |
|  |  |  | BABOON | AATACTCATATAGCTTTGGGATTTTGAATTGGTAAATATTCATGATGTGTGAAAAATCATGATACATACTGTACA | 6mer |
|  |  |  | DOG | TTGTAAATAGCTTTTAAA | 6mer |
|  |  |  | DOG | TTGTAAATAGCTTTT | 6mer |
|  |  |  | DOG | TGTAAATAGCTTTT | 6mer |
|  |  |  | DOG | AACATCTAAGCTTT | 6mer |
|  |  |  | COW | TTGTAAATAGCTTTT | 6mer |
|  |  |  | COW | TGTAAATAGCTTTT | 6mer |
|  |  |  | SHEEP | TTGTAAATAGCTTTT | 6mer |
|  |  |  | SHEEP | TGTAAATAGCTTTT | 6mer |
|  |  |  | PIG | TTGTAAATAGCTTTT | 6mer |
|  |  |  | PIG | TGTAAATAGCTTTT | 6mer |
|  |  |  | ARMADILLO | TGTAAATAGCTTTT | 6mer |

  
  
  
  

| Seed Matches to the miRNA miR-28-5p/708-5p | | | | | |
| --- | --- | --- | --- | --- | --- |
| Seed | Conservation | Species | Matches | | |
|  |  |  | Sequence | Motif | Type |
| AGGAGCU | Conserved | Human (Homo sapiens)  Mouse (Mus musculus)  Rat (Rattus norvegicus) | HUMAN | GCTCCT | 6mer |
|  |  |  | HUMAN | GGGTTCTGTGCTCCTGAGATTAGTTCAGATGGTCTAACCATTGTTCTATATGTGCATTTTAGTTAATATTGTGTATTAAAGGATAAGTCTTAATGCTCAAAGTATGTTAAAAATAGATGTAGTAAA | 6mer |
|  |  |  | BABOON | GCTCCT | 6mer |
|  |  |  | BABOON | GGGTTCTGTGCTCCTGAGATTAGTTCAGATGGTCTAACCATTGTTCTATATGTGCATTTTAGTTAATATTGTGTATTAAAGGATAAGTCTTAATGCTCAAAGTATGTTAAAAATAGATGTAGTAAA | 6mer |

  
  
  
  

| Seed Matches to the miRNA miR-194-5p | | | | | |
| --- | --- | --- | --- | --- | --- |
| Seed | Conservation | Species | Matches | | |
|  |  |  | Sequence | Motif | Type |
| GUAACAG | Broadly Conserved | Human (Homo sapiens)  Rhesus (Macaca mulatta)  Mouse (Mus musculus)  Rat (Rattus norvegicus) | HUMAN | CTTTTTGTTACAATTCATAGGAAAT | 7mer-A1 |
|  |  |  | HUMAN | TTGTTACATGTTTGCAGTTTATTCAAGACTGCT | 7mer-A1 |
|  |  |  | HUMAN | TGTTACA | 7mer-A1 |
|  |  |  | HUMAN | TATGTTACATGCATTTCATTTAACTTTGCTATACTGTATATATTGT | 7mer-A1 |
|  |  |  | HUMAN | AGATATAACCATTGTTAC | 7mer-A1 |
|  |  |  | HUMAN | CCATTGTTAC | 7mer-A1 |
|  |  |  | BABOON | CTTTTTGTTACAATTCATAGGAAAT | 7mer-A1 |
|  |  |  | BABOON | TTGTTACATGTTTGCAGTTTATTCAAGACTGCT | 7mer-A1 |
|  |  |  | BABOON | TGTTACA | 7mer-A1 |
|  |  |  | BABOON | TATGTTACATGCATTTCATTTAACTTTGCTATACTGTATATATTGT | 7mer-A1 |
|  |  |  | BABOON | AGATATAACCATTGTTAC | 6mer |
|  |  |  | BABOON | CCATTGTTAC | 6mer |
|  |  |  | DOG | TGTTACA | 8mer |
|  |  |  | DOG | CCATTGTTAC | 7mer-A1 |
|  |  |  | COW | TGTTACA | 8mer |
|  |  |  | COW | CCATTGTTAC | 7mer-A1 |
|  |  |  | SHEEP | TGTTACA | 8mer |
|  |  |  | SHEEP | CCATTGTTAC | 7mer-A1 |
|  |  |  | PIG | TGTTACA | 8mer |

  
  
  
  

| Seed Matches to the miRNA miR-25-3p/32-5p/92-3p/363-3p/367-3p | | | | | |
| --- | --- | --- | --- | --- | --- |
| Seed | Conservation | Species | Matches | | |
|  |  |  | Sequence | Motif | Type |
| AUUGCAC | Broadly Conserved | Human (Homo sapiens)  Mouse (Mus musculus) | HUMAN | TGTGTGCAATTTTATTTAACAGTGCT | 7mer-m8 |
|  |  |  | HUMAN | TGTGTGCAATT | 7mer-m8 |
|  |  |  | HUMAN | GTGTGCAATT | 7mer-m8 |
|  |  |  | BABOON | TGTGTGCAATTTTATTTAACAGTGCT | 7mer-m8 |
|  |  |  | BABOON | TGTGTGCAATT | 7mer-m8 |
|  |  |  | BABOON | GTGTGCAATT | 7mer-m8 |
|  |  |  | DOG | TGTGTGCAATT | 7mer-m8 |
|  |  |  | DOG | GTGTGCAATT | 7mer-m8 |
|  |  |  | COW | TGTGTGCAATT | 7mer-m8 |
|  |  |  | COW | GTGTGCAATT | 7mer-m8 |
|  |  |  | SHEEP | TGTGTGCAATT | 7mer-m8 |
|  |  |  | SHEEP | GTGTGCAATT | 7mer-m8 |
|  |  |  | PIG | GTGTGCAATT | 7mer-m8 |

  
  
  
  

| Seed Matches to the miRNA miR-21-5p/590-5p | | | | | |
| --- | --- | --- | --- | --- | --- |
| Seed | Conservation | Species | Matches | | |
|  |  |  | Sequence | Motif | Type |
| AGCUUAU | Broadly Conserved | Human (Homo sapiens)  Rhesus (Macaca mulatta) | HUMAN | TTGACAATGCTAAGCTTTTTTGA | 6mer |
|  |  |  | HUMAN | AACATCTAAGCTTTA | 6mer |
|  |  |  | HUMAN | AACATCTAAGCTTT | 6mer |
|  |  |  | BABOON | TTGACAATGCTAAGCTTTTTTGA | 6mer |
|  |  |  | BABOON | AACATCTAAGCTTTA | 6mer |
|  |  |  | BABOON | AACATCTAAGCTTT | 6mer |
|  |  |  | DOG | AACATCTAAGCTTT | 6mer |

  
  
  
  

| Seed Matches to the miRNA miR-496.2 | | | | | |
| --- | --- | --- | --- | --- | --- |
| Seed | Conservation | Species | Matches | | |
|  |  |  | Sequence | Motif | Type |
| GUAUUAC | Conserved | Human (Homo sapiens) | HUMAN | GGGGGGAATGGCCAAATCACCTGTTGAGTAATACTCATTGTGTTTGTGCAGTGGTTC | 7mer-m8 |
|  |  |  | BABOON | GGGGGGAATGGCCAAATCACCTGTTGAGTAATACTCATTGTGTTTGTGCAGTGGTTC | 7mer-m8 |

  
  
  
  

| Seed Matches to the miRNA miR-183-5p.1 | | | | | |
| --- | --- | --- | --- | --- | --- |
| Seed | Conservation | Species | Matches | | |
|  |  |  | Sequence | Motif | Type |
| AUGGCAC | Broadly Conserved | Human (Homo sapiens) | HUMAN | TATGCCATC | 6mer |
|  |  |  | HUMAN | GGTGTTTTGCCATTGTTTATTAGAAAATTTCAGCTTAATCCATTG | 6mer |
|  |  |  | HUMAN | GAACTCTGCCAATGCTTTTATCTAGAGGCGTGTTGCCATTTTTGTCTT | 6mer |
|  |  |  | HUMAN | TGTTGCCATTTTTGTC | 6mer |
|  |  |  | BABOON | TATGCCATC | 6mer |
|  |  |  | BABOON | GGTGTTTTGCCATTGTTTATTAGAAAATTTCAGCTTAATCCATTG | 6mer |
|  |  |  | BABOON | GAACTCTGCCAATGCTTTTATCTAGAGGCGTGTTGCCATTTTTGTCTT | 6mer |
|  |  |  | BABOON | TGTTGCCATTTTTGTC | 6mer |
|  |  |  | DOG | TGTTGCCATTTTTGTC | 6mer |
|  |  |  | COW | TGTTGCCATTTTTGTC | 6mer |
|  |  |  | SHEEP | TGTTGCCATTTTTGTC | 6mer |

  
  
  
  

| Seed Matches to the miRNA miR-199-3p | | | | | |
| --- | --- | --- | --- | --- | --- |
| Seed | Conservation | Species | Matches | | |
|  |  |  | Sequence | Motif | Type |
| CAGUAGU | Broadly Conserved | Human (Homo sapiens)  Rhesus (Macaca mulatta)  Cow (Bos taurus)  Mouse (Mus musculus)  Rat (Rattus norvegicus)  Opossum (Monodelphis domestica) | HUMAN | AGAAGGTTCTATGCTAGACTGGTCATATTTAGAAGACATTTTCATATTCTATCCATTGTTTTGTGTGCATTTTATTCCTCACTACTGTGTATATA | 7mer-m8 |
|  |  |  | HUMAN | ACTACTGTGTATATA | 7mer-m8 |
|  |  |  | HUMAN | CTACTGTGTATATAGC | 6mer |
|  |  |  | HUMAN | CTACTGTGTATATAG | 6mer |
|  |  |  | HUMAN | CTACTGTGTATATA | 6mer |
|  |  |  | HUMAN | AGTCTGTGTTATGTATGTATACCATTTATTCAATGCTACTGTGTATATAATGGAAAACTT | 6mer |
|  |  |  | BABOON | AGAAGGTTCTATGCTAGACTGGTCATATTTAGAAGACATTTTCATATTCTATCCATTGTTTTGTGTGCATTTTATTCCTCACTACTGTGTATATA | 7mer-m8 |
|  |  |  | BABOON | ACTACTGTGTATATA | 7mer-m8 |
|  |  |  | BABOON | CTACTGTGTATATAGC | 7mer-m8 |
|  |  |  | BABOON | CTACTGTGTATATAG | 7mer-m8 |
|  |  |  | BABOON | CTACTGTGTATATA | 7mer-m8 |
|  |  |  | BABOON | AGTCTGTGTTATGTATGTATACCATTTATTCAATGCTACTGTGTATATAATGGAAAACTT | 6mer |
|  |  |  | DOG | ACTACTGTGTATATA | 7mer-m8 |
|  |  |  | DOG | CTACTGTGTATATAG | 7mer-m8 |
|  |  |  | DOG | CTACTGTGTATATA | 7mer-m8 |
|  |  |  | COW | CTACTGTGTATATAG | 7mer-m8 |
|  |  |  | COW | CTACTGTGTATATA | 7mer-m8 |
|  |  |  | SHEEP | CTACTGTGTATATAG | 7mer-m8 |
|  |  |  | SHEEP | CTACTGTGTATATA | 7mer-m8 |
|  |  |  | PIG | CTACTGTGTATATA | 7mer-m8 |
|  |  |  | ARMADILLO | CTACTGTGTATATA | 7mer-m8 |
|  |  |  | GUINEAPIG | CTACTGTGTATATA | 6mer |

  
  
  
  

| Seed Matches to the miRNA miR-126-3p.2 | | | | | |
| --- | --- | --- | --- | --- | --- |
| Seed | Conservation | Species | Matches | | |
|  |  |  | Sequence | Motif | Type |
| GUACCGU | Broadly Conserved | Human (Homo sapiens)  Mouse (Mus musculus) | HUMAN | GGCACTTCCGGTAC | 6mer |
|  |  |  | BABOON | GGCACTTCCGGTAC | 6mer |

  
  
  
  

| Seed Matches to the miRNA miR-542-3p | | | | | |
| --- | --- | --- | --- | --- | --- |
| Seed | Conservation | Species | Matches | | |
|  |  |  | Sequence | Motif | Type |
| GUGACAG | Conserved | Human (Homo sapiens)  Rhesus (Macaca mulatta)  Mouse (Mus musculus)  Rat (Rattus norvegicus) | HUMAN | TGGAAGGCCTGTGTATATAATATGAAAAAGCTGCTCTCAACTCCACCCCAACCTTTTAATAGAAAACATTTGTCACATCTAGCCCTT | 7mer-A1 |
|  |  |  | BABOON | TGGAAGGCCTGTGTATATAATATGAAAAAGCTGCTCTCAACTCCACCCCAACCTTTTAATAGAAAACATTTGTCACATCTAGCCCTT | 7mer-A1 |

  
  
  
  

| Seed Matches to the miRNA miR-652-3p | | | | | |
| --- | --- | --- | --- | --- | --- |
| Seed | Conservation | Species | Matches | | |
|  |  |  | Sequence | Motif | Type |
| AUGGCGC | Conserved | Human (Homo sapiens)  Mouse (Mus musculus)  Rat (Rattus norvegicus) | HUMAN | CCACGGAGCCGCGCAGATCCGGTTCCCGGGTGACCACTCTGTCGCCATTGGGCGA | 6mer |
|  |  |  | BABOON | CCACGGAGCCGCGCAGATCCGGTTCCCGGGTGACCACTCTGTCGCCATTGGGCGA | 6mer |

  
  
  
  

| Seed Matches to the miRNA miR-409-3p | | | | | |
| --- | --- | --- | --- | --- | --- |
| Seed | Conservation | Species | Matches | | |
|  |  |  | Sequence | Motif | Type |
| AAUGUUG | Conserved | Human (Homo sapiens)  Rhesus (Macaca mulatta)  Mouse (Mus musculus) | HUMAN | TGGAAGGCCTGTGTATATAATATGAAAAAGCTGCTCTCAACTCCACCCCAACCTTTTAATAGAAAACATTTGTCACATCTAGCCCTT | 6mer |
|  |  |  | BABOON | TGGAAGGCCTGTGTATATAATATGAAAAAGCTGCTCTCAACTCCACCCCAACCTTTTAATAGAAAACATTTGTCACATCTAGCCCTT | 6mer |

  
  
  
  

| Seed Matches to the miRNA miR-17-5p/20-5p/93-5p/106-5p/519-3p | | | | | |
| --- | --- | --- | --- | --- | --- |
| Seed | Conservation | Species | Matches | | |
|  |  |  | Sequence | Motif | Type |
| AAAGUGC | Broadly Conserved | Human (Homo sapiens) | HUMAN | AGGTAGTAAAATACCACTTTGTAAATATCTTTTTGCTAAAATTCATAGGAAAT | 6mer |
|  |  |  | HUMAN | TAATATCTAGTCTCTAGATATTAAAGAGGTTGCCAATGTATGACAGAAGTAGAGTTAGTAAACTAACACATTTTGTACACTTTGTTAAAATTTGTAGAAAGGCTGTCTTCTGAAAAGGACTTTTGGAAGTGA | 6mer |
|  |  |  | HUMAN | AAGTAGAGTTAGTAAACTAACACATTTTGTACACTTTGT | 6mer |
|  |  |  | HUMAN | GTTAGTAAACTAACACATTTTGTACACTTTGT | 6mer |
|  |  |  | HUMAN | ATGCCTGTAATCCCAGCACTTTGGGAGGCCGAGGCAGGC | 7mer-m8 |
|  |  |  | BABOON | AGGTAGTAAAATACCACTTTGTAAATATCTTTTTGCTAAAATTCATAGGAAAT | 6mer |
|  |  |  | BABOON | TAATATCTAGTCTCTAGATATTAAAGAGGTTGCCAATGTATGACAGAAGTAGAGTTAGTAAACTAACACATTTTGTACACTTTGTTAAAATTTGTAGAAAGGCTGTCTTCTGAAAAGGACTTTTGGAAGTGA | 6mer |
|  |  |  | BABOON | AAGTAGAGTTAGTAAACTAACACATTTTGTACACTTTGT | 6mer |
|  |  |  | BABOON | GTTAGTAAACTAACACATTTTGTACACTTTGT | 6mer |
|  |  |  | BABOON | ATGCCTGTAATCCCAGCACTTTGGGAGGCCGAGGCAGGC | 7mer-m8 |
|  |  |  | DOG | AAGTAGAGTTAGTAAACTAACACATTTTGTACACTTTGT | 6mer |
|  |  |  | DOG | GTTAGTAAACTAACACATTTTGTACACTTTGT | 6mer |
|  |  |  | COW | GTTAGTAAACTAACACATTTTGTACACTTTGT | 6mer |
|  |  |  | SHEEP | GTTAGTAAACTAACACATTTTGTACACTTTGT | 6mer |

  
  
  
  

| Seed Matches to the miRNA miR-379-5p | | | | | |
| --- | --- | --- | --- | --- | --- |
| Seed | Conservation | Species | Matches | | |
|  |  |  | Sequence | Motif | Type |
| GGUAGAC | Conserved | Human (Homo sapiens)  Rhesus (Macaca mulatta)  Rat (Rattus norvegicus) | HUMAN | TTCTACCAGACTTT | 7mer-A1 |
|  |  |  | HUMAN | TATTTGTGCTGGTTCAGGGGGAAGGAGGAGCACAAAGTGCAAAGGGCTTTCTACCAGTGTCCAGTGTGTTTA | 7mer-A1 |
|  |  |  | BABOON | TTCTACCAGACTTT | 7mer-A1 |
|  |  |  | BABOON | TATTTGTGCTGGTTCAGGGGGAAGGAGGAGCACAAAGTGCAAAGGGCTTTCTACCAGTGTCCAGTGTGTTTA | 7mer-A1 |

  
  
  
  

| Seed Matches to the miRNA miR-1193 | | | | | |
| --- | --- | --- | --- | --- | --- |
| Seed | Conservation | Species | Matches | | |
|  |  |  | Sequence | Motif | Type |
| AGGUCAC | Conserved | Human (Homo sapiens)  Cow (Bos taurus) | HUMAN | TCTGGGAGTGACCTT | 7mer-m8 |
|  |  |  | BABOON | TCTGGGAGTGACCTT | 7mer-m8 |

  
  
  
  

| Seed Matches to the miRNA miR-196-5p | | | | | |
| --- | --- | --- | --- | --- | --- |
| Seed | Conservation | Species | Matches | | |
|  |  |  | Sequence | Motif | Type |
| AGGUAGU | Broadly Conserved | Human (Homo sapiens)  Chicken (Gallus gallus)  Rhesus (Macaca mulatta)  Mouse (Mus musculus)  Rat (Rattus norvegicus)  Opossum (Monodelphis domestica) | HUMAN | ACCTACCTAGTCCTGACGACAACGGACAAAGGCCTTAA | 7mer-A1 |
|  |  |  | BABOON | ACCTACCTAGTCCTGACGACAACGGACAAAGGCCTTAA | 7mer-A1 |

  
  
  
  

| Seed Matches to the miRNA miR-129-5p | | | | | |
| --- | --- | --- | --- | --- | --- |
| Seed | Conservation | Species | Matches | | |
|  |  |  | Sequence | Motif | Type |
| UUUUUGC | Broadly Conserved | Human (Homo sapiens)  Rhesus (Macaca mulatta)  Cow (Bos taurus)  Mouse (Mus musculus)  Rat (Rattus norvegicus)  Opossum (Monodelphis domestica) | HUMAN | AAAATACAAAAATTAGCTGGTCGT | 6mer |
|  |  |  | HUMAN | TTTCAAAAA | 7mer-A1 |
|  |  |  | BABOON | AAAATACAAAAATTAGCTGGTCGT | 6mer |
|  |  |  | BABOON | TTTCAAAAA | 6mer |

  
  
  
  

| Seed Matches to the miRNA miR-653-5p | | | | | |
| --- | --- | --- | --- | --- | --- |
| Seed | Conservation | Species | Matches | | |
|  |  |  | Sequence | Motif | Type |
| UGAAACA | Conserved | Human (Homo sapiens)  Rhesus (Macaca mulatta)  Mouse (Mus musculus)  Rat (Rattus norvegicus) | HUMAN | TATTCTGTTGCTGTGTGTTTCATTTTAAATTGAGCATTAAGGGAATGCAGCATTTAAATC | 7mer-m8 |
|  |  |  | BABOON | TATTCTGTTGCTGTGTGTTTCATTTTAAATTGAGCATTAAGGGAATGCAGCATTTAAATC | 7mer-m8 |

  
  
  
  

| Seed Matches to the miRNA miR-455-3p.2 | | | | | |
| --- | --- | --- | --- | --- | --- |
| Seed | Conservation | Species | Matches | | |
|  |  |  | Sequence | Motif | Type |
| UGCAGUC | Broadly Conserved | Human (Homo sapiens)  Mouse (Mus musculus) | HUMAN | CACCACTGCACTCCA | 6mer |
|  |  |  | BABOON | CACCACTGCACTCCA | 6mer |

  
  
  
  

| Seed Matches to the miRNA miR-362-5p/500b-5p | | | | | |
| --- | --- | --- | --- | --- | --- |
| Seed | Conservation | Species | Matches | | |
|  |  |  | Sequence | Motif | Type |
| AUCCUUG | Conserved | Human (Homo sapiens) | HUMAN | GGGTTCTGTGCTCCTGAGATTAGTTCAGATGGTCTAACCATTGTTCTATATGTGCATTTTAGTTAATATTGTGTATTAAAGGATAAGTCTTAATGCTCAAAGTATGTTAAAAATAGATGTAGTAAA | 7mer-A1 |
|  |  |  | BABOON | GGGTTCTGTGCTCCTGAGATTAGTTCAGATGGTCTAACCATTGTTCTATATGTGCATTTTAGTTAATATTGTGTATTAAAGGATAAGTCTTAATGCTCAAAGTATGTTAAAAATAGATGTAGTAAA | 7mer-A1 |

  
  
  
  

| Seed Matches to the miRNA miR-302-3p/372-3p/373-3p/520-3p | | | | | |
| --- | --- | --- | --- | --- | --- |
| Seed | Conservation | Species | Matches | | |
|  |  |  | Sequence | Motif | Type |
| AAGUGCU | Broadly Conserved | Human (Homo sapiens) | HUMAN | GGCACTTCCGGTAC | 6mer |
|  |  |  | HUMAN | GGCACTTCCGGT | 6mer |
|  |  |  | HUMAN | ATGCCTGTAATCCCAGCACTTTGGGAGGCCGAGGCAGGC | 7mer-m8 |
|  |  |  | BABOON | GGCACTTCCGGTAC | 6mer |
|  |  |  | BABOON | GGCACTTCCGGT | 6mer |
|  |  |  | BABOON | ATGCCTGTAATCCCAGCACTTTGGGAGGCCGAGGCAGGC | 7mer-m8 |
|  |  |  | DOG | GGCACTTCCGGT | 6mer |

  
  
  
  

| Seed Matches to the miRNA miR-96-5p/1271-5p | | | | | |
| --- | --- | --- | --- | --- | --- |
| Seed | Conservation | Species | Matches | | |
|  |  |  | Sequence | Motif | Type |
| UUGGCAC | Broadly Conserved | Human (Homo sapiens) | HUMAN | CAGAGAACTGCCAAGTCAGTTCCGGTC | 6mer |
|  |  |  | HUMAN | AGAGAACTGCCAAGTCAGTTCCGG | 6mer |
|  |  |  | HUMAN | AGAGAACTGCCAA | 6mer |
|  |  |  | HUMAN | TGCCAA | 6mer |
|  |  |  | HUMAN | TAATATCTAGTCTCTAGATATTAAAGAGGTTGCCAATGTATGACAGAAGTAGAGTTAGTAAACTAACACATTTTGTACACTTTGTTAAAATTTGTAGAAAGGCTGTCTTCTGAAAAGGACTTTTGGAAGTGA | 6mer |
|  |  |  | HUMAN | TAATATCTAGTCTCTAGATATTAAAGAGGTTGCCAATGTATGACA | 6mer |
|  |  |  | HUMAN | AAAGAGGTTGCCAATGTATGACA | 6mer |
|  |  |  | HUMAN | GAACTCTGCCAATGCTTTTATCTAGAGGCGTGTTGCCATTTTTGTCTT | 6mer |
|  |  |  | BABOON | CAGAGAACTGCCAAGTCAGTTCCGGTC | 6mer |
|  |  |  | BABOON | AGAGAACTGCCAAGTCAGTTCCGG | 6mer |
|  |  |  | BABOON | AGAGAACTGCCAA | 6mer |
|  |  |  | BABOON | TGCCAA | 6mer |
|  |  |  | BABOON | TAATATCTAGTCTCTAGATATTAAAGAGGTTGCCAATGTATGACAGAAGTAGAGTTAGTAAACTAACACATTTTGTACACTTTGTTAAAATTTGTAGAAAGGCTGTCTTCTGAAAAGGACTTTTGGAAGTGA | 6mer |
|  |  |  | BABOON | TAATATCTAGTCTCTAGATATTAAAGAGGTTGCCAATGTATGACA | 6mer |
|  |  |  | BABOON | AAAGAGGTTGCCAATGTATGACA | 6mer |
|  |  |  | BABOON | GAACTCTGCCAATGCTTTTATCTAGAGGCGTGTTGCCATTTTTGTCTT | 6mer |
|  |  |  | DOG | AGAGAACTGCCAAGTCAGTTCCGG | 6mer |
|  |  |  | DOG | AGAGAACTGCCAA | 6mer |
|  |  |  | DOG | TGCCAA | 6mer |
|  |  |  | DOG | TAATATCTAGTCTCTAGATATTAAAGAGGTTGCCAATGTATGACA | 6mer |
|  |  |  | DOG | AAAGAGGTTGCCAATGTATGACA | 6mer |
|  |  |  | COW | AGAGAACTGCCAAGTCAGTTCCGG | 6mer |
|  |  |  | COW | AGAGAACTGCCAA | 6mer |
|  |  |  | COW | TGCCAA | 6mer |
|  |  |  | COW | TAATATCTAGTCTCTAGATATTAAAGAGGTTGCCAATGTATGACA | 6mer |
|  |  |  | COW | AAAGAGGTTGCCAATGTATGACA | 6mer |
|  |  |  | SHEEP | AGAGAACTGCCAA | 6mer |
|  |  |  | SHEEP | TGCCAA | 6mer |
|  |  |  | SHEEP | TAATATCTAGTCTCTAGATATTAAAGAGGTTGCCAATGTATGACA | 6mer |
|  |  |  | SHEEP | AAAGAGGTTGCCAATGTATGACA | 6mer |
|  |  |  | PIG | AGAGAACTGCCAA | 6mer |
|  |  |  | PIG | TGCCAA | 6mer |
|  |  |  | PIG | AAAGAGGTTGCCAATGTATGACA | 6mer |
|  |  |  | ARMADILLO | AGAGAACTGCCAA | 6mer |
|  |  |  | ARMADILLO | TGCCAA | 6mer |
|  |  |  | ARMADILLO | AAAGAGGTTGCCAATGTATGACA | 6mer |
|  |  |  | GUINEAPIG | TGCCAA | 7mer-m8 |
|  |  |  | GUINEAPIG | AAAGAGGTTGCCAATGTATGACA | 6mer |
|  |  |  | MOUSE | TGCCAA | 6mer |
|  |  |  | MOUSE | AAAGAGGTTGCCAATGTATGACA | 6mer |

  
  
  
  

| Seed Matches to the miRNA miR-182-5p | | | | | |
| --- | --- | --- | --- | --- | --- |
| Seed | Conservation | Species | Matches | | |
|  |  |  | Sequence | Motif | Type |
| UUGGCAA | Broadly Conserved | Human (Homo sapiens)  X. tropicalis (Xenopus tropicalis)  Mouse (Mus musculus)  Opossum (Monodelphis domestica) | HUMAN | CAGAGAACTGCCAAGTCAGTTCCGGTC | 6mer |
|  |  |  | HUMAN | AGAGAACTGCCAAGTCAGTTCCGG | 6mer |
|  |  |  | HUMAN | AGAGAACTGCCAA | 6mer |
|  |  |  | HUMAN | TGCCAA | 6mer |
|  |  |  | HUMAN | TAATATCTAGTCTCTAGATATTAAAGAGGTTGCCAATGTATGACAGAAGTAGAGTTAGTAAACTAACACATTTTGTACACTTTGTTAAAATTTGTAGAAAGGCTGTCTTCTGAAAAGGACTTTTGGAAGTGA | 7mer-m8 |
|  |  |  | HUMAN | TAATATCTAGTCTCTAGATATTAAAGAGGTTGCCAATGTATGACA | 7mer-m8 |
|  |  |  | HUMAN | AAAGAGGTTGCCAATGTATGACA | 7mer-m8 |
|  |  |  | HUMAN | GAACTCTGCCAATGCTTTTATCTAGAGGCGTGTTGCCATTTTTGTCTT | 6mer |
|  |  |  | BABOON | CAGAGAACTGCCAAGTCAGTTCCGGTC | 6mer |
|  |  |  | BABOON | AGAGAACTGCCAAGTCAGTTCCGG | 6mer |
|  |  |  | BABOON | AGAGAACTGCCAA | 6mer |
|  |  |  | BABOON | TGCCAA | 6mer |
|  |  |  | BABOON | TAATATCTAGTCTCTAGATATTAAAGAGGTTGCCAATGTATGACAGAAGTAGAGTTAGTAAACTAACACATTTTGTACACTTTGTTAAAATTTGTAGAAAGGCTGTCTTCTGAAAAGGACTTTTGGAAGTGA | 7mer-m8 |
|  |  |  | BABOON | TAATATCTAGTCTCTAGATATTAAAGAGGTTGCCAATGTATGACA | 7mer-m8 |
|  |  |  | BABOON | AAAGAGGTTGCCAATGTATGACA | 7mer-m8 |
|  |  |  | BABOON | GAACTCTGCCAATGCTTTTATCTAGAGGCGTGTTGCCATTTTTGTCTT | 6mer |
|  |  |  | DOG | AGAGAACTGCCAAGTCAGTTCCGG | 6mer |
|  |  |  | DOG | AGAGAACTGCCAA | 6mer |
|  |  |  | DOG | TGCCAA | 6mer |
|  |  |  | DOG | TAATATCTAGTCTCTAGATATTAAAGAGGTTGCCAATGTATGACA | 7mer-m8 |
|  |  |  | DOG | AAAGAGGTTGCCAATGTATGACA | 7mer-m8 |
|  |  |  | COW | AGAGAACTGCCAAGTCAGTTCCGG | 6mer |
|  |  |  | COW | AGAGAACTGCCAA | 6mer |
|  |  |  | COW | TGCCAA | 6mer |
|  |  |  | COW | TAATATCTAGTCTCTAGATATTAAAGAGGTTGCCAATGTATGACA | 7mer-m8 |
|  |  |  | COW | AAAGAGGTTGCCAATGTATGACA | 7mer-m8 |
|  |  |  | SHEEP | AGAGAACTGCCAA | 6mer |
|  |  |  | SHEEP | TGCCAA | 6mer |
|  |  |  | SHEEP | TAATATCTAGTCTCTAGATATTAAAGAGGTTGCCAATGTATGACA | 7mer-m8 |
|  |  |  | SHEEP | AAAGAGGTTGCCAATGTATGACA | 7mer-m8 |
|  |  |  | PIG | AGAGAACTGCCAA | 6mer |
|  |  |  | PIG | TGCCAA | 6mer |
|  |  |  | PIG | AAAGAGGTTGCCAATGTATGACA | 7mer-m8 |
|  |  |  | ARMADILLO | AGAGAACTGCCAA | 6mer |
|  |  |  | ARMADILLO | TGCCAA | 6mer |
|  |  |  | ARMADILLO | AAAGAGGTTGCCAATGTATGACA | 7mer-m8 |
|  |  |  | GUINEAPIG | TGCCAA | 6mer |
|  |  |  | GUINEAPIG | AAAGAGGTTGCCAATGTATGACA | 7mer-m8 |
|  |  |  | MOUSE | TGCCAA | 7mer-m8 |
|  |  |  | MOUSE | AAAGAGGTTGCCAATGTATGACA | 7mer-m8 |

  
  
  
  

| Seed Matches to the miRNA miR-33-5p | | | | | |
| --- | --- | --- | --- | --- | --- |
| Seed | Conservation | Species | Matches | | |
|  |  |  | Sequence | Motif | Type |
| UGCAUUG | Broadly Conserved | Human (Homo sapiens)  Chicken (Gallus gallus)  Rhesus (Macaca mulatta)  Mouse (Mus musculus)  Rat (Rattus norvegicus)  Opossum (Monodelphis domestica) | HUMAN | TTAGGAGCGAATGCAGACT | 6mer |
|  |  |  | HUMAN | TATTCTGTTGCTGTGTGTTTCATTTTAAATTGAGCATTAAGGGAATGCAGCATTTAAATC | 6mer |
|  |  |  | HUMAN | CATTAAGGGAATGCAG | 6mer |
|  |  |  | HUMAN | TAATGCACATA | 6mer |
|  |  |  | BABOON | TTAGGAGCGAATGCAGACT | 6mer |
|  |  |  | BABOON | TATTCTGTTGCTGTGTGTTTCATTTTAAATTGAGCATTAAGGGAATGCAGCATTTAAATC | 6mer |
|  |  |  | BABOON | CATTAAGGGAATGCAG | 6mer |
|  |  |  | BABOON | TAATGCACATA | 6mer |
|  |  |  | DOG | CATTAAGGGAATGCAG | 6mer |

  
  
  
  

| Seed Matches to the miRNA miR-216b-5p | | | | | |
| --- | --- | --- | --- | --- | --- |
| Seed | Conservation | Species | Matches | | |
|  |  |  | Sequence | Motif | Type |
| AAUCUCU | Broadly Conserved | Human (Homo sapiens)  Mouse (Mus musculus)  Rat (Rattus norvegicus) | HUMAN | GGGTTCTGTGCTCCTGAGATTAGTTCAGATGGTCTAACCATTGTTCTATATGTGCATTTTAGTTAATATTGTGTATTAAAGGATAAGTCTTAATGCTCAAAGTATGTTAAAAATAGATGTAGTAAA | 7mer-A1 |
|  |  |  | BABOON | GGGTTCTGTGCTCCTGAGATTAGTTCAGATGGTCTAACCATTGTTCTATATGTGCATTTTAGTTAATATTGTGTATTAAAGGATAAGTCTTAATGCTCAAAGTATGTTAAAAATAGATGTAGTAAA | 7mer-A1 |

  
  
  
  

| Seed Matches to the miRNA miR-221-3p/222-3p | | | | | |
| --- | --- | --- | --- | --- | --- |
| Seed | Conservation | Species | Matches | | |
|  |  |  | Sequence | Motif | Type |
| GCUACAU | Broadly Conserved | Human (Homo sapiens)  Chicken (Gallus gallus)  Rhesus (Macaca mulatta)  Mouse (Mus musculus)  Rat (Rattus norvegicus) | HUMAN | GACAAACTTAAGTCCTTATTTGAAACATCTAGTCTTTCTAGATGTTTAGAAGTGCACAAAGTATGTTAAAAGTAGAGGTAGTAAATAACACATTTTGTAGCTATCCTTTTGATATGAAATATTGTCTTGGAAA | 6mer |
|  |  |  | BABOON | GACAAACTTAAGTCCTTATTTGAAACATCTAGTCTTTCTAGATGTTTAGAAGTGCACAAAGTATGTTAAAAGTAGAGGTAGTAAATAACACATTTTGTAGCTATCCTTTTGATATGAAATATTGTCTTGGAAA | 6mer |

  
  
  
  

| Seed Matches to the miRNA miR-205-5p | | | | | |
| --- | --- | --- | --- | --- | --- |
| Seed | Conservation | Species | Matches | | |
|  |  |  | Sequence | Motif | Type |
| CCUUCAU | Broadly Conserved | Human (Homo sapiens)  Mouse (Mus musculus) | HUMAN | CAGGTTGGTGGTGGAGAGGAGTTGGAAGGAATGAAGGGTTCTAGACCAGAATGTTC | 7mer-m8 |
|  |  |  | HUMAN | AATGAAGGGTTCTAGAC | 7mer-m8 |
|  |  |  | HUMAN | GAGGAAGGGTGAAGGGAAGGGCTCTTTGCTAGTATCT | 6mer |
|  |  |  | HUMAN | GGGTGAAGGGAAGGGCT | 6mer |
|  |  |  | BABOON | CAGGTTGGTGGTGGAGAGGAGTTGGAAGGAATGAAGGGTTCTAGACCAGAATGTTC | 7mer-m8 |
|  |  |  | BABOON | AATGAAGGGTTCTAGAC | 7mer-m8 |
|  |  |  | BABOON | GAGGAAGGGTGAAGGGAAGGGCTCTTTGCTAGTATCT | 6mer |
|  |  |  | BABOON | GGGTGAAGGGAAGGGCT | 6mer |
|  |  |  | DOG | AATGAAGGGTTCTAGAC | 7mer-m8 |
|  |  |  | DOG | GGGTGAAGGGAAGGGCT | 6mer |

  
  
  
  

| Seed Matches to the miRNA miR-203a-3p.2 | | | | | |
| --- | --- | --- | --- | --- | --- |
| Seed | Conservation | Species | Matches | | |
|  |  |  | Sequence | Motif | Type |
| UGAAAUG | Broadly Conserved | Human (Homo sapiens) | HUMAN | GGTGTTTTGCCATTGTTTATTAGAAAATTTCAGCTTAATCCATTG | 6mer |
|  |  |  | HUMAN | AAATTTCAGCTTA | 6mer |
|  |  |  | HUMAN | ATTTCAGCTTA | 6mer |
|  |  |  | HUMAN | ATTTCA | 6mer |
|  |  |  | HUMAN | TATGTTACATGCATTTCATTTAACTTTGCTATACTGTATATATTGT | 7mer-m8 |
|  |  |  | BABOON | GGTGTTTTGCCATTGTTTATTAGAAAATTTCAGCTTAATCCATTG | 6mer |
|  |  |  | BABOON | AAATTTCAGCTTA | 6mer |
|  |  |  | BABOON | ATTTCAGCTTA | 6mer |
|  |  |  | BABOON | ATTTCA | 6mer |
|  |  |  | BABOON | TATGTTACATGCATTTCATTTAACTTTGCTATACTGTATATATTGT | 7mer-m8 |
|  |  |  | DOG | AAATTTCAGCTTA | 6mer |
|  |  |  | DOG | ATTTCAGCTTA | 6mer |
|  |  |  | DOG | ATTTCA | 6mer |
|  |  |  | COW | ATTTCAGCTTA | 7mer-m8 |
|  |  |  | COW | ATTTCA | 7mer-m8 |
|  |  |  | SHEEP | ATTTCAGCTTA | 7mer-m8 |
|  |  |  | SHEEP | ATTTCA | 7mer-m8 |
|  |  |  | PIG | ATTTCA | 6mer |

  
  
  
  

| Seed Matches to the miRNA miR-216a-5p | | | | | |
| --- | --- | --- | --- | --- | --- |
| Seed | Conservation | Species | Matches | | |
|  |  |  | Sequence | Motif | Type |
| AAUCUCA | Broadly Conserved | Human (Homo sapiens)  Mouse (Mus musculus)  Rat (Rattus norvegicus) | HUMAN | GGGTTCTGTGCTCCTGAGATTAGTTCAGATGGTCTAACCATTGTTCTATATGTGCATTTTAGTTAATATTGTGTATTAAAGGATAAGTCTTAATGCTCAAAGTATGTTAAAAATAGATGTAGTAAA | 8mer |
|  |  |  | BABOON | GGGTTCTGTGCTCCTGAGATTAGTTCAGATGGTCTAACCATTGTTCTATATGTGCATTTTAGTTAATATTGTGTATTAAAGGATAAGTCTTAATGCTCAAAGTATGTTAAAAATAGATGTAGTAAA | 8mer |

  
  
  
  

| Seed Matches to the miRNA miR-483-3p.1 | | | | | |
| --- | --- | --- | --- | --- | --- |
| Seed | Conservation | Species | Matches | | |
|  |  |  | Sequence | Motif | Type |
| ACUCCUC | Conserved | Human (Homo sapiens)  Mouse (Mus musculus) | HUMAN | CAGGTTGGTGGTGGAGAGGAGTTGGAAGGAATGAAGGGTTCTAGACCAGAATGTTC | 7mer-m8 |
|  |  |  | HUMAN | AGGAGTCCTAATTTAC | 6mer |
|  |  |  | HUMAN | CTAGGAGTGGTTGCATTTGGGAATGGAATTGTTAAAACTTGATG | 6mer |
|  |  |  | HUMAN | TAGGAGT | 6mer |
|  |  |  | BABOON | CAGGTTGGTGGTGGAGAGGAGTTGGAAGGAATGAAGGGTTCTAGACCAGAATGTTC | 7mer-m8 |
|  |  |  | BABOON | AGGAGTCCTAATTTAC | 6mer |
|  |  |  | BABOON | CTAGGAGTGGTTGCATTTGGGAATGGAATTGTTAAAACTTGATG | 6mer |
|  |  |  | BABOON | TAGGAGT | 6mer |
|  |  |  | DOG | TAGGAGT | 7mer-A1 |

  
  
  
  

| Seed Matches to the miRNA miR-15-5p/16-5p/195-5p/424-5p/497-5p | | | | | |
| --- | --- | --- | --- | --- | --- |
| Seed | Conservation | Species | Matches | | |
|  |  |  | Sequence | Motif | Type |
| AGCAGCA | Broadly Conserved | Human (Homo sapiens)  Rhesus (Macaca mulatta) | HUMAN | TGGAAGGCCTGTGTATATAATATGAAAAAGCTGCTCTCAACTCCACCCCAACCTTTTAATAGAAAACATTTGTCACATCTAGCCCTT | 6mer |
|  |  |  | HUMAN | GAAGGCCTGTGTATATAATATGAAAAAGCTGCTCTCAACT | 6mer |
|  |  |  | HUMAN | AAGGCCTGTGTATATAATATGAAAAAGCTGCTCTCAACT | 6mer |
|  |  |  | HUMAN | AAGGCCTGTGTATATAATATGAAAAAGCTGCT | 6mer |
|  |  |  | HUMAN | CCTGTGTATATAATATGAAAAAGCTGCT | 6mer |
|  |  |  | BABOON | TGGAAGGCCTGTGTATATAATATGAAAAAGCTGCTCTCAACTCCACCCCAACCTTTTAATAGAAAACATTTGTCACATCTAGCCCTT | 6mer |
|  |  |  | BABOON | GAAGGCCTGTGTATATAATATGAAAAAGCTGCTCTCAACT | 6mer |
|  |  |  | BABOON | AAGGCCTGTGTATATAATATGAAAAAGCTGCTCTCAACT | 6mer |
|  |  |  | BABOON | AAGGCCTGTGTATATAATATGAAAAAGCTGCT | 6mer |
|  |  |  | BABOON | CCTGTGTATATAATATGAAAAAGCTGCT | 6mer |
|  |  |  | DOG | GAAGGCCTGTGTATATAATATGAAAAAGCTGCTCTCAACT | 6mer |
|  |  |  | DOG | AAGGCCTGTGTATATAATATGAAAAAGCTGCTCTCAACT | 6mer |
|  |  |  | DOG | AAGGCCTGTGTATATAATATGAAAAAGCTGCT | 6mer |
|  |  |  | DOG | CCTGTGTATATAATATGAAAAAGCTGCT | 6mer |
|  |  |  | COW | AAGGCCTGTGTATATAATATGAAAAAGCTGCTCTCAACT | 6mer |
|  |  |  | COW | AAGGCCTGTGTATATAATATGAAAAAGCTGCT | 6mer |
|  |  |  | COW | CCTGTGTATATAATATGAAAAAGCTGCT | 6mer |
|  |  |  | SHEEP | AAGGCCTGTGTATATAATATGAAAAAGCTGCTCTCAACT | 6mer |
|  |  |  | SHEEP | AAGGCCTGTGTATATAATATGAAAAAGCTGCT | 6mer |
|  |  |  | SHEEP | CCTGTGTATATAATATGAAAAAGCTGCT | 6mer |
|  |  |  | PIG | AAGGCCTGTGTATATAATATGAAAAAGCTGCTCTCAACT | 6mer |
|  |  |  | PIG | AAGGCCTGTGTATATAATATGAAAAAGCTGCT | 6mer |
|  |  |  | PIG | CCTGTGTATATAATATGAAAAAGCTGCT | 6mer |
|  |  |  | ARMADILLO | AAGGCCTGTGTATATAATATGAAAAAGCTGCT | 7mer-A1 |
|  |  |  | ARMADILLO | CCTGTGTATATAATATGAAAAAGCTGCT | 7mer-A1 |
|  |  |  | GUINEAPIG | AAGGCCTGTGTATATAATATGAAAAAGCTGCT | 6mer |
|  |  |  | GUINEAPIG | CCTGTGTATATAATATGAAAAAGCTGCT | 6mer |
|  |  |  | MOUSE | CCTGTGTATATAATATGAAAAAGCTGCT | 6mer |

  
  
  
  

| Seed Matches to the miRNA miR-139-5p | | | | | |
| --- | --- | --- | --- | --- | --- |
| Seed | Conservation | Species | Matches | | |
|  |  |  | Sequence | Motif | Type |
| CUACAGU | Broadly Conserved | Human (Homo sapiens)  Rhesus (Macaca mulatta)  Mouse (Mus musculus)  Rat (Rattus norvegicus)  Opossum (Monodelphis domestica) | HUMAN | CACCTGTAG | 6mer |
|  |  |  | BABOON | CACCTGTAG | 6mer |

  
  
  
  

| Seed Matches to the miRNA miR-539-3p | | | | | |
| --- | --- | --- | --- | --- | --- |
| Seed | Conservation | Species | Matches | | |
|  |  |  | Sequence | Motif | Type |
| UCAUACA | Conserved | Human (Homo sapiens) | HUMAN | TAGATGGAAAGAGGTTGCCGACGTATGATAAA | 6mer |
|  |  |  | HUMAN | TCTGTGCATTTTGTTTTACTTATCTGTGTATATAGTGTACATAAAGGACAGACGAGTCCTAATTGACAACATCTAGTCTTTCTGGATGTTAAAGAGGTTGCCAGTGTATGACAAAAGTAGAGTT | 7mer-m8 |
|  |  |  | HUMAN | GATGTTAAAGAGGTTGCCAGTGTATGACAAAA | 7mer-m8 |
|  |  |  | HUMAN | GATGTTAAAGAGGTTGCCAGTGTATGA | 7mer-m8 |
|  |  |  | HUMAN | AAAGAGGTTGCCAGTGTATGA | 7mer-m8 |
|  |  |  | HUMAN | TATATAATGGACAAATAGTCCTAATTTTTCAACATCTAGTCTCTAGATGTTAAAGAGGTTGCCAGTGTATGACAAAG | 7mer-m8 |
|  |  |  | HUMAN | TGTATGACAAAG | 7mer-m8 |
|  |  |  | HUMAN | TGTATGACAAA | 7mer-m8 |
|  |  |  | HUMAN | TAATATCTAGTCTCTAGATATTAAAGAGGTTGCCAATGTATGACAGAAGTAGAGTTAGTAAACTAACACATTTTGTACACTTTGTTAAAATTTGTAGAAAGGCTGTCTTCTGAAAAGGACTTTTGGAAGTGA | 7mer-m8 |
|  |  |  | HUMAN | TAATATCTAGTCTCTAGATATTAAAGAGGTTGCCAATGTATGACA | 7mer-m8 |
|  |  |  | HUMAN | AAAGAGGTTGCCAATGTATGACA | 7mer-m8 |
|  |  |  | BABOON | TAGATGGAAAGAGGTTGCCGACGTATGATAAA | 6mer |
|  |  |  | BABOON | TCTGTGCATTTTGTTTTACTTATCTGTGTATATAGTGTACATAAAGGACAGACGAGTCCTAATTGACAACATCTAGTCTTTCTGGATGTTAAAGAGGTTGCCAGTGTATGACAAAAGTAGAGTT | 7mer-m8 |
|  |  |  | BABOON | GATGTTAAAGAGGTTGCCAGTGTATGACAAAA | 7mer-m8 |
|  |  |  | BABOON | GATGTTAAAGAGGTTGCCAGTGTATGA | 7mer-m8 |
|  |  |  | BABOON | AAAGAGGTTGCCAGTGTATGA | 7mer-m8 |
|  |  |  | BABOON | TATATAATGGACAAATAGTCCTAATTTTTCAACATCTAGTCTCTAGATGTTAAAGAGGTTGCCAGTGTATGACAAAG | 7mer-m8 |
|  |  |  | BABOON | TGTATGACAAAG | 7mer-m8 |
|  |  |  | BABOON | TGTATGACAAA | 7mer-m8 |
|  |  |  | BABOON | TAATATCTAGTCTCTAGATATTAAAGAGGTTGCCAATGTATGACAGAAGTAGAGTTAGTAAACTAACACATTTTGTACACTTTGTTAAAATTTGTAGAAAGGCTGTCTTCTGAAAAGGACTTTTGGAAGTGA | 7mer-m8 |
|  |  |  | BABOON | TAATATCTAGTCTCTAGATATTAAAGAGGTTGCCAATGTATGACA | 7mer-m8 |
|  |  |  | BABOON | AAAGAGGTTGCCAATGTATGACA | 7mer-m8 |
|  |  |  | DOG | GATGTTAAAGAGGTTGCCAGTGTATGACAAAA | 7mer-m8 |
|  |  |  | DOG | GATGTTAAAGAGGTTGCCAGTGTATGA | 7mer-m8 |
|  |  |  | DOG | AAAGAGGTTGCCAGTGTATGA | 7mer-m8 |
|  |  |  | DOG | TGTATGACAAAG | 7mer-m8 |
|  |  |  | DOG | TGTATGACAAA | 7mer-m8 |
|  |  |  | DOG | TAATATCTAGTCTCTAGATATTAAAGAGGTTGCCAATGTATGACA | 7mer-m8 |
|  |  |  | DOG | AAAGAGGTTGCCAATGTATGACA | 7mer-m8 |
|  |  |  | COW | GATGTTAAAGAGGTTGCCAGTGTATGACAAAA | 7mer-m8 |
|  |  |  | COW | GATGTTAAAGAGGTTGCCAGTGTATGA | 7mer-m8 |
|  |  |  | COW | AAAGAGGTTGCCAGTGTATGA | 7mer-m8 |
|  |  |  | COW | TGTATGACAAAG | 7mer-m8 |
|  |  |  | COW | TGTATGACAAA | 7mer-m8 |
|  |  |  | COW | TAATATCTAGTCTCTAGATATTAAAGAGGTTGCCAATGTATGACA | 7mer-m8 |
|  |  |  | COW | AAAGAGGTTGCCAATGTATGACA | 7mer-m8 |
|  |  |  | SHEEP | GATGTTAAAGAGGTTGCCAGTGTATGACAAAA | 7mer-m8 |
|  |  |  | SHEEP | GATGTTAAAGAGGTTGCCAGTGTATGA | 7mer-m8 |
|  |  |  | SHEEP | AAAGAGGTTGCCAGTGTATGA | 7mer-m8 |
|  |  |  | SHEEP | TGTATGACAAAG | 7mer-m8 |
|  |  |  | SHEEP | TGTATGACAAA | 7mer-m8 |
|  |  |  | SHEEP | TAATATCTAGTCTCTAGATATTAAAGAGGTTGCCAATGTATGACA | 7mer-m8 |
|  |  |  | SHEEP | AAAGAGGTTGCCAATGTATGACA | 7mer-m8 |
|  |  |  | PIG | GATGTTAAAGAGGTTGCCAGTGTATGA | 7mer-m8 |
|  |  |  | PIG | AAAGAGGTTGCCAGTGTATGA | 7mer-m8 |
|  |  |  | PIG | TGTATGACAAA | 7mer-m8 |
|  |  |  | PIG | AAAGAGGTTGCCAATGTATGACA | 7mer-m8 |
|  |  |  | ARMADILLO | AAAGAGGTTGCCAGTGTATGA | 8mer |
|  |  |  | ARMADILLO | AAAGAGGTTGCCAATGTATGACA | 7mer-m8 |
|  |  |  | GUINEAPIG | AAAGAGGTTGCCAATGTATGACA | 7mer-m8 |
|  |  |  | MOUSE | AAAGAGGTTGCCAATGTATGACA | 7mer-m8 |

  
  
  
  

| Seed Matches to the miRNA miR-503-5p | | | | | |
| --- | --- | --- | --- | --- | --- |
| Seed | Conservation | Species | Matches | | |
|  |  |  | Sequence | Motif | Type |
| AGCAGCG | Conserved | Human (Homo sapiens)  Rhesus (Macaca mulatta)  Mouse (Mus musculus)  Rat (Rattus norvegicus) | HUMAN | TGGAAGGCCTGTGTATATAATATGAAAAAGCTGCTCTCAACTCCACCCCAACCTTTTAATAGAAAACATTTGTCACATCTAGCCCTT | 6mer |
|  |  |  | HUMAN | GAAGGCCTGTGTATATAATATGAAAAAGCTGCTCTCAACT | 6mer |
|  |  |  | HUMAN | AAGGCCTGTGTATATAATATGAAAAAGCTGCTCTCAACT | 6mer |
|  |  |  | HUMAN | AAGGCCTGTGTATATAATATGAAAAAGCTGCT | 6mer |
|  |  |  | HUMAN | CCTGTGTATATAATATGAAAAAGCTGCT | 6mer |
|  |  |  | BABOON | TGGAAGGCCTGTGTATATAATATGAAAAAGCTGCTCTCAACTCCACCCCAACCTTTTAATAGAAAACATTTGTCACATCTAGCCCTT | 6mer |
|  |  |  | BABOON | GAAGGCCTGTGTATATAATATGAAAAAGCTGCTCTCAACT | 6mer |
|  |  |  | BABOON | AAGGCCTGTGTATATAATATGAAAAAGCTGCTCTCAACT | 6mer |
|  |  |  | BABOON | AAGGCCTGTGTATATAATATGAAAAAGCTGCT | 6mer |
|  |  |  | BABOON | CCTGTGTATATAATATGAAAAAGCTGCT | 6mer |
|  |  |  | DOG | GAAGGCCTGTGTATATAATATGAAAAAGCTGCTCTCAACT | 6mer |
|  |  |  | DOG | AAGGCCTGTGTATATAATATGAAAAAGCTGCTCTCAACT | 6mer |
|  |  |  | DOG | AAGGCCTGTGTATATAATATGAAAAAGCTGCT | 6mer |
|  |  |  | DOG | CCTGTGTATATAATATGAAAAAGCTGCT | 6mer |
|  |  |  | COW | AAGGCCTGTGTATATAATATGAAAAAGCTGCTCTCAACT | 6mer |
|  |  |  | COW | AAGGCCTGTGTATATAATATGAAAAAGCTGCT | 6mer |
|  |  |  | COW | CCTGTGTATATAATATGAAAAAGCTGCT | 6mer |
|  |  |  | SHEEP | AAGGCCTGTGTATATAATATGAAAAAGCTGCTCTCAACT | 6mer |
|  |  |  | SHEEP | AAGGCCTGTGTATATAATATGAAAAAGCTGCT | 6mer |
|  |  |  | SHEEP | CCTGTGTATATAATATGAAAAAGCTGCT | 6mer |
|  |  |  | PIG | AAGGCCTGTGTATATAATATGAAAAAGCTGCTCTCAACT | 6mer |
|  |  |  | PIG | AAGGCCTGTGTATATAATATGAAAAAGCTGCT | 6mer |
|  |  |  | PIG | CCTGTGTATATAATATGAAAAAGCTGCT | 6mer |
|  |  |  | ARMADILLO | AAGGCCTGTGTATATAATATGAAAAAGCTGCT | 7mer-A1 |
|  |  |  | ARMADILLO | CCTGTGTATATAATATGAAAAAGCTGCT | 7mer-A1 |
|  |  |  | GUINEAPIG | AAGGCCTGTGTATATAATATGAAAAAGCTGCT | 6mer |
|  |  |  | GUINEAPIG | CCTGTGTATATAATATGAAAAAGCTGCT | 6mer |
|  |  |  | MOUSE | CCTGTGTATATAATATGAAAAAGCTGCT | 6mer |

  
  
  
  

| Seed Matches to the miRNA miR-127-3p | | | | | |
| --- | --- | --- | --- | --- | --- |
| Seed | Conservation | Species | Matches | | |
|  |  |  | Sequence | Motif | Type |
| CGGAUCC | Conserved | Human (Homo sapiens)  Rhesus (Macaca mulatta)  Mouse (Mus musculus)  Rat (Rattus norvegicus) | HUMAN | CCACGGAGCCGCGCAGATCCGGTTCCCGGGTGACCACTCTGTCGCCATTGGGCGA | 6mer |
|  |  |  | BABOON | CCACGGAGCCGCGCAGATCCGGTTCCCGGGTGACCACTCTGTCGCCATTGGGCGA | 6mer |

  
  
  
  

| Seed Matches to the miRNA miR-99-5p/100-5p | | | | | |
| --- | --- | --- | --- | --- | --- |
| Seed | Conservation | Species | Matches | | |
|  |  |  | Sequence | Motif | Type |
| ACCCGUA | Broadly Conserved | Human (Homo sapiens)  Chicken (Gallus gallus)  Rhesus (Macaca mulatta)  Mouse (Mus musculus)  Rat (Rattus norvegicus) | HUMAN | CCCGAACGACGACGGGTGGAACG | 6mer |
|  |  |  | HUMAN | CGACGGGTGGAAC | 6mer |
|  |  |  | BABOON | CCCGAACGACGACGGGTGGAACG | 6mer |
|  |  |  | BABOON | CGACGGGTGGAAC | 6mer |
|  |  |  | DOG | CGACGGGTGGAAC | 6mer |
|  |  |  | COW | CGACGGGTGGAAC | 6mer |

  
  
  
  

| Seed Matches to the miRNA miR-202-5p | | | | | |
| --- | --- | --- | --- | --- | --- |
| Seed | Conservation | Species | Matches | | |
|  |  |  | Sequence | Motif | Type |
| UCCUAUG | Broadly Conserved | Human (Homo sapiens)  Mouse (Mus musculus)  Rat (Rattus norvegicus) | HUMAN | CTTTTTGTTACAATTCATAGGAAAT | 8mer |
|  |  |  | HUMAN | ATTCATAGGAAAT | 8mer |
|  |  |  | HUMAN | AGGTAGTAAAATACCACTTTGTAAATATCTTTTTGCTAAAATTCATAGGAAAT | 8mer |
|  |  |  | HUMAN | TGTAAATATCTTTTTGCTAAAATTCATAGGAAAT | 8mer |
|  |  |  | HUMAN | AAATTCATAGGAA | 8mer |
|  |  |  | HUMAN | TTTGCATAGGAATTTGTT | 8mer |
|  |  |  | BABOON | CTTTTTGTTACAATTCATAGGAAAT | 8mer |
|  |  |  | BABOON | ATTCATAGGAAAT | 8mer |
|  |  |  | BABOON | AGGTAGTAAAATACCACTTTGTAAATATCTTTTTGCTAAAATTCATAGGAAAT | 8mer |
|  |  |  | BABOON | TGTAAATATCTTTTTGCTAAAATTCATAGGAAAT | 8mer |
|  |  |  | BABOON | AAATTCATAGGAA | 8mer |
|  |  |  | BABOON | TTTGCATAGGAATTTGTT | 8mer |
|  |  |  | DOG | ATTCATAGGAAAT | 8mer |
|  |  |  | DOG | TGTAAATATCTTTTTGCTAAAATTCATAGGAAAT | 8mer |
|  |  |  | DOG | AAATTCATAGGAA | 8mer |
|  |  |  | COW | AAATTCATAGGAA | 8mer |

  
  
  
  

| Seed Matches to the miRNA miR-193-3p | | | | | |
| --- | --- | --- | --- | --- | --- |
| Seed | Conservation | Species | Matches | | |
|  |  |  | Sequence | Motif | Type |
| ACUGGCC | Broadly Conserved | Human (Homo sapiens)  Chicken (Gallus gallus)  Rhesus (Macaca mulatta)  Cow (Bos taurus)  Mouse (Mus musculus)  Rat (Rattus norvegicus)  Opossum (Monodelphis domestica) | HUMAN | TCTGTGCATTTTGTTTTACTTATCTGTGTATATAGTGTACATAAAGGACAGACGAGTCCTAATTGACAACATCTAGTCTTTCTGGATGTTAAAGAGGTTGCCAGTGTATGACAAAAGTAGAGTT | 6mer |
|  |  |  | HUMAN | GATGTTAAAGAGGTTGCCAGTGTATGACAAAA | 6mer |
|  |  |  | HUMAN | GATGTTAAAGAGGTTGCCAGTGTATGA | 6mer |
|  |  |  | HUMAN | AAAGAGGTTGCCAGTGTATGA | 6mer |
|  |  |  | HUMAN | TATATAATGGACAAATAGTCCTAATTTTTCAACATCTAGTCTCTAGATGTTAAAGAGGTTGCCAGTGTATGACAAAG | 6mer |
|  |  |  | HUMAN | AGGTTGCCAGT | 7mer-A1 |
|  |  |  | BABOON | TCTGTGCATTTTGTTTTACTTATCTGTGTATATAGTGTACATAAAGGACAGACGAGTCCTAATTGACAACATCTAGTCTTTCTGGATGTTAAAGAGGTTGCCAGTGTATGACAAAAGTAGAGTT | 6mer |
|  |  |  | BABOON | GATGTTAAAGAGGTTGCCAGTGTATGACAAAA | 6mer |
|  |  |  | BABOON | GATGTTAAAGAGGTTGCCAGTGTATGA | 6mer |
|  |  |  | BABOON | AAAGAGGTTGCCAGTGTATGA | 6mer |
|  |  |  | BABOON | TATATAATGGACAAATAGTCCTAATTTTTCAACATCTAGTCTCTAGATGTTAAAGAGGTTGCCAGTGTATGACAAAG | 6mer |
|  |  |  | BABOON | AGGTTGCCAGT | 6mer |
|  |  |  | DOG | GATGTTAAAGAGGTTGCCAGTGTATGACAAAA | 6mer |
|  |  |  | DOG | GATGTTAAAGAGGTTGCCAGTGTATGA | 6mer |
|  |  |  | DOG | AAAGAGGTTGCCAGTGTATGA | 6mer |
|  |  |  | COW | GATGTTAAAGAGGTTGCCAGTGTATGACAAAA | 6mer |
|  |  |  | COW | GATGTTAAAGAGGTTGCCAGTGTATGA | 6mer |
|  |  |  | COW | AAAGAGGTTGCCAGTGTATGA | 6mer |
|  |  |  | SHEEP | GATGTTAAAGAGGTTGCCAGTGTATGACAAAA | 6mer |
|  |  |  | SHEEP | GATGTTAAAGAGGTTGCCAGTGTATGA | 6mer |
|  |  |  | SHEEP | AAAGAGGTTGCCAGTGTATGA | 6mer |
|  |  |  | PIG | GATGTTAAAGAGGTTGCCAGTGTATGA | 6mer |
|  |  |  | PIG | AAAGAGGTTGCCAGTGTATGA | 6mer |
|  |  |  | ARMADILLO | AAAGAGGTTGCCAGTGTATGA | 6mer |

  
  
  
  

| Seed Matches to the miRNA miR-199-5p | | | | | |
| --- | --- | --- | --- | --- | --- |
| Seed | Conservation | Species | Matches | | |
|  |  |  | Sequence | Motif | Type |
| CCAGUGU | Broadly Conserved | Human (Homo sapiens)  X. tropicalis (Xenopus tropicalis)  Chicken (Gallus gallus)  Rhesus (Macaca mulatta)  Chimp (Pan troglodytes)  Cow (Bos taurus)  Mouse (Mus musculus)  Rat (Rattus norvegicus) | HUMAN | GGCTGCCCACTGGCTGTGCCC | 6mer |
|  |  |  | HUMAN | CCTGTACTTGTCCACTGGATTGAAG | 7mer-A1 |
|  |  |  | BABOON | GGCTGCCCACTGGCTGTGCCC | 6mer |
|  |  |  | BABOON | CCTGTACTTGTCCACTGGATTGAAG | 7mer-A1 |

  
  
  
  

| Seed Matches to the miRNA miR-494-3p | | | | | |
| --- | --- | --- | --- | --- | --- |
| Seed | Conservation | Species | Matches | | |
|  |  |  | Sequence | Motif | Type |
| GAAACAU | Conserved | Human (Homo sapiens)  Rhesus (Macaca mulatta)  Mouse (Mus musculus)  Rat (Rattus norvegicus) | HUMAN | TATTCTGTTGCTGTGTGTTTCATTTTAAATTGAGCATTAAGGGAATGCAGCATTTAAATC | 7mer-A1 |
|  |  |  | BABOON | TATTCTGTTGCTGTGTGTTTCATTTTAAATTGAGCATTAAGGGAATGCAGCATTTAAATC | 7mer-A1 |

  
  
  
  

| Seed Matches to the miRNA miR-143-3p | | | | | |
| --- | --- | --- | --- | --- | --- |
| Seed | Conservation | Species | Matches | | |
|  |  |  | Sequence | Motif | Type |
| GAGAUGA | Broadly Conserved | Human (Homo sapiens)  Rhesus (Macaca mulatta)  Mouse (Mus musculus)  Rat (Rattus norvegicus)  Opossum (Monodelphis domestica) | HUMAN | ACCATCTCT | 6mer |
|  |  |  | HUMAN | TATGTCCATATTTACATTTTGATAGCCATTGATGTATGCATCTCTT | 6mer |
|  |  |  | BABOON | ACCATCTCT | 6mer |
|  |  |  | BABOON | TATGTCCATATTTACATTTTGATAGCCATTGATGTATGCATCTCTT | 6mer |

  
  
  
  

| Seed Matches to the miRNA miR-129-3p | | | | | |
| --- | --- | --- | --- | --- | --- |
| Seed | Conservation | Species | Matches | | |
|  |  |  | Sequence | Motif | Type |
| AGCCCUU | Broadly Conserved | Human (Homo sapiens)  Rhesus (Macaca mulatta)  Cow (Bos taurus)  Mouse (Mus musculus)  Rat (Rattus norvegicus) | HUMAN | TATTTGTGCTGGTTCAGGGGGAAGGAGGAGCACAAAGTGCAAAGGGCTTTCTACCAGTGTCCAGTGTGTTTA | 7mer-m8 |
|  |  |  | HUMAN | AAAGTGCAAAGGGCTTT | 7mer-m8 |
|  |  |  | HUMAN | AGTAGAGGGCTTAAGTAACACCCCTCTAAGCATTTGTTTTCA | 6mer |
|  |  |  | HUMAN | GAGGAAGGGTGAAGGGAAGGGCTCTTTGCTAGTATCT | 7mer-m8 |
|  |  |  | HUMAN | GGGTGAAGGGAAGGGCT | 7mer-m8 |
|  |  |  | HUMAN | GGGAAGGGCT | 7mer-m8 |
|  |  |  | BABOON | TATTTGTGCTGGTTCAGGGGGAAGGAGGAGCACAAAGTGCAAAGGGCTTTCTACCAGTGTCCAGTGTGTTTA | 7mer-m8 |
|  |  |  | BABOON | AAAGTGCAAAGGGCTTT | 7mer-m8 |
|  |  |  | BABOON | AGTAGAGGGCTTAAGTAACACCCCTCTAAGCATTTGTTTTCA | 6mer |
|  |  |  | BABOON | GAGGAAGGGTGAAGGGAAGGGCTCTTTGCTAGTATCT | 7mer-m8 |
|  |  |  | BABOON | GGGTGAAGGGAAGGGCT | 7mer-m8 |
|  |  |  | BABOON | GGGAAGGGCT | 7mer-m8 |
|  |  |  | DOG | AAAGTGCAAAGGGCTTT | 7mer-m8 |
|  |  |  | DOG | GGGTGAAGGGAAGGGCT | 7mer-m8 |
|  |  |  | DOG | GGGAAGGGCT | 7mer-m8 |
|  |  |  | COW | GGGAAGGGCT | 7mer-m8 |

  
  
  
  

| Seed Matches to the miRNA miR-187-3p | | | | | |
| --- | --- | --- | --- | --- | --- |
| Seed | Conservation | Species | Matches | | |
|  |  |  | Sequence | Motif | Type |
| CGUGUCU | Broadly Conserved | Human (Homo sapiens)  Chicken (Gallus gallus)  Rhesus (Macaca mulatta)  Mouse (Mus musculus)  Rat (Rattus norvegicus)  Opossum (Monodelphis domestica) | HUMAN | GATAACATCAGCTCTAAGTGACACGTGCCTATAT | 6mer |
|  |  |  | BABOON | GATAACATCAGCTCTAAGTGACACGTGCCTATAT | 6mer |

  
  
  
  

| Seed Matches to the miRNA miR-490-3p | | | | | |
| --- | --- | --- | --- | --- | --- |
| Seed | Conservation | Species | Matches | | |
|  |  |  | Sequence | Motif | Type |
| AACCUGG | Broadly Conserved | Human (Homo sapiens)  Chicken (Gallus gallus)  Rhesus (Macaca mulatta)  Mouse (Mus musculus)  Rat (Rattus norvegicus) | HUMAN | CAGGTTGGTGGTGGAGAGGAGTTGGAAGGAATGAAGGGTTCTAGACCAGAATGTTC | 6mer |
|  |  |  | BABOON | CAGGTTGGTGGTGGAGAGGAGTTGGAAGGAATGAAGGGTTCTAGACCAGAATGTTC | 7mer-m8 |

  
  
  
  

| Seed Matches to the miRNA miR-224-5p | | | | | |
| --- | --- | --- | --- | --- | --- |
| Seed | Conservation | Species | Matches | | |
|  |  |  | Sequence | Motif | Type |
| AAGUCAC | Conserved | Human (Homo sapiens)  Rhesus (Macaca mulatta)  Mouse (Mus musculus)  Rat (Rattus norvegicus) | HUMAN | TGTCTTCTGGAGATGACTTTTGGAAATG | 6mer |
|  |  |  | HUMAN | AATGACTTTGTTCTTTGCTT | 6mer |
|  |  |  | HUMAN | ATGACTTTGTTCTTTGCTT | 6mer |
|  |  |  | HUMAN | ATGACTT | 6mer |
|  |  |  | HUMAN | TGACTT | 6mer |
|  |  |  | BABOON | TGTCTTCTGGAGATGACTTTTGGAAATG | 6mer |
|  |  |  | BABOON | AATGACTTTGTTCTTTGCTT | 6mer |
|  |  |  | BABOON | ATGACTTTGTTCTTTGCTT | 6mer |
|  |  |  | BABOON | ATGACTT | 6mer |
|  |  |  | BABOON | TGACTT | 6mer |
|  |  |  | DOG | ATGACTTTGTTCTTTGCTT | 6mer |
|  |  |  | DOG | ATGACTT | 6mer |
|  |  |  | DOG | TGACTT | 6mer |
|  |  |  | COW | ATGACTT | 6mer |
|  |  |  | COW | TGACTT | 6mer |
|  |  |  | SHEEP | ATGACTT | 6mer |
|  |  |  | SHEEP | TGACTT | 6mer |
|  |  |  | PIG | ATGACTT | 6mer |
|  |  |  | PIG | TGACTT | 6mer |
|  |  |  | ARMADILLO | ATGACTT | 6mer |
|  |  |  | ARMADILLO | TGACTT | 6mer |
|  |  |  | GUINEAPIG | ATGACTT | 6mer |
|  |  |  | GUINEAPIG | TGACTT | 6mer |
|  |  |  | MOUSE | TGACTT | 7mer-m8 |

  
  
  
  

| Seed Matches to the miRNA miR-668-3p | | | | | |
| --- | --- | --- | --- | --- | --- |
| Seed | Conservation | Species | Matches | | |
|  |  |  | Sequence | Motif | Type |
| GUCACUC | Conserved | Human (Homo sapiens)  Mouse (Mus musculus) | HUMAN | GATAACATCAGCTCTAAGTGACACGTGCCTATAT | 7mer-A1 |
|  |  |  | HUMAN | AGTGACA | 7mer-A1 |
|  |  |  | HUMAN | TCTGGGAGTGACCTT | 7mer-m8 |
|  |  |  | BABOON | GATAACATCAGCTCTAAGTGACACGTGCCTATAT | 7mer-A1 |
|  |  |  | BABOON | AGTGACA | 7mer-A1 |
|  |  |  | BABOON | TCTGGGAGTGACCTT | 7mer-m8 |
|  |  |  | DOG | AGTGACA | 7mer-A1 |

  
  
  
  

| Seed Matches to the miRNA miR-655-3p | | | | | |
| --- | --- | --- | --- | --- | --- |
| Seed | Conservation | Species | Matches | | |
|  |  |  | Sequence | Motif | Type |
| UAAUACA | Conserved | Human (Homo sapiens) | HUMAN | GGGTTCTGTGCTCCTGAGATTAGTTCAGATGGTCTAACCATTGTTCTATATGTGCATTTTAGTTAATATTGTGTATTAAAGGATAAGTCTTAATGCTCAAAGTATGTTAAAAATAGATGTAGTAAA | 8mer |
|  |  |  | HUMAN | TAACTTTTAAAAAATAAAGGTTATTTTAAAAGCCTGTATTAAGCCCTCGTTGCTTGTAGAATAGAGT | 8mer |
|  |  |  | BABOON | GGGTTCTGTGCTCCTGAGATTAGTTCAGATGGTCTAACCATTGTTCTATATGTGCATTTTAGTTAATATTGTGTATTAAAGGATAAGTCTTAATGCTCAAAGTATGTTAAAAATAGATGTAGTAAA | 8mer |
|  |  |  | BABOON | TAACTTTTAAAAAATAAAGGTTATTTTAAAAGCCTGTATTAAGCCCTCGTTGCTTGTAGAATAGAGT | 8mer |

  
  
  
  

| Seed Matches to the miRNA miR-144-3p | | | | | |
| --- | --- | --- | --- | --- | --- |
| Seed | Conservation | Species | Matches | | |
|  |  |  | Sequence | Motif | Type |
| ACAGUAU | Broadly Conserved | Human (Homo sapiens)  Mouse (Mus musculus)  Rat (Rattus norvegicus)  Opossum (Monodelphis domestica) | HUMAN | AGAAGGTTCTATGCTAGACTGGTCATATTTAGAAGACATTTTCATATTCTATCCATTGTTTTGTGTGCATTTTATTCCTCACTACTGTGTATATA | 6mer |
|  |  |  | HUMAN | ACTACTGTGTATATA | 6mer |
|  |  |  | HUMAN | TATGTTACATGCATTTCATTTAACTTTGCTATACTGTATATATTGT | 8mer |
|  |  |  | HUMAN | CTACTGTGTATATAGC | 6mer |
|  |  |  | HUMAN | CTACTGTGTATATAG | 6mer |
|  |  |  | HUMAN | CTACTGTGTATATA | 6mer |
|  |  |  | HUMAN | TGTCTGCATTTTCATTTACTGTGCTGTGTATATAGTGTATATAAG | 6mer |
|  |  |  | HUMAN | AGTCTGTGTTATGTATGTATACCATTTATTCAATGCTACTGTGTATATAATGGAAAACTT | 6mer |
|  |  |  | HUMAN | AATACTCATATAGCTTTGGGATTTTGAATTGGTAAATATTCATGATGTGTGAAAAATCATGATACATACTGTACA | 8mer |
|  |  |  | BABOON | AGAAGGTTCTATGCTAGACTGGTCATATTTAGAAGACATTTTCATATTCTATCCATTGTTTTGTGTGCATTTTATTCCTCACTACTGTGTATATA | 6mer |
|  |  |  | BABOON | ACTACTGTGTATATA | 6mer |
|  |  |  | BABOON | TATGTTACATGCATTTCATTTAACTTTGCTATACTGTATATATTGT | 8mer |
|  |  |  | BABOON | CTACTGTGTATATAGC | 6mer |
|  |  |  | BABOON | CTACTGTGTATATAG | 6mer |
|  |  |  | BABOON | CTACTGTGTATATA | 6mer |
|  |  |  | BABOON | TGTCTGCATTTTCATTTACTGTGCTGTGTATATAGTGTATATAAG | 6mer |
|  |  |  | BABOON | AGTCTGTGTTATGTATGTATACCATTTATTCAATGCTACTGTGTATATAATGGAAAACTT | 6mer |
|  |  |  | BABOON | AATACTCATATAGCTTTGGGATTTTGAATTGGTAAATATTCATGATGTGTGAAAAATCATGATACATACTGTACA | 8mer |
|  |  |  | DOG | ACTACTGTGTATATA | 6mer |
|  |  |  | DOG | CTACTGTGTATATAG | 6mer |
|  |  |  | DOG | CTACTGTGTATATA | 6mer |
|  |  |  | COW | CTACTGTGTATATAG | 6mer |
|  |  |  | COW | CTACTGTGTATATA | 6mer |
|  |  |  | SHEEP | CTACTGTGTATATAG | 6mer |
|  |  |  | SHEEP | CTACTGTGTATATA | 6mer |
|  |  |  | PIG | CTACTGTGTATATA | 6mer |
|  |  |  | ARMADILLO | CTACTGTGTATATA | 6mer |
|  |  |  | GUINEAPIG | CTACTGTGTATATA | 6mer |

  
  
  
  

| Seed Matches to the miRNA miR-128-3p | | | | | |
| --- | --- | --- | --- | --- | --- |
| Seed | Conservation | Species | Matches | | |
|  |  |  | Sequence | Motif | Type |
| CACAGUG | Broadly Conserved | Human (Homo sapiens)  Chicken (Gallus gallus)  Rhesus (Macaca mulatta)  Mouse (Mus musculus)  Rat (Rattus norvegicus)  Opossum (Monodelphis domestica) | HUMAN | AGAAGGTTCTATGCTAGACTGGTCATATTTAGAAGACATTTTCATATTCTATCCATTGTTTTGTGTGCATTTTATTCCTCACTACTGTGTATATA | 6mer |
|  |  |  | HUMAN | ACTACTGTGTATATA | 6mer |
|  |  |  | HUMAN | CTACTGTGTATATAGC | 6mer |
|  |  |  | HUMAN | CTACTGTGTATATAG | 6mer |
|  |  |  | HUMAN | CTACTGTGTATATA | 6mer |
|  |  |  | HUMAN | TGTCTGCATTTTCATTTACTGTGCTGTGTATATAGTGTATATAAG | 6mer |
|  |  |  | HUMAN | AGTCTGTGTTATGTATGTATACCATTTATTCAATGCTACTGTGTATATAATGGAAAACTT | 6mer |
|  |  |  | BABOON | AGAAGGTTCTATGCTAGACTGGTCATATTTAGAAGACATTTTCATATTCTATCCATTGTTTTGTGTGCATTTTATTCCTCACTACTGTGTATATA | 6mer |
|  |  |  | BABOON | ACTACTGTGTATATA | 6mer |
|  |  |  | BABOON | CTACTGTGTATATAGC | 6mer |
|  |  |  | BABOON | CTACTGTGTATATAG | 6mer |
|  |  |  | BABOON | CTACTGTGTATATA | 6mer |
|  |  |  | BABOON | TGTCTGCATTTTCATTTACTGTGCTGTGTATATAGTGTATATAAG | 6mer |
|  |  |  | BABOON | AGTCTGTGTTATGTATGTATACCATTTATTCAATGCTACTGTGTATATAATGGAAAACTT | 6mer |
|  |  |  | DOG | ACTACTGTGTATATA | 6mer |
|  |  |  | DOG | CTACTGTGTATATAG | 6mer |
|  |  |  | DOG | CTACTGTGTATATA | 6mer |
|  |  |  | COW | CTACTGTGTATATAG | 6mer |
|  |  |  | COW | CTACTGTGTATATA | 6mer |
|  |  |  | SHEEP | CTACTGTGTATATAG | 6mer |
|  |  |  | SHEEP | CTACTGTGTATATA | 6mer |
|  |  |  | PIG | CTACTGTGTATATA | 6mer |
|  |  |  | ARMADILLO | CTACTGTGTATATA | 6mer |
|  |  |  | GUINEAPIG | CTACTGTGTATATA | 6mer |

  
  
  
  

| Seed Matches to the miRNA miR-299-3p | | | | | |
| --- | --- | --- | --- | --- | --- |
| Seed | Conservation | Species | Matches | | |
|  |  |  | Sequence | Motif | Type |
| AUGUGGG | Conserved | Human (Homo sapiens)  Rhesus (Macaca mulatta)  Mouse (Mus musculus)  Rat (Rattus norvegicus) | HUMAN | GAGGAGGGAATGATTCAAGGCCAAAATGGCCACATTTAGAAGATACCTCAGATGATAACCATTGTTAT | 6mer |
|  |  |  | BABOON | GAGGAGGGAATGATTCAAGGCCAAAATGGCCACATTTAGAAGATACCTCAGATGATAACCATTGTTAT | 6mer |

  
  
  
  

| Seed Matches to the miRNA miR-330-3p | | | | | |
| --- | --- | --- | --- | --- | --- |
| Seed | Conservation | Species | Matches | | |
|  |  |  | Sequence | Motif | Type |
| CAAAGCA | Conserved | Human (Homo sapiens)  Rhesus (Macaca mulatta)  Rat (Rattus norvegicus) | HUMAN | AATACTCATATAGCTTTGGGATTTTGAATTGGTAAATATTCATGATGTGTGAAAAATCATGATACATACTGTACA | 6mer |
|  |  |  | BABOON | AATACTCATATAGCTTTGGGATTTTGAATTGGTAAATATTCATGATGTGTGAAAAATCATGATACATACTGTACA | 6mer |

  
  
  
  

| Seed Matches to the miRNA miR-151-3p | | | | | |
| --- | --- | --- | --- | --- | --- |
| Seed | Conservation | Species | Matches | | |
|  |  |  | Sequence | Motif | Type |
| UAGACUG | Conserved | Human (Homo sapiens)  Rhesus (Macaca mulatta)  Cow (Bos taurus)  Mouse (Mus musculus)  Rat (Rattus norvegicus) | HUMAN | GACAAATTAACTCCTTACTTGAAACATCTAGTCTATCTAGATGTTTAGAAGTGCCC | 6mer |
|  |  |  | BABOON | GACAAATTAACTCCTTACTTGAAACATCTAGTCTATCTAGATGTTTAGAAGTGCCC | 6mer |

  
  
  
  

| Seed Matches to the miRNA miR-582-5p | | | | | |
| --- | --- | --- | --- | --- | --- |
| Seed | Conservation | Species | Matches | | |
|  |  |  | Sequence | Motif | Type |
| UACAGUU | Conserved | Human (Homo sapiens)  Rhesus (Macaca mulatta)  Mouse (Mus musculus) | HUMAN | TATGTTACATGCATTTCATTTAACTTTGCTATACTGTATATATTGT | 6mer |
|  |  |  | HUMAN | ACTGTATATATTGT | 6mer |
|  |  |  | HUMAN | AATACTCATATAGCTTTGGGATTTTGAATTGGTAAATATTCATGATGTGTGAAAAATCATGATACATACTGTACA | 6mer |
|  |  |  | BABOON | TATGTTACATGCATTTCATTTAACTTTGCTATACTGTATATATTGT | 6mer |
|  |  |  | BABOON | ACTGTATATATTGT | 6mer |
|  |  |  | BABOON | AATACTCATATAGCTTTGGGATTTTGAATTGGTAAATATTCATGATGTGTGAAAAATCATGATACATACTGTACA | 6mer |
|  |  |  | DOG | ACTGTATATATTGT | 6mer |

  
  
  
  

| Seed Matches to the miRNA miR-101-3p.2 | | | | | |
| --- | --- | --- | --- | --- | --- |
| Seed | Conservation | Species | Matches | | |
|  |  |  | Sequence | Motif | Type |
| UACAGUA | Broadly Conserved | Human (Homo sapiens) | HUMAN | TATGTTACATGCATTTCATTTAACTTTGCTATACTGTATATATTGT | 7mer-m8 |
|  |  |  | HUMAN | ACTGTATATATTGT | 7mer-m8 |
|  |  |  | HUMAN | AATACTCATATAGCTTTGGGATTTTGAATTGGTAAATATTCATGATGTGTGAAAAATCATGATACATACTGTACA | 7mer-m8 |
|  |  |  | BABOON | TATGTTACATGCATTTCATTTAACTTTGCTATACTGTATATATTGT | 7mer-m8 |
|  |  |  | BABOON | ACTGTATATATTGT | 7mer-m8 |
|  |  |  | BABOON | AATACTCATATAGCTTTGGGATTTTGAATTGGTAAATATTCATGATGTGTGAAAAATCATGATACATACTGTACA | 7mer-m8 |
|  |  |  | DOG | ACTGTATATATTGT | 6mer |

  
  
  
  

| Seed Matches to the miRNA miR-346 | | | | | |
| --- | --- | --- | --- | --- | --- |
| Seed | Conservation | Species | Matches | | |
|  |  |  | Sequence | Motif | Type |
| GUCUGCC | Conserved | Human (Homo sapiens)  Rhesus (Macaca mulatta)  Chimp (Pan troglodytes)  Dog (Canis lupus familiaris)  Cow (Bos taurus)  Rat (Rattus norvegicus) | HUMAN | TTAGGAGCGAATGCAGACT | 6mer |
|  |  |  | BABOON | TTAGGAGCGAATGCAGACT | 6mer |

  
  
  
  

| Seed Matches to the miRNA let-7-5p/98-5p | | | | | |
| --- | --- | --- | --- | --- | --- |
| Seed | Conservation | Species | Matches | | |
|  |  |  | Sequence | Motif | Type |
| GAGGUAG | Broadly Conserved | Human (Homo sapiens) | HUMAN | GAGGAGGGAATGATTCAAGGCCAAAATGGCCACATTTAGAAGATACCTCAGATGATAACCATTGTTAT | 7mer-A1 |
|  |  |  | HUMAN | ATACCTCAGAT | 7mer-A1 |
|  |  |  | HUMAN | ATACCTCA | 7mer-A1 |
|  |  |  | BABOON | GAGGAGGGAATGATTCAAGGCCAAAATGGCCACATTTAGAAGATACCTCAGATGATAACCATTGTTAT | 7mer-A1 |
|  |  |  | BABOON | ATACCTCAGAT | 7mer-A1 |
|  |  |  | BABOON | ATACCTCA | 7mer-A1 |
|  |  |  | DOG | ATACCTCAGAT | 7mer-A1 |
|  |  |  | DOG | ATACCTCA | 7mer-A1 |
|  |  |  | COW | ATACCTCA | 7mer-A1 |
|  |  |  | SHEEP | ATACCTCA | 7mer-A1 |
|  |  |  | PIG | ATACCTCA | 7mer-A1 |
|  |  |  | ARMADILLO | ATACCTCA | 7mer-A1 |

  
  
  
  

| Seed Matches to the miRNA miR-190-5p | | | | | |
| --- | --- | --- | --- | --- | --- |
| Seed | Conservation | Species | Matches | | |
|  |  |  | Sequence | Motif | Type |
| GAUAUGU | Broadly Conserved | Human (Homo sapiens)  Chicken (Gallus gallus)  Rhesus (Macaca mulatta)  Mouse (Mus musculus)  Rat (Rattus norvegicus)  Opossum (Monodelphis domestica) | HUMAN | CATATCTAGACGATGGTTTTAGATGATAACCACAGGTCTA | 6mer |
|  |  |  | BABOON | CATATCTAGACGATGGTTTTAGATGATAACCACAGGTCTA | 6mer |

  
  
  
  

| Seed Matches to the miRNA miR-377-3p | | | | | |
| --- | --- | --- | --- | --- | --- |
| Seed | Conservation | Species | Matches | | |
|  |  |  | Sequence | Motif | Type |
| UCACACA | Conserved | Human (Homo sapiens)  Rhesus (Macaca mulatta)  Mouse (Mus musculus) | HUMAN | AATACTCATATAGCTTTGGGATTTTGAATTGGTAAATATTCATGATGTGTGAAAAATCATGATACATACTGTACA | 8mer |
|  |  |  | BABOON | AATACTCATATAGCTTTGGGATTTTGAATTGGTAAATATTCATGATGTGTGAAAAATCATGATACATACTGTACA | 8mer |

  
  
  
  

| Seed Matches to the miRNA miR-452-5p/892-3p | | | | | |
| --- | --- | --- | --- | --- | --- |
| Seed | Conservation | Species | Matches | | |
|  |  |  | Sequence | Motif | Type |
| ACUGUUU | Conserved | Human (Homo sapiens)  Rhesus (Macaca mulatta) | HUMAN | TGTGTGCAATTTTATTTAACAGTGCT | 6mer |
|  |  |  | HUMAN | TATTTAACAGTGCT | 6mer |
|  |  |  | HUMAN | TTAACAGT | 6mer |
|  |  |  | BABOON | TGTGTGCAATTTTATTTAACAGTGCT | 6mer |
|  |  |  | BABOON | TATTTAACAGTGCT | 6mer |
|  |  |  | BABOON | TTAACAGT | 6mer |
|  |  |  | DOG | TATTTAACAGTGCT | 6mer |
|  |  |  | DOG | TTAACAGT | 6mer |
|  |  |  | COW | TTAACAGT | 7mer-A1 |
|  |  |  | SHEEP | TTAACAGT | 7mer-A1 |
|  |  |  | PIG | TTAACAGT | 6mer |

  
  
  
  

| Seed Matches to the miRNA miR-142-3p.1 | | | | | |
| --- | --- | --- | --- | --- | --- |
| Seed | Conservation | Species | Matches | | |
|  |  |  | Sequence | Motif | Type |
| GUAGUGU | Broadly Conserved | Human (Homo sapiens)  Mouse (Mus musculus) | HUMAN | AGAAGGTTCTATGCTAGACTGGTCATATTTAGAAGACATTTTCATATTCTATCCATTGTTTTGTGTGCATTTTATTCCTCACTACTGTGTATATA | 6mer |
|  |  |  | BABOON | AGAAGGTTCTATGCTAGACTGGTCATATTTAGAAGACATTTTCATATTCTATCCATTGTTTTGTGTGCATTTTATTCCTCACTACTGTGTATATA | 6mer |

  
  
  
  

| Seed Matches to the miRNA miR-1197 | | | | | |
| --- | --- | --- | --- | --- | --- |
| Seed | Conservation | Species | Matches | | |
|  |  |  | Sequence | Motif | Type |
| AGGACAC | Conserved | Human (Homo sapiens)  Chimp (Pan troglodytes)  Cow (Bos taurus) | HUMAN | CAGTCCCTTTGTGAATGTCCTTTTGTTA | 6mer |
|  |  |  | HUMAN | AATGTCCTTTTGTTA | 6mer |
|  |  |  | BABOON | CAGTCCCTTTGTGAATGTCCTTTTGTTA | 6mer |
|  |  |  | BABOON | AATGTCCTTTTGTTA | 6mer |
|  |  |  | DOG | AATGTCCTTTTGTTA | 6mer |

  
  
  
  

| Seed Matches to the miRNA miR-186-5p | | | | | |
| --- | --- | --- | --- | --- | --- |
| Seed | Conservation | Species | Matches | | |
|  |  |  | Sequence | Motif | Type |
| AAAGAAU | Conserved | Human (Homo sapiens)  Rhesus (Macaca mulatta)  Mouse (Mus musculus)  Rat (Rattus norvegicus)  Opossum (Monodelphis domestica) | HUMAN | TTCTTTTTAGATGTTCTGAAGTGCCTGA | 6mer |
|  |  |  | HUMAN | AATGACTTTGTTCTTTGCTT | 6mer |
|  |  |  | HUMAN | ATGACTTTGTTCTTTGCTT | 6mer |
|  |  |  | BABOON | TTCTTTTTAGATGTTCTGAAGTGCCTGA | 7mer-m8 |
|  |  |  | BABOON | AATGACTTTGTTCTTTGCTT | 6mer |
|  |  |  | BABOON | ATGACTTTGTTCTTTGCTT | 6mer |
|  |  |  | DOG | ATGACTTTGTTCTTTGCTT | 6mer |

  
  
  
  
