# Supplementary material for: Uncovering deeply conserved motif combinations in rapidly evolving noncoding sequences
Source: Genome Biol. 2021 Jan 11;22:29. doi: 10.1186/s13059-020-02247-1 (PMC7798263; doi:10.1186/s13059-020-02247-1)
Supplement: Supplementary file 3 — Additional file 3. LncLOOM output results for NORAD sequences from nine mammals. [file 13059_2020_2247_MOESM3_ESM.gz › AdditionalFile3/Html_Files/eCLIP_results_BLAT.html]

 eCLIP Matches (BLAT)

# eCLIP Annotation Results (BLAT)

  
  

| Match 1 in HUMAN | | | | | | | |
| --- | --- | --- | --- | --- | --- | --- | --- |
| Motif | Start in Seq (1 Indexed) | End in Seq (1 Indexed) | Strand | Chrm | Exon | Start in Chrm (0 Indexed) | End in Chrm (1 Indexed) |
| CAGAGAACTGCCAAGTCAGTTCCGGTC | 34 | 60 | - | chr20 | 1 | 34638872 | 34638899 |
| eCLIP Fold-Enrichment | Binding Protein | Cell Line | Strand | Chrm | | Start in Chrm (0 Indexed) | End in Chrm (1 Indexed) |
| 4.16525548317 | ddx3x (bg=13.89%) | K562 | - | chr20 | | 34638787 | 34638873 |
| 2.73720405992 | EIF3G (bg=2.39%) | K562 | - | chr20 | | 34638788 | 34638875 |

  
  

| Match 2 in HUMAN | | | | | | | |
| --- | --- | --- | --- | --- | --- | --- | --- |
| Motif | Start in Seq (1 Indexed) | End in Seq (1 Indexed) | Strand | Chrm | Exon | Start in Chrm (0 Indexed) | End in Chrm (1 Indexed) |
| AGAGAACTGCCAAGTCAGTTCCGG | 35 | 58 | - | chr20 | 1 | 34638874 | 34638898 |
| eCLIP Fold-Enrichment | Binding Protein | Cell Line | Strand | Chrm | | Start in Chrm (0 Indexed) | End in Chrm (1 Indexed) |
| 2.73720405992 | EIF3G (bg=2.39%) | K562 | - | chr20 | | 34638788 | 34638875 |

  
  

| Match 3 in HUMAN | | | | | | | |
| --- | --- | --- | --- | --- | --- | --- | --- |
| Motif | Start in Seq (1 Indexed) | End in Seq (1 Indexed) | Strand | Chrm | Exon | Start in Chrm (0 Indexed) | End in Chrm (1 Indexed) |
| TCAGTTCCGG | 49 | 58 | - | chr20 | 1 | 34638874 | 34638884 |
| eCLIP Fold-Enrichment | Binding Protein | Cell Line | Strand | Chrm | | Start in Chrm (0 Indexed) | End in Chrm (1 Indexed) |
| 2.73720405992 | EIF3G (bg=2.39%) | K562 | - | chr20 | | 34638788 | 34638875 |

  
  

| Match 4 in HUMAN | | | | | | | |
| --- | --- | --- | --- | --- | --- | --- | --- |
| Motif | Start in Seq (1 Indexed) | End in Seq (1 Indexed) | Strand | Chrm | Exon | Start in Chrm (0 Indexed) | End in Chrm (1 Indexed) |
| GGCAGAGA | 62 | 69 | - | chr20 | 1 | 34638863 | 34638871 |
| eCLIP Fold-Enrichment | Binding Protein | Cell Line | Strand | Chrm | | Start in Chrm (0 Indexed) | End in Chrm (1 Indexed) |
| 4.16525548317 | ddx3x (bg=13.89%) | K562 | - | chr20 | | 34638787 | 34638873 |
| 3.85540971786 | ddx3x (bg=13.89%) | K562 | - | chr20 | | 34638839 | 34638867 |
| 2.73720405992 | EIF3G (bg=2.39%) | K562 | - | chr20 | | 34638788 | 34638875 |
| 2.49727091402 | ybx3 (bg=22.82%) | K562 | - | chr20 | | 34638800 | 34638865 |

  
  

| Match 5 in HUMAN | | | | | | | |
| --- | --- | --- | --- | --- | --- | --- | --- |
| Motif | Start in Seq (1 Indexed) | End in Seq (1 Indexed) | Strand | Chrm | Exon | Start in Chrm (0 Indexed) | End in Chrm (1 Indexed) |
| CGCGGAGAGACGCAGAACGC | 71 | 90 | - | chr20 | 1 | 34638842 | 34638862 |
| eCLIP Fold-Enrichment | Binding Protein | Cell Line | Strand | Chrm | | Start in Chrm (0 Indexed) | End in Chrm (1 Indexed) |
| 4.16525548317 | ddx3x (bg=13.89%) | K562 | - | chr20 | | 34638787 | 34638873 |
| 6.60725052175 | ddx3x (bg=13.89%) | HepG2 | - | chr20 | | 34638789 | 34638844 |
| 6.50233673689 | ddx3x (bg=13.89%) | HepG2 | - | chr20 | | 34638804 | 34638847 |
| 3.85540971786 | ddx3x (bg=13.89%) | K562 | - | chr20 | | 34638839 | 34638867 |
| 4.31494711407 | EIF3G (bg=2.39%) | K562 | - | chr20 | | 34638745 | 34638846 |
| 2.73720405992 | EIF3G (bg=2.39%) | K562 | - | chr20 | | 34638788 | 34638875 |
| 2.13276454614 | EIF3H (bg=7.83%) | HepG2 | - | chr20 | | 34638749 | 34638846 |
| 2.08134599223 | FMR1 (bg=6.3%) | K562 | - | chr20 | | 34638794 | 34638851 |
| 2.0543993622 | METAP2 (bg=6.14%) | K562 | - | chr20 | | 34638692 | 34638859 |
| 4.0529564641 | RPS3 (bg=4.15%) | HepG2 | - | chr20 | | 34638753 | 34638853 |
| 3.55970359824 | RPS3 (bg=4.15%) | K562 | - | chr20 | | 34638782 | 34638849 |
| 3.57513601707 | RPS3 (bg=4.15%) | HepG2 | - | chr20 | | 34638785 | 34638853 |
| 2.95573402664 | SDAD1 (bg=5.99%) | K562 | - | chr20 | | 34638779 | 34638851 |
| 5.4139677122 | SERBP1 (bg=0.81%) | K562 | - | chr20 | | 34638801 | 34638845 |
| 2.28414767494 | WRN (bg=3.31%) | K562 | - | chr20 | | 34638776 | 34638844 |
| 2.49727091402 | ybx3 (bg=22.82%) | K562 | - | chr20 | | 34638800 | 34638865 |

  
  

| Match 6 in HUMAN | | | | | | | |
| --- | --- | --- | --- | --- | --- | --- | --- |
| Motif | Start in Seq (1 Indexed) | End in Seq (1 Indexed) | Strand | Chrm | Exon | Start in Chrm (0 Indexed) | End in Chrm (1 Indexed) |
| GCTCCT | 96 | 101 | - | chr20 | 1 | 34638831 | 34638837 |
| eCLIP Fold-Enrichment | Binding Protein | Cell Line | Strand | Chrm | | Start in Chrm (0 Indexed) | End in Chrm (1 Indexed) |
| 4.16525548317 | ddx3x (bg=13.89%) | K562 | - | chr20 | | 34638787 | 34638873 |
| 6.60725052175 | ddx3x (bg=13.89%) | HepG2 | - | chr20 | | 34638789 | 34638844 |
| 6.50233673689 | ddx3x (bg=13.89%) | HepG2 | - | chr20 | | 34638804 | 34638847 |
| 4.80478364479 | ddx3x (bg=13.89%) | K562 | - | chr20 | | 34638808 | 34638839 |
| 3.74226397535 | drosha (bg=11.37%) | K562 | - | chr20 | | 34638765 | 34638841 |
| 4.31494711407 | EIF3G (bg=2.39%) | K562 | - | chr20 | | 34638745 | 34638846 |
| 2.73720405992 | EIF3G (bg=2.39%) | K562 | - | chr20 | | 34638788 | 34638875 |
| 2.13276454614 | EIF3H (bg=7.83%) | HepG2 | - | chr20 | | 34638749 | 34638846 |
| 2.08134599223 | FMR1 (bg=6.3%) | K562 | - | chr20 | | 34638794 | 34638851 |
| 2.0543993622 | METAP2 (bg=6.14%) | K562 | - | chr20 | | 34638692 | 34638859 |
| 2.33540589762 | PUS1 (bg=2.87%) | K562 | - | chr20 | | 34638791 | 34638838 |
| 4.0529564641 | RPS3 (bg=4.15%) | HepG2 | - | chr20 | | 34638753 | 34638853 |
| 3.55970359824 | RPS3 (bg=4.15%) | K562 | - | chr20 | | 34638782 | 34638849 |
| 3.57513601707 | RPS3 (bg=4.15%) | HepG2 | - | chr20 | | 34638785 | 34638853 |
| 2.27293773084 | SDAD1 (bg=5.99%) | HepG2 | - | chr20 | | 34638636 | 34638838 |
| 2.95573402664 | SDAD1 (bg=5.99%) | K562 | - | chr20 | | 34638779 | 34638851 |
| 5.4139677122 | SERBP1 (bg=0.81%) | K562 | - | chr20 | | 34638801 | 34638845 |
| 2.28414767494 | WRN (bg=3.31%) | K562 | - | chr20 | | 34638776 | 34638844 |
| 2.49727091402 | ybx3 (bg=22.82%) | K562 | - | chr20 | | 34638800 | 34638865 |

  
  

| Match 7 in HUMAN | | | | | | | |
| --- | --- | --- | --- | --- | --- | --- | --- |
| Motif | Start in Seq (1 Indexed) | End in Seq (1 Indexed) | Strand | Chrm | Exon | Start in Chrm (0 Indexed) | End in Chrm (1 Indexed) |
| CAGGGCCCTCCAGGCCCTCCGGCCC | 103 | 127 | - | chr20 | 1 | 34638805 | 34638830 |
| eCLIP Fold-Enrichment | Binding Protein | Cell Line | Strand | Chrm | | Start in Chrm (0 Indexed) | End in Chrm (1 Indexed) |
| 4.16525548317 | ddx3x (bg=13.89%) | K562 | - | chr20 | | 34638787 | 34638873 |
| 6.60725052175 | ddx3x (bg=13.89%) | HepG2 | - | chr20 | | 34638789 | 34638844 |
| 5.66985406471 | ddx3x (bg=13.89%) | K562 | - | chr20 | | 34638792 | 34638808 |
| 6.50233673689 | ddx3x (bg=13.89%) | HepG2 | - | chr20 | | 34638804 | 34638847 |
| 4.80478364479 | ddx3x (bg=13.89%) | K562 | - | chr20 | | 34638808 | 34638839 |
| 2.12735172601 | ddx6 (bg=23.92%) | K562 | - | chr20 | | 34638802 | 34638821 |
| 3.74226397535 | drosha (bg=11.37%) | K562 | - | chr20 | | 34638765 | 34638841 |
| 2.08369907785 | drosha (bg=11.37%) | K562 | - | chr20 | | 34638786 | 34638820 |
| 4.31494711407 | EIF3G (bg=2.39%) | K562 | - | chr20 | | 34638745 | 34638846 |
| 2.73720405992 | EIF3G (bg=2.39%) | K562 | - | chr20 | | 34638788 | 34638875 |
| 2.13276454614 | EIF3H (bg=7.83%) | HepG2 | - | chr20 | | 34638749 | 34638846 |
| 2.08134599223 | FMR1 (bg=6.3%) | K562 | - | chr20 | | 34638794 | 34638851 |
| 2.0543993622 | METAP2 (bg=6.14%) | K562 | - | chr20 | | 34638692 | 34638859 |
| 2.33540589762 | PUS1 (bg=2.87%) | K562 | - | chr20 | | 34638791 | 34638838 |
| 4.0529564641 | RPS3 (bg=4.15%) | HepG2 | - | chr20 | | 34638753 | 34638853 |
| 3.55970359824 | RPS3 (bg=4.15%) | K562 | - | chr20 | | 34638782 | 34638849 |
| 3.57513601707 | RPS3 (bg=4.15%) | HepG2 | - | chr20 | | 34638785 | 34638853 |
| 2.27293773084 | SDAD1 (bg=5.99%) | HepG2 | - | chr20 | | 34638636 | 34638838 |
| 2.95573402664 | SDAD1 (bg=5.99%) | K562 | - | chr20 | | 34638779 | 34638851 |
| 5.4139677122 | SERBP1 (bg=0.81%) | K562 | - | chr20 | | 34638801 | 34638845 |
| 3.60836987779 | SF3B1 (bg=6.74%) | K562 | - | chr20 | | 34638793 | 34638829 |
| 2.28414767494 | WRN (bg=3.31%) | K562 | - | chr20 | | 34638776 | 34638844 |
| 2.49727091402 | ybx3 (bg=22.82%) | K562 | - | chr20 | | 34638800 | 34638865 |

  
  

| Match 8 in HUMAN | | | | | | | |
| --- | --- | --- | --- | --- | --- | --- | --- |
| Motif | Start in Seq (1 Indexed) | End in Seq (1 Indexed) | Strand | Chrm | Exon | Start in Chrm (0 Indexed) | End in Chrm (1 Indexed) |
| GGGCCCTCCAGGCCCTCCGGCC | 105 | 126 | - | chr20 | 1 | 34638806 | 34638828 |
| eCLIP Fold-Enrichment | Binding Protein | Cell Line | Strand | Chrm | | Start in Chrm (0 Indexed) | End in Chrm (1 Indexed) |
| 4.16525548317 | ddx3x (bg=13.89%) | K562 | - | chr20 | | 34638787 | 34638873 |
| 6.60725052175 | ddx3x (bg=13.89%) | HepG2 | - | chr20 | | 34638789 | 34638844 |
| 5.66985406471 | ddx3x (bg=13.89%) | K562 | - | chr20 | | 34638792 | 34638808 |
| 6.50233673689 | ddx3x (bg=13.89%) | HepG2 | - | chr20 | | 34638804 | 34638847 |
| 4.80478364479 | ddx3x (bg=13.89%) | K562 | - | chr20 | | 34638808 | 34638839 |
| 2.12735172601 | ddx6 (bg=23.92%) | K562 | - | chr20 | | 34638802 | 34638821 |
| 3.74226397535 | drosha (bg=11.37%) | K562 | - | chr20 | | 34638765 | 34638841 |
| 2.08369907785 | drosha (bg=11.37%) | K562 | - | chr20 | | 34638786 | 34638820 |
| 4.31494711407 | EIF3G (bg=2.39%) | K562 | - | chr20 | | 34638745 | 34638846 |
| 2.73720405992 | EIF3G (bg=2.39%) | K562 | - | chr20 | | 34638788 | 34638875 |
| 2.13276454614 | EIF3H (bg=7.83%) | HepG2 | - | chr20 | | 34638749 | 34638846 |
| 2.08134599223 | FMR1 (bg=6.3%) | K562 | - | chr20 | | 34638794 | 34638851 |
| 2.0543993622 | METAP2 (bg=6.14%) | K562 | - | chr20 | | 34638692 | 34638859 |
| 2.33540589762 | PUS1 (bg=2.87%) | K562 | - | chr20 | | 34638791 | 34638838 |
| 4.0529564641 | RPS3 (bg=4.15%) | HepG2 | - | chr20 | | 34638753 | 34638853 |
| 3.55970359824 | RPS3 (bg=4.15%) | K562 | - | chr20 | | 34638782 | 34638849 |
| 3.57513601707 | RPS3 (bg=4.15%) | HepG2 | - | chr20 | | 34638785 | 34638853 |
| 2.27293773084 | SDAD1 (bg=5.99%) | HepG2 | - | chr20 | | 34638636 | 34638838 |
| 2.95573402664 | SDAD1 (bg=5.99%) | K562 | - | chr20 | | 34638779 | 34638851 |
| 5.4139677122 | SERBP1 (bg=0.81%) | K562 | - | chr20 | | 34638801 | 34638845 |
| 3.60836987779 | SF3B1 (bg=6.74%) | K562 | - | chr20 | | 34638793 | 34638829 |
| 2.28414767494 | WRN (bg=3.31%) | K562 | - | chr20 | | 34638776 | 34638844 |
| 2.49727091402 | ybx3 (bg=22.82%) | K562 | - | chr20 | | 34638800 | 34638865 |

  
  

| Match 9 in HUMAN | | | | | | | |
| --- | --- | --- | --- | --- | --- | --- | --- |
| Motif | Start in Seq (1 Indexed) | End in Seq (1 Indexed) | Strand | Chrm | Exon | Start in Chrm (0 Indexed) | End in Chrm (1 Indexed) |
| GGCCCTCCGG | 115 | 124 | - | chr20 | 1 | 34638808 | 34638818 |
| eCLIP Fold-Enrichment | Binding Protein | Cell Line | Strand | Chrm | | Start in Chrm (0 Indexed) | End in Chrm (1 Indexed) |
| 4.16525548317 | ddx3x (bg=13.89%) | K562 | - | chr20 | | 34638787 | 34638873 |
| 6.60725052175 | ddx3x (bg=13.89%) | HepG2 | - | chr20 | | 34638789 | 34638844 |
| 5.66985406471 | ddx3x (bg=13.89%) | K562 | - | chr20 | | 34638792 | 34638808 |
| 6.50233673689 | ddx3x (bg=13.89%) | HepG2 | - | chr20 | | 34638804 | 34638847 |
| 4.80478364479 | ddx3x (bg=13.89%) | K562 | - | chr20 | | 34638808 | 34638839 |
| 2.12735172601 | ddx6 (bg=23.92%) | K562 | - | chr20 | | 34638802 | 34638821 |
| 3.74226397535 | drosha (bg=11.37%) | K562 | - | chr20 | | 34638765 | 34638841 |
| 2.08369907785 | drosha (bg=11.37%) | K562 | - | chr20 | | 34638786 | 34638820 |
| 4.31494711407 | EIF3G (bg=2.39%) | K562 | - | chr20 | | 34638745 | 34638846 |
| 2.73720405992 | EIF3G (bg=2.39%) | K562 | - | chr20 | | 34638788 | 34638875 |
| 2.13276454614 | EIF3H (bg=7.83%) | HepG2 | - | chr20 | | 34638749 | 34638846 |
| 2.08134599223 | FMR1 (bg=6.3%) | K562 | - | chr20 | | 34638794 | 34638851 |
| 2.0543993622 | METAP2 (bg=6.14%) | K562 | - | chr20 | | 34638692 | 34638859 |
| 2.33540589762 | PUS1 (bg=2.87%) | K562 | - | chr20 | | 34638791 | 34638838 |
| 4.0529564641 | RPS3 (bg=4.15%) | HepG2 | - | chr20 | | 34638753 | 34638853 |
| 3.55970359824 | RPS3 (bg=4.15%) | K562 | - | chr20 | | 34638782 | 34638849 |
| 3.57513601707 | RPS3 (bg=4.15%) | HepG2 | - | chr20 | | 34638785 | 34638853 |
| 2.27293773084 | SDAD1 (bg=5.99%) | HepG2 | - | chr20 | | 34638636 | 34638838 |
| 2.95573402664 | SDAD1 (bg=5.99%) | K562 | - | chr20 | | 34638779 | 34638851 |
| 5.4139677122 | SERBP1 (bg=0.81%) | K562 | - | chr20 | | 34638801 | 34638845 |
| 3.60836987779 | SF3B1 (bg=6.74%) | K562 | - | chr20 | | 34638793 | 34638829 |
| 2.28414767494 | WRN (bg=3.31%) | K562 | - | chr20 | | 34638776 | 34638844 |
| 2.49727091402 | ybx3 (bg=22.82%) | K562 | - | chr20 | | 34638800 | 34638865 |

  
  

| Match 10 in HUMAN | | | | | | | |
| --- | --- | --- | --- | --- | --- | --- | --- |
| Motif | Start in Seq (1 Indexed) | End in Seq (1 Indexed) | Strand | Chrm | Exon | Start in Chrm (0 Indexed) | End in Chrm (1 Indexed) |
| GGCCCTCC | 115 | 122 | - | chr20 | 1 | 34638810 | 34638818 |
| eCLIP Fold-Enrichment | Binding Protein | Cell Line | Strand | Chrm | | Start in Chrm (0 Indexed) | End in Chrm (1 Indexed) |
| 4.16525548317 | ddx3x (bg=13.89%) | K562 | - | chr20 | | 34638787 | 34638873 |
| 6.60725052175 | ddx3x (bg=13.89%) | HepG2 | - | chr20 | | 34638789 | 34638844 |
| 6.50233673689 | ddx3x (bg=13.89%) | HepG2 | - | chr20 | | 34638804 | 34638847 |
| 4.80478364479 | ddx3x (bg=13.89%) | K562 | - | chr20 | | 34638808 | 34638839 |
| 2.12735172601 | ddx6 (bg=23.92%) | K562 | - | chr20 | | 34638802 | 34638821 |
| 3.74226397535 | drosha (bg=11.37%) | K562 | - | chr20 | | 34638765 | 34638841 |
| 2.08369907785 | drosha (bg=11.37%) | K562 | - | chr20 | | 34638786 | 34638820 |
| 4.31494711407 | EIF3G (bg=2.39%) | K562 | - | chr20 | | 34638745 | 34638846 |
| 2.73720405992 | EIF3G (bg=2.39%) | K562 | - | chr20 | | 34638788 | 34638875 |
| 2.13276454614 | EIF3H (bg=7.83%) | HepG2 | - | chr20 | | 34638749 | 34638846 |
| 2.08134599223 | FMR1 (bg=6.3%) | K562 | - | chr20 | | 34638794 | 34638851 |
| 2.0543993622 | METAP2 (bg=6.14%) | K562 | - | chr20 | | 34638692 | 34638859 |
| 2.33540589762 | PUS1 (bg=2.87%) | K562 | - | chr20 | | 34638791 | 34638838 |
| 4.0529564641 | RPS3 (bg=4.15%) | HepG2 | - | chr20 | | 34638753 | 34638853 |
| 3.55970359824 | RPS3 (bg=4.15%) | K562 | - | chr20 | | 34638782 | 34638849 |
| 3.57513601707 | RPS3 (bg=4.15%) | HepG2 | - | chr20 | | 34638785 | 34638853 |
| 2.27293773084 | SDAD1 (bg=5.99%) | HepG2 | - | chr20 | | 34638636 | 34638838 |
| 2.95573402664 | SDAD1 (bg=5.99%) | K562 | - | chr20 | | 34638779 | 34638851 |
| 5.4139677122 | SERBP1 (bg=0.81%) | K562 | - | chr20 | | 34638801 | 34638845 |
| 3.60836987779 | SF3B1 (bg=6.74%) | K562 | - | chr20 | | 34638793 | 34638829 |
| 2.28414767494 | WRN (bg=3.31%) | K562 | - | chr20 | | 34638776 | 34638844 |
| 2.49727091402 | ybx3 (bg=22.82%) | K562 | - | chr20 | | 34638800 | 34638865 |

  
  

| Match 11 in HUMAN | | | | | | | |
| --- | --- | --- | --- | --- | --- | --- | --- |
| Motif | Start in Seq (1 Indexed) | End in Seq (1 Indexed) | Strand | Chrm | Exon | Start in Chrm (0 Indexed) | End in Chrm (1 Indexed) |
| GCCCTC | 116 | 121 | - | chr20 | 1 | 34638811 | 34638817 |
| eCLIP Fold-Enrichment | Binding Protein | Cell Line | Strand | Chrm | | Start in Chrm (0 Indexed) | End in Chrm (1 Indexed) |
| 4.16525548317 | ddx3x (bg=13.89%) | K562 | - | chr20 | | 34638787 | 34638873 |
| 6.60725052175 | ddx3x (bg=13.89%) | HepG2 | - | chr20 | | 34638789 | 34638844 |
| 6.50233673689 | ddx3x (bg=13.89%) | HepG2 | - | chr20 | | 34638804 | 34638847 |
| 4.80478364479 | ddx3x (bg=13.89%) | K562 | - | chr20 | | 34638808 | 34638839 |
| 2.12735172601 | ddx6 (bg=23.92%) | K562 | - | chr20 | | 34638802 | 34638821 |
| 3.74226397535 | drosha (bg=11.37%) | K562 | - | chr20 | | 34638765 | 34638841 |
| 2.08369907785 | drosha (bg=11.37%) | K562 | - | chr20 | | 34638786 | 34638820 |
| 4.31494711407 | EIF3G (bg=2.39%) | K562 | - | chr20 | | 34638745 | 34638846 |
| 2.73720405992 | EIF3G (bg=2.39%) | K562 | - | chr20 | | 34638788 | 34638875 |
| 2.13276454614 | EIF3H (bg=7.83%) | HepG2 | - | chr20 | | 34638749 | 34638846 |
| 2.08134599223 | FMR1 (bg=6.3%) | K562 | - | chr20 | | 34638794 | 34638851 |
| 2.0543993622 | METAP2 (bg=6.14%) | K562 | - | chr20 | | 34638692 | 34638859 |
| 2.33540589762 | PUS1 (bg=2.87%) | K562 | - | chr20 | | 34638791 | 34638838 |
| 4.0529564641 | RPS3 (bg=4.15%) | HepG2 | - | chr20 | | 34638753 | 34638853 |
| 3.55970359824 | RPS3 (bg=4.15%) | K562 | - | chr20 | | 34638782 | 34638849 |
| 3.57513601707 | RPS3 (bg=4.15%) | HepG2 | - | chr20 | | 34638785 | 34638853 |
| 2.27293773084 | SDAD1 (bg=5.99%) | HepG2 | - | chr20 | | 34638636 | 34638838 |
| 2.95573402664 | SDAD1 (bg=5.99%) | K562 | - | chr20 | | 34638779 | 34638851 |
| 5.4139677122 | SERBP1 (bg=0.81%) | K562 | - | chr20 | | 34638801 | 34638845 |
| 3.60836987779 | SF3B1 (bg=6.74%) | K562 | - | chr20 | | 34638793 | 34638829 |
| 2.28414767494 | WRN (bg=3.31%) | K562 | - | chr20 | | 34638776 | 34638844 |
| 2.49727091402 | ybx3 (bg=22.82%) | K562 | - | chr20 | | 34638800 | 34638865 |

  
  

| Match 12 in HUMAN | | | | | | | |
| --- | --- | --- | --- | --- | --- | --- | --- |
| Motif | Start in Seq (1 Indexed) | End in Seq (1 Indexed) | Strand | Chrm | Exon | Start in Chrm (0 Indexed) | End in Chrm (1 Indexed) |
| GGGCCGGCGGGTGAACTGGGGGGCCCCGGGACAGGCCGAGCCCT | 129 | 172 | - | chr20 | 1 | 34638760 | 34638804 |
| eCLIP Fold-Enrichment | Binding Protein | Cell Line | Strand | Chrm | | Start in Chrm (0 Indexed) | End in Chrm (1 Indexed) |
| 2.02606301023 | AKAP1 (bg=3.4%) | HepG2 | - | chr20 | | 34638696 | 34638763 |
| 2.15980585748 | DDX24 (bg=2.31%) | K562 | - | chr20 | | 34638716 | 34638787 |
| 5.14427011107 | ddx3x (bg=13.89%) | K562 | - | chr20 | | 34638739 | 34638762 |
| 4.50936149965 | ddx3x (bg=13.89%) | K562 | - | chr20 | | 34638739 | 34638787 |
| 6.57570732084 | ddx3x (bg=13.89%) | HepG2 | - | chr20 | | 34638747 | 34638774 |
| 7.54753615632 | ddx3x (bg=13.89%) | HepG2 | - | chr20 | | 34638749 | 34638760 |
| 7.21388698388 | ddx3x (bg=13.89%) | HepG2 | - | chr20 | | 34638760 | 34638770 |
| 5.9247834209 | ddx3x (bg=13.89%) | K562 | - | chr20 | | 34638762 | 34638792 |
| 7.11811473888 | ddx3x (bg=13.89%) | HepG2 | - | chr20 | | 34638770 | 34638786 |
| 6.18495210183 | ddx3x (bg=13.89%) | HepG2 | - | chr20 | | 34638774 | 34638789 |
| 6.89162066486 | ddx3x (bg=13.89%) | HepG2 | - | chr20 | | 34638786 | 34638797 |
| 4.16525548317 | ddx3x (bg=13.89%) | K562 | - | chr20 | | 34638787 | 34638873 |
| 6.60725052175 | ddx3x (bg=13.89%) | HepG2 | - | chr20 | | 34638789 | 34638844 |
| 5.66985406471 | ddx3x (bg=13.89%) | K562 | - | chr20 | | 34638792 | 34638808 |
| 6.6826957396 | ddx3x (bg=13.89%) | HepG2 | - | chr20 | | 34638797 | 34638804 |
| 6.50233673689 | ddx3x (bg=13.89%) | HepG2 | - | chr20 | | 34638804 | 34638847 |
| 2.12735172601 | ddx6 (bg=23.92%) | K562 | - | chr20 | | 34638802 | 34638821 |
| 2.72971054112 | DHX30 (bg=5.05%) | K562 | - | chr20 | | 34638707 | 34638760 |
| 3.04432613533 | DHX30 (bg=5.05%) | K562 | - | chr20 | | 34638710 | 34638778 |
| 2.68468691476 | drosha (bg=11.37%) | HepG2 | - | chr20 | | 34638672 | 34638771 |
| 2.68468691476 | drosha (bg=11.37%) | HepG2 | - | chr20 | | 34638672 | 34638771 |
| 4.33287303922 | drosha (bg=11.37%) | K562 | - | chr20 | | 34638696 | 34638760 |
| 3.74226397535 | drosha (bg=11.37%) | K562 | - | chr20 | | 34638765 | 34638841 |
| 2.08369907785 | drosha (bg=11.37%) | K562 | - | chr20 | | 34638786 | 34638820 |
| 4.31494711407 | EIF3G (bg=2.39%) | K562 | - | chr20 | | 34638745 | 34638846 |
| 2.73720405992 | EIF3G (bg=2.39%) | K562 | - | chr20 | | 34638788 | 34638875 |
| 2.13276454614 | EIF3H (bg=7.83%) | HepG2 | - | chr20 | | 34638749 | 34638846 |
| 2.58999404054 | fam120a (bg=18.43%) | K562 | - | chr20 | | 34638719 | 34638779 |
| 2.08134599223 | FMR1 (bg=6.3%) | K562 | - | chr20 | | 34638794 | 34638851 |
| 4.51359332856 | GEMIN5 (bg=2.46%) | K562 | - | chr20 | | 34638776 | 34638794 |
| 2.0543993622 | METAP2 (bg=6.14%) | K562 | - | chr20 | | 34638692 | 34638859 |
| 2.27019016827 | NCBP2 (bg=2.92%) | HepG2 | - | chr20 | | 34638732 | 34638788 |
| 3.10617873433 | NCBP2 (bg=2.92%) | HepG2 | - | chr20 | | 34638733 | 34638786 |
| 3.54037631567 | PHF6 (bg=1.71%) | K562 | - | chr20 | | 34638744 | 34638800 |
| 2.33540589762 | PUS1 (bg=2.87%) | K562 | - | chr20 | | 34638791 | 34638838 |
| 2.81813333372 | rbm15 (bg=11.81%) | K562 | - | chr20 | | 34638691 | 34638761 |
| 3.72991753225 | RPS3 (bg=4.15%) | HepG2 | - | chr20 | | 34638752 | 34638785 |
| 4.0529564641 | RPS3 (bg=4.15%) | HepG2 | - | chr20 | | 34638753 | 34638853 |
| 3.55970359824 | RPS3 (bg=4.15%) | K562 | - | chr20 | | 34638782 | 34638849 |
| 3.57513601707 | RPS3 (bg=4.15%) | HepG2 | - | chr20 | | 34638785 | 34638853 |
| 2.27293773084 | SDAD1 (bg=5.99%) | HepG2 | - | chr20 | | 34638636 | 34638838 |
| 2.95573402664 | SDAD1 (bg=5.99%) | K562 | - | chr20 | | 34638779 | 34638851 |
| 5.4139677122 | SERBP1 (bg=0.81%) | K562 | - | chr20 | | 34638801 | 34638845 |
| 3.60836987779 | SF3B1 (bg=6.74%) | K562 | - | chr20 | | 34638793 | 34638829 |
| 2.07982202421 | SUB1 (bg=9.24%) | HepG2 | - | chr20 | | 34638714 | 34638788 |
| 2.14205711854 | TBRG4 (bg=7.0%) | HepG2 | - | chr20 | | 34638690 | 34638777 |
| 3.55702557402 | UTP3 (bg=2.81%) | K562 | - | chr20 | | 34638729 | 34638772 |
| 2.28414767494 | WRN (bg=3.31%) | K562 | - | chr20 | | 34638776 | 34638844 |
| 2.49727091402 | ybx3 (bg=22.82%) | K562 | - | chr20 | | 34638800 | 34638865 |

  
  

| Match 13 in HUMAN | | | | | | | |
| --- | --- | --- | --- | --- | --- | --- | --- |
| Motif | Start in Seq (1 Indexed) | End in Seq (1 Indexed) | Strand | Chrm | Exon | Start in Chrm (0 Indexed) | End in Chrm (1 Indexed) |
| GGCCGG | 130 | 135 | - | chr20 | 1 | 34638797 | 34638803 |
| eCLIP Fold-Enrichment | Binding Protein | Cell Line | Strand | Chrm | | Start in Chrm (0 Indexed) | End in Chrm (1 Indexed) |
| 6.89162066486 | ddx3x (bg=13.89%) | HepG2 | - | chr20 | | 34638786 | 34638797 |
| 4.16525548317 | ddx3x (bg=13.89%) | K562 | - | chr20 | | 34638787 | 34638873 |
| 6.60725052175 | ddx3x (bg=13.89%) | HepG2 | - | chr20 | | 34638789 | 34638844 |
| 5.66985406471 | ddx3x (bg=13.89%) | K562 | - | chr20 | | 34638792 | 34638808 |
| 6.6826957396 | ddx3x (bg=13.89%) | HepG2 | - | chr20 | | 34638797 | 34638804 |
| 2.12735172601 | ddx6 (bg=23.92%) | K562 | - | chr20 | | 34638802 | 34638821 |
| 3.74226397535 | drosha (bg=11.37%) | K562 | - | chr20 | | 34638765 | 34638841 |
| 2.08369907785 | drosha (bg=11.37%) | K562 | - | chr20 | | 34638786 | 34638820 |
| 4.31494711407 | EIF3G (bg=2.39%) | K562 | - | chr20 | | 34638745 | 34638846 |
| 2.73720405992 | EIF3G (bg=2.39%) | K562 | - | chr20 | | 34638788 | 34638875 |
| 2.13276454614 | EIF3H (bg=7.83%) | HepG2 | - | chr20 | | 34638749 | 34638846 |
| 2.08134599223 | FMR1 (bg=6.3%) | K562 | - | chr20 | | 34638794 | 34638851 |
| 2.0543993622 | METAP2 (bg=6.14%) | K562 | - | chr20 | | 34638692 | 34638859 |
| 3.54037631567 | PHF6 (bg=1.71%) | K562 | - | chr20 | | 34638744 | 34638800 |
| 2.33540589762 | PUS1 (bg=2.87%) | K562 | - | chr20 | | 34638791 | 34638838 |
| 4.0529564641 | RPS3 (bg=4.15%) | HepG2 | - | chr20 | | 34638753 | 34638853 |
| 3.55970359824 | RPS3 (bg=4.15%) | K562 | - | chr20 | | 34638782 | 34638849 |
| 3.57513601707 | RPS3 (bg=4.15%) | HepG2 | - | chr20 | | 34638785 | 34638853 |
| 2.27293773084 | SDAD1 (bg=5.99%) | HepG2 | - | chr20 | | 34638636 | 34638838 |
| 2.95573402664 | SDAD1 (bg=5.99%) | K562 | - | chr20 | | 34638779 | 34638851 |
| 5.4139677122 | SERBP1 (bg=0.81%) | K562 | - | chr20 | | 34638801 | 34638845 |
| 3.60836987779 | SF3B1 (bg=6.74%) | K562 | - | chr20 | | 34638793 | 34638829 |
| 2.28414767494 | WRN (bg=3.31%) | K562 | - | chr20 | | 34638776 | 34638844 |
| 2.49727091402 | ybx3 (bg=22.82%) | K562 | - | chr20 | | 34638800 | 34638865 |

  
  

| Match 14 in HUMAN | | | | | | | |
| --- | --- | --- | --- | --- | --- | --- | --- |
| Motif | Start in Seq (1 Indexed) | End in Seq (1 Indexed) | Strand | Chrm | Exon | Start in Chrm (0 Indexed) | End in Chrm (1 Indexed) |
| GGGTGAACTGGGGGGCCC | 137 | 154 | - | chr20 | 1 | 34638778 | 34638796 |
| eCLIP Fold-Enrichment | Binding Protein | Cell Line | Strand | Chrm | | Start in Chrm (0 Indexed) | End in Chrm (1 Indexed) |
| 2.15980585748 | DDX24 (bg=2.31%) | K562 | - | chr20 | | 34638716 | 34638787 |
| 4.50936149965 | ddx3x (bg=13.89%) | K562 | - | chr20 | | 34638739 | 34638787 |
| 5.9247834209 | ddx3x (bg=13.89%) | K562 | - | chr20 | | 34638762 | 34638792 |
| 7.11811473888 | ddx3x (bg=13.89%) | HepG2 | - | chr20 | | 34638770 | 34638786 |
| 6.18495210183 | ddx3x (bg=13.89%) | HepG2 | - | chr20 | | 34638774 | 34638789 |
| 6.89162066486 | ddx3x (bg=13.89%) | HepG2 | - | chr20 | | 34638786 | 34638797 |
| 4.16525548317 | ddx3x (bg=13.89%) | K562 | - | chr20 | | 34638787 | 34638873 |
| 6.60725052175 | ddx3x (bg=13.89%) | HepG2 | - | chr20 | | 34638789 | 34638844 |
| 5.66985406471 | ddx3x (bg=13.89%) | K562 | - | chr20 | | 34638792 | 34638808 |
| 3.04432613533 | DHX30 (bg=5.05%) | K562 | - | chr20 | | 34638710 | 34638778 |
| 3.74226397535 | drosha (bg=11.37%) | K562 | - | chr20 | | 34638765 | 34638841 |
| 2.08369907785 | drosha (bg=11.37%) | K562 | - | chr20 | | 34638786 | 34638820 |
| 4.31494711407 | EIF3G (bg=2.39%) | K562 | - | chr20 | | 34638745 | 34638846 |
| 2.73720405992 | EIF3G (bg=2.39%) | K562 | - | chr20 | | 34638788 | 34638875 |
| 2.13276454614 | EIF3H (bg=7.83%) | HepG2 | - | chr20 | | 34638749 | 34638846 |
| 2.58999404054 | fam120a (bg=18.43%) | K562 | - | chr20 | | 34638719 | 34638779 |
| 2.08134599223 | FMR1 (bg=6.3%) | K562 | - | chr20 | | 34638794 | 34638851 |
| 4.51359332856 | GEMIN5 (bg=2.46%) | K562 | - | chr20 | | 34638776 | 34638794 |
| 2.0543993622 | METAP2 (bg=6.14%) | K562 | - | chr20 | | 34638692 | 34638859 |
| 2.27019016827 | NCBP2 (bg=2.92%) | HepG2 | - | chr20 | | 34638732 | 34638788 |
| 3.10617873433 | NCBP2 (bg=2.92%) | HepG2 | - | chr20 | | 34638733 | 34638786 |
| 3.54037631567 | PHF6 (bg=1.71%) | K562 | - | chr20 | | 34638744 | 34638800 |
| 2.33540589762 | PUS1 (bg=2.87%) | K562 | - | chr20 | | 34638791 | 34638838 |
| 3.72991753225 | RPS3 (bg=4.15%) | HepG2 | - | chr20 | | 34638752 | 34638785 |
| 4.0529564641 | RPS3 (bg=4.15%) | HepG2 | - | chr20 | | 34638753 | 34638853 |
| 3.55970359824 | RPS3 (bg=4.15%) | K562 | - | chr20 | | 34638782 | 34638849 |
| 3.57513601707 | RPS3 (bg=4.15%) | HepG2 | - | chr20 | | 34638785 | 34638853 |
| 2.27293773084 | SDAD1 (bg=5.99%) | HepG2 | - | chr20 | | 34638636 | 34638838 |
| 2.95573402664 | SDAD1 (bg=5.99%) | K562 | - | chr20 | | 34638779 | 34638851 |
| 3.60836987779 | SF3B1 (bg=6.74%) | K562 | - | chr20 | | 34638793 | 34638829 |
| 2.07982202421 | SUB1 (bg=9.24%) | HepG2 | - | chr20 | | 34638714 | 34638788 |
| 2.28414767494 | WRN (bg=3.31%) | K562 | - | chr20 | | 34638776 | 34638844 |

  
  

| Match 15 in HUMAN | | | | | | | |
| --- | --- | --- | --- | --- | --- | --- | --- |
| Motif | Start in Seq (1 Indexed) | End in Seq (1 Indexed) | Strand | Chrm | Exon | Start in Chrm (0 Indexed) | End in Chrm (1 Indexed) |
| GGTGAACTGGGGGGCCC | 138 | 154 | - | chr20 | 1 | 34638778 | 34638795 |
| eCLIP Fold-Enrichment | Binding Protein | Cell Line | Strand | Chrm | | Start in Chrm (0 Indexed) | End in Chrm (1 Indexed) |
| 2.15980585748 | DDX24 (bg=2.31%) | K562 | - | chr20 | | 34638716 | 34638787 |
| 4.50936149965 | ddx3x (bg=13.89%) | K562 | - | chr20 | | 34638739 | 34638787 |
| 5.9247834209 | ddx3x (bg=13.89%) | K562 | - | chr20 | | 34638762 | 34638792 |
| 7.11811473888 | ddx3x (bg=13.89%) | HepG2 | - | chr20 | | 34638770 | 34638786 |
| 6.18495210183 | ddx3x (bg=13.89%) | HepG2 | - | chr20 | | 34638774 | 34638789 |
| 6.89162066486 | ddx3x (bg=13.89%) | HepG2 | - | chr20 | | 34638786 | 34638797 |
| 4.16525548317 | ddx3x (bg=13.89%) | K562 | - | chr20 | | 34638787 | 34638873 |
| 6.60725052175 | ddx3x (bg=13.89%) | HepG2 | - | chr20 | | 34638789 | 34638844 |
| 5.66985406471 | ddx3x (bg=13.89%) | K562 | - | chr20 | | 34638792 | 34638808 |
| 3.04432613533 | DHX30 (bg=5.05%) | K562 | - | chr20 | | 34638710 | 34638778 |
| 3.74226397535 | drosha (bg=11.37%) | K562 | - | chr20 | | 34638765 | 34638841 |
| 2.08369907785 | drosha (bg=11.37%) | K562 | - | chr20 | | 34638786 | 34638820 |
| 4.31494711407 | EIF3G (bg=2.39%) | K562 | - | chr20 | | 34638745 | 34638846 |
| 2.73720405992 | EIF3G (bg=2.39%) | K562 | - | chr20 | | 34638788 | 34638875 |
| 2.13276454614 | EIF3H (bg=7.83%) | HepG2 | - | chr20 | | 34638749 | 34638846 |
| 2.58999404054 | fam120a (bg=18.43%) | K562 | - | chr20 | | 34638719 | 34638779 |
| 2.08134599223 | FMR1 (bg=6.3%) | K562 | - | chr20 | | 34638794 | 34638851 |
| 4.51359332856 | GEMIN5 (bg=2.46%) | K562 | - | chr20 | | 34638776 | 34638794 |
| 2.0543993622 | METAP2 (bg=6.14%) | K562 | - | chr20 | | 34638692 | 34638859 |
| 2.27019016827 | NCBP2 (bg=2.92%) | HepG2 | - | chr20 | | 34638732 | 34638788 |
| 3.10617873433 | NCBP2 (bg=2.92%) | HepG2 | - | chr20 | | 34638733 | 34638786 |
| 3.54037631567 | PHF6 (bg=1.71%) | K562 | - | chr20 | | 34638744 | 34638800 |
| 2.33540589762 | PUS1 (bg=2.87%) | K562 | - | chr20 | | 34638791 | 34638838 |
| 3.72991753225 | RPS3 (bg=4.15%) | HepG2 | - | chr20 | | 34638752 | 34638785 |
| 4.0529564641 | RPS3 (bg=4.15%) | HepG2 | - | chr20 | | 34638753 | 34638853 |
| 3.55970359824 | RPS3 (bg=4.15%) | K562 | - | chr20 | | 34638782 | 34638849 |
| 3.57513601707 | RPS3 (bg=4.15%) | HepG2 | - | chr20 | | 34638785 | 34638853 |
| 2.27293773084 | SDAD1 (bg=5.99%) | HepG2 | - | chr20 | | 34638636 | 34638838 |
| 2.95573402664 | SDAD1 (bg=5.99%) | K562 | - | chr20 | | 34638779 | 34638851 |
| 3.60836987779 | SF3B1 (bg=6.74%) | K562 | - | chr20 | | 34638793 | 34638829 |
| 2.07982202421 | SUB1 (bg=9.24%) | HepG2 | - | chr20 | | 34638714 | 34638788 |
| 2.28414767494 | WRN (bg=3.31%) | K562 | - | chr20 | | 34638776 | 34638844 |

  
  

| Match 16 in HUMAN | | | | | | | |
| --- | --- | --- | --- | --- | --- | --- | --- |
| Motif | Start in Seq (1 Indexed) | End in Seq (1 Indexed) | Strand | Chrm | Exon | Start in Chrm (0 Indexed) | End in Chrm (1 Indexed) |
| GGGGGGCCC | 146 | 154 | - | chr20 | 1 | 34638778 | 34638787 |
| eCLIP Fold-Enrichment | Binding Protein | Cell Line | Strand | Chrm | | Start in Chrm (0 Indexed) | End in Chrm (1 Indexed) |
| 2.15980585748 | DDX24 (bg=2.31%) | K562 | - | chr20 | | 34638716 | 34638787 |
| 4.50936149965 | ddx3x (bg=13.89%) | K562 | - | chr20 | | 34638739 | 34638787 |
| 5.9247834209 | ddx3x (bg=13.89%) | K562 | - | chr20 | | 34638762 | 34638792 |
| 7.11811473888 | ddx3x (bg=13.89%) | HepG2 | - | chr20 | | 34638770 | 34638786 |
| 6.18495210183 | ddx3x (bg=13.89%) | HepG2 | - | chr20 | | 34638774 | 34638789 |
| 6.89162066486 | ddx3x (bg=13.89%) | HepG2 | - | chr20 | | 34638786 | 34638797 |
| 4.16525548317 | ddx3x (bg=13.89%) | K562 | - | chr20 | | 34638787 | 34638873 |
| 3.04432613533 | DHX30 (bg=5.05%) | K562 | - | chr20 | | 34638710 | 34638778 |
| 3.74226397535 | drosha (bg=11.37%) | K562 | - | chr20 | | 34638765 | 34638841 |
| 2.08369907785 | drosha (bg=11.37%) | K562 | - | chr20 | | 34638786 | 34638820 |
| 4.31494711407 | EIF3G (bg=2.39%) | K562 | - | chr20 | | 34638745 | 34638846 |
| 2.13276454614 | EIF3H (bg=7.83%) | HepG2 | - | chr20 | | 34638749 | 34638846 |
| 2.58999404054 | fam120a (bg=18.43%) | K562 | - | chr20 | | 34638719 | 34638779 |
| 4.51359332856 | GEMIN5 (bg=2.46%) | K562 | - | chr20 | | 34638776 | 34638794 |
| 2.0543993622 | METAP2 (bg=6.14%) | K562 | - | chr20 | | 34638692 | 34638859 |
| 2.27019016827 | NCBP2 (bg=2.92%) | HepG2 | - | chr20 | | 34638732 | 34638788 |
| 3.10617873433 | NCBP2 (bg=2.92%) | HepG2 | - | chr20 | | 34638733 | 34638786 |
| 3.54037631567 | PHF6 (bg=1.71%) | K562 | - | chr20 | | 34638744 | 34638800 |
| 3.72991753225 | RPS3 (bg=4.15%) | HepG2 | - | chr20 | | 34638752 | 34638785 |
| 4.0529564641 | RPS3 (bg=4.15%) | HepG2 | - | chr20 | | 34638753 | 34638853 |
| 3.55970359824 | RPS3 (bg=4.15%) | K562 | - | chr20 | | 34638782 | 34638849 |
| 3.57513601707 | RPS3 (bg=4.15%) | HepG2 | - | chr20 | | 34638785 | 34638853 |
| 2.27293773084 | SDAD1 (bg=5.99%) | HepG2 | - | chr20 | | 34638636 | 34638838 |
| 2.95573402664 | SDAD1 (bg=5.99%) | K562 | - | chr20 | | 34638779 | 34638851 |
| 2.07982202421 | SUB1 (bg=9.24%) | HepG2 | - | chr20 | | 34638714 | 34638788 |
| 2.28414767494 | WRN (bg=3.31%) | K562 | - | chr20 | | 34638776 | 34638844 |

  
  

| Match 17 in HUMAN | | | | | | | |
| --- | --- | --- | --- | --- | --- | --- | --- |
| Motif | Start in Seq (1 Indexed) | End in Seq (1 Indexed) | Strand | Chrm | Exon | Start in Chrm (0 Indexed) | End in Chrm (1 Indexed) |
| GGGCCC | 149 | 154 | - | chr20 | 1 | 34638778 | 34638784 |
| eCLIP Fold-Enrichment | Binding Protein | Cell Line | Strand | Chrm | | Start in Chrm (0 Indexed) | End in Chrm (1 Indexed) |
| 2.15980585748 | DDX24 (bg=2.31%) | K562 | - | chr20 | | 34638716 | 34638787 |
| 4.50936149965 | ddx3x (bg=13.89%) | K562 | - | chr20 | | 34638739 | 34638787 |
| 5.9247834209 | ddx3x (bg=13.89%) | K562 | - | chr20 | | 34638762 | 34638792 |
| 7.11811473888 | ddx3x (bg=13.89%) | HepG2 | - | chr20 | | 34638770 | 34638786 |
| 6.18495210183 | ddx3x (bg=13.89%) | HepG2 | - | chr20 | | 34638774 | 34638789 |
| 3.04432613533 | DHX30 (bg=5.05%) | K562 | - | chr20 | | 34638710 | 34638778 |
| 3.74226397535 | drosha (bg=11.37%) | K562 | - | chr20 | | 34638765 | 34638841 |
| 4.31494711407 | EIF3G (bg=2.39%) | K562 | - | chr20 | | 34638745 | 34638846 |
| 2.13276454614 | EIF3H (bg=7.83%) | HepG2 | - | chr20 | | 34638749 | 34638846 |
| 2.58999404054 | fam120a (bg=18.43%) | K562 | - | chr20 | | 34638719 | 34638779 |
| 4.51359332856 | GEMIN5 (bg=2.46%) | K562 | - | chr20 | | 34638776 | 34638794 |
| 2.0543993622 | METAP2 (bg=6.14%) | K562 | - | chr20 | | 34638692 | 34638859 |
| 2.27019016827 | NCBP2 (bg=2.92%) | HepG2 | - | chr20 | | 34638732 | 34638788 |
| 3.10617873433 | NCBP2 (bg=2.92%) | HepG2 | - | chr20 | | 34638733 | 34638786 |
| 3.54037631567 | PHF6 (bg=1.71%) | K562 | - | chr20 | | 34638744 | 34638800 |
| 3.72991753225 | RPS3 (bg=4.15%) | HepG2 | - | chr20 | | 34638752 | 34638785 |
| 4.0529564641 | RPS3 (bg=4.15%) | HepG2 | - | chr20 | | 34638753 | 34638853 |
| 3.55970359824 | RPS3 (bg=4.15%) | K562 | - | chr20 | | 34638782 | 34638849 |
| 2.27293773084 | SDAD1 (bg=5.99%) | HepG2 | - | chr20 | | 34638636 | 34638838 |
| 2.95573402664 | SDAD1 (bg=5.99%) | K562 | - | chr20 | | 34638779 | 34638851 |
| 2.07982202421 | SUB1 (bg=9.24%) | HepG2 | - | chr20 | | 34638714 | 34638788 |
| 2.28414767494 | WRN (bg=3.31%) | K562 | - | chr20 | | 34638776 | 34638844 |

  
  

| Match 18 in HUMAN | | | | | | | |
| --- | --- | --- | --- | --- | --- | --- | --- |
| Motif | Start in Seq (1 Indexed) | End in Seq (1 Indexed) | Strand | Chrm | Exon | Start in Chrm (0 Indexed) | End in Chrm (1 Indexed) |
| TGCAGA | 179 | 184 | - | chr20 | 1 | 34638748 | 34638754 |
| eCLIP Fold-Enrichment | Binding Protein | Cell Line | Strand | Chrm | | Start in Chrm (0 Indexed) | End in Chrm (1 Indexed) |
| 2.02606301023 | AKAP1 (bg=3.4%) | HepG2 | - | chr20 | | 34638696 | 34638763 |
| 3.16376610925 | BCCIP (bg=4.61%) | HepG2 | - | chr20 | | 34638686 | 34638752 |
| 2.15980585748 | DDX24 (bg=2.31%) | K562 | - | chr20 | | 34638716 | 34638787 |
| 6.91610497633 | ddx3x (bg=13.89%) | HepG2 | - | chr20 | | 34638720 | 34638749 |
| 5.14427011107 | ddx3x (bg=13.89%) | K562 | - | chr20 | | 34638739 | 34638762 |
| 4.50936149965 | ddx3x (bg=13.89%) | K562 | - | chr20 | | 34638739 | 34638787 |
| 6.57570732084 | ddx3x (bg=13.89%) | HepG2 | - | chr20 | | 34638747 | 34638774 |
| 7.54753615632 | ddx3x (bg=13.89%) | HepG2 | - | chr20 | | 34638749 | 34638760 |
| 2.81197426856 | ddx6 (bg=23.92%) | HepG2 | - | chr20 | | 34638710 | 34638756 |
| 2.72971054112 | DHX30 (bg=5.05%) | K562 | - | chr20 | | 34638707 | 34638760 |
| 3.04432613533 | DHX30 (bg=5.05%) | K562 | - | chr20 | | 34638710 | 34638778 |
| 2.68468691476 | drosha (bg=11.37%) | HepG2 | - | chr20 | | 34638672 | 34638771 |
| 2.68468691476 | drosha (bg=11.37%) | HepG2 | - | chr20 | | 34638672 | 34638771 |
| 4.33287303922 | drosha (bg=11.37%) | K562 | - | chr20 | | 34638696 | 34638760 |
| 4.31494711407 | EIF3G (bg=2.39%) | K562 | - | chr20 | | 34638745 | 34638846 |
| 2.13276454614 | EIF3H (bg=7.83%) | HepG2 | - | chr20 | | 34638749 | 34638846 |
| 2.40159921113 | fam120a (bg=18.43%) | HepG2 | - | chr20 | | 34638700 | 34638753 |
| 2.58999404054 | fam120a (bg=18.43%) | K562 | - | chr20 | | 34638719 | 34638779 |
| 2.92112239198 | FMR1 (bg=6.3%) | K562 | - | chr20 | | 34638657 | 34638759 |
| 2.37618729963 | FMR1 (bg=6.3%) | K562 | - | chr20 | | 34638674 | 34638757 |
| 3.66210453802 | FXR2 (bg=7.51%) | K562 | - | chr20 | | 34638675 | 34638750 |
| 2.0543993622 | METAP2 (bg=6.14%) | K562 | - | chr20 | | 34638692 | 34638859 |
| 2.27019016827 | NCBP2 (bg=2.92%) | HepG2 | - | chr20 | | 34638732 | 34638788 |
| 3.10617873433 | NCBP2 (bg=2.92%) | HepG2 | - | chr20 | | 34638733 | 34638786 |
| 2.14852044292 | NIPBL (bg=2.83%) | K562 | - | chr20 | | 34638695 | 34638759 |
| 3.37675025123 | NIPBL (bg=2.83%) | K562 | - | chr20 | | 34638701 | 34638752 |
| 4.55180985306 | PCBP1 (bg=2.3%) | HepG2 | - | chr20 | | 34638733 | 34638759 |
| 3.54037631567 | PHF6 (bg=1.71%) | K562 | - | chr20 | | 34638744 | 34638800 |
| 2.92350144381 | PTBP1 (bg=1.43%) | HepG2 | - | chr20 | | 34638674 | 34638752 |
| 2.81813333372 | rbm15 (bg=11.81%) | K562 | - | chr20 | | 34638691 | 34638761 |
| 3.72991753225 | RPS3 (bg=4.15%) | HepG2 | - | chr20 | | 34638752 | 34638785 |
| 4.0529564641 | RPS3 (bg=4.15%) | HepG2 | - | chr20 | | 34638753 | 34638853 |
| 2.27293773084 | SDAD1 (bg=5.99%) | HepG2 | - | chr20 | | 34638636 | 34638838 |
| 2.07982202421 | SUB1 (bg=9.24%) | HepG2 | - | chr20 | | 34638714 | 34638788 |
| 2.14205711854 | TBRG4 (bg=7.0%) | HepG2 | - | chr20 | | 34638690 | 34638777 |
| 2.69561744349 | TROVE2 (bg=3.49%) | HepG2 | - | chr20 | | 34638685 | 34638755 |
| 3.55702557402 | UTP3 (bg=2.81%) | K562 | - | chr20 | | 34638729 | 34638772 |
| 2.0308924365 | ybx3 (bg=22.82%) | K562 | - | chr20 | | 34638666 | 34638755 |

  
  

| Match 19 in HUMAN | | | | | | | |
| --- | --- | --- | --- | --- | --- | --- | --- |
| Motif | Start in Seq (1 Indexed) | End in Seq (1 Indexed) | Strand | Chrm | Exon | Start in Chrm (0 Indexed) | End in Chrm (1 Indexed) |
| TGCAGATA | 179 | 186 | - | chr20 | 1 | 34638746 | 34638754 |
| eCLIP Fold-Enrichment | Binding Protein | Cell Line | Strand | Chrm | | Start in Chrm (0 Indexed) | End in Chrm (1 Indexed) |
| 2.02606301023 | AKAP1 (bg=3.4%) | HepG2 | - | chr20 | | 34638696 | 34638763 |
| 3.16376610925 | BCCIP (bg=4.61%) | HepG2 | - | chr20 | | 34638686 | 34638752 |
| 2.15980585748 | DDX24 (bg=2.31%) | K562 | - | chr20 | | 34638716 | 34638787 |
| 6.2868402545 | ddx3x (bg=13.89%) | HepG2 | - | chr20 | | 34638678 | 34638747 |
| 6.91610497633 | ddx3x (bg=13.89%) | HepG2 | - | chr20 | | 34638720 | 34638749 |
| 5.14427011107 | ddx3x (bg=13.89%) | K562 | - | chr20 | | 34638739 | 34638762 |
| 4.50936149965 | ddx3x (bg=13.89%) | K562 | - | chr20 | | 34638739 | 34638787 |
| 6.57570732084 | ddx3x (bg=13.89%) | HepG2 | - | chr20 | | 34638747 | 34638774 |
| 7.54753615632 | ddx3x (bg=13.89%) | HepG2 | - | chr20 | | 34638749 | 34638760 |
| 2.81197426856 | ddx6 (bg=23.92%) | HepG2 | - | chr20 | | 34638710 | 34638756 |
| 2.72971054112 | DHX30 (bg=5.05%) | K562 | - | chr20 | | 34638707 | 34638760 |
| 3.04432613533 | DHX30 (bg=5.05%) | K562 | - | chr20 | | 34638710 | 34638778 |
| 2.68468691476 | drosha (bg=11.37%) | HepG2 | - | chr20 | | 34638672 | 34638771 |
| 2.68468691476 | drosha (bg=11.37%) | HepG2 | - | chr20 | | 34638672 | 34638771 |
| 4.33287303922 | drosha (bg=11.37%) | K562 | - | chr20 | | 34638696 | 34638760 |
| 4.31494711407 | EIF3G (bg=2.39%) | K562 | - | chr20 | | 34638745 | 34638846 |
| 2.13276454614 | EIF3H (bg=7.83%) | HepG2 | - | chr20 | | 34638749 | 34638846 |
| 2.40159921113 | fam120a (bg=18.43%) | HepG2 | - | chr20 | | 34638700 | 34638753 |
| 2.58999404054 | fam120a (bg=18.43%) | K562 | - | chr20 | | 34638719 | 34638779 |
| 2.92112239198 | FMR1 (bg=6.3%) | K562 | - | chr20 | | 34638657 | 34638759 |
| 2.37618729963 | FMR1 (bg=6.3%) | K562 | - | chr20 | | 34638674 | 34638757 |
| 3.45365768331 | fubp3 (bg=23.31%) | HepG2 | - | chr20 | | 34638732 | 34638747 |
| 3.66210453802 | FXR2 (bg=7.51%) | K562 | - | chr20 | | 34638675 | 34638750 |
| 2.0543993622 | METAP2 (bg=6.14%) | K562 | - | chr20 | | 34638692 | 34638859 |
| 2.27019016827 | NCBP2 (bg=2.92%) | HepG2 | - | chr20 | | 34638732 | 34638788 |
| 3.10617873433 | NCBP2 (bg=2.92%) | HepG2 | - | chr20 | | 34638733 | 34638786 |
| 2.14852044292 | NIPBL (bg=2.83%) | K562 | - | chr20 | | 34638695 | 34638759 |
| 3.37675025123 | NIPBL (bg=2.83%) | K562 | - | chr20 | | 34638701 | 34638752 |
| 4.55180985306 | PCBP1 (bg=2.3%) | HepG2 | - | chr20 | | 34638733 | 34638759 |
| 3.54037631567 | PHF6 (bg=1.71%) | K562 | - | chr20 | | 34638744 | 34638800 |
| 2.92350144381 | PTBP1 (bg=1.43%) | HepG2 | - | chr20 | | 34638674 | 34638752 |
| 2.81813333372 | rbm15 (bg=11.81%) | K562 | - | chr20 | | 34638691 | 34638761 |
| 3.72991753225 | RPS3 (bg=4.15%) | HepG2 | - | chr20 | | 34638752 | 34638785 |
| 4.0529564641 | RPS3 (bg=4.15%) | HepG2 | - | chr20 | | 34638753 | 34638853 |
| 2.27293773084 | SDAD1 (bg=5.99%) | HepG2 | - | chr20 | | 34638636 | 34638838 |
| 2.07982202421 | SUB1 (bg=9.24%) | HepG2 | - | chr20 | | 34638714 | 34638788 |
| 2.14205711854 | TBRG4 (bg=7.0%) | HepG2 | - | chr20 | | 34638690 | 34638777 |
| 2.69561744349 | TROVE2 (bg=3.49%) | HepG2 | - | chr20 | | 34638685 | 34638755 |
| 3.55702557402 | UTP3 (bg=2.81%) | K562 | - | chr20 | | 34638729 | 34638772 |
| 2.0308924365 | ybx3 (bg=22.82%) | K562 | - | chr20 | | 34638666 | 34638755 |

  
  

| Match 20 in HUMAN | | | | | | | |
| --- | --- | --- | --- | --- | --- | --- | --- |
| Motif | Start in Seq (1 Indexed) | End in Seq (1 Indexed) | Strand | Chrm | Exon | Start in Chrm (0 Indexed) | End in Chrm (1 Indexed) |
| CGGAGGCCTCTGCTG | 188 | 202 | - | chr20 | 1 | 34638730 | 34638745 |
| eCLIP Fold-Enrichment | Binding Protein | Cell Line | Strand | Chrm | | Start in Chrm (0 Indexed) | End in Chrm (1 Indexed) |
| 2.02606301023 | AKAP1 (bg=3.4%) | HepG2 | - | chr20 | | 34638696 | 34638763 |
| 3.16376610925 | BCCIP (bg=4.61%) | HepG2 | - | chr20 | | 34638686 | 34638752 |
| 2.15980585748 | DDX24 (bg=2.31%) | K562 | - | chr20 | | 34638716 | 34638787 |
| 6.2868402545 | ddx3x (bg=13.89%) | HepG2 | - | chr20 | | 34638678 | 34638747 |
| 3.6579819537 | ddx3x (bg=13.89%) | K562 | - | chr20 | | 34638716 | 34638739 |
| 4.67120049041 | ddx3x (bg=13.89%) | K562 | - | chr20 | | 34638718 | 34638739 |
| 6.91610497633 | ddx3x (bg=13.89%) | HepG2 | - | chr20 | | 34638720 | 34638749 |
| 5.14427011107 | ddx3x (bg=13.89%) | K562 | - | chr20 | | 34638739 | 34638762 |
| 4.50936149965 | ddx3x (bg=13.89%) | K562 | - | chr20 | | 34638739 | 34638787 |
| 3.55631259438 | ddx6 (bg=23.92%) | HepG2 | - | chr20 | | 34638697 | 34638735 |
| 2.81197426856 | ddx6 (bg=23.92%) | HepG2 | - | chr20 | | 34638710 | 34638756 |
| 2.60597642722 | dgcr8 (bg=19.31%) | K562 | - | chr20 | | 34638582 | 34638744 |
| 2.72971054112 | DHX30 (bg=5.05%) | K562 | - | chr20 | | 34638707 | 34638760 |
| 3.04432613533 | DHX30 (bg=5.05%) | K562 | - | chr20 | | 34638710 | 34638778 |
| 2.68468691476 | drosha (bg=11.37%) | HepG2 | - | chr20 | | 34638672 | 34638771 |
| 2.68468691476 | drosha (bg=11.37%) | HepG2 | - | chr20 | | 34638672 | 34638771 |
| 4.33287303922 | drosha (bg=11.37%) | K562 | - | chr20 | | 34638696 | 34638760 |
| 4.31494711407 | EIF3G (bg=2.39%) | K562 | - | chr20 | | 34638745 | 34638846 |
| 2.9103721248 | EIF3H (bg=7.83%) | HepG2 | - | chr20 | | 34638704 | 34638741 |
| 2.40159921113 | fam120a (bg=18.43%) | HepG2 | - | chr20 | | 34638700 | 34638753 |
| 2.58999404054 | fam120a (bg=18.43%) | K562 | - | chr20 | | 34638719 | 34638779 |
| 2.92112239198 | FMR1 (bg=6.3%) | K562 | - | chr20 | | 34638657 | 34638759 |
| 2.37618729963 | FMR1 (bg=6.3%) | K562 | - | chr20 | | 34638674 | 34638757 |
| 3.45365768331 | fubp3 (bg=23.31%) | HepG2 | - | chr20 | | 34638732 | 34638747 |
| 3.66210453802 | FXR2 (bg=7.51%) | K562 | - | chr20 | | 34638675 | 34638750 |
| 2.0543993622 | METAP2 (bg=6.14%) | K562 | - | chr20 | | 34638692 | 34638859 |
| 3.16091738521 | METAP2 (bg=6.14%) | K562 | - | chr20 | | 34638702 | 34638736 |
| 2.27019016827 | NCBP2 (bg=2.92%) | HepG2 | - | chr20 | | 34638732 | 34638788 |
| 3.10617873433 | NCBP2 (bg=2.92%) | HepG2 | - | chr20 | | 34638733 | 34638786 |
| 2.14852044292 | NIPBL (bg=2.83%) | K562 | - | chr20 | | 34638695 | 34638759 |
| 3.37675025123 | NIPBL (bg=2.83%) | K562 | - | chr20 | | 34638701 | 34638752 |
| 4.55180985306 | PCBP1 (bg=2.3%) | HepG2 | - | chr20 | | 34638733 | 34638759 |
| 3.54037631567 | PHF6 (bg=1.71%) | K562 | - | chr20 | | 34638744 | 34638800 |
| 2.92350144381 | PTBP1 (bg=1.43%) | HepG2 | - | chr20 | | 34638674 | 34638752 |
| 4.59532243639 | pum2 (bg=21.55%) | K562 | - | chr20 | | 34638716 | 34638744 |
| 4.21073815409 | rbm15 (bg=11.81%) | HepG2 | - | chr20 | | 34638652 | 34638730 |
| 3.19493478759 | rbm15 (bg=11.81%) | HepG2 | - | chr20 | | 34638678 | 34638741 |
| 2.81813333372 | rbm15 (bg=11.81%) | K562 | - | chr20 | | 34638691 | 34638761 |
| 2.54263008489 | RPS3 (bg=4.15%) | K562 | - | chr20 | | 34638702 | 34638735 |
| 2.89036414179 | SBDS (bg=1.91%) | K562 | - | chr20 | | 34638683 | 34638741 |
| 2.27293773084 | SDAD1 (bg=5.99%) | HepG2 | - | chr20 | | 34638636 | 34638838 |
| 5.60836987779 | SF3B1 (bg=6.74%) | K562 | - | chr20 | | 34638711 | 34638732 |
| 2.07982202421 | SUB1 (bg=9.24%) | HepG2 | - | chr20 | | 34638714 | 34638788 |
| 2.14205711854 | TBRG4 (bg=7.0%) | HepG2 | - | chr20 | | 34638690 | 34638777 |
| 2.69561744349 | TROVE2 (bg=3.49%) | HepG2 | - | chr20 | | 34638685 | 34638755 |
| 3.55702557402 | UTP3 (bg=2.81%) | K562 | - | chr20 | | 34638729 | 34638772 |
| 3.3197514998 | WRN (bg=3.31%) | K562 | - | chr20 | | 34638693 | 34638732 |
| 2.0308924365 | ybx3 (bg=22.82%) | K562 | - | chr20 | | 34638666 | 34638755 |

  
  

| Match 21 in HUMAN | | | | | | | |
| --- | --- | --- | --- | --- | --- | --- | --- |
| Motif | Start in Seq (1 Indexed) | End in Seq (1 Indexed) | Strand | Chrm | Exon | Start in Chrm (0 Indexed) | End in Chrm (1 Indexed) |
| GGCCTCT | 192 | 198 | - | chr20 | 1 | 34638734 | 34638741 |
| eCLIP Fold-Enrichment | Binding Protein | Cell Line | Strand | Chrm | | Start in Chrm (0 Indexed) | End in Chrm (1 Indexed) |
| 2.02606301023 | AKAP1 (bg=3.4%) | HepG2 | - | chr20 | | 34638696 | 34638763 |
| 3.16376610925 | BCCIP (bg=4.61%) | HepG2 | - | chr20 | | 34638686 | 34638752 |
| 2.15980585748 | DDX24 (bg=2.31%) | K562 | - | chr20 | | 34638716 | 34638787 |
| 6.2868402545 | ddx3x (bg=13.89%) | HepG2 | - | chr20 | | 34638678 | 34638747 |
| 3.6579819537 | ddx3x (bg=13.89%) | K562 | - | chr20 | | 34638716 | 34638739 |
| 4.67120049041 | ddx3x (bg=13.89%) | K562 | - | chr20 | | 34638718 | 34638739 |
| 6.91610497633 | ddx3x (bg=13.89%) | HepG2 | - | chr20 | | 34638720 | 34638749 |
| 5.14427011107 | ddx3x (bg=13.89%) | K562 | - | chr20 | | 34638739 | 34638762 |
| 4.50936149965 | ddx3x (bg=13.89%) | K562 | - | chr20 | | 34638739 | 34638787 |
| 3.55631259438 | ddx6 (bg=23.92%) | HepG2 | - | chr20 | | 34638697 | 34638735 |
| 2.81197426856 | ddx6 (bg=23.92%) | HepG2 | - | chr20 | | 34638710 | 34638756 |
| 2.60597642722 | dgcr8 (bg=19.31%) | K562 | - | chr20 | | 34638582 | 34638744 |
| 2.72971054112 | DHX30 (bg=5.05%) | K562 | - | chr20 | | 34638707 | 34638760 |
| 3.04432613533 | DHX30 (bg=5.05%) | K562 | - | chr20 | | 34638710 | 34638778 |
| 2.68468691476 | drosha (bg=11.37%) | HepG2 | - | chr20 | | 34638672 | 34638771 |
| 2.68468691476 | drosha (bg=11.37%) | HepG2 | - | chr20 | | 34638672 | 34638771 |
| 4.33287303922 | drosha (bg=11.37%) | K562 | - | chr20 | | 34638696 | 34638760 |
| 2.9103721248 | EIF3H (bg=7.83%) | HepG2 | - | chr20 | | 34638704 | 34638741 |
| 2.40159921113 | fam120a (bg=18.43%) | HepG2 | - | chr20 | | 34638700 | 34638753 |
| 2.58999404054 | fam120a (bg=18.43%) | K562 | - | chr20 | | 34638719 | 34638779 |
| 2.92112239198 | FMR1 (bg=6.3%) | K562 | - | chr20 | | 34638657 | 34638759 |
| 2.37618729963 | FMR1 (bg=6.3%) | K562 | - | chr20 | | 34638674 | 34638757 |
| 3.45365768331 | fubp3 (bg=23.31%) | HepG2 | - | chr20 | | 34638732 | 34638747 |
| 3.66210453802 | FXR2 (bg=7.51%) | K562 | - | chr20 | | 34638675 | 34638750 |
| 2.0543993622 | METAP2 (bg=6.14%) | K562 | - | chr20 | | 34638692 | 34638859 |
| 3.16091738521 | METAP2 (bg=6.14%) | K562 | - | chr20 | | 34638702 | 34638736 |
| 2.27019016827 | NCBP2 (bg=2.92%) | HepG2 | - | chr20 | | 34638732 | 34638788 |
| 3.10617873433 | NCBP2 (bg=2.92%) | HepG2 | - | chr20 | | 34638733 | 34638786 |
| 2.14852044292 | NIPBL (bg=2.83%) | K562 | - | chr20 | | 34638695 | 34638759 |
| 3.37675025123 | NIPBL (bg=2.83%) | K562 | - | chr20 | | 34638701 | 34638752 |
| 4.55180985306 | PCBP1 (bg=2.3%) | HepG2 | - | chr20 | | 34638733 | 34638759 |
| 2.92350144381 | PTBP1 (bg=1.43%) | HepG2 | - | chr20 | | 34638674 | 34638752 |
| 4.59532243639 | pum2 (bg=21.55%) | K562 | - | chr20 | | 34638716 | 34638744 |
| 3.19493478759 | rbm15 (bg=11.81%) | HepG2 | - | chr20 | | 34638678 | 34638741 |
| 2.81813333372 | rbm15 (bg=11.81%) | K562 | - | chr20 | | 34638691 | 34638761 |
| 2.54263008489 | RPS3 (bg=4.15%) | K562 | - | chr20 | | 34638702 | 34638735 |
| 2.89036414179 | SBDS (bg=1.91%) | K562 | - | chr20 | | 34638683 | 34638741 |
| 2.27293773084 | SDAD1 (bg=5.99%) | HepG2 | - | chr20 | | 34638636 | 34638838 |
| 2.07982202421 | SUB1 (bg=9.24%) | HepG2 | - | chr20 | | 34638714 | 34638788 |
| 2.14205711854 | TBRG4 (bg=7.0%) | HepG2 | - | chr20 | | 34638690 | 34638777 |
| 2.69561744349 | TROVE2 (bg=3.49%) | HepG2 | - | chr20 | | 34638685 | 34638755 |
| 3.55702557402 | UTP3 (bg=2.81%) | K562 | - | chr20 | | 34638729 | 34638772 |
| 2.0308924365 | ybx3 (bg=22.82%) | K562 | - | chr20 | | 34638666 | 34638755 |

  
  

| Match 22 in HUMAN | | | | | | | |
| --- | --- | --- | --- | --- | --- | --- | --- |
| Motif | Start in Seq (1 Indexed) | End in Seq (1 Indexed) | Strand | Chrm | Exon | Start in Chrm (0 Indexed) | End in Chrm (1 Indexed) |
| GGCTGCCCACT | 204 | 214 | - | chr20 | 1 | 34638718 | 34638729 |
| eCLIP Fold-Enrichment | Binding Protein | Cell Line | Strand | Chrm | | Start in Chrm (0 Indexed) | End in Chrm (1 Indexed) |
| 2.02606301023 | AKAP1 (bg=3.4%) | HepG2 | - | chr20 | | 34638696 | 34638763 |
| 3.16376610925 | BCCIP (bg=4.61%) | HepG2 | - | chr20 | | 34638686 | 34638752 |
| 2.15980585748 | DDX24 (bg=2.31%) | K562 | - | chr20 | | 34638716 | 34638787 |
| 4.11012885549 | ddx3x (bg=13.89%) | K562 | - | chr20 | | 34638598 | 34638718 |
| 6.55497448798 | ddx3x (bg=13.89%) | HepG2 | - | chr20 | | 34638672 | 34638720 |
| 6.2868402545 | ddx3x (bg=13.89%) | HepG2 | - | chr20 | | 34638678 | 34638747 |
| 3.6579819537 | ddx3x (bg=13.89%) | K562 | - | chr20 | | 34638716 | 34638739 |
| 4.67120049041 | ddx3x (bg=13.89%) | K562 | - | chr20 | | 34638718 | 34638739 |
| 6.91610497633 | ddx3x (bg=13.89%) | HepG2 | - | chr20 | | 34638720 | 34638749 |
| 3.55631259438 | ddx6 (bg=23.92%) | HepG2 | - | chr20 | | 34638697 | 34638735 |
| 2.81197426856 | ddx6 (bg=23.92%) | HepG2 | - | chr20 | | 34638710 | 34638756 |
| 2.60597642722 | dgcr8 (bg=19.31%) | K562 | - | chr20 | | 34638582 | 34638744 |
| 2.72971054112 | DHX30 (bg=5.05%) | K562 | - | chr20 | | 34638707 | 34638760 |
| 3.04432613533 | DHX30 (bg=5.05%) | K562 | - | chr20 | | 34638710 | 34638778 |
| 2.68468691476 | drosha (bg=11.37%) | HepG2 | - | chr20 | | 34638672 | 34638771 |
| 2.68468691476 | drosha (bg=11.37%) | HepG2 | - | chr20 | | 34638672 | 34638771 |
| 4.33287303922 | drosha (bg=11.37%) | K562 | - | chr20 | | 34638696 | 34638760 |
| 2.9103721248 | EIF3H (bg=7.83%) | HepG2 | - | chr20 | | 34638704 | 34638741 |
| 2.40159921113 | fam120a (bg=18.43%) | HepG2 | - | chr20 | | 34638700 | 34638753 |
| 2.58999404054 | fam120a (bg=18.43%) | K562 | - | chr20 | | 34638719 | 34638779 |
| 2.92112239198 | FMR1 (bg=6.3%) | K562 | - | chr20 | | 34638657 | 34638759 |
| 2.37618729963 | FMR1 (bg=6.3%) | K562 | - | chr20 | | 34638674 | 34638757 |
| 3.66210453802 | FXR2 (bg=7.51%) | K562 | - | chr20 | | 34638675 | 34638750 |
| 2.0543993622 | METAP2 (bg=6.14%) | K562 | - | chr20 | | 34638692 | 34638859 |
| 3.16091738521 | METAP2 (bg=6.14%) | K562 | - | chr20 | | 34638702 | 34638736 |
| 2.14852044292 | NIPBL (bg=2.83%) | K562 | - | chr20 | | 34638695 | 34638759 |
| 3.37675025123 | NIPBL (bg=2.83%) | K562 | - | chr20 | | 34638701 | 34638752 |
| 2.92350144381 | PTBP1 (bg=1.43%) | HepG2 | - | chr20 | | 34638674 | 34638752 |
| 4.59532243639 | pum2 (bg=21.55%) | K562 | - | chr20 | | 34638716 | 34638744 |
| 4.21073815409 | rbm15 (bg=11.81%) | HepG2 | - | chr20 | | 34638652 | 34638730 |
| 3.19493478759 | rbm15 (bg=11.81%) | HepG2 | - | chr20 | | 34638678 | 34638741 |
| 2.81813333372 | rbm15 (bg=11.81%) | K562 | - | chr20 | | 34638691 | 34638761 |
| 2.54263008489 | RPS3 (bg=4.15%) | K562 | - | chr20 | | 34638702 | 34638735 |
| 2.89036414179 | SBDS (bg=1.91%) | K562 | - | chr20 | | 34638683 | 34638741 |
| 2.27293773084 | SDAD1 (bg=5.99%) | HepG2 | - | chr20 | | 34638636 | 34638838 |
| 5.60836987779 | SF3B1 (bg=6.74%) | K562 | - | chr20 | | 34638711 | 34638732 |
| 2.07982202421 | SUB1 (bg=9.24%) | HepG2 | - | chr20 | | 34638714 | 34638788 |
| 2.14205711854 | TBRG4 (bg=7.0%) | HepG2 | - | chr20 | | 34638690 | 34638777 |
| 2.69561744349 | TROVE2 (bg=3.49%) | HepG2 | - | chr20 | | 34638685 | 34638755 |
| 3.55702557402 | UTP3 (bg=2.81%) | K562 | - | chr20 | | 34638729 | 34638772 |
| 3.3197514998 | WRN (bg=3.31%) | K562 | - | chr20 | | 34638693 | 34638732 |
| 2.0308924365 | ybx3 (bg=22.82%) | K562 | - | chr20 | | 34638666 | 34638755 |
| 3.12353769592 | ZC3H11A (bg=6.25%) | K562 | - | chr20 | | 34638706 | 34638725 |
| 3.12353769592 | ZC3H11A (bg=6.25%) | K562 | - | chr20 | | 34638706 | 34638725 |

  
  

| Match 23 in HUMAN | | | | | | | |
| --- | --- | --- | --- | --- | --- | --- | --- |
| Motif | Start in Seq (1 Indexed) | End in Seq (1 Indexed) | Strand | Chrm | Exon | Start in Chrm (0 Indexed) | End in Chrm (1 Indexed) |
| GGCTGCCCACTGGCTGTGCCC | 204 | 224 | - | chr20 | 1 | 34638708 | 34638729 |
| eCLIP Fold-Enrichment | Binding Protein | Cell Line | Strand | Chrm | | Start in Chrm (0 Indexed) | End in Chrm (1 Indexed) |
| 2.02606301023 | AKAP1 (bg=3.4%) | HepG2 | - | chr20 | | 34638696 | 34638763 |
| 3.16376610925 | BCCIP (bg=4.61%) | HepG2 | - | chr20 | | 34638686 | 34638752 |
| 2.15980585748 | DDX24 (bg=2.31%) | K562 | - | chr20 | | 34638716 | 34638787 |
| 4.11012885549 | ddx3x (bg=13.89%) | K562 | - | chr20 | | 34638598 | 34638718 |
| 3.54964509511 | ddx3x (bg=13.89%) | K562 | - | chr20 | | 34638670 | 34638716 |
| 6.55497448798 | ddx3x (bg=13.89%) | HepG2 | - | chr20 | | 34638672 | 34638720 |
| 6.2868402545 | ddx3x (bg=13.89%) | HepG2 | - | chr20 | | 34638678 | 34638747 |
| 3.6579819537 | ddx3x (bg=13.89%) | K562 | - | chr20 | | 34638716 | 34638739 |
| 4.67120049041 | ddx3x (bg=13.89%) | K562 | - | chr20 | | 34638718 | 34638739 |
| 6.91610497633 | ddx3x (bg=13.89%) | HepG2 | - | chr20 | | 34638720 | 34638749 |
| 3.55631259438 | ddx6 (bg=23.92%) | HepG2 | - | chr20 | | 34638697 | 34638735 |
| 2.81197426856 | ddx6 (bg=23.92%) | HepG2 | - | chr20 | | 34638710 | 34638756 |
| 2.60597642722 | dgcr8 (bg=19.31%) | K562 | - | chr20 | | 34638582 | 34638744 |
| 2.72971054112 | DHX30 (bg=5.05%) | K562 | - | chr20 | | 34638707 | 34638760 |
| 3.04432613533 | DHX30 (bg=5.05%) | K562 | - | chr20 | | 34638710 | 34638778 |
| 2.68468691476 | drosha (bg=11.37%) | HepG2 | - | chr20 | | 34638672 | 34638771 |
| 2.68468691476 | drosha (bg=11.37%) | HepG2 | - | chr20 | | 34638672 | 34638771 |
| 4.33287303922 | drosha (bg=11.37%) | K562 | - | chr20 | | 34638696 | 34638760 |
| 2.9103721248 | EIF3H (bg=7.83%) | HepG2 | - | chr20 | | 34638704 | 34638741 |
| 2.40159921113 | fam120a (bg=18.43%) | HepG2 | - | chr20 | | 34638700 | 34638753 |
| 2.58999404054 | fam120a (bg=18.43%) | K562 | - | chr20 | | 34638719 | 34638779 |
| 2.92112239198 | FMR1 (bg=6.3%) | K562 | - | chr20 | | 34638657 | 34638759 |
| 2.37618729963 | FMR1 (bg=6.3%) | K562 | - | chr20 | | 34638674 | 34638757 |
| 3.66210453802 | FXR2 (bg=7.51%) | K562 | - | chr20 | | 34638675 | 34638750 |
| 2.0543993622 | METAP2 (bg=6.14%) | K562 | - | chr20 | | 34638692 | 34638859 |
| 3.16091738521 | METAP2 (bg=6.14%) | K562 | - | chr20 | | 34638702 | 34638736 |
| 2.14852044292 | NIPBL (bg=2.83%) | K562 | - | chr20 | | 34638695 | 34638759 |
| 3.37675025123 | NIPBL (bg=2.83%) | K562 | - | chr20 | | 34638701 | 34638752 |
| 2.92350144381 | PTBP1 (bg=1.43%) | HepG2 | - | chr20 | | 34638674 | 34638752 |
| 4.59532243639 | pum2 (bg=21.55%) | K562 | - | chr20 | | 34638716 | 34638744 |
| 4.21073815409 | rbm15 (bg=11.81%) | HepG2 | - | chr20 | | 34638652 | 34638730 |
| 3.19493478759 | rbm15 (bg=11.81%) | HepG2 | - | chr20 | | 34638678 | 34638741 |
| 2.81813333372 | rbm15 (bg=11.81%) | K562 | - | chr20 | | 34638691 | 34638761 |
| 2.54263008489 | RPS3 (bg=4.15%) | K562 | - | chr20 | | 34638702 | 34638735 |
| 2.89036414179 | SBDS (bg=1.91%) | K562 | - | chr20 | | 34638683 | 34638741 |
| 2.27293773084 | SDAD1 (bg=5.99%) | HepG2 | - | chr20 | | 34638636 | 34638838 |
| 5.60836987779 | SF3B1 (bg=6.74%) | K562 | - | chr20 | | 34638704 | 34638711 |
| 5.60836987779 | SF3B1 (bg=6.74%) | K562 | - | chr20 | | 34638711 | 34638732 |
| 2.07982202421 | SUB1 (bg=9.24%) | HepG2 | - | chr20 | | 34638714 | 34638788 |
| 2.14205711854 | TBRG4 (bg=7.0%) | HepG2 | - | chr20 | | 34638690 | 34638777 |
| 2.69561744349 | TROVE2 (bg=3.49%) | HepG2 | - | chr20 | | 34638685 | 34638755 |
| 3.55702557402 | UTP3 (bg=2.81%) | K562 | - | chr20 | | 34638729 | 34638772 |
| 3.3197514998 | WRN (bg=3.31%) | K562 | - | chr20 | | 34638693 | 34638732 |
| 2.0308924365 | ybx3 (bg=22.82%) | K562 | - | chr20 | | 34638666 | 34638755 |
| 3.12353769592 | ZC3H11A (bg=6.25%) | K562 | - | chr20 | | 34638706 | 34638725 |
| 3.12353769592 | ZC3H11A (bg=6.25%) | K562 | - | chr20 | | 34638706 | 34638725 |

  
  

| Match 24 in HUMAN | | | | | | | |
| --- | --- | --- | --- | --- | --- | --- | --- |
| Motif | Start in Seq (1 Indexed) | End in Seq (1 Indexed) | Strand | Chrm | Exon | Start in Chrm (0 Indexed) | End in Chrm (1 Indexed) |
| CCTTGAAGCCGCAGCGAACCTCTCTT | 245 | 270 | - | chr20 | 1 | 34638662 | 34638688 |
| eCLIP Fold-Enrichment | Binding Protein | Cell Line | Strand | Chrm | | Start in Chrm (0 Indexed) | End in Chrm (1 Indexed) |
| 2.31060749757 | BCCIP (bg=4.61%) | HepG2 | - | chr20 | | 34638558 | 34638666 |
| 3.16376610925 | BCCIP (bg=4.61%) | HepG2 | - | chr20 | | 34638686 | 34638752 |
| 2.57192950951 | ddx3x (bg=13.89%) | K562 | - | chr20 | | 34638594 | 34638670 |
| 5.58567128521 | ddx3x (bg=13.89%) | HepG2 | - | chr20 | | 34638596 | 34638663 |
| 4.11012885549 | ddx3x (bg=13.89%) | K562 | - | chr20 | | 34638598 | 34638718 |
| 3.54964509511 | ddx3x (bg=13.89%) | K562 | - | chr20 | | 34638670 | 34638716 |
| 6.55497448798 | ddx3x (bg=13.89%) | HepG2 | - | chr20 | | 34638672 | 34638720 |
| 6.2868402545 | ddx3x (bg=13.89%) | HepG2 | - | chr20 | | 34638678 | 34638747 |
| 2.60597642722 | dgcr8 (bg=19.31%) | K562 | - | chr20 | | 34638582 | 34638744 |
| 2.54302009491 | dgcr8 (bg=19.31%) | HepG2 | - | chr20 | | 34638587 | 34638667 |
| 2.45675630989 | drosha (bg=11.37%) | HepG2 | - | chr20 | | 34638571 | 34638672 |
| 2.45675630989 | drosha (bg=11.37%) | HepG2 | - | chr20 | | 34638571 | 34638672 |
| 2.68468691476 | drosha (bg=11.37%) | HepG2 | - | chr20 | | 34638672 | 34638771 |
| 2.68468691476 | drosha (bg=11.37%) | HepG2 | - | chr20 | | 34638672 | 34638771 |
| 2.92112239198 | FMR1 (bg=6.3%) | K562 | - | chr20 | | 34638657 | 34638759 |
| 2.37618729963 | FMR1 (bg=6.3%) | K562 | - | chr20 | | 34638674 | 34638757 |
| 2.53069347253 | FTO (bg=7.53%) | HepG2 | - | chr20 | | 34638587 | 34638672 |
| 2.49695843524 | FTO (bg=7.53%) | HepG2 | - | chr20 | | 34638605 | 34638667 |
| 2.20135547148 | FXR2 (bg=7.51%) | K562 | - | chr20 | | 34638566 | 34638679 |
| 3.62648062829 | FXR2 (bg=7.51%) | K562 | - | chr20 | | 34638590 | 34638675 |
| 3.66210453802 | FXR2 (bg=7.51%) | K562 | - | chr20 | | 34638675 | 34638750 |
| 2.99902015573 | GEMIN5 (bg=2.46%) | K562 | - | chr20 | | 34638603 | 34638675 |
| 2.28644826729 | METAP2 (bg=6.14%) | K562 | - | chr20 | | 34638590 | 34638666 |
| 2.54576340698 | NIP7 (bg=2.54%) | HepG2 | - | chr20 | | 34638594 | 34638679 |
| 3.59076180613 | PCBP1 (bg=2.3%) | K562 | - | chr20 | | 34638588 | 34638671 |
| 2.80331914555 | PCBP1 (bg=2.3%) | K562 | - | chr20 | | 34638591 | 34638664 |
| 2.92350144381 | PTBP1 (bg=1.43%) | HepG2 | - | chr20 | | 34638674 | 34638752 |
| 2.40138566506 | rbm15 (bg=11.81%) | HepG2 | - | chr20 | | 34638590 | 34638664 |
| 2.51067342866 | rbm15 (bg=11.81%) | K562 | - | chr20 | | 34638601 | 34638662 |
| 2.46046205374 | rbm15 (bg=11.81%) | K562 | - | chr20 | | 34638601 | 34638665 |
| 4.21073815409 | rbm15 (bg=11.81%) | HepG2 | - | chr20 | | 34638652 | 34638730 |
| 3.19493478759 | rbm15 (bg=11.81%) | HepG2 | - | chr20 | | 34638678 | 34638741 |
| 2.89036414179 | SBDS (bg=1.91%) | K562 | - | chr20 | | 34638683 | 34638741 |
| 2.54069652736 | SDAD1 (bg=5.99%) | K562 | - | chr20 | | 34638589 | 34638681 |
| 2.27293773084 | SDAD1 (bg=5.99%) | HepG2 | - | chr20 | | 34638636 | 34638838 |
| 2.69561744349 | TROVE2 (bg=3.49%) | HepG2 | - | chr20 | | 34638685 | 34638755 |
| 2.0308924365 | ybx3 (bg=22.82%) | K562 | - | chr20 | | 34638666 | 34638755 |

  
  

| Match 25 in HUMAN | | | | | | | |
| --- | --- | --- | --- | --- | --- | --- | --- |
| Motif | Start in Seq (1 Indexed) | End in Seq (1 Indexed) | Strand | Chrm | Exon | Start in Chrm (0 Indexed) | End in Chrm (1 Indexed) |
| GAAGCC | 249 | 254 | - | chr20 | 1 | 34638678 | 34638684 |
| eCLIP Fold-Enrichment | Binding Protein | Cell Line | Strand | Chrm | | Start in Chrm (0 Indexed) | End in Chrm (1 Indexed) |
| 4.11012885549 | ddx3x (bg=13.89%) | K562 | - | chr20 | | 34638598 | 34638718 |
| 3.54964509511 | ddx3x (bg=13.89%) | K562 | - | chr20 | | 34638670 | 34638716 |
| 6.55497448798 | ddx3x (bg=13.89%) | HepG2 | - | chr20 | | 34638672 | 34638720 |
| 6.2868402545 | ddx3x (bg=13.89%) | HepG2 | - | chr20 | | 34638678 | 34638747 |
| 2.60597642722 | dgcr8 (bg=19.31%) | K562 | - | chr20 | | 34638582 | 34638744 |
| 2.68468691476 | drosha (bg=11.37%) | HepG2 | - | chr20 | | 34638672 | 34638771 |
| 2.68468691476 | drosha (bg=11.37%) | HepG2 | - | chr20 | | 34638672 | 34638771 |
| 2.92112239198 | FMR1 (bg=6.3%) | K562 | - | chr20 | | 34638657 | 34638759 |
| 2.37618729963 | FMR1 (bg=6.3%) | K562 | - | chr20 | | 34638674 | 34638757 |
| 2.20135547148 | FXR2 (bg=7.51%) | K562 | - | chr20 | | 34638566 | 34638679 |
| 3.66210453802 | FXR2 (bg=7.51%) | K562 | - | chr20 | | 34638675 | 34638750 |
| 2.54576340698 | NIP7 (bg=2.54%) | HepG2 | - | chr20 | | 34638594 | 34638679 |
| 2.92350144381 | PTBP1 (bg=1.43%) | HepG2 | - | chr20 | | 34638674 | 34638752 |
| 4.21073815409 | rbm15 (bg=11.81%) | HepG2 | - | chr20 | | 34638652 | 34638730 |
| 3.19493478759 | rbm15 (bg=11.81%) | HepG2 | - | chr20 | | 34638678 | 34638741 |
| 2.89036414179 | SBDS (bg=1.91%) | K562 | - | chr20 | | 34638683 | 34638741 |
| 2.54069652736 | SDAD1 (bg=5.99%) | K562 | - | chr20 | | 34638589 | 34638681 |
| 2.27293773084 | SDAD1 (bg=5.99%) | HepG2 | - | chr20 | | 34638636 | 34638838 |
| 2.0308924365 | ybx3 (bg=22.82%) | K562 | - | chr20 | | 34638666 | 34638755 |

  
  

| Match 26 in HUMAN | | | | | | | |
| --- | --- | --- | --- | --- | --- | --- | --- |
| Motif | Start in Seq (1 Indexed) | End in Seq (1 Indexed) | Strand | Chrm | Exon | Start in Chrm (0 Indexed) | End in Chrm (1 Indexed) |
| CCCACCCCACCTCGGTGACT | 272 | 291 | - | chr20 | 1 | 34638641 | 34638661 |
| eCLIP Fold-Enrichment | Binding Protein | Cell Line | Strand | Chrm | | Start in Chrm (0 Indexed) | End in Chrm (1 Indexed) |
| 2.31060749757 | BCCIP (bg=4.61%) | HepG2 | - | chr20 | | 34638558 | 34638666 |
| 4.93017974172 | ddx3x (bg=13.89%) | HepG2 | - | chr20 | | 34638580 | 34638654 |
| 2.57192950951 | ddx3x (bg=13.89%) | K562 | - | chr20 | | 34638594 | 34638670 |
| 5.58567128521 | ddx3x (bg=13.89%) | HepG2 | - | chr20 | | 34638596 | 34638663 |
| 4.11012885549 | ddx3x (bg=13.89%) | K562 | - | chr20 | | 34638598 | 34638718 |
| 2.39207101411 | ddx6 (bg=23.92%) | HepG2 | - | chr20 | | 34638597 | 34638646 |
| 2.62342679024 | ddx6 (bg=23.92%) | HepG2 | - | chr20 | | 34638609 | 34638661 |
| 2.60597642722 | dgcr8 (bg=19.31%) | K562 | - | chr20 | | 34638582 | 34638744 |
| 2.54302009491 | dgcr8 (bg=19.31%) | HepG2 | - | chr20 | | 34638587 | 34638667 |
| 2.12738187506 | DHX30 (bg=5.05%) | K562 | - | chr20 | | 34638594 | 34638647 |
| 2.50731811978 | DHX30 (bg=5.05%) | K562 | - | chr20 | | 34638602 | 34638653 |
| 2.45675630989 | drosha (bg=11.37%) | HepG2 | - | chr20 | | 34638571 | 34638672 |
| 2.45675630989 | drosha (bg=11.37%) | HepG2 | - | chr20 | | 34638571 | 34638672 |
| 2.52180683689 | EIF3H (bg=7.83%) | HepG2 | - | chr20 | | 34638510 | 34638656 |
| 2.34680713545 | FMR1 (bg=6.3%) | K562 | - | chr20 | | 34638590 | 34638657 |
| 2.92112239198 | FMR1 (bg=6.3%) | K562 | - | chr20 | | 34638657 | 34638759 |
| 2.53069347253 | FTO (bg=7.53%) | HepG2 | - | chr20 | | 34638587 | 34638672 |
| 2.49695843524 | FTO (bg=7.53%) | HepG2 | - | chr20 | | 34638605 | 34638667 |
| 2.66010856078 | fubp3 (bg=23.31%) | HepG2 | - | chr20 | | 34638628 | 34638642 |
| 2.20135547148 | FXR2 (bg=7.51%) | K562 | - | chr20 | | 34638566 | 34638679 |
| 3.62648062829 | FXR2 (bg=7.51%) | K562 | - | chr20 | | 34638590 | 34638675 |
| 2.99902015573 | GEMIN5 (bg=2.46%) | K562 | - | chr20 | | 34638603 | 34638675 |
| 2.02549898345 | IGF2BP3 (bg=4.26%) | HepG2 | - | chr20 | | 34638575 | 34638656 |
| 2.28644826729 | METAP2 (bg=6.14%) | K562 | - | chr20 | | 34638590 | 34638666 |
| 2.54576340698 | NIP7 (bg=2.54%) | HepG2 | - | chr20 | | 34638594 | 34638679 |
| 3.36738528192 | PCBP1 (bg=2.3%) | HepG2 | - | chr20 | | 34638572 | 34638655 |
| 3.59076180613 | PCBP1 (bg=2.3%) | K562 | - | chr20 | | 34638588 | 34638671 |
| 2.80331914555 | PCBP1 (bg=2.3%) | K562 | - | chr20 | | 34638591 | 34638664 |
| 3.06789113969 | PCBP1 (bg=2.3%) | HepG2 | - | chr20 | | 34638595 | 34638657 |
| 5.07003205176 | pum1 (bg=29.85%) | K562 | - | chr20 | | 34638591 | 34638658 |
| 4.87487192901 | pum2 (bg=21.55%) | K562 | - | chr20 | | 34638601 | 34638650 |
| 2.40138566506 | rbm15 (bg=11.81%) | HepG2 | - | chr20 | | 34638590 | 34638664 |
| 2.51067342866 | rbm15 (bg=11.81%) | K562 | - | chr20 | | 34638601 | 34638662 |
| 2.46046205374 | rbm15 (bg=11.81%) | K562 | - | chr20 | | 34638601 | 34638665 |
| 4.21073815409 | rbm15 (bg=11.81%) | HepG2 | - | chr20 | | 34638652 | 34638730 |
| 2.54069652736 | SDAD1 (bg=5.99%) | K562 | - | chr20 | | 34638589 | 34638681 |
| 2.27293773084 | SDAD1 (bg=5.99%) | HepG2 | - | chr20 | | 34638636 | 34638838 |
| 3.38368334849 | WRN (bg=3.31%) | K562 | - | chr20 | | 34638605 | 34638644 |

  
  

| Match 27 in HUMAN | | | | | | | |
| --- | --- | --- | --- | --- | --- | --- | --- |
| Motif | Start in Seq (1 Indexed) | End in Seq (1 Indexed) | Strand | Chrm | Exon | Start in Chrm (0 Indexed) | End in Chrm (1 Indexed) |
| ATGGCGGC | 293 | 300 | - | chr20 | 1 | 34638632 | 34638640 |
| eCLIP Fold-Enrichment | Binding Protein | Cell Line | Strand | Chrm | | Start in Chrm (0 Indexed) | End in Chrm (1 Indexed) |
| 2.31060749757 | BCCIP (bg=4.61%) | HepG2 | - | chr20 | | 34638558 | 34638666 |
| 4.93017974172 | ddx3x (bg=13.89%) | HepG2 | - | chr20 | | 34638580 | 34638654 |
| 2.57192950951 | ddx3x (bg=13.89%) | K562 | - | chr20 | | 34638594 | 34638670 |
| 5.58567128521 | ddx3x (bg=13.89%) | HepG2 | - | chr20 | | 34638596 | 34638663 |
| 4.11012885549 | ddx3x (bg=13.89%) | K562 | - | chr20 | | 34638598 | 34638718 |
| 2.39207101411 | ddx6 (bg=23.92%) | HepG2 | - | chr20 | | 34638597 | 34638646 |
| 2.62342679024 | ddx6 (bg=23.92%) | HepG2 | - | chr20 | | 34638609 | 34638661 |
| 2.60597642722 | dgcr8 (bg=19.31%) | K562 | - | chr20 | | 34638582 | 34638744 |
| 2.54302009491 | dgcr8 (bg=19.31%) | HepG2 | - | chr20 | | 34638587 | 34638667 |
| 2.12738187506 | DHX30 (bg=5.05%) | K562 | - | chr20 | | 34638594 | 34638647 |
| 2.50731811978 | DHX30 (bg=5.05%) | K562 | - | chr20 | | 34638602 | 34638653 |
| 2.45675630989 | drosha (bg=11.37%) | HepG2 | - | chr20 | | 34638571 | 34638672 |
| 2.45675630989 | drosha (bg=11.37%) | HepG2 | - | chr20 | | 34638571 | 34638672 |
| 2.52180683689 | EIF3H (bg=7.83%) | HepG2 | - | chr20 | | 34638510 | 34638656 |
| 2.34680713545 | FMR1 (bg=6.3%) | K562 | - | chr20 | | 34638590 | 34638657 |
| 2.53069347253 | FTO (bg=7.53%) | HepG2 | - | chr20 | | 34638587 | 34638672 |
| 2.49695843524 | FTO (bg=7.53%) | HepG2 | - | chr20 | | 34638605 | 34638667 |
| 2.66010856078 | fubp3 (bg=23.31%) | HepG2 | - | chr20 | | 34638628 | 34638642 |
| 2.20135547148 | FXR2 (bg=7.51%) | K562 | - | chr20 | | 34638566 | 34638679 |
| 3.62648062829 | FXR2 (bg=7.51%) | K562 | - | chr20 | | 34638590 | 34638675 |
| 2.99902015573 | GEMIN5 (bg=2.46%) | K562 | - | chr20 | | 34638603 | 34638675 |
| 2.02549898345 | IGF2BP3 (bg=4.26%) | HepG2 | - | chr20 | | 34638575 | 34638656 |
| 2.28644826729 | METAP2 (bg=6.14%) | K562 | - | chr20 | | 34638590 | 34638666 |
| 2.54576340698 | NIP7 (bg=2.54%) | HepG2 | - | chr20 | | 34638594 | 34638679 |
| 3.36738528192 | PCBP1 (bg=2.3%) | HepG2 | - | chr20 | | 34638572 | 34638655 |
| 3.59076180613 | PCBP1 (bg=2.3%) | K562 | - | chr20 | | 34638588 | 34638671 |
| 2.80331914555 | PCBP1 (bg=2.3%) | K562 | - | chr20 | | 34638591 | 34638664 |
| 3.06789113969 | PCBP1 (bg=2.3%) | HepG2 | - | chr20 | | 34638595 | 34638657 |
| 5.07003205176 | pum1 (bg=29.85%) | K562 | - | chr20 | | 34638591 | 34638658 |
| 4.87487192901 | pum2 (bg=21.55%) | K562 | - | chr20 | | 34638601 | 34638650 |
| 2.40138566506 | rbm15 (bg=11.81%) | HepG2 | - | chr20 | | 34638590 | 34638664 |
| 2.51067342866 | rbm15 (bg=11.81%) | K562 | - | chr20 | | 34638601 | 34638662 |
| 2.46046205374 | rbm15 (bg=11.81%) | K562 | - | chr20 | | 34638601 | 34638665 |
| 2.54069652736 | SDAD1 (bg=5.99%) | K562 | - | chr20 | | 34638589 | 34638681 |
| 2.27293773084 | SDAD1 (bg=5.99%) | HepG2 | - | chr20 | | 34638636 | 34638838 |
| 3.38368334849 | WRN (bg=3.31%) | K562 | - | chr20 | | 34638605 | 34638644 |

  
  

| Match 28 in HUMAN | | | | | | | |
| --- | --- | --- | --- | --- | --- | --- | --- |
| Motif | Start in Seq (1 Indexed) | End in Seq (1 Indexed) | Strand | Chrm | Exon | Start in Chrm (0 Indexed) | End in Chrm (1 Indexed) |
| TCTCCCAGCCCGGACCC | 308 | 324 | - | chr20 | 1 | 34638608 | 34638625 |
| eCLIP Fold-Enrichment | Binding Protein | Cell Line | Strand | Chrm | | Start in Chrm (0 Indexed) | End in Chrm (1 Indexed) |
| 2.31060749757 | BCCIP (bg=4.61%) | HepG2 | - | chr20 | | 34638558 | 34638666 |
| 4.93017974172 | ddx3x (bg=13.89%) | HepG2 | - | chr20 | | 34638580 | 34638654 |
| 2.57192950951 | ddx3x (bg=13.89%) | K562 | - | chr20 | | 34638594 | 34638670 |
| 5.58567128521 | ddx3x (bg=13.89%) | HepG2 | - | chr20 | | 34638596 | 34638663 |
| 4.11012885549 | ddx3x (bg=13.89%) | K562 | - | chr20 | | 34638598 | 34638718 |
| 2.39207101411 | ddx6 (bg=23.92%) | HepG2 | - | chr20 | | 34638597 | 34638646 |
| 2.62342679024 | ddx6 (bg=23.92%) | HepG2 | - | chr20 | | 34638609 | 34638661 |
| 2.60597642722 | dgcr8 (bg=19.31%) | K562 | - | chr20 | | 34638582 | 34638744 |
| 2.54302009491 | dgcr8 (bg=19.31%) | HepG2 | - | chr20 | | 34638587 | 34638667 |
| 2.12738187506 | DHX30 (bg=5.05%) | K562 | - | chr20 | | 34638594 | 34638647 |
| 2.50731811978 | DHX30 (bg=5.05%) | K562 | - | chr20 | | 34638602 | 34638653 |
| 2.45675630989 | drosha (bg=11.37%) | HepG2 | - | chr20 | | 34638571 | 34638672 |
| 2.45675630989 | drosha (bg=11.37%) | HepG2 | - | chr20 | | 34638571 | 34638672 |
| 2.52180683689 | EIF3H (bg=7.83%) | HepG2 | - | chr20 | | 34638510 | 34638656 |
| 2.34680713545 | FMR1 (bg=6.3%) | K562 | - | chr20 | | 34638590 | 34638657 |
| 2.53069347253 | FTO (bg=7.53%) | HepG2 | - | chr20 | | 34638587 | 34638672 |
| 2.49695843524 | FTO (bg=7.53%) | HepG2 | - | chr20 | | 34638605 | 34638667 |
| 2.20135547148 | FXR2 (bg=7.51%) | K562 | - | chr20 | | 34638566 | 34638679 |
| 3.62648062829 | FXR2 (bg=7.51%) | K562 | - | chr20 | | 34638590 | 34638675 |
| 2.99902015573 | GEMIN5 (bg=2.46%) | K562 | - | chr20 | | 34638603 | 34638675 |
| 2.02549898345 | IGF2BP3 (bg=4.26%) | HepG2 | - | chr20 | | 34638575 | 34638656 |
| 2.28644826729 | METAP2 (bg=6.14%) | K562 | - | chr20 | | 34638590 | 34638666 |
| 2.54576340698 | NIP7 (bg=2.54%) | HepG2 | - | chr20 | | 34638594 | 34638679 |
| 3.36738528192 | PCBP1 (bg=2.3%) | HepG2 | - | chr20 | | 34638572 | 34638655 |
| 3.59076180613 | PCBP1 (bg=2.3%) | K562 | - | chr20 | | 34638588 | 34638671 |
| 2.80331914555 | PCBP1 (bg=2.3%) | K562 | - | chr20 | | 34638591 | 34638664 |
| 3.06789113969 | PCBP1 (bg=2.3%) | HepG2 | - | chr20 | | 34638595 | 34638657 |
| 5.07003205176 | pum1 (bg=29.85%) | K562 | - | chr20 | | 34638591 | 34638658 |
| 4.87487192901 | pum2 (bg=21.55%) | K562 | - | chr20 | | 34638601 | 34638650 |
| 2.40138566506 | rbm15 (bg=11.81%) | HepG2 | - | chr20 | | 34638590 | 34638664 |
| 2.51067342866 | rbm15 (bg=11.81%) | K562 | - | chr20 | | 34638601 | 34638662 |
| 2.46046205374 | rbm15 (bg=11.81%) | K562 | - | chr20 | | 34638601 | 34638665 |
| 2.54069652736 | SDAD1 (bg=5.99%) | K562 | - | chr20 | | 34638589 | 34638681 |
| 3.38368334849 | WRN (bg=3.31%) | K562 | - | chr20 | | 34638605 | 34638644 |

  
  

| Match 29 in HUMAN | | | | | | | |
| --- | --- | --- | --- | --- | --- | --- | --- |
| Motif | Start in Seq (1 Indexed) | End in Seq (1 Indexed) | Strand | Chrm | Exon | Start in Chrm (0 Indexed) | End in Chrm (1 Indexed) |
| GCCGGC | 326 | 331 | - | chr20 | 1 | 34638601 | 34638607 |
| eCLIP Fold-Enrichment | Binding Protein | Cell Line | Strand | Chrm | | Start in Chrm (0 Indexed) | End in Chrm (1 Indexed) |
| 2.31060749757 | BCCIP (bg=4.61%) | HepG2 | - | chr20 | | 34638558 | 34638666 |
| 4.93017974172 | ddx3x (bg=13.89%) | HepG2 | - | chr20 | | 34638580 | 34638654 |
| 2.57192950951 | ddx3x (bg=13.89%) | K562 | - | chr20 | | 34638594 | 34638670 |
| 5.58567128521 | ddx3x (bg=13.89%) | HepG2 | - | chr20 | | 34638596 | 34638663 |
| 4.11012885549 | ddx3x (bg=13.89%) | K562 | - | chr20 | | 34638598 | 34638718 |
| 2.39207101411 | ddx6 (bg=23.92%) | HepG2 | - | chr20 | | 34638597 | 34638646 |
| 2.60597642722 | dgcr8 (bg=19.31%) | K562 | - | chr20 | | 34638582 | 34638744 |
| 2.54302009491 | dgcr8 (bg=19.31%) | HepG2 | - | chr20 | | 34638587 | 34638667 |
| 2.12738187506 | DHX30 (bg=5.05%) | K562 | - | chr20 | | 34638594 | 34638647 |
| 2.50731811978 | DHX30 (bg=5.05%) | K562 | - | chr20 | | 34638602 | 34638653 |
| 2.45675630989 | drosha (bg=11.37%) | HepG2 | - | chr20 | | 34638571 | 34638672 |
| 2.45675630989 | drosha (bg=11.37%) | HepG2 | - | chr20 | | 34638571 | 34638672 |
| 2.52180683689 | EIF3H (bg=7.83%) | HepG2 | - | chr20 | | 34638510 | 34638656 |
| 2.34680713545 | FMR1 (bg=6.3%) | K562 | - | chr20 | | 34638590 | 34638657 |
| 2.53069347253 | FTO (bg=7.53%) | HepG2 | - | chr20 | | 34638587 | 34638672 |
| 2.49695843524 | FTO (bg=7.53%) | HepG2 | - | chr20 | | 34638605 | 34638667 |
| 2.20135547148 | FXR2 (bg=7.51%) | K562 | - | chr20 | | 34638566 | 34638679 |
| 3.62648062829 | FXR2 (bg=7.51%) | K562 | - | chr20 | | 34638590 | 34638675 |
| 2.99902015573 | GEMIN5 (bg=2.46%) | K562 | - | chr20 | | 34638603 | 34638675 |
| 2.02549898345 | IGF2BP3 (bg=4.26%) | HepG2 | - | chr20 | | 34638575 | 34638656 |
| 2.28644826729 | METAP2 (bg=6.14%) | K562 | - | chr20 | | 34638590 | 34638666 |
| 2.54576340698 | NIP7 (bg=2.54%) | HepG2 | - | chr20 | | 34638594 | 34638679 |
| 3.36738528192 | PCBP1 (bg=2.3%) | HepG2 | - | chr20 | | 34638572 | 34638655 |
| 3.59076180613 | PCBP1 (bg=2.3%) | K562 | - | chr20 | | 34638588 | 34638671 |
| 2.80331914555 | PCBP1 (bg=2.3%) | K562 | - | chr20 | | 34638591 | 34638664 |
| 3.06789113969 | PCBP1 (bg=2.3%) | HepG2 | - | chr20 | | 34638595 | 34638657 |
| 5.07003205176 | pum1 (bg=29.85%) | K562 | - | chr20 | | 34638591 | 34638658 |
| 4.87487192901 | pum2 (bg=21.55%) | K562 | - | chr20 | | 34638601 | 34638650 |
| 2.40138566506 | rbm15 (bg=11.81%) | HepG2 | - | chr20 | | 34638590 | 34638664 |
| 2.51067342866 | rbm15 (bg=11.81%) | K562 | - | chr20 | | 34638601 | 34638662 |
| 2.46046205374 | rbm15 (bg=11.81%) | K562 | - | chr20 | | 34638601 | 34638665 |
| 2.54069652736 | SDAD1 (bg=5.99%) | K562 | - | chr20 | | 34638589 | 34638681 |
| 3.38368334849 | WRN (bg=3.31%) | K562 | - | chr20 | | 34638605 | 34638644 |

  
  

| Match 30 in HUMAN | | | | | | | |
| --- | --- | --- | --- | --- | --- | --- | --- |
| Motif | Start in Seq (1 Indexed) | End in Seq (1 Indexed) | Strand | Chrm | Exon | Start in Chrm (0 Indexed) | End in Chrm (1 Indexed) |
| CCGGGTCTCCCG | 334 | 345 | - | chr20 | 1 | 34638587 | 34638599 |
| eCLIP Fold-Enrichment | Binding Protein | Cell Line | Strand | Chrm | | Start in Chrm (0 Indexed) | End in Chrm (1 Indexed) |
| 2.31060749757 | BCCIP (bg=4.61%) | HepG2 | - | chr20 | | 34638558 | 34638666 |
| 5.03112163667 | ddx3x (bg=13.89%) | HepG2 | - | chr20 | | 34638573 | 34638596 |
| 4.93017974172 | ddx3x (bg=13.89%) | HepG2 | - | chr20 | | 34638580 | 34638654 |
| 2.57192950951 | ddx3x (bg=13.89%) | K562 | - | chr20 | | 34638594 | 34638670 |
| 5.58567128521 | ddx3x (bg=13.89%) | HepG2 | - | chr20 | | 34638596 | 34638663 |
| 4.11012885549 | ddx3x (bg=13.89%) | K562 | - | chr20 | | 34638598 | 34638718 |
| 2.39207101411 | ddx6 (bg=23.92%) | HepG2 | - | chr20 | | 34638597 | 34638646 |
| 2.60597642722 | dgcr8 (bg=19.31%) | K562 | - | chr20 | | 34638582 | 34638744 |
| 2.54302009491 | dgcr8 (bg=19.31%) | HepG2 | - | chr20 | | 34638587 | 34638667 |
| 2.12738187506 | DHX30 (bg=5.05%) | K562 | - | chr20 | | 34638594 | 34638647 |
| 2.45675630989 | drosha (bg=11.37%) | HepG2 | - | chr20 | | 34638571 | 34638672 |
| 2.45675630989 | drosha (bg=11.37%) | HepG2 | - | chr20 | | 34638571 | 34638672 |
| 2.52180683689 | EIF3H (bg=7.83%) | HepG2 | - | chr20 | | 34638510 | 34638656 |
| 2.34680713545 | FMR1 (bg=6.3%) | K562 | - | chr20 | | 34638590 | 34638657 |
| 2.53069347253 | FTO (bg=7.53%) | HepG2 | - | chr20 | | 34638587 | 34638672 |
| 2.20135547148 | FXR2 (bg=7.51%) | K562 | - | chr20 | | 34638566 | 34638679 |
| 3.62648062829 | FXR2 (bg=7.51%) | K562 | - | chr20 | | 34638590 | 34638675 |
| 2.02549898345 | IGF2BP3 (bg=4.26%) | HepG2 | - | chr20 | | 34638575 | 34638656 |
| 5.25176815835 | LARP7 (bg=0.81%) | HepG2 | - | chr20 | | 34638559 | 34638593 |
| 2.28644826729 | METAP2 (bg=6.14%) | K562 | - | chr20 | | 34638590 | 34638666 |
| 2.54576340698 | NIP7 (bg=2.54%) | HepG2 | - | chr20 | | 34638594 | 34638679 |
| 3.36738528192 | PCBP1 (bg=2.3%) | HepG2 | - | chr20 | | 34638572 | 34638655 |
| 3.59076180613 | PCBP1 (bg=2.3%) | K562 | - | chr20 | | 34638588 | 34638671 |
| 2.80331914555 | PCBP1 (bg=2.3%) | K562 | - | chr20 | | 34638591 | 34638664 |
| 3.06789113969 | PCBP1 (bg=2.3%) | HepG2 | - | chr20 | | 34638595 | 34638657 |
| 5.07003205176 | pum1 (bg=29.85%) | K562 | - | chr20 | | 34638591 | 34638658 |
| 2.40138566506 | rbm15 (bg=11.81%) | HepG2 | - | chr20 | | 34638590 | 34638664 |
| 2.54069652736 | SDAD1 (bg=5.99%) | K562 | - | chr20 | | 34638589 | 34638681 |

  
  

| Match 31 in HUMAN | | | | | | | |
| --- | --- | --- | --- | --- | --- | --- | --- |
| Motif | Start in Seq (1 Indexed) | End in Seq (1 Indexed) | Strand | Chrm | Exon | Start in Chrm (0 Indexed) | End in Chrm (1 Indexed) |
| CGGGTCTCC | 335 | 343 | - | chr20 | 1 | 34638589 | 34638598 |
| eCLIP Fold-Enrichment | Binding Protein | Cell Line | Strand | Chrm | | Start in Chrm (0 Indexed) | End in Chrm (1 Indexed) |
| 2.31060749757 | BCCIP (bg=4.61%) | HepG2 | - | chr20 | | 34638558 | 34638666 |
| 5.03112163667 | ddx3x (bg=13.89%) | HepG2 | - | chr20 | | 34638573 | 34638596 |
| 4.93017974172 | ddx3x (bg=13.89%) | HepG2 | - | chr20 | | 34638580 | 34638654 |
| 2.57192950951 | ddx3x (bg=13.89%) | K562 | - | chr20 | | 34638594 | 34638670 |
| 5.58567128521 | ddx3x (bg=13.89%) | HepG2 | - | chr20 | | 34638596 | 34638663 |
| 4.11012885549 | ddx3x (bg=13.89%) | K562 | - | chr20 | | 34638598 | 34638718 |
| 2.39207101411 | ddx6 (bg=23.92%) | HepG2 | - | chr20 | | 34638597 | 34638646 |
| 2.60597642722 | dgcr8 (bg=19.31%) | K562 | - | chr20 | | 34638582 | 34638744 |
| 2.54302009491 | dgcr8 (bg=19.31%) | HepG2 | - | chr20 | | 34638587 | 34638667 |
| 2.12738187506 | DHX30 (bg=5.05%) | K562 | - | chr20 | | 34638594 | 34638647 |
| 2.45675630989 | drosha (bg=11.37%) | HepG2 | - | chr20 | | 34638571 | 34638672 |
| 2.45675630989 | drosha (bg=11.37%) | HepG2 | - | chr20 | | 34638571 | 34638672 |
| 2.52180683689 | EIF3H (bg=7.83%) | HepG2 | - | chr20 | | 34638510 | 34638656 |
| 2.34680713545 | FMR1 (bg=6.3%) | K562 | - | chr20 | | 34638590 | 34638657 |
| 2.53069347253 | FTO (bg=7.53%) | HepG2 | - | chr20 | | 34638587 | 34638672 |
| 2.20135547148 | FXR2 (bg=7.51%) | K562 | - | chr20 | | 34638566 | 34638679 |
| 3.62648062829 | FXR2 (bg=7.51%) | K562 | - | chr20 | | 34638590 | 34638675 |
| 2.02549898345 | IGF2BP3 (bg=4.26%) | HepG2 | - | chr20 | | 34638575 | 34638656 |
| 5.25176815835 | LARP7 (bg=0.81%) | HepG2 | - | chr20 | | 34638559 | 34638593 |
| 2.28644826729 | METAP2 (bg=6.14%) | K562 | - | chr20 | | 34638590 | 34638666 |
| 2.54576340698 | NIP7 (bg=2.54%) | HepG2 | - | chr20 | | 34638594 | 34638679 |
| 3.36738528192 | PCBP1 (bg=2.3%) | HepG2 | - | chr20 | | 34638572 | 34638655 |
| 3.59076180613 | PCBP1 (bg=2.3%) | K562 | - | chr20 | | 34638588 | 34638671 |
| 2.80331914555 | PCBP1 (bg=2.3%) | K562 | - | chr20 | | 34638591 | 34638664 |
| 3.06789113969 | PCBP1 (bg=2.3%) | HepG2 | - | chr20 | | 34638595 | 34638657 |
| 5.07003205176 | pum1 (bg=29.85%) | K562 | - | chr20 | | 34638591 | 34638658 |
| 2.40138566506 | rbm15 (bg=11.81%) | HepG2 | - | chr20 | | 34638590 | 34638664 |
| 2.54069652736 | SDAD1 (bg=5.99%) | K562 | - | chr20 | | 34638589 | 34638681 |

  
  

| Match 32 in HUMAN | | | | | | | |
| --- | --- | --- | --- | --- | --- | --- | --- |
| Motif | Start in Seq (1 Indexed) | End in Seq (1 Indexed) | Strand | Chrm | Exon | Start in Chrm (0 Indexed) | End in Chrm (1 Indexed) |
| CCCAAGCCT | 347 | 355 | - | chr20 | 1 | 34638577 | 34638586 |
| eCLIP Fold-Enrichment | Binding Protein | Cell Line | Strand | Chrm | | Start in Chrm (0 Indexed) | End in Chrm (1 Indexed) |
| 2.31060749757 | BCCIP (bg=4.61%) | HepG2 | - | chr20 | | 34638558 | 34638666 |
| 5.03112163667 | ddx3x (bg=13.89%) | HepG2 | - | chr20 | | 34638573 | 34638596 |
| 4.93017974172 | ddx3x (bg=13.89%) | HepG2 | - | chr20 | | 34638580 | 34638654 |
| 2.60597642722 | dgcr8 (bg=19.31%) | K562 | - | chr20 | | 34638582 | 34638744 |
| 2.45675630989 | drosha (bg=11.37%) | HepG2 | - | chr20 | | 34638571 | 34638672 |
| 2.45675630989 | drosha (bg=11.37%) | HepG2 | - | chr20 | | 34638571 | 34638672 |
| 2.52180683689 | EIF3H (bg=7.83%) | HepG2 | - | chr20 | | 34638510 | 34638656 |
| 2.20135547148 | FXR2 (bg=7.51%) | K562 | - | chr20 | | 34638566 | 34638679 |
| 2.12000705475 | GRWD1 (bg=4.85%) | HepG2 | - | chr20 | | 34638408 | 34638584 |
| 2.02549898345 | IGF2BP3 (bg=4.26%) | HepG2 | - | chr20 | | 34638575 | 34638656 |
| 5.25176815835 | LARP7 (bg=0.81%) | HepG2 | - | chr20 | | 34638559 | 34638593 |
| 3.36738528192 | PCBP1 (bg=2.3%) | HepG2 | - | chr20 | | 34638572 | 34638655 |
| 2.64927400746 | ybx3 (bg=22.82%) | K562 | - | chr20 | | 34638521 | 34638584 |

  
  

| Match 33 in HUMAN | | | | | | | |
| --- | --- | --- | --- | --- | --- | --- | --- |
| Motif | Start in Seq (1 Indexed) | End in Seq (1 Indexed) | Strand | Chrm | Exon | Start in Chrm (0 Indexed) | End in Chrm (1 Indexed) |
| ACGAAACCCCCGCAGAGCCGCCGGGACGCAGCGC | 358 | 391 | - | chr20 | 1 | 34638541 | 34638575 |
| eCLIP Fold-Enrichment | Binding Protein | Cell Line | Strand | Chrm | | Start in Chrm (0 Indexed) | End in Chrm (1 Indexed) |
| 2.31060749757 | BCCIP (bg=4.61%) | HepG2 | - | chr20 | | 34638558 | 34638666 |
| 4.98934278401 | ddx3x (bg=13.89%) | HepG2 | - | chr20 | | 34638478 | 34638557 |
| 6.09332805976 | ddx3x (bg=13.89%) | HepG2 | - | chr20 | | 34638523 | 34638542 |
| 5.03209676008 | ddx3x (bg=13.89%) | HepG2 | - | chr20 | | 34638542 | 34638573 |
| 5.03112163667 | ddx3x (bg=13.89%) | HepG2 | - | chr20 | | 34638573 | 34638596 |
| 2.7026767609 | dgcr8 (bg=19.31%) | HepG2 | - | chr20 | | 34638555 | 34638558 |
| 2.40381580631 | DHX30 (bg=5.05%) | HepG2 | - | chr20 | | 34638449 | 34638562 |
| 2.30079198364 | drosha (bg=11.37%) | HepG2 | - | chr20 | | 34638407 | 34638544 |
| 2.30079198364 | drosha (bg=11.37%) | HepG2 | - | chr20 | | 34638407 | 34638544 |
| 2.73359651524 | drosha (bg=11.37%) | HepG2 | - | chr20 | | 34638434 | 34638559 |
| 2.73359651524 | drosha (bg=11.37%) | HepG2 | - | chr20 | | 34638434 | 34638559 |
| 2.45675630989 | drosha (bg=11.37%) | HepG2 | - | chr20 | | 34638571 | 34638672 |
| 2.45675630989 | drosha (bg=11.37%) | HepG2 | - | chr20 | | 34638571 | 34638672 |
| 2.52921178508 | EIF3H (bg=7.83%) | HepG2 | - | chr20 | | 34638481 | 34638563 |
| 2.52180683689 | EIF3H (bg=7.83%) | HepG2 | - | chr20 | | 34638510 | 34638656 |
| 2.01423179637 | FMR1 (bg=6.3%) | K562 | - | chr20 | | 34638502 | 34638565 |
| 2.76418360275 | FTO (bg=7.53%) | HepG2 | - | chr20 | | 34638477 | 34638557 |
| 3.08192093596 | FTO (bg=7.53%) | HepG2 | - | chr20 | | 34638481 | 34638549 |
| 2.70665097697 | FXR2 (bg=7.51%) | K562 | - | chr20 | | 34638474 | 34638569 |
| 2.20135547148 | FXR2 (bg=7.51%) | K562 | - | chr20 | | 34638566 | 34638679 |
| 2.12000705475 | GRWD1 (bg=4.85%) | HepG2 | - | chr20 | | 34638408 | 34638584 |
| 2.02549898345 | IGF2BP3 (bg=4.26%) | HepG2 | - | chr20 | | 34638575 | 34638656 |
| 5.25176815835 | LARP7 (bg=0.81%) | HepG2 | - | chr20 | | 34638549 | 34638559 |
| 5.25176815835 | LARP7 (bg=0.81%) | HepG2 | - | chr20 | | 34638559 | 34638593 |
| 3.36738528192 | PCBP1 (bg=2.3%) | HepG2 | - | chr20 | | 34638572 | 34638655 |
| 4.72093288641 | rbm15 (bg=11.81%) | HepG2 | - | chr20 | | 34638443 | 34638542 |
| 2.61758863258 | rbm15 (bg=11.81%) | K562 | - | chr20 | | 34638542 | 34638558 |
| 3.08035714764 | SDAD1 (bg=5.99%) | HepG2 | - | chr20 | | 34638494 | 34638558 |
| 2.64927400746 | ybx3 (bg=22.82%) | K562 | - | chr20 | | 34638521 | 34638584 |
| 2.43143036609 | ybx3 (bg=22.82%) | K562 | - | chr20 | | 34638522 | 34638570 |

  
  

| Match 34 in HUMAN | | | | | | | |
| --- | --- | --- | --- | --- | --- | --- | --- |
| Motif | Start in Seq (1 Indexed) | End in Seq (1 Indexed) | Strand | Chrm | Exon | Start in Chrm (0 Indexed) | End in Chrm (1 Indexed) |
| TTTGGGC | 393 | 399 | - | chr20 | 1 | 34638533 | 34638540 |
| eCLIP Fold-Enrichment | Binding Protein | Cell Line | Strand | Chrm | | Start in Chrm (0 Indexed) | End in Chrm (1 Indexed) |
| 4.98934278401 | ddx3x (bg=13.89%) | HepG2 | - | chr20 | | 34638478 | 34638557 |
| 6.09332805976 | ddx3x (bg=13.89%) | HepG2 | - | chr20 | | 34638523 | 34638542 |
| 2.40381580631 | DHX30 (bg=5.05%) | HepG2 | - | chr20 | | 34638449 | 34638562 |
| 2.30079198364 | drosha (bg=11.37%) | HepG2 | - | chr20 | | 34638407 | 34638544 |
| 2.30079198364 | drosha (bg=11.37%) | HepG2 | - | chr20 | | 34638407 | 34638544 |
| 2.73359651524 | drosha (bg=11.37%) | HepG2 | - | chr20 | | 34638434 | 34638559 |
| 2.73359651524 | drosha (bg=11.37%) | HepG2 | - | chr20 | | 34638434 | 34638559 |
| 2.52921178508 | EIF3H (bg=7.83%) | HepG2 | - | chr20 | | 34638481 | 34638563 |
| 2.52180683689 | EIF3H (bg=7.83%) | HepG2 | - | chr20 | | 34638510 | 34638656 |
| 2.01423179637 | FMR1 (bg=6.3%) | K562 | - | chr20 | | 34638502 | 34638565 |
| 2.76418360275 | FTO (bg=7.53%) | HepG2 | - | chr20 | | 34638477 | 34638557 |
| 3.08192093596 | FTO (bg=7.53%) | HepG2 | - | chr20 | | 34638481 | 34638549 |
| 2.70665097697 | FXR2 (bg=7.51%) | K562 | - | chr20 | | 34638474 | 34638569 |
| 2.12000705475 | GRWD1 (bg=4.85%) | HepG2 | - | chr20 | | 34638408 | 34638584 |
| 3.0655399198 | METAP2 (bg=6.14%) | K562 | - | chr20 | | 34638454 | 34638537 |
| 4.72093288641 | rbm15 (bg=11.81%) | HepG2 | - | chr20 | | 34638443 | 34638542 |
| 3.59159342405 | rbm15 (bg=11.81%) | K562 | - | chr20 | | 34638492 | 34638540 |
| 3.6323400999 | rbm15 (bg=11.81%) | HepG2 | - | chr20 | | 34638525 | 34638538 |
| 4.76021335779 | rbm15 (bg=11.81%) | K562 | - | chr20 | | 34638525 | 34638540 |
| 3.08035714764 | SDAD1 (bg=5.99%) | HepG2 | - | chr20 | | 34638494 | 34638558 |
| 2.04832617426 | TBRG4 (bg=7.0%) | HepG2 | - | chr20 | | 34638474 | 34638534 |
| 2.64927400746 | ybx3 (bg=22.82%) | K562 | - | chr20 | | 34638521 | 34638584 |
| 2.43143036609 | ybx3 (bg=22.82%) | K562 | - | chr20 | | 34638522 | 34638570 |

  
  

| Match 35 in HUMAN | | | | | | | |
| --- | --- | --- | --- | --- | --- | --- | --- |
| Motif | Start in Seq (1 Indexed) | End in Seq (1 Indexed) | Strand | Chrm | Exon | Start in Chrm (0 Indexed) | End in Chrm (1 Indexed) |
| GGGCGTGG | 406 | 413 | - | chr20 | 1 | 34638519 | 34638527 |
| eCLIP Fold-Enrichment | Binding Protein | Cell Line | Strand | Chrm | | Start in Chrm (0 Indexed) | End in Chrm (1 Indexed) |
| 4.98934278401 | ddx3x (bg=13.89%) | HepG2 | - | chr20 | | 34638478 | 34638557 |
| 6.46264725873 | ddx3x (bg=13.89%) | HepG2 | - | chr20 | | 34638505 | 34638523 |
| 6.09332805976 | ddx3x (bg=13.89%) | HepG2 | - | chr20 | | 34638523 | 34638542 |
| 2.40381580631 | DHX30 (bg=5.05%) | HepG2 | - | chr20 | | 34638449 | 34638562 |
| 2.19632922878 | DHX30 (bg=5.05%) | K562 | - | chr20 | | 34638471 | 34638525 |
| 2.49524528748 | DHX30 (bg=5.05%) | K562 | - | chr20 | | 34638472 | 34638521 |
| 2.30079198364 | drosha (bg=11.37%) | HepG2 | - | chr20 | | 34638407 | 34638544 |
| 2.30079198364 | drosha (bg=11.37%) | HepG2 | - | chr20 | | 34638407 | 34638544 |
| 2.73359651524 | drosha (bg=11.37%) | HepG2 | - | chr20 | | 34638434 | 34638559 |
| 2.73359651524 | drosha (bg=11.37%) | HepG2 | - | chr20 | | 34638434 | 34638559 |
| 2.91783553994 | drosha (bg=11.37%) | K562 | - | chr20 | | 34638513 | 34638525 |
| 2.52921178508 | EIF3H (bg=7.83%) | HepG2 | - | chr20 | | 34638481 | 34638563 |
| 2.52180683689 | EIF3H (bg=7.83%) | HepG2 | - | chr20 | | 34638510 | 34638656 |
| 2.01423179637 | FMR1 (bg=6.3%) | K562 | - | chr20 | | 34638502 | 34638565 |
| 2.76418360275 | FTO (bg=7.53%) | HepG2 | - | chr20 | | 34638477 | 34638557 |
| 3.08192093596 | FTO (bg=7.53%) | HepG2 | - | chr20 | | 34638481 | 34638549 |
| 2.70665097697 | FXR2 (bg=7.51%) | K562 | - | chr20 | | 34638474 | 34638569 |
| 2.12000705475 | GRWD1 (bg=4.85%) | HepG2 | - | chr20 | | 34638408 | 34638584 |
| 3.0655399198 | METAP2 (bg=6.14%) | K562 | - | chr20 | | 34638454 | 34638537 |
| 2.63978465706 | NIPBL (bg=2.83%) | K562 | - | chr20 | | 34638435 | 34638525 |
| 4.72093288641 | rbm15 (bg=11.81%) | HepG2 | - | chr20 | | 34638443 | 34638542 |
| 4.19868692245 | rbm15 (bg=11.81%) | HepG2 | - | chr20 | | 34638491 | 34638525 |
| 3.59159342405 | rbm15 (bg=11.81%) | K562 | - | chr20 | | 34638492 | 34638540 |
| 4.52267745019 | rbm15 (bg=11.81%) | K562 | - | chr20 | | 34638503 | 34638525 |
| 3.6323400999 | rbm15 (bg=11.81%) | HepG2 | - | chr20 | | 34638525 | 34638538 |
| 4.76021335779 | rbm15 (bg=11.81%) | K562 | - | chr20 | | 34638525 | 34638540 |
| 3.08035714764 | SDAD1 (bg=5.99%) | HepG2 | - | chr20 | | 34638494 | 34638558 |
| 2.16104423517 | sf3a3 (bg=12.13%) | HepG2 | - | chr20 | | 34638456 | 34638527 |
| 2.04622054079 | SLTM (bg=1.1%) | K562 | - | chr20 | | 34638460 | 34638520 |
| 2.03501246429 | SRSF1 (bg=2.06%) | K562 | - | chr20 | | 34638415 | 34638527 |
| 2.04832617426 | TBRG4 (bg=7.0%) | HepG2 | - | chr20 | | 34638474 | 34638534 |
| 3.47213667643 | UTP3 (bg=2.81%) | K562 | - | chr20 | | 34638508 | 34638531 |
| 2.70754372143 | WDR43 (bg=0.9%) | HepG2 | - | chr20 | | 34638479 | 34638528 |
| 3.11875929076 | ybx3 (bg=22.82%) | K562 | - | chr20 | | 34638475 | 34638521 |
| 3.36431617023 | ybx3 (bg=22.82%) | K562 | - | chr20 | | 34638495 | 34638522 |
| 2.64927400746 | ybx3 (bg=22.82%) | K562 | - | chr20 | | 34638521 | 34638584 |
| 2.43143036609 | ybx3 (bg=22.82%) | K562 | - | chr20 | | 34638522 | 34638570 |

  
  

| Match 36 in HUMAN | | | | | | | |
| --- | --- | --- | --- | --- | --- | --- | --- |
| Motif | Start in Seq (1 Indexed) | End in Seq (1 Indexed) | Strand | Chrm | Exon | Start in Chrm (0 Indexed) | End in Chrm (1 Indexed) |
| GGGCCGGGAAG | 415 | 425 | - | chr20 | 1 | 34638507 | 34638518 |
| eCLIP Fold-Enrichment | Binding Protein | Cell Line | Strand | Chrm | | Start in Chrm (0 Indexed) | End in Chrm (1 Indexed) |
| 4.98934278401 | ddx3x (bg=13.89%) | HepG2 | - | chr20 | | 34638478 | 34638557 |
| 6.46264725873 | ddx3x (bg=13.89%) | HepG2 | - | chr20 | | 34638505 | 34638523 |
| 2.04772932362 | DHX30 (bg=5.05%) | HepG2 | - | chr20 | | 34638417 | 34638507 |
| 2.40381580631 | DHX30 (bg=5.05%) | HepG2 | - | chr20 | | 34638449 | 34638562 |
| 2.19632922878 | DHX30 (bg=5.05%) | K562 | - | chr20 | | 34638471 | 34638525 |
| 2.49524528748 | DHX30 (bg=5.05%) | K562 | - | chr20 | | 34638472 | 34638521 |
| 2.30079198364 | drosha (bg=11.37%) | HepG2 | - | chr20 | | 34638407 | 34638544 |
| 2.30079198364 | drosha (bg=11.37%) | HepG2 | - | chr20 | | 34638407 | 34638544 |
| 2.73359651524 | drosha (bg=11.37%) | HepG2 | - | chr20 | | 34638434 | 34638559 |
| 2.73359651524 | drosha (bg=11.37%) | HepG2 | - | chr20 | | 34638434 | 34638559 |
| 2.91783553994 | drosha (bg=11.37%) | K562 | - | chr20 | | 34638513 | 34638525 |
| 2.52921178508 | EIF3H (bg=7.83%) | HepG2 | - | chr20 | | 34638481 | 34638563 |
| 2.52180683689 | EIF3H (bg=7.83%) | HepG2 | - | chr20 | | 34638510 | 34638656 |
| 2.01423179637 | FMR1 (bg=6.3%) | K562 | - | chr20 | | 34638502 | 34638565 |
| 2.76418360275 | FTO (bg=7.53%) | HepG2 | - | chr20 | | 34638477 | 34638557 |
| 3.08192093596 | FTO (bg=7.53%) | HepG2 | - | chr20 | | 34638481 | 34638549 |
| 2.70665097697 | FXR2 (bg=7.51%) | K562 | - | chr20 | | 34638474 | 34638569 |
| 2.12000705475 | GRWD1 (bg=4.85%) | HepG2 | - | chr20 | | 34638408 | 34638584 |
| 3.0655399198 | METAP2 (bg=6.14%) | K562 | - | chr20 | | 34638454 | 34638537 |
| 3.60837636218 | METAP2 (bg=6.14%) | K562 | - | chr20 | | 34638471 | 34638512 |
| 2.63978465706 | NIPBL (bg=2.83%) | K562 | - | chr20 | | 34638435 | 34638525 |
| 4.72093288641 | rbm15 (bg=11.81%) | HepG2 | - | chr20 | | 34638443 | 34638542 |
| 4.19868692245 | rbm15 (bg=11.81%) | HepG2 | - | chr20 | | 34638491 | 34638525 |
| 3.59159342405 | rbm15 (bg=11.81%) | K562 | - | chr20 | | 34638492 | 34638540 |
| 4.52267745019 | rbm15 (bg=11.81%) | K562 | - | chr20 | | 34638503 | 34638525 |
| 3.08035714764 | SDAD1 (bg=5.99%) | HepG2 | - | chr20 | | 34638494 | 34638558 |
| 2.16104423517 | sf3a3 (bg=12.13%) | HepG2 | - | chr20 | | 34638456 | 34638527 |
| 2.04622054079 | SLTM (bg=1.1%) | K562 | - | chr20 | | 34638460 | 34638520 |
| 2.03501246429 | SRSF1 (bg=2.06%) | K562 | - | chr20 | | 34638415 | 34638527 |
| 2.04832617426 | TBRG4 (bg=7.0%) | HepG2 | - | chr20 | | 34638474 | 34638534 |
| 2.23948572672 | TRA2A (bg=1.38%) | HepG2 | - | chr20 | | 34638432 | 34638507 |
| 3.2091022706 | UTP3 (bg=2.81%) | K562 | - | chr20 | | 34638500 | 34638508 |
| 3.47213667643 | UTP3 (bg=2.81%) | K562 | - | chr20 | | 34638508 | 34638531 |
| 2.70754372143 | WDR43 (bg=0.9%) | HepG2 | - | chr20 | | 34638479 | 34638528 |
| 3.11875929076 | ybx3 (bg=22.82%) | K562 | - | chr20 | | 34638475 | 34638521 |
| 3.36431617023 | ybx3 (bg=22.82%) | K562 | - | chr20 | | 34638495 | 34638522 |

  
  

| Match 37 in HUMAN | | | | | | | |
| --- | --- | --- | --- | --- | --- | --- | --- |
| Motif | Start in Seq (1 Indexed) | End in Seq (1 Indexed) | Strand | Chrm | Exon | Start in Chrm (0 Indexed) | End in Chrm (1 Indexed) |
| ATGGCGGC | 427 | 434 | - | chr20 | 1 | 34638498 | 34638506 |
| eCLIP Fold-Enrichment | Binding Protein | Cell Line | Strand | Chrm | | Start in Chrm (0 Indexed) | End in Chrm (1 Indexed) |
| 4.98934278401 | ddx3x (bg=13.89%) | HepG2 | - | chr20 | | 34638478 | 34638557 |
| 5.78457535362 | ddx3x (bg=13.89%) | HepG2 | - | chr20 | | 34638488 | 34638505 |
| 6.46264725873 | ddx3x (bg=13.89%) | HepG2 | - | chr20 | | 34638505 | 34638523 |
| 2.04772932362 | DHX30 (bg=5.05%) | HepG2 | - | chr20 | | 34638417 | 34638507 |
| 2.40381580631 | DHX30 (bg=5.05%) | HepG2 | - | chr20 | | 34638449 | 34638562 |
| 2.19632922878 | DHX30 (bg=5.05%) | K562 | - | chr20 | | 34638471 | 34638525 |
| 2.49524528748 | DHX30 (bg=5.05%) | K562 | - | chr20 | | 34638472 | 34638521 |
| 2.30079198364 | drosha (bg=11.37%) | HepG2 | - | chr20 | | 34638407 | 34638544 |
| 2.30079198364 | drosha (bg=11.37%) | HepG2 | - | chr20 | | 34638407 | 34638544 |
| 2.73359651524 | drosha (bg=11.37%) | HepG2 | - | chr20 | | 34638434 | 34638559 |
| 2.73359651524 | drosha (bg=11.37%) | HepG2 | - | chr20 | | 34638434 | 34638559 |
| 2.52921178508 | EIF3H (bg=7.83%) | HepG2 | - | chr20 | | 34638481 | 34638563 |
| 2.01423179637 | FMR1 (bg=6.3%) | K562 | - | chr20 | | 34638502 | 34638565 |
| 2.76418360275 | FTO (bg=7.53%) | HepG2 | - | chr20 | | 34638477 | 34638557 |
| 3.08192093596 | FTO (bg=7.53%) | HepG2 | - | chr20 | | 34638481 | 34638549 |
| 2.70665097697 | FXR2 (bg=7.51%) | K562 | - | chr20 | | 34638474 | 34638569 |
| 2.12000705475 | GRWD1 (bg=4.85%) | HepG2 | - | chr20 | | 34638408 | 34638584 |
| 3.0655399198 | METAP2 (bg=6.14%) | K562 | - | chr20 | | 34638454 | 34638537 |
| 3.60837636218 | METAP2 (bg=6.14%) | K562 | - | chr20 | | 34638471 | 34638512 |
| 2.63978465706 | NIPBL (bg=2.83%) | K562 | - | chr20 | | 34638435 | 34638525 |
| 4.72093288641 | rbm15 (bg=11.81%) | HepG2 | - | chr20 | | 34638443 | 34638542 |
| 3.21299295106 | rbm15 (bg=11.81%) | K562 | - | chr20 | | 34638481 | 34638503 |
| 4.19868692245 | rbm15 (bg=11.81%) | HepG2 | - | chr20 | | 34638491 | 34638525 |
| 3.59159342405 | rbm15 (bg=11.81%) | K562 | - | chr20 | | 34638492 | 34638540 |
| 4.52267745019 | rbm15 (bg=11.81%) | K562 | - | chr20 | | 34638503 | 34638525 |
| 3.08035714764 | SDAD1 (bg=5.99%) | HepG2 | - | chr20 | | 34638494 | 34638558 |
| 2.16104423517 | sf3a3 (bg=12.13%) | HepG2 | - | chr20 | | 34638456 | 34638527 |
| 2.04622054079 | SLTM (bg=1.1%) | K562 | - | chr20 | | 34638460 | 34638520 |
| 2.03501246429 | SRSF1 (bg=2.06%) | K562 | - | chr20 | | 34638415 | 34638527 |
| 2.04832617426 | TBRG4 (bg=7.0%) | HepG2 | - | chr20 | | 34638474 | 34638534 |
| 2.23948572672 | TRA2A (bg=1.38%) | HepG2 | - | chr20 | | 34638432 | 34638507 |
| 3.2091022706 | UTP3 (bg=2.81%) | K562 | - | chr20 | | 34638500 | 34638508 |
| 2.70754372143 | WDR43 (bg=0.9%) | HepG2 | - | chr20 | | 34638479 | 34638528 |
| 3.11875929076 | ybx3 (bg=22.82%) | K562 | - | chr20 | | 34638475 | 34638521 |
| 3.36431617023 | ybx3 (bg=22.82%) | K562 | - | chr20 | | 34638495 | 34638522 |

  
  

| Match 38 in HUMAN | | | | | | | |
| --- | --- | --- | --- | --- | --- | --- | --- |
| Motif | Start in Seq (1 Indexed) | End in Seq (1 Indexed) | Strand | Chrm | Exon | Start in Chrm (0 Indexed) | End in Chrm (1 Indexed) |
| GCTCGAACGCCGCGCGGCGGAGGCCATTA | 436 | 464 | - | chr20 | 1 | 34638468 | 34638497 |
| eCLIP Fold-Enrichment | Binding Protein | Cell Line | Strand | Chrm | | Start in Chrm (0 Indexed) | End in Chrm (1 Indexed) |
| 3.39433831549 | AATF (bg=0.85%) | K562 | - | chr20 | | 34638427 | 34638473 |
| 5.60836830054 | ddx3x (bg=13.89%) | HepG2 | - | chr20 | | 34638430 | 34638472 |
| 4.96785398016 | ddx3x (bg=13.89%) | HepG2 | - | chr20 | | 34638431 | 34638478 |
| 5.75810314226 | ddx3x (bg=13.89%) | HepG2 | - | chr20 | | 34638472 | 34638488 |
| 4.98934278401 | ddx3x (bg=13.89%) | HepG2 | - | chr20 | | 34638478 | 34638557 |
| 5.78457535362 | ddx3x (bg=13.89%) | HepG2 | - | chr20 | | 34638488 | 34638505 |
| 2.04886625959 | ddx6 (bg=23.92%) | HepG2 | - | chr20 | | 34638456 | 34638473 |
| 2.55067366745 | dgcr8 (bg=19.31%) | HepG2 | - | chr20 | | 34638438 | 34638472 |
| 2.04772932362 | DHX30 (bg=5.05%) | HepG2 | - | chr20 | | 34638417 | 34638507 |
| 2.40381580631 | DHX30 (bg=5.05%) | HepG2 | - | chr20 | | 34638449 | 34638562 |
| 2.19632922878 | DHX30 (bg=5.05%) | K562 | - | chr20 | | 34638471 | 34638525 |
| 2.49524528748 | DHX30 (bg=5.05%) | K562 | - | chr20 | | 34638472 | 34638521 |
| 2.30079198364 | drosha (bg=11.37%) | HepG2 | - | chr20 | | 34638407 | 34638544 |
| 2.30079198364 | drosha (bg=11.37%) | HepG2 | - | chr20 | | 34638407 | 34638544 |
| 2.73359651524 | drosha (bg=11.37%) | HepG2 | - | chr20 | | 34638434 | 34638559 |
| 2.73359651524 | drosha (bg=11.37%) | HepG2 | - | chr20 | | 34638434 | 34638559 |
| 2.52921178508 | EIF3H (bg=7.83%) | HepG2 | - | chr20 | | 34638481 | 34638563 |
| 2.76418360275 | FTO (bg=7.53%) | HepG2 | - | chr20 | | 34638477 | 34638557 |
| 3.08192093596 | FTO (bg=7.53%) | HepG2 | - | chr20 | | 34638481 | 34638549 |
| 4.25874884379 | FXR2 (bg=7.51%) | K562 | - | chr20 | | 34638395 | 34638474 |
| 2.70665097697 | FXR2 (bg=7.51%) | K562 | - | chr20 | | 34638474 | 34638569 |
| 2.12000705475 | GRWD1 (bg=4.85%) | HepG2 | - | chr20 | | 34638408 | 34638584 |
| 3.0655399198 | METAP2 (bg=6.14%) | K562 | - | chr20 | | 34638454 | 34638537 |
| 3.60837636218 | METAP2 (bg=6.14%) | K562 | - | chr20 | | 34638471 | 34638512 |
| 2.63978465706 | NIPBL (bg=2.83%) | K562 | - | chr20 | | 34638435 | 34638525 |
| 2.89769655177 | rbm15 (bg=11.81%) | K562 | - | chr20 | | 34638437 | 34638472 |
| 3.57499848532 | rbm15 (bg=11.81%) | K562 | - | chr20 | | 34638437 | 34638481 |
| 3.65982083632 | rbm15 (bg=11.81%) | HepG2 | - | chr20 | | 34638438 | 34638491 |
| 4.72093288641 | rbm15 (bg=11.81%) | HepG2 | - | chr20 | | 34638443 | 34638542 |
| 3.21299295106 | rbm15 (bg=11.81%) | K562 | - | chr20 | | 34638481 | 34638503 |
| 4.19868692245 | rbm15 (bg=11.81%) | HepG2 | - | chr20 | | 34638491 | 34638525 |
| 3.59159342405 | rbm15 (bg=11.81%) | K562 | - | chr20 | | 34638492 | 34638540 |
| 3.08035714764 | SDAD1 (bg=5.99%) | HepG2 | - | chr20 | | 34638494 | 34638558 |
| 2.16104423517 | sf3a3 (bg=12.13%) | HepG2 | - | chr20 | | 34638456 | 34638527 |
| 2.04622054079 | SLTM (bg=1.1%) | K562 | - | chr20 | | 34638460 | 34638520 |
| 2.03501246429 | SRSF1 (bg=2.06%) | K562 | - | chr20 | | 34638415 | 34638527 |
| 2.04832617426 | TBRG4 (bg=7.0%) | HepG2 | - | chr20 | | 34638474 | 34638534 |
| 2.23948572672 | TRA2A (bg=1.38%) | HepG2 | - | chr20 | | 34638432 | 34638507 |
| 2.45735583679 | UCHL5 (bg=1.08%) | HepG2 | - | chr20 | | 34638420 | 34638479 |
| 2.70754372143 | WDR43 (bg=0.9%) | HepG2 | - | chr20 | | 34638479 | 34638528 |
| 3.19953447206 | ybx3 (bg=22.82%) | K562 | - | chr20 | | 34638459 | 34638495 |
| 2.75138362467 | ybx3 (bg=22.82%) | K562 | - | chr20 | | 34638463 | 34638475 |
| 3.11875929076 | ybx3 (bg=22.82%) | K562 | - | chr20 | | 34638475 | 34638521 |
| 3.36431617023 | ybx3 (bg=22.82%) | K562 | - | chr20 | | 34638495 | 34638522 |

  
  

| Match 39 in HUMAN | | | | | | | |
| --- | --- | --- | --- | --- | --- | --- | --- |
| Motif | Start in Seq (1 Indexed) | End in Seq (1 Indexed) | Strand | Chrm | Exon | Start in Chrm (0 Indexed) | End in Chrm (1 Indexed) |
| GGCGTG | 466 | 471 | - | chr20 | 1 | 34638461 | 34638467 |
| eCLIP Fold-Enrichment | Binding Protein | Cell Line | Strand | Chrm | | Start in Chrm (0 Indexed) | End in Chrm (1 Indexed) |
| 3.39433831549 | AATF (bg=0.85%) | K562 | - | chr20 | | 34638427 | 34638473 |
| 5.60836830054 | ddx3x (bg=13.89%) | HepG2 | - | chr20 | | 34638430 | 34638472 |
| 4.96785398016 | ddx3x (bg=13.89%) | HepG2 | - | chr20 | | 34638431 | 34638478 |
| 2.04886625959 | ddx6 (bg=23.92%) | HepG2 | - | chr20 | | 34638456 | 34638473 |
| 2.55067366745 | dgcr8 (bg=19.31%) | HepG2 | - | chr20 | | 34638438 | 34638472 |
| 2.04772932362 | DHX30 (bg=5.05%) | HepG2 | - | chr20 | | 34638417 | 34638507 |
| 2.40381580631 | DHX30 (bg=5.05%) | HepG2 | - | chr20 | | 34638449 | 34638562 |
| 2.30079198364 | drosha (bg=11.37%) | HepG2 | - | chr20 | | 34638407 | 34638544 |
| 2.30079198364 | drosha (bg=11.37%) | HepG2 | - | chr20 | | 34638407 | 34638544 |
| 2.73359651524 | drosha (bg=11.37%) | HepG2 | - | chr20 | | 34638434 | 34638559 |
| 2.73359651524 | drosha (bg=11.37%) | HepG2 | - | chr20 | | 34638434 | 34638559 |
| 3.09223430837 | FMR1 (bg=6.3%) | K562 | - | chr20 | | 34638411 | 34638465 |
| 4.25874884379 | FXR2 (bg=7.51%) | K562 | - | chr20 | | 34638395 | 34638474 |
| 2.12000705475 | GRWD1 (bg=4.85%) | HepG2 | - | chr20 | | 34638408 | 34638584 |
| 3.0655399198 | METAP2 (bg=6.14%) | K562 | - | chr20 | | 34638454 | 34638537 |
| 3.06771611018 | NIP7 (bg=2.54%) | HepG2 | - | chr20 | | 34638413 | 34638466 |
| 2.63978465706 | NIPBL (bg=2.83%) | K562 | - | chr20 | | 34638435 | 34638525 |
| 2.89769655177 | rbm15 (bg=11.81%) | K562 | - | chr20 | | 34638437 | 34638472 |
| 3.57499848532 | rbm15 (bg=11.81%) | K562 | - | chr20 | | 34638437 | 34638481 |
| 3.65982083632 | rbm15 (bg=11.81%) | HepG2 | - | chr20 | | 34638438 | 34638491 |
| 4.72093288641 | rbm15 (bg=11.81%) | HepG2 | - | chr20 | | 34638443 | 34638542 |
| 2.16104423517 | sf3a3 (bg=12.13%) | HepG2 | - | chr20 | | 34638456 | 34638527 |
| 2.04622054079 | SLTM (bg=1.1%) | K562 | - | chr20 | | 34638460 | 34638520 |
| 2.03501246429 | SRSF1 (bg=2.06%) | K562 | - | chr20 | | 34638415 | 34638527 |
| 2.23948572672 | TRA2A (bg=1.38%) | HepG2 | - | chr20 | | 34638432 | 34638507 |
| 2.45735583679 | UCHL5 (bg=1.08%) | HepG2 | - | chr20 | | 34638420 | 34638479 |
| 2.06020710841 | ybx3 (bg=22.82%) | K562 | - | chr20 | | 34638447 | 34638463 |
| 3.19953447206 | ybx3 (bg=22.82%) | K562 | - | chr20 | | 34638459 | 34638495 |
| 2.75138362467 | ybx3 (bg=22.82%) | K562 | - | chr20 | | 34638463 | 34638475 |

  
  

| Match 40 in HUMAN | | | | | | | |
| --- | --- | --- | --- | --- | --- | --- | --- |
| Motif | Start in Seq (1 Indexed) | End in Seq (1 Indexed) | Strand | Chrm | Exon | Start in Chrm (0 Indexed) | End in Chrm (1 Indexed) |
| GGAAGGCGGCCTAGGGACGCA | 481 | 501 | - | chr20 | 1 | 34638431 | 34638452 |
| eCLIP Fold-Enrichment | Binding Protein | Cell Line | Strand | Chrm | | Start in Chrm (0 Indexed) | End in Chrm (1 Indexed) |
| 3.39433831549 | AATF (bg=0.85%) | K562 | - | chr20 | | 34638427 | 34638473 |
| 5.60836830054 | ddx3x (bg=13.89%) | HepG2 | - | chr20 | | 34638430 | 34638472 |
| 4.96785398016 | ddx3x (bg=13.89%) | HepG2 | - | chr20 | | 34638431 | 34638478 |
| 2.55067366745 | dgcr8 (bg=19.31%) | HepG2 | - | chr20 | | 34638438 | 34638472 |
| 2.04772932362 | DHX30 (bg=5.05%) | HepG2 | - | chr20 | | 34638417 | 34638507 |
| 2.40381580631 | DHX30 (bg=5.05%) | HepG2 | - | chr20 | | 34638449 | 34638562 |
| 2.30079198364 | drosha (bg=11.37%) | HepG2 | - | chr20 | | 34638407 | 34638544 |
| 2.30079198364 | drosha (bg=11.37%) | HepG2 | - | chr20 | | 34638407 | 34638544 |
| 2.73359651524 | drosha (bg=11.37%) | HepG2 | - | chr20 | | 34638434 | 34638559 |
| 2.73359651524 | drosha (bg=11.37%) | HepG2 | - | chr20 | | 34638434 | 34638559 |
| 3.09223430837 | FMR1 (bg=6.3%) | K562 | - | chr20 | | 34638411 | 34638465 |
| 4.25874884379 | FXR2 (bg=7.51%) | K562 | - | chr20 | | 34638395 | 34638474 |
| 2.12000705475 | GRWD1 (bg=4.85%) | HepG2 | - | chr20 | | 34638408 | 34638584 |
| 3.06771611018 | NIP7 (bg=2.54%) | HepG2 | - | chr20 | | 34638413 | 34638466 |
| 2.63978465706 | NIPBL (bg=2.83%) | K562 | - | chr20 | | 34638435 | 34638525 |
| 2.89769655177 | rbm15 (bg=11.81%) | K562 | - | chr20 | | 34638437 | 34638472 |
| 3.57499848532 | rbm15 (bg=11.81%) | K562 | - | chr20 | | 34638437 | 34638481 |
| 3.65982083632 | rbm15 (bg=11.81%) | HepG2 | - | chr20 | | 34638438 | 34638491 |
| 4.72093288641 | rbm15 (bg=11.81%) | HepG2 | - | chr20 | | 34638443 | 34638542 |
| 2.03501246429 | SRSF1 (bg=2.06%) | K562 | - | chr20 | | 34638415 | 34638527 |
| 2.23948572672 | TRA2A (bg=1.38%) | HepG2 | - | chr20 | | 34638432 | 34638507 |
| 2.45735583679 | UCHL5 (bg=1.08%) | HepG2 | - | chr20 | | 34638420 | 34638479 |
| 3.33657117974 | ybx3 (bg=22.82%) | K562 | - | chr20 | | 34638421 | 34638450 |
| 2.06020710841 | ybx3 (bg=22.82%) | K562 | - | chr20 | | 34638447 | 34638463 |
| 3.21992626089 | ybx3 (bg=22.82%) | K562 | - | chr20 | | 34638450 | 34638459 |

  
  

| Match 41 in HUMAN | | | | | | | |
| --- | --- | --- | --- | --- | --- | --- | --- |
| Motif | Start in Seq (1 Indexed) | End in Seq (1 Indexed) | Strand | Chrm | Exon | Start in Chrm (0 Indexed) | End in Chrm (1 Indexed) |
| GCAGGCTCGGC | 503 | 513 | - | chr20 | 1 | 34638419 | 34638430 |
| eCLIP Fold-Enrichment | Binding Protein | Cell Line | Strand | Chrm | | Start in Chrm (0 Indexed) | End in Chrm (1 Indexed) |
| 3.39433831549 | AATF (bg=0.85%) | K562 | - | chr20 | | 34638427 | 34638473 |
| 5.60836830054 | ddx3x (bg=13.89%) | HepG2 | - | chr20 | | 34638430 | 34638472 |
| 2.04772932362 | DHX30 (bg=5.05%) | HepG2 | - | chr20 | | 34638417 | 34638507 |
| 2.30079198364 | drosha (bg=11.37%) | HepG2 | - | chr20 | | 34638407 | 34638544 |
| 2.30079198364 | drosha (bg=11.37%) | HepG2 | - | chr20 | | 34638407 | 34638544 |
| 3.09223430837 | FMR1 (bg=6.3%) | K562 | - | chr20 | | 34638411 | 34638465 |
| 4.25874884379 | FXR2 (bg=7.51%) | K562 | - | chr20 | | 34638395 | 34638474 |
| 2.12000705475 | GRWD1 (bg=4.85%) | HepG2 | - | chr20 | | 34638408 | 34638584 |
| 3.06771611018 | NIP7 (bg=2.54%) | HepG2 | - | chr20 | | 34638413 | 34638466 |
| 2.03501246429 | SRSF1 (bg=2.06%) | K562 | - | chr20 | | 34638415 | 34638527 |
| 2.45735583679 | UCHL5 (bg=1.08%) | HepG2 | - | chr20 | | 34638420 | 34638479 |
| 2.74458825135 | ybx3 (bg=22.82%) | K562 | - | chr20 | | 34638384 | 34638421 |
| 2.96604039723 | ybx3 (bg=22.82%) | K562 | - | chr20 | | 34638394 | 34638420 |
| 2.52955022368 | ybx3 (bg=22.82%) | HepG2 | - | chr20 | | 34638395 | 34638419 |
| 3.33657117974 | ybx3 (bg=22.82%) | K562 | - | chr20 | | 34638421 | 34638450 |

  
  

| Match 42 in HUMAN | | | | | | | |
| --- | --- | --- | --- | --- | --- | --- | --- |
| Motif | Start in Seq (1 Indexed) | End in Seq (1 Indexed) | Strand | Chrm | Exon | Start in Chrm (0 Indexed) | End in Chrm (1 Indexed) |
| GCTCGGC | 507 | 513 | - | chr20 | 1 | 34638419 | 34638426 |
| eCLIP Fold-Enrichment | Binding Protein | Cell Line | Strand | Chrm | | Start in Chrm (0 Indexed) | End in Chrm (1 Indexed) |
| 2.04772932362 | DHX30 (bg=5.05%) | HepG2 | - | chr20 | | 34638417 | 34638507 |
| 2.30079198364 | drosha (bg=11.37%) | HepG2 | - | chr20 | | 34638407 | 34638544 |
| 2.30079198364 | drosha (bg=11.37%) | HepG2 | - | chr20 | | 34638407 | 34638544 |
| 3.09223430837 | FMR1 (bg=6.3%) | K562 | - | chr20 | | 34638411 | 34638465 |
| 4.25874884379 | FXR2 (bg=7.51%) | K562 | - | chr20 | | 34638395 | 34638474 |
| 2.12000705475 | GRWD1 (bg=4.85%) | HepG2 | - | chr20 | | 34638408 | 34638584 |
| 3.06771611018 | NIP7 (bg=2.54%) | HepG2 | - | chr20 | | 34638413 | 34638466 |
| 2.03501246429 | SRSF1 (bg=2.06%) | K562 | - | chr20 | | 34638415 | 34638527 |
| 2.45735583679 | UCHL5 (bg=1.08%) | HepG2 | - | chr20 | | 34638420 | 34638479 |
| 2.74458825135 | ybx3 (bg=22.82%) | K562 | - | chr20 | | 34638384 | 34638421 |
| 2.96604039723 | ybx3 (bg=22.82%) | K562 | - | chr20 | | 34638394 | 34638420 |
| 2.52955022368 | ybx3 (bg=22.82%) | HepG2 | - | chr20 | | 34638395 | 34638419 |
| 3.33657117974 | ybx3 (bg=22.82%) | K562 | - | chr20 | | 34638421 | 34638450 |

  
  

| Match 43 in HUMAN | | | | | | | |
| --- | --- | --- | --- | --- | --- | --- | --- |
| Motif | Start in Seq (1 Indexed) | End in Seq (1 Indexed) | Strand | Chrm | Exon | Start in Chrm (0 Indexed) | End in Chrm (1 Indexed) |
| GCCTCTTTAG | 515 | 524 | - | chr20 | 1 | 34638408 | 34638418 |
| eCLIP Fold-Enrichment | Binding Protein | Cell Line | Strand | Chrm | | Start in Chrm (0 Indexed) | End in Chrm (1 Indexed) |
| 2.04772932362 | DHX30 (bg=5.05%) | HepG2 | - | chr20 | | 34638417 | 34638507 |
| 2.30079198364 | drosha (bg=11.37%) | HepG2 | - | chr20 | | 34638407 | 34638544 |
| 2.30079198364 | drosha (bg=11.37%) | HepG2 | - | chr20 | | 34638407 | 34638544 |
| 3.09223430837 | FMR1 (bg=6.3%) | K562 | - | chr20 | | 34638411 | 34638465 |
| 4.25874884379 | FXR2 (bg=7.51%) | K562 | - | chr20 | | 34638395 | 34638474 |
| 2.12000705475 | GRWD1 (bg=4.85%) | HepG2 | - | chr20 | | 34638408 | 34638584 |
| 3.06771611018 | NIP7 (bg=2.54%) | HepG2 | - | chr20 | | 34638413 | 34638466 |
| 2.03501246429 | SRSF1 (bg=2.06%) | K562 | - | chr20 | | 34638415 | 34638527 |
| 2.74458825135 | ybx3 (bg=22.82%) | K562 | - | chr20 | | 34638384 | 34638421 |
| 2.96604039723 | ybx3 (bg=22.82%) | K562 | - | chr20 | | 34638394 | 34638420 |
| 2.52955022368 | ybx3 (bg=22.82%) | HepG2 | - | chr20 | | 34638395 | 34638419 |

  
  

| Match 44 in HUMAN | | | | | | | |
| --- | --- | --- | --- | --- | --- | --- | --- |
| Motif | Start in Seq (1 Indexed) | End in Seq (1 Indexed) | Strand | Chrm | Exon | Start in Chrm (0 Indexed) | End in Chrm (1 Indexed) |
| CCACGGAGCCGCGCAGATCCGGTTCCCGGGTGACCACTCTGTCGCCATTGGGCGA | 526 | 580 | - | chr20 | 1 | 34638352 | 34638407 |
| eCLIP Fold-Enrichment | Binding Protein | Cell Line | Strand | Chrm | | Start in Chrm (0 Indexed) | End in Chrm (1 Indexed) |
| 2.30079198364 | drosha (bg=11.37%) | HepG2 | - | chr20 | | 34638407 | 34638544 |
| 2.30079198364 | drosha (bg=11.37%) | HepG2 | - | chr20 | | 34638407 | 34638544 |
| 4.25874884379 | FXR2 (bg=7.51%) | K562 | - | chr20 | | 34638395 | 34638474 |
| 3.70148576657 | METAP2 (bg=6.14%) | K562 | - | chr20 | | 34638350 | 34638358 |
| 2.71849674365 | rbm15 (bg=11.81%) | HepG2 | - | chr20 | | 34638268 | 34638355 |
| 3.68091720756 | rbm15 (bg=11.81%) | HepG2 | - | chr20 | | 34638294 | 34638361 |
| 2.99436656048 | ybx3 (bg=22.82%) | K562 | - | chr20 | | 34638282 | 34638366 |
| 2.39266587079 | ybx3 (bg=22.82%) | K562 | - | chr20 | | 34638314 | 34638360 |
| 2.29195200604 | ybx3 (bg=22.82%) | K562 | - | chr20 | | 34638360 | 34638394 |
| 2.48296066673 | ybx3 (bg=22.82%) | K562 | - | chr20 | | 34638366 | 34638384 |
| 2.74458825135 | ybx3 (bg=22.82%) | K562 | - | chr20 | | 34638384 | 34638421 |
| 2.96604039723 | ybx3 (bg=22.82%) | K562 | - | chr20 | | 34638394 | 34638420 |
| 2.52955022368 | ybx3 (bg=22.82%) | HepG2 | - | chr20 | | 34638395 | 34638419 |

  
  

| Match 45 in HUMAN | | | | | | | |
| --- | --- | --- | --- | --- | --- | --- | --- |
| Motif | Start in Seq (1 Indexed) | End in Seq (1 Indexed) | Strand | Chrm | Exon | Start in Chrm (0 Indexed) | End in Chrm (1 Indexed) |
| CCCGGG | 550 | 555 | - | chr20 | 1 | 34638377 | 34638383 |
| eCLIP Fold-Enrichment | Binding Protein | Cell Line | Strand | Chrm | | Start in Chrm (0 Indexed) | End in Chrm (1 Indexed) |
| 2.29195200604 | ybx3 (bg=22.82%) | K562 | - | chr20 | | 34638360 | 34638394 |
| 2.48296066673 | ybx3 (bg=22.82%) | K562 | - | chr20 | | 34638366 | 34638384 |

  
  

| Match 46 in HUMAN | | | | | | | |
| --- | --- | --- | --- | --- | --- | --- | --- |
| Motif | Start in Seq (1 Indexed) | End in Seq (1 Indexed) | Strand | Chrm | Exon | Start in Chrm (0 Indexed) | End in Chrm (1 Indexed) |
| TGACCACTC | 556 | 564 | - | chr20 | 1 | 34638368 | 34638377 |
| eCLIP Fold-Enrichment | Binding Protein | Cell Line | Strand | Chrm | | Start in Chrm (0 Indexed) | End in Chrm (1 Indexed) |
| 2.29195200604 | ybx3 (bg=22.82%) | K562 | - | chr20 | | 34638360 | 34638394 |
| 2.48296066673 | ybx3 (bg=22.82%) | K562 | - | chr20 | | 34638366 | 34638384 |

  
  

| Match 47 in HUMAN | | | | | | | |
| --- | --- | --- | --- | --- | --- | --- | --- |
| Motif | Start in Seq (1 Indexed) | End in Seq (1 Indexed) | Strand | Chrm | Exon | Start in Chrm (0 Indexed) | End in Chrm (1 Indexed) |
| ACCTACCTAGTCCTGACGACAACGGACAAAGGCCTTAA | 582 | 619 | - | chr20 | 1 | 34638313 | 34638351 |
| eCLIP Fold-Enrichment | Binding Protein | Cell Line | Strand | Chrm | | Start in Chrm (0 Indexed) | End in Chrm (1 Indexed) |
| 3.75347093395 | BUD13 (bg=0.7%) | K562 | - | chr20 | | 34638288 | 34638326 |
| 4.77517665562 | ddx3x (bg=13.89%) | HepG2 | - | chr20 | | 34638298 | 34638324 |
| 2.30678256797 | DDX52 (bg=1.27%) | HepG2 | - | chr20 | | 34638285 | 34638324 |
| 2.57676654417 | FTO (bg=7.53%) | K562 | - | chr20 | | 34638266 | 34638315 |
| 2.57087860296 | lin28b (bg=16.96%) | K562 | - | chr20 | | 34638297 | 34638347 |
| 3.70148576657 | METAP2 (bg=6.14%) | K562 | - | chr20 | | 34638350 | 34638358 |
| 2.71849674365 | rbm15 (bg=11.81%) | HepG2 | - | chr20 | | 34638268 | 34638355 |
| 3.09563592939 | rbm15 (bg=11.81%) | K562 | - | chr20 | | 34638281 | 34638338 |
| 2.48951058625 | rbm15 (bg=11.81%) | K562 | - | chr20 | | 34638292 | 34638344 |
| 3.68091720756 | rbm15 (bg=11.81%) | HepG2 | - | chr20 | | 34638294 | 34638361 |
| 2.35101828622 | SRSF7 (bg=1.29%) | K562 | - | chr20 | | 34638273 | 34638343 |
| 2.97531821082 | ybx3 (bg=22.82%) | K562 | - | chr20 | | 34638275 | 34638314 |
| 2.99436656048 | ybx3 (bg=22.82%) | K562 | - | chr20 | | 34638282 | 34638366 |
| 2.39266587079 | ybx3 (bg=22.82%) | K562 | - | chr20 | | 34638314 | 34638360 |

  
  

| Match 48 in HUMAN | | | | | | | |
| --- | --- | --- | --- | --- | --- | --- | --- |
| Motif | Start in Seq (1 Indexed) | End in Seq (1 Indexed) | Strand | Chrm | Exon | Start in Chrm (0 Indexed) | End in Chrm (1 Indexed) |
| ACCTAC | 582 | 587 | - | chr20 | 1 | 34638345 | 34638351 |
| eCLIP Fold-Enrichment | Binding Protein | Cell Line | Strand | Chrm | | Start in Chrm (0 Indexed) | End in Chrm (1 Indexed) |
| 2.57087860296 | lin28b (bg=16.96%) | K562 | - | chr20 | | 34638297 | 34638347 |
| 3.70148576657 | METAP2 (bg=6.14%) | K562 | - | chr20 | | 34638350 | 34638358 |
| 2.71849674365 | rbm15 (bg=11.81%) | HepG2 | - | chr20 | | 34638268 | 34638355 |
| 3.68091720756 | rbm15 (bg=11.81%) | HepG2 | - | chr20 | | 34638294 | 34638361 |
| 2.99436656048 | ybx3 (bg=22.82%) | K562 | - | chr20 | | 34638282 | 34638366 |
| 2.39266587079 | ybx3 (bg=22.82%) | K562 | - | chr20 | | 34638314 | 34638360 |

  
  

| Match 49 in HUMAN | | | | | | | |
| --- | --- | --- | --- | --- | --- | --- | --- |
| Motif | Start in Seq (1 Indexed) | End in Seq (1 Indexed) | Strand | Chrm | Exon | Start in Chrm (0 Indexed) | End in Chrm (1 Indexed) |
| GGGCCTGG | 621 | 628 | - | chr20 | 1 | 34638304 | 34638312 |
| eCLIP Fold-Enrichment | Binding Protein | Cell Line | Strand | Chrm | | Start in Chrm (0 Indexed) | End in Chrm (1 Indexed) |
| 3.75347093395 | BUD13 (bg=0.7%) | K562 | - | chr20 | | 34638288 | 34638326 |
| 4.77517665562 | ddx3x (bg=13.89%) | HepG2 | - | chr20 | | 34638298 | 34638324 |
| 2.30678256797 | DDX52 (bg=1.27%) | HepG2 | - | chr20 | | 34638285 | 34638324 |
| 2.30718200679 | dgcr8 (bg=19.31%) | K562 | - | chr20 | | 34638296 | 34638308 |
| 2.39852300929 | FTO (bg=7.53%) | K562 | - | chr20 | | 34638256 | 34638308 |
| 2.57676654417 | FTO (bg=7.53%) | K562 | - | chr20 | | 34638266 | 34638315 |
| 2.57087860296 | lin28b (bg=16.96%) | K562 | - | chr20 | | 34638297 | 34638347 |
| 2.71849674365 | rbm15 (bg=11.81%) | HepG2 | - | chr20 | | 34638268 | 34638355 |
| 3.09563592939 | rbm15 (bg=11.81%) | K562 | - | chr20 | | 34638281 | 34638338 |
| 2.48951058625 | rbm15 (bg=11.81%) | K562 | - | chr20 | | 34638292 | 34638344 |
| 3.68091720756 | rbm15 (bg=11.81%) | HepG2 | - | chr20 | | 34638294 | 34638361 |
| 2.35101828622 | SRSF7 (bg=1.29%) | K562 | - | chr20 | | 34638273 | 34638343 |
| 2.97531821082 | ybx3 (bg=22.82%) | K562 | - | chr20 | | 34638275 | 34638314 |
| 2.99436656048 | ybx3 (bg=22.82%) | K562 | - | chr20 | | 34638282 | 34638366 |

  
  

| Match 50 in HUMAN | | | | | | | |
| --- | --- | --- | --- | --- | --- | --- | --- |
| Motif | Start in Seq (1 Indexed) | End in Seq (1 Indexed) | Strand | Chrm | Exon | Start in Chrm (0 Indexed) | End in Chrm (1 Indexed) |
| AGGTGAGCG | 630 | 638 | - | chr20 | 1 | 34638294 | 34638303 |
| eCLIP Fold-Enrichment | Binding Protein | Cell Line | Strand | Chrm | | Start in Chrm (0 Indexed) | End in Chrm (1 Indexed) |
| 3.75347093395 | BUD13 (bg=0.7%) | K562 | - | chr20 | | 34638288 | 34638326 |
| 4.77517665562 | ddx3x (bg=13.89%) | HepG2 | - | chr20 | | 34638298 | 34638324 |
| 2.30678256797 | DDX52 (bg=1.27%) | HepG2 | - | chr20 | | 34638285 | 34638324 |
| 2.30718200679 | dgcr8 (bg=19.31%) | K562 | - | chr20 | | 34638296 | 34638308 |
| 2.39852300929 | FTO (bg=7.53%) | K562 | - | chr20 | | 34638256 | 34638308 |
| 2.57676654417 | FTO (bg=7.53%) | K562 | - | chr20 | | 34638266 | 34638315 |
| 2.57087860296 | lin28b (bg=16.96%) | K562 | - | chr20 | | 34638297 | 34638347 |
| 2.71849674365 | rbm15 (bg=11.81%) | HepG2 | - | chr20 | | 34638268 | 34638355 |
| 3.09563592939 | rbm15 (bg=11.81%) | K562 | - | chr20 | | 34638281 | 34638338 |
| 2.48951058625 | rbm15 (bg=11.81%) | K562 | - | chr20 | | 34638292 | 34638344 |
| 3.68091720756 | rbm15 (bg=11.81%) | HepG2 | - | chr20 | | 34638294 | 34638361 |
| 2.35101828622 | SRSF7 (bg=1.29%) | K562 | - | chr20 | | 34638273 | 34638343 |
| 2.97531821082 | ybx3 (bg=22.82%) | K562 | - | chr20 | | 34638275 | 34638314 |
| 2.99436656048 | ybx3 (bg=22.82%) | K562 | - | chr20 | | 34638282 | 34638366 |

  
  

| Match 51 in HUMAN | | | | | | | |
| --- | --- | --- | --- | --- | --- | --- | --- |
| Motif | Start in Seq (1 Indexed) | End in Seq (1 Indexed) | Strand | Chrm | Exon | Start in Chrm (0 Indexed) | End in Chrm (1 Indexed) |
| AGGTGAGCGAAG | 630 | 641 | - | chr20 | 1 | 34638291 | 34638303 |
| eCLIP Fold-Enrichment | Binding Protein | Cell Line | Strand | Chrm | | Start in Chrm (0 Indexed) | End in Chrm (1 Indexed) |
| 3.75347093395 | BUD13 (bg=0.7%) | K562 | - | chr20 | | 34638288 | 34638326 |
| 4.77517665562 | ddx3x (bg=13.89%) | HepG2 | - | chr20 | | 34638298 | 34638324 |
| 2.30678256797 | DDX52 (bg=1.27%) | HepG2 | - | chr20 | | 34638285 | 34638324 |
| 2.30718200679 | dgcr8 (bg=19.31%) | K562 | - | chr20 | | 34638296 | 34638308 |
| 2.39852300929 | FTO (bg=7.53%) | K562 | - | chr20 | | 34638256 | 34638308 |
| 2.57676654417 | FTO (bg=7.53%) | K562 | - | chr20 | | 34638266 | 34638315 |
| 2.57087860296 | lin28b (bg=16.96%) | K562 | - | chr20 | | 34638297 | 34638347 |
| 2.71849674365 | rbm15 (bg=11.81%) | HepG2 | - | chr20 | | 34638268 | 34638355 |
| 3.09563592939 | rbm15 (bg=11.81%) | K562 | - | chr20 | | 34638281 | 34638338 |
| 2.48951058625 | rbm15 (bg=11.81%) | K562 | - | chr20 | | 34638292 | 34638344 |
| 3.68091720756 | rbm15 (bg=11.81%) | HepG2 | - | chr20 | | 34638294 | 34638361 |
| 2.35101828622 | SRSF7 (bg=1.29%) | K562 | - | chr20 | | 34638273 | 34638343 |
| 2.97531821082 | ybx3 (bg=22.82%) | K562 | - | chr20 | | 34638275 | 34638314 |
| 2.99436656048 | ybx3 (bg=22.82%) | K562 | - | chr20 | | 34638282 | 34638366 |

  
  

| Match 52 in HUMAN | | | | | | | |
| --- | --- | --- | --- | --- | --- | --- | --- |
| Motif | Start in Seq (1 Indexed) | End in Seq (1 Indexed) | Strand | Chrm | Exon | Start in Chrm (0 Indexed) | End in Chrm (1 Indexed) |
| CCCGAACGACGACGGGTGGAACG | 643 | 665 | - | chr20 | 1 | 34638267 | 34638290 |
| eCLIP Fold-Enrichment | Binding Protein | Cell Line | Strand | Chrm | | Start in Chrm (0 Indexed) | End in Chrm (1 Indexed) |
| 3.75347093395 | BUD13 (bg=0.7%) | K562 | - | chr20 | | 34638288 | 34638326 |
| 2.30678256797 | DDX52 (bg=1.27%) | HepG2 | - | chr20 | | 34638285 | 34638324 |
| 2.39852300929 | FTO (bg=7.53%) | K562 | - | chr20 | | 34638256 | 34638308 |
| 2.57676654417 | FTO (bg=7.53%) | K562 | - | chr20 | | 34638266 | 34638315 |
| 2.71849674365 | rbm15 (bg=11.81%) | HepG2 | - | chr20 | | 34638268 | 34638355 |
| 3.09563592939 | rbm15 (bg=11.81%) | K562 | - | chr20 | | 34638281 | 34638338 |
| 2.35101828622 | SRSF7 (bg=1.29%) | K562 | - | chr20 | | 34638273 | 34638343 |
| 2.97531821082 | ybx3 (bg=22.82%) | K562 | - | chr20 | | 34638275 | 34638314 |
| 2.99436656048 | ybx3 (bg=22.82%) | K562 | - | chr20 | | 34638282 | 34638366 |

  
  

| Match 53 in HUMAN | | | | | | | |
| --- | --- | --- | --- | --- | --- | --- | --- |
| Motif | Start in Seq (1 Indexed) | End in Seq (1 Indexed) | Strand | Chrm | Exon | Start in Chrm (0 Indexed) | End in Chrm (1 Indexed) |
| CGACGGGTGGAAC | 652 | 664 | - | chr20 | 1 | 34638268 | 34638281 |
| eCLIP Fold-Enrichment | Binding Protein | Cell Line | Strand | Chrm | | Start in Chrm (0 Indexed) | End in Chrm (1 Indexed) |
| 2.39852300929 | FTO (bg=7.53%) | K562 | - | chr20 | | 34638256 | 34638308 |
| 2.57676654417 | FTO (bg=7.53%) | K562 | - | chr20 | | 34638266 | 34638315 |
| 2.71849674365 | rbm15 (bg=11.81%) | HepG2 | - | chr20 | | 34638268 | 34638355 |
| 3.09563592939 | rbm15 (bg=11.81%) | K562 | - | chr20 | | 34638281 | 34638338 |
| 2.35101828622 | SRSF7 (bg=1.29%) | K562 | - | chr20 | | 34638273 | 34638343 |
| 2.97531821082 | ybx3 (bg=22.82%) | K562 | - | chr20 | | 34638275 | 34638314 |

  
  

| Match 54 in HUMAN | | | | | | | |
| --- | --- | --- | --- | --- | --- | --- | --- |
| Motif | Start in Seq (1 Indexed) | End in Seq (1 Indexed) | Strand | Chrm | Exon | Start in Chrm (0 Indexed) | End in Chrm (1 Indexed) |
| CGGGTGGAAC | 655 | 664 | - | chr20 | 1 | 34638268 | 34638278 |
| eCLIP Fold-Enrichment | Binding Protein | Cell Line | Strand | Chrm | | Start in Chrm (0 Indexed) | End in Chrm (1 Indexed) |
| 2.39852300929 | FTO (bg=7.53%) | K562 | - | chr20 | | 34638256 | 34638308 |
| 2.57676654417 | FTO (bg=7.53%) | K562 | - | chr20 | | 34638266 | 34638315 |
| 2.71849674365 | rbm15 (bg=11.81%) | HepG2 | - | chr20 | | 34638268 | 34638355 |
| 2.35101828622 | SRSF7 (bg=1.29%) | K562 | - | chr20 | | 34638273 | 34638343 |
| 2.97531821082 | ybx3 (bg=22.82%) | K562 | - | chr20 | | 34638275 | 34638314 |

  
  

| Match 55 in HUMAN | | | | | | | |
| --- | --- | --- | --- | --- | --- | --- | --- |
| Motif | Start in Seq (1 Indexed) | End in Seq (1 Indexed) | Strand | Chrm | Exon | Start in Chrm (0 Indexed) | End in Chrm (1 Indexed) |
| GTGGAA | 658 | 663 | - | chr20 | 1 | 34638269 | 34638275 |
| eCLIP Fold-Enrichment | Binding Protein | Cell Line | Strand | Chrm | | Start in Chrm (0 Indexed) | End in Chrm (1 Indexed) |
| 2.39852300929 | FTO (bg=7.53%) | K562 | - | chr20 | | 34638256 | 34638308 |
| 2.57676654417 | FTO (bg=7.53%) | K562 | - | chr20 | | 34638266 | 34638315 |
| 2.71849674365 | rbm15 (bg=11.81%) | HepG2 | - | chr20 | | 34638268 | 34638355 |
| 2.35101828622 | SRSF7 (bg=1.29%) | K562 | - | chr20 | | 34638273 | 34638343 |
| 2.97531821082 | ybx3 (bg=22.82%) | K562 | - | chr20 | | 34638275 | 34638314 |

  
  

| Match 56 in HUMAN | | | | | | | |
| --- | --- | --- | --- | --- | --- | --- | --- |
| Motif | Start in Seq (1 Indexed) | End in Seq (1 Indexed) | Strand | Chrm | Exon | Start in Chrm (0 Indexed) | End in Chrm (1 Indexed) |
| TTAGCGGCCATCGGGC | 667 | 682 | - | chr20 | 1 | 34638250 | 34638266 |
| eCLIP Fold-Enrichment | Binding Protein | Cell Line | Strand | Chrm | | Start in Chrm (0 Indexed) | End in Chrm (1 Indexed) |
| 2.39852300929 | FTO (bg=7.53%) | K562 | - | chr20 | | 34638256 | 34638308 |
| 2.57676654417 | FTO (bg=7.53%) | K562 | - | chr20 | | 34638266 | 34638315 |
| 3.8127614692 | UTP18 (bg=0.53%) | K562 | - | chr20 | | 34638226 | 34638255 |

  
  

| Match 57 in HUMAN | | | | | | | |
| --- | --- | --- | --- | --- | --- | --- | --- |
| Motif | Start in Seq (1 Indexed) | End in Seq (1 Indexed) | Strand | Chrm | Exon | Start in Chrm (0 Indexed) | End in Chrm (1 Indexed) |
| GTTGGTCTTC | 684 | 693 | - | chr20 | 1 | 34638239 | 34638249 |
| eCLIP Fold-Enrichment | Binding Protein | Cell Line | Strand | Chrm | | Start in Chrm (0 Indexed) | End in Chrm (1 Indexed) |
| 3.8127614692 | UTP18 (bg=0.53%) | K562 | - | chr20 | | 34638226 | 34638255 |

  
  

| Match 58 in HUMAN | | | | | | | |
| --- | --- | --- | --- | --- | --- | --- | --- |
| Motif | Start in Seq (1 Indexed) | End in Seq (1 Indexed) | Strand | Chrm | Exon | Start in Chrm (0 Indexed) | End in Chrm (1 Indexed) |
| TTCTAC | 695 | 700 | - | chr20 | 1 | 34638232 | 34638238 |
| eCLIP Fold-Enrichment | Binding Protein | Cell Line | Strand | Chrm | | Start in Chrm (0 Indexed) | End in Chrm (1 Indexed) |
| 3.8127614692 | UTP18 (bg=0.53%) | K562 | - | chr20 | | 34638226 | 34638255 |

  
  

| Match 59 in HUMAN | | | | | | | |
| --- | --- | --- | --- | --- | --- | --- | --- |
| Motif | Start in Seq (1 Indexed) | End in Seq (1 Indexed) | Strand | Chrm | Exon | Start in Chrm (0 Indexed) | End in Chrm (1 Indexed) |
| TTCTACCAGACTTT | 695 | 708 | - | chr20 | 1 | 34638224 | 34638238 |
| eCLIP Fold-Enrichment | Binding Protein | Cell Line | Strand | Chrm | | Start in Chrm (0 Indexed) | End in Chrm (1 Indexed) |
| 3.8127614692 | UTP18 (bg=0.53%) | K562 | - | chr20 | | 34638226 | 34638255 |

  
  

| Match 60 in HUMAN | | | | | | | |
| --- | --- | --- | --- | --- | --- | --- | --- |
| Motif | Start in Seq (1 Indexed) | End in Seq (1 Indexed) | Strand | Chrm | Exon | Start in Chrm (0 Indexed) | End in Chrm (1 Indexed) |
| CTGTCGGAAGAGAGAAATGGTAGAATGACAGGCCACGTTTGGCCCGTTGGAAATGCCC | 710 | 767 | - | chr20 | 1 | 34638165 | 34638223 |
| eCLIP Fold-Enrichment | Binding Protein | Cell Line | Strand | Chrm | | Start in Chrm (0 Indexed) | End in Chrm (1 Indexed) |
| 4.08829485808 | ddx55 (bg=12.35%) | HepG2 | - | chr20 | | 34638165 | 34638173 |
| 4.08829485808 | ddx55 (bg=12.35%) | HepG2 | - | chr20 | | 34638173 | 34638203 |

  
  

| Match 61 in HUMAN | | | | | | | |
| --- | --- | --- | --- | --- | --- | --- | --- |
| Motif | Start in Seq (1 Indexed) | End in Seq (1 Indexed) | Strand | Chrm | Exon | Start in Chrm (0 Indexed) | End in Chrm (1 Indexed) |
| GAAATGG | 723 | 729 | - | chr20 | 1 | 34638203 | 34638210 |
| eCLIP Fold-Enrichment | Binding Protein | Cell Line | Strand | Chrm | | Start in Chrm (0 Indexed) | End in Chrm (1 Indexed) |
| 4.08829485808 | ddx55 (bg=12.35%) | HepG2 | - | chr20 | | 34638173 | 34638203 |

  
  

| Match 62 in HUMAN | | | | | | | |
| --- | --- | --- | --- | --- | --- | --- | --- |
| Motif | Start in Seq (1 Indexed) | End in Seq (1 Indexed) | Strand | Chrm | Exon | Start in Chrm (0 Indexed) | End in Chrm (1 Indexed) |
| CGTTTGGCC | 745 | 753 | - | chr20 | 1 | 34638179 | 34638188 |
| eCLIP Fold-Enrichment | Binding Protein | Cell Line | Strand | Chrm | | Start in Chrm (0 Indexed) | End in Chrm (1 Indexed) |
| 4.08829485808 | ddx55 (bg=12.35%) | HepG2 | - | chr20 | | 34638173 | 34638203 |

  
  

| Match 63 in HUMAN | | | | | | | |
| --- | --- | --- | --- | --- | --- | --- | --- |
| Motif | Start in Seq (1 Indexed) | End in Seq (1 Indexed) | Strand | Chrm | Exon | Start in Chrm (0 Indexed) | End in Chrm (1 Indexed) |
| TGGAAGGCCTGTGTATATAATATGAAAAAGCTGCTCTCAACTCCACCCCAACCTTTTAATAGAAAACATTTGTCACATCTAGCCCTT | 800 | 886 | - | chr20 | 1 | 34638046 | 34638133 |
| eCLIP Fold-Enrichment | Binding Protein | Cell Line | Strand | Chrm | | Start in Chrm (0 Indexed) | End in Chrm (1 Indexed) |
| 2.08456334663 | KHDRBS1 (bg=1.16%) | K562 | - | chr20 | | 34637987 | 34638050 |
| 4.85852794657 | pum1 (bg=29.85%) | K562 | - | chr20 | | 34638011 | 34638052 |
| 4.70360898728 | pum1 (bg=29.85%) | K562 | - | chr20 | | 34638020 | 34638050 |
| 5.73988345007 | pum1 (bg=29.85%) | K562 | - | chr20 | | 34638052 | 34638099 |
| 4.77399831518 | pum1 (bg=29.85%) | K562 | - | chr20 | | 34638080 | 34638095 |
| 3.78988137689 | rbm15 (bg=11.81%) | HepG2 | - | chr20 | | 34637999 | 34638056 |
| 3.33379566658 | rbm15 (bg=11.81%) | K562 | - | chr20 | | 34638004 | 34638047 |

  
  

| Match 64 in HUMAN | | | | | | | |
| --- | --- | --- | --- | --- | --- | --- | --- |
| Motif | Start in Seq (1 Indexed) | End in Seq (1 Indexed) | Strand | Chrm | Exon | Start in Chrm (0 Indexed) | End in Chrm (1 Indexed) |
| GAAGGCCTGTGTATATAATATGAAAAAGCTGCTCTCAACT | 802 | 841 | - | chr20 | 1 | 34638091 | 34638131 |
| eCLIP Fold-Enrichment | Binding Protein | Cell Line | Strand | Chrm | | Start in Chrm (0 Indexed) | End in Chrm (1 Indexed) |
| 5.73988345007 | pum1 (bg=29.85%) | K562 | - | chr20 | | 34638052 | 34638099 |
| 4.77399831518 | pum1 (bg=29.85%) | K562 | - | chr20 | | 34638080 | 34638095 |

  
  

| Match 65 in HUMAN | | | | | | | |
| --- | --- | --- | --- | --- | --- | --- | --- |
| Motif | Start in Seq (1 Indexed) | End in Seq (1 Indexed) | Strand | Chrm | Exon | Start in Chrm (0 Indexed) | End in Chrm (1 Indexed) |
| AAGGCCTGTGTATATAATATGAAAAAGCTGCT | 803 | 834 | - | chr20 | 1 | 34638098 | 34638130 |
| eCLIP Fold-Enrichment | Binding Protein | Cell Line | Strand | Chrm | | Start in Chrm (0 Indexed) | End in Chrm (1 Indexed) |
| 5.73988345007 | pum1 (bg=29.85%) | K562 | - | chr20 | | 34638052 | 34638099 |

  
  

| Match 66 in HUMAN | | | | | | | |
| --- | --- | --- | --- | --- | --- | --- | --- |
| Motif | Start in Seq (1 Indexed) | End in Seq (1 Indexed) | Strand | Chrm | Exon | Start in Chrm (0 Indexed) | End in Chrm (1 Indexed) |
| AAGGCCTGTGTATATAATATGAAAAAGCTGCTCTCAACT | 803 | 841 | - | chr20 | 1 | 34638091 | 34638130 |
| eCLIP Fold-Enrichment | Binding Protein | Cell Line | Strand | Chrm | | Start in Chrm (0 Indexed) | End in Chrm (1 Indexed) |
| 5.73988345007 | pum1 (bg=29.85%) | K562 | - | chr20 | | 34638052 | 34638099 |
| 4.77399831518 | pum1 (bg=29.85%) | K562 | - | chr20 | | 34638080 | 34638095 |

  
  

| Match 67 in HUMAN | | | | | | | |
| --- | --- | --- | --- | --- | --- | --- | --- |
| Motif | Start in Seq (1 Indexed) | End in Seq (1 Indexed) | Strand | Chrm | Exon | Start in Chrm (0 Indexed) | End in Chrm (1 Indexed) |
| CCTGTGTATATAATATGAAAAAGCTGCT | 807 | 834 | - | chr20 | 1 | 34638098 | 34638126 |
| eCLIP Fold-Enrichment | Binding Protein | Cell Line | Strand | Chrm | | Start in Chrm (0 Indexed) | End in Chrm (1 Indexed) |
| 5.73988345007 | pum1 (bg=29.85%) | K562 | - | chr20 | | 34638052 | 34638099 |

  
  

| Match 68 in HUMAN | | | | | | | |
| --- | --- | --- | --- | --- | --- | --- | --- |
| Motif | Start in Seq (1 Indexed) | End in Seq (1 Indexed) | Strand | Chrm | Exon | Start in Chrm (0 Indexed) | End in Chrm (1 Indexed) |
| TCAACT | 836 | 841 | - | chr20 | 1 | 34638091 | 34638097 |
| eCLIP Fold-Enrichment | Binding Protein | Cell Line | Strand | Chrm | | Start in Chrm (0 Indexed) | End in Chrm (1 Indexed) |
| 5.73988345007 | pum1 (bg=29.85%) | K562 | - | chr20 | | 34638052 | 34638099 |
| 4.77399831518 | pum1 (bg=29.85%) | K562 | - | chr20 | | 34638080 | 34638095 |

  
  

| Match 69 in HUMAN | | | | | | | |
| --- | --- | --- | --- | --- | --- | --- | --- |
| Motif | Start in Seq (1 Indexed) | End in Seq (1 Indexed) | Strand | Chrm | Exon | Start in Chrm (0 Indexed) | End in Chrm (1 Indexed) |
| CCCCAACCTTT | 845 | 855 | - | chr20 | 1 | 34638077 | 34638088 |
| eCLIP Fold-Enrichment | Binding Protein | Cell Line | Strand | Chrm | | Start in Chrm (0 Indexed) | End in Chrm (1 Indexed) |
| 5.73988345007 | pum1 (bg=29.85%) | K562 | - | chr20 | | 34638052 | 34638099 |
| 4.77399831518 | pum1 (bg=29.85%) | K562 | - | chr20 | | 34638080 | 34638095 |

  
  

| Match 70 in HUMAN | | | | | | | |
| --- | --- | --- | --- | --- | --- | --- | --- |
| Motif | Start in Seq (1 Indexed) | End in Seq (1 Indexed) | Strand | Chrm | Exon | Start in Chrm (0 Indexed) | End in Chrm (1 Indexed) |
| CCCCAACCTTTT | 845 | 856 | - | chr20 | 1 | 34638076 | 34638088 |
| eCLIP Fold-Enrichment | Binding Protein | Cell Line | Strand | Chrm | | Start in Chrm (0 Indexed) | End in Chrm (1 Indexed) |
| 5.73988345007 | pum1 (bg=29.85%) | K562 | - | chr20 | | 34638052 | 34638099 |
| 4.77399831518 | pum1 (bg=29.85%) | K562 | - | chr20 | | 34638080 | 34638095 |

  
  

| Match 71 in HUMAN | | | | | | | |
| --- | --- | --- | --- | --- | --- | --- | --- |
| Motif | Start in Seq (1 Indexed) | End in Seq (1 Indexed) | Strand | Chrm | Exon | Start in Chrm (0 Indexed) | End in Chrm (1 Indexed) |
| CAACCTTT | 848 | 855 | - | chr20 | 1 | 34638077 | 34638085 |
| eCLIP Fold-Enrichment | Binding Protein | Cell Line | Strand | Chrm | | Start in Chrm (0 Indexed) | End in Chrm (1 Indexed) |
| 5.73988345007 | pum1 (bg=29.85%) | K562 | - | chr20 | | 34638052 | 34638099 |
| 4.77399831518 | pum1 (bg=29.85%) | K562 | - | chr20 | | 34638080 | 34638095 |

  
  

| Match 72 in HUMAN | | | | | | | |
| --- | --- | --- | --- | --- | --- | --- | --- |
| Motif | Start in Seq (1 Indexed) | End in Seq (1 Indexed) | Strand | Chrm | Exon | Start in Chrm (0 Indexed) | End in Chrm (1 Indexed) |
| AGAAAAC | 860 | 866 | - | chr20 | 1 | 34638066 | 34638073 |
| eCLIP Fold-Enrichment | Binding Protein | Cell Line | Strand | Chrm | | Start in Chrm (0 Indexed) | End in Chrm (1 Indexed) |
| 5.73988345007 | pum1 (bg=29.85%) | K562 | - | chr20 | | 34638052 | 34638099 |

  
  

| Match 73 in HUMAN | | | | | | | |
| --- | --- | --- | --- | --- | --- | --- | --- |
| Motif | Start in Seq (1 Indexed) | End in Seq (1 Indexed) | Strand | Chrm | Exon | Start in Chrm (0 Indexed) | End in Chrm (1 Indexed) |
| CACATCTAG | 873 | 881 | - | chr20 | 1 | 34638051 | 34638060 |
| eCLIP Fold-Enrichment | Binding Protein | Cell Line | Strand | Chrm | | Start in Chrm (0 Indexed) | End in Chrm (1 Indexed) |
| 4.85852794657 | pum1 (bg=29.85%) | K562 | - | chr20 | | 34638011 | 34638052 |
| 5.73988345007 | pum1 (bg=29.85%) | K562 | - | chr20 | | 34638052 | 34638099 |
| 3.78988137689 | rbm15 (bg=11.81%) | HepG2 | - | chr20 | | 34637999 | 34638056 |

  
  

| Match 74 in HUMAN | | | | | | | |
| --- | --- | --- | --- | --- | --- | --- | --- |
| Motif | Start in Seq (1 Indexed) | End in Seq (1 Indexed) | Strand | Chrm | Exon | Start in Chrm (0 Indexed) | End in Chrm (1 Indexed) |
| ACATCTAG | 874 | 881 | - | chr20 | 1 | 34638051 | 34638059 |
| eCLIP Fold-Enrichment | Binding Protein | Cell Line | Strand | Chrm | | Start in Chrm (0 Indexed) | End in Chrm (1 Indexed) |
| 4.85852794657 | pum1 (bg=29.85%) | K562 | - | chr20 | | 34638011 | 34638052 |
| 5.73988345007 | pum1 (bg=29.85%) | K562 | - | chr20 | | 34638052 | 34638099 |
| 3.78988137689 | rbm15 (bg=11.81%) | HepG2 | - | chr20 | | 34637999 | 34638056 |

  
  

| Match 75 in HUMAN | | | | | | | |
| --- | --- | --- | --- | --- | --- | --- | --- |
| Motif | Start in Seq (1 Indexed) | End in Seq (1 Indexed) | Strand | Chrm | Exon | Start in Chrm (0 Indexed) | End in Chrm (1 Indexed) |
| TAGATG | 888 | 893 | - | chr20 | 1 | 34638039 | 34638045 |
| eCLIP Fold-Enrichment | Binding Protein | Cell Line | Strand | Chrm | | Start in Chrm (0 Indexed) | End in Chrm (1 Indexed) |
| 2.40398834513 | dgcr8 (bg=19.31%) | SM-9MVZL | - | chr20 | | 34638010 | 34638040 |
| 2.08456334663 | KHDRBS1 (bg=1.16%) | K562 | - | chr20 | | 34637987 | 34638050 |
| 4.85852794657 | pum1 (bg=29.85%) | K562 | - | chr20 | | 34638011 | 34638052 |
| 4.70360898728 | pum1 (bg=29.85%) | K562 | - | chr20 | | 34638020 | 34638050 |
| 4.48827212962 | rbm15 (bg=11.81%) | HepG2 | - | chr20 | | 34637996 | 34638044 |
| 3.32377338178 | rbm15 (bg=11.81%) | K562 | - | chr20 | | 34637997 | 34638043 |
| 3.78988137689 | rbm15 (bg=11.81%) | HepG2 | - | chr20 | | 34637999 | 34638056 |
| 3.33379566658 | rbm15 (bg=11.81%) | K562 | - | chr20 | | 34638004 | 34638047 |

  
  

| Match 76 in HUMAN | | | | | | | |
| --- | --- | --- | --- | --- | --- | --- | --- |
| Motif | Start in Seq (1 Indexed) | End in Seq (1 Indexed) | Strand | Chrm | Exon | Start in Chrm (0 Indexed) | End in Chrm (1 Indexed) |
| TAGATGGAAAGAGGTTGCCGACGTATGATAAA | 888 | 919 | - | chr20 | 1 | 34638013 | 34638045 |
| eCLIP Fold-Enrichment | Binding Protein | Cell Line | Strand | Chrm | | Start in Chrm (0 Indexed) | End in Chrm (1 Indexed) |
| 2.40398834513 | dgcr8 (bg=19.31%) | SM-9MVZL | - | chr20 | | 34638010 | 34638040 |
| 2.638641711 | IGF2BP2 (bg=3.44%) | K562 | - | chr20 | | 34638008 | 34638030 |
| 2.08456334663 | KHDRBS1 (bg=1.16%) | K562 | - | chr20 | | 34637987 | 34638050 |
| 2.25893459665 | lin28b (bg=16.96%) | K562 | - | chr20 | | 34637979 | 34638036 |
| 3.16065543545 | lin28b (bg=16.96%) | HepG2 | - | chr20 | | 34637984 | 34638032 |
| 3.26552090847 | lin28b (bg=16.96%) | HepG2 | - | chr20 | | 34637997 | 34638037 |
| 4.85852794657 | pum1 (bg=29.85%) | K562 | - | chr20 | | 34638011 | 34638052 |
| 4.70360898728 | pum1 (bg=29.85%) | K562 | - | chr20 | | 34638020 | 34638050 |
| 3.69734337333 | pum2 (bg=21.55%) | K562 | - | chr20 | | 34638009 | 34638035 |
| 4.48827212962 | rbm15 (bg=11.81%) | HepG2 | - | chr20 | | 34637996 | 34638044 |
| 3.32377338178 | rbm15 (bg=11.81%) | K562 | - | chr20 | | 34637997 | 34638043 |
| 3.78988137689 | rbm15 (bg=11.81%) | HepG2 | - | chr20 | | 34637999 | 34638056 |
| 3.33379566658 | rbm15 (bg=11.81%) | K562 | - | chr20 | | 34638004 | 34638047 |
| 2.65759961637 | tia1 (bg=16.04%) | K562 | - | chr20 | | 34638008 | 34638032 |

  
  

| Match 77 in HUMAN | | | | | | | |
| --- | --- | --- | --- | --- | --- | --- | --- |
| Motif | Start in Seq (1 Indexed) | End in Seq (1 Indexed) | Strand | Chrm | Exon | Start in Chrm (0 Indexed) | End in Chrm (1 Indexed) |
| AAAGAGGTTGCCGAC | 895 | 909 | - | chr20 | 1 | 34638023 | 34638038 |
| eCLIP Fold-Enrichment | Binding Protein | Cell Line | Strand | Chrm | | Start in Chrm (0 Indexed) | End in Chrm (1 Indexed) |
| 2.40398834513 | dgcr8 (bg=19.31%) | SM-9MVZL | - | chr20 | | 34638010 | 34638040 |
| 2.638641711 | IGF2BP2 (bg=3.44%) | K562 | - | chr20 | | 34638008 | 34638030 |
| 2.08456334663 | KHDRBS1 (bg=1.16%) | K562 | - | chr20 | | 34637987 | 34638050 |
| 2.25893459665 | lin28b (bg=16.96%) | K562 | - | chr20 | | 34637979 | 34638036 |
| 3.16065543545 | lin28b (bg=16.96%) | HepG2 | - | chr20 | | 34637984 | 34638032 |
| 3.26552090847 | lin28b (bg=16.96%) | HepG2 | - | chr20 | | 34637997 | 34638037 |
| 4.85852794657 | pum1 (bg=29.85%) | K562 | - | chr20 | | 34638011 | 34638052 |
| 4.70360898728 | pum1 (bg=29.85%) | K562 | - | chr20 | | 34638020 | 34638050 |
| 3.69734337333 | pum2 (bg=21.55%) | K562 | - | chr20 | | 34638009 | 34638035 |
| 4.48827212962 | rbm15 (bg=11.81%) | HepG2 | - | chr20 | | 34637996 | 34638044 |
| 3.32377338178 | rbm15 (bg=11.81%) | K562 | - | chr20 | | 34637997 | 34638043 |
| 3.78988137689 | rbm15 (bg=11.81%) | HepG2 | - | chr20 | | 34637999 | 34638056 |
| 3.33379566658 | rbm15 (bg=11.81%) | K562 | - | chr20 | | 34638004 | 34638047 |
| 2.65759961637 | tia1 (bg=16.04%) | K562 | - | chr20 | | 34638008 | 34638032 |

  
  

| Match 78 in HUMAN | | | | | | | |
| --- | --- | --- | --- | --- | --- | --- | --- |
| Motif | Start in Seq (1 Indexed) | End in Seq (1 Indexed) | Strand | Chrm | Exon | Start in Chrm (0 Indexed) | End in Chrm (1 Indexed) |
| AAGAGGTTGCCGAC | 896 | 909 | - | chr20 | 1 | 34638023 | 34638037 |
| eCLIP Fold-Enrichment | Binding Protein | Cell Line | Strand | Chrm | | Start in Chrm (0 Indexed) | End in Chrm (1 Indexed) |
| 2.40398834513 | dgcr8 (bg=19.31%) | SM-9MVZL | - | chr20 | | 34638010 | 34638040 |
| 2.638641711 | IGF2BP2 (bg=3.44%) | K562 | - | chr20 | | 34638008 | 34638030 |
| 2.08456334663 | KHDRBS1 (bg=1.16%) | K562 | - | chr20 | | 34637987 | 34638050 |
| 2.25893459665 | lin28b (bg=16.96%) | K562 | - | chr20 | | 34637979 | 34638036 |
| 3.16065543545 | lin28b (bg=16.96%) | HepG2 | - | chr20 | | 34637984 | 34638032 |
| 3.26552090847 | lin28b (bg=16.96%) | HepG2 | - | chr20 | | 34637997 | 34638037 |
| 4.85852794657 | pum1 (bg=29.85%) | K562 | - | chr20 | | 34638011 | 34638052 |
| 4.70360898728 | pum1 (bg=29.85%) | K562 | - | chr20 | | 34638020 | 34638050 |
| 3.69734337333 | pum2 (bg=21.55%) | K562 | - | chr20 | | 34638009 | 34638035 |
| 4.48827212962 | rbm15 (bg=11.81%) | HepG2 | - | chr20 | | 34637996 | 34638044 |
| 3.32377338178 | rbm15 (bg=11.81%) | K562 | - | chr20 | | 34637997 | 34638043 |
| 3.78988137689 | rbm15 (bg=11.81%) | HepG2 | - | chr20 | | 34637999 | 34638056 |
| 3.33379566658 | rbm15 (bg=11.81%) | K562 | - | chr20 | | 34638004 | 34638047 |
| 2.65759961637 | tia1 (bg=16.04%) | K562 | - | chr20 | | 34638008 | 34638032 |

  
  

| Match 79 in HUMAN | | | | | | | |
| --- | --- | --- | --- | --- | --- | --- | --- |
| Motif | Start in Seq (1 Indexed) | End in Seq (1 Indexed) | Strand | Chrm | Exon | Start in Chrm (0 Indexed) | End in Chrm (1 Indexed) |
| AAGAGGT | 896 | 902 | - | chr20 | 1 | 34638030 | 34638037 |
| eCLIP Fold-Enrichment | Binding Protein | Cell Line | Strand | Chrm | | Start in Chrm (0 Indexed) | End in Chrm (1 Indexed) |
| 2.40398834513 | dgcr8 (bg=19.31%) | SM-9MVZL | - | chr20 | | 34638010 | 34638040 |
| 2.638641711 | IGF2BP2 (bg=3.44%) | K562 | - | chr20 | | 34638008 | 34638030 |
| 2.08456334663 | KHDRBS1 (bg=1.16%) | K562 | - | chr20 | | 34637987 | 34638050 |
| 2.25893459665 | lin28b (bg=16.96%) | K562 | - | chr20 | | 34637979 | 34638036 |
| 3.16065543545 | lin28b (bg=16.96%) | HepG2 | - | chr20 | | 34637984 | 34638032 |
| 3.26552090847 | lin28b (bg=16.96%) | HepG2 | - | chr20 | | 34637997 | 34638037 |
| 4.85852794657 | pum1 (bg=29.85%) | K562 | - | chr20 | | 34638011 | 34638052 |
| 4.70360898728 | pum1 (bg=29.85%) | K562 | - | chr20 | | 34638020 | 34638050 |
| 3.69734337333 | pum2 (bg=21.55%) | K562 | - | chr20 | | 34638009 | 34638035 |
| 4.48827212962 | rbm15 (bg=11.81%) | HepG2 | - | chr20 | | 34637996 | 34638044 |
| 3.32377338178 | rbm15 (bg=11.81%) | K562 | - | chr20 | | 34637997 | 34638043 |
| 3.78988137689 | rbm15 (bg=11.81%) | HepG2 | - | chr20 | | 34637999 | 34638056 |
| 3.33379566658 | rbm15 (bg=11.81%) | K562 | - | chr20 | | 34638004 | 34638047 |
| 2.65759961637 | tia1 (bg=16.04%) | K562 | - | chr20 | | 34638008 | 34638032 |

  
  

| Match 80 in HUMAN | | | | | | | |
| --- | --- | --- | --- | --- | --- | --- | --- |
| Motif | Start in Seq (1 Indexed) | End in Seq (1 Indexed) | Strand | Chrm | Exon | Start in Chrm (0 Indexed) | End in Chrm (1 Indexed) |
| AGAGGT | 897 | 902 | - | chr20 | 1 | 34638030 | 34638036 |
| eCLIP Fold-Enrichment | Binding Protein | Cell Line | Strand | Chrm | | Start in Chrm (0 Indexed) | End in Chrm (1 Indexed) |
| 2.40398834513 | dgcr8 (bg=19.31%) | SM-9MVZL | - | chr20 | | 34638010 | 34638040 |
| 2.638641711 | IGF2BP2 (bg=3.44%) | K562 | - | chr20 | | 34638008 | 34638030 |
| 2.08456334663 | KHDRBS1 (bg=1.16%) | K562 | - | chr20 | | 34637987 | 34638050 |
| 2.25893459665 | lin28b (bg=16.96%) | K562 | - | chr20 | | 34637979 | 34638036 |
| 3.16065543545 | lin28b (bg=16.96%) | HepG2 | - | chr20 | | 34637984 | 34638032 |
| 3.26552090847 | lin28b (bg=16.96%) | HepG2 | - | chr20 | | 34637997 | 34638037 |
| 4.85852794657 | pum1 (bg=29.85%) | K562 | - | chr20 | | 34638011 | 34638052 |
| 4.70360898728 | pum1 (bg=29.85%) | K562 | - | chr20 | | 34638020 | 34638050 |
| 3.69734337333 | pum2 (bg=21.55%) | K562 | - | chr20 | | 34638009 | 34638035 |
| 4.48827212962 | rbm15 (bg=11.81%) | HepG2 | - | chr20 | | 34637996 | 34638044 |
| 3.32377338178 | rbm15 (bg=11.81%) | K562 | - | chr20 | | 34637997 | 34638043 |
| 3.78988137689 | rbm15 (bg=11.81%) | HepG2 | - | chr20 | | 34637999 | 34638056 |
| 3.33379566658 | rbm15 (bg=11.81%) | K562 | - | chr20 | | 34638004 | 34638047 |
| 2.65759961637 | tia1 (bg=16.04%) | K562 | - | chr20 | | 34638008 | 34638032 |

  
  

| Match 81 in HUMAN | | | | | | | |
| --- | --- | --- | --- | --- | --- | --- | --- |
| Motif | Start in Seq (1 Indexed) | End in Seq (1 Indexed) | Strand | Chrm | Exon | Start in Chrm (0 Indexed) | End in Chrm (1 Indexed) |
| GCCGAC | 904 | 909 | - | chr20 | 1 | 34638023 | 34638029 |
| eCLIP Fold-Enrichment | Binding Protein | Cell Line | Strand | Chrm | | Start in Chrm (0 Indexed) | End in Chrm (1 Indexed) |
| 2.40398834513 | dgcr8 (bg=19.31%) | SM-9MVZL | - | chr20 | | 34638010 | 34638040 |
| 2.638641711 | IGF2BP2 (bg=3.44%) | K562 | - | chr20 | | 34638008 | 34638030 |
| 2.08456334663 | KHDRBS1 (bg=1.16%) | K562 | - | chr20 | | 34637987 | 34638050 |
| 2.25893459665 | lin28b (bg=16.96%) | K562 | - | chr20 | | 34637979 | 34638036 |
| 3.16065543545 | lin28b (bg=16.96%) | HepG2 | - | chr20 | | 34637984 | 34638032 |
| 3.26552090847 | lin28b (bg=16.96%) | HepG2 | - | chr20 | | 34637997 | 34638037 |
| 4.85852794657 | pum1 (bg=29.85%) | K562 | - | chr20 | | 34638011 | 34638052 |
| 4.70360898728 | pum1 (bg=29.85%) | K562 | - | chr20 | | 34638020 | 34638050 |
| 3.69734337333 | pum2 (bg=21.55%) | K562 | - | chr20 | | 34638009 | 34638035 |
| 4.48827212962 | rbm15 (bg=11.81%) | HepG2 | - | chr20 | | 34637996 | 34638044 |
| 3.32377338178 | rbm15 (bg=11.81%) | K562 | - | chr20 | | 34637997 | 34638043 |
| 3.78988137689 | rbm15 (bg=11.81%) | HepG2 | - | chr20 | | 34637999 | 34638056 |
| 3.33379566658 | rbm15 (bg=11.81%) | K562 | - | chr20 | | 34638004 | 34638047 |
| 2.65759961637 | tia1 (bg=16.04%) | K562 | - | chr20 | | 34638008 | 34638032 |

  
  

| Match 82 in HUMAN | | | | | | | |
| --- | --- | --- | --- | --- | --- | --- | --- |
| Motif | Start in Seq (1 Indexed) | End in Seq (1 Indexed) | Strand | Chrm | Exon | Start in Chrm (0 Indexed) | End in Chrm (1 Indexed) |
| TATGATAAA | 911 | 919 | - | chr20 | 1 | 34638013 | 34638022 |
| eCLIP Fold-Enrichment | Binding Protein | Cell Line | Strand | Chrm | | Start in Chrm (0 Indexed) | End in Chrm (1 Indexed) |
| 2.40398834513 | dgcr8 (bg=19.31%) | SM-9MVZL | - | chr20 | | 34638010 | 34638040 |
| 2.638641711 | IGF2BP2 (bg=3.44%) | K562 | - | chr20 | | 34638008 | 34638030 |
| 2.08456334663 | KHDRBS1 (bg=1.16%) | K562 | - | chr20 | | 34637987 | 34638050 |
| 2.25893459665 | lin28b (bg=16.96%) | K562 | - | chr20 | | 34637979 | 34638036 |
| 3.16065543545 | lin28b (bg=16.96%) | HepG2 | - | chr20 | | 34637984 | 34638032 |
| 3.26552090847 | lin28b (bg=16.96%) | HepG2 | - | chr20 | | 34637997 | 34638037 |
| 4.85852794657 | pum1 (bg=29.85%) | K562 | - | chr20 | | 34638011 | 34638052 |
| 4.70360898728 | pum1 (bg=29.85%) | K562 | - | chr20 | | 34638020 | 34638050 |
| 3.69734337333 | pum2 (bg=21.55%) | K562 | - | chr20 | | 34638009 | 34638035 |
| 4.48827212962 | rbm15 (bg=11.81%) | HepG2 | - | chr20 | | 34637996 | 34638044 |
| 3.32377338178 | rbm15 (bg=11.81%) | K562 | - | chr20 | | 34637997 | 34638043 |
| 3.78988137689 | rbm15 (bg=11.81%) | HepG2 | - | chr20 | | 34637999 | 34638056 |
| 3.33379566658 | rbm15 (bg=11.81%) | K562 | - | chr20 | | 34638004 | 34638047 |
| 2.65759961637 | tia1 (bg=16.04%) | K562 | - | chr20 | | 34638008 | 34638032 |

  
  

| Match 83 in HUMAN | | | | | | | |
| --- | --- | --- | --- | --- | --- | --- | --- |
| Motif | Start in Seq (1 Indexed) | End in Seq (1 Indexed) | Strand | Chrm | Exon | Start in Chrm (0 Indexed) | End in Chrm (1 Indexed) |
| TAGAGTTAGAAA | 921 | 932 | - | chr20 | 1 | 34638000 | 34638012 |
| eCLIP Fold-Enrichment | Binding Protein | Cell Line | Strand | Chrm | | Start in Chrm (0 Indexed) | End in Chrm (1 Indexed) |
| 3.62638076647 | dgcr8 (bg=19.31%) | SM-9MVZL | - | chr20 | | 34638001 | 34638010 |
| 2.40398834513 | dgcr8 (bg=19.31%) | SM-9MVZL | - | chr20 | | 34638010 | 34638040 |
| 2.638641711 | IGF2BP2 (bg=3.44%) | K562 | - | chr20 | | 34638008 | 34638030 |
| 2.08456334663 | KHDRBS1 (bg=1.16%) | K562 | - | chr20 | | 34637987 | 34638050 |
| 2.25893459665 | lin28b (bg=16.96%) | K562 | - | chr20 | | 34637979 | 34638036 |
| 3.16065543545 | lin28b (bg=16.96%) | HepG2 | - | chr20 | | 34637984 | 34638032 |
| 3.26552090847 | lin28b (bg=16.96%) | HepG2 | - | chr20 | | 34637997 | 34638037 |
| 4.85852794657 | pum1 (bg=29.85%) | K562 | - | chr20 | | 34638011 | 34638052 |
| 3.69734337333 | pum2 (bg=21.55%) | K562 | - | chr20 | | 34638009 | 34638035 |
| 4.48827212962 | rbm15 (bg=11.81%) | HepG2 | - | chr20 | | 34637996 | 34638044 |
| 3.32377338178 | rbm15 (bg=11.81%) | K562 | - | chr20 | | 34637997 | 34638043 |
| 3.78988137689 | rbm15 (bg=11.81%) | HepG2 | - | chr20 | | 34637999 | 34638056 |
| 3.33379566658 | rbm15 (bg=11.81%) | K562 | - | chr20 | | 34638004 | 34638047 |
| 2.65759961637 | tia1 (bg=16.04%) | K562 | - | chr20 | | 34638008 | 34638032 |

  
  

| Match 84 in HUMAN | | | | | | | |
| --- | --- | --- | --- | --- | --- | --- | --- |
| Motif | Start in Seq (1 Indexed) | End in Seq (1 Indexed) | Strand | Chrm | Exon | Start in Chrm (0 Indexed) | End in Chrm (1 Indexed) |
| TAGAGTTAGAAAGT | 921 | 934 | - | chr20 | 1 | 34637998 | 34638012 |
| eCLIP Fold-Enrichment | Binding Protein | Cell Line | Strand | Chrm | | Start in Chrm (0 Indexed) | End in Chrm (1 Indexed) |
| 3.62638076647 | dgcr8 (bg=19.31%) | SM-9MVZL | - | chr20 | | 34638001 | 34638010 |
| 2.40398834513 | dgcr8 (bg=19.31%) | SM-9MVZL | - | chr20 | | 34638010 | 34638040 |
| 2.638641711 | IGF2BP2 (bg=3.44%) | K562 | - | chr20 | | 34638008 | 34638030 |
| 2.08456334663 | KHDRBS1 (bg=1.16%) | K562 | - | chr20 | | 34637987 | 34638050 |
| 2.25893459665 | lin28b (bg=16.96%) | K562 | - | chr20 | | 34637979 | 34638036 |
| 3.16065543545 | lin28b (bg=16.96%) | HepG2 | - | chr20 | | 34637984 | 34638032 |
| 3.26552090847 | lin28b (bg=16.96%) | HepG2 | - | chr20 | | 34637997 | 34638037 |
| 4.85852794657 | pum1 (bg=29.85%) | K562 | - | chr20 | | 34638011 | 34638052 |
| 3.69734337333 | pum2 (bg=21.55%) | K562 | - | chr20 | | 34638009 | 34638035 |
| 4.48827212962 | rbm15 (bg=11.81%) | HepG2 | - | chr20 | | 34637996 | 34638044 |
| 3.32377338178 | rbm15 (bg=11.81%) | K562 | - | chr20 | | 34637997 | 34638043 |
| 3.78988137689 | rbm15 (bg=11.81%) | HepG2 | - | chr20 | | 34637999 | 34638056 |
| 3.33379566658 | rbm15 (bg=11.81%) | K562 | - | chr20 | | 34638004 | 34638047 |
| 2.65759961637 | tia1 (bg=16.04%) | K562 | - | chr20 | | 34638008 | 34638032 |

  
  

| Match 85 in HUMAN | | | | | | | |
| --- | --- | --- | --- | --- | --- | --- | --- |
| Motif | Start in Seq (1 Indexed) | End in Seq (1 Indexed) | Strand | Chrm | Exon | Start in Chrm (0 Indexed) | End in Chrm (1 Indexed) |
| AGTTAGAAA | 924 | 932 | - | chr20 | 1 | 34638000 | 34638009 |
| eCLIP Fold-Enrichment | Binding Protein | Cell Line | Strand | Chrm | | Start in Chrm (0 Indexed) | End in Chrm (1 Indexed) |
| 3.62638076647 | dgcr8 (bg=19.31%) | SM-9MVZL | - | chr20 | | 34638001 | 34638010 |
| 2.638641711 | IGF2BP2 (bg=3.44%) | K562 | - | chr20 | | 34638008 | 34638030 |
| 2.08456334663 | KHDRBS1 (bg=1.16%) | K562 | - | chr20 | | 34637987 | 34638050 |
| 2.25893459665 | lin28b (bg=16.96%) | K562 | - | chr20 | | 34637979 | 34638036 |
| 3.16065543545 | lin28b (bg=16.96%) | HepG2 | - | chr20 | | 34637984 | 34638032 |
| 3.26552090847 | lin28b (bg=16.96%) | HepG2 | - | chr20 | | 34637997 | 34638037 |
| 3.69734337333 | pum2 (bg=21.55%) | K562 | - | chr20 | | 34638009 | 34638035 |
| 4.48827212962 | rbm15 (bg=11.81%) | HepG2 | - | chr20 | | 34637996 | 34638044 |
| 3.32377338178 | rbm15 (bg=11.81%) | K562 | - | chr20 | | 34637997 | 34638043 |
| 3.78988137689 | rbm15 (bg=11.81%) | HepG2 | - | chr20 | | 34637999 | 34638056 |
| 3.33379566658 | rbm15 (bg=11.81%) | K562 | - | chr20 | | 34638004 | 34638047 |
| 2.65759961637 | tia1 (bg=16.04%) | K562 | - | chr20 | | 34638008 | 34638032 |

  
  

| Match 86 in HUMAN | | | | | | | |
| --- | --- | --- | --- | --- | --- | --- | --- |
| Motif | Start in Seq (1 Indexed) | End in Seq (1 Indexed) | Strand | Chrm | Exon | Start in Chrm (0 Indexed) | End in Chrm (1 Indexed) |
| AGTTAG | 924 | 929 | - | chr20 | 1 | 34638003 | 34638009 |
| eCLIP Fold-Enrichment | Binding Protein | Cell Line | Strand | Chrm | | Start in Chrm (0 Indexed) | End in Chrm (1 Indexed) |
| 3.62638076647 | dgcr8 (bg=19.31%) | SM-9MVZL | - | chr20 | | 34638001 | 34638010 |
| 2.638641711 | IGF2BP2 (bg=3.44%) | K562 | - | chr20 | | 34638008 | 34638030 |
| 2.08456334663 | KHDRBS1 (bg=1.16%) | K562 | - | chr20 | | 34637987 | 34638050 |
| 2.25893459665 | lin28b (bg=16.96%) | K562 | - | chr20 | | 34637979 | 34638036 |
| 3.16065543545 | lin28b (bg=16.96%) | HepG2 | - | chr20 | | 34637984 | 34638032 |
| 3.26552090847 | lin28b (bg=16.96%) | HepG2 | - | chr20 | | 34637997 | 34638037 |
| 3.69734337333 | pum2 (bg=21.55%) | K562 | - | chr20 | | 34638009 | 34638035 |
| 4.48827212962 | rbm15 (bg=11.81%) | HepG2 | - | chr20 | | 34637996 | 34638044 |
| 3.32377338178 | rbm15 (bg=11.81%) | K562 | - | chr20 | | 34637997 | 34638043 |
| 3.78988137689 | rbm15 (bg=11.81%) | HepG2 | - | chr20 | | 34637999 | 34638056 |
| 3.33379566658 | rbm15 (bg=11.81%) | K562 | - | chr20 | | 34638004 | 34638047 |
| 2.65759961637 | tia1 (bg=16.04%) | K562 | - | chr20 | | 34638008 | 34638032 |

  
  

| Match 87 in HUMAN | | | | | | | |
| --- | --- | --- | --- | --- | --- | --- | --- |
| Motif | Start in Seq (1 Indexed) | End in Seq (1 Indexed) | Strand | Chrm | Exon | Start in Chrm (0 Indexed) | End in Chrm (1 Indexed) |
| ACACATCTTGTAAATTCTCATTTGTTTAAAAGAAATCATAGAAAATAC | 936 | 983 | - | chr20 | 1 | 34637949 | 34637997 |
| eCLIP Fold-Enrichment | Binding Protein | Cell Line | Strand | Chrm | | Start in Chrm (0 Indexed) | End in Chrm (1 Indexed) |
| 3.11622088669 | fubp3 (bg=23.31%) | HepG2 | - | chr20 | | 34637905 | 34637964 |
| 2.15760822025 | fubp3 (bg=23.31%) | HepG2 | - | chr20 | | 34637927 | 34637962 |
| 2.0537229917 | GRWD1 (bg=4.85%) | HepG2 | - | chr20 | | 34637879 | 34637955 |
| 2.08456334663 | KHDRBS1 (bg=1.16%) | K562 | - | chr20 | | 34637987 | 34638050 |
| 2.25893459665 | lin28b (bg=16.96%) | K562 | - | chr20 | | 34637979 | 34638036 |
| 3.16065543545 | lin28b (bg=16.96%) | HepG2 | - | chr20 | | 34637984 | 34638032 |
| 3.26552090847 | lin28b (bg=16.96%) | HepG2 | - | chr20 | | 34637997 | 34638037 |
| 3.27897894204 | pum1 (bg=29.85%) | K562 | - | chr20 | | 34637920 | 34637962 |
| 2.96664339312 | pum1 (bg=29.85%) | K562 | - | chr20 | | 34637927 | 34637968 |
| 3.25828744911 | pum2 (bg=21.55%) | K562 | - | chr20 | | 34637920 | 34637967 |
| 4.17932008679 | pum2 (bg=21.55%) | K562 | - | chr20 | | 34637928 | 34637969 |
| 2.55248679221 | rbm15 (bg=11.81%) | HepG2 | - | chr20 | | 34637930 | 34637967 |
| 4.48827212962 | rbm15 (bg=11.81%) | HepG2 | - | chr20 | | 34637996 | 34638044 |
| 3.32377338178 | rbm15 (bg=11.81%) | K562 | - | chr20 | | 34637997 | 34638043 |
| 2.24350639536 | sf3a3 (bg=12.13%) | HepG2 | - | chr20 | | 34637933 | 34637963 |
| 2.49087812115 | ZNF622 (bg=1.54%) | K562 | - | chr20 | | 34637875 | 34637959 |

  
  

| Match 88 in HUMAN | | | | | | | |
| --- | --- | --- | --- | --- | --- | --- | --- |
| Motif | Start in Seq (1 Indexed) | End in Seq (1 Indexed) | Strand | Chrm | Exon | Start in Chrm (0 Indexed) | End in Chrm (1 Indexed) |
| TCTTGTAAAT | 941 | 950 | - | chr20 | 1 | 34637982 | 34637992 |
| eCLIP Fold-Enrichment | Binding Protein | Cell Line | Strand | Chrm | | Start in Chrm (0 Indexed) | End in Chrm (1 Indexed) |
| 2.08456334663 | KHDRBS1 (bg=1.16%) | K562 | - | chr20 | | 34637987 | 34638050 |
| 2.25893459665 | lin28b (bg=16.96%) | K562 | - | chr20 | | 34637979 | 34638036 |
| 3.16065543545 | lin28b (bg=16.96%) | HepG2 | - | chr20 | | 34637984 | 34638032 |

  
  

| Match 89 in HUMAN | | | | | | | |
| --- | --- | --- | --- | --- | --- | --- | --- |
| Motif | Start in Seq (1 Indexed) | End in Seq (1 Indexed) | Strand | Chrm | Exon | Start in Chrm (0 Indexed) | End in Chrm (1 Indexed) |
| TTGTAA | 943 | 948 | - | chr20 | 1 | 34637984 | 34637990 |
| eCLIP Fold-Enrichment | Binding Protein | Cell Line | Strand | Chrm | | Start in Chrm (0 Indexed) | End in Chrm (1 Indexed) |
| 2.08456334663 | KHDRBS1 (bg=1.16%) | K562 | - | chr20 | | 34637987 | 34638050 |
| 2.25893459665 | lin28b (bg=16.96%) | K562 | - | chr20 | | 34637979 | 34638036 |
| 3.16065543545 | lin28b (bg=16.96%) | HepG2 | - | chr20 | | 34637984 | 34638032 |

  
  

| Match 90 in HUMAN | | | | | | | |
| --- | --- | --- | --- | --- | --- | --- | --- |
| Motif | Start in Seq (1 Indexed) | End in Seq (1 Indexed) | Strand | Chrm | Exon | Start in Chrm (0 Indexed) | End in Chrm (1 Indexed) |
| AAATCATAGAAA | 968 | 979 | - | chr20 | 1 | 34637953 | 34637965 |
| eCLIP Fold-Enrichment | Binding Protein | Cell Line | Strand | Chrm | | Start in Chrm (0 Indexed) | End in Chrm (1 Indexed) |
| 3.11622088669 | fubp3 (bg=23.31%) | HepG2 | - | chr20 | | 34637905 | 34637964 |
| 2.15760822025 | fubp3 (bg=23.31%) | HepG2 | - | chr20 | | 34637927 | 34637962 |
| 2.0537229917 | GRWD1 (bg=4.85%) | HepG2 | - | chr20 | | 34637879 | 34637955 |
| 3.27897894204 | pum1 (bg=29.85%) | K562 | - | chr20 | | 34637920 | 34637962 |
| 2.96664339312 | pum1 (bg=29.85%) | K562 | - | chr20 | | 34637927 | 34637968 |
| 3.25828744911 | pum2 (bg=21.55%) | K562 | - | chr20 | | 34637920 | 34637967 |
| 4.17932008679 | pum2 (bg=21.55%) | K562 | - | chr20 | | 34637928 | 34637969 |
| 2.55248679221 | rbm15 (bg=11.81%) | HepG2 | - | chr20 | | 34637930 | 34637967 |
| 2.24350639536 | sf3a3 (bg=12.13%) | HepG2 | - | chr20 | | 34637933 | 34637963 |
| 2.49087812115 | ZNF622 (bg=1.54%) | K562 | - | chr20 | | 34637875 | 34637959 |

  
  

| Match 91 in HUMAN | | | | | | | |
| --- | --- | --- | --- | --- | --- | --- | --- |
| Motif | Start in Seq (1 Indexed) | End in Seq (1 Indexed) | Strand | Chrm | Exon | Start in Chrm (0 Indexed) | End in Chrm (1 Indexed) |
| ATAGAAA | 973 | 979 | - | chr20 | 1 | 34637953 | 34637960 |
| eCLIP Fold-Enrichment | Binding Protein | Cell Line | Strand | Chrm | | Start in Chrm (0 Indexed) | End in Chrm (1 Indexed) |
| 3.11622088669 | fubp3 (bg=23.31%) | HepG2 | - | chr20 | | 34637905 | 34637964 |
| 2.15760822025 | fubp3 (bg=23.31%) | HepG2 | - | chr20 | | 34637927 | 34637962 |
| 2.0537229917 | GRWD1 (bg=4.85%) | HepG2 | - | chr20 | | 34637879 | 34637955 |
| 3.27897894204 | pum1 (bg=29.85%) | K562 | - | chr20 | | 34637920 | 34637962 |
| 2.96664339312 | pum1 (bg=29.85%) | K562 | - | chr20 | | 34637927 | 34637968 |
| 3.25828744911 | pum2 (bg=21.55%) | K562 | - | chr20 | | 34637920 | 34637967 |
| 4.17932008679 | pum2 (bg=21.55%) | K562 | - | chr20 | | 34637928 | 34637969 |
| 2.55248679221 | rbm15 (bg=11.81%) | HepG2 | - | chr20 | | 34637930 | 34637967 |
| 2.24350639536 | sf3a3 (bg=12.13%) | HepG2 | - | chr20 | | 34637933 | 34637963 |
| 2.49087812115 | ZNF622 (bg=1.54%) | K562 | - | chr20 | | 34637875 | 34637959 |

  
  

| Match 92 in HUMAN | | | | | | | |
| --- | --- | --- | --- | --- | --- | --- | --- |
| Motif | Start in Seq (1 Indexed) | End in Seq (1 Indexed) | Strand | Chrm | Exon | Start in Chrm (0 Indexed) | End in Chrm (1 Indexed) |
| TGTCTTCTGGAGATGACTTTTGGAAATG | 985 | 1012 | - | chr20 | 1 | 34637920 | 34637948 |
| eCLIP Fold-Enrichment | Binding Protein | Cell Line | Strand | Chrm | | Start in Chrm (0 Indexed) | End in Chrm (1 Indexed) |
| 3.11622088669 | fubp3 (bg=23.31%) | HepG2 | - | chr20 | | 34637905 | 34637964 |
| 2.15760822025 | fubp3 (bg=23.31%) | HepG2 | - | chr20 | | 34637927 | 34637962 |
| 2.0537229917 | GRWD1 (bg=4.85%) | HepG2 | - | chr20 | | 34637879 | 34637955 |
| 2.37906836964 | PPIG (bg=0.88%) | HepG2 | - | chr20 | | 34637873 | 34637921 |
| 3.47567085304 | pum1 (bg=29.85%) | K562 | - | chr20 | | 34637875 | 34637920 |
| 2.94588483295 | pum1 (bg=29.85%) | K562 | - | chr20 | | 34637883 | 34637927 |
| 3.27897894204 | pum1 (bg=29.85%) | K562 | - | chr20 | | 34637920 | 34637962 |
| 2.96664339312 | pum1 (bg=29.85%) | K562 | - | chr20 | | 34637927 | 34637968 |
| 3.25828744911 | pum2 (bg=21.55%) | K562 | - | chr20 | | 34637920 | 34637967 |
| 4.17932008679 | pum2 (bg=21.55%) | K562 | - | chr20 | | 34637928 | 34637969 |
| 2.58780780317 | rbm15 (bg=11.81%) | HepG2 | - | chr20 | | 34637893 | 34637923 |
| 2.3533641502 | rbm15 (bg=11.81%) | HepG2 | - | chr20 | | 34637898 | 34637930 |
| 2.55248679221 | rbm15 (bg=11.81%) | HepG2 | - | chr20 | | 34637930 | 34637967 |
| 2.24350639536 | sf3a3 (bg=12.13%) | HepG2 | - | chr20 | | 34637933 | 34637963 |
| 3.9837301591 | SND1 (bg=1.27%) | HepG2 | - | chr20 | | 34637911 | 34637943 |
| 2.49087812115 | ZNF622 (bg=1.54%) | K562 | - | chr20 | | 34637875 | 34637959 |

  
  

| Match 93 in HUMAN | | | | | | | |
| --- | --- | --- | --- | --- | --- | --- | --- |
| Motif | Start in Seq (1 Indexed) | End in Seq (1 Indexed) | Strand | Chrm | Exon | Start in Chrm (0 Indexed) | End in Chrm (1 Indexed) |
| CTTTTGGAAATG | 1001 | 1012 | - | chr20 | 1 | 34637920 | 34637932 |
| eCLIP Fold-Enrichment | Binding Protein | Cell Line | Strand | Chrm | | Start in Chrm (0 Indexed) | End in Chrm (1 Indexed) |
| 3.11622088669 | fubp3 (bg=23.31%) | HepG2 | - | chr20 | | 34637905 | 34637964 |
| 2.15760822025 | fubp3 (bg=23.31%) | HepG2 | - | chr20 | | 34637927 | 34637962 |
| 2.0537229917 | GRWD1 (bg=4.85%) | HepG2 | - | chr20 | | 34637879 | 34637955 |
| 2.37906836964 | PPIG (bg=0.88%) | HepG2 | - | chr20 | | 34637873 | 34637921 |
| 3.47567085304 | pum1 (bg=29.85%) | K562 | - | chr20 | | 34637875 | 34637920 |
| 2.94588483295 | pum1 (bg=29.85%) | K562 | - | chr20 | | 34637883 | 34637927 |
| 3.27897894204 | pum1 (bg=29.85%) | K562 | - | chr20 | | 34637920 | 34637962 |
| 2.96664339312 | pum1 (bg=29.85%) | K562 | - | chr20 | | 34637927 | 34637968 |
| 3.25828744911 | pum2 (bg=21.55%) | K562 | - | chr20 | | 34637920 | 34637967 |
| 4.17932008679 | pum2 (bg=21.55%) | K562 | - | chr20 | | 34637928 | 34637969 |
| 2.58780780317 | rbm15 (bg=11.81%) | HepG2 | - | chr20 | | 34637893 | 34637923 |
| 2.3533641502 | rbm15 (bg=11.81%) | HepG2 | - | chr20 | | 34637898 | 34637930 |
| 2.55248679221 | rbm15 (bg=11.81%) | HepG2 | - | chr20 | | 34637930 | 34637967 |
| 3.9837301591 | SND1 (bg=1.27%) | HepG2 | - | chr20 | | 34637911 | 34637943 |
| 2.49087812115 | ZNF622 (bg=1.54%) | K562 | - | chr20 | | 34637875 | 34637959 |

  
  

| Match 94 in HUMAN | | | | | | | |
| --- | --- | --- | --- | --- | --- | --- | --- |
| Motif | Start in Seq (1 Indexed) | End in Seq (1 Indexed) | Strand | Chrm | Exon | Start in Chrm (0 Indexed) | End in Chrm (1 Indexed) |
| TGGAAATG | 1005 | 1012 | - | chr20 | 1 | 34637920 | 34637928 |
| eCLIP Fold-Enrichment | Binding Protein | Cell Line | Strand | Chrm | | Start in Chrm (0 Indexed) | End in Chrm (1 Indexed) |
| 3.11622088669 | fubp3 (bg=23.31%) | HepG2 | - | chr20 | | 34637905 | 34637964 |
| 2.15760822025 | fubp3 (bg=23.31%) | HepG2 | - | chr20 | | 34637927 | 34637962 |
| 2.0537229917 | GRWD1 (bg=4.85%) | HepG2 | - | chr20 | | 34637879 | 34637955 |
| 2.37906836964 | PPIG (bg=0.88%) | HepG2 | - | chr20 | | 34637873 | 34637921 |
| 3.47567085304 | pum1 (bg=29.85%) | K562 | - | chr20 | | 34637875 | 34637920 |
| 2.94588483295 | pum1 (bg=29.85%) | K562 | - | chr20 | | 34637883 | 34637927 |
| 3.27897894204 | pum1 (bg=29.85%) | K562 | - | chr20 | | 34637920 | 34637962 |
| 2.96664339312 | pum1 (bg=29.85%) | K562 | - | chr20 | | 34637927 | 34637968 |
| 3.25828744911 | pum2 (bg=21.55%) | K562 | - | chr20 | | 34637920 | 34637967 |
| 4.17932008679 | pum2 (bg=21.55%) | K562 | - | chr20 | | 34637928 | 34637969 |
| 2.58780780317 | rbm15 (bg=11.81%) | HepG2 | - | chr20 | | 34637893 | 34637923 |
| 2.3533641502 | rbm15 (bg=11.81%) | HepG2 | - | chr20 | | 34637898 | 34637930 |
| 3.9837301591 | SND1 (bg=1.27%) | HepG2 | - | chr20 | | 34637911 | 34637943 |
| 2.49087812115 | ZNF622 (bg=1.54%) | K562 | - | chr20 | | 34637875 | 34637959 |

  
  

| Match 95 in HUMAN | | | | | | | |
| --- | --- | --- | --- | --- | --- | --- | --- |
| Motif | Start in Seq (1 Indexed) | End in Seq (1 Indexed) | Strand | Chrm | Exon | Start in Chrm (0 Indexed) | End in Chrm (1 Indexed) |
| GAAATG | 1007 | 1012 | - | chr20 | 1 | 34637920 | 34637926 |
| eCLIP Fold-Enrichment | Binding Protein | Cell Line | Strand | Chrm | | Start in Chrm (0 Indexed) | End in Chrm (1 Indexed) |
| 3.11622088669 | fubp3 (bg=23.31%) | HepG2 | - | chr20 | | 34637905 | 34637964 |
| 2.0537229917 | GRWD1 (bg=4.85%) | HepG2 | - | chr20 | | 34637879 | 34637955 |
| 2.37906836964 | PPIG (bg=0.88%) | HepG2 | - | chr20 | | 34637873 | 34637921 |
| 3.47567085304 | pum1 (bg=29.85%) | K562 | - | chr20 | | 34637875 | 34637920 |
| 2.94588483295 | pum1 (bg=29.85%) | K562 | - | chr20 | | 34637883 | 34637927 |
| 3.27897894204 | pum1 (bg=29.85%) | K562 | - | chr20 | | 34637920 | 34637962 |
| 3.25828744911 | pum2 (bg=21.55%) | K562 | - | chr20 | | 34637920 | 34637967 |
| 2.58780780317 | rbm15 (bg=11.81%) | HepG2 | - | chr20 | | 34637893 | 34637923 |
| 2.3533641502 | rbm15 (bg=11.81%) | HepG2 | - | chr20 | | 34637898 | 34637930 |
| 3.9837301591 | SND1 (bg=1.27%) | HepG2 | - | chr20 | | 34637911 | 34637943 |
| 2.49087812115 | ZNF622 (bg=1.54%) | K562 | - | chr20 | | 34637875 | 34637959 |

  
  

| Match 96 in HUMAN | | | | | | | |
| --- | --- | --- | --- | --- | --- | --- | --- |
| Motif | Start in Seq (1 Indexed) | End in Seq (1 Indexed) | Strand | Chrm | Exon | Start in Chrm (0 Indexed) | End in Chrm (1 Indexed) |
| AGTTGTT | 1014 | 1020 | - | chr20 | 1 | 34637912 | 34637919 |
| eCLIP Fold-Enrichment | Binding Protein | Cell Line | Strand | Chrm | | Start in Chrm (0 Indexed) | End in Chrm (1 Indexed) |
| 3.11622088669 | fubp3 (bg=23.31%) | HepG2 | - | chr20 | | 34637905 | 34637964 |
| 2.0537229917 | GRWD1 (bg=4.85%) | HepG2 | - | chr20 | | 34637879 | 34637955 |
| 2.37906836964 | PPIG (bg=0.88%) | HepG2 | - | chr20 | | 34637873 | 34637921 |
| 3.47567085304 | pum1 (bg=29.85%) | K562 | - | chr20 | | 34637875 | 34637920 |
| 2.94588483295 | pum1 (bg=29.85%) | K562 | - | chr20 | | 34637883 | 34637927 |
| 2.58780780317 | rbm15 (bg=11.81%) | HepG2 | - | chr20 | | 34637893 | 34637923 |
| 2.3533641502 | rbm15 (bg=11.81%) | HepG2 | - | chr20 | | 34637898 | 34637930 |
| 3.9837301591 | SND1 (bg=1.27%) | HepG2 | - | chr20 | | 34637911 | 34637943 |
| 2.49087812115 | ZNF622 (bg=1.54%) | K562 | - | chr20 | | 34637875 | 34637959 |

  
  

| Match 97 in HUMAN | | | | | | | |
| --- | --- | --- | --- | --- | --- | --- | --- |
| Motif | Start in Seq (1 Indexed) | End in Seq (1 Indexed) | Strand | Chrm | Exon | Start in Chrm (0 Indexed) | End in Chrm (1 Indexed) |
| AGACGGCCTCTGGAAGCGATACGTCCACG | 1022 | 1050 | - | chr20 | 1 | 34637882 | 34637911 |
| eCLIP Fold-Enrichment | Binding Protein | Cell Line | Strand | Chrm | | Start in Chrm (0 Indexed) | End in Chrm (1 Indexed) |
| 4.19062327747 | fubp3 (bg=23.31%) | HepG2 | - | chr20 | | 34637890 | 34637898 |
| 3.11622088669 | fubp3 (bg=23.31%) | HepG2 | - | chr20 | | 34637905 | 34637964 |
| 3.59698808722 | GNL3 (bg=0.85%) | K562 | - | chr20 | | 34637858 | 34637904 |
| 2.0537229917 | GRWD1 (bg=4.85%) | HepG2 | - | chr20 | | 34637879 | 34637955 |
| 2.19382229939 | lin28b (bg=16.96%) | HepG2 | - | chr20 | | 34637883 | 34637909 |
| 2.37906836964 | PPIG (bg=0.88%) | HepG2 | - | chr20 | | 34637873 | 34637921 |
| 3.47567085304 | pum1 (bg=29.85%) | K562 | - | chr20 | | 34637875 | 34637920 |
| 2.94588483295 | pum1 (bg=29.85%) | K562 | - | chr20 | | 34637883 | 34637927 |
| 2.58780780317 | rbm15 (bg=11.81%) | HepG2 | - | chr20 | | 34637893 | 34637923 |
| 2.3533641502 | rbm15 (bg=11.81%) | HepG2 | - | chr20 | | 34637898 | 34637930 |
| 3.9837301591 | SND1 (bg=1.27%) | HepG2 | - | chr20 | | 34637911 | 34637943 |
| 2.49087812115 | ZNF622 (bg=1.54%) | K562 | - | chr20 | | 34637875 | 34637959 |

  
  

| Match 98 in HUMAN | | | | | | | |
| --- | --- | --- | --- | --- | --- | --- | --- |
| Motif | Start in Seq (1 Indexed) | End in Seq (1 Indexed) | Strand | Chrm | Exon | Start in Chrm (0 Indexed) | End in Chrm (1 Indexed) |
| GAAGCGA | 1034 | 1040 | - | chr20 | 1 | 34637892 | 34637899 |
| eCLIP Fold-Enrichment | Binding Protein | Cell Line | Strand | Chrm | | Start in Chrm (0 Indexed) | End in Chrm (1 Indexed) |
| 4.19062327747 | fubp3 (bg=23.31%) | HepG2 | - | chr20 | | 34637890 | 34637898 |
| 3.59698808722 | GNL3 (bg=0.85%) | K562 | - | chr20 | | 34637858 | 34637904 |
| 2.0537229917 | GRWD1 (bg=4.85%) | HepG2 | - | chr20 | | 34637879 | 34637955 |
| 2.19382229939 | lin28b (bg=16.96%) | HepG2 | - | chr20 | | 34637883 | 34637909 |
| 2.37906836964 | PPIG (bg=0.88%) | HepG2 | - | chr20 | | 34637873 | 34637921 |
| 3.47567085304 | pum1 (bg=29.85%) | K562 | - | chr20 | | 34637875 | 34637920 |
| 2.94588483295 | pum1 (bg=29.85%) | K562 | - | chr20 | | 34637883 | 34637927 |
| 2.58780780317 | rbm15 (bg=11.81%) | HepG2 | - | chr20 | | 34637893 | 34637923 |
| 2.3533641502 | rbm15 (bg=11.81%) | HepG2 | - | chr20 | | 34637898 | 34637930 |
| 2.49087812115 | ZNF622 (bg=1.54%) | K562 | - | chr20 | | 34637875 | 34637959 |

  
  

| Match 99 in HUMAN | | | | | | | |
| --- | --- | --- | --- | --- | --- | --- | --- |
| Motif | Start in Seq (1 Indexed) | End in Seq (1 Indexed) | Strand | Chrm | Exon | Start in Chrm (0 Indexed) | End in Chrm (1 Indexed) |
| TTAAGTGGGTTAGATGACATGGAGCTGGAAGAC | 1055 | 1087 | - | chr20 | 1 | 34637845 | 34637878 |
| eCLIP Fold-Enrichment | Binding Protein | Cell Line | Strand | Chrm | | Start in Chrm (0 Indexed) | End in Chrm (1 Indexed) |
| 3.59698808722 | GNL3 (bg=0.85%) | K562 | - | chr20 | | 34637858 | 34637904 |
| 2.37906836964 | PPIG (bg=0.88%) | HepG2 | - | chr20 | | 34637873 | 34637921 |
| 3.47567085304 | pum1 (bg=29.85%) | K562 | - | chr20 | | 34637875 | 34637920 |
| 2.49087812115 | ZNF622 (bg=1.54%) | K562 | - | chr20 | | 34637875 | 34637959 |

  
  

| Match 100 in HUMAN | | | | | | | |
| --- | --- | --- | --- | --- | --- | --- | --- |
| Motif | Start in Seq (1 Indexed) | End in Seq (1 Indexed) | Strand | Chrm | Exon | Start in Chrm (0 Indexed) | End in Chrm (1 Indexed) |
| AGTGGGT | 1058 | 1064 | - | chr20 | 1 | 34637868 | 34637875 |
| eCLIP Fold-Enrichment | Binding Protein | Cell Line | Strand | Chrm | | Start in Chrm (0 Indexed) | End in Chrm (1 Indexed) |
| 3.59698808722 | GNL3 (bg=0.85%) | K562 | - | chr20 | | 34637858 | 34637904 |
| 2.37906836964 | PPIG (bg=0.88%) | HepG2 | - | chr20 | | 34637873 | 34637921 |
| 3.47567085304 | pum1 (bg=29.85%) | K562 | - | chr20 | | 34637875 | 34637920 |
| 2.49087812115 | ZNF622 (bg=1.54%) | K562 | - | chr20 | | 34637875 | 34637959 |

  
  

| Match 101 in HUMAN | | | | | | | |
| --- | --- | --- | --- | --- | --- | --- | --- |
| Motif | Start in Seq (1 Indexed) | End in Seq (1 Indexed) | Strand | Chrm | Exon | Start in Chrm (0 Indexed) | End in Chrm (1 Indexed) |
| GTGGGT | 1059 | 1064 | - | chr20 | 1 | 34637868 | 34637874 |
| eCLIP Fold-Enrichment | Binding Protein | Cell Line | Strand | Chrm | | Start in Chrm (0 Indexed) | End in Chrm (1 Indexed) |
| 3.59698808722 | GNL3 (bg=0.85%) | K562 | - | chr20 | | 34637858 | 34637904 |
| 2.37906836964 | PPIG (bg=0.88%) | HepG2 | - | chr20 | | 34637873 | 34637921 |

  
  

| Match 102 in HUMAN | | | | | | | |
| --- | --- | --- | --- | --- | --- | --- | --- |
| Motif | Start in Seq (1 Indexed) | End in Seq (1 Indexed) | Strand | Chrm | Exon | Start in Chrm (0 Indexed) | End in Chrm (1 Indexed) |
| GACATG | 1070 | 1075 | - | chr20 | 1 | 34637857 | 34637863 |
| eCLIP Fold-Enrichment | Binding Protein | Cell Line | Strand | Chrm | | Start in Chrm (0 Indexed) | End in Chrm (1 Indexed) |
| 3.59698808722 | GNL3 (bg=0.85%) | K562 | - | chr20 | | 34637858 | 34637904 |

  
  

| Match 103 in HUMAN | | | | | | | |
| --- | --- | --- | --- | --- | --- | --- | --- |
| Motif | Start in Seq (1 Indexed) | End in Seq (1 Indexed) | Strand | Chrm | Exon | Start in Chrm (0 Indexed) | End in Chrm (1 Indexed) |
| TTCTTTTTAGATGTTCTGAAGTGCCTGA | 1230 | 1257 | - | chr20 | 1 | 34637675 | 34637703 |
| eCLIP Fold-Enrichment | Binding Protein | Cell Line | Strand | Chrm | | Start in Chrm (0 Indexed) | End in Chrm (1 Indexed) |
| 4.95637526997 | pum1 (bg=29.85%) | K562 | - | chr20 | | 34637643 | 34637680 |
| 3.21799156929 | PUS1 (bg=2.87%) | K562 | - | chr20 | | 34637623 | 34637687 |
| 4.22747120573 | tial1 (bg=14.09%) | HepG2 | - | chr20 | | 34637649 | 34637689 |

  
  

| Match 104 in HUMAN | | | | | | | |
| --- | --- | --- | --- | --- | --- | --- | --- |
| Motif | Start in Seq (1 Indexed) | End in Seq (1 Indexed) | Strand | Chrm | Exon | Start in Chrm (0 Indexed) | End in Chrm (1 Indexed) |
| TAGATGTTCTGAAGTGCCTGA | 1237 | 1257 | - | chr20 | 1 | 34637675 | 34637696 |
| eCLIP Fold-Enrichment | Binding Protein | Cell Line | Strand | Chrm | | Start in Chrm (0 Indexed) | End in Chrm (1 Indexed) |
| 4.95637526997 | pum1 (bg=29.85%) | K562 | - | chr20 | | 34637643 | 34637680 |
| 3.21799156929 | PUS1 (bg=2.87%) | K562 | - | chr20 | | 34637623 | 34637687 |
| 4.22747120573 | tial1 (bg=14.09%) | HepG2 | - | chr20 | | 34637649 | 34637689 |

  
  

| Match 105 in HUMAN | | | | | | | |
| --- | --- | --- | --- | --- | --- | --- | --- |
| Motif | Start in Seq (1 Indexed) | End in Seq (1 Indexed) | Strand | Chrm | Exon | Start in Chrm (0 Indexed) | End in Chrm (1 Indexed) |
| TTCTGAAGTGCCTGA | 1243 | 1257 | - | chr20 | 1 | 34637675 | 34637690 |
| eCLIP Fold-Enrichment | Binding Protein | Cell Line | Strand | Chrm | | Start in Chrm (0 Indexed) | End in Chrm (1 Indexed) |
| 4.95637526997 | pum1 (bg=29.85%) | K562 | - | chr20 | | 34637643 | 34637680 |
| 3.21799156929 | PUS1 (bg=2.87%) | K562 | - | chr20 | | 34637623 | 34637687 |
| 4.22747120573 | tial1 (bg=14.09%) | HepG2 | - | chr20 | | 34637649 | 34637689 |

  
  

| Match 106 in HUMAN | | | | | | | |
| --- | --- | --- | --- | --- | --- | --- | --- |
| Motif | Start in Seq (1 Indexed) | End in Seq (1 Indexed) | Strand | Chrm | Exon | Start in Chrm (0 Indexed) | End in Chrm (1 Indexed) |
| AAGTGCCTGA | 1248 | 1257 | - | chr20 | 1 | 34637675 | 34637685 |
| eCLIP Fold-Enrichment | Binding Protein | Cell Line | Strand | Chrm | | Start in Chrm (0 Indexed) | End in Chrm (1 Indexed) |
| 4.95637526997 | pum1 (bg=29.85%) | K562 | - | chr20 | | 34637643 | 34637680 |
| 3.21799156929 | PUS1 (bg=2.87%) | K562 | - | chr20 | | 34637623 | 34637687 |
| 4.22747120573 | tial1 (bg=14.09%) | HepG2 | - | chr20 | | 34637649 | 34637689 |

  
  

| Match 107 in HUMAN | | | | | | | |
| --- | --- | --- | --- | --- | --- | --- | --- |
| Motif | Start in Seq (1 Indexed) | End in Seq (1 Indexed) | Strand | Chrm | Exon | Start in Chrm (0 Indexed) | End in Chrm (1 Indexed) |
| AAGTGCCTG | 1248 | 1256 | - | chr20 | 1 | 34637676 | 34637685 |
| eCLIP Fold-Enrichment | Binding Protein | Cell Line | Strand | Chrm | | Start in Chrm (0 Indexed) | End in Chrm (1 Indexed) |
| 4.95637526997 | pum1 (bg=29.85%) | K562 | - | chr20 | | 34637643 | 34637680 |
| 3.21799156929 | PUS1 (bg=2.87%) | K562 | - | chr20 | | 34637623 | 34637687 |
| 4.22747120573 | tial1 (bg=14.09%) | HepG2 | - | chr20 | | 34637649 | 34637689 |

  
  

| Match 108 in HUMAN | | | | | | | |
| --- | --- | --- | --- | --- | --- | --- | --- |
| Motif | Start in Seq (1 Indexed) | End in Seq (1 Indexed) | Strand | Chrm | Exon | Start in Chrm (0 Indexed) | End in Chrm (1 Indexed) |
| TATGTTAAAATTAGAGGTAGCAAAAT | 1258 | 1283 | - | chr20 | 1 | 34637649 | 34637675 |
| eCLIP Fold-Enrichment | Binding Protein | Cell Line | Strand | Chrm | | Start in Chrm (0 Indexed) | End in Chrm (1 Indexed) |
| 2.17688888899 | CPEB4 (bg=2.3%) | K562 | - | chr20 | | 34637573 | 34637650 |
| 2.65016917728 | ddx55 (bg=12.35%) | HepG2 | - | chr20 | | 34637647 | 34637665 |
| 4.31005121542 | pum1 (bg=29.85%) | K562 | - | chr20 | | 34637563 | 34637664 |
| 4.95637526997 | pum1 (bg=29.85%) | K562 | - | chr20 | | 34637643 | 34637680 |
| 3.21799156929 | PUS1 (bg=2.87%) | K562 | - | chr20 | | 34637623 | 34637687 |
| 3.4424840967 | tial1 (bg=14.09%) | HepG2 | - | chr20 | | 34637568 | 34637649 |
| 4.22747120573 | tial1 (bg=14.09%) | HepG2 | - | chr20 | | 34637649 | 34637689 |

  
  

| Match 109 in HUMAN | | | | | | | |
| --- | --- | --- | --- | --- | --- | --- | --- |
| Motif | Start in Seq (1 Indexed) | End in Seq (1 Indexed) | Strand | Chrm | Exon | Start in Chrm (0 Indexed) | End in Chrm (1 Indexed) |
| ATGTTAAAA | 1259 | 1267 | - | chr20 | 1 | 34637665 | 34637674 |
| eCLIP Fold-Enrichment | Binding Protein | Cell Line | Strand | Chrm | | Start in Chrm (0 Indexed) | End in Chrm (1 Indexed) |
| 2.65016917728 | ddx55 (bg=12.35%) | HepG2 | - | chr20 | | 34637647 | 34637665 |
| 4.95637526997 | pum1 (bg=29.85%) | K562 | - | chr20 | | 34637643 | 34637680 |
| 3.21799156929 | PUS1 (bg=2.87%) | K562 | - | chr20 | | 34637623 | 34637687 |
| 4.22747120573 | tial1 (bg=14.09%) | HepG2 | - | chr20 | | 34637649 | 34637689 |

  
  

| Match 110 in HUMAN | | | | | | | |
| --- | --- | --- | --- | --- | --- | --- | --- |
| Motif | Start in Seq (1 Indexed) | End in Seq (1 Indexed) | Strand | Chrm | Exon | Start in Chrm (0 Indexed) | End in Chrm (1 Indexed) |
| TGTTAAAA | 1260 | 1267 | - | chr20 | 1 | 34637665 | 34637673 |
| eCLIP Fold-Enrichment | Binding Protein | Cell Line | Strand | Chrm | | Start in Chrm (0 Indexed) | End in Chrm (1 Indexed) |
| 2.65016917728 | ddx55 (bg=12.35%) | HepG2 | - | chr20 | | 34637647 | 34637665 |
| 4.95637526997 | pum1 (bg=29.85%) | K562 | - | chr20 | | 34637643 | 34637680 |
| 3.21799156929 | PUS1 (bg=2.87%) | K562 | - | chr20 | | 34637623 | 34637687 |
| 4.22747120573 | tial1 (bg=14.09%) | HepG2 | - | chr20 | | 34637649 | 34637689 |

  
  

| Match 111 in HUMAN | | | | | | | |
| --- | --- | --- | --- | --- | --- | --- | --- |
| Motif | Start in Seq (1 Indexed) | End in Seq (1 Indexed) | Strand | Chrm | Exon | Start in Chrm (0 Indexed) | End in Chrm (1 Indexed) |
| GTTAAAA | 1261 | 1267 | - | chr20 | 1 | 34637665 | 34637672 |
| eCLIP Fold-Enrichment | Binding Protein | Cell Line | Strand | Chrm | | Start in Chrm (0 Indexed) | End in Chrm (1 Indexed) |
| 2.65016917728 | ddx55 (bg=12.35%) | HepG2 | - | chr20 | | 34637647 | 34637665 |
| 4.95637526997 | pum1 (bg=29.85%) | K562 | - | chr20 | | 34637643 | 34637680 |
| 3.21799156929 | PUS1 (bg=2.87%) | K562 | - | chr20 | | 34637623 | 34637687 |
| 4.22747120573 | tial1 (bg=14.09%) | HepG2 | - | chr20 | | 34637649 | 34637689 |

  
  

| Match 112 in HUMAN | | | | | | | |
| --- | --- | --- | --- | --- | --- | --- | --- |
| Motif | Start in Seq (1 Indexed) | End in Seq (1 Indexed) | Strand | Chrm | Exon | Start in Chrm (0 Indexed) | End in Chrm (1 Indexed) |
| TAGAGGTAG | 1269 | 1277 | - | chr20 | 1 | 34637655 | 34637664 |
| eCLIP Fold-Enrichment | Binding Protein | Cell Line | Strand | Chrm | | Start in Chrm (0 Indexed) | End in Chrm (1 Indexed) |
| 2.65016917728 | ddx55 (bg=12.35%) | HepG2 | - | chr20 | | 34637647 | 34637665 |
| 4.31005121542 | pum1 (bg=29.85%) | K562 | - | chr20 | | 34637563 | 34637664 |
| 4.95637526997 | pum1 (bg=29.85%) | K562 | - | chr20 | | 34637643 | 34637680 |
| 3.21799156929 | PUS1 (bg=2.87%) | K562 | - | chr20 | | 34637623 | 34637687 |
| 4.22747120573 | tial1 (bg=14.09%) | HepG2 | - | chr20 | | 34637649 | 34637689 |

  
  

| Match 113 in HUMAN | | | | | | | |
| --- | --- | --- | --- | --- | --- | --- | --- |
| Motif | Start in Seq (1 Indexed) | End in Seq (1 Indexed) | Strand | Chrm | Exon | Start in Chrm (0 Indexed) | End in Chrm (1 Indexed) |
| GAGGTAG | 1271 | 1277 | - | chr20 | 1 | 34637655 | 34637662 |
| eCLIP Fold-Enrichment | Binding Protein | Cell Line | Strand | Chrm | | Start in Chrm (0 Indexed) | End in Chrm (1 Indexed) |
| 2.65016917728 | ddx55 (bg=12.35%) | HepG2 | - | chr20 | | 34637647 | 34637665 |
| 4.31005121542 | pum1 (bg=29.85%) | K562 | - | chr20 | | 34637563 | 34637664 |
| 4.95637526997 | pum1 (bg=29.85%) | K562 | - | chr20 | | 34637643 | 34637680 |
| 3.21799156929 | PUS1 (bg=2.87%) | K562 | - | chr20 | | 34637623 | 34637687 |
| 4.22747120573 | tial1 (bg=14.09%) | HepG2 | - | chr20 | | 34637649 | 34637689 |

  
  

| Match 114 in HUMAN | | | | | | | |
| --- | --- | --- | --- | --- | --- | --- | --- |
| Motif | Start in Seq (1 Indexed) | End in Seq (1 Indexed) | Strand | Chrm | Exon | Start in Chrm (0 Indexed) | End in Chrm (1 Indexed) |
| ACATTTTGTAAATA | 1285 | 1298 | - | chr20 | 1 | 34637634 | 34637648 |
| eCLIP Fold-Enrichment | Binding Protein | Cell Line | Strand | Chrm | | Start in Chrm (0 Indexed) | End in Chrm (1 Indexed) |
| 2.17688888899 | CPEB4 (bg=2.3%) | K562 | - | chr20 | | 34637573 | 34637650 |
| 2.65016917728 | ddx55 (bg=12.35%) | HepG2 | - | chr20 | | 34637647 | 34637665 |
| 2.41166842036 | drosha (bg=11.37%) | HepG2 | - | chr20 | | 34637559 | 34637634 |
| 2.41166842036 | drosha (bg=11.37%) | HepG2 | - | chr20 | | 34637559 | 34637634 |
| 4.31005121542 | pum1 (bg=29.85%) | K562 | - | chr20 | | 34637563 | 34637664 |
| 4.95637526997 | pum1 (bg=29.85%) | K562 | - | chr20 | | 34637643 | 34637680 |
| 3.21799156929 | PUS1 (bg=2.87%) | K562 | - | chr20 | | 34637623 | 34637687 |
| 3.4424840967 | tial1 (bg=14.09%) | HepG2 | - | chr20 | | 34637568 | 34637649 |

  
  

| Match 115 in HUMAN | | | | | | | |
| --- | --- | --- | --- | --- | --- | --- | --- |
| Motif | Start in Seq (1 Indexed) | End in Seq (1 Indexed) | Strand | Chrm | Exon | Start in Chrm (0 Indexed) | End in Chrm (1 Indexed) |
| TTTTGT | 1288 | 1293 | - | chr20 | 1 | 34637639 | 34637645 |
| eCLIP Fold-Enrichment | Binding Protein | Cell Line | Strand | Chrm | | Start in Chrm (0 Indexed) | End in Chrm (1 Indexed) |
| 2.17688888899 | CPEB4 (bg=2.3%) | K562 | - | chr20 | | 34637573 | 34637650 |
| 4.31005121542 | pum1 (bg=29.85%) | K562 | - | chr20 | | 34637563 | 34637664 |
| 4.95637526997 | pum1 (bg=29.85%) | K562 | - | chr20 | | 34637643 | 34637680 |
| 3.21799156929 | PUS1 (bg=2.87%) | K562 | - | chr20 | | 34637623 | 34637687 |
| 3.4424840967 | tial1 (bg=14.09%) | HepG2 | - | chr20 | | 34637568 | 34637649 |

  
  

| Match 116 in HUMAN | | | | | | | |
| --- | --- | --- | --- | --- | --- | --- | --- |
| Motif | Start in Seq (1 Indexed) | End in Seq (1 Indexed) | Strand | Chrm | Exon | Start in Chrm (0 Indexed) | End in Chrm (1 Indexed) |
| CTTTTTGTTACAATTCATAGGAAAT | 1300 | 1324 | - | chr20 | 1 | 34637608 | 34637633 |
| eCLIP Fold-Enrichment | Binding Protein | Cell Line | Strand | Chrm | | Start in Chrm (0 Indexed) | End in Chrm (1 Indexed) |
| 2.0938301576 | AGGF1 (bg=0.88%) | HepG2 | - | chr20 | | 34637578 | 34637626 |
| 2.17688888899 | CPEB4 (bg=2.3%) | K562 | - | chr20 | | 34637573 | 34637650 |
| 2.09722209672 | ddx55 (bg=12.35%) | HepG2 | - | chr20 | | 34637566 | 34637620 |
| 2.33801271334 | ddx6 (bg=23.92%) | K562 | - | chr20 | | 34637574 | 34637622 |
| 2.53019981628 | ddx6 (bg=23.92%) | K562 | - | chr20 | | 34637575 | 34637622 |
| 2.80701342071 | dgcr8 (bg=19.31%) | HepG2 | - | chr20 | | 34637569 | 34637620 |
| 3.8894151723 | dgcr8 (bg=19.31%) | SM-9MVZL | - | chr20 | | 34637569 | 34637625 |
| 2.41166842036 | drosha (bg=11.37%) | HepG2 | - | chr20 | | 34637559 | 34637634 |
| 2.41166842036 | drosha (bg=11.37%) | HepG2 | - | chr20 | | 34637559 | 34637634 |
| 2.00194750186 | drosha (bg=11.37%) | HepG2 | - | chr20 | | 34637568 | 34637633 |
| 2.00194750186 | drosha (bg=11.37%) | HepG2 | - | chr20 | | 34637568 | 34637633 |
| 2.37424065865 | fam120a (bg=18.43%) | K562 | - | chr20 | | 34637564 | 34637622 |
| 2.37576014259 | fam120a (bg=18.43%) | K562 | - | chr20 | | 34637570 | 34637625 |
| 2.41465872263 | FASTKD2 (bg=7.73%) | K562 | - | chr20 | | 34637568 | 34637625 |
| 2.34605558939 | FTO (bg=7.53%) | K562 | - | chr20 | | 34637569 | 34637613 |
| 4.34104012003 | fubp3 (bg=23.31%) | HepG2 | - | chr20 | | 34637569 | 34637628 |
| 5.20611750672 | fubp3 (bg=23.31%) | HepG2 | - | chr20 | | 34637570 | 34637626 |
| 2.08882384234 | FXR2 (bg=7.51%) | K562 | - | chr20 | | 34637561 | 34637611 |
| 2.87890330771 | igf2bp1 (bg=11.67%) | K562 | - | chr20 | | 34637559 | 34637622 |
| 2.12201058463 | igf2bp1 (bg=11.67%) | K562 | - | chr20 | | 34637564 | 34637620 |
| 3.70575590686 | IGF2BP2 (bg=3.44%) | K562 | - | chr20 | | 34637559 | 34637612 |
| 2.58398927281 | IGF2BP3 (bg=4.26%) | HepG2 | - | chr20 | | 34637562 | 34637609 |
| 4.46145699382 | pum1 (bg=29.85%) | K562 | - | chr20 | | 34637559 | 34637620 |
| 4.31005121542 | pum1 (bg=29.85%) | K562 | - | chr20 | | 34637563 | 34637664 |
| 7.29756905346 | pum2 (bg=21.55%) | K562 | - | chr20 | | 34637567 | 34637611 |
| 5.502213032 | pum2 (bg=21.55%) | K562 | - | chr20 | | 34637568 | 34637610 |
| 4.98763985917 | pum2 (bg=21.55%) | K562 | - | chr20 | | 34637610 | 34637623 |
| 6.98839148144 | pum2 (bg=21.55%) | K562 | - | chr20 | | 34637611 | 34637624 |
| 3.21799156929 | PUS1 (bg=2.87%) | K562 | - | chr20 | | 34637623 | 34637687 |
| 2.58780780317 | rbm15 (bg=11.81%) | HepG2 | - | chr20 | | 34637579 | 34637631 |
| 3.3054341445 | sf3a3 (bg=12.13%) | HepG2 | - | chr20 | | 34637569 | 34637614 |
| 2.54011071178 | sf3a3 (bg=12.13%) | HepG2 | - | chr20 | | 34637572 | 34637618 |
| 2.76190805194 | SF3B1 (bg=6.74%) | K562 | - | chr20 | | 34637568 | 34637615 |
| 4.88473919131 | tia1 (bg=16.04%) | HepG2 | - | chr20 | | 34637558 | 34637621 |
| 3.8639368292 | tia1 (bg=16.04%) | HepG2 | - | chr20 | | 34637594 | 34637612 |
| 3.4424840967 | tial1 (bg=14.09%) | HepG2 | - | chr20 | | 34637568 | 34637649 |
| 3.7674384469 | tial1 (bg=14.09%) | HepG2 | - | chr20 | | 34637574 | 34637632 |
| 2.67189325399 | UPF1 (bg=3.62%) | HepG2 | - | chr20 | | 34637562 | 34637633 |

  
  

| Match 117 in HUMAN | | | | | | | |
| --- | --- | --- | --- | --- | --- | --- | --- |
| Motif | Start in Seq (1 Indexed) | End in Seq (1 Indexed) | Strand | Chrm | Exon | Start in Chrm (0 Indexed) | End in Chrm (1 Indexed) |
| ATTCATA | 1312 | 1318 | - | chr20 | 1 | 34637614 | 34637621 |
| eCLIP Fold-Enrichment | Binding Protein | Cell Line | Strand | Chrm | | Start in Chrm (0 Indexed) | End in Chrm (1 Indexed) |
| 2.0938301576 | AGGF1 (bg=0.88%) | HepG2 | - | chr20 | | 34637578 | 34637626 |
| 2.17688888899 | CPEB4 (bg=2.3%) | K562 | - | chr20 | | 34637573 | 34637650 |
| 2.09722209672 | ddx55 (bg=12.35%) | HepG2 | - | chr20 | | 34637566 | 34637620 |
| 2.33801271334 | ddx6 (bg=23.92%) | K562 | - | chr20 | | 34637574 | 34637622 |
| 2.53019981628 | ddx6 (bg=23.92%) | K562 | - | chr20 | | 34637575 | 34637622 |
| 2.80701342071 | dgcr8 (bg=19.31%) | HepG2 | - | chr20 | | 34637569 | 34637620 |
| 3.8894151723 | dgcr8 (bg=19.31%) | SM-9MVZL | - | chr20 | | 34637569 | 34637625 |
| 2.41166842036 | drosha (bg=11.37%) | HepG2 | - | chr20 | | 34637559 | 34637634 |
| 2.41166842036 | drosha (bg=11.37%) | HepG2 | - | chr20 | | 34637559 | 34637634 |
| 2.00194750186 | drosha (bg=11.37%) | HepG2 | - | chr20 | | 34637568 | 34637633 |
| 2.00194750186 | drosha (bg=11.37%) | HepG2 | - | chr20 | | 34637568 | 34637633 |
| 2.37424065865 | fam120a (bg=18.43%) | K562 | - | chr20 | | 34637564 | 34637622 |
| 2.37576014259 | fam120a (bg=18.43%) | K562 | - | chr20 | | 34637570 | 34637625 |
| 2.41465872263 | FASTKD2 (bg=7.73%) | K562 | - | chr20 | | 34637568 | 34637625 |
| 4.34104012003 | fubp3 (bg=23.31%) | HepG2 | - | chr20 | | 34637569 | 34637628 |
| 5.20611750672 | fubp3 (bg=23.31%) | HepG2 | - | chr20 | | 34637570 | 34637626 |
| 2.87890330771 | igf2bp1 (bg=11.67%) | K562 | - | chr20 | | 34637559 | 34637622 |
| 2.12201058463 | igf2bp1 (bg=11.67%) | K562 | - | chr20 | | 34637564 | 34637620 |
| 4.46145699382 | pum1 (bg=29.85%) | K562 | - | chr20 | | 34637559 | 34637620 |
| 4.31005121542 | pum1 (bg=29.85%) | K562 | - | chr20 | | 34637563 | 34637664 |
| 4.98763985917 | pum2 (bg=21.55%) | K562 | - | chr20 | | 34637610 | 34637623 |
| 6.98839148144 | pum2 (bg=21.55%) | K562 | - | chr20 | | 34637611 | 34637624 |
| 2.58780780317 | rbm15 (bg=11.81%) | HepG2 | - | chr20 | | 34637579 | 34637631 |
| 3.3054341445 | sf3a3 (bg=12.13%) | HepG2 | - | chr20 | | 34637569 | 34637614 |
| 2.54011071178 | sf3a3 (bg=12.13%) | HepG2 | - | chr20 | | 34637572 | 34637618 |
| 2.76190805194 | SF3B1 (bg=6.74%) | K562 | - | chr20 | | 34637568 | 34637615 |
| 4.88473919131 | tia1 (bg=16.04%) | HepG2 | - | chr20 | | 34637558 | 34637621 |
| 3.4424840967 | tial1 (bg=14.09%) | HepG2 | - | chr20 | | 34637568 | 34637649 |
| 3.7674384469 | tial1 (bg=14.09%) | HepG2 | - | chr20 | | 34637574 | 34637632 |
| 2.67189325399 | UPF1 (bg=3.62%) | HepG2 | - | chr20 | | 34637562 | 34637633 |

  
  

| Match 118 in HUMAN | | | | | | | |
| --- | --- | --- | --- | --- | --- | --- | --- |
| Motif | Start in Seq (1 Indexed) | End in Seq (1 Indexed) | Strand | Chrm | Exon | Start in Chrm (0 Indexed) | End in Chrm (1 Indexed) |
| ATTCATAGGAAAT | 1312 | 1324 | - | chr20 | 1 | 34637608 | 34637621 |
| eCLIP Fold-Enrichment | Binding Protein | Cell Line | Strand | Chrm | | Start in Chrm (0 Indexed) | End in Chrm (1 Indexed) |
| 2.0938301576 | AGGF1 (bg=0.88%) | HepG2 | - | chr20 | | 34637578 | 34637626 |
| 2.17688888899 | CPEB4 (bg=2.3%) | K562 | - | chr20 | | 34637573 | 34637650 |
| 2.09722209672 | ddx55 (bg=12.35%) | HepG2 | - | chr20 | | 34637566 | 34637620 |
| 2.33801271334 | ddx6 (bg=23.92%) | K562 | - | chr20 | | 34637574 | 34637622 |
| 2.53019981628 | ddx6 (bg=23.92%) | K562 | - | chr20 | | 34637575 | 34637622 |
| 2.80701342071 | dgcr8 (bg=19.31%) | HepG2 | - | chr20 | | 34637569 | 34637620 |
| 3.8894151723 | dgcr8 (bg=19.31%) | SM-9MVZL | - | chr20 | | 34637569 | 34637625 |
| 2.41166842036 | drosha (bg=11.37%) | HepG2 | - | chr20 | | 34637559 | 34637634 |
| 2.41166842036 | drosha (bg=11.37%) | HepG2 | - | chr20 | | 34637559 | 34637634 |
| 2.00194750186 | drosha (bg=11.37%) | HepG2 | - | chr20 | | 34637568 | 34637633 |
| 2.00194750186 | drosha (bg=11.37%) | HepG2 | - | chr20 | | 34637568 | 34637633 |
| 2.37424065865 | fam120a (bg=18.43%) | K562 | - | chr20 | | 34637564 | 34637622 |
| 2.37576014259 | fam120a (bg=18.43%) | K562 | - | chr20 | | 34637570 | 34637625 |
| 2.41465872263 | FASTKD2 (bg=7.73%) | K562 | - | chr20 | | 34637568 | 34637625 |
| 2.34605558939 | FTO (bg=7.53%) | K562 | - | chr20 | | 34637569 | 34637613 |
| 4.34104012003 | fubp3 (bg=23.31%) | HepG2 | - | chr20 | | 34637569 | 34637628 |
| 5.20611750672 | fubp3 (bg=23.31%) | HepG2 | - | chr20 | | 34637570 | 34637626 |
| 2.08882384234 | FXR2 (bg=7.51%) | K562 | - | chr20 | | 34637561 | 34637611 |
| 2.87890330771 | igf2bp1 (bg=11.67%) | K562 | - | chr20 | | 34637559 | 34637622 |
| 2.12201058463 | igf2bp1 (bg=11.67%) | K562 | - | chr20 | | 34637564 | 34637620 |
| 3.70575590686 | IGF2BP2 (bg=3.44%) | K562 | - | chr20 | | 34637559 | 34637612 |
| 2.58398927281 | IGF2BP3 (bg=4.26%) | HepG2 | - | chr20 | | 34637562 | 34637609 |
| 4.46145699382 | pum1 (bg=29.85%) | K562 | - | chr20 | | 34637559 | 34637620 |
| 4.31005121542 | pum1 (bg=29.85%) | K562 | - | chr20 | | 34637563 | 34637664 |
| 7.29756905346 | pum2 (bg=21.55%) | K562 | - | chr20 | | 34637567 | 34637611 |
| 5.502213032 | pum2 (bg=21.55%) | K562 | - | chr20 | | 34637568 | 34637610 |
| 4.98763985917 | pum2 (bg=21.55%) | K562 | - | chr20 | | 34637610 | 34637623 |
| 6.98839148144 | pum2 (bg=21.55%) | K562 | - | chr20 | | 34637611 | 34637624 |
| 2.58780780317 | rbm15 (bg=11.81%) | HepG2 | - | chr20 | | 34637579 | 34637631 |
| 3.3054341445 | sf3a3 (bg=12.13%) | HepG2 | - | chr20 | | 34637569 | 34637614 |
| 2.54011071178 | sf3a3 (bg=12.13%) | HepG2 | - | chr20 | | 34637572 | 34637618 |
| 2.76190805194 | SF3B1 (bg=6.74%) | K562 | - | chr20 | | 34637568 | 34637615 |
| 4.88473919131 | tia1 (bg=16.04%) | HepG2 | - | chr20 | | 34637558 | 34637621 |
| 3.8639368292 | tia1 (bg=16.04%) | HepG2 | - | chr20 | | 34637594 | 34637612 |
| 3.4424840967 | tial1 (bg=14.09%) | HepG2 | - | chr20 | | 34637568 | 34637649 |
| 3.7674384469 | tial1 (bg=14.09%) | HepG2 | - | chr20 | | 34637574 | 34637632 |
| 2.67189325399 | UPF1 (bg=3.62%) | HepG2 | - | chr20 | | 34637562 | 34637633 |

  
  

| Match 119 in HUMAN | | | | | | | |
| --- | --- | --- | --- | --- | --- | --- | --- |
| Motif | Start in Seq (1 Indexed) | End in Seq (1 Indexed) | Strand | Chrm | Exon | Start in Chrm (0 Indexed) | End in Chrm (1 Indexed) |
| GGGGGGAATGGCCAAATCACCTGTTGAGTAATACTCATTGTGTTTGTGCAGTGGTTC | 1332 | 1388 | - | chr20 | 1 | 34637544 | 34637601 |
| eCLIP Fold-Enrichment | Binding Protein | Cell Line | Strand | Chrm | | Start in Chrm (0 Indexed) | End in Chrm (1 Indexed) |
| 2.0938301576 | AGGF1 (bg=0.88%) | HepG2 | - | chr20 | | 34637578 | 34637626 |
| 2.17688888899 | CPEB4 (bg=2.3%) | K562 | - | chr20 | | 34637573 | 34637650 |
| 2.09722209672 | ddx55 (bg=12.35%) | HepG2 | - | chr20 | | 34637566 | 34637620 |
| 2.33801271334 | ddx6 (bg=23.92%) | K562 | - | chr20 | | 34637574 | 34637622 |
| 2.53019981628 | ddx6 (bg=23.92%) | K562 | - | chr20 | | 34637575 | 34637622 |
| 3.2316052267 | dgcr8 (bg=19.31%) | HepG2 | - | chr20 | | 34637487 | 34637545 |
| 2.80701342071 | dgcr8 (bg=19.31%) | HepG2 | - | chr20 | | 34637569 | 34637620 |
| 3.8894151723 | dgcr8 (bg=19.31%) | SM-9MVZL | - | chr20 | | 34637569 | 34637625 |
| 2.41166842036 | drosha (bg=11.37%) | HepG2 | - | chr20 | | 34637559 | 34637634 |
| 2.41166842036 | drosha (bg=11.37%) | HepG2 | - | chr20 | | 34637559 | 34637634 |
| 2.00194750186 | drosha (bg=11.37%) | HepG2 | - | chr20 | | 34637568 | 34637633 |
| 2.00194750186 | drosha (bg=11.37%) | HepG2 | - | chr20 | | 34637568 | 34637633 |
| 3.56693694308 | fam120a (bg=18.43%) | HepG2 | - | chr20 | | 34637476 | 34637546 |
| 2.79752788746 | fam120a (bg=18.43%) | HepG2 | - | chr20 | | 34637562 | 34637605 |
| 2.37424065865 | fam120a (bg=18.43%) | K562 | - | chr20 | | 34637564 | 34637622 |
| 2.37576014259 | fam120a (bg=18.43%) | K562 | - | chr20 | | 34637570 | 34637625 |
| 2.41465872263 | FASTKD2 (bg=7.73%) | K562 | - | chr20 | | 34637568 | 34637625 |
| 2.34605558939 | FTO (bg=7.53%) | K562 | - | chr20 | | 34637569 | 34637613 |
| 4.34104012003 | fubp3 (bg=23.31%) | HepG2 | - | chr20 | | 34637569 | 34637628 |
| 5.20611750672 | fubp3 (bg=23.31%) | HepG2 | - | chr20 | | 34637570 | 34637626 |
| 2.08882384234 | FXR2 (bg=7.51%) | K562 | - | chr20 | | 34637561 | 34637611 |
| 2.87890330771 | igf2bp1 (bg=11.67%) | K562 | - | chr20 | | 34637559 | 34637622 |
| 2.12201058463 | igf2bp1 (bg=11.67%) | K562 | - | chr20 | | 34637564 | 34637620 |
| 2.41592749342 | IGF2BP2 (bg=3.44%) | K562 | - | chr20 | | 34637559 | 34637576 |
| 3.70575590686 | IGF2BP2 (bg=3.44%) | K562 | - | chr20 | | 34637559 | 34637612 |
| 2.33911189637 | IGF2BP2 (bg=3.44%) | K562 | - | chr20 | | 34637576 | 34637607 |
| 3.2772293678 | IGF2BP3 (bg=4.26%) | HepG2 | - | chr20 | | 34637559 | 34637607 |
| 2.58398927281 | IGF2BP3 (bg=4.26%) | HepG2 | - | chr20 | | 34637562 | 34637609 |
| 2.59119744835 | KHSRP (bg=8.1%) | HepG2 | - | chr20 | | 34637475 | 34637545 |
| 4.64622434286 | pum1 (bg=29.85%) | K562 | - | chr20 | | 34637499 | 34637559 |
| 4.46145699382 | pum1 (bg=29.85%) | K562 | - | chr20 | | 34637559 | 34637620 |
| 4.31005121542 | pum1 (bg=29.85%) | K562 | - | chr20 | | 34637563 | 34637664 |
| 7.29756905346 | pum2 (bg=21.55%) | K562 | - | chr20 | | 34637567 | 34637611 |
| 5.502213032 | pum2 (bg=21.55%) | K562 | - | chr20 | | 34637568 | 34637610 |
| 2.58780780317 | rbm15 (bg=11.81%) | HepG2 | - | chr20 | | 34637579 | 34637631 |
| 3.3054341445 | sf3a3 (bg=12.13%) | HepG2 | - | chr20 | | 34637569 | 34637614 |
| 2.54011071178 | sf3a3 (bg=12.13%) | HepG2 | - | chr20 | | 34637572 | 34637618 |
| 2.76190805194 | SF3B1 (bg=6.74%) | K562 | - | chr20 | | 34637568 | 34637615 |
| 4.88473919131 | tia1 (bg=16.04%) | HepG2 | - | chr20 | | 34637558 | 34637621 |
| 3.8639368292 | tia1 (bg=16.04%) | HepG2 | - | chr20 | | 34637560 | 34637594 |
| 3.8639368292 | tia1 (bg=16.04%) | HepG2 | - | chr20 | | 34637594 | 34637612 |
| 3.4424840967 | tial1 (bg=14.09%) | HepG2 | - | chr20 | | 34637568 | 34637649 |
| 3.7674384469 | tial1 (bg=14.09%) | HepG2 | - | chr20 | | 34637574 | 34637632 |
| 2.67189325399 | UPF1 (bg=3.62%) | HepG2 | - | chr20 | | 34637562 | 34637633 |

  
  

| Match 120 in HUMAN | | | | | | | |
| --- | --- | --- | --- | --- | --- | --- | --- |
| Motif | Start in Seq (1 Indexed) | End in Seq (1 Indexed) | Strand | Chrm | Exon | Start in Chrm (0 Indexed) | End in Chrm (1 Indexed) |
| GGGGAATGGCCAAA | 1334 | 1347 | - | chr20 | 1 | 34637585 | 34637599 |
| eCLIP Fold-Enrichment | Binding Protein | Cell Line | Strand | Chrm | | Start in Chrm (0 Indexed) | End in Chrm (1 Indexed) |
| 2.0938301576 | AGGF1 (bg=0.88%) | HepG2 | - | chr20 | | 34637578 | 34637626 |
| 2.17688888899 | CPEB4 (bg=2.3%) | K562 | - | chr20 | | 34637573 | 34637650 |
| 2.09722209672 | ddx55 (bg=12.35%) | HepG2 | - | chr20 | | 34637566 | 34637620 |
| 2.33801271334 | ddx6 (bg=23.92%) | K562 | - | chr20 | | 34637574 | 34637622 |
| 2.53019981628 | ddx6 (bg=23.92%) | K562 | - | chr20 | | 34637575 | 34637622 |
| 2.80701342071 | dgcr8 (bg=19.31%) | HepG2 | - | chr20 | | 34637569 | 34637620 |
| 3.8894151723 | dgcr8 (bg=19.31%) | SM-9MVZL | - | chr20 | | 34637569 | 34637625 |
| 2.41166842036 | drosha (bg=11.37%) | HepG2 | - | chr20 | | 34637559 | 34637634 |
| 2.41166842036 | drosha (bg=11.37%) | HepG2 | - | chr20 | | 34637559 | 34637634 |
| 2.00194750186 | drosha (bg=11.37%) | HepG2 | - | chr20 | | 34637568 | 34637633 |
| 2.00194750186 | drosha (bg=11.37%) | HepG2 | - | chr20 | | 34637568 | 34637633 |
| 2.79752788746 | fam120a (bg=18.43%) | HepG2 | - | chr20 | | 34637562 | 34637605 |
| 2.37424065865 | fam120a (bg=18.43%) | K562 | - | chr20 | | 34637564 | 34637622 |
| 2.37576014259 | fam120a (bg=18.43%) | K562 | - | chr20 | | 34637570 | 34637625 |
| 2.41465872263 | FASTKD2 (bg=7.73%) | K562 | - | chr20 | | 34637568 | 34637625 |
| 2.34605558939 | FTO (bg=7.53%) | K562 | - | chr20 | | 34637569 | 34637613 |
| 4.34104012003 | fubp3 (bg=23.31%) | HepG2 | - | chr20 | | 34637569 | 34637628 |
| 5.20611750672 | fubp3 (bg=23.31%) | HepG2 | - | chr20 | | 34637570 | 34637626 |
| 2.08882384234 | FXR2 (bg=7.51%) | K562 | - | chr20 | | 34637561 | 34637611 |
| 2.87890330771 | igf2bp1 (bg=11.67%) | K562 | - | chr20 | | 34637559 | 34637622 |
| 2.12201058463 | igf2bp1 (bg=11.67%) | K562 | - | chr20 | | 34637564 | 34637620 |
| 3.70575590686 | IGF2BP2 (bg=3.44%) | K562 | - | chr20 | | 34637559 | 34637612 |
| 2.33911189637 | IGF2BP2 (bg=3.44%) | K562 | - | chr20 | | 34637576 | 34637607 |
| 3.2772293678 | IGF2BP3 (bg=4.26%) | HepG2 | - | chr20 | | 34637559 | 34637607 |
| 2.58398927281 | IGF2BP3 (bg=4.26%) | HepG2 | - | chr20 | | 34637562 | 34637609 |
| 4.46145699382 | pum1 (bg=29.85%) | K562 | - | chr20 | | 34637559 | 34637620 |
| 4.31005121542 | pum1 (bg=29.85%) | K562 | - | chr20 | | 34637563 | 34637664 |
| 7.29756905346 | pum2 (bg=21.55%) | K562 | - | chr20 | | 34637567 | 34637611 |
| 5.502213032 | pum2 (bg=21.55%) | K562 | - | chr20 | | 34637568 | 34637610 |
| 2.58780780317 | rbm15 (bg=11.81%) | HepG2 | - | chr20 | | 34637579 | 34637631 |
| 3.3054341445 | sf3a3 (bg=12.13%) | HepG2 | - | chr20 | | 34637569 | 34637614 |
| 2.54011071178 | sf3a3 (bg=12.13%) | HepG2 | - | chr20 | | 34637572 | 34637618 |
| 2.76190805194 | SF3B1 (bg=6.74%) | K562 | - | chr20 | | 34637568 | 34637615 |
| 4.88473919131 | tia1 (bg=16.04%) | HepG2 | - | chr20 | | 34637558 | 34637621 |
| 3.8639368292 | tia1 (bg=16.04%) | HepG2 | - | chr20 | | 34637560 | 34637594 |
| 3.8639368292 | tia1 (bg=16.04%) | HepG2 | - | chr20 | | 34637594 | 34637612 |
| 3.4424840967 | tial1 (bg=14.09%) | HepG2 | - | chr20 | | 34637568 | 34637649 |
| 3.7674384469 | tial1 (bg=14.09%) | HepG2 | - | chr20 | | 34637574 | 34637632 |
| 2.67189325399 | UPF1 (bg=3.62%) | HepG2 | - | chr20 | | 34637562 | 34637633 |

  
  

| Match 121 in HUMAN | | | | | | | |
| --- | --- | --- | --- | --- | --- | --- | --- |
| Motif | Start in Seq (1 Indexed) | End in Seq (1 Indexed) | Strand | Chrm | Exon | Start in Chrm (0 Indexed) | End in Chrm (1 Indexed) |
| TGGCCA | 1340 | 1345 | - | chr20 | 1 | 34637587 | 34637593 |
| eCLIP Fold-Enrichment | Binding Protein | Cell Line | Strand | Chrm | | Start in Chrm (0 Indexed) | End in Chrm (1 Indexed) |
| 2.0938301576 | AGGF1 (bg=0.88%) | HepG2 | - | chr20 | | 34637578 | 34637626 |
| 2.17688888899 | CPEB4 (bg=2.3%) | K562 | - | chr20 | | 34637573 | 34637650 |
| 2.09722209672 | ddx55 (bg=12.35%) | HepG2 | - | chr20 | | 34637566 | 34637620 |
| 2.33801271334 | ddx6 (bg=23.92%) | K562 | - | chr20 | | 34637574 | 34637622 |
| 2.53019981628 | ddx6 (bg=23.92%) | K562 | - | chr20 | | 34637575 | 34637622 |
| 2.80701342071 | dgcr8 (bg=19.31%) | HepG2 | - | chr20 | | 34637569 | 34637620 |
| 3.8894151723 | dgcr8 (bg=19.31%) | SM-9MVZL | - | chr20 | | 34637569 | 34637625 |
| 2.41166842036 | drosha (bg=11.37%) | HepG2 | - | chr20 | | 34637559 | 34637634 |
| 2.41166842036 | drosha (bg=11.37%) | HepG2 | - | chr20 | | 34637559 | 34637634 |
| 2.00194750186 | drosha (bg=11.37%) | HepG2 | - | chr20 | | 34637568 | 34637633 |
| 2.00194750186 | drosha (bg=11.37%) | HepG2 | - | chr20 | | 34637568 | 34637633 |
| 2.79752788746 | fam120a (bg=18.43%) | HepG2 | - | chr20 | | 34637562 | 34637605 |
| 2.37424065865 | fam120a (bg=18.43%) | K562 | - | chr20 | | 34637564 | 34637622 |
| 2.37576014259 | fam120a (bg=18.43%) | K562 | - | chr20 | | 34637570 | 34637625 |
| 2.41465872263 | FASTKD2 (bg=7.73%) | K562 | - | chr20 | | 34637568 | 34637625 |
| 2.34605558939 | FTO (bg=7.53%) | K562 | - | chr20 | | 34637569 | 34637613 |
| 4.34104012003 | fubp3 (bg=23.31%) | HepG2 | - | chr20 | | 34637569 | 34637628 |
| 5.20611750672 | fubp3 (bg=23.31%) | HepG2 | - | chr20 | | 34637570 | 34637626 |
| 2.08882384234 | FXR2 (bg=7.51%) | K562 | - | chr20 | | 34637561 | 34637611 |
| 2.87890330771 | igf2bp1 (bg=11.67%) | K562 | - | chr20 | | 34637559 | 34637622 |
| 2.12201058463 | igf2bp1 (bg=11.67%) | K562 | - | chr20 | | 34637564 | 34637620 |
| 3.70575590686 | IGF2BP2 (bg=3.44%) | K562 | - | chr20 | | 34637559 | 34637612 |
| 2.33911189637 | IGF2BP2 (bg=3.44%) | K562 | - | chr20 | | 34637576 | 34637607 |
| 3.2772293678 | IGF2BP3 (bg=4.26%) | HepG2 | - | chr20 | | 34637559 | 34637607 |
| 2.58398927281 | IGF2BP3 (bg=4.26%) | HepG2 | - | chr20 | | 34637562 | 34637609 |
| 4.46145699382 | pum1 (bg=29.85%) | K562 | - | chr20 | | 34637559 | 34637620 |
| 4.31005121542 | pum1 (bg=29.85%) | K562 | - | chr20 | | 34637563 | 34637664 |
| 7.29756905346 | pum2 (bg=21.55%) | K562 | - | chr20 | | 34637567 | 34637611 |
| 5.502213032 | pum2 (bg=21.55%) | K562 | - | chr20 | | 34637568 | 34637610 |
| 2.58780780317 | rbm15 (bg=11.81%) | HepG2 | - | chr20 | | 34637579 | 34637631 |
| 3.3054341445 | sf3a3 (bg=12.13%) | HepG2 | - | chr20 | | 34637569 | 34637614 |
| 2.54011071178 | sf3a3 (bg=12.13%) | HepG2 | - | chr20 | | 34637572 | 34637618 |
| 2.76190805194 | SF3B1 (bg=6.74%) | K562 | - | chr20 | | 34637568 | 34637615 |
| 4.88473919131 | tia1 (bg=16.04%) | HepG2 | - | chr20 | | 34637558 | 34637621 |
| 3.8639368292 | tia1 (bg=16.04%) | HepG2 | - | chr20 | | 34637560 | 34637594 |
| 3.4424840967 | tial1 (bg=14.09%) | HepG2 | - | chr20 | | 34637568 | 34637649 |
| 3.7674384469 | tial1 (bg=14.09%) | HepG2 | - | chr20 | | 34637574 | 34637632 |
| 2.67189325399 | UPF1 (bg=3.62%) | HepG2 | - | chr20 | | 34637562 | 34637633 |

  
  

| Match 122 in HUMAN | | | | | | | |
| --- | --- | --- | --- | --- | --- | --- | --- |
| Motif | Start in Seq (1 Indexed) | End in Seq (1 Indexed) | Strand | Chrm | Exon | Start in Chrm (0 Indexed) | End in Chrm (1 Indexed) |
| CATTGTGTTTGTGCA | 1367 | 1381 | - | chr20 | 1 | 34637551 | 34637566 |
| eCLIP Fold-Enrichment | Binding Protein | Cell Line | Strand | Chrm | | Start in Chrm (0 Indexed) | End in Chrm (1 Indexed) |
| 2.09722209672 | ddx55 (bg=12.35%) | HepG2 | - | chr20 | | 34637566 | 34637620 |
| 2.41166842036 | drosha (bg=11.37%) | HepG2 | - | chr20 | | 34637559 | 34637634 |
| 2.41166842036 | drosha (bg=11.37%) | HepG2 | - | chr20 | | 34637559 | 34637634 |
| 2.79752788746 | fam120a (bg=18.43%) | HepG2 | - | chr20 | | 34637562 | 34637605 |
| 2.37424065865 | fam120a (bg=18.43%) | K562 | - | chr20 | | 34637564 | 34637622 |
| 2.08882384234 | FXR2 (bg=7.51%) | K562 | - | chr20 | | 34637561 | 34637611 |
| 2.87890330771 | igf2bp1 (bg=11.67%) | K562 | - | chr20 | | 34637559 | 34637622 |
| 2.12201058463 | igf2bp1 (bg=11.67%) | K562 | - | chr20 | | 34637564 | 34637620 |
| 2.41592749342 | IGF2BP2 (bg=3.44%) | K562 | - | chr20 | | 34637559 | 34637576 |
| 3.70575590686 | IGF2BP2 (bg=3.44%) | K562 | - | chr20 | | 34637559 | 34637612 |
| 3.2772293678 | IGF2BP3 (bg=4.26%) | HepG2 | - | chr20 | | 34637559 | 34637607 |
| 2.58398927281 | IGF2BP3 (bg=4.26%) | HepG2 | - | chr20 | | 34637562 | 34637609 |
| 4.64622434286 | pum1 (bg=29.85%) | K562 | - | chr20 | | 34637499 | 34637559 |
| 4.46145699382 | pum1 (bg=29.85%) | K562 | - | chr20 | | 34637559 | 34637620 |
| 4.31005121542 | pum1 (bg=29.85%) | K562 | - | chr20 | | 34637563 | 34637664 |
| 4.88473919131 | tia1 (bg=16.04%) | HepG2 | - | chr20 | | 34637558 | 34637621 |
| 3.8639368292 | tia1 (bg=16.04%) | HepG2 | - | chr20 | | 34637560 | 34637594 |
| 2.67189325399 | UPF1 (bg=3.62%) | HepG2 | - | chr20 | | 34637562 | 34637633 |

  
  

| Match 123 in HUMAN | | | | | | | |
| --- | --- | --- | --- | --- | --- | --- | --- |
| Motif | Start in Seq (1 Indexed) | End in Seq (1 Indexed) | Strand | Chrm | Exon | Start in Chrm (0 Indexed) | End in Chrm (1 Indexed) |
| TTTGTGCA | 1374 | 1381 | - | chr20 | 1 | 34637551 | 34637559 |
| eCLIP Fold-Enrichment | Binding Protein | Cell Line | Strand | Chrm | | Start in Chrm (0 Indexed) | End in Chrm (1 Indexed) |
| 2.41166842036 | drosha (bg=11.37%) | HepG2 | - | chr20 | | 34637559 | 34637634 |
| 2.41166842036 | drosha (bg=11.37%) | HepG2 | - | chr20 | | 34637559 | 34637634 |
| 2.87890330771 | igf2bp1 (bg=11.67%) | K562 | - | chr20 | | 34637559 | 34637622 |
| 2.41592749342 | IGF2BP2 (bg=3.44%) | K562 | - | chr20 | | 34637559 | 34637576 |
| 3.70575590686 | IGF2BP2 (bg=3.44%) | K562 | - | chr20 | | 34637559 | 34637612 |
| 3.2772293678 | IGF2BP3 (bg=4.26%) | HepG2 | - | chr20 | | 34637559 | 34637607 |
| 4.64622434286 | pum1 (bg=29.85%) | K562 | - | chr20 | | 34637499 | 34637559 |
| 4.46145699382 | pum1 (bg=29.85%) | K562 | - | chr20 | | 34637559 | 34637620 |
| 4.88473919131 | tia1 (bg=16.04%) | HepG2 | - | chr20 | | 34637558 | 34637621 |

  
  

| Match 124 in HUMAN | | | | | | | |
| --- | --- | --- | --- | --- | --- | --- | --- |
| Motif | Start in Seq (1 Indexed) | End in Seq (1 Indexed) | Strand | Chrm | Exon | Start in Chrm (0 Indexed) | End in Chrm (1 Indexed) |
| TTGTGC | 1375 | 1380 | - | chr20 | 1 | 34637552 | 34637558 |
| eCLIP Fold-Enrichment | Binding Protein | Cell Line | Strand | Chrm | | Start in Chrm (0 Indexed) | End in Chrm (1 Indexed) |
| 4.64622434286 | pum1 (bg=29.85%) | K562 | - | chr20 | | 34637499 | 34637559 |
| 4.88473919131 | tia1 (bg=16.04%) | HepG2 | - | chr20 | | 34637558 | 34637621 |

  
  

| Match 125 in HUMAN | | | | | | | |
| --- | --- | --- | --- | --- | --- | --- | --- |
| Motif | Start in Seq (1 Indexed) | End in Seq (1 Indexed) | Strand | Chrm | Exon | Start in Chrm (0 Indexed) | End in Chrm (1 Indexed) |
| TTGTGCA | 1375 | 1381 | - | chr20 | 1 | 34637551 | 34637558 |
| eCLIP Fold-Enrichment | Binding Protein | Cell Line | Strand | Chrm | | Start in Chrm (0 Indexed) | End in Chrm (1 Indexed) |
| 4.64622434286 | pum1 (bg=29.85%) | K562 | - | chr20 | | 34637499 | 34637559 |
| 4.88473919131 | tia1 (bg=16.04%) | HepG2 | - | chr20 | | 34637558 | 34637621 |

  
  

| Match 126 in HUMAN | | | | | | | |
| --- | --- | --- | --- | --- | --- | --- | --- |
| Motif | Start in Seq (1 Indexed) | End in Seq (1 Indexed) | Strand | Chrm | Exon | Start in Chrm (0 Indexed) | End in Chrm (1 Indexed) |
| TGGTTC | 1383 | 1388 | - | chr20 | 1 | 34637544 | 34637550 |
| eCLIP Fold-Enrichment | Binding Protein | Cell Line | Strand | Chrm | | Start in Chrm (0 Indexed) | End in Chrm (1 Indexed) |
| 3.2316052267 | dgcr8 (bg=19.31%) | HepG2 | - | chr20 | | 34637487 | 34637545 |
| 3.56693694308 | fam120a (bg=18.43%) | HepG2 | - | chr20 | | 34637476 | 34637546 |
| 2.59119744835 | KHSRP (bg=8.1%) | HepG2 | - | chr20 | | 34637475 | 34637545 |
| 4.64622434286 | pum1 (bg=29.85%) | K562 | - | chr20 | | 34637499 | 34637559 |

  
  

| Match 127 in HUMAN | | | | | | | |
| --- | --- | --- | --- | --- | --- | --- | --- |
| Motif | Start in Seq (1 Indexed) | End in Seq (1 Indexed) | Strand | Chrm | Exon | Start in Chrm (0 Indexed) | End in Chrm (1 Indexed) |
| AGGAGAGAGGAGGGGGAGGTGCAGAGAGCT | 1394 | 1423 | - | chr20 | 1 | 34637509 | 34637539 |
| eCLIP Fold-Enrichment | Binding Protein | Cell Line | Strand | Chrm | | Start in Chrm (0 Indexed) | End in Chrm (1 Indexed) |
| 2.66353927519 | AKAP1 (bg=3.4%) | HepG2 | - | chr20 | | 34637495 | 34637518 |
| 2.31618719386 | CDC40 (bg=1.71%) | HepG2 | - | chr20 | | 34637477 | 34637534 |
| 2.13842841681 | CDC40 (bg=1.71%) | HepG2 | - | chr20 | | 34637488 | 34637543 |
| 2.59444912925 | ddx3x (bg=13.89%) | K562 | - | chr20 | | 34637461 | 34637526 |
| 2.71389345717 | ddx55 (bg=12.35%) | HepG2 | - | chr20 | | 34637481 | 34637538 |
| 2.38575354105 | ddx55 (bg=12.35%) | HepG2 | - | chr20 | | 34637491 | 34637526 |
| 2.26990108805 | DDX59 (bg=0.72%) | HepG2 | - | chr20 | | 34637485 | 34637524 |
| 2.00731440883 | ddx6 (bg=23.92%) | K562 | - | chr20 | | 34637477 | 34637524 |
| 3.03436668989 | ddx6 (bg=23.92%) | HepG2 | - | chr20 | | 34637491 | 34637511 |
| 2.82554280447 | dgcr8 (bg=19.31%) | K562 | - | chr20 | | 34637483 | 34637527 |
| 3.2316052267 | dgcr8 (bg=19.31%) | HepG2 | - | chr20 | | 34637487 | 34637545 |
| 2.30718200679 | dgcr8 (bg=19.31%) | K562 | - | chr20 | | 34637508 | 34637522 |
| 2.41166842036 | drosha (bg=11.37%) | HepG2 | - | chr20 | | 34637467 | 34637519 |
| 2.41166842036 | drosha (bg=11.37%) | HepG2 | - | chr20 | | 34637467 | 34637519 |
| 3.56693694308 | fam120a (bg=18.43%) | HepG2 | - | chr20 | | 34637476 | 34637546 |
| 2.07977348143 | FASTKD2 (bg=7.73%) | HepG2 | - | chr20 | | 34637480 | 34637531 |
| 2.04492173601 | FASTKD2 (bg=7.73%) | HepG2 | - | chr20 | | 34637486 | 34637542 |
| 2.0386270642 | FTO (bg=7.53%) | K562 | - | chr20 | | 34637442 | 34637513 |
| 2.49876403216 | FTO (bg=7.53%) | K562 | - | chr20 | | 34637482 | 34637533 |
| 3.55431389675 | fubp3 (bg=23.31%) | HepG2 | - | chr20 | | 34637477 | 34637526 |
| 2.34791999174 | GTF2F1 (bg=2.31%) | K562 | - | chr20 | | 34637483 | 34637527 |
| 2.28622437506 | GTF2F1 (bg=2.31%) | HepG2 | - | chr20 | | 34637486 | 34637532 |
| 2.53208273863 | igf2bp1 (bg=11.67%) | HepG2 | - | chr20 | | 34637466 | 34637543 |
| 2.33355404339 | igf2bp1 (bg=11.67%) | HepG2 | - | chr20 | | 34637475 | 34637539 |
| 2.59119744835 | KHSRP (bg=8.1%) | HepG2 | - | chr20 | | 34637475 | 34637545 |
| 2.2032009189 | NOLC1 (bg=6.58%) | HepG2 | - | chr20 | | 34637465 | 34637521 |
| 4.64622434286 | pum1 (bg=29.85%) | K562 | - | chr20 | | 34637499 | 34637559 |
| 4.92221678417 | pum2 (bg=21.55%) | K562 | - | chr20 | | 34637476 | 34637528 |
| 3.31389572325 | sf3a3 (bg=12.13%) | HepG2 | - | chr20 | | 34637469 | 34637542 |
| 2.2030757245 | sf3a3 (bg=12.13%) | HepG2 | - | chr20 | | 34637475 | 34637528 |
| 2.32586014979 | TBRG4 (bg=7.0%) | HepG2 | - | chr20 | | 34637467 | 34637534 |
| 2.68269059733 | tia1 (bg=16.04%) | K562 | - | chr20 | | 34637490 | 34637533 |
| 2.3917853348 | UPF1 (bg=3.62%) | HepG2 | - | chr20 | | 34637475 | 34637542 |
| 2.12552149591 | UPF1 (bg=3.62%) | K562 | - | chr20 | | 34637482 | 34637538 |
| 2.56011441113 | XRCC6 (bg=1.65%) | HepG2 | - | chr20 | | 34637433 | 34637523 |

  
  

| Match 128 in HUMAN | | | | | | | |
| --- | --- | --- | --- | --- | --- | --- | --- |
| Motif | Start in Seq (1 Indexed) | End in Seq (1 Indexed) | Strand | Chrm | Exon | Start in Chrm (0 Indexed) | End in Chrm (1 Indexed) |
| GGAGAG | 1395 | 1400 | - | chr20 | 1 | 34637532 | 34637538 |
| eCLIP Fold-Enrichment | Binding Protein | Cell Line | Strand | Chrm | | Start in Chrm (0 Indexed) | End in Chrm (1 Indexed) |
| 2.31618719386 | CDC40 (bg=1.71%) | HepG2 | - | chr20 | | 34637477 | 34637534 |
| 2.13842841681 | CDC40 (bg=1.71%) | HepG2 | - | chr20 | | 34637488 | 34637543 |
| 2.71389345717 | ddx55 (bg=12.35%) | HepG2 | - | chr20 | | 34637481 | 34637538 |
| 3.2316052267 | dgcr8 (bg=19.31%) | HepG2 | - | chr20 | | 34637487 | 34637545 |
| 3.56693694308 | fam120a (bg=18.43%) | HepG2 | - | chr20 | | 34637476 | 34637546 |
| 2.04492173601 | FASTKD2 (bg=7.73%) | HepG2 | - | chr20 | | 34637486 | 34637542 |
| 2.49876403216 | FTO (bg=7.53%) | K562 | - | chr20 | | 34637482 | 34637533 |
| 2.28622437506 | GTF2F1 (bg=2.31%) | HepG2 | - | chr20 | | 34637486 | 34637532 |
| 2.53208273863 | igf2bp1 (bg=11.67%) | HepG2 | - | chr20 | | 34637466 | 34637543 |
| 2.33355404339 | igf2bp1 (bg=11.67%) | HepG2 | - | chr20 | | 34637475 | 34637539 |
| 2.59119744835 | KHSRP (bg=8.1%) | HepG2 | - | chr20 | | 34637475 | 34637545 |
| 4.64622434286 | pum1 (bg=29.85%) | K562 | - | chr20 | | 34637499 | 34637559 |
| 3.31389572325 | sf3a3 (bg=12.13%) | HepG2 | - | chr20 | | 34637469 | 34637542 |
| 2.32586014979 | TBRG4 (bg=7.0%) | HepG2 | - | chr20 | | 34637467 | 34637534 |
| 2.68269059733 | tia1 (bg=16.04%) | K562 | - | chr20 | | 34637490 | 34637533 |
| 2.3917853348 | UPF1 (bg=3.62%) | HepG2 | - | chr20 | | 34637475 | 34637542 |
| 2.12552149591 | UPF1 (bg=3.62%) | K562 | - | chr20 | | 34637482 | 34637538 |

  
  

| Match 129 in HUMAN | | | | | | | |
| --- | --- | --- | --- | --- | --- | --- | --- |
| Motif | Start in Seq (1 Indexed) | End in Seq (1 Indexed) | Strand | Chrm | Exon | Start in Chrm (0 Indexed) | End in Chrm (1 Indexed) |
| TATGCCATC | 1425 | 1433 | - | chr20 | 1 | 34637499 | 34637508 |
| eCLIP Fold-Enrichment | Binding Protein | Cell Line | Strand | Chrm | | Start in Chrm (0 Indexed) | End in Chrm (1 Indexed) |
| 2.66353927519 | AKAP1 (bg=3.4%) | HepG2 | - | chr20 | | 34637495 | 34637518 |
| 2.31618719386 | CDC40 (bg=1.71%) | HepG2 | - | chr20 | | 34637477 | 34637534 |
| 2.13842841681 | CDC40 (bg=1.71%) | HepG2 | - | chr20 | | 34637488 | 34637543 |
| 2.59444912925 | ddx3x (bg=13.89%) | K562 | - | chr20 | | 34637461 | 34637526 |
| 2.71389345717 | ddx55 (bg=12.35%) | HepG2 | - | chr20 | | 34637481 | 34637538 |
| 2.38575354105 | ddx55 (bg=12.35%) | HepG2 | - | chr20 | | 34637491 | 34637526 |
| 2.26990108805 | DDX59 (bg=0.72%) | HepG2 | - | chr20 | | 34637485 | 34637524 |
| 2.00731440883 | ddx6 (bg=23.92%) | K562 | - | chr20 | | 34637477 | 34637524 |
| 3.03436668989 | ddx6 (bg=23.92%) | HepG2 | - | chr20 | | 34637491 | 34637511 |
| 2.82554280447 | dgcr8 (bg=19.31%) | K562 | - | chr20 | | 34637483 | 34637527 |
| 3.2316052267 | dgcr8 (bg=19.31%) | HepG2 | - | chr20 | | 34637487 | 34637545 |
| 2.30718200679 | dgcr8 (bg=19.31%) | K562 | - | chr20 | | 34637508 | 34637522 |
| 2.41166842036 | drosha (bg=11.37%) | HepG2 | - | chr20 | | 34637467 | 34637519 |
| 2.41166842036 | drosha (bg=11.37%) | HepG2 | - | chr20 | | 34637467 | 34637519 |
| 3.56693694308 | fam120a (bg=18.43%) | HepG2 | - | chr20 | | 34637476 | 34637546 |
| 2.26078260886 | fam120a (bg=18.43%) | K562 | - | chr20 | | 34637477 | 34637504 |
| 2.07977348143 | FASTKD2 (bg=7.73%) | HepG2 | - | chr20 | | 34637480 | 34637531 |
| 2.04492173601 | FASTKD2 (bg=7.73%) | HepG2 | - | chr20 | | 34637486 | 34637542 |
| 2.0386270642 | FTO (bg=7.53%) | K562 | - | chr20 | | 34637442 | 34637513 |
| 2.49876403216 | FTO (bg=7.53%) | K562 | - | chr20 | | 34637482 | 34637533 |
| 4.00432800662 | fubp3 (bg=23.31%) | HepG2 | - | chr20 | | 34637475 | 34637507 |
| 3.55431389675 | fubp3 (bg=23.31%) | HepG2 | - | chr20 | | 34637477 | 34637526 |
| 2.34791999174 | GTF2F1 (bg=2.31%) | K562 | - | chr20 | | 34637483 | 34637527 |
| 2.28622437506 | GTF2F1 (bg=2.31%) | HepG2 | - | chr20 | | 34637486 | 34637532 |
| 2.53208273863 | igf2bp1 (bg=11.67%) | HepG2 | - | chr20 | | 34637466 | 34637543 |
| 2.33355404339 | igf2bp1 (bg=11.67%) | HepG2 | - | chr20 | | 34637475 | 34637539 |
| 2.59119744835 | KHSRP (bg=8.1%) | HepG2 | - | chr20 | | 34637475 | 34637545 |
| 2.2032009189 | NOLC1 (bg=6.58%) | HepG2 | - | chr20 | | 34637465 | 34637521 |
| 4.91802895832 | pum1 (bg=29.85%) | K562 | - | chr20 | | 34637477 | 34637499 |
| 4.64622434286 | pum1 (bg=29.85%) | K562 | - | chr20 | | 34637499 | 34637559 |
| 4.92221678417 | pum2 (bg=21.55%) | K562 | - | chr20 | | 34637476 | 34637528 |
| 3.31389572325 | sf3a3 (bg=12.13%) | HepG2 | - | chr20 | | 34637469 | 34637542 |
| 2.2030757245 | sf3a3 (bg=12.13%) | HepG2 | - | chr20 | | 34637475 | 34637528 |
| 2.32586014979 | TBRG4 (bg=7.0%) | HepG2 | - | chr20 | | 34637467 | 34637534 |
| 2.68269059733 | tia1 (bg=16.04%) | K562 | - | chr20 | | 34637490 | 34637533 |
| 2.3917853348 | UPF1 (bg=3.62%) | HepG2 | - | chr20 | | 34637475 | 34637542 |
| 2.12552149591 | UPF1 (bg=3.62%) | K562 | - | chr20 | | 34637482 | 34637538 |
| 2.56011441113 | XRCC6 (bg=1.65%) | HepG2 | - | chr20 | | 34637433 | 34637523 |

  
  

| Match 130 in HUMAN | | | | | | | |
| --- | --- | --- | --- | --- | --- | --- | --- |
| Motif | Start in Seq (1 Indexed) | End in Seq (1 Indexed) | Strand | Chrm | Exon | Start in Chrm (0 Indexed) | End in Chrm (1 Indexed) |
| CAGCGAGGCAAGATGAATCATTAT | 1441 | 1464 | - | chr20 | 1 | 34637468 | 34637492 |
| eCLIP Fold-Enrichment | Binding Protein | Cell Line | Strand | Chrm | | Start in Chrm (0 Indexed) | End in Chrm (1 Indexed) |
| 2.31618719386 | CDC40 (bg=1.71%) | HepG2 | - | chr20 | | 34637477 | 34637534 |
| 2.13842841681 | CDC40 (bg=1.71%) | HepG2 | - | chr20 | | 34637488 | 34637543 |
| 2.59444912925 | ddx3x (bg=13.89%) | K562 | - | chr20 | | 34637461 | 34637526 |
| 2.71389345717 | ddx55 (bg=12.35%) | HepG2 | - | chr20 | | 34637481 | 34637538 |
| 2.38575354105 | ddx55 (bg=12.35%) | HepG2 | - | chr20 | | 34637491 | 34637526 |
| 2.26990108805 | DDX59 (bg=0.72%) | HepG2 | - | chr20 | | 34637485 | 34637524 |
| 2.00731440883 | ddx6 (bg=23.92%) | K562 | - | chr20 | | 34637477 | 34637524 |
| 3.03436668989 | ddx6 (bg=23.92%) | HepG2 | - | chr20 | | 34637491 | 34637511 |
| 4.28939140163 | dgcr8 (bg=19.31%) | SM-9MVZL | - | chr20 | | 34637463 | 34637479 |
| 2.82554280447 | dgcr8 (bg=19.31%) | K562 | - | chr20 | | 34637483 | 34637527 |
| 3.2316052267 | dgcr8 (bg=19.31%) | HepG2 | - | chr20 | | 34637487 | 34637545 |
| 2.41166842036 | drosha (bg=11.37%) | HepG2 | - | chr20 | | 34637467 | 34637519 |
| 2.41166842036 | drosha (bg=11.37%) | HepG2 | - | chr20 | | 34637467 | 34637519 |
| 2.74620943603 | fam120a (bg=18.43%) | K562 | - | chr20 | | 34637449 | 34637477 |
| 3.56693694308 | fam120a (bg=18.43%) | HepG2 | - | chr20 | | 34637476 | 34637546 |
| 2.26078260886 | fam120a (bg=18.43%) | K562 | - | chr20 | | 34637477 | 34637504 |
| 2.07977348143 | FASTKD2 (bg=7.73%) | HepG2 | - | chr20 | | 34637480 | 34637531 |
| 2.04492173601 | FASTKD2 (bg=7.73%) | HepG2 | - | chr20 | | 34637486 | 34637542 |
| 2.0386270642 | FTO (bg=7.53%) | K562 | - | chr20 | | 34637442 | 34637513 |
| 2.49876403216 | FTO (bg=7.53%) | K562 | - | chr20 | | 34637482 | 34637533 |
| 4.39707415494 | fubp3 (bg=23.31%) | HepG2 | - | chr20 | | 34637455 | 34637477 |
| 4.00432800662 | fubp3 (bg=23.31%) | HepG2 | - | chr20 | | 34637475 | 34637507 |
| 3.55431389675 | fubp3 (bg=23.31%) | HepG2 | - | chr20 | | 34637477 | 34637526 |
| 2.34791999174 | GTF2F1 (bg=2.31%) | K562 | - | chr20 | | 34637483 | 34637527 |
| 2.28622437506 | GTF2F1 (bg=2.31%) | HepG2 | - | chr20 | | 34637486 | 34637532 |
| 2.53208273863 | igf2bp1 (bg=11.67%) | HepG2 | - | chr20 | | 34637466 | 34637543 |
| 2.33355404339 | igf2bp1 (bg=11.67%) | HepG2 | - | chr20 | | 34637475 | 34637539 |
| 2.59119744835 | KHSRP (bg=8.1%) | HepG2 | - | chr20 | | 34637475 | 34637545 |
| 2.2032009189 | NOLC1 (bg=6.58%) | HepG2 | - | chr20 | | 34637465 | 34637521 |
| 4.98911205638 | pum1 (bg=29.85%) | K562 | - | chr20 | | 34637447 | 34637477 |
| 4.91802895832 | pum1 (bg=29.85%) | K562 | - | chr20 | | 34637477 | 34637499 |
| 6.08258826322 | pum2 (bg=21.55%) | K562 | - | chr20 | | 34637451 | 34637476 |
| 7.10684201517 | pum2 (bg=21.55%) | K562 | - | chr20 | | 34637453 | 34637494 |
| 4.92221678417 | pum2 (bg=21.55%) | K562 | - | chr20 | | 34637476 | 34637528 |
| 3.31389572325 | sf3a3 (bg=12.13%) | HepG2 | - | chr20 | | 34637469 | 34637542 |
| 2.2030757245 | sf3a3 (bg=12.13%) | HepG2 | - | chr20 | | 34637475 | 34637528 |
| 2.40263197644 | SUB1 (bg=9.24%) | HepG2 | - | chr20 | | 34637406 | 34637472 |
| 2.32586014979 | TBRG4 (bg=7.0%) | HepG2 | - | chr20 | | 34637467 | 34637534 |
| 2.57959710436 | tia1 (bg=16.04%) | K562 | - | chr20 | | 34637460 | 34637490 |
| 2.68269059733 | tia1 (bg=16.04%) | K562 | - | chr20 | | 34637490 | 34637533 |
| 2.3917853348 | UPF1 (bg=3.62%) | HepG2 | - | chr20 | | 34637475 | 34637542 |
| 2.12552149591 | UPF1 (bg=3.62%) | K562 | - | chr20 | | 34637482 | 34637538 |
| 2.56011441113 | XRCC6 (bg=1.65%) | HepG2 | - | chr20 | | 34637433 | 34637523 |

  
  

| Match 131 in HUMAN | | | | | | | |
| --- | --- | --- | --- | --- | --- | --- | --- |
| Motif | Start in Seq (1 Indexed) | End in Seq (1 Indexed) | Strand | Chrm | Exon | Start in Chrm (0 Indexed) | End in Chrm (1 Indexed) |
| TCTGTGCATTTTGTTTTACTTATCTGTGTATATAGTGTACATAAAGGACAGACGAGTCCTAATTGACAACATCTAGTCTTTCTGGATGTTAAAGAGGTTGCCAGTGTATGACAAAAGTAGAGTT | 1466 | 1589 | - | chr20 | 1 | 34637343 | 34637467 |
| eCLIP Fold-Enrichment | Binding Protein | Cell Line | Strand | Chrm | | Start in Chrm (0 Indexed) | End in Chrm (1 Indexed) |
| 2.72012280355 | AKAP1 (bg=3.4%) | HepG2 | - | chr20 | | 34637401 | 34637456 |
| 2.59444912925 | ddx3x (bg=13.89%) | K562 | - | chr20 | | 34637461 | 34637526 |
| 2.41750045439 | ddx55 (bg=12.35%) | HepG2 | - | chr20 | | 34637402 | 34637452 |
| 2.16657972231 | dgcr8 (bg=19.31%) | K562 | - | chr20 | | 34637403 | 34637443 |
| 2.89214450752 | dgcr8 (bg=19.31%) | K562 | - | chr20 | | 34637403 | 34637466 |
| 2.6881771912 | dgcr8 (bg=19.31%) | HepG2 | - | chr20 | | 34637406 | 34637452 |
| 4.28939140163 | dgcr8 (bg=19.31%) | SM-9MVZL | - | chr20 | | 34637459 | 34637463 |
| 4.28939140163 | dgcr8 (bg=19.31%) | SM-9MVZL | - | chr20 | | 34637463 | 34637479 |
| 2.35433524529 | drosha (bg=11.37%) | HepG2 | - | chr20 | | 34637403 | 34637467 |
| 2.35433524529 | drosha (bg=11.37%) | HepG2 | - | chr20 | | 34637403 | 34637467 |
| 2.41166842036 | drosha (bg=11.37%) | HepG2 | - | chr20 | | 34637467 | 34637519 |
| 2.41166842036 | drosha (bg=11.37%) | HepG2 | - | chr20 | | 34637467 | 34637519 |
| 2.78015676796 | fam120a (bg=18.43%) | K562 | - | chr20 | | 34637406 | 34637421 |
| 3.1560852302 | fam120a (bg=18.43%) | K562 | - | chr20 | | 34637421 | 34637449 |
| 2.74620943603 | fam120a (bg=18.43%) | K562 | - | chr20 | | 34637449 | 34637477 |
| 2.0386270642 | FTO (bg=7.53%) | K562 | - | chr20 | | 34637442 | 34637513 |
| 4.61946657628 | fubp3 (bg=23.31%) | HepG2 | - | chr20 | | 34637402 | 34637419 |
| 4.82209271176 | fubp3 (bg=23.31%) | HepG2 | - | chr20 | | 34637404 | 34637447 |
| 5.2271491535 | fubp3 (bg=23.31%) | HepG2 | - | chr20 | | 34637419 | 34637455 |
| 4.39707415494 | fubp3 (bg=23.31%) | HepG2 | - | chr20 | | 34637455 | 34637477 |
| 2.76586677441 | igf2bp1 (bg=11.67%) | K562 | - | chr20 | | 34637409 | 34637459 |
| 2.53208273863 | igf2bp1 (bg=11.67%) | HepG2 | - | chr20 | | 34637466 | 34637543 |
| 2.94271748233 | NKRF (bg=3.64%) | HepG2 | - | chr20 | | 34637408 | 34637444 |
| 2.2032009189 | NOLC1 (bg=6.58%) | HepG2 | - | chr20 | | 34637465 | 34637521 |
| 4.65753933859 | pum1 (bg=29.85%) | K562 | - | chr20 | | 34637400 | 34637445 |
| 4.17828494242 | pum1 (bg=29.85%) | K562 | - | chr20 | | 34637408 | 34637447 |
| 4.98911205638 | pum1 (bg=29.85%) | K562 | - | chr20 | | 34637447 | 34637477 |
| 7.46711393388 | pum2 (bg=21.55%) | K562 | - | chr20 | | 34637401 | 34637453 |
| 5.81835877429 | pum2 (bg=21.55%) | K562 | - | chr20 | | 34637403 | 34637420 |
| 6.06366877983 | pum2 (bg=21.55%) | K562 | - | chr20 | | 34637420 | 34637451 |
| 6.08258826322 | pum2 (bg=21.55%) | K562 | - | chr20 | | 34637451 | 34637476 |
| 7.10684201517 | pum2 (bg=21.55%) | K562 | - | chr20 | | 34637453 | 34637494 |
| 2.74766978511 | sf3a3 (bg=12.13%) | HepG2 | - | chr20 | | 34637409 | 34637463 |
| 2.40263197644 | SUB1 (bg=9.24%) | HepG2 | - | chr20 | | 34637406 | 34637472 |
| 2.32586014979 | TBRG4 (bg=7.0%) | HepG2 | - | chr20 | | 34637467 | 34637534 |
| 3.75964397133 | tia1 (bg=16.04%) | HepG2 | - | chr20 | | 34637404 | 34637446 |
| 2.57959710436 | tia1 (bg=16.04%) | K562 | - | chr20 | | 34637460 | 34637490 |
| 3.26141853765 | tial1 (bg=14.09%) | HepG2 | - | chr20 | | 34637400 | 34637448 |
| 3.20519602268 | tial1 (bg=14.09%) | HepG2 | - | chr20 | | 34637406 | 34637447 |
| 2.51761914151 | TROVE2 (bg=3.49%) | HepG2 | - | chr20 | | 34637402 | 34637446 |
| 2.56011441113 | XRCC6 (bg=1.65%) | HepG2 | - | chr20 | | 34637433 | 34637523 |

  
  

| Match 132 in HUMAN | | | | | | | |
| --- | --- | --- | --- | --- | --- | --- | --- |
| Motif | Start in Seq (1 Indexed) | End in Seq (1 Indexed) | Strand | Chrm | Exon | Start in Chrm (0 Indexed) | End in Chrm (1 Indexed) |
| TGCATTTTGTTTTACTTA | 1470 | 1487 | - | chr20 | 1 | 34637445 | 34637463 |
| eCLIP Fold-Enrichment | Binding Protein | Cell Line | Strand | Chrm | | Start in Chrm (0 Indexed) | End in Chrm (1 Indexed) |
| 2.72012280355 | AKAP1 (bg=3.4%) | HepG2 | - | chr20 | | 34637401 | 34637456 |
| 2.59444912925 | ddx3x (bg=13.89%) | K562 | - | chr20 | | 34637461 | 34637526 |
| 2.41750045439 | ddx55 (bg=12.35%) | HepG2 | - | chr20 | | 34637402 | 34637452 |
| 2.89214450752 | dgcr8 (bg=19.31%) | K562 | - | chr20 | | 34637403 | 34637466 |
| 2.6881771912 | dgcr8 (bg=19.31%) | HepG2 | - | chr20 | | 34637406 | 34637452 |
| 4.28939140163 | dgcr8 (bg=19.31%) | SM-9MVZL | - | chr20 | | 34637459 | 34637463 |
| 4.28939140163 | dgcr8 (bg=19.31%) | SM-9MVZL | - | chr20 | | 34637463 | 34637479 |
| 2.35433524529 | drosha (bg=11.37%) | HepG2 | - | chr20 | | 34637403 | 34637467 |
| 2.35433524529 | drosha (bg=11.37%) | HepG2 | - | chr20 | | 34637403 | 34637467 |
| 3.1560852302 | fam120a (bg=18.43%) | K562 | - | chr20 | | 34637421 | 34637449 |
| 2.74620943603 | fam120a (bg=18.43%) | K562 | - | chr20 | | 34637449 | 34637477 |
| 2.0386270642 | FTO (bg=7.53%) | K562 | - | chr20 | | 34637442 | 34637513 |
| 4.82209271176 | fubp3 (bg=23.31%) | HepG2 | - | chr20 | | 34637404 | 34637447 |
| 5.2271491535 | fubp3 (bg=23.31%) | HepG2 | - | chr20 | | 34637419 | 34637455 |
| 4.39707415494 | fubp3 (bg=23.31%) | HepG2 | - | chr20 | | 34637455 | 34637477 |
| 2.76586677441 | igf2bp1 (bg=11.67%) | K562 | - | chr20 | | 34637409 | 34637459 |
| 4.65753933859 | pum1 (bg=29.85%) | K562 | - | chr20 | | 34637400 | 34637445 |
| 4.17828494242 | pum1 (bg=29.85%) | K562 | - | chr20 | | 34637408 | 34637447 |
| 4.98911205638 | pum1 (bg=29.85%) | K562 | - | chr20 | | 34637447 | 34637477 |
| 7.46711393388 | pum2 (bg=21.55%) | K562 | - | chr20 | | 34637401 | 34637453 |
| 6.06366877983 | pum2 (bg=21.55%) | K562 | - | chr20 | | 34637420 | 34637451 |
| 6.08258826322 | pum2 (bg=21.55%) | K562 | - | chr20 | | 34637451 | 34637476 |
| 7.10684201517 | pum2 (bg=21.55%) | K562 | - | chr20 | | 34637453 | 34637494 |
| 2.74766978511 | sf3a3 (bg=12.13%) | HepG2 | - | chr20 | | 34637409 | 34637463 |
| 2.40263197644 | SUB1 (bg=9.24%) | HepG2 | - | chr20 | | 34637406 | 34637472 |
| 3.75964397133 | tia1 (bg=16.04%) | HepG2 | - | chr20 | | 34637404 | 34637446 |
| 2.57959710436 | tia1 (bg=16.04%) | K562 | - | chr20 | | 34637460 | 34637490 |
| 3.26141853765 | tial1 (bg=14.09%) | HepG2 | - | chr20 | | 34637400 | 34637448 |
| 3.20519602268 | tial1 (bg=14.09%) | HepG2 | - | chr20 | | 34637406 | 34637447 |
| 2.51761914151 | TROVE2 (bg=3.49%) | HepG2 | - | chr20 | | 34637402 | 34637446 |
| 2.56011441113 | XRCC6 (bg=1.65%) | HepG2 | - | chr20 | | 34637433 | 34637523 |

  
  

| Match 133 in HUMAN | | | | | | | |
| --- | --- | --- | --- | --- | --- | --- | --- |
| Motif | Start in Seq (1 Indexed) | End in Seq (1 Indexed) | Strand | Chrm | Exon | Start in Chrm (0 Indexed) | End in Chrm (1 Indexed) |
| TGCATTTTGTTT | 1470 | 1481 | - | chr20 | 1 | 34637451 | 34637463 |
| eCLIP Fold-Enrichment | Binding Protein | Cell Line | Strand | Chrm | | Start in Chrm (0 Indexed) | End in Chrm (1 Indexed) |
| 2.72012280355 | AKAP1 (bg=3.4%) | HepG2 | - | chr20 | | 34637401 | 34637456 |
| 2.59444912925 | ddx3x (bg=13.89%) | K562 | - | chr20 | | 34637461 | 34637526 |
| 2.41750045439 | ddx55 (bg=12.35%) | HepG2 | - | chr20 | | 34637402 | 34637452 |
| 2.89214450752 | dgcr8 (bg=19.31%) | K562 | - | chr20 | | 34637403 | 34637466 |
| 2.6881771912 | dgcr8 (bg=19.31%) | HepG2 | - | chr20 | | 34637406 | 34637452 |
| 4.28939140163 | dgcr8 (bg=19.31%) | SM-9MVZL | - | chr20 | | 34637459 | 34637463 |
| 4.28939140163 | dgcr8 (bg=19.31%) | SM-9MVZL | - | chr20 | | 34637463 | 34637479 |
| 2.35433524529 | drosha (bg=11.37%) | HepG2 | - | chr20 | | 34637403 | 34637467 |
| 2.35433524529 | drosha (bg=11.37%) | HepG2 | - | chr20 | | 34637403 | 34637467 |
| 2.74620943603 | fam120a (bg=18.43%) | K562 | - | chr20 | | 34637449 | 34637477 |
| 2.0386270642 | FTO (bg=7.53%) | K562 | - | chr20 | | 34637442 | 34637513 |
| 5.2271491535 | fubp3 (bg=23.31%) | HepG2 | - | chr20 | | 34637419 | 34637455 |
| 4.39707415494 | fubp3 (bg=23.31%) | HepG2 | - | chr20 | | 34637455 | 34637477 |
| 2.76586677441 | igf2bp1 (bg=11.67%) | K562 | - | chr20 | | 34637409 | 34637459 |
| 4.98911205638 | pum1 (bg=29.85%) | K562 | - | chr20 | | 34637447 | 34637477 |
| 7.46711393388 | pum2 (bg=21.55%) | K562 | - | chr20 | | 34637401 | 34637453 |
| 6.06366877983 | pum2 (bg=21.55%) | K562 | - | chr20 | | 34637420 | 34637451 |
| 6.08258826322 | pum2 (bg=21.55%) | K562 | - | chr20 | | 34637451 | 34637476 |
| 7.10684201517 | pum2 (bg=21.55%) | K562 | - | chr20 | | 34637453 | 34637494 |
| 2.74766978511 | sf3a3 (bg=12.13%) | HepG2 | - | chr20 | | 34637409 | 34637463 |
| 2.40263197644 | SUB1 (bg=9.24%) | HepG2 | - | chr20 | | 34637406 | 34637472 |
| 2.57959710436 | tia1 (bg=16.04%) | K562 | - | chr20 | | 34637460 | 34637490 |
| 2.56011441113 | XRCC6 (bg=1.65%) | HepG2 | - | chr20 | | 34637433 | 34637523 |

  
  

| Match 134 in HUMAN | | | | | | | |
| --- | --- | --- | --- | --- | --- | --- | --- |
| Motif | Start in Seq (1 Indexed) | End in Seq (1 Indexed) | Strand | Chrm | Exon | Start in Chrm (0 Indexed) | End in Chrm (1 Indexed) |
| TTTTGTTT | 1474 | 1481 | - | chr20 | 1 | 34637451 | 34637459 |
| eCLIP Fold-Enrichment | Binding Protein | Cell Line | Strand | Chrm | | Start in Chrm (0 Indexed) | End in Chrm (1 Indexed) |
| 2.72012280355 | AKAP1 (bg=3.4%) | HepG2 | - | chr20 | | 34637401 | 34637456 |
| 2.41750045439 | ddx55 (bg=12.35%) | HepG2 | - | chr20 | | 34637402 | 34637452 |
| 2.89214450752 | dgcr8 (bg=19.31%) | K562 | - | chr20 | | 34637403 | 34637466 |
| 2.6881771912 | dgcr8 (bg=19.31%) | HepG2 | - | chr20 | | 34637406 | 34637452 |
| 4.28939140163 | dgcr8 (bg=19.31%) | SM-9MVZL | - | chr20 | | 34637459 | 34637463 |
| 2.35433524529 | drosha (bg=11.37%) | HepG2 | - | chr20 | | 34637403 | 34637467 |
| 2.35433524529 | drosha (bg=11.37%) | HepG2 | - | chr20 | | 34637403 | 34637467 |
| 2.74620943603 | fam120a (bg=18.43%) | K562 | - | chr20 | | 34637449 | 34637477 |
| 2.0386270642 | FTO (bg=7.53%) | K562 | - | chr20 | | 34637442 | 34637513 |
| 5.2271491535 | fubp3 (bg=23.31%) | HepG2 | - | chr20 | | 34637419 | 34637455 |
| 4.39707415494 | fubp3 (bg=23.31%) | HepG2 | - | chr20 | | 34637455 | 34637477 |
| 2.76586677441 | igf2bp1 (bg=11.67%) | K562 | - | chr20 | | 34637409 | 34637459 |
| 4.98911205638 | pum1 (bg=29.85%) | K562 | - | chr20 | | 34637447 | 34637477 |
| 7.46711393388 | pum2 (bg=21.55%) | K562 | - | chr20 | | 34637401 | 34637453 |
| 6.06366877983 | pum2 (bg=21.55%) | K562 | - | chr20 | | 34637420 | 34637451 |
| 6.08258826322 | pum2 (bg=21.55%) | K562 | - | chr20 | | 34637451 | 34637476 |
| 7.10684201517 | pum2 (bg=21.55%) | K562 | - | chr20 | | 34637453 | 34637494 |
| 2.74766978511 | sf3a3 (bg=12.13%) | HepG2 | - | chr20 | | 34637409 | 34637463 |
| 2.40263197644 | SUB1 (bg=9.24%) | HepG2 | - | chr20 | | 34637406 | 34637472 |
| 2.56011441113 | XRCC6 (bg=1.65%) | HepG2 | - | chr20 | | 34637433 | 34637523 |

  
  

| Match 135 in HUMAN | | | | | | | |
| --- | --- | --- | --- | --- | --- | --- | --- |
| Motif | Start in Seq (1 Indexed) | End in Seq (1 Indexed) | Strand | Chrm | Exon | Start in Chrm (0 Indexed) | End in Chrm (1 Indexed) |
| CTGTGTATATAGTGTA | 1489 | 1504 | - | chr20 | 1 | 34637428 | 34637444 |
| eCLIP Fold-Enrichment | Binding Protein | Cell Line | Strand | Chrm | | Start in Chrm (0 Indexed) | End in Chrm (1 Indexed) |
| 2.72012280355 | AKAP1 (bg=3.4%) | HepG2 | - | chr20 | | 34637401 | 34637456 |
| 2.41750045439 | ddx55 (bg=12.35%) | HepG2 | - | chr20 | | 34637402 | 34637452 |
| 2.16657972231 | dgcr8 (bg=19.31%) | K562 | - | chr20 | | 34637403 | 34637443 |
| 2.89214450752 | dgcr8 (bg=19.31%) | K562 | - | chr20 | | 34637403 | 34637466 |
| 2.6881771912 | dgcr8 (bg=19.31%) | HepG2 | - | chr20 | | 34637406 | 34637452 |
| 2.35433524529 | drosha (bg=11.37%) | HepG2 | - | chr20 | | 34637403 | 34637467 |
| 2.35433524529 | drosha (bg=11.37%) | HepG2 | - | chr20 | | 34637403 | 34637467 |
| 3.1560852302 | fam120a (bg=18.43%) | K562 | - | chr20 | | 34637421 | 34637449 |
| 2.0386270642 | FTO (bg=7.53%) | K562 | - | chr20 | | 34637442 | 34637513 |
| 4.82209271176 | fubp3 (bg=23.31%) | HepG2 | - | chr20 | | 34637404 | 34637447 |
| 5.2271491535 | fubp3 (bg=23.31%) | HepG2 | - | chr20 | | 34637419 | 34637455 |
| 2.76586677441 | igf2bp1 (bg=11.67%) | K562 | - | chr20 | | 34637409 | 34637459 |
| 2.94271748233 | NKRF (bg=3.64%) | HepG2 | - | chr20 | | 34637408 | 34637444 |
| 4.65753933859 | pum1 (bg=29.85%) | K562 | - | chr20 | | 34637400 | 34637445 |
| 4.17828494242 | pum1 (bg=29.85%) | K562 | - | chr20 | | 34637408 | 34637447 |
| 7.46711393388 | pum2 (bg=21.55%) | K562 | - | chr20 | | 34637401 | 34637453 |
| 6.06366877983 | pum2 (bg=21.55%) | K562 | - | chr20 | | 34637420 | 34637451 |
| 2.74766978511 | sf3a3 (bg=12.13%) | HepG2 | - | chr20 | | 34637409 | 34637463 |
| 2.40263197644 | SUB1 (bg=9.24%) | HepG2 | - | chr20 | | 34637406 | 34637472 |
| 3.75964397133 | tia1 (bg=16.04%) | HepG2 | - | chr20 | | 34637404 | 34637446 |
| 3.26141853765 | tial1 (bg=14.09%) | HepG2 | - | chr20 | | 34637400 | 34637448 |
| 3.20519602268 | tial1 (bg=14.09%) | HepG2 | - | chr20 | | 34637406 | 34637447 |
| 2.51761914151 | TROVE2 (bg=3.49%) | HepG2 | - | chr20 | | 34637402 | 34637446 |
| 2.56011441113 | XRCC6 (bg=1.65%) | HepG2 | - | chr20 | | 34637433 | 34637523 |

  
  

| Match 136 in HUMAN | | | | | | | |
| --- | --- | --- | --- | --- | --- | --- | --- |
| Motif | Start in Seq (1 Indexed) | End in Seq (1 Indexed) | Strand | Chrm | Exon | Start in Chrm (0 Indexed) | End in Chrm (1 Indexed) |
| ATATAGTGTA | 1495 | 1504 | - | chr20 | 1 | 34637428 | 34637438 |
| eCLIP Fold-Enrichment | Binding Protein | Cell Line | Strand | Chrm | | Start in Chrm (0 Indexed) | End in Chrm (1 Indexed) |
| 2.72012280355 | AKAP1 (bg=3.4%) | HepG2 | - | chr20 | | 34637401 | 34637456 |
| 2.41750045439 | ddx55 (bg=12.35%) | HepG2 | - | chr20 | | 34637402 | 34637452 |
| 2.16657972231 | dgcr8 (bg=19.31%) | K562 | - | chr20 | | 34637403 | 34637443 |
| 2.89214450752 | dgcr8 (bg=19.31%) | K562 | - | chr20 | | 34637403 | 34637466 |
| 2.6881771912 | dgcr8 (bg=19.31%) | HepG2 | - | chr20 | | 34637406 | 34637452 |
| 2.35433524529 | drosha (bg=11.37%) | HepG2 | - | chr20 | | 34637403 | 34637467 |
| 2.35433524529 | drosha (bg=11.37%) | HepG2 | - | chr20 | | 34637403 | 34637467 |
| 3.1560852302 | fam120a (bg=18.43%) | K562 | - | chr20 | | 34637421 | 34637449 |
| 4.82209271176 | fubp3 (bg=23.31%) | HepG2 | - | chr20 | | 34637404 | 34637447 |
| 5.2271491535 | fubp3 (bg=23.31%) | HepG2 | - | chr20 | | 34637419 | 34637455 |
| 2.76586677441 | igf2bp1 (bg=11.67%) | K562 | - | chr20 | | 34637409 | 34637459 |
| 2.94271748233 | NKRF (bg=3.64%) | HepG2 | - | chr20 | | 34637408 | 34637444 |
| 4.65753933859 | pum1 (bg=29.85%) | K562 | - | chr20 | | 34637400 | 34637445 |
| 4.17828494242 | pum1 (bg=29.85%) | K562 | - | chr20 | | 34637408 | 34637447 |
| 7.46711393388 | pum2 (bg=21.55%) | K562 | - | chr20 | | 34637401 | 34637453 |
| 6.06366877983 | pum2 (bg=21.55%) | K562 | - | chr20 | | 34637420 | 34637451 |
| 2.74766978511 | sf3a3 (bg=12.13%) | HepG2 | - | chr20 | | 34637409 | 34637463 |
| 2.40263197644 | SUB1 (bg=9.24%) | HepG2 | - | chr20 | | 34637406 | 34637472 |
| 3.75964397133 | tia1 (bg=16.04%) | HepG2 | - | chr20 | | 34637404 | 34637446 |
| 3.26141853765 | tial1 (bg=14.09%) | HepG2 | - | chr20 | | 34637400 | 34637448 |
| 3.20519602268 | tial1 (bg=14.09%) | HepG2 | - | chr20 | | 34637406 | 34637447 |
| 2.51761914151 | TROVE2 (bg=3.49%) | HepG2 | - | chr20 | | 34637402 | 34637446 |
| 2.56011441113 | XRCC6 (bg=1.65%) | HepG2 | - | chr20 | | 34637433 | 34637523 |

  
  

| Match 137 in HUMAN | | | | | | | |
| --- | --- | --- | --- | --- | --- | --- | --- |
| Motif | Start in Seq (1 Indexed) | End in Seq (1 Indexed) | Strand | Chrm | Exon | Start in Chrm (0 Indexed) | End in Chrm (1 Indexed) |
| TAGTGT | 1498 | 1503 | - | chr20 | 1 | 34637429 | 34637435 |
| eCLIP Fold-Enrichment | Binding Protein | Cell Line | Strand | Chrm | | Start in Chrm (0 Indexed) | End in Chrm (1 Indexed) |
| 2.72012280355 | AKAP1 (bg=3.4%) | HepG2 | - | chr20 | | 34637401 | 34637456 |
| 2.41750045439 | ddx55 (bg=12.35%) | HepG2 | - | chr20 | | 34637402 | 34637452 |
| 2.16657972231 | dgcr8 (bg=19.31%) | K562 | - | chr20 | | 34637403 | 34637443 |
| 2.89214450752 | dgcr8 (bg=19.31%) | K562 | - | chr20 | | 34637403 | 34637466 |
| 2.6881771912 | dgcr8 (bg=19.31%) | HepG2 | - | chr20 | | 34637406 | 34637452 |
| 2.35433524529 | drosha (bg=11.37%) | HepG2 | - | chr20 | | 34637403 | 34637467 |
| 2.35433524529 | drosha (bg=11.37%) | HepG2 | - | chr20 | | 34637403 | 34637467 |
| 3.1560852302 | fam120a (bg=18.43%) | K562 | - | chr20 | | 34637421 | 34637449 |
| 4.82209271176 | fubp3 (bg=23.31%) | HepG2 | - | chr20 | | 34637404 | 34637447 |
| 5.2271491535 | fubp3 (bg=23.31%) | HepG2 | - | chr20 | | 34637419 | 34637455 |
| 2.76586677441 | igf2bp1 (bg=11.67%) | K562 | - | chr20 | | 34637409 | 34637459 |
| 2.94271748233 | NKRF (bg=3.64%) | HepG2 | - | chr20 | | 34637408 | 34637444 |
| 4.65753933859 | pum1 (bg=29.85%) | K562 | - | chr20 | | 34637400 | 34637445 |
| 4.17828494242 | pum1 (bg=29.85%) | K562 | - | chr20 | | 34637408 | 34637447 |
| 7.46711393388 | pum2 (bg=21.55%) | K562 | - | chr20 | | 34637401 | 34637453 |
| 6.06366877983 | pum2 (bg=21.55%) | K562 | - | chr20 | | 34637420 | 34637451 |
| 2.74766978511 | sf3a3 (bg=12.13%) | HepG2 | - | chr20 | | 34637409 | 34637463 |
| 2.40263197644 | SUB1 (bg=9.24%) | HepG2 | - | chr20 | | 34637406 | 34637472 |
| 3.75964397133 | tia1 (bg=16.04%) | HepG2 | - | chr20 | | 34637404 | 34637446 |
| 3.26141853765 | tial1 (bg=14.09%) | HepG2 | - | chr20 | | 34637400 | 34637448 |
| 3.20519602268 | tial1 (bg=14.09%) | HepG2 | - | chr20 | | 34637406 | 34637447 |
| 2.51761914151 | TROVE2 (bg=3.49%) | HepG2 | - | chr20 | | 34637402 | 34637446 |
| 2.56011441113 | XRCC6 (bg=1.65%) | HepG2 | - | chr20 | | 34637433 | 34637523 |

  
  

| Match 138 in HUMAN | | | | | | | |
| --- | --- | --- | --- | --- | --- | --- | --- |
| Motif | Start in Seq (1 Indexed) | End in Seq (1 Indexed) | Strand | Chrm | Exon | Start in Chrm (0 Indexed) | End in Chrm (1 Indexed) |
| GAGTCCTAATT | 1519 | 1529 | - | chr20 | 1 | 34637403 | 34637414 |
| eCLIP Fold-Enrichment | Binding Protein | Cell Line | Strand | Chrm | | Start in Chrm (0 Indexed) | End in Chrm (1 Indexed) |
| 2.72012280355 | AKAP1 (bg=3.4%) | HepG2 | - | chr20 | | 34637401 | 34637456 |
| 2.41750045439 | ddx55 (bg=12.35%) | HepG2 | - | chr20 | | 34637402 | 34637452 |
| 2.16657972231 | dgcr8 (bg=19.31%) | K562 | - | chr20 | | 34637403 | 34637443 |
| 2.89214450752 | dgcr8 (bg=19.31%) | K562 | - | chr20 | | 34637403 | 34637466 |
| 2.6881771912 | dgcr8 (bg=19.31%) | HepG2 | - | chr20 | | 34637406 | 34637452 |
| 2.35433524529 | drosha (bg=11.37%) | HepG2 | - | chr20 | | 34637403 | 34637467 |
| 2.35433524529 | drosha (bg=11.37%) | HepG2 | - | chr20 | | 34637403 | 34637467 |
| 2.78015676796 | fam120a (bg=18.43%) | K562 | - | chr20 | | 34637406 | 34637421 |
| 4.61946657628 | fubp3 (bg=23.31%) | HepG2 | - | chr20 | | 34637402 | 34637419 |
| 4.82209271176 | fubp3 (bg=23.31%) | HepG2 | - | chr20 | | 34637404 | 34637447 |
| 2.76586677441 | igf2bp1 (bg=11.67%) | K562 | - | chr20 | | 34637409 | 34637459 |
| 2.94271748233 | NKRF (bg=3.64%) | HepG2 | - | chr20 | | 34637408 | 34637444 |
| 4.65753933859 | pum1 (bg=29.85%) | K562 | - | chr20 | | 34637400 | 34637445 |
| 4.17828494242 | pum1 (bg=29.85%) | K562 | - | chr20 | | 34637408 | 34637447 |
| 7.46711393388 | pum2 (bg=21.55%) | K562 | - | chr20 | | 34637401 | 34637453 |
| 5.81835877429 | pum2 (bg=21.55%) | K562 | - | chr20 | | 34637403 | 34637420 |
| 2.74766978511 | sf3a3 (bg=12.13%) | HepG2 | - | chr20 | | 34637409 | 34637463 |
| 2.40263197644 | SUB1 (bg=9.24%) | HepG2 | - | chr20 | | 34637406 | 34637472 |
| 3.75964397133 | tia1 (bg=16.04%) | HepG2 | - | chr20 | | 34637404 | 34637446 |
| 3.26141853765 | tial1 (bg=14.09%) | HepG2 | - | chr20 | | 34637400 | 34637448 |
| 3.20519602268 | tial1 (bg=14.09%) | HepG2 | - | chr20 | | 34637406 | 34637447 |
| 2.51761914151 | TROVE2 (bg=3.49%) | HepG2 | - | chr20 | | 34637402 | 34637446 |

  
  

| Match 139 in HUMAN | | | | | | | |
| --- | --- | --- | --- | --- | --- | --- | --- |
| Motif | Start in Seq (1 Indexed) | End in Seq (1 Indexed) | Strand | Chrm | Exon | Start in Chrm (0 Indexed) | End in Chrm (1 Indexed) |
| GTCCTAATT | 1521 | 1529 | - | chr20 | 1 | 34637403 | 34637412 |
| eCLIP Fold-Enrichment | Binding Protein | Cell Line | Strand | Chrm | | Start in Chrm (0 Indexed) | End in Chrm (1 Indexed) |
| 2.72012280355 | AKAP1 (bg=3.4%) | HepG2 | - | chr20 | | 34637401 | 34637456 |
| 2.41750045439 | ddx55 (bg=12.35%) | HepG2 | - | chr20 | | 34637402 | 34637452 |
| 2.16657972231 | dgcr8 (bg=19.31%) | K562 | - | chr20 | | 34637403 | 34637443 |
| 2.89214450752 | dgcr8 (bg=19.31%) | K562 | - | chr20 | | 34637403 | 34637466 |
| 2.6881771912 | dgcr8 (bg=19.31%) | HepG2 | - | chr20 | | 34637406 | 34637452 |
| 2.35433524529 | drosha (bg=11.37%) | HepG2 | - | chr20 | | 34637403 | 34637467 |
| 2.35433524529 | drosha (bg=11.37%) | HepG2 | - | chr20 | | 34637403 | 34637467 |
| 2.78015676796 | fam120a (bg=18.43%) | K562 | - | chr20 | | 34637406 | 34637421 |
| 4.61946657628 | fubp3 (bg=23.31%) | HepG2 | - | chr20 | | 34637402 | 34637419 |
| 4.82209271176 | fubp3 (bg=23.31%) | HepG2 | - | chr20 | | 34637404 | 34637447 |
| 2.76586677441 | igf2bp1 (bg=11.67%) | K562 | - | chr20 | | 34637409 | 34637459 |
| 2.94271748233 | NKRF (bg=3.64%) | HepG2 | - | chr20 | | 34637408 | 34637444 |
| 4.65753933859 | pum1 (bg=29.85%) | K562 | - | chr20 | | 34637400 | 34637445 |
| 4.17828494242 | pum1 (bg=29.85%) | K562 | - | chr20 | | 34637408 | 34637447 |
| 7.46711393388 | pum2 (bg=21.55%) | K562 | - | chr20 | | 34637401 | 34637453 |
| 5.81835877429 | pum2 (bg=21.55%) | K562 | - | chr20 | | 34637403 | 34637420 |
| 2.74766978511 | sf3a3 (bg=12.13%) | HepG2 | - | chr20 | | 34637409 | 34637463 |
| 2.40263197644 | SUB1 (bg=9.24%) | HepG2 | - | chr20 | | 34637406 | 34637472 |
| 3.75964397133 | tia1 (bg=16.04%) | HepG2 | - | chr20 | | 34637404 | 34637446 |
| 3.26141853765 | tial1 (bg=14.09%) | HepG2 | - | chr20 | | 34637400 | 34637448 |
| 3.20519602268 | tial1 (bg=14.09%) | HepG2 | - | chr20 | | 34637406 | 34637447 |
| 2.51761914151 | TROVE2 (bg=3.49%) | HepG2 | - | chr20 | | 34637402 | 34637446 |

  
  

| Match 140 in HUMAN | | | | | | | |
| --- | --- | --- | --- | --- | --- | --- | --- |
| Motif | Start in Seq (1 Indexed) | End in Seq (1 Indexed) | Strand | Chrm | Exon | Start in Chrm (0 Indexed) | End in Chrm (1 Indexed) |
| CAACATCT | 1532 | 1539 | - | chr20 | 1 | 34637393 | 34637401 |
| eCLIP Fold-Enrichment | Binding Protein | Cell Line | Strand | Chrm | | Start in Chrm (0 Indexed) | End in Chrm (1 Indexed) |
| 2.72012280355 | AKAP1 (bg=3.4%) | HepG2 | - | chr20 | | 34637401 | 34637456 |
| 4.65753933859 | pum1 (bg=29.85%) | K562 | - | chr20 | | 34637400 | 34637445 |
| 7.46711393388 | pum2 (bg=21.55%) | K562 | - | chr20 | | 34637401 | 34637453 |
| 3.26141853765 | tial1 (bg=14.09%) | HepG2 | - | chr20 | | 34637400 | 34637448 |

  
  

| Match 141 in HUMAN | | | | | | | |
| --- | --- | --- | --- | --- | --- | --- | --- |
| Motif | Start in Seq (1 Indexed) | End in Seq (1 Indexed) | Strand | Chrm | Exon | Start in Chrm (0 Indexed) | End in Chrm (1 Indexed) |
| CAACATCTAGTCTTT | 1532 | 1546 | - | chr20 | 1 | 34637386 | 34637401 |
| eCLIP Fold-Enrichment | Binding Protein | Cell Line | Strand | Chrm | | Start in Chrm (0 Indexed) | End in Chrm (1 Indexed) |
| 2.72012280355 | AKAP1 (bg=3.4%) | HepG2 | - | chr20 | | 34637401 | 34637456 |
| 4.65753933859 | pum1 (bg=29.85%) | K562 | - | chr20 | | 34637400 | 34637445 |
| 7.46711393388 | pum2 (bg=21.55%) | K562 | - | chr20 | | 34637401 | 34637453 |
| 3.26141853765 | tial1 (bg=14.09%) | HepG2 | - | chr20 | | 34637400 | 34637448 |

  
  

| Match 142 in HUMAN | | | | | | | |
| --- | --- | --- | --- | --- | --- | --- | --- |
| Motif | Start in Seq (1 Indexed) | End in Seq (1 Indexed) | Strand | Chrm | Exon | Start in Chrm (0 Indexed) | End in Chrm (1 Indexed) |
| TAAACTAATATATTTTGTACATTTTGTTTTACAAGTCCTAGGAAAGATTGTCTTCTGAAAATTTGATGTCTTCTGGGTTGATGGAGATGGGAAGGGTTCTAGGCCAGAATGTTCACATTTGGAAGACT | 1592 | 1719 | - | chr20 | 1 | 34637213 | 34637341 |
| eCLIP Fold-Enrichment | Binding Protein | Cell Line | Strand | Chrm | | Start in Chrm (0 Indexed) | End in Chrm (1 Indexed) |
| 2.43466314153 | ddx55 (bg=12.35%) | HepG2 | - | chr20 | | 34637249 | 34637286 |
| 2.25521778393 | dgcr8 (bg=19.31%) | HepG2 | - | chr20 | | 34637245 | 34637299 |
| 2.81814392607 | dgcr8 (bg=19.31%) | K562 | - | chr20 | | 34637251 | 34637290 |
| 2.42428134115 | fam120a (bg=18.43%) | K562 | - | chr20 | | 34637255 | 34637294 |
| 4.72785133787 | fubp3 (bg=23.31%) | HepG2 | - | chr20 | | 34637240 | 34637285 |
| 3.99797819953 | fubp3 (bg=23.31%) | HepG2 | - | chr20 | | 34637254 | 34637280 |
| 2.08233792671 | KHSRP (bg=8.1%) | HepG2 | - | chr20 | | 34637240 | 34637287 |
| 3.43070094492 | lin28b (bg=16.96%) | K562 | - | chr20 | | 34637237 | 34637261 |
| 2.48791332981 | lin28b (bg=16.96%) | HepG2 | - | chr20 | | 34637240 | 34637281 |
| 2.36374730083 | lin28b (bg=16.96%) | HepG2 | - | chr20 | | 34637242 | 34637279 |
| 3.07337894349 | lin28b (bg=16.96%) | K562 | - | chr20 | | 34637261 | 34637281 |
| 2.23135583374 | NOLC1 (bg=6.58%) | K562 | - | chr20 | | 34637251 | 34637296 |
| 3.64622434286 | pum1 (bg=29.85%) | K562 | - | chr20 | | 34637250 | 34637310 |
| 3.57071471679 | pum1 (bg=29.85%) | K562 | - | chr20 | | 34637260 | 34637293 |
| 4.18884695061 | pum2 (bg=21.55%) | K562 | - | chr20 | | 34637250 | 34637311 |
| 5.14789791304 | pum2 (bg=21.55%) | K562 | - | chr20 | | 34637266 | 34637287 |
| 5.23620515033 | pum2 (bg=21.55%) | K562 | - | chr20 | | 34637287 | 34637305 |
| 3.16104423517 | sf3a3 (bg=12.13%) | HepG2 | - | chr20 | | 34637248 | 34637303 |
| 2.27868551896 | tia1 (bg=16.04%) | K562 | - | chr20 | | 34637244 | 34637299 |
| 3.14297261088 | tia1 (bg=16.04%) | HepG2 | - | chr20 | | 34637256 | 34637273 |
| 3.59751344546 | tial1 (bg=14.09%) | HepG2 | - | chr20 | | 34637280 | 34637294 |
| 3.22747120573 | tial1 (bg=14.09%) | HepG2 | - | chr20 | | 34637296 | 34637306 |
| 2.21052167302 | ZC3H11A (bg=6.25%) | K562 | - | chr20 | | 34637256 | 34637274 |
| 2.21052167302 | ZC3H11A (bg=6.25%) | K562 | - | chr20 | | 34637256 | 34637274 |

  
  

| Match 143 in HUMAN | | | | | | | |
| --- | --- | --- | --- | --- | --- | --- | --- |
| Motif | Start in Seq (1 Indexed) | End in Seq (1 Indexed) | Strand | Chrm | Exon | Start in Chrm (0 Indexed) | End in Chrm (1 Indexed) |
| AAGATTGTCTTCTGAAAATT | 1635 | 1654 | - | chr20 | 1 | 34637278 | 34637298 |
| eCLIP Fold-Enrichment | Binding Protein | Cell Line | Strand | Chrm | | Start in Chrm (0 Indexed) | End in Chrm (1 Indexed) |
| 2.43466314153 | ddx55 (bg=12.35%) | HepG2 | - | chr20 | | 34637249 | 34637286 |
| 2.25521778393 | dgcr8 (bg=19.31%) | HepG2 | - | chr20 | | 34637245 | 34637299 |
| 2.81814392607 | dgcr8 (bg=19.31%) | K562 | - | chr20 | | 34637251 | 34637290 |
| 2.42428134115 | fam120a (bg=18.43%) | K562 | - | chr20 | | 34637255 | 34637294 |
| 4.72785133787 | fubp3 (bg=23.31%) | HepG2 | - | chr20 | | 34637240 | 34637285 |
| 3.99797819953 | fubp3 (bg=23.31%) | HepG2 | - | chr20 | | 34637254 | 34637280 |
| 2.08233792671 | KHSRP (bg=8.1%) | HepG2 | - | chr20 | | 34637240 | 34637287 |
| 2.48791332981 | lin28b (bg=16.96%) | HepG2 | - | chr20 | | 34637240 | 34637281 |
| 2.36374730083 | lin28b (bg=16.96%) | HepG2 | - | chr20 | | 34637242 | 34637279 |
| 3.07337894349 | lin28b (bg=16.96%) | K562 | - | chr20 | | 34637261 | 34637281 |
| 2.23135583374 | NOLC1 (bg=6.58%) | K562 | - | chr20 | | 34637251 | 34637296 |
| 3.64622434286 | pum1 (bg=29.85%) | K562 | - | chr20 | | 34637250 | 34637310 |
| 3.57071471679 | pum1 (bg=29.85%) | K562 | - | chr20 | | 34637260 | 34637293 |
| 4.18884695061 | pum2 (bg=21.55%) | K562 | - | chr20 | | 34637250 | 34637311 |
| 5.14789791304 | pum2 (bg=21.55%) | K562 | - | chr20 | | 34637266 | 34637287 |
| 5.23620515033 | pum2 (bg=21.55%) | K562 | - | chr20 | | 34637287 | 34637305 |
| 3.16104423517 | sf3a3 (bg=12.13%) | HepG2 | - | chr20 | | 34637248 | 34637303 |
| 2.27868551896 | tia1 (bg=16.04%) | K562 | - | chr20 | | 34637244 | 34637299 |
| 3.59751344546 | tial1 (bg=14.09%) | HepG2 | - | chr20 | | 34637280 | 34637294 |
| 3.22747120573 | tial1 (bg=14.09%) | HepG2 | - | chr20 | | 34637296 | 34637306 |

  
  

| Match 144 in HUMAN | | | | | | | |
| --- | --- | --- | --- | --- | --- | --- | --- |
| Motif | Start in Seq (1 Indexed) | End in Seq (1 Indexed) | Strand | Chrm | Exon | Start in Chrm (0 Indexed) | End in Chrm (1 Indexed) |
| AAGATTGTCTT | 1635 | 1645 | - | chr20 | 1 | 34637287 | 34637298 |
| eCLIP Fold-Enrichment | Binding Protein | Cell Line | Strand | Chrm | | Start in Chrm (0 Indexed) | End in Chrm (1 Indexed) |
| 2.25521778393 | dgcr8 (bg=19.31%) | HepG2 | - | chr20 | | 34637245 | 34637299 |
| 2.81814392607 | dgcr8 (bg=19.31%) | K562 | - | chr20 | | 34637251 | 34637290 |
| 2.42428134115 | fam120a (bg=18.43%) | K562 | - | chr20 | | 34637255 | 34637294 |
| 2.08233792671 | KHSRP (bg=8.1%) | HepG2 | - | chr20 | | 34637240 | 34637287 |
| 2.23135583374 | NOLC1 (bg=6.58%) | K562 | - | chr20 | | 34637251 | 34637296 |
| 3.64622434286 | pum1 (bg=29.85%) | K562 | - | chr20 | | 34637250 | 34637310 |
| 3.57071471679 | pum1 (bg=29.85%) | K562 | - | chr20 | | 34637260 | 34637293 |
| 4.18884695061 | pum2 (bg=21.55%) | K562 | - | chr20 | | 34637250 | 34637311 |
| 5.14789791304 | pum2 (bg=21.55%) | K562 | - | chr20 | | 34637266 | 34637287 |
| 5.23620515033 | pum2 (bg=21.55%) | K562 | - | chr20 | | 34637287 | 34637305 |
| 3.16104423517 | sf3a3 (bg=12.13%) | HepG2 | - | chr20 | | 34637248 | 34637303 |
| 2.27868551896 | tia1 (bg=16.04%) | K562 | - | chr20 | | 34637244 | 34637299 |
| 3.59751344546 | tial1 (bg=14.09%) | HepG2 | - | chr20 | | 34637280 | 34637294 |
| 3.22747120573 | tial1 (bg=14.09%) | HepG2 | - | chr20 | | 34637296 | 34637306 |

  
  

| Match 145 in HUMAN | | | | | | | |
| --- | --- | --- | --- | --- | --- | --- | --- |
| Motif | Start in Seq (1 Indexed) | End in Seq (1 Indexed) | Strand | Chrm | Exon | Start in Chrm (0 Indexed) | End in Chrm (1 Indexed) |
| AAAATT | 1649 | 1654 | - | chr20 | 1 | 34637278 | 34637284 |
| eCLIP Fold-Enrichment | Binding Protein | Cell Line | Strand | Chrm | | Start in Chrm (0 Indexed) | End in Chrm (1 Indexed) |
| 2.43466314153 | ddx55 (bg=12.35%) | HepG2 | - | chr20 | | 34637249 | 34637286 |
| 2.25521778393 | dgcr8 (bg=19.31%) | HepG2 | - | chr20 | | 34637245 | 34637299 |
| 2.81814392607 | dgcr8 (bg=19.31%) | K562 | - | chr20 | | 34637251 | 34637290 |
| 2.42428134115 | fam120a (bg=18.43%) | K562 | - | chr20 | | 34637255 | 34637294 |
| 4.72785133787 | fubp3 (bg=23.31%) | HepG2 | - | chr20 | | 34637240 | 34637285 |
| 3.99797819953 | fubp3 (bg=23.31%) | HepG2 | - | chr20 | | 34637254 | 34637280 |
| 2.08233792671 | KHSRP (bg=8.1%) | HepG2 | - | chr20 | | 34637240 | 34637287 |
| 2.48791332981 | lin28b (bg=16.96%) | HepG2 | - | chr20 | | 34637240 | 34637281 |
| 2.36374730083 | lin28b (bg=16.96%) | HepG2 | - | chr20 | | 34637242 | 34637279 |
| 3.07337894349 | lin28b (bg=16.96%) | K562 | - | chr20 | | 34637261 | 34637281 |
| 2.23135583374 | NOLC1 (bg=6.58%) | K562 | - | chr20 | | 34637251 | 34637296 |
| 3.64622434286 | pum1 (bg=29.85%) | K562 | - | chr20 | | 34637250 | 34637310 |
| 3.57071471679 | pum1 (bg=29.85%) | K562 | - | chr20 | | 34637260 | 34637293 |
| 4.18884695061 | pum2 (bg=21.55%) | K562 | - | chr20 | | 34637250 | 34637311 |
| 5.14789791304 | pum2 (bg=21.55%) | K562 | - | chr20 | | 34637266 | 34637287 |
| 3.16104423517 | sf3a3 (bg=12.13%) | HepG2 | - | chr20 | | 34637248 | 34637303 |
| 2.27868551896 | tia1 (bg=16.04%) | K562 | - | chr20 | | 34637244 | 34637299 |
| 3.59751344546 | tial1 (bg=14.09%) | HepG2 | - | chr20 | | 34637280 | 34637294 |

  
  

| Match 146 in HUMAN | | | | | | | |
| --- | --- | --- | --- | --- | --- | --- | --- |
| Motif | Start in Seq (1 Indexed) | End in Seq (1 Indexed) | Strand | Chrm | Exon | Start in Chrm (0 Indexed) | End in Chrm (1 Indexed) |
| TGGAGA | 1673 | 1678 | - | chr20 | 1 | 34637254 | 34637260 |
| eCLIP Fold-Enrichment | Binding Protein | Cell Line | Strand | Chrm | | Start in Chrm (0 Indexed) | End in Chrm (1 Indexed) |
| 2.43466314153 | ddx55 (bg=12.35%) | HepG2 | - | chr20 | | 34637249 | 34637286 |
| 2.25521778393 | dgcr8 (bg=19.31%) | HepG2 | - | chr20 | | 34637245 | 34637299 |
| 2.81814392607 | dgcr8 (bg=19.31%) | K562 | - | chr20 | | 34637251 | 34637290 |
| 2.42428134115 | fam120a (bg=18.43%) | K562 | - | chr20 | | 34637255 | 34637294 |
| 4.72785133787 | fubp3 (bg=23.31%) | HepG2 | - | chr20 | | 34637240 | 34637285 |
| 3.99797819953 | fubp3 (bg=23.31%) | HepG2 | - | chr20 | | 34637254 | 34637280 |
| 2.08233792671 | KHSRP (bg=8.1%) | HepG2 | - | chr20 | | 34637240 | 34637287 |
| 3.43070094492 | lin28b (bg=16.96%) | K562 | - | chr20 | | 34637237 | 34637261 |
| 2.48791332981 | lin28b (bg=16.96%) | HepG2 | - | chr20 | | 34637240 | 34637281 |
| 2.36374730083 | lin28b (bg=16.96%) | HepG2 | - | chr20 | | 34637242 | 34637279 |
| 2.23135583374 | NOLC1 (bg=6.58%) | K562 | - | chr20 | | 34637251 | 34637296 |
| 3.64622434286 | pum1 (bg=29.85%) | K562 | - | chr20 | | 34637250 | 34637310 |
| 3.57071471679 | pum1 (bg=29.85%) | K562 | - | chr20 | | 34637260 | 34637293 |
| 4.18884695061 | pum2 (bg=21.55%) | K562 | - | chr20 | | 34637250 | 34637311 |
| 3.16104423517 | sf3a3 (bg=12.13%) | HepG2 | - | chr20 | | 34637248 | 34637303 |
| 2.27868551896 | tia1 (bg=16.04%) | K562 | - | chr20 | | 34637244 | 34637299 |
| 3.14297261088 | tia1 (bg=16.04%) | HepG2 | - | chr20 | | 34637256 | 34637273 |
| 2.21052167302 | ZC3H11A (bg=6.25%) | K562 | - | chr20 | | 34637256 | 34637274 |
| 2.21052167302 | ZC3H11A (bg=6.25%) | K562 | - | chr20 | | 34637256 | 34637274 |

  
  

| Match 147 in HUMAN | | | | | | | |
| --- | --- | --- | --- | --- | --- | --- | --- |
| Motif | Start in Seq (1 Indexed) | End in Seq (1 Indexed) | Strand | Chrm | Exon | Start in Chrm (0 Indexed) | End in Chrm (1 Indexed) |
| AGGTAGTAAAATACCACTTTGTAAATATCTTTTTGCTAAAATTCATAGGAAAT | 1856 | 1908 | - | chr20 | 1 | 34637024 | 34637077 |
| eCLIP Fold-Enrichment | Binding Protein | Cell Line | Strand | Chrm | | Start in Chrm (0 Indexed) | End in Chrm (1 Indexed) |
| 4.43360003097 | fubp3 (bg=23.31%) | HepG2 | - | chr20 | | 34636975 | 34637034 |
| 5.01147007586 | fubp3 (bg=23.31%) | HepG2 | - | chr20 | | 34636990 | 34637033 |
| 3.51572885751 | pum1 (bg=29.85%) | K562 | - | chr20 | | 34636977 | 34637047 |
| 2.88361892753 | pum1 (bg=29.85%) | K562 | - | chr20 | | 34636979 | 34637041 |
| 4.68434609141 | pum2 (bg=21.55%) | K562 | - | chr20 | | 34636996 | 34637029 |
| 2.05319292923 | tial1 (bg=14.09%) | HepG2 | - | chr20 | | 34636979 | 34637036 |

  
  

| Match 148 in HUMAN | | | | | | | |
| --- | --- | --- | --- | --- | --- | --- | --- |
| Motif | Start in Seq (1 Indexed) | End in Seq (1 Indexed) | Strand | Chrm | Exon | Start in Chrm (0 Indexed) | End in Chrm (1 Indexed) |
| TGTAAATATCTTTTTGCTAAAATTCATAGGAAAT | 1875 | 1908 | - | chr20 | 1 | 34637024 | 34637058 |
| eCLIP Fold-Enrichment | Binding Protein | Cell Line | Strand | Chrm | | Start in Chrm (0 Indexed) | End in Chrm (1 Indexed) |
| 4.43360003097 | fubp3 (bg=23.31%) | HepG2 | - | chr20 | | 34636975 | 34637034 |
| 5.01147007586 | fubp3 (bg=23.31%) | HepG2 | - | chr20 | | 34636990 | 34637033 |
| 3.51572885751 | pum1 (bg=29.85%) | K562 | - | chr20 | | 34636977 | 34637047 |
| 2.88361892753 | pum1 (bg=29.85%) | K562 | - | chr20 | | 34636979 | 34637041 |
| 4.68434609141 | pum2 (bg=21.55%) | K562 | - | chr20 | | 34636996 | 34637029 |
| 2.05319292923 | tial1 (bg=14.09%) | HepG2 | - | chr20 | | 34636979 | 34637036 |

  
  

| Match 149 in HUMAN | | | | | | | |
| --- | --- | --- | --- | --- | --- | --- | --- |
| Motif | Start in Seq (1 Indexed) | End in Seq (1 Indexed) | Strand | Chrm | Exon | Start in Chrm (0 Indexed) | End in Chrm (1 Indexed) |
| CTTTTTGCT | 1884 | 1892 | - | chr20 | 1 | 34637040 | 34637049 |
| eCLIP Fold-Enrichment | Binding Protein | Cell Line | Strand | Chrm | | Start in Chrm (0 Indexed) | End in Chrm (1 Indexed) |
| 3.51572885751 | pum1 (bg=29.85%) | K562 | - | chr20 | | 34636977 | 34637047 |
| 2.88361892753 | pum1 (bg=29.85%) | K562 | - | chr20 | | 34636979 | 34637041 |

  
  

| Match 150 in HUMAN | | | | | | | |
| --- | --- | --- | --- | --- | --- | --- | --- |
| Motif | Start in Seq (1 Indexed) | End in Seq (1 Indexed) | Strand | Chrm | Exon | Start in Chrm (0 Indexed) | End in Chrm (1 Indexed) |
| AAATTCATAGGAA | 1894 | 1906 | - | chr20 | 1 | 34637026 | 34637039 |
| eCLIP Fold-Enrichment | Binding Protein | Cell Line | Strand | Chrm | | Start in Chrm (0 Indexed) | End in Chrm (1 Indexed) |
| 4.43360003097 | fubp3 (bg=23.31%) | HepG2 | - | chr20 | | 34636975 | 34637034 |
| 5.01147007586 | fubp3 (bg=23.31%) | HepG2 | - | chr20 | | 34636990 | 34637033 |
| 3.51572885751 | pum1 (bg=29.85%) | K562 | - | chr20 | | 34636977 | 34637047 |
| 2.88361892753 | pum1 (bg=29.85%) | K562 | - | chr20 | | 34636979 | 34637041 |
| 4.68434609141 | pum2 (bg=21.55%) | K562 | - | chr20 | | 34636996 | 34637029 |
| 2.05319292923 | tial1 (bg=14.09%) | HepG2 | - | chr20 | | 34636979 | 34637036 |

  
  

| Match 151 in HUMAN | | | | | | | |
| --- | --- | --- | --- | --- | --- | --- | --- |
| Motif | Start in Seq (1 Indexed) | End in Seq (1 Indexed) | Strand | Chrm | Exon | Start in Chrm (0 Indexed) | End in Chrm (1 Indexed) |
| CTTTTGGAAATTGAATTGTGAAGCCACCTTTG | 1910 | 1941 | - | chr20 | 1 | 34636991 | 34637023 |
| eCLIP Fold-Enrichment | Binding Protein | Cell Line | Strand | Chrm | | Start in Chrm (0 Indexed) | End in Chrm (1 Indexed) |
| 2.76243580573 | DDX21 (bg=1.64%) | K562 | - | chr20 | | 34636965 | 34637014 |
| 2.7555073169 | ddx6 (bg=23.92%) | HepG2 | - | chr20 | | 34636978 | 34637022 |
| 2.88646119607 | ddx6 (bg=23.92%) | HepG2 | - | chr20 | | 34636981 | 34637015 |
| 4.21134326719 | dgcr8 (bg=19.31%) | SM-9MVZL | - | chr20 | | 34636974 | 34637012 |
| 4.43360003097 | fubp3 (bg=23.31%) | HepG2 | - | chr20 | | 34636975 | 34637034 |
| 5.01147007586 | fubp3 (bg=23.31%) | HepG2 | - | chr20 | | 34636990 | 34637033 |
| 3.51572885751 | pum1 (bg=29.85%) | K562 | - | chr20 | | 34636977 | 34637047 |
| 2.88361892753 | pum1 (bg=29.85%) | K562 | - | chr20 | | 34636979 | 34637041 |
| 3.5083391348 | pum2 (bg=21.55%) | K562 | - | chr20 | | 34636985 | 34637001 |
| 4.68434609141 | pum2 (bg=21.55%) | K562 | - | chr20 | | 34636996 | 34637029 |
| 2.64277657878 | sf3a3 (bg=12.13%) | HepG2 | - | chr20 | | 34636975 | 34637022 |
| 2.32586014979 | TBRG4 (bg=7.0%) | HepG2 | - | chr20 | | 34636969 | 34637023 |
| 2.62839943805 | tia1 (bg=16.04%) | HepG2 | - | chr20 | | 34636972 | 34637016 |
| 2.05319292923 | tial1 (bg=14.09%) | HepG2 | - | chr20 | | 34636979 | 34637036 |

  
  

| Match 152 in HUMAN | | | | | | | |
| --- | --- | --- | --- | --- | --- | --- | --- |
| Motif | Start in Seq (1 Indexed) | End in Seq (1 Indexed) | Strand | Chrm | Exon | Start in Chrm (0 Indexed) | End in Chrm (1 Indexed) |
| GAATTGT | 1922 | 1928 | - | chr20 | 1 | 34637004 | 34637011 |
| eCLIP Fold-Enrichment | Binding Protein | Cell Line | Strand | Chrm | | Start in Chrm (0 Indexed) | End in Chrm (1 Indexed) |
| 2.76243580573 | DDX21 (bg=1.64%) | K562 | - | chr20 | | 34636965 | 34637014 |
| 2.7555073169 | ddx6 (bg=23.92%) | HepG2 | - | chr20 | | 34636978 | 34637022 |
| 2.88646119607 | ddx6 (bg=23.92%) | HepG2 | - | chr20 | | 34636981 | 34637015 |
| 4.21134326719 | dgcr8 (bg=19.31%) | SM-9MVZL | - | chr20 | | 34636974 | 34637012 |
| 4.43360003097 | fubp3 (bg=23.31%) | HepG2 | - | chr20 | | 34636975 | 34637034 |
| 5.01147007586 | fubp3 (bg=23.31%) | HepG2 | - | chr20 | | 34636990 | 34637033 |
| 3.51572885751 | pum1 (bg=29.85%) | K562 | - | chr20 | | 34636977 | 34637047 |
| 2.88361892753 | pum1 (bg=29.85%) | K562 | - | chr20 | | 34636979 | 34637041 |
| 4.68434609141 | pum2 (bg=21.55%) | K562 | - | chr20 | | 34636996 | 34637029 |
| 2.64277657878 | sf3a3 (bg=12.13%) | HepG2 | - | chr20 | | 34636975 | 34637022 |
| 2.32586014979 | TBRG4 (bg=7.0%) | HepG2 | - | chr20 | | 34636969 | 34637023 |
| 2.62839943805 | tia1 (bg=16.04%) | HepG2 | - | chr20 | | 34636972 | 34637016 |
| 2.05319292923 | tial1 (bg=14.09%) | HepG2 | - | chr20 | | 34636979 | 34637036 |

  
  

| Match 153 in HUMAN | | | | | | | |
| --- | --- | --- | --- | --- | --- | --- | --- |
| Motif | Start in Seq (1 Indexed) | End in Seq (1 Indexed) | Strand | Chrm | Exon | Start in Chrm (0 Indexed) | End in Chrm (1 Indexed) |
| CCACCT | 1933 | 1938 | - | chr20 | 1 | 34636994 | 34637000 |
| eCLIP Fold-Enrichment | Binding Protein | Cell Line | Strand | Chrm | | Start in Chrm (0 Indexed) | End in Chrm (1 Indexed) |
| 2.76243580573 | DDX21 (bg=1.64%) | K562 | - | chr20 | | 34636965 | 34637014 |
| 2.7555073169 | ddx6 (bg=23.92%) | HepG2 | - | chr20 | | 34636978 | 34637022 |
| 2.88646119607 | ddx6 (bg=23.92%) | HepG2 | - | chr20 | | 34636981 | 34637015 |
| 4.21134326719 | dgcr8 (bg=19.31%) | SM-9MVZL | - | chr20 | | 34636974 | 34637012 |
| 4.43360003097 | fubp3 (bg=23.31%) | HepG2 | - | chr20 | | 34636975 | 34637034 |
| 5.01147007586 | fubp3 (bg=23.31%) | HepG2 | - | chr20 | | 34636990 | 34637033 |
| 3.51572885751 | pum1 (bg=29.85%) | K562 | - | chr20 | | 34636977 | 34637047 |
| 2.88361892753 | pum1 (bg=29.85%) | K562 | - | chr20 | | 34636979 | 34637041 |
| 3.5083391348 | pum2 (bg=21.55%) | K562 | - | chr20 | | 34636985 | 34637001 |
| 4.68434609141 | pum2 (bg=21.55%) | K562 | - | chr20 | | 34636996 | 34637029 |
| 2.64277657878 | sf3a3 (bg=12.13%) | HepG2 | - | chr20 | | 34636975 | 34637022 |
| 2.32586014979 | TBRG4 (bg=7.0%) | HepG2 | - | chr20 | | 34636969 | 34637023 |
| 2.62839943805 | tia1 (bg=16.04%) | HepG2 | - | chr20 | | 34636972 | 34637016 |
| 2.05319292923 | tial1 (bg=14.09%) | HepG2 | - | chr20 | | 34636979 | 34637036 |

  
  

| Match 154 in HUMAN | | | | | | | |
| --- | --- | --- | --- | --- | --- | --- | --- |
| Motif | Start in Seq (1 Indexed) | End in Seq (1 Indexed) | Strand | Chrm | Exon | Start in Chrm (0 Indexed) | End in Chrm (1 Indexed) |
| CAGTATAGTAATGTCTATACTTGTTCAAT | 1946 | 1974 | - | chr20 | 1 | 34636958 | 34636987 |
| eCLIP Fold-Enrichment | Binding Protein | Cell Line | Strand | Chrm | | Start in Chrm (0 Indexed) | End in Chrm (1 Indexed) |
| 2.76243580573 | DDX21 (bg=1.64%) | K562 | - | chr20 | | 34636965 | 34637014 |
| 2.7555073169 | ddx6 (bg=23.92%) | HepG2 | - | chr20 | | 34636978 | 34637022 |
| 2.88646119607 | ddx6 (bg=23.92%) | HepG2 | - | chr20 | | 34636981 | 34637015 |
| 4.21134326719 | dgcr8 (bg=19.31%) | SM-9MVZL | - | chr20 | | 34636974 | 34637012 |
| 5.40138646239 | fubp3 (bg=23.31%) | HepG2 | - | chr20 | | 34636975 | 34636990 |
| 4.43360003097 | fubp3 (bg=23.31%) | HepG2 | - | chr20 | | 34636975 | 34637034 |
| 3.51572885751 | pum1 (bg=29.85%) | K562 | - | chr20 | | 34636977 | 34637047 |
| 2.88361892753 | pum1 (bg=29.85%) | K562 | - | chr20 | | 34636979 | 34637041 |
| 3.5083391348 | pum2 (bg=21.55%) | K562 | - | chr20 | | 34636985 | 34637001 |
| 2.64277657878 | sf3a3 (bg=12.13%) | HepG2 | - | chr20 | | 34636975 | 34637022 |
| 2.32586014979 | TBRG4 (bg=7.0%) | HepG2 | - | chr20 | | 34636969 | 34637023 |
| 2.62839943805 | tia1 (bg=16.04%) | HepG2 | - | chr20 | | 34636972 | 34637016 |
| 2.05319292923 | tial1 (bg=14.09%) | HepG2 | - | chr20 | | 34636979 | 34637036 |

  
  

| Match 155 in HUMAN | | | | | | | |
| --- | --- | --- | --- | --- | --- | --- | --- |
| Motif | Start in Seq (1 Indexed) | End in Seq (1 Indexed) | Strand | Chrm | Exon | Start in Chrm (0 Indexed) | End in Chrm (1 Indexed) |
| GTTTAGAGGAGGT | 1976 | 1988 | - | chr20 | 1 | 34636944 | 34636957 |
| eCLIP Fold-Enrichment | Binding Protein | Cell Line | Strand | Chrm | | Start in Chrm (0 Indexed) | End in Chrm (1 Indexed) |
| 2.68632868806 | fam120a (bg=18.43%) | HepG2 | - | chr20 | | 34636911 | 34636953 |
| 2.41624928967 | IGF2BP2 (bg=3.44%) | K562 | - | chr20 | | 34636909 | 34636953 |
| 3.03509826165 | pum1 (bg=29.85%) | K562 | - | chr20 | | 34636864 | 34636954 |
| 2.46589881669 | sf3a3 (bg=12.13%) | HepG2 | - | chr20 | | 34636935 | 34636953 |

  
  

| Match 156 in HUMAN | | | | | | | |
| --- | --- | --- | --- | --- | --- | --- | --- |
| Motif | Start in Seq (1 Indexed) | End in Seq (1 Indexed) | Strand | Chrm | Exon | Start in Chrm (0 Indexed) | End in Chrm (1 Indexed) |
| GAGGAGGT | 1981 | 1988 | - | chr20 | 1 | 34636944 | 34636952 |
| eCLIP Fold-Enrichment | Binding Protein | Cell Line | Strand | Chrm | | Start in Chrm (0 Indexed) | End in Chrm (1 Indexed) |
| 2.68632868806 | fam120a (bg=18.43%) | HepG2 | - | chr20 | | 34636911 | 34636953 |
| 2.41624928967 | IGF2BP2 (bg=3.44%) | K562 | - | chr20 | | 34636909 | 34636953 |
| 3.03509826165 | pum1 (bg=29.85%) | K562 | - | chr20 | | 34636864 | 34636954 |
| 2.46589881669 | sf3a3 (bg=12.13%) | HepG2 | - | chr20 | | 34636935 | 34636953 |

  
  

| Match 157 in HUMAN | | | | | | | |
| --- | --- | --- | --- | --- | --- | --- | --- |
| Motif | Start in Seq (1 Indexed) | End in Seq (1 Indexed) | Strand | Chrm | Exon | Start in Chrm (0 Indexed) | End in Chrm (1 Indexed) |
| GGAGGGAAGAAATTGCAAAAGGTAATAT | 1990 | 2017 | - | chr20 | 1 | 34636915 | 34636943 |
| eCLIP Fold-Enrichment | Binding Protein | Cell Line | Strand | Chrm | | Start in Chrm (0 Indexed) | End in Chrm (1 Indexed) |
| 2.14983518721 | AARS (bg=2.33%) | K562 | - | chr20 | | 34636924 | 34636939 |
| 2.91293340743 | ddx6 (bg=23.92%) | HepG2 | - | chr20 | | 34636898 | 34636918 |
| 2.68632868806 | fam120a (bg=18.43%) | HepG2 | - | chr20 | | 34636911 | 34636953 |
| 2.41624928967 | IGF2BP2 (bg=3.44%) | K562 | - | chr20 | | 34636909 | 34636953 |
| 3.03509826165 | pum1 (bg=29.85%) | K562 | - | chr20 | | 34636864 | 34636954 |
| 2.46589881669 | sf3a3 (bg=12.13%) | HepG2 | - | chr20 | | 34636935 | 34636953 |

  
  

| Match 158 in HUMAN | | | | | | | |
| --- | --- | --- | --- | --- | --- | --- | --- |
| Motif | Start in Seq (1 Indexed) | End in Seq (1 Indexed) | Strand | Chrm | Exon | Start in Chrm (0 Indexed) | End in Chrm (1 Indexed) |
| AGGGAA | 1992 | 1997 | - | chr20 | 1 | 34636935 | 34636941 |
| eCLIP Fold-Enrichment | Binding Protein | Cell Line | Strand | Chrm | | Start in Chrm (0 Indexed) | End in Chrm (1 Indexed) |
| 2.14983518721 | AARS (bg=2.33%) | K562 | - | chr20 | | 34636924 | 34636939 |
| 2.68632868806 | fam120a (bg=18.43%) | HepG2 | - | chr20 | | 34636911 | 34636953 |
| 2.41624928967 | IGF2BP2 (bg=3.44%) | K562 | - | chr20 | | 34636909 | 34636953 |
| 3.03509826165 | pum1 (bg=29.85%) | K562 | - | chr20 | | 34636864 | 34636954 |
| 2.46589881669 | sf3a3 (bg=12.13%) | HepG2 | - | chr20 | | 34636935 | 34636953 |

  
  

| Match 159 in HUMAN | | | | | | | |
| --- | --- | --- | --- | --- | --- | --- | --- |
| Motif | Start in Seq (1 Indexed) | End in Seq (1 Indexed) | Strand | Chrm | Exon | Start in Chrm (0 Indexed) | End in Chrm (1 Indexed) |
| AAAAGGTAAT | 2006 | 2015 | - | chr20 | 1 | 34636917 | 34636927 |
| eCLIP Fold-Enrichment | Binding Protein | Cell Line | Strand | Chrm | | Start in Chrm (0 Indexed) | End in Chrm (1 Indexed) |
| 2.14983518721 | AARS (bg=2.33%) | K562 | - | chr20 | | 34636924 | 34636939 |
| 2.91293340743 | ddx6 (bg=23.92%) | HepG2 | - | chr20 | | 34636898 | 34636918 |
| 2.68632868806 | fam120a (bg=18.43%) | HepG2 | - | chr20 | | 34636911 | 34636953 |
| 2.41624928967 | IGF2BP2 (bg=3.44%) | K562 | - | chr20 | | 34636909 | 34636953 |
| 3.03509826165 | pum1 (bg=29.85%) | K562 | - | chr20 | | 34636864 | 34636954 |

  
  

| Match 160 in HUMAN | | | | | | | |
| --- | --- | --- | --- | --- | --- | --- | --- |
| Motif | Start in Seq (1 Indexed) | End in Seq (1 Indexed) | Strand | Chrm | Exon | Start in Chrm (0 Indexed) | End in Chrm (1 Indexed) |
| AAAAGGTAATAT | 2006 | 2017 | - | chr20 | 1 | 34636915 | 34636927 |
| eCLIP Fold-Enrichment | Binding Protein | Cell Line | Strand | Chrm | | Start in Chrm (0 Indexed) | End in Chrm (1 Indexed) |
| 2.14983518721 | AARS (bg=2.33%) | K562 | - | chr20 | | 34636924 | 34636939 |
| 2.91293340743 | ddx6 (bg=23.92%) | HepG2 | - | chr20 | | 34636898 | 34636918 |
| 2.68632868806 | fam120a (bg=18.43%) | HepG2 | - | chr20 | | 34636911 | 34636953 |
| 2.41624928967 | IGF2BP2 (bg=3.44%) | K562 | - | chr20 | | 34636909 | 34636953 |
| 3.03509826165 | pum1 (bg=29.85%) | K562 | - | chr20 | | 34636864 | 34636954 |

  
  

| Match 161 in HUMAN | | | | | | | |
| --- | --- | --- | --- | --- | --- | --- | --- |
| Motif | Start in Seq (1 Indexed) | End in Seq (1 Indexed) | Strand | Chrm | Exon | Start in Chrm (0 Indexed) | End in Chrm (1 Indexed) |
| ACTAGTGTGTTCATACTTGGACATTTTCAGACA | 2019 | 2051 | - | chr20 | 1 | 34636881 | 34636914 |
| eCLIP Fold-Enrichment | Binding Protein | Cell Line | Strand | Chrm | | Start in Chrm (0 Indexed) | End in Chrm (1 Indexed) |
| 2.91293340743 | ddx6 (bg=23.92%) | HepG2 | - | chr20 | | 34636898 | 34636918 |
| 2.68632868806 | fam120a (bg=18.43%) | HepG2 | - | chr20 | | 34636911 | 34636953 |
| 2.41624928967 | IGF2BP2 (bg=3.44%) | K562 | - | chr20 | | 34636909 | 34636953 |
| 3.03509826165 | pum1 (bg=29.85%) | K562 | - | chr20 | | 34636864 | 34636954 |
| 5.7313145309 | pum2 (bg=21.55%) | K562 | - | chr20 | | 34636880 | 34636883 |

  
  

| Match 162 in HUMAN | | | | | | | |
| --- | --- | --- | --- | --- | --- | --- | --- |
| Motif | Start in Seq (1 Indexed) | End in Seq (1 Indexed) | Strand | Chrm | Exon | Start in Chrm (0 Indexed) | End in Chrm (1 Indexed) |
| GTGTGTTCATACTTGGACATTTTCAGA | 2023 | 2049 | - | chr20 | 1 | 34636883 | 34636910 |
| eCLIP Fold-Enrichment | Binding Protein | Cell Line | Strand | Chrm | | Start in Chrm (0 Indexed) | End in Chrm (1 Indexed) |
| 2.91293340743 | ddx6 (bg=23.92%) | HepG2 | - | chr20 | | 34636898 | 34636918 |
| 2.41624928967 | IGF2BP2 (bg=3.44%) | K562 | - | chr20 | | 34636909 | 34636953 |
| 3.03509826165 | pum1 (bg=29.85%) | K562 | - | chr20 | | 34636864 | 34636954 |
| 5.7313145309 | pum2 (bg=21.55%) | K562 | - | chr20 | | 34636880 | 34636883 |

  
  

| Match 163 in HUMAN | | | | | | | |
| --- | --- | --- | --- | --- | --- | --- | --- |
| Motif | Start in Seq (1 Indexed) | End in Seq (1 Indexed) | Strand | Chrm | Exon | Start in Chrm (0 Indexed) | End in Chrm (1 Indexed) |
| GACATTTTCAGA | 2038 | 2049 | - | chr20 | 1 | 34636883 | 34636895 |
| eCLIP Fold-Enrichment | Binding Protein | Cell Line | Strand | Chrm | | Start in Chrm (0 Indexed) | End in Chrm (1 Indexed) |
| 3.03509826165 | pum1 (bg=29.85%) | K562 | - | chr20 | | 34636864 | 34636954 |
| 5.7313145309 | pum2 (bg=21.55%) | K562 | - | chr20 | | 34636880 | 34636883 |

  
  

| Match 164 in HUMAN | | | | | | | |
| --- | --- | --- | --- | --- | --- | --- | --- |
| Motif | Start in Seq (1 Indexed) | End in Seq (1 Indexed) | Strand | Chrm | Exon | Start in Chrm (0 Indexed) | End in Chrm (1 Indexed) |
| TTTTCAGA | 2042 | 2049 | - | chr20 | 1 | 34636883 | 34636891 |
| eCLIP Fold-Enrichment | Binding Protein | Cell Line | Strand | Chrm | | Start in Chrm (0 Indexed) | End in Chrm (1 Indexed) |
| 3.03509826165 | pum1 (bg=29.85%) | K562 | - | chr20 | | 34636864 | 34636954 |
| 5.7313145309 | pum2 (bg=21.55%) | K562 | - | chr20 | | 34636880 | 34636883 |

  
  

| Match 165 in HUMAN | | | | | | | |
| --- | --- | --- | --- | --- | --- | --- | --- |
| Motif | Start in Seq (1 Indexed) | End in Seq (1 Indexed) | Strand | Chrm | Exon | Start in Chrm (0 Indexed) | End in Chrm (1 Indexed) |
| TTTCAG | 2043 | 2048 | - | chr20 | 1 | 34636884 | 34636890 |
| eCLIP Fold-Enrichment | Binding Protein | Cell Line | Strand | Chrm | | Start in Chrm (0 Indexed) | End in Chrm (1 Indexed) |
| 3.03509826165 | pum1 (bg=29.85%) | K562 | - | chr20 | | 34636864 | 34636954 |

  
  

| Match 166 in HUMAN | | | | | | | |
| --- | --- | --- | --- | --- | --- | --- | --- |
| Motif | Start in Seq (1 Indexed) | End in Seq (1 Indexed) | Strand | Chrm | Exon | Start in Chrm (0 Indexed) | End in Chrm (1 Indexed) |
| TTTTTCT | 2055 | 2061 | - | chr20 | 1 | 34636871 | 34636878 |
| eCLIP Fold-Enrichment | Binding Protein | Cell Line | Strand | Chrm | | Start in Chrm (0 Indexed) | End in Chrm (1 Indexed) |
| 3.03509826165 | pum1 (bg=29.85%) | K562 | - | chr20 | | 34636864 | 34636954 |

  
  

| Match 167 in HUMAN | | | | | | | |
| --- | --- | --- | --- | --- | --- | --- | --- |
| Motif | Start in Seq (1 Indexed) | End in Seq (1 Indexed) | Strand | Chrm | Exon | Start in Chrm (0 Indexed) | End in Chrm (1 Indexed) |
| TATGTTTTGTGCATTTTGTTTTGCTCTGTATATAGT | 2063 | 2098 | - | chr20 | 1 | 34636834 | 34636870 |
| eCLIP Fold-Enrichment | Binding Protein | Cell Line | Strand | Chrm | | Start in Chrm (0 Indexed) | End in Chrm (1 Indexed) |
| 2.30138112578 | ddx6 (bg=23.92%) | K562 | - | chr20 | | 34636787 | 34636836 |
| 4.83595839617 | fubp3 (bg=23.31%) | HepG2 | - | chr20 | | 34636811 | 34636852 |
| 2.46169476031 | GEMIN5 (bg=2.46%) | K562 | - | chr20 | | 34636809 | 34636853 |
| 2.95974999675 | pum1 (bg=29.85%) | K562 | - | chr20 | | 34636784 | 34636837 |
| 3.58017504604 | pum1 (bg=29.85%) | K562 | - | chr20 | | 34636810 | 34636863 |
| 3.03509826165 | pum1 (bg=29.85%) | K562 | - | chr20 | | 34636864 | 34636954 |
| 5.76671986703 | pum2 (bg=21.55%) | K562 | - | chr20 | | 34636813 | 34636835 |
| 6.47833378973 | pum2 (bg=21.55%) | K562 | - | chr20 | | 34636841 | 34636852 |
| 6.02988361035 | pum2 (bg=21.55%) | K562 | - | chr20 | | 34636860 | 34636865 |
| 3.41063664723 | PUS1 (bg=2.87%) | K562 | - | chr20 | | 34636790 | 34636835 |
| 4.52864074045 | tial1 (bg=14.09%) | HepG2 | - | chr20 | | 34636759 | 34636835 |

  
  

| Match 168 in HUMAN | | | | | | | |
| --- | --- | --- | --- | --- | --- | --- | --- |
| Motif | Start in Seq (1 Indexed) | End in Seq (1 Indexed) | Strand | Chrm | Exon | Start in Chrm (0 Indexed) | End in Chrm (1 Indexed) |
| TGTGCATTTT | 2070 | 2079 | - | chr20 | 1 | 34636853 | 34636863 |
| eCLIP Fold-Enrichment | Binding Protein | Cell Line | Strand | Chrm | | Start in Chrm (0 Indexed) | End in Chrm (1 Indexed) |
| 2.46169476031 | GEMIN5 (bg=2.46%) | K562 | - | chr20 | | 34636809 | 34636853 |
| 3.58017504604 | pum1 (bg=29.85%) | K562 | - | chr20 | | 34636810 | 34636863 |
| 6.02988361035 | pum2 (bg=21.55%) | K562 | - | chr20 | | 34636860 | 34636865 |

  
  

| Match 169 in HUMAN | | | | | | | |
| --- | --- | --- | --- | --- | --- | --- | --- |
| Motif | Start in Seq (1 Indexed) | End in Seq (1 Indexed) | Strand | Chrm | Exon | Start in Chrm (0 Indexed) | End in Chrm (1 Indexed) |
| GTGCATTTT | 2071 | 2079 | - | chr20 | 1 | 34636853 | 34636862 |
| eCLIP Fold-Enrichment | Binding Protein | Cell Line | Strand | Chrm | | Start in Chrm (0 Indexed) | End in Chrm (1 Indexed) |
| 2.46169476031 | GEMIN5 (bg=2.46%) | K562 | - | chr20 | | 34636809 | 34636853 |
| 3.58017504604 | pum1 (bg=29.85%) | K562 | - | chr20 | | 34636810 | 34636863 |
| 6.02988361035 | pum2 (bg=21.55%) | K562 | - | chr20 | | 34636860 | 34636865 |

  
  

| Match 170 in HUMAN | | | | | | | |
| --- | --- | --- | --- | --- | --- | --- | --- |
| Motif | Start in Seq (1 Indexed) | End in Seq (1 Indexed) | Strand | Chrm | Exon | Start in Chrm (0 Indexed) | End in Chrm (1 Indexed) |
| TGCATTTT | 2072 | 2079 | - | chr20 | 1 | 34636853 | 34636861 |
| eCLIP Fold-Enrichment | Binding Protein | Cell Line | Strand | Chrm | | Start in Chrm (0 Indexed) | End in Chrm (1 Indexed) |
| 2.46169476031 | GEMIN5 (bg=2.46%) | K562 | - | chr20 | | 34636809 | 34636853 |
| 3.58017504604 | pum1 (bg=29.85%) | K562 | - | chr20 | | 34636810 | 34636863 |
| 6.02988361035 | pum2 (bg=21.55%) | K562 | - | chr20 | | 34636860 | 34636865 |

  
  

| Match 171 in HUMAN | | | | | | | |
| --- | --- | --- | --- | --- | --- | --- | --- |
| Motif | Start in Seq (1 Indexed) | End in Seq (1 Indexed) | Strand | Chrm | Exon | Start in Chrm (0 Indexed) | End in Chrm (1 Indexed) |
| TTTTGCT | 2081 | 2087 | - | chr20 | 1 | 34636845 | 34636852 |
| eCLIP Fold-Enrichment | Binding Protein | Cell Line | Strand | Chrm | | Start in Chrm (0 Indexed) | End in Chrm (1 Indexed) |
| 4.83595839617 | fubp3 (bg=23.31%) | HepG2 | - | chr20 | | 34636811 | 34636852 |
| 2.46169476031 | GEMIN5 (bg=2.46%) | K562 | - | chr20 | | 34636809 | 34636853 |
| 3.58017504604 | pum1 (bg=29.85%) | K562 | - | chr20 | | 34636810 | 34636863 |
| 6.47833378973 | pum2 (bg=21.55%) | K562 | - | chr20 | | 34636841 | 34636852 |

  
  

| Match 172 in HUMAN | | | | | | | |
| --- | --- | --- | --- | --- | --- | --- | --- |
| Motif | Start in Seq (1 Indexed) | End in Seq (1 Indexed) | Strand | Chrm | Exon | Start in Chrm (0 Indexed) | End in Chrm (1 Indexed) |
| TGTATATAGT | 2089 | 2098 | - | chr20 | 1 | 34636834 | 34636844 |
| eCLIP Fold-Enrichment | Binding Protein | Cell Line | Strand | Chrm | | Start in Chrm (0 Indexed) | End in Chrm (1 Indexed) |
| 2.30138112578 | ddx6 (bg=23.92%) | K562 | - | chr20 | | 34636787 | 34636836 |
| 4.83595839617 | fubp3 (bg=23.31%) | HepG2 | - | chr20 | | 34636811 | 34636852 |
| 2.46169476031 | GEMIN5 (bg=2.46%) | K562 | - | chr20 | | 34636809 | 34636853 |
| 2.95974999675 | pum1 (bg=29.85%) | K562 | - | chr20 | | 34636784 | 34636837 |
| 3.58017504604 | pum1 (bg=29.85%) | K562 | - | chr20 | | 34636810 | 34636863 |
| 5.76671986703 | pum2 (bg=21.55%) | K562 | - | chr20 | | 34636813 | 34636835 |
| 6.47833378973 | pum2 (bg=21.55%) | K562 | - | chr20 | | 34636841 | 34636852 |
| 3.41063664723 | PUS1 (bg=2.87%) | K562 | - | chr20 | | 34636790 | 34636835 |
| 4.52864074045 | tial1 (bg=14.09%) | HepG2 | - | chr20 | | 34636759 | 34636835 |

  
  

| Match 173 in HUMAN | | | | | | | |
| --- | --- | --- | --- | --- | --- | --- | --- |
| Motif | Start in Seq (1 Indexed) | End in Seq (1 Indexed) | Strand | Chrm | Exon | Start in Chrm (0 Indexed) | End in Chrm (1 Indexed) |
| TATATAATGGACAAAT | 2100 | 2115 | - | chr20 | 1 | 34636817 | 34636833 |
| eCLIP Fold-Enrichment | Binding Protein | Cell Line | Strand | Chrm | | Start in Chrm (0 Indexed) | End in Chrm (1 Indexed) |
| 2.30138112578 | ddx6 (bg=23.92%) | K562 | - | chr20 | | 34636787 | 34636836 |
| 4.83595839617 | fubp3 (bg=23.31%) | HepG2 | - | chr20 | | 34636811 | 34636852 |
| 2.46169476031 | GEMIN5 (bg=2.46%) | K562 | - | chr20 | | 34636809 | 34636853 |
| 2.39485456602 | NOLC1 (bg=6.58%) | K562 | - | chr20 | | 34636772 | 34636821 |
| 2.95974999675 | pum1 (bg=29.85%) | K562 | - | chr20 | | 34636784 | 34636837 |
| 3.58017504604 | pum1 (bg=29.85%) | K562 | - | chr20 | | 34636810 | 34636863 |
| 5.76671986703 | pum2 (bg=21.55%) | K562 | - | chr20 | | 34636813 | 34636835 |
| 3.41063664723 | PUS1 (bg=2.87%) | K562 | - | chr20 | | 34636790 | 34636835 |
| 4.52864074045 | tial1 (bg=14.09%) | HepG2 | - | chr20 | | 34636759 | 34636835 |

  
  

| Match 174 in HUMAN | | | | | | | |
| --- | --- | --- | --- | --- | --- | --- | --- |
| Motif | Start in Seq (1 Indexed) | End in Seq (1 Indexed) | Strand | Chrm | Exon | Start in Chrm (0 Indexed) | End in Chrm (1 Indexed) |
| TATATAATGGACAAATAGTCCTAATTTTTCAACATCTAGTCTCTAGATGTTAAAGAGGTTGCCAGTGTATGACAAAG | 2100 | 2176 | - | chr20 | 1 | 34636756 | 34636833 |
| eCLIP Fold-Enrichment | Binding Protein | Cell Line | Strand | Chrm | | Start in Chrm (0 Indexed) | End in Chrm (1 Indexed) |
| 2.06322138859 | ddx6 (bg=23.92%) | K562 | - | chr20 | | 34636723 | 34636779 |
| 2.49247291106 | ddx6 (bg=23.92%) | HepG2 | - | chr20 | | 34636774 | 34636797 |
| 2.30138112578 | ddx6 (bg=23.92%) | K562 | - | chr20 | | 34636787 | 34636836 |
| 3.12125066776 | FASTKD2 (bg=7.73%) | K562 | - | chr20 | | 34636748 | 34636799 |
| 5.03767995519 | fubp3 (bg=23.31%) | HepG2 | - | chr20 | | 34636744 | 34636787 |
| 4.73049788867 | fubp3 (bg=23.31%) | HepG2 | - | chr20 | | 34636756 | 34636793 |
| 4.84454756258 | fubp3 (bg=23.31%) | HepG2 | - | chr20 | | 34636787 | 34636805 |
| 4.82237998967 | fubp3 (bg=23.31%) | HepG2 | - | chr20 | | 34636793 | 34636807 |
| 4.83595839617 | fubp3 (bg=23.31%) | HepG2 | - | chr20 | | 34636811 | 34636852 |
| 2.46169476031 | GEMIN5 (bg=2.46%) | K562 | - | chr20 | | 34636809 | 34636853 |
| 2.70212313624 | lin28b (bg=16.96%) | K562 | - | chr20 | | 34636694 | 34636778 |
| 2.39485456602 | NOLC1 (bg=6.58%) | K562 | - | chr20 | | 34636772 | 34636821 |
| 4.18349010155 | pum1 (bg=29.85%) | K562 | - | chr20 | | 34636740 | 34636784 |
| 4.41025004459 | pum1 (bg=29.85%) | K562 | - | chr20 | | 34636753 | 34636783 |
| 4.1387041389 | pum1 (bg=29.85%) | K562 | - | chr20 | | 34636783 | 34636810 |
| 2.95974999675 | pum1 (bg=29.85%) | K562 | - | chr20 | | 34636784 | 34636837 |
| 3.58017504604 | pum1 (bg=29.85%) | K562 | - | chr20 | | 34636810 | 34636863 |
| 5.35280290764 | pum2 (bg=21.55%) | K562 | - | chr20 | | 34636744 | 34636761 |
| 3.96878083192 | pum2 (bg=21.55%) | K562 | - | chr20 | | 34636752 | 34636768 |
| 5.64137240558 | pum2 (bg=21.55%) | K562 | - | chr20 | | 34636761 | 34636786 |
| 5.6919140142 | pum2 (bg=21.55%) | K562 | - | chr20 | | 34636786 | 34636807 |
| 5.76671986703 | pum2 (bg=21.55%) | K562 | - | chr20 | | 34636813 | 34636835 |
| 3.41063664723 | PUS1 (bg=2.87%) | K562 | - | chr20 | | 34636790 | 34636835 |
| 2.36044236434 | SF3B1 (bg=6.74%) | K562 | - | chr20 | | 34636755 | 34636809 |
| 4.52864074045 | tial1 (bg=14.09%) | HepG2 | - | chr20 | | 34636759 | 34636835 |
| 3.31372047946 | tial1 (bg=14.09%) | HepG2 | - | chr20 | | 34636770 | 34636803 |

  
  

| Match 175 in HUMAN | | | | | | | |
| --- | --- | --- | --- | --- | --- | --- | --- |
| Motif | Start in Seq (1 Indexed) | End in Seq (1 Indexed) | Strand | Chrm | Exon | Start in Chrm (0 Indexed) | End in Chrm (1 Indexed) |
| GGACAAAT | 2108 | 2115 | - | chr20 | 1 | 34636817 | 34636825 |
| eCLIP Fold-Enrichment | Binding Protein | Cell Line | Strand | Chrm | | Start in Chrm (0 Indexed) | End in Chrm (1 Indexed) |
| 2.30138112578 | ddx6 (bg=23.92%) | K562 | - | chr20 | | 34636787 | 34636836 |
| 4.83595839617 | fubp3 (bg=23.31%) | HepG2 | - | chr20 | | 34636811 | 34636852 |
| 2.46169476031 | GEMIN5 (bg=2.46%) | K562 | - | chr20 | | 34636809 | 34636853 |
| 2.39485456602 | NOLC1 (bg=6.58%) | K562 | - | chr20 | | 34636772 | 34636821 |
| 2.95974999675 | pum1 (bg=29.85%) | K562 | - | chr20 | | 34636784 | 34636837 |
| 3.58017504604 | pum1 (bg=29.85%) | K562 | - | chr20 | | 34636810 | 34636863 |
| 5.76671986703 | pum2 (bg=21.55%) | K562 | - | chr20 | | 34636813 | 34636835 |
| 3.41063664723 | PUS1 (bg=2.87%) | K562 | - | chr20 | | 34636790 | 34636835 |
| 4.52864074045 | tial1 (bg=14.09%) | HepG2 | - | chr20 | | 34636759 | 34636835 |

  
  

| Match 176 in HUMAN | | | | | | | |
| --- | --- | --- | --- | --- | --- | --- | --- |
| Motif | Start in Seq (1 Indexed) | End in Seq (1 Indexed) | Strand | Chrm | Exon | Start in Chrm (0 Indexed) | End in Chrm (1 Indexed) |
| AGTCCTA | 2116 | 2122 | - | chr20 | 1 | 34636810 | 34636817 |
| eCLIP Fold-Enrichment | Binding Protein | Cell Line | Strand | Chrm | | Start in Chrm (0 Indexed) | End in Chrm (1 Indexed) |
| 2.30138112578 | ddx6 (bg=23.92%) | K562 | - | chr20 | | 34636787 | 34636836 |
| 4.83595839617 | fubp3 (bg=23.31%) | HepG2 | - | chr20 | | 34636811 | 34636852 |
| 2.46169476031 | GEMIN5 (bg=2.46%) | K562 | - | chr20 | | 34636809 | 34636853 |
| 2.39485456602 | NOLC1 (bg=6.58%) | K562 | - | chr20 | | 34636772 | 34636821 |
| 4.1387041389 | pum1 (bg=29.85%) | K562 | - | chr20 | | 34636783 | 34636810 |
| 2.95974999675 | pum1 (bg=29.85%) | K562 | - | chr20 | | 34636784 | 34636837 |
| 3.58017504604 | pum1 (bg=29.85%) | K562 | - | chr20 | | 34636810 | 34636863 |
| 5.76671986703 | pum2 (bg=21.55%) | K562 | - | chr20 | | 34636813 | 34636835 |
| 3.41063664723 | PUS1 (bg=2.87%) | K562 | - | chr20 | | 34636790 | 34636835 |
| 4.52864074045 | tial1 (bg=14.09%) | HepG2 | - | chr20 | | 34636759 | 34636835 |

  
  

| Match 177 in HUMAN | | | | | | | |
| --- | --- | --- | --- | --- | --- | --- | --- |
| Motif | Start in Seq (1 Indexed) | End in Seq (1 Indexed) | Strand | Chrm | Exon | Start in Chrm (0 Indexed) | End in Chrm (1 Indexed) |
| CAACATCTAGTCTCTAGATGTTAAAGAGGTTGCCA | 2129 | 2163 | - | chr20 | 1 | 34636769 | 34636804 |
| eCLIP Fold-Enrichment | Binding Protein | Cell Line | Strand | Chrm | | Start in Chrm (0 Indexed) | End in Chrm (1 Indexed) |
| 2.06322138859 | ddx6 (bg=23.92%) | K562 | - | chr20 | | 34636723 | 34636779 |
| 2.49247291106 | ddx6 (bg=23.92%) | HepG2 | - | chr20 | | 34636774 | 34636797 |
| 2.30138112578 | ddx6 (bg=23.92%) | K562 | - | chr20 | | 34636787 | 34636836 |
| 3.12125066776 | FASTKD2 (bg=7.73%) | K562 | - | chr20 | | 34636748 | 34636799 |
| 5.03767995519 | fubp3 (bg=23.31%) | HepG2 | - | chr20 | | 34636744 | 34636787 |
| 4.73049788867 | fubp3 (bg=23.31%) | HepG2 | - | chr20 | | 34636756 | 34636793 |
| 4.84454756258 | fubp3 (bg=23.31%) | HepG2 | - | chr20 | | 34636787 | 34636805 |
| 4.82237998967 | fubp3 (bg=23.31%) | HepG2 | - | chr20 | | 34636793 | 34636807 |
| 2.70212313624 | lin28b (bg=16.96%) | K562 | - | chr20 | | 34636694 | 34636778 |
| 2.39485456602 | NOLC1 (bg=6.58%) | K562 | - | chr20 | | 34636772 | 34636821 |
| 4.18349010155 | pum1 (bg=29.85%) | K562 | - | chr20 | | 34636740 | 34636784 |
| 4.41025004459 | pum1 (bg=29.85%) | K562 | - | chr20 | | 34636753 | 34636783 |
| 4.1387041389 | pum1 (bg=29.85%) | K562 | - | chr20 | | 34636783 | 34636810 |
| 2.95974999675 | pum1 (bg=29.85%) | K562 | - | chr20 | | 34636784 | 34636837 |
| 5.64137240558 | pum2 (bg=21.55%) | K562 | - | chr20 | | 34636761 | 34636786 |
| 5.6919140142 | pum2 (bg=21.55%) | K562 | - | chr20 | | 34636786 | 34636807 |
| 3.41063664723 | PUS1 (bg=2.87%) | K562 | - | chr20 | | 34636790 | 34636835 |
| 2.36044236434 | SF3B1 (bg=6.74%) | K562 | - | chr20 | | 34636755 | 34636809 |
| 4.52864074045 | tial1 (bg=14.09%) | HepG2 | - | chr20 | | 34636759 | 34636835 |
| 3.31372047946 | tial1 (bg=14.09%) | HepG2 | - | chr20 | | 34636770 | 34636803 |

  
  

| Match 178 in HUMAN | | | | | | | |
| --- | --- | --- | --- | --- | --- | --- | --- |
| Motif | Start in Seq (1 Indexed) | End in Seq (1 Indexed) | Strand | Chrm | Exon | Start in Chrm (0 Indexed) | End in Chrm (1 Indexed) |
| ACATCTA | 2131 | 2137 | - | chr20 | 1 | 34636795 | 34636802 |
| eCLIP Fold-Enrichment | Binding Protein | Cell Line | Strand | Chrm | | Start in Chrm (0 Indexed) | End in Chrm (1 Indexed) |
| 2.49247291106 | ddx6 (bg=23.92%) | HepG2 | - | chr20 | | 34636774 | 34636797 |
| 2.30138112578 | ddx6 (bg=23.92%) | K562 | - | chr20 | | 34636787 | 34636836 |
| 3.12125066776 | FASTKD2 (bg=7.73%) | K562 | - | chr20 | | 34636748 | 34636799 |
| 4.84454756258 | fubp3 (bg=23.31%) | HepG2 | - | chr20 | | 34636787 | 34636805 |
| 4.82237998967 | fubp3 (bg=23.31%) | HepG2 | - | chr20 | | 34636793 | 34636807 |
| 2.39485456602 | NOLC1 (bg=6.58%) | K562 | - | chr20 | | 34636772 | 34636821 |
| 4.1387041389 | pum1 (bg=29.85%) | K562 | - | chr20 | | 34636783 | 34636810 |
| 2.95974999675 | pum1 (bg=29.85%) | K562 | - | chr20 | | 34636784 | 34636837 |
| 5.6919140142 | pum2 (bg=21.55%) | K562 | - | chr20 | | 34636786 | 34636807 |
| 3.41063664723 | PUS1 (bg=2.87%) | K562 | - | chr20 | | 34636790 | 34636835 |
| 2.36044236434 | SF3B1 (bg=6.74%) | K562 | - | chr20 | | 34636755 | 34636809 |
| 4.52864074045 | tial1 (bg=14.09%) | HepG2 | - | chr20 | | 34636759 | 34636835 |
| 3.31372047946 | tial1 (bg=14.09%) | HepG2 | - | chr20 | | 34636770 | 34636803 |

  
  

| Match 179 in HUMAN | | | | | | | |
| --- | --- | --- | --- | --- | --- | --- | --- |
| Motif | Start in Seq (1 Indexed) | End in Seq (1 Indexed) | Strand | Chrm | Exon | Start in Chrm (0 Indexed) | End in Chrm (1 Indexed) |
| TCTCTAGATGTT | 2139 | 2150 | - | chr20 | 1 | 34636782 | 34636794 |
| eCLIP Fold-Enrichment | Binding Protein | Cell Line | Strand | Chrm | | Start in Chrm (0 Indexed) | End in Chrm (1 Indexed) |
| 2.49247291106 | ddx6 (bg=23.92%) | HepG2 | - | chr20 | | 34636774 | 34636797 |
| 2.30138112578 | ddx6 (bg=23.92%) | K562 | - | chr20 | | 34636787 | 34636836 |
| 3.12125066776 | FASTKD2 (bg=7.73%) | K562 | - | chr20 | | 34636748 | 34636799 |
| 5.03767995519 | fubp3 (bg=23.31%) | HepG2 | - | chr20 | | 34636744 | 34636787 |
| 4.73049788867 | fubp3 (bg=23.31%) | HepG2 | - | chr20 | | 34636756 | 34636793 |
| 4.84454756258 | fubp3 (bg=23.31%) | HepG2 | - | chr20 | | 34636787 | 34636805 |
| 4.82237998967 | fubp3 (bg=23.31%) | HepG2 | - | chr20 | | 34636793 | 34636807 |
| 2.39485456602 | NOLC1 (bg=6.58%) | K562 | - | chr20 | | 34636772 | 34636821 |
| 4.18349010155 | pum1 (bg=29.85%) | K562 | - | chr20 | | 34636740 | 34636784 |
| 4.41025004459 | pum1 (bg=29.85%) | K562 | - | chr20 | | 34636753 | 34636783 |
| 4.1387041389 | pum1 (bg=29.85%) | K562 | - | chr20 | | 34636783 | 34636810 |
| 2.95974999675 | pum1 (bg=29.85%) | K562 | - | chr20 | | 34636784 | 34636837 |
| 5.64137240558 | pum2 (bg=21.55%) | K562 | - | chr20 | | 34636761 | 34636786 |
| 5.6919140142 | pum2 (bg=21.55%) | K562 | - | chr20 | | 34636786 | 34636807 |
| 3.41063664723 | PUS1 (bg=2.87%) | K562 | - | chr20 | | 34636790 | 34636835 |
| 2.36044236434 | SF3B1 (bg=6.74%) | K562 | - | chr20 | | 34636755 | 34636809 |
| 4.52864074045 | tial1 (bg=14.09%) | HepG2 | - | chr20 | | 34636759 | 34636835 |
| 3.31372047946 | tial1 (bg=14.09%) | HepG2 | - | chr20 | | 34636770 | 34636803 |

  
  

| Match 180 in HUMAN | | | | | | | |
| --- | --- | --- | --- | --- | --- | --- | --- |
| Motif | Start in Seq (1 Indexed) | End in Seq (1 Indexed) | Strand | Chrm | Exon | Start in Chrm (0 Indexed) | End in Chrm (1 Indexed) |
| AAAGAGGTTGCCA | 2151 | 2163 | - | chr20 | 1 | 34636769 | 34636782 |
| eCLIP Fold-Enrichment | Binding Protein | Cell Line | Strand | Chrm | | Start in Chrm (0 Indexed) | End in Chrm (1 Indexed) |
| 2.06322138859 | ddx6 (bg=23.92%) | K562 | - | chr20 | | 34636723 | 34636779 |
| 2.49247291106 | ddx6 (bg=23.92%) | HepG2 | - | chr20 | | 34636774 | 34636797 |
| 3.12125066776 | FASTKD2 (bg=7.73%) | K562 | - | chr20 | | 34636748 | 34636799 |
| 5.03767995519 | fubp3 (bg=23.31%) | HepG2 | - | chr20 | | 34636744 | 34636787 |
| 4.73049788867 | fubp3 (bg=23.31%) | HepG2 | - | chr20 | | 34636756 | 34636793 |
| 2.70212313624 | lin28b (bg=16.96%) | K562 | - | chr20 | | 34636694 | 34636778 |
| 2.39485456602 | NOLC1 (bg=6.58%) | K562 | - | chr20 | | 34636772 | 34636821 |
| 4.18349010155 | pum1 (bg=29.85%) | K562 | - | chr20 | | 34636740 | 34636784 |
| 4.41025004459 | pum1 (bg=29.85%) | K562 | - | chr20 | | 34636753 | 34636783 |
| 5.64137240558 | pum2 (bg=21.55%) | K562 | - | chr20 | | 34636761 | 34636786 |
| 2.36044236434 | SF3B1 (bg=6.74%) | K562 | - | chr20 | | 34636755 | 34636809 |
| 4.52864074045 | tial1 (bg=14.09%) | HepG2 | - | chr20 | | 34636759 | 34636835 |
| 3.31372047946 | tial1 (bg=14.09%) | HepG2 | - | chr20 | | 34636770 | 34636803 |

  
  

| Match 181 in HUMAN | | | | | | | |
| --- | --- | --- | --- | --- | --- | --- | --- |
| Motif | Start in Seq (1 Indexed) | End in Seq (1 Indexed) | Strand | Chrm | Exon | Start in Chrm (0 Indexed) | End in Chrm (1 Indexed) |
| TGTATGACAAAG | 2165 | 2176 | - | chr20 | 1 | 34636756 | 34636768 |
| eCLIP Fold-Enrichment | Binding Protein | Cell Line | Strand | Chrm | | Start in Chrm (0 Indexed) | End in Chrm (1 Indexed) |
| 2.06322138859 | ddx6 (bg=23.92%) | K562 | - | chr20 | | 34636723 | 34636779 |
| 3.12125066776 | FASTKD2 (bg=7.73%) | K562 | - | chr20 | | 34636748 | 34636799 |
| 5.03767995519 | fubp3 (bg=23.31%) | HepG2 | - | chr20 | | 34636744 | 34636787 |
| 4.73049788867 | fubp3 (bg=23.31%) | HepG2 | - | chr20 | | 34636756 | 34636793 |
| 2.70212313624 | lin28b (bg=16.96%) | K562 | - | chr20 | | 34636694 | 34636778 |
| 4.18349010155 | pum1 (bg=29.85%) | K562 | - | chr20 | | 34636740 | 34636784 |
| 4.41025004459 | pum1 (bg=29.85%) | K562 | - | chr20 | | 34636753 | 34636783 |
| 5.35280290764 | pum2 (bg=21.55%) | K562 | - | chr20 | | 34636744 | 34636761 |
| 3.96878083192 | pum2 (bg=21.55%) | K562 | - | chr20 | | 34636752 | 34636768 |
| 5.64137240558 | pum2 (bg=21.55%) | K562 | - | chr20 | | 34636761 | 34636786 |
| 2.36044236434 | SF3B1 (bg=6.74%) | K562 | - | chr20 | | 34636755 | 34636809 |
| 4.52864074045 | tial1 (bg=14.09%) | HepG2 | - | chr20 | | 34636759 | 34636835 |

  
  

| Match 182 in HUMAN | | | | | | | |
| --- | --- | --- | --- | --- | --- | --- | --- |
| Motif | Start in Seq (1 Indexed) | End in Seq (1 Indexed) | Strand | Chrm | Exon | Start in Chrm (0 Indexed) | End in Chrm (1 Indexed) |
| TGTATGACAAA | 2165 | 2175 | - | chr20 | 1 | 34636757 | 34636768 |
| eCLIP Fold-Enrichment | Binding Protein | Cell Line | Strand | Chrm | | Start in Chrm (0 Indexed) | End in Chrm (1 Indexed) |
| 2.06322138859 | ddx6 (bg=23.92%) | K562 | - | chr20 | | 34636723 | 34636779 |
| 3.12125066776 | FASTKD2 (bg=7.73%) | K562 | - | chr20 | | 34636748 | 34636799 |
| 5.03767995519 | fubp3 (bg=23.31%) | HepG2 | - | chr20 | | 34636744 | 34636787 |
| 4.73049788867 | fubp3 (bg=23.31%) | HepG2 | - | chr20 | | 34636756 | 34636793 |
| 2.70212313624 | lin28b (bg=16.96%) | K562 | - | chr20 | | 34636694 | 34636778 |
| 4.18349010155 | pum1 (bg=29.85%) | K562 | - | chr20 | | 34636740 | 34636784 |
| 4.41025004459 | pum1 (bg=29.85%) | K562 | - | chr20 | | 34636753 | 34636783 |
| 5.35280290764 | pum2 (bg=21.55%) | K562 | - | chr20 | | 34636744 | 34636761 |
| 3.96878083192 | pum2 (bg=21.55%) | K562 | - | chr20 | | 34636752 | 34636768 |
| 5.64137240558 | pum2 (bg=21.55%) | K562 | - | chr20 | | 34636761 | 34636786 |
| 2.36044236434 | SF3B1 (bg=6.74%) | K562 | - | chr20 | | 34636755 | 34636809 |
| 4.52864074045 | tial1 (bg=14.09%) | HepG2 | - | chr20 | | 34636759 | 34636835 |

  
  

| Match 183 in HUMAN | | | | | | | |
| --- | --- | --- | --- | --- | --- | --- | --- |
| Motif | Start in Seq (1 Indexed) | End in Seq (1 Indexed) | Strand | Chrm | Exon | Start in Chrm (0 Indexed) | End in Chrm (1 Indexed) |
| TATGACAAA | 2167 | 2175 | - | chr20 | 1 | 34636757 | 34636766 |
| eCLIP Fold-Enrichment | Binding Protein | Cell Line | Strand | Chrm | | Start in Chrm (0 Indexed) | End in Chrm (1 Indexed) |
| 2.06322138859 | ddx6 (bg=23.92%) | K562 | - | chr20 | | 34636723 | 34636779 |
| 3.12125066776 | FASTKD2 (bg=7.73%) | K562 | - | chr20 | | 34636748 | 34636799 |
| 5.03767995519 | fubp3 (bg=23.31%) | HepG2 | - | chr20 | | 34636744 | 34636787 |
| 4.73049788867 | fubp3 (bg=23.31%) | HepG2 | - | chr20 | | 34636756 | 34636793 |
| 2.70212313624 | lin28b (bg=16.96%) | K562 | - | chr20 | | 34636694 | 34636778 |
| 4.18349010155 | pum1 (bg=29.85%) | K562 | - | chr20 | | 34636740 | 34636784 |
| 4.41025004459 | pum1 (bg=29.85%) | K562 | - | chr20 | | 34636753 | 34636783 |
| 5.35280290764 | pum2 (bg=21.55%) | K562 | - | chr20 | | 34636744 | 34636761 |
| 3.96878083192 | pum2 (bg=21.55%) | K562 | - | chr20 | | 34636752 | 34636768 |
| 5.64137240558 | pum2 (bg=21.55%) | K562 | - | chr20 | | 34636761 | 34636786 |
| 2.36044236434 | SF3B1 (bg=6.74%) | K562 | - | chr20 | | 34636755 | 34636809 |
| 4.52864074045 | tial1 (bg=14.09%) | HepG2 | - | chr20 | | 34636759 | 34636835 |

  
  

| Match 184 in HUMAN | | | | | | | |
| --- | --- | --- | --- | --- | --- | --- | --- |
| Motif | Start in Seq (1 Indexed) | End in Seq (1 Indexed) | Strand | Chrm | Exon | Start in Chrm (0 Indexed) | End in Chrm (1 Indexed) |
| AGTAAAATTAGCATATTTTGTAC | 2178 | 2200 | - | chr20 | 1 | 34636732 | 34636755 |
| eCLIP Fold-Enrichment | Binding Protein | Cell Line | Strand | Chrm | | Start in Chrm (0 Indexed) | End in Chrm (1 Indexed) |
| 2.98997257053 | ddx6 (bg=23.92%) | HepG2 | - | chr20 | | 34636689 | 34636743 |
| 2.06322138859 | ddx6 (bg=23.92%) | K562 | - | chr20 | | 34636723 | 34636779 |
| 3.12125066776 | FASTKD2 (bg=7.73%) | K562 | - | chr20 | | 34636748 | 34636799 |
| 4.86391288745 | fubp3 (bg=23.31%) | HepG2 | - | chr20 | | 34636690 | 34636744 |
| 3.90972245085 | fubp3 (bg=23.31%) | HepG2 | - | chr20 | | 34636728 | 34636737 |
| 5.03767995519 | fubp3 (bg=23.31%) | HepG2 | - | chr20 | | 34636744 | 34636787 |
| 3.8922820029 | fubp3 (bg=23.31%) | HepG2 | - | chr20 | | 34636745 | 34636750 |
| 2.12201058463 | igf2bp1 (bg=11.67%) | K562 | - | chr20 | | 34636652 | 34636734 |
| 2.70212313624 | lin28b (bg=16.96%) | K562 | - | chr20 | | 34636694 | 34636778 |
| 4.91999329982 | pum1 (bg=29.85%) | K562 | - | chr20 | | 34636691 | 34636753 |
| 4.32209533978 | pum1 (bg=29.85%) | K562 | - | chr20 | | 34636695 | 34636740 |
| 4.18349010155 | pum1 (bg=29.85%) | K562 | - | chr20 | | 34636740 | 34636784 |
| 4.41025004459 | pum1 (bg=29.85%) | K562 | - | chr20 | | 34636753 | 34636783 |
| 4.68278527764 | pum2 (bg=21.55%) | K562 | - | chr20 | | 34636691 | 34636736 |
| 5.75189886305 | pum2 (bg=21.55%) | K562 | - | chr20 | | 34636729 | 34636738 |
| 5.35280290764 | pum2 (bg=21.55%) | K562 | - | chr20 | | 34636744 | 34636761 |
| 3.96878083192 | pum2 (bg=21.55%) | K562 | - | chr20 | | 34636752 | 34636768 |
| 2.17044293317 | sf3a3 (bg=12.13%) | HepG2 | - | chr20 | | 34636693 | 34636732 |
| 2.36044236434 | SF3B1 (bg=6.74%) | K562 | - | chr20 | | 34636755 | 34636809 |
| 2.21883480041 | SUB1 (bg=9.24%) | HepG2 | - | chr20 | | 34636674 | 34636745 |

  
  

| Match 185 in HUMAN | | | | | | | |
| --- | --- | --- | --- | --- | --- | --- | --- |
| Motif | Start in Seq (1 Indexed) | End in Seq (1 Indexed) | Strand | Chrm | Exon | Start in Chrm (0 Indexed) | End in Chrm (1 Indexed) |
| AAATTAGCA | 2182 | 2190 | - | chr20 | 1 | 34636742 | 34636751 |
| eCLIP Fold-Enrichment | Binding Protein | Cell Line | Strand | Chrm | | Start in Chrm (0 Indexed) | End in Chrm (1 Indexed) |
| 2.98997257053 | ddx6 (bg=23.92%) | HepG2 | - | chr20 | | 34636689 | 34636743 |
| 2.06322138859 | ddx6 (bg=23.92%) | K562 | - | chr20 | | 34636723 | 34636779 |
| 3.12125066776 | FASTKD2 (bg=7.73%) | K562 | - | chr20 | | 34636748 | 34636799 |
| 4.86391288745 | fubp3 (bg=23.31%) | HepG2 | - | chr20 | | 34636690 | 34636744 |
| 5.03767995519 | fubp3 (bg=23.31%) | HepG2 | - | chr20 | | 34636744 | 34636787 |
| 3.8922820029 | fubp3 (bg=23.31%) | HepG2 | - | chr20 | | 34636745 | 34636750 |
| 2.70212313624 | lin28b (bg=16.96%) | K562 | - | chr20 | | 34636694 | 34636778 |
| 4.91999329982 | pum1 (bg=29.85%) | K562 | - | chr20 | | 34636691 | 34636753 |
| 4.18349010155 | pum1 (bg=29.85%) | K562 | - | chr20 | | 34636740 | 34636784 |
| 5.35280290764 | pum2 (bg=21.55%) | K562 | - | chr20 | | 34636744 | 34636761 |
| 2.21883480041 | SUB1 (bg=9.24%) | HepG2 | - | chr20 | | 34636674 | 34636745 |

  
  

| Match 186 in HUMAN | | | | | | | |
| --- | --- | --- | --- | --- | --- | --- | --- |
| Motif | Start in Seq (1 Indexed) | End in Seq (1 Indexed) | Strand | Chrm | Exon | Start in Chrm (0 Indexed) | End in Chrm (1 Indexed) |
| CTTTGTGTTGAAATTC | 2202 | 2217 | - | chr20 | 1 | 34636715 | 34636731 |
| eCLIP Fold-Enrichment | Binding Protein | Cell Line | Strand | Chrm | | Start in Chrm (0 Indexed) | End in Chrm (1 Indexed) |
| 3.62342679024 | ddx6 (bg=23.92%) | HepG2 | - | chr20 | | 34636689 | 34636728 |
| 2.98997257053 | ddx6 (bg=23.92%) | HepG2 | - | chr20 | | 34636689 | 34636743 |
| 2.06322138859 | ddx6 (bg=23.92%) | K562 | - | chr20 | | 34636723 | 34636779 |
| 4.29753848139 | fubp3 (bg=23.31%) | HepG2 | - | chr20 | | 34636688 | 34636728 |
| 4.86391288745 | fubp3 (bg=23.31%) | HepG2 | - | chr20 | | 34636690 | 34636744 |
| 3.90972245085 | fubp3 (bg=23.31%) | HepG2 | - | chr20 | | 34636728 | 34636737 |
| 2.12201058463 | igf2bp1 (bg=11.67%) | K562 | - | chr20 | | 34636652 | 34636734 |
| 2.70212313624 | lin28b (bg=16.96%) | K562 | - | chr20 | | 34636694 | 34636778 |
| 2.00983167819 | NKRF (bg=3.64%) | HepG2 | - | chr20 | | 34636699 | 34636729 |
| 4.91999329982 | pum1 (bg=29.85%) | K562 | - | chr20 | | 34636691 | 34636753 |
| 4.32209533978 | pum1 (bg=29.85%) | K562 | - | chr20 | | 34636695 | 34636740 |
| 5.74127485047 | pum2 (bg=21.55%) | K562 | - | chr20 | | 34636689 | 34636729 |
| 4.68278527764 | pum2 (bg=21.55%) | K562 | - | chr20 | | 34636691 | 34636736 |
| 5.75189886305 | pum2 (bg=21.55%) | K562 | - | chr20 | | 34636729 | 34636738 |
| 2.17044293317 | sf3a3 (bg=12.13%) | HepG2 | - | chr20 | | 34636693 | 34636732 |
| 2.21883480041 | SUB1 (bg=9.24%) | HepG2 | - | chr20 | | 34636674 | 34636745 |
| 3.70318334749 | tia1 (bg=16.04%) | K562 | - | chr20 | | 34636686 | 34636725 |
| 2.8047801339 | tial1 (bg=14.09%) | HepG2 | - | chr20 | | 34636622 | 34636729 |

  
  

| Match 187 in HUMAN | | | | | | | |
| --- | --- | --- | --- | --- | --- | --- | --- |
| Motif | Start in Seq (1 Indexed) | End in Seq (1 Indexed) | Strand | Chrm | Exon | Start in Chrm (0 Indexed) | End in Chrm (1 Indexed) |
| TTGTGTTGAAATT | 2204 | 2216 | - | chr20 | 1 | 34636716 | 34636729 |
| eCLIP Fold-Enrichment | Binding Protein | Cell Line | Strand | Chrm | | Start in Chrm (0 Indexed) | End in Chrm (1 Indexed) |
| 3.62342679024 | ddx6 (bg=23.92%) | HepG2 | - | chr20 | | 34636689 | 34636728 |
| 2.98997257053 | ddx6 (bg=23.92%) | HepG2 | - | chr20 | | 34636689 | 34636743 |
| 2.06322138859 | ddx6 (bg=23.92%) | K562 | - | chr20 | | 34636723 | 34636779 |
| 4.29753848139 | fubp3 (bg=23.31%) | HepG2 | - | chr20 | | 34636688 | 34636728 |
| 4.86391288745 | fubp3 (bg=23.31%) | HepG2 | - | chr20 | | 34636690 | 34636744 |
| 3.90972245085 | fubp3 (bg=23.31%) | HepG2 | - | chr20 | | 34636728 | 34636737 |
| 2.12201058463 | igf2bp1 (bg=11.67%) | K562 | - | chr20 | | 34636652 | 34636734 |
| 2.70212313624 | lin28b (bg=16.96%) | K562 | - | chr20 | | 34636694 | 34636778 |
| 2.00983167819 | NKRF (bg=3.64%) | HepG2 | - | chr20 | | 34636699 | 34636729 |
| 4.91999329982 | pum1 (bg=29.85%) | K562 | - | chr20 | | 34636691 | 34636753 |
| 4.32209533978 | pum1 (bg=29.85%) | K562 | - | chr20 | | 34636695 | 34636740 |
| 5.74127485047 | pum2 (bg=21.55%) | K562 | - | chr20 | | 34636689 | 34636729 |
| 4.68278527764 | pum2 (bg=21.55%) | K562 | - | chr20 | | 34636691 | 34636736 |
| 5.75189886305 | pum2 (bg=21.55%) | K562 | - | chr20 | | 34636729 | 34636738 |
| 2.17044293317 | sf3a3 (bg=12.13%) | HepG2 | - | chr20 | | 34636693 | 34636732 |
| 2.21883480041 | SUB1 (bg=9.24%) | HepG2 | - | chr20 | | 34636674 | 34636745 |
| 3.70318334749 | tia1 (bg=16.04%) | K562 | - | chr20 | | 34636686 | 34636725 |
| 2.8047801339 | tial1 (bg=14.09%) | HepG2 | - | chr20 | | 34636622 | 34636729 |

  
  

| Match 188 in HUMAN | | | | | | | |
| --- | --- | --- | --- | --- | --- | --- | --- |
| Motif | Start in Seq (1 Indexed) | End in Seq (1 Indexed) | Strand | Chrm | Exon | Start in Chrm (0 Indexed) | End in Chrm (1 Indexed) |
| TTGTGTTGAAATTC | 2204 | 2217 | - | chr20 | 1 | 34636715 | 34636729 |
| eCLIP Fold-Enrichment | Binding Protein | Cell Line | Strand | Chrm | | Start in Chrm (0 Indexed) | End in Chrm (1 Indexed) |
| 3.62342679024 | ddx6 (bg=23.92%) | HepG2 | - | chr20 | | 34636689 | 34636728 |
| 2.98997257053 | ddx6 (bg=23.92%) | HepG2 | - | chr20 | | 34636689 | 34636743 |
| 2.06322138859 | ddx6 (bg=23.92%) | K562 | - | chr20 | | 34636723 | 34636779 |
| 4.29753848139 | fubp3 (bg=23.31%) | HepG2 | - | chr20 | | 34636688 | 34636728 |
| 4.86391288745 | fubp3 (bg=23.31%) | HepG2 | - | chr20 | | 34636690 | 34636744 |
| 3.90972245085 | fubp3 (bg=23.31%) | HepG2 | - | chr20 | | 34636728 | 34636737 |
| 2.12201058463 | igf2bp1 (bg=11.67%) | K562 | - | chr20 | | 34636652 | 34636734 |
| 2.70212313624 | lin28b (bg=16.96%) | K562 | - | chr20 | | 34636694 | 34636778 |
| 2.00983167819 | NKRF (bg=3.64%) | HepG2 | - | chr20 | | 34636699 | 34636729 |
| 4.91999329982 | pum1 (bg=29.85%) | K562 | - | chr20 | | 34636691 | 34636753 |
| 4.32209533978 | pum1 (bg=29.85%) | K562 | - | chr20 | | 34636695 | 34636740 |
| 5.74127485047 | pum2 (bg=21.55%) | K562 | - | chr20 | | 34636689 | 34636729 |
| 4.68278527764 | pum2 (bg=21.55%) | K562 | - | chr20 | | 34636691 | 34636736 |
| 5.75189886305 | pum2 (bg=21.55%) | K562 | - | chr20 | | 34636729 | 34636738 |
| 2.17044293317 | sf3a3 (bg=12.13%) | HepG2 | - | chr20 | | 34636693 | 34636732 |
| 2.21883480041 | SUB1 (bg=9.24%) | HepG2 | - | chr20 | | 34636674 | 34636745 |
| 3.70318334749 | tia1 (bg=16.04%) | K562 | - | chr20 | | 34636686 | 34636725 |
| 2.8047801339 | tial1 (bg=14.09%) | HepG2 | - | chr20 | | 34636622 | 34636729 |

  
  

| Match 189 in HUMAN | | | | | | | |
| --- | --- | --- | --- | --- | --- | --- | --- |
| Motif | Start in Seq (1 Indexed) | End in Seq (1 Indexed) | Strand | Chrm | Exon | Start in Chrm (0 Indexed) | End in Chrm (1 Indexed) |
| TGAAAT | 2210 | 2215 | - | chr20 | 1 | 34636717 | 34636723 |
| eCLIP Fold-Enrichment | Binding Protein | Cell Line | Strand | Chrm | | Start in Chrm (0 Indexed) | End in Chrm (1 Indexed) |
| 3.62342679024 | ddx6 (bg=23.92%) | HepG2 | - | chr20 | | 34636689 | 34636728 |
| 2.98997257053 | ddx6 (bg=23.92%) | HepG2 | - | chr20 | | 34636689 | 34636743 |
| 2.06322138859 | ddx6 (bg=23.92%) | K562 | - | chr20 | | 34636723 | 34636779 |
| 4.29753848139 | fubp3 (bg=23.31%) | HepG2 | - | chr20 | | 34636688 | 34636728 |
| 4.86391288745 | fubp3 (bg=23.31%) | HepG2 | - | chr20 | | 34636690 | 34636744 |
| 2.12201058463 | igf2bp1 (bg=11.67%) | K562 | - | chr20 | | 34636652 | 34636734 |
| 2.70212313624 | lin28b (bg=16.96%) | K562 | - | chr20 | | 34636694 | 34636778 |
| 2.00983167819 | NKRF (bg=3.64%) | HepG2 | - | chr20 | | 34636699 | 34636729 |
| 4.91999329982 | pum1 (bg=29.85%) | K562 | - | chr20 | | 34636691 | 34636753 |
| 4.32209533978 | pum1 (bg=29.85%) | K562 | - | chr20 | | 34636695 | 34636740 |
| 5.74127485047 | pum2 (bg=21.55%) | K562 | - | chr20 | | 34636689 | 34636729 |
| 4.68278527764 | pum2 (bg=21.55%) | K562 | - | chr20 | | 34636691 | 34636736 |
| 2.17044293317 | sf3a3 (bg=12.13%) | HepG2 | - | chr20 | | 34636693 | 34636732 |
| 2.21883480041 | SUB1 (bg=9.24%) | HepG2 | - | chr20 | | 34636674 | 34636745 |
| 3.70318334749 | tia1 (bg=16.04%) | K562 | - | chr20 | | 34636686 | 34636725 |
| 2.8047801339 | tial1 (bg=14.09%) | HepG2 | - | chr20 | | 34636622 | 34636729 |

  
  

| Match 190 in HUMAN | | | | | | | |
| --- | --- | --- | --- | --- | --- | --- | --- |
| Motif | Start in Seq (1 Indexed) | End in Seq (1 Indexed) | Strand | Chrm | Exon | Start in Chrm (0 Indexed) | End in Chrm (1 Indexed) |
| TAGGAAAACTTGTCTTCTGTAAA | 2219 | 2241 | - | chr20 | 1 | 34636691 | 34636714 |
| eCLIP Fold-Enrichment | Binding Protein | Cell Line | Strand | Chrm | | Start in Chrm (0 Indexed) | End in Chrm (1 Indexed) |
| 2.89096374245 | AQR (bg=1.27%) | HepG2 | - | chr20 | | 34636632 | 34636701 |
| 3.62342679024 | ddx6 (bg=23.92%) | HepG2 | - | chr20 | | 34636689 | 34636728 |
| 2.98997257053 | ddx6 (bg=23.92%) | HepG2 | - | chr20 | | 34636689 | 34636743 |
| 4.29753848139 | fubp3 (bg=23.31%) | HepG2 | - | chr20 | | 34636688 | 34636728 |
| 4.86391288745 | fubp3 (bg=23.31%) | HepG2 | - | chr20 | | 34636690 | 34636744 |
| 2.12201058463 | igf2bp1 (bg=11.67%) | K562 | - | chr20 | | 34636652 | 34636734 |
| 2.70212313624 | lin28b (bg=16.96%) | K562 | - | chr20 | | 34636694 | 34636778 |
| 2.00983167819 | NKRF (bg=3.64%) | HepG2 | - | chr20 | | 34636699 | 34636729 |
| 3.99392907268 | pum1 (bg=29.85%) | K562 | - | chr20 | | 34636617 | 34636695 |
| 4.54144602762 | pum1 (bg=29.85%) | K562 | - | chr20 | | 34636656 | 34636691 |
| 4.91999329982 | pum1 (bg=29.85%) | K562 | - | chr20 | | 34636691 | 34636753 |
| 4.32209533978 | pum1 (bg=29.85%) | K562 | - | chr20 | | 34636695 | 34636740 |
| 4.27461231966 | pum2 (bg=21.55%) | K562 | - | chr20 | | 34636677 | 34636691 |
| 5.74127485047 | pum2 (bg=21.55%) | K562 | - | chr20 | | 34636689 | 34636729 |
| 4.68278527764 | pum2 (bg=21.55%) | K562 | - | chr20 | | 34636691 | 34636736 |
| 2.17044293317 | sf3a3 (bg=12.13%) | HepG2 | - | chr20 | | 34636693 | 34636732 |
| 2.21883480041 | SUB1 (bg=9.24%) | HepG2 | - | chr20 | | 34636674 | 34636745 |
| 3.70318334749 | tia1 (bg=16.04%) | K562 | - | chr20 | | 34636686 | 34636725 |
| 2.8047801339 | tial1 (bg=14.09%) | HepG2 | - | chr20 | | 34636622 | 34636729 |

  
  

| Match 191 in HUMAN | | | | | | | |
| --- | --- | --- | --- | --- | --- | --- | --- |
| Motif | Start in Seq (1 Indexed) | End in Seq (1 Indexed) | Strand | Chrm | Exon | Start in Chrm (0 Indexed) | End in Chrm (1 Indexed) |
| AGGAAA | 2220 | 2225 | - | chr20 | 1 | 34636707 | 34636713 |
| eCLIP Fold-Enrichment | Binding Protein | Cell Line | Strand | Chrm | | Start in Chrm (0 Indexed) | End in Chrm (1 Indexed) |
| 3.62342679024 | ddx6 (bg=23.92%) | HepG2 | - | chr20 | | 34636689 | 34636728 |
| 2.98997257053 | ddx6 (bg=23.92%) | HepG2 | - | chr20 | | 34636689 | 34636743 |
| 4.29753848139 | fubp3 (bg=23.31%) | HepG2 | - | chr20 | | 34636688 | 34636728 |
| 4.86391288745 | fubp3 (bg=23.31%) | HepG2 | - | chr20 | | 34636690 | 34636744 |
| 2.12201058463 | igf2bp1 (bg=11.67%) | K562 | - | chr20 | | 34636652 | 34636734 |
| 2.70212313624 | lin28b (bg=16.96%) | K562 | - | chr20 | | 34636694 | 34636778 |
| 2.00983167819 | NKRF (bg=3.64%) | HepG2 | - | chr20 | | 34636699 | 34636729 |
| 4.91999329982 | pum1 (bg=29.85%) | K562 | - | chr20 | | 34636691 | 34636753 |
| 4.32209533978 | pum1 (bg=29.85%) | K562 | - | chr20 | | 34636695 | 34636740 |
| 5.74127485047 | pum2 (bg=21.55%) | K562 | - | chr20 | | 34636689 | 34636729 |
| 4.68278527764 | pum2 (bg=21.55%) | K562 | - | chr20 | | 34636691 | 34636736 |
| 2.17044293317 | sf3a3 (bg=12.13%) | HepG2 | - | chr20 | | 34636693 | 34636732 |
| 2.21883480041 | SUB1 (bg=9.24%) | HepG2 | - | chr20 | | 34636674 | 34636745 |
| 3.70318334749 | tia1 (bg=16.04%) | K562 | - | chr20 | | 34636686 | 34636725 |
| 2.8047801339 | tial1 (bg=14.09%) | HepG2 | - | chr20 | | 34636622 | 34636729 |

  
  

| Match 192 in HUMAN | | | | | | | |
| --- | --- | --- | --- | --- | --- | --- | --- |
| Motif | Start in Seq (1 Indexed) | End in Seq (1 Indexed) | Strand | Chrm | Exon | Start in Chrm (0 Indexed) | End in Chrm (1 Indexed) |
| TTCTGTA | 2233 | 2239 | - | chr20 | 1 | 34636693 | 34636700 |
| eCLIP Fold-Enrichment | Binding Protein | Cell Line | Strand | Chrm | | Start in Chrm (0 Indexed) | End in Chrm (1 Indexed) |
| 2.89096374245 | AQR (bg=1.27%) | HepG2 | - | chr20 | | 34636632 | 34636701 |
| 3.62342679024 | ddx6 (bg=23.92%) | HepG2 | - | chr20 | | 34636689 | 34636728 |
| 2.98997257053 | ddx6 (bg=23.92%) | HepG2 | - | chr20 | | 34636689 | 34636743 |
| 4.29753848139 | fubp3 (bg=23.31%) | HepG2 | - | chr20 | | 34636688 | 34636728 |
| 4.86391288745 | fubp3 (bg=23.31%) | HepG2 | - | chr20 | | 34636690 | 34636744 |
| 2.12201058463 | igf2bp1 (bg=11.67%) | K562 | - | chr20 | | 34636652 | 34636734 |
| 2.70212313624 | lin28b (bg=16.96%) | K562 | - | chr20 | | 34636694 | 34636778 |
| 2.00983167819 | NKRF (bg=3.64%) | HepG2 | - | chr20 | | 34636699 | 34636729 |
| 3.99392907268 | pum1 (bg=29.85%) | K562 | - | chr20 | | 34636617 | 34636695 |
| 4.91999329982 | pum1 (bg=29.85%) | K562 | - | chr20 | | 34636691 | 34636753 |
| 4.32209533978 | pum1 (bg=29.85%) | K562 | - | chr20 | | 34636695 | 34636740 |
| 5.74127485047 | pum2 (bg=21.55%) | K562 | - | chr20 | | 34636689 | 34636729 |
| 4.68278527764 | pum2 (bg=21.55%) | K562 | - | chr20 | | 34636691 | 34636736 |
| 2.17044293317 | sf3a3 (bg=12.13%) | HepG2 | - | chr20 | | 34636693 | 34636732 |
| 2.21883480041 | SUB1 (bg=9.24%) | HepG2 | - | chr20 | | 34636674 | 34636745 |
| 3.70318334749 | tia1 (bg=16.04%) | K562 | - | chr20 | | 34636686 | 34636725 |
| 2.8047801339 | tial1 (bg=14.09%) | HepG2 | - | chr20 | | 34636622 | 34636729 |

  
  

| Match 193 in HUMAN | | | | | | | |
| --- | --- | --- | --- | --- | --- | --- | --- |
| Motif | Start in Seq (1 Indexed) | End in Seq (1 Indexed) | Strand | Chrm | Exon | Start in Chrm (0 Indexed) | End in Chrm (1 Indexed) |
| TTCTGT | 2233 | 2238 | - | chr20 | 1 | 34636694 | 34636700 |
| eCLIP Fold-Enrichment | Binding Protein | Cell Line | Strand | Chrm | | Start in Chrm (0 Indexed) | End in Chrm (1 Indexed) |
| 2.89096374245 | AQR (bg=1.27%) | HepG2 | - | chr20 | | 34636632 | 34636701 |
| 3.62342679024 | ddx6 (bg=23.92%) | HepG2 | - | chr20 | | 34636689 | 34636728 |
| 2.98997257053 | ddx6 (bg=23.92%) | HepG2 | - | chr20 | | 34636689 | 34636743 |
| 4.29753848139 | fubp3 (bg=23.31%) | HepG2 | - | chr20 | | 34636688 | 34636728 |
| 4.86391288745 | fubp3 (bg=23.31%) | HepG2 | - | chr20 | | 34636690 | 34636744 |
| 2.12201058463 | igf2bp1 (bg=11.67%) | K562 | - | chr20 | | 34636652 | 34636734 |
| 2.70212313624 | lin28b (bg=16.96%) | K562 | - | chr20 | | 34636694 | 34636778 |
| 2.00983167819 | NKRF (bg=3.64%) | HepG2 | - | chr20 | | 34636699 | 34636729 |
| 3.99392907268 | pum1 (bg=29.85%) | K562 | - | chr20 | | 34636617 | 34636695 |
| 4.91999329982 | pum1 (bg=29.85%) | K562 | - | chr20 | | 34636691 | 34636753 |
| 4.32209533978 | pum1 (bg=29.85%) | K562 | - | chr20 | | 34636695 | 34636740 |
| 5.74127485047 | pum2 (bg=21.55%) | K562 | - | chr20 | | 34636689 | 34636729 |
| 4.68278527764 | pum2 (bg=21.55%) | K562 | - | chr20 | | 34636691 | 34636736 |
| 2.17044293317 | sf3a3 (bg=12.13%) | HepG2 | - | chr20 | | 34636693 | 34636732 |
| 2.21883480041 | SUB1 (bg=9.24%) | HepG2 | - | chr20 | | 34636674 | 34636745 |
| 3.70318334749 | tia1 (bg=16.04%) | K562 | - | chr20 | | 34636686 | 34636725 |
| 2.8047801339 | tial1 (bg=14.09%) | HepG2 | - | chr20 | | 34636622 | 34636729 |

  
  

| Match 194 in HUMAN | | | | | | | |
| --- | --- | --- | --- | --- | --- | --- | --- |
| Motif | Start in Seq (1 Indexed) | End in Seq (1 Indexed) | Strand | Chrm | Exon | Start in Chrm (0 Indexed) | End in Chrm (1 Indexed) |
| TTCTGTAAA | 2233 | 2241 | - | chr20 | 1 | 34636691 | 34636700 |
| eCLIP Fold-Enrichment | Binding Protein | Cell Line | Strand | Chrm | | Start in Chrm (0 Indexed) | End in Chrm (1 Indexed) |
| 2.89096374245 | AQR (bg=1.27%) | HepG2 | - | chr20 | | 34636632 | 34636701 |
| 3.62342679024 | ddx6 (bg=23.92%) | HepG2 | - | chr20 | | 34636689 | 34636728 |
| 2.98997257053 | ddx6 (bg=23.92%) | HepG2 | - | chr20 | | 34636689 | 34636743 |
| 4.29753848139 | fubp3 (bg=23.31%) | HepG2 | - | chr20 | | 34636688 | 34636728 |
| 4.86391288745 | fubp3 (bg=23.31%) | HepG2 | - | chr20 | | 34636690 | 34636744 |
| 2.12201058463 | igf2bp1 (bg=11.67%) | K562 | - | chr20 | | 34636652 | 34636734 |
| 2.70212313624 | lin28b (bg=16.96%) | K562 | - | chr20 | | 34636694 | 34636778 |
| 2.00983167819 | NKRF (bg=3.64%) | HepG2 | - | chr20 | | 34636699 | 34636729 |
| 3.99392907268 | pum1 (bg=29.85%) | K562 | - | chr20 | | 34636617 | 34636695 |
| 4.54144602762 | pum1 (bg=29.85%) | K562 | - | chr20 | | 34636656 | 34636691 |
| 4.91999329982 | pum1 (bg=29.85%) | K562 | - | chr20 | | 34636691 | 34636753 |
| 4.32209533978 | pum1 (bg=29.85%) | K562 | - | chr20 | | 34636695 | 34636740 |
| 4.27461231966 | pum2 (bg=21.55%) | K562 | - | chr20 | | 34636677 | 34636691 |
| 5.74127485047 | pum2 (bg=21.55%) | K562 | - | chr20 | | 34636689 | 34636729 |
| 4.68278527764 | pum2 (bg=21.55%) | K562 | - | chr20 | | 34636691 | 34636736 |
| 2.17044293317 | sf3a3 (bg=12.13%) | HepG2 | - | chr20 | | 34636693 | 34636732 |
| 2.21883480041 | SUB1 (bg=9.24%) | HepG2 | - | chr20 | | 34636674 | 34636745 |
| 3.70318334749 | tia1 (bg=16.04%) | K562 | - | chr20 | | 34636686 | 34636725 |
| 2.8047801339 | tial1 (bg=14.09%) | HepG2 | - | chr20 | | 34636622 | 34636729 |

  
  

| Match 195 in HUMAN | | | | | | | |
| --- | --- | --- | --- | --- | --- | --- | --- |
| Motif | Start in Seq (1 Indexed) | End in Seq (1 Indexed) | Strand | Chrm | Exon | Start in Chrm (0 Indexed) | End in Chrm (1 Indexed) |
| TTTGCATAGGAATTTGTT | 2246 | 2263 | - | chr20 | 1 | 34636669 | 34636687 |
| eCLIP Fold-Enrichment | Binding Protein | Cell Line | Strand | Chrm | | Start in Chrm (0 Indexed) | End in Chrm (1 Indexed) |
| 2.89096374245 | AQR (bg=1.27%) | HepG2 | - | chr20 | | 34636632 | 34636701 |
| 2.28275431976 | ddx6 (bg=23.92%) | HepG2 | - | chr20 | | 34636625 | 34636680 |
| 2.25419298057 | ddx6 (bg=23.92%) | HepG2 | - | chr20 | | 34636635 | 34636684 |
| 4.49018355933 | fubp3 (bg=23.31%) | HepG2 | - | chr20 | | 34636628 | 34636674 |
| 4.75403526968 | fubp3 (bg=23.31%) | HepG2 | - | chr20 | | 34636632 | 34636677 |
| 4.66010856078 | fubp3 (bg=23.31%) | HepG2 | - | chr20 | | 34636674 | 34636688 |
| 4.98255738395 | fubp3 (bg=23.31%) | HepG2 | - | chr20 | | 34636677 | 34636690 |
| 2.12201058463 | igf2bp1 (bg=11.67%) | K562 | - | chr20 | | 34636652 | 34636734 |
| 3.99392907268 | pum1 (bg=29.85%) | K562 | - | chr20 | | 34636617 | 34636695 |
| 4.54144602762 | pum1 (bg=29.85%) | K562 | - | chr20 | | 34636656 | 34636691 |
| 4.82587252829 | pum2 (bg=21.55%) | K562 | - | chr20 | | 34636637 | 34636676 |
| 3.82155487873 | pum2 (bg=21.55%) | K562 | - | chr20 | | 34636638 | 34636677 |
| 5.40477289794 | pum2 (bg=21.55%) | K562 | - | chr20 | | 34636676 | 34636689 |
| 4.27461231966 | pum2 (bg=21.55%) | K562 | - | chr20 | | 34636677 | 34636691 |
| 2.21883480041 | SUB1 (bg=9.24%) | HepG2 | - | chr20 | | 34636674 | 34636745 |
| 2.37060800841 | tia1 (bg=16.04%) | K562 | - | chr20 | | 34636630 | 34636686 |
| 2.64154440786 | tia1 (bg=16.04%) | HepG2 | - | chr20 | | 34636634 | 34636687 |
| 3.70318334749 | tia1 (bg=16.04%) | K562 | - | chr20 | | 34636686 | 34636725 |
| 3.03808603684 | tial1 (bg=14.09%) | HepG2 | - | chr20 | | 34636620 | 34636683 |
| 2.8047801339 | tial1 (bg=14.09%) | HepG2 | - | chr20 | | 34636622 | 34636729 |

  
  

| Match 196 in HUMAN | | | | | | | |
| --- | --- | --- | --- | --- | --- | --- | --- |
| Motif | Start in Seq (1 Indexed) | End in Seq (1 Indexed) | Strand | Chrm | Exon | Start in Chrm (0 Indexed) | End in Chrm (1 Indexed) |
| GAATTTGTT | 2255 | 2263 | - | chr20 | 1 | 34636669 | 34636678 |
| eCLIP Fold-Enrichment | Binding Protein | Cell Line | Strand | Chrm | | Start in Chrm (0 Indexed) | End in Chrm (1 Indexed) |
| 2.89096374245 | AQR (bg=1.27%) | HepG2 | - | chr20 | | 34636632 | 34636701 |
| 2.28275431976 | ddx6 (bg=23.92%) | HepG2 | - | chr20 | | 34636625 | 34636680 |
| 2.25419298057 | ddx6 (bg=23.92%) | HepG2 | - | chr20 | | 34636635 | 34636684 |
| 4.49018355933 | fubp3 (bg=23.31%) | HepG2 | - | chr20 | | 34636628 | 34636674 |
| 4.75403526968 | fubp3 (bg=23.31%) | HepG2 | - | chr20 | | 34636632 | 34636677 |
| 4.66010856078 | fubp3 (bg=23.31%) | HepG2 | - | chr20 | | 34636674 | 34636688 |
| 4.98255738395 | fubp3 (bg=23.31%) | HepG2 | - | chr20 | | 34636677 | 34636690 |
| 2.12201058463 | igf2bp1 (bg=11.67%) | K562 | - | chr20 | | 34636652 | 34636734 |
| 3.99392907268 | pum1 (bg=29.85%) | K562 | - | chr20 | | 34636617 | 34636695 |
| 4.54144602762 | pum1 (bg=29.85%) | K562 | - | chr20 | | 34636656 | 34636691 |
| 4.82587252829 | pum2 (bg=21.55%) | K562 | - | chr20 | | 34636637 | 34636676 |
| 3.82155487873 | pum2 (bg=21.55%) | K562 | - | chr20 | | 34636638 | 34636677 |
| 5.40477289794 | pum2 (bg=21.55%) | K562 | - | chr20 | | 34636676 | 34636689 |
| 4.27461231966 | pum2 (bg=21.55%) | K562 | - | chr20 | | 34636677 | 34636691 |
| 2.21883480041 | SUB1 (bg=9.24%) | HepG2 | - | chr20 | | 34636674 | 34636745 |
| 2.37060800841 | tia1 (bg=16.04%) | K562 | - | chr20 | | 34636630 | 34636686 |
| 2.64154440786 | tia1 (bg=16.04%) | HepG2 | - | chr20 | | 34636634 | 34636687 |
| 3.03808603684 | tial1 (bg=14.09%) | HepG2 | - | chr20 | | 34636620 | 34636683 |
| 2.8047801339 | tial1 (bg=14.09%) | HepG2 | - | chr20 | | 34636622 | 34636729 |

  
  

| Match 197 in HUMAN | | | | | | | |
| --- | --- | --- | --- | --- | --- | --- | --- |
| Motif | Start in Seq (1 Indexed) | End in Seq (1 Indexed) | Strand | Chrm | Exon | Start in Chrm (0 Indexed) | End in Chrm (1 Indexed) |
| ACCATCTCT | 2266 | 2274 | - | chr20 | 1 | 34636658 | 34636667 |
| eCLIP Fold-Enrichment | Binding Protein | Cell Line | Strand | Chrm | | Start in Chrm (0 Indexed) | End in Chrm (1 Indexed) |
| 2.89096374245 | AQR (bg=1.27%) | HepG2 | - | chr20 | | 34636632 | 34636701 |
| 2.28275431976 | ddx6 (bg=23.92%) | HepG2 | - | chr20 | | 34636625 | 34636680 |
| 2.25419298057 | ddx6 (bg=23.92%) | HepG2 | - | chr20 | | 34636635 | 34636684 |
| 4.49018355933 | fubp3 (bg=23.31%) | HepG2 | - | chr20 | | 34636628 | 34636674 |
| 4.75403526968 | fubp3 (bg=23.31%) | HepG2 | - | chr20 | | 34636632 | 34636677 |
| 2.12201058463 | igf2bp1 (bg=11.67%) | K562 | - | chr20 | | 34636652 | 34636734 |
| 3.99392907268 | pum1 (bg=29.85%) | K562 | - | chr20 | | 34636617 | 34636695 |
| 4.54144602762 | pum1 (bg=29.85%) | K562 | - | chr20 | | 34636656 | 34636691 |
| 4.82587252829 | pum2 (bg=21.55%) | K562 | - | chr20 | | 34636637 | 34636676 |
| 3.82155487873 | pum2 (bg=21.55%) | K562 | - | chr20 | | 34636638 | 34636677 |
| 3.29438675834 | SUPV3L1 (bg=0.85%) | K562 | - | chr20 | | 34636619 | 34636665 |
| 2.37060800841 | tia1 (bg=16.04%) | K562 | - | chr20 | | 34636630 | 34636686 |
| 2.64154440786 | tia1 (bg=16.04%) | HepG2 | - | chr20 | | 34636634 | 34636687 |
| 3.03808603684 | tial1 (bg=14.09%) | HepG2 | - | chr20 | | 34636620 | 34636683 |
| 2.8047801339 | tial1 (bg=14.09%) | HepG2 | - | chr20 | | 34636622 | 34636729 |

  
  

| Match 198 in HUMAN | | | | | | | |
| --- | --- | --- | --- | --- | --- | --- | --- |
| Motif | Start in Seq (1 Indexed) | End in Seq (1 Indexed) | Strand | Chrm | Exon | Start in Chrm (0 Indexed) | End in Chrm (1 Indexed) |
| AGCATTA | 2276 | 2282 | - | chr20 | 1 | 34636650 | 34636657 |
| eCLIP Fold-Enrichment | Binding Protein | Cell Line | Strand | Chrm | | Start in Chrm (0 Indexed) | End in Chrm (1 Indexed) |
| 2.89096374245 | AQR (bg=1.27%) | HepG2 | - | chr20 | | 34636632 | 34636701 |
| 2.28275431976 | ddx6 (bg=23.92%) | HepG2 | - | chr20 | | 34636625 | 34636680 |
| 2.25419298057 | ddx6 (bg=23.92%) | HepG2 | - | chr20 | | 34636635 | 34636684 |
| 4.49018355933 | fubp3 (bg=23.31%) | HepG2 | - | chr20 | | 34636628 | 34636674 |
| 4.75403526968 | fubp3 (bg=23.31%) | HepG2 | - | chr20 | | 34636632 | 34636677 |
| 2.12201058463 | igf2bp1 (bg=11.67%) | K562 | - | chr20 | | 34636652 | 34636734 |
| 3.99392907268 | pum1 (bg=29.85%) | K562 | - | chr20 | | 34636617 | 34636695 |
| 4.46414305259 | pum1 (bg=29.85%) | K562 | - | chr20 | | 34636622 | 34636656 |
| 4.54144602762 | pum1 (bg=29.85%) | K562 | - | chr20 | | 34636656 | 34636691 |
| 4.82587252829 | pum2 (bg=21.55%) | K562 | - | chr20 | | 34636637 | 34636676 |
| 3.82155487873 | pum2 (bg=21.55%) | K562 | - | chr20 | | 34636638 | 34636677 |
| 3.29438675834 | SUPV3L1 (bg=0.85%) | K562 | - | chr20 | | 34636619 | 34636665 |
| 2.37060800841 | tia1 (bg=16.04%) | K562 | - | chr20 | | 34636630 | 34636686 |
| 2.64154440786 | tia1 (bg=16.04%) | HepG2 | - | chr20 | | 34636634 | 34636687 |
| 3.03808603684 | tial1 (bg=14.09%) | HepG2 | - | chr20 | | 34636620 | 34636683 |
| 2.8047801339 | tial1 (bg=14.09%) | HepG2 | - | chr20 | | 34636622 | 34636729 |

  
  

| Match 199 in HUMAN | | | | | | | |
| --- | --- | --- | --- | --- | --- | --- | --- |
| Motif | Start in Seq (1 Indexed) | End in Seq (1 Indexed) | Strand | Chrm | Exon | Start in Chrm (0 Indexed) | End in Chrm (1 Indexed) |
| CCTGTACTTGTCCACTGGATTGAAG | 2289 | 2313 | - | chr20 | 1 | 34636619 | 34636644 |
| eCLIP Fold-Enrichment | Binding Protein | Cell Line | Strand | Chrm | | Start in Chrm (0 Indexed) | End in Chrm (1 Indexed) |
| 2.89096374245 | AQR (bg=1.27%) | HepG2 | - | chr20 | | 34636632 | 34636701 |
| 2.28275431976 | ddx6 (bg=23.92%) | HepG2 | - | chr20 | | 34636625 | 34636680 |
| 2.25419298057 | ddx6 (bg=23.92%) | HepG2 | - | chr20 | | 34636635 | 34636684 |
| 4.49018355933 | fubp3 (bg=23.31%) | HepG2 | - | chr20 | | 34636628 | 34636674 |
| 4.75403526968 | fubp3 (bg=23.31%) | HepG2 | - | chr20 | | 34636632 | 34636677 |
| 3.99392907268 | pum1 (bg=29.85%) | K562 | - | chr20 | | 34636617 | 34636695 |
| 4.46414305259 | pum1 (bg=29.85%) | K562 | - | chr20 | | 34636622 | 34636656 |
| 4.82587252829 | pum2 (bg=21.55%) | K562 | - | chr20 | | 34636637 | 34636676 |
| 3.82155487873 | pum2 (bg=21.55%) | K562 | - | chr20 | | 34636638 | 34636677 |
| 3.29438675834 | SUPV3L1 (bg=0.85%) | K562 | - | chr20 | | 34636619 | 34636665 |
| 2.37060800841 | tia1 (bg=16.04%) | K562 | - | chr20 | | 34636630 | 34636686 |
| 2.64154440786 | tia1 (bg=16.04%) | HepG2 | - | chr20 | | 34636634 | 34636687 |
| 3.03808603684 | tial1 (bg=14.09%) | HepG2 | - | chr20 | | 34636620 | 34636683 |
| 2.8047801339 | tial1 (bg=14.09%) | HepG2 | - | chr20 | | 34636622 | 34636729 |

  
  

| Match 200 in HUMAN | | | | | | | |
| --- | --- | --- | --- | --- | --- | --- | --- |
| Motif | Start in Seq (1 Indexed) | End in Seq (1 Indexed) | Strand | Chrm | Exon | Start in Chrm (0 Indexed) | End in Chrm (1 Indexed) |
| CTGTAC | 2290 | 2295 | - | chr20 | 1 | 34636637 | 34636643 |
| eCLIP Fold-Enrichment | Binding Protein | Cell Line | Strand | Chrm | | Start in Chrm (0 Indexed) | End in Chrm (1 Indexed) |
| 2.89096374245 | AQR (bg=1.27%) | HepG2 | - | chr20 | | 34636632 | 34636701 |
| 2.28275431976 | ddx6 (bg=23.92%) | HepG2 | - | chr20 | | 34636625 | 34636680 |
| 2.25419298057 | ddx6 (bg=23.92%) | HepG2 | - | chr20 | | 34636635 | 34636684 |
| 4.49018355933 | fubp3 (bg=23.31%) | HepG2 | - | chr20 | | 34636628 | 34636674 |
| 4.75403526968 | fubp3 (bg=23.31%) | HepG2 | - | chr20 | | 34636632 | 34636677 |
| 3.99392907268 | pum1 (bg=29.85%) | K562 | - | chr20 | | 34636617 | 34636695 |
| 4.46414305259 | pum1 (bg=29.85%) | K562 | - | chr20 | | 34636622 | 34636656 |
| 4.82587252829 | pum2 (bg=21.55%) | K562 | - | chr20 | | 34636637 | 34636676 |
| 3.82155487873 | pum2 (bg=21.55%) | K562 | - | chr20 | | 34636638 | 34636677 |
| 3.29438675834 | SUPV3L1 (bg=0.85%) | K562 | - | chr20 | | 34636619 | 34636665 |
| 2.37060800841 | tia1 (bg=16.04%) | K562 | - | chr20 | | 34636630 | 34636686 |
| 2.64154440786 | tia1 (bg=16.04%) | HepG2 | - | chr20 | | 34636634 | 34636687 |
| 3.03808603684 | tial1 (bg=14.09%) | HepG2 | - | chr20 | | 34636620 | 34636683 |
| 2.8047801339 | tial1 (bg=14.09%) | HepG2 | - | chr20 | | 34636622 | 34636729 |

  
  

| Match 201 in HUMAN | | | | | | | |
| --- | --- | --- | --- | --- | --- | --- | --- |
| Motif | Start in Seq (1 Indexed) | End in Seq (1 Indexed) | Strand | Chrm | Exon | Start in Chrm (0 Indexed) | End in Chrm (1 Indexed) |
| ACTGGATT | 2302 | 2309 | - | chr20 | 1 | 34636623 | 34636631 |
| eCLIP Fold-Enrichment | Binding Protein | Cell Line | Strand | Chrm | | Start in Chrm (0 Indexed) | End in Chrm (1 Indexed) |
| 2.28275431976 | ddx6 (bg=23.92%) | HepG2 | - | chr20 | | 34636625 | 34636680 |
| 4.49018355933 | fubp3 (bg=23.31%) | HepG2 | - | chr20 | | 34636628 | 34636674 |
| 3.99392907268 | pum1 (bg=29.85%) | K562 | - | chr20 | | 34636617 | 34636695 |
| 4.46414305259 | pum1 (bg=29.85%) | K562 | - | chr20 | | 34636622 | 34636656 |
| 3.29438675834 | SUPV3L1 (bg=0.85%) | K562 | - | chr20 | | 34636619 | 34636665 |
| 2.37060800841 | tia1 (bg=16.04%) | K562 | - | chr20 | | 34636630 | 34636686 |
| 3.03808603684 | tial1 (bg=14.09%) | HepG2 | - | chr20 | | 34636620 | 34636683 |
| 2.8047801339 | tial1 (bg=14.09%) | HepG2 | - | chr20 | | 34636622 | 34636729 |

  
  

| Match 202 in HUMAN | | | | | | | |
| --- | --- | --- | --- | --- | --- | --- | --- |
| Motif | Start in Seq (1 Indexed) | End in Seq (1 Indexed) | Strand | Chrm | Exon | Start in Chrm (0 Indexed) | End in Chrm (1 Indexed) |
| GAGGAGGGAATGATTCAAGGCCAAAATGGCCACATTTAGAAGATACCTCAGATGATAACCATTGTTAT | 2328 | 2395 | - | chr20 | 1 | 34636537 | 34636605 |
| eCLIP Fold-Enrichment | Binding Protein | Cell Line | Strand | Chrm | | Start in Chrm (0 Indexed) | End in Chrm (1 Indexed) |
| 2.77988557746 | AARS (bg=2.33%) | K562 | - | chr20 | | 34636579 | 34636593 |
| 2.93147538642 | lin28b (bg=16.96%) | K562 | - | chr20 | | 34636482 | 34636544 |

  
  

| Match 203 in HUMAN | | | | | | | |
| --- | --- | --- | --- | --- | --- | --- | --- |
| Motif | Start in Seq (1 Indexed) | End in Seq (1 Indexed) | Strand | Chrm | Exon | Start in Chrm (0 Indexed) | End in Chrm (1 Indexed) |
| AGGCCAAAATGG | 2345 | 2356 | - | chr20 | 1 | 34636576 | 34636588 |
| eCLIP Fold-Enrichment | Binding Protein | Cell Line | Strand | Chrm | | Start in Chrm (0 Indexed) | End in Chrm (1 Indexed) |
| 2.77988557746 | AARS (bg=2.33%) | K562 | - | chr20 | | 34636579 | 34636593 |

  
  

| Match 204 in HUMAN | | | | | | | |
| --- | --- | --- | --- | --- | --- | --- | --- |
| Motif | Start in Seq (1 Indexed) | End in Seq (1 Indexed) | Strand | Chrm | Exon | Start in Chrm (0 Indexed) | End in Chrm (1 Indexed) |
| AGGCCAAAAT | 2345 | 2354 | - | chr20 | 1 | 34636578 | 34636588 |
| eCLIP Fold-Enrichment | Binding Protein | Cell Line | Strand | Chrm | | Start in Chrm (0 Indexed) | End in Chrm (1 Indexed) |
| 2.77988557746 | AARS (bg=2.33%) | K562 | - | chr20 | | 34636579 | 34636593 |

  
  

| Match 205 in HUMAN | | | | | | | |
| --- | --- | --- | --- | --- | --- | --- | --- |
| Motif | Start in Seq (1 Indexed) | End in Seq (1 Indexed) | Strand | Chrm | Exon | Start in Chrm (0 Indexed) | End in Chrm (1 Indexed) |
| AGGCCAA | 2345 | 2351 | - | chr20 | 1 | 34636581 | 34636588 |
| eCLIP Fold-Enrichment | Binding Protein | Cell Line | Strand | Chrm | | Start in Chrm (0 Indexed) | End in Chrm (1 Indexed) |
| 2.77988557746 | AARS (bg=2.33%) | K562 | - | chr20 | | 34636579 | 34636593 |

  
  

| Match 206 in HUMAN | | | | | | | |
| --- | --- | --- | --- | --- | --- | --- | --- |
| Motif | Start in Seq (1 Indexed) | End in Seq (1 Indexed) | Strand | Chrm | Exon | Start in Chrm (0 Indexed) | End in Chrm (1 Indexed) |
| TTGTTA | 2389 | 2394 | - | chr20 | 1 | 34636538 | 34636544 |
| eCLIP Fold-Enrichment | Binding Protein | Cell Line | Strand | Chrm | | Start in Chrm (0 Indexed) | End in Chrm (1 Indexed) |
| 2.93147538642 | lin28b (bg=16.96%) | K562 | - | chr20 | | 34636482 | 34636544 |

  
  

| Match 207 in HUMAN | | | | | | | |
| --- | --- | --- | --- | --- | --- | --- | --- |
| Motif | Start in Seq (1 Indexed) | End in Seq (1 Indexed) | Strand | Chrm | Exon | Start in Chrm (0 Indexed) | End in Chrm (1 Indexed) |
| TGTGTGCAATTTTATTTAACAGTGCT | 2397 | 2422 | - | chr20 | 1 | 34636510 | 34636536 |
| eCLIP Fold-Enrichment | Binding Protein | Cell Line | Strand | Chrm | | Start in Chrm (0 Indexed) | End in Chrm (1 Indexed) |
| 2.42908125027 | CDC40 (bg=1.71%) | HepG2 | - | chr20 | | 34636490 | 34636517 |
| 2.4168583432 | ddx6 (bg=23.92%) | K562 | - | chr20 | | 34636485 | 34636513 |
| 4.15989757198 | ddx6 (bg=23.92%) | HepG2 | - | chr20 | | 34636486 | 34636525 |
| 2.81299275308 | ddx6 (bg=23.92%) | K562 | - | chr20 | | 34636486 | 34636526 |
| 4.68232047929 | ddx6 (bg=23.92%) | HepG2 | - | chr20 | | 34636495 | 34636523 |
| 5.28939140163 | dgcr8 (bg=19.31%) | SM-9MVZL | - | chr20 | | 34636485 | 34636525 |
| 4.11180759364 | dgcr8 (bg=19.31%) | SM-9MVZL | - | chr20 | | 34636488 | 34636533 |
| 2.05060006432 | dgcr8 (bg=19.31%) | HepG2 | - | chr20 | | 34636491 | 34636520 |
| 3.87378700654 | fam120a (bg=18.43%) | K562 | - | chr20 | | 34636494 | 34636517 |
| 2.79932257287 | FASTKD2 (bg=7.73%) | K562 | - | chr20 | | 34636496 | 34636528 |
| 7.49618110295 | fubp3 (bg=23.31%) | HepG2 | - | chr20 | | 34636486 | 34636523 |
| 6.94139467117 | fubp3 (bg=23.31%) | HepG2 | - | chr20 | | 34636493 | 34636522 |
| 2.02006659366 | FUS (bg=2.59%) | K562 | - | chr20 | | 34636485 | 34636523 |
| 2.20907868643 | G3BP1 (bg=1.51%) | HepG2 | - | chr20 | | 34636491 | 34636522 |
| 2.26818329545 | HNRNPL (bg=3.4%) | HepG2 | - | chr20 | | 34636497 | 34636528 |
| 2.51432800741 | igf2bp1 (bg=11.67%) | K562 | - | chr20 | | 34636489 | 34636519 |
| 2.93147538642 | lin28b (bg=16.96%) | K562 | - | chr20 | | 34636482 | 34636544 |
| 3.98591610224 | lin28b (bg=16.96%) | K562 | - | chr20 | | 34636483 | 34636524 |
| 2.41752400192 | lin28b (bg=16.96%) | HepG2 | - | chr20 | | 34636488 | 34636528 |
| 4.01979289961 | lin28b (bg=16.96%) | HepG2 | - | chr20 | | 34636498 | 34636528 |
| 2.63464817023 | NCBP2 (bg=2.92%) | K562 | - | chr20 | | 34636489 | 34636524 |
| 2.66587727697 | NKRF (bg=3.64%) | HepG2 | - | chr20 | | 34636489 | 34636528 |
| 2.9968905801 | NOLC1 (bg=6.58%) | K562 | - | chr20 | | 34636484 | 34636531 |
| 2.62748422106 | NOLC1 (bg=6.58%) | K562 | - | chr20 | | 34636484 | 34636533 |
| 3.67446264163 | pum1 (bg=29.85%) | K562 | - | chr20 | | 34636490 | 34636511 |
| 3.19556293385 | pum1 (bg=29.85%) | K562 | - | chr20 | | 34636496 | 34636513 |
| 4.96691175402 | pum2 (bg=21.55%) | K562 | - | chr20 | | 34636498 | 34636517 |
| 3.79919476976 | pum2 (bg=21.55%) | K562 | - | chr20 | | 34636508 | 34636517 |
| 2.36560711525 | SF3B1 (bg=6.74%) | K562 | - | chr20 | | 34636481 | 34636524 |
| 2.79856065277 | SUB1 (bg=9.24%) | HepG2 | - | chr20 | | 34636488 | 34636528 |

  
  

| Match 208 in HUMAN | | | | | | | |
| --- | --- | --- | --- | --- | --- | --- | --- |
| Motif | Start in Seq (1 Indexed) | End in Seq (1 Indexed) | Strand | Chrm | Exon | Start in Chrm (0 Indexed) | End in Chrm (1 Indexed) |
| TGTGTGCAATT | 2397 | 2407 | - | chr20 | 1 | 34636525 | 34636536 |
| eCLIP Fold-Enrichment | Binding Protein | Cell Line | Strand | Chrm | | Start in Chrm (0 Indexed) | End in Chrm (1 Indexed) |
| 4.15989757198 | ddx6 (bg=23.92%) | HepG2 | - | chr20 | | 34636486 | 34636525 |
| 2.81299275308 | ddx6 (bg=23.92%) | K562 | - | chr20 | | 34636486 | 34636526 |
| 5.28939140163 | dgcr8 (bg=19.31%) | SM-9MVZL | - | chr20 | | 34636485 | 34636525 |
| 4.11180759364 | dgcr8 (bg=19.31%) | SM-9MVZL | - | chr20 | | 34636488 | 34636533 |
| 2.79932257287 | FASTKD2 (bg=7.73%) | K562 | - | chr20 | | 34636496 | 34636528 |
| 2.26818329545 | HNRNPL (bg=3.4%) | HepG2 | - | chr20 | | 34636497 | 34636528 |
| 2.93147538642 | lin28b (bg=16.96%) | K562 | - | chr20 | | 34636482 | 34636544 |
| 2.41752400192 | lin28b (bg=16.96%) | HepG2 | - | chr20 | | 34636488 | 34636528 |
| 4.01979289961 | lin28b (bg=16.96%) | HepG2 | - | chr20 | | 34636498 | 34636528 |
| 2.66587727697 | NKRF (bg=3.64%) | HepG2 | - | chr20 | | 34636489 | 34636528 |
| 2.9968905801 | NOLC1 (bg=6.58%) | K562 | - | chr20 | | 34636484 | 34636531 |
| 2.62748422106 | NOLC1 (bg=6.58%) | K562 | - | chr20 | | 34636484 | 34636533 |
| 2.79856065277 | SUB1 (bg=9.24%) | HepG2 | - | chr20 | | 34636488 | 34636528 |

  
  

| Match 209 in HUMAN | | | | | | | |
| --- | --- | --- | --- | --- | --- | --- | --- |
| Motif | Start in Seq (1 Indexed) | End in Seq (1 Indexed) | Strand | Chrm | Exon | Start in Chrm (0 Indexed) | End in Chrm (1 Indexed) |
| GTGTGCAATT | 2398 | 2407 | - | chr20 | 1 | 34636525 | 34636535 |
| eCLIP Fold-Enrichment | Binding Protein | Cell Line | Strand | Chrm | | Start in Chrm (0 Indexed) | End in Chrm (1 Indexed) |
| 4.15989757198 | ddx6 (bg=23.92%) | HepG2 | - | chr20 | | 34636486 | 34636525 |
| 2.81299275308 | ddx6 (bg=23.92%) | K562 | - | chr20 | | 34636486 | 34636526 |
| 5.28939140163 | dgcr8 (bg=19.31%) | SM-9MVZL | - | chr20 | | 34636485 | 34636525 |
| 4.11180759364 | dgcr8 (bg=19.31%) | SM-9MVZL | - | chr20 | | 34636488 | 34636533 |
| 2.79932257287 | FASTKD2 (bg=7.73%) | K562 | - | chr20 | | 34636496 | 34636528 |
| 2.26818329545 | HNRNPL (bg=3.4%) | HepG2 | - | chr20 | | 34636497 | 34636528 |
| 2.93147538642 | lin28b (bg=16.96%) | K562 | - | chr20 | | 34636482 | 34636544 |
| 2.41752400192 | lin28b (bg=16.96%) | HepG2 | - | chr20 | | 34636488 | 34636528 |
| 4.01979289961 | lin28b (bg=16.96%) | HepG2 | - | chr20 | | 34636498 | 34636528 |
| 2.66587727697 | NKRF (bg=3.64%) | HepG2 | - | chr20 | | 34636489 | 34636528 |
| 2.9968905801 | NOLC1 (bg=6.58%) | K562 | - | chr20 | | 34636484 | 34636531 |
| 2.62748422106 | NOLC1 (bg=6.58%) | K562 | - | chr20 | | 34636484 | 34636533 |
| 2.79856065277 | SUB1 (bg=9.24%) | HepG2 | - | chr20 | | 34636488 | 34636528 |

  
  

| Match 210 in HUMAN | | | | | | | |
| --- | --- | --- | --- | --- | --- | --- | --- |
| Motif | Start in Seq (1 Indexed) | End in Seq (1 Indexed) | Strand | Chrm | Exon | Start in Chrm (0 Indexed) | End in Chrm (1 Indexed) |
| TATTTAACAGTGCT | 2409 | 2422 | - | chr20 | 1 | 34636510 | 34636524 |
| eCLIP Fold-Enrichment | Binding Protein | Cell Line | Strand | Chrm | | Start in Chrm (0 Indexed) | End in Chrm (1 Indexed) |
| 2.42908125027 | CDC40 (bg=1.71%) | HepG2 | - | chr20 | | 34636490 | 34636517 |
| 2.4168583432 | ddx6 (bg=23.92%) | K562 | - | chr20 | | 34636485 | 34636513 |
| 4.15989757198 | ddx6 (bg=23.92%) | HepG2 | - | chr20 | | 34636486 | 34636525 |
| 2.81299275308 | ddx6 (bg=23.92%) | K562 | - | chr20 | | 34636486 | 34636526 |
| 4.68232047929 | ddx6 (bg=23.92%) | HepG2 | - | chr20 | | 34636495 | 34636523 |
| 5.28939140163 | dgcr8 (bg=19.31%) | SM-9MVZL | - | chr20 | | 34636485 | 34636525 |
| 4.11180759364 | dgcr8 (bg=19.31%) | SM-9MVZL | - | chr20 | | 34636488 | 34636533 |
| 2.05060006432 | dgcr8 (bg=19.31%) | HepG2 | - | chr20 | | 34636491 | 34636520 |
| 3.87378700654 | fam120a (bg=18.43%) | K562 | - | chr20 | | 34636494 | 34636517 |
| 2.79932257287 | FASTKD2 (bg=7.73%) | K562 | - | chr20 | | 34636496 | 34636528 |
| 7.49618110295 | fubp3 (bg=23.31%) | HepG2 | - | chr20 | | 34636486 | 34636523 |
| 6.94139467117 | fubp3 (bg=23.31%) | HepG2 | - | chr20 | | 34636493 | 34636522 |
| 2.02006659366 | FUS (bg=2.59%) | K562 | - | chr20 | | 34636485 | 34636523 |
| 2.20907868643 | G3BP1 (bg=1.51%) | HepG2 | - | chr20 | | 34636491 | 34636522 |
| 2.26818329545 | HNRNPL (bg=3.4%) | HepG2 | - | chr20 | | 34636497 | 34636528 |
| 2.51432800741 | igf2bp1 (bg=11.67%) | K562 | - | chr20 | | 34636489 | 34636519 |
| 2.93147538642 | lin28b (bg=16.96%) | K562 | - | chr20 | | 34636482 | 34636544 |
| 3.98591610224 | lin28b (bg=16.96%) | K562 | - | chr20 | | 34636483 | 34636524 |
| 2.41752400192 | lin28b (bg=16.96%) | HepG2 | - | chr20 | | 34636488 | 34636528 |
| 4.01979289961 | lin28b (bg=16.96%) | HepG2 | - | chr20 | | 34636498 | 34636528 |
| 2.63464817023 | NCBP2 (bg=2.92%) | K562 | - | chr20 | | 34636489 | 34636524 |
| 2.66587727697 | NKRF (bg=3.64%) | HepG2 | - | chr20 | | 34636489 | 34636528 |
| 2.9968905801 | NOLC1 (bg=6.58%) | K562 | - | chr20 | | 34636484 | 34636531 |
| 2.62748422106 | NOLC1 (bg=6.58%) | K562 | - | chr20 | | 34636484 | 34636533 |
| 3.67446264163 | pum1 (bg=29.85%) | K562 | - | chr20 | | 34636490 | 34636511 |
| 3.19556293385 | pum1 (bg=29.85%) | K562 | - | chr20 | | 34636496 | 34636513 |
| 4.96691175402 | pum2 (bg=21.55%) | K562 | - | chr20 | | 34636498 | 34636517 |
| 3.79919476976 | pum2 (bg=21.55%) | K562 | - | chr20 | | 34636508 | 34636517 |
| 2.36560711525 | SF3B1 (bg=6.74%) | K562 | - | chr20 | | 34636481 | 34636524 |
| 2.79856065277 | SUB1 (bg=9.24%) | HepG2 | - | chr20 | | 34636488 | 34636528 |

  
  

| Match 211 in HUMAN | | | | | | | |
| --- | --- | --- | --- | --- | --- | --- | --- |
| Motif | Start in Seq (1 Indexed) | End in Seq (1 Indexed) | Strand | Chrm | Exon | Start in Chrm (0 Indexed) | End in Chrm (1 Indexed) |
| TTAACA | 2412 | 2417 | - | chr20 | 1 | 34636515 | 34636521 |
| eCLIP Fold-Enrichment | Binding Protein | Cell Line | Strand | Chrm | | Start in Chrm (0 Indexed) | End in Chrm (1 Indexed) |
| 2.42908125027 | CDC40 (bg=1.71%) | HepG2 | - | chr20 | | 34636490 | 34636517 |
| 4.15989757198 | ddx6 (bg=23.92%) | HepG2 | - | chr20 | | 34636486 | 34636525 |
| 2.81299275308 | ddx6 (bg=23.92%) | K562 | - | chr20 | | 34636486 | 34636526 |
| 4.68232047929 | ddx6 (bg=23.92%) | HepG2 | - | chr20 | | 34636495 | 34636523 |
| 5.28939140163 | dgcr8 (bg=19.31%) | SM-9MVZL | - | chr20 | | 34636485 | 34636525 |
| 4.11180759364 | dgcr8 (bg=19.31%) | SM-9MVZL | - | chr20 | | 34636488 | 34636533 |
| 2.05060006432 | dgcr8 (bg=19.31%) | HepG2 | - | chr20 | | 34636491 | 34636520 |
| 3.87378700654 | fam120a (bg=18.43%) | K562 | - | chr20 | | 34636494 | 34636517 |
| 2.79932257287 | FASTKD2 (bg=7.73%) | K562 | - | chr20 | | 34636496 | 34636528 |
| 7.49618110295 | fubp3 (bg=23.31%) | HepG2 | - | chr20 | | 34636486 | 34636523 |
| 6.94139467117 | fubp3 (bg=23.31%) | HepG2 | - | chr20 | | 34636493 | 34636522 |
| 2.02006659366 | FUS (bg=2.59%) | K562 | - | chr20 | | 34636485 | 34636523 |
| 2.20907868643 | G3BP1 (bg=1.51%) | HepG2 | - | chr20 | | 34636491 | 34636522 |
| 2.26818329545 | HNRNPL (bg=3.4%) | HepG2 | - | chr20 | | 34636497 | 34636528 |
| 2.51432800741 | igf2bp1 (bg=11.67%) | K562 | - | chr20 | | 34636489 | 34636519 |
| 2.93147538642 | lin28b (bg=16.96%) | K562 | - | chr20 | | 34636482 | 34636544 |
| 3.98591610224 | lin28b (bg=16.96%) | K562 | - | chr20 | | 34636483 | 34636524 |
| 2.41752400192 | lin28b (bg=16.96%) | HepG2 | - | chr20 | | 34636488 | 34636528 |
| 4.01979289961 | lin28b (bg=16.96%) | HepG2 | - | chr20 | | 34636498 | 34636528 |
| 2.63464817023 | NCBP2 (bg=2.92%) | K562 | - | chr20 | | 34636489 | 34636524 |
| 2.66587727697 | NKRF (bg=3.64%) | HepG2 | - | chr20 | | 34636489 | 34636528 |
| 2.9968905801 | NOLC1 (bg=6.58%) | K562 | - | chr20 | | 34636484 | 34636531 |
| 2.62748422106 | NOLC1 (bg=6.58%) | K562 | - | chr20 | | 34636484 | 34636533 |
| 4.96691175402 | pum2 (bg=21.55%) | K562 | - | chr20 | | 34636498 | 34636517 |
| 3.79919476976 | pum2 (bg=21.55%) | K562 | - | chr20 | | 34636508 | 34636517 |
| 2.36560711525 | SF3B1 (bg=6.74%) | K562 | - | chr20 | | 34636481 | 34636524 |
| 2.79856065277 | SUB1 (bg=9.24%) | HepG2 | - | chr20 | | 34636488 | 34636528 |

  
  

| Match 212 in HUMAN | | | | | | | |
| --- | --- | --- | --- | --- | --- | --- | --- |
| Motif | Start in Seq (1 Indexed) | End in Seq (1 Indexed) | Strand | Chrm | Exon | Start in Chrm (0 Indexed) | End in Chrm (1 Indexed) |
| TTAACAGT | 2412 | 2419 | - | chr20 | 1 | 34636513 | 34636521 |
| eCLIP Fold-Enrichment | Binding Protein | Cell Line | Strand | Chrm | | Start in Chrm (0 Indexed) | End in Chrm (1 Indexed) |
| 2.42908125027 | CDC40 (bg=1.71%) | HepG2 | - | chr20 | | 34636490 | 34636517 |
| 2.4168583432 | ddx6 (bg=23.92%) | K562 | - | chr20 | | 34636485 | 34636513 |
| 4.15989757198 | ddx6 (bg=23.92%) | HepG2 | - | chr20 | | 34636486 | 34636525 |
| 2.81299275308 | ddx6 (bg=23.92%) | K562 | - | chr20 | | 34636486 | 34636526 |
| 4.68232047929 | ddx6 (bg=23.92%) | HepG2 | - | chr20 | | 34636495 | 34636523 |
| 5.28939140163 | dgcr8 (bg=19.31%) | SM-9MVZL | - | chr20 | | 34636485 | 34636525 |
| 4.11180759364 | dgcr8 (bg=19.31%) | SM-9MVZL | - | chr20 | | 34636488 | 34636533 |
| 2.05060006432 | dgcr8 (bg=19.31%) | HepG2 | - | chr20 | | 34636491 | 34636520 |
| 3.87378700654 | fam120a (bg=18.43%) | K562 | - | chr20 | | 34636494 | 34636517 |
| 2.79932257287 | FASTKD2 (bg=7.73%) | K562 | - | chr20 | | 34636496 | 34636528 |
| 7.49618110295 | fubp3 (bg=23.31%) | HepG2 | - | chr20 | | 34636486 | 34636523 |
| 6.94139467117 | fubp3 (bg=23.31%) | HepG2 | - | chr20 | | 34636493 | 34636522 |
| 2.02006659366 | FUS (bg=2.59%) | K562 | - | chr20 | | 34636485 | 34636523 |
| 2.20907868643 | G3BP1 (bg=1.51%) | HepG2 | - | chr20 | | 34636491 | 34636522 |
| 2.26818329545 | HNRNPL (bg=3.4%) | HepG2 | - | chr20 | | 34636497 | 34636528 |
| 2.51432800741 | igf2bp1 (bg=11.67%) | K562 | - | chr20 | | 34636489 | 34636519 |
| 2.93147538642 | lin28b (bg=16.96%) | K562 | - | chr20 | | 34636482 | 34636544 |
| 3.98591610224 | lin28b (bg=16.96%) | K562 | - | chr20 | | 34636483 | 34636524 |
| 2.41752400192 | lin28b (bg=16.96%) | HepG2 | - | chr20 | | 34636488 | 34636528 |
| 4.01979289961 | lin28b (bg=16.96%) | HepG2 | - | chr20 | | 34636498 | 34636528 |
| 2.63464817023 | NCBP2 (bg=2.92%) | K562 | - | chr20 | | 34636489 | 34636524 |
| 2.66587727697 | NKRF (bg=3.64%) | HepG2 | - | chr20 | | 34636489 | 34636528 |
| 2.9968905801 | NOLC1 (bg=6.58%) | K562 | - | chr20 | | 34636484 | 34636531 |
| 2.62748422106 | NOLC1 (bg=6.58%) | K562 | - | chr20 | | 34636484 | 34636533 |
| 3.19556293385 | pum1 (bg=29.85%) | K562 | - | chr20 | | 34636496 | 34636513 |
| 4.96691175402 | pum2 (bg=21.55%) | K562 | - | chr20 | | 34636498 | 34636517 |
| 3.79919476976 | pum2 (bg=21.55%) | K562 | - | chr20 | | 34636508 | 34636517 |
| 2.36560711525 | SF3B1 (bg=6.74%) | K562 | - | chr20 | | 34636481 | 34636524 |
| 2.79856065277 | SUB1 (bg=9.24%) | HepG2 | - | chr20 | | 34636488 | 34636528 |

  
  

| Match 213 in HUMAN | | | | | | | |
| --- | --- | --- | --- | --- | --- | --- | --- |
| Motif | Start in Seq (1 Indexed) | End in Seq (1 Indexed) | Strand | Chrm | Exon | Start in Chrm (0 Indexed) | End in Chrm (1 Indexed) |
| GTGGTG | 2429 | 2434 | - | chr20 | 1 | 34636498 | 34636504 |
| eCLIP Fold-Enrichment | Binding Protein | Cell Line | Strand | Chrm | | Start in Chrm (0 Indexed) | End in Chrm (1 Indexed) |
| 2.42908125027 | CDC40 (bg=1.71%) | HepG2 | - | chr20 | | 34636490 | 34636517 |
| 2.4168583432 | ddx6 (bg=23.92%) | K562 | - | chr20 | | 34636485 | 34636513 |
| 4.15989757198 | ddx6 (bg=23.92%) | HepG2 | - | chr20 | | 34636486 | 34636525 |
| 2.81299275308 | ddx6 (bg=23.92%) | K562 | - | chr20 | | 34636486 | 34636526 |
| 4.68232047929 | ddx6 (bg=23.92%) | HepG2 | - | chr20 | | 34636495 | 34636523 |
| 5.28939140163 | dgcr8 (bg=19.31%) | SM-9MVZL | - | chr20 | | 34636485 | 34636525 |
| 4.11180759364 | dgcr8 (bg=19.31%) | SM-9MVZL | - | chr20 | | 34636488 | 34636533 |
| 2.05060006432 | dgcr8 (bg=19.31%) | HepG2 | - | chr20 | | 34636491 | 34636520 |
| 3.87378700654 | fam120a (bg=18.43%) | K562 | - | chr20 | | 34636494 | 34636517 |
| 2.79932257287 | FASTKD2 (bg=7.73%) | K562 | - | chr20 | | 34636496 | 34636528 |
| 7.49618110295 | fubp3 (bg=23.31%) | HepG2 | - | chr20 | | 34636486 | 34636523 |
| 6.94139467117 | fubp3 (bg=23.31%) | HepG2 | - | chr20 | | 34636493 | 34636522 |
| 2.02006659366 | FUS (bg=2.59%) | K562 | - | chr20 | | 34636485 | 34636523 |
| 2.20907868643 | G3BP1 (bg=1.51%) | HepG2 | - | chr20 | | 34636491 | 34636522 |
| 2.26818329545 | HNRNPL (bg=3.4%) | HepG2 | - | chr20 | | 34636497 | 34636528 |
| 2.51432800741 | igf2bp1 (bg=11.67%) | K562 | - | chr20 | | 34636489 | 34636519 |
| 2.93147538642 | lin28b (bg=16.96%) | K562 | - | chr20 | | 34636482 | 34636544 |
| 3.98591610224 | lin28b (bg=16.96%) | K562 | - | chr20 | | 34636483 | 34636524 |
| 4.19382229939 | lin28b (bg=16.96%) | HepG2 | - | chr20 | | 34636488 | 34636498 |
| 2.41752400192 | lin28b (bg=16.96%) | HepG2 | - | chr20 | | 34636488 | 34636528 |
| 4.01979289961 | lin28b (bg=16.96%) | HepG2 | - | chr20 | | 34636498 | 34636528 |
| 2.63464817023 | NCBP2 (bg=2.92%) | K562 | - | chr20 | | 34636489 | 34636524 |
| 2.66587727697 | NKRF (bg=3.64%) | HepG2 | - | chr20 | | 34636489 | 34636528 |
| 2.9968905801 | NOLC1 (bg=6.58%) | K562 | - | chr20 | | 34636484 | 34636531 |
| 2.62748422106 | NOLC1 (bg=6.58%) | K562 | - | chr20 | | 34636484 | 34636533 |
| 3.67446264163 | pum1 (bg=29.85%) | K562 | - | chr20 | | 34636490 | 34636511 |
| 3.19556293385 | pum1 (bg=29.85%) | K562 | - | chr20 | | 34636496 | 34636513 |
| 4.96691175402 | pum2 (bg=21.55%) | K562 | - | chr20 | | 34636498 | 34636517 |
| 2.36560711525 | SF3B1 (bg=6.74%) | K562 | - | chr20 | | 34636481 | 34636524 |
| 2.79856065277 | SUB1 (bg=9.24%) | HepG2 | - | chr20 | | 34636488 | 34636528 |

  
  

| Match 214 in HUMAN | | | | | | | |
| --- | --- | --- | --- | --- | --- | --- | --- |
| Motif | Start in Seq (1 Indexed) | End in Seq (1 Indexed) | Strand | Chrm | Exon | Start in Chrm (0 Indexed) | End in Chrm (1 Indexed) |
| ACAAGTTATATGAAATATCTAGTCTTTCTAGATATTTGGAAG | 2436 | 2477 | - | chr20 | 1 | 34636455 | 34636497 |
| eCLIP Fold-Enrichment | Binding Protein | Cell Line | Strand | Chrm | | Start in Chrm (0 Indexed) | End in Chrm (1 Indexed) |
| 2.42908125027 | CDC40 (bg=1.71%) | HepG2 | - | chr20 | | 34636490 | 34636517 |
| 2.4168583432 | ddx6 (bg=23.92%) | K562 | - | chr20 | | 34636485 | 34636513 |
| 4.15989757198 | ddx6 (bg=23.92%) | HepG2 | - | chr20 | | 34636486 | 34636525 |
| 2.81299275308 | ddx6 (bg=23.92%) | K562 | - | chr20 | | 34636486 | 34636526 |
| 4.68232047929 | ddx6 (bg=23.92%) | HepG2 | - | chr20 | | 34636495 | 34636523 |
| 5.28939140163 | dgcr8 (bg=19.31%) | SM-9MVZL | - | chr20 | | 34636485 | 34636525 |
| 4.11180759364 | dgcr8 (bg=19.31%) | SM-9MVZL | - | chr20 | | 34636488 | 34636533 |
| 2.05060006432 | dgcr8 (bg=19.31%) | HepG2 | - | chr20 | | 34636491 | 34636520 |
| 3.87378700654 | fam120a (bg=18.43%) | K562 | - | chr20 | | 34636494 | 34636517 |
| 2.79932257287 | FASTKD2 (bg=7.73%) | K562 | - | chr20 | | 34636496 | 34636528 |
| 6.37282660869 | fubp3 (bg=23.31%) | HepG2 | - | chr20 | | 34636480 | 34636493 |
| 5.83476654179 | fubp3 (bg=23.31%) | HepG2 | - | chr20 | | 34636482 | 34636483 |
| 7.49618110295 | fubp3 (bg=23.31%) | HepG2 | - | chr20 | | 34636486 | 34636523 |
| 6.94139467117 | fubp3 (bg=23.31%) | HepG2 | - | chr20 | | 34636493 | 34636522 |
| 2.02006659366 | FUS (bg=2.59%) | K562 | - | chr20 | | 34636485 | 34636523 |
| 2.20907868643 | G3BP1 (bg=1.51%) | HepG2 | - | chr20 | | 34636491 | 34636522 |
| 2.26818329545 | HNRNPL (bg=3.4%) | HepG2 | - | chr20 | | 34636497 | 34636528 |
| 2.51432800741 | igf2bp1 (bg=11.67%) | K562 | - | chr20 | | 34636489 | 34636519 |
| 2.93147538642 | lin28b (bg=16.96%) | K562 | - | chr20 | | 34636482 | 34636544 |
| 3.98591610224 | lin28b (bg=16.96%) | K562 | - | chr20 | | 34636483 | 34636524 |
| 4.19382229939 | lin28b (bg=16.96%) | HepG2 | - | chr20 | | 34636488 | 34636498 |
| 2.41752400192 | lin28b (bg=16.96%) | HepG2 | - | chr20 | | 34636488 | 34636528 |
| 2.63464817023 | NCBP2 (bg=2.92%) | K562 | - | chr20 | | 34636489 | 34636524 |
| 2.66587727697 | NKRF (bg=3.64%) | HepG2 | - | chr20 | | 34636489 | 34636528 |
| 2.9968905801 | NOLC1 (bg=6.58%) | K562 | - | chr20 | | 34636484 | 34636531 |
| 2.62748422106 | NOLC1 (bg=6.58%) | K562 | - | chr20 | | 34636484 | 34636533 |
| 3.67446264163 | pum1 (bg=29.85%) | K562 | - | chr20 | | 34636490 | 34636511 |
| 3.19556293385 | pum1 (bg=29.85%) | K562 | - | chr20 | | 34636496 | 34636513 |
| 2.36560711525 | SF3B1 (bg=6.74%) | K562 | - | chr20 | | 34636481 | 34636524 |
| 2.79856065277 | SUB1 (bg=9.24%) | HepG2 | - | chr20 | | 34636488 | 34636528 |

  
  

| Match 215 in HUMAN | | | | | | | |
| --- | --- | --- | --- | --- | --- | --- | --- |
| Motif | Start in Seq (1 Indexed) | End in Seq (1 Indexed) | Strand | Chrm | Exon | Start in Chrm (0 Indexed) | End in Chrm (1 Indexed) |
| ACAAGT | 2436 | 2441 | - | chr20 | 1 | 34636491 | 34636497 |
| eCLIP Fold-Enrichment | Binding Protein | Cell Line | Strand | Chrm | | Start in Chrm (0 Indexed) | End in Chrm (1 Indexed) |
| 2.42908125027 | CDC40 (bg=1.71%) | HepG2 | - | chr20 | | 34636490 | 34636517 |
| 2.4168583432 | ddx6 (bg=23.92%) | K562 | - | chr20 | | 34636485 | 34636513 |
| 4.15989757198 | ddx6 (bg=23.92%) | HepG2 | - | chr20 | | 34636486 | 34636525 |
| 2.81299275308 | ddx6 (bg=23.92%) | K562 | - | chr20 | | 34636486 | 34636526 |
| 4.68232047929 | ddx6 (bg=23.92%) | HepG2 | - | chr20 | | 34636495 | 34636523 |
| 5.28939140163 | dgcr8 (bg=19.31%) | SM-9MVZL | - | chr20 | | 34636485 | 34636525 |
| 4.11180759364 | dgcr8 (bg=19.31%) | SM-9MVZL | - | chr20 | | 34636488 | 34636533 |
| 2.05060006432 | dgcr8 (bg=19.31%) | HepG2 | - | chr20 | | 34636491 | 34636520 |
| 3.87378700654 | fam120a (bg=18.43%) | K562 | - | chr20 | | 34636494 | 34636517 |
| 2.79932257287 | FASTKD2 (bg=7.73%) | K562 | - | chr20 | | 34636496 | 34636528 |
| 6.37282660869 | fubp3 (bg=23.31%) | HepG2 | - | chr20 | | 34636480 | 34636493 |
| 7.49618110295 | fubp3 (bg=23.31%) | HepG2 | - | chr20 | | 34636486 | 34636523 |
| 6.94139467117 | fubp3 (bg=23.31%) | HepG2 | - | chr20 | | 34636493 | 34636522 |
| 2.02006659366 | FUS (bg=2.59%) | K562 | - | chr20 | | 34636485 | 34636523 |
| 2.20907868643 | G3BP1 (bg=1.51%) | HepG2 | - | chr20 | | 34636491 | 34636522 |
| 2.26818329545 | HNRNPL (bg=3.4%) | HepG2 | - | chr20 | | 34636497 | 34636528 |
| 2.51432800741 | igf2bp1 (bg=11.67%) | K562 | - | chr20 | | 34636489 | 34636519 |
| 2.93147538642 | lin28b (bg=16.96%) | K562 | - | chr20 | | 34636482 | 34636544 |
| 3.98591610224 | lin28b (bg=16.96%) | K562 | - | chr20 | | 34636483 | 34636524 |
| 4.19382229939 | lin28b (bg=16.96%) | HepG2 | - | chr20 | | 34636488 | 34636498 |
| 2.41752400192 | lin28b (bg=16.96%) | HepG2 | - | chr20 | | 34636488 | 34636528 |
| 2.63464817023 | NCBP2 (bg=2.92%) | K562 | - | chr20 | | 34636489 | 34636524 |
| 2.66587727697 | NKRF (bg=3.64%) | HepG2 | - | chr20 | | 34636489 | 34636528 |
| 2.9968905801 | NOLC1 (bg=6.58%) | K562 | - | chr20 | | 34636484 | 34636531 |
| 2.62748422106 | NOLC1 (bg=6.58%) | K562 | - | chr20 | | 34636484 | 34636533 |
| 3.67446264163 | pum1 (bg=29.85%) | K562 | - | chr20 | | 34636490 | 34636511 |
| 3.19556293385 | pum1 (bg=29.85%) | K562 | - | chr20 | | 34636496 | 34636513 |
| 2.36560711525 | SF3B1 (bg=6.74%) | K562 | - | chr20 | | 34636481 | 34636524 |
| 2.79856065277 | SUB1 (bg=9.24%) | HepG2 | - | chr20 | | 34636488 | 34636528 |

  
  

| Match 216 in HUMAN | | | | | | | |
| --- | --- | --- | --- | --- | --- | --- | --- |
| Motif | Start in Seq (1 Indexed) | End in Seq (1 Indexed) | Strand | Chrm | Exon | Start in Chrm (0 Indexed) | End in Chrm (1 Indexed) |
| ACAAGTTA | 2436 | 2443 | - | chr20 | 1 | 34636489 | 34636497 |
| eCLIP Fold-Enrichment | Binding Protein | Cell Line | Strand | Chrm | | Start in Chrm (0 Indexed) | End in Chrm (1 Indexed) |
| 2.42908125027 | CDC40 (bg=1.71%) | HepG2 | - | chr20 | | 34636490 | 34636517 |
| 2.4168583432 | ddx6 (bg=23.92%) | K562 | - | chr20 | | 34636485 | 34636513 |
| 4.15989757198 | ddx6 (bg=23.92%) | HepG2 | - | chr20 | | 34636486 | 34636525 |
| 2.81299275308 | ddx6 (bg=23.92%) | K562 | - | chr20 | | 34636486 | 34636526 |
| 4.68232047929 | ddx6 (bg=23.92%) | HepG2 | - | chr20 | | 34636495 | 34636523 |
| 5.28939140163 | dgcr8 (bg=19.31%) | SM-9MVZL | - | chr20 | | 34636485 | 34636525 |
| 4.11180759364 | dgcr8 (bg=19.31%) | SM-9MVZL | - | chr20 | | 34636488 | 34636533 |
| 2.05060006432 | dgcr8 (bg=19.31%) | HepG2 | - | chr20 | | 34636491 | 34636520 |
| 3.87378700654 | fam120a (bg=18.43%) | K562 | - | chr20 | | 34636494 | 34636517 |
| 2.79932257287 | FASTKD2 (bg=7.73%) | K562 | - | chr20 | | 34636496 | 34636528 |
| 6.37282660869 | fubp3 (bg=23.31%) | HepG2 | - | chr20 | | 34636480 | 34636493 |
| 7.49618110295 | fubp3 (bg=23.31%) | HepG2 | - | chr20 | | 34636486 | 34636523 |
| 6.94139467117 | fubp3 (bg=23.31%) | HepG2 | - | chr20 | | 34636493 | 34636522 |
| 2.02006659366 | FUS (bg=2.59%) | K562 | - | chr20 | | 34636485 | 34636523 |
| 2.20907868643 | G3BP1 (bg=1.51%) | HepG2 | - | chr20 | | 34636491 | 34636522 |
| 2.26818329545 | HNRNPL (bg=3.4%) | HepG2 | - | chr20 | | 34636497 | 34636528 |
| 2.51432800741 | igf2bp1 (bg=11.67%) | K562 | - | chr20 | | 34636489 | 34636519 |
| 2.93147538642 | lin28b (bg=16.96%) | K562 | - | chr20 | | 34636482 | 34636544 |
| 3.98591610224 | lin28b (bg=16.96%) | K562 | - | chr20 | | 34636483 | 34636524 |
| 4.19382229939 | lin28b (bg=16.96%) | HepG2 | - | chr20 | | 34636488 | 34636498 |
| 2.41752400192 | lin28b (bg=16.96%) | HepG2 | - | chr20 | | 34636488 | 34636528 |
| 2.63464817023 | NCBP2 (bg=2.92%) | K562 | - | chr20 | | 34636489 | 34636524 |
| 2.66587727697 | NKRF (bg=3.64%) | HepG2 | - | chr20 | | 34636489 | 34636528 |
| 2.9968905801 | NOLC1 (bg=6.58%) | K562 | - | chr20 | | 34636484 | 34636531 |
| 2.62748422106 | NOLC1 (bg=6.58%) | K562 | - | chr20 | | 34636484 | 34636533 |
| 3.67446264163 | pum1 (bg=29.85%) | K562 | - | chr20 | | 34636490 | 34636511 |
| 3.19556293385 | pum1 (bg=29.85%) | K562 | - | chr20 | | 34636496 | 34636513 |
| 2.36560711525 | SF3B1 (bg=6.74%) | K562 | - | chr20 | | 34636481 | 34636524 |
| 2.79856065277 | SUB1 (bg=9.24%) | HepG2 | - | chr20 | | 34636488 | 34636528 |

  
  

| Match 217 in HUMAN | | | | | | | |
| --- | --- | --- | --- | --- | --- | --- | --- |
| Motif | Start in Seq (1 Indexed) | End in Seq (1 Indexed) | Strand | Chrm | Exon | Start in Chrm (0 Indexed) | End in Chrm (1 Indexed) |
| TGAAATATCTAGTCTT | 2446 | 2461 | - | chr20 | 1 | 34636471 | 34636487 |
| eCLIP Fold-Enrichment | Binding Protein | Cell Line | Strand | Chrm | | Start in Chrm (0 Indexed) | End in Chrm (1 Indexed) |
| 2.4168583432 | ddx6 (bg=23.92%) | K562 | - | chr20 | | 34636485 | 34636513 |
| 4.15989757198 | ddx6 (bg=23.92%) | HepG2 | - | chr20 | | 34636486 | 34636525 |
| 2.81299275308 | ddx6 (bg=23.92%) | K562 | - | chr20 | | 34636486 | 34636526 |
| 5.28939140163 | dgcr8 (bg=19.31%) | SM-9MVZL | - | chr20 | | 34636485 | 34636525 |
| 6.37282660869 | fubp3 (bg=23.31%) | HepG2 | - | chr20 | | 34636480 | 34636493 |
| 5.83476654179 | fubp3 (bg=23.31%) | HepG2 | - | chr20 | | 34636482 | 34636483 |
| 7.49618110295 | fubp3 (bg=23.31%) | HepG2 | - | chr20 | | 34636486 | 34636523 |
| 2.02006659366 | FUS (bg=2.59%) | K562 | - | chr20 | | 34636485 | 34636523 |
| 2.93147538642 | lin28b (bg=16.96%) | K562 | - | chr20 | | 34636482 | 34636544 |
| 3.98591610224 | lin28b (bg=16.96%) | K562 | - | chr20 | | 34636483 | 34636524 |
| 2.9968905801 | NOLC1 (bg=6.58%) | K562 | - | chr20 | | 34636484 | 34636531 |
| 2.62748422106 | NOLC1 (bg=6.58%) | K562 | - | chr20 | | 34636484 | 34636533 |
| 2.36560711525 | SF3B1 (bg=6.74%) | K562 | - | chr20 | | 34636481 | 34636524 |

  
  

| Match 218 in HUMAN | | | | | | | |
| --- | --- | --- | --- | --- | --- | --- | --- |
| Motif | Start in Seq (1 Indexed) | End in Seq (1 Indexed) | Strand | Chrm | Exon | Start in Chrm (0 Indexed) | End in Chrm (1 Indexed) |
| TGAAATATCTAGTCTTTCTAGATA | 2446 | 2469 | - | chr20 | 1 | 34636463 | 34636487 |
| eCLIP Fold-Enrichment | Binding Protein | Cell Line | Strand | Chrm | | Start in Chrm (0 Indexed) | End in Chrm (1 Indexed) |
| 2.4168583432 | ddx6 (bg=23.92%) | K562 | - | chr20 | | 34636485 | 34636513 |
| 4.15989757198 | ddx6 (bg=23.92%) | HepG2 | - | chr20 | | 34636486 | 34636525 |
| 2.81299275308 | ddx6 (bg=23.92%) | K562 | - | chr20 | | 34636486 | 34636526 |
| 5.28939140163 | dgcr8 (bg=19.31%) | SM-9MVZL | - | chr20 | | 34636485 | 34636525 |
| 6.37282660869 | fubp3 (bg=23.31%) | HepG2 | - | chr20 | | 34636480 | 34636493 |
| 5.83476654179 | fubp3 (bg=23.31%) | HepG2 | - | chr20 | | 34636482 | 34636483 |
| 7.49618110295 | fubp3 (bg=23.31%) | HepG2 | - | chr20 | | 34636486 | 34636523 |
| 2.02006659366 | FUS (bg=2.59%) | K562 | - | chr20 | | 34636485 | 34636523 |
| 2.93147538642 | lin28b (bg=16.96%) | K562 | - | chr20 | | 34636482 | 34636544 |
| 3.98591610224 | lin28b (bg=16.96%) | K562 | - | chr20 | | 34636483 | 34636524 |
| 2.9968905801 | NOLC1 (bg=6.58%) | K562 | - | chr20 | | 34636484 | 34636531 |
| 2.62748422106 | NOLC1 (bg=6.58%) | K562 | - | chr20 | | 34636484 | 34636533 |
| 2.36560711525 | SF3B1 (bg=6.74%) | K562 | - | chr20 | | 34636481 | 34636524 |

  
  

| Match 219 in HUMAN | | | | | | | |
| --- | --- | --- | --- | --- | --- | --- | --- |
| Motif | Start in Seq (1 Indexed) | End in Seq (1 Indexed) | Strand | Chrm | Exon | Start in Chrm (0 Indexed) | End in Chrm (1 Indexed) |
| TTGTAAATAGCTTTTAAAAACTGATGGGAAATGCTGTTTGGAAGTGGAATTGTTGAACCA | 2515 | 2574 | - | chr20 | 1 | 34636358 | 34636418 |
| eCLIP Fold-Enrichment | Binding Protein | Cell Line | Strand | Chrm | | Start in Chrm (0 Indexed) | End in Chrm (1 Indexed) |
| 2.26022265897 | ddx55 (bg=12.35%) | HepG2 | - | chr20 | | 34636324 | 34636363 |
| 3.42984423938 | FTO (bg=7.53%) | HepG2 | - | chr20 | | 34636328 | 34636379 |
| 2.8161630582 | GRSF1 (bg=3.8%) | HepG2 | - | chr20 | | 34636296 | 34636387 |
| 3.21493573025 | GRSF1 (bg=3.8%) | HepG2 | - | chr20 | | 34636297 | 34636392 |
| 2.56685847973 | KHSRP (bg=8.1%) | HepG2 | - | chr20 | | 34636314 | 34636373 |
| 2.50219244229 | KHSRP (bg=8.1%) | HepG2 | - | chr20 | | 34636337 | 34636385 |
| 3.51918441615 | pum1 (bg=29.85%) | K562 | - | chr20 | | 34636330 | 34636377 |
| 3.76814171011 | pum1 (bg=29.85%) | K562 | - | chr20 | | 34636342 | 34636396 |
| 2.58386452955 | SUB1 (bg=9.24%) | HepG2 | - | chr20 | | 34636316 | 34636364 |
| 2.41773886883 | SUB1 (bg=9.24%) | HepG2 | - | chr20 | | 34636329 | 34636369 |

  
  

| Match 220 in HUMAN | | | | | | | |
| --- | --- | --- | --- | --- | --- | --- | --- |
| Motif | Start in Seq (1 Indexed) | End in Seq (1 Indexed) | Strand | Chrm | Exon | Start in Chrm (0 Indexed) | End in Chrm (1 Indexed) |
| ACTGATGGGAAAT | 2534 | 2546 | - | chr20 | 1 | 34636386 | 34636399 |
| eCLIP Fold-Enrichment | Binding Protein | Cell Line | Strand | Chrm | | Start in Chrm (0 Indexed) | End in Chrm (1 Indexed) |
| 2.8161630582 | GRSF1 (bg=3.8%) | HepG2 | - | chr20 | | 34636296 | 34636387 |
| 3.21493573025 | GRSF1 (bg=3.8%) | HepG2 | - | chr20 | | 34636297 | 34636392 |
| 3.76814171011 | pum1 (bg=29.85%) | K562 | - | chr20 | | 34636342 | 34636396 |

  
  

| Match 221 in HUMAN | | | | | | | |
| --- | --- | --- | --- | --- | --- | --- | --- |
| Motif | Start in Seq (1 Indexed) | End in Seq (1 Indexed) | Strand | Chrm | Exon | Start in Chrm (0 Indexed) | End in Chrm (1 Indexed) |
| ATGGGAAAT | 2538 | 2546 | - | chr20 | 1 | 34636386 | 34636395 |
| eCLIP Fold-Enrichment | Binding Protein | Cell Line | Strand | Chrm | | Start in Chrm (0 Indexed) | End in Chrm (1 Indexed) |
| 2.8161630582 | GRSF1 (bg=3.8%) | HepG2 | - | chr20 | | 34636296 | 34636387 |
| 3.21493573025 | GRSF1 (bg=3.8%) | HepG2 | - | chr20 | | 34636297 | 34636392 |
| 3.76814171011 | pum1 (bg=29.85%) | K562 | - | chr20 | | 34636342 | 34636396 |

  
  

| Match 222 in HUMAN | | | | | | | |
| --- | --- | --- | --- | --- | --- | --- | --- |
| Motif | Start in Seq (1 Indexed) | End in Seq (1 Indexed) | Strand | Chrm | Exon | Start in Chrm (0 Indexed) | End in Chrm (1 Indexed) |
| GGAAAT | 2541 | 2546 | - | chr20 | 1 | 34636386 | 34636392 |
| eCLIP Fold-Enrichment | Binding Protein | Cell Line | Strand | Chrm | | Start in Chrm (0 Indexed) | End in Chrm (1 Indexed) |
| 2.8161630582 | GRSF1 (bg=3.8%) | HepG2 | - | chr20 | | 34636296 | 34636387 |
| 3.21493573025 | GRSF1 (bg=3.8%) | HepG2 | - | chr20 | | 34636297 | 34636392 |
| 3.76814171011 | pum1 (bg=29.85%) | K562 | - | chr20 | | 34636342 | 34636396 |

  
  

| Match 223 in HUMAN | | | | | | | |
| --- | --- | --- | --- | --- | --- | --- | --- |
| Motif | Start in Seq (1 Indexed) | End in Seq (1 Indexed) | Strand | Chrm | Exon | Start in Chrm (0 Indexed) | End in Chrm (1 Indexed) |
| GAATTG | 2561 | 2566 | - | chr20 | 1 | 34636366 | 34636372 |
| eCLIP Fold-Enrichment | Binding Protein | Cell Line | Strand | Chrm | | Start in Chrm (0 Indexed) | End in Chrm (1 Indexed) |
| 3.42984423938 | FTO (bg=7.53%) | HepG2 | - | chr20 | | 34636328 | 34636379 |
| 2.8161630582 | GRSF1 (bg=3.8%) | HepG2 | - | chr20 | | 34636296 | 34636387 |
| 3.21493573025 | GRSF1 (bg=3.8%) | HepG2 | - | chr20 | | 34636297 | 34636392 |
| 2.56685847973 | KHSRP (bg=8.1%) | HepG2 | - | chr20 | | 34636314 | 34636373 |
| 2.50219244229 | KHSRP (bg=8.1%) | HepG2 | - | chr20 | | 34636337 | 34636385 |
| 3.51918441615 | pum1 (bg=29.85%) | K562 | - | chr20 | | 34636330 | 34636377 |
| 3.76814171011 | pum1 (bg=29.85%) | K562 | - | chr20 | | 34636342 | 34636396 |
| 2.41773886883 | SUB1 (bg=9.24%) | HepG2 | - | chr20 | | 34636329 | 34636369 |

  
  

| Match 224 in HUMAN | | | | | | | |
| --- | --- | --- | --- | --- | --- | --- | --- |
| Motif | Start in Seq (1 Indexed) | End in Seq (1 Indexed) | Strand | Chrm | Exon | Start in Chrm (0 Indexed) | End in Chrm (1 Indexed) |
| CTGGGAGGTGGGAGGGAA | 2576 | 2593 | - | chr20 | 1 | 34636339 | 34636357 |
| eCLIP Fold-Enrichment | Binding Protein | Cell Line | Strand | Chrm | | Start in Chrm (0 Indexed) | End in Chrm (1 Indexed) |
| 2.26022265897 | ddx55 (bg=12.35%) | HepG2 | - | chr20 | | 34636324 | 34636363 |
| 3.42984423938 | FTO (bg=7.53%) | HepG2 | - | chr20 | | 34636328 | 34636379 |
| 2.8161630582 | GRSF1 (bg=3.8%) | HepG2 | - | chr20 | | 34636296 | 34636387 |
| 3.21493573025 | GRSF1 (bg=3.8%) | HepG2 | - | chr20 | | 34636297 | 34636392 |
| 2.56685847973 | KHSRP (bg=8.1%) | HepG2 | - | chr20 | | 34636314 | 34636373 |
| 2.50219244229 | KHSRP (bg=8.1%) | HepG2 | - | chr20 | | 34636337 | 34636385 |
| 4.31104015127 | pum1 (bg=29.85%) | K562 | - | chr20 | | 34636305 | 34636342 |
| 3.51918441615 | pum1 (bg=29.85%) | K562 | - | chr20 | | 34636330 | 34636377 |
| 3.76814171011 | pum1 (bg=29.85%) | K562 | - | chr20 | | 34636342 | 34636396 |
| 2.58386452955 | SUB1 (bg=9.24%) | HepG2 | - | chr20 | | 34636316 | 34636364 |
| 2.41773886883 | SUB1 (bg=9.24%) | HepG2 | - | chr20 | | 34636329 | 34636369 |

  
  

| Match 225 in HUMAN | | | | | | | |
| --- | --- | --- | --- | --- | --- | --- | --- |
| Motif | Start in Seq (1 Indexed) | End in Seq (1 Indexed) | Strand | Chrm | Exon | Start in Chrm (0 Indexed) | End in Chrm (1 Indexed) |
| GGGAGGTGGGAGGGAA | 2578 | 2593 | - | chr20 | 1 | 34636339 | 34636355 |
| eCLIP Fold-Enrichment | Binding Protein | Cell Line | Strand | Chrm | | Start in Chrm (0 Indexed) | End in Chrm (1 Indexed) |
| 2.26022265897 | ddx55 (bg=12.35%) | HepG2 | - | chr20 | | 34636324 | 34636363 |
| 3.42984423938 | FTO (bg=7.53%) | HepG2 | - | chr20 | | 34636328 | 34636379 |
| 2.8161630582 | GRSF1 (bg=3.8%) | HepG2 | - | chr20 | | 34636296 | 34636387 |
| 3.21493573025 | GRSF1 (bg=3.8%) | HepG2 | - | chr20 | | 34636297 | 34636392 |
| 2.56685847973 | KHSRP (bg=8.1%) | HepG2 | - | chr20 | | 34636314 | 34636373 |
| 2.50219244229 | KHSRP (bg=8.1%) | HepG2 | - | chr20 | | 34636337 | 34636385 |
| 4.31104015127 | pum1 (bg=29.85%) | K562 | - | chr20 | | 34636305 | 34636342 |
| 3.51918441615 | pum1 (bg=29.85%) | K562 | - | chr20 | | 34636330 | 34636377 |
| 3.76814171011 | pum1 (bg=29.85%) | K562 | - | chr20 | | 34636342 | 34636396 |
| 2.58386452955 | SUB1 (bg=9.24%) | HepG2 | - | chr20 | | 34636316 | 34636364 |
| 2.41773886883 | SUB1 (bg=9.24%) | HepG2 | - | chr20 | | 34636329 | 34636369 |

  
  

| Match 226 in HUMAN | | | | | | | |
| --- | --- | --- | --- | --- | --- | --- | --- |
| Motif | Start in Seq (1 Indexed) | End in Seq (1 Indexed) | Strand | Chrm | Exon | Start in Chrm (0 Indexed) | End in Chrm (1 Indexed) |
| GTGGGAGGGAA | 2583 | 2593 | - | chr20 | 1 | 34636339 | 34636350 |
| eCLIP Fold-Enrichment | Binding Protein | Cell Line | Strand | Chrm | | Start in Chrm (0 Indexed) | End in Chrm (1 Indexed) |
| 2.26022265897 | ddx55 (bg=12.35%) | HepG2 | - | chr20 | | 34636324 | 34636363 |
| 3.42984423938 | FTO (bg=7.53%) | HepG2 | - | chr20 | | 34636328 | 34636379 |
| 2.8161630582 | GRSF1 (bg=3.8%) | HepG2 | - | chr20 | | 34636296 | 34636387 |
| 3.21493573025 | GRSF1 (bg=3.8%) | HepG2 | - | chr20 | | 34636297 | 34636392 |
| 2.56685847973 | KHSRP (bg=8.1%) | HepG2 | - | chr20 | | 34636314 | 34636373 |
| 2.50219244229 | KHSRP (bg=8.1%) | HepG2 | - | chr20 | | 34636337 | 34636385 |
| 4.31104015127 | pum1 (bg=29.85%) | K562 | - | chr20 | | 34636305 | 34636342 |
| 3.51918441615 | pum1 (bg=29.85%) | K562 | - | chr20 | | 34636330 | 34636377 |
| 3.76814171011 | pum1 (bg=29.85%) | K562 | - | chr20 | | 34636342 | 34636396 |
| 2.58386452955 | SUB1 (bg=9.24%) | HepG2 | - | chr20 | | 34636316 | 34636364 |
| 2.41773886883 | SUB1 (bg=9.24%) | HepG2 | - | chr20 | | 34636329 | 34636369 |

  
  

| Match 227 in HUMAN | | | | | | | |
| --- | --- | --- | --- | --- | --- | --- | --- |
| Motif | Start in Seq (1 Indexed) | End in Seq (1 Indexed) | Strand | Chrm | Exon | Start in Chrm (0 Indexed) | End in Chrm (1 Indexed) |
| TGCAAA | 2599 | 2604 | - | chr20 | 1 | 34636328 | 34636334 |
| eCLIP Fold-Enrichment | Binding Protein | Cell Line | Strand | Chrm | | Start in Chrm (0 Indexed) | End in Chrm (1 Indexed) |
| 2.26022265897 | ddx55 (bg=12.35%) | HepG2 | - | chr20 | | 34636324 | 34636363 |
| 3.42984423938 | FTO (bg=7.53%) | HepG2 | - | chr20 | | 34636328 | 34636379 |
| 4.46746348283 | fubp3 (bg=23.31%) | HepG2 | - | chr20 | | 34636323 | 34636334 |
| 2.8161630582 | GRSF1 (bg=3.8%) | HepG2 | - | chr20 | | 34636296 | 34636387 |
| 3.21493573025 | GRSF1 (bg=3.8%) | HepG2 | - | chr20 | | 34636297 | 34636392 |
| 3.05062906699 | KHSRP (bg=8.1%) | HepG2 | - | chr20 | | 34636310 | 34636337 |
| 2.56685847973 | KHSRP (bg=8.1%) | HepG2 | - | chr20 | | 34636314 | 34636373 |
| 4.31104015127 | pum1 (bg=29.85%) | K562 | - | chr20 | | 34636305 | 34636342 |
| 3.51918441615 | pum1 (bg=29.85%) | K562 | - | chr20 | | 34636330 | 34636377 |
| 2.58386452955 | SUB1 (bg=9.24%) | HepG2 | - | chr20 | | 34636316 | 34636364 |
| 2.41773886883 | SUB1 (bg=9.24%) | HepG2 | - | chr20 | | 34636329 | 34636369 |

  
  

| Match 228 in HUMAN | | | | | | | |
| --- | --- | --- | --- | --- | --- | --- | --- |
| Motif | Start in Seq (1 Indexed) | End in Seq (1 Indexed) | Strand | Chrm | Exon | Start in Chrm (0 Indexed) | End in Chrm (1 Indexed) |
| GGTGTTTTGCCATTGTTTATTAGAAAATTTCAGCTTAATCCATTG | 2606 | 2650 | - | chr20 | 1 | 34636282 | 34636327 |
| eCLIP Fold-Enrichment | Binding Protein | Cell Line | Strand | Chrm | | Start in Chrm (0 Indexed) | End in Chrm (1 Indexed) |
| 2.85027490535 | AARS (bg=2.33%) | K562 | - | chr20 | | 34636248 | 34636302 |
| 2.52238257884 | AARS (bg=2.33%) | K562 | - | chr20 | | 34636256 | 34636298 |
| 2.47360770411 | CPEB4 (bg=2.3%) | K562 | - | chr20 | | 34636256 | 34636304 |
| 2.30913225945 | ddx55 (bg=12.35%) | HepG2 | - | chr20 | | 34636284 | 34636286 |
| 2.26022265897 | ddx55 (bg=12.35%) | HepG2 | - | chr20 | | 34636324 | 34636363 |
| 2.11464845294 | ddx6 (bg=23.92%) | K562 | - | chr20 | | 34636248 | 34636297 |
| 2.18810358807 | dgcr8 (bg=19.31%) | HepG2 | - | chr20 | | 34636256 | 34636300 |
| 4.7044289009 | dgcr8 (bg=19.31%) | SM-9MVZL | - | chr20 | | 34636256 | 34636300 |
| 2.79225712105 | fam120a (bg=18.43%) | K562 | - | chr20 | | 34636256 | 34636306 |
| 3.08744795966 | FASTKD2 (bg=7.73%) | K562 | - | chr20 | | 34636224 | 34636296 |
| 2.04019932576 | FASTKD2 (bg=7.73%) | HepG2 | - | chr20 | | 34636247 | 34636298 |
| 5.05865793727 | fubp3 (bg=23.31%) | HepG2 | - | chr20 | | 34636230 | 34636303 |
| 5.27895038673 | fubp3 (bg=23.31%) | HepG2 | - | chr20 | | 34636255 | 34636301 |
| 4.46746348283 | fubp3 (bg=23.31%) | HepG2 | - | chr20 | | 34636323 | 34636334 |
| 2.80929692898 | GRSF1 (bg=3.8%) | HepG2 | - | chr20 | | 34636260 | 34636297 |
| 2.8161630582 | GRSF1 (bg=3.8%) | HepG2 | - | chr20 | | 34636296 | 34636387 |
| 3.21493573025 | GRSF1 (bg=3.8%) | HepG2 | - | chr20 | | 34636297 | 34636392 |
| 4.29394080699 | igf2bp1 (bg=11.67%) | K562 | - | chr20 | | 34636220 | 34636289 |
| 4.61386368096 | igf2bp1 (bg=11.67%) | K562 | - | chr20 | | 34636244 | 34636298 |
| 2.77859916528 | KHSRP (bg=8.1%) | K562 | - | chr20 | | 34636234 | 34636308 |
| 3.06126059463 | KHSRP (bg=8.1%) | K562 | - | chr20 | | 34636236 | 34636309 |
| 4.50797737112 | KHSRP (bg=8.1%) | HepG2 | - | chr20 | | 34636236 | 34636310 |
| 4.00157834487 | KHSRP (bg=8.1%) | HepG2 | - | chr20 | | 34636244 | 34636309 |
| 3.05062906699 | KHSRP (bg=8.1%) | HepG2 | - | chr20 | | 34636310 | 34636337 |
| 2.56685847973 | KHSRP (bg=8.1%) | HepG2 | - | chr20 | | 34636314 | 34636373 |
| 3.31272007535 | NCBP2 (bg=2.92%) | K562 | - | chr20 | | 34636246 | 34636290 |
| 2.55040005955 | NKRF (bg=3.64%) | HepG2 | - | chr20 | | 34636265 | 34636288 |
| 4.52512901379 | NOLC1 (bg=6.58%) | HepG2 | - | chr20 | | 34636229 | 34636286 |
| 4.28616548263 | pum1 (bg=29.85%) | K562 | - | chr20 | | 34636247 | 34636305 |
| 4.0895001409 | pum1 (bg=29.85%) | K562 | - | chr20 | | 34636255 | 34636296 |
| 4.31104015127 | pum1 (bg=29.85%) | K562 | - | chr20 | | 34636305 | 34636342 |
| 5.36962739238 | pum2 (bg=21.55%) | K562 | - | chr20 | | 34636257 | 34636303 |
| 6.53500623886 | pum2 (bg=21.55%) | K562 | - | chr20 | | 34636258 | 34636302 |
| 3.56899230966 | sf3a3 (bg=12.13%) | HepG2 | - | chr20 | | 34636250 | 34636290 |
| 2.35507881794 | sf3a3 (bg=12.13%) | HepG2 | - | chr20 | | 34636266 | 34636296 |
| 2.58386452955 | SUB1 (bg=9.24%) | HepG2 | - | chr20 | | 34636316 | 34636364 |
| 2.52852546562 | TBRG4 (bg=7.0%) | HepG2 | - | chr20 | | 34636244 | 34636291 |
| 2.42539582334 | TBRG4 (bg=7.0%) | HepG2 | - | chr20 | | 34636255 | 34636302 |
| 4.52148423413 | tia1 (bg=16.04%) | HepG2 | - | chr20 | | 34636250 | 34636298 |
| 3.16510636392 | tia1 (bg=16.04%) | HepG2 | - | chr20 | | 34636263 | 34636298 |
| 2.4230754283 | tia1 (bg=16.04%) | K562 | - | chr20 | | 34636265 | 34636294 |
| 4.31372047946 | tial1 (bg=14.09%) | HepG2 | - | chr20 | | 34636258 | 34636296 |
| 4.37431259406 | tial1 (bg=14.09%) | HepG2 | - | chr20 | | 34636258 | 34636298 |
| 2.21199590194 | ZC3H11A (bg=6.25%) | HepG2 | - | chr20 | | 34636239 | 34636297 |
| 2.21199590194 | ZC3H11A (bg=6.25%) | HepG2 | - | chr20 | | 34636239 | 34636297 |
| 2.51210298384 | ZC3H11A (bg=6.25%) | K562 | - | chr20 | | 34636264 | 34636300 |
| 2.51210298384 | ZC3H11A (bg=6.25%) | K562 | - | chr20 | | 34636264 | 34636300 |

  
  

| Match 229 in HUMAN | | | | | | | |
| --- | --- | --- | --- | --- | --- | --- | --- |
| Motif | Start in Seq (1 Indexed) | End in Seq (1 Indexed) | Strand | Chrm | Exon | Start in Chrm (0 Indexed) | End in Chrm (1 Indexed) |
| TTTTGC | 2610 | 2615 | - | chr20 | 1 | 34636317 | 34636323 |
| eCLIP Fold-Enrichment | Binding Protein | Cell Line | Strand | Chrm | | Start in Chrm (0 Indexed) | End in Chrm (1 Indexed) |
| 4.46746348283 | fubp3 (bg=23.31%) | HepG2 | - | chr20 | | 34636323 | 34636334 |
| 2.8161630582 | GRSF1 (bg=3.8%) | HepG2 | - | chr20 | | 34636296 | 34636387 |
| 3.21493573025 | GRSF1 (bg=3.8%) | HepG2 | - | chr20 | | 34636297 | 34636392 |
| 3.05062906699 | KHSRP (bg=8.1%) | HepG2 | - | chr20 | | 34636310 | 34636337 |
| 2.56685847973 | KHSRP (bg=8.1%) | HepG2 | - | chr20 | | 34636314 | 34636373 |
| 4.31104015127 | pum1 (bg=29.85%) | K562 | - | chr20 | | 34636305 | 34636342 |
| 2.58386452955 | SUB1 (bg=9.24%) | HepG2 | - | chr20 | | 34636316 | 34636364 |

  
  

| Match 230 in HUMAN | | | | | | | |
| --- | --- | --- | --- | --- | --- | --- | --- |
| Motif | Start in Seq (1 Indexed) | End in Seq (1 Indexed) | Strand | Chrm | Exon | Start in Chrm (0 Indexed) | End in Chrm (1 Indexed) |
| AAATTTCAGCTTA | 2630 | 2642 | - | chr20 | 1 | 34636290 | 34636303 |
| eCLIP Fold-Enrichment | Binding Protein | Cell Line | Strand | Chrm | | Start in Chrm (0 Indexed) | End in Chrm (1 Indexed) |
| 2.85027490535 | AARS (bg=2.33%) | K562 | - | chr20 | | 34636248 | 34636302 |
| 2.52238257884 | AARS (bg=2.33%) | K562 | - | chr20 | | 34636256 | 34636298 |
| 2.47360770411 | CPEB4 (bg=2.3%) | K562 | - | chr20 | | 34636256 | 34636304 |
| 2.11464845294 | ddx6 (bg=23.92%) | K562 | - | chr20 | | 34636248 | 34636297 |
| 2.18810358807 | dgcr8 (bg=19.31%) | HepG2 | - | chr20 | | 34636256 | 34636300 |
| 4.7044289009 | dgcr8 (bg=19.31%) | SM-9MVZL | - | chr20 | | 34636256 | 34636300 |
| 2.79225712105 | fam120a (bg=18.43%) | K562 | - | chr20 | | 34636256 | 34636306 |
| 3.08744795966 | FASTKD2 (bg=7.73%) | K562 | - | chr20 | | 34636224 | 34636296 |
| 2.04019932576 | FASTKD2 (bg=7.73%) | HepG2 | - | chr20 | | 34636247 | 34636298 |
| 5.05865793727 | fubp3 (bg=23.31%) | HepG2 | - | chr20 | | 34636230 | 34636303 |
| 5.27895038673 | fubp3 (bg=23.31%) | HepG2 | - | chr20 | | 34636255 | 34636301 |
| 2.80929692898 | GRSF1 (bg=3.8%) | HepG2 | - | chr20 | | 34636260 | 34636297 |
| 2.8161630582 | GRSF1 (bg=3.8%) | HepG2 | - | chr20 | | 34636296 | 34636387 |
| 3.21493573025 | GRSF1 (bg=3.8%) | HepG2 | - | chr20 | | 34636297 | 34636392 |
| 4.61386368096 | igf2bp1 (bg=11.67%) | K562 | - | chr20 | | 34636244 | 34636298 |
| 2.77859916528 | KHSRP (bg=8.1%) | K562 | - | chr20 | | 34636234 | 34636308 |
| 3.06126059463 | KHSRP (bg=8.1%) | K562 | - | chr20 | | 34636236 | 34636309 |
| 4.50797737112 | KHSRP (bg=8.1%) | HepG2 | - | chr20 | | 34636236 | 34636310 |
| 4.00157834487 | KHSRP (bg=8.1%) | HepG2 | - | chr20 | | 34636244 | 34636309 |
| 3.31272007535 | NCBP2 (bg=2.92%) | K562 | - | chr20 | | 34636246 | 34636290 |
| 4.28616548263 | pum1 (bg=29.85%) | K562 | - | chr20 | | 34636247 | 34636305 |
| 4.0895001409 | pum1 (bg=29.85%) | K562 | - | chr20 | | 34636255 | 34636296 |
| 5.36962739238 | pum2 (bg=21.55%) | K562 | - | chr20 | | 34636257 | 34636303 |
| 6.53500623886 | pum2 (bg=21.55%) | K562 | - | chr20 | | 34636258 | 34636302 |
| 3.56899230966 | sf3a3 (bg=12.13%) | HepG2 | - | chr20 | | 34636250 | 34636290 |
| 2.35507881794 | sf3a3 (bg=12.13%) | HepG2 | - | chr20 | | 34636266 | 34636296 |
| 2.52852546562 | TBRG4 (bg=7.0%) | HepG2 | - | chr20 | | 34636244 | 34636291 |
| 2.42539582334 | TBRG4 (bg=7.0%) | HepG2 | - | chr20 | | 34636255 | 34636302 |
| 4.52148423413 | tia1 (bg=16.04%) | HepG2 | - | chr20 | | 34636250 | 34636298 |
| 3.16510636392 | tia1 (bg=16.04%) | HepG2 | - | chr20 | | 34636263 | 34636298 |
| 2.4230754283 | tia1 (bg=16.04%) | K562 | - | chr20 | | 34636265 | 34636294 |
| 4.31372047946 | tial1 (bg=14.09%) | HepG2 | - | chr20 | | 34636258 | 34636296 |
| 4.37431259406 | tial1 (bg=14.09%) | HepG2 | - | chr20 | | 34636258 | 34636298 |
| 2.21199590194 | ZC3H11A (bg=6.25%) | HepG2 | - | chr20 | | 34636239 | 34636297 |
| 2.21199590194 | ZC3H11A (bg=6.25%) | HepG2 | - | chr20 | | 34636239 | 34636297 |
| 2.51210298384 | ZC3H11A (bg=6.25%) | K562 | - | chr20 | | 34636264 | 34636300 |
| 2.51210298384 | ZC3H11A (bg=6.25%) | K562 | - | chr20 | | 34636264 | 34636300 |

  
  

| Match 231 in HUMAN | | | | | | | |
| --- | --- | --- | --- | --- | --- | --- | --- |
| Motif | Start in Seq (1 Indexed) | End in Seq (1 Indexed) | Strand | Chrm | Exon | Start in Chrm (0 Indexed) | End in Chrm (1 Indexed) |
| ATTTCA | 2632 | 2637 | - | chr20 | 1 | 34636295 | 34636301 |
| eCLIP Fold-Enrichment | Binding Protein | Cell Line | Strand | Chrm | | Start in Chrm (0 Indexed) | End in Chrm (1 Indexed) |
| 2.85027490535 | AARS (bg=2.33%) | K562 | - | chr20 | | 34636248 | 34636302 |
| 2.52238257884 | AARS (bg=2.33%) | K562 | - | chr20 | | 34636256 | 34636298 |
| 2.47360770411 | CPEB4 (bg=2.3%) | K562 | - | chr20 | | 34636256 | 34636304 |
| 2.11464845294 | ddx6 (bg=23.92%) | K562 | - | chr20 | | 34636248 | 34636297 |
| 2.18810358807 | dgcr8 (bg=19.31%) | HepG2 | - | chr20 | | 34636256 | 34636300 |
| 4.7044289009 | dgcr8 (bg=19.31%) | SM-9MVZL | - | chr20 | | 34636256 | 34636300 |
| 2.79225712105 | fam120a (bg=18.43%) | K562 | - | chr20 | | 34636256 | 34636306 |
| 3.08744795966 | FASTKD2 (bg=7.73%) | K562 | - | chr20 | | 34636224 | 34636296 |
| 2.04019932576 | FASTKD2 (bg=7.73%) | HepG2 | - | chr20 | | 34636247 | 34636298 |
| 5.05865793727 | fubp3 (bg=23.31%) | HepG2 | - | chr20 | | 34636230 | 34636303 |
| 5.27895038673 | fubp3 (bg=23.31%) | HepG2 | - | chr20 | | 34636255 | 34636301 |
| 2.80929692898 | GRSF1 (bg=3.8%) | HepG2 | - | chr20 | | 34636260 | 34636297 |
| 2.8161630582 | GRSF1 (bg=3.8%) | HepG2 | - | chr20 | | 34636296 | 34636387 |
| 3.21493573025 | GRSF1 (bg=3.8%) | HepG2 | - | chr20 | | 34636297 | 34636392 |
| 4.61386368096 | igf2bp1 (bg=11.67%) | K562 | - | chr20 | | 34636244 | 34636298 |
| 2.77859916528 | KHSRP (bg=8.1%) | K562 | - | chr20 | | 34636234 | 34636308 |
| 3.06126059463 | KHSRP (bg=8.1%) | K562 | - | chr20 | | 34636236 | 34636309 |
| 4.50797737112 | KHSRP (bg=8.1%) | HepG2 | - | chr20 | | 34636236 | 34636310 |
| 4.00157834487 | KHSRP (bg=8.1%) | HepG2 | - | chr20 | | 34636244 | 34636309 |
| 4.28616548263 | pum1 (bg=29.85%) | K562 | - | chr20 | | 34636247 | 34636305 |
| 4.0895001409 | pum1 (bg=29.85%) | K562 | - | chr20 | | 34636255 | 34636296 |
| 5.36962739238 | pum2 (bg=21.55%) | K562 | - | chr20 | | 34636257 | 34636303 |
| 6.53500623886 | pum2 (bg=21.55%) | K562 | - | chr20 | | 34636258 | 34636302 |
| 2.35507881794 | sf3a3 (bg=12.13%) | HepG2 | - | chr20 | | 34636266 | 34636296 |
| 2.42539582334 | TBRG4 (bg=7.0%) | HepG2 | - | chr20 | | 34636255 | 34636302 |
| 4.52148423413 | tia1 (bg=16.04%) | HepG2 | - | chr20 | | 34636250 | 34636298 |
| 3.16510636392 | tia1 (bg=16.04%) | HepG2 | - | chr20 | | 34636263 | 34636298 |
| 4.31372047946 | tial1 (bg=14.09%) | HepG2 | - | chr20 | | 34636258 | 34636296 |
| 4.37431259406 | tial1 (bg=14.09%) | HepG2 | - | chr20 | | 34636258 | 34636298 |
| 2.21199590194 | ZC3H11A (bg=6.25%) | HepG2 | - | chr20 | | 34636239 | 34636297 |
| 2.21199590194 | ZC3H11A (bg=6.25%) | HepG2 | - | chr20 | | 34636239 | 34636297 |
| 2.51210298384 | ZC3H11A (bg=6.25%) | K562 | - | chr20 | | 34636264 | 34636300 |
| 2.51210298384 | ZC3H11A (bg=6.25%) | K562 | - | chr20 | | 34636264 | 34636300 |

  
  

| Match 232 in HUMAN | | | | | | | |
| --- | --- | --- | --- | --- | --- | --- | --- |
| Motif | Start in Seq (1 Indexed) | End in Seq (1 Indexed) | Strand | Chrm | Exon | Start in Chrm (0 Indexed) | End in Chrm (1 Indexed) |
| ATTTCAGCTTA | 2632 | 2642 | - | chr20 | 1 | 34636290 | 34636301 |
| eCLIP Fold-Enrichment | Binding Protein | Cell Line | Strand | Chrm | | Start in Chrm (0 Indexed) | End in Chrm (1 Indexed) |
| 2.85027490535 | AARS (bg=2.33%) | K562 | - | chr20 | | 34636248 | 34636302 |
| 2.52238257884 | AARS (bg=2.33%) | K562 | - | chr20 | | 34636256 | 34636298 |
| 2.47360770411 | CPEB4 (bg=2.3%) | K562 | - | chr20 | | 34636256 | 34636304 |
| 2.11464845294 | ddx6 (bg=23.92%) | K562 | - | chr20 | | 34636248 | 34636297 |
| 2.18810358807 | dgcr8 (bg=19.31%) | HepG2 | - | chr20 | | 34636256 | 34636300 |
| 4.7044289009 | dgcr8 (bg=19.31%) | SM-9MVZL | - | chr20 | | 34636256 | 34636300 |
| 2.79225712105 | fam120a (bg=18.43%) | K562 | - | chr20 | | 34636256 | 34636306 |
| 3.08744795966 | FASTKD2 (bg=7.73%) | K562 | - | chr20 | | 34636224 | 34636296 |
| 2.04019932576 | FASTKD2 (bg=7.73%) | HepG2 | - | chr20 | | 34636247 | 34636298 |
| 5.05865793727 | fubp3 (bg=23.31%) | HepG2 | - | chr20 | | 34636230 | 34636303 |
| 5.27895038673 | fubp3 (bg=23.31%) | HepG2 | - | chr20 | | 34636255 | 34636301 |
| 2.80929692898 | GRSF1 (bg=3.8%) | HepG2 | - | chr20 | | 34636260 | 34636297 |
| 2.8161630582 | GRSF1 (bg=3.8%) | HepG2 | - | chr20 | | 34636296 | 34636387 |
| 3.21493573025 | GRSF1 (bg=3.8%) | HepG2 | - | chr20 | | 34636297 | 34636392 |
| 4.61386368096 | igf2bp1 (bg=11.67%) | K562 | - | chr20 | | 34636244 | 34636298 |
| 2.77859916528 | KHSRP (bg=8.1%) | K562 | - | chr20 | | 34636234 | 34636308 |
| 3.06126059463 | KHSRP (bg=8.1%) | K562 | - | chr20 | | 34636236 | 34636309 |
| 4.50797737112 | KHSRP (bg=8.1%) | HepG2 | - | chr20 | | 34636236 | 34636310 |
| 4.00157834487 | KHSRP (bg=8.1%) | HepG2 | - | chr20 | | 34636244 | 34636309 |
| 3.31272007535 | NCBP2 (bg=2.92%) | K562 | - | chr20 | | 34636246 | 34636290 |
| 4.28616548263 | pum1 (bg=29.85%) | K562 | - | chr20 | | 34636247 | 34636305 |
| 4.0895001409 | pum1 (bg=29.85%) | K562 | - | chr20 | | 34636255 | 34636296 |
| 5.36962739238 | pum2 (bg=21.55%) | K562 | - | chr20 | | 34636257 | 34636303 |
| 6.53500623886 | pum2 (bg=21.55%) | K562 | - | chr20 | | 34636258 | 34636302 |
| 3.56899230966 | sf3a3 (bg=12.13%) | HepG2 | - | chr20 | | 34636250 | 34636290 |
| 2.35507881794 | sf3a3 (bg=12.13%) | HepG2 | - | chr20 | | 34636266 | 34636296 |
| 2.52852546562 | TBRG4 (bg=7.0%) | HepG2 | - | chr20 | | 34636244 | 34636291 |
| 2.42539582334 | TBRG4 (bg=7.0%) | HepG2 | - | chr20 | | 34636255 | 34636302 |
| 4.52148423413 | tia1 (bg=16.04%) | HepG2 | - | chr20 | | 34636250 | 34636298 |
| 3.16510636392 | tia1 (bg=16.04%) | HepG2 | - | chr20 | | 34636263 | 34636298 |
| 2.4230754283 | tia1 (bg=16.04%) | K562 | - | chr20 | | 34636265 | 34636294 |
| 4.31372047946 | tial1 (bg=14.09%) | HepG2 | - | chr20 | | 34636258 | 34636296 |
| 4.37431259406 | tial1 (bg=14.09%) | HepG2 | - | chr20 | | 34636258 | 34636298 |
| 2.21199590194 | ZC3H11A (bg=6.25%) | HepG2 | - | chr20 | | 34636239 | 34636297 |
| 2.21199590194 | ZC3H11A (bg=6.25%) | HepG2 | - | chr20 | | 34636239 | 34636297 |
| 2.51210298384 | ZC3H11A (bg=6.25%) | K562 | - | chr20 | | 34636264 | 34636300 |
| 2.51210298384 | ZC3H11A (bg=6.25%) | K562 | - | chr20 | | 34636264 | 34636300 |

  
  

| Match 233 in HUMAN | | | | | | | |
| --- | --- | --- | --- | --- | --- | --- | --- |
| Motif | Start in Seq (1 Indexed) | End in Seq (1 Indexed) | Strand | Chrm | Exon | Start in Chrm (0 Indexed) | End in Chrm (1 Indexed) |
| TCCATTG | 2644 | 2650 | - | chr20 | 1 | 34636282 | 34636289 |
| eCLIP Fold-Enrichment | Binding Protein | Cell Line | Strand | Chrm | | Start in Chrm (0 Indexed) | End in Chrm (1 Indexed) |
| 2.85027490535 | AARS (bg=2.33%) | K562 | - | chr20 | | 34636248 | 34636302 |
| 2.52238257884 | AARS (bg=2.33%) | K562 | - | chr20 | | 34636256 | 34636298 |
| 2.47360770411 | CPEB4 (bg=2.3%) | K562 | - | chr20 | | 34636256 | 34636304 |
| 2.30913225945 | ddx55 (bg=12.35%) | HepG2 | - | chr20 | | 34636284 | 34636286 |
| 2.11464845294 | ddx6 (bg=23.92%) | K562 | - | chr20 | | 34636248 | 34636297 |
| 2.18810358807 | dgcr8 (bg=19.31%) | HepG2 | - | chr20 | | 34636256 | 34636300 |
| 4.7044289009 | dgcr8 (bg=19.31%) | SM-9MVZL | - | chr20 | | 34636256 | 34636300 |
| 2.79225712105 | fam120a (bg=18.43%) | K562 | - | chr20 | | 34636256 | 34636306 |
| 3.08744795966 | FASTKD2 (bg=7.73%) | K562 | - | chr20 | | 34636224 | 34636296 |
| 2.04019932576 | FASTKD2 (bg=7.73%) | HepG2 | - | chr20 | | 34636247 | 34636298 |
| 5.05865793727 | fubp3 (bg=23.31%) | HepG2 | - | chr20 | | 34636230 | 34636303 |
| 5.27895038673 | fubp3 (bg=23.31%) | HepG2 | - | chr20 | | 34636255 | 34636301 |
| 2.80929692898 | GRSF1 (bg=3.8%) | HepG2 | - | chr20 | | 34636260 | 34636297 |
| 4.29394080699 | igf2bp1 (bg=11.67%) | K562 | - | chr20 | | 34636220 | 34636289 |
| 4.61386368096 | igf2bp1 (bg=11.67%) | K562 | - | chr20 | | 34636244 | 34636298 |
| 2.77859916528 | KHSRP (bg=8.1%) | K562 | - | chr20 | | 34636234 | 34636308 |
| 3.06126059463 | KHSRP (bg=8.1%) | K562 | - | chr20 | | 34636236 | 34636309 |
| 4.50797737112 | KHSRP (bg=8.1%) | HepG2 | - | chr20 | | 34636236 | 34636310 |
| 4.00157834487 | KHSRP (bg=8.1%) | HepG2 | - | chr20 | | 34636244 | 34636309 |
| 3.31272007535 | NCBP2 (bg=2.92%) | K562 | - | chr20 | | 34636246 | 34636290 |
| 2.55040005955 | NKRF (bg=3.64%) | HepG2 | - | chr20 | | 34636265 | 34636288 |
| 4.52512901379 | NOLC1 (bg=6.58%) | HepG2 | - | chr20 | | 34636229 | 34636286 |
| 4.28616548263 | pum1 (bg=29.85%) | K562 | - | chr20 | | 34636247 | 34636305 |
| 4.0895001409 | pum1 (bg=29.85%) | K562 | - | chr20 | | 34636255 | 34636296 |
| 5.36962739238 | pum2 (bg=21.55%) | K562 | - | chr20 | | 34636257 | 34636303 |
| 6.53500623886 | pum2 (bg=21.55%) | K562 | - | chr20 | | 34636258 | 34636302 |
| 3.56899230966 | sf3a3 (bg=12.13%) | HepG2 | - | chr20 | | 34636250 | 34636290 |
| 2.35507881794 | sf3a3 (bg=12.13%) | HepG2 | - | chr20 | | 34636266 | 34636296 |
| 2.52852546562 | TBRG4 (bg=7.0%) | HepG2 | - | chr20 | | 34636244 | 34636291 |
| 2.42539582334 | TBRG4 (bg=7.0%) | HepG2 | - | chr20 | | 34636255 | 34636302 |
| 4.52148423413 | tia1 (bg=16.04%) | HepG2 | - | chr20 | | 34636250 | 34636298 |
| 3.16510636392 | tia1 (bg=16.04%) | HepG2 | - | chr20 | | 34636263 | 34636298 |
| 2.4230754283 | tia1 (bg=16.04%) | K562 | - | chr20 | | 34636265 | 34636294 |
| 4.31372047946 | tial1 (bg=14.09%) | HepG2 | - | chr20 | | 34636258 | 34636296 |
| 4.37431259406 | tial1 (bg=14.09%) | HepG2 | - | chr20 | | 34636258 | 34636298 |
| 2.21199590194 | ZC3H11A (bg=6.25%) | HepG2 | - | chr20 | | 34636239 | 34636297 |
| 2.21199590194 | ZC3H11A (bg=6.25%) | HepG2 | - | chr20 | | 34636239 | 34636297 |
| 2.51210298384 | ZC3H11A (bg=6.25%) | K562 | - | chr20 | | 34636264 | 34636300 |
| 2.51210298384 | ZC3H11A (bg=6.25%) | K562 | - | chr20 | | 34636264 | 34636300 |

  
  

| Match 234 in HUMAN | | | | | | | |
| --- | --- | --- | --- | --- | --- | --- | --- |
| Motif | Start in Seq (1 Indexed) | End in Seq (1 Indexed) | Strand | Chrm | Exon | Start in Chrm (0 Indexed) | End in Chrm (1 Indexed) |
| CCATTG | 2645 | 2650 | - | chr20 | 1 | 34636282 | 34636288 |
| eCLIP Fold-Enrichment | Binding Protein | Cell Line | Strand | Chrm | | Start in Chrm (0 Indexed) | End in Chrm (1 Indexed) |
| 2.85027490535 | AARS (bg=2.33%) | K562 | - | chr20 | | 34636248 | 34636302 |
| 2.52238257884 | AARS (bg=2.33%) | K562 | - | chr20 | | 34636256 | 34636298 |
| 2.47360770411 | CPEB4 (bg=2.3%) | K562 | - | chr20 | | 34636256 | 34636304 |
| 2.30913225945 | ddx55 (bg=12.35%) | HepG2 | - | chr20 | | 34636284 | 34636286 |
| 2.11464845294 | ddx6 (bg=23.92%) | K562 | - | chr20 | | 34636248 | 34636297 |
| 2.18810358807 | dgcr8 (bg=19.31%) | HepG2 | - | chr20 | | 34636256 | 34636300 |
| 4.7044289009 | dgcr8 (bg=19.31%) | SM-9MVZL | - | chr20 | | 34636256 | 34636300 |
| 2.79225712105 | fam120a (bg=18.43%) | K562 | - | chr20 | | 34636256 | 34636306 |
| 3.08744795966 | FASTKD2 (bg=7.73%) | K562 | - | chr20 | | 34636224 | 34636296 |
| 2.04019932576 | FASTKD2 (bg=7.73%) | HepG2 | - | chr20 | | 34636247 | 34636298 |
| 5.05865793727 | fubp3 (bg=23.31%) | HepG2 | - | chr20 | | 34636230 | 34636303 |
| 5.27895038673 | fubp3 (bg=23.31%) | HepG2 | - | chr20 | | 34636255 | 34636301 |
| 2.80929692898 | GRSF1 (bg=3.8%) | HepG2 | - | chr20 | | 34636260 | 34636297 |
| 4.29394080699 | igf2bp1 (bg=11.67%) | K562 | - | chr20 | | 34636220 | 34636289 |
| 4.61386368096 | igf2bp1 (bg=11.67%) | K562 | - | chr20 | | 34636244 | 34636298 |
| 2.77859916528 | KHSRP (bg=8.1%) | K562 | - | chr20 | | 34636234 | 34636308 |
| 3.06126059463 | KHSRP (bg=8.1%) | K562 | - | chr20 | | 34636236 | 34636309 |
| 4.50797737112 | KHSRP (bg=8.1%) | HepG2 | - | chr20 | | 34636236 | 34636310 |
| 4.00157834487 | KHSRP (bg=8.1%) | HepG2 | - | chr20 | | 34636244 | 34636309 |
| 3.31272007535 | NCBP2 (bg=2.92%) | K562 | - | chr20 | | 34636246 | 34636290 |
| 2.55040005955 | NKRF (bg=3.64%) | HepG2 | - | chr20 | | 34636265 | 34636288 |
| 4.52512901379 | NOLC1 (bg=6.58%) | HepG2 | - | chr20 | | 34636229 | 34636286 |
| 4.28616548263 | pum1 (bg=29.85%) | K562 | - | chr20 | | 34636247 | 34636305 |
| 4.0895001409 | pum1 (bg=29.85%) | K562 | - | chr20 | | 34636255 | 34636296 |
| 5.36962739238 | pum2 (bg=21.55%) | K562 | - | chr20 | | 34636257 | 34636303 |
| 6.53500623886 | pum2 (bg=21.55%) | K562 | - | chr20 | | 34636258 | 34636302 |
| 3.56899230966 | sf3a3 (bg=12.13%) | HepG2 | - | chr20 | | 34636250 | 34636290 |
| 2.35507881794 | sf3a3 (bg=12.13%) | HepG2 | - | chr20 | | 34636266 | 34636296 |
| 2.52852546562 | TBRG4 (bg=7.0%) | HepG2 | - | chr20 | | 34636244 | 34636291 |
| 2.42539582334 | TBRG4 (bg=7.0%) | HepG2 | - | chr20 | | 34636255 | 34636302 |
| 4.52148423413 | tia1 (bg=16.04%) | HepG2 | - | chr20 | | 34636250 | 34636298 |
| 3.16510636392 | tia1 (bg=16.04%) | HepG2 | - | chr20 | | 34636263 | 34636298 |
| 2.4230754283 | tia1 (bg=16.04%) | K562 | - | chr20 | | 34636265 | 34636294 |
| 4.31372047946 | tial1 (bg=14.09%) | HepG2 | - | chr20 | | 34636258 | 34636296 |
| 4.37431259406 | tial1 (bg=14.09%) | HepG2 | - | chr20 | | 34636258 | 34636298 |
| 2.21199590194 | ZC3H11A (bg=6.25%) | HepG2 | - | chr20 | | 34636239 | 34636297 |
| 2.21199590194 | ZC3H11A (bg=6.25%) | HepG2 | - | chr20 | | 34636239 | 34636297 |
| 2.51210298384 | ZC3H11A (bg=6.25%) | K562 | - | chr20 | | 34636264 | 34636300 |
| 2.51210298384 | ZC3H11A (bg=6.25%) | K562 | - | chr20 | | 34636264 | 34636300 |

  
  

| Match 235 in HUMAN | | | | | | | |
| --- | --- | --- | --- | --- | --- | --- | --- |
| Motif | Start in Seq (1 Indexed) | End in Seq (1 Indexed) | Strand | Chrm | Exon | Start in Chrm (0 Indexed) | End in Chrm (1 Indexed) |
| TATGTTACATGCATTTCATTTAACTTTGCTATACTGTATATATTGT | 2655 | 2700 | - | chr20 | 1 | 34636232 | 34636278 |
| eCLIP Fold-Enrichment | Binding Protein | Cell Line | Strand | Chrm | | Start in Chrm (0 Indexed) | End in Chrm (1 Indexed) |
| 2.85027490535 | AARS (bg=2.33%) | K562 | - | chr20 | | 34636248 | 34636302 |
| 2.52238257884 | AARS (bg=2.33%) | K562 | - | chr20 | | 34636256 | 34636298 |
| 2.3416111803 | AKAP1 (bg=3.4%) | HepG2 | - | chr20 | | 34636194 | 34636234 |
| 2.47360770411 | CPEB4 (bg=2.3%) | K562 | - | chr20 | | 34636256 | 34636304 |
| 2.45433017499 | ddx55 (bg=12.35%) | HepG2 | - | chr20 | | 34636180 | 34636233 |
| 2.11464845294 | ddx6 (bg=23.92%) | K562 | - | chr20 | | 34636248 | 34636297 |
| 4.47437767302 | dgcr8 (bg=19.31%) | SM-9MVZL | - | chr20 | | 34636178 | 34636281 |
| 3.15569140617 | dgcr8 (bg=19.31%) | K562 | - | chr20 | | 34636207 | 34636233 |
| 3.2700452016 | dgcr8 (bg=19.31%) | HepG2 | - | chr20 | | 34636214 | 34636235 |
| 2.43386000247 | dgcr8 (bg=19.31%) | HepG2 | - | chr20 | | 34636235 | 34636256 |
| 2.18810358807 | dgcr8 (bg=19.31%) | HepG2 | - | chr20 | | 34636256 | 34636300 |
| 4.7044289009 | dgcr8 (bg=19.31%) | SM-9MVZL | - | chr20 | | 34636256 | 34636300 |
| 3.13784604159 | EIF4G2 (bg=0.7%) | K562 | - | chr20 | | 34636209 | 34636247 |
| 2.68792046123 | fam120a (bg=18.43%) | K562 | - | chr20 | | 34636191 | 34636242 |
| 2.79225712105 | fam120a (bg=18.43%) | K562 | - | chr20 | | 34636256 | 34636306 |
| 3.08744795966 | FASTKD2 (bg=7.73%) | K562 | - | chr20 | | 34636224 | 34636296 |
| 2.04019932576 | FASTKD2 (bg=7.73%) | HepG2 | - | chr20 | | 34636247 | 34636298 |
| 5.30751953893 | fubp3 (bg=23.31%) | HepG2 | - | chr20 | | 34636209 | 34636236 |
| 5.05865793727 | fubp3 (bg=23.31%) | HepG2 | - | chr20 | | 34636230 | 34636303 |
| 6.23500703914 | fubp3 (bg=23.31%) | HepG2 | - | chr20 | | 34636236 | 34636255 |
| 5.27895038673 | fubp3 (bg=23.31%) | HepG2 | - | chr20 | | 34636255 | 34636301 |
| 2.80929692898 | GRSF1 (bg=3.8%) | HepG2 | - | chr20 | | 34636260 | 34636297 |
| 3.37670775222 | HNRNPL (bg=3.4%) | HepG2 | - | chr20 | | 34636224 | 34636262 |
| 4.29193558607 | igf2bp1 (bg=11.67%) | K562 | - | chr20 | | 34636191 | 34636244 |
| 4.29394080699 | igf2bp1 (bg=11.67%) | K562 | - | chr20 | | 34636220 | 34636289 |
| 4.61386368096 | igf2bp1 (bg=11.67%) | K562 | - | chr20 | | 34636244 | 34636298 |
| 2.21773905282 | KHSRP (bg=8.1%) | HepG2 | - | chr20 | | 34636187 | 34636236 |
| 2.77859916528 | KHSRP (bg=8.1%) | K562 | - | chr20 | | 34636234 | 34636308 |
| 3.06126059463 | KHSRP (bg=8.1%) | K562 | - | chr20 | | 34636236 | 34636309 |
| 4.50797737112 | KHSRP (bg=8.1%) | HepG2 | - | chr20 | | 34636236 | 34636310 |
| 4.00157834487 | KHSRP (bg=8.1%) | HepG2 | - | chr20 | | 34636244 | 34636309 |
| 3.48264507679 | NCBP2 (bg=2.92%) | K562 | - | chr20 | | 34636214 | 34636238 |
| 3.31272007535 | NCBP2 (bg=2.92%) | K562 | - | chr20 | | 34636246 | 34636290 |
| 3.87232815444 | NKRF (bg=3.64%) | HepG2 | - | chr20 | | 34636198 | 34636232 |
| 2.55040005955 | NKRF (bg=3.64%) | HepG2 | - | chr20 | | 34636265 | 34636288 |
| 4.52512901379 | NOLC1 (bg=6.58%) | HepG2 | - | chr20 | | 34636229 | 34636286 |
| 4.76711192959 | pum1 (bg=29.85%) | K562 | - | chr20 | | 34636178 | 34636255 |
| 4.33549319499 | pum1 (bg=29.85%) | K562 | - | chr20 | | 34636179 | 34636240 |
| 4.28616548263 | pum1 (bg=29.85%) | K562 | - | chr20 | | 34636247 | 34636305 |
| 4.0895001409 | pum1 (bg=29.85%) | K562 | - | chr20 | | 34636255 | 34636296 |
| 6.11439700145 | pum2 (bg=21.55%) | K562 | - | chr20 | | 34636198 | 34636237 |
| 7.59182792087 | pum2 (bg=21.55%) | K562 | - | chr20 | | 34636199 | 34636233 |
| 6.93776540837 | pum2 (bg=21.55%) | K562 | - | chr20 | | 34636233 | 34636258 |
| 5.30440624894 | pum2 (bg=21.55%) | K562 | - | chr20 | | 34636237 | 34636257 |
| 5.36962739238 | pum2 (bg=21.55%) | K562 | - | chr20 | | 34636257 | 34636303 |
| 6.53500623886 | pum2 (bg=21.55%) | K562 | - | chr20 | | 34636258 | 34636302 |
| 3.74343279222 | sf3a3 (bg=12.13%) | HepG2 | - | chr20 | | 34636196 | 34636250 |
| 3.56899230966 | sf3a3 (bg=12.13%) | HepG2 | - | chr20 | | 34636250 | 34636290 |
| 2.35507881794 | sf3a3 (bg=12.13%) | HepG2 | - | chr20 | | 34636266 | 34636296 |
| 2.97278130399 | SF3B1 (bg=6.74%) | K562 | - | chr20 | | 34636196 | 34636272 |
| 2.52852546562 | TBRG4 (bg=7.0%) | HepG2 | - | chr20 | | 34636244 | 34636291 |
| 2.42539582334 | TBRG4 (bg=7.0%) | HepG2 | - | chr20 | | 34636255 | 34636302 |
| 4.1392824623 | tia1 (bg=16.04%) | K562 | - | chr20 | | 34636183 | 34636249 |
| 3.45406622228 | tia1 (bg=16.04%) | K562 | - | chr20 | | 34636193 | 34636249 |
| 4.52148423413 | tia1 (bg=16.04%) | HepG2 | - | chr20 | | 34636250 | 34636298 |
| 3.16510636392 | tia1 (bg=16.04%) | HepG2 | - | chr20 | | 34636263 | 34636298 |
| 2.4230754283 | tia1 (bg=16.04%) | K562 | - | chr20 | | 34636265 | 34636294 |
| 5.18247594618 | tial1 (bg=14.09%) | HepG2 | - | chr20 | | 34636201 | 34636258 |
| 5.14803673823 | tial1 (bg=14.09%) | HepG2 | - | chr20 | | 34636204 | 34636258 |
| 4.31372047946 | tial1 (bg=14.09%) | HepG2 | - | chr20 | | 34636258 | 34636296 |
| 4.37431259406 | tial1 (bg=14.09%) | HepG2 | - | chr20 | | 34636258 | 34636298 |
| 2.23168025505 | WRN (bg=3.31%) | K562 | - | chr20 | | 34636198 | 34636232 |
| 3.47355607885 | ZC3H11A (bg=6.25%) | K562 | - | chr20 | | 34636185 | 34636233 |
| 3.47355607885 | ZC3H11A (bg=6.25%) | K562 | - | chr20 | | 34636185 | 34636233 |
| 3.14953290445 | ZC3H11A (bg=6.25%) | K562 | - | chr20 | | 34636186 | 34636235 |
| 3.14953290445 | ZC3H11A (bg=6.25%) | K562 | - | chr20 | | 34636186 | 34636235 |
| 2.61299556781 | ZC3H11A (bg=6.25%) | HepG2 | - | chr20 | | 34636193 | 34636239 |
| 2.61299556781 | ZC3H11A (bg=6.25%) | HepG2 | - | chr20 | | 34636193 | 34636239 |
| 2.18450287254 | ZC3H11A (bg=6.25%) | HepG2 | - | chr20 | | 34636202 | 34636235 |
| 2.18450287254 | ZC3H11A (bg=6.25%) | HepG2 | - | chr20 | | 34636202 | 34636235 |
| 2.21199590194 | ZC3H11A (bg=6.25%) | HepG2 | - | chr20 | | 34636239 | 34636297 |
| 2.21199590194 | ZC3H11A (bg=6.25%) | HepG2 | - | chr20 | | 34636239 | 34636297 |
| 2.51210298384 | ZC3H11A (bg=6.25%) | K562 | - | chr20 | | 34636264 | 34636300 |
| 2.51210298384 | ZC3H11A (bg=6.25%) | K562 | - | chr20 | | 34636264 | 34636300 |

  
  

| Match 236 in HUMAN | | | | | | | |
| --- | --- | --- | --- | --- | --- | --- | --- |
| Motif | Start in Seq (1 Indexed) | End in Seq (1 Indexed) | Strand | Chrm | Exon | Start in Chrm (0 Indexed) | End in Chrm (1 Indexed) |
| TATGTTA | 2655 | 2661 | - | chr20 | 1 | 34636271 | 34636278 |
| eCLIP Fold-Enrichment | Binding Protein | Cell Line | Strand | Chrm | | Start in Chrm (0 Indexed) | End in Chrm (1 Indexed) |
| 2.85027490535 | AARS (bg=2.33%) | K562 | - | chr20 | | 34636248 | 34636302 |
| 2.52238257884 | AARS (bg=2.33%) | K562 | - | chr20 | | 34636256 | 34636298 |
| 2.47360770411 | CPEB4 (bg=2.3%) | K562 | - | chr20 | | 34636256 | 34636304 |
| 2.11464845294 | ddx6 (bg=23.92%) | K562 | - | chr20 | | 34636248 | 34636297 |
| 4.47437767302 | dgcr8 (bg=19.31%) | SM-9MVZL | - | chr20 | | 34636178 | 34636281 |
| 2.18810358807 | dgcr8 (bg=19.31%) | HepG2 | - | chr20 | | 34636256 | 34636300 |
| 4.7044289009 | dgcr8 (bg=19.31%) | SM-9MVZL | - | chr20 | | 34636256 | 34636300 |
| 2.79225712105 | fam120a (bg=18.43%) | K562 | - | chr20 | | 34636256 | 34636306 |
| 3.08744795966 | FASTKD2 (bg=7.73%) | K562 | - | chr20 | | 34636224 | 34636296 |
| 2.04019932576 | FASTKD2 (bg=7.73%) | HepG2 | - | chr20 | | 34636247 | 34636298 |
| 5.05865793727 | fubp3 (bg=23.31%) | HepG2 | - | chr20 | | 34636230 | 34636303 |
| 5.27895038673 | fubp3 (bg=23.31%) | HepG2 | - | chr20 | | 34636255 | 34636301 |
| 2.80929692898 | GRSF1 (bg=3.8%) | HepG2 | - | chr20 | | 34636260 | 34636297 |
| 4.29394080699 | igf2bp1 (bg=11.67%) | K562 | - | chr20 | | 34636220 | 34636289 |
| 4.61386368096 | igf2bp1 (bg=11.67%) | K562 | - | chr20 | | 34636244 | 34636298 |
| 2.77859916528 | KHSRP (bg=8.1%) | K562 | - | chr20 | | 34636234 | 34636308 |
[truncated: 319,750 more chars]
