# Supplementary material for: Uncovering deeply conserved motif combinations in rapidly evolving noncoding sequences
Source: Genome Biol. 2021 Jan 11;22:29. doi: 10.1186/s13059-020-02247-1 (PMC7798263; doi:10.1186/s13059-020-02247-1)
Supplement: Supplementary file 3 — Additional file 3. LncLOOM output results for NORAD sequences from nine mammals. [file 13059_2020_2247_MOESM3_ESM.gz › AdditionalFile3/Html_Files/kmers_in_seqs_layer_conservation.html]

 MOTIF CONSERVATION

# MOTIF CONSERVATION

## Motifs mapped to anchor sequence

  

NAVIGATE ▼

▶BABOON▶DOG▶COW▶SHEEP▶PIG▶ARMADILLO▶GUINEAPIG▶MOUSE

  
  
  
  

## >HUMAN TO BABOON (5443 bases)

```
GGC

GGCACTTCCGGT  
Depth:3 (DOG)  
Ei-value:0.000, Pi-value:0.000  
Er-value:0.000, Pr-value:0.000  
No matches to eCLIP DataMATCHES To TargetScan▶ miR-302-3p/372-3p/373-3p/520-3p:AAGUGCU


ACTTCCGG

ACTTCCGG  
Depth:8 (GUINEAPIG)  
Ei-value:0.000, Pi-value:0.000  
Er-value:0.000, Pr-value:0.000  
No matches to eCLIP DataNo matches to TargetScan


T

GGCACTTCCGGT  
Depth:3 (DOG)  
Ei-value:0.000, Pi-value:0.000  
Er-value:0.000, Pr-value:0.000  
No matches to eCLIP DataMATCHES To TargetScan▶ miR-302-3p/372-3p/373-3p/520-3p:AAGUGCU


ac

ggcacttccggtac  
Depth:2 (BABOON)  
Ei-value:1.000, Pi-value:0.000  
Er-value:0.000, Pr-value:0.000  
No matches to eCLIP DataMATCHES To TargetScan▶ miR-126-3p.2:GUACCGU▶ miR-302-3p/372-3p/373-3p/520-3p:AAGUGCU

CG

CTCCTCTC

CTCCTCTC  
Depth:5 (SHEEP)  
Ei-value:0.000, Pi-value:0.000  
Er-value:0.000, Pr-value:0.000  
No matches to eCLIP DataNo matches to TargetScan


t

ctcctctct  
Depth:2 (BABOON)  
Ei-value:1.000, Pi-value:0.000  
Er-value:0.000, Pr-value:0.000  
No matches to eCLIP DataNo matches to TargetScan

T

GCCAGC

GCCAGC  
Depth:5 (SHEEP)  
Ei-value:0.000, Pi-value:0.000  
Er-value:0.000, Pr-value:0.000  
No matches to eCLIP DataNo matches to TargetScan

G

C

CAGAGAACTGCCAAGTCAGTTCCGGTC  
Depth:2 (BABOON)  
Ei-value:0.000, Pi-value:0.000  
Er-value:0.000, Pr-value:0.000  
eCLIP MATCHES▶ddx3x (bg=13.89%)▶EIF3G (bg=2.39%)MATCHES To TargetScan▶ miR-34-5p/449-5p:GGCAGUG▶ miR-182-5p:UUGGCAA▶ miR-96-5p/1271-5p:UUGGCAC


AGAGAA

AGAGAA  
Depth:8 (GUINEAPIG)  
Ei-value:0.000, Pi-value:0.000  
Er-value:0.000, Pr-value:0.000  
No matches to eCLIP DataNo matches to TargetScan


C

AGAGAACTGCCAA  
Depth:7 (ARMADILLO)  
Ei-value:0.000, Pi-value:0.000  
Er-value:0.000, Pr-value:0.000  
No matches to eCLIP DataMATCHES To TargetScan▶ miR-34-5p/449-5p:GGCAGUG▶ miR-182-5p:UUGGCAA▶ miR-96-5p/1271-5p:UUGGCAC


TGCCAA

TGCCAA  
Depth:9 (MOUSE)  
Ei-value:0.000, Pi-value:0.000  
Er-value:0.000, Pr-value:0.000  
No matches to eCLIP DataMATCHES To TargetScan▶ miR-182-5p:UUGGCAA▶ miR-96-5p/1271-5p:UUGGCAC


G

AGAGAACTGCCAAGTCAGTTCCGG  
Depth:4 (COW)  
Ei-value:0.000, Pi-value:0.000  
Er-value:0.000, Pr-value:0.000  
eCLIP MATCHES▶EIF3G (bg=2.39%)MATCHES To TargetScan▶ miR-34-5p/449-5p:GGCAGUG▶ miR-182-5p:UUGGCAA▶ miR-96-5p/1271-5p:UUGGCAC


TCAGTTCCGG

TCAGTTCCGG  
Depth:9 (MOUSE)  
Ei-value:0.000, Pi-value:0.000  
Er-value:0.000, Pr-value:0.000  
eCLIP MATCHES▶EIF3G (bg=2.39%)No matches to TargetScan


TC

CAGAGAACTGCCAAGTCAGTTCCGGTC  
Depth:2 (BABOON)  
Ei-value:0.000, Pi-value:0.000  
Er-value:0.000, Pr-value:0.000  
eCLIP MATCHES▶ddx3x (bg=13.89%)▶EIF3G (bg=2.39%)MATCHES To TargetScan▶ miR-34-5p/449-5p:GGCAGUG▶ miR-182-5p:UUGGCAA▶ miR-96-5p/1271-5p:UUGGCAC

C

ggcagaga

ggcagaga  
Depth:2 (BABOON)  
Ei-value:1.000, Pi-value:0.000  
Er-value:0.000, Pr-value:0.000  
eCLIP MATCHES▶ddx3x (bg=13.89%)▶EIF3G (bg=2.39%)▶ybx3 (bg=22.82%)No matches to TargetScan

T

cgcggagagacgcagaacgc

cgcggagagacgcagaacgc  
Depth:2 (BABOON)  
Ei-value:0.620, Pi-value:0.000  
Er-value:0.000, Pr-value:0.000  
eCLIP MATCHES▶ddx3x (bg=13.89%)▶EIF3G (bg=2.39%)▶EIF3H (bg=7.83%)▶FMR1 (bg=6.3%)▶METAP2 (bg=6.14%)▶RPS3 (bg=4.15%)▶SDAD1 (bg=5.99%)▶SERBP1 (bg=0.81%)▶WRN (bg=3.31%)▶ybx3 (bg=22.82%)No matches to TargetScan

AGCCC

gctcct

gctcct  
Depth:2 (BABOON)  
Ei-value:1.000, Pi-value:0.020  
Er-value:0.000, Pr-value:0.020  
eCLIP MATCHES▶ddx3x (bg=13.89%)▶drosha (bg=11.37%)▶EIF3G (bg=2.39%)▶EIF3H (bg=7.83%)▶FMR1 (bg=6.3%)▶METAP2 (bg=6.14%)▶PUS1 (bg=2.87%)▶RPS3 (bg=4.15%)▶SDAD1 (bg=5.99%)▶SERBP1 (bg=0.81%)▶WRN (bg=3.31%)▶ybx3 (bg=22.82%)MATCHES To TargetScan▶ miR-28-5p/708-5p:AGGAGCU

C

CA

CAGGGCCCTCCAGGCCCTCCGGCCC  
Depth:2 (BABOON)  
Ei-value:0.000, Pi-value:0.000  
Er-value:0.000, Pr-value:0.000  
eCLIP MATCHES▶ddx3x (bg=13.89%)▶ddx6 (bg=23.92%)▶drosha (bg=11.37%)▶EIF3G (bg=2.39%)▶EIF3H (bg=7.83%)▶FMR1 (bg=6.3%)▶METAP2 (bg=6.14%)▶PUS1 (bg=2.87%)▶RPS3 (bg=4.15%)▶SDAD1 (bg=5.99%)▶SERBP1 (bg=0.81%)▶SF3B1 (bg=6.74%)▶WRN (bg=3.31%)▶ybx3 (bg=22.82%)MATCHES To TargetScan▶ miR-296-5p:GGGCCCC


GGGCCCTCCA

GGGCCCTCCAGGCCCTCCGGCC  
Depth:5 (SHEEP)  
Ei-value:0.000, Pi-value:0.000  
Er-value:0.000, Pr-value:0.000  
eCLIP MATCHES▶ddx3x (bg=13.89%)▶ddx6 (bg=23.92%)▶drosha (bg=11.37%)▶EIF3G (bg=2.39%)▶EIF3H (bg=7.83%)▶FMR1 (bg=6.3%)▶METAP2 (bg=6.14%)▶PUS1 (bg=2.87%)▶RPS3 (bg=4.15%)▶SDAD1 (bg=5.99%)▶SERBP1 (bg=0.81%)▶SF3B1 (bg=6.74%)▶WRN (bg=3.31%)▶ybx3 (bg=22.82%)MATCHES To TargetScan▶ miR-296-5p:GGGCCCC


G

GGCCCTCC  
Depth:8 (GUINEAPIG)  
Ei-value:0.000, Pi-value:0.000  
Er-value:0.000, Pr-value:0.000  
eCLIP MATCHES▶ddx3x (bg=13.89%)▶ddx6 (bg=23.92%)▶drosha (bg=11.37%)▶EIF3G (bg=2.39%)▶EIF3H (bg=7.83%)▶FMR1 (bg=6.3%)▶METAP2 (bg=6.14%)▶PUS1 (bg=2.87%)▶RPS3 (bg=4.15%)▶SDAD1 (bg=5.99%)▶SERBP1 (bg=0.81%)▶SF3B1 (bg=6.74%)▶WRN (bg=3.31%)▶ybx3 (bg=22.82%)No matches to TargetScan


GCCCT

GCCCTC  
Depth:9 (MOUSE)  
Ei-value:0.000, Pi-value:0.000  
Er-value:0.000, Pr-value:0.000  
eCLIP MATCHES▶ddx3x (bg=13.89%)▶ddx6 (bg=23.92%)▶drosha (bg=11.37%)▶EIF3G (bg=2.39%)▶EIF3H (bg=7.83%)▶FMR1 (bg=6.3%)▶METAP2 (bg=6.14%)▶PUS1 (bg=2.87%)▶RPS3 (bg=4.15%)▶SDAD1 (bg=5.99%)▶SERBP1 (bg=0.81%)▶SF3B1 (bg=6.74%)▶WRN (bg=3.31%)▶ybx3 (bg=22.82%)No matches to TargetScan

 120  


C

GCCCTC  
Depth:9 (MOUSE)  
Ei-value:0.000, Pi-value:0.000  
Er-value:0.000, Pr-value:0.000  
eCLIP MATCHES▶ddx3x (bg=13.89%)▶ddx6 (bg=23.92%)▶drosha (bg=11.37%)▶EIF3G (bg=2.39%)▶EIF3H (bg=7.83%)▶FMR1 (bg=6.3%)▶METAP2 (bg=6.14%)▶PUS1 (bg=2.87%)▶RPS3 (bg=4.15%)▶SDAD1 (bg=5.99%)▶SERBP1 (bg=0.81%)▶SF3B1 (bg=6.74%)▶WRN (bg=3.31%)▶ybx3 (bg=22.82%)No matches to TargetScan


C

GGCCCTCC  
Depth:8 (GUINEAPIG)  
Ei-value:0.000, Pi-value:0.000  
Er-value:0.000, Pr-value:0.000  
eCLIP MATCHES▶ddx3x (bg=13.89%)▶ddx6 (bg=23.92%)▶drosha (bg=11.37%)▶EIF3G (bg=2.39%)▶EIF3H (bg=7.83%)▶FMR1 (bg=6.3%)▶METAP2 (bg=6.14%)▶PUS1 (bg=2.87%)▶RPS3 (bg=4.15%)▶SDAD1 (bg=5.99%)▶SERBP1 (bg=0.81%)▶SF3B1 (bg=6.74%)▶WRN (bg=3.31%)▶ybx3 (bg=22.82%)No matches to TargetScan


GG

GGCCCTCCGG  
Depth:7 (ARMADILLO)  
Ei-value:0.000, Pi-value:0.000  
Er-value:0.000, Pr-value:0.000  
eCLIP MATCHES▶ddx3x (bg=13.89%)▶ddx6 (bg=23.92%)▶drosha (bg=11.37%)▶EIF3G (bg=2.39%)▶EIF3H (bg=7.83%)▶FMR1 (bg=6.3%)▶METAP2 (bg=6.14%)▶PUS1 (bg=2.87%)▶RPS3 (bg=4.15%)▶SDAD1 (bg=5.99%)▶SERBP1 (bg=0.81%)▶SF3B1 (bg=6.74%)▶WRN (bg=3.31%)▶ybx3 (bg=22.82%)No matches to TargetScan


CC

GGGCCCTCCAGGCCCTCCGGCC  
Depth:5 (SHEEP)  
Ei-value:0.000, Pi-value:0.000  
Er-value:0.000, Pr-value:0.000  
eCLIP MATCHES▶ddx3x (bg=13.89%)▶ddx6 (bg=23.92%)▶drosha (bg=11.37%)▶EIF3G (bg=2.39%)▶EIF3H (bg=7.83%)▶FMR1 (bg=6.3%)▶METAP2 (bg=6.14%)▶PUS1 (bg=2.87%)▶RPS3 (bg=4.15%)▶SDAD1 (bg=5.99%)▶SERBP1 (bg=0.81%)▶SF3B1 (bg=6.74%)▶WRN (bg=3.31%)▶ybx3 (bg=22.82%)MATCHES To TargetScan▶ miR-296-5p:GGGCCCC


C

CAGGGCCCTCCAGGCCCTCCGGCCC  
Depth:2 (BABOON)  
Ei-value:0.000, Pi-value:0.000  
Er-value:0.000, Pr-value:0.000  
eCLIP MATCHES▶ddx3x (bg=13.89%)▶ddx6 (bg=23.92%)▶drosha (bg=11.37%)▶EIF3G (bg=2.39%)▶EIF3H (bg=7.83%)▶FMR1 (bg=6.3%)▶METAP2 (bg=6.14%)▶PUS1 (bg=2.87%)▶RPS3 (bg=4.15%)▶SDAD1 (bg=5.99%)▶SERBP1 (bg=0.81%)▶SF3B1 (bg=6.74%)▶WRN (bg=3.31%)▶ybx3 (bg=22.82%)MATCHES To TargetScan▶ miR-296-5p:GGGCCCC

C

G

GGGCCGGCGGGTGAACTGGGGGGCCCCGGGACAGGCCGAGCCCT  
Depth:2 (BABOON)  
Ei-value:0.000, Pi-value:0.000  
Er-value:0.000, Pr-value:0.000  
eCLIP MATCHES▶AKAP1 (bg=3.4%)▶DDX24 (bg=2.31%)▶ddx3x (bg=13.89%)▶ddx6 (bg=23.92%)▶DHX30 (bg=5.05%)▶drosha (bg=11.37%)▶EIF3G (bg=2.39%)▶EIF3H (bg=7.83%)▶fam120a (bg=18.43%)▶FMR1 (bg=6.3%)▶GEMIN5 (bg=2.46%)▶METAP2 (bg=6.14%)▶NCBP2 (bg=2.92%)▶PHF6 (bg=1.71%)▶PUS1 (bg=2.87%)▶rbm15 (bg=11.81%)▶RPS3 (bg=4.15%)▶SDAD1 (bg=5.99%)▶SERBP1 (bg=0.81%)▶SF3B1 (bg=6.74%)▶SUB1 (bg=9.24%)▶TBRG4 (bg=7.0%)▶UTP3 (bg=2.81%)▶WRN (bg=3.31%)▶ybx3 (bg=22.82%)MATCHES To TargetScan▶ miR-423-3p:GCUCGGU▶ miR-296-5p:GGGCCCC▶ miR-214-5p:GCCUGUC


ggccgg

ggccgg  
Depth:3 (DOG)  
Ei-value:0.080, Pi-value:0.000  
Er-value:0.000, Pr-value:0.000  
eCLIP MATCHES▶ddx3x (bg=13.89%)▶ddx6 (bg=23.92%)▶drosha (bg=11.37%)▶EIF3G (bg=2.39%)▶EIF3H (bg=7.83%)▶FMR1 (bg=6.3%)▶METAP2 (bg=6.14%)▶PHF6 (bg=1.71%)▶PUS1 (bg=2.87%)▶RPS3 (bg=4.15%)▶SDAD1 (bg=5.99%)▶SERBP1 (bg=0.81%)▶SF3B1 (bg=6.74%)▶WRN (bg=3.31%)▶ybx3 (bg=22.82%)No matches to TargetScan


C

GGGCCGGCGGGTGAACTGGGGGGCCCCGGGACAGGCCGAGCCCT  
Depth:2 (BABOON)  
Ei-value:0.000, Pi-value:0.000  
Er-value:0.000, Pr-value:0.000  
eCLIP MATCHES▶AKAP1 (bg=3.4%)▶DDX24 (bg=2.31%)▶ddx3x (bg=13.89%)▶ddx6 (bg=23.92%)▶DHX30 (bg=5.05%)▶drosha (bg=11.37%)▶EIF3G (bg=2.39%)▶EIF3H (bg=7.83%)▶fam120a (bg=18.43%)▶FMR1 (bg=6.3%)▶GEMIN5 (bg=2.46%)▶METAP2 (bg=6.14%)▶NCBP2 (bg=2.92%)▶PHF6 (bg=1.71%)▶PUS1 (bg=2.87%)▶rbm15 (bg=11.81%)▶RPS3 (bg=4.15%)▶SDAD1 (bg=5.99%)▶SERBP1 (bg=0.81%)▶SF3B1 (bg=6.74%)▶SUB1 (bg=9.24%)▶TBRG4 (bg=7.0%)▶UTP3 (bg=2.81%)▶WRN (bg=3.31%)▶ybx3 (bg=22.82%)MATCHES To TargetScan▶ miR-423-3p:GCUCGGU▶ miR-296-5p:GGGCCCC▶ miR-214-5p:GCCUGUC


G

GGGTGAACTGGGGGGCCC  
Depth:3 (DOG)  
Ei-value:0.000, Pi-value:0.000  
Er-value:0.000, Pr-value:0.000  
eCLIP MATCHES▶DDX24 (bg=2.31%)▶ddx3x (bg=13.89%)▶DHX30 (bg=5.05%)▶drosha (bg=11.37%)▶EIF3G (bg=2.39%)▶EIF3H (bg=7.83%)▶fam120a (bg=18.43%)▶FMR1 (bg=6.3%)▶GEMIN5 (bg=2.46%)▶METAP2 (bg=6.14%)▶NCBP2 (bg=2.92%)▶PHF6 (bg=1.71%)▶PUS1 (bg=2.87%)▶RPS3 (bg=4.15%)▶SDAD1 (bg=5.99%)▶SF3B1 (bg=6.74%)▶SUB1 (bg=9.24%)▶WRN (bg=3.31%)MATCHES To TargetScan▶ miR-296-5p:GGGCCCC


GGTGAACT

GGTGAACTGGGGGGCCC  
Depth:6 (PIG)  
Ei-value:0.000, Pi-value:0.000  
Er-value:0.000, Pr-value:0.000  
eCLIP MATCHES▶DDX24 (bg=2.31%)▶ddx3x (bg=13.89%)▶DHX30 (bg=5.05%)▶drosha (bg=11.37%)▶EIF3G (bg=2.39%)▶EIF3H (bg=7.83%)▶fam120a (bg=18.43%)▶FMR1 (bg=6.3%)▶GEMIN5 (bg=2.46%)▶METAP2 (bg=6.14%)▶NCBP2 (bg=2.92%)▶PHF6 (bg=1.71%)▶PUS1 (bg=2.87%)▶RPS3 (bg=4.15%)▶SDAD1 (bg=5.99%)▶SF3B1 (bg=6.74%)▶SUB1 (bg=9.24%)▶WRN (bg=3.31%)MATCHES To TargetScan▶ miR-296-5p:GGGCCCC


GGG

GGGGGGCCC  
Depth:7 (ARMADILLO)  
Ei-value:0.000, Pi-value:0.000  
Er-value:0.000, Pr-value:0.000  
eCLIP MATCHES▶DDX24 (bg=2.31%)▶ddx3x (bg=13.89%)▶DHX30 (bg=5.05%)▶drosha (bg=11.37%)▶EIF3G (bg=2.39%)▶EIF3H (bg=7.83%)▶fam120a (bg=18.43%)▶GEMIN5 (bg=2.46%)▶METAP2 (bg=6.14%)▶NCBP2 (bg=2.92%)▶PHF6 (bg=1.71%)▶RPS3 (bg=4.15%)▶SDAD1 (bg=5.99%)▶SUB1 (bg=9.24%)▶WRN (bg=3.31%)MATCHES To TargetScan▶ miR-296-5p:GGGCCCC


GGGCCC

GGGCCC  
Depth:8 (GUINEAPIG)  
Ei-value:0.000, Pi-value:0.000  
Er-value:0.000, Pr-value:0.000  
eCLIP MATCHES▶DDX24 (bg=2.31%)▶ddx3x (bg=13.89%)▶DHX30 (bg=5.05%)▶drosha (bg=11.37%)▶EIF3G (bg=2.39%)▶EIF3H (bg=7.83%)▶fam120a (bg=18.43%)▶GEMIN5 (bg=2.46%)▶METAP2 (bg=6.14%)▶NCBP2 (bg=2.92%)▶PHF6 (bg=1.71%)▶RPS3 (bg=4.15%)▶SDAD1 (bg=5.99%)▶SUB1 (bg=9.24%)▶WRN (bg=3.31%)MATCHES To TargetScan▶ miR-296-5p:GGGCCCC


CGGGACAGGCCGAGCCCT

GGGCCGGCGGGTGAACTGGGGGGCCCCGGGACAGGCCGAGCCCT  
Depth:2 (BABOON)  
Ei-value:0.000, Pi-value:0.000  
Er-value:0.000, Pr-value:0.000  
eCLIP MATCHES▶AKAP1 (bg=3.4%)▶DDX24 (bg=2.31%)▶ddx3x (bg=13.89%)▶ddx6 (bg=23.92%)▶DHX30 (bg=5.05%)▶drosha (bg=11.37%)▶EIF3G (bg=2.39%)▶EIF3H (bg=7.83%)▶fam120a (bg=18.43%)▶FMR1 (bg=6.3%)▶GEMIN5 (bg=2.46%)▶METAP2 (bg=6.14%)▶NCBP2 (bg=2.92%)▶PHF6 (bg=1.71%)▶PUS1 (bg=2.87%)▶rbm15 (bg=11.81%)▶RPS3 (bg=4.15%)▶SDAD1 (bg=5.99%)▶SERBP1 (bg=0.81%)▶SF3B1 (bg=6.74%)▶SUB1 (bg=9.24%)▶TBRG4 (bg=7.0%)▶UTP3 (bg=2.81%)▶WRN (bg=3.31%)▶ybx3 (bg=22.82%)MATCHES To TargetScan▶ miR-423-3p:GCUCGGU▶ miR-296-5p:GGGCCCC▶ miR-214-5p:GCCUGUC

CTGCCC

tgcaga

tgcaga  
Depth:4 (COW)  
Ei-value:1.000, Pi-value:0.010  
Er-value:0.000, Pr-value:0.000  
eCLIP MATCHES▶AKAP1 (bg=3.4%)▶BCCIP (bg=4.61%)▶DDX24 (bg=2.31%)▶ddx3x (bg=13.89%)▶ddx6 (bg=23.92%)▶DHX30 (bg=5.05%)▶drosha (bg=11.37%)▶EIF3G (bg=2.39%)▶EIF3H (bg=7.83%)▶fam120a (bg=18.43%)▶FMR1 (bg=6.3%)▶FXR2 (bg=7.51%)▶METAP2 (bg=6.14%)▶NCBP2 (bg=2.92%)▶NIPBL (bg=2.83%)▶PCBP1 (bg=2.3%)▶PHF6 (bg=1.71%)▶PTBP1 (bg=1.43%)▶rbm15 (bg=11.81%)▶RPS3 (bg=4.15%)▶SDAD1 (bg=5.99%)▶SUB1 (bg=9.24%)▶TBRG4 (bg=7.0%)▶TROVE2 (bg=3.49%)▶UTP3 (bg=2.81%)▶ybx3 (bg=22.82%)No matches to TargetScan


ta

tgcagata  
Depth:2 (BABOON)  
Ei-value:1.000, Pi-value:0.010  
Er-value:0.000, Pr-value:0.000  
eCLIP MATCHES▶AKAP1 (bg=3.4%)▶BCCIP (bg=4.61%)▶DDX24 (bg=2.31%)▶ddx3x (bg=13.89%)▶ddx6 (bg=23.92%)▶DHX30 (bg=5.05%)▶drosha (bg=11.37%)▶EIF3G (bg=2.39%)▶EIF3H (bg=7.83%)▶fam120a (bg=18.43%)▶FMR1 (bg=6.3%)▶fubp3 (bg=23.31%)▶FXR2 (bg=7.51%)▶METAP2 (bg=6.14%)▶NCBP2 (bg=2.92%)▶NIPBL (bg=2.83%)▶PCBP1 (bg=2.3%)▶PHF6 (bg=1.71%)▶PTBP1 (bg=1.43%)▶rbm15 (bg=11.81%)▶RPS3 (bg=4.15%)▶SDAD1 (bg=5.99%)▶SUB1 (bg=9.24%)▶TBRG4 (bg=7.0%)▶TROVE2 (bg=3.49%)▶UTP3 (bg=2.81%)▶ybx3 (bg=22.82%)No matches to TargetScan

A

cgga

cggaggcctctgctg  
Depth:2 (BABOON)  
Ei-value:1.000, Pi-value:0.000  
Er-value:0.000, Pr-value:0.000  
eCLIP MATCHES▶AKAP1 (bg=3.4%)▶BCCIP (bg=4.61%)▶DDX24 (bg=2.31%)▶ddx3x (bg=13.89%)▶ddx6 (bg=23.92%)▶dgcr8 (bg=19.31%)▶DHX30 (bg=5.05%)▶drosha (bg=11.37%)▶EIF3G (bg=2.39%)▶EIF3H (bg=7.83%)▶fam120a (bg=18.43%)▶FMR1 (bg=6.3%)▶fubp3 (bg=23.31%)▶FXR2 (bg=7.51%)▶METAP2 (bg=6.14%)▶NCBP2 (bg=2.92%)▶NIPBL (bg=2.83%)▶PCBP1 (bg=2.3%)▶PHF6 (bg=1.71%)▶PTBP1 (bg=1.43%)▶pum2 (bg=21.55%)▶rbm15 (bg=11.81%)▶RPS3 (bg=4.15%)▶SBDS (bg=1.91%)▶SDAD1 (bg=5.99%)▶SF3B1 (bg=6.74%)▶SUB1 (bg=9.24%)▶TBRG4 (bg=7.0%)▶TROVE2 (bg=3.49%)▶UTP3 (bg=2.81%)▶WRN (bg=3.31%)▶ybx3 (bg=22.82%)No matches to TargetScan


GGCCTCT

GGCCTCT  
Depth:3 (DOG)  
Ei-value:0.000, Pi-value:0.010  
Er-value:0.000, Pr-value:0.000  
eCLIP MATCHES▶AKAP1 (bg=3.4%)▶BCCIP (bg=4.61%)▶DDX24 (bg=2.31%)▶ddx3x (bg=13.89%)▶ddx6 (bg=23.92%)▶dgcr8 (bg=19.31%)▶DHX30 (bg=5.05%)▶drosha (bg=11.37%)▶EIF3H (bg=7.83%)▶fam120a (bg=18.43%)▶FMR1 (bg=6.3%)▶fubp3 (bg=23.31%)▶FXR2 (bg=7.51%)▶METAP2 (bg=6.14%)▶NCBP2 (bg=2.92%)▶NIPBL (bg=2.83%)▶PCBP1 (bg=2.3%)▶PTBP1 (bg=1.43%)▶pum2 (bg=21.55%)▶rbm15 (bg=11.81%)▶RPS3 (bg=4.15%)▶SBDS (bg=1.91%)▶SDAD1 (bg=5.99%)▶SUB1 (bg=9.24%)▶TBRG4 (bg=7.0%)▶TROVE2 (bg=3.49%)▶UTP3 (bg=2.81%)▶ybx3 (bg=22.82%)No matches to TargetScan


gctg

cggaggcctctgctg  
Depth:2 (BABOON)  
Ei-value:1.000, Pi-value:0.000  
Er-value:0.000, Pr-value:0.000  
eCLIP MATCHES▶AKAP1 (bg=3.4%)▶BCCIP (bg=4.61%)▶DDX24 (bg=2.31%)▶ddx3x (bg=13.89%)▶ddx6 (bg=23.92%)▶dgcr8 (bg=19.31%)▶DHX30 (bg=5.05%)▶drosha (bg=11.37%)▶EIF3G (bg=2.39%)▶EIF3H (bg=7.83%)▶fam120a (bg=18.43%)▶FMR1 (bg=6.3%)▶fubp3 (bg=23.31%)▶FXR2 (bg=7.51%)▶METAP2 (bg=6.14%)▶NCBP2 (bg=2.92%)▶NIPBL (bg=2.83%)▶PCBP1 (bg=2.3%)▶PHF6 (bg=1.71%)▶PTBP1 (bg=1.43%)▶pum2 (bg=21.55%)▶rbm15 (bg=11.81%)▶RPS3 (bg=4.15%)▶SBDS (bg=1.91%)▶SDAD1 (bg=5.99%)▶SF3B1 (bg=6.74%)▶SUB1 (bg=9.24%)▶TBRG4 (bg=7.0%)▶TROVE2 (bg=3.49%)▶UTP3 (bg=2.81%)▶WRN (bg=3.31%)▶ybx3 (bg=22.82%)No matches to TargetScan

T

GGCTGCCCACT

GGCTGCCCACT  
Depth:3 (DOG)  
Ei-value:0.000, Pi-value:0.000  
Er-value:0.000, Pr-value:0.000  
eCLIP MATCHES▶AKAP1 (bg=3.4%)▶BCCIP (bg=4.61%)▶DDX24 (bg=2.31%)▶ddx3x (bg=13.89%)▶ddx6 (bg=23.92%)▶dgcr8 (bg=19.31%)▶DHX30 (bg=5.05%)▶drosha (bg=11.37%)▶EIF3H (bg=7.83%)▶fam120a (bg=18.43%)▶FMR1 (bg=6.3%)▶FXR2 (bg=7.51%)▶METAP2 (bg=6.14%)▶NIPBL (bg=2.83%)▶PTBP1 (bg=1.43%)▶pum2 (bg=21.55%)▶rbm15 (bg=11.81%)▶RPS3 (bg=4.15%)▶SBDS (bg=1.91%)▶SDAD1 (bg=5.99%)▶SF3B1 (bg=6.74%)▶SUB1 (bg=9.24%)▶TBRG4 (bg=7.0%)▶TROVE2 (bg=3.49%)▶UTP3 (bg=2.81%)▶WRN (bg=3.31%)▶ybx3 (bg=22.82%)▶ZC3H11A (bg=6.25%)No matches to TargetScan


ggctgtgccc

ggctgcccactggctgtgccc  
Depth:2 (BABOON)  
Ei-value:0.490, Pi-value:0.000  
Er-value:0.000, Pr-value:0.000  
eCLIP MATCHES▶AKAP1 (bg=3.4%)▶BCCIP (bg=4.61%)▶DDX24 (bg=2.31%)▶ddx3x (bg=13.89%)▶ddx6 (bg=23.92%)▶dgcr8 (bg=19.31%)▶DHX30 (bg=5.05%)▶drosha (bg=11.37%)▶EIF3H (bg=7.83%)▶fam120a (bg=18.43%)▶FMR1 (bg=6.3%)▶FXR2 (bg=7.51%)▶METAP2 (bg=6.14%)▶NIPBL (bg=2.83%)▶PTBP1 (bg=1.43%)▶pum2 (bg=21.55%)▶rbm15 (bg=11.81%)▶RPS3 (bg=4.15%)▶SBDS (bg=1.91%)▶SDAD1 (bg=5.99%)▶SF3B1 (bg=6.74%)▶SUB1 (bg=9.24%)▶TBRG4 (bg=7.0%)▶TROVE2 (bg=3.49%)▶UTP3 (bg=2.81%)▶WRN (bg=3.31%)▶ybx3 (bg=22.82%)▶ZC3H11A (bg=6.25%)MATCHES To TargetScan▶ miR-199-5p:CCAGUGU

GCCCACTGGCTGTGCC 240  
 CAGA

CCTT

CCTTGAAGCCGCAGCGAACCTCTCTT  
Depth:2 (BABOON)  
Ei-value:0.000, Pi-value:0.000  
Er-value:0.000, Pr-value:0.000  
eCLIP MATCHES▶BCCIP (bg=4.61%)▶ddx3x (bg=13.89%)▶dgcr8 (bg=19.31%)▶drosha (bg=11.37%)▶FMR1 (bg=6.3%)▶FTO (bg=7.53%)▶FXR2 (bg=7.51%)▶GEMIN5 (bg=2.46%)▶METAP2 (bg=6.14%)▶NIP7 (bg=2.54%)▶PCBP1 (bg=2.3%)▶PTBP1 (bg=1.43%)▶rbm15 (bg=11.81%)▶SBDS (bg=1.91%)▶SDAD1 (bg=5.99%)▶TROVE2 (bg=3.49%)▶ybx3 (bg=22.82%)No matches to TargetScan


gaagcc

gaagcc  
Depth:4 (COW)  
Ei-value:1.000, Pi-value:0.010  
Er-value:0.000, Pr-value:0.000  
eCLIP MATCHES▶ddx3x (bg=13.89%)▶dgcr8 (bg=19.31%)▶drosha (bg=11.37%)▶FMR1 (bg=6.3%)▶FXR2 (bg=7.51%)▶NIP7 (bg=2.54%)▶PTBP1 (bg=1.43%)▶rbm15 (bg=11.81%)▶SBDS (bg=1.91%)▶SDAD1 (bg=5.99%)▶ybx3 (bg=22.82%)No matches to TargetScan


GCAGCGAACCTCTCTT

CCTTGAAGCCGCAGCGAACCTCTCTT  
Depth:2 (BABOON)  
Ei-value:0.000, Pi-value:0.000  
Er-value:0.000, Pr-value:0.000  
eCLIP MATCHES▶BCCIP (bg=4.61%)▶ddx3x (bg=13.89%)▶dgcr8 (bg=19.31%)▶drosha (bg=11.37%)▶FMR1 (bg=6.3%)▶FTO (bg=7.53%)▶FXR2 (bg=7.51%)▶GEMIN5 (bg=2.46%)▶METAP2 (bg=6.14%)▶NIP7 (bg=2.54%)▶PCBP1 (bg=2.3%)▶PTBP1 (bg=1.43%)▶rbm15 (bg=11.81%)▶SBDS (bg=1.91%)▶SDAD1 (bg=5.99%)▶TROVE2 (bg=3.49%)▶ybx3 (bg=22.82%)No matches to TargetScan

T

cccaccccacctcggtgact

cccaccccacctcggtgact  
Depth:2 (BABOON)  
Ei-value:0.620, Pi-value:0.000  
Er-value:0.000, Pr-value:0.000  
eCLIP MATCHES▶BCCIP (bg=4.61%)▶ddx3x (bg=13.89%)▶ddx6 (bg=23.92%)▶dgcr8 (bg=19.31%)▶DHX30 (bg=5.05%)▶drosha (bg=11.37%)▶EIF3H (bg=7.83%)▶FMR1 (bg=6.3%)▶FTO (bg=7.53%)▶fubp3 (bg=23.31%)▶FXR2 (bg=7.51%)▶GEMIN5 (bg=2.46%)▶IGF2BP3 (bg=4.26%)▶METAP2 (bg=6.14%)▶NIP7 (bg=2.54%)▶PCBP1 (bg=2.3%)▶pum1 (bg=29.85%)▶pum2 (bg=21.55%)▶rbm15 (bg=11.81%)▶SDAD1 (bg=5.99%)▶WRN (bg=3.31%)MATCHES To TargetScan▶ miR-491-5p:GUGGGGA

A

ATGGCGGC

ATGGCGGC  
Depth:9 (MOUSE)  
Ei-value:0.000, Pi-value:0.000  
Er-value:0.000, Pr-value:0.000  
eCLIP MATCHES▶BCCIP (bg=4.61%)▶ddx3x (bg=13.89%)▶ddx6 (bg=23.92%)▶dgcr8 (bg=19.31%)▶DHX30 (bg=5.05%)▶drosha (bg=11.37%)▶EIF3H (bg=7.83%)▶FMR1 (bg=6.3%)▶FTO (bg=7.53%)▶fubp3 (bg=23.31%)▶FXR2 (bg=7.51%)▶GEMIN5 (bg=2.46%)▶IGF2BP3 (bg=4.26%)▶METAP2 (bg=6.14%)▶NIP7 (bg=2.54%)▶PCBP1 (bg=2.3%)▶pum1 (bg=29.85%)▶pum2 (bg=21.55%)▶rbm15 (bg=11.81%)▶SDAD1 (bg=5.99%)▶WRN (bg=3.31%)No matches to TargetScan

CGTGGCG

tctcccagcccggaccc

tctcccagcccggaccc  
Depth:2 (BABOON)  
Ei-value:1.000, Pi-value:0.000  
Er-value:0.000, Pr-value:0.000  
eCLIP MATCHES▶BCCIP (bg=4.61%)▶ddx3x (bg=13.89%)▶ddx6 (bg=23.92%)▶dgcr8 (bg=19.31%)▶DHX30 (bg=5.05%)▶drosha (bg=11.37%)▶EIF3H (bg=7.83%)▶FMR1 (bg=6.3%)▶FTO (bg=7.53%)▶FXR2 (bg=7.51%)▶GEMIN5 (bg=2.46%)▶IGF2BP3 (bg=4.26%)▶METAP2 (bg=6.14%)▶NIP7 (bg=2.54%)▶PCBP1 (bg=2.3%)▶pum1 (bg=29.85%)▶pum2 (bg=21.55%)▶rbm15 (bg=11.81%)▶SDAD1 (bg=5.99%)▶WRN (bg=3.31%)No matches to TargetScan

C

gccggc

gccggc  
Depth:3 (DOG)  
Ei-value:0.080, Pi-value:0.000  
Er-value:0.000, Pr-value:0.000  
eCLIP MATCHES▶BCCIP (bg=4.61%)▶ddx3x (bg=13.89%)▶ddx6 (bg=23.92%)▶dgcr8 (bg=19.31%)▶DHX30 (bg=5.05%)▶drosha (bg=11.37%)▶EIF3H (bg=7.83%)▶FMR1 (bg=6.3%)▶FTO (bg=7.53%)▶FXR2 (bg=7.51%)▶GEMIN5 (bg=2.46%)▶IGF2BP3 (bg=4.26%)▶METAP2 (bg=6.14%)▶NIP7 (bg=2.54%)▶PCBP1 (bg=2.3%)▶pum1 (bg=29.85%)▶pum2 (bg=21.55%)▶rbm15 (bg=11.81%)▶SDAD1 (bg=5.99%)▶WRN (bg=3.31%)No matches to TargetScan

AC

c

ccgggtctcccg  
Depth:2 (BABOON)  
Ei-value:1.000, Pi-value:0.000  
Er-value:0.000, Pr-value:0.000  
eCLIP MATCHES▶BCCIP (bg=4.61%)▶ddx3x (bg=13.89%)▶ddx6 (bg=23.92%)▶dgcr8 (bg=19.31%)▶DHX30 (bg=5.05%)▶drosha (bg=11.37%)▶EIF3H (bg=7.83%)▶FMR1 (bg=6.3%)▶FTO (bg=7.53%)▶FXR2 (bg=7.51%)▶IGF2BP3 (bg=4.26%)▶LARP7 (bg=0.81%)▶METAP2 (bg=6.14%)▶NIP7 (bg=2.54%)▶PCBP1 (bg=2.3%)▶pum1 (bg=29.85%)▶rbm15 (bg=11.81%)▶SDAD1 (bg=5.99%)No matches to TargetScan


CGGGTCTCC

CGGGTCTCC  
Depth:3 (DOG)  
Ei-value:0.000, Pi-value:0.000  
Er-value:0.000, Pr-value:0.000  
eCLIP MATCHES▶BCCIP (bg=4.61%)▶ddx3x (bg=13.89%)▶ddx6 (bg=23.92%)▶dgcr8 (bg=19.31%)▶DHX30 (bg=5.05%)▶drosha (bg=11.37%)▶EIF3H (bg=7.83%)▶FMR1 (bg=6.3%)▶FTO (bg=7.53%)▶FXR2 (bg=7.51%)▶IGF2BP3 (bg=4.26%)▶LARP7 (bg=0.81%)▶METAP2 (bg=6.14%)▶NIP7 (bg=2.54%)▶PCBP1 (bg=2.3%)▶pum1 (bg=29.85%)▶rbm15 (bg=11.81%)▶SDAD1 (bg=5.99%)No matches to TargetScan


cg

ccgggtctcccg  
Depth:2 (BABOON)  
Ei-value:1.000, Pi-value:0.000  
Er-value:0.000, Pr-value:0.000  
eCLIP MATCHES▶BCCIP (bg=4.61%)▶ddx3x (bg=13.89%)▶ddx6 (bg=23.92%)▶dgcr8 (bg=19.31%)▶DHX30 (bg=5.05%)▶drosha (bg=11.37%)▶EIF3H (bg=7.83%)▶FMR1 (bg=6.3%)▶FTO (bg=7.53%)▶FXR2 (bg=7.51%)▶IGF2BP3 (bg=4.26%)▶LARP7 (bg=0.81%)▶METAP2 (bg=6.14%)▶NIP7 (bg=2.54%)▶PCBP1 (bg=2.3%)▶pum1 (bg=29.85%)▶rbm15 (bg=11.81%)▶SDAD1 (bg=5.99%)No matches to TargetScan

A

cccaagcct

cccaagcct  
Depth:2 (BABOON)  
Ei-value:1.000, Pi-value:0.000  
Er-value:0.000, Pr-value:0.000  
eCLIP MATCHES▶BCCIP (bg=4.61%)▶ddx3x (bg=13.89%)▶dgcr8 (bg=19.31%)▶drosha (bg=11.37%)▶EIF3H (bg=7.83%)▶FXR2 (bg=7.51%)▶GRWD1 (bg=4.85%)▶IGF2BP3 (bg=4.26%)▶LARP7 (bg=0.81%)▶PCBP1 (bg=2.3%)▶ybx3 (bg=22.82%)No matches to TargetScan

CG

ACG

ACGAAACCCCCGCAGAGCCGCCGGGACGCAGCGC  
Depth:2 (BABOON)  
Ei-value:0.000, Pi-value:0.000  
Er-value:0.000, Pr-value:0.000  
eCLIP MATCHES▶BCCIP (bg=4.61%)▶ddx3x (bg=13.89%)▶dgcr8 (bg=19.31%)▶DHX30 (bg=5.05%)▶drosha (bg=11.37%)▶EIF3H (bg=7.83%)▶FMR1 (bg=6.3%)▶FTO (bg=7.53%)▶FXR2 (bg=7.51%)▶GRWD1 (bg=4.85%)▶IGF2BP3 (bg=4.26%)▶LARP7 (bg=0.81%)▶PCBP1 (bg=2.3%)▶rbm15 (bg=11.81%)▶SDAD1 (bg=5.99%)▶ybx3 (bg=22.82%)MATCHES To TargetScan▶ miR-760:GGCUCUG▶ miR-744-5p:GCGGGGC

 360  


AAACCCCCGCAGAGCCGCCGGGACGCAGCGC

ACGAAACCCCCGCAGAGCCGCCGGGACGCAGCGC  
Depth:2 (BABOON)  
Ei-value:0.000, Pi-value:0.000  
Er-value:0.000, Pr-value:0.000  
eCLIP MATCHES▶BCCIP (bg=4.61%)▶ddx3x (bg=13.89%)▶dgcr8 (bg=19.31%)▶DHX30 (bg=5.05%)▶drosha (bg=11.37%)▶EIF3H (bg=7.83%)▶FMR1 (bg=6.3%)▶FTO (bg=7.53%)▶FXR2 (bg=7.51%)▶GRWD1 (bg=4.85%)▶IGF2BP3 (bg=4.26%)▶LARP7 (bg=0.81%)▶PCBP1 (bg=2.3%)▶rbm15 (bg=11.81%)▶SDAD1 (bg=5.99%)▶ybx3 (bg=22.82%)MATCHES To TargetScan▶ miR-760:GGCUCUG▶ miR-744-5p:GCGGGGC

C

tttgggc

tttgggc  
Depth:2 (BABOON)  
Ei-value:1.000, Pi-value:0.000  
Er-value:0.000, Pr-value:0.000  
eCLIP MATCHES▶ddx3x (bg=13.89%)▶DHX30 (bg=5.05%)▶drosha (bg=11.37%)▶EIF3H (bg=7.83%)▶FMR1 (bg=6.3%)▶FTO (bg=7.53%)▶FXR2 (bg=7.51%)▶GRWD1 (bg=4.85%)▶METAP2 (bg=6.14%)▶rbm15 (bg=11.81%)▶SDAD1 (bg=5.99%)▶TBRG4 (bg=7.0%)▶ybx3 (bg=22.82%)No matches to TargetScan

GGCGCT

gggcgtgg

gggcgtgg  
Depth:2 (BABOON)  
Ei-value:1.000, Pi-value:0.000  
Er-value:0.000, Pr-value:0.000  
eCLIP MATCHES▶ddx3x (bg=13.89%)▶DHX30 (bg=5.05%)▶drosha (bg=11.37%)▶EIF3H (bg=7.83%)▶FMR1 (bg=6.3%)▶FTO (bg=7.53%)▶FXR2 (bg=7.51%)▶GRWD1 (bg=4.85%)▶METAP2 (bg=6.14%)▶NIPBL (bg=2.83%)▶rbm15 (bg=11.81%)▶SDAD1 (bg=5.99%)▶sf3a3 (bg=12.13%)▶SLTM (bg=1.1%)▶SRSF1 (bg=2.06%)▶TBRG4 (bg=7.0%)▶UTP3 (bg=2.81%)▶WDR43 (bg=0.9%)▶ybx3 (bg=22.82%)No matches to TargetScan

T

gggccgggaag

gggccgggaag  
Depth:2 (BABOON)  
Ei-value:1.000, Pi-value:0.000  
Er-value:0.000, Pr-value:0.000  
eCLIP MATCHES▶ddx3x (bg=13.89%)▶DHX30 (bg=5.05%)▶drosha (bg=11.37%)▶EIF3H (bg=7.83%)▶FMR1 (bg=6.3%)▶FTO (bg=7.53%)▶FXR2 (bg=7.51%)▶GRWD1 (bg=4.85%)▶METAP2 (bg=6.14%)▶NIPBL (bg=2.83%)▶rbm15 (bg=11.81%)▶SDAD1 (bg=5.99%)▶sf3a3 (bg=12.13%)▶SLTM (bg=1.1%)▶SRSF1 (bg=2.06%)▶TBRG4 (bg=7.0%)▶TRA2A (bg=1.38%)▶UTP3 (bg=2.81%)▶WDR43 (bg=0.9%)▶ybx3 (bg=22.82%)No matches to TargetScan

T

ATGGCGGC

ATGGCGGC  
Depth:9 (MOUSE)  
Ei-value:0.000, Pi-value:0.000  
Er-value:0.000, Pr-value:0.000  
eCLIP MATCHES▶ddx3x (bg=13.89%)▶DHX30 (bg=5.05%)▶drosha (bg=11.37%)▶EIF3H (bg=7.83%)▶FMR1 (bg=6.3%)▶FTO (bg=7.53%)▶FXR2 (bg=7.51%)▶GRWD1 (bg=4.85%)▶METAP2 (bg=6.14%)▶NIPBL (bg=2.83%)▶rbm15 (bg=11.81%)▶SDAD1 (bg=5.99%)▶sf3a3 (bg=12.13%)▶SLTM (bg=1.1%)▶SRSF1 (bg=2.06%)▶TBRG4 (bg=7.0%)▶TRA2A (bg=1.38%)▶UTP3 (bg=2.81%)▶WDR43 (bg=0.9%)▶ybx3 (bg=22.82%)No matches to TargetScan

A

GCTCGAACGCCGCGCGGCGGAGGCCATTA

GCTCGAACGCCGCGCGGCGGAGGCCATTA  
Depth:2 (BABOON)  
Ei-value:0.000, Pi-value:0.000  
Er-value:0.000, Pr-value:0.000  
eCLIP MATCHES▶AATF (bg=0.85%)▶ddx3x (bg=13.89%)▶ddx6 (bg=23.92%)▶dgcr8 (bg=19.31%)▶DHX30 (bg=5.05%)▶drosha (bg=11.37%)▶EIF3H (bg=7.83%)▶FTO (bg=7.53%)▶FXR2 (bg=7.51%)▶GRWD1 (bg=4.85%)▶METAP2 (bg=6.14%)▶NIPBL (bg=2.83%)▶rbm15 (bg=11.81%)▶SDAD1 (bg=5.99%)▶sf3a3 (bg=12.13%)▶SLTM (bg=1.1%)▶SRSF1 (bg=2.06%)▶TBRG4 (bg=7.0%)▶TRA2A (bg=1.38%)▶UCHL5 (bg=1.08%)▶WDR43 (bg=0.9%)▶ybx3 (bg=22.82%)No matches to TargetScan

A

ggcgtg

ggcgtg  
Depth:2 (BABOON)  
Ei-value:1.000, Pi-value:0.020  
Er-value:0.000, Pr-value:0.000  
eCLIP MATCHES▶AATF (bg=0.85%)▶ddx3x (bg=13.89%)▶ddx6 (bg=23.92%)▶dgcr8 (bg=19.31%)▶DHX30 (bg=5.05%)▶drosha (bg=11.37%)▶FMR1 (bg=6.3%)▶FXR2 (bg=7.51%)▶GRWD1 (bg=4.85%)▶METAP2 (bg=6.14%)▶NIP7 (bg=2.54%)▶NIPBL (bg=2.83%)▶rbm15 (bg=11.81%)▶sf3a3 (bg=12.13%)▶SLTM (bg=1.1%)▶SRSF1 (bg=2.06%)▶TRA2A (bg=1.38%)▶UCHL5 (bg=1.08%)▶ybx3 (bg=22.82%)No matches to TargetScan

GACGGCCCG 480  


ggaaggcggcctagggacgca

ggaaggcggcctagggacgca  
Depth:2 (BABOON)  
Ei-value:0.490, Pi-value:0.000  
Er-value:0.000, Pr-value:0.000  
eCLIP MATCHES▶AATF (bg=0.85%)▶ddx3x (bg=13.89%)▶dgcr8 (bg=19.31%)▶DHX30 (bg=5.05%)▶drosha (bg=11.37%)▶FMR1 (bg=6.3%)▶FXR2 (bg=7.51%)▶GRWD1 (bg=4.85%)▶NIP7 (bg=2.54%)▶NIPBL (bg=2.83%)▶rbm15 (bg=11.81%)▶SRSF1 (bg=2.06%)▶TRA2A (bg=1.38%)▶UCHL5 (bg=1.08%)▶ybx3 (bg=22.82%)No matches to TargetScan

A

gcag

gcaggctcggc  
Depth:2 (BABOON)  
Ei-value:1.000, Pi-value:0.000  
Er-value:0.000, Pr-value:0.000  
eCLIP MATCHES▶AATF (bg=0.85%)▶ddx3x (bg=13.89%)▶DHX30 (bg=5.05%)▶drosha (bg=11.37%)▶FMR1 (bg=6.3%)▶FXR2 (bg=7.51%)▶GRWD1 (bg=4.85%)▶NIP7 (bg=2.54%)▶SRSF1 (bg=2.06%)▶UCHL5 (bg=1.08%)▶ybx3 (bg=22.82%)MATCHES To TargetScan▶ miR-615-3p:CCGAGCC


GCTCGGC

GCTCGGC  
Depth:3 (DOG)  
Ei-value:0.000, Pi-value:0.000  
Er-value:0.000, Pr-value:0.000  
eCLIP MATCHES▶DHX30 (bg=5.05%)▶drosha (bg=11.37%)▶FMR1 (bg=6.3%)▶FXR2 (bg=7.51%)▶GRWD1 (bg=4.85%)▶NIP7 (bg=2.54%)▶SRSF1 (bg=2.06%)▶UCHL5 (bg=1.08%)▶ybx3 (bg=22.82%)MATCHES To TargetScan▶ miR-615-3p:CCGAGCC

C

gcctctttag

gcctctttag  
Depth:2 (BABOON)  
Ei-value:1.000, Pi-value:0.000  
Er-value:0.000, Pr-value:0.000  
eCLIP MATCHES▶DHX30 (bg=5.05%)▶drosha (bg=11.37%)▶FMR1 (bg=6.3%)▶FXR2 (bg=7.51%)▶GRWD1 (bg=4.85%)▶NIP7 (bg=2.54%)▶SRSF1 (bg=2.06%)▶ybx3 (bg=22.82%)No matches to TargetScan

G

CCACGGAGCCGCGCAGATCCGGTT

CCACGGAGCCGCGCAGATCCGGTTCCCGGGTGACCACTCTGTCGCCATTGGGCGA  
Depth:2 (BABOON)  
Ei-value:0.000, Pi-value:0.000  
Er-value:0.000, Pr-value:0.000  
eCLIP MATCHES▶drosha (bg=11.37%)▶FXR2 (bg=7.51%)▶METAP2 (bg=6.14%)▶rbm15 (bg=11.81%)▶ybx3 (bg=22.82%)MATCHES To TargetScan▶ miR-140-5p:AGUGGUU▶ miR-127-3p:CGGAUCC▶ miR-652-3p:AUGGCGC


cccggg

cccggg  
Depth:4 (COW)  
Ei-value:1.000, Pi-value:0.010  
Er-value:0.000, Pr-value:0.000  
eCLIP MATCHES▶ybx3 (bg=22.82%)No matches to TargetScan


TGACCACTC

TGACCACTC  
Depth:3 (DOG)  
Ei-value:0.000, Pi-value:0.000  
Er-value:0.000, Pr-value:0.000  
eCLIP MATCHES▶ybx3 (bg=22.82%)MATCHES To TargetScan▶ miR-140-5p:AGUGGUU


TGTCGCCATTGGGCGA

CCACGGAGCCGCGCAGATCCGGTTCCCGGGTGACCACTCTGTCGCCATTGGGCGA  
Depth:2 (BABOON)  
Ei-value:0.000, Pi-value:0.000  
Er-value:0.000, Pr-value:0.000  
eCLIP MATCHES▶drosha (bg=11.37%)▶FXR2 (bg=7.51%)▶METAP2 (bg=6.14%)▶rbm15 (bg=11.81%)▶ybx3 (bg=22.82%)MATCHES To TargetScan▶ miR-140-5p:AGUGGUU▶ miR-127-3p:CGGAUCC▶ miR-652-3p:AUGGCGC

G

acctac

acctac  
Depth:3 (DOG)  
Ei-value:0.080, Pi-value:0.000  
Er-value:0.000, Pr-value:0.000  
eCLIP MATCHES▶lin28b (bg=16.96%)▶METAP2 (bg=6.14%)▶rbm15 (bg=11.81%)▶ybx3 (bg=22.82%)No matches to TargetScan


CTAGTCCTGACGA

ACCTACCTAGTCCTGACGACAACGGACAAAGGCCTTAA  
Depth:2 (BABOON)  
Ei-value:0.000, Pi-value:0.000  
Er-value:0.000, Pr-value:0.000  
eCLIP MATCHES▶BUD13 (bg=0.7%)▶ddx3x (bg=13.89%)▶DDX52 (bg=1.27%)▶FTO (bg=7.53%)▶lin28b (bg=16.96%)▶METAP2 (bg=6.14%)▶rbm15 (bg=11.81%)▶SRSF7 (bg=1.29%)▶ybx3 (bg=22.82%)MATCHES To TargetScan▶ miR-124-3p.2/506-3p:UAAGGCA▶ miR-196-5p:AGGUAGU

 600  


CAACGGACAAAGGCCTTAA

ACCTACCTAGTCCTGACGACAACGGACAAAGGCCTTAA  
Depth:2 (BABOON)  
Ei-value:0.000, Pi-value:0.000  
Er-value:0.000, Pr-value:0.000  
eCLIP MATCHES▶BUD13 (bg=0.7%)▶ddx3x (bg=13.89%)▶DDX52 (bg=1.27%)▶FTO (bg=7.53%)▶lin28b (bg=16.96%)▶METAP2 (bg=6.14%)▶rbm15 (bg=11.81%)▶SRSF7 (bg=1.29%)▶ybx3 (bg=22.82%)MATCHES To TargetScan▶ miR-124-3p.2/506-3p:UAAGGCA▶ miR-196-5p:AGGUAGU

G

gggcctgg

gggcctgg  
Depth:2 (BABOON)  
Ei-value:1.000, Pi-value:0.000  
Er-value:0.000, Pr-value:0.000  
eCLIP MATCHES▶BUD13 (bg=0.7%)▶ddx3x (bg=13.89%)▶DDX52 (bg=1.27%)▶dgcr8 (bg=19.31%)▶FTO (bg=7.53%)▶lin28b (bg=16.96%)▶rbm15 (bg=11.81%)▶SRSF7 (bg=1.29%)▶ybx3 (bg=22.82%)No matches to TargetScan

A

aggtgagcg

aggtgagcg  
Depth:4 (COW)  
Ei-value:0.150, Pi-value:0.000  
Er-value:0.000, Pr-value:0.000  
eCLIP MATCHES▶BUD13 (bg=0.7%)▶ddx3x (bg=13.89%)▶DDX52 (bg=1.27%)▶dgcr8 (bg=19.31%)▶FTO (bg=7.53%)▶lin28b (bg=16.96%)▶rbm15 (bg=11.81%)▶SRSF7 (bg=1.29%)▶ybx3 (bg=22.82%)No matches to TargetScan


AAG

AGGTGAGCGAAG  
Depth:3 (DOG)  
Ei-value:0.000, Pi-value:0.000  
Er-value:0.000, Pr-value:0.000  
eCLIP MATCHES▶BUD13 (bg=0.7%)▶ddx3x (bg=13.89%)▶DDX52 (bg=1.27%)▶dgcr8 (bg=19.31%)▶FTO (bg=7.53%)▶lin28b (bg=16.96%)▶rbm15 (bg=11.81%)▶SRSF7 (bg=1.29%)▶ybx3 (bg=22.82%)No matches to TargetScan

T

cccgaacga

cccgaacgacgacgggtggaacg  
Depth:2 (BABOON)  
Ei-value:0.080, Pi-value:0.000  
Er-value:0.000, Pr-value:0.000  
eCLIP MATCHES▶BUD13 (bg=0.7%)▶DDX52 (bg=1.27%)▶FTO (bg=7.53%)▶rbm15 (bg=11.81%)▶SRSF7 (bg=1.29%)▶ybx3 (bg=22.82%)MATCHES To TargetScan▶ miR-99-5p/100-5p:ACCCGUA


CGA

CGACGGGTGGAAC  
Depth:4 (COW)  
Ei-value:0.000, Pi-value:0.000  
Er-value:0.000, Pr-value:0.000  
eCLIP MATCHES▶FTO (bg=7.53%)▶rbm15 (bg=11.81%)▶SRSF7 (bg=1.29%)▶ybx3 (bg=22.82%)MATCHES To TargetScan▶ miR-99-5p/100-5p:ACCCGUA


CGG

CGGGTGGAAC  
Depth:7 (ARMADILLO)  
Ei-value:0.000, Pi-value:0.000  
Er-value:0.000, Pr-value:0.000  
eCLIP MATCHES▶FTO (bg=7.53%)▶rbm15 (bg=11.81%)▶SRSF7 (bg=1.29%)▶ybx3 (bg=22.82%)No matches to TargetScan


GTGGAA

GTGGAA  
Depth:8 (GUINEAPIG)  
Ei-value:0.000, Pi-value:0.000  
Er-value:0.000, Pr-value:0.000  
eCLIP MATCHES▶FTO (bg=7.53%)▶rbm15 (bg=11.81%)▶SRSF7 (bg=1.29%)▶ybx3 (bg=22.82%)No matches to TargetScan


C

CGGGTGGAAC  
Depth:7 (ARMADILLO)  
Ei-value:0.000, Pi-value:0.000  
Er-value:0.000, Pr-value:0.000  
eCLIP MATCHES▶FTO (bg=7.53%)▶rbm15 (bg=11.81%)▶SRSF7 (bg=1.29%)▶ybx3 (bg=22.82%)No matches to TargetScan


g

cccgaacgacgacgggtggaacg  
Depth:2 (BABOON)  
Ei-value:0.080, Pi-value:0.000  
Er-value:0.000, Pr-value:0.000  
eCLIP MATCHES▶BUD13 (bg=0.7%)▶DDX52 (bg=1.27%)▶FTO (bg=7.53%)▶rbm15 (bg=11.81%)▶SRSF7 (bg=1.29%)▶ybx3 (bg=22.82%)MATCHES To TargetScan▶ miR-99-5p/100-5p:ACCCGUA

G

ttagcggccatcgggc

ttagcggccatcgggc  
Depth:2 (BABOON)  
Ei-value:1.000, Pi-value:0.000  
Er-value:0.000, Pr-value:0.000  
eCLIP MATCHES▶FTO (bg=7.53%)▶UTP18 (bg=0.53%)No matches to TargetScan

G

GTTGGTCTTC

GTTGGTCTTC  
Depth:3 (DOG)  
Ei-value:0.000, Pi-value:0.000  
Er-value:0.000, Pr-value:0.000  
eCLIP MATCHES▶UTP18 (bg=0.53%)No matches to TargetScan

A

TTCTAC

TTCTAC  
Depth:6 (PIG)  
Ei-value:0.000, Pi-value:0.000  
Er-value:0.000, Pr-value:0.000  
eCLIP MATCHES▶UTP18 (bg=0.53%)No matches to TargetScan


cagacttt

ttctaccagacttt  
Depth:2 (BABOON)  
Ei-value:1.000, Pi-value:0.000  
Er-value:0.000, Pr-value:0.000  
eCLIP MATCHES▶UTP18 (bg=0.53%)MATCHES To TargetScan▶ miR-379-5p:GGUAGAC

G

C

CTGTCGGAAGA  
Depth:3 (DOG)  
Ei-value:0.000, Pi-value:0.000  
Er-value:0.000, Pr-value:0.000  
No matches to eCLIP DataNo matches to TargetScan


TGTCGGAAGA

TGTCGGAAGA  
Depth:5 (SHEEP)  
Ei-value:0.000, Pi-value:0.000  
Er-value:0.000, Pr-value:0.000  
No matches to eCLIP DataNo matches to TargetScan

 720  


TGTCGGAAGA  
Depth:5 (SHEEP)  
Ei-value:0.000, Pi-value:0.000  
Er-value:0.000, Pr-value:0.000  
No matches to eCLIP DataNo matches to TargetScan


GA

CTGTCGGAAGAGAGAAATGGTAGAATGACAGGCCACGTTTGGCCCGTTGGAAATGCCC  
Depth:2 (BABOON)  
Ei-value:0.000, Pi-value:0.000  
Er-value:0.000, Pr-value:0.000  
eCLIP MATCHES▶ddx55 (bg=12.35%)MATCHES To TargetScan▶ miR-1298-5p:UCAUUCG▶ miR-214-5p:GCCUGUC


GAAATGG

GAAATGG  
Depth:6 (PIG)  
Ei-value:0.000, Pi-value:0.000  
Er-value:0.000, Pr-value:0.000  
eCLIP MATCHES▶ddx55 (bg=12.35%)No matches to TargetScan


TAGAATGACAGGCCA

CTGTCGGAAGAGAGAAATGGTAGAATGACAGGCCACGTTTGGCCCGTTGGAAATGCCC  
Depth:2 (BABOON)  
Ei-value:0.000, Pi-value:0.000  
Er-value:0.000, Pr-value:0.000  
eCLIP MATCHES▶ddx55 (bg=12.35%)MATCHES To TargetScan▶ miR-1298-5p:UCAUUCG▶ miR-214-5p:GCCUGUC


CGTTTGGCC

CGTTTGGCC  
Depth:3 (DOG)  
Ei-value:0.000, Pi-value:0.000  
Er-value:0.000, Pr-value:0.000  
eCLIP MATCHES▶ddx55 (bg=12.35%)No matches to TargetScan


CGTTGGAAATGCCC

CTGTCGGAAGAGAGAAATGGTAGAATGACAGGCCACGTTTGGCCCGTTGGAAATGCCC  
Depth:2 (BABOON)  
Ei-value:0.000, Pi-value:0.000  
Er-value:0.000, Pr-value:0.000  
eCLIP MATCHES▶ddx55 (bg=12.35%)MATCHES To TargetScan▶ miR-1298-5p:UCAUUCG▶ miR-214-5p:GCCUGUC

A

CCACCCT

CCACCCT  
Depth:3 (DOG)  
Ei-value:0.000, Pi-value:0.000  
Er-value:0.000, Pr-value:0.000  
No matches to eCLIP DataNo matches to TargetScan


C

CCACCCTCTGGGAAGATTTACTGGCC  
Depth:2 (BABOON)  
Ei-value:0.000, Pi-value:0.000  
Er-value:0.000, Pr-value:0.000  
No matches to eCLIP DataMATCHES To TargetScan▶ miR-802:CAGUAAC


T

TGGGAAGATTTA  
Depth:8 (GUINEAPIG)  
Ei-value:0.000, Pi-value:0.000  
Er-value:0.000, Pr-value:0.000  
No matches to eCLIP DataNo matches to TargetScan


GGGAAGA

GGGAAGA  
Depth:9 (MOUSE)  
Ei-value:0.000, Pi-value:0.000  
Er-value:0.000, Pr-value:0.000  
No matches to eCLIP DataNo matches to TargetScan


TTTA

TGGGAAGATTTA  
Depth:8 (GUINEAPIG)  
Ei-value:0.000, Pi-value:0.000  
Er-value:0.000, Pr-value:0.000  
No matches to eCLIP DataNo matches to TargetScan


CTGGCC

TGGGAAGATTTACTGGCC  
Depth:5 (SHEEP)  
Ei-value:0.000, Pi-value:0.000  
Er-value:0.000, Pr-value:0.000  
No matches to eCLIP DataMATCHES To TargetScan▶ miR-802:CAGUAAC

GTTTA

TG

TGGAAGGCCTGTGTATATAATATGAAAAAGCTGCTCTCAACTCCACCCCAACCTTTTAATAGAAAACATTTGTCACATCTAGCCCTT  
Depth:2 (BABOON)  
Ei-value:0.000, Pi-value:0.000  
Er-value:0.000, Pr-value:0.000  
eCLIP MATCHES▶KHDRBS1 (bg=1.16%)▶pum1 (bg=29.85%)▶rbm15 (bg=11.81%)MATCHES To TargetScan▶ miR-542-3p:GUGACAG▶ miR-409-3p:AAUGUUG▶ miR-15-5p/16-5p/195-5p/424-5p/497-5p:AGCAGCA▶ miR-503-5p:AGCAGCG


G

GAAGGCCTGTGTATATAATATGAAAAAGCTGCTCTCAACT  
Depth:3 (DOG)  
Ei-value:0.000, Pi-value:0.000  
Er-value:0.000, Pr-value:0.000  
eCLIP MATCHES▶pum1 (bg=29.85%)MATCHES To TargetScan▶ miR-15-5p/16-5p/195-5p/424-5p/497-5p:AGCAGCA▶ miR-503-5p:AGCAGCG


AAGG

AAGGCCTGTGTATATAATATGAAAAAGCTGCT  
Depth:8 (GUINEAPIG)  
Ei-value:0.000, Pi-value:0.000  
Er-value:0.000, Pr-value:0.000  
eCLIP MATCHES▶pum1 (bg=29.85%)MATCHES To TargetScan▶ miR-15-5p/16-5p/195-5p/424-5p/497-5p:AGCAGCA▶ miR-503-5p:AGCAGCG


CCTGTGTATATAATATGAAAAAGCTGCT

CCTGTGTATATAATATGAAAAAGCTGCT  
Depth:9 (MOUSE)  
Ei-value:0.000, Pi-value:0.000  
Er-value:0.000, Pr-value:0.000  
eCLIP MATCHES▶pum1 (bg=29.85%)MATCHES To TargetScan▶ miR-15-5p/16-5p/195-5p/424-5p/497-5p:AGCAGCA▶ miR-503-5p:AGCAGCG


C

AAGGCCTGTGTATATAATATGAAAAAGCTGCTCTCAACT  
Depth:6 (PIG)  
Ei-value:0.000, Pi-value:0.000  
Er-value:0.000, Pr-value:0.000  
eCLIP MATCHES▶pum1 (bg=29.85%)MATCHES To TargetScan▶ miR-15-5p/16-5p/195-5p/424-5p/497-5p:AGCAGCA▶ miR-503-5p:AGCAGCG


TCAAC

TCAACT  
Depth:7 (ARMADILLO)  
Ei-value:0.000, Pi-value:0.000  
Er-value:0.000, Pr-value:0.000  
eCLIP MATCHES▶pum1 (bg=29.85%)No matches to TargetScan

 840  


T

TCAACT  
Depth:7 (ARMADILLO)  
Ei-value:0.000, Pi-value:0.000  
Er-value:0.000, Pr-value:0.000  
eCLIP MATCHES▶pum1 (bg=29.85%)No matches to TargetScan


CCA

TGGAAGGCCTGTGTATATAATATGAAAAAGCTGCTCTCAACTCCACCCCAACCTTTTAATAGAAAACATTTGTCACATCTAGCCCTT  
Depth:2 (BABOON)  
Ei-value:0.000, Pi-value:0.000  
Er-value:0.000, Pr-value:0.000  
eCLIP MATCHES▶KHDRBS1 (bg=1.16%)▶pum1 (bg=29.85%)▶rbm15 (bg=11.81%)MATCHES To TargetScan▶ miR-542-3p:GUGACAG▶ miR-409-3p:AAUGUUG▶ miR-15-5p/16-5p/195-5p/424-5p/497-5p:AGCAGCA▶ miR-503-5p:AGCAGCG


CCC

CCCCAACCTTT  
Depth:7 (ARMADILLO)  
Ei-value:0.000, Pi-value:0.000  
Er-value:0.000, Pr-value:0.000  
eCLIP MATCHES▶pum1 (bg=29.85%)No matches to TargetScan


CAACCTTT

CAACCTTT  
Depth:9 (MOUSE)  
Ei-value:0.000, Pi-value:0.000  
Er-value:0.000, Pr-value:0.000  
eCLIP MATCHES▶pum1 (bg=29.85%)No matches to TargetScan


T

CCCCAACCTTTT  
Depth:3 (DOG)  
Ei-value:0.000, Pi-value:0.000  
Er-value:0.000, Pr-value:0.000  
eCLIP MATCHES▶pum1 (bg=29.85%)No matches to TargetScan


AAT

TGGAAGGCCTGTGTATATAATATGAAAAAGCTGCTCTCAACTCCACCCCAACCTTTTAATAGAAAACATTTGTCACATCTAGCCCTT  
Depth:2 (BABOON)  
Ei-value:0.000, Pi-value:0.000  
Er-value:0.000, Pr-value:0.000  
eCLIP MATCHES▶KHDRBS1 (bg=1.16%)▶pum1 (bg=29.85%)▶rbm15 (bg=11.81%)MATCHES To TargetScan▶ miR-542-3p:GUGACAG▶ miR-409-3p:AAUGUUG▶ miR-15-5p/16-5p/195-5p/424-5p/497-5p:AGCAGCA▶ miR-503-5p:AGCAGCG


AGAAAAC

AGAAAAC  
Depth:8 (GUINEAPIG)  
Ei-value:0.000, Pi-value:0.000  
Er-value:0.000, Pr-value:0.000  
eCLIP MATCHES▶pum1 (bg=29.85%)No matches to TargetScan


ATTTGT

TGGAAGGCCTGTGTATATAATATGAAAAAGCTGCTCTCAACTCCACCCCAACCTTTTAATAGAAAACATTTGTCACATCTAGCCCTT  
Depth:2 (BABOON)  
Ei-value:0.000, Pi-value:0.000  
Er-value:0.000, Pr-value:0.000  
eCLIP MATCHES▶KHDRBS1 (bg=1.16%)▶pum1 (bg=29.85%)▶rbm15 (bg=11.81%)MATCHES To TargetScan▶ miR-542-3p:GUGACAG▶ miR-409-3p:AAUGUUG▶ miR-15-5p/16-5p/195-5p/424-5p/497-5p:AGCAGCA▶ miR-503-5p:AGCAGCG


C

CACATCTAG  
Depth:3 (DOG)  
Ei-value:0.000, Pi-value:0.000  
Er-value:0.000, Pr-value:0.000  
eCLIP MATCHES▶pum1 (bg=29.85%)▶rbm15 (bg=11.81%)No matches to TargetScan


ACATCTAG

ACATCTAG  
Depth:7 (ARMADILLO)  
Ei-value:0.000, Pi-value:0.000  
Er-value:0.000, Pr-value:0.000  
eCLIP MATCHES▶pum1 (bg=29.85%)▶rbm15 (bg=11.81%)No matches to TargetScan


CCCTT

TGGAAGGCCTGTGTATATAATATGAAAAAGCTGCTCTCAACTCCACCCCAACCTTTTAATAGAAAACATTTGTCACATCTAGCCCTT  
Depth:2 (BABOON)  
Ei-value:0.000, Pi-value:0.000  
Er-value:0.000, Pr-value:0.000  
eCLIP MATCHES▶KHDRBS1 (bg=1.16%)▶pum1 (bg=29.85%)▶rbm15 (bg=11.81%)MATCHES To TargetScan▶ miR-542-3p:GUGACAG▶ miR-409-3p:AAUGUUG▶ miR-15-5p/16-5p/195-5p/424-5p/497-5p:AGCAGCA▶ miR-503-5p:AGCAGCG

C

TAGATG

TAGATG  
Depth:8 (GUINEAPIG)  
Ei-value:0.000, Pi-value:0.000  
Er-value:0.000, Pr-value:0.000  
eCLIP MATCHES▶dgcr8 (bg=19.31%)▶KHDRBS1 (bg=1.16%)▶pum1 (bg=29.85%)▶rbm15 (bg=11.81%)No matches to TargetScan


G

TAGATGGAAAGAGGTTGCCGACGTATGATAAA  
Depth:2 (BABOON)  
Ei-value:0.000, Pi-value:0.000  
Er-value:0.000, Pr-value:0.000  
eCLIP MATCHES▶dgcr8 (bg=19.31%)▶IGF2BP2 (bg=3.44%)▶KHDRBS1 (bg=1.16%)▶lin28b (bg=16.96%)▶pum1 (bg=29.85%)▶pum2 (bg=21.55%)▶rbm15 (bg=11.81%)▶tia1 (bg=16.04%)MATCHES To TargetScan▶ miR-154-3p/487-3p:AUCAUAC▶ miR-539-3p:UCAUACA


A

AAAGAGGTTGCCGAC  
Depth:4 (COW)  
Ei-value:0.020, Pi-value:0.000  
Er-value:0.000, Pr-value:0.000  
eCLIP MATCHES▶dgcr8 (bg=19.31%)▶IGF2BP2 (bg=3.44%)▶KHDRBS1 (bg=1.16%)▶lin28b (bg=16.96%)▶pum1 (bg=29.85%)▶pum2 (bg=21.55%)▶rbm15 (bg=11.81%)▶tia1 (bg=16.04%)No matches to TargetScan


A

AAGAGGT  
Depth:8 (GUINEAPIG)  
Ei-value:0.000, Pi-value:0.000  
Er-value:0.000, Pr-value:0.000  
eCLIP MATCHES▶dgcr8 (bg=19.31%)▶IGF2BP2 (bg=3.44%)▶KHDRBS1 (bg=1.16%)▶lin28b (bg=16.96%)▶pum1 (bg=29.85%)▶pum2 (bg=21.55%)▶rbm15 (bg=11.81%)▶tia1 (bg=16.04%)No matches to TargetScan


AGAGGT

AGAGGT  
Depth:9 (MOUSE)  
Ei-value:0.000, Pi-value:0.000  
Er-value:0.000, Pr-value:0.000  
eCLIP MATCHES▶dgcr8 (bg=19.31%)▶IGF2BP2 (bg=3.44%)▶KHDRBS1 (bg=1.16%)▶lin28b (bg=16.96%)▶pum1 (bg=29.85%)▶pum2 (bg=21.55%)▶rbm15 (bg=11.81%)▶tia1 (bg=16.04%)No matches to TargetScan


T

AAGAGGTTGCCGAC  
Depth:6 (PIG)  
Ei-value:0.000, Pi-value:0.000  
Er-value:0.000, Pr-value:0.000  
eCLIP MATCHES▶dgcr8 (bg=19.31%)▶IGF2BP2 (bg=3.44%)▶KHDRBS1 (bg=1.16%)▶lin28b (bg=16.96%)▶pum1 (bg=29.85%)▶pum2 (bg=21.55%)▶rbm15 (bg=11.81%)▶tia1 (bg=16.04%)No matches to TargetScan


GCCGAC

GCCGAC  
Depth:7 (ARMADILLO)  
Ei-value:0.000, Pi-value:0.000  
Er-value:0.000, Pr-value:0.000  
eCLIP MATCHES▶dgcr8 (bg=19.31%)▶IGF2BP2 (bg=3.44%)▶KHDRBS1 (bg=1.16%)▶lin28b (bg=16.96%)▶pum1 (bg=29.85%)▶pum2 (bg=21.55%)▶rbm15 (bg=11.81%)▶tia1 (bg=16.04%)No matches to TargetScan


G

TAGATGGAAAGAGGTTGCCGACGTATGATAAA  
Depth:2 (BABOON)  
Ei-value:0.000, Pi-value:0.000  
Er-value:0.000, Pr-value:0.000  
eCLIP MATCHES▶dgcr8 (bg=19.31%)▶IGF2BP2 (bg=3.44%)▶KHDRBS1 (bg=1.16%)▶lin28b (bg=16.96%)▶pum1 (bg=29.85%)▶pum2 (bg=21.55%)▶rbm15 (bg=11.81%)▶tia1 (bg=16.04%)MATCHES To TargetScan▶ miR-154-3p/487-3p:AUCAUAC▶ miR-539-3p:UCAUACA


TATGATAAA

TATGATAAA  
Depth:8 (GUINEAPIG)  
Ei-value:0.000, Pi-value:0.000  
Er-value:0.000, Pr-value:0.000  
eCLIP MATCHES▶dgcr8 (bg=19.31%)▶IGF2BP2 (bg=3.44%)▶KHDRBS1 (bg=1.16%)▶lin28b (bg=16.96%)▶pum1 (bg=29.85%)▶pum2 (bg=21.55%)▶rbm15 (bg=11.81%)▶tia1 (bg=16.04%)MATCHES To TargetScan▶ miR-154-3p/487-3p:AUCAUAC

A

TAG

TAGAGTTAGAAA  
Depth:3 (DOG)  
Ei-value:0.000, Pi-value:0.000  
Er-value:0.000, Pr-value:0.000  
eCLIP MATCHES▶dgcr8 (bg=19.31%)▶IGF2BP2 (bg=3.44%)▶KHDRBS1 (bg=1.16%)▶lin28b (bg=16.96%)▶pum1 (bg=29.85%)▶pum2 (bg=21.55%)▶rbm15 (bg=11.81%)▶tia1 (bg=16.04%)No matches to TargetScan


AGTTAG

AGTTAG  
Depth:8 (GUINEAPIG)  
Ei-value:0.000, Pi-value:0.000  
Er-value:0.000, Pr-value:0.000  
eCLIP MATCHES▶dgcr8 (bg=19.31%)▶IGF2BP2 (bg=3.44%)▶KHDRBS1 (bg=1.16%)▶lin28b (bg=16.96%)▶pum2 (bg=21.55%)▶rbm15 (bg=11.81%)▶tia1 (bg=16.04%)No matches to TargetScan


AAA

AGTTAGAAA  
Depth:7 (ARMADILLO)  
Ei-value:0.000, Pi-value:0.000  
Er-value:0.000, Pr-value:0.000  
eCLIP MATCHES▶dgcr8 (bg=19.31%)▶IGF2BP2 (bg=3.44%)▶KHDRBS1 (bg=1.16%)▶lin28b (bg=16.96%)▶pum2 (bg=21.55%)▶rbm15 (bg=11.81%)▶tia1 (bg=16.04%)No matches to TargetScan


gt

tagagttagaaagt  
Depth:2 (BABOON)  
Ei-value:1.000, Pi-value:0.000  
Er-value:0.000, Pr-value:0.000  
eCLIP MATCHES▶dgcr8 (bg=19.31%)▶IGF2BP2 (bg=3.44%)▶KHDRBS1 (bg=1.16%)▶lin28b (bg=16.96%)▶pum1 (bg=29.85%)▶pum2 (bg=21.55%)▶rbm15 (bg=11.81%)▶tia1 (bg=16.04%)No matches to TargetScan

T

ACACA

ACACATCTTGTAAATTCTCATTTGTTTAAAAGAAATCATAGAAAATAC  
Depth:2 (BABOON)  
Ei-value:0.000, Pi-value:0.000  
Er-value:0.000, Pr-value:0.000  
eCLIP MATCHES▶fubp3 (bg=23.31%)▶GRWD1 (bg=4.85%)▶KHDRBS1 (bg=1.16%)▶lin28b (bg=16.96%)▶pum1 (bg=29.85%)▶pum2 (bg=21.55%)▶rbm15 (bg=11.81%)▶sf3a3 (bg=12.13%)▶ZNF622 (bg=1.54%)MATCHES To TargetScan▶ miR-495-3p:AACAAAC


TC

TCTTGTAAAT  
Depth:6 (PIG)  
Ei-value:0.000, Pi-value:0.000  
Er-value:0.000, Pr-value:0.000  
eCLIP MATCHES▶KHDRBS1 (bg=1.16%)▶lin28b (bg=16.96%)No matches to TargetScan


TTGTAA

TTGTAA  
Depth:7 (ARMADILLO)  
Ei-value:0.000, Pi-value:0.020  
Er-value:0.000, Pr-value:0.020  
eCLIP MATCHES▶KHDRBS1 (bg=1.16%)▶lin28b (bg=16.96%)No matches to TargetScan


AT

TCTTGTAAAT  
Depth:6 (PIG)  
Ei-value:0.000, Pi-value:0.000  
Er-value:0.000, Pr-value:0.000  
eCLIP MATCHES▶KHDRBS1 (bg=1.16%)▶lin28b (bg=16.96%)No matches to TargetScan


TCTCA

ACACATCTTGTAAATTCTCATTTGTTTAAAAGAAATCATAGAAAATAC  
Depth:2 (BABOON)  
Ei-value:0.000, Pi-value:0.000  
Er-value:0.000, Pr-value:0.000  
eCLIP MATCHES▶fubp3 (bg=23.31%)▶GRWD1 (bg=4.85%)▶KHDRBS1 (bg=1.16%)▶lin28b (bg=16.96%)▶pum1 (bg=29.85%)▶pum2 (bg=21.55%)▶rbm15 (bg=11.81%)▶sf3a3 (bg=12.13%)▶ZNF622 (bg=1.54%)MATCHES To TargetScan▶ miR-495-3p:AACAAAC


TTTGT

TTTGTTT  
Depth:5 (SHEEP)  
Ei-value:0.000, Pi-value:0.020  
Er-value:0.000, Pr-value:0.010  
No matches to eCLIP DataMATCHES To TargetScan▶ miR-495-3p:AACAAAC

 960  


TT

TTTGTTT  
Depth:5 (SHEEP)  
Ei-value:0.000, Pi-value:0.020  
Er-value:0.000, Pr-value:0.010  
No matches to eCLIP DataMATCHES To TargetScan▶ miR-495-3p:AACAAAC


AAAAG

ACACATCTTGTAAATTCTCATTTGTTTAAAAGAAATCATAGAAAATAC  
Depth:2 (BABOON)  
Ei-value:0.000, Pi-value:0.000  
Er-value:0.000, Pr-value:0.000  
eCLIP MATCHES▶fubp3 (bg=23.31%)▶GRWD1 (bg=4.85%)▶KHDRBS1 (bg=1.16%)▶lin28b (bg=16.96%)▶pum1 (bg=29.85%)▶pum2 (bg=21.55%)▶rbm15 (bg=11.81%)▶sf3a3 (bg=12.13%)▶ZNF622 (bg=1.54%)MATCHES To TargetScan▶ miR-495-3p:AACAAAC


AAATC

AAATCATAGAAA  
Depth:3 (DOG)  
Ei-value:0.000, Pi-value:0.000  
Er-value:0.000, Pr-value:0.000  
eCLIP MATCHES▶fubp3 (bg=23.31%)▶GRWD1 (bg=4.85%)▶pum1 (bg=29.85%)▶pum2 (bg=21.55%)▶rbm15 (bg=11.81%)▶sf3a3 (bg=12.13%)▶ZNF622 (bg=1.54%)No matches to TargetScan


ATAGAAA

ATAGAAA  
Depth:5 (SHEEP)  
Ei-value:0.000, Pi-value:0.000  
Er-value:0.000, Pr-value:0.000  
eCLIP MATCHES▶fubp3 (bg=23.31%)▶GRWD1 (bg=4.85%)▶pum1 (bg=29.85%)▶pum2 (bg=21.55%)▶rbm15 (bg=11.81%)▶sf3a3 (bg=12.13%)▶ZNF622 (bg=1.54%)No matches to TargetScan


ATAC

ACACATCTTGTAAATTCTCATTTGTTTAAAAGAAATCATAGAAAATAC  
Depth:2 (BABOON)  
Ei-value:0.000, Pi-value:0.000  
Er-value:0.000, Pr-value:0.000  
eCLIP MATCHES▶fubp3 (bg=23.31%)▶GRWD1 (bg=4.85%)▶KHDRBS1 (bg=1.16%)▶lin28b (bg=16.96%)▶pum1 (bg=29.85%)▶pum2 (bg=21.55%)▶rbm15 (bg=11.81%)▶sf3a3 (bg=12.13%)▶ZNF622 (bg=1.54%)MATCHES To TargetScan▶ miR-495-3p:AACAAAC

A

TGTCTTCTGGAGATGA

TGTCTTCTGGAGATGACTTTTGGAAATG  
Depth:2 (BABOON)  
Ei-value:0.000, Pi-value:0.000  
Er-value:0.000, Pr-value:0.000  
eCLIP MATCHES▶fubp3 (bg=23.31%)▶GRWD1 (bg=4.85%)▶PPIG (bg=0.88%)▶pum1 (bg=29.85%)▶pum2 (bg=21.55%)▶rbm15 (bg=11.81%)▶sf3a3 (bg=12.13%)▶SND1 (bg=1.27%)▶ZNF622 (bg=1.54%)MATCHES To TargetScan▶ miR-224-5p:AAGUCAC


CTTT

CTTTTGGAAATG  
Depth:3 (DOG)  
Ei-value:0.000, Pi-value:0.000  
Er-value:0.000, Pr-value:0.000  
eCLIP MATCHES▶fubp3 (bg=23.31%)▶GRWD1 (bg=4.85%)▶PPIG (bg=0.88%)▶pum1 (bg=29.85%)▶pum2 (bg=21.55%)▶rbm15 (bg=11.81%)▶SND1 (bg=1.27%)▶ZNF622 (bg=1.54%)No matches to TargetScan


TG

TGGAAATG  
Depth:8 (GUINEAPIG)  
Ei-value:0.000, Pi-value:0.000  
Er-value:0.000, Pr-value:0.000  
eCLIP MATCHES▶fubp3 (bg=23.31%)▶GRWD1 (bg=4.85%)▶PPIG (bg=0.88%)▶pum1 (bg=29.85%)▶pum2 (bg=21.55%)▶rbm15 (bg=11.81%)▶SND1 (bg=1.27%)▶ZNF622 (bg=1.54%)No matches to TargetScan


GAAATG

GAAATG  
Depth:9 (MOUSE)  
Ei-value:0.000, Pi-value:0.000  
Er-value:0.000, Pr-value:0.000  
eCLIP MATCHES▶fubp3 (bg=23.31%)▶GRWD1 (bg=4.85%)▶PPIG (bg=0.88%)▶pum1 (bg=29.85%)▶pum2 (bg=21.55%)▶rbm15 (bg=11.81%)▶SND1 (bg=1.27%)▶ZNF622 (bg=1.54%)No matches to TargetScan

G

agttgtt

agttgtt  
Depth:2 (BABOON)  
Ei-value:1.000, Pi-value:0.020  
Er-value:0.000, Pr-value:0.010  
eCLIP MATCHES▶fubp3 (bg=23.31%)▶GRWD1 (bg=4.85%)▶PPIG (bg=0.88%)▶pum1 (bg=29.85%)▶rbm15 (bg=11.81%)▶SND1 (bg=1.27%)▶ZNF622 (bg=1.54%)No matches to TargetScan

A

AGACGGCCTCTG

AGACGGCCTCTGGAAGCGATACGTCCACG  
Depth:2 (BABOON)  
Ei-value:0.000, Pi-value:0.000  
Er-value:0.000, Pr-value:0.000  
eCLIP MATCHES▶fubp3 (bg=23.31%)▶GNL3 (bg=0.85%)▶GRWD1 (bg=4.85%)▶lin28b (bg=16.96%)▶PPIG (bg=0.88%)▶pum1 (bg=29.85%)▶rbm15 (bg=11.81%)▶SND1 (bg=1.27%)▶ZNF622 (bg=1.54%)No matches to TargetScan


GAAGCGA

GAAGCGA  
Depth:3 (DOG)  
Ei-value:0.000, Pi-value:0.000  
Er-value:0.000, Pr-value:0.000  
eCLIP MATCHES▶fubp3 (bg=23.31%)▶GNL3 (bg=0.85%)▶GRWD1 (bg=4.85%)▶lin28b (bg=16.96%)▶PPIG (bg=0.88%)▶pum1 (bg=29.85%)▶rbm15 (bg=11.81%)▶ZNF622 (bg=1.54%)No matches to TargetScan


TACGTCCACG

AGACGGCCTCTGGAAGCGATACGTCCACG  
Depth:2 (BABOON)  
Ei-value:0.000, Pi-value:0.000  
Er-value:0.000, Pr-value:0.000  
eCLIP MATCHES▶fubp3 (bg=23.31%)▶GNL3 (bg=0.85%)▶GRWD1 (bg=4.85%)▶lin28b (bg=16.96%)▶PPIG (bg=0.88%)▶pum1 (bg=29.85%)▶rbm15 (bg=11.81%)▶SND1 (bg=1.27%)▶ZNF622 (bg=1.54%)No matches to TargetScan

TTTG

TTA

TTAAGTGGGTTAGATGACATGGAGCTGGAAGAC  
Depth:2 (BABOON)  
Ei-value:0.000, Pi-value:0.000  
Er-value:0.000, Pr-value:0.000  
eCLIP MATCHES▶GNL3 (bg=0.85%)▶PPIG (bg=0.88%)▶pum1 (bg=29.85%)▶ZNF622 (bg=1.54%)MATCHES To TargetScan▶ miR-136-5p:CUCCAUU


A

AGTGGGT  
Depth:6 (PIG)  
Ei-value:0.000, Pi-value:0.000  
Er-value:0.000, Pr-value:0.000  
eCLIP MATCHES▶GNL3 (bg=0.85%)▶PPIG (bg=0.88%)▶pum1 (bg=29.85%)▶ZNF622 (bg=1.54%)No matches to TargetScan


GTGGGT

GTGGGT  
Depth:7 (ARMADILLO)  
Ei-value:0.000, Pi-value:0.000  
Er-value:0.000, Pr-value:0.000  
eCLIP MATCHES▶GNL3 (bg=0.85%)▶PPIG (bg=0.88%)No matches to TargetScan


TAGAT

TTAAGTGGGTTAGATGACATGGAGCTGGAAGAC  
Depth:2 (BABOON)  
Ei-value:0.000, Pi-value:0.000  
Er-value:0.000, Pr-value:0.000  
eCLIP MATCHES▶GNL3 (bg=0.85%)▶PPIG (bg=0.88%)▶pum1 (bg=29.85%)▶ZNF622 (bg=1.54%)MATCHES To TargetScan▶ miR-136-5p:CUCCAUU


gacatg

gacatg  
Depth:3 (DOG)  
Ei-value:0.080, Pi-value:0.010  
Er-value:0.000, Pr-value:0.000  
eCLIP MATCHES▶GNL3 (bg=0.85%)No matches to TargetScan


GAGCT

TTAAGTGGGTTAGATGACATGGAGCTGGAAGAC  
Depth:2 (BABOON)  
Ei-value:0.000, Pi-value:0.000  
Er-value:0.000, Pr-value:0.000  
eCLIP MATCHES▶GNL3 (bg=0.85%)▶PPIG (bg=0.88%)▶pum1 (bg=29.85%)▶ZNF622 (bg=1.54%)MATCHES To TargetScan▶ miR-136-5p:CUCCAUU

 1080  


TTAAGTGGGTTAGATGACATGGAGCTGGAAGAC  
Depth:2 (BABOON)  
Ei-value:0.000, Pi-value:0.000  
Er-value:0.000, Pr-value:0.000  
eCLIP MATCHES▶GNL3 (bg=0.85%)▶PPIG (bg=0.88%)▶pum1 (bg=29.85%)▶ZNF622 (bg=1.54%)MATCHES To TargetScan▶ miR-136-5p:CUCCAUU


GGAAGA

GGAAGA  
Depth:6 (PIG)  
Ei-value:0.000, Pi-value:0.000  
Er-value:0.000, Pr-value:0.000  
No matches to eCLIP DataNo matches to TargetScan


C

TTAAGTGGGTTAGATGACATGGAGCTGGAAGAC  
Depth:2 (BABOON)  
Ei-value:0.000, Pi-value:0.000  
Er-value:0.000, Pr-value:0.000  
eCLIP MATCHES▶GNL3 (bg=0.85%)▶PPIG (bg=0.88%)▶pum1 (bg=29.85%)▶ZNF622 (bg=1.54%)MATCHES To TargetScan▶ miR-136-5p:CUCCAUU

C

tgagaaggaag

tgagaaggaag  
Depth:2 (BABOON)  
Ei-value:1.000, Pi-value:0.000  
Er-value:0.000, Pr-value:0.000  
No matches to eCLIP DataNo matches to TargetScan

AGA

AGAAGGTTCT

AGAAGGTTCT  
Depth:3 (DOG)  
Ei-value:0.000, Pi-value:0.000  
Er-value:0.000, Pr-value:0.000  
No matches to eCLIP DataNo matches to TargetScan


ATGCT

AGAAGGTTCTATGCTAGACTGGTCATATTTAGAAGACATTTTCATATTCTATCCATTGTTTTGTGTGCATTTTATTCCTCACTACTGTGTATATA  
Depth:2 (BABOON)  
Ei-value:0.000, Pi-value:0.000  
Er-value:0.000, Pr-value:0.000  
No matches to eCLIP DataMATCHES To TargetScan▶ miR-1224-5p:UGAGGAC▶ miR-199-3p:CAGUAGU▶ miR-501-3p/502-3p:AUGCACC▶ miR-144-3p:ACAGUAU▶ miR-128-3p:CACAGUG▶ miR-101-3p.1:ACAGUAC▶ miR-142-3p.1:GUAGUGU


AGACTG

AGACTGGTCATA  
Depth:5 (SHEEP)  
Ei-value:0.000, Pi-value:0.000  
Er-value:0.000, Pr-value:0.000  
No matches to eCLIP DataNo matches to TargetScan


GTCATA

GTCATA  
Depth:7 (ARMADILLO)  
Ei-value:0.000, Pi-value:0.000  
Er-value:0.000, Pr-value:0.000  
No matches to eCLIP DataNo matches to TargetScan


T

AGAAGGTTCTATGCTAGACTGGTCATATTTAGAAGACATTTTCATATTCTATCCATTGTTTTGTGTGCATTTTATTCCTCACTACTGTGTATATA  
Depth:2 (BABOON)  
Ei-value:0.000, Pi-value:0.000  
Er-value:0.000, Pr-value:0.000  
No matches to eCLIP DataMATCHES To TargetScan▶ miR-1224-5p:UGAGGAC▶ miR-199-3p:CAGUAGU▶ miR-501-3p/502-3p:AUGCACC▶ miR-144-3p:ACAGUAU▶ miR-128-3p:CACAGUG▶ miR-101-3p.1:ACAGUAC▶ miR-142-3p.1:GUAGUGU


T

TTAGAAGA  
Depth:7 (ARMADILLO)  
Ei-value:0.000, Pi-value:0.000  
Er-value:0.000, Pr-value:0.000  
No matches to eCLIP DataNo matches to TargetScan


T

TAGAAGA  
Depth:8 (GUINEAPIG)  
Ei-value:0.000, Pi-value:0.000  
Er-value:0.000, Pr-value:0.000  
No matches to eCLIP DataNo matches to TargetScan


AGAAGA

AGAAGA  
Depth:9 (MOUSE)  
Ei-value:0.000, Pi-value:0.000  
Er-value:0.000, Pr-value:0.000  
No matches to eCLIP DataNo matches to TargetScan


CATTT

TTAGAAGACATTT  
Depth:6 (PIG)  
Ei-value:0.000, Pi-value:0.000  
Er-value:0.000, Pr-value:0.000  
No matches to eCLIP DataNo matches to TargetScan


TCA

TTAGAAGACATTTTCA  
Depth:3 (DOG)  
Ei-value:0.000, Pi-value:0.000  
Er-value:0.000, Pr-value:0.000  
No matches to eCLIP DataNo matches to TargetScan


TATTCTAT

AGAAGGTTCTATGCTAGACTGGTCATATTTAGAAGACATTTTCATATTCTATCCATTGTTTTGTGTGCATTTTATTCCTCACTACTGTGTATATA  
Depth:2 (BABOON)  
Ei-value:0.000, Pi-value:0.000  
Er-value:0.000, Pr-value:0.000  
No matches to eCLIP DataMATCHES To TargetScan▶ miR-1224-5p:UGAGGAC▶ miR-199-3p:CAGUAGU▶ miR-501-3p/502-3p:AUGCACC▶ miR-144-3p:ACAGUAU▶ miR-128-3p:CACAGUG▶ miR-101-3p.1:ACAGUAC▶ miR-142-3p.1:GUAGUGU


CCATTGTTT

CCATTGTTT  
Depth:3 (DOG)  
Ei-value:0.000, Pi-value:0.010  
Er-value:0.000, Pr-value:0.000  
No matches to eCLIP DataNo matches to TargetScan


TGTGTGCATTTT

TGTGTGCATTTT  
Depth:6 (PIG)  
Ei-value:0.000, Pi-value:0.000  
Er-value:0.000, Pr-value:0.000  
No matches to eCLIP DataMATCHES To TargetScan▶ miR-501-3p/502-3p:AUGCACC


A

TGTGTGCATTTTATTCCTC  
Depth:3 (DOG)  
Ei-value:0.000, Pi-value:0.000  
Er-value:0.000, Pr-value:0.000  
No matches to eCLIP DataMATCHES To TargetScan▶ miR-501-3p/502-3p:AUGCACC


TTCCTC

TTCCTC  
Depth:7 (ARMADILLO)  
Ei-value:0.000, Pi-value:0.000  
Er-value:0.000, Pr-value:0.000  
No matches to eCLIP DataNo matches to TargetScan


ACTACTG

ACTACTGTGTATATA  
Depth:3 (DOG)  
Ei-value:0.000, Pi-value:0.000  
Er-value:0.000, Pr-value:0.000  
No matches to eCLIP DataMATCHES To TargetScan▶ miR-199-3p:CAGUAGU▶ miR-144-3p:ACAGUAU▶ miR-128-3p:CACAGUG▶ miR-101-3p.1:ACAGUAC


TG

TGTATATA  
Depth:9 (MOUSE)  
Ei-value:0.000, Pi-value:0.000  
Er-value:0.000, Pr-value:0.000  
No matches to eCLIP DataNo matches to TargetScan


TATATA

TATATA  
Depth:6 (PIG)  
Ei-value:0.000, Pi-value:0.030  
Er-value:0.000, Pr-value:0.000  
No matches to eCLIP DataNo matches to TargetScan

G

TT

TTGACAATGCTAAG  
Depth:3 (DOG)  
Ei-value:0.000, Pi-value:0.000  
Er-value:0.000, Pr-value:0.000  
No matches to eCLIP DataNo matches to TargetScan

 1200  


GACAATGCTAAG

TTGACAATGCTAAG  
Depth:3 (DOG)  
Ei-value:0.000, Pi-value:0.000  
Er-value:0.000, Pr-value:0.000  
No matches to eCLIP DataNo matches to TargetScan


cttttttga

ttgacaatgctaagcttttttga  
Depth:2 (BABOON)  
Ei-value:0.080, Pi-value:0.000  
Er-value:0.000, Pr-value:0.000  
No matches to eCLIP DataMATCHES To TargetScan▶ miR-320:AAAGCUG▶ miR-21-5p/590-5p:AGCUUAU

A

atgtct

atgtct  
Depth:2 (BABOON)  
Ei-value:1.000, Pi-value:0.030  
Er-value:0.000, Pr-value:0.010  
No matches to eCLIP DataNo matches to TargetScan

C

TTCTTTT

TTCTTTTTAGATGTTCTGAAGTGCCTGA  
Depth:2 (BABOON)  
Ei-value:0.000, Pi-value:0.000  
Er-value:0.000, Pr-value:0.000  
eCLIP MATCHES▶pum1 (bg=29.85%)▶PUS1 (bg=2.87%)▶tial1 (bg=14.09%)MATCHES To TargetScan▶ miR-186-5p:AAAGAAU


TAGATG

TAGATGTTCTGAAGTGCCTGA  
Depth:5 (SHEEP)  
Ei-value:0.000, Pi-value:0.000  
Er-value:0.000, Pr-value:0.000  
eCLIP MATCHES▶pum1 (bg=29.85%)▶PUS1 (bg=2.87%)▶tial1 (bg=14.09%)No matches to TargetScan


TTCTG

TTCTGAAGTGCCTGA  
Depth:7 (ARMADILLO)  
Ei-value:0.000, Pi-value:0.000  
Er-value:0.000, Pr-value:0.000  
eCLIP MATCHES▶pum1 (bg=29.85%)▶PUS1 (bg=2.87%)▶tial1 (bg=14.09%)No matches to TargetScan


AAGTGCCTG

AAGTGCCTG  
Depth:9 (MOUSE)  
Ei-value:0.000, Pi-value:0.000  
Er-value:0.000, Pr-value:0.000  
eCLIP MATCHES▶pum1 (bg=29.85%)▶PUS1 (bg=2.87%)▶tial1 (bg=14.09%)No matches to TargetScan


A

AAGTGCCTGA  
Depth:8 (GUINEAPIG)  
Ei-value:0.000, Pi-value:0.000  
Er-value:0.000, Pr-value:0.000  
eCLIP MATCHES▶pum1 (bg=29.85%)▶PUS1 (bg=2.87%)▶tial1 (bg=14.09%)No matches to TargetScan


T

TATGTTAAAATTAGAGGTAGCAAAAT  
Depth:2 (BABOON)  
Ei-value:0.000, Pi-value:0.000  
Er-value:0.000, Pr-value:0.000  
eCLIP MATCHES▶CPEB4 (bg=2.3%)▶ddx55 (bg=12.35%)▶pum1 (bg=29.85%)▶PUS1 (bg=2.87%)▶tial1 (bg=14.09%)No matches to TargetScan


A

ATGTTAAAA  
Depth:6 (PIG)  
Ei-value:0.000, Pi-value:0.000  
Er-value:0.000, Pr-value:0.000  
eCLIP MATCHES▶ddx55 (bg=12.35%)▶pum1 (bg=29.85%)▶PUS1 (bg=2.87%)▶tial1 (bg=14.09%)No matches to TargetScan


T

TGTTAAAA  
Depth:7 (ARMADILLO)  
Ei-value:0.000, Pi-value:0.000  
Er-value:0.000, Pr-value:0.000  
eCLIP MATCHES▶ddx55 (bg=12.35%)▶pum1 (bg=29.85%)▶PUS1 (bg=2.87%)▶tial1 (bg=14.09%)No matches to TargetScan


GTTAAAA

GTTAAAA  
Depth:8 (GUINEAPIG)  
Ei-value:0.000, Pi-value:0.000  
Er-value:0.000, Pr-value:0.000  
eCLIP MATCHES▶ddx55 (bg=12.35%)▶pum1 (bg=29.85%)▶PUS1 (bg=2.87%)▶tial1 (bg=14.09%)No matches to TargetScan


T

TATGTTAAAATTAGAGGTAGCAAAAT  
Depth:2 (BABOON)  
Ei-value:0.000, Pi-value:0.000  
Er-value:0.000, Pr-value:0.000  
eCLIP MATCHES▶CPEB4 (bg=2.3%)▶ddx55 (bg=12.35%)▶pum1 (bg=29.85%)▶PUS1 (bg=2.87%)▶tial1 (bg=14.09%)No matches to TargetScan


TA

TAGAGGTAG  
Depth:3 (DOG)  
Ei-value:0.000, Pi-value:0.000  
Er-value:0.000, Pr-value:0.000  
eCLIP MATCHES▶ddx55 (bg=12.35%)▶pum1 (bg=29.85%)▶PUS1 (bg=2.87%)▶tial1 (bg=14.09%)No matches to TargetScan


GAGGTAG

GAGGTAG  
Depth:6 (PIG)  
Ei-value:0.000, Pi-value:0.000  
Er-value:0.000, Pr-value:0.000  
eCLIP MATCHES▶ddx55 (bg=12.35%)▶pum1 (bg=29.85%)▶PUS1 (bg=2.87%)▶tial1 (bg=14.09%)No matches to TargetScan


CAAAAT

TATGTTAAAATTAGAGGTAGCAAAAT  
Depth:2 (BABOON)  
Ei-value:0.000, Pi-value:0.000  
Er-value:0.000, Pr-value:0.000  
eCLIP MATCHES▶CPEB4 (bg=2.3%)▶ddx55 (bg=12.35%)▶pum1 (bg=29.85%)▶PUS1 (bg=2.87%)▶tial1 (bg=14.09%)No matches to TargetScan

C

ACA

ACATTTTGTAAATA  
Depth:5 (SHEEP)  
Ei-value:0.000, Pi-value:0.000  
Er-value:0.000, Pr-value:0.000  
eCLIP MATCHES▶CPEB4 (bg=2.3%)▶ddx55 (bg=12.35%)▶drosha (bg=11.37%)▶pum1 (bg=29.85%)▶PUS1 (bg=2.87%)▶tial1 (bg=14.09%)No matches to TargetScan


TTTTGT

TTTTGT  
Depth:6 (PIG)  
Ei-value:0.000, Pi-value:0.020  
Er-value:0.000, Pr-value:0.040  
eCLIP MATCHES▶CPEB4 (bg=2.3%)▶pum1 (bg=29.85%)▶PUS1 (bg=2.87%)▶tial1 (bg=14.09%)No matches to TargetScan


AAATA

ACATTTTGTAAATA  
Depth:5 (SHEEP)  
Ei-value:0.000, Pi-value:0.000  
Er-value:0.000, Pr-value:0.000  
eCLIP MATCHES▶CPEB4 (bg=2.3%)▶ddx55 (bg=12.35%)▶drosha (bg=11.37%)▶pum1 (bg=29.85%)▶PUS1 (bg=2.87%)▶tial1 (bg=14.09%)No matches to TargetScan

C

CTTTTTGTTACA

CTTTTTGTTACAATTCATAGGAAAT  
Depth:2 (BABOON)  
Ei-value:0.000, Pi-value:0.000  
Er-value:0.000, Pr-value:0.000  
eCLIP MATCHES▶AGGF1 (bg=0.88%)▶CPEB4 (bg=2.3%)▶ddx55 (bg=12.35%)▶ddx6 (bg=23.92%)▶dgcr8 (bg=19.31%)▶drosha (bg=11.37%)▶fam120a (bg=18.43%)▶FASTKD2 (bg=7.73%)▶FTO (bg=7.53%)▶fubp3 (bg=23.31%)▶FXR2 (bg=7.51%)▶igf2bp1 (bg=11.67%)▶IGF2BP2 (bg=3.44%)▶IGF2BP3 (bg=4.26%)▶pum1 (bg=29.85%)▶pum2 (bg=21.55%)▶PUS1 (bg=2.87%)▶rbm15 (bg=11.81%)▶sf3a3 (bg=12.13%)▶SF3B1 (bg=6.74%)▶tia1 (bg=16.04%)▶tial1 (bg=14.09%)▶UPF1 (bg=3.62%)MATCHES To TargetScan▶ miR-219a-2-3p:GAAUUGU▶ miR-194-5p:GUAACAG▶ miR-495-3p:AACAAAC▶ miR-202-5p:UCCUAUG


ATTCATA

ATTCATA  
Depth:5 (SHEEP)  
Ei-value:0.000, Pi-value:0.000  
Er-value:0.000, Pr-value:0.000  
eCLIP MATCHES▶AGGF1 (bg=0.88%)▶CPEB4 (bg=2.3%)▶ddx55 (bg=12.35%)▶ddx6 (bg=23.92%)▶dgcr8 (bg=19.31%)▶drosha (bg=11.37%)▶fam120a (bg=18.43%)▶FASTKD2 (bg=7.73%)▶fubp3 (bg=23.31%)▶igf2bp1 (bg=11.67%)▶pum1 (bg=29.85%)▶pum2 (bg=21.55%)▶rbm15 (bg=11.81%)▶sf3a3 (bg=12.13%)▶SF3B1 (bg=6.74%)▶tia1 (bg=16.04%)▶tial1 (bg=14.09%)▶UPF1 (bg=3.62%)No matches to TargetScan


GG

ATTCATAGGAAAT  
Depth:3 (DOG)  
Ei-value:0.000, Pi-value:0.000  
Er-value:0.000, Pr-value:0.000  
eCLIP MATCHES▶AGGF1 (bg=0.88%)▶CPEB4 (bg=2.3%)▶ddx55 (bg=12.35%)▶ddx6 (bg=23.92%)▶dgcr8 (bg=19.31%)▶drosha (bg=11.37%)▶fam120a (bg=18.43%)▶FASTKD2 (bg=7.73%)▶FTO (bg=7.53%)▶fubp3 (bg=23.31%)▶FXR2 (bg=7.51%)▶igf2bp1 (bg=11.67%)▶IGF2BP2 (bg=3.44%)▶IGF2BP3 (bg=4.26%)▶pum1 (bg=29.85%)▶pum2 (bg=21.55%)▶rbm15 (bg=11.81%)▶sf3a3 (bg=12.13%)▶SF3B1 (bg=6.74%)▶tia1 (bg=16.04%)▶tial1 (bg=14.09%)▶UPF1 (bg=3.62%)MATCHES To TargetScan▶ miR-202-5p:UCCUAUG

 1320  


AAAT

ATTCATAGGAAAT  
Depth:3 (DOG)  
Ei-value:0.000, Pi-value:0.000  
Er-value:0.000, Pr-value:0.000  
eCLIP MATCHES▶AGGF1 (bg=0.88%)▶CPEB4 (bg=2.3%)▶ddx55 (bg=12.35%)▶ddx6 (bg=23.92%)▶dgcr8 (bg=19.31%)▶drosha (bg=11.37%)▶fam120a (bg=18.43%)▶FASTKD2 (bg=7.73%)▶FTO (bg=7.53%)▶fubp3 (bg=23.31%)▶FXR2 (bg=7.51%)▶igf2bp1 (bg=11.67%)▶IGF2BP2 (bg=3.44%)▶IGF2BP3 (bg=4.26%)▶pum1 (bg=29.85%)▶pum2 (bg=21.55%)▶rbm15 (bg=11.81%)▶sf3a3 (bg=12.13%)▶SF3B1 (bg=6.74%)▶tia1 (bg=16.04%)▶tial1 (bg=14.09%)▶UPF1 (bg=3.62%)MATCHES To TargetScan▶ miR-202-5p:UCCUAUG

ATTTTTG

GG

GGGGGGAATGGCCAAATCACCTGTTGAGTAATACTCATTGTGTTTGTGCAGTGGTTC  
Depth:2 (BABOON)  
Ei-value:0.000, Pi-value:0.000  
Er-value:0.000, Pr-value:0.000  
eCLIP MATCHES▶AGGF1 (bg=0.88%)▶CPEB4 (bg=2.3%)▶ddx55 (bg=12.35%)▶ddx6 (bg=23.92%)▶dgcr8 (bg=19.31%)▶drosha (bg=11.37%)▶fam120a (bg=18.43%)▶FASTKD2 (bg=7.73%)▶FTO (bg=7.53%)▶fubp3 (bg=23.31%)▶FXR2 (bg=7.51%)▶igf2bp1 (bg=11.67%)▶IGF2BP2 (bg=3.44%)▶IGF2BP3 (bg=4.26%)▶KHSRP (bg=8.1%)▶pum1 (bg=29.85%)▶pum2 (bg=21.55%)▶rbm15 (bg=11.81%)▶sf3a3 (bg=12.13%)▶SF3B1 (bg=6.74%)▶tia1 (bg=16.04%)▶tial1 (bg=14.09%)▶UPF1 (bg=3.62%)MATCHES To TargetScan▶ miR-421:UCAACAG▶ miR-217:ACUGCAU▶ miR-505-3p.2:UCAACAC▶ miR-496.1:GAGUAUU▶ miR-496.2:GUAUUAC


GGGGAA

GGGGAATGGCCAAA  
Depth:3 (DOG)  
Ei-value:0.000, Pi-value:0.000  
Er-value:0.000, Pr-value:0.000  
eCLIP MATCHES▶AGGF1 (bg=0.88%)▶CPEB4 (bg=2.3%)▶ddx55 (bg=12.35%)▶ddx6 (bg=23.92%)▶dgcr8 (bg=19.31%)▶drosha (bg=11.37%)▶fam120a (bg=18.43%)▶FASTKD2 (bg=7.73%)▶FTO (bg=7.53%)▶fubp3 (bg=23.31%)▶FXR2 (bg=7.51%)▶igf2bp1 (bg=11.67%)▶IGF2BP2 (bg=3.44%)▶IGF2BP3 (bg=4.26%)▶pum1 (bg=29.85%)▶pum2 (bg=21.55%)▶rbm15 (bg=11.81%)▶sf3a3 (bg=12.13%)▶SF3B1 (bg=6.74%)▶tia1 (bg=16.04%)▶tial1 (bg=14.09%)▶UPF1 (bg=3.62%)No matches to TargetScan


TGGCCA

TGGCCA  
Depth:6 (PIG)  
Ei-value:0.000, Pi-value:0.000  
Er-value:0.000, Pr-value:0.000  
eCLIP MATCHES▶AGGF1 (bg=0.88%)▶CPEB4 (bg=2.3%)▶ddx55 (bg=12.35%)▶ddx6 (bg=23.92%)▶dgcr8 (bg=19.31%)▶drosha (bg=11.37%)▶fam120a (bg=18.43%)▶FASTKD2 (bg=7.73%)▶FTO (bg=7.53%)▶fubp3 (bg=23.31%)▶FXR2 (bg=7.51%)▶igf2bp1 (bg=11.67%)▶IGF2BP2 (bg=3.44%)▶IGF2BP3 (bg=4.26%)▶pum1 (bg=29.85%)▶pum2 (bg=21.55%)▶rbm15 (bg=11.81%)▶sf3a3 (bg=12.13%)▶SF3B1 (bg=6.74%)▶tia1 (bg=16.04%)▶tial1 (bg=14.09%)▶UPF1 (bg=3.62%)No matches to TargetScan


AA

GGGGAATGGCCAAA  
Depth:3 (DOG)  
Ei-value:0.000, Pi-value:0.000  
Er-value:0.000, Pr-value:0.000  
eCLIP MATCHES▶AGGF1 (bg=0.88%)▶CPEB4 (bg=2.3%)▶ddx55 (bg=12.35%)▶ddx6 (bg=23.92%)▶dgcr8 (bg=19.31%)▶drosha (bg=11.37%)▶fam120a (bg=18.43%)▶FASTKD2 (bg=7.73%)▶FTO (bg=7.53%)▶fubp3 (bg=23.31%)▶FXR2 (bg=7.51%)▶igf2bp1 (bg=11.67%)▶IGF2BP2 (bg=3.44%)▶IGF2BP3 (bg=4.26%)▶pum1 (bg=29.85%)▶pum2 (bg=21.55%)▶rbm15 (bg=11.81%)▶sf3a3 (bg=12.13%)▶SF3B1 (bg=6.74%)▶tia1 (bg=16.04%)▶tial1 (bg=14.09%)▶UPF1 (bg=3.62%)No matches to TargetScan


TCACCTGTTGAGTAATACT

GGGGGGAATGGCCAAATCACCTGTTGAGTAATACTCATTGTGTTTGTGCAGTGGTTC  
Depth:2 (BABOON)  
Ei-value:0.000, Pi-value:0.000  
Er-value:0.000, Pr-value:0.000  
eCLIP MATCHES▶AGGF1 (bg=0.88%)▶CPEB4 (bg=2.3%)▶ddx55 (bg=12.35%)▶ddx6 (bg=23.92%)▶dgcr8 (bg=19.31%)▶drosha (bg=11.37%)▶fam120a (bg=18.43%)▶FASTKD2 (bg=7.73%)▶FTO (bg=7.53%)▶fubp3 (bg=23.31%)▶FXR2 (bg=7.51%)▶igf2bp1 (bg=11.67%)▶IGF2BP2 (bg=3.44%)▶IGF2BP3 (bg=4.26%)▶KHSRP (bg=8.1%)▶pum1 (bg=29.85%)▶pum2 (bg=21.55%)▶rbm15 (bg=11.81%)▶sf3a3 (bg=12.13%)▶SF3B1 (bg=6.74%)▶tia1 (bg=16.04%)▶tial1 (bg=14.09%)▶UPF1 (bg=3.62%)MATCHES To TargetScan▶ miR-421:UCAACAG▶ miR-217:ACUGCAU▶ miR-505-3p.2:UCAACAC▶ miR-496.1:GAGUAUU▶ miR-496.2:GUAUUAC


CATTGTG

CATTGTGTTTGTGCA  
Depth:3 (DOG)  
Ei-value:0.000, Pi-value:0.000  
Er-value:0.000, Pr-value:0.000  
eCLIP MATCHES▶ddx55 (bg=12.35%)▶drosha (bg=11.37%)▶fam120a (bg=18.43%)▶FXR2 (bg=7.51%)▶igf2bp1 (bg=11.67%)▶IGF2BP2 (bg=3.44%)▶IGF2BP3 (bg=4.26%)▶pum1 (bg=29.85%)▶tia1 (bg=16.04%)▶UPF1 (bg=3.62%)No matches to TargetScan


T

TTTGTGCA  
Depth:7 (ARMADILLO)  
Ei-value:0.000, Pi-value:0.000  
Er-value:0.000, Pr-value:0.000  
eCLIP MATCHES▶drosha (bg=11.37%)▶igf2bp1 (bg=11.67%)▶IGF2BP2 (bg=3.44%)▶IGF2BP3 (bg=4.26%)▶pum1 (bg=29.85%)▶tia1 (bg=16.04%)No matches to TargetScan


TTGTGC

TTGTGC  
Depth:9 (MOUSE)  
Ei-value:0.000, Pi-value:0.000  
Er-value:0.000, Pr-value:0.000  
eCLIP MATCHES▶pum1 (bg=29.85%)▶tia1 (bg=16.04%)No matches to TargetScan


A

TTGTGCA  
Depth:8 (GUINEAPIG)  
Ei-value:0.000, Pi-value:0.000  
Er-value:0.000, Pr-value:0.000  
eCLIP MATCHES▶pum1 (bg=29.85%)▶tia1 (bg=16.04%)No matches to TargetScan


G

GGGGGGAATGGCCAAATCACCTGTTGAGTAATACTCATTGTGTTTGTGCAGTGGTTC  
Depth:2 (BABOON)  
Ei-value:0.000, Pi-value:0.000  
Er-value:0.000, Pr-value:0.000  
eCLIP MATCHES▶AGGF1 (bg=0.88%)▶CPEB4 (bg=2.3%)▶ddx55 (bg=12.35%)▶ddx6 (bg=23.92%)▶dgcr8 (bg=19.31%)▶drosha (bg=11.37%)▶fam120a (bg=18.43%)▶FASTKD2 (bg=7.73%)▶FTO (bg=7.53%)▶fubp3 (bg=23.31%)▶FXR2 (bg=7.51%)▶igf2bp1 (bg=11.67%)▶IGF2BP2 (bg=3.44%)▶IGF2BP3 (bg=4.26%)▶KHSRP (bg=8.1%)▶pum1 (bg=29.85%)▶pum2 (bg=21.55%)▶rbm15 (bg=11.81%)▶sf3a3 (bg=12.13%)▶SF3B1 (bg=6.74%)▶tia1 (bg=16.04%)▶tial1 (bg=14.09%)▶UPF1 (bg=3.62%)MATCHES To TargetScan▶ miR-421:UCAACAG▶ miR-217:ACUGCAU▶ miR-505-3p.2:UCAACAC▶ miR-496.1:GAGUAUU▶ miR-496.2:GUAUUAC


tggttc

tggttc  
Depth:3 (DOG)  
Ei-value:0.080, Pi-value:0.030  
Er-value:0.000, Pr-value:0.000  
eCLIP MATCHES▶dgcr8 (bg=19.31%)▶fam120a (bg=18.43%)▶KHSRP (bg=8.1%)▶pum1 (bg=29.85%)No matches to TargetScan

AGGGG

A

AGGAGAGAGGAGGGGGAGGTGCAGAGAGCT  
Depth:2 (BABOON)  
Ei-value:0.000, Pi-value:0.000  
Er-value:0.000, Pr-value:0.000  
eCLIP MATCHES▶AKAP1 (bg=3.4%)▶CDC40 (bg=1.71%)▶ddx3x (bg=13.89%)▶ddx55 (bg=12.35%)▶DDX59 (bg=0.72%)▶ddx6 (bg=23.92%)▶dgcr8 (bg=19.31%)▶drosha (bg=11.37%)▶fam120a (bg=18.43%)▶FASTKD2 (bg=7.73%)▶FTO (bg=7.53%)▶fubp3 (bg=23.31%)▶GTF2F1 (bg=2.31%)▶igf2bp1 (bg=11.67%)▶KHSRP (bg=8.1%)▶NOLC1 (bg=6.58%)▶pum1 (bg=29.85%)▶pum2 (bg=21.55%)▶sf3a3 (bg=12.13%)▶TBRG4 (bg=7.0%)▶tia1 (bg=16.04%)▶UPF1 (bg=3.62%)▶XRCC6 (bg=1.65%)MATCHES To TargetScan▶ miR-1306-5p:CACCUCC


GGAGAG

GGAGAG  
Depth:5 (SHEEP)  
Ei-value:0.000, Pi-value:0.030  
Er-value:0.000, Pr-value:0.000  
eCLIP MATCHES▶CDC40 (bg=1.71%)▶ddx55 (bg=12.35%)▶dgcr8 (bg=19.31%)▶fam120a (bg=18.43%)▶FASTKD2 (bg=7.73%)▶FTO (bg=7.53%)▶GTF2F1 (bg=2.31%)▶igf2bp1 (bg=11.67%)▶KHSRP (bg=8.1%)▶pum1 (bg=29.85%)▶sf3a3 (bg=12.13%)▶TBRG4 (bg=7.0%)▶tia1 (bg=16.04%)▶UPF1 (bg=3.62%)No matches to TargetScan


AGGAGGGGGAGGTGCAGAGAGCT

AGGAGAGAGGAGGGGGAGGTGCAGAGAGCT  
Depth:2 (BABOON)  
Ei-value:0.000, Pi-value:0.000  
Er-value:0.000, Pr-value:0.000  
eCLIP MATCHES▶AKAP1 (bg=3.4%)▶CDC40 (bg=1.71%)▶ddx3x (bg=13.89%)▶ddx55 (bg=12.35%)▶DDX59 (bg=0.72%)▶ddx6 (bg=23.92%)▶dgcr8 (bg=19.31%)▶drosha (bg=11.37%)▶fam120a (bg=18.43%)▶FASTKD2 (bg=7.73%)▶FTO (bg=7.53%)▶fubp3 (bg=23.31%)▶GTF2F1 (bg=2.31%)▶igf2bp1 (bg=11.67%)▶KHSRP (bg=8.1%)▶NOLC1 (bg=6.58%)▶pum1 (bg=29.85%)▶pum2 (bg=21.55%)▶sf3a3 (bg=12.13%)▶TBRG4 (bg=7.0%)▶tia1 (bg=16.04%)▶UPF1 (bg=3.62%)▶XRCC6 (bg=1.65%)MATCHES To TargetScan▶ miR-1306-5p:CACCUCC

C

tatgccatc

tatgccatc  
Depth:2 (BABOON)  
Ei-value:1.000, Pi-value:0.000  
Er-value:0.000, Pr-value:0.000  
eCLIP MATCHES▶AKAP1 (bg=3.4%)▶CDC40 (bg=1.71%)▶ddx3x (bg=13.89%)▶ddx55 (bg=12.35%)▶DDX59 (bg=0.72%)▶ddx6 (bg=23.92%)▶dgcr8 (bg=19.31%)▶drosha (bg=11.37%)▶fam120a (bg=18.43%)▶FASTKD2 (bg=7.73%)▶FTO (bg=7.53%)▶fubp3 (bg=23.31%)▶GTF2F1 (bg=2.31%)▶igf2bp1 (bg=11.67%)▶KHSRP (bg=8.1%)▶NOLC1 (bg=6.58%)▶pum1 (bg=29.85%)▶pum2 (bg=21.55%)▶sf3a3 (bg=12.13%)▶TBRG4 (bg=7.0%)▶tia1 (bg=16.04%)▶UPF1 (bg=3.62%)▶XRCC6 (bg=1.65%)MATCHES To TargetScan▶ miR-183-5p.1:AUGGCAC

CTGTTTA 1440  


CAGCGAGGCAAGATGAATCATTAT

CAGCGAGGCAAGATGAATCATTAT  
Depth:2 (BABOON)  
Ei-value:0.030, Pi-value:0.000  
Er-value:0.000, Pr-value:0.000  
eCLIP MATCHES▶CDC40 (bg=1.71%)▶ddx3x (bg=13.89%)▶ddx55 (bg=12.35%)▶DDX59 (bg=0.72%)▶ddx6 (bg=23.92%)▶dgcr8 (bg=19.31%)▶drosha (bg=11.37%)▶fam120a (bg=18.43%)▶FASTKD2 (bg=7.73%)▶FTO (bg=7.53%)▶fubp3 (bg=23.31%)▶GTF2F1 (bg=2.31%)▶igf2bp1 (bg=11.67%)▶KHSRP (bg=8.1%)▶NOLC1 (bg=6.58%)▶pum1 (bg=29.85%)▶pum2 (bg=21.55%)▶sf3a3 (bg=12.13%)▶SUB1 (bg=9.24%)▶TBRG4 (bg=7.0%)▶tia1 (bg=16.04%)▶UPF1 (bg=3.62%)▶XRCC6 (bg=1.65%)No matches to TargetScan

G

TCTG

TCTGTGCATTTTGTTTTACTTATCTGTGTATATAGTGTACATAAAGGACAGACGAGTCCTAATTGACAACATCTAGTCTTTCTGGATGTTAAAGAGGTTGCCAGTGTATGACAAAAGTAGAGTT  
Depth:2 (BABOON)  
Ei-value:0.000, Pi-value:0.000  
Er-value:0.000, Pr-value:0.000  
eCLIP MATCHES▶AKAP1 (bg=3.4%)▶ddx3x (bg=13.89%)▶ddx55 (bg=12.35%)▶dgcr8 (bg=19.31%)▶drosha (bg=11.37%)▶fam120a (bg=18.43%)▶FTO (bg=7.53%)▶fubp3 (bg=23.31%)▶igf2bp1 (bg=11.67%)▶NKRF (bg=3.64%)▶NOLC1 (bg=6.58%)▶pum1 (bg=29.85%)▶pum2 (bg=21.55%)▶sf3a3 (bg=12.13%)▶SUB1 (bg=9.24%)▶TBRG4 (bg=7.0%)▶tia1 (bg=16.04%)▶tial1 (bg=14.09%)▶TROVE2 (bg=3.49%)▶XRCC6 (bg=1.65%)MATCHES To TargetScan▶ miR-493-5p:UGUACAU▶ miR-28-3p:ACUAGAU▶ miR-495-3p:AACAAAC▶ miR-539-3p:UCAUACA▶ miR-501-3p/502-3p:AUGCACC▶ miR-193-3p:ACUGGCC


TGCA

TGCATTTTGTTT  
Depth:4 (COW)  
Ei-value:0.000, Pi-value:0.000  
Er-value:0.000, Pr-value:0.000  
eCLIP MATCHES▶AKAP1 (bg=3.4%)▶ddx3x (bg=13.89%)▶ddx55 (bg=12.35%)▶dgcr8 (bg=19.31%)▶drosha (bg=11.37%)▶fam120a (bg=18.43%)▶FTO (bg=7.53%)▶fubp3 (bg=23.31%)▶igf2bp1 (bg=11.67%)▶pum1 (bg=29.85%)▶pum2 (bg=21.55%)▶sf3a3 (bg=12.13%)▶SUB1 (bg=9.24%)▶tia1 (bg=16.04%)▶XRCC6 (bg=1.65%)MATCHES To TargetScan▶ miR-495-3p:AACAAAC


TTTTGTTT

TTTTGTTT  
Depth:5 (SHEEP)  
Ei-value:0.000, Pi-value:0.000  
Er-value:0.000, Pr-value:0.000  
eCLIP MATCHES▶AKAP1 (bg=3.4%)▶ddx55 (bg=12.35%)▶dgcr8 (bg=19.31%)▶drosha (bg=11.37%)▶fam120a (bg=18.43%)▶FTO (bg=7.53%)▶fubp3 (bg=23.31%)▶igf2bp1 (bg=11.67%)▶pum1 (bg=29.85%)▶pum2 (bg=21.55%)▶sf3a3 (bg=12.13%)▶SUB1 (bg=9.24%)▶XRCC6 (bg=1.65%)MATCHES To TargetScan▶ miR-495-3p:AACAAAC


TACTTA

TGCATTTTGTTTTACTTA  
Depth:3 (DOG)  
Ei-value:0.000, Pi-value:0.000  
Er-value:0.000, Pr-value:0.000  
eCLIP MATCHES▶AKAP1 (bg=3.4%)▶ddx3x (bg=13.89%)▶ddx55 (bg=12.35%)▶dgcr8 (bg=19.31%)▶drosha (bg=11.37%)▶fam120a (bg=18.43%)▶FTO (bg=7.53%)▶fubp3 (bg=23.31%)▶igf2bp1 (bg=11.67%)▶pum1 (bg=29.85%)▶pum2 (bg=21.55%)▶sf3a3 (bg=12.13%)▶SUB1 (bg=9.24%)▶tia1 (bg=16.04%)▶tial1 (bg=14.09%)▶TROVE2 (bg=3.49%)▶XRCC6 (bg=1.65%)MATCHES To TargetScan▶ miR-495-3p:AACAAAC


T

TCTGTGCATTTTGTTTTACTTATCTGTGTATATAGTGTACATAAAGGACAGACGAGTCCTAATTGACAACATCTAGTCTTTCTGGATGTTAAAGAGGTTGCCAGTGTATGACAAAAGTAGAGTT  
Depth:2 (BABOON)  
Ei-value:0.000, Pi-value:0.000  
Er-value:0.000, Pr-value:0.000  
eCLIP MATCHES▶AKAP1 (bg=3.4%)▶ddx3x (bg=13.89%)▶ddx55 (bg=12.35%)▶dgcr8 (bg=19.31%)▶drosha (bg=11.37%)▶fam120a (bg=18.43%)▶FTO (bg=7.53%)▶fubp3 (bg=23.31%)▶igf2bp1 (bg=11.67%)▶NKRF (bg=3.64%)▶NOLC1 (bg=6.58%)▶pum1 (bg=29.85%)▶pum2 (bg=21.55%)▶sf3a3 (bg=12.13%)▶SUB1 (bg=9.24%)▶TBRG4 (bg=7.0%)▶tia1 (bg=16.04%)▶tial1 (bg=14.09%)▶TROVE2 (bg=3.49%)▶XRCC6 (bg=1.65%)MATCHES To TargetScan▶ miR-493-5p:UGUACAU▶ miR-28-3p:ACUAGAU▶ miR-495-3p:AACAAAC▶ miR-539-3p:UCAUACA▶ miR-501-3p/502-3p:AUGCACC▶ miR-193-3p:ACUGGCC


CTGTGT

CTGTGTATATAGTGTA  
Depth:3 (DOG)  
Ei-value:0.000, Pi-value:0.000  
Er-value:0.000, Pr-value:0.000  
eCLIP MATCHES▶AKAP1 (bg=3.4%)▶ddx55 (bg=12.35%)▶dgcr8 (bg=19.31%)▶drosha (bg=11.37%)▶fam120a (bg=18.43%)▶FTO (bg=7.53%)▶fubp3 (bg=23.31%)▶igf2bp1 (bg=11.67%)▶NKRF (bg=3.64%)▶pum1 (bg=29.85%)▶pum2 (bg=21.55%)▶sf3a3 (bg=12.13%)▶SUB1 (bg=9.24%)▶tia1 (bg=16.04%)▶tial1 (bg=14.09%)▶TROVE2 (bg=3.49%)▶XRCC6 (bg=1.65%)No matches to TargetScan


ATA

ATATAGTGTA  
Depth:6 (PIG)  
Ei-value:0.000, Pi-value:0.000  
Er-value:0.000, Pr-value:0.000  
eCLIP MATCHES▶AKAP1 (bg=3.4%)▶ddx55 (bg=12.35%)▶dgcr8 (bg=19.31%)▶drosha (bg=11.37%)▶fam120a (bg=18.43%)▶fubp3 (bg=23.31%)▶igf2bp1 (bg=11.67%)▶NKRF (bg=3.64%)▶pum1 (bg=29.85%)▶pum2 (bg=21.55%)▶sf3a3 (bg=12.13%)▶SUB1 (bg=9.24%)▶tia1 (bg=16.04%)▶tial1 (bg=14.09%)▶TROVE2 (bg=3.49%)▶XRCC6 (bg=1.65%)No matches to TargetScan


TAGTGT

TAGTGT  
Depth:7 (ARMADILLO)  
Ei-value:0.000, Pi-value:0.000  
Er-value:0.000, Pr-value:0.010  
eCLIP MATCHES▶AKAP1 (bg=3.4%)▶ddx55 (bg=12.35%)▶dgcr8 (bg=19.31%)▶drosha (bg=11.37%)▶fam120a (bg=18.43%)▶fubp3 (bg=23.31%)▶igf2bp1 (bg=11.67%)▶NKRF (bg=3.64%)▶pum1 (bg=29.85%)▶pum2 (bg=21.55%)▶sf3a3 (bg=12.13%)▶SUB1 (bg=9.24%)▶tia1 (bg=16.04%)▶tial1 (bg=14.09%)▶TROVE2 (bg=3.49%)▶XRCC6 (bg=1.65%)No matches to TargetScan


A

ATATAGTGTA  
Depth:6 (PIG)  
Ei-value:0.000, Pi-value:0.000  
Er-value:0.000, Pr-value:0.000  
eCLIP MATCHES▶AKAP1 (bg=3.4%)▶ddx55 (bg=12.35%)▶dgcr8 (bg=19.31%)▶drosha (bg=11.37%)▶fam120a (bg=18.43%)▶fubp3 (bg=23.31%)▶igf2bp1 (bg=11.67%)▶NKRF (bg=3.64%)▶pum1 (bg=29.85%)▶pum2 (bg=21.55%)▶sf3a3 (bg=12.13%)▶SUB1 (bg=9.24%)▶tia1 (bg=16.04%)▶tial1 (bg=14.09%)▶TROVE2 (bg=3.49%)▶XRCC6 (bg=1.65%)No matches to TargetScan


CATAAAGGACAGAC

TCTGTGCATTTTGTTTTACTTATCTGTGTATATAGTGTACATAAAGGACAGACGAGTCCTAATTGACAACATCTAGTCTTTCTGGATGTTAAAGAGGTTGCCAGTGTATGACAAAAGTAGAGTT  
Depth:2 (BABOON)  
Ei-value:0.000, Pi-value:0.000  
Er-value:0.000, Pr-value:0.000  
eCLIP MATCHES▶AKAP1 (bg=3.4%)▶ddx3x (bg=13.89%)▶ddx55 (bg=12.35%)▶dgcr8 (bg=19.31%)▶drosha (bg=11.37%)▶fam120a (bg=18.43%)▶FTO (bg=7.53%)▶fubp3 (bg=23.31%)▶igf2bp1 (bg=11.67%)▶NKRF (bg=3.64%)▶NOLC1 (bg=6.58%)▶pum1 (bg=29.85%)▶pum2 (bg=21.55%)▶sf3a3 (bg=12.13%)▶SUB1 (bg=9.24%)▶TBRG4 (bg=7.0%)▶tia1 (bg=16.04%)▶tial1 (bg=14.09%)▶TROVE2 (bg=3.49%)▶XRCC6 (bg=1.65%)MATCHES To TargetScan▶ miR-493-5p:UGUACAU▶ miR-28-3p:ACUAGAU▶ miR-495-3p:AACAAAC▶ miR-539-3p:UCAUACA▶ miR-501-3p/502-3p:AUGCACC▶ miR-193-3p:ACUGGCC


GA

GAGTCCTAATT  
Depth:6 (PIG)  
Ei-value:0.000, Pi-value:0.000  
Er-value:0.000, Pr-value:0.000  
eCLIP MATCHES▶AKAP1 (bg=3.4%)▶ddx55 (bg=12.35%)▶dgcr8 (bg=19.31%)▶drosha (bg=11.37%)▶fam120a (bg=18.43%)▶fubp3 (bg=23.31%)▶igf2bp1 (bg=11.67%)▶NKRF (bg=3.64%)▶pum1 (bg=29.85%)▶pum2 (bg=21.55%)▶sf3a3 (bg=12.13%)▶SUB1 (bg=9.24%)▶tia1 (bg=16.04%)▶tial1 (bg=14.09%)▶TROVE2 (bg=3.49%)No matches to TargetScan


GTCCTAATT

GTCCTAATT  
Depth:7 (ARMADILLO)  
Ei-value:0.000, Pi-value:0.000  
Er-value:0.000, Pr-value:0.000  
eCLIP MATCHES▶AKAP1 (bg=3.4%)▶ddx55 (bg=12.35%)▶dgcr8 (bg=19.31%)▶drosha (bg=11.37%)▶fam120a (bg=18.43%)▶fubp3 (bg=23.31%)▶igf2bp1 (bg=11.67%)▶NKRF (bg=3.64%)▶pum1 (bg=29.85%)▶pum2 (bg=21.55%)▶sf3a3 (bg=12.13%)▶SUB1 (bg=9.24%)▶tia1 (bg=16.04%)▶tial1 (bg=14.09%)▶TROVE2 (bg=3.49%)No matches to TargetScan


GA

TCTGTGCATTTTGTTTTACTTATCTGTGTATATAGTGTACATAAAGGACAGACGAGTCCTAATTGACAACATCTAGTCTTTCTGGATGTTAAAGAGGTTGCCAGTGTATGACAAAAGTAGAGTT  
Depth:2 (BABOON)  
Ei-value:0.000, Pi-value:0.000  
Er-value:0.000, Pr-value:0.000  
eCLIP MATCHES▶AKAP1 (bg=3.4%)▶ddx3x (bg=13.89%)▶ddx55 (bg=12.35%)▶dgcr8 (bg=19.31%)▶drosha (bg=11.37%)▶fam120a (bg=18.43%)▶FTO (bg=7.53%)▶fubp3 (bg=23.31%)▶igf2bp1 (bg=11.67%)▶NKRF (bg=3.64%)▶NOLC1 (bg=6.58%)▶pum1 (bg=29.85%)▶pum2 (bg=21.55%)▶sf3a3 (bg=12.13%)▶SUB1 (bg=9.24%)▶TBRG4 (bg=7.0%)▶tia1 (bg=16.04%)▶tial1 (bg=14.09%)▶TROVE2 (bg=3.49%)▶XRCC6 (bg=1.65%)MATCHES To TargetScan▶ miR-493-5p:UGUACAU▶ miR-28-3p:ACUAGAU▶ miR-495-3p:AACAAAC▶ miR-539-3p:UCAUACA▶ miR-501-3p/502-3p:AUGCACC▶ miR-193-3p:ACUGGCC


CA

CAACATCT  
Depth:6 (PIG)  
Ei-value:0.000, Pi-value:0.000  
Er-value:0.000, Pr-value:0.000  
eCLIP MATCHES▶AKAP1 (bg=3.4%)▶pum1 (bg=29.85%)▶pum2 (bg=21.55%)▶tial1 (bg=14.09%)No matches to TargetScan


ACATCT

ACATCT  
Depth:7 (ARMADILLO)  
Ei-value:0.000, Pi-value:0.000  
Er-value:0.000, Pr-value:0.000  
No matches to eCLIP DataNo matches to TargetScan


AGTCTTT

CAACATCTAGTCTTT  
Depth:5 (SHEEP)  
Ei-value:0.000, Pi-value:0.000  
Er-value:0.000, Pr-value:0.000  
eCLIP MATCHES▶AKAP1 (bg=3.4%)▶pum1 (bg=29.85%)▶pum2 (bg=21.55%)▶tial1 (bg=14.09%)MATCHES To TargetScan▶ miR-28-3p:ACUAGAU


CTG

TCTGTGCATTTTGTTTTACTTATCTGTGTATATAGTGTACATAAAGGACAGACGAGTCCTAATTGACAACATCTAGTCTTTCTGGATGTTAAAGAGGTTGCCAGTGTATGACAAAAGTAGAGTT  
Depth:2 (BABOON)  
Ei-value:0.000, Pi-value:0.000  
Er-value:0.000, Pr-value:0.000  
eCLIP MATCHES▶AKAP1 (bg=3.4%)▶ddx3x (bg=13.89%)▶ddx55 (bg=12.35%)▶dgcr8 (bg=19.31%)▶drosha (bg=11.37%)▶fam120a (bg=18.43%)▶FTO (bg=7.53%)▶fubp3 (bg=23.31%)▶igf2bp1 (bg=11.67%)▶NKRF (bg=3.64%)▶NOLC1 (bg=6.58%)▶pum1 (bg=29.85%)▶pum2 (bg=21.55%)▶sf3a3 (bg=12.13%)▶SUB1 (bg=9.24%)▶TBRG4 (bg=7.0%)▶tia1 (bg=16.04%)▶tial1 (bg=14.09%)▶TROVE2 (bg=3.49%)▶XRCC6 (bg=1.65%)MATCHES To TargetScan▶ miR-493-5p:UGUACAU▶ miR-28-3p:ACUAGAU▶ miR-495-3p:AACAAAC▶ miR-539-3p:UCAUACA▶ miR-501-3p/502-3p:AUGCACC▶ miR-193-3p:ACUGGCC


GATGTT

GATGTT  
Depth:9 (MOUSE)  
Ei-value:0.000, Pi-value:0.000  
Er-value:0.000, Pr-value:0.000  
No matches to eCLIP DataNo matches to TargetScan


AAAGA

AAAGAGGTTGCCA  
Depth:9 (MOUSE)  
Ei-value:0.000, Pi-value:0.000  
Er-value:0.000, Pr-value:0.000  
No matches to eCLIP DataNo matches to TargetScan

 1560  


GGTTGCCA

AAAGAGGTTGCCA  
Depth:9 (MOUSE)  
Ei-value:0.000, Pi-value:0.000  
Er-value:0.000, Pr-value:0.000  
No matches to eCLIP DataNo matches to TargetScan


GTGTATGA

AAAGAGGTTGCCAGTGTATGA  
Depth:7 (ARMADILLO)  
Ei-value:0.000, Pi-value:0.000  
Er-value:0.000, Pr-value:0.000  
No matches to eCLIP DataMATCHES To TargetScan▶ miR-539-3p:UCAUACA▶ miR-193-3p:ACUGGCC


CAAAA

GATGTTAAAGAGGTTGCCAGTGTATGACAAAA  
Depth:5 (SHEEP)  
Ei-value:0.000, Pi-value:0.000  
Er-value:0.000, Pr-value:0.000  
No matches to eCLIP DataMATCHES To TargetScan▶ miR-539-3p:UCAUACA▶ miR-193-3p:ACUGGCC


GTAGAGTT

TCTGTGCATTTTGTTTTACTTATCTGTGTATATAGTGTACATAAAGGACAGACGAGTCCTAATTGACAACATCTAGTCTTTCTGGATGTTAAAGAGGTTGCCAGTGTATGACAAAAGTAGAGTT  
Depth:2 (BABOON)  
Ei-value:0.000, Pi-value:0.000  
Er-value:0.000, Pr-value:0.000  
eCLIP MATCHES▶AKAP1 (bg=3.4%)▶ddx3x (bg=13.89%)▶ddx55 (bg=12.35%)▶dgcr8 (bg=19.31%)▶drosha (bg=11.37%)▶fam120a (bg=18.43%)▶FTO (bg=7.53%)▶fubp3 (bg=23.31%)▶igf2bp1 (bg=11.67%)▶NKRF (bg=3.64%)▶NOLC1 (bg=6.58%)▶pum1 (bg=29.85%)▶pum2 (bg=21.55%)▶sf3a3 (bg=12.13%)▶SUB1 (bg=9.24%)▶TBRG4 (bg=7.0%)▶tia1 (bg=16.04%)▶tial1 (bg=14.09%)▶TROVE2 (bg=3.49%)▶XRCC6 (bg=1.65%)MATCHES To TargetScan▶ miR-493-5p:UGUACAU▶ miR-28-3p:ACUAGAU▶ miR-495-3p:AACAAAC▶ miR-539-3p:UCAUACA▶ miR-501-3p/502-3p:AUGCACC▶ miR-193-3p:ACUGGCC

AG

TAAACTAATAT

TAAACTAATAT  
Depth:3 (DOG)  
Ei-value:0.000, Pi-value:0.000  
Er-value:0.000, Pr-value:0.000  
No matches to eCLIP DataNo matches to TargetScan


A

TAAACTAATATATTTTGTACATTTTGTTTTACAAGTCCTAGGAAAGATTGTCTTCTGAAAATTTGATGTCTTCTGGGTTGATGGAGATGGGAAGGGTTCTAGGCCAGAATGTTCACATTTGGAAGACT  
Depth:2 (BABOON)  
Ei-value:0.000, Pi-value:0.000  
Er-value:0.000, Pr-value:0.000  
eCLIP MATCHES▶ddx55 (bg=12.35%)▶dgcr8 (bg=19.31%)▶fam120a (bg=18.43%)▶fubp3 (bg=23.31%)▶KHSRP (bg=8.1%)▶lin28b (bg=16.96%)▶NOLC1 (bg=6.58%)▶pum1 (bg=29.85%)▶pum2 (bg=21.55%)▶sf3a3 (bg=12.13%)▶tia1 (bg=16.04%)▶tial1 (bg=14.09%)▶ZC3H11A (bg=6.25%)MATCHES To TargetScan▶ miR-181-5p:ACAUUCA▶ miR-136-5p:CUCCAUU▶ miR-493-5p:UGUACAU▶ miR-543:AACAUUC▶ miR-495-3p:AACAAAC


TTTTG

TTTTGTACATTTTGT  
Depth:5 (SHEEP)  
Ei-value:0.000, Pi-value:0.000  
Er-value:0.000, Pr-value:0.000  
No matches to eCLIP DataMATCHES To TargetScan▶ miR-493-5p:UGUACAU


TAC

TACATTTTGT  
Depth:7 (ARMADILLO)  
Ei-value:0.000, Pi-value:0.000  
Er-value:0.000, Pr-value:0.000  
No matches to eCLIP DataNo matches to TargetScan


ATTTTGT

ATTTTGT  
Depth:9 (MOUSE)  
Ei-value:0.000, Pi-value:0.000  
Er-value:0.000, Pr-value:0.000  
No matches to eCLIP DataNo matches to TargetScan


TTTACAAGTCCTAGGA

TAAACTAATATATTTTGTACATTTTGTTTTACAAGTCCTAGGAAAGATTGTCTTCTGAAAATTTGATGTCTTCTGGGTTGATGGAGATGGGAAGGGTTCTAGGCCAGAATGTTCACATTTGGAAGACT  
Depth:2 (BABOON)  
Ei-value:0.000, Pi-value:0.000  
Er-value:0.000, Pr-value:0.000  
eCLIP MATCHES▶ddx55 (bg=12.35%)▶dgcr8 (bg=19.31%)▶fam120a (bg=18.43%)▶fubp3 (bg=23.31%)▶KHSRP (bg=8.1%)▶lin28b (bg=16.96%)▶NOLC1 (bg=6.58%)▶pum1 (bg=29.85%)▶pum2 (bg=21.55%)▶sf3a3 (bg=12.13%)▶tia1 (bg=16.04%)▶tial1 (bg=14.09%)▶ZC3H11A (bg=6.25%)MATCHES To TargetScan▶ miR-181-5p:ACAUUCA▶ miR-136-5p:CUCCAUU▶ miR-493-5p:UGUACAU▶ miR-543:AACAUUC▶ miR-495-3p:AACAAAC


AAGATTGTCTT

AAGATTGTCTT  
Depth:5 (SHEEP)  
Ei-value:0.000, Pi-value:0.000  
Er-value:0.000, Pr-value:0.000  
eCLIP MATCHES▶dgcr8 (bg=19.31%)▶fam120a (bg=18.43%)▶KHSRP (bg=8.1%)▶NOLC1 (bg=6.58%)▶pum1 (bg=29.85%)▶pum2 (bg=21.55%)▶sf3a3 (bg=12.13%)▶tia1 (bg=16.04%)▶tial1 (bg=14.09%)No matches to TargetScan


CTG

AAGATTGTCTTCTGAAAATT  
Depth:3 (DOG)  
Ei-value:0.000, Pi-value:0.000  
Er-value:0.000, Pr-value:0.000  
eCLIP MATCHES▶ddx55 (bg=12.35%)▶dgcr8 (bg=19.31%)▶fam120a (bg=18.43%)▶fubp3 (bg=23.31%)▶KHSRP (bg=8.1%)▶lin28b (bg=16.96%)▶NOLC1 (bg=6.58%)▶pum1 (bg=29.85%)▶pum2 (bg=21.55%)▶sf3a3 (bg=12.13%)▶tia1 (bg=16.04%)▶tial1 (bg=14.09%)No matches to TargetScan


AAAATT

AAAATT  
Depth:5 (SHEEP)  
Ei-value:0.000, Pi-value:0.010  
Er-value:0.000, Pr-value:0.020  
eCLIP MATCHES▶ddx55 (bg=12.35%)▶dgcr8 (bg=19.31%)▶fam120a (bg=18.43%)▶fubp3 (bg=23.31%)▶KHSRP (bg=8.1%)▶lin28b (bg=16.96%)▶NOLC1 (bg=6.58%)▶pum1 (bg=29.85%)▶pum2 (bg=21.55%)▶sf3a3 (bg=12.13%)▶tia1 (bg=16.04%)▶tial1 (bg=14.09%)No matches to TargetScan


TGATGTCTTCTGGGTTGA

TAAACTAATATATTTTGTACATTTTGTTTTACAAGTCCTAGGAAAGATTGTCTTCTGAAAATTTGATGTCTTCTGGGTTGATGGAGATGGGAAGGGTTCTAGGCCAGAATGTTCACATTTGGAAGACT  
Depth:2 (BABOON)  
Ei-value:0.000, Pi-value:0.000  
Er-value:0.000, Pr-value:0.000  
eCLIP MATCHES▶ddx55 (bg=12.35%)▶dgcr8 (bg=19.31%)▶fam120a (bg=18.43%)▶fubp3 (bg=23.31%)▶KHSRP (bg=8.1%)▶lin28b (bg=16.96%)▶NOLC1 (bg=6.58%)▶pum1 (bg=29.85%)▶pum2 (bg=21.55%)▶sf3a3 (bg=12.13%)▶tia1 (bg=16.04%)▶tial1 (bg=14.09%)▶ZC3H11A (bg=6.25%)MATCHES To TargetScan▶ miR-181-5p:ACAUUCA▶ miR-136-5p:CUCCAUU▶ miR-493-5p:UGUACAU▶ miR-543:AACAUUC▶ miR-495-3p:AACAAAC


tggaga

tggaga  
Depth:3 (DOG)  
Ei-value:0.080, Pi-value:0.030  
Er-value:0.000, Pr-value:0.000  
eCLIP MATCHES▶ddx55 (bg=12.35%)▶dgcr8 (bg=19.31%)▶fam120a (bg=18.43%)▶fubp3 (bg=23.31%)▶KHSRP (bg=8.1%)▶lin28b (bg=16.96%)▶NOLC1 (bg=6.58%)▶pum1 (bg=29.85%)▶pum2 (bg=21.55%)▶sf3a3 (bg=12.13%)▶tia1 (bg=16.04%)▶ZC3H11A (bg=6.25%)No matches to TargetScan


TG

TAAACTAATATATTTTGTACATTTTGTTTTACAAGTCCTAGGAAAGATTGTCTTCTGAAAATTTGATGTCTTCTGGGTTGATGGAGATGGGAAGGGTTCTAGGCCAGAATGTTCACATTTGGAAGACT  
Depth:2 (BABOON)  
Ei-value:0.000, Pi-value:0.000  
Er-value:0.000, Pr-value:0.000  
eCLIP MATCHES▶ddx55 (bg=12.35%)▶dgcr8 (bg=19.31%)▶fam120a (bg=18.43%)▶fubp3 (bg=23.31%)▶KHSRP (bg=8.1%)▶lin28b (bg=16.96%)▶NOLC1 (bg=6.58%)▶pum1 (bg=29.85%)▶pum2 (bg=21.55%)▶sf3a3 (bg=12.13%)▶tia1 (bg=16.04%)▶tial1 (bg=14.09%)▶ZC3H11A (bg=6.25%)MATCHES To TargetScan▶ miR-181-5p:ACAUUCA▶ miR-136-5p:CUCCAUU▶ miR-493-5p:UGUACAU▶ miR-543:AACAUUC▶ miR-495-3p:AACAAAC

 1680  


GGAAGGGTTCTAGGCC

TAAACTAATATATTTTGTACATTTTGTTTTACAAGTCCTAGGAAAGATTGTCTTCTGAAAATTTGATGTCTTCTGGGTTGATGGAGATGGGAAGGGTTCTAGGCCAGAATGTTCACATTTGGAAGACT  
Depth:2 (BABOON)  
Ei-value:0.000, Pi-value:0.000  
Er-value:0.000, Pr-value:0.000  
eCLIP MATCHES▶ddx55 (bg=12.35%)▶dgcr8 (bg=19.31%)▶fam120a (bg=18.43%)▶fubp3 (bg=23.31%)▶KHSRP (bg=8.1%)▶lin28b (bg=16.96%)▶NOLC1 (bg=6.58%)▶pum1 (bg=29.85%)▶pum2 (bg=21.55%)▶sf3a3 (bg=12.13%)▶tia1 (bg=16.04%)▶tial1 (bg=14.09%)▶ZC3H11A (bg=6.25%)MATCHES To TargetScan▶ miR-181-5p:ACAUUCA▶ miR-136-5p:CUCCAUU▶ miR-493-5p:UGUACAU▶ miR-543:AACAUUC▶ miR-495-3p:AACAAAC


AGAATGTTC

AGAATGTTC  
Depth:5 (SHEEP)  
Ei-value:0.000, Pi-value:0.000  
Er-value:0.000, Pr-value:0.000  
No matches to eCLIP DataMATCHES To TargetScan▶ miR-181-5p:ACAUUCA▶ miR-543:AACAUUC


ACATTTG

TAAACTAATATATTTTGTACATTTTGTTTTACAAGTCCTAGGAAAGATTGTCTTCTGAAAATTTGATGTCTTCTGGGTTGATGGAGATGGGAAGGGTTCTAGGCCAGAATGTTCACATTTGGAAGACT  
Depth:2 (BABOON)  
Ei-value:0.000, Pi-value:0.000  
Er-value:0.000, Pr-value:0.000  
eCLIP MATCHES▶ddx55 (bg=12.35%)▶dgcr8 (bg=19.31%)▶fam120a (bg=18.43%)▶fubp3 (bg=23.31%)▶KHSRP (bg=8.1%)▶lin28b (bg=16.96%)▶NOLC1 (bg=6.58%)▶pum1 (bg=29.85%)▶pum2 (bg=21.55%)▶sf3a3 (bg=12.13%)▶tia1 (bg=16.04%)▶tial1 (bg=14.09%)▶ZC3H11A (bg=6.25%)MATCHES To TargetScan▶ miR-181-5p:ACAUUCA▶ miR-136-5p:CUCCAUU▶ miR-493-5p:UGUACAU▶ miR-543:AACAUUC▶ miR-495-3p:AACAAAC


GAAGAC

GAAGAC  
Depth:7 (ARMADILLO)  
Ei-value:0.000, Pi-value:0.000  
Er-value:0.000, Pr-value:0.000  
No matches to eCLIP DataNo matches to TargetScan


T

TAAACTAATATATTTTGTACATTTTGTTTTACAAGTCCTAGGAAAGATTGTCTTCTGAAAATTTGATGTCTTCTGGGTTGATGGAGATGGGAAGGGTTCTAGGCCAGAATGTTCACATTTGGAAGACT  
Depth:2 (BABOON)  
Ei-value:0.000, Pi-value:0.000  
Er-value:0.000, Pr-value:0.000  
eCLIP MATCHES▶ddx55 (bg=12.35%)▶dgcr8 (bg=19.31%)▶fam120a (bg=18.43%)▶fubp3 (bg=23.31%)▶KHSRP (bg=8.1%)▶lin28b (bg=16.96%)▶NOLC1 (bg=6.58%)▶pum1 (bg=29.85%)▶pum2 (bg=21.55%)▶sf3a3 (bg=12.13%)▶tia1 (bg=16.04%)▶tial1 (bg=14.09%)▶ZC3H11A (bg=6.25%)MATCHES To TargetScan▶ miR-181-5p:ACAUUCA▶ miR-136-5p:CUCCAUU▶ miR-493-5p:UGUACAU▶ miR-543:AACAUUC▶ miR-495-3p:AACAAAC

CTTTC

aa

aaattataa  
Depth:2 (BABOON)  
Ei-value:1.000, Pi-value:0.000  
Er-value:0.000, Pr-value:0.000  
No matches to eCLIP DataMATCHES To TargetScan▶ miR-374-5p:UAUAAUA


ATTATAA

ATTATAA  
Depth:3 (DOG)  
Ei-value:0.000, Pi-value:0.020  
Er-value:0.000, Pr-value:0.000  
No matches to eCLIP DataMATCHES To TargetScan▶ miR-374-5p:UAUAAUA

CTG

T

TTGTTACATGTTTGCAGTTTATTCAAGACTGCT  
Depth:2 (BABOON)  
Ei-value:0.000, Pi-value:0.000  
Er-value:0.000, Pr-value:0.000  
No matches to eCLIP DataMATCHES To TargetScan▶ miR-431-5p:GUCUUGC▶ miR-217:ACUGCAU▶ miR-194-5p:GUAACAG▶ miR-411-3p:AUGUAAC


TGTTACA

TGTTACA  
Depth:6 (PIG)  
Ei-value:0.000, Pi-value:0.000  
Er-value:0.000, Pr-value:0.000  
No matches to eCLIP DataMATCHES To TargetScan▶ miR-194-5p:GUAACAG


TGTT

TTGTTACATGTTTGCAGTTTATTCAAGACTGCT  
Depth:2 (BABOON)  
Ei-value:0.000, Pi-value:0.000  
Er-value:0.000, Pr-value:0.000  
No matches to eCLIP DataMATCHES To TargetScan▶ miR-431-5p:GUCUUGC▶ miR-217:ACUGCAU▶ miR-194-5p:GUAACAG▶ miR-411-3p:AUGUAAC


T

TGCAGTTTATTCAAGACTGCT  
Depth:3 (DOG)  
Ei-value:0.000, Pi-value:0.000  
Er-value:0.000, Pr-value:0.000  
No matches to eCLIP DataMATCHES To TargetScan▶ miR-431-5p:GUCUUGC▶ miR-217:ACUGCAU


GCAGTTTATT

GCAGTTTATT  
Depth:6 (PIG)  
Ei-value:0.000, Pi-value:0.000  
Er-value:0.000, Pr-value:0.000  
No matches to eCLIP DataNo matches to TargetScan


CA

GCAGTTTATTCAAGACTGCT  
Depth:5 (SHEEP)  
Ei-value:0.000, Pi-value:0.000  
Er-value:0.000, Pr-value:0.000  
No matches to eCLIP DataMATCHES To TargetScan▶ miR-431-5p:GUCUUGC


AGACTGCT

AGACTGCT  
Depth:6 (PIG)  
Ei-value:0.000, Pi-value:0.000  
Er-value:0.000, Pr-value:0.000  
No matches to eCLIP DataNo matches to TargetScan

GTATACATAGTA

GACAAATTAAC

GACAAATTAACTCCTTACTTGAAACATCTAGTCTATCTAGATGTTTAGAAGTGCCC  
Depth:2 (BABOON)  
Ei-value:0.000, Pi-value:0.000  
Er-value:0.000, Pr-value:0.000  
No matches to eCLIP DataMATCHES To TargetScan▶ miR-28-3p:ACUAGAU▶ miR-26-5p:UCAAGUA▶ miR-151-3p:UAGACUG


TCCTTA

TCCTTA  
Depth:9 (MOUSE)  
Ei-value:0.000, Pi-value:0.000  
Er-value:0.000, Pr-value:0.000  
No matches to eCLIP DataNo matches to TargetScan


CT

GACAAATTAACTCCTTACTTGAAACATCTAGTCTATCTAGATGTTTAGAAGTGCCC  
Depth:2 (BABOON)  
Ei-value:0.000, Pi-value:0.000  
Er-value:0.000, Pr-value:0.000  
No matches to eCLIP DataMATCHES To TargetScan▶ miR-28-3p:ACUAGAU▶ miR-26-5p:UCAAGUA▶ miR-151-3p:UAGACUG

 1800  


TG

GACAAATTAACTCCTTACTTGAAACATCTAGTCTATCTAGATGTTTAGAAGTGCCC  
Depth:2 (BABOON)  
Ei-value:0.000, Pi-value:0.000  
Er-value:0.000, Pr-value:0.000  
No matches to eCLIP DataMATCHES To TargetScan▶ miR-28-3p:ACUAGAU▶ miR-26-5p:UCAAGUA▶ miR-151-3p:UAGACUG


AAACATCT

AAACATCT  
Depth:9 (MOUSE)  
Ei-value:0.000, Pi-value:0.000  
Er-value:0.000, Pr-value:0.000  
No matches to eCLIP DataNo matches to TargetScan


AG

AAACATCTAG  
Depth:8 (GUINEAPIG)  
Ei-value:0.000, Pi-value:0.000  
Er-value:0.000, Pr-value:0.000  
No matches to eCLIP DataNo matches to TargetScan


TCT

AAACATCTAGTCT  
Depth:3 (DOG)  
Ei-value:0.000, Pi-value:0.000  
Er-value:0.000, Pr-value:0.000  
No matches to eCLIP DataMATCHES To TargetScan▶ miR-28-3p:ACUAGAU


A

GACAAATTAACTCCTTACTTGAAACATCTAGTCTATCTAGATGTTTAGAAGTGCCC  
Depth:2 (BABOON)  
Ei-value:0.000, Pi-value:0.000  
Er-value:0.000, Pr-value:0.000  
No matches to eCLIP DataMATCHES To TargetScan▶ miR-28-3p:ACUAGAU▶ miR-26-5p:UCAAGUA▶ miR-151-3p:UAGACUG


T

TCTAGATGTTTAGAAGTGCCC  
Depth:3 (DOG)  
Ei-value:0.000, Pi-value:0.000  
Er-value:0.000, Pr-value:0.000  
No matches to eCLIP DataNo matches to TargetScan


CTAGATGTTTAGAAGTGCCC

CTAGATGTTTAGAAGTGCCC  
Depth:9 (MOUSE)  
Ei-value:0.000, Pi-value:0.000  
Er-value:0.000, Pr-value:0.000  
No matches to eCLIP DataNo matches to TargetScan

G

at

atgtatgttaaatgta  
Depth:2 (BABOON)  
Ei-value:1.000, Pi-value:0.000  
Er-value:0.000, Pr-value:0.000  
No matches to eCLIP DataNo matches to TargetScan


GTATGTTAAA

GTATGTTAAA  
Depth:9 (MOUSE)  
Ei-value:0.000, Pi-value:0.000  
Er-value:0.000, Pr-value:0.000  
No matches to eCLIP DataNo matches to TargetScan


TGTA

GTATGTTAAATGTA  
Depth:6 (PIG)  
Ei-value:0.000, Pi-value:0.000  
Er-value:0.000, Pr-value:0.000  
No matches to eCLIP DataNo matches to TargetScan

T

AGGTAGT

AGGTAGT  
Depth:6 (PIG)  
Ei-value:0.000, Pi-value:0.000  
Er-value:0.000, Pr-value:0.000  
No matches to eCLIP DataNo matches to TargetScan


AAAATA

AGGTAGTAAAATA  
Depth:3 (DOG)  
Ei-value:0.000, Pi-value:0.000  
Er-value:0.000, Pr-value:0.000  
No matches to eCLIP DataNo matches to TargetScan


CCACTT

AGGTAGTAAAATACCACTTTGTAAATATCTTTTTGCTAAAATTCATAGGAAAT  
Depth:2 (BABOON)  
Ei-value:0.000, Pi-value:0.000  
Er-value:0.000, Pr-value:0.000  
eCLIP MATCHES▶fubp3 (bg=23.31%)▶pum1 (bg=29.85%)▶pum2 (bg=21.55%)▶tial1 (bg=14.09%)MATCHES To TargetScan▶ miR-140-5p:AGUGGUU▶ miR-17-5p/20-5p/93-5p/106-5p/519-3p:AAAGUGC▶ miR-202-5p:UCCUAUG


TGTAAATA

TGTAAATA  
Depth:9 (MOUSE)  
Ei-value:0.000, Pi-value:0.000  
Er-value:0.000, Pr-value:0.000  
No matches to eCLIP DataNo matches to TargetScan


T

TGTAAATATCTTTTTGCTAAAATTCATAGGAAAT  
Depth:3 (DOG)  
Ei-value:0.000, Pi-value:0.000  
Er-value:0.000, Pr-value:0.000  
eCLIP MATCHES▶fubp3 (bg=23.31%)▶pum1 (bg=29.85%)▶pum2 (bg=21.55%)▶tial1 (bg=14.09%)MATCHES To TargetScan▶ miR-202-5p:UCCUAUG


CTTTTTGCT

CTTTTTGCT  
Depth:5 (SHEEP)  
Ei-value:0.000, Pi-value:0.000  
Er-value:0.000, Pr-value:0.000  
eCLIP MATCHES▶pum1 (bg=29.85%)No matches to TargetScan


A

TGTAAATATCTTTTTGCTAAAATTCATAGGAAAT  
Depth:3 (DOG)  
Ei-value:0.000, Pi-value:0.000  
Er-value:0.000, Pr-value:0.000  
eCLIP MATCHES▶fubp3 (bg=23.31%)▶pum1 (bg=29.85%)▶pum2 (bg=21.55%)▶tial1 (bg=14.09%)MATCHES To TargetScan▶ miR-202-5p:UCCUAUG


AAATTCATAGGAA

AAATTCATAGGAA  
Depth:4 (COW)  
Ei-value:0.000, Pi-value:0.000  
Er-value:0.000, Pr-value:0.000  
eCLIP MATCHES▶fubp3 (bg=23.31%)▶pum1 (bg=29.85%)▶pum2 (bg=21.55%)▶tial1 (bg=14.09%)MATCHES To TargetScan▶ miR-202-5p:UCCUAUG


AT

TGTAAATATCTTTTTGCTAAAATTCATAGGAAAT  
Depth:3 (DOG)  
Ei-value:0.000, Pi-value:0.000  
Er-value:0.000, Pr-value:0.000  
eCLIP MATCHES▶fubp3 (bg=23.31%)▶pum1 (bg=29.85%)▶pum2 (bg=21.55%)▶tial1 (bg=14.09%)MATCHES To TargetScan▶ miR-202-5p:UCCUAUG

G

CTTTTGGAAAT

CTTTTGGAAATTGAATTGTGAAGCCACCTTTG  
Depth:2 (BABOON)  
Ei-value:0.000, Pi-value:0.000  
Er-value:0.000, Pr-value:0.000  
eCLIP MATCHES▶DDX21 (bg=1.64%)▶ddx6 (bg=23.92%)▶dgcr8 (bg=19.31%)▶fubp3 (bg=23.31%)▶pum1 (bg=29.85%)▶pum2 (bg=21.55%)▶sf3a3 (bg=12.13%)▶TBRG4 (bg=7.0%)▶tia1 (bg=16.04%)▶tial1 (bg=14.09%)MATCHES To TargetScan▶ miR-18-5p:AAGGUGC

 1920  


T

CTTTTGGAAATTGAATTGTGAAGCCACCTTTG  
Depth:2 (BABOON)  
Ei-value:0.000, Pi-value:0.000  
Er-value:0.000, Pr-value:0.000  
eCLIP MATCHES▶DDX21 (bg=1.64%)▶ddx6 (bg=23.92%)▶dgcr8 (bg=19.31%)▶fubp3 (bg=23.31%)▶pum1 (bg=29.85%)▶pum2 (bg=21.55%)▶sf3a3 (bg=12.13%)▶TBRG4 (bg=7.0%)▶tia1 (bg=16.04%)▶tial1 (bg=14.09%)MATCHES To TargetScan▶ miR-18-5p:AAGGUGC


GAATTGT

GAATTGT  
Depth:3 (DOG)  
Ei-value:0.000, Pi-value:0.000  
Er-value:0.000, Pr-value:0.000  
eCLIP MATCHES▶DDX21 (bg=1.64%)▶ddx6 (bg=23.92%)▶dgcr8 (bg=19.31%)▶fubp3 (bg=23.31%)▶pum1 (bg=29.85%)▶pum2 (bg=21.55%)▶sf3a3 (bg=12.13%)▶TBRG4 (bg=7.0%)▶tia1 (bg=16.04%)▶tial1 (bg=14.09%)No matches to TargetScan


GAAG

CTTTTGGAAATTGAATTGTGAAGCCACCTTTG  
Depth:2 (BABOON)  
Ei-value:0.000, Pi-value:0.000  
Er-value:0.000, Pr-value:0.000  
eCLIP MATCHES▶DDX21 (bg=1.64%)▶ddx6 (bg=23.92%)▶dgcr8 (bg=19.31%)▶fubp3 (bg=23.31%)▶pum1 (bg=29.85%)▶pum2 (bg=21.55%)▶sf3a3 (bg=12.13%)▶TBRG4 (bg=7.0%)▶tia1 (bg=16.04%)▶tial1 (bg=14.09%)MATCHES To TargetScan▶ miR-18-5p:AAGGUGC


ccacct

ccacct  
Depth:3 (DOG)  
Ei-value:0.080, Pi-value:0.000  
Er-value:0.000, Pr-value:0.000  
eCLIP MATCHES▶DDX21 (bg=1.64%)▶ddx6 (bg=23.92%)▶dgcr8 (bg=19.31%)▶fubp3 (bg=23.31%)▶pum1 (bg=29.85%)▶pum2 (bg=21.55%)▶sf3a3 (bg=12.13%)▶TBRG4 (bg=7.0%)▶tia1 (bg=16.04%)▶tial1 (bg=14.09%)No matches to TargetScan


TTG

CTTTTGGAAATTGAATTGTGAAGCCACCTTTG  
Depth:2 (BABOON)  
Ei-value:0.000, Pi-value:0.000  
Er-value:0.000, Pr-value:0.000  
eCLIP MATCHES▶DDX21 (bg=1.64%)▶ddx6 (bg=23.92%)▶dgcr8 (bg=19.31%)▶fubp3 (bg=23.31%)▶pum1 (bg=29.85%)▶pum2 (bg=21.55%)▶sf3a3 (bg=12.13%)▶TBRG4 (bg=7.0%)▶tia1 (bg=16.04%)▶tial1 (bg=14.09%)MATCHES To TargetScan▶ miR-18-5p:AAGGUGC

TGAA

CAGTATAGTAATGTCTATACTTGTTCAAT

CAGTATAGTAATGTCTATACTTGTTCAAT  
Depth:2 (BABOON)  
Ei-value:0.000, Pi-value:0.000  
Er-value:0.000, Pr-value:0.000  
eCLIP MATCHES▶DDX21 (bg=1.64%)▶ddx6 (bg=23.92%)▶dgcr8 (bg=19.31%)▶fubp3 (bg=23.31%)▶pum1 (bg=29.85%)▶pum2 (bg=21.55%)▶sf3a3 (bg=12.13%)▶TBRG4 (bg=7.0%)▶tia1 (bg=16.04%)▶tial1 (bg=14.09%)MATCHES To TargetScan▶ miR-323-3p:ACAUUAC

A

gttta

gtttagaggaggt  
Depth:2 (BABOON)  
Ei-value:1.000, Pi-value:0.000  
Er-value:0.000, Pr-value:0.000  
eCLIP MATCHES▶fam120a (bg=18.43%)▶IGF2BP2 (bg=3.44%)▶pum1 (bg=29.85%)▶sf3a3 (bg=12.13%)No matches to TargetScan


GAGGAGGT

GAGGAGGT  
Depth:5 (SHEEP)  
Ei-value:0.000, Pi-value:0.000  
Er-value:0.000, Pr-value:0.000  
eCLIP MATCHES▶fam120a (bg=18.43%)▶IGF2BP2 (bg=3.44%)▶pum1 (bg=29.85%)▶sf3a3 (bg=12.13%)No matches to TargetScan

A

GG

GGAGGGAAGAAATTGCAAAAGGTAATAT  
Depth:2 (BABOON)  
Ei-value:0.000, Pi-value:0.000  
Er-value:0.000, Pr-value:0.000  
eCLIP MATCHES▶AARS (bg=2.33%)▶ddx6 (bg=23.92%)▶fam120a (bg=18.43%)▶IGF2BP2 (bg=3.44%)▶pum1 (bg=29.85%)▶sf3a3 (bg=12.13%)No matches to TargetScan


AGGGAA

AGGGAA  
Depth:5 (SHEEP)  
Ei-value:0.000, Pi-value:0.010  
Er-value:0.000, Pr-value:0.000  
eCLIP MATCHES▶AARS (bg=2.33%)▶fam120a (bg=18.43%)▶IGF2BP2 (bg=3.44%)▶pum1 (bg=29.85%)▶sf3a3 (bg=12.13%)No matches to TargetScan


GAAATTGC

GGAGGGAAGAAATTGCAAAAGGTAATAT  
Depth:2 (BABOON)  
Ei-value:0.000, Pi-value:0.000  
Er-value:0.000, Pr-value:0.000  
eCLIP MATCHES▶AARS (bg=2.33%)▶ddx6 (bg=23.92%)▶fam120a (bg=18.43%)▶IGF2BP2 (bg=3.44%)▶pum1 (bg=29.85%)▶sf3a3 (bg=12.13%)No matches to TargetScan


AAAAGGTAAT

AAAAGGTAAT  
Depth:7 (ARMADILLO)  
Ei-value:0.000, Pi-value:0.000  
Er-value:0.000, Pr-value:0.000  
eCLIP MATCHES▶AARS (bg=2.33%)▶ddx6 (bg=23.92%)▶fam120a (bg=18.43%)▶IGF2BP2 (bg=3.44%)▶pum1 (bg=29.85%)No matches to TargetScan


AT

AAAAGGTAATAT  
Depth:6 (PIG)  
Ei-value:0.000, Pi-value:0.000  
Er-value:0.000, Pr-value:0.000  
eCLIP MATCHES▶AARS (bg=2.33%)▶ddx6 (bg=23.92%)▶fam120a (bg=18.43%)▶IGF2BP2 (bg=3.44%)▶pum1 (bg=29.85%)No matches to TargetScan

T

ACTA

ACTAGTGTGTTCATACTTGGACATTTTCAGACA  
Depth:2 (BABOON)  
Ei-value:0.000, Pi-value:0.000  
Er-value:0.000, Pr-value:0.000  
eCLIP MATCHES▶ddx6 (bg=23.92%)▶fam120a (bg=18.43%)▶IGF2BP2 (bg=3.44%)▶pum1 (bg=29.85%)▶pum2 (bg=21.55%)MATCHES To TargetScan▶ miR-329-3p/362-3p:ACACACC


GTGTGTTCATACTTG

GTGTGTTCATACTTGGACATTTTCAGA  
Depth:3 (DOG)  
Ei-value:0.000, Pi-value:0.000  
Er-value:0.000, Pr-value:0.000  
eCLIP MATCHES▶ddx6 (bg=23.92%)▶IGF2BP2 (bg=3.44%)▶pum1 (bg=29.85%)▶pum2 (bg=21.55%)MATCHES To TargetScan▶ miR-329-3p/362-3p:ACACACC


GAC

GACATTTTCAGA  
Depth:5 (SHEEP)  
Ei-value:0.000, Pi-value:0.000  
Er-value:0.000, Pr-value:0.000  
eCLIP MATCHES▶pum1 (bg=29.85%)▶pum2 (bg=21.55%)No matches to TargetScan

 2040  


A

GACATTTTCAGA  
Depth:5 (SHEEP)  
Ei-value:0.000, Pi-value:0.000  
Er-value:0.000, Pr-value:0.000  
eCLIP MATCHES▶pum1 (bg=29.85%)▶pum2 (bg=21.55%)No matches to TargetScan


T

TTTTCAGA  
Depth:6 (PIG)  
Ei-value:0.000, Pi-value:0.000  
Er-value:0.000, Pr-value:0.000  
eCLIP MATCHES▶pum1 (bg=29.85%)▶pum2 (bg=21.55%)No matches to TargetScan


TTTCAG

TTTCAG  
Depth:7 (ARMADILLO)  
Ei-value:0.000, Pi-value:0.000  
Er-value:0.000, Pr-value:0.000  
eCLIP MATCHES▶pum1 (bg=29.85%)No matches to TargetScan


A

TTTTCAGA  
Depth:6 (PIG)  
Ei-value:0.000, Pi-value:0.000  
Er-value:0.000, Pr-value:0.000  
eCLIP MATCHES▶pum1 (bg=29.85%)▶pum2 (bg=21.55%)No matches to TargetScan


CA

ACTAGTGTGTTCATACTTGGACATTTTCAGACA  
Depth:2 (BABOON)  
Ei-value:0.000, Pi-value:0.000  
Er-value:0.000, Pr-value:0.000  
eCLIP MATCHES▶ddx6 (bg=23.92%)▶fam120a (bg=18.43%)▶IGF2BP2 (bg=3.44%)▶pum1 (bg=29.85%)▶pum2 (bg=21.55%)MATCHES To TargetScan▶ miR-329-3p/362-3p:ACACACC

CCA

tttttct

tttttct  
Depth:2 (BABOON)  
Ei-value:1.000, Pi-value:0.020  
Er-value:0.000, Pr-value:0.000  
eCLIP MATCHES▶pum1 (bg=29.85%)No matches to TargetScan

A

TATGTTT

TATGTTTTGTGCATTTTGTTTTGCTCTGTATATAGT  
Depth:2 (BABOON)  
Ei-value:0.000, Pi-value:0.000  
Er-value:0.000, Pr-value:0.000  
eCLIP MATCHES▶ddx6 (bg=23.92%)▶fubp3 (bg=23.31%)▶GEMIN5 (bg=2.46%)▶pum1 (bg=29.85%)▶pum2 (bg=21.55%)▶PUS1 (bg=2.87%)▶tial1 (bg=14.09%)MATCHES To TargetScan▶ miR-495-3p:AACAAAC▶ miR-501-3p/502-3p:AUGCACC


T

TGTGCATTTT  
Depth:4 (COW)  
Ei-value:0.020, Pi-value:0.000  
Er-value:0.000, Pr-value:0.000  
eCLIP MATCHES▶GEMIN5 (bg=2.46%)▶pum1 (bg=29.85%)▶pum2 (bg=21.55%)MATCHES To TargetScan▶ miR-501-3p/502-3p:AUGCACC


G

GTGCATTTT  
Depth:6 (PIG)  
Ei-value:0.000, Pi-value:0.000  
Er-value:0.000, Pr-value:0.000  
eCLIP MATCHES▶GEMIN5 (bg=2.46%)▶pum1 (bg=29.85%)▶pum2 (bg=21.55%)MATCHES To TargetScan▶ miR-501-3p/502-3p:AUGCACC


TGCATTTT

TGCATTTT  
Depth:7 (ARMADILLO)  
Ei-value:0.000, Pi-value:0.000  
Er-value:0.000, Pr-value:0.000  
eCLIP MATCHES▶GEMIN5 (bg=2.46%)▶pum1 (bg=29.85%)▶pum2 (bg=21.55%)No matches to TargetScan


G

TATGTTTTGTGCATTTTGTTTTGCTCTGTATATAGT  
Depth:2 (BABOON)  
Ei-value:0.000, Pi-value:0.000  
Er-value:0.000, Pr-value:0.000  
eCLIP MATCHES▶ddx6 (bg=23.92%)▶fubp3 (bg=23.31%)▶GEMIN5 (bg=2.46%)▶pum1 (bg=29.85%)▶pum2 (bg=21.55%)▶PUS1 (bg=2.87%)▶tial1 (bg=14.09%)MATCHES To TargetScan▶ miR-495-3p:AACAAAC▶ miR-501-3p/502-3p:AUGCACC


TTTTGCT

TTTTGCT  
Depth:5 (SHEEP)  
Ei-value:0.000, Pi-value:0.000  
Er-value:0.000, Pr-value:0.000  
eCLIP MATCHES▶fubp3 (bg=23.31%)▶GEMIN5 (bg=2.46%)▶pum1 (bg=29.85%)▶pum2 (bg=21.55%)No matches to TargetScan


C

TATGTTTTGTGCATTTTGTTTTGCTCTGTATATAGT  
Depth:2 (BABOON)  
Ei-value:0.000, Pi-value:0.000  
Er-value:0.000, Pr-value:0.000  
eCLIP MATCHES▶ddx6 (bg=23.92%)▶fubp3 (bg=23.31%)▶GEMIN5 (bg=2.46%)▶pum1 (bg=29.85%)▶pum2 (bg=21.55%)▶PUS1 (bg=2.87%)▶tial1 (bg=14.09%)MATCHES To TargetScan▶ miR-495-3p:AACAAAC▶ miR-501-3p/502-3p:AUGCACC


TGTATATAGT

TGTATATAGT  
Depth:7 (ARMADILLO)  
Ei-value:0.000, Pi-value:0.000  
Er-value:0.000, Pr-value:0.000  
eCLIP MATCHES▶ddx6 (bg=23.92%)▶fubp3 (bg=23.31%)▶GEMIN5 (bg=2.46%)▶pum1 (bg=29.85%)▶pum2 (bg=21.55%)▶PUS1 (bg=2.87%)▶tial1 (bg=14.09%)No matches to TargetScan

A

TATATAAT

TATATAATGGACAAAT  
Depth:6 (PIG)  
Ei-value:0.000, Pi-value:0.000  
Er-value:0.000, Pr-value:0.000  
eCLIP MATCHES▶ddx6 (bg=23.92%)▶fubp3 (bg=23.31%)▶GEMIN5 (bg=2.46%)▶NOLC1 (bg=6.58%)▶pum1 (bg=29.85%)▶pum2 (bg=21.55%)▶PUS1 (bg=2.87%)▶tial1 (bg=14.09%)No matches to TargetScan


GGACAAAT

GGACAAAT  
Depth:7 (ARMADILLO)  
Ei-value:0.000, Pi-value:0.000  
Er-value:0.000, Pr-value:0.000  
eCLIP MATCHES▶ddx6 (bg=23.92%)▶fubp3 (bg=23.31%)▶GEMIN5 (bg=2.46%)▶NOLC1 (bg=6.58%)▶pum1 (bg=29.85%)▶pum2 (bg=21.55%)▶PUS1 (bg=2.87%)▶tial1 (bg=14.09%)No matches to TargetScan


AGTCCTA

AGTCCTA  
Depth:7 (ARMADILLO)  
Ei-value:0.000, Pi-value:0.000  
Er-value:0.000, Pr-value:0.000  
eCLIP MATCHES▶ddx6 (bg=23.92%)▶fubp3 (bg=23.31%)▶GEMIN5 (bg=2.46%)▶NOLC1 (bg=6.58%)▶pum1 (bg=29.85%)▶pum2 (bg=21.55%)▶PUS1 (bg=2.87%)▶tial1 (bg=14.09%)No matches to TargetScan


ATTTTT

TATATAATGGACAAATAGTCCTAATTTTTCAACATCTAGTCTCTAGATGTTAAAGAGGTTGCCAGTGTATGACAAAG  
Depth:2 (BABOON)  
Ei-value:0.000, Pi-value:0.000  
Er-value:0.000, Pr-value:0.000  
eCLIP MATCHES▶ddx6 (bg=23.92%)▶FASTKD2 (bg=7.73%)▶fubp3 (bg=23.31%)▶GEMIN5 (bg=2.46%)▶lin28b (bg=16.96%)▶NOLC1 (bg=6.58%)▶pum1 (bg=29.85%)▶pum2 (bg=21.55%)▶PUS1 (bg=2.87%)▶SF3B1 (bg=6.74%)▶tial1 (bg=14.09%)MATCHES To TargetScan▶ miR-28-3p:ACUAGAU▶ miR-539-3p:UCAUACA▶ miR-193-3p:ACUGGCC


CA

CAACATCTAGTCTCTAGATGTTAAAGAGGTTGCCA  
Depth:3 (DOG)  
Ei-value:0.000, Pi-value:0.000  
Er-value:0.000, Pr-value:0.000  
eCLIP MATCHES▶ddx6 (bg=23.92%)▶FASTKD2 (bg=7.73%)▶fubp3 (bg=23.31%)▶lin28b (bg=16.96%)▶NOLC1 (bg=6.58%)▶pum1 (bg=29.85%)▶pum2 (bg=21.55%)▶PUS1 (bg=2.87%)▶SF3B1 (bg=6.74%)▶tial1 (bg=14.09%)MATCHES To TargetScan▶ miR-28-3p:ACUAGAU


ACATCTA

ACATCTA  
Depth:7 (ARMADILLO)  
Ei-value:0.000, Pi-value:0.000  
Er-value:0.000, Pr-value:0.000  
eCLIP MATCHES▶ddx6 (bg=23.92%)▶FASTKD2 (bg=7.73%)▶fubp3 (bg=23.31%)▶NOLC1 (bg=6.58%)▶pum1 (bg=29.85%)▶pum2 (bg=21.55%)▶PUS1 (bg=2.87%)▶SF3B1 (bg=6.74%)▶tial1 (bg=14.09%)No matches to TargetScan


G

CAACATCTAGTCTCTAGATGTTAAAGAGGTTGCCA  
Depth:3 (DOG)  
Ei-value:0.000, Pi-value:0.000  
Er-value:0.000, Pr-value:0.000  
eCLIP MATCHES▶ddx6 (bg=23.92%)▶FASTKD2 (bg=7.73%)▶fubp3 (bg=23.31%)▶lin28b (bg=16.96%)▶NOLC1 (bg=6.58%)▶pum1 (bg=29.85%)▶pum2 (bg=21.55%)▶PUS1 (bg=2.87%)▶SF3B1 (bg=6.74%)▶tial1 (bg=14.09%)MATCHES To TargetScan▶ miR-28-3p:ACUAGAU


TCTCTAGATGTT

TCTCTAGATGTT  
Depth:6 (PIG)  
Ei-value:0.000, Pi-value:0.000  
Er-value:0.000, Pr-value:0.000  
eCLIP MATCHES▶ddx6 (bg=23.92%)▶FASTKD2 (bg=7.73%)▶fubp3 (bg=23.31%)▶NOLC1 (bg=6.58%)▶pum1 (bg=29.85%)▶pum2 (bg=21.55%)▶PUS1 (bg=2.87%)▶SF3B1 (bg=6.74%)▶tial1 (bg=14.09%)No matches to TargetScan


AAAGAGGTTG

AAAGAGGTTGCCA  
Depth:9 (MOUSE)  
Ei-value:0.000, Pi-value:0.000  
Er-value:0.000, Pr-value:0.000  
eCLIP MATCHES▶ddx6 (bg=23.92%)▶FASTKD2 (bg=7.73%)▶fubp3 (bg=23.31%)▶lin28b (bg=16.96%)▶NOLC1 (bg=6.58%)▶pum1 (bg=29.85%)▶pum2 (bg=21.55%)▶SF3B1 (bg=6.74%)▶tial1 (bg=14.09%)No matches to TargetScan

 2160  


CCA

AAAGAGGTTGCCA  
Depth:9 (MOUSE)  
Ei-value:0.000, Pi-value:0.000  
Er-value:0.000, Pr-value:0.000  
eCLIP MATCHES▶ddx6 (bg=23.92%)▶FASTKD2 (bg=7.73%)▶fubp3 (bg=23.31%)▶lin28b (bg=16.96%)▶NOLC1 (bg=6.58%)▶pum1 (bg=29.85%)▶pum2 (bg=21.55%)▶SF3B1 (bg=6.74%)▶tial1 (bg=14.09%)No matches to TargetScan


G

TATATAATGGACAAATAGTCCTAATTTTTCAACATCTAGTCTCTAGATGTTAAAGAGGTTGCCAGTGTATGACAAAG  
Depth:2 (BABOON)  
Ei-value:0.000, Pi-value:0.000  
Er-value:0.000, Pr-value:0.000  
eCLIP MATCHES▶ddx6 (bg=23.92%)▶FASTKD2 (bg=7.73%)▶fubp3 (bg=23.31%)▶GEMIN5 (bg=2.46%)▶lin28b (bg=16.96%)▶NOLC1 (bg=6.58%)▶pum1 (bg=29.85%)▶pum2 (bg=21.55%)▶PUS1 (bg=2.87%)▶SF3B1 (bg=6.74%)▶tial1 (bg=14.09%)MATCHES To TargetScan▶ miR-28-3p:ACUAGAU▶ miR-539-3p:UCAUACA▶ miR-193-3p:ACUGGCC


TG

TGTATGACAAA  
Depth:6 (PIG)  
Ei-value:0.000, Pi-value:0.000  
Er-value:0.000, Pr-value:0.000  
eCLIP MATCHES▶ddx6 (bg=23.92%)▶FASTKD2 (bg=7.73%)▶fubp3 (bg=23.31%)▶lin28b (bg=16.96%)▶pum1 (bg=29.85%)▶pum2 (bg=21.55%)▶SF3B1 (bg=6.74%)▶tial1 (bg=14.09%)MATCHES To TargetScan▶ miR-539-3p:UCAUACA


TATGACAAA

TATGACAAA  
Depth:7 (ARMADILLO)  
Ei-value:0.000, Pi-value:0.000  
Er-value:0.000, Pr-value:0.000  
eCLIP MATCHES▶ddx6 (bg=23.92%)▶FASTKD2 (bg=7.73%)▶fubp3 (bg=23.31%)▶lin28b (bg=16.96%)▶pum1 (bg=29.85%)▶pum2 (bg=21.55%)▶SF3B1 (bg=6.74%)▶tial1 (bg=14.09%)No matches to TargetScan


G

TGTATGACAAAG  
Depth:5 (SHEEP)  
Ei-value:0.000, Pi-value:0.000  
Er-value:0.000, Pr-value:0.000  
eCLIP MATCHES▶ddx6 (bg=23.92%)▶FASTKD2 (bg=7.73%)▶fubp3 (bg=23.31%)▶lin28b (bg=16.96%)▶pum1 (bg=29.85%)▶pum2 (bg=21.55%)▶SF3B1 (bg=6.74%)▶tial1 (bg=14.09%)MATCHES To TargetScan▶ miR-539-3p:UCAUACA

G

agta

agtaaaattagcatattttgtac  
Depth:2 (BABOON)  
Ei-value:0.080, Pi-value:0.000  
Er-value:0.000, Pr-value:0.000  
eCLIP MATCHES▶ddx6 (bg=23.92%)▶FASTKD2 (bg=7.73%)▶fubp3 (bg=23.31%)▶igf2bp1 (bg=11.67%)▶lin28b (bg=16.96%)▶pum1 (bg=29.85%)▶pum2 (bg=21.55%)▶sf3a3 (bg=12.13%)▶SF3B1 (bg=6.74%)▶SUB1 (bg=9.24%)No matches to TargetScan


AAATTAGCA

AAATTAGCA  
Depth:5 (SHEEP)  
Ei-value:0.000, Pi-value:0.000  
Er-value:0.000, Pr-value:0.000  
eCLIP MATCHES▶ddx6 (bg=23.92%)▶FASTKD2 (bg=7.73%)▶fubp3 (bg=23.31%)▶lin28b (bg=16.96%)▶pum1 (bg=29.85%)▶pum2 (bg=21.55%)▶SUB1 (bg=9.24%)No matches to TargetScan


tattttgtac

agtaaaattagcatattttgtac  
Depth:2 (BABOON)  
Ei-value:0.080, Pi-value:0.000  
Er-value:0.000, Pr-value:0.000  
eCLIP MATCHES▶ddx6 (bg=23.92%)▶FASTKD2 (bg=7.73%)▶fubp3 (bg=23.31%)▶igf2bp1 (bg=11.67%)▶lin28b (bg=16.96%)▶pum1 (bg=29.85%)▶pum2 (bg=21.55%)▶sf3a3 (bg=12.13%)▶SF3B1 (bg=6.74%)▶SUB1 (bg=9.24%)No matches to TargetScan

A

ct

ctttgtgttgaaattc  
Depth:2 (BABOON)  
Ei-value:1.000, Pi-value:0.000  
Er-value:0.000, Pr-value:0.000  
eCLIP MATCHES▶ddx6 (bg=23.92%)▶fubp3 (bg=23.31%)▶igf2bp1 (bg=11.67%)▶lin28b (bg=16.96%)▶NKRF (bg=3.64%)▶pum1 (bg=29.85%)▶pum2 (bg=21.55%)▶sf3a3 (bg=12.13%)▶SUB1 (bg=9.24%)▶tia1 (bg=16.04%)▶tial1 (bg=14.09%)MATCHES To TargetScan▶ miR-421:UCAACAG▶ miR-505-3p.2:UCAACAC


TTGTGT

TTGTGTTGAAATT  
Depth:7 (ARMADILLO)  
Ei-value:0.000, Pi-value:0.000  
Er-value:0.000, Pr-value:0.000  
eCLIP MATCHES▶ddx6 (bg=23.92%)▶fubp3 (bg=23.31%)▶igf2bp1 (bg=11.67%)▶lin28b (bg=16.96%)▶NKRF (bg=3.64%)▶pum1 (bg=29.85%)▶pum2 (bg=21.55%)▶sf3a3 (bg=12.13%)▶SUB1 (bg=9.24%)▶tia1 (bg=16.04%)▶tial1 (bg=14.09%)MATCHES To TargetScan▶ miR-421:UCAACAG▶ miR-505-3p.2:UCAACAC


TGAAAT

TGAAAT  
Depth:8 (GUINEAPIG)  
Ei-value:0.000, Pi-value:0.010  
Er-value:0.000, Pr-value:0.010  
eCLIP MATCHES▶ddx6 (bg=23.92%)▶fubp3 (bg=23.31%)▶igf2bp1 (bg=11.67%)▶lin28b (bg=16.96%)▶NKRF (bg=3.64%)▶pum1 (bg=29.85%)▶pum2 (bg=21.55%)▶sf3a3 (bg=12.13%)▶SUB1 (bg=9.24%)▶tia1 (bg=16.04%)▶tial1 (bg=14.09%)No matches to TargetScan


T

TTGTGTTGAAATT  
Depth:7 (ARMADILLO)  
Ei-value:0.000, Pi-value:0.000  
Er-value:0.000, Pr-value:0.000  
eCLIP MATCHES▶ddx6 (bg=23.92%)▶fubp3 (bg=23.31%)▶igf2bp1 (bg=11.67%)▶lin28b (bg=16.96%)▶NKRF (bg=3.64%)▶pum1 (bg=29.85%)▶pum2 (bg=21.55%)▶sf3a3 (bg=12.13%)▶SUB1 (bg=9.24%)▶tia1 (bg=16.04%)▶tial1 (bg=14.09%)MATCHES To TargetScan▶ miR-421:UCAACAG▶ miR-505-3p.2:UCAACAC


C

TTGTGTTGAAATTC  
Depth:5 (SHEEP)  
Ei-value:0.000, Pi-value:0.000  
Er-value:0.000, Pr-value:0.000  
eCLIP MATCHES▶ddx6 (bg=23.92%)▶fubp3 (bg=23.31%)▶igf2bp1 (bg=11.67%)▶lin28b (bg=16.96%)▶NKRF (bg=3.64%)▶pum1 (bg=29.85%)▶pum2 (bg=21.55%)▶sf3a3 (bg=12.13%)▶SUB1 (bg=9.24%)▶tia1 (bg=16.04%)▶tial1 (bg=14.09%)MATCHES To TargetScan▶ miR-421:UCAACAG▶ miR-505-3p.2:UCAACAC

G

t

taggaaaacttgtcttctgtaaa  
Depth:2 (BABOON)  
Ei-value:0.080, Pi-value:0.000  
Er-value:0.000, Pr-value:0.000  
eCLIP MATCHES▶AQR (bg=1.27%)▶ddx6 (bg=23.92%)▶fubp3 (bg=23.31%)▶igf2bp1 (bg=11.67%)▶lin28b (bg=16.96%)▶NKRF (bg=3.64%)▶pum1 (bg=29.85%)▶pum2 (bg=21.55%)▶sf3a3 (bg=12.13%)▶SUB1 (bg=9.24%)▶tia1 (bg=16.04%)▶tial1 (bg=14.09%)No matches to TargetScan


aggaaa

aggaaa  
Depth:3 (DOG)  
Ei-value:0.080, Pi-value:0.050  
Er-value:0.000, Pr-value:0.010  
eCLIP MATCHES▶ddx6 (bg=23.92%)▶fubp3 (bg=23.31%)▶igf2bp1 (bg=11.67%)▶lin28b (bg=16.96%)▶NKRF (bg=3.64%)▶pum1 (bg=29.85%)▶pum2 (bg=21.55%)▶sf3a3 (bg=12.13%)▶SUB1 (bg=9.24%)▶tia1 (bg=16.04%)▶tial1 (bg=14.09%)No matches to TargetScan


acttgtc

taggaaaacttgtcttctgtaaa  
Depth:2 (BABOON)  
Ei-value:0.080, Pi-value:0.000  
Er-value:0.000, Pr-value:0.000  
eCLIP MATCHES▶AQR (bg=1.27%)▶ddx6 (bg=23.92%)▶fubp3 (bg=23.31%)▶igf2bp1 (bg=11.67%)▶lin28b (bg=16.96%)▶NKRF (bg=3.64%)▶pum1 (bg=29.85%)▶pum2 (bg=21.55%)▶sf3a3 (bg=12.13%)▶SUB1 (bg=9.24%)▶tia1 (bg=16.04%)▶tial1 (bg=14.09%)No matches to TargetScan


TTCTGT

TTCTGT  
Depth:7 (ARMADILLO)  
Ei-value:0.000, Pi-value:0.000  
Er-value:0.000, Pr-value:0.000  
eCLIP MATCHES▶AQR (bg=1.27%)▶ddx6 (bg=23.92%)▶fubp3 (bg=23.31%)▶igf2bp1 (bg=11.67%)▶lin28b (bg=16.96%)▶NKRF (bg=3.64%)▶pum1 (bg=29.85%)▶pum2 (bg=21.55%)▶sf3a3 (bg=12.13%)▶SUB1 (bg=9.24%)▶tia1 (bg=16.04%)▶tial1 (bg=14.09%)No matches to TargetScan


A

TTCTGTA  
Depth:6 (PIG)  
Ei-value:0.000, Pi-value:0.000  
Er-value:0.000, Pr-value:0.000  
eCLIP MATCHES▶AQR (bg=1.27%)▶ddx6 (bg=23.92%)▶fubp3 (bg=23.31%)▶igf2bp1 (bg=11.67%)▶lin28b (bg=16.96%)▶NKRF (bg=3.64%)▶pum1 (bg=29.85%)▶pum2 (bg=21.55%)▶sf3a3 (bg=12.13%)▶SUB1 (bg=9.24%)▶tia1 (bg=16.04%)▶tial1 (bg=14.09%)No matches to TargetScan


AA

TTCTGTAAA  
Depth:5 (SHEEP)  
Ei-value:0.000, Pi-value:0.000  
Er-value:0.000, Pr-value:0.000  
eCLIP MATCHES▶AQR (bg=1.27%)▶ddx6 (bg=23.92%)▶fubp3 (bg=23.31%)▶igf2bp1 (bg=11.67%)▶lin28b (bg=16.96%)▶NKRF (bg=3.64%)▶pum1 (bg=29.85%)▶pum2 (bg=21.55%)▶sf3a3 (bg=12.13%)▶SUB1 (bg=9.24%)▶tia1 (bg=16.04%)▶tial1 (bg=14.09%)No matches to TargetScan

GACT

tttgcatag

tttgcataggaatttgtt  
Depth:2 (BABOON)  
Ei-value:0.990, Pi-value:0.000  
Er-value:0.000, Pr-value:0.000  
eCLIP MATCHES▶AQR (bg=1.27%)▶ddx6 (bg=23.92%)▶fubp3 (bg=23.31%)▶igf2bp1 (bg=11.67%)▶pum1 (bg=29.85%)▶pum2 (bg=21.55%)▶SUB1 (bg=9.24%)▶tia1 (bg=16.04%)▶tial1 (bg=14.09%)MATCHES To TargetScan▶ miR-495-3p:AACAAAC▶ miR-202-5p:UCCUAUG


GAATTTGTT

GAATTTGTT  
Depth:3 (DOG)  
Ei-value:0.000, Pi-value:0.000  
Er-value:0.000, Pr-value:0.000  
eCLIP MATCHES▶AQR (bg=1.27%)▶ddx6 (bg=23.92%)▶fubp3 (bg=23.31%)▶igf2bp1 (bg=11.67%)▶pum1 (bg=29.85%)▶pum2 (bg=21.55%)▶SUB1 (bg=9.24%)▶tia1 (bg=16.04%)▶tial1 (bg=14.09%)MATCHES To TargetScan▶ miR-495-3p:AACAAAC

TG

accatctct

accatctct  
Depth:2 (BABOON)  
Ei-value:1.000, Pi-value:0.000  
Er-value:0.000, Pr-value:0.000  
eCLIP MATCHES▶AQR (bg=1.27%)▶ddx6 (bg=23.92%)▶fubp3 (bg=23.31%)▶igf2bp1 (bg=11.67%)▶pum1 (bg=29.85%)▶pum2 (bg=21.55%)▶SUPV3L1 (bg=0.85%)▶tia1 (bg=16.04%)▶tial1 (bg=14.09%)MATCHES To TargetScan▶ miR-143-3p:GAGAUGA

A

AGCAT

AGCATTA  
Depth:3 (DOG)  
Ei-value:0.000, Pi-value:0.000  
Er-value:0.000, Pr-value:0.000  
eCLIP MATCHES▶AQR (bg=1.27%)▶ddx6 (bg=23.92%)▶fubp3 (bg=23.31%)▶igf2bp1 (bg=11.67%)▶pum1 (bg=29.85%)▶pum2 (bg=21.55%)▶SUPV3L1 (bg=0.85%)▶tia1 (bg=16.04%)▶tial1 (bg=14.09%)MATCHES To TargetScan▶ miR-155-5p:UAAUGCU

 2280  


TA

AGCATTA  
Depth:3 (DOG)  
Ei-value:0.000, Pi-value:0.000  
Er-value:0.000, Pr-value:0.000  
eCLIP MATCHES▶AQR (bg=1.27%)▶ddx6 (bg=23.92%)▶fubp3 (bg=23.31%)▶igf2bp1 (bg=11.67%)▶pum1 (bg=29.85%)▶pum2 (bg=21.55%)▶SUPV3L1 (bg=0.85%)▶tia1 (bg=16.04%)▶tial1 (bg=14.09%)MATCHES To TargetScan▶ miR-155-5p:UAAUGCU

CACGTG

C

CCTGTACTTGTCCACTGGATTGAAG  
Depth:2 (BABOON)  
Ei-value:0.000, Pi-value:0.000  
Er-value:0.000, Pr-value:0.000  
eCLIP MATCHES▶AQR (bg=1.27%)▶ddx6 (bg=23.92%)▶fubp3 (bg=23.31%)▶pum1 (bg=29.85%)▶pum2 (bg=21.55%)▶SUPV3L1 (bg=0.85%)▶tia1 (bg=16.04%)▶tial1 (bg=14.09%)MATCHES To TargetScan▶ miR-145-5p:UCCAGUU▶ miR-199-5p:CCAGUGU


CTGTAC

CTGTAC  
Depth:7 (ARMADILLO)  
Ei-value:0.000, Pi-value:0.000  
Er-value:0.000, Pr-value:0.000  
eCLIP MATCHES▶AQR (bg=1.27%)▶ddx6 (bg=23.92%)▶fubp3 (bg=23.31%)▶pum1 (bg=29.85%)▶pum2 (bg=21.55%)▶SUPV3L1 (bg=0.85%)▶tia1 (bg=16.04%)▶tial1 (bg=14.09%)No matches to TargetScan


TTGTCC

CCTGTACTTGTCCACTGGATTGAAG  
Depth:2 (BABOON)  
Ei-value:0.000, Pi-value:0.000  
Er-value:0.000, Pr-value:0.000  
eCLIP MATCHES▶AQR (bg=1.27%)▶ddx6 (bg=23.92%)▶fubp3 (bg=23.31%)▶pum1 (bg=29.85%)▶pum2 (bg=21.55%)▶SUPV3L1 (bg=0.85%)▶tia1 (bg=16.04%)▶tial1 (bg=14.09%)MATCHES To TargetScan▶ miR-145-5p:UCCAGUU▶ miR-199-5p:CCAGUGU


ACTGGATT

ACTGGATT  
Depth:5 (SHEEP)  
Ei-value:0.000, Pi-value:0.000  
Er-value:0.000, Pr-value:0.000  
eCLIP MATCHES▶ddx6 (bg=23.92%)▶fubp3 (bg=23.31%)▶pum1 (bg=29.85%)▶SUPV3L1 (bg=0.85%)▶tia1 (bg=16.04%)▶tial1 (bg=14.09%)MATCHES To TargetScan▶ miR-145-5p:UCCAGUU


GAAG

CCTGTACTTGTCCACTGGATTGAAG  
Depth:2 (BABOON)  
Ei-value:0.000, Pi-value:0.000  
Er-value:0.000, Pr-value:0.000  
eCLIP MATCHES▶AQR (bg=1.27%)▶ddx6 (bg=23.92%)▶fubp3 (bg=23.31%)▶pum1 (bg=29.85%)▶pum2 (bg=21.55%)▶SUPV3L1 (bg=0.85%)▶tia1 (bg=16.04%)▶tial1 (bg=14.09%)MATCHES To TargetScan▶ miR-145-5p:UCCAGUU▶ miR-199-5p:CCAGUGU

GCAG

AG

AGAAGGAAG  
Depth:3 (DOG)  
Ei-value:0.000, Pi-value:0.000  
Er-value:0.000, Pr-value:0.000  
No matches to eCLIP DataNo matches to TargetScan


AAGGAAG

AAGGAAG  
Depth:6 (PIG)  
Ei-value:0.000, Pi-value:0.010  
Er-value:0.000, Pr-value:0.000  
No matches to eCLIP DataNo matches to TargetScan


g

agaaggaagg  
Depth:2 (BABOON)  
Ei-value:1.000, Pi-value:0.000  
Er-value:0.000, Pr-value:0.000  
No matches to eCLIP DataNo matches to TargetScan


GA

GAGGAGGGAATGATTCAAGGCCAAAATGGCCACATTTAGAAGATACCTCAGATGATAACCATTGTTAT  
Depth:2 (BABOON)  
Ei-value:0.000, Pi-value:0.000  
Er-value:0.000, Pr-value:0.000  
eCLIP MATCHES▶AARS (bg=2.33%)▶lin28b (bg=16.96%)MATCHES To TargetScan▶ miR-382-3p:AUCAUUC▶ miR-1298-5p:UCAUUCG▶ miR-299-3p:AUGUGGG▶ let-7-5p/98-5p:GAGGUAG


GGA

GGAGGGAAT  
Depth:3 (DOG)  
Ei-value:0.000, Pi-value:0.000  
Er-value:0.000, Pr-value:0.000  
No matches to eCLIP DataNo matches to TargetScan


GGGAAT

GGGAAT  
Depth:7 (ARMADILLO)  
Ei-value:0.000, Pi-value:0.000  
Er-value:0.000, Pr-value:0.000  
No matches to eCLIP DataNo matches to TargetScan


GATTCA

GAGGAGGGAATGATTCAAGGCCAAAATGGCCACATTTAGAAGATACCTCAGATGATAACCATTGTTAT  
Depth:2 (BABOON)  
Ei-value:0.000, Pi-value:0.000  
Er-value:0.000, Pr-value:0.000  
eCLIP MATCHES▶AARS (bg=2.33%)▶lin28b (bg=16.96%)MATCHES To TargetScan▶ miR-382-3p:AUCAUUC▶ miR-1298-5p:UCAUUCG▶ miR-299-3p:AUGUGGG▶ let-7-5p/98-5p:GAGGUAG


AGGCCAA

AGGCCAA  
Depth:7 (ARMADILLO)  
Ei-value:0.000, Pi-value:0.000  
Er-value:0.000, Pr-value:0.000  
eCLIP MATCHES▶AARS (bg=2.33%)No matches to TargetScan


AAT

AGGCCAAAAT  
Depth:6 (PIG)  
Ei-value:0.000, Pi-value:0.000  
Er-value:0.000, Pr-value:0.000  
eCLIP MATCHES▶AARS (bg=2.33%)No matches to TargetScan


GG

AGGCCAAAATGG  
Depth:3 (DOG)  
Ei-value:0.000, Pi-value:0.000  
Er-value:0.000, Pr-value:0.000  
eCLIP MATCHES▶AARS (bg=2.33%)No matches to TargetScan


CCAC

GAGGAGGGAATGATTCAAGGCCAAAATGGCCACATTTAGAAGATACCTCAGATGATAACCATTGTTAT  
Depth:2 (BABOON)  
Ei-value:0.000, Pi-value:0.000  
Er-value:0.000, Pr-value:0.000  
eCLIP MATCHES▶AARS (bg=2.33%)▶lin28b (bg=16.96%)MATCHES To TargetScan▶ miR-382-3p:AUCAUUC▶ miR-1298-5p:UCAUUCG▶ miR-299-3p:AUGUGGG▶ let-7-5p/98-5p:GAGGUAG


ATTTAG

ATTTAG  
Depth:6 (PIG)  
Ei-value:0.000, Pi-value:0.010  
Er-value:0.000, Pr-value:0.000  
No matches to eCLIP DataNo matches to TargetScan


AA

ATTTAGAA  
Depth:3 (DOG)  
Ei-value:0.000, Pi-value:0.000  
Er-value:0.000, Pr-value:0.000  
No matches to eCLIP DataNo matches to TargetScan


G

GAGGAGGGAATGATTCAAGGCCAAAATGGCCACATTTAGAAGATACCTCAGATGATAACCATTGTTAT  
Depth:2 (BABOON)  
Ei-value:0.000, Pi-value:0.000  
Er-value:0.000, Pr-value:0.000  
eCLIP MATCHES▶AARS (bg=2.33%)▶lin28b (bg=16.96%)MATCHES To TargetScan▶ miR-382-3p:AUCAUUC▶ miR-1298-5p:UCAUUCG▶ miR-299-3p:AUGUGGG▶ let-7-5p/98-5p:GAGGUAG


ATACCTCA

ATACCTCA  
Depth:7 (ARMADILLO)  
Ei-value:0.000, Pi-value:0.000  
Er-value:0.000, Pr-value:0.000  
No matches to eCLIP DataMATCHES To TargetScan▶ let-7-5p/98-5p:GAGGUAG


GAT

ATACCTCAGAT  
Depth:3 (DOG)  
Ei-value:0.000, Pi-value:0.000  
Er-value:0.000, Pr-value:0.000  
No matches to eCLIP DataMATCHES To TargetScan▶ let-7-5p/98-5p:GAGGUAG


GATAACCA

GAGGAGGGAATGATTCAAGGCCAAAATGGCCACATTTAGAAGATACCTCAGATGATAACCATTGTTAT  
Depth:2 (BABOON)  
Ei-value:0.000, Pi-value:0.000  
Er-value:0.000, Pr-value:0.000  
eCLIP MATCHES▶AARS (bg=2.33%)▶lin28b (bg=16.96%)MATCHES To TargetScan▶ miR-382-3p:AUCAUUC▶ miR-1298-5p:UCAUUCG▶ miR-299-3p:AUGUGGG▶ let-7-5p/98-5p:GAGGUAG


TTGTTA

TTGTTA  
Depth:7 (ARMADILLO)  
Ei-value:0.000, Pi-value:0.020  
Er-value:0.000, Pr-value:0.010  
eCLIP MATCHES▶lin28b (bg=16.96%)No matches to TargetScan


T

GAGGAGGGAATGATTCAAGGCCAAAATGGCCACATTTAGAAGATACCTCAGATGATAACCATTGTTAT  
Depth:2 (BABOON)  
Ei-value:0.000, Pi-value:0.000  
Er-value:0.000, Pr-value:0.000  
eCLIP MATCHES▶AARS (bg=2.33%)▶lin28b (bg=16.96%)MATCHES To TargetScan▶ miR-382-3p:AUCAUUC▶ miR-1298-5p:UCAUUCG▶ miR-299-3p:AUGUGGG▶ let-7-5p/98-5p:GAGGUAG

G

T

TGTGTGCAATT  
Depth:5 (SHEEP)  
Ei-value:0.000, Pi-value:0.000  
Er-value:0.000, Pr-value:0.000  
eCLIP MATCHES▶ddx6 (bg=23.92%)▶dgcr8 (bg=19.31%)▶FASTKD2 (bg=7.73%)▶HNRNPL (bg=3.4%)▶lin28b (bg=16.96%)▶NKRF (bg=3.64%)▶NOLC1 (bg=6.58%)▶SUB1 (bg=9.24%)MATCHES To TargetScan▶ miR-25-3p/32-5p/92-3p/363-3p/367-3p:AUUGCAC


GTG

GTGTGCAATT  
Depth:6 (PIG)  
Ei-value:0.000, Pi-value:0.000  
Er-value:0.000, Pr-value:0.000  
eCLIP MATCHES▶ddx6 (bg=23.92%)▶dgcr8 (bg=19.31%)▶FASTKD2 (bg=7.73%)▶HNRNPL (bg=3.4%)▶lin28b (bg=16.96%)▶NKRF (bg=3.64%)▶NOLC1 (bg=6.58%)▶SUB1 (bg=9.24%)MATCHES To TargetScan▶ miR-25-3p/32-5p/92-3p/363-3p/367-3p:AUUGCAC

 2400  


TGCAATT

GTGTGCAATT  
Depth:6 (PIG)  
Ei-value:0.000, Pi-value:0.000  
Er-value:0.000, Pr-value:0.000  
eCLIP MATCHES▶ddx6 (bg=23.92%)▶dgcr8 (bg=19.31%)▶FASTKD2 (bg=7.73%)▶HNRNPL (bg=3.4%)▶lin28b (bg=16.96%)▶NKRF (bg=3.64%)▶NOLC1 (bg=6.58%)▶SUB1 (bg=9.24%)MATCHES To TargetScan▶ miR-25-3p/32-5p/92-3p/363-3p/367-3p:AUUGCAC


T

TGTGTGCAATTTTATTTAACAGTGCT  
Depth:2 (BABOON)  
Ei-value:0.000, Pi-value:0.000  
Er-value:0.000, Pr-value:0.000  
eCLIP MATCHES▶CDC40 (bg=1.71%)▶ddx6 (bg=23.92%)▶dgcr8 (bg=19.31%)▶fam120a (bg=18.43%)▶FASTKD2 (bg=7.73%)▶fubp3 (bg=23.31%)▶FUS (bg=2.59%)▶G3BP1 (bg=1.51%)▶HNRNPL (bg=3.4%)▶igf2bp1 (bg=11.67%)▶lin28b (bg=16.96%)▶NCBP2 (bg=2.92%)▶NKRF (bg=3.64%)▶NOLC1 (bg=6.58%)▶pum1 (bg=29.85%)▶pum2 (bg=21.55%)▶SF3B1 (bg=6.74%)▶SUB1 (bg=9.24%)MATCHES To TargetScan▶ miR-25-3p/32-5p/92-3p/363-3p/367-3p:AUUGCAC▶ miR-452-5p/892-3p:ACUGUUU


TAT

TATTTAACAGTGCT  
Depth:3 (DOG)  
Ei-value:0.000, Pi-value:0.000  
Er-value:0.000, Pr-value:0.000  
eCLIP MATCHES▶CDC40 (bg=1.71%)▶ddx6 (bg=23.92%)▶dgcr8 (bg=19.31%)▶fam120a (bg=18.43%)▶FASTKD2 (bg=7.73%)▶fubp3 (bg=23.31%)▶FUS (bg=2.59%)▶G3BP1 (bg=1.51%)▶HNRNPL (bg=3.4%)▶igf2bp1 (bg=11.67%)▶lin28b (bg=16.96%)▶NCBP2 (bg=2.92%)▶NKRF (bg=3.64%)▶NOLC1 (bg=6.58%)▶pum1 (bg=29.85%)▶pum2 (bg=21.55%)▶SF3B1 (bg=6.74%)▶SUB1 (bg=9.24%)MATCHES To TargetScan▶ miR-452-5p/892-3p:ACUGUUU


TTAACA

TTAACA  
Depth:7 (ARMADILLO)  
Ei-value:0.000, Pi-value:0.010  
Er-value:0.000, Pr-value:0.000  
eCLIP MATCHES▶CDC40 (bg=1.71%)▶ddx6 (bg=23.92%)▶dgcr8 (bg=19.31%)▶fam120a (bg=18.43%)▶FASTKD2 (bg=7.73%)▶fubp3 (bg=23.31%)▶FUS (bg=2.59%)▶G3BP1 (bg=1.51%)▶HNRNPL (bg=3.4%)▶igf2bp1 (bg=11.67%)▶lin28b (bg=16.96%)▶NCBP2 (bg=2.92%)▶NKRF (bg=3.64%)▶NOLC1 (bg=6.58%)▶pum2 (bg=21.55%)▶SF3B1 (bg=6.74%)▶SUB1 (bg=9.24%)No matches to TargetScan


GT

TTAACAGT  
Depth:6 (PIG)  
Ei-value:0.000, Pi-value:0.000  
Er-value:0.000, Pr-value:0.000  
eCLIP MATCHES▶CDC40 (bg=1.71%)▶ddx6 (bg=23.92%)▶dgcr8 (bg=19.31%)▶fam120a (bg=18.43%)▶FASTKD2 (bg=7.73%)▶fubp3 (bg=23.31%)▶FUS (bg=2.59%)▶G3BP1 (bg=1.51%)▶HNRNPL (bg=3.4%)▶igf2bp1 (bg=11.67%)▶lin28b (bg=16.96%)▶NCBP2 (bg=2.92%)▶NKRF (bg=3.64%)▶NOLC1 (bg=6.58%)▶pum1 (bg=29.85%)▶pum2 (bg=21.55%)▶SF3B1 (bg=6.74%)▶SUB1 (bg=9.24%)MATCHES To TargetScan▶ miR-452-5p/892-3p:ACUGUUU


GCT

TATTTAACAGTGCT  
Depth:3 (DOG)  
Ei-value:0.000, Pi-value:0.000  
Er-value:0.000, Pr-value:0.000  
eCLIP MATCHES▶CDC40 (bg=1.71%)▶ddx6 (bg=23.92%)▶dgcr8 (bg=19.31%)▶fam120a (bg=18.43%)▶FASTKD2 (bg=7.73%)▶fubp3 (bg=23.31%)▶FUS (bg=2.59%)▶G3BP1 (bg=1.51%)▶HNRNPL (bg=3.4%)▶igf2bp1 (bg=11.67%)▶lin28b (bg=16.96%)▶NCBP2 (bg=2.92%)▶NKRF (bg=3.64%)▶NOLC1 (bg=6.58%)▶pum1 (bg=29.85%)▶pum2 (bg=21.55%)▶SF3B1 (bg=6.74%)▶SUB1 (bg=9.24%)MATCHES To TargetScan▶ miR-452-5p/892-3p:ACUGUUU

GTGTAT

gtggtg

gtggtg  
Depth:2 (BABOON)  
Ei-value:1.000, Pi-value:0.060  
Er-value:0.000, Pr-value:0.030  
eCLIP MATCHES▶CDC40 (bg=1.71%)▶ddx6 (bg=23.92%)▶dgcr8 (bg=19.31%)▶fam120a (bg=18.43%)▶FASTKD2 (bg=7.73%)▶fubp3 (bg=23.31%)▶FUS (bg=2.59%)▶G3BP1 (bg=1.51%)▶HNRNPL (bg=3.4%)▶igf2bp1 (bg=11.67%)▶lin28b (bg=16.96%)▶NCBP2 (bg=2.92%)▶NKRF (bg=3.64%)▶NOLC1 (bg=6.58%)▶pum1 (bg=29.85%)▶pum2 (bg=21.55%)▶SF3B1 (bg=6.74%)▶SUB1 (bg=9.24%)No matches to TargetScan

G

ACAAGT

ACAAGT  
Depth:7 (ARMADILLO)  
Ei-value:0.000, Pi-value:0.000  
Er-value:0.000, Pr-value:0.000  
eCLIP MATCHES▶CDC40 (bg=1.71%)▶ddx6 (bg=23.92%)▶dgcr8 (bg=19.31%)▶fam120a (bg=18.43%)▶FASTKD2 (bg=7.73%)▶fubp3 (bg=23.31%)▶FUS (bg=2.59%)▶G3BP1 (bg=1.51%)▶HNRNPL (bg=3.4%)▶igf2bp1 (bg=11.67%)▶lin28b (bg=16.96%)▶NCBP2 (bg=2.92%)▶NKRF (bg=3.64%)▶NOLC1 (bg=6.58%)▶pum1 (bg=29.85%)▶SF3B1 (bg=6.74%)▶SUB1 (bg=9.24%)No matches to TargetScan


TA

ACAAGTTA  
Depth:3 (DOG)  
Ei-value:0.000, Pi-value:0.000  
Er-value:0.000, Pr-value:0.000  
eCLIP MATCHES▶CDC40 (bg=1.71%)▶ddx6 (bg=23.92%)▶dgcr8 (bg=19.31%)▶fam120a (bg=18.43%)▶FASTKD2 (bg=7.73%)▶fubp3 (bg=23.31%)▶FUS (bg=2.59%)▶G3BP1 (bg=1.51%)▶HNRNPL (bg=3.4%)▶igf2bp1 (bg=11.67%)▶lin28b (bg=16.96%)▶NCBP2 (bg=2.92%)▶NKRF (bg=3.64%)▶NOLC1 (bg=6.58%)▶pum1 (bg=29.85%)▶SF3B1 (bg=6.74%)▶SUB1 (bg=9.24%)No matches to TargetScan


TA

ACAAGTTATATGAAATATCTAGTCTTTCTAGATATTTGGAAG  
Depth:2 (BABOON)  
Ei-value:0.000, Pi-value:0.000  
Er-value:0.000, Pr-value:0.000  
eCLIP MATCHES▶CDC40 (bg=1.71%)▶ddx6 (bg=23.92%)▶dgcr8 (bg=19.31%)▶fam120a (bg=18.43%)▶FASTKD2 (bg=7.73%)▶fubp3 (bg=23.31%)▶FUS (bg=2.59%)▶G3BP1 (bg=1.51%)▶HNRNPL (bg=3.4%)▶igf2bp1 (bg=11.67%)▶lin28b (bg=16.96%)▶NCBP2 (bg=2.92%)▶NKRF (bg=3.64%)▶NOLC1 (bg=6.58%)▶pum1 (bg=29.85%)▶SF3B1 (bg=6.74%)▶SUB1 (bg=9.24%)MATCHES To TargetScan▶ miR-410-3p:AUAUAAC▶ miR-28-3p:ACUAGAU


TGAAATATCTAGTCTT

TGAAATATCTAGTCTT  
Depth:7 (ARMADILLO)  
Ei-value:0.000, Pi-value:0.000  
Er-value:0.000, Pr-value:0.000  
eCLIP MATCHES▶ddx6 (bg=23.92%)▶dgcr8 (bg=19.31%)▶fubp3 (bg=23.31%)▶FUS (bg=2.59%)▶lin28b (bg=16.96%)▶NOLC1 (bg=6.58%)▶SF3B1 (bg=6.74%)MATCHES To TargetScan▶ miR-28-3p:ACUAGAU


TCTAGATA

TGAAATATCTAGTCTTTCTAGATA  
Depth:5 (SHEEP)  
Ei-value:0.000, Pi-value:0.000  
Er-value:0.000, Pr-value:0.000  
eCLIP MATCHES▶ddx6 (bg=23.92%)▶dgcr8 (bg=19.31%)▶fubp3 (bg=23.31%)▶FUS (bg=2.59%)▶lin28b (bg=16.96%)▶NOLC1 (bg=6.58%)▶SF3B1 (bg=6.74%)MATCHES To TargetScan▶ miR-28-3p:ACUAGAU


TTTGGAAG

ACAAGTTATATGAAATATCTAGTCTTTCTAGATATTTGGAAG  
Depth:2 (BABOON)  
Ei-value:0.000, Pi-value:0.000  
Er-value:0.000, Pr-value:0.000  
eCLIP MATCHES▶CDC40 (bg=1.71%)▶ddx6 (bg=23.92%)▶dgcr8 (bg=19.31%)▶fam120a (bg=18.43%)▶FASTKD2 (bg=7.73%)▶fubp3 (bg=23.31%)▶FUS (bg=2.59%)▶G3BP1 (bg=1.51%)▶HNRNPL (bg=3.4%)▶igf2bp1 (bg=11.67%)▶lin28b (bg=16.96%)▶NCBP2 (bg=2.92%)▶NKRF (bg=3.64%)▶NOLC1 (bg=6.58%)▶pum1 (bg=29.85%)▶SF3B1 (bg=6.74%)▶SUB1 (bg=9.24%)MATCHES To TargetScan▶ miR-410-3p:AUAUAAC▶ miR-28-3p:ACUAGAU


TGCTTGATG

TGCTTGATG  
Depth:7 (ARMADILLO)  
Ei-value:0.000, Pi-value:0.000  
Er-value:0.000, Pr-value:0.000  
No matches to eCLIP DataNo matches to TargetScan


TATTTA

TGCTTGATGTATTTA  
Depth:3 (DOG)  
Ei-value:0.000, Pi-value:0.000  
Er-value:0.000, Pr-value:0.000  
No matches to eCLIP DataNo matches to TargetScan


AAAGTGGT

TGCTTGATGTATTTAAAAGTGGTAGTAGAATAACACT  
Depth:2 (BABOON)  
Ei-value:0.000, Pi-value:0.000  
Er-value:0.000, Pr-value:0.000  
No matches to eCLIP DataNo matches to TargetScan


AGTAGA

AGTAGA  
Depth:5 (SHEEP)  
Ei-value:0.000, Pi-value:0.010  
Er-value:0.000, Pr-value:0.000  
No matches to eCLIP DataNo matches to TargetScan


ATAACACT

TGCTTGATGTATTTAAAAGTGGTAGTAGAATAACACT  
Depth:2 (BABOON)  
Ei-value:0.000, Pi-value:0.000  
Er-value:0.000, Pr-value:0.000  
No matches to eCLIP DataNo matches to TargetScan


T

TTGTAAATAGCTTTT  
Depth:6 (PIG)  
Ei-value:0.000, Pi-value:0.000  
Er-value:0.000, Pr-value:0.000  
No matches to eCLIP DataMATCHES To TargetScan▶ miR-320:AAAGCUG


TGTAA

TGTAAATAGCTTTT  
Depth:7 (ARMADILLO)  
Ei-value:0.000, Pi-value:0.000  
Er-value:0.000, Pr-value:0.000  
No matches to eCLIP DataMATCHES To TargetScan▶ miR-320:AAAGCUG

 2520  


ATAGCTTTT

TGTAAATAGCTTTT  
Depth:7 (ARMADILLO)  
Ei-value:0.000, Pi-value:0.000  
Er-value:0.000, Pr-value:0.000  
No matches to eCLIP DataMATCHES To TargetScan▶ miR-320:AAAGCUG


AAA

TTGTAAATAGCTTTTAAA  
Depth:3 (DOG)  
Ei-value:0.000, Pi-value:0.000  
Er-value:0.000, Pr-value:0.000  
No matches to eCLIP DataMATCHES To TargetScan▶ miR-320:AAAGCUG


A

TTGTAAATAGCTTTTAAAAACTGATGGGAAATGCTGTTTGGAAGTGGAATTGTTGAACCA  
Depth:2 (BABOON)  
Ei-value:0.000, Pi-value:0.000  
Er-value:0.000, Pr-value:0.000  
eCLIP MATCHES▶ddx55 (bg=12.35%)▶FTO (bg=7.53%)▶GRSF1 (bg=3.8%)▶KHSRP (bg=8.1%)▶pum1 (bg=29.85%)▶SUB1 (bg=9.24%)MATCHES To TargetScan▶ miR-421:UCAACAG▶ miR-505-3p.2:UCAACAC▶ miR-320:AAAGCUG


ACTG

ACTGATGGGAAAT  
Depth:5 (SHEEP)  
Ei-value:0.000, Pi-value:0.000  
Er-value:0.000, Pr-value:0.000  
eCLIP MATCHES▶GRSF1 (bg=3.8%)▶pum1 (bg=29.85%)No matches to TargetScan


ATG

ATGGGAAAT  
Depth:6 (PIG)  
Ei-value:0.000, Pi-value:0.000  
Er-value:0.000, Pr-value:0.000  
eCLIP MATCHES▶GRSF1 (bg=3.8%)▶pum1 (bg=29.85%)No matches to TargetScan


GGAAAT

GGAAAT  
Depth:7 (ARMADILLO)  
Ei-value:0.000, Pi-value:0.000  
Er-value:0.000, Pr-value:0.000  
eCLIP MATCHES▶GRSF1 (bg=3.8%)▶pum1 (bg=29.85%)No matches to TargetScan


GCTGTTTGGAAGTG

TTGTAAATAGCTTTTAAAAACTGATGGGAAATGCTGTTTGGAAGTGGAATTGTTGAACCA  
Depth:2 (BABOON)  
Ei-value:0.000, Pi-value:0.000  
Er-value:0.000, Pr-value:0.000  
eCLIP MATCHES▶ddx55 (bg=12.35%)▶FTO (bg=7.53%)▶GRSF1 (bg=3.8%)▶KHSRP (bg=8.1%)▶pum1 (bg=29.85%)▶SUB1 (bg=9.24%)MATCHES To TargetScan▶ miR-421:UCAACAG▶ miR-505-3p.2:UCAACAC▶ miR-320:AAAGCUG


GAATTG

GAATTG  
Depth:6 (PIG)  
Ei-value:0.000, Pi-value:0.010  
Er-value:0.000, Pr-value:0.000  
eCLIP MATCHES▶FTO (bg=7.53%)▶GRSF1 (bg=3.8%)▶KHSRP (bg=8.1%)▶pum1 (bg=29.85%)▶SUB1 (bg=9.24%)No matches to TargetScan


TTGAACCA

TTGTAAATAGCTTTTAAAAACTGATGGGAAATGCTGTTTGGAAGTGGAATTGTTGAACCA  
Depth:2 (BABOON)  
Ei-value:0.000, Pi-value:0.000  
Er-value:0.000, Pr-value:0.000  
eCLIP MATCHES▶ddx55 (bg=12.35%)▶FTO (bg=7.53%)▶GRSF1 (bg=3.8%)▶KHSRP (bg=8.1%)▶pum1 (bg=29.85%)▶SUB1 (bg=9.24%)MATCHES To TargetScan▶ miR-421:UCAACAG▶ miR-505-3p.2:UCAACAC▶ miR-320:AAAGCUG

C

ct

ctgggaggtgggagggaa  
Depth:2 (BABOON)  
Ei-value:0.990, Pi-value:0.000  
Er-value:0.000, Pr-value:0.000  
eCLIP MATCHES▶ddx55 (bg=12.35%)▶FTO (bg=7.53%)▶GRSF1 (bg=3.8%)▶KHSRP (bg=8.1%)▶pum1 (bg=29.85%)▶SUB1 (bg=9.24%)MATCHES To TargetScan▶ miR-1306-5p:CACCUCC▶ miR-150-5p:CUCCCAA▶ miR-532-3p:CUCCCAC


GGGAG

GGGAGGTGGGAGGGAA  
Depth:5 (SHEEP)  
Ei-value:0.000, Pi-value:0.000  
Er-value:0.000, Pr-value:0.000  
eCLIP MATCHES▶ddx55 (bg=12.35%)▶FTO (bg=7.53%)▶GRSF1 (bg=3.8%)▶KHSRP (bg=8.1%)▶pum1 (bg=29.85%)▶SUB1 (bg=9.24%)MATCHES To TargetScan▶ miR-1306-5p:CACCUCC▶ miR-150-5p:CUCCCAA▶ miR-532-3p:CUCCCAC


GTGGGAGGGAA

GTGGGAGGGAA  
Depth:9 (MOUSE)  
Ei-value:0.000, Pi-value:0.000  
Er-value:0.000, Pr-value:0.000  
eCLIP MATCHES▶ddx55 (bg=12.35%)▶FTO (bg=7.53%)▶GRSF1 (bg=3.8%)▶KHSRP (bg=8.1%)▶pum1 (bg=29.85%)▶SUB1 (bg=9.24%)MATCHES To TargetScan▶ miR-150-5p:CUCCCAA▶ miR-532-3p:CUCCCAC

GAAAT

TGCAAA

TGCAAA  
Depth:9 (MOUSE)  
Ei-value:0.000, Pi-value:0.000  
Er-value:0.000, Pr-value:0.000  
eCLIP MATCHES▶ddx55 (bg=12.35%)▶FTO (bg=7.53%)▶fubp3 (bg=23.31%)▶GRSF1 (bg=3.8%)▶KHSRP (bg=8.1%)▶pum1 (bg=29.85%)▶SUB1 (bg=9.24%)No matches to TargetScan

T

GGTG

GGTGTTTTGCCATTGTTTATTAGAAAATTTCAGCTTAATCCATTG  
Depth:2 (BABOON)  
Ei-value:0.000, Pi-value:0.000  
Er-value:0.000, Pr-value:0.000  
eCLIP MATCHES▶AARS (bg=2.33%)▶CPEB4 (bg=2.3%)▶ddx55 (bg=12.35%)▶ddx6 (bg=23.92%)▶dgcr8 (bg=19.31%)▶fam120a (bg=18.43%)▶FASTKD2 (bg=7.73%)▶fubp3 (bg=23.31%)▶GRSF1 (bg=3.8%)▶igf2bp1 (bg=11.67%)▶KHSRP (bg=8.1%)▶NCBP2 (bg=2.92%)▶NKRF (bg=3.64%)▶NOLC1 (bg=6.58%)▶pum1 (bg=29.85%)▶pum2 (bg=21.55%)▶sf3a3 (bg=12.13%)▶SUB1 (bg=9.24%)▶TBRG4 (bg=7.0%)▶tia1 (bg=16.04%)▶tial1 (bg=14.09%)▶ZC3H11A (bg=6.25%)MATCHES To TargetScan▶ miR-183-5p.1:AUGGCAC▶ miR-203a-3p.2:UGAAAUG


TTTTGC

TTTTGC  
Depth:8 (GUINEAPIG)  
Ei-value:0.000, Pi-value:0.010  
Er-value:0.000, Pr-value:0.000  
eCLIP MATCHES▶fubp3 (bg=23.31%)▶GRSF1 (bg=3.8%)▶KHSRP (bg=8.1%)▶pum1 (bg=29.85%)▶SUB1 (bg=9.24%)No matches to TargetScan


CATTGTTTATTAGA

GGTGTTTTGCCATTGTTTATTAGAAAATTTCAGCTTAATCCATTG  
Depth:2 (BABOON)  
Ei-value:0.000, Pi-value:0.000  
Er-value:0.000, Pr-value:0.000  
eCLIP MATCHES▶AARS (bg=2.33%)▶CPEB4 (bg=2.3%)▶ddx55 (bg=12.35%)▶ddx6 (bg=23.92%)▶dgcr8 (bg=19.31%)▶fam120a (bg=18.43%)▶FASTKD2 (bg=7.73%)▶fubp3 (bg=23.31%)▶GRSF1 (bg=3.8%)▶igf2bp1 (bg=11.67%)▶KHSRP (bg=8.1%)▶NCBP2 (bg=2.92%)▶NKRF (bg=3.64%)▶NOLC1 (bg=6.58%)▶pum1 (bg=29.85%)▶pum2 (bg=21.55%)▶sf3a3 (bg=12.13%)▶SUB1 (bg=9.24%)▶TBRG4 (bg=7.0%)▶tia1 (bg=16.04%)▶tial1 (bg=14.09%)▶ZC3H11A (bg=6.25%)MATCHES To TargetScan▶ miR-183-5p.1:AUGGCAC▶ miR-203a-3p.2:UGAAAUG


AA

AAATTTCAGCTTA  
Depth:3 (DOG)  
Ei-value:0.000, Pi-value:0.000  
Er-value:0.000, Pr-value:0.000  
eCLIP MATCHES▶AARS (bg=2.33%)▶CPEB4 (bg=2.3%)▶ddx6 (bg=23.92%)▶dgcr8 (bg=19.31%)▶fam120a (bg=18.43%)▶FASTKD2 (bg=7.73%)▶fubp3 (bg=23.31%)▶GRSF1 (bg=3.8%)▶igf2bp1 (bg=11.67%)▶KHSRP (bg=8.1%)▶NCBP2 (bg=2.92%)▶pum1 (bg=29.85%)▶pum2 (bg=21.55%)▶sf3a3 (bg=12.13%)▶TBRG4 (bg=7.0%)▶tia1 (bg=16.04%)▶tial1 (bg=14.09%)▶ZC3H11A (bg=6.25%)MATCHES To TargetScan▶ miR-203a-3p.2:UGAAAUG


ATTTCA

ATTTCA  
Depth:6 (PIG)  
Ei-value:0.000, Pi-value:0.010  
Er-value:0.000, Pr-value:0.010  
eCLIP MATCHES▶AARS (bg=2.33%)▶CPEB4 (bg=2.3%)▶ddx6 (bg=23.92%)▶dgcr8 (bg=19.31%)▶fam120a (bg=18.43%)▶FASTKD2 (bg=7.73%)▶fubp3 (bg=23.31%)▶GRSF1 (bg=3.8%)▶igf2bp1 (bg=11.67%)▶KHSRP (bg=8.1%)▶pum1 (bg=29.85%)▶pum2 (bg=21.55%)▶sf3a3 (bg=12.13%)▶TBRG4 (bg=7.0%)▶tia1 (bg=16.04%)▶tial1 (bg=14.09%)▶ZC3H11A (bg=6.25%)MATCHES To TargetScan▶ miR-203a-3p.2:UGAAAUG


GCT

ATTTCAGCTTA  
Depth:5 (SHEEP)  
Ei-value:0.000, Pi-value:0.000  
Er-value:0.000, Pr-value:0.000  
eCLIP MATCHES▶AARS (bg=2.33%)▶CPEB4 (bg=2.3%)▶ddx6 (bg=23.92%)▶dgcr8 (bg=19.31%)▶fam120a (bg=18.43%)▶FASTKD2 (bg=7.73%)▶fubp3 (bg=23.31%)▶GRSF1 (bg=3.8%)▶igf2bp1 (bg=11.67%)▶KHSRP (bg=8.1%)▶NCBP2 (bg=2.92%)▶pum1 (bg=29.85%)▶pum2 (bg=21.55%)▶sf3a3 (bg=12.13%)▶TBRG4 (bg=7.0%)▶tia1 (bg=16.04%)▶tial1 (bg=14.09%)▶ZC3H11A (bg=6.25%)MATCHES To TargetScan▶ miR-203a-3p.2:UGAAAUG

 2640  


TA

ATTTCAGCTTA  
Depth:5 (SHEEP)  
Ei-value:0.000, Pi-value:0.000  
Er-value:0.000, Pr-value:0.000  
eCLIP MATCHES▶AARS (bg=2.33%)▶CPEB4 (bg=2.3%)▶ddx6 (bg=23.92%)▶dgcr8 (bg=19.31%)▶fam120a (bg=18.43%)▶FASTKD2 (bg=7.73%)▶fubp3 (bg=23.31%)▶GRSF1 (bg=3.8%)▶igf2bp1 (bg=11.67%)▶KHSRP (bg=8.1%)▶NCBP2 (bg=2.92%)▶pum1 (bg=29.85%)▶pum2 (bg=21.55%)▶sf3a3 (bg=12.13%)▶TBRG4 (bg=7.0%)▶tia1 (bg=16.04%)▶tial1 (bg=14.09%)▶ZC3H11A (bg=6.25%)MATCHES To TargetScan▶ miR-203a-3p.2:UGAAAUG


A

GGTGTTTTGCCATTGTTTATTAGAAAATTTCAGCTTAATCCATTG  
Depth:2 (BABOON)  
Ei-value:0.000, Pi-value:0.000  
Er-value:0.000, Pr-value:0.000  
eCLIP MATCHES▶AARS (bg=2.33%)▶CPEB4 (bg=2.3%)▶ddx55 (bg=12.35%)▶ddx6 (bg=23.92%)▶dgcr8 (bg=19.31%)▶fam120a (bg=18.43%)▶FASTKD2 (bg=7.73%)▶fubp3 (bg=23.31%)▶GRSF1 (bg=3.8%)▶igf2bp1 (bg=11.67%)▶KHSRP (bg=8.1%)▶NCBP2 (bg=2.92%)▶NKRF (bg=3.64%)▶NOLC1 (bg=6.58%)▶pum1 (bg=29.85%)▶pum2 (bg=21.55%)▶sf3a3 (bg=12.13%)▶SUB1 (bg=9.24%)▶TBRG4 (bg=7.0%)▶tia1 (bg=16.04%)▶tial1 (bg=14.09%)▶ZC3H11A (bg=6.25%)MATCHES To TargetScan▶ miR-183-5p.1:AUGGCAC▶ miR-203a-3p.2:UGAAAUG


T

TCCATTG  
Depth:3 (DOG)  
Ei-value:0.000, Pi-value:0.000  
Er-value:0.000, Pr-value:0.000  
eCLIP MATCHES▶AARS (bg=2.33%)▶CPEB4 (bg=2.3%)▶ddx55 (bg=12.35%)▶ddx6 (bg=23.92%)▶dgcr8 (bg=19.31%)▶fam120a (bg=18.43%)▶FASTKD2 (bg=7.73%)▶fubp3 (bg=23.31%)▶GRSF1 (bg=3.8%)▶igf2bp1 (bg=11.67%)▶KHSRP (bg=8.1%)▶NCBP2 (bg=2.92%)▶NKRF (bg=3.64%)▶NOLC1 (bg=6.58%)▶pum1 (bg=29.85%)▶pum2 (bg=21.55%)▶sf3a3 (bg=12.13%)▶TBRG4 (bg=7.0%)▶tia1 (bg=16.04%)▶tial1 (bg=14.09%)▶ZC3H11A (bg=6.25%)No matches to TargetScan


CCATTG

CCATTG  
Depth:5 (SHEEP)  
Ei-value:0.000, Pi-value:0.000  
Er-value:0.000, Pr-value:0.000  
eCLIP MATCHES▶AARS (bg=2.33%)▶CPEB4 (bg=2.3%)▶ddx55 (bg=12.35%)▶ddx6 (bg=23.92%)▶dgcr8 (bg=19.31%)▶fam120a (bg=18.43%)▶FASTKD2 (bg=7.73%)▶fubp3 (bg=23.31%)▶GRSF1 (bg=3.8%)▶igf2bp1 (bg=11.67%)▶KHSRP (bg=8.1%)▶NCBP2 (bg=2.92%)▶NKRF (bg=3.64%)▶NOLC1 (bg=6.58%)▶pum1 (bg=29.85%)▶pum2 (bg=21.55%)▶sf3a3 (bg=12.13%)▶TBRG4 (bg=7.0%)▶tia1 (bg=16.04%)▶tial1 (bg=14.09%)▶ZC3H11A (bg=6.25%)No matches to TargetScan

TGTA

TATGTTA

TATGTTA  
Depth:6 (PIG)  
Ei-value:0.000, Pi-value:0.000  
Er-value:0.000, Pr-value:0.000  
eCLIP MATCHES▶AARS (bg=2.33%)▶CPEB4 (bg=2.3%)▶ddx6 (bg=23.92%)▶dgcr8 (bg=19.31%)▶fam120a (bg=18.43%)▶FASTKD2 (bg=7.73%)▶fubp3 (bg=23.31%)▶GRSF1 (bg=3.8%)▶igf2bp1 (bg=11.67%)▶KHSRP (bg=8.1%)▶NCBP2 (bg=2.92%)▶NKRF (bg=3.64%)▶NOLC1 (bg=6.58%)▶pum1 (bg=29.85%)▶pum2 (bg=21.55%)▶sf3a3 (bg=12.13%)▶SF3B1 (bg=6.74%)▶TBRG4 (bg=7.0%)▶tia1 (bg=16.04%)▶tial1 (bg=14.09%)▶ZC3H11A (bg=6.25%)No matches to TargetScan


CA

TATGTTACATGCATTTCATTTAACTTTGCTATACTGTATATATTGT  
Depth:2 (BABOON)  
Ei-value:0.000, Pi-value:0.000  
Er-value:0.000, Pr-value:0.000  
eCLIP MATCHES▶AARS (bg=2.33%)▶AKAP1 (bg=3.4%)▶CPEB4 (bg=2.3%)▶ddx55 (bg=12.35%)▶ddx6 (bg=23.92%)▶dgcr8 (bg=19.31%)▶EIF4G2 (bg=0.7%)▶fam120a (bg=18.43%)▶FASTKD2 (bg=7.73%)▶fubp3 (bg=23.31%)▶GRSF1 (bg=3.8%)▶HNRNPL (bg=3.4%)▶igf2bp1 (bg=11.67%)▶KHSRP (bg=8.1%)▶NCBP2 (bg=2.92%)▶NKRF (bg=3.64%)▶NOLC1 (bg=6.58%)▶pum1 (bg=29.85%)▶pum2 (bg=21.55%)▶sf3a3 (bg=12.13%)▶SF3B1 (bg=6.74%)▶TBRG4 (bg=7.0%)▶tia1 (bg=16.04%)▶tial1 (bg=14.09%)▶WRN (bg=3.31%)▶ZC3H11A (bg=6.25%)MATCHES To TargetScan▶ miR-203a-3p.1:GAAAUGU▶ miR-194-5p:GUAACAG▶ miR-203a-3p.2:UGAAAUG▶ miR-144-3p:ACAGUAU▶ miR-101-3p.1:ACAGUAC▶ miR-411-3p:AUGUAAC▶ miR-582-5p:UACAGUU▶ miR-101-3p.2:UACAGUA


tgcatt

tgcatt  
Depth:4 (COW)  
Ei-value:1.000, Pi-value:0.010  
Er-value:0.000, Pr-value:0.000  
eCLIP MATCHES▶AARS (bg=2.33%)▶CPEB4 (bg=2.3%)▶ddx6 (bg=23.92%)▶dgcr8 (bg=19.31%)▶fam120a (bg=18.43%)▶FASTKD2 (bg=7.73%)▶fubp3 (bg=23.31%)▶GRSF1 (bg=3.8%)▶igf2bp1 (bg=11.67%)▶KHSRP (bg=8.1%)▶NCBP2 (bg=2.92%)▶NKRF (bg=3.64%)▶NOLC1 (bg=6.58%)▶pum1 (bg=29.85%)▶pum2 (bg=21.55%)▶sf3a3 (bg=12.13%)▶SF3B1 (bg=6.74%)▶TBRG4 (bg=7.0%)▶tia1 (bg=16.04%)▶tial1 (bg=14.09%)▶ZC3H11A (bg=6.25%)No matches to TargetScan


TCATTTAACTTTGCTAT

TATGTTACATGCATTTCATTTAACTTTGCTATACTGTATATATTGT  
Depth:2 (BABOON)  
Ei-value:0.000, Pi-value:0.000  
Er-value:0.000, Pr-value:0.000  
eCLIP MATCHES▶AARS (bg=2.33%)▶AKAP1 (bg=3.4%)▶CPEB4 (bg=2.3%)▶ddx55 (bg=12.35%)▶ddx6 (bg=23.92%)▶dgcr8 (bg=19.31%)▶EIF4G2 (bg=0.7%)▶fam120a (bg=18.43%)▶FASTKD2 (bg=7.73%)▶fubp3 (bg=23.31%)▶GRSF1 (bg=3.8%)▶HNRNPL (bg=3.4%)▶igf2bp1 (bg=11.67%)▶KHSRP (bg=8.1%)▶NCBP2 (bg=2.92%)▶NKRF (bg=3.64%)▶NOLC1 (bg=6.58%)▶pum1 (bg=29.85%)▶pum2 (bg=21.55%)▶sf3a3 (bg=12.13%)▶SF3B1 (bg=6.74%)▶TBRG4 (bg=7.0%)▶tia1 (bg=16.04%)▶tial1 (bg=14.09%)▶WRN (bg=3.31%)▶ZC3H11A (bg=6.25%)MATCHES To TargetScan▶ miR-203a-3p.1:GAAAUGU▶ miR-194-5p:GUAACAG▶ miR-203a-3p.2:UGAAAUG▶ miR-144-3p:ACAGUAU▶ miR-101-3p.1:ACAGUAC▶ miR-411-3p:AUGUAAC▶ miR-582-5p:UACAGUU▶ miR-101-3p.2:UACAGUA


AC

ACTGTATATATTGT  
Depth:3 (DOG)  
Ei-value:0.000, Pi-value:0.000  
Er-value:0.000, Pr-value:0.000  
eCLIP MATCHES▶AKAP1 (bg=3.4%)▶ddx55 (bg=12.35%)▶dgcr8 (bg=19.31%)▶EIF4G2 (bg=0.7%)▶fam120a (bg=18.43%)▶FASTKD2 (bg=7.73%)▶fubp3 (bg=23.31%)▶HNRNPL (bg=3.4%)▶igf2bp1 (bg=11.67%)▶KHSRP (bg=8.1%)▶NCBP2 (bg=2.92%)▶NKRF (bg=3.64%)▶NOLC1 (bg=6.58%)▶pum1 (bg=29.85%)▶pum2 (bg=21.55%)▶sf3a3 (bg=12.13%)▶SF3B1 (bg=6.74%)▶TBRG4 (bg=7.0%)▶tia1 (bg=16.04%)▶tial1 (bg=14.09%)▶WRN (bg=3.31%)▶ZC3H11A (bg=6.25%)MATCHES To TargetScan▶ miR-582-5p:UACAGUU▶ miR-101-3p.2:UACAGUA


TGTATATA

TGTATATA  
Depth:9 (MOUSE)  
Ei-value:0.000, Pi-value:0.000  
Er-value:0.000, Pr-value:0.000  
eCLIP MATCHES▶dgcr8 (bg=19.31%)▶EIF4G2 (bg=0.7%)▶fam120a (bg=18.43%)▶FASTKD2 (bg=7.73%)▶fubp3 (bg=23.31%)▶HNRNPL (bg=3.4%)▶igf2bp1 (bg=11.67%)▶KHSRP (bg=8.1%)▶NCBP2 (bg=2.92%)▶NOLC1 (bg=6.58%)▶pum1 (bg=29.85%)▶pum2 (bg=21.55%)▶sf3a3 (bg=12.13%)▶SF3B1 (bg=6.74%)▶TBRG4 (bg=7.0%)▶tia1 (bg=16.04%)▶tial1 (bg=14.09%)▶ZC3H11A (bg=6.25%)No matches to TargetScan


TTG

TGTATATATTG  
Depth:4 (COW)  
Ei-value:0.000, Pi-value:0.000  
Er-value:0.000, Pr-value:0.000  
eCLIP MATCHES▶AKAP1 (bg=3.4%)▶ddx55 (bg=12.35%)▶dgcr8 (bg=19.31%)▶EIF4G2 (bg=0.7%)▶fam120a (bg=18.43%)▶FASTKD2 (bg=7.73%)▶fubp3 (bg=23.31%)▶HNRNPL (bg=3.4%)▶igf2bp1 (bg=11.67%)▶KHSRP (bg=8.1%)▶NCBP2 (bg=2.92%)▶NOLC1 (bg=6.58%)▶pum1 (bg=29.85%)▶pum2 (bg=21.55%)▶sf3a3 (bg=12.13%)▶SF3B1 (bg=6.74%)▶TBRG4 (bg=7.0%)▶tia1 (bg=16.04%)▶tial1 (bg=14.09%)▶ZC3H11A (bg=6.25%)No matches to TargetScan


T

ACTGTATATATTGT  
Depth:3 (DOG)  
Ei-value:0.000, Pi-value:0.000  
Er-value:0.000, Pr-value:0.000  
eCLIP MATCHES▶AKAP1 (bg=3.4%)▶ddx55 (bg=12.35%)▶dgcr8 (bg=19.31%)▶EIF4G2 (bg=0.7%)▶fam120a (bg=18.43%)▶FASTKD2 (bg=7.73%)▶fubp3 (bg=23.31%)▶HNRNPL (bg=3.4%)▶igf2bp1 (bg=11.67%)▶KHSRP (bg=8.1%)▶NCBP2 (bg=2.92%)▶NKRF (bg=3.64%)▶NOLC1 (bg=6.58%)▶pum1 (bg=29.85%)▶pum2 (bg=21.55%)▶sf3a3 (bg=12.13%)▶SF3B1 (bg=6.74%)▶TBRG4 (bg=7.0%)▶tia1 (bg=16.04%)▶tial1 (bg=14.09%)▶WRN (bg=3.31%)▶ZC3H11A (bg=6.25%)MATCHES To TargetScan▶ miR-582-5p:UACAGUU▶ miR-101-3p.2:UACAGUA

A

TATATA

TATATA  
Depth:6 (PIG)  
Ei-value:0.000, Pi-value:0.030  
Er-value:0.000, Pr-value:0.000  
eCLIP MATCHES▶AKAP1 (bg=3.4%)▶ddx55 (bg=12.35%)▶dgcr8 (bg=19.31%)▶EIF4G2 (bg=0.7%)▶fam120a (bg=18.43%)▶FASTKD2 (bg=7.73%)▶fubp3 (bg=23.31%)▶HNRNPL (bg=3.4%)▶igf2bp1 (bg=11.67%)▶KHSRP (bg=8.1%)▶NCBP2 (bg=2.92%)▶NKRF (bg=3.64%)▶NOLC1 (bg=6.58%)▶PHF6 (bg=1.71%)▶pum1 (bg=29.85%)▶pum2 (bg=21.55%)▶sf3a3 (bg=12.13%)▶SF3B1 (bg=6.74%)▶tia1 (bg=16.04%)▶tial1 (bg=14.09%)▶TROVE2 (bg=3.49%)▶WRN (bg=3.31%)▶ZC3H11A (bg=6.25%)No matches to TargetScan

ACGGACAAATTAGTCCC

gatttt

gatttt  
Depth:2 (BABOON)  
Ei-value:1.000, Pi-value:0.030  
Er-value:0.000, Pr-value:0.030  
eCLIP MATCHES▶AKAP1 (bg=3.4%)▶ddx55 (bg=12.35%)▶dgcr8 (bg=19.31%)▶fam120a (bg=18.43%)▶fubp3 (bg=23.31%)▶GRSF1 (bg=3.8%)▶igf2bp1 (bg=11.67%)▶KHSRP (bg=8.1%)▶NKRF (bg=3.64%)▶PHF6 (bg=1.71%)▶pum1 (bg=29.85%)▶pum2 (bg=21.55%)▶sf3a3 (bg=12.13%)▶SF3B1 (bg=6.74%)▶tia1 (bg=16.04%)▶tial1 (bg=14.09%)▶WRN (bg=3.31%)▶ZC3H11A (bg=6.25%)No matches to TargetScan

A

T

TAATATCTAGTCTCTAGATATT  
Depth:6 (PIG)  
Ei-value:0.000, Pi-value:0.000  
Er-value:0.000, Pr-value:0.000  
eCLIP MATCHES▶AKAP1 (bg=3.4%)▶ddx55 (bg=12.35%)▶dgcr8 (bg=19.31%)▶fam120a (bg=18.43%)▶FASTKD2 (bg=7.73%)▶fubp3 (bg=23.31%)▶GRSF1 (bg=3.8%)▶igf2bp1 (bg=11.67%)▶KHSRP (bg=8.1%)▶NKRF (bg=3.64%)▶PHF6 (bg=1.71%)▶pum1 (bg=29.85%)▶pum2 (bg=21.55%)▶sf3a3 (bg=12.13%)▶SF3B1 (bg=6.74%)▶tia1 (bg=16.04%)▶tial1 (bg=14.09%)▶WRN (bg=3.31%)▶ZC3H11A (bg=6.25%)MATCHES To TargetScan▶ miR-28-3p:ACUAGAU


A

AATATCTAG  
Depth:8 (GUINEAPIG)  
Ei-value:0.000, Pi-value:0.000  
Er-value:0.000, Pr-value:0.000  
eCLIP MATCHES▶AKAP1 (bg=3.4%)▶ddx55 (bg=12.35%)▶dgcr8 (bg=19.31%)▶fam120a (bg=18.43%)▶fubp3 (bg=23.31%)▶GRSF1 (bg=3.8%)▶igf2bp1 (bg=11.67%)▶KHSRP (bg=8.1%)▶NKRF (bg=3.64%)▶PHF6 (bg=1.71%)▶pum1 (bg=29.85%)▶pum2 (bg=21.55%)▶sf3a3 (bg=12.13%)▶SF3B1 (bg=6.74%)▶tia1 (bg=16.04%)▶WRN (bg=3.31%)▶ZC3H11A (bg=6.25%)No matches to TargetScan


ATATCTAG

ATATCTAG  
Depth:9 (MOUSE)  
Ei-value:0.000, Pi-value:0.000  
Er-value:0.000, Pr-value:0.000  
eCLIP MATCHES▶AKAP1 (bg=3.4%)▶ddx55 (bg=12.35%)▶dgcr8 (bg=19.31%)▶fam120a (bg=18.43%)▶fubp3 (bg=23.31%)▶GRSF1 (bg=3.8%)▶igf2bp1 (bg=11.67%)▶KHSRP (bg=8.1%)▶NKRF (bg=3.64%)▶PHF6 (bg=1.71%)▶pum1 (bg=29.85%)▶pum2 (bg=21.55%)▶sf3a3 (bg=12.13%)▶SF3B1 (bg=6.74%)▶tia1 (bg=16.04%)▶WRN (bg=3.31%)▶ZC3H11A (bg=6.25%)No matches to TargetScan


T

AATATCTAGTCTCTAGATATT  
Depth:7 (ARMADILLO)  
Ei-value:0.000, Pi-value:0.000  
Er-value:0.000, Pr-value:0.000  
eCLIP MATCHES▶AKAP1 (bg=3.4%)▶ddx55 (bg=12.35%)▶dgcr8 (bg=19.31%)▶fam120a (bg=18.43%)▶FASTKD2 (bg=7.73%)▶fubp3 (bg=23.31%)▶GRSF1 (bg=3.8%)▶igf2bp1 (bg=11.67%)▶KHSRP (bg=8.1%)▶NKRF (bg=3.64%)▶PHF6 (bg=1.71%)▶pum1 (bg=29.85%)▶pum2 (bg=21.55%)▶sf3a3 (bg=12.13%)▶SF3B1 (bg=6.74%)▶tia1 (bg=16.04%)▶tial1 (bg=14.09%)▶WRN (bg=3.31%)▶ZC3H11A (bg=6.25%)MATCHES To TargetScan▶ miR-28-3p:ACUAGAU


CTCTAG

CTCTAG  
Depth:9 (MOUSE)  
Ei-value:0.000, Pi-value:0.000  
Er-value:0.000, Pr-value:0.000  
eCLIP MATCHES▶ddx55 (bg=12.35%)▶dgcr8 (bg=19.31%)▶FASTKD2 (bg=7.73%)▶fubp3 (bg=23.31%)▶GRSF1 (bg=3.8%)▶KHSRP (bg=8.1%)▶pum1 (bg=29.85%)▶pum2 (bg=21.55%)▶tia1 (bg=16.04%)▶ZC3H11A (bg=6.25%)No matches to TargetScan


ATATT

CTCTAGATATT  
Depth:8 (GUINEAPIG)  
Ei-value:0.000, Pi-value:0.000  
Er-value:0.000, Pr-value:0.000  
eCLIP MATCHES▶ddx55 (bg=12.35%)▶dgcr8 (bg=19.31%)▶FASTKD2 (bg=7.73%)▶fubp3 (bg=23.31%)▶GRSF1 (bg=3.8%)▶KHSRP (bg=8.1%)▶pum1 (bg=29.85%)▶pum2 (bg=21.55%)▶tia1 (bg=16.04%)▶tial1 (bg=14.09%)▶ZC3H11A (bg=6.25%)No matches to TargetScan


AAAGAGG

AAAGAGGTTGCCAATGTATGACA  
Depth:9 (MOUSE)  
Ei-value:0.000, Pi-value:0.000  
Er-value:0.000, Pr-value:0.000  
eCLIP MATCHES▶ddx55 (bg=12.35%)▶dgcr8 (bg=19.31%)▶FASTKD2 (bg=7.73%)▶fubp3 (bg=23.31%)▶GRSF1 (bg=3.8%)▶NOLC1 (bg=6.58%)▶pum1 (bg=29.85%)▶pum2 (bg=21.55%)▶tial1 (bg=14.09%)MATCHES To TargetScan▶ miR-182-5p:UUGGCAA▶ miR-96-5p/1271-5p:UUGGCAC▶ miR-539-3p:UCAUACA

 2760  


TTGCCAATGTATGACA

AAAGAGGTTGCCAATGTATGACA  
Depth:9 (MOUSE)  
Ei-value:0.000, Pi-value:0.000  
Er-value:0.000, Pr-value:0.000  
eCLIP MATCHES▶ddx55 (bg=12.35%)▶dgcr8 (bg=19.31%)▶FASTKD2 (bg=7.73%)▶fubp3 (bg=23.31%)▶GRSF1 (bg=3.8%)▶NOLC1 (bg=6.58%)▶pum1 (bg=29.85%)▶pum2 (bg=21.55%)▶tial1 (bg=14.09%)MATCHES To TargetScan▶ miR-182-5p:UUGGCAA▶ miR-96-5p/1271-5p:UUGGCAC▶ miR-539-3p:UCAUACA


G

TAATATCTAGTCTCTAGATATTAAAGAGGTTGCCAATGTATGACAGAAGTAGAGTTAGTAAACTAACACATTTTGTACACTTTGTTAAAATTTGTAGAAAGGCTGTCTTCTGAAAAGGACTTTTGGAAGTGA  
Depth:2 (BABOON)  
Ei-value:0.000, Pi-value:0.000  
Er-value:0.000, Pr-value:0.000  
eCLIP MATCHES▶AKAP1 (bg=3.4%)▶ddx55 (bg=12.35%)▶dgcr8 (bg=19.31%)▶fam120a (bg=18.43%)▶FASTKD2 (bg=7.73%)▶fubp3 (bg=23.31%)▶GRSF1 (bg=3.8%)▶igf2bp1 (bg=11.67%)▶KHSRP (bg=8.1%)▶NKRF (bg=3.64%)▶NOLC1 (bg=6.58%)▶PHF6 (bg=1.71%)▶pum1 (bg=29.85%)▶pum2 (bg=21.55%)▶sf3a3 (bg=12.13%)▶SF3B1 (bg=6.74%)▶tia1 (bg=16.04%)▶tial1 (bg=14.09%)▶WRN (bg=3.31%)▶ybx3 (bg=22.82%)▶ZC3H11A (bg=6.25%)MATCHES To TargetScan▶ miR-493-5p:UGUACAU▶ miR-28-3p:ACUAGAU▶ miR-182-5p:UUGGCAA▶ miR-17-5p/20-5p/93-5p/106-5p/519-3p:AAAGUGC▶ miR-96-5p/1271-5p:UUGGCAC▶ miR-495-3p:AACAAAC▶ miR-539-3p:UCAUACA


AAGTAG

AAGTAG  
Depth:8 (GUINEAPIG)  
Ei-value:0.000, Pi-value:0.000  
Er-value:0.000, Pr-value:0.000  
eCLIP MATCHES▶ddx55 (bg=12.35%)▶dgcr8 (bg=19.31%)▶FASTKD2 (bg=7.73%)▶NOLC1 (bg=6.58%)▶pum1 (bg=29.85%)▶pum2 (bg=21.55%)▶tial1 (bg=14.09%)No matches to TargetScan


A

AAGTAGAGTTAGTAAACTAACACATTTTGTACACTTTGT  
Depth:3 (DOG)  
Ei-value:0.000, Pi-value:0.000  
Er-value:0.000, Pr-value:0.000  
eCLIP MATCHES▶ddx55 (bg=12.35%)▶dgcr8 (bg=19.31%)▶FASTKD2 (bg=7.73%)▶NOLC1 (bg=6.58%)▶pum1 (bg=29.85%)▶pum2 (bg=21.55%)▶tial1 (bg=14.09%)MATCHES To TargetScan▶ miR-493-5p:UGUACAU▶ miR-17-5p/20-5p/93-5p/106-5p/519-3p:AAAGUGC


GTTAGTAAACT

GTTAGTAAACT  
Depth:9 (MOUSE)  
Ei-value:0.000, Pi-value:0.000  
Er-value:0.000, Pr-value:0.000  
eCLIP MATCHES▶ddx55 (bg=12.35%)▶dgcr8 (bg=19.31%)▶FASTKD2 (bg=7.73%)▶NOLC1 (bg=6.58%)▶pum1 (bg=29.85%)▶pum2 (bg=21.55%)No matches to TargetScan


A

GTTAGTAAACTAACACATTTTGTACACTT  
Depth:8 (GUINEAPIG)  
Ei-value:0.000, Pi-value:0.000  
Er-value:0.000, Pr-value:0.000  
eCLIP MATCHES▶ddx55 (bg=12.35%)▶dgcr8 (bg=19.31%)▶FASTKD2 (bg=7.73%)▶NOLC1 (bg=6.58%)▶pum1 (bg=29.85%)▶pum2 (bg=21.55%)MATCHES To TargetScan▶ miR-493-5p:UGUACAU


ACACATT

ACACATT  
Depth:9 (MOUSE)  
Ei-value:0.000, Pi-value:0.000  
Er-value:0.000, Pr-value:0.000  
eCLIP MATCHES▶NOLC1 (bg=6.58%)No matches to TargetScan


TTGTACACTT

GTTAGTAAACTAACACATTTTGTACACTT  
Depth:8 (GUINEAPIG)  
Ei-value:0.000, Pi-value:0.000  
Er-value:0.000, Pr-value:0.000  
eCLIP MATCHES▶ddx55 (bg=12.35%)▶dgcr8 (bg=19.31%)▶FASTKD2 (bg=7.73%)▶NOLC1 (bg=6.58%)▶pum1 (bg=29.85%)▶pum2 (bg=21.55%)MATCHES To TargetScan▶ miR-493-5p:UGUACAU


TGT

GTTAGTAAACTAACACATTTTGTACACTTTGT  
Depth:5 (SHEEP)  
Ei-value:0.000, Pi-value:0.000  
Er-value:0.000, Pr-value:0.000  
eCLIP MATCHES▶ddx55 (bg=12.35%)▶dgcr8 (bg=19.31%)▶FASTKD2 (bg=7.73%)▶NOLC1 (bg=6.58%)▶pum1 (bg=29.85%)▶pum2 (bg=21.55%)MATCHES To TargetScan▶ miR-493-5p:UGUACAU▶ miR-17-5p/20-5p/93-5p/106-5p/519-3p:AAAGUGC


TAAAATT

TAAAATT  
Depth:3 (DOG)  
Ei-value:0.000, Pi-value:0.050  
Er-value:0.000, Pr-value:0.000  
eCLIP MATCHES▶dgcr8 (bg=19.31%)No matches to TargetScan


TGTA

TAATATCTAGTCTCTAGATATTAAAGAGGTTGCCAATGTATGACAGAAGTAGAGTTAGTAAACTAACACATTTTGTACACTTTGTTAAAATTTGTAGAAAGGCTGTCTTCTGAAAAGGACTTTTGGAAGTGA  
Depth:2 (BABOON)  
Ei-value:0.000, Pi-value:0.000  
Er-value:0.000, Pr-value:0.000  
eCLIP MATCHES▶AKAP1 (bg=3.4%)▶ddx55 (bg=12.35%)▶dgcr8 (bg=19.31%)▶fam120a (bg=18.43%)▶FASTKD2 (bg=7.73%)▶fubp3 (bg=23.31%)▶GRSF1 (bg=3.8%)▶igf2bp1 (bg=11.67%)▶KHSRP (bg=8.1%)▶NKRF (bg=3.64%)▶NOLC1 (bg=6.58%)▶PHF6 (bg=1.71%)▶pum1 (bg=29.85%)▶pum2 (bg=21.55%)▶sf3a3 (bg=12.13%)▶SF3B1 (bg=6.74%)▶tia1 (bg=16.04%)▶tial1 (bg=14.09%)▶WRN (bg=3.31%)▶ybx3 (bg=22.82%)▶ZC3H11A (bg=6.25%)MATCHES To TargetScan▶ miR-493-5p:UGUACAU▶ miR-28-3p:ACUAGAU▶ miR-182-5p:UUGGCAA▶ miR-17-5p/20-5p/93-5p/106-5p/519-3p:AAAGUGC▶ miR-96-5p/1271-5p:UUGGCAC▶ miR-495-3p:AACAAAC▶ miR-539-3p:UCAUACA


GAAAG

GAAAGGCTGTCTTCTGAAAAGGAC  
Depth:6 (PIG)  
Ei-value:0.000, Pi-value:0.000  
Er-value:0.000, Pr-value:0.000  
eCLIP MATCHES▶dgcr8 (bg=19.31%)▶pum2 (bg=21.55%)▶ybx3 (bg=22.82%)No matches to TargetScan


GC

GCTGTCTTCTGAAAA  
Depth:7 (ARMADILLO)  
Ei-value:0.000, Pi-value:0.000  
Er-value:0.000, Pr-value:0.000  
eCLIP MATCHES▶dgcr8 (bg=19.31%)▶pum2 (bg=21.55%)▶ybx3 (bg=22.82%)No matches to TargetScan


TGTCTTCTGAA

TGTCTTCTGAA  
Depth:9 (MOUSE)  
Ei-value:0.000, Pi-value:0.000  
Er-value:0.000, Pr-value:0.000  
eCLIP MATCHES▶dgcr8 (bg=19.31%)▶pum2 (bg=21.55%)▶ybx3 (bg=22.82%)No matches to TargetScan


AA

TGTCTTCTGAAAA  
Depth:8 (GUINEAPIG)  
Ei-value:0.000, Pi-value:0.000  
Er-value:0.000, Pr-value:0.000  
eCLIP MATCHES▶dgcr8 (bg=19.31%)▶pum2 (bg=21.55%)▶ybx3 (bg=22.82%)No matches to TargetScan


GGAC

GAAAGGCTGTCTTCTGAAAAGGAC  
Depth:6 (PIG)  
Ei-value:0.000, Pi-value:0.000  
Er-value:0.000, Pr-value:0.000  
eCLIP MATCHES▶dgcr8 (bg=19.31%)▶pum2 (bg=21.55%)▶ybx3 (bg=22.82%)No matches to TargetScan


TTT

GAAAGGCTGTCTTCTGAAAAGGACTTT  
Depth:3 (DOG)  
Ei-value:0.000, Pi-value:0.000  
Er-value:0.000, Pr-value:0.000  
eCLIP MATCHES▶dgcr8 (bg=19.31%)▶pum2 (bg=21.55%)▶ybx3 (bg=22.82%)No matches to TargetScan


TGGAAGTGA

TAATATCTAGTCTCTAGATATTAAAGAGGTTGCCAATGTATGACAGAAGTAGAGTTAGTAAACTAACACATTTTGTACACTTTGTTAAAATTTGTAGAAAGGCTGTCTTCTGAAAAGGACTTTTGGAAGTGA  
Depth:2 (BABOON)  
Ei-value:0.000, Pi-value:0.000  
Er-value:0.000, Pr-value:0.000  
eCLIP MATCHES▶AKAP1 (bg=3.4%)▶ddx55 (bg=12.35%)▶dgcr8 (bg=19.31%)▶fam120a (bg=18.43%)▶FASTKD2 (bg=7.73%)▶fubp3 (bg=23.31%)▶GRSF1 (bg=3.8%)▶igf2bp1 (bg=11.67%)▶KHSRP (bg=8.1%)▶NKRF (bg=3.64%)▶NOLC1 (bg=6.58%)▶PHF6 (bg=1.71%)▶pum1 (bg=29.85%)▶pum2 (bg=21.55%)▶sf3a3 (bg=12.13%)▶SF3B1 (bg=6.74%)▶tia1 (bg=16.04%)▶tial1 (bg=14.09%)▶WRN (bg=3.31%)▶ybx3 (bg=22.82%)▶ZC3H11A (bg=6.25%)MATCHES To TargetScan▶ miR-493-5p:UGUACAU▶ miR-28-3p:ACUAGAU▶ miR-182-5p:UUGGCAA▶ miR-17-5p/20-5p/93-5p/106-5p/519-3p:AAAGUGC▶ miR-96-5p/1271-5p:UUGGCAC▶ miR-495-3p:AACAAAC▶ miR-539-3p:UCAUACA


GATAACATCAGCTCTA

GATAACATCAGCTCTAAGTGACACGTGCCTATAT  
Depth:2 (BABOON)  
Ei-value:0.000, Pi-value:0.000  
Er-value:0.000, Pr-value:0.000  
eCLIP MATCHES▶lin28b (bg=16.96%)▶ybx3 (bg=22.82%)MATCHES To TargetScan▶ miR-187-3p:CGUGUCU▶ miR-668-3p:GUCACUC


A

AGTGACA  
Depth:3 (DOG)  
Ei-value:0.000, Pi-value:0.000  
Er-value:0.000, Pr-value:0.000  
eCLIP MATCHES▶lin28b (bg=16.96%)▶ybx3 (bg=22.82%)MATCHES To TargetScan▶ miR-668-3p:GUCACUC

 2880  


GTGACA

AGTGACA  
Depth:3 (DOG)  
Ei-value:0.000, Pi-value:0.000  
Er-value:0.000, Pr-value:0.000  
eCLIP MATCHES▶lin28b (bg=16.96%)▶ybx3 (bg=22.82%)MATCHES To TargetScan▶ miR-668-3p:GUCACUC


C

GATAACATCAGCTCTAAGTGACACGTGCCTATAT  
Depth:2 (BABOON)  
Ei-value:0.000, Pi-value:0.000  
Er-value:0.000, Pr-value:0.000  
eCLIP MATCHES▶lin28b (bg=16.96%)▶ybx3 (bg=22.82%)MATCHES To TargetScan▶ miR-187-3p:CGUGUCU▶ miR-668-3p:GUCACUC


GTGCCT

GTGCCT  
Depth:9 (MOUSE)  
Ei-value:0.000, Pi-value:0.000  
Er-value:0.000, Pr-value:0.000  
eCLIP MATCHES▶lin28b (bg=16.96%)▶ybx3 (bg=22.82%)No matches to TargetScan


ATAT

GATAACATCAGCTCTAAGTGACACGTGCCTATAT  
Depth:2 (BABOON)  
Ei-value:0.000, Pi-value:0.000  
Er-value:0.000, Pr-value:0.000  
eCLIP MATCHES▶lin28b (bg=16.96%)▶ybx3 (bg=22.82%)MATCHES To TargetScan▶ miR-187-3p:CGUGUCU▶ miR-668-3p:GUCACUC

CCAT

CA

CAGGTTGGTGGTGGAGAGGAGTTGGAAGGAATGAAGGGTTCTAGACCAGAATGTTC  
Depth:2 (BABOON)  
Ei-value:0.000, Pi-value:0.000  
Er-value:0.000, Pr-value:0.000  
eCLIP MATCHES▶AARS (bg=2.33%)▶EIF3H (bg=7.83%)▶FUS (bg=2.59%)▶lin28b (bg=16.96%)▶NOLC1 (bg=6.58%)▶UTP3 (bg=2.81%)▶ybx3 (bg=22.82%)MATCHES To TargetScan▶ miR-181-5p:ACAUUCA▶ miR-1298-5p:UCAUUCG▶ miR-543:AACAUUC▶ miR-483-3p.1:ACUCCUC▶ miR-205-5p:CCUUCAU▶ miR-490-3p:AACCUGG


ggttgg

ggttgg  
Depth:3 (DOG)  
Ei-value:0.080, Pi-value:0.020  
Er-value:0.000, Pr-value:0.020  
eCLIP MATCHES▶EIF3H (bg=7.83%)▶lin28b (bg=16.96%)▶ybx3 (bg=22.82%)No matches to TargetScan


TG

CAGGTTGGTGGTGGAGAGGAGTTGGAAGGAATGAAGGGTTCTAGACCAGAATGTTC  
Depth:2 (BABOON)  
Ei-value:0.000, Pi-value:0.000  
Er-value:0.000, Pr-value:0.000  
eCLIP MATCHES▶AARS (bg=2.33%)▶EIF3H (bg=7.83%)▶FUS (bg=2.59%)▶lin28b (bg=16.96%)▶NOLC1 (bg=6.58%)▶UTP3 (bg=2.81%)▶ybx3 (bg=22.82%)MATCHES To TargetScan▶ miR-181-5p:ACAUUCA▶ miR-1298-5p:UCAUUCG▶ miR-543:AACAUUC▶ miR-483-3p.1:ACUCCUC▶ miR-205-5p:CCUUCAU▶ miR-490-3p:AACCUGG


GTGGAGAG

GTGGAGAG  
Depth:3 (DOG)  
Ei-value:0.000, Pi-value:0.000  
Er-value:0.000, Pr-value:0.000  
eCLIP MATCHES▶EIF3H (bg=7.83%)▶lin28b (bg=16.96%)▶UTP3 (bg=2.81%)▶ybx3 (bg=22.82%)No matches to TargetScan


GAGTTGGAAGG

CAGGTTGGTGGTGGAGAGGAGTTGGAAGGAATGAAGGGTTCTAGACCAGAATGTTC  
Depth:2 (BABOON)  
Ei-value:0.000, Pi-value:0.000  
Er-value:0.000, Pr-value:0.000  
eCLIP MATCHES▶AARS (bg=2.33%)▶EIF3H (bg=7.83%)▶FUS (bg=2.59%)▶lin28b (bg=16.96%)▶NOLC1 (bg=6.58%)▶UTP3 (bg=2.81%)▶ybx3 (bg=22.82%)MATCHES To TargetScan▶ miR-181-5p:ACAUUCA▶ miR-1298-5p:UCAUUCG▶ miR-543:AACAUUC▶ miR-483-3p.1:ACUCCUC▶ miR-205-5p:CCUUCAU▶ miR-490-3p:AACCUGG


AATGAA

AATGAA  
Depth:8 (GUINEAPIG)  
Ei-value:0.000, Pi-value:0.020  
Er-value:0.000, Pr-value:0.000  
eCLIP MATCHES▶EIF3H (bg=7.83%)▶lin28b (bg=16.96%)▶UTP3 (bg=2.81%)▶ybx3 (bg=22.82%)No matches to TargetScan


GG

AATGAAGGGTTCTAGAC  
Depth:3 (DOG)  
Ei-value:0.000, Pi-value:0.000  
Er-value:0.000, Pr-value:0.000  
eCLIP MATCHES▶EIF3H (bg=7.83%)▶FUS (bg=2.59%)▶lin28b (bg=16.96%)▶UTP3 (bg=2.81%)▶ybx3 (bg=22.82%)MATCHES To TargetScan▶ miR-205-5p:CCUUCAU


GTTCTAGAC

GTTCTAGAC  
Depth:5 (SHEEP)  
Ei-value:0.000, Pi-value:0.000  
Er-value:0.000, Pr-value:0.000  
eCLIP MATCHES▶FUS (bg=2.59%)▶lin28b (bg=16.96%)▶UTP3 (bg=2.81%)▶ybx3 (bg=22.82%)No matches to TargetScan


C

CAGGTTGGTGGTGGAGAGGAGTTGGAAGGAATGAAGGGTTCTAGACCAGAATGTTC  
Depth:2 (BABOON)  
Ei-value:0.000, Pi-value:0.000  
Er-value:0.000, Pr-value:0.000  
eCLIP MATCHES▶AARS (bg=2.33%)▶EIF3H (bg=7.83%)▶FUS (bg=2.59%)▶lin28b (bg=16.96%)▶NOLC1 (bg=6.58%)▶UTP3 (bg=2.81%)▶ybx3 (bg=22.82%)MATCHES To TargetScan▶ miR-181-5p:ACAUUCA▶ miR-1298-5p:UCAUUCG▶ miR-543:AACAUUC▶ miR-483-3p.1:ACUCCUC▶ miR-205-5p:CCUUCAU▶ miR-490-3p:AACCUGG


AGAATG

AGAATG  
Depth:9 (MOUSE)  
Ei-value:0.000, Pi-value:0.000  
Er-value:0.000, Pr-value:0.000  
eCLIP MATCHES▶AARS (bg=2.33%)▶FUS (bg=2.59%)▶lin28b (bg=16.96%)▶UTP3 (bg=2.81%)▶ybx3 (bg=22.82%)No matches to TargetScan


TT

AGAATGTT  
Depth:7 (ARMADILLO)  
Ei-value:0.000, Pi-value:0.000  
Er-value:0.000, Pr-value:0.000  
eCLIP MATCHES▶AARS (bg=2.33%)▶FUS (bg=2.59%)▶lin28b (bg=16.96%)▶UTP3 (bg=2.81%)▶ybx3 (bg=22.82%)MATCHES To TargetScan▶ miR-181-5p:ACAUUCA▶ miR-543:AACAUUC


C

AGAATGTTC  
Depth:5 (SHEEP)  
Ei-value:0.000, Pi-value:0.000  
Er-value:0.000, Pr-value:0.000  
eCLIP MATCHES▶AARS (bg=2.33%)▶FUS (bg=2.59%)▶lin28b (bg=16.96%)▶UTP3 (bg=2.81%)▶ybx3 (bg=22.82%)MATCHES To TargetScan▶ miR-181-5p:ACAUUCA▶ miR-543:AACAUUC

G

tattt

tatttagaagacact  
Depth:2 (BABOON)  
Ei-value:1.000, Pi-value:0.000  
Er-value:0.000, Pr-value:0.000  
eCLIP MATCHES▶AARS (bg=2.33%)▶FUS (bg=2.59%)▶lin28b (bg=16.96%)No matches to TargetScan


AGAAGA

AGAAGA  
Depth:9 (MOUSE)  
Ei-value:0.000, Pi-value:0.000  
Er-value:0.000, Pr-value:0.000  
eCLIP MATCHES▶AARS (bg=2.33%)▶FUS (bg=2.59%)▶lin28b (bg=16.96%)No matches to TargetScan


CACT

AGAAGACACT  
Depth:3 (DOG)  
Ei-value:0.000, Pi-value:0.000  
Er-value:0.000, Pr-value:0.000  
eCLIP MATCHES▶AARS (bg=2.33%)▶FUS (bg=2.59%)▶lin28b (bg=16.96%)No matches to TargetScan

ATC

AGATATAA

AGATATAA  
Depth:3 (DOG)  
Ei-value:0.000, Pi-value:0.000  
Er-value:0.000, Pr-value:0.000  
eCLIP MATCHES▶AARS (bg=2.33%)▶FUS (bg=2.59%)▶HNRNPL (bg=3.4%)▶pum2 (bg=21.55%)No matches to TargetScan


CCATTGTTAC

CCATTGTTAC  
Depth:5 (SHEEP)  
Ei-value:0.000, Pi-value:0.000  
Er-value:0.000, Pr-value:0.000  
eCLIP MATCHES▶AARS (bg=2.33%)▶DDX21 (bg=1.64%)▶ddx6 (bg=23.92%)▶dgcr8 (bg=19.31%)▶FUS (bg=2.59%)▶HNRNPL (bg=3.4%)▶igf2bp1 (bg=11.67%)▶pum2 (bg=21.55%)MATCHES To TargetScan▶ miR-194-5p:GUAACAG

A

TGTGT

TGTGTGT  
Depth:6 (PIG)  
Ei-value:0.000, Pi-value:0.010  
Er-value:0.000, Pr-value:0.000  
eCLIP MATCHES▶AARS (bg=2.33%)▶DDX21 (bg=1.64%)▶ddx6 (bg=23.92%)▶dgcr8 (bg=19.31%)▶fam120a (bg=18.43%)▶HNRNPL (bg=3.4%)▶igf2bp1 (bg=11.67%)▶pum2 (bg=21.55%)▶sf3a3 (bg=12.13%)▶ZC3H11A (bg=6.25%)MATCHES To TargetScan▶ miR-329-3p/362-3p:ACACACC

 3000  


GT

TGTGTGT  
Depth:6 (PIG)  
Ei-value:0.000, Pi-value:0.010  
Er-value:0.000, Pr-value:0.000  
eCLIP MATCHES▶AARS (bg=2.33%)▶DDX21 (bg=1.64%)▶ddx6 (bg=23.92%)▶dgcr8 (bg=19.31%)▶fam120a (bg=18.43%)▶HNRNPL (bg=3.4%)▶igf2bp1 (bg=11.67%)▶pum2 (bg=21.55%)▶sf3a3 (bg=12.13%)▶ZC3H11A (bg=6.25%)MATCHES To TargetScan▶ miR-329-3p/362-3p:ACACACC


A

TGTGTGTAGTTTATTCAAC  
Depth:5 (SHEEP)  
Ei-value:0.000, Pi-value:0.000  
Er-value:0.000, Pr-value:0.000  
eCLIP MATCHES▶AARS (bg=2.33%)▶DDX21 (bg=1.64%)▶ddx6 (bg=23.92%)▶dgcr8 (bg=19.31%)▶fam120a (bg=18.43%)▶HNRNPL (bg=3.4%)▶igf2bp1 (bg=11.67%)▶pum2 (bg=21.55%)▶sf3a3 (bg=12.13%)▶ZC3H11A (bg=6.25%)MATCHES To TargetScan▶ miR-329-3p/362-3p:ACACACC


GTTTATT

GTTTATT  
Depth:8 (GUINEAPIG)  
Ei-value:0.000, Pi-value:0.000  
Er-value:0.000, Pr-value:0.000  
eCLIP MATCHES▶DDX21 (bg=1.64%)▶ddx6 (bg=23.92%)▶dgcr8 (bg=19.31%)▶fam120a (bg=18.43%)▶HNRNPL (bg=3.4%)▶igf2bp1 (bg=11.67%)▶pum2 (bg=21.55%)▶sf3a3 (bg=12.13%)▶ZC3H11A (bg=6.25%)No matches to TargetScan


CAAC

GTTTATTCAAC  
Depth:6 (PIG)  
Ei-value:0.000, Pi-value:0.000  
Er-value:0.000, Pr-value:0.000  
eCLIP MATCHES▶DDX21 (bg=1.64%)▶ddx6 (bg=23.92%)▶dgcr8 (bg=19.31%)▶fam120a (bg=18.43%)▶HNRNPL (bg=3.4%)▶igf2bp1 (bg=11.67%)▶pum2 (bg=21.55%)▶sf3a3 (bg=12.13%)▶ZC3H11A (bg=6.25%)No matches to TargetScan

C

CTACTG

CTACTGTGTATATA  
Depth:8 (GUINEAPIG)  
Ei-value:0.000, Pi-value:0.000  
Er-value:0.000, Pr-value:0.000  
eCLIP MATCHES▶DDX21 (bg=1.64%)▶ddx6 (bg=23.92%)▶dgcr8 (bg=19.31%)▶fam120a (bg=18.43%)▶HNRNPL (bg=3.4%)▶igf2bp1 (bg=11.67%)▶pum2 (bg=21.55%)▶sf3a3 (bg=12.13%)▶ZC3H11A (bg=6.25%)MATCHES To TargetScan▶ miR-199-3p:CAGUAGU▶ miR-144-3p:ACAGUAU▶ miR-128-3p:CACAGUG▶ miR-101-3p.1:ACAGUAC


TGTATATA

TGTATATA  
Depth:9 (MOUSE)  
Ei-value:0.000, Pi-value:0.000  
Er-value:0.000, Pr-value:0.000  
eCLIP MATCHES▶DDX21 (bg=1.64%)▶dgcr8 (bg=19.31%)▶fam120a (bg=18.43%)▶HNRNPL (bg=3.4%)▶igf2bp1 (bg=11.67%)▶pum2 (bg=21.55%)▶ZC3H11A (bg=6.25%)No matches to TargetScan


G

CTACTGTGTATATAG  
Depth:5 (SHEEP)  
Ei-value:0.000, Pi-value:0.000  
Er-value:0.000, Pr-value:0.000  
eCLIP MATCHES▶DDX21 (bg=1.64%)▶ddx6 (bg=23.92%)▶dgcr8 (bg=19.31%)▶fam120a (bg=18.43%)▶HNRNPL (bg=3.4%)▶igf2bp1 (bg=11.67%)▶pum2 (bg=21.55%)▶sf3a3 (bg=12.13%)▶ZC3H11A (bg=6.25%)MATCHES To TargetScan▶ miR-199-3p:CAGUAGU▶ miR-144-3p:ACAGUAU▶ miR-128-3p:CACAGUG▶ miR-101-3p.1:ACAGUAC


c

ctactgtgtatatagc  
Depth:2 (BABOON)  
Ei-value:1.000, Pi-value:0.000  
Er-value:0.000, Pr-value:0.000  
eCLIP MATCHES▶DDX21 (bg=1.64%)▶ddx6 (bg=23.92%)▶dgcr8 (bg=19.31%)▶fam120a (bg=18.43%)▶HNRNPL (bg=3.4%)▶igf2bp1 (bg=11.67%)▶pum2 (bg=21.55%)▶sf3a3 (bg=12.13%)▶ZC3H11A (bg=6.25%)MATCHES To TargetScan▶ miR-199-3p:CAGUAGU▶ miR-144-3p:ACAGUAU▶ miR-128-3p:CACAGUG▶ miR-101-3p.1:ACAGUAC

G

GACAAAC

GACAAAC  
Depth:6 (PIG)  
Ei-value:0.000, Pi-value:0.000  
Er-value:0.000, Pr-value:0.000  
eCLIP MATCHES▶DDX21 (bg=1.64%)▶pum2 (bg=21.55%)No matches to TargetScan


TT

GACAAACTTAAGTCCTTATTTGAAACATCTAG  
Depth:4 (COW)  
Ei-value:0.000, Pi-value:0.000  
Er-value:0.000, Pr-value:0.000  
eCLIP MATCHES▶DDX21 (bg=1.64%)▶pum2 (bg=21.55%)No matches to TargetScan


AAGTCCT

AAGTCCTTATTTGAAACATCTAG  
Depth:7 (ARMADILLO)  
Ei-value:0.000, Pi-value:0.000  
Er-value:0.000, Pr-value:0.000  
No matches to eCLIP DataNo matches to TargetScan


TATT

TATTTGAAACATCTAG  
Depth:8 (GUINEAPIG)  
Ei-value:0.000, Pi-value:0.000  
Er-value:0.000, Pr-value:0.000  
No matches to eCLIP DataNo matches to TargetScan


TGAAACATCTAG

TGAAACATCTAG  
Depth:9 (MOUSE)  
Ei-value:0.000, Pi-value:0.000  
Er-value:0.000, Pr-value:0.000  
No matches to eCLIP DataNo matches to TargetScan


T

GACAAACTTAAGTCCTTATTTGAAACATCTAGTCTTTCTAGATGTTTAGAAGTGCACAAAGTATGTTAAAAGTAGAGGTAGTAAATAACACATTTTGTAGCTATCCTTTTGATATGAAATATTGTCTTGGAAA  
Depth:2 (BABOON)  
Ei-value:0.000, Pi-value:0.000  
Er-value:0.000, Pr-value:0.000  
eCLIP MATCHES▶DDX21 (bg=1.64%)▶ddx55 (bg=12.35%)▶fam120a (bg=18.43%)▶FASTKD2 (bg=7.73%)▶fubp3 (bg=23.31%)▶pum2 (bg=21.55%)▶SUB1 (bg=9.24%)▶tia1 (bg=16.04%)▶tial1 (bg=14.09%)MATCHES To TargetScan▶ miR-28-3p:ACUAGAU▶ miR-221-3p/222-3p:GCUACAU


CT

CTTTCTAGATGTTTAGAAGTGCACAAAGTATGTTAAAAGTAGAGGTAGT  
Depth:8 (GUINEAPIG)  
Ei-value:0.000, Pi-value:0.000  
Er-value:0.000, Pr-value:0.000  
No matches to eCLIP DataNo matches to TargetScan


TTCTAG

TTCTAG  
Depth:9 (MOUSE)  
Ei-value:0.000, Pi-value:0.000  
Er-value:0.000, Pr-value:0.000  
No matches to eCLIP DataNo matches to TargetScan


A

CTTTCTAGATGTTTAGAAGTGCACAAAGTATGTTAAAAGTAGAGGTAGT  
Depth:8 (GUINEAPIG)  
Ei-value:0.000, Pi-value:0.000  
Er-value:0.000, Pr-value:0.000  
No matches to eCLIP DataNo matches to TargetScan


TGTTTAGAAGTGCACAAAGTATGTTAAAAGTAGA

TGTTTAGAAGTGCACAAAGTATGTTAAAAGTAGA  
Depth:9 (MOUSE)  
Ei-value:0.000, Pi-value:0.000  
Er-value:0.000, Pr-value:0.000  
No matches to eCLIP DataNo matches to TargetScan


GGTAGT

CTTTCTAGATGTTTAGAAGTGCACAAAGTATGTTAAAAGTAGAGGTAGT  
Depth:8 (GUINEAPIG)  
Ei-value:0.000, Pi-value:0.000  
Er-value:0.000, Pr-value:0.000  
No matches to eCLIP DataNo matches to TargetScan


AAA

CTTTCTAGATGTTTAGAAGTGCACAAAGTATGTTAAAAGTAGAGGTAGTAAA  
Depth:3 (DOG)  
Ei-value:0.000, Pi-value:0.000  
Er-value:0.000, Pr-value:0.000  
No matches to eCLIP DataNo matches to TargetScan


TAA

TAACACAT  
Depth:3 (DOG)  
Ei-value:0.000, Pi-value:0.000  
Er-value:0.000, Pr-value:0.000  
No matches to eCLIP DataNo matches to TargetScan

 3120  


CACAT

TAACACAT  
Depth:3 (DOG)  
Ei-value:0.000, Pi-value:0.000  
Er-value:0.000, Pr-value:0.000  
No matches to eCLIP DataNo matches to TargetScan


T

GACAAACTTAAGTCCTTATTTGAAACATCTAGTCTTTCTAGATGTTTAGAAGTGCACAAAGTATGTTAAAAGTAGAGGTAGTAAATAACACATTTTGTAGCTATCCTTTTGATATGAAATATTGTCTTGGAAA  
Depth:2 (BABOON)  
Ei-value:0.000, Pi-value:0.000  
Er-value:0.000, Pr-value:0.000  
eCLIP MATCHES▶DDX21 (bg=1.64%)▶ddx55 (bg=12.35%)▶fam120a (bg=18.43%)▶FASTKD2 (bg=7.73%)▶fubp3 (bg=23.31%)▶pum2 (bg=21.55%)▶SUB1 (bg=9.24%)▶tia1 (bg=16.04%)▶tial1 (bg=14.09%)MATCHES To TargetScan▶ miR-28-3p:ACUAGAU▶ miR-221-3p/222-3p:GCUACAU


TTGTAG

TTGTAG  
Depth:6 (PIG)  
Ei-value:0.000, Pi-value:0.010  
Er-value:0.000, Pr-value:0.010  
No matches to eCLIP DataNo matches to TargetScan


CT

GACAAACTTAAGTCCTTATTTGAAACATCTAGTCTTTCTAGATGTTTAGAAGTGCACAAAGTATGTTAAAAGTAGAGGTAGTAAATAACACATTTTGTAGCTATCCTTTTGATATGAAATATTGTCTTGGAAA  
Depth:2 (BABOON)  
Ei-value:0.000, Pi-value:0.000  
Er-value:0.000, Pr-value:0.000  
eCLIP MATCHES▶DDX21 (bg=1.64%)▶ddx55 (bg=12.35%)▶fam120a (bg=18.43%)▶FASTKD2 (bg=7.73%)▶fubp3 (bg=23.31%)▶pum2 (bg=21.55%)▶SUB1 (bg=9.24%)▶tia1 (bg=16.04%)▶tial1 (bg=14.09%)MATCHES To TargetScan▶ miR-28-3p:ACUAGAU▶ miR-221-3p/222-3p:GCUACAU


ATCCTTT

ATCCTTT  
Depth:5 (SHEEP)  
Ei-value:0.000, Pi-value:0.000  
Er-value:0.000, Pr-value:0.000  
No matches to eCLIP DataNo matches to TargetScan


TG

ATCCTTTTG  
Depth:3 (DOG)  
Ei-value:0.000, Pi-value:0.000  
Er-value:0.000, Pr-value:0.000  
eCLIP MATCHES▶SUB1 (bg=9.24%)No matches to TargetScan


ATAT

ATATGAAATA  
Depth:3 (DOG)  
Ei-value:0.000, Pi-value:0.000  
Er-value:0.000, Pr-value:0.000  
eCLIP MATCHES▶SUB1 (bg=9.24%)No matches to TargetScan


GAAATA

GAAATA  
Depth:8 (GUINEAPIG)  
Ei-value:0.000, Pi-value:0.000  
Er-value:0.000, Pr-value:0.010  
eCLIP MATCHES▶SUB1 (bg=9.24%)No matches to TargetScan


TTGTCTTGGAAA

GACAAACTTAAGTCCTTATTTGAAACATCTAGTCTTTCTAGATGTTTAGAAGTGCACAAAGTATGTTAAAAGTAGAGGTAGTAAATAACACATTTTGTAGCTATCCTTTTGATATGAAATATTGTCTTGGAAA  
Depth:2 (BABOON)  
Ei-value:0.000, Pi-value:0.000  
Er-value:0.000, Pr-value:0.000  
eCLIP MATCHES▶DDX21 (bg=1.64%)▶ddx55 (bg=12.35%)▶fam120a (bg=18.43%)▶FASTKD2 (bg=7.73%)▶fubp3 (bg=23.31%)▶pum2 (bg=21.55%)▶SUB1 (bg=9.24%)▶tia1 (bg=16.04%)▶tial1 (bg=14.09%)MATCHES To TargetScan▶ miR-28-3p:ACUAGAU▶ miR-221-3p/222-3p:GCUACAU

T

TGATCAA

TGATCAA  
Depth:5 (SHEEP)  
Ei-value:0.000, Pi-value:0.000  
Er-value:0.000, Pr-value:0.000  
eCLIP MATCHES▶DDX52 (bg=1.27%)▶ddx55 (bg=12.35%)▶dgcr8 (bg=19.31%)▶fam120a (bg=18.43%)▶FASTKD2 (bg=7.73%)▶fubp3 (bg=23.31%)▶SUB1 (bg=9.24%)▶tia1 (bg=16.04%)▶tial1 (bg=14.09%)No matches to TargetScan


TTCTCT

TGATCAATTCTCTGAGCAGTACCCATTTTGA  
Depth:2 (BABOON)  
Ei-value:0.000, Pi-value:0.000  
Er-value:0.000, Pr-value:0.000  
eCLIP MATCHES▶DDX52 (bg=1.27%)▶ddx55 (bg=12.35%)▶ddx6 (bg=23.92%)▶dgcr8 (bg=19.31%)▶fam120a (bg=18.43%)▶FASTKD2 (bg=7.73%)▶fubp3 (bg=23.31%)▶SUB1 (bg=9.24%)▶tia1 (bg=16.04%)▶tial1 (bg=14.09%)MATCHES To TargetScan▶ miR-219a-2-3p:GAAUUGU


GAGCAG

GAGCAG  
Depth:6 (PIG)  
Ei-value:0.000, Pi-value:0.000  
Er-value:0.000, Pr-value:0.000  
eCLIP MATCHES▶DDX52 (bg=1.27%)▶ddx55 (bg=12.35%)▶ddx6 (bg=23.92%)▶dgcr8 (bg=19.31%)▶fam120a (bg=18.43%)▶FASTKD2 (bg=7.73%)▶fubp3 (bg=23.31%)▶SUB1 (bg=9.24%)▶tia1 (bg=16.04%)▶tial1 (bg=14.09%)No matches to TargetScan


TACCCATTTTGA

TGATCAATTCTCTGAGCAGTACCCATTTTGA  
Depth:2 (BABOON)  
Ei-value:0.000, Pi-value:0.000  
Er-value:0.000, Pr-value:0.000  
eCLIP MATCHES▶DDX52 (bg=1.27%)▶ddx55 (bg=12.35%)▶ddx6 (bg=23.92%)▶dgcr8 (bg=19.31%)▶fam120a (bg=18.43%)▶FASTKD2 (bg=7.73%)▶fubp3 (bg=23.31%)▶SUB1 (bg=9.24%)▶tia1 (bg=16.04%)▶tial1 (bg=14.09%)MATCHES To TargetScan▶ miR-219a-2-3p:GAAUUGU


TATT

TATTTGTGCTGGTTCAGGGGGAAGGAGGAGCACAAAGTGCAAAGGGCTTTCTACCAGTGTCCAGTGTGTTTA  
Depth:2 (BABOON)  
Ei-value:0.000, Pi-value:0.000  
Er-value:0.000, Pr-value:0.000  
eCLIP MATCHES▶DDX52 (bg=1.27%)▶ddx55 (bg=12.35%)▶FASTKD2 (bg=7.73%)▶SUB1 (bg=9.24%)▶tia1 (bg=16.04%)▶tial1 (bg=14.09%)▶ybx3 (bg=22.82%)MATCHES To TargetScan▶ miR-218-5p:UGUGCUU▶ miR-125-5p:CCCUGAG▶ miR-329-3p/362-3p:ACACACC▶ miR-379-5p:GGUAGAC▶ miR-378-3p:CUGGACU▶ miR-331-3p:CCCCUGG▶ miR-129-3p:AGCCCUU▶ miR-338-3p:CCAGCAU


TGTGCTG

TGTGCTG  
Depth:7 (ARMADILLO)  
Ei-value:0.000, Pi-value:0.000  
Er-value:0.000, Pr-value:0.000  
eCLIP MATCHES▶ddx55 (bg=12.35%)▶FASTKD2 (bg=7.73%)▶tial1 (bg=14.09%)▶ybx3 (bg=22.82%)No matches to TargetScan


GTTCA

TGTGCTGGTTCA  
Depth:6 (PIG)  
Ei-value:0.000, Pi-value:0.000  
Er-value:0.000, Pr-value:0.000  
eCLIP MATCHES▶ddx55 (bg=12.35%)▶FASTKD2 (bg=7.73%)▶tial1 (bg=14.09%)▶ybx3 (bg=22.82%)MATCHES To TargetScan▶ miR-338-3p:CCAGCAU


GGG

TGTGCTGGTTCAGGG  
Depth:5 (SHEEP)  
Ei-value:0.000, Pi-value:0.000  
Er-value:0.000, Pr-value:0.000  
eCLIP MATCHES▶ddx55 (bg=12.35%)▶FASTKD2 (bg=7.73%)▶tial1 (bg=14.09%)▶ybx3 (bg=22.82%)MATCHES To TargetScan▶ miR-125-5p:CCCUGAG▶ miR-338-3p:CCAGCAU


GG

TATTTGTGCTGGTTCAGGGGGAAGGAGGAGCACAAAGTGCAAAGGGCTTTCTACCAGTGTCCAGTGTGTTTA  
Depth:2 (BABOON)  
Ei-value:0.000, Pi-value:0.000  
Er-value:0.000, Pr-value:0.000  
eCLIP MATCHES▶DDX52 (bg=1.27%)▶ddx55 (bg=12.35%)▶FASTKD2 (bg=7.73%)▶SUB1 (bg=9.24%)▶tia1 (bg=16.04%)▶tial1 (bg=14.09%)▶ybx3 (bg=22.82%)MATCHES To TargetScan▶ miR-218-5p:UGUGCUU▶ miR-125-5p:CCCUGAG▶ miR-329-3p/362-3p:ACACACC▶ miR-379-5p:GGUAGAC▶ miR-378-3p:CUGGACU▶ miR-331-3p:CCCCUGG▶ miR-129-3p:AGCCCUU▶ miR-338-3p:CCAGCAU


aaggag

aaggag  
Depth:3 (DOG)  
Ei-value:0.080, Pi-value:0.000  
Er-value:0.000, Pr-value:0.000  
No matches to eCLIP DataNo matches to TargetScan


GAGCAC

TATTTGTGCTGGTTCAGGGGGAAGGAGGAGCACAAAGTGCAAAGGGCTTTCTACCAGTGTCCAGTGTGTTTA  
Depth:2 (BABOON)  
Ei-value:0.000, Pi-value:0.000  
Er-value:0.000, Pr-value:0.000  
eCLIP MATCHES▶DDX52 (bg=1.27%)▶ddx55 (bg=12.35%)▶FASTKD2 (bg=7.73%)▶SUB1 (bg=9.24%)▶tia1 (bg=16.04%)▶tial1 (bg=14.09%)▶ybx3 (bg=22.82%)MATCHES To TargetScan▶ miR-218-5p:UGUGCUU▶ miR-125-5p:CCCUGAG▶ miR-329-3p/362-3p:ACACACC▶ miR-379-5p:GGUAGAC▶ miR-378-3p:CUGGACU▶ miR-331-3p:CCCCUGG▶ miR-129-3p:AGCCCUU▶ miR-338-3p:CCAGCAU


AAAGTGCAAA

AAAGTGCAAAGGGCTTT  
Depth:3 (DOG)  
Ei-value:0.000, Pi-value:0.000  
Er-value:0.000, Pr-value:0.000  
No matches to eCLIP DataMATCHES To TargetScan▶ miR-129-3p:AGCCCUU

 3240  


GGGCTTT

AAAGTGCAAAGGGCTTT  
Depth:3 (DOG)  
Ei-value:0.000, Pi-value:0.000  
Er-value:0.000, Pr-value:0.000  
No matches to eCLIP DataMATCHES To TargetScan▶ miR-129-3p:AGCCCUU


CT

TATTTGTGCTGGTTCAGGGGGAAGGAGGAGCACAAAGTGCAAAGGGCTTTCTACCAGTGTCCAGTGTGTTTA  
Depth:2 (BABOON)  
Ei-value:0.000, Pi-value:0.000  
Er-value:0.000, Pr-value:0.000  
eCLIP MATCHES▶DDX52 (bg=1.27%)▶ddx55 (bg=12.35%)▶FASTKD2 (bg=7.73%)▶SUB1 (bg=9.24%)▶tia1 (bg=16.04%)▶tial1 (bg=14.09%)▶ybx3 (bg=22.82%)MATCHES To TargetScan▶ miR-218-5p:UGUGCUU▶ miR-125-5p:CCCUGAG▶ miR-329-3p/362-3p:ACACACC▶ miR-379-5p:GGUAGAC▶ miR-378-3p:CUGGACU▶ miR-331-3p:CCCCUGG▶ miR-129-3p:AGCCCUU▶ miR-338-3p:CCAGCAU


ACCAGT

ACCAGT  
Depth:5 (SHEEP)  
Ei-value:0.000, Pi-value:0.010  
Er-value:0.000, Pr-value:0.000  
No matches to eCLIP DataNo matches to TargetScan


GTCCAGTG

TATTTGTGCTGGTTCAGGGGGAAGGAGGAGCACAAAGTGCAAAGGGCTTTCTACCAGTGTCCAGTGTGTTTA  
Depth:2 (BABOON)  
Ei-value:0.000, Pi-value:0.000  
Er-value:0.000, Pr-value:0.000  
eCLIP MATCHES▶DDX52 (bg=1.27%)▶ddx55 (bg=12.35%)▶FASTKD2 (bg=7.73%)▶SUB1 (bg=9.24%)▶tia1 (bg=16.04%)▶tial1 (bg=14.09%)▶ybx3 (bg=22.82%)MATCHES To TargetScan▶ miR-218-5p:UGUGCUU▶ miR-125-5p:CCCUGAG▶ miR-329-3p/362-3p:ACACACC▶ miR-379-5p:GGUAGAC▶ miR-378-3p:CUGGACU▶ miR-331-3p:CCCCUGG▶ miR-129-3p:AGCCCUU▶ miR-338-3p:CCAGCAU


TGTTTA

TGTTTA  
Depth:6 (PIG)  
Ei-value:0.000, Pi-value:0.020  
Er-value:0.000, Pr-value:0.010  
No matches to eCLIP DataNo matches to TargetScan

T

GAGGAGGCACATTG

GAGGAGGCACATTGACCATTGTCCCTT  
Depth:2 (BABOON)  
Ei-value:0.000, Pi-value:0.000  
Er-value:0.000, Pr-value:0.000  
eCLIP MATCHES▶WDR3 (bg=0.75%)MATCHES To TargetScan▶ miR-455-5p:AUGUGCC


ACCA

ACCATTGTCC  
Depth:3 (DOG)  
Ei-value:0.000, Pi-value:0.000  
Er-value:0.000, Pr-value:0.000  
eCLIP MATCHES▶WDR3 (bg=0.75%)No matches to TargetScan


TTGTCC

TTGTCC  
Depth:5 (SHEEP)  
Ei-value:0.000, Pi-value:0.020  
Er-value:0.000, Pr-value:0.000  
eCLIP MATCHES▶WDR3 (bg=0.75%)No matches to TargetScan


CTT

GAGGAGGCACATTGACCATTGTCCCTT  
Depth:2 (BABOON)  
Ei-value:0.000, Pi-value:0.000  
Er-value:0.000, Pr-value:0.000  
eCLIP MATCHES▶WDR3 (bg=0.75%)MATCHES To TargetScan▶ miR-455-5p:AUGUGCC

A

T

TGTCTGCATTTTCATTTACTGTGCTGTGTATATAGTGTATATAAG  
Depth:2 (BABOON)  
Ei-value:0.000, Pi-value:0.000  
Er-value:0.000, Pr-value:0.000  
eCLIP MATCHES▶APOBEC3C (bg=3.95%)▶ddx6 (bg=23.92%)▶dgcr8 (bg=19.31%)▶fam120a (bg=18.43%)▶fubp3 (bg=23.31%)▶pum1 (bg=29.85%)▶pum2 (bg=21.55%)▶sf3a3 (bg=12.13%)▶SF3B1 (bg=6.74%)▶WDR3 (bg=0.75%)MATCHES To TargetScan▶ miR-802:CAGUAAC▶ miR-144-3p:ACAGUAU▶ miR-128-3p:CACAGUG▶ miR-101-3p.1:ACAGUAC


GTCTGC

GTCTGC  
Depth:5 (SHEEP)  
Ei-value:0.000, Pi-value:0.020  
Er-value:0.000, Pr-value:0.000  
eCLIP MATCHES▶WDR3 (bg=0.75%)No matches to TargetScan


ATTTT

GTCTGCATTTT  
Depth:3 (DOG)  
Ei-value:0.000, Pi-value:0.000  
Er-value:0.000, Pr-value:0.000  
eCLIP MATCHES▶WDR3 (bg=0.75%)No matches to TargetScan


CATTTACTGTGCTG

TGTCTGCATTTTCATTTACTGTGCTGTGTATATAGTGTATATAAG  
Depth:2 (BABOON)  
Ei-value:0.000, Pi-value:0.000  
Er-value:0.000, Pr-value:0.000  
eCLIP MATCHES▶APOBEC3C (bg=3.95%)▶ddx6 (bg=23.92%)▶dgcr8 (bg=19.31%)▶fam120a (bg=18.43%)▶fubp3 (bg=23.31%)▶pum1 (bg=29.85%)▶pum2 (bg=21.55%)▶sf3a3 (bg=12.13%)▶SF3B1 (bg=6.74%)▶WDR3 (bg=0.75%)MATCHES To TargetScan▶ miR-802:CAGUAAC▶ miR-144-3p:ACAGUAU▶ miR-128-3p:CACAGUG▶ miR-101-3p.1:ACAGUAC


TGTATA

TGTATA  
Depth:9 (MOUSE)  
Ei-value:0.000, Pi-value:0.000  
Er-value:0.000, Pr-value:0.000  
eCLIP MATCHES▶APOBEC3C (bg=3.95%)▶pum1 (bg=29.85%)▶pum2 (bg=21.55%)No matches to TargetScan


TA

TGTATATA  
Depth:9 (MOUSE)  
Ei-value:0.000, Pi-value:0.000  
Er-value:0.000, Pr-value:0.000  
eCLIP MATCHES▶APOBEC3C (bg=3.95%)▶dgcr8 (bg=19.31%)▶pum1 (bg=29.85%)▶pum2 (bg=21.55%)No matches to TargetScan


GTGTA

TGTATATAGTGTA  
Depth:6 (PIG)  
Ei-value:0.000, Pi-value:0.000  
Er-value:0.000, Pr-value:0.000  
eCLIP MATCHES▶APOBEC3C (bg=3.95%)▶ddx6 (bg=23.92%)▶dgcr8 (bg=19.31%)▶pum1 (bg=29.85%)▶pum2 (bg=21.55%)▶SF3B1 (bg=6.74%)No matches to TargetScan


TATAA

TGTATATAGTGTATATAA  
Depth:3 (DOG)  
Ei-value:0.000, Pi-value:0.000  
Er-value:0.000, Pr-value:0.000  
eCLIP MATCHES▶APOBEC3C (bg=3.95%)▶ddx6 (bg=23.92%)▶dgcr8 (bg=19.31%)▶fam120a (bg=18.43%)▶fubp3 (bg=23.31%)▶pum1 (bg=29.85%)▶pum2 (bg=21.55%)▶sf3a3 (bg=12.13%)▶SF3B1 (bg=6.74%)No matches to TargetScan


G

TGTCTGCATTTTCATTTACTGTGCTGTGTATATAGTGTATATAAG  
Depth:2 (BABOON)  
Ei-value:0.000, Pi-value:0.000  
Er-value:0.000, Pr-value:0.000  
eCLIP MATCHES▶APOBEC3C (bg=3.95%)▶ddx6 (bg=23.92%)▶dgcr8 (bg=19.31%)▶fam120a (bg=18.43%)▶fubp3 (bg=23.31%)▶pum1 (bg=29.85%)▶pum2 (bg=21.55%)▶sf3a3 (bg=12.13%)▶SF3B1 (bg=6.74%)▶WDR3 (bg=0.75%)MATCHES To TargetScan▶ miR-802:CAGUAAC▶ miR-144-3p:ACAGUAU▶ miR-128-3p:CACAGUG▶ miR-101-3p.1:ACAGUAC

CGGACAT

ag

aggagtcctaatttac  
Depth:2 (BABOON)  
Ei-value:1.000, Pi-value:0.000  
Er-value:0.000, Pr-value:0.000  
eCLIP MATCHES▶APOBEC3C (bg=3.95%)▶ddx6 (bg=23.92%)▶dgcr8 (bg=19.31%)▶fam120a (bg=18.43%)▶fubp3 (bg=23.31%)▶pum1 (bg=29.85%)▶pum2 (bg=21.55%)▶sf3a3 (bg=12.13%)▶SF3B1 (bg=6.74%)▶tial1 (bg=14.09%)MATCHES To TargetScan▶ miR-483-3p.1:ACUCCUC


GAGTCCT

GAGTCCTAATTTAC  
Depth:3 (DOG)  
Ei-value:0.000, Pi-value:0.000  
Er-value:0.000, Pr-value:0.000  
eCLIP MATCHES▶APOBEC3C (bg=3.95%)▶ddx6 (bg=23.92%)▶dgcr8 (bg=19.31%)▶fam120a (bg=18.43%)▶fubp3 (bg=23.31%)▶pum1 (bg=29.85%)▶pum2 (bg=21.55%)▶sf3a3 (bg=12.13%)▶SF3B1 (bg=6.74%)▶tial1 (bg=14.09%)No matches to TargetScan


A

AATTTAC  
Depth:6 (PIG)  
Ei-value:0.000, Pi-value:0.000  
Er-value:0.000, Pr-value:0.000  
eCLIP MATCHES▶APOBEC3C (bg=3.95%)▶ddx6 (bg=23.92%)▶dgcr8 (bg=19.31%)▶fam120a (bg=18.43%)▶fubp3 (bg=23.31%)▶pum1 (bg=29.85%)▶pum2 (bg=21.55%)▶sf3a3 (bg=12.13%)▶SF3B1 (bg=6.74%)▶tial1 (bg=14.09%)No matches to TargetScan

 3360  


ATTTAC

AATTTAC  
Depth:6 (PIG)  
Ei-value:0.000, Pi-value:0.000  
Er-value:0.000, Pr-value:0.000  
eCLIP MATCHES▶APOBEC3C (bg=3.95%)▶ddx6 (bg=23.92%)▶dgcr8 (bg=19.31%)▶fam120a (bg=18.43%)▶fubp3 (bg=23.31%)▶pum1 (bg=29.85%)▶pum2 (bg=21.55%)▶sf3a3 (bg=12.13%)▶SF3B1 (bg=6.74%)▶tial1 (bg=14.09%)No matches to TargetScan

G

tctagtcgatgttaaa

tctagtcgatgttaaa  
Depth:2 (BABOON)  
Ei-value:1.000, Pi-value:0.000  
Er-value:0.000, Pr-value:0.000  
eCLIP MATCHES▶APOBEC3C (bg=3.95%)▶ddx6 (bg=23.92%)▶dgcr8 (bg=19.31%)▶fam120a (bg=18.43%)▶fubp3 (bg=23.31%)▶pum1 (bg=29.85%)▶pum2 (bg=21.55%)▶sf3a3 (bg=12.13%)▶SF3B1 (bg=6.74%)▶tial1 (bg=14.09%)MATCHES To TargetScan▶ miR-28-3p:ACUAGAU

A

aggttgccagt

aggttgccagt  
Depth:2 (BABOON)  
Ei-value:1.000, Pi-value:0.000  
Er-value:0.000, Pr-value:0.000  
eCLIP MATCHES▶APOBEC3C (bg=3.95%)▶ddx6 (bg=23.92%)▶dgcr8 (bg=19.31%)▶fam120a (bg=18.43%)▶fubp3 (bg=23.31%)▶pum1 (bg=29.85%)▶pum2 (bg=21.55%)▶tial1 (bg=14.09%)MATCHES To TargetScan▶ miR-193-3p:ACUGGCC

A

TATGACAAAAGTAGAATTAGTAAACT

TATGACAAAAGTAGAATTAGTAAACT  
Depth:2 (BABOON)  
Ei-value:0.000, Pi-value:0.000  
Er-value:0.000, Pr-value:0.000  
eCLIP MATCHES▶ddx6 (bg=23.92%)▶dgcr8 (bg=19.31%)▶fubp3 (bg=23.31%)▶pum1 (bg=29.85%)▶pum2 (bg=21.55%)No matches to TargetScan

ACTACATTGAGTAC

actttgtgttaaaattca

actttgtgttaaaattca  
Depth:2 (BABOON)  
Ei-value:0.990, Pi-value:0.000  
Er-value:0.000, Pr-value:0.000  
eCLIP MATCHES▶ddx6 (bg=23.92%)No matches to TargetScan

T

agggaagacttcttaaaaaca

agggaagacttcttaaaaaca  
Depth:2 (BABOON)  
Ei-value:0.490, Pi-value:0.000  
Er-value:0.000, Pr-value:0.000  
eCLIP MATCHES▶ddx6 (bg=23.92%)▶fam120a (bg=18.43%)▶pum1 (bg=29.85%)▶tial1 (bg=14.09%)No matches to TargetScan

AGT

g

gaaattgttaaaa  
Depth:2 (BABOON)  
Ei-value:1.000, Pi-value:0.000  
Er-value:0.000, Pr-value:0.000  
eCLIP MATCHES▶ddx6 (bg=23.92%)▶fam120a (bg=18.43%)▶pum1 (bg=29.85%)▶tial1 (bg=14.09%)No matches to TargetScan

 3480  


aaattgttaaaa

gaaattgttaaaa  
Depth:2 (BABOON)  
Ei-value:1.000, Pi-value:0.000  
Er-value:0.000, Pr-value:0.000  
eCLIP MATCHES▶ddx6 (bg=23.92%)▶fam120a (bg=18.43%)▶pum1 (bg=29.85%)▶tial1 (bg=14.09%)No matches to TargetScan


CCCCCCCTAAGCATTACAGATGGCTTATAGCTGTCCAC

CCCCCCCTAAGCATTACAGATGGCTTATAGCTGTCCAC  
Depth:2 (BABOON)  
Ei-value:0.000, Pi-value:0.000  
Er-value:0.000, Pr-value:0.000  
eCLIP MATCHES▶ddx6 (bg=23.92%)▶fam120a (bg=18.43%)▶pum1 (bg=29.85%)▶tial1 (bg=14.09%)MATCHES To TargetScan▶ miR-155-5p:UAAUGCU

GG

GGTTGGTAGAGGTGGGAAAGGGAAGGGTTCTAGGCCAGAATGTTCCTATTTAGAAGACACTCAAATTA

GGTTGGTAGAGGTGGGAAAGGGAAGGGTTCTAGGCCAGAATGTTCCTATTTAGAAGACACTCAAATTA  
Depth:2 (BABOON)  
Ei-value:0.000, Pi-value:0.000  
Er-value:0.000, Pr-value:0.000  
No matches to eCLIP DataMATCHES To TargetScan▶ miR-204-5p/211-5p:UCCCUUU▶ miR-1306-5p:CACCUCC▶ miR-181-5p:ACAUUCA▶ miR-543:AACAUUC

 3600  


GGTTGGTAGAGGTGGGAAAGGGAAGGGTTCTAGGCCAGAATGTTCCTATTTAGAAGACACTCAAATTA  
Depth:2 (BABOON)  
Ei-value:0.000, Pi-value:0.000  
Er-value:0.000, Pr-value:0.000  
No matches to eCLIP DataMATCHES To TargetScan▶ miR-204-5p/211-5p:UCCCUUU▶ miR-1306-5p:CACCUCC▶ miR-181-5p:ACAUUCA▶ miR-543:AACAUUC

C

AGTCTGTGTTATGTATGTATACCATTTATTCAATGCTACTGTGTATATAATGGAAAACTT

AGTCTGTGTTATGTATGTATACCATTTATTCAATGCTACTGTGTATATAATGGAAAACTT  
Depth:2 (BABOON)  
Ei-value:0.000, Pi-value:0.000  
Er-value:0.000, Pr-value:0.000  
eCLIP MATCHES▶fam120a (bg=18.43%)▶pum1 (bg=29.85%)▶pum2 (bg=21.55%)▶ybx3 (bg=22.82%)▶ZC3H11A (bg=6.25%)MATCHES To TargetScan▶ miR-199-3p:CAGUAGU▶ miR-144-3p:ACAGUAU▶ miR-128-3p:CACAGUG▶ miR-101-3p.1:ACAGUAC

A

AGTCCAGTTTGA

AGTCCAGTTTGAAACATCTAGTCTTTCTAGGTGTTTAAAAGTG  
Depth:2 (BABOON)  
Ei-value:0.000, Pi-value:0.000  
Er-value:0.000, Pr-value:0.000  
eCLIP MATCHES▶ddx6 (bg=23.92%)▶fam120a (bg=18.43%)▶igf2bp1 (bg=11.67%)▶IGF2BP3 (bg=4.26%)▶pum1 (bg=29.85%)▶pum2 (bg=21.55%)▶RPS3 (bg=4.15%)▶ybx3 (bg=22.82%)▶ZC3H11A (bg=6.25%)MATCHES To TargetScan▶ miR-28-3p:ACUAGAU▶ miR-378-3p:CUGGACU


AA

AACATCTAGTCTT  
Depth:6 (PIG)  
Ei-value:0.000, Pi-value:0.000  
Er-value:0.000, Pr-value:0.000  
eCLIP MATCHES▶fam120a (bg=18.43%)▶pum1 (bg=29.85%)▶pum2 (bg=21.55%)MATCHES To TargetScan▶ miR-28-3p:ACUAGAU


CATCT

CATCTAGTCTT  
Depth:8 (GUINEAPIG)  
Ei-value:0.000, Pi-value:0.000  
Er-value:0.000, Pr-value:0.000  
eCLIP MATCHES▶fam120a (bg=18.43%)▶pum1 (bg=29.85%)▶pum2 (bg=21.55%)MATCHES To TargetScan▶ miR-28-3p:ACUAGAU


AGTCTT

AGTCTT  
Depth:9 (MOUSE)  
Ei-value:0.000, Pi-value:0.000  
Er-value:0.000, Pr-value:0.000  
eCLIP MATCHES▶fam120a (bg=18.43%)▶pum1 (bg=29.85%)▶pum2 (bg=21.55%)No matches to TargetScan


TCTAG

AACATCTAGTCTTTCTAG  
Depth:3 (DOG)  
Ei-value:0.000, Pi-value:0.000  
Er-value:0.000, Pr-value:0.000  
eCLIP MATCHES▶fam120a (bg=18.43%)▶pum1 (bg=29.85%)▶pum2 (bg=21.55%)▶RPS3 (bg=4.15%)MATCHES To TargetScan▶ miR-28-3p:ACUAGAU


GTGTTTAAA

GTGTTTAAA  
Depth:3 (DOG)  
Ei-value:0.000, Pi-value:0.000  
Er-value:0.000, Pr-value:0.000  
eCLIP MATCHES▶ddx6 (bg=23.92%)▶fam120a (bg=18.43%)▶igf2bp1 (bg=11.67%)▶RPS3 (bg=4.15%)No matches to TargetScan


AGTG

AGTCCAGTTTGAAACATCTAGTCTTTCTAGGTGTTTAAAAGTG  
Depth:2 (BABOON)  
Ei-value:0.000, Pi-value:0.000  
Er-value:0.000, Pr-value:0.000  
eCLIP MATCHES▶ddx6 (bg=23.92%)▶fam120a (bg=18.43%)▶igf2bp1 (bg=11.67%)▶IGF2BP3 (bg=4.26%)▶pum1 (bg=29.85%)▶pum2 (bg=21.55%)▶RPS3 (bg=4.15%)▶ybx3 (bg=22.82%)▶ZC3H11A (bg=6.25%)MATCHES To TargetScan▶ miR-28-3p:ACUAGAU▶ miR-378-3p:CUGGACU

T

acaacggcc

acaacggcc  
Depth:2 (BABOON)  
Ei-value:1.000, Pi-value:0.000  
Er-value:0.000, Pr-value:0.000  
eCLIP MATCHES▶ddx6 (bg=23.92%)▶fam120a (bg=18.43%)▶igf2bp1 (bg=11.67%)▶IGF2BP3 (bg=4.26%)▶pum2 (bg=21.55%)▶RPS3 (bg=4.15%)No matches to TargetScan

TGTCG 3720  


cagtgg

cagtgg  
Depth:2 (BABOON)  
Ei-value:1.000, Pi-value:0.010  
Er-value:0.000, Pr-value:0.000  
eCLIP MATCHES▶ddx6 (bg=23.92%)▶fam120a (bg=18.43%)▶igf2bp1 (bg=11.67%)▶IGF2BP3 (bg=4.26%)▶pum2 (bg=21.55%)▶RPS3 (bg=4.15%)No matches to TargetScan

CGC

ATGCCTGTAATCCCAGCACTTTGGGAGGCCGAGGCAGGC

ATGCCTGTAATCCCAGCACTTTGGGAGGCCGAGGCAGGC  
Depth:2 (BABOON)  
Ei-value:0.000, Pi-value:0.000  
Er-value:0.000, Pr-value:0.000  
eCLIP MATCHES▶ddx6 (bg=23.92%)▶fam120a (bg=18.43%)▶igf2bp1 (bg=11.67%)▶IGF2BP3 (bg=4.26%)▶RPS3 (bg=4.15%)MATCHES To TargetScan▶ miR-302c-3p.2/520-3p:AGUGCUU▶ miR-150-5p:CUCCCAA▶ miR-532-3p:CUCCCAC▶ miR-17-5p/20-5p/93-5p/106-5p/519-3p:AAAGUGC▶ miR-302-3p/372-3p/373-3p/520-3p:AAGUGCU

G

gatcacgaggtc

gatcacgaggtc  
Depth:2 (BABOON)  
Ei-value:1.000, Pi-value:0.000  
Er-value:0.000, Pr-value:0.000  
No matches to eCLIP DataNo matches to TargetScan

A

agagatc

agagatc  
Depth:2 (BABOON)  
Ei-value:1.000, Pi-value:0.010  
Er-value:0.000, Pr-value:0.000  
No matches to eCLIP DataNo matches to TargetScan

AG

gaccatcttggcca

gaccatcttggcca  
Depth:2 (BABOON)  
Ei-value:1.000, Pi-value:0.000  
Er-value:0.000, Pr-value:0.000  
No matches to eCLIP DataNo matches to TargetScan

A

catg

catggtgaaacc  
Depth:2 (BABOON)  
Ei-value:1.000, Pi-value:0.000  
Er-value:0.000, Pr-value:0.000  
No matches to eCLIP DataNo matches to TargetScan


gtgaaa

gtgaaa  
Depth:3 (DOG)  
Ei-value:0.080, Pi-value:0.030  
Er-value:0.000, Pr-value:0.000  
No matches to eCLIP DataNo matches to TargetScan


cc

catggtgaaacc  
Depth:2 (BABOON)  
Ei-value:1.000, Pi-value:0.000  
Er-value:0.000, Pr-value:0.000  
No matches to eCLIP DataNo matches to TargetScan

CCATCTTTACTA

AAAATACAAA

AAAATACAAAAATTAGCTGGTCGT  
Depth:2 (BABOON)  
Ei-value:0.030, Pi-value:0.000  
Er-value:0.000, Pr-value:0.000  
eCLIP MATCHES▶ddx6 (bg=23.92%)▶dgcr8 (bg=19.31%)▶fam120a (bg=18.43%)▶igf2bp1 (bg=11.67%)▶IGF2BP3 (bg=4.26%)▶PABPC4 (bg=2.72%)▶SND1 (bg=1.27%)▶ybx3 (bg=22.82%)MATCHES To TargetScan▶ miR-129-5p:UUUUUGC

 3840  


AATTAGCTGGTCGT

AAAATACAAAAATTAGCTGGTCGT  
Depth:2 (BABOON)  
Ei-value:0.030, Pi-value:0.000  
Er-value:0.000, Pr-value:0.000  
eCLIP MATCHES▶ddx6 (bg=23.92%)▶dgcr8 (bg=19.31%)▶fam120a (bg=18.43%)▶igf2bp1 (bg=11.67%)▶IGF2BP3 (bg=4.26%)▶PABPC4 (bg=2.72%)▶SND1 (bg=1.27%)▶ybx3 (bg=22.82%)MATCHES To TargetScan▶ miR-129-5p:UUUUUGC


ggtggtg

ggtggtg  
Depth:2 (BABOON)  
Ei-value:1.000, Pi-value:0.000  
Er-value:0.000, Pr-value:0.010  
eCLIP MATCHES▶ddx6 (bg=23.92%)▶dgcr8 (bg=19.31%)▶fam120a (bg=18.43%)▶igf2bp1 (bg=11.67%)▶IGF2BP3 (bg=4.26%)▶PABPC4 (bg=2.72%)▶pum2 (bg=21.55%)▶SND1 (bg=1.27%)▶ybx3 (bg=22.82%)No matches to TargetScan

CC

cacctgtag

cacctgtag  
Depth:2 (BABOON)  
Ei-value:1.000, Pi-value:0.000  
Er-value:0.000, Pr-value:0.000  
eCLIP MATCHES▶ddx6 (bg=23.92%)▶dgcr8 (bg=19.31%)▶fam120a (bg=18.43%)▶igf2bp1 (bg=11.67%)▶IGF2BP3 (bg=4.26%)▶PABPC4 (bg=2.72%)▶pum2 (bg=21.55%)▶SND1 (bg=1.27%)▶ybx3 (bg=22.82%)MATCHES To TargetScan▶ miR-139-5p:CUACAGU

CCCCAGTTACTCGAGAG

gctgaggcaggagaat

gctgaggcaggagaat  
Depth:2 (BABOON)  
Ei-value:1.000, Pi-value:0.000  
Er-value:0.000, Pr-value:0.000  
eCLIP MATCHES▶igf2bp1 (bg=11.67%)▶IGF2BP3 (bg=4.26%)▶SUGP2 (bg=1.49%)▶ybx3 (bg=22.82%)No matches to TargetScan

C

gcttgaacttgggaggcgga

gcttgaacttgggaggcgga  
Depth:2 (BABOON)  
Ei-value:0.620, Pi-value:0.000  
Er-value:0.000, Pr-value:0.000  
eCLIP MATCHES▶SUGP2 (bg=1.49%)▶ybx3 (bg=22.82%)MATCHES To TargetScan▶ miR-150-5p:CUCCCAA▶ miR-532-3p:CUCCCAC

AGTTGCAGTGAGCCAAGATCG

caccactgcactc

caccactgcactcca  
Depth:2 (BABOON)  
Ei-value:1.000, Pi-value:0.000  
Er-value:0.000, Pr-value:0.000  
eCLIP MATCHES▶SUGP2 (bg=1.49%)▶ybx3 (bg=22.82%)MATCHES To TargetScan▶ miR-122-5p:GGAGUGU▶ miR-140-5p:AGUGGUU▶ miR-130-3p/301-3p/454-3p:AGUGCAA▶ miR-455-3p.2:UGCAGUC

 3960  


ca

caccactgcactcca  
Depth:2 (BABOON)  
Ei-value:1.000, Pi-value:0.000  
Er-value:0.000, Pr-value:0.000  
eCLIP MATCHES▶SUGP2 (bg=1.49%)▶ybx3 (bg=22.82%)MATCHES To TargetScan▶ miR-122-5p:GGAGUGU▶ miR-140-5p:AGUGGUU▶ miR-130-3p/301-3p/454-3p:AGUGCAA▶ miR-455-3p.2:UGCAGUC

G

cctggcgacagagc

cctggcgacagagc  
Depth:2 (BABOON)  
Ei-value:1.000, Pi-value:0.000  
Er-value:0.000, Pr-value:0.000  
eCLIP MATCHES▶ybx3 (bg=22.82%)No matches to TargetScan

GAGGCTCCG

tttcaaaaa

tttcaaaaa  
Depth:2 (BABOON)  
Ei-value:1.000, Pi-value:0.000  
Er-value:0.000, Pr-value:0.000  
No matches to eCLIP DataMATCHES To TargetScan▶ miR-129-5p:UUUUUGC

AAAAA

gtgcacaat

gtgcacaat  
Depth:2 (BABOON)  
Ei-value:1.000, Pi-value:0.000  
Er-value:0.000, Pr-value:0.000  
No matches to eCLIP DataNo matches to TargetScan

G

taggttaa

taggttaa  
Depth:2 (BABOON)  
Ei-value:1.000, Pi-value:0.010  
Er-value:0.000, Pr-value:0.000  
eCLIP MATCHES▶FUS (bg=2.59%)No matches to TargetScan

C

AGTAGAGGGCTTAAGTAACA

AGTAGAGGGCTTAAGTAACACCCCTCTAAGCATTTGTTTTCA  
Depth:2 (BABOON)  
Ei-value:0.000, Pi-value:0.000  
Er-value:0.000, Pr-value:0.000  
eCLIP MATCHES▶FUS (bg=2.59%)MATCHES To TargetScan▶ miR-423-5p:GAGGGGC▶ miR-877-5p:UAGAGGA▶ miR-495-3p:AACAAAC▶ miR-129-3p:AGCCCUU


CCCCTCTAAG

CCCCTCTAAG  
Depth:3 (DOG)  
Ei-value:0.000, Pi-value:0.000  
Er-value:0.000, Pr-value:0.000  
eCLIP MATCHES▶FUS (bg=2.59%)MATCHES To TargetScan▶ miR-423-5p:GAGGGGC▶ miR-877-5p:UAGAGGA


CATTTG

AGTAGAGGGCTTAAGTAACACCCCTCTAAGCATTTGTTTTCA  
Depth:2 (BABOON)  
Ei-value:0.000, Pi-value:0.000  
Er-value:0.000, Pr-value:0.000  
eCLIP MATCHES▶FUS (bg=2.59%)MATCHES To TargetScan▶ miR-423-5p:GAGGGGC▶ miR-877-5p:UAGAGGA▶ miR-495-3p:AACAAAC▶ miR-129-3p:AGCCCUU


ttttca

ttttca  
Depth:3 (DOG)  
Ei-value:0.080, Pi-value:0.060  
Er-value:0.000, Pr-value:0.020  
No matches to eCLIP DataNo matches to TargetScan

GTACTTC

C

CTAGGAGTGGTTGCATTTGGGAATGGAATTGTTAAAACTTGATG  
Depth:2 (BABOON)  
Ei-value:0.000, Pi-value:0.000  
Er-value:0.000, Pr-value:0.000  
eCLIP MATCHES▶GRSF1 (bg=3.8%)MATCHES To TargetScan▶ miR-26-5p:UCAAGUA▶ miR-483-3p.2:CACUCCU▶ miR-483-3p.1:ACUCCUC


TAGGAGT

TAGGAGT  
Depth:3 (DOG)  
Ei-value:0.000, Pi-value:0.000  
Er-value:0.000, Pr-value:0.000  
No matches to eCLIP DataMATCHES To TargetScan▶ miR-483-3p.1:ACUCCUC


GGTT

CTAGGAGTGGTTGCATTTGGGAATGGAATTGTTAAAACTTGATG  
Depth:2 (BABOON)  
Ei-value:0.000, Pi-value:0.000  
Er-value:0.000, Pr-value:0.000  
eCLIP MATCHES▶GRSF1 (bg=3.8%)MATCHES To TargetScan▶ miR-26-5p:UCAAGUA▶ miR-483-3p.2:CACUCCU▶ miR-483-3p.1:ACUCCUC

 4080  


GCA

CTAGGAGTGGTTGCATTTGGGAATGGAATTGTTAAAACTTGATG  
Depth:2 (BABOON)  
Ei-value:0.000, Pi-value:0.000  
Er-value:0.000, Pr-value:0.000  
eCLIP MATCHES▶GRSF1 (bg=3.8%)MATCHES To TargetScan▶ miR-26-5p:UCAAGUA▶ miR-483-3p.2:CACUCCU▶ miR-483-3p.1:ACUCCUC


TTTGGGAATGG

TTTGGGAATGGAATTGTTAAA  
Depth:3 (DOG)  
Ei-value:0.000, Pi-value:0.000  
Er-value:0.000, Pr-value:0.000  
No matches to eCLIP DataNo matches to TargetScan


AATTGTTA

AATTGTTA  
Depth:9 (MOUSE)  
Ei-value:0.000, Pi-value:0.000  
Er-value:0.000, Pr-value:0.000  
No matches to eCLIP DataNo matches to TargetScan


AA

AATTGTTAAA  
Depth:7 (ARMADILLO)  
Ei-value:0.000, Pi-value:0.000  
Er-value:0.000, Pr-value:0.000  
No matches to eCLIP DataNo matches to TargetScan


ACTTGATG

CTAGGAGTGGTTGCATTTGGGAATGGAATTGTTAAAACTTGATG  
Depth:2 (BABOON)  
Ei-value:0.000, Pi-value:0.000  
Er-value:0.000, Pr-value:0.000  
eCLIP MATCHES▶GRSF1 (bg=3.8%)MATCHES To TargetScan▶ miR-26-5p:UCAAGUA▶ miR-483-3p.2:CACUCCU▶ miR-483-3p.1:ACUCCUC

C

ttaggagcgaatgcagact

ttaggagcgaatgcagact  
Depth:2 (BABOON)  
Ei-value:0.940, Pi-value:0.000  
Er-value:0.000, Pr-value:0.000  
eCLIP MATCHES▶GRSF1 (bg=3.8%)▶PCBP2 (bg=1.64%)MATCHES To TargetScan▶ miR-33-5p:UGCAUUG▶ miR-346:GUCUGCC

AT

tcattgg

tcattgg  
Depth:2 (BABOON)  
Ei-value:1.000, Pi-value:0.010  
Er-value:0.000, Pr-value:0.000  
eCLIP MATCHES▶PCBP2 (bg=1.64%)▶PRPF4 (bg=2.33%)No matches to TargetScan

GTGT

ttgggg

ttggggtggggga  
Depth:2 (BABOON)  
Ei-value:1.000, Pi-value:0.000  
Er-value:0.000, Pr-value:0.000  
eCLIP MATCHES▶BCCIP (bg=4.61%)▶fam120a (bg=18.43%)▶FASTKD2 (bg=7.73%)▶GTF2F1 (bg=2.31%)▶HNRNPM (bg=1.27%)▶IGF2BP2 (bg=3.44%)▶PCBP2 (bg=1.64%)▶PRPF4 (bg=2.33%)▶sf3a3 (bg=12.13%)▶tia1 (bg=16.04%)▶TROVE2 (bg=3.49%)▶ZRANB2 (bg=0.99%)No matches to TargetScan


tggggg

tggggg  
Depth:4 (COW)  
Ei-value:1.000, Pi-value:0.010  
Er-value:0.000, Pr-value:0.000  
eCLIP MATCHES▶BCCIP (bg=4.61%)▶fam120a (bg=18.43%)▶FASTKD2 (bg=7.73%)▶GTF2F1 (bg=2.31%)▶HNRNPM (bg=1.27%)▶IGF2BP2 (bg=3.44%)▶PCBP2 (bg=1.64%)▶PRPF4 (bg=2.33%)▶sf3a3 (bg=12.13%)▶tia1 (bg=16.04%)No matches to TargetScan


A

TGGGGGA  
Depth:3 (DOG)  
Ei-value:0.000, Pi-value:0.000  
Er-value:0.000, Pr-value:0.000  
eCLIP MATCHES▶BCCIP (bg=4.61%)▶fam120a (bg=18.43%)▶FASTKD2 (bg=7.73%)▶GTF2F1 (bg=2.31%)▶HNRNPM (bg=1.27%)▶IGF2BP2 (bg=3.44%)▶PCBP2 (bg=1.64%)▶PRPF4 (bg=2.33%)▶sf3a3 (bg=12.13%)▶tia1 (bg=16.04%)▶TROVE2 (bg=3.49%)▶ZRANB2 (bg=0.99%)No matches to TargetScan

AGGGGGGGTGGGCA

gaggaggtatgca

gaggaggtatgca  
Depth:2 (BABOON)  
Ei-value:1.000, Pi-value:0.000  
Er-value:0.000, Pr-value:0.000  
eCLIP MATCHES▶APOBEC3C (bg=3.95%)▶BCCIP (bg=4.61%)▶CSTF2T (bg=1.12%)▶ddx3x (bg=13.89%)▶ddx55 (bg=12.35%)▶ddx6 (bg=23.92%)▶EWSR1 (bg=1.16%)▶fam120a (bg=18.43%)▶FASTKD2 (bg=7.73%)▶fubp3 (bg=23.31%)▶G3BP1 (bg=1.51%)▶GTF2F1 (bg=2.31%)▶HNRNPM (bg=1.27%)▶HNRNPU (bg=0.96%)▶IGF2BP2 (bg=3.44%)▶PABPC4 (bg=2.72%)▶PCBP2 (bg=1.64%)▶PRPF4 (bg=2.33%)▶pum1 (bg=29.85%)▶pum2 (bg=21.55%)▶RPS11 (bg=1.07%)▶SBDS (bg=1.91%)▶sf3a3 (bg=12.13%)▶TBRG4 (bg=7.0%)▶tia1 (bg=16.04%)▶TROVE2 (bg=3.49%)▶UPF1 (bg=3.62%)▶UTP3 (bg=2.81%)▶ZRANB2 (bg=0.99%)MATCHES To TargetScan▶ miR-875-5p:AUACCUC▶ miR-448:UGCAUAU▶ miR-153-3p:UGCAUAG

GGGAGAG

GG

GGGTTCTGTGC  
Depth:3 (DOG)  
Ei-value:0.000, Pi-value:0.000  
Er-value:0.000, Pr-value:0.000  
eCLIP MATCHES▶APOBEC3C (bg=3.95%)▶BCCIP (bg=4.61%)▶CSTF2T (bg=1.12%)▶ddx3x (bg=13.89%)▶ddx55 (bg=12.35%)▶ddx6 (bg=23.92%)▶EWSR1 (bg=1.16%)▶fam120a (bg=18.43%)▶FASTKD2 (bg=7.73%)▶fubp3 (bg=23.31%)▶G3BP1 (bg=1.51%)▶GTF2F1 (bg=2.31%)▶HNRNPM (bg=1.27%)▶HNRNPU (bg=0.96%)▶IGF2BP2 (bg=3.44%)▶PABPC4 (bg=2.72%)▶PCBP2 (bg=1.64%)▶PRPF4 (bg=2.33%)▶pum1 (bg=29.85%)▶pum2 (bg=21.55%)▶RPS11 (bg=1.07%)▶SBDS (bg=1.91%)▶sf3a3 (bg=12.13%)▶TBRG4 (bg=7.0%)▶tia1 (bg=16.04%)▶TROVE2 (bg=3.49%)▶UPF1 (bg=3.62%)▶UTP3 (bg=2.81%)▶ZRANB2 (bg=0.99%)No matches to TargetScan


gttctg

gttctgt  
Depth:4 (COW)  
Ei-value:0.960, Pi-value:0.000  
Er-value:0.000, Pr-value:0.000  
eCLIP MATCHES▶APOBEC3C (bg=3.95%)▶BCCIP (bg=4.61%)▶CSTF2T (bg=1.12%)▶ddx3x (bg=13.89%)▶ddx55 (bg=12.35%)▶ddx6 (bg=23.92%)▶EWSR1 (bg=1.16%)▶fam120a (bg=18.43%)▶FASTKD2 (bg=7.73%)▶fubp3 (bg=23.31%)▶G3BP1 (bg=1.51%)▶GTF2F1 (bg=2.31%)▶HNRNPM (bg=1.27%)▶HNRNPU (bg=0.96%)▶IGF2BP2 (bg=3.44%)▶PABPC4 (bg=2.72%)▶PCBP2 (bg=1.64%)▶PRPF4 (bg=2.33%)▶pum1 (bg=29.85%)▶pum2 (bg=21.55%)▶RPS11 (bg=1.07%)▶SBDS (bg=1.91%)▶sf3a3 (bg=12.13%)▶TBRG4 (bg=7.0%)▶tia1 (bg=16.04%)▶TROVE2 (bg=3.49%)▶UPF1 (bg=3.62%)▶UTP3 (bg=2.81%)▶ZRANB2 (bg=0.99%)No matches to TargetScan

 4200  


t

gttctgt  
Depth:4 (COW)  
Ei-value:0.960, Pi-value:0.000  
Er-value:0.000, Pr-value:0.000  
eCLIP MATCHES▶APOBEC3C (bg=3.95%)▶BCCIP (bg=4.61%)▶CSTF2T (bg=1.12%)▶ddx3x (bg=13.89%)▶ddx55 (bg=12.35%)▶ddx6 (bg=23.92%)▶EWSR1 (bg=1.16%)▶fam120a (bg=18.43%)▶FASTKD2 (bg=7.73%)▶fubp3 (bg=23.31%)▶G3BP1 (bg=1.51%)▶GTF2F1 (bg=2.31%)▶HNRNPM (bg=1.27%)▶HNRNPU (bg=0.96%)▶IGF2BP2 (bg=3.44%)▶PABPC4 (bg=2.72%)▶PCBP2 (bg=1.64%)▶PRPF4 (bg=2.33%)▶pum1 (bg=29.85%)▶pum2 (bg=21.55%)▶RPS11 (bg=1.07%)▶SBDS (bg=1.91%)▶sf3a3 (bg=12.13%)▶TBRG4 (bg=7.0%)▶tia1 (bg=16.04%)▶TROVE2 (bg=3.49%)▶UPF1 (bg=3.62%)▶UTP3 (bg=2.81%)▶ZRANB2 (bg=0.99%)No matches to TargetScan


GC

GGGTTCTGTGC  
Depth:3 (DOG)  
Ei-value:0.000, Pi-value:0.000  
Er-value:0.000, Pr-value:0.000  
eCLIP MATCHES▶APOBEC3C (bg=3.95%)▶BCCIP (bg=4.61%)▶CSTF2T (bg=1.12%)▶ddx3x (bg=13.89%)▶ddx55 (bg=12.35%)▶ddx6 (bg=23.92%)▶EWSR1 (bg=1.16%)▶fam120a (bg=18.43%)▶FASTKD2 (bg=7.73%)▶fubp3 (bg=23.31%)▶G3BP1 (bg=1.51%)▶GTF2F1 (bg=2.31%)▶HNRNPM (bg=1.27%)▶HNRNPU (bg=0.96%)▶IGF2BP2 (bg=3.44%)▶PABPC4 (bg=2.72%)▶PCBP2 (bg=1.64%)▶PRPF4 (bg=2.33%)▶pum1 (bg=29.85%)▶pum2 (bg=21.55%)▶RPS11 (bg=1.07%)▶SBDS (bg=1.91%)▶sf3a3 (bg=12.13%)▶TBRG4 (bg=7.0%)▶tia1 (bg=16.04%)▶TROVE2 (bg=3.49%)▶UPF1 (bg=3.62%)▶UTP3 (bg=2.81%)▶ZRANB2 (bg=0.99%)No matches to TargetScan


TCCTGAGATTAGTTCAGATGGTCTAACCATTGTTCTATATGTGCATTTTAGTTAATATTGTGTATTAAAGGATA

GGGTTCTGTGCTCCTGAGATTAGTTCAGATGGTCTAACCATTGTTCTATATGTGCATTTTAGTTAATATTGTGTATTAAAGGATAAGTCTTAATGCTCAAAGTATGTTAAAAATAGATGTAGTAAA  
Depth:2 (BABOON)  
Ei-value:0.000, Pi-value:0.000  
Er-value:0.000, Pr-value:0.000  
eCLIP MATCHES▶APOBEC3C (bg=3.95%)▶BCCIP (bg=4.61%)▶CSTF2T (bg=1.12%)▶DDX24 (bg=2.31%)▶ddx3x (bg=13.89%)▶ddx55 (bg=12.35%)▶ddx6 (bg=23.92%)▶EWSR1 (bg=1.16%)▶fam120a (bg=18.43%)▶FASTKD2 (bg=7.73%)▶fubp3 (bg=23.31%)▶G3BP1 (bg=1.51%)▶GRWD1 (bg=4.85%)▶GTF2F1 (bg=2.31%)▶HNRNPM (bg=1.27%)▶HNRNPU (bg=0.96%)▶IGF2BP2 (bg=3.44%)▶PABPC4 (bg=2.72%)▶PCBP2 (bg=1.64%)▶PRPF4 (bg=2.33%)▶pum1 (bg=29.85%)▶pum2 (bg=21.55%)▶RPS11 (bg=1.07%)▶SBDS (bg=1.91%)▶sf3a3 (bg=12.13%)▶TBRG4 (bg=7.0%)▶tia1 (bg=16.04%)▶TROVE2 (bg=3.49%)▶UPF1 (bg=3.62%)▶UTP3 (bg=2.81%)▶ZRANB2 (bg=0.99%)MATCHES To TargetScan▶ miR-499a-5p:UAAGACU▶ miR-208-3p:UAAGACG▶ miR-216a-5p:AAUCUCA▶ miR-216b-5p:AAUCUCU▶ miR-28-5p/708-5p:AGGAGCU▶ miR-655-3p:UAAUACA▶ miR-362-5p/500b-5p:AUCCUUG▶ miR-501-3p/502-3p:AUGCACC


AGTCTT

AGTCTT  
Depth:9 (MOUSE)  
Ei-value:0.000, Pi-value:0.000  
Er-value:0.000, Pr-value:0.000  
eCLIP MATCHES▶DDX24 (bg=2.31%)No matches to TargetScan


AATGCTC

GGGTTCTGTGCTCCTGAGATTAGTTCAGATGGTCTAACCATTGTTCTATATGTGCATTTTAGTTAATATTGTGTATTAAAGGATAAGTCTTAATGCTCAAAGTATGTTAAAAATAGATGTAGTAAA  
Depth:2 (BABOON)  
Ei-value:0.000, Pi-value:0.000  
Er-value:0.000, Pr-value:0.000  
eCLIP MATCHES▶APOBEC3C (bg=3.95%)▶BCCIP (bg=4.61%)▶CSTF2T (bg=1.12%)▶DDX24 (bg=2.31%)▶ddx3x (bg=13.89%)▶ddx55 (bg=12.35%)▶ddx6 (bg=23.92%)▶EWSR1 (bg=1.16%)▶fam120a (bg=18.43%)▶FASTKD2 (bg=7.73%)▶fubp3 (bg=23.31%)▶G3BP1 (bg=1.51%)▶GRWD1 (bg=4.85%)▶GTF2F1 (bg=2.31%)▶HNRNPM (bg=1.27%)▶HNRNPU (bg=0.96%)▶IGF2BP2 (bg=3.44%)▶PABPC4 (bg=2.72%)▶PCBP2 (bg=1.64%)▶PRPF4 (bg=2.33%)▶pum1 (bg=29.85%)▶pum2 (bg=21.55%)▶RPS11 (bg=1.07%)▶SBDS (bg=1.91%)▶sf3a3 (bg=12.13%)▶TBRG4 (bg=7.0%)▶tia1 (bg=16.04%)▶TROVE2 (bg=3.49%)▶UPF1 (bg=3.62%)▶UTP3 (bg=2.81%)▶ZRANB2 (bg=0.99%)MATCHES To TargetScan▶ miR-499a-5p:UAAGACU▶ miR-208-3p:UAAGACG▶ miR-216a-5p:AAUCUCA▶ miR-216b-5p:AAUCUCU▶ miR-28-5p/708-5p:AGGAGCU▶ miR-655-3p:UAAUACA▶ miR-362-5p/500b-5p:AUCCUUG▶ miR-501-3p/502-3p:AUGCACC


aaagta

aaagta  
Depth:4 (COW)  
Ei-value:1.000, Pi-value:0.020  
Er-value:0.000, Pr-value:0.000  
eCLIP MATCHES▶DDX24 (bg=2.31%)No matches to TargetScan


TGTTAAAAATAGAT

GGGTTCTGTGCTCCTGAGATTAGTTCAGATGGTCTAACCATTGTTCTATATGTGCATTTTAGTTAATATTGTGTATTAAAGGATAAGTCTTAATGCTCAAAGTATGTTAAAAATAGATGTAGTAAA  
Depth:2 (BABOON)  
Ei-value:0.000, Pi-value:0.000  
Er-value:0.000, Pr-value:0.000  
eCLIP MATCHES▶APOBEC3C (bg=3.95%)▶BCCIP (bg=4.61%)▶CSTF2T (bg=1.12%)▶DDX24 (bg=2.31%)▶ddx3x (bg=13.89%)▶ddx55 (bg=12.35%)▶ddx6 (bg=23.92%)▶EWSR1 (bg=1.16%)▶fam120a (bg=18.43%)▶FASTKD2 (bg=7.73%)▶fubp3 (bg=23.31%)▶G3BP1 (bg=1.51%)▶GRWD1 (bg=4.85%)▶GTF2F1 (bg=2.31%)▶HNRNPM (bg=1.27%)▶HNRNPU (bg=0.96%)▶IGF2BP2 (bg=3.44%)▶PABPC4 (bg=2.72%)▶PCBP2 (bg=1.64%)▶PRPF4 (bg=2.33%)▶pum1 (bg=29.85%)▶pum2 (bg=21.55%)▶RPS11 (bg=1.07%)▶SBDS (bg=1.91%)▶sf3a3 (bg=12.13%)▶TBRG4 (bg=7.0%)▶tia1 (bg=16.04%)▶TROVE2 (bg=3.49%)▶UPF1 (bg=3.62%)▶UTP3 (bg=2.81%)▶ZRANB2 (bg=0.99%)MATCHES To TargetScan▶ miR-499a-5p:UAAGACU▶ miR-208-3p:UAAGACG▶ miR-216a-5p:AAUCUCA▶ miR-216b-5p:AAUCUCU▶ miR-28-5p/708-5p:AGGAGCU▶ miR-655-3p:UAAUACA▶ miR-362-5p/500b-5p:AUCCUUG▶ miR-501-3p/502-3p:AUGCACC


gtagtaaa

gtagtaaa  
Depth:4 (COW)  
Ei-value:0.670, Pi-value:0.000  
Er-value:0.000, Pr-value:0.000  
eCLIP MATCHES▶ddx6 (bg=23.92%)No matches to TargetScan

T

C

CAGTCCCTTTGTGAATGTCCTTTTGTTA  
Depth:2 (BABOON)  
Ei-value:0.000, Pi-value:0.000  
Er-value:0.000, Pr-value:0.000  
eCLIP MATCHES▶ddx55 (bg=12.35%)▶ddx6 (bg=23.92%)▶fam120a (bg=18.43%)▶lin28b (bg=16.96%)▶SUB1 (bg=9.24%)MATCHES To TargetScan▶ miR-181-5p:ACAUUCA▶ miR-495-3p:AACAAAC▶ miR-1197:AGGACAC

 4320  


AG

CAGTCCCTTTGTGAATGTCCTTTTGTTA  
Depth:2 (BABOON)  
Ei-value:0.000, Pi-value:0.000  
Er-value:0.000, Pr-value:0.000  
eCLIP MATCHES▶ddx55 (bg=12.35%)▶ddx6 (bg=23.92%)▶fam120a (bg=18.43%)▶lin28b (bg=16.96%)▶SUB1 (bg=9.24%)MATCHES To TargetScan▶ miR-181-5p:ACAUUCA▶ miR-495-3p:AACAAAC▶ miR-1197:AGGACAC


TCCCTTT

TCCCTTT  
Depth:3 (DOG)  
Ei-value:0.000, Pi-value:0.000  
Er-value:0.000, Pr-value:0.000  
eCLIP MATCHES▶ddx55 (bg=12.35%)▶ddx6 (bg=23.92%)▶SUB1 (bg=9.24%)No matches to TargetScan


GTG

CAGTCCCTTTGTGAATGTCCTTTTGTTA  
Depth:2 (BABOON)  
Ei-value:0.000, Pi-value:0.000  
Er-value:0.000, Pr-value:0.000  
eCLIP MATCHES▶ddx55 (bg=12.35%)▶ddx6 (bg=23.92%)▶fam120a (bg=18.43%)▶lin28b (bg=16.96%)▶SUB1 (bg=9.24%)MATCHES To TargetScan▶ miR-181-5p:ACAUUCA▶ miR-495-3p:AACAAAC▶ miR-1197:AGGACAC


AATGT

AATGTCCTTTTGTTA  
Depth:3 (DOG)  
Ei-value:0.000, Pi-value:0.000  
Er-value:0.000, Pr-value:0.000  
eCLIP MATCHES▶ddx55 (bg=12.35%)▶ddx6 (bg=23.92%)▶fam120a (bg=18.43%)▶lin28b (bg=16.96%)▶SUB1 (bg=9.24%)MATCHES To TargetScan▶ miR-495-3p:AACAAAC▶ miR-1197:AGGACAC


ccttttgtt

ccttttgtt  
Depth:4 (COW)  
Ei-value:0.150, Pi-value:0.000  
Er-value:0.000, Pr-value:0.000  
eCLIP MATCHES▶ddx55 (bg=12.35%)▶ddx6 (bg=23.92%)▶fam120a (bg=18.43%)▶lin28b (bg=16.96%)▶SUB1 (bg=9.24%)MATCHES To TargetScan▶ miR-495-3p:AACAAAC


A

AATGTCCTTTTGTTA  
Depth:3 (DOG)  
Ei-value:0.000, Pi-value:0.000  
Er-value:0.000, Pr-value:0.000  
eCLIP MATCHES▶ddx55 (bg=12.35%)▶ddx6 (bg=23.92%)▶fam120a (bg=18.43%)▶lin28b (bg=16.96%)▶SUB1 (bg=9.24%)MATCHES To TargetScan▶ miR-495-3p:AACAAAC▶ miR-1197:AGGACAC

GT

TTTTAGGAAGG

TTTTAGGAAGG  
Depth:4 (COW)  
Ei-value:0.000, Pi-value:0.000  
Er-value:0.000, Pr-value:0.000  
eCLIP MATCHES▶ddx55 (bg=12.35%)▶ddx6 (bg=23.92%)▶fam120a (bg=18.43%)▶fubp3 (bg=23.31%)▶lin28b (bg=16.96%)▶pum1 (bg=29.85%)▶SUB1 (bg=9.24%)▶tia1 (bg=16.04%)No matches to TargetScan


cctgt

ttttaggaaggcctgt  
Depth:2 (BABOON)  
Ei-value:1.000, Pi-value:0.000  
Er-value:0.000, Pr-value:0.000  
eCLIP MATCHES▶APOBEC3C (bg=3.95%)▶ddx55 (bg=12.35%)▶ddx6 (bg=23.92%)▶fam120a (bg=18.43%)▶fubp3 (bg=23.31%)▶lin28b (bg=16.96%)▶pum1 (bg=29.85%)▶SUB1 (bg=9.24%)▶tia1 (bg=16.04%)No matches to TargetScan

CC

tctgggagt

tctgggagt  
Depth:4 (COW)  
Ei-value:0.150, Pi-value:0.000  
Er-value:0.000, Pr-value:0.000  
eCLIP MATCHES▶APOBEC3C (bg=3.95%)▶ddx55 (bg=12.35%)▶ddx6 (bg=23.92%)▶fam120a (bg=18.43%)▶fubp3 (bg=23.31%)▶lin28b (bg=16.96%)▶pum1 (bg=29.85%)▶SUB1 (bg=9.24%)▶tia1 (bg=16.04%)MATCHES To TargetScan▶ miR-150-5p:CUCCCAA▶ miR-532-3p:CUCCCAC


G

TCTGGGAGTG  
Depth:3 (DOG)  
Ei-value:0.000, Pi-value:0.000  
Er-value:0.000, Pr-value:0.000  
eCLIP MATCHES▶APOBEC3C (bg=3.95%)▶ddx55 (bg=12.35%)▶ddx6 (bg=23.92%)▶fam120a (bg=18.43%)▶fubp3 (bg=23.31%)▶lin28b (bg=16.96%)▶pum1 (bg=29.85%)▶SUB1 (bg=9.24%)▶tia1 (bg=16.04%)MATCHES To TargetScan▶ miR-150-5p:CUCCCAA▶ miR-532-3p:CUCCCAC▶ miR-483-3p.2:CACUCCU


acctt

tctgggagtgacctt  
Depth:2 (BABOON)  
Ei-value:1.000, Pi-value:0.000  
Er-value:0.000, Pr-value:0.000  
eCLIP MATCHES▶APOBEC3C (bg=3.95%)▶ddx55 (bg=12.35%)▶ddx6 (bg=23.92%)▶fam120a (bg=18.43%)▶fubp3 (bg=23.31%)▶lin28b (bg=16.96%)▶pum1 (bg=29.85%)▶SUB1 (bg=9.24%)▶tia1 (bg=16.04%)MATCHES To TargetScan▶ miR-150-5p:CUCCCAA▶ miR-532-3p:CUCCCAC▶ miR-483-3p.2:CACUCCU▶ miR-1193:AGGUCAC▶ miR-668-3p:GUCACUC

TATTAGTCCACCC

cttg

cttggagctagacatcct  
Depth:2 (BABOON)  
Ei-value:0.990, Pi-value:0.000  
Er-value:0.000, Pr-value:0.000  
eCLIP MATCHES▶ddx55 (bg=12.35%)▶ddx6 (bg=23.92%)▶fam120a (bg=18.43%)▶fubp3 (bg=23.31%)▶lin28b (bg=16.96%)▶pum1 (bg=29.85%)▶SUB1 (bg=9.24%)▶tia1 (bg=16.04%)▶ybx3 (bg=22.82%)No matches to TargetScan


GAGCTAGA

GAGCTAGA  
Depth:3 (DOG)  
Ei-value:0.000, Pi-value:0.000  
Er-value:0.000, Pr-value:0.000  
eCLIP MATCHES▶ddx55 (bg=12.35%)▶ddx6 (bg=23.92%)▶fam120a (bg=18.43%)▶lin28b (bg=16.96%)▶pum1 (bg=29.85%)▶SUB1 (bg=9.24%)▶tia1 (bg=16.04%)▶ybx3 (bg=22.82%)No matches to TargetScan


catcct

cttggagctagacatcct  
Depth:2 (BABOON)  
Ei-value:0.990, Pi-value:0.000  
Er-value:0.000, Pr-value:0.000  
eCLIP MATCHES▶ddx55 (bg=12.35%)▶ddx6 (bg=23.92%)▶fam120a (bg=18.43%)▶fubp3 (bg=23.31%)▶lin28b (bg=16.96%)▶pum1 (bg=29.85%)▶SUB1 (bg=9.24%)▶tia1 (bg=16.04%)▶ybx3 (bg=22.82%)No matches to TargetScan

G

tacttagtc

tacttagtc  
Depth:4 (COW)  
Ei-value:0.150, Pi-value:0.000  
Er-value:0.000, Pr-value:0.000  
eCLIP MATCHES▶ddx55 (bg=12.35%)▶ddx6 (bg=23.92%)▶lin28b (bg=16.96%)▶ybx3 (bg=22.82%)No matches to TargetScan


ac

tacttagtcac  
Depth:2 (BABOON)  
Ei-value:1.000, Pi-value:0.000  
Er-value:0.000, Pr-value:0.000  
eCLIP MATCHES▶ddx55 (bg=12.35%)▶ddx6 (bg=23.92%)▶lin28b (bg=16.96%)▶ybx3 (bg=22.82%)MATCHES To TargetScan▶ miR-134-5p:GUGACUG

G

ggg

gggatggtggaagagggaga  
Depth:2 (BABOON)  
Ei-value:0.620, Pi-value:0.000  
Er-value:0.000, Pr-value:0.000  
eCLIP MATCHES▶ddx55 (bg=12.35%)▶ddx6 (bg=23.92%)▶lin28b (bg=16.96%)No matches to TargetScan


ATGGTGGA

ATGGTGGA  
Depth:3 (DOG)  
Ei-value:0.000, Pi-value:0.000  
Er-value:0.000, Pr-value:0.010  
eCLIP MATCHES▶ddx55 (bg=12.35%)▶lin28b (bg=16.96%)No matches to TargetScan


aga

gggatggtggaagagggaga  
Depth:2 (BABOON)  
Ei-value:0.620, Pi-value:0.000  
Er-value:0.000, Pr-value:0.000  
eCLIP MATCHES▶ddx55 (bg=12.35%)▶ddx6 (bg=23.92%)▶lin28b (bg=16.96%)No matches to TargetScan

 4440  


gggaga

gggatggtggaagagggaga  
Depth:2 (BABOON)  
Ei-value:0.620, Pi-value:0.000  
Er-value:0.000, Pr-value:0.000  
eCLIP MATCHES▶ddx55 (bg=12.35%)▶ddx6 (bg=23.92%)▶lin28b (bg=16.96%)No matches to TargetScan

A

GAGGAA

GAGGAAGGGTGAAGGGAAGGGCTCTTTGCTAGTATCT  
Depth:2 (BABOON)  
Ei-value:0.000, Pi-value:0.000  
Er-value:0.000, Pr-value:0.000  
eCLIP MATCHES▶lin28b (bg=16.96%)MATCHES To TargetScan▶ miR-204-5p/211-5p:UCCCUUU▶ miR-670-3p:UUCCUCA▶ miR-205-5p:CCUUCAU▶ miR-129-3p:AGCCCUU


gggtga

gggtga  
Depth:4 (COW)  
Ei-value:1.000, Pi-value:0.010  
Er-value:0.000, Pr-value:0.000  
No matches to eCLIP DataNo matches to TargetScan


A

GGGTGAAGGGAAGGGCT  
Depth:3 (DOG)  
Ei-value:0.000, Pi-value:0.000  
Er-value:0.000, Pr-value:0.000  
No matches to eCLIP DataMATCHES To TargetScan▶ miR-204-5p/211-5p:UCCCUUU▶ miR-205-5p:CCUUCAU▶ miR-129-3p:AGCCCUU


GGGAAGGG

GGGAAGGG  
Depth:5 (SHEEP)  
Ei-value:0.000, Pi-value:0.000  
Er-value:0.000, Pr-value:0.000  
No matches to eCLIP DataNo matches to TargetScan


CT

GGGAAGGGCT  
Depth:4 (COW)  
Ei-value:0.020, Pi-value:0.000  
Er-value:0.000, Pr-value:0.000  
No matches to eCLIP DataMATCHES To TargetScan▶ miR-129-3p:AGCCCUU


C

GAGGAAGGGTGAAGGGAAGGGCTCTTTGCTAGTATCT  
Depth:2 (BABOON)  
Ei-value:0.000, Pi-value:0.000  
Er-value:0.000, Pr-value:0.000  
eCLIP MATCHES▶lin28b (bg=16.96%)MATCHES To TargetScan▶ miR-204-5p/211-5p:UCCCUUU▶ miR-670-3p:UUCCUCA▶ miR-205-5p:CCUUCAU▶ miR-129-3p:AGCCCUU


TTTGCTAGTATCT

TTTGCTAGTATCT  
Depth:3 (DOG)  
Ei-value:0.000, Pi-value:0.000  
Er-value:0.000, Pr-value:0.000  
No matches to eCLIP DataNo matches to TargetScan

C

CAT

CATATCTAGACGATGGTTTTAGATGATAACCACAGGTCTA  
Depth:2 (BABOON)  
Ei-value:0.000, Pi-value:0.000  
Er-value:0.000, Pr-value:0.000  
eCLIP MATCHES▶lin28b (bg=16.96%)MATCHES To TargetScan▶ miR-190-5p:GAUAUGU


ATCTAGA

ATCTAGA  
Depth:3 (DOG)  
Ei-value:0.000, Pi-value:0.010  
Er-value:0.000, Pr-value:0.000  
No matches to eCLIP DataNo matches to TargetScan


CGATG

CATATCTAGACGATGGTTTTAGATGATAACCACAGGTCTA  
Depth:2 (BABOON)  
Ei-value:0.000, Pi-value:0.000  
Er-value:0.000, Pr-value:0.000  
eCLIP MATCHES▶lin28b (bg=16.96%)MATCHES To TargetScan▶ miR-190-5p:GAUAUGU


gttttag

gttttag  
Depth:4 (COW)  
Ei-value:0.960, Pi-value:0.010  
Er-value:0.000, Pr-value:0.000  
eCLIP MATCHES▶lin28b (bg=16.96%)No matches to TargetScan


ATG

CATATCTAGACGATGGTTTTAGATGATAACCACAGGTCTA  
Depth:2 (BABOON)  
Ei-value:0.000, Pi-value:0.000  
Er-value:0.000, Pr-value:0.000  
eCLIP MATCHES▶lin28b (bg=16.96%)MATCHES To TargetScan▶ miR-190-5p:GAUAUGU


ATAACCA

ATAACCA  
Depth:3 (DOG)  
Ei-value:0.000, Pi-value:0.010  
Er-value:0.000, Pr-value:0.000  
eCLIP MATCHES▶lin28b (bg=16.96%)No matches to TargetScan


CAGGTCTA

CATATCTAGACGATGGTTTTAGATGATAACCACAGGTCTA  
Depth:2 (BABOON)  
Ei-value:0.000, Pi-value:0.000  
Er-value:0.000, Pr-value:0.000  
eCLIP MATCHES▶lin28b (bg=16.96%)MATCHES To TargetScan▶ miR-190-5p:GAUAUGU

CAAGAGCG

ttttta

ttttta  
Depth:3 (DOG)  
Ei-value:0.080, Pi-value:0.120  
Er-value:0.000, Pr-value:0.120  
eCLIP MATCHES▶lin28b (bg=16.96%)No matches to TargetScan


GTAAAGTG

TTTTTAGTAAAGTGCCTGTGTTCATTGTGGACAAAGTT  
Depth:2 (BABOON)  
Ei-value:0.000, Pi-value:0.000  
Er-value:0.000, Pr-value:0.000  
eCLIP MATCHES▶lin28b (bg=16.96%)▶ybx3 (bg=22.82%)No matches to TargetScan


CCTGTGTT

CCTGTGTT  
Depth:3 (DOG)  
Ei-value:0.000, Pi-value:0.000  
Er-value:0.000, Pr-value:0.000  
eCLIP MATCHES▶lin28b (bg=16.96%)No matches to TargetScan


CA

TTTTTAGTAAAGTGCCTGTGTTCATTGTGGACAAAGTT  
Depth:2 (BABOON)  
Ei-value:0.000, Pi-value:0.000  
Er-value:0.000, Pr-value:0.000  
eCLIP MATCHES▶lin28b (bg=16.96%)▶ybx3 (bg=22.82%)No matches to TargetScan


ttg

ttgtgga  
Depth:4 (COW)  
Ei-value:0.960, Pi-value:0.000  
Er-value:0.000, Pr-value:0.000  
eCLIP MATCHES▶lin28b (bg=16.96%)No matches to TargetScan

 4560  


tgga

ttgtgga  
Depth:4 (COW)  
Ei-value:0.960, Pi-value:0.000  
Er-value:0.000, Pr-value:0.000  
eCLIP MATCHES▶lin28b (bg=16.96%)No matches to TargetScan


CAAAGTT

TTTTTAGTAAAGTGCCTGTGTTCATTGTGGACAAAGTT  
Depth:2 (BABOON)  
Ei-value:0.000, Pi-value:0.000  
Er-value:0.000, Pr-value:0.000  
eCLIP MATCHES▶lin28b (bg=16.96%)▶ybx3 (bg=22.82%)No matches to TargetScan


a

attattttg  
Depth:2 (BABOON)  
Ei-value:1.000, Pi-value:0.000  
Er-value:0.000, Pr-value:0.000  
eCLIP MATCHES▶lin28b (bg=16.96%)▶ybx3 (bg=22.82%)No matches to TargetScan


TTATTTTG

TTATTTTG  
Depth:3 (DOG)  
Ei-value:0.000, Pi-value:0.000  
Er-value:0.000, Pr-value:0.010  
eCLIP MATCHES▶lin28b (bg=16.96%)▶ybx3 (bg=22.82%)No matches to TargetScan

C

AACATCTA

AACATCTA  
Depth:5 (SHEEP)  
Ei-value:0.000, Pi-value:0.000  
Er-value:0.000, Pr-value:0.000  
eCLIP MATCHES▶lin28b (bg=16.96%)▶ybx3 (bg=22.82%)No matches to TargetScan


AGCTTT

AACATCTAAGCTTT  
Depth:3 (DOG)  
Ei-value:0.000, Pi-value:0.000  
Er-value:0.000, Pr-value:0.000  
eCLIP MATCHES▶lin28b (bg=16.96%)▶ybx3 (bg=22.82%)MATCHES To TargetScan▶ miR-320:AAAGCUG▶ miR-21-5p/590-5p:AGCUUAU


a

aacatctaagcttta  
Depth:2 (BABOON)  
Ei-value:1.000, Pi-value:0.000  
Er-value:0.000, Pr-value:0.000  
eCLIP MATCHES▶ddx6 (bg=23.92%)▶lin28b (bg=16.96%)▶ybx3 (bg=22.82%)MATCHES To TargetScan▶ miR-320:AAAGCUG▶ miR-21-5p/590-5p:AGCUUAU

C

GAATGG

GAATGGGGTGACAACTTATGATAAAAACTAGAGCTAGTGAATTAGCC  
Depth:2 (BABOON)  
Ei-value:0.000, Pi-value:0.000  
Er-value:0.000, Pr-value:0.000  
eCLIP MATCHES▶ddx6 (bg=23.92%)▶lin28b (bg=16.96%)▶ybx3 (bg=22.82%)MATCHES To TargetScan▶ miR-382-5p:AAGUUGU▶ miR-1251-5p:CUCUAGC▶ miR-154-3p/487-3p:AUCAUAC


ggtgacaa

ggtgacaa  
Depth:4 (COW)  
Ei-value:0.670, Pi-value:0.000  
Er-value:0.000, Pr-value:0.000  
eCLIP MATCHES▶ddx6 (bg=23.92%)▶lin28b (bg=16.96%)▶ybx3 (bg=22.82%)No matches to TargetScan


CT

GAATGGGGTGACAACTTATGATAAAAACTAGAGCTAGTGAATTAGCC  
Depth:2 (BABOON)  
Ei-value:0.000, Pi-value:0.000  
Er-value:0.000, Pr-value:0.000  
eCLIP MATCHES▶ddx6 (bg=23.92%)▶lin28b (bg=16.96%)▶ybx3 (bg=22.82%)MATCHES To TargetScan▶ miR-382-5p:AAGUUGU▶ miR-1251-5p:CUCUAGC▶ miR-154-3p/487-3p:AUCAUAC


tatgata

tatgata  
Depth:4 (COW)  
Ei-value:0.960, Pi-value:0.020  
Er-value:0.000, Pr-value:0.000  
eCLIP MATCHES▶ddx6 (bg=23.92%)▶lin28b (bg=16.96%)▶ybx3 (bg=22.82%)MATCHES To TargetScan▶ miR-154-3p/487-3p:AUCAUAC


AAA

TATGATAAAA  
Depth:3 (DOG)  
Ei-value:0.000, Pi-value:0.000  
Er-value:0.000, Pr-value:0.000  
eCLIP MATCHES▶ddx6 (bg=23.92%)▶lin28b (bg=16.96%)▶ybx3 (bg=22.82%)MATCHES To TargetScan▶ miR-154-3p/487-3p:AUCAUAC


ACTAG

GAATGGGGTGACAACTTATGATAAAAACTAGAGCTAGTGAATTAGCC  
Depth:2 (BABOON)  
Ei-value:0.000, Pi-value:0.000  
Er-value:0.000, Pr-value:0.000  
eCLIP MATCHES▶ddx6 (bg=23.92%)▶lin28b (bg=16.96%)▶ybx3 (bg=22.82%)MATCHES To TargetScan▶ miR-382-5p:AAGUUGU▶ miR-1251-5p:CUCUAGC▶ miR-154-3p/487-3p:AUCAUAC


AGCTA

AGCTAGTGAATTA  
Depth:4 (COW)  
Ei-value:0.000, Pi-value:0.000  
Er-value:0.000, Pr-value:0.000  
eCLIP MATCHES▶lin28b (bg=16.96%)▶ybx3 (bg=22.82%)No matches to TargetScan


GTGAATT

GTGAATT  
Depth:5 (SHEEP)  
Ei-value:0.000, Pi-value:0.010  
Er-value:0.000, Pr-value:0.000  
eCLIP MATCHES▶lin28b (bg=16.96%)▶ybx3 (bg=22.82%)No matches to TargetScan


A

AGCTAGTGAATTA  
Depth:4 (COW)  
Ei-value:0.000, Pi-value:0.000  
Er-value:0.000, Pr-value:0.000  
eCLIP MATCHES▶lin28b (bg=16.96%)▶ybx3 (bg=22.82%)No matches to TargetScan


GCC

GAATGGGGTGACAACTTATGATAAAAACTAGAGCTAGTGAATTAGCC  
Depth:2 (BABOON)  
Ei-value:0.000, Pi-value:0.000  
Er-value:0.000, Pr-value:0.000  
eCLIP MATCHES▶ddx6 (bg=23.92%)▶lin28b (bg=16.96%)▶ybx3 (bg=22.82%)MATCHES To TargetScan▶ miR-382-5p:AAGUUGU▶ miR-1251-5p:CUCUAGC▶ miR-154-3p/487-3p:AUCAUAC

T

ATTTGTAAATAC

ATTTGTAAATACCTTTGTTATAATTGATAG  
Depth:2 (BABOON)  
Ei-value:0.000, Pi-value:0.000  
Er-value:0.000, Pr-value:0.000  
eCLIP MATCHES▶dgcr8 (bg=19.31%)▶fubp3 (bg=23.31%)▶lin28b (bg=16.96%)▶NKRF (bg=3.64%)▶ybx3 (bg=22.82%)MATCHES To TargetScan▶ miR-495-3p:AACAAAC


c

ctttgtta  
Depth:4 (COW)  
Ei-value:0.670, Pi-value:0.010  
Er-value:0.000, Pr-value:0.000  
eCLIP MATCHES▶fubp3 (bg=23.31%)▶lin28b (bg=16.96%)▶ybx3 (bg=22.82%)MATCHES To TargetScan▶ miR-495-3p:AACAAAC


TTTGTTA

TTTGTTA  
Depth:5 (SHEEP)  
Ei-value:0.000, Pi-value:0.010  
Er-value:0.000, Pr-value:0.000  
eCLIP MATCHES▶fubp3 (bg=23.31%)▶lin28b (bg=16.96%)▶ybx3 (bg=22.82%)MATCHES To TargetScan▶ miR-495-3p:AACAAAC


T

CTTTGTTAT  
Depth:3 (DOG)  
Ei-value:0.000, Pi-value:0.000  
Er-value:0.000, Pr-value:0.000  
eCLIP MATCHES▶fubp3 (bg=23.31%)▶lin28b (bg=16.96%)▶NKRF (bg=3.64%)▶ybx3 (bg=22.82%)MATCHES To TargetScan▶ miR-495-3p:AACAAAC


A

ATTTGTAAATACCTTTGTTATAATTGATAG  
Depth:2 (BABOON)  
Ei-value:0.000, Pi-value:0.000  
Er-value:0.000, Pr-value:0.000  
eCLIP MATCHES▶dgcr8 (bg=19.31%)▶fubp3 (bg=23.31%)▶lin28b (bg=16.96%)▶NKRF (bg=3.64%)▶ybx3 (bg=22.82%)MATCHES To TargetScan▶ miR-495-3p:AACAAAC


attgat

attgat  
Depth:4 (COW)  
Ei-value:1.000, Pi-value:0.040  
Er-value:0.000, Pr-value:0.000  
eCLIP MATCHES▶dgcr8 (bg=19.31%)▶fubp3 (bg=23.31%)▶lin28b (bg=16.96%)▶NKRF (bg=3.64%)▶ybx3 (bg=22.82%)No matches to TargetScan


AG

ATTGATAG  
Depth:3 (DOG)  
Ei-value:0.000, Pi-value:0.000  
Er-value:0.000, Pr-value:0.000  
eCLIP MATCHES▶dgcr8 (bg=19.31%)▶fubp3 (bg=23.31%)▶lin28b (bg=16.96%)▶NKRF (bg=3.64%)▶ybx3 (bg=22.82%)No matches to TargetScan

GATA

C

CATCTTGGAC  
Depth:4 (COW)  
Ei-value:0.020, Pi-value:0.000  
Er-value:0.000, Pr-value:0.000  
eCLIP MATCHES▶ddx6 (bg=23.92%)▶dgcr8 (bg=19.31%)▶fubp3 (bg=23.31%)▶lin28b (bg=16.96%)▶NKRF (bg=3.64%)▶ybx3 (bg=22.82%)No matches to TargetScan

 4680  


ATCTTGGAC

CATCTTGGAC  
Depth:4 (COW)  
Ei-value:0.020, Pi-value:0.000  
Er-value:0.000, Pr-value:0.000  
eCLIP MATCHES▶ddx6 (bg=23.92%)▶dgcr8 (bg=19.31%)▶fubp3 (bg=23.31%)▶lin28b (bg=16.96%)▶NKRF (bg=3.64%)▶ybx3 (bg=22.82%)No matches to TargetScan


ATG

CATCTTGGACATG  
Depth:3 (DOG)  
Ei-value:0.000, Pi-value:0.000  
Er-value:0.000, Pr-value:0.000  
eCLIP MATCHES▶ddx6 (bg=23.92%)▶dgcr8 (bg=19.31%)▶fubp3 (bg=23.31%)▶lin28b (bg=16.96%)▶NKRF (bg=3.64%)▶ybx3 (bg=22.82%)No matches to TargetScan


GA

CATCTTGGACATGGAATTGTTAAGCCACCTCTGAGCAGTGTATGTCAGGACTT  
Depth:2 (BABOON)  
Ei-value:0.000, Pi-value:0.000  
Er-value:0.000, Pr-value:0.000  
eCLIP MATCHES▶ddx6 (bg=23.92%)▶dgcr8 (bg=19.31%)▶fubp3 (bg=23.31%)▶lin28b (bg=16.96%)▶NKRF (bg=3.64%)▶ybx3 (bg=22.82%)MATCHES To TargetScan▶ miR-489-3p:UGACAUC


ATTGTTAAGC

ATTGTTAAGC  
Depth:3 (DOG)  
Ei-value:0.000, Pi-value:0.000  
Er-value:0.000, Pr-value:0.000  
eCLIP MATCHES▶ddx6 (bg=23.92%)▶fubp3 (bg=23.31%)▶lin28b (bg=16.96%)▶NKRF (bg=3.64%)▶ybx3 (bg=22.82%)No matches to TargetScan


CAC

CATCTTGGACATGGAATTGTTAAGCCACCTCTGAGCAGTGTATGTCAGGACTT  
Depth:2 (BABOON)  
Ei-value:0.000, Pi-value:0.000  
Er-value:0.000, Pr-value:0.000  
eCLIP MATCHES▶ddx6 (bg=23.92%)▶dgcr8 (bg=19.31%)▶fubp3 (bg=23.31%)▶lin28b (bg=16.96%)▶NKRF (bg=3.64%)▶ybx3 (bg=22.82%)MATCHES To TargetScan▶ miR-489-3p:UGACAUC


ctctgag

ctctgag  
Depth:4 (COW)  
Ei-value:0.960, Pi-value:0.000  
Er-value:0.000, Pr-value:0.000  
eCLIP MATCHES▶ddx6 (bg=23.92%)▶lin28b (bg=16.96%)▶ybx3 (bg=22.82%)No matches to TargetScan


CAGTGT

CATCTTGGACATGGAATTGTTAAGCCACCTCTGAGCAGTGTATGTCAGGACTT  
Depth:2 (BABOON)  
Ei-value:0.000, Pi-value:0.000  
Er-value:0.000, Pr-value:0.000  
eCLIP MATCHES▶ddx6 (bg=23.92%)▶dgcr8 (bg=19.31%)▶fubp3 (bg=23.31%)▶lin28b (bg=16.96%)▶NKRF (bg=3.64%)▶ybx3 (bg=22.82%)MATCHES To TargetScan▶ miR-489-3p:UGACAUC


ATGTCAG

ATGTCAG  
Depth:3 (DOG)  
Ei-value:0.000, Pi-value:0.020  
Er-value:0.000, Pr-value:0.000  
eCLIP MATCHES▶ddx6 (bg=23.92%)▶lin28b (bg=16.96%)▶ybx3 (bg=22.82%)MATCHES To TargetScan▶ miR-489-3p:UGACAUC


GACTT

CATCTTGGACATGGAATTGTTAAGCCACCTCTGAGCAGTGTATGTCAGGACTT  
Depth:2 (BABOON)  
Ei-value:0.000, Pi-value:0.000  
Er-value:0.000, Pr-value:0.000  
eCLIP MATCHES▶ddx6 (bg=23.92%)▶dgcr8 (bg=19.31%)▶fubp3 (bg=23.31%)▶lin28b (bg=16.96%)▶NKRF (bg=3.64%)▶ybx3 (bg=22.82%)MATCHES To TargetScan▶ miR-489-3p:UGACAUC


gttcat

gttcattaggttggcagcagag  
Depth:2 (BABOON)  
Ei-value:0.260, Pi-value:0.000  
Er-value:0.000, Pr-value:0.000  
eCLIP MATCHES▶lin28b (bg=16.96%)▶ybx3 (bg=22.82%)No matches to TargetScan


TAGGTTGGCAGC

TAGGTTGGCAGC  
Depth:4 (COW)  
Ei-value:0.000, Pi-value:0.000  
Er-value:0.000, Pr-value:0.000  
eCLIP MATCHES▶lin28b (bg=16.96%)No matches to TargetScan


AGAG

TAGGTTGGCAGCAGAG  
Depth:3 (DOG)  
Ei-value:0.000, Pi-value:0.000  
Er-value:0.000, Pr-value:0.000  
eCLIP MATCHES▶lin28b (bg=16.96%)No matches to TargetScan

G

GGCA

GGCAGAAGGAA  
Depth:3 (DOG)  
Ei-value:0.000, Pi-value:0.000  
Er-value:0.000, Pr-value:0.000  
eCLIP MATCHES▶ddx6 (bg=23.92%)▶lin28b (bg=16.96%)No matches to TargetScan


gaaggaa

gaaggaa  
Depth:4 (COW)  
Ei-value:0.960, Pi-value:0.010  
Er-value:0.000, Pr-value:0.000  
eCLIP MATCHES▶ddx6 (bg=23.92%)▶lin28b (bg=16.96%)No matches to TargetScan

T

tatacagg

tatacagg  
Depth:2 (BABOON)  
Ei-value:1.000, Pi-value:0.000  
Er-value:0.000, Pr-value:0.000  
eCLIP MATCHES▶ddx6 (bg=23.92%)▶lin28b (bg=16.96%)MATCHES To TargetScan▶ miR-486-5p:CCUGUAC

TAGAGA

TGTATGC

TGTATGC  
Depth:3 (DOG)  
Ei-value:0.000, Pi-value:0.010  
Er-value:0.000, Pr-value:0.000  
eCLIP MATCHES▶ddx6 (bg=23.92%)▶lin28b (bg=16.96%)No matches to TargetScan


agatgtgtc

tgtatgcagatgtgtc  
Depth:2 (BABOON)  
Ei-value:1.000, Pi-value:0.000  
Er-value:0.000, Pr-value:0.000  
eCLIP MATCHES▶ddx6 (bg=23.92%)▶lin28b (bg=16.96%)MATCHES To TargetScan▶ miR-448:UGCAUAU▶ miR-153-3p:UGCAUAG

CAT 4800  
 A

TATGTC

TATGTCCATATTTACATTTTGATAGCCATTGATGTATGCATCTCTT  
Depth:2 (BABOON)  
Ei-value:0.000, Pi-value:0.000  
Er-value:0.000, Pr-value:0.000  
eCLIP MATCHES▶lin28b (bg=16.96%)▶tia1 (bg=16.04%)▶ybx3 (bg=22.82%)MATCHES To TargetScan▶ miR-135-5p:AUGGCUU▶ miR-448:UGCAUAU▶ miR-153-3p:UGCAUAG▶ miR-143-3p:GAGAUGA▶ miR-411-3p:AUGUAAC


C

CATATTTACATTTT  
Depth:3 (DOG)  
Ei-value:0.000, Pi-value:0.000  
Er-value:0.000, Pr-value:0.000  
eCLIP MATCHES▶lin28b (bg=16.96%)▶ybx3 (bg=22.82%)MATCHES To TargetScan▶ miR-411-3p:AUGUAAC


atattt

atattt  
Depth:4 (COW)  
Ei-value:1.000, Pi-value:0.080  
Er-value:0.000, Pr-value:0.010  
eCLIP MATCHES▶lin28b (bg=16.96%)No matches to TargetScan


ACATTTT

CATATTTACATTTT  
Depth:3 (DOG)  
Ei-value:0.000, Pi-value:0.000  
Er-value:0.000, Pr-value:0.000  
eCLIP MATCHES▶lin28b (bg=16.96%)▶ybx3 (bg=22.82%)MATCHES To TargetScan▶ miR-411-3p:AUGUAAC


GATAGCCATTGATGTATGCA

TATGTCCATATTTACATTTTGATAGCCATTGATGTATGCATCTCTT  
Depth:2 (BABOON)  
Ei-value:0.000, Pi-value:0.000  
Er-value:0.000, Pr-value:0.000  
eCLIP MATCHES▶lin28b (bg=16.96%)▶tia1 (bg=16.04%)▶ybx3 (bg=22.82%)MATCHES To TargetScan▶ miR-135-5p:AUGGCUU▶ miR-448:UGCAUAU▶ miR-153-3p:UGCAUAG▶ miR-143-3p:GAGAUGA▶ miR-411-3p:AUGUAAC


tctctt

tctctt  
Depth:3 (DOG)  
Ei-value:0.080, Pi-value:0.040  
Er-value:0.000, Pr-value:0.000  
eCLIP MATCHES▶tia1 (bg=16.04%)▶ybx3 (bg=22.82%)No matches to TargetScan

G

GCTGTACTATA

GCTGTACTATA  
Depth:4 (COW)  
Ei-value:0.000, Pi-value:0.000  
Er-value:0.000, Pr-value:0.000  
eCLIP MATCHES▶tia1 (bg=16.04%)▶ybx3 (bg=22.82%)No matches to TargetScan

AGAACACAT

ta

taattcaatggaaata  
Depth:2 (BABOON)  
Ei-value:1.000, Pi-value:0.000  
Er-value:0.000, Pr-value:0.000  
eCLIP MATCHES▶HNRNPL (bg=3.4%)No matches to TargetScan


ATTCAATGGAAA

ATTCAATGGAAA  
Depth:3 (DOG)  
Ei-value:0.000, Pi-value:0.000  
Er-value:0.000, Pr-value:0.000  
eCLIP MATCHES▶HNRNPL (bg=3.4%)No matches to TargetScan


ta

taattcaatggaaata  
Depth:2 (BABOON)  
Ei-value:1.000, Pi-value:0.000  
Er-value:0.000, Pr-value:0.000  
eCLIP MATCHES▶HNRNPL (bg=3.4%)No matches to TargetScan

C

A

ACTTTGCTAATATTTTAATGGTATAGATCTGCTAATGAATTCTCTTAAAAAC  
Depth:2 (BABOON)  
Ei-value:0.000, Pi-value:0.000  
Er-value:0.000, Pr-value:0.000  
eCLIP MATCHES▶dgcr8 (bg=19.31%)▶HNRNPL (bg=3.4%)▶igf2bp1 (bg=11.67%)No matches to TargetScan


CT

CTTTGCTAAT  
Depth:3 (DOG)  
Ei-value:0.000, Pi-value:0.000  
Er-value:0.000, Pr-value:0.000  
eCLIP MATCHES▶HNRNPL (bg=3.4%)No matches to TargetScan


ttgctaa

ttgctaa  
Depth:4 (COW)  
Ei-value:0.960, Pi-value:0.000  
Er-value:0.000, Pr-value:0.000  
eCLIP MATCHES▶HNRNPL (bg=3.4%)No matches to TargetScan


T

CTTTGCTAAT  
Depth:3 (DOG)  
Ei-value:0.000, Pi-value:0.000  
Er-value:0.000, Pr-value:0.000  
eCLIP MATCHES▶HNRNPL (bg=3.4%)No matches to TargetScan


ATTTTAATGG

ACTTTGCTAATATTTTAATGGTATAGATCTGCTAATGAATTCTCTTAAAAAC  
Depth:2 (BABOON)  
Ei-value:0.000, Pi-value:0.000  
Er-value:0.000, Pr-value:0.000  
eCLIP MATCHES▶dgcr8 (bg=19.31%)▶HNRNPL (bg=3.4%)▶igf2bp1 (bg=11.67%)No matches to TargetScan


TATAGAT

TATAGAT  
Depth:3 (DOG)  
Ei-value:0.000, Pi-value:0.010  
Er-value:0.000, Pr-value:0.000  
eCLIP MATCHES▶dgcr8 (bg=19.31%)▶HNRNPL (bg=3.4%)▶igf2bp1 (bg=11.67%)No matches to TargetScan


CTGCTA

ACTTTGCTAATATTTTAATGGTATAGATCTGCTAATGAATTCTCTTAAAAAC  
Depth:2 (BABOON)  
Ei-value:0.000, Pi-value:0.000  
Er-value:0.000, Pr-value:0.000  
eCLIP MATCHES▶dgcr8 (bg=19.31%)▶HNRNPL (bg=3.4%)▶igf2bp1 (bg=11.67%)No matches to TargetScan


A

ATGAATTCT  
Depth:3 (DOG)  
Ei-value:0.000, Pi-value:0.000  
Er-value:0.000, Pr-value:0.000  
eCLIP MATCHES▶dgcr8 (bg=19.31%)▶HNRNPL (bg=3.4%)▶igf2bp1 (bg=11.67%)No matches to TargetScan

 4920  


TGAATTCT

ATGAATTCT  
Depth:3 (DOG)  
Ei-value:0.000, Pi-value:0.000  
Er-value:0.000, Pr-value:0.000  
eCLIP MATCHES▶dgcr8 (bg=19.31%)▶HNRNPL (bg=3.4%)▶igf2bp1 (bg=11.67%)No matches to TargetScan


CTTAAAAAC

ACTTTGCTAATATTTTAATGGTATAGATCTGCTAATGAATTCTCTTAAAAAC  
Depth:2 (BABOON)  
Ei-value:0.000, Pi-value:0.000  
Er-value:0.000, Pr-value:0.000  
eCLIP MATCHES▶dgcr8 (bg=19.31%)▶HNRNPL (bg=3.4%)▶igf2bp1 (bg=11.67%)No matches to TargetScan

ATACTG

TAT

TATTCTGTTGCTGTGTGTTTCATTTTAAATTGAGCATTAAGGGAATGCAGCATTTAAATC  
Depth:2 (BABOON)  
Ei-value:0.000, Pi-value:0.000  
Er-value:0.000, Pr-value:0.000  
eCLIP MATCHES▶dgcr8 (bg=19.31%)▶HNRNPL (bg=3.4%)▶igf2bp1 (bg=11.67%)▶SF3B1 (bg=6.74%)MATCHES To TargetScan▶ miR-494-3p:GAAACAU▶ miR-204-5p/211-5p:UCCCUUU▶ miR-329-3p/362-3p:ACACACC▶ miR-653-5p:UGAAACA▶ miR-33-5p:UGCAUUG▶ miR-155-5p:UAAUGCU


tctgtt

tctgtt  
Depth:4 (COW)  
Ei-value:1.000, Pi-value:0.000  
Er-value:0.000, Pr-value:0.000  
eCLIP MATCHES▶dgcr8 (bg=19.31%)▶HNRNPL (bg=3.4%)▶igf2bp1 (bg=11.67%)No matches to TargetScan


GCTGTGTGTTTCATTTTA

TATTCTGTTGCTGTGTGTTTCATTTTAAATTGAGCATTAAGGGAATGCAGCATTTAAATC  
Depth:2 (BABOON)  
Ei-value:0.000, Pi-value:0.000  
Er-value:0.000, Pr-value:0.000  
eCLIP MATCHES▶dgcr8 (bg=19.31%)▶HNRNPL (bg=3.4%)▶igf2bp1 (bg=11.67%)▶SF3B1 (bg=6.74%)MATCHES To TargetScan▶ miR-494-3p:GAAACAU▶ miR-204-5p/211-5p:UCCCUUU▶ miR-329-3p/362-3p:ACACACC▶ miR-653-5p:UGAAACA▶ miR-33-5p:UGCAUUG▶ miR-155-5p:UAAUGCU


aattga

aattga  
Depth:4 (COW)  
Ei-value:1.000, Pi-value:0.010  
Er-value:0.000, Pr-value:0.010  
eCLIP MATCHES▶SF3B1 (bg=6.74%)No matches to TargetScan


G

TATTCTGTTGCTGTGTGTTTCATTTTAAATTGAGCATTAAGGGAATGCAGCATTTAAATC  
Depth:2 (BABOON)  
Ei-value:0.000, Pi-value:0.000  
Er-value:0.000, Pr-value:0.000  
eCLIP MATCHES▶dgcr8 (bg=19.31%)▶HNRNPL (bg=3.4%)▶igf2bp1 (bg=11.67%)▶SF3B1 (bg=6.74%)MATCHES To TargetScan▶ miR-494-3p:GAAACAU▶ miR-204-5p/211-5p:UCCCUUU▶ miR-329-3p/362-3p:ACACACC▶ miR-653-5p:UGAAACA▶ miR-33-5p:UGCAUUG▶ miR-155-5p:UAAUGCU


CATTAAG

CATTAAG  
Depth:5 (SHEEP)  
Ei-value:0.000, Pi-value:0.010  
Er-value:0.000, Pr-value:0.000  
eCLIP MATCHES▶SF3B1 (bg=6.74%)No matches to TargetScan


GGAATG

CATTAAGGGAATG  
Depth:4 (COW)  
Ei-value:0.000, Pi-value:0.000  
Er-value:0.000, Pr-value:0.000  
eCLIP MATCHES▶SF3B1 (bg=6.74%)MATCHES To TargetScan▶ miR-204-5p/211-5p:UCCCUUU


CAG

CATTAAGGGAATGCAG  
Depth:3 (DOG)  
Ei-value:0.000, Pi-value:0.000  
Er-value:0.000, Pr-value:0.000  
eCLIP MATCHES▶SF3B1 (bg=6.74%)MATCHES To TargetScan▶ miR-204-5p/211-5p:UCCCUUU▶ miR-33-5p:UGCAUUG


CATTTAAATC

TATTCTGTTGCTGTGTGTTTCATTTTAAATTGAGCATTAAGGGAATGCAGCATTTAAATC  
Depth:2 (BABOON)  
Ei-value:0.000, Pi-value:0.000  
Er-value:0.000, Pr-value:0.000  
eCLIP MATCHES▶dgcr8 (bg=19.31%)▶HNRNPL (bg=3.4%)▶igf2bp1 (bg=11.67%)▶SF3B1 (bg=6.74%)MATCHES To TargetScan▶ miR-494-3p:GAAACAU▶ miR-204-5p/211-5p:UCCCUUU▶ miR-329-3p/362-3p:ACACACC▶ miR-653-5p:UGAAACA▶ miR-33-5p:UGCAUUG▶ miR-155-5p:UAAUGCU

A

G

GAACTCTGCCAATGCTTTTATCTAGAGGCGTGTTGCCATTTTTGTCTT  
Depth:2 (BABOON)  
Ei-value:0.000, Pi-value:0.000  
Er-value:0.000, Pr-value:0.000  
eCLIP MATCHES▶EIF3H (bg=7.83%)▶HNRNPC (bg=1.49%)▶PABPC4 (bg=2.72%)▶tia1 (bg=16.04%)MATCHES To TargetScan▶ miR-1251-5p:CUCUAGC▶ miR-182-5p:UUGGCAA▶ miR-330-3p.2:AAAGCAC▶ miR-183-5p.1:AUGGCAC▶ miR-96-5p/1271-5p:UUGGCAC


AACTCTG

AACTCTG  
Depth:3 (DOG)  
Ei-value:0.000, Pi-value:0.010  
Er-value:0.000, Pr-value:0.000  
No matches to eCLIP DataNo matches to TargetScan


CCAATGCT

GAACTCTGCCAATGCTTTTATCTAGAGGCGTGTTGCCATTTTTGTCTT  
Depth:2 (BABOON)  
Ei-value:0.000, Pi-value:0.000  
Er-value:0.000, Pr-value:0.000  
eCLIP MATCHES▶EIF3H (bg=7.83%)▶HNRNPC (bg=1.49%)▶PABPC4 (bg=2.72%)▶tia1 (bg=16.04%)MATCHES To TargetScan▶ miR-1251-5p:CUCUAGC▶ miR-182-5p:UUGGCAA▶ miR-330-3p.2:AAAGCAC▶ miR-183-5p.1:AUGGCAC▶ miR-96-5p/1271-5p:UUGGCAC


tttatc

tttatc  
Depth:3 (DOG)  
Ei-value:0.080, Pi-value:0.050  
Er-value:0.000, Pr-value:0.000  
eCLIP MATCHES▶EIF3H (bg=7.83%)▶HNRNPC (bg=1.49%)No matches to TargetScan


TAGAGGCG

GAACTCTGCCAATGCTTTTATCTAGAGGCGTGTTGCCATTTTTGTCTT  
Depth:2 (BABOON)  
Ei-value:0.000, Pi-value:0.000  
Er-value:0.000, Pr-value:0.000  
eCLIP MATCHES▶EIF3H (bg=7.83%)▶HNRNPC (bg=1.49%)▶PABPC4 (bg=2.72%)▶tia1 (bg=16.04%)MATCHES To TargetScan▶ miR-1251-5p:CUCUAGC▶ miR-182-5p:UUGGCAA▶ miR-330-3p.2:AAAGCAC▶ miR-183-5p.1:AUGGCAC▶ miR-96-5p/1271-5p:UUGGCAC


TGTTGC

TGTTGC  
Depth:6 (PIG)  
Ei-value:0.000, Pi-value:0.010  
Er-value:0.000, Pr-value:0.000  
eCLIP MATCHES▶EIF3H (bg=7.83%)▶HNRNPC (bg=1.49%)No matches to TargetScan

 5040  


TGTTGC  
Depth:6 (PIG)  
Ei-value:0.000, Pi-value:0.010  
Er-value:0.000, Pr-value:0.000  
eCLIP MATCHES▶EIF3H (bg=7.83%)▶HNRNPC (bg=1.49%)No matches to TargetScan


C

TGTTGCCATTTTTGTC  
Depth:5 (SHEEP)  
Ei-value:0.000, Pi-value:0.000  
Er-value:0.000, Pr-value:0.000  
eCLIP MATCHES▶EIF3H (bg=7.83%)▶HNRNPC (bg=1.49%)▶tia1 (bg=16.04%)MATCHES To TargetScan▶ miR-183-5p.1:AUGGCAC


ATTTTTGT

ATTTTTGT  
Depth:9 (MOUSE)  
Ei-value:0.000, Pi-value:0.000  
Er-value:0.000, Pr-value:0.000  
eCLIP MATCHES▶EIF3H (bg=7.83%)▶HNRNPC (bg=1.49%)No matches to TargetScan


C

ATTTTTGTC  
Depth:7 (ARMADILLO)  
Ei-value:0.000, Pi-value:0.000  
Er-value:0.000, Pr-value:0.000  
eCLIP MATCHES▶EIF3H (bg=7.83%)▶HNRNPC (bg=1.49%)▶tia1 (bg=16.04%)No matches to TargetScan


TT

GAACTCTGCCAATGCTTTTATCTAGAGGCGTGTTGCCATTTTTGTCTT  
Depth:2 (BABOON)  
Ei-value:0.000, Pi-value:0.000  
Er-value:0.000, Pr-value:0.000  
eCLIP MATCHES▶EIF3H (bg=7.83%)▶HNRNPC (bg=1.49%)▶PABPC4 (bg=2.72%)▶tia1 (bg=16.04%)MATCHES To TargetScan▶ miR-1251-5p:CUCUAGC▶ miR-182-5p:UUGGCAA▶ miR-330-3p.2:AAAGCAC▶ miR-183-5p.1:AUGGCAC▶ miR-96-5p/1271-5p:UUGGCAC

A

tatgaaattt

tatgaaattt  
Depth:2 (BABOON)  
Ei-value:1.000, Pi-value:0.000  
Er-value:0.000, Pr-value:0.000  
eCLIP MATCHES▶HNRNPC (bg=1.49%)▶PABPC4 (bg=2.72%)▶tia1 (bg=16.04%)No matches to TargetScan

C

tgtccc

tgtccc  
Depth:2 (BABOON)  
Ei-value:1.000, Pi-value:0.000  
Er-value:0.000, Pr-value:0.000  
eCLIP MATCHES▶HNRNPC (bg=1.49%)▶PABPC4 (bg=2.72%)▶tia1 (bg=16.04%)No matches to TargetScan

A

AGA

AGAAAGGCAGGATTACAT  
Depth:3 (DOG)  
Ei-value:0.000, Pi-value:0.000  
Er-value:0.000, Pr-value:0.000  
eCLIP MATCHES▶HNRNPC (bg=1.49%)▶PABPC4 (bg=2.72%)▶tia1 (bg=16.04%)MATCHES To TargetScan▶ miR-411-3p:AUGUAAC


AAGGCA

AAGGCA  
Depth:6 (PIG)  
Ei-value:0.000, Pi-value:0.010  
Er-value:0.000, Pr-value:0.000  
eCLIP MATCHES▶HNRNPC (bg=1.49%)▶tia1 (bg=16.04%)No matches to TargetScan


G

AGAAAGGCAGGATTACAT  
Depth:3 (DOG)  
Ei-value:0.000, Pi-value:0.000  
Er-value:0.000, Pr-value:0.000  
eCLIP MATCHES▶HNRNPC (bg=1.49%)▶PABPC4 (bg=2.72%)▶tia1 (bg=16.04%)MATCHES To TargetScan▶ miR-411-3p:AUGUAAC


GATTACAT

GATTACAT  
Depth:6 (PIG)  
Ei-value:0.000, Pi-value:0.000  
Er-value:0.000, Pr-value:0.000  
eCLIP MATCHES▶HNRNPC (bg=1.49%)▶tia1 (bg=16.04%)MATCHES To TargetScan▶ miR-411-3p:AUGUAAC

C

TTTTTTTTTTTTTTT

TTTTTTTTTTTTTTT  
Depth:3 (DOG)  
Ei-value:0.000, Pi-value:0.000  
Er-value:0.000, Pr-value:0.000  
eCLIP MATCHES▶APOBEC3C (bg=3.95%)▶ddx55 (bg=12.35%)▶ddx6 (bg=23.92%)▶EIF3H (bg=7.83%)▶fubp3 (bg=23.31%)▶HNRNPC (bg=1.49%)▶tia1 (bg=16.04%)▶tial1 (bg=14.09%)▶ZC3H11A (bg=6.25%)No matches to TargetScan


AGCAGTTT

AGCAGTTTGAGTTGGTGTAGTGTATTCTTGGTTATCA  
Depth:2 (BABOON)  
Ei-value:0.000, Pi-value:0.000  
Er-value:0.000, Pr-value:0.000  
eCLIP MATCHES▶APOBEC3C (bg=3.95%)▶ddx55 (bg=12.35%)▶ddx6 (bg=23.92%)▶EIF3H (bg=7.83%)▶fam120a (bg=18.43%)▶fubp3 (bg=23.31%)▶FUS (bg=2.59%)▶tia1 (bg=16.04%)▶tial1 (bg=14.09%)▶ZC3H11A (bg=6.25%)MATCHES To TargetScan▶ miR-371-5p:CUCAAAC


GAGTTG

GAGTTG  
Depth:6 (PIG)  
Ei-value:0.000, Pi-value:0.020  
Er-value:0.000, Pr-value:0.000  
eCLIP MATCHES▶APOBEC3C (bg=3.95%)▶ddx55 (bg=12.35%)▶ddx6 (bg=23.92%)▶EIF3H (bg=7.83%)▶fam120a (bg=18.43%)▶fubp3 (bg=23.31%)▶FUS (bg=2.59%)▶tia1 (bg=16.04%)▶tial1 (bg=14.09%)▶ZC3H11A (bg=6.25%)No matches to TargetScan


GTGTA

AGCAGTTTGAGTTGGTGTAGTGTATTCTTGGTTATCA  
Depth:2 (BABOON)  
Ei-value:0.000, Pi-value:0.000  
Er-value:0.000, Pr-value:0.000  
eCLIP MATCHES▶APOBEC3C (bg=3.95%)▶ddx55 (bg=12.35%)▶ddx6 (bg=23.92%)▶EIF3H (bg=7.83%)▶fam120a (bg=18.43%)▶fubp3 (bg=23.31%)▶FUS (bg=2.59%)▶tia1 (bg=16.04%)▶tial1 (bg=14.09%)▶ZC3H11A (bg=6.25%)MATCHES To TargetScan▶ miR-371-5p:CUCAAAC


GTG

GTGTATTCTTGGTTATCA  
Depth:3 (DOG)  
Ei-value:0.000, Pi-value:0.000  
Er-value:0.000, Pr-value:0.000  
eCLIP MATCHES▶APOBEC3C (bg=3.95%)▶ddx55 (bg=12.35%)▶ddx6 (bg=23.92%)▶EIF3H (bg=7.83%)▶fam120a (bg=18.43%)▶fubp3 (bg=23.31%)▶FUS (bg=2.59%)▶tia1 (bg=16.04%)▶tial1 (bg=14.09%)▶ZC3H11A (bg=6.25%)No matches to TargetScan


TA

TATTCTTGGT  
Depth:7 (ARMADILLO)  
Ei-value:0.000, Pi-value:0.000  
Er-value:0.000, Pr-value:0.000  
eCLIP MATCHES▶APOBEC3C (bg=3.95%)▶ddx55 (bg=12.35%)▶ddx6 (bg=23.92%)▶EIF3H (bg=7.83%)▶fam120a (bg=18.43%)▶fubp3 (bg=23.31%)▶FUS (bg=2.59%)▶tia1 (bg=16.04%)▶tial1 (bg=14.09%)▶ZC3H11A (bg=6.25%)No matches to TargetScan


TTCTTGGT

TTCTTGGT  
Depth:9 (MOUSE)  
Ei-value:0.000, Pi-value:0.000  
Er-value:0.000, Pr-value:0.000  
eCLIP MATCHES▶APOBEC3C (bg=3.95%)▶ddx55 (bg=12.35%)▶ddx6 (bg=23.92%)▶EIF3H (bg=7.83%)▶fam120a (bg=18.43%)▶fubp3 (bg=23.31%)▶FUS (bg=2.59%)▶tia1 (bg=16.04%)▶tial1 (bg=14.09%)▶ZC3H11A (bg=6.25%)No matches to TargetScan


TATCA

TATTCTTGGTTATCA  
Depth:6 (PIG)  
Ei-value:0.000, Pi-value:0.000  
Er-value:0.000, Pr-value:0.000  
eCLIP MATCHES▶APOBEC3C (bg=3.95%)▶ddx55 (bg=12.35%)▶ddx6 (bg=23.92%)▶EIF3H (bg=7.83%)▶fam120a (bg=18.43%)▶fubp3 (bg=23.31%)▶FUS (bg=2.59%)▶tia1 (bg=16.04%)▶tial1 (bg=14.09%)▶ZC3H11A (bg=6.25%)No matches to TargetScan

G

AATACTCATA

AATACTCATA  
Depth:6 (PIG)  
Ei-value:0.000, Pi-value:0.000  
Er-value:0.000, Pr-value:0.000  
eCLIP MATCHES▶APOBEC3C (bg=3.95%)▶ddx55 (bg=12.35%)▶ddx6 (bg=23.92%)▶EIF3H (bg=7.83%)▶fam120a (bg=18.43%)▶fubp3 (bg=23.31%)▶FUS (bg=2.59%)▶tia1 (bg=16.04%)▶tial1 (bg=14.09%)▶ZC3H11A (bg=6.25%)MATCHES To TargetScan▶ miR-496.1:GAGUAUU


TAGC

AATACTCATATAGCTTTGGGATTTTGAATTGGTAAATATTCATGATGTGTGAAAAATCATGATACATACTGTACA  
Depth:2 (BABOON)  
Ei-value:0.000, Pi-value:0.000  
Er-value:0.000, Pr-value:0.000  
eCLIP MATCHES▶APOBEC3C (bg=3.95%)▶ddx55 (bg=12.35%)▶ddx6 (bg=23.92%)▶EIF3H (bg=7.83%)▶fam120a (bg=18.43%)▶fubp3 (bg=23.31%)▶FUS (bg=2.59%)▶PABPC4 (bg=2.72%)▶tia1 (bg=16.04%)▶tial1 (bg=14.09%)▶ZC3H11A (bg=6.25%)MATCHES To TargetScan▶ miR-433-3p:UCAUGAU▶ miR-493-5p:UGUACAU▶ miR-330-3p:CAAAGCA▶ miR-496.1:GAGUAUU▶ miR-320:AAAGCUG▶ miR-377-3p:UCACACA▶ miR-144-3p:ACAGUAU▶ miR-101-3p.1:ACAGUAC▶ miR-582-5p:UACAGUU▶ miR-101-3p.2:UACAGUA


TTT

TTTGGG  
Depth:7 (ARMADILLO)  
Ei-value:0.000, Pi-value:0.010  
Er-value:0.000, Pr-value:0.000  
eCLIP MATCHES▶APOBEC3C (bg=3.95%)▶ddx55 (bg=12.35%)▶ddx6 (bg=23.92%)▶fam120a (bg=18.43%)▶fubp3 (bg=23.31%)▶tia1 (bg=16.04%)▶ZC3H11A (bg=6.25%)No matches to TargetScan

 5160  


GGG

TTTGGG  
Depth:7 (ARMADILLO)  
Ei-value:0.000, Pi-value:0.010  
Er-value:0.000, Pr-value:0.000  
eCLIP MATCHES▶APOBEC3C (bg=3.95%)▶ddx55 (bg=12.35%)▶ddx6 (bg=23.92%)▶fam120a (bg=18.43%)▶fubp3 (bg=23.31%)▶tia1 (bg=16.04%)▶ZC3H11A (bg=6.25%)No matches to TargetScan


ATTTTGAATTGG

AATACTCATATAGCTTTGGGATTTTGAATTGGTAAATATTCATGATGTGTGAAAAATCATGATACATACTGTACA  
Depth:2 (BABOON)  
Ei-value:0.000, Pi-value:0.000  
Er-value:0.000, Pr-value:0.000  
eCLIP MATCHES▶APOBEC3C (bg=3.95%)▶ddx55 (bg=12.35%)▶ddx6 (bg=23.92%)▶EIF3H (bg=7.83%)▶fam120a (bg=18.43%)▶fubp3 (bg=23.31%)▶FUS (bg=2.59%)▶PABPC4 (bg=2.72%)▶tia1 (bg=16.04%)▶tial1 (bg=14.09%)▶ZC3H11A (bg=6.25%)MATCHES To TargetScan▶ miR-433-3p:UCAUGAU▶ miR-493-5p:UGUACAU▶ miR-330-3p:CAAAGCA▶ miR-496.1:GAGUAUU▶ miR-320:AAAGCUG▶ miR-377-3p:UCACACA▶ miR-144-3p:ACAGUAU▶ miR-101-3p.1:ACAGUAC▶ miR-582-5p:UACAGUU▶ miR-101-3p.2:UACAGUA


TA

TAAATATTCATG  
Depth:7 (ARMADILLO)  
Ei-value:0.000, Pi-value:0.000  
Er-value:0.000, Pr-value:0.000  
No matches to eCLIP DataNo matches to TargetScan


AATATTCA

AATATTCA  
Depth:9 (MOUSE)  
Ei-value:0.000, Pi-value:0.000  
Er-value:0.000, Pr-value:0.000  
No matches to eCLIP DataNo matches to TargetScan


TG

AATATTCATG  
Depth:8 (GUINEAPIG)  
Ei-value:0.000, Pi-value:0.000  
Er-value:0.000, Pr-value:0.000  
No matches to eCLIP DataNo matches to TargetScan


ATGTGTGAAAAAT

AATACTCATATAGCTTTGGGATTTTGAATTGGTAAATATTCATGATGTGTGAAAAATCATGATACATACTGTACA  
Depth:2 (BABOON)  
Ei-value:0.000, Pi-value:0.000  
Er-value:0.000, Pr-value:0.000  
eCLIP MATCHES▶APOBEC3C (bg=3.95%)▶ddx55 (bg=12.35%)▶ddx6 (bg=23.92%)▶EIF3H (bg=7.83%)▶fam120a (bg=18.43%)▶fubp3 (bg=23.31%)▶FUS (bg=2.59%)▶PABPC4 (bg=2.72%)▶tia1 (bg=16.04%)▶tial1 (bg=14.09%)▶ZC3H11A (bg=6.25%)MATCHES To TargetScan▶ miR-433-3p:UCAUGAU▶ miR-493-5p:UGUACAU▶ miR-330-3p:CAAAGCA▶ miR-496.1:GAGUAUU▶ miR-320:AAAGCUG▶ miR-377-3p:UCACACA▶ miR-144-3p:ACAGUAU▶ miR-101-3p.1:ACAGUAC▶ miR-582-5p:UACAGUU▶ miR-101-3p.2:UACAGUA


CATGATACAT

CATGATACAT  
Depth:7 (ARMADILLO)  
Ei-value:0.000, Pi-value:0.000  
Er-value:0.000, Pr-value:0.000  
eCLIP MATCHES▶PABPC4 (bg=2.72%)No matches to TargetScan


A

CATGATACATA  
Depth:6 (PIG)  
Ei-value:0.000, Pi-value:0.000  
Er-value:0.000, Pr-value:0.000  
eCLIP MATCHES▶PABPC4 (bg=2.72%)No matches to TargetScan


CTGTACA

CTGTACA  
Depth:3 (DOG)  
Ei-value:0.000, Pi-value:0.000  
Er-value:0.000, Pr-value:0.000  
eCLIP MATCHES▶PABPC4 (bg=2.72%)MATCHES To TargetScan▶ miR-493-5p:UGUACAU

G

TCTCAGTCCCA

TCTCAGTCCCATAAAATTGGATGTT  
Depth:2 (BABOON)  
Ei-value:0.000, Pi-value:0.000  
Er-value:0.000, Pr-value:0.000  
eCLIP MATCHES▶KHSRP (bg=8.1%)▶PABPC4 (bg=2.72%)No matches to TargetScan


taaaat

taaaat  
Depth:3 (DOG)  
Ei-value:0.080, Pi-value:0.060  
Er-value:0.000, Pr-value:0.000  
eCLIP MATCHES▶KHSRP (bg=8.1%)▶PABPC4 (bg=2.72%)No matches to TargetScan


TGGATGTT

TCTCAGTCCCATAAAATTGGATGTT  
Depth:2 (BABOON)  
Ei-value:0.000, Pi-value:0.000  
Er-value:0.000, Pr-value:0.000  
eCLIP MATCHES▶KHSRP (bg=8.1%)▶PABPC4 (bg=2.72%)No matches to TargetScan

G

tgcc

tgcctacacaca  
Depth:2 (BABOON)  
Ei-value:1.000, Pi-value:0.000  
Er-value:0.000, Pr-value:0.000  
eCLIP MATCHES▶KHSRP (bg=8.1%)No matches to TargetScan


TACACA

TACACA  
Depth:8 (GUINEAPIG)  
Ei-value:0.000, Pi-value:0.000  
Er-value:0.000, Pr-value:0.000  
eCLIP MATCHES▶KHSRP (bg=8.1%)No matches to TargetScan


CA

TACACACA  
Depth:3 (DOG)  
Ei-value:0.000, Pi-value:0.000  
Er-value:0.000, Pr-value:0.000  
eCLIP MATCHES▶KHSRP (bg=8.1%)No matches to TargetScan

G

gat

gatctagaagaa  
Depth:2 (BABOON)  
Ei-value:1.000, Pi-value:0.000  
Er-value:0.000, Pr-value:0.000  
eCLIP MATCHES▶KHSRP (bg=8.1%)▶TARDBP (bg=1.75%)No matches to TargetScan


CTAGAA

CTAGAA  
Depth:6 (PIG)  
Ei-value:0.000, Pi-value:0.000  
Er-value:0.000, Pr-value:0.000  
eCLIP MATCHES▶KHSRP (bg=8.1%)▶TARDBP (bg=1.75%)No matches to TargetScan


gaa

gatctagaagaa  
Depth:2 (BABOON)  
Ei-value:1.000, Pi-value:0.000  
Er-value:0.000, Pr-value:0.000  
eCLIP MATCHES▶KHSRP (bg=8.1%)▶TARDBP (bg=1.75%)No matches to TargetScan

T

AT

ATGTCAAACTATAAACTGCTTGTGATT  
Depth:2 (BABOON)  
Ei-value:0.000, Pi-value:0.000  
Er-value:0.000, Pr-value:0.000  
eCLIP MATCHES▶fubp3 (bg=23.31%)▶KHSRP (bg=8.1%)▶NOLC1 (bg=6.58%)▶SUB1 (bg=9.24%)▶TARDBP (bg=1.75%)▶ZC3H11A (bg=6.25%)MATCHES To TargetScan▶ miR-489-3p:UGACAUC


GTCAAAC

GTCAAACT  
Depth:3 (DOG)  
Ei-value:0.000, Pi-value:0.000  
Er-value:0.000, Pr-value:0.000  
eCLIP MATCHES▶KHSRP (bg=8.1%)▶NOLC1 (bg=6.58%)▶TARDBP (bg=1.75%)No matches to TargetScan

 5280  


T

GTCAAACT  
Depth:3 (DOG)  
Ei-value:0.000, Pi-value:0.000  
Er-value:0.000, Pr-value:0.000  
eCLIP MATCHES▶KHSRP (bg=8.1%)▶NOLC1 (bg=6.58%)▶TARDBP (bg=1.75%)No matches to TargetScan


AT

ATGTCAAACTATAAACTGCTTGTGATT  
Depth:2 (BABOON)  
Ei-value:0.000, Pi-value:0.000  
Er-value:0.000, Pr-value:0.000  
eCLIP MATCHES▶fubp3 (bg=23.31%)▶KHSRP (bg=8.1%)▶NOLC1 (bg=6.58%)▶SUB1 (bg=9.24%)▶TARDBP (bg=1.75%)▶ZC3H11A (bg=6.25%)MATCHES To TargetScan▶ miR-489-3p:UGACAUC


AAA

AAACTGCTTG  
Depth:7 (ARMADILLO)  
Ei-value:0.000, Pi-value:0.000  
Er-value:0.000, Pr-value:0.000  
eCLIP MATCHES▶fubp3 (bg=23.31%)▶KHSRP (bg=8.1%)▶NOLC1 (bg=6.58%)▶SUB1 (bg=9.24%)▶TARDBP (bg=1.75%)No matches to TargetScan


CTGCTT

CTGCTT  
Depth:9 (MOUSE)  
Ei-value:0.000, Pi-value:0.000  
Er-value:0.000, Pr-value:0.000  
eCLIP MATCHES▶fubp3 (bg=23.31%)▶KHSRP (bg=8.1%)▶NOLC1 (bg=6.58%)▶TARDBP (bg=1.75%)No matches to TargetScan


G

AAACTGCTTG  
Depth:7 (ARMADILLO)  
Ei-value:0.000, Pi-value:0.000  
Er-value:0.000, Pr-value:0.000  
eCLIP MATCHES▶fubp3 (bg=23.31%)▶KHSRP (bg=8.1%)▶NOLC1 (bg=6.58%)▶SUB1 (bg=9.24%)▶TARDBP (bg=1.75%)No matches to TargetScan


TGATT

AAACTGCTTGTGATT  
Depth:5 (SHEEP)  
Ei-value:0.000, Pi-value:0.000  
Er-value:0.000, Pr-value:0.000  
eCLIP MATCHES▶fubp3 (bg=23.31%)▶KHSRP (bg=8.1%)▶NOLC1 (bg=6.58%)▶SUB1 (bg=9.24%)▶TARDBP (bg=1.75%)▶ZC3H11A (bg=6.25%)No matches to TargetScan

GTG

a

aatgactttgttctttgctt  
Depth:2 (BABOON)  
Ei-value:0.620, Pi-value:0.000  
Er-value:0.000, Pr-value:0.000  
eCLIP MATCHES▶ddx55 (bg=12.35%)▶ddx6 (bg=23.92%)▶fubp3 (bg=23.31%)▶KHSRP (bg=8.1%)▶NOLC1 (bg=6.58%)▶SUB1 (bg=9.24%)▶TARDBP (bg=1.75%)▶tia1 (bg=16.04%)▶tial1 (bg=14.09%)▶ZC3H11A (bg=6.25%)MATCHES To TargetScan▶ miR-495-3p:AACAAAC▶ miR-224-5p:AAGUCAC▶ miR-186-5p:AAAGAAU


A

ATGACTT  
Depth:8 (GUINEAPIG)  
Ei-value:0.000, Pi-value:0.000  
Er-value:0.000, Pr-value:0.000  
eCLIP MATCHES▶fubp3 (bg=23.31%)▶KHSRP (bg=8.1%)▶NOLC1 (bg=6.58%)▶SUB1 (bg=9.24%)▶TARDBP (bg=1.75%)▶ZC3H11A (bg=6.25%)MATCHES To TargetScan▶ miR-224-5p:AAGUCAC


TGACTT

TGACTT  
Depth:9 (MOUSE)  
Ei-value:0.000, Pi-value:0.000  
Er-value:0.000, Pr-value:0.000  
eCLIP MATCHES▶fubp3 (bg=23.31%)▶KHSRP (bg=8.1%)▶NOLC1 (bg=6.58%)▶SUB1 (bg=9.24%)▶TARDBP (bg=1.75%)▶ZC3H11A (bg=6.25%)MATCHES To TargetScan▶ miR-224-5p:AAGUCAC


TGTTC

ATGACTTTGTTCTTTGCTT  
Depth:3 (DOG)  
Ei-value:0.000, Pi-value:0.000  
Er-value:0.000, Pr-value:0.000  
eCLIP MATCHES▶ddx55 (bg=12.35%)▶ddx6 (bg=23.92%)▶fubp3 (bg=23.31%)▶KHSRP (bg=8.1%)▶NOLC1 (bg=6.58%)▶SUB1 (bg=9.24%)▶TARDBP (bg=1.75%)▶tia1 (bg=16.04%)▶tial1 (bg=14.09%)▶ZC3H11A (bg=6.25%)MATCHES To TargetScan▶ miR-495-3p:AACAAAC▶ miR-224-5p:AAGUCAC▶ miR-186-5p:AAAGAAU


T

TTTGCTT  
Depth:8 (GUINEAPIG)  
Ei-value:0.000, Pi-value:0.000  
Er-value:0.000, Pr-value:0.000  
eCLIP MATCHES▶ddx55 (bg=12.35%)▶ddx6 (bg=23.92%)▶fubp3 (bg=23.31%)▶KHSRP (bg=8.1%)▶NOLC1 (bg=6.58%)▶SUB1 (bg=9.24%)▶TARDBP (bg=1.75%)▶tia1 (bg=16.04%)▶tial1 (bg=14.09%)▶ZC3H11A (bg=6.25%)No matches to TargetScan


TTGCTT

TTGCTT  
Depth:9 (MOUSE)  
Ei-value:0.000, Pi-value:0.000  
Er-value:0.000, Pr-value:0.010  
eCLIP MATCHES▶ddx55 (bg=12.35%)▶ddx6 (bg=23.92%)▶fubp3 (bg=23.31%)▶KHSRP (bg=8.1%)▶NOLC1 (bg=6.58%)▶SUB1 (bg=9.24%)▶TARDBP (bg=1.75%)▶tia1 (bg=16.04%)▶tial1 (bg=14.09%)▶ZC3H11A (bg=6.25%)No matches to TargetScan


GTGTTTTTCA

GTGTTTTTCA  
Depth:3 (DOG)  
Ei-value:0.000, Pi-value:0.000  
Er-value:0.000, Pr-value:0.000  
eCLIP MATCHES▶ddx55 (bg=12.35%)▶ddx6 (bg=23.92%)▶fubp3 (bg=23.31%)▶igf2bp1 (bg=11.67%)▶KHSRP (bg=8.1%)▶NOLC1 (bg=6.58%)▶RPS3 (bg=4.15%)▶SUB1 (bg=9.24%)▶TARDBP (bg=1.75%)▶tia1 (bg=16.04%)▶tial1 (bg=14.09%)▶ZC3H11A (bg=6.25%)No matches to TargetScan

A

TTTCCT

TTTCCT  
Depth:6 (PIG)  
Ei-value:0.000, Pi-value:0.000  
Er-value:0.000, Pr-value:0.000  
eCLIP MATCHES▶ddx55 (bg=12.35%)▶ddx6 (bg=23.92%)▶fubp3 (bg=23.31%)▶igf2bp1 (bg=11.67%)▶KHSRP (bg=8.1%)▶NOLC1 (bg=6.58%)▶RPS3 (bg=4.15%)▶SUB1 (bg=9.24%)▶TARDBP (bg=1.75%)▶tia1 (bg=16.04%)▶tial1 (bg=14.09%)▶ZC3H11A (bg=6.25%)No matches to TargetScan

A

ta

taatgcacata  
Depth:2 (BABOON)  
Ei-value:1.000, Pi-value:0.000  
Er-value:0.000, Pr-value:0.000  
eCLIP MATCHES▶ddx55 (bg=12.35%)▶ddx6 (bg=23.92%)▶fubp3 (bg=23.31%)▶igf2bp1 (bg=11.67%)▶KHSRP (bg=8.1%)▶RPS3 (bg=4.15%)▶SUB1 (bg=9.24%)▶TARDBP (bg=1.75%)▶tia1 (bg=16.04%)▶tial1 (bg=14.09%)▶ZC3H11A (bg=6.25%)MATCHES To TargetScan▶ miR-455-5p:AUGUGCC▶ miR-33-5p:UGCAUUG


atgcacat

atgcacat  
Depth:4 (COW)  
Ei-value:0.670, Pi-value:0.000  
Er-value:0.000, Pr-value:0.000  
eCLIP MATCHES▶ddx6 (bg=23.92%)▶fubp3 (bg=23.31%)▶igf2bp1 (bg=11.67%)▶KHSRP (bg=8.1%)▶RPS3 (bg=4.15%)▶SUB1 (bg=9.24%)▶TARDBP (bg=1.75%)▶tia1 (bg=16.04%)▶tial1 (bg=14.09%)▶ZC3H11A (bg=6.25%)MATCHES To TargetScan▶ miR-455-5p:AUGUGCC


A

ATGCACATA  
Depth:3 (DOG)  
Ei-value:0.000, Pi-value:0.000  
Er-value:0.000, Pr-value:0.000  
eCLIP MATCHES▶ddx6 (bg=23.92%)▶fubp3 (bg=23.31%)▶igf2bp1 (bg=11.67%)▶KHSRP (bg=8.1%)▶RPS3 (bg=4.15%)▶SUB1 (bg=9.24%)▶TARDBP (bg=1.75%)▶tia1 (bg=16.04%)▶tial1 (bg=14.09%)▶ZC3H11A (bg=6.25%)MATCHES To TargetScan▶ miR-455-5p:AUGUGCC

C

TAACTTTTAA

TAACTTTTAA  
Depth:6 (PIG)  
Ei-value:0.000, Pi-value:0.000  
Er-value:0.000, Pr-value:0.000  
eCLIP MATCHES▶fubp3 (bg=23.31%)▶igf2bp1 (bg=11.67%)▶RPS3 (bg=4.15%)▶SUB1 (bg=9.24%)▶TARDBP (bg=1.75%)▶tia1 (bg=16.04%)▶tial1 (bg=14.09%)▶ZC3H11A (bg=6.25%)No matches to TargetScan


A

TAACTTTTAAAAAATAAA  
Depth:3 (DOG)  
Ei-value:0.000, Pi-value:0.000  
Er-value:0.000, Pr-value:0.000  
eCLIP MATCHES▶fubp3 (bg=23.31%)▶igf2bp1 (bg=11.67%)▶RPS3 (bg=4.15%)▶SUB1 (bg=9.24%)▶TARDBP (bg=1.75%)▶tia1 (bg=16.04%)▶tial1 (bg=14.09%)▶ZC3H11A (bg=6.25%)No matches to TargetScan


AAATAAA

AAATAAA  
Depth:9 (MOUSE)  
Ei-value:0.000, Pi-value:0.000  
Er-value:0.000, Pr-value:0.000  
eCLIP MATCHES▶ZC3H11A (bg=6.25%)No matches to TargetScan


GGTTA

TAACTTTTAAAAAATAAAGGTTATTTTAAAAGCCTGTATTAAGCCCTCGTTGCTTGTAGAATAGAGT  
Depth:2 (BABOON)  
Ei-value:0.000, Pi-value:0.000  
Er-value:0.000, Pr-value:0.000  
eCLIP MATCHES▶fubp3 (bg=23.31%)▶igf2bp1 (bg=11.67%)▶RPS3 (bg=4.15%)▶SUB1 (bg=9.24%)▶TARDBP (bg=1.75%)▶tia1 (bg=16.04%)▶tial1 (bg=14.09%)▶ZC3H11A (bg=6.25%)MATCHES To TargetScan▶ miR-655-3p:UAAUACA


TT

TTTTAAAA  
Depth:5 (SHEEP)  
Ei-value:0.000, Pi-value:0.010  
Er-value:0.000, Pr-value:0.000  
No matches to eCLIP DataNo matches to TargetScan


TTAAAA

TTAAAA  
Depth:8 (GUINEAPIG)  
Ei-value:0.000, Pi-value:0.010  
Er-value:0.000, Pr-value:0.000  
No matches to eCLIP DataNo matches to TargetScan


GCCTGTATTAAGCCCTCG

TAACTTTTAAAAAATAAAGGTTATTTTAAAAGCCTGTATTAAGCCCTCGTTGCTTGTAGAATAGAGT  
Depth:2 (BABOON)  
Ei-value:0.000, Pi-value:0.000  
Er-value:0.000, Pr-value:0.000  
eCLIP MATCHES▶fubp3 (bg=23.31%)▶igf2bp1 (bg=11.67%)▶RPS3 (bg=4.15%)▶SUB1 (bg=9.24%)▶TARDBP (bg=1.75%)▶tia1 (bg=16.04%)▶tial1 (bg=14.09%)▶ZC3H11A (bg=6.25%)MATCHES To TargetScan▶ miR-655-3p:UAAUACA

 5400  


T

TAACTTTTAAAAAATAAAGGTTATTTTAAAAGCCTGTATTAAGCCCTCGTTGCTTGTAGAATAGAGT  
Depth:2 (BABOON)  
Ei-value:0.000, Pi-value:0.000  
Er-value:0.000, Pr-value:0.000  
eCLIP MATCHES▶fubp3 (bg=23.31%)▶igf2bp1 (bg=11.67%)▶RPS3 (bg=4.15%)▶SUB1 (bg=9.24%)▶TARDBP (bg=1.75%)▶tia1 (bg=16.04%)▶tial1 (bg=14.09%)▶ZC3H11A (bg=6.25%)MATCHES To TargetScan▶ miR-655-3p:UAAUACA


tgcttg

tgcttg  
Depth:3 (DOG)  
Ei-value:0.080, Pi-value:0.020  
Er-value:0.000, Pr-value:0.000  
No matches to eCLIP DataNo matches to TargetScan


TAGAATAGAGT

TAACTTTTAAAAAATAAAGGTTATTTTAAAAGCCTGTATTAAGCCCTCGTTGCTTGTAGAATAGAGT  
Depth:2 (BABOON)  
Ei-value:0.000, Pi-value:0.000  
Er-value:0.000, Pr-value:0.000  
eCLIP MATCHES▶fubp3 (bg=23.31%)▶igf2bp1 (bg=11.67%)▶RPS3 (bg=4.15%)▶SUB1 (bg=9.24%)▶TARDBP (bg=1.75%)▶tia1 (bg=16.04%)▶tial1 (bg=14.09%)▶ZC3H11A (bg=6.25%)MATCHES To TargetScan▶ miR-655-3p:UAAUACA

TAGTATACG

ctacagaagcacaggt

ctacagaagcacaggt  
Depth:2 (BABOON)  
Ei-value:1.000, Pi-value:0.000  
Er-value:0.000, Pr-value:0.000  
No matches to eCLIP DataMATCHES To TargetScan▶ miR-218-5p:UGUGCUU

5443
```

|  |  |  |  |  |  |  |  |
| --- | --- | --- | --- | --- | --- | --- | --- |
| | | | | | | | | | | | | | | | |
| 2 |  |  |  | 6 |  |  | 9 |
| Depth of motif conservation (number of species) | | | | | | | |

  
  

---

  

## >HUMAN TO DOG (5443 bases)

```
GGC

GGCACTTCCGGT  
Depth:3 (DOG)  
Ei-value:0.000, Pi-value:0.000  
Er-value:0.000, Pr-value:0.000  
No matches to eCLIP DataMATCHES To TargetScan▶ miR-302-3p/372-3p/373-3p/520-3p:AAGUGCU


ACTTCCGG

ACTTCCGG  
Depth:8 (GUINEAPIG)  
Ei-value:0.000, Pi-value:0.000  
Er-value:0.000, Pr-value:0.000  
No matches to eCLIP DataNo matches to TargetScan


T

GGCACTTCCGGT  
Depth:3 (DOG)  
Ei-value:0.000, Pi-value:0.000  
Er-value:0.000, Pr-value:0.000  
No matches to eCLIP DataMATCHES To TargetScan▶ miR-302-3p/372-3p/373-3p/520-3p:AAGUGCU

ACCG

CTCCTCTC

CTCCTCTC  
Depth:5 (SHEEP)  
Ei-value:0.000, Pi-value:0.000  
Er-value:0.000, Pr-value:0.000  
No matches to eCLIP DataNo matches to TargetScan

TT

GCCAGC

GCCAGC  
Depth:5 (SHEEP)  
Ei-value:0.000, Pi-value:0.000  
Er-value:0.000, Pr-value:0.000  
No matches to eCLIP DataNo matches to TargetScan

GC

AGAGAA

AGAGAA  
Depth:8 (GUINEAPIG)  
Ei-value:0.000, Pi-value:0.000  
Er-value:0.000, Pr-value:0.000  
No matches to eCLIP DataNo matches to TargetScan


C

AGAGAACTGCCAA  
Depth:7 (ARMADILLO)  
Ei-value:0.000, Pi-value:0.000  
Er-value:0.000, Pr-value:0.000  
No matches to eCLIP DataMATCHES To TargetScan▶ miR-34-5p/449-5p:GGCAGUG▶ miR-182-5p:UUGGCAA▶ miR-96-5p/1271-5p:UUGGCAC


TGCCAA

TGCCAA  
Depth:9 (MOUSE)  
Ei-value:0.000, Pi-value:0.000  
Er-value:0.000, Pr-value:0.000  
No matches to eCLIP DataMATCHES To TargetScan▶ miR-182-5p:UUGGCAA▶ miR-96-5p/1271-5p:UUGGCAC


G

AGAGAACTGCCAAGTCAGTTCCGG  
Depth:4 (COW)  
Ei-value:0.000, Pi-value:0.000  
Er-value:0.000, Pr-value:0.000  
eCLIP MATCHES▶EIF3G (bg=2.39%)MATCHES To TargetScan▶ miR-34-5p/449-5p:GGCAGUG▶ miR-182-5p:UUGGCAA▶ miR-96-5p/1271-5p:UUGGCAC


TCAGTTCCGG

TCAGTTCCGG  
Depth:9 (MOUSE)  
Ei-value:0.000, Pi-value:0.000  
Er-value:0.000, Pr-value:0.000  
eCLIP MATCHES▶EIF3G (bg=2.39%)No matches to TargetScan

TCCGGCAGAGATCGCGGAGAGACGCAGAACGCAGCCCGCTCCTCCA

GGGCCCTCCA

GGGCCCTCCAGGCCCTCCGGCC  
Depth:5 (SHEEP)  
Ei-value:0.000, Pi-value:0.000  
Er-value:0.000, Pr-value:0.000  
eCLIP MATCHES▶ddx3x (bg=13.89%)▶ddx6 (bg=23.92%)▶drosha (bg=11.37%)▶EIF3G (bg=2.39%)▶EIF3H (bg=7.83%)▶FMR1 (bg=6.3%)▶METAP2 (bg=6.14%)▶PUS1 (bg=2.87%)▶RPS3 (bg=4.15%)▶SDAD1 (bg=5.99%)▶SERBP1 (bg=0.81%)▶SF3B1 (bg=6.74%)▶WRN (bg=3.31%)▶ybx3 (bg=22.82%)MATCHES To TargetScan▶ miR-296-5p:GGGCCCC


G

GGCCCTCC  
Depth:8 (GUINEAPIG)  
Ei-value:0.000, Pi-value:0.000  
Er-value:0.000, Pr-value:0.000  
eCLIP MATCHES▶ddx3x (bg=13.89%)▶ddx6 (bg=23.92%)▶drosha (bg=11.37%)▶EIF3G (bg=2.39%)▶EIF3H (bg=7.83%)▶FMR1 (bg=6.3%)▶METAP2 (bg=6.14%)▶PUS1 (bg=2.87%)▶RPS3 (bg=4.15%)▶SDAD1 (bg=5.99%)▶SERBP1 (bg=0.81%)▶SF3B1 (bg=6.74%)▶WRN (bg=3.31%)▶ybx3 (bg=22.82%)No matches to TargetScan


GCCCT

GCCCTC  
Depth:9 (MOUSE)  
Ei-value:0.000, Pi-value:0.000  
Er-value:0.000, Pr-value:0.000  
eCLIP MATCHES▶ddx3x (bg=13.89%)▶ddx6 (bg=23.92%)▶drosha (bg=11.37%)▶EIF3G (bg=2.39%)▶EIF3H (bg=7.83%)▶FMR1 (bg=6.3%)▶METAP2 (bg=6.14%)▶PUS1 (bg=2.87%)▶RPS3 (bg=4.15%)▶SDAD1 (bg=5.99%)▶SERBP1 (bg=0.81%)▶SF3B1 (bg=6.74%)▶WRN (bg=3.31%)▶ybx3 (bg=22.82%)No matches to TargetScan

 120  


C

GCCCTC  
Depth:9 (MOUSE)  
Ei-value:0.000, Pi-value:0.000  
Er-value:0.000, Pr-value:0.000  
eCLIP MATCHES▶ddx3x (bg=13.89%)▶ddx6 (bg=23.92%)▶drosha (bg=11.37%)▶EIF3G (bg=2.39%)▶EIF3H (bg=7.83%)▶FMR1 (bg=6.3%)▶METAP2 (bg=6.14%)▶PUS1 (bg=2.87%)▶RPS3 (bg=4.15%)▶SDAD1 (bg=5.99%)▶SERBP1 (bg=0.81%)▶SF3B1 (bg=6.74%)▶WRN (bg=3.31%)▶ybx3 (bg=22.82%)No matches to TargetScan


C

GGCCCTCC  
Depth:8 (GUINEAPIG)  
Ei-value:0.000, Pi-value:0.000  
Er-value:0.000, Pr-value:0.000  
eCLIP MATCHES▶ddx3x (bg=13.89%)▶ddx6 (bg=23.92%)▶drosha (bg=11.37%)▶EIF3G (bg=2.39%)▶EIF3H (bg=7.83%)▶FMR1 (bg=6.3%)▶METAP2 (bg=6.14%)▶PUS1 (bg=2.87%)▶RPS3 (bg=4.15%)▶SDAD1 (bg=5.99%)▶SERBP1 (bg=0.81%)▶SF3B1 (bg=6.74%)▶WRN (bg=3.31%)▶ybx3 (bg=22.82%)No matches to TargetScan


GG

GGCCCTCCGG  
Depth:7 (ARMADILLO)  
Ei-value:0.000, Pi-value:0.000  
Er-value:0.000, Pr-value:0.000  
eCLIP MATCHES▶ddx3x (bg=13.89%)▶ddx6 (bg=23.92%)▶drosha (bg=11.37%)▶EIF3G (bg=2.39%)▶EIF3H (bg=7.83%)▶FMR1 (bg=6.3%)▶METAP2 (bg=6.14%)▶PUS1 (bg=2.87%)▶RPS3 (bg=4.15%)▶SDAD1 (bg=5.99%)▶SERBP1 (bg=0.81%)▶SF3B1 (bg=6.74%)▶WRN (bg=3.31%)▶ybx3 (bg=22.82%)No matches to TargetScan


CC

GGGCCCTCCAGGCCCTCCGGCC  
Depth:5 (SHEEP)  
Ei-value:0.000, Pi-value:0.000  
Er-value:0.000, Pr-value:0.000  
eCLIP MATCHES▶ddx3x (bg=13.89%)▶ddx6 (bg=23.92%)▶drosha (bg=11.37%)▶EIF3G (bg=2.39%)▶EIF3H (bg=7.83%)▶FMR1 (bg=6.3%)▶METAP2 (bg=6.14%)▶PUS1 (bg=2.87%)▶RPS3 (bg=4.15%)▶SDAD1 (bg=5.99%)▶SERBP1 (bg=0.81%)▶SF3B1 (bg=6.74%)▶WRN (bg=3.31%)▶ybx3 (bg=22.82%)MATCHES To TargetScan▶ miR-296-5p:GGGCCCC

CCG

ggccgg

ggccgg  
Depth:3 (DOG)  
Ei-value:0.080, Pi-value:0.000  
Er-value:0.000, Pr-value:0.000  
eCLIP MATCHES▶ddx3x (bg=13.89%)▶ddx6 (bg=23.92%)▶drosha (bg=11.37%)▶EIF3G (bg=2.39%)▶EIF3H (bg=7.83%)▶FMR1 (bg=6.3%)▶METAP2 (bg=6.14%)▶PHF6 (bg=1.71%)▶PUS1 (bg=2.87%)▶RPS3 (bg=4.15%)▶SDAD1 (bg=5.99%)▶SERBP1 (bg=0.81%)▶SF3B1 (bg=6.74%)▶WRN (bg=3.31%)▶ybx3 (bg=22.82%)No matches to TargetScan

C

G

GGGTGAACTGGGGGGCCC  
Depth:3 (DOG)  
Ei-value:0.000, Pi-value:0.000  
Er-value:0.000, Pr-value:0.000  
eCLIP MATCHES▶DDX24 (bg=2.31%)▶ddx3x (bg=13.89%)▶DHX30 (bg=5.05%)▶drosha (bg=11.37%)▶EIF3G (bg=2.39%)▶EIF3H (bg=7.83%)▶fam120a (bg=18.43%)▶FMR1 (bg=6.3%)▶GEMIN5 (bg=2.46%)▶METAP2 (bg=6.14%)▶NCBP2 (bg=2.92%)▶PHF6 (bg=1.71%)▶PUS1 (bg=2.87%)▶RPS3 (bg=4.15%)▶SDAD1 (bg=5.99%)▶SF3B1 (bg=6.74%)▶SUB1 (bg=9.24%)▶WRN (bg=3.31%)MATCHES To TargetScan▶ miR-296-5p:GGGCCCC


GGTGAACT

GGTGAACTGGGGGGCCC  
Depth:6 (PIG)  
Ei-value:0.000, Pi-value:0.000  
Er-value:0.000, Pr-value:0.000  
eCLIP MATCHES▶DDX24 (bg=2.31%)▶ddx3x (bg=13.89%)▶DHX30 (bg=5.05%)▶drosha (bg=11.37%)▶EIF3G (bg=2.39%)▶EIF3H (bg=7.83%)▶fam120a (bg=18.43%)▶FMR1 (bg=6.3%)▶GEMIN5 (bg=2.46%)▶METAP2 (bg=6.14%)▶NCBP2 (bg=2.92%)▶PHF6 (bg=1.71%)▶PUS1 (bg=2.87%)▶RPS3 (bg=4.15%)▶SDAD1 (bg=5.99%)▶SF3B1 (bg=6.74%)▶SUB1 (bg=9.24%)▶WRN (bg=3.31%)MATCHES To TargetScan▶ miR-296-5p:GGGCCCC


GGG

GGGGGGCCC  
Depth:7 (ARMADILLO)  
Ei-value:0.000, Pi-value:0.000  
Er-value:0.000, Pr-value:0.000  
eCLIP MATCHES▶DDX24 (bg=2.31%)▶ddx3x (bg=13.89%)▶DHX30 (bg=5.05%)▶drosha (bg=11.37%)▶EIF3G (bg=2.39%)▶EIF3H (bg=7.83%)▶fam120a (bg=18.43%)▶GEMIN5 (bg=2.46%)▶METAP2 (bg=6.14%)▶NCBP2 (bg=2.92%)▶PHF6 (bg=1.71%)▶RPS3 (bg=4.15%)▶SDAD1 (bg=5.99%)▶SUB1 (bg=9.24%)▶WRN (bg=3.31%)MATCHES To TargetScan▶ miR-296-5p:GGGCCCC


GGGCCC

GGGCCC  
Depth:8 (GUINEAPIG)  
Ei-value:0.000, Pi-value:0.000  
Er-value:0.000, Pr-value:0.000  
eCLIP MATCHES▶DDX24 (bg=2.31%)▶ddx3x (bg=13.89%)▶DHX30 (bg=5.05%)▶drosha (bg=11.37%)▶EIF3G (bg=2.39%)▶EIF3H (bg=7.83%)▶fam120a (bg=18.43%)▶GEMIN5 (bg=2.46%)▶METAP2 (bg=6.14%)▶NCBP2 (bg=2.92%)▶PHF6 (bg=1.71%)▶RPS3 (bg=4.15%)▶SDAD1 (bg=5.99%)▶SUB1 (bg=9.24%)▶WRN (bg=3.31%)MATCHES To TargetScan▶ miR-296-5p:GGGCCCC

CGGGACAGGCCGAGCCCTCTGCCC

tgcaga

tgcaga  
Depth:4 (COW)  
Ei-value:1.000, Pi-value:0.010  
Er-value:0.000, Pr-value:0.000  
eCLIP MATCHES▶AKAP1 (bg=3.4%)▶BCCIP (bg=4.61%)▶DDX24 (bg=2.31%)▶ddx3x (bg=13.89%)▶ddx6 (bg=23.92%)▶DHX30 (bg=5.05%)▶drosha (bg=11.37%)▶EIF3G (bg=2.39%)▶EIF3H (bg=7.83%)▶fam120a (bg=18.43%)▶FMR1 (bg=6.3%)▶FXR2 (bg=7.51%)▶METAP2 (bg=6.14%)▶NCBP2 (bg=2.92%)▶NIPBL (bg=2.83%)▶PCBP1 (bg=2.3%)▶PHF6 (bg=1.71%)▶PTBP1 (bg=1.43%)▶rbm15 (bg=11.81%)▶RPS3 (bg=4.15%)▶SDAD1 (bg=5.99%)▶SUB1 (bg=9.24%)▶TBRG4 (bg=7.0%)▶TROVE2 (bg=3.49%)▶UTP3 (bg=2.81%)▶ybx3 (bg=22.82%)No matches to TargetScan

TAACGGA

GGCCTCT

GGCCTCT  
Depth:3 (DOG)  
Ei-value:0.000, Pi-value:0.010  
Er-value:0.000, Pr-value:0.000  
eCLIP MATCHES▶AKAP1 (bg=3.4%)▶BCCIP (bg=4.61%)▶DDX24 (bg=2.31%)▶ddx3x (bg=13.89%)▶ddx6 (bg=23.92%)▶dgcr8 (bg=19.31%)▶DHX30 (bg=5.05%)▶drosha (bg=11.37%)▶EIF3H (bg=7.83%)▶fam120a (bg=18.43%)▶FMR1 (bg=6.3%)▶fubp3 (bg=23.31%)▶FXR2 (bg=7.51%)▶METAP2 (bg=6.14%)▶NCBP2 (bg=2.92%)▶NIPBL (bg=2.83%)▶PCBP1 (bg=2.3%)▶PTBP1 (bg=1.43%)▶pum2 (bg=21.55%)▶rbm15 (bg=11.81%)▶RPS3 (bg=4.15%)▶SBDS (bg=1.91%)▶SDAD1 (bg=5.99%)▶SUB1 (bg=9.24%)▶TBRG4 (bg=7.0%)▶TROVE2 (bg=3.49%)▶UTP3 (bg=2.81%)▶ybx3 (bg=22.82%)No matches to TargetScan

GCTGT

GGCTGCCCACT

GGCTGCCCACT  
Depth:3 (DOG)  
Ei-value:0.000, Pi-value:0.000  
Er-value:0.000, Pr-value:0.000  
eCLIP MATCHES▶AKAP1 (bg=3.4%)▶BCCIP (bg=4.61%)▶DDX24 (bg=2.31%)▶ddx3x (bg=13.89%)▶ddx6 (bg=23.92%)▶dgcr8 (bg=19.31%)▶DHX30 (bg=5.05%)▶drosha (bg=11.37%)▶EIF3H (bg=7.83%)▶fam120a (bg=18.43%)▶FMR1 (bg=6.3%)▶FXR2 (bg=7.51%)▶METAP2 (bg=6.14%)▶NIPBL (bg=2.83%)▶PTBP1 (bg=1.43%)▶pum2 (bg=21.55%)▶rbm15 (bg=11.81%)▶RPS3 (bg=4.15%)▶SBDS (bg=1.91%)▶SDAD1 (bg=5.99%)▶SF3B1 (bg=6.74%)▶SUB1 (bg=9.24%)▶TBRG4 (bg=7.0%)▶TROVE2 (bg=3.49%)▶UTP3 (bg=2.81%)▶WRN (bg=3.31%)▶ybx3 (bg=22.82%)▶ZC3H11A (bg=6.25%)No matches to TargetScan

GGCTGTGCCCGCCCACTGGCTGTGCC 240  
 CAGACCTT

gaagcc

gaagcc  
Depth:4 (COW)  
Ei-value:1.000, Pi-value:0.010  
Er-value:0.000, Pr-value:0.000  
eCLIP MATCHES▶ddx3x (bg=13.89%)▶dgcr8 (bg=19.31%)▶drosha (bg=11.37%)▶FMR1 (bg=6.3%)▶FXR2 (bg=7.51%)▶NIP7 (bg=2.54%)▶PTBP1 (bg=1.43%)▶rbm15 (bg=11.81%)▶SBDS (bg=1.91%)▶SDAD1 (bg=5.99%)▶ybx3 (bg=22.82%)No matches to TargetScan

GCAGCGAACCTCTCTTTCCCACCCCACCTCGGTGACTA

ATGGCGGC

ATGGCGGC  
Depth:9 (MOUSE)  
Ei-value:0.000, Pi-value:0.000  
Er-value:0.000, Pr-value:0.000  
eCLIP MATCHES▶BCCIP (bg=4.61%)▶ddx3x (bg=13.89%)▶ddx6 (bg=23.92%)▶dgcr8 (bg=19.31%)▶DHX30 (bg=5.05%)▶drosha (bg=11.37%)▶EIF3H (bg=7.83%)▶FMR1 (bg=6.3%)▶FTO (bg=7.53%)▶fubp3 (bg=23.31%)▶FXR2 (bg=7.51%)▶GEMIN5 (bg=2.46%)▶IGF2BP3 (bg=4.26%)▶METAP2 (bg=6.14%)▶NIP7 (bg=2.54%)▶PCBP1 (bg=2.3%)▶pum1 (bg=29.85%)▶pum2 (bg=21.55%)▶rbm15 (bg=11.81%)▶SDAD1 (bg=5.99%)▶WRN (bg=3.31%)No matches to TargetScan

CGTGGCGTCTCCCAGCCCGGACCCC

gccggc

gccggc  
Depth:3 (DOG)  
Ei-value:0.080, Pi-value:0.000  
Er-value:0.000, Pr-value:0.000  
eCLIP MATCHES▶BCCIP (bg=4.61%)▶ddx3x (bg=13.89%)▶ddx6 (bg=23.92%)▶dgcr8 (bg=19.31%)▶DHX30 (bg=5.05%)▶drosha (bg=11.37%)▶EIF3H (bg=7.83%)▶FMR1 (bg=6.3%)▶FTO (bg=7.53%)▶FXR2 (bg=7.51%)▶GEMIN5 (bg=2.46%)▶IGF2BP3 (bg=4.26%)▶METAP2 (bg=6.14%)▶NIP7 (bg=2.54%)▶PCBP1 (bg=2.3%)▶pum1 (bg=29.85%)▶pum2 (bg=21.55%)▶rbm15 (bg=11.81%)▶SDAD1 (bg=5.99%)▶WRN (bg=3.31%)No matches to TargetScan

ACC

CGGGTCTCC

CGGGTCTCC  
Depth:3 (DOG)  
Ei-value:0.000, Pi-value:0.000  
Er-value:0.000, Pr-value:0.000  
eCLIP MATCHES▶BCCIP (bg=4.61%)▶ddx3x (bg=13.89%)▶ddx6 (bg=23.92%)▶dgcr8 (bg=19.31%)▶DHX30 (bg=5.05%)▶drosha (bg=11.37%)▶EIF3H (bg=7.83%)▶FMR1 (bg=6.3%)▶FTO (bg=7.53%)▶FXR2 (bg=7.51%)▶IGF2BP3 (bg=4.26%)▶LARP7 (bg=0.81%)▶METAP2 (bg=6.14%)▶NIP7 (bg=2.54%)▶PCBP1 (bg=2.3%)▶pum1 (bg=29.85%)▶rbm15 (bg=11.81%)▶SDAD1 (bg=5.99%)No matches to TargetScan

CGACCCAAGCCTCGACG 360  
 AAACCCCCGCAGAGCCGCCGGGACGCAGCGCCTTTGGGCGGCGCTGGGCGTGGTGGGCCGGGAAGTATGGCGGCAGCTCGAACGCCGCGCGGCGGAGGCCATTAAGGCGTGGACGGCCCG 480  
 GGAAGGCGGCCTAGGGACGCAAGCAG

GCTCGGC

GCTCGGC  
Depth:3 (DOG)  
Ei-value:0.000, Pi-value:0.000  
Er-value:0.000, Pr-value:0.000  
eCLIP MATCHES▶DHX30 (bg=5.05%)▶drosha (bg=11.37%)▶FMR1 (bg=6.3%)▶FXR2 (bg=7.51%)▶GRWD1 (bg=4.85%)▶NIP7 (bg=2.54%)▶SRSF1 (bg=2.06%)▶UCHL5 (bg=1.08%)▶ybx3 (bg=22.82%)MATCHES To TargetScan▶ miR-615-3p:CCGAGCC

CGCCTCTTTAGGCCACGGAGCCGCGCAGATCCGGTT

cccggg

cccggg  
Depth:4 (COW)  
Ei-value:1.000, Pi-value:0.010  
Er-value:0.000, Pr-value:0.000  
eCLIP MATCHES▶ybx3 (bg=22.82%)No matches to TargetScan


TGACCACTC

TGACCACTC  
Depth:3 (DOG)  
Ei-value:0.000, Pi-value:0.000  
Er-value:0.000, Pr-value:0.000  
eCLIP MATCHES▶ybx3 (bg=22.82%)MATCHES To TargetScan▶ miR-140-5p:AGUGGUU

TGTCGCCATTGGGCGAG

acctac

acctac  
Depth:3 (DOG)  
Ei-value:0.080, Pi-value:0.000  
Er-value:0.000, Pr-value:0.000  
eCLIP MATCHES▶lin28b (bg=16.96%)▶METAP2 (bg=6.14%)▶rbm15 (bg=11.81%)▶ybx3 (bg=22.82%)No matches to TargetScan

CTAGTCCTGACGA 600  
 CAACGGACAAAGGCCTTAAGGGGCCTGGA

aggtgagcg

aggtgagcg  
Depth:4 (COW)  
Ei-value:0.150, Pi-value:0.000  
Er-value:0.000, Pr-value:0.000  
eCLIP MATCHES▶BUD13 (bg=0.7%)▶ddx3x (bg=13.89%)▶DDX52 (bg=1.27%)▶dgcr8 (bg=19.31%)▶FTO (bg=7.53%)▶lin28b (bg=16.96%)▶rbm15 (bg=11.81%)▶SRSF7 (bg=1.29%)▶ybx3 (bg=22.82%)No matches to TargetScan


AAG

AGGTGAGCGAAG  
Depth:3 (DOG)  
Ei-value:0.000, Pi-value:0.000  
Er-value:0.000, Pr-value:0.000  
eCLIP MATCHES▶BUD13 (bg=0.7%)▶ddx3x (bg=13.89%)▶DDX52 (bg=1.27%)▶dgcr8 (bg=19.31%)▶FTO (bg=7.53%)▶lin28b (bg=16.96%)▶rbm15 (bg=11.81%)▶SRSF7 (bg=1.29%)▶ybx3 (bg=22.82%)No matches to TargetScan

TCCCGAACGA

CGA

CGACGGGTGGAAC  
Depth:4 (COW)  
Ei-value:0.000, Pi-value:0.000  
Er-value:0.000, Pr-value:0.000  
eCLIP MATCHES▶FTO (bg=7.53%)▶rbm15 (bg=11.81%)▶SRSF7 (bg=1.29%)▶ybx3 (bg=22.82%)MATCHES To TargetScan▶ miR-99-5p/100-5p:ACCCGUA


CGG

CGGGTGGAAC  
Depth:7 (ARMADILLO)  
Ei-value:0.000, Pi-value:0.000  
Er-value:0.000, Pr-value:0.000  
eCLIP MATCHES▶FTO (bg=7.53%)▶rbm15 (bg=11.81%)▶SRSF7 (bg=1.29%)▶ybx3 (bg=22.82%)No matches to TargetScan


GTGGAA

GTGGAA  
Depth:8 (GUINEAPIG)  
Ei-value:0.000, Pi-value:0.000  
Er-value:0.000, Pr-value:0.000  
eCLIP MATCHES▶FTO (bg=7.53%)▶rbm15 (bg=11.81%)▶SRSF7 (bg=1.29%)▶ybx3 (bg=22.82%)No matches to TargetScan


C

CGGGTGGAAC  
Depth:7 (ARMADILLO)  
Ei-value:0.000, Pi-value:0.000  
Er-value:0.000, Pr-value:0.000  
eCLIP MATCHES▶FTO (bg=7.53%)▶rbm15 (bg=11.81%)▶SRSF7 (bg=1.29%)▶ybx3 (bg=22.82%)No matches to TargetScan

GGTTAGCGGCCATCGGGCG

GTTGGTCTTC

GTTGGTCTTC  
Depth:3 (DOG)  
Ei-value:0.000, Pi-value:0.000  
Er-value:0.000, Pr-value:0.000  
eCLIP MATCHES▶UTP18 (bg=0.53%)No matches to TargetScan

A

TTCTAC

TTCTAC  
Depth:6 (PIG)  
Ei-value:0.000, Pi-value:0.000  
Er-value:0.000, Pr-value:0.000  
eCLIP MATCHES▶UTP18 (bg=0.53%)No matches to TargetScan

CAGACTTTG

C

CTGTCGGAAGA  
Depth:3 (DOG)  
Ei-value:0.000, Pi-value:0.000  
Er-value:0.000, Pr-value:0.000  
No matches to eCLIP DataNo matches to TargetScan


TGTCGGAAGA

TGTCGGAAGA  
Depth:5 (SHEEP)  
Ei-value:0.000, Pi-value:0.000  
Er-value:0.000, Pr-value:0.000  
No matches to eCLIP DataNo matches to TargetScan

 720  


TGTCGGAAGA  
Depth:5 (SHEEP)  
Ei-value:0.000, Pi-value:0.000  
Er-value:0.000, Pr-value:0.000  
No matches to eCLIP DataNo matches to TargetScan

GA

GAAATGG

GAAATGG  
Depth:6 (PIG)  
Ei-value:0.000, Pi-value:0.000  
Er-value:0.000, Pr-value:0.000  
eCLIP MATCHES▶ddx55 (bg=12.35%)No matches to TargetScan

TAGAATGACAGGCCA

CGTTTGGCC

CGTTTGGCC  
Depth:3 (DOG)  
Ei-value:0.000, Pi-value:0.000  
Er-value:0.000, Pr-value:0.000  
eCLIP MATCHES▶ddx55 (bg=12.35%)No matches to TargetScan

CGTTGGAAATGCCCA

CCACCCT

CCACCCT  
Depth:3 (DOG)  
Ei-value:0.000, Pi-value:0.000  
Er-value:0.000, Pr-value:0.000  
No matches to eCLIP DataNo matches to TargetScan

C

T

TGGGAAGATTTA  
Depth:8 (GUINEAPIG)  
Ei-value:0.000, Pi-value:0.000  
Er-value:0.000, Pr-value:0.000  
No matches to eCLIP DataNo matches to TargetScan


GGGAAGA

GGGAAGA  
Depth:9 (MOUSE)  
Ei-value:0.000, Pi-value:0.000  
Er-value:0.000, Pr-value:0.000  
No matches to eCLIP DataNo matches to TargetScan


TTTA

TGGGAAGATTTA  
Depth:8 (GUINEAPIG)  
Ei-value:0.000, Pi-value:0.000  
Er-value:0.000, Pr-value:0.000  
No matches to eCLIP DataNo matches to TargetScan


CTGGCC

TGGGAAGATTTACTGGCC  
Depth:5 (SHEEP)  
Ei-value:0.000, Pi-value:0.000  
Er-value:0.000, Pr-value:0.000  
No matches to eCLIP DataMATCHES To TargetScan▶ miR-802:CAGUAAC

GTTTATG

G

GAAGGCCTGTGTATATAATATGAAAAAGCTGCTCTCAACT  
Depth:3 (DOG)  
Ei-value:0.000, Pi-value:0.000  
Er-value:0.000, Pr-value:0.000  
eCLIP MATCHES▶pum1 (bg=29.85%)MATCHES To TargetScan▶ miR-15-5p/16-5p/195-5p/424-5p/497-5p:AGCAGCA▶ miR-503-5p:AGCAGCG


AAGG

AAGGCCTGTGTATATAATATGAAAAAGCTGCT  
Depth:8 (GUINEAPIG)  
Ei-value:0.000, Pi-value:0.000  
Er-value:0.000, Pr-value:0.000  
eCLIP MATCHES▶pum1 (bg=29.85%)MATCHES To TargetScan▶ miR-15-5p/16-5p/195-5p/424-5p/497-5p:AGCAGCA▶ miR-503-5p:AGCAGCG


CCTGTGTATATAATATGAAAAAGCTGCT

CCTGTGTATATAATATGAAAAAGCTGCT  
Depth:9 (MOUSE)  
Ei-value:0.000, Pi-value:0.000  
Er-value:0.000, Pr-value:0.000  
eCLIP MATCHES▶pum1 (bg=29.85%)MATCHES To TargetScan▶ miR-15-5p/16-5p/195-5p/424-5p/497-5p:AGCAGCA▶ miR-503-5p:AGCAGCG


C

AAGGCCTGTGTATATAATATGAAAAAGCTGCTCTCAACT  
Depth:6 (PIG)  
Ei-value:0.000, Pi-value:0.000  
Er-value:0.000, Pr-value:0.000  
eCLIP MATCHES▶pum1 (bg=29.85%)MATCHES To TargetScan▶ miR-15-5p/16-5p/195-5p/424-5p/497-5p:AGCAGCA▶ miR-503-5p:AGCAGCG


TCAAC

TCAACT  
Depth:7 (ARMADILLO)  
Ei-value:0.000, Pi-value:0.000  
Er-value:0.000, Pr-value:0.000  
eCLIP MATCHES▶pum1 (bg=29.85%)No matches to TargetScan

 840  


T

TCAACT  
Depth:7 (ARMADILLO)  
Ei-value:0.000, Pi-value:0.000  
Er-value:0.000, Pr-value:0.000  
eCLIP MATCHES▶pum1 (bg=29.85%)No matches to TargetScan

CCA

CCC

CCCCAACCTTT  
Depth:7 (ARMADILLO)  
Ei-value:0.000, Pi-value:0.000  
Er-value:0.000, Pr-value:0.000  
eCLIP MATCHES▶pum1 (bg=29.85%)No matches to TargetScan


CAACCTTT

CAACCTTT  
Depth:9 (MOUSE)  
Ei-value:0.000, Pi-value:0.000  
Er-value:0.000, Pr-value:0.000  
eCLIP MATCHES▶pum1 (bg=29.85%)No matches to TargetScan


T

CCCCAACCTTTT  
Depth:3 (DOG)  
Ei-value:0.000, Pi-value:0.000  
Er-value:0.000, Pr-value:0.000  
eCLIP MATCHES▶pum1 (bg=29.85%)No matches to TargetScan

AAT

AGAAAAC

AGAAAAC  
Depth:8 (GUINEAPIG)  
Ei-value:0.000, Pi-value:0.000  
Er-value:0.000, Pr-value:0.000  
eCLIP MATCHES▶pum1 (bg=29.85%)No matches to TargetScan

ATTTGT

C

CACATCTAG  
Depth:3 (DOG)  
Ei-value:0.000, Pi-value:0.000  
Er-value:0.000, Pr-value:0.000  
eCLIP MATCHES▶pum1 (bg=29.85%)▶rbm15 (bg=11.81%)No matches to TargetScan


ACATCTAG

ACATCTAG  
Depth:7 (ARMADILLO)  
Ei-value:0.000, Pi-value:0.000  
Er-value:0.000, Pr-value:0.000  
eCLIP MATCHES▶pum1 (bg=29.85%)▶rbm15 (bg=11.81%)No matches to TargetScan

CCCTTC

TAGATG

TAGATG  
Depth:8 (GUINEAPIG)  
Ei-value:0.000, Pi-value:0.000  
Er-value:0.000, Pr-value:0.000  
eCLIP MATCHES▶dgcr8 (bg=19.31%)▶KHDRBS1 (bg=1.16%)▶pum1 (bg=29.85%)▶rbm15 (bg=11.81%)No matches to TargetScan

G

A

AAAGAGGTTGCCGAC  
Depth:4 (COW)  
Ei-value:0.020, Pi-value:0.000  
Er-value:0.000, Pr-value:0.000  
eCLIP MATCHES▶dgcr8 (bg=19.31%)▶IGF2BP2 (bg=3.44%)▶KHDRBS1 (bg=1.16%)▶lin28b (bg=16.96%)▶pum1 (bg=29.85%)▶pum2 (bg=21.55%)▶rbm15 (bg=11.81%)▶tia1 (bg=16.04%)No matches to TargetScan


A

AAGAGGT  
Depth:8 (GUINEAPIG)  
Ei-value:0.000, Pi-value:0.000  
Er-value:0.000, Pr-value:0.000  
eCLIP MATCHES▶dgcr8 (bg=19.31%)▶IGF2BP2 (bg=3.44%)▶KHDRBS1 (bg=1.16%)▶lin28b (bg=16.96%)▶pum1 (bg=29.85%)▶pum2 (bg=21.55%)▶rbm15 (bg=11.81%)▶tia1 (bg=16.04%)No matches to TargetScan


AGAGGT

AGAGGT  
Depth:9 (MOUSE)  
Ei-value:0.000, Pi-value:0.000  
Er-value:0.000, Pr-value:0.000  
eCLIP MATCHES▶dgcr8 (bg=19.31%)▶IGF2BP2 (bg=3.44%)▶KHDRBS1 (bg=1.16%)▶lin28b (bg=16.96%)▶pum1 (bg=29.85%)▶pum2 (bg=21.55%)▶rbm15 (bg=11.81%)▶tia1 (bg=16.04%)No matches to TargetScan


T

AAGAGGTTGCCGAC  
Depth:6 (PIG)  
Ei-value:0.000, Pi-value:0.000  
Er-value:0.000, Pr-value:0.000  
eCLIP MATCHES▶dgcr8 (bg=19.31%)▶IGF2BP2 (bg=3.44%)▶KHDRBS1 (bg=1.16%)▶lin28b (bg=16.96%)▶pum1 (bg=29.85%)▶pum2 (bg=21.55%)▶rbm15 (bg=11.81%)▶tia1 (bg=16.04%)No matches to TargetScan


GCCGAC

GCCGAC  
Depth:7 (ARMADILLO)  
Ei-value:0.000, Pi-value:0.000  
Er-value:0.000, Pr-value:0.000  
eCLIP MATCHES▶dgcr8 (bg=19.31%)▶IGF2BP2 (bg=3.44%)▶KHDRBS1 (bg=1.16%)▶lin28b (bg=16.96%)▶pum1 (bg=29.85%)▶pum2 (bg=21.55%)▶rbm15 (bg=11.81%)▶tia1 (bg=16.04%)No matches to TargetScan

G

TATGATAAA

TATGATAAA  
Depth:8 (GUINEAPIG)  
Ei-value:0.000, Pi-value:0.000  
Er-value:0.000, Pr-value:0.000  
eCLIP MATCHES▶dgcr8 (bg=19.31%)▶IGF2BP2 (bg=3.44%)▶KHDRBS1 (bg=1.16%)▶lin28b (bg=16.96%)▶pum1 (bg=29.85%)▶pum2 (bg=21.55%)▶rbm15 (bg=11.81%)▶tia1 (bg=16.04%)MATCHES To TargetScan▶ miR-154-3p/487-3p:AUCAUAC

A

TAG

TAGAGTTAGAAA  
Depth:3 (DOG)  
Ei-value:0.000, Pi-value:0.000  
Er-value:0.000, Pr-value:0.000  
eCLIP MATCHES▶dgcr8 (bg=19.31%)▶IGF2BP2 (bg=3.44%)▶KHDRBS1 (bg=1.16%)▶lin28b (bg=16.96%)▶pum1 (bg=29.85%)▶pum2 (bg=21.55%)▶rbm15 (bg=11.81%)▶tia1 (bg=16.04%)No matches to TargetScan


AGTTAG

AGTTAG  
Depth:8 (GUINEAPIG)  
Ei-value:0.000, Pi-value:0.000  
Er-value:0.000, Pr-value:0.000  
eCLIP MATCHES▶dgcr8 (bg=19.31%)▶IGF2BP2 (bg=3.44%)▶KHDRBS1 (bg=1.16%)▶lin28b (bg=16.96%)▶pum2 (bg=21.55%)▶rbm15 (bg=11.81%)▶tia1 (bg=16.04%)No matches to TargetScan


AAA

AGTTAGAAA  
Depth:7 (ARMADILLO)  
Ei-value:0.000, Pi-value:0.000  
Er-value:0.000, Pr-value:0.000  
eCLIP MATCHES▶dgcr8 (bg=19.31%)▶IGF2BP2 (bg=3.44%)▶KHDRBS1 (bg=1.16%)▶lin28b (bg=16.96%)▶pum2 (bg=21.55%)▶rbm15 (bg=11.81%)▶tia1 (bg=16.04%)No matches to TargetScan

GTTACACA

TC

TCTTGTAAAT  
Depth:6 (PIG)  
Ei-value:0.000, Pi-value:0.000  
Er-value:0.000, Pr-value:0.000  
eCLIP MATCHES▶KHDRBS1 (bg=1.16%)▶lin28b (bg=16.96%)No matches to TargetScan


TTGTAA

TTGTAA  
Depth:7 (ARMADILLO)  
Ei-value:0.000, Pi-value:0.020  
Er-value:0.000, Pr-value:0.020  
eCLIP MATCHES▶KHDRBS1 (bg=1.16%)▶lin28b (bg=16.96%)No matches to TargetScan


AT

TCTTGTAAAT  
Depth:6 (PIG)  
Ei-value:0.000, Pi-value:0.000  
Er-value:0.000, Pr-value:0.000  
eCLIP MATCHES▶KHDRBS1 (bg=1.16%)▶lin28b (bg=16.96%)No matches to TargetScan

TCTCA

TTTGT

TTTGTTT  
Depth:5 (SHEEP)  
Ei-value:0.000, Pi-value:0.020  
Er-value:0.000, Pr-value:0.010  
No matches to eCLIP DataMATCHES To TargetScan▶ miR-495-3p:AACAAAC

 960  


TT

TTTGTTT  
Depth:5 (SHEEP)  
Ei-value:0.000, Pi-value:0.020  
Er-value:0.000, Pr-value:0.010  
No matches to eCLIP DataMATCHES To TargetScan▶ miR-495-3p:AACAAAC

AAAAG

AAATC

AAATCATAGAAA  
Depth:3 (DOG)  
Ei-value:0.000, Pi-value:0.000  
Er-value:0.000, Pr-value:0.000  
eCLIP MATCHES▶fubp3 (bg=23.31%)▶GRWD1 (bg=4.85%)▶pum1 (bg=29.85%)▶pum2 (bg=21.55%)▶rbm15 (bg=11.81%)▶sf3a3 (bg=12.13%)▶ZNF622 (bg=1.54%)No matches to TargetScan


ATAGAAA

ATAGAAA  
Depth:5 (SHEEP)  
Ei-value:0.000, Pi-value:0.000  
Er-value:0.000, Pr-value:0.000  
eCLIP MATCHES▶fubp3 (bg=23.31%)▶GRWD1 (bg=4.85%)▶pum1 (bg=29.85%)▶pum2 (bg=21.55%)▶rbm15 (bg=11.81%)▶sf3a3 (bg=12.13%)▶ZNF622 (bg=1.54%)No matches to TargetScan

ATACATGTCTTCTGGAGATGA

CTTT

CTTTTGGAAATG  
Depth:3 (DOG)  
Ei-value:0.000, Pi-value:0.000  
Er-value:0.000, Pr-value:0.000  
eCLIP MATCHES▶fubp3 (bg=23.31%)▶GRWD1 (bg=4.85%)▶PPIG (bg=0.88%)▶pum1 (bg=29.85%)▶pum2 (bg=21.55%)▶rbm15 (bg=11.81%)▶SND1 (bg=1.27%)▶ZNF622 (bg=1.54%)No matches to TargetScan


TG

TGGAAATG  
Depth:8 (GUINEAPIG)  
Ei-value:0.000, Pi-value:0.000  
Er-value:0.000, Pr-value:0.000  
eCLIP MATCHES▶fubp3 (bg=23.31%)▶GRWD1 (bg=4.85%)▶PPIG (bg=0.88%)▶pum1 (bg=29.85%)▶pum2 (bg=21.55%)▶rbm15 (bg=11.81%)▶SND1 (bg=1.27%)▶ZNF622 (bg=1.54%)No matches to TargetScan


GAAATG

GAAATG  
Depth:9 (MOUSE)  
Ei-value:0.000, Pi-value:0.000  
Er-value:0.000, Pr-value:0.000  
eCLIP MATCHES▶fubp3 (bg=23.31%)▶GRWD1 (bg=4.85%)▶PPIG (bg=0.88%)▶pum1 (bg=29.85%)▶pum2 (bg=21.55%)▶rbm15 (bg=11.81%)▶SND1 (bg=1.27%)▶ZNF622 (bg=1.54%)No matches to TargetScan

GAGTTGTTAAGACGGCCTCTG

GAAGCGA

GAAGCGA  
Depth:3 (DOG)  
Ei-value:0.000, Pi-value:0.000  
Er-value:0.000, Pr-value:0.000  
eCLIP MATCHES▶fubp3 (bg=23.31%)▶GNL3 (bg=0.85%)▶GRWD1 (bg=4.85%)▶lin28b (bg=16.96%)▶PPIG (bg=0.88%)▶pum1 (bg=29.85%)▶rbm15 (bg=11.81%)▶ZNF622 (bg=1.54%)No matches to TargetScan

TACGTCCACGTTTGTTA

A

AGTGGGT  
Depth:6 (PIG)  
Ei-value:0.000, Pi-value:0.000  
Er-value:0.000, Pr-value:0.000  
eCLIP MATCHES▶GNL3 (bg=0.85%)▶PPIG (bg=0.88%)▶pum1 (bg=29.85%)▶ZNF622 (bg=1.54%)No matches to TargetScan


GTGGGT

GTGGGT  
Depth:7 (ARMADILLO)  
Ei-value:0.000, Pi-value:0.000  
Er-value:0.000, Pr-value:0.000  
eCLIP MATCHES▶GNL3 (bg=0.85%)▶PPIG (bg=0.88%)No matches to TargetScan

TAGAT

gacatg

gacatg  
Depth:3 (DOG)  
Ei-value:0.080, Pi-value:0.010  
Er-value:0.000, Pr-value:0.000  
eCLIP MATCHES▶GNL3 (bg=0.85%)No matches to TargetScan

GAGCT 1080  


GGAAGA

GGAAGA  
Depth:6 (PIG)  
Ei-value:0.000, Pi-value:0.000  
Er-value:0.000, Pr-value:0.000  
No matches to eCLIP DataNo matches to TargetScan

CCTGAGAAGGAAGAGA

AGAAGGTTCT

AGAAGGTTCT  
Depth:3 (DOG)  
Ei-value:0.000, Pi-value:0.000  
Er-value:0.000, Pr-value:0.000  
No matches to eCLIP DataNo matches to TargetScan

ATGCT

AGACTG

AGACTGGTCATA  
Depth:5 (SHEEP)  
Ei-value:0.000, Pi-value:0.000  
Er-value:0.000, Pr-value:0.000  
No matches to eCLIP DataNo matches to TargetScan


GTCATA

GTCATA  
Depth:7 (ARMADILLO)  
Ei-value:0.000, Pi-value:0.000  
Er-value:0.000, Pr-value:0.000  
No matches to eCLIP DataNo matches to TargetScan

T

T

TTAGAAGA  
Depth:7 (ARMADILLO)  
Ei-value:0.000, Pi-value:0.000  
Er-value:0.000, Pr-value:0.000  
No matches to eCLIP DataNo matches to TargetScan


T

TAGAAGA  
Depth:8 (GUINEAPIG)  
Ei-value:0.000, Pi-value:0.000  
Er-value:0.000, Pr-value:0.000  
No matches to eCLIP DataNo matches to TargetScan


AGAAGA

AGAAGA  
Depth:9 (MOUSE)  
Ei-value:0.000, Pi-value:0.000  
Er-value:0.000, Pr-value:0.000  
No matches to eCLIP DataNo matches to TargetScan


CATTT

TTAGAAGACATTT  
Depth:6 (PIG)  
Ei-value:0.000, Pi-value:0.000  
Er-value:0.000, Pr-value:0.000  
No matches to eCLIP DataNo matches to TargetScan


TCA

TTAGAAGACATTTTCA  
Depth:3 (DOG)  
Ei-value:0.000, Pi-value:0.000  
Er-value:0.000, Pr-value:0.000  
No matches to eCLIP DataNo matches to TargetScan

TATTCTAT

CCATTGTTT

CCATTGTTT  
Depth:3 (DOG)  
Ei-value:0.000, Pi-value:0.010  
Er-value:0.000, Pr-value:0.000  
No matches to eCLIP DataNo matches to TargetScan


TGTGTGCATTTT

TGTGTGCATTTT  
Depth:6 (PIG)  
Ei-value:0.000, Pi-value:0.000  
Er-value:0.000, Pr-value:0.000  
No matches to eCLIP DataMATCHES To TargetScan▶ miR-501-3p/502-3p:AUGCACC


A

TGTGTGCATTTTATTCCTC  
Depth:3 (DOG)  
Ei-value:0.000, Pi-value:0.000  
Er-value:0.000, Pr-value:0.000  
No matches to eCLIP DataMATCHES To TargetScan▶ miR-501-3p/502-3p:AUGCACC


TTCCTC

TTCCTC  
Depth:7 (ARMADILLO)  
Ei-value:0.000, Pi-value:0.000  
Er-value:0.000, Pr-value:0.000  
No matches to eCLIP DataNo matches to TargetScan


ACTACTG

ACTACTGTGTATATA  
Depth:3 (DOG)  
Ei-value:0.000, Pi-value:0.000  
Er-value:0.000, Pr-value:0.000  
No matches to eCLIP DataMATCHES To TargetScan▶ miR-199-3p:CAGUAGU▶ miR-144-3p:ACAGUAU▶ miR-128-3p:CACAGUG▶ miR-101-3p.1:ACAGUAC


TG

TGTATATA  
Depth:9 (MOUSE)  
Ei-value:0.000, Pi-value:0.000  
Er-value:0.000, Pr-value:0.000  
No matches to eCLIP DataNo matches to TargetScan


TATATA

TATATA  
Depth:6 (PIG)  
Ei-value:0.000, Pi-value:0.030  
Er-value:0.000, Pr-value:0.000  
No matches to eCLIP DataNo matches to TargetScan

G

TT

TTGACAATGCTAAG  
Depth:3 (DOG)  
Ei-value:0.000, Pi-value:0.000  
Er-value:0.000, Pr-value:0.000  
No matches to eCLIP DataNo matches to TargetScan

 1200  


GACAATGCTAAG

TTGACAATGCTAAG  
Depth:3 (DOG)  
Ei-value:0.000, Pi-value:0.000  
Er-value:0.000, Pr-value:0.000  
No matches to eCLIP DataNo matches to TargetScan

CTTTTTTGAAATGTCTCTTCTTTT

TAGATG

TAGATGTTCTGAAGTGCCTGA  
Depth:5 (SHEEP)  
Ei-value:0.000, Pi-value:0.000  
Er-value:0.000, Pr-value:0.000  
eCLIP MATCHES▶pum1 (bg=29.85%)▶PUS1 (bg=2.87%)▶tial1 (bg=14.09%)No matches to TargetScan


TTCTG

TTCTGAAGTGCCTGA  
Depth:7 (ARMADILLO)  
Ei-value:0.000, Pi-value:0.000  
Er-value:0.000, Pr-value:0.000  
eCLIP MATCHES▶pum1 (bg=29.85%)▶PUS1 (bg=2.87%)▶tial1 (bg=14.09%)No matches to TargetScan


AAGTGCCTG

AAGTGCCTG  
Depth:9 (MOUSE)  
Ei-value:0.000, Pi-value:0.000  
Er-value:0.000, Pr-value:0.000  
eCLIP MATCHES▶pum1 (bg=29.85%)▶PUS1 (bg=2.87%)▶tial1 (bg=14.09%)No matches to TargetScan


A

AAGTGCCTGA  
Depth:8 (GUINEAPIG)  
Ei-value:0.000, Pi-value:0.000  
Er-value:0.000, Pr-value:0.000  
eCLIP MATCHES▶pum1 (bg=29.85%)▶PUS1 (bg=2.87%)▶tial1 (bg=14.09%)No matches to TargetScan

T

A

ATGTTAAAA  
Depth:6 (PIG)  
Ei-value:0.000, Pi-value:0.000  
Er-value:0.000, Pr-value:0.000  
eCLIP MATCHES▶ddx55 (bg=12.35%)▶pum1 (bg=29.85%)▶PUS1 (bg=2.87%)▶tial1 (bg=14.09%)No matches to TargetScan


T

TGTTAAAA  
Depth:7 (ARMADILLO)  
Ei-value:0.000, Pi-value:0.000  
Er-value:0.000, Pr-value:0.000  
eCLIP MATCHES▶ddx55 (bg=12.35%)▶pum1 (bg=29.85%)▶PUS1 (bg=2.87%)▶tial1 (bg=14.09%)No matches to TargetScan


GTTAAAA

GTTAAAA  
Depth:8 (GUINEAPIG)  
Ei-value:0.000, Pi-value:0.000  
Er-value:0.000, Pr-value:0.000  
eCLIP MATCHES▶ddx55 (bg=12.35%)▶pum1 (bg=29.85%)▶PUS1 (bg=2.87%)▶tial1 (bg=14.09%)No matches to TargetScan

T

TA

TAGAGGTAG  
Depth:3 (DOG)  
Ei-value:0.000, Pi-value:0.000  
Er-value:0.000, Pr-value:0.000  
eCLIP MATCHES▶ddx55 (bg=12.35%)▶pum1 (bg=29.85%)▶PUS1 (bg=2.87%)▶tial1 (bg=14.09%)No matches to TargetScan


GAGGTAG

GAGGTAG  
Depth:6 (PIG)  
Ei-value:0.000, Pi-value:0.000  
Er-value:0.000, Pr-value:0.000  
eCLIP MATCHES▶ddx55 (bg=12.35%)▶pum1 (bg=29.85%)▶PUS1 (bg=2.87%)▶tial1 (bg=14.09%)No matches to TargetScan

CAAAATC

ACA

ACATTTTGTAAATA  
Depth:5 (SHEEP)  
Ei-value:0.000, Pi-value:0.000  
Er-value:0.000, Pr-value:0.000  
eCLIP MATCHES▶CPEB4 (bg=2.3%)▶ddx55 (bg=12.35%)▶drosha (bg=11.37%)▶pum1 (bg=29.85%)▶PUS1 (bg=2.87%)▶tial1 (bg=14.09%)No matches to TargetScan


TTTTGT

TTTTGT  
Depth:6 (PIG)  
Ei-value:0.000, Pi-value:0.020  
Er-value:0.000, Pr-value:0.040  
eCLIP MATCHES▶CPEB4 (bg=2.3%)▶pum1 (bg=29.85%)▶PUS1 (bg=2.87%)▶tial1 (bg=14.09%)No matches to TargetScan


AAATA

ACATTTTGTAAATA  
Depth:5 (SHEEP)  
Ei-value:0.000, Pi-value:0.000  
Er-value:0.000, Pr-value:0.000  
eCLIP MATCHES▶CPEB4 (bg=2.3%)▶ddx55 (bg=12.35%)▶drosha (bg=11.37%)▶pum1 (bg=29.85%)▶PUS1 (bg=2.87%)▶tial1 (bg=14.09%)No matches to TargetScan

CCTTTTTGTTACA

ATTCATA

ATTCATA  
Depth:5 (SHEEP)  
Ei-value:0.000, Pi-value:0.000  
Er-value:0.000, Pr-value:0.000  
eCLIP MATCHES▶AGGF1 (bg=0.88%)▶CPEB4 (bg=2.3%)▶ddx55 (bg=12.35%)▶ddx6 (bg=23.92%)▶dgcr8 (bg=19.31%)▶drosha (bg=11.37%)▶fam120a (bg=18.43%)▶FASTKD2 (bg=7.73%)▶fubp3 (bg=23.31%)▶igf2bp1 (bg=11.67%)▶pum1 (bg=29.85%)▶pum2 (bg=21.55%)▶rbm15 (bg=11.81%)▶sf3a3 (bg=12.13%)▶SF3B1 (bg=6.74%)▶tia1 (bg=16.04%)▶tial1 (bg=14.09%)▶UPF1 (bg=3.62%)No matches to TargetScan


GG

ATTCATAGGAAAT  
Depth:3 (DOG)  
Ei-value:0.000, Pi-value:0.000  
Er-value:0.000, Pr-value:0.000  
eCLIP MATCHES▶AGGF1 (bg=0.88%)▶CPEB4 (bg=2.3%)▶ddx55 (bg=12.35%)▶ddx6 (bg=23.92%)▶dgcr8 (bg=19.31%)▶drosha (bg=11.37%)▶fam120a (bg=18.43%)▶FASTKD2 (bg=7.73%)▶FTO (bg=7.53%)▶fubp3 (bg=23.31%)▶FXR2 (bg=7.51%)▶igf2bp1 (bg=11.67%)▶IGF2BP2 (bg=3.44%)▶IGF2BP3 (bg=4.26%)▶pum1 (bg=29.85%)▶pum2 (bg=21.55%)▶rbm15 (bg=11.81%)▶sf3a3 (bg=12.13%)▶SF3B1 (bg=6.74%)▶tia1 (bg=16.04%)▶tial1 (bg=14.09%)▶UPF1 (bg=3.62%)MATCHES To TargetScan▶ miR-202-5p:UCCUAUG

 1320  


AAAT

ATTCATAGGAAAT  
Depth:3 (DOG)  
Ei-value:0.000, Pi-value:0.000  
Er-value:0.000, Pr-value:0.000  
eCLIP MATCHES▶AGGF1 (bg=0.88%)▶CPEB4 (bg=2.3%)▶ddx55 (bg=12.35%)▶ddx6 (bg=23.92%)▶dgcr8 (bg=19.31%)▶drosha (bg=11.37%)▶fam120a (bg=18.43%)▶FASTKD2 (bg=7.73%)▶FTO (bg=7.53%)▶fubp3 (bg=23.31%)▶FXR2 (bg=7.51%)▶igf2bp1 (bg=11.67%)▶IGF2BP2 (bg=3.44%)▶IGF2BP3 (bg=4.26%)▶pum1 (bg=29.85%)▶pum2 (bg=21.55%)▶rbm15 (bg=11.81%)▶sf3a3 (bg=12.13%)▶SF3B1 (bg=6.74%)▶tia1 (bg=16.04%)▶tial1 (bg=14.09%)▶UPF1 (bg=3.62%)MATCHES To TargetScan▶ miR-202-5p:UCCUAUG

ATTTTTGGG

GGGGAA

GGGGAATGGCCAAA  
Depth:3 (DOG)  
Ei-value:0.000, Pi-value:0.000  
Er-value:0.000, Pr-value:0.000  
eCLIP MATCHES▶AGGF1 (bg=0.88%)▶CPEB4 (bg=2.3%)▶ddx55 (bg=12.35%)▶ddx6 (bg=23.92%)▶dgcr8 (bg=19.31%)▶drosha (bg=11.37%)▶fam120a (bg=18.43%)▶FASTKD2 (bg=7.73%)▶FTO (bg=7.53%)▶fubp3 (bg=23.31%)▶FXR2 (bg=7.51%)▶igf2bp1 (bg=11.67%)▶IGF2BP2 (bg=3.44%)▶IGF2BP3 (bg=4.26%)▶pum1 (bg=29.85%)▶pum2 (bg=21.55%)▶rbm15 (bg=11.81%)▶sf3a3 (bg=12.13%)▶SF3B1 (bg=6.74%)▶tia1 (bg=16.04%)▶tial1 (bg=14.09%)▶UPF1 (bg=3.62%)No matches to TargetScan


TGGCCA

TGGCCA  
Depth:6 (PIG)  
Ei-value:0.000, Pi-value:0.000  
Er-value:0.000, Pr-value:0.000  
eCLIP MATCHES▶AGGF1 (bg=0.88%)▶CPEB4 (bg=2.3%)▶ddx55 (bg=12.35%)▶ddx6 (bg=23.92%)▶dgcr8 (bg=19.31%)▶drosha (bg=11.37%)▶fam120a (bg=18.43%)▶FASTKD2 (bg=7.73%)▶FTO (bg=7.53%)▶fubp3 (bg=23.31%)▶FXR2 (bg=7.51%)▶igf2bp1 (bg=11.67%)▶IGF2BP2 (bg=3.44%)▶IGF2BP3 (bg=4.26%)▶pum1 (bg=29.85%)▶pum2 (bg=21.55%)▶rbm15 (bg=11.81%)▶sf3a3 (bg=12.13%)▶SF3B1 (bg=6.74%)▶tia1 (bg=16.04%)▶tial1 (bg=14.09%)▶UPF1 (bg=3.62%)No matches to TargetScan


AA

GGGGAATGGCCAAA  
Depth:3 (DOG)  
Ei-value:0.000, Pi-value:0.000  
Er-value:0.000, Pr-value:0.000  
eCLIP MATCHES▶AGGF1 (bg=0.88%)▶CPEB4 (bg=2.3%)▶ddx55 (bg=12.35%)▶ddx6 (bg=23.92%)▶dgcr8 (bg=19.31%)▶drosha (bg=11.37%)▶fam120a (bg=18.43%)▶FASTKD2 (bg=7.73%)▶FTO (bg=7.53%)▶fubp3 (bg=23.31%)▶FXR2 (bg=7.51%)▶igf2bp1 (bg=11.67%)▶IGF2BP2 (bg=3.44%)▶IGF2BP3 (bg=4.26%)▶pum1 (bg=29.85%)▶pum2 (bg=21.55%)▶rbm15 (bg=11.81%)▶sf3a3 (bg=12.13%)▶SF3B1 (bg=6.74%)▶tia1 (bg=16.04%)▶tial1 (bg=14.09%)▶UPF1 (bg=3.62%)No matches to TargetScan

TCACCTGTTGAGTAATACT

CATTGTG

CATTGTGTTTGTGCA  
Depth:3 (DOG)  
Ei-value:0.000, Pi-value:0.000  
Er-value:0.000, Pr-value:0.000  
eCLIP MATCHES▶ddx55 (bg=12.35%)▶drosha (bg=11.37%)▶fam120a (bg=18.43%)▶FXR2 (bg=7.51%)▶igf2bp1 (bg=11.67%)▶IGF2BP2 (bg=3.44%)▶IGF2BP3 (bg=4.26%)▶pum1 (bg=29.85%)▶tia1 (bg=16.04%)▶UPF1 (bg=3.62%)No matches to TargetScan


T

TTTGTGCA  
Depth:7 (ARMADILLO)  
Ei-value:0.000, Pi-value:0.000  
Er-value:0.000, Pr-value:0.000  
eCLIP MATCHES▶drosha (bg=11.37%)▶igf2bp1 (bg=11.67%)▶IGF2BP2 (bg=3.44%)▶IGF2BP3 (bg=4.26%)▶pum1 (bg=29.85%)▶tia1 (bg=16.04%)No matches to TargetScan


TTGTGC

TTGTGC  
Depth:9 (MOUSE)  
Ei-value:0.000, Pi-value:0.000  
Er-value:0.000, Pr-value:0.000  
eCLIP MATCHES▶pum1 (bg=29.85%)▶tia1 (bg=16.04%)No matches to TargetScan


A

TTGTGCA  
Depth:8 (GUINEAPIG)  
Ei-value:0.000, Pi-value:0.000  
Er-value:0.000, Pr-value:0.000  
eCLIP MATCHES▶pum1 (bg=29.85%)▶tia1 (bg=16.04%)No matches to TargetScan

G

tggttc

tggttc  
Depth:3 (DOG)  
Ei-value:0.080, Pi-value:0.030  
Er-value:0.000, Pr-value:0.000  
eCLIP MATCHES▶dgcr8 (bg=19.31%)▶fam120a (bg=18.43%)▶KHSRP (bg=8.1%)▶pum1 (bg=29.85%)No matches to TargetScan

AGGGGA

GGAGAG

GGAGAG  
Depth:5 (SHEEP)  
Ei-value:0.000, Pi-value:0.030  
Er-value:0.000, Pr-value:0.000  
eCLIP MATCHES▶CDC40 (bg=1.71%)▶ddx55 (bg=12.35%)▶dgcr8 (bg=19.31%)▶fam120a (bg=18.43%)▶FASTKD2 (bg=7.73%)▶FTO (bg=7.53%)▶GTF2F1 (bg=2.31%)▶igf2bp1 (bg=11.67%)▶KHSRP (bg=8.1%)▶pum1 (bg=29.85%)▶sf3a3 (bg=12.13%)▶TBRG4 (bg=7.0%)▶tia1 (bg=16.04%)▶UPF1 (bg=3.62%)No matches to TargetScan

AGGAGGGGGAGGTGCAGAGAGCTCTATGCCATCCTGTTTA 1440  
 CAGCGAGGCAAGATGAATCATTATGTCTG

TGCA

TGCATTTTGTTT  
Depth:4 (COW)  
Ei-value:0.000, Pi-value:0.000  
Er-value:0.000, Pr-value:0.000  
eCLIP MATCHES▶AKAP1 (bg=3.4%)▶ddx3x (bg=13.89%)▶ddx55 (bg=12.35%)▶dgcr8 (bg=19.31%)▶drosha (bg=11.37%)▶fam120a (bg=18.43%)▶FTO (bg=7.53%)▶fubp3 (bg=23.31%)▶igf2bp1 (bg=11.67%)▶pum1 (bg=29.85%)▶pum2 (bg=21.55%)▶sf3a3 (bg=12.13%)▶SUB1 (bg=9.24%)▶tia1 (bg=16.04%)▶XRCC6 (bg=1.65%)MATCHES To TargetScan▶ miR-495-3p:AACAAAC


TTTTGTTT

TTTTGTTT  
Depth:5 (SHEEP)  
Ei-value:0.000, Pi-value:0.000  
Er-value:0.000, Pr-value:0.000  
eCLIP MATCHES▶AKAP1 (bg=3.4%)▶ddx55 (bg=12.35%)▶dgcr8 (bg=19.31%)▶drosha (bg=11.37%)▶fam120a (bg=18.43%)▶FTO (bg=7.53%)▶fubp3 (bg=23.31%)▶igf2bp1 (bg=11.67%)▶pum1 (bg=29.85%)▶pum2 (bg=21.55%)▶sf3a3 (bg=12.13%)▶SUB1 (bg=9.24%)▶XRCC6 (bg=1.65%)MATCHES To TargetScan▶ miR-495-3p:AACAAAC


TACTTA

TGCATTTTGTTTTACTTA  
Depth:3 (DOG)  
Ei-value:0.000, Pi-value:0.000  
Er-value:0.000, Pr-value:0.000  
eCLIP MATCHES▶AKAP1 (bg=3.4%)▶ddx3x (bg=13.89%)▶ddx55 (bg=12.35%)▶dgcr8 (bg=19.31%)▶drosha (bg=11.37%)▶fam120a (bg=18.43%)▶FTO (bg=7.53%)▶fubp3 (bg=23.31%)▶igf2bp1 (bg=11.67%)▶pum1 (bg=29.85%)▶pum2 (bg=21.55%)▶sf3a3 (bg=12.13%)▶SUB1 (bg=9.24%)▶tia1 (bg=16.04%)▶tial1 (bg=14.09%)▶TROVE2 (bg=3.49%)▶XRCC6 (bg=1.65%)MATCHES To TargetScan▶ miR-495-3p:AACAAAC

T

CTGTGT

CTGTGTATATAGTGTA  
Depth:3 (DOG)  
Ei-value:0.000, Pi-value:0.000  
Er-value:0.000, Pr-value:0.000  
eCLIP MATCHES▶AKAP1 (bg=3.4%)▶ddx55 (bg=12.35%)▶dgcr8 (bg=19.31%)▶drosha (bg=11.37%)▶fam120a (bg=18.43%)▶FTO (bg=7.53%)▶fubp3 (bg=23.31%)▶igf2bp1 (bg=11.67%)▶NKRF (bg=3.64%)▶pum1 (bg=29.85%)▶pum2 (bg=21.55%)▶sf3a3 (bg=12.13%)▶SUB1 (bg=9.24%)▶tia1 (bg=16.04%)▶tial1 (bg=14.09%)▶TROVE2 (bg=3.49%)▶XRCC6 (bg=1.65%)No matches to TargetScan


ATA

ATATAGTGTA  
Depth:6 (PIG)  
Ei-value:0.000, Pi-value:0.000  
Er-value:0.000, Pr-value:0.000  
eCLIP MATCHES▶AKAP1 (bg=3.4%)▶ddx55 (bg=12.35%)▶dgcr8 (bg=19.31%)▶drosha (bg=11.37%)▶fam120a (bg=18.43%)▶fubp3 (bg=23.31%)▶igf2bp1 (bg=11.67%)▶NKRF (bg=3.64%)▶pum1 (bg=29.85%)▶pum2 (bg=21.55%)▶sf3a3 (bg=12.13%)▶SUB1 (bg=9.24%)▶tia1 (bg=16.04%)▶tial1 (bg=14.09%)▶TROVE2 (bg=3.49%)▶XRCC6 (bg=1.65%)No matches to TargetScan


TAGTGT

TAGTGT  
Depth:7 (ARMADILLO)  
Ei-value:0.000, Pi-value:0.000  
Er-value:0.000, Pr-value:0.010  
eCLIP MATCHES▶AKAP1 (bg=3.4%)▶ddx55 (bg=12.35%)▶dgcr8 (bg=19.31%)▶drosha (bg=11.37%)▶fam120a (bg=18.43%)▶fubp3 (bg=23.31%)▶igf2bp1 (bg=11.67%)▶NKRF (bg=3.64%)▶pum1 (bg=29.85%)▶pum2 (bg=21.55%)▶sf3a3 (bg=12.13%)▶SUB1 (bg=9.24%)▶tia1 (bg=16.04%)▶tial1 (bg=14.09%)▶TROVE2 (bg=3.49%)▶XRCC6 (bg=1.65%)No matches to TargetScan


A

ATATAGTGTA  
Depth:6 (PIG)  
Ei-value:0.000, Pi-value:0.000  
Er-value:0.000, Pr-value:0.000  
eCLIP MATCHES▶AKAP1 (bg=3.4%)▶ddx55 (bg=12.35%)▶dgcr8 (bg=19.31%)▶drosha (bg=11.37%)▶fam120a (bg=18.43%)▶fubp3 (bg=23.31%)▶igf2bp1 (bg=11.67%)▶NKRF (bg=3.64%)▶pum1 (bg=29.85%)▶pum2 (bg=21.55%)▶sf3a3 (bg=12.13%)▶SUB1 (bg=9.24%)▶tia1 (bg=16.04%)▶tial1 (bg=14.09%)▶TROVE2 (bg=3.49%)▶XRCC6 (bg=1.65%)No matches to TargetScan

CATAAAGGACAGAC

GA

GAGTCCTAATT  
Depth:6 (PIG)  
Ei-value:0.000, Pi-value:0.000  
Er-value:0.000, Pr-value:0.000  
eCLIP MATCHES▶AKAP1 (bg=3.4%)▶ddx55 (bg=12.35%)▶dgcr8 (bg=19.31%)▶drosha (bg=11.37%)▶fam120a (bg=18.43%)▶fubp3 (bg=23.31%)▶igf2bp1 (bg=11.67%)▶NKRF (bg=3.64%)▶pum1 (bg=29.85%)▶pum2 (bg=21.55%)▶sf3a3 (bg=12.13%)▶SUB1 (bg=9.24%)▶tia1 (bg=16.04%)▶tial1 (bg=14.09%)▶TROVE2 (bg=3.49%)No matches to TargetScan


GTCCTAATT

GTCCTAATT  
Depth:7 (ARMADILLO)  
Ei-value:0.000, Pi-value:0.000  
Er-value:0.000, Pr-value:0.000  
eCLIP MATCHES▶AKAP1 (bg=3.4%)▶ddx55 (bg=12.35%)▶dgcr8 (bg=19.31%)▶drosha (bg=11.37%)▶fam120a (bg=18.43%)▶fubp3 (bg=23.31%)▶igf2bp1 (bg=11.67%)▶NKRF (bg=3.64%)▶pum1 (bg=29.85%)▶pum2 (bg=21.55%)▶sf3a3 (bg=12.13%)▶SUB1 (bg=9.24%)▶tia1 (bg=16.04%)▶tial1 (bg=14.09%)▶TROVE2 (bg=3.49%)No matches to TargetScan

GA

CA

CAACATCT  
Depth:6 (PIG)  
Ei-value:0.000, Pi-value:0.000  
Er-value:0.000, Pr-value:0.000  
eCLIP MATCHES▶AKAP1 (bg=3.4%)▶pum1 (bg=29.85%)▶pum2 (bg=21.55%)▶tial1 (bg=14.09%)No matches to TargetScan


ACATCT

ACATCT  
Depth:7 (ARMADILLO)  
Ei-value:0.000, Pi-value:0.000  
Er-value:0.000, Pr-value:0.000  
No matches to eCLIP DataNo matches to TargetScan


AGTCTTT

CAACATCTAGTCTTT  
Depth:5 (SHEEP)  
Ei-value:0.000, Pi-value:0.000  
Er-value:0.000, Pr-value:0.000  
eCLIP MATCHES▶AKAP1 (bg=3.4%)▶pum1 (bg=29.85%)▶pum2 (bg=21.55%)▶tial1 (bg=14.09%)MATCHES To TargetScan▶ miR-28-3p:ACUAGAU

CTG

GATGTT

GATGTT  
Depth:9 (MOUSE)  
Ei-value:0.000, Pi-value:0.000  
Er-value:0.000, Pr-value:0.000  
No matches to eCLIP DataNo matches to TargetScan


AAAGA

AAAGAGGTTGCCA  
Depth:9 (MOUSE)  
Ei-value:0.000, Pi-value:0.000  
Er-value:0.000, Pr-value:0.000  
No matches to eCLIP DataNo matches to TargetScan

 1560  


GGTTGCCA

AAAGAGGTTGCCA  
Depth:9 (MOUSE)  
Ei-value:0.000, Pi-value:0.000  
Er-value:0.000, Pr-value:0.000  
No matches to eCLIP DataNo matches to TargetScan


GTGTATGA

AAAGAGGTTGCCAGTGTATGA  
Depth:7 (ARMADILLO)  
Ei-value:0.000, Pi-value:0.000  
Er-value:0.000, Pr-value:0.000  
No matches to eCLIP DataMATCHES To TargetScan▶ miR-539-3p:UCAUACA▶ miR-193-3p:ACUGGCC


CAAAA

GATGTTAAAGAGGTTGCCAGTGTATGACAAAA  
Depth:5 (SHEEP)  
Ei-value:0.000, Pi-value:0.000  
Er-value:0.000, Pr-value:0.000  
No matches to eCLIP DataMATCHES To TargetScan▶ miR-539-3p:UCAUACA▶ miR-193-3p:ACUGGCC

GTAGAGTTAG

TAAACTAATAT

TAAACTAATAT  
Depth:3 (DOG)  
Ei-value:0.000, Pi-value:0.000  
Er-value:0.000, Pr-value:0.000  
No matches to eCLIP DataNo matches to TargetScan

A

TTTTG

TTTTGTACATTTTGT  
Depth:5 (SHEEP)  
Ei-value:0.000, Pi-value:0.000  
Er-value:0.000, Pr-value:0.000  
No matches to eCLIP DataMATCHES To TargetScan▶ miR-493-5p:UGUACAU


TAC

TACATTTTGT  
Depth:7 (ARMADILLO)  
Ei-value:0.000, Pi-value:0.000  
Er-value:0.000, Pr-value:0.000  
No matches to eCLIP DataNo matches to TargetScan


ATTTTGT

ATTTTGT  
Depth:9 (MOUSE)  
Ei-value:0.000, Pi-value:0.000  
Er-value:0.000, Pr-value:0.000  
No matches to eCLIP DataNo matches to TargetScan

TTTACAAGTCCTAGGA

AAGATTGTCTT

AAGATTGTCTT  
Depth:5 (SHEEP)  
Ei-value:0.000, Pi-value:0.000  
Er-value:0.000, Pr-value:0.000  
eCLIP MATCHES▶dgcr8 (bg=19.31%)▶fam120a (bg=18.43%)▶KHSRP (bg=8.1%)▶NOLC1 (bg=6.58%)▶pum1 (bg=29.85%)▶pum2 (bg=21.55%)▶sf3a3 (bg=12.13%)▶tia1 (bg=16.04%)▶tial1 (bg=14.09%)No matches to TargetScan


CTG

AAGATTGTCTTCTGAAAATT  
Depth:3 (DOG)  
Ei-value:0.000, Pi-value:0.000  
Er-value:0.000, Pr-value:0.000  
eCLIP MATCHES▶ddx55 (bg=12.35%)▶dgcr8 (bg=19.31%)▶fam120a (bg=18.43%)▶fubp3 (bg=23.31%)▶KHSRP (bg=8.1%)▶lin28b (bg=16.96%)▶NOLC1 (bg=6.58%)▶pum1 (bg=29.85%)▶pum2 (bg=21.55%)▶sf3a3 (bg=12.13%)▶tia1 (bg=16.04%)▶tial1 (bg=14.09%)No matches to TargetScan


AAAATT

AAAATT  
Depth:5 (SHEEP)  
Ei-value:0.000, Pi-value:0.010  
Er-value:0.000, Pr-value:0.020  
eCLIP MATCHES▶ddx55 (bg=12.35%)▶dgcr8 (bg=19.31%)▶fam120a (bg=18.43%)▶fubp3 (bg=23.31%)▶KHSRP (bg=8.1%)▶lin28b (bg=16.96%)▶NOLC1 (bg=6.58%)▶pum1 (bg=29.85%)▶pum2 (bg=21.55%)▶sf3a3 (bg=12.13%)▶tia1 (bg=16.04%)▶tial1 (bg=14.09%)No matches to TargetScan

TGATGTCTTCTGGGTTGA

tggaga

tggaga  
Depth:3 (DOG)  
Ei-value:0.080, Pi-value:0.030  
Er-value:0.000, Pr-value:0.000  
eCLIP MATCHES▶ddx55 (bg=12.35%)▶dgcr8 (bg=19.31%)▶fam120a (bg=18.43%)▶fubp3 (bg=23.31%)▶KHSRP (bg=8.1%)▶lin28b (bg=16.96%)▶NOLC1 (bg=6.58%)▶pum1 (bg=29.85%)▶pum2 (bg=21.55%)▶sf3a3 (bg=12.13%)▶tia1 (bg=16.04%)▶ZC3H11A (bg=6.25%)No matches to TargetScan

TG 1680  
 GGAAGGGTTCTAGGCC

AGAATGTTC

AGAATGTTC  
Depth:5 (SHEEP)  
Ei-value:0.000, Pi-value:0.000  
Er-value:0.000, Pr-value:0.000  
No matches to eCLIP DataMATCHES To TargetScan▶ miR-181-5p:ACAUUCA▶ miR-543:AACAUUC

ACATTTG

GAAGAC

GAAGAC  
Depth:7 (ARMADILLO)  
Ei-value:0.000, Pi-value:0.000  
Er-value:0.000, Pr-value:0.000  
No matches to eCLIP DataNo matches to TargetScan

TCTTTCAA

ATTATAA

ATTATAA  
Depth:3 (DOG)  
Ei-value:0.000, Pi-value:0.020  
Er-value:0.000, Pr-value:0.000  
No matches to eCLIP DataMATCHES To TargetScan▶ miR-374-5p:UAUAAUA

CTGT

TGTTACA

TGTTACA  
Depth:6 (PIG)  
Ei-value:0.000, Pi-value:0.000  
Er-value:0.000, Pr-value:0.000  
No matches to eCLIP DataMATCHES To TargetScan▶ miR-194-5p:GUAACAG

TGTT

T

TGCAGTTTATTCAAGACTGCT  
Depth:3 (DOG)  
Ei-value:0.000, Pi-value:0.000  
Er-value:0.000, Pr-value:0.000  
No matches to eCLIP DataMATCHES To TargetScan▶ miR-431-5p:GUCUUGC▶ miR-217:ACUGCAU


GCAGTTTATT

GCAGTTTATT  
Depth:6 (PIG)  
Ei-value:0.000, Pi-value:0.000  
Er-value:0.000, Pr-value:0.000  
No matches to eCLIP DataNo matches to TargetScan


CA

GCAGTTTATTCAAGACTGCT  
Depth:5 (SHEEP)  
Ei-value:0.000, Pi-value:0.000  
Er-value:0.000, Pr-value:0.000  
No matches to eCLIP DataMATCHES To TargetScan▶ miR-431-5p:GUCUUGC


AGACTGCT

AGACTGCT  
Depth:6 (PIG)  
Ei-value:0.000, Pi-value:0.000  
Er-value:0.000, Pr-value:0.000  
No matches to eCLIP DataNo matches to TargetScan

GTATACATAGTAGACAAATTAAC

TCCTTA

TCCTTA  
Depth:9 (MOUSE)  
Ei-value:0.000, Pi-value:0.000  
Er-value:0.000, Pr-value:0.000  
No matches to eCLIP DataNo matches to TargetScan

CT 1800  
 TG

AAACATCT

AAACATCT  
Depth:9 (MOUSE)  
Ei-value:0.000, Pi-value:0.000  
Er-value:0.000, Pr-value:0.000  
No matches to eCLIP DataNo matches to TargetScan


AG

AAACATCTAG  
Depth:8 (GUINEAPIG)  
Ei-value:0.000, Pi-value:0.000  
Er-value:0.000, Pr-value:0.000  
No matches to eCLIP DataNo matches to TargetScan


TCT

AAACATCTAGTCT  
Depth:3 (DOG)  
Ei-value:0.000, Pi-value:0.000  
Er-value:0.000, Pr-value:0.000  
No matches to eCLIP DataMATCHES To TargetScan▶ miR-28-3p:ACUAGAU

A

T

TCTAGATGTTTAGAAGTGCCC  
Depth:3 (DOG)  
Ei-value:0.000, Pi-value:0.000  
Er-value:0.000, Pr-value:0.000  
No matches to eCLIP DataNo matches to TargetScan


CTAGATGTTTAGAAGTGCCC

CTAGATGTTTAGAAGTGCCC  
Depth:9 (MOUSE)  
Ei-value:0.000, Pi-value:0.000  
Er-value:0.000, Pr-value:0.000  
No matches to eCLIP DataNo matches to TargetScan

GAT

GTATGTTAAA

GTATGTTAAA  
Depth:9 (MOUSE)  
Ei-value:0.000, Pi-value:0.000  
Er-value:0.000, Pr-value:0.000  
No matches to eCLIP DataNo matches to TargetScan


TGTA

GTATGTTAAATGTA  
Depth:6 (PIG)  
Ei-value:0.000, Pi-value:0.000  
Er-value:0.000, Pr-value:0.000  
No matches to eCLIP DataNo matches to TargetScan

T

AGGTAGT

AGGTAGT  
Depth:6 (PIG)  
Ei-value:0.000, Pi-value:0.000  
Er-value:0.000, Pr-value:0.000  
No matches to eCLIP DataNo matches to TargetScan


AAAATA

AGGTAGTAAAATA  
Depth:3 (DOG)  
Ei-value:0.000, Pi-value:0.000  
Er-value:0.000, Pr-value:0.000  
No matches to eCLIP DataNo matches to TargetScan

CCACTT

TGTAAATA

TGTAAATA  
Depth:9 (MOUSE)  
Ei-value:0.000, Pi-value:0.000  
Er-value:0.000, Pr-value:0.000  
No matches to eCLIP DataNo matches to TargetScan


T

TGTAAATATCTTTTTGCTAAAATTCATAGGAAAT  
Depth:3 (DOG)  
Ei-value:0.000, Pi-value:0.000  
Er-value:0.000, Pr-value:0.000  
eCLIP MATCHES▶fubp3 (bg=23.31%)▶pum1 (bg=29.85%)▶pum2 (bg=21.55%)▶tial1 (bg=14.09%)MATCHES To TargetScan▶ miR-202-5p:UCCUAUG


CTTTTTGCT

CTTTTTGCT  
Depth:5 (SHEEP)  
Ei-value:0.000, Pi-value:0.000  
Er-value:0.000, Pr-value:0.000  
eCLIP MATCHES▶pum1 (bg=29.85%)No matches to TargetScan


A

TGTAAATATCTTTTTGCTAAAATTCATAGGAAAT  
Depth:3 (DOG)  
Ei-value:0.000, Pi-value:0.000  
Er-value:0.000, Pr-value:0.000  
eCLIP MATCHES▶fubp3 (bg=23.31%)▶pum1 (bg=29.85%)▶pum2 (bg=21.55%)▶tial1 (bg=14.09%)MATCHES To TargetScan▶ miR-202-5p:UCCUAUG


AAATTCATAGGAA

AAATTCATAGGAA  
Depth:4 (COW)  
Ei-value:0.000, Pi-value:0.000  
Er-value:0.000, Pr-value:0.000  
eCLIP MATCHES▶fubp3 (bg=23.31%)▶pum1 (bg=29.85%)▶pum2 (bg=21.55%)▶tial1 (bg=14.09%)MATCHES To TargetScan▶ miR-202-5p:UCCUAUG


AT

TGTAAATATCTTTTTGCTAAAATTCATAGGAAAT  
Depth:3 (DOG)  
Ei-value:0.000, Pi-value:0.000  
Er-value:0.000, Pr-value:0.000  
eCLIP MATCHES▶fubp3 (bg=23.31%)▶pum1 (bg=29.85%)▶pum2 (bg=21.55%)▶tial1 (bg=14.09%)MATCHES To TargetScan▶ miR-202-5p:UCCUAUG

GCTTTTGGAAAT 1920  
 T

GAATTGT

GAATTGT  
Depth:3 (DOG)  
Ei-value:0.000, Pi-value:0.000  
Er-value:0.000, Pr-value:0.000  
eCLIP MATCHES▶DDX21 (bg=1.64%)▶ddx6 (bg=23.92%)▶dgcr8 (bg=19.31%)▶fubp3 (bg=23.31%)▶pum1 (bg=29.85%)▶pum2 (bg=21.55%)▶sf3a3 (bg=12.13%)▶TBRG4 (bg=7.0%)▶tia1 (bg=16.04%)▶tial1 (bg=14.09%)No matches to TargetScan

GAAG

ccacct

ccacct  
Depth:3 (DOG)  
Ei-value:0.080, Pi-value:0.000  
Er-value:0.000, Pr-value:0.000  
eCLIP MATCHES▶DDX21 (bg=1.64%)▶ddx6 (bg=23.92%)▶dgcr8 (bg=19.31%)▶fubp3 (bg=23.31%)▶pum1 (bg=29.85%)▶pum2 (bg=21.55%)▶sf3a3 (bg=12.13%)▶TBRG4 (bg=7.0%)▶tia1 (bg=16.04%)▶tial1 (bg=14.09%)No matches to TargetScan

TTGTGAACAGTATAGTAATGTCTATACTTGTTCAATAGTTTA

GAGGAGGT

GAGGAGGT  
Depth:5 (SHEEP)  
Ei-value:0.000, Pi-value:0.000  
Er-value:0.000, Pr-value:0.000  
eCLIP MATCHES▶fam120a (bg=18.43%)▶IGF2BP2 (bg=3.44%)▶pum1 (bg=29.85%)▶sf3a3 (bg=12.13%)No matches to TargetScan

AGG

AGGGAA

AGGGAA  
Depth:5 (SHEEP)  
Ei-value:0.000, Pi-value:0.010  
Er-value:0.000, Pr-value:0.000  
eCLIP MATCHES▶AARS (bg=2.33%)▶fam120a (bg=18.43%)▶IGF2BP2 (bg=3.44%)▶pum1 (bg=29.85%)▶sf3a3 (bg=12.13%)No matches to TargetScan

GAAATTGC

AAAAGGTAAT

AAAAGGTAAT  
Depth:7 (ARMADILLO)  
Ei-value:0.000, Pi-value:0.000  
Er-value:0.000, Pr-value:0.000  
eCLIP MATCHES▶AARS (bg=2.33%)▶ddx6 (bg=23.92%)▶fam120a (bg=18.43%)▶IGF2BP2 (bg=3.44%)▶pum1 (bg=29.85%)No matches to TargetScan


AT

AAAAGGTAATAT  
Depth:6 (PIG)  
Ei-value:0.000, Pi-value:0.000  
Er-value:0.000, Pr-value:0.000  
eCLIP MATCHES▶AARS (bg=2.33%)▶ddx6 (bg=23.92%)▶fam120a (bg=18.43%)▶IGF2BP2 (bg=3.44%)▶pum1 (bg=29.85%)No matches to TargetScan

TACTA

GTGTGTTCATACTTG

GTGTGTTCATACTTGGACATTTTCAGA  
Depth:3 (DOG)  
Ei-value:0.000, Pi-value:0.000  
Er-value:0.000, Pr-value:0.000  
eCLIP MATCHES▶ddx6 (bg=23.92%)▶IGF2BP2 (bg=3.44%)▶pum1 (bg=29.85%)▶pum2 (bg=21.55%)MATCHES To TargetScan▶ miR-329-3p/362-3p:ACACACC


GAC

GACATTTTCAGA  
Depth:5 (SHEEP)  
Ei-value:0.000, Pi-value:0.000  
Er-value:0.000, Pr-value:0.000  
eCLIP MATCHES▶pum1 (bg=29.85%)▶pum2 (bg=21.55%)No matches to TargetScan

 2040  


A

GACATTTTCAGA  
Depth:5 (SHEEP)  
Ei-value:0.000, Pi-value:0.000  
Er-value:0.000, Pr-value:0.000  
eCLIP MATCHES▶pum1 (bg=29.85%)▶pum2 (bg=21.55%)No matches to TargetScan


T

TTTTCAGA  
Depth:6 (PIG)  
Ei-value:0.000, Pi-value:0.000  
Er-value:0.000, Pr-value:0.000  
eCLIP MATCHES▶pum1 (bg=29.85%)▶pum2 (bg=21.55%)No matches to TargetScan


TTTCAG

TTTCAG  
Depth:7 (ARMADILLO)  
Ei-value:0.000, Pi-value:0.000  
Er-value:0.000, Pr-value:0.000  
eCLIP MATCHES▶pum1 (bg=29.85%)No matches to TargetScan


A

TTTTCAGA  
Depth:6 (PIG)  
Ei-value:0.000, Pi-value:0.000  
Er-value:0.000, Pr-value:0.000  
eCLIP MATCHES▶pum1 (bg=29.85%)▶pum2 (bg=21.55%)No matches to TargetScan

CACCATTTTTCTATATGTTT

T

TGTGCATTTT  
Depth:4 (COW)  
Ei-value:0.020, Pi-value:0.000  
Er-value:0.000, Pr-value:0.000  
eCLIP MATCHES▶GEMIN5 (bg=2.46%)▶pum1 (bg=29.85%)▶pum2 (bg=21.55%)MATCHES To TargetScan▶ miR-501-3p/502-3p:AUGCACC


G

GTGCATTTT  
Depth:6 (PIG)  
Ei-value:0.000, Pi-value:0.000  
Er-value:0.000, Pr-value:0.000  
eCLIP MATCHES▶GEMIN5 (bg=2.46%)▶pum1 (bg=29.85%)▶pum2 (bg=21.55%)MATCHES To TargetScan▶ miR-501-3p/502-3p:AUGCACC


TGCATTTT

TGCATTTT  
Depth:7 (ARMADILLO)  
Ei-value:0.000, Pi-value:0.000  
Er-value:0.000, Pr-value:0.000  
eCLIP MATCHES▶GEMIN5 (bg=2.46%)▶pum1 (bg=29.85%)▶pum2 (bg=21.55%)No matches to TargetScan

G

TTTTGCT

TTTTGCT  
Depth:5 (SHEEP)  
Ei-value:0.000, Pi-value:0.000  
Er-value:0.000, Pr-value:0.000  
eCLIP MATCHES▶fubp3 (bg=23.31%)▶GEMIN5 (bg=2.46%)▶pum1 (bg=29.85%)▶pum2 (bg=21.55%)No matches to TargetScan

C

TGTATATAGT

TGTATATAGT  
Depth:7 (ARMADILLO)  
Ei-value:0.000, Pi-value:0.000  
Er-value:0.000, Pr-value:0.000  
eCLIP MATCHES▶ddx6 (bg=23.92%)▶fubp3 (bg=23.31%)▶GEMIN5 (bg=2.46%)▶pum1 (bg=29.85%)▶pum2 (bg=21.55%)▶PUS1 (bg=2.87%)▶tial1 (bg=14.09%)No matches to TargetScan

A

TATATAAT

TATATAATGGACAAAT  
Depth:6 (PIG)  
Ei-value:0.000, Pi-value:0.000  
Er-value:0.000, Pr-value:0.000  
eCLIP MATCHES▶ddx6 (bg=23.92%)▶fubp3 (bg=23.31%)▶GEMIN5 (bg=2.46%)▶NOLC1 (bg=6.58%)▶pum1 (bg=29.85%)▶pum2 (bg=21.55%)▶PUS1 (bg=2.87%)▶tial1 (bg=14.09%)No matches to TargetScan


GGACAAAT

GGACAAAT  
Depth:7 (ARMADILLO)  
Ei-value:0.000, Pi-value:0.000  
Er-value:0.000, Pr-value:0.000  
eCLIP MATCHES▶ddx6 (bg=23.92%)▶fubp3 (bg=23.31%)▶GEMIN5 (bg=2.46%)▶NOLC1 (bg=6.58%)▶pum1 (bg=29.85%)▶pum2 (bg=21.55%)▶PUS1 (bg=2.87%)▶tial1 (bg=14.09%)No matches to TargetScan


AGTCCTA

AGTCCTA  
Depth:7 (ARMADILLO)  
Ei-value:0.000, Pi-value:0.000  
Er-value:0.000, Pr-value:0.000  
eCLIP MATCHES▶ddx6 (bg=23.92%)▶fubp3 (bg=23.31%)▶GEMIN5 (bg=2.46%)▶NOLC1 (bg=6.58%)▶pum1 (bg=29.85%)▶pum2 (bg=21.55%)▶PUS1 (bg=2.87%)▶tial1 (bg=14.09%)No matches to TargetScan

ATTTTT

CA

CAACATCTAGTCTCTAGATGTTAAAGAGGTTGCCA  
Depth:3 (DOG)  
Ei-value:0.000, Pi-value:0.000  
Er-value:0.000, Pr-value:0.000  
eCLIP MATCHES▶ddx6 (bg=23.92%)▶FASTKD2 (bg=7.73%)▶fubp3 (bg=23.31%)▶lin28b (bg=16.96%)▶NOLC1 (bg=6.58%)▶pum1 (bg=29.85%)▶pum2 (bg=21.55%)▶PUS1 (bg=2.87%)▶SF3B1 (bg=6.74%)▶tial1 (bg=14.09%)MATCHES To TargetScan▶ miR-28-3p:ACUAGAU


ACATCTA

ACATCTA  
Depth:7 (ARMADILLO)  
Ei-value:0.000, Pi-value:0.000  
Er-value:0.000, Pr-value:0.000  
eCLIP MATCHES▶ddx6 (bg=23.92%)▶FASTKD2 (bg=7.73%)▶fubp3 (bg=23.31%)▶NOLC1 (bg=6.58%)▶pum1 (bg=29.85%)▶pum2 (bg=21.55%)▶PUS1 (bg=2.87%)▶SF3B1 (bg=6.74%)▶tial1 (bg=14.09%)No matches to TargetScan


G

CAACATCTAGTCTCTAGATGTTAAAGAGGTTGCCA  
Depth:3 (DOG)  
Ei-value:0.000, Pi-value:0.000  
Er-value:0.000, Pr-value:0.000  
eCLIP MATCHES▶ddx6 (bg=23.92%)▶FASTKD2 (bg=7.73%)▶fubp3 (bg=23.31%)▶lin28b (bg=16.96%)▶NOLC1 (bg=6.58%)▶pum1 (bg=29.85%)▶pum2 (bg=21.55%)▶PUS1 (bg=2.87%)▶SF3B1 (bg=6.74%)▶tial1 (bg=14.09%)MATCHES To TargetScan▶ miR-28-3p:ACUAGAU


TCTCTAGATGTT

TCTCTAGATGTT  
Depth:6 (PIG)  
Ei-value:0.000, Pi-value:0.000  
Er-value:0.000, Pr-value:0.000  
eCLIP MATCHES▶ddx6 (bg=23.92%)▶FASTKD2 (bg=7.73%)▶fubp3 (bg=23.31%)▶NOLC1 (bg=6.58%)▶pum1 (bg=29.85%)▶pum2 (bg=21.55%)▶PUS1 (bg=2.87%)▶SF3B1 (bg=6.74%)▶tial1 (bg=14.09%)No matches to TargetScan


AAAGAGGTTG

AAAGAGGTTGCCA  
Depth:9 (MOUSE)  
Ei-value:0.000, Pi-value:0.000  
Er-value:0.000, Pr-value:0.000  
eCLIP MATCHES▶ddx6 (bg=23.92%)▶FASTKD2 (bg=7.73%)▶fubp3 (bg=23.31%)▶lin28b (bg=16.96%)▶NOLC1 (bg=6.58%)▶pum1 (bg=29.85%)▶pum2 (bg=21.55%)▶SF3B1 (bg=6.74%)▶tial1 (bg=14.09%)No matches to TargetScan

 2160  


CCA

AAAGAGGTTGCCA  
Depth:9 (MOUSE)  
Ei-value:0.000, Pi-value:0.000  
Er-value:0.000, Pr-value:0.000  
eCLIP MATCHES▶ddx6 (bg=23.92%)▶FASTKD2 (bg=7.73%)▶fubp3 (bg=23.31%)▶lin28b (bg=16.96%)▶NOLC1 (bg=6.58%)▶pum1 (bg=29.85%)▶pum2 (bg=21.55%)▶SF3B1 (bg=6.74%)▶tial1 (bg=14.09%)No matches to TargetScan

G

TG

TGTATGACAAA  
Depth:6 (PIG)  
Ei-value:0.000, Pi-value:0.000  
Er-value:0.000, Pr-value:0.000  
eCLIP MATCHES▶ddx6 (bg=23.92%)▶FASTKD2 (bg=7.73%)▶fubp3 (bg=23.31%)▶lin28b (bg=16.96%)▶pum1 (bg=29.85%)▶pum2 (bg=21.55%)▶SF3B1 (bg=6.74%)▶tial1 (bg=14.09%)MATCHES To TargetScan▶ miR-539-3p:UCAUACA


TATGACAAA

TATGACAAA  
Depth:7 (ARMADILLO)  
Ei-value:0.000, Pi-value:0.000  
Er-value:0.000, Pr-value:0.000  
eCLIP MATCHES▶ddx6 (bg=23.92%)▶FASTKD2 (bg=7.73%)▶fubp3 (bg=23.31%)▶lin28b (bg=16.96%)▶pum1 (bg=29.85%)▶pum2 (bg=21.55%)▶SF3B1 (bg=6.74%)▶tial1 (bg=14.09%)No matches to TargetScan


G

TGTATGACAAAG  
Depth:5 (SHEEP)  
Ei-value:0.000, Pi-value:0.000  
Er-value:0.000, Pr-value:0.000  
eCLIP MATCHES▶ddx6 (bg=23.92%)▶FASTKD2 (bg=7.73%)▶fubp3 (bg=23.31%)▶lin28b (bg=16.96%)▶pum1 (bg=29.85%)▶pum2 (bg=21.55%)▶SF3B1 (bg=6.74%)▶tial1 (bg=14.09%)MATCHES To TargetScan▶ miR-539-3p:UCAUACA

GAGTA

AAATTAGCA

AAATTAGCA  
Depth:5 (SHEEP)  
Ei-value:0.000, Pi-value:0.000  
Er-value:0.000, Pr-value:0.000  
eCLIP MATCHES▶ddx6 (bg=23.92%)▶FASTKD2 (bg=7.73%)▶fubp3 (bg=23.31%)▶lin28b (bg=16.96%)▶pum1 (bg=29.85%)▶pum2 (bg=21.55%)▶SUB1 (bg=9.24%)No matches to TargetScan

TATTTTGTACACT

TTGTGT

TTGTGTTGAAATT  
Depth:7 (ARMADILLO)  
Ei-value:0.000, Pi-value:0.000  
Er-value:0.000, Pr-value:0.000  
eCLIP MATCHES▶ddx6 (bg=23.92%)▶fubp3 (bg=23.31%)▶igf2bp1 (bg=11.67%)▶lin28b (bg=16.96%)▶NKRF (bg=3.64%)▶pum1 (bg=29.85%)▶pum2 (bg=21.55%)▶sf3a3 (bg=12.13%)▶SUB1 (bg=9.24%)▶tia1 (bg=16.04%)▶tial1 (bg=14.09%)MATCHES To TargetScan▶ miR-421:UCAACAG▶ miR-505-3p.2:UCAACAC


TGAAAT

TGAAAT  
Depth:8 (GUINEAPIG)  
Ei-value:0.000, Pi-value:0.010  
Er-value:0.000, Pr-value:0.010  
eCLIP MATCHES▶ddx6 (bg=23.92%)▶fubp3 (bg=23.31%)▶igf2bp1 (bg=11.67%)▶lin28b (bg=16.96%)▶NKRF (bg=3.64%)▶pum1 (bg=29.85%)▶pum2 (bg=21.55%)▶sf3a3 (bg=12.13%)▶SUB1 (bg=9.24%)▶tia1 (bg=16.04%)▶tial1 (bg=14.09%)No matches to TargetScan


T

TTGTGTTGAAATT  
Depth:7 (ARMADILLO)  
Ei-value:0.000, Pi-value:0.000  
Er-value:0.000, Pr-value:0.000  
eCLIP MATCHES▶ddx6 (bg=23.92%)▶fubp3 (bg=23.31%)▶igf2bp1 (bg=11.67%)▶lin28b (bg=16.96%)▶NKRF (bg=3.64%)▶pum1 (bg=29.85%)▶pum2 (bg=21.55%)▶sf3a3 (bg=12.13%)▶SUB1 (bg=9.24%)▶tia1 (bg=16.04%)▶tial1 (bg=14.09%)MATCHES To TargetScan▶ miR-421:UCAACAG▶ miR-505-3p.2:UCAACAC


C

TTGTGTTGAAATTC  
Depth:5 (SHEEP)  
Ei-value:0.000, Pi-value:0.000  
Er-value:0.000, Pr-value:0.000  
eCLIP MATCHES▶ddx6 (bg=23.92%)▶fubp3 (bg=23.31%)▶igf2bp1 (bg=11.67%)▶lin28b (bg=16.96%)▶NKRF (bg=3.64%)▶pum1 (bg=29.85%)▶pum2 (bg=21.55%)▶sf3a3 (bg=12.13%)▶SUB1 (bg=9.24%)▶tia1 (bg=16.04%)▶tial1 (bg=14.09%)MATCHES To TargetScan▶ miR-421:UCAACAG▶ miR-505-3p.2:UCAACAC

GT

aggaaa

aggaaa  
Depth:3 (DOG)  
Ei-value:0.080, Pi-value:0.050  
Er-value:0.000, Pr-value:0.010  
eCLIP MATCHES▶ddx6 (bg=23.92%)▶fubp3 (bg=23.31%)▶igf2bp1 (bg=11.67%)▶lin28b (bg=16.96%)▶NKRF (bg=3.64%)▶pum1 (bg=29.85%)▶pum2 (bg=21.55%)▶sf3a3 (bg=12.13%)▶SUB1 (bg=9.24%)▶tia1 (bg=16.04%)▶tial1 (bg=14.09%)No matches to TargetScan

ACTTGTC

TTCTGT

TTCTGT  
Depth:7 (ARMADILLO)  
Ei-value:0.000, Pi-value:0.000  
Er-value:0.000, Pr-value:0.000  
eCLIP MATCHES▶AQR (bg=1.27%)▶ddx6 (bg=23.92%)▶fubp3 (bg=23.31%)▶igf2bp1 (bg=11.67%)▶lin28b (bg=16.96%)▶NKRF (bg=3.64%)▶pum1 (bg=29.85%)▶pum2 (bg=21.55%)▶sf3a3 (bg=12.13%)▶SUB1 (bg=9.24%)▶tia1 (bg=16.04%)▶tial1 (bg=14.09%)No matches to TargetScan


A

TTCTGTA  
Depth:6 (PIG)  
Ei-value:0.000, Pi-value:0.000  
Er-value:0.000, Pr-value:0.000  
eCLIP MATCHES▶AQR (bg=1.27%)▶ddx6 (bg=23.92%)▶fubp3 (bg=23.31%)▶igf2bp1 (bg=11.67%)▶lin28b (bg=16.96%)▶NKRF (bg=3.64%)▶pum1 (bg=29.85%)▶pum2 (bg=21.55%)▶sf3a3 (bg=12.13%)▶SUB1 (bg=9.24%)▶tia1 (bg=16.04%)▶tial1 (bg=14.09%)No matches to TargetScan


AA

TTCTGTAAA  
Depth:5 (SHEEP)  
Ei-value:0.000, Pi-value:0.000  
Er-value:0.000, Pr-value:0.000  
eCLIP MATCHES▶AQR (bg=1.27%)▶ddx6 (bg=23.92%)▶fubp3 (bg=23.31%)▶igf2bp1 (bg=11.67%)▶lin28b (bg=16.96%)▶NKRF (bg=3.64%)▶pum1 (bg=29.85%)▶pum2 (bg=21.55%)▶sf3a3 (bg=12.13%)▶SUB1 (bg=9.24%)▶tia1 (bg=16.04%)▶tial1 (bg=14.09%)No matches to TargetScan

GACTTTTGCATAG

GAATTTGTT

GAATTTGTT  
Depth:3 (DOG)  
Ei-value:0.000, Pi-value:0.000  
Er-value:0.000, Pr-value:0.000  
eCLIP MATCHES▶AQR (bg=1.27%)▶ddx6 (bg=23.92%)▶fubp3 (bg=23.31%)▶igf2bp1 (bg=11.67%)▶pum1 (bg=29.85%)▶pum2 (bg=21.55%)▶SUB1 (bg=9.24%)▶tia1 (bg=16.04%)▶tial1 (bg=14.09%)MATCHES To TargetScan▶ miR-495-3p:AACAAAC

TGACCATCTCTA

AGCAT

AGCATTA  
Depth:3 (DOG)  
Ei-value:0.000, Pi-value:0.000  
Er-value:0.000, Pr-value:0.000  
eCLIP MATCHES▶AQR (bg=1.27%)▶ddx6 (bg=23.92%)▶fubp3 (bg=23.31%)▶igf2bp1 (bg=11.67%)▶pum1 (bg=29.85%)▶pum2 (bg=21.55%)▶SUPV3L1 (bg=0.85%)▶tia1 (bg=16.04%)▶tial1 (bg=14.09%)MATCHES To TargetScan▶ miR-155-5p:UAAUGCU

 2280  


TA

AGCATTA  
Depth:3 (DOG)  
Ei-value:0.000, Pi-value:0.000  
Er-value:0.000, Pr-value:0.000  
eCLIP MATCHES▶AQR (bg=1.27%)▶ddx6 (bg=23.92%)▶fubp3 (bg=23.31%)▶igf2bp1 (bg=11.67%)▶pum1 (bg=29.85%)▶pum2 (bg=21.55%)▶SUPV3L1 (bg=0.85%)▶tia1 (bg=16.04%)▶tial1 (bg=14.09%)MATCHES To TargetScan▶ miR-155-5p:UAAUGCU

CACGTGC

CTGTAC

CTGTAC  
Depth:7 (ARMADILLO)  
Ei-value:0.000, Pi-value:0.000  
Er-value:0.000, Pr-value:0.000  
eCLIP MATCHES▶AQR (bg=1.27%)▶ddx6 (bg=23.92%)▶fubp3 (bg=23.31%)▶pum1 (bg=29.85%)▶pum2 (bg=21.55%)▶SUPV3L1 (bg=0.85%)▶tia1 (bg=16.04%)▶tial1 (bg=14.09%)No matches to TargetScan

TTGTCC

ACTGGATT

ACTGGATT  
Depth:5 (SHEEP)  
Ei-value:0.000, Pi-value:0.000  
Er-value:0.000, Pr-value:0.000  
eCLIP MATCHES▶ddx6 (bg=23.92%)▶fubp3 (bg=23.31%)▶pum1 (bg=29.85%)▶SUPV3L1 (bg=0.85%)▶tia1 (bg=16.04%)▶tial1 (bg=14.09%)MATCHES To TargetScan▶ miR-145-5p:UCCAGUU

GAAGGCAG

AG

AGAAGGAAG  
Depth:3 (DOG)  
Ei-value:0.000, Pi-value:0.000  
Er-value:0.000, Pr-value:0.000  
No matches to eCLIP DataNo matches to TargetScan


AAGGAAG

AAGGAAG  
Depth:6 (PIG)  
Ei-value:0.000, Pi-value:0.010  
Er-value:0.000, Pr-value:0.000  
No matches to eCLIP DataNo matches to TargetScan

GGA

GGA

GGAGGGAAT  
Depth:3 (DOG)  
Ei-value:0.000, Pi-value:0.000  
Er-value:0.000, Pr-value:0.000  
No matches to eCLIP DataNo matches to TargetScan


GGGAAT

GGGAAT  
Depth:7 (ARMADILLO)  
Ei-value:0.000, Pi-value:0.000  
Er-value:0.000, Pr-value:0.000  
No matches to eCLIP DataNo matches to TargetScan

GATTCA

AGGCCAA

AGGCCAA  
Depth:7 (ARMADILLO)  
Ei-value:0.000, Pi-value:0.000  
Er-value:0.000, Pr-value:0.000  
eCLIP MATCHES▶AARS (bg=2.33%)No matches to TargetScan


AAT

AGGCCAAAAT  
Depth:6 (PIG)  
Ei-value:0.000, Pi-value:0.000  
Er-value:0.000, Pr-value:0.000  
eCLIP MATCHES▶AARS (bg=2.33%)No matches to TargetScan


GG

AGGCCAAAATGG  
Depth:3 (DOG)  
Ei-value:0.000, Pi-value:0.000  
Er-value:0.000, Pr-value:0.000  
eCLIP MATCHES▶AARS (bg=2.33%)No matches to TargetScan

CCAC

ATTTAG

ATTTAG  
Depth:6 (PIG)  
Ei-value:0.000, Pi-value:0.010  
Er-value:0.000, Pr-value:0.000  
No matches to eCLIP DataNo matches to TargetScan


AA

ATTTAGAA  
Depth:3 (DOG)  
Ei-value:0.000, Pi-value:0.000  
Er-value:0.000, Pr-value:0.000  
No matches to eCLIP DataNo matches to TargetScan

G

ATACCTCA

ATACCTCA  
Depth:7 (ARMADILLO)  
Ei-value:0.000, Pi-value:0.000  
Er-value:0.000, Pr-value:0.000  
No matches to eCLIP DataMATCHES To TargetScan▶ let-7-5p/98-5p:GAGGUAG


GAT

ATACCTCAGAT  
Depth:3 (DOG)  
Ei-value:0.000, Pi-value:0.000  
Er-value:0.000, Pr-value:0.000  
No matches to eCLIP DataMATCHES To TargetScan▶ let-7-5p/98-5p:GAGGUAG

GATAACCA

TTGTTA

TTGTTA  
Depth:7 (ARMADILLO)  
Ei-value:0.000, Pi-value:0.020  
Er-value:0.000, Pr-value:0.010  
eCLIP MATCHES▶lin28b (bg=16.96%)No matches to TargetScan

TG

T

TGTGTGCAATT  
Depth:5 (SHEEP)  
Ei-value:0.000, Pi-value:0.000  
Er-value:0.000, Pr-value:0.000  
eCLIP MATCHES▶ddx6 (bg=23.92%)▶dgcr8 (bg=19.31%)▶FASTKD2 (bg=7.73%)▶HNRNPL (bg=3.4%)▶lin28b (bg=16.96%)▶NKRF (bg=3.64%)▶NOLC1 (bg=6.58%)▶SUB1 (bg=9.24%)MATCHES To TargetScan▶ miR-25-3p/32-5p/92-3p/363-3p/367-3p:AUUGCAC


GTG

GTGTGCAATT  
Depth:6 (PIG)  
Ei-value:0.000, Pi-value:0.000  
Er-value:0.000, Pr-value:0.000  
eCLIP MATCHES▶ddx6 (bg=23.92%)▶dgcr8 (bg=19.31%)▶FASTKD2 (bg=7.73%)▶HNRNPL (bg=3.4%)▶lin28b (bg=16.96%)▶NKRF (bg=3.64%)▶NOLC1 (bg=6.58%)▶SUB1 (bg=9.24%)MATCHES To TargetScan▶ miR-25-3p/32-5p/92-3p/363-3p/367-3p:AUUGCAC

 2400  


TGCAATT

GTGTGCAATT  
Depth:6 (PIG)  
Ei-value:0.000, Pi-value:0.000  
Er-value:0.000, Pr-value:0.000  
eCLIP MATCHES▶ddx6 (bg=23.92%)▶dgcr8 (bg=19.31%)▶FASTKD2 (bg=7.73%)▶HNRNPL (bg=3.4%)▶lin28b (bg=16.96%)▶NKRF (bg=3.64%)▶NOLC1 (bg=6.58%)▶SUB1 (bg=9.24%)MATCHES To TargetScan▶ miR-25-3p/32-5p/92-3p/363-3p/367-3p:AUUGCAC

T

TAT

TATTTAACAGTGCT  
Depth:3 (DOG)  
Ei-value:0.000, Pi-value:0.000  
Er-value:0.000, Pr-value:0.000  
eCLIP MATCHES▶CDC40 (bg=1.71%)▶ddx6 (bg=23.92%)▶dgcr8 (bg=19.31%)▶fam120a (bg=18.43%)▶FASTKD2 (bg=7.73%)▶fubp3 (bg=23.31%)▶FUS (bg=2.59%)▶G3BP1 (bg=1.51%)▶HNRNPL (bg=3.4%)▶igf2bp1 (bg=11.67%)▶lin28b (bg=16.96%)▶NCBP2 (bg=2.92%)▶NKRF (bg=3.64%)▶NOLC1 (bg=6.58%)▶pum1 (bg=29.85%)▶pum2 (bg=21.55%)▶SF3B1 (bg=6.74%)▶SUB1 (bg=9.24%)MATCHES To TargetScan▶ miR-452-5p/892-3p:ACUGUUU


TTAACA

TTAACA  
Depth:7 (ARMADILLO)  
Ei-value:0.000, Pi-value:0.010  
Er-value:0.000, Pr-value:0.000  
eCLIP MATCHES▶CDC40 (bg=1.71%)▶ddx6 (bg=23.92%)▶dgcr8 (bg=19.31%)▶fam120a (bg=18.43%)▶FASTKD2 (bg=7.73%)▶fubp3 (bg=23.31%)▶FUS (bg=2.59%)▶G3BP1 (bg=1.51%)▶HNRNPL (bg=3.4%)▶igf2bp1 (bg=11.67%)▶lin28b (bg=16.96%)▶NCBP2 (bg=2.92%)▶NKRF (bg=3.64%)▶NOLC1 (bg=6.58%)▶pum2 (bg=21.55%)▶SF3B1 (bg=6.74%)▶SUB1 (bg=9.24%)No matches to TargetScan


GT

TTAACAGT  
Depth:6 (PIG)  
Ei-value:0.000, Pi-value:0.000  
Er-value:0.000, Pr-value:0.000  
eCLIP MATCHES▶CDC40 (bg=1.71%)▶ddx6 (bg=23.92%)▶dgcr8 (bg=19.31%)▶fam120a (bg=18.43%)▶FASTKD2 (bg=7.73%)▶fubp3 (bg=23.31%)▶FUS (bg=2.59%)▶G3BP1 (bg=1.51%)▶HNRNPL (bg=3.4%)▶igf2bp1 (bg=11.67%)▶lin28b (bg=16.96%)▶NCBP2 (bg=2.92%)▶NKRF (bg=3.64%)▶NOLC1 (bg=6.58%)▶pum1 (bg=29.85%)▶pum2 (bg=21.55%)▶SF3B1 (bg=6.74%)▶SUB1 (bg=9.24%)MATCHES To TargetScan▶ miR-452-5p/892-3p:ACUGUUU


GCT

TATTTAACAGTGCT  
Depth:3 (DOG)  
Ei-value:0.000, Pi-value:0.000  
Er-value:0.000, Pr-value:0.000  
eCLIP MATCHES▶CDC40 (bg=1.71%)▶ddx6 (bg=23.92%)▶dgcr8 (bg=19.31%)▶fam120a (bg=18.43%)▶FASTKD2 (bg=7.73%)▶fubp3 (bg=23.31%)▶FUS (bg=2.59%)▶G3BP1 (bg=1.51%)▶HNRNPL (bg=3.4%)▶igf2bp1 (bg=11.67%)▶lin28b (bg=16.96%)▶NCBP2 (bg=2.92%)▶NKRF (bg=3.64%)▶NOLC1 (bg=6.58%)▶pum1 (bg=29.85%)▶pum2 (bg=21.55%)▶SF3B1 (bg=6.74%)▶SUB1 (bg=9.24%)MATCHES To TargetScan▶ miR-452-5p/892-3p:ACUGUUU

GTGTATGTGGTGG

ACAAGT

ACAAGT  
Depth:7 (ARMADILLO)  
Ei-value:0.000, Pi-value:0.000  
Er-value:0.000, Pr-value:0.000  
eCLIP MATCHES▶CDC40 (bg=1.71%)▶ddx6 (bg=23.92%)▶dgcr8 (bg=19.31%)▶fam120a (bg=18.43%)▶FASTKD2 (bg=7.73%)▶fubp3 (bg=23.31%)▶FUS (bg=2.59%)▶G3BP1 (bg=1.51%)▶HNRNPL (bg=3.4%)▶igf2bp1 (bg=11.67%)▶lin28b (bg=16.96%)▶NCBP2 (bg=2.92%)▶NKRF (bg=3.64%)▶NOLC1 (bg=6.58%)▶pum1 (bg=29.85%)▶SF3B1 (bg=6.74%)▶SUB1 (bg=9.24%)No matches to TargetScan


TA

ACAAGTTA  
Depth:3 (DOG)  
Ei-value:0.000, Pi-value:0.000  
Er-value:0.000, Pr-value:0.000  
eCLIP MATCHES▶CDC40 (bg=1.71%)▶ddx6 (bg=23.92%)▶dgcr8 (bg=19.31%)▶fam120a (bg=18.43%)▶FASTKD2 (bg=7.73%)▶fubp3 (bg=23.31%)▶FUS (bg=2.59%)▶G3BP1 (bg=1.51%)▶HNRNPL (bg=3.4%)▶igf2bp1 (bg=11.67%)▶lin28b (bg=16.96%)▶NCBP2 (bg=2.92%)▶NKRF (bg=3.64%)▶NOLC1 (bg=6.58%)▶pum1 (bg=29.85%)▶SF3B1 (bg=6.74%)▶SUB1 (bg=9.24%)No matches to TargetScan

TA

TGAAATATCTAGTCTT

TGAAATATCTAGTCTT  
Depth:7 (ARMADILLO)  
Ei-value:0.000, Pi-value:0.000  
Er-value:0.000, Pr-value:0.000  
eCLIP MATCHES▶ddx6 (bg=23.92%)▶dgcr8 (bg=19.31%)▶fubp3 (bg=23.31%)▶FUS (bg=2.59%)▶lin28b (bg=16.96%)▶NOLC1 (bg=6.58%)▶SF3B1 (bg=6.74%)MATCHES To TargetScan▶ miR-28-3p:ACUAGAU


TCTAGATA

TGAAATATCTAGTCTTTCTAGATA  
Depth:5 (SHEEP)  
Ei-value:0.000, Pi-value:0.000  
Er-value:0.000, Pr-value:0.000  
eCLIP MATCHES▶ddx6 (bg=23.92%)▶dgcr8 (bg=19.31%)▶fubp3 (bg=23.31%)▶FUS (bg=2.59%)▶lin28b (bg=16.96%)▶NOLC1 (bg=6.58%)▶SF3B1 (bg=6.74%)MATCHES To TargetScan▶ miR-28-3p:ACUAGAU

TTTGGAAG

TGCTTGATG

TGCTTGATG  
Depth:7 (ARMADILLO)  
Ei-value:0.000, Pi-value:0.000  
Er-value:0.000, Pr-value:0.000  
No matches to eCLIP DataNo matches to TargetScan


TATTTA

TGCTTGATGTATTTA  
Depth:3 (DOG)  
Ei-value:0.000, Pi-value:0.000  
Er-value:0.000, Pr-value:0.000  
No matches to eCLIP DataNo matches to TargetScan

AAAGTGGT

AGTAGA

AGTAGA  
Depth:5 (SHEEP)  
Ei-value:0.000, Pi-value:0.010  
Er-value:0.000, Pr-value:0.000  
No matches to eCLIP DataNo matches to TargetScan

ATAACACT

T

TTGTAAATAGCTTTT  
Depth:6 (PIG)  
Ei-value:0.000, Pi-value:0.000  
Er-value:0.000, Pr-value:0.000  
No matches to eCLIP DataMATCHES To TargetScan▶ miR-320:AAAGCUG


TGTAA

TGTAAATAGCTTTT  
Depth:7 (ARMADILLO)  
Ei-value:0.000, Pi-value:0.000  
Er-value:0.000, Pr-value:0.000  
No matches to eCLIP DataMATCHES To TargetScan▶ miR-320:AAAGCUG

 2520  


ATAGCTTTT

TGTAAATAGCTTTT  
Depth:7 (ARMADILLO)  
Ei-value:0.000, Pi-value:0.000  
Er-value:0.000, Pr-value:0.000  
No matches to eCLIP DataMATCHES To TargetScan▶ miR-320:AAAGCUG


AAA

TTGTAAATAGCTTTTAAA  
Depth:3 (DOG)  
Ei-value:0.000, Pi-value:0.000  
Er-value:0.000, Pr-value:0.000  
No matches to eCLIP DataMATCHES To TargetScan▶ miR-320:AAAGCUG

A

ACTG

ACTGATGGGAAAT  
Depth:5 (SHEEP)  
Ei-value:0.000, Pi-value:0.000  
Er-value:0.000, Pr-value:0.000  
eCLIP MATCHES▶GRSF1 (bg=3.8%)▶pum1 (bg=29.85%)No matches to TargetScan


ATG

ATGGGAAAT  
Depth:6 (PIG)  
Ei-value:0.000, Pi-value:0.000  
Er-value:0.000, Pr-value:0.000  
eCLIP MATCHES▶GRSF1 (bg=3.8%)▶pum1 (bg=29.85%)No matches to TargetScan


GGAAAT

GGAAAT  
Depth:7 (ARMADILLO)  
Ei-value:0.000, Pi-value:0.000  
Er-value:0.000, Pr-value:0.000  
eCLIP MATCHES▶GRSF1 (bg=3.8%)▶pum1 (bg=29.85%)No matches to TargetScan

GCTGTTTGGAAGTG

GAATTG

GAATTG  
Depth:6 (PIG)  
Ei-value:0.000, Pi-value:0.010  
Er-value:0.000, Pr-value:0.000  
eCLIP MATCHES▶FTO (bg=7.53%)▶GRSF1 (bg=3.8%)▶KHSRP (bg=8.1%)▶pum1 (bg=29.85%)▶SUB1 (bg=9.24%)No matches to TargetScan

TTGAACCACCT

GGGAG

GGGAGGTGGGAGGGAA  
Depth:5 (SHEEP)  
Ei-value:0.000, Pi-value:0.000  
Er-value:0.000, Pr-value:0.000  
eCLIP MATCHES▶ddx55 (bg=12.35%)▶FTO (bg=7.53%)▶GRSF1 (bg=3.8%)▶KHSRP (bg=8.1%)▶pum1 (bg=29.85%)▶SUB1 (bg=9.24%)MATCHES To TargetScan▶ miR-1306-5p:CACCUCC▶ miR-150-5p:CUCCCAA▶ miR-532-3p:CUCCCAC


GTGGGAGGGAA

GTGGGAGGGAA  
Depth:9 (MOUSE)  
Ei-value:0.000, Pi-value:0.000  
Er-value:0.000, Pr-value:0.000  
eCLIP MATCHES▶ddx55 (bg=12.35%)▶FTO (bg=7.53%)▶GRSF1 (bg=3.8%)▶KHSRP (bg=8.1%)▶pum1 (bg=29.85%)▶SUB1 (bg=9.24%)MATCHES To TargetScan▶ miR-150-5p:CUCCCAA▶ miR-532-3p:CUCCCAC

GAAAT

TGCAAA

TGCAAA  
Depth:9 (MOUSE)  
Ei-value:0.000, Pi-value:0.000  
Er-value:0.000, Pr-value:0.000  
eCLIP MATCHES▶ddx55 (bg=12.35%)▶FTO (bg=7.53%)▶fubp3 (bg=23.31%)▶GRSF1 (bg=3.8%)▶KHSRP (bg=8.1%)▶pum1 (bg=29.85%)▶SUB1 (bg=9.24%)No matches to TargetScan

TGGTG

TTTTGC

TTTTGC  
Depth:8 (GUINEAPIG)  
Ei-value:0.000, Pi-value:0.010  
Er-value:0.000, Pr-value:0.000  
eCLIP MATCHES▶fubp3 (bg=23.31%)▶GRSF1 (bg=3.8%)▶KHSRP (bg=8.1%)▶pum1 (bg=29.85%)▶SUB1 (bg=9.24%)No matches to TargetScan

CATTGTTTATTAGA

AA

AAATTTCAGCTTA  
Depth:3 (DOG)  
Ei-value:0.000, Pi-value:0.000  
Er-value:0.000, Pr-value:0.000  
eCLIP MATCHES▶AARS (bg=2.33%)▶CPEB4 (bg=2.3%)▶ddx6 (bg=23.92%)▶dgcr8 (bg=19.31%)▶fam120a (bg=18.43%)▶FASTKD2 (bg=7.73%)▶fubp3 (bg=23.31%)▶GRSF1 (bg=3.8%)▶igf2bp1 (bg=11.67%)▶KHSRP (bg=8.1%)▶NCBP2 (bg=2.92%)▶pum1 (bg=29.85%)▶pum2 (bg=21.55%)▶sf3a3 (bg=12.13%)▶TBRG4 (bg=7.0%)▶tia1 (bg=16.04%)▶tial1 (bg=14.09%)▶ZC3H11A (bg=6.25%)MATCHES To TargetScan▶ miR-203a-3p.2:UGAAAUG


ATTTCA

ATTTCA  
Depth:6 (PIG)  
Ei-value:0.000, Pi-value:0.010  
Er-value:0.000, Pr-value:0.010  
eCLIP MATCHES▶AARS (bg=2.33%)▶CPEB4 (bg=2.3%)▶ddx6 (bg=23.92%)▶dgcr8 (bg=19.31%)▶fam120a (bg=18.43%)▶FASTKD2 (bg=7.73%)▶fubp3 (bg=23.31%)▶GRSF1 (bg=3.8%)▶igf2bp1 (bg=11.67%)▶KHSRP (bg=8.1%)▶pum1 (bg=29.85%)▶pum2 (bg=21.55%)▶sf3a3 (bg=12.13%)▶TBRG4 (bg=7.0%)▶tia1 (bg=16.04%)▶tial1 (bg=14.09%)▶ZC3H11A (bg=6.25%)MATCHES To TargetScan▶ miR-203a-3p.2:UGAAAUG


GCT

ATTTCAGCTTA  
Depth:5 (SHEEP)  
Ei-value:0.000, Pi-value:0.000  
Er-value:0.000, Pr-value:0.000  
eCLIP MATCHES▶AARS (bg=2.33%)▶CPEB4 (bg=2.3%)▶ddx6 (bg=23.92%)▶dgcr8 (bg=19.31%)▶fam120a (bg=18.43%)▶FASTKD2 (bg=7.73%)▶fubp3 (bg=23.31%)▶GRSF1 (bg=3.8%)▶igf2bp1 (bg=11.67%)▶KHSRP (bg=8.1%)▶NCBP2 (bg=2.92%)▶pum1 (bg=29.85%)▶pum2 (bg=21.55%)▶sf3a3 (bg=12.13%)▶TBRG4 (bg=7.0%)▶tia1 (bg=16.04%)▶tial1 (bg=14.09%)▶ZC3H11A (bg=6.25%)MATCHES To TargetScan▶ miR-203a-3p.2:UGAAAUG

 2640  


TA

ATTTCAGCTTA  
Depth:5 (SHEEP)  
Ei-value:0.000, Pi-value:0.000  
Er-value:0.000, Pr-value:0.000  
eCLIP MATCHES▶AARS (bg=2.33%)▶CPEB4 (bg=2.3%)▶ddx6 (bg=23.92%)▶dgcr8 (bg=19.31%)▶fam120a (bg=18.43%)▶FASTKD2 (bg=7.73%)▶fubp3 (bg=23.31%)▶GRSF1 (bg=3.8%)▶igf2bp1 (bg=11.67%)▶KHSRP (bg=8.1%)▶NCBP2 (bg=2.92%)▶pum1 (bg=29.85%)▶pum2 (bg=21.55%)▶sf3a3 (bg=12.13%)▶TBRG4 (bg=7.0%)▶tia1 (bg=16.04%)▶tial1 (bg=14.09%)▶ZC3H11A (bg=6.25%)MATCHES To TargetScan▶ miR-203a-3p.2:UGAAAUG

A

T

TCCATTG  
Depth:3 (DOG)  
Ei-value:0.000, Pi-value:0.000  
Er-value:0.000, Pr-value:0.000  
eCLIP MATCHES▶AARS (bg=2.33%)▶CPEB4 (bg=2.3%)▶ddx55 (bg=12.35%)▶ddx6 (bg=23.92%)▶dgcr8 (bg=19.31%)▶fam120a (bg=18.43%)▶FASTKD2 (bg=7.73%)▶fubp3 (bg=23.31%)▶GRSF1 (bg=3.8%)▶igf2bp1 (bg=11.67%)▶KHSRP (bg=8.1%)▶NCBP2 (bg=2.92%)▶NKRF (bg=3.64%)▶NOLC1 (bg=6.58%)▶pum1 (bg=29.85%)▶pum2 (bg=21.55%)▶sf3a3 (bg=12.13%)▶TBRG4 (bg=7.0%)▶tia1 (bg=16.04%)▶tial1 (bg=14.09%)▶ZC3H11A (bg=6.25%)No matches to TargetScan


CCATTG

CCATTG  
Depth:5 (SHEEP)  
Ei-value:0.000, Pi-value:0.000  
Er-value:0.000, Pr-value:0.000  
eCLIP MATCHES▶AARS (bg=2.33%)▶CPEB4 (bg=2.3%)▶ddx55 (bg=12.35%)▶ddx6 (bg=23.92%)▶dgcr8 (bg=19.31%)▶fam120a (bg=18.43%)▶FASTKD2 (bg=7.73%)▶fubp3 (bg=23.31%)▶GRSF1 (bg=3.8%)▶igf2bp1 (bg=11.67%)▶KHSRP (bg=8.1%)▶NCBP2 (bg=2.92%)▶NKRF (bg=3.64%)▶NOLC1 (bg=6.58%)▶pum1 (bg=29.85%)▶pum2 (bg=21.55%)▶sf3a3 (bg=12.13%)▶TBRG4 (bg=7.0%)▶tia1 (bg=16.04%)▶tial1 (bg=14.09%)▶ZC3H11A (bg=6.25%)No matches to TargetScan

TGTA

TATGTTA

TATGTTA  
Depth:6 (PIG)  
Ei-value:0.000, Pi-value:0.000  
Er-value:0.000, Pr-value:0.000  
eCLIP MATCHES▶AARS (bg=2.33%)▶CPEB4 (bg=2.3%)▶ddx6 (bg=23.92%)▶dgcr8 (bg=19.31%)▶fam120a (bg=18.43%)▶FASTKD2 (bg=7.73%)▶fubp3 (bg=23.31%)▶GRSF1 (bg=3.8%)▶igf2bp1 (bg=11.67%)▶KHSRP (bg=8.1%)▶NCBP2 (bg=2.92%)▶NKRF (bg=3.64%)▶NOLC1 (bg=6.58%)▶pum1 (bg=29.85%)▶pum2 (bg=21.55%)▶sf3a3 (bg=12.13%)▶SF3B1 (bg=6.74%)▶TBRG4 (bg=7.0%)▶tia1 (bg=16.04%)▶tial1 (bg=14.09%)▶ZC3H11A (bg=6.25%)No matches to TargetScan

CA

tgcatt

tgcatt  
Depth:4 (COW)  
Ei-value:1.000, Pi-value:0.010  
Er-value:0.000, Pr-value:0.000  
eCLIP MATCHES▶AARS (bg=2.33%)▶CPEB4 (bg=2.3%)▶ddx6 (bg=23.92%)▶dgcr8 (bg=19.31%)▶fam120a (bg=18.43%)▶FASTKD2 (bg=7.73%)▶fubp3 (bg=23.31%)▶GRSF1 (bg=3.8%)▶igf2bp1 (bg=11.67%)▶KHSRP (bg=8.1%)▶NCBP2 (bg=2.92%)▶NKRF (bg=3.64%)▶NOLC1 (bg=6.58%)▶pum1 (bg=29.85%)▶pum2 (bg=21.55%)▶sf3a3 (bg=12.13%)▶SF3B1 (bg=6.74%)▶TBRG4 (bg=7.0%)▶tia1 (bg=16.04%)▶tial1 (bg=14.09%)▶ZC3H11A (bg=6.25%)No matches to TargetScan

TCATTTAACTTTGCTAT

AC

ACTGTATATATTGT  
Depth:3 (DOG)  
Ei-value:0.000, Pi-value:0.000  
Er-value:0.000, Pr-value:0.000  
eCLIP MATCHES▶AKAP1 (bg=3.4%)▶ddx55 (bg=12.35%)▶dgcr8 (bg=19.31%)▶EIF4G2 (bg=0.7%)▶fam120a (bg=18.43%)▶FASTKD2 (bg=7.73%)▶fubp3 (bg=23.31%)▶HNRNPL (bg=3.4%)▶igf2bp1 (bg=11.67%)▶KHSRP (bg=8.1%)▶NCBP2 (bg=2.92%)▶NKRF (bg=3.64%)▶NOLC1 (bg=6.58%)▶pum1 (bg=29.85%)▶pum2 (bg=21.55%)▶sf3a3 (bg=12.13%)▶SF3B1 (bg=6.74%)▶TBRG4 (bg=7.0%)▶tia1 (bg=16.04%)▶tial1 (bg=14.09%)▶WRN (bg=3.31%)▶ZC3H11A (bg=6.25%)MATCHES To TargetScan▶ miR-582-5p:UACAGUU▶ miR-101-3p.2:UACAGUA


TGTATATA

TGTATATA  
Depth:9 (MOUSE)  
Ei-value:0.000, Pi-value:0.000  
Er-value:0.000, Pr-value:0.000  
eCLIP MATCHES▶dgcr8 (bg=19.31%)▶EIF4G2 (bg=0.7%)▶fam120a (bg=18.43%)▶FASTKD2 (bg=7.73%)▶fubp3 (bg=23.31%)▶HNRNPL (bg=3.4%)▶igf2bp1 (bg=11.67%)▶KHSRP (bg=8.1%)▶NCBP2 (bg=2.92%)▶NOLC1 (bg=6.58%)▶pum1 (bg=29.85%)▶pum2 (bg=21.55%)▶sf3a3 (bg=12.13%)▶SF3B1 (bg=6.74%)▶TBRG4 (bg=7.0%)▶tia1 (bg=16.04%)▶tial1 (bg=14.09%)▶ZC3H11A (bg=6.25%)No matches to TargetScan


TTG

TGTATATATTG  
Depth:4 (COW)  
Ei-value:0.000, Pi-value:0.000  
Er-value:0.000, Pr-value:0.000  
eCLIP MATCHES▶AKAP1 (bg=3.4%)▶ddx55 (bg=12.35%)▶dgcr8 (bg=19.31%)▶EIF4G2 (bg=0.7%)▶fam120a (bg=18.43%)▶FASTKD2 (bg=7.73%)▶fubp3 (bg=23.31%)▶HNRNPL (bg=3.4%)▶igf2bp1 (bg=11.67%)▶KHSRP (bg=8.1%)▶NCBP2 (bg=2.92%)▶NOLC1 (bg=6.58%)▶pum1 (bg=29.85%)▶pum2 (bg=21.55%)▶sf3a3 (bg=12.13%)▶SF3B1 (bg=6.74%)▶TBRG4 (bg=7.0%)▶tia1 (bg=16.04%)▶tial1 (bg=14.09%)▶ZC3H11A (bg=6.25%)No matches to TargetScan


T

ACTGTATATATTGT  
Depth:3 (DOG)  
Ei-value:0.000, Pi-value:0.000  
Er-value:0.000, Pr-value:0.000  
eCLIP MATCHES▶AKAP1 (bg=3.4%)▶ddx55 (bg=12.35%)▶dgcr8 (bg=19.31%)▶EIF4G2 (bg=0.7%)▶fam120a (bg=18.43%)▶FASTKD2 (bg=7.73%)▶fubp3 (bg=23.31%)▶HNRNPL (bg=3.4%)▶igf2bp1 (bg=11.67%)▶KHSRP (bg=8.1%)▶NCBP2 (bg=2.92%)▶NKRF (bg=3.64%)▶NOLC1 (bg=6.58%)▶pum1 (bg=29.85%)▶pum2 (bg=21.55%)▶sf3a3 (bg=12.13%)▶SF3B1 (bg=6.74%)▶TBRG4 (bg=7.0%)▶tia1 (bg=16.04%)▶tial1 (bg=14.09%)▶WRN (bg=3.31%)▶ZC3H11A (bg=6.25%)MATCHES To TargetScan▶ miR-582-5p:UACAGUU▶ miR-101-3p.2:UACAGUA

A

TATATA

TATATA  
Depth:6 (PIG)  
Ei-value:0.000, Pi-value:0.030  
Er-value:0.000, Pr-value:0.000  
eCLIP MATCHES▶AKAP1 (bg=3.4%)▶ddx55 (bg=12.35%)▶dgcr8 (bg=19.31%)▶EIF4G2 (bg=0.7%)▶fam120a (bg=18.43%)▶FASTKD2 (bg=7.73%)▶fubp3 (bg=23.31%)▶HNRNPL (bg=3.4%)▶igf2bp1 (bg=11.67%)▶KHSRP (bg=8.1%)▶NCBP2 (bg=2.92%)▶NKRF (bg=3.64%)▶NOLC1 (bg=6.58%)▶PHF6 (bg=1.71%)▶pum1 (bg=29.85%)▶pum2 (bg=21.55%)▶sf3a3 (bg=12.13%)▶SF3B1 (bg=6.74%)▶tia1 (bg=16.04%)▶tial1 (bg=14.09%)▶TROVE2 (bg=3.49%)▶WRN (bg=3.31%)▶ZC3H11A (bg=6.25%)No matches to TargetScan

ACGGACAAATTAGTCCCGATTTTA

T

TAATATCTAGTCTCTAGATATT  
Depth:6 (PIG)  
Ei-value:0.000, Pi-value:0.000  
Er-value:0.000, Pr-value:0.000  
eCLIP MATCHES▶AKAP1 (bg=3.4%)▶ddx55 (bg=12.35%)▶dgcr8 (bg=19.31%)▶fam120a (bg=18.43%)▶FASTKD2 (bg=7.73%)▶fubp3 (bg=23.31%)▶GRSF1 (bg=3.8%)▶igf2bp1 (bg=11.67%)▶KHSRP (bg=8.1%)▶NKRF (bg=3.64%)▶PHF6 (bg=1.71%)▶pum1 (bg=29.85%)▶pum2 (bg=21.55%)▶sf3a3 (bg=12.13%)▶SF3B1 (bg=6.74%)▶tia1 (bg=16.04%)▶tial1 (bg=14.09%)▶WRN (bg=3.31%)▶ZC3H11A (bg=6.25%)MATCHES To TargetScan▶ miR-28-3p:ACUAGAU


A

AATATCTAG  
Depth:8 (GUINEAPIG)  
Ei-value:0.000, Pi-value:0.000  
Er-value:0.000, Pr-value:0.000  
eCLIP MATCHES▶AKAP1 (bg=3.4%)▶ddx55 (bg=12.35%)▶dgcr8 (bg=19.31%)▶fam120a (bg=18.43%)▶fubp3 (bg=23.31%)▶GRSF1 (bg=3.8%)▶igf2bp1 (bg=11.67%)▶KHSRP (bg=8.1%)▶NKRF (bg=3.64%)▶PHF6 (bg=1.71%)▶pum1 (bg=29.85%)▶pum2 (bg=21.55%)▶sf3a3 (bg=12.13%)▶SF3B1 (bg=6.74%)▶tia1 (bg=16.04%)▶WRN (bg=3.31%)▶ZC3H11A (bg=6.25%)No matches to TargetScan


ATATCTAG

ATATCTAG  
Depth:9 (MOUSE)  
Ei-value:0.000, Pi-value:0.000  
Er-value:0.000, Pr-value:0.000  
eCLIP MATCHES▶AKAP1 (bg=3.4%)▶ddx55 (bg=12.35%)▶dgcr8 (bg=19.31%)▶fam120a (bg=18.43%)▶fubp3 (bg=23.31%)▶GRSF1 (bg=3.8%)▶igf2bp1 (bg=11.67%)▶KHSRP (bg=8.1%)▶NKRF (bg=3.64%)▶PHF6 (bg=1.71%)▶pum1 (bg=29.85%)▶pum2 (bg=21.55%)▶sf3a3 (bg=12.13%)▶SF3B1 (bg=6.74%)▶tia1 (bg=16.04%)▶WRN (bg=3.31%)▶ZC3H11A (bg=6.25%)No matches to TargetScan


T

AATATCTAGTCTCTAGATATT  
Depth:7 (ARMADILLO)  
Ei-value:0.000, Pi-value:0.000  
Er-value:0.000, Pr-value:0.000  
eCLIP MATCHES▶AKAP1 (bg=3.4%)▶ddx55 (bg=12.35%)▶dgcr8 (bg=19.31%)▶fam120a (bg=18.43%)▶FASTKD2 (bg=7.73%)▶fubp3 (bg=23.31%)▶GRSF1 (bg=3.8%)▶igf2bp1 (bg=11.67%)▶KHSRP (bg=8.1%)▶NKRF (bg=3.64%)▶PHF6 (bg=1.71%)▶pum1 (bg=29.85%)▶pum2 (bg=21.55%)▶sf3a3 (bg=12.13%)▶SF3B1 (bg=6.74%)▶tia1 (bg=16.04%)▶tial1 (bg=14.09%)▶WRN (bg=3.31%)▶ZC3H11A (bg=6.25%)MATCHES To TargetScan▶ miR-28-3p:ACUAGAU


CTCTAG

CTCTAG  
Depth:9 (MOUSE)  
Ei-value:0.000, Pi-value:0.000  
Er-value:0.000, Pr-value:0.000  
eCLIP MATCHES▶ddx55 (bg=12.35%)▶dgcr8 (bg=19.31%)▶FASTKD2 (bg=7.73%)▶fubp3 (bg=23.31%)▶GRSF1 (bg=3.8%)▶KHSRP (bg=8.1%)▶pum1 (bg=29.85%)▶pum2 (bg=21.55%)▶tia1 (bg=16.04%)▶ZC3H11A (bg=6.25%)No matches to TargetScan


ATATT

CTCTAGATATT  
Depth:8 (GUINEAPIG)  
Ei-value:0.000, Pi-value:0.000  
Er-value:0.000, Pr-value:0.000  
eCLIP MATCHES▶ddx55 (bg=12.35%)▶dgcr8 (bg=19.31%)▶FASTKD2 (bg=7.73%)▶fubp3 (bg=23.31%)▶GRSF1 (bg=3.8%)▶KHSRP (bg=8.1%)▶pum1 (bg=29.85%)▶pum2 (bg=21.55%)▶tia1 (bg=16.04%)▶tial1 (bg=14.09%)▶ZC3H11A (bg=6.25%)No matches to TargetScan


AAAGAGG

AAAGAGGTTGCCAATGTATGACA  
Depth:9 (MOUSE)  
Ei-value:0.000, Pi-value:0.000  
Er-value:0.000, Pr-value:0.000  
eCLIP MATCHES▶ddx55 (bg=12.35%)▶dgcr8 (bg=19.31%)▶FASTKD2 (bg=7.73%)▶fubp3 (bg=23.31%)▶GRSF1 (bg=3.8%)▶NOLC1 (bg=6.58%)▶pum1 (bg=29.85%)▶pum2 (bg=21.55%)▶tial1 (bg=14.09%)MATCHES To TargetScan▶ miR-182-5p:UUGGCAA▶ miR-96-5p/1271-5p:UUGGCAC▶ miR-539-3p:UCAUACA

 2760  


TTGCCAATGTATGACA

AAAGAGGTTGCCAATGTATGACA  
Depth:9 (MOUSE)  
Ei-value:0.000, Pi-value:0.000  
Er-value:0.000, Pr-value:0.000  
eCLIP MATCHES▶ddx55 (bg=12.35%)▶dgcr8 (bg=19.31%)▶FASTKD2 (bg=7.73%)▶fubp3 (bg=23.31%)▶GRSF1 (bg=3.8%)▶NOLC1 (bg=6.58%)▶pum1 (bg=29.85%)▶pum2 (bg=21.55%)▶tial1 (bg=14.09%)MATCHES To TargetScan▶ miR-182-5p:UUGGCAA▶ miR-96-5p/1271-5p:UUGGCAC▶ miR-539-3p:UCAUACA

G

AAGTAG

AAGTAG  
Depth:8 (GUINEAPIG)  
Ei-value:0.000, Pi-value:0.000  
Er-value:0.000, Pr-value:0.000  
eCLIP MATCHES▶ddx55 (bg=12.35%)▶dgcr8 (bg=19.31%)▶FASTKD2 (bg=7.73%)▶NOLC1 (bg=6.58%)▶pum1 (bg=29.85%)▶pum2 (bg=21.55%)▶tial1 (bg=14.09%)No matches to TargetScan


A

AAGTAGAGTTAGTAAACTAACACATTTTGTACACTTTGT  
Depth:3 (DOG)  
Ei-value:0.000, Pi-value:0.000  
Er-value:0.000, Pr-value:0.000  
eCLIP MATCHES▶ddx55 (bg=12.35%)▶dgcr8 (bg=19.31%)▶FASTKD2 (bg=7.73%)▶NOLC1 (bg=6.58%)▶pum1 (bg=29.85%)▶pum2 (bg=21.55%)▶tial1 (bg=14.09%)MATCHES To TargetScan▶ miR-493-5p:UGUACAU▶ miR-17-5p/20-5p/93-5p/106-5p/519-3p:AAAGUGC


GTTAGTAAACT

GTTAGTAAACT  
Depth:9 (MOUSE)  
Ei-value:0.000, Pi-value:0.000  
Er-value:0.000, Pr-value:0.000  
eCLIP MATCHES▶ddx55 (bg=12.35%)▶dgcr8 (bg=19.31%)▶FASTKD2 (bg=7.73%)▶NOLC1 (bg=6.58%)▶pum1 (bg=29.85%)▶pum2 (bg=21.55%)No matches to TargetScan


A

GTTAGTAAACTAACACATTTTGTACACTT  
Depth:8 (GUINEAPIG)  
Ei-value:0.000, Pi-value:0.000  
Er-value:0.000, Pr-value:0.000  
eCLIP MATCHES▶ddx55 (bg=12.35%)▶dgcr8 (bg=19.31%)▶FASTKD2 (bg=7.73%)▶NOLC1 (bg=6.58%)▶pum1 (bg=29.85%)▶pum2 (bg=21.55%)MATCHES To TargetScan▶ miR-493-5p:UGUACAU


ACACATT

ACACATT  
Depth:9 (MOUSE)  
Ei-value:0.000, Pi-value:0.000  
Er-value:0.000, Pr-value:0.000  
eCLIP MATCHES▶NOLC1 (bg=6.58%)No matches to TargetScan


TTGTACACTT

GTTAGTAAACTAACACATTTTGTACACTT  
Depth:8 (GUINEAPIG)  
Ei-value:0.000, Pi-value:0.000  
Er-value:0.000, Pr-value:0.000  
eCLIP MATCHES▶ddx55 (bg=12.35%)▶dgcr8 (bg=19.31%)▶FASTKD2 (bg=7.73%)▶NOLC1 (bg=6.58%)▶pum1 (bg=29.85%)▶pum2 (bg=21.55%)MATCHES To TargetScan▶ miR-493-5p:UGUACAU


TGT

GTTAGTAAACTAACACATTTTGTACACTTTGT  
Depth:5 (SHEEP)  
Ei-value:0.000, Pi-value:0.000  
Er-value:0.000, Pr-value:0.000  
eCLIP MATCHES▶ddx55 (bg=12.35%)▶dgcr8 (bg=19.31%)▶FASTKD2 (bg=7.73%)▶NOLC1 (bg=6.58%)▶pum1 (bg=29.85%)▶pum2 (bg=21.55%)MATCHES To TargetScan▶ miR-493-5p:UGUACAU▶ miR-17-5p/20-5p/93-5p/106-5p/519-3p:AAAGUGC


TAAAATT

TAAAATT  
Depth:3 (DOG)  
Ei-value:0.000, Pi-value:0.050  
Er-value:0.000, Pr-value:0.000  
eCLIP MATCHES▶dgcr8 (bg=19.31%)No matches to TargetScan

TGTA

GAAAG

GAAAGGCTGTCTTCTGAAAAGGAC  
Depth:6 (PIG)  
Ei-value:0.000, Pi-value:0.000  
Er-value:0.000, Pr-value:0.000  
eCLIP MATCHES▶dgcr8 (bg=19.31%)▶pum2 (bg=21.55%)▶ybx3 (bg=22.82%)No matches to TargetScan


GC

GCTGTCTTCTGAAAA  
Depth:7 (ARMADILLO)  
Ei-value:0.000, Pi-value:0.000  
Er-value:0.000, Pr-value:0.000  
eCLIP MATCHES▶dgcr8 (bg=19.31%)▶pum2 (bg=21.55%)▶ybx3 (bg=22.82%)No matches to TargetScan


TGTCTTCTGAA

TGTCTTCTGAA  
Depth:9 (MOUSE)  
Ei-value:0.000, Pi-value:0.000  
Er-value:0.000, Pr-value:0.000  
eCLIP MATCHES▶dgcr8 (bg=19.31%)▶pum2 (bg=21.55%)▶ybx3 (bg=22.82%)No matches to TargetScan


AA

TGTCTTCTGAAAA  
Depth:8 (GUINEAPIG)  
Ei-value:0.000, Pi-value:0.000  
Er-value:0.000, Pr-value:0.000  
eCLIP MATCHES▶dgcr8 (bg=19.31%)▶pum2 (bg=21.55%)▶ybx3 (bg=22.82%)No matches to TargetScan


GGAC

GAAAGGCTGTCTTCTGAAAAGGAC  
Depth:6 (PIG)  
Ei-value:0.000, Pi-value:0.000  
Er-value:0.000, Pr-value:0.000  
eCLIP MATCHES▶dgcr8 (bg=19.31%)▶pum2 (bg=21.55%)▶ybx3 (bg=22.82%)No matches to TargetScan


TTT

GAAAGGCTGTCTTCTGAAAAGGACTTT  
Depth:3 (DOG)  
Ei-value:0.000, Pi-value:0.000  
Er-value:0.000, Pr-value:0.000  
eCLIP MATCHES▶dgcr8 (bg=19.31%)▶pum2 (bg=21.55%)▶ybx3 (bg=22.82%)No matches to TargetScan

TGGAAGTGAGATAACATCAGCTCTA

A

AGTGACA  
Depth:3 (DOG)  
Ei-value:0.000, Pi-value:0.000  
Er-value:0.000, Pr-value:0.000  
eCLIP MATCHES▶lin28b (bg=16.96%)▶ybx3 (bg=22.82%)MATCHES To TargetScan▶ miR-668-3p:GUCACUC

 2880  


GTGACA

AGTGACA  
Depth:3 (DOG)  
Ei-value:0.000, Pi-value:0.000  
Er-value:0.000, Pr-value:0.000  
eCLIP MATCHES▶lin28b (bg=16.96%)▶ybx3 (bg=22.82%)MATCHES To TargetScan▶ miR-668-3p:GUCACUC

C

GTGCCT

GTGCCT  
Depth:9 (MOUSE)  
Ei-value:0.000, Pi-value:0.000  
Er-value:0.000, Pr-value:0.000  
eCLIP MATCHES▶lin28b (bg=16.96%)▶ybx3 (bg=22.82%)No matches to TargetScan

ATATCCATCA

ggttgg

ggttgg  
Depth:3 (DOG)  
Ei-value:0.080, Pi-value:0.020  
Er-value:0.000, Pr-value:0.020  
eCLIP MATCHES▶EIF3H (bg=7.83%)▶lin28b (bg=16.96%)▶ybx3 (bg=22.82%)No matches to TargetScan

TG

GTGGAGAG

GTGGAGAG  
Depth:3 (DOG)  
Ei-value:0.000, Pi-value:0.000  
Er-value:0.000, Pr-value:0.000  
eCLIP MATCHES▶EIF3H (bg=7.83%)▶lin28b (bg=16.96%)▶UTP3 (bg=2.81%)▶ybx3 (bg=22.82%)No matches to TargetScan

GAGTTGGAAGG

AATGAA

AATGAA  
Depth:8 (GUINEAPIG)  
Ei-value:0.000, Pi-value:0.020  
Er-value:0.000, Pr-value:0.000  
eCLIP MATCHES▶EIF3H (bg=7.83%)▶lin28b (bg=16.96%)▶UTP3 (bg=2.81%)▶ybx3 (bg=22.82%)No matches to TargetScan


GG

AATGAAGGGTTCTAGAC  
Depth:3 (DOG)  
Ei-value:0.000, Pi-value:0.000  
Er-value:0.000, Pr-value:0.000  
eCLIP MATCHES▶EIF3H (bg=7.83%)▶FUS (bg=2.59%)▶lin28b (bg=16.96%)▶UTP3 (bg=2.81%)▶ybx3 (bg=22.82%)MATCHES To TargetScan▶ miR-205-5p:CCUUCAU


GTTCTAGAC

GTTCTAGAC  
Depth:5 (SHEEP)  
Ei-value:0.000, Pi-value:0.000  
Er-value:0.000, Pr-value:0.000  
eCLIP MATCHES▶FUS (bg=2.59%)▶lin28b (bg=16.96%)▶UTP3 (bg=2.81%)▶ybx3 (bg=22.82%)No matches to TargetScan

C

AGAATG

AGAATG  
Depth:9 (MOUSE)  
Ei-value:0.000, Pi-value:0.000  
Er-value:0.000, Pr-value:0.000  
eCLIP MATCHES▶AARS (bg=2.33%)▶FUS (bg=2.59%)▶lin28b (bg=16.96%)▶UTP3 (bg=2.81%)▶ybx3 (bg=22.82%)No matches to TargetScan


TT

AGAATGTT  
Depth:7 (ARMADILLO)  
Ei-value:0.000, Pi-value:0.000  
Er-value:0.000, Pr-value:0.000  
eCLIP MATCHES▶AARS (bg=2.33%)▶FUS (bg=2.59%)▶lin28b (bg=16.96%)▶UTP3 (bg=2.81%)▶ybx3 (bg=22.82%)MATCHES To TargetScan▶ miR-181-5p:ACAUUCA▶ miR-543:AACAUUC


C

AGAATGTTC  
Depth:5 (SHEEP)  
Ei-value:0.000, Pi-value:0.000  
Er-value:0.000, Pr-value:0.000  
eCLIP MATCHES▶AARS (bg=2.33%)▶FUS (bg=2.59%)▶lin28b (bg=16.96%)▶UTP3 (bg=2.81%)▶ybx3 (bg=22.82%)MATCHES To TargetScan▶ miR-181-5p:ACAUUCA▶ miR-543:AACAUUC

GTATTT

AGAAGA

AGAAGA  
Depth:9 (MOUSE)  
Ei-value:0.000, Pi-value:0.000  
Er-value:0.000, Pr-value:0.000  
eCLIP MATCHES▶AARS (bg=2.33%)▶FUS (bg=2.59%)▶lin28b (bg=16.96%)No matches to TargetScan


CACT

AGAAGACACT  
Depth:3 (DOG)  
Ei-value:0.000, Pi-value:0.000  
Er-value:0.000, Pr-value:0.000  
eCLIP MATCHES▶AARS (bg=2.33%)▶FUS (bg=2.59%)▶lin28b (bg=16.96%)No matches to TargetScan

ATC

AGATATAA

AGATATAA  
Depth:3 (DOG)  
Ei-value:0.000, Pi-value:0.000  
Er-value:0.000, Pr-value:0.000  
eCLIP MATCHES▶AARS (bg=2.33%)▶FUS (bg=2.59%)▶HNRNPL (bg=3.4%)▶pum2 (bg=21.55%)No matches to TargetScan


CCATTGTTAC

CCATTGTTAC  
Depth:5 (SHEEP)  
Ei-value:0.000, Pi-value:0.000  
Er-value:0.000, Pr-value:0.000  
eCLIP MATCHES▶AARS (bg=2.33%)▶DDX21 (bg=1.64%)▶ddx6 (bg=23.92%)▶dgcr8 (bg=19.31%)▶FUS (bg=2.59%)▶HNRNPL (bg=3.4%)▶igf2bp1 (bg=11.67%)▶pum2 (bg=21.55%)MATCHES To TargetScan▶ miR-194-5p:GUAACAG

A

TGTGT

TGTGTGT  
Depth:6 (PIG)  
Ei-value:0.000, Pi-value:0.010  
Er-value:0.000, Pr-value:0.000  
eCLIP MATCHES▶AARS (bg=2.33%)▶DDX21 (bg=1.64%)▶ddx6 (bg=23.92%)▶dgcr8 (bg=19.31%)▶fam120a (bg=18.43%)▶HNRNPL (bg=3.4%)▶igf2bp1 (bg=11.67%)▶pum2 (bg=21.55%)▶sf3a3 (bg=12.13%)▶ZC3H11A (bg=6.25%)MATCHES To TargetScan▶ miR-329-3p/362-3p:ACACACC

 3000  


GT

TGTGTGT  
Depth:6 (PIG)  
Ei-value:0.000, Pi-value:0.010  
Er-value:0.000, Pr-value:0.000  
eCLIP MATCHES▶AARS (bg=2.33%)▶DDX21 (bg=1.64%)▶ddx6 (bg=23.92%)▶dgcr8 (bg=19.31%)▶fam120a (bg=18.43%)▶HNRNPL (bg=3.4%)▶igf2bp1 (bg=11.67%)▶pum2 (bg=21.55%)▶sf3a3 (bg=12.13%)▶ZC3H11A (bg=6.25%)MATCHES To TargetScan▶ miR-329-3p/362-3p:ACACACC


A

TGTGTGTAGTTTATTCAAC  
Depth:5 (SHEEP)  
Ei-value:0.000, Pi-value:0.000  
Er-value:0.000, Pr-value:0.000  
eCLIP MATCHES▶AARS (bg=2.33%)▶DDX21 (bg=1.64%)▶ddx6 (bg=23.92%)▶dgcr8 (bg=19.31%)▶fam120a (bg=18.43%)▶HNRNPL (bg=3.4%)▶igf2bp1 (bg=11.67%)▶pum2 (bg=21.55%)▶sf3a3 (bg=12.13%)▶ZC3H11A (bg=6.25%)MATCHES To TargetScan▶ miR-329-3p/362-3p:ACACACC


GTTTATT

GTTTATT  
Depth:8 (GUINEAPIG)  
Ei-value:0.000, Pi-value:0.000  
Er-value:0.000, Pr-value:0.000  
eCLIP MATCHES▶DDX21 (bg=1.64%)▶ddx6 (bg=23.92%)▶dgcr8 (bg=19.31%)▶fam120a (bg=18.43%)▶HNRNPL (bg=3.4%)▶igf2bp1 (bg=11.67%)▶pum2 (bg=21.55%)▶sf3a3 (bg=12.13%)▶ZC3H11A (bg=6.25%)No matches to TargetScan


CAAC

GTTTATTCAAC  
Depth:6 (PIG)  
Ei-value:0.000, Pi-value:0.000  
Er-value:0.000, Pr-value:0.000  
eCLIP MATCHES▶DDX21 (bg=1.64%)▶ddx6 (bg=23.92%)▶dgcr8 (bg=19.31%)▶fam120a (bg=18.43%)▶HNRNPL (bg=3.4%)▶igf2bp1 (bg=11.67%)▶pum2 (bg=21.55%)▶sf3a3 (bg=12.13%)▶ZC3H11A (bg=6.25%)No matches to TargetScan

C

CTACTG

CTACTGTGTATATA  
Depth:8 (GUINEAPIG)  
Ei-value:0.000, Pi-value:0.000  
Er-value:0.000, Pr-value:0.000  
eCLIP MATCHES▶DDX21 (bg=1.64%)▶ddx6 (bg=23.92%)▶dgcr8 (bg=19.31%)▶fam120a (bg=18.43%)▶HNRNPL (bg=3.4%)▶igf2bp1 (bg=11.67%)▶pum2 (bg=21.55%)▶sf3a3 (bg=12.13%)▶ZC3H11A (bg=6.25%)MATCHES To TargetScan▶ miR-199-3p:CAGUAGU▶ miR-144-3p:ACAGUAU▶ miR-128-3p:CACAGUG▶ miR-101-3p.1:ACAGUAC


TGTATATA

TGTATATA  
Depth:9 (MOUSE)  
Ei-value:0.000, Pi-value:0.000  
Er-value:0.000, Pr-value:0.000  
eCLIP MATCHES▶DDX21 (bg=1.64%)▶dgcr8 (bg=19.31%)▶fam120a (bg=18.43%)▶HNRNPL (bg=3.4%)▶igf2bp1 (bg=11.67%)▶pum2 (bg=21.55%)▶ZC3H11A (bg=6.25%)No matches to TargetScan


G

CTACTGTGTATATAG  
Depth:5 (SHEEP)  
Ei-value:0.000, Pi-value:0.000  
Er-value:0.000, Pr-value:0.000  
eCLIP MATCHES▶DDX21 (bg=1.64%)▶ddx6 (bg=23.92%)▶dgcr8 (bg=19.31%)▶fam120a (bg=18.43%)▶HNRNPL (bg=3.4%)▶igf2bp1 (bg=11.67%)▶pum2 (bg=21.55%)▶sf3a3 (bg=12.13%)▶ZC3H11A (bg=6.25%)MATCHES To TargetScan▶ miR-199-3p:CAGUAGU▶ miR-144-3p:ACAGUAU▶ miR-128-3p:CACAGUG▶ miR-101-3p.1:ACAGUAC

CG

GACAAAC

GACAAAC  
Depth:6 (PIG)  
Ei-value:0.000, Pi-value:0.000  
Er-value:0.000, Pr-value:0.000  
eCLIP MATCHES▶DDX21 (bg=1.64%)▶pum2 (bg=21.55%)No matches to TargetScan


TT

GACAAACTTAAGTCCTTATTTGAAACATCTAG  
Depth:4 (COW)  
Ei-value:0.000, Pi-value:0.000  
Er-value:0.000, Pr-value:0.000  
eCLIP MATCHES▶DDX21 (bg=1.64%)▶pum2 (bg=21.55%)No matches to TargetScan


AAGTCCT

AAGTCCTTATTTGAAACATCTAG  
Depth:7 (ARMADILLO)  
Ei-value:0.000, Pi-value:0.000  
Er-value:0.000, Pr-value:0.000  
No matches to eCLIP DataNo matches to TargetScan


TATT

TATTTGAAACATCTAG  
Depth:8 (GUINEAPIG)  
Ei-value:0.000, Pi-value:0.000  
Er-value:0.000, Pr-value:0.000  
No matches to eCLIP DataNo matches to TargetScan


TGAAACATCTAG

TGAAACATCTAG  
Depth:9 (MOUSE)  
Ei-value:0.000, Pi-value:0.000  
Er-value:0.000, Pr-value:0.000  
No matches to eCLIP DataNo matches to TargetScan

T

CT

CTTTCTAGATGTTTAGAAGTGCACAAAGTATGTTAAAAGTAGAGGTAGT  
Depth:8 (GUINEAPIG)  
Ei-value:0.000, Pi-value:0.000  
Er-value:0.000, Pr-value:0.000  
No matches to eCLIP DataNo matches to TargetScan


TTCTAG

TTCTAG  
Depth:9 (MOUSE)  
Ei-value:0.000, Pi-value:0.000  
Er-value:0.000, Pr-value:0.000  
No matches to eCLIP DataNo matches to TargetScan


A

CTTTCTAGATGTTTAGAAGTGCACAAAGTATGTTAAAAGTAGAGGTAGT  
Depth:8 (GUINEAPIG)  
Ei-value:0.000, Pi-value:0.000  
Er-value:0.000, Pr-value:0.000  
No matches to eCLIP DataNo matches to TargetScan


TGTTTAGAAGTGCACAAAGTATGTTAAAAGTAGA

TGTTTAGAAGTGCACAAAGTATGTTAAAAGTAGA  
Depth:9 (MOUSE)  
Ei-value:0.000, Pi-value:0.000  
Er-value:0.000, Pr-value:0.000  
No matches to eCLIP DataNo matches to TargetScan


GGTAGT

CTTTCTAGATGTTTAGAAGTGCACAAAGTATGTTAAAAGTAGAGGTAGT  
Depth:8 (GUINEAPIG)  
Ei-value:0.000, Pi-value:0.000  
Er-value:0.000, Pr-value:0.000  
No matches to eCLIP DataNo matches to TargetScan


AAA

CTTTCTAGATGTTTAGAAGTGCACAAAGTATGTTAAAAGTAGAGGTAGTAAA  
Depth:3 (DOG)  
Ei-value:0.000, Pi-value:0.000  
Er-value:0.000, Pr-value:0.000  
No matches to eCLIP DataNo matches to TargetScan


TAA

TAACACAT  
Depth:3 (DOG)  
Ei-value:0.000, Pi-value:0.000  
Er-value:0.000, Pr-value:0.000  
No matches to eCLIP DataNo matches to TargetScan

 3120  


CACAT

TAACACAT  
Depth:3 (DOG)  
Ei-value:0.000, Pi-value:0.000  
Er-value:0.000, Pr-value:0.000  
No matches to eCLIP DataNo matches to TargetScan

T

TTGTAG

TTGTAG  
Depth:6 (PIG)  
Ei-value:0.000, Pi-value:0.010  
Er-value:0.000, Pr-value:0.010  
No matches to eCLIP DataNo matches to TargetScan

CT

ATCCTTT

ATCCTTT  
Depth:5 (SHEEP)  
Ei-value:0.000, Pi-value:0.000  
Er-value:0.000, Pr-value:0.000  
No matches to eCLIP DataNo matches to TargetScan


TG

ATCCTTTTG  
Depth:3 (DOG)  
Ei-value:0.000, Pi-value:0.000  
Er-value:0.000, Pr-value:0.000  
eCLIP MATCHES▶SUB1 (bg=9.24%)No matches to TargetScan


ATAT

ATATGAAATA  
Depth:3 (DOG)  
Ei-value:0.000, Pi-value:0.000  
Er-value:0.000, Pr-value:0.000  
eCLIP MATCHES▶SUB1 (bg=9.24%)No matches to TargetScan


GAAATA

GAAATA  
Depth:8 (GUINEAPIG)  
Ei-value:0.000, Pi-value:0.000  
Er-value:0.000, Pr-value:0.010  
eCLIP MATCHES▶SUB1 (bg=9.24%)No matches to TargetScan

TTGTCTTGGAAAT

TGATCAA

TGATCAA  
Depth:5 (SHEEP)  
Ei-value:0.000, Pi-value:0.000  
Er-value:0.000, Pr-value:0.000  
eCLIP MATCHES▶DDX52 (bg=1.27%)▶ddx55 (bg=12.35%)▶dgcr8 (bg=19.31%)▶fam120a (bg=18.43%)▶FASTKD2 (bg=7.73%)▶fubp3 (bg=23.31%)▶SUB1 (bg=9.24%)▶tia1 (bg=16.04%)▶tial1 (bg=14.09%)No matches to TargetScan

TTCTCT

GAGCAG

GAGCAG  
Depth:6 (PIG)  
Ei-value:0.000, Pi-value:0.000  
Er-value:0.000, Pr-value:0.000  
eCLIP MATCHES▶DDX52 (bg=1.27%)▶ddx55 (bg=12.35%)▶ddx6 (bg=23.92%)▶dgcr8 (bg=19.31%)▶fam120a (bg=18.43%)▶FASTKD2 (bg=7.73%)▶fubp3 (bg=23.31%)▶SUB1 (bg=9.24%)▶tia1 (bg=16.04%)▶tial1 (bg=14.09%)No matches to TargetScan

TACCCATTTTGATATT

TGTGCTG

TGTGCTG  
Depth:7 (ARMADILLO)  
Ei-value:0.000, Pi-value:0.000  
Er-value:0.000, Pr-value:0.000  
eCLIP MATCHES▶ddx55 (bg=12.35%)▶FASTKD2 (bg=7.73%)▶tial1 (bg=14.09%)▶ybx3 (bg=22.82%)No matches to TargetScan


GTTCA

TGTGCTGGTTCA  
Depth:6 (PIG)  
Ei-value:0.000, Pi-value:0.000  
Er-value:0.000, Pr-value:0.000  
eCLIP MATCHES▶ddx55 (bg=12.35%)▶FASTKD2 (bg=7.73%)▶tial1 (bg=14.09%)▶ybx3 (bg=22.82%)MATCHES To TargetScan▶ miR-338-3p:CCAGCAU


GGG

TGTGCTGGTTCAGGG  
Depth:5 (SHEEP)  
Ei-value:0.000, Pi-value:0.000  
Er-value:0.000, Pr-value:0.000  
eCLIP MATCHES▶ddx55 (bg=12.35%)▶FASTKD2 (bg=7.73%)▶tial1 (bg=14.09%)▶ybx3 (bg=22.82%)MATCHES To TargetScan▶ miR-125-5p:CCCUGAG▶ miR-338-3p:CCAGCAU

GG

aaggag

aaggag  
Depth:3 (DOG)  
Ei-value:0.080, Pi-value:0.000  
Er-value:0.000, Pr-value:0.000  
No matches to eCLIP DataNo matches to TargetScan

GAGCAC

AAAGTGCAAA

AAAGTGCAAAGGGCTTT  
Depth:3 (DOG)  
Ei-value:0.000, Pi-value:0.000  
Er-value:0.000, Pr-value:0.000  
No matches to eCLIP DataMATCHES To TargetScan▶ miR-129-3p:AGCCCUU

 3240  


GGGCTTT

AAAGTGCAAAGGGCTTT  
Depth:3 (DOG)  
Ei-value:0.000, Pi-value:0.000  
Er-value:0.000, Pr-value:0.000  
No matches to eCLIP DataMATCHES To TargetScan▶ miR-129-3p:AGCCCUU

CT

ACCAGT

ACCAGT  
Depth:5 (SHEEP)  
Ei-value:0.000, Pi-value:0.010  
Er-value:0.000, Pr-value:0.000  
No matches to eCLIP DataNo matches to TargetScan

GTCCAGTG

TGTTTA

TGTTTA  
Depth:6 (PIG)  
Ei-value:0.000, Pi-value:0.020  
Er-value:0.000, Pr-value:0.010  
No matches to eCLIP DataNo matches to TargetScan

TGAGGAGGCACATTG

ACCA

ACCATTGTCC  
Depth:3 (DOG)  
Ei-value:0.000, Pi-value:0.000  
Er-value:0.000, Pr-value:0.000  
eCLIP MATCHES▶WDR3 (bg=0.75%)No matches to TargetScan


TTGTCC

TTGTCC  
Depth:5 (SHEEP)  
Ei-value:0.000, Pi-value:0.020  
Er-value:0.000, Pr-value:0.000  
eCLIP MATCHES▶WDR3 (bg=0.75%)No matches to TargetScan

CTTAT

GTCTGC

GTCTGC  
Depth:5 (SHEEP)  
Ei-value:0.000, Pi-value:0.020  
Er-value:0.000, Pr-value:0.000  
eCLIP MATCHES▶WDR3 (bg=0.75%)No matches to TargetScan


ATTTT

GTCTGCATTTT  
Depth:3 (DOG)  
Ei-value:0.000, Pi-value:0.000  
Er-value:0.000, Pr-value:0.000  
eCLIP MATCHES▶WDR3 (bg=0.75%)No matches to TargetScan

CATTTACTGTGCTG

TGTATA

TGTATA  
Depth:9 (MOUSE)  
Ei-value:0.000, Pi-value:0.000  
Er-value:0.000, Pr-value:0.000  
eCLIP MATCHES▶APOBEC3C (bg=3.95%)▶pum1 (bg=29.85%)▶pum2 (bg=21.55%)No matches to TargetScan


TA

TGTATATA  
Depth:9 (MOUSE)  
Ei-value:0.000, Pi-value:0.000  
Er-value:0.000, Pr-value:0.000  
eCLIP MATCHES▶APOBEC3C (bg=3.95%)▶dgcr8 (bg=19.31%)▶pum1 (bg=29.85%)▶pum2 (bg=21.55%)No matches to TargetScan


GTGTA

TGTATATAGTGTA  
Depth:6 (PIG)  
Ei-value:0.000, Pi-value:0.000  
Er-value:0.000, Pr-value:0.000  
eCLIP MATCHES▶APOBEC3C (bg=3.95%)▶ddx6 (bg=23.92%)▶dgcr8 (bg=19.31%)▶pum1 (bg=29.85%)▶pum2 (bg=21.55%)▶SF3B1 (bg=6.74%)No matches to TargetScan


TATAA

TGTATATAGTGTATATAA  
Depth:3 (DOG)  
Ei-value:0.000, Pi-value:0.000  
Er-value:0.000, Pr-value:0.000  
eCLIP MATCHES▶APOBEC3C (bg=3.95%)▶ddx6 (bg=23.92%)▶dgcr8 (bg=19.31%)▶fam120a (bg=18.43%)▶fubp3 (bg=23.31%)▶pum1 (bg=29.85%)▶pum2 (bg=21.55%)▶sf3a3 (bg=12.13%)▶SF3B1 (bg=6.74%)No matches to TargetScan

GCGGACATAG

GAGTCCT

GAGTCCTAATTTAC  
Depth:3 (DOG)  
Ei-value:0.000, Pi-value:0.000  
Er-value:0.000, Pr-value:0.000  
eCLIP MATCHES▶APOBEC3C (bg=3.95%)▶ddx6 (bg=23.92%)▶dgcr8 (bg=19.31%)▶fam120a (bg=18.43%)▶fubp3 (bg=23.31%)▶pum1 (bg=29.85%)▶pum2 (bg=21.55%)▶sf3a3 (bg=12.13%)▶SF3B1 (bg=6.74%)▶tial1 (bg=14.09%)No matches to TargetScan


A

AATTTAC  
Depth:6 (PIG)  
Ei-value:0.000, Pi-value:0.000  
Er-value:0.000, Pr-value:0.000  
eCLIP MATCHES▶APOBEC3C (bg=3.95%)▶ddx6 (bg=23.92%)▶dgcr8 (bg=19.31%)▶fam120a (bg=18.43%)▶fubp3 (bg=23.31%)▶pum1 (bg=29.85%)▶pum2 (bg=21.55%)▶sf3a3 (bg=12.13%)▶SF3B1 (bg=6.74%)▶tial1 (bg=14.09%)No matches to TargetScan

 3360  


ATTTAC

AATTTAC  
Depth:6 (PIG)  
Ei-value:0.000, Pi-value:0.000  
Er-value:0.000, Pr-value:0.000  
eCLIP MATCHES▶APOBEC3C (bg=3.95%)▶ddx6 (bg=23.92%)▶dgcr8 (bg=19.31%)▶fam120a (bg=18.43%)▶fubp3 (bg=23.31%)▶pum1 (bg=29.85%)▶pum2 (bg=21.55%)▶sf3a3 (bg=12.13%)▶SF3B1 (bg=6.74%)▶tial1 (bg=14.09%)No matches to TargetScan

GTCTAGTCGATGTTAAAAAGGTTGCCAGTATATGACAAAAGTAGAATTAGTAAACTACTACATTGAGTACACTTTGTGTTAAAATTCATAGGGAAGACTTCTTAAAAACAAGTG 3480  
 AAATTGTTAAAACCCCCCCTAAGCATTACAGATGGCTTATAGCTGTCCACGGGGTTGGTAGAGGTGGGAAAGGGAAGGGTTCTAGGCCAGAATGTTCCTATTTAGAAGACACTCAAATTA 3600  
 CAGTCTGTGTTATGTATGTATACCATTTATTCAATGCTACTGTGTATATAATGGAAAACTTAAGTCCAGTTTGA

AA

AACATCTAGTCTT  
Depth:6 (PIG)  
Ei-value:0.000, Pi-value:0.000  
Er-value:0.000, Pr-value:0.000  
eCLIP MATCHES▶fam120a (bg=18.43%)▶pum1 (bg=29.85%)▶pum2 (bg=21.55%)MATCHES To TargetScan▶ miR-28-3p:ACUAGAU


CATCT

CATCTAGTCTT  
Depth:8 (GUINEAPIG)  
Ei-value:0.000, Pi-value:0.000  
Er-value:0.000, Pr-value:0.000  
eCLIP MATCHES▶fam120a (bg=18.43%)▶pum1 (bg=29.85%)▶pum2 (bg=21.55%)MATCHES To TargetScan▶ miR-28-3p:ACUAGAU


AGTCTT

AGTCTT  
Depth:9 (MOUSE)  
Ei-value:0.000, Pi-value:0.000  
Er-value:0.000, Pr-value:0.000  
eCLIP MATCHES▶fam120a (bg=18.43%)▶pum1 (bg=29.85%)▶pum2 (bg=21.55%)No matches to TargetScan


TCTAG

AACATCTAGTCTTTCTAG  
Depth:3 (DOG)  
Ei-value:0.000, Pi-value:0.000  
Er-value:0.000, Pr-value:0.000  
eCLIP MATCHES▶fam120a (bg=18.43%)▶pum1 (bg=29.85%)▶pum2 (bg=21.55%)▶RPS3 (bg=4.15%)MATCHES To TargetScan▶ miR-28-3p:ACUAGAU


GTGTTTAAA

GTGTTTAAA  
Depth:3 (DOG)  
Ei-value:0.000, Pi-value:0.000  
Er-value:0.000, Pr-value:0.000  
eCLIP MATCHES▶ddx6 (bg=23.92%)▶fam120a (bg=18.43%)▶igf2bp1 (bg=11.67%)▶RPS3 (bg=4.15%)No matches to TargetScan

AGTGTACAACGGCCTGTCG 3720  
 CAGTGGCGCATGCCTGTAATCCCAGCACTTTGGGAGGCCGAGGCAGGCGGATCACGAGGTCAAGAGATCAGGACCATCTTGGCCAACATG

gtgaaa

gtgaaa  
Depth:3 (DOG)  
Ei-value:0.080, Pi-value:0.030  
Er-value:0.000, Pr-value:0.000  
No matches to eCLIP DataNo matches to TargetScan

CCCCATCTTTACTAAAAATACAAA 3840  
 AATTAGCTGGTCGTGGTGGTGCCCACCTGTAGCCCCAGTTACTCGAGAGGCTGAGGCAGGAGAATCGCTTGAACTTGGGAGGCGGAAGTTGCAGTGAGCCAAGATCGCACCACTGCACTC 3960  
 CAGCCTGGCGACAGAGCGAGGCTCCGTTTCAAAAAAAAAAGTGCACAATGTAGGTTAACAGTAGAGGGCTTAAGTAACA

CCCCTCTAAG

CCCCTCTAAG  
Depth:3 (DOG)  
Ei-value:0.000, Pi-value:0.000  
Er-value:0.000, Pr-value:0.000  
eCLIP MATCHES▶FUS (bg=2.59%)MATCHES To TargetScan▶ miR-423-5p:GAGGGGC▶ miR-877-5p:UAGAGGA

CATTTG

ttttca

ttttca  
Depth:3 (DOG)  
Ei-value:0.080, Pi-value:0.060  
Er-value:0.000, Pr-value:0.020  
No matches to eCLIP DataNo matches to TargetScan

GTACTTCC

TAGGAGT

TAGGAGT  
Depth:3 (DOG)  
Ei-value:0.000, Pi-value:0.000  
Er-value:0.000, Pr-value:0.000  
No matches to eCLIP DataMATCHES To TargetScan▶ miR-483-3p.1:ACUCCUC

GGTT 4080  
 GCA

TTTGGGAATGG

TTTGGGAATGGAATTGTTAAA  
Depth:3 (DOG)  
Ei-value:0.000, Pi-value:0.000  
Er-value:0.000, Pr-value:0.000  
No matches to eCLIP DataNo matches to TargetScan


AATTGTTA

AATTGTTA  
Depth:9 (MOUSE)  
Ei-value:0.000, Pi-value:0.000  
Er-value:0.000, Pr-value:0.000  
No matches to eCLIP DataNo matches to TargetScan


AA

AATTGTTAAA  
Depth:7 (ARMADILLO)  
Ei-value:0.000, Pi-value:0.000  
Er-value:0.000, Pr-value:0.000  
No matches to eCLIP DataNo matches to TargetScan

ACTTGATGCTTAGGAGCGAATGCAGACTATTCATTGGGTGTTTGGGG

tggggg

tggggg  
Depth:4 (COW)  
Ei-value:1.000, Pi-value:0.010  
Er-value:0.000, Pr-value:0.000  
eCLIP MATCHES▶BCCIP (bg=4.61%)▶fam120a (bg=18.43%)▶FASTKD2 (bg=7.73%)▶GTF2F1 (bg=2.31%)▶HNRNPM (bg=1.27%)▶IGF2BP2 (bg=3.44%)▶PCBP2 (bg=1.64%)▶PRPF4 (bg=2.33%)▶sf3a3 (bg=12.13%)▶tia1 (bg=16.04%)No matches to TargetScan


A

TGGGGGA  
Depth:3 (DOG)  
Ei-value:0.000, Pi-value:0.000  
Er-value:0.000, Pr-value:0.000  
eCLIP MATCHES▶BCCIP (bg=4.61%)▶fam120a (bg=18.43%)▶FASTKD2 (bg=7.73%)▶GTF2F1 (bg=2.31%)▶HNRNPM (bg=1.27%)▶IGF2BP2 (bg=3.44%)▶PCBP2 (bg=1.64%)▶PRPF4 (bg=2.33%)▶sf3a3 (bg=12.13%)▶tia1 (bg=16.04%)▶TROVE2 (bg=3.49%)▶ZRANB2 (bg=0.99%)No matches to TargetScan

AGGGGGGGTGGGCAGAGGAGGTATGCAGGGAGAG

GG

GGGTTCTGTGC  
Depth:3 (DOG)  
Ei-value:0.000, Pi-value:0.000  
Er-value:0.000, Pr-value:0.000  
eCLIP MATCHES▶APOBEC3C (bg=3.95%)▶BCCIP (bg=4.61%)▶CSTF2T (bg=1.12%)▶ddx3x (bg=13.89%)▶ddx55 (bg=12.35%)▶ddx6 (bg=23.92%)▶EWSR1 (bg=1.16%)▶fam120a (bg=18.43%)▶FASTKD2 (bg=7.73%)▶fubp3 (bg=23.31%)▶G3BP1 (bg=1.51%)▶GTF2F1 (bg=2.31%)▶HNRNPM (bg=1.27%)▶HNRNPU (bg=0.96%)▶IGF2BP2 (bg=3.44%)▶PABPC4 (bg=2.72%)▶PCBP2 (bg=1.64%)▶PRPF4 (bg=2.33%)▶pum1 (bg=29.85%)▶pum2 (bg=21.55%)▶RPS11 (bg=1.07%)▶SBDS (bg=1.91%)▶sf3a3 (bg=12.13%)▶TBRG4 (bg=7.0%)▶tia1 (bg=16.04%)▶TROVE2 (bg=3.49%)▶UPF1 (bg=3.62%)▶UTP3 (bg=2.81%)▶ZRANB2 (bg=0.99%)No matches to TargetScan


gttctg

gttctgt  
Depth:4 (COW)  
Ei-value:0.960, Pi-value:0.000  
Er-value:0.000, Pr-value:0.000  
eCLIP MATCHES▶APOBEC3C (bg=3.95%)▶BCCIP (bg=4.61%)▶CSTF2T (bg=1.12%)▶ddx3x (bg=13.89%)▶ddx55 (bg=12.35%)▶ddx6 (bg=23.92%)▶EWSR1 (bg=1.16%)▶fam120a (bg=18.43%)▶FASTKD2 (bg=7.73%)▶fubp3 (bg=23.31%)▶G3BP1 (bg=1.51%)▶GTF2F1 (bg=2.31%)▶HNRNPM (bg=1.27%)▶HNRNPU (bg=0.96%)▶IGF2BP2 (bg=3.44%)▶PABPC4 (bg=2.72%)▶PCBP2 (bg=1.64%)▶PRPF4 (bg=2.33%)▶pum1 (bg=29.85%)▶pum2 (bg=21.55%)▶RPS11 (bg=1.07%)▶SBDS (bg=1.91%)▶sf3a3 (bg=12.13%)▶TBRG4 (bg=7.0%)▶tia1 (bg=16.04%)▶TROVE2 (bg=3.49%)▶UPF1 (bg=3.62%)▶UTP3 (bg=2.81%)▶ZRANB2 (bg=0.99%)No matches to TargetScan

 4200  


t

gttctgt  
Depth:4 (COW)  
Ei-value:0.960, Pi-value:0.000  
Er-value:0.000, Pr-value:0.000  
eCLIP MATCHES▶APOBEC3C (bg=3.95%)▶BCCIP (bg=4.61%)▶CSTF2T (bg=1.12%)▶ddx3x (bg=13.89%)▶ddx55 (bg=12.35%)▶ddx6 (bg=23.92%)▶EWSR1 (bg=1.16%)▶fam120a (bg=18.43%)▶FASTKD2 (bg=7.73%)▶fubp3 (bg=23.31%)▶G3BP1 (bg=1.51%)▶GTF2F1 (bg=2.31%)▶HNRNPM (bg=1.27%)▶HNRNPU (bg=0.96%)▶IGF2BP2 (bg=3.44%)▶PABPC4 (bg=2.72%)▶PCBP2 (bg=1.64%)▶PRPF4 (bg=2.33%)▶pum1 (bg=29.85%)▶pum2 (bg=21.55%)▶RPS11 (bg=1.07%)▶SBDS (bg=1.91%)▶sf3a3 (bg=12.13%)▶TBRG4 (bg=7.0%)▶tia1 (bg=16.04%)▶TROVE2 (bg=3.49%)▶UPF1 (bg=3.62%)▶UTP3 (bg=2.81%)▶ZRANB2 (bg=0.99%)No matches to TargetScan


GC

GGGTTCTGTGC  
Depth:3 (DOG)  
Ei-value:0.000, Pi-value:0.000  
Er-value:0.000, Pr-value:0.000  
eCLIP MATCHES▶APOBEC3C (bg=3.95%)▶BCCIP (bg=4.61%)▶CSTF2T (bg=1.12%)▶ddx3x (bg=13.89%)▶ddx55 (bg=12.35%)▶ddx6 (bg=23.92%)▶EWSR1 (bg=1.16%)▶fam120a (bg=18.43%)▶FASTKD2 (bg=7.73%)▶fubp3 (bg=23.31%)▶G3BP1 (bg=1.51%)▶GTF2F1 (bg=2.31%)▶HNRNPM (bg=1.27%)▶HNRNPU (bg=0.96%)▶IGF2BP2 (bg=3.44%)▶PABPC4 (bg=2.72%)▶PCBP2 (bg=1.64%)▶PRPF4 (bg=2.33%)▶pum1 (bg=29.85%)▶pum2 (bg=21.55%)▶RPS11 (bg=1.07%)▶SBDS (bg=1.91%)▶sf3a3 (bg=12.13%)▶TBRG4 (bg=7.0%)▶tia1 (bg=16.04%)▶TROVE2 (bg=3.49%)▶UPF1 (bg=3.62%)▶UTP3 (bg=2.81%)▶ZRANB2 (bg=0.99%)No matches to TargetScan

TCCTGAGATTAGTTCAGATGGTCTAACCATTGTTCTATATGTGCATTTTAGTTAATATTGTGTATTAAAGGATA

AGTCTT

AGTCTT  
Depth:9 (MOUSE)  
Ei-value:0.000, Pi-value:0.000  
Er-value:0.000, Pr-value:0.000  
eCLIP MATCHES▶DDX24 (bg=2.31%)No matches to TargetScan

AATGCTC

aaagta

aaagta  
Depth:4 (COW)  
Ei-value:1.000, Pi-value:0.020  
Er-value:0.000, Pr-value:0.000  
eCLIP MATCHES▶DDX24 (bg=2.31%)No matches to TargetScan

TGTTAAAAATAGAT

gtagtaaa

gtagtaaa  
Depth:4 (COW)  
Ei-value:0.670, Pi-value:0.000  
Er-value:0.000, Pr-value:0.000  
eCLIP MATCHES▶ddx6 (bg=23.92%)No matches to TargetScan

TC 4320  
 AG

TCCCTTT

TCCCTTT  
Depth:3 (DOG)  
Ei-value:0.000, Pi-value:0.000  
Er-value:0.000, Pr-value:0.000  
eCLIP MATCHES▶ddx55 (bg=12.35%)▶ddx6 (bg=23.92%)▶SUB1 (bg=9.24%)No matches to TargetScan

GTG

AATGT

AATGTCCTTTTGTTA  
Depth:3 (DOG)  
Ei-value:0.000, Pi-value:0.000  
Er-value:0.000, Pr-value:0.000  
eCLIP MATCHES▶ddx55 (bg=12.35%)▶ddx6 (bg=23.92%)▶fam120a (bg=18.43%)▶lin28b (bg=16.96%)▶SUB1 (bg=9.24%)MATCHES To TargetScan▶ miR-495-3p:AACAAAC▶ miR-1197:AGGACAC


ccttttgtt

ccttttgtt  
Depth:4 (COW)  
Ei-value:0.150, Pi-value:0.000  
Er-value:0.000, Pr-value:0.000  
eCLIP MATCHES▶ddx55 (bg=12.35%)▶ddx6 (bg=23.92%)▶fam120a (bg=18.43%)▶lin28b (bg=16.96%)▶SUB1 (bg=9.24%)MATCHES To TargetScan▶ miR-495-3p:AACAAAC


A

AATGTCCTTTTGTTA  
Depth:3 (DOG)  
Ei-value:0.000, Pi-value:0.000  
Er-value:0.000, Pr-value:0.000  
eCLIP MATCHES▶ddx55 (bg=12.35%)▶ddx6 (bg=23.92%)▶fam120a (bg=18.43%)▶lin28b (bg=16.96%)▶SUB1 (bg=9.24%)MATCHES To TargetScan▶ miR-495-3p:AACAAAC▶ miR-1197:AGGACAC

GT

TTTTAGGAAGG

TTTTAGGAAGG  
Depth:4 (COW)  
Ei-value:0.000, Pi-value:0.000  
Er-value:0.000, Pr-value:0.000  
eCLIP MATCHES▶ddx55 (bg=12.35%)▶ddx6 (bg=23.92%)▶fam120a (bg=18.43%)▶fubp3 (bg=23.31%)▶lin28b (bg=16.96%)▶pum1 (bg=29.85%)▶SUB1 (bg=9.24%)▶tia1 (bg=16.04%)No matches to TargetScan

CCTGTCC

tctgggagt

tctgggagt  
Depth:4 (COW)  
Ei-value:0.150, Pi-value:0.000  
Er-value:0.000, Pr-value:0.000  
eCLIP MATCHES▶APOBEC3C (bg=3.95%)▶ddx55 (bg=12.35%)▶ddx6 (bg=23.92%)▶fam120a (bg=18.43%)▶fubp3 (bg=23.31%)▶lin28b (bg=16.96%)▶pum1 (bg=29.85%)▶SUB1 (bg=9.24%)▶tia1 (bg=16.04%)MATCHES To TargetScan▶ miR-150-5p:CUCCCAA▶ miR-532-3p:CUCCCAC


G

TCTGGGAGTG  
Depth:3 (DOG)  
Ei-value:0.000, Pi-value:0.000  
Er-value:0.000, Pr-value:0.000  
eCLIP MATCHES▶APOBEC3C (bg=3.95%)▶ddx55 (bg=12.35%)▶ddx6 (bg=23.92%)▶fam120a (bg=18.43%)▶fubp3 (bg=23.31%)▶lin28b (bg=16.96%)▶pum1 (bg=29.85%)▶SUB1 (bg=9.24%)▶tia1 (bg=16.04%)MATCHES To TargetScan▶ miR-150-5p:CUCCCAA▶ miR-532-3p:CUCCCAC▶ miR-483-3p.2:CACUCCU

ACCTTTATTAGTCCACCCCTTG

GAGCTAGA

GAGCTAGA  
Depth:3 (DOG)  
Ei-value:0.000, Pi-value:0.000  
Er-value:0.000, Pr-value:0.000  
eCLIP MATCHES▶ddx55 (bg=12.35%)▶ddx6 (bg=23.92%)▶fam120a (bg=18.43%)▶lin28b (bg=16.96%)▶pum1 (bg=29.85%)▶SUB1 (bg=9.24%)▶tia1 (bg=16.04%)▶ybx3 (bg=22.82%)No matches to TargetScan

CATCCTG

tacttagtc

tacttagtc  
Depth:4 (COW)  
Ei-value:0.150, Pi-value:0.000  
Er-value:0.000, Pr-value:0.000  
eCLIP MATCHES▶ddx55 (bg=12.35%)▶ddx6 (bg=23.92%)▶lin28b (bg=16.96%)▶ybx3 (bg=22.82%)No matches to TargetScan

ACGGGG

ATGGTGGA

ATGGTGGA  
Depth:3 (DOG)  
Ei-value:0.000, Pi-value:0.000  
Er-value:0.000, Pr-value:0.010  
eCLIP MATCHES▶ddx55 (bg=12.35%)▶lin28b (bg=16.96%)No matches to TargetScan

AGA 4440  
 GGGAGAAGAGGAA

gggtga

gggtga  
Depth:4 (COW)  
Ei-value:1.000, Pi-value:0.010  
Er-value:0.000, Pr-value:0.000  
No matches to eCLIP DataNo matches to TargetScan


A

GGGTGAAGGGAAGGGCT  
Depth:3 (DOG)  
Ei-value:0.000, Pi-value:0.000  
Er-value:0.000, Pr-value:0.000  
No matches to eCLIP DataMATCHES To TargetScan▶ miR-204-5p/211-5p:UCCCUUU▶ miR-205-5p:CCUUCAU▶ miR-129-3p:AGCCCUU


GGGAAGGG

GGGAAGGG  
Depth:5 (SHEEP)  
Ei-value:0.000, Pi-value:0.000  
Er-value:0.000, Pr-value:0.000  
No matches to eCLIP DataNo matches to TargetScan


CT

GGGAAGGGCT  
Depth:4 (COW)  
Ei-value:0.020, Pi-value:0.000  
Er-value:0.000, Pr-value:0.000  
No matches to eCLIP DataMATCHES To TargetScan▶ miR-129-3p:AGCCCUU

C

TTTGCTAGTATCT

TTTGCTAGTATCT  
Depth:3 (DOG)  
Ei-value:0.000, Pi-value:0.000  
Er-value:0.000, Pr-value:0.000  
No matches to eCLIP DataNo matches to TargetScan

CCAT

ATCTAGA

ATCTAGA  
Depth:3 (DOG)  
Ei-value:0.000, Pi-value:0.010  
Er-value:0.000, Pr-value:0.000  
No matches to eCLIP DataNo matches to TargetScan

CGATG

gttttag

gttttag  
Depth:4 (COW)  
Ei-value:0.960, Pi-value:0.010  
Er-value:0.000, Pr-value:0.000  
eCLIP MATCHES▶lin28b (bg=16.96%)No matches to TargetScan

ATG

ATAACCA

ATAACCA  
Depth:3 (DOG)  
Ei-value:0.000, Pi-value:0.010  
Er-value:0.000, Pr-value:0.000  
eCLIP MATCHES▶lin28b (bg=16.96%)No matches to TargetScan

CAGGTCTACAAGAGCG

ttttta

ttttta  
Depth:3 (DOG)  
Ei-value:0.080, Pi-value:0.120  
Er-value:0.000, Pr-value:0.120  
eCLIP MATCHES▶lin28b (bg=16.96%)No matches to TargetScan

GTAAAGTG

CCTGTGTT

CCTGTGTT  
Depth:3 (DOG)  
Ei-value:0.000, Pi-value:0.000  
Er-value:0.000, Pr-value:0.000  
eCLIP MATCHES▶lin28b (bg=16.96%)No matches to TargetScan

CA

ttg

ttgtgga  
Depth:4 (COW)  
Ei-value:0.960, Pi-value:0.000  
Er-value:0.000, Pr-value:0.000  
eCLIP MATCHES▶lin28b (bg=16.96%)No matches to TargetScan

 4560  


tgga

ttgtgga  
Depth:4 (COW)  
Ei-value:0.960, Pi-value:0.000  
Er-value:0.000, Pr-value:0.000  
eCLIP MATCHES▶lin28b (bg=16.96%)No matches to TargetScan

CAAAGTTA

TTATTTTG

TTATTTTG  
Depth:3 (DOG)  
Ei-value:0.000, Pi-value:0.000  
Er-value:0.000, Pr-value:0.010  
eCLIP MATCHES▶lin28b (bg=16.96%)▶ybx3 (bg=22.82%)No matches to TargetScan

C

AACATCTA

AACATCTA  
Depth:5 (SHEEP)  
Ei-value:0.000, Pi-value:0.000  
Er-value:0.000, Pr-value:0.000  
eCLIP MATCHES▶lin28b (bg=16.96%)▶ybx3 (bg=22.82%)No matches to TargetScan


AGCTTT

AACATCTAAGCTTT  
Depth:3 (DOG)  
Ei-value:0.000, Pi-value:0.000  
Er-value:0.000, Pr-value:0.000  
eCLIP MATCHES▶lin28b (bg=16.96%)▶ybx3 (bg=22.82%)MATCHES To TargetScan▶ miR-320:AAAGCUG▶ miR-21-5p/590-5p:AGCUUAU

ACGAATGG

ggtgacaa

ggtgacaa  
Depth:4 (COW)  
Ei-value:0.670, Pi-value:0.000  
Er-value:0.000, Pr-value:0.000  
eCLIP MATCHES▶ddx6 (bg=23.92%)▶lin28b (bg=16.96%)▶ybx3 (bg=22.82%)No matches to TargetScan

CT

tatgata

tatgata  
Depth:4 (COW)  
Ei-value:0.960, Pi-value:0.020  
Er-value:0.000, Pr-value:0.000  
eCLIP MATCHES▶ddx6 (bg=23.92%)▶lin28b (bg=16.96%)▶ybx3 (bg=22.82%)MATCHES To TargetScan▶ miR-154-3p/487-3p:AUCAUAC


AAA

TATGATAAAA  
Depth:3 (DOG)  
Ei-value:0.000, Pi-value:0.000  
Er-value:0.000, Pr-value:0.000  
eCLIP MATCHES▶ddx6 (bg=23.92%)▶lin28b (bg=16.96%)▶ybx3 (bg=22.82%)MATCHES To TargetScan▶ miR-154-3p/487-3p:AUCAUAC

ACTAG

AGCTA

AGCTAGTGAATTA  
Depth:4 (COW)  
Ei-value:0.000, Pi-value:0.000  
Er-value:0.000, Pr-value:0.000  
eCLIP MATCHES▶lin28b (bg=16.96%)▶ybx3 (bg=22.82%)No matches to TargetScan


GTGAATT

GTGAATT  
Depth:5 (SHEEP)  
Ei-value:0.000, Pi-value:0.010  
Er-value:0.000, Pr-value:0.000  
eCLIP MATCHES▶lin28b (bg=16.96%)▶ybx3 (bg=22.82%)No matches to TargetScan


A

AGCTAGTGAATTA  
Depth:4 (COW)  
Ei-value:0.000, Pi-value:0.000  
Er-value:0.000, Pr-value:0.000  
eCLIP MATCHES▶lin28b (bg=16.96%)▶ybx3 (bg=22.82%)No matches to TargetScan

GCCTATTTGTAAATAC

c

ctttgtta  
Depth:4 (COW)  
Ei-value:0.670, Pi-value:0.010  
Er-value:0.000, Pr-value:0.000  
eCLIP MATCHES▶fubp3 (bg=23.31%)▶lin28b (bg=16.96%)▶ybx3 (bg=22.82%)MATCHES To TargetScan▶ miR-495-3p:AACAAAC


TTTGTTA

TTTGTTA  
Depth:5 (SHEEP)  
Ei-value:0.000, Pi-value:0.010  
Er-value:0.000, Pr-value:0.000  
eCLIP MATCHES▶fubp3 (bg=23.31%)▶lin28b (bg=16.96%)▶ybx3 (bg=22.82%)MATCHES To TargetScan▶ miR-495-3p:AACAAAC


T

CTTTGTTAT  
Depth:3 (DOG)  
Ei-value:0.000, Pi-value:0.000  
Er-value:0.000, Pr-value:0.000  
eCLIP MATCHES▶fubp3 (bg=23.31%)▶lin28b (bg=16.96%)▶NKRF (bg=3.64%)▶ybx3 (bg=22.82%)MATCHES To TargetScan▶ miR-495-3p:AACAAAC

A

attgat

attgat  
Depth:4 (COW)  
Ei-value:1.000, Pi-value:0.040  
Er-value:0.000, Pr-value:0.000  
eCLIP MATCHES▶dgcr8 (bg=19.31%)▶fubp3 (bg=23.31%)▶lin28b (bg=16.96%)▶NKRF (bg=3.64%)▶ybx3 (bg=22.82%)No matches to TargetScan


AG

ATTGATAG  
Depth:3 (DOG)  
Ei-value:0.000, Pi-value:0.000  
Er-value:0.000, Pr-value:0.000  
eCLIP MATCHES▶dgcr8 (bg=19.31%)▶fubp3 (bg=23.31%)▶lin28b (bg=16.96%)▶NKRF (bg=3.64%)▶ybx3 (bg=22.82%)No matches to TargetScan

GATA

C

CATCTTGGAC  
Depth:4 (COW)  
Ei-value:0.020, Pi-value:0.000  
Er-value:0.000, Pr-value:0.000  
eCLIP MATCHES▶ddx6 (bg=23.92%)▶dgcr8 (bg=19.31%)▶fubp3 (bg=23.31%)▶lin28b (bg=16.96%)▶NKRF (bg=3.64%)▶ybx3 (bg=22.82%)No matches to TargetScan

 4680  


ATCTTGGAC

CATCTTGGAC  
Depth:4 (COW)  
Ei-value:0.020, Pi-value:0.000  
Er-value:0.000, Pr-value:0.000  
eCLIP MATCHES▶ddx6 (bg=23.92%)▶dgcr8 (bg=19.31%)▶fubp3 (bg=23.31%)▶lin28b (bg=16.96%)▶NKRF (bg=3.64%)▶ybx3 (bg=22.82%)No matches to TargetScan


ATG

CATCTTGGACATG  
Depth:3 (DOG)  
Ei-value:0.000, Pi-value:0.000  
Er-value:0.000, Pr-value:0.000  
eCLIP MATCHES▶ddx6 (bg=23.92%)▶dgcr8 (bg=19.31%)▶fubp3 (bg=23.31%)▶lin28b (bg=16.96%)▶NKRF (bg=3.64%)▶ybx3 (bg=22.82%)No matches to TargetScan

GA

ATTGTTAAGC

ATTGTTAAGC  
Depth:3 (DOG)  
Ei-value:0.000, Pi-value:0.000  
Er-value:0.000, Pr-value:0.000  
eCLIP MATCHES▶ddx6 (bg=23.92%)▶fubp3 (bg=23.31%)▶lin28b (bg=16.96%)▶NKRF (bg=3.64%)▶ybx3 (bg=22.82%)No matches to TargetScan

CAC

ctctgag

ctctgag  
Depth:4 (COW)  
Ei-value:0.960, Pi-value:0.000  
Er-value:0.000, Pr-value:0.000  
eCLIP MATCHES▶ddx6 (bg=23.92%)▶lin28b (bg=16.96%)▶ybx3 (bg=22.82%)No matches to TargetScan

CAGTGT

ATGTCAG

ATGTCAG  
Depth:3 (DOG)  
Ei-value:0.000, Pi-value:0.020  
Er-value:0.000, Pr-value:0.000  
eCLIP MATCHES▶ddx6 (bg=23.92%)▶lin28b (bg=16.96%)▶ybx3 (bg=22.82%)MATCHES To TargetScan▶ miR-489-3p:UGACAUC

GACTTGTTCAT

TAGGTTGGCAGC

TAGGTTGGCAGC  
Depth:4 (COW)  
Ei-value:0.000, Pi-value:0.000  
Er-value:0.000, Pr-value:0.000  
eCLIP MATCHES▶lin28b (bg=16.96%)No matches to TargetScan


AGAG

TAGGTTGGCAGCAGAG  
Depth:3 (DOG)  
Ei-value:0.000, Pi-value:0.000  
Er-value:0.000, Pr-value:0.000  
eCLIP MATCHES▶lin28b (bg=16.96%)No matches to TargetScan

G

GGCA

GGCAGAAGGAA  
Depth:3 (DOG)  
Ei-value:0.000, Pi-value:0.000  
Er-value:0.000, Pr-value:0.000  
eCLIP MATCHES▶ddx6 (bg=23.92%)▶lin28b (bg=16.96%)No matches to TargetScan


gaaggaa

gaaggaa  
Depth:4 (COW)  
Ei-value:0.960, Pi-value:0.010  
Er-value:0.000, Pr-value:0.000  
eCLIP MATCHES▶ddx6 (bg=23.92%)▶lin28b (bg=16.96%)No matches to TargetScan

TTATACAGGTAGAGA

TGTATGC

TGTATGC  
Depth:3 (DOG)  
Ei-value:0.000, Pi-value:0.010  
Er-value:0.000, Pr-value:0.000  
eCLIP MATCHES▶ddx6 (bg=23.92%)▶lin28b (bg=16.96%)No matches to TargetScan

AGATGTGTCCAT 4800  
 ATATGTC

C

CATATTTACATTTT  
Depth:3 (DOG)  
Ei-value:0.000, Pi-value:0.000  
Er-value:0.000, Pr-value:0.000  
eCLIP MATCHES▶lin28b (bg=16.96%)▶ybx3 (bg=22.82%)MATCHES To TargetScan▶ miR-411-3p:AUGUAAC


atattt

atattt  
Depth:4 (COW)  
Ei-value:1.000, Pi-value:0.080  
Er-value:0.000, Pr-value:0.010  
eCLIP MATCHES▶lin28b (bg=16.96%)No matches to TargetScan


ACATTTT

CATATTTACATTTT  
Depth:3 (DOG)  
Ei-value:0.000, Pi-value:0.000  
Er-value:0.000, Pr-value:0.000  
eCLIP MATCHES▶lin28b (bg=16.96%)▶ybx3 (bg=22.82%)MATCHES To TargetScan▶ miR-411-3p:AUGUAAC

GATAGCCATTGATGTATGCA

tctctt

tctctt  
Depth:3 (DOG)  
Ei-value:0.080, Pi-value:0.040  
Er-value:0.000, Pr-value:0.000  
eCLIP MATCHES▶tia1 (bg=16.04%)▶ybx3 (bg=22.82%)No matches to TargetScan

G

GCTGTACTATA

GCTGTACTATA  
Depth:4 (COW)  
Ei-value:0.000, Pi-value:0.000  
Er-value:0.000, Pr-value:0.000  
eCLIP MATCHES▶tia1 (bg=16.04%)▶ybx3 (bg=22.82%)No matches to TargetScan

AGAACACATTA

ATTCAATGGAAA

ATTCAATGGAAA  
Depth:3 (DOG)  
Ei-value:0.000, Pi-value:0.000  
Er-value:0.000, Pr-value:0.000  
eCLIP MATCHES▶HNRNPL (bg=3.4%)No matches to TargetScan

TACA

CT

CTTTGCTAAT  
Depth:3 (DOG)  
Ei-value:0.000, Pi-value:0.000  
Er-value:0.000, Pr-value:0.000  
eCLIP MATCHES▶HNRNPL (bg=3.4%)No matches to TargetScan


ttgctaa

ttgctaa  
Depth:4 (COW)  
Ei-value:0.960, Pi-value:0.000  
Er-value:0.000, Pr-value:0.000  
eCLIP MATCHES▶HNRNPL (bg=3.4%)No matches to TargetScan


T

CTTTGCTAAT  
Depth:3 (DOG)  
Ei-value:0.000, Pi-value:0.000  
Er-value:0.000, Pr-value:0.000  
eCLIP MATCHES▶HNRNPL (bg=3.4%)No matches to TargetScan

ATTTTAATGG

TATAGAT

TATAGAT  
Depth:3 (DOG)  
Ei-value:0.000, Pi-value:0.010  
Er-value:0.000, Pr-value:0.000  
eCLIP MATCHES▶dgcr8 (bg=19.31%)▶HNRNPL (bg=3.4%)▶igf2bp1 (bg=11.67%)No matches to TargetScan

CTGCTA

A

ATGAATTCT  
Depth:3 (DOG)  
Ei-value:0.000, Pi-value:0.000  
Er-value:0.000, Pr-value:0.000  
eCLIP MATCHES▶dgcr8 (bg=19.31%)▶HNRNPL (bg=3.4%)▶igf2bp1 (bg=11.67%)No matches to TargetScan

 4920  


TGAATTCT

ATGAATTCT  
Depth:3 (DOG)  
Ei-value:0.000, Pi-value:0.000  
Er-value:0.000, Pr-value:0.000  
eCLIP MATCHES▶dgcr8 (bg=19.31%)▶HNRNPL (bg=3.4%)▶igf2bp1 (bg=11.67%)No matches to TargetScan

CTTAAAAACATACTGTAT

tctgtt

tctgtt  
Depth:4 (COW)  
Ei-value:1.000, Pi-value:0.000  
Er-value:0.000, Pr-value:0.000  
eCLIP MATCHES▶dgcr8 (bg=19.31%)▶HNRNPL (bg=3.4%)▶igf2bp1 (bg=11.67%)No matches to TargetScan

GCTGTGTGTTTCATTTTA

aattga

aattga  
Depth:4 (COW)  
Ei-value:1.000, Pi-value:0.010  
Er-value:0.000, Pr-value:0.010  
eCLIP MATCHES▶SF3B1 (bg=6.74%)No matches to TargetScan

G

CATTAAG

CATTAAG  
Depth:5 (SHEEP)  
Ei-value:0.000, Pi-value:0.010  
Er-value:0.000, Pr-value:0.000  
eCLIP MATCHES▶SF3B1 (bg=6.74%)No matches to TargetScan


GGAATG

CATTAAGGGAATG  
Depth:4 (COW)  
Ei-value:0.000, Pi-value:0.000  
Er-value:0.000, Pr-value:0.000  
eCLIP MATCHES▶SF3B1 (bg=6.74%)MATCHES To TargetScan▶ miR-204-5p/211-5p:UCCCUUU


CAG

CATTAAGGGAATGCAG  
Depth:3 (DOG)  
Ei-value:0.000, Pi-value:0.000  
Er-value:0.000, Pr-value:0.000  
eCLIP MATCHES▶SF3B1 (bg=6.74%)MATCHES To TargetScan▶ miR-204-5p/211-5p:UCCCUUU▶ miR-33-5p:UGCAUUG

CATTTAAATCAG

AACTCTG

AACTCTG  
Depth:3 (DOG)  
Ei-value:0.000, Pi-value:0.010  
Er-value:0.000, Pr-value:0.000  
No matches to eCLIP DataNo matches to TargetScan

CCAATGCT

tttatc

tttatc  
Depth:3 (DOG)  
Ei-value:0.080, Pi-value:0.050  
Er-value:0.000, Pr-value:0.000  
eCLIP MATCHES▶EIF3H (bg=7.83%)▶HNRNPC (bg=1.49%)No matches to TargetScan

TAGAGGCG

TGTTGC

TGTTGC  
Depth:6 (PIG)  
Ei-value:0.000, Pi-value:0.010  
Er-value:0.000, Pr-value:0.000  
eCLIP MATCHES▶EIF3H (bg=7.83%)▶HNRNPC (bg=1.49%)No matches to TargetScan

 5040  


TGTTGC  
Depth:6 (PIG)  
Ei-value:0.000, Pi-value:0.010  
Er-value:0.000, Pr-value:0.000  
eCLIP MATCHES▶EIF3H (bg=7.83%)▶HNRNPC (bg=1.49%)No matches to TargetScan


C

TGTTGCCATTTTTGTC  
Depth:5 (SHEEP)  
Ei-value:0.000, Pi-value:0.000  
Er-value:0.000, Pr-value:0.000  
eCLIP MATCHES▶EIF3H (bg=7.83%)▶HNRNPC (bg=1.49%)▶tia1 (bg=16.04%)MATCHES To TargetScan▶ miR-183-5p.1:AUGGCAC


ATTTTTGT

ATTTTTGT  
Depth:9 (MOUSE)  
Ei-value:0.000, Pi-value:0.000  
Er-value:0.000, Pr-value:0.000  
eCLIP MATCHES▶EIF3H (bg=7.83%)▶HNRNPC (bg=1.49%)No matches to TargetScan


C

ATTTTTGTC  
Depth:7 (ARMADILLO)  
Ei-value:0.000, Pi-value:0.000  
Er-value:0.000, Pr-value:0.000  
eCLIP MATCHES▶EIF3H (bg=7.83%)▶HNRNPC (bg=1.49%)▶tia1 (bg=16.04%)No matches to TargetScan

TTATATGAAATTTCTGTCCCA

AGA

AGAAAGGCAGGATTACAT  
Depth:3 (DOG)  
Ei-value:0.000, Pi-value:0.000  
Er-value:0.000, Pr-value:0.000  
eCLIP MATCHES▶HNRNPC (bg=1.49%)▶PABPC4 (bg=2.72%)▶tia1 (bg=16.04%)MATCHES To TargetScan▶ miR-411-3p:AUGUAAC


AAGGCA

AAGGCA  
Depth:6 (PIG)  
Ei-value:0.000, Pi-value:0.010  
Er-value:0.000, Pr-value:0.000  
eCLIP MATCHES▶HNRNPC (bg=1.49%)▶tia1 (bg=16.04%)No matches to TargetScan


G

AGAAAGGCAGGATTACAT  
Depth:3 (DOG)  
Ei-value:0.000, Pi-value:0.000  
Er-value:0.000, Pr-value:0.000  
eCLIP MATCHES▶HNRNPC (bg=1.49%)▶PABPC4 (bg=2.72%)▶tia1 (bg=16.04%)MATCHES To TargetScan▶ miR-411-3p:AUGUAAC


GATTACAT

GATTACAT  
Depth:6 (PIG)  
Ei-value:0.000, Pi-value:0.000  
Er-value:0.000, Pr-value:0.000  
eCLIP MATCHES▶HNRNPC (bg=1.49%)▶tia1 (bg=16.04%)MATCHES To TargetScan▶ miR-411-3p:AUGUAAC

C

TTTTTTTTTTTTTTT

TTTTTTTTTTTTTTT  
Depth:3 (DOG)  
Ei-value:0.000, Pi-value:0.000  
Er-value:0.000, Pr-value:0.000  
eCLIP MATCHES▶APOBEC3C (bg=3.95%)▶ddx55 (bg=12.35%)▶ddx6 (bg=23.92%)▶EIF3H (bg=7.83%)▶fubp3 (bg=23.31%)▶HNRNPC (bg=1.49%)▶tia1 (bg=16.04%)▶tial1 (bg=14.09%)▶ZC3H11A (bg=6.25%)No matches to TargetScan

AGCAGTTT

GAGTTG

GAGTTG  
Depth:6 (PIG)  
Ei-value:0.000, Pi-value:0.020  
Er-value:0.000, Pr-value:0.000  
eCLIP MATCHES▶APOBEC3C (bg=3.95%)▶ddx55 (bg=12.35%)▶ddx6 (bg=23.92%)▶EIF3H (bg=7.83%)▶fam120a (bg=18.43%)▶fubp3 (bg=23.31%)▶FUS (bg=2.59%)▶tia1 (bg=16.04%)▶tial1 (bg=14.09%)▶ZC3H11A (bg=6.25%)No matches to TargetScan

GTGTA

GTG

GTGTATTCTTGGTTATCA  
Depth:3 (DOG)  
Ei-value:0.000, Pi-value:0.000  
Er-value:0.000, Pr-value:0.000  
eCLIP MATCHES▶APOBEC3C (bg=3.95%)▶ddx55 (bg=12.35%)▶ddx6 (bg=23.92%)▶EIF3H (bg=7.83%)▶fam120a (bg=18.43%)▶fubp3 (bg=23.31%)▶FUS (bg=2.59%)▶tia1 (bg=16.04%)▶tial1 (bg=14.09%)▶ZC3H11A (bg=6.25%)No matches to TargetScan


TA

TATTCTTGGT  
Depth:7 (ARMADILLO)  
Ei-value:0.000, Pi-value:0.000  
Er-value:0.000, Pr-value:0.000  
eCLIP MATCHES▶APOBEC3C (bg=3.95%)▶ddx55 (bg=12.35%)▶ddx6 (bg=23.92%)▶EIF3H (bg=7.83%)▶fam120a (bg=18.43%)▶fubp3 (bg=23.31%)▶FUS (bg=2.59%)▶tia1 (bg=16.04%)▶tial1 (bg=14.09%)▶ZC3H11A (bg=6.25%)No matches to TargetScan


TTCTTGGT

TTCTTGGT  
Depth:9 (MOUSE)  
Ei-value:0.000, Pi-value:0.000  
Er-value:0.000, Pr-value:0.000  
eCLIP MATCHES▶APOBEC3C (bg=3.95%)▶ddx55 (bg=12.35%)▶ddx6 (bg=23.92%)▶EIF3H (bg=7.83%)▶fam120a (bg=18.43%)▶fubp3 (bg=23.31%)▶FUS (bg=2.59%)▶tia1 (bg=16.04%)▶tial1 (bg=14.09%)▶ZC3H11A (bg=6.25%)No matches to TargetScan


TATCA

TATTCTTGGTTATCA  
Depth:6 (PIG)  
Ei-value:0.000, Pi-value:0.000  
Er-value:0.000, Pr-value:0.000  
eCLIP MATCHES▶APOBEC3C (bg=3.95%)▶ddx55 (bg=12.35%)▶ddx6 (bg=23.92%)▶EIF3H (bg=7.83%)▶fam120a (bg=18.43%)▶fubp3 (bg=23.31%)▶FUS (bg=2.59%)▶tia1 (bg=16.04%)▶tial1 (bg=14.09%)▶ZC3H11A (bg=6.25%)No matches to TargetScan

G

AATACTCATA

AATACTCATA  
Depth:6 (PIG)  
Ei-value:0.000, Pi-value:0.000  
Er-value:0.000, Pr-value:0.000  
eCLIP MATCHES▶APOBEC3C (bg=3.95%)▶ddx55 (bg=12.35%)▶ddx6 (bg=23.92%)▶EIF3H (bg=7.83%)▶fam120a (bg=18.43%)▶fubp3 (bg=23.31%)▶FUS (bg=2.59%)▶tia1 (bg=16.04%)▶tial1 (bg=14.09%)▶ZC3H11A (bg=6.25%)MATCHES To TargetScan▶ miR-496.1:GAGUAUU

TAGC

TTT

TTTGGG  
Depth:7 (ARMADILLO)  
Ei-value:0.000, Pi-value:0.010  
Er-value:0.000, Pr-value:0.000  
eCLIP MATCHES▶APOBEC3C (bg=3.95%)▶ddx55 (bg=12.35%)▶ddx6 (bg=23.92%)▶fam120a (bg=18.43%)▶fubp3 (bg=23.31%)▶tia1 (bg=16.04%)▶ZC3H11A (bg=6.25%)No matches to TargetScan

 5160  


GGG

TTTGGG  
Depth:7 (ARMADILLO)  
Ei-value:0.000, Pi-value:0.010  
Er-value:0.000, Pr-value:0.000  
eCLIP MATCHES▶APOBEC3C (bg=3.95%)▶ddx55 (bg=12.35%)▶ddx6 (bg=23.92%)▶fam120a (bg=18.43%)▶fubp3 (bg=23.31%)▶tia1 (bg=16.04%)▶ZC3H11A (bg=6.25%)No matches to TargetScan

ATTTTGAATTGG

TA

TAAATATTCATG  
Depth:7 (ARMADILLO)  
Ei-value:0.000, Pi-value:0.000  
Er-value:0.000, Pr-value:0.000  
No matches to eCLIP DataNo matches to TargetScan


AATATTCA

AATATTCA  
Depth:9 (MOUSE)  
Ei-value:0.000, Pi-value:0.000  
Er-value:0.000, Pr-value:0.000  
No matches to eCLIP DataNo matches to TargetScan


TG

AATATTCATG  
Depth:8 (GUINEAPIG)  
Ei-value:0.000, Pi-value:0.000  
Er-value:0.000, Pr-value:0.000  
No matches to eCLIP DataNo matches to TargetScan

ATGTGTGAAAAAT

CATGATACAT

CATGATACAT  
Depth:7 (ARMADILLO)  
Ei-value:0.000, Pi-value:0.000  
Er-value:0.000, Pr-value:0.000  
eCLIP MATCHES▶PABPC4 (bg=2.72%)No matches to TargetScan


A

CATGATACATA  
Depth:6 (PIG)  
Ei-value:0.000, Pi-value:0.000  
Er-value:0.000, Pr-value:0.000  
eCLIP MATCHES▶PABPC4 (bg=2.72%)No matches to TargetScan


CTGTACA

CTGTACA  
Depth:3 (DOG)  
Ei-value:0.000, Pi-value:0.000  
Er-value:0.000, Pr-value:0.000  
eCLIP MATCHES▶PABPC4 (bg=2.72%)MATCHES To TargetScan▶ miR-493-5p:UGUACAU

GTCTCAGTCCCA

taaaat

taaaat  
Depth:3 (DOG)  
Ei-value:0.080, Pi-value:0.060  
Er-value:0.000, Pr-value:0.000  
eCLIP MATCHES▶KHSRP (bg=8.1%)▶PABPC4 (bg=2.72%)No matches to TargetScan

TGGATGTTGTGCC

TACACA

TACACA  
Depth:8 (GUINEAPIG)  
Ei-value:0.000, Pi-value:0.000  
Er-value:0.000, Pr-value:0.000  
eCLIP MATCHES▶KHSRP (bg=8.1%)No matches to TargetScan


CA

TACACACA  
Depth:3 (DOG)  
Ei-value:0.000, Pi-value:0.000  
Er-value:0.000, Pr-value:0.000  
eCLIP MATCHES▶KHSRP (bg=8.1%)No matches to TargetScan

GGAT

CTAGAA

CTAGAA  
Depth:6 (PIG)  
Ei-value:0.000, Pi-value:0.000  
Er-value:0.000, Pr-value:0.000  
eCLIP MATCHES▶KHSRP (bg=8.1%)▶TARDBP (bg=1.75%)No matches to TargetScan

GAATAT

GTCAAAC

GTCAAACT  
Depth:3 (DOG)  
Ei-value:0.000, Pi-value:0.000  
Er-value:0.000, Pr-value:0.000  
eCLIP MATCHES▶KHSRP (bg=8.1%)▶NOLC1 (bg=6.58%)▶TARDBP (bg=1.75%)No matches to TargetScan

 5280  


T

GTCAAACT  
Depth:3 (DOG)  
Ei-value:0.000, Pi-value:0.000  
Er-value:0.000, Pr-value:0.000  
eCLIP MATCHES▶KHSRP (bg=8.1%)▶NOLC1 (bg=6.58%)▶TARDBP (bg=1.75%)No matches to TargetScan

AT

AAA

AAACTGCTTG  
Depth:7 (ARMADILLO)  
Ei-value:0.000, Pi-value:0.000  
Er-value:0.000, Pr-value:0.000  
eCLIP MATCHES▶fubp3 (bg=23.31%)▶KHSRP (bg=8.1%)▶NOLC1 (bg=6.58%)▶SUB1 (bg=9.24%)▶TARDBP (bg=1.75%)No matches to TargetScan


CTGCTT

CTGCTT  
Depth:9 (MOUSE)  
Ei-value:0.000, Pi-value:0.000  
Er-value:0.000, Pr-value:0.000  
eCLIP MATCHES▶fubp3 (bg=23.31%)▶KHSRP (bg=8.1%)▶NOLC1 (bg=6.58%)▶TARDBP (bg=1.75%)No matches to TargetScan


G

AAACTGCTTG  
Depth:7 (ARMADILLO)  
Ei-value:0.000, Pi-value:0.000  
Er-value:0.000, Pr-value:0.000  
eCLIP MATCHES▶fubp3 (bg=23.31%)▶KHSRP (bg=8.1%)▶NOLC1 (bg=6.58%)▶SUB1 (bg=9.24%)▶TARDBP (bg=1.75%)No matches to TargetScan


TGATT

AAACTGCTTGTGATT  
Depth:5 (SHEEP)  
Ei-value:0.000, Pi-value:0.000  
Er-value:0.000, Pr-value:0.000  
eCLIP MATCHES▶fubp3 (bg=23.31%)▶KHSRP (bg=8.1%)▶NOLC1 (bg=6.58%)▶SUB1 (bg=9.24%)▶TARDBP (bg=1.75%)▶ZC3H11A (bg=6.25%)No matches to TargetScan

GTGA

A

ATGACTT  
Depth:8 (GUINEAPIG)  
Ei-value:0.000, Pi-value:0.000  
Er-value:0.000, Pr-value:0.000  
eCLIP MATCHES▶fubp3 (bg=23.31%)▶KHSRP (bg=8.1%)▶NOLC1 (bg=6.58%)▶SUB1 (bg=9.24%)▶TARDBP (bg=1.75%)▶ZC3H11A (bg=6.25%)MATCHES To TargetScan▶ miR-224-5p:AAGUCAC


TGACTT

TGACTT  
Depth:9 (MOUSE)  
Ei-value:0.000, Pi-value:0.000  
Er-value:0.000, Pr-value:0.000  
eCLIP MATCHES▶fubp3 (bg=23.31%)▶KHSRP (bg=8.1%)▶NOLC1 (bg=6.58%)▶SUB1 (bg=9.24%)▶TARDBP (bg=1.75%)▶ZC3H11A (bg=6.25%)MATCHES To TargetScan▶ miR-224-5p:AAGUCAC


TGTTC

ATGACTTTGTTCTTTGCTT  
Depth:3 (DOG)  
Ei-value:0.000, Pi-value:0.000  
Er-value:0.000, Pr-value:0.000  
eCLIP MATCHES▶ddx55 (bg=12.35%)▶ddx6 (bg=23.92%)▶fubp3 (bg=23.31%)▶KHSRP (bg=8.1%)▶NOLC1 (bg=6.58%)▶SUB1 (bg=9.24%)▶TARDBP (bg=1.75%)▶tia1 (bg=16.04%)▶tial1 (bg=14.09%)▶ZC3H11A (bg=6.25%)MATCHES To TargetScan▶ miR-495-3p:AACAAAC▶ miR-224-5p:AAGUCAC▶ miR-186-5p:AAAGAAU


T

TTTGCTT  
Depth:8 (GUINEAPIG)  
Ei-value:0.000, Pi-value:0.000  
Er-value:0.000, Pr-value:0.000  
eCLIP MATCHES▶ddx55 (bg=12.35%)▶ddx6 (bg=23.92%)▶fubp3 (bg=23.31%)▶KHSRP (bg=8.1%)▶NOLC1 (bg=6.58%)▶SUB1 (bg=9.24%)▶TARDBP (bg=1.75%)▶tia1 (bg=16.04%)▶tial1 (bg=14.09%)▶ZC3H11A (bg=6.25%)No matches to TargetScan


TTGCTT

TTGCTT  
Depth:9 (MOUSE)  
Ei-value:0.000, Pi-value:0.000  
Er-value:0.000, Pr-value:0.010  
eCLIP MATCHES▶ddx55 (bg=12.35%)▶ddx6 (bg=23.92%)▶fubp3 (bg=23.31%)▶KHSRP (bg=8.1%)▶NOLC1 (bg=6.58%)▶SUB1 (bg=9.24%)▶TARDBP (bg=1.75%)▶tia1 (bg=16.04%)▶tial1 (bg=14.09%)▶ZC3H11A (bg=6.25%)No matches to TargetScan


GTGTTTTTCA

GTGTTTTTCA  
Depth:3 (DOG)  
Ei-value:0.000, Pi-value:0.000  
Er-value:0.000, Pr-value:0.000  
eCLIP MATCHES▶ddx55 (bg=12.35%)▶ddx6 (bg=23.92%)▶fubp3 (bg=23.31%)▶igf2bp1 (bg=11.67%)▶KHSRP (bg=8.1%)▶NOLC1 (bg=6.58%)▶RPS3 (bg=4.15%)▶SUB1 (bg=9.24%)▶TARDBP (bg=1.75%)▶tia1 (bg=16.04%)▶tial1 (bg=14.09%)▶ZC3H11A (bg=6.25%)No matches to TargetScan

A

TTTCCT

TTTCCT  
Depth:6 (PIG)  
Ei-value:0.000, Pi-value:0.000  
Er-value:0.000, Pr-value:0.000  
eCLIP MATCHES▶ddx55 (bg=12.35%)▶ddx6 (bg=23.92%)▶fubp3 (bg=23.31%)▶igf2bp1 (bg=11.67%)▶KHSRP (bg=8.1%)▶NOLC1 (bg=6.58%)▶RPS3 (bg=4.15%)▶SUB1 (bg=9.24%)▶TARDBP (bg=1.75%)▶tia1 (bg=16.04%)▶tial1 (bg=14.09%)▶ZC3H11A (bg=6.25%)No matches to TargetScan

ATA

atgcacat

atgcacat  
Depth:4 (COW)  
Ei-value:0.670, Pi-value:0.000  
Er-value:0.000, Pr-value:0.000  
eCLIP MATCHES▶ddx6 (bg=23.92%)▶fubp3 (bg=23.31%)▶igf2bp1 (bg=11.67%)▶KHSRP (bg=8.1%)▶RPS3 (bg=4.15%)▶SUB1 (bg=9.24%)▶TARDBP (bg=1.75%)▶tia1 (bg=16.04%)▶tial1 (bg=14.09%)▶ZC3H11A (bg=6.25%)MATCHES To TargetScan▶ miR-455-5p:AUGUGCC


A

ATGCACATA  
Depth:3 (DOG)  
Ei-value:0.000, Pi-value:0.000  
Er-value:0.000, Pr-value:0.000  
eCLIP MATCHES▶ddx6 (bg=23.92%)▶fubp3 (bg=23.31%)▶igf2bp1 (bg=11.67%)▶KHSRP (bg=8.1%)▶RPS3 (bg=4.15%)▶SUB1 (bg=9.24%)▶TARDBP (bg=1.75%)▶tia1 (bg=16.04%)▶tial1 (bg=14.09%)▶ZC3H11A (bg=6.25%)MATCHES To TargetScan▶ miR-455-5p:AUGUGCC

C

TAACTTTTAA

TAACTTTTAA  
Depth:6 (PIG)  
Ei-value:0.000, Pi-value:0.000  
Er-value:0.000, Pr-value:0.000  
eCLIP MATCHES▶fubp3 (bg=23.31%)▶igf2bp1 (bg=11.67%)▶RPS3 (bg=4.15%)▶SUB1 (bg=9.24%)▶TARDBP (bg=1.75%)▶tia1 (bg=16.04%)▶tial1 (bg=14.09%)▶ZC3H11A (bg=6.25%)No matches to TargetScan


A

TAACTTTTAAAAAATAAA  
Depth:3 (DOG)  
Ei-value:0.000, Pi-value:0.000  
Er-value:0.000, Pr-value:0.000  
eCLIP MATCHES▶fubp3 (bg=23.31%)▶igf2bp1 (bg=11.67%)▶RPS3 (bg=4.15%)▶SUB1 (bg=9.24%)▶TARDBP (bg=1.75%)▶tia1 (bg=16.04%)▶tial1 (bg=14.09%)▶ZC3H11A (bg=6.25%)No matches to TargetScan


AAATAAA

AAATAAA  
Depth:9 (MOUSE)  
Ei-value:0.000, Pi-value:0.000  
Er-value:0.000, Pr-value:0.000  
eCLIP MATCHES▶ZC3H11A (bg=6.25%)No matches to TargetScan

GGTTA

TT

TTTTAAAA  
Depth:5 (SHEEP)  
Ei-value:0.000, Pi-value:0.010  
Er-value:0.000, Pr-value:0.000  
No matches to eCLIP DataNo matches to TargetScan


TTAAAA

TTAAAA  
Depth:8 (GUINEAPIG)  
Ei-value:0.000, Pi-value:0.010  
Er-value:0.000, Pr-value:0.000  
No matches to eCLIP DataNo matches to TargetScan

GCCTGTATTAAGCCCTCG 5400  
 T

tgcttg

tgcttg  
Depth:3 (DOG)  
Ei-value:0.080, Pi-value:0.020  
Er-value:0.000, Pr-value:0.000  
No matches to eCLIP DataNo matches to TargetScan

TAGAATAGAGTTAGTATACGCTACAGAAGCACAGGT                                                                              5443
```

|  |  |  |  |  |  |  |  |
| --- | --- | --- | --- | --- | --- | --- | --- |
| | | | | | | | | | | | | | | | |
| 2 |  |  |  | 6 |  |  | 9 |
| Depth of motif conservation (number of species) | | | | | | | |

  
  

---

  

## >HUMAN TO COW (5443 bases)

```
 GGC

ACTTCCGG

ACTTCCGG  
Depth:8 (GUINEAPIG)  
Ei-value:0.000, Pi-value:0.000  
Er-value:0.000, Pr-value:0.000  
No matches to eCLIP DataNo matches to TargetScan

TACCG

CTCCTCTC

CTCCTCTC  
Depth:5 (SHEEP)  
Ei-value:0.000, Pi-value:0.000  
Er-value:0.000, Pr-value:0.000  
No matches to eCLIP DataNo matches to TargetScan

TT

GCCAGC

GCCAGC  
Depth:5 (SHEEP)  
Ei-value:0.000, Pi-value:0.000  
Er-value:0.000, Pr-value:0.000  
No matches to eCLIP DataNo matches to TargetScan

GC

AGAGAA

AGAGAA  
Depth:8 (GUINEAPIG)  
Ei-value:0.000, Pi-value:0.000  
Er-value:0.000, Pr-value:0.000  
No matches to eCLIP DataNo matches to TargetScan


C

AGAGAACTGCCAA  
Depth:7 (ARMADILLO)  
Ei-value:0.000, Pi-value:0.000  
Er-value:0.000, Pr-value:0.000  
No matches to eCLIP DataMATCHES To TargetScan▶ miR-34-5p/449-5p:GGCAGUG▶ miR-182-5p:UUGGCAA▶ miR-96-5p/1271-5p:UUGGCAC


TGCCAA

TGCCAA  
Depth:9 (MOUSE)  
Ei-value:0.000, Pi-value:0.000  
Er-value:0.000, Pr-value:0.000  
No matches to eCLIP DataMATCHES To TargetScan▶ miR-182-5p:UUGGCAA▶ miR-96-5p/1271-5p:UUGGCAC


G

AGAGAACTGCCAAGTCAGTTCCGG  
Depth:4 (COW)  
Ei-value:0.000, Pi-value:0.000  
Er-value:0.000, Pr-value:0.000  
eCLIP MATCHES▶EIF3G (bg=2.39%)MATCHES To TargetScan▶ miR-34-5p/449-5p:GGCAGUG▶ miR-182-5p:UUGGCAA▶ miR-96-5p/1271-5p:UUGGCAC


TCAGTTCCGG

TCAGTTCCGG  
Depth:9 (MOUSE)  
Ei-value:0.000, Pi-value:0.000  
Er-value:0.000, Pr-value:0.000  
eCLIP MATCHES▶EIF3G (bg=2.39%)No matches to TargetScan

TCCGGCAGAGATCGCGGAGAGACGCAGAACGCAGCCCGCTCCTCCA

GGGCCCTCCA

GGGCCCTCCAGGCCCTCCGGCC  
Depth:5 (SHEEP)  
Ei-value:0.000, Pi-value:0.000  
Er-value:0.000, Pr-value:0.000  
eCLIP MATCHES▶ddx3x (bg=13.89%)▶ddx6 (bg=23.92%)▶drosha (bg=11.37%)▶EIF3G (bg=2.39%)▶EIF3H (bg=7.83%)▶FMR1 (bg=6.3%)▶METAP2 (bg=6.14%)▶PUS1 (bg=2.87%)▶RPS3 (bg=4.15%)▶SDAD1 (bg=5.99%)▶SERBP1 (bg=0.81%)▶SF3B1 (bg=6.74%)▶WRN (bg=3.31%)▶ybx3 (bg=22.82%)MATCHES To TargetScan▶ miR-296-5p:GGGCCCC


G

GGCCCTCC  
Depth:8 (GUINEAPIG)  
Ei-value:0.000, Pi-value:0.000  
Er-value:0.000, Pr-value:0.000  
eCLIP MATCHES▶ddx3x (bg=13.89%)▶ddx6 (bg=23.92%)▶drosha (bg=11.37%)▶EIF3G (bg=2.39%)▶EIF3H (bg=7.83%)▶FMR1 (bg=6.3%)▶METAP2 (bg=6.14%)▶PUS1 (bg=2.87%)▶RPS3 (bg=4.15%)▶SDAD1 (bg=5.99%)▶SERBP1 (bg=0.81%)▶SF3B1 (bg=6.74%)▶WRN (bg=3.31%)▶ybx3 (bg=22.82%)No matches to TargetScan


GCCCT

GCCCTC  
Depth:9 (MOUSE)  
Ei-value:0.000, Pi-value:0.000  
Er-value:0.000, Pr-value:0.000  
eCLIP MATCHES▶ddx3x (bg=13.89%)▶ddx6 (bg=23.92%)▶drosha (bg=11.37%)▶EIF3G (bg=2.39%)▶EIF3H (bg=7.83%)▶FMR1 (bg=6.3%)▶METAP2 (bg=6.14%)▶PUS1 (bg=2.87%)▶RPS3 (bg=4.15%)▶SDAD1 (bg=5.99%)▶SERBP1 (bg=0.81%)▶SF3B1 (bg=6.74%)▶WRN (bg=3.31%)▶ybx3 (bg=22.82%)No matches to TargetScan

 120  


C

GCCCTC  
Depth:9 (MOUSE)  
Ei-value:0.000, Pi-value:0.000  
Er-value:0.000, Pr-value:0.000  
eCLIP MATCHES▶ddx3x (bg=13.89%)▶ddx6 (bg=23.92%)▶drosha (bg=11.37%)▶EIF3G (bg=2.39%)▶EIF3H (bg=7.83%)▶FMR1 (bg=6.3%)▶METAP2 (bg=6.14%)▶PUS1 (bg=2.87%)▶RPS3 (bg=4.15%)▶SDAD1 (bg=5.99%)▶SERBP1 (bg=0.81%)▶SF3B1 (bg=6.74%)▶WRN (bg=3.31%)▶ybx3 (bg=22.82%)No matches to TargetScan


C

GGCCCTCC  
Depth:8 (GUINEAPIG)  
Ei-value:0.000, Pi-value:0.000  
Er-value:0.000, Pr-value:0.000  
eCLIP MATCHES▶ddx3x (bg=13.89%)▶ddx6 (bg=23.92%)▶drosha (bg=11.37%)▶EIF3G (bg=2.39%)▶EIF3H (bg=7.83%)▶FMR1 (bg=6.3%)▶METAP2 (bg=6.14%)▶PUS1 (bg=2.87%)▶RPS3 (bg=4.15%)▶SDAD1 (bg=5.99%)▶SERBP1 (bg=0.81%)▶SF3B1 (bg=6.74%)▶WRN (bg=3.31%)▶ybx3 (bg=22.82%)No matches to TargetScan


GG

GGCCCTCCGG  
Depth:7 (ARMADILLO)  
Ei-value:0.000, Pi-value:0.000  
Er-value:0.000, Pr-value:0.000  
eCLIP MATCHES▶ddx3x (bg=13.89%)▶ddx6 (bg=23.92%)▶drosha (bg=11.37%)▶EIF3G (bg=2.39%)▶EIF3H (bg=7.83%)▶FMR1 (bg=6.3%)▶METAP2 (bg=6.14%)▶PUS1 (bg=2.87%)▶RPS3 (bg=4.15%)▶SDAD1 (bg=5.99%)▶SERBP1 (bg=0.81%)▶SF3B1 (bg=6.74%)▶WRN (bg=3.31%)▶ybx3 (bg=22.82%)No matches to TargetScan


CC

GGGCCCTCCAGGCCCTCCGGCC  
Depth:5 (SHEEP)  
Ei-value:0.000, Pi-value:0.000  
Er-value:0.000, Pr-value:0.000  
eCLIP MATCHES▶ddx3x (bg=13.89%)▶ddx6 (bg=23.92%)▶drosha (bg=11.37%)▶EIF3G (bg=2.39%)▶EIF3H (bg=7.83%)▶FMR1 (bg=6.3%)▶METAP2 (bg=6.14%)▶PUS1 (bg=2.87%)▶RPS3 (bg=4.15%)▶SDAD1 (bg=5.99%)▶SERBP1 (bg=0.81%)▶SF3B1 (bg=6.74%)▶WRN (bg=3.31%)▶ybx3 (bg=22.82%)MATCHES To TargetScan▶ miR-296-5p:GGGCCCC

CCGGGCCGGCG

GGTGAACT

GGTGAACTGGGGGGCCC  
Depth:6 (PIG)  
Ei-value:0.000, Pi-value:0.000  
Er-value:0.000, Pr-value:0.000  
eCLIP MATCHES▶DDX24 (bg=2.31%)▶ddx3x (bg=13.89%)▶DHX30 (bg=5.05%)▶drosha (bg=11.37%)▶EIF3G (bg=2.39%)▶EIF3H (bg=7.83%)▶fam120a (bg=18.43%)▶FMR1 (bg=6.3%)▶GEMIN5 (bg=2.46%)▶METAP2 (bg=6.14%)▶NCBP2 (bg=2.92%)▶PHF6 (bg=1.71%)▶PUS1 (bg=2.87%)▶RPS3 (bg=4.15%)▶SDAD1 (bg=5.99%)▶SF3B1 (bg=6.74%)▶SUB1 (bg=9.24%)▶WRN (bg=3.31%)MATCHES To TargetScan▶ miR-296-5p:GGGCCCC


GGG

GGGGGGCCC  
Depth:7 (ARMADILLO)  
Ei-value:0.000, Pi-value:0.000  
Er-value:0.000, Pr-value:0.000  
eCLIP MATCHES▶DDX24 (bg=2.31%)▶ddx3x (bg=13.89%)▶DHX30 (bg=5.05%)▶drosha (bg=11.37%)▶EIF3G (bg=2.39%)▶EIF3H (bg=7.83%)▶fam120a (bg=18.43%)▶GEMIN5 (bg=2.46%)▶METAP2 (bg=6.14%)▶NCBP2 (bg=2.92%)▶PHF6 (bg=1.71%)▶RPS3 (bg=4.15%)▶SDAD1 (bg=5.99%)▶SUB1 (bg=9.24%)▶WRN (bg=3.31%)MATCHES To TargetScan▶ miR-296-5p:GGGCCCC


GGGCCC

GGGCCC  
Depth:8 (GUINEAPIG)  
Ei-value:0.000, Pi-value:0.000  
Er-value:0.000, Pr-value:0.000  
eCLIP MATCHES▶DDX24 (bg=2.31%)▶ddx3x (bg=13.89%)▶DHX30 (bg=5.05%)▶drosha (bg=11.37%)▶EIF3G (bg=2.39%)▶EIF3H (bg=7.83%)▶fam120a (bg=18.43%)▶GEMIN5 (bg=2.46%)▶METAP2 (bg=6.14%)▶NCBP2 (bg=2.92%)▶PHF6 (bg=1.71%)▶RPS3 (bg=4.15%)▶SDAD1 (bg=5.99%)▶SUB1 (bg=9.24%)▶WRN (bg=3.31%)MATCHES To TargetScan▶ miR-296-5p:GGGCCCC

CGGGACAGGCCGAGCCCTCTGCCC

tgcaga

tgcaga  
Depth:4 (COW)  
Ei-value:1.000, Pi-value:0.010  
Er-value:0.000, Pr-value:0.000  
eCLIP MATCHES▶AKAP1 (bg=3.4%)▶BCCIP (bg=4.61%)▶DDX24 (bg=2.31%)▶ddx3x (bg=13.89%)▶ddx6 (bg=23.92%)▶DHX30 (bg=5.05%)▶drosha (bg=11.37%)▶EIF3G (bg=2.39%)▶EIF3H (bg=7.83%)▶fam120a (bg=18.43%)▶FMR1 (bg=6.3%)▶FXR2 (bg=7.51%)▶METAP2 (bg=6.14%)▶NCBP2 (bg=2.92%)▶NIPBL (bg=2.83%)▶PCBP1 (bg=2.3%)▶PHF6 (bg=1.71%)▶PTBP1 (bg=1.43%)▶rbm15 (bg=11.81%)▶RPS3 (bg=4.15%)▶SDAD1 (bg=5.99%)▶SUB1 (bg=9.24%)▶TBRG4 (bg=7.0%)▶TROVE2 (bg=3.49%)▶UTP3 (bg=2.81%)▶ybx3 (bg=22.82%)No matches to TargetScan

TAACGGAGGCCTCTGCTGTGGCTGCCCACTGGCTGTGCCCGCCCACTGGCTGTGCC 240  
 CAGACCTT

gaagcc

gaagcc  
Depth:4 (COW)  
Ei-value:1.000, Pi-value:0.010  
Er-value:0.000, Pr-value:0.000  
eCLIP MATCHES▶ddx3x (bg=13.89%)▶dgcr8 (bg=19.31%)▶drosha (bg=11.37%)▶FMR1 (bg=6.3%)▶FXR2 (bg=7.51%)▶NIP7 (bg=2.54%)▶PTBP1 (bg=1.43%)▶rbm15 (bg=11.81%)▶SBDS (bg=1.91%)▶SDAD1 (bg=5.99%)▶ybx3 (bg=22.82%)No matches to TargetScan

GCAGCGAACCTCTCTTTCCCACCCCACCTCGGTGACTA

ATGGCGGC

ATGGCGGC  
Depth:9 (MOUSE)  
Ei-value:0.000, Pi-value:0.000  
Er-value:0.000, Pr-value:0.000  
eCLIP MATCHES▶BCCIP (bg=4.61%)▶ddx3x (bg=13.89%)▶ddx6 (bg=23.92%)▶dgcr8 (bg=19.31%)▶DHX30 (bg=5.05%)▶drosha (bg=11.37%)▶EIF3H (bg=7.83%)▶FMR1 (bg=6.3%)▶FTO (bg=7.53%)▶fubp3 (bg=23.31%)▶FXR2 (bg=7.51%)▶GEMIN5 (bg=2.46%)▶IGF2BP3 (bg=4.26%)▶METAP2 (bg=6.14%)▶NIP7 (bg=2.54%)▶PCBP1 (bg=2.3%)▶pum1 (bg=29.85%)▶pum2 (bg=21.55%)▶rbm15 (bg=11.81%)▶SDAD1 (bg=5.99%)▶WRN (bg=3.31%)No matches to TargetScan

CGTGGCGTCTCCCAGCCCGGACCCCGCCGGCACCCGGGTCTCCCGACCCAAGCCTCGACG 360  
 AAACCCCCGCAGAGCCGCCGGGACGCAGCGCCTTTGGGCGGCGCTGGGCGTGGTGGGCCGGGAAGTATGGCGGCAGCTCGAACGCCGCGCGGCGGAGGCCATTAAGGCGTGGACGGCCCG 480  
 GGAAGGCGGCCTAGGGACGCAAGCAGGCTCGGCCGCCTCTTTAGGCCACGGAGCCGCGCAGATCCGGTT

cccggg

cccggg  
Depth:4 (COW)  
Ei-value:1.000, Pi-value:0.010  
Er-value:0.000, Pr-value:0.000  
eCLIP MATCHES▶ybx3 (bg=22.82%)No matches to TargetScan

TGACCACTCTGTCGCCATTGGGCGAGACCTACCTAGTCCTGACGA 600  
 CAACGGACAAAGGCCTTAAGGGGCCTGGA

aggtgagcg

aggtgagcg  
Depth:4 (COW)  
Ei-value:0.150, Pi-value:0.000  
Er-value:0.000, Pr-value:0.000  
eCLIP MATCHES▶BUD13 (bg=0.7%)▶ddx3x (bg=13.89%)▶DDX52 (bg=1.27%)▶dgcr8 (bg=19.31%)▶FTO (bg=7.53%)▶lin28b (bg=16.96%)▶rbm15 (bg=11.81%)▶SRSF7 (bg=1.29%)▶ybx3 (bg=22.82%)No matches to TargetScan

AAGTCCCGAACGA

CGA

CGACGGGTGGAAC  
Depth:4 (COW)  
Ei-value:0.000, Pi-value:0.000  
Er-value:0.000, Pr-value:0.000  
eCLIP MATCHES▶FTO (bg=7.53%)▶rbm15 (bg=11.81%)▶SRSF7 (bg=1.29%)▶ybx3 (bg=22.82%)MATCHES To TargetScan▶ miR-99-5p/100-5p:ACCCGUA


CGG

CGGGTGGAAC  
Depth:7 (ARMADILLO)  
Ei-value:0.000, Pi-value:0.000  
Er-value:0.000, Pr-value:0.000  
eCLIP MATCHES▶FTO (bg=7.53%)▶rbm15 (bg=11.81%)▶SRSF7 (bg=1.29%)▶ybx3 (bg=22.82%)No matches to TargetScan


GTGGAA

GTGGAA  
Depth:8 (GUINEAPIG)  
Ei-value:0.000, Pi-value:0.000  
Er-value:0.000, Pr-value:0.000  
eCLIP MATCHES▶FTO (bg=7.53%)▶rbm15 (bg=11.81%)▶SRSF7 (bg=1.29%)▶ybx3 (bg=22.82%)No matches to TargetScan


C

CGGGTGGAAC  
Depth:7 (ARMADILLO)  
Ei-value:0.000, Pi-value:0.000  
Er-value:0.000, Pr-value:0.000  
eCLIP MATCHES▶FTO (bg=7.53%)▶rbm15 (bg=11.81%)▶SRSF7 (bg=1.29%)▶ybx3 (bg=22.82%)No matches to TargetScan

GGTTAGCGGCCATCGGGCGGTTGGTCTTCA

TTCTAC

TTCTAC  
Depth:6 (PIG)  
Ei-value:0.000, Pi-value:0.000  
Er-value:0.000, Pr-value:0.000  
eCLIP MATCHES▶UTP18 (bg=0.53%)No matches to TargetScan

CAGACTTTGC

TGTCGGAAGA

TGTCGGAAGA  
Depth:5 (SHEEP)  
Ei-value:0.000, Pi-value:0.000  
Er-value:0.000, Pr-value:0.000  
No matches to eCLIP DataNo matches to TargetScan

 720  


TGTCGGAAGA  
Depth:5 (SHEEP)  
Ei-value:0.000, Pi-value:0.000  
Er-value:0.000, Pr-value:0.000  
No matches to eCLIP DataNo matches to TargetScan

GA

GAAATGG

GAAATGG  
Depth:6 (PIG)  
Ei-value:0.000, Pi-value:0.000  
Er-value:0.000, Pr-value:0.000  
eCLIP MATCHES▶ddx55 (bg=12.35%)No matches to TargetScan

TAGAATGACAGGCCACGTTTGGCCCGTTGGAAATGCCCACCACCCTC

T

TGGGAAGATTTA  
Depth:8 (GUINEAPIG)  
Ei-value:0.000, Pi-value:0.000  
Er-value:0.000, Pr-value:0.000  
No matches to eCLIP DataNo matches to TargetScan


GGGAAGA

GGGAAGA  
Depth:9 (MOUSE)  
Ei-value:0.000, Pi-value:0.000  
Er-value:0.000, Pr-value:0.000  
No matches to eCLIP DataNo matches to TargetScan


TTTA

TGGGAAGATTTA  
Depth:8 (GUINEAPIG)  
Ei-value:0.000, Pi-value:0.000  
Er-value:0.000, Pr-value:0.000  
No matches to eCLIP DataNo matches to TargetScan


CTGGCC

TGGGAAGATTTACTGGCC  
Depth:5 (SHEEP)  
Ei-value:0.000, Pi-value:0.000  
Er-value:0.000, Pr-value:0.000  
No matches to eCLIP DataMATCHES To TargetScan▶ miR-802:CAGUAAC

GTTTATGG

AAGG

AAGGCCTGTGTATATAATATGAAAAAGCTGCT  
Depth:8 (GUINEAPIG)  
Ei-value:0.000, Pi-value:0.000  
Er-value:0.000, Pr-value:0.000  
eCLIP MATCHES▶pum1 (bg=29.85%)MATCHES To TargetScan▶ miR-15-5p/16-5p/195-5p/424-5p/497-5p:AGCAGCA▶ miR-503-5p:AGCAGCG


CCTGTGTATATAATATGAAAAAGCTGCT

CCTGTGTATATAATATGAAAAAGCTGCT  
Depth:9 (MOUSE)  
Ei-value:0.000, Pi-value:0.000  
Er-value:0.000, Pr-value:0.000  
eCLIP MATCHES▶pum1 (bg=29.85%)MATCHES To TargetScan▶ miR-15-5p/16-5p/195-5p/424-5p/497-5p:AGCAGCA▶ miR-503-5p:AGCAGCG


C

AAGGCCTGTGTATATAATATGAAAAAGCTGCTCTCAACT  
Depth:6 (PIG)  
Ei-value:0.000, Pi-value:0.000  
Er-value:0.000, Pr-value:0.000  
eCLIP MATCHES▶pum1 (bg=29.85%)MATCHES To TargetScan▶ miR-15-5p/16-5p/195-5p/424-5p/497-5p:AGCAGCA▶ miR-503-5p:AGCAGCG


TCAAC

TCAACT  
Depth:7 (ARMADILLO)  
Ei-value:0.000, Pi-value:0.000  
Er-value:0.000, Pr-value:0.000  
eCLIP MATCHES▶pum1 (bg=29.85%)No matches to TargetScan

 840  


T

TCAACT  
Depth:7 (ARMADILLO)  
Ei-value:0.000, Pi-value:0.000  
Er-value:0.000, Pr-value:0.000  
eCLIP MATCHES▶pum1 (bg=29.85%)No matches to TargetScan

CCA

CCC

CCCCAACCTTT  
Depth:7 (ARMADILLO)  
Ei-value:0.000, Pi-value:0.000  
Er-value:0.000, Pr-value:0.000  
eCLIP MATCHES▶pum1 (bg=29.85%)No matches to TargetScan


CAACCTTT

CAACCTTT  
Depth:9 (MOUSE)  
Ei-value:0.000, Pi-value:0.000  
Er-value:0.000, Pr-value:0.000  
eCLIP MATCHES▶pum1 (bg=29.85%)No matches to TargetScan

TAAT

AGAAAAC

AGAAAAC  
Depth:8 (GUINEAPIG)  
Ei-value:0.000, Pi-value:0.000  
Er-value:0.000, Pr-value:0.000  
eCLIP MATCHES▶pum1 (bg=29.85%)No matches to TargetScan

ATTTGTC

ACATCTAG

ACATCTAG  
Depth:7 (ARMADILLO)  
Ei-value:0.000, Pi-value:0.000  
Er-value:0.000, Pr-value:0.000  
eCLIP MATCHES▶pum1 (bg=29.85%)▶rbm15 (bg=11.81%)No matches to TargetScan

CCCTTC

TAGATG

TAGATG  
Depth:8 (GUINEAPIG)  
Ei-value:0.000, Pi-value:0.000  
Er-value:0.000, Pr-value:0.000  
eCLIP MATCHES▶dgcr8 (bg=19.31%)▶KHDRBS1 (bg=1.16%)▶pum1 (bg=29.85%)▶rbm15 (bg=11.81%)No matches to TargetScan

G

A

AAAGAGGTTGCCGAC  
Depth:4 (COW)  
Ei-value:0.020, Pi-value:0.000  
Er-value:0.000, Pr-value:0.000  
eCLIP MATCHES▶dgcr8 (bg=19.31%)▶IGF2BP2 (bg=3.44%)▶KHDRBS1 (bg=1.16%)▶lin28b (bg=16.96%)▶pum1 (bg=29.85%)▶pum2 (bg=21.55%)▶rbm15 (bg=11.81%)▶tia1 (bg=16.04%)No matches to TargetScan


A

AAGAGGT  
Depth:8 (GUINEAPIG)  
Ei-value:0.000, Pi-value:0.000  
Er-value:0.000, Pr-value:0.000  
eCLIP MATCHES▶dgcr8 (bg=19.31%)▶IGF2BP2 (bg=3.44%)▶KHDRBS1 (bg=1.16%)▶lin28b (bg=16.96%)▶pum1 (bg=29.85%)▶pum2 (bg=21.55%)▶rbm15 (bg=11.81%)▶tia1 (bg=16.04%)No matches to TargetScan


AGAGGT

AGAGGT  
Depth:9 (MOUSE)  
Ei-value:0.000, Pi-value:0.000  
Er-value:0.000, Pr-value:0.000  
eCLIP MATCHES▶dgcr8 (bg=19.31%)▶IGF2BP2 (bg=3.44%)▶KHDRBS1 (bg=1.16%)▶lin28b (bg=16.96%)▶pum1 (bg=29.85%)▶pum2 (bg=21.55%)▶rbm15 (bg=11.81%)▶tia1 (bg=16.04%)No matches to TargetScan


T

AAGAGGTTGCCGAC  
Depth:6 (PIG)  
Ei-value:0.000, Pi-value:0.000  
Er-value:0.000, Pr-value:0.000  
eCLIP MATCHES▶dgcr8 (bg=19.31%)▶IGF2BP2 (bg=3.44%)▶KHDRBS1 (bg=1.16%)▶lin28b (bg=16.96%)▶pum1 (bg=29.85%)▶pum2 (bg=21.55%)▶rbm15 (bg=11.81%)▶tia1 (bg=16.04%)No matches to TargetScan


GCCGAC

GCCGAC  
Depth:7 (ARMADILLO)  
Ei-value:0.000, Pi-value:0.000  
Er-value:0.000, Pr-value:0.000  
eCLIP MATCHES▶dgcr8 (bg=19.31%)▶IGF2BP2 (bg=3.44%)▶KHDRBS1 (bg=1.16%)▶lin28b (bg=16.96%)▶pum1 (bg=29.85%)▶pum2 (bg=21.55%)▶rbm15 (bg=11.81%)▶tia1 (bg=16.04%)No matches to TargetScan

G

TATGATAAA

TATGATAAA  
Depth:8 (GUINEAPIG)  
Ei-value:0.000, Pi-value:0.000  
Er-value:0.000, Pr-value:0.000  
eCLIP MATCHES▶dgcr8 (bg=19.31%)▶IGF2BP2 (bg=3.44%)▶KHDRBS1 (bg=1.16%)▶lin28b (bg=16.96%)▶pum1 (bg=29.85%)▶pum2 (bg=21.55%)▶rbm15 (bg=11.81%)▶tia1 (bg=16.04%)MATCHES To TargetScan▶ miR-154-3p/487-3p:AUCAUAC

ATAG

AGTTAG

AGTTAG  
Depth:8 (GUINEAPIG)  
Ei-value:0.000, Pi-value:0.000  
Er-value:0.000, Pr-value:0.000  
eCLIP MATCHES▶dgcr8 (bg=19.31%)▶IGF2BP2 (bg=3.44%)▶KHDRBS1 (bg=1.16%)▶lin28b (bg=16.96%)▶pum2 (bg=21.55%)▶rbm15 (bg=11.81%)▶tia1 (bg=16.04%)No matches to TargetScan


AAA

AGTTAGAAA  
Depth:7 (ARMADILLO)  
Ei-value:0.000, Pi-value:0.000  
Er-value:0.000, Pr-value:0.000  
eCLIP MATCHES▶dgcr8 (bg=19.31%)▶IGF2BP2 (bg=3.44%)▶KHDRBS1 (bg=1.16%)▶lin28b (bg=16.96%)▶pum2 (bg=21.55%)▶rbm15 (bg=11.81%)▶tia1 (bg=16.04%)No matches to TargetScan

GTTACACA

TC

TCTTGTAAAT  
Depth:6 (PIG)  
Ei-value:0.000, Pi-value:0.000  
Er-value:0.000, Pr-value:0.000  
eCLIP MATCHES▶KHDRBS1 (bg=1.16%)▶lin28b (bg=16.96%)No matches to TargetScan


TTGTAA

TTGTAA  
Depth:7 (ARMADILLO)  
Ei-value:0.000, Pi-value:0.020  
Er-value:0.000, Pr-value:0.020  
eCLIP MATCHES▶KHDRBS1 (bg=1.16%)▶lin28b (bg=16.96%)No matches to TargetScan


AT

TCTTGTAAAT  
Depth:6 (PIG)  
Ei-value:0.000, Pi-value:0.000  
Er-value:0.000, Pr-value:0.000  
eCLIP MATCHES▶KHDRBS1 (bg=1.16%)▶lin28b (bg=16.96%)No matches to TargetScan

TCTCA

TTTGT

TTTGTTT  
Depth:5 (SHEEP)  
Ei-value:0.000, Pi-value:0.020  
Er-value:0.000, Pr-value:0.010  
No matches to eCLIP DataMATCHES To TargetScan▶ miR-495-3p:AACAAAC

 960  


TT

TTTGTTT  
Depth:5 (SHEEP)  
Ei-value:0.000, Pi-value:0.020  
Er-value:0.000, Pr-value:0.010  
No matches to eCLIP DataMATCHES To TargetScan▶ miR-495-3p:AACAAAC

AAAAGAAATC

ATAGAAA

ATAGAAA  
Depth:5 (SHEEP)  
Ei-value:0.000, Pi-value:0.000  
Er-value:0.000, Pr-value:0.000  
eCLIP MATCHES▶fubp3 (bg=23.31%)▶GRWD1 (bg=4.85%)▶pum1 (bg=29.85%)▶pum2 (bg=21.55%)▶rbm15 (bg=11.81%)▶sf3a3 (bg=12.13%)▶ZNF622 (bg=1.54%)No matches to TargetScan

ATACATGTCTTCTGGAGATGACTTT

TG

TGGAAATG  
Depth:8 (GUINEAPIG)  
Ei-value:0.000, Pi-value:0.000  
Er-value:0.000, Pr-value:0.000  
eCLIP MATCHES▶fubp3 (bg=23.31%)▶GRWD1 (bg=4.85%)▶PPIG (bg=0.88%)▶pum1 (bg=29.85%)▶pum2 (bg=21.55%)▶rbm15 (bg=11.81%)▶SND1 (bg=1.27%)▶ZNF622 (bg=1.54%)No matches to TargetScan


GAAATG

GAAATG  
Depth:9 (MOUSE)  
Ei-value:0.000, Pi-value:0.000  
Er-value:0.000, Pr-value:0.000  
eCLIP MATCHES▶fubp3 (bg=23.31%)▶GRWD1 (bg=4.85%)▶PPIG (bg=0.88%)▶pum1 (bg=29.85%)▶pum2 (bg=21.55%)▶rbm15 (bg=11.81%)▶SND1 (bg=1.27%)▶ZNF622 (bg=1.54%)No matches to TargetScan

GAGTTGTTAAGACGGCCTCTGGAAGCGATACGTCCACGTTTGTTA

A

AGTGGGT  
Depth:6 (PIG)  
Ei-value:0.000, Pi-value:0.000  
Er-value:0.000, Pr-value:0.000  
eCLIP MATCHES▶GNL3 (bg=0.85%)▶PPIG (bg=0.88%)▶pum1 (bg=29.85%)▶ZNF622 (bg=1.54%)No matches to TargetScan


GTGGGT

GTGGGT  
Depth:7 (ARMADILLO)  
Ei-value:0.000, Pi-value:0.000  
Er-value:0.000, Pr-value:0.000  
eCLIP MATCHES▶GNL3 (bg=0.85%)▶PPIG (bg=0.88%)No matches to TargetScan

TAGATGACATGGAGCT 1080  


GGAAGA

GGAAGA  
Depth:6 (PIG)  
Ei-value:0.000, Pi-value:0.000  
Er-value:0.000, Pr-value:0.000  
No matches to eCLIP DataNo matches to TargetScan

CCTGAGAAGGAAGAGAAGAAGGTTCTATGCT

AGACTG

AGACTGGTCATA  
Depth:5 (SHEEP)  
Ei-value:0.000, Pi-value:0.000  
Er-value:0.000, Pr-value:0.000  
No matches to eCLIP DataNo matches to TargetScan


GTCATA

GTCATA  
Depth:7 (ARMADILLO)  
Ei-value:0.000, Pi-value:0.000  
Er-value:0.000, Pr-value:0.000  
No matches to eCLIP DataNo matches to TargetScan

T

T

TTAGAAGA  
Depth:7 (ARMADILLO)  
Ei-value:0.000, Pi-value:0.000  
Er-value:0.000, Pr-value:0.000  
No matches to eCLIP DataNo matches to TargetScan


T

TAGAAGA  
Depth:8 (GUINEAPIG)  
Ei-value:0.000, Pi-value:0.000  
Er-value:0.000, Pr-value:0.000  
No matches to eCLIP DataNo matches to TargetScan


AGAAGA

AGAAGA  
Depth:9 (MOUSE)  
Ei-value:0.000, Pi-value:0.000  
Er-value:0.000, Pr-value:0.000  
No matches to eCLIP DataNo matches to TargetScan


CATTT

TTAGAAGACATTT  
Depth:6 (PIG)  
Ei-value:0.000, Pi-value:0.000  
Er-value:0.000, Pr-value:0.000  
No matches to eCLIP DataNo matches to TargetScan

TCATATTCTATCCATTGTTT

TGTGTGCATTTT

TGTGTGCATTTT  
Depth:6 (PIG)  
Ei-value:0.000, Pi-value:0.000  
Er-value:0.000, Pr-value:0.000  
No matches to eCLIP DataMATCHES To TargetScan▶ miR-501-3p/502-3p:AUGCACC

A

TTCCTC

TTCCTC  
Depth:7 (ARMADILLO)  
Ei-value:0.000, Pi-value:0.000  
Er-value:0.000, Pr-value:0.000  
No matches to eCLIP DataNo matches to TargetScan

ACTACTG

TG

TGTATATA  
Depth:9 (MOUSE)  
Ei-value:0.000, Pi-value:0.000  
Er-value:0.000, Pr-value:0.000  
No matches to eCLIP DataNo matches to TargetScan


TATATA

TATATA  
Depth:6 (PIG)  
Ei-value:0.000, Pi-value:0.030  
Er-value:0.000, Pr-value:0.000  
No matches to eCLIP DataNo matches to TargetScan

GTT 1200  
 GACAATGCTAAGCTTTTTTGAAATGTCTCTTCTTTT

TAGATG

TAGATGTTCTGAAGTGCCTGA  
Depth:5 (SHEEP)  
Ei-value:0.000, Pi-value:0.000  
Er-value:0.000, Pr-value:0.000  
eCLIP MATCHES▶pum1 (bg=29.85%)▶PUS1 (bg=2.87%)▶tial1 (bg=14.09%)No matches to TargetScan


TTCTG

TTCTGAAGTGCCTGA  
Depth:7 (ARMADILLO)  
Ei-value:0.000, Pi-value:0.000  
Er-value:0.000, Pr-value:0.000  
eCLIP MATCHES▶pum1 (bg=29.85%)▶PUS1 (bg=2.87%)▶tial1 (bg=14.09%)No matches to TargetScan


AAGTGCCTG

AAGTGCCTG  
Depth:9 (MOUSE)  
Ei-value:0.000, Pi-value:0.000  
Er-value:0.000, Pr-value:0.000  
eCLIP MATCHES▶pum1 (bg=29.85%)▶PUS1 (bg=2.87%)▶tial1 (bg=14.09%)No matches to TargetScan


A

AAGTGCCTGA  
Depth:8 (GUINEAPIG)  
Ei-value:0.000, Pi-value:0.000  
Er-value:0.000, Pr-value:0.000  
eCLIP MATCHES▶pum1 (bg=29.85%)▶PUS1 (bg=2.87%)▶tial1 (bg=14.09%)No matches to TargetScan

T

A

ATGTTAAAA  
Depth:6 (PIG)  
Ei-value:0.000, Pi-value:0.000  
Er-value:0.000, Pr-value:0.000  
eCLIP MATCHES▶ddx55 (bg=12.35%)▶pum1 (bg=29.85%)▶PUS1 (bg=2.87%)▶tial1 (bg=14.09%)No matches to TargetScan


T

TGTTAAAA  
Depth:7 (ARMADILLO)  
Ei-value:0.000, Pi-value:0.000  
Er-value:0.000, Pr-value:0.000  
eCLIP MATCHES▶ddx55 (bg=12.35%)▶pum1 (bg=29.85%)▶PUS1 (bg=2.87%)▶tial1 (bg=14.09%)No matches to TargetScan


GTTAAAA

GTTAAAA  
Depth:8 (GUINEAPIG)  
Ei-value:0.000, Pi-value:0.000  
Er-value:0.000, Pr-value:0.000  
eCLIP MATCHES▶ddx55 (bg=12.35%)▶pum1 (bg=29.85%)▶PUS1 (bg=2.87%)▶tial1 (bg=14.09%)No matches to TargetScan

TTA

GAGGTAG

GAGGTAG  
Depth:6 (PIG)  
Ei-value:0.000, Pi-value:0.000  
Er-value:0.000, Pr-value:0.000  
eCLIP MATCHES▶ddx55 (bg=12.35%)▶pum1 (bg=29.85%)▶PUS1 (bg=2.87%)▶tial1 (bg=14.09%)No matches to TargetScan

CAAAATC

ACA

ACATTTTGTAAATA  
Depth:5 (SHEEP)  
Ei-value:0.000, Pi-value:0.000  
Er-value:0.000, Pr-value:0.000  
eCLIP MATCHES▶CPEB4 (bg=2.3%)▶ddx55 (bg=12.35%)▶drosha (bg=11.37%)▶pum1 (bg=29.85%)▶PUS1 (bg=2.87%)▶tial1 (bg=14.09%)No matches to TargetScan


TTTTGT

TTTTGT  
Depth:6 (PIG)  
Ei-value:0.000, Pi-value:0.020  
Er-value:0.000, Pr-value:0.040  
eCLIP MATCHES▶CPEB4 (bg=2.3%)▶pum1 (bg=29.85%)▶PUS1 (bg=2.87%)▶tial1 (bg=14.09%)No matches to TargetScan


AAATA

ACATTTTGTAAATA  
Depth:5 (SHEEP)  
Ei-value:0.000, Pi-value:0.000  
Er-value:0.000, Pr-value:0.000  
eCLIP MATCHES▶CPEB4 (bg=2.3%)▶ddx55 (bg=12.35%)▶drosha (bg=11.37%)▶pum1 (bg=29.85%)▶PUS1 (bg=2.87%)▶tial1 (bg=14.09%)No matches to TargetScan

CCTTTTTGTTACA

ATTCATA

ATTCATA  
Depth:5 (SHEEP)  
Ei-value:0.000, Pi-value:0.000  
Er-value:0.000, Pr-value:0.000  
eCLIP MATCHES▶AGGF1 (bg=0.88%)▶CPEB4 (bg=2.3%)▶ddx55 (bg=12.35%)▶ddx6 (bg=23.92%)▶dgcr8 (bg=19.31%)▶drosha (bg=11.37%)▶fam120a (bg=18.43%)▶FASTKD2 (bg=7.73%)▶fubp3 (bg=23.31%)▶igf2bp1 (bg=11.67%)▶pum1 (bg=29.85%)▶pum2 (bg=21.55%)▶rbm15 (bg=11.81%)▶sf3a3 (bg=12.13%)▶SF3B1 (bg=6.74%)▶tia1 (bg=16.04%)▶tial1 (bg=14.09%)▶UPF1 (bg=3.62%)No matches to TargetScan

GG 1320  
 AAATATTTTTGGGGGGGAA

TGGCCA

TGGCCA  
Depth:6 (PIG)  
Ei-value:0.000, Pi-value:0.000  
Er-value:0.000, Pr-value:0.000  
eCLIP MATCHES▶AGGF1 (bg=0.88%)▶CPEB4 (bg=2.3%)▶ddx55 (bg=12.35%)▶ddx6 (bg=23.92%)▶dgcr8 (bg=19.31%)▶drosha (bg=11.37%)▶fam120a (bg=18.43%)▶FASTKD2 (bg=7.73%)▶FTO (bg=7.53%)▶fubp3 (bg=23.31%)▶FXR2 (bg=7.51%)▶igf2bp1 (bg=11.67%)▶IGF2BP2 (bg=3.44%)▶IGF2BP3 (bg=4.26%)▶pum1 (bg=29.85%)▶pum2 (bg=21.55%)▶rbm15 (bg=11.81%)▶sf3a3 (bg=12.13%)▶SF3B1 (bg=6.74%)▶tia1 (bg=16.04%)▶tial1 (bg=14.09%)▶UPF1 (bg=3.62%)No matches to TargetScan

AATCACCTGTTGAGTAATACTCATTGTG

T

TTTGTGCA  
Depth:7 (ARMADILLO)  
Ei-value:0.000, Pi-value:0.000  
Er-value:0.000, Pr-value:0.000  
eCLIP MATCHES▶drosha (bg=11.37%)▶igf2bp1 (bg=11.67%)▶IGF2BP2 (bg=3.44%)▶IGF2BP3 (bg=4.26%)▶pum1 (bg=29.85%)▶tia1 (bg=16.04%)No matches to TargetScan


TTGTGC

TTGTGC  
Depth:9 (MOUSE)  
Ei-value:0.000, Pi-value:0.000  
Er-value:0.000, Pr-value:0.000  
eCLIP MATCHES▶pum1 (bg=29.85%)▶tia1 (bg=16.04%)No matches to TargetScan


A

TTGTGCA  
Depth:8 (GUINEAPIG)  
Ei-value:0.000, Pi-value:0.000  
Er-value:0.000, Pr-value:0.000  
eCLIP MATCHES▶pum1 (bg=29.85%)▶tia1 (bg=16.04%)No matches to TargetScan

GTGGTTCAGGGGA

GGAGAG

GGAGAG  
Depth:5 (SHEEP)  
Ei-value:0.000, Pi-value:0.030  
Er-value:0.000, Pr-value:0.000  
eCLIP MATCHES▶CDC40 (bg=1.71%)▶ddx55 (bg=12.35%)▶dgcr8 (bg=19.31%)▶fam120a (bg=18.43%)▶FASTKD2 (bg=7.73%)▶FTO (bg=7.53%)▶GTF2F1 (bg=2.31%)▶igf2bp1 (bg=11.67%)▶KHSRP (bg=8.1%)▶pum1 (bg=29.85%)▶sf3a3 (bg=12.13%)▶TBRG4 (bg=7.0%)▶tia1 (bg=16.04%)▶UPF1 (bg=3.62%)No matches to TargetScan

AGGAGGGGGAGGTGCAGAGAGCTCTATGCCATCCTGTTTA 1440  
 CAGCGAGGCAAGATGAATCATTATGTCTG

TGCA

TGCATTTTGTTT  
Depth:4 (COW)  
Ei-value:0.000, Pi-value:0.000  
Er-value:0.000, Pr-value:0.000  
eCLIP MATCHES▶AKAP1 (bg=3.4%)▶ddx3x (bg=13.89%)▶ddx55 (bg=12.35%)▶dgcr8 (bg=19.31%)▶drosha (bg=11.37%)▶fam120a (bg=18.43%)▶FTO (bg=7.53%)▶fubp3 (bg=23.31%)▶igf2bp1 (bg=11.67%)▶pum1 (bg=29.85%)▶pum2 (bg=21.55%)▶sf3a3 (bg=12.13%)▶SUB1 (bg=9.24%)▶tia1 (bg=16.04%)▶XRCC6 (bg=1.65%)MATCHES To TargetScan▶ miR-495-3p:AACAAAC


TTTTGTTT

TTTTGTTT  
Depth:5 (SHEEP)  
Ei-value:0.000, Pi-value:0.000  
Er-value:0.000, Pr-value:0.000  
eCLIP MATCHES▶AKAP1 (bg=3.4%)▶ddx55 (bg=12.35%)▶dgcr8 (bg=19.31%)▶drosha (bg=11.37%)▶fam120a (bg=18.43%)▶FTO (bg=7.53%)▶fubp3 (bg=23.31%)▶igf2bp1 (bg=11.67%)▶pum1 (bg=29.85%)▶pum2 (bg=21.55%)▶sf3a3 (bg=12.13%)▶SUB1 (bg=9.24%)▶XRCC6 (bg=1.65%)MATCHES To TargetScan▶ miR-495-3p:AACAAAC

TACTTATCTGTGT

ATA

ATATAGTGTA  
Depth:6 (PIG)  
Ei-value:0.000, Pi-value:0.000  
Er-value:0.000, Pr-value:0.000  
eCLIP MATCHES▶AKAP1 (bg=3.4%)▶ddx55 (bg=12.35%)▶dgcr8 (bg=19.31%)▶drosha (bg=11.37%)▶fam120a (bg=18.43%)▶fubp3 (bg=23.31%)▶igf2bp1 (bg=11.67%)▶NKRF (bg=3.64%)▶pum1 (bg=29.85%)▶pum2 (bg=21.55%)▶sf3a3 (bg=12.13%)▶SUB1 (bg=9.24%)▶tia1 (bg=16.04%)▶tial1 (bg=14.09%)▶TROVE2 (bg=3.49%)▶XRCC6 (bg=1.65%)No matches to TargetScan


TAGTGT

TAGTGT  
Depth:7 (ARMADILLO)  
Ei-value:0.000, Pi-value:0.000  
Er-value:0.000, Pr-value:0.010  
eCLIP MATCHES▶AKAP1 (bg=3.4%)▶ddx55 (bg=12.35%)▶dgcr8 (bg=19.31%)▶drosha (bg=11.37%)▶fam120a (bg=18.43%)▶fubp3 (bg=23.31%)▶igf2bp1 (bg=11.67%)▶NKRF (bg=3.64%)▶pum1 (bg=29.85%)▶pum2 (bg=21.55%)▶sf3a3 (bg=12.13%)▶SUB1 (bg=9.24%)▶tia1 (bg=16.04%)▶tial1 (bg=14.09%)▶TROVE2 (bg=3.49%)▶XRCC6 (bg=1.65%)No matches to TargetScan


A

ATATAGTGTA  
Depth:6 (PIG)  
Ei-value:0.000, Pi-value:0.000  
Er-value:0.000, Pr-value:0.000  
eCLIP MATCHES▶AKAP1 (bg=3.4%)▶ddx55 (bg=12.35%)▶dgcr8 (bg=19.31%)▶drosha (bg=11.37%)▶fam120a (bg=18.43%)▶fubp3 (bg=23.31%)▶igf2bp1 (bg=11.67%)▶NKRF (bg=3.64%)▶pum1 (bg=29.85%)▶pum2 (bg=21.55%)▶sf3a3 (bg=12.13%)▶SUB1 (bg=9.24%)▶tia1 (bg=16.04%)▶tial1 (bg=14.09%)▶TROVE2 (bg=3.49%)▶XRCC6 (bg=1.65%)No matches to TargetScan

CATAAAGGACAGAC

GA

GAGTCCTAATT  
Depth:6 (PIG)  
Ei-value:0.000, Pi-value:0.000  
Er-value:0.000, Pr-value:0.000  
eCLIP MATCHES▶AKAP1 (bg=3.4%)▶ddx55 (bg=12.35%)▶dgcr8 (bg=19.31%)▶drosha (bg=11.37%)▶fam120a (bg=18.43%)▶fubp3 (bg=23.31%)▶igf2bp1 (bg=11.67%)▶NKRF (bg=3.64%)▶pum1 (bg=29.85%)▶pum2 (bg=21.55%)▶sf3a3 (bg=12.13%)▶SUB1 (bg=9.24%)▶tia1 (bg=16.04%)▶tial1 (bg=14.09%)▶TROVE2 (bg=3.49%)No matches to TargetScan


GTCCTAATT

GTCCTAATT  
Depth:7 (ARMADILLO)  
Ei-value:0.000, Pi-value:0.000  
Er-value:0.000, Pr-value:0.000  
eCLIP MATCHES▶AKAP1 (bg=3.4%)▶ddx55 (bg=12.35%)▶dgcr8 (bg=19.31%)▶drosha (bg=11.37%)▶fam120a (bg=18.43%)▶fubp3 (bg=23.31%)▶igf2bp1 (bg=11.67%)▶NKRF (bg=3.64%)▶pum1 (bg=29.85%)▶pum2 (bg=21.55%)▶sf3a3 (bg=12.13%)▶SUB1 (bg=9.24%)▶tia1 (bg=16.04%)▶tial1 (bg=14.09%)▶TROVE2 (bg=3.49%)No matches to TargetScan

GA

CA

CAACATCT  
Depth:6 (PIG)  
Ei-value:0.000, Pi-value:0.000  
Er-value:0.000, Pr-value:0.000  
eCLIP MATCHES▶AKAP1 (bg=3.4%)▶pum1 (bg=29.85%)▶pum2 (bg=21.55%)▶tial1 (bg=14.09%)No matches to TargetScan


ACATCT

ACATCT  
Depth:7 (ARMADILLO)  
Ei-value:0.000, Pi-value:0.000  
Er-value:0.000, Pr-value:0.000  
No matches to eCLIP DataNo matches to TargetScan


AGTCTTT

CAACATCTAGTCTTT  
Depth:5 (SHEEP)  
Ei-value:0.000, Pi-value:0.000  
Er-value:0.000, Pr-value:0.000  
eCLIP MATCHES▶AKAP1 (bg=3.4%)▶pum1 (bg=29.85%)▶pum2 (bg=21.55%)▶tial1 (bg=14.09%)MATCHES To TargetScan▶ miR-28-3p:ACUAGAU

CTG

GATGTT

GATGTT  
Depth:9 (MOUSE)  
Ei-value:0.000, Pi-value:0.000  
Er-value:0.000, Pr-value:0.000  
No matches to eCLIP DataNo matches to TargetScan


AAAGA

AAAGAGGTTGCCA  
Depth:9 (MOUSE)  
Ei-value:0.000, Pi-value:0.000  
Er-value:0.000, Pr-value:0.000  
No matches to eCLIP DataNo matches to TargetScan

 1560  


GGTTGCCA

AAAGAGGTTGCCA  
Depth:9 (MOUSE)  
Ei-value:0.000, Pi-value:0.000  
Er-value:0.000, Pr-value:0.000  
No matches to eCLIP DataNo matches to TargetScan


GTGTATGA

AAAGAGGTTGCCAGTGTATGA  
Depth:7 (ARMADILLO)  
Ei-value:0.000, Pi-value:0.000  
Er-value:0.000, Pr-value:0.000  
No matches to eCLIP DataMATCHES To TargetScan▶ miR-539-3p:UCAUACA▶ miR-193-3p:ACUGGCC


CAAAA

GATGTTAAAGAGGTTGCCAGTGTATGACAAAA  
Depth:5 (SHEEP)  
Ei-value:0.000, Pi-value:0.000  
Er-value:0.000, Pr-value:0.000  
No matches to eCLIP DataMATCHES To TargetScan▶ miR-539-3p:UCAUACA▶ miR-193-3p:ACUGGCC

GTAGAGTTAGTAAACTAATATA

TTTTG

TTTTGTACATTTTGT  
Depth:5 (SHEEP)  
Ei-value:0.000, Pi-value:0.000  
Er-value:0.000, Pr-value:0.000  
No matches to eCLIP DataMATCHES To TargetScan▶ miR-493-5p:UGUACAU


TAC

TACATTTTGT  
Depth:7 (ARMADILLO)  
Ei-value:0.000, Pi-value:0.000  
Er-value:0.000, Pr-value:0.000  
No matches to eCLIP DataNo matches to TargetScan


ATTTTGT

ATTTTGT  
Depth:9 (MOUSE)  
Ei-value:0.000, Pi-value:0.000  
Er-value:0.000, Pr-value:0.000  
No matches to eCLIP DataNo matches to TargetScan

TTTACAAGTCCTAGGA

AAGATTGTCTT

AAGATTGTCTT  
Depth:5 (SHEEP)  
Ei-value:0.000, Pi-value:0.000  
Er-value:0.000, Pr-value:0.000  
eCLIP MATCHES▶dgcr8 (bg=19.31%)▶fam120a (bg=18.43%)▶KHSRP (bg=8.1%)▶NOLC1 (bg=6.58%)▶pum1 (bg=29.85%)▶pum2 (bg=21.55%)▶sf3a3 (bg=12.13%)▶tia1 (bg=16.04%)▶tial1 (bg=14.09%)No matches to TargetScan

CTG

AAAATT

AAAATT  
Depth:5 (SHEEP)  
Ei-value:0.000, Pi-value:0.010  
Er-value:0.000, Pr-value:0.020  
eCLIP MATCHES▶ddx55 (bg=12.35%)▶dgcr8 (bg=19.31%)▶fam120a (bg=18.43%)▶fubp3 (bg=23.31%)▶KHSRP (bg=8.1%)▶lin28b (bg=16.96%)▶NOLC1 (bg=6.58%)▶pum1 (bg=29.85%)▶pum2 (bg=21.55%)▶sf3a3 (bg=12.13%)▶tia1 (bg=16.04%)▶tial1 (bg=14.09%)No matches to TargetScan

TGATGTCTTCTGGGTTGATGGAGATG 1680  
 GGAAGGGTTCTAGGCCAGAATGTTCACATTTG

GAAGAC

GAAGAC  
Depth:7 (ARMADILLO)  
Ei-value:0.000, Pi-value:0.000  
Er-value:0.000, Pr-value:0.000  
No matches to eCLIP DataNo matches to TargetScan

TCTTTCAAATTATAACTGT

TGTTACA

TGTTACA  
Depth:6 (PIG)  
Ei-value:0.000, Pi-value:0.000  
Er-value:0.000, Pr-value:0.000  
No matches to eCLIP DataMATCHES To TargetScan▶ miR-194-5p:GUAACAG

TGTTT

GCAGTTTATT

GCAGTTTATT  
Depth:6 (PIG)  
Ei-value:0.000, Pi-value:0.000  
Er-value:0.000, Pr-value:0.000  
No matches to eCLIP DataNo matches to TargetScan


CA

GCAGTTTATTCAAGACTGCT  
Depth:5 (SHEEP)  
Ei-value:0.000, Pi-value:0.000  
Er-value:0.000, Pr-value:0.000  
No matches to eCLIP DataMATCHES To TargetScan▶ miR-431-5p:GUCUUGC


AGACTGCT

AGACTGCT  
Depth:6 (PIG)  
Ei-value:0.000, Pi-value:0.000  
Er-value:0.000, Pr-value:0.000  
No matches to eCLIP DataNo matches to TargetScan

GTATACATAGTAGACAAATTAAC

TCCTTA

TCCTTA  
Depth:9 (MOUSE)  
Ei-value:0.000, Pi-value:0.000  
Er-value:0.000, Pr-value:0.000  
No matches to eCLIP DataNo matches to TargetScan

CT 1800  
 TG

AAACATCT

AAACATCT  
Depth:9 (MOUSE)  
Ei-value:0.000, Pi-value:0.000  
Er-value:0.000, Pr-value:0.000  
No matches to eCLIP DataNo matches to TargetScan


AG

AAACATCTAG  
Depth:8 (GUINEAPIG)  
Ei-value:0.000, Pi-value:0.000  
Er-value:0.000, Pr-value:0.000  
No matches to eCLIP DataNo matches to TargetScan

TCTAT

CTAGATGTTTAGAAGTGCCC

CTAGATGTTTAGAAGTGCCC  
Depth:9 (MOUSE)  
Ei-value:0.000, Pi-value:0.000  
Er-value:0.000, Pr-value:0.000  
No matches to eCLIP DataNo matches to TargetScan

GAT

GTATGTTAAA

GTATGTTAAA  
Depth:9 (MOUSE)  
Ei-value:0.000, Pi-value:0.000  
Er-value:0.000, Pr-value:0.000  
No matches to eCLIP DataNo matches to TargetScan


TGTA

GTATGTTAAATGTA  
Depth:6 (PIG)  
Ei-value:0.000, Pi-value:0.000  
Er-value:0.000, Pr-value:0.000  
No matches to eCLIP DataNo matches to TargetScan

T

AGGTAGT

AGGTAGT  
Depth:6 (PIG)  
Ei-value:0.000, Pi-value:0.000  
Er-value:0.000, Pr-value:0.000  
No matches to eCLIP DataNo matches to TargetScan

AAAATACCACTT

TGTAAATA

TGTAAATA  
Depth:9 (MOUSE)  
Ei-value:0.000, Pi-value:0.000  
Er-value:0.000, Pr-value:0.000  
No matches to eCLIP DataNo matches to TargetScan

T

CTTTTTGCT

CTTTTTGCT  
Depth:5 (SHEEP)  
Ei-value:0.000, Pi-value:0.000  
Er-value:0.000, Pr-value:0.000  
eCLIP MATCHES▶pum1 (bg=29.85%)No matches to TargetScan

A

AAATTCATAGGAA

AAATTCATAGGAA  
Depth:4 (COW)  
Ei-value:0.000, Pi-value:0.000  
Er-value:0.000, Pr-value:0.000  
eCLIP MATCHES▶fubp3 (bg=23.31%)▶pum1 (bg=29.85%)▶pum2 (bg=21.55%)▶tial1 (bg=14.09%)MATCHES To TargetScan▶ miR-202-5p:UCCUAUG

ATGCTTTTGGAAAT 1920  
 TGAATTGTGAAGCCACCTTTGTGAACAGTATAGTAATGTCTATACTTGTTCAATAGTTTA

GAGGAGGT

GAGGAGGT  
Depth:5 (SHEEP)  
Ei-value:0.000, Pi-value:0.000  
Er-value:0.000, Pr-value:0.000  
eCLIP MATCHES▶fam120a (bg=18.43%)▶IGF2BP2 (bg=3.44%)▶pum1 (bg=29.85%)▶sf3a3 (bg=12.13%)No matches to TargetScan

AGG

AGGGAA

AGGGAA  
Depth:5 (SHEEP)  
Ei-value:0.000, Pi-value:0.010  
Er-value:0.000, Pr-value:0.000  
eCLIP MATCHES▶AARS (bg=2.33%)▶fam120a (bg=18.43%)▶IGF2BP2 (bg=3.44%)▶pum1 (bg=29.85%)▶sf3a3 (bg=12.13%)No matches to TargetScan

GAAATTGC

AAAAGGTAAT

AAAAGGTAAT  
Depth:7 (ARMADILLO)  
Ei-value:0.000, Pi-value:0.000  
Er-value:0.000, Pr-value:0.000  
eCLIP MATCHES▶AARS (bg=2.33%)▶ddx6 (bg=23.92%)▶fam120a (bg=18.43%)▶IGF2BP2 (bg=3.44%)▶pum1 (bg=29.85%)No matches to TargetScan


AT

AAAAGGTAATAT  
Depth:6 (PIG)  
Ei-value:0.000, Pi-value:0.000  
Er-value:0.000, Pr-value:0.000  
eCLIP MATCHES▶AARS (bg=2.33%)▶ddx6 (bg=23.92%)▶fam120a (bg=18.43%)▶IGF2BP2 (bg=3.44%)▶pum1 (bg=29.85%)No matches to TargetScan

TACTAGTGTGTTCATACTTG

GAC

GACATTTTCAGA  
Depth:5 (SHEEP)  
Ei-value:0.000, Pi-value:0.000  
Er-value:0.000, Pr-value:0.000  
eCLIP MATCHES▶pum1 (bg=29.85%)▶pum2 (bg=21.55%)No matches to TargetScan

 2040  


A

GACATTTTCAGA  
Depth:5 (SHEEP)  
Ei-value:0.000, Pi-value:0.000  
Er-value:0.000, Pr-value:0.000  
eCLIP MATCHES▶pum1 (bg=29.85%)▶pum2 (bg=21.55%)No matches to TargetScan


T

TTTTCAGA  
Depth:6 (PIG)  
Ei-value:0.000, Pi-value:0.000  
Er-value:0.000, Pr-value:0.000  
eCLIP MATCHES▶pum1 (bg=29.85%)▶pum2 (bg=21.55%)No matches to TargetScan


TTTCAG

TTTCAG  
Depth:7 (ARMADILLO)  
Ei-value:0.000, Pi-value:0.000  
Er-value:0.000, Pr-value:0.000  
eCLIP MATCHES▶pum1 (bg=29.85%)No matches to TargetScan


A

TTTTCAGA  
Depth:6 (PIG)  
Ei-value:0.000, Pi-value:0.000  
Er-value:0.000, Pr-value:0.000  
eCLIP MATCHES▶pum1 (bg=29.85%)▶pum2 (bg=21.55%)No matches to TargetScan

CACCATTTTTCTATATGTTT

T

TGTGCATTTT  
Depth:4 (COW)  
Ei-value:0.020, Pi-value:0.000  
Er-value:0.000, Pr-value:0.000  
eCLIP MATCHES▶GEMIN5 (bg=2.46%)▶pum1 (bg=29.85%)▶pum2 (bg=21.55%)MATCHES To TargetScan▶ miR-501-3p/502-3p:AUGCACC


G

GTGCATTTT  
Depth:6 (PIG)  
Ei-value:0.000, Pi-value:0.000  
Er-value:0.000, Pr-value:0.000  
eCLIP MATCHES▶GEMIN5 (bg=2.46%)▶pum1 (bg=29.85%)▶pum2 (bg=21.55%)MATCHES To TargetScan▶ miR-501-3p/502-3p:AUGCACC


TGCATTTT

TGCATTTT  
Depth:7 (ARMADILLO)  
Ei-value:0.000, Pi-value:0.000  
Er-value:0.000, Pr-value:0.000  
eCLIP MATCHES▶GEMIN5 (bg=2.46%)▶pum1 (bg=29.85%)▶pum2 (bg=21.55%)No matches to TargetScan

G

TTTTGCT

TTTTGCT  
Depth:5 (SHEEP)  
Ei-value:0.000, Pi-value:0.000  
Er-value:0.000, Pr-value:0.000  
eCLIP MATCHES▶fubp3 (bg=23.31%)▶GEMIN5 (bg=2.46%)▶pum1 (bg=29.85%)▶pum2 (bg=21.55%)No matches to TargetScan

C

TGTATATAGT

TGTATATAGT  
Depth:7 (ARMADILLO)  
Ei-value:0.000, Pi-value:0.000  
Er-value:0.000, Pr-value:0.000  
eCLIP MATCHES▶ddx6 (bg=23.92%)▶fubp3 (bg=23.31%)▶GEMIN5 (bg=2.46%)▶pum1 (bg=29.85%)▶pum2 (bg=21.55%)▶PUS1 (bg=2.87%)▶tial1 (bg=14.09%)No matches to TargetScan

A

TATATAAT

TATATAATGGACAAAT  
Depth:6 (PIG)  
Ei-value:0.000, Pi-value:0.000  
Er-value:0.000, Pr-value:0.000  
eCLIP MATCHES▶ddx6 (bg=23.92%)▶fubp3 (bg=23.31%)▶GEMIN5 (bg=2.46%)▶NOLC1 (bg=6.58%)▶pum1 (bg=29.85%)▶pum2 (bg=21.55%)▶PUS1 (bg=2.87%)▶tial1 (bg=14.09%)No matches to TargetScan


GGACAAAT

GGACAAAT  
Depth:7 (ARMADILLO)  
Ei-value:0.000, Pi-value:0.000  
Er-value:0.000, Pr-value:0.000  
eCLIP MATCHES▶ddx6 (bg=23.92%)▶fubp3 (bg=23.31%)▶GEMIN5 (bg=2.46%)▶NOLC1 (bg=6.58%)▶pum1 (bg=29.85%)▶pum2 (bg=21.55%)▶PUS1 (bg=2.87%)▶tial1 (bg=14.09%)No matches to TargetScan


AGTCCTA

AGTCCTA  
Depth:7 (ARMADILLO)  
Ei-value:0.000, Pi-value:0.000  
Er-value:0.000, Pr-value:0.000  
eCLIP MATCHES▶ddx6 (bg=23.92%)▶fubp3 (bg=23.31%)▶GEMIN5 (bg=2.46%)▶NOLC1 (bg=6.58%)▶pum1 (bg=29.85%)▶pum2 (bg=21.55%)▶PUS1 (bg=2.87%)▶tial1 (bg=14.09%)No matches to TargetScan

ATTTTTCA

ACATCTA

ACATCTA  
Depth:7 (ARMADILLO)  
Ei-value:0.000, Pi-value:0.000  
Er-value:0.000, Pr-value:0.000  
eCLIP MATCHES▶ddx6 (bg=23.92%)▶FASTKD2 (bg=7.73%)▶fubp3 (bg=23.31%)▶NOLC1 (bg=6.58%)▶pum1 (bg=29.85%)▶pum2 (bg=21.55%)▶PUS1 (bg=2.87%)▶SF3B1 (bg=6.74%)▶tial1 (bg=14.09%)No matches to TargetScan

G

TCTCTAGATGTT

TCTCTAGATGTT  
Depth:6 (PIG)  
Ei-value:0.000, Pi-value:0.000  
Er-value:0.000, Pr-value:0.000  
eCLIP MATCHES▶ddx6 (bg=23.92%)▶FASTKD2 (bg=7.73%)▶fubp3 (bg=23.31%)▶NOLC1 (bg=6.58%)▶pum1 (bg=29.85%)▶pum2 (bg=21.55%)▶PUS1 (bg=2.87%)▶SF3B1 (bg=6.74%)▶tial1 (bg=14.09%)No matches to TargetScan


AAAGAGGTTG

AAAGAGGTTGCCA  
Depth:9 (MOUSE)  
Ei-value:0.000, Pi-value:0.000  
Er-value:0.000, Pr-value:0.000  
eCLIP MATCHES▶ddx6 (bg=23.92%)▶FASTKD2 (bg=7.73%)▶fubp3 (bg=23.31%)▶lin28b (bg=16.96%)▶NOLC1 (bg=6.58%)▶pum1 (bg=29.85%)▶pum2 (bg=21.55%)▶SF3B1 (bg=6.74%)▶tial1 (bg=14.09%)No matches to TargetScan

 2160  


CCA

AAAGAGGTTGCCA  
Depth:9 (MOUSE)  
Ei-value:0.000, Pi-value:0.000  
Er-value:0.000, Pr-value:0.000  
eCLIP MATCHES▶ddx6 (bg=23.92%)▶FASTKD2 (bg=7.73%)▶fubp3 (bg=23.31%)▶lin28b (bg=16.96%)▶NOLC1 (bg=6.58%)▶pum1 (bg=29.85%)▶pum2 (bg=21.55%)▶SF3B1 (bg=6.74%)▶tial1 (bg=14.09%)No matches to TargetScan

G

TG

TGTATGACAAA  
Depth:6 (PIG)  
Ei-value:0.000, Pi-value:0.000  
Er-value:0.000, Pr-value:0.000  
eCLIP MATCHES▶ddx6 (bg=23.92%)▶FASTKD2 (bg=7.73%)▶fubp3 (bg=23.31%)▶lin28b (bg=16.96%)▶pum1 (bg=29.85%)▶pum2 (bg=21.55%)▶SF3B1 (bg=6.74%)▶tial1 (bg=14.09%)MATCHES To TargetScan▶ miR-539-3p:UCAUACA


TATGACAAA

TATGACAAA  
Depth:7 (ARMADILLO)  
Ei-value:0.000, Pi-value:0.000  
Er-value:0.000, Pr-value:0.000
[truncated: 273,178 more chars]
