# Supplementary material for: Uncovering deeply conserved motif combinations in rapidly evolving noncoding sequences
Source: Genome Biol. 2021 Jan 11;22:29. doi: 10.1186/s13059-020-02247-1 (PMC7798263; doi:10.1186/s13059-020-02247-1)
Supplement: Supplementary file 4 — Additional file 4. LncLOOM output results for XIST sequences from six mammals. [file 13059_2020_2247_MOESM4_ESM.gz › AdditionalFile4/Html_Files/Modules.html]

 MODULES


# MODULES

## start-MOTIF-end--(number of bases in between)--start-MOTIF-end

  

NAVIGATE ▼

▶MOUSE (depth:6)▶RABBIT (depth:5)▶DOG (depth:4)▶COW (depth:3)▶PIG (depth:2)

  
  
  
  
  
  

|  |  |  |  |  |
| --- | --- | --- | --- | --- |
| | | | | | | | | | |
| 2 |  | 4 |  | 6 |
| Depth of motif conservation (number of species) | | | | |

  
  

# Modules conserved to MOUSE (Depth: 6)

## Modules in Main Graph (All sequences considered):

```
>HUMAN  
      5034-

AATGTGCAT

AATGTGCAT  
Depth:6 (MOUSE)  
Ei-value:0.000, Pi-value:0.000  
Er-value:0.000, Pr-value:0.000  
No matches to eCLIP DataMATCHES To TargetScan▶ miR-501-3p/502-3p:AUGCACC

-5042--(862)--5905-

TTAAGGCC

TTAAGGCC  
Depth:6 (MOUSE)  
Ei-value:0.000, Pi-value:0.000  
Er-value:0.000, Pr-value:0.000  
eCLIP MATCHES▶HNRNPL (bg=0.64%)No matches to TargetScan

-5912--(3693)--9606-

GCACAATG

GCACAATG  
Depth:6 (MOUSE)  
Ei-value:0.000, Pi-value:0.000  
Er-value:0.000, Pr-value:0.000  
No matches to eCLIP DataNo matches to TargetScan

-9613--(133)--9747-

CTCCCA

CTCCCA  
Depth:6 (MOUSE)  
Ei-value:0.000, Pi-value:0.000  
Er-value:0.000, Pr-value:0.000  
eCLIP MATCHES▶hnrnpk (bg=12.88%)No matches to TargetScan

-9752--(456)--10209-

AAAAGCAG

AAAAGCAG  
Depth:6 (MOUSE)  
Ei-value:0.000, Pi-value:0.000  
Er-value:0.000, Pr-value:0.000  
No matches to eCLIP DataNo matches to TargetScan

-10216--(123)--10340-

GCAAAAT

GCAAAAT  
Depth:6 (MOUSE)  
Ei-value:0.000, Pi-value:0.000  
Er-value:0.000, Pr-value:0.000  
No matches to eCLIP DataNo matches to TargetScan

-10346--(127)--10474-

GATTGCCTGG

GATTGCCTGG  
Depth:6 (MOUSE)  
Ei-value:0.000, Pi-value:0.000  
Er-value:0.000, Pr-value:0.000  
No matches to eCLIP DataNo matches to TargetScan

-10483--(804)--11288-

AAAGATC

AAAGATC  
Depth:6 (MOUSE)  
Ei-value:0.000, Pi-value:0.000  
Er-value:0.000, Pr-value:0.000  
eCLIP MATCHES▶SRSF1 (bg=8.47%)▶U2AF2 (bg=1.76%)▶uchl5 (bg=11.16%)No matches to TargetScan

-11294--(327)--11622-

TTCCCTTTGA

TTCCCTTTGA  
Depth:6 (MOUSE)  
Ei-value:0.000, Pi-value:0.000  
Er-value:0.000, Pr-value:0.000  
eCLIP MATCHES▶ILF3 (bg=3.0%)▶RBM15 (bg=7.27%)▶SRSF7 (bg=2.32%)▶ZNF622 (bg=6.58%)No matches to TargetScan

-11631--(7)--11639-

TAGGTGGAGATGGGGCATGAGGATCCTCCAGGGGAA

TAGGTGGAGATGGGGCATGAGGATCCTCCAGGGGAA  
Depth:6 (MOUSE)  
Ei-value:0.000, Pi-value:0.000  
Er-value:0.000, Pr-value:0.000  
eCLIP MATCHES▶ILF3 (bg=3.0%)▶NOLC1 (bg=9.43%)▶RBM15 (bg=7.27%)▶SRSF7 (bg=2.32%)▶ZNF622 (bg=6.58%)MATCHES To TargetScan▶ miR-331-3p:CCCCUGG

-11674--(17)--11692-

GCAACA

GCAACA  
Depth:6 (MOUSE)  
Ei-value:0.000, Pi-value:0.000  
Er-value:0.000, Pr-value:0.000  
eCLIP MATCHES▶ILF3 (bg=3.0%)No matches to TargetScan

-11697--(105)--11803-

CCAAAT

CCAAAT  
Depth:6 (MOUSE)  
Ei-value:0.000, Pi-value:0.000  
Er-value:0.000, Pr-value:0.000  
eCLIP MATCHES▶GRWD1 (bg=5.13%)▶NOLC1 (bg=9.43%)No matches to TargetScan

-11808--(93)--11902-

GATCAACATGC

GATCAACATGC  
Depth:6 (MOUSE)  
Ei-value:0.000, Pi-value:0.000  
Er-value:0.000, Pr-value:0.000  
eCLIP MATCHES▶GRWD1 (bg=5.13%)▶NOLC1 (bg=9.43%)▶PTBP1 (bg=3.74%)▶RBM15 (bg=7.27%)▶TRA2A (bg=4.8%)▶uchl5 (bg=11.16%)▶ZNF622 (bg=6.58%)No matches to TargetScan

-11912--(34)--11947-

TGTGTAT

TGTGTAT  
Depth:6 (MOUSE)  
Ei-value:0.000, Pi-value:0.000  
Er-value:0.000, Pr-value:0.000  
eCLIP MATCHES▶TARDBP (bg=2.79%)▶ZC3H11A (bg=6.55%)No matches to TargetScan

-11953--(313)--12267-

TTCTCTTTG

TTCTCTTTG  
Depth:6 (MOUSE)  
Ei-value:0.000, Pi-value:0.000  
Er-value:0.000, Pr-value:0.000  
eCLIP MATCHES▶MATR3 (bg=2.98%)▶PTBP1 (bg=3.74%)▶SMNDC1 (bg=0.63%)▶TIA1 (bg=4.07%)No matches to TargetScan

-12275--(84)--12360-

TTTCTAC

TTTCTAC  
Depth:6 (MOUSE)  
Ei-value:0.000, Pi-value:0.000  
Er-value:0.000, Pr-value:0.000  
eCLIP MATCHES▶MATR3 (bg=2.98%)▶PTBP1 (bg=3.74%)▶TIA1 (bg=4.07%)No matches to TargetScan

-12366--(12)--12379-

ATTTCTC

ATTTCTC  
Depth:6 (MOUSE)  
Ei-value:0.000, Pi-value:0.000  
Er-value:0.000, Pr-value:0.000  
eCLIP MATCHES▶MATR3 (bg=2.98%)▶PTBP1 (bg=3.74%)▶TIA1 (bg=4.07%)No matches to TargetScan

-12385--(1312)--13698-

TCTAGAGAAAA

TCTAGAGAAAA  
Depth:6 (MOUSE)  
Ei-value:0.000, Pi-value:0.000  
Er-value:0.000, Pr-value:0.000  
eCLIP MATCHES▶CPSF6 (bg=0.4%)▶LARP4 (bg=4.72%)▶UTP3 (bg=3.66%)▶WDR43 (bg=3.37%)MATCHES To TargetScan▶ miR-1251-5p:CUCUAGC

-13708--(24)--13733-

TGAGAAGAATTAGACA

TGAGAAGAATTAGACA  
Depth:6 (MOUSE)  
Ei-value:0.000, Pi-value:0.000  
Er-value:0.000, Pr-value:0.000  
eCLIP MATCHES▶LARP4 (bg=4.72%)▶NOLC1 (bg=9.43%)▶SRSF7 (bg=2.32%)No matches to TargetScan

-13748--(386)--14135-

ATTGGCA

ATTGGCA  
Depth:6 (MOUSE)  
Ei-value:0.000, Pi-value:0.000  
Er-value:0.000, Pr-value:0.000  
eCLIP MATCHES▶HNRNPA1 (bg=2.57%)No matches to TargetScan

-14141--(34)--14176-

TTGTGAAG

TTGTGAAG  
Depth:6 (MOUSE)  
Ei-value:0.000, Pi-value:0.000  
Er-value:0.000, Pr-value:0.000  
eCLIP MATCHES▶HNRNPA1 (bg=2.57%)No matches to TargetScan

-14183--(4081)--18265-

AAAAGGT

AAAAGGT  
Depth:6 (MOUSE)  
Ei-value:0.000, Pi-value:0.000  
Er-value:0.000, Pr-value:0.000  
eCLIP MATCHES▶ILF3 (bg=3.0%)▶SF3B1 (bg=2.48%)▶ZC3H11A (bg=6.55%)No matches to TargetScan

-18271  
  
>PIG  
     13513-

AATGTGCAT

AATGTGCAT  
Depth:6 (MOUSE)  
Ei-value:0.000, Pi-value:0.000  
Er-value:0.000, Pr-value:0.000  
MATCHES To TargetScan▶ miR-501-3p/502-3p:AUGCACC

-13521--(624)--14146-

TTAAGGCC

TTAAGGCC  
Depth:6 (MOUSE)  
Ei-value:0.000, Pi-value:0.000  
Er-value:0.000, Pr-value:0.000  
No matches to TargetScan

-14153--(1192)--15346-

GCACAATG

GCACAATG  
Depth:6 (MOUSE)  
Ei-value:0.000, Pi-value:0.000  
Er-value:0.000, Pr-value:0.000  
No matches to TargetScan

-15353--(132)--15486-

CTCCCA

CTCCCA  
Depth:6 (MOUSE)  
Ei-value:0.000, Pi-value:0.000  
Er-value:0.000, Pr-value:0.000  
No matches to TargetScan

-15491--(496)--15988-

AAAAGCAG

AAAAGCAG  
Depth:6 (MOUSE)  
Ei-value:0.000, Pi-value:0.000  
Er-value:0.000, Pr-value:0.000  
No matches to TargetScan

-15995--(121)--16117-

GCAAAAT

GCAAAAT  
Depth:6 (MOUSE)  
Ei-value:0.000, Pi-value:0.000  
Er-value:0.000, Pr-value:0.000  
No matches to TargetScan

-16123--(140)--16264-

GATTGCCTGG

GATTGCCTGG  
Depth:6 (MOUSE)  
Ei-value:0.000, Pi-value:0.000  
Er-value:0.000, Pr-value:0.000  
No matches to TargetScan

-16273--(827)--17101-

AAAGATC

AAAGATC  
Depth:6 (MOUSE)  
Ei-value:0.000, Pi-value:0.000  
Er-value:0.000, Pr-value:0.000  
No matches to TargetScan

-17107--(350)--17458-

TTCCCTTTGA

TTCCCTTTGA  
Depth:6 (MOUSE)  
Ei-value:0.000, Pi-value:0.000  
Er-value:0.000, Pr-value:0.000  
No matches to TargetScan

-17467--(7)--17475-

TAGGTGGAGATGGGGCATGAGGATCCTCCAGGGGAA

TAGGTGGAGATGGGGCATGAGGATCCTCCAGGGGAA  
Depth:6 (MOUSE)  
Ei-value:0.000, Pi-value:0.000  
Er-value:0.000, Pr-value:0.000  
MATCHES To TargetScan▶ miR-331-3p:CCCCUGG

-17510--(17)--17528-

GCAACA

GCAACA  
Depth:6 (MOUSE)  
Ei-value:0.000, Pi-value:0.000  
Er-value:0.000, Pr-value:0.000  
No matches to TargetScan

-17533--(439)--17973-

CCAAAT

CCAAAT  
Depth:6 (MOUSE)  
Ei-value:0.000, Pi-value:0.000  
Er-value:0.000, Pr-value:0.000  
No matches to TargetScan

-17978--(56)--18035-

GATCAACATGC

GATCAACATGC  
Depth:6 (MOUSE)  
Ei-value:0.000, Pi-value:0.000  
Er-value:0.000, Pr-value:0.000  
No matches to TargetScan

-18045--(66)--18112-

TGTGTAT

TGTGTAT  
Depth:6 (MOUSE)  
Ei-value:0.000, Pi-value:0.000  
Er-value:0.000, Pr-value:0.000  
No matches to TargetScan

-18118--(305)--18424-

TTCTCTTTG

TTCTCTTTG  
Depth:6 (MOUSE)  
Ei-value:0.000, Pi-value:0.000  
Er-value:0.000, Pr-value:0.000  
No matches to TargetScan

-18432--(82)--18515-

TTTCTAC

TTTCTAC  
Depth:6 (MOUSE)  
Ei-value:0.000, Pi-value:0.000  
Er-value:0.000, Pr-value:0.000  
No matches to TargetScan

-18521--(12)--18534-

ATTTCTC

ATTTCTC  
Depth:6 (MOUSE)  
Ei-value:0.000, Pi-value:0.000  
Er-value:0.000, Pr-value:0.000  
No matches to TargetScan

-18540--(951)--19492-

TCTAGAGAAAA

TCTAGAGAAAA  
Depth:6 (MOUSE)  
Ei-value:0.000, Pi-value:0.000  
Er-value:0.000, Pr-value:0.000  
MATCHES To TargetScan▶ miR-1251-5p:CUCUAGC

-19502--(23)--19526-

TGAGAAGAATTAGACA

TGAGAAGAATTAGACA  
Depth:6 (MOUSE)  
Ei-value:0.000, Pi-value:0.000  
Er-value:0.000, Pr-value:0.000  
No matches to TargetScan

-19541--(393)--19935-

ATTGGCA

ATTGGCA  
Depth:6 (MOUSE)  
Ei-value:0.000, Pi-value:0.000  
Er-value:0.000, Pr-value:0.000  
No matches to TargetScan

-19941--(36)--19978-

TTGTGAAG

TTGTGAAG  
Depth:6 (MOUSE)  
Ei-value:0.000, Pi-value:0.000  
Er-value:0.000, Pr-value:0.000  
No matches to TargetScan

-19985--(4105)--24091-

AAAAGGT

AAAAGGT  
Depth:6 (MOUSE)  
Ei-value:0.000, Pi-value:0.000  
Er-value:0.000, Pr-value:0.000  
No matches to TargetScan

-24097  
  
>COW  
      3091-

AATGTGCAT

AATGTGCAT  
Depth:6 (MOUSE)  
Ei-value:0.000, Pi-value:0.000  
Er-value:0.000, Pr-value:0.000  
MATCHES To TargetScan▶ miR-501-3p/502-3p:AUGCACC

-3099--(630)--3730-

TTAAGGCC

TTAAGGCC  
Depth:6 (MOUSE)  
Ei-value:0.000, Pi-value:0.000  
Er-value:0.000, Pr-value:0.000  
No matches to TargetScan

-3737--(699)--4437-

GCACAATG

GCACAATG  
Depth:6 (MOUSE)  
Ei-value:0.000, Pi-value:0.000  
Er-value:0.000, Pr-value:0.000  
No matches to TargetScan

-4444--(45)--4490-

CTCCCA

CTCCCA  
Depth:6 (MOUSE)  
Ei-value:0.000, Pi-value:0.000  
Er-value:0.000, Pr-value:0.000  
No matches to TargetScan

-4495--(570)--5066-

AAAAGCAG

AAAAGCAG  
Depth:6 (MOUSE)  
Ei-value:0.000, Pi-value:0.000  
Er-value:0.000, Pr-value:0.000  
No matches to TargetScan

-5073--(121)--5195-

GCAAAAT

GCAAAAT  
Depth:6 (MOUSE)  
Ei-value:0.000, Pi-value:0.000  
Er-value:0.000, Pr-value:0.000  
No matches to TargetScan

-5201--(134)--5336-

GATTGCCTGG

GATTGCCTGG  
Depth:6 (MOUSE)  
Ei-value:0.000, Pi-value:0.000  
Er-value:0.000, Pr-value:0.000  
No matches to TargetScan

-5345--(837)--6183-

AAAGATC

AAAGATC  
Depth:6 (MOUSE)  
Ei-value:0.000, Pi-value:0.000  
Er-value:0.000, Pr-value:0.000  
No matches to TargetScan

-6189--(352)--6542-

TTCCCTTTGA

TTCCCTTTGA  
Depth:6 (MOUSE)  
Ei-value:0.000, Pi-value:0.000  
Er-value:0.000, Pr-value:0.000  
No matches to TargetScan

-6551--(7)--6559-

TAGGTGGAGATGGGGCATGAGGATCCTCCAGGGGAA

TAGGTGGAGATGGGGCATGAGGATCCTCCAGGGGAA  
Depth:6 (MOUSE)  
Ei-value:0.000, Pi-value:0.000  
Er-value:0.000, Pr-value:0.000  
MATCHES To TargetScan▶ miR-331-3p:CCCCUGG

-6594--(17)--6612-

GCAACA

GCAACA  
Depth:6 (MOUSE)  
Ei-value:0.000, Pi-value:0.000  
Er-value:0.000, Pr-value:0.000  
No matches to TargetScan

-6617--(111)--6729-

CCAAAT

CCAAAT  
Depth:6 (MOUSE)  
Ei-value:0.000, Pi-value:0.000  
Er-value:0.000, Pr-value:0.000  
No matches to TargetScan

-6734--(55)--6790-

GATCAACATGC

GATCAACATGC  
Depth:6 (MOUSE)  
Ei-value:0.000, Pi-value:0.000  
Er-value:0.000, Pr-value:0.000  
No matches to TargetScan

-6800--(97)--6898-

TGTGTAT

TGTGTAT  
Depth:6 (MOUSE)  
Ei-value:0.000, Pi-value:0.000  
Er-value:0.000, Pr-value:0.000  
No matches to TargetScan

-6904--(296)--7201-

TTCTCTTTG

TTCTCTTTG  
Depth:6 (MOUSE)  
Ei-value:0.000, Pi-value:0.000  
Er-value:0.000, Pr-value:0.000  
No matches to TargetScan

-7209--(71)--7281-

TTTCTAC

TTTCTAC  
Depth:6 (MOUSE)  
Ei-value:0.000, Pi-value:0.000  
Er-value:0.000, Pr-value:0.000  
No matches to TargetScan

-7287--(14)--7302-

ATTTCTC

ATTTCTC  
Depth:6 (MOUSE)  
Ei-value:0.000, Pi-value:0.000  
Er-value:0.000, Pr-value:0.000  
No matches to TargetScan

-7308--(1030)--8339-

TCTAGAGAAAA

TCTAGAGAAAA  
Depth:6 (MOUSE)  
Ei-value:0.000, Pi-value:0.000  
Er-value:0.000, Pr-value:0.000  
MATCHES To TargetScan▶ miR-1251-5p:CUCUAGC

-8349--(25)--8375-

TGAGAAGAATTAGACA

TGAGAAGAATTAGACA  
Depth:6 (MOUSE)  
Ei-value:0.000, Pi-value:0.000  
Er-value:0.000, Pr-value:0.000  
No matches to TargetScan

-8390--(387)--8778-

ATTGGCA

ATTGGCA  
Depth:6 (MOUSE)  
Ei-value:0.000, Pi-value:0.000  
Er-value:0.000, Pr-value:0.000  
No matches to TargetScan

-8784--(36)--8821-

TTGTGAAG

TTGTGAAG  
Depth:6 (MOUSE)  
Ei-value:0.000, Pi-value:0.000  
Er-value:0.000, Pr-value:0.000  
No matches to TargetScan

-8828--(4284)--13113-

AAAAGGT

AAAAGGT  
Depth:6 (MOUSE)  
Ei-value:0.000, Pi-value:0.000  
Er-value:0.000, Pr-value:0.000  
No matches to TargetScan

-13119  
  
>DOG  
      3833-

AATGTGCAT

AATGTGCAT  
Depth:6 (MOUSE)  
Ei-value:0.000, Pi-value:0.000  
Er-value:0.000, Pr-value:0.000  
MATCHES To TargetScan▶ miR-501-3p/502-3p:AUGCACC

-3841--(446)--4288-

TTAAGGCC

TTAAGGCC  
Depth:6 (MOUSE)  
Ei-value:0.000, Pi-value:0.000  
Er-value:0.000, Pr-value:0.000  
No matches to TargetScan

-4295--(1629)--5925-

GCACAATG

GCACAATG  
Depth:6 (MOUSE)  
Ei-value:0.000, Pi-value:0.000  
Er-value:0.000, Pr-value:0.000  
No matches to TargetScan

-5932--(44)--5977-

CTCCCA

CTCCCA  
Depth:6 (MOUSE)  
Ei-value:0.000, Pi-value:0.000  
Er-value:0.000, Pr-value:0.000  
No matches to TargetScan

-5982--(549)--6532-

AAAAGCAG

AAAAGCAG  
Depth:6 (MOUSE)  
Ei-value:0.000, Pi-value:0.000  
Er-value:0.000, Pr-value:0.000  
No matches to TargetScan

-6539--(119)--6659-

GCAAAAT

GCAAAAT  
Depth:6 (MOUSE)  
Ei-value:0.000, Pi-value:0.000  
Er-value:0.000, Pr-value:0.000  
No matches to TargetScan

-6665--(133)--6799-

GATTGCCTGG

GATTGCCTGG  
Depth:6 (MOUSE)  
Ei-value:0.000, Pi-value:0.000  
Er-value:0.000, Pr-value:0.000  
No matches to TargetScan

-6808--(1044)--7853-

AAAGATC

AAAGATC  
Depth:6 (MOUSE)  
Ei-value:0.000, Pi-value:0.000  
Er-value:0.000, Pr-value:0.000  
No matches to TargetScan

-7859--(222)--8082-

TTCCCTTTGA

TTCCCTTTGA  
Depth:6 (MOUSE)  
Ei-value:0.000, Pi-value:0.000  
Er-value:0.000, Pr-value:0.000  
No matches to TargetScan

-8091--(7)--8099-

TAGGTGGAGATGGGGCATGAGGATCCTCCAGGGGAA

TAGGTGGAGATGGGGCATGAGGATCCTCCAGGGGAA  
Depth:6 (MOUSE)  
Ei-value:0.000, Pi-value:0.000  
Er-value:0.000, Pr-value:0.000  
MATCHES To TargetScan▶ miR-331-3p:CCCCUGG

-8134--(17)--8152-

GCAACA

GCAACA  
Depth:6 (MOUSE)  
Ei-value:0.000, Pi-value:0.000  
Er-value:0.000, Pr-value:0.000  
No matches to TargetScan

-8157--(377)--8535-

CCAAAT

CCAAAT  
Depth:6 (MOUSE)  
Ei-value:0.000, Pi-value:0.000  
Er-value:0.000, Pr-value:0.000  
No matches to TargetScan

-8540--(54)--8595-

GATCAACATGC

GATCAACATGC  
Depth:6 (MOUSE)  
Ei-value:0.000, Pi-value:0.000  
Er-value:0.000, Pr-value:0.000  
No matches to TargetScan

-8605--(34)--8640-

TGTGTAT

TGTGTAT  
Depth:6 (MOUSE)  
Ei-value:0.000, Pi-value:0.000  
Er-value:0.000, Pr-value:0.000  
No matches to TargetScan

-8646--(190)--8837-

TTCTCTTTG

TTCTCTTTG  
Depth:6 (MOUSE)  
Ei-value:0.000, Pi-value:0.000  
Er-value:0.000, Pr-value:0.000  
No matches to TargetScan

-8845--(195)--9041-

TTTCTAC

TTTCTAC  
Depth:6 (MOUSE)  
Ei-value:0.000, Pi-value:0.000  
Er-value:0.000, Pr-value:0.000  
No matches to TargetScan

-9047--(12)--9060-

ATTTCTC

ATTTCTC  
Depth:6 (MOUSE)  
Ei-value:0.000, Pi-value:0.000  
Er-value:0.000, Pr-value:0.000  
No matches to TargetScan

-9066--(1233)--10300-

TCTAGAGAAAA

TCTAGAGAAAA  
Depth:6 (MOUSE)  
Ei-value:0.000, Pi-value:0.000  
Er-value:0.000, Pr-value:0.000  
MATCHES To TargetScan▶ miR-1251-5p:CUCUAGC

-10310--(23)--10334-

TGAGAAGAATTAGACA

TGAGAAGAATTAGACA  
Depth:6 (MOUSE)  
Ei-value:0.000, Pi-value:0.000  
Er-value:0.000, Pr-value:0.000  
No matches to TargetScan

-10349--(392)--10742-

ATTGGCA

ATTGGCA  
Depth:6 (MOUSE)  
Ei-value:0.000, Pi-value:0.000  
Er-value:0.000, Pr-value:0.000  
No matches to TargetScan

-10748--(35)--10784-

TTGTGAAG

TTGTGAAG  
Depth:6 (MOUSE)  
Ei-value:0.000, Pi-value:0.000  
Er-value:0.000, Pr-value:0.000  
No matches to TargetScan

-10791--(4243)--15035-

AAAAGGT

AAAAGGT  
Depth:6 (MOUSE)  
Ei-value:0.000, Pi-value:0.000  
Er-value:0.000, Pr-value:0.000  
No matches to TargetScan

-15041  
  
>RABBIT  
      1648-

AATGTGCAT

AATGTGCAT  
Depth:6 (MOUSE)  
Ei-value:0.000, Pi-value:0.000  
Er-value:0.000, Pr-value:0.000  
MATCHES To TargetScan▶ miR-501-3p/502-3p:AUGCACC

-1656--(526)--2183-

TTAAGGCC

TTAAGGCC  
Depth:6 (MOUSE)  
Ei-value:0.000, Pi-value:0.000  
Er-value:0.000, Pr-value:0.000  
No matches to TargetScan

-2190--(3405)--5596-

GCACAATG

GCACAATG  
Depth:6 (MOUSE)  
Ei-value:0.000, Pi-value:0.000  
Er-value:0.000, Pr-value:0.000  
No matches to TargetScan

-5603--(51)--5655-

GCACAATG

GCACAATG  
Depth:6 (MOUSE)  
Ei-value:0.000, Pi-value:0.000  
Er-value:0.000, Pr-value:0.000  
No matches to TargetScan

-5662--(425)--6088-

CTCCCA

CTCCCA  
Depth:6 (MOUSE)  
Ei-value:0.000, Pi-value:0.000  
Er-value:0.000, Pr-value:0.000  
No matches to TargetScan

-6093--(148)--6242-

AAAAGCAG

AAAAGCAG  
Depth:6 (MOUSE)  
Ei-value:0.000, Pi-value:0.000  
Er-value:0.000, Pr-value:0.000  
No matches to TargetScan

-6249--(123)--6373-

GCAAAAT

GCAAAAT  
Depth:6 (MOUSE)  
Ei-value:0.000, Pi-value:0.000  
Er-value:0.000, Pr-value:0.000  
No matches to TargetScan

-6379--(120)--6500-

GATTGCCTGG

GATTGCCTGG  
Depth:6 (MOUSE)  
Ei-value:0.000, Pi-value:0.000  
Er-value:0.000, Pr-value:0.000  
No matches to TargetScan

-6509--(800)--7310-

AAAGATC

AAAGATC  
Depth:6 (MOUSE)  
Ei-value:0.000, Pi-value:0.000  
Er-value:0.000, Pr-value:0.000  
No matches to TargetScan

-7316--(263)--7580-

TTCCCTTTGA

TTCCCTTTGA  
Depth:6 (MOUSE)  
Ei-value:0.000, Pi-value:0.000  
Er-value:0.000, Pr-value:0.000  
No matches to TargetScan

-7589--(7)--7597-

TAGGTGGAGATGGGGCATGAGGATCCTCCAGGGGAA

TAGGTGGAGATGGGGCATGAGGATCCTCCAGGGGAA  
Depth:6 (MOUSE)  
Ei-value:0.000, Pi-value:0.000  
Er-value:0.000, Pr-value:0.000  
MATCHES To TargetScan▶ miR-331-3p:CCCCUGG

-7632--(17)--7650-

GCAACA

GCAACA  
Depth:6 (MOUSE)  
Ei-value:0.000, Pi-value:0.000  
Er-value:0.000, Pr-value:0.000  
No matches to TargetScan

-7655--(327)--7983-

CCAAAT

CCAAAT  
Depth:6 (MOUSE)  
Ei-value:0.000, Pi-value:0.000  
Er-value:0.000, Pr-value:0.000  
No matches to TargetScan

-7988--(86)--8075-

GATCAACATGC

GATCAACATGC  
Depth:6 (MOUSE)  
Ei-value:0.000, Pi-value:0.000  
Er-value:0.000, Pr-value:0.000  
No matches to TargetScan

-8085--(37)--8123-

TGTGTAT

TGTGTAT  
Depth:6 (MOUSE)  
Ei-value:0.000, Pi-value:0.000  
Er-value:0.000, Pr-value:0.000  
No matches to TargetScan

-8129--(0)--8130-

TTCTCTTTG

TTCTCTTTG  
Depth:6 (MOUSE)  
Ei-value:0.000, Pi-value:0.000  
Er-value:0.000, Pr-value:0.000  
No matches to TargetScan

-8138--(228)--8367-

TTTCTAC

TTTCTAC  
Depth:6 (MOUSE)  
Ei-value:0.000, Pi-value:0.000  
Er-value:0.000, Pr-value:0.000  
No matches to TargetScan

-8373--(152)--8526-

ATTTCTC

ATTTCTC  
Depth:6 (MOUSE)  
Ei-value:0.000, Pi-value:0.000  
Er-value:0.000, Pr-value:0.000  
No matches to TargetScan

-8532--(1285)--9818-

TCTAGAGAAAA

TCTAGAGAAAA  
Depth:6 (MOUSE)  
Ei-value:0.000, Pi-value:0.000  
Er-value:0.000, Pr-value:0.000  
MATCHES To TargetScan▶ miR-1251-5p:CUCUAGC

-9828--(22)--9851-

TGAGAAGAATTAGACA

TGAGAAGAATTAGACA  
Depth:6 (MOUSE)  
Ei-value:0.000, Pi-value:0.000  
Er-value:0.000, Pr-value:0.000  
No matches to TargetScan

-9866--(380)--10247-

ATTGGCA

ATTGGCA  
Depth:6 (MOUSE)  
Ei-value:0.000, Pi-value:0.000  
Er-value:0.000, Pr-value:0.000  
No matches to TargetScan

-10253--(36)--10290-

TTGTGAAG

TTGTGAAG  
Depth:6 (MOUSE)  
Ei-value:0.000, Pi-value:0.000  
Er-value:0.000, Pr-value:0.000  
No matches to TargetScan

-10297--(106)--10404-

AAAAGGT

AAAAGGT  
Depth:6 (MOUSE)  
Ei-value:0.000, Pi-value:0.000  
Er-value:0.000, Pr-value:0.000  
No matches to TargetScan

-10410  
  
>MOUSE  
      1718-

AATGTGCAT

AATGTGCAT  
Depth:6 (MOUSE)  
Ei-value:0.000, Pi-value:0.000  
Er-value:0.000, Pr-value:0.000  
MATCHES To TargetScan▶ miR-501-3p/502-3p:AUGCACC

-1726--(1020)--2747-

TTAAGGCC

TTAAGGCC  
Depth:6 (MOUSE)  
Ei-value:0.000, Pi-value:0.000  
Er-value:0.000, Pr-value:0.000  
No matches to TargetScan

-2754--(4865)--7620-

GCACAATG

GCACAATG  
Depth:6 (MOUSE)  
Ei-value:0.000, Pi-value:0.000  
Er-value:0.000, Pr-value:0.000  
No matches to TargetScan

-7627--(405)--8033-

CTCCCA

CTCCCA  
Depth:6 (MOUSE)  
Ei-value:0.000, Pi-value:0.000  
Er-value:0.000, Pr-value:0.000  
No matches to TargetScan

-8038--(106)--8145-

AAAAGCAG

AAAAGCAG  
Depth:6 (MOUSE)  
Ei-value:0.000, Pi-value:0.000  
Er-value:0.000, Pr-value:0.000  
No matches to TargetScan

-8152--(123)--8276-

GCAAAAT

GCAAAAT  
Depth:6 (MOUSE)  
Ei-value:0.000, Pi-value:0.000  
Er-value:0.000, Pr-value:0.000  
No matches to TargetScan

-8282--(1280)--9563-

GATTGCCTGG

GATTGCCTGG  
Depth:6 (MOUSE)  
Ei-value:0.000, Pi-value:0.000  
Er-value:0.000, Pr-value:0.000  
No matches to TargetScan

-9572--(165)--9738-

AAAGATC

AAAGATC  
Depth:6 (MOUSE)  
Ei-value:0.000, Pi-value:0.000  
Er-value:0.000, Pr-value:0.000  
No matches to TargetScan

-9744--(46)--9791-

TTCCCTTTGA

TTCCCTTTGA  
Depth:6 (MOUSE)  
Ei-value:0.000, Pi-value:0.000  
Er-value:0.000, Pr-value:0.000  
No matches to TargetScan

-9800--(7)--9808-

TAGGTGGAGATGGGGCATGAGGATCCTCCAGGGGAA

TAGGTGGAGATGGGGCATGAGGATCCTCCAGGGGAA  
Depth:6 (MOUSE)  
Ei-value:0.000, Pi-value:0.000  
Er-value:0.000, Pr-value:0.000  
MATCHES To TargetScan▶ miR-331-3p:CCCCUGG

-9843--(17)--9861-

GCAACA

GCAACA  
Depth:6 (MOUSE)  
Ei-value:0.000, Pi-value:0.000  
Er-value:0.000, Pr-value:0.000  
No matches to TargetScan

-9866--(242)--10109-

CCAAAT

CCAAAT  
Depth:6 (MOUSE)  
Ei-value:0.000, Pi-value:0.000  
Er-value:0.000, Pr-value:0.000  
No matches to TargetScan

-10114--(96)--10211-

GATCAACATGC

GATCAACATGC  
Depth:6 (MOUSE)  
Ei-value:0.000, Pi-value:0.000  
Er-value:0.000, Pr-value:0.000  
No matches to TargetScan

-10221--(33)--10255-

TGTGTAT

TGTGTAT  
Depth:6 (MOUSE)  
Ei-value:0.000, Pi-value:0.000  
Er-value:0.000, Pr-value:0.000  
No matches to TargetScan

-10261--(669)--10931-

TGTGTAT

TGTGTAT  
Depth:6 (MOUSE)  
Ei-value:0.000, Pi-value:0.000  
Er-value:0.000, Pr-value:0.000  
No matches to TargetScan

-10937--(345)--11283-

TTCTCTTTG

TTCTCTTTG  
Depth:6 (MOUSE)  
Ei-value:0.000, Pi-value:0.000  
Er-value:0.000, Pr-value:0.000  
No matches to TargetScan

-11291--(580)--11872-

TTTCTAC

TTTCTAC  
Depth:6 (MOUSE)  
Ei-value:0.000, Pi-value:0.000  
Er-value:0.000, Pr-value:0.000  
No matches to TargetScan

-11878--(391)--12270-

ATTTCTC

ATTTCTC  
Depth:6 (MOUSE)  
Ei-value:0.000, Pi-value:0.000  
Er-value:0.000, Pr-value:0.000  
No matches to TargetScan

-12276--(184)--12461-

TCTAGAGAAAA

TCTAGAGAAAA  
Depth:6 (MOUSE)  
Ei-value:0.000, Pi-value:0.000  
Er-value:0.000, Pr-value:0.000  
MATCHES To TargetScan▶ miR-1251-5p:CUCUAGC

-12471--(23)--12495-

TGAGAAGAATTAGACA

TGAGAAGAATTAGACA  
Depth:6 (MOUSE)  
Ei-value:0.000, Pi-value:0.000  
Er-value:0.000, Pr-value:0.000  
No matches to TargetScan

-12510--(318)--12829-

ATTGGCA

ATTGGCA  
Depth:6 (MOUSE)  
Ei-value:0.000, Pi-value:0.000  
Er-value:0.000, Pr-value:0.000  
No matches to TargetScan

-12835--(1699)--14535-

TTGTGAAG

TTGTGAAG  
Depth:6 (MOUSE)  
Ei-value:0.000, Pi-value:0.000  
Er-value:0.000, Pr-value:0.000  
No matches to TargetScan

-14542--(340)--14883-

AAAAGGT

AAAAGGT  
Depth:6 (MOUSE)  
Ei-value:0.000, Pi-value:0.000  
Er-value:0.000, Pr-value:0.000  
No matches to TargetScan

-14889
```

---

# Modules conserved to RABBIT (Depth: 5)

## Modules in Main Graph (All sequences considered):

```
>HUMAN  
      4489-

TGTTAGTC

TGTTAGTC  
Depth:5 (RABBIT)  
Ei-value:0.000, Pi-value:0.000  
Er-value:0.000, Pr-value:0.000  
eCLIP MATCHES▶AKAP8L (bg=2.19%)No matches to TargetScan

-4496--(171)--4668-

TTAATGTGCT

TTAATGTGCT  
Depth:5 (RABBIT)  
Ei-value:0.000, Pi-value:0.000  
Er-value:0.000, Pr-value:0.000  
No matches to eCLIP DataMATCHES To TargetScan▶ miR-323-3p:ACAUUAC

-4677--(356)--5034-

AATGTGCAT

AATGTGCAT  
Depth:6 (MOUSE)  
Ei-value:0.000, Pi-value:0.000  
Er-value:0.000, Pr-value:0.000  
No matches to eCLIP DataMATCHES To TargetScan▶ miR-501-3p/502-3p:AUGCACC

-5042--(563)--5606-

GTAAGGA

GTAAGGA  
Depth:5 (RABBIT)  
Ei-value:0.000, Pi-value:0.000  
Er-value:0.000, Pr-value:0.000  
No matches to eCLIP DataNo matches to TargetScan

-5612--(141)--5754-

ACTTAT

ACTTAT  
Depth:5 (RABBIT)  
Ei-value:0.000, Pi-value:0.000  
Er-value:0.000, Pr-value:0.000  
eCLIP MATCHES▶HNRNPU (bg=5.92%)No matches to TargetScan

-5759--(145)--5905-

TTAAGGCC

TTAAGGCC  
Depth:6 (MOUSE)  
Ei-value:0.000, Pi-value:0.000  
Er-value:0.000, Pr-value:0.000  
eCLIP MATCHES▶HNRNPL (bg=0.64%)No matches to TargetScan


CCTTT

TTAAGGCCCCTTT  
Depth:5 (RABBIT)  
Ei-value:0.000, Pi-value:0.000  
Er-value:0.000, Pr-value:0.000  
eCLIP MATCHES▶HNRNPL (bg=0.64%)No matches to TargetScan

-5917--(3440)--9358-

CTCAGCTCTTGG

CTCAGCTCTTGG  
Depth:5 (RABBIT)  
Ei-value:0.000, Pi-value:0.000  
Er-value:0.000, Pr-value:0.000  
No matches to eCLIP DataMATCHES To TargetScan▶ miR-335-5p:CAAGAGC

-9369--(236)--9606-

GCACAATG

GCACAATG  
Depth:6 (MOUSE)  
Ei-value:0.000, Pi-value:0.000  
Er-value:0.000, Pr-value:0.000  
No matches to eCLIP DataNo matches to TargetScan

-9613--(133)--9747-

CTCCCA

CTCCCA  
Depth:6 (MOUSE)  
Ei-value:0.000, Pi-value:0.000  
Er-value:0.000, Pr-value:0.000  
eCLIP MATCHES▶hnrnpk (bg=12.88%)No matches to TargetScan

-9752--(456)--10209-

AAAAGCAG

AAAAGCAG  
Depth:6 (MOUSE)  
Ei-value:0.000, Pi-value:0.000  
Er-value:0.000, Pr-value:0.000  
No matches to eCLIP DataNo matches to TargetScan

-10216--(123)--10340-

GCAAAAT

GCAAAAT  
Depth:6 (MOUSE)  
Ei-value:0.000, Pi-value:0.000  
Er-value:0.000, Pr-value:0.000  
No matches to eCLIP DataNo matches to TargetScan

-10346--(106)--10453-

TGCATTCTTC

TGCATTCTTC  
Depth:5 (RABBIT)  
Ei-value:0.000, Pi-value:0.000  
Er-value:0.000, Pr-value:0.000  
eCLIP MATCHES▶SF3B1 (bg=2.48%)No matches to TargetScan

-10462--(10)--10473-

A

AGATTGCCTGG  
Depth:5 (RABBIT)  
Ei-value:0.000, Pi-value:0.000  
Er-value:0.000, Pr-value:0.000  
No matches to eCLIP DataNo matches to TargetScan


GATTGCCTGG

GATTGCCTGG  
Depth:6 (MOUSE)  
Ei-value:0.000, Pi-value:0.000  
Er-value:0.000, Pr-value:0.000  
No matches to eCLIP DataNo matches to TargetScan

-10483--(139)--10623-

CTGGTCATT

CTGGTCATT  
Depth:5 (RABBIT)  
Ei-value:0.000, Pi-value:0.000  
Er-value:0.000, Pr-value:0.000  
No matches to eCLIP DataNo matches to TargetScan

-10631--(21)--10653-

CCATTTAT

CCATTTAT  
Depth:5 (RABBIT)  
Ei-value:0.000, Pi-value:0.000  
Er-value:0.000, Pr-value:0.000  
No matches to eCLIP DataNo matches to TargetScan

-10660--(17)--10678-

CAGTGTCTCTCATTT

CAGTGTCTCTCATTT  
Depth:5 (RABBIT)  
Ei-value:0.000, Pi-value:0.000  
Er-value:0.000, Pr-value:0.000  
eCLIP MATCHES▶SUPV3L1 (bg=1.57%)No matches to TargetScan

-10692--(9)--10702-

GTGGTG

GTGGTG  
Depth:5 (RABBIT)  
Ei-value:0.000, Pi-value:0.000  
Er-value:0.000, Pr-value:0.000  
eCLIP MATCHES▶SUPV3L1 (bg=1.57%)No matches to TargetScan

-10707--(1)--10709-

GTCTGTGGATA

GTCTGTGGATA  
Depth:5 (RABBIT)  
Ei-value:0.000, Pi-value:0.000  
Er-value:0.000, Pr-value:0.000  
eCLIP MATCHES▶SUPV3L1 (bg=1.57%)MATCHES To TargetScan▶ miR-140-3p.1:CCACAGG

-10719--(125)--10845-

AATTTCTTCATCTGGAGC

AATTTCTTCATCTGGAGC  
Depth:5 (RABBIT)  
Ei-value:0.000, Pi-value:0.000  
Er-value:0.000, Pr-value:0.000  
eCLIP MATCHES▶SUPV3L1 (bg=1.57%)▶U2AF2 (bg=1.76%)No matches to TargetScan

-10862--(209)--11072-

AAAAATAAGCCA

AAAAATAAGCCA  
Depth:5 (RABBIT)  
Ei-value:0.000, Pi-value:0.000  
Er-value:0.000, Pr-value:0.000  
No matches to eCLIP DataNo matches to TargetScan

-11083--(149)--11233-

CAGCAGTTC

CAGCAGTTC  
Depth:5 (RABBIT)  
Ei-value:0.000, Pi-value:0.000  
Er-value:0.000, Pr-value:0.000  
No matches to eCLIP DataNo matches to TargetScan

-11241--(9)--11251-

ACTGAAAA

ACTGAAAA  
Depth:5 (RABBIT)  
Ei-value:0.000, Pi-value:0.000  
Er-value:0.000, Pr-value:0.000  
No matches to eCLIP DataNo matches to TargetScan

-11258--(19)--11278-

AAGGATG

AAGGATG  
Depth:5 (RABBIT)  
Ei-value:0.000, Pi-value:0.000  
Er-value:0.000, Pr-value:0.000  
eCLIP MATCHES▶SRSF1 (bg=8.47%)▶U2AF2 (bg=1.76%)▶uchl5 (bg=11.16%)MATCHES To TargetScan▶ miR-362-5p/500b-5p:AUCCUUG

-11284--(3)--11288-

AAAGATC

AAAGATC  
Depth:6 (MOUSE)  
Ei-value:0.000, Pi-value:0.000  
Er-value:0.000, Pr-value:0.000  
eCLIP MATCHES▶SRSF1 (bg=8.47%)▶U2AF2 (bg=1.76%)▶uchl5 (bg=11.16%)No matches to TargetScan

-11294--(38)--11333-

GGACAGCTG

GGACAGCTG  
Depth:5 (RABBIT)  
Ei-value:0.000, Pi-value:0.000  
Er-value:0.000, Pr-value:0.000  
eCLIP MATCHES▶SRSF1 (bg=8.47%)▶SRSF7 (bg=2.32%)▶U2AF2 (bg=1.76%)▶ZNF622 (bg=6.58%)No matches to TargetScan

-11341--(3)--11345-

AGAAGAGTCTCTGGCTCTTTA

AGAAGAGTCTCTGGCTCTTTA  
Depth:5 (RABBIT)  
Ei-value:0.000, Pi-value:0.000  
Er-value:0.000, Pr-value:0.000  
eCLIP MATCHES▶DDX24 (bg=2.97%)▶SRSF1 (bg=8.47%)▶SRSF7 (bg=2.32%)▶U2AF2 (bg=1.76%)▶ZNF622 (bg=6.58%)No matches to TargetScan

-11365--(99)--11465-

CTGCAA

CTGCAA  
Depth:5 (RABBIT)  
Ei-value:0.000, Pi-value:0.000  
Er-value:0.000, Pr-value:0.000  
eCLIP MATCHES▶DDX24 (bg=2.97%)▶GRWD1 (bg=5.13%)▶MTPAP (bg=2.21%)▶NOLC1 (bg=9.43%)▶SRSF1 (bg=8.47%)▶UTP3 (bg=3.66%)▶ZNF622 (bg=6.58%)No matches to TargetScan

-11470--(114)--11585-

AAGAATAGGC

AAGAATAGGC  
Depth:5 (RABBIT)  
Ei-value:0.000, Pi-value:0.000  
Er-value:0.000, Pr-value:0.000  
eCLIP MATCHES▶NOLC1 (bg=9.43%)▶SRSF7 (bg=2.32%)▶uchl5 (bg=11.16%)No matches to TargetScan

-11594--(9)--11604-

TACAGTGTTAGTGA

TACAGTGTTAGTGA  
Depth:5 (RABBIT)  
Ei-value:0.000, Pi-value:0.000  
Er-value:0.000, Pr-value:0.000  
eCLIP MATCHES▶ILF3 (bg=3.0%)▶NOLC1 (bg=9.43%)▶RBM15 (bg=7.27%)▶SRSF7 (bg=2.32%)▶ZNF622 (bg=6.58%)MATCHES To TargetScan▶ miR-141-3p/200a-3p:AACACUG

-11617--(4)--11622-

TTCCCTTTGA

TTCCCTTTGA  
Depth:6 (MOUSE)  
Ei-value:0.000, Pi-value:0.000  
Er-value:0.000, Pr-value:0.000  
eCLIP MATCHES▶ILF3 (bg=3.0%)▶RBM15 (bg=7.27%)▶SRSF7 (bg=2.32%)▶ZNF622 (bg=6.58%)No matches to TargetScan

-11631--(7)--11639-

TAGGTGGAGATGGGGCATGAGGATCCTCCAGGGGAA

TAGGTGGAGATGGGGCATGAGGATCCTCCAGGGGAA  
Depth:6 (MOUSE)  
Ei-value:0.000, Pi-value:0.000  
Er-value:0.000, Pr-value:0.000  
eCLIP MATCHES▶ILF3 (bg=3.0%)▶NOLC1 (bg=9.43%)▶RBM15 (bg=7.27%)▶SRSF7 (bg=2.32%)▶ZNF622 (bg=6.58%)MATCHES To TargetScan▶ miR-331-3p:CCCCUGG


A

TAGGTGGAGATGGGGCATGAGGATCCTCCAGGGGAAA  
Depth:5 (RABBIT)  
Ei-value:0.000, Pi-value:0.000  
Er-value:0.000, Pr-value:0.000  
eCLIP MATCHES▶ILF3 (bg=3.0%)▶NOLC1 (bg=9.43%)▶RBM15 (bg=7.27%)▶SRSF7 (bg=2.32%)▶ZNF622 (bg=6.58%)MATCHES To TargetScan▶ miR-331-3p:CCCCUGG

-11675--(3)--11679-

TCACTA

TCACTA  
Depth:5 (RABBIT)  
Ei-value:0.000, Pi-value:0.000  
Er-value:0.000, Pr-value:0.000  
eCLIP MATCHES▶ILF3 (bg=3.0%)No matches to TargetScan

-11684--(7)--11692-

GCAACA

GCAACA  
Depth:6 (MOUSE)  
Ei-value:0.000, Pi-value:0.000  
Er-value:0.000, Pr-value:0.000  
eCLIP MATCHES▶ILF3 (bg=3.0%)No matches to TargetScan


AC

GCAACAAC  
Depth:5 (RABBIT)  
Ei-value:0.000, Pi-value:0.000  
Er-value:0.000, Pr-value:0.000  
eCLIP MATCHES▶ILF3 (bg=3.0%)No matches to TargetScan

-11699--(64)--11764-

ACAACCACC

ACAACCACC  
Depth:5 (RABBIT)  
Ei-value:0.000, Pi-value:0.000  
Er-value:0.000, Pr-value:0.000  
eCLIP MATCHES▶PRPF8 (bg=0.26%)No matches to TargetScan

-11772--(30)--11803-

CCAAAT

CCAAAT  
Depth:6 (MOUSE)  
Ei-value:0.000, Pi-value:0.000  
Er-value:0.000, Pr-value:0.000  
eCLIP MATCHES▶GRWD1 (bg=5.13%)▶NOLC1 (bg=9.43%)No matches to TargetScan


C

CCAAATC  
Depth:5 (RABBIT)  
Ei-value:0.000, Pi-value:0.000  
Er-value:0.000, Pr-value:0.000  
eCLIP MATCHES▶GRWD1 (bg=5.13%)▶NOLC1 (bg=9.43%)No matches to TargetScan

-11809--(47)--11857-

CAAGAAA

CAAGAAA  
Depth:5 (RABBIT)  
Ei-value:0.000, Pi-value:0.000  
Er-value:0.000, Pr-value:0.000  
eCLIP MATCHES▶GRWD1 (bg=5.13%)▶NOLC1 (bg=9.43%)▶uchl5 (bg=11.16%)▶ZNF622 (bg=6.58%)No matches to TargetScan

-11863--(36)--11900-

AA

AAGATCAACATGC  
Depth:5 (RABBIT)  
Ei-value:0.000, Pi-value:0.000  
Er-value:0.000, Pr-value:0.000  
eCLIP MATCHES▶GRWD1 (bg=5.13%)▶NOLC1 (bg=9.43%)▶PTBP1 (bg=3.74%)▶RBM15 (bg=7.27%)▶TRA2A (bg=4.8%)▶uchl5 (bg=11.16%)▶ZNF622 (bg=6.58%)No matches to TargetScan


GATCAACATGC

GATCAACATGC  
Depth:6 (MOUSE)  
Ei-value:0.000, Pi-value:0.000  
Er-value:0.000, Pr-value:0.000  
eCLIP MATCHES▶GRWD1 (bg=5.13%)▶NOLC1 (bg=9.43%)▶PTBP1 (bg=3.74%)▶RBM15 (bg=7.27%)▶TRA2A (bg=4.8%)▶uchl5 (bg=11.16%)▶ZNF622 (bg=6.58%)No matches to TargetScan

-11912--(34)--11947-

TGTGTAT

TGTGTAT  
Depth:6 (MOUSE)  
Ei-value:0.000, Pi-value:0.000  
Er-value:0.000, Pr-value:0.000  
eCLIP MATCHES▶TARDBP (bg=2.79%)▶ZC3H11A (bg=6.55%)No matches to TargetScan

-11953--(313)--12267-

TTCTCTTTG

TTCTCTTTG  
Depth:6 (MOUSE)  
Ei-value:0.000, Pi-value:0.000  
Er-value:0.000, Pr-value:0.000  
eCLIP MATCHES▶MATR3 (bg=2.98%)▶PTBP1 (bg=3.74%)▶SMNDC1 (bg=0.63%)▶TIA1 (bg=4.07%)No matches to TargetScan

-12275--(84)--12360-

TTTCTAC

TTTCTAC  
Depth:6 (MOUSE)  
Ei-value:0.000, Pi-value:0.000  
Er-value:0.000, Pr-value:0.000  
eCLIP MATCHES▶MATR3 (bg=2.98%)▶PTBP1 (bg=3.74%)▶TIA1 (bg=4.07%)No matches to TargetScan


T

TTTCTACT  
Depth:5 (RABBIT)  
Ei-value:0.000, Pi-value:0.000  
Er-value:0.000, Pr-value:0.000  
eCLIP MATCHES▶MATR3 (bg=2.98%)▶PTBP1 (bg=3.74%)▶TIA1 (bg=4.07%)MATCHES To TargetScan▶ miR-411-5p.1:AGUAGAC

-12367--(11)--12379-

ATTTCTC

ATTTCTC  
Depth:6 (MOUSE)  
Ei-value:0.000, Pi-value:0.000  
Er-value:0.000, Pr-value:0.000  
eCLIP MATCHES▶MATR3 (bg=2.98%)▶PTBP1 (bg=3.74%)▶TIA1 (bg=4.07%)No matches to TargetScan

-12385--(24)--12410-

TCTTGGG

TCTTGGG  
Depth:5 (RABBIT)  
Ei-value:0.000, Pi-value:0.000  
Er-value:0.000, Pr-value:0.000  
eCLIP MATCHES▶MATR3 (bg=2.98%)▶PTBP1 (bg=3.74%)▶SMNDC1 (bg=0.63%)▶TIA1 (bg=4.07%)No matches to TargetScan

-12416--(245)--12662-

TCCTAACCCCT

TCCTAACCCCT  
Depth:5 (RABBIT)  
Ei-value:0.000, Pi-value:0.000  
Er-value:0.000, Pr-value:0.000  
eCLIP MATCHES▶AATF (bg=0.64%)▶DDX24 (bg=2.97%)▶NCBP2 (bg=1.49%)▶NOLC1 (bg=9.43%)▶PTBP1 (bg=3.74%)▶SND1 (bg=0.45%)▶SRSF7 (bg=2.32%)▶TARDBP (bg=2.79%)▶UTP3 (bg=3.66%)▶WDR43 (bg=3.37%)▶XRCC6 (bg=2.91%)▶ZC3H8 (bg=0.29%)No matches to TargetScan

-12672--(53)--12726-

TTATGCCA

TTATGCCA  
Depth:5 (RABBIT)  
Ei-value:0.000, Pi-value:0.000  
Er-value:0.000, Pr-value:0.000  
eCLIP MATCHES▶DDX24 (bg=2.97%)▶FASTKD2 (bg=1.99%)▶LARP4 (bg=4.72%)▶NOLC1 (bg=9.43%)▶NPM1 (bg=1.21%)▶RBFOX2 (bg=4.63%)▶RBM15 (bg=7.27%)▶RPS3 (bg=0.76%)▶SRSF1 (bg=8.47%)▶SRSF7 (bg=2.32%)▶TARDBP (bg=2.79%)▶TRA2A (bg=4.8%)▶U2AF2 (bg=1.76%)▶uchl5 (bg=11.16%)▶WDR43 (bg=3.37%)▶YWHAG (bg=1.87%)▶ZC3H11A (bg=6.55%)▶ZNF622 (bg=6.58%)▶ZNF800 (bg=1.92%)No matches to TargetScan

-12733--(100)--12834-

AGGCCCAA

AGGCCCAA  
Depth:5 (RABBIT)  
Ei-value:0.000, Pi-value:0.000  
Er-value:0.000, Pr-value:0.000  
eCLIP MATCHES▶DDX24 (bg=2.97%)▶LARP4 (bg=4.72%)▶MTPAP (bg=2.21%)▶NOLC1 (bg=9.43%)▶SRSF1 (bg=8.47%)▶SRSF7 (bg=2.32%)▶TRA2A (bg=4.8%)▶uchl5 (bg=11.16%)▶UTP3 (bg=3.66%)▶ZNF622 (bg=6.58%)▶ZNF800 (bg=1.92%)No matches to TargetScan

-12841--(361)--13203-

GACTAA

GACTAA  
Depth:5 (RABBIT)  
Ei-value:0.000, Pi-value:0.000  
Er-value:0.000, Pr-value:0.000  
eCLIP MATCHES▶CPEB4 (bg=1.89%)▶FASTKD2 (bg=1.99%)▶GRWD1 (bg=5.13%)▶LARP4 (bg=4.72%)▶MTPAP (bg=2.21%)▶NOLC1 (bg=9.43%)▶RBFOX2 (bg=4.63%)▶SRSF1 (bg=8.47%)▶TRA2A (bg=4.8%)▶uchl5 (bg=11.16%)▶UTP18 (bg=0.72%)▶UTP3 (bg=3.66%)▶WDR43 (bg=3.37%)▶ZNF622 (bg=6.58%)No matches to TargetScan

-13208--(70)--13279-

AAGATGA

AAGATGA  
Depth:5 (RABBIT)  
Ei-value:0.000, Pi-value:0.000  
Er-value:0.000, Pr-value:0.000  
eCLIP MATCHES▶CPEB4 (bg=1.89%)▶FTO (bg=0.32%)▶GRWD1 (bg=5.13%)▶LARP4 (bg=4.72%)▶MTPAP (bg=2.21%)▶SRSF1 (bg=8.47%)▶TRA2A (bg=4.8%)▶uchl5 (bg=11.16%)▶ZNF622 (bg=6.58%)No matches to TargetScan

-13285--(412)--13698-

TCTAGAGAAAA

TCTAGAGAAAA  
Depth:6 (MOUSE)  
Ei-value:0.000, Pi-value:0.000  
Er-value:0.000, Pr-value:0.000  
eCLIP MATCHES▶CPSF6 (bg=0.4%)▶LARP4 (bg=4.72%)▶UTP3 (bg=3.66%)▶WDR43 (bg=3.37%)MATCHES To TargetScan▶ miR-1251-5p:CUCUAGC

-13708--(3)--13712-

TGAAGAGATG

TGAAGAGATG  
Depth:5 (RABBIT)  
Ei-value:0.000, Pi-value:0.000  
Er-value:0.000, Pr-value:0.000  
eCLIP MATCHES▶CPSF6 (bg=0.4%)▶LARP4 (bg=4.72%)▶SRSF7 (bg=2.32%)▶UTP3 (bg=3.66%)▶WDR43 (bg=3.37%)No matches to TargetScan

-13721--(11)--13733-

TGAGAAGAATTAGACA

TGAGAAGAATTAGACA  
Depth:6 (MOUSE)  
Ei-value:0.000, Pi-value:0.000  
Er-value:0.000, Pr-value:0.000  
eCLIP MATCHES▶LARP4 (bg=4.72%)▶NOLC1 (bg=9.43%)▶SRSF7 (bg=2.32%)No matches to TargetScan

-13748--(103)--13852-

TGGTTA

TGGTTA  
Depth:5 (RABBIT)  
Ei-value:0.000, Pi-value:0.000  
Er-value:0.000, Pr-value:0.000  
eCLIP MATCHES▶AKAP8L (bg=2.19%)▶NOLC1 (bg=9.43%)▶PUS1 (bg=1.04%)▶SF3B1 (bg=2.48%)No matches to TargetScan

-13857--(129)--13987-

AGGCTTA

AGGCTTA  
Depth:5 (RABBIT)  
Ei-value:0.000, Pi-value:0.000  
Er-value:0.000, Pr-value:0.000  
No matches to eCLIP DataNo matches to TargetScan

-13993--(30)--14024-

GTTTAAT

GTTTAAT  
Depth:5 (RABBIT)  
Ei-value:0.000, Pi-value:0.000  
Er-value:0.000, Pr-value:0.000  
No matches to eCLIP DataNo matches to TargetScan

-14030--(103)--14134-

T

TATTGGCA  
Depth:5 (RABBIT)  
Ei-value:0.000, Pi-value:0.000  
Er-value:0.000, Pr-value:0.000  
eCLIP MATCHES▶HNRNPA1 (bg=2.57%)No matches to TargetScan


ATTGGCA

ATTGGCA  
Depth:6 (MOUSE)  
Ei-value:0.000, Pi-value:0.000  
Er-value:0.000, Pr-value:0.000  
eCLIP MATCHES▶HNRNPA1 (bg=2.57%)No matches to TargetScan

-14141--(34)--14176-

TTGTGAAG

TTGTGAAG  
Depth:6 (MOUSE)  
Ei-value:0.000, Pi-value:0.000  
Er-value:0.000, Pr-value:0.000  
eCLIP MATCHES▶HNRNPA1 (bg=2.57%)No matches to TargetScan

-14183--(4)--14188-

ATGTAAAT

ATGTAAAT  
Depth:5 (RABBIT)  
Ei-value:0.000, Pi-value:0.000  
Er-value:0.000, Pr-value:0.000  
No matches to eCLIP DataNo matches to TargetScan

-14195--(3436)--17632-

ATAAGC

ATAAGC  
Depth:5 (RABBIT)  
Ei-value:0.000, Pi-value:0.010  
Er-value:0.000, Pr-value:0.000  
No matches to eCLIP DataNo matches to TargetScan

-17637--(627)--18265-

AAAAGGT

AAAAGGT  
Depth:6 (MOUSE)  
Ei-value:0.000, Pi-value:0.000  
Er-value:0.000, Pr-value:0.000  
eCLIP MATCHES▶ILF3 (bg=3.0%)▶SF3B1 (bg=2.48%)▶ZC3H11A (bg=6.55%)No matches to TargetScan

-18271  
  
>PIG  
      7682-

TGTTAGTC

TGTTAGTC  
Depth:5 (RABBIT)  
Ei-value:0.000, Pi-value:0.000  
Er-value:0.000, Pr-value:0.000  
No matches to TargetScan

-7689--(2060)--9750-

TTAATGTGCT

TTAATGTGCT  
Depth:5 (RABBIT)  
Ei-value:0.000, Pi-value:0.000  
Er-value:0.000, Pr-value:0.000  
MATCHES To TargetScan▶ miR-323-3p:ACAUUAC

-9759--(3753)--13513-

AATGTGCAT

AATGTGCAT  
Depth:6 (MOUSE)  
Ei-value:0.000, Pi-value:0.000  
Er-value:0.000, Pr-value:0.000  
MATCHES To TargetScan▶ miR-501-3p/502-3p:AUGCACC

-13521--(327)--13849-

GTAAGGA

GTAAGGA  
Depth:5 (RABBIT)  
Ei-value:0.000, Pi-value:0.000  
Er-value:0.000, Pr-value:0.000  
No matches to TargetScan

-13855--(141)--13997-

ACTTAT

ACTTAT  
Depth:5 (RABBIT)  
Ei-value:0.000, Pi-value:0.000  
Er-value:0.000, Pr-value:0.000  
No matches to TargetScan

-14002--(143)--14146-

TTAAGGCC

TTAAGGCC  
Depth:6 (MOUSE)  
Ei-value:0.000, Pi-value:0.000  
Er-value:0.000, Pr-value:0.000  
No matches to TargetScan


CCTTT

TTAAGGCCCCTTT  
Depth:5 (RABBIT)  
Ei-value:0.000, Pi-value:0.000  
Er-value:0.000, Pr-value:0.000  
No matches to TargetScan

-14158--(939)--15098-

CTCAGCTCTTGG

CTCAGCTCTTGG  
Depth:5 (RABBIT)  
Ei-value:0.000, Pi-value:0.000  
Er-value:0.000, Pr-value:0.000  
MATCHES To TargetScan▶ miR-335-5p:CAAGAGC

-15109--(236)--15346-

GCACAATG

GCACAATG  
Depth:6 (MOUSE)  
Ei-value:0.000, Pi-value:0.000  
Er-value:0.000, Pr-value:0.000  
No matches to TargetScan

-15353--(132)--15486-

CTCCCA

CTCCCA  
Depth:6 (MOUSE)  
Ei-value:0.000, Pi-value:0.000  
Er-value:0.000, Pr-value:0.000  
No matches to TargetScan

-15491--(496)--15988-

AAAAGCAG

AAAAGCAG  
Depth:6 (MOUSE)  
Ei-value:0.000, Pi-value:0.000  
Er-value:0.000, Pr-value:0.000  
No matches to TargetScan

-15995--(121)--16117-

GCAAAAT

GCAAAAT  
Depth:6 (MOUSE)  
Ei-value:0.000, Pi-value:0.000  
Er-value:0.000, Pr-value:0.000  
No matches to TargetScan

-16123--(119)--16243-

TGCATTCTTC

TGCATTCTTC  
Depth:5 (RABBIT)  
Ei-value:0.000, Pi-value:0.000  
Er-value:0.000, Pr-value:0.000  
No matches to TargetScan

-16252--(10)--16263-

A

AGATTGCCTGG  
Depth:5 (RABBIT)  
Ei-value:0.000, Pi-value:0.000  
Er-value:0.000, Pr-value:0.000  
No matches to TargetScan


GATTGCCTGG

GATTGCCTGG  
Depth:6 (MOUSE)  
Ei-value:0.000, Pi-value:0.000  
Er-value:0.000, Pr-value:0.000  
No matches to TargetScan

-16273--(128)--16402-

CTGGTCATT

CTGGTCATT  
Depth:5 (RABBIT)  
Ei-value:0.000, Pi-value:0.000  
Er-value:0.000, Pr-value:0.000  
No matches to TargetScan

-16410--(22)--16433-

CCATTTAT

CCATTTAT  
Depth:5 (RABBIT)  
Ei-value:0.000, Pi-value:0.000  
Er-value:0.000, Pr-value:0.000  
No matches to TargetScan

-16440--(17)--16458-

CAGTGTCTCTCATTT

CAGTGTCTCTCATTT  
Depth:5 (RABBIT)  
Ei-value:0.000, Pi-value:0.000  
Er-value:0.000, Pr-value:0.000  
No matches to TargetScan

-16472--(7)--16480-

GTGGTG

GTGGTG  
Depth:5 (RABBIT)  
Ei-value:0.000, Pi-value:0.000  
Er-value:0.000, Pr-value:0.000  
No matches to TargetScan

-16485--(1)--16487-

GTCTGTGGATA

GTCTGTGGATA  
Depth:5 (RABBIT)  
Ei-value:0.000, Pi-value:0.000  
Er-value:0.000, Pr-value:0.000  
MATCHES To TargetScan▶ miR-140-3p.1:CCACAGG

-16497--(162)--16660-

AATTTCTTCATCTGGAGC

AATTTCTTCATCTGGAGC  
Depth:5 (RABBIT)  
Ei-value:0.000, Pi-value:0.000  
Er-value:0.000, Pr-value:0.000  
No matches to TargetScan

-16677--(208)--16886-

AAAAATAAGCCA

AAAAATAAGCCA  
Depth:5 (RABBIT)  
Ei-value:0.000, Pi-value:0.000  
Er-value:0.000, Pr-value:0.000  
No matches to TargetScan

-16897--(150)--17048-

CAGCAGTTC

CAGCAGTTC  
Depth:5 (RABBIT)  
Ei-value:0.000, Pi-value:0.000  
Er-value:0.000, Pr-value:0.000  
No matches to TargetScan

-17056--(9)--17066-

ACTGAAAA

ACTGAAAA  
Depth:5 (RABBIT)  
Ei-value:0.000, Pi-value:0.000  
Er-value:0.000, Pr-value:0.000  
No matches to TargetScan

-17073--(17)--17091-

AAGGATG

AAGGATG  
Depth:5 (RABBIT)  
Ei-value:0.000, Pi-value:0.000  
Er-value:0.000, Pr-value:0.000  
MATCHES To TargetScan▶ miR-362-5p/500b-5p:AUCCUUG

-17097--(3)--17101-

AAAGATC

AAAGATC  
Depth:6 (MOUSE)  
Ei-value:0.000, Pi-value:0.000  
Er-value:0.000, Pr-value:0.000  
No matches to TargetScan

-17107--(38)--17146-

GGACAGCTG

GGACAGCTG  
Depth:5 (RABBIT)  
Ei-value:0.000, Pi-value:0.000  
Er-value:0.000, Pr-value:0.000  
No matches to TargetScan

-17154--(2)--17157-

AGAAGAGTCTCTGGCTCTTTA

AGAAGAGTCTCTGGCTCTTTA  
Depth:5 (RABBIT)  
Ei-value:0.000, Pi-value:0.000  
Er-value:0.000, Pr-value:0.000  
No matches to TargetScan

-17177--(123)--17301-

CTGCAA

CTGCAA  
Depth:5 (RABBIT)  
Ei-value:0.000, Pi-value:0.000  
Er-value:0.000, Pr-value:0.000  
No matches to TargetScan

-17306--(114)--17421-

AAGAATAGGC

AAGAATAGGC  
Depth:5 (RABBIT)  
Ei-value:0.000, Pi-value:0.000  
Er-value:0.000, Pr-value:0.000  
No matches to TargetScan

-17430--(9)--17440-

TACAGTGTTAGTGA

TACAGTGTTAGTGA  
Depth:5 (RABBIT)  
Ei-value:0.000, Pi-value:0.000  
Er-value:0.000, Pr-value:0.000  
MATCHES To TargetScan▶ miR-141-3p/200a-3p:AACACUG

-17453--(4)--17458-

TTCCCTTTGA

TTCCCTTTGA  
Depth:6 (MOUSE)  
Ei-value:0.000, Pi-value:0.000  
Er-value:0.000, Pr-value:0.000  
No matches to TargetScan

-17467--(7)--17475-

TAGGTGGAGATGGGGCATGAGGATCCTCCAGGGGAA

TAGGTGGAGATGGGGCATGAGGATCCTCCAGGGGAA  
Depth:6 (MOUSE)  
Ei-value:0.000, Pi-value:0.000  
Er-value:0.000, Pr-value:0.000  
MATCHES To TargetScan▶ miR-331-3p:CCCCUGG


A

TAGGTGGAGATGGGGCATGAGGATCCTCCAGGGGAAA  
Depth:5 (RABBIT)  
Ei-value:0.000, Pi-value:0.000  
Er-value:0.000, Pr-value:0.000  
MATCHES To TargetScan▶ miR-331-3p:CCCCUGG

-17511--(3)--17515-

TCACTA

TCACTA  
Depth:5 (RABBIT)  
Ei-value:0.000, Pi-value:0.000  
Er-value:0.000, Pr-value:0.000  
No matches to TargetScan

-17520--(7)--17528-

GCAACA

GCAACA  
Depth:6 (MOUSE)  
Ei-value:0.000, Pi-value:0.000  
Er-value:0.000, Pr-value:0.000  
No matches to TargetScan


AC

GCAACAAC  
Depth:5 (RABBIT)  
Ei-value:0.000, Pi-value:0.000  
Er-value:0.000, Pr-value:0.000  
No matches to TargetScan

-17535--(63)--17599-

ACAACCACC

ACAACCACC  
Depth:5 (RABBIT)  
Ei-value:0.000, Pi-value:0.000  
Er-value:0.000, Pr-value:0.000  
No matches to TargetScan

-17607--(365)--17973-

CCAAAT

CCAAAT  
Depth:6 (MOUSE)  
Ei-value:0.000, Pi-value:0.000  
Er-value:0.000, Pr-value:0.000  
No matches to TargetScan


C

CCAAATC  
Depth:5 (RABBIT)  
Ei-value:0.000, Pi-value:0.000  
Er-value:0.000, Pr-value:0.000  
No matches to TargetScan

-17979--(29)--18009-

CAAGAAA

CAAGAAA  
Depth:5 (RABBIT)  
Ei-value:0.000, Pi-value:0.000  
Er-value:0.000, Pr-value:0.000  
No matches to TargetScan

-18015--(17)--18033-

AA

AAGATCAACATGC  
Depth:5 (RABBIT)  
Ei-value:0.000, Pi-value:0.000  
Er-value:0.000, Pr-value:0.000  
No matches to TargetScan


GATCAACATGC

GATCAACATGC  
Depth:6 (MOUSE)  
Ei-value:0.000, Pi-value:0.000  
Er-value:0.000, Pr-value:0.000  
No matches to TargetScan

-18045--(66)--18112-

TGTGTAT

TGTGTAT  
Depth:6 (MOUSE)  
Ei-value:0.000, Pi-value:0.000  
Er-value:0.000, Pr-value:0.000  
No matches to TargetScan

-18118--(305)--18424-

TTCTCTTTG

TTCTCTTTG  
Depth:6 (MOUSE)  
Ei-value:0.000, Pi-value:0.000  
Er-value:0.000, Pr-value:0.000  
No matches to TargetScan

-18432--(82)--18515-

TTTCTAC

TTTCTAC  
Depth:6 (MOUSE)  
Ei-value:0.000, Pi-value:0.000  
Er-value:0.000, Pr-value:0.000  
No matches to TargetScan


T

TTTCTACT  
Depth:5 (RABBIT)  
Ei-value:0.000, Pi-value:0.000  
Er-value:0.000, Pr-value:0.000  
MATCHES To TargetScan▶ miR-411-5p.1:AGUAGAC

-18522--(11)--18534-

ATTTCTC

ATTTCTC  
Depth:6 (MOUSE)  
Ei-value:0.000, Pi-value:0.000  
Er-value:0.000, Pr-value:0.000  
No matches to TargetScan

-18540--(23)--18564-

TCTTGGG

TCTTGGG  
Depth:5 (RABBIT)  
Ei-value:0.000, Pi-value:0.000  
Er-value:0.000, Pr-value:0.000  
No matches to TargetScan

-18570--(239)--18810-

TCCTAACCCCT

TCCTAACCCCT  
Depth:5 (RABBIT)  
Ei-value:0.000, Pi-value:0.000  
Er-value:0.000, Pr-value:0.000  
No matches to TargetScan

-18820--(55)--18876-

TTATGCCA

TTATGCCA  
Depth:5 (RABBIT)  
Ei-value:0.000, Pi-value:0.000  
Er-value:0.000, Pr-value:0.000  
No matches to TargetScan

-18883--(56)--18940-

AGGCCCAA

AGGCCCAA  
Depth:5 (RABBIT)  
Ei-value:0.000, Pi-value:0.000  
Er-value:0.000, Pr-value:0.000  
No matches to TargetScan

-18947--(114)--19062-

GACTAA

GACTAA  
Depth:5 (RABBIT)  
Ei-value:0.000, Pi-value:0.000  
Er-value:0.000, Pr-value:0.000  
No matches to TargetScan

-19067--(73)--19141-

AAGATGA

AAGATGA  
Depth:5 (RABBIT)  
Ei-value:0.000, Pi-value:0.000  
Er-value:0.000, Pr-value:0.000  
No matches to TargetScan

-19147--(344)--19492-

TCTAGAGAAAA

TCTAGAGAAAA  
Depth:6 (MOUSE)  
Ei-value:0.000, Pi-value:0.000  
Er-value:0.000, Pr-value:0.000  
MATCHES To TargetScan▶ miR-1251-5p:CUCUAGC

-19502--(2)--19505-

TGAAGAGATG

TGAAGAGATG  
Depth:5 (RABBIT)  
Ei-value:0.000, Pi-value:0.000  
Er-value:0.000, Pr-value:0.000  
No matches to TargetScan

-19514--(11)--19526-

TGAGAAGAATTAGACA

TGAGAAGAATTAGACA  
Depth:6 (MOUSE)  
Ei-value:0.000, Pi-value:0.000  
Er-value:0.000, Pr-value:0.000  
No matches to TargetScan

-19541--(102)--19644-

TGGTTA

TGGTTA  
Depth:5 (RABBIT)  
Ei-value:0.000, Pi-value:0.000  
Er-value:0.000, Pr-value:0.000  
No matches to TargetScan

-19649--(133)--19783-

AGGCTTA

AGGCTTA  
Depth:5 (RABBIT)  
Ei-value:0.000, Pi-value:0.000  
Er-value:0.000, Pr-value:0.000  
No matches to TargetScan

-19789--(30)--19820-

GTTTAAT

GTTTAAT  
Depth:5 (RABBIT)  
Ei-value:0.000, Pi-value:0.000  
Er-value:0.000, Pr-value:0.000  
No matches to TargetScan

-19826--(107)--19934-

T

TATTGGCA  
Depth:5 (RABBIT)  
Ei-value:0.000, Pi-value:0.000  
Er-value:0.000, Pr-value:0.000  
No matches to TargetScan


ATTGGCA

ATTGGCA  
Depth:6 (MOUSE)  
Ei-value:0.000, Pi-value:0.000  
Er-value:0.000, Pr-value:0.000  
No matches to TargetScan

-19941--(36)--19978-

TTGTGAAG

TTGTGAAG  
Depth:6 (MOUSE)  
Ei-value:0.000, Pi-value:0.000  
Er-value:0.000, Pr-value:0.000  
No matches to TargetScan

-19985--(4)--19990-

ATGTAAAT

ATGTAAAT  
Depth:5 (RABBIT)  
Ei-value:0.000, Pi-value:0.000  
Er-value:0.000, Pr-value:0.000  
No matches to TargetScan

-19997--(3513)--23511-

ATAAGC

ATAAGC  
Depth:5 (RABBIT)  
Ei-value:0.000, Pi-value:0.010  
Er-value:0.000, Pr-value:0.000  
No matches to TargetScan

-23516--(574)--24091-

AAAAGGT

AAAAGGT  
Depth:6 (MOUSE)  
Ei-value:0.000, Pi-value:0.000  
Er-value:0.000, Pr-value:0.000  
No matches to TargetScan

-24097  
  
>COW  
       248-

TGTTAGTC

TGTTAGTC  
Depth:5 (RABBIT)  
Ei-value:0.000, Pi-value:0.000  
Er-value:0.000, Pr-value:0.000  
No matches to TargetScan

-255--(1900)--2156-

TTAATGTGCT

TTAATGTGCT  
Depth:5 (RABBIT)  
Ei-value:0.000, Pi-value:0.000  
Er-value:0.000, Pr-value:0.000  
MATCHES To TargetScan▶ miR-323-3p:ACAUUAC

-2165--(925)--3091-

AATGTGCAT

AATGTGCAT  
Depth:6 (MOUSE)  
Ei-value:0.000, Pi-value:0.000  
Er-value:0.000, Pr-value:0.000  
MATCHES To TargetScan▶ miR-501-3p/502-3p:AUGCACC

-3099--(328)--3428-

GTAAGGA

GTAAGGA  
Depth:5 (RABBIT)  
Ei-value:0.000, Pi-value:0.000  
Er-value:0.000, Pr-value:0.000  
No matches to TargetScan

-3434--(144)--3579-

ACTTAT

ACTTAT  
Depth:5 (RABBIT)  
Ei-value:0.000, Pi-value:0.000  
Er-value:0.000, Pr-value:0.000  
No matches to TargetScan

-3584--(145)--3730-

TTAAGGCC

TTAAGGCC  
Depth:6 (MOUSE)  
Ei-value:0.000, Pi-value:0.000  
Er-value:0.000, Pr-value:0.000  
No matches to TargetScan


CCTTT

TTAAGGCCCCTTT  
Depth:5 (RABBIT)  
Ei-value:0.000, Pi-value:0.000  
Er-value:0.000, Pr-value:0.000  
No matches to TargetScan

-3742--(449)--4192-

CTCAGCTCTTGG

CTCAGCTCTTGG  
Depth:5 (RABBIT)  
Ei-value:0.000, Pi-value:0.000  
Er-value:0.000, Pr-value:0.000  
MATCHES To TargetScan▶ miR-335-5p:CAAGAGC

-4203--(233)--4437-

GCACAATG

GCACAATG  
Depth:6 (MOUSE)  
Ei-value:0.000, Pi-value:0.000  
Er-value:0.000, Pr-value:0.000  
No matches to TargetScan

-4444--(45)--4490-

CTCCCA

CTCCCA  
Depth:6 (MOUSE)  
Ei-value:0.000, Pi-value:0.000  
Er-value:0.000, Pr-value:0.000  
No matches to TargetScan

-4495--(570)--5066-

AAAAGCAG

AAAAGCAG  
Depth:6 (MOUSE)  
Ei-value:0.000, Pi-value:0.000  
Er-value:0.000, Pr-value:0.000  
No matches to TargetScan

-5073--(121)--5195-

GCAAAAT

GCAAAAT  
Depth:6 (MOUSE)  
Ei-value:0.000, Pi-value:0.000  
Er-value:0.000, Pr-value:0.000  
No matches to TargetScan

-5201--(113)--5315-

TGCATTCTTC

TGCATTCTTC  
Depth:5 (RABBIT)  
Ei-value:0.000, Pi-value:0.000  
Er-value:0.000, Pr-value:0.000  
No matches to TargetScan

-5324--(10)--5335-

A

AGATTGCCTGG  
Depth:5 (RABBIT)  
Ei-value:0.000, Pi-value:0.000  
Er-value:0.000, Pr-value:0.000  
No matches to TargetScan


GATTGCCTGG

GATTGCCTGG  
Depth:6 (MOUSE)  
Ei-value:0.000, Pi-value:0.000  
Er-value:0.000, Pr-value:0.000  
No matches to TargetScan

-5345--(135)--5481-

CTGGTCATT

CTGGTCATT  
Depth:5 (RABBIT)  
Ei-value:0.000, Pi-value:0.000  
Er-value:0.000, Pr-value:0.000  
No matches to TargetScan

-5489--(21)--5511-

CCATTTAT

CCATTTAT  
Depth:5 (RABBIT)  
Ei-value:0.000, Pi-value:0.000  
Er-value:0.000, Pr-value:0.000  
No matches to TargetScan

-5518--(17)--5536-

CAGTGTCTCTCATTT

CAGTGTCTCTCATTT  
Depth:5 (RABBIT)  
Ei-value:0.000, Pi-value:0.000  
Er-value:0.000, Pr-value:0.000  
No matches to TargetScan

-5550--(8)--5559-

GTGGTG

GTGGTG  
Depth:5 (RABBIT)  
Ei-value:0.000, Pi-value:0.000  
Er-value:0.000, Pr-value:0.000  
No matches to TargetScan

-5564--(1)--5566-

GTCTGTGGATA

GTCTGTGGATA  
Depth:5 (RABBIT)  
Ei-value:0.000, Pi-value:0.000  
Er-value:0.000, Pr-value:0.000  
MATCHES To TargetScan▶ miR-140-3p.1:CCACAGG

-5576--(163)--5740-

AATTTCTTCATCTGGAGC

AATTTCTTCATCTGGAGC  
Depth:5 (RABBIT)  
Ei-value:0.000, Pi-value:0.000  
Er-value:0.000, Pr-value:0.000  
No matches to TargetScan

-5757--(218)--5976-

AAAAATAAGCCA

AAAAATAAGCCA  
Depth:5 (RABBIT)  
Ei-value:0.000, Pi-value:0.000  
Er-value:0.000, Pr-value:0.000  
No matches to TargetScan

-5987--(141)--6129-

CAGCAGTTC

CAGCAGTTC  
Depth:5 (RABBIT)  
Ei-value:0.000, Pi-value:0.000  
Er-value:0.000, Pr-value:0.000  
No matches to TargetScan

-6137--(9)--6147-

ACTGAAAA

ACTGAAAA  
Depth:5 (RABBIT)  
Ei-value:0.000, Pi-value:0.000  
Er-value:0.000, Pr-value:0.000  
No matches to TargetScan

-6154--(18)--6173-

AAGGATG

AAGGATG  
Depth:5 (RABBIT)  
Ei-value:0.000, Pi-value:0.000  
Er-value:0.000, Pr-value:0.000  
MATCHES To TargetScan▶ miR-362-5p/500b-5p:AUCCUUG

-6179--(3)--6183-

AAAGATC

AAAGATC  
Depth:6 (MOUSE)  
Ei-value:0.000, Pi-value:0.000  
Er-value:0.000, Pr-value:0.000  
No matches to TargetScan

-6189--(38)--6228-

GGACAGCTG

GGACAGCTG  
Depth:5 (RABBIT)  
Ei-value:0.000, Pi-value:0.000  
Er-value:0.000, Pr-value:0.000  
No matches to TargetScan

-6236--(2)--6239-

AGAAGAGTCTCTGGCTCTTTA

AGAAGAGTCTCTGGCTCTTTA  
Depth:5 (RABBIT)  
Ei-value:0.000, Pi-value:0.000  
Er-value:0.000, Pr-value:0.000  
No matches to TargetScan

-6259--(125)--6385-

CTGCAA

CTGCAA  
Depth:5 (RABBIT)  
Ei-value:0.000, Pi-value:0.000  
Er-value:0.000, Pr-value:0.000  
No matches to TargetScan

-6390--(114)--6505-

AAGAATAGGC

AAGAATAGGC  
Depth:5 (RABBIT)  
Ei-value:0.000, Pi-value:0.000  
Er-value:0.000, Pr-value:0.000  
No matches to TargetScan

-6514--(9)--6524-

TACAGTGTTAGTGA

TACAGTGTTAGTGA  
Depth:5 (RABBIT)  
Ei-value:0.000, Pi-value:0.000  
Er-value:0.000, Pr-value:0.000  
MATCHES To TargetScan▶ miR-141-3p/200a-3p:AACACUG

-6537--(4)--6542-

TTCCCTTTGA

TTCCCTTTGA  
Depth:6 (MOUSE)  
Ei-value:0.000, Pi-value:0.000  
Er-value:0.000, Pr-value:0.000  
No matches to TargetScan

-6551--(7)--6559-

TAGGTGGAGATGGGGCATGAGGATCCTCCAGGGGAA

TAGGTGGAGATGGGGCATGAGGATCCTCCAGGGGAA  
Depth:6 (MOUSE)  
Ei-value:0.000, Pi-value:0.000  
Er-value:0.000, Pr-value:0.000  
MATCHES To TargetScan▶ miR-331-3p:CCCCUGG


A

TAGGTGGAGATGGGGCATGAGGATCCTCCAGGGGAAA  
Depth:5 (RABBIT)  
Ei-value:0.000, Pi-value:0.000  
Er-value:0.000, Pr-value:0.000  
MATCHES To TargetScan▶ miR-331-3p:CCCCUGG

-6595--(3)--6599-

TCACTA

TCACTA  
Depth:5 (RABBIT)  
Ei-value:0.000, Pi-value:0.000  
Er-value:0.000, Pr-value:0.000  
No matches to TargetScan

-6604--(7)--6612-

GCAACA

GCAACA  
Depth:6 (MOUSE)  
Ei-value:0.000, Pi-value:0.000  
Er-value:0.000, Pr-value:0.000  
No matches to TargetScan


AC

GCAACAAC  
Depth:5 (RABBIT)  
Ei-value:0.000, Pi-value:0.000  
Er-value:0.000, Pr-value:0.000  
No matches to TargetScan

-6619--(63)--6683-

ACAACCACC

ACAACCACC  
Depth:5 (RABBIT)  
Ei-value:0.000, Pi-value:0.000  
Er-value:0.000, Pr-value:0.000  
No matches to TargetScan

-6691--(37)--6729-

CCAAAT

CCAAAT  
Depth:6 (MOUSE)  
Ei-value:0.000, Pi-value:0.000  
Er-value:0.000, Pr-value:0.000  
No matches to TargetScan


C

CCAAATC  
Depth:5 (RABBIT)  
Ei-value:0.000, Pi-value:0.000  
Er-value:0.000, Pr-value:0.000  
No matches to TargetScan

-6735--(29)--6765-

CAAGAAA

CAAGAAA  
Depth:5 (RABBIT)  
Ei-value:0.000, Pi-value:0.000  
Er-value:0.000, Pr-value:0.000  
No matches to TargetScan

-6771--(16)--6788-

AA

AAGATCAACATGC  
Depth:5 (RABBIT)  
Ei-value:0.000, Pi-value:0.000  
Er-value:0.000, Pr-value:0.000  
No matches to TargetScan


GATCAACATGC

GATCAACATGC  
Depth:6 (MOUSE)  
Ei-value:0.000, Pi-value:0.000  
Er-value:0.000, Pr-value:0.000  
No matches to TargetScan

-6800--(97)--6898-

TGTGTAT

TGTGTAT  
Depth:6 (MOUSE)  
Ei-value:0.000, Pi-value:0.000  
Er-value:0.000, Pr-value:0.000  
No matches to TargetScan

-6904--(296)--7201-

TTCTCTTTG

TTCTCTTTG  
Depth:6 (MOUSE)  
Ei-value:0.000, Pi-value:0.000  
Er-value:0.000, Pr-value:0.000  
No matches to TargetScan

-7209--(71)--7281-

TTTCTAC

TTTCTAC  
Depth:6 (MOUSE)  
Ei-value:0.000, Pi-value:0.000  
Er-value:0.000, Pr-value:0.000  
No matches to TargetScan


T

TTTCTACT  
Depth:5 (RABBIT)  
Ei-value:0.000, Pi-value:0.000  
Er-value:0.000, Pr-value:0.000  
MATCHES To TargetScan▶ miR-411-5p.1:AGUAGAC

-7288--(13)--7302-

ATTTCTC

ATTTCTC  
Depth:6 (MOUSE)  
Ei-value:0.000, Pi-value:0.000  
Er-value:0.000, Pr-value:0.000  
No matches to TargetScan

-7308--(24)--7333-

TCTTGGG

TCTTGGG  
Depth:5 (RABBIT)  
Ei-value:0.000, Pi-value:0.000  
Er-value:0.000, Pr-value:0.000  
No matches to TargetScan

-7339--(240)--7580-

TCCTAACCCCT

TCCTAACCCCT  
Depth:5 (RABBIT)  
Ei-value:0.000, Pi-value:0.000  
Er-value:0.000, Pr-value:0.000  
No matches to TargetScan

-7590--(52)--7643-

TTATGCCA

TTATGCCA  
Depth:5 (RABBIT)  
Ei-value:0.000, Pi-value:0.000  
Er-value:0.000, Pr-value:0.000  
No matches to TargetScan

-7650--(206)--7857-

AGGCCCAA

AGGCCCAA  
Depth:5 (RABBIT)  
Ei-value:0.000, Pi-value:0.000  
Er-value:0.000, Pr-value:0.000  
No matches to TargetScan

-7864--(18)--7883-

GACTAA

GACTAA  
Depth:5 (RABBIT)  
Ei-value:0.000, Pi-value:0.000  
Er-value:0.000, Pr-value:0.000  
No matches to TargetScan

-7888--(73)--7962-

AAGATGA

AAGATGA  
Depth:5 (RABBIT)  
Ei-value:0.000, Pi-value:0.000  
Er-value:0.000, Pr-value:0.000  
No matches to TargetScan

-7968--(370)--8339-

TCTAGAGAAAA

TCTAGAGAAAA  
Depth:6 (MOUSE)  
Ei-value:0.000, Pi-value:0.000  
Er-value:0.000, Pr-value:0.000  
MATCHES To TargetScan▶ miR-1251-5p:CUCUAGC

-8349--(2)--8352-

TGAAGAGATG

TGAAGAGATG  
Depth:5 (RABBIT)  
Ei-value:0.000, Pi-value:0.000  
Er-value:0.000, Pr-value:0.000  
No matches to TargetScan

-8361--(13)--8375-

TGAGAAGAATTAGACA

TGAGAAGAATTAGACA  
Depth:6 (MOUSE)  
Ei-value:0.000, Pi-value:0.000  
Er-value:0.000, Pr-value:0.000  
No matches to TargetScan

-8390--(104)--8495-

TGGTTA

TGGTTA  
Depth:5 (RABBIT)  
Ei-value:0.000, Pi-value:0.000  
Er-value:0.000, Pr-value:0.000  
No matches to TargetScan

-8500--(121)--8622-

AGGCTTA

AGGCTTA  
Depth:5 (RABBIT)  
Ei-value:0.000, Pi-value:0.000  
Er-value:0.000, Pr-value:0.000  
No matches to TargetScan

-8628--(30)--8659-

GTTTAAT

GTTTAAT  
Depth:5 (RABBIT)  
Ei-value:0.000, Pi-value:0.000  
Er-value:0.000, Pr-value:0.000  
No matches to TargetScan

-8665--(111)--8777-

T

TATTGGCA  
Depth:5 (RABBIT)  
Ei-value:0.000, Pi-value:0.000  
Er-value:0.000, Pr-value:0.000  
No matches to TargetScan


ATTGGCA

ATTGGCA  
Depth:6 (MOUSE)  
Ei-value:0.000, Pi-value:0.000  
Er-value:0.000, Pr-value:0.000  
No matches to TargetScan

-8784--(36)--8821-

TTGTGAAG

TTGTGAAG  
Depth:6 (MOUSE)  
Ei-value:0.000, Pi-value:0.000  
Er-value:0.000, Pr-value:0.000  
No matches to TargetScan

-8828--(4)--8833-

ATGTAAAT

ATGTAAAT  
Depth:5 (RABBIT)  
Ei-value:0.000, Pi-value:0.000  
Er-value:0.000, Pr-value:0.000  
No matches to TargetScan

-8840--(3708)--12549-

ATAAGC

ATAAGC  
Depth:5 (RABBIT)  
Ei-value:0.000, Pi-value:0.010  
Er-value:0.000, Pr-value:0.000  
No matches to TargetScan

-12554--(558)--13113-

AAAAGGT

AAAAGGT  
Depth:6 (MOUSE)  
Ei-value:0.000, Pi-value:0.000  
Er-value:0.000, Pr-value:0.000  
No matches to TargetScan

-13119  
  
>DOG  
      1695-

TGTTAGTC

TGTTAGTC  
Depth:5 (RABBIT)  
Ei-value:0.000, Pi-value:0.000  
Er-value:0.000, Pr-value:0.000  
No matches to TargetScan

-1702--(1265)--2968-

TTAATGTGCT

TTAATGTGCT  
Depth:5 (RABBIT)  
Ei-value:0.000, Pi-value:0.000  
Er-value:0.000, Pr-value:0.000  
MATCHES To TargetScan▶ miR-323-3p:ACAUUAC

-2977--(855)--3833-

AATGTGCAT

AATGTGCAT  
Depth:6 (MOUSE)  
Ei-value:0.000, Pi-value:0.000  
Er-value:0.000, Pr-value:0.000  
MATCHES To TargetScan▶ miR-501-3p/502-3p:AUGCACC

-3841--(148)--3990-

GTAAGGA

GTAAGGA  
Depth:5 (RABBIT)  
Ei-value:0.000, Pi-value:0.000  
Er-value:0.000, Pr-value:0.000  
No matches to TargetScan

-3996--(141)--4138-

ACTTAT

ACTTAT  
Depth:5 (RABBIT)  
Ei-value:0.000, Pi-value:0.000  
Er-value:0.000, Pr-value:0.000  
No matches to TargetScan

-4143--(144)--4288-

TTAAGGCC

TTAAGGCC  
Depth:6 (MOUSE)  
Ei-value:0.000, Pi-value:0.000  
Er-value:0.000, Pr-value:0.000  
No matches to TargetScan


CCTTT

TTAAGGCCCCTTT  
Depth:5 (RABBIT)  
Ei-value:0.000, Pi-value:0.000  
Er-value:0.000, Pr-value:0.000  
No matches to TargetScan

-4300--(1405)--5706-

CTCAGCTCTTGG

CTCAGCTCTTGG  
Depth:5 (RABBIT)  
Ei-value:0.000, Pi-value:0.000  
Er-value:0.000, Pr-value:0.000  
MATCHES To TargetScan▶ miR-335-5p:CAAGAGC

-5717--(207)--5925-

GCACAATG

GCACAATG  
Depth:6 (MOUSE)  
Ei-value:0.000, Pi-value:0.000  
Er-value:0.000, Pr-value:0.000  
No matches to TargetScan

-5932--(44)--5977-

CTCCCA

CTCCCA  
Depth:6 (MOUSE)  
Ei-value:0.000, Pi-value:0.000  
Er-value:0.000, Pr-value:0.000  
No matches to TargetScan

-5982--(549)--6532-

AAAAGCAG

AAAAGCAG  
Depth:6 (MOUSE)  
Ei-value:0.000, Pi-value:0.000  
Er-value:0.000, Pr-value:0.000  
No matches to TargetScan

-6539--(119)--6659-

GCAAAAT

GCAAAAT  
Depth:6 (MOUSE)  
Ei-value:0.000, Pi-value:0.000  
Er-value:0.000, Pr-value:0.000  
No matches to TargetScan

-6665--(112)--6778-

TGCATTCTTC

TGCATTCTTC  
Depth:5 (RABBIT)  
Ei-value:0.000, Pi-value:0.000  
Er-value:0.000, Pr-value:0.000  
No matches to TargetScan

-6787--(10)--6798-

A

AGATTGCCTGG  
Depth:5 (RABBIT)  
Ei-value:0.000, Pi-value:0.000  
Er-value:0.000, Pr-value:0.000  
No matches to TargetScan


GATTGCCTGG

GATTGCCTGG  
Depth:6 (MOUSE)  
Ei-value:0.000, Pi-value:0.000  
Er-value:0.000, Pr-value:0.000  
No matches to TargetScan

-6808--(156)--6965-

CTGGTCATT

CTGGTCATT  
Depth:5 (RABBIT)  
Ei-value:0.000, Pi-value:0.000  
Er-value:0.000, Pr-value:0.000  
No matches to TargetScan

-6973--(21)--6995-

CCATTTAT

CCATTTAT  
Depth:5 (RABBIT)  
Ei-value:0.000, Pi-value:0.000  
Er-value:0.000, Pr-value:0.000  
No matches to TargetScan

-7002--(17)--7020-

CAGTGTCTCTCATTT

CAGTGTCTCTCATTT  
Depth:5 (RABBIT)  
Ei-value:0.000, Pi-value:0.000  
Er-value:0.000, Pr-value:0.000  
No matches to TargetScan

-7034--(8)--7043-

GTGGTG

GTGGTG  
Depth:5 (RABBIT)  
Ei-value:0.000, Pi-value:0.000  
Er-value:0.000, Pr-value:0.000  
No matches to TargetScan

-7048--(1)--7050-

GTCTGTGGATA

GTCTGTGGATA  
Depth:5 (RABBIT)  
Ei-value:0.000, Pi-value:0.000  
Er-value:0.000, Pr-value:0.000  
MATCHES To TargetScan▶ miR-140-3p.1:CCACAGG

-7060--(140)--7201-

AATTTCTTCATCTGGAGC

AATTTCTTCATCTGGAGC  
Depth:5 (RABBIT)  
Ei-value:0.000, Pi-value:0.000  
Er-value:0.000, Pr-value:0.000  
No matches to TargetScan

-7218--(423)--7642-

AAAAATAAGCCA

AAAAATAAGCCA  
Depth:5 (RABBIT)  
Ei-value:0.000, Pi-value:0.000  
Er-value:0.000, Pr-value:0.000  
No matches to TargetScan

-7653--(144)--7798-

CAGCAGTTC

CAGCAGTTC  
Depth:5 (RABBIT)  
Ei-value:0.000, Pi-value:0.000  
Er-value:0.000, Pr-value:0.000  
No matches to TargetScan

-7806--(9)--7816-

ACTGAAAA

ACTGAAAA  
Depth:5 (RABBIT)  
Ei-value:0.000, Pi-value:0.000  
Er-value:0.000, Pr-value:0.000  
No matches to TargetScan

-7823--(19)--7843-

AAGGATG

AAGGATG  
Depth:5 (RABBIT)  
Ei-value:0.000, Pi-value:0.000  
Er-value:0.000, Pr-value:0.000  
MATCHES To TargetScan▶ miR-362-5p/500b-5p:AUCCUUG

-7849--(3)--7853-

AAAGATC

AAAGATC  
Depth:6 (MOUSE)  
Ei-value:0.000, Pi-value:0.000  
Er-value:0.000, Pr-value:0.000  
No matches to TargetScan

-7859--(39)--7899-

GGACAGCTG

GGACAGCTG  
Depth:5 (RABBIT)  
Ei-value:0.000, Pi-value:0.000  
Er-value:0.000, Pr-value:0.000  
No matches to TargetScan

-7907--(2)--7910-

AGAAGAGTCTCTGGCTCTTTA

AGAAGAGTCTCTGGCTCTTTA  
Depth:5 (RABBIT)  
Ei-value:0.000, Pi-value:0.000  
Er-value:0.000, Pr-value:0.000  
No matches to TargetScan

-7930--(78)--8009-

CTGCAA

CTGCAA  
Depth:5 (RABBIT)  
Ei-value:0.000, Pi-value:0.000  
Er-value:0.000, Pr-value:0.000  
No matches to TargetScan

-8014--(30)--8045-

AAGAATAGGC

AAGAATAGGC  
Depth:5 (RABBIT)  
Ei-value:0.000, Pi-value:0.000  
Er-value:0.000, Pr-value:0.000  
No matches to TargetScan

-8054--(9)--8064-

TACAGTGTTAGTGA

TACAGTGTTAGTGA  
Depth:5 (RABBIT)  
Ei-value:0.000, Pi-value:0.000  
Er-value:0.000, Pr-value:0.000  
MATCHES To TargetScan▶ miR-141-3p/200a-3p:AACACUG

-8077--(4)--8082-

TTCCCTTTGA

TTCCCTTTGA  
Depth:6 (MOUSE)  
Ei-value:0.000, Pi-value:0.000  
Er-value:0.000, Pr-value:0.000  
No matches to TargetScan

-8091--(7)--8099-

TAGGTGGAGATGGGGCATGAGGATCCTCCAGGGGAA

TAGGTGGAGATGGGGCATGAGGATCCTCCAGGGGAA  
Depth:6 (MOUSE)  
Ei-value:0.000, Pi-value:0.000  
Er-value:0.000, Pr-value:0.000  
MATCHES To TargetScan▶ miR-331-3p:CCCCUGG


A

TAGGTGGAGATGGGGCATGAGGATCCTCCAGGGGAAA  
Depth:5 (RABBIT)  
Ei-value:0.000, Pi-value:0.000  
Er-value:0.000, Pr-value:0.000  
MATCHES To TargetScan▶ miR-331-3p:CCCCUGG

-8135--(3)--8139-

TCACTA

TCACTA  
Depth:5 (RABBIT)  
Ei-value:0.000, Pi-value:0.000  
Er-value:0.000, Pr-value:0.000  
No matches to TargetScan

-8144--(7)--8152-

GCAACA

GCAACA  
Depth:6 (MOUSE)  
Ei-value:0.000, Pi-value:0.000  
Er-value:0.000, Pr-value:0.000  
No matches to TargetScan


AC

GCAACAAC  
Depth:5 (RABBIT)  
Ei-value:0.000, Pi-value:0.000  
Er-value:0.000, Pr-value:0.000  
No matches to TargetScan

-8159--(66)--8226-

ACAACCACC

ACAACCACC  
Depth:5 (RABBIT)  
Ei-value:0.000, Pi-value:0.000  
Er-value:0.000, Pr-value:0.000  
No matches to TargetScan

-8234--(300)--8535-

CCAAAT

CCAAAT  
Depth:6 (MOUSE)  
Ei-value:0.000, Pi-value:0.000  
Er-value:0.000, Pr-value:0.000  
No matches to TargetScan


C

CCAAATC  
Depth:5 (RABBIT)  
Ei-value:0.000, Pi-value:0.000  
Er-value:0.000, Pr-value:0.000  
No matches to TargetScan

-8541--(27)--8569-

CAAGAAA

CAAGAAA  
Depth:5 (RABBIT)  
Ei-value:0.000, Pi-value:0.000  
Er-value:0.000, Pr-value:0.000  
No matches to TargetScan

-8575--(17)--8593-

AA

AAGATCAACATGC  
Depth:5 (RABBIT)  
Ei-value:0.000, Pi-value:0.000  
Er-value:0.000, Pr-value:0.000  
No matches to TargetScan


GATCAACATGC

GATCAACATGC  
Depth:6 (MOUSE)  
Ei-value:0.000, Pi-value:0.000  
Er-value:0.000, Pr-value:0.000  
No matches to TargetScan

-8605--(34)--8640-

TGTGTAT

TGTGTAT  
Depth:6 (MOUSE)  
Ei-value:0.000, Pi-value:0.000  
Er-value:0.000, Pr-value:0.000  
No matches to TargetScan

-8646--(190)--8837-

TTCTCTTTG

TTCTCTTTG  
Depth:6 (MOUSE)  
Ei-value:0.000, Pi-value:0.000  
Er-value:0.000, Pr-value:0.000  
No matches to TargetScan

-8845--(195)--9041-

TTTCTAC

TTTCTAC  
Depth:6 (MOUSE)  
Ei-value:0.000, Pi-value:0.000  
Er-value:0.000, Pr-value:0.000  
No matches to TargetScan


T

TTTCTACT  
Depth:5 (RABBIT)  
Ei-value:0.000, Pi-value:0.000  
Er-value:0.000, Pr-value:0.000  
MATCHES To TargetScan▶ miR-411-5p.1:AGUAGAC

-9048--(11)--9060-

ATTTCTC

ATTTCTC  
Depth:6 (MOUSE)  
Ei-value:0.000, Pi-value:0.000  
Er-value:0.000, Pr-value:0.000  
No matches to TargetScan

-9066--(25)--9092-

TCTTGGG

TCTTGGG  
Depth:5 (RABBIT)  
Ei-value:0.000, Pi-value:0.000  
Er-value:0.000, Pr-value:0.000  
No matches to TargetScan

-9098--(237)--9336-

TCCTAACCCCT

TCCTAACCCCT  
Depth:5 (RABBIT)  
Ei-value:0.000, Pi-value:0.000  
Er-value:0.000, Pr-value:0.000  
No matches to TargetScan

-9346--(53)--9400-

TTATGCCA

TTATGCCA  
Depth:5 (RABBIT)  
Ei-value:0.000, Pi-value:0.000  
Er-value:0.000, Pr-value:0.000  
No matches to TargetScan

-9407--(65)--9473-

AGGCCCAA

AGGCCCAA  
Depth:5 (RABBIT)  
Ei-value:0.000, Pi-value:0.000  
Er-value:0.000, Pr-value:0.000  
No matches to TargetScan

-9480--(359)--9840-

GACTAA

GACTAA  
Depth:5 (RABBIT)  
Ei-value:0.000, Pi-value:0.000  
Er-value:0.000, Pr-value:0.000  
No matches to TargetScan

-9845--(72)--9918-

AAGATGA

AAGATGA  
Depth:5 (RABBIT)  
Ei-value:0.000, Pi-value:0.000  
Er-value:0.000, Pr-value:0.000  
No matches to TargetScan

-9924--(375)--10300-

TCTAGAGAAAA

TCTAGAGAAAA  
Depth:6 (MOUSE)  
Ei-value:0.000, Pi-value:0.000  
Er-value:0.000, Pr-value:0.000  
MATCHES To TargetScan▶ miR-1251-5p:CUCUAGC

-10310--(2)--10313-

TGAAGAGATG

TGAAGAGATG  
Depth:5 (RABBIT)  
Ei-value:0.000, Pi-value:0.000  
Er-value:0.000, Pr-value:0.000  
No matches to TargetScan

-10322--(11)--10334-

TGAGAAGAATTAGACA

TGAGAAGAATTAGACA  
Depth:6 (MOUSE)  
Ei-value:0.000, Pi-value:0.000  
Er-value:0.000, Pr-value:0.000  
No matches to TargetScan

-10349--(104)--10454-

TGGTTA

TGGTTA  
Depth:5 (RABBIT)  
Ei-value:0.000, Pi-value:0.000  
Er-value:0.000, Pr-value:0.000  
No matches to TargetScan

-10459--(122)--10582-

AGGCTTA

AGGCTTA  
Depth:5 (RABBIT)  
Ei-value:0.000, Pi-value:0.000  
Er-value:0.000, Pr-value:0.000  
No matches to TargetScan

-10588--(30)--10619-

GTTTAAT

GTTTAAT  
Depth:5 (RABBIT)  
Ei-value:0.000, Pi-value:0.000  
Er-value:0.000, Pr-value:0.000  
No matches to TargetScan

-10625--(115)--10741-

T

TATTGGCA  
Depth:5 (RABBIT)  
Ei-value:0.000, Pi-value:0.000  
Er-value:0.000, Pr-value:0.000  
No matches to TargetScan


ATTGGCA

ATTGGCA  
Depth:6 (MOUSE)  
Ei-value:0.000, Pi-value:0.000  
Er-value:0.000, Pr-value:0.000  
No matches to TargetScan

-10748--(35)--10784-

TTGTGAAG

TTGTGAAG  
Depth:6 (MOUSE)  
Ei-value:0.000, Pi-value:0.000  
Er-value:0.000, Pr-value:0.000  
No matches to TargetScan

-10791--(186)--10978-

ATGTAAAT

ATGTAAAT  
Depth:5 (RABBIT)  
Ei-value:0.000, Pi-value:0.000  
Er-value:0.000, Pr-value:0.000  
No matches to TargetScan

-10985--(3388)--14374-

ATAAGC

ATAAGC  
Depth:5 (RABBIT)  
Ei-value:0.000, Pi-value:0.010  
Er-value:0.000, Pr-value:0.000  
No matches to TargetScan

-14379--(655)--15035-

AAAAGGT

AAAAGGT  
Depth:6 (MOUSE)  
Ei-value:0.000, Pi-value:0.000  
Er-value:0.000, Pr-value:0.000  
No matches to TargetScan

-15041  
  
>RABBIT  
       511-

TGTTAGTC

TGTTAGTC  
Depth:5 (RABBIT)  
Ei-value:0.000, Pi-value:0.000  
Er-value:0.000, Pr-value:0.000  
No matches to TargetScan

-518--(54)--573-

TTAATGTGCT

TTAATGTGCT  
Depth:5 (RABBIT)  
Ei-value:0.000, Pi-value:0.000  
Er-value:0.000, Pr-value:0.000  
MATCHES To TargetScan▶ miR-323-3p:ACAUUAC

-582--(1065)--1648-

AATGTGCAT

AATGTGCAT  
Depth:6 (MOUSE)  
Ei-value:0.000, Pi-value:0.000  
Er-value:0.000, Pr-value:0.000  
MATCHES To TargetScan▶ miR-501-3p/502-3p:AUGCACC

-1656--(251)--1908-

GTAAGGA

GTAAGGA  
Depth:5 (RABBIT)  
Ei-value:0.000, Pi-value:0.000  
Er-value:0.000, Pr-value:0.000  
No matches to TargetScan

-1914--(117)--2032-

ACTTAT

ACTTAT  
Depth:5 (RABBIT)  
Ei-value:0.000, Pi-value:0.000  
Er-value:0.000, Pr-value:0.000  
No matches to TargetScan

-2037--(145)--2183-

TTAAGGCC

TTAAGGCC  
Depth:6 (MOUSE)  
Ei-value:0.000, Pi-value:0.000  
Er-value:0.000, Pr-value:0.000  
No matches to TargetScan


CCTTT

TTAAGGCCCCTTT  
Depth:5 (RABBIT)  
Ei-value:0.000, Pi-value:0.000  
Er-value:0.000, Pr-value:0.000  
No matches to TargetScan

-2195--(3152)--5348-

CTCAGCTCTTGG

CTCAGCTCTTGG  
Depth:5 (RABBIT)  
Ei-value:0.000, Pi-value:0.000  
Er-value:0.000, Pr-value:0.000  
MATCHES To TargetScan▶ miR-335-5p:CAAGAGC

-5359--(236)--5596-

GCACAATG

GCACAATG  
Depth:6 (MOUSE)  
Ei-value:0.000, Pi-value:0.000  
Er-value:0.000, Pr-value:0.000  
No matches to TargetScan

-5603--(51)--5655-

GCACAATG

GCACAATG  
Depth:6 (MOUSE)  
Ei-value:0.000, Pi-value:0.000  
Er-value:0.000, Pr-value:0.000  
No matches to TargetScan

-5662--(425)--6088-

CTCCCA

CTCCCA  
Depth:6 (MOUSE)  
Ei-value:0.000, Pi-value:0.000  
Er-value:0.000, Pr-value:0.000  
No matches to TargetScan

-6093--(148)--6242-

AAAAGCAG

AAAAGCAG  
Depth:6 (MOUSE)  
Ei-value:0.000, Pi-value:0.000  
Er-value:0.000, Pr-value:0.000  
No matches to TargetScan

-6249--(123)--6373-

GCAAAAT

GCAAAAT  
Depth:6 (MOUSE)  
Ei-value:0.000, Pi-value:0.000  
Er-value:0.000, Pr-value:0.000  
No matches to TargetScan

-6379--(100)--6480-

TGCATTCTTC

TGCATTCTTC  
Depth:5 (RABBIT)  
Ei-value:0.000, Pi-value:0.000  
Er-value:0.000, Pr-value:0.000  
No matches to TargetScan

-6489--(9)--6499-

A

AGATTGCCTGG  
Depth:5 (RABBIT)  
Ei-value:0.000, Pi-value:0.000  
Er-value:0.000, Pr-value:0.000  
No matches to TargetScan


GATTGCCTGG

GATTGCCTGG  
Depth:6 (MOUSE)  
Ei-value:0.000, Pi-value:0.000  
Er-value:0.000, Pr-value:0.000  
No matches to TargetScan

-6509--(122)--6632-

CTGGTCATT

CTGGTCATT  
Depth:5 (RABBIT)  
Ei-value:0.000, Pi-value:0.000  
Er-value:0.000, Pr-value:0.000  
No matches to TargetScan

-6640--(21)--6662-

CCATTTAT

CCATTTAT  
Depth:5 (RABBIT)  
Ei-value:0.000, Pi-value:0.000  
Er-value:0.000, Pr-value:0.000  
No matches to TargetScan

-6669--(17)--6687-

CAGTGTCTCTCATTT

CAGTGTCTCTCATTT  
Depth:5 (RABBIT)  
Ei-value:0.000, Pi-value:0.000  
Er-value:0.000, Pr-value:0.000  
No matches to TargetScan

-6701--(8)--6710-

GTGGTG

GTGGTG  
Depth:5 (RABBIT)  
Ei-value:0.000, Pi-value:0.000  
Er-value:0.000, Pr-value:0.000  
No matches to TargetScan

-6715--(1)--6717-

GTCTGTGGATA

GTCTGTGGATA  
Depth:5 (RABBIT)  
Ei-value:0.000, Pi-value:0.000  
Er-value:0.000, Pr-value:0.000  
MATCHES To TargetScan▶ miR-140-3p.1:CCACAGG

-6727--(171)--6899-

AATTTCTTCATCTGGAGC

AATTTCTTCATCTGGAGC  
Depth:5 (RABBIT)  
Ei-value:0.000, Pi-value:0.000  
Er-value:0.000, Pr-value:0.000  
No matches to TargetScan

-6916--(178)--7095-

AAAAATAAGCCA

AAAAATAAGCCA  
Depth:5 (RABBIT)  
Ei-value:0.000, Pi-value:0.000  
Er-value:0.000, Pr-value:0.000  
No matches to TargetScan

-7106--(148)--7255-

CAGCAGTTC

CAGCAGTTC  
Depth:5 (RABBIT)  
Ei-value:0.000, Pi-value:0.000  
Er-value:0.000, Pr-value:0.000  
No matches to TargetScan

-7263--(9)--7273-

ACTGAAAA

ACTGAAAA  
Depth:5 (RABBIT)  
Ei-value:0.000, Pi-value:0.000  
Er-value:0.000, Pr-value:0.000  
No matches to TargetScan

-7280--(19)--7300-

AAGGATG

AAGGATG  
Depth:5 (RABBIT)  
Ei-value:0.000, Pi-value:0.000  
Er-value:0.000, Pr-value:0.000  
MATCHES To TargetScan▶ miR-362-5p/500b-5p:AUCCUUG

-7306--(3)--7310-

AAAGATC

AAAGATC  
Depth:6 (MOUSE)  
Ei-value:0.000, Pi-value:0.000  
Er-value:0.000, Pr-value:0.000  
No matches to TargetScan

-7316--(38)--7355-

GGACAGCTG

GGACAGCTG  
Depth:5 (RABBIT)  
Ei-value:0.000, Pi-value:0.000  
Er-value:0.000, Pr-value:0.000  
No matches to TargetScan

-7363--(2)--7366-

AGAAGAGTCTCTGGCTCTTTA

AGAAGAGTCTCTGGCTCTTTA  
Depth:5 (RABBIT)  
Ei-value:0.000, Pi-value:0.000  
Er-value:0.000, Pr-value:0.000  
No matches to TargetScan

-7386--(47)--7434-

CTGCAA

CTGCAA  
Depth:5 (RABBIT)  
Ei-value:0.000, Pi-value:0.000  
Er-value:0.000, Pr-value:0.000  
No matches to TargetScan

-7439--(103)--7543-

AAGAATAGGC

AAGAATAGGC  
Depth:5 (RABBIT)  
Ei-value:0.000, Pi-value:0.000  
Er-value:0.000, Pr-value:0.000  
No matches to TargetScan

-7552--(9)--7562-

TACAGTGTTAGTGA

TACAGTGTTAGTGA  
Depth:5 (RABBIT)  
Ei-value:0.000, Pi-value:0.000  
Er-value:0.000, Pr-value:0.000  
MATCHES To TargetScan▶ miR-141-3p/200a-3p:AACACUG

-7575--(4)--7580-

TTCCCTTTGA

TTCCCTTTGA  
Depth:6 (MOUSE)  
Ei-value:0.000, Pi-value:0.000  
Er-value:0.000, Pr-value:0.000  
No matches to TargetScan

-7589--(7)--7597-

TAGGTGGAGATGGGGCATGAGGATCCTCCAGGGGAA

TAGGTGGAGATGGGGCATGAGGATCCTCCAGGGGAA  
Depth:6 (MOUSE)  
Ei-value:0.000, Pi-value:0.000  
Er-value:0.000, Pr-value:0.000  
MATCHES To TargetScan▶ miR-331-3p:CCCCUGG


A

TAGGTGGAGATGGGGCATGAGGATCCTCCAGGGGAAA  
Depth:5 (RABBIT)  
Ei-value:0.000, Pi-value:0.000  
Er-value:0.000, Pr-value:0.000  
MATCHES To TargetScan▶ miR-331-3p:CCCCUGG

-7633--(3)--7637-

TCACTA

TCACTA  
Depth:5 (RABBIT)  
Ei-value:0.000, Pi-value:0.000  
Er-value:0.000, Pr-value:0.000  
No matches to TargetScan

-7642--(7)--7650-

GCAACA

GCAACA  
Depth:6 (MOUSE)  
Ei-value:0.000, Pi-value:0.000  
Er-value:0.000, Pr-value:0.000  
No matches to TargetScan


AC

GCAACAAC  
Depth:5 (RABBIT)  
Ei-value:0.000, Pi-value:0.000  
Er-value:0.000, Pr-value:0.000  
No matches to TargetScan

-7657--(64)--7722-

ACAACCACC

ACAACCACC  
Depth:5 (RABBIT)  
Ei-value:0.000, Pi-value:0.000  
Er-value:0.000, Pr-value:0.000  
No matches to TargetScan

-7730--(252)--7983-

CCAAAT

CCAAAT  
Depth:6 (MOUSE)  
Ei-value:0.000, Pi-value:0.000  
Er-value:0.000, Pr-value:0.000  
No matches to TargetScan


C

CCAAATC  
Depth:5 (RABBIT)  
Ei-value:0.000, Pi-value:0.000  
Er-value:0.000, Pr-value:0.000  
No matches to TargetScan

-7989--(44)--8034-

CAAGAAA

CAAGAAA  
Depth:5 (RABBIT)  
Ei-value:0.000, Pi-value:0.000  
Er-value:0.000, Pr-value:0.000  
No matches to TargetScan

-8040--(32)--8073-

AA

AAGATCAACATGC  
Depth:5 (RABBIT)  
Ei-value:0.000, Pi-value:0.000  
Er-value:0.000, Pr-value:0.000  
No matches to TargetScan


GATCAACATGC

GATCAACATGC  
Depth:6 (MOUSE)  
Ei-value:0.000, Pi-value:0.000  
Er-value:0.000, Pr-value:0.000  
No matches to TargetScan

-8085--(37)--8123-

TGTGTAT

TGTGTAT  
Depth:6 (MOUSE)  
Ei-value:0.000, Pi-value:0.000  
Er-value:0.000, Pr-value:0.000  
No matches to TargetScan

-8129--(0)--8130-

TTCTCTTTG

TTCTCTTTG  
Depth:6 (MOUSE)  
Ei-value:0.000, Pi-value:0.000  
Er-value:0.000, Pr-value:0.000  
No matches to TargetScan

-8138--(228)--8367-

TTTCTAC

TTTCTAC  
Depth:6 (MOUSE)  
Ei-value:0.000, Pi-value:0.000  
Er-value:0.000, Pr-value:0.000  
No matches to TargetScan


T

TTTCTACT  
Depth:5 (RABBIT)  
Ei-value:0.000, Pi-value:0.000  
Er-value:0.000, Pr-value:0.000  
MATCHES To TargetScan▶ miR-411-5p.1:AGUAGAC

-8374--(151)--8526-

ATTTCTC

ATTTCTC  
Depth:6 (MOUSE)  
Ei-value:0.000, Pi-value:0.000  
Er-value:0.000, Pr-value:0.000  
No matches to TargetScan

-8532--(24)--8557-

TCTTGGG

TCTTGGG  
Depth:5 (RABBIT)  
Ei-value:0.000, Pi-value:0.000  
Er-value:0.000, Pr-value:0.000  
No matches to TargetScan

-8563--(243)--8807-

TCCTAACCCCT

TCCTAACCCCT  
Depth:5 (RABBIT)  
Ei-value:0.000, Pi-value:0.000  
Er-value:0.000, Pr-value:0.000  
No matches to TargetScan

-8817--(54)--8872-

TTATGCCA

TTATGCCA  
Depth:5 (RABBIT)  
Ei-value:0.000, Pi-value:0.000  
Er-value:0.000, Pr-value:0.000  
No matches to TargetScan

-8879--(287)--9167-

AGGCCCAA

AGGCCCAA  
Depth:5 (RABBIT)  
Ei-value:0.000, Pi-value:0.000  
Er-value:0.000, Pr-value:0.000  
No matches to TargetScan

-9174--(175)--9350-

GACTAA

GACTAA  
Depth:5 (RABBIT)  
Ei-value:0.000, Pi-value:0.000  
Er-value:0.000, Pr-value:0.000  
No matches to TargetScan

-9355--(61)--9417-

AAGATGA

AAGATGA  
Depth:5 (RABBIT)  
Ei-value:0.000, Pi-value:0.000  
Er-value:0.000, Pr-value:0.000  
No matches to TargetScan

-9423--(394)--9818-

TCTAGAGAAAA

TCTAGAGAAAA  
Depth:6 (MOUSE)  
Ei-value:0.000, Pi-value:0.000  
Er-value:0.000, Pr-value:0.000  
MATCHES To TargetScan▶ miR-1251-5p:CUCUAGC

-9828--(2)--9831-

TGAAGAGATG

TGAAGAGATG  
Depth:5 (RABBIT)  
Ei-value:0.000, Pi-value:0.000  
Er-value:0.000, Pr-value:0.000  
No matches to TargetScan

-9840--(10)--9851-

TGAGAAGAATTAGACA

TGAGAAGAATTAGACA  
Depth:6 (MOUSE)  
Ei-value:0.000, Pi-value:0.000  
Er-value:0.000, Pr-value:0.000  
No matches to TargetScan

-9866--(97)--9964-

TGGTTA

TGGTTA  
Depth:5 (RABBIT)  
Ei-value:0.000, Pi-value:0.000  
Er-value:0.000, Pr-value:0.000  
No matches to TargetScan

-9969--(27)--9997-

AGGCTTA

AGGCTTA  
Depth:5 (RABBIT)  
Ei-value:0.000, Pi-value:0.000  
Er-value:0.000, Pr-value:0.000  
No matches to TargetScan

-10003--(113)--10117-

GTTTAAT

GTTTAAT  
Depth:5 (RABBIT)  
Ei-value:0.000, Pi-value:0.000  
Er-value:0.000, Pr-value:0.000  
No matches to TargetScan

-10123--(122)--10246-

T

TATTGGCA  
Depth:5 (RABBIT)  
Ei-value:0.000, Pi-value:0.000  
Er-value:0.000, Pr-value:0.000  
No matches to TargetScan


ATTGGCA

ATTGGCA  
Depth:6 (MOUSE)  
Ei-value:0.000, Pi-value:0.000  
Er-value:0.000, Pr-value:0.000  
No matches to TargetScan

-10253--(36)--10290-

TTGTGAAG

TTGTGAAG  
Depth:6 (MOUSE)  
Ei-value:0.000, Pi-value:0.000  
Er-value:0.000, Pr-value:0.000  
No matches to TargetScan

-10297--(4)--10302-

ATGTAAAT

ATGTAAAT  
Depth:5 (RABBIT)  
Ei-value:0.000, Pi-value:0.000  
Er-value:0.000, Pr-value:0.000  
No matches to TargetScan

-10309--(55)--10365-

ATAAGC

ATAAGC  
Depth:5 (RABBIT)  
Ei-value:0.000, Pi-value:0.010  
Er-value:0.000, Pr-value:0.000  
No matches to TargetScan

-10370--(33)--10404-

AAAAGGT

AAAAGGT  
Depth:6 (MOUSE)  
Ei-value:0.000, Pi-value:0.000  
Er-value:0.000, Pr-value:0.000  
No matches to TargetScan

-10410
```

---

# Modules conserved to DOG (Depth: 4)

## Modules in Main Graph (All sequences considered):

```
>HUMAN  
      1403-

AAACATG

AAACATG  
Depth:4 (DOG)  
Ei-value:0.000, Pi-value:0.000  
Er-value:0.000, Pr-value:0.000  
eCLIP MATCHES▶HNRNPM (bg=4.29%)No matches to TargetScan

-1409--(1251)--2661-

TTCCCATC

TTCCCATC  
Depth:4 (DOG)  
Ei-value:0.000, Pi-value:0.000  
Er-value:0.000, Pr-value:0.000  
eCLIP MATCHES▶ILF3 (bg=3.0%)No matches to TargetScan

-2668--(1819)--4488-

C

CTGTTAGTCT  
Depth:4 (DOG)  
Ei-value:0.000, Pi-value:0.000  
Er-value:0.000, Pr-value:0.000  
eCLIP MATCHES▶AKAP8L (bg=2.19%)No matches to TargetScan


TGTTAGTC

TGTTAGTC  
Depth:5 (RABBIT)  
Ei-value:0.000, Pi-value:0.000  
Er-value:0.000, Pr-value:0.000  
eCLIP MATCHES▶AKAP8L (bg=2.19%)No matches to TargetScan


T

CTGTTAGTCT  
Depth:4 (DOG)  
Ei-value:0.000, Pi-value:0.000  
Er-value:0.000, Pr-value:0.000  
eCLIP MATCHES▶AKAP8L (bg=2.19%)No matches to TargetScan

-4497--(6)--4504-

TCATCC

TCATCC  
Depth:4 (DOG)  
Ei-value:0.000, Pi-value:0.020  
Er-value:0.000, Pr-value:0.000  
eCLIP MATCHES▶AKAP8L (bg=2.19%)No matches to TargetScan

-4509--(31)--4541-

TACTTGGGACTGTTAAT

TACTTGGGACTGTTAAT  
Depth:4 (DOG)  
Ei-value:0.000, Pi-value:0.000  
Er-value:0.000, Pr-value:0.000  
eCLIP MATCHES▶AKAP8L (bg=2.19%)MATCHES To TargetScan▶ miR-132-3p/212-3p:AACAGUC▶ miR-455-3p.1:CAGUCCA

-4557--(106)--4664-

ACTG

ACTGTTAATGTGCT  
Depth:4 (DOG)  
Ei-value:0.000, Pi-value:0.000  
Er-value:0.000, Pr-value:0.000  
No matches to eCLIP DataMATCHES To TargetScan▶ miR-132-3p/212-3p:AACAGUC▶ miR-323-3p:ACAUUAC


TTAATGTGCT

TTAATGTGCT  
Depth:5 (RABBIT)  
Ei-value:0.000, Pi-value:0.000  
Er-value:0.000, Pr-value:0.000  
No matches to eCLIP DataMATCHES To TargetScan▶ miR-323-3p:ACAUUAC

-4677--(356)--5034-

AATGTGCAT

AATGTGCAT  
Depth:6 (MOUSE)  
Ei-value:0.000, Pi-value:0.000  
Er-value:0.000, Pr-value:0.000  
No matches to eCLIP DataMATCHES To TargetScan▶ miR-501-3p/502-3p:AUGCACC

-5042--(436)--5479-

TATGTTAGA

TATGTTAGA  
Depth:4 (DOG)  
Ei-value:0.000, Pi-value:0.000  
Er-value:0.000, Pr-value:0.000  
eCLIP MATCHES▶HNRNPU (bg=5.92%)No matches to TargetScan

-5487--(72)--5560-

ACTGTTAATGT

ACTGTTAATGT  
Depth:4 (DOG)  
Ei-value:0.000, Pi-value:0.000  
Er-value:0.000, Pr-value:0.000  
No matches to eCLIP DataMATCHES To TargetScan▶ miR-132-3p/212-3p:AACAGUC▶ miR-323-3p:ACAUUAC

-5570--(10)--5581-

ATTTGCT

ATTTGCT  
Depth:4 (DOG)  
Ei-value:0.000, Pi-value:0.000  
Er-value:0.000, Pr-value:0.000  
No matches to eCLIP DataNo matches to TargetScan

-5587--(18)--5606-

GTAAGGA

GTAAGGA  
Depth:5 (RABBIT)  
Ei-value:0.000, Pi-value:0.000  
Er-value:0.000, Pr-value:0.000  
No matches to eCLIP DataNo matches to TargetScan

-5612--(141)--5754-

ACTTAT

ACTTAT  
Depth:5 (RABBIT)  
Ei-value:0.000, Pi-value:0.000  
Er-value:0.000, Pr-value:0.000  
eCLIP MATCHES▶HNRNPU (bg=5.92%)No matches to TargetScan

-5759--(145)--5905-

TTAAGGCC

TTAAGGCC  
Depth:6 (MOUSE)  
Ei-value:0.000, Pi-value:0.000  
Er-value:0.000, Pr-value:0.000  
eCLIP MATCHES▶HNRNPL (bg=0.64%)No matches to TargetScan


CCTTT

TTAAGGCCCCTTT  
Depth:5 (RABBIT)  
Ei-value:0.000, Pi-value:0.000  
Er-value:0.000, Pr-value:0.000  
eCLIP MATCHES▶HNRNPL (bg=0.64%)No matches to TargetScan


CTCAA

TTAAGGCCCCTTTCTCAA  
Depth:4 (DOG)  
Ei-value:0.000, Pi-value:0.000  
Er-value:0.000, Pr-value:0.000  
eCLIP MATCHES▶HNRNPL (bg=0.64%)No matches to TargetScan

-5922--(2772)--8695-

ACAGTTAATGTG

ACAGTTAATGTG  
Depth:4 (DOG)  
Ei-value:0.000, Pi-value:0.000  
Er-value:0.000, Pr-value:0.000  
eCLIP MATCHES▶HNRNPU (bg=5.92%)MATCHES To TargetScan▶ miR-323-3p:ACAUUAC

-8706--(651)--9358-

CTCAGCTCTTGG

CTCAGCTCTTGG  
Depth:5 (RABBIT)  
Ei-value:0.000, Pi-value:0.000  
Er-value:0.000, Pr-value:0.000  
No matches to eCLIP DataMATCHES To TargetScan▶ miR-335-5p:CAAGAGC


ACA

CTCAGCTCTTGGACA  
Depth:4 (DOG)  
Ei-value:0.000, Pi-value:0.000  
Er-value:0.000, Pr-value:0.000  
No matches to eCLIP DataMATCHES To TargetScan▶ miR-335-5p:CAAGAGC

-9372--(206)--9579-

CAAAACTT

CAAAACTT  
Depth:4 (DOG)  
Ei-value:0.000, Pi-value:0.000  
Er-value:0.000, Pr-value:0.000  
eCLIP MATCHES▶SF3B1 (bg=2.48%)No matches to TargetScan

-9586--(19)--9606-

GCACAATG

GCACAATG  
Depth:6 (MOUSE)  
Ei-value:0.000, Pi-value:0.000  
Er-value:0.000, Pr-value:0.000  
No matches to eCLIP DataNo matches to TargetScan

-9613--(132)--9746-

A

ACTCCCA  
Depth:4 (DOG)  
Ei-value:0.000, Pi-value:0.000  
Er-value:0.000, Pr-value:0.000  
eCLIP MATCHES▶hnrnpk (bg=12.88%)No matches to TargetScan


CTCCCA

CTCCCA  
Depth:6 (MOUSE)  
Ei-value:0.000, Pi-value:0.000  
Er-value:0.000, Pr-value:0.000  
eCLIP MATCHES▶hnrnpk (bg=12.88%)No matches to TargetScan

-9752--(1)--9754-

CCCTTTTGCATT

CCCTTTTGCATT  
Depth:4 (DOG)  
Ei-value:0.000, Pi-value:0.000  
Er-value:0.000, Pr-value:0.000  
eCLIP MATCHES▶hnrnpk (bg=12.88%)No matches to TargetScan

-9765--(376)--10142-

CCATTTTT

CCATTTTT  
Depth:4 (DOG)  
Ei-value:0.000, Pi-value:0.000  
Er-value:0.000, Pr-value:0.000  
eCLIP MATCHES▶hnrnpk (bg=12.88%)No matches to TargetScan

-10149--(0)--10150-

CAGCCCA

CAGCCCA  
Depth:4 (DOG)  
Ei-value:0.000, Pi-value:0.000  
Er-value:0.000, Pr-value:0.000  
eCLIP MATCHES▶hnrnpk (bg=12.88%)No matches to TargetScan

-10156--(15)--10172-

CCTACCA

CCTACCA  
Depth:4 (DOG)  
Ei-value:0.000, Pi-value:0.000  
Er-value:0.000, Pr-value:0.000  
eCLIP MATCHES▶hnrnpk (bg=12.88%)No matches to TargetScan

-10178--(30)--10209-

AAAAGCAG

AAAAGCAG  
Depth:6 (MOUSE)  
Ei-value:0.000, Pi-value:0.000  
Er-value:0.000, Pr-value:0.000  
No matches to eCLIP DataNo matches to TargetScan

-10216--(44)--10261-

TTAATGATCC

TTAATGATCC  
Depth:4 (DOG)  
Ei-value:0.000, Pi-value:0.000  
Er-value:0.000, Pr-value:0.000  
No matches to eCLIP DataMATCHES To TargetScan▶ miR-382-3p:AUCAUUC

-10270--(19)--10290-

ATTCTGGG

ATTCTGGG  
Depth:4 (DOG)  
Ei-value:0.000, Pi-value:0.000  
Er-value:0.000, Pr-value:0.000  
No matches to eCLIP DataNo matches to TargetScan

-10297--(33)--10331-

CTTTACT

CTTTACT  
Depth:4 (DOG)  
Ei-value:0.000, Pi-value:0.000  
Er-value:0.000, Pr-value:0.000  
No matches to eCLIP DataNo matches to TargetScan

-10337--(2)--10340-

GCAAAAT

GCAAAAT  
Depth:6 (MOUSE)  
Ei-value:0.000, Pi-value:0.000  
Er-value:0.000, Pr-value:0.000  
No matches to eCLIP DataNo matches to TargetScan

-10346--(4)--10351-

AAGGCAA

AAGGCAA  
Depth:4 (DOG)  
Ei-value:0.000, Pi-value:0.000  
Er-value:0.000, Pr-value:0.000  
No matches to eCLIP DataNo matches to TargetScan

-10357--(16)--10374-

TGGATTGC

TGGATTGC  
Depth:4 (DOG)  
Ei-value:0.000, Pi-value:0.000  
Er-value:0.000, Pr-value:0.000  
No matches to eCLIP DataNo matches to TargetScan

-10381--(71)--10453-

TGCATTCTTC

TGCATTCTTC  
Depth:5 (RABBIT)  
Ei-value:0.000, Pi-value:0.000  
Er-value:0.000, Pr-value:0.000  
eCLIP MATCHES▶SF3B1 (bg=2.48%)No matches to TargetScan

-10462--(7)--10470-

AGC

AGCAGATTGCCTGG  
Depth:4 (DOG)  
Ei-value:0.000, Pi-value:0.000  
Er-value:0.000, Pr-value:0.000  
eCLIP MATCHES▶SF3B1 (bg=2.48%)No matches to TargetScan


A

AGATTGCCTGG  
Depth:5 (RABBIT)  
Ei-value:0.000, Pi-value:0.000  
Er-value:0.000, Pr-value:0.000  
No matches to eCLIP DataNo matches to TargetScan


GATTGCCTGG

GATTGCCTGG  
Depth:6 (MOUSE)  
Ei-value:0.000, Pi-value:0.000  
Er-value:0.000, Pr-value:0.000  
No matches to eCLIP DataNo matches to TargetScan

-10483--(23)--10507-

TTGTATATT

TTGTATATT  
Depth:4 (DOG)  
Ei-value:0.000, Pi-value:0.000  
Er-value:0.000, Pr-value:0.000  
No matches to eCLIP DataMATCHES To TargetScan▶ miR-381-3p:AUACAAG

-10515--(104)--10620-

AAC

AACCTGGTCATT  
Depth:4 (DOG)  
Ei-value:0.000, Pi-value:0.000  
Er-value:0.000, Pr-value:0.000  
No matches to eCLIP DataNo matches to TargetScan


CTGGTCATT

CTGGTCATT  
Depth:5 (RABBIT)  
Ei-value:0.000, Pi-value:0.000  
Er-value:0.000, Pr-value:0.000  
No matches to eCLIP DataNo matches to TargetScan

-10631--(21)--10653-

CCATTTAT

CCATTTAT  
Depth:5 (RABBIT)  
Ei-value:0.000, Pi-value:0.000  
Er-value:0.000, Pr-value:0.000  
No matches to eCLIP DataNo matches to TargetScan

-10660--(13)--10674-

TGAC

TGACCAGTGTCTCTCATTT  
Depth:4 (DOG)  
Ei-value:0.000, Pi-value:0.000  
Er-value:0.000, Pr-value:0.000  
eCLIP MATCHES▶SUPV3L1 (bg=1.57%)No matches to TargetScan


CAGTGTCTCTCATTT

CAGTGTCTCTCATTT  
Depth:5 (RABBIT)  
Ei-value:0.000, Pi-value:0.000  
Er-value:0.000, Pr-value:0.000  
eCLIP MATCHES▶SUPV3L1 (bg=1.57%)No matches to TargetScan

-10692--(6)--10699-

AGG

AGGGTGGTG  
Depth:4 (DOG)  
Ei-value:0.000, Pi-value:0.000  
Er-value:0.000, Pr-value:0.000  
eCLIP MATCHES▶SUPV3L1 (bg=1.57%)No matches to TargetScan


GTGGTG

GTGGTG  
Depth:5 (RABBIT)  
Ei-value:0.000, Pi-value:0.000  
Er-value:0.000, Pr-value:0.000  
eCLIP MATCHES▶SUPV3L1 (bg=1.57%)No matches to TargetScan

-10707--(1)--10709-

GTCTGTGGATA

GTCTGTGGATA  
Depth:5 (RABBIT)  
Ei-value:0.000, Pi-value:0.000  
Er-value:0.000, Pr-value:0.000  
eCLIP MATCHES▶SUPV3L1 (bg=1.57%)MATCHES To TargetScan▶ miR-140-3p.1:CCACAGG

-10719--(35)--10755-

TTCTAGA

TTCTAGA  
Depth:4 (DOG)  
Ei-value:0.000, Pi-value:0.000  
Er-value:0.000, Pr-value:0.000  
No matches to eCLIP DataNo matches to TargetScan

-10761--(43)--10805-

ATTCACTT

ATTCACTT  
Depth:4 (DOG)  
Ei-value:0.000, Pi-value:0.000  
Er-value:0.000, Pr-value:0.000  
No matches to eCLIP DataNo matches to TargetScan

-10812--(3)--10816-

GAAAAAC

GAAAAAC  
Depth:4 (DOG)  
Ei-value:0.000, Pi-value:0.000  
Er-value:0.000, Pr-value:0.000  
No matches to eCLIP DataNo matches to TargetScan

-10822--(22)--10845-

AATTTCTTCATCTGGAGC

AATTTCTTCATCTGGAGC  
Depth:5 (RABBIT)  
Ei-value:0.000, Pi-value:0.000  
Er-value:0.000, Pr-value:0.000  
eCLIP MATCHES▶SUPV3L1 (bg=1.57%)▶U2AF2 (bg=1.76%)No matches to TargetScan

-10862--(11)--10874-

CTTATTT

CTTATTT  
Depth:4 (DOG)  
Ei-value:0.000, Pi-value:0.000  
Er-value:0.000, Pr-value:0.010  
eCLIP MATCHES▶SUPV3L1 (bg=1.57%)▶U2AF2 (bg=1.76%)No matches to TargetScan

-10880--(89)--10970-

ATAAAATG

ATAAAATG  
Depth:4 (DOG)  
Ei-value:0.000, Pi-value:0.000  
Er-value:0.000, Pr-value:0.000  
No matches to eCLIP DataNo matches to TargetScan

-10977--(94)--11072-

AAAAATAAGCCA

AAAAATAAGCCA  
Depth:5 (RABBIT)  
Ei-value:0.000, Pi-value:0.000  
Er-value:0.000, Pr-value:0.000  
No matches to eCLIP DataNo matches to TargetScan


A

AAAAATAAGCCAA  
Depth:4 (DOG)  
Ei-value:0.000, Pi-value:0.000  
Er-value:0.000, Pr-value:0.000  
No matches to eCLIP DataNo matches to TargetScan

-11084--(55)--11140-

ATGAATAATA

ATGAATAATA  
Depth:4 (DOG)  
Ei-value:0.000, Pi-value:0.000  
Er-value:0.000, Pr-value:0.000  
No matches to eCLIP DataNo matches to TargetScan

-11149--(49)--11199-

TGGAACTGCT

TGGAACTGCT  
Depth:4 (DOG)  
Ei-value:0.000, Pi-value:0.000  
Er-value:0.000, Pr-value:0.000  
No matches to eCLIP DataNo matches to TargetScan

-11208--(8)--11217-

TAACTA

TAACTA  
Depth:4 (DOG)  
Ei-value:0.000, Pi-value:0.000  
Er-value:0.000, Pr-value:0.000  
No matches to eCLIP DataNo matches to TargetScan

-11222--(10)--11233-

CAGCAGTTC

CAGCAGTTC  
Depth:5 (RABBIT)  
Ei-value:0.000, Pi-value:0.000  
Er-value:0.000, Pr-value:0.000  
No matches to eCLIP DataNo matches to TargetScan

-11241--(1)--11243-

TTGTAAT

TTGTAAT  
Depth:4 (DOG)  
Ei-value:0.000, Pi-value:0.000  
Er-value:0.000, Pr-value:0.000  
No matches to eCLIP DataNo matches to TargetScan

-11249--(1)--11251-

ACTGAAAA

ACTGAAAA  
Depth:5 (RABBIT)  
Ei-value:0.000, Pi-value:0.000  
Er-value:0.000, Pr-value:0.000  
No matches to eCLIP DataNo matches to TargetScan

-11258--(19)--11278-

AAGGATG

AAGGATG  
Depth:5 (RABBIT)  
Ei-value:0.000, Pi-value:0.000  
Er-value:0.000, Pr-value:0.000  
eCLIP MATCHES▶SRSF1 (bg=8.47%)▶U2AF2 (bg=1.76%)▶uchl5 (bg=11.16%)MATCHES To TargetScan▶ miR-362-5p/500b-5p:AUCCUUG


TCA

AAGGATGTCAAAAGATC  
Depth:4 (DOG)  
Ei-value:0.000, Pi-value:0.000  
Er-value:0.000, Pr-value:0.000  
eCLIP MATCHES▶SRSF1 (bg=8.47%)▶U2AF2 (bg=1.76%)▶uchl5 (bg=11.16%)MATCHES To TargetScan▶ miR-362-5p/500b-5p:AUCCUUG▶ miR-489-3p:UGACAUC


AAAGATC

AAAGATC  
Depth:6 (MOUSE)  
Ei-value:0.000, Pi-value:0.000  
Er-value:0.000, Pr-value:0.000  
eCLIP MATCHES▶SRSF1 (bg=8.47%)▶U2AF2 (bg=1.76%)▶uchl5 (bg=11.16%)No matches to TargetScan

-11294--(4)--11299-

CAGCTCAGGG

CAGCTCAGGG  
Depth:4 (DOG)  
Ei-value:0.000, Pi-value:0.000  
Er-value:0.000, Pr-value:0.000  
eCLIP MATCHES▶SRSF1 (bg=8.47%)▶U2AF2 (bg=1.76%)▶uchl5 (bg=11.16%)MATCHES To TargetScan▶ miR-125-5p:CCCUGAG

-11308--(11)--11320-

CTACTAGCTCCT

CTACTAGCTCCT  
Depth:4 (DOG)  
Ei-value:0.000, Pi-value:0.000  
Er-value:0.000, Pr-value:0.000  
eCLIP MATCHES▶SRSF1 (bg=8.47%)▶U2AF2 (bg=1.76%)▶uchl5 (bg=11.16%)MATCHES To TargetScan▶ miR-28-5p/708-5p:AGGAGCU▶ miR-411-5p.2:UAGUAGA

-11331--(1)--11333-

GGACAGCTG

GGACAGCTG  
Depth:5 (RABBIT)  
Ei-value:0.000, Pi-value:0.000  
Er-value:0.000, Pr-value:0.000  
eCLIP MATCHES▶SRSF1 (bg=8.47%)▶SRSF7 (bg=2.32%)▶U2AF2 (bg=1.76%)▶ZNF622 (bg=6.58%)No matches to TargetScan


T

GGACAGCTGT  
Depth:4 (DOG)  
Ei-value:0.000, Pi-value:0.000  
Er-value:0.000, Pr-value:0.000  
eCLIP MATCHES▶SRSF1 (bg=8.47%)▶SRSF7 (bg=2.32%)▶U2AF2 (bg=1.76%)▶ZNF622 (bg=6.58%)No matches to TargetScan

-11342--(2)--11345-

AGAAGAGTCTCTGGCTCTTTA

AGAAGAGTCTCTGGCTCTTTA  
Depth:5 (RABBIT)  
Ei-value:0.000, Pi-value:0.000  
Er-value:0.000, Pr-value:0.000  
eCLIP MATCHES▶DDX24 (bg=2.97%)▶SRSF1 (bg=8.47%)▶SRSF7 (bg=2.32%)▶U2AF2 (bg=1.76%)▶ZNF622 (bg=6.58%)No matches to TargetScan


GA

AGAAGAGTCTCTGGCTCTTTAGA  
Depth:4 (DOG)  
Ei-value:0.000, Pi-value:0.000  
Er-value:0.000, Pr-value:0.000  
eCLIP MATCHES▶DDX24 (bg=2.97%)▶SRSF1 (bg=8.47%)▶SRSF7 (bg=2.32%)▶U2AF2 (bg=1.76%)▶ZNF622 (bg=6.58%)No matches to TargetScan

-11367--(72)--11440-

ATTCTGAGC

ATTCTGAGC  
Depth:4 (DOG)  
Ei-value:0.000, Pi-value:0.000  
Er-value:0.000, Pr-value:0.000  
eCLIP MATCHES▶DDX24 (bg=2.97%)▶GRWD1 (bg=5.13%)▶MTPAP (bg=2.21%)▶NOLC1 (bg=9.43%)▶SRSF1 (bg=8.47%)▶ZNF622 (bg=6.58%)No matches to TargetScan

-11448--(16)--11465-

CTGCAA

CTGCAA  
Depth:5 (RABBIT)  
Ei-value:0.000, Pi-value:0.000  
Er-value:0.000, Pr-value:0.000  
eCLIP MATCHES▶DDX24 (bg=2.97%)▶GRWD1 (bg=5.13%)▶MTPAP (bg=2.21%)▶NOLC1 (bg=9.43%)▶SRSF1 (bg=8.47%)▶UTP3 (bg=3.66%)▶ZNF622 (bg=6.58%)No matches to TargetScan

-11470--(114)--11585-

AAGAATAGGC

AAGAATAGGC  
Depth:5 (RABBIT)  
Ei-value:0.000, Pi-value:0.000  
Er-value:0.000, Pr-value:0.000  
eCLIP MATCHES▶NOLC1 (bg=9.43%)▶SRSF7 (bg=2.32%)▶uchl5 (bg=11.16%)No matches to TargetScan

-11594--(9)--11604-

TACAGTGTTAGTGA

TACAGTGTTAGTGA  
Depth:5 (RABBIT)  
Ei-value:0.000, Pi-value:0.000  
Er-value:0.000, Pr-value:0.000  
eCLIP MATCHES▶ILF3 (bg=3.0%)▶NOLC1 (bg=9.43%)▶RBM15 (bg=7.27%)▶SRSF7 (bg=2.32%)▶ZNF622 (bg=6.58%)MATCHES To TargetScan▶ miR-141-3p/200a-3p:AACACUG

-11617--(4)--11622-

TTCCCTTTGA

TTCCCTTTGA  
Depth:6 (MOUSE)  
Ei-value:0.000, Pi-value:0.000  
Er-value:0.000, Pr-value:0.000  
eCLIP MATCHES▶ILF3 (bg=3.0%)▶RBM15 (bg=7.27%)▶SRSF7 (bg=2.32%)▶ZNF622 (bg=6.58%)No matches to TargetScan

-11631--(7)--11639-

TAGGTGGAGATGGGGCATGAGGATCCTCCAGGGGAA

TAGGTGGAGATGGGGCATGAGGATCCTCCAGGGGAA  
Depth:6 (MOUSE)  
Ei-value:0.000, Pi-value:0.000  
Er-value:0.000, Pr-value:0.000  
eCLIP MATCHES▶ILF3 (bg=3.0%)▶NOLC1 (bg=9.43%)▶RBM15 (bg=7.27%)▶SRSF7 (bg=2.32%)▶ZNF622 (bg=6.58%)MATCHES To TargetScan▶ miR-331-3p:CCCCUGG


A

TAGGTGGAGATGGGGCATGAGGATCCTCCAGGGGAAA  
Depth:5 (RABBIT)  
Ei-value:0.000, Pi-value:0.000  
Er-value:0.000, Pr-value:0.000  
eCLIP MATCHES▶ILF3 (bg=3.0%)▶NOLC1 (bg=9.43%)▶RBM15 (bg=7.27%)▶SRSF7 (bg=2.32%)▶ZNF622 (bg=6.58%)MATCHES To TargetScan▶ miR-331-3p:CCCCUGG

-11675--(3)--11679-

TCACTA

TCACTA  
Depth:5 (RABBIT)  
Ei-value:0.000, Pi-value:0.000  
Er-value:0.000, Pr-value:0.000  
eCLIP MATCHES▶ILF3 (bg=3.0%)No matches to TargetScan


CCACT

TCACTACCACT  
Depth:4 (DOG)  
Ei-value:0.000, Pi-value:0.000  
Er-value:0.000, Pr-value:0.000  
eCLIP MATCHES▶ILF3 (bg=3.0%)MATCHES To TargetScan▶ miR-140-5p:AGUGGUU▶ miR-142-3p.1:GUAGUGU

-11689--(2)--11692-

GCAACA

GCAACA  
Depth:6 (MOUSE)  
Ei-value:0.000, Pi-value:0.000  
Er-value:0.000, Pr-value:0.000  
eCLIP MATCHES▶ILF3 (bg=3.0%)No matches to TargetScan


AC

GCAACAAC  
Depth:5 (RABBIT)  
Ei-value:0.000, Pi-value:0.000  
Er-value:0.000, Pr-value:0.000  
eCLIP MATCHES▶ILF3 (bg=3.0%)No matches to TargetScan

-11699--(64)--11764-

ACAACCACC

ACAACCACC  
Depth:5 (RABBIT)  
Ei-value:0.000, Pi-value:0.000  
Er-value:0.000, Pr-value:0.000  
eCLIP MATCHES▶PRPF8 (bg=0.26%)No matches to TargetScan


ACAC

ACAACCACCACAC  
Depth:4 (DOG)  
Ei-value:0.000, Pi-value:0.000  
Er-value:0.000, Pr-value:0.000  
eCLIP MATCHES▶PRPF8 (bg=0.26%)No matches to TargetScan

-11776--(13)--11790-

TTGTTCC

TTGTTCC  
Depth:4 (DOG)  
Ei-value:0.000, Pi-value:0.000  
Er-value:0.000, Pr-value:0.000  
eCLIP MATCHES▶GRWD1 (bg=5.13%)▶SF3B4 (bg=0.05%)No matches to TargetScan

-11796--(6)--11803-

CCAAAT

CCAAAT  
Depth:6 (MOUSE)  
Ei-value:0.000, Pi-value:0.000  
Er-value:0.000, Pr-value:0.000  
eCLIP MATCHES▶GRWD1 (bg=5.13%)▶NOLC1 (bg=9.43%)No matches to TargetScan


C

CCAAATC  
Depth:5 (RABBIT)  
Ei-value:0.000, Pi-value:0.000  
Er-value:0.000, Pr-value:0.000  
eCLIP MATCHES▶GRWD1 (bg=5.13%)▶NOLC1 (bg=9.43%)No matches to TargetScan

-11809--(47)--11857-

CAAGAAA

CAAGAAA  
Depth:5 (RABBIT)  
Ei-value:0.000, Pi-value:0.000  
Er-value:0.000, Pr-value:0.000  
eCLIP MATCHES▶GRWD1 (bg=5.13%)▶NOLC1 (bg=9.43%)▶uchl5 (bg=11.16%)▶ZNF622 (bg=6.58%)No matches to TargetScan

-11863--(35)--11899-

G

GAAGATCAACATGCCTG  
Depth:4 (DOG)  
Ei-value:0.000, Pi-value:0.000  
Er-value:0.000, Pr-value:0.000  
eCLIP MATCHES▶GRWD1 (bg=5.13%)▶NOLC1 (bg=9.43%)▶PTBP1 (bg=3.74%)▶RBM15 (bg=7.27%)▶TRA2A (bg=4.8%)▶uchl5 (bg=11.16%)▶ZNF622 (bg=6.58%)No matches to TargetScan


AA

AAGATCAACATGC  
Depth:5 (RABBIT)  
Ei-value:0.000, Pi-value:0.000  
Er-value:0.000, Pr-value:0.000  
eCLIP MATCHES▶GRWD1 (bg=5.13%)▶NOLC1 (bg=9.43%)▶PTBP1 (bg=3.74%)▶RBM15 (bg=7.27%)▶TRA2A (bg=4.8%)▶uchl5 (bg=11.16%)▶ZNF622 (bg=6.58%)No matches to TargetScan


GATCAACATGC

GATCAACATGC  
Depth:6 (MOUSE)  
Ei-value:0.000, Pi-value:0.000  
Er-value:0.000, Pr-value:0.000  
eCLIP MATCHES▶GRWD1 (bg=5.13%)▶NOLC1 (bg=9.43%)▶PTBP1 (bg=3.74%)▶RBM15 (bg=7.27%)▶TRA2A (bg=4.8%)▶uchl5 (bg=11.16%)▶ZNF622 (bg=6.58%)No matches to TargetScan


CTG

GAAGATCAACATGCCTG  
Depth:4 (DOG)  
Ei-value:0.000, Pi-value:0.000  
Er-value:0.000, Pr-value:0.000  
eCLIP MATCHES▶GRWD1 (bg=5.13%)▶NOLC1 (bg=9.43%)▶PTBP1 (bg=3.74%)▶RBM15 (bg=7.27%)▶TRA2A (bg=4.8%)▶uchl5 (bg=11.16%)▶ZNF622 (bg=6.58%)No matches to TargetScan

-11915--(31)--11947-

TGTGTAT

TGTGTAT  
Depth:6 (MOUSE)  
Ei-value:0.000, Pi-value:0.000  
Er-value:0.000, Pr-value:0.000  
eCLIP MATCHES▶TARDBP (bg=2.79%)▶ZC3H11A (bg=6.55%)No matches to TargetScan


TT

TGTGTATTT  
Depth:4 (DOG)  
Ei-value:0.000, Pi-value:0.000  
Er-value:0.000, Pr-value:0.000  
eCLIP MATCHES▶TARDBP (bg=2.79%)▶ZC3H11A (bg=6.55%)No matches to TargetScan

-11955--(49)--12005-

TGTCTTA

TGTCTTA  
Depth:4 (DOG)  
Ei-value:0.000, Pi-value:0.000  
Er-value:0.000, Pr-value:0.000  
eCLIP MATCHES▶MATR3 (bg=2.98%)▶PTBP1 (bg=3.74%)▶TARDBP (bg=2.79%)▶ZC3H11A (bg=6.55%)MATCHES To TargetScan▶ miR-208-3p:UAAGACG▶ miR-499a-5p:UAAGACU

-12011--(58)--12070-

TTTTTGT

TTTTTGT  
Depth:4 (DOG)  
Ei-value:0.000, Pi-value:0.000  
Er-value:0.000, Pr-value:0.000  
eCLIP MATCHES▶MATR3 (bg=2.98%)▶PTBP1 (bg=3.74%)▶TARDBP (bg=2.79%)▶TIA1 (bg=4.07%)▶ZC3H11A (bg=6.55%)No matches to TargetScan

-12076--(44)--12121-

TTCATTTTGTT

TTCATTTTGTT  
Depth:4 (DOG)  
Ei-value:0.000, Pi-value:0.000  
Er-value:0.000, Pr-value:0.000  
No matches to eCLIP DataMATCHES To TargetScan▶ miR-495-3p:AACAAAC

-12131--(135)--12267-

TTCTCTTTG

TTCTCTTTG  
Depth:6 (MOUSE)  
Ei-value:0.000, Pi-value:0.000  
Er-value:0.000, Pr-value:0.000  
eCLIP MATCHES▶MATR3 (bg=2.98%)▶PTBP1 (bg=3.74%)▶SMNDC1 (bg=0.63%)▶TIA1 (bg=4.07%)No matches to TargetScan

-12275--(46)--12322-

ATTTCACCT

ATTTCACCT  
Depth:4 (DOG)  
Ei-value:0.000, Pi-value:0.000  
Er-value:0.000, Pr-value:0.000  
eCLIP MATCHES▶TIA1 (bg=4.07%)MATCHES To TargetScan▶ miR-203a-3p.2:UGAAAUG

-12330--(29)--12360-

TTTCTAC

TTTCTAC  
Depth:6 (MOUSE)  
Ei-value:0.000, Pi-value:0.000  
Er-value:0.000, Pr-value:0.000  
eCLIP MATCHES▶MATR3 (bg=2.98%)▶PTBP1 (bg=3.74%)▶TIA1 (bg=4.07%)No matches to TargetScan


T

TTTCTACT  
Depth:5 (RABBIT)  
Ei-value:0.000, Pi-value:0.000  
Er-value:0.000, Pr-value:0.000  
eCLIP MATCHES▶MATR3 (bg=2.98%)▶PTBP1 (bg=3.74%)▶TIA1 (bg=4.07%)MATCHES To TargetScan▶ miR-411-5p.1:AGUAGAC

-12367--(11)--12379-

ATTTCTC

ATTTCTC  
Depth:6 (MOUSE)  
Ei-value:0.000, Pi-value:0.000  
Er-value:0.000, Pr-value:0.000  
eCLIP MATCHES▶MATR3 (bg=2.98%)▶PTBP1 (bg=3.74%)▶TIA1 (bg=4.07%)No matches to TargetScan

-12385--(24)--12410-

TCTTGGG

TCTTGGG  
Depth:5 (RABBIT)  
Ei-value:0.000, Pi-value:0.000  
Er-value:0.000, Pr-value:0.000  
eCLIP MATCHES▶MATR3 (bg=2.98%)▶PTBP1 (bg=3.74%)▶SMNDC1 (bg=0.63%)▶TIA1 (bg=4.07%)No matches to TargetScan

-12416--(44)--12461-

TTTGTGA

TTTGTGA  
Depth:4 (DOG)  
Ei-value:0.000, Pi-value:0.010  
Er-value:0.000, Pr-value:0.000  
eCLIP MATCHES▶MATR3 (bg=2.98%)▶PTBP1 (bg=3.74%)▶TIA1 (bg=4.07%)No matches to TargetScan

-12467--(19)--12487-

TCTCTGTT

TCTCTGTT  
Depth:4 (DOG)  
Ei-value:0.000, Pi-value:0.000  
Er-value:0.000, Pr-value:0.000  
eCLIP MATCHES▶MATR3 (bg=2.98%)▶PTBP1 (bg=3.74%)No matches to TargetScan

-12494--(40)--12535-

TTTGAGTATTT

TTTGAGTATTT  
Depth:4 (DOG)  
Ei-value:0.000, Pi-value:0.000  
Er-value:0.000, Pr-value:0.000  
eCLIP MATCHES▶MATR3 (bg=2.98%)▶PTBP1 (bg=3.74%)▶TIA1 (bg=4.07%)MATCHES To TargetScan▶ miR-200bc-3p/429:AAUACUG▶ miR-371-5p:CUCAAAC

-12545--(86)--12632-

TGTGTGTG

TGTGTGTG  
Depth:4 (DOG)  
Ei-value:0.000, Pi-value:0.000  
Er-value:0.000, Pr-value:0.000  
eCLIP MATCHES▶AATF (bg=0.64%)▶DDX24 (bg=2.97%)▶NCBP2 (bg=1.49%)▶NOLC1 (bg=9.43%)▶PTBP1 (bg=3.74%)▶SND1 (bg=0.45%)▶SRSF7 (bg=2.32%)▶TARDBP (bg=2.79%)▶WDR43 (bg=3.37%)▶XRCC6 (bg=2.91%)▶ZC3H8 (bg=0.29%)MATCHES To TargetScan▶ miR-329-3p/362-3p:ACACACC

-12639--(22)--12662-

TCCTAACCCCT

TCCTAACCCCT  
Depth:5 (RABBIT)  
Ei-value:0.000, Pi-value:0.000  
Er-value:0.000, Pr-value:0.000  
eCLIP MATCHES▶AATF (bg=0.64%)▶DDX24 (bg=2.97%)▶NCBP2 (bg=1.49%)▶NOLC1 (bg=9.43%)▶PTBP1 (bg=3.74%)▶SND1 (bg=0.45%)▶SRSF7 (bg=2.32%)▶TARDBP (bg=2.79%)▶UTP3 (bg=3.66%)▶WDR43 (bg=3.37%)▶XRCC6 (bg=2.91%)▶ZC3H8 (bg=0.29%)No matches to TargetScan

-12672--(33)--12706-

AAGCATTG

AAGCATTG  
Depth:4 (DOG)  
Ei-value:0.000, Pi-value:0.000  
Er-value:0.000, Pr-value:0.000  
eCLIP MATCHES▶DDX24 (bg=2.97%)▶NOLC1 (bg=9.43%)▶NPM1 (bg=1.21%)▶RBFOX2 (bg=4.63%)▶RPS3 (bg=0.76%)▶SRSF1 (bg=8.47%)▶SRSF7 (bg=2.32%)▶TARDBP (bg=2.79%)▶TRA2A (bg=4.8%)▶U2AF2 (bg=1.76%)▶uchl5 (bg=11.16%)▶YWHAG (bg=1.87%)▶ZNF622 (bg=6.58%)No matches to TargetScan

-12713--(12)--12726-

TTATGCCA

TTATGCCA  
Depth:5 (RABBIT)  
Ei-value:0.000, Pi-value:0.000  
Er-value:0.000, Pr-value:0.000  
eCLIP MATCHES▶DDX24 (bg=2.97%)▶FASTKD2 (bg=1.99%)▶LARP4 (bg=4.72%)▶NOLC1 (bg=9.43%)▶NPM1 (bg=1.21%)▶RBFOX2 (bg=4.63%)▶RBM15 (bg=7.27%)▶RPS3 (bg=0.76%)▶SRSF1 (bg=8.47%)▶SRSF7 (bg=2.32%)▶TARDBP (bg=2.79%)▶TRA2A (bg=4.8%)▶U2AF2 (bg=1.76%)▶uchl5 (bg=11.16%)▶WDR43 (bg=3.37%)▶YWHAG (bg=1.87%)▶ZC3H11A (bg=6.55%)▶ZNF622 (bg=6.58%)▶ZNF800 (bg=1.92%)No matches to TargetScan


G

TTATGCCAG  
Depth:4 (DOG)  
Ei-value:0.000, Pi-value:0.000  
Er-value:0.000, Pr-value:0.000  
eCLIP MATCHES▶DDX24 (bg=2.97%)▶FASTKD2 (bg=1.99%)▶LARP4 (bg=4.72%)▶NOLC1 (bg=9.43%)▶NPM1 (bg=1.21%)▶RBFOX2 (bg=4.63%)▶RBM15 (bg=7.27%)▶RPS3 (bg=0.76%)▶SRSF1 (bg=8.47%)▶SRSF7 (bg=2.32%)▶TARDBP (bg=2.79%)▶TRA2A (bg=4.8%)▶U2AF2 (bg=1.76%)▶uchl5 (bg=11.16%)▶WDR43 (bg=3.37%)▶YWHAG (bg=1.87%)▶ZC3H11A (bg=6.55%)▶ZNF622 (bg=6.58%)▶ZNF800 (bg=1.92%)No matches to TargetScan

-12734--(96)--12831-

AGA

AGAAGGCCCAA  
Depth:4 (DOG)  
Ei-value:0.000, Pi-value:0.000  
Er-value:0.000, Pr-value:0.000  
eCLIP MATCHES▶DDX24 (bg=2.97%)▶LARP4 (bg=4.72%)▶MTPAP (bg=2.21%)▶NOLC1 (bg=9.43%)▶SRSF1 (bg=8.47%)▶SRSF7 (bg=2.32%)▶TRA2A (bg=4.8%)▶uchl5 (bg=11.16%)▶UTP3 (bg=3.66%)▶ZNF622 (bg=6.58%)▶ZNF800 (bg=1.92%)No matches to TargetScan


AGGCCCAA

AGGCCCAA  
Depth:5 (RABBIT)  
Ei-value:0.000, Pi-value:0.000  
Er-value:0.000, Pr-value:0.000  
eCLIP MATCHES▶DDX24 (bg=2.97%)▶LARP4 (bg=4.72%)▶MTPAP (bg=2.21%)▶NOLC1 (bg=9.43%)▶SRSF1 (bg=8.47%)▶SRSF7 (bg=2.32%)▶TRA2A (bg=4.8%)▶uchl5 (bg=11.16%)▶UTP3 (bg=3.66%)▶ZNF622 (bg=6.58%)▶ZNF800 (bg=1.92%)No matches to TargetScan

-12841--(357)--13199-

TCAA

TCAAGACTAA  
Depth:4 (DOG)  
Ei-value:0.000, Pi-value:0.000  
Er-value:0.000, Pr-value:0.000  
eCLIP MATCHES▶CPEB4 (bg=1.89%)▶FASTKD2 (bg=1.99%)▶GRWD1 (bg=5.13%)▶LARP4 (bg=4.72%)▶MTPAP (bg=2.21%)▶NOLC1 (bg=9.43%)▶RBFOX2 (bg=4.63%)▶SRSF1 (bg=8.47%)▶TRA2A (bg=4.8%)▶uchl5 (bg=11.16%)▶UTP18 (bg=0.72%)▶UTP3 (bg=3.66%)▶WDR43 (bg=3.37%)▶ZNF622 (bg=6.58%)MATCHES To TargetScan▶ miR-431-5p:GUCUUGC


GACTAA

GACTAA  
Depth:5 (RABBIT)  
Ei-value:0.000, Pi-value:0.000  
Er-value:0.000, Pr-value:0.000  
eCLIP MATCHES▶CPEB4 (bg=1.89%)▶FASTKD2 (bg=1.99%)▶GRWD1 (bg=5.13%)▶LARP4 (bg=4.72%)▶MTPAP (bg=2.21%)▶NOLC1 (bg=9.43%)▶RBFOX2 (bg=4.63%)▶SRSF1 (bg=8.47%)▶TRA2A (bg=4.8%)▶uchl5 (bg=11.16%)▶UTP18 (bg=0.72%)▶UTP3 (bg=3.66%)▶WDR43 (bg=3.37%)▶ZNF622 (bg=6.58%)No matches to TargetScan

-13208--(43)--13252-

AGAAGC

AGAAGC  
Depth:4 (DOG)  
Ei-value:0.000, Pi-value:0.000  
Er-value:0.000, Pr-value:0.010  
eCLIP MATCHES▶CPEB4 (bg=1.89%)▶GRWD1 (bg=5.13%)▶LARP4 (bg=4.72%)▶MTPAP (bg=2.21%)▶NOLC1 (bg=9.43%)▶PCBP1 (bg=1.07%)▶RBFOX2 (bg=4.63%)▶SRSF1 (bg=8.47%)▶TRA2A (bg=4.8%)▶uchl5 (bg=11.16%)▶ZNF622 (bg=6.58%)No matches to TargetScan

-13257--(21)--13279-

AAGATGA

AAGATGA  
Depth:5 (RABBIT)  
Ei-value:0.000, Pi-value:0.000  
Er-value:0.000, Pr-value:0.000  
eCLIP MATCHES▶CPEB4 (bg=1.89%)▶FTO (bg=0.32%)▶GRWD1 (bg=5.13%)▶LARP4 (bg=4.72%)▶MTPAP (bg=2.21%)▶SRSF1 (bg=8.47%)▶TRA2A (bg=4.8%)▶uchl5 (bg=11.16%)▶ZNF622 (bg=6.58%)No matches to TargetScan

-13285--(99)--13385-

CTTTTTGATGTT

CTTTTTGATGTT  
Depth:4 (DOG)  
Ei-value:0.000, Pi-value:0.000  
Er-value:0.000, Pr-value:0.000  
eCLIP MATCHES▶TIA1 (bg=4.07%)No matches to TargetScan

-13396--(23)--13420-

TATTATGC

TATTATGC  
Depth:4 (DOG)  
Ei-value:0.000, Pi-value:0.000  
Er-value:0.000, Pr-value:0.000  
No matches to eCLIP DataMATCHES To TargetScan▶ miR-369-3p:AUAAUAC

-13427--(183)--13611-

TATTTCAGT

TATTTCAGT  
Depth:4 (DOG)  
Ei-value:0.000, Pi-value:0.000  
Er-value:0.000, Pr-value:0.000  
eCLIP MATCHES▶NOLC1 (bg=9.43%)▶ZC3H11A (bg=6.55%)MATCHES To TargetScan▶ miR-203a-3p.2:UGAAAUG

-13619--(55)--13675-

GGGGAAA

GGGGAAA  
Depth:4 (DOG)  
Ei-value:0.000, Pi-value:0.000  
Er-value:0.000, Pr-value:0.000  
eCLIP MATCHES▶CPSF6 (bg=0.4%)▶LARP4 (bg=4.72%)▶WDR43 (bg=3.37%)▶ZC3H11A (bg=6.55%)No matches to TargetScan

-13681--(16)--13698-

TCTAGAGAAAA

TCTAGAGAAAA  
Depth:6 (MOUSE)  
Ei-value:0.000, Pi-value:0.000  
Er-value:0.000, Pr-value:0.000  
eCLIP MATCHES▶CPSF6 (bg=0.4%)▶LARP4 (bg=4.72%)▶UTP3 (bg=3.66%)▶WDR43 (bg=3.37%)MATCHES To TargetScan▶ miR-1251-5p:CUCUAGC

-13708--(3)--13712-

TGAAGAGATG

TGAAGAGATG  
Depth:5 (RABBIT)  
Ei-value:0.000, Pi-value:0.000  
Er-value:0.000, Pr-value:0.000  
eCLIP MATCHES▶CPSF6 (bg=0.4%)▶LARP4 (bg=4.72%)▶SRSF7 (bg=2.32%)▶UTP3 (bg=3.66%)▶WDR43 (bg=3.37%)No matches to TargetScan

-13721--(5)--13727-

GGCCAA

GGCCAATGAGAAGAATTAGACA  
Depth:4 (DOG)  
Ei-value:0.000, Pi-value:0.000  
Er-value:0.000, Pr-value:0.000  
eCLIP MATCHES▶LARP4 (bg=4.72%)▶NOLC1 (bg=9.43%)▶SRSF7 (bg=2.32%)▶UTP3 (bg=3.66%)No matches to TargetScan


TGAGAAGAATTAGACA

TGAGAAGAATTAGACA  
Depth:6 (MOUSE)  
Ei-value:0.000, Pi-value:0.000  
Er-value:0.000, Pr-value:0.000  
eCLIP MATCHES▶LARP4 (bg=4.72%)▶NOLC1 (bg=9.43%)▶SRSF7 (bg=2.32%)No matches to TargetScan

-13748--(26)--13775-

TGAGAAG

TGAGAAG  
Depth:4 (DOG)  
Ei-value:0.000, Pi-value:0.000  
Er-value:0.000, Pr-value:0.010  
eCLIP MATCHES▶AARS (bg=2.18%)▶NOLC1 (bg=9.43%)▶PUS1 (bg=1.04%)▶SRSF7 (bg=2.32%)▶ZC3H11A (bg=6.55%)No matches to TargetScan

-13781--(9)--13791-

GCAACA

GCAACA  
Depth:6 (MOUSE)  
Ei-value:0.000, Pi-value:0.000  
Er-value:0.000, Pr-value:0.000  
eCLIP MATCHES▶AARS (bg=2.18%)▶NOLC1 (bg=9.43%)▶PUS1 (bg=1.04%)▶ZC3H11A (bg=6.55%)No matches to TargetScan

-13796--(19)--13816-

GGTGAGC

GGTGAGC  
Depth:4 (DOG)  
Ei-value:0.000, Pi-value:0.000  
Er-value:0.000, Pr-value:0.000  
eCLIP MATCHES▶AARS (bg=2.18%)▶NOLC1 (bg=9.43%)▶PUS1 (bg=1.04%)▶ZC3H11A (bg=6.55%)No matches to TargetScan

-13822--(10)--13833-

GGTTTGGG

GGTTTGGG  
Depth:4 (DOG)  
Ei-value:0.000, Pi-value:0.000  
Er-value:0.000, Pr-value:0.000  
eCLIP MATCHES▶AARS (bg=2.18%)▶AKAP8L (bg=2.19%)▶NOLC1 (bg=9.43%)▶PUS1 (bg=1.04%)No matches to TargetScan

-13840--(11)--13852-

TGGTTA

TGGTTA  
Depth:5 (RABBIT)  
Ei-value:0.000, Pi-value:0.000  
Er-value:0.000, Pr-value:0.000  
eCLIP MATCHES▶AKAP8L (bg=2.19%)▶NOLC1 (bg=9.43%)▶PUS1 (bg=1.04%)▶SF3B1 (bg=2.48%)No matches to TargetScan


T

TGGTTAT  
Depth:4 (DOG)  
Ei-value:0.000, Pi-value:0.000  
Er-value:0.000, Pr-value:0.000  
eCLIP MATCHES▶AKAP8L (bg=2.19%)▶NOLC1 (bg=9.43%)▶PUS1 (bg=1.04%)▶SF3B1 (bg=2.48%)No matches to TargetScan

-13858--(34)--13893-

CCCAAGG

CCCAAGG  
Depth:4 (DOG)  
Ei-value:0.000, Pi-value:0.000  
Er-value:0.000, Pr-value:0.000  
eCLIP MATCHES▶PUS1 (bg=1.04%)▶UTP3 (bg=3.66%)MATCHES To TargetScan▶ miR-212-5p:CCUUGGC

-13899--(8)--13908-

TGAACTCCCTGCT

TGAACTCCCTGCT  
Depth:4 (DOG)  
Ei-value:0.000, Pi-value:0.000  
Er-value:0.000, Pr-value:0.000  
eCLIP MATCHES▶UTP3 (bg=3.66%)No matches to TargetScan

-13920--(1)--13922-

ATAGTAGTGGCC

ATAGTAGTGGCC  
Depth:4 (DOG)  
Ei-value:0.000, Pi-value:0.000  
Er-value:0.000, Pr-value:0.000  
No matches to eCLIP DataNo matches to TargetScan

-13933--(37)--13971-

TTTAATAC

TTTAATAC  
Depth:4 (DOG)  
Ei-value:0.000, Pi-value:0.000  
Er-value:0.000, Pr-value:0.000  
eCLIP MATCHES▶WRN (bg=0.77%)MATCHES To TargetScan▶ miR-496.2:GUAUUAC

-13978--(6)--13985-

CT

CTAGGCTTAAAG  
Depth:4 (DOG)  
Ei-value:0.000, Pi-value:0.000  
Er-value:0.000, Pr-value:0.000  
No matches to eCLIP DataNo matches to TargetScan


AGGCTTA

AGGCTTA  
Depth:5 (RABBIT)  
Ei-value:0.000, Pi-value:0.000  
Er-value:0.000, Pr-value:0.000  
No matches to eCLIP DataNo matches to TargetScan


AAG

CTAGGCTTAAAG  
Depth:4 (DOG)  
Ei-value:0.000, Pi-value:0.000  
Er-value:0.000, Pr-value:0.000  
No matches to eCLIP DataNo matches to TargetScan

-13996--(27)--14024-

GTTTAAT

GTTTAAT  
Depth:5 (RABBIT)  
Ei-value:0.000, Pi-value:0.000  
Er-value:0.000, Pr-value:0.000  
No matches to eCLIP DataNo matches to TargetScan

-14030--(103)--14134-

T

TATTGGCA  
Depth:5 (RABBIT)  
Ei-value:0.000, Pi-value:0.000  
Er-value:0.000, Pr-value:0.000  
eCLIP MATCHES▶HNRNPA1 (bg=2.57%)No matches to TargetScan


ATTGGCA

ATTGGCA  
Depth:6 (MOUSE)  
Ei-value:0.000, Pi-value:0.000  
Er-value:0.000, Pr-value:0.000  
eCLIP MATCHES▶HNRNPA1 (bg=2.57%)No matches to TargetScan

-14141--(34)--14176-

TTGTGAAG

TTGTGAAG  
Depth:6 (MOUSE)  
Ei-value:0.000, Pi-value:0.000  
Er-value:0.000, Pr-value:0.000  
eCLIP MATCHES▶HNRNPA1 (bg=2.57%)No matches to TargetScan

-14183--(4)--14188-

ATGTAAAT

ATGTAAAT  
Depth:5 (RABBIT)  
Ei-value:0.000, Pi-value:0.000  
Er-value:0.000, Pr-value:0.000  
No matches to eCLIP DataNo matches to TargetScan

-14195--(230)--14426-

CTGTCCCT

CTGTCCCT  
Depth:4 (DOG)  
Ei-value:0.000, Pi-value:0.000  
Er-value:0.000, Pr-value:0.000  
No matches to eCLIP DataNo matches to TargetScan

-14433--(4)--14438-

TAGGCACT

TAGGCACT  
Depth:4 (DOG)  
Ei-value:0.000, Pi-value:0.000  
Er-value:0.000, Pr-value:0.000  
No matches to eCLIP DataNo matches to TargetScan

-14445--(66)--14512-

TAAAGCA

TAAAGCA  
Depth:4 (DOG)  
Ei-value:0.000, Pi-value:0.000  
Er-value:0.000, Pr-value:0.000  
eCLIP MATCHES▶LIN28B (bg=0.74%)No matches to TargetScan

-14518--(277)--14796-

AATGTGCCAGATA

AATGTGCCAGATA  
Depth:4 (DOG)  
Ei-value:0.000, Pi-value:0.000  
Er-value:0.000, Pr-value:0.000  
No matches to eCLIP DataMATCHES To TargetScan▶ miR-183-5p.2:UGGCACU

-14808--(73)--14882-

TTAAAGTG

TTAAAGTG  
Depth:4 (DOG)  
Ei-value:0.000, Pi-value:0.000  
Er-value:0.000, Pr-value:0.000  
eCLIP MATCHES▶SF3B1 (bg=2.48%)No matches to TargetScan

-14889--(9)--14899-

CTAAAGCA

CTAAAGCA  
Depth:4 (DOG)  
Ei-value:0.000, Pi-value:0.000  
Er-value:0.000, Pr-value:0.000  
eCLIP MATCHES▶SF3B1 (bg=2.48%)No matches to TargetScan

-14906--(49)--14956-

ATGAATA

ATGAATA  
Depth:4 (DOG)  
Ei-value:0.000, Pi-value:0.000  
Er-value:0.000, Pr-value:0.000  
eCLIP MATCHES▶DROSHA (bg=2.49%)▶TARDBP (bg=2.79%)▶ZC3H11A (bg=6.55%)No matches to TargetScan

-14962--(30)--14993-

GGTACTGT

GGTACTGT  
Depth:4 (DOG)  
Ei-value:0.000, Pi-value:0.000  
Er-value:0.000, Pr-value:0.000  
eCLIP MATCHES▶AARS (bg=2.18%)▶DROSHA (bg=2.49%)▶ILF3 (bg=3.0%)▶TARDBP (bg=2.79%)▶ZC3H11A (bg=6.55%)MATCHES To TargetScan▶ miR-101-3p.1:ACAGUAC▶ miR-144-3p:ACAGUAU

-15000--(27)--15028-

ATAAGAGG

ATAAGAGG  
Depth:4 (DOG)  
Ei-value:0.000, Pi-value:0.000  
Er-value:0.000, Pr-value:0.000  
eCLIP MATCHES▶ILF3 (bg=3.0%)No matches to TargetScan

-15035--(86)--15122-

TAAATTAT

TAAATTAT  
Depth:4 (DOG)  
Ei-value:0.000, Pi-value:0.010  
Er-value:0.000, Pr-value:0.000  
No matches to eCLIP DataNo matches to TargetScan

-15129--(158)--15288-

ATAAAAC

ATAAAAC  
Depth:4 (DOG)  
Ei-value:0.000, Pi-value:0.010  
Er-value:0.000, Pr-value:0.000  
No matches to eCLIP DataNo matches to TargetScan

-15294--(27)--15322-

AAAATTCTCA

AAAATTCTCA  
Depth:4 (DOG)  
Ei-value:0.000, Pi-value:0.000  
Er-value:0.000, Pr-value:0.000  
eCLIP MATCHES▶HNRNPU (bg=5.92%)No matches to TargetScan

-15331--(41)--15373-

TATACAAAC

TATACAAAC  
Depth:4 (DOG)  
Ei-value:0.000, Pi-value:0.000  
Er-value:0.000, Pr-value:0.000  
No matches to eCLIP DataNo matches to TargetScan

-15381--(48)--15430-

GTGAACTCA

GTGAACTCA  
Depth:4 (DOG)  
Ei-value:0.000, Pi-value:0.000  
Er-value:0.000, Pr-value:0.000  
No matches to eCLIP DataNo matches to TargetScan

-15438--(274)--15713-

TGTGCCA

TGTGCCA  
Depth:4 (DOG)  
Ei-value:0.000, Pi-value:0.000  
Er-value:0.000, Pr-value:0.000  
No matches to eCLIP DataMATCHES To TargetScan▶ miR-183-5p.2:UGGCACU

-15719--(27)--15747-

AAGATAA

AAGATAA  
Depth:4 (DOG)  
Ei-value:0.000, Pi-value:0.000  
Er-value:0.000, Pr-value:0.000  
No matches to eCLIP DataNo matches to TargetScan

-15753--(61)--15815-

TAAACTG

TAAACTG  
Depth:4 (DOG)  
Ei-value:0.000, Pi-value:0.000  
Er-value:0.000, Pr-value:0.000  
eCLIP MATCHES▶HNRNPU (bg=5.92%)No matches to TargetScan

-15821--(34)--15856-

ATTGATTA

ATTGATTA  
Depth:4 (DOG)  
Ei-value:0.000, Pi-value:0.000  
Er-value:0.000, Pr-value:0.010  
No matches to eCLIP DataNo matches to TargetScan

-15863--(11)--15875-

AGAGATA

AGAGATA  
Depth:4 (DOG)  
Ei-value:0.000, Pi-value:0.000  
Er-value:0.000, Pr-value:0.000  
No matches to eCLIP DataNo matches to TargetScan

-15881--(44)--15926-

CAGAGATCT

CAGAGATCT  
Depth:4 (DOG)  
Ei-value:0.000, Pi-value:0.000  
Er-value:0.000, Pr-value:0.000  
eCLIP MATCHES▶HNRNPA1 (bg=2.57%)▶HNRNPU (bg=5.92%)No matches to TargetScan

-15934--(49)--15984-

TTGGCT

TTGGCT  
Depth:4 (DOG)  
Ei-value:0.000, Pi-value:0.000  
Er-value:0.000, Pr-value:0.000  
eCLIP MATCHES▶HNRNPA1 (bg=2.57%)No matches to TargetScan

-15989--(13)--16003-

TTACTTTCT

TTACTTTCT  
Depth:4 (DOG)  
Ei-value:0.000, Pi-value:0.000  
Er-value:0.000, Pr-value:0.010  
eCLIP MATCHES▶UTP3 (bg=3.66%)No matches to TargetScan

-16011--(341)--16353-

GAGTAAAAA

GAGTAAAAA  
Depth:4 (DOG)  
Ei-value:0.000, Pi-value:0.000  
Er-value:0.000, Pr-value:0.000  
No matches to eCLIP DataNo matches to TargetScan

-16361--(28)--16390-

ATTTGAT

ATTTGAT  
Depth:4 (DOG)  
Ei-value:0.000, Pi-value:0.010  
Er-value:0.000, Pr-value:0.000  
No matches to eCLIP DataNo matches to TargetScan

-16396--(12)--16409-

TTTTATGT

TTTTATGT  
Depth:4 (DOG)  
Ei-value:0.000, Pi-value:0.000  
Er-value:0.000, Pr-value:0.000  
eCLIP MATCHES▶SAFB (bg=2.69%)No matches to TargetScan

-16416--(295)--16712-

AAGCCAG

AAGCCAG  
Depth:4 (DOG)  
Ei-value:0.000, Pi-value:0.000  
Er-value:0.000, Pr-value:0.000  
eCLIP MATCHES▶SAFB (bg=2.69%)MATCHES To TargetScan▶ miR-149-5p:CUGGCUC▶ miR-3064-5p:CUGGCUG

-16718--(7)--16726-

ATAAAAG

ATAAAAG  
Depth:4 (DOG)  
Ei-value:0.000, Pi-value:0.000  
Er-value:0.000, Pr-value:0.000  
No matches to eCLIP DataNo matches to TargetScan

-16732--(42)--16775-

TTTTATTA

TTTTATTA  
Depth:4 (DOG)  
Ei-value:0.000, Pi-value:0.010  
Er-value:0.000, Pr-value:0.000  
No matches to eCLIP DataNo matches to TargetScan

-16782--(3)--16786-

TTAAATGG

TTAAATGG  
Depth:4 (DOG)  
Ei-value:0.000, Pi-value:0.000  
Er-value:0.000, Pr-value:0.000  
No matches to eCLIP DataNo matches to TargetScan

-16793--(524)--17318-

ACAGAAAACAAAA

ACAGAAAACAAAA  
Depth:4 (DOG)  
Ei-value:0.000, Pi-value:0.000  
Er-value:0.000, Pr-value:0.000  
No matches to eCLIP DataNo matches to TargetScan

-17330--(60)--17391-

AGGTTA

AGGTTA  
Depth:4 (DOG)  
Ei-value:0.000, Pi-value:0.000  
Er-value:0.000, Pr-value:0.000  
No matches to eCLIP DataNo matches to TargetScan

-17396--(25)--17422-

TTCATTCT

TTCATTCT  
Depth:4 (DOG)  
Ei-value:0.000, Pi-value:0.000  
Er-value:0.000, Pr-value:0.000  
No matches to eCLIP DataNo matches to TargetScan

-17429--(50)--17480-

AGAGACA

AGAGACA  
Depth:4 (DOG)  
Ei-value:0.000, Pi-value:0.000  
Er-value:0.000, Pr-value:0.000  
No matches to eCLIP DataNo matches to TargetScan

-17486--(43)--17530-

CCTTTTGG

CCTTTTGG  
Depth:4 (DOG)  
Ei-value:0.000, Pi-value:0.000  
Er-value:0.000, Pr-value:0.000  
No matches to eCLIP DataNo matches to TargetScan

-17537--(51)--17589-

TTAATTC

TTAATTC  
Depth:4 (DOG)  
Ei-value:0.000, Pi-value:0.000  
Er-value:0.000, Pr-value:0.000  
No matches to eCLIP DataNo matches to TargetScan

-17595--(21)--17617-

ACTCTGGCCACTAC

ACTCTGGCCACTAC  
Depth:4 (DOG)  
Ei-value:0.000, Pi-value:0.000  
Er-value:0.000, Pr-value:0.000  
No matches to eCLIP DataMATCHES To TargetScan▶ miR-142-3p.1:GUAGUGU

-17630--(1)--17632-

ATAAGC

ATAAGC  
Depth:5 (RABBIT)  
Ei-value:0.000, Pi-value:0.010  
Er-value:0.000, Pr-value:0.000  
No matches to eCLIP DataNo matches to TargetScan


AGG

ATAAGCAGG  
Depth:4 (DOG)  
Ei-value:0.000, Pi-value:0.000  
Er-value:0.000, Pr-value:0.000  
No matches to eCLIP DataNo matches to TargetScan

-17640--(86)--17727-

GAAAAATG

GAAAAATG  
Depth:4 (DOG)  
Ei-value:0.000, Pi-value:0.000  
Er-value:0.000, Pr-value:0.000  
No matches to eCLIP DataNo matches to TargetScan

-17734--(54)--17789-

AGTCTCA

AGTCTCA  
Depth:4 (DOG)  
Ei-value:0.000, Pi-value:0.000  
Er-value:0.000, Pr-value:0.000  
eCLIP MATCHES▶ZC3H11A (bg=6.55%)No matches to TargetScan

-17795--(42)--17838-

TGGTTTTGAA

TGGTTTTGAA  
Depth:4 (DOG)  
Ei-value:0.000, Pi-value:0.000  
Er-value:0.000, Pr-value:0.000  
No matches to eCLIP DataNo matches to TargetScan

-17847--(163)--18011-

TTAGAAAT

TTAGAAAT  
Depth:4 (DOG)  
Ei-value:0.000, Pi-value:0.000  
Er-value:0.000, Pr-value:0.000  
eCLIP MATCHES▶WDR3 (bg=0.25%)No matches to TargetScan

-18018--(14)--18033-

CATCAAA

CATCAAA  
Depth:4 (DOG)  
Ei-value:0.000, Pi-value:0.000  
Er-value:0.000, Pr-value:0.000  
eCLIP MATCHES▶ZC3H11A (bg=6.55%)No matches to TargetScan

-18039--(180)--18220-

TTTTTAAATCACTCA

TTTTTAAATCACTCA  
Depth:4 (DOG)  
Ei-value:0.000, Pi-value:0.000  
Er-value:0.000, Pr-value:0.000  
eCLIP MATCHES▶ILF3 (bg=3.0%)▶NOLC1 (bg=9.43%)▶PPIL4 (bg=0.52%)No matches to TargetScan

-18234--(1)--18236-

AGAGGGTGGGA

AGAGGGTGGGA  
Depth:4 (DOG)  
Ei-value:0.000, Pi-value:0.000  
Er-value:0.000, Pr-value:0.000  
eCLIP MATCHES▶ILF3 (bg=3.0%)▶ZC3H11A (bg=6.55%)No matches to TargetScan

-18246--(1)--18248-

AGGAGGAAGAGTGAA

AGGAGGAAGAGTGAA  
Depth:4 (DOG)  
Ei-value:0.000, Pi-value:0.000  
Er-value:0.000, Pr-value:0.000  
eCLIP MATCHES▶ILF3 (bg=3.0%)▶ZC3H11A (bg=6.55%)MATCHES To TargetScan▶ miR-670-3p:UUCCUCA

-18262--(1)--18264-

G

GAAAAGGTCA  
Depth:4 (DOG)  
Ei-value:0.000, Pi-value:0.000  
Er-value:0.000, Pr-value:0.000  
eCLIP MATCHES▶ILF3 (bg=3.0%)▶SF3B1 (bg=2.48%)▶ZC3H11A (bg=6.55%)MATCHES To TargetScan▶ miR-192-5p/215-5p:UGACCUA


AAAAGGT

AAAAGGT  
Depth:6 (MOUSE)  
Ei-value:0.000, Pi-value:0.000  
Er-value:0.000, Pr-value:0.000  
eCLIP MATCHES▶ILF3 (bg=3.0%)▶SF3B1 (bg=2.48%)▶ZC3H11A (bg=6.55%)No matches to TargetScan


CA

GAAAAGGTCA  
Depth:4 (DOG)  
Ei-value:0.000, Pi-value:0.000  
Er-value:0.000, Pr-value:0.000  
eCLIP MATCHES▶ILF3 (bg=3.0%)▶SF3B1 (bg=2.48%)▶ZC3H11A (bg=6.55%)MATCHES To TargetScan▶ miR-192-5p/215-5p:UGACCUA

-18273--(503)--18777-

TAATGTTT

TAATGTTT  
Depth:4 (DOG)  
Ei-value:0.000, Pi-value:0.000  
Er-value:0.000, Pr-value:0.000  
eCLIP MATCHES▶CPEB4 (bg=1.89%)▶KHDRBS1 (bg=1.71%)▶LARP4 (bg=4.72%)▶LSM11 (bg=2.28%)▶NOLC1 (bg=9.43%)▶RBFOX2 (bg=4.63%)▶SAFB (bg=2.69%)▶SAFB2 (bg=0.8%)▶WDR43 (bg=3.37%)▶ZC3H11A (bg=6.55%)MATCHES To TargetScan▶ miR-323-3p:ACAUUAC▶ miR-543:AACAUUC

-18784--(20)--18805-

AGCTGGA

AGCTGGA  
Depth:4 (DOG)  
Ei-value:0.000, Pi-value:0.000  
Er-value:0.000, Pr-value:0.000  
eCLIP MATCHES▶CPEB4 (bg=1.89%)▶KHDRBS1 (bg=1.71%)▶LSM11 (bg=2.28%)▶NOLC1 (bg=9.43%)▶RBFOX2 (bg=4.63%)▶SAFB (bg=2.69%)▶SAFB2 (bg=0.8%)▶SF3B1 (bg=2.48%)▶TRA2A (bg=4.8%)▶WDR43 (bg=3.37%)▶ZC3H11A (bg=6.55%)No matches to TargetScan

-18811--(40)--18852-

ATTATTGGAAA

ATTATTGGAAA  
Depth:4 (DOG)  
Ei-value:0.000, Pi-value:0.000  
Er-value:0.000, Pr-value:0.000  
eCLIP MATCHES▶FASTKD2 (bg=1.99%)▶FUS (bg=2.21%)▶LARP4 (bg=4.72%)▶NOLC1 (bg=9.43%)▶RBFOX2 (bg=4.63%)▶SAFB (bg=2.69%)▶SAFB2 (bg=0.8%)▶TRA2A (bg=4.8%)▶WDR43 (bg=3.37%)▶ZC3H11A (bg=6.55%)No matches to TargetScan

-18862--(9)--18872-

AGAAAGTAAC

AGAAAGTAAC  
Depth:4 (DOG)  
Ei-value:0.000, Pi-value:0.000  
Er-value:0.000, Pr-value:0.000  
eCLIP MATCHES▶FASTKD2 (bg=1.99%)▶FUS (bg=2.21%)▶LARP4 (bg=4.72%)▶NIPBL (bg=5.39%)▶NOLC1 (bg=9.43%)▶RBFOX2 (bg=4.63%)▶SAFB (bg=2.69%)▶SAFB2 (bg=0.8%)▶TRA2A (bg=4.8%)▶uchl5 (bg=11.16%)▶WDR43 (bg=3.37%)▶ZC3H11A (bg=6.55%)▶ZNF800 (bg=1.92%)No matches to TargetScan

-18881--(12)--18894-

TTTCACAGTTTCTGGCATC

TTTCACAGTTTCTGGCATC  
Depth:4 (DOG)  
Ei-value:0.000, Pi-value:0.000  
Er-value:0.000, Pr-value:0.000  
eCLIP MATCHES▶FASTKD2 (bg=1.99%)▶FUS (bg=2.21%)▶LARP4 (bg=4.72%)▶NIPBL (bg=5.39%)▶NOLC1 (bg=9.43%)▶RBFOX2 (bg=4.63%)▶SAFB (bg=2.69%)▶SAFB2 (bg=0.8%)▶uchl5 (bg=11.16%)▶WDR43 (bg=3.37%)▶ZC3H11A (bg=6.55%)▶ZNF800 (bg=1.92%)No matches to TargetScan

-18912--(7)--18920-

CTACTGAT

CTACTGAT  
Depth:4 (DOG)  
Ei-value:0.000, Pi-value:0.000  
Er-value:0.000, Pr-value:0.000  
eCLIP MATCHES▶FASTKD2 (bg=1.99%)▶FUS (bg=2.21%)▶LARP4 (bg=4.72%)▶NIPBL (bg=5.39%)▶NOLC1 (bg=9.43%)▶RBFOX2 (bg=4.63%)▶SAFB (bg=2.69%)▶SAFB2 (bg=0.8%)▶uchl5 (bg=11.16%)▶ZC3H11A (bg=6.55%)▶ZNF800 (bg=1.92%)MATCHES To TargetScan▶ miR-199-3p:CAGUAGU

-18927--(14)--18942-

AGAACAT

AGAACAT  
Depth:4 (DOG)  
Ei-value:0.000, Pi-value:0.000  
Er-value:0.000, Pr-value:0.000  
eCLIP MATCHES▶FASTKD2 (bg=1.99%)▶FUS (bg=2.21%)▶LARP4 (bg=4.72%)▶NIPBL (bg=5.39%)▶NOLC1 (bg=9.43%)▶RBFOX2 (bg=4.63%)▶SAFB2 (bg=0.8%)▶uchl5 (bg=11.16%)▶ZNF622 (bg=6.58%)No matches to TargetScan

-18948--(7)--18956-

TCATCTG

TCATCTG  
Depth:4 (DOG)  
Ei-value:0.000, Pi-value:0.010  
Er-value:0.000, Pr-value:0.000  
eCLIP MATCHES▶FUS (bg=2.21%)▶LARP4 (bg=4.72%)▶NOLC1 (bg=9.43%)▶RBFOX2 (bg=4.63%)▶RPS3 (bg=0.76%)▶uchl5 (bg=11.16%)▶ZNF622 (bg=6.58%)No matches to TargetScan

-18962--(9)--18972-

CATAAATGAA

CATAAATGAA  
Depth:4 (DOG)  
Ei-value:0.000, Pi-value:0.000  
Er-value:0.000, Pr-value:0.000  
eCLIP MATCHES▶FUS (bg=2.21%)▶NOLC1 (bg=9.43%)▶RPS3 (bg=0.76%)▶uchl5 (bg=11.16%)▶ZNF622 (bg=6.58%)No matches to TargetScan

-18981--(121)--19103-

TGAACTGATGTGAAA

TGAACTGATGTGAAA  
Depth:4 (DOG)  
Ei-value:0.000, Pi-value:0.000  
Er-value:0.000, Pr-value:0.000  
eCLIP MATCHES▶FUS (bg=2.21%)▶NOLC1 (bg=9.43%)▶RBFOX2 (bg=4.63%)▶TRA2A (bg=4.8%)MATCHES To TargetScan▶ miR-23-3p:UCACAUU

-19117--(134)--19252-

AAATAAAA

AAATAAAA  
Depth:4 (DOG)  
Ei-value:0.000, Pi-value:0.000  
Er-value:0.000, Pr-value:0.000  
eCLIP MATCHES▶WDR43 (bg=3.37%)▶ZC3H11A (bg=6.55%)No matches to TargetScan

-19259  
  
>PIG  
      1366-

AAACATG

AAACATG  
Depth:4 (DOG)  
Ei-value:0.000, Pi-value:0.000  
Er-value:0.000, Pr-value:0.000  
No matches to TargetScan

-1372--(1158)--2531-

TTCCCATC

TTCCCATC  
Depth:4 (DOG)  
Ei-value:0.000, Pi-value:0.000  
Er-value:0.000, Pr-value:0.000  
No matches to TargetScan

-2538--(5142)--7681-

C

CTGTTAGTCT  
Depth:4 (DOG)  
Ei-value:0.000, Pi-value:0.000  
Er-value:0.000, Pr-value:0.000  
No matches to TargetScan


TGTTAGTC

TGTTAGTC  
Depth:5 (RABBIT)  
Ei-value:0.000, Pi-value:0.000  
Er-value:0.000, Pr-value:0.000  
No matches to TargetScan


T

CTGTTAGTCT  
Depth:4 (DOG)  
Ei-value:0.000, Pi-value:0.000  
Er-value:0.000, Pr-value:0.000  
No matches to TargetScan

-7690--(961)--8652-

TCATCC

TCATCC  
Depth:4 (DOG)  
Ei-value:0.000, Pi-value:0.020  
Er-value:0.000, Pr-value:0.000  
No matches to TargetScan

-8657--(605)--9263-

TACTTGGGACTGTTAAT

TACTTGGGACTGTTAAT  
Depth:4 (DOG)  
Ei-value:0.000, Pi-value:0.000  
Er-value:0.000, Pr-value:0.000  
MATCHES To TargetScan▶ miR-132-3p/212-3p:AACAGUC▶ miR-455-3p.1:CAGUCCA

-9279--(466)--9746-

ACTG

ACTGTTAATGTGCT  
Depth:4 (DOG)  
Ei-value:0.000, Pi-value:0.000  
Er-value:0.000, Pr-value:0.000  
MATCHES To TargetScan▶ miR-132-3p/212-3p:AACAGUC▶ miR-323-3p:ACAUUAC


TTAATGTGCT

TTAATGTGCT  
Depth:5 (RABBIT)  
Ei-value:0.000, Pi-value:0.000  
Er-value:0.000, Pr-value:0.000  
MATCHES To TargetScan▶ miR-323-3p:ACAUUAC

-9759--(3753)--13513-

AATGTGCAT

AATGTGCAT  
Depth:6 (MOUSE)  
Ei-value:0.000, Pi-value:0.000  
Er-value:0.000, Pr-value:0.000  
MATCHES To TargetScan▶ miR-501-3p/502-3p:AUGCACC

-13521--(206)--13728-

TATGTTAGA

TATGTTAGA  
Depth:4 (DOG)  
Ei-value:0.000, Pi-value:0.000  
Er-value:0.000, Pr-value:0.000  
No matches to TargetScan

-13736--(72)--13809-

ACTGTTAATGT

ACTGTTAATGT  
Depth:4 (DOG)  
Ei-value:0.000, Pi-value:0.000  
Er-value:0.000, Pr-value:0.000  
MATCHES To TargetScan▶ miR-132-3p/212-3p:AACAGUC▶ miR-323-3p:ACAUUAC

-13819--(10)--13830-

ATTTGCT

ATTTGCT  
Depth:4 (DOG)  
Ei-value:0.000, Pi-value:0.000  
Er-value:0.000, Pr-value:0.000  
No matches to TargetScan

-13836--(12)--13849-

GTAAGGA

GTAAGGA  
Depth:5 (RABBIT)  
Ei-value:0.000, Pi-value:0.000  
Er-value:0.000, Pr-value:0.000  
No matches to TargetScan

-13855--(141)--13997-

ACTTAT

ACTTAT  
Depth:5 (RABBIT)  
Ei-value:0.000, Pi-value:0.000  
Er-value:0.000, Pr-value:0.000  
No matches to TargetScan

-14002--(143)--14146-

TTAAGGCC

TTAAGGCC  
Depth:6 (MOUSE)  
Ei-value:0.000, Pi-value:0.000  
Er-value:0.000, Pr-value:0.000  
No matches to TargetScan


CCTTT

TTAAGGCCCCTTT  
Depth:5 (RABBIT)  
Ei-value:0.000, Pi-value:0.000  
Er-value:0.000, Pr-value:0.000  
No matches to TargetScan


CTCAA

TTAAGGCCCCTTTCTCAA  
Depth:4 (DOG)  
Ei-value:0.000, Pi-value:0.000  
Er-value:0.000, Pr-value:0.000  
No matches to TargetScan

-14163--(744)--14908-

ACAGTTAATGTG

ACAGTTAATGTG  
Depth:4 (DOG)  
Ei-value:0.000, Pi-value:0.000  
Er-value:0.000, Pr-value:0.000  
MATCHES To TargetScan▶ miR-323-3p:ACAUUAC

-14919--(178)--15098-

CTCAGCTCTTGG

CTCAGCTCTTGG  
Depth:5 (RABBIT)  
Ei-value:0.000, Pi-value:0.000  
Er-value:0.000, Pr-value:0.000  
MATCHES To TargetScan▶ miR-335-5p:CAAGAGC


ACA

CTCAGCTCTTGGACA  
Depth:4 (DOG)  
Ei-value:0.000, Pi-value:0.000  
Er-value:0.000, Pr-value:0.000  
MATCHES To TargetScan▶ miR-335-5p:CAAGAGC

-15112--(206)--15319-

CAAAACTT

CAAAACTT  
Depth:4 (DOG)  
Ei-value:0.000, Pi-value:0.000  
Er-value:0.000, Pr-value:0.000  
No matches to TargetScan

-15326--(19)--15346-

GCACAATG

GCACAATG  
Depth:6 (MOUSE)  
Ei-value:0.000, Pi-value:0.000  
Er-value:0.000, Pr-value:0.000  
No matches to TargetScan

-15353--(131)--15485-

A

ACTCCCA  
Depth:4 (DOG)  
Ei-value:0.000, Pi-value:0.000  
Er-value:0.000, Pr-value:0.000  
No matches to TargetScan


CTCCCA

CTCCCA  
Depth:6 (MOUSE)  
Ei-value:0.000, Pi-value:0.000  
Er-value:0.000, Pr-value:0.000  
No matches to TargetScan

-15491--(191)--15683-

CCCTTTTGCATT

CCCTTTTGCATT  
Depth:4 (DOG)  
Ei-value:0.000, Pi-value:0.000  
Er-value:0.000, Pr-value:0.000  
No matches to TargetScan

-15694--(222)--15917-

CCATTTTT

CCATTTTT  
Depth:4 (DOG)  
Ei-value:0.000, Pi-value:0.000  
Er-value:0.000, Pr-value:0.000  
No matches to TargetScan

-15924--(1)--15926-

CAGCCCA

CAGCCCA  
Depth:4 (DOG)  
Ei-value:0.000, Pi-value:0.000  
Er-value:0.000, Pr-value:0.000  
No matches to TargetScan

-15932--(1)--15934-

CAGCCCA

CAGCCCA  
Depth:4 (DOG)  
Ei-value:0.000, Pi-value:0.000  
Er-value:0.000, Pr-value:0.000  
No matches to TargetScan

-15940--(7)--15948-

CCTACCA

CCTACCA  
Depth:4 (DOG)  
Ei-value:0.000, Pi-value:0.000  
Er-value:0.000, Pr-value:0.000  
No matches to TargetScan

-15954--(33)--15988-

AAAAGCAG

AAAAGCAG  
Depth:6 (MOUSE)  
Ei-value:0.000, Pi-value:0.000  
Er-value:0.000, Pr-value:0.000  
No matches to TargetScan

-15995--(44)--16040-

TTAATGATCC

TTAATGATCC  
Depth:4 (DOG)  
Ei-value:0.000, Pi-value:0.000  
Er-value:0.000, Pr-value:0.000  
MATCHES To TargetScan▶ miR-382-3p:AUCAUUC

-16049--(19)--16069-

ATTCTGGG

ATTCTGGG  
Depth:4 (DOG)  
Ei-value:0.000, Pi-value:0.000  
Er-value:0.000, Pr-value:0.000  
No matches to TargetScan

-16076--(31)--16108-

CTTTACT

CTTTACT  
Depth:4 (DOG)  
Ei-value:0.000, Pi-value:0.000  
Er-value:0.000, Pr-value:0.000  
No matches to TargetScan

-16114--(2)--16117-

GCAAAAT

GCAAAAT  
Depth:6 (MOUSE)  
Ei-value:0.000, Pi-value:0.000  
Er-value:0.000, Pr-value:0.000  
No matches to TargetScan

-16123--(4)--16128-

AAGGCAA

AAGGCAA  
Depth:4 (DOG)  
Ei-value:0.000, Pi-value:0.000  
Er-value:0.000, Pr-value:0.000  
No matches to TargetScan

-16134--(18)--16153-

TGGATTGC

TGGATTGC  
Depth:4 (DOG)  
Ei-value:0.000, Pi-value:0.000  
Er-value:0.000, Pr-value:0.000  
No matches to TargetScan

-16160--(82)--16243-

TGCATTCTTC

TGCATTCTTC  
Depth:5 (RABBIT)  
Ei-value:0.000, Pi-value:0.000  
Er-value:0.000, Pr-value:0.000  
No matches to TargetScan

-16252--(7)--16260-

AGC

AGCAGATTGCCTGG  
Depth:4 (DOG)  
Ei-value:0.000, Pi-value:0.000  
Er-value:0.000, Pr-value:0.000  
No matches to TargetScan


A

AGATTGCCTGG  
Depth:5 (RABBIT)  
Ei-value:0.000, Pi-value:0.000  
Er-value:0.000, Pr-value:0.000  
No matches to TargetScan


GATTGCCTGG

GATTGCCTGG  
Depth:6 (MOUSE)  
Ei-value:0.000, Pi-value:0.000  
Er-value:0.000, Pr-value:0.000  
No matches to TargetScan

-16273--(21)--16295-

TTGTATATT

TTGTATATT  
Depth:4 (DOG)  
Ei-value:0.000, Pi-value:0.000  
Er-value:0.000, Pr-value:0.000  
MATCHES To TargetScan▶ miR-381-3p:AUACAAG

-16303--(95)--16399-

AAC

AACCTGGTCATT  
Depth:4 (DOG)  
Ei-value:0.000, Pi-value:0.000  
Er-value:0.000, Pr-value:0.000  
No matches to TargetScan


CTGGTCATT

CTGGTCATT  
Depth:5 (RABBIT)  
Ei-value:0.000, Pi-value:0.000  
Er-value:0.000, Pr-value:0.000  
No matches to TargetScan

-16410--(22)--16433-

CCATTTAT

CCATTTAT  
Depth:5 (RABBIT)  
Ei-value:0.000, Pi-value:0.000  
Er-value:0.000, Pr-value:0.000  
No matches to TargetScan

-16440--(13)--16454-

TGAC

TGACCAGTGTCTCTCATTT  
Depth:4 (DOG)  
Ei-value:0.000, Pi-value:0.000  
Er-value:0.000, Pr-value:0.000  
No matches to TargetScan


CAGTGTCTCTCATTT

CAGTGTCTCTCATTT  
Depth:5 (RABBIT)  
Ei-value:0.000, Pi-value:0.000  
Er-value:0.000, Pr-value:0.000  
No matches to TargetScan

-16472--(4)--16477-

AGG

AGGGTGGTG  
Depth:4 (DOG)  
Ei-value:0.000, Pi-value:0.000  
Er-value:0.000, Pr-value:0.000  
No matches to TargetScan


GTGGTG

GTGGTG  
Depth:5 (RABBIT)  
Ei-value:0.000, Pi-value:0.000  
Er-value:0.000, Pr-value:0.000  
No matches to TargetScan

-16485--(1)--16487-

GTCTGTGGATA

GTCTGTGGATA  
Depth:5 (RABBIT)  
Ei-value:0.000, Pi-value:0.000  
Er-value:0.000, Pr-value:0.000  
MATCHES To TargetScan▶ miR-140-3p.1:CCACAGG

-16497--(42)--16540-

TTCTAGA

TTCTAGA  
Depth:4 (DOG)  
Ei-value:0.000, Pi-value:0.000  
Er-value:0.000, Pr-value:0.000  
No matches to TargetScan

-16546--(73)--16620-

ATTCACTT

ATTCACTT  
Depth:4 (DOG)  
Ei-value:0.000, Pi-value:0.000  
Er-value:0.000, Pr-value:0.000  
No matches to TargetScan

-16627--(3)--16631-

GAAAAAC

GAAAAAC  
Depth:4 (DOG)  
Ei-value:0.000, Pi-value:0.000  
Er-value:0.000, Pr-value:0.000  
No matches to TargetScan

-16637--(22)--16660-

AATTTCTTCATCTGGAGC

AATTTCTTCATCTGGAGC  
Depth:5 (RABBIT)  
Ei-value:0.000, Pi-value:0.000  
Er-value:0.000, Pr-value:0.000  
No matches to TargetScan

-16677--(15)--16693-

CTTATTT

CTTATTT  
Depth:4 (DOG)  
Ei-value:0.000, Pi-value:0.000  
Er-value:0.000, Pr-value:0.010  
No matches to TargetScan

-16699--(21)--16721-

ATAAAATG

ATAAAATG  
Depth:4 (DOG)  
Ei-value:0.000, Pi-value:0.000  
Er-value:0.000, Pr-value:0.000  
No matches to TargetScan

-16728--(157)--16886-

AAAAATAAGCCA

AAAAATAAGCCA  
Depth:5 (RABBIT)  
Ei-value:0.000, Pi-value:0.000  
Er-value:0.000, Pr-value:0.000  
No matches to TargetScan


A

AAAAATAAGCCAA  
Depth:4 (DOG)  
Ei-value:0.000, Pi-value:0.000  
Er-value:0.000, Pr-value:0.000  
No matches to TargetScan

-16898--(57)--16956-

ATGAATAATA

ATGAATAATA  
Depth:4 (DOG)  
Ei-value:0.000, Pi-value:0.000  
Er-value:0.000, Pr-value:0.000  
No matches to TargetScan

-16965--(48)--17014-

TGGAACTGCT

TGGAACTGCT  
Depth:4 (DOG)  
Ei-value:0.000, Pi-value:0.000  
Er-value:0.000, Pr-value:0.000  
No matches to TargetScan

-17023--(8)--17032-

TAACTA

TAACTA  
Depth:4 (DOG)  
Ei-value:0.000, Pi-value:0.000  
Er-value:0.000, Pr-value:0.000  
No matches to TargetScan

-17037--(10)--17048-

CAGCAGTTC

CAGCAGTTC  
Depth:5 (RABBIT)  
Ei-value:0.000, Pi-value:0.000  
Er-value:0.000, Pr-value:0.000  
No matches to TargetScan

-17056--(1)--17058-

TTGTAAT

TTGTAAT  
Depth:4 (DOG)  
Ei-value:0.000, Pi-value:0.000  
Er-value:0.000, Pr-value:0.000  
No matches to TargetScan

-17064--(1)--17066-

ACTGAAAA

ACTGAAAA  
Depth:5 (RABBIT)  
Ei-value:0.000, Pi-value:0.000  
Er-value:0.000, Pr-value:0.000  
No matches to TargetScan

-17073--(17)--17091-

AAGGATG

AAGGATG  
Depth:5 (RABBIT)  
Ei-value:0.000, Pi-value:0.000  
Er-value:0.000, Pr-value:0.000  
MATCHES To TargetScan▶ miR-362-5p/500b-5p:AUCCUUG


TCA

AAGGATGTCAAAAGATC  
Depth:4 (DOG)  
Ei-value:0.000, Pi-value:0.000  
Er-value:0.000, Pr-value:0.000  
MATCHES To TargetScan▶ miR-362-5p/500b-5p:AUCCUUG▶ miR-489-3p:UGACAUC


AAAGATC

AAAGATC  
Depth:6 (MOUSE)  
Ei-value:0.000, Pi-value:0.000  
Er-value:0.000, Pr-value:0.000  
No matches to TargetScan

-17107--(4)--17112-

CAGCTCAGGG

CAGCTCAGGG  
Depth:4 (DOG)  
Ei-value:0.000, Pi-value:0.000  
Er-value:0.000, Pr-value:0.000  
MATCHES To TargetScan▶ miR-125-5p:CCCUGAG

-17121--(11)--17133-

CTACTAGCTCCT

CTACTAGCTCCT  
Depth:4 (DOG)  
Ei-value:0.000, Pi-value:0.000  
Er-value:0.000, Pr-value:0.000  
MATCHES To TargetScan▶ miR-28-5p/708-5p:AGGAGCU▶ miR-411-5p.2:UAGUAGA

-17144--(1)--17146-

GGACAGCTG

GGACAGCTG  
Depth:5 (RABBIT)  
Ei-value:0.000, Pi-value:0.000  
Er-value:0.000, Pr-value:0.000  
No matches to TargetScan


T

GGACAGCTGT  
Depth:4 (DOG)  
Ei-value:0.000, Pi-value:0.000  
Er-value:0.000, Pr-value:0.000  
No matches to TargetScan

-17155--(1)--17157-

AGAAGAGTCTCTGGCTCTTTA

AGAAGAGTCTCTGGCTCTTTA  
Depth:5 (RABBIT)  
Ei-value:0.000, Pi-value:0.000  
Er-value:0.000, Pr-value:0.000  
No matches to TargetScan


GA

AGAAGAGTCTCTGGCTCTTTAGA  
Depth:4 (DOG)  
Ei-value:0.000, Pi-value:0.000  
Er-value:0.000, Pr-value:0.000  
No matches to TargetScan

-17179--(11)--17191-

ATTCTGAGC

ATTCTGAGC  
Depth:4 (DOG)  
Ei-value:0.000, Pi-value:0.000  
Er-value:0.000, Pr-value:0.000  
No matches to TargetScan

-17199--(101)--17301-

CTGCAA

CTGCAA  
Depth:5 (RABBIT)  
Ei-value:0.000, Pi-value:0.000  
Er-value:0.000, Pr-value:0.000  
No matches to TargetScan

-17306--(114)--17421-

AAGAATAGGC

AAGAATAGGC  
Depth:5 (RABBIT)  
Ei-value:0.000, Pi-value:0.000  
Er-value:0.000, Pr-value:0.000  
No matches to TargetScan

-17430--(9)--17440-

TACAGTGTTAGTGA

TACAGTGTTAGTGA  
Depth:5 (RABBIT)  
Ei-value:0.000, Pi-value:0.000  
Er-value:0.000, Pr-value:0.000  
MATCHES To TargetScan▶ miR-141-3p/200a-3p:AACACUG

-17453--(4)--17458-

TTCCCTTTGA

TTCCCTTTGA  
Depth:6 (MOUSE)  
Ei-value:0.000, Pi-value:0.000  
Er-value:0.000, Pr-value:0.000  
No matches to TargetScan

-17467--(7)--17475-

TAGGTGGAGATGGGGCATGAGGATCCTCCAGGGGAA

TAGGTGGAGATGGGGCATGAGGATCCTCCAGGGGAA  
Depth:6 (MOUSE)  
Ei-value:0.000, Pi-value:0.000  
Er-value:0.000, Pr-value:0.000  
MATCHES To TargetScan▶ miR-331-3p:CCCCUGG


A

TAGGTGGAGATGGGGCATGAGGATCCTCCAGGGGAAA  
Depth:5 (RABBIT)  
Ei-value:0.000, Pi-value:0.000  
Er-value:0.000, Pr-value:0.000  
MATCHES To TargetScan▶ miR-331-3p:CCCCUGG

-17511--(3)--17515-

TCACTA

TCACTA  
Depth:5 (RABBIT)  
Ei-value:0.000, Pi-value:0.000  
Er-value:0.000, Pr-value:0.000  
No matches to TargetScan


CCACT

TCACTACCACT  
Depth:4 (DOG)  
Ei-value:0.000, Pi-value:0.000  
Er-value:0.000, Pr-value:0.000  
MATCHES To TargetScan▶ miR-140-5p:AGUGGUU▶ miR-142-3p.1:GUAGUGU

-17525--(2)--17528-

GCAACA

GCAACA  
Depth:6 (MOUSE)  
Ei-value:0.000, Pi-value:0.000  
Er-value:0.000, Pr-value:0.000  
No matches to TargetScan


AC

GCAACAAC  
Depth:5 (RABBIT)  
Ei-value:0.000, Pi-value:0.000  
Er-value:0.000, Pr-value:0.000  
No matches to TargetScan

-17535--(63)--17599-

ACAACCACC

ACAACCACC  
Depth:5 (RABBIT)  
Ei-value:0.000, Pi-value:0.000  
Er-value:0.000, Pr-value:0.000  
No matches to TargetScan


ACAC

ACAACCACCACAC  
Depth:4 (DOG)  
Ei-value:0.000, Pi-value:0.000  
Er-value:0.000, Pr-value:0.000  
No matches to TargetScan

-17611--(343)--17955-

TTGTTCC

TTGTTCC  
Depth:4 (DOG)  
Ei-value:0.000, Pi-value:0.000  
Er-value:0.000, Pr-value:0.000  
No matches to TargetScan

-17961--(11)--17973-

CCAAAT

CCAAAT  
Depth:6 (MOUSE)  
Ei-value:0.000, Pi-value:0.000  
Er-value:0.000, Pr-value:0.000  
No matches to TargetScan


C

CCAAATC  
Depth:5 (RABBIT)  
Ei-value:0.000, Pi-value:0.000  
Er-value:0.000, Pr-value:0.000  
No matches to TargetScan

-17979--(29)--18009-

CAAGAAA

CAAGAAA  
Depth:5 (RABBIT)  
Ei-value:0.000, Pi-value:0.000  
Er-value:0.000, Pr-value:0.000  
No matches to TargetScan

-18015--(16)--18032-

G

GAAGATCAACATGCCTG  
Depth:4 (DOG)  
Ei-value:0.000, Pi-value:0.000  
Er-value:0.000, Pr-value:0.000  
No matches to TargetScan


AA

AAGATCAACATGC  
Depth:5 (RABBIT)  
Ei-value:0.000, Pi-value:0.000  
Er-value:0.000, Pr-value:0.000  
No matches to TargetScan


GATCAACATGC

GATCAACATGC  
Depth:6 (MOUSE)  
Ei-value:0.000, Pi-value:0.000  
Er-value:0.000, Pr-value:0.000  
No matches to TargetScan


CTG

GAAGATCAACATGCCTG  
Depth:4 (DOG)  
Ei-value:0.000, Pi-value:0.000  
Er-value:0.000, Pr-value:0.000  
No matches to TargetScan

-18048--(63)--18112-

TGTGTAT

TGTGTAT  
Depth:6 (MOUSE)  
Ei-value:0.000, Pi-value:0.000  
Er-value:0.000, Pr-value:0.000  
No matches to TargetScan


TT

TGTGTATTT  
Depth:4 (DOG)  
Ei-value:0.000, Pi-value:0.000  
Er-value:0.000, Pr-value:0.000  
No matches to TargetScan

-18120--(56)--18177-

TGTCTTA

TGTCTTA  
Depth:4 (DOG)  
Ei-value:0.000, Pi-value:0.000  
Er-value:0.000, Pr-value:0.000  
MATCHES To TargetScan▶ miR-208-3p:UAAGACG▶ miR-499a-5p:UAAGACU

-18183--(52)--18236-

TTTTTGT

TTTTTGT  
Depth:4 (DOG)  
Ei-value:0.000, Pi-value:0.000  
Er-value:0.000, Pr-value:0.000  
No matches to TargetScan

-18242--(54)--18297-

TTCATTTTGTT

TTCATTTTGTT  
Depth:4 (DOG)  
Ei-value:0.000, Pi-value:0.000  
Er-value:0.000, Pr-value:0.000  
MATCHES To TargetScan▶ miR-495-3p:AACAAAC

-18307--(116)--18424-

TTCTCTTTG

TTCTCTTTG  
Depth:6 (MOUSE)  
Ei-value:0.000, Pi-value:0.000  
Er-value:0.000, Pr-value:0.000  
No matches to TargetScan

-18432--(46)--18479-

ATTTCACCT

ATTTCACCT  
Depth:4 (DOG)  
Ei-value:0.000, Pi-value:0.000  
Er-value:0.000, Pr-value:0.000  
MATCHES To TargetScan▶ miR-203a-3p.2:UGAAAUG

-18487--(27)--18515-

TTTCTAC

TTTCTAC  
Depth:6 (MOUSE)  
Ei-value:0.000, Pi-value:0.000  
Er-value:0.000, Pr-value:0.000  
No matches to TargetScan


T

TTTCTACT  
Depth:5 (RABBIT)  
Ei-value:0.000, Pi-value:0.000  
Er-value:0.000, Pr-value:0.000  
MATCHES To TargetScan▶ miR-411-5p.1:AGUAGAC

-18522--(11)--18534-

ATTTCTC

ATTTCTC  
Depth:6 (MOUSE)  
Ei-value:0.000, Pi-value:0.000  
Er-value:0.000, Pr-value:0.000  
No matches to TargetScan

-18540--(23)--18564-

TCTTGGG

TCTTGGG  
Depth:5 (RABBIT)  
Ei-value:0.000, Pi-value:0.000  
Er-value:0.000, Pr-value:0.000  
No matches to TargetScan

-18570--(47)--18618-

TTTGTGA

TTTGTGA  
Depth:4 (DOG)  
Ei-value:0.000, Pi-value:0.010  
Er-value:0.000, Pr-value:0.000  
No matches to TargetScan

-18624--(21)--18646-

TCTCTGTT

TCTCTGTT  
Depth:4 (DOG)  
Ei-value:0.000, Pi-value:0.000  
Er-value:0.000, Pr-value:0.000  
No matches to TargetScan

-18653--(37)--18691-

TTTGAGTATTT

TTTGAGTATTT  
Depth:4 (DOG)  
Ei-value:0.000, Pi-value:0.000  
Er-value:0.000, Pr-value:0.000  
MATCHES To TargetScan▶ miR-200bc-3p/429:AAUACUG▶ miR-371-5p:CUCAAAC

-18701--(71)--18773-

TGTGTGTG

TGTGTGTG  
Depth:4 (DOG)  
Ei-value:0.000, Pi-value:0.000  
Er-value:0.000, Pr-value:0.000  
MATCHES To TargetScan▶ miR-329-3p/362-3p:ACACACC

-18780--(29)--18810-

TCCTAACCCCT

TCCTAACCCCT  
Depth:5 (RABBIT)  
Ei-value:0.000, Pi-value:0.000  
Er-value:0.000, Pr-value:0.000  
No matches to TargetScan

-18820--(35)--18856-

AAGCATTG

AAGCATTG  
Depth:4 (DOG)  
Ei-value:0.000, Pi-value:0.000  
Er-value:0.000, Pr-value:0.000  
No matches to TargetScan

-18863--(12)--18876-

TTATGCCA

TTATGCCA  
Depth:5 (RABBIT)  
Ei-value:0.000, Pi-value:0.000  
Er-value:0.000, Pr-value:0.000  
No matches to TargetScan


G

TTATGCCAG  
Depth:4 (DOG)  
Ei-value:0.000, Pi-value:0.000  
Er-value:0.000, Pr-value:0.000  
No matches to TargetScan

-18884--(52)--18937-

AGA

AGAAGGCCCAA  
Depth:4 (DOG)  
Ei-value:0.000, Pi-value:0.000  
Er-value:0.000, Pr-value:0.000  
No matches to TargetScan


AGGCCCAA

AGGCCCAA  
Depth:5 (RABBIT)  
Ei-value:0.000, Pi-value:0.000  
Er-value:0.000, Pr-value:0.000  
No matches to TargetScan

-18947--(110)--19058-

TCAA

TCAAGACTAA  
Depth:4 (DOG)  
Ei-value:0.000, Pi-value:0.000  
Er-value:0.000, Pr-value:0.000  
MATCHES To TargetScan▶ miR-431-5p:GUCUUGC


GACTAA

GACTAA  
Depth:5 (RABBIT)  
Ei-value:0.000, Pi-value:0.000  
Er-value:0.000, Pr-value:0.000  
No matches to TargetScan

-19067--(46)--19114-

AGAAGC

AGAAGC  
Depth:4 (DOG)  
Ei-value:0.000, Pi-value:0.000  
Er-value:0.000, Pr-value:0.010  
No matches to TargetScan

-19119--(21)--19141-

AAGATGA

AAGATGA  
Depth:5 (RABBIT)  
Ei-value:0.000, Pi-value:0.000  
Er-value:0.000, Pr-value:0.000  
No matches to TargetScan

-19147--(109)--19257-

CTTTTTGATGTT

CTTTTTGATGTT  
Depth:4 (DOG)  
Ei-value:0.000, Pi-value:0.000  
Er-value:0.000, Pr-value:0.000  
No matches to TargetScan

-19268--(43)--19312-

TATTATGC

TATTATGC  
Depth:4 (DOG)  
Ei-value:0.000, Pi-value:0.000  
Er-value:0.000, Pr-value:0.000  
MATCHES To TargetScan▶ miR-369-3p:AUAAUAC

-19319--(82)--19402-

TATTTCAGT

TATTTCAGT  
Depth:4 (DOG)  
Ei-value:0.000, Pi-value:0.000  
Er-value:0.000, Pr-value:0.000  
MATCHES To TargetScan▶ miR-203a-3p.2:UGAAAUG

-19410--(59)--19470-

GGGGAAA

GGGGAAA  
Depth:4 (DOG)  
Ei-value:0.000, Pi-value:0.000  
Er-value:0.000, Pr-value:0.000  
No matches to TargetScan

-19476--(15)--19492-

TCTAGAGAAAA

TCTAGAGAAAA  
Depth:6 (MOUSE)  
Ei-value:0.000, Pi-value:0.000  
Er-value:0.000, Pr-value:0.000  
MATCHES To TargetScan▶ miR-1251-5p:CUCUAGC

-19502--(2)--19505-

TGAAGAGATG

TGAAGAGATG  
Depth:5 (RABBIT)  
Ei-value:0.000, Pi-value:0.000  
Er-value:0.000, Pr-value:0.000  
No matches to TargetScan

-19514--(5)--19520-

GGCCAA

GGCCAATGAGAAGAATTAGACA  
Depth:4 (DOG)  
Ei-value:0.000, Pi-value:0.000  
Er-value:0.000, Pr-value:0.000  
No matches to TargetScan


TGAGAAGAATTAGACA

TGAGAAGAATTAGACA  
Depth:6 (MOUSE)  
Ei-value:0.000, Pi-value:0.000  
Er-value:0.000, Pr-value:0.000  
No matches to TargetScan

-19541--(26)--19568-

TGAGAAG

TGAGAAG  
Depth:4 (DOG)  
Ei-value:0.000, Pi-value:0.000  
Er-value:0.000, Pr-value:0.010  
No matches to TargetScan

-19574--(9)--19584-

GCAACA

GCAACA  
Depth:6 (MOUSE)  
Ei-value:0.000, Pi-value:0.000  
Er-value:0.000, Pr-value:0.000  
No matches to TargetScan

-19589--(19)--19609-

GGTGAGC

GGTGAGC  
Depth:4 (DOG)  
Ei-value:0.000, Pi-value:0.000  
Er-value:0.000, Pr-value:0.000  
No matches to TargetScan

-19615--(11)--19627-

GGTTTGGG

GGTTTGGG  
Depth:4 (DOG)  
Ei-value:0.000, Pi-value:0.000  
Er-value:0.000, Pr-value:0.000  
No matches to TargetScan

-19634--(9)--19644-

TGGTTA

TGGTTA  
Depth:5 (RABBIT)  
Ei-value:0.000, Pi-value:0.000  
Er-value:0.000, Pr-value:0.000  
No matches to TargetScan


T

TGGTTAT  
Depth:4 (DOG)  
Ei-value:0.000, Pi-value:0.000  
Er-value:0.000, Pr-value:0.000  
No matches to TargetScan

-19650--(38)--19689-

CCCAAGG

CCCAAGG  
Depth:4 (DOG)  
Ei-value:0.000, Pi-value:0.000  
Er-value:0.000, Pr-value:0.000  
MATCHES To TargetScan▶ miR-212-5p:CCUUGGC

-19695--(8)--19704-

TGAACTCCCTGCT

TGAACTCCCTGCT  
Depth:4 (DOG)  
Ei-value:0.000, Pi-value:0.000  
Er-value:0.000, Pr-value:0.000  
No matches to TargetScan

-19716--(1)--19718-

ATAGTAGTGGCC

ATAGTAGTGGCC  
Depth:4 (DOG)  
Ei-value:0.000, Pi-value:0.000  
Er-value:0.000, Pr-value:0.000  
No matches to TargetScan

-19729--(37)--19767-

TTTAATAC

TTTAATAC  
Depth:4 (DOG)  
Ei-value:0.000, Pi-value:0.000  
Er-value:0.000, Pr-value:0.000  
MATCHES To TargetScan▶ miR-496.2:GUAUUAC

-19774--(6)--19781-

CT

CTAGGCTTAAAG  
Depth:4 (DOG)  
Ei-value:0.000, Pi-value:0.000  
Er-value:0.000, Pr-value:0.000  
No matches to TargetScan


AGGCTTA

AGGCTTA  
Depth:5 (RABBIT)  
Ei-value:0.000, Pi-value:0.000  
Er-value:0.000, Pr-value:0.000  
No matches to TargetScan


AAG

CTAGGCTTAAAG  
Depth:4 (DOG)  
Ei-value:0.000, Pi-value:0.000  
Er-value:0.000, Pr-value:0.000  
No matches to TargetScan

-19792--(27)--19820-

GTTTAAT

GTTTAAT  
Depth:5 (RABBIT)  
Ei-value:0.000, Pi-value:0.000  
Er-value:0.000, Pr-value:0.000  
No matches to TargetScan

-19826--(107)--19934-

T

TATTGGCA  
Depth:5 (RABBIT)  
Ei-value:0.000, Pi-value:0.000  
Er-value:0.000, Pr-value:0.000  
No matches to TargetScan


ATTGGCA

ATTGGCA  
Depth:6 (MOUSE)  
Ei-value:0.000, Pi-value:0.000  
Er-value:0.000, Pr-value:0.000  
No matches to TargetScan

-19941--(36)--19978-

TTGTGAAG

TTGTGAAG  
Depth:6 (MOUSE)  
Ei-value:0.000, Pi-value:0.000  
Er-value:0.000, Pr-value:0.000  
No matches to TargetScan

-19985--(4)--19990-

ATGTAAAT

ATGTAAAT  
Depth:5 (RABBIT)  
Ei-value:0.000, Pi-value:0.000  
Er-value:0.000, Pr-value:0.000  
No matches to TargetScan

-19997--(164)--20162-

CTGTCCCT

CTGTCCCT  
Depth:4 (DOG)  
Ei-value:0.000, Pi-value:0.000  
Er-value:0.000, Pr-value:0.000  
No matches to TargetScan

-20169--(4)--20174-

TAGGCACT

TAGGCACT  
Depth:4 (DOG)  
Ei-value:0.000, Pi-value:0.000  
Er-value:0.000, Pr-value:0.000  
No matches to TargetScan

-20181--(73)--20255-

TAAAGCA

TAAAGCA  
Depth:4 (DOG)  
Ei-value:0.000, Pi-value:0.000  
Er-value:0.000, Pr-value:0.000  
No matches to TargetScan

-20261--(120)--20382-

AATGTGCCAGATA

AATGTGCCAGATA  
Depth:4 (DOG)  
Ei-value:0.000, Pi-value:0.000  
Er-value:0.000, Pr-value:0.000  
MATCHES To TargetScan▶ miR-183-5p.2:UGGCACU

-20394--(79)--20474-

TTAAAGTG

TTAAAGTG  
Depth:4 (DOG)  
Ei-value:0.000, Pi-value:0.000  
Er-value:0.000, Pr-value:0.000  
No matches to TargetScan

-20481--(292)--20774-

CTAAAGCA

CTAAAGCA  
Depth:4 (DOG)  
Ei-value:0.000, Pi-value:0.000  
Er-value:0.000, Pr-value:0.000  
No matches to TargetScan

-20781--(35)--20817-

ATGAATA

ATGAATA  
Depth:4 (DOG)  
Ei-value:0.000, Pi-value:0.000  
Er-value:0.000, Pr-value:0.000  
No matches to TargetScan

-20823--(41)--20865-

GGTACTGT

GGTACTGT  
Depth:4 (DOG)  
Ei-value:0.000, Pi-value:0.000  
Er-value:0.000, Pr-value:0.000  
MATCHES To TargetScan▶ miR-101-3p.1:ACAGUAC▶ miR-144-3p:ACAGUAU

-20872--(26)--20899-

ATAAGAGG

ATAAGAGG  
Depth:4 (DOG)  
Ei-value:0.000, Pi-value:0.000  
Er-value:0.000, Pr-value:0.000  
No matches to TargetScan

-20906--(84)--20991-

TAAATTAT

TAAATTAT  
Depth:4 (DOG)  
Ei-value:0.000, Pi-value:0.010  
Er-value:0.000, Pr-value:0.000  
No matches to TargetScan

-20998--(106)--21105-

ATAAAAC

ATAAAAC  
Depth:4 (DOG)  
Ei-value:0.000, Pi-value:0.010  
Er-value:0.000, Pr-value:0.000  
No matches to TargetScan

-21111--(26)--21138-

AAAATTCTCA

AAAATTCTCA  
Depth:4 (DOG)  
Ei-value:0.000, Pi-value:0.000  
Er-value:0.000, Pr-value:0.000  
No matches to TargetScan

-21147--(41)--21189-

TATACAAAC

TATACAAAC  
Depth:4 (DOG)  
Ei-value:0.000, Pi-value:0.000  
Er-value:0.000, Pr-value:0.000  
No matches to TargetScan

-21197--(48)--21246-

GTGAACTCA

GTGAACTCA  
Depth:4 (DOG)  
Ei-value:0.000, Pi-value:0.000  
Er-value:0.000, Pr-value:0.000  
No matches to TargetScan

-21254--(291)--21546-

TGTGCCA

TGTGCCA  
Depth:4 (DOG)  
Ei-value:0.000, Pi-value:0.000  
Er-value:0.000, Pr-value:0.000  
MATCHES To TargetScan▶ miR-183-5p.2:UGGCACU

-21552--(21)--21574-

AAGATAA

AAGATAA  
Depth:4 (DOG)  
Ei-value:0.000, Pi-value:0.000  
Er-value:0.000, Pr-value:0.000  
No matches to TargetScan

-21580--(61)--21642-

TAAACTG

TAAACTG  
Depth:4 (DOG)  
Ei-value:0.000, Pi-value:0.000  
Er-value:0.000, Pr-value:0.000  
No matches to TargetScan

-21648--(23)--21672-

ATTGATTA

ATTGATTA  
Depth:4 (DOG)  
Ei-value:0.000, Pi-value:0.000  
Er-value:0.000, Pr-value:0.010  
No matches to TargetScan

-21679--(19)--21699-

AGAGATA

AGAGATA  
Depth:4 (DOG)  
Ei-value:0.000, Pi-value:0.000  
Er-value:0.000, Pr-value:0.000  
No matches to TargetScan

-21705--(46)--21752-

CAGAGATCT

CAGAGATCT  
Depth:4 (DOG)  
Ei-value:0.000, Pi-value:0.000  
Er-value:0.000, Pr-value:0.000  
No matches to TargetScan

-21760--(52)--21813-

TTGGCT

TTGGCT  
Depth:4 (DOG)  
Ei-value:0.000, Pi-value:0.000  
Er-value:0.000, Pr-value:0.000  
No matches to TargetScan

-21818--(17)--21836-

TTACTTTCT

TTACTTTCT  
Depth:4 (DOG)  
Ei-value:0.000, Pi-value:0.000  
Er-value:0.000, Pr-value:0.010  
No matches to TargetScan

-21844--(337)--22182-

GAGTAAAAA

GAGTAAAAA  
Depth:4 (DOG)  
Ei-value:0.000, Pi-value:0.000  
Er-value:0.000, Pr-value:0.000  
No matches to TargetScan

-22190--(27)--22218-

ATTTGAT

ATTTGAT  
Depth:4 (DOG)  
Ei-value:0.000, Pi-value:0.010  
Er-value:0.000, Pr-value:0.000  
No matches to TargetScan

-22224--(10)--22235-

TTTTATGT

TTTTATGT  
Depth:4 (DOG)  
Ei-value:0.000, Pi-value:0.000  
Er-value:0.000, Pr-value:0.000  
No matches to TargetScan

-22242--(289)--22532-

AAGCCAG

AAGCCAG  
Depth:4 (DOG)  
Ei-value:0.000, Pi-value:0.000  
Er-value:0.000, Pr-value:0.000  
MATCHES To TargetScan▶ miR-149-5p:CUGGCUC▶ miR-3064-5p:CUGGCUG

-22538--(7)--22546-

ATAAAAG

ATAAAAG  
Depth:4 (DOG)  
Ei-value:0.000, Pi-value:0.000  
Er-value:0.000, Pr-value:0.000  
No matches to TargetScan

-22552--(37)--22590-

TTTTATTA

TTTTATTA  
Depth:4 (DOG)  
Ei-value:0.000, Pi-value:0.010  
Er-value:0.000, Pr-value:0.000  
No matches to TargetScan

-22597--(3)--22601-

TTAAATGG

TTAAATGG  
Depth:4 (DOG)  
Ei-value:0.000, Pi-value:0.000  
Er-value:0.000, Pr-value:0.000  
No matches to TargetScan

-22608--(579)--23188-

ACAGAAAACAAAA

ACAGAAAACAAAA  
Depth:4 (DOG)  
Ei-value:0.000, Pi-value:0.000  
Er-value:0.000, Pr-value:0.000  
No matches to TargetScan

-23200--(52)--23253-

AGGTTA

AGGTTA  
Depth:4 (DOG)  
Ei-value:0.000, Pi-value:0.000  
Er-value:0.000, Pr-value:0.000  
No matches to TargetScan

-23258--(27)--23286-

TTCATTCT

TTCATTCT  
Depth:4 (DOG)  
Ei-value:0.000, Pi-value:0.000  
Er-value:0.000, Pr-value:0.000  
No matches to TargetScan

-23293--(61)--23355-

AGAGACA

AGAGACA  
Depth:4 (DOG)  
Ei-value:0.000, Pi-value:0.000  
Er-value:0.000, Pr-value:0.000  
No matches to TargetScan

-23361--(47)--23409-

CCTTTTGG

CCTTTTGG  
Depth:4 (DOG)  
Ei-value:0.000, Pi-value:0.000  
Er-value:0.000, Pr-value:0.000  
No matches to TargetScan

-23416--(51)--23468-

TTAATTC

TTAATTC  
Depth:4 (DOG)  
Ei-value:0.000, Pi-value:0.000  
Er-value:0.000, Pr-value:0.000  
No matches to TargetScan

-23474--(21)--23496-

ACTCTGGCCACTAC

ACTCTGGCCACTAC  
Depth:4 (DOG)  
Ei-value:0.000, Pi-value:0.000  
Er-value:0.000, Pr-value:0.000  
MATCHES To TargetScan▶ miR-142-3p.1:GUAGUGU

-23509--(1)--23511-

ATAAGC

ATAAGC  
Depth:5 (RABBIT)  
Ei-value:0.000, Pi-value:0.010  
Er-value:0.000, Pr-value:0.000  
No matches to TargetScan


AGG

ATAAGCAGG  
Depth:4 (DOG)  
Ei-value:0.000, Pi-value:0.000  
Er-value:0.000, Pr-value:0.000  
No matches to TargetScan

-23519--(64)--23584-

GAAAAATG

GAAAAATG  
Depth:4 (DOG)  
Ei-value:0.000, Pi-value:0.000  
Er-value:0.000, Pr-value:0.000  
No matches to TargetScan

-23591--(53)--23645-

AGTCTCA

AGTCTCA  
Depth:4 (DOG)  
Ei-value:0.000, Pi-value:0.000  
Er-value:0.000, Pr-value:0.000  
No matches to TargetScan

-23651--(41)--23693-

TGGTTTTGAA

TGGTTTTGAA  
Depth:4 (DOG)  
Ei-value:0.000, Pi-value:0.000  
Er-value:0.000, Pr-value:0.000  
No matches to TargetScan

-23702--(134)--23837-

TTAGAAAT

TTAGAAAT  
Depth:4 (DOG)  
Ei-value:0.000, Pi-value:0.000  
Er-value:0.000, Pr-value:0.000  
No matches to TargetScan

-23844--(14)--23859-

CATCAAA

CATCAAA  
Depth:4 (DOG)  
Ei-value:0.000, Pi-value:0.000  
Er-value:0.000, Pr-value:0.000  
No matches to TargetScan

-23865--(180)--24046-

TTTTTAAATCACTCA

TTTTTAAATCACTCA  
Depth:4 (DOG)  
Ei-value:0.000, Pi-value:0.000  
Er-value:0.000, Pr-value:0.000  
No matches to TargetScan

-24060--(1)--24062-

AGAGGGTGGGA

AGAGGGTGGGA  
Depth:4 (DOG)  
Ei-value:0.000, Pi-value:0.000  
Er-value:0.000, Pr-value:0.000  
No matches to TargetScan

-24072--(1)--24074-

AGGAGGAAGAGTGAA

AGGAGGAAGAGTGAA  
Depth:4 (DOG)  
Ei-value:0.000, Pi-value:0.000  
Er-value:0.000, Pr-value:0.000  
MATCHES To TargetScan▶ miR-670-3p:UUCCUCA

-24088--(1)--24090-

G

GAAAAGGTCA  
Depth:4 (DOG)  
Ei-value:0.000, Pi-value:0.000  
Er-value:0.000, Pr-value:0.000  
MATCHES To TargetScan▶ miR-192-5p/215-5p:UGACCUA


AAAAGGT

AAAAGGT  
Depth:6 (MOUSE)  
Ei-value:0.000, Pi-value:0.000  
Er-value:0.000, Pr-value:0.000  
No matches to TargetScan


CA

GAAAAGGTCA  
Depth:4 (DOG)  
Ei-value:0.000, Pi-value:0.000  
Er-value:0.000, Pr-value:0.000  
MATCHES To TargetScan▶ miR-192-5p/215-5p:UGACCUA

-24099--(606)--24706-

TAATGTTT

TAATGTTT  
Depth:4 (DOG)  
Ei-value:0.000, Pi-value:0.000  
Er-value:0.000, Pr-value:0.000  
MATCHES To TargetScan▶ miR-323-3p:ACAUUAC▶ miR-543:AACAUUC

-24713--(11)--24725-

TAATGTTT

TAATGTTT  
Depth:4 (DOG)  
Ei-value:0.000, Pi-value:0.000  
Er-value:0.000, Pr-value:0.000  
MATCHES To TargetScan▶ miR-323-3p:ACAUUAC▶ miR-543:AACAUUC

-24732--(13)--24746-

AGCTGGA

AGCTGGA  
Depth:4 (DOG)  
Ei-value:0.000, Pi-value:0.000  
Er-value:0.000, Pr-value:0.000  
No matches to TargetScan

-24752--(39)--24792-

ATTATTGGAAA

ATTATTGGAAA  
Depth:4 (DOG)  
Ei-value:0.000, Pi-value:0.000  
Er-value:0.000, Pr-value:0.000  
No matches to TargetScan

-24802--(10)--24813-

AGAAAGTAAC

AGAAAGTAAC  
Depth:4 (DOG)  
Ei-value:0.000, Pi-value:0.000  
Er-value:0.000, Pr-value:0.000  
No matches to TargetScan

-24822--(12)--24835-

TTTCACAGTTTCTGGCATC

TTTCACAGTTTCTGGCATC  
Depth:4 (DOG)  
Ei-value:0.000, Pi-value:0.000  
Er-value:0.000, Pr-value:0.000  
No matches to TargetScan

-24853--(11)--24865-

CTACTGAT

CTACTGAT  
Depth:4 (DOG)  
Ei-value:0.000, Pi-value:0.000  
Er-value:0.000, Pr-value:0.000  
MATCHES To TargetScan▶ miR-199-3p:CAGUAGU

-24872--(13)--24886-

AGAACAT

AGAACAT  
Depth:4 (DOG)  
Ei-value:0.000, Pi-value:0.000  
Er-value:0.000, Pr-value:0.000  
No matches to TargetScan

-24892--(4)--24897-

TCATCTG

TCATCTG  
Depth:4 (DOG)  
Ei-value:0.000, Pi-value:0.010  
Er-value:0.000, Pr-value:0.000  
No matches to TargetScan

-24903--(10)--24914-

CATAAATGAA

CATAAATGAA  
Depth:4 (DOG)  
Ei-value:0.000, Pi-value:0.000  
Er-value:0.000, Pr-value:0.000  
No matches to TargetScan

-24923--(117)--25041-

TGAACTGATGTGAAA

TGAACTGATGTGAAA  
Depth:4 (DOG)  
Ei-value:0.000, Pi-value:0.000  
Er-value:0.000, Pr-value:0.000  
MATCHES To TargetScan▶ miR-23-3p:UCACAUU

-25055--(132)--25188-

AAATAAAA

AAATAAAA  
Depth:4 (DOG)  
Ei-value:0.000, Pi-value:0.000  
Er-value:0.000, Pr-value:0.000  
No matches to TargetScan

-25195  
  
>COW  
       121-

AAACATG

AAACATG  
Depth:4 (DOG)  
Ei-value:0.000, Pi-value:0.000  
Er-value:0.000, Pr-value:0.000  
No matches to TargetScan

-127--(42)--170-

TTCCCATC

TTCCCATC  
Depth:4 (DOG)  
Ei-value:0.000, Pi-value:0.000  
Er-value:0.000, Pr-value:0.000  
No matches to TargetScan

-177--(69)--247-

C

CTGTTAGTCT  
Depth:4 (DOG)  
Ei-value:0.000, Pi-value:0.000  
Er-value:0.000, Pr-value:0.000  
No matches to TargetScan


TGTTAGTC

TGTTAGTC  
Depth:5 (RABBIT)  
Ei-value:0.000, Pi-value:0.000  
Er-value:0.000, Pr-value:0.000  
No matches to TargetScan


T

CTGTTAGTCT  
Depth:4 (DOG)  
Ei-value:0.000, Pi-value:0.000  
Er-value:0.000, Pr-value:0.000  
No matches to TargetScan

-256--(1463)--1720-

TCATCC

TCATCC  
Depth:4 (DOG)  
Ei-value:0.000, Pi-value:0.020  
Er-value:0.000, Pr-value:0.000  
No matches to TargetScan

-1725--(320)--2046-

TACTTGGGACTGTTAAT

TACTTGGGACTGTTAAT  
Depth:4 (DOG)  
Ei-value:0.000, Pi-value:0.000  
Er-value:0.000, Pr-value:0.000  
MATCHES To TargetScan▶ miR-132-3p/212-3p:AACAGUC▶ miR-455-3p.1:CAGUCCA

-2062--(89)--2152-

ACTG

ACTGTTAATGTGCT  
Depth:4 (DOG)  
Ei-value:0.000, Pi-value:0.000  
Er-value:0.000, Pr-value:0.000  
MATCHES To TargetScan▶ miR-132-3p/212-3p:AACAGUC▶ miR-323-3p:ACAUUAC


TTAATGTGCT

TTAATGTGCT  
Depth:5 (RABBIT)  
Ei-value:0.000, Pi-value:0.000  
Er-value:0.000, Pr-value:0.000  
MATCHES To TargetScan▶ miR-323-3p:ACAUUAC

-2165--(925)--3091-

AATGTGCAT

AATGTGCAT  
Depth:6 (MOUSE)  
Ei-value:0.000, Pi-value:0.000  
Er-value:0.000, Pr-value:0.000  
MATCHES To TargetScan▶ miR-501-3p/502-3p:AUGCACC

-3099--(201)--3301-

TATGTTAGA

TATGTTAGA  
Depth:4 (DOG)  
Ei-value:0.000, Pi-value:0.000  
Er-value:0.000, Pr-value:0.000  
No matches to TargetScan

-3309--(72)--3382-

ACTGTTAATGT

ACTGTTAATGT  
Depth:4 (DOG)  
Ei-value:0.000, Pi-value:0.000  
Er-value:0.000, Pr-value:0.000  
MATCHES To TargetScan▶ miR-132-3p/212-3p:AACAGUC▶ miR-323-3p:ACAUUAC

-3392--(10)--3403-

ATTTGCT

ATTTGCT  
Depth:4 (DOG)  
Ei-value:0.000, Pi-value:0.000  
Er-value:0.000, Pr-value:0.000  
No matches to TargetScan

-3409--(18)--3428-

GTAAGGA

GTAAGGA  
Depth:5 (RABBIT)  
Ei-value:0.000, Pi-value:0.000  
Er-value:0.000, Pr-value:0.000  
No matches to TargetScan

-3434--(144)--3579-

ACTTAT

ACTTAT  
Depth:5 (RABBIT)  
Ei-value:0.000, Pi-value:0.000  
Er-value:0.000, Pr-value:0.000  
No matches to TargetScan

-3584--(145)--3730-

TTAAGGCC

TTAAGGCC  
Depth:6 (MOUSE)  
Ei-value:0.000, Pi-value:0.000  
Er-value:0.000, Pr-value:0.000  
No matches to TargetScan


CCTTT

TTAAGGCCCCTTT  
Depth:5 (RABBIT)  
Ei-value:0.000, Pi-value:0.000  
Er-value:0.000, Pr-value:0.000  
No matches to TargetScan


CTCAA

TTAAGGCCCCTTTCTCAA  
Depth:4 (DOG)  
Ei-value:0.000, Pi-value:0.000  
Er-value:0.000, Pr-value:0.000  
No matches to TargetScan

-3747--(260)--4008-

ACAGTTAATGTG

ACAGTTAATGTG  
Depth:4 (DOG)  
Ei-value:0.000, Pi-value:0.000  
Er-value:0.000, Pr-value:0.000  
MATCHES To TargetScan▶ miR-323-3p:ACAUUAC

-4019--(172)--4192-

CTCAGCTCTTGG

CTCAGCTCTTGG  
Depth:5 (RABBIT)  
Ei-value:0.000, Pi-value:0.000  
Er-value:0.000, Pr-value:0.000  
MATCHES To TargetScan▶ miR-335-5p:CAAGAGC


ACA

CTCAGCTCTTGGACA  
Depth:4 (DOG)  
Ei-value:0.000, Pi-value:0.000  
Er-value:0.000, Pr-value:0.000  
MATCHES To TargetScan▶ miR-335-5p:CAAGAGC

-4206--(203)--4410-

CAAAACTT

CAAAACTT  
Depth:4 (DOG)  
Ei-value:0.000, Pi-value:0.000  
Er-value:0.000, Pr-value:0.000  
No matches to TargetScan

-4417--(19)--4437-

GCACAATG

GCACAATG  
Depth:6 (MOUSE)  
Ei-value:0.000, Pi-value:0.000  
Er-value:0.000, Pr-value:0.000  
No matches to TargetScan

-4444--(44)--4489-

A

ACTCCCA  
Depth:4 (DOG)  
Ei-value:0.000, Pi-value:0.000  
Er-value:0.000, Pr-value:0.000  
No matches to TargetScan


CTCCCA

CTCCCA  
Depth:6 (MOUSE)  
Ei-value:0.000, Pi-value:0.000  
Er-value:0.000, Pr-value:0.000  
No matches to TargetScan

-4495--(265)--4761-

CCCTTTTGCATT

CCCTTTTGCATT  
Depth:4 (DOG)  
Ei-value:0.000, Pi-value:0.000  
Er-value:0.000, Pr-value:0.000  
No matches to TargetScan

-4772--(223)--4996-

CCATTTTT

CCATTTTT  
Depth:4 (DOG)  
Ei-value:0.000, Pi-value:0.000  
Er-value:0.000, Pr-value:0.000  
No matches to TargetScan

-5003--(8)--5012-

CAGCCCA

CAGCCCA  
Depth:4 (DOG)  
Ei-value:0.000, Pi-value:0.000  
Er-value:0.000, Pr-value:0.000  
No matches to TargetScan

-5018--(7)--5026-

CCTACCA

CCTACCA  
Depth:4 (DOG)  
Ei-value:0.000, Pi-value:0.000  
Er-value:0.000, Pr-value:0.000  
No matches to TargetScan

-5032--(33)--5066-

AAAAGCAG

AAAAGCAG  
Depth:6 (MOUSE)  
Ei-value:0.000, Pi-value:0.000  
Er-value:0.000, Pr-value:0.000  
No matches to TargetScan

-5073--(43)--5117-

TTAATGATCC

TTAATGATCC  
Depth:4 (DOG)  
Ei-value:0.000, Pi-value:0.000  
Er-value:0.000, Pr-value:0.000  
MATCHES To TargetScan▶ miR-382-3p:AUCAUUC

-5126--(19)--5146-

ATTCTGGG

ATTCTGGG  
Depth:4 (DOG)  
Ei-value:0.000, Pi-value:0.000  
Er-value:0.000, Pr-value:0.000  
No matches to TargetScan

-5153--(32)--5186-

CTTTACT

CTTTACT  
Depth:4 (DOG)  
Ei-value:0.000, Pi-value:0.000  
Er-value:0.000, Pr-value:0.000  
No matches to TargetScan

-5192--(2)--5195-

GCAAAAT

GCAAAAT  
Depth:6 (MOUSE)  
Ei-value:0.000, Pi-value:0.000  
Er-value:0.000, Pr-value:0.000  
No matches to TargetScan

-5201--(4)--5206-

AAGGCAA

AAGGCAA  
Depth:4 (DOG)  
Ei-value:0.000, Pi-value:0.000  
Er-value:0.000, Pr-value:0.000  
No matches to TargetScan

-5212--(17)--5230-

TGGATTGC

TGGATTGC  
Depth:4 (DOG)  
Ei-value:0.000, Pi-value:0.000  
Er-value:0.000, Pr-value:0.000  
No matches to TargetScan

-5237--(77)--5315-

TGCATTCTTC

TGCATTCTTC  
Depth:5 (RABBIT)  
Ei-value:0.000, Pi-value:0.000  
Er-value:0.000, Pr-value:0.000  
No matches to TargetScan

-5324--(7)--5332-

AGC

AGCAGATTGCCTGG  
Depth:4 (DOG)  
Ei-value:0.000, Pi-value:0.000  
Er-value:0.000, Pr-value:0.000  
No matches to TargetScan


A

AGATTGCCTGG  
Depth:5 (RABBIT)  
Ei-value:0.000, Pi-value:0.000  
Er-value:0.000, Pr-value:0.000  
No matches to TargetScan


GATTGCCTGG

GATTGCCTGG  
Depth:6 (MOUSE)  
Ei-value:0.000, Pi-value:0.000  
Er-value:0.000, Pr-value:0.000  
No matches to TargetScan

-5345--(19)--5365-

TTGTATATT

TTGTATATT  
Depth:4 (DOG)  
Ei-value:0.000, Pi-value:0.000  
Er-value:0.000, Pr-value:0.000  
MATCHES To TargetScan▶ miR-381-3p:AUACAAG

-5373--(104)--5478-

AAC

AACCTGGTCATT  
Depth:4 (DOG)  
Ei-value:0.000, Pi-value:0.000  
Er-value:0.000, Pr-value:0.000  
No matches to TargetScan


CTGGTCATT

CTGGTCATT  
Depth:5 (RABBIT)  
Ei-value:0.000, Pi-value:0.000  
Er-value:0.000, Pr-value:0.000  
No matches to TargetScan

-5489--(21)--5511-

CCATTTAT

CCATTTAT  
Depth:5 (RABBIT)  
Ei-value:0.000, Pi-value:0.000  
Er-value:0.000, Pr-value:0.000  
No matches to TargetScan

-5518--(13)--5532-

TGAC

TGACCAGTGTCTCTCATTT  
Depth:4 (DOG)  
Ei-value:0.000, Pi-value:0.000  
Er-value:0.000, Pr-value:0.000  
No matches to TargetScan


CAGTGTCTCTCATTT

CAGTGTCTCTCATTT  
Depth:5 (RABBIT)  
Ei-value:0.000, Pi-value:0.000  
Er-value:0.000, Pr-value:0.000  
No matches to TargetScan

-5550--(5)--5556-

AGG

AGGGTGGTG  
Depth:4 (DOG)  
Ei-value:0.000, Pi-value:0.000  
Er-value:0.000, Pr-value:0.000  
No matches to TargetScan


GTGGTG

GTGGTG  
Depth:5 (RABBIT)  
Ei-value:0.000, Pi-value:0.000  
Er-value:0.000, Pr-value:0.000  
No matches to TargetScan

-5564--(1)--5566-

GTCTGTGGATA

GTCTGTGGATA  
Depth:5 (RABBIT)  
Ei-value:0.000, Pi-value:0.000  
Er-value:0.000, Pr-value:0.000  
MATCHES To TargetScan▶ miR-140-3p.1:CCACAGG

-5576--(41)--5618-

TTCTAGA

TTCTAGA  
Depth:4 (DOG)  
Ei-value:0.000, Pi-value:0.000  
Er-value:0.000, Pr-value:0.000  
No matches to TargetScan

-5624--(74)--5699-

ATTCACTT

ATTCACTT  
Depth:4 (DOG)  
Ei-value:0.000, Pi-value:0.000  
Er-value:0.000, Pr-value:0.000  
No matches to TargetScan

-5706--(4)--5711-

GAAAAAC

GAAAAAC  
Depth:4 (DOG)  
Ei-value:0.000, Pi-value:0.000  
Er-value:0.000, Pr-value:0.000  
No matches to TargetScan

-5717--(22)--5740-

AATTTCTTCATCTGGAGC

AATTTCTTCATCTGGAGC  
Depth:5 (RABBIT)  
Ei-value:0.000, Pi-value:0.000  
Er-value:0.000, Pr-value:0.000  
No matches to TargetScan

-5757--(15)--5773-

CTTATTT

CTTATTT  
Depth:4 (DOG)  
Ei-value:0.000, Pi-value:0.000  
Er-value:0.000, Pr-value:0.010  
No matches to TargetScan

-5779--(21)--5801-

ATAAAATG

ATAAAATG  
Depth:4 (DOG)  
Ei-value:0.000, Pi-value:0.000  
Er-value:0.000, Pr-value:0.000  
No matches to TargetScan

-5808--(167)--5976-

AAAAATAAGCCA

AAAAATAAGCCA  
Depth:5 (RABBIT)  
Ei-value:0.000, Pi-value:0.000  
Er-value:0.000, Pr-value:0.000  
No matches to TargetScan


A

AAAAATAAGCCAA  
Depth:4 (DOG)  
Ei-value:0.000, Pi-value:0.000  
Er-value:0.000, Pr-value:0.000  
No matches to TargetScan

-5988--(50)--6039-

ATGAATAATA

ATGAATAATA  
Depth:4 (DOG)  
Ei-value:0.000, Pi-value:0.000  
Er-value:0.000, Pr-value:0.000  
No matches to TargetScan

-6048--(46)--6095-

TGGAACTGCT

TGGAACTGCT  
Depth:4 (DOG)  
Ei-value:0.000, Pi-value:0.000  
Er-value:0.000, Pr-value:0.000  
No matches to TargetScan

-6104--(8)--6113-

TAACTA

TAACTA  
Depth:4 (DOG)  
Ei-value:0.000, Pi-value:0.000  
Er-value:0.000, Pr-value:0.000  
No matches to TargetScan

-6118--(10)--6129-

CAGCAGTTC

CAGCAGTTC  
Depth:5 (RABBIT)  
Ei-value:0.000, Pi-value:0.000  
Er-value:0.000, Pr-value:0.000  
No matches to TargetScan

-6137--(1)--6139-

TTGTAAT

TTGTAAT  
Depth:4 (DOG)  
Ei-value:0.000, Pi-value:0.000  
Er-value:0.000, Pr-value:0.000  
No matches to TargetScan

-6145--(1)--6147-

ACTGAAAA

ACTGAAAA  
Depth:5 (RABBIT)  
Ei-value:0.000, Pi-value:0.000  
Er-value:0.000, Pr-value:0.000  
No matches to TargetScan

-6154--(18)--6173-

AAGGATG

AAGGATG  
Depth:5 (RABBIT)  
Ei-value:0.000, Pi-value:0.000  
Er-value:0.000, Pr-value:0.000  
MATCHES To TargetScan▶ miR-362-5p/500b-5p:AUCCUUG


TCA

AAGGATGTCAAAAGATC  
Depth:4 (DOG)  
Ei-value:0.000, Pi-value:0.000  
Er-value:0.000, Pr-value:0.000  
MATCHES To TargetScan▶ miR-362-5p/500b-5p:AUCCUUG▶ miR-489-3p:UGACAUC


AAAGATC

AAAGATC  
Depth:6 (MOUSE)  
Ei-value:0.000, Pi-value:0.000  
Er-value:0.000, Pr-value:0.000  
No matches to TargetScan

-6189--(4)--6194-

CAGCTCAGGG

CAGCTCAGGG  
Depth:4 (DOG)  
Ei-value:0.000, Pi-value:0.000  
Er-value:0.000, Pr-value:0.000  
MATCHES To TargetScan▶ miR-125-5p:CCCUGAG

-6203--(11)--6215-

CTACTAGCTCCT

CTACTAGCTCCT  
Depth:4 (DOG)  
Ei-value:0.000, Pi-value:0.000  
Er-value:0.000, Pr-value:0.000  
MATCHES To TargetScan▶ miR-28-5p/708-5p:AGGAGCU▶ miR-411-5p.2:UAGUAGA

-6226--(1)--6228-

GGACAGCTG

GGACAGCTG  
Depth:5 (RABBIT)  
Ei-value:0.000, Pi-value:0.000  
Er-value:0.000, Pr-value:0.000  
No matches to TargetScan


T

GGACAGCTGT  
Depth:4 (DOG)  
Ei-value:0.000, Pi-value:0.000  
Er-value:0.000, Pr-value:0.000  
No matches to TargetScan

-6237--(1)--6239-

AGAAGAGTCTCTGGCTCTTTA

AGAAGAGTCTCTGGCTCTTTA  
Depth:5 (RABBIT)  
Ei-value:0.000, Pi-value:0.000  
Er-value:0.000, Pr-value:0.000  
No matches to TargetScan


GA

AGAAGAGTCTCTGGCTCTTTAGA  
Depth:4 (DOG)  
Ei-value:0.000, Pi-value:0.000  
Er-value:0.000, Pr-value:0.000  
No matches to TargetScan

-6261--(11)--6273-

ATTCTGAGC

ATTCTGAGC  
Depth:4 (DOG)  
Ei-value:0.000, Pi-value:0.000  
Er-value:0.000, Pr-value:0.000  
No matches to TargetScan

-6281--(103)--6385-

CTGCAA

CTGCAA  
Depth:5 (RABBIT)  
Ei-value:0.000, Pi-value:0.000  
Er-value:0.000, Pr-value:0.000  
No matches to TargetScan

-6390--(114)--6505-

AAGAATAGGC

AAGAATAGGC  
Depth:5 (RABBIT)  
Ei-value:0.000, Pi-value:0.000  
Er-value:0.000, Pr-value:0.000  
No matches to TargetScan

-6514--(9)--6524-

TACAGTGTTAGTGA

TACAGTGTTAGTGA  
Depth:5 (RABBIT)  
Ei-value:0.000, Pi-value:0.000  
Er-value:0.000, Pr-value:0.000  
MATCHES To TargetScan▶ miR-141-3p/200a-3p:AACACUG

-6537--(4)--6542-

TTCCCTTTGA

TTCCCTTTGA  
Depth:6 (MOUSE)  
Ei-value:0.000, Pi-value:0.000  
Er-value:0.000, Pr-value:0.000  
No matches to TargetScan

-6551--(7)--6559-

TAGGTGGAGATGGGGCATGAGGATCCTCCAGGGGAA

TAGGTGGAGATGGGGCATGAGGATCCTCCAGGGGAA  
Depth:6 (MOUSE)  
Ei-value:0.000, Pi-value:0.000  
Er-value:0.000, Pr-value:0.000  
MATCHES To TargetScan▶ miR-331-3p:CCCCUGG


A

TAGGTGGAGATGGGGCATGAGGATCCTCCAGGGGAAA  
Depth:5 (RABBIT)  
Ei-value:0.000, Pi-value:0.000  
Er-value:0.000, Pr-value:0.000  
MATCHES To TargetScan▶ miR-331-3p:CCCCUGG

-6595--(3)--6599-

TCACTA

TCACTA  
Depth:5 (RABBIT)  
Ei-value:0.000, Pi-value:0.000  
Er-value:0.000, Pr-value:0.000  
No matches to TargetScan


CCACT

TCACTACCACT  
Depth:4 (DOG)  
Ei-value:0.000, Pi-value:0.000  
Er-value:0.000, Pr-value:0.000  
MATCHES To TargetScan▶ miR-140-5p:AGUGGUU▶ miR-142-3p.1:GUAGUGU

-6609--(2)--6612-

GCAACA

GCAACA  
Depth:6 (MOUSE)  
Ei-value:0.000, Pi-value:0.000  
Er-value:0.000, Pr-value:0.000  
No matches to TargetScan


AC

GCAACAAC  
Depth:5 (RABBIT)  
Ei-value:0.000, Pi-value:0.000  
Er-value:0.000, Pr-value:0.000  
No matches to TargetScan

-6619--(63)--6683-

ACAACCACC

ACAACCACC  
Depth:5 (RABBIT)  
Ei-value:0.000, Pi-value:0.000  
Er-value:0.000, Pr-value:0.000  
No matches to TargetScan


ACAC

ACAACCACCACAC  
Depth:4 (DOG)  
Ei-value:0.000, Pi-value:0.000  
Er-value:0.000, Pr-value:0.000  
No matches to TargetScan

-6695--(15)--6711-

TTGTTCC

TTGTTCC  
Depth:4 (DOG)  
Ei-value:0.000, Pi-value:0.000  
Er-value:0.000, Pr-value:0.000  
No matches to TargetScan

-6717--(11)--6729-

CCAAAT

CCAAAT  
Depth:6 (MOUSE)  
Ei-value:0.000, Pi-value:0.000  
Er-value:0.000, Pr-value:0.000  
No matches to TargetScan


C

CCAAATC  
Depth:5 (RABBIT)  
Ei-value:0.000, Pi-value:0.000  
Er-value:0.000, Pr-value:0.000  
No matches to TargetScan

-6735--(29)--6765-

CAAGAAA

CAAGAAA  
Depth:5 (RABBIT)  
Ei-value:0.000, Pi-value:0.000  
Er-value:0.000, Pr-value:0.000  
No matches to TargetScan

-6771--(15)--6787-

G

GAAGATCAACATGCCTG  
Depth:4 (DOG)  
Ei-value:0.000, Pi-value:0.000  
Er-value:0.000, Pr-value:0.000  
No matches to TargetScan


AA

AAGATCAACATGC  
Depth:5 (RABBIT)  
Ei-value:0.000, Pi-value:0.000  
Er-value:0.000, Pr-value:0.000  
No matches to TargetScan


GATCAACATGC

GATCAACATGC  
Depth:6 (MOUSE)  
Ei-value:0.000, Pi-value:0.000  
Er-value:0.000, Pr-value:0.000  
No matches to TargetScan


CTG

GAAGATCAACATGCCTG  
Depth:4 (DOG)  
Ei-value:0.000, Pi-value:0.000  
Er-value:0.000, Pr-value:0.000  
No matches to TargetScan

-6803--(94)--6898-

TGTGTAT

TGTGTAT  
Depth:6 (MOUSE)  
Ei-value:0.000, Pi-value:0.000  
Er-value:0.000, Pr-value:0.000  
No matches to TargetScan


TT

TGTGTATTT  
Depth:4 (DOG)  
Ei-value:0.000, Pi-value:0.000  
Er-value:0.000, Pr-value:0.000  
No matches to TargetScan

-6906--(53)--6960-

TGTCTTA

TGTCTTA  
Depth:4 (DOG)  
Ei-value:0.000, Pi-value:0.000  
Er-value:0.000, Pr-value:0.000  
MATCHES To TargetScan▶ miR-208-3p:UAAGACG▶ miR-499a-5p:UAAGACU

-6966--(57)--7024-

TTTTTGT

TTTTTGT  
Depth:4 (DOG)  
Ei-value:0.000, Pi-value:0.000  
Er-value:0.000, Pr-value:0.000  
No matches to TargetScan

-7030--(48)--7079-

TTCATTTTGTT

TTCATTTTGTT  
Depth:4 (DOG)  
Ei-value:0.000, Pi-value:0.000  
Er-value:0.000, Pr-value:0.000  
MATCHES To TargetScan▶ miR-495-3p:AACAAAC

-7089--(111)--7201-

TTCTCTTTG

TTCTCTTTG  
Depth:6 (MOUSE)  
Ei-value:0.000, Pi-value:0.000  
Er-value:0.000, Pr-value:0.000  
No matches to TargetScan

-7209--(35)--7245-

ATTTCACCT

ATTTCACCT  
Depth:4 (DOG)  
Ei-value:0.000, Pi-value:0.000  
Er-value:0.000, Pr-value:0.000  
MATCHES To TargetScan▶ miR-203a-3p.2:UGAAAUG

-7253--(27)--7281-

TTTCTAC

TTTCTAC  
Depth:6 (MOUSE)  
Ei-value:0.000, Pi-value:0.000  
Er-value:0.000, Pr-value:0.000  
No matches to TargetScan


T

TTTCTACT  
Depth:5 (RABBIT)  
Ei-value:0.000, Pi-value:0.000  
Er-value:0.000, Pr-value:0.000  
MATCHES To TargetScan▶ miR-411-5p.1:AGUAGAC

-7288--(13)--7302-

ATTTCTC

ATTTCTC  
Depth:6 (MOUSE)  
Ei-value:0.000, Pi-value:0.000  
Er-value:0.000, Pr-value:0.000  
No matches to TargetScan

-7308--(24)--7333-

TCTTGGG

TCTTGGG  
Depth:5 (RABBIT)  
Ei-value:0.000, Pi-value:0.000  
Er-value:0.000, Pr-value:0.000  
No matches to TargetScan

-7339--(52)--7392-

TTTGTGA

TTTGTGA  
Depth:4 (DOG)  
Ei-value:0.000, Pi-value:0.010  
Er-value:0.000, Pr-value:0.000  
No matches to TargetScan

-7398--(19)--7418-

TCTCTGTT

TCTCTGTT  
Depth:4 (DOG)  
Ei-value:0.000, Pi-value:0.000  
Er-value:0.000, Pr-value:0.000  
No matches to TargetScan

-7425--(37)--7463-

TTTGAGTATTT

TTTGAGTATTT  
Depth:4 (DOG)  
Ei-value:0.000, Pi-value:0.000  
Er-value:0.000, Pr-value:0.000  
MATCHES To TargetScan▶ miR-200bc-3p/429:AAUACUG▶ miR-371-5p:CUCAAAC

-7473--(70)--7544-

TGTGTGTG

TGTGTGTG  
Depth:4 (DOG)  
Ei-value:0.000, Pi-value:0.000  
Er-value:0.000, Pr-value:0.000  
MATCHES To TargetScan▶ miR-329-3p/362-3p:ACACACC

-7551--(28)--7580-

TCCTAACCCCT

TCCTAACCCCT  
Depth:5 (RABBIT)  
Ei-value:0.000, Pi-value:0.000  
Er-value:0.000, Pr-value:0.000  
No matches to TargetScan

-7590--(32)--7623-

AAGCATTG

AAGCATTG  
Depth:4 (DOG)  
Ei-value:0.000, Pi-value:0.000  
Er-value:0.000, Pr-value:0.000  
No matches to TargetScan

-7630--(12)--7643-

TTATGCCA

TTATGCCA  
Depth:5 (RABBIT)  
Ei-value:0.000, Pi-value:0.000  
Er-value:0.000, Pr-value:0.000  
No matches to TargetScan


G

TTATGCCAG  
Depth:4 (DOG)  
Ei-value:0.000, Pi-value:0.000  
Er-value:0.000, Pr-value:0.000  
No matches to TargetScan

-7651--(202)--7854-

AGA

AGAAGGCCCAA  
Depth:4 (DOG)  
Ei-value:0.000, Pi-value:0.000  
Er-value:0.000, Pr-value:0.000  
No matches to TargetScan


AGGCCCAA

AGGCCCAA  
Depth:5 (RABBIT)  
Ei-value:0.000, Pi-value:0.000  
Er-value:0.000, Pr-value:0.000  
No matches to TargetScan

-7864--(14)--7879-

TCAA

TCAAGACTAA  
Depth:4 (DOG)  
Ei-value:0.000, Pi-value:0.000  
Er-value:0.000, Pr-value:0.000  
MATCHES To TargetScan▶ miR-431-5p:GUCUUGC


GACTAA

GACTAA  
Depth:5 (RABBIT)  
Ei-value:0.000, Pi-value:0.000  
Er-value:0.000, Pr-value:0.000  
No matches to TargetScan

-7888--(46)--7935-

AGAAGC

AGAAGC  
Depth:4 (DOG)  
Ei-value:0.000, Pi-value:0.000  
Er-value:0.000, Pr-value:0.010  
No matches to TargetScan

-7940--(21)--7962-

AAGATGA

AAGATGA  
Depth:5 (RABBIT)  
Ei-value:0.000, Pi-value:0.000  
Er-value:0.000, Pr-value:0.000  
No matches to TargetScan

-7968--(108)--8077-

CTTTTTGATGTT

CTTTTTGATGTT  
Depth:4 (DOG)  
Ei-value:0.000, Pi-value:0.000  
Er-value:0.000, Pr-value:0.000  
No matches to TargetScan

-8088--(39)--8128-

TATTATGC

TATTATGC  
Depth:4 (DOG)  
Ei-value:0.000, Pi-value:0.000  
Er-value:0.000, Pr-value:0.000  
MATCHES To TargetScan▶ miR-369-3p:AUAAUAC

-8135--(110)--8246-

TATTTCAGT

TATTTCAGT  
Depth:4 (DOG)  
Ei-value:0.000, Pi-value:0.000  
Er-value:0.000, Pr-value:0.000  
MATCHES To TargetScan▶ miR-203a-3p.2:UGAAAUG

-8254--(62)--8317-

GGGGAAA

GGGGAAA  
Depth:4 (DOG)  
Ei-value:0.000, Pi-value:0.000  
Er-value:0.000, Pr-value:0.000  
No matches to TargetScan

-8323--(15)--8339-

TCTAGAGAAAA

TCTAGAGAAAA  
Depth:6 (MOUSE)  
Ei-value:0.000, Pi-value:0.000  
Er-value:0.000, Pr-value:0.000  
MATCHES To TargetScan▶ miR-1251-5p:CUCUAGC

-8349--(2)--8352-

TGAAGAGATG

TGAAGAGATG  
Depth:5 (RABBIT)  
Ei-value:0.000, Pi-value:0.000  
Er-value:0.000, Pr-value:0.000  
No matches to TargetScan

-8361--(7)--8369-

GGCCAA

GGCCAATGAGAAGAATTAGACA  
Depth:4 (DOG)  
Ei-value:0.000, Pi-value:0.000  
Er-value:0.000, Pr-value:0.000  
No matches to TargetScan


TGAGAAGAATTAGACA

TGAGAAGAATTAGACA  
Depth:6 (MOUSE)  
Ei-value:0.000, Pi-value:0.000  
Er-value:0.000, Pr-value:0.000  
No matches to TargetScan

-8390--(26)--8417-

TGAGAAG

TGAGAAG  
Depth:4 (DOG)  
Ei-value:0.000, Pi-value:0.000  
Er-value:0.000, Pr-value:0.010  
No matches to TargetScan

-8423--(9)--8433-

GCAACA

GCAACA  
Depth:6 (MOUSE)  
Ei-value:0.000, Pi-value:0.000  
Er-value:0.000, Pr-value:0.000  
No matches to TargetScan

-8438--(19)--8458-

GGTGAGC

GGTGAGC  
Depth:4 (DOG)  
Ei-value:0.000, Pi-value:0.000  
Er-value:0.000, Pr-value:0.000  
No matches to TargetScan

-8464--(11)--8476-

GGTTTGGG

GGTTTGGG  
Depth:4 (DOG)  
Ei-value:0.000, Pi-value:0.000  
Er-value:0.000, Pr-value:0.000  
No matches to TargetScan

-8483--(11)--8495-

TGGTTA

TGGTTA  
Depth:5 (RABBIT)  
Ei-value:0.000, Pi-value:0.000  
Er-value:0.000, Pr-value:0.000  
No matches to TargetScan


T

TGGTTAT  
Depth:4 (DOG)  
Ei-value:0.000, Pi-value:0.000  
Er-value:0.000, Pr-value:0.000  
No matches to TargetScan

-8501--(32)--8534-

CCCAAGG

CCCAAGG  
Depth:4 (DOG)  
Ei-value:0.000, Pi-value:0.000  
Er-value:0.000, Pr-value:0.000  
MATCHES To TargetScan▶ miR-212-5p:CCUUGGC

-8540--(8)--8549-

TGAACTCCCTGCT

TGAACTCCCTGCT  
Depth:4 (DOG)  
Ei-value:0.000, Pi-value:0.000  
Er-value:0.000, Pr-value:0.000  
No matches to TargetScan

-8561--(1)--8563-

ATAGTAGTGGCC

ATAGTAGTGGCC  
Depth:4 (DOG)  
Ei-value:0.000, Pi-value:0.000  
Er-value:0.000, Pr-value:0.000  
No matches to TargetScan

-8574--(37)--8612-

TTTAATAC

TTTAATAC  
Depth:4 (DOG)  
Ei-value:0.000, Pi-value:0.000  
Er-value:0.000, Pr-value:0.000  
MATCHES To TargetScan▶ miR-496.2:GUAUUAC

-8619--(0)--8620-

CT

CTAGGCTTAAAG  
Depth:4 (DOG)  
Ei-value:0.000, Pi-value:0.000  
Er-value:0.000, Pr-value:0.000  
No matches to TargetScan


AGGCTTA

AGGCTTA  
Depth:5 (RABBIT)  
Ei-value:0.000, Pi-value:0.000  
Er-value:0.000, Pr-value:0.000  
No matches to TargetScan


AAG

CTAGGCTTAAAG  
Depth:4 (DOG)  
Ei-value:0.000, Pi-value:0.000  
Er-value:0.000, Pr-value:0.000  
No matches to TargetScan

-8631--(27)--8659-

GTTTAAT

GTTTAAT  
Depth:5 (RABBIT)  
Ei-value:0.000, Pi-value:0.000  
Er-value:0.000, Pr-value:0.000  
No matches to TargetScan

-8665--(111)--8777-

T

TATTGGCA  
Depth:5 (RABBIT)  
Ei-value:0.000, Pi-value:0.000  
Er-value:0.000, Pr-value:0.000  
No matches to TargetScan


ATTGGCA

ATTGGCA  
Depth:6 (MOUSE)  
Ei-value:0.000, Pi-value:0.000  
Er-value:0.000, Pr-value:0.000  
No matches to TargetScan

-8784--(36)--8821-

TTGTGAAG

TTGTGAAG  
Depth:6 (MOUSE)  
Ei-value:0.000, Pi-value:0.000  
Er-value:0.000, Pr-value:0.000  
No matches to TargetScan

-8828--(4)--8833-

ATGTAAAT

ATGTAAAT  
Depth:5 (RABBIT)  
Ei-value:0.000, Pi-value:0.000  
Er-value:0.000, Pr-value:0.000  
No matches to TargetScan

-8840--(185)--9026-

CTGTCCCT

CTGTCCCT  
Depth:4 (DOG)  
Ei-value:0.000, Pi-value:0.000  
Er-value:0.000, Pr-value:0.000  
No matches to TargetScan

-9033--(155)--9189-

TAGGCACT

TAGGCACT  
Depth:4 (DOG)  
Ei-value:0.000, Pi-value:0.000  
Er-value:0.000, Pr-value:0.000  
No matches to TargetScan

-9196--(259)--9456-

TAAAGCA

TAAAGCA  
Depth:4 (DOG)  
Ei-value:0.000, Pi-value:0.000  
Er-value:0.000, Pr-value:0.000  
No matches to TargetScan

-9462--(41)--9504-

AATGTGCCAGATA

AATGTGCCAGATA  
Depth:4 (DOG)  
Ei-value:0.000, Pi-value:0.000  
Er-value:0.000, Pr-value:0.000  
MATCHES To TargetScan▶ miR-183-5p.2:UGGCACU

-9516--(81)--9598-

TTAAAGTG

TTAAAGTG  
Depth:4 (DOG)  
Ei-value:0.000, Pi-value:0.000  
Er-value:0.000, Pr-value:0.000  
No matches to TargetScan

-9605--(7)--9613-

CTAAAGCA

CTAAAGCA  
Depth:4 (DOG)  
Ei-value:0.000, Pi-value:0.000  
Er-value:0.000, Pr-value:0.000  
No matches to TargetScan

-9620--(37)--9658-

ATGAATA

ATGAATA  
Depth:4 (DOG)  
Ei-value:0.000, Pi-value:0.000  
Er-value:0.000, Pr-value:0.000  
No matches to TargetScan

-9664--(37)--9702-

GGTACTGT

GGTACTGT  
Depth:4 (DOG)  
Ei-value:0.000, Pi-value:0.000  
Er-value:0.000, Pr-value:0.000  
MATCHES To TargetScan▶ miR-101-3p.1:ACAGUAC▶ miR-144-3p:ACAGUAU

-9709--(26)--9736-

ATAAGAGG

ATAAGAGG  
Depth:4 (DOG)  
Ei-value:0.000, Pi-value:0.000  
Er-value:0.000, Pr-value:0.000  
No matches to TargetScan

-9743--(85)--9829-

TAAATTAT

TAAATTAT  
Depth:4 (DOG)  
Ei-value:0.000, Pi-value:0.010  
Er-value:0.000, Pr-value:0.000  
No matches to TargetScan

-9836--(78)--9915-

ATAAAAC

ATAAAAC  
Depth:4 (DOG)  
Ei-value:0.000, Pi-value:0.010  
Er-value:0.000, Pr-value:0.000  
No matches to TargetScan

-9921--(291)--10213-

AAAATTCTCA

AAAATTCTCA  
Depth:4 (DOG)  
Ei-value:0.000, Pi-value:0.000  
Er-value:0.000, Pr-value:0.000  
No matches to TargetScan

-10222--(1)--10224-

TATACAAAC

TATACAAAC  
Depth:4 (DOG)  
Ei-value:0.000, Pi-value:0.000  
Er-value:0.000, Pr-value:0.000  
No matches to TargetScan

-10232--(55)--10288-

GTGAACTCA

GTGAACTCA  
Depth:4 (DOG)  
Ei-value:0.000, Pi-value:0.000  
Er-value:0.000, Pr-value:0.000  
No matches to TargetScan

-10296--(291)--10588-

TGTGCCA

TGTGCCA  
Depth:4 (DOG)  
Ei-value:0.000, Pi-value:0.000  
Er-value:0.000, Pr-value:0.000  
MATCHES To TargetScan▶ miR-183-5p.2:UGGCACU

-10594--(27)--10622-

AAGATAA

AAGATAA  
Depth:4 (DOG)  
Ei-value:0.000, Pi-value:0.000  
Er-value:0.000, Pr-value:0.000  
No matches to TargetScan

-10628--(59)--10688-

TAAACTG

TAAACTG  
Depth:4 (DOG)  
Ei-value:0.000, Pi-value:0.000  
Er-value:0.000, Pr-value:0.000  
No matches to TargetScan

-10694--(34)--10729-

ATTGATTA

ATTGATTA  
Depth:4 (DOG)  
Ei-value:0.000, Pi-value:0.000  
Er-value:0.000, Pr-value:0.010  
No matches to TargetScan

-10736--(19)--10756-

AGAGATA

AGAGATA  
Depth:4 (DOG)  
Ei-value:0.000, Pi-value:0.000  
Er-value:0.000, Pr-value:0.000  
No matches to TargetScan

-10762--(47)--10810-

CAGAGATCT

CAGAGATCT  
Depth:4 (DOG)  
Ei-value:0.000, Pi-value:0.000  
Er-value:0.000, Pr-value:0.000  
No matches to TargetScan

-10818--(51)--10870-

TTGGCT

TTGGCT  
Depth:4 (DOG)  
Ei-value:0.000, Pi-value:0.000  
Er-value:0.000, Pr-value:0.000  
No matches to TargetScan

-10875--(17)--10893-

TTACTTTCT

TTACTTTCT  
Depth:4 (DOG)  
Ei-value:0.000, Pi-value:0.000  
Er-value:0.000, Pr-value:0.010  
No matches to TargetScan

-10901--(364)--11266-

GAGTAAAAA

GAGTAAAAA  
Depth:4 (DOG)  
Ei-value:0.000, Pi-value:0.000  
Er-value:0.000, Pr-value:0.000  
No matches to TargetScan

-11274--(25)--11300-

ATTTGAT

ATTTGAT  
Depth:4 (DOG)  
Ei-value:0.000, Pi-value:0.010  
Er-value:0.000, Pr-value:0.000  
No matches to TargetScan

-11306--(10)--11317-

TTTTATGT

TTTTATGT  
Depth:4 (DOG)  
Ei-value:0.000, Pi-value:0.000  
Er-value:0.000, Pr-value:0.000  
No matches to TargetScan

-11324--(324)--11649-

AAGCCAG

AAGCCAG  
Depth:4 (DOG)  
Ei-value:0.000, Pi-value:0.000  
Er-value:0.000, Pr-value:0.000  
MATCHES To TargetScan▶ miR-149-5p:CUGGCUC▶ miR-3064-5p:CUGGCUG

-11655--(7)--11663-

ATAAAAG

ATAAAAG  
Depth:4 (DOG)  
Ei-value:0.000, Pi-value:0.000  
Er-value:0.000, Pr-value:0.000  
No matches to TargetScan

-11669--(37)--11707-

TTTTATTA

TTTTATTA  
Depth:4 (DOG)  
Ei-value:0.000, Pi-value:0.010  
Er-value:0.000, Pr-value:0.000  
No matches to TargetScan

-11714--(3)--11718-

TTAAATGG

TTAAATGG  
Depth:4 (DOG)  
Ei-value:0.000, Pi-value:0.000  
Er-value:0.000, Pr-value:0.000  
No matches to TargetScan

-11725--(496)--12222-

ACAGAAAACAAAA

ACAGAAAACAAAA  
Depth:4 (DOG)  
Ei-value:0.000, Pi-value:0.000  
Er-value:0.000, Pr-value:0.000  
No matches to TargetScan

-12234--(54)--12289-

AGGTTA

AGGTTA  
Depth:4 (DOG)  
Ei-value:0.000, Pi-value:0.000  
Er-value:0.000, Pr-value:0.000  
No matches to TargetScan

-12294--(24)--12319-

TTCATTCT

TTCATTCT  
Depth:4 (DOG)  
Ei-value:0.000, Pi-value:0.000  
Er-value:0.000, Pr-value:0.000  
No matches to TargetScan

-12326--(77)--12404-

AGAGACA

AGAGACA  
Depth:4 (DOG)  
Ei-value:0.000, Pi-value:0.000  
Er-value:0.000, Pr-value:0.000  
No matches to TargetScan

-12410--(38)--12449-

CCTTTTGG

CCTTTTGG  
Depth:4 (DOG)  
Ei-value:0.000, Pi-value:0.000  
Er-value:0.000, Pr-value:0.000  
No matches to TargetScan

-12456--(53)--12510-

TTAATTC

TTAATTC  
Depth:4 (DOG)  
Ei-value:0.000, Pi-value:0.000  
Er-value:0.000, Pr-value:0.000  
No matches to TargetScan

-12516--(17)--12534-

ACTCTGGCCACTAC

ACTCTGGCCACTAC  
Depth:4 (DOG)  
Ei-value:0.000, Pi-value:0.000  
Er-value:0.000, Pr-value:0.000  
MATCHES To TargetScan▶ miR-142-3p.1:GUAGUGU

-12547--(1)--12549-

ATAAGC

ATAAGC  
Depth:5 (RABBIT)  
Ei-value:0.000, Pi-value:0.010  
Er-value:0.000, Pr-value:0.000  
No matches to TargetScan


AGG

ATAAGCAGG  
Depth:4 (DOG)  
Ei-value:0.000, Pi-value:0.000  
Er-value:0.000, Pr-value:0.000  
No matches to TargetScan

-12557--(80)--12638-

GAAAAATG

GAAAAATG  
Depth:4 (DOG)  
Ei-value:0.000, Pi-value:0.000  
Er-value:0.000, Pr-value:0.000  
No matches to TargetScan

-12645--(55)--12701-

AGTCTCA

AGTCTCA  
Depth:4 (DOG)  
Ei-value:0.000, Pi-value:0.000  
Er-value:0.000, Pr-value:0.000  
No matches to TargetScan

-12707--(42)--12750-

TGGTTTTGAA

TGGTTTTGAA  
Depth:4 (DOG)  
Ei-value:0.000, Pi-value:0.000  
Er-value:0.000, Pr-value:0.000  
No matches to TargetScan

-12759--(136)--12896-

TTAGAAAT

TTAGAAAT  
Depth:4 (DOG)  
Ei-value:0.000, Pi-value:0.000  
Er-value:0.000, Pr-value:0.000  
No matches to TargetScan

-12903--(14)--12918-

CATCAAA

CATCAAA  
Depth:4 (DOG)  
Ei-value:0.000, Pi-value:0.000  
Er-value:0.000, Pr-value:0.000  
No matches to TargetScan

-12924--(143)--13068-

TTTTTAAATCACTCA

TTTTTAAATCACTCA  
Depth:4 (DOG)  
Ei-value:0.000, Pi-value:0.000  
Er-value:0.000, Pr-value:0.000  
No matches to TargetScan

-13082--(1)--13084-

AGAGGGTGGGA

AGAGGGTGGGA  
Depth:4 (DOG)  
Ei-value:0.000, Pi-value:0.000  
Er-value:0.000, Pr-value:0.000  
No matches to TargetScan

-13094--(1)--13096-

AGGAGGAAGAGTGAA

AGGAGGAAGAGTGAA  
Depth:4 (DOG)  
Ei-value:0.000, Pi-value:0.000  
Er-value:0.000, Pr-value:0.000  
MATCHES To TargetScan▶ miR-670-3p:UUCCUCA

-13110--(1)--13112-

G

GAAAAGGTCA  
Depth:4 (DOG)  
Ei-value:0.000, Pi-value:0.000  
Er-value:0.000, Pr-value:0.000  
MATCHES To TargetScan▶ miR-192-5p/215-5p:UGACCUA


AAAAGGT

AAAAGGT  
Depth:6 (MOUSE)  
Ei-value:0.000, Pi-value:0.000  
Er-value:0.000, Pr-value:0.000  
No matches to TargetScan


CA

GAAAAGGTCA  
Depth:4 (DOG)  
Ei-value:0.000, Pi-value:0.000  
Er-value:0.000, Pr-value:0.000  
MATCHES To TargetScan▶ miR-192-5p/215-5p:UGACCUA

-13121--(392)--13514-

TAATGTTT

TAATGTTT  
Depth:4 (DOG)  
Ei-value:0.000, Pi-value:0.000  
Er-value:0.000, Pr-value:0.000  
MATCHES To TargetScan▶ miR-323-3p:ACAUUAC▶ miR-543:AACAUUC

-13521--(53)--13575-

TAATGTTT

TAATGTTT  
Depth:4 (DOG)  
Ei-value:0.000, Pi-value:0.000  
Er-value:0.000, Pr-value:0.000  
MATCHES To TargetScan▶ miR-323-3p:ACAUUAC▶ miR-543:AACAUUC

-13582--(20)--13603-

AGCTGGA

AGCTGGA  
Depth:4 (DOG)  
Ei-value:0.000, Pi-value:0.000  
Er-value:0.000, Pr-value:0.000  
No matches to TargetScan

-13609--(40)--13650-

ATTATTGGAAA

ATTATTGGAAA  
Depth:4 (DOG)  
Ei-value:0.000, Pi-value:0.000  
Er-value:0.000, Pr-value:0.000  
No matches to TargetScan

-13660--(2)--13663-

AGAAAGTAAC

AGAAAGTAAC  
Depth:4 (DOG)  
Ei-value:0.000, Pi-value:0.000  
Er-value:0.000, Pr-value:0.000  
No matches to TargetScan

-13672--(8)--13681-

TTTCACAGTTTCTGGCATC

TTTCACAGTTTCTGGCATC  
Depth:4 (DOG)  
Ei-value:0.000, Pi-value:0.000  
Er-value:0.000, Pr-value:0.000  
No matches to TargetScan

-13699--(11)--13711-

CTACTGAT

CTACTGAT  
Depth:4 (DOG)  
Ei-value:0.000, Pi-value:0.000  
Er-value:0.000, Pr-value:0.000  
MATCHES To TargetScan▶ miR-199-3p:CAGUAGU

-13718--(13)--13732-

AGAACAT

AGAACAT  
Depth:4 (DOG)  
Ei-value:0.000, Pi-value:0.000  
Er-value:0.000, Pr-value:0.000  
No matches to TargetScan

-13738--(4)--13743-

TCATCTG

TCATCTG  
Depth:4 (DOG)  
Ei-value:0.000, Pi-value:0.010  
Er-value:0.000, Pr-value:0.000  
No matches to TargetScan

-13749--(10)--13760-

CATAAATGAA

CATAAATGAA  
Depth:4 (DOG)  
Ei-value:0.000, Pi-value:0.000  
Er-value:0.000, Pr-value:0.000  
No matches to TargetScan

-13769--(114)--13884-

TGAACTGATGTGAAA

TGAACTGATGTGAAA  
Depth:4 (DOG)  
Ei-value:0.000, Pi-value:0.000  
Er-value:0.000, Pr-value:0.000  
MATCHES To TargetScan▶ miR-23-3p:UCACAUU

-13898--(133)--14032-

AAATAAAA

AAATAAAA  
Depth:4 (DOG)  
Ei-value:0.000, Pi-value:0.000  
Er-value:0.000, Pr-value:0.000  
No matches to TargetScan

-14039  
  
>DOG  
       482-

AAACATG

AAACATG  
Depth:4 (DOG)  
Ei-value:0.000, Pi-value:0.000  
Er-value:0.000, Pr-value:0.000  
No matches to TargetScan

-488--(1126)--1615-

TTCCCATC

TTCCCATC  
Depth:4 (DOG)  
Ei-value:0.000, Pi-value:0.000  
Er-value:0.000, Pr-value:0.000  
No matches to TargetScan

-1622--(71)--1694-

C

CTGTTAGTCT  
Depth:4 (DOG)  
Ei-value:0.000, Pi-value:0.000  
Er-value:0.000, Pr-value:0.000  
No matches to TargetScan


TGTTAGTC

TGTTAGTC  
Depth:5 (RABBIT)  
Ei-value:0.000, Pi-value:0.000  
Er-value:0.000, Pr-value:0.000  
No matches to TargetScan


T

CTGTTAGTCT  
Depth:4 (DOG)  
Ei-value:0.000, Pi-value:0.000  
Er-value:0.000, Pr-value:0.000  
No matches to TargetScan

-1703--(101)--1805-

TCATCC

TCATCC  
Depth:4 (DOG)  
Ei-value:0.000, Pi-value:0.020  
Er-value:0.000, Pr-value:0.000  
No matches to TargetScan

-1810--(780)--2591-

TACTTGGGACTGTTAAT

TACTTGGGACTGTTAAT  
Depth:4 (DOG)  
Ei-value:0.000, Pi-value:0.000  
Er-value:0.000, Pr-value:0.000  
MATCHES To TargetScan▶ miR-132-3p/212-3p:AACAGUC▶ miR-455-3p.1:CAGUCCA

-2607--(356)--2964-

ACTG

ACTGTTAATGTGCT  
Depth:4 (DOG)  
Ei-value:0.000, Pi-value:0.000  
Er-value:0.000, Pr-value:0.000  
MATCHES To TargetScan▶ miR-132-3p/212-3p:AACAGUC▶ miR-323-3p:ACAUUAC


TTAATGTGCT

TTAATGTGCT  
Depth:5 (RABBIT)  
Ei-value:0.000, Pi-value:0.000  
Er-value:0.000, Pr-value:0.000  
MATCHES To TargetScan▶ miR-323-3p:ACAUUAC

-2977--(855)--3833-

AATGTGCAT

AATGTGCAT  
Depth:6 (MOUSE)  
Ei-value:0.000, Pi-value:0.000  
Er-value:0.000, Pr-value:0.000  
MATCHES To TargetScan▶ miR-501-3p/502-3p:AUGCACC

-3841--(23)--3865-

TATGTTAGA

TATGTTAGA  
Depth:4 (DOG)  
Ei-value:0.000, Pi-value:0.000  
Er-value:0.000, Pr-value:0.000  
No matches to TargetScan

-3873--(72)--3946-

ACTGTTAATGT

ACTGTTAATGT  
Depth:4 (DOG)  
Ei-value:0.000, Pi-value:0.000  
Er-value:0.000, Pr-value:0.000  
MATCHES To TargetScan▶ miR-132-3p/212-3p:AACAGUC▶ miR-323-3p:ACAUUAC

-3956--(10)--3967-

ATTTGCT

ATTTGCT  
Depth:4 (DOG)  
Ei-value:0.000, Pi-value:0.000  
Er-value:0.000, Pr-value:0.000  
No matches to TargetScan

-3973--(16)--3990-

GTAAGGA

GTAAGGA  
Depth:5 (RABBIT)  
Ei-value:0.000, Pi-value:0.000  
Er-value:0.000, Pr-value:0.000  
No matches to TargetScan

-3996--(141)--4138-

ACTTAT

ACTTAT  
Depth:5 (RABBIT)  
Ei-value:0.000, Pi-value:0.000  
Er-value:0.000, Pr-value:0.000  
No matches to TargetScan

-4143--(144)--4288-

TTAAGGCC

TTAAGGCC  
Depth:6 (MOUSE)  
Ei-value:0.000, Pi-value:0.000  
Er-value:0.000, Pr-value:0.000  
No matches to TargetScan


CCTTT

TTAAGGCCCCTTT  
Depth:5 (RABBIT)  
Ei-value:0.000, Pi-value:0.000  
Er-value:0.000, Pr-value:0.000  
No matches to TargetScan


CTCAA

TTAAGGCCCCTTTCTCAA  
Depth:4 (DOG)  
Ei-value:0.000, Pi-value:0.000  
Er-value:0.000, Pr-value:0.000  
No matches to TargetScan

-4305--(0)--4306-

ACAGTTAATGTG

ACAGTTAATGTG  
Depth:4 (DOG)  
Ei-value:0.000, Pi-value:0.000  
Er-value:0.000, Pr-value:0.000  
MATCHES To TargetScan▶ miR-323-3p:ACAUUAC

-4317--(1388)--5706-

CTCAGCTCTTGG

CTCAGCTCTTGG  
Depth:5 (RABBIT)  
Ei-value:0.000, Pi-value:0.000  
Er-value:0.000, Pr-value:0.000  
MATCHES To TargetScan▶ miR-335-5p:CAAGAGC


ACA

CTCAGCTCTTGGACA  
Depth:4 (DOG)  
Ei-value:0.000, Pi-value:0.000  
Er-value:0.000, Pr-value:0.000  
MATCHES To TargetScan▶ miR-335-5p:CAAGAGC

-5720--(177)--5898-

CAAAACTT

CAAAACTT  
Depth:4 (DOG)  
Ei-value:0.000, Pi-value:0.000  
Er-value:0.000, Pr-value:0.000  
No matches to TargetScan

-5905--(19)--5925-

GCACAATG

GCACAATG  
Depth:6 (MOUSE)  
Ei-value:0.000, Pi-value:0.000  
Er-value:0.000, Pr-value:0.000  
No matches to TargetScan

-5932--(43)--5976-

A

ACTCCCA  
Depth:4 (DOG)  
Ei-value:0.000, Pi-value:0.000  
Er-value:0.000, Pr-value:0.000  
No matches to TargetScan


CTCCCA

CTCCCA  
Depth:6 (MOUSE)  
Ei-value:0.000, Pi-value:0.000  
Er-value:0.000, Pr-value:0.000  
No matches to TargetScan

-5982--(249)--6232-

CCCTTTTGCATT

CCCTTTTGCATT  
Depth:4 (DOG)  
Ei-value:0.000, Pi-value:0.000  
Er-value:0.000, Pr-value:0.000  
No matches to TargetScan

-6243--(145)--6389-

CCATTTTT

CCATTTTT  
Depth:4 (DOG)  
Ei-value:0.000, Pi-value:0.000  
Er-value:0.000, Pr-value:0.000  
No matches to TargetScan

-6396--(85)--6482-

CAGCCCA

CAGCCCA  
Depth:4 (DOG)  
Ei-value:0.000, Pi-value:0.000  
Er-value:0.000, Pr-value:0.000  
No matches to TargetScan

-6488--(7)--6496-

CCTACCA

CCTACCA  
Depth:4 (DOG)  
Ei-value:0.000, Pi-value:0.000  
Er-value:0.000, Pr-value:0.000  
No matches to TargetScan

-6502--(29)--6532-

AAAAGCAG

AAAAGCAG  
Depth:6 (MOUSE)  
Ei-value:0.000, Pi-value:0.000  
Er-value:0.000, Pr-value:0.000  
No matches to TargetScan

-6539--(44)--6584-

TTAATGATCC

TTAATGATCC  
Depth:4 (DOG)  
Ei-value:0.000, Pi-value:0.000  
Er-value:0.000, Pr-value:0.000  
MATCHES To TargetScan▶ miR-382-3p:AUCAUUC

-6593--(16)--6610-

ATTCTGGG

ATTCTGGG  
Depth:4 (DOG)  
Ei-value:0.000, Pi-value:0.000  
Er-value:0.000, Pr-value:0.000  
No matches to TargetScan

-6617--(32)--6650-

CTTTACT

CTTTACT  
Depth:4 (DOG)  
Ei-value:0.000, Pi-value:0.000  
Er-value:0.000, Pr-value:0.000  
No matches to TargetScan

-6656--(2)--6659-

GCAAAAT

GCAAAAT  
Depth:6 (MOUSE)  
Ei-value:0.000, Pi-value:0.000  
Er-value:0.000, Pr-value:0.000  
No matches to TargetScan

-6665--(4)--6670-

AAGGCAA

AAGGCAA  
Depth:4 (DOG)  
Ei-value:0.000, Pi-value:0.000  
Er-value:0.000, Pr-value:0.000  
No matches to TargetScan

-6676--(17)--6694-

TGGATTGC

TGGATTGC  
Depth:4 (DOG)  
Ei-value:0.000, Pi-value:0.000  
Er-value:0.000, Pr-value:0.000  
No matches to TargetScan

-6701--(76)--6778-

TGCATTCTTC

TGCATTCTTC  
Depth:5 (RABBIT)  
Ei-value:0.000, Pi-value:0.000  
Er-value:0.000, Pr-value:0.000  
No matches to TargetScan

-6787--(7)--6795-

AGC

AGCAGATTGCCTGG  
Depth:4 (DOG)  
Ei-value:0.000, Pi-value:0.000  
Er-value:0.000, Pr-value:0.000  
No matches to TargetScan


A

AGATTGCCTGG  
Depth:5 (RABBIT)  
Ei-value:0.000, Pi-value:0.000  
Er-value:0.000, Pr-value:0.000  
No matches to TargetScan


GATTGCCTGG

GATTGCCTGG  
Depth:6 (MOUSE)  
Ei-value:0.000, Pi-value:0.000  
Er-value:0.000, Pr-value:0.000  
No matches to TargetScan

-6808--(18)--6827-

TTGTATATT

TTGTATATT  
Depth:4 (DOG)  
Ei-value:0.000, Pi-value:0.000  
Er-value:0.000, Pr-value:0.000  
MATCHES To TargetScan▶ miR-381-3p:AUACAAG

-6835--(126)--6962-

AAC

AACCTGGTCATT  
Depth:4 (DOG)  
Ei-value:0.000, Pi-value:0.000  
Er-value:0.000, Pr-value:0.000  
No matches to TargetScan


CTGGTCATT

CTGGTCATT  
Depth:5 (RABBIT)  
Ei-value:0.000, Pi-value:0.000  
Er-value:0.000, Pr-value:0.000  
No matches to TargetScan

-6973--(21)--6995-

CCATTTAT

CCATTTAT  
Depth:5 (RABBIT)  
Ei-value:0.000, Pi-value:0.000  
Er-value:0.000, Pr-value:0.000  
No matches to TargetScan

-7002--(13)--7016-

TGAC

TGACCAGTGTCTCTCATTT  
Depth:4 (DOG)  
Ei-value:0.000, Pi-value:0.000  
Er-value:0.000, Pr-value:0.000  
No matches to TargetScan


CAGTGTCTCTCATTT

CAGTGTCTCTCATTT  
Depth:5 (RABBIT)  
Ei-value:0.000, Pi-value:0.000  
Er-value:0.000, Pr-value:0.000  
No matches to TargetScan

-7034--(5)--7040-

AGG

AGGGTGGTG  
Depth:4 (DOG)  
Ei-value:0.000, Pi-value:0.000  
Er-value:0.000, Pr-value:0.000  
No matches to TargetScan


GTGGTG

GTGGTG  
Depth:5 (RABBIT)  
Ei-value:0.000, Pi-value:0.000  
Er-value:0.000, Pr-value:0.000  
No matches to TargetScan

-7048--(1)--7050-

GTCTGTGGATA

GTCTGTGGATA  
Depth:5 (RABBIT)  
Ei-value:0.000, Pi-value:0.000  
Er-value:0.000, Pr-value:0.000  
MATCHES To TargetScan▶ miR-140-3p.1:CCACAGG

-7060--(23)--7084-

TTCTAGA

TTCTAGA  
Depth:4 (DOG)  
Ei-value:0.000, Pi-value:0.000  
Er-value:0.000, Pr-value:0.000  
No matches to TargetScan

-7090--(70)--7161-

ATTCACTT

ATTCACTT  
Depth:4 (DOG)  
Ei-value:0.000, Pi-value:0.000  
Er-value:0.000, Pr-value:0.000  
No matches to TargetScan

-7168--(3)--7172-

GAAAAAC

GAAAAAC  
Depth:4 (DOG)  
Ei-value:0.000, Pi-value:0.000  
Er-value:0.000, Pr-value:0.000  
No matches to TargetScan

-7178--(22)--7201-

AATTTCTTCATCTGGAGC

AATTTCTTCATCTGGAGC  
Depth:5 (RABBIT)  
Ei-value:0.000, Pi-value:0.000  
Er-value:0.000, Pr-value:0.000  
No matches to TargetScan

-7218--(18)--7237-

CTTATTT

CTTATTT  
Depth:4 (DOG)  
Ei-value:0.000, Pi-value:0.000  
Er-value:0.000, Pr-value:0.010  
No matches to TargetScan

-7243--(91)--7335-

ATAAAATG

ATAAAATG  
Depth:4 (DOG)  
Ei-value:0.000, Pi-value:0.000  
Er-value:0.000, Pr-value:0.000  
No matches to TargetScan

-7342--(299)--7642-

AAAAATAAGCCA

AAAAATAAGCCA  
Depth:5 (RABBIT)  
Ei-value:0.000, Pi-value:0.000  
Er-value:0.000, Pr-value:0.000  
No matches to TargetScan


A

AAAAATAAGCCAA  
Depth:4 (DOG)  
Ei-value:0.000, Pi-value:0.000  
Er-value:0.000, Pr-value:0.000  
No matches to TargetScan

-7654--(55)--7710-

ATGAATAATA

ATGAATAATA  
Depth:4 (DOG)  
Ei-value:0.000, Pi-value:0.000  
Er-value:0.000, Pr-value:0.000  
No matches to TargetScan

-7719--(44)--7764-

TGGAACTGCT

TGGAACTGCT  
Depth:4 (DOG)  
Ei-value:0.000, Pi-value:0.000  
Er-value:0.000, Pr-value:0.000  
No matches to TargetScan

-7773--(8)--7782-

TAACTA

TAACTA  
Depth:4 (DOG)  
Ei-value:0.000, Pi-value:0.000  
Er-value:0.000, Pr-value:0.000  
No matches to TargetScan

-7787--(10)--7798-

CAGCAGTTC

CAGCAGTTC  
Depth:5 (RABBIT)  
Ei-value:0.000, Pi-value:0.000  
Er-value:0.000, Pr-value:0.000  
No matches to TargetScan

-7806--(1)--7808-

TTGTAAT

TTGTAAT  
Depth:4 (DOG)  
Ei-value:0.000, Pi-value:0.000  
Er-value:0.000, Pr-value:0.000  
No matches to TargetScan

-7814--(1)--7816-

ACTGAAAA

ACTGAAAA  
Depth:5 (RABBIT)  
Ei-value:0.000, Pi-value:0.000  
Er-value:0.000, Pr-value:0.000  
No matches to TargetScan

-7823--(19)--7843-

AAGGATG

AAGGATG  
Depth:5 (RABBIT)  
Ei-value:0.000, Pi-value:0.000  
Er-value:0.000, Pr-value:0.000  
MATCHES To TargetScan▶ miR-362-5p/500b-5p:AUCCUUG


TCA

AAGGATGTCAAAAGATC  
Depth:4 (DOG)  
Ei-value:0.000, Pi-value:0.000  
Er-value:0.000, Pr-value:0.000  
MATCHES To TargetScan▶ miR-362-5p/500b-5p:AUCCUUG▶ miR-489-3p:UGACAUC


AAAGATC

AAAGATC  
Depth:6 (MOUSE)  
Ei-value:0.000, Pi-value:0.000  
Er-value:0.000, Pr-value:0.000  
No matches to TargetScan

-7859--(4)--7864-

CAGCTCAGGG

CAGCTCAGGG  
Depth:4 (DOG)  
Ei-value:0.000, Pi-value:0.000  
Er-value:0.000, Pr-value:0.000  
MATCHES To TargetScan▶ miR-125-5p:CCCUGAG

-7873--(12)--7886-

CTACTAGCTCCT

CTACTAGCTCCT  
Depth:4 (DOG)  
Ei-value:0.000, Pi-value:0.000  
Er-value:0.000, Pr-value:0.000  
MATCHES To TargetScan▶ miR-28-5p/708-5p:AGGAGCU▶ miR-411-5p.2:UAGUAGA

-7897--(1)--7899-

GGACAGCTG

GGACAGCTG  
Depth:5 (RABBIT)  
Ei-value:0.000, Pi-value:0.000  
Er-value:0.000, Pr-value:0.000  
No matches to TargetScan


T

GGACAGCTGT  
Depth:4 (DOG)  
Ei-value:0.000, Pi-value:0.000  
Er-value:0.000, Pr-value:0.000  
No matches to TargetScan

-7908--(1)--7910-

AGAAGAGTCTCTGGCTCTTTA

AGAAGAGTCTCTGGCTCTTTA  
Depth:5 (RABBIT)  
Ei-value:0.000, Pi-value:0.000  
Er-value:0.000, Pr-value:0.000  
No matches to TargetScan


GA

AGAAGAGTCTCTGGCTCTTTAGA  
Depth:4 (DOG)  
Ei-value:0.000, Pi-value:0.000  
Er-value:0.000, Pr-value:0.000  
No matches to TargetScan

-7932--(11)--7944-

ATTCTGAGC

ATTCTGAGC  
Depth:4 (DOG)  
Ei-value:0.000, Pi-value:0.000  
Er-value:0.000, Pr-value:0.000  
No matches to TargetScan

-7952--(56)--8009-

CTGCAA

CTGCAA  
Depth:5 (RABBIT)  
Ei-value:0.000, Pi-value:0.000  
Er-value:0.000, Pr-value:0.000  
No matches to TargetScan

-8014--(30)--8045-

AAGAATAGGC

AAGAATAGGC  
Depth:5 (RABBIT)  
Ei-value:0.000, Pi-value:0.000  
Er-value:0.000, Pr-value:0.000  
No matches to TargetScan

-8054--(9)--8064-

TACAGTGTTAGTGA

TACAGTGTTAGTGA  
Depth:5 (RABBIT)  
Ei-value:0.000, Pi-value:0.000  
Er-value:0.000, Pr-value:0.000  
MATCHES To TargetScan▶ miR-141-3p/200a-3p:AACACUG

-8077--(4)--8082-

TTCCCTTTGA

TTCCCTTTGA  
Depth:6 (MOUSE)  
Ei-value:0.000, Pi-value:0.000  
Er-value:0.000, Pr-value:0.000  
No matches to TargetScan

-8091--(7)--8099-

TAGGTGGAGATGGGGCATGAGGATCCTCCAGGGGAA

TAGGTGGAGATGGGGCATGAGGATCCTCCAGGGGAA  
Depth:6 (MOUSE)  
Ei-value:0.000, Pi-value:0.000  
Er-value:0.000, Pr-value:0.000  
MATCHES To TargetScan▶ miR-331-3p:CCCCUGG


A

TAGGTGGAGATGGGGCATGAGGATCCTCCAGGGGAAA  
Depth:5 (RABBIT)  
Ei-value:0.000, Pi-value:0.000  
Er-value:0.000, Pr-value:0.000  
MATCHES To TargetScan▶ miR-331-3p:CCCCUGG

-8135--(3)--8139-

TCACTA

TCACTA  
Depth:5 (RABBIT)  
Ei-value:0.000, Pi-value:0.000  
Er-value:0.000, Pr-value:0.000  
No matches to TargetScan


CCACT

TCACTACCACT  
Depth:4 (DOG)  
Ei-value:0.000, Pi-value:0.000  
Er-value:0.000, Pr-value:0.000  
MATCHES To TargetScan▶ miR-140-5p:AGUGGUU▶ miR-142-3p.1:GUAGUGU

-8149--(2)--8152-

GCAACA

GCAACA  
Depth:6 (MOUSE)  
Ei-value:0.000, Pi-value:0.000  
Er-value:0.000, Pr-value:0.000  
No matches to TargetScan


AC

GCAACAAC  
Depth:5 (RABBIT)  
Ei-value:0.000, Pi-value:0.000  
Er-value:0.000, Pr-value:0.000  
No matches to TargetScan

-8159--(66)--8226-

ACAACCACC

ACAACCACC  
Depth:5 (RABBIT)  
Ei-value:0.000, Pi-value:0.000  
Er-value:0.000, Pr-value:0.000  
No matches to TargetScan


ACAC

ACAACCACCACAC  
Depth:4 (DOG)  
Ei-value:0.000, Pi-value:0.000  
Er-value:0.000, Pr-value:0.000  
No matches to TargetScan

-8238--(278)--8517-

TTGTTCC

TTGTTCC  
Depth:4 (DOG)  
Ei-value:0.000, Pi-value:0.000  
Er-value:0.000, Pr-value:0.000  
No matches to TargetScan

-8523--(11)--8535-

CCAAAT

CCAAAT  
Depth:6 (MOUSE)  
Ei-value:0.000, Pi-value:0.000  
Er-value:0.000, Pr-value:0.000  
No matches to TargetScan


C

CCAAATC  
Depth:5 (RABBIT)  
Ei-value:0.000, Pi-value:0.000  
Er-value:0.000, Pr-value:0.000  
No matches to TargetScan

-8541--(27)--8569-

CAAGAAA

CAAGAAA  
Depth:5 (RABBIT)  
Ei-value:0.000, Pi-value:0.000  
Er-value:0.000, Pr-value:0.000  
No matches to TargetScan

-8575--(16)--8592-

G

GAAGATCAACATGCCTG  
Depth:4 (DOG)  
Ei-value:0.000, Pi-value:0.000  
Er-value:0.000, Pr-value:0.000  
No matches to TargetScan


AA

AAGATCAACATGC  
Depth:5 (RABBIT)  
Ei-value:0.000, Pi-value:0.000  
Er-value:0.000, Pr-value:0.000  
No matches to TargetScan


GATCAACATGC

GATCAACATGC  
Depth:6 (MOUSE)  
Ei-value:0.000, Pi-value:0.000  
Er-value:0.000, Pr-value:0.000  
No matches to TargetScan


CTG

GAAGATCAACATGCCTG  
Depth:4 (DOG)  
Ei-value:0.000, Pi-value:0.000  
Er-value:0.000, Pr-value:0.000  
No matches to TargetScan

-8608--(31)--8640-

TGTGTAT

TGTGTAT  
Depth:6 (MOUSE)  
Ei-value:0.000, Pi-value:0.000  
Er-value:0.000, Pr-value:0.000  
No matches to TargetScan


TT

TGTGTATTT  
Depth:4 (DOG)  
Ei-value:0.000, Pi-value:0.000  
Er-value:0.000, Pr-value:0.000  
No matches to TargetScan

-8648--(55)--8704-

TGTCTTA

TGTCTTA  
Depth:4 (DOG)  
Ei-value:0.000, Pi-value:0.000  
Er-value:0.000, Pr-value:0.000  
MATCHES To TargetScan▶ miR-208-3p:UAAGACG▶ miR-499a-5p:UAAGACU

-8710--(56)--8767-

TTTTTGT

TTTTTGT  
Depth:4 (DOG)  
Ei-value:0.000, Pi-value:0.000  
Er-value:0.000, Pr-value:0.000  
No matches to TargetScan

-8773--(47)--8821-

TTCATTTTGTT

TTCATTTTGTT  
Depth:4 (DOG)  
Ei-value:0.000, Pi-value:0.000  
Er-value:0.000, Pr-value:0.000  
MATCHES To TargetScan▶ miR-495-3p:AACAAAC

-8831--(5)--8837-

TTCTCTTTG

TTCTCTTTG  
Depth:6 (MOUSE)  
Ei-value:0.000, Pi-value:0.000  
Er-value:0.000, Pr-value:0.000  
No matches to TargetScan

-8845--(156)--9002-

ATTTCACCT

ATTTCACCT  
Depth:4 (DOG)  
Ei-value:0.000, Pi-value:0.000  
Er-value:0.000, Pr-value:0.000  
MATCHES To TargetScan▶ miR-203a-3p.2:UGAAAUG

-9010--(30)--9041-

TTTCTAC

TTTCTAC  
Depth:6 (MOUSE)  
Ei-value:0.000, Pi-value:0.000  
Er-value:0.000, Pr-value:0.000  
No matches to TargetScan


T

TTTCTACT  
Depth:5 (RABBIT)  
Ei-value:0.000, Pi-value:0.000  
Er-value:0.000, Pr-value:0.000  
MATCHES To TargetScan▶ miR-411-5p.1:AGUAGAC

-9048--(11)--9060-

ATTTCTC

ATTTCTC  
Depth:6 (MOUSE)  
Ei-value:0.000, Pi-value:0.000  
Er-value:0.000, Pr-value:0.000  
No matches to TargetScan

-9066--(25)--9092-

TCTTGGG

TCTTGGG  
Depth:5 (RABBIT)  
Ei-value:0.000, Pi-value:0.000  
Er-value:0.000, Pr-value:0.000  
No matches to TargetScan

-9098--(42)--9141-

TTTGTGA

TTTGTGA  
Depth:4 (DOG)  
Ei-value:0.000, Pi-value:0.010  
Er-value:0.000, Pr-value:0.000  
No matches to TargetScan

-9147--(19)--9167-

TCTCTGTT

TCTCTGTT  
Depth:4 (DOG)  
Ei-value:0.000, Pi-value:0.000  
Er-value:0.000, Pr-value:0.000  
No matches to TargetScan

-9174--(37)--9212-

TTTGAGTATTT

TTTGAGTATTT  
Depth:4 (DOG)  
Ei-value:0.000, Pi-value:0.000  
Er-value:0.000, Pr-value:0.000  
MATCHES To TargetScan▶ miR-200bc-3p/429:AAUACUG▶ miR-371-5p:CUCAAAC

-9222--(88)--9311-

TGTGTGTG

TGTGTGTG  
Depth:4 (DOG)  
Ei-value:0.000, Pi-value:0.000  
Er-value:0.000, Pr-value:0.000  
MATCHES To TargetScan▶ miR-329-3p/362-3p:ACACACC

-9318--(17)--9336-

TCCTAACCCCT

TCCTAACCCCT  
Depth:5 (RABBIT)  
Ei-value:0.000, Pi-value:0.000  
Er-value:0.000, Pr-value:0.000  
No matches to TargetScan

-9346--(33)--9380-

AAGCATTG

AAGCATTG  
Depth:4 (DOG)  
Ei-value:0.000, Pi-value:0.000  
Er-value:0.000, Pr-value:0.000  
No matches to TargetScan

-9387--(12)--9400-

TTATGCCA

TTATGCCA  
Depth:5 (RABBIT)  
Ei-value:0.000, Pi-value:0.000  
Er-value:0.000, Pr-value:0.000  
No matches to TargetScan


G

TTATGCCAG  
Depth:4 (DOG)  
Ei-value:0.000, Pi-value:0.000  
Er-value:0.000, Pr-value:0.000  
No matches to TargetScan

-9408--(61)--9470-

AGA

AGAAGGCCCAA  
Depth:4 (DOG)  
Ei-value:0.000, Pi-value:0.000  
Er-value:0.000, Pr-value:0.000  
No matches to TargetScan


AGGCCCAA

AGGCCCAA  
Depth:5 (RABBIT)  
Ei-value:0.000, Pi-value:0.000  
Er-value:0.000, Pr-value:0.000  
No matches to TargetScan

-9480--(355)--9836-

TCAA

TCAAGACTAA  
Depth:4 (DOG)  
Ei-value:0.000, Pi-value:0.000  
Er-value:0.000, Pr-value:0.000  
MATCHES To TargetScan▶ miR-431-5p:GUCUUGC


GACTAA

GACTAA  
Depth:5 (RABBIT)  
Ei-value:0.000, Pi-value:0.000  
Er-value:0.000, Pr-value:0.000  
No matches to TargetScan

-9845--(14)--9860-

AGAAGC

AGAAGC  
Depth:4 (DOG)  
Ei-value:0.000, Pi-value:0.000  
Er-value:0.000, Pr-value:0.010  
No matches to TargetScan

-9865--(25)--9891-

AGAAGC

AGAAGC  
Depth:4 (DOG)  
Ei-value:0.000, Pi-value:0.000  
Er-value:0.000, Pr-value:0.010  
No matches to TargetScan

-9896--(21)--9918-

AAGATGA

AAGATGA  
Depth:5 (RABBIT)  
Ei-value:0.000, Pi-value:0.000  
Er-value:0.000, Pr-value:0.000  
No matches to TargetScan

-9924--(112)--10037-

CTTTTTGATGTT

CTTTTTGATGTT  
Depth:4 (DOG)  
Ei-value:0.000, Pi-value:0.000  
Er-value:0.000, Pr-value:0.000  
No matches to TargetScan

-10048--(37)--10086-

TATTATGC

TATTATGC  
Depth:4 (DOG)  
Ei-value:0.000, Pi-value:0.000  
Er-value:0.000, Pr-value:0.000  
MATCHES To TargetScan▶ miR-369-3p:AUAAUAC

-10093--(118)--10212-

TATTTCAGT

TATTTCAGT  
Depth:4 (DOG)  
Ei-value:0.000, Pi-value:0.000  
Er-value:0.000, Pr-value:0.000  
MATCHES To TargetScan▶ miR-203a-3p.2:UGAAAUG

-10220--(57)--10278-

GGGGAAA

GGGGAAA  
Depth:4 (DOG)  
Ei-value:0.000, Pi-value:0.000  
Er-value:0.000, Pr-value:0.000  
No matches to TargetScan

-10284--(15)--10300-

TCTAGAGAAAA

TCTAGAGAAAA  
Depth:6 (MOUSE)  
Ei-value:0.000, Pi-value:0.000  
Er-value:0.000, Pr-value:0.000  
MATCHES To TargetScan▶ miR-1251-5p:CUCUAGC

-10310--(2)--10313-

TGAAGAGATG

TGAAGAGATG  
Depth:5 (RABBIT)  
Ei-value:0.000, Pi-value:0.000  
Er-value:0.000, Pr-value:0.000  
No matches to TargetScan

-10322--(5)--10328-

GGCCAA

GGCCAATGAGAAGAATTAGACA  
Depth:4 (DOG)  
Ei-value:0.000, Pi-value:0.000  
Er-value:0.000, Pr-value:0.000  
No matches to TargetScan


TGAGAAGAATTAGACA

TGAGAAGAATTAGACA  
Depth:6 (MOUSE)  
Ei-value:0.000, Pi-value:0.000  
Er-value:0.000, Pr-value:0.000  
No matches to TargetScan

-10349--(26)--10376-

TGAGAAG

TGAGAAG  
Depth:4 (DOG)  
Ei-value:0.000, Pi-value:0.000  
Er-value:0.000, Pr-value:0.010  
No matches to TargetScan

-10382--(9)--10392-

GCAACA

GCAACA  
Depth:6 (MOUSE)  
Ei-value:0.000, Pi-value:0.000  
Er-value:0.000, Pr-value:0.000  
No matches to TargetScan

-10397--(19)--10417-

GGTGAGC

GGTGAGC  
Depth:4 (DOG)  
Ei-value:0.000, Pi-value:0.000  
Er-value:0.000, Pr-value:0.000  
No matches to TargetScan

-10423--(11)--10435-

GGTTTGGG

GGTTTGGG  
Depth:4 (DOG)  
Ei-value:0.000, Pi-value:0.000  
Er-value:0.000, Pr-value:0.000  
No matches to TargetScan

-10442--(11)--10454-

TGGTTA

TGGTTA  
Depth:5 (RABBIT)  
Ei-value:0.000, Pi-value:0.000  
Er-value:0.000, Pr-value:0.000  
No matches to TargetScan


T

TGGTTAT  
Depth:4 (DOG)  
Ei-value:0.000, Pi-value:0.000  
Er-value:0.000, Pr-value:0.000  
No matches to TargetScan

-10460--(27)--10488-

CCCAAGG

CCCAAGG  
Depth:4 (DOG)  
Ei-value:0.000, Pi-value:0.000  
Er-value:0.000, Pr-value:0.000  
MATCHES To TargetScan▶ miR-212-5p:CCUUGGC

-10494--(8)--10503-

TGAACTCCCTGCT

TGAACTCCCTGCT  
Depth:4 (DOG)  
Ei-value:0.000, Pi-value:0.000  
Er-value:0.000, Pr-value:0.000  
No matches to TargetScan

-10515--(1)--10517-

ATAGTAGTGGCC

ATAGTAGTGGCC  
Depth:4 (DOG)  
Ei-value:0.000, Pi-value:0.000  
Er-value:0.000, Pr-value:0.000  
No matches to TargetScan

-10528--(37)--10566-

TTTAATAC

TTTAATAC  
Depth:4 (DOG)  
Ei-value:0.000, Pi-value:0.000  
Er-value:0.000, Pr-value:0.000  
MATCHES To TargetScan▶ miR-496.2:GUAUUAC

-10573--(6)--10580-

CT

CTAGGCTTAAAG  
Depth:4 (DOG)  
Ei-value:0.000, Pi-value:0.000  
Er-value:0.000, Pr-value:0.000  
No matches to TargetScan


AGGCTTA

AGGCTTA  
Depth:5 (RABBIT)  
Ei-value:0.000, Pi-value:0.000  
Er-value:0.000, Pr-value:0.000  
No matches to TargetScan


AAG

CTAGGCTTAAAG  
Depth:4 (DOG)  
Ei-value:0.000, Pi-value:0.000  
Er-value:0.000, Pr-value:0.000  
No matches to TargetScan

-10591--(27)--10619-

GTTTAAT

GTTTAAT  
Depth:5 (RABBIT)  
Ei-value:0.000, Pi-value:0.000  
Er-value:0.000, Pr-value:0.000  
No matches to TargetScan

-10625--(115)--10741-

T

TATTGGCA  
Depth:5 (RABBIT)  
Ei-value:0.000, Pi-value:0.000  
Er-value:0.000, Pr-value:0.000  
No matches to TargetScan


ATTGGCA

ATTGGCA  
Depth:6 (MOUSE)  
Ei-value:0.000, Pi-value:0.000  
Er-value:0.000, Pr-value:0.000  
No matches to TargetScan

-10748--(35)--10784-

TTGTGAAG

TTGTGAAG  
Depth:6 (MOUSE)  
Ei-value:0.000, Pi-value:0.000  
Er-value:0.000, Pr-value:0.000  
No matches to TargetScan

-10791--(186)--10978-

ATGTAAAT

ATGTAAAT  
Depth:5 (RABBIT)  
Ei-value:0.000, Pi-value:0.000  
Er-value:0.000, Pr-value:0.000  
No matches to TargetScan

-10985--(39)--11025-

CTGTCCCT

CTGTCCCT  
Depth:4 (DOG)  
Ei-value:0.000, Pi-value:0.000  
Er-value:0.000, Pr-value:0.000  
No matches to TargetScan

-11032--(4)--11037-

TAGGCACT

TAGGCACT  
Depth:4 (DOG)  
Ei-value:0.000, Pi-value:0.000  
Er-value:0.000, Pr-value:0.000  
No matches to TargetScan

-11044--(73)--11118-

TAAAGCA

TAAAGCA  
Depth:4 (DOG)  
Ei-value:0.000, Pi-value:0.000  
Er-value:0.000, Pr-value:0.000  
No matches to TargetScan

-11124--(328)--11453-

AATGTGCCAGATA

AATGTGCCAGATA  
Depth:4 (DOG)  
Ei-value:0.000, Pi-value:0.000  
Er-value:0.000, Pr-value:0.000  
MATCHES To TargetScan▶ miR-183-5p.2:UGGCACU

-11465--(76)--11542-

TTAAAGTG

TTAAAGTG  
Depth:4 (DOG)  
Ei-value:0.000, Pi-value:0.000  
Er-value:0.000, Pr-value:0.000  
No matches to TargetScan

-11549--(7)--11557-

CTAAAGCA

CTAAAGCA  
Depth:4 (DOG)  
Ei-value:0.000, Pi-value:0.000  
Er-value:0.000, Pr-value:0.000  
No matches to TargetScan

-11564--(37)--11602-

ATGAATA

ATGAATA  
Depth:4 (DOG)  
Ei-value:0.000, Pi-value:0.000  
Er-value:0.000, Pr-value:0.000  
No matches to TargetScan

-11608--(17)--11626-

GGTACTGT

GGTACTGT  
Depth:4 (DOG)  
Ei-value:0.000, Pi-value:0.000  
Er-value:0.000, Pr-value:0.000  
MATCHES To TargetScan▶ miR-101-3p.1:ACAGUAC▶ miR-144-3p:ACAGUAU

-11633--(37)--11671-

ATAAGAGG

ATAAGAGG  
Depth:4 (DOG)  
Ei-value:0.000, Pi-value:0.000  
Er-value:0.000, Pr-value:0.000  
No matches to TargetScan

-11678--(81)--11760-

TAAATTAT

TAAATTAT  
Depth:4 (DOG)  
Ei-value:0.000, Pi-value:0.010  
Er-value:0.000, Pr-value:0.000  
No matches to TargetScan

-11767--(307)--12075-

ATAAAAC

ATAAAAC  
Depth:4 (DOG)  
Ei-value:0.000, Pi-value:0.010  
Er-value:0.000, Pr-value:0.000  
No matches to TargetScan

-12081--(28)--12110-

AAAATTCTCA

AAAATTCTCA  
Depth:4 (DOG)  
Ei-value:0.000, Pi-value:0.000  
Er-value:0.000, Pr-value:0.000  
No matches to TargetScan

-12119--(43)--12163-

TATACAAAC

TATACAAAC  
Depth:4 (DOG)  
Ei-value:0.000, Pi-value:0.000  
Er-value:0.000, Pr-value:0.000  
No matches to TargetScan

-12171--(51)--12223-

GTGAACTCA

GTGAACTCA  
Depth:4 (DOG)  
Ei-value:0.000, Pi-value:0.000  
Er-value:0.000, Pr-value:0.000  
No matches to TargetScan

-12231--(288)--12520-

TGTGCCA

TGTGCCA  
Depth:4 (DOG)  
Ei-value:0.000, Pi-value:0.000  
Er-value:0.000, Pr-value:0.000  
MATCHES To TargetScan▶ miR-183-5p.2:UGGCACU

-12526--(27)--12554-

AAGATAA

AAGATAA  
Depth:4 (DOG)  
Ei-value:0.000, Pi-value:0.000  
Er-value:0.000, Pr-value:0.000  
No matches to TargetScan

-12560--(60)--12621-

TAAACTG

TAAACTG  
Depth:4 (DOG)  
Ei-value:0.000, Pi-value:0.000  
Er-value:0.000, Pr-value:0.000  
No matches to TargetScan

-12627--(40)--12668-

ATTGATTA

ATTGATTA  
Depth:4 (DOG)  
Ei-value:0.000, Pi-value:0.000  
Er-value:0.000, Pr-value:0.010  
No matches to TargetScan

-12675--(19)--12695-

AGAGATA

AGAGATA  
Depth:4 (DOG)  
Ei-value:0.000, Pi-value:0.000  
Er-value:0.000, Pr-value:0.000  
No matches to TargetScan

-12701--(27)--12729-

CAGAGATCT

CAGAGATCT  
Depth:4 (DOG)  
Ei-value:0.000, Pi-value:0.000  
Er-value:0.000, Pr-value:0.000  
No matches to TargetScan

-12737--(40)--12778-

TTGGCT

TTGGCT  
Depth:4 (DOG)  
Ei-value:0.000, Pi-value:0.000  
Er-value:0.000, Pr-value:0.000  
No matches to TargetScan

-12783--(17)--12801-

TTACTTTCT

TTACTTTCT  
Depth:4 (DOG)  
Ei-value:0.000, Pi-value:0.000  
Er-value:0.000, Pr-value:0.010  
No matches to TargetScan

-12809--(240)--13050-

GAGTAAAAA

GAGTAAAAA  
Depth:4 (DOG)  
Ei-value:0.000, Pi-value:0.000  
Er-value:0.000, Pr-value:0.000  
No matches to TargetScan

-13058--(28)--13087-

ATTTGAT

ATTTGAT  
Depth:4 (DOG)  
Ei-value:0.000, Pi-value:0.010  
Er-value:0.000, Pr-value:0.000  
No matches to TargetScan

-13093--(12)--13106-

TTTTATGT

TTTTATGT  
Depth:4 (DOG)  
Ei-value:0.000, Pi-value:0.000  
Er-value:0.000, Pr-value:0.000  
No matches to TargetScan

-13113--(304)--13418-

AAGCCAG

AAGCCAG  
Depth:4 (DOG)  
Ei-value:0.000, Pi-value:0.000  
Er-value:0.000, Pr-value:0.000  
MATCHES To TargetScan▶ miR-149-5p:CUGGCUC▶ miR-3064-5p:CUGGCUG

-13424--(7)--13432-

ATAAAAG

ATAAAAG  
Depth:4 (DOG)  
Ei-value:0.000, Pi-value:0.000  
Er-value:0.000, Pr-value:0.000  
No matches to TargetScan

-13438--(44)--13483-

TTTTATTA

TTTTATTA  
Depth:4 (DOG)  
Ei-value:0.000, Pi-value:0.010  
Er-value:0.000, Pr-value:0.000  
No matches to TargetScan

-13490--(524)--14015-

TTAAATGG

TTAAATGG  
Depth:4 (DOG)  
Ei-value:0.000, Pi-value:0.000  
Er-value:0.000, Pr-value:0.000  
No matches to TargetScan

-14022--(35)--14058-

ACAGAAAACAAAA

ACAGAAAACAAAA  
Depth:4 (DOG)  
Ei-value:0.000, Pi-value:0.000  
Er-value:0.000, Pr-value:0.000  
No matches to TargetScan

-14070--(59)--14130-

AGGTTA

AGGTTA  
Depth:4 (DOG)  
Ei-value:0.000, Pi-value:0.000  
Er-value:0.000, Pr-value:0.000  
No matches to TargetScan

-14135--(19)--14155-

TTCATTCT

TTCATTCT  
Depth:4 (DOG)  
Ei-value:0.000, Pi-value:0.000  
Er-value:0.000, Pr-value:0.000  
No matches to TargetScan

-14162--(56)--14219-

AGAGACA

AGAGACA  
Depth:4 (DOG)  
Ei-value:0.000, Pi-value:0.000  
Er-value:0.000, Pr-value:0.000  
No matches to TargetScan

-14225--(40)--14266-

CCTTTTGG

CCTTTTGG  
Depth:4 (DOG)  
Ei-value:0.000, Pi-value:0.000  
Er-value:0.000, Pr-value:0.000  
No matches to TargetScan

-14273--(57)--14331-

TTAATTC

TTAATTC  
Depth:4 (DOG)  
Ei-value:0.000, Pi-value:0.000  
Er-value:0.000, Pr-value:0.000  
No matches to TargetScan

-14337--(21)--14359-

ACTCTGGCCACTAC

ACTCTGGCCACTAC  
Depth:4 (DOG)  
Ei-value:0.000, Pi-value:0.000  
Er-value:0.000, Pr-value:0.000  
MATCHES To TargetScan▶ miR-142-3p.1:GUAGUGU

-14372--(1)--14374-

ATAAGC

ATAAGC  
Depth:5 (RABBIT)  
Ei-value:0.000, Pi-value:0.010  
Er-value:0.000, Pr-value:0.000  
No matches to TargetScan


AGG

ATAAGCAGG  
Depth:4 (DOG)  
Ei-value:0.000, Pi-value:0.000  
Er-value:0.000, Pr-value:0.000  
No matches to TargetScan

-14382--(80)--14463-

GAAAAATG

GAAAAATG  
Depth:4 (DOG)  
Ei-value:0.000, Pi-value:0.000  
Er-value:0.000, Pr-value:0.000  
No matches to TargetScan

-14470--(103)--14574-

AGTCTCA

AGTCTCA  
Depth:4 (DOG)  
Ei-value:0.000, Pi-value:0.000  
Er-value:0.000, Pr-value:0.000  
No matches to TargetScan

-14580--(42)--14623-

TGGTTTTGAA

TGGTTTTGAA  
Depth:4 (DOG)  
Ei-value:0.000, Pi-value:0.000  
Er-value:0.000, Pr-value:0.000  
No matches to TargetScan

-14632--(160)--14793-

TTAGAAAT

TTAGAAAT  
Depth:4 (DOG)  
Ei-value:0.000, Pi-value:0.000  
Er-value:0.000, Pr-value:0.000  
No matches to TargetScan

-14800--(5)--14806-

CATCAAA

CATCAAA  
Depth:4 (DOG)  
Ei-value:0.000, Pi-value:0.000  
Er-value:0.000, Pr-value:0.000  
No matches to TargetScan

-14812--(176)--14989-

TTTTTAAATCACTCA

TTTTTAAATCACTCA  
Depth:4 (DOG)  
Ei-value:0.000, Pi-value:0.000  
Er-value:0.000, Pr-value:0.000  
No matches to TargetScan

-15003--(1)--15005-

AGAGGGTGGGA

AGAGGGTGGGA  
Depth:4 (DOG)  
Ei-value:0.000, Pi-value:0.000  
Er-value:0.000, Pr-value:0.000  
No matches to TargetScan

-15015--(2)--15018-

AGGAGGAAGAGTGAA

AGGAGGAAGAGTGAA  
Depth:4 (DOG)  
Ei-value:0.000, Pi-value:0.000  
Er-value:0.000, Pr-value:0.000  
MATCHES To TargetScan▶ miR-670-3p:UUCCUCA

-15032--(1)--15034-

G

GAAAAGGTCA  
Depth:4 (DOG)  
Ei-value:0.000, Pi-value:0.000  
Er-value:0.000, Pr-value:0.000  
MATCHES To TargetScan▶ miR-192-5p/215-5p:UGACCUA


AAAAGGT

AAAAGGT  
Depth:6 (MOUSE)  
Ei-value:0.000, Pi-value:0.000  
Er-value:0.000, Pr-value:0.000  
No matches to TargetScan


CA

GAAAAGGTCA  
Depth:4 (DOG)  
Ei-value:0.000, Pi-value:0.000  
Er-value:0.000, Pr-value:0.000  
MATCHES To TargetScan▶ miR-192-5p/215-5p:UGACCUA

-15043--(209)--15253-

TAATGTTT

TAATGTTT  
Depth:4 (DOG)  
Ei-value:0.000, Pi-value:0.000  
Er-value:0.000, Pr-value:0.000  
MATCHES To TargetScan▶ miR-323-3p:ACAUUAC▶ miR-543:AACAUUC

-15260--(20)--15281-

AGCTGGA

AGCTGGA  
Depth:4 (DOG)  
Ei-value:0.000, Pi-value:0.000  
Er-value:0.000, Pr-value:0.000  
No matches to TargetScan

-15287--(41)--15329-

ATTATTGGAAA

ATTATTGGAAA  
Depth:4 (DOG)  
Ei-value:0.000, Pi-value:0.000  
Er-value:0.000, Pr-value:0.000  
No matches to TargetScan

-15339--(9)--15349-

AGAAAGTAAC

AGAAAGTAAC  
Depth:4 (DOG)  
Ei-value:0.000, Pi-value:0.000  
Er-value:0.000, Pr-value:0.000  
No matches to TargetScan

-15358--(12)--15371-

TTTCACAGTTTCTGGCATC

TTTCACAGTTTCTGGCATC  
Depth:4 (DOG)  
Ei-value:0.000, Pi-value:0.000  
Er-value:0.000, Pr-value:0.000  
No matches to TargetScan

-15389--(12)--15402-

CTACTGAT

CTACTGAT  
Depth:4 (DOG)  
Ei-value:0.000, Pi-value:0.000  
Er-value:0.000, Pr-value:0.000  
MATCHES To TargetScan▶ miR-199-3p:CAGUAGU

-15409--(7)--15417-

AGAACAT

AGAACAT  
Depth:4 (DOG)  
Ei-value:0.000, Pi-value:0.000  
Er-value:0.000, Pr-value:0.000  
No matches to TargetScan

-15423--(4)--15428-

TCATCTG

TCATCTG  
Depth:4 (DOG)  
Ei-value:0.000, Pi-value:0.010  
Er-value:0.000, Pr-value:0.000  
No matches to TargetScan

-15434--(10)--15445-

CATAAATGAA

CATAAATGAA  
Depth:4 (DOG)  
Ei-value:0.000, Pi-value:0.000  
Er-value:0.000, Pr-value:0.000  
No matches to TargetScan

-15454--(121)--15576-

TGAACTGATGTGAAA

TGAACTGATGTGAAA  
Depth:4 (DOG)  
Ei-value:0.000, Pi-value:0.000  
Er-value:0.000, Pr-value:0.000  
MATCHES To TargetScan▶ miR-23-3p:UCACAUU

-15590--(130)--15721-

AAATAAAA

AAATAAAA  
Depth:4 (DOG)  
Ei-value:0.000, Pi-value:0.000  
Er-value:0.000, Pr-value:0.000  
No matches to TargetScan

-15728
```

---

# Modules conserved to COW (Depth: 3)

## Modules in Main Graph (All sequences considered):

```
>HUMAN  
       995-

AGGCAAGA

AGGCAAGA  
Depth:3 (COW)  
Ei-value:0.000, Pi-value:0.000  
Er-value:0.000, Pr-value:0.000  
eCLIP MATCHES▶AARS (bg=2.18%)▶EXOSC5 (bg=5.38%)▶HNRNPA1 (bg=2.57%)▶NIPBL (bg=5.39%)▶RBM15 (bg=7.27%)▶RBM22 (bg=4.62%)▶SDAD1 (bg=2.97%)▶uchl5 (bg=11.16%)No matches to TargetScan

-1002--(400)--1403-

AAACATG

AAACATG  
Depth:4 (DOG)  
Ei-value:0.000, Pi-value:0.000  
Er-value:0.000, Pr-value:0.000  
eCLIP MATCHES▶HNRNPM (bg=4.29%)No matches to TargetScan

-1409--(775)--2185-

CAACAG

CAACAG  
Depth:3 (COW)  
Ei-value:0.000, Pi-value:0.000  
Er-value:0.000, Pr-value:0.000  
eCLIP MATCHES▶CSTF2T (bg=0.82%)▶GRWD1 (bg=5.13%)▶HNRNPM (bg=4.29%)▶MTPAP (bg=2.21%)▶NCBP2 (bg=1.49%)▶NIPBL (bg=5.39%)▶PUM1 (bg=1.56%)▶RBM15 (bg=7.27%)▶SRSF1 (bg=8.47%)▶TRA2A (bg=4.8%)▶uchl5 (bg=11.16%)▶XRCC6 (bg=2.91%)▶ZNF622 (bg=6.58%)▶ZNF800 (bg=1.92%)No matches to TargetScan

-2190--(470)--2661-

TTCCCATC

TTCCCATC  
Depth:4 (DOG)  
Ei-value:0.000, Pi-value:0.000  
Er-value:0.000, Pr-value:0.000  
eCLIP MATCHES▶ILF3 (bg=3.0%)No matches to TargetScan

-2668--(587)--3256-

CTCTGT

CTCTGT  
Depth:3 (COW)  
Ei-value:0.000, Pi-value:0.000  
Er-value:0.000, Pr-value:0.000  
eCLIP MATCHES▶EIF3G (bg=0.32%)▶hnrnpk (bg=12.88%)No matches to TargetScan

-3261--(1226)--4488-

C

CTGTTAGTCT  
Depth:4 (DOG)  
Ei-value:0.000, Pi-value:0.000  
Er-value:0.000, Pr-value:0.000  
eCLIP MATCHES▶AKAP8L (bg=2.19%)No matches to TargetScan


TGTTAGTC

TGTTAGTC  
Depth:5 (RABBIT)  
Ei-value:0.000, Pi-value:0.000  
Er-value:0.000, Pr-value:0.000  
eCLIP MATCHES▶AKAP8L (bg=2.19%)No matches to TargetScan


T

CTGTTAGTCT  
Depth:4 (DOG)  
Ei-value:0.000, Pi-value:0.000  
Er-value:0.000, Pr-value:0.000  
eCLIP MATCHES▶AKAP8L (bg=2.19%)No matches to TargetScan

-4497--(6)--4504-

TCATCC

TCATCC  
Depth:4 (DOG)  
Ei-value:0.000, Pi-value:0.020  
Er-value:0.000, Pr-value:0.000  
eCLIP MATCHES▶AKAP8L (bg=2.19%)No matches to TargetScan

-4509--(28)--4538-

GGG

GGGTACTTGGGACTGTTAAT  
Depth:3 (COW)  
Ei-value:0.000, Pi-value:0.000  
Er-value:0.000, Pr-value:0.000  
eCLIP MATCHES▶AKAP8L (bg=2.19%)MATCHES To TargetScan▶ miR-132-3p/212-3p:AACAGUC▶ miR-455-3p.1:CAGUCCA


TACTTGGGACTGTTAAT

TACTTGGGACTGTTAAT  
Depth:4 (DOG)  
Ei-value:0.000, Pi-value:0.000  
Er-value:0.000, Pr-value:0.000  
eCLIP MATCHES▶AKAP8L (bg=2.19%)MATCHES To TargetScan▶ miR-132-3p/212-3p:AACAGUC▶ miR-455-3p.1:CAGUCCA

-4557--(106)--4664-

ACTG

ACTGTTAATGTGCT  
Depth:4 (DOG)  
Ei-value:0.000, Pi-value:0.000  
Er-value:0.000, Pr-value:0.000  
No matches to eCLIP DataMATCHES To TargetScan▶ miR-132-3p/212-3p:AACAGUC▶ miR-323-3p:ACAUUAC


TTAATGTGCT

TTAATGTGCT  
Depth:5 (RABBIT)  
Ei-value:0.000, Pi-value:0.000  
Er-value:0.000, Pr-value:0.000  
No matches to eCLIP DataMATCHES To TargetScan▶ miR-323-3p:ACAUUAC

-4677--(314)--4992-

CTTGGGACTC

CTTGGGACTC  
Depth:3 (COW)  
Ei-value:0.000, Pi-value:0.000  
Er-value:0.000, Pr-value:0.000  
No matches to eCLIP DataNo matches to TargetScan

-5001--(32)--5034-

AATGTGCAT

AATGTGCAT  
Depth:6 (MOUSE)  
Ei-value:0.000, Pi-value:0.000  
Er-value:0.000, Pr-value:0.000  
No matches to eCLIP DataMATCHES To TargetScan▶ miR-501-3p/502-3p:AUGCACC

-5042--(81)--5124-

CTAATA

CTAATA  
Depth:3 (COW)  
Ei-value:0.000, Pi-value:0.000  
Er-value:0.000, Pr-value:0.000  
No matches to eCLIP DataNo matches to TargetScan

-5129--(18)--5148-

CTAATA

CTAATA  
Depth:3 (COW)  
Ei-value:0.000, Pi-value:0.000  
Er-value:0.000, Pr-value:0.000  
No matches to eCLIP DataNo matches to TargetScan

-5153--(269)--5423-

TGCTTCT

TGCTTCT  
Depth:3 (COW)  
Ei-value:0.000, Pi-value:0.000  
Er-value:0.000, Pr-value:0.010  
No matches to eCLIP DataNo matches to TargetScan

-5429--(49)--5479-

TATGTTAGA

TATGTTAGA  
Depth:4 (DOG)  
Ei-value:0.000, Pi-value:0.000  
Er-value:0.000, Pr-value:0.000  
eCLIP MATCHES▶HNRNPU (bg=5.92%)No matches to TargetScan

-5487--(66)--5554-

TCTTGG

TCTTGGACTGTTAATGT  
Depth:3 (COW)  
Ei-value:0.000, Pi-value:0.000  
Er-value:0.000, Pr-value:0.000  
No matches to eCLIP DataMATCHES To TargetScan▶ miR-132-3p/212-3p:AACAGUC▶ miR-323-3p:ACAUUAC▶ miR-455-3p.1:CAGUCCA


ACTGTTAATGT

ACTGTTAATGT  
Depth:4 (DOG)  
Ei-value:0.000, Pi-value:0.000  
Er-value:0.000, Pr-value:0.000  
No matches to eCLIP DataMATCHES To TargetScan▶ miR-132-3p/212-3p:AACAGUC▶ miR-323-3p:ACAUUAC

-5570--(10)--5581-

ATTTGCT

ATTTGCT  
Depth:4 (DOG)  
Ei-value:0.000, Pi-value:0.000  
Er-value:0.000, Pr-value:0.000  
No matches to eCLIP DataNo matches to TargetScan

-5587--(18)--5606-

GTAAGGA

GTAAGGA  
Depth:5 (RABBIT)  
Ei-value:0.000, Pi-value:0.000  
Er-value:0.000, Pr-value:0.000  
No matches to eCLIP DataNo matches to TargetScan


CCC

GTAAGGACCC  
Depth:3 (COW)  
Ei-value:0.000, Pi-value:0.000  
Er-value:0.000, Pr-value:0.000  
No matches to eCLIP DataNo matches to TargetScan

-5615--(75)--5691-

ATCTTAG

ATCTTAG  
Depth:3 (COW)  
Ei-value:0.000, Pi-value:0.000  
Er-value:0.000, Pr-value:0.000  
eCLIP MATCHES▶HNRNPU (bg=5.92%)No matches to TargetScan

-5697--(8)--5706-

TACACATT

TACACATT  
Depth:3 (COW)  
Ei-value:0.000, Pi-value:0.000  
Er-value:0.000, Pr-value:0.000  
eCLIP MATCHES▶HNRNPU (bg=5.92%)No matches to TargetScan

-5713--(40)--5754-

ACTTAT

ACTTAT  
Depth:5 (RABBIT)  
Ei-value:0.000, Pi-value:0.000  
Er-value:0.000, Pr-value:0.000  
eCLIP MATCHES▶HNRNPU (bg=5.92%)No matches to TargetScan

-5759--(55)--5815-

TGTAATT

TGTAATT  
Depth:3 (COW)  
Ei-value:0.000, Pi-value:0.000  
Er-value:0.000, Pr-value:0.000  
No matches to eCLIP DataNo matches to TargetScan

-5821--(10)--5832-

ATGGTC

ATGGTC  
Depth:3 (COW)  
Ei-value:0.000, Pi-value:0.020  
Er-value:0.000, Pr-value:0.000  
No matches to eCLIP DataNo matches to TargetScan

-5837--(51)--5889-

ATGGGGTACT

ATGGGGTACT  
Depth:3 (COW)  
Ei-value:0.000, Pi-value:0.000  
Er-value:0.000, Pr-value:0.000  
eCLIP MATCHES▶HNRNPL (bg=0.64%)No matches to TargetScan

-5898--(3)--5902-

CAC

CACTTAAGGCCCCTTTCTCAA  
Depth:3 (COW)  
Ei-value:0.000, Pi-value:0.000  
Er-value:0.000, Pr-value:0.000  
eCLIP MATCHES▶HNRNPL (bg=0.64%)No matches to TargetScan


TTAAGGCC

TTAAGGCC  
Depth:6 (MOUSE)  
Ei-value:0.000, Pi-value:0.000  
Er-value:0.000, Pr-value:0.000  
eCLIP MATCHES▶HNRNPL (bg=0.64%)No matches to TargetScan


CCTTT

TTAAGGCCCCTTT  
Depth:5 (RABBIT)  
Ei-value:0.000, Pi-value:0.000  
Er-value:0.000, Pr-value:0.000  
eCLIP MATCHES▶HNRNPL (bg=0.64%)No matches to TargetScan


CTCAA

TTAAGGCCCCTTTCTCAA  
Depth:4 (DOG)  
Ei-value:0.000, Pi-value:0.000  
Er-value:0.000, Pr-value:0.000  
eCLIP MATCHES▶HNRNPL (bg=0.64%)No matches to TargetScan

-5922--(14)--5937-

TAATGACAATTACAT

TAATGACAATTACAT  
Depth:3 (COW)  
Ei-value:0.000, Pi-value:0.000  
Er-value:0.000, Pr-value:0.000  
eCLIP MATCHES▶HNRNPL (bg=0.64%)MATCHES To TargetScan▶ miR-411-3p:AUGUAAC

-5951--(2469)--8421-

CTTATATTT

CTTATATTT  
Depth:3 (COW)  
Ei-value:0.000, Pi-value:0.000  
Er-value:0.000, Pr-value:0.000  
eCLIP MATCHES▶DDX21 (bg=0.25%)MATCHES To TargetScan▶ miR-410-3p:AUAUAAC

-8429--(15)--8445-

TTTTAATTGACCA

TTTTAATTGACCA  
Depth:3 (COW)  
Ei-value:0.000, Pi-value:0.000  
Er-value:0.000, Pr-value:0.000  
No matches to eCLIP DataNo matches to TargetScan

-8457--(16)--8474-

ACATTAAT

ACATTAAT  
Depth:3 (COW)  
Ei-value:0.000, Pi-value:0.000  
Er-value:0.000, Pr-value:0.000  
No matches to eCLIP DataNo matches to TargetScan

-8481--(118)--8600-

CATAATTGCA

CATAATTGCA  
Depth:3 (COW)  
Ei-value:0.000, Pi-value:0.000  
Er-value:0.000, Pr-value:0.000  
eCLIP MATCHES▶hnrnpk (bg=12.88%)▶TIA1 (bg=4.07%)No matches to TargetScan

-8609--(20)--8630-

CTAGACAAGGA

CTAGACAAGGA  
Depth:3 (COW)  
Ei-value:0.000, Pi-value:0.000  
Er-value:0.000, Pr-value:0.000  
eCLIP MATCHES▶UTP3 (bg=3.66%)No matches to TargetScan

-8640--(54)--8695-

ACAGTTAATGTG

ACAGTTAATGTG  
Depth:4 (DOG)  
Ei-value:0.000, Pi-value:0.000  
Er-value:0.000, Pr-value:0.000  
eCLIP MATCHES▶HNRNPU (bg=5.92%)MATCHES To TargetScan▶ miR-323-3p:ACAUUAC

-8706--(69)--8776-

ATACTGTTT

ATACTGTTT  
Depth:3 (COW)  
Ei-value:0.000, Pi-value:0.000  
Er-value:0.000, Pr-value:0.000  
No matches to eCLIP DataMATCHES To TargetScan▶ miR-101-3p.1:ACAGUAC▶ miR-132-3p/212-3p:AACAGUC▶ miR-144-3p:ACAGUAU

-8784--(51)--8836-

TTGTCTT

TTGTCTT  
Depth:3 (COW)  
Ei-value:0.000, Pi-value:0.000  
Er-value:0.000, Pr-value:0.010  
No matches to eCLIP DataNo matches to TargetScan

-8842--(515)--9358-

CTCAGCTCTTGG

CTCAGCTCTTGG  
Depth:5 (RABBIT)  
Ei-value:0.000, Pi-value:0.000  
Er-value:0.000, Pr-value:0.000  
No matches to eCLIP DataMATCHES To TargetScan▶ miR-335-5p:CAAGAGC


ACA

CTCAGCTCTTGGACA  
Depth:4 (DOG)  
Ei-value:0.000, Pi-value:0.000  
Er-value:0.000, Pr-value:0.000  
No matches to eCLIP DataMATCHES To TargetScan▶ miR-335-5p:CAAGAGC


ATTAATA

CTCAGCTCTTGGACAATTAATA  
Depth:3 (COW)  
Ei-value:0.000, Pi-value:0.000  
Er-value:0.000, Pr-value:0.000  
No matches to eCLIP DataMATCHES To TargetScan▶ miR-335-5p:CAAGAGC

-9379--(27)--9407-

GATCAT

GATCAT  
Depth:3 (COW)  
Ei-value:0.000, Pi-value:0.000  
Er-value:0.000, Pr-value:0.000  
eCLIP MATCHES▶HNRNPU (bg=5.92%)No matches to TargetScan

-9412--(50)--9463-

TAAGGC

TAAGGC  
Depth:3 (COW)  
Ei-value:0.000, Pi-value:0.000  
Er-value:0.000, Pr-value:0.000  
No matches to eCLIP DataNo matches to TargetScan

-9468--(18)--9487-

GAATATTTGCA

GAATATTTGCA  
Depth:3 (COW)  
Ei-value:0.000, Pi-value:0.000  
Er-value:0.000, Pr-value:0.000  
No matches to eCLIP DataNo matches to TargetScan

-9497--(56)--9554-

ATTACTG

ATTACTG  
Depth:3 (COW)  
Ei-value:0.000, Pi-value:0.010  
Er-value:0.000, Pr-value:0.020  
No matches to eCLIP DataMATCHES To TargetScan▶ miR-802:CAGUAAC

-9560--(3)--9564-

GGGCTGCTGA

GGGCTGCTGA  
Depth:3 (COW)  
Ei-value:0.000, Pi-value:0.000  
Er-value:0.000, Pr-value:0.000  
No matches to eCLIP DataMATCHES To TargetScan▶ miR-15-5p/16-5p/195-5p/424-5p/497-5p:AGCAGCA▶ miR-503-5p:AGCAGCG

-9573--(5)--9579-

CAAAACTT

CAAAACTT  
Depth:4 (DOG)  
Ei-value:0.000, Pi-value:0.000  
Er-value:0.000, Pr-value:0.000  
eCLIP MATCHES▶SF3B1 (bg=2.48%)No matches to TargetScan

-9586--(3)--9590-

CTGGGACTG

CTGGGACTG  
Depth:3 (COW)  
Ei-value:0.000, Pi-value:0.000  
Er-value:0.000, Pr-value:0.000  
eCLIP MATCHES▶SF3B1 (bg=2.48%)MATCHES To TargetScan▶ miR-455-3p.1:CAGUCCA

-9598--(7)--9606-

GCACAATG

GCACAATG  
Depth:6 (MOUSE)  
Ei-value:0.000, Pi-value:0.000  
Er-value:0.000, Pr-value:0.000  
No matches to eCLIP DataNo matches to TargetScan

-9613--(22)--9636-

CTCCCTG

CTCCCTG  
Depth:3 (COW)  
Ei-value:0.000, Pi-value:0.000  
Er-value:0.000, Pr-value:0.000  
eCLIP MATCHES▶DDX42 (bg=0.58%)No matches to TargetScan

-9642--(10)--9653-

GCAAGC

GCAAGC  
Depth:3 (COW)  
Ei-value:0.000, Pi-value:0.000  
Er-value:0.000, Pr-value:0.000  
eCLIP MATCHES▶DDX42 (bg=0.58%)▶hnrnpk (bg=12.88%)No matches to TargetScan

-9658--(87)--9746-

A

ACTCCCA  
Depth:4 (DOG)  
Ei-value:0.000, Pi-value:0.000  
Er-value:0.000, Pr-value:0.000  
eCLIP MATCHES▶hnrnpk (bg=12.88%)No matches to TargetScan


CTCCCA

CTCCCA  
Depth:6 (MOUSE)  
Ei-value:0.000, Pi-value:0.000  
Er-value:0.000, Pr-value:0.000  
eCLIP MATCHES▶hnrnpk (bg=12.88%)No matches to TargetScan

-9752--(1)--9754-

CCCTTTTGCATT

CCCTTTTGCATT  
Depth:4 (DOG)  
Ei-value:0.000, Pi-value:0.000  
Er-value:0.000, Pr-value:0.000  
eCLIP MATCHES▶hnrnpk (bg=12.88%)No matches to TargetScan


G

CCCTTTTGCATTG  
Depth:3 (COW)  
Ei-value:0.000, Pi-value:0.000  
Er-value:0.000, Pr-value:0.000  
eCLIP MATCHES▶hnrnpk (bg=12.88%)No matches to TargetScan

-9766--(295)--10062-

ACTTCCTT

ACTTCCTT  
Depth:3 (COW)  
Ei-value:0.000, Pi-value:0.000  
Er-value:0.000, Pr-value:0.000  
eCLIP MATCHES▶hnrnpk (bg=12.88%)No matches to TargetScan

-10069--(32)--10102-

AGCCCCTTCT

AGCCCCTTCT  
Depth:3 (COW)  
Ei-value:0.000, Pi-value:0.000  
Er-value:0.000, Pr-value:0.000  
eCLIP MATCHES▶hnrnpk (bg=12.88%)No matches to TargetScan

-10111--(14)--10126-

CACAGTA

CACAGTA  
Depth:3 (COW)  
Ei-value:0.000, Pi-value:0.000  
Er-value:0.000, Pr-value:0.000  
eCLIP MATCHES▶hnrnpk (bg=12.88%)No matches to TargetScan

-10132--(1)--10134-

TGATTGTC

TGATTGTCCCATTTTT  
Depth:3 (COW)  
Ei-value:0.000, Pi-value:0.000  
Er-value:0.000, Pr-value:0.000  
eCLIP MATCHES▶hnrnpk (bg=12.88%)No matches to TargetScan


CCATTTTT

CCATTTTT  
Depth:4 (DOG)  
Ei-value:0.000, Pi-value:0.000  
Er-value:0.000, Pr-value:0.000  
eCLIP MATCHES▶hnrnpk (bg=12.88%)No matches to TargetScan

-10149--(0)--10150-

CAGCCCA

CAGCCCA  
Depth:4 (DOG)  
Ei-value:0.000, Pi-value:0.000  
Er-value:0.000, Pr-value:0.000  
eCLIP MATCHES▶hnrnpk (bg=12.88%)No matches to TargetScan

-10156--(11)--10168-

TCTC

TCTCCCTACCA  
Depth:3 (COW)  
Ei-value:0.000, Pi-value:0.000  
Er-value:0.000, Pr-value:0.000  
eCLIP MATCHES▶hnrnpk (bg=12.88%)No matches to TargetScan


CCTACCA

CCTACCA  
Depth:4 (DOG)  
Ei-value:0.000, Pi-value:0.000  
Er-value:0.000, Pr-value:0.000  
eCLIP MATCHES▶hnrnpk (bg=12.88%)No matches to TargetScan

-10178--(13)--10192-

GTGCAGT

GTGCAGT  
Depth:3 (COW)  
Ei-value:0.000, Pi-value:0.000  
Er-value:0.000, Pr-value:0.000  
eCLIP MATCHES▶hnrnpk (bg=12.88%)MATCHES To TargetScan▶ miR-217:ACUGCAU

-10198--(10)--10209-

AAAAGCAG

AAAAGCAG  
Depth:6 (MOUSE)  
Ei-value:0.000, Pi-value:0.000  
Er-value:0.000, Pr-value:0.000  
No matches to eCLIP DataNo matches to TargetScan

-10216--(4)--10221-

GAACTA

GAACTA  
Depth:3 (COW)  
Ei-value:0.000, Pi-value:0.000  
Er-value:0.000, Pr-value:0.000  
No matches to eCLIP DataNo matches to TargetScan

-10226--(34)--10261-

TTAATGATCC

TTAATGATCC  
Depth:4 (DOG)  
Ei-value:0.000, Pi-value:0.000  
Er-value:0.000, Pr-value:0.000  
No matches to eCLIP DataMATCHES To TargetScan▶ miR-382-3p:AUCAUUC

-10270--(8)--10279-

ATTATTGT

ATTATTGT  
Depth:3 (COW)  
Ei-value:0.000, Pi-value:0.000  
Er-value:0.000, Pr-value:0.000  
No matches to eCLIP DataNo matches to TargetScan

-10286--(3)--10290-

ATTCTGGG

ATTCTGGG  
Depth:4 (DOG)  
Ei-value:0.000, Pi-value:0.000  
Er-value:0.000, Pr-value:0.000  
No matches to eCLIP DataNo matches to TargetScan

-10297--(31)--10329-

TG

TGCTTTACT  
Depth:3 (COW)  
Ei-value:0.000, Pi-value:0.000  
Er-value:0.000, Pr-value:0.000  
No matches to eCLIP DataMATCHES To TargetScan▶ miR-330-3p.2:AAAGCAC


CTTTACT

CTTTACT  
Depth:4 (DOG)  
Ei-value:0.000, Pi-value:0.000  
Er-value:0.000, Pr-value:0.000  
No matches to eCLIP DataNo matches to TargetScan

-10337--(2)--10340-

GCAAAAT

GCAAAAT  
Depth:6 (MOUSE)  
Ei-value:0.000, Pi-value:0.000  
Er-value:0.000, Pr-value:0.000  
No matches to eCLIP DataNo matches to TargetScan

-10346--(4)--10351-

AAGGCAA

AAGGCAA  
Depth:4 (DOG)  
Ei-value:0.000, Pi-value:0.000  
Er-value:0.000, Pr-value:0.000  
No matches to eCLIP DataNo matches to TargetScan


GTCAGACCCA

AAGGCAAGTCAGACCCA  
Depth:3 (COW)  
Ei-value:0.000, Pi-value:0.000  
Er-value:0.000, Pr-value:0.000  
No matches to eCLIP DataMATCHES To TargetScan▶ miR-193a-5p:GGGUCUU

-10367--(6)--10374-

TGGATTGC

TGGATTGC  
Depth:4 (DOG)  
Ei-value:0.000, Pi-value:0.000  
Er-value:0.000, Pr-value:0.000  
No matches to eCLIP DataNo matches to TargetScan

-10381--(49)--10431-

GAAGGAAG

GAAGGAAG  
Depth:3 (COW)  
Ei-value:0.000, Pi-value:0.000  
Er-value:0.000, Pr-value:0.000  
eCLIP MATCHES▶SF3B1 (bg=2.48%)No matches to TargetScan

-10438--(14)--10453-

TGCATTCTTC

TGCATTCTTC  
Depth:5 (RABBIT)  
Ei-value:0.000, Pi-value:0.000  
Er-value:0.000, Pr-value:0.000  
eCLIP MATCHES▶SF3B1 (bg=2.48%)No matches to TargetScan

-10462--(7)--10470-

AGC

AGCAGATTGCCTGG  
Depth:4 (DOG)  
Ei-value:0.000, Pi-value:0.000  
Er-value:0.000, Pr-value:0.000  
eCLIP MATCHES▶SF3B1 (bg=2.48%)No matches to TargetScan


A

AGATTGCCTGG  
Depth:5 (RABBIT)  
Ei-value:0.000, Pi-value:0.000  
Er-value:0.000, Pr-value:0.000  
No matches to eCLIP DataNo matches to TargetScan


GATTGCCTGG

GATTGCCTGG  
Depth:6 (MOUSE)  
Ei-value:0.000, Pi-value:0.000  
Er-value:0.000, Pr-value:0.000  
No matches to eCLIP DataNo matches to TargetScan

-10483--(23)--10507-

TTGTATATT

TTGTATATT  
Depth:4 (DOG)  
Ei-value:0.000, Pi-value:0.000  
Er-value:0.000, Pr-value:0.000  
No matches to eCLIP DataMATCHES To TargetScan▶ miR-381-3p:AUACAAG

-10515--(12)--10528-

TGCCAA

TGCCAA  
Depth:3 (COW)  
Ei-value:0.000, Pi-value:0.000  
Er-value:0.000, Pr-value:0.000  
No matches to eCLIP DataMATCHES To TargetScan▶ miR-182-5p:UUGGCAA▶ miR-96-5p/1271-5p:UUGGCAC

-10533--(1)--10535-

TGCCAGGATACA

TGCCAGGATACA  
Depth:3 (COW)  
Ei-value:0.000, Pi-value:0.000  
Er-value:0.000, Pr-value:0.000  
No matches to eCLIP DataNo matches to TargetScan

-10546--(46)--10593-

ACATCTGG

ACATCTGG  
Depth:3 (COW)  
Ei-value:0.000, Pi-value:0.000  
Er-value:0.000, Pr-value:0.000  
No matches to eCLIP DataNo matches to TargetScan

-10600--(16)--10617-

GAT

GATAACCTGGTCATT  
Depth:3 (COW)  
Ei-value:0.000, Pi-value:0.000  
Er-value:0.000, Pr-value:0.000  
No matches to eCLIP DataMATCHES To TargetScan▶ miR-154-5p:AGGUUAU


AAC

AACCTGGTCATT  
Depth:4 (DOG)  
Ei-value:0.000, Pi-value:0.000  
Er-value:0.000, Pr-value:0.000  
No matches to eCLIP DataNo matches to TargetScan


CTGGTCATT

CTGGTCATT  
Depth:5 (RABBIT)  
Ei-value:0.000, Pi-value:0.000  
Er-value:0.000, Pr-value:0.000  
No matches to eCLIP DataNo matches to TargetScan

-10631--(4)--10636-

TTTTGAA

TTTTGAA  
Depth:3 (COW)  
Ei-value:0.000, Pi-value:0.000  
Er-value:0.000, Pr-value:0.010  
No matches to eCLIP DataNo matches to TargetScan

-10642--(10)--10653-

CCATTTAT

CCATTTAT  
Depth:5 (RABBIT)  
Ei-value:0.000, Pi-value:0.000  
Er-value:0.000, Pr-value:0.000  
No matches to eCLIP DataNo matches to TargetScan

-10660--(13)--10674-

TGAC

TGACCAGTGTCTCTCATTT  
Depth:4 (DOG)  
Ei-value:0.000, Pi-value:0.000  
Er-value:0.000, Pr-value:0.000  
eCLIP MATCHES▶SUPV3L1 (bg=1.57%)No matches to TargetScan


CAGTGTCTCTCATTT

CAGTGTCTCTCATTT  
Depth:5 (RABBIT)  
Ei-value:0.000, Pi-value:0.000  
Er-value:0.000, Pr-value:0.000  
eCLIP MATCHES▶SUPV3L1 (bg=1.57%)No matches to TargetScan

-10692--(6)--10699-

AGG

AGGGTGGTG  
Depth:4 (DOG)  
Ei-value:0.000, Pi-value:0.000  
Er-value:0.000, Pr-value:0.000  
eCLIP MATCHES▶SUPV3L1 (bg=1.57%)No matches to TargetScan


GTGGTG

GTGGTG  
Depth:5 (RABBIT)  
Ei-value:0.000, Pi-value:0.000  
Er-value:0.000, Pr-value:0.000  
eCLIP MATCHES▶SUPV3L1 (bg=1.57%)No matches to TargetScan

-10707--(1)--10709-

GTCTGTGGATA

GTCTGTGGATA  
Depth:5 (RABBIT)  
Ei-value:0.000, Pi-value:0.000  
Er-value:0.000, Pr-value:0.000  
eCLIP MATCHES▶SUPV3L1 (bg=1.57%)MATCHES To TargetScan▶ miR-140-3p.1:CCACAGG


GA

GTCTGTGGATAGA  
Depth:3 (COW)  
Ei-value:0.000, Pi-value:0.000  
Er-value:0.000, Pr-value:0.000  
eCLIP MATCHES▶SUPV3L1 (bg=1.57%)MATCHES To TargetScan▶ miR-140-3p.1:CCACAGG

-10721--(14)--10736-

TATTTTA

TATTTTA  
Depth:3 (COW)  
Ei-value:0.000, Pi-value:0.040  
Er-value:0.000, Pr-value:0.020  
eCLIP MATCHES▶SUPV3L1 (bg=1.57%)No matches to TargetScan

-10742--(12)--10755-

TTCTAGA

TTCTAGA  
Depth:4 (DOG)  
Ei-value:0.000, Pi-value:0.000  
Er-value:0.000, Pr-value:0.000  
No matches to eCLIP DataNo matches to TargetScan

-10761--(17)--10779-

AGTATCTTTG

AGTATCTTTG  
Depth:3 (COW)  
Ei-value:0.000, Pi-value:0.000  
Er-value:0.000, Pr-value:0.000  
No matches to eCLIP DataNo matches to TargetScan

-10788--(16)--10805-

ATTCACTT

ATTCACTT  
Depth:4 (DOG)  
Ei-value:0.000, Pi-value:0.000  
Er-value:0.000, Pr-value:0.000  
No matches to eCLIP DataNo matches to TargetScan

-10812--(3)--10816-

GAAAAAC

GAAAAAC  
Depth:4 (DOG)  
Ei-value:0.000, Pi-value:0.000  
Er-value:0.000, Pr-value:0.000  
No matches to eCLIP DataNo matches to TargetScan

-10822--(22)--10845-

AATTTCTTCATCTGGAGC

AATTTCTTCATCTGGAGC  
Depth:5 (RABBIT)  
Ei-value:0.000, Pi-value:0.000  
Er-value:0.000, Pr-value:0.000  
eCLIP MATCHES▶SUPV3L1 (bg=1.57%)▶U2AF2 (bg=1.76%)No matches to TargetScan

-10862--(11)--10874-

CTTATTT

CTTATTT  
Depth:4 (DOG)  
Ei-value:0.000, Pi-value:0.000  
Er-value:0.000, Pr-value:0.010  
eCLIP MATCHES▶SUPV3L1 (bg=1.57%)▶U2AF2 (bg=1.76%)No matches to TargetScan


CAAGAA

CTTATTTCAAGAA  
Depth:3 (COW)  
Ei-value:0.000, Pi-value:0.000  
Er-value:0.000, Pr-value:0.000  
eCLIP MATCHES▶SUPV3L1 (bg=1.57%)▶U2AF2 (bg=1.76%)MATCHES To TargetScan▶ miR-203a-3p.2:UGAAAUG

-10886--(83)--10970-

ATAAAATG

ATAAAATG  
Depth:4 (DOG)  
Ei-value:0.000, Pi-value:0.000  
Er-value:0.000, Pr-value:0.000  
No matches to eCLIP DataNo matches to TargetScan


A

ATAAAATGA  
Depth:3 (COW)  
Ei-value:0.000, Pi-value:0.000  
Er-value:0.000, Pr-value:0.000  
No matches to eCLIP DataNo matches to TargetScan

-10978--(39)--11018-

ACCACACT

ACCACACT  
Depth:3 (COW)  
Ei-value:0.000, Pi-value:0.000  
Er-value:0.000, Pr-value:0.000  
No matches to eCLIP DataNo matches to TargetScan

-11025--(3)--11029-

GTGAGG

GTGAGG  
Depth:3 (COW)  
Ei-value:0.000, Pi-value:0.000  
Er-value:0.000, Pr-value:0.000  
No matches to eCLIP DataNo matches to TargetScan

-11034--(28)--11063-

TTTTATA

TTTTATA  
Depth:3 (COW)  
Ei-value:0.000, Pi-value:0.000  
Er-value:0.000, Pr-value:0.010  
No matches to eCLIP DataMATCHES To TargetScan▶ miR-340-5p:UAUAAAG

-11069--(2)--11072-

AAAAATAAGCCA

AAAAATAAGCCA  
Depth:5 (RABBIT)  
Ei-value:0.000, Pi-value:0.000  
Er-value:0.000, Pr-value:0.000  
No matches to eCLIP DataNo matches to TargetScan


A

AAAAATAAGCCAA  
Depth:4 (DOG)  
Ei-value:0.000, Pi-value:0.000  
Er-value:0.000, Pr-value:0.000  
No matches to eCLIP DataNo matches to TargetScan

-11084--(10)--11095-

TCTTTTGGATATA

TCTTTTGGATATA  
Depth:3 (COW)  
Ei-value:0.000, Pi-value:0.000  
Er-value:0.000, Pr-value:0.000  
No matches to eCLIP DataNo matches to TargetScan

-11107--(32)--11140-

ATGAATAATA

ATGAATAATA  
Depth:4 (DOG)  
Ei-value:0.000, Pi-value:0.000  
Er-value:0.000, Pr-value:0.000  
No matches to eCLIP DataNo matches to TargetScan

-11149--(11)--11161-

AGTGTACA

AGTGTACA  
Depth:3 (COW)  
Ei-value:0.000, Pi-value:0.000  
Er-value:0.000, Pr-value:0.000  
No matches to eCLIP DataMATCHES To TargetScan▶ miR-493-5p:UGUACAU

-11168--(1)--11170-

GGTGTTT

GGTGTTT  
Depth:3 (COW)  
Ei-value:0.000, Pi-value:0.000  
Er-value:0.000, Pr-value:0.000  
No matches to eCLIP DataNo matches to TargetScan

-11176--(22)--11199-

TGGAACTGCT

TGGAACTGCT  
Depth:4 (DOG)  
Ei-value:0.000, Pi-value:0.000  
Er-value:0.000, Pr-value:0.000  
No matches to eCLIP DataNo matches to TargetScan

-11208--(8)--11217-

TAACTA

TAACTA  
Depth:4 (DOG)  
Ei-value:0.000, Pi-value:0.000  
Er-value:0.000, Pr-value:0.000  
No matches to eCLIP DataNo matches to TargetScan

-11222--(10)--11233-

CAGCAGTTC

CAGCAGTTC  
Depth:5 (RABBIT)  
Ei-value:0.000, Pi-value:0.000  
Er-value:0.000, Pr-value:0.000  
No matches to eCLIP DataNo matches to TargetScan

-11241--(1)--11243-

TTGTAAT

TTGTAAT  
Depth:4 (DOG)  
Ei-value:0.000, Pi-value:0.000  
Er-value:0.000, Pr-value:0.000  
No matches to eCLIP DataNo matches to TargetScan

-11249--(1)--11251-

ACTGAAAA

ACTGAAAA  
Depth:5 (RABBIT)  
Ei-value:0.000, Pi-value:0.000  
Er-value:0.000, Pr-value:0.000  
No matches to eCLIP DataNo matches to TargetScan

-11258--(16)--11275-

GAG

GAGAAGGATGTCAAAAGATCGGC  
Depth:3 (COW)  
Ei-value:0.000, Pi-value:0.000  
Er-value:0.000, Pr-value:0.000  
eCLIP MATCHES▶SRSF1 (bg=8.47%)▶U2AF2 (bg=1.76%)▶uchl5 (bg=11.16%)MATCHES To TargetScan▶ miR-362-5p/500b-5p:AUCCUUG▶ miR-489-3p:UGACAUC


AAGGATG

AAGGATG  
Depth:5 (RABBIT)  
Ei-value:0.000, Pi-value:0.000  
Er-value:0.000, Pr-value:0.000  
eCLIP MATCHES▶SRSF1 (bg=8.47%)▶U2AF2 (bg=1.76%)▶uchl5 (bg=11.16%)MATCHES To TargetScan▶ miR-362-5p/500b-5p:AUCCUUG


TCA

AAGGATGTCAAAAGATC  
Depth:4 (DOG)  
Ei-value:0.000, Pi-value:0.000  
Er-value:0.000, Pr-value:0.000  
eCLIP MATCHES▶SRSF1 (bg=8.47%)▶U2AF2 (bg=1.76%)▶uchl5 (bg=11.16%)MATCHES To TargetScan▶ miR-362-5p/500b-5p:AUCCUUG▶ miR-489-3p:UGACAUC


AAAGATC

AAAGATC  
Depth:6 (MOUSE)  
Ei-value:0.000, Pi-value:0.000  
Er-value:0.000, Pr-value:0.000  
eCLIP MATCHES▶SRSF1 (bg=8.47%)▶U2AF2 (bg=1.76%)▶uchl5 (bg=11.16%)No matches to TargetScan


GGC

GAGAAGGATGTCAAAAGATCGGC  
Depth:3 (COW)  
Ei-value:0.000, Pi-value:0.000  
Er-value:0.000, Pr-value:0.000  
eCLIP MATCHES▶SRSF1 (bg=8.47%)▶U2AF2 (bg=1.76%)▶uchl5 (bg=11.16%)MATCHES To TargetScan▶ miR-362-5p/500b-5p:AUCCUUG▶ miR-489-3p:UGACAUC

-11297--(1)--11299-

CAGCTCAGGG

CAGCTCAGGG  
Depth:4 (DOG)  
Ei-value:0.000, Pi-value:0.000  
Er-value:0.000, Pr-value:0.000  
eCLIP MATCHES▶SRSF1 (bg=8.47%)▶U2AF2 (bg=1.76%)▶uchl5 (bg=11.16%)MATCHES To TargetScan▶ miR-125-5p:CCCUGAG

-11308--(1)--11310-

GCAGTTTGC

GCAGTTTGC  
Depth:3 (COW)  
Ei-value:0.000, Pi-value:0.000  
Er-value:0.000, Pr-value:0.000  
eCLIP MATCHES▶SRSF1 (bg=8.47%)▶U2AF2 (bg=1.76%)▶uchl5 (bg=11.16%)No matches to TargetScan

-11318--(1)--11320-

CTACTAGCTCCT

CTACTAGCTCCT  
Depth:4 (DOG)  
Ei-value:0.000, Pi-value:0.000  
Er-value:0.000, Pr-value:0.000  
eCLIP MATCHES▶SRSF1 (bg=8.47%)▶U2AF2 (bg=1.76%)▶uchl5 (bg=11.16%)MATCHES To TargetScan▶ miR-28-5p/708-5p:AGGAGCU▶ miR-411-5p.2:UAGUAGA

-11331--(1)--11333-

GGACAGCTG

GGACAGCTG  
Depth:5 (RABBIT)  
Ei-value:0.000, Pi-value:0.000  
Er-value:0.000, Pr-value:0.000  
eCLIP MATCHES▶SRSF1 (bg=8.47%)▶SRSF7 (bg=2.32%)▶U2AF2 (bg=1.76%)▶ZNF622 (bg=6.58%)No matches to TargetScan


T

GGACAGCTGT  
Depth:4 (DOG)  
Ei-value:0.000, Pi-value:0.000  
Er-value:0.000, Pr-value:0.000  
eCLIP MATCHES▶SRSF1 (bg=8.47%)▶SRSF7 (bg=2.32%)▶U2AF2 (bg=1.76%)▶ZNF622 (bg=6.58%)No matches to TargetScan

-11342--(1)--11344-

A

AAGAAGAGTCTCTGGCTCTTTAGA  
Depth:3 (COW)  
Ei-value:0.000, Pi-value:0.000  
Er-value:0.000, Pr-value:0.000  
eCLIP MATCHES▶DDX24 (bg=2.97%)▶SRSF1 (bg=8.47%)▶SRSF7 (bg=2.32%)▶U2AF2 (bg=1.76%)▶ZNF622 (bg=6.58%)No matches to TargetScan


AGAAGAGTCTCTGGCTCTTTA

AGAAGAGTCTCTGGCTCTTTA  
Depth:5 (RABBIT)  
Ei-value:0.000, Pi-value:0.000  
Er-value:0.000, Pr-value:0.000  
eCLIP MATCHES▶DDX24 (bg=2.97%)▶SRSF1 (bg=8.47%)▶SRSF7 (bg=2.32%)▶U2AF2 (bg=1.76%)▶ZNF622 (bg=6.58%)No matches to TargetScan


GA

AGAAGAGTCTCTGGCTCTTTAGA  
Depth:4 (DOG)  
Ei-value:0.000, Pi-value:0.000  
Er-value:0.000, Pr-value:0.000  
eCLIP MATCHES▶DDX24 (bg=2.97%)▶SRSF1 (bg=8.47%)▶SRSF7 (bg=2.32%)▶U2AF2 (bg=1.76%)▶ZNF622 (bg=6.58%)No matches to TargetScan

-11367--(72)--11440-

ATTCTGAGC

ATTCTGAGC  
Depth:4 (DOG)  
Ei-value:0.000, Pi-value:0.000  
Er-value:0.000, Pr-value:0.000  
eCLIP MATCHES▶DDX24 (bg=2.97%)▶GRWD1 (bg=5.13%)▶MTPAP (bg=2.21%)▶NOLC1 (bg=9.43%)▶SRSF1 (bg=8.47%)▶ZNF622 (bg=6.58%)No matches to TargetScan

-11448--(14)--11463-

GA

GACTGCAA  
Depth:3 (COW)  
Ei-value:0.000, Pi-value:0.000  
Er-value:0.000, Pr-value:0.000  
eCLIP MATCHES▶DDX24 (bg=2.97%)▶GRWD1 (bg=5.13%)▶MTPAP (bg=2.21%)▶NOLC1 (bg=9.43%)▶SRSF1 (bg=8.47%)▶UTP3 (bg=3.66%)▶ZNF622 (bg=6.58%)MATCHES To TargetScan▶ miR-455-3p.2:UGCAGUC


CTGCAA

CTGCAA  
Depth:5 (RABBIT)  
Ei-value:0.000, Pi-value:0.000  
Er-value:0.000, Pr-value:0.000  
eCLIP MATCHES▶DDX24 (bg=2.97%)▶GRWD1 (bg=5.13%)▶MTPAP (bg=2.21%)▶NOLC1 (bg=9.43%)▶SRSF1 (bg=8.47%)▶UTP3 (bg=3.66%)▶ZNF622 (bg=6.58%)No matches to TargetScan

-11470--(38)--11509-

TTTGAGAATCTGG

TTTGAGAATCTGG  
Depth:3 (COW)  
Ei-value:0.000, Pi-value:0.000  
Er-value:0.000, Pr-value:0.000  
eCLIP MATCHES▶DDX24 (bg=2.97%)▶GRWD1 (bg=5.13%)▶NOLC1 (bg=9.43%)▶SRSF1 (bg=8.47%)▶uchl5 (bg=11.16%)▶ZNF622 (bg=6.58%)MATCHES To TargetScan▶ miR-371-5p:CUCAAAC

-11521--(2)--11524-

AAGCTCCA

AAGCTCCA  
Depth:3 (COW)  
Ei-value:0.000, Pi-value:0.000  
Er-value:0.000, Pr-value:0.000  
eCLIP MATCHES▶DDX24 (bg=2.97%)▶GRWD1 (bg=5.13%)▶NOLC1 (bg=9.43%)▶RBM15 (bg=7.27%)▶SRSF1 (bg=8.47%)▶uchl5 (bg=11.16%)▶ZNF622 (bg=6.58%)No matches to TargetScan

-11531--(12)--11544-

GGATGG

GGATGG  
Depth:3 (COW)  
Ei-value:0.000, Pi-value:0.000  
Er-value:0.000, Pr-value:0.010  
eCLIP MATCHES▶DDX24 (bg=2.97%)▶GRWD1 (bg=5.13%)▶NIPBL (bg=5.39%)▶NOLC1 (bg=9.43%)▶RBM15 (bg=7.27%)▶SRSF1 (bg=8.47%)▶TARDBP (bg=2.79%)▶uchl5 (bg=11.16%)▶ZNF622 (bg=6.58%)No matches to TargetScan

-11549--(12)--11562-

CTGGAGAAAAAGATCT

CTGGAGAAAAAGATCT  
Depth:3 (COW)  
Ei-value:0.000, Pi-value:0.000  
Er-value:0.000, Pr-value:0.000  
eCLIP MATCHES▶DDX24 (bg=2.97%)▶GRWD1 (bg=5.13%)▶NIPBL (bg=5.39%)▶NOLC1 (bg=9.43%)▶SRSF1 (bg=8.47%)▶SRSF7 (bg=2.32%)▶TARDBP (bg=2.79%)▶uchl5 (bg=11.16%)▶ZNF622 (bg=6.58%)No matches to TargetScan

-11577--(7)--11585-

AAGAATAGGC

AAGAATAGGC  
Depth:5 (RABBIT)  
Ei-value:0.000, Pi-value:0.000  
Er-value:0.000, Pr-value:0.000  
eCLIP MATCHES▶NOLC1 (bg=9.43%)▶SRSF7 (bg=2.32%)▶uchl5 (bg=11.16%)No matches to TargetScan

-11594--(8)--11603-

T

TTACAGTGTTAGTGA  
Depth:3 (COW)  
Ei-value:0.000, Pi-value:0.000  
Er-value:0.000, Pr-value:0.000  
eCLIP MATCHES▶ILF3 (bg=3.0%)▶NOLC1 (bg=9.43%)▶RBM15 (bg=7.27%)▶SRSF7 (bg=2.32%)▶ZNF622 (bg=6.58%)MATCHES To TargetScan▶ miR-141-3p/200a-3p:AACACUG


TACAGTGTTAGTGA

TACAGTGTTAGTGA  
Depth:5 (RABBIT)  
Ei-value:0.000, Pi-value:0.000  
Er-value:0.000, Pr-value:0.000  
eCLIP MATCHES▶ILF3 (bg=3.0%)▶NOLC1 (bg=9.43%)▶RBM15 (bg=7.27%)▶SRSF7 (bg=2.32%)▶ZNF622 (bg=6.58%)MATCHES To TargetScan▶ miR-141-3p/200a-3p:AACACUG

-11617--(2)--11620-

CA

CATTCCCTTTGA  
Depth:3 (COW)  
Ei-value:0.000, Pi-value:0.000  
Er-value:0.000, Pr-value:0.000  
eCLIP MATCHES▶ILF3 (bg=3.0%)▶RBM15 (bg=7.27%)▶SRSF7 (bg=2.32%)▶ZNF622 (bg=6.58%)MATCHES To TargetScan▶ miR-1-3p/206:GGAAUGU


TTCCCTTTGA

TTCCCTTTGA  
Depth:6 (MOUSE)  
Ei-value:0.000, Pi-value:0.000  
Er-value:0.000, Pr-value:0.000  
eCLIP MATCHES▶ILF3 (bg=3.0%)▶RBM15 (bg=7.27%)▶SRSF7 (bg=2.32%)▶ZNF622 (bg=6.58%)No matches to TargetScan

-11631--(7)--11639-

TAGGTGGAGATGGGGCATGAGGATCCTCCAGGGGAA

TAGGTGGAGATGGGGCATGAGGATCCTCCAGGGGAA  
Depth:6 (MOUSE)  
Ei-value:0.000, Pi-value:0.000  
Er-value:0.000, Pr-value:0.000  
eCLIP MATCHES▶ILF3 (bg=3.0%)▶NOLC1 (bg=9.43%)▶RBM15 (bg=7.27%)▶SRSF7 (bg=2.32%)▶ZNF622 (bg=6.58%)MATCHES To TargetScan▶ miR-331-3p:CCCCUGG


A

TAGGTGGAGATGGGGCATGAGGATCCTCCAGGGGAAA  
Depth:5 (RABBIT)  
Ei-value:0.000, Pi-value:0.000  
Er-value:0.000, Pr-value:0.000  
eCLIP MATCHES▶ILF3 (bg=3.0%)▶NOLC1 (bg=9.43%)▶RBM15 (bg=7.27%)▶SRSF7 (bg=2.32%)▶ZNF622 (bg=6.58%)MATCHES To TargetScan▶ miR-331-3p:CCCCUGG

-11675--(3)--11679-

TCACTA

TCACTA  
Depth:5 (RABBIT)  
Ei-value:0.000, Pi-value:0.000  
Er-value:0.000, Pr-value:0.000  
eCLIP MATCHES▶ILF3 (bg=3.0%)No matches to TargetScan


CCACT

TCACTACCACT  
Depth:4 (DOG)  
Ei-value:0.000, Pi-value:0.000  
Er-value:0.000, Pr-value:0.000  
eCLIP MATCHES▶ILF3 (bg=3.0%)MATCHES To TargetScan▶ miR-140-5p:AGUGGUU▶ miR-142-3p.1:GUAGUGU


G

TCACTACCACTG  
Depth:3 (COW)  
Ei-value:0.000, Pi-value:0.000  
Er-value:0.000, Pr-value:0.000  
eCLIP MATCHES▶ILF3 (bg=3.0%)MATCHES To TargetScan▶ miR-140-5p:AGUGGUU▶ miR-142-3p.1:GUAGUGU

-11690--(1)--11692-

GCAACA

GCAACA  
Depth:6 (MOUSE)  
Ei-value:0.000, Pi-value:0.000  
Er-value:0.000, Pr-value:0.000  
eCLIP MATCHES▶ILF3 (bg=3.0%)No matches to TargetScan


AC

GCAACAAC  
Depth:5 (RABBIT)  
Ei-value:0.000, Pi-value:0.000  
Er-value:0.000, Pr-value:0.000  
eCLIP MATCHES▶ILF3 (bg=3.0%)No matches to TargetScan

-11699--(27)--11727-

CTTTCCTGG

CTTTCCTGG  
Depth:3 (COW)  
Ei-value:0.000, Pi-value:0.000  
Er-value:0.000, Pr-value:0.000  
eCLIP MATCHES▶ILF3 (bg=3.0%)MATCHES To TargetScan▶ miR-665:CCAGGAG▶ miR-873-5p.1:CAGGAAC

-11735--(28)--11764-

ACAACCACC

ACAACCACC  
Depth:5 (RABBIT)  
Ei-value:0.000, Pi-value:0.000  
Er-value:0.000, Pr-value:0.000  
eCLIP MATCHES▶PRPF8 (bg=0.26%)No matches to TargetScan


ACAC

ACAACCACCACAC  
Depth:4 (DOG)  
Ei-value:0.000, Pi-value:0.000  
Er-value:0.000, Pr-value:0.000  
eCLIP MATCHES▶PRPF8 (bg=0.26%)No matches to TargetScan

-11776--(13)--11790-

TTGTTCC

TTGTTCC  
Depth:4 (DOG)  
Ei-value:0.000, Pi-value:0.000  
Er-value:0.000, Pr-value:0.000  
eCLIP MATCHES▶GRWD1 (bg=5.13%)▶SF3B4 (bg=0.05%)No matches to TargetScan

-11796--(4)--11801-

TG

TGCCAAATC  
Depth:3 (COW)  
Ei-value:0.000, Pi-value:0.000  
Er-value:0.000, Pr-value:0.000  
eCLIP MATCHES▶GRWD1 (bg=5.13%)▶NOLC1 (bg=9.43%)MATCHES To TargetScan▶ miR-182-5p:UUGGCAA▶ miR-96-5p/1271-5p:UUGGCAC


CCAAAT

CCAAAT  
Depth:6 (MOUSE)  
Ei-value:0.000, Pi-value:0.000  
Er-value:0.000, Pr-value:0.000  
eCLIP MATCHES▶GRWD1 (bg=5.13%)▶NOLC1 (bg=9.43%)No matches to TargetScan


C

CCAAATC  
Depth:5 (RABBIT)  
Ei-value:0.000, Pi-value:0.000  
Er-value:0.000, Pr-value:0.000  
eCLIP MATCHES▶GRWD1 (bg=5.13%)▶NOLC1 (bg=9.43%)No matches to TargetScan

-11809--(47)--11857-

CAAGAAA

CAAGAAA  
Depth:5 (RABBIT)  
Ei-value:0.000, Pi-value:0.000  
Er-value:0.000, Pr-value:0.000  
eCLIP MATCHES▶GRWD1 (bg=5.13%)▶NOLC1 (bg=9.43%)▶uchl5 (bg=11.16%)▶ZNF622 (bg=6.58%)No matches to TargetScan


T

CAAGAAAT  
Depth:3 (COW)  
Ei-value:0.000, Pi-value:0.000  
Er-value:0.000, Pr-value:0.000  
eCLIP MATCHES▶GRWD1 (bg=5.13%)▶NOLC1 (bg=9.43%)▶TRA2A (bg=4.8%)▶uchl5 (bg=11.16%)▶ZNF622 (bg=6.58%)No matches to TargetScan

-11864--(1)--11866-

TGAACACAC

TGAACACAC  
Depth:3 (COW)  
Ei-value:0.000, Pi-value:0.000  
Er-value:0.000, Pr-value:0.000  
eCLIP MATCHES▶GRWD1 (bg=5.13%)▶NOLC1 (bg=9.43%)▶PTBP1 (bg=3.74%)▶RBM15 (bg=7.27%)▶TRA2A (bg=4.8%)▶uchl5 (bg=11.16%)▶ZNF622 (bg=6.58%)No matches to TargetScan

-11874--(24)--11899-

G

GAAGATCAACATGCCTG  
Depth:4 (DOG)  
Ei-value:0.000, Pi-value:0.000  
Er-value:0.000, Pr-value:0.000  
eCLIP MATCHES▶GRWD1 (bg=5.13%)▶NOLC1 (bg=9.43%)▶PTBP1 (bg=3.74%)▶RBM15 (bg=7.27%)▶TRA2A (bg=4.8%)▶uchl5 (bg=11.16%)▶ZNF622 (bg=6.58%)No matches to TargetScan


AA

AAGATCAACATGC  
Depth:5 (RABBIT)  
Ei-value:0.000, Pi-value:0.000  
Er-value:0.000, Pr-value:0.000  
eCLIP MATCHES▶GRWD1 (bg=5.13%)▶NOLC1 (bg=9.43%)▶PTBP1 (bg=3.74%)▶RBM15 (bg=7.27%)▶TRA2A (bg=4.8%)▶uchl5 (bg=11.16%)▶ZNF622 (bg=6.58%)No matches to TargetScan


GATCAACATGC

GATCAACATGC  
Depth:6 (MOUSE)  
Ei-value:0.000, Pi-value:0.000  
Er-value:0.000, Pr-value:0.000  
eCLIP MATCHES▶GRWD1 (bg=5.13%)▶NOLC1 (bg=9.43%)▶PTBP1 (bg=3.74%)▶RBM15 (bg=7.27%)▶TRA2A (bg=4.8%)▶uchl5 (bg=11.16%)▶ZNF622 (bg=6.58%)No matches to TargetScan


CTG

GAAGATCAACATGCCTG  
Depth:4 (DOG)  
Ei-value:0.000, Pi-value:0.000  
Er-value:0.000, Pr-value:0.000  
eCLIP MATCHES▶GRWD1 (bg=5.13%)▶NOLC1 (bg=9.43%)▶PTBP1 (bg=3.74%)▶RBM15 (bg=7.27%)▶TRA2A (bg=4.8%)▶uchl5 (bg=11.16%)▶ZNF622 (bg=6.58%)No matches to TargetScan

-11915--(31)--11947-

TGTGTAT

TGTGTAT  
Depth:6 (MOUSE)  
Ei-value:0.000, Pi-value:0.000  
Er-value:0.000, Pr-value:0.000  
eCLIP MATCHES▶TARDBP (bg=2.79%)▶ZC3H11A (bg=6.55%)No matches to TargetScan


TT

TGTGTATTT  
Depth:4 (DOG)  
Ei-value:0.000, Pi-value:0.000  
Er-value:0.000, Pr-value:0.000  
eCLIP MATCHES▶TARDBP (bg=2.79%)▶ZC3H11A (bg=6.55%)No matches to TargetScan

-11955--(7)--11963-

TCTTTCTT

TCTTTCTT  
Depth:3 (COW)  
Ei-value:0.000, Pi-value:0.000  
Er-value:0.000, Pr-value:0.000  
eCLIP MATCHES▶TARDBP (bg=2.79%)▶ZC3H11A (bg=6.55%)No matches to TargetScan

-11970--(34)--12005-

TGTCTTA

TGTCTTA  
Depth:4 (DOG)  
Ei-value:0.000, Pi-value:0.000  
Er-value:0.000, Pr-value:0.000  
eCLIP MATCHES▶MATR3 (bg=2.98%)▶PTBP1 (bg=3.74%)▶TARDBP (bg=2.79%)▶ZC3H11A (bg=6.55%)MATCHES To TargetScan▶ miR-208-3p:UAAGACG▶ miR-499a-5p:UAAGACU


CCCATTTCCATG

TGTCTTACCCATTTCCATG  
Depth:3 (COW)  
Ei-value:0.000, Pi-value:0.000  
Er-value:0.000, Pr-value:0.000  
eCLIP MATCHES▶MATR3 (bg=2.98%)▶PTBP1 (bg=3.74%)▶TARDBP (bg=2.79%)▶ZC3H11A (bg=6.55%)MATCHES To TargetScan▶ miR-203a-3p.1:GAAAUGU▶ miR-208-3p:UAAGACG▶ miR-499a-5p:UAAGACU

-12023--(46)--12070-

TTTTTGT

TTTTTGT  
Depth:4 (DOG)  
Ei-value:0.000, Pi-value:0.000  
Er-value:0.000, Pr-value:0.000  
eCLIP MATCHES▶MATR3 (bg=2.98%)▶PTBP1 (bg=3.74%)▶TARDBP (bg=2.79%)▶TIA1 (bg=4.07%)▶ZC3H11A (bg=6.55%)No matches to TargetScan

-12076--(44)--12121-

TTCATTTTGTT

TTCATTTTGTT  
Depth:4 (DOG)  
Ei-value:0.000, Pi-value:0.000  
Er-value:0.000, Pr-value:0.000  
No matches to eCLIP DataMATCHES To TargetScan▶ miR-495-3p:AACAAAC

-12131--(10)--12142-

TTTGCTC

TTTGCTC  
Depth:3 (COW)  
Ei-value:0.000, Pi-value:0.000  
Er-value:0.000, Pr-value:0.000  
eCLIP MATCHES▶MATR3 (bg=2.98%)▶PTBP1 (bg=3.74%)▶TIA1 (bg=4.07%)No matches to TargetScan

-12148--(116)--12265-

TT

TTTTCTCTTTGTGAA  
Depth:3 (COW)  
Ei-value:0.000, Pi-value:0.000  
Er-value:0.000, Pr-value:0.000  
eCLIP MATCHES▶MATR3 (bg=2.98%)▶PTBP1 (bg=3.74%)▶SMNDC1 (bg=0.63%)▶TIA1 (bg=4.07%)No matches to TargetScan


TTCTCTTTG

TTCTCTTTG  
Depth:6 (MOUSE)  
Ei-value:0.000, Pi-value:0.000  
Er-value:0.000, Pr-value:0.000  
eCLIP MATCHES▶MATR3 (bg=2.98%)▶PTBP1 (bg=3.74%)▶SMNDC1 (bg=0.63%)▶TIA1 (bg=4.07%)No matches to TargetScan


TGAA

TTTTCTCTTTGTGAA  
Depth:3 (COW)  
Ei-value:0.000, Pi-value:0.000  
Er-value:0.000, Pr-value:0.000  
eCLIP MATCHES▶MATR3 (bg=2.98%)▶PTBP1 (bg=3.74%)▶SMNDC1 (bg=0.63%)▶TIA1 (bg=4.07%)No matches to TargetScan

-12279--(20)--12300-

TTCCCCTT

TTCCCCTT  
Depth:3 (COW)  
Ei-value:0.000, Pi-value:0.000  
Er-value:0.000, Pr-value:0.000  
eCLIP MATCHES▶MATR3 (bg=2.98%)▶PTBP1 (bg=3.74%)▶TIA1 (bg=4.07%)No matches to TargetScan

-12307--(14)--12322-

ATTTCACCT

ATTTCACCT  
Depth:4 (DOG)  
Ei-value:0.000, Pi-value:0.000  
Er-value:0.000, Pr-value:0.000  
eCLIP MATCHES▶TIA1 (bg=4.07%)MATCHES To TargetScan▶ miR-203a-3p.2:UGAAAUG

-12330--(24)--12355-

TGCTG

TGCTGTTTCTACT  
Depth:3 (COW)  
Ei-value:0.000, Pi-value:0.000  
Er-value:0.000, Pr-value:0.000  
eCLIP MATCHES▶MATR3 (bg=2.98%)▶PTBP1 (bg=3.74%)▶TIA1 (bg=4.07%)MATCHES To TargetScan▶ miR-411-5p.1:AGUAGAC▶ miR-494-3p:GAAACAU


TTTCTAC

TTTCTAC  
Depth:6 (MOUSE)  
Ei-value:0.000, Pi-value:0.000  
Er-value:0.000, Pr-value:0.000  
eCLIP MATCHES▶MATR3 (bg=2.98%)▶PTBP1 (bg=3.74%)▶TIA1 (bg=4.07%)No matches to TargetScan


T

TTTCTACT  
Depth:5 (RABBIT)  
Ei-value:0.000, Pi-value:0.000  
Er-value:0.000, Pr-value:0.000  
eCLIP MATCHES▶MATR3 (bg=2.98%)▶PTBP1 (bg=3.74%)▶TIA1 (bg=4.07%)MATCHES To TargetScan▶ miR-411-5p.1:AGUAGAC

-12367--(11)--12379-

ATTTCTC

ATTTCTC  
Depth:6 (MOUSE)  
Ei-value:0.000, Pi-value:0.000  
Er-value:0.000, Pr-value:0.000  
eCLIP MATCHES▶MATR3 (bg=2.98%)▶PTBP1 (bg=3.74%)▶TIA1 (bg=4.07%)No matches to TargetScan

-12385--(24)--12410-

TCTTGGG

TCTTGGG  
Depth:5 (RABBIT)  
Ei-value:0.000, Pi-value:0.000  
Er-value:0.000, Pr-value:0.000  
eCLIP MATCHES▶MATR3 (bg=2.98%)▶PTBP1 (bg=3.74%)▶SMNDC1 (bg=0.63%)▶TIA1 (bg=4.07%)No matches to TargetScan


C

TCTTGGGC  
Depth:3 (COW)  
Ei-value:0.000, Pi-value:0.000  
Er-value:0.000, Pr-value:0.000  
eCLIP MATCHES▶MATR3 (bg=2.98%)▶PTBP1 (bg=3.74%)▶SMNDC1 (bg=0.63%)▶TIA1 (bg=4.07%)No matches to TargetScan

-12417--(43)--12461-

TTTGTGA

TTTGTGA  
Depth:4 (DOG)  
Ei-value:0.000, Pi-value:0.010  
Er-value:0.000, Pr-value:0.000  
eCLIP MATCHES▶MATR3 (bg=2.98%)▶PTBP1 (bg=3.74%)▶TIA1 (bg=4.07%)No matches to TargetScan


TTTTC

TTTGTGATTTTC  
Depth:3 (COW)  
Ei-value:0.000, Pi-value:0.000  
Er-value:0.000, Pr-value:0.000  
eCLIP MATCHES▶MATR3 (bg=2.98%)▶PTBP1 (bg=3.74%)▶TIA1 (bg=4.07%)No matches to TargetScan

-12472--(14)--12487-

TCTCTGTT

TCTCTGTT  
Depth:4 (DOG)  
Ei-value:0.000, Pi-value:0.000  
Er-value:0.000, Pr-value:0.000  
eCLIP MATCHES▶MATR3 (bg=2.98%)▶PTBP1 (bg=3.74%)No matches to TargetScan

-12494--(40)--12535-

TTTGAGTATTT

TTTGAGTATTT  
Depth:4 (DOG)  
Ei-value:0.000, Pi-value:0.000  
Er-value:0.000, Pr-value:0.000  
eCLIP MATCHES▶MATR3 (bg=2.98%)▶PTBP1 (bg=3.74%)▶TIA1 (bg=4.07%)MATCHES To TargetScan▶ miR-200bc-3p/429:AAUACUG▶ miR-371-5p:CUCAAAC

-12545--(28)--12574-

CTTTGATT

CTTTGATT  
Depth:3 (COW)  
Ei-value:0.000, Pi-value:0.000  
Er-value:0.000, Pr-value:0.000  
eCLIP MATCHES▶MATR3 (bg=2.98%)▶PTBP1 (bg=3.74%)▶TIA1 (bg=4.07%)No matches to TargetScan

-12581--(50)--12632-

TGTGTGTG

TGTGTGTG  
Depth:4 (DOG)  
Ei-value:0.000, Pi-value:0.000  
Er-value:0.000, Pr-value:0.000  
eCLIP MATCHES▶AATF (bg=0.64%)▶DDX24 (bg=2.97%)▶NCBP2 (bg=1.49%)▶NOLC1 (bg=9.43%)▶PTBP1 (bg=3.74%)▶SND1 (bg=0.45%)▶SRSF7 (bg=2.32%)▶TARDBP (bg=2.79%)▶WDR43 (bg=3.37%)▶XRCC6 (bg=2.91%)▶ZC3H8 (bg=0.29%)MATCHES To TargetScan▶ miR-329-3p/362-3p:ACACACC

-12639--(22)--12662-

TCCTAACCCCT

TCCTAACCCCT  
Depth:5 (RABBIT)  
Ei-value:0.000, Pi-value:0.000  
Er-value:0.000, Pr-value:0.000  
eCLIP MATCHES▶AATF (bg=0.64%)▶DDX24 (bg=2.97%)▶NCBP2 (bg=1.49%)▶NOLC1 (bg=9.43%)▶PTBP1 (bg=3.74%)▶SND1 (bg=0.45%)▶SRSF7 (bg=2.32%)▶TARDBP (bg=2.79%)▶UTP3 (bg=3.66%)▶WDR43 (bg=3.37%)▶XRCC6 (bg=2.91%)▶ZC3H8 (bg=0.29%)No matches to TargetScan

-12672--(6)--12679-

TAGGTGCA

TAGGTGCA  
Depth:3 (COW)  
Ei-value:0.000, Pi-value:0.000  
Er-value:0.000, Pr-value:0.000  
eCLIP MATCHES▶DDX24 (bg=2.97%)▶NOLC1 (bg=9.43%)▶SND1 (bg=0.45%)▶SRSF7 (bg=2.32%)▶TARDBP (bg=2.79%)▶UTP3 (bg=3.66%)▶WDR43 (bg=3.37%)▶XRCC6 (bg=2.91%)▶ZC3H8 (bg=0.29%)No matches to TargetScan

-12686--(19)--12706-

AAGCATTG

AAGCATTG  
Depth:4 (DOG)  
Ei-value:0.000, Pi-value:0.000  
Er-value:0.000, Pr-value:0.000  
eCLIP MATCHES▶DDX24 (bg=2.97%)▶NOLC1 (bg=9.43%)▶NPM1 (bg=1.21%)▶RBFOX2 (bg=4.63%)▶RPS3 (bg=0.76%)▶SRSF1 (bg=8.47%)▶SRSF7 (bg=2.32%)▶TARDBP (bg=2.79%)▶TRA2A (bg=4.8%)▶U2AF2 (bg=1.76%)▶uchl5 (bg=11.16%)▶YWHAG (bg=1.87%)▶ZNF622 (bg=6.58%)No matches to TargetScan

-12713--(12)--12726-

TTATGCCA

TTATGCCA  
Depth:5 (RABBIT)  
Ei-value:0.000, Pi-value:0.000  
Er-value:0.000, Pr-value:0.000  
eCLIP MATCHES▶DDX24 (bg=2.97%)▶FASTKD2 (bg=1.99%)▶LARP4 (bg=4.72%)▶NOLC1 (bg=9.43%)▶NPM1 (bg=1.21%)▶RBFOX2 (bg=4.63%)▶RBM15 (bg=7.27%)▶RPS3 (bg=0.76%)▶SRSF1 (bg=8.47%)▶SRSF7 (bg=2.32%)▶TARDBP (bg=2.79%)▶TRA2A (bg=4.8%)▶U2AF2 (bg=1.76%)▶uchl5 (bg=11.16%)▶WDR43 (bg=3.37%)▶YWHAG (bg=1.87%)▶ZC3H11A (bg=6.55%)▶ZNF622 (bg=6.58%)▶ZNF800 (bg=1.92%)No matches to TargetScan


G

TTATGCCAG  
Depth:4 (DOG)  
Ei-value:0.000, Pi-value:0.000  
Er-value:0.000, Pr-value:0.000  
eCLIP MATCHES▶DDX24 (bg=2.97%)▶FASTKD2 (bg=1.99%)▶LARP4 (bg=4.72%)▶NOLC1 (bg=9.43%)▶NPM1 (bg=1.21%)▶RBFOX2 (bg=4.63%)▶RBM15 (bg=7.27%)▶RPS3 (bg=0.76%)▶SRSF1 (bg=8.47%)▶SRSF7 (bg=2.32%)▶TARDBP (bg=2.79%)▶TRA2A (bg=4.8%)▶U2AF2 (bg=1.76%)▶uchl5 (bg=11.16%)▶WDR43 (bg=3.37%)▶YWHAG (bg=1.87%)▶ZC3H11A (bg=6.55%)▶ZNF622 (bg=6.58%)▶ZNF800 (bg=1.92%)No matches to TargetScan

-12734--(17)--12752-

TCCAAG

TCCAAG  
Depth:3 (COW)  
Ei-value:0.000, Pi-value:0.000  
Er-value:0.000, Pr-value:0.000  
eCLIP MATCHES▶DDX24 (bg=2.97%)▶FASTKD2 (bg=1.99%)▶LARP4 (bg=4.72%)▶NOLC1 (bg=9.43%)▶NPM1 (bg=1.21%)▶RBFOX2 (bg=4.63%)▶RBM15 (bg=7.27%)▶SRSF1 (bg=8.47%)▶SRSF7 (bg=2.32%)▶TARDBP (bg=2.79%)▶TRA2A (bg=4.8%)▶U2AF2 (bg=1.76%)▶uchl5 (bg=11.16%)▶WDR43 (bg=3.37%)▶YWHAG (bg=1.87%)▶ZC3H11A (bg=6.55%)▶ZNF622 (bg=6.58%)▶ZNF800 (bg=1.92%)No matches to TargetScan

-12757--(73)--12831-

AGA

AGAAGGCCCAA  
Depth:4 (DOG)  
Ei-value:0.000, Pi-value:0.000  
Er-value:0.000, Pr-value:0.000  
eCLIP MATCHES▶DDX24 (bg=2.97%)▶LARP4 (bg=4.72%)▶MTPAP (bg=2.21%)▶NOLC1 (bg=9.43%)▶SRSF1 (bg=8.47%)▶SRSF7 (bg=2.32%)▶TRA2A (bg=4.8%)▶uchl5 (bg=11.16%)▶UTP3 (bg=3.66%)▶ZNF622 (bg=6.58%)▶ZNF800 (bg=1.92%)No matches to TargetScan


AGGCCCAA

AGGCCCAA  
Depth:5 (RABBIT)  
Ei-value:0.000, Pi-value:0.000  
Er-value:0.000, Pr-value:0.000  
eCLIP MATCHES▶DDX24 (bg=2.97%)▶LARP4 (bg=4.72%)▶MTPAP (bg=2.21%)▶NOLC1 (bg=9.43%)▶SRSF1 (bg=8.47%)▶SRSF7 (bg=2.32%)▶TRA2A (bg=4.8%)▶uchl5 (bg=11.16%)▶UTP3 (bg=3.66%)▶ZNF622 (bg=6.58%)▶ZNF800 (bg=1.92%)No matches to TargetScan

-12841--(357)--13199-

TCAA

TCAAGACTAA  
Depth:4 (DOG)  
Ei-value:0.000, Pi-value:0.000  
Er-value:0.000, Pr-value:0.000  
eCLIP MATCHES▶CPEB4 (bg=1.89%)▶FASTKD2 (bg=1.99%)▶GRWD1 (bg=5.13%)▶LARP4 (bg=4.72%)▶MTPAP (bg=2.21%)▶NOLC1 (bg=9.43%)▶RBFOX2 (bg=4.63%)▶SRSF1 (bg=8.47%)▶TRA2A (bg=4.8%)▶uchl5 (bg=11.16%)▶UTP18 (bg=0.72%)▶UTP3 (bg=3.66%)▶WDR43 (bg=3.37%)▶ZNF622 (bg=6.58%)MATCHES To TargetScan▶ miR-431-5p:GUCUUGC


GACTAA

GACTAA  
Depth:5 (RABBIT)  
Ei-value:0.000, Pi-value:0.000  
Er-value:0.000, Pr-value:0.000  
eCLIP MATCHES▶CPEB4 (bg=1.89%)▶FASTKD2 (bg=1.99%)▶GRWD1 (bg=5.13%)▶LARP4 (bg=4.72%)▶MTPAP (bg=2.21%)▶NOLC1 (bg=9.43%)▶RBFOX2 (bg=4.63%)▶SRSF1 (bg=8.47%)▶TRA2A (bg=4.8%)▶uchl5 (bg=11.16%)▶UTP18 (bg=0.72%)▶UTP3 (bg=3.66%)▶WDR43 (bg=3.37%)▶ZNF622 (bg=6.58%)No matches to TargetScan

-13208--(43)--13252-

AGAAGC

AGAAGC  
Depth:4 (DOG)  
Ei-value:0.000, Pi-value:0.000  
Er-value:0.000, Pr-value:0.010  
eCLIP MATCHES▶CPEB4 (bg=1.89%)▶GRWD1 (bg=5.13%)▶LARP4 (bg=4.72%)▶MTPAP (bg=2.21%)▶NOLC1 (bg=9.43%)▶PCBP1 (bg=1.07%)▶RBFOX2 (bg=4.63%)▶SRSF1 (bg=8.47%)▶TRA2A (bg=4.8%)▶uchl5 (bg=11.16%)▶ZNF622 (bg=6.58%)No matches to TargetScan

-13257--(20)--13278-

C

CAAGATGA  
Depth:3 (COW)  
Ei-value:0.000, Pi-value:0.000  
Er-value:0.000, Pr-value:0.000  
eCLIP MATCHES▶CPEB4 (bg=1.89%)▶FTO (bg=0.32%)▶GRWD1 (bg=5.13%)▶LARP4 (bg=4.72%)▶MTPAP (bg=2.21%)▶SRSF1 (bg=8.47%)▶TRA2A (bg=4.8%)▶uchl5 (bg=11.16%)▶ZNF622 (bg=6.58%)No matches to TargetScan


AAGATGA

AAGATGA  
Depth:5 (RABBIT)  
Ei-value:0.000, Pi-value:0.000  
Er-value:0.000, Pr-value:0.000  
eCLIP MATCHES▶CPEB4 (bg=1.89%)▶FTO (bg=0.32%)▶GRWD1 (bg=5.13%)▶LARP4 (bg=4.72%)▶MTPAP (bg=2.21%)▶SRSF1 (bg=8.47%)▶TRA2A (bg=4.8%)▶uchl5 (bg=11.16%)▶ZNF622 (bg=6.58%)No matches to TargetScan

-13285--(20)--13306-

TTTCTATTG

TTTCTATTG  
Depth:3 (COW)  
Ei-value:0.000, Pi-value:0.000  
Er-value:0.000, Pr-value:0.000  
No matches to eCLIP DataNo matches to TargetScan

-13314--(5)--13320-

ACTTCTT

ACTTCTT  
Depth:3 (COW)  
Ei-value:0.000, Pi-value:0.020  
Er-value:0.000, Pr-value:0.000  
eCLIP MATCHES▶NOLC1 (bg=9.43%)No matches to TargetScan

-13326--(58)--13385-

CTTTTTGATGTT

CTTTTTGATGTT  
Depth:4 (DOG)  
Ei-value:0.000, Pi-value:0.000  
Er-value:0.000, Pr-value:0.000  
eCLIP MATCHES▶TIA1 (bg=4.07%)No matches to TargetScan

-13396--(23)--13420-

TATTATGC

TATTATGC  
Depth:4 (DOG)  
Ei-value:0.000, Pi-value:0.000  
Er-value:0.000, Pr-value:0.000  
No matches to eCLIP DataMATCHES To TargetScan▶ miR-369-3p:AUAAUAC

-13427--(65)--13493-

TAAACTTC

TAAACTTC  
Depth:3 (COW)  
Ei-value:0.000, Pi-value:0.000  
Er-value:0.000, Pr-value:0.000  
eCLIP MATCHES▶NIPBL (bg=5.39%)▶NOLC1 (bg=9.43%)▶ZC3H11A (bg=6.55%)No matches to TargetScan

-13500--(18)--13519-

CTCCACTTGAGAG

CTCCACTTGAGAG  
Depth:3 (COW)  
Ei-value:0.000, Pi-value:0.000  
Er-value:0.000, Pr-value:0.000  
eCLIP MATCHES▶NIPBL (bg=5.39%)▶NOLC1 (bg=9.43%)▶ZC3H11A (bg=6.55%)MATCHES To TargetScan▶ miR-26-5p:UCAAGUA

-13531--(79)--13611-

TATTTCAGT

TATTTCAGT  
Depth:4 (DOG)  
Ei-value:0.000, Pi-value:0.000  
Er-value:0.000, Pr-value:0.000  
eCLIP MATCHES▶NOLC1 (bg=9.43%)▶ZC3H11A (bg=6.55%)MATCHES To TargetScan▶ miR-203a-3p.2:UGAAAUG


CC

TATTTCAGTCC  
Depth:3 (COW)  
Ei-value:0.000, Pi-value:0.000  
Er-value:0.000, Pr-value:0.000  
eCLIP MATCHES▶NOLC1 (bg=9.43%)▶ZC3H11A (bg=6.55%)MATCHES To TargetScan▶ miR-203a-3p.2:UGAAAUG

-13621--(53)--13675-

GGGGAAA

GGGGAAA  
Depth:4 (DOG)  
Ei-value:0.000, Pi-value:0.000  
Er-value:0.000, Pr-value:0.000  
eCLIP MATCHES▶CPSF6 (bg=0.4%)▶LARP4 (bg=4.72%)▶WDR43 (bg=3.37%)▶ZC3H11A (bg=6.55%)No matches to TargetScan

-13681--(16)--13698-

TCTAGAGAAAA

TCTAGAGAAAA  
Depth:6 (MOUSE)  
Ei-value:0.000, Pi-value:0.000  
Er-value:0.000, Pr-value:0.000  
eCLIP MATCHES▶CPSF6 (bg=0.4%)▶LARP4 (bg=4.72%)▶UTP3 (bg=3.66%)▶WDR43 (bg=3.37%)MATCHES To TargetScan▶ miR-1251-5p:CUCUAGC

-13708--(3)--13712-

TGAAGAGATG

TGAAGAGATG  
Depth:5 (RABBIT)  
Ei-value:0.000, Pi-value:0.000  
Er-value:0.000, Pr-value:0.000  
eCLIP MATCHES▶CPSF6 (bg=0.4%)▶LARP4 (bg=4.72%)▶SRSF7 (bg=2.32%)▶UTP3 (bg=3.66%)▶WDR43 (bg=3.37%)No matches to TargetScan


CTCCA

TGAAGAGATGCTCCA  
Depth:3 (COW)  
Ei-value:0.000, Pi-value:0.000  
Er-value:0.000, Pr-value:0.000  
eCLIP MATCHES▶CPSF6 (bg=0.4%)▶LARP4 (bg=4.72%)▶SRSF7 (bg=2.32%)▶UTP3 (bg=3.66%)▶WDR43 (bg=3.37%)No matches to TargetScan

-13726--(0)--13727-

GGCCAA

GGCCAATGAGAAGAATTAGACA  
Depth:4 (DOG)  
Ei-value:0.000, Pi-value:0.000  
Er-value:0.000, Pr-value:0.000  
eCLIP MATCHES▶LARP4 (bg=4.72%)▶NOLC1 (bg=9.43%)▶SRSF7 (bg=2.32%)▶UTP3 (bg=3.66%)No matches to TargetScan


TGAGAAGAATTAGACA

TGAGAAGAATTAGACA  
Depth:6 (MOUSE)  
Ei-value:0.000, Pi-value:0.000  
Er-value:0.000, Pr-value:0.000  
eCLIP MATCHES▶LARP4 (bg=4.72%)▶NOLC1 (bg=9.43%)▶SRSF7 (bg=2.32%)No matches to TargetScan

-13748--(1)--13750-

GAAATACACAGATG

GAAATACACAGATG  
Depth:3 (COW)  
Ei-value:0.000, Pi-value:0.000  
Er-value:0.000, Pr-value:0.000  
eCLIP MATCHES▶LARP4 (bg=4.72%)▶NOLC1 (bg=9.43%)▶SRSF7 (bg=2.32%)No matches to TargetScan

-13763--(10)--13774-

C

CTGAGAAG  
Depth:3 (COW)  
Ei-value:0.000, Pi-value:0.000  
Er-value:0.000, Pr-value:0.000  
eCLIP MATCHES▶AARS (bg=2.18%)▶NOLC1 (bg=9.43%)▶PUS1 (bg=1.04%)▶SRSF7 (bg=2.32%)▶ZC3H11A (bg=6.55%)No matches to TargetScan


TGAGAAG

TGAGAAG  
Depth:4 (DOG)  
Ei-value:0.000, Pi-value:0.000  
Er-value:0.000, Pr-value:0.010  
eCLIP MATCHES▶AARS (bg=2.18%)▶NOLC1 (bg=9.43%)▶PUS1 (bg=1.04%)▶SRSF7 (bg=2.32%)▶ZC3H11A (bg=6.55%)No matches to TargetScan

-13781--(5)--13787-

GCCA

GCCAGCAACA  
Depth:3 (COW)  
Ei-value:0.000, Pi-value:0.000  
Er-value:0.000, Pr-value:0.000  
eCLIP MATCHES▶AARS (bg=2.18%)▶NOLC1 (bg=9.43%)▶PUS1 (bg=1.04%)▶SRSF7 (bg=2.32%)▶ZC3H11A (bg=6.55%)No matches to TargetScan


GCAACA

GCAACA  
Depth:6 (MOUSE)  
Ei-value:0.000, Pi-value:0.000  
Er-value:0.000, Pr-value:0.000  
eCLIP MATCHES▶AARS (bg=2.18%)▶NOLC1 (bg=9.43%)▶PUS1 (bg=1.04%)▶ZC3H11A (bg=6.55%)No matches to TargetScan

-13796--(9)--13806-

TTTGAGCTT

TTTGAGCTT  
Depth:3 (COW)  
Ei-value:0.000, Pi-value:0.000  
Er-value:0.000, Pr-value:0.000  
eCLIP MATCHES▶AARS (bg=2.18%)▶NOLC1 (bg=9.43%)▶PUS1 (bg=1.04%)▶ZC3H11A (bg=6.55%)MATCHES To TargetScan▶ miR-371-5p:CUCAAAC

-13814--(1)--13816-

GGTGAGC

GGTGAGC  
Depth:4 (DOG)  
Ei-value:0.000, Pi-value:0.000  
Er-value:0.000, Pr-value:0.000  
eCLIP MATCHES▶AARS (bg=2.18%)▶NOLC1 (bg=9.43%)▶PUS1 (bg=1.04%)▶ZC3H11A (bg=6.55%)No matches to TargetScan


AGGAT

GGTGAGCAGGAT  
Depth:3 (COW)  
Ei-value:0.000, Pi-value:0.000  
Er-value:0.000, Pr-value:0.000  
eCLIP MATCHES▶AARS (bg=2.18%)▶AKAP8L (bg=2.19%)▶NOLC1 (bg=9.43%)▶PUS1 (bg=1.04%)▶ZC3H11A (bg=6.55%)No matches to TargetScan

-13827--(5)--13833-

GGTTTGGG

GGTTTGGG  
Depth:4 (DOG)  
Ei-value:0.000, Pi-value:0.000  
Er-value:0.000, Pr-value:0.000  
eCLIP MATCHES▶AARS (bg=2.18%)▶AKAP8L (bg=2.19%)▶NOLC1 (bg=9.43%)▶PUS1 (bg=1.04%)No matches to TargetScan

-13840--(11)--13852-

TGGTTA

TGGTTA  
Depth:5 (RABBIT)  
Ei-value:0.000, Pi-value:0.000  
Er-value:0.000, Pr-value:0.000  
eCLIP MATCHES▶AKAP8L (bg=2.19%)▶NOLC1 (bg=9.43%)▶PUS1 (bg=1.04%)▶SF3B1 (bg=2.48%)No matches to TargetScan


T

TGGTTAT  
Depth:4 (DOG)  
Ei-value:0.000, Pi-value:0.000  
Er-value:0.000, Pr-value:0.000  
eCLIP MATCHES▶AKAP8L (bg=2.19%)▶NOLC1 (bg=9.43%)▶PUS1 (bg=1.04%)▶SF3B1 (bg=2.48%)No matches to TargetScan


G

TGGTTATG  
Depth:3 (COW)  
Ei-value:0.000, Pi-value:0.000  
Er-value:0.000, Pr-value:0.000  
eCLIP MATCHES▶AKAP8L (bg=2.19%)▶NOLC1 (bg=9.43%)▶PUS1 (bg=1.04%)▶SF3B1 (bg=2.48%)No matches to TargetScan

-13859--(33)--13893-

CCCAAGG

CCCAAGG  
Depth:4 (DOG)  
Ei-value:0.000, Pi-value:0.000  
Er-value:0.000, Pr-value:0.000  
eCLIP MATCHES▶PUS1 (bg=1.04%)▶UTP3 (bg=3.66%)MATCHES To TargetScan▶ miR-212-5p:CCUUGGC

-13899--(8)--13908-

TGAACTCCCTGCT

TGAACTCCCTGCT  
Depth:4 (DOG)  
Ei-value:0.000, Pi-value:0.000  
Er-value:0.000, Pr-value:0.000  
eCLIP MATCHES▶UTP3 (bg=3.66%)No matches to TargetScan


C

TGAACTCCCTGCTCATAGTAGTGGCC  
Depth:3 (COW)  
Ei-value:0.000, Pi-value:0.000  
Er-value:0.000, Pr-value:0.000  
eCLIP MATCHES▶UTP3 (bg=3.66%)No matches to TargetScan


ATAGTAGTGGCC

ATAGTAGTGGCC  
Depth:4 (DOG)  
Ei-value:0.000, Pi-value:0.000  
Er-value:0.000, Pr-value:0.000  
No matches to eCLIP DataNo matches to TargetScan

-13933--(37)--13971-

TTTAATAC

TTTAATAC  
Depth:4 (DOG)  
Ei-value:0.000, Pi-value:0.000  
Er-value:0.000, Pr-value:0.000  
eCLIP MATCHES▶WRN (bg=0.77%)MATCHES To TargetScan▶ miR-496.2:GUAUUAC

-13978--(6)--13985-

CT

CTAGGCTTAAAG  
Depth:4 (DOG)  
Ei-value:0.000, Pi-value:0.000  
Er-value:0.000, Pr-value:0.000  
No matches to eCLIP DataNo matches to TargetScan


AGGCTTA

AGGCTTA  
Depth:5 (RABBIT)  
Ei-value:0.000, Pi-value:0.000  
Er-value:0.000, Pr-value:0.000  
No matches to eCLIP DataNo matches to TargetScan


AAG

CTAGGCTTAAAG  
Depth:4 (DOG)  
Ei-value:0.000, Pi-value:0.000  
Er-value:0.000, Pr-value:0.000  
No matches to eCLIP DataNo matches to TargetScan

-13996--(27)--14024-

GTTTAAT

GTTTAAT  
Depth:5 (RABBIT)  
Ei-value:0.000, Pi-value:0.000  
Er-value:0.000, Pr-value:0.000  
No matches to eCLIP DataNo matches to TargetScan

-14030--(73)--14104-

TGTAAAACA

TGTAAAACA  
Depth:3 (COW)  
Ei-value:0.000, Pi-value:0.000  
Er-value:0.000, Pr-value:0.000  
eCLIP MATCHES▶TARDBP (bg=2.79%)▶WDR43 (bg=3.37%)No matches to TargetScan

-14112--(21)--14134-

T

TATTGGCA  
Depth:5 (RABBIT)  
Ei-value:0.000, Pi-value:0.000  
Er-value:0.000, Pr-value:0.000  
eCLIP MATCHES▶HNRNPA1 (bg=2.57%)No matches to TargetScan


ATTGGCA

ATTGGCA  
Depth:6 (MOUSE)  
Ei-value:0.000, Pi-value:0.000  
Er-value:0.000, Pr-value:0.000  
eCLIP MATCHES▶HNRNPA1 (bg=2.57%)No matches to TargetScan

-14141--(34)--14176-

TTGTGAAG

TTGTGAAG  
Depth:6 (MOUSE)  
Ei-value:0.000, Pi-value:0.000  
Er-value:0.000, Pr-value:0.000  
eCLIP MATCHES▶HNRNPA1 (bg=2.57%)No matches to TargetScan

-14183--(3)--14187-

T

TATGTAAATCA  
Depth:3 (COW)  
Ei-value:0.000, Pi-value:0.000  
Er-value:0.000, Pr-value:0.000  
No matches to eCLIP DataNo matches to TargetScan


ATGTAAAT

ATGTAAAT  
Depth:5 (RABBIT)  
Ei-value:0.000, Pi-value:0.000  
Er-value:0.000, Pr-value:0.000  
No matches to eCLIP DataNo matches to TargetScan


CA

TATGTAAATCA  
Depth:3 (COW)  
Ei-value:0.000, Pi-value:0.000  
Er-value:0.000, Pr-value:0.000  
No matches to eCLIP DataNo matches to TargetScan

-14197--(53)--14251-

GCCATATGGT

GCCATATGGT  
Depth:3 (COW)  
Ei-value:0.000, Pi-value:0.000  
Er-value:0.000, Pr-value:0.000  
No matches to eCLIP DataNo matches to TargetScan

-14260--(160)--14421-

TGTGC

TGTGCCTGTCCCTGT  
Depth:3 (COW)  
Ei-value:0.000, Pi-value:0.000  
Er-value:0.000, Pr-value:0.000  
No matches to eCLIP DataNo matches to TargetScan


CTGTCCCT

CTGTCCCT  
Depth:4 (DOG)  
Ei-value:0.000, Pi-value:0.000  
Er-value:0.000, Pr-value:0.000  
No matches to eCLIP DataNo matches to TargetScan


GT

TGTGCCTGTCCCTGT  
Depth:3 (COW)  
Ei-value:0.000, Pi-value:0.000  
Er-value:0.000, Pr-value:0.000  
No matches to eCLIP DataNo matches to TargetScan

-14435--(2)--14438-

TAGGCACT

TAGGCACT  
Depth:4 (DOG)  
Ei-value:0.000, Pi-value:0.000  
Er-value:0.000, Pr-value:0.000  
No matches to eCLIP DataNo matches to TargetScan

-14445--(66)--14512-

TAAAGCA

TAAAGCA  
Depth:4 (DOG)  
Ei-value:0.000, Pi-value:0.000  
Er-value:0.000, Pr-value:0.000  
eCLIP MATCHES▶LIN28B (bg=0.74%)No matches to TargetScan

-14518--(274)--14793-

TAT

TATAATGTGCCAGATA  
Depth:3 (COW)  
Ei-value:0.000, Pi-value:0.000  
Er-value:0.000, Pr-value:0.000  
No matches to eCLIP DataMATCHES To TargetScan▶ miR-183-5p.2:UGGCACU▶ miR-323-3p:ACAUUAC


AATGTGCCAGATA

AATGTGCCAGATA  
Depth:4 (DOG)  
Ei-value:0.000, Pi-value:0.000  
Er-value:0.000, Pr-value:0.000  
No matches to eCLIP DataMATCHES To TargetScan▶ miR-183-5p.2:UGGCACU

-14808--(73)--14882-

TTAAAGTG

TTAAAGTG  
Depth:4 (DOG)  
Ei-value:0.000, Pi-value:0.000  
Er-value:0.000, Pr-value:0.000  
eCLIP MATCHES▶SF3B1 (bg=2.48%)No matches to TargetScan


CTTTGTA

TTAAAGTGCTTTGTA  
Depth:3 (COW)  
Ei-value:0.000, Pi-value:0.000  
Er-value:0.000, Pr-value:0.000  
eCLIP MATCHES▶SF3B1 (bg=2.48%)MATCHES To TargetScan▶ miR-330-3p:CAAAGCA▶ miR-330-3p.2:AAAGCAC

-14896--(2)--14899-

CTAAAGCA

CTAAAGCA  
Depth:4 (DOG)  
Ei-value:0.000, Pi-value:0.000  
Er-value:0.000, Pr-value:0.000  
eCLIP MATCHES▶SF3B1 (bg=2.48%)No matches to TargetScan

-14906--(12)--14919-

CAATGGGCTA

CAATGGGCTA  
Depth:3 (COW)  
Ei-value:0.000, Pi-value:0.000  
Er-value:0.000, Pr-value:0.000  
No matches to eCLIP DataNo matches to TargetScan

-14928--(25)--14954-

GA

GAATGAATA  
Depth:3 (COW)  
Ei-value:0.000, Pi-value:0.000  
Er-value:0.000, Pr-value:0.000  
eCLIP MATCHES▶DROSHA (bg=2.49%)▶TARDBP (bg=2.79%)▶ZC3H11A (bg=6.55%)MATCHES To TargetScan▶ miR-1298-5p:UCAUUCG


ATGAATA

ATGAATA  
Depth:4 (DOG)  
Ei-value:0.000, Pi-value:0.000  
Er-value:0.000, Pr-value:0.000  
eCLIP MATCHES▶DROSHA (bg=2.49%)▶TARDBP (bg=2.79%)▶ZC3H11A (bg=6.55%)No matches to TargetScan

-14962--(18)--14981-

CCAGCTATT

CCAGCTATT  
Depth:3 (COW)  
Ei-value:0.000, Pi-value:0.000  
Er-value:0.000, Pr-value:0.000  
eCLIP MATCHES▶AARS (bg=2.18%)▶DROSHA (bg=2.49%)▶ILF3 (bg=3.0%)▶TARDBP (bg=2.79%)▶ZC3H11A (bg=6.55%)No matches to TargetScan

-14989--(3)--14993-

GGTACTGT

GGTACTGT  
Depth:4 (DOG)  
Ei-value:0.000, Pi-value:0.000  
Er-value:0.000, Pr-value:0.000  
eCLIP MATCHES▶AARS (bg=2.18%)▶DROSHA (bg=2.49%)▶ILF3 (bg=3.0%)▶TARDBP (bg=2.79%)▶ZC3H11A (bg=6.55%)MATCHES To TargetScan▶ miR-101-3p.1:ACAGUAC▶ miR-144-3p:ACAGUAU

-15000--(27)--15028-

ATAAGAGG

ATAAGAGG  
Depth:4 (DOG)  
Ei-value:0.000, Pi-value:0.000  
Er-value:0.000, Pr-value:0.000  
eCLIP MATCHES▶ILF3 (bg=3.0%)No matches to TargetScan

-15035--(62)--15098-

AAGACTTTAC

AAGACTTTAC  
Depth:3 (COW)  
Ei-value:0.000, Pi-value:0.000  
Er-value:0.000, Pr-value:0.000  
No matches to eCLIP DataNo matches to TargetScan

-15107--(14)--15122-

TAAATTAT

TAAATTAT  
Depth:4 (DOG)  
Ei-value:0.000, Pi-value:0.010  
Er-value:0.000, Pr-value:0.000  
No matches to eCLIP DataNo matches to TargetScan


TAC

TAAATTATTAC  
Depth:3 (COW)  
Ei-value:0.000, Pi-value:0.000  
Er-value:0.000, Pr-value:0.000  
No matches to eCLIP DataNo matches to TargetScan

-15132--(14)--15147-

AGGTAA

AGGTAA  
Depth:3 (COW)  
Ei-value:0.000, Pi-value:0.000  
Er-value:0.000, Pr-value:0.000  
No matches to eCLIP DataNo matches to TargetScan

-15152--(83)--15236-

TTTCTAA

TTTCTAA  
Depth:3 (COW)  
Ei-value:0.000, Pi-value:0.000  
Er-value:0.000, Pr-value:0.010  
No matches to eCLIP DataNo matches to TargetScan

-15242--(45)--15288-

ATAAAAC

ATAAAAC  
Depth:4 (DOG)  
Ei-value:0.000, Pi-value:0.010  
Er-value:0.000, Pr-value:0.000  
No matches to eCLIP DataNo matches to TargetScan

-15294--(27)--15322-

AAAATTCTCA

AAAATTCTCA  
Depth:4 (DOG)  
Ei-value:0.000, Pi-value:0.000  
Er-value:0.000, Pr-value:0.000  
eCLIP MATCHES▶HNRNPU (bg=5.92%)No matches to TargetScan

-15331--(41)--15373-

TATACAAAC

TATACAAAC  
Depth:4 (DOG)  
Ei-value:0.000, Pi-value:0.000  
Er-value:0.000, Pr-value:0.000  
No matches to eCLIP DataNo matches to TargetScan

-15381--(5)--15387-

GTTTAAATAC

GTTTAAATAC  
Depth:3 (COW)  
Ei-value:0.000, Pi-value:0.000  
Er-value:0.000, Pr-value:0.000  
No matches to eCLIP DataNo matches to TargetScan

-15396--(22)--15419-

TTGCCTACTAT

TTGCCTACTATGTGAACTCACTGTTA  
Depth:3 (COW)  
Ei-value:0.000, Pi-value:0.000  
Er-value:0.000, Pr-value:0.000  
No matches to eCLIP DataMATCHES To TargetScan▶ miR-132-3p/212-3p:AACAGUC▶ miR-23-3p:UCACAUU▶ miR-376c-3p:ACAUAGA▶ miR-411-5p.2:UAGUAGA


GTGAACTCA

GTGAACTCA  
Depth:4 (DOG)  
Ei-value:0.000, Pi-value:0.000  
Er-value:0.000, Pr-value:0.000  
No matches to eCLIP DataNo matches to TargetScan


CTGTTA

TTGCCTACTATGTGAACTCACTGTTA  
Depth:3 (COW)  
Ei-value:0.000, Pi-value:0.000  
Er-value:0.000, Pr-value:0.000  
No matches to eCLIP DataMATCHES To TargetScan▶ miR-132-3p/212-3p:AACAGUC▶ miR-23-3p:UCACAUU▶ miR-376c-3p:ACAUAGA▶ miR-411-5p.2:UAGUAGA

-15444--(12)--15457-

ATTTATCAT

ATTTATCAT  
Depth:3 (COW)  
Ei-value:0.000, Pi-value:0.000  
Er-value:0.000, Pr-value:0.000  
No matches to eCLIP DataNo matches to TargetScan

-15465--(45)--15511-

ATTTTGTGAACTCTAA

ATTTTGTGAACTCTAA  
Depth:3 (COW)  
Ei-value:0.000, Pi-value:0.000  
Er-value:0.000, Pr-value:0.000  
No matches to eCLIP DataNo matches to TargetScan

-15526--(57)--15584-

AAAATTG

AAAATTG  
Depth:3 (COW)  
Ei-value:0.000, Pi-value:0.000  
Er-value:0.000, Pr-value:0.000  
No matches to eCLIP DataNo matches to TargetScan

-15590--(122)--15713-

TGTGCCA

TGTGCCA  
Depth:4 (DOG)  
Ei-value:0.000, Pi-value:0.000  
Er-value:0.000, Pr-value:0.000  
No matches to eCLIP DataMATCHES To TargetScan▶ miR-183-5p.2:UGGCACU

-15719--(27)--15747-

AAGATAA

AAGATAA  
Depth:4 (DOG)  
Ei-value:0.000, Pi-value:0.000  
Er-value:0.000, Pr-value:0.000  
No matches to eCLIP DataNo matches to TargetScan

-15753--(23)--15777-

AGCAGAA

AGCAGAA  
Depth:3 (COW)  
Ei-value:0.000, Pi-value:0.000  
Er-value:0.000, Pr-value:0.000  
eCLIP MATCHES▶UTP3 (bg=3.66%)No matches to TargetScan

-15783--(11)--15795-

TAAAATCAATTT

TAAAATCAATTT  
Depth:3 (COW)  
Ei-value:0.000, Pi-value:0.000  
Er-value:0.000, Pr-value:0.000  
eCLIP MATCHES▶UTP3 (bg=3.66%)No matches to TargetScan

-15806--(8)--15815-

TAAACTG

TAAACTG  
Depth:4 (DOG)  
Ei-value:0.000, Pi-value:0.000  
Er-value:0.000, Pr-value:0.000  
eCLIP MATCHES▶HNRNPU (bg=5.92%)No matches to TargetScan

-15821--(20)--15842-

TCTGCTGAATGA

TCTGCTGAATGA  
Depth:3 (COW)  
Ei-value:0.000, Pi-value:0.000  
Er-value:0.000, Pr-value:0.000  
No matches to eCLIP DataMATCHES To TargetScan▶ miR-1298-5p:UCAUUCG

-15853--(1)--15855-

C

CATTGATTA  
Depth:3 (COW)  
Ei-value:0.000, Pi-value:0.000  
Er-value:0.000, Pr-value:0.000  
No matches to eCLIP DataNo matches to TargetScan


ATTGATTA

ATTGATTA  
Depth:4 (DOG)  
Ei-value:0.000, Pi-value:0.000  
Er-value:0.000, Pr-value:0.010  
No matches to eCLIP DataNo matches to TargetScan

-15863--(11)--15875-

AGAGATA

AGAGATA  
Depth:4 (DOG)  
Ei-value:0.000, Pi-value:0.000  
Er-value:0.000, Pr-value:0.000  
No matches to eCLIP DataNo matches to TargetScan

-15881--(33)--15915-

TGAACCT

TGAACCT  
Depth:3 (COW)  
Ei-value:0.000, Pi-value:0.000  
Er-value:0.000, Pr-value:0.010  
eCLIP MATCHES▶HNRNPU (bg=5.92%)No matches to TargetScan

-15921--(2)--15924-

AA

AACAGAGATCT  
Depth:3 (COW)  
Ei-value:0.000, Pi-value:0.000  
Er-value:0.000, Pr-value:0.000  
eCLIP MATCHES▶HNRNPA1 (bg=2.57%)▶HNRNPU (bg=5.92%)No matches to TargetScan


CAGAGATCT

CAGAGATCT  
Depth:4 (DOG)  
Ei-value:0.000, Pi-value:0.000  
Er-value:0.000, Pr-value:0.000  
eCLIP MATCHES▶HNRNPA1 (bg=2.57%)▶HNRNPU (bg=5.92%)No matches to TargetScan

-15934--(9)--15944-

TTTACAAAGC

TTTACAAAGC  
Depth:3 (COW)  
Ei-value:0.000, Pi-value:0.000  
Er-value:0.000, Pr-value:0.000  
eCLIP MATCHES▶HNRNPA1 (bg=2.57%)▶HNRNPU (bg=5.92%)No matches to TargetScan

-15953--(7)--15961-

TCTATACA

TCTATACA  
Depth:3 (COW)  
Ei-value:0.000, Pi-value:0.000  
Er-value:0.000, Pr-value:0.000  
eCLIP MATCHES▶HNRNPA1 (bg=2.57%)▶HNRNPU (bg=5.92%)No matches to TargetScan

-15968--(15)--15984-

TTGGCT

TTGGCT  
Depth:4 (DOG)  
Ei-value:0.000, Pi-value:0.000  
Er-value:0.000, Pr-value:0.000  
eCLIP MATCHES▶HNRNPA1 (bg=2.57%)No matches to TargetScan

-15989--(13)--16003-

TTACTTTCT

TTACTTTCT  
Depth:4 (DOG)  
Ei-value:0.000, Pi-value:0.000  
Er-value:0.000, Pr-value:0.010  
eCLIP MATCHES▶UTP3 (bg=3.66%)No matches to TargetScan

-16011--(22)--16034-

CTAGGATAT

CTAGGATAT  
Depth:3 (COW)  
Ei-value:0.000, Pi-value:0.000  
Er-value:0.000, Pr-value:0.000  
No matches to eCLIP DataNo matches to TargetScan

-16042--(1)--16044-

AAAATGA

AAAATGA  
Depth:3 (COW)  
Ei-value:0.000, Pi-value:0.000  
Er-value:0.000, Pr-value:0.000  
No matches to eCLIP DataNo matches to TargetScan

-16050--(52)--16103-

ATCCAGACCA

ATCCAGACCA  
Depth:3 (COW)  
Ei-value:0.000, Pi-value:0.000  
Er-value:0.000, Pr-value:0.000  
eCLIP MATCHES▶KHDRBS1 (bg=1.71%)No matches to TargetScan

-16112--(129)--16242-

AGATGGA

AGATGGA  
Depth:3 (COW)  
Ei-value:0.000, Pi-value:0.000  
Er-value:0.000, Pr-value:0.000  
No matches to eCLIP DataNo matches to TargetScan

-16248--(104)--16353-

GAGTAAAAA

GAGTAAAAA  
Depth:4 (DOG)  
Ei-value:0.000, Pi-value:0.000  
Er-value:0.000, Pr-value:0.000  
No matches to eCLIP DataNo matches to TargetScan

-16361--(28)--16390-

ATTTGAT

ATTTGAT  
Depth:4 (DOG)  
Ei-value:0.000, Pi-value:0.010  
Er-value:0.000, Pr-value:0.000  
No matches to eCLIP DataNo matches to TargetScan

-16396--(9)--16406-

ATC

ATCTTTTATGT  
Depth:3 (COW)  
Ei-value:0.000, Pi-value:0.000  
Er-value:0.000, Pr-value:0.000  
eCLIP MATCHES▶SAFB (bg=2.69%)No matches to TargetScan


TTTTATGT

TTTTATGT  
Depth:4 (DOG)  
Ei-value:0.000, Pi-value:0.000  
Er-value:0.000, Pr-value:0.000  
eCLIP MATCHES▶SAFB (bg=2.69%)No matches to TargetScan

-16416--(16)--16433-

GGTCCTGAG

GGTCCTGAG  
Depth:3 (COW)  
Ei-value:0.000, Pi-value:0.000  
Er-value:0.000, Pr-value:0.000  
eCLIP MATCHES▶SAFB (bg=2.69%)No matches to TargetScan

-16441--(149)--16591-

TTGCCTT

TTGCCTT  
Depth:3 (COW)  
Ei-value:0.000, Pi-value:0.000  
Er-value:0.000, Pr-value:0.000  
eCLIP MATCHES▶KHDRBS1 (bg=1.71%)▶UTP18 (bg=0.72%)MATCHES To TargetScan▶ miR-124-3p.1:AAGGCAC

-16597--(114)--16712-

AAGCCAG

AAGCCAG  
Depth:4 (DOG)  
Ei-value:0.000, Pi-value:0.000  
Er-value:0.000, Pr-value:0.000  
eCLIP MATCHES▶SAFB (bg=2.69%)MATCHES To TargetScan▶ miR-149-5p:CUGGCUC▶ miR-3064-5p:CUGGCUG

-16718--(7)--16726-

ATAAAAG

ATAAAAG  
Depth:4 (DOG)  
Ei-value:0.000, Pi-value:0.000  
Er-value:0.000, Pr-value:0.000  
No matches to eCLIP DataNo matches to TargetScan

-16732--(15)--16748-

CTTTAATTC

CTTTAATTC  
Depth:3 (COW)  
Ei-value:0.000, Pi-value:0.000  
Er-value:0.000, Pr-value:0.000  
No matches to eCLIP DataNo matches to TargetScan

-16756--(18)--16775-

TTTTATTA

TTTTATTA  
Depth:4 (DOG)  
Ei-value:0.000, Pi-value:0.010  
Er-value:0.000, Pr-value:0.000  
No matches to eCLIP DataNo matches to TargetScan

-16782--(2)--16785-

G

GTTAAATGG  
Depth:3 (COW)  
Ei-value:0.000, Pi-value:0.000  
Er-value:0.000, Pr-value:0.000  
No matches to eCLIP DataNo matches to TargetScan


TTAAATGG

TTAAATGG  
Depth:4 (DOG)  
Ei-value:0.000, Pi-value:0.000  
Er-value:0.000, Pr-value:0.000  
No matches to eCLIP DataNo matches to TargetScan

-16793--(136)--16930-

CTGTTCTTAAGT

CTGTTCTTAAGT  
Depth:3 (COW)  
Ei-value:0.000, Pi-value:0.000  
Er-value:0.000, Pr-value:0.000  
eCLIP MATCHES▶KHDRBS1 (bg=1.71%)No matches to TargetScan

-16941--(59)--17001-

GAACAAATT

GAACAAATT  
Depth:3 (COW)  
Ei-value:0.000, Pi-value:0.000  
Er-value:0.000, Pr-value:0.000  
No matches to eCLIP DataMATCHES To TargetScan▶ miR-375:UUGUUCG

-17009--(20)--17030-

TTAGTTG

TTAGTTG  
Depth:3 (COW)  
Ei-value:0.000, Pi-value:0.000  
Er-value:0.000, Pr-value:0.010  
No matches to eCLIP DataNo matches to TargetScan

-17036--(5)--17042-

AAACTTCATTGA

AAACTTCATTGA  
Depth:3 (COW)  
Ei-value:0.000, Pi-value:0.000  
Er-value:0.000, Pr-value:0.000  
eCLIP MATCHES▶HNRNPA1 (bg=2.57%)No matches to TargetScan

-17053--(264)--17318-

ACAGAAAACAAAA

ACAGAAAACAAAA  
Depth:4 (DOG)  
Ei-value:0.000, Pi-value:0.000  
Er-value:0.000, Pr-value:0.000  
No matches to eCLIP DataNo matches to TargetScan

-17330--(36)--17367-

CTTGGAAA

CTTGGAAA  
Depth:3 (COW)  
Ei-value:0.000, Pi-value:0.000  
Er-value:0.000, Pr-value:0.000  
No matches to eCLIP DataNo matches to TargetScan

-17374--(16)--17391-

AGGTTA

AGGTTA  
Depth:4 (DOG)  
Ei-value:0.000, Pi-value:0.000  
Er-value:0.000, Pr-value:0.000  
No matches to eCLIP DataNo matches to TargetScan

-17396--(25)--17422-

TTCATTCT

TTCATTCT  
Depth:4 (DOG)  
Ei-value:0.000, Pi-value:0.000  
Er-value:0.000, Pr-value:0.000  
No matches to eCLIP DataNo matches to TargetScan

-17429--(50)--17480-

AGAGACA

AGAGACA  
Depth:4 (DOG)  
Ei-value:0.000, Pi-value:0.000  
Er-value:0.000, Pr-value:0.000  
No matches to eCLIP DataNo matches to TargetScan


TG

AGAGACATG  
Depth:3 (COW)  
Ei-value:0.000, Pi-value:0.000  
Er-value:0.000, Pr-value:0.000  
No matches to eCLIP DataNo matches to TargetScan

-17488--(41)--17530-

CCTTTTGG

CCTTTTGG  
Depth:4 (DOG)  
Ei-value:0.000, Pi-value:0.000  
Er-value:0.000, Pr-value:0.000  
No matches to eCLIP DataNo matches to TargetScan


C

CCTTTTGGC  
Depth:3 (COW)  
Ei-value:0.000, Pi-value:0.000  
Er-value:0.000, Pr-value:0.000  
No matches to eCLIP DataNo matches to TargetScan

-17538--(40)--17579-

ATTGTGT

ATTGTGT  
Depth:3 (COW)  
Ei-value:0.000, Pi-value:0.000  
Er-value:0.000, Pr-value:0.010  
No matches to eCLIP DataNo matches to TargetScan

-17585--(3)--17589-

TTAATTC

TTAATTC  
Depth:4 (DOG)  
Ei-value:0.000, Pi-value:0.000  
Er-value:0.000, Pr-value:0.000  
No matches to eCLIP DataNo matches to TargetScan

-17595--(21)--17617-

ACTCTGGCCACTAC

ACTCTGGCCACTAC  
Depth:4 (DOG)  
Ei-value:0.000, Pi-value:0.000  
Er-value:0.000, Pr-value:0.000  
No matches to eCLIP DataMATCHES To TargetScan▶ miR-142-3p.1:GUAGUGU

-17630--(1)--17632-

ATAAGC

ATAAGC  
Depth:5 (RABBIT)  
Ei-value:0.000, Pi-value:0.010  
Er-value:0.000, Pr-value:0.000  
No matches to eCLIP DataNo matches to TargetScan


AGG

ATAAGCAGG  
Depth:4 (DOG)  
Ei-value:0.000, Pi-value:0.000  
Er-value:0.000, Pr-value:0.000  
No matches to eCLIP DataNo matches to TargetScan

-17640--(22)--17663-

TGCTCCTT

TGCTCCTT  
Depth:3 (COW)  
Ei-value:0.000, Pi-value:0.000  
Er-value:0.000, Pr-value:0.000  
No matches to eCLIP DataMATCHES To TargetScan▶ miR-28-5p/708-5p:AGGAGCU

-17670--(29)--17700-

ACTTCA

ACTTCA  
Depth:3 (COW)  
Ei-value:0.000, Pi-value:0.000  
Er-value:0.000, Pr-value:0.000  
No matches to eCLIP DataNo matches to TargetScan

-17705--(0)--17706-

TTTTCCTA

TTTTCCTA  
Depth:3 (COW)  
Ei-value:0.000, Pi-value:0.000  
Er-value:0.000, Pr-value:0.000  
No matches to eCLIP DataNo matches to TargetScan

-17713--(11)--17725-

AT

ATGAAAAATG  
Depth:3 (COW)  
Ei-value:0.000, Pi-value:0.000  
Er-value:0.000, Pr-value:0.000  
No matches to eCLIP DataNo matches to TargetScan


GAAAAATG

GAAAAATG  
Depth:4 (DOG)  
Ei-value:0.000, Pi-value:0.000  
Er-value:0.000, Pr-value:0.000  
No matches to eCLIP DataNo matches to TargetScan

-17734--(54)--17789-

AGTCTCA

AGTCTCA  
Depth:4 (DOG)  
Ei-value:0.000, Pi-value:0.000  
Er-value:0.000, Pr-value:0.000  
eCLIP MATCHES▶ZC3H11A (bg=6.55%)No matches to TargetScan


TTGGTACCA

AGTCTCATTGGTACCA  
Depth:3 (COW)  
Ei-value:0.000, Pi-value:0.000  
Er-value:0.000, Pr-value:0.000  
eCLIP MATCHES▶NOLC1 (bg=9.43%)▶ZC3H11A (bg=6.55%)No matches to TargetScan

-17804--(33)--17838-

TGGTTTTGAA

TGGTTTTGAA  
Depth:4 (DOG)  
Ei-value:0.000, Pi-value:0.000  
Er-value:0.000, Pr-value:0.000  
No matches to eCLIP DataNo matches to TargetScan

-17847--(88)--17936-

ACAATCC

ACAATCC  
Depth:3 (COW)  
Ei-value:0.000, Pi-value:0.000  
Er-value:0.000, Pr-value:0.000  
No matches to eCLIP DataMATCHES To TargetScan▶ miR-219-5p:GAUUGUC

-17942--(20)--17963-

TGGAGATG

TGGAGATG  
Depth:3 (COW)  
Ei-value:0.000, Pi-value:0.000  
Er-value:0.000, Pr-value:0.000  
No matches to eCLIP DataNo matches to TargetScan

-17970--(22)--17993-

AGCTTCTC

AGCTTCTC  
Depth:3 (COW)  
Ei-value:0.000, Pi-value:0.000  
Er-value:0.000, Pr-value:0.000  
No matches to eCLIP DataNo matches to TargetScan

-18000--(10)--18011-

TTAGAAAT

TTAGAAAT  
Depth:4 (DOG)  
Ei-value:0.000, Pi-value:0.000  
Er-value:0.000, Pr-value:0.000  
eCLIP MATCHES▶WDR3 (bg=0.25%)No matches to TargetScan

-18018--(14)--18033-

CATCAAA

CATCAAA  
Depth:4 (DOG)  
Ei-value:0.000, Pi-value:0.000  
Er-value:0.000, Pr-value:0.000  
eCLIP MATCHES▶ZC3H11A (bg=6.55%)No matches to TargetScan

-18039--(121)--18161-

GGAAAAA

GGAAAAA  
Depth:3 (COW)  
Ei-value:0.000, Pi-value:0.000  
Er-value:0.000, Pr-value:0.000  
No matches to eCLIP DataNo matches to TargetScan

-18167--(33)--18201-

TGGGCTTTG

TGGGCTTTG  
Depth:3 (COW)  
Ei-value:0.000, Pi-value:0.000  
Er-value:0.000, Pr-value:0.000  
eCLIP MATCHES▶NOLC1 (bg=9.43%)▶PPIL4 (bg=0.52%)MATCHES To TargetScan▶ miR-330-3p:CAAAGCA

-18209--(10)--18220-

TTTTTAAATCACTCA

TTTTTAAATCACTCA  
Depth:4 (DOG)  
Ei-value:0.000, Pi-value:0.000  
Er-value:0.000, Pr-value:0.000  
eCLIP MATCHES▶ILF3 (bg=3.0%)▶NOLC1 (bg=9.43%)▶PPIL4 (bg=0.52%)No matches to TargetScan

-18234--(1)--18236-

AGAGGGTGGGA

AGAGGGTGGGA  
Depth:4 (DOG)  
Ei-value:0.000, Pi-value:0.000  
Er-value:0.000, Pr-value:0.000  
eCLIP MATCHES▶ILF3 (bg=3.0%)▶ZC3H11A (bg=6.55%)No matches to TargetScan

-18246--(1)--18248-

AGGAGGAAGAGTGAA

AGGAGGAAGAGTGAA  
Depth:4 (DOG)  
Ei-value:0.000, Pi-value:0.000  
Er-value:0.000, Pr-value:0.000  
eCLIP MATCHES▶ILF3 (bg=3.0%)▶ZC3H11A (bg=6.55%)MATCHES To TargetScan▶ miR-670-3p:UUCCUCA

-18262--(1)--18264-

G

GAAAAGGTCA  
Depth:4 (DOG)  
Ei-value:0.000, Pi-value:0.000  
Er-value:0.000, Pr-value:0.000  
eCLIP MATCHES▶ILF3 (bg=3.0%)▶SF3B1 (bg=2.48%)▶ZC3H11A (bg=6.55%)MATCHES To TargetScan▶ miR-192-5p/215-5p:UGACCUA


AAAAGGT

AAAAGGT  
Depth:6 (MOUSE)  
Ei-value:0.000, Pi-value:0.000  
Er-value:0.000, Pr-value:0.000  
eCLIP MATCHES▶ILF3 (bg=3.0%)▶SF3B1 (bg=2.48%)▶ZC3H11A (bg=6.55%)No matches to TargetScan


CA

GAAAAGGTCA  
Depth:4 (DOG)  
Ei-value:0.000, Pi-value:0.000  
Er-value:0.000, Pr-value:0.000  
eCLIP MATCHES▶ILF3 (bg=3.0%)▶SF3B1 (bg=2.48%)▶ZC3H11A (bg=6.55%)MATCHES To TargetScan▶ miR-192-5p/215-5p:UGACCUA

-18273--(35)--18309-

TTGGTCTTAA

TTGGTCTTAA  
Depth:3 (COW)  
Ei-value:0.000, Pi-value:0.000  
Er-value:0.000, Pr-value:0.000  
eCLIP MATCHES▶ILF3 (bg=3.0%)▶ZC3H11A (bg=6.55%)MATCHES To TargetScan▶ miR-208-3p:UAAGACG▶ miR-499a-5p:UAAGACU

-18318--(65)--18384-

AGATGAGGACAAA

AGATGAGGACAAA  
Depth:3 (COW)  
Ei-value:0.000, Pi-value:0.000  
Er-value:0.000, Pr-value:0.000  
eCLIP MATCHES▶HNRNPA1 (bg=2.57%)No matches to TargetScan

-18396--(1)--18398-

TCCTTTGT

TCCTTTGT  
Depth:3 (COW)  
Ei-value:0.000, Pi-value:0.000  
Er-value:0.000, Pr-value:0.000  
eCLIP MATCHES▶HNRNPA1 (bg=2.57%)No matches to TargetScan

-18405--(131)--18537-

TAAAGC

TAAAGC  
Depth:3 (COW)  
Ei-value:0.000, Pi-value:0.000  
Er-value:0.000, Pr-value:0.000  
eCLIP MATCHES▶NOLC1 (bg=9.43%)No matches to TargetScan

-18542--(49)--18592-

TTCATACA

TTCATACA  
Depth:3 (COW)  
Ei-value:0.000, Pi-value:0.000  
Er-value:0.000, Pr-value:0.000  
No matches to eCLIP DataNo matches to TargetScan

-18599--(0)--18600-

TTCAAAGCATC

TTCAAAGCATC  
Depth:3 (COW)  
Ei-value:0.000, Pi-value:0.000  
Er-value:0.000, Pr-value:0.000  
No matches to eCLIP DataNo matches to TargetScan

-18610--(43)--18654-

AGAAGGAAATAGA

AGAAGGAAATAGA  
Depth:3 (COW)  
Ei-value:0.000, Pi-value:0.000  
Er-value:0.000, Pr-value:0.000  
eCLIP MATCHES▶ZC3H11A (bg=6.55%)No matches to TargetScan

-18666--(21)--18688-

TGGAGGGAGC

TGGAGGGAGC  
Depth:3 (COW)  
Ei-value:0.000, Pi-value:0.000  
Er-value:0.000, Pr-value:0.000  
eCLIP MATCHES▶FTO (bg=0.32%)▶LARP4 (bg=4.72%)▶LSM11 (bg=2.28%)▶NOLC1 (bg=9.43%)▶XRCC6 (bg=2.91%)▶ZC3H11A (bg=6.55%)No matches to TargetScan

-18697--(79)--18777-

TAATGTTT

TAATGTTT  
Depth:4 (DOG)  
Ei-value:0.000, Pi-value:0.000  
Er-value:0.000, Pr-value:0.000  
eCLIP MATCHES▶CPEB4 (bg=1.89%)▶KHDRBS1 (bg=1.71%)▶LARP4 (bg=4.72%)▶LSM11 (bg=2.28%)▶NOLC1 (bg=9.43%)▶RBFOX2 (bg=4.63%)▶SAFB (bg=2.69%)▶SAFB2 (bg=0.8%)▶WDR43 (bg=3.37%)▶ZC3H11A (bg=6.55%)MATCHES To TargetScan▶ miR-323-3p:ACAUUAC▶ miR-543:AACAUUC

-18784--(20)--18805-

AGCTGGA

AGCTGGA  
Depth:4 (DOG)  
Ei-value:0.000, Pi-value:0.000  
Er-value:0.000, Pr-value:0.000  
eCLIP MATCHES▶CPEB4 (bg=1.89%)▶KHDRBS1 (bg=1.71%)▶LSM11 (bg=2.28%)▶NOLC1 (bg=9.43%)▶RBFOX2 (bg=4.63%)▶SAFB (bg=2.69%)▶SAFB2 (bg=0.8%)▶SF3B1 (bg=2.48%)▶TRA2A (bg=4.8%)▶WDR43 (bg=3.37%)▶ZC3H11A (bg=6.55%)No matches to TargetScan

-18811--(40)--18852-

ATTATTGGAAA

ATTATTGGAAA  
Depth:4 (DOG)  
Ei-value:0.000, Pi-value:0.000  
Er-value:0.000, Pr-value:0.000  
eCLIP MATCHES▶FASTKD2 (bg=1.99%)▶FUS (bg=2.21%)▶LARP4 (bg=4.72%)▶NOLC1 (bg=9.43%)▶RBFOX2 (bg=4.63%)▶SAFB (bg=2.69%)▶SAFB2 (bg=0.8%)▶TRA2A (bg=4.8%)▶WDR43 (bg=3.37%)▶ZC3H11A (bg=6.55%)No matches to TargetScan

-18862--(9)--18872-

AGAAAGTAAC

AGAAAGTAAC  
Depth:4 (DOG)  
Ei-value:0.000, Pi-value:0.000  
Er-value:0.000, Pr-value:0.000  
eCLIP MATCHES▶FASTKD2 (bg=1.99%)▶FUS (bg=2.21%)▶LARP4 (bg=4.72%)▶NIPBL (bg=5.39%)▶NOLC1 (bg=9.43%)▶RBFOX2 (bg=4.63%)▶SAFB (bg=2.69%)▶SAFB2 (bg=0.8%)▶TRA2A (bg=4.8%)▶uchl5 (bg=11.16%)▶WDR43 (bg=3.37%)▶ZC3H11A (bg=6.55%)▶ZNF800 (bg=1.92%)No matches to TargetScan

-18881--(12)--18894-

TTTCACAGTTTCTGGCATC

TTTCACAGTTTCTGGCATC  
Depth:4 (DOG)  
Ei-value:0.000, Pi-value:0.000  
Er-value:0.000, Pr-value:0.000  
eCLIP MATCHES▶FASTKD2 (bg=1.99%)▶FUS (bg=2.21%)▶LARP4 (bg=4.72%)▶NIPBL (bg=5.39%)▶NOLC1 (bg=9.43%)▶RBFOX2 (bg=4.63%)▶SAFB (bg=2.69%)▶SAFB2 (bg=0.8%)▶uchl5 (bg=11.16%)▶WDR43 (bg=3.37%)▶ZC3H11A (bg=6.55%)▶ZNF800 (bg=1.92%)No matches to TargetScan

-18912--(5)--18918-

CA

CACTACTGAT  
Depth:3 (COW)  
Ei-value:0.000, Pi-value:0.000  
Er-value:0.000, Pr-value:0.000  
eCLIP MATCHES▶FASTKD2 (bg=1.99%)▶FUS (bg=2.21%)▶LARP4 (bg=4.72%)▶NIPBL (bg=5.39%)▶NOLC1 (bg=9.43%)▶RBFOX2 (bg=4.63%)▶SAFB (bg=2.69%)▶SAFB2 (bg=0.8%)▶uchl5 (bg=11.16%)▶ZC3H11A (bg=6.55%)▶ZNF800 (bg=1.92%)MATCHES To TargetScan▶ miR-142-3p.1:GUAGUGU▶ miR-199-3p:CAGUAGU


CTACTGAT

CTACTGAT  
Depth:4 (DOG)  
Ei-value:0.000, Pi-value:0.000  
Er-value:0.000, Pr-value:0.000  
eCLIP MATCHES▶FASTKD2 (bg=1.99%)▶FUS (bg=2.21%)▶LARP4 (bg=4.72%)▶NIPBL (bg=5.39%)▶NOLC1 (bg=9.43%)▶RBFOX2 (bg=4.63%)▶SAFB (bg=2.69%)▶SAFB2 (bg=0.8%)▶uchl5 (bg=11.16%)▶ZC3H11A (bg=6.55%)▶ZNF800 (bg=1.92%)MATCHES To TargetScan▶ miR-199-3p:CAGUAGU

-18927--(1)--18929-

AAACAAGAATAA

AAACAAGAATAA  
Depth:3 (COW)  
Ei-value:0.000, Pi-value:0.000  
Er-value:0.000, Pr-value:0.000  
eCLIP MATCHES▶FASTKD2 (bg=1.99%)▶FUS (bg=2.21%)▶LARP4 (bg=4.72%)▶NIPBL (bg=5.39%)▶NOLC1 (bg=9.43%)▶RBFOX2 (bg=4.63%)▶SAFB2 (bg=0.8%)▶uchl5 (bg=11.16%)▶ZC3H11A (bg=6.55%)MATCHES To TargetScan▶ miR-544a-5p:CUUGUUA

-18940--(1)--18942-

AGAACAT

AGAACAT  
Depth:4 (DOG)  
Ei-value:0.000, Pi-value:0.000  
Er-value:0.000, Pr-value:0.000  
eCLIP MATCHES▶FASTKD2 (bg=1.99%)▶FUS (bg=2.21%)▶LARP4 (bg=4.72%)▶NIPBL (bg=5.39%)▶NOLC1 (bg=9.43%)▶RBFOX2 (bg=4.63%)▶SAFB2 (bg=0.8%)▶uchl5 (bg=11.16%)▶ZNF622 (bg=6.58%)No matches to TargetScan

-18948--(7)--18956-

TCATCTG

TCATCTG  
Depth:4 (DOG)  
Ei-value:0.000, Pi-value:0.010  
Er-value:0.000, Pr-value:0.000  
eCLIP MATCHES▶FUS (bg=2.21%)▶LARP4 (bg=4.72%)▶NOLC1 (bg=9.43%)▶RBFOX2 (bg=4.63%)▶RPS3 (bg=0.76%)▶uchl5 (bg=11.16%)▶ZNF622 (bg=6.58%)No matches to TargetScan

-18962--(9)--18972-

CATAAATGAA

CATAAATGAA  
Depth:4 (DOG)  
Ei-value:0.000, Pi-value:0.000  
Er-value:0.000, Pr-value:0.000  
eCLIP MATCHES▶FUS (bg=2.21%)▶NOLC1 (bg=9.43%)▶RPS3 (bg=0.76%)▶uchl5 (bg=11.16%)▶ZNF622 (bg=6.58%)No matches to TargetScan


GTTGTGA

CATAAATGAAGTTGTGA  
Depth:3 (COW)  
Ei-value:0.000, Pi-value:0.000  
Er-value:0.000, Pr-value:0.000  
eCLIP MATCHES▶FUS (bg=2.21%)▶NOLC1 (bg=9.43%)▶RPS3 (bg=0.76%)▶uchl5 (bg=11.16%)▶ZNF622 (bg=6.58%)No matches to TargetScan

-18988--(109)--19098-

ACTTG

ACTTGTGAACTGATGTGAAA  
Depth:3 (COW)  
Ei-value:0.000, Pi-value:0.000  
Er-value:0.000, Pr-value:0.000  
eCLIP MATCHES▶FUS (bg=2.21%)▶NOLC1 (bg=9.43%)▶RBFOX2 (bg=4.63%)▶TRA2A (bg=4.8%)MATCHES To TargetScan▶ miR-23-3p:UCACAUU


TGAACTGATGTGAAA

TGAACTGATGTGAAA  
Depth:4 (DOG)  
Ei-value:0.000, Pi-value:0.000  
Er-value:0.000, Pr-value:0.000  
eCLIP MATCHES▶FUS (bg=2.21%)▶NOLC1 (bg=9.43%)▶RBFOX2 (bg=4.63%)▶TRA2A (bg=4.8%)MATCHES To TargetScan▶ miR-23-3p:UCACAUU

-19117--(46)--19164-

TTGTTCA

TTGTTCA  
Depth:3 (COW)  
Ei-value:0.000, Pi-value:0.000  
Er-value:0.000, Pr-value:0.000  
eCLIP MATCHES▶AARS (bg=2.18%)▶AATF (bg=0.64%)▶CPEB4 (bg=1.89%)▶DROSHA (bg=2.49%)▶FASTKD2 (bg=1.99%)▶FUS (bg=2.21%)▶GRWD1 (bg=5.13%)▶LARP4 (bg=4.72%)▶LSM11 (bg=2.28%)▶NOLC1 (bg=9.43%)▶RBFOX2 (bg=4.63%)▶TRA2A (bg=4.8%)▶uchl5 (bg=11.16%)▶UTP3 (bg=3.66%)▶WDR43 (bg=3.37%)▶XRCC6 (bg=2.91%)▶ZC3H11A (bg=6.55%)▶ZNF622 (bg=6.58%)No matches to TargetScan

-19170--(9)--19180-

ACCACCA

ACCACCA  
Depth:3 (COW)  
Ei-value:0.000, Pi-value:0.000  
Er-value:0.000, Pr-value:0.000  
eCLIP MATCHES▶AARS (bg=2.18%)▶AATF (bg=0.64%)▶AKAP8L (bg=2.19%)▶CPEB4 (bg=1.89%)▶DROSHA (bg=2.49%)▶FASTKD2 (bg=1.99%)▶FUS (bg=2.21%)▶GRWD1 (bg=5.13%)▶KHDRBS1 (bg=1.71%)▶LARP4 (bg=4.72%)▶LSM11 (bg=2.28%)▶NIPBL (bg=5.39%)▶NOLC1 (bg=9.43%)▶RBFOX2 (bg=4.63%)▶RPS3 (bg=0.76%)▶TRA2A (bg=4.8%)▶uchl5 (bg=11.16%)▶UTP3 (bg=3.66%)▶WDR43 (bg=3.37%)▶XRCC6 (bg=2.91%)▶ZC3H11A (bg=6.55%)▶ZNF622 (bg=6.58%)No matches to TargetScan

-19186--(65)--19252-

AAATAAAA

AAATAAAA  
Depth:4 (DOG)  
Ei-value:0.000, Pi-value:0.000  
Er-value:0.000, Pr-value:0.000  
eCLIP MATCHES▶WDR43 (bg=3.37%)▶ZC3H11A (bg=6.55%)No matches to TargetScan

-19259  
  
>PIG  
       965-

AGGCAAGA

AGGCAAGA  
Depth:3 (COW)  
Ei-value:0.000, Pi-value:0.000  
Er-value:0.000, Pr-value:0.000  
No matches to TargetScan

-972--(393)--1366-

AAACATG

AAACATG  
Depth:4 (DOG)  
Ei-value:0.000, Pi-value:0.000  
Er-value:0.000, Pr-value:0.000  
No matches to TargetScan

-1372--(671)--2044-

CAACAG

CAACAG  
Depth:3 (COW)  
Ei-value:0.000, Pi-value:0.000  
Er-value:0.000, Pr-value:0.000  
No matches to TargetScan

-2049--(481)--2531-

TTCCCATC

TTCCCATC  
Depth:4 (DOG)  
Ei-value:0.000, Pi-value:0.000  
Er-value:0.000, Pr-value:0.000  
No matches to TargetScan

-2538--(647)--3186-

CTCTGT

CTCTGT  
Depth:3 (COW)  
Ei-value:0.000, Pi-value:0.000  
Er-value:0.000, Pr-value:0.000  
No matches to TargetScan

-3191--(4489)--7681-

C

CTGTTAGTCT  
Depth:4 (DOG)  
Ei-value:0.000, Pi-value:0.000  
Er-value:0.000, Pr-value:0.000  
No matches to TargetScan


TGTTAGTC

TGTTAGTC  
Depth:5 (RABBIT)  
Ei-value:0.000, Pi-value:0.000  
Er-value:0.000, Pr-value:0.000  
No matches to TargetScan


T

CTGTTAGTCT  
Depth:4 (DOG)  
Ei-value:0.000, Pi-value:0.000  
Er-value:0.000, Pr-value:0.000  
No matches to TargetScan

-7690--(961)--8652-

TCATCC

TCATCC  
Depth:4 (DOG)  
Ei-value:0.000, Pi-value:0.020  
Er-value:0.000, Pr-value:0.000  
No matches to TargetScan

-8657--(602)--9260-

GGG

GGGTACTTGGGACTGTTAAT  
Depth:3 (COW)  
Ei-value:0.000, Pi-value:0.000  
Er-value:0.000, Pr-value:0.000  
MATCHES To TargetScan▶ miR-132-3p/212-3p:AACAGUC▶ miR-455-3p.1:CAGUCCA


TACTTGGGACTGTTAAT

TACTTGGGACTGTTAAT  
Depth:4 (DOG)  
Ei-value:0.000, Pi-value:0.000  
Er-value:0.000, Pr-value:0.000  
MATCHES To TargetScan▶ miR-132-3p/212-3p:AACAGUC▶ miR-455-3p.1:CAGUCCA

-9279--(466)--9746-

ACTG

ACTGTTAATGTGCT  
Depth:4 (DOG)  
Ei-value:0.000, Pi-value:0.000  
Er-value:0.000, Pr-value:0.000  
MATCHES To TargetScan▶ miR-132-3p/212-3p:AACAGUC▶ miR-323-3p:ACAUUAC


TTAATGTGCT

TTAATGTGCT  
Depth:5 (RABBIT)  
Ei-value:0.000, Pi-value:0.000  
Er-value:0.000, Pr-value:0.000  
MATCHES To TargetScan▶ miR-323-3p:ACAUUAC

-9759--(78)--9838-

CTTGGGACTC

CTTGGGACTC  
Depth:3 (COW)  
Ei-value:0.000, Pi-value:0.000  
Er-value:0.000, Pr-value:0.000  
No matches to TargetScan

-9847--(3665)--13513-

AATGTGCAT

AATGTGCAT  
Depth:6 (MOUSE)  
Ei-value:0.000, Pi-value:0.000  
Er-value:0.000, Pr-value:0.000  
MATCHES To TargetScan▶ miR-501-3p/502-3p:AUGCACC

-13521--(16)--13538-

CTAATA

CTAATA  
Depth:3 (COW)  
Ei-value:0.000, Pi-value:0.000  
Er-value:0.000, Pr-value:0.000  
No matches to TargetScan

-13543--(132)--13676-

TGCTTCT

TGCTTCT  
Depth:3 (COW)  
Ei-value:0.000, Pi-value:0.000  
Er-value:0.000, Pr-value:0.010  
No matches to TargetScan

-13682--(45)--13728-

TATGTTAGA

TATGTTAGA  
Depth:4 (DOG)  
Ei-value:0.000, Pi-value:0.000  
Er-value:0.000, Pr-value:0.000  
No matches to TargetScan

-13736--(66)--13803-

TCTTGG

TCTTGGACTGTTAATGT  
Depth:3 (COW)  
Ei-value:0.000, Pi-value:0.000  
Er-value:0.000, Pr-value:0.000  
MATCHES To TargetScan▶ miR-132-3p/212-3p:AACAGUC▶ miR-323-3p:ACAUUAC▶ miR-455-3p.1:CAGUCCA


ACTGTTAATGT

ACTGTTAATGT  
Depth:4 (DOG)  
Ei-value:0.000, Pi-value:0.000  
Er-value:0.000, Pr-value:0.000  
MATCHES To TargetScan▶ miR-132-3p/212-3p:AACAGUC▶ miR-323-3p:ACAUUAC

-13819--(10)--13830-

ATTTGCT

ATTTGCT  
Depth:4 (DOG)  
Ei-value:0.000, Pi-value:0.000  
Er-value:0.000, Pr-value:0.000  
No matches to TargetScan

-13836--(12)--13849-

GTAAGGA

GTAAGGA  
Depth:5 (RABBIT)  
Ei-value:0.000, Pi-value:0.000  
Er-value:0.000, Pr-value:0.000  
No matches to TargetScan


CCC

GTAAGGACCC  
Depth:3 (COW)  
Ei-value:0.000, Pi-value:0.000  
Er-value:0.000, Pr-value:0.000  
No matches to TargetScan

-13858--(75)--13934-

ATCTTAG

ATCTTAG  
Depth:3 (COW)  
Ei-value:0.000, Pi-value:0.000  
Er-value:0.000, Pr-value:0.000  
No matches to TargetScan

-13940--(8)--13949-

TACACATT

TACACATT  
Depth:3 (COW)  
Ei-value:0.000, Pi-value:0.000  
Er-value:0.000, Pr-value:0.000  
No matches to TargetScan

-13956--(40)--13997-

ACTTAT

ACTTAT  
Depth:5 (RABBIT)  
Ei-value:0.000, Pi-value:0.000  
Er-value:0.000, Pr-value:0.000  
No matches to TargetScan

-14002--(54)--14057-

TGTAATT

TGTAATT  
Depth:3 (COW)  
Ei-value:0.000, Pi-value:0.000  
Er-value:0.000, Pr-value:0.000  
No matches to TargetScan

-14063--(10)--14074-

ATGGTC

ATGGTC  
Depth:3 (COW)  
Ei-value:0.000, Pi-value:0.020  
Er-value:0.000, Pr-value:0.000  
No matches to TargetScan

-14079--(50)--14130-

ATGGGGTACT

ATGGGGTACT  
Depth:3 (COW)  
Ei-value:0.000, Pi-value:0.000  
Er-value:0.000, Pr-value:0.000  
No matches to TargetScan

-14139--(3)--14143-

CAC

CACTTAAGGCCCCTTTCTCAA  
Depth:3 (COW)  
Ei-value:0.000, Pi-value:0.000  
Er-value:0.000, Pr-value:0.000  
No matches to TargetScan


TTAAGGCC

TTAAGGCC  
Depth:6 (MOUSE)  
Ei-value:0.000, Pi-value:0.000  
Er-value:0.000, Pr-value:0.000  
No matches to TargetScan


CCTTT

TTAAGGCCCCTTT  
Depth:5 (RABBIT)  
Ei-value:0.000, Pi-value:0.000  
Er-value:0.000, Pr-value:0.000  
No matches to TargetScan


CTCAA

TTAAGGCCCCTTTCTCAA  
Depth:4 (DOG)  
Ei-value:0.000, Pi-value:0.000  
Er-value:0.000, Pr-value:0.000  
No matches to TargetScan

-14163--(13)--14177-

TAATGACAATTACAT

TAATGACAATTACAT  
Depth:3 (COW)  
Ei-value:0.000, Pi-value:0.000  
Er-value:0.000, Pr-value:0.000  
MATCHES To TargetScan▶ miR-411-3p:AUGUAAC

-14191--(433)--14625-

CTTATATTT

CTTATATTT  
Depth:3 (COW)  
Ei-value:0.000, Pi-value:0.000  
Er-value:0.000, Pr-value:0.000  
MATCHES To TargetScan▶ miR-410-3p:AUAUAAC

-14633--(19)--14653-

TTTTAATTGACCA

TTTTAATTGACCA  
Depth:3 (COW)  
Ei-value:0.000, Pi-value:0.000  
Er-value:0.000, Pr-value:0.000  
No matches to TargetScan

-14665--(15)--14681-

ACATTAAT

ACATTAAT  
Depth:3 (COW)  
Ei-value:0.000, Pi-value:0.000  
Er-value:0.000, Pr-value:0.000  
No matches to TargetScan

-14688--(102)--14791-

CATAATTGCA

CATAATTGCA  
Depth:3 (COW)  
Ei-value:0.000, Pi-value:0.000  
Er-value:0.000, Pr-value:0.000  
No matches to TargetScan

-14800--(20)--14821-

CTAGACAAGGA

CTAGACAAGGA  
Depth:3 (COW)  
Ei-value:0.000, Pi-value:0.000  
Er-value:0.000, Pr-value:0.000  
No matches to TargetScan

-14831--(76)--14908-

ACAGTTAATGTG

ACAGTTAATGTG  
Depth:4 (DOG)  
Ei-value:0.000, Pi-value:0.000  
Er-value:0.000, Pr-value:0.000  
MATCHES To TargetScan▶ miR-323-3p:ACAUUAC

-14919--(69)--14989-

ATACTGTTT

ATACTGTTT  
Depth:3 (COW)  
Ei-value:0.000, Pi-value:0.000  
Er-value:0.000, Pr-value:0.000  
MATCHES To TargetScan▶ miR-101-3p.1:ACAGUAC▶ miR-132-3p/212-3p:AACAGUC▶ miR-144-3p:ACAGUAU

-14997--(49)--15047-

TTGTCTT

TTGTCTT  
Depth:3 (COW)  
Ei-value:0.000, Pi-value:0.000  
Er-value:0.000, Pr-value:0.010  
No matches to TargetScan

-15053--(44)--15098-

CTCAGCTCTTGG

CTCAGCTCTTGG  
Depth:5 (RABBIT)  
Ei-value:0.000, Pi-value:0.000  
Er-value:0.000, Pr-value:0.000  
MATCHES To TargetScan▶ miR-335-5p:CAAGAGC


ACA

CTCAGCTCTTGGACA  
Depth:4 (DOG)  
Ei-value:0.000, Pi-value:0.000  
Er-value:0.000, Pr-value:0.000  
MATCHES To TargetScan▶ miR-335-5p:CAAGAGC


ATTAATA

CTCAGCTCTTGGACAATTAATA  
Depth:3 (COW)  
Ei-value:0.000, Pi-value:0.000  
Er-value:0.000, Pr-value:0.000  
MATCHES To TargetScan▶ miR-335-5p:CAAGAGC

-15119--(26)--15146-

GATCAT

GATCAT  
Depth:3 (COW)  
Ei-value:0.000, Pi-value:0.000  
Er-value:0.000, Pr-value:0.000  
No matches to TargetScan

-15151--(46)--15198-

TAAGGC

TAAGGC  
Depth:3 (COW)  
Ei-value:0.000, Pi-value:0.000  
Er-value:0.000, Pr-value:0.000  
No matches to TargetScan

-15203--(22)--15226-

GAATATTTGCA

GAATATTTGCA  
Depth:3 (COW)  
Ei-value:0.000, Pi-value:0.000  
Er-value:0.000, Pr-value:0.000  
No matches to TargetScan

-15236--(56)--15293-

ATTACTG

ATTACTG  
Depth:3 (COW)  
Ei-value:0.000, Pi-value:0.010  
Er-value:0.000, Pr-value:0.020  
MATCHES To TargetScan▶ miR-802:CAGUAAC

-15299--(4)--15304-

GGGCTGCTGA

GGGCTGCTGA  
Depth:3 (COW)  
Ei-value:0.000, Pi-value:0.000  
Er-value:0.000, Pr-value:0.000  
MATCHES To TargetScan▶ miR-15-5p/16-5p/195-5p/424-5p/497-5p:AGCAGCA▶ miR-503-5p:AGCAGCG

-15313--(5)--15319-

CAAAACTT

CAAAACTT  
Depth:4 (DOG)  
Ei-value:0.000, Pi-value:0.000  
Er-value:0.000, Pr-value:0.000  
No matches to TargetScan

-15326--(3)--15330-

CTGGGACTG

CTGGGACTG  
Depth:3 (COW)  
Ei-value:0.000, Pi-value:0.000  
Er-value:0.000, Pr-value:0.000  
MATCHES To TargetScan▶ miR-455-3p.1:CAGUCCA

-15338--(7)--15346-

GCACAATG

GCACAATG  
Depth:6 (MOUSE)  
Ei-value:0.000, Pi-value:0.000  
Er-value:0.000, Pr-value:0.000  
No matches to TargetScan

-15353--(21)--15375-

CTCCCTG

CTCCCTG  
Depth:3 (COW)  
Ei-value:0.000, Pi-value:0.000  
Er-value:0.000, Pr-value:0.000  
No matches to TargetScan

-15381--(10)--15392-

GCAAGC

GCAAGC  
Depth:3 (COW)  
Ei-value:0.000, Pi-value:0.000  
Er-value:0.000, Pr-value:0.000  
No matches to TargetScan

-15397--(87)--15485-

A

ACTCCCA  
Depth:4 (DOG)  
Ei-value:0.000, Pi-value:0.000  
Er-value:0.000, Pr-value:0.000  
No matches to TargetScan


CTCCCA

CTCCCA  
Depth:6 (MOUSE)  
Ei-value:0.000, Pi-value:0.000  
Er-value:0.000, Pr-value:0.000  
No matches to TargetScan

-15491--(191)--15683-

CCCTTTTGCATT

CCCTTTTGCATT  
Depth:4 (DOG)  
Ei-value:0.000, Pi-value:0.000  
Er-value:0.000, Pr-value:0.000  
No matches to TargetScan


G

CCCTTTTGCATTG  
Depth:3 (COW)  
Ei-value:0.000, Pi-value:0.000  
Er-value:0.000, Pr-value:0.000  
No matches to TargetScan

-15695--(142)--15838-

ACTTCCTT

ACTTCCTT  
Depth:3 (COW)  
Ei-value:0.000, Pi-value:0.000  
Er-value:0.000, Pr-value:0.000  
No matches to TargetScan

-15845--(35)--15881-

AGCCCCTTCT

AGCCCCTTCT  
Depth:3 (COW)  
Ei-value:0.000, Pi-value:0.000  
Er-value:0.000, Pr-value:0.000  
No matches to TargetScan

-15890--(10)--15901-

CACAGTA

CACAGTA  
Depth:3 (COW)  
Ei-value:0.000, Pi-value:0.000  
Er-value:0.000, Pr-value:0.000  
No matches to TargetScan

-15907--(1)--15909-

TGATTGTC

TGATTGTCCCATTTTT  
Depth:3 (COW)  
Ei-value:0.000, Pi-value:0.000  
Er-value:0.000, Pr-value:0.000  
No matches to TargetScan


CCATTTTT

CCATTTTT  
Depth:4 (DOG)  
Ei-value:0.000, Pi-value:0.000  
Er-value:0.000, Pr-value:0.000  
No matches to TargetScan

-15924--(1)--15926-

CAGCCCA

CAGCCCA  
Depth:4 (DOG)  
Ei-value:0.000, Pi-value:0.000  
Er-value:0.000, Pr-value:0.000  
No matches to TargetScan

-15932--(1)--15934-

CAGCCCA

CAGCCCA  
Depth:4 (DOG)  
Ei-value:0.000, Pi-value:0.000  
Er-value:0.000, Pr-value:0.000  
No matches to TargetScan

-15940--(3)--15944-

TCTC

TCTCCCTACCA  
Depth:3 (COW)  
Ei-value:0.000, Pi-value:0.000  
Er-value:0.000, Pr-value:0.000  
No matches to TargetScan


CCTACCA

CCTACCA  
Depth:4 (DOG)  
Ei-value:0.000, Pi-value:0.000  
Er-value:0.000, Pr-value:0.000  
No matches to TargetScan

-15954--(16)--15971-

GTGCAGT

GTGCAGT  
Depth:3 (COW)  
Ei-value:0.000, Pi-value:0.000  
Er-value:0.000, Pr-value:0.000  
MATCHES To TargetScan▶ miR-217:ACUGCAU

-15977--(10)--15988-

AAAAGCAG

AAAAGCAG  
Depth:6 (MOUSE)  
Ei-value:0.000, Pi-value:0.000  
Er-value:0.000, Pr-value:0.000  
No matches to TargetScan

-15995--(4)--16000-

GAACTA

GAACTA  
Depth:3 (COW)  
Ei-value:0.000, Pi-value:0.000  
Er-value:0.000, Pr-value:0.000  
No matches to TargetScan

-16005--(34)--16040-

TTAATGATCC

TTAATGATCC  
Depth:4 (DOG)  
Ei-value:0.000, Pi-value:0.000  
Er-value:0.000, Pr-value:0.000  
MATCHES To TargetScan▶ miR-382-3p:AUCAUUC

-16049--(8)--16058-

ATTATTGT

ATTATTGT  
Depth:3 (COW)  
Ei-value:0.000, Pi-value:0.000  
Er-value:0.000, Pr-value:0.000  
No matches to TargetScan

-16065--(3)--16069-

ATTCTGGG

ATTCTGGG  
Depth:4 (DOG)  
Ei-value:0.000, Pi-value:0.000  
Er-value:0.000, Pr-value:0.000  
No matches to TargetScan

-16076--(29)--16106-

TG

TGCTTTACT  
Depth:3 (COW)  
Ei-value:0.000, Pi-value:0.000  
Er-value:0.000, Pr-value:0.000  
MATCHES To TargetScan▶ miR-330-3p.2:AAAGCAC


CTTTACT

CTTTACT  
Depth:4 (DOG)  
Ei-value:0.000, Pi-value:0.000  
Er-value:0.000, Pr-value:0.000  
No matches to TargetScan

-16114--(2)--16117-

GCAAAAT

GCAAAAT  
Depth:6 (MOUSE)  
Ei-value:0.000, Pi-value:0.000  
Er-value:0.000, Pr-value:0.000  
No matches to TargetScan

-16123--(4)--16128-

AAGGCAA

AAGGCAA  
Depth:4 (DOG)  
Ei-value:0.000, Pi-value:0.000  
Er-value:0.000, Pr-value:0.000  
No matches to TargetScan


GTCAGACCCA

AAGGCAAGTCAGACCCA  
Depth:3 (COW)  
Ei-value:0.000, Pi-value:0.000  
Er-value:0.000, Pr-value:0.000  
MATCHES To TargetScan▶ miR-193a-5p:GGGUCUU

-16144--(8)--16153-

TGGATTGC

TGGATTGC  
Depth:4 (DOG)  
Ei-value:0.000, Pi-value:0.000  
Er-value:0.000, Pr-value:0.000  
No matches to TargetScan

-16160--(60)--16221-

GAAGGAAG

GAAGGAAG  
Depth:3 (COW)  
Ei-value:0.000, Pi-value:0.000  
Er-value:0.000, Pr-value:0.000  
No matches to TargetScan

-16228--(14)--16243-

TGCATTCTTC

TGCATTCTTC  
Depth:5 (RABBIT)  
Ei-value:0.000, Pi-value:0.000  
Er-value:0.000, Pr-value:0.000  
No matches to TargetScan

-16252--(7)--16260-

AGC

AGCAGATTGCCTGG  
Depth:4 (DOG)  
Ei-value:0.000, Pi-value:0.000  
Er-value:0.000, Pr-value:0.000  
No matches to TargetScan


A

AGATTGCCTGG  
Depth:5 (RABBIT)  
Ei-value:0.000, Pi-value:0.000  
Er-value:0.000, Pr-value:0.000  
No matches to TargetScan


GATTGCCTGG

GATTGCCTGG  
Depth:6 (MOUSE)  
Ei-value:0.000, Pi-value:0.000  
Er-value:0.000, Pr-value:0.000  
No matches to TargetScan

-16273--(21)--16295-

TTGTATATT

TTGTATATT  
Depth:4 (DOG)  
Ei-value:0.000, Pi-value:0.000  
Er-value:0.000, Pr-value:0.000  
MATCHES To TargetScan▶ miR-381-3p:AUACAAG

-16303--(12)--16316-

TGCCAA

TGCCAA  
Depth:3 (COW)  
Ei-value:0.000, Pi-value:0.000  
Er-value:0.000, Pr-value:0.000  
MATCHES To TargetScan▶ miR-182-5p:UUGGCAA▶ miR-96-5p/1271-5p:UUGGCAC

-16321--(1)--16323-

TGCCAGGATACA

TGCCAGGATACA  
Depth:3 (COW)  
Ei-value:0.000, Pi-value:0.000  
Er-value:0.000, Pr-value:0.000  
No matches to TargetScan

-16334--(37)--16372-

ACATCTGG

ACATCTGG  
Depth:3 (COW)  
Ei-value:0.000, Pi-value:0.000  
Er-value:0.000, Pr-value:0.000  
No matches to TargetScan

-16379--(16)--16396-

GAT

GATAACCTGGTCATT  
Depth:3 (COW)  
Ei-value:0.000, Pi-value:0.000  
Er-value:0.000, Pr-value:0.000  
MATCHES To TargetScan▶ miR-154-5p:AGGUUAU


AAC

AACCTGGTCATT  
Depth:4 (DOG)  
Ei-value:0.000, Pi-value:0.000  
Er-value:0.000, Pr-value:0.000  
No matches to TargetScan


CTGGTCATT

CTGGTCATT  
Depth:5 (RABBIT)  
Ei-value:0.000, Pi-value:0.000  
Er-value:0.000, Pr-value:0.000  
No matches to TargetScan

-16410--(4)--16415-

TTTTGAA

TTTTGAA  
Depth:3 (COW)  
Ei-value:0.000, Pi-value:0.000  
Er-value:0.000, Pr-value:0.010  
No matches to TargetScan

-16421--(11)--16433-

CCATTTAT

CCATTTAT  
Depth:5 (RABBIT)  
Ei-value:0.000, Pi-value:0.000  
Er-value:0.000, Pr-value:0.000  
No matches to TargetScan

-16440--(13)--16454-

TGAC

TGACCAGTGTCTCTCATTT  
Depth:4 (DOG)  
Ei-value:0.000, Pi-value:0.000  
Er-value:0.000, Pr-value:0.000  
No matches to TargetScan


CAGTGTCTCTCATTT

CAGTGTCTCTCATTT  
Depth:5 (RABBIT)  
Ei-value:0.000, Pi-value:0.000  
Er-value:0.000, Pr-value:0.000  
No matches to TargetScan

-16472--(4)--16477-

AGG

AGGGTGGTG  
Depth:4 (DOG)  
Ei-value:0.000, Pi-value:0.000  
Er-value:0.000, Pr-value:0.000  
No matches to TargetScan


GTGGTG

GTGGTG  
Depth:5 (RABBIT)  
Ei-value:0.000, Pi-value:0.000  
Er-value:0.000, Pr-value:0.000  
No matches to TargetScan

-16485--(1)--16487-

GTCTGTGGATA

GTCTGTGGATA  
Depth:5 (RABBIT)  
Ei-value:0.000, Pi-value:0.000  
Er-value:0.000, Pr-value:0.000  
MATCHES To TargetScan▶ miR-140-3p.1:CCACAGG


GA

GTCTGTGGATAGA  
Depth:3 (COW)  
Ei-value:0.000, Pi-value:0.000  
Er-value:0.000, Pr-value:0.000  
MATCHES To TargetScan▶ miR-140-3p.1:CCACAGG

-16499--(15)--16515-

TATTTTA

TATTTTA  
Depth:3 (COW)  
Ei-value:0.000, Pi-value:0.040  
Er-value:0.000, Pr-value:0.020  
No matches to TargetScan

-16521--(18)--16540-

TTCTAGA

TTCTAGA  
Depth:4 (DOG)  
Ei-value:0.000, Pi-value:0.000  
Er-value:0.000, Pr-value:0.000  
No matches to TargetScan

-16546--(17)--16564-

AGTATCTTTG

AGTATCTTTG  
Depth:3 (COW)  
Ei-value:0.000, Pi-value:0.000  
Er-value:0.000, Pr-value:0.000  
No matches to TargetScan

-16573--(46)--16620-

ATTCACTT

ATTCACTT  
Depth:4 (DOG)  
Ei-value:0.000, Pi-value:0.000  
Er-value:0.000, Pr-value:0.000  
No matches to TargetScan

-16627--(3)--16631-

GAAAAAC

GAAAAAC  
Depth:4 (DOG)  
Ei-value:0.000, Pi-value:0.000  
Er-value:0.000, Pr-value:0.000  
No matches to TargetScan

-16637--(22)--16660-

AATTTCTTCATCTGGAGC

AATTTCTTCATCTGGAGC  
Depth:5 (RABBIT)  
Ei-value:0.000, Pi-value:0.000  
Er-value:0.000, Pr-value:0.000  
No matches to TargetScan

-16677--(15)--16693-

CTTATTT

CTTATTT  
Depth:4 (DOG)  
Ei-value:0.000, Pi-value:0.000  
Er-value:0.000, Pr-value:0.010  
No matches to TargetScan


CAAGAA

CTTATTTCAAGAA  
Depth:3 (COW)  
Ei-value:0.000, Pi-value:0.000  
Er-value:0.000, Pr-value:0.000  
MATCHES To TargetScan▶ miR-203a-3p.2:UGAAAUG

-16705--(15)--16721-

ATAAAATG

ATAAAATG  
Depth:4 (DOG)  
Ei-value:0.000, Pi-value:0.000  
Er-value:0.000, Pr-value:0.000  
No matches to TargetScan


A

ATAAAATGA  
Depth:3 (COW)  
Ei-value:0.000, Pi-value:0.000  
Er-value:0.000, Pr-value:0.000  
No matches to TargetScan

-16729--(97)--16827-

ACCACACT

ACCACACT  
Depth:3 (COW)  
Ei-value:0.000, Pi-value:0.000  
Er-value:0.000, Pr-value:0.000  
No matches to TargetScan

-16834--(3)--16838-

GTGAGG

GTGAGG  
Depth:3 (COW)  
Ei-value:0.000, Pi-value:0.000  
Er-value:0.000, Pr-value:0.000  
No matches to TargetScan

-16843--(28)--16872-

TTTTATA

TTTTATA  
Depth:3 (COW)  
Ei-value:0.000, Pi-value:0.000  
Er-value:0.000, Pr-value:0.010  
MATCHES To TargetScan▶ miR-340-5p:UAUAAAG

-16878--(7)--16886-

AAAAATAAGCCA

AAAAATAAGCCA  
Depth:5 (RABBIT)  
Ei-value:0.000, Pi-value:0.000  
Er-value:0.000, Pr-value:0.000  
No matches to TargetScan


A

AAAAATAAGCCAA  
Depth:4 (DOG)  
Ei-value:0.000, Pi-value:0.000  
Er-value:0.000, Pr-value:0.000  
No matches to TargetScan

-16898--(11)--16910-

TCTTTTGGATATA

TCTTTTGGATATA  
Depth:3 (COW)  
Ei-value:0.000, Pi-value:0.000  
Er-value:0.000, Pr-value:0.000  
No matches to TargetScan

-16922--(33)--16956-

ATGAATAATA

ATGAATAATA  
Depth:4 (DOG)  
Ei-value:0.000, Pi-value:0.000  
Er-value:0.000, Pr-value:0.000  
No matches to TargetScan

-16965--(11)--16977-

AGTGTACA

AGTGTACA  
Depth:3 (COW)  
Ei-value:0.000, Pi-value:0.000  
Er-value:0.000, Pr-value:0.000  
MATCHES To TargetScan▶ miR-493-5p:UGUACAU

-16984--(1)--16986-

GGTGTTT

GGTGTTT  
Depth:3 (COW)  
Ei-value:0.000, Pi-value:0.000  
Er-value:0.000, Pr-value:0.000  
No matches to TargetScan

-16992--(21)--17014-

TGGAACTGCT

TGGAACTGCT  
Depth:4 (DOG)  
Ei-value:0.000, Pi-value:0.000  
Er-value:0.000, Pr-value:0.000  
No matches to TargetScan

-17023--(8)--17032-

TAACTA

TAACTA  
Depth:4 (DOG)  
Ei-value:0.000, Pi-value:0.000  
Er-value:0.000, Pr-value:0.000  
No matches to TargetScan

-17037--(10)--17048-

CAGCAGTTC

CAGCAGTTC  
Depth:5 (RABBIT)  
Ei-value:0.000, Pi-value:0.000  
Er-value:0.000, Pr-value:0.000  
No matches to TargetScan

-17056--(1)--17058-

TTGTAAT

TTGTAAT  
Depth:4 (DOG)  
Ei-value:0.000, Pi-value:0.000  
Er-value:0.000, Pr-value:0.000  
No matches to TargetScan

-17064--(1)--17066-

ACTGAAAA

ACTGAAAA  
Depth:5 (RABBIT)  
Ei-value:0.000, Pi-value:0.000  
Er-value:0.000, Pr-value:0.000  
No matches to TargetScan

-17073--(14)--17088-

GAG

GAGAAGGATGTCAAAAGATCGGC  
Depth:3 (COW)  
Ei-value:0.000, Pi-value:0.000  
Er-value:0.000, Pr-value:0.000  
MATCHES To TargetScan▶ miR-362-5p/500b-5p:AUCCUUG▶ miR-489-3p:UGACAUC


AAGGATG

AAGGATG  
Depth:5 (RABBIT)  
Ei-value:0.000, Pi-value:0.000  
Er-value:0.000, Pr-value:0.000  
MATCHES To TargetScan▶ miR-362-5p/500b-5p:AUCCUUG


TCA

AAGGATGTCAAAAGATC  
Depth:4 (DOG)  
Ei-value:0.000, Pi-value:0.000  
Er-value:0.000, Pr-value:0.000  
MATCHES To TargetScan▶ miR-362-5p/500b-5p:AUCCUUG▶ miR-489-3p:UGACAUC


AAAGATC

AAAGATC  
Depth:6 (MOUSE)  
Ei-value:0.000, Pi-value:0.000  
Er-value:0.000, Pr-value:0.000  
No matches to TargetScan


GGC

GAGAAGGATGTCAAAAGATCGGC  
Depth:3 (COW)  
Ei-value:0.000, Pi-value:0.000  
Er-value:0.000, Pr-value:0.000  
MATCHES To TargetScan▶ miR-362-5p/500b-5p:AUCCUUG▶ miR-489-3p:UGACAUC

-17110--(1)--17112-

CAGCTCAGGG

CAGCTCAGGG  
Depth:4 (DOG)  
Ei-value:0.000, Pi-value:0.000  
Er-value:0.000, Pr-value:0.000  
MATCHES To TargetScan▶ miR-125-5p:CCCUGAG

-17121--(1)--17123-

GCAGTTTGC

GCAGTTTGC  
Depth:3 (COW)  
Ei-value:0.000, Pi-value:0.000  
Er-value:0.000, Pr-value:0.000  
No matches to TargetScan

-17131--(1)--17133-

CTACTAGCTCCT

CTACTAGCTCCT  
Depth:4 (DOG)  
Ei-value:0.000, Pi-value:0.000  
Er-value:0.000, Pr-value:0.000  
MATCHES To TargetScan▶ miR-28-5p/708-5p:AGGAGCU▶ miR-411-5p.2:UAGUAGA

-17144--(1)--17146-

GGACAGCTG

GGACAGCTG  
Depth:5 (RABBIT)  
Ei-value:0.000, Pi-value:0.000  
Er-value:0.000, Pr-value:0.000  
No matches to TargetScan


T

GGACAGCTGT  
Depth:4 (DOG)  
Ei-value:0.000, Pi-value:0.000  
Er-value:0.000, Pr-value:0.000  
No matches to TargetScan

-17155--(0)--17156-

A

AAGAAGAGTCTCTGGCTCTTTAGA  
Depth:3 (COW)  
Ei-value:0.000, Pi-value:0.000  
Er-value:0.000, Pr-value:0.000  
No matches to TargetScan


AGAAGAGTCTCTGGCTCTTTA

AGAAGAGTCTCTGGCTCTTTA  
Depth:5 (RABBIT)  
Ei-value:0.000, Pi-value:0.000  
Er-value:0.000, Pr-value:0.000  
No matches to TargetScan


GA

AGAAGAGTCTCTGGCTCTTTAGA  
Depth:4 (DOG)  
Ei-value:0.000, Pi-value:0.000  
Er-value:0.000, Pr-value:0.000  
No matches to TargetScan

-17179--(11)--17191-

ATTCTGAGC

ATTCTGAGC  
Depth:4 (DOG)  
Ei-value:0.000, Pi-value:0.000  
Er-value:0.000, Pr-value:0.000  
No matches to TargetScan

-17199--(99)--17299-

GA

GACTGCAA  
Depth:3 (COW)  
Ei-value:0.000, Pi-value:0.000  
Er-value:0.000, Pr-value:0.000  
MATCHES To TargetScan▶ miR-455-3p.2:UGCAGUC


CTGCAA

CTGCAA  
Depth:5 (RABBIT)  
Ei-value:0.000, Pi-value:0.000  
Er-value:0.000, Pr-value:0.000  
No matches to TargetScan

-17306--(38)--17345-

TTTGAGAATCTGG

TTTGAGAATCTGG  
Depth:3 (COW)  
Ei-value:0.000, Pi-value:0.000  
Er-value:0.000, Pr-value:0.000  
MATCHES To TargetScan▶ miR-371-5p:CUCAAAC

-17357--(2)--17360-

AAGCTCCA

AAGCTCCA  
Depth:3 (COW)  
Ei-value:0.000, Pi-value:0.000  
Er-value:0.000, Pr-value:0.000  
No matches to TargetScan

-17367--(12)--17380-

GGATGG

GGATGG  
Depth:3 (COW)  
Ei-value:0.000, Pi-value:0.000  
Er-value:0.000, Pr-value:0.010  
No matches to TargetScan

-17385--(12)--17398-

CTGGAGAAAAAGATCT

CTGGAGAAAAAGATCT  
Depth:3 (COW)  
Ei-value:0.000, Pi-value:0.000  
Er-value:0.000, Pr-value:0.000  
No matches to TargetScan

-17413--(7)--17421-

AAGAATAGGC

AAGAATAGGC  
Depth:5 (RABBIT)  
Ei-value:0.000, Pi-value:0.000  
Er-value:0.000, Pr-value:0.000  
No matches to TargetScan

-17430--(8)--17439-

T

TTACAGTGTTAGTGA  
Depth:3 (COW)  
Ei-value:0.000, Pi-value:0.000  
Er-value:0.000, Pr-value:0.000  
MATCHES To TargetScan▶ miR-141-3p/200a-3p:AACACUG


TACAGTGTTAGTGA

TACAGTGTTAGTGA  
Depth:5 (RABBIT)  
Ei-value:0.000, Pi-value:0.000  
Er-value:0.000, Pr-value:0.000  
MATCHES To TargetScan▶ miR-141-3p/200a-3p:AACACUG

-17453--(2)--17456-

CA

CATTCCCTTTGA  
Depth:3 (COW)  
Ei-value:0.000, Pi-value:0.000  
Er-value:0.000, Pr-value:0.000  
MATCHES To TargetScan▶ miR-1-3p/206:GGAAUGU


TTCCCTTTGA

TTCCCTTTGA  
Depth:6 (MOUSE)  
Ei-value:0.000, Pi-value:0.000  
Er-value:0.000, Pr-value:0.000  
No matches to TargetScan

-17467--(7)--17475-

TAGGTGGAGATGGGGCATGAGGATCCTCCAGGGGAA

TAGGTGGAGATGGGGCATGAGGATCCTCCAGGGGAA  
Depth:6 (MOUSE)  
Ei-value:0.000, Pi-value:0.000  
Er-value:0.000, Pr-value:0.000  
MATCHES To TargetScan▶ miR-331-3p:CCCCUGG


A

TAGGTGGAGATGGGGCATGAGGATCCTCCAGGGGAAA  
Depth:5 (RABBIT)  
Ei-value:0.000, Pi-value:0.000  
Er-value:0.000, Pr-value:0.000  
MATCHES To TargetScan▶ miR-331-3p:CCCCUGG

-17511--(3)--17515-

TCACTA

TCACTA  
Depth:5 (RABBIT)  
Ei-value:0.000, Pi-value:0.000  
Er-value:0.000, Pr-value:0.000  
No matches to TargetScan


CCACT

TCACTACCACT  
Depth:4 (DOG)  
Ei-value:0.000, Pi-value:0.000  
Er-value:0.000, Pr-value:0.000  
MATCHES To TargetScan▶ miR-140-5p:AGUGGUU▶ miR-142-3p.1:GUAGUGU


G

TCACTACCACTG  
Depth:3 (COW)  
Ei-value:0.000, Pi-value:0.000  
Er-value:0.000, Pr-value:0.000  
MATCHES To TargetScan▶ miR-140-5p:AGUGGUU▶ miR-142-3p.1:GUAGUGU

-17526--(1)--17528-

GCAACA

GCAACA  
Depth:6 (MOUSE)  
Ei-value:0.000, Pi-value:0.000  
Er-value:0.000, Pr-value:0.000  
No matches to TargetScan


AC

GCAACAAC  
Depth:5 (RABBIT)  
Ei-value:0.000, Pi-value:0.000  
Er-value:0.000, Pr-value:0.000  
No matches to TargetScan

-17535--(27)--17563-

CTTTCCTGG

CTTTCCTGG  
Depth:3 (COW)  
Ei-value:0.000, Pi-value:0.000  
Er-value:0.000, Pr-value:0.000  
MATCHES To TargetScan▶ miR-665:CCAGGAG▶ miR-873-5p.1:CAGGAAC

-17571--(27)--17599-

ACAACCACC

ACAACCACC  
Depth:5 (RABBIT)  
Ei-value:0.000, Pi-value:0.000  
Er-value:0.000, Pr-value:0.000  
No matches to TargetScan


ACAC

ACAACCACCACAC  
Depth:4 (DOG)  
Ei-value:0.000, Pi-value:0.000  
Er-value:0.000, Pr-value:0.000  
No matches to TargetScan

-17611--(343)--17955-

TTGTTCC

TTGTTCC  
Depth:4 (DOG)  
Ei-value:0.000, Pi-value:0.000  
Er-value:0.000, Pr-value:0.000  
No matches to TargetScan

-17961--(9)--17971-

TG

TGCCAAATC  
Depth:3 (COW)  
Ei-value:0.000, Pi-value:0.000  
Er-value:0.000, Pr-value:0.000  
MATCHES To TargetScan▶ miR-182-5p:UUGGCAA▶ miR-96-5p/1271-5p:UUGGCAC


CCAAAT

CCAAAT  
Depth:6 (MOUSE)  
Ei-value:0.000, Pi-value:0.000  
Er-value:0.000, Pr-value:0.000  
No matches to TargetScan


C

CCAAATC  
Depth:5 (RABBIT)  
Ei-value:0.000, Pi-value:0.000  
Er-value:0.000, Pr-value:0.000  
No matches to TargetScan

-17979--(29)--18009-

CAAGAAA

CAAGAAA  
Depth:5 (RABBIT)  
Ei-value:0.000, Pi-value:0.000  
Er-value:0.000, Pr-value:0.000  
No matches to TargetScan


T

CAAGAAAT  
Depth:3 (COW)  
Ei-value:0.000, Pi-value:0.000  
Er-value:0.000, Pr-value:0.000  
No matches to TargetScan

-18016--(1)--18018-

TGAACACAC

TGAACACAC  
Depth:3 (COW)  
Ei-value:0.000, Pi-value:0.000  
Er-value:0.000, Pr-value:0.000  
No matches to TargetScan

-18026--(5)--18032-

G

GAAGATCAACATGCCTG  
Depth:4 (DOG)  
Ei-value:0.000, Pi-value:0.000  
Er-value:0.000, Pr-value:0.000  
No matches to TargetScan


AA

AAGATCAACATGC  
Depth:5 (RABBIT)  
Ei-value:0.000, Pi-value:0.000  
Er-value:0.000, Pr-value:0.000  
No matches to TargetScan


GATCAACATGC

GATCAACATGC  
Depth:6 (MOUSE)  
Ei-value:0.000, Pi-value:0.000  
Er-value:0.000, Pr-value:0.000  
No matches to TargetScan


CTG

GAAGATCAACATGCCTG  
Depth:4 (DOG)  
Ei-value:0.000, Pi-value:0.000  
Er-value:0.000, Pr-value:0.000  
No matches to TargetScan

-18048--(63)--18112-

TGTGTAT

TGTGTAT  
Depth:6 (MOUSE)  
Ei-value:0.000, Pi-value:0.000  
Er-value:0.000, Pr-value:0.000  
No matches to TargetScan


TT

TGTGTATTT  
Depth:4 (DOG)  
Ei-value:0.000, Pi-value:0.000  
Er-value:0.000, Pr-value:0.000  
No matches to TargetScan

-18120--(11)--18132-

TCTTTCTT

TCTTTCTT  
Depth:3 (COW)  
Ei-value:0.000, Pi-value:0.000  
Er-value:0.000, Pr-value:0.000  
No matches to TargetScan

-18139--(37)--18177-

TGTCTTA

TGTCTTA  
Depth:4 (DOG)  
Ei-value:0.000, Pi-value:0.000  
Er-value:0.000, Pr-value:0.000  
MATCHES To TargetScan▶ miR-208-3p:UAAGACG▶ miR-499a-5p:UAAGACU


CCCATTTCCATG

TGTCTTACCCATTTCCATG  
Depth:3 (COW)  
Ei-value:0.000, Pi-value:0.000  
Er-value:0.000, Pr-value:0.000  
MATCHES To TargetScan▶ miR-203a-3p.1:GAAAUGU▶ miR-208-3p:UAAGACG▶ miR-499a-5p:UAAGACU

-18195--(40)--18236-

TTTTTGT

TTTTTGT  
Depth:4 (DOG)  
Ei-value:0.000, Pi-value:0.000  
Er-value:0.000, Pr-value:0.000  
No matches to TargetScan

-18242--(54)--18297-

TTCATTTTGTT

TTCATTTTGTT  
Depth:4 (DOG)  
Ei-value:0.000, Pi-value:0.000  
Er-value:0.000, Pr-value:0.000  
MATCHES To TargetScan▶ miR-495-3p:AACAAAC

-18307--(80)--18388-

TTTGCTC

TTTGCTC  
Depth:3 (COW)  
Ei-value:0.000, Pi-value:0.000  
Er-value:0.000, Pr-value:0.000  
No matches to TargetScan

-18394--(27)--18422-

TT

TTTTCTCTTTGTGAA  
Depth:3 (COW)  
Ei-value:0.000, Pi-value:0.000  
Er-value:0.000, Pr-value:0.000  
No matches to TargetScan


TTCTCTTTG

TTCTCTTTG  
Depth:6 (MOUSE)  
Ei-value:0.000, Pi-value:0.000  
Er-value:0.000, Pr-value:0.000  
No matches to TargetScan


TGAA

TTTTCTCTTTGTGAA  
Depth:3 (COW)  
Ei-value:0.000, Pi-value:0.000  
Er-value:0.000, Pr-value:0.000  
No matches to TargetScan

-18436--(20)--18457-

TTCCCCTT

TTCCCCTT  
Depth:3 (COW)  
Ei-value:0.000, Pi-value:0.000  
Er-value:0.000, Pr-value:0.000  
No matches to TargetScan

-18464--(14)--18479-

ATTTCACCT

ATTTCACCT  
Depth:4 (DOG)  
Ei-value:0.000, Pi-value:0.000  
Er-value:0.000, Pr-value:0.000  
MATCHES To TargetScan▶ miR-203a-3p.2:UGAAAUG

-18487--(22)--18510-

TGCTG

TGCTGTTTCTACT  
Depth:3 (COW)  
Ei-value:0.000, Pi-value:0.000  
Er-value:0.000, Pr-value:0.000  
MATCHES To TargetScan▶ miR-411-5p.1:AGUAGAC▶ miR-494-3p:GAAACAU


TTTCTAC

TTTCTAC  
Depth:6 (MOUSE)  
Ei-value:0.000, Pi-value:0.000  
Er-value:0.000, Pr-value:0.000  
No matches to TargetScan


T

TTTCTACT  
Depth:5 (RABBIT)  
Ei-value:0.000, Pi-value:0.000  
Er-value:0.000, Pr-value:0.000  
MATCHES To TargetScan▶ miR-411-5p.1:AGUAGAC

-18522--(11)--18534-

ATTTCTC

ATTTCTC  
Depth:6 (MOUSE)  
Ei-value:0.000, Pi-value:0.000  
Er-value:0.000, Pr-value:0.000  
No matches to TargetScan

-18540--(23)--18564-

TCTTGGG

TCTTGGG  
Depth:5 (RABBIT)  
Ei-value:0.000, Pi-value:0.000  
Er-value:0.000, Pr-value:0.000  
No matches to TargetScan


C

TCTTGGGC  
Depth:3 (COW)  
Ei-value:0.000, Pi-value:0.000  
Er-value:0.000, Pr-value:0.000  
No matches to TargetScan

-18571--(46)--18618-

TTTGTGA

TTTGTGA  
Depth:4 (DOG)  
Ei-value:0.000, Pi-value:0.010  
Er-value:0.000, Pr-value:0.000  
No matches to TargetScan


TTTTC

TTTGTGATTTTC  
Depth:3 (COW)  
Ei-value:0.000, Pi-value:0.000  
Er-value:0.000, Pr-value:0.000  
No matches to TargetScan

-18629--(16)--18646-

TCTCTGTT

TCTCTGTT  
Depth:4 (DOG)  
Ei-value:0.000, Pi-value:0.000  
Er-value:0.000, Pr-value:0.000  
No matches to TargetScan

-18653--(37)--18691-

TTTGAGTATTT

TTTGAGTATTT  
Depth:4 (DOG)  
Ei-value:0.000, Pi-value:0.000  
Er-value:0.000, Pr-value:0.000  
MATCHES To TargetScan▶ miR-200bc-3p/429:AAUACUG▶ miR-371-5p:CUCAAAC

-18701--(27)--18729-

CTTTGATT

CTTTGATT  
Depth:3 (COW)  
Ei-value:0.000, Pi-value:0.000  
Er-value:0.000, Pr-value:0.000  
No matches to TargetScan

-18736--(36)--18773-

TGTGTGTG

TGTGTGTG  
Depth:4 (DOG)  
Ei-value:0.000, Pi-value:0.000  
Er-value:0.000, Pr-value:0.000  
MATCHES To TargetScan▶ miR-329-3p/362-3p:ACACACC

-18780--(29)--18810-

TCCTAACCCCT

TCCTAACCCCT  
Depth:5 (RABBIT)  
Ei-value:0.000, Pi-value:0.000  
Er-value:0.000, Pr-value:0.000  
No matches to TargetScan

-18820--(6)--18827-

TAGGTGCA

TAGGTGCA  
Depth:3 (COW)  
Ei-value:0.000, Pi-value:0.000  
Er-value:0.000, Pr-value:0.000  
No matches to TargetScan

-18834--(21)--18856-

AAGCATTG

AAGCATTG  
Depth:4 (DOG)  
Ei-value:0.000, Pi-value:0.000  
Er-value:0.000, Pr-value:0.000  
No matches to TargetScan

-18863--(12)--18876-

TTATGCCA

TTATGCCA  
Depth:5 (RABBIT)  
Ei-value:0.000, Pi-value:0.000  
Er-value:0.000, Pr-value:0.000  
No matches to TargetScan


G

TTATGCCAG  
Depth:4 (DOG)  
Ei-value:0.000, Pi-value:0.000  
Er-value:0.000, Pr-value:0.000  
No matches to TargetScan

-18884--(40)--18925-

TCCAAG

TCCAAG  
Depth:3 (COW)  
Ei-value:0.000, Pi-value:0.000  
Er-value:0.000, Pr-value:0.000  
No matches to TargetScan

-18930--(6)--18937-

AGA

AGAAGGCCCAA  
Depth:4 (DOG)  
Ei-value:0.000, Pi-value:0.000  
Er-value:0.000, Pr-value:0.000  
No matches to TargetScan


AGGCCCAA

AGGCCCAA  
Depth:5 (RABBIT)  
Ei-value:0.000, Pi-value:0.000  
Er-value:0.000, Pr-value:0.000  
No matches to TargetScan

-18947--(110)--19058-

TCAA

TCAAGACTAA  
Depth:4 (DOG)  
Ei-value:0.000, Pi-value:0.000  
Er-value:0.000, Pr-value:0.000  
MATCHES To TargetScan▶ miR-431-5p:GUCUUGC


GACTAA

GACTAA  
Depth:5 (RABBIT)  
Ei-value:0.000, Pi-value:0.000  
Er-value:0.000, Pr-value:0.000  
No matches to TargetScan

-19067--(46)--19114-

AGAAGC

AGAAGC  
Depth:4 (DOG)  
Ei-value:0.000, Pi-value:0.000  
Er-value:0.000, Pr-value:0.010  
No matches to TargetScan

-19119--(20)--19140-

C

CAAGATGA  
Depth:3 (COW)  
Ei-value:0.000, Pi-value:0.000  
Er-value:0.000, Pr-value:0.000  
No matches to TargetScan


AAGATGA

AAGATGA  
Depth:5 (RABBIT)  
Ei-value:0.000, Pi-value:0.000  
Er-value:0.000, Pr-value:0.000  
No matches to TargetScan

-19147--(20)--19168-

TTTCTATTG

TTTCTATTG  
Depth:3 (COW)  
Ei-value:0.000, Pi-value:0.000  
Er-value:0.000, Pr-value:0.000  
No matches to TargetScan

-19176--(27)--19204-

ACTTCTT

ACTTCTT  
Depth:3 (COW)  
Ei-value:0.000, Pi-value:0.020  
Er-value:0.000, Pr-value:0.000  
No matches to TargetScan

-19210--(46)--19257-

CTTTTTGATGTT

CTTTTTGATGTT  
Depth:4 (DOG)  
Ei-value:0.000, Pi-value:0.000  
Er-value:0.000, Pr-value:0.000  
No matches to TargetScan

-19268--(43)--19312-

TATTATGC

TATTATGC  
Depth:4 (DOG)  
Ei-value:0.000, Pi-value:0.000  
Er-value:0.000, Pr-value:0.000  
MATCHES To TargetScan▶ miR-369-3p:AUAAUAC

-19319--(26)--19346-

TAAACTTC

TAAACTTC  
Depth:3 (COW)  
Ei-value:0.000, Pi-value:0.000  
Er-value:0.000, Pr-value:0.000  
No matches to TargetScan

-19353--(18)--19372-

CTCCACTTGAGAG

CTCCACTTGAGAG  
Depth:3 (COW)  
Ei-value:0.000, Pi-value:0.000  
Er-value:0.000, Pr-value:0.000  
MATCHES To TargetScan▶ miR-26-5p:UCAAGUA

-19384--(17)--19402-

TATTTCAGT

TATTTCAGT  
Depth:4 (DOG)  
Ei-value:0.000, Pi-value:0.000  
Er-value:0.000, Pr-value:0.000  
MATCHES To TargetScan▶ miR-203a-3p.2:UGAAAUG


CC

TATTTCAGTCC  
Depth:3 (COW)  
Ei-value:0.000, Pi-value:0.000  
Er-value:0.000, Pr-value:0.000  
MATCHES To TargetScan▶ miR-203a-3p.2:UGAAAUG

-19412--(57)--19470-

GGGGAAA

GGGGAAA  
Depth:4 (DOG)  
Ei-value:0.000, Pi-value:0.000  
Er-value:0.000, Pr-value:0.000  
No matches to TargetScan

-19476--(15)--19492-

TCTAGAGAAAA

TCTAGAGAAAA  
Depth:6 (MOUSE)  
Ei-value:0.000, Pi-value:0.000  
Er-value:0.000, Pr-value:0.000  
MATCHES To TargetScan▶ miR-1251-5p:CUCUAGC

-19502--(2)--19505-

TGAAGAGATG

TGAAGAGATG  
Depth:5 (RABBIT)  
Ei-value:0.000, Pi-value:0.000  
Er-value:0.000, Pr-value:0.000  
No matches to TargetScan


CTCCA

TGAAGAGATGCTCCA  
Depth:3 (COW)  
Ei-value:0.000, Pi-value:0.000  
Er-value:0.000, Pr-value:0.000  
No matches to TargetScan

-19519--(0)--19520-

GGCCAA

GGCCAATGAGAAGAATTAGACA  
Depth:4 (DOG)  
Ei-value:0.000, Pi-value:0.000  
Er-value:0.000, Pr-value:0.000  
No matches to TargetScan


TGAGAAGAATTAGACA

TGAGAAGAATTAGACA  
Depth:6 (MOUSE)  
Ei-value:0.000, Pi-value:0.000  
Er-value:0.000, Pr-value:0.000  
No matches to TargetScan

-19541--(1)--19543-

GAAATACACAGATG

GAAATACACAGATG  
Depth:3 (COW)  
Ei-value:0.000, Pi-value:0.000  
Er-value:0.000, Pr-value:0.000  
No matches to TargetScan

-19556--(10)--19567-

C

CTGAGAAG  
Depth:3 (COW)  
Ei-value:0.000, Pi-value:0.000  
Er-value:0.000, Pr-value:0.000  
No matches to TargetScan


TGAGAAG

TGAGAAG  
Depth:4 (DOG)  
Ei-value:0.000, Pi-value:0.000  
Er-value:0.000, Pr-value:0.010  
No matches to TargetScan

-19574--(5)--19580-

GCCA

GCCAGCAACA  
Depth:3 (COW)  
Ei-value:0.000, Pi-value:0.000  
Er-value:0.000, Pr-value:0.000  
No matches to TargetScan


GCAACA

GCAACA  
Depth:6 (MOUSE)  
Ei-value:0.000, Pi-value:0.000  
Er-value:0.000, Pr-value:0.000  
No matches to TargetScan

-19589--(9)--19599-

TTTGAGCTT

TTTGAGCTT  
Depth:3 (COW)  
Ei-value:0.000, Pi-value:0.000  
Er-value:0.000, Pr-value:0.000  
MATCHES To TargetScan▶ miR-371-5p:CUCAAAC

-19607--(1)--19609-

GGTGAGC

GGTGAGC  
Depth:4 (DOG)  
Ei-value:0.000, Pi-value:0.000  
Er-value:0.000, Pr-value:0.000  
No matches to TargetScan


AGGAT

GGTGAGCAGGAT  
Depth:3 (COW)  
Ei-value:0.000, Pi-value:0.000  
Er-value:0.000, Pr-value:0.000  
No matches to TargetScan

-19620--(6)--19627-

GGTTTGGG

GGTTTGGG  
Depth:4 (DOG)  
Ei-value:0.000, Pi-value:0.000  
Er-value:0.000, Pr-value:0.000  
No matches to TargetScan

-19634--(9)--19644-

TGGTTA

TGGTTA  
Depth:5 (RABBIT)  
Ei-value:0.000, Pi-value:0.000  
Er-value:0.000, Pr-value:0.000  
No matches to TargetScan


T

TGGTTAT  
Depth:4 (DOG)  
Ei-value:0.000, Pi-value:0.000  
Er-value:0.000, Pr-value:0.000  
No matches to TargetScan


G

TGGTTATG  
Depth:3 (COW)  
Ei-value:0.000, Pi-value:0.000  
Er-value:0.000, Pr-value:0.000  
No matches to TargetScan

-19651--(37)--19689-

CCCAAGG

CCCAAGG  
Depth:4 (DOG)  
Ei-value:0.000, Pi-value:0.000  
Er-value:0.000, Pr-value:0.000  
MATCHES To TargetScan▶ miR-212-5p:CCUUGGC

-19695--(8)--19704-

TGAACTCCCTGCT

TGAACTCCCTGCT  
Depth:4 (DOG)  
Ei-value:0.000, Pi-value:0.000  
Er-value:0.000, Pr-value:0.000  
No matches to TargetScan


C

TGAACTCCCTGCTCATAGTAGTGGCC  
Depth:3 (COW)  
Ei-value:0.000, Pi-value:0.000  
Er-value:0.000, Pr-value:0.000  
No matches to TargetScan


ATAGTAGTGGCC

ATAGTAGTGGCC  
Depth:4 (DOG)  
Ei-value:0.000, Pi-value:0.000  
Er-value:0.000, Pr-value:0.000  
No matches to TargetScan

-19729--(37)--19767-

TTTAATAC

TTTAATAC  
Depth:4 (DOG)  
Ei-value:0.000, Pi-value:0.000  
Er-value:0.000, Pr-value:0.000  
MATCHES To TargetScan▶ miR-496.2:GUAUUAC

-19774--(6)--19781-

CT

CTAGGCTTAAAG  
Depth:4 (DOG)  
Ei-value:0.000, Pi-value:0.000  
Er-value:0.000, Pr-value:0.000  
No matches to TargetScan


AGGCTTA

AGGCTTA  
Depth:5 (RABBIT)  
Ei-value:0.000, Pi-value:0.000  
Er-value:0.000, Pr-value:0.000  
No matches to TargetScan


AAG

CTAGGCTTAAAG  
Depth:4 (DOG)  
Ei-value:0.000, Pi-value:0.000  
Er-value:0.000, Pr-value:0.000  
No matches to TargetScan

-19792--(27)--19820-

GTTTAAT

GTTTAAT  
Depth:5 (RABBIT)  
Ei-value:0.000, Pi-value:0.000  
Er-value:0.000, Pr-value:0.000  
No matches to TargetScan

-19826--(68)--19895-

TGTAAAACA

TGTAAAACA  
Depth:3 (COW)  
Ei-value:0.000, Pi-value:0.000  
Er-value:0.000, Pr-value:0.000  
No matches to TargetScan

-19903--(30)--19934-

T

TATTGGCA  
Depth:5 (RABBIT)  
Ei-value:0.000, Pi-value:0.000  
Er-value:0.000, Pr-value:0.000  
No matches to TargetScan


ATTGGCA

ATTGGCA  
Depth:6 (MOUSE)  
Ei-value:0.000, Pi-value:0.000  
Er-value:0.000, Pr-value:0.000  
No matches to TargetScan

-19941--(36)--19978-

TTGTGAAG

TTGTGAAG  
Depth:6 (MOUSE)  
Ei-value:0.000, Pi-value:0.000  
Er-value:0.000, Pr-value:0.000  
No matches to TargetScan

-19985--(3)--19989-

T

TATGTAAATCA  
Depth:3 (COW)  
Ei-value:0.000, Pi-value:0.000  
Er-value:0.000, Pr-value:0.000  
No matches to TargetScan


ATGTAAAT

ATGTAAAT  
Depth:5 (RABBIT)  
Ei-value:0.000, Pi-value:0.000  
Er-value:0.000, Pr-value:0.000  
No matches to TargetScan


CA

TATGTAAATCA  
Depth:3 (COW)  
Ei-value:0.000, Pi-value:0.000  
Er-value:0.000, Pr-value:0.000  
No matches to TargetScan

-19999--(48)--20048-

GCCATATGGT

GCCATATGGT  
Depth:3 (COW)  
Ei-value:0.000, Pi-value:0.000  
Er-value:0.000, Pr-value:0.000  
No matches to TargetScan

-20057--(99)--20157-

TGTGC

TGTGCCTGTCCCTGT  
Depth:3 (COW)  
Ei-value:0.000, Pi-value:0.000  
Er-value:0.000, Pr-value:0.000  
No matches to TargetScan


CTGTCCCT

CTGTCCCT  
Depth:4 (DOG)  
Ei-value:0.000, Pi-value:0.000  
Er-value:0.000, Pr-value:0.000  
No matches to TargetScan


GT

TGTGCCTGTCCCTGT  
Depth:3 (COW)  
Ei-value:0.000, Pi-value:0.000  
Er-value:0.000, Pr-value:0.000  
No matches to TargetScan

-20171--(2)--20174-

TAGGCACT

TAGGCACT  
Depth:4 (DOG)  
Ei-value:0.000, Pi-value:0.000  
Er-value:0.000, Pr-value:0.000  
No matches to TargetScan

-20181--(73)--20255-

TAAAGCA

TAAAGCA  
Depth:4 (DOG)  
Ei-value:0.000, Pi-value:0.000  
Er-value:0.000, Pr-value:0.000  
No matches to TargetScan

-20261--(117)--20379-

TAT

TATAATGTGCCAGATA  
Depth:3 (COW)  
Ei-value:0.000, Pi-value:0.000  
Er-value:0.000, Pr-value:0.000  
MATCHES To TargetScan▶ miR-183-5p.2:UGGCACU▶ miR-323-3p:ACAUUAC


AATGTGCCAGATA

AATGTGCCAGATA  
Depth:4 (DOG)  
Ei-value:0.000, Pi-value:0.000  
Er-value:0.000, Pr-value:0.000  
MATCHES To TargetScan▶ miR-183-5p.2:UGGCACU

-20394--(79)--20474-

TTAAAGTG

TTAAAGTG  
Depth:4 (DOG)  
Ei-value:0.000, Pi-value:0.000  
Er-value:0.000, Pr-value:0.000  
No matches to TargetScan


CTTTGTA

TTAAAGTGCTTTGTA  
Depth:3 (COW)  
Ei-value:0.000, Pi-value:0.000  
Er-value:0.000, Pr-value:0.000  
MATCHES To TargetScan▶ miR-330-3p:CAAAGCA▶ miR-330-3p.2:AAAGCAC

-20488--(285)--20774-

CTAAAGCA

CTAAAGCA  
Depth:4 (DOG)  
Ei-value:0.000, Pi-value:0.000  
Er-value:0.000, Pr-value:0.000  
No matches to TargetScan

-20781--(12)--20794-

CAATGGGCTA

CAATGGGCTA  
Depth:3 (COW)  
Ei-value:0.000, Pi-value:0.000  
Er-value:0.000, Pr-value:0.000  
No matches to TargetScan

-20803--(11)--20815-

GA

GAATGAATA  
Depth:3 (COW)  
Ei-value:0.000, Pi-value:0.000  
Er-value:0.000, Pr-value:0.000  
MATCHES To TargetScan▶ miR-1298-5p:UCAUUCG


ATGAATA

ATGAATA  
Depth:4 (DOG)  
Ei-value:0.000, Pi-value:0.000  
Er-value:0.000, Pr-value:0.000  
No matches to TargetScan

-20823--(26)--20850-

CCAGCTATT

CCAGCTATT  
Depth:3 (COW)  
Ei-value:0.000, Pi-value:0.000  
Er-value:0.000, Pr-value:0.000  
No matches to TargetScan

-20858--(6)--20865-

GGTACTGT

GGTACTGT  
Depth:4 (DOG)  
Ei-value:0.000, Pi-value:0.000  
Er-value:0.000, Pr-value:0.000  
MATCHES To TargetScan▶ miR-101-3p.1:ACAGUAC▶ miR-144-3p:ACAGUAU

-20872--(26)--20899-

ATAAGAGG

ATAAGAGG  
Depth:4 (DOG)  
Ei-value:0.000, Pi-value:0.000  
Er-value:0.000, Pr-value:0.000  
No matches to TargetScan

-20906--(60)--20967-

AAGACTTTAC

AAGACTTTAC  
Depth:3 (COW)  
Ei-value:0.000, Pi-value:0.000  
Er-value:0.000, Pr-value:0.000  
No matches to TargetScan

-20976--(14)--20991-

TAAATTAT

TAAATTAT  
Depth:4 (DOG)  
Ei-value:0.000, Pi-value:0.010  
Er-value:0.000, Pr-value:0.000  
No matches to TargetScan


TAC

TAAATTATTAC  
Depth:3 (COW)  
Ei-value:0.000, Pi-value:0.000  
Er-value:0.000, Pr-value:0.000  
No matches to TargetScan

-21001--(14)--21016-

AGGTAA

AGGTAA  
Depth:3 (COW)  
Ei-value:0.000, Pi-value:0.000  
Er-value:0.000, Pr-value:0.000  
No matches to TargetScan

-21021--(28)--21050-

TTTCTAA

TTTCTAA  
Depth:3 (COW)  
Ei-value:0.000, Pi-value:0.000  
Er-value:0.000, Pr-value:0.010  
No matches to TargetScan

-21056--(48)--21105-

ATAAAAC

ATAAAAC  
Depth:4 (DOG)  
Ei-value:0.000, Pi-value:0.010  
Er-value:0.000, Pr-value:0.000  
No matches to TargetScan

-21111--(26)--21138-

AAAATTCTCA

AAAATTCTCA  
Depth:4 (DOG)  
Ei-value:0.000, Pi-value:0.000  
Er-value:0.000, Pr-value:0.000  
No matches to TargetScan

-21147--(41)--21189-

TATACAAAC

TATACAAAC  
Depth:4 (DOG)  
Ei-value:0.000, Pi-value:0.000  
Er-value:0.000, Pr-value:0.000  
No matches to TargetScan

-21197--(1)--21199-

GTTTAAATAC

GTTTAAATAC  
Depth:3 (COW)  
Ei-value:0.000, Pi-value:0.000  
Er-value:0.000, Pr-value:0.000  
No matches to TargetScan

-21208--(26)--21235-

TTGCCTACTAT

TTGCCTACTATGTGAACTCACTGTTA  
Depth:3 (COW)  
Ei-value:0.000, Pi-value:0.000  
Er-value:0.000, Pr-value:0.000  
MATCHES To TargetScan▶ miR-132-3p/212-3p:AACAGUC▶ miR-23-3p:UCACAUU▶ miR-376c-3p:ACAUAGA▶ miR-411-5p.2:UAGUAGA


GTGAACTCA

GTGAACTCA  
Depth:4 (DOG)  
Ei-value:0.000, Pi-value:0.000  
Er-value:0.000, Pr-value:0.000  
No matches to TargetScan


CTGTTA

TTGCCTACTATGTGAACTCACTGTTA  
Depth:3 (COW)  
Ei-value:0.000, Pi-value:0.000  
Er-value:0.000, Pr-value:0.000  
MATCHES To TargetScan▶ miR-132-3p/212-3p:AACAGUC▶ miR-23-3p:UCACAUU▶ miR-376c-3p:ACAUAGA▶ miR-411-5p.2:UAGUAGA

-21260--(22)--21283-

ATTTATCAT

ATTTATCAT  
Depth:3 (COW)  
Ei-value:0.000, Pi-value:0.000  
Er-value:0.000, Pr-value:0.000  
No matches to TargetScan

-21291--(44)--21336-

ATTTTGTGAACTCTAA

ATTTTGTGAACTCTAA  
Depth:3 (COW)  
Ei-value:0.000, Pi-value:0.000  
Er-value:0.000, Pr-value:0.000  
No matches to TargetScan

-21351--(57)--21409-

AAAATTG

AAAATTG  
Depth:3 (COW)  
Ei-value:0.000, Pi-value:0.000  
Er-value:0.000, Pr-value:0.000  
No matches to TargetScan

-21415--(130)--21546-

TGTGCCA

TGTGCCA  
Depth:4 (DOG)  
Ei-value:0.000, Pi-value:0.000  
Er-value:0.000, Pr-value:0.000  
MATCHES To TargetScan▶ miR-183-5p.2:UGGCACU

-21552--(21)--21574-

AAGATAA

AAGATAA  
Depth:4 (DOG)  
Ei-value:0.000, Pi-value:0.000  
Er-value:0.000, Pr-value:0.000  
No matches to TargetScan

-21580--(25)--21606-

AGCAGAA

AGCAGAA  
Depth:3 (COW)  
Ei-value:0.000, Pi-value:0.000  
Er-value:0.000, Pr-value:0.000  
No matches to TargetScan

-21612--(9)--21622-

TAAAATCAATTT

TAAAATCAATTT  
Depth:3 (COW)  
Ei-value:0.000, Pi-value:0.000  
Er-value:0.000, Pr-value:0.000  
No matches to TargetScan

-21633--(8)--21642-

TAAACTG

TAAACTG  
Depth:4 (DOG)  
Ei-value:0.000, Pi-value:0.000  
Er-value:0.000, Pr-value:0.000  
No matches to TargetScan

-21648--(9)--21658-

TCTGCTGAATGA

TCTGCTGAATGA  
Depth:3 (COW)  
Ei-value:0.000, Pi-value:0.000  
Er-value:0.000, Pr-value:0.000  
MATCHES To TargetScan▶ miR-1298-5p:UCAUUCG

-21669--(1)--21671-

C

CATTGATTA  
Depth:3 (COW)  
Ei-value:0.000, Pi-value:0.000  
Er-value:0.000, Pr-value:0.000  
No matches to TargetScan


ATTGATTA

ATTGATTA  
Depth:4 (DOG)  
Ei-value:0.000, Pi-value:0.000  
Er-value:0.000, Pr-value:0.010  
No matches to TargetScan

-21679--(19)--21699-

AGAGATA

AGAGATA  
Depth:4 (DOG)  
Ei-value:0.000, Pi-value:0.000  
Er-value:0.000, Pr-value:0.000  
No matches to TargetScan

-21705--(35)--21741-

TGAACCT

TGAACCT  
Depth:3 (COW)  
Ei-value:0.000, Pi-value:0.000  
Er-value:0.000, Pr-value:0.010  
No matches to TargetScan

-21747--(2)--21750-

AA

AACAGAGATCT  
Depth:3 (COW)  
Ei-value:0.000, Pi-value:0.000  
Er-value:0.000, Pr-value:0.000  
No matches to TargetScan


CAGAGATCT

CAGAGATCT  
Depth:4 (DOG)  
Ei-value:0.000, Pi-value:0.000  
Er-value:0.000, Pr-value:0.000  
No matches to TargetScan

-21760--(9)--21770-

TTTACAAAGC

TTTACAAAGC  
Depth:3 (COW)  
Ei-value:0.000, Pi-value:0.000  
Er-value:0.000, Pr-value:0.000  
No matches to TargetScan

-21779--(7)--21787-

TCTATACA

TCTATACA  
Depth:3 (COW)  
Ei-value:0.000, Pi-value:0.000  
Er-value:0.000, Pr-value:0.000  
No matches to TargetScan

-21794--(18)--21813-

TTGGCT

TTGGCT  
Depth:4 (DOG)  
Ei-value:0.000, Pi-value:0.000  
Er-value:0.000, Pr-value:0.000  
No matches to TargetScan

-21818--(17)--21836-

TTACTTTCT

TTACTTTCT  
Depth:4 (DOG)  
Ei-value:0.000, Pi-value:0.000  
Er-value:0.000, Pr-value:0.010  
No matches to TargetScan

-21844--(24)--21869-

CTAGGATAT

CTAGGATAT  
Depth:3 (COW)  
Ei-value:0.000, Pi-value:0.000  
Er-value:0.000, Pr-value:0.000  
No matches to TargetScan

-21877--(1)--21879-

AAAATGA

AAAATGA  
Depth:3 (COW)  
Ei-value:0.000, Pi-value:0.000  
Er-value:0.000, Pr-value:0.000  
No matches to TargetScan

-21885--(162)--22048-

ATCCAGACCA

ATCCAGACCA  
Depth:3 (COW)  
Ei-value:0.000, Pi-value:0.000  
Er-value:0.000, Pr-value:0.000  
No matches to TargetScan

-22057--(83)--22141-

AGATGGA

AGATGGA  
Depth:3 (COW)  
Ei-value:0.000, Pi-value:0.000  
Er-value:0.000, Pr-value:0.000  
No matches to TargetScan

-22147--(34)--22182-

GAGTAAAAA

GAGTAAAAA  
Depth:4 (DOG)  
Ei-value:0.000, Pi-value:0.000  
Er-value:0.000, Pr-value:0.000  
No matches to TargetScan

-22190--(27)--22218-

ATTTGAT

ATTTGAT  
Depth:4 (DOG)  
Ei-value:0.000, Pi-value:0.010  
Er-value:0.000, Pr-value:0.000  
No matches to TargetScan

-22224--(7)--22232-

ATC

ATCTTTTATGT  
Depth:3 (COW)  
Ei-value:0.000, Pi-value:0.000  
Er-value:0.000, Pr-value:0.000  
No matches to TargetScan


TTTTATGT

TTTTATGT  
Depth:4 (DOG)  
Ei-value:0.000, Pi-value:0.000  
Er-value:0.000, Pr-value:0.000  
No matches to TargetScan

-22242--(16)--22259-

GGTCCTGAG

GGTCCTGAG  
Depth:3 (COW)  
Ei-value:0.000, Pi-value:0.000  
Er-value:0.000, Pr-value:0.000  
No matches to TargetScan

-22267--(151)--22419-

TTGCCTT

TTGCCTT  
Depth:3 (COW)  
Ei-value:0.000, Pi-value:0.000  
Er-value:0.000, Pr-value:0.000  
MATCHES To TargetScan▶ miR-124-3p.1:AAGGCAC

-22425--(106)--22532-

AAGCCAG

AAGCCAG  
Depth:4 (DOG)  
Ei-value:0.000, Pi-value:0.000  
Er-value:0.000, Pr-value:0.000  
MATCHES To TargetScan▶ miR-149-5p:CUGGCUC▶ miR-3064-5p:CUGGCUG

-22538--(7)--22546-

ATAAAAG

ATAAAAG  
Depth:4 (DOG)  
Ei-value:0.000, Pi-value:0.000  
Er-value:0.000, Pr-value:0.000  
No matches to TargetScan

-22552--(15)--22568-

CTTTAATTC

CTTTAATTC  
Depth:3 (COW)  
Ei-value:0.000, Pi-value:0.000  
Er-value:0.000, Pr-value:0.000  
No matches to TargetScan

-22576--(13)--22590-

TTTTATTA

TTTTATTA  
Depth:4 (DOG)  
Ei-value:0.000, Pi-value:0.010  
Er-value:0.000, Pr-value:0.000  
No matches to TargetScan

-22597--(2)--22600-

G

GTTAAATGG  
Depth:3 (COW)  
Ei-value:0.000, Pi-value:0.000  
Er-value:0.000, Pr-value:0.000  
No matches to TargetScan


TTAAATGG

TTAAATGG  
Depth:4 (DOG)  
Ei-value:0.000, Pi-value:0.000  
Er-value:0.000, Pr-value:0.000  
No matches to TargetScan

-22608--(187)--22796-

CTGTTCTTAAGT

CTGTTCTTAAGT  
Depth:3 (COW)  
Ei-value:0.000, Pi-value:0.000  
Er-value:0.000, Pr-value:0.000  
No matches to TargetScan

-22807--(67)--22875-

GAACAAATT

GAACAAATT  
Depth:3 (COW)  
Ei-value:0.000, Pi-value:0.000  
Er-value:0.000, Pr-value:0.000  
MATCHES To TargetScan▶ miR-375:UUGUUCG

-22883--(24)--22908-

TTAGTTG

TTAGTTG  
Depth:3 (COW)  
Ei-value:0.000, Pi-value:0.000  
Er-value:0.000, Pr-value:0.010  
No matches to TargetScan

-22914--(2)--22917-

AAACTTCATTGA

AAACTTCATTGA  
Depth:3 (COW)  
Ei-value:0.000, Pi-value:0.000  
Er-value:0.000, Pr-value:0.000  
No matches to TargetScan

-22928--(259)--23188-

ACAGAAAACAAAA

ACAGAAAACAAAA  
Depth:4 (DOG)  
Ei-value:0.000, Pi-value:0.000  
Er-value:0.000, Pr-value:0.000  
No matches to TargetScan

-23200--(27)--23228-

CTTGGAAA

CTTGGAAA  
Depth:3 (COW)  
Ei-value:0.000, Pi-value:0.000  
Er-value:0.000, Pr-value:0.000  
No matches to TargetScan

-23235--(17)--23253-

AGGTTA

AGGTTA  
Depth:4 (DOG)  
Ei-value:0.000, Pi-value:0.000  
Er-value:0.000, Pr-value:0.000  
No matches to TargetScan

-23258--(27)--23286-

TTCATTCT

TTCATTCT  
Depth:4 (DOG)  
Ei-value:0.000, Pi-value:0.000  
Er-value:0.000, Pr-value:0.000  
No matches to TargetScan

-23293--(61)--23355-

AGAGACA

AGAGACA  
Depth:4 (DOG)  
Ei-value:0.000, Pi-value:0.000  
Er-value:0.000, Pr-value:0.000  
No matches to TargetScan


TG

AGAGACATG  
Depth:3 (COW)  
Ei-value:0.000, Pi-value:0.000  
Er-value:0.000, Pr-value:0.000  
No matches to TargetScan

-23363--(45)--23409-

CCTTTTGG

CCTTTTGG  
Depth:4 (DOG)  
Ei-value:0.000, Pi-value:0.000  
Er-value:0.000, Pr-value:0.000  
No matches to TargetScan


C

CCTTTTGGC  
Depth:3 (COW)  
Ei-value:0.000, Pi-value:0.000  
Er-value:0.000, Pr-value:0.000  
No matches to TargetScan

-23417--(40)--23458-

ATTGTGT

ATTGTGT  
Depth:3 (COW)  
Ei-value:0.000, Pi-value:0.000  
Er-value:0.000, Pr-value:0.010  
No matches to TargetScan

-23464--(3)--23468-

TTAATTC

TTAATTC  
Depth:4 (DOG)  
Ei-value:0.000, Pi-value:0.000  
Er-value:0.000, Pr-value:0.000  
No matches to TargetScan

-23474--(21)--23496-

ACTCTGGCCACTAC

ACTCTGGCCACTAC  
Depth:4 (DOG)  
Ei-value:0.000, Pi-value:0.000  
Er-value:0.000, Pr-value:0.000  
MATCHES To TargetScan▶ miR-142-3p.1:GUAGUGU

-23509--(1)--23511-

ATAAGC

ATAAGC  
Depth:5 (RABBIT)  
Ei-value:0.000, Pi-value:0.010  
Er-value:0.000, Pr-value:0.000  
No matches to TargetScan


AGG

ATAAGCAGG  
Depth:4 (DOG)  
Ei-value:0.000, Pi-value:0.000  
Er-value:0.000, Pr-value:0.000  
No matches to TargetScan

-23519--(15)--23535-

TGCTCCTT

TGCTCCTT  
Depth:3 (COW)  
Ei-value:0.000, Pi-value:0.000  
Er-value:0.000, Pr-value:0.000  
MATCHES To TargetScan▶ miR-28-5p/708-5p:AGGAGCU

-23542--(13)--23556-

ACTTCA

ACTTCA  
Depth:3 (COW)  
Ei-value:0.000, Pi-value:0.000  
Er-value:0.000, Pr-value:0.000  
No matches to TargetScan

-23561--(1)--23563-

TTTTCCTA

TTTTCCTA  
Depth:3 (COW)  
Ei-value:0.000, Pi-value:0.000  
Er-value:0.000, Pr-value:0.000  
No matches to TargetScan

-23570--(11)--23582-

AT

ATGAAAAATG  
Depth:3 (COW)  
Ei-value:0.000, Pi-value:0.000  
Er-value:0.000, Pr-value:0.000  
No matches to TargetScan


GAAAAATG

GAAAAATG  
Depth:4 (DOG)  
Ei-value:0.000, Pi-value:0.000  
Er-value:0.000, Pr-value:0.000  
No matches to TargetScan

-23591--(53)--23645-

AGTCTCA

AGTCTCA  
Depth:4 (DOG)  
Ei-value:0.000, Pi-value:0.000  
Er-value:0.000, Pr-value:0.000  
No matches to TargetScan


TTGGTACCA

AGTCTCATTGGTACCA  
Depth:3 (COW)  
Ei-value:0.000, Pi-value:0.000  
Er-value:0.000, Pr-value:0.000  
No matches to TargetScan

-23660--(32)--23693-

TGGTTTTGAA

TGGTTTTGAA  
Depth:4 (DOG)  
Ei-value:0.000, Pi-value:0.000  
Er-value:0.000, Pr-value:0.000  
No matches to TargetScan

-23702--(66)--23769-

ACAATCC

ACAATCC  
Depth:3 (COW)  
Ei-value:0.000, Pi-value:0.000  
Er-value:0.000, Pr-value:0.000  
MATCHES To TargetScan▶ miR-219-5p:GAUUGUC

-23775--(21)--23797-

TGGAGATG

TGGAGATG  
Depth:3 (COW)  
Ei-value:0.000, Pi-value:0.000  
Er-value:0.000, Pr-value:0.000  
No matches to TargetScan

-23804--(15)--23820-

AGCTTCTC

AGCTTCTC  
Depth:3 (COW)  
Ei-value:0.000, Pi-value:0.000  
Er-value:0.000, Pr-value:0.000  
No matches to TargetScan

-23827--(9)--23837-

TTAGAAAT

TTAGAAAT  
Depth:4 (DOG)  
Ei-value:0.000, Pi-value:0.000  
Er-value:0.000, Pr-value:0.000  
No matches to TargetScan

-23844--(14)--23859-

CATCAAA

CATCAAA  
Depth:4 (DOG)  
Ei-value:0.000, Pi-value:0.000  
Er-value:0.000, Pr-value:0.000  
No matches to TargetScan

-23865--(103)--23969-

GGAAAAA

GGAAAAA  
Depth:3 (COW)  
Ei-value:0.000, Pi-value:0.000  
Er-value:0.000, Pr-value:0.000  
No matches to TargetScan

-23975--(51)--24027-

TGGGCTTTG

TGGGCTTTG  
Depth:3 (COW)  
Ei-value:0.000, Pi-value:0.000  
Er-value:0.000, Pr-value:0.000  
MATCHES To TargetScan▶ miR-330-3p:CAAAGCA

-24035--(10)--24046-

TTTTTAAATCACTCA

TTTTTAAATCACTCA  
Depth:4 (DOG)  
Ei-value:0.000, Pi-value:0.000  
Er-value:0.000, Pr-value:0.000  
No matches to TargetScan

-24060--(1)--24062-

AGAGGGTGGGA

AGAGGGTGGGA  
Depth:4 (DOG)  
Ei-value:0.000, Pi-value:0.000  
Er-value:0.000, Pr-value:0.000  
No matches to TargetScan

-24072--(1)--24074-

AGGAGGAAGAGTGAA

AGGAGGAAGAGTGAA  
Depth:4 (DOG)  
Ei-value:0.000, Pi-value:0.000  
Er-value:0.000, Pr-value:0.000  
MATCHES To TargetScan▶ miR-670-3p:UUCCUCA

-24088--(1)--24090-

G

GAAAAGGTCA  
Depth:4 (DOG)  
Ei-value:0.000, Pi-value:0.000  
Er-value:0.000, Pr-value:0.000  
MATCHES To TargetScan▶ miR-192-5p/215-5p:UGACCUA


AAAAGGT

AAAAGGT  
Depth:6 (MOUSE)  
Ei-value:0.000, Pi-value:0.000  
Er-value:0.000, Pr-value:0.000  
No matches to TargetScan


CA

GAAAAGGTCA  
Depth:4 (DOG)  
Ei-value:0.000, Pi-value:0.000  
Er-value:0.000, Pr-value:0.000  
MATCHES To TargetScan▶ miR-192-5p/215-5p:UGACCUA

-24099--(185)--24285-

TTGGTCTTAA

TTGGTCTTAA  
Depth:3 (COW)  
Ei-value:0.000, Pi-value:0.000  
Er-value:0.000, Pr-value:0.000  
MATCHES To TargetScan▶ miR-208-3p:UAAGACG▶ miR-499a-5p:UAAGACU

-24294--(78)--24373-

AGATGAGGACAAA

AGATGAGGACAAA  
Depth:3 (COW)  
Ei-value:0.000, Pi-value:0.000  
Er-value:0.000, Pr-value:0.000  
No matches to TargetScan

-24385--(1)--24387-

TCCTTTGT

TCCTTTGT  
Depth:3 (COW)  
Ei-value:0.000, Pi-value:0.000  
Er-value:0.000, Pr-value:0.000  
No matches to TargetScan

-24394--(110)--24505-

TAAAGC

TAAAGC  
Depth:3 (COW)  
Ei-value:0.000, Pi-value:0.000  
Er-value:0.000, Pr-value:0.000  
No matches to TargetScan

-24510--(54)--24565-

TTCATACA

TTCATACA  
Depth:3 (COW)  
Ei-value:0.000, Pi-value:0.000  
Er-value:0.000, Pr-value:0.000  
No matches to TargetScan

-24572--(3)--24576-

TTCAAAGCATC

TTCAAAGCATC  
Depth:3 (COW)  
Ei-value:0.000, Pi-value:0.000  
Er-value:0.000, Pr-value:0.000  
No matches to TargetScan

-24586--(45)--24632-

AGAAGGAAATAGA

AGAAGGAAATAGA  
Depth:3 (COW)  
Ei-value:0.000, Pi-value:0.000  
Er-value:0.000, Pr-value:0.000  
No matches to TargetScan

-24644--(21)--24666-

TGGAGGGAGC

TGGAGGGAGC  
Depth:3 (COW)  
Ei-value:0.000, Pi-value:0.000  
Er-value:0.000, Pr-value:0.000  
No matches to TargetScan

-24675--(30)--24706-

TAATGTTT

TAATGTTT  
Depth:4 (DOG)  
Ei-value:0.000, Pi-value:0.000  
Er-value:0.000, Pr-value:0.000  
MATCHES To TargetScan▶ miR-323-3p:ACAUUAC▶ miR-543:AACAUUC

-24713--(11)--24725-

TAATGTTT

TAATGTTT  
Depth:4 (DOG)  
Ei-value:0.000, Pi-value:0.000  
Er-value:0.000, Pr-value:0.000  
MATCHES To TargetScan▶ miR-323-3p:ACAUUAC▶ miR-543:AACAUUC

-24732--(13)--24746-

AGCTGGA

AGCTGGA  
Depth:4 (DOG)  
Ei-value:0.000, Pi-value:0.000  
Er-value:0.000, Pr-value:0.000  
No matches to TargetScan

-24752--(39)--24792-

ATTATTGGAAA

ATTATTGGAAA  
Depth:4 (DOG)  
Ei-value:0.000, Pi-value:0.000  
Er-value:0.000, Pr-value:0.000  
No matches to TargetScan

-24802--(10)--24813-

AGAAAGTAAC

AGAAAGTAAC  
Depth:4 (DOG)  
Ei-value:0.000, Pi-value:0.000  
Er-value:0.000, Pr-value:0.000  
No matches to TargetScan

-24822--(12)--24835-

TTTCACAGTTTCTGGCATC

TTTCACAGTTTCTGGCATC  
Depth:4 (DOG)  
Ei-value:0.000, Pi-value:0.000  
Er-value:0.000, Pr-value:0.000  
No matches to TargetScan

-24853--(9)--24863-

CA

CACTACTGAT  
Depth:3 (COW)  
Ei-value:0.000, Pi-value:0.000  
Er-value:0.000, Pr-value:0.000  
MATCHES To TargetScan▶ miR-142-3p.1:GUAGUGU▶ miR-199-3p:CAGUAGU


CTACTGAT

CTACTGAT  
Depth:4 (DOG)  
Ei-value:0.000, Pi-value:0.000  
Er-value:0.000, Pr-value:0.000  
MATCHES To TargetScan▶ miR-199-3p:CAGUAGU

-24872--(0)--24873-

AAACAAGAATAA

AAACAAGAATAA  
Depth:3 (COW)  
Ei-value:0.000, Pi-value:0.000  
Er-value:0.000, Pr-value:0.000  
MATCHES To TargetScan▶ miR-544a-5p:CUUGUUA

-24884--(1)--24886-

AGAACAT

AGAACAT  
Depth:4 (DOG)  
Ei-value:0.000, Pi-value:0.000  
Er-value:0.000, Pr-value:0.000  
No matches to TargetScan

-24892--(4)--24897-

TCATCTG

TCATCTG  
Depth:4 (DOG)  
Ei-value:0.000, Pi-value:0.010  
Er-value:0.000, Pr-value:0.000  
No matches to TargetScan

-24903--(10)--24914-

CATAAATGAA

CATAAATGAA  
Depth:4 (DOG)  
Ei-value:0.000, Pi-value:0.000  
Er-value:0.000, Pr-value:0.000  
No matches to TargetScan


GTTGTGA

CATAAATGAAGTTGTGA  
Depth:3 (COW)  
Ei-value:0.000, Pi-value:0.000  
Er-value:0.000, Pr-value:0.000  
No matches to TargetScan

-24930--(105)--25036-

ACTTG

ACTTGTGAACTGATGTGAAA  
Depth:3 (COW)  
Ei-value:0.000, Pi-value:0.000  
Er-value:0.000, Pr-value:0.000  
MATCHES To TargetScan▶ miR-23-3p:UCACAUU


TGAACTGATGTGAAA

TGAACTGATGTGAAA  
Depth:4 (DOG)  
Ei-value:0.000, Pi-value:0.000  
Er-value:0.000, Pr-value:0.000  
MATCHES To TargetScan▶ miR-23-3p:UCACAUU

-25055--(43)--25099-

TTGTTCA

TTGTTCA  
Depth:3 (COW)  
Ei-value:0.000, Pi-value:0.000  
Er-value:0.000, Pr-value:0.000  
No matches to TargetScan

-25105--(9)--25115-

ACCACCA

ACCACCA  
Depth:3 (COW)  
Ei-value:0.000, Pi-value:0.000  
Er-value:0.000, Pr-value:0.000  
No matches to TargetScan

-25121--(66)--25188-

AAATAAAA

AAATAAAA  
Depth:4 (DOG)  
Ei-value:0.000, Pi-value:0.000  
Er-value:0.000, Pr-value:0.000  
No matches to TargetScan

-25195  
  
>COW  
        69-

AGGCAAGA

AGGCAAGA  
Depth:3 (COW)  
Ei-value:0.000, Pi-value:0.000  
Er-value:0.000, Pr-value:0.000  
No matches to TargetScan

-76--(44)--121-

AAACATG

AAACATG  
Depth:4 (DOG)  
Ei-value:0.000, Pi-value:0.000  
Er-value:0.000, Pr-value:0.000  
No matches to TargetScan

-127--(8)--136-

CAACAG

CAACAG  
Depth:3 (COW)  
Ei-value:0.000, Pi-value:0.000  
Er-value:0.000, Pr-value:0.000  
No matches to TargetScan

-141--(28)--170-

TTCCCATC

TTCCCATC  
Depth:4 (DOG)  
Ei-value:0.000, Pi-value:0.000  
Er-value:0.000, Pr-value:0.000  
No matches to TargetScan

-177--(3)--181-

CTCTGT

CTCTGT  
Depth:3 (COW)  
Ei-value:0.000, Pi-value:0.000  
Er-value:0.000, Pr-value:0.000  
No matches to TargetScan

-186--(60)--247-

C

CTGTTAGTCT  
Depth:4 (DOG)  
Ei-value:0.000, Pi-value:0.000  
Er-value:0.000, Pr-value:0.000  
No matches to TargetScan


TGTTAGTC

TGTTAGTC  
Depth:5 (RABBIT)  
Ei-value:0.000, Pi-value:0.000  
Er-value:0.000, Pr-value:0.000  
No matches to TargetScan


T

CTGTTAGTCT  
Depth:4 (DOG)  
Ei-value:0.000, Pi-value:0.000  
Er-value:0.000, Pr-value:0.000  
No matches to TargetScan

-256--(1463)--1720-

TCATCC

TCATCC  
Depth:4 (DOG)  
Ei-value:0.000, Pi-value:0.020  
Er-value:0.000, Pr-value:0.000  
No matches to TargetScan

-1725--(317)--2043-

GGG

GGGTACTTGGGACTGTTAAT  
Depth:3 (COW)  
Ei-value:0.000, Pi-value:0.000  
Er-value:0.000, Pr-value:0.000  
MATCHES To TargetScan▶ miR-132-3p/212-3p:AACAGUC▶ miR-455-3p.1:CAGUCCA


TACTTGGGACTGTTAAT

TACTTGGGACTGTTAAT  
Depth:4 (DOG)  
Ei-value:0.000, Pi-value:0.000  
Er-value:0.000, Pr-value:0.000  
MATCHES To TargetScan▶ miR-132-3p/212-3p:AACAGUC▶ miR-455-3p.1:CAGUCCA

-2062--(89)--2152-

ACTG

ACTGTTAATGTGCT  
Depth:4 (DOG)  
Ei-value:0.000, Pi-value:0.000  
Er-value:0.000, Pr-value:0.000  
MATCHES To TargetScan▶ miR-132-3p/212-3p:AACAGUC▶ miR-323-3p:ACAUUAC


TTAATGTGCT

TTAATGTGCT  
Depth:5 (RABBIT)  
Ei-value:0.000, Pi-value:0.000  
Er-value:0.000, Pr-value:0.000  
MATCHES To TargetScan▶ miR-323-3p:ACAUUAC

-2165--(915)--3081-

CTTGGGACTC

CTTGGGACTC  
Depth:3 (COW)  
Ei-value:0.000, Pi-value:0.000  
Er-value:0.000, Pr-value:0.000  
No matches to TargetScan

-3090--(0)--3091-

AATGTGCAT

AATGTGCAT  
Depth:6 (MOUSE)  
Ei-value:0.000, Pi-value:0.000  
Er-value:0.000, Pr-value:0.000  
MATCHES To TargetScan▶ miR-501-3p/502-3p:AUGCACC

-3099--(16)--3116-

CTAATA

CTAATA  
Depth:3 (COW)  
Ei-value:0.000, Pi-value:0.000  
Er-value:0.000, Pr-value:0.000  
No matches to TargetScan

-3121--(130)--3252-

TGCTTCT

TGCTTCT  
Depth:3 (COW)  
Ei-value:0.000, Pi-value:0.000  
Er-value:0.000, Pr-value:0.010  
No matches to TargetScan

-3258--(42)--3301-

TATGTTAGA

TATGTTAGA  
Depth:4 (DOG)  
Ei-value:0.000, Pi-value:0.000  
Er-value:0.000, Pr-value:0.000  
No matches to TargetScan

-3309--(66)--3376-

TCTTGG

TCTTGGACTGTTAATGT  
Depth:3 (COW)  
Ei-value:0.000, Pi-value:0.000  
Er-value:0.000, Pr-value:0.000  
MATCHES To TargetScan▶ miR-132-3p/212-3p:AACAGUC▶ miR-323-3p:ACAUUAC▶ miR-455-3p.1:CAGUCCA


ACTGTTAATGT

ACTGTTAATGT  
Depth:4 (DOG)  
Ei-value:0.000, Pi-value:0.000  
Er-value:0.000, Pr-value:0.000  
MATCHES To TargetScan▶ miR-132-3p/212-3p:AACAGUC▶ miR-323-3p:ACAUUAC

-3392--(10)--3403-

ATTTGCT

ATTTGCT  
Depth:4 (DOG)  
Ei-value:0.000, Pi-value:0.000  
Er-value:0.000, Pr-value:0.000  
No matches to TargetScan

-3409--(18)--3428-

GTAAGGA

GTAAGGA  
Depth:5 (RABBIT)  
Ei-value:0.000, Pi-value:0.000  
Er-value:0.000, Pr-value:0.000  
No matches to TargetScan


CCC

GTAAGGACCC  
Depth:3 (COW)  
Ei-value:0.000, Pi-value:0.000  
Er-value:0.000, Pr-value:0.000  
No matches to TargetScan

-3437--(76)--3514-

ATCTTAG

ATCTTAG  
Depth:3 (COW)  
Ei-value:0.000, Pi-value:0.000  
Er-value:0.000, Pr-value:0.000  
No matches to TargetScan

-3520--(8)--3529-

TACACATT

TACACATT  
Depth:3 (COW)  
Ei-value:0.000, Pi-value:0.000  
Er-value:0.000, Pr-value:0.000  
No matches to TargetScan

-3536--(42)--3579-

ACTTAT

ACTTAT  
Depth:5 (RABBIT)  
Ei-value:0.000, Pi-value:0.000  
Er-value:0.000, Pr-value:0.000  
No matches to TargetScan

-3584--(55)--3640-

TGTAATT

TGTAATT  
Depth:3 (COW)  
Ei-value:0.000, Pi-value:0.000  
Er-value:0.000, Pr-value:0.000  
No matches to TargetScan

-3646--(10)--3657-

ATGGTC

ATGGTC  
Depth:3 (COW)  
Ei-value:0.000, Pi-value:0.020  
Er-value:0.000, Pr-value:0.000  
No matches to TargetScan

-3662--(51)--3714-

ATGGGGTACT

ATGGGGTACT  
Depth:3 (COW)  
Ei-value:0.000, Pi-value:0.000  
Er-value:0.000, Pr-value:0.000  
No matches to TargetScan

-3723--(3)--3727-

CAC

CACTTAAGGCCCCTTTCTCAA  
Depth:3 (COW)  
Ei-value:0.000, Pi-value:0.000  
Er-value:0.000, Pr-value:0.000  
No matches to TargetScan


TTAAGGCC

TTAAGGCC  
Depth:6 (MOUSE)  
Ei-value:0.000, Pi-value:0.000  
Er-value:0.000, Pr-value:0.000  
No matches to TargetScan


CCTTT

TTAAGGCCCCTTT  
Depth:5 (RABBIT)  
Ei-value:0.000, Pi-value:0.000  
Er-value:0.000, Pr-value:0.000  
No matches to TargetScan


CTCAA

TTAAGGCCCCTTTCTCAA  
Depth:4 (DOG)  
Ei-value:0.000, Pi-value:0.000  
Er-value:0.000, Pr-value:0.000  
No matches to TargetScan

-3747--(7)--3755-

TAATGACAATTACAT

TAATGACAATTACAT  
Depth:3 (COW)  
Ei-value:0.000, Pi-value:0.000  
Er-value:0.000, Pr-value:0.000  
MATCHES To TargetScan▶ miR-411-3p:AUGUAAC

-3769--(29)--3799-

CTTATATTT

CTTATATTT  
Depth:3 (COW)  
Ei-value:0.000, Pi-value:0.000  
Er-value:0.000, Pr-value:0.000  
MATCHES To TargetScan▶ miR-410-3p:AUAUAAC

-3807--(15)--3823-

TTTTAATTGACCA

TTTTAATTGACCA  
Depth:3 (COW)  
Ei-value:0.000, Pi-value:0.000  
Er-value:0.000, Pr-value:0.000  
No matches to TargetScan

-3835--(15)--3851-

ACATTAAT

ACATTAAT  
Depth:3 (COW)  
Ei-value:0.000, Pi-value:0.000  
Er-value:0.000, Pr-value:0.000  
No matches to TargetScan

-3858--(3)--3862-

CATAATTGCA

CATAATTGCA  
Depth:3 (COW)  
Ei-value:0.000, Pi-value:0.000  
Er-value:0.000, Pr-value:0.000  
No matches to TargetScan

-3871--(63)--3935-

CTAGACAAGGA

CTAGACAAGGA  
Depth:3 (COW)  
Ei-value:0.000, Pi-value:0.000  
Er-value:0.000, Pr-value:0.000  
No matches to TargetScan

-3945--(62)--4008-

ACAGTTAATGTG

ACAGTTAATGTG  
Depth:4 (DOG)  
Ei-value:0.000, Pi-value:0.000  
Er-value:0.000, Pr-value:0.000  
MATCHES To TargetScan▶ miR-323-3p:ACAUUAC

-4019--(69)--4089-

ATACTGTTT

ATACTGTTT  
Depth:3 (COW)  
Ei-value:0.000, Pi-value:0.000  
Er-value:0.000, Pr-value:0.000  
MATCHES To TargetScan▶ miR-101-3p.1:ACAGUAC▶ miR-132-3p/212-3p:AACAGUC▶ miR-144-3p:ACAGUAU

-4097--(43)--4141-

TTGTCTT

TTGTCTT  
Depth:3 (COW)  
Ei-value:0.000, Pi-value:0.000  
Er-value:0.000, Pr-value:0.010  
No matches to TargetScan

-4147--(44)--4192-

CTCAGCTCTTGG

CTCAGCTCTTGG  
Depth:5 (RABBIT)  
Ei-value:0.000, Pi-value:0.000  
Er-value:0.000, Pr-value:0.000  
MATCHES To TargetScan▶ miR-335-5p:CAAGAGC


ACA

CTCAGCTCTTGGACA  
Depth:4 (DOG)  
Ei-value:0.000, Pi-value:0.000  
Er-value:0.000, Pr-value:0.000  
MATCHES To TargetScan▶ miR-335-5p:CAAGAGC


ATTAATA

CTCAGCTCTTGGACAATTAATA  
Depth:3 (COW)  
Ei-value:0.000, Pi-value:0.000  
Er-value:0.000, Pr-value:0.000  
MATCHES To TargetScan▶ miR-335-5p:CAAGAGC

-4213--(59)--4273-

GATCAT

GATCAT  
Depth:3 (COW)  
Ei-value:0.000, Pi-value:0.000  
Er-value:0.000, Pr-value:0.000  
No matches to TargetScan

-4278--(11)--4290-

TAAGGC

TAAGGC  
Depth:3 (COW)  
Ei-value:0.000, Pi-value:0.000  
Er-value:0.000, Pr-value:0.000  
No matches to TargetScan

-4295--(22)--4318-

GAATATTTGCA

GAATATTTGCA  
Depth:3 (COW)  
Ei-value:0.000, Pi-value:0.000  
Er-value:0.000, Pr-value:0.000  
No matches to TargetScan

-4328--(56)--4385-

ATTACTG

ATTACTG  
Depth:3 (COW)  
Ei-value:0.000, Pi-value:0.010  
Er-value:0.000, Pr-value:0.020  
MATCHES To TargetScan▶ miR-802:CAGUAAC

-4391--(3)--4395-

GGGCTGCTGA

GGGCTGCTGA  
Depth:3 (COW)  
Ei-value:0.000, Pi-value:0.000  
Er-value:0.000, Pr-value:0.000  
MATCHES To TargetScan▶ miR-15-5p/16-5p/195-5p/424-5p/497-5p:AGCAGCA▶ miR-503-5p:AGCAGCG

-4404--(5)--4410-

CAAAACTT

CAAAACTT  
Depth:4 (DOG)  
Ei-value:0.000, Pi-value:0.000  
Er-value:0.000, Pr-value:0.000  
No matches to TargetScan

-4417--(3)--4421-

CTGGGACTG

CTGGGACTG  
Depth:3 (COW)  
Ei-value:0.000, Pi-value:0.000  
Er-value:0.000, Pr-value:0.000  
MATCHES To TargetScan▶ miR-455-3p.1:CAGUCCA

-4429--(7)--4437-

GCACAATG

GCACAATG  
Depth:6 (MOUSE)  
Ei-value:0.000, Pi-value:0.000  
Er-value:0.000, Pr-value:0.000  
No matches to TargetScan

-4444--(21)--4466-

CTCCCTG

CTCCCTG  
Depth:3 (COW)  
Ei-value:0.000, Pi-value:0.000  
Er-value:0.000, Pr-value:0.000  
No matches to TargetScan

-4472--(10)--4483-

GCAAGC

GCAAGC  
Depth:3 (COW)  
Ei-value:0.000, Pi-value:0.000  
Er-value:0.000, Pr-value:0.000  
No matches to TargetScan

-4488--(0)--4489-

A

ACTCCCA  
Depth:4 (DOG)  
Ei-value:0.000, Pi-value:0.000  
Er-value:0.000, Pr-value:0.000  
No matches to TargetScan


CTCCCA

CTCCCA  
Depth:6 (MOUSE)  
Ei-value:0.000, Pi-value:0.000  
Er-value:0.000, Pr-value:0.000  
No matches to TargetScan

-4495--(265)--4761-

CCCTTTTGCATT

CCCTTTTGCATT  
Depth:4 (DOG)  
Ei-value:0.000, Pi-value:0.000  
Er-value:0.000, Pr-value:0.000  
No matches to TargetScan


G

CCCTTTTGCATTG  
Depth:3 (COW)  
Ei-value:0.000, Pi-value:0.000  
Er-value:0.000, Pr-value:0.000  
No matches to TargetScan

-4773--(143)--4917-

ACTTCCTT

ACTTCCTT  
Depth:3 (COW)  
Ei-value:0.000, Pi-value:0.000  
Er-value:0.000, Pr-value:0.000  
No matches to TargetScan

-4924--(35)--4960-

AGCCCCTTCT

AGCCCCTTCT  
Depth:3 (COW)  
Ei-value:0.000, Pi-value:0.000  
Er-value:0.000, Pr-value:0.000  
No matches to TargetScan

-4969--(10)--4980-

CACAGTA

CACAGTA  
Depth:3 (COW)  
Ei-value:0.000, Pi-value:0.000  
Er-value:0.000, Pr-value:0.000  
No matches to TargetScan

-4986--(1)--4988-

TGATTGTC

TGATTGTCCCATTTTT  
Depth:3 (COW)  
Ei-value:0.000, Pi-value:0.000  
Er-value:0.000, Pr-value:0.000  
No matches to TargetScan


CCATTTTT

CCATTTTT  
Depth:4 (DOG)  
Ei-value:0.000, Pi-value:0.000  
Er-value:0.000, Pr-value:0.000  
No matches to TargetScan

-5003--(8)--5012-

CAGCCCA

CAGCCCA  
Depth:4 (DOG)  
Ei-value:0.000, Pi-value:0.000  
Er-value:0.000, Pr-value:0.000  
No matches to TargetScan

-5018--(3)--5022-

TCTC

TCTCCCTACCA  
Depth:3 (COW)  
Ei-value:0.000, Pi-value:0.000  
Er-value:0.000, Pr-value:0.000  
No matches to TargetScan


CCTACCA

CCTACCA  
Depth:4 (DOG)  
Ei-value:0.000, Pi-value:0.000  
Er-value:0.000, Pr-value:0.000  
No matches to TargetScan

-5032--(16)--5049-

GTGCAGT

GTGCAGT  
Depth:3 (COW)  
Ei-value:0.000, Pi-value:0.000  
Er-value:0.000, Pr-value:0.000  
MATCHES To TargetScan▶ miR-217:ACUGCAU

-5055--(10)--5066-

AAAAGCAG

AAAAGCAG  
Depth:6 (MOUSE)  
Ei-value:0.000, Pi-value:0.000  
Er-value:0.000, Pr-value:0.000  
No matches to TargetScan

-5073--(4)--5078-

GAACTA

GAACTA  
Depth:3 (COW)  
Ei-value:0.000, Pi-value:0.000  
Er-value:0.000, Pr-value:0.000  
No matches to TargetScan

-5083--(33)--5117-

TTAATGATCC

TTAATGATCC  
Depth:4 (DOG)  
Ei-value:0.000, Pi-value:0.000  
Er-value:0.000, Pr-value:0.000  
MATCHES To TargetScan▶ miR-382-3p:AUCAUUC

-5126--(8)--5135-

ATTATTGT

ATTATTGT  
Depth:3 (COW)  
Ei-value:0.000, Pi-value:0.000  
Er-value:0.000, Pr-value:0.000  
No matches to TargetScan

-5142--(3)--5146-

ATTCTGGG

ATTCTGGG  
Depth:4 (DOG)  
Ei-value:0.000, Pi-value:0.000  
Er-value:0.000, Pr-value:0.000  
No matches to TargetScan

-5153--(30)--5184-

TG

TGCTTTACT  
Depth:3 (COW)  
Ei-value:0.000, Pi-value:0.000  
Er-value:0.000, Pr-value:0.000  
MATCHES To TargetScan▶ miR-330-3p.2:AAAGCAC


CTTTACT

CTTTACT  
Depth:4 (DOG)  
Ei-value:0.000, Pi-value:0.000  
Er-value:0.000, Pr-value:0.000  
No matches to TargetScan

-5192--(2)--5195-

GCAAAAT

GCAAAAT  
Depth:6 (MOUSE)  
Ei-value:0.000, Pi-value:0.000  
Er-value:0.000, Pr-value:0.000  
No matches to TargetScan

-5201--(4)--5206-

AAGGCAA

AAGGCAA  
Depth:4 (DOG)  
Ei-value:0.000, Pi-value:0.000  
Er-value:0.000, Pr-value:0.000  
No matches to TargetScan


GTCAGACCCA

AAGGCAAGTCAGACCCA  
Depth:3 (COW)  
Ei-value:0.000, Pi-value:0.000  
Er-value:0.000, Pr-value:0.000  
MATCHES To TargetScan▶ miR-193a-5p:GGGUCUU

-5222--(7)--5230-

TGGATTGC

TGGATTGC  
Depth:4 (DOG)  
Ei-value:0.000, Pi-value:0.000  
Er-value:0.000, Pr-value:0.000  
No matches to TargetScan

-5237--(55)--5293-

GAAGGAAG

GAAGGAAG  
Depth:3 (COW)  
Ei-value:0.000, Pi-value:0.000  
Er-value:0.000, Pr-value:0.000  
No matches to TargetScan

-5300--(14)--5315-

TGCATTCTTC

TGCATTCTTC  
Depth:5 (RABBIT)  
Ei-value:0.000, Pi-value:0.000  
Er-value:0.000, Pr-value:0.000  
No matches to TargetScan

-5324--(7)--5332-

AGC

AGCAGATTGCCTGG  
Depth:4 (DOG)  
Ei-value:0.000, Pi-value:0.000  
Er-value:0.000, Pr-value:0.000  
No matches to TargetScan


A

AGATTGCCTGG  
Depth:5 (RABBIT)  
Ei-value:0.000, Pi-value:0.000  
Er-value:0.000, Pr-value:0.000  
No matches to TargetScan


GATTGCCTGG

GATTGCCTGG  
Depth:6 (MOUSE)  
Ei-value:0.000, Pi-value:0.000  
Er-value:0.000, Pr-value:0.000  
No matches to TargetScan

-5345--(19)--5365-

TTGTATATT

TTGTATATT  
Depth:4 (DOG)  
Ei-value:0.000, Pi-value:0.000  
Er-value:0.000, Pr-value:0.000  
MATCHES To TargetScan▶ miR-381-3p:AUACAAG

-5373--(12)--5386-

TGCCAA

TGCCAA  
Depth:3 (COW)  
Ei-value:0.000, Pi-value:0.000  
Er-value:0.000, Pr-value:0.000  
MATCHES To TargetScan▶ miR-182-5p:UUGGCAA▶ miR-96-5p/1271-5p:UUGGCAC

-5391--(1)--5393-

TGCCAGGATACA

TGCCAGGATACA  
Depth:3 (COW)  
Ei-value:0.000, Pi-value:0.000  
Er-value:0.000, Pr-value:0.000  
No matches to TargetScan

-5404--(46)--5451-

ACATCTGG

ACATCTGG  
Depth:3 (COW)  
Ei-value:0.000, Pi-value:0.000  
Er-value:0.000, Pr-value:0.000  
No matches to TargetScan

-5458--(16)--5475-

GAT

GATAACCTGGTCATT  
Depth:3 (COW)  
Ei-value:0.000, Pi-value:0.000  
Er-value:0.000, Pr-value:0.000  
MATCHES To TargetScan▶ miR-154-5p:AGGUUAU


AAC

AACCTGGTCATT  
Depth:4 (DOG)  
Ei-value:0.000, Pi-value:0.000  
Er-value:0.000, Pr-value:0.000  
No matches to TargetScan


CTGGTCATT

CTGGTCATT  
Depth:5 (RABBIT)  
Ei-value:0.000, Pi-value:0.000  
Er-value:0.000, Pr-value:0.000  
No matches to TargetScan

-5489--(4)--5494-

TTTTGAA

TTTTGAA  
Depth:3 (COW)  
Ei-value:0.000, Pi-value:0.000  
Er-value:0.000, Pr-value:0.010  
No matches to TargetScan

-5500--(10)--5511-

CCATTTAT

CCATTTAT  
Depth:5 (RABBIT)  
Ei-value:0.000, Pi-value:0.000  
Er-value:0.000, Pr-value:0.000  
No matches to TargetScan

-5518--(13)--5532-

TGAC

TGACCAGTGTCTCTCATTT  
Depth:4 (DOG)  
Ei-value:0.000, Pi-value:0.000  
Er-value:0.000, Pr-value:0.000  
No matches to TargetScan


CAGTGTCTCTCATTT

CAGTGTCTCTCATTT  
Depth:5 (RABBIT)  
Ei-value:0.000, Pi-value:0.000  
Er-value:0.000, Pr-value:0.000  
No matches to TargetScan

-5550--(5)--5556-

AGG

AGGGTGGTG  
Depth:4 (DOG)  
Ei-value:0.000, Pi-value:0.000  
Er-value:0.000, Pr-value:0.000  
No matches to TargetScan


GTGGTG

GTGGTG  
Depth:5 (RABBIT)  
Ei-value:0.000, Pi-value:0.000  
Er-value:0.000, Pr-value:0.000  
No matches to TargetScan

-5564--(1)--5566-

GTCTGTGGATA

GTCTGTGGATA  
Depth:5 (RABBIT)  
Ei-value:0.000, Pi-value:0.000  
Er-value:0.000, Pr-value:0.000  
MATCHES To TargetScan▶ miR-140-3p.1:CCACAGG


GA

GTCTGTGGATAGA  
Depth:3 (COW)  
Ei-value:0.000, Pi-value:0.000  
Er-value:0.000, Pr-value:0.000  
MATCHES To TargetScan▶ miR-140-3p.1:CCACAGG

-5578--(15)--5594-

TATTTTA

TATTTTA  
Depth:3 (COW)  
Ei-value:0.000, Pi-value:0.040  
Er-value:0.000, Pr-value:0.020  
No matches to TargetScan

-5600--(17)--5618-

TTCTAGA

TTCTAGA  
Depth:4 (DOG)  
Ei-value:0.000, Pi-value:0.000  
Er-value:0.000, Pr-value:0.000  
No matches to TargetScan

-5624--(17)--5642-

AGTATCTTTG

AGTATCTTTG  
Depth:3 (COW)  
Ei-value:0.000, Pi-value:0.000  
Er-value:0.000, Pr-value:0.000  
No matches to TargetScan

-5651--(47)--5699-

ATTCACTT

ATTCACTT  
Depth:4 (DOG)  
Ei-value:0.000, Pi-value:0.000  
Er-value:0.000, Pr-value:0.000  
No matches to TargetScan

-5706--(4)--5711-

GAAAAAC

GAAAAAC  
Depth:4 (DOG)  
Ei-value:0.000, Pi-value:0.000  
Er-value:0.000, Pr-value:0.000  
No matches to TargetScan

-5717--(22)--5740-

AATTTCTTCATCTGGAGC

AATTTCTTCATCTGGAGC  
Depth:5 (RABBIT)  
Ei-value:0.000, Pi-value:0.000  
Er-value:0.000, Pr-value:0.000  
No matches to TargetScan

-5757--(15)--5773-

CTTATTT

CTTATTT  
Depth:4 (DOG)  
Ei-value:0.000, Pi-value:0.000  
Er-value:0.000, Pr-value:0.010  
No matches to TargetScan


CAAGAA

CTTATTTCAAGAA  
Depth:3 (COW)  
Ei-value:0.000, Pi-value:0.000  
Er-value:0.000, Pr-value:0.000  
MATCHES To TargetScan▶ miR-203a-3p.2:UGAAAUG

-5785--(15)--5801-

ATAAAATG

ATAAAATG  
Depth:4 (DOG)  
Ei-value:0.000, Pi-value:0.000  
Er-value:0.000, Pr-value:0.000  
No matches to TargetScan


A

ATAAAATGA  
Depth:3 (COW)  
Ei-value:0.000, Pi-value:0.000  
Er-value:0.000, Pr-value:0.000  
No matches to TargetScan

-5809--(106)--5916-

ACCACACT

ACCACACT  
Depth:3 (COW)  
Ei-value:0.000, Pi-value:0.000  
Er-value:0.000, Pr-value:0.000  
No matches to TargetScan

-5923--(3)--5927-

GTGAGG

GTGAGG  
Depth:3 (COW)  
Ei-value:0.000, Pi-value:0.000  
Er-value:0.000, Pr-value:0.000  
No matches to TargetScan

-5932--(29)--5962-

TTTTATA

TTTTATA  
Depth:3 (COW)  
Ei-value:0.000, Pi-value:0.000  
Er-value:0.000, Pr-value:0.010  
MATCHES To TargetScan▶ miR-340-5p:UAUAAAG

-5968--(7)--5976-

AAAAATAAGCCA

AAAAATAAGCCA  
Depth:5 (RABBIT)  
Ei-value:0.000, Pi-value:0.000  
Er-value:0.000, Pr-value:0.000  
No matches to TargetScan


A

AAAAATAAGCCAA  
Depth:4 (DOG)  
Ei-value:0.000, Pi-value:0.000  
Er-value:0.000, Pr-value:0.000  
No matches to TargetScan

-5988--(11)--6000-

TCTTTTGGATATA

TCTTTTGGATATA  
Depth:3 (COW)  
Ei-value:0.000, Pi-value:0.000  
Er-value:0.000, Pr-value:0.000  
No matches to TargetScan

-6012--(26)--6039-

ATGAATAATA

ATGAATAATA  
Depth:4 (DOG)  
Ei-value:0.000, Pi-value:0.000  
Er-value:0.000, Pr-value:0.000  
No matches to TargetScan

-6048--(11)--6060-

AGTGTACA

AGTGTACA  
Depth:3 (COW)  
Ei-value:0.000, Pi-value:0.000  
Er-value:0.000, Pr-value:0.000  
MATCHES To TargetScan▶ miR-493-5p:UGUACAU

-6067--(1)--6069-

GGTGTTT

GGTGTTT  
Depth:3 (COW)  
Ei-value:0.000, Pi-value:0.000  
Er-value:0.000, Pr-value:0.000  
No matches to TargetScan

-6075--(19)--6095-

TGGAACTGCT

TGGAACTGCT  
Depth:4 (DOG)  
Ei-value:0.000, Pi-value:0.000  
Er-value:0.000, Pr-value:0.000  
No matches to TargetScan

-6104--(8)--6113-

TAACTA

TAACTA  
Depth:4 (DOG)  
Ei-value:0.000, Pi-value:0.000  
Er-value:0.000, Pr-value:0.000  
No matches to TargetScan

-6118--(10)--6129-

CAGCAGTTC

CAGCAGTTC  
Depth:5 (RABBIT)  
Ei-value:0.000, Pi-value:0.000  
Er-value:0.000, Pr-value:0.000  
No matches to TargetScan

-6137--(1)--6139-

TTGTAAT

TTGTAAT  
Depth:4 (DOG)  
Ei-value:0.000, Pi-value:0.000  
Er-value:0.000, Pr-value:0.000  
No matches to TargetScan

-6145--(1)--6147-

ACTGAAAA

ACTGAAAA  
Depth:5 (RABBIT)  
Ei-value:0.000, Pi-value:0.000  
Er-value:0.000, Pr-value:0.000  
No matches to TargetScan

-6154--(15)--6170-

GAG

GAGAAGGATGTCAAAAGATCGGC  
Depth:3 (COW)  
Ei-value:0.000, Pi-value:0.000  
Er-value:0.000, Pr-value:0.000  
MATCHES To TargetScan▶ miR-362-5p/500b-5p:AUCCUUG▶ miR-489-3p:UGACAUC


AAGGATG

AAGGATG  
Depth:5 (RABBIT)  
Ei-value:0.000, Pi-value:0.000  
Er-value:0.000, Pr-value:0.000  
MATCHES To TargetScan▶ miR-362-5p/500b-5p:AUCCUUG


TCA

AAGGATGTCAAAAGATC  
Depth:4 (DOG)  
Ei-value:0.000, Pi-value:0.000  
Er-value:0.000, Pr-value:0.000  
MATCHES To TargetScan▶ miR-362-5p/500b-5p:AUCCUUG▶ miR-489-3p:UGACAUC


AAAGATC

AAAGATC  
Depth:6 (MOUSE)  
Ei-value:0.000, Pi-value:0.000  
Er-value:0.000, Pr-value:0.000  
No matches to TargetScan


GGC

GAGAAGGATGTCAAAAGATCGGC  
Depth:3 (COW)  
Ei-value:0.000, Pi-value:0.000  
Er-value:0.000, Pr-value:0.000  
MATCHES To TargetScan▶ miR-362-5p/500b-5p:AUCCUUG▶ miR-489-3p:UGACAUC

-6192--(1)--6194-

CAGCTCAGGG

CAGCTCAGGG  
Depth:4 (DOG)  
Ei-value:0.000, Pi-value:0.000  
Er-value:0.000, Pr-value:0.000  
MATCHES To TargetScan▶ miR-125-5p:CCCUGAG

-6203--(1)--6205-

GCAGTTTGC

GCAGTTTGC  
Depth:3 (COW)  
Ei-value:0.000, Pi-value:0.000  
Er-value:0.000, Pr-value:0.000  
No matches to TargetScan

-6213--(1)--6215-

CTACTAGCTCCT

CTACTAGCTCCT  
Depth:4 (DOG)  
Ei-value:0.000, Pi-value:0.000  
Er-value:0.000, Pr-value:0.000  
MATCHES To TargetScan▶ miR-28-5p/708-5p:AGGAGCU▶ miR-411-5p.2:UAGUAGA

-6226--(1)--6228-

GGACAGCTG

GGACAGCTG  
Depth:5 (RABBIT)  
Ei-value:0.000, Pi-value:0.000  
Er-value:0.000, Pr-value:0.000  
No matches to TargetScan


T

GGACAGCTGT  
Depth:4 (DOG)  
Ei-value:0.000, Pi-value:0.000  
Er-value:0.000, Pr-value:0.000  
No matches to TargetScan

-6237--(0)--6238-

A

AAGAAGAGTCTCTGGCTCTTTAGA  
Depth:3 (COW)  
Ei-value:0.000, Pi-value:0.000  
Er-value:0.000, Pr-value:0.000  
No matches to TargetScan


AGAAGAGTCTCTGGCTCTTTA

AGAAGAGTCTCTGGCTCTTTA  
Depth:5 (RABBIT)  
Ei-value:0.000, Pi-value:0.000  
Er-value:0.000, Pr-value:0.000  
No matches to TargetScan


GA

AGAAGAGTCTCTGGCTCTTTAGA  
Depth:4 (DOG)  
Ei-value:0.000, Pi-value:0.000  
Er-value:0.000, Pr-value:0.000  
No matches to TargetScan

-6261--(11)--6273-

ATTCTGAGC

ATTCTGAGC  
Depth:4 (DOG)  
Ei-value:0.000, Pi-value:0.000  
Er-value:0.000, Pr-value:0.000  
No matches to TargetScan

-6281--(101)--6383-

GA

GACTGCAA  
Depth:3 (COW)  
Ei-value:0.000, Pi-value:0.000  
Er-value:0.000, Pr-value:0.000  
MATCHES To TargetScan▶ miR-455-3p.2:UGCAGUC


CTGCAA

CTGCAA  
Depth:5 (RABBIT)  
Ei-value:0.000, Pi-value:0.000  
Er-value:0.000, Pr-value:0.000  
No matches to TargetScan

-6390--(38)--6429-

TTTGAGAATCTGG

TTTGAGAATCTGG  
Depth:3 (COW)  
Ei-value:0.000, Pi-value:0.000  
Er-value:0.000, Pr-value:0.000  
MATCHES To TargetScan▶ miR-371-5p:CUCAAAC

-6441--(2)--6444-

AAGCTCCA

AAGCTCCA  
Depth:3 (COW)  
Ei-value:0.000, Pi-value:0.000  
Er-value:0.000, Pr-value:0.000  
No matches to TargetScan

-6451--(12)--6464-

GGATGG

GGATGG  
Depth:3 (COW)  
Ei-value:0.000, Pi-value:0.000  
Er-value:0.000, Pr-value:0.010  
No matches to TargetScan

-6469--(12)--6482-

CTGGAGAAAAAGATCT

CTGGAGAAAAAGATCT  
Depth:3 (COW)  
Ei-value:0.000, Pi-value:0.000  
Er-value:0.000, Pr-value:0.000  
No matches to TargetScan

-6497--(7)--6505-

AAGAATAGGC

AAGAATAGGC  
Depth:5 (RABBIT)  
Ei-value:0.000, Pi-value:0.000  
Er-value:0.000, Pr-value:0.000  
No matches to TargetScan

-6514--(8)--6523-

T

TTACAGTGTTAGTGA  
Depth:3 (COW)  
Ei-value:0.000, Pi-value:0.000  
Er-value:0.000, Pr-value:0.000  
MATCHES To TargetScan▶ miR-141-3p/200a-3p:AACACUG


TACAGTGTTAGTGA

TACAGTGTTAGTGA  
Depth:5 (RABBIT)  
Ei-value:0.000, Pi-value:0.000  
Er-value:0.000, Pr-value:0.000  
MATCHES To TargetScan▶ miR-141-3p/200a-3p:AACACUG

-6537--(2)--6540-

CA

CATTCCCTTTGA  
Depth:3 (COW)  
Ei-value:0.000, Pi-value:0.000  
Er-value:0.000, Pr-value:0.000  
MATCHES To TargetScan▶ miR-1-3p/206:GGAAUGU


TTCCCTTTGA

TTCCCTTTGA  
Depth:6 (MOUSE)  
Ei-value:0.000, Pi-value:0.000  
Er-value:0.000, Pr-value:0.000  
No matches to TargetScan

-6551--(7)--6559-

TAGGTGGAGATGGGGCATGAGGATCCTCCAGGGGAA

TAGGTGGAGATGGGGCATGAGGATCCTCCAGGGGAA  
Depth:6 (MOUSE)  
Ei-value:0.000, Pi-value:0.000  
Er-value:0.000, Pr-value:0.000  
MATCHES To TargetScan▶ miR-331-3p:CCCCUGG


A

TAGGTGGAGATGGGGCATGAGGATCCTCCAGGGGAAA  
Depth:5 (RABBIT)  
Ei-value:0.000, Pi-value:0.000  
Er-value:0.000, Pr-value:0.000  
MATCHES To TargetScan▶ miR-331-3p:CCCCUGG

-6595--(3)--6599-

TCACTA

TCACTA  
Depth:5 (RABBIT)  
Ei-value:0.000, Pi-value:0.000  
Er-value:0.000, Pr-value:0.000  
No matches to TargetScan


CCACT

TCACTACCACT  
Depth:4 (DOG)  
Ei-value:0.000, Pi-value:0.000  
Er-value:0.000, Pr-value:0.000  
MATCHES To TargetScan▶ miR-140-5p:AGUGGUU▶ miR-142-3p.1:GUAGUGU


G

TCACTACCACTG  
Depth:3 (COW)  
Ei-value:0.000, Pi-value:0.000  
Er-value:0.000, Pr-value:0.000  
MATCHES To TargetScan▶ miR-140-5p:AGUGGUU▶ miR-142-3p.1:GUAGUGU

-6610--(1)--6612-

GCAACA

GCAACA  
Depth:6 (MOUSE)  
Ei-value:0.000, Pi-value:0.000  
Er-value:0.000, Pr-value:0.000  
No matches to TargetScan


AC

GCAACAAC  
Depth:5 (RABBIT)  
Ei-value:0.000, Pi-value:0.000  
Er-value:0.000, Pr-value:0.000  
No matches to TargetScan

-6619--(27)--6647-

CTTTCCTGG

CTTTCCTGG  
Depth:3 (COW)  
Ei-value:0.000, Pi-value:0.000  
Er-value:0.000, Pr-value:0.000  
MATCHES To TargetScan▶ miR-665:CCAGGAG▶ miR-873-5p.1:CAGGAAC

-6655--(27)--6683-

ACAACCACC

ACAACCACC  
Depth:5 (RABBIT)  
Ei-value:0.000, Pi-value:0.000  
Er-value:0.000, Pr-value:0.000  
No matches to TargetScan


ACAC

ACAACCACCACAC  
Depth:4 (DOG)  
Ei-value:0.000, Pi-value:0.000  
Er-value:0.000, Pr-value:0.000  
No matches to TargetScan

-6695--(15)--6711-

TTGTTCC

TTGTTCC  
Depth:4 (DOG)  
Ei-value:0.000, Pi-value:0.000  
Er-value:0.000, Pr-value:0.000  
No matches to TargetScan

-6717--(9)--6727-

TG

TGCCAAATC  
Depth:3 (COW)  
Ei-value:0.000, Pi-value:0.000  
Er-value:0.000, Pr-value:0.000  
MATCHES To TargetScan▶ miR-182-5p:UUGGCAA▶ miR-96-5p/1271-5p:UUGGCAC


CCAAAT

CCAAAT  
Depth:6 (MOUSE)  
Ei-value:0.000, Pi-value:0.000  
Er-value:0.000, Pr-value:0.000  
No matches to TargetScan


C

CCAAATC  
Depth:5 (RABBIT)  
Ei-value:0.000, Pi-value:0.000  
Er-value:0.000, Pr-value:0.000  
No matches to TargetScan

-6735--(29)--6765-

CAAGAAA

CAAGAAA  
Depth:5 (RABBIT)  
Ei-value:0.000, Pi-value:0.000  
Er-value:0.000, Pr-value:0.000  
No matches to TargetScan


T

CAAGAAAT  
Depth:3 (COW)  
Ei-value:0.000, Pi-value:0.000  
Er-value:0.000, Pr-value:0.000  
No matches to TargetScan

-6772--(1)--6774-

TGAACACAC

TGAACACAC  
Depth:3 (COW)  
Ei-value:0.000, Pi-value:0.000  
Er-value:0.000, Pr-value:0.000  
No matches to TargetScan

-6782--(4)--6787-

G

GAAGATCAACATGCCTG  
Depth:4 (DOG)  
Ei-value:0.000, Pi-value:0.000  
Er-value:0.000, Pr-value:0.000  
No matches to TargetScan


AA

AAGATCAACATGC  
Depth:5 (RABBIT)  
Ei-value:0.000, Pi-value:0.000  
Er-value:0.000, Pr-value:0.000  
No matches to TargetScan


GATCAACATGC

GATCAACATGC  
Depth:6 (MOUSE)  
Ei-value:0.000, Pi-value:0.000  
Er-value:0.000, Pr-value:0.000  
No matches to TargetScan


CTG

GAAGATCAACATGCCTG  
Depth:4 (DOG)  
Ei-value:0.000, Pi-value:0.000  
Er-value:0.000, Pr-value:0.000  
No matches to TargetScan

-6803--(94)--6898-

TGTGTAT

TGTGTAT  
Depth:6 (MOUSE)  
Ei-value:0.000, Pi-value:0.000  
Er-value:0.000, Pr-value:0.000  
No matches to TargetScan


TT

TGTGTATTT  
Depth:4 (DOG)  
Ei-value:0.000, Pi-value:0.000  
Er-value:0.000, Pr-value:0.000  
No matches to TargetScan

-6906--(35)--6942-

TCTTTCTT

TCTTTCTT  
Depth:3 (COW)  
Ei-value:0.000, Pi-value:0.000  
Er-value:0.000, Pr-value:0.000  
No matches to TargetScan

-6949--(10)--6960-

TGTCTTA

TGTCTTA  
Depth:4 (DOG)  
Ei-value:0.000, Pi-value:0.000  
Er-value:0.000, Pr-value:0.000  
MATCHES To TargetScan▶ miR-208-3p:UAAGACG▶ miR-499a-5p:UAAGACU


CCCATTTCCATG

TGTCTTACCCATTTCCATG  
Depth:3 (COW)  
Ei-value:0.000, Pi-value:0.000  
Er-value:0.000, Pr-value:0.000  
MATCHES To TargetScan▶ miR-203a-3p.1:GAAAUGU▶ miR-208-3p:UAAGACG▶ miR-499a-5p:UAAGACU

-6978--(45)--7024-

TTTTTGT

TTTTTGT  
Depth:4 (DOG)  
Ei-value:0.000, Pi-value:0.000  
Er-value:0.000, Pr-value:0.000  
No matches to TargetScan

-7030--(48)--7079-

TTCATTTTGTT

TTCATTTTGTT  
Depth:4 (DOG)  
Ei-value:0.000, Pi-value:0.000  
Er-value:0.000, Pr-value:0.000  
MATCHES To TargetScan▶ miR-495-3p:AACAAAC

-7089--(75)--7165-

TTTGCTC

TTTGCTC  
Depth:3 (COW)  
Ei-value:0.000, Pi-value:0.000  
Er-value:0.000, Pr-value:0.000  
No matches to TargetScan

-7171--(27)--7199-

TT

TTTTCTCTTTGTGAA  
Depth:3 (COW)  
Ei-value:0.000, Pi-value:0.000  
Er-value:0.000, Pr-value:0.000  
No matches to TargetScan


TTCTCTTTG

TTCTCTTTG  
Depth:6 (MOUSE)  
Ei-value:0.000, Pi-value:0.000  
Er-value:0.000, Pr-value:0.000  
No matches to TargetScan


TGAA

TTTTCTCTTTGTGAA  
Depth:3 (COW)  
Ei-value:0.000, Pi-value:0.000  
Er-value:0.000, Pr-value:0.000  
No matches to TargetScan

-7213--(12)--7226-

TTCCCCTT

TTCCCCTT  
Depth:3 (COW)  
Ei-value:0.000, Pi-value:0.000  
Er-value:0.000, Pr-value:0.000  
No matches to TargetScan

-7233--(11)--7245-

ATTTCACCT

ATTTCACCT  
Depth:4 (DOG)  
Ei-value:0.000, Pi-value:0.000  
Er-value:0.000, Pr-value:0.000  
MATCHES To TargetScan▶ miR-203a-3p.2:UGAAAUG

-7253--(22)--7276-

TGCTG

TGCTGTTTCTACT  
Depth:3 (COW)  
Ei-value:0.000, Pi-value:0.000  
Er-value:0.000, Pr-value:0.000  
MATCHES To TargetScan▶ miR-411-5p.1:AGUAGAC▶ miR-494-3p:GAAACAU


TTTCTAC

TTTCTAC  
Depth:6 (MOUSE)  
Ei-value:0.000, Pi-value:0.000  
Er-value:0.000, Pr-value:0.000  
No matches to TargetScan


T

TTTCTACT  
Depth:5 (RABBIT)  
Ei-value:0.000, Pi-value:0.000  
Er-value:0.000, Pr-value:0.000  
MATCHES To TargetScan▶ miR-411-5p.1:AGUAGAC

-7288--(13)--7302-

ATTTCTC

ATTTCTC  
Depth:6 (MOUSE)  
Ei-value:0.000, Pi-value:0.000  
Er-value:0.000, Pr-value:0.000  
No matches to TargetScan

-7308--(24)--7333-

TCTTGGG

TCTTGGG  
Depth:5 (RABBIT)  
Ei-value:0.000, Pi-value:0.000  
Er-value:0.000, Pr-value:0.000  
No matches to TargetScan


C

TCTTGGGC  
Depth:3 (COW)  
Ei-value:0.000, Pi-value:0.000  
Er-value:0.000, Pr-value:0.000  
No matches to TargetScan

-7340--(51)--7392-

TTTGTGA

TTTGTGA  
Depth:4 (DOG)  
Ei-value:0.000, Pi-value:0.010  
Er-value:0.000, Pr-value:0.000  
No matches to TargetScan


TTTTC

TTTGTGATTTTC  
Depth:3 (COW)  
Ei-value:0.000, Pi-value:0.000  
Er-value:0.000, Pr-value:0.000  
No matches to TargetScan

-7403--(14)--7418-

TCTCTGTT

TCTCTGTT  
Depth:4 (DOG)  
Ei-value:0.000, Pi-value:0.000  
Er-value:0.000, Pr-value:0.000  
No matches to TargetScan

-7425--(37)--7463-

TTTGAGTATTT

TTTGAGTATTT  
Depth:4 (DOG)  
Ei-value:0.000, Pi-value:0.000  
Er-value:0.000, Pr-value:0.000  
MATCHES To TargetScan▶ miR-200bc-3p/429:AAUACUG▶ miR-371-5p:CUCAAAC

-7473--(21)--7495-

CTTTGATT

CTTTGATT  
Depth:3 (COW)  
Ei-value:0.000, Pi-value:0.000  
Er-value:0.000, Pr-value:0.000  
No matches to TargetScan

-7502--(41)--7544-

TGTGTGTG

TGTGTGTG  
Depth:4 (DOG)  
Ei-value:0.000, Pi-value:0.000  
Er-value:0.000, Pr-value:0.000  
MATCHES To TargetScan▶ miR-329-3p/362-3p:ACACACC

-7551--(28)--7580-

TCCTAACCCCT

TCCTAACCCCT  
Depth:5 (RABBIT)  
Ei-value:0.000, Pi-value:0.000  
Er-value:0.000, Pr-value:0.000  
No matches to TargetScan

-7590--(5)--7596-

TAGGTGCA

TAGGTGCA  
Depth:3 (COW)  
Ei-value:0.000, Pi-value:0.000  
Er-value:0.000, Pr-value:0.000  
No matches to TargetScan

-7603--(19)--7623-

AAGCATTG

AAGCATTG  
Depth:4 (DOG)  
Ei-value:0.000, Pi-value:0.000  
Er-value:0.000, Pr-value:0.000  
No matches to TargetScan

-7630--(12)--7643-

TTATGCCA

TTATGCCA  
Depth:5 (RABBIT)  
Ei-value:0.000, Pi-value:0.000  
Er-value:0.000, Pr-value:0.000  
No matches to TargetScan


G

TTATGCCAG  
Depth:4 (DOG)  
Ei-value:0.000, Pi-value:0.000  
Er-value:0.000, Pr-value:0.000  
No matches to TargetScan

-7651--(34)--7686-

TCCAAG

TCCAAG  
Depth:3 (COW)  
Ei-value:0.000, Pi-value:0.000  
Er-value:0.000, Pr-value:0.000  
No matches to TargetScan

-7691--(162)--7854-

AGA

AGAAGGCCCAA  
Depth:4 (DOG)  
Ei-value:0.000, Pi-value:0.000  
Er-value:0.000, Pr-value:0.000  
No matches to TargetScan


AGGCCCAA

AGGCCCAA  
Depth:5 (RABBIT)  
Ei-value:0.000, Pi-value:0.000  
Er-value:0.000, Pr-value:0.000  
No matches to TargetScan

-7864--(14)--7879-

TCAA

TCAAGACTAA  
Depth:4 (DOG)  
Ei-value:0.000, Pi-value:0.000  
Er-value:0.000, Pr-value:0.000  
MATCHES To TargetScan▶ miR-431-5p:GUCUUGC


GACTAA

GACTAA  
Depth:5 (RABBIT)  
Ei-value:0.000, Pi-value:0.000  
Er-value:0.000, Pr-value:0.000  
No matches to TargetScan

-7888--(46)--7935-

AGAAGC

AGAAGC  
Depth:4 (DOG)  
Ei-value:0.000, Pi-value:0.000  
Er-value:0.000, Pr-value:0.010  
No matches to TargetScan

-7940--(20)--7961-

C

CAAGATGA  
Depth:3 (COW)  
Ei-value:0.000, Pi-value:0.000  
Er-value:0.000, Pr-value:0.000  
No matches to TargetScan


AAGATGA

AAGATGA  
Depth:5 (RABBIT)  
Ei-value:0.000, Pi-value:0.000  
Er-value:0.000, Pr-value:0.000  
No matches to TargetScan

-7968--(19)--7988-

TTTCTATTG

TTTCTATTG  
Depth:3 (COW)  
Ei-value:0.000, Pi-value:0.000  
Er-value:0.000, Pr-value:0.000  
No matches to TargetScan

-7996--(63)--8060-

ACTTCTT

ACTTCTT  
Depth:3 (COW)  
Ei-value:0.000, Pi-value:0.020  
Er-value:0.000, Pr-value:0.000  
No matches to TargetScan

-8066--(10)--8077-

CTTTTTGATGTT

CTTTTTGATGTT  
Depth:4 (DOG)  
Ei-value:0.000, Pi-value:0.000  
Er-value:0.000, Pr-value:0.000  
No matches to TargetScan

-8088--(39)--8128-

TATTATGC

TATTATGC  
Depth:4 (DOG)  
Ei-value:0.000, Pi-value:0.000  
Er-value:0.000, Pr-value:0.000  
MATCHES To TargetScan▶ miR-369-3p:AUAAUAC

-8135--(52)--8188-

TAAACTTC

TAAACTTC  
Depth:3 (COW)  
Ei-value:0.000, Pi-value:0.000  
Er-value:0.000, Pr-value:0.000  
No matches to TargetScan

-8195--(18)--8214-

CTCCACTTGAGAG

CTCCACTTGAGAG  
Depth:3 (COW)  
Ei-value:0.000, Pi-value:0.000  
Er-value:0.000, Pr-value:0.000  
MATCHES To TargetScan▶ miR-26-5p:UCAAGUA

-8226--(19)--8246-

TATTTCAGT

TATTTCAGT  
Depth:4 (DOG)  
Ei-value:0.000, Pi-value:0.000  
Er-value:0.000, Pr-value:0.000  
MATCHES To TargetScan▶ miR-203a-3p.2:UGAAAUG


CC

TATTTCAGTCC  
Depth:3 (COW)  
Ei-value:0.000, Pi-value:0.000  
Er-value:0.000, Pr-value:0.000  
MATCHES To TargetScan▶ miR-203a-3p.2:UGAAAUG

-8256--(60)--8317-

GGGGAAA

GGGGAAA  
Depth:4 (DOG)  
Ei-value:0.000, Pi-value:0.000  
Er-value:0.000, Pr-value:0.000  
No matches to TargetScan

-8323--(15)--8339-

TCTAGAGAAAA

TCTAGAGAAAA  
Depth:6 (MOUSE)  
Ei-value:0.000, Pi-value:0.000  
Er-value:0.000, Pr-value:0.000  
MATCHES To TargetScan▶ miR-1251-5p:CUCUAGC

-8349--(2)--8352-

TGAAGAGATG

TGAAGAGATG  
Depth:5 (RABBIT)  
Ei-value:0.000, Pi-value:0.000  
Er-value:0.000, Pr-value:0.000  
No matches to TargetScan


CTCCA

TGAAGAGATGCTCCA  
Depth:3 (COW)  
Ei-value:0.000, Pi-value:0.000  
Er-value:0.000, Pr-value:0.000  
No matches to TargetScan

-8366--(2)--8369-

GGCCAA

GGCCAATGAGAAGAATTAGACA  
Depth:4 (DOG)  
Ei-value:0.000, Pi-value:0.000  
Er-value:0.000, Pr-value:0.000  
No matches to TargetScan


TGAGAAGAATTAGACA

TGAGAAGAATTAGACA  
Depth:6 (MOUSE)  
Ei-value:0.000, Pi-value:0.000  
Er-value:0.000, Pr-value:0.000  
No matches to TargetScan

-8390--(1)--8392-

GAAATACACAGATG

GAAATACACAGATG  
Depth:3 (COW)  
Ei-value:0.000, Pi-value:0.000  
Er-value:0.000, Pr-value:0.000  
No matches to TargetScan

-8405--(10)--8416-

C

CTGAGAAG  
Depth:3 (COW)  
Ei-value:0.000, Pi-value:0.000  
Er-value:0.000, Pr-value:0.000  
No matches to TargetScan


TGAGAAG

TGAGAAG  
Depth:4 (DOG)  
Ei-value:0.000, Pi-value:0.000  
Er-value:0.000, Pr-value:0.010  
No matches to TargetScan

-8423--(5)--8429-

GCCA

GCCAGCAACA  
Depth:3 (COW)  
Ei-value:0.000, Pi-value:0.000  
Er-value:0.000, Pr-value:0.000  
No matches to TargetScan


GCAACA

GCAACA  
Depth:6 (MOUSE)  
Ei-value:0.000, Pi-value:0.000  
Er-value:0.000, Pr-value:0.000  
No matches to TargetScan

-8438--(9)--8448-

TTTGAGCTT

TTTGAGCTT  
Depth:3 (COW)  
Ei-value:0.000, Pi-value:0.000  
Er-value:0.000, Pr-value:0.000  
MATCHES To TargetScan▶ miR-371-5p:CUCAAAC

-8456--(1)--8458-

GGTGAGC

GGTGAGC  
Depth:4 (DOG)  
Ei-value:0.000, Pi-value:0.000  
Er-value:0.000, Pr-value:0.000  
No matches to TargetScan


AGGAT

GGTGAGCAGGAT  
Depth:3 (COW)  
Ei-value:0.000, Pi-value:0.000  
Er-value:0.000, Pr-value:0.000  
No matches to TargetScan

-8469--(6)--8476-

GGTTTGGG

GGTTTGGG  
Depth:4 (DOG)  
Ei-value:0.000, Pi-value:0.000  
Er-value:0.000, Pr-value:0.000  
No matches to TargetScan

-8483--(11)--8495-

TGGTTA

TGGTTA  
Depth:5 (RABBIT)  
Ei-value:0.000, Pi-value:0.000  
Er-value:0.000, Pr-value:0.000  
No matches to TargetScan


T

TGGTTAT  
Depth:4 (DOG)  
Ei-value:0.000, Pi-value:0.000  
Er-value:0.000, Pr-value:0.000  
No matches to TargetScan


G

TGGTTATG  
Depth:3 (COW)  
Ei-value:0.000, Pi-value:0.000  
Er-value:0.000, Pr-value:0.000  
No matches to TargetScan

-8502--(31)--8534-

CCCAAGG

CCCAAGG  
Depth:4 (DOG)  
Ei-value:0.000, Pi-value:0.000  
Er-value:0.000, Pr-value:0.000  
MATCHES To TargetScan▶ miR-212-5p:CCUUGGC

-8540--(8)--8549-

TGAACTCCCTGCT

TGAACTCCCTGCT  
Depth:4 (DOG)  
Ei-value:0.000, Pi-value:0.000  
Er-value:0.000, Pr-value:0.000  
No matches to TargetScan


C

TGAACTCCCTGCTCATAGTAGTGGCC  
Depth:3 (COW)  
Ei-value:0.000, Pi-value:0.000  
Er-value:0.000, Pr-value:0.000  
No matches to TargetScan


ATAGTAGTGGCC

ATAGTAGTGGCC  
Depth:4 (DOG)  
Ei-value:0.000, Pi-value:0.000  
Er-value:0.000, Pr-value:0.000  
No matches to TargetScan

-8574--(37)--8612-

TTTAATAC

TTTAATAC  
Depth:4 (DOG)  
Ei-value:0.000, Pi-value:0.000  
Er-value:0.000, Pr-value:0.000  
MATCHES To TargetScan▶ miR-496.2:GUAUUAC

-8619--(0)--8620-

CT

CTAGGCTTAAAG  
Depth:4 (DOG)  
Ei-value:0.000, Pi-value:0.000  
Er-value:0.000, Pr-value:0.000  
No matches to TargetScan


AGGCTTA

AGGCTTA  
Depth:5 (RABBIT)  
Ei-value:0.000, Pi-value:0.000  
Er-value:0.000, Pr-value:0.000  
No matches to TargetScan


AAG

CTAGGCTTAAAG  
Depth:4 (DOG)  
Ei-value:0.000, Pi-value:0.000  
Er-value:0.000, Pr-value:0.000  
No matches to TargetScan

-8631--(27)--8659-

GTTTAAT

GTTTAAT  
Depth:5 (RABBIT)  
Ei-value:0.000, Pi-value:0.000  
Er-value:0.000, Pr-value:0.000  
No matches to TargetScan

-8665--(68)--8734-

TGTAAAACA

TGTAAAACA  
Depth:3 (COW)  
Ei-value:0.000, Pi-value:0.000  
Er-value:0.000, Pr-value:0.000  
No matches to TargetScan

-8742--(34)--8777-

T

TATTGGCA  
Depth:5 (RABBIT)  
Ei-value:0.000, Pi-value:0.000  
Er-value:0.000, Pr-value:0.000  
No matches to TargetScan


ATTGGCA

ATTGGCA  
Depth:6 (MOUSE)  
Ei-value:0.000, Pi-value:0.000  
Er-value:0.000, Pr-value:0.000  
No matches to TargetScan

-8784--(36)--8821-

TTGTGAAG

TTGTGAAG  
Depth:6 (MOUSE)  
Ei-value:0.000, Pi-value:0.000  
Er-value:0.000, Pr-value:0.000  
No matches to TargetScan

-8828--(3)--8832-

T

TATGTAAATCA  
Depth:3 (COW)  
Ei-value:0.000, Pi-value:0.000  
Er-value:0.000, Pr-value:0.000  
No matches to TargetScan


ATGTAAAT

ATGTAAAT  
Depth:5 (RABBIT)  
Ei-value:0.000, Pi-value:0.000  
Er-value:0.000, Pr-value:0.000  
No matches to TargetScan


CA

TATGTAAATCA  
Depth:3 (COW)  
Ei-value:0.000, Pi-value:0.000  
Er-value:0.000, Pr-value:0.000  
No matches to TargetScan

-8842--(57)--8900-

GCCATATGGT

GCCATATGGT  
Depth:3 (COW)  
Ei-value:0.000, Pi-value:0.000  
Er-value:0.000, Pr-value:0.000  
No matches to TargetScan

-8909--(111)--9021-

TGTGC

TGTGCCTGTCCCTGT  
Depth:3 (COW)  
Ei-value:0.000, Pi-value:0.000  
Er-value:0.000, Pr-value:0.000  
No matches to TargetScan


CTGTCCCT

CTGTCCCT  
Depth:4 (DOG)  
Ei-value:0.000, Pi-value:0.000  
Er-value:0.000, Pr-value:0.000  
No matches to TargetScan


GT

TGTGCCTGTCCCTGT  
Depth:3 (COW)  
Ei-value:0.000, Pi-value:0.000  
Er-value:0.000, Pr-value:0.000  
No matches to TargetScan

-9035--(153)--9189-

TAGGCACT

TAGGCACT  
Depth:4 (DOG)  
Ei-value:0.000, Pi-value:0.000  
Er-value:0.000, Pr-value:0.000  
No matches to TargetScan

-9196--(259)--9456-

TAAAGCA

TAAAGCA  
Depth:4 (DOG)  
Ei-value:0.000, Pi-value:0.000  
Er-value:0.000, Pr-value:0.000  
No matches to TargetScan

-9462--(38)--9501-

TAT

TATAATGTGCCAGATA  
Depth:3 (COW)  
Ei-value:0.000, Pi-value:0.000  
Er-value:0.000, Pr-value:0.000  
MATCHES To TargetScan▶ miR-183-5p.2:UGGCACU▶ miR-323-3p:ACAUUAC


AATGTGCCAGATA

AATGTGCCAGATA  
Depth:4 (DOG)  
Ei-value:0.000, Pi-value:0.000  
Er-value:0.000, Pr-value:0.000  
MATCHES To TargetScan▶ miR-183-5p.2:UGGCACU

-9516--(81)--9598-

TTAAAGTG

TTAAAGTG  
Depth:4 (DOG)  
Ei-value:0.000, Pi-value:0.000  
Er-value:0.000, Pr-value:0.000  
No matches to TargetScan


CTTTGTA

TTAAAGTGCTTTGTA  
Depth:3 (COW)  
Ei-value:0.000, Pi-value:0.000  
Er-value:0.000, Pr-value:0.000  
MATCHES To TargetScan▶ miR-330-3p:CAAAGCA▶ miR-330-3p.2:AAAGCAC

-9612--(0)--9613-

CTAAAGCA

CTAAAGCA  
Depth:4 (DOG)  
Ei-value:0.000, Pi-value:0.000  
Er-value:0.000, Pr-value:0.000  
No matches to TargetScan

-9620--(12)--9633-

CAATGGGCTA

CAATGGGCTA  
Depth:3 (COW)  
Ei-value:0.000, Pi-value:0.000  
Er-value:0.000, Pr-value:0.000  
No matches to TargetScan

-9642--(13)--9656-

GA

GAATGAATA  
Depth:3 (COW)  
Ei-value:0.000, Pi-value:0.000  
Er-value:0.000, Pr-value:0.000  
MATCHES To TargetScan▶ miR-1298-5p:UCAUUCG


ATGAATA

ATGAATA  
Depth:4 (DOG)  
Ei-value:0.000, Pi-value:0.000  
Er-value:0.000, Pr-value:0.000  
No matches to TargetScan

-9664--(22)--9687-

CCAGCTATT

CCAGCTATT  
Depth:3 (COW)  
Ei-value:0.000, Pi-value:0.000  
Er-value:0.000, Pr-value:0.000  
No matches to TargetScan

-9695--(6)--9702-

GGTACTGT

GGTACTGT  
Depth:4 (DOG)  
Ei-value:0.000, Pi-value:0.000  
Er-value:0.000, Pr-value:0.000  
MATCHES To TargetScan▶ miR-101-3p.1:ACAGUAC▶ miR-144-3p:ACAGUAU

-9709--(26)--9736-

ATAAGAGG

ATAAGAGG  
Depth:4 (DOG)  
Ei-value:0.000, Pi-value:0.000  
Er-value:0.000, Pr-value:0.000  
No matches to TargetScan

-9743--(61)--9805-

AAGACTTTAC

AAGACTTTAC  
Depth:3 (COW)  
Ei-value:0.000, Pi-value:0.000  
Er-value:0.000, Pr-value:0.000  
No matches to TargetScan

-9814--(14)--9829-

TAAATTAT

TAAATTAT  
Depth:4 (DOG)  
Ei-value:0.000, Pi-value:0.010  
Er-value:0.000, Pr-value:0.000  
No matches to TargetScan


TAC

TAAATTATTAC  
Depth:3 (COW)  
Ei-value:0.000, Pi-value:0.000  
Er-value:0.000, Pr-value:0.000  
No matches to TargetScan

-9839--(14)--9854-

AGGTAA

AGGTAA  
Depth:3 (COW)  
Ei-value:0.000, Pi-value:0.000  
Er-value:0.000, Pr-value:0.000  
No matches to TargetScan

-9859--(28)--9888-

TTTCTAA

TTTCTAA  
Depth:3 (COW)  
Ei-value:0.000, Pi-value:0.000  
Er-value:0.000, Pr-value:0.010  
No matches to TargetScan

-9894--(20)--9915-

ATAAAAC

ATAAAAC  
Depth:4 (DOG)  
Ei-value:0.000, Pi-value:0.010  
Er-value:0.000, Pr-value:0.000  
No matches to TargetScan

-9921--(291)--10213-

AAAATTCTCA

AAAATTCTCA  
Depth:4 (DOG)  
Ei-value:0.000, Pi-value:0.000  
Er-value:0.000, Pr-value:0.000  
No matches to TargetScan

-10222--(1)--10224-

TATACAAAC

TATACAAAC  
Depth:4 (DOG)  
Ei-value:0.000, Pi-value:0.000  
Er-value:0.000, Pr-value:0.000  
No matches to TargetScan

-10232--(5)--10238-

GTTTAAATAC

GTTTAAATAC  
Depth:3 (COW)  
Ei-value:0.000, Pi-value:0.000  
Er-value:0.000, Pr-value:0.000  
No matches to TargetScan

-10247--(29)--10277-

TTGCCTACTAT

TTGCCTACTATGTGAACTCACTGTTA  
Depth:3 (COW)  
Ei-value:0.000, Pi-value:0.000  
Er-value:0.000, Pr-value:0.000  
MATCHES To TargetScan▶ miR-132-3p/212-3p:AACAGUC▶ miR-23-3p:UCACAUU▶ miR-376c-3p:ACAUAGA▶ miR-411-5p.2:UAGUAGA


GTGAACTCA

GTGAACTCA  
Depth:4 (DOG)  
Ei-value:0.000, Pi-value:0.000  
Er-value:0.000, Pr-value:0.000  
No matches to TargetScan


CTGTTA

TTGCCTACTATGTGAACTCACTGTTA  
Depth:3 (COW)  
Ei-value:0.000, Pi-value:0.000  
Er-value:0.000, Pr-value:0.000  
MATCHES To TargetScan▶ miR-132-3p/212-3p:AACAGUC▶ miR-23-3p:UCACAUU▶ miR-376c-3p:ACAUAGA▶ miR-411-5p.2:UAGUAGA

-10302--(22)--10325-

ATTTATCAT

ATTTATCAT  
Depth:3 (COW)  
Ei-value:0.000, Pi-value:0.000  
Er-value:0.000, Pr-value:0.000  
No matches to TargetScan

-10333--(44)--10378-

ATTTTGTGAACTCTAA

ATTTTGTGAACTCTAA  
Depth:3 (COW)  
Ei-value:0.000, Pi-value:0.000  
Er-value:0.000, Pr-value:0.000  
No matches to TargetScan

-10393--(60)--10454-

AAAATTG

AAAATTG  
Depth:3 (COW)  
Ei-value:0.000, Pi-value:0.000  
Er-value:0.000, Pr-value:0.000  
No matches to TargetScan

-10460--(127)--10588-

TGTGCCA

TGTGCCA  
Depth:4 (DOG)  
Ei-value:0.000, Pi-value:0.000  
Er-value:0.000, Pr-value:0.000  
MATCHES To TargetScan▶ miR-183-5p.2:UGGCACU

-10594--(27)--10622-

AAGATAA

AAGATAA  
Depth:4 (DOG)  
Ei-value:0.000, Pi-value:0.000  
Er-value:0.000, Pr-value:0.000  
No matches to TargetScan

-10628--(23)--10652-

AGCAGAA

AGCAGAA  
Depth:3 (COW)  
Ei-value:0.000, Pi-value:0.000  
Er-value:0.000, Pr-value:0.000  
No matches to TargetScan

-10658--(9)--10668-

TAAAATCAATTT

TAAAATCAATTT  
Depth:3 (COW)  
Ei-value:0.000, Pi-value:0.000  
Er-value:0.000, Pr-value:0.000  
No matches to TargetScan

-10679--(8)--10688-

TAAACTG

TAAACTG  
Depth:4 (DOG)  
Ei-value:0.000, Pi-value:0.000  
Er-value:0.000, Pr-value:0.000  
No matches to TargetScan

-10694--(20)--10715-

TCTGCTGAATGA

TCTGCTGAATGA  
Depth:3 (COW)  
Ei-value:0.000, Pi-value:0.000  
Er-value:0.000, Pr-value:0.000  
MATCHES To TargetScan▶ miR-1298-5p:UCAUUCG

-10726--(1)--10728-

C

CATTGATTA  
Depth:3 (COW)  
Ei-value:0.000, Pi-value:0.000  
Er-value:0.000, Pr-value:0.000  
No matches to TargetScan


ATTGATTA

ATTGATTA  
Depth:4 (DOG)  
Ei-value:0.000, Pi-value:0.000  
Er-value:0.000, Pr-value:0.010  
No matches to TargetScan

-10736--(19)--10756-

AGAGATA

AGAGATA  
Depth:4 (DOG)  
Ei-value:0.000, Pi-value:0.000  
Er-value:0.000, Pr-value:0.000  
No matches to TargetScan

-10762--(36)--10799-

TGAACCT

TGAACCT  
Depth:3 (COW)  
Ei-value:0.000, Pi-value:0.000  
Er-value:0.000, Pr-value:0.010  
No matches to TargetScan

-10805--(2)--10808-

AA

AACAGAGATCT  
Depth:3 (COW)  
Ei-value:0.000, Pi-value:0.000  
Er-value:0.000, Pr-value:0.000  
No matches to TargetScan


CAGAGATCT

CAGAGATCT  
Depth:4 (DOG)  
Ei-value:0.000, Pi-value:0.000  
Er-value:0.000, Pr-value:0.000  
No matches to TargetScan

-10818--(8)--10827-

TTTACAAAGC

TTTACAAAGC  
Depth:3 (COW)  
Ei-value:0.000, Pi-value:0.000  
Er-value:0.000, Pr-value:0.000  
No matches to TargetScan

-10836--(7)--10844-

TCTATACA

TCTATACA  
Depth:3 (COW)  
Ei-value:0.000, Pi-value:0.000  
Er-value:0.000, Pr-value:0.000  
No matches to TargetScan

-10851--(18)--10870-

TTGGCT

TTGGCT  
Depth:4 (DOG)  
Ei-value:0.000, Pi-value:0.000
[truncated: 338,789 more chars]
