# Supplementary material for: Uncovering deeply conserved motif combinations in rapidly evolving noncoding sequences
Source: Genome Biol. 2021 Jan 11;22:29. doi: 10.1186/s13059-020-02247-1 (PMC7798263; doi:10.1186/s13059-020-02247-1)
Supplement: Supplementary file 4 — Additional file 4. LncLOOM output results for XIST sequences from six mammals. [file 13059_2020_2247_MOESM4_ESM.gz › AdditionalFile4/Html_Files/kmers_in_blocks.html]

 MOTIFS IN BLOCKS

# MOTIFS IN BLOCK DIAGRAMS

  

NAVIGATE ▼

▶HUMAN (depth:1)▶PIG (depth:2)▶COW (depth:3)▶DOG (depth:4)▶RABBIT (depth:5)▶MOUSE (depth:6)

  
  
  

## >HUMAN (19280 bases)

```
 -------------------------------------------------------------

TGGAAGCTT

TGGAAGCTT  
Depth:2 (PIG)  
Ei-value:0.000, Pi-value:0.000  
Er-value:0.000, Pr-value:0.000  
eCLIP MATCHES▶DGCR8 (bg=1.84%)▶HNRNPC (bg=3.65%)▶LSM11 (bg=2.28%)▶NCBP2 (bg=1.49%)▶RBM15 (bg=7.27%)▶RBM22 (bg=4.62%)▶SLTM (bg=2.2%)▶SRSF1 (bg=8.47%)▶uchl5 (bg=11.16%)▶YWHAG (bg=1.87%)No matches to TargetScan

----------

GATCTCT

GATCTCT  
Depth:2 (PIG)  
Ei-value:0.000, Pi-value:0.010  
Er-value:0.000, Pr-value:0.000  
eCLIP MATCHES▶DGCR8 (bg=1.84%)▶HNRNPC (bg=3.65%)▶LSM11 (bg=2.28%)▶NCBP2 (bg=1.49%)▶RBM15 (bg=7.27%)▶RBM22 (bg=4.62%)▶SLTM (bg=2.2%)▶SRSF1 (bg=8.47%)▶uchl5 (bg=11.16%)▶YWHAG (bg=1.87%)No matches to TargetScan

----------

GGTTCTTTCT

GGTTCTTTCT  
Depth:2 (PIG)  
Ei-value:0.000, Pi-value:0.000  
Er-value:0.000, Pr-value:0.000  
eCLIP MATCHES▶DGCR8 (bg=1.84%)▶LSM11 (bg=2.28%)▶SRSF1 (bg=8.47%)MATCHES To TargetScan▶ miR-186-5p:AAAGAAU

-

GAACATTTTC

GAACATTTTC  
Depth:2 (PIG)  
Ei-value:0.000, Pi-value:0.000  
Er-value:0.000, Pr-value:0.000  
eCLIP MATCHES▶LSM11 (bg=2.28%)▶RBM15 (bg=7.27%)▶RBM22 (bg=4.62%)▶SRSF1 (bg=8.47%)▶uchl5 (bg=11.16%)MATCHES To TargetScan▶ miR-409-3p:AAUGUUG

-- 120  
 ---------------

TTATGGC

TTATGGC  
Depth:2 (PIG)  
Ei-value:0.000, Pi-value:0.000  
Er-value:0.000, Pr-value:0.010  
eCLIP MATCHES▶HNRNPC (bg=3.65%)▶LSM11 (bg=2.28%)▶RBM15 (bg=7.27%)▶RBM22 (bg=4.62%)▶SRSF1 (bg=8.47%)▶uchl5 (bg=11.16%)No matches to TargetScan

-

TATTTCTTTAAAAAAA

TATTTCTTTAAAAAAA  
Depth:2 (PIG)  
Ei-value:0.000, Pi-value:0.000  
Er-value:0.000, Pr-value:0.000  
eCLIP MATCHES▶HNRNPC (bg=3.65%)▶LSM11 (bg=2.28%)▶RBM15 (bg=7.27%)▶RBM22 (bg=4.62%)▶SRSF1 (bg=8.47%)▶uchl5 (bg=11.16%)MATCHES To TargetScan▶ miR-186-5p:AAAGAAU

------------

CATAAAAT

CATAAAAT  
Depth:2 (PIG)  
Ei-value:0.000, Pi-value:0.000  
Er-value:0.000, Pr-value:0.000  
No matches to eCLIP DataNo matches to TargetScan

-----------------

ACTTTCTCCTA

ACTTTCTCCTA  
Depth:2 (PIG)  
Ei-value:0.000, Pi-value:0.000  
Er-value:0.000, Pr-value:0.000  
eCLIP MATCHES▶DGCR8 (bg=1.84%)▶HNRNPC (bg=3.65%)▶LSM11 (bg=2.28%)▶RBFOX2 (bg=4.63%)▶RBM15 (bg=7.27%)▶RBM22 (bg=4.62%)▶SRSF1 (bg=8.47%)▶uchl5 (bg=11.16%)▶YWHAG (bg=1.87%)No matches to TargetScan

-----

TTCTTGACAC

TTCTTGACAC  
Depth:2 (PIG)  
Ei-value:0.000, Pi-value:0.000  
Er-value:0.000, Pr-value:0.000  
eCLIP MATCHES▶DGCR8 (bg=1.84%)▶HNRNPC (bg=3.65%)▶LSM11 (bg=2.28%)▶RBFOX2 (bg=4.63%)▶RBM15 (bg=7.27%)▶RBM22 (bg=4.62%)▶SRSF1 (bg=8.47%)▶uchl5 (bg=11.16%)▶YWHAG (bg=1.87%)No matches to TargetScan

------------------ 240  
 -------

TATTTGG

TATTTGG  
Depth:2 (PIG)  
Ei-value:0.000, Pi-value:0.010  
Er-value:0.000, Pr-value:0.000  
No matches to eCLIP DataNo matches to TargetScan

------------------------

TTTAAGG

TTTAAGG  
Depth:2 (PIG)  
Ei-value:0.000, Pi-value:0.000  
Er-value:0.000, Pr-value:0.010  
eCLIP MATCHES▶HNRNPC (bg=3.65%)No matches to TargetScan


AATTTTTCTTTGGAAT

AATTTTTCTTTGGAAT  
Depth:2 (PIG)  
Ei-value:0.000, Pi-value:0.000  
Er-value:0.000, Pr-value:0.000  
eCLIP MATCHES▶DGCR8 (bg=1.84%)▶HNRNPC (bg=3.65%)▶PUS1 (bg=1.04%)▶RBM15 (bg=7.27%)▶SRSF1 (bg=8.47%)▶uchl5 (bg=11.16%)▶UTP18 (bg=0.72%)MATCHES To TargetScan▶ miR-186-5p:AAAGAAU

--

TTTTTGGTTGAC

TTTTTGGTTGAC  
Depth:2 (PIG)  
Ei-value:0.000, Pi-value:0.000  
Er-value:0.000, Pr-value:0.000  
eCLIP MATCHES▶DGCR8 (bg=1.84%)▶HNRNPC (bg=3.65%)▶NIPBL (bg=5.39%)▶PUS1 (bg=1.04%)▶RBM15 (bg=7.27%)▶SDAD1 (bg=2.97%)▶SRSF1 (bg=8.47%)▶uchl5 (bg=11.16%)▶UTP18 (bg=0.72%)MATCHES To TargetScan▶ miR-505-3p.1:GUCAACA

------

GTTTTTT

GTTTTTT  
Depth:2 (PIG)  
Ei-value:0.000, Pi-value:0.020  
Er-value:0.000, Pr-value:0.020  
eCLIP MATCHES▶DGCR8 (bg=1.84%)▶HNRNPC (bg=3.65%)▶NIPBL (bg=5.39%)▶PUS1 (bg=1.04%)▶RBM15 (bg=7.27%)▶SDAD1 (bg=2.97%)▶SRSF1 (bg=8.47%)▶uchl5 (bg=11.16%)▶UTP18 (bg=0.72%)▶YWHAG (bg=1.87%)No matches to TargetScan

--------

GTTTTTT

GTTTTTT  
Depth:2 (PIG)  
Ei-value:0.000, Pi-value:0.020  
Er-value:0.000, Pr-value:0.020  
eCLIP MATCHES▶DGCR8 (bg=1.84%)▶HNRNPC (bg=3.65%)▶NIPBL (bg=5.39%)▶RBM15 (bg=7.27%)▶RBM22 (bg=4.62%)▶SDAD1 (bg=2.97%)▶SRSF1 (bg=8.47%)▶uchl5 (bg=11.16%)▶UTP18 (bg=0.72%)▶YWHAG (bg=1.87%)No matches to TargetScan

----------------- 360  
 ---------

TGCCCATCGGGGCTG

TGCCCATCGGGGCTG  
Depth:2 (PIG)  
Ei-value:0.000, Pi-value:0.000  
Er-value:0.000, Pr-value:0.000  
eCLIP MATCHES▶DDX51 (bg=1.63%)▶HNRNPC (bg=3.65%)▶NIPBL (bg=5.39%)▶RBM15 (bg=7.27%)▶SDAD1 (bg=2.97%)▶SRSF1 (bg=8.47%)▶U2AF1 (bg=1.17%)▶uchl5 (bg=11.16%)No matches to TargetScan

-

GGATACCTGGTTTTA

GGATACCTGGTTTTA  
Depth:2 (PIG)  
Ei-value:0.000, Pi-value:0.000  
Er-value:0.000, Pr-value:0.000  
eCLIP MATCHES▶DDX51 (bg=1.63%)▶HNRNPC (bg=3.65%)▶NIPBL (bg=5.39%)▶RBM15 (bg=7.27%)▶SDAD1 (bg=2.97%)▶SRSF1 (bg=8.47%)▶U2AF1 (bg=1.17%)▶uchl5 (bg=11.16%)No matches to TargetScan


TTATTTT

TTATTTT  
Depth:2 (PIG)  
Ei-value:0.000, Pi-value:0.010  
Er-value:0.000, Pr-value:0.010  
eCLIP MATCHES▶DDX51 (bg=1.63%)▶HNRNPC (bg=3.65%)▶NIPBL (bg=5.39%)▶RBM15 (bg=7.27%)▶SDAD1 (bg=2.97%)▶SRSF1 (bg=8.47%)▶U2AF1 (bg=1.17%)No matches to TargetScan

---

TTTGCCCAACGGGGCCGTGGATACCTGCCTTTTAATTCTTTTTT

TTTGCCCAACGGGGCCGTGGATACCTGCCTTTTAATTCTTTTTT  
Depth:2 (PIG)  
Ei-value:0.000, Pi-value:0.000  
Er-value:0.000, Pr-value:0.000  
eCLIP MATCHES▶DDX51 (bg=1.63%)▶HNRNPC (bg=3.65%)▶NIPBL (bg=5.39%)▶RBM15 (bg=7.27%)▶SDAD1 (bg=2.97%)▶SRSF1 (bg=8.47%)▶U2AF1 (bg=1.17%)▶uchl5 (bg=11.16%)MATCHES To TargetScan▶ miR-124-3p.1:AAGGCAC▶ miR-186-5p:AAAGAAU

----

GCCCATCGGGGCCGCGGATACC

GCCCATCGGGGCCGCGGATACCTGCTTTT  
Depth:2 (PIG)  
Ei-value:0.000, Pi-value:0.000  
Er-value:0.000, Pr-value:0.000  
eCLIP MATCHES▶DDX51 (bg=1.63%)▶HNRNPC (bg=3.65%)▶NIPBL (bg=5.39%)▶RBM15 (bg=7.27%)▶SDAD1 (bg=2.97%)▶SRSF1 (bg=8.47%)▶U2AF1 (bg=1.17%)▶uchl5 (bg=11.16%)MATCHES To TargetScan▶ miR-330-3p.2:AAAGCAC

 480  


TGCTTTT

GCCCATCGGGGCCGCGGATACCTGCTTTT  
Depth:2 (PIG)  
Ei-value:0.000, Pi-value:0.000  
Er-value:0.000, Pr-value:0.000  
eCLIP MATCHES▶DDX51 (bg=1.63%)▶HNRNPC (bg=3.65%)▶NIPBL (bg=5.39%)▶RBM15 (bg=7.27%)▶SDAD1 (bg=2.97%)▶SRSF1 (bg=8.47%)▶U2AF1 (bg=1.17%)▶uchl5 (bg=11.16%)MATCHES To TargetScan▶ miR-330-3p.2:AAAGCAC

-

ATTTTTTTTT

ATTTTTTTTT  
Depth:2 (PIG)  
Ei-value:0.000, Pi-value:0.000  
Er-value:0.000, Pr-value:0.000  
eCLIP MATCHES▶DDX51 (bg=1.63%)▶HNRNPC (bg=3.65%)▶NIPBL (bg=5.39%)▶RBM15 (bg=7.27%)▶SDAD1 (bg=2.97%)▶SRSF1 (bg=8.47%)▶U2AF1 (bg=1.17%)▶uchl5 (bg=11.16%)▶YWHAG (bg=1.87%)No matches to TargetScan


CCTTAGCCCATCGGGG

CCTTAGCCCATCGGGG  
Depth:2 (PIG)  
Ei-value:0.000, Pi-value:0.000  
Er-value:0.000, Pr-value:0.000  
eCLIP MATCHES▶DDX51 (bg=1.63%)▶HNRNPC (bg=3.65%)▶NIPBL (bg=5.39%)▶RBM15 (bg=7.27%)▶SDAD1 (bg=2.97%)▶SRSF1 (bg=8.47%)▶uchl5 (bg=11.16%)▶YWHAG (bg=1.87%)No matches to TargetScan

--

TCGGATACCTGCTG

TCGGATACCTGCTG  
Depth:2 (PIG)  
Ei-value:0.000, Pi-value:0.000  
Er-value:0.000, Pr-value:0.000  
eCLIP MATCHES▶DDX51 (bg=1.63%)▶HNRNPC (bg=3.65%)▶NIPBL (bg=5.39%)▶RBM15 (bg=7.27%)▶SDAD1 (bg=2.97%)▶SRSF1 (bg=8.47%)▶uchl5 (bg=11.16%)▶YWHAG (bg=1.87%)No matches to TargetScan

--------

CCCCTCT

CCCCTCT  
Depth:2 (PIG)  
Ei-value:0.000, Pi-value:0.000  
Er-value:0.000, Pr-value:0.010  
eCLIP MATCHES▶HNRNPC (bg=3.65%)▶NIPBL (bg=5.39%)▶RBM15 (bg=7.27%)▶SDAD1 (bg=2.97%)▶SRSF1 (bg=8.47%)▶uchl5 (bg=11.16%)MATCHES To TargetScan▶ miR-423-5p:GAGGGGC

-

AACCCC

AACCCC  
Depth:2 (PIG)  
Ei-value:0.000, Pi-value:0.010  
Er-value:0.000, Pr-value:0.010  
eCLIP MATCHES▶HNRNPC (bg=3.65%)▶NIPBL (bg=5.39%)▶RBM15 (bg=7.27%)▶SDAD1 (bg=2.97%)▶SRSF1 (bg=8.47%)▶uchl5 (bg=11.16%)No matches to TargetScan

--------

TGGCCCATC

TGGCCCATC  
Depth:2 (PIG)  
Ei-value:0.000, Pi-value:0.000  
Er-value:0.000, Pr-value:0.000  
eCLIP MATCHES▶HNRNPC (bg=3.65%)▶NIPBL (bg=5.39%)▶RBM15 (bg=7.27%)▶SDAD1 (bg=2.97%)▶SRSF1 (bg=8.47%)No matches to TargetScan

--------------

CTGCTTTTT

CTGCTTTTT  
Depth:2 (PIG)  
Ei-value:0.000, Pi-value:0.000  
Er-value:0.000, Pr-value:0.000  
eCLIP MATCHES▶CPEB4 (bg=1.89%)▶RBM15 (bg=7.27%)MATCHES To TargetScan▶ miR-330-3p.2:AAAGCAC

-------- 600  
 --

TTTTTTTGGCCCATCGGGGC

TTTTTTTGGCCCATCGGGGC  
Depth:2 (PIG)  
Ei-value:0.000, Pi-value:0.000  
Er-value:0.000, Pr-value:0.000  
eCLIP MATCHES▶FASTKD2 (bg=1.99%)▶HNRNPC (bg=3.65%)▶RBM15 (bg=7.27%)▶SRSF1 (bg=8.47%)▶U2AF1 (bg=1.17%)▶YWHAG (bg=1.87%)No matches to TargetScan

-

TCGGATACCTGCTTT

TCGGATACCTGCTTT  
Depth:2 (PIG)  
Ei-value:0.000, Pi-value:0.000  
Er-value:0.000, Pr-value:0.000  
eCLIP MATCHES▶HNRNPC (bg=3.65%)▶RBM15 (bg=7.27%)▶SRSF1 (bg=8.47%)▶U2AF1 (bg=1.17%)MATCHES To TargetScan▶ miR-330-3p.2:AAAGCAC

-----------

TTTTTCCTTGCCCATCGGGGCCTCGGATACCTGCTTTA

TTTTTCCTTGCCCATCGGGGCCTCGGATACCTGCTTTA  
Depth:2 (PIG)  
Ei-value:0.000, Pi-value:0.000  
Er-value:0.000, Pr-value:0.000  
eCLIP MATCHES▶AARS (bg=2.18%)▶AKAP1 (bg=0.21%)▶HNRNPC (bg=3.65%)▶NIPBL (bg=5.39%)▶RBM15 (bg=7.27%)▶SDAD1 (bg=2.97%)▶SRSF1 (bg=8.47%)▶uchl5 (bg=11.16%)MATCHES To TargetScan▶ miR-31-5p:GGCAAGA▶ miR-330-3p.2:AAAGCAC

---------------

GCCCATCGGGGCCG

GCCCATCGGGGCCG  
Depth:2 (PIG)  
Ei-value:0.000, Pi-value:0.000  
Er-value:0.000, Pr-value:0.000  
eCLIP MATCHES▶AATF (bg=0.64%)▶HNRNPC (bg=3.65%)▶LSM11 (bg=2.28%)▶NIPBL (bg=5.39%)▶RBM15 (bg=7.27%)▶SDAD1 (bg=2.97%)▶SRSF1 (bg=8.47%)▶U2AF1 (bg=1.17%)No matches to TargetScan

-

GGA

GGATACCTGCTT  
Depth:2 (PIG)  
Ei-value:0.000, Pi-value:0.000  
Er-value:0.000, Pr-value:0.000  
eCLIP MATCHES▶AATF (bg=0.64%)▶HNRNPC (bg=3.65%)▶LSM11 (bg=2.28%)▶NIPBL (bg=5.39%)▶RBM15 (bg=7.27%)▶SRSF1 (bg=8.47%)▶U2AF1 (bg=1.17%)No matches to TargetScan

 720  


TACCTGCTT

GGATACCTGCTT  
Depth:2 (PIG)  
Ei-value:0.000, Pi-value:0.000  
Er-value:0.000, Pr-value:0.000  
eCLIP MATCHES▶AATF (bg=0.64%)▶HNRNPC (bg=3.65%)▶LSM11 (bg=2.28%)▶NIPBL (bg=5.39%)▶RBM15 (bg=7.27%)▶SRSF1 (bg=8.47%)▶U2AF1 (bg=1.17%)No matches to TargetScan

-

GATTTTTTTTTTTCATC

GATTTTTTTTTTTCATC  
Depth:2 (PIG)  
Ei-value:0.000, Pi-value:0.000  
Er-value:0.000, Pr-value:0.000  
eCLIP MATCHES▶HNRNPC (bg=3.65%)▶NIPBL (bg=5.39%)▶RBM15 (bg=7.27%)▶SDAD1 (bg=2.97%)▶SRSF1 (bg=8.47%)▶U2AF1 (bg=1.17%)▶UTP3 (bg=3.66%)No matches to TargetScan

-

CCCATCGG

CCCATCGG  
Depth:2 (PIG)  
Ei-value:0.000, Pi-value:0.000  
Er-value:0.000, Pr-value:0.000  
eCLIP MATCHES▶HNRNPC (bg=3.65%)▶NIPBL (bg=5.39%)▶RBM15 (bg=7.27%)▶SDAD1 (bg=2.97%)▶SRSF1 (bg=8.47%)▶UTP3 (bg=3.66%)▶YWHAG (bg=1.87%)No matches to TargetScan

-------

TATGGATG

TATGGATG  
Depth:2 (PIG)  
Ei-value:0.000, Pi-value:0.000  
Er-value:0.000, Pr-value:0.000  
eCLIP MATCHES▶HNRNPC (bg=3.65%)▶NIPBL (bg=5.39%)▶RBM15 (bg=7.27%)▶SDAD1 (bg=2.97%)▶SRSF1 (bg=8.47%)▶uchl5 (bg=11.16%)▶UTP3 (bg=3.66%)▶YWHAG (bg=1.87%)No matches to TargetScan

----------

GGTTTTGTGG

GGTTTTGTGG  
Depth:2 (PIG)  
Ei-value:0.000, Pi-value:0.000  
Er-value:0.000, Pr-value:0.000  
eCLIP MATCHES▶DGCR8 (bg=1.84%)▶EXOSC5 (bg=5.38%)▶GTF2F1 (bg=0.51%)▶NIPBL (bg=5.39%)▶RBM15 (bg=7.27%)▶SRSF1 (bg=8.47%)▶uchl5 (bg=11.16%)▶YWHAG (bg=1.87%)No matches to TargetScan

------------

TCTGGAAT

TCTGGAAT  
Depth:2 (PIG)  
Ei-value:0.000, Pi-value:0.000  
Er-value:0.000, Pr-value:0.000  
eCLIP MATCHES▶DGCR8 (bg=1.84%)▶EXOSC5 (bg=5.38%)▶GTF2F1 (bg=0.51%)▶NIPBL (bg=5.39%)▶RBM15 (bg=7.27%)▶RBM22 (bg=4.62%)▶SDAD1 (bg=2.97%)▶SRSF1 (bg=8.47%)▶uchl5 (bg=11.16%)▶YWHAG (bg=1.87%)No matches to TargetScan

-

TCTACA

TCTACA  
Depth:2 (PIG)  
Ei-value:0.000, Pi-value:0.000  
Er-value:0.000, Pr-value:0.010  
eCLIP MATCHES▶DGCR8 (bg=1.84%)▶NIPBL (bg=5.39%)▶RBM15 (bg=7.27%)▶RBM22 (bg=4.62%)▶SDAD1 (bg=2.97%)▶SRSF1 (bg=8.47%)▶YWHAG (bg=1.87%)No matches to TargetScan

-----

TTTTGCTGCT

TTTTGCTGCT  
Depth:2 (PIG)  
Ei-value:0.000, Pi-value:0.000  
Er-value:0.000, Pr-value:0.010  
eCLIP MATCHES▶DDX52 (bg=0.46%)▶DGCR8 (bg=1.84%)▶EXOSC5 (bg=5.38%)▶NCBP2 (bg=1.49%)▶NIPBL (bg=5.39%)▶RBM15 (bg=7.27%)▶RBM22 (bg=4.62%)▶SDAD1 (bg=2.97%)▶SRSF1 (bg=8.47%)▶uchl5 (bg=11.16%)▶WDR3 (bg=0.25%)▶YWHAG (bg=1.87%)MATCHES To TargetScan▶ miR-103-3p/107:GCAGCAU▶ miR-15-5p/16-5p/195-5p/424-5p/497-5p:AGCAGCA▶ miR-503-5p:AGCAGCG

-----

TT

TTTGGTG  
Depth:2 (PIG)  
Ei-value:0.000, Pi-value:0.000  
Er-value:0.000, Pr-value:0.010  
eCLIP MATCHES▶DDX52 (bg=0.46%)▶DGCR8 (bg=1.84%)▶EXOSC5 (bg=5.38%)▶NCBP2 (bg=1.49%)▶NIPBL (bg=5.39%)▶RBM15 (bg=7.27%)▶RBM22 (bg=4.62%)▶SDAD1 (bg=2.97%)▶SLTM (bg=2.2%)▶SRSF1 (bg=8.47%)▶uchl5 (bg=11.16%)▶UTP3 (bg=3.66%)▶WDR3 (bg=0.25%)▶YWHAG (bg=1.87%)No matches to TargetScan

 840  


TGGTG

TTTGGTG  
Depth:2 (PIG)  
Ei-value:0.000, Pi-value:0.000  
Er-value:0.000, Pr-value:0.010  
eCLIP MATCHES▶DDX52 (bg=0.46%)▶DGCR8 (bg=1.84%)▶EXOSC5 (bg=5.38%)▶NCBP2 (bg=1.49%)▶NIPBL (bg=5.39%)▶RBM15 (bg=7.27%)▶RBM22 (bg=4.62%)▶SDAD1 (bg=2.97%)▶SLTM (bg=2.2%)▶SRSF1 (bg=8.47%)▶uchl5 (bg=11.16%)▶UTP3 (bg=3.66%)▶WDR3 (bg=0.25%)▶YWHAG (bg=1.87%)No matches to TargetScan

-

TGTGTGAGTG

TGTGTGAGTG  
Depth:2 (PIG)  
Ei-value:0.000, Pi-value:0.000  
Er-value:0.000, Pr-value:0.000  
eCLIP MATCHES▶DDX52 (bg=0.46%)▶DGCR8 (bg=1.84%)▶EXOSC5 (bg=5.38%)▶NCBP2 (bg=1.49%)▶NIPBL (bg=5.39%)▶RBM15 (bg=7.27%)▶RBM22 (bg=4.62%)▶SDAD1 (bg=2.97%)▶SLTM (bg=2.2%)▶SRSF1 (bg=8.47%)▶uchl5 (bg=11.16%)▶UTP3 (bg=3.66%)▶WDR3 (bg=0.25%)▶YWHAG (bg=1.87%)MATCHES To TargetScan▶ miR-342-3p:CUCACAC▶ miR-377-3p:UCACACA

--------

GCTTTGG

GCTTTGG  
Depth:2 (PIG)  
Ei-value:0.000, Pi-value:0.000  
Er-value:0.000, Pr-value:0.010  
eCLIP MATCHES▶DGCR8 (bg=1.84%)▶EXOSC5 (bg=5.38%)▶NCBP2 (bg=1.49%)▶NIPBL (bg=5.39%)▶RBM15 (bg=7.27%)▶RBM22 (bg=4.62%)▶SLTM (bg=2.2%)▶SRSF1 (bg=8.47%)▶uchl5 (bg=11.16%)▶YWHAG (bg=1.87%)MATCHES To TargetScan▶ miR-330-3p:CAAAGCA

-------------

TGCAGTTA

TGCAGTTA  
Depth:2 (PIG)  
Ei-value:0.000, Pi-value:0.000  
Er-value:0.000, Pr-value:0.000  
eCLIP MATCHES▶DGCR8 (bg=1.84%)▶NIPBL (bg=5.39%)▶RBM15 (bg=7.27%)▶RBM22 (bg=4.62%)▶SF3B1 (bg=2.48%)▶SLTM (bg=2.2%)▶SRSF1 (bg=8.47%)▶uchl5 (bg=11.16%)MATCHES To TargetScan▶ miR-217:ACUGCAU

-----------------------

GGAGGAAA

GGAGGAAA  
Depth:2 (PIG)  
Ei-value:0.000, Pi-value:0.000  
Er-value:0.000, Pr-value:0.000  
eCLIP MATCHES▶DDX51 (bg=1.63%)▶DHX30 (bg=0.14%)▶EXOSC5 (bg=5.38%)▶HNRNPM (bg=4.29%)▶NIPBL (bg=5.39%)▶RBM15 (bg=7.27%)▶SF3B1 (bg=2.48%)▶SLTM (bg=2.2%)▶uchl5 (bg=11.16%)MATCHES To TargetScan▶ miR-670-3p:UUCCUCA

-------------------

TTGCCGC

TTGCCGC  
Depth:2 (PIG)  
Ei-value:0.000, Pi-value:0.000  
Er-value:0.000, Pr-value:0.010  
eCLIP MATCHES▶EXOSC5 (bg=5.38%)▶HNRNPM (bg=4.29%)▶NIPBL (bg=5.39%)▶RBM15 (bg=7.27%)▶RBM22 (bg=4.62%)▶uchl5 (bg=11.16%)No matches to TargetScan

----

CTCGGCT

CTCGGCT  
Depth:2 (PIG)  
Ei-value:0.000, Pi-value:0.000  
Er-value:0.000, Pr-value:0.000  
eCLIP MATCHES▶EXOSC5 (bg=5.38%)▶NIPBL (bg=5.39%)▶RBM15 (bg=7.27%)▶RBM22 (bg=4.62%)▶SDAD1 (bg=2.97%)▶uchl5 (bg=11.16%)No matches to TargetScan

 960  


CTCGGCT  
Depth:2 (PIG)  
Ei-value:0.000, Pi-value:0.000  
Er-value:0.000, Pr-value:0.000  
eCLIP MATCHES▶EXOSC5 (bg=5.38%)▶NIPBL (bg=5.39%)▶RBM15 (bg=7.27%)▶RBM22 (bg=4.62%)▶SDAD1 (bg=2.97%)▶uchl5 (bg=11.16%)No matches to TargetScan

-

AGGGCTA

AGGGCTA  
Depth:2 (PIG)  
Ei-value:0.000, Pi-value:0.000  
Er-value:0.000, Pr-value:0.010  
eCLIP MATCHES▶EXOSC5 (bg=5.38%)▶HNRNPA1 (bg=2.57%)▶NIPBL (bg=5.39%)▶RBM15 (bg=7.27%)▶RBM22 (bg=4.62%)▶SDAD1 (bg=2.97%)▶uchl5 (bg=11.16%)MATCHES To TargetScan▶ miR-129-3p:AGCCCUU

--------

TGCTAAGT

TGCTAAGT  
Depth:2 (PIG)  
Ei-value:0.000, Pi-value:0.000  
Er-value:0.000, Pr-value:0.000  
eCLIP MATCHES▶AARS (bg=2.18%)▶EXOSC5 (bg=5.38%)▶HNRNPA1 (bg=2.57%)▶NIPBL (bg=5.39%)▶RBM15 (bg=7.27%)▶RBM22 (bg=4.62%)▶SDAD1 (bg=2.97%)▶uchl5 (bg=11.16%)No matches to TargetScan


TAAACTAGGG

TAAACTAGGGAGGCAAGATG  
Depth:2 (PIG)  
Ei-value:0.000, Pi-value:0.000  
Er-value:0.000, Pr-value:0.000  
eCLIP MATCHES▶AARS (bg=2.18%)▶EXOSC5 (bg=5.38%)▶HNRNPA1 (bg=2.57%)▶NIPBL (bg=5.39%)▶RBM15 (bg=7.27%)▶RBM22 (bg=4.62%)▶SDAD1 (bg=2.97%)▶uchl5 (bg=11.16%)No matches to TargetScan


AGGCAAGA

AGGCAAGA  
Depth:3 (COW)  
Ei-value:0.000, Pi-value:0.000  
Er-value:0.000, Pr-value:0.000  
eCLIP MATCHES▶AARS (bg=2.18%)▶EXOSC5 (bg=5.38%)▶HNRNPA1 (bg=2.57%)▶NIPBL (bg=5.39%)▶RBM15 (bg=7.27%)▶RBM22 (bg=4.62%)▶SDAD1 (bg=2.97%)▶uchl5 (bg=11.16%)No matches to TargetScan


TG

TAAACTAGGGAGGCAAGATG  
Depth:2 (PIG)  
Ei-value:0.000, Pi-value:0.000  
Er-value:0.000, Pr-value:0.000  
eCLIP MATCHES▶AARS (bg=2.18%)▶EXOSC5 (bg=5.38%)▶HNRNPA1 (bg=2.57%)▶NIPBL (bg=5.39%)▶RBM15 (bg=7.27%)▶RBM22 (bg=4.62%)▶SDAD1 (bg=2.97%)▶uchl5 (bg=11.16%)No matches to TargetScan

-------------

CAGGCAGAGGAA

CAGGCAGAGGAA  
Depth:2 (PIG)  
Ei-value:0.000, Pi-value:0.000  
Er-value:0.000, Pr-value:0.000  
eCLIP MATCHES▶AARS (bg=2.18%)▶EXOSC5 (bg=5.38%)▶HNRNPA1 (bg=2.57%)▶HNRNPM (bg=4.29%)▶NIPBL (bg=5.39%)▶RBM15 (bg=7.27%)▶RBM22 (bg=4.62%)▶SDAD1 (bg=2.97%)▶uchl5 (bg=11.16%)MATCHES To TargetScan▶ miR-670-3p:UUCCUCA

------

TGCATTG

TGCATTG  
Depth:2 (PIG)  
Ei-value:0.000, Pi-value:0.010  
Er-value:0.000, Pr-value:0.020  
eCLIP MATCHES▶EXOSC5 (bg=5.38%)▶HNRNPA1 (bg=2.57%)▶HNRNPM (bg=4.29%)▶RBM15 (bg=7.27%)▶RBM22 (bg=4.62%)▶uchl5 (bg=11.16%)No matches to TargetScan

-

ATGAGCTA

ATGAGCTA  
Depth:2 (PIG)  
Ei-value:0.000, Pi-value:0.000  
Er-value:0.000, Pr-value:0.000  
eCLIP MATCHES▶HNRNPA1 (bg=2.57%)▶HNRNPM (bg=4.29%)▶RBM22 (bg=4.62%)▶uchl5 (bg=11.16%)No matches to TargetScan

------------------

GATTTGGG

GATTTGGG  
Depth:2 (PIG)  
Ei-value:0.000, Pi-value:0.000  
Er-value:0.000, Pr-value:0.000  
eCLIP MATCHES▶CPEB4 (bg=1.89%)▶EXOSC5 (bg=5.38%)▶GNL3 (bg=0.43%)▶HNRNPM (bg=4.29%)▶TARDBP (bg=2.79%)No matches to TargetScan

-

CT

CTTGTTAGGA  
Depth:2 (PIG)  
Ei-value:0.000, Pi-value:0.000  
Er-value:0.000, Pr-value:0.000  
eCLIP MATCHES▶CPEB4 (bg=1.89%)▶EXOSC5 (bg=5.38%)▶GNL3 (bg=0.43%)▶HNRNPM (bg=4.29%)▶TARDBP (bg=2.79%)▶uchl5 (bg=11.16%)No matches to TargetScan

 1080  


TGTTAGGA

CTTGTTAGGA  
Depth:2 (PIG)  
Ei-value:0.000, Pi-value:0.000  
Er-value:0.000, Pr-value:0.000  
eCLIP MATCHES▶CPEB4 (bg=1.89%)▶EXOSC5 (bg=5.38%)▶GNL3 (bg=0.43%)▶HNRNPM (bg=4.29%)▶TARDBP (bg=2.79%)▶uchl5 (bg=11.16%)No matches to TargetScan

------------------------------------------------------------------------

GAAGGTT

GAAGGTT  
Depth:2 (PIG)  
Ei-value:0.000, Pi-value:0.000  
Er-value:0.000, Pr-value:0.000  
eCLIP MATCHES▶EXOSC5 (bg=5.38%)▶NPM1 (bg=1.21%)▶RBFOX2 (bg=4.63%)▶RBM15 (bg=7.27%)▶RBM22 (bg=4.62%)▶uchl5 (bg=11.16%)No matches to TargetScan

------------------------

AAAATGGCG

AAAATGGCGATTTTGAC  
Depth:2 (PIG)  
Ei-value:0.000, Pi-value:0.000  
Er-value:0.000, Pr-value:0.000  
eCLIP MATCHES▶EXOSC5 (bg=5.38%)▶GNL3 (bg=0.43%)▶NPM1 (bg=1.21%)▶RBFOX2 (bg=4.63%)▶RBM15 (bg=7.27%)▶RBM22 (bg=4.62%)▶uchl5 (bg=11.16%)No matches to TargetScan

 1200  


ATTTTGAC

AAAATGGCGATTTTGAC  
Depth:2 (PIG)  
Ei-value:0.000, Pi-value:0.000  
Er-value:0.000, Pr-value:0.000  
eCLIP MATCHES▶EXOSC5 (bg=5.38%)▶GNL3 (bg=0.43%)▶NPM1 (bg=1.21%)▶RBFOX2 (bg=4.63%)▶RBM15 (bg=7.27%)▶RBM22 (bg=4.62%)▶uchl5 (bg=11.16%)No matches to TargetScan

------

GCATTGCT

GCATTGCT  
Depth:2 (PIG)  
Ei-value:0.000, Pi-value:0.000  
Er-value:0.000, Pr-value:0.000  
eCLIP MATCHES▶EXOSC5 (bg=5.38%)▶HNRNPM (bg=4.29%)▶RBFOX2 (bg=4.63%)▶RBM15 (bg=7.27%)▶RBM22 (bg=4.62%)▶uchl5 (bg=11.16%)No matches to TargetScan

-

AGCATGGC

AGCATGGC  
Depth:2 (PIG)  
Ei-value:0.000, Pi-value:0.000  
Er-value:0.000, Pr-value:0.010  
eCLIP MATCHES▶EXOSC5 (bg=5.38%)▶HNRNPM (bg=4.29%)▶RBFOX2 (bg=4.63%)▶RBM15 (bg=7.27%)▶RBM22 (bg=4.62%)▶uchl5 (bg=11.16%)No matches to TargetScan

------

TGCTTTGTTAG

TGCTTTGTTAG  
Depth:2 (PIG)  
Ei-value:0.000, Pi-value:0.000  
Er-value:0.000, Pr-value:0.000  
eCLIP MATCHES▶EXOSC5 (bg=5.38%)▶HNRNPM (bg=4.29%)▶RBFOX2 (bg=4.63%)▶RBM15 (bg=7.27%)▶RBM22 (bg=4.62%)▶SDAD1 (bg=2.97%)▶uchl5 (bg=11.16%)MATCHES To TargetScan▶ miR-330-3p:CAAAGCA▶ miR-330-3p.2:AAAGCAC▶ miR-495-3p:AACAAAC

------

CAAAATGGCGGA

CAAAATGGCGGA  
Depth:2 (PIG)  
Ei-value:0.000, Pi-value:0.000  
Er-value:0.000, Pr-value:0.000  
eCLIP MATCHES▶HNRNPM (bg=4.29%)▶RBFOX2 (bg=4.63%)▶RBM22 (bg=4.62%)▶uchl5 (bg=11.16%)No matches to TargetScan

-----------

CGCAGTGTTC

CGCAGTGTTC  
Depth:2 (PIG)  
Ei-value:0.000, Pi-value:0.000  
Er-value:0.000, Pr-value:0.000  
eCLIP MATCHES▶EXOSC5 (bg=5.38%)▶HNRNPM (bg=4.29%)▶RBM22 (bg=4.62%)MATCHES To TargetScan▶ miR-141-3p/200a-3p:AACACUG

-

AGTGGCGGGAAG

AGTGGCGGGAAG  
Depth:2 (PIG)  
Ei-value:0.000, Pi-value:0.000  
Er-value:0.000, Pr-value:0.000  
eCLIP MATCHES▶EXOSC5 (bg=5.38%)▶HNRNPM (bg=4.29%)▶LARP4 (bg=4.72%)▶RBM22 (bg=4.62%)No matches to TargetScan

-

CCACAT

CCACAT  
Depth:2 (PIG)  
Ei-value:0.000, Pi-value:0.030  
Er-value:0.000, Pr-value:0.020  
eCLIP MATCHES▶EXOSC5 (bg=5.38%)▶HNRNPM (bg=4.29%)▶LARP4 (bg=4.72%)▶RBM22 (bg=4.62%)▶SDAD1 (bg=2.97%)MATCHES To TargetScan▶ miR-299-3p:AUGUGGG

------------- 1320  
 ----------------

AGCATGG

AGCATGG  
Depth:2 (PIG)  
Ei-value:0.000, Pi-value:0.000  
Er-value:0.000, Pr-value:0.000  
eCLIP MATCHES▶AARS (bg=2.18%)▶EXOSC5 (bg=5.38%)▶HNRNPM (bg=4.29%)▶NIPBL (bg=5.39%)▶RBM15 (bg=7.27%)▶RBM22 (bg=4.62%)▶SDAD1 (bg=2.97%)▶uchl5 (bg=11.16%)No matches to TargetScan

-------------------------

AAAAGATGGCGGCT

AAAAGATGGCGGCT  
Depth:2 (PIG)  
Ei-value:0.000, Pi-value:0.000  
Er-value:0.000, Pr-value:0.000  
eCLIP MATCHES▶AARS (bg=2.18%)▶EXOSC5 (bg=5.38%)▶HNRNPM (bg=4.29%)▶NIPBL (bg=5.39%)▶RBM15 (bg=7.27%)▶RBM22 (bg=4.62%)▶uchl5 (bg=11.16%)No matches to TargetScan

------

CTTGCCGCA

CTTGCCGCA  
Depth:2 (PIG)  
Ei-value:0.000, Pi-value:0.000  
Er-value:0.000, Pr-value:0.000  
eCLIP MATCHES▶AARS (bg=2.18%)▶EXOSC5 (bg=5.38%)▶HNRNPM (bg=4.29%)▶uchl5 (bg=11.16%)MATCHES To TargetScan▶ miR-31-5p:GGCAAGA

----

A

AAAACATGGCGGGCCT  
Depth:2 (PIG)  
Ei-value:0.000, Pi-value:0.000  
Er-value:0.000, Pr-value:0.000  
eCLIP MATCHES▶HNRNPM (bg=4.29%)No matches to TargetScan


AAACATG

AAACATG  
Depth:4 (DOG)  
Ei-value:0.000, Pi-value:0.000  
Er-value:0.000, Pr-value:0.000  
eCLIP MATCHES▶HNRNPM (bg=4.29%)No matches to TargetScan


GCGGGCCT

AAAACATGGCGGGCCT  
Depth:2 (PIG)  
Ei-value:0.000, Pi-value:0.000  
Er-value:0.000, Pr-value:0.000  
eCLIP MATCHES▶HNRNPM (bg=4.29%)No matches to TargetScan

--

TTGTCTTTGC

TTGTCTTTGC  
Depth:2 (PIG)  
Ei-value:0.000, Pi-value:0.000  
Er-value:0.000, Pr-value:0.000  
eCLIP MATCHES▶HNRNPM (bg=4.29%)No matches to TargetScan

----------- 1440  
 ---------

TTTTGCCGCAGGGACAATATGGC

TTTTGCCGCAGGGACAATATGGC  
Depth:2 (PIG)  
Ei-value:0.000, Pi-value:0.000  
Er-value:0.000, Pr-value:0.000  
eCLIP MATCHES▶AKAP8L (bg=2.19%)▶DDX51 (bg=1.63%)▶DDX52 (bg=0.46%)▶EXOSC5 (bg=5.38%)▶GNL3 (bg=0.43%)▶HNRNPM (bg=4.29%)▶HNRNPUL1 (bg=1.16%)▶LARP4 (bg=4.72%)▶METAP2 (bg=0.78%)▶NCBP2 (bg=1.49%)▶RBM22 (bg=4.62%)▶SDAD1 (bg=2.97%)▶SLTM (bg=2.2%)▶uchl5 (bg=11.16%)▶WRN (bg=0.77%)▶XRCC6 (bg=2.91%)No matches to TargetScan

-----

TTGTCAT

TTGTCAT  
Depth:2 (PIG)  
Ei-value:0.000, Pi-value:0.020  
Er-value:0.000, Pr-value:0.040  
eCLIP MATCHES▶DDX51 (bg=1.63%)▶DDX52 (bg=0.46%)▶EXOSC5 (bg=5.38%)▶GNL3 (bg=0.43%)▶HNRNPM (bg=4.29%)▶HNRNPUL1 (bg=1.16%)▶METAP2 (bg=0.78%)▶NCBP2 (bg=1.49%)▶RBM22 (bg=4.62%)▶SLTM (bg=2.2%)▶uchl5 (bg=11.16%)▶WRN (bg=0.77%)▶XRCC6 (bg=2.91%)MATCHES To TargetScan▶ miR-425-5p:AUGACAC

-----------------

TGTCACGTGGAC

TGTCACGTGGAC  
Depth:2 (PIG)  
Ei-value:0.000, Pi-value:0.000  
Er-value:0.000, Pr-value:0.000  
eCLIP MATCHES▶DDX51 (bg=1.63%)▶EXOSC5 (bg=5.38%)▶HNRNPM (bg=4.29%)MATCHES To TargetScan▶ miR-542-3p:GUGACAG

----

TGGCGGGCT

TGGCGGGCT  
Depth:2 (PIG)  
Ei-value:0.000, Pi-value:0.000  
Er-value:0.000, Pr-value:0.000  
eCLIP MATCHES▶DDX51 (bg=1.63%)▶EXOSC5 (bg=5.38%)▶HNRNPM (bg=4.29%)No matches to TargetScan


TGCCGCATTGTT

TGCCGCATTGTT  
Depth:2 (PIG)  
Ei-value:0.000, Pi-value:0.000  
Er-value:0.000, Pr-value:0.000  
eCLIP MATCHES▶DDX51 (bg=1.63%)▶EXOSC5 (bg=5.38%)▶HNRNPM (bg=4.29%)No matches to TargetScan

-

AAGATGGCGGG

AAGATGGCGGG  
Depth:2 (PIG)  
Ei-value:0.000, Pi-value:0.000  
Er-value:0.000, Pr-value:0.000  
eCLIP MATCHES▶HNRNPM (bg=4.29%)▶RBFOX2 (bg=4.63%)No matches to TargetScan

-

TTTGCCGC

TTTGCCGC  
Depth:2 (PIG)  
Ei-value:0.000, Pi-value:0.000  
Er-value:0.000, Pr-value:0.000  
eCLIP MATCHES▶EXOSC5 (bg=5.38%)▶HNRNPM (bg=4.29%)▶RBFOX2 (bg=4.63%)No matches to TargetScan

- 1560  
 ------------------------------------------

TGGATTGC

TGGATTGC  
Depth:4 (DOG)  
Ei-value:0.000, Pi-value:0.000  
Er-value:0.000, Pr-value:0.000  
eCLIP MATCHES▶AARS (bg=2.18%)▶EXOSC5 (bg=5.38%)▶HNRNPM (bg=4.29%)▶RBFOX2 (bg=4.63%)▶RBM22 (bg=4.62%)▶SUPV3L1 (bg=1.57%)▶uchl5 (bg=11.16%)No matches to TargetScan

---------------------------

GGTGGAATTG

GGTGGAATTG  
Depth:2 (PIG)  
Ei-value:0.000, Pi-value:0.000  
Er-value:0.000, Pr-value:0.000  
eCLIP MATCHES▶EXOSC5 (bg=5.38%)▶NPM1 (bg=1.21%)▶RBM22 (bg=4.62%)▶SDAD1 (bg=2.97%)▶SUPV3L1 (bg=1.57%)▶uchl5 (bg=11.16%)No matches to TargetScan

-

TCACAG

TCACAG  
Depth:2 (PIG)  
Ei-value:0.000, Pi-value:0.010  
Er-value:0.000, Pr-value:0.020  
eCLIP MATCHES▶EXOSC5 (bg=5.38%)▶NPM1 (bg=1.21%)▶RBM22 (bg=4.62%)▶SDAD1 (bg=2.97%)▶uchl5 (bg=11.16%)No matches to TargetScan

---------------

GATGGAATTAG

GATGGAATTAG  
Depth:2 (PIG)  
Ei-value:0.000, Pi-value:0.000  
Er-value:0.000, Pr-value:0.000  
eCLIP MATCHES▶EXOSC5 (bg=5.38%)▶NPM1 (bg=1.21%)▶RBM22 (bg=4.62%)▶SDAD1 (bg=2.97%)▶uchl5 (bg=11.16%)No matches to TargetScan

 1680  


GATGGAATTAG  
Depth:2 (PIG)  
Ei-value:0.000, Pi-value:0.000  
Er-value:0.000, Pr-value:0.000  
eCLIP MATCHES▶EXOSC5 (bg=5.38%)▶NPM1 (bg=1.21%)▶RBM22 (bg=4.62%)▶SDAD1 (bg=2.97%)▶uchl5 (bg=11.16%)No matches to TargetScan

--------------------------------------------

TTAGCAT

TTAGCAT  
Depth:2 (PIG)  
Ei-value:0.000, Pi-value:0.020  
Er-value:0.000, Pr-value:0.010  
eCLIP MATCHES▶SLTM (bg=2.2%)No matches to TargetScan

--------------------

AGCCAGTCAG

AGCCAGTCAG  
Depth:2 (PIG)  
Ei-value:0.000, Pi-value:0.000  
Er-value:0.000, Pr-value:0.000  
eCLIP MATCHES▶DDX51 (bg=1.63%)▶HNRNPM (bg=4.29%)▶WRN (bg=0.77%)MATCHES To TargetScan▶ miR-149-5p:CUGGCUC▶ miR-193-3p:ACUGGCC▶ miR-3064-5p:CUGGCUG

----------------

GGCCACGT

GGCCACGT  
Depth:2 (PIG)  
Ei-value:0.000, Pi-value:0.000  
Er-value:0.000, Pr-value:0.000  
eCLIP MATCHES▶DDX51 (bg=1.63%)▶DROSHA (bg=2.49%)▶EXOSC5 (bg=5.38%)▶HNRNPM (bg=4.29%)▶NCBP2 (bg=1.49%)▶RBM22 (bg=4.62%)▶SLTM (bg=2.2%)▶uchl5 (bg=11.16%)▶UTP3 (bg=3.66%)▶WRN (bg=0.77%)No matches to TargetScan

------

CTCCCAGTG

CTCCCAGTGGG  
Depth:2 (PIG)  
Ei-value:0.000, Pi-value:0.000  
Er-value:0.000, Pr-value:0.000  
eCLIP MATCHES▶DROSHA (bg=2.49%)▶EXOSC5 (bg=5.38%)▶HNRNPM (bg=4.29%)▶NCBP2 (bg=1.49%)▶RBM22 (bg=4.62%)▶SLTM (bg=2.2%)▶uchl5 (bg=11.16%)▶UTP3 (bg=3.66%)▶WRN (bg=0.77%)No matches to TargetScan

 1800  


GG

CTCCCAGTGGG  
Depth:2 (PIG)  
Ei-value:0.000, Pi-value:0.000  
Er-value:0.000, Pr-value:0.000  
eCLIP MATCHES▶DROSHA (bg=2.49%)▶EXOSC5 (bg=5.38%)▶HNRNPM (bg=4.29%)▶NCBP2 (bg=1.49%)▶RBM22 (bg=4.62%)▶SLTM (bg=2.2%)▶uchl5 (bg=11.16%)▶UTP3 (bg=3.66%)▶WRN (bg=0.77%)No matches to TargetScan

------------------

CAAGGTCTTT

CAAGGTCTTT  
Depth:2 (PIG)  
Ei-value:0.000, Pi-value:0.000  
Er-value:0.000, Pr-value:0.000  
eCLIP MATCHES▶EXOSC5 (bg=5.38%)No matches to TargetScan

-

CAAGGAC

CAAGGAC  
Depth:2 (PIG)  
Ei-value:0.000, Pi-value:0.000  
Er-value:0.000, Pr-value:0.010  
No matches to eCLIP DataNo matches to TargetScan

-----

GCCTTTCCACCTC

GCCTTTCCACCTC  
Depth:2 (PIG)  
Ei-value:0.000, Pi-value:0.000  
Er-value:0.000, Pr-value:0.000  
eCLIP MATCHES▶EXOSC5 (bg=5.38%)▶PCBP1 (bg=1.07%)▶SDAD1 (bg=2.97%)▶TIA1 (bg=4.07%)▶uchl5 (bg=11.16%)No matches to TargetScan

--

TCCCCTCT

TCCCCTCT  
Depth:2 (PIG)  
Ei-value:0.000, Pi-value:0.000  
Er-value:0.000, Pr-value:0.000  
eCLIP MATCHES▶EXOSC5 (bg=5.38%)▶PCBP1 (bg=1.07%)▶SDAD1 (bg=2.97%)▶TIA1 (bg=4.07%)▶uchl5 (bg=11.16%)MATCHES To TargetScan▶ miR-423-5p:GAGGGGC

------

TCCCCTCC

TCCCCTCC  
Depth:2 (PIG)  
Ei-value:0.000, Pi-value:0.000  
Er-value:0.000, Pr-value:0.000  
eCLIP MATCHES▶EXOSC5 (bg=5.38%)▶HNRNPM (bg=4.29%)▶PCBP1 (bg=1.07%)▶SDAD1 (bg=2.97%)▶uchl5 (bg=11.16%)MATCHES To TargetScan▶ miR-423-5p:GAGGGGC

---------------------------------------- 1920  
 -----

CTGAACCTC

CTGAACCTC  
Depth:2 (PIG)  
Ei-value:0.000, Pi-value:0.000  
Er-value:0.000, Pr-value:0.000  
eCLIP MATCHES▶EXOSC5 (bg=5.38%)▶hnrnpk (bg=12.88%)▶PCBP1 (bg=1.07%)No matches to TargetScan

---

CCATTCCTCTG

CCATTCCTCTG  
Depth:2 (PIG)  
Ei-value:0.000, Pi-value:0.000  
Er-value:0.000, Pr-value:0.000  
eCLIP MATCHES▶EXOSC5 (bg=5.38%)▶hnrnpk (bg=12.88%)▶PCBP1 (bg=1.07%)MATCHES To TargetScan▶ miR-1-3p/206:GGAAUGU

-

ATTGGTG

ATTGGTG  
Depth:2 (PIG)  
Ei-value:0.000, Pi-value:0.000  
Er-value:0.000, Pr-value:0.010  
eCLIP MATCHES▶EXOSC5 (bg=5.38%)▶hnrnpk (bg=12.88%)No matches to TargetScan

-----

CCTAAGGCTAA

CCTAAGGCTAA  
Depth:2 (PIG)  
Ei-value:0.000, Pi-value:0.000  
Er-value:0.000, Pr-value:0.000  
No matches to eCLIP DataNo matches to TargetScan

-----

CCTCCCCC

CCTCCCCC  
Depth:2 (PIG)  
Ei-value:0.000, Pi-value:0.000  
Er-value:0.000, Pr-value:0.000  
No matches to eCLIP DataNo matches to TargetScan

------------------------------------------------------- 2040  
 -----------------------------

CCTCTG

CCTCTG  
Depth:2 (PIG)  
Ei-value:0.000, Pi-value:0.020  
Er-value:0.000, Pr-value:0.000  
eCLIP MATCHES▶CSTF2T (bg=0.82%)▶DDX51 (bg=1.63%)▶DROSHA (bg=2.49%)▶GTF2F1 (bg=0.51%)▶hnrnpk (bg=12.88%)▶ILF3 (bg=3.0%)▶PCBP1 (bg=1.07%)▶PUM1 (bg=1.56%)No matches to TargetScan

------------

CTGCACTGT

CTGCACTGT  
Depth:2 (PIG)  
Ei-value:0.000, Pi-value:0.000  
Er-value:0.000, Pr-value:0.000  
eCLIP MATCHES▶CSTF2T (bg=0.82%)▶DGCR8 (bg=1.84%)▶DROSHA (bg=2.49%)▶GTF2F1 (bg=0.51%)▶hnrnpk (bg=12.88%)▶ILF3 (bg=3.0%)▶PCBP1 (bg=1.07%)▶PUM1 (bg=1.56%)▶RBM15 (bg=7.27%)MATCHES To TargetScan▶ miR-130-3p/301-3p/454-3p:AGUGCAA▶ miR-148-3p/152-3p:CAGUGCA

------

GGGCAGTGCTCCA

GGGCAGTGCTCCA  
Depth:2 (PIG)  
Ei-value:0.000, Pi-value:0.000  
Er-value:0.000, Pr-value:0.000  
eCLIP MATCHES▶CSTF2T (bg=0.82%)▶DGCR8 (bg=1.84%)▶DROSHA (bg=2.49%)▶GTF2F1 (bg=0.51%)▶hnrnpk (bg=12.88%)▶HNRNPM (bg=4.29%)▶ILF3 (bg=3.0%)▶NCBP2 (bg=1.49%)▶PCBP1 (bg=1.07%)▶PUM1 (bg=1.56%)▶RBM15 (bg=7.27%)No matches to TargetScan

-

GCCTGC

GCCTGC  
Depth:2 (PIG)  
Ei-value:0.000, Pi-value:0.000  
Er-value:0.000, Pr-value:0.000  
eCLIP MATCHES▶CSTF2T (bg=0.82%)▶DGCR8 (bg=1.84%)▶DROSHA (bg=2.49%)▶GTF2F1 (bg=0.51%)▶hnrnpk (bg=12.88%)▶HNRNPM (bg=4.29%)▶HNRNPUL1 (bg=1.16%)▶ILF3 (bg=3.0%)▶NCBP2 (bg=1.49%)▶PCBP1 (bg=1.07%)▶PUM1 (bg=1.56%)▶RBM15 (bg=7.27%)▶SRSF1 (bg=8.47%)No matches to TargetScan

----------------

GGTGAG

GGTGAG  
Depth:2 (PIG)  
Ei-value:0.000, Pi-value:0.000  
Er-value:0.000, Pr-value:0.010  
eCLIP MATCHES▶CSTF2T (bg=0.82%)▶DDX52 (bg=0.46%)▶DGCR8 (bg=1.84%)▶DROSHA (bg=2.49%)▶EXOSC5 (bg=5.38%)▶GRWD1 (bg=5.13%)▶GTF2F1 (bg=0.51%)▶hnrnpk (bg=12.88%)▶HNRNPM (bg=4.29%)▶HNRNPUL1 (bg=1.16%)▶ILF3 (bg=3.0%)▶NCBP2 (bg=1.49%)▶PUM1 (bg=1.56%)▶RBM15 (bg=7.27%)▶SF3B1 (bg=2.48%)▶SRSF1 (bg=8.47%)▶SUPV3L1 (bg=1.57%)▶ZNF622 (bg=6.58%)▶ZNF800 (bg=1.92%)No matches to TargetScan

-

CGTGGCAAGGACCAG

CGTGGCAAGGACCAGAATGGATC  
Depth:2 (PIG)  
Ei-value:0.000, Pi-value:0.000  
Er-value:0.000, Pr-value:0.000  
eCLIP MATCHES▶CSTF2T (bg=0.82%)▶DDX52 (bg=0.46%)▶DGCR8 (bg=1.84%)▶DROSHA (bg=2.49%)▶EXOSC5 (bg=5.38%)▶GRWD1 (bg=5.13%)▶GTF2F1 (bg=0.51%)▶hnrnpk (bg=12.88%)▶HNRNPM (bg=4.29%)▶HNRNPUL1 (bg=1.16%)▶ILF3 (bg=3.0%)▶NCBP2 (bg=1.49%)▶PUM1 (bg=1.56%)▶RBM15 (bg=7.27%)▶SF3B1 (bg=2.48%)▶SRSF1 (bg=8.47%)▶SUPV3L1 (bg=1.57%)▶TRA2A (bg=4.8%)▶ZNF622 (bg=6.58%)▶ZNF800 (bg=1.92%)MATCHES To TargetScan▶ miR-133a-3p.1:UGGUCCC

 2160  


AATGGATC

CGTGGCAAGGACCAGAATGGATC  
Depth:2 (PIG)  
Ei-value:0.000, Pi-value:0.000  
Er-value:0.000, Pr-value:0.000  
eCLIP MATCHES▶CSTF2T (bg=0.82%)▶DDX52 (bg=0.46%)▶DGCR8 (bg=1.84%)▶DROSHA (bg=2.49%)▶EXOSC5 (bg=5.38%)▶GRWD1 (bg=5.13%)▶GTF2F1 (bg=0.51%)▶hnrnpk (bg=12.88%)▶HNRNPM (bg=4.29%)▶HNRNPUL1 (bg=1.16%)▶ILF3 (bg=3.0%)▶NCBP2 (bg=1.49%)▶PUM1 (bg=1.56%)▶RBM15 (bg=7.27%)▶SF3B1 (bg=2.48%)▶SRSF1 (bg=8.47%)▶SUPV3L1 (bg=1.57%)▶TRA2A (bg=4.8%)▶ZNF622 (bg=6.58%)▶ZNF800 (bg=1.92%)MATCHES To TargetScan▶ miR-133a-3p.1:UGGUCCC

-

CAGATGATCGTTGGC

CAGATGATCGTTGGCCAACAGGTGGC  
Depth:2 (PIG)  
Ei-value:0.000, Pi-value:0.000  
Er-value:0.000, Pr-value:0.000  
eCLIP MATCHES▶CSTF2T (bg=0.82%)▶GRWD1 (bg=5.13%)▶hnrnpk (bg=12.88%)▶HNRNPM (bg=4.29%)▶ILF3 (bg=3.0%)▶MTPAP (bg=2.21%)▶NCBP2 (bg=1.49%)▶NIPBL (bg=5.39%)▶PUM1 (bg=1.56%)▶RBM15 (bg=7.27%)▶SRSF1 (bg=8.47%)▶TRA2A (bg=4.8%)▶uchl5 (bg=11.16%)▶XRCC6 (bg=2.91%)▶ZNF622 (bg=6.58%)▶ZNF800 (bg=1.92%)No matches to TargetScan


CAACAG

CAACAG  
Depth:3 (COW)  
Ei-value:0.000, Pi-value:0.000  
Er-value:0.000, Pr-value:0.000  
eCLIP MATCHES▶CSTF2T (bg=0.82%)▶GRWD1 (bg=5.13%)▶HNRNPM (bg=4.29%)▶MTPAP (bg=2.21%)▶NCBP2 (bg=1.49%)▶NIPBL (bg=5.39%)▶PUM1 (bg=1.56%)▶RBM15 (bg=7.27%)▶SRSF1 (bg=8.47%)▶TRA2A (bg=4.8%)▶uchl5 (bg=11.16%)▶XRCC6 (bg=2.91%)▶ZNF622 (bg=6.58%)▶ZNF800 (bg=1.92%)No matches to TargetScan


GTGGC

CAGATGATCGTTGGCCAACAGGTGGC  
Depth:2 (PIG)  
Ei-value:0.000, Pi-value:0.000  
Er-value:0.000, Pr-value:0.000  
eCLIP MATCHES▶CSTF2T (bg=0.82%)▶GRWD1 (bg=5.13%)▶hnrnpk (bg=12.88%)▶HNRNPM (bg=4.29%)▶ILF3 (bg=3.0%)▶MTPAP (bg=2.21%)▶NCBP2 (bg=1.49%)▶NIPBL (bg=5.39%)▶PUM1 (bg=1.56%)▶RBM15 (bg=7.27%)▶SRSF1 (bg=8.47%)▶TRA2A (bg=4.8%)▶uchl5 (bg=11.16%)▶XRCC6 (bg=2.91%)▶ZNF622 (bg=6.58%)▶ZNF800 (bg=1.92%)No matches to TargetScan

-

GAAGAGGAAT

GAAGAGGAAT  
Depth:2 (PIG)  
Ei-value:0.000, Pi-value:0.000  
Er-value:0.000, Pr-value:0.000  
eCLIP MATCHES▶CSTF2T (bg=0.82%)▶GRWD1 (bg=5.13%)▶HNRNPM (bg=4.29%)▶MTPAP (bg=2.21%)▶NCBP2 (bg=1.49%)▶NIPBL (bg=5.39%)▶PUM1 (bg=1.56%)▶RBM15 (bg=7.27%)▶SRSF1 (bg=8.47%)▶TRA2A (bg=4.8%)▶uchl5 (bg=11.16%)▶UTP3 (bg=3.66%)▶XRCC6 (bg=2.91%)▶ZNF622 (bg=6.58%)▶ZNF800 (bg=1.92%)MATCHES To TargetScan▶ miR-670-3p:UUCCUCA

------

CTTCCTCAAGAGGAACACCTACCCC

CTTCCTCAAGAGGAACACCTACCCC  
Depth:2 (PIG)  
Ei-value:0.000, Pi-value:0.000  
Er-value:0.000, Pr-value:0.000  
eCLIP MATCHES▶CSTF2T (bg=0.82%)▶GRWD1 (bg=5.13%)▶HNRNPM (bg=4.29%)▶MTPAP (bg=2.21%)▶NCBP2 (bg=1.49%)▶NIPBL (bg=5.39%)▶PUM1 (bg=1.56%)▶RBM15 (bg=7.27%)▶SRSF1 (bg=8.47%)▶TRA2A (bg=4.8%)▶uchl5 (bg=11.16%)▶UTP3 (bg=3.66%)▶ZNF622 (bg=6.58%)MATCHES To TargetScan▶ miR-1224-5p:UGAGGAC▶ miR-670-3p:UUCCUCA

-

TGGCTAATGCTGGGGTCGGATTTTGATTT

TGGCTAATGCTGGGGTCGGATTTTGATTT  
Depth:2 (PIG)  
Ei-value:0.000, Pi-value:0.000  
Er-value:0.000, Pr-value:0.000  
eCLIP MATCHES▶GRWD1 (bg=5.13%)▶SRSF1 (bg=8.47%)▶ZNF622 (bg=6.58%)MATCHES To TargetScan▶ miR-338-3p:CCAGCAU▶ miR-551-3p:CGACCCA

-----------

TT

TTGGATGTCAGTCATA  
Depth:2 (PIG)  
Ei-value:0.000, Pi-value:0.000  
Er-value:0.000, Pr-value:0.000  
eCLIP MATCHES▶CPSF6 (bg=0.4%)▶EXOSC5 (bg=5.38%)▶HNRNPUL1 (bg=1.16%)▶KHSRP (bg=0.67%)▶RBM15 (bg=7.27%)▶TIA1 (bg=4.07%)▶XRCC6 (bg=2.91%)MATCHES To TargetScan▶ miR-489-3p:UGACAUC

 2280  


GGATGTCAGTCATA

TTGGATGTCAGTCATA  
Depth:2 (PIG)  
Ei-value:0.000, Pi-value:0.000  
Er-value:0.000, Pr-value:0.000  
eCLIP MATCHES▶CPSF6 (bg=0.4%)▶EXOSC5 (bg=5.38%)▶HNRNPUL1 (bg=1.16%)▶KHSRP (bg=0.67%)▶RBM15 (bg=7.27%)▶TIA1 (bg=4.07%)▶XRCC6 (bg=2.91%)MATCHES To TargetScan▶ miR-489-3p:UGACAUC

-----------

TGTGGTTTGCTAGTGTT

TGTGGTTTGCTAGTGTT  
Depth:2 (PIG)  
Ei-value:0.000, Pi-value:0.000  
Er-value:0.000, Pr-value:0.000  
eCLIP MATCHES▶CPSF6 (bg=0.4%)▶EXOSC5 (bg=5.38%)▶HNRNPM (bg=4.29%)▶HNRNPUL1 (bg=1.16%)▶KHSRP (bg=0.67%)▶NIPBL (bg=5.39%)▶RBM22 (bg=4.62%)▶TIA1 (bg=4.07%)▶XRCC6 (bg=2.91%)MATCHES To TargetScan▶ miR-140-3p.2:ACCACAG▶ miR-141-3p/200a-3p:AACACUG

---

ATTTAAG

ATTTAAG  
Depth:2 (PIG)  
Ei-value:0.000, Pi-value:0.000  
Er-value:0.000, Pr-value:0.000  
eCLIP MATCHES▶EXOSC5 (bg=5.38%)▶KHSRP (bg=0.67%)▶NIPBL (bg=5.39%)▶TIA1 (bg=4.07%)No matches to TargetScan

-

CTTAAGTGACTA

CTTAAGTGACTA  
Depth:2 (PIG)  
Ei-value:0.000, Pi-value:0.000  
Er-value:0.000, Pr-value:0.000  
eCLIP MATCHES▶EXOSC5 (bg=5.38%)▶KHSRP (bg=0.67%)▶NIPBL (bg=5.39%)▶TIA1 (bg=4.07%)MATCHES To TargetScan▶ miR-668-3p:GUCACUC

-------

AATGTATT

AATGTATT  
Depth:2 (PIG)  
Ei-value:0.000, Pi-value:0.000  
Er-value:0.000, Pr-value:0.000  
eCLIP MATCHES▶KHSRP (bg=0.67%)▶TIA1 (bg=4.07%)No matches to TargetScan

--------

TTATTTGTAGAATTCA

TTATTTGTAGAATTCA  
Depth:2 (PIG)  
Ei-value:0.000, Pi-value:0.000  
Er-value:0.000, Pr-value:0.000  
eCLIP MATCHES▶KHSRP (bg=0.67%)▶TIA1 (bg=4.07%)No matches to TargetScan

------

TTACATTTA

TTACATTTA  
Depth:2 (PIG)  
Ei-value:0.000, Pi-value:0.000  
Er-value:0.000, Pr-value:0.000  
eCLIP MATCHES▶KHSRP (bg=0.67%)▶TIA1 (bg=4.07%)MATCHES To TargetScan▶ miR-411-3p:AUGUAAC

- 2400  
 ----------------

GTTCCTT

GTTCCTT  
Depth:2 (PIG)  
Ei-value:0.000, Pi-value:0.010  
Er-value:0.000, Pr-value:0.010  
eCLIP MATCHES▶U2AF2 (bg=1.76%)No matches to TargetScan

-

AAATTCCTTAAAGTTTT

AAATTCCTTAAAGTTTT  
Depth:2 (PIG)  
Ei-value:0.000, Pi-value:0.000  
Er-value:0.000, Pr-value:0.000  
eCLIP MATCHES▶U2AF2 (bg=1.76%)No matches to TargetScan

----------

TTACAAAT

TTACAAAT  
Depth:2 (PIG)  
Ei-value:0.000, Pi-value:0.000  
Er-value:0.000, Pr-value:0.000  
eCLIP MATCHES▶U2AF2 (bg=1.76%)No matches to TargetScan

------------------------

ATAGTCAAAGTCAA

ATAGTCAAAGTCAA  
Depth:2 (PIG)  
Ei-value:0.000, Pi-value:0.000  
Er-value:0.000, Pr-value:0.000  
eCLIP MATCHES▶EXOSC5 (bg=5.38%)▶LSM11 (bg=2.28%)▶SUPV3L1 (bg=1.57%)▶U2AF2 (bg=1.76%)No matches to TargetScan

----------------------- 2520  
 --------------

CTTTGAAATTGACTTAA

CTTTGAAATTGACTTAA  
Depth:2 (PIG)  
Ei-value:0.000, Pi-value:0.000  
Er-value:0.000, Pr-value:0.000  
eCLIP MATCHES▶LSM11 (bg=2.28%)▶PUS1 (bg=1.04%)▶SF3B1 (bg=2.48%)MATCHES To TargetScan▶ miR-224-5p:AAGUCAC

---------

TTTGAAG

TTTGAAG  
Depth:2 (PIG)  
Ei-value:0.000, Pi-value:0.010  
Er-value:0.000, Pr-value:0.000  
eCLIP MATCHES▶LSM11 (bg=2.28%)No matches to TargetScan

-----------------------

AAAATTTAAC

AAAATTTAAC  
Depth:2 (PIG)  
Ei-value:0.000, Pi-value:0.000  
Er-value:0.000, Pr-value:0.000  
eCLIP MATCHES▶LSM11 (bg=2.28%)No matches to TargetScan

---

ATGACC

ATGACC  
Depth:2 (PIG)  
Ei-value:0.000, Pi-value:0.000  
Er-value:0.000, Pr-value:0.000  
eCLIP MATCHES▶ILF3 (bg=3.0%)▶LSM11 (bg=2.28%)No matches to TargetScan

-----------------

TTTGAAGGT

TTTGAAGGT  
Depth:2 (PIG)  
Ei-value:0.000, Pi-value:0.000  
Er-value:0.000, Pr-value:0.000  
eCLIP MATCHES▶ILF3 (bg=3.0%)▶LSM11 (bg=2.28%)MATCHES To TargetScan▶ miR-205-5p:CCUUCAU

----- 2640  
 ---

GTCCAGG

GTCCAGG  
Depth:2 (PIG)  
Ei-value:0.000, Pi-value:0.000  
Er-value:0.000, Pr-value:0.000  
eCLIP MATCHES▶ILF3 (bg=3.0%)MATCHES To TargetScan▶ miR-378-3p:CUGGACU

-

CTTGCTTTG

CTTGCTTTGTTCCCATCCTT  
Depth:2 (PIG)  
Ei-value:0.000, Pi-value:0.000  
Er-value:0.000, Pr-value:0.000  
eCLIP MATCHES▶ILF3 (bg=3.0%)MATCHES To TargetScan▶ miR-330-3p:CAAAGCA▶ miR-330-3p.2:AAAGCAC▶ miR-495-3p:AACAAAC


TTCCCATC

TTCCCATC  
Depth:4 (DOG)  
Ei-value:0.000, Pi-value:0.000  
Er-value:0.000, Pr-value:0.000  
eCLIP MATCHES▶ILF3 (bg=3.0%)No matches to TargetScan


CTT

CTTGCTTTGTTCCCATCCTT  
Depth:2 (PIG)  
Ei-value:0.000, Pi-value:0.000  
Er-value:0.000, Pr-value:0.000  
eCLIP MATCHES▶ILF3 (bg=3.0%)MATCHES To TargetScan▶ miR-330-3p:CAAAGCA▶ miR-330-3p.2:AAAGCAC▶ miR-495-3p:AACAAAC

-

ATGCTGCACT

ATGCTGCACT  
Depth:2 (PIG)  
Ei-value:0.000, Pi-value:0.000  
Er-value:0.000, Pr-value:0.000  
No matches to eCLIP DataMATCHES To TargetScan▶ miR-103-3p/107:GCAGCAU▶ miR-130-3p/301-3p/454-3p:AGUGCAA

------------------------------

AAACTTGAATTGCTGTGG

AAACTTGAATTGCTGTGG  
Depth:2 (PIG)  
Ei-value:0.000, Pi-value:0.000  
Er-value:0.000, Pr-value:0.000  
No matches to eCLIP DataMATCHES To TargetScan▶ miR-140-3p.1:CCACAGG▶ miR-26-5p:UCAAGUA

---------------------

TTATTATAT

TTATTATATTGGAGTATT  
Depth:2 (PIG)  
Ei-value:0.000, Pi-value:0.000  
Er-value:0.000, Pr-value:0.000  
No matches to eCLIP DataMATCHES To TargetScan▶ miR-200bc-3p/429:AAUACUG▶ miR-369-3p:AUAAUAC▶ miR-374-5p:UAUAAUA▶ miR-410-3p:AUAUAAC

 2760  


TGGAGTATT

TTATTATATTGGAGTATT  
Depth:2 (PIG)  
Ei-value:0.000, Pi-value:0.000  
Er-value:0.000, Pr-value:0.000  
No matches to eCLIP DataMATCHES To TargetScan▶ miR-200bc-3p/429:AAUACUG▶ miR-369-3p:AUAAUAC▶ miR-374-5p:UAUAAUA▶ miR-410-3p:AUAUAAC


TCAATTTT

TCAATTTT  
Depth:2 (PIG)  
Ei-value:0.000, Pi-value:0.010  
Er-value:0.000, Pr-value:0.010  
No matches to eCLIP DataNo matches to TargetScan

----------------------------------------------

TCCCAGCAAACCC

TCCCAGCAAACCC  
Depth:2 (PIG)  
Ei-value:0.000, Pi-value:0.000  
Er-value:0.000, Pr-value:0.000  
eCLIP MATCHES▶hnrnpk (bg=12.88%)No matches to TargetScan

------

TAGCCCCAGCCC

TAGCCCCAGCCC  
Depth:2 (PIG)  
Ei-value:0.000, Pi-value:0.000  
Er-value:0.000, Pr-value:0.000  
No matches to eCLIP DataNo matches to TargetScan

-------------------

CCCTGCC

CCCTGCCCCAGCCCCAG  
Depth:2 (PIG)  
Ei-value:0.000, Pi-value:0.000  
Er-value:0.000, Pr-value:0.000  
eCLIP MATCHES▶DROSHA (bg=2.49%)No matches to TargetScan

 2880  


CCAGCCCCAG

CCCTGCCCCAGCCCCAG  
Depth:2 (PIG)  
Ei-value:0.000, Pi-value:0.000  
Er-value:0.000, Pr-value:0.000  
eCLIP MATCHES▶DROSHA (bg=2.49%)No matches to TargetScan

--------------------

AGCCCCAG

AGCCCCAG  
Depth:2 (PIG)  
Ei-value:0.000, Pi-value:0.000  
Er-value:0.000, Pr-value:0.000  
eCLIP MATCHES▶DGCR8 (bg=1.84%)▶DROSHA (bg=2.49%)▶hnrnpk (bg=12.88%)▶SDAD1 (bg=2.97%)▶XRN2 (bg=0.39%)No matches to TargetScan

--

CCAGTCC

CCAGTCC  
Depth:2 (PIG)  
Ei-value:0.000, Pi-value:0.000  
Er-value:0.000, Pr-value:0.010  
eCLIP MATCHES▶DGCR8 (bg=1.84%)▶DROSHA (bg=2.49%)▶hnrnpk (bg=12.88%)▶SDAD1 (bg=2.97%)▶XRN2 (bg=0.39%)No matches to TargetScan

------------------------------------------------

ATTGATT

ATTGATT  
Depth:2 (PIG)  
Ei-value:0.000, Pi-value:0.000  
Er-value:0.000, Pr-value:0.030  
eCLIP MATCHES▶DROSHA (bg=2.49%)▶hnrnpk (bg=12.88%)No matches to TargetScan

--------

AAAATAAGTT

AAAATAAGTT  
Depth:2 (PIG)  
Ei-value:0.000, Pi-value:0.000  
Er-value:0.000, Pr-value:0.000  
eCLIP MATCHES▶DROSHA (bg=2.49%)▶hnrnpk (bg=12.88%)No matches to TargetScan

 3000  


AAAATAAGTT  
Depth:2 (PIG)  
Ei-value:0.000, Pi-value:0.000  
Er-value:0.000, Pr-value:0.000  
eCLIP MATCHES▶DROSHA (bg=2.49%)▶hnrnpk (bg=12.88%)No matches to TargetScan

---------------------

ACTGGGATA

ACTGGGATA  
Depth:2 (PIG)  
Ei-value:0.000, Pi-value:0.000  
Er-value:0.000, Pr-value:0.000  
eCLIP MATCHES▶DROSHA (bg=2.49%)▶hnrnpk (bg=12.88%)▶ILF3 (bg=3.0%)No matches to TargetScan

---------------

GCATTGCTGATCTT

GCATTGCTGATCTT  
Depth:2 (PIG)  
Ei-value:0.000, Pi-value:0.000  
Er-value:0.000, Pr-value:0.000  
eCLIP MATCHES▶hnrnpk (bg=12.88%)▶ILF3 (bg=3.0%)MATCHES To TargetScan▶ miR-383-5p.1:GAUCAGA▶ miR-383-5p.2:AGAUCAG

----------------

ACCATTTTCA

ACCATTTTCA  
Depth:2 (PIG)  
Ei-value:0.000, Pi-value:0.000  
Er-value:0.000, Pr-value:0.000  
eCLIP MATCHES▶hnrnpk (bg=12.88%)▶ILF3 (bg=3.0%)No matches to TargetScan

----------

ACAATCCCATTTG

ACAATCCCATTTG  
Depth:2 (PIG)  
Ei-value:0.000, Pi-value:0.000  
Er-value:0.000, Pr-value:0.000  
eCLIP MATCHES▶hnrnpk (bg=12.88%)▶HNRNPU (bg=5.92%)▶ILF3 (bg=3.0%)MATCHES To TargetScan▶ miR-219-5p:GAUUGUC

------------ 3120  
 --

ACAAAGAATTT

ACAAAGAATTT  
Depth:2 (PIG)  
Ei-value:0.000, Pi-value:0.000  
Er-value:0.000, Pr-value:0.000  
eCLIP MATCHES▶hnrnpk (bg=12.88%)No matches to TargetScan

-------------------

GTTAGG

GTTAGG  
Depth:2 (PIG)  
Ei-value:0.000, Pi-value:0.010  
Er-value:0.000, Pr-value:0.010  
No matches to eCLIP DataNo matches to TargetScan

-------------

GAGCTT

GAGCTT  
Depth:2 (PIG)  
Ei-value:0.000, Pi-value:0.010  
Er-value:0.000, Pr-value:0.010  
No matches to eCLIP DataNo matches to TargetScan

--

TATCAGA

TATCAGA  
Depth:2 (PIG)  
Ei-value:0.000, Pi-value:0.000  
Er-value:0.000, Pr-value:0.000  
No matches to eCLIP DataNo matches to TargetScan

--

ATTATTG

ATTATTG  
Depth:2 (PIG)  
Ei-value:0.000, Pi-value:0.000  
Er-value:0.000, Pr-value:0.010  
No matches to eCLIP DataNo matches to TargetScan

--------------------------------------------- 3240  


AAGGAGAAACCATT

AAGGAGAAACCATT  
Depth:2 (PIG)  
Ei-value:0.000, Pi-value:0.000  
Er-value:0.000, Pr-value:0.000  
eCLIP MATCHES▶EIF3G (bg=0.32%)▶hnrnpk (bg=12.88%)No matches to TargetScan

-

CTCTGT

CTCTGT  
Depth:3 (COW)  
Ei-value:0.000, Pi-value:0.000  
Er-value:0.000, Pr-value:0.000  
eCLIP MATCHES▶EIF3G (bg=0.32%)▶hnrnpk (bg=12.88%)No matches to TargetScan


CATTGCT

CTCTGTCATTGCT  
Depth:2 (PIG)  
Ei-value:0.000, Pi-value:0.000  
Er-value:0.000, Pr-value:0.000  
eCLIP MATCHES▶EIF3G (bg=0.32%)▶hnrnpk (bg=12.88%)MATCHES To TargetScan▶ miR-425-5p:AUGACAC

---

GTAGTCA

GTAGTCA  
Depth:2 (PIG)  
Ei-value:0.000, Pi-value:0.000  
Er-value:0.000, Pr-value:0.000  
eCLIP MATCHES▶hnrnpk (bg=12.88%)▶SUPV3L1 (bg=1.57%)No matches to TargetScan

----------------------------------

TACTGTG

TACTGTG  
Depth:2 (PIG)  
Ei-value:0.000, Pi-value:0.000  
Er-value:0.000, Pr-value:0.000  
eCLIP MATCHES▶HNRNPU (bg=5.92%)▶WRN (bg=0.77%)MATCHES To TargetScan▶ miR-101-3p.1:ACAGUAC▶ miR-128-3p:CACAGUG▶ miR-144-3p:ACAGUAU

-----------

AAACTCTTTGCA

AAACTCTTTGCA  
Depth:2 (PIG)  
Ei-value:0.000, Pi-value:0.000  
Er-value:0.000, Pr-value:0.000  
eCLIP MATCHES▶HNRNPU (bg=5.92%)▶WRN (bg=0.77%)No matches to TargetScan

-------

AAATTCTAATT

AAATTCTAATTG  
Depth:2 (PIG)  
Ei-value:0.000, Pi-value:0.000  
Er-value:0.000, Pr-value:0.000  
No matches to eCLIP DataNo matches to TargetScan

 3360  


G

AAATTCTAATTG  
Depth:2 (PIG)  
Ei-value:0.000, Pi-value:0.000  
Er-value:0.000, Pr-value:0.000  
No matches to eCLIP DataNo matches to TargetScan

---

ATAATCCT

ATAATCCT  
Depth:2 (PIG)  
Ei-value:0.000, Pi-value:0.000  
Er-value:0.000, Pr-value:0.000  
eCLIP MATCHES▶HNRNPU (bg=5.92%)No matches to TargetScan

---

CATTGGA

CATTGGA  
Depth:2 (PIG)  
Ei-value:0.000, Pi-value:0.010  
Er-value:0.000, Pr-value:0.010  
eCLIP MATCHES▶HNRNPU (bg=5.92%)No matches to TargetScan

----------------------

TTTGCATTCAGCAG

TTTGCATTCAGCAG  
Depth:2 (PIG)  
Ei-value:0.000, Pi-value:0.000  
Er-value:0.000, Pr-value:0.000  
eCLIP MATCHES▶HNRNPU (bg=5.92%)No matches to TargetScan

----------------------------------------------------

TTGTCATA

TTGTCATA  
Depth:2 (PIG)  
Ei-value:0.000, Pi-value:0.000  
Er-value:0.000, Pr-value:0.000  
eCLIP MATCHES▶HNRNPU (bg=5.92%)MATCHES To TargetScan▶ miR-425-5p:AUGACAC

-- 3480  
 --------

TTAAACAAAGGCA

TTAAACAAAGGCA  
Depth:2 (PIG)  
Ei-value:0.000, Pi-value:0.000  
Er-value:0.000, Pr-value:0.000  
eCLIP MATCHES▶HNRNPU (bg=5.92%)No matches to TargetScan

--------------------------------------

CTGTTCTTGGACAATTAAAG

CTGTTCTTGGACAATTAAAG  
Depth:2 (PIG)  
Ei-value:0.000, Pi-value:0.000  
Er-value:0.000, Pr-value:0.000  
eCLIP MATCHES▶HNRNPU (bg=5.92%)No matches to TargetScan

---------

GTAATTG

GTAATTG  
Depth:2 (PIG)  
Ei-value:0.000, Pi-value:0.000  
Er-value:0.000, Pr-value:0.000  
No matches to eCLIP DataNo matches to TargetScan

---

ATTGTCTCAC

ATTGTCTCAC  
Depth:2 (PIG)  
Ei-value:0.000, Pi-value:0.000  
Er-value:0.000, Pr-value:0.000  
No matches to eCLIP DataNo matches to TargetScan

-

CATTAATCA

CATTAATCA  
Depth:2 (PIG)  
Ei-value:0.000, Pi-value:0.000  
Er-value:0.000, Pr-value:0.000  
No matches to eCLIP DataNo matches to TargetScan

-- 3600  
 -------------------------------

AGGCAG

AGGCAG  
Depth:2 (PIG)  
Ei-value:0.000, Pi-value:0.010  
Er-value:0.000, Pr-value:0.020  
No matches to eCLIP DataNo matches to TargetScan

-

GCTGACTA

GCTGACTA  
Depth:2 (PIG)  
Ei-value:0.000, Pi-value:0.000  
Er-value:0.000, Pr-value:0.000  
No matches to eCLIP DataNo matches to TargetScan

-----------------------

ATTATTGA

ATTATTGA  
Depth:2 (PIG)  
Ei-value:0.000, Pi-value:0.010  
Er-value:0.000, Pr-value:0.000  
No matches to eCLIP DataNo matches to TargetScan

-------------------------------

TTACAC

TTACAC  
Depth:2 (PIG)  
Ei-value:0.000, Pi-value:0.010  
Er-value:0.000, Pr-value:0.000  
No matches to eCLIP DataNo matches to TargetScan

------ 3720  
 -----------

TCTTTGCA

TCTTTGCA  
Depth:2 (PIG)  
Ei-value:0.000, Pi-value:0.000  
Er-value:0.000, Pr-value:0.000  
No matches to eCLIP DataNo matches to TargetScan

------------------------------------------

GGAATATTAATGGATACAAT

GGAATATTAATGGATACAAT  
Depth:2 (PIG)  
Ei-value:0.000, Pi-value:0.000  
Er-value:0.000, Pr-value:0.000  
No matches to eCLIP DataNo matches to TargetScan

---------

TCATGGT

TCATGGT  
Depth:2 (PIG)  
Ei-value:0.000, Pi-value:0.000  
Er-value:0.000, Pr-value:0.020  
No matches to eCLIP DataNo matches to TargetScan

--------------

AAGACCCAC

AAGACCCAC  
Depth:2 (PIG)  
Ei-value:0.000, Pi-value:0.000  
Er-value:0.000, Pr-value:0.000  
eCLIP MATCHES▶HNRNPUL1 (bg=1.16%)MATCHES To TargetScan▶ miR-193a-5p:GGGUCUU

 3840  


AAGACCCAC  
Depth:2 (PIG)  
Ei-value:0.000, Pi-value:0.000  
Er-value:0.000, Pr-value:0.000  
eCLIP MATCHES▶HNRNPUL1 (bg=1.16%)MATCHES To TargetScan▶ miR-193a-5p:GGGUCUU

-------------------------------

TAGTGTG

TAGTGTG  
Depth:2 (PIG)  
Ei-value:0.000, Pi-value:0.000  
Er-value:0.000, Pr-value:0.000  
No matches to eCLIP DataNo matches to TargetScan

-

ACTACCTACCACCTTGCATTAATAT

ACTACCTACCACCTTGCATTAATAT  
Depth:2 (PIG)  
Ei-value:0.000, Pi-value:0.000  
Er-value:0.000, Pr-value:0.000  
No matches to eCLIP DataMATCHES To TargetScan▶ miR-155-5p:UAAUGCU▶ miR-18-5p:AAGGUGC▶ miR-196-5p:AGGUAGU

---------------------

GTCCCACT

GTCCCACT  
Depth:2 (PIG)  
Ei-value:0.000, Pi-value:0.000  
Er-value:0.000, Pr-value:0.000  
eCLIP MATCHES▶hnrnpk (bg=12.88%)No matches to TargetScan

----------------------

TGCCA

TGCCATT  
Depth:2 (PIG)  
Ei-value:0.000, Pi-value:0.010  
Er-value:0.000, Pr-value:0.000  
eCLIP MATCHES▶hnrnpk (bg=12.88%)MATCHES To TargetScan▶ miR-183-5p.1:AUGGCAC

 3960  


TT

TGCCATT  
Depth:2 (PIG)  
Ei-value:0.000, Pi-value:0.010  
Er-value:0.000, Pr-value:0.000  
eCLIP MATCHES▶hnrnpk (bg=12.88%)MATCHES To TargetScan▶ miR-183-5p.1:AUGGCAC

------

GAGTTCTGA

GAGTTCTGA  
Depth:2 (PIG)  
Ei-value:0.000, Pi-value:0.000  
Er-value:0.000, Pr-value:0.000  
eCLIP MATCHES▶hnrnpk (bg=12.88%)No matches to TargetScan

--------------

TTCTCTTAAACA

TTCTCTTAAACA  
Depth:2 (PIG)  
Ei-value:0.000, Pi-value:0.000  
Er-value:0.000, Pr-value:0.000  
No matches to eCLIP DataNo matches to TargetScan

-------

TGCATAATTGCATATA

TGCATAATTGCATATA  
Depth:2 (PIG)  
Ei-value:0.000, Pi-value:0.000  
Er-value:0.000, Pr-value:0.000  
No matches to eCLIP DataNo matches to TargetScan

----------------------------------------------

TCTGTGG

TCTGTGG  
Depth:2 (PIG)  
Ei-value:0.000, Pi-value:0.000  
Er-value:0.000, Pr-value:0.010  
eCLIP MATCHES▶HNRNPL (bg=0.64%)▶HNRNPU (bg=5.92%)MATCHES To TargetScan▶ miR-140-3p.1:CCACAGG

- 4080  
 ------------

AGTGCA

AGTGCA  
Depth:2 (PIG)  
Ei-value:0.000, Pi-value:0.020  
Er-value:0.000, Pr-value:0.000  
eCLIP MATCHES▶HNRNPU (bg=5.92%)No matches to TargetScan

-

ATTATTCAA

ATTATTCAA  
Depth:2 (PIG)  
Ei-value:0.000, Pi-value:0.000  
Er-value:0.000, Pr-value:0.000  
eCLIP MATCHES▶HNRNPA1 (bg=2.57%)▶HNRNPU (bg=5.92%)No matches to TargetScan

-------------

ACAGTTAAT

ACAGTTAAT  
Depth:2 (PIG)  
Ei-value:0.000, Pi-value:0.000  
Er-value:0.000, Pr-value:0.010  
eCLIP MATCHES▶HNRNPA1 (bg=2.57%)No matches to TargetScan

--

GCACAGTTGC

GCACAGTTGC  
Depth:2 (PIG)  
Ei-value:0.000, Pi-value:0.000  
Er-value:0.000, Pr-value:0.000  
eCLIP MATCHES▶HNRNPA1 (bg=2.57%)No matches to TargetScan

--

TTGTCCAGAGTCC

TTGTCCAGAGTCC  
Depth:2 (PIG)  
Ei-value:0.000, Pi-value:0.000  
Er-value:0.000, Pr-value:0.000  
eCLIP MATCHES▶HNRNPA1 (bg=2.57%)MATCHES To TargetScan▶ miR-326:CUCUGGG▶ miR-378-3p:CUGGACU

------------------------------------------- 4200  
 -

GTGGGC

GTGGGC  
Depth:2 (PIG)  
Ei-value:0.000, Pi-value:0.000  
Er-value:0.000, Pr-value:0.010  
eCLIP MATCHES▶HNRNPU (bg=5.92%)No matches to TargetScan

-----------------------------------

CATAATTG

CATAATTG  
Depth:2 (PIG)  
Ei-value:0.000, Pi-value:0.000  
Er-value:0.000, Pr-value:0.000  
eCLIP MATCHES▶HNRNPU (bg=5.92%)No matches to TargetScan

--------------------------------------

CTTTGTATTCCAGCAGGGGACCCTT

CTTTGTATTCCAGCAGGGGACCCTT  
Depth:2 (PIG)  
Ei-value:0.000, Pi-value:0.000  
Er-value:0.000, Pr-value:0.000  
eCLIP MATCHES▶hnrnpk (bg=12.88%)▶HNRNPU (bg=5.92%)MATCHES To TargetScan▶ miR-331-3p:CCCCUGG▶ miR-381-3p:AUACAAG

------- 4320  
 -----------------------------------

CCTAATTGATTAGA

CCTAATTGATTAGA  
Depth:2 (PIG)  
Ei-value:0.000, Pi-value:0.000  
Er-value:0.000, Pr-value:0.000  
eCLIP MATCHES▶HNRNPA1 (bg=2.57%)No matches to TargetScan

------

TCTTTTAT

TCTTTTAT  
Depth:2 (PIG)  
Ei-value:0.000, Pi-value:0.000  
Er-value:0.000, Pr-value:0.010  
eCLIP MATCHES▶HNRNPA1 (bg=2.57%)▶hnrnpk (bg=12.88%)▶HNRNPU (bg=5.92%)▶UTP3 (bg=3.66%)No matches to TargetScan

-----------

TGCATC

TGCATC  
Depth:2 (PIG)  
Ei-value:0.000, Pi-value:0.030  
Er-value:0.000, Pr-value:0.020  
eCLIP MATCHES▶HNRNPA1 (bg=2.57%)▶hnrnpk (bg=12.88%)▶HNRNPU (bg=5.92%)▶UTP3 (bg=3.66%)No matches to TargetScan

-----------------------------

TTACCTA

TTACCTA  
Depth:2 (PIG)  
Ei-value:0.000, Pi-value:0.000  
Er-value:0.000, Pr-value:0.020  
eCLIP MATCHES▶HNRNPU (bg=5.92%)▶UTP3 (bg=3.66%)No matches to TargetScan

---- 4440  
 ---------------------------

AAATGCAATT

AAATGCAATT  
Depth:2 (PIG)  
Ei-value:0.000, Pi-value:0.000  
Er-value:0.000, Pr-value:0.000  
eCLIP MATCHES▶AKAP8L (bg=2.19%)MATCHES To TargetScan▶ miR-25-3p/32-5p/92-3p/363-3p/367-3p:AUUGCAC▶ miR-33-5p:UGCAUUG

----------

C

CTGTTAGTCT  
Depth:4 (DOG)  
Ei-value:0.000, Pi-value:0.000  
Er-value:0.000, Pr-value:0.000  
eCLIP MATCHES▶AKAP8L (bg=2.19%)No matches to TargetScan


TGTTAGTC

TGTTAGTC  
Depth:5 (RABBIT)  
Ei-value:0.000, Pi-value:0.000  
Er-value:0.000, Pr-value:0.000  
eCLIP MATCHES▶AKAP8L (bg=2.19%)No matches to TargetScan


T

CTGTTAGTCT  
Depth:4 (DOG)  
Ei-value:0.000, Pi-value:0.000  
Er-value:0.000, Pr-value:0.000  
eCLIP MATCHES▶AKAP8L (bg=2.19%)No matches to TargetScan

----

TC

TCTCATCCCC  
Depth:2 (PIG)  
Ei-value:0.000, Pi-value:0.000  
Er-value:0.000, Pr-value:0.010  
eCLIP MATCHES▶AKAP8L (bg=2.19%)No matches to TargetScan


TCATCC

TCATCC  
Depth:4 (DOG)  
Ei-value:0.000, Pi-value:0.020  
Er-value:0.000, Pr-value:0.000  
eCLIP MATCHES▶AKAP8L (bg=2.19%)No matches to TargetScan


CC

TCTCATCCCC  
Depth:2 (PIG)  
Ei-value:0.000, Pi-value:0.000  
Er-value:0.000, Pr-value:0.010  
eCLIP MATCHES▶AKAP8L (bg=2.19%)No matches to TargetScan

-------

CCTTTTGT

CCTTTTGT  
Depth:2 (PIG)  
Ei-value:0.000, Pi-value:0.000  
Er-value:0.000, Pr-value:0.010  
eCLIP MATCHES▶AKAP8L (bg=2.19%)No matches to TargetScan

-----------

GGG

GGGTACTTGGGACTGTTAAT  
Depth:3 (COW)  
Ei-value:0.000, Pi-value:0.000  
Er-value:0.000, Pr-value:0.000  
eCLIP MATCHES▶AKAP8L (bg=2.19%)MATCHES To TargetScan▶ miR-132-3p/212-3p:AACAGUC▶ miR-455-3p.1:CAGUCCA


TACTTGGGACTGTTAAT

TACTTGGGACTGTTAAT  
Depth:4 (DOG)  
Ei-value:0.000, Pi-value:0.000  
Er-value:0.000, Pr-value:0.000  
eCLIP MATCHES▶AKAP8L (bg=2.19%)MATCHES To TargetScan▶ miR-132-3p/212-3p:AACAGUC▶ miR-455-3p.1:CAGUCCA


G

GGGTACTTGGGACTGTTAATG  
Depth:2 (PIG)  
Ei-value:0.000, Pi-value:0.000  
Er-value:0.000, Pr-value:0.000  
eCLIP MATCHES▶AKAP8L (bg=2.19%)MATCHES To TargetScan▶ miR-132-3p/212-3p:AACAGUC▶ miR-455-3p.1:CAGUCCA

-- 4560  
 -------------------------------------------------------------------------------------------------------

ACTG

ACTGTTAATGTGCT  
Depth:4 (DOG)  
Ei-value:0.000, Pi-value:0.000  
Er-value:0.000, Pr-value:0.000  
No matches to eCLIP DataMATCHES To TargetScan▶ miR-132-3p/212-3p:AACAGUC▶ miR-323-3p:ACAUUAC


TTAATGTGCT

TTAATGTGCT  
Depth:5 (RABBIT)  
Ei-value:0.000, Pi-value:0.000  
Er-value:0.000, Pr-value:0.000  
No matches to eCLIP DataMATCHES To TargetScan▶ miR-323-3p:ACAUUAC


A

ACTGTTAATGTGCTA  
Depth:2 (PIG)  
Ei-value:0.000, Pi-value:0.000  
Er-value:0.000, Pr-value:0.000  
No matches to eCLIP DataMATCHES To TargetScan▶ miR-132-3p/212-3p:AACAGUC▶ miR-323-3p:ACAUUAC

-- 4680  
 ------------------------------------------------------------------------------------------------------------------------ 4800  
 ------------------------------------------------------------------------------------------------------------------------ 4920  
 -----------------------------------------------------------------------

CTTGGGACTC

CTTGGGACTC  
Depth:3 (COW)  
Ei-value:0.000, Pi-value:0.000  
Er-value:0.000, Pr-value:0.000  
No matches to eCLIP DataNo matches to TargetScan


TTAATG

CTTGGGACTCTTAATG  
Depth:2 (PIG)  
Ei-value:0.000, Pi-value:0.000  
Er-value:0.000, Pr-value:0.000  
No matches to eCLIP DataNo matches to TargetScan

-------------

ATCCATG

ATCCATG  
Depth:2 (PIG)  
Ei-value:0.000, Pi-value:0.010  
Er-value:0.000, Pr-value:0.000  
No matches to eCLIP DataNo matches to TargetScan

-----

T

TAATGTGCAT  
Depth:2 (PIG)  
Ei-value:0.000, Pi-value:0.000  
Er-value:0.000, Pr-value:0.000  
No matches to eCLIP DataMATCHES To TargetScan▶ miR-323-3p:ACAUUAC▶ miR-501-3p/502-3p:AUGCACC


AATGTGC

AATGTGCAT  
Depth:6 (MOUSE)  
Ei-value:0.000, Pi-value:0.000  
Er-value:0.000, Pr-value:0.000  
No matches to eCLIP DataMATCHES To TargetScan▶ miR-501-3p/502-3p:AUGCACC

 5040  


AT

AATGTGCAT  
Depth:6 (MOUSE)  
Ei-value:0.000, Pi-value:0.000  
Er-value:0.000, Pr-value:0.000  
No matches to eCLIP DataMATCHES To TargetScan▶ miR-501-3p/502-3p:AUGCACC

---------------------------------------------------------------------------------

CTAATA

CTAATA  
Depth:3 (COW)  
Ei-value:0.000, Pi-value:0.000  
Er-value:0.000, Pr-value:0.000  
No matches to eCLIP DataNo matches to TargetScan

------------------

CTAATA

CTAATA  
Depth:3 (COW)  
Ei-value:0.000, Pi-value:0.000  
Er-value:0.000, Pr-value:0.000  
No matches to eCLIP DataNo matches to TargetScan

------- 5160  
 -----------------------------------

AAGTCTTT

AAGTCTTT  
Depth:2 (PIG)  
Ei-value:0.000, Pi-value:0.000  
Er-value:0.000, Pr-value:0.000  
No matches to eCLIP DataNo matches to TargetScan

---------------

TGCAAAATT

TGCAAAATT  
Depth:2 (PIG)  
Ei-value:0.000, Pi-value:0.000  
Er-value:0.000, Pr-value:0.000  
eCLIP MATCHES▶SUPV3L1 (bg=1.57%)No matches to TargetScan

----------------------------------------------------- 5280  
 ------------------------------------------------------------------------------------------------------------------------ 5400  
 ----------------------

TGCTTCT

TGCTTCT  
Depth:3 (COW)  
Ei-value:0.000, Pi-value:0.000  
Er-value:0.000, Pr-value:0.010  
No matches to eCLIP DataNo matches to TargetScan

-------------------------------------------------

TATGTTAGA

TATGTTAGA  
Depth:4 (DOG)  
Ei-value:0.000, Pi-value:0.000  
Er-value:0.000, Pr-value:0.000  
eCLIP MATCHES▶HNRNPU (bg=5.92%)No matches to TargetScan

-

TAGAATCCC

TAGAATCCC  
Depth:2 (PIG)  
Ei-value:0.000, Pi-value:0.000  
Er-value:0.000, Pr-value:0.000  
eCLIP MATCHES▶HNRNPU (bg=5.92%)No matches to TargetScan

----------------------- 5520  
 -----

GGAGCTTCT

GGAGCTTCT  
Depth:2 (PIG)  
Ei-value:0.000, Pi-value:0.000  
Er-value:0.000, Pr-value:0.000  
No matches to eCLIP DataNo matches to TargetScan

-------------------

TCTTGG

TCTTGGACTGTTAATGT  
Depth:3 (COW)  
Ei-value:0.000, Pi-value:0.000  
Er-value:0.000, Pr-value:0.000  
No matches to eCLIP DataMATCHES To TargetScan▶ miR-132-3p/212-3p:AACAGUC▶ miR-323-3p:ACAUUAC▶ miR-455-3p.1:CAGUCCA


ACTGTTAATGT

ACTGTTAATGT  
Depth:4 (DOG)  
Ei-value:0.000, Pi-value:0.000  
Er-value:0.000, Pr-value:0.000  
No matches to eCLIP DataMATCHES To TargetScan▶ miR-132-3p/212-3p:AACAGUC▶ miR-323-3p:ACAUUAC


G

TCTTGGACTGTTAATGTG  
Depth:2 (PIG)  
Ei-value:0.000, Pi-value:0.000  
Er-value:0.000, Pr-value:0.000  
No matches to eCLIP DataMATCHES To TargetScan▶ miR-132-3p/212-3p:AACAGUC▶ miR-323-3p:ACAUUAC▶ miR-455-3p.1:CAGUCCA

---------

ATTTGCT

ATTTGCT  
Depth:4 (DOG)  
Ei-value:0.000, Pi-value:0.000  
Er-value:0.000, Pr-value:0.000  
No matches to eCLIP DataNo matches to TargetScan

------------------

GTAAGGA

GTAAGGA  
Depth:5 (RABBIT)  
Ei-value:0.000, Pi-value:0.000  
Er-value:0.000, Pr-value:0.000  
No matches to eCLIP DataNo matches to TargetScan


CCC

GTAAGGACCC  
Depth:3 (COW)  
Ei-value:0.000, Pi-value:0.000  
Er-value:0.000, Pr-value:0.000  
No matches to eCLIP DataNo matches to TargetScan

------------------------- 5640  
 --------------

CTTAAGA

CTTAAGA  
Depth:2 (PIG)  
Ei-value:0.000, Pi-value:0.000  
Er-value:0.000, Pr-value:0.000  
No matches to eCLIP DataNo matches to TargetScan

-----------------------

TGCATA

TGCATAATCTTAG  
Depth:2 (PIG)  
Ei-value:0.000, Pi-value:0.000  
Er-value:0.000, Pr-value:0.000  
eCLIP MATCHES▶HNRNPU (bg=5.92%)No matches to TargetScan


ATCTTAG

ATCTTAG  
Depth:3 (COW)  
Ei-value:0.000, Pi-value:0.000  
Er-value:0.000, Pr-value:0.000  
eCLIP MATCHES▶HNRNPU (bg=5.92%)No matches to TargetScan

--------

TACACATT

TACACATT  
Depth:3 (COW)  
Ei-value:0.000, Pi-value:0.000  
Er-value:0.000, Pr-value:0.000  
eCLIP MATCHES▶HNRNPU (bg=5.92%)No matches to TargetScan

-----------------------------

AGGACTCCT

AGGACTCCT  
Depth:2 (PIG)  
Ei-value:0.000, Pi-value:0.000  
Er-value:0.000, Pr-value:0.000  
eCLIP MATCHES▶HNRNPU (bg=5.92%)No matches to TargetScan

-

T

TACTTAT  
Depth:2 (PIG)  
Ei-value:0.000, Pi-value:0.010  
Er-value:0.000, Pr-value:0.030  
eCLIP MATCHES▶HNRNPU (bg=5.92%)No matches to TargetScan


ACTTAT

ACTTAT  
Depth:5 (RABBIT)  
Ei-value:0.000, Pi-value:0.000  
Er-value:0.000, Pr-value:0.000  
eCLIP MATCHES▶HNRNPU (bg=5.92%)No matches to TargetScan

- 5760  
 -----------------------

TTAAGC

TTAAGC  
Depth:2 (PIG)  
Ei-value:0.000, Pi-value:0.000  
Er-value:0.000, Pr-value:0.010  
No matches to eCLIP DataNo matches to TargetScan

-------------------------

TGTAATT

TGTAATT  
Depth:3 (COW)  
Ei-value:0.000, Pi-value:0.000  
Er-value:0.000, Pr-value:0.000  
No matches to eCLIP DataNo matches to TargetScan

----------

ATGGTC

ATGGTC  
Depth:3 (COW)  
Ei-value:0.000, Pi-value:0.020  
Er-value:0.000, Pr-value:0.000  
No matches to eCLIP DataNo matches to TargetScan


CTT

ATGGTCCTT  
Depth:2 (PIG)  
Ei-value:0.000, Pi-value:0.000  
Er-value:0.000, Pr-value:0.000  
No matches to eCLIP DataNo matches to TargetScan

-------------------------

TCACAC

TCACAC  
Depth:2 (PIG)  
Ei-value:0.000, Pi-value:0.010  
Er-value:0.000, Pr-value:0.020  
No matches to eCLIP DataNo matches to TargetScan

--------

C

CTTTATTGC  
Depth:2 (PIG)  
Ei-value:0.000, Pi-value:0.000  
Er-value:0.000, Pr-value:0.000  
eCLIP MATCHES▶HNRNPL (bg=0.64%)MATCHES To TargetScan▶ miR-142-5p:AUAAAGU

 5880  


TTTATTGC

CTTTATTGC  
Depth:2 (PIG)  
Ei-value:0.000, Pi-value:0.000  
Er-value:0.000, Pr-value:0.000  
eCLIP MATCHES▶HNRNPL (bg=0.64%)MATCHES To TargetScan▶ miR-142-5p:AUAAAGU


ATGGGGTACT

ATGGGGTACT  
Depth:3 (COW)  
Ei-value:0.000, Pi-value:0.000  
Er-value:0.000, Pr-value:0.000  
eCLIP MATCHES▶HNRNPL (bg=0.64%)No matches to TargetScan

-

TT

TTCACTTAAGGCCCCTTTCTCAAAC  
Depth:2 (PIG)  
Ei-value:0.000, Pi-value:0.000  
Er-value:0.000, Pr-value:0.000  
eCLIP MATCHES▶HNRNPL (bg=0.64%)No matches to TargetScan


CAC

CACTTAAGGCCCCTTTCTCAA  
Depth:3 (COW)  
Ei-value:0.000, Pi-value:0.000  
Er-value:0.000, Pr-value:0.000  
eCLIP MATCHES▶HNRNPL (bg=0.64%)No matches to TargetScan


TTAAGGCC

TTAAGGCC  
Depth:6 (MOUSE)  
Ei-value:0.000, Pi-value:0.000  
Er-value:0.000, Pr-value:0.000  
eCLIP MATCHES▶HNRNPL (bg=0.64%)No matches to TargetScan


CCTTT

TTAAGGCCCCTTT  
Depth:5 (RABBIT)  
Ei-value:0.000, Pi-value:0.000  
Er-value:0.000, Pr-value:0.000  
eCLIP MATCHES▶HNRNPL (bg=0.64%)No matches to TargetScan


CTCAA

TTAAGGCCCCTTTCTCAA  
Depth:4 (DOG)  
Ei-value:0.000, Pi-value:0.000  
Er-value:0.000, Pr-value:0.000  
eCLIP MATCHES▶HNRNPL (bg=0.64%)No matches to TargetScan


AC

TTCACTTAAGGCCCCTTTCTCAAAC  
Depth:2 (PIG)  
Ei-value:0.000, Pi-value:0.000  
Er-value:0.000, Pr-value:0.000  
eCLIP MATCHES▶HNRNPL (bg=0.64%)No matches to TargetScan

------------

TAATGACAATTACAT

TAATGACAATTACAT  
Depth:3 (COW)  
Ei-value:0.000, Pi-value:0.000  
Er-value:0.000, Pr-value:0.000  
eCLIP MATCHES▶HNRNPL (bg=0.64%)MATCHES To TargetScan▶ miR-411-3p:AUGUAAC

----

ATCCTTCC

ATCCTTCC  
Depth:2 (PIG)  
Ei-value:0.000, Pi-value:0.010  
Er-value:0.000, Pr-value:0.000  
No matches to eCLIP DataNo matches to TargetScan

-

TTTGAAG

TTTGAAG  
Depth:2 (PIG)  
Ei-value:0.000, Pi-value:0.010  
Er-value:0.000, Pr-value:0.000  
eCLIP MATCHES▶HNRNPC (bg=3.65%)No matches to TargetScan

-------------------

CCTAAGG

CCTAAGG  
Depth:2 (PIG)  
Ei-value:0.000, Pi-value:0.010  
Er-value:0.000, Pr-value:0.000  
eCLIP MATCHES▶DDX51 (bg=1.63%)▶HNRNPC (bg=3.65%)No matches to TargetScan

-

CC

CCCATTTCTTG  
Depth:2 (PIG)  
Ei-value:0.000, Pi-value:0.000  
Er-value:0.000, Pr-value:0.000  
eCLIP MATCHES▶DDX51 (bg=1.63%)▶HNRNPC (bg=3.65%)MATCHES To TargetScan▶ miR-203a-3p.1:GAAAUGU

 6000  


CATTTCTTG

CCCATTTCTTG  
Depth:2 (PIG)  
Ei-value:0.000, Pi-value:0.000  
Er-value:0.000, Pr-value:0.000  
eCLIP MATCHES▶DDX51 (bg=1.63%)▶HNRNPC (bg=3.65%)MATCHES To TargetScan▶ miR-203a-3p.1:GAAAUGU

-------------------

TGTATTTGTC

TGTATTTGTC  
Depth:2 (PIG)  
Ei-value:0.000, Pi-value:0.000  
Er-value:0.000, Pr-value:0.000  
eCLIP MATCHES▶DDX51 (bg=1.63%)No matches to TargetScan

----------------------------------------------------

CTTCCAGCAGGAAGTGCCC

CTTCCAGCAGGAAGTGCCC  
Depth:2 (PIG)  
Ei-value:0.000, Pi-value:0.000  
Er-value:0.000, Pr-value:0.000  
eCLIP MATCHES▶hnrnpk (bg=12.88%)No matches to TargetScan

----------- 6120  
 ----------------------------------------

CCACAAG

CCACAAG  
Depth:2 (PIG)  
Ei-value:0.000, Pi-value:0.000  
Er-value:0.000, Pr-value:0.000  
eCLIP MATCHES▶HNRNPU (bg=5.92%)▶UTP3 (bg=3.66%)No matches to TargetScan

------------------------------------------------------------------------- 6240  
 ------------------------------------------------------------------------------------------------------------------------ 6360  
 ---------------------------------------------------

TTTGGACAGTCAAG

TTTGGACAGTCAAG  
Depth:2 (PIG)  
Ei-value:0.000, Pi-value:0.000  
Er-value:0.000, Pr-value:0.000  
eCLIP MATCHES▶hnrnpk (bg=12.88%)No matches to TargetScan

---------------------------

ATGCAC

ATGCAC  
Depth:2 (PIG)  
Ei-value:0.000, Pi-value:0.030  
Er-value:0.000, Pr-value:0.010  
No matches to eCLIP DataNo matches to TargetScan

---------------------- 6480  
 ------------------------------------------------------------------------------------------------------------------------ 6600  
 ------------------------------------------------------------------------------------------------------------------------ 6720  
 ------------------------------------------------------------------------------------------------------------------------ 6840  
 ------------------------------------------------------------------------------------------------------------------------ 6960  
 --------------------------------------------------------------------------

ATGCAC

ATGCAC  
Depth:2 (PIG)  
Ei-value:0.000, Pi-value:0.030  
Er-value:0.000, Pr-value:0.010  
eCLIP MATCHES▶hnrnpk (bg=12.88%)▶HNRNPU (bg=5.92%)No matches to TargetScan

---------------------------

GTAACTG

GTAACTG  
Depth:2 (PIG)  
Ei-value:0.000, Pi-value:0.000  
Er-value:0.000, Pr-value:0.000  
eCLIP MATCHES▶hnrnpk (bg=12.88%)No matches to TargetScan

------ 7080  
 ------------------------------------------------------------------------------------------------------------------------ 7200  
 ------------------------------------------------------------------------------------------------------------------------ 7320  
 -----------------

CACCTTGGA

CACCTTGGA  
Depth:2 (PIG)  
Ei-value:0.000, Pi-value:0.000  
Er-value:0.000, Pr-value:0.000  
eCLIP MATCHES▶hnrnpk (bg=12.88%)▶HNRNPU (bg=5.92%)MATCHES To TargetScan▶ miR-18-5p:AAGGUGC

---------------------------------------------------------------------------------------------- 7440  
 ------------------------------------------------------------------------------------------------------------------------ 7560  
 ------------------------------------------------------------------------------------------------------------------------ 7680  
 ------------------------------------------------------------------------------------------------------------------------ 7800  
 ------------------------------------------------------------------------------------------------------------------------ 7920  
 ----------

TAATGTGT

TAATGTGT  
Depth:2 (PIG)  
Ei-value:0.000, Pi-value:0.010  
Er-value:0.000, Pr-value:0.000  
No matches to eCLIP DataMATCHES To TargetScan▶ miR-323-3p:ACAUUAC

------------------------------------------------------------------------------------------------------ 8040  
 ------------------------------------------------------------------------------------------------------------------------ 8160  
 ------------------------------------------------------------------------

ATAACTGCACATGGCT

ATAACTGCACATGGCT  
Depth:2 (PIG)  
Ei-value:0.000, Pi-value:0.000  
Er-value:0.000, Pr-value:0.000  
No matches to eCLIP DataMATCHES To TargetScan▶ miR-455-3p.2:UGCAGUC▶ miR-455-5p:AUGUGCC

-----------------------

CTCTCAGAC

CTCTCAGACCCC  
Depth:2 (PIG)  
Ei-value:0.000, Pi-value:0.000  
Er-value:0.000, Pr-value:0.000  
eCLIP MATCHES▶SF3B1 (bg=2.48%)MATCHES To TargetScan▶ miR-193a-5p:GGGUCUU

 8280  


CCC

CTCTCAGACCCC  
Depth:2 (PIG)  
Ei-value:0.000, Pi-value:0.000  
Er-value:0.000, Pr-value:0.000  
eCLIP MATCHES▶SF3B1 (bg=2.48%)MATCHES To TargetScan▶ miR-193a-5p:GGGUCUU

------

AGTATAGC

AGTATAGC  
Depth:2 (PIG)  
Ei-value:0.000, Pi-value:0.000  
Er-value:0.000, Pr-value:0.000  
eCLIP MATCHES▶SF3B1 (bg=2.48%)No matches to TargetScan

--------------------

CCTCTTT

CCTCTTT  
Depth:2 (PIG)  
Ei-value:0.000, Pi-value:0.010  
Er-value:0.000, Pr-value:0.020  
No matches to eCLIP DataNo matches to TargetScan

--------------------------------------------------

ACTCCCTTTG

ACTCCCTTTG  
Depth:2 (PIG)  
Ei-value:0.000, Pi-value:0.000  
Er-value:0.000, Pr-value:0.000  
eCLIP MATCHES▶DDX21 (bg=0.25%)No matches to TargetScan

---------------- 8400  
 ---

CACTACTTT

CACTACTTT  
Depth:2 (PIG)  
Ei-value:0.000, Pi-value:0.000  
Er-value:0.000, Pr-value:0.000  
eCLIP MATCHES▶DDX21 (bg=0.25%)MATCHES To TargetScan▶ miR-142-3p.1:GUAGUGU

--------

CTTATATTT

CTTATATTT  
Depth:3 (COW)  
Ei-value:0.000, Pi-value:0.000  
Er-value:0.000, Pr-value:0.000  
eCLIP MATCHES▶DDX21 (bg=0.25%)MATCHES To TargetScan▶ miR-410-3p:AUAUAAC

---

CAAAGTACATG

CAAAGTACATG  
Depth:2 (PIG)  
Ei-value:0.000, Pi-value:0.000  
Er-value:0.000, Pr-value:0.000  
No matches to eCLIP DataNo matches to TargetScan

-

TTTTAATTGACCA

TTTTAATTGACCA  
Depth:3 (COW)  
Ei-value:0.000, Pi-value:0.000  
Er-value:0.000, Pr-value:0.000  
No matches to eCLIP DataNo matches to TargetScan

------------

TTGG

TTGGACATTAATGTA  
Depth:2 (PIG)  
Ei-value:0.000, Pi-value:0.000  
Er-value:0.000, Pr-value:0.000  
No matches to eCLIP DataMATCHES To TargetScan▶ miR-323-3p:ACAUUAC


ACATTAAT

ACATTAAT  
Depth:3 (COW)  
Ei-value:0.000, Pi-value:0.000  
Er-value:0.000, Pr-value:0.000  
No matches to eCLIP DataNo matches to TargetScan


GTA

TTGGACATTAATGTA  
Depth:2 (PIG)  
Ei-value:0.000, Pi-value:0.000  
Er-value:0.000, Pr-value:0.000  
No matches to eCLIP DataMATCHES To TargetScan▶ miR-323-3p:ACAUUAC

------------------------------------ 8520  
 ---------------

CTCCATTTGCAGTATA

CTCCATTTGCAGTATA  
Depth:2 (PIG)  
Ei-value:0.000, Pi-value:0.000  
Er-value:0.000, Pr-value:0.000  
eCLIP MATCHES▶hnrnpk (bg=12.88%)▶TIA1 (bg=4.07%)MATCHES To TargetScan▶ miR-217:ACUGCAU

-

CAGGGTT

CAGGGTT  
Depth:2 (PIG)  
Ei-value:0.000, Pi-value:0.000  
Er-value:0.000, Pr-value:0.000  
eCLIP MATCHES▶hnrnpk (bg=12.88%)▶TIA1 (bg=4.07%)MATCHES To TargetScan▶ miR-10-5p:ACCCUGU▶ miR-504-5p.1:ACCCUGG

--

TGACCC

TGACCC  
Depth:2 (PIG)  
Ei-value:0.000, Pi-value:0.030  
Er-value:0.000, Pr-value:0.000  
eCLIP MATCHES▶hnrnpk (bg=12.88%)▶TIA1 (bg=4.07%)No matches to TargetScan

------------------------------

TG

TGCATAATTGCATTT  
Depth:2 (PIG)  
Ei-value:0.000, Pi-value:0.000  
Er-value:0.000, Pr-value:0.000  
eCLIP MATCHES▶hnrnpk (bg=12.88%)▶TIA1 (bg=4.07%)No matches to TargetScan


CATAATTGCA

CATAATTGCA  
Depth:3 (COW)  
Ei-value:0.000, Pi-value:0.000  
Er-value:0.000, Pr-value:0.000  
eCLIP MATCHES▶hnrnpk (bg=12.88%)▶TIA1 (bg=4.07%)No matches to TargetScan


TTT

TGCATAATTGCATTT  
Depth:2 (PIG)  
Ei-value:0.000, Pi-value:0.000  
Er-value:0.000, Pr-value:0.000  
eCLIP MATCHES▶hnrnpk (bg=12.88%)▶TIA1 (bg=4.07%)No matches to TargetScan

-----

GGTTCTTG

GGTTCTTG  
Depth:2 (PIG)  
Ei-value:0.000, Pi-value:0.000  
Er-value:0.000, Pr-value:0.000  
eCLIP MATCHES▶UTP3 (bg=3.66%)No matches to TargetScan

----

CTAGACAAGGA

CTAGACAAGGA  
Depth:3 (COW)  
Ei-value:0.000, Pi-value:0.000  
Er-value:0.000, Pr-value:0.000  
eCLIP MATCHES▶UTP3 (bg=3.66%)No matches to TargetScan

 8640  


CTAGACAAGGA  
Depth:3 (COW)  
Ei-value:0.000, Pi-value:0.000  
Er-value:0.000, Pr-value:0.000  
eCLIP MATCHES▶UTP3 (bg=3.66%)No matches to TargetScan

------------------------------------------------------

ACAGTTAATGTG

ACAGTTAATGTG  
Depth:4 (DOG)  
Ei-value:0.000, Pi-value:0.000  
Er-value:0.000, Pr-value:0.000  
eCLIP MATCHES▶HNRNPU (bg=5.92%)MATCHES To TargetScan▶ miR-323-3p:ACAUUAC

---

AATTGCAGTT

AATTGCAGTT  
Depth:2 (PIG)  
Ei-value:0.000, Pi-value:0.000  
Er-value:0.000, Pr-value:0.000  
eCLIP MATCHES▶HNRNPU (bg=5.92%)MATCHES To TargetScan▶ miR-217:ACUGCAU

-

TCCACAACCC

TCCACAACCC  
Depth:2 (PIG)  
Ei-value:0.000, Pi-value:0.000  
Er-value:0.000, Pr-value:0.000  
eCLIP MATCHES▶hnrnpk (bg=12.88%)▶HNRNPU (bg=5.92%)No matches to TargetScan

-------------------

ATACCTC

ATACCTC  
Depth:2 (PIG)  
Ei-value:0.000, Pi-value:0.000  
Er-value:0.000, Pr-value:0.000  
No matches to eCLIP DataMATCHES To TargetScan▶ let-7-5p/98-5p:GAGGUAG

---- 8760  
 ---------------

ATACTGTTT

ATACTGTTT  
Depth:3 (COW)  
Ei-value:0.000, Pi-value:0.000  
Er-value:0.000, Pr-value:0.000  
No matches to eCLIP DataMATCHES To TargetScan▶ miR-101-3p.1:ACAGUAC▶ miR-132-3p/212-3p:AACAGUC▶ miR-144-3p:ACAGUAU

-------------------

GTGGAC

GTGGAC  
Depth:2 (PIG)  
Ei-value:0.000, Pi-value:0.000  
Er-value:0.000, Pr-value:0.010  
No matches to eCLIP DataNo matches to TargetScan

----------

TAATTGAAAT

TAATTGAAAT  
Depth:2 (PIG)  
Ei-value:0.000, Pi-value:0.000  
Er-value:0.000, Pr-value:0.000  
No matches to eCLIP DataNo matches to TargetScan

------

TTGTCTT

TTGTCTT  
Depth:3 (COW)  
Ei-value:0.000, Pi-value:0.000  
Er-value:0.000, Pr-value:0.010  
No matches to eCLIP DataNo matches to TargetScan

-------------------------------------- 8880  
 ------------------------------------------------------------------------------------------------------------------------ 9000  
 ------------------------------------------------------------------------------------------------------------------------ 9120  
 --------------------------------------------------------

TAGACT

TAGACT  
Depth:2 (PIG)  
Ei-value:0.000, Pi-value:0.000  
Er-value:0.000, Pr-value:0.000  
No matches to eCLIP DataNo matches to TargetScan

---------------------------------------------------------- 9240  
 -------------------------------------------------------------------------------------------------------

TAACTA

TAACTA  
Depth:4 (DOG)  
Ei-value:0.000, Pi-value:0.000  
Er-value:0.000, Pr-value:0.000  
No matches to eCLIP DataNo matches to TargetScan

--------

CTC

CTCAGCTCTTGG  
Depth:5 (RABBIT)  
Ei-value:0.000, Pi-value:0.000  
Er-value:0.000, Pr-value:0.000  
No matches to eCLIP DataMATCHES To TargetScan▶ miR-335-5p:CAAGAGC

 9360  


AGCTCTTGG

CTCAGCTCTTGG  
Depth:5 (RABBIT)  
Ei-value:0.000, Pi-value:0.000  
Er-value:0.000, Pr-value:0.000  
No matches to eCLIP DataMATCHES To TargetScan▶ miR-335-5p:CAAGAGC


ACA

CTCAGCTCTTGGACA  
Depth:4 (DOG)  
Ei-value:0.000, Pi-value:0.000  
Er-value:0.000, Pr-value:0.000  
No matches to eCLIP DataMATCHES To TargetScan▶ miR-335-5p:CAAGAGC


ATTAATA

CTCAGCTCTTGGACAATTAATA  
Depth:3 (COW)  
Ei-value:0.000, Pi-value:0.000  
Er-value:0.000, Pr-value:0.000  
No matches to eCLIP DataMATCHES To TargetScan▶ miR-335-5p:CAAGAGC

-----

AATAACA

AATAACA  
Depth:2 (PIG)  
Ei-value:0.000, Pi-value:0.010  
Er-value:0.000, Pr-value:0.000  
No matches to eCLIP DataNo matches to TargetScan

------------

ACT

ACTGATCATTAGATA  
Depth:2 (PIG)  
Ei-value:0.000, Pi-value:0.000  
Er-value:0.000, Pr-value:0.000  
eCLIP MATCHES▶HNRNPU (bg=5.92%)MATCHES To TargetScan▶ miR-383-5p.1:GAUCAGA


GATCAT

GATCAT  
Depth:3 (COW)  
Ei-value:0.000, Pi-value:0.000  
Er-value:0.000, Pr-value:0.000  
eCLIP MATCHES▶HNRNPU (bg=5.92%)No matches to TargetScan


TAGATA

ACTGATCATTAGATA  
Depth:2 (PIG)  
Ei-value:0.000, Pi-value:0.000  
Er-value:0.000, Pr-value:0.000  
eCLIP MATCHES▶HNRNPU (bg=5.92%)MATCHES To TargetScan▶ miR-383-5p.1:GAUCAGA

------------------------------------------

AC

ACTAAGGCCCC  
Depth:2 (PIG)  
Ei-value:0.000, Pi-value:0.000  
Er-value:0.000, Pr-value:0.000  
No matches to eCLIP DataNo matches to TargetScan


TAAGGC

TAAGGC  
Depth:3 (COW)  
Ei-value:0.000, Pi-value:0.000  
Er-value:0.000, Pr-value:0.000  
No matches to eCLIP DataNo matches to TargetScan


CCC

ACTAAGGCCCC  
Depth:2 (PIG)  
Ei-value:0.000, Pi-value:0.000  
Er-value:0.000, Pr-value:0.000  
No matches to eCLIP DataNo matches to TargetScan

--------- 9480  
 ------

GAATATTTGCA

GAATATTTGCA  
Depth:3 (COW)  
Ei-value:0.000, Pi-value:0.000  
Er-value:0.000, Pr-value:0.000  
No matches to eCLIP DataNo matches to TargetScan


ATTAT

GAATATTTGCAATTAT  
Depth:2 (PIG)  
Ei-value:0.000, Pi-value:0.000  
Er-value:0.000, Pr-value:0.000  
No matches to eCLIP DataMATCHES To TargetScan▶ miR-25-3p/32-5p/92-3p/363-3p/367-3p:AUUGCAC

-----------------------------------------

TCCCCTT

TCCCCTT  
Depth:2 (PIG)  
Ei-value:0.000, Pi-value:0.000  
Er-value:0.000, Pr-value:0.030  
No matches to eCLIP DataNo matches to TargetScan

---

ATTACTG

ATTACTG  
Depth:3 (COW)  
Ei-value:0.000, Pi-value:0.010  
Er-value:0.000, Pr-value:0.020  
No matches to eCLIP DataMATCHES To TargetScan▶ miR-802:CAGUAAC

-

AG

AGGGGCTGCTGAC  
Depth:2 (PIG)  
Ei-value:0.000, Pi-value:0.000  
Er-value:0.000, Pr-value:0.000  
No matches to eCLIP DataMATCHES To TargetScan▶ miR-15-5p/16-5p/195-5p/424-5p/497-5p:AGCAGCA▶ miR-503-5p:AGCAGCG


GGGCTGCTGA

GGGCTGCTGA  
Depth:3 (COW)  
Ei-value:0.000, Pi-value:0.000  
Er-value:0.000, Pr-value:0.000  
No matches to eCLIP DataMATCHES To TargetScan▶ miR-15-5p/16-5p/195-5p/424-5p/497-5p:AGCAGCA▶ miR-503-5p:AGCAGCG


C

AGGGGCTGCTGAC  
Depth:2 (PIG)  
Ei-value:0.000, Pi-value:0.000  
Er-value:0.000, Pr-value:0.000  
No matches to eCLIP DataMATCHES To TargetScan▶ miR-15-5p/16-5p/195-5p/424-5p/497-5p:AGCAGCA▶ miR-503-5p:AGCAGCG

----

CAAAACTT

CAAAACTT  
Depth:4 (DOG)  
Ei-value:0.000, Pi-value:0.000  
Er-value:0.000, Pr-value:0.000  
eCLIP MATCHES▶SF3B1 (bg=2.48%)No matches to TargetScan


CT

CAAAACTTCT  
Depth:2 (PIG)  
Ei-value:0.000, Pi-value:0.000  
Er-value:0.000, Pr-value:0.000  
eCLIP MATCHES▶SF3B1 (bg=2.48%)No matches to TargetScan

-

CTGGGACTG

CTGGGACTG  
Depth:3 (COW)  
Ei-value:0.000, Pi-value:0.000  
Er-value:0.000, Pr-value:0.000  
eCLIP MATCHES▶SF3B1 (bg=2.48%)MATCHES To TargetScan▶ miR-455-3p.1:CAGUCCA

-- 9600  
 -----

GCACAATG

GCACAATG  
Depth:6 (MOUSE)  
Ei-value:0.000, Pi-value:0.000  
Er-value:0.000, Pr-value:0.000  
No matches to eCLIP DataNo matches to TargetScan

----------------------

CTCCCTG

CTCCCTG  
Depth:3 (COW)  
Ei-value:0.000, Pi-value:0.000  
Er-value:0.000, Pr-value:0.000  
eCLIP MATCHES▶DDX42 (bg=0.58%)No matches to TargetScan

-

CCTTGTT

CCTTGTT  
Depth:2 (PIG)  
Ei-value:0.000, Pi-value:0.000  
Er-value:0.000, Pr-value:0.040  
eCLIP MATCHES▶DDX42 (bg=0.58%)No matches to TargetScan

--

GCAAGC

GCAAGC  
Depth:3 (COW)  
Ei-value:0.000, Pi-value:0.000  
Er-value:0.000, Pr-value:0.000  
eCLIP MATCHES▶DDX42 (bg=0.58%)▶hnrnpk (bg=12.88%)No matches to TargetScan


GC

GCAAGCGC  
Depth:2 (PIG)  
Ei-value:0.000, Pi-value:0.000  
Er-value:0.000, Pr-value:0.000  
eCLIP MATCHES▶DDX42 (bg=0.58%)▶hnrnpk (bg=12.88%)No matches to TargetScan

----------------

TTTCCCATGG

TTTCCCATGG  
Depth:2 (PIG)  
Ei-value:0.000, Pi-value:0.000  
Er-value:0.000, Pr-value:0.000  
eCLIP MATCHES▶DDX42 (bg=0.58%)▶hnrnpk (bg=12.88%)No matches to TargetScan

-

ATAATAAAGTATAA

ATAATAAAGTATAA  
Depth:2 (PIG)  
Ei-value:0.000, Pi-value:0.000  
Er-value:0.000, Pr-value:0.000  
eCLIP MATCHES▶DDX42 (bg=0.58%)▶KHDRBS1 (bg=1.71%)No matches to TargetScan

------------------- 9720  
 --------

ATCAAACAG

ATCAAACAG  
Depth:2 (PIG)  
Ei-value:0.000, Pi-value:0.000  
Er-value:0.000, Pr-value:0.000  
No matches to eCLIP DataNo matches to TargetScan

----

CCAT

CCATACTCCCA  
Depth:2 (PIG)  
Ei-value:0.000, Pi-value:0.000  
Er-value:0.000, Pr-value:0.000  
eCLIP MATCHES▶hnrnpk (bg=12.88%)MATCHES To TargetScan▶ miR-496.1:GAGUAUU


A

ACTCCCA  
Depth:4 (DOG)  
Ei-value:0.000, Pi-value:0.000  
Er-value:0.000, Pr-value:0.000  
eCLIP MATCHES▶hnrnpk (bg=12.88%)No matches to TargetScan


CTCCCA

CTCCCA  
Depth:6 (MOUSE)  
Ei-value:0.000, Pi-value:0.000  
Er-value:0.000, Pr-value:0.000  
eCLIP MATCHES▶hnrnpk (bg=12.88%)No matches to TargetScan

-

CCCTTTTGCATT

CCCTTTTGCATT  
Depth:4 (DOG)  
Ei-value:0.000, Pi-value:0.000  
Er-value:0.000, Pr-value:0.000  
eCLIP MATCHES▶hnrnpk (bg=12.88%)No matches to TargetScan


G

CCCTTTTGCATTG  
Depth:3 (COW)  
Ei-value:0.000, Pi-value:0.000  
Er-value:0.000, Pr-value:0.000  
eCLIP MATCHES▶hnrnpk (bg=12.88%)No matches to TargetScan

-------------------------------------------------------------------------- 9840  
 ------------------------------------------------------------------------------------------------------------------------ 9960  
 --

TTGCTGGG

TTGCTGGG  
Depth:2 (PIG)  
Ei-value:0.000, Pi-value:0.000  
Er-value:0.000, Pr-value:0.010  
eCLIP MATCHES▶hnrnpk (bg=12.88%)MATCHES To TargetScan▶ miR-338-3p:CCAGCAU

-------------------

CCCTTTCT

CCCTTTCT  
Depth:2 (PIG)  
Ei-value:0.000, Pi-value:0.000  
Er-value:0.000, Pr-value:0.000  
eCLIP MATCHES▶hnrnpk (bg=12.88%)No matches to TargetScan

----------------------------------------------------------------

ACTTCCTT

ACTTCCTT  
Depth:3 (COW)  
Ei-value:0.000, Pi-value:0.000  
Er-value:0.000, Pr-value:0.000  
eCLIP MATCHES▶hnrnpk (bg=12.88%)No matches to TargetScan

----------- 10080  


CTGAGTA

CTGAGTA  
Depth:2 (PIG)  
Ei-value:0.000, Pi-value:0.010  
Er-value:0.000, Pr-value:0.000  
eCLIP MATCHES▶hnrnpk (bg=12.88%)No matches to TargetScan

--

CTGACTACCCA

CTGACTACCCA  
Depth:2 (PIG)  
Ei-value:0.000, Pi-value:0.000  
Er-value:0.000, Pr-value:0.000  
eCLIP MATCHES▶hnrnpk (bg=12.88%)No matches to TargetScan

-

AGCCCCTTCT

AGCCCCTTCT  
Depth:3 (COW)  
Ei-value:0.000, Pi-value:0.000  
Er-value:0.000, Pr-value:0.000  
eCLIP MATCHES▶hnrnpk (bg=12.88%)No matches to TargetScan


GTGTTATTAA

AGCCCCTTCTGTGTTATTAA  
Depth:2 (PIG)  
Ei-value:0.000, Pi-value:0.000  
Er-value:0.000, Pr-value:0.000  
eCLIP MATCHES▶hnrnpk (bg=12.88%)No matches to TargetScan

----

CACAGTA

CACAGTA  
Depth:3 (COW)  
Ei-value:0.000, Pi-value:0.000  
Er-value:0.000, Pr-value:0.000  
eCLIP MATCHES▶hnrnpk (bg=12.88%)No matches to TargetScan

-

TGATTGTC

TGATTGTCCCATTTTT  
Depth:3 (COW)  
Ei-value:0.000, Pi-value:0.000  
Er-value:0.000, Pr-value:0.000  
eCLIP MATCHES▶hnrnpk (bg=12.88%)No matches to TargetScan


CCATTTTT

CCATTTTT  
Depth:4 (DOG)  
Ei-value:0.000, Pi-value:0.000  
Er-value:0.000, Pr-value:0.000  
eCLIP MATCHES▶hnrnpk (bg=12.88%)No matches to TargetScan


CAGCCCA

CAGCCCA  
Depth:4 (DOG)  
Ei-value:0.000, Pi-value:0.000  
Er-value:0.000, Pr-value:0.000  
eCLIP MATCHES▶hnrnpk (bg=12.88%)No matches to TargetScan

-----------

TCTC

TCTCCCTACCA  
Depth:3 (COW)  
Ei-value:0.000, Pi-value:0.000  
Er-value:0.000, Pr-value:0.000  
eCLIP MATCHES▶hnrnpk (bg=12.88%)No matches to TargetScan


CCTACCA

CCTACCA  
Depth:4 (DOG)  
Ei-value:0.000, Pi-value:0.000  
Er-value:0.000, Pr-value:0.000  
eCLIP MATCHES▶hnrnpk (bg=12.88%)No matches to TargetScan


CTTTG

TCTCCCTACCACTTTG  
Depth:2 (PIG)  
Ei-value:0.000, Pi-value:0.000  
Er-value:0.000, Pr-value:0.000  
eCLIP MATCHES▶hnrnpk (bg=12.88%)MATCHES To TargetScan▶ miR-140-5p:AGUGGUU▶ miR-17-5p/20-5p/93-5p/106-5p/519-3p:AAAGUGC

--------

GTGCAGT

GTGCAGT  
Depth:3 (COW)  
Ei-value:0.000, Pi-value:0.000  
Er-value:0.000, Pr-value:0.000  
eCLIP MATCHES▶hnrnpk (bg=12.88%)MATCHES To TargetScan▶ miR-217:ACUGCAU

-

T

TTGACTA  
Depth:2 (PIG)  
Ei-value:0.000, Pi-value:0.020  
Er-value:0.000, Pr-value:0.000  
No matches to eCLIP DataNo matches to TargetScan

 10200  


TGACTA

TTGACTA  
Depth:2 (PIG)  
Ei-value:0.000, Pi-value:0.020  
Er-value:0.000, Pr-value:0.000  
No matches to eCLIP DataNo matches to TargetScan

--

AAAAGCAG

AAAAGCAG  
Depth:6 (MOUSE)  
Ei-value:0.000, Pi-value:0.000  
Er-value:0.000, Pr-value:0.000  
No matches to eCLIP DataNo matches to TargetScan

-

CCT

CCTGAACTA  
Depth:2 (PIG)  
Ei-value:0.000, Pi-value:0.000  
Er-value:0.000, Pr-value:0.000  
No matches to eCLIP DataNo matches to TargetScan


GAACTA

GAACTA  
Depth:3 (COW)  
Ei-value:0.000, Pi-value:0.000  
Er-value:0.000, Pr-value:0.000  
No matches to eCLIP DataNo matches to TargetScan

---------

GCCTTCACTC

GCCTTCACTC  
Depth:2 (PIG)  
Ei-value:0.000, Pi-value:0.000  
Er-value:0.000, Pr-value:0.000  
No matches to eCLIP DataNo matches to TargetScan

---------------

TTAATGATCC

TTAATGATCC  
Depth:4 (DOG)  
Ei-value:0.000, Pi-value:0.000  
Er-value:0.000, Pr-value:0.000  
No matches to eCLIP DataMATCHES To TargetScan▶ miR-382-3p:AUCAUUC

------

CA

CAATTATTGT  
Depth:2 (PIG)  
Ei-value:0.000, Pi-value:0.000  
Er-value:0.000, Pr-value:0.000  
No matches to eCLIP DataNo matches to TargetScan


ATTATTGT

ATTATTGT  
Depth:3 (COW)  
Ei-value:0.000, Pi-value:0.000  
Er-value:0.000, Pr-value:0.000  
No matches to eCLIP DataNo matches to TargetScan

---

ATTCTGGG

ATTCTGGG  
Depth:4 (DOG)  
Ei-value:0.000, Pi-value:0.000  
Er-value:0.000, Pr-value:0.000  
No matches to eCLIP DataNo matches to TargetScan


GACA

ATTCTGGGGACA  
Depth:2 (PIG)  
Ei-value:0.000, Pi-value:0.000  
Er-value:0.000, Pr-value:0.000  
No matches to eCLIP DataNo matches to TargetScan

-

GAACCATTC

GAACCATTC  
Depth:2 (PIG)  
Ei-value:0.000, Pi-value:0.000  
Er-value:0.000, Pr-value:0.000  
No matches to eCLIP DataNo matches to TargetScan

--------- 10320  
 ----

TTAC

TTACTGCTTTACT  
Depth:2 (PIG)  
Ei-value:0.000, Pi-value:0.000  
Er-value:0.000, Pr-value:0.000  
No matches to eCLIP DataMATCHES To TargetScan▶ miR-330-3p.2:AAAGCAC▶ miR-802:CAGUAAC


TG

TGCTTTACT  
Depth:3 (COW)  
Ei-value:0.000, Pi-value:0.000  
Er-value:0.000, Pr-value:0.000  
No matches to eCLIP DataMATCHES To TargetScan▶ miR-330-3p.2:AAAGCAC


CTTTACT

CTTTACT  
Depth:4 (DOG)  
Ei-value:0.000, Pi-value:0.000  
Er-value:0.000, Pr-value:0.000  
No matches to eCLIP DataNo matches to TargetScan

--

GCAAAAT

GCAAAAT  
Depth:6 (MOUSE)  
Ei-value:0.000, Pi-value:0.000  
Er-value:0.000, Pr-value:0.000  
No matches to eCLIP DataNo matches to TargetScan

----

AAGGCAA

AAGGCAA  
Depth:4 (DOG)  
Ei-value:0.000, Pi-value:0.000  
Er-value:0.000, Pr-value:0.000  
No matches to eCLIP DataNo matches to TargetScan


GTCAGACCCA

AAGGCAAGTCAGACCCA  
Depth:3 (COW)  
Ei-value:0.000, Pi-value:0.000  
Er-value:0.000, Pr-value:0.000  
No matches to eCLIP DataMATCHES To TargetScan▶ miR-193a-5p:GGGUCUU

------

TGGATTGC

TGGATTGC  
Depth:4 (DOG)  
Ei-value:0.000, Pi-value:0.000  
Er-value:0.000, Pr-value:0.000  
No matches to eCLIP DataNo matches to TargetScan

----------------------------------------

TCTCCCCAG

TCTCCCCAGGAAGGAAG  
Depth:2 (PIG)  
Ei-value:0.000, Pi-value:0.000  
Er-value:0.000, Pr-value:0.000  
eCLIP MATCHES▶SF3B1 (bg=2.48%)No matches to TargetScan


GAAGGAAG

GAAGGAAG  
Depth:3 (COW)  
Ei-value:0.000, Pi-value:0.000  
Er-value:0.000, Pr-value:0.000  
eCLIP MATCHES▶SF3B1 (bg=2.48%)No matches to TargetScan

-- 10440  
 --------

TCTC

TCTCTGCATTCTTC  
Depth:2 (PIG)  
Ei-value:0.000, Pi-value:0.000  
Er-value:0.000, Pr-value:0.000  
eCLIP MATCHES▶SF3B1 (bg=2.48%)No matches to TargetScan


TGCATTCTTC

TGCATTCTTC  
Depth:5 (RABBIT)  
Ei-value:0.000, Pi-value:0.000  
Er-value:0.000, Pr-value:0.000  
eCLIP MATCHES▶SF3B1 (bg=2.48%)No matches to TargetScan

----

CAG

CAGAGCAGATTGCCTGG  
Depth:2 (PIG)  
Ei-value:0.000, Pi-value:0.000  
Er-value:0.000, Pr-value:0.000  
eCLIP MATCHES▶SF3B1 (bg=2.48%)No matches to TargetScan


AGC

AGCAGATTGCCTGG  
Depth:4 (DOG)  
Ei-value:0.000, Pi-value:0.000  
Er-value:0.000, Pr-value:0.000  
eCLIP MATCHES▶SF3B1 (bg=2.48%)No matches to TargetScan


A

AGATTGCCTGG  
Depth:5 (RABBIT)  
Ei-value:0.000, Pi-value:0.000  
Er-value:0.000, Pr-value:0.000  
No matches to eCLIP DataNo matches to TargetScan


GATTGCCTGG

GATTGCCTGG  
Depth:6 (MOUSE)  
Ei-value:0.000, Pi-value:0.000  
Er-value:0.000, Pr-value:0.000  
No matches to eCLIP DataNo matches to TargetScan

-

TAAGAATCTCT

TAAGAATCTCT  
Depth:2 (PIG)  
Ei-value:0.000, Pi-value:0.000  
Er-value:0.000, Pr-value:0.000  
No matches to eCLIP DataNo matches to TargetScan

-----------

TTGTATATT

TTGTATATT  
Depth:4 (DOG)  
Ei-value:0.000, Pi-value:0.000  
Er-value:0.000, Pr-value:0.000  
No matches to eCLIP DataMATCHES To TargetScan▶ miR-381-3p:AUACAAG

------------

TGCCAA

TGCCAA  
Depth:3 (COW)  
Ei-value:0.000, Pi-value:0.000  
Er-value:0.000, Pr-value:0.000  
No matches to eCLIP DataMATCHES To TargetScan▶ miR-182-5p:UUGGCAA▶ miR-96-5p/1271-5p:UUGGCAC

-

TGCCAGGATACA

TGCCAGGATACA  
Depth:3 (COW)  
Ei-value:0.000, Pi-value:0.000  
Er-value:0.000, Pr-value:0.000  
No matches to eCLIP DataNo matches to TargetScan

-------------- 10560  
 ---------------

AAATTTTTT

AAATTTTTT  
Depth:2 (PIG)  
Ei-value:0.000, Pi-value:0.000  
Er-value:0.000, Pr-value:0.000  
No matches to eCLIP DataNo matches to TargetScan

--------

ACATCTGG

ACATCTGG  
Depth:3 (COW)  
Ei-value:0.000, Pi-value:0.000  
Er-value:0.000, Pr-value:0.000  
No matches to eCLIP DataNo matches to TargetScan

----------------

GAT

GATAACCTGGTCATT  
Depth:3 (COW)  
Ei-value:0.000, Pi-value:0.000  
Er-value:0.000, Pr-value:0.000  
No matches to eCLIP DataMATCHES To TargetScan▶ miR-154-5p:AGGUUAU


AAC

AACCTGGTCATT  
Depth:4 (DOG)  
Ei-value:0.000, Pi-value:0.000  
Er-value:0.000, Pr-value:0.000  
No matches to eCLIP DataNo matches to TargetScan


CTGGTCATT

CTGGTCATT  
Depth:5 (RABBIT)  
Ei-value:0.000, Pi-value:0.000  
Er-value:0.000, Pr-value:0.000  
No matches to eCLIP DataNo matches to TargetScan

---

T

TTTTTGAAG  
Depth:2 (PIG)  
Ei-value:0.000, Pi-value:0.000  
Er-value:0.000, Pr-value:0.000  
No matches to eCLIP DataNo matches to TargetScan


TTTTGAA

TTTTGAA  
Depth:3 (COW)  
Ei-value:0.000, Pi-value:0.000  
Er-value:0.000, Pr-value:0.010  
No matches to eCLIP DataNo matches to TargetScan


G

TTTTTGAAG  
Depth:2 (PIG)  
Ei-value:0.000, Pi-value:0.000  
Er-value:0.000, Pr-value:0.000  
No matches to eCLIP DataNo matches to TargetScan

---------

CCATTTAT

CCATTTAT  
Depth:5 (RABBIT)  
Ei-value:0.000, Pi-value:0.000  
Er-value:0.000, Pr-value:0.000  
No matches to eCLIP DataNo matches to TargetScan

-------------

TGAC

TGACCAGTGTCTCTCATTT  
Depth:4 (DOG)  
Ei-value:0.000, Pi-value:0.000  
Er-value:0.000, Pr-value:0.000  
eCLIP MATCHES▶SUPV3L1 (bg=1.57%)No matches to TargetScan


CAG

CAGTGTCTCTCATTT  
Depth:5 (RABBIT)  
Ei-value:0.000, Pi-value:0.000  
Er-value:0.000, Pr-value:0.000  
eCLIP MATCHES▶SUPV3L1 (bg=1.57%)No matches to TargetScan

 10680  


TGTCTCTCATTT

CAGTGTCTCTCATTT  
Depth:5 (RABBIT)  
Ei-value:0.000, Pi-value:0.000  
Er-value:0.000, Pr-value:0.000  
eCLIP MATCHES▶SUPV3L1 (bg=1.57%)No matches to TargetScan

------

AGG

AGGGTGGTG  
Depth:4 (DOG)  
Ei-value:0.000, Pi-value:0.000  
Er-value:0.000, Pr-value:0.000  
eCLIP MATCHES▶SUPV3L1 (bg=1.57%)No matches to TargetScan


GTGGTG

GTGGTG  
Depth:5 (RABBIT)  
Ei-value:0.000, Pi-value:0.000  
Er-value:0.000, Pr-value:0.000  
eCLIP MATCHES▶SUPV3L1 (bg=1.57%)No matches to TargetScan


G

AGGGTGGTGGGTCTGTGGATAGA  
Depth:2 (PIG)  
Ei-value:0.000, Pi-value:0.000  
Er-value:0.000, Pr-value:0.000  
eCLIP MATCHES▶SUPV3L1 (bg=1.57%)MATCHES To TargetScan▶ miR-140-3p.1:CCACAGG


GTCTGTGGATA

GTCTGTGGATA  
Depth:5 (RABBIT)  
Ei-value:0.000, Pi-value:0.000  
Er-value:0.000, Pr-value:0.000  
eCLIP MATCHES▶SUPV3L1 (bg=1.57%)MATCHES To TargetScan▶ miR-140-3p.1:CCACAGG


GA

GTCTGTGGATAGA  
Depth:3 (COW)  
Ei-value:0.000, Pi-value:0.000  
Er-value:0.000, Pr-value:0.000  
eCLIP MATCHES▶SUPV3L1 (bg=1.57%)MATCHES To TargetScan▶ miR-140-3p.1:CCACAGG

--------------

TATTTTA

TATTTTA  
Depth:3 (COW)  
Ei-value:0.000, Pi-value:0.040  
Er-value:0.000, Pr-value:0.020  
eCLIP MATCHES▶SUPV3L1 (bg=1.57%)No matches to TargetScan

------------

TTCTAGA

TTCTAGA  
Depth:4 (DOG)  
Ei-value:0.000, Pi-value:0.000  
Er-value:0.000, Pr-value:0.000  
No matches to eCLIP DataNo matches to TargetScan

-----------------

AGTATCTTTG

AGTATCTTTG  
Depth:3 (COW)  
Ei-value:0.000, Pi-value:0.000  
Er-value:0.000, Pr-value:0.000  
No matches to eCLIP DataNo matches to TargetScan

------------ 10800  
 ----

ATTCACTT

ATTCACTT  
Depth:4 (DOG)  
Ei-value:0.000, Pi-value:0.000  
Er-value:0.000, Pr-value:0.000  
No matches to eCLIP DataNo matches to TargetScan


TTA

ATTCACTTTTAGAAAAAC  
Depth:2 (PIG)  
Ei-value:0.000, Pi-value:0.000  
Er-value:0.000, Pr-value:0.000  
No matches to eCLIP DataMATCHES To TargetScan▶ miR-17-5p/20-5p/93-5p/106-5p/519-3p:AAAGUGC


GAAAAAC

GAAAAAC  
Depth:4 (DOG)  
Ei-value:0.000, Pi-value:0.000  
Er-value:0.000, Pr-value:0.000  
No matches to eCLIP DataNo matches to TargetScan

-------------

TAATCCTT

TAATCCTT  
Depth:2 (PIG)  
Ei-value:0.000, Pi-value:0.010  
Er-value:0.000, Pr-value:0.000  
eCLIP MATCHES▶SUPV3L1 (bg=1.57%)No matches to TargetScan

-

AATTTCTTCATCTGGAGC

AATTTCTTCATCTGGAGC  
Depth:5 (RABBIT)  
Ei-value:0.000, Pi-value:0.000  
Er-value:0.000, Pr-value:0.000  
eCLIP MATCHES▶SUPV3L1 (bg=1.57%)▶U2AF2 (bg=1.76%)No matches to TargetScan


A

AATTTCTTCATCTGGAGCA  
Depth:2 (PIG)  
Ei-value:0.000, Pi-value:0.000  
Er-value:0.000, Pr-value:0.000  
eCLIP MATCHES▶SUPV3L1 (bg=1.57%)▶U2AF2 (bg=1.76%)No matches to TargetScan

----------

CTTATTT

CTTATTT  
Depth:4 (DOG)  
Ei-value:0.000, Pi-value:0.000  
Er-value:0.000, Pr-value:0.010  
eCLIP MATCHES▶SUPV3L1 (bg=1.57%)▶U2AF2 (bg=1.76%)No matches to TargetScan


CAAGAA

CTTATTTCAAGAA  
Depth:3 (COW)  
Ei-value:0.000, Pi-value:0.000  
Er-value:0.000, Pr-value:0.000  
eCLIP MATCHES▶SUPV3L1 (bg=1.57%)▶U2AF2 (bg=1.76%)MATCHES To TargetScan▶ miR-203a-3p.2:UGAAAUG

---------------------------------- 10920  
 -------------------------------------------------

ATAAAATG

ATAAAATG  
Depth:4 (DOG)  
Ei-value:0.000, Pi-value:0.000  
Er-value:0.000, Pr-value:0.000  
No matches to eCLIP DataNo matches to TargetScan


A

ATAAAATGA  
Depth:3 (COW)  
Ei-value:0.000, Pi-value:0.000  
Er-value:0.000, Pr-value:0.000  
No matches to eCLIP DataNo matches to TargetScan

---------------------------------------

ACCACACT

ACCACACT  
Depth:3 (COW)  
Ei-value:0.000, Pi-value:0.000  
Er-value:0.000, Pr-value:0.000  
No matches to eCLIP DataNo matches to TargetScan


GA

ACCACACTGA  
Depth:2 (PIG)  
Ei-value:0.000, Pi-value:0.000  
Er-value:0.000, Pr-value:0.000  
No matches to eCLIP DataNo matches to TargetScan

-

GTGAGG

GTGAGG  
Depth:3 (COW)  
Ei-value:0.000, Pi-value:0.000  
Er-value:0.000, Pr-value:0.000  
No matches to eCLIP DataNo matches to TargetScan

------ 11040  


AAATGAT

AAATGAT  
Depth:2 (PIG)  
Ei-value:0.000, Pi-value:0.020  
Er-value:0.000, Pr-value:0.020  
No matches to eCLIP DataMATCHES To TargetScan▶ miR-382-3p:AUCAUUC

---------------

TTTTATA

TTTTATA  
Depth:3 (COW)  
Ei-value:0.000, Pi-value:0.000  
Er-value:0.000, Pr-value:0.010  
No matches to eCLIP DataMATCHES To TargetScan▶ miR-340-5p:UAUAAAG

--

AAAAATAAGCCA

AAAAATAAGCCA  
Depth:5 (RABBIT)  
Ei-value:0.000, Pi-value:0.000  
Er-value:0.000, Pr-value:0.000  
No matches to eCLIP DataNo matches to TargetScan


A

AAAAATAAGCCAA  
Depth:4 (DOG)  
Ei-value:0.000, Pi-value:0.000  
Er-value:0.000, Pr-value:0.000  
No matches to eCLIP DataNo matches to TargetScan

--------

AT

ATTCTTTTGGATATA  
Depth:2 (PIG)  
Ei-value:0.000, Pi-value:0.000  
Er-value:0.000, Pr-value:0.000  
No matches to eCLIP DataMATCHES To TargetScan▶ miR-186-5p:AAAGAAU


TCTTTTGGATATA

TCTTTTGGATATA  
Depth:3 (COW)  
Ei-value:0.000, Pi-value:0.000  
Er-value:0.000, Pr-value:0.000  
No matches to eCLIP DataNo matches to TargetScan

---------

AGTGAGATAGCTGCCT

AGTGAGATAGCTGCCT  
Depth:2 (PIG)  
Ei-value:0.000, Pi-value:0.000  
Er-value:0.000, Pr-value:0.000  
No matches to eCLIP DataNo matches to TargetScan

-------

ATGAATAATA

ATGAATAATA  
Depth:4 (DOG)  
Ei-value:0.000, Pi-value:0.000  
Er-value:0.000, Pr-value:0.000  
No matches to eCLIP DataNo matches to TargetScan

----------- 11160  


AGTGTACA

AGTGTACA  
Depth:3 (COW)  
Ei-value:0.000, Pi-value:0.000  
Er-value:0.000, Pr-value:0.000  
No matches to eCLIP DataMATCHES To TargetScan▶ miR-493-5p:UGUACAU


G

AGTGTACAGGGTGTTT  
Depth:2 (PIG)  
Ei-value:0.000, Pi-value:0.000  
Er-value:0.000, Pr-value:0.000  
No matches to eCLIP DataMATCHES To TargetScan▶ miR-10-5p:ACCCUGU▶ miR-339-5p:CCCUGUC▶ miR-486-5p:CCUGUAC▶ miR-493-5p:UGUACAU▶ miR-504-5p.1:ACCCUGG


GGTGTTT

GGTGTTT  
Depth:3 (COW)  
Ei-value:0.000, Pi-value:0.000  
Er-value:0.000, Pr-value:0.000  
No matches to eCLIP DataNo matches to TargetScan

----------------------

TGGAACTGCT

TGGAACTGCT  
Depth:4 (DOG)  
Ei-value:0.000, Pi-value:0.000  
Er-value:0.000, Pr-value:0.000  
No matches to eCLIP DataNo matches to TargetScan

-----

AAA

AAATAACTAGT  
Depth:2 (PIG)  
Ei-value:0.000, Pi-value:0.000  
Er-value:0.000, Pr-value:0.000  
No matches to eCLIP DataNo matches to TargetScan


TAACTA

TAACTA  
Depth:4 (DOG)  
Ei-value:0.000, Pi-value:0.000  
Er-value:0.000, Pr-value:0.000  
No matches to eCLIP DataNo matches to TargetScan


GT

AAATAACTAGT  
Depth:2 (PIG)  
Ei-value:0.000, Pi-value:0.000  
Er-value:0.000, Pr-value:0.000  
No matches to eCLIP DataNo matches to TargetScan

--------

CAGCAGTTC

CAGCAGTTC  
Depth:5 (RABBIT)  
Ei-value:0.000, Pi-value:0.000  
Er-value:0.000, Pr-value:0.000  
No matches to eCLIP DataNo matches to TargetScan

-

TTGTAAT

TTGTAAT  
Depth:4 (DOG)  
Ei-value:0.000, Pi-value:0.000  
Er-value:0.000, Pr-value:0.000  
No matches to eCLIP DataNo matches to TargetScan

-

ACTGAAAA

ACTGAAAA  
Depth:5 (RABBIT)  
Ei-value:0.000, Pi-value:0.000  
Er-value:0.000, Pr-value:0.000  
No matches to eCLIP DataNo matches to TargetScan

----------------

GAG

GAGAAGGATGTCAAAAGATCGGC  
Depth:3 (COW)  
Ei-value:0.000, Pi-value:0.000  
Er-value:0.000, Pr-value:0.000  
eCLIP MATCHES▶SRSF1 (bg=8.47%)▶U2AF2 (bg=1.76%)▶uchl5 (bg=11.16%)MATCHES To TargetScan▶ miR-362-5p/500b-5p:AUCCUUG▶ miR-489-3p:UGACAUC


AAG

AAGGATG  
Depth:5 (RABBIT)  
Ei-value:0.000, Pi-value:0.000  
Er-value:0.000, Pr-value:0.000  
eCLIP MATCHES▶SRSF1 (bg=8.47%)▶U2AF2 (bg=1.76%)▶uchl5 (bg=11.16%)MATCHES To TargetScan▶ miR-362-5p/500b-5p:AUCCUUG

 11280  


GATG

AAGGATG  
Depth:5 (RABBIT)  
Ei-value:0.000, Pi-value:0.000  
Er-value:0.000, Pr-value:0.000  
eCLIP MATCHES▶SRSF1 (bg=8.47%)▶U2AF2 (bg=1.76%)▶uchl5 (bg=11.16%)MATCHES To TargetScan▶ miR-362-5p/500b-5p:AUCCUUG


TCA

AAGGATGTCAAAAGATC  
Depth:4 (DOG)  
Ei-value:0.000, Pi-value:0.000  
Er-value:0.000, Pr-value:0.000  
eCLIP MATCHES▶SRSF1 (bg=8.47%)▶U2AF2 (bg=1.76%)▶uchl5 (bg=11.16%)MATCHES To TargetScan▶ miR-362-5p/500b-5p:AUCCUUG▶ miR-489-3p:UGACAUC


AAAGATC

AAAGATC  
Depth:6 (MOUSE)  
Ei-value:0.000, Pi-value:0.000  
Er-value:0.000, Pr-value:0.000  
eCLIP MATCHES▶SRSF1 (bg=8.47%)▶U2AF2 (bg=1.76%)▶uchl5 (bg=11.16%)No matches to TargetScan


GGC

GAGAAGGATGTCAAAAGATCGGC  
Depth:3 (COW)  
Ei-value:0.000, Pi-value:0.000  
Er-value:0.000, Pr-value:0.000  
eCLIP MATCHES▶SRSF1 (bg=8.47%)▶U2AF2 (bg=1.76%)▶uchl5 (bg=11.16%)MATCHES To TargetScan▶ miR-362-5p/500b-5p:AUCCUUG▶ miR-489-3p:UGACAUC

-

CAGCTCAGGG

CAGCTCAGGG  
Depth:4 (DOG)  
Ei-value:0.000, Pi-value:0.000  
Er-value:0.000, Pr-value:0.000  
eCLIP MATCHES▶SRSF1 (bg=8.47%)▶U2AF2 (bg=1.76%)▶uchl5 (bg=11.16%)MATCHES To TargetScan▶ miR-125-5p:CCCUGAG

-

GCAGTTTGC

GCAGTTTGC  
Depth:3 (COW)  
Ei-value:0.000, Pi-value:0.000  
Er-value:0.000, Pr-value:0.000  
eCLIP MATCHES▶SRSF1 (bg=8.47%)▶U2AF2 (bg=1.76%)▶uchl5 (bg=11.16%)No matches to TargetScan

-

CTACTAGCTCCT

CTACTAGCTCCT  
Depth:4 (DOG)  
Ei-value:0.000, Pi-value:0.000  
Er-value:0.000, Pr-value:0.000  
eCLIP MATCHES▶SRSF1 (bg=8.47%)▶U2AF2 (bg=1.76%)▶uchl5 (bg=11.16%)MATCHES To TargetScan▶ miR-28-5p/708-5p:AGGAGCU▶ miR-411-5p.2:UAGUAGA

-

GGACAGCTG

GGACAGCTG  
Depth:5 (RABBIT)  
Ei-value:0.000, Pi-value:0.000  
Er-value:0.000, Pr-value:0.000  
eCLIP MATCHES▶SRSF1 (bg=8.47%)▶SRSF7 (bg=2.32%)▶U2AF2 (bg=1.76%)▶ZNF622 (bg=6.58%)No matches to TargetScan


T

GGACAGCTGT  
Depth:4 (DOG)  
Ei-value:0.000, Pi-value:0.000  
Er-value:0.000, Pr-value:0.000  
eCLIP MATCHES▶SRSF1 (bg=8.47%)▶SRSF7 (bg=2.32%)▶U2AF2 (bg=1.76%)▶ZNF622 (bg=6.58%)No matches to TargetScan

-

A

AAGAAGAGTCTCTGGCTCTTTAGA  
Depth:3 (COW)  
Ei-value:0.000, Pi-value:0.000  
Er-value:0.000, Pr-value:0.000  
eCLIP MATCHES▶DDX24 (bg=2.97%)▶SRSF1 (bg=8.47%)▶SRSF7 (bg=2.32%)▶U2AF2 (bg=1.76%)▶ZNF622 (bg=6.58%)No matches to TargetScan


AGAAGAGTCTCTGGCTCTTTA

AGAAGAGTCTCTGGCTCTTTA  
Depth:5 (RABBIT)  
Ei-value:0.000, Pi-value:0.000  
Er-value:0.000, Pr-value:0.000  
eCLIP MATCHES▶DDX24 (bg=2.97%)▶SRSF1 (bg=8.47%)▶SRSF7 (bg=2.32%)▶U2AF2 (bg=1.76%)▶ZNF622 (bg=6.58%)No matches to TargetScan


GA

AGAAGAGTCTCTGGCTCTTTAGA  
Depth:4 (DOG)  
Ei-value:0.000, Pi-value:0.000  
Er-value:0.000, Pr-value:0.000  
eCLIP MATCHES▶DDX24 (bg=2.97%)▶SRSF1 (bg=8.47%)▶SRSF7 (bg=2.32%)▶U2AF2 (bg=1.76%)▶ZNF622 (bg=6.58%)No matches to TargetScan

-----||-------------------------- 11398  
 --------------------------------------||---

ATTCTGAGC

ATTCTGAGC  
Depth:4 (DOG)  
Ei-value:0.000, Pi-value:0.000  
Er-value:0.000, Pr-value:0.000  
eCLIP MATCHES▶DDX24 (bg=2.97%)▶GRWD1 (bg=5.13%)▶MTPAP (bg=2.21%)▶NOLC1 (bg=9.43%)▶SRSF1 (bg=8.47%)▶ZNF622 (bg=6.58%)No matches to TargetScan

--------------

GA

GACTGCAA  
Depth:3 (COW)  
Ei-value:0.000, Pi-value:0.000  
Er-value:0.000, Pr-value:0.000  
eCLIP MATCHES▶DDX24 (bg=2.97%)▶GRWD1 (bg=5.13%)▶MTPAP (bg=2.21%)▶NOLC1 (bg=9.43%)▶SRSF1 (bg=8.47%)▶UTP3 (bg=3.66%)▶ZNF622 (bg=6.58%)MATCHES To TargetScan▶ miR-455-3p.2:UGCAGUC


CTGCAA

CTGCAA  
Depth:5 (RABBIT)  
Ei-value:0.000, Pi-value:0.000  
Er-value:0.000, Pr-value:0.000  
eCLIP MATCHES▶DDX24 (bg=2.97%)▶GRWD1 (bg=5.13%)▶MTPAP (bg=2.21%)▶NOLC1 (bg=9.43%)▶SRSF1 (bg=8.47%)▶UTP3 (bg=3.66%)▶ZNF622 (bg=6.58%)No matches to TargetScan

--------------------------------------

TTTGAGAA

TTTGAGAATCTGG  
Depth:3 (COW)  
Ei-value:0.000, Pi-value:0.000  
Er-value:0.000, Pr-value:0.000  
eCLIP MATCHES▶DDX24 (bg=2.97%)▶GRWD1 (bg=5.13%)▶NOLC1 (bg=9.43%)▶SRSF1 (bg=8.47%)▶uchl5 (bg=11.16%)▶ZNF622 (bg=6.58%)MATCHES To TargetScan▶ miR-371-5p:CUCAAAC

 11516  


TCTGG

TTTGAGAATCTGG  
Depth:3 (COW)  
Ei-value:0.000, Pi-value:0.000  
Er-value:0.000, Pr-value:0.000  
eCLIP MATCHES▶DDX24 (bg=2.97%)▶GRWD1 (bg=5.13%)▶NOLC1 (bg=9.43%)▶SRSF1 (bg=8.47%)▶uchl5 (bg=11.16%)▶ZNF622 (bg=6.58%)MATCHES To TargetScan▶ miR-371-5p:CUCAAAC

--

AAGCTCCA

AAGCTCCA  
Depth:3 (COW)  
Ei-value:0.000, Pi-value:0.000  
Er-value:0.000, Pr-value:0.000  
eCLIP MATCHES▶DDX24 (bg=2.97%)▶GRWD1 (bg=5.13%)▶NOLC1 (bg=9.43%)▶RBM15 (bg=7.27%)▶SRSF1 (bg=8.47%)▶uchl5 (bg=11.16%)▶ZNF622 (bg=6.58%)No matches to TargetScan

---

AATCTA

AATCTA  
Depth:2 (PIG)  
Ei-value:0.000, Pi-value:0.000  
Er-value:0.000, Pr-value:0.000  
eCLIP MATCHES▶DDX24 (bg=2.97%)▶GRWD1 (bg=5.13%)▶NOLC1 (bg=9.43%)▶RBM15 (bg=7.27%)▶SRSF1 (bg=8.47%)▶TARDBP (bg=2.79%)▶uchl5 (bg=11.16%)▶ZNF622 (bg=6.58%)No matches to TargetScan

---

GGATGG

GGATGG  
Depth:3 (COW)  
Ei-value:0.000, Pi-value:0.000  
Er-value:0.000, Pr-value:0.010  
eCLIP MATCHES▶DDX24 (bg=2.97%)▶GRWD1 (bg=5.13%)▶NIPBL (bg=5.39%)▶NOLC1 (bg=9.43%)▶RBM15 (bg=7.27%)▶SRSF1 (bg=8.47%)▶TARDBP (bg=2.79%)▶uchl5 (bg=11.16%)▶ZNF622 (bg=6.58%)No matches to TargetScan

-----------

T

TCTGGAGAAAAAGATCTTCCTCAGAAGAATAGGCTTGTTG  
Depth:2 (PIG)  
Ei-value:0.000, Pi-value:0.000  
Er-value:0.000, Pr-value:0.000  
eCLIP MATCHES▶DDX24 (bg=2.97%)▶GRWD1 (bg=5.13%)▶NIPBL (bg=5.39%)▶NOLC1 (bg=9.43%)▶SRSF1 (bg=8.47%)▶SRSF7 (bg=2.32%)▶TARDBP (bg=2.79%)▶uchl5 (bg=11.16%)▶ZNF622 (bg=6.58%)MATCHES To TargetScan▶ miR-1224-5p:UGAGGAC▶ miR-7-5p:GGAAGAC


CTGGAGAAAAAG||ATCT

CTGGAGAAAAAGATCT  
Depth:3 (COW)  
Ei-value:0.000, Pi-value:0.000  
Er-value:0.000, Pr-value:0.000  
eCLIP MATCHES▶DDX24 (bg=2.97%)▶GRWD1 (bg=5.13%)▶NIPBL (bg=5.39%)▶NOLC1 (bg=9.43%)▶SRSF1 (bg=8.47%)▶SRSF7 (bg=2.32%)▶TARDBP (bg=2.79%)▶uchl5 (bg=11.16%)▶ZNF622 (bg=6.58%)No matches to TargetScan


TCCTCAG

TCTGGAGAAAAAGATCTTCCTCAGAAGAATAGGCTTGTTG  
Depth:2 (PIG)  
Ei-value:0.000, Pi-value:0.000  
Er-value:0.000, Pr-value:0.000  
eCLIP MATCHES▶DDX24 (bg=2.97%)▶GRWD1 (bg=5.13%)▶NIPBL (bg=5.39%)▶NOLC1 (bg=9.43%)▶SRSF1 (bg=8.47%)▶SRSF7 (bg=2.32%)▶TARDBP (bg=2.79%)▶uchl5 (bg=11.16%)▶ZNF622 (bg=6.58%)MATCHES To TargetScan▶ miR-1224-5p:UGAGGAC▶ miR-7-5p:GGAAGAC


AAGAATAGGC

AAGAATAGGC  
Depth:5 (RABBIT)  
Ei-value:0.000, Pi-value:0.000  
Er-value:0.000, Pr-value:0.000  
eCLIP MATCHES▶NOLC1 (bg=9.43%)▶SRSF7 (bg=2.32%)▶uchl5 (bg=11.16%)No matches to TargetScan


TTGTTG

TCTGGAGAAAAAGATCTTCCTCAGAAGAATAGGCTTGTTG  
Depth:2 (PIG)  
Ei-value:0.000, Pi-value:0.000  
Er-value:0.000, Pr-value:0.000  
eCLIP MATCHES▶DDX24 (bg=2.97%)▶GRWD1 (bg=5.13%)▶NIPBL (bg=5.39%)▶NOLC1 (bg=9.43%)▶SRSF1 (bg=8.47%)▶SRSF7 (bg=2.32%)▶TARDBP (bg=2.79%)▶uchl5 (bg=11.16%)▶ZNF622 (bg=6.58%)MATCHES To TargetScan▶ miR-1224-5p:UGAGGAC▶ miR-7-5p:GGAAGAC

--

T

TTACAGTGTTAGTGA  
Depth:3 (COW)  
Ei-value:0.000, Pi-value:0.000  
Er-value:0.000, Pr-value:0.000  
eCLIP MATCHES▶ILF3 (bg=3.0%)▶NOLC1 (bg=9.43%)▶RBM15 (bg=7.27%)▶SRSF7 (bg=2.32%)▶ZNF622 (bg=6.58%)MATCHES To TargetScan▶ miR-141-3p/200a-3p:AACACUG


TACAGTGTTAGTGA

TACAGTGTTAGTGA  
Depth:5 (RABBIT)  
Ei-value:0.000, Pi-value:0.000  
Er-value:0.000, Pr-value:0.000  
eCLIP MATCHES▶ILF3 (bg=3.0%)▶NOLC1 (bg=9.43%)▶RBM15 (bg=7.27%)▶SRSF7 (bg=2.32%)▶ZNF622 (bg=6.58%)MATCHES To TargetScan▶ miR-141-3p/200a-3p:AACACUG

--

CA

CATTCCCTTTGA  
Depth:3 (COW)  
Ei-value:0.000, Pi-value:0.000  
Er-value:0.000, Pr-value:0.000  
eCLIP MATCHES▶ILF3 (bg=3.0%)▶RBM15 (bg=7.27%)▶SRSF7 (bg=2.32%)▶ZNF622 (bg=6.58%)MATCHES To TargetScan▶ miR-1-3p/206:GGAAUGU


TTCCCTTTGA

TTCCCTTTGA  
Depth:6 (MOUSE)  
Ei-value:0.000, Pi-value:0.000  
Er-value:0.000, Pr-value:0.000  
eCLIP MATCHES▶ILF3 (bg=3.0%)▶RBM15 (bg=7.27%)▶SRSF7 (bg=2.32%)▶ZNF622 (bg=6.58%)No matches to TargetScan

--- 11634  
 ----

TAGGTGGAGATGGGGCATGAGGATCCTCCAGGGGAA

TAGGTGGAGATGGGGCATGAGGATCCTCCAGGGGAA  
Depth:6 (MOUSE)  
Ei-value:0.000, Pi-value:0.000  
Er-value:0.000, Pr-value:0.000  
eCLIP MATCHES▶ILF3 (bg=3.0%)▶NOLC1 (bg=9.43%)▶RBM15 (bg=7.27%)▶SRSF7 (bg=2.32%)▶ZNF622 (bg=6.58%)MATCHES To TargetScan▶ miR-331-3p:CCCCUGG


A

TAGGTGGAGATGGGGCATGAGGATCCTCCAGGGGAAA  
Depth:5 (RABBIT)  
Ei-value:0.000, Pi-value:0.000  
Er-value:0.000, Pr-value:0.000  
eCLIP MATCHES▶ILF3 (bg=3.0%)▶NOLC1 (bg=9.43%)▶RBM15 (bg=7.27%)▶SRSF7 (bg=2.32%)▶ZNF622 (bg=6.58%)MATCHES To TargetScan▶ miR-331-3p:CCCCUGG


AGC

TAGGTGGAGATGGGGCATGAGGATCCTCCAGGGGAAAAGCTCACTACCACTGGGCAACAACCCTAGGTCAGGAG  
Depth:2 (PIG)  
Ei-value:0.000, Pi-value:0.000  
Er-value:0.000, Pr-value:0.000  
eCLIP MATCHES▶ILF3 (bg=3.0%)▶NOLC1 (bg=9.43%)▶RBM15 (bg=7.27%)▶SRSF7 (bg=2.32%)▶ZNF622 (bg=6.58%)MATCHES To TargetScan▶ miR-140-5p:AGUGGUU▶ miR-142-3p.1:GUAGUGU▶ miR-192-5p/215-5p:UGACCUA▶ miR-199-5p:CCAGUGU▶ miR-296-3p:AGGGUUG▶ miR-331-3p:CCCCUGG


TCACTA

TCACTA  
Depth:5 (RABBIT)  
Ei-value:0.000, Pi-value:0.000  
Er-value:0.000, Pr-value:0.000  
eCLIP MATCHES▶ILF3 (bg=3.0%)No matches to TargetScan


CCACT

TCACTACCACT  
Depth:4 (DOG)  
Ei-value:0.000, Pi-value:0.000  
Er-value:0.000, Pr-value:0.000  
eCLIP MATCHES▶ILF3 (bg=3.0%)MATCHES To TargetScan▶ miR-140-5p:AGUGGUU▶ miR-142-3p.1:GUAGUGU


G

TCACTACCACTG  
Depth:3 (COW)  
Ei-value:0.000, Pi-value:0.000  
Er-value:0.000, Pr-value:0.000  
eCLIP MATCHES▶ILF3 (bg=3.0%)MATCHES To TargetScan▶ miR-140-5p:AGUGGUU▶ miR-142-3p.1:GUAGUGU


G

TAGGTGGAGATGGGGCATGAGGATCCTCCAGGGGAAAAGCTCACTACCACTGGGCAACAACCCTAGGTCAGGAG  
Depth:2 (PIG)  
Ei-value:0.000, Pi-value:0.000  
Er-value:0.000, Pr-value:0.000  
eCLIP MATCHES▶ILF3 (bg=3.0%)▶NOLC1 (bg=9.43%)▶RBM15 (bg=7.27%)▶SRSF7 (bg=2.32%)▶ZNF622 (bg=6.58%)MATCHES To TargetScan▶ miR-140-5p:AGUGGUU▶ miR-142-3p.1:GUAGUGU▶ miR-192-5p/215-5p:UGACCUA▶ miR-199-5p:CCAGUGU▶ miR-296-3p:AGGGUUG▶ miR-331-3p:CCCCUGG


GCAACA

GCAACA  
Depth:6 (MOUSE)  
Ei-value:0.000, Pi-value:0.000  
Er-value:0.000, Pr-value:0.000  
eCLIP MATCHES▶ILF3 (bg=3.0%)No matches to TargetScan


AC

GCAACAAC  
Depth:5 (RABBIT)  
Ei-value:0.000, Pi-value:0.000  
Er-value:0.000, Pr-value:0.000  
eCLIP MATCHES▶ILF3 (bg=3.0%)No matches to TargetScan


CCTAGGTCAGGAG

TAGGTGGAGATGGGGCATGAGGATCCTCCAGGGGAAAAGCTCACTACCACTGGGCAACAACCCTAGGTCAGGAG  
Depth:2 (PIG)  
Ei-value:0.000, Pi-value:0.000  
Er-value:0.000, Pr-value:0.000  
eCLIP MATCHES▶ILF3 (bg=3.0%)▶NOLC1 (bg=9.43%)▶RBM15 (bg=7.27%)▶SRSF7 (bg=2.32%)▶ZNF622 (bg=6.58%)MATCHES To TargetScan▶ miR-140-5p:AGUGGUU▶ miR-142-3p.1:GUAGUGU▶ miR-192-5p/215-5p:UGACCUA▶ miR-199-5p:CCAGUGU▶ miR-296-3p:AGGGUUG▶ miR-331-3p:CCCCUGG

--------------

CTTTCCTGG

CTTTCCTGG  
Depth:3 (COW)  
Ei-value:0.000, Pi-value:0.000  
Er-value:0.000, Pr-value:0.000  
eCLIP MATCHES▶ILF3 (bg=3.0%)MATCHES To TargetScan▶ miR-665:CCAGGAG▶ miR-873-5p.1:CAGGAAC

--

CCAGATAGGAAGAT

CCAGATAGGAAGAT  
Depth:2 (PIG)  
Ei-value:0.000, Pi-value:0.000  
Er-value:0.000, Pr-value:0.000  
eCLIP MATCHES▶ILF3 (bg=3.0%)MATCHES To TargetScan▶ miR-202-5p:UCCUAUG

-

AA

AAGTCTCAA  
Depth:2 (PIG)  
Ei-value:0.000, Pi-value:0.000  
Er-value:0.000, Pr-value:0.000  
No matches to eCLIP DataNo matches to TargetScan

 11754  


GTCTCAA

AAGTCTCAA  
Depth:2 (PIG)  
Ei-value:0.000, Pi-value:0.000  
Er-value:0.000, Pr-value:0.000  
No matches to eCLIP DataNo matches to TargetScan

--

ACAACCACC

ACAACCACC  
Depth:5 (RABBIT)  
Ei-value:0.000, Pi-value:0.000  
Er-value:0.000, Pr-value:0.000  
eCLIP MATCHES▶PRPF8 (bg=0.26%)No matches to TargetScan


ACAC

ACAACCACCACAC  
Depth:4 (DOG)  
Ei-value:0.000, Pi-value:0.000  
Er-value:0.000, Pr-value:0.000  
eCLIP MATCHES▶PRPF8 (bg=0.26%)No matches to TargetScan

------||------

A

ATTGTTCC  
Depth:2 (PIG)  
Ei-value:0.000, Pi-value:0.000  
Er-value:0.000, Pr-value:0.000  
eCLIP MATCHES▶GRWD1 (bg=5.13%)▶SF3B4 (bg=0.05%)No matches to TargetScan


TTGTTCC

TTGTTCC  
Depth:4 (DOG)  
Ei-value:0.000, Pi-value:0.000  
Er-value:0.000, Pr-value:0.000  
eCLIP MATCHES▶GRWD1 (bg=5.13%)▶SF3B4 (bg=0.05%)No matches to TargetScan

----

TG

TGCCAAATC  
Depth:3 (COW)  
Ei-value:0.000, Pi-value:0.000  
Er-value:0.000, Pr-value:0.000  
eCLIP MATCHES▶GRWD1 (bg=5.13%)▶NOLC1 (bg=9.43%)MATCHES To TargetScan▶ miR-182-5p:UUGGCAA▶ miR-96-5p/1271-5p:UUGGCAC


CCAAAT

CCAAAT  
Depth:6 (MOUSE)  
Ei-value:0.000, Pi-value:0.000  
Er-value:0.000, Pr-value:0.000  
eCLIP MATCHES▶GRWD1 (bg=5.13%)▶NOLC1 (bg=9.43%)No matches to TargetScan


C

CCAAATC  
Depth:5 (RABBIT)  
Ei-value:0.000, Pi-value:0.000  
Er-value:0.000, Pr-value:0.000  
eCLIP MATCHES▶GRWD1 (bg=5.13%)▶NOLC1 (bg=9.43%)No matches to TargetScan

--------------

AAGCAGTG

AAGCAGTG  
Depth:2 (PIG)  
Ei-value:0.000, Pi-value:0.000  
Er-value:0.000, Pr-value:0.000  
eCLIP MATCHES▶GRWD1 (bg=5.13%)▶NOLC1 (bg=9.43%)▶uchl5 (bg=11.16%)No matches to TargetScan

-

AGAGAG

AGAGAG  
Depth:2 (PIG)  
Ei-value:0.000, Pi-value:0.010  
Er-value:0.000, Pr-value:0.000  
eCLIP MATCHES▶GRWD1 (bg=5.13%)▶NOLC1 (bg=9.43%)▶uchl5 (bg=11.16%)▶ZNF622 (bg=6.58%)No matches to TargetScan

------------------

CAAGAAA

CAAGAAA  
Depth:5 (RABBIT)  
Ei-value:0.000, Pi-value:0.000  
Er-value:0.000, Pr-value:0.000  
eCLIP MATCHES▶GRWD1 (bg=5.13%)▶NOLC1 (bg=9.43%)▶uchl5 (bg=11.16%)▶ZNF622 (bg=6.58%)No matches to TargetScan


T

CAAGAAAT  
Depth:3 (COW)  
Ei-value:0.000, Pi-value:0.000  
Er-value:0.000, Pr-value:0.000  
eCLIP MATCHES▶GRWD1 (bg=5.13%)▶NOLC1 (bg=9.43%)▶TRA2A (bg=4.8%)▶uchl5 (bg=11.16%)▶ZNF622 (bg=6.58%)No matches to TargetScan


T

CAAGAAATTTGAACACAC  
Depth:2 (PIG)  
Ei-value:0.000, Pi-value:0.000  
Er-value:0.000, Pr-value:0.000  
eCLIP MATCHES▶GRWD1 (bg=5.13%)▶NOLC1 (bg=9.43%)▶PTBP1 (bg=3.74%)▶RBM15 (bg=7.27%)▶TRA2A (bg=4.8%)▶uchl5 (bg=11.16%)▶ZNF622 (bg=6.58%)No matches to TargetScan


TGAACAC

TGAACACAC  
Depth:3 (COW)  
Ei-value:0.000, Pi-value:0.000  
Er-value:0.000, Pr-value:0.000  
eCLIP MATCHES▶GRWD1 (bg=5.13%)▶NOLC1 (bg=9.43%)▶PTBP1 (bg=3.74%)▶RBM15 (bg=7.27%)▶TRA2A (bg=4.8%)▶uchl5 (bg=11.16%)▶ZNF622 (bg=6.58%)No matches to TargetScan

 11872  


AC

TGAACACAC  
Depth:3 (COW)  
Ei-value:0.000, Pi-value:0.000  
Er-value:0.000, Pr-value:0.000  
eCLIP MATCHES▶GRWD1 (bg=5.13%)▶NOLC1 (bg=9.43%)▶PTBP1 (bg=3.74%)▶RBM15 (bg=7.27%)▶TRA2A (bg=4.8%)▶uchl5 (bg=11.16%)▶ZNF622 (bg=6.58%)No matches to TargetScan

------------------------

G

GAAGATCAACATGCCTG  
Depth:4 (DOG)  
Ei-value:0.000, Pi-value:0.000  
Er-value:0.000, Pr-value:0.000  
eCLIP MATCHES▶GRWD1 (bg=5.13%)▶NOLC1 (bg=9.43%)▶PTBP1 (bg=3.74%)▶RBM15 (bg=7.27%)▶TRA2A (bg=4.8%)▶uchl5 (bg=11.16%)▶ZNF622 (bg=6.58%)No matches to TargetScan


AA

AAGATCAACATGC  
Depth:5 (RABBIT)  
Ei-value:0.000, Pi-value:0.000  
Er-value:0.000, Pr-value:0.000  
eCLIP MATCHES▶GRWD1 (bg=5.13%)▶NOLC1 (bg=9.43%)▶PTBP1 (bg=3.74%)▶RBM15 (bg=7.27%)▶TRA2A (bg=4.8%)▶uchl5 (bg=11.16%)▶ZNF622 (bg=6.58%)No matches to TargetScan


GATCAACATGC

GATCAACATGC  
Depth:6 (MOUSE)  
Ei-value:0.000, Pi-value:0.000  
Er-value:0.000, Pr-value:0.000  
eCLIP MATCHES▶GRWD1 (bg=5.13%)▶NOLC1 (bg=9.43%)▶PTBP1 (bg=3.74%)▶RBM15 (bg=7.27%)▶TRA2A (bg=4.8%)▶uchl5 (bg=11.16%)▶ZNF622 (bg=6.58%)No matches to TargetScan


CTG

GAAGATCAACATGCCTG  
Depth:4 (DOG)  
Ei-value:0.000, Pi-value:0.000  
Er-value:0.000, Pr-value:0.000  
eCLIP MATCHES▶GRWD1 (bg=5.13%)▶NOLC1 (bg=9.43%)▶PTBP1 (bg=3.74%)▶RBM15 (bg=7.27%)▶TRA2A (bg=4.8%)▶uchl5 (bg=11.16%)▶ZNF622 (bg=6.58%)No matches to TargetScan


GC

GAAGATCAACATGCCTGGC  
Depth:2 (PIG)  
Ei-value:0.000, Pi-value:0.000  
Er-value:0.000, Pr-value:0.000  
eCLIP MATCHES▶GRWD1 (bg=5.13%)▶NOLC1 (bg=9.43%)▶PTBP1 (bg=3.74%)▶RBM15 (bg=7.27%)▶TRA2A (bg=4.8%)▶uchl5 (bg=11.16%)▶ZNF622 (bg=6.58%)No matches to TargetScan

---------------------

TGAATGA

TGAATGA  
Depth:2 (PIG)  
Ei-value:0.000, Pi-value:0.010  
Er-value:0.000, Pr-value:0.020  
eCLIP MATCHES▶AQR (bg=0.33%)▶GRWD1 (bg=5.13%)▶TRA2A (bg=4.8%)MATCHES To TargetScan▶ miR-1298-5p:UCAUUCG

-||

TGTGTAT

TGTGTAT  
Depth:6 (MOUSE)  
Ei-value:0.000, Pi-value:0.000  
Er-value:0.000, Pr-value:0.000  
eCLIP MATCHES▶TARDBP (bg=2.79%)▶ZC3H11A (bg=6.55%)No matches to TargetScan


TT

TGTGTATTT  
Depth:4 (DOG)  
Ei-value:0.000, Pi-value:0.000  
Er-value:0.000, Pr-value:0.000  
eCLIP MATCHES▶TARDBP (bg=2.79%)▶ZC3H11A (bg=6.55%)No matches to TargetScan

--

TTGTC

TTGTCTCTTTCTTTCTT  
Depth:2 (PIG)  
Ei-value:0.000, Pi-value:0.000  
Er-value:0.000, Pr-value:0.000  
eCLIP MATCHES▶PTBP1 (bg=3.74%)▶TARDBP (bg=2.79%)▶ZC3H11A (bg=6.55%)MATCHES To TargetScan▶ miR-186-5p:AAAGAAU


TCTTTCTT

TCTTTCTT  
Depth:3 (COW)  
Ei-value:0.000, Pi-value:0.000  
Er-value:0.000, Pr-value:0.000  
eCLIP MATCHES▶TARDBP (bg=2.79%)▶ZC3H11A (bg=6.55%)No matches to TargetScan


TCTT

TTGTCTCTTTCTTTCTT  
Depth:2 (PIG)  
Ei-value:0.000, Pi-value:0.000  
Er-value:0.000, Pr-value:0.000  
eCLIP MATCHES▶PTBP1 (bg=3.74%)▶TARDBP (bg=2.79%)▶ZC3H11A (bg=6.55%)MATCHES To TargetScan▶ miR-186-5p:AAAGAAU

--------------

TT

TTCTCTA  
Depth:2 (PIG)  
Ei-value:0.000, Pi-value:0.010  
Er-value:0.000, Pr-value:0.000  
eCLIP MATCHES▶MATR3 (bg=2.98%)▶PTBP1 (bg=3.74%)▶TARDBP (bg=2.79%)▶ZC3H11A (bg=6.55%)No matches to TargetScan

 11990  


CTCTA

TTCTCTA  
Depth:2 (PIG)  
Ei-value:0.000, Pi-value:0.010  
Er-value:0.000, Pr-value:0.000  
eCLIP MATCHES▶MATR3 (bg=2.98%)▶PTBP1 (bg=3.74%)▶TARDBP (bg=2.79%)▶ZC3H11A (bg=6.55%)No matches to TargetScan

-------

TG

TGTGTCTTACCCATTTCCATG  
Depth:2 (PIG)  
Ei-value:0.000, Pi-value:0.000  
Er-value:0.000, Pr-value:0.000  
eCLIP MATCHES▶MATR3 (bg=2.98%)▶PTBP1 (bg=3.74%)▶TARDBP (bg=2.79%)▶ZC3H11A (bg=6.55%)MATCHES To TargetScan▶ miR-203a-3p.1:GAAAUGU▶ miR-208-3p:UAAGACG▶ miR-499a-5p:UAAGACU


TGTCTTA

TGTCTTA  
Depth:4 (DOG)  
Ei-value:0.000, Pi-value:0.000  
Er-value:0.000, Pr-value:0.000  
eCLIP MATCHES▶MATR3 (bg=2.98%)▶PTBP1 (bg=3.74%)▶TARDBP (bg=2.79%)▶ZC3H11A (bg=6.55%)MATCHES To TargetScan▶ miR-208-3p:UAAGACG▶ miR-499a-5p:UAAGACU


CCCATTTCCATG

TGTCTTACCCATTTCCATG  
Depth:3 (COW)  
Ei-value:0.000, Pi-value:0.000  
Er-value:0.000, Pr-value:0.000  
eCLIP MATCHES▶MATR3 (bg=2.98%)▶PTBP1 (bg=3.74%)▶TARDBP (bg=2.79%)▶ZC3H11A (bg=6.55%)MATCHES To TargetScan▶ miR-203a-3p.1:GAAAUGU▶ miR-208-3p:UAAGACG▶ miR-499a-5p:UAAGACU

----------------------------------------------

TTTTTGT

TTTTTGT  
Depth:4 (DOG)  
Ei-value:0.000, Pi-value:0.000  
Er-value:0.000, Pr-value:0.000  
eCLIP MATCHES▶MATR3 (bg=2.98%)▶PTBP1 (bg=3.74%)▶TARDBP (bg=2.79%)▶TIA1 (bg=4.07%)▶ZC3H11A (bg=6.55%)No matches to TargetScan

----------

GGTCTGTGTCT

GGTCTGTGTCT  
Depth:2 (PIG)  
Ei-value:0.000, Pi-value:0.000  
Er-value:0.000, Pr-value:0.000  
eCLIP MATCHES▶MATR3 (bg=2.98%)▶PTBP1 (bg=3.74%)▶TIA1 (bg=4.07%)▶ZC3H11A (bg=6.55%)No matches to TargetScan

-

GTCTTAGA

GTCTTAGA  
Depth:2 (PIG)  
Ei-value:0.000, Pi-value:0.000  
Er-value:0.000, Pr-value:0.000  
eCLIP MATCHES▶MATR3 (bg=2.98%)▶PTBP1 (bg=3.74%)▶TIA1 (bg=4.07%)▶ZC3H11A (bg=6.55%)MATCHES To TargetScan▶ miR-208-3p:UAAGACG▶ miR-499a-5p:UAAGACU

---- 12110  
 -------

TTT

TTTTTCATTTTGTT  
Depth:2 (PIG)  
Ei-value:0.000, Pi-value:0.000  
Er-value:0.000, Pr-value:0.000  
No matches to eCLIP DataMATCHES To TargetScan▶ miR-495-3p:AACAAAC


TTCATTTTGTT

TTCATTTTGTT  
Depth:4 (DOG)  
Ei-value:0.000, Pi-value:0.000  
Er-value:0.000, Pr-value:0.000  
No matches to eCLIP DataMATCHES To TargetScan▶ miR-495-3p:AACAAAC

-------

CTC

CTCTTTGCTC  
Depth:2 (PIG)  
Ei-value:0.000, Pi-value:0.000  
Er-value:0.000, Pr-value:0.000  
eCLIP MATCHES▶MATR3 (bg=2.98%)▶PTBP1 (bg=3.74%)▶TIA1 (bg=4.07%)No matches to TargetScan


TTTGCTC

TTTGCTC  
Depth:3 (COW)  
Ei-value:0.000, Pi-value:0.000  
Er-value:0.000, Pr-value:0.000  
eCLIP MATCHES▶MATR3 (bg=2.98%)▶PTBP1 (bg=3.74%)▶TIA1 (bg=4.07%)No matches to TargetScan

---------------------------------------------------------------------------

TTTCTTG

TTTCTTGTT  
Depth:2 (PIG)  
Ei-value:0.000, Pi-value:0.000  
Er-value:0.000, Pr-value:0.000  
eCLIP MATCHES▶MATR3 (bg=2.98%)▶PTBP1 (bg=3.74%)▶SMNDC1 (bg=0.63%)▶TIA1 (bg=4.07%)No matches to TargetScan

 12230  


TT

TTTCTTGTT  
Depth:2 (PIG)  
Ei-value:0.000, Pi-value:0.000  
Er-value:0.000, Pr-value:0.000  
eCLIP MATCHES▶MATR3 (bg=2.98%)▶PTBP1 (bg=3.74%)▶SMNDC1 (bg=0.63%)▶TIA1 (bg=4.07%)No matches to TargetScan

--------------------

TGCCTACCT

TGCCTACCT  
Depth:2 (PIG)  
Ei-value:0.000, Pi-value:0.000  
Er-value:0.000, Pr-value:0.000  
eCLIP MATCHES▶MATR3 (bg=2.98%)▶PTBP1 (bg=3.74%)▶SMNDC1 (bg=0.63%)▶TIA1 (bg=4.07%)MATCHES To TargetScan▶ miR-196-5p:AGGUAGU

---

TT

TTTTCTCTTTGTGAA  
Depth:3 (COW)  
Ei-value:0.000, Pi-value:0.000  
Er-value:0.000, Pr-value:0.000  
eCLIP MATCHES▶MATR3 (bg=2.98%)▶PTBP1 (bg=3.74%)▶SMNDC1 (bg=0.63%)▶TIA1 (bg=4.07%)No matches to TargetScan


TTCTCTTTG

TTCTCTTTG  
Depth:6 (MOUSE)  
Ei-value:0.000, Pi-value:0.000  
Er-value:0.000, Pr-value:0.000  
eCLIP MATCHES▶MATR3 (bg=2.98%)▶PTBP1 (bg=3.74%)▶SMNDC1 (bg=0.63%)▶TIA1 (bg=4.07%)No matches to TargetScan


TGAA

TTTTCTCTTTGTGAA  
Depth:3 (COW)  
Ei-value:0.000, Pi-value:0.000  
Er-value:0.000, Pr-value:0.000  
eCLIP MATCHES▶MATR3 (bg=2.98%)▶PTBP1 (bg=3.74%)▶SMNDC1 (bg=0.63%)▶TIA1 (bg=4.07%)No matches to TargetScan

--------------------

TTCCCCTT

TTCCCCTT  
Depth:3 (COW)  
Ei-value:0.000, Pi-value:0.000  
Er-value:0.000, Pr-value:0.000  
eCLIP MATCHES▶MATR3 (bg=2.98%)▶PTBP1 (bg=3.74%)▶TIA1 (bg=4.07%)No matches to TargetScan


CT

TTCCCCTTCT  
Depth:2 (PIG)  
Ei-value:0.000, Pi-value:0.000  
Er-value:0.000, Pr-value:0.000  
eCLIP MATCHES▶MATR3 (bg=2.98%)▶PTBP1 (bg=3.74%)▶TIA1 (bg=4.07%)No matches to TargetScan

-

GTTCGTTT

GTTCGTTT  
Depth:2 (PIG)  
Ei-value:0.000, Pi-value:0.000  
Er-value:0.000, Pr-value:0.000  
eCLIP MATCHES▶MATR3 (bg=2.98%)▶PTBP1 (bg=3.74%)▶TIA1 (bg=4.07%)No matches to TargetScan

---

ATTTCACCT

ATTTCACCT  
Depth:4 (DOG)  
Ei-value:0.000, Pi-value:0.000  
Er-value:0.000, Pr-value:0.000  
eCLIP MATCHES▶TIA1 (bg=4.07%)MATCHES To TargetScan▶ miR-203a-3p.2:UGAAAUG

-------------------- 12350  
 ----

TGCTG

TGCTGTTTCTACT  
Depth:3 (COW)  
Ei-value:0.000, Pi-value:0.000  
Er-value:0.000, Pr-value:0.000  
eCLIP MATCHES▶MATR3 (bg=2.98%)▶PTBP1 (bg=3.74%)▶TIA1 (bg=4.07%)MATCHES To TargetScan▶ miR-411-5p.1:AGUAGAC▶ miR-494-3p:GAAACAU


TTTCTAC

TTTCTAC  
Depth:6 (MOUSE)  
Ei-value:0.000, Pi-value:0.000  
Er-value:0.000, Pr-value:0.000  
eCLIP MATCHES▶MATR3 (bg=2.98%)▶PTBP1 (bg=3.74%)▶TIA1 (bg=4.07%)No matches to TargetScan


T

TTTCTACT  
Depth:5 (RABBIT)  
Ei-value:0.000, Pi-value:0.000  
Er-value:0.000, Pr-value:0.000  
eCLIP MATCHES▶MATR3 (bg=2.98%)▶PTBP1 (bg=3.74%)▶TIA1 (bg=4.07%)MATCHES To TargetScan▶ miR-411-5p.1:AGUAGAC

----

ATCTCAC

ATCTCACATTTCTC  
Depth:2 (PIG)  
Ei-value:0.000, Pi-value:0.000  
Er-value:0.000, Pr-value:0.000  
eCLIP MATCHES▶MATR3 (bg=2.98%)▶PTBP1 (bg=3.74%)▶TIA1 (bg=4.07%)MATCHES To TargetScan▶ miR-203a-3p.1:GAAAUGU


ATTTCTC

ATTTCTC  
Depth:6 (MOUSE)  
Ei-value:0.000, Pi-value:0.000  
Er-value:0.000, Pr-value:0.000  
eCLIP MATCHES▶MATR3 (bg=2.98%)▶PTBP1 (bg=3.74%)▶TIA1 (bg=4.07%)No matches to TargetScan

------------------

TGCCTC

TGCCTCTCTTGGGC  
Depth:2 (PIG)  
Ei-value:0.000, Pi-value:0.000  
Er-value:0.000, Pr-value:0.000  
eCLIP MATCHES▶MATR3 (bg=2.98%)▶PTBP1 (bg=3.74%)▶SMNDC1 (bg=0.63%)▶TIA1 (bg=4.07%)MATCHES To TargetScan▶ miR-335-5p:CAAGAGC


TCTTGGG

TCTTGGG  
Depth:5 (RABBIT)  
Ei-value:0.000, Pi-value:0.000  
Er-value:0.000, Pr-value:0.000  
eCLIP MATCHES▶MATR3 (bg=2.98%)▶PTBP1 (bg=3.74%)▶SMNDC1 (bg=0.63%)▶TIA1 (bg=4.07%)No matches to TargetScan


C

TCTTGGGC  
Depth:3 (COW)  
Ei-value:0.000, Pi-value:0.000  
Er-value:0.000, Pr-value:0.000  
eCLIP MATCHES▶MATR3 (bg=2.98%)▶PTBP1 (bg=3.74%)▶SMNDC1 (bg=0.63%)▶TIA1 (bg=4.07%)No matches to TargetScan

-------------------------------------------

TTTGTGA

TTTGTGA  
Depth:4 (DOG)  
Ei-value:0.000, Pi-value:0.010  
Er-value:0.000, Pr-value:0.000  
eCLIP MATCHES▶MATR3 (bg=2.98%)▶PTBP1 (bg=3.74%)▶TIA1 (bg=4.07%)No matches to TargetScan


TTT

TTTGTGATTTTC  
Depth:3 (COW)  
Ei-value:0.000, Pi-value:0.000  
Er-value:0.000, Pr-value:0.000  
eCLIP MATCHES▶MATR3 (bg=2.98%)▶PTBP1 (bg=3.74%)▶TIA1 (bg=4.07%)No matches to TargetScan

 12470  


TC

TTTGTGATTTTC  
Depth:3 (COW)  
Ei-value:0.000, Pi-value:0.000  
Er-value:0.000, Pr-value:0.000  
eCLIP MATCHES▶MATR3 (bg=2.98%)▶PTBP1 (bg=3.74%)▶TIA1 (bg=4.07%)No matches to TargetScan

--------------

TCTCTGTT

TCTCTGTT  
Depth:4 (DOG)  
Ei-value:0.000, Pi-value:0.000  
Er-value:0.000, Pr-value:0.000  
eCLIP MATCHES▶MATR3 (bg=2.98%)▶PTBP1 (bg=3.74%)No matches to TargetScan

-----------------------------------

TCACC

TCACCTTTGAGTATTT  
Depth:2 (PIG)  
Ei-value:0.000, Pi-value:0.000  
Er-value:0.000, Pr-value:0.000  
eCLIP MATCHES▶MATR3 (bg=2.98%)▶PTBP1 (bg=3.74%)▶TIA1 (bg=4.07%)MATCHES To TargetScan▶ miR-18-5p:AAGGUGC▶ miR-200bc-3p/429:AAUACUG▶ miR-371-5p:CUCAAAC


TTTGAGTATTT

TTTGAGTATTT  
Depth:4 (DOG)  
Ei-value:0.000, Pi-value:0.000  
Er-value:0.000, Pr-value:0.000  
eCLIP MATCHES▶MATR3 (bg=2.98%)▶PTBP1 (bg=3.74%)▶TIA1 (bg=4.07%)MATCHES To TargetScan▶ miR-200bc-3p/429:AAUACUG▶ miR-371-5p:CUCAAAC

--

GCCTCTTC

GCCTCTTC  
Depth:2 (PIG)  
Ei-value:0.000, Pi-value:0.000  
Er-value:0.000, Pr-value:0.000  
eCLIP MATCHES▶MATR3 (bg=2.98%)▶PTBP1 (bg=3.74%)▶TIA1 (bg=4.07%)No matches to TargetScan

------------------

CTTTGATT

CTTTGATT  
Depth:3 (COW)  
Ei-value:0.000, Pi-value:0.000  
Er-value:0.000, Pr-value:0.000  
eCLIP MATCHES▶MATR3 (bg=2.98%)▶PTBP1 (bg=3.74%)▶TIA1 (bg=4.07%)No matches to TargetScan

--------- 12590  
 -----------------------------------------

TGTGTGTG

TGTGTGTG  
Depth:4 (DOG)  
Ei-value:0.000, Pi-value:0.000  
Er-value:0.000, Pr-value:0.000  
eCLIP MATCHES▶AATF (bg=0.64%)▶DDX24 (bg=2.97%)▶NCBP2 (bg=1.49%)▶NOLC1 (bg=9.43%)▶PTBP1 (bg=3.74%)▶SND1 (bg=0.45%)▶SRSF7 (bg=2.32%)▶TARDBP (bg=2.79%)▶WDR43 (bg=3.37%)▶XRCC6 (bg=2.91%)▶ZC3H8 (bg=0.29%)MATCHES To TargetScan▶ miR-329-3p/362-3p:ACACACC

---------------

AGGGGCT

AGGGGCTTCCTAACCCCT  
Depth:2 (PIG)  
Ei-value:0.000, Pi-value:0.000  
Er-value:0.000, Pr-value:0.000  
eCLIP MATCHES▶AATF (bg=0.64%)▶DDX24 (bg=2.97%)▶NCBP2 (bg=1.49%)▶NOLC1 (bg=9.43%)▶PTBP1 (bg=3.74%)▶SND1 (bg=0.45%)▶SRSF7 (bg=2.32%)▶TARDBP (bg=2.79%)▶UTP3 (bg=3.66%)▶WDR43 (bg=3.37%)▶XRCC6 (bg=2.91%)▶ZC3H8 (bg=0.29%)No matches to TargetScan


TCCTAACCCCT

TCCTAACCCCT  
Depth:5 (RABBIT)  
Ei-value:0.000, Pi-value:0.000  
Er-value:0.000, Pr-value:0.000  
eCLIP MATCHES▶AATF (bg=0.64%)▶DDX24 (bg=2.97%)▶NCBP2 (bg=1.49%)▶NOLC1 (bg=9.43%)▶PTBP1 (bg=3.74%)▶SND1 (bg=0.45%)▶SRSF7 (bg=2.32%)▶TARDBP (bg=2.79%)▶UTP3 (bg=3.66%)▶WDR43 (bg=3.37%)▶XRCC6 (bg=2.91%)▶ZC3H8 (bg=0.29%)No matches to TargetScan

------

TAGGTGCA

TAGGTGCA  
Depth:3 (COW)  
Ei-value:0.000, Pi-value:0.000  
Er-value:0.000, Pr-value:0.000  
eCLIP MATCHES▶DDX24 (bg=2.97%)▶NOLC1 (bg=9.43%)▶SND1 (bg=0.45%)▶SRSF7 (bg=2.32%)▶TARDBP (bg=2.79%)▶UTP3 (bg=3.66%)▶WDR43 (bg=3.37%)▶XRCC6 (bg=2.91%)▶ZC3H8 (bg=0.29%)No matches to TargetScan

-------------------

AAGCA

AAGCATTG  
Depth:4 (DOG)  
Ei-value:0.000, Pi-value:0.000  
Er-value:0.000, Pr-value:0.000  
eCLIP MATCHES▶DDX24 (bg=2.97%)▶NOLC1 (bg=9.43%)▶NPM1 (bg=1.21%)▶RBFOX2 (bg=4.63%)▶RPS3 (bg=0.76%)▶SRSF1 (bg=8.47%)▶SRSF7 (bg=2.32%)▶TARDBP (bg=2.79%)▶TRA2A (bg=4.8%)▶U2AF2 (bg=1.76%)▶uchl5 (bg=11.16%)▶YWHAG (bg=1.87%)▶ZNF622 (bg=6.58%)No matches to TargetScan

 12710  


TTG

AAGCATTG  
Depth:4 (DOG)  
Ei-value:0.000, Pi-value:0.000  
Er-value:0.000, Pr-value:0.000  
eCLIP MATCHES▶DDX24 (bg=2.97%)▶NOLC1 (bg=9.43%)▶NPM1 (bg=1.21%)▶RBFOX2 (bg=4.63%)▶RPS3 (bg=0.76%)▶SRSF1 (bg=8.47%)▶SRSF7 (bg=2.32%)▶TARDBP (bg=2.79%)▶TRA2A (bg=4.8%)▶U2AF2 (bg=1.76%)▶uchl5 (bg=11.16%)▶YWHAG (bg=1.87%)▶ZNF622 (bg=6.58%)No matches to TargetScan

-------

GTTCC

GTTCCTTATGCCAG  
Depth:2 (PIG)  
Ei-value:0.000, Pi-value:0.000  
Er-value:0.000, Pr-value:0.000  
eCLIP MATCHES▶DDX24 (bg=2.97%)▶FASTKD2 (bg=1.99%)▶LARP4 (bg=4.72%)▶NOLC1 (bg=9.43%)▶NPM1 (bg=1.21%)▶RBFOX2 (bg=4.63%)▶RBM15 (bg=7.27%)▶RPS3 (bg=0.76%)▶SRSF1 (bg=8.47%)▶SRSF7 (bg=2.32%)▶TARDBP (bg=2.79%)▶TRA2A (bg=4.8%)▶U2AF2 (bg=1.76%)▶uchl5 (bg=11.16%)▶WDR43 (bg=3.37%)▶YWHAG (bg=1.87%)▶ZC3H11A (bg=6.55%)▶ZNF622 (bg=6.58%)▶ZNF800 (bg=1.92%)No matches to TargetScan


TTATGCCA

TTATGCCA  
Depth:5 (RABBIT)  
Ei-value:0.000, Pi-value:0.000  
Er-value:0.000, Pr-value:0.000  
eCLIP MATCHES▶DDX24 (bg=2.97%)▶FASTKD2 (bg=1.99%)▶LARP4 (bg=4.72%)▶NOLC1 (bg=9.43%)▶NPM1 (bg=1.21%)▶RBFOX2 (bg=4.63%)▶RBM15 (bg=7.27%)▶RPS3 (bg=0.76%)▶SRSF1 (bg=8.47%)▶SRSF7 (bg=2.32%)▶TARDBP (bg=2.79%)▶TRA2A (bg=4.8%)▶U2AF2 (bg=1.76%)▶uchl5 (bg=11.16%)▶WDR43 (bg=3.37%)▶YWHAG (bg=1.87%)▶ZC3H11A (bg=6.55%)▶ZNF622 (bg=6.58%)▶ZNF800 (bg=1.92%)No matches to TargetScan


G

TTATGCCAG  
Depth:4 (DOG)  
Ei-value:0.000, Pi-value:0.000  
Er-value:0.000, Pr-value:0.000  
eCLIP MATCHES▶DDX24 (bg=2.97%)▶FASTKD2 (bg=1.99%)▶LARP4 (bg=4.72%)▶NOLC1 (bg=9.43%)▶NPM1 (bg=1.21%)▶RBFOX2 (bg=4.63%)▶RBM15 (bg=7.27%)▶RPS3 (bg=0.76%)▶SRSF1 (bg=8.47%)▶SRSF7 (bg=2.32%)▶TARDBP (bg=2.79%)▶TRA2A (bg=4.8%)▶U2AF2 (bg=1.76%)▶uchl5 (bg=11.16%)▶WDR43 (bg=3.37%)▶YWHAG (bg=1.87%)▶ZC3H11A (bg=6.55%)▶ZNF622 (bg=6.58%)▶ZNF800 (bg=1.92%)No matches to TargetScan

-------------

ATGA

ATGATCCAAGACCAA  
Depth:2 (PIG)  
Ei-value:0.000, Pi-value:0.000  
Er-value:0.000, Pr-value:0.000  
eCLIP MATCHES▶DDX24 (bg=2.97%)▶FASTKD2 (bg=1.99%)▶LARP4 (bg=4.72%)▶NOLC1 (bg=9.43%)▶NPM1 (bg=1.21%)▶RBFOX2 (bg=4.63%)▶RBM15 (bg=7.27%)▶SRSF1 (bg=8.47%)▶SRSF7 (bg=2.32%)▶TARDBP (bg=2.79%)▶TRA2A (bg=4.8%)▶U2AF2 (bg=1.76%)▶uchl5 (bg=11.16%)▶WDR43 (bg=3.37%)▶YWHAG (bg=1.87%)▶ZC3H11A (bg=6.55%)▶ZNF622 (bg=6.58%)▶ZNF800 (bg=1.92%)MATCHES To TargetScan▶ miR-133a-3p.2/133b:UUGGUCC▶ miR-431-5p:GUCUUGC


TCCAAG

TCCAAG  
Depth:3 (COW)  
Ei-value:0.000, Pi-value:0.000  
Er-value:0.000, Pr-value:0.000  
eCLIP MATCHES▶DDX24 (bg=2.97%)▶FASTKD2 (bg=1.99%)▶LARP4 (bg=4.72%)▶NOLC1 (bg=9.43%)▶NPM1 (bg=1.21%)▶RBFOX2 (bg=4.63%)▶RBM15 (bg=7.27%)▶SRSF1 (bg=8.47%)▶SRSF7 (bg=2.32%)▶TARDBP (bg=2.79%)▶TRA2A (bg=4.8%)▶U2AF2 (bg=1.76%)▶uchl5 (bg=11.16%)▶WDR43 (bg=3.37%)▶YWHAG (bg=1.87%)▶ZC3H11A (bg=6.55%)▶ZNF622 (bg=6.58%)▶ZNF800 (bg=1.92%)No matches to TargetScan


ACCAA

ATGATCCAAGACCAA  
Depth:2 (PIG)  
Ei-value:0.000, Pi-value:0.000  
Er-value:0.000, Pr-value:0.000  
eCLIP MATCHES▶DDX24 (bg=2.97%)▶FASTKD2 (bg=1.99%)▶LARP4 (bg=4.72%)▶NOLC1 (bg=9.43%)▶NPM1 (bg=1.21%)▶RBFOX2 (bg=4.63%)▶RBM15 (bg=7.27%)▶SRSF1 (bg=8.47%)▶SRSF7 (bg=2.32%)▶TARDBP (bg=2.79%)▶TRA2A (bg=4.8%)▶U2AF2 (bg=1.76%)▶uchl5 (bg=11.16%)▶WDR43 (bg=3.37%)▶YWHAG (bg=1.87%)▶ZC3H11A (bg=6.55%)▶ZNF622 (bg=6.58%)▶ZNF800 (bg=1.92%)MATCHES To TargetScan▶ miR-133a-3p.2/133b:UUGGUCC▶ miR-431-5p:GUCUUGC

-------------------------------------------------------------------

T

TAGAAGGCCCAA  
Depth:2 (PIG)  
Ei-value:0.000, Pi-value:0.000  
Er-value:0.000, Pr-value:0.000  
eCLIP MATCHES▶DDX24 (bg=2.97%)▶LARP4 (bg=4.72%)▶MTPAP (bg=2.21%)▶NOLC1 (bg=9.43%)▶SRSF1 (bg=8.47%)▶SRSF7 (bg=2.32%)▶TRA2A (bg=4.8%)▶uchl5 (bg=11.16%)▶UTP3 (bg=3.66%)▶ZNF622 (bg=6.58%)▶ZNF800 (bg=1.92%)No matches to TargetScan

 12830  


TAGAAGGCCCAA  
Depth:2 (PIG)  
Ei-value:0.000, Pi-value:0.000  
Er-value:0.000, Pr-value:0.000  
eCLIP MATCHES▶DDX24 (bg=2.97%)▶LARP4 (bg=4.72%)▶MTPAP (bg=2.21%)▶NOLC1 (bg=9.43%)▶SRSF1 (bg=8.47%)▶SRSF7 (bg=2.32%)▶TRA2A (bg=4.8%)▶uchl5 (bg=11.16%)▶UTP3 (bg=3.66%)▶ZNF622 (bg=6.58%)▶ZNF800 (bg=1.92%)No matches to TargetScan


AGA

AGAAGGCCCAA  
Depth:4 (DOG)  
Ei-value:0.000, Pi-value:0.000  
Er-value:0.000, Pr-value:0.000  
eCLIP MATCHES▶DDX24 (bg=2.97%)▶LARP4 (bg=4.72%)▶MTPAP (bg=2.21%)▶NOLC1 (bg=9.43%)▶SRSF1 (bg=8.47%)▶SRSF7 (bg=2.32%)▶TRA2A (bg=4.8%)▶uchl5 (bg=11.16%)▶UTP3 (bg=3.66%)▶ZNF622 (bg=6.58%)▶ZNF800 (bg=1.92%)No matches to TargetScan


AGGCCCAA

AGGCCCAA  
Depth:5 (RABBIT)  
Ei-value:0.000, Pi-value:0.000  
Er-value:0.000, Pr-value:0.000  
eCLIP MATCHES▶DDX24 (bg=2.97%)▶LARP4 (bg=4.72%)▶MTPAP (bg=2.21%)▶NOLC1 (bg=9.43%)▶SRSF1 (bg=8.47%)▶SRSF7 (bg=2.32%)▶TRA2A (bg=4.8%)▶uchl5 (bg=11.16%)▶UTP3 (bg=3.66%)▶ZNF622 (bg=6.58%)▶ZNF800 (bg=1.92%)No matches to TargetScan

------------------------------------------------------------------------------------------------------------- 12950  
 ---------------------------------------

GAGTTGGATGGAAG

GAGTTGGATGGAAG  
Depth:2 (PIG)  
Ei-value:0.000, Pi-value:0.000  
Er-value:0.000, Pr-value:0.000  
eCLIP MATCHES▶AARS (bg=2.18%)▶CPEB4 (bg=1.89%)▶DDX24 (bg=2.97%)▶FASTKD2 (bg=1.99%)▶GRWD1 (bg=5.13%)▶HLTF (bg=0.4%)▶LARP4 (bg=4.72%)▶METAP2 (bg=0.78%)▶MTPAP (bg=2.21%)▶NOLC1 (bg=9.43%)▶PUM1 (bg=1.56%)▶RBFOX2 (bg=4.63%)▶RPS11 (bg=0.63%)▶SAFB (bg=2.69%)▶SLTM (bg=2.2%)▶SRSF1 (bg=8.47%)▶TRA2A (bg=4.8%)▶uchl5 (bg=11.16%)▶XRCC6 (bg=2.91%)▶ZNF622 (bg=6.58%)▶ZNF800 (bg=1.92%)No matches to TargetScan

------------------

AAGTCT

AAGTCT  
Depth:2 (PIG)  
Ei-value:0.000, Pi-value:0.010  
Er-value:0.000, Pr-value:0.010  
eCLIP MATCHES▶CPEB4 (bg=1.89%)▶DDX24 (bg=2.97%)▶DROSHA (bg=2.49%)▶FASTKD2 (bg=1.99%)▶GRWD1 (bg=5.13%)▶HLTF (bg=0.4%)▶LARP4 (bg=4.72%)▶METAP2 (bg=0.78%)▶MTPAP (bg=2.21%)▶NOLC1 (bg=9.43%)▶NPM1 (bg=1.21%)▶PPIL4 (bg=0.52%)▶PUM1 (bg=1.56%)▶RBFOX2 (bg=4.63%)▶RPS11 (bg=0.63%)▶SAFB (bg=2.69%)▶SLTM (bg=2.2%)▶SRSF1 (bg=8.47%)▶TRA2A (bg=4.8%)▶uchl5 (bg=11.16%)▶WDR43 (bg=3.37%)▶XRCC6 (bg=2.91%)▶ZC3H11A (bg=6.55%)▶ZNF622 (bg=6.58%)▶ZNF800 (bg=1.92%)No matches to TargetScan

------------------------------------------- 13070  
 ---------

AAGGCCAA

AAGGCCAA  
Depth:2 (PIG)  
Ei-value:0.000, Pi-value:0.000  
Er-value:0.000, Pr-value:0.000  
eCLIP MATCHES▶GRWD1 (bg=5.13%)▶MTPAP (bg=2.21%)▶SAFB (bg=2.69%)▶SLTM (bg=2.2%)▶SRSF1 (bg=8.47%)▶TRA2A (bg=4.8%)▶uchl5 (bg=11.16%)▶UTP3 (bg=3.66%)▶WDR43 (bg=3.37%)▶XRCC6 (bg=2.91%)No matches to TargetScan

-

GACCTAAGA

GACCTAAGA  
Depth:2 (PIG)  
Ei-value:0.000, Pi-value:0.000  
Er-value:0.000, Pr-value:0.000  
eCLIP MATCHES▶GRWD1 (bg=5.13%)▶NOLC1 (bg=9.43%)▶SAFB (bg=2.69%)▶SRSF1 (bg=8.47%)▶TRA2A (bg=4.8%)▶UTP3 (bg=3.66%)▶WDR43 (bg=3.37%)▶XRCC6 (bg=2.91%)▶ZNF622 (bg=6.58%)No matches to TargetScan

------------------------------------------

GAAGGCCC

GAAGGCCC  
Depth:2 (PIG)  
Ei-value:0.000, Pi-value:0.000  
Er-value:0.000, Pr-value:0.000  
eCLIP MATCHES▶RBFOX2 (bg=4.63%)▶SLTM (bg=2.2%)▶SRSF1 (bg=8.47%)▶TRA2A (bg=4.8%)▶ZNF622 (bg=6.58%)No matches to TargetScan

------------------------------------------- 13190  
 ----

TATC

TATCTCAAGACTAA  
Depth:2 (PIG)  
Ei-value:0.000, Pi-value:0.000  
Er-value:0.000, Pr-value:0.000  
eCLIP MATCHES▶CPEB4 (bg=1.89%)▶FASTKD2 (bg=1.99%)▶GRWD1 (bg=5.13%)▶LARP4 (bg=4.72%)▶MTPAP (bg=2.21%)▶NOLC1 (bg=9.43%)▶RBFOX2 (bg=4.63%)▶SRSF1 (bg=8.47%)▶TRA2A (bg=4.8%)▶uchl5 (bg=11.16%)▶UTP18 (bg=0.72%)▶UTP3 (bg=3.66%)▶WDR43 (bg=3.37%)▶ZNF622 (bg=6.58%)MATCHES To TargetScan▶ miR-431-5p:GUCUUGC


TCAA

TCAAGACTAA  
Depth:4 (DOG)  
Ei-value:0.000, Pi-value:0.000  
Er-value:0.000, Pr-value:0.000  
eCLIP MATCHES▶CPEB4 (bg=1.89%)▶FASTKD2 (bg=1.99%)▶GRWD1 (bg=5.13%)▶LARP4 (bg=4.72%)▶MTPAP (bg=2.21%)▶NOLC1 (bg=9.43%)▶RBFOX2 (bg=4.63%)▶SRSF1 (bg=8.47%)▶TRA2A (bg=4.8%)▶uchl5 (bg=11.16%)▶UTP18 (bg=0.72%)▶UTP3 (bg=3.66%)▶WDR43 (bg=3.37%)▶ZNF622 (bg=6.58%)MATCHES To TargetScan▶ miR-431-5p:GUCUUGC


GACTAA

GACTAA  
Depth:5 (RABBIT)  
Ei-value:0.000, Pi-value:0.000  
Er-value:0.000, Pr-value:0.000  
eCLIP MATCHES▶CPEB4 (bg=1.89%)▶FASTKD2 (bg=1.99%)▶GRWD1 (bg=5.13%)▶LARP4 (bg=4.72%)▶MTPAP (bg=2.21%)▶NOLC1 (bg=9.43%)▶RBFOX2 (bg=4.63%)▶SRSF1 (bg=8.47%)▶TRA2A (bg=4.8%)▶uchl5 (bg=11.16%)▶UTP18 (bg=0.72%)▶UTP3 (bg=3.66%)▶WDR43 (bg=3.37%)▶ZNF622 (bg=6.58%)No matches to TargetScan

------

GAATCTGG

GAATCTGG  
Depth:2 (PIG)  
Ei-value:0.000, Pi-value:0.000  
Er-value:0.000, Pr-value:0.000  
eCLIP MATCHES▶CPEB4 (bg=1.89%)▶FASTKD2 (bg=1.99%)▶GRWD1 (bg=5.13%)▶LARP4 (bg=4.72%)▶MTPAP (bg=2.21%)▶NOLC1 (bg=9.43%)▶PCBP1 (bg=1.07%)▶RBFOX2 (bg=4.63%)▶SRSF1 (bg=8.47%)▶TRA2A (bg=4.8%)▶uchl5 (bg=11.16%)▶UTP18 (bg=0.72%)▶UTP3 (bg=3.66%)▶WDR43 (bg=3.37%)▶ZNF622 (bg=6.58%)No matches to TargetScan

--------------------------

GAT

GATAGAAGC  
Depth:2 (PIG)  
Ei-value:0.000, Pi-value:0.000  
Er-value:0.000, Pr-value:0.000  
eCLIP MATCHES▶CPEB4 (bg=1.89%)▶GRWD1 (bg=5.13%)▶LARP4 (bg=4.72%)▶MTPAP (bg=2.21%)▶NOLC1 (bg=9.43%)▶PCBP1 (bg=1.07%)▶RBFOX2 (bg=4.63%)▶SRSF1 (bg=8.47%)▶TRA2A (bg=4.8%)▶uchl5 (bg=11.16%)▶ZNF622 (bg=6.58%)No matches to TargetScan


AGAAGC

AGAAGC  
Depth:4 (DOG)  
Ei-value:0.000, Pi-value:0.000  
Er-value:0.000, Pr-value:0.010  
eCLIP MATCHES▶CPEB4 (bg=1.89%)▶GRWD1 (bg=5.13%)▶LARP4 (bg=4.72%)▶MTPAP (bg=2.21%)▶NOLC1 (bg=9.43%)▶PCBP1 (bg=1.07%)▶RBFOX2 (bg=4.63%)▶SRSF1 (bg=8.47%)▶TRA2A (bg=4.8%)▶uchl5 (bg=11.16%)▶ZNF622 (bg=6.58%)No matches to TargetScan

-----------

GGGAAAT

GGGAAAT  
Depth:2 (PIG)  
Ei-value:0.000, Pi-value:0.000  
Er-value:0.000, Pr-value:0.010  
eCLIP MATCHES▶CPEB4 (bg=1.89%)▶FTO (bg=0.32%)▶GRWD1 (bg=5.13%)▶LARP4 (bg=4.72%)▶MTPAP (bg=2.21%)▶SRSF1 (bg=8.47%)▶TRA2A (bg=4.8%)▶uchl5 (bg=11.16%)▶ZNF622 (bg=6.58%)No matches to TargetScan

--

C

CAAGATGA  
Depth:3 (COW)  
Ei-value:0.000, Pi-value:0.000  
Er-value:0.000, Pr-value:0.000  
eCLIP MATCHES▶CPEB4 (bg=1.89%)▶FTO (bg=0.32%)▶GRWD1 (bg=5.13%)▶LARP4 (bg=4.72%)▶MTPAP (bg=2.21%)▶SRSF1 (bg=8.47%)▶TRA2A (bg=4.8%)▶uchl5 (bg=11.16%)▶ZNF622 (bg=6.58%)No matches to TargetScan


AAGATGA

AAGATGA  
Depth:5 (RABBIT)  
Ei-value:0.000, Pi-value:0.000  
Er-value:0.000, Pr-value:0.000  
eCLIP MATCHES▶CPEB4 (bg=1.89%)▶FTO (bg=0.32%)▶GRWD1 (bg=5.13%)▶LARP4 (bg=4.72%)▶MTPAP (bg=2.21%)▶SRSF1 (bg=8.47%)▶TRA2A (bg=4.8%)▶uchl5 (bg=11.16%)▶ZNF622 (bg=6.58%)No matches to TargetScan

-

AACCCTAAA

AACCCTAAA  
Depth:2 (PIG)  
Ei-value:0.000, Pi-value:0.000  
Er-value:0.000, Pr-value:0.000  
eCLIP MATCHES▶FTO (bg=0.32%)▶GRWD1 (bg=5.13%)▶LARP4 (bg=4.72%)▶MTPAP (bg=2.21%)▶ZNF622 (bg=6.58%)MATCHES To TargetScan▶ miR-296-3p:AGGGUUG

------

CTCT

CTCTTTTCTATTGTT  
Depth:2 (PIG)  
Ei-value:0.000, Pi-value:0.000  
Er-value:0.000, Pr-value:0.000  
eCLIP MATCHES▶FTO (bg=0.32%)▶LARP4 (bg=4.72%)No matches to TargetScan


TTTCT

TTTCTATTG  
Depth:3 (COW)  
Ei-value:0.000, Pi-value:0.000  
Er-value:0.000, Pr-value:0.000  
No matches to eCLIP DataNo matches to TargetScan

 13310  


ATTG

TTTCTATTG  
Depth:3 (COW)  
Ei-value:0.000, Pi-value:0.000  
Er-value:0.000, Pr-value:0.000  
No matches to eCLIP DataNo matches to TargetScan


TT

CTCTTTTCTATTGTT  
Depth:2 (PIG)  
Ei-value:0.000, Pi-value:0.000  
Er-value:0.000, Pr-value:0.000  
eCLIP MATCHES▶FTO (bg=0.32%)▶LARP4 (bg=4.72%)No matches to TargetScan

--

C

CACTTCTT  
Depth:2 (PIG)  
Ei-value:0.000, Pi-value:0.010  
Er-value:0.000, Pr-value:0.000  
eCLIP MATCHES▶NOLC1 (bg=9.43%)No matches to TargetScan


ACTTCTT

ACTTCTT  
Depth:3 (COW)  
Ei-value:0.000, Pi-value:0.020  
Er-value:0.000, Pr-value:0.000  
eCLIP MATCHES▶NOLC1 (bg=9.43%)No matches to TargetScan

---------------------

TCCTGTT

TCCTGTT  
Depth:2 (PIG)  
Ei-value:0.000, Pi-value:0.020  
Er-value:0.000, Pr-value:0.000  
eCLIP MATCHES▶NOLC1 (bg=9.43%)▶TIA1 (bg=4.07%)▶ZC3H11A (bg=6.55%)No matches to TargetScan

------------------------------

CTTTTTGATGTT

CTTTTTGATGTT  
Depth:4 (DOG)  
Ei-value:0.000, Pi-value:0.000  
Er-value:0.000, Pr-value:0.000  
eCLIP MATCHES▶TIA1 (bg=4.07%)No matches to TargetScan


GC

CTTTTTGATGTTGC  
Depth:2 (PIG)  
Ei-value:0.000, Pi-value:0.000  
Er-value:0.000, Pr-value:0.000  
eCLIP MATCHES▶TIA1 (bg=4.07%)No matches to TargetScan

--

GTTACCTT

GTTACCTT  
Depth:2 (PIG)  
Ei-value:0.000, Pi-value:0.000  
Er-value:0.000, Pr-value:0.010  
No matches to eCLIP DataNo matches to TargetScan

-------

ACAG

ACAGTATTATGCCTGGGCCAGTCTT  
Depth:2 (PIG)  
Ei-value:0.000, Pi-value:0.000  
Er-value:0.000, Pr-value:0.000  
No matches to eCLIP DataMATCHES To TargetScan▶ miR-193-3p:ACUGGCC▶ miR-200bc-3p/429:AAUACUG▶ miR-328-3p:UGGCCCU▶ miR-369-3p:AUAAUAC▶ miR-655-3p:UAAUACA


TATTATGC

TATTATGC  
Depth:4 (DOG)  
Ei-value:0.000, Pi-value:0.000  
Er-value:0.000, Pr-value:0.000  
No matches to eCLIP DataMATCHES To TargetScan▶ miR-369-3p:AUAAUAC


CTG

ACAGTATTATGCCTGGGCCAGTCTT  
Depth:2 (PIG)  
Ei-value:0.000, Pi-value:0.000  
Er-value:0.000, Pr-value:0.000  
No matches to eCLIP DataMATCHES To TargetScan▶ miR-193-3p:ACUGGCC▶ miR-200bc-3p/429:AAUACUG▶ miR-328-3p:UGGCCCU▶ miR-369-3p:AUAAUAC▶ miR-655-3p:UAAUACA

 13430  


GGCCAGTCTT

ACAGTATTATGCCTGGGCCAGTCTT  
Depth:2 (PIG)  
Ei-value:0.000, Pi-value:0.000  
Er-value:0.000, Pr-value:0.000  
No matches to eCLIP DataMATCHES To TargetScan▶ miR-193-3p:ACUGGCC▶ miR-200bc-3p/429:AAUACUG▶ miR-328-3p:UGGCCCU▶ miR-369-3p:AUAAUAC▶ miR-655-3p:UAAUACA

----------------------------------------------------

TAAACTTC

TAAACTTC  
Depth:3 (COW)  
Ei-value:0.000, Pi-value:0.000  
Er-value:0.000, Pr-value:0.000  
eCLIP MATCHES▶NIPBL (bg=5.39%)▶NOLC1 (bg=9.43%)▶ZC3H11A (bg=6.55%)No matches to TargetScan

------------------

CTCCACTTGAGAG

CTCCACTTGAGAG  
Depth:3 (COW)  
Ei-value:0.000, Pi-value:0.000  
Er-value:0.000, Pr-value:0.000  
eCLIP MATCHES▶NIPBL (bg=5.39%)▶NOLC1 (bg=9.43%)▶ZC3H11A (bg=6.55%)MATCHES To TargetScan▶ miR-26-5p:UCAAGUA


A

CTCCACTTGAGAGA  
Depth:2 (PIG)  
Ei-value:0.000, Pi-value:0.000  
Er-value:0.000, Pr-value:0.000  
eCLIP MATCHES▶NIPBL (bg=5.39%)▶NOLC1 (bg=9.43%)▶ZC3H11A (bg=6.55%)MATCHES To TargetScan▶ miR-26-5p:UCAAGUA

------------------ 13550  
 -----------------------------------------------

ATAGGTGA

ATAGGTGA  
Depth:2 (PIG)  
Ei-value:0.000, Pi-value:0.000  
Er-value:0.000, Pr-value:0.010  
eCLIP MATCHES▶NOLC1 (bg=9.43%)▶ZC3H11A (bg=6.55%)No matches to TargetScan

-----

TATTTCAGT

TATTTCAGT  
Depth:4 (DOG)  
Ei-value:0.000, Pi-value:0.000  
Er-value:0.000, Pr-value:0.000  
eCLIP MATCHES▶NOLC1 (bg=9.43%)▶ZC3H11A (bg=6.55%)MATCHES To TargetScan▶ miR-203a-3p.2:UGAAAUG


CC

TATTTCAGTCC  
Depth:3 (COW)  
Ei-value:0.000, Pi-value:0.000  
Er-value:0.000, Pr-value:0.000  
eCLIP MATCHES▶NOLC1 (bg=9.43%)▶ZC3H11A (bg=6.55%)MATCHES To TargetScan▶ miR-203a-3p.2:UGAAAUG


T

TATTTCAGTCCT  
Depth:2 (PIG)  
Ei-value:0.000, Pi-value:0.000  
Er-value:0.000, Pr-value:0.000  
eCLIP MATCHES▶NOLC1 (bg=9.43%)▶ZC3H11A (bg=6.55%)MATCHES To TargetScan▶ miR-203a-3p.2:UGAAAUG

--------------------------

TGAGAAGA

TGAGAAGA  
Depth:2 (PIG)  
Ei-value:0.000, Pi-value:0.000  
Er-value:0.000, Pr-value:0.000  
eCLIP MATCHES▶LARP4 (bg=4.72%)▶NIPBL (bg=5.39%)▶NOLC1 (bg=9.43%)▶WDR43 (bg=3.37%)▶ZC3H11A (bg=6.55%)No matches to TargetScan

-------------- 13670  
 ----

GGGGAAA

GGGGAAA  
Depth:4 (DOG)  
Ei-value:0.000, Pi-value:0.000  
Er-value:0.000, Pr-value:0.000  
eCLIP MATCHES▶CPSF6 (bg=0.4%)▶LARP4 (bg=4.72%)▶WDR43 (bg=3.37%)▶ZC3H11A (bg=6.55%)No matches to TargetScan


AAA

GGGGAAAAAA  
Depth:2 (PIG)  
Ei-value:0.000, Pi-value:0.000  
Er-value:0.000, Pr-value:0.000  
eCLIP MATCHES▶CPSF6 (bg=0.4%)▶LARP4 (bg=4.72%)▶WDR43 (bg=3.37%)▶ZC3H11A (bg=6.55%)No matches to TargetScan

--

GTGCCAGGCT

GTGCCAGGCT  
Depth:2 (PIG)  
Ei-value:0.000, Pi-value:0.000  
Er-value:0.000, Pr-value:0.000  
eCLIP MATCHES▶CPSF6 (bg=0.4%)▶LARP4 (bg=4.72%)▶WDR43 (bg=3.37%)MATCHES To TargetScan▶ miR-183-5p.2:UGGCACU

-

TCTAGAGAAAA

TCTAGAGAAAA  
Depth:6 (MOUSE)  
Ei-value:0.000, Pi-value:0.000  
Er-value:0.000, Pr-value:0.000  
eCLIP MATCHES▶CPSF6 (bg=0.4%)▶LARP4 (bg=4.72%)▶UTP3 (bg=3.66%)▶WDR43 (bg=3.37%)MATCHES To TargetScan▶ miR-1251-5p:CUCUAGC

---

TGAAGAGATG

TGAAGAGATG  
Depth:5 (RABBIT)  
Ei-value:0.000, Pi-value:0.000  
Er-value:0.000, Pr-value:0.000  
eCLIP MATCHES▶CPSF6 (bg=0.4%)▶LARP4 (bg=4.72%)▶SRSF7 (bg=2.32%)▶UTP3 (bg=3.66%)▶WDR43 (bg=3.37%)No matches to TargetScan


CTCCA

TGAAGAGATGCTCCA  
Depth:3 (COW)  
Ei-value:0.000, Pi-value:0.000  
Er-value:0.000, Pr-value:0.000  
eCLIP MATCHES▶CPSF6 (bg=0.4%)▶LARP4 (bg=4.72%)▶SRSF7 (bg=2.32%)▶UTP3 (bg=3.66%)▶WDR43 (bg=3.37%)No matches to TargetScan


GGCCAA

GGCCAATGAGAAGAATTAGACA  
Depth:4 (DOG)  
Ei-value:0.000, Pi-value:0.000  
Er-value:0.000, Pr-value:0.000  
eCLIP MATCHES▶LARP4 (bg=4.72%)▶NOLC1 (bg=9.43%)▶SRSF7 (bg=2.32%)▶UTP3 (bg=3.66%)No matches to TargetScan


TGAGAAGAATTAGACA

TGAGAAGAATTAGACA  
Depth:6 (MOUSE)  
Ei-value:0.000, Pi-value:0.000  
Er-value:0.000, Pr-value:0.000  
eCLIP MATCHES▶LARP4 (bg=4.72%)▶NOLC1 (bg=9.43%)▶SRSF7 (bg=2.32%)No matches to TargetScan

-

GAAATACACAGATG

GAAATACACAGATG  
Depth:3 (COW)  
Ei-value:0.000, Pi-value:0.000  
Er-value:0.000, Pr-value:0.000  
eCLIP MATCHES▶LARP4 (bg=4.72%)▶NOLC1 (bg=9.43%)▶SRSF7 (bg=2.32%)No matches to TargetScan

----------

C

CTGAGAAG  
Depth:3 (COW)  
Ei-value:0.000, Pi-value:0.000  
Er-value:0.000, Pr-value:0.000  
eCLIP MATCHES▶AARS (bg=2.18%)▶NOLC1 (bg=9.43%)▶PUS1 (bg=1.04%)▶SRSF7 (bg=2.32%)▶ZC3H11A (bg=6.55%)No matches to TargetScan


TGAGAAG

TGAGAAG  
Depth:4 (DOG)  
Ei-value:0.000, Pi-value:0.000  
Er-value:0.000, Pr-value:0.010  
eCLIP MATCHES▶AARS (bg=2.18%)▶NOLC1 (bg=9.43%)▶PUS1 (bg=1.04%)▶SRSF7 (bg=2.32%)▶ZC3H11A (bg=6.55%)No matches to TargetScan


CA

CTGAGAAGCA  
Depth:2 (PIG)  
Ei-value:0.000, Pi-value:0.000  
Er-value:0.000, Pr-value:0.000  
eCLIP MATCHES▶AARS (bg=2.18%)▶NOLC1 (bg=9.43%)▶PUS1 (bg=1.04%)▶SRSF7 (bg=2.32%)▶ZC3H11A (bg=6.55%)No matches to TargetScan

---

GCCA

GCCAGCAACA  
Depth:3 (COW)  
Ei-value:0.000, Pi-value:0.000  
Er-value:0.000, Pr-value:0.000  
eCLIP MATCHES▶AARS (bg=2.18%)▶NOLC1 (bg=9.43%)▶PUS1 (bg=1.04%)▶SRSF7 (bg=2.32%)▶ZC3H11A (bg=6.55%)No matches to TargetScan

 13790  


GCCAGCAACA  
Depth:3 (COW)  
Ei-value:0.000, Pi-value:0.000  
Er-value:0.000, Pr-value:0.000  
eCLIP MATCHES▶AARS (bg=2.18%)▶NOLC1 (bg=9.43%)▶PUS1 (bg=1.04%)▶SRSF7 (bg=2.32%)▶ZC3H11A (bg=6.55%)No matches to TargetScan


GCAACA

GCAACA  
Depth:6 (MOUSE)  
Ei-value:0.000, Pi-value:0.000  
Er-value:0.000, Pr-value:0.000  
eCLIP MATCHES▶AARS (bg=2.18%)▶NOLC1 (bg=9.43%)▶PUS1 (bg=1.04%)▶ZC3H11A (bg=6.55%)No matches to TargetScan

--------

C

CTTTGAGCTTAGGTGAGCAGGATTC  
Depth:2 (PIG)  
Ei-value:0.000, Pi-value:0.000  
Er-value:0.000, Pr-value:0.000  
eCLIP MATCHES▶AARS (bg=2.18%)▶AKAP8L (bg=2.19%)▶NOLC1 (bg=9.43%)▶PUS1 (bg=1.04%)▶ZC3H11A (bg=6.55%)MATCHES To TargetScan▶ miR-371-5p:CUCAAAC


TTTGAGCTT

TTTGAGCTT  
Depth:3 (COW)  
Ei-value:0.000, Pi-value:0.000  
Er-value:0.000, Pr-value:0.000  
eCLIP MATCHES▶AARS (bg=2.18%)▶NOLC1 (bg=9.43%)▶PUS1 (bg=1.04%)▶ZC3H11A (bg=6.55%)MATCHES To TargetScan▶ miR-371-5p:CUCAAAC


A

CTTTGAGCTTAGGTGAGCAGGATTC  
Depth:2 (PIG)  
Ei-value:0.000, Pi-value:0.000  
Er-value:0.000, Pr-value:0.000  
eCLIP MATCHES▶AARS (bg=2.18%)▶AKAP8L (bg=2.19%)▶NOLC1 (bg=9.43%)▶PUS1 (bg=1.04%)▶ZC3H11A (bg=6.55%)MATCHES To TargetScan▶ miR-371-5p:CUCAAAC


GGTGAGC

GGTGAGC  
Depth:4 (DOG)  
Ei-value:0.000, Pi-value:0.000  
Er-value:0.000, Pr-value:0.000  
eCLIP MATCHES▶AARS (bg=2.18%)▶NOLC1 (bg=9.43%)▶PUS1 (bg=1.04%)▶ZC3H11A (bg=6.55%)No matches to TargetScan


AGGAT

GGTGAGCAGGAT  
Depth:3 (COW)  
Ei-value:0.000, Pi-value:0.000  
Er-value:0.000, Pr-value:0.000  
eCLIP MATCHES▶AARS (bg=2.18%)▶AKAP8L (bg=2.19%)▶NOLC1 (bg=9.43%)▶PUS1 (bg=1.04%)▶ZC3H11A (bg=6.55%)No matches to TargetScan


TC

CTTTGAGCTTAGGTGAGCAGGATTC  
Depth:2 (PIG)  
Ei-value:0.000, Pi-value:0.000  
Er-value:0.000, Pr-value:0.000  
eCLIP MATCHES▶AARS (bg=2.18%)▶AKAP8L (bg=2.19%)▶NOLC1 (bg=9.43%)▶PUS1 (bg=1.04%)▶ZC3H11A (bg=6.55%)MATCHES To TargetScan▶ miR-371-5p:CUCAAAC

---

GGTTTGGG

GGTTTGGG  
Depth:4 (DOG)  
Ei-value:0.000, Pi-value:0.000  
Er-value:0.000, Pr-value:0.000  
eCLIP MATCHES▶AARS (bg=2.18%)▶AKAP8L (bg=2.19%)▶NOLC1 (bg=9.43%)▶PUS1 (bg=1.04%)No matches to TargetScan

----

CTAGTGA

CTAGTGATGGTTATG  
Depth:2 (PIG)  
Ei-value:0.000, Pi-value:0.000  
Er-value:0.000, Pr-value:0.000  
eCLIP MATCHES▶AKAP8L (bg=2.19%)▶NOLC1 (bg=9.43%)▶PUS1 (bg=1.04%)▶SF3B1 (bg=2.48%)No matches to TargetScan


TGGTTA

TGGTTA  
Depth:5 (RABBIT)  
Ei-value:0.000, Pi-value:0.000  
Er-value:0.000, Pr-value:0.000  
eCLIP MATCHES▶AKAP8L (bg=2.19%)▶NOLC1 (bg=9.43%)▶PUS1 (bg=1.04%)▶SF3B1 (bg=2.48%)No matches to TargetScan


T

TGGTTAT  
Depth:4 (DOG)  
Ei-value:0.000, Pi-value:0.000  
Er-value:0.000, Pr-value:0.000  
eCLIP MATCHES▶AKAP8L (bg=2.19%)▶NOLC1 (bg=9.43%)▶PUS1 (bg=1.04%)▶SF3B1 (bg=2.48%)No matches to TargetScan


G

TGGTTATG  
Depth:3 (COW)  
Ei-value:0.000, Pi-value:0.000  
Er-value:0.000, Pr-value:0.000  
eCLIP MATCHES▶AKAP8L (bg=2.19%)▶NOLC1 (bg=9.43%)▶PUS1 (bg=1.04%)▶SF3B1 (bg=2.48%)No matches to TargetScan

----------------

CTGGGACA

CTGGGACA  
Depth:2 (PIG)  
Ei-value:0.000, Pi-value:0.000  
Er-value:0.000, Pr-value:0.000  
eCLIP MATCHES▶AKAP8L (bg=2.19%)▶NOLC1 (bg=9.43%)▶PUS1 (bg=1.04%)▶SF3B1 (bg=2.48%)No matches to TargetScan

----

GAGGT

GAGGTCCCAAGG  
Depth:2 (PIG)  
Ei-value:0.000, Pi-value:0.000  
Er-value:0.000, Pr-value:0.000  
eCLIP MATCHES▶AKAP8L (bg=2.19%)▶PUS1 (bg=1.04%)▶UTP3 (bg=3.66%)MATCHES To TargetScan▶ miR-212-5p:CCUUGGC


CCCAAGG

CCCAAGG  
Depth:4 (DOG)  
Ei-value:0.000, Pi-value:0.000  
Er-value:0.000, Pr-value:0.000  
eCLIP MATCHES▶PUS1 (bg=1.04%)▶UTP3 (bg=3.66%)MATCHES To TargetScan▶ miR-212-5p:CCUUGGC

----

AGCC

AGCCTGAACTCCCTGCTCATAGTAGTGGCC  
Depth:2 (PIG)  
Ei-value:0.000, Pi-value:0.000  
Er-value:0.000, Pr-value:0.000  
eCLIP MATCHES▶UTP3 (bg=3.66%)No matches to TargetScan


TGA

TGAACTCCCTGCT  
Depth:4 (DOG)  
Ei-value:0.000, Pi-value:0.000  
Er-value:0.000, Pr-value:0.000  
eCLIP MATCHES▶UTP3 (bg=3.66%)No matches to TargetScan

 13910  


ACTCCCTGCT

TGAACTCCCTGCT  
Depth:4 (DOG)  
Ei-value:0.000, Pi-value:0.000  
Er-value:0.000, Pr-value:0.000  
eCLIP MATCHES▶UTP3 (bg=3.66%)No matches to TargetScan


C

TGAACTCCCTGCTCATAGTAGTGGCC  
Depth:3 (COW)  
Ei-value:0.000, Pi-value:0.000  
Er-value:0.000, Pr-value:0.000  
eCLIP MATCHES▶UTP3 (bg=3.66%)No matches to TargetScan


ATAGTAGTGGCC

ATAGTAGTGGCC  
Depth:4 (DOG)  
Ei-value:0.000, Pi-value:0.000  
Er-value:0.000, Pr-value:0.000  
No matches to eCLIP DataNo matches to TargetScan

-

AATAATTTGG

AATAATTTGG  
Depth:2 (PIG)  
Ei-value:0.000, Pi-value:0.000  
Er-value:0.000, Pr-value:0.000  
No matches to eCLIP DataNo matches to TargetScan

--------------------------

TTTAATAC

TTTAATAC  
Depth:4 (DOG)  
Ei-value:0.000, Pi-value:0.000  
Er-value:0.000, Pr-value:0.000  
eCLIP MATCHES▶WRN (bg=0.77%)MATCHES To TargetScan▶ miR-496.2:GUAUUAC


CCA

TTTAATACCCA  
Depth:2 (PIG)  
Ei-value:0.000, Pi-value:0.000  
Er-value:0.000, Pr-value:0.000  
eCLIP MATCHES▶WRN (bg=0.77%)MATCHES To TargetScan▶ miR-496.2:GUAUUAC

-

CT

CTCTAGGCTTAAAG  
Depth:2 (PIG)  
Ei-value:0.000, Pi-value:0.000  
Er-value:0.000, Pr-value:0.000  
No matches to eCLIP DataNo matches to TargetScan


CT

CTAGGCTTAAAG  
Depth:4 (DOG)  
Ei-value:0.000, Pi-value:0.000  
Er-value:0.000, Pr-value:0.000  
No matches to eCLIP DataNo matches to TargetScan


AGGCTTA

AGGCTTA  
Depth:5 (RABBIT)  
Ei-value:0.000, Pi-value:0.000  
Er-value:0.000, Pr-value:0.000  
No matches to eCLIP DataNo matches to TargetScan


AAG

CTAGGCTTAAAG  
Depth:4 (DOG)  
Ei-value:0.000, Pi-value:0.000  
Er-value:0.000, Pr-value:0.000  
No matches to eCLIP DataNo matches to TargetScan

---------------------------

GTTTAAT

GTTTAAT  
Depth:5 (RABBIT)  
Ei-value:0.000, Pi-value:0.000  
Er-value:0.000, Pr-value:0.000  
No matches to eCLIP DataNo matches to TargetScan

 14030  


GTTTAAT  
Depth:5 (RABBIT)  
Ei-value:0.000, Pi-value:0.000  
Er-value:0.000, Pr-value:0.000  
No matches to eCLIP DataNo matches to TargetScan


ACTTTCCTT

GTTTAATACTTTCCTT  
Depth:2 (PIG)  
Ei-value:0.000, Pi-value:0.000  
Er-value:0.000, Pr-value:0.000  
No matches to eCLIP DataMATCHES To TargetScan▶ miR-496.2:GUAUUAC

-----------------------

GGGAAG

GGGAAG  
Depth:2 (PIG)  
Ei-value:0.000, Pi-value:0.020  
Er-value:0.000, Pr-value:0.020  
eCLIP MATCHES▶UTP3 (bg=3.66%)No matches to TargetScan

---

ATTTAAATGA

ATTTAAATGA  
Depth:2 (PIG)  
Ei-value:0.000, Pi-value:0.000  
Er-value:0.000, Pr-value:0.000  
eCLIP MATCHES▶TARDBP (bg=2.79%)▶UTP3 (bg=3.66%)No matches to TargetScan

----------------------

TGTAAAACA

TGTAAAACA  
Depth:3 (COW)  
Ei-value:0.000, Pi-value:0.000  
Er-value:0.000, Pr-value:0.000  
eCLIP MATCHES▶TARDBP (bg=2.79%)▶WDR43 (bg=3.37%)No matches to TargetScan

---------------------

T

TATTGGCA  
Depth:5 (RABBIT)  
Ei-value:0.000, Pi-value:0.000  
Er-value:0.000, Pr-value:0.000  
eCLIP MATCHES▶HNRNPA1 (bg=2.57%)No matches to TargetScan


ATTGGCA

ATTGGCA  
Depth:6 (MOUSE)  
Ei-value:0.000, Pi-value:0.000  
Er-value:0.000, Pr-value:0.000  
eCLIP MATCHES▶HNRNPA1 (bg=2.57%)No matches to TargetScan

--------- 14150  
 -------------------------

TTGTGAAG

TTGTGAAG  
Depth:6 (MOUSE)  
Ei-value:0.000, Pi-value:0.000  
Er-value:0.000, Pr-value:0.000  
eCLIP MATCHES▶HNRNPA1 (bg=2.57%)No matches to TargetScan

---

T

TATGTAAATCA  
Depth:3 (COW)  
Ei-value:0.000, Pi-value:0.000  
Er-value:0.000, Pr-value:0.000  
No matches to eCLIP DataNo matches to TargetScan


ATGTAAAT

ATGTAAAT  
Depth:5 (RABBIT)  
Ei-value:0.000, Pi-value:0.000  
Er-value:0.000, Pr-value:0.000  
No matches to eCLIP DataNo matches to TargetScan


CA

TATGTAAATCA  
Depth:3 (COW)  
Ei-value:0.000, Pi-value:0.000  
Er-value:0.000, Pr-value:0.000  
No matches to eCLIP DataNo matches to TargetScan


GGGGTC

TATGTAAATCAGGGGTC  
Depth:2 (PIG)  
Ei-value:0.000, Pi-value:0.000  
Er-value:0.000, Pr-value:0.000  
No matches to eCLIP DataMATCHES To TargetScan▶ miR-125-5p:CCCUGAG▶ miR-331-3p:CCCCUGG

-------

TTTCTGTAA

TTTCTGTAA  
Depth:2 (PIG)  
Ei-value:0.000, Pi-value:0.000  
Er-value:0.000, Pr-value:0.000  
No matches to eCLIP DataNo matches to TargetScan

-----------------------------

GG

GGGCCATATGGTTTC  
Depth:2 (PIG)  
Ei-value:0.000, Pi-value:0.000  
Er-value:0.000, Pr-value:0.000  
No matches to eCLIP DataMATCHES To TargetScan▶ miR-328-3p:UGGCCCU


GCCATATGGT

GCCATATGGT  
Depth:3 (COW)  
Ei-value:0.000, Pi-value:0.000  
Er-value:0.000, Pr-value:0.000  
No matches to eCLIP DataNo matches to TargetScan


TTC

GGGCCATATGGTTTC  
Depth:2 (PIG)  
Ei-value:0.000, Pi-value:0.000  
Er-value:0.000, Pr-value:0.000  
No matches to eCLIP DataMATCHES To TargetScan▶ miR-328-3p:UGGCCCU

------- 14270  
 -------------------------------------------------------------------------------------------

CCCCTGATGTA

CCCCTGATGTA  
Depth:2 (PIG)  
Ei-value:0.000, Pi-value:0.000  
Er-value:0.000, Pr-value:0.000  
No matches to eCLIP DataNo matches to TargetScan

------------------ 14390  
 ----

TTCTGAA

TTCTGAA  
Depth:2 (PIG)  
Ei-value:0.000, Pi-value:0.020  
Er-value:0.000, Pr-value:0.020  
No matches to eCLIP DataNo matches to TargetScan

-----------------

TC

TCTGTGCCTGTCCCTGT  
Depth:2 (PIG)  
Ei-value:0.000, Pi-value:0.000  
Er-value:0.000, Pr-value:0.000  
No matches to eCLIP DataNo matches to TargetScan


TGTGC

TGTGCCTGTCCCTGT  
Depth:3 (COW)  
Ei-value:0.000, Pi-value:0.000  
Er-value:0.000, Pr-value:0.000  
No matches to eCLIP DataNo matches to TargetScan


CTGTCCCT

CTGTCCCT  
Depth:4 (DOG)  
Ei-value:0.000, Pi-value:0.000  
Er-value:0.000, Pr-value:0.000  
No matches to eCLIP DataNo matches to TargetScan


GT

TGTGCCTGTCCCTGT  
Depth:3 (COW)  
Ei-value:0.000, Pi-value:0.000  
Er-value:0.000, Pr-value:0.000  
No matches to eCLIP DataNo matches to TargetScan

--

TAGGCACT

TAGGCACT  
Depth:4 (DOG)  
Ei-value:0.000, Pi-value:0.000  
Er-value:0.000, Pr-value:0.000  
No matches to eCLIP DataNo matches to TargetScan


AA

TAGGCACTAA  
Depth:2 (PIG)  
Ei-value:0.000, Pi-value:0.000  
Er-value:0.000, Pr-value:0.000  
No matches to eCLIP DataNo matches to TargetScan

------

AATGATTA

AATGATTA  
Depth:2 (PIG)  
Ei-value:0.000, Pi-value:0.000  
Er-value:0.000, Pr-value:0.000  
No matches to eCLIP DataMATCHES To TargetScan▶ miR-382-3p:AUCAUUC

---

ATATCTAGGTGA

ATATCTAGGTGA  
Depth:2 (PIG)  
Ei-value:0.000, Pi-value:0.000  
Er-value:0.000, Pr-value:0.000  
eCLIP MATCHES▶HNRNPU (bg=5.92%)No matches to TargetScan

-----------------

AATGTGCTTTGTAAACT

AATGTGCTTTGTAAACT  
Depth:2 (PIG)  
Ei-value:0.000, Pi-value:0.000  
Er-value:0.000, Pr-value:0.000  
eCLIP MATCHES▶HNRNPU (bg=5.92%)MATCHES To TargetScan▶ miR-330-3p:CAAAGCA▶ miR-330-3p.2:AAAGCAC

 14510  


AATGTGCTTTGTAAACT  
Depth:2 (PIG)  
Ei-value:0.000, Pi-value:0.000  
Er-value:0.000, Pr-value:0.000  
eCLIP MATCHES▶HNRNPU (bg=5.92%)MATCHES To TargetScan▶ miR-330-3p:CAAAGCA▶ miR-330-3p.2:AAAGCAC

-

TAAAGCA

TAAAGCA  
Depth:4 (DOG)  
Ei-value:0.000, Pi-value:0.000  
Er-value:0.000, Pr-value:0.000  
eCLIP MATCHES▶LIN28B (bg=0.74%)No matches to TargetScan


CTT

TAAAGCACTT  
Depth:2 (PIG)  
Ei-value:0.000, Pi-value:0.000  
Er-value:0.000, Pr-value:0.000  
eCLIP MATCHES▶LIN28B (bg=0.74%)MATCHES To TargetScan▶ miR-302-3p/372-3p/373-3p/520-3p:AAGUGCU▶ miR-302c-3p.2/520-3p:AGUGCUU

---------------------

TGTGGATACAAA

TGTGGATACAAA  
Depth:2 (PIG)  
Ei-value:0.000, Pi-value:0.000  
Er-value:0.000, Pr-value:0.000  
eCLIP MATCHES▶LIN28B (bg=0.74%)▶UTP3 (bg=3.66%)No matches to TargetScan

---------------------------------------------------------------------------- 14630  
 ------------------------------------------------------------------------------------------------------------------------ 14750  
 ------------------------------------------

TAT

TATAATGTGCCAGATA  
Depth:3 (COW)  
Ei-value:0.000, Pi-value:0.000  
Er-value:0.000, Pr-value:0.000  
No matches to eCLIP DataMATCHES To TargetScan▶ miR-183-5p.2:UGGCACU▶ miR-323-3p:ACAUUAC


AATGTGCCAGATA

AATGTGCCAGATA  
Depth:4 (DOG)  
Ei-value:0.000, Pi-value:0.000  
Er-value:0.000, Pr-value:0.000  
No matches to eCLIP DataMATCHES To TargetScan▶ miR-183-5p.2:UGGCACU

--------------------------------------

TTCTCAT

TTCTCAT  
Depth:2 (PIG)  
Ei-value:0.000, Pi-value:0.000  
Er-value:0.000, Pr-value:0.010  
No matches to eCLIP DataNo matches to TargetScan

----------------- 14870  
 -------

CATA

CATATTAAAGTGCTTTGTA  
Depth:2 (PIG)  
Ei-value:0.000, Pi-value:0.000  
Er-value:0.000, Pr-value:0.000  
eCLIP MATCHES▶SF3B1 (bg=2.48%)MATCHES To TargetScan▶ miR-330-3p:CAAAGCA▶ miR-330-3p.2:AAAGCAC


TTAAAGTG

TTAAAGTG  
Depth:4 (DOG)  
Ei-value:0.000, Pi-value:0.000  
Er-value:0.000, Pr-value:0.000  
eCLIP MATCHES▶SF3B1 (bg=2.48%)No matches to TargetScan


CTTTGTA

TTAAAGTGCTTTGTA  
Depth:3 (COW)  
Ei-value:0.000, Pi-value:0.000  
Er-value:0.000, Pr-value:0.000  
eCLIP MATCHES▶SF3B1 (bg=2.48%)MATCHES To TargetScan▶ miR-330-3p:CAAAGCA▶ miR-330-3p.2:AAAGCAC


AA

AACTAAAGCA  
Depth:2 (PIG)  
Ei-value:0.000, Pi-value:0.000  
Er-value:0.000, Pr-value:0.000  
eCLIP MATCHES▶SF3B1 (bg=2.48%)No matches to TargetScan


CTAAAGCA

CTAAAGCA  
Depth:4 (DOG)  
Ei-value:0.000, Pi-value:0.000  
Er-value:0.000, Pr-value:0.000  
eCLIP MATCHES▶SF3B1 (bg=2.48%)No matches to TargetScan

------------

CAATGGGCTA

CAATGGGCTA  
Depth:3 (COW)  
Ei-value:0.000, Pi-value:0.000  
Er-value:0.000, Pr-value:0.000  
No matches to eCLIP DataNo matches to TargetScan

-------------------------

GA

GAATGAATA  
Depth:3 (COW)  
Ei-value:0.000, Pi-value:0.000  
Er-value:0.000, Pr-value:0.000  
eCLIP MATCHES▶DROSHA (bg=2.49%)▶TARDBP (bg=2.79%)▶ZC3H11A (bg=6.55%)MATCHES To TargetScan▶ miR-1298-5p:UCAUUCG


ATGAATA

ATGAATA  
Depth:4 (DOG)  
Ei-value:0.000, Pi-value:0.000  
Er-value:0.000, Pr-value:0.000  
eCLIP MATCHES▶DROSHA (bg=2.49%)▶TARDBP (bg=2.79%)▶ZC3H11A (bg=6.55%)No matches to TargetScan

------------------

CCAGCTATT

CCAGCTATT  
Depth:3 (COW)  
Ei-value:0.000, Pi-value:0.000  
Er-value:0.000, Pr-value:0.000  
eCLIP MATCHES▶AARS (bg=2.18%)▶DROSHA (bg=2.49%)▶ILF3 (bg=3.0%)▶TARDBP (bg=2.79%)▶ZC3H11A (bg=6.55%)No matches to TargetScan

- 14990  
 --

GGTACTGT

GGTACTGT  
Depth:4 (DOG)  
Ei-value:0.000, Pi-value:0.000  
Er-value:0.000, Pr-value:0.000  
eCLIP MATCHES▶AARS (bg=2.18%)▶DROSHA (bg=2.49%)▶ILF3 (bg=3.0%)▶TARDBP (bg=2.79%)▶ZC3H11A (bg=6.55%)MATCHES To TargetScan▶ miR-101-3p.1:ACAGUAC▶ miR-144-3p:ACAGUAU

------------------------

GTA

GTAATAAGAGG  
Depth:2 (PIG)  
Ei-value:0.000, Pi-value:0.000  
Er-value:0.000, Pr-value:0.000  
eCLIP MATCHES▶ILF3 (bg=3.0%)No matches to TargetScan


ATAAGAGG

ATAAGAGG  
Depth:4 (DOG)  
Ei-value:0.000, Pi-value:0.000  
Er-value:0.000, Pr-value:0.000  
eCLIP MATCHES▶ILF3 (bg=3.0%)No matches to TargetScan

---------------------------------------

ATATTGCTTA

ATATTGCTTA  
Depth:2 (PIG)  
Ei-value:0.000, Pi-value:0.000  
Er-value:0.000, Pr-value:0.000  
No matches to eCLIP DataNo matches to TargetScan

--------

CAATC

CAATCAAGACTTTAC  
Depth:2 (PIG)  
Ei-value:0.000, Pi-value:0.000  
Er-value:0.000, Pr-value:0.000  
No matches to eCLIP DataMATCHES To TargetScan▶ miR-431-5p:GUCUUGC


AAGACTTTAC

AAGACTTTAC  
Depth:3 (COW)  
Ei-value:0.000, Pi-value:0.000  
Er-value:0.000, Pr-value:0.000  
No matches to eCLIP DataNo matches to TargetScan

--

G

GTGAGGT  
Depth:2 (PIG)  
Ei-value:0.000, Pi-value:0.000  
Er-value:0.000, Pr-value:0.000  
No matches to eCLIP DataNo matches to TargetScan

 15110  


TGAGGT

GTGAGGT  
Depth:2 (PIG)  
Ei-value:0.000, Pi-value:0.000  
Er-value:0.000, Pr-value:0.000  
No matches to eCLIP DataNo matches to TargetScan

----

T

TTAAATTATTAC  
Depth:2 (PIG)  
Ei-value:0.000, Pi-value:0.000  
Er-value:0.000, Pr-value:0.000  
No matches to eCLIP DataNo matches to TargetScan


TAAATTAT

TAAATTAT  
Depth:4 (DOG)  
Ei-value:0.000, Pi-value:0.010  
Er-value:0.000, Pr-value:0.000  
No matches to eCLIP DataNo matches to TargetScan


TAC

TAAATTATTAC  
Depth:3 (COW)  
Ei-value:0.000, Pi-value:0.000  
Er-value:0.000, Pr-value:0.000  
No matches to eCLIP DataNo matches to TargetScan

------------

CC

CCAGGTAAC  
Depth:2 (PIG)  
Ei-value:0.000, Pi-value:0.000  
Er-value:0.000, Pr-value:0.000  
No matches to eCLIP DataNo matches to TargetScan


AGGTAA

AGGTAA  
Depth:3 (COW)  
Ei-value:0.000, Pi-value:0.000  
Er-value:0.000, Pr-value:0.000  
No matches to eCLIP DataNo matches to TargetScan


C

CCAGGTAAC  
Depth:2 (PIG)  
Ei-value:0.000, Pi-value:0.000  
Er-value:0.000, Pr-value:0.000  
No matches to eCLIP DataNo matches to TargetScan

----------------------------------------------------------------------------- 15230  
 -----

TTTCTAA

TTTCTAA  
Depth:3 (COW)  
Ei-value:0.000, Pi-value:0.000  
Er-value:0.000, Pr-value:0.010  
No matches to eCLIP DataNo matches to TargetScan


A

TTTCTAAA  
Depth:2 (PIG)  
Ei-value:0.000, Pi-value:0.000  
Er-value:0.000, Pr-value:0.000  
No matches to eCLIP DataNo matches to TargetScan

-----------------------------------------

TGA

TGAATAAAACTT  
Depth:2 (PIG)  
Ei-value:0.000, Pi-value:0.000  
Er-value:0.000, Pr-value:0.000  
No matches to eCLIP DataNo matches to TargetScan


ATAAAAC

ATAAAAC  
Depth:4 (DOG)  
Ei-value:0.000, Pi-value:0.010  
Er-value:0.000, Pr-value:0.000  
No matches to eCLIP DataNo matches to TargetScan


TT

TGAATAAAACTT  
Depth:2 (PIG)  
Ei-value:0.000, Pi-value:0.000  
Er-value:0.000, Pr-value:0.000  
No matches to eCLIP DataNo matches to TargetScan

------------------

TCTCTAC

TCTCTACAAAATTCTCATTGT  
Depth:2 (PIG)  
Ei-value:0.000, Pi-value:0.000  
Er-value:0.000, Pr-value:0.000  
eCLIP MATCHES▶HNRNPU (bg=5.92%)No matches to TargetScan


AAAATTCTCA

AAAATTCTCA  
Depth:4 (DOG)  
Ei-value:0.000, Pi-value:0.000  
Er-value:0.000, Pr-value:0.000  
eCLIP MATCHES▶HNRNPU (bg=5.92%)No matches to TargetScan


TTGT

TCTCTACAAAATTCTCATTGT  
Depth:2 (PIG)  
Ei-value:0.000, Pi-value:0.000  
Er-value:0.000, Pr-value:0.000  
eCLIP MATCHES▶HNRNPU (bg=5.92%)No matches to TargetScan

--------------- 15350  
 ---------------------

T

TTATACAAAC  
Depth:2 (PIG)  
Ei-value:0.000, Pi-value:0.000  
Er-value:0.000, Pr-value:0.000  
No matches to eCLIP DataNo matches to TargetScan


TATACAAAC

TATACAAAC  
Depth:4 (DOG)  
Ei-value:0.000, Pi-value:0.000  
Er-value:0.000, Pr-value:0.000  
No matches to eCLIP DataNo matches to TargetScan

-----

GTTTAAATAC

GTTTAAATAC  
Depth:3 (COW)  
Ei-value:0.000, Pi-value:0.000  
Er-value:0.000, Pr-value:0.000  
No matches to eCLIP DataNo matches to TargetScan

---------------------

A

ATTGCCTACTATGTGAACTCACTGTTA  
Depth:2 (PIG)  
Ei-value:0.000, Pi-value:0.000  
Er-value:0.000, Pr-value:0.000  
No matches to eCLIP DataMATCHES To TargetScan▶ miR-132-3p/212-3p:AACAGUC▶ miR-23-3p:UCACAUU▶ miR-376c-3p:ACAUAGA▶ miR-411-5p.2:UAGUAGA


TTGCCTACTAT

TTGCCTACTATGTGAACTCACTGTTA  
Depth:3 (COW)  
Ei-value:0.000, Pi-value:0.000  
Er-value:0.000, Pr-value:0.000  
No matches to eCLIP DataMATCHES To TargetScan▶ miR-132-3p/212-3p:AACAGUC▶ miR-23-3p:UCACAUU▶ miR-376c-3p:ACAUAGA▶ miR-411-5p.2:UAGUAGA


GTGAACTCA

GTGAACTCA  
Depth:4 (DOG)  
Ei-value:0.000, Pi-value:0.000  
Er-value:0.000, Pr-value:0.000  
No matches to eCLIP DataNo matches to TargetScan


CTGTTA

TTGCCTACTATGTGAACTCACTGTTA  
Depth:3 (COW)  
Ei-value:0.000, Pi-value:0.000  
Er-value:0.000, Pr-value:0.000  
No matches to eCLIP DataMATCHES To TargetScan▶ miR-132-3p/212-3p:AACAGUC▶ miR-23-3p:UCACAUU▶ miR-376c-3p:ACAUAGA▶ miR-411-5p.2:UAGUAGA

------------

ATTTATCAT

ATTTATCAT  
Depth:3 (COW)  
Ei-value:0.000, Pi-value:0.000  
Er-value:0.000, Pr-value:0.000  
No matches to eCLIP DataNo matches to TargetScan

----- 15470  
 -------------------------------

TATGTTAGC

TATGTTAGCATTTTGTGAACTCTAA  
Depth:2 (PIG)  
Ei-value:0.000, Pi-value:0.000  
Er-value:0.000, Pr-value:0.000  
No matches to eCLIP DataNo matches to TargetScan


ATTTTGTGAACTCTAA

ATTTTGTGAACTCTAA  
Depth:3 (COW)  
Ei-value:0.000, Pi-value:0.000  
Er-value:0.000, Pr-value:0.000  
No matches to eCLIP DataNo matches to TargetScan

-

GCACCAT

GCACCAT  
Depth:2 (PIG)  
Ei-value:0.000, Pi-value:0.000  
Er-value:0.000, Pr-value:0.000  
No matches to eCLIP DataNo matches to TargetScan

------------------------------------

GTACTAG

GTACTAG  
Depth:2 (PIG)  
Ei-value:0.000, Pi-value:0.000  
Er-value:0.000, Pr-value:0.000  
No matches to eCLIP DataNo matches to TargetScan

------

AAAATTG

AAAATTG  
Depth:3 (COW)  
Ei-value:0.000, Pi-value:0.000  
Er-value:0.000, Pr-value:0.000  
No matches to eCLIP DataNo matches to TargetScan

 15590  


AAAATTG  
Depth:3 (COW)  
Ei-value:0.000, Pi-value:0.000  
Er-value:0.000, Pr-value:0.000  
No matches to eCLIP DataNo matches to TargetScan

----

ATAGTTAT

ATAGTTAT  
Depth:2 (PIG)  
Ei-value:0.000, Pi-value:0.000  
Er-value:0.000, Pr-value:0.000  
No matches to eCLIP DataNo matches to TargetScan

--------------------------

TTTGAATA

TTTGAATA  
Depth:2 (PIG)  
Ei-value:0.000, Pi-value:0.000  
Er-value:0.000, Pr-value:0.010  
eCLIP MATCHES▶HNRNPUL1 (bg=1.16%)No matches to TargetScan

-------------

TTTGAATA

TTTGAATA  
Depth:2 (PIG)  
Ei-value:0.000, Pi-value:0.000  
Er-value:0.000, Pr-value:0.010  
eCLIP MATCHES▶HNRNPUL1 (bg=1.16%)No matches to TargetScan

-----------------------------

AATGCC

AATGCC  
Depth:2 (PIG)  
Ei-value:0.000, Pi-value:0.000  
Er-value:0.000, Pr-value:0.000  
eCLIP MATCHES▶SAFB (bg=2.69%)No matches to TargetScan

------------------ 15710  
 --

TGTGCCA

TGTGCCA  
Depth:4 (DOG)  
Ei-value:0.000, Pi-value:0.000  
Er-value:0.000, Pr-value:0.000  
No matches to eCLIP DataMATCHES To TargetScan▶ miR-183-5p.2:UGGCACU

---------------------------

AAGATAA

AAGATAA  
Depth:4 (DOG)  
Ei-value:0.000, Pi-value:0.000  
Er-value:0.000, Pr-value:0.000  
No matches to eCLIP DataNo matches to TargetScan

------

TTTATTGTGT

TTTATTGTGT  
Depth:2 (PIG)  
Ei-value:0.000, Pi-value:0.000  
Er-value:0.000, Pr-value:0.000  
eCLIP MATCHES▶UTP3 (bg=3.66%)No matches to TargetScan

----

GGT

GGTAGCAGAA  
Depth:2 (PIG)  
Ei-value:0.000, Pi-value:0.000  
Er-value:0.000, Pr-value:0.000  
eCLIP MATCHES▶UTP3 (bg=3.66%)No matches to TargetScan


AGCAGAA

AGCAGAA  
Depth:3 (COW)  
Ei-value:0.000, Pi-value:0.000  
Er-value:0.000, Pr-value:0.000  
eCLIP MATCHES▶UTP3 (bg=3.66%)No matches to TargetScan

------

ATGTG

ATGTGTAAAATCAATTT  
Depth:2 (PIG)  
Ei-value:0.000, Pi-value:0.000  
Er-value:0.000, Pr-value:0.000  
eCLIP MATCHES▶UTP3 (bg=3.66%)No matches to TargetScan


TAAAATCAATTT

TAAAATCAATTT  
Depth:3 (COW)  
Ei-value:0.000, Pi-value:0.000  
Er-value:0.000, Pr-value:0.000  
eCLIP MATCHES▶UTP3 (bg=3.66%)No matches to TargetScan

--------

TAAACTG

TAAACTG  
Depth:4 (DOG)  
Ei-value:0.000, Pi-value:0.000  
Er-value:0.000, Pr-value:0.000  
eCLIP MATCHES▶HNRNPU (bg=5.92%)No matches to TargetScan

--------- 15830  
 -----------

TCTGCTGAATGA

TCTGCTGAATGA  
Depth:3 (COW)  
Ei-value:0.000, Pi-value:0.000  
Er-value:0.000, Pr-value:0.000  
No matches to eCLIP DataMATCHES To TargetScan▶ miR-1298-5p:UCAUUCG

-

C

CATTGATTA  
Depth:3 (COW)  
Ei-value:0.000, Pi-value:0.000  
Er-value:0.000, Pr-value:0.000  
No matches to eCLIP DataNo matches to TargetScan


ATTGATTA

ATTGATTA  
Depth:4 (DOG)  
Ei-value:0.000, Pi-value:0.000  
Er-value:0.000, Pr-value:0.010  
No matches to eCLIP DataNo matches to TargetScan

-

TCTTATCC

TCTTATCC  
Depth:2 (PIG)  
Ei-value:0.000, Pi-value:0.000  
Er-value:0.000, Pr-value:0.000  
No matches to eCLIP DataNo matches to TargetScan

--

AGAGATA

AGAGATA  
Depth:4 (DOG)  
Ei-value:0.000, Pi-value:0.000  
Er-value:0.000, Pr-value:0.000  
No matches to eCLIP DataNo matches to TargetScan

---------------------------------

TGAACCT

TGAACCT  
Depth:3 (COW)  
Ei-value:0.000, Pi-value:0.000  
Er-value:0.000, Pr-value:0.010  
eCLIP MATCHES▶HNRNPU (bg=5.92%)No matches to TargetScan

-

C

CAACAGAGATCT  
Depth:2 (PIG)  
Ei-value:0.000, Pi-value:0.000  
Er-value:0.000, Pr-value:0.000  
eCLIP MATCHES▶HNRNPA1 (bg=2.57%)▶HNRNPU (bg=5.92%)No matches to TargetScan


AA

AACAGAGATCT  
Depth:3 (COW)  
Ei-value:0.000, Pi-value:0.000  
Er-value:0.000, Pr-value:0.000  
eCLIP MATCHES▶HNRNPA1 (bg=2.57%)▶HNRNPU (bg=5.92%)No matches to TargetScan


CAGAGATCT

CAGAGATCT  
Depth:4 (DOG)  
Ei-value:0.000, Pi-value:0.000  
Er-value:0.000, Pr-value:0.000  
eCLIP MATCHES▶HNRNPA1 (bg=2.57%)▶HNRNPU (bg=5.92%)No matches to TargetScan

--------

A

ATTTACAAAGCCTA  
Depth:2 (PIG)  
Ei-value:0.000, Pi-value:0.000  
Er-value:0.000, Pr-value:0.000  
eCLIP MATCHES▶HNRNPA1 (bg=2.57%)▶HNRNPU (bg=5.92%)No matches to TargetScan


TTTACAA

TTTACAAAGC  
Depth:3 (COW)  
Ei-value:0.000, Pi-value:0.000  
Er-value:0.000, Pr-value:0.000  
eCLIP MATCHES▶HNRNPA1 (bg=2.57%)▶HNRNPU (bg=5.92%)No matches to TargetScan

 15950  


AGC

TTTACAAAGC  
Depth:3 (COW)  
Ei-value:0.000, Pi-value:0.000  
Er-value:0.000, Pr-value:0.000  
eCLIP MATCHES▶HNRNPA1 (bg=2.57%)▶HNRNPU (bg=5.92%)No matches to TargetScan


CTA

ATTTACAAAGCCTA  
Depth:2 (PIG)  
Ei-value:0.000, Pi-value:0.000  
Er-value:0.000, Pr-value:0.000  
eCLIP MATCHES▶HNRNPA1 (bg=2.57%)▶HNRNPU (bg=5.92%)No matches to TargetScan

----

TCTATACA

TCTATACA  
Depth:3 (COW)  
Ei-value:0.000, Pi-value:0.000  
Er-value:0.000, Pr-value:0.000  
eCLIP MATCHES▶HNRNPA1 (bg=2.57%)▶HNRNPU (bg=5.92%)No matches to TargetScan

--

TAGGAAT

TAGGAAT  
Depth:2 (PIG)  
Ei-value:0.000, Pi-value:0.000  
Er-value:0.000, Pr-value:0.000  
eCLIP MATCHES▶HNRNPA1 (bg=2.57%)No matches to TargetScan

------

TTGGCT

TTGGCT  
Depth:4 (DOG)  
Ei-value:0.000, Pi-value:0.000  
Er-value:0.000, Pr-value:0.000  
eCLIP MATCHES▶HNRNPA1 (bg=2.57%)No matches to TargetScan

-------------

TTACTTTCT

TTACTTTCT  
Depth:4 (DOG)  
Ei-value:0.000, Pi-value:0.000  
Er-value:0.000, Pr-value:0.010  
eCLIP MATCHES▶UTP3 (bg=3.66%)No matches to TargetScan

---------------

ACATGC

ACATGC  
Depth:2 (PIG)  
Ei-value:0.000, Pi-value:0.010  
Er-value:0.000, Pr-value:0.020  
No matches to eCLIP DataNo matches to TargetScan

-

CTAGGATAT

CTAGGATAT  
Depth:3 (COW)  
Ei-value:0.000, Pi-value:0.000  
Er-value:0.000, Pr-value:0.000  
No matches to eCLIP DataNo matches to TargetScan

-

AAAATGA

AAAATGA  
Depth:3 (COW)  
Ei-value:0.000, Pi-value:0.000  
Er-value:0.000, Pr-value:0.000  
No matches to eCLIP DataNo matches to TargetScan


T

AAAATGAT  
Depth:2 (PIG)  
Ei-value:0.000, Pi-value:0.010  
Er-value:0.000, Pr-value:0.000  
No matches to eCLIP DataMATCHES To TargetScan▶ miR-382-3p:AUCAUUC

------------------- 16070  
 ------

AGGAAATGA

AGGAAATGA  
Depth:2 (PIG)  
Ei-value:0.000, Pi-value:0.000  
Er-value:0.000, Pr-value:0.000  
eCLIP MATCHES▶KHDRBS1 (bg=1.71%)No matches to TargetScan

------

ATAGGTGTG

ATAGGTGTG  
Depth:2 (PIG)  
Ei-value:0.000, Pi-value:0.000  
Er-value:0.000, Pr-value:0.000  
eCLIP MATCHES▶KHDRBS1 (bg=1.71%)No matches to TargetScan

--

ATCCAGACCA

ATCCAGACCA  
Depth:3 (COW)  
Ei-value:0.000, Pi-value:0.000  
Er-value:0.000, Pr-value:0.000  
eCLIP MATCHES▶KHDRBS1 (bg=1.71%)No matches to TargetScan

------------------------------------------------------------------------------ 16190  
 ---------------------------------------

GCCTGATTGA

GCCTGATTGA  
Depth:2 (PIG)  
Ei-value:0.000, Pi-value:0.000  
Er-value:0.000, Pr-value:0.000  
eCLIP MATCHES▶HNRNPA1 (bg=2.57%)No matches to TargetScan

--

AGATGGA

AGATGGA  
Depth:3 (COW)  
Ei-value:0.000, Pi-value:0.000  
Er-value:0.000, Pr-value:0.000  
No matches to eCLIP DataNo matches to TargetScan

-------------------------------------------------------------- 16310  
 -------------------------------------

TAAAG

TAAAGGAGTAAAAAT  
Depth:2 (PIG)  
Ei-value:0.000, Pi-value:0.000  
Er-value:0.000, Pr-value:0.000  
No matches to eCLIP DataMATCHES To TargetScan▶ miR-483-3p.1:ACUCCUC


GAGTAAAAA

GAGTAAAAA  
Depth:4 (DOG)  
Ei-value:0.000, Pi-value:0.000  
Er-value:0.000, Pr-value:0.000  
No matches to eCLIP DataNo matches to TargetScan


T

TAAAGGAGTAAAAAT  
Depth:2 (PIG)  
Ei-value:0.000, Pi-value:0.000  
Er-value:0.000, Pr-value:0.000  
No matches to eCLIP DataMATCHES To TargetScan▶ miR-483-3p.1:ACUCCUC

---------------------------

ATTTGAT

ATTTGAT  
Depth:4 (DOG)  
Ei-value:0.000, Pi-value:0.010  
Er-value:0.000, Pr-value:0.000  
No matches to eCLIP DataNo matches to TargetScan


AAACA

ATTTGATAAACA  
Depth:2 (PIG)  
Ei-value:0.000, Pi-value:0.000  
Er-value:0.000, Pr-value:0.000  
No matches to eCLIP DataNo matches to TargetScan

----

ATC

ATCTTTTATGT  
Depth:3 (COW)  
Ei-value:0.000, Pi-value:0.000  
Er-value:0.000, Pr-value:0.000  
eCLIP MATCHES▶SAFB (bg=2.69%)No matches to TargetScan


TTTTATGT

TTTTATGT  
Depth:4 (DOG)  
Ei-value:0.000, Pi-value:0.000  
Er-value:0.000, Pr-value:0.000  
eCLIP MATCHES▶SAFB (bg=2.69%)No matches to TargetScan


GGAATA

ATCTTTTATGTGGAATA  
Depth:2 (PIG)  
Ei-value:0.000, Pi-value:0.000  
Er-value:0.000, Pr-value:0.000  
eCLIP MATCHES▶SAFB (bg=2.69%)No matches to TargetScan

-------- 16430  
 --

GGTCCTGAG

GGTCCTGAG  
Depth:3 (COW)  
Ei-value:0.000, Pi-value:0.000  
Er-value:0.000, Pr-value:0.000  
eCLIP MATCHES▶SAFB (bg=2.69%)No matches to TargetScan

-------------

AGGGCATTAG

AGGGCATTAG  
Depth:2 (PIG)  
Ei-value:0.000, Pi-value:0.000  
Er-value:0.000, Pr-value:0.000  
eCLIP MATCHES▶SAFB (bg=2.69%)MATCHES To TargetScan▶ miR-155-5p:UAAUGCU▶ miR-365-3p:AAUGCCC▶ miR-874-3p:UGCCCUG

----------

GCTGAA

GCTGAA  
Depth:2 (PIG)  
Ei-value:0.000, Pi-value:0.010  
Er-value:0.000, Pr-value:0.000  
No matches to eCLIP DataNo matches to TargetScan

-------------------

TTTGATT

TTTGATT  
Depth:2 (PIG)  
Ei-value:0.000, Pi-value:0.020  
Er-value:0.000, Pr-value:0.010  
No matches to eCLIP DataNo matches to TargetScan

-------------------------------------------- 16550  
 ----------------------------------------

TTGCCTT

TTGCCTT  
Depth:3 (COW)  
Ei-value:0.000, Pi-value:0.000  
Er-value:0.000, Pr-value:0.000  
eCLIP MATCHES▶KHDRBS1 (bg=1.71%)▶UTP18 (bg=0.72%)MATCHES To TargetScan▶ miR-124-3p.1:AAGGCAC

-----------------

AAAAAAAGA

AAAAAAAGA  
Depth:2 (PIG)  
Ei-value:0.000, Pi-value:0.000  
Er-value:0.000, Pr-value:0.000  
eCLIP MATCHES▶KHDRBS1 (bg=1.71%)No matches to TargetScan

--------------------------------

GACATTTTTCCTAG

GACATTTTTCCTAG  
Depth:2 (PIG)  
Ei-value:0.000, Pi-value:0.000  
Er-value:0.000, Pr-value:0.000  
eCLIP MATCHES▶AARS (bg=2.18%)▶HNRNPU (bg=5.92%)▶SAFB (bg=2.69%)No matches to TargetScan

- 16670  
 -----------------------------------------

AAGCCAG

AAGCCAG  
Depth:4 (DOG)  
Ei-value:0.000, Pi-value:0.000  
Er-value:0.000, Pr-value:0.000  
eCLIP MATCHES▶SAFB (bg=2.69%)MATCHES To TargetScan▶ miR-149-5p:CUGGCUC▶ miR-3064-5p:CUGGCUG

-----

TG

TGATAAAAG  
Depth:2 (PIG)  
Ei-value:0.000, Pi-value:0.000  
Er-value:0.000, Pr-value:0.000  
No matches to eCLIP DataNo matches to TargetScan


ATAAAAG

ATAAAAG  
Depth:4 (DOG)  
Ei-value:0.000, Pi-value:0.000  
Er-value:0.000, Pr-value:0.000  
No matches to eCLIP DataNo matches to TargetScan

---------------

CTTTAATTC

CTTTAATTC  
Depth:3 (COW)  
Ei-value:0.000, Pi-value:0.000  
Er-value:0.000, Pr-value:0.000  
No matches to eCLIP DataNo matches to TargetScan

--------

AACATTCTGC

AACATTCTGCTTTTATTA  
Depth:2 (PIG)  
Ei-value:0.000, Pi-value:0.000  
Er-value:0.000, Pr-value:0.000  
No matches to eCLIP DataMATCHES To TargetScan▶ miR-330-3p.2:AAAGCAC▶ miR-409-3p:AAUGUUG


TTTTATTA

TTTTATTA  
Depth:4 (DOG)  
Ei-value:0.000, Pi-value:0.010  
Er-value:0.000, Pr-value:0.000  
No matches to eCLIP DataNo matches to TargetScan

--

G

GTTAAATGG  
Depth:3 (COW)  
Ei-value:0.000, Pi-value:0.000  
Er-value:0.000, Pr-value:0.000  
No matches to eCLIP DataNo matches to TargetScan


TTAAA

TTAAATGG  
Depth:4 (DOG)  
Ei-value:0.000, Pi-value:0.000  
Er-value:0.000, Pr-value:0.000  
No matches to eCLIP DataNo matches to TargetScan

 16790  


TGG

TTAAATGG  
Depth:4 (DOG)  
Ei-value:0.000, Pi-value:0.000  
Er-value:0.000, Pr-value:0.000  
No matches to eCLIP DataNo matches to TargetScan


TT

GTTAAATGGTT  
Depth:2 (PIG)  
Ei-value:0.000, Pi-value:0.000  
Er-value:0.000, Pr-value:0.000  
No matches to eCLIP DataNo matches to TargetScan

--------

AACAACTAGTT

AACAACTAGTT  
Depth:2 (PIG)  
Ei-value:0.000, Pi-value:0.000  
Er-value:0.000, Pr-value:0.000  
No matches to eCLIP DataNo matches to TargetScan

-------------

CTCATTGGTCTG

CTCATTGGTCTG  
Depth:2 (PIG)  
Ei-value:0.000, Pi-value:0.000  
Er-value:0.000, Pr-value:0.000  
No matches to eCLIP DataNo matches to TargetScan

----------------------------

TAAAAAAAA

TAAAAAAAA  
Depth:2 (PIG)  
Ei-value:0.000, Pi-value:0.000  
Er-value:0.000, Pr-value:0.000  
No matches to eCLIP DataNo matches to TargetScan

------------------------

GTAAAAA

GTAAAAA  
Depth:2 (PIG)  
Ei-value:0.000, Pi-value:0.010  
Er-value:0.000, Pr-value:0.000  
eCLIP MATCHES▶KHDRBS1 (bg=1.71%)No matches to TargetScan

--- 16910  
 -------------------

CTGTTCTTAAGT

CTGTTCTTAAGT  
Depth:3 (COW)  
Ei-value:0.000, Pi-value:0.000  
Er-value:0.000, Pr-value:0.000  
eCLIP MATCHES▶KHDRBS1 (bg=1.71%)No matches to TargetScan

-------------------------------------------------

GGAAACA

GGAAACA  
Depth:2 (PIG)  
Ei-value:0.000, Pi-value:0.000  
Er-value:0.000, Pr-value:0.000  
No matches to eCLIP DataNo matches to TargetScan

---

GAACAAATT

GAACAAATT  
Depth:3 (COW)  
Ei-value:0.000, Pi-value:0.000  
Er-value:0.000, Pr-value:0.000  
No matches to eCLIP DataMATCHES To TargetScan▶ miR-375:UUGUUCG

---

TAAGAGACTG

TAAGAGACTG  
Depth:2 (PIG)  
Ei-value:0.000, Pi-value:0.000  
Er-value:0.000, Pr-value:0.000  
No matches to eCLIP DataNo matches to TargetScan

-------

T

TTAGTTG  
Depth:3 (COW)  
Ei-value:0.000, Pi-value:0.000  
Er-value:0.000, Pr-value:0.010  
No matches to eCLIP DataNo matches to TargetScan

 17030  


TAGTTG

TTAGTTG  
Depth:3 (COW)  
Ei-value:0.000, Pi-value:0.000  
Er-value:0.000, Pr-value:0.010  
No matches to eCLIP DataNo matches to TargetScan


A

TTAGTTGA  
Depth:2 (PIG)  
Ei-value:0.000, Pi-value:0.000  
Er-value:0.000, Pr-value:0.000  
No matches to eCLIP DataNo matches to TargetScan

----

AAACTTCATTGA

AAACTTCATTGA  
Depth:3 (COW)  
Ei-value:0.000, Pi-value:0.000  
Er-value:0.000, Pr-value:0.000  
eCLIP MATCHES▶HNRNPA1 (bg=2.57%)No matches to TargetScan


G

AAACTTCATTGAG  
Depth:2 (PIG)  
Ei-value:0.000, Pi-value:0.000  
Er-value:0.000, Pr-value:0.000  
eCLIP MATCHES▶HNRNPA1 (bg=2.57%)No matches to TargetScan

------

TGATAT

TGATAT  
Depth:2 (PIG)  
Ei-value:0.000, Pi-value:0.000  
Er-value:0.000, Pr-value:0.010  
eCLIP MATCHES▶HNRNPA1 (bg=2.57%)No matches to TargetScan

------------------------------------------------------

GGAATAAGAGA

GGAATAAGAGA  
Depth:2 (PIG)  
Ei-value:0.000, Pi-value:0.000  
Er-value:0.000, Pr-value:0.000  
No matches to eCLIP DataNo matches to TargetScan

------------------- 17150  
 ----------------

GAAGATGAT

GAAGATGAT  
Depth:2 (PIG)  
Ei-value:0.000, Pi-value:0.000  
Er-value:0.000, Pr-value:0.000  
No matches to eCLIP DataNo matches to TargetScan

-------

GCTAAAT

GCTAAAT  
Depth:2 (PIG)  
Ei-value:0.000, Pi-value:0.020  
Er-value:0.000, Pr-value:0.000  
No matches to eCLIP DataNo matches to TargetScan

---------------

CAAAAG

CAAAAG  
Depth:2 (PIG)  
Ei-value:0.000, Pi-value:0.010  
Er-value:0.000, Pr-value:0.000  
No matches to eCLIP DataNo matches to TargetScan

-------------

CTTGGAGATG

CTTGGAGATG  
Depth:2 (PIG)  
Ei-value:0.000, Pi-value:0.000  
Er-value:0.000, Pr-value:0.000  
No matches to eCLIP DataNo matches to TargetScan

--------------

AAATGGA

AAATGGA  
Depth:2 (PIG)  
Ei-value:0.000, Pi-value:0.000  
Er-value:0.000, Pr-value:0.010  
No matches to eCLIP DataNo matches to TargetScan

-----

GTGATACTC

GTGATACTC  
Depth:2 (PIG)  
Ei-value:0.000, Pi-value:0.000  
Er-value:0.000, Pr-value:0.000  
No matches to eCLIP DataMATCHES To TargetScan▶ miR-496.1:GAGUAUU

-- 17270  
 -----------------------------------------------

ACAGAAAACAAAA

ACAGAAAACAAAA  
Depth:4 (DOG)  
Ei-value:0.000, Pi-value:0.000  
Er-value:0.000, Pr-value:0.000  
No matches to eCLIP DataNo matches to TargetScan

-------------------

ACTTGC

ACTTGC  
Depth:2 (PIG)  
Ei-value:0.000, Pi-value:0.020  
Er-value:0.000, Pr-value:0.000  
No matches to eCLIP DataNo matches to TargetScan

-------

TTGG

TTGGCTTGGAAA  
Depth:2 (PIG)  
Ei-value:0.000, Pi-value:0.000  
Er-value:0.000, Pr-value:0.000  
No matches to eCLIP DataNo matches to TargetScan


CTTGGAAA

CTTGGAAA  
Depth:3 (COW)  
Ei-value:0.000, Pi-value:0.000  
Er-value:0.000, Pr-value:0.000  
No matches to eCLIP DataNo matches to TargetScan

---------------- 17390  


AGGTTA

AGGTTA  
Depth:4 (DOG)  
Ei-value:0.000, Pi-value:0.000  
Er-value:0.000, Pr-value:0.000  
No matches to eCLIP DataNo matches to TargetScan


CTGTTTATT

AGGTTACTGTTTATT  
Depth:2 (PIG)  
Ei-value:0.000, Pi-value:0.000  
Er-value:0.000, Pr-value:0.000  
No matches to eCLIP DataMATCHES To TargetScan▶ miR-101-3p.1:ACAGUAC▶ miR-132-3p/212-3p:AACAGUC▶ miR-144-3p:ACAGUAU▶ miR-802:CAGUAAC

----------------

TTCATTCT

TTCATTCT  
Depth:4 (DOG)  
Ei-value:0.000, Pi-value:0.000  
Er-value:0.000, Pr-value:0.000  
No matches to eCLIP DataNo matches to TargetScan

------------------------------------------

TTGGCCCC

TTGGCCCCAGAGACATG  
Depth:2 (PIG)  
Ei-value:0.000, Pi-value:0.000  
Er-value:0.000, Pr-value:0.000  
No matches to eCLIP DataMATCHES To TargetScan▶ miR-326:CUCUGGG


AGAGACA

AGAGACA  
Depth:4 (DOG)  
Ei-value:0.000, Pi-value:0.000  
Er-value:0.000, Pr-value:0.000  
No matches to eCLIP DataNo matches to TargetScan


TG

AGAGACATG  
Depth:3 (COW)  
Ei-value:0.000, Pi-value:0.000  
Er-value:0.000, Pr-value:0.000  
No matches to eCLIP DataNo matches to TargetScan

---

AAAAAATG

AAAAAATG  
Depth:2 (PIG)  
Ei-value:0.000, Pi-value:0.000  
Er-value:0.000, Pr-value:0.000  
No matches to eCLIP DataNo matches to TargetScan

----------- 17510  
 -------------------

CCTTTTGG

CCTTTTGG  
Depth:4 (DOG)  
Ei-value:0.000, Pi-value:0.000  
Er-value:0.000, Pr-value:0.000  
No matches to eCLIP DataNo matches to TargetScan


C

CCTTTTGGC  
Depth:3 (COW)  
Ei-value:0.000, Pi-value:0.000  
Er-value:0.000, Pr-value:0.000  
No matches to eCLIP DataNo matches to TargetScan

--

GTTTTCT

GTTTTCT  
Depth:2 (PIG)  
Ei-value:0.000, Pi-value:0.010  
Er-value:0.000, Pr-value:0.010  
No matches to eCLIP DataNo matches to TargetScan

-

GTTAGGGGCA

GTTAGGGGCA  
Depth:2 (PIG)  
Ei-value:0.000, Pi-value:0.000  
Er-value:0.000, Pr-value:0.000  
No matches to eCLIP DataNo matches to TargetScan

-

GGCTTAGT

GGCTTAGT  
Depth:2 (PIG)  
Ei-value:0.000, Pi-value:0.000  
Er-value:0.000, Pr-value:0.000  
No matches to eCLIP DataNo matches to TargetScan

-------

TAAC

TAACATTGTGT  
Depth:2 (PIG)  
Ei-value:0.000, Pi-value:0.000  
Er-value:0.000, Pr-value:0.000  
No matches to eCLIP DataMATCHES To TargetScan▶ miR-409-3p:AAUGUUG


ATTGTGT

ATTGTGT  
Depth:3 (COW)  
Ei-value:0.000, Pi-value:0.000  
Er-value:0.000, Pr-value:0.010  
No matches to eCLIP DataNo matches to TargetScan

---

TTAATTC

TTAATTC  
Depth:4 (DOG)  
Ei-value:0.000, Pi-value:0.000  
Er-value:0.000, Pr-value:0.000  
No matches to eCLIP DataNo matches to TargetScan

---------------------

ACTCTGGCCACTAC

ACTCTGGCCACTAC  
Depth:4 (DOG)  
Ei-value:0.000, Pi-value:0.000  
Er-value:0.000, Pr-value:0.000  
No matches to eCLIP DataMATCHES To TargetScan▶ miR-142-3p.1:GUAGUGU

 17630  


ACTCTGGCCACTAC  
Depth:4 (DOG)  
Ei-value:0.000, Pi-value:0.000  
Er-value:0.000, Pr-value:0.000  
No matches to eCLIP DataMATCHES To TargetScan▶ miR-142-3p.1:GUAGUGU

-

ATAAGC

ATAAGC  
Depth:5 (RABBIT)  
Ei-value:0.000, Pi-value:0.010  
Er-value:0.000, Pr-value:0.000  
No matches to eCLIP DataNo matches to TargetScan


AGG

ATAAGCAGG  
Depth:4 (DOG)  
Ei-value:0.000, Pi-value:0.000  
Er-value:0.000, Pr-value:0.000  
No matches to eCLIP DataNo matches to TargetScan

-------------------

GCT

GCTTGCTCCTT  
Depth:2 (PIG)  
Ei-value:0.000, Pi-value:0.000  
Er-value:0.000, Pr-value:0.000  
No matches to eCLIP DataMATCHES To TargetScan▶ miR-28-5p/708-5p:AGGAGCU


TGCTCCTT

TGCTCCTT  
Depth:3 (COW)  
Ei-value:0.000, Pi-value:0.000  
Er-value:0.000, Pr-value:0.000  
No matches to eCLIP DataMATCHES To TargetScan▶ miR-28-5p/708-5p:AGGAGCU

-----------------------------

ACTTCA

ACTTCA  
Depth:3 (COW)  
Ei-value:0.000, Pi-value:0.000  
Er-value:0.000, Pr-value:0.000  
No matches to eCLIP DataNo matches to TargetScan


TTTTCCTA

TTTTCCTA  
Depth:3 (COW)  
Ei-value:0.000, Pi-value:0.000  
Er-value:0.000, Pr-value:0.000  
No matches to eCLIP DataNo matches to TargetScan


GTCCATCC

TTTTCCTAGTCCATCC  
Depth:2 (PIG)  
Ei-value:0.000, Pi-value:0.000  
Er-value:0.000, Pr-value:0.000  
No matches to eCLIP DataNo matches to TargetScan

---

AT

ATGAAAAATG  
Depth:3 (COW)  
Ei-value:0.000, Pi-value:0.000  
Er-value:0.000, Pr-value:0.000  
No matches to eCLIP DataNo matches to TargetScan


GAAAAATG

GAAAAATG  
Depth:4 (DOG)  
Ei-value:0.000, Pi-value:0.000  
Er-value:0.000, Pr-value:0.000  
No matches to eCLIP DataNo matches to TargetScan

------------

TGGG

TGGGCAG  
Depth:2 (PIG)  
Ei-value:0.000, Pi-value:0.000  
Er-value:0.000, Pr-value:0.000  
No matches to eCLIP DataNo matches to TargetScan

 17750  


CAG

TGGGCAG  
Depth:2 (PIG)  
Ei-value:0.000, Pi-value:0.000  
Er-value:0.000, Pr-value:0.000  
No matches to eCLIP DataNo matches to TargetScan

-----------------------------------

AGTCTCA

AGTCTCA  
Depth:4 (DOG)  
Ei-value:0.000, Pi-value:0.000  
Er-value:0.000, Pr-value:0.000  
eCLIP MATCHES▶ZC3H11A (bg=6.55%)No matches to TargetScan


TTGGTACCA

AGTCTCATTGGTACCA  
Depth:3 (COW)  
Ei-value:0.000, Pi-value:0.000  
Er-value:0.000, Pr-value:0.000  
eCLIP MATCHES▶NOLC1 (bg=9.43%)▶ZC3H11A (bg=6.55%)No matches to TargetScan


GC

AGTCTCATTGGTACCAGC  
Depth:2 (PIG)  
Ei-value:0.000, Pi-value:0.000  
Er-value:0.000, Pr-value:0.000  
eCLIP MATCHES▶NOLC1 (bg=9.43%)▶RPS3 (bg=0.76%)▶ZC3H11A (bg=6.55%)MATCHES To TargetScan▶ miR-138-5p:GCUGGUG

----

GGGAAC

GGGAAC  
Depth:2 (PIG)  
Ei-value:0.000, Pi-value:0.000  
Er-value:0.000, Pr-value:0.010  
eCLIP MATCHES▶NOLC1 (bg=9.43%)▶RBFOX2 (bg=4.63%)▶RPS3 (bg=0.76%)▶ZC3H11A (bg=6.55%)No matches to TargetScan

------------

AGCCACAA

AGCCACAA  
Depth:2 (PIG)  
Ei-value:0.000, Pi-value:0.000  
Er-value:0.000, Pr-value:0.000  
eCLIP MATCHES▶RBFOX2 (bg=4.63%)▶RPS3 (bg=0.76%)▶ZC3H11A (bg=6.55%)No matches to TargetScan

-

TGGTTTTGAA

TGGTTTTGAA  
Depth:4 (DOG)  
Ei-value:0.000, Pi-value:0.000  
Er-value:0.000, Pr-value:0.000  
No matches to eCLIP DataNo matches to TargetScan

---

CATTTAC

CATTTAC  
Depth:2 (PIG)  
Ei-value:0.000, Pi-value:0.000  
Er-value:0.000, Pr-value:0.000  
No matches to eCLIP DataNo matches to TargetScan

------------- 17870  
 --

TCAGTTC

TCAGTTC  
Depth:2 (PIG)  
Ei-value:0.000, Pi-value:0.010  
Er-value:0.000, Pr-value:0.000  
eCLIP MATCHES▶HNRNPL (bg=0.64%)No matches to TargetScan

--------------------------------------------------------

ACAATCC

ACAATCC  
Depth:3 (COW)  
Ei-value:0.000, Pi-value:0.000  
Er-value:0.000, Pr-value:0.000  
No matches to eCLIP DataMATCHES To TargetScan▶ miR-219-5p:GAUUGUC

--------------------

TGGAGATG

TGGAGATG  
Depth:3 (COW)  
Ei-value:0.000, Pi-value:0.000  
Er-value:0.000, Pr-value:0.000  
No matches to eCLIP DataNo matches to TargetScan

-------------------- 17990  
 --

AGCTTCTC

AGCTTCTC  
Depth:3 (COW)  
Ei-value:0.000, Pi-value:0.000  
Er-value:0.000, Pr-value:0.000  
No matches to eCLIP DataNo matches to TargetScan

---------

C

CTTAGAAAT  
Depth:2 (PIG)  
Ei-value:0.000, Pi-value:0.000  
Er-value:0.000, Pr-value:0.000  
eCLIP MATCHES▶WDR3 (bg=0.25%)No matches to TargetScan


TTAGAAAT

TTAGAAAT  
Depth:4 (DOG)  
Ei-value:0.000, Pi-value:0.000  
Er-value:0.000, Pr-value:0.000  
eCLIP MATCHES▶WDR3 (bg=0.25%)No matches to TargetScan

--------------

CATCAAA

CATCAAA  
Depth:4 (DOG)  
Ei-value:0.000, Pi-value:0.000  
Er-value:0.000, Pr-value:0.000  
eCLIP MATCHES▶ZC3H11A (bg=6.55%)No matches to TargetScan

------------------------------------------

ATGAAAA

ATGAAAA  
Depth:2 (PIG)  
Ei-value:0.000, Pi-value:0.030  
Er-value:0.000, Pr-value:0.010  
eCLIP MATCHES▶ZC3H11A (bg=6.55%)No matches to TargetScan

--------------------

CT

CTTAAGTTTTA  
Depth:2 (PIG)  
Ei-value:0.000, Pi-value:0.000  
Er-value:0.000, Pr-value:0.000  
No matches to eCLIP DataNo matches to TargetScan

 18110  


TAAGTTTTA

CTTAAGTTTTA  
Depth:2 (PIG)  
Ei-value:0.000, Pi-value:0.000  
Er-value:0.000, Pr-value:0.000  
No matches to eCLIP DataNo matches to TargetScan

-------

AATAAAAATTGGAA

AATAAAAATTGGAA  
Depth:2 (PIG)  
Ei-value:0.000, Pi-value:0.000  
Er-value:0.000, Pr-value:0.000  
No matches to eCLIP DataNo matches to TargetScan

--------------------

GGAAAAA

GGAAAAA  
Depth:3 (COW)  
Ei-value:0.000, Pi-value:0.000  
Er-value:0.000, Pr-value:0.000  
No matches to eCLIP DataNo matches to TargetScan

---------------

TCTAAAT

TCTAAAT  
Depth:2 (PIG)  
Ei-value:0.000, Pi-value:0.000  
Er-value:0.000, Pr-value:0.010  
eCLIP MATCHES▶PPIL4 (bg=0.52%)No matches to TargetScan

-----------

TGGGCTTTG

TGGGCTTTG  
Depth:3 (COW)  
Ei-value:0.000, Pi-value:0.000  
Er-value:0.000, Pr-value:0.000  
eCLIP MATCHES▶NOLC1 (bg=9.43%)▶PPIL4 (bg=0.52%)MATCHES To TargetScan▶ miR-330-3p:CAAAGCA

----------

TTTTTAAATCA

TTTTTAAATCACTCA  
Depth:4 (DOG)  
Ei-value:0.000, Pi-value:0.000  
Er-value:0.000, Pr-value:0.000  
eCLIP MATCHES▶ILF3 (bg=3.0%)▶NOLC1 (bg=9.43%)▶PPIL4 (bg=0.52%)No matches to TargetScan

 18230  


CTCA

TTTTTAAATCACTCA  
Depth:4 (DOG)  
Ei-value:0.000, Pi-value:0.000  
Er-value:0.000, Pr-value:0.000  
eCLIP MATCHES▶ILF3 (bg=3.0%)▶NOLC1 (bg=9.43%)▶PPIL4 (bg=0.52%)No matches to TargetScan

-

AGAGGGTGGGA

AGAGGGTGGGA  
Depth:4 (DOG)  
Ei-value:0.000, Pi-value:0.000  
Er-value:0.000, Pr-value:0.000  
eCLIP MATCHES▶ILF3 (bg=3.0%)▶ZC3H11A (bg=6.55%)No matches to TargetScan

-

AGGAGGAAGAGTGAA

AGGAGGAAGAGTGAA  
Depth:4 (DOG)  
Ei-value:0.000, Pi-value:0.000  
Er-value:0.000, Pr-value:0.000  
eCLIP MATCHES▶ILF3 (bg=3.0%)▶ZC3H11A (bg=6.55%)MATCHES To TargetScan▶ miR-670-3p:UUCCUCA

-

G

GAAAAGGTCA  
Depth:4 (DOG)  
Ei-value:0.000, Pi-value:0.000  
Er-value:0.000, Pr-value:0.000  
eCLIP MATCHES▶ILF3 (bg=3.0%)▶SF3B1 (bg=2.48%)▶ZC3H11A (bg=6.55%)MATCHES To TargetScan▶ miR-192-5p/215-5p:UGACCUA


AAAAGGT

AAAAGGT  
Depth:6 (MOUSE)  
Ei-value:0.000, Pi-value:0.000  
Er-value:0.000, Pr-value:0.000  
eCLIP MATCHES▶ILF3 (bg=3.0%)▶SF3B1 (bg=2.48%)▶ZC3H11A (bg=6.55%)No matches to TargetScan


CA

GAAAAGGTCA  
Depth:4 (DOG)  
Ei-value:0.000, Pi-value:0.000  
Er-value:0.000, Pr-value:0.000  
eCLIP MATCHES▶ILF3 (bg=3.0%)▶SF3B1 (bg=2.48%)▶ZC3H11A (bg=6.55%)MATCHES To TargetScan▶ miR-192-5p/215-5p:UGACCUA

------------

GGGCAACCTGCCTTTGTTCTG

GGGCAACCTGCCTTTGTTCTG  
Depth:2 (PIG)  
Ei-value:0.000, Pi-value:0.000  
Er-value:0.000, Pr-value:0.000  
eCLIP MATCHES▶ILF3 (bg=3.0%)▶ZC3H11A (bg=6.55%)MATCHES To TargetScan▶ miR-124-3p.1:AAGGCAC▶ miR-495-3p:AACAAAC

-

A

ATTGGTCTTAA  
Depth:2 (PIG)  
Ei-value:0.000, Pi-value:0.000  
Er-value:0.000, Pr-value:0.000  
eCLIP MATCHES▶ILF3 (bg=3.0%)▶ZC3H11A (bg=6.55%)MATCHES To TargetScan▶ miR-208-3p:UAAGACG▶ miR-499a-5p:UAAGACU


TTGGTCTTAA

TTGGTCTTAA  
Depth:3 (COW)  
Ei-value:0.000, Pi-value:0.000  
Er-value:0.000, Pr-value:0.000  
eCLIP MATCHES▶ILF3 (bg=3.0%)▶ZC3H11A (bg=6.55%)MATCHES To TargetScan▶ miR-208-3p:UAAGACG▶ miR-499a-5p:UAAGACU

------------------

GTTTAAAT

GTTTAAAT  
Depth:2 (PIG)  
Ei-value:0.000, Pi-value:0.000  
Er-value:0.000, Pr-value:0.000  
eCLIP MATCHES▶ILF3 (bg=3.0%)No matches to TargetScan

------ 18350  
 ---------------------------------

AGATGAGGACAAA

AGATGAGGACAAA  
Depth:3 (COW)  
Ei-value:0.000, Pi-value:0.000  
Er-value:0.000, Pr-value:0.000  
eCLIP MATCHES▶HNRNPA1 (bg=2.57%)No matches to TargetScan

-

TCCTTTGT

TCCTTTGT  
Depth:3 (COW)  
Ei-value:0.000, Pi-value:0.000  
Er-value:0.000, Pr-value:0.000  
eCLIP MATCHES▶HNRNPA1 (bg=2.57%)No matches to TargetScan

-----------------

ACATTTTT

ACATTTTT  
Depth:2 (PIG)  
Ei-value:0.000, Pi-value:0.020  
Er-value:0.000, Pr-value:0.000  
No matches to eCLIP DataNo matches to TargetScan

--------------------------------------

CC

CCTTGTG  
Depth:2 (PIG)  
Ei-value:0.000, Pi-value:0.000  
Er-value:0.000, Pr-value:0.020  
eCLIP MATCHES▶NOLC1 (bg=9.43%)▶ZC3H11A (bg=6.55%)No matches to TargetScan

 18470  


TTGTG

CCTTGTG  
Depth:2 (PIG)  
Ei-value:0.000, Pi-value:0.000  
Er-value:0.000, Pr-value:0.020  
eCLIP MATCHES▶NOLC1 (bg=9.43%)▶ZC3H11A (bg=6.55%)No matches to TargetScan

-------------------------------------------------------------

TAAAGC

TAAAGC  
Depth:3 (COW)  
Ei-value:0.000, Pi-value:0.000  
Er-value:0.000, Pr-value:0.000  
eCLIP MATCHES▶NOLC1 (bg=9.43%)No matches to TargetScan

------------------

CAAAAGAAGAGG

CAAAAGAAGAGG  
Depth:2 (PIG)  
Ei-value:0.000, Pi-value:0.000  
Er-value:0.000, Pr-value:0.000  
No matches to eCLIP DataNo matches to TargetScan

------------------ 18590  
 -

TTCATACA

TTCATACA  
Depth:3 (COW)  
Ei-value:0.000, Pi-value:0.000  
Er-value:0.000, Pr-value:0.000  
No matches to eCLIP DataNo matches to TargetScan


TTCAAAGCATC

TTCAAAGCATC  
Depth:3 (COW)  
Ei-value:0.000, Pi-value:0.000  
Er-value:0.000, Pr-value:0.000  
No matches to eCLIP DataNo matches to TargetScan

------------------

GCAAGCCAA

GCAAGCCAA  
Depth:2 (PIG)  
Ei-value:0.000, Pi-value:0.000  
Er-value:0.000, Pr-value:0.000  
No matches to eCLIP DataNo matches to TargetScan

-------------

TGG

TGGAGAAGGAAATAGAT  
Depth:2 (PIG)  
Ei-value:0.000, Pi-value:0.000  
Er-value:0.000, Pr-value:0.000  
eCLIP MATCHES▶ZC3H11A (bg=6.55%)No matches to TargetScan


AGAAGGAAATAGA

AGAAGGAAATAGA  
Depth:3 (COW)  
Ei-value:0.000, Pi-value:0.000  
Er-value:0.000, Pr-value:0.000  
eCLIP MATCHES▶ZC3H11A (bg=6.55%)No matches to TargetScan


T

TGGAGAAGGAAATAGAT  
Depth:2 (PIG)  
Ei-value:0.000, Pi-value:0.000  
Er-value:0.000, Pr-value:0.000  
eCLIP MATCHES▶ZC3H11A (bg=6.55%)No matches to TargetScan

--------------------

TGGAGGGAGC

TGGAGGGAGC  
Depth:3 (COW)  
Ei-value:0.000, Pi-value:0.000  
Er-value:0.000, Pr-value:0.000  
eCLIP MATCHES▶FTO (bg=0.32%)▶LARP4 (bg=4.72%)▶LSM11 (bg=2.28%)▶NOLC1 (bg=9.43%)▶XRCC6 (bg=2.91%)▶ZC3H11A (bg=6.55%)No matches to TargetScan


AA

TGGAGGGAGCAA  
Depth:2 (PIG)  
Ei-value:0.000, Pi-value:0.000  
Er-value:0.000, Pr-value:0.000  
eCLIP MATCHES▶FTO (bg=0.32%)▶LARP4 (bg=4.72%)▶LSM11 (bg=2.28%)▶NOLC1 (bg=9.43%)▶XRCC6 (bg=2.91%)▶ZC3H11A (bg=6.55%)No matches to TargetScan

-

GTTGAAGA

GTTGAAGA  
Depth:2 (PIG)  
Ei-value:0.000, Pi-value:0.000  
Er-value:0.000, Pr-value:0.000  
eCLIP MATCHES▶FTO (bg=0.32%)▶LARP4 (bg=4.72%)▶LSM11 (bg=2.28%)▶NOLC1 (bg=9.43%)▶XRCC6 (bg=2.91%)▶ZC3H11A (bg=6.55%)No matches to TargetScan

-- 18710  
 ------------------------------------------------------------------

TAATGTTT

TAATGTTT  
Depth:4 (DOG)  
Ei-value:0.000, Pi-value:0.000  
Er-value:0.000, Pr-value:0.000  
eCLIP MATCHES▶CPEB4 (bg=1.89%)▶KHDRBS1 (bg=1.71%)▶LARP4 (bg=4.72%)▶LSM11 (bg=2.28%)▶NOLC1 (bg=9.43%)▶RBFOX2 (bg=4.63%)▶SAFB (bg=2.69%)▶SAFB2 (bg=0.8%)▶WDR43 (bg=3.37%)▶ZC3H11A (bg=6.55%)MATCHES To TargetScan▶ miR-323-3p:ACAUUAC▶ miR-543:AACAUUC

----------------

TATC

TATCAGCTGGA  
Depth:2 (PIG)  
Ei-value:0.000, Pi-value:0.000  
Er-value:0.000, Pr-value:0.000  
eCLIP MATCHES▶CPEB4 (bg=1.89%)▶KHDRBS1 (bg=1.71%)▶LSM11 (bg=2.28%)▶NOLC1 (bg=9.43%)▶RBFOX2 (bg=4.63%)▶SAFB (bg=2.69%)▶SAFB2 (bg=0.8%)▶SF3B1 (bg=2.48%)▶TRA2A (bg=4.8%)▶WDR43 (bg=3.37%)▶ZC3H11A (bg=6.55%)No matches to TargetScan


AGCTGGA

AGCTGGA  
Depth:4 (DOG)  
Ei-value:0.000, Pi-value:0.000  
Er-value:0.000, Pr-value:0.000  
eCLIP MATCHES▶CPEB4 (bg=1.89%)▶KHDRBS1 (bg=1.71%)▶LSM11 (bg=2.28%)▶NOLC1 (bg=9.43%)▶RBFOX2 (bg=4.63%)▶SAFB (bg=2.69%)▶SAFB2 (bg=0.8%)▶SF3B1 (bg=2.48%)▶TRA2A (bg=4.8%)▶WDR43 (bg=3.37%)▶ZC3H11A (bg=6.55%)No matches to TargetScan

------------------- 18830  
 ---------------------

ATTATTGGAAA

ATTATTGGAAA  
Depth:4 (DOG)  
Ei-value:0.000, Pi-value:0.000  
Er-value:0.000, Pr-value:0.000  
eCLIP MATCHES▶FASTKD2 (bg=1.99%)▶FUS (bg=2.21%)▶LARP4 (bg=4.72%)▶NOLC1 (bg=9.43%)▶RBFOX2 (bg=4.63%)▶SAFB (bg=2.69%)▶SAFB2 (bg=0.8%)▶TRA2A (bg=4.8%)▶WDR43 (bg=3.37%)▶ZC3H11A (bg=6.55%)No matches to TargetScan


A

ATTATTGGAAAA  
Depth:2 (PIG)  
Ei-value:0.000, Pi-value:0.000  
Er-value:0.000, Pr-value:0.000  
eCLIP MATCHES▶FASTKD2 (bg=1.99%)▶FUS (bg=2.21%)▶LARP4 (bg=4.72%)▶NOLC1 (bg=9.43%)▶RBFOX2 (bg=4.63%)▶SAFB (bg=2.69%)▶SAFB2 (bg=0.8%)▶TRA2A (bg=4.8%)▶WDR43 (bg=3.37%)▶ZC3H11A (bg=6.55%)No matches to TargetScan


TGGAAAG

TGGAAAG  
Depth:2 (PIG)  
Ei-value:0.000, Pi-value:0.000  
Er-value:0.000, Pr-value:0.010  
eCLIP MATCHES▶FASTKD2 (bg=1.99%)▶FUS (bg=2.21%)▶LARP4 (bg=4.72%)▶NIPBL (bg=5.39%)▶NOLC1 (bg=9.43%)▶RBFOX2 (bg=4.63%)▶SAFB (bg=2.69%)▶SAFB2 (bg=0.8%)▶TRA2A (bg=4.8%)▶WDR43 (bg=3.37%)▶ZC3H11A (bg=6.55%)No matches to TargetScan

-

AGAAAGTAAC

AGAAAGTAAC  
Depth:4 (DOG)  
Ei-value:0.000, Pi-value:0.000  
Er-value:0.000, Pr-value:0.000  
eCLIP MATCHES▶FASTKD2 (bg=1.99%)▶FUS (bg=2.21%)▶LARP4 (bg=4.72%)▶NIPBL (bg=5.39%)▶NOLC1 (bg=9.43%)▶RBFOX2 (bg=4.63%)▶SAFB (bg=2.69%)▶SAFB2 (bg=0.8%)▶TRA2A (bg=4.8%)▶uchl5 (bg=11.16%)▶WDR43 (bg=3.37%)▶ZC3H11A (bg=6.55%)▶ZNF800 (bg=1.92%)No matches to TargetScan


TAAAAGCCTTCC

AGAAAGTAACTAAAAGCCTTCCTTTCACAGTTTCTGGCATC  
Depth:2 (PIG)  
Ei-value:0.000, Pi-value:0.000  
Er-value:0.000, Pr-value:0.000  
eCLIP MATCHES▶FASTKD2 (bg=1.99%)▶FUS (bg=2.21%)▶LARP4 (bg=4.72%)▶NIPBL (bg=5.39%)▶NOLC1 (bg=9.43%)▶RBFOX2 (bg=4.63%)▶SAFB (bg=2.69%)▶SAFB2 (bg=0.8%)▶TRA2A (bg=4.8%)▶uchl5 (bg=11.16%)▶WDR43 (bg=3.37%)▶ZC3H11A (bg=6.55%)▶ZNF800 (bg=1.92%)MATCHES To TargetScan▶ miR-488-3p:UGAAAGG


TTTCACAGTTTCTGGCATC

TTTCACAGTTTCTGGCATC  
Depth:4 (DOG)  
Ei-value:0.000, Pi-value:0.000  
Er-value:0.000, Pr-value:0.000  
eCLIP MATCHES▶FASTKD2 (bg=1.99%)▶FUS (bg=2.21%)▶LARP4 (bg=4.72%)▶NIPBL (bg=5.39%)▶NOLC1 (bg=9.43%)▶RBFOX2 (bg=4.63%)▶SAFB (bg=2.69%)▶SAFB2 (bg=0.8%)▶uchl5 (bg=11.16%)▶WDR43 (bg=3.37%)▶ZC3H11A (bg=6.55%)▶ZNF800 (bg=1.92%)No matches to TargetScan


ACTAC

ACTACCACTACTGAT  
Depth:2 (PIG)  
Ei-value:0.000, Pi-value:0.000  
Er-value:0.000, Pr-value:0.000  
eCLIP MATCHES▶FASTKD2 (bg=1.99%)▶FUS (bg=2.21%)▶LARP4 (bg=4.72%)▶NIPBL (bg=5.39%)▶NOLC1 (bg=9.43%)▶RBFOX2 (bg=4.63%)▶RBM15 (bg=7.27%)▶SAFB (bg=2.69%)▶SAFB2 (bg=0.8%)▶uchl5 (bg=11.16%)▶ZC3H11A (bg=6.55%)▶ZNF800 (bg=1.92%)MATCHES To TargetScan▶ miR-140-5p:AGUGGUU▶ miR-142-3p.1:GUAGUGU▶ miR-199-3p:CAGUAGU


CA

CACTACTGAT  
Depth:3 (COW)  
Ei-value:0.000, Pi-value:0.000  
Er-value:0.000, Pr-value:0.000  
eCLIP MATCHES▶FASTKD2 (bg=1.99%)▶FUS (bg=2.21%)▶LARP4 (bg=4.72%)▶NIPBL (bg=5.39%)▶NOLC1 (bg=9.43%)▶RBFOX2 (bg=4.63%)▶SAFB (bg=2.69%)▶SAFB2 (bg=0.8%)▶uchl5 (bg=11.16%)▶ZC3H11A (bg=6.55%)▶ZNF800 (bg=1.92%)MATCHES To TargetScan▶ miR-142-3p.1:GUAGUGU▶ miR-199-3p:CAGUAGU


CTACTGAT

CTACTGAT  
Depth:4 (DOG)  
Ei-value:0.000, Pi-value:0.000  
Er-value:0.000, Pr-value:0.000  
eCLIP MATCHES▶FASTKD2 (bg=1.99%)▶FUS (bg=2.21%)▶LARP4 (bg=4.72%)▶NIPBL (bg=5.39%)▶NOLC1 (bg=9.43%)▶RBFOX2 (bg=4.63%)▶SAFB (bg=2.69%)▶SAFB2 (bg=0.8%)▶uchl5 (bg=11.16%)▶ZC3H11A (bg=6.55%)▶ZNF800 (bg=1.92%)MATCHES To TargetScan▶ miR-199-3p:CAGUAGU

-

AAACAAGAATAA

AAACAAGAATAA  
Depth:3 (COW)  
Ei-value:0.000, Pi-value:0.000  
Er-value:0.000, Pr-value:0.000  
eCLIP MATCHES▶FASTKD2 (bg=1.99%)▶FUS (bg=2.21%)▶LARP4 (bg=4.72%)▶NIPBL (bg=5.39%)▶NOLC1 (bg=9.43%)▶RBFOX2 (bg=4.63%)▶SAFB2 (bg=0.8%)▶uchl5 (bg=11.16%)▶ZC3H11A (bg=6.55%)MATCHES To TargetScan▶ miR-544a-5p:CUUGUUA


G

AAACAAGAATAAGAGAACAT  
Depth:2 (PIG)  
Ei-value:0.000, Pi-value:0.000  
Er-value:0.000, Pr-value:0.000  
eCLIP MATCHES▶FASTKD2 (bg=1.99%)▶FUS (bg=2.21%)▶LARP4 (bg=4.72%)▶NIPBL (bg=5.39%)▶NOLC1 (bg=9.43%)▶RBFOX2 (bg=4.63%)▶SAFB2 (bg=0.8%)▶uchl5 (bg=11.16%)▶ZC3H11A (bg=6.55%)▶ZNF622 (bg=6.58%)MATCHES To TargetScan▶ miR-544a-5p:CUUGUUA


AGAACAT

AGAACAT  
Depth:4 (DOG)  
Ei-value:0.000, Pi-value:0.000  
Er-value:0.000, Pr-value:0.000  
eCLIP MATCHES▶FASTKD2 (bg=1.99%)▶FUS (bg=2.21%)▶LARP4 (bg=4.72%)▶NIPBL (bg=5.39%)▶NOLC1 (bg=9.43%)▶RBFOX2 (bg=4.63%)▶SAFB2 (bg=0.8%)▶uchl5 (bg=11.16%)▶ZNF622 (bg=6.58%)No matches to TargetScan

-- 18950  
 ----

A

ATCATCTG  
Depth:2 (PIG)  
Ei-value:0.000, Pi-value:0.000  
Er-value:0.000, Pr-value:0.000  
eCLIP MATCHES▶FUS (bg=2.21%)▶LARP4 (bg=4.72%)▶NOLC1 (bg=9.43%)▶RBFOX2 (bg=4.63%)▶RPS3 (bg=0.76%)▶uchl5 (bg=11.16%)▶ZNF622 (bg=6.58%)No matches to TargetScan


TCATCTG

TCATCTG  
Depth:4 (DOG)  
Ei-value:0.000, Pi-value:0.010  
Er-value:0.000, Pr-value:0.000  
eCLIP MATCHES▶FUS (bg=2.21%)▶LARP4 (bg=4.72%)▶NOLC1 (bg=9.43%)▶RBFOX2 (bg=4.63%)▶RPS3 (bg=0.76%)▶uchl5 (bg=11.16%)▶ZNF622 (bg=6.58%)No matches to TargetScan

---------

CATAAATGAA

CATAAATGAA  
Depth:4 (DOG)  
Ei-value:0.000, Pi-value:0.000  
Er-value:0.000, Pr-value:0.000  
eCLIP MATCHES▶FUS (bg=2.21%)▶NOLC1 (bg=9.43%)▶RPS3 (bg=0.76%)▶uchl5 (bg=11.16%)▶ZNF622 (bg=6.58%)No matches to TargetScan


GTTGTGA

CATAAATGAAGTTGTGA  
Depth:3 (COW)  
Ei-value:0.000, Pi-value:0.000  
Er-value:0.000, Pr-value:0.000  
eCLIP MATCHES▶FUS (bg=2.21%)▶NOLC1 (bg=9.43%)▶RPS3 (bg=0.76%)▶uchl5 (bg=11.16%)▶ZNF622 (bg=6.58%)No matches to TargetScan

-----

AAATCT

AAATCT  
Depth:2 (PIG)  
Ei-value:0.000, Pi-value:0.000  
Er-value:0.000, Pr-value:0.000  
eCLIP MATCHES▶FUS (bg=2.21%)▶NOLC1 (bg=9.43%)▶RBFOX2 (bg=4.63%)▶ZNF622 (bg=6.58%)No matches to TargetScan

---------------------------

TGGCTTC

TGGCTTC  
Depth:2 (PIG)  
Ei-value:0.000, Pi-value:0.000  
Er-value:0.000, Pr-value:0.000  
eCLIP MATCHES▶BUD13 (bg=0.18%)▶FUS (bg=2.21%)▶NOLC1 (bg=9.43%)▶RBFOX2 (bg=4.63%)▶XRCC6 (bg=2.91%)No matches to TargetScan

------------

CTACCTCAAAG

CTACCTCAAAG  
Depth:2 (PIG)  
Ei-value:0.000, Pi-value:0.000  
Er-value:0.000, Pr-value:0.000  
eCLIP MATCHES▶BUD13 (bg=0.18%)▶CPEB4 (bg=1.89%)▶FUS (bg=2.21%)▶NOLC1 (bg=9.43%)▶RBFOX2 (bg=4.63%)▶uchl5 (bg=11.16%)MATCHES To TargetScan▶ let-7-5p/98-5p:GAGGUAG▶ miR-196-5p:AGGUAGU

-------------- 19070  
 ---------------------------

ACTTG

ACTTGTGAACTGATGTGAAA  
Depth:3 (COW)  
Ei-value:0.000, Pi-value:0.000  
Er-value:0.000, Pr-value:0.000  
eCLIP MATCHES▶FUS (bg=2.21%)▶NOLC1 (bg=9.43%)▶RBFOX2 (bg=4.63%)▶TRA2A (bg=4.8%)MATCHES To TargetScan▶ miR-23-3p:UCACAUU


TGAACTGATGTGAAA

TGAACTGATGTGAAA  
Depth:4 (DOG)  
Ei-value:0.000, Pi-value:0.000  
Er-value:0.000, Pr-value:0.000  
eCLIP MATCHES▶FUS (bg=2.21%)▶NOLC1 (bg=9.43%)▶RBFOX2 (bg=4.63%)▶TRA2A (bg=4.8%)MATCHES To TargetScan▶ miR-23-3p:UCACAUU

---

AGAATCTCT

AGAATCTCT  
Depth:2 (PIG)  
Ei-value:0.000, Pi-value:0.000  
Er-value:0.000, Pr-value:0.000  
eCLIP MATCHES▶FUS (bg=2.21%)▶NOLC1 (bg=9.43%)▶RBFOX2 (bg=4.63%)▶TRA2A (bg=4.8%)▶ZC3H11A (bg=6.55%)▶ZNF622 (bg=6.58%)No matches to TargetScan

-------------------

GAAGATTGAAAAAT

GAAGATTGAAAAAT  
Depth:2 (PIG)  
Ei-value:0.000, Pi-value:0.000  
Er-value:0.000, Pr-value:0.000  
eCLIP MATCHES▶AARS (bg=2.18%)▶CPEB4 (bg=1.89%)▶DROSHA (bg=2.49%)▶FUS (bg=2.21%)▶GRWD1 (bg=5.13%)▶LARP4 (bg=4.72%)▶NOLC1 (bg=9.43%)▶RBFOX2 (bg=4.63%)▶TRA2A (bg=4.8%)▶WDR43 (bg=3.37%)▶XRCC6 (bg=2.91%)▶ZC3H11A (bg=6.55%)▶ZNF622 (bg=6.58%)No matches to TargetScan

-

TTGTTCA

TTGTTCA  
Depth:3 (COW)  
Ei-value:0.000, Pi-value:0.000  
Er-value:0.000, Pr-value:0.000  
eCLIP MATCHES▶AARS (bg=2.18%)▶AATF (bg=0.64%)▶CPEB4 (bg=1.89%)▶DROSHA (bg=2.49%)▶FASTKD2 (bg=1.99%)▶FUS (bg=2.21%)▶GRWD1 (bg=5.13%)▶LARP4 (bg=4.72%)▶LSM11 (bg=2.28%)▶NOLC1 (bg=9.43%)▶RBFOX2 (bg=4.63%)▶TRA2A (bg=4.8%)▶uchl5 (bg=11.16%)▶UTP3 (bg=3.66%)▶WDR43 (bg=3.37%)▶XRCC6 (bg=2.91%)▶ZC3H11A (bg=6.55%)▶ZNF622 (bg=6.58%)No matches to TargetScan

-------

TG

TGACCACCA  
Depth:2 (PIG)  
Ei-value:0.000, Pi-value:0.000  
Er-value:0.000, Pr-value:0.000  
eCLIP MATCHES▶AARS (bg=2.18%)▶AATF (bg=0.64%)▶AKAP8L (bg=2.19%)▶CPEB4 (bg=1.89%)▶DROSHA (bg=2.49%)▶FASTKD2 (bg=1.99%)▶FUS (bg=2.21%)▶GRWD1 (bg=5.13%)▶KHDRBS1 (bg=1.71%)▶LARP4 (bg=4.72%)▶LSM11 (bg=2.28%)▶NIPBL (bg=5.39%)▶NOLC1 (bg=9.43%)▶RBFOX2 (bg=4.63%)▶RPS3 (bg=0.76%)▶TRA2A (bg=4.8%)▶uchl5 (bg=11.16%)▶UTP3 (bg=3.66%)▶WDR43 (bg=3.37%)▶XRCC6 (bg=2.91%)▶ZC3H11A (bg=6.55%)▶ZNF622 (bg=6.58%)No matches to TargetScan


ACCACCA

ACCACCA  
Depth:3 (COW)  
Ei-value:0.000, Pi-value:0.000  
Er-value:0.000, Pr-value:0.000  
eCLIP MATCHES▶AARS (bg=2.18%)▶AATF (bg=0.64%)▶AKAP8L (bg=2.19%)▶CPEB4 (bg=1.89%)▶DROSHA (bg=2.49%)▶FASTKD2 (bg=1.99%)▶FUS (bg=2.21%)▶GRWD1 (bg=5.13%)▶KHDRBS1 (bg=1.71%)▶LARP4 (bg=4.72%)▶LSM11 (bg=2.28%)▶NIPBL (bg=5.39%)▶NOLC1 (bg=9.43%)▶RBFOX2 (bg=4.63%)▶RPS3 (bg=0.76%)▶TRA2A (bg=4.8%)▶uchl5 (bg=11.16%)▶UTP3 (bg=3.66%)▶WDR43 (bg=3.37%)▶XRCC6 (bg=2.91%)▶ZC3H11A (bg=6.55%)▶ZNF622 (bg=6.58%)No matches to TargetScan

---- 19190  
 --------------------------

AATTGAA

AATTGAA  
Depth:2 (PIG)  
Ei-value:0.000, Pi-value:0.020  
Er-value:0.000, Pr-value:0.010  
eCLIP MATCHES▶FASTKD2 (bg=1.99%)▶FUS (bg=2.21%)▶GRWD1 (bg=5.13%)▶LARP4 (bg=4.72%)▶RPS3 (bg=0.76%)▶WDR43 (bg=3.37%)▶ZC3H11A (bg=6.55%)▶ZNF622 (bg=6.58%)No matches to TargetScan

----------------------------

AAATAAAA

AAATAAAA  
Depth:4 (DOG)  
Ei-value:0.000, Pi-value:0.000  
Er-value:0.000, Pr-value:0.000  
eCLIP MATCHES▶WDR43 (bg=3.37%)▶ZC3H11A (bg=6.55%)No matches to TargetScan


TA

AAATAAAATA  
Depth:2 (PIG)  
Ei-value:0.000, Pi-value:0.000  
Er-value:0.000, Pr-value:0.000  
eCLIP MATCHES▶WDR43 (bg=3.37%)▶ZC3H11A (bg=6.55%)No matches to TargetScan

-----

TTTTGAAAACTT

TTTTGAAAACTT  
Depth:2 (PIG)  
Ei-value:0.000, Pi-value:0.000  
Er-value:0.000, Pr-value:0.000  
No matches to eCLIP DataNo matches to TargetScan

--                               19280
```

---

## >PIG (25215 bases)

```
 --------------------

TGGAAGCTT

TGGAAGCTT  
Depth:2 (PIG)  
Ei-value:0.000, Pi-value:0.000  
Er-value:0.000, Pr-value:0.000  
No matches to TargetScan

----------

GATCTCT

GATCTCT  
Depth:2 (PIG)  
Ei-value:0.000, Pi-value:0.010  
Er-value:0.000, Pr-value:0.000  
No matches to TargetScan

----------

GGTTCTTTCT

GGTTCTTTCT  
Depth:2 (PIG)  
Ei-value:0.000, Pi-value:0.000  
Er-value:0.000, Pr-value:0.000  
MATCHES To TargetScan▶ miR-186-5p:AAAGAAU

-

GAACATTTTC

GAACATTTTC  
Depth:2 (PIG)  
Ei-value:0.000, Pi-value:0.000  
Er-value:0.000, Pr-value:0.000  
MATCHES To TargetScan▶ miR-409-3p:AAUGUUG

-----------------

TTATGGC

TTATGGC  
Depth:2 (PIG)  
Ei-value:0.000, Pi-value:0.000  
Er-value:0.000, Pr-value:0.010  
No matches to TargetScan

-

TATTTCTTTAAAAAAA

TATTTCTTTAAAAAAA  
Depth:2 (PIG)  
Ei-value:0.000, Pi-value:0.000  
Er-value:0.000, Pr-value:0.000  
MATCHES To TargetScan▶ miR-186-5p:AAAGAAU

-- 120  
 -------------

CATAAAAT

CATAAAAT  
Depth:2 (PIG)  
Ei-value:0.000, Pi-value:0.000  
Er-value:0.000, Pr-value:0.000  
No matches to TargetScan

----------------

ACTTTCTCCTA

ACTTTCTCCTA  
Depth:2 (PIG)  
Ei-value:0.000, Pi-value:0.000  
Er-value:0.000, Pr-value:0.000  
No matches to TargetScan

-----

TTCTTGACAC

TTCTTGACAC  
Depth:2 (PIG)  
Ei-value:0.000, Pi-value:0.000  
Er-value:0.000, Pr-value:0.000  
No matches to TargetScan

-------------------

TATTTGG

TATTTGG  
Depth:2 (PIG)  
Ei-value:0.000, Pi-value:0.010  
Er-value:0.000, Pr-value:0.000  
No matches to TargetScan

-----

TTTAAGG

TTTAAGG  
Depth:2 (PIG)  
Ei-value:0.000, Pi-value:0.000  
Er-value:0.000, Pr-value:0.010  
No matches to TargetScan

------------------- 240  


AATTTTTCTTTGGAAT

AATTTTTCTTTGGAAT  
Depth:2 (PIG)  
Ei-value:0.000, Pi-value:0.000  
Er-value:0.000, Pr-value:0.000  
MATCHES To TargetScan▶ miR-186-5p:AAAGAAU

--

TTTTTGGTTGAC

TTTTTGGTTGAC  
Depth:2 (PIG)  
Ei-value:0.000, Pi-value:0.000  
Er-value:0.000, Pr-value:0.000  
MATCHES To TargetScan▶ miR-505-3p.1:GUCAACA

-------

GTTTTTT

GTTTTTT  
Depth:2 (PIG)  
Ei-value:0.000, Pi-value:0.020  
Er-value:0.000, Pr-value:0.020  
No matches to TargetScan

-----------------------------------------

TGCCCATCGGGGCTG

TGCCCATCGGGGCTG  
Depth:2 (PIG)  
Ei-value:0.000, Pi-value:0.000  
Er-value:0.000, Pr-value:0.000  
No matches to TargetScan

-

GGATACCTGGTTTTA

GGATACCTGGTTTTA  
Depth:2 (PIG)  
Ei-value:0.000, Pi-value:0.000  
Er-value:0.000, Pr-value:0.000  
No matches to TargetScan

---- 360  
 -------

TTATTTT

TTATTTT  
Depth:2 (PIG)  
Ei-value:0.000, Pi-value:0.010  
Er-value:0.000, Pr-value:0.010  
No matches to TargetScan


TTTGCCCAACGGGGCCGTGGATACCTGCCTTTTAATTCTTTTTT

TTTGCCCAACGGGGCCGTGGATACCTGCCTTTTAATTCTTTTTT  
Depth:2 (PIG)  
Ei-value:0.000, Pi-value:0.000  
Er-value:0.000, Pr-value:0.000  
MATCHES To TargetScan▶ miR-124-3p.1:AAGGCAC▶ miR-186-5p:AAAGAAU

----------

GCCCATCGGGGCCGCGGATACCTGCTTTT

GCCCATCGGGGCCGCGGATACCTGCTTTT  
Depth:2 (PIG)  
Ei-value:0.000, Pi-value:0.000  
Er-value:0.000, Pr-value:0.000  
MATCHES To TargetScan▶ miR-330-3p.2:AAAGCAC

-

ATTTTTTTTT

ATTTTTTTTT  
Depth:2 (PIG)  
Ei-value:0.000, Pi-value:0.000  
Er-value:0.000, Pr-value:0.000  
No matches to TargetScan

--

CCTTAGCCCA

CCTTAGCCCATCGGGG  
Depth:2 (PIG)  
Ei-value:0.000, Pi-value:0.000  
Er-value:0.000, Pr-value:0.000  
No matches to TargetScan

 480  


TCGGGG

CCTTAGCCCATCGGGG  
Depth:2 (PIG)  
Ei-value:0.000, Pi-value:0.000  
Er-value:0.000, Pr-value:0.000  
No matches to TargetScan

--

TCGGATACCTGCTG

TCGGATACCTGCTG  
Depth:2 (PIG)  
Ei-value:0.000, Pi-value:0.000  
Er-value:0.000, Pr-value:0.000  
No matches to TargetScan

----

CCCCTCT

CCCCTCT  
Depth:2 (PIG)  
Ei-value:0.000, Pi-value:0.000  
Er-value:0.000, Pr-value:0.010  
MATCHES To TargetScan▶ miR-423-5p:GAGGGGC

------

AACCCC

AACCCC  
Depth:2 (PIG)  
Ei-value:0.000, Pi-value:0.010  
Er-value:0.000, Pr-value:0.010  
No matches to TargetScan

-

TGGCCCATC

TGGCCCATC  
Depth:2 (PIG)  
Ei-value:0.000, Pi-value:0.000  
Er-value:0.000, Pr-value:0.000  
No matches to TargetScan

--------------

CTGCTTTTT

CTGCTTTTT  
Depth:2 (PIG)  
Ei-value:0.000, Pi-value:0.000  
Er-value:0.000, Pr-value:0.000  
MATCHES To TargetScan▶ miR-330-3p.2:AAAGCAC

---------------

TTTTTTTGGCCCATCGGGGC

TTTTTTTGGCCCATCGGGGC  
Depth:2 (PIG)  
Ei-value:0.000, Pi-value:0.000  
Er-value:0.000, Pr-value:0.000  
No matches to TargetScan

-

TCGGAT

TCGGATACCTGCTTT  
Depth:2 (PIG)  
Ei-value:0.000, Pi-value:0.000  
Er-value:0.000, Pr-value:0.000  
MATCHES To TargetScan▶ miR-330-3p.2:AAAGCAC

 600  


ACCTGCTTT

TCGGATACCTGCTTT  
Depth:2 (PIG)  
Ei-value:0.000, Pi-value:0.000  
Er-value:0.000, Pr-value:0.000  
MATCHES To TargetScan▶ miR-330-3p.2:AAAGCAC

--------

TTTTTCCTTGCCCATCGGGGCCTCGGATACCTGCTTTA

TTTTTCCTTGCCCATCGGGGCCTCGGATACCTGCTTTA  
Depth:2 (PIG)  
Ei-value:0.000, Pi-value:0.000  
Er-value:0.000, Pr-value:0.000  
MATCHES To TargetScan▶ miR-31-5p:GGCAAGA▶ miR-330-3p.2:AAAGCAC

----------------

GCCCATCGGGGCCG

GCCCATCGGGGCCG  
Depth:2 (PIG)  
Ei-value:0.000, Pi-value:0.000  
Er-value:0.000, Pr-value:0.000  
No matches to TargetScan

-

GGATACCTGCTT

GGATACCTGCTT  
Depth:2 (PIG)  
Ei-value:0.000, Pi-value:0.000  
Er-value:0.000, Pr-value:0.000  
No matches to TargetScan

-

GATTTTTTTTTTTCATC

GATTTTTTTTTTTCATC  
Depth:2 (PIG)  
Ei-value:0.000, Pi-value:0.000  
Er-value:0.000, Pr-value:0.000  
No matches to TargetScan

-

CCC

CCCATCGG  
Depth:2 (PIG)  
Ei-value:0.000, Pi-value:0.000  
Er-value:0.000, Pr-value:0.000  
No matches to TargetScan

 720  


ATCGG

CCCATCGG  
Depth:2 (PIG)  
Ei-value:0.000, Pi-value:0.000  
Er-value:0.000, Pr-value:0.000  
No matches to TargetScan

-------

TATGGATG

TATGGATG  
Depth:2 (PIG)  
Ei-value:0.000, Pi-value:0.000  
Er-value:0.000, Pr-value:0.000  
No matches to TargetScan

-----------

GGTTTTGTGG

GGTTTTGTGG  
Depth:2 (PIG)  
Ei-value:0.000, Pi-value:0.000  
Er-value:0.000, Pr-value:0.000  
No matches to TargetScan

------------

TCTGGAAT

TCTGGAAT  
Depth:2 (PIG)  
Ei-value:0.000, Pi-value:0.000  
Er-value:0.000, Pr-value:0.000  
No matches to TargetScan

-

TCTACA

TCTACA  
Depth:2 (PIG)  
Ei-value:0.000, Pi-value:0.000  
Er-value:0.000, Pr-value:0.010  
No matches to TargetScan

---

TTTTGCTGCT

TTTTGCTGCT  
Depth:2 (PIG)  
Ei-value:0.000, Pi-value:0.000  
Er-value:0.000, Pr-value:0.010  
MATCHES To TargetScan▶ miR-103-3p/107:GCAGCAU▶ miR-15-5p/16-5p/195-5p/424-5p/497-5p:AGCAGCA▶ miR-503-5p:AGCAGCG

-----

TTTGGTG

TTTGGTG  
Depth:2 (PIG)  
Ei-value:0.000, Pi-value:0.000  
Er-value:0.000, Pr-value:0.010  
No matches to TargetScan

-

TGTGTGAGTG

TGTGTGAGTG  
Depth:2 (PIG)  
Ei-value:0.000, Pi-value:0.000  
Er-value:0.000, Pr-value:0.000  
MATCHES To TargetScan▶ miR-342-3p:CUCACAC▶ miR-377-3p:UCACACA

--------

GCTTTGG

GCTTTGG  
Depth:2 (PIG)  
Ei-value:0.000, Pi-value:0.000  
Er-value:0.000, Pr-value:0.010  
MATCHES To TargetScan▶ miR-330-3p:CAAAGCA

- 840  
 -------------

TGCAGTTA

TGCAGTTA  
Depth:2 (PIG)  
Ei-value:0.000, Pi-value:0.000  
Er-value:0.000, Pr-value:0.000  
MATCHES To TargetScan▶ miR-217:ACUGCAU

-----------------------

GGAGGAAA

GGAGGAAA  
Depth:2 (PIG)  
Ei-value:0.000, Pi-value:0.000  
Er-value:0.000, Pr-value:0.000  
MATCHES To TargetScan▶ miR-670-3p:UUCCUCA

-------------------

TTGCCGC

TTGCCGC  
Depth:2 (PIG)  
Ei-value:0.000, Pi-value:0.000  
Er-value:0.000, Pr-value:0.010  
No matches to TargetScan

----

CTCGGCT

CTCGGCT  
Depth:2 (PIG)  
Ei-value:0.000, Pi-value:0.000  
Er-value:0.000, Pr-value:0.000  
No matches to TargetScan

-

AGGGCTA

AGGGCTA  
Depth:2 (PIG)  
Ei-value:0.000, Pi-value:0.000  
Er-value:0.000, Pr-value:0.010  
MATCHES To TargetScan▶ miR-129-3p:AGCCCUU

--------

TGCTAAGT

TGCTAAGT  
Depth:2 (PIG)  
Ei-value:0.000, Pi-value:0.000  
Er-value:0.000, Pr-value:0.000  
No matches to TargetScan

-

TAAACT

TAAACTAGGGAGGCAAGATG  
Depth:2 (PIG)  
Ei-value:0.000, Pi-value:0.000  
Er-value:0.000, Pr-value:0.000  
No matches to TargetScan

 960  


AGGG

TAAACTAGGGAGGCAAGATG  
Depth:2 (PIG)  
Ei-value:0.000, Pi-value:0.000  
Er-value:0.000, Pr-value:0.000  
No matches to TargetScan


AGGCAAGA

AGGCAAGA  
Depth:3 (COW)  
Ei-value:0.000, Pi-value:0.000  
Er-value:0.000, Pr-value:0.000  
No matches to TargetScan


TG

TAAACTAGGGAGGCAAGATG  
Depth:2 (PIG)  
Ei-value:0.000, Pi-value:0.000  
Er-value:0.000, Pr-value:0.000  
No matches to TargetScan

----------

CAGGCAGAGGAA

CAGGCAGAGGAA  
Depth:2 (PIG)  
Ei-value:0.000, Pi-value:0.000  
Er-value:0.000, Pr-value:0.000  
MATCHES To TargetScan▶ miR-670-3p:UUCCUCA

-------

TGCATTG

TGCATTG  
Depth:2 (PIG)  
Ei-value:0.000, Pi-value:0.010  
Er-value:0.000, Pr-value:0.020  
No matches to TargetScan

-

ATGAGCTA

ATGAGCTA  
Depth:2 (PIG)  
Ei-value:0.000, Pi-value:0.000  
Er-value:0.000, Pr-value:0.000  
No matches to TargetScan

-----------------

GATTTGGG

GATTTGGG  
Depth:2 (PIG)  
Ei-value:0.000, Pi-value:0.000  
Er-value:0.000, Pr-value:0.000  
No matches to TargetScan

-

CTTGTTAGGA

CTTGTTAGGA  
Depth:2 (PIG)  
Ei-value:0.000, Pi-value:0.000  
Er-value:0.000, Pr-value:0.000  
No matches to TargetScan

------------------------- 1080  
 -------------------------------------------------------------

GAAGGTT

GAAGGTT  
Depth:2 (PIG)  
Ei-value:0.000, Pi-value:0.000  
Er-value:0.000, Pr-value:0.000  
No matches to TargetScan

--------------

AAAATGGCGATTTTGAC

AAAATGGCGATTTTGAC  
Depth:2 (PIG)  
Ei-value:0.000, Pi-value:0.000  
Er-value:0.000, Pr-value:0.000  
No matches to TargetScan

------

GCATTGCT

GCATTGCT  
Depth:2 (PIG)  
Ei-value:0.000, Pi-value:0.000  
Er-value:0.000, Pr-value:0.000  
No matches to TargetScan

-

AGCATG

AGCATGGC  
Depth:2 (PIG)  
Ei-value:0.000, Pi-value:0.000  
Er-value:0.000, Pr-value:0.010  
No matches to TargetScan

 1200  


GC

AGCATGGC  
Depth:2 (PIG)  
Ei-value:0.000, Pi-value:0.000  
Er-value:0.000, Pr-value:0.010  
No matches to TargetScan

----

TGCTTTGTTAG

TGCTTTGTTAG  
Depth:2 (PIG)  
Ei-value:0.000, Pi-value:0.000  
Er-value:0.000, Pr-value:0.000  
MATCHES To TargetScan▶ miR-330-3p:CAAAGCA▶ miR-330-3p.2:AAAGCAC▶ miR-495-3p:AACAAAC

------

CAAAATGGCGGA

CAAAATGGCGGA  
Depth:2 (PIG)  
Ei-value:0.000, Pi-value:0.000  
Er-value:0.000, Pr-value:0.000  
No matches to TargetScan

----------

CGCAGTGTTC

CGCAGTGTTC  
Depth:2 (PIG)  
Ei-value:0.000, Pi-value:0.000  
Er-value:0.000, Pr-value:0.000  
MATCHES To TargetScan▶ miR-141-3p/200a-3p:AACACUG

-

AGTGGCGGGAAG

AGTGGCGGGAAG  
Depth:2 (PIG)  
Ei-value:0.000, Pi-value:0.000  
Er-value:0.000, Pr-value:0.000  
No matches to TargetScan


CCACAT

CCACAT  
Depth:2 (PIG)  
Ei-value:0.000, Pi-value:0.030  
Er-value:0.000, Pr-value:0.020  
MATCHES To TargetScan▶ miR-299-3p:AUGUGGG

-------------------------

AGCATGG

AGCATGG  
Depth:2 (PIG)  
Ei-value:0.000, Pi-value:0.000  
Er-value:0.000, Pr-value:0.000  
No matches to TargetScan

-------------- 1320  
 -----------

AAAAGATGGCGGCT

AAAAGATGGCGGCT  
Depth:2 (PIG)  
Ei-value:0.000, Pi-value:0.000  
Er-value:0.000, Pr-value:0.000  
No matches to TargetScan

------

CTTGCCGCA

CTTGCCGCA  
Depth:2 (PIG)  
Ei-value:0.000, Pi-value:0.000  
Er-value:0.000, Pr-value:0.000  
MATCHES To TargetScan▶ miR-31-5p:GGCAAGA

----

A

AAAACATGGCGGGCCT  
Depth:2 (PIG)  
Ei-value:0.000, Pi-value:0.000  
Er-value:0.000, Pr-value:0.000  
No matches to TargetScan


AAACATG

AAACATG  
Depth:4 (DOG)  
Ei-value:0.000, Pi-value:0.000  
Er-value:0.000, Pr-value:0.000  
No matches to TargetScan


GCGGGCCT

AAAACATGGCGGGCCT  
Depth:2 (PIG)  
Ei-value:0.000, Pi-value:0.000  
Er-value:0.000, Pr-value:0.000  
No matches to TargetScan


TTGTCTTTGC

TTGTCTTTGC  
Depth:2 (PIG)  
Ei-value:0.000, Pi-value:0.000  
Er-value:0.000, Pr-value:0.000  
No matches to TargetScan

--------------------

TTTTGCCGCAGGGACAATATGGC

TTTTGCCGCAGGGACAATATGGC  
Depth:2 (PIG)  
Ei-value:0.000, Pi-value:0.000  
Er-value:0.000, Pr-value:0.000  
No matches to TargetScan

-----

TT

TTGTCAT  
Depth:2 (PIG)  
Ei-value:0.000, Pi-value:0.020  
Er-value:0.000, Pr-value:0.040  
MATCHES To TargetScan▶ miR-425-5p:AUGACAC

 1440  


GTCAT

TTGTCAT  
Depth:2 (PIG)  
Ei-value:0.000, Pi-value:0.020  
Er-value:0.000, Pr-value:0.040  
MATCHES To TargetScan▶ miR-425-5p:AUGACAC

------------------

TGTCACGTGGAC

TGTCACGTGGAC  
Depth:2 (PIG)  
Ei-value:0.000, Pi-value:0.000  
Er-value:0.000, Pr-value:0.000  
MATCHES To TargetScan▶ miR-542-3p:GUGACAG

--------------------------------

TGGCGGGCT

TGGCGGGCT  
Depth:2 (PIG)  
Ei-value:0.000, Pi-value:0.000  
Er-value:0.000, Pr-value:0.000  
No matches to TargetScan

----------------

TGCCGCATTGTT

TGCCGCATTGTT  
Depth:2 (PIG)  
Ei-value:0.000, Pi-value:0.000  
Er-value:0.000, Pr-value:0.000  
No matches to TargetScan

-

AAGATGGCGGG

AAGATGGCGGG  
Depth:2 (PIG)  
Ei-value:0.000, Pi-value:0.000  
Er-value:0.000, Pr-value:0.000  
No matches to TargetScan

-

TTT

TTTGCCGC  
Depth:2 (PIG)  
Ei-value:0.000, Pi-value:0.000  
Er-value:0.000, Pr-value:0.000  
No matches to TargetScan

 1560  


GCCGC

TTTGCCGC  
Depth:2 (PIG)  
Ei-value:0.000, Pi-value:0.000  
Er-value:0.000, Pr-value:0.000  
No matches to TargetScan

----------------------------

TGGATTGC

TGGATTGC  
Depth:4 (DOG)  
Ei-value:0.000, Pi-value:0.000  
Er-value:0.000, Pr-value:0.000  
No matches to TargetScan

---------------------------

GGTGGAATTG

GGTGGAATTG  
Depth:2 (PIG)  
Ei-value:0.000, Pi-value:0.000  
Er-value:0.000, Pr-value:0.000  
No matches to TargetScan

-

TCACAG

TCACAG  
Depth:2 (PIG)  
Ei-value:0.000, Pi-value:0.010  
Er-value:0.000, Pr-value:0.020  
No matches to TargetScan

-------------

GATGGAATTAG

GATGGAATTAG  
Depth:2 (PIG)  
Ei-value:0.000, Pi-value:0.000  
Er-value:0.000, Pr-value:0.000  
No matches to TargetScan

------

TTAGC

TTAGCAT  
Depth:2 (PIG)  
Ei-value:0.000, Pi-value:0.020  
Er-value:0.000, Pr-value:0.010  
No matches to TargetScan

 1680  


AT

TTAGCAT  
Depth:2 (PIG)  
Ei-value:0.000, Pi-value:0.020  
Er-value:0.000, Pr-value:0.010  
No matches to TargetScan

------------------------

AGCCAGTCAG

AGCCAGTCAG  
Depth:2 (PIG)  
Ei-value:0.000, Pi-value:0.000  
Er-value:0.000, Pr-value:0.000  
MATCHES To TargetScan▶ miR-149-5p:CUGGCUC▶ miR-193-3p:ACUGGCC▶ miR-3064-5p:CUGGCUG

----

GGCCACGT

GGCCACGT  
Depth:2 (PIG)  
Ei-value:0.000, Pi-value:0.000  
Er-value:0.000, Pr-value:0.000  
No matches to TargetScan

--------

CTCCCAGTGGG

CTCCCAGTGGG  
Depth:2 (PIG)  
Ei-value:0.000, Pi-value:0.000  
Er-value:0.000, Pr-value:0.000  
No matches to TargetScan

-------

CAAGGTCTTT

CAAGGTCTTT  
Depth:2 (PIG)  
Ei-value:0.000, Pi-value:0.000  
Er-value:0.000, Pr-value:0.000  
No matches to TargetScan

-

CAAGGAC

CAAGGAC  
Depth:2 (PIG)  
Ei-value:0.000, Pi-value:0.000  
Er-value:0.000, Pr-value:0.010  
No matches to TargetScan

-----

GCCTTTCCACCTC

GCCTTTCCACCTC  
Depth:2 (PIG)  
Ei-value:0.000, Pi-value:0.000  
Er-value:0.000, Pr-value:0.000  
No matches to TargetScan

--

TCCCCTCT

TCCCCTCT  
Depth:2 (PIG)  
Ei-value:0.000, Pi-value:0.000  
Er-value:0.000, Pr-value:0.000  
MATCHES To TargetScan▶ miR-423-5p:GAGGGGC

 1800  


TCCCCTCT  
Depth:2 (PIG)  
Ei-value:0.000, Pi-value:0.000  
Er-value:0.000, Pr-value:0.000  
MATCHES To TargetScan▶ miR-423-5p:GAGGGGC

-----------

TCCCCTCC

TCCCCTCC  
Depth:2 (PIG)  
Ei-value:0.000, Pi-value:0.000  
Er-value:0.000, Pr-value:0.000  
MATCHES To TargetScan▶ miR-423-5p:GAGGGGC

----------------------------------------

CTGAACCTC

CTGAACCTC  
Depth:2 (PIG)  
Ei-value:0.000, Pi-value:0.000  
Er-value:0.000, Pr-value:0.000  
No matches to TargetScan

---

CCATTCCTCTG

CCATTCCTCTG  
Depth:2 (PIG)  
Ei-value:0.000, Pi-value:0.000  
Er-value:0.000, Pr-value:0.000  
MATCHES To TargetScan▶ miR-1-3p/206:GGAAUGU

-

ATTGGTG

ATTGGTG  
Depth:2 (PIG)  
Ei-value:0.000, Pi-value:0.000  
Er-value:0.000, Pr-value:0.010  
No matches to TargetScan

-

CCTAAGGCTAA

CCTAAGGCTAA  
Depth:2 (PIG)  
Ei-value:0.000, Pi-value:0.000  
Er-value:0.000, Pr-value:0.000  
No matches to TargetScan

------------------ 1920  


CCTCCCCC

CCTCCCCC  
Depth:2 (PIG)  
Ei-value:0.000, Pi-value:0.000  
Er-value:0.000, Pr-value:0.000  
No matches to TargetScan

-------

CCTCTG

CCTCTG  
Depth:2 (PIG)  
Ei-value:0.000, Pi-value:0.020  
Er-value:0.000, Pr-value:0.000  
No matches to TargetScan

-----

CTGCACTGT

CTGCACTGT  
Depth:2 (PIG)  
Ei-value:0.000, Pi-value:0.000  
Er-value:0.000, Pr-value:0.000  
MATCHES To TargetScan▶ miR-130-3p/301-3p/454-3p:AGUGCAA▶ miR-148-3p/152-3p:CAGUGCA

------

GGGCAGTGCTCCA

GGGCAGTGCTCCA  
Depth:2 (PIG)  
Ei-value:0.000, Pi-value:0.000  
Er-value:0.000, Pr-value:0.000  
No matches to TargetScan

-

GCCTGC

GCCTGC  
Depth:2 (PIG)  
Ei-value:0.000, Pi-value:0.000  
Er-value:0.000, Pr-value:0.000  
No matches to TargetScan

----------------

GGTGAG

GGTGAG  
Depth:2 (PIG)  
Ei-value:0.000, Pi-value:0.000  
Er-value:0.000, Pr-value:0.010  
No matches to TargetScan

-

CGTGGCAAGGACCAGAATGGATC

CGTGGCAAGGACCAGAATGGATC  
Depth:2 (PIG)  
Ei-value:0.000, Pi-value:0.000  
Er-value:0.000, Pr-value:0.000  
MATCHES To TargetScan▶ miR-133a-3p.1:UGGUCCC

-

CAGATGATCGTT

CAGATGATCGTTGGCCAACAGGTGGC  
Depth:2 (PIG)  
Ei-value:0.000, Pi-value:0.000  
Er-value:0.000, Pr-value:0.000  
No matches to TargetScan

 2040  


GGC

CAGATGATCGTTGGCCAACAGGTGGC  
Depth:2 (PIG)  
Ei-value:0.000, Pi-value:0.000  
Er-value:0.000, Pr-value:0.000  
No matches to TargetScan


CAACAG

CAACAG  
Depth:3 (COW)  
Ei-value:0.000, Pi-value:0.000  
Er-value:0.000, Pr-value:0.000  
No matches to TargetScan


GTGGC

CAGATGATCGTTGGCCAACAGGTGGC  
Depth:2 (PIG)  
Ei-value:0.000, Pi-value:0.000  
Er-value:0.000, Pr-value:0.000  
No matches to TargetScan

-

GAAGAGGAAT

GAAGAGGAAT  
Depth:2 (PIG)  
Ei-value:0.000, Pi-value:0.000  
Er-value:0.000, Pr-value:0.000  
MATCHES To TargetScan▶ miR-670-3p:UUCCUCA

------

CTTCCTCAAGAGGAACACCTACCCC

CTTCCTCAAGAGGAACACCTACCCC  
Depth:2 (PIG)  
Ei-value:0.000, Pi-value:0.000  
Er-value:0.000, Pr-value:0.000  
MATCHES To TargetScan▶ miR-1224-5p:UGAGGAC▶ miR-670-3p:UUCCUCA

-

TGGCTAATGCTGGGGTCGGATTTTGATTT

TGGCTAATGCTGGGGTCGGATTTTGATTT  
Depth:2 (PIG)  
Ei-value:0.000, Pi-value:0.000  
Er-value:0.000, Pr-value:0.000  
MATCHES To TargetScan▶ miR-338-3p:CCAGCAU▶ miR-551-3p:CGACCCA

-----------

TTGGATGTCAGTCATA

TTGGATGTCAGTCATA  
Depth:2 (PIG)  
Ei-value:0.000, Pi-value:0.000  
Er-value:0.000, Pr-value:0.000  
MATCHES To TargetScan▶ miR-489-3p:UGACAUC

------- 2160  
 ------

TGTGGTTTGCTAGTGTT

TGTGGTTTGCTAGTGTT  
Depth:2 (PIG)  
Ei-value:0.000, Pi-value:0.000  
Er-value:0.000, Pr-value:0.000  
MATCHES To TargetScan▶ miR-140-3p.2:ACCACAG▶ miR-141-3p/200a-3p:AACACUG

--

ATTTAAG

ATTTAAG  
Depth:2 (PIG)  
Ei-value:0.000, Pi-value:0.000  
Er-value:0.000, Pr-value:0.000  
No matches to TargetScan

-

CTTAAGTGACTA

CTTAAGTGACTA  
Depth:2 (PIG)  
Ei-value:0.000, Pi-value:0.000  
Er-value:0.000, Pr-value:0.000  
MATCHES To TargetScan▶ miR-668-3p:GUCACUC

-------

AATGTATT

AATGTATT  
Depth:2 (PIG)  
Ei-value:0.000, Pi-value:0.000  
Er-value:0.000, Pr-value:0.000  
No matches to TargetScan

-----------

TTATTTGTAGAATTCA

TTATTTGTAGAATTCA  
Depth:2 (PIG)  
Ei-value:0.000, Pi-value:0.000  
Er-value:0.000, Pr-value:0.000  
No matches to TargetScan

------

TTACATTTA

TTACATTTA  
Depth:2 (PIG)  
Ei-value:0.000, Pi-value:0.000  
Er-value:0.000, Pr-value:0.000  
MATCHES To TargetScan▶ miR-411-3p:AUGUAAC

------------------ 2280  


GTTCCTT

GTTCCTT  
Depth:2 (PIG)  
Ei-value:0.000, Pi-value:0.010  
Er-value:0.000, Pr-value:0.010  
No matches to TargetScan

--

AAATTCCTTAAAGTTTT

AAATTCCTTAAAGTTTT  
Depth:2 (PIG)  
Ei-value:0.000, Pi-value:0.000  
Er-value:0.000, Pr-value:0.000  
No matches to TargetScan

-------------

TTACAAAT

TTACAAAT  
Depth:2 (PIG)  
Ei-value:0.000, Pi-value:0.000  
Er-value:0.000, Pr-value:0.000  
No matches to TargetScan

----------------------

ATAGTCAAAGTCAA

ATAGTCAAAGTCAA  
Depth:2 (PIG)  
Ei-value:0.000, Pi-value:0.000  
Er-value:0.000, Pr-value:0.000  
No matches to TargetScan

-----------------------------------

CT

CTTTGAAATTGACTTAA  
Depth:2 (PIG)  
Ei-value:0.000, Pi-value:0.000  
Er-value:0.000, Pr-value:0.000  
MATCHES To TargetScan▶ miR-224-5p:AAGUCAC

 2400  


TTGAAATTGACTTAA

CTTTGAAATTGACTTAA  
Depth:2 (PIG)  
Ei-value:0.000, Pi-value:0.000  
Er-value:0.000, Pr-value:0.000  
MATCHES To TargetScan▶ miR-224-5p:AAGUCAC

---------------------

TTTGAAG

TTTGAAG  
Depth:2 (PIG)  
Ei-value:0.000, Pi-value:0.010  
Er-value:0.000, Pr-value:0.000  
No matches to TargetScan

--------------------------

AAAATTTAAC

AAAATTTAAC  
Depth:2 (PIG)  
Ei-value:0.000, Pi-value:0.000  
Er-value:0.000, Pr-value:0.000  
No matches to TargetScan

---

ATGACC

ATGACC  
Depth:2 (PIG)  
Ei-value:0.000, Pi-value:0.000  
Er-value:0.000, Pr-value:0.000  
No matches to TargetScan

--------

TTTGAAGGT

TTTGAAGGT  
Depth:2 (PIG)  
Ei-value:0.000, Pi-value:0.000  
Er-value:0.000, Pr-value:0.000  
MATCHES To TargetScan▶ miR-205-5p:CCUUCAU

--------

GTCCAGG

GTCCAGG  
Depth:2 (PIG)  
Ei-value:0.000, Pi-value:0.000  
Er-value:0.000, Pr-value:0.000  
MATCHES To TargetScan▶ miR-378-3p:CUGGACU

 2520  


GTCCAGG  
Depth:2 (PIG)  
Ei-value:0.000, Pi-value:0.000  
Er-value:0.000, Pr-value:0.000  
MATCHES To TargetScan▶ miR-378-3p:CUGGACU

-

CTTGCTTTG

CTTGCTTTGTTCCCATCCTT  
Depth:2 (PIG)  
Ei-value:0.000, Pi-value:0.000  
Er-value:0.000, Pr-value:0.000  
MATCHES To TargetScan▶ miR-330-3p:CAAAGCA▶ miR-330-3p.2:AAAGCAC▶ miR-495-3p:AACAAAC


TTCCCATC

TTCCCATC  
Depth:4 (DOG)  
Ei-value:0.000, Pi-value:0.000  
Er-value:0.000, Pr-value:0.000  
No matches to TargetScan


CTT

CTTGCTTTGTTCCCATCCTT  
Depth:2 (PIG)  
Ei-value:0.000, Pi-value:0.000  
Er-value:0.000, Pr-value:0.000  
MATCHES To TargetScan▶ miR-330-3p:CAAAGCA▶ miR-330-3p.2:AAAGCAC▶ miR-495-3p:AACAAAC

-

ATGCTGCACT

ATGCTGCACT  
Depth:2 (PIG)  
Ei-value:0.000, Pi-value:0.000  
Er-value:0.000, Pr-value:0.000  
MATCHES To TargetScan▶ miR-103-3p/107:GCAGCAU▶ miR-130-3p/301-3p/454-3p:AGUGCAA

-----------------------------

AAACTTGAATTGCTGTGG

AAACTTGAATTGCTGTGG  
Depth:2 (PIG)  
Ei-value:0.000, Pi-value:0.000  
Er-value:0.000, Pr-value:0.000  
MATCHES To TargetScan▶ miR-140-3p.1:CCACAGG▶ miR-26-5p:UCAAGUA

-----------------

TTATTATATTGGAGTATT

TTATTATATTGGAGTATT  
Depth:2 (PIG)  
Ei-value:0.000, Pi-value:0.000  
Er-value:0.000, Pr-value:0.000  
MATCHES To TargetScan▶ miR-200bc-3p/429:AAUACUG▶ miR-369-3p:AUAAUAC▶ miR-374-5p:UAUAAUA▶ miR-410-3p:AUAUAAC

------ 2640  
 -------

TCAATTTT

TCAATTTT  
Depth:2 (PIG)  
Ei-value:0.000, Pi-value:0.010  
Er-value:0.000, Pr-value:0.010  
No matches to TargetScan

----------------------------------------------

TCCCAGCAAACCC

TCCCAGCAAACCC  
Depth:2 (PIG)  
Ei-value:0.000, Pi-value:0.000  
Er-value:0.000, Pr-value:0.000  
No matches to TargetScan

-----

TAGCCCCAGCCC

TAGCCCCAGCCC  
Depth:2 (PIG)  
Ei-value:0.000, Pi-value:0.000  
Er-value:0.000, Pr-value:0.000  
No matches to TargetScan

----------------------------- 2760  
 -----------------------------------------------------

CCCTGCCCCAGCCCCAG

CCCTGCCCCAGCCCCAG  
Depth:2 (PIG)  
Ei-value:0.000, Pi-value:0.000  
Er-value:0.000, Pr-value:0.000  
No matches to TargetScan

---------

AGCCCCAG

AGCCCCAG  
Depth:2 (PIG)  
Ei-value:0.000, Pi-value:0.000  
Er-value:0.000, Pr-value:0.000  
No matches to TargetScan

----

AGCCCCAG

AGCCCCAG  
Depth:2 (PIG)  
Ei-value:0.000, Pi-value:0.000  
Er-value:0.000, Pr-value:0.000  
No matches to TargetScan

--------------------- 2880  
 --------------

CCAGTCC

CCAGTCC  
Depth:2 (PIG)  
Ei-value:0.000, Pi-value:0.000  
Er-value:0.000, Pr-value:0.010  
No matches to TargetScan

-------

ATTGATT

ATTGATT  
Depth:2 (PIG)  
Ei-value:0.000, Pi-value:0.000  
Er-value:0.000, Pr-value:0.030  
No matches to TargetScan

--------

AAAATAAGTT

AAAATAAGTT  
Depth:2 (PIG)  
Ei-value:0.000, Pi-value:0.000  
Er-value:0.000, Pr-value:0.000  
No matches to TargetScan

-------------------

ACTGGGATA

ACTGGGATA  
Depth:2 (PIG)  
Ei-value:0.000, Pi-value:0.000  
Er-value:0.000, Pr-value:0.000  
No matches to TargetScan

---------------

GCATTGCTGATCTT

GCATTGCTGATCTT  
Depth:2 (PIG)  
Ei-value:0.000, Pi-value:0.000  
Er-value:0.000, Pr-value:0.000  
MATCHES To TargetScan▶ miR-383-5p.1:GAUCAGA▶ miR-383-5p.2:AGAUCAG

---------- 3000  
 ----------

ACCATTTTCA

ACCATTTTCA  
Depth:2 (PIG)  
Ei-value:0.000, Pi-value:0.000  
Er-value:0.000, Pr-value:0.000  
No matches to TargetScan

----------

ACAATCCCATTTG

ACAATCCCATTTG  
Depth:2 (PIG)  
Ei-value:0.000, Pi-value:0.000  
Er-value:0.000, Pr-value:0.000  
MATCHES To TargetScan▶ miR-219-5p:GAUUGUC

-------------------

ACAAAGAATTT

ACAAAGAATTT  
Depth:2 (PIG)  
Ei-value:0.000, Pi-value:0.000  
Er-value:0.000, Pr-value:0.000  
No matches to TargetScan

----------

GTTAGG

GTTAGG  
Depth:2 (PIG)  
Ei-value:0.000, Pi-value:0.010  
Er-value:0.000, Pr-value:0.010  
No matches to TargetScan

------------

GAGCTT

GAGCTT  
Depth:2 (PIG)  
Ei-value:0.000, Pi-value:0.010  
Er-value:0.000, Pr-value:0.010  
No matches to TargetScan

--

TATCAGA

TATCAGA  
Depth:2 (PIG)  
Ei-value:0.000, Pi-value:0.000  
Er-value:0.000, Pr-value:0.000  
No matches to TargetScan

--

AT

ATTATTG  
Depth:2 (PIG)  
Ei-value:0.000, Pi-value:0.000  
Er-value:0.000, Pr-value:0.010  
No matches to TargetScan

 3120  


TATTG

ATTATTG  
Depth:2 (PIG)  
Ei-value:0.000, Pi-value:0.000  
Er-value:0.000, Pr-value:0.010  
No matches to TargetScan

---------------------------------------------

AAGGAGAAACCATT

AAGGAGAAACCATT  
Depth:2 (PIG)  
Ei-value:0.000, Pi-value:0.000  
Er-value:0.000, Pr-value:0.000  
No matches to TargetScan

-

CTCTGT

CTCTGT  
Depth:3 (COW)  
Ei-value:0.000, Pi-value:0.000  
Er-value:0.000, Pr-value:0.000  
No matches to TargetScan


CATTGCT

CTCTGTCATTGCT  
Depth:2 (PIG)  
Ei-value:0.000, Pi-value:0.000  
Er-value:0.000, Pr-value:0.000  
MATCHES To TargetScan▶ miR-425-5p:AUGACAC

---

GTAGTCA

GTAGTCA  
Depth:2 (PIG)  
Ei-value:0.000, Pi-value:0.000  
Er-value:0.000, Pr-value:0.000  
No matches to TargetScan

-----------------------------

TAC

TACTGTG  
Depth:2 (PIG)  
Ei-value:0.000, Pi-value:0.000  
Er-value:0.000, Pr-value:0.000  
MATCHES To TargetScan▶ miR-101-3p.1:ACAGUAC▶ miR-128-3p:CACAGUG▶ miR-144-3p:ACAGUAU

 3240  


TGTG

TACTGTG  
Depth:2 (PIG)  
Ei-value:0.000, Pi-value:0.000  
Er-value:0.000, Pr-value:0.000  
MATCHES To TargetScan▶ miR-101-3p.1:ACAGUAC▶ miR-128-3p:CACAGUG▶ miR-144-3p:ACAGUAU

----------

AAACTCTTTGCA

AAACTCTTTGCA  
Depth:2 (PIG)  
Ei-value:0.000, Pi-value:0.000  
Er-value:0.000, Pr-value:0.000  
No matches to TargetScan

-------

AAATTCTAATTG

AAATTCTAATTG  
Depth:2 (PIG)  
Ei-value:0.000, Pi-value:0.000  
Er-value:0.000, Pr-value:0.000  
No matches to TargetScan

---

ATAATCCT

ATAATCCT  
Depth:2 (PIG)  
Ei-value:0.000, Pi-value:0.000  
Er-value:0.000, Pr-value:0.000  
No matches to TargetScan

-

CATTGGA

CATTGGA  
Depth:2 (PIG)  
Ei-value:0.000, Pi-value:0.010  
Er-value:0.000, Pr-value:0.010  
No matches to TargetScan

-----------------------

TTTGCATTCAGCAG

TTTGCATTCAGCAG  
Depth:2 (PIG)  
Ei-value:0.000, Pi-value:0.000  
Er-value:0.000, Pr-value:0.000  
No matches to TargetScan

------

TTGTCATA

TTGTCATA  
Depth:2 (PIG)  
Ei-value:0.000, Pi-value:0.000  
Er-value:0.000, Pr-value:0.000  
MATCHES To TargetScan▶ miR-425-5p:AUGACAC

----- 3360  
 -----

TTAAACAAAGGCA

TTAAACAAAGGCA  
Depth:2 (PIG)  
Ei-value:0.000, Pi-value:0.000  
Er-value:0.000, Pr-value:0.000  
No matches to TargetScan

-------------------------------

CTGTTCTTGGACAATTAAAG

CTGTTCTTGGACAATTAAAG  
Depth:2 (PIG)  
Ei-value:0.000, Pi-value:0.000  
Er-value:0.000, Pr-value:0.000  
No matches to TargetScan

---------

GTAATTG

GTAATTG  
Depth:2 (PIG)  
Ei-value:0.000, Pi-value:0.000  
Er-value:0.000, Pr-value:0.000  
No matches to TargetScan

---

ATTGTCTCAC

ATTGTCTCAC  
Depth:2 (PIG)  
Ei-value:0.000, Pi-value:0.000  
Er-value:0.000, Pr-value:0.000  
No matches to TargetScan

-

CATTAATCA

CATTAATCA  
Depth:2 (PIG)  
Ei-value:0.000, Pi-value:0.000  
Er-value:0.000, Pr-value:0.000  
No matches to TargetScan

------------ 3480  
 ------------------

AGGCAG

AGGCAG  
Depth:2 (PIG)  
Ei-value:0.000, Pi-value:0.010  
Er-value:0.000, Pr-value:0.020  
No matches to TargetScan

-

GCTGACTA

GCTGACTA  
Depth:2 (PIG)  
Ei-value:0.000, Pi-value:0.000  
Er-value:0.000, Pr-value:0.000  
No matches to TargetScan

--------------------------

ATTATTGA

ATTATTGA  
Depth:2 (PIG)  
Ei-value:0.000, Pi-value:0.010  
Er-value:0.000, Pr-value:0.000  
No matches to TargetScan

-------------------

TTACAC

TTACAC  
Depth:2 (PIG)  
Ei-value:0.000, Pi-value:0.010  
Er-value:0.000, Pr-value:0.000  
No matches to TargetScan

---------------

TCTTTGCA

TCTTTGCA  
Depth:2 (PIG)  
Ei-value:0.000, Pi-value:0.000  
Er-value:0.000, Pr-value:0.000  
No matches to TargetScan

----- 3600  
 ---------------------------------

GGAATATTAATGGATACAAT

GGAATATTAATGGATACAAT  
Depth:2 (PIG)  
Ei-value:0.000, Pi-value:0.000  
Er-value:0.000, Pr-value:0.000  
No matches to TargetScan

---------

TCATGGT

TCATGGT  
Depth:2 (PIG)  
Ei-value:0.000, Pi-value:0.000  
Er-value:0.000, Pr-value:0.020  
No matches to TargetScan

--------------

AAGACCCAC

AAGACCCAC  
Depth:2 (PIG)  
Ei-value:0.000, Pi-value:0.000  
Er-value:0.000, Pr-value:0.000  
MATCHES To TargetScan▶ miR-193a-5p:GGGUCUU

-------------------------

TAG

TAGTGTG  
Depth:2 (PIG)  
Ei-value:0.000, Pi-value:0.000  
Er-value:0.000, Pr-value:0.000  
No matches to TargetScan

 3720  


TGTG

TAGTGTG  
Depth:2 (PIG)  
Ei-value:0.000, Pi-value:0.000  
Er-value:0.000, Pr-value:0.000  
No matches to TargetScan

-

ACTACCTACCACCTTGCATTAATAT

ACTACCTACCACCTTGCATTAATAT  
Depth:2 (PIG)  
Ei-value:0.000, Pi-value:0.000  
Er-value:0.000, Pr-value:0.000  
MATCHES To TargetScan▶ miR-155-5p:UAAUGCU▶ miR-18-5p:AAGGUGC▶ miR-196-5p:AGGUAGU

---------------------

GTCCCACT

GTCCCACT  
Depth:2 (PIG)  
Ei-value:0.000, Pi-value:0.000  
Er-value:0.000, Pr-value:0.000  
No matches to TargetScan

---------------------------

TGCCATT

TGCCATT  
Depth:2 (PIG)  
Ei-value:0.000, Pi-value:0.010  
Er-value:0.000, Pr-value:0.000  
MATCHES To TargetScan▶ miR-183-5p.1:AUGGCAC

---------

GAGTTCTGA

GAGTTCTGA  
Depth:2 (PIG)  
Ei-value:0.000, Pi-value:0.000  
Er-value:0.000, Pr-value:0.000  
No matches to TargetScan

--------- 3840  
 -----

TTCTCTTAAACA

TTCTCTTAAACA  
Depth:2 (PIG)  
Ei-value:0.000, Pi-value:0.000  
Er-value:0.000, Pr-value:0.000  
No matches to TargetScan

-------

TGCATAATTGCATATA

TGCATAATTGCATATA  
Depth:2 (PIG)  
Ei-value:0.000, Pi-value:0.000  
Er-value:0.000, Pr-value:0.000  
No matches to TargetScan

-------------------------------------

TCTGTGG

TCTGTGG  
Depth:2 (PIG)  
Ei-value:0.000, Pi-value:0.000  
Er-value:0.000, Pr-value:0.010  
MATCHES To TargetScan▶ miR-140-3p.1:CCACAGG

-------------

AGTGCA

AGTGCA  
Depth:2 (PIG)  
Ei-value:0.000, Pi-value:0.020  
Er-value:0.000, Pr-value:0.000  
No matches to TargetScan

-

ATTATTCAA

ATTATTCAA  
Depth:2 (PIG)  
Ei-value:0.000, Pi-value:0.000  
Er-value:0.000, Pr-value:0.000  
No matches to TargetScan

------- 3960  
 --------

ACAGTTAAT

ACAGTTAAT  
Depth:2 (PIG)  
Ei-value:0.000, Pi-value:0.000  
Er-value:0.000, Pr-value:0.010  
No matches to TargetScan

--

GCACAGTTGC

GCACAGTTGC  
Depth:2 (PIG)  
Ei-value:0.000, Pi-value:0.000  
Er-value:0.000, Pr-value:0.000  
No matches to TargetScan

--

TTGTCCAGAGTCC

TTGTCCAGAGTCC  
Depth:2 (PIG)  
Ei-value:0.000, Pi-value:0.000  
Er-value:0.000, Pr-value:0.000  
MATCHES To TargetScan▶ miR-326:CUCUGGG▶ miR-378-3p:CUGGACU

-----------

GTGGGC

GTGGGC  
Depth:2 (PIG)  
Ei-value:0.000, Pi-value:0.000  
Er-value:0.000, Pr-value:0.010  
No matches to TargetScan

----------------------------------------------------------- 4080  
 ---

CATAATTG

CATAATTG  
Depth:2 (PIG)  
Ei-value:0.000, Pi-value:0.000  
Er-value:0.000, Pr-value:0.000  
No matches to TargetScan

--------------------------------------

CTTTGTATTCCAGCAGGGGACCCTT

CTTTGTATTCCAGCAGGGGACCCTT  
Depth:2 (PIG)  
Ei-value:0.000, Pi-value:0.000  
Er-value:0.000, Pr-value:0.000  
MATCHES To TargetScan▶ miR-331-3p:CCCCUGG▶ miR-381-3p:AUACAAG

-------------------------------------------

CCT

CCTAATTGATTAGA  
Depth:2 (PIG)  
Ei-value:0.000, Pi-value:0.000  
Er-value:0.000, Pr-value:0.000  
No matches to TargetScan

 4200  


AATTGATTAGA

CCTAATTGATTAGA  
Depth:2 (PIG)  
Ei-value:0.000, Pi-value:0.000  
Er-value:0.000, Pr-value:0.000  
No matches to TargetScan

------------------------------------------------------------------------------------------------------------- 4320  
 ------------------------------------------------------------------------------------------------------------------------ 4440  
 ------------------------------------------------------------------------------------------------------------------------ 4560  
 ------------------------------------------------------------------------------------------------------------------------ 4680  
 ------------------------------------------------------------------------------------------------------------------------ 4800  
 ------------------------------------------------------------------------------------------------------------------------ 4920  
 -------------------------------------------

TCTTTTAT

TCTTTTAT  
Depth:2 (PIG)  
Ei-value:0.000, Pi-value:0.000  
Er-value:0.000, Pr-value:0.010  
No matches to TargetScan

--------------------------------------------------------------------- 5040  
 ------------------------------------------------------------------------------------------------------------------------ 5160  
 ------------------------------------------------------------------------------------------------------------------------ 5280  
 ------------------------------------------------------------------------------------------------------------------------ 5400  
 ------------------------------------------------------------------------------------------------------------------------ 5520  
 ------------------------------------------------------------------------------------------------------------------------ 5640  
 ------------------------------------------------------------------------------------------------------------------------ 5760  
 ------------------------------------------------------------------------------------------------------------------------ 5880  
 ------------------------------------------------------------------------------------------------------------------------ 6000  
 ------------------------------------------------------------------------------------------------------------------------ 6120  
 --------------------------------------------------------------------------------------------------------------

TGCATC

TGCATC  
Depth:2 (PIG)  
Ei-value:0.000, Pi-value:0.030  
Er-value:0.000, Pr-value:0.020  
No matches to TargetScan

---- 6240  
 --------

TTACCTA

TTACCTA  
Depth:2 (PIG)  
Ei-value:0.000, Pi-value:0.000  
Er-value:0.000, Pr-value:0.020  
No matches to TargetScan

--------------------------------------------------------------------------------------------------------- 6360  
 ------------------------------------------------------------------------------------------------------------------------ 6480  
 ------------------------------------------------------------------------------------------------------------------------ 6600  
 ------------------------------------------------------------------------------------------------------------------------ 6720  
 ------------------------------------------------------------------------------------------------------------------------ 6840  
 -------------------------------------------------

AAATGCAATT

AAATGCAATT  
Depth:2 (PIG)  
Ei-value:0.000, Pi-value:0.000  
Er-value:0.000, Pr-value:0.000  
MATCHES To TargetScan▶ miR-25-3p/32-5p/92-3p/363-3p/367-3p:AUUGCAC▶ miR-33-5p:UGCAUUG

------------------------------------------------------------- 6960  
 ------------------------------------------------------------------------------------------------------------------------ 7080  
 ------------------------------------------------------------------------------------------------------------------------ 7200  
 ------------------------------------------------------------------------------------------------------------------------ 7320  
 ------------------------------------------------------------------------------------------------------------------------ 7440  
 ------------------------------------------------------------------------------------------------------------------------ 7560  
 ------------------------------------------------------------------------------------------------------------------------ 7680  


C

CTGTTAGTCT  
Depth:4 (DOG)  
Ei-value:0.000, Pi-value:0.000  
Er-value:0.000, Pr-value:0.000  
No matches to TargetScan


TGTTAGTC

TGTTAGTC  
Depth:5 (RABBIT)  
Ei-value:0.000, Pi-value:0.000  
Er-value:0.000, Pr-value:0.000  
No matches to TargetScan


T

CTGTTAGTCT  
Depth:4 (DOG)  
Ei-value:0.000, Pi-value:0.000  
Er-value:0.000, Pr-value:0.000  
No matches to TargetScan

-------------------------------------------------------------------------------------------------------------- 7800  
 ------------------------------------------------------------------------------------------------------------------------ 7920  
 ------------------------------------------------------------------------------------------------------------------------ 8040  
 ------------------------------------------------------------------------------------------------------------------------ 8160  
 ------------------------------------------------------------------------------------------------------------------------ 8280  
 ------------------------------------------------------------------------------------------------------------------------ 8400  
 ------------------------------------------------------------------------------------------------------------------------ 8520  
 ------------------------------------------------------------------------------------------------------------------------ 8640  
 ---------

TC

TCTCATCCCC  
Depth:2 (PIG)  
Ei-value:0.000, Pi-value:0.000  
Er-value:0.000, Pr-value:0.010  
No matches to TargetScan


TCATCC

TCATCC  
Depth:4 (DOG)  
Ei-value:0.000, Pi-value:0.020  
Er-value:0.000, Pr-value:0.000  
No matches to TargetScan


CC

TCTCATCCCC  
Depth:2 (PIG)  
Ei-value:0.000, Pi-value:0.000  
Er-value:0.000, Pr-value:0.010  
No matches to TargetScan

---------------------------------------------------------------------

CCTTTTGT

CCTTTTGT  
Depth:2 (PIG)  
Ei-value:0.000, Pi-value:0.000  
Er-value:0.000, Pr-value:0.010  
No matches to TargetScan

------------------------ 8760  
 ------------------------------------------------------------------------------------------------------------------------ 8880  
 ------------------------------------------------------------------------------------------------------------------------ 9000  
 ------------------------------------------------------------------------------------------------------------------------ 9120  
 ------------------------------------------------------------------------------------------------------------------------ 9240  
 -------------------

GGG

GGGTACTTGGGACTGTTAAT  
Depth:3 (COW)  
Ei-value:0.000, Pi-value:0.000  
Er-value:0.000, Pr-value:0.000  
MATCHES To TargetScan▶ miR-132-3p/212-3p:AACAGUC▶ miR-455-3p.1:CAGUCCA


TACTTGGGACTGTTAAT

TACTTGGGACTGTTAAT  
Depth:4 (DOG)  
Ei-value:0.000, Pi-value:0.000  
Er-value:0.000, Pr-value:0.000  
MATCHES To TargetScan▶ miR-132-3p/212-3p:AACAGUC▶ miR-455-3p.1:CAGUCCA


G

GGGTACTTGGGACTGTTAATG  
Depth:2 (PIG)  
Ei-value:0.000, Pi-value:0.000  
Er-value:0.000, Pr-value:0.000  
MATCHES To TargetScan▶ miR-132-3p/212-3p:AACAGUC▶ miR-455-3p.1:CAGUCCA

-------------------------------------------------------------------------------- 9360  
 ------------------------------------------------------------------------------------------------------------------------ 9480  
 ------------------------------------------------------------------------------------------------------------------------ 9600  
 ------------------------------------------------------------------------------------------------------------------------ 9720  
 -------------------------

ACTG

ACTGTTAATGTGCT  
Depth:4 (DOG)  
Ei-value:0.000, Pi-value:0.000  
Er-value:0.000, Pr-value:0.000  
MATCHES To TargetScan▶ miR-132-3p/212-3p:AACAGUC▶ miR-323-3p:ACAUUAC


TTAATGTGCT

TTAATGTGCT  
Depth:5 (RABBIT)  
Ei-value:0.000, Pi-value:0.000  
Er-value:0.000, Pr-value:0.000  
MATCHES To TargetScan▶ miR-323-3p:ACAUUAC


A

ACTGTTAATGTGCTA  
Depth:2 (PIG)  
Ei-value:0.000, Pi-value:0.000  
Er-value:0.000, Pr-value:0.000  
MATCHES To TargetScan▶ miR-132-3p/212-3p:AACAGUC▶ miR-323-3p:ACAUUAC

-----------------------------------------------------------------------------

CTT

CTTGGGACTC  
Depth:3 (COW)  
Ei-value:0.000, Pi-value:0.000  
Er-value:0.000, Pr-value:0.000  
No matches to TargetScan

 9840  


GGGACTC

CTTGGGACTC  
Depth:3 (COW)  
Ei-value:0.000, Pi-value:0.000  
Er-value:0.000, Pr-value:0.000  
No matches to TargetScan


TTAATG

CTTGGGACTCTTAATG  
Depth:2 (PIG)  
Ei-value:0.000, Pi-value:0.000  
Er-value:0.000, Pr-value:0.000  
No matches to TargetScan

----------------------------------------------------------------------------------------------------------- 9960  
 ------------------------------------------------------------------------------------------------------------------------ 10080  
 ------------------------------------------------------------------------------------------------------------------------ 10200  
 --------------------------------------------------------------------

ATCCATG

ATCCATG  
Depth:2 (PIG)  
Ei-value:0.000, Pi-value:0.010  
Er-value:0.000, Pr-value:0.000  
No matches to TargetScan

--------------------------------------------- 10320  
 ------------------------------------------------------------------------------------------------------------------------ 10440  
 ------------------------------------------------------------------------------------------------------------------------ 10560  
 ------------------------------------------------------------------------------------------------------------------------ 10680  
 ------------------------------------------------------------------------------------------------------------------------ 10800  
 ------------------------------------------------------------------------------------------------------------------------ 10920  
 ------------------------------------------------------------------------------------------------------------------------ 11040  
 ------------------------------------------------------------------------------------------------------------------------ 11160  
 ------------------------------------------------------------------------------------------------------------------------ 11280  
 ------------------------------------------------------------------------------------------------------------------------ 11400  
 ------------------------------------------------------------------------------------------------------------------------ 11520  
 ------------------------------------------------------------------------------------------------------------------------ 11640  
 ------------------------------------------------------------------------------------------------------------------------ 11760  
 ------------------------------------------------------------------------------------------------------------------------ 11880  
 ------------------------------------------------------------------------------------------------------------------------ 12000  
 ------------------------------------------------------------------------------------------------------------------------ 12120  
 ------------------------------------------------------------------------------------------------------------------------ 12240  
 ------------------------------------------------------------------------------------------------------------------------ 12360  
 ------------------------------------------------------------------------------------------------------------------------ 12480  
 ------------------------------------------------------------------------------------------------------------------------ 12600  
 ------------------------------------------------------------------------------------------------------------------------ 12720  
 ------------------------------------------------------------------------------------------------------------------------ 12840  
 ------------------------------------------------------------------------------------------------------------------------ 12960  
 ------------------------------------------------------------------------------------------------------------------------ 13080  
 ------------------------------------------------------------------------------------------------------------------------ 13200  
 ------------------------------------------------------------------------------------------------------------------------ 13320  
 ------------------------------------------------------------------------------------------------------------------------ 13440  
 -----------------------------------------------------------------------

T

TAATGTGCAT  
Depth:2 (PIG)  
Ei-value:0.000, Pi-value:0.000  
Er-value:0.000, Pr-value:0.000  
MATCHES To TargetScan▶ miR-323-3p:ACAUUAC▶ miR-501-3p/502-3p:AUGCACC


AATGTGCAT

AATGTGCAT  
Depth:6 (MOUSE)  
Ei-value:0.000, Pi-value:0.000  
Er-value:0.000, Pr-value:0.000  
MATCHES To TargetScan▶ miR-501-3p/502-3p:AUGCACC

----------------

CTAATA

CTAATA  
Depth:3 (COW)  
Ei-value:0.000, Pi-value:0.000  
Er-value:0.000, Pr-value:0.000  
No matches to TargetScan

----------------- 13560  
 -----------

AAGTCTTT

AAGTCTTT  
Depth:2 (PIG)  
Ei-value:0.000, Pi-value:0.000  
Er-value:0.000, Pr-value:0.000  
No matches to TargetScan

----------------------------

TGCAAAATT

TGCAAAATT  
Depth:2 (PIG)  
Ei-value:0.000, Pi-value:0.000  
Er-value:0.000, Pr-value:0.000  
No matches to TargetScan

-----------------------------------------------------------

TGCTT

TGCTTCT  
Depth:3 (COW)  
Ei-value:0.000, Pi-value:0.000  
Er-value:0.000, Pr-value:0.010  
No matches to TargetScan

 13680  


CT

TGCTTCT  
Depth:3 (COW)  
Ei-value:0.000, Pi-value:0.000  
Er-value:0.000, Pr-value:0.010  
No matches to TargetScan

---------------------------------------------

TATGTTAGA

TATGTTAGA  
Depth:4 (DOG)  
Ei-value:0.000, Pi-value:0.000  
Er-value:0.000, Pr-value:0.000  
No matches to TargetScan

-

TAGAATCCC

TAGAATCCC  
Depth:2 (PIG)  
Ei-value:0.000, Pi-value:0.000  
Er-value:0.000, Pr-value:0.000  
No matches to TargetScan

----------------------------

GGAGCTTCT

GGAGCTTCT  
Depth:2 (PIG)  
Ei-value:0.000, Pi-value:0.000  
Er-value:0.000, Pr-value:0.000  
No matches to TargetScan

----------------- 13800  
 --

TCTTGG

TCTTGGACTGTTAATGT  
Depth:3 (COW)  
Ei-value:0.000, Pi-value:0.000  
Er-value:0.000, Pr-value:0.000  
MATCHES To TargetScan▶ miR-132-3p/212-3p:AACAGUC▶ miR-323-3p:ACAUUAC▶ miR-455-3p.1:CAGUCCA


ACTGTTAATGT

ACTGTTAATGT  
Depth:4 (DOG)  
Ei-value:0.000, Pi-value:0.000  
Er-value:0.000, Pr-value:0.000  
MATCHES To TargetScan▶ miR-132-3p/212-3p:AACAGUC▶ miR-323-3p:ACAUUAC


G

TCTTGGACTGTTAATGTG  
Depth:2 (PIG)  
Ei-value:0.000, Pi-value:0.000  
Er-value:0.000, Pr-value:0.000  
MATCHES To TargetScan▶ miR-132-3p/212-3p:AACAGUC▶ miR-323-3p:ACAUUAC▶ miR-455-3p.1:CAGUCCA

---------

ATTTGCT

ATTTGCT  
Depth:4 (DOG)  
Ei-value:0.000, Pi-value:0.000  
Er-value:0.000, Pr-value:0.000  
No matches to TargetScan

------------

GTAAGGA

GTAAGGA  
Depth:5 (RABBIT)  
Ei-value:0.000, Pi-value:0.000  
Er-value:0.000, Pr-value:0.000  
No matches to TargetScan


CCC

GTAAGGACCC  
Depth:3 (COW)  
Ei-value:0.000, Pi-value:0.000  
Er-value:0.000, Pr-value:0.000  
No matches to TargetScan

---------------------------------------

CTTAAGA

CTTAAGA  
Depth:2 (PIG)  
Ei-value:0.000, Pi-value:0.000  
Er-value:0.000, Pr-value:0.000  
No matches to TargetScan

---------------- 13920  
 -------

TGCATA

TGCATAATCTTAG  
Depth:2 (PIG)  
Ei-value:0.000, Pi-value:0.000  
Er-value:0.000, Pr-value:0.000  
No matches to TargetScan


ATCTTAG

ATCTTAG  
Depth:3 (COW)  
Ei-value:0.000, Pi-value:0.000  
Er-value:0.000, Pr-value:0.000  
No matches to TargetScan

--------

TACACATT

TACACATT  
Depth:3 (COW)  
Ei-value:0.000, Pi-value:0.000  
Er-value:0.000, Pr-value:0.000  
No matches to TargetScan

-----------------------------

AGGACTCCT

AGGACTCCT  
Depth:2 (PIG)  
Ei-value:0.000, Pi-value:0.000  
Er-value:0.000, Pr-value:0.000  
No matches to TargetScan

-

T

TACTTAT  
Depth:2 (PIG)  
Ei-value:0.000, Pi-value:0.010  
Er-value:0.000, Pr-value:0.030  
No matches to TargetScan


ACTTAT

ACTTAT  
Depth:5 (RABBIT)  
Ei-value:0.000, Pi-value:0.000  
Er-value:0.000, Pr-value:0.000  
No matches to TargetScan


TTAAGC

TTAAGC  
Depth:2 (PIG)  
Ei-value:0.000, Pi-value:0.000  
Er-value:0.000, Pr-value:0.010  
No matches to TargetScan

-------------------------------- 14040  
 ----------------

TGTAATT

TGTAATT  
Depth:3 (COW)  
Ei-value:0.000, Pi-value:0.000  
Er-value:0.000, Pr-value:0.000  
No matches to TargetScan

----------

ATGGTC

ATGGTC  
Depth:3 (COW)  
Ei-value:0.000, Pi-value:0.020  
Er-value:0.000, Pr-value:0.000  
No matches to TargetScan


CTT

ATGGTCCTT  
Depth:2 (PIG)  
Ei-value:0.000, Pi-value:0.000  
Er-value:0.000, Pr-value:0.000  
No matches to TargetScan

-----------------------

TCACAC

TCACAC  
Depth:2 (PIG)  
Ei-value:0.000, Pi-value:0.010  
Er-value:0.000, Pr-value:0.020  
No matches to TargetScan

--------

CTTTATTGC

CTTTATTGC  
Depth:2 (PIG)  
Ei-value:0.000, Pi-value:0.000  
Er-value:0.000, Pr-value:0.000  
MATCHES To TargetScan▶ miR-142-5p:AUAAAGU

-

ATGGGGTACT

ATGGGGTACT  
Depth:3 (COW)  
Ei-value:0.000, Pi-value:0.000  
Er-value:0.000, Pr-value:0.000  
No matches to TargetScan

-

TT

TTCACTTAAGGCCCCTTTCTCAAAC  
Depth:2 (PIG)  
Ei-value:0.000, Pi-value:0.000  
Er-value:0.000, Pr-value:0.000  
No matches to TargetScan


CAC

CACTTAAGGCCCCTTTCTCAA  
Depth:3 (COW)  
Ei-value:0.000, Pi-value:0.000  
Er-value:0.000, Pr-value:0.000  
No matches to TargetScan


TTAAGGCC

TTAAGGCC  
Depth:6 (MOUSE)  
Ei-value:0.000, Pi-value:0.000  
Er-value:0.000, Pr-value:0.000  
No matches to TargetScan


CCTTT

TTAAGGCCCCTTT  
Depth:5 (RABBIT)  
Ei-value:0.000, Pi-value:0.000  
Er-value:0.000, Pr-value:0.000  
No matches to TargetScan


CT

TTAAGGCCCCTTTCTCAA  
Depth:4 (DOG)  
Ei-value:0.000, Pi-value:0.000  
Er-value:0.000, Pr-value:0.000  
No matches to TargetScan

 14160  


CAA

TTAAGGCCCCTTTCTCAA  
Depth:4 (DOG)  
Ei-value:0.000, Pi-value:0.000  
Er-value:0.000, Pr-value:0.000  
No matches to TargetScan


AC

TTCACTTAAGGCCCCTTTCTCAAAC  
Depth:2 (PIG)  
Ei-value:0.000, Pi-value:0.000  
Er-value:0.000, Pr-value:0.000  
No matches to TargetScan

-----------

TAATGACAATTACAT

TAATGACAATTACAT  
Depth:3 (COW)  
Ei-value:0.000, Pi-value:0.000  
Er-value:0.000, Pr-value:0.000  
MATCHES To TargetScan▶ miR-411-3p:AUGUAAC

----

ATCCTTCC

ATCCTTCC  
Depth:2 (PIG)  
Ei-value:0.000, Pi-value:0.010  
Er-value:0.000, Pr-value:0.000  
No matches to TargetScan

-------------

TTTGAAG

TTTGAAG  
Depth:2 (PIG)  
Ei-value:0.000, Pi-value:0.010  
Er-value:0.000, Pr-value:0.000  
No matches to TargetScan

-------------------

CCTAAGG

CCTAAGG  
Depth:2 (PIG)  
Ei-value:0.000, Pi-value:0.010  
Er-value:0.000, Pr-value:0.000  
No matches to TargetScan

-

CCCATTTCTTG

CCCATTTCTTG  
Depth:2 (PIG)  
Ei-value:0.000, Pi-value:0.000  
Er-value:0.000, Pr-value:0.000  
MATCHES To TargetScan▶ miR-203a-3p.1:GAAAUGU

------------------

T

TGTATTTGTC  
Depth:2 (PIG)  
Ei-value:0.000, Pi-value:0.000  
Er-value:0.000, Pr-value:0.000  
No matches to TargetScan

 14280  


GTATTTGTC

TGTATTTGTC  
Depth:2 (PIG)  
Ei-value:0.000, Pi-value:0.000  
Er-value:0.000, Pr-value:0.000  
No matches to TargetScan

------------------------

CTTCCAGCAGGAAGTGCCC

CTTCCAGCAGGAAGTGCCC  
Depth:2 (PIG)  
Ei-value:0.000, Pi-value:0.000  
Er-value:0.000, Pr-value:0.000  
No matches to TargetScan

---

CCACAAG

CCACAAG  
Depth:2 (PIG)  
Ei-value:0.000, Pi-value:0.000  
Er-value:0.000, Pr-value:0.000  
No matches to TargetScan

---------

TTTGGACAGTCAAG

TTTGGACAGTCAAG  
Depth:2 (PIG)  
Ei-value:0.000, Pi-value:0.000  
Er-value:0.000, Pr-value:0.000  
No matches to TargetScan


ATGCAC

ATGCAC  
Depth:2 (PIG)  
Ei-value:0.000, Pi-value:0.030  
Er-value:0.000, Pr-value:0.010  
No matches to TargetScan

----

GTAACTG

GTAACTG  
Depth:2 (PIG)  
Ei-value:0.000, Pi-value:0.000  
Er-value:0.000, Pr-value:0.000  
No matches to TargetScan

------------

CACCTT

CACCTTGGA  
Depth:2 (PIG)  
Ei-value:0.000, Pi-value:0.000  
Er-value:0.000, Pr-value:0.000  
MATCHES To TargetScan▶ miR-18-5p:AAGGUGC

 14400  


GGA

CACCTTGGA  
Depth:2 (PIG)  
Ei-value:0.000, Pi-value:0.000  
Er-value:0.000, Pr-value:0.000  
MATCHES To TargetScan▶ miR-18-5p:AAGGUGC

---

TAATGTGT

TAATGTGT  
Depth:2 (PIG)  
Ei-value:0.000, Pi-value:0.010  
Er-value:0.000, Pr-value:0.000  
MATCHES To TargetScan▶ miR-323-3p:ACAUUAC


ATAACTGCACATGGCT

ATAACTGCACATGGCT  
Depth:2 (PIG)  
Ei-value:0.000, Pi-value:0.000  
Er-value:0.000, Pr-value:0.000  
MATCHES To TargetScan▶ miR-455-3p.2:UGCAGUC▶ miR-455-5p:AUGUGCC

-------------------------

CTCTCAGACCCC

CTCTCAGACCCC  
Depth:2 (PIG)  
Ei-value:0.000, Pi-value:0.000  
Er-value:0.000, Pr-value:0.000  
MATCHES To TargetScan▶ miR-193a-5p:GGGUCUU

------

AGTATAGC

AGTATAGC  
Depth:2 (PIG)  
Ei-value:0.000, Pi-value:0.000  
Er-value:0.000, Pr-value:0.000  
No matches to TargetScan

--------------------

CCTCTTT

CCTCTTT  
Depth:2 (PIG)  
Ei-value:0.000, Pi-value:0.010  
Er-value:0.000, Pr-value:0.020  
No matches to TargetScan

------------ 14520  
 -------------------------------------------------------------

ACTCCCTTTG

ACTCCCTTTG  
Depth:2 (PIG)  
Ei-value:0.000, Pi-value:0.000  
Er-value:0.000, Pr-value:0.000  
No matches to TargetScan

------------------

CACTACTTT

CACTACTTT  
Depth:2 (PIG)  
Ei-value:0.000, Pi-value:0.000  
Er-value:0.000, Pr-value:0.000  
MATCHES To TargetScan▶ miR-142-3p.1:GUAGUGU

------

CTTATATTT

CTTATATTT  
Depth:3 (COW)  
Ei-value:0.000, Pi-value:0.000  
Er-value:0.000, Pr-value:0.000  
MATCHES To TargetScan▶ miR-410-3p:AUAUAAC

------- 14640  


CAAAGTACATG

CAAAGTACATG  
Depth:2 (PIG)  
Ei-value:0.000, Pi-value:0.000  
Er-value:0.000, Pr-value:0.000  
No matches to TargetScan

-

TTTTAATTGACCA

TTTTAATTGACCA  
Depth:3 (COW)  
Ei-value:0.000, Pi-value:0.000  
Er-value:0.000, Pr-value:0.000  
No matches to TargetScan

-----------

TTGG

TTGGACATTAATGTA  
Depth:2 (PIG)  
Ei-value:0.000, Pi-value:0.000  
Er-value:0.000, Pr-value:0.000  
MATCHES To TargetScan▶ miR-323-3p:ACAUUAC


ACATTAAT

ACATTAAT  
Depth:3 (COW)  
Ei-value:0.000, Pi-value:0.000  
Er-value:0.000, Pr-value:0.000  
No matches to TargetScan


GTA

TTGGACATTAATGTA  
Depth:2 (PIG)  
Ei-value:0.000, Pi-value:0.000  
Er-value:0.000, Pr-value:0.000  
MATCHES To TargetScan▶ miR-323-3p:ACAUUAC

-----------------------------------

CTCCATTTGCAGTATA

CTCCATTTGCAGTATA  
Depth:2 (PIG)  
Ei-value:0.000, Pi-value:0.000  
Er-value:0.000, Pr-value:0.000  
MATCHES To TargetScan▶ miR-217:ACUGCAU

-

CAGGGTT

CAGGGTT  
Depth:2 (PIG)  
Ei-value:0.000, Pi-value:0.000  
Er-value:0.000, Pr-value:0.000  
MATCHES To TargetScan▶ miR-10-5p:ACCCUGU▶ miR-504-5p.1:ACCCUGG

--

TGACCC

TGACCC  
Depth:2 (PIG)  
Ei-value:0.000, Pi-value:0.030  
Er-value:0.000, Pr-value:0.000  
No matches to TargetScan

-- 14760  
 ----------------------------

TG

TGCATAATTGCATTT  
Depth:2 (PIG)  
Ei-value:0.000, Pi-value:0.000  
Er-value:0.000, Pr-value:0.000  
No matches to TargetScan


CATAATTGCA

CATAATTGCA  
Depth:3 (COW)  
Ei-value:0.000, Pi-value:0.000  
Er-value:0.000, Pr-value:0.000  
No matches to TargetScan


TTT

TGCATAATTGCATTT  
Depth:2 (PIG)  
Ei-value:0.000, Pi-value:0.000  
Er-value:0.000, Pr-value:0.000  
No matches to TargetScan

-----

GGTTCTTG

GGTTCTTG  
Depth:2 (PIG)  
Ei-value:0.000, Pi-value:0.000  
Er-value:0.000, Pr-value:0.000  
No matches to TargetScan

----

CTAGACAAGGA

CTAGACAAGGA  
Depth:3 (COW)  
Ei-value:0.000, Pi-value:0.000  
Er-value:0.000, Pr-value:0.000  
No matches to TargetScan

------------------------------------------------- 14880  
 ---------------------------

ACAGTTAATGTG

ACAGTTAATGTG  
Depth:4 (DOG)  
Ei-value:0.000, Pi-value:0.000  
Er-value:0.000, Pr-value:0.000  
MATCHES To TargetScan▶ miR-323-3p:ACAUUAC

---

AATTGCAGTT

AATTGCAGTT  
Depth:2 (PIG)  
Ei-value:0.000, Pi-value:0.000  
Er-value:0.000, Pr-value:0.000  
MATCHES To TargetScan▶ miR-217:ACUGCAU

-

TCCACAACCC

TCCACAACCC  
Depth:2 (PIG)  
Ei-value:0.000, Pi-value:0.000  
Er-value:0.000, Pr-value:0.000  
No matches to TargetScan

---------------------------------

ATACCTC

ATACCTC  
Depth:2 (PIG)  
Ei-value:0.000, Pi-value:0.000  
Er-value:0.000, Pr-value:0.000  
MATCHES To TargetScan▶ let-7-5p/98-5p:GAGGUAG

-----

ATACTGTTT

ATACTGTTT  
Depth:3 (COW)  
Ei-value:0.000, Pi-value:0.000  
Er-value:0.000, Pr-value:0.000  
MATCHES To TargetScan▶ miR-101-3p.1:ACAGUAC▶ miR-132-3p/212-3p:AACAGUC▶ miR-144-3p:ACAGUAU

--- 15000  
 ----------------

GTGGAC

GTGGAC  
Depth:2 (PIG)  
Ei-value:0.000, Pi-value:0.000  
Er-value:0.000, Pr-value:0.010  
No matches to TargetScan

------------

TAATTGAAAT

TAATTGAAAT  
Depth:2 (PIG)  
Ei-value:0.000, Pi-value:0.000  
Er-value:0.000, Pr-value:0.000  
No matches to TargetScan

--

TTGTCTT

TTGTCTT  
Depth:3 (COW)  
Ei-value:0.000, Pi-value:0.000  
Er-value:0.000, Pr-value:0.010  
No matches to TargetScan

------

TAGACT

TAGACT  
Depth:2 (PIG)  
Ei-value:0.000, Pi-value:0.000  
Er-value:0.000, Pr-value:0.000  
No matches to TargetScan

-------------------

TAACTA

TAACTA  
Depth:4 (DOG)  
Ei-value:0.000, Pi-value:0.000  
Er-value:0.000, Pr-value:0.000  
No matches to TargetScan

-------

CTCAGCTCTTGG

CTCAGCTCTTGG  
Depth:5 (RABBIT)  
Ei-value:0.000, Pi-value:0.000  
Er-value:0.000, Pr-value:0.000  
MATCHES To TargetScan▶ miR-335-5p:CAAGAGC


ACA

CTCAGCTCTTGGACA  
Depth:4 (DOG)  
Ei-value:0.000, Pi-value:0.000  
Er-value:0.000, Pr-value:0.000  
MATCHES To TargetScan▶ miR-335-5p:CAAGAGC


ATTAATA

CTCAGCTCTTGGACAATTAATA  
Depth:3 (COW)  
Ei-value:0.000, Pi-value:0.000  
Er-value:0.000, Pr-value:0.000  
MATCHES To TargetScan▶ miR-335-5p:CAAGAGC

- 15120  
 -----

AATAACA

AATAACA  
Depth:2 (PIG)  
Ei-value:0.000, Pi-value:0.010  
Er-value:0.000, Pr-value:0.000  
No matches to TargetScan

----------

ACT

ACTGATCATTAGATA  
Depth:2 (PIG)  
Ei-value:0.000, Pi-value:0.000  
Er-value:0.000, Pr-value:0.000  
MATCHES To TargetScan▶ miR-383-5p.1:GAUCAGA


GATCAT

GATCAT  
Depth:3 (COW)  
Ei-value:0.000, Pi-value:0.000  
Er-value:0.000, Pr-value:0.000  
No matches to TargetScan


TAGATA

ACTGATCATTAGATA  
Depth:2 (PIG)  
Ei-value:0.000, Pi-value:0.000  
Er-value:0.000, Pr-value:0.000  
MATCHES To TargetScan▶ miR-383-5p.1:GAUCAGA

--------------------------------------

AC

ACTAAGGCCCC  
Depth:2 (PIG)  
Ei-value:0.000, Pi-value:0.000  
Er-value:0.000, Pr-value:0.000  
No matches to TargetScan


TAAGGC

TAAGGC  
Depth:3 (COW)  
Ei-value:0.000, Pi-value:0.000  
Er-value:0.000, Pr-value:0.000  
No matches to TargetScan


CCC

ACTAAGGCCCC  
Depth:2 (PIG)  
Ei-value:0.000, Pi-value:0.000  
Er-value:0.000, Pr-value:0.000  
No matches to TargetScan

-------------------

GAATATTTGCA

GAATATTTGCA  
Depth:3 (COW)  
Ei-value:0.000, Pi-value:0.000  
Er-value:0.000, Pr-value:0.000  
No matches to TargetScan


ATTA

GAATATTTGCAATTAT  
Depth:2 (PIG)  
Ei-value:0.000, Pi-value:0.000  
Er-value:0.000, Pr-value:0.000  
MATCHES To TargetScan▶ miR-25-3p/32-5p/92-3p/363-3p/367-3p:AUUGCAC

 15240  


T

GAATATTTGCAATTAT  
Depth:2 (PIG)  
Ei-value:0.000, Pi-value:0.000  
Er-value:0.000, Pr-value:0.000  
MATCHES To TargetScan▶ miR-25-3p/32-5p/92-3p/363-3p/367-3p:AUUGCAC

-----

TCCCCTT

TCCCCTT  
Depth:2 (PIG)  
Ei-value:0.000, Pi-value:0.000  
Er-value:0.000, Pr-value:0.030  
No matches to TargetScan

---------------------------------------

ATTACTG

ATTACTG  
Depth:3 (COW)  
Ei-value:0.000, Pi-value:0.010  
Er-value:0.000, Pr-value:0.020  
MATCHES To TargetScan▶ miR-802:CAGUAAC

--

AG

AGGGGCTGCTGAC  
Depth:2 (PIG)  
Ei-value:0.000, Pi-value:0.000  
Er-value:0.000, Pr-value:0.000  
MATCHES To TargetScan▶ miR-15-5p/16-5p/195-5p/424-5p/497-5p:AGCAGCA▶ miR-503-5p:AGCAGCG


GGGCTGCTGA

GGGCTGCTGA  
Depth:3 (COW)  
Ei-value:0.000, Pi-value:0.000  
Er-value:0.000, Pr-value:0.000  
MATCHES To TargetScan▶ miR-15-5p/16-5p/195-5p/424-5p/497-5p:AGCAGCA▶ miR-503-5p:AGCAGCG


C

AGGGGCTGCTGAC  
Depth:2 (PIG)  
Ei-value:0.000, Pi-value:0.000  
Er-value:0.000, Pr-value:0.000  
MATCHES To TargetScan▶ miR-15-5p/16-5p/195-5p/424-5p/497-5p:AGCAGCA▶ miR-503-5p:AGCAGCG

----

CAAAACTT

CAAAACTT  
Depth:4 (DOG)  
Ei-value:0.000, Pi-value:0.000  
Er-value:0.000, Pr-value:0.000  
No matches to TargetScan


CT

CAAAACTTCT  
Depth:2 (PIG)  
Ei-value:0.000, Pi-value:0.000  
Er-value:0.000, Pr-value:0.000  
No matches to TargetScan

-

CTGGGACTG

CTGGGACTG  
Depth:3 (COW)  
Ei-value:0.000, Pi-value:0.000  
Er-value:0.000, Pr-value:0.000  
MATCHES To TargetScan▶ miR-455-3p.1:CAGUCCA

-------

GCACAATG

GCACAATG  
Depth:6 (MOUSE)  
Ei-value:0.000, Pi-value:0.000  
Er-value:0.000, Pr-value:0.000  
No matches to TargetScan

------- 15360  
 --------------

CTCCCTG

CTCCCTG  
Depth:3 (COW)  
Ei-value:0.000, Pi-value:0.000  
Er-value:0.000, Pr-value:0.000  
No matches to TargetScan

-

CCTTGTT

CCTTGTT  
Depth:2 (PIG)  
Ei-value:0.000, Pi-value:0.000  
Er-value:0.000, Pr-value:0.040  
No matches to TargetScan

--

GCAAGC

GCAAGC  
Depth:3 (COW)  
Ei-value:0.000, Pi-value:0.000  
Er-value:0.000, Pr-value:0.000  
No matches to TargetScan


GC

GCAAGCGC  
Depth:2 (PIG)  
Ei-value:0.000, Pi-value:0.000  
Er-value:0.000, Pr-value:0.000  
No matches to TargetScan

-----------------

TTTCCCATGG

TTTCCCATGG  
Depth:2 (PIG)  
Ei-value:0.000, Pi-value:0.000  
Er-value:0.000, Pr-value:0.000  
No matches to TargetScan

-

ATAATAAAGTATAA

ATAATAAAGTATAA  
Depth:2 (PIG)  
Ei-value:0.000, Pi-value:0.000  
Er-value:0.000, Pr-value:0.000  
No matches to TargetScan

--------------------------

ATCAAACAG

ATCAAACAG  
Depth:2 (PIG)  
Ei-value:0.000, Pi-value:0.000  
Er-value:0.000, Pr-value:0.000  
No matches to TargetScan

---- 15480  


CCAT

CCATACTCCCA  
Depth:2 (PIG)  
Ei-value:0.000, Pi-value:0.000  
Er-value:0.000, Pr-value:0.000  
MATCHES To TargetScan▶ miR-496.1:GAGUAUU


A

ACTCCCA  
Depth:4 (DOG)  
Ei-value:0.000, Pi-value:0.000  
Er-value:0.000, Pr-value:0.000  
No matches to TargetScan


CTCCCA

CTCCCA  
Depth:6 (MOUSE)  
Ei-value:0.000, Pi-value:0.000  
Er-value:0.000, Pr-value:0.000  
No matches to TargetScan

------------------------------------------------------------------------------------------------------------- 15600  
 ----------------------------------------------------------------------------------

CCCTTTTGCATT

CCCTTTTGCATT  
Depth:4 (DOG)  
Ei-value:0.000, Pi-value:0.000  
Er-value:0.000, Pr-value:0.000  
No matches to TargetScan


G

CCCTTTTGCATTG  
Depth:3 (COW)  
Ei-value:0.000, Pi-value:0.000  
Er-value:0.000, Pr-value:0.000  
No matches to TargetScan


TTGCTGGG

TTGCTGGG  
Depth:2 (PIG)  
Ei-value:0.000, Pi-value:0.000  
Er-value:0.000, Pr-value:0.010  
MATCHES To TargetScan▶ miR-338-3p:CCAGCAU

----------------- 15720  
 ------------------------------------------------------------------------------

CCCTTTCT

CCCTTTCT  
Depth:2 (PIG)  
Ei-value:0.000, Pi-value:0.000  
Er-value:0.000, Pr-value:0.000  
No matches to TargetScan

-------------------------------

ACT

ACTTCCTT  
Depth:3 (COW)  
Ei-value:0.000, Pi-value:0.000  
Er-value:0.000, Pr-value:0.000  
No matches to TargetScan

 15840  


TCCTT

ACTTCCTT  
Depth:3 (COW)  
Ei-value:0.000, Pi-value:0.000  
Er-value:0.000, Pr-value:0.000  
No matches to TargetScan

-------------

CTGAGTA

CTGAGTA  
Depth:2 (PIG)  
Ei-value:0.000, Pi-value:0.010  
Er-value:0.000, Pr-value:0.000  
No matches to TargetScan

---

CTGACTACCCA

CTGACTACCCA  
Depth:2 (PIG)  
Ei-value:0.000, Pi-value:0.000  
Er-value:0.000, Pr-value:0.000  
No matches to TargetScan

-

AGCCCCTTCT

AGCCCCTTCT  
Depth:3 (COW)  
Ei-value:0.000, Pi-value:0.000  
Er-value:0.000, Pr-value:0.000  
No matches to TargetScan


GTGTTATTAA

AGCCCCTTCTGTGTTATTAA  
Depth:2 (PIG)  
Ei-value:0.000, Pi-value:0.000  
Er-value:0.000, Pr-value:0.000  
No matches to TargetScan


CACAGTA

CACAGTA  
Depth:3 (COW)  
Ei-value:0.000, Pi-value:0.000  
Er-value:0.000, Pr-value:0.000  
No matches to TargetScan

-

TGATTGTC

TGATTGTCCCATTTTT  
Depth:3 (COW)  
Ei-value:0.000, Pi-value:0.000  
Er-value:0.000, Pr-value:0.000  
No matches to TargetScan


CCATTTTT

CCATTTTT  
Depth:4 (DOG)  
Ei-value:0.000, Pi-value:0.000  
Er-value:0.000, Pr-value:0.000  
No matches to TargetScan

-

CAGCCCA

CAGCCCA  
Depth:4 (DOG)  
Ei-value:0.000, Pi-value:0.000  
Er-value:0.000, Pr-value:0.000  
No matches to TargetScan

-

CAGCCCA

CAGCCCA  
Depth:4 (DOG)  
Ei-value:0.000, Pi-value:0.000  
Er-value:0.000, Pr-value:0.000  
No matches to TargetScan

---

TCTC

TCTCCCTACCA  
Depth:3 (COW)  
Ei-value:0.000, Pi-value:0.000  
Er-value:0.000, Pr-value:0.000  
No matches to TargetScan


CCTACCA

CCTACCA  
Depth:4 (DOG)  
Ei-value:0.000, Pi-value:0.000  
Er-value:0.000, Pr-value:0.000  
No matches to TargetScan


CTTTG

TCTCCCTACCACTTTG  
Depth:2 (PIG)  
Ei-value:0.000, Pi-value:0.000  
Er-value:0.000, Pr-value:0.000  
MATCHES To TargetScan▶ miR-140-5p:AGUGGUU▶ miR-17-5p/20-5p/93-5p/106-5p/519-3p:AAAGUGC

- 15960  
 ----------

GTGCAGT

GTGCAGT  
Depth:3 (COW)  
Ei-value:0.000, Pi-value:0.000  
Er-value:0.000, Pr-value:0.000  
MATCHES To TargetScan▶ miR-217:ACUGCAU

-

TTGACTA

TTGACTA  
Depth:2 (PIG)  
Ei-value:0.000, Pi-value:0.020  
Er-value:0.000, Pr-value:0.000  
No matches to TargetScan

--

AAAAGCAG

AAAAGCAG  
Depth:6 (MOUSE)  
Ei-value:0.000, Pi-value:0.000  
Er-value:0.000, Pr-value:0.000  
No matches to TargetScan

-

CCT

CCTGAACTA  
Depth:2 (PIG)  
Ei-value:0.000, Pi-value:0.000  
Er-value:0.000, Pr-value:0.000  
No matches to TargetScan


GAACTA

GAACTA  
Depth:3 (COW)  
Ei-value:0.000, Pi-value:0.000  
Er-value:0.000, Pr-value:0.000  
No matches to TargetScan

---------

GCCTTCACTC

GCCTTCACTC  
Depth:2 (PIG)  
Ei-value:0.000, Pi-value:0.000  
Er-value:0.000, Pr-value:0.000  
No matches to TargetScan

---------------

TTAATGATCC

TTAATGATCC  
Depth:4 (DOG)  
Ei-value:0.000, Pi-value:0.000  
Er-value:0.000, Pr-value:0.000  
MATCHES To TargetScan▶ miR-382-3p:AUCAUUC

------

CA

CAATTATTGT  
Depth:2 (PIG)  
Ei-value:0.000, Pi-value:0.000  
Er-value:0.000, Pr-value:0.000  
No matches to TargetScan


ATTATTGT

ATTATTGT  
Depth:3 (COW)  
Ei-value:0.000, Pi-value:0.000  
Er-value:0.000, Pr-value:0.000  
No matches to TargetScan

---

ATTCTGGG

ATTCTGGG  
Depth:4 (DOG)  
Ei-value:0.000, Pi-value:0.000  
Er-value:0.000, Pr-value:0.000  
No matches to TargetScan


GACA

ATTCTGGGGACA  
Depth:2 (PIG)  
Ei-value:0.000, Pi-value:0.000  
Er-value:0.000, Pr-value:0.000  
No matches to TargetScan

 16080  


ATTCTGGGGACA  
Depth:2 (PIG)  
Ei-value:0.000, Pi-value:0.000  
Er-value:0.000, Pr-value:0.000  
No matches to TargetScan

-

GAACCATTC

GAACCATTC  
Depth:2 (PIG)  
Ei-value:0.000, Pi-value:0.000  
Er-value:0.000, Pr-value:0.000  
No matches to TargetScan

-----------

TTAC

TTACTGCTTTACT  
Depth:2 (PIG)  
Ei-value:0.000, Pi-value:0.000  
Er-value:0.000, Pr-value:0.000  
MATCHES To TargetScan▶ miR-330-3p.2:AAAGCAC▶ miR-802:CAGUAAC


TG

TGCTTTACT  
Depth:3 (COW)  
Ei-value:0.000, Pi-value:0.000  
Er-value:0.000, Pr-value:0.000  
MATCHES To TargetScan▶ miR-330-3p.2:AAAGCAC


CTTTACT

CTTTACT  
Depth:4 (DOG)  
Ei-value:0.000, Pi-value:0.000  
Er-value:0.000, Pr-value:0.000  
No matches to TargetScan

--

GCAAAAT

GCAAAAT  
Depth:6 (MOUSE)  
Ei-value:0.000, Pi-value:0.000  
Er-value:0.000, Pr-value:0.000  
No matches to TargetScan

----

AAGGCAA

AAGGCAA  
Depth:4 (DOG)  
Ei-value:0.000, Pi-value:0.000  
Er-value:0.000, Pr-value:0.000  
No matches to TargetScan


GTCAGACCCA

AAGGCAAGTCAGACCCA  
Depth:3 (COW)  
Ei-value:0.000, Pi-value:0.000  
Er-value:0.000, Pr-value:0.000  
MATCHES To TargetScan▶ miR-193a-5p:GGGUCUU

--------

TGGATTGC

TGGATTGC  
Depth:4 (DOG)  
Ei-value:0.000, Pi-value:0.000  
Er-value:0.000, Pr-value:0.000  
No matches to TargetScan

---------------------------------------- 16200  
 -----------

TCTCCCCAG

TCTCCCCAGGAAGGAAG  
Depth:2 (PIG)  
Ei-value:0.000, Pi-value:0.000  
Er-value:0.000, Pr-value:0.000  
No matches to TargetScan


GAAGGAAG

GAAGGAAG  
Depth:3 (COW)  
Ei-value:0.000, Pi-value:0.000  
Er-value:0.000, Pr-value:0.000  
No matches to TargetScan

----------

TCTC

TCTCTGCATTCTTC  
Depth:2 (PIG)  
Ei-value:0.000, Pi-value:0.000  
Er-value:0.000, Pr-value:0.000  
No matches to TargetScan


TGCATTCTTC

TGCATTCTTC  
Depth:5 (RABBIT)  
Ei-value:0.000, Pi-value:0.000  
Er-value:0.000, Pr-value:0.000  
No matches to TargetScan

----

CAG

CAGAGCAGATTGCCTGG  
Depth:2 (PIG)  
Ei-value:0.000, Pi-value:0.000  
Er-value:0.000, Pr-value:0.000  
No matches to TargetScan


AGC

AGCAGATTGCCTGG  
Depth:4 (DOG)  
Ei-value:0.000, Pi-value:0.000  
Er-value:0.000, Pr-value:0.000  
No matches to TargetScan


A

AGATTGCCTGG  
Depth:5 (RABBIT)  
Ei-value:0.000, Pi-value:0.000  
Er-value:0.000, Pr-value:0.000  
No matches to TargetScan


GATTGCCTGG

GATTGCCTGG  
Depth:6 (MOUSE)  
Ei-value:0.000, Pi-value:0.000  
Er-value:0.000, Pr-value:0.000  
No matches to TargetScan

-

TAAGAATCTCT

TAAGAATCTCT  
Depth:2 (PIG)  
Ei-value:0.000, Pi-value:0.000  
Er-value:0.000, Pr-value:0.000  
No matches to TargetScan

---------

TTGTATATT

TTGTATATT  
Depth:4 (DOG)  
Ei-value:0.000, Pi-value:0.000  
Er-value:0.000, Pr-value:0.000  
MATCHES To TargetScan▶ miR-381-3p:AUACAAG

------------

TGCCA

TGCCAA  
Depth:3 (COW)  
Ei-value:0.000, Pi-value:0.000  
Er-value:0.000, Pr-value:0.000  
MATCHES To TargetScan▶ miR-182-5p:UUGGCAA▶ miR-96-5p/1271-5p:UUGGCAC

 16320  


A

TGCCAA  
Depth:3 (COW)  
Ei-value:0.000, Pi-value:0.000  
Er-value:0.000, Pr-value:0.000  
MATCHES To TargetScan▶ miR-182-5p:UUGGCAA▶ miR-96-5p/1271-5p:UUGGCAC

-

TGCCAGGATACA

TGCCAGGATACA  
Depth:3 (COW)  
Ei-value:0.000, Pi-value:0.000  
Er-value:0.000, Pr-value:0.000  
No matches to TargetScan

---------------------

AAATTTTTT

AAATTTTTT  
Depth:2 (PIG)  
Ei-value:0.000, Pi-value:0.000  
Er-value:0.000, Pr-value:0.000  
No matches to TargetScan

-------

ACATCTGG

ACATCTGG  
Depth:3 (COW)  
Ei-value:0.000, Pi-value:0.000  
Er-value:0.000, Pr-value:0.000  
No matches to TargetScan

----------------

GAT

GATAACCTGGTCATT  
Depth:3 (COW)  
Ei-value:0.000, Pi-value:0.000  
Er-value:0.000, Pr-value:0.000  
MATCHES To TargetScan▶ miR-154-5p:AGGUUAU


AAC

AACCTGGTCATT  
Depth:4 (DOG)  
Ei-value:0.000, Pi-value:0.000  
Er-value:0.000, Pr-value:0.000  
No matches to TargetScan


CTGGTCATT

CTGGTCATT  
Depth:5 (RABBIT)  
Ei-value:0.000, Pi-value:0.000  
Er-value:0.000, Pr-value:0.000  
No matches to TargetScan

---

T

TTTTTGAAG  
Depth:2 (PIG)  
Ei-value:0.000, Pi-value:0.000  
Er-value:0.000, Pr-value:0.000  
No matches to TargetScan


TTTTGAA

TTTTGAA  
Depth:3 (COW)  
Ei-value:0.000, Pi-value:0.000  
Er-value:0.000, Pr-value:0.010  
No matches to TargetScan


G

TTTTTGAAG  
Depth:2 (PIG)  
Ei-value:0.000, Pi-value:0.000  
Er-value:0.000, Pr-value:0.000  
No matches to TargetScan

----------

CCATTTAT

CCATTTAT  
Depth:5 (RABBIT)  
Ei-value:0.000, Pi-value:0.000  
Er-value:0.000, Pr-value:0.000  
No matches to TargetScan

 16440  


CCATTTAT  
Depth:5 (RABBIT)  
Ei-value:0.000, Pi-value:0.000  
Er-value:0.000, Pr-value:0.000  
No matches to TargetScan

-------------

TGAC

TGACCAGTGTCTCTCATTT  
Depth:4 (DOG)  
Ei-value:0.000, Pi-value:0.000  
Er-value:0.000, Pr-value:0.000  
No matches to TargetScan


CAGTGTCTCTCATTT

CAGTGTCTCTCATTT  
Depth:5 (RABBIT)  
Ei-value:0.000, Pi-value:0.000  
Er-value:0.000, Pr-value:0.000  
No matches to TargetScan

----

AGG

AGGGTGGTG  
Depth:4 (DOG)  
Ei-value:0.000, Pi-value:0.000  
Er-value:0.000, Pr-value:0.000  
No matches to TargetScan


GTGGTG

GTGGTG  
Depth:5 (RABBIT)  
Ei-value:0.000, Pi-value:0.000  
Er-value:0.000, Pr-value:0.000  
No matches to TargetScan


G

AGGGTGGTGGGTCTGTGGATAGA  
Depth:2 (PIG)  
Ei-value:0.000, Pi-value:0.000  
Er-value:0.000, Pr-value:0.000  
MATCHES To TargetScan▶ miR-140-3p.1:CCACAGG


GTCTGTGGATA

GTCTGTGGATA  
Depth:5 (RABBIT)  
Ei-value:0.000, Pi-value:0.000  
Er-value:0.000, Pr-value:0.000  
MATCHES To TargetScan▶ miR-140-3p.1:CCACAGG


GA

GTCTGTGGATAGA  
Depth:3 (COW)  
Ei-value:0.000, Pi-value:0.000  
Er-value:0.000, Pr-value:0.000  
MATCHES To TargetScan▶ miR-140-3p.1:CCACAGG

---------------

TATTTTA

TATTTTA  
Depth:3 (COW)  
Ei-value:0.000, Pi-value:0.040  
Er-value:0.000, Pr-value:0.020  
No matches to TargetScan

------------------

TTCTAGA

TTCTAGA  
Depth:4 (DOG)  
Ei-value:0.000, Pi-value:0.000  
Er-value:0.000, Pr-value:0.000  
No matches to TargetScan

-------------- 16560  
 ---

AGTATCTTTG

AGTATCTTTG  
Depth:3 (COW)  
Ei-value:0.000, Pi-value:0.000  
Er-value:0.000, Pr-value:0.000  
No matches to TargetScan

----------------------------------------------

ATTCACTT

ATTCACTT  
Depth:4 (DOG)  
Ei-value:0.000, Pi-value:0.000  
Er-value:0.000, Pr-value:0.000  
No matches to TargetScan


TTA

ATTCACTTTTAGAAAAAC  
Depth:2 (PIG)  
Ei-value:0.000, Pi-value:0.000  
Er-value:0.000, Pr-value:0.000  
MATCHES To TargetScan▶ miR-17-5p/20-5p/93-5p/106-5p/519-3p:AAAGUGC


GAAAAAC

GAAAAAC  
Depth:4 (DOG)  
Ei-value:0.000, Pi-value:0.000  
Er-value:0.000, Pr-value:0.000  
No matches to TargetScan

-------------

TAATCCTT

TAATCCTT  
Depth:2 (PIG)  
Ei-value:0.000, Pi-value:0.010  
Er-value:0.000, Pr-value:0.000  
No matches to TargetScan

-

AATTTCTTCATCTGGAGC

AATTTCTTCATCTGGAGC  
Depth:5 (RABBIT)  
Ei-value:0.000, Pi-value:0.000  
Er-value:0.000, Pr-value:0.000  
No matches to TargetScan


A

AATTTCTTCATCTGGAGCA  
Depth:2 (PIG)  
Ei-value:0.000, Pi-value:0.000  
Er-value:0.000, Pr-value:0.000  
No matches to TargetScan

-- 16680  
 ------------

CTTATTT

CTTATTT  
Depth:4 (DOG)  
Ei-value:0.000, Pi-value:0.000  
Er-value:0.000, Pr-value:0.010  
No matches to TargetScan


CAAGAA

CTTATTTCAAGAA  
Depth:3 (COW)  
Ei-value:0.000, Pi-value:0.000  
Er-value:0.000, Pr-value:0.000  
MATCHES To TargetScan▶ miR-203a-3p.2:UGAAAUG

---------------

ATAAAATG

ATAAAATG  
Depth:4 (DOG)  
Ei-value:0.000, Pi-value:0.000  
Er-value:0.000, Pr-value:0.000  
No matches to TargetScan


A

ATAAAATGA  
Depth:3 (COW)  
Ei-value:0.000, Pi-value:0.000  
Er-value:0.000, Pr-value:0.000  
No matches to TargetScan

----------------------------------------------------------------------- 16800  
 --------------------------

ACCACACT

ACCACACT  
Depth:3 (COW)  
Ei-value:0.000, Pi-value:0.000  
Er-value:0.000, Pr-value:0.000  
No matches to TargetScan


GA

ACCACACTGA  
Depth:2 (PIG)  
Ei-value:0.000, Pi-value:0.000  
Er-value:0.000, Pr-value:0.000  
No matches to TargetScan

-

GTGAGG

GTGAGG  
Depth:3 (COW)  
Ei-value:0.000, Pi-value:0.000  
Er-value:0.000, Pr-value:0.000  
No matches to TargetScan

------

AAATGAT

AAATGAT  
Depth:2 (PIG)  
Ei-value:0.000, Pi-value:0.020  
Er-value:0.000, Pr-value:0.020  
MATCHES To TargetScan▶ miR-382-3p:AUCAUUC

---------------

TTTTATA

TTTTATA  
Depth:3 (COW)  
Ei-value:0.000, Pi-value:0.000  
Er-value:0.000, Pr-value:0.010  
MATCHES To TargetScan▶ miR-340-5p:UAUAAAG

-------

AAAAATAAGCCA

AAAAATAAGCCA  
Depth:5 (RABBIT)  
Ei-value:0.000, Pi-value:0.000  
Er-value:0.000, Pr-value:0.000  
No matches to TargetScan


A

AAAAATAAGCCAA  
Depth:4 (DOG)  
Ei-value:0.000, Pi-value:0.000  
Er-value:0.000, Pr-value:0.000  
No matches to TargetScan

---------

AT

ATTCTTTTGGATATA  
Depth:2 (PIG)  
Ei-value:0.000, Pi-value:0.000  
Er-value:0.000, Pr-value:0.000  
MATCHES To TargetScan▶ miR-186-5p:AAAGAAU


TCTTTTGGATA

TCTTTTGGATATA  
Depth:3 (COW)  
Ei-value:0.000, Pi-value:0.000  
Er-value:0.000, Pr-value:0.000  
No matches to TargetScan

 16920  


TA

TCTTTTGGATATA  
Depth:3 (COW)  
Ei-value:0.000, Pi-value:0.000  
Er-value:0.000, Pr-value:0.000  
No matches to TargetScan

----------

AGTGAGATAGCTGCCT

AGTGAGATAGCTGCCT  
Depth:2 (PIG)  
Ei-value:0.000, Pi-value:0.000  
Er-value:0.000, Pr-value:0.000  
No matches to TargetScan

-------

ATGAATAATA

ATGAATAATA  
Depth:4 (DOG)  
Ei-value:0.000, Pi-value:0.000  
Er-value:0.000, Pr-value:0.000  
No matches to TargetScan

-----------

AGTGTACA

AGTGTACA  
Depth:3 (COW)  
Ei-value:0.000, Pi-value:0.000  
Er-value:0.000, Pr-value:0.000  
MATCHES To TargetScan▶ miR-493-5p:UGUACAU


G

AGTGTACAGGGTGTTT  
Depth:2 (PIG)  
Ei-value:0.000, Pi-value:0.000  
Er-value:0.000, Pr-value:0.000  
MATCHES To TargetScan▶ miR-10-5p:ACCCUGU▶ miR-339-5p:CCCUGUC▶ miR-486-5p:CCUGUAC▶ miR-493-5p:UGUACAU▶ miR-504-5p.1:ACCCUGG


GGTGTTT

GGTGTTT  
Depth:3 (COW)  
Ei-value:0.000, Pi-value:0.000  
Er-value:0.000, Pr-value:0.000  
No matches to TargetScan

---------------------

TGGAACTGCT

TGGAACTGCT  
Depth:4 (DOG)  
Ei-value:0.000, Pi-value:0.000  
Er-value:0.000, Pr-value:0.000  
No matches to TargetScan

-----

AAA

AAATAACTAGT  
Depth:2 (PIG)  
Ei-value:0.000, Pi-value:0.000  
Er-value:0.000, Pr-value:0.000  
No matches to TargetScan


TAACTA

TAACTA  
Depth:4 (DOG)  
Ei-value:0.000, Pi-value:0.000  
Er-value:0.000, Pr-value:0.000  
No matches to TargetScan


GT

AAATAACTAGT  
Depth:2 (PIG)  
Ei-value:0.000, Pi-value:0.000  
Er-value:0.000, Pr-value:0.000  
No matches to TargetScan

- 17040  
 -------

CAGCAGTTC

CAGCAGTTC  
Depth:5 (RABBIT)  
Ei-value:0.000, Pi-value:0.000  
Er-value:0.000, Pr-value:0.000  
No matches to TargetScan

-

TTGTAAT

TTGTAAT  
Depth:4 (DOG)  
Ei-value:0.000, Pi-value:0.000  
Er-value:0.000, Pr-value:0.000  
No matches to TargetScan

-

ACTGAAAA

ACTGAAAA  
Depth:5 (RABBIT)  
Ei-value:0.000, Pi-value:0.000  
Er-value:0.000, Pr-value:0.000  
No matches to TargetScan

--------------

GAG

GAGAAGGATGTCAAAAGATCGGC  
Depth:3 (COW)  
Ei-value:0.000, Pi-value:0.000  
Er-value:0.000, Pr-value:0.000  
MATCHES To TargetScan▶ miR-362-5p/500b-5p:AUCCUUG▶ miR-489-3p:UGACAUC


AAGGATG

AAGGATG  
Depth:5 (RABBIT)  
Ei-value:0.000, Pi-value:0.000  
Er-value:0.000, Pr-value:0.000  
MATCHES To TargetScan▶ miR-362-5p/500b-5p:AUCCUUG


TCA

AAGGATGTCAAAAGATC  
Depth:4 (DOG)  
Ei-value:0.000, Pi-value:0.000  
Er-value:0.000, Pr-value:0.000  
MATCHES To TargetScan▶ miR-362-5p/500b-5p:AUCCUUG▶ miR-489-3p:UGACAUC


AAAGATC

AAAGATC  
Depth:6 (MOUSE)  
Ei-value:0.000, Pi-value:0.000  
Er-value:0.000, Pr-value:0.000  
No matches to TargetScan


GGC

GAGAAGGATGTCAAAAGATCGGC  
Depth:3 (COW)  
Ei-value:0.000, Pi-value:0.000  
Er-value:0.000, Pr-value:0.000  
MATCHES To TargetScan▶ miR-362-5p/500b-5p:AUCCUUG▶ miR-489-3p:UGACAUC

-

CAGCTCAGGG

CAGCTCAGGG  
Depth:4 (DOG)  
Ei-value:0.000, Pi-value:0.000  
Er-value:0.000, Pr-value:0.000  
MATCHES To TargetScan▶ miR-125-5p:CCCUGAG

-

GCAGTTTGC

GCAGTTTGC  
Depth:3 (COW)  
Ei-value:0.000, Pi-value:0.000  
Er-value:0.000, Pr-value:0.000  
No matches to TargetScan

-

CTACTAGCTCCT

CTACTAGCTCCT  
Depth:4 (DOG)  
Ei-value:0.000, Pi-value:0.000  
Er-value:0.000, Pr-value:0.000  
MATCHES To TargetScan▶ miR-28-5p/708-5p:AGGAGCU▶ miR-411-5p.2:UAGUAGA

-

GGACAGCTG

GGACAGCTG  
Depth:5 (RABBIT)  
Ei-value:0.000, Pi-value:0.000  
Er-value:0.000, Pr-value:0.000  
No matches to TargetScan


T

GGACAGCTGT  
Depth:4 (DOG)  
Ei-value:0.000, Pi-value:0.000  
Er-value:0.000, Pr-value:0.000  
No matches to TargetScan


A

AAGAAGAGTCTCTGGCTCTTTAGA  
Depth:3 (COW)  
Ei-value:0.000, Pi-value:0.000  
Er-value:0.000, Pr-value:0.000  
No matches to TargetScan


AGAA

AGAAGAGTCTCTGGCTCTTTA  
Depth:5 (RABBIT)  
Ei-value:0.000, Pi-value:0.000  
Er-value:0.000, Pr-value:0.000  
No matches to TargetScan

 17160  


GAGTCTCTGGCTCTTTA

AGAAGAGTCTCTGGCTCTTTA  
Depth:5 (RABBIT)  
Ei-value:0.000, Pi-value:0.000  
Er-value:0.000, Pr-value:0.000  
No matches to TargetScan


GA

AGAAGAGTCTCTGGCTCTTTAGA  
Depth:4 (DOG)  
Ei-value:0.000, Pi-value:0.000  
Er-value:0.000, Pr-value:0.000  
No matches to TargetScan

-----------

ATTCTGAGC

ATTCTGAGC  
Depth:4 (DOG)  
Ei-value:0.000, Pi-value:0.000  
Er-value:0.000, Pr-value:0.000  
No matches to TargetScan

--------------------------------------------------------------------------------- 17280  
 ------------------

GA

GACTGCAA  
Depth:3 (COW)  
Ei-value:0.000, Pi-value:0.000  
Er-value:0.000, Pr-value:0.000  
MATCHES To TargetScan▶ miR-455-3p.2:UGCAGUC


CTGCAA

CTGCAA  
Depth:5 (RABBIT)  
Ei-value:0.000, Pi-value:0.000  
Er-value:0.000, Pr-value:0.000  
No matches to TargetScan

--------------------------------------

TTTGAGAATCTGG

TTTGAGAATCTGG  
Depth:3 (COW)  
Ei-value:0.000, Pi-value:0.000  
Er-value:0.000, Pr-value:0.000  
MATCHES To TargetScan▶ miR-371-5p:CUCAAAC

--

AAGCTCCA

AAGCTCCA  
Depth:3 (COW)  
Ei-value:0.000, Pi-value:0.000  
Er-value:0.000, Pr-value:0.000  
No matches to TargetScan

---

AATCTA

AATCTA  
Depth:2 (PIG)  
Ei-value:0.000, Pi-value:0.000  
Er-value:0.000, Pr-value:0.000  
No matches to TargetScan

---

GGATGG

GGATGG  
Depth:3 (COW)  
Ei-value:0.000, Pi-value:0.000  
Er-value:0.000, Pr-value:0.010  
No matches to TargetScan

-----------

T

TCTGGAGAAAAAGATCTTCCTCAGAAGAATAGGCTTGTTG  
Depth:2 (PIG)  
Ei-value:0.000, Pi-value:0.000  
Er-value:0.000, Pr-value:0.000  
MATCHES To TargetScan▶ miR-1224-5p:UGAGGAC▶ miR-7-5p:GGAAGAC


CTG

CTGGAGAAAAAGATCT  
Depth:3 (COW)  
Ei-value:0.000, Pi-value:0.000  
Er-value:0.000, Pr-value:0.000  
No matches to TargetScan

 17400  


GAGAAAAAGATCT

CTGGAGAAAAAGATCT  
Depth:3 (COW)  
Ei-value:0.000, Pi-value:0.000  
Er-value:0.000, Pr-value:0.000  
No matches to TargetScan


TCCTCAG

TCTGGAGAAAAAGATCTTCCTCAGAAGAATAGGCTTGTTG  
Depth:2 (PIG)  
Ei-value:0.000, Pi-value:0.000  
Er-value:0.000, Pr-value:0.000  
MATCHES To TargetScan▶ miR-1224-5p:UGAGGAC▶ miR-7-5p:GGAAGAC


AAGAATAGGC

AAGAATAGGC  
Depth:5 (RABBIT)  
Ei-value:0.000, Pi-value:0.000  
Er-value:0.000, Pr-value:0.000  
No matches to TargetScan


TTGTTG

TCTGGAGAAAAAGATCTTCCTCAGAAGAATAGGCTTGTTG  
Depth:2 (PIG)  
Ei-value:0.000, Pi-value:0.000  
Er-value:0.000, Pr-value:0.000  
MATCHES To TargetScan▶ miR-1224-5p:UGAGGAC▶ miR-7-5p:GGAAGAC

--

T

TTACAGTGTTAGTGA  
Depth:3 (COW)  
Ei-value:0.000, Pi-value:0.000  
Er-value:0.000, Pr-value:0.000  
MATCHES To TargetScan▶ miR-141-3p/200a-3p:AACACUG


TACAGTGTTAGTGA

TACAGTGTTAGTGA  
Depth:5 (RABBIT)  
Ei-value:0.000, Pi-value:0.000  
Er-value:0.000, Pr-value:0.000  
MATCHES To TargetScan▶ miR-141-3p/200a-3p:AACACUG

--

CA

CATTCCCTTTGA  
Depth:3 (COW)  
Ei-value:0.000, Pi-value:0.000  
Er-value:0.000, Pr-value:0.000  
MATCHES To TargetScan▶ miR-1-3p/206:GGAAUGU


TTCCCTTTGA

TTCCCTTTGA  
Depth:6 (MOUSE)  
Ei-value:0.000, Pi-value:0.000  
Er-value:0.000, Pr-value:0.000  
No matches to TargetScan

-------

TAGGTGGAGATGGGGCATGAGGATCCTCCAGGGGAA

TAGGTGGAGATGGGGCATGAGGATCCTCCAGGGGAA  
Depth:6 (MOUSE)  
Ei-value:0.000, Pi-value:0.000  
Er-value:0.000, Pr-value:0.000  
MATCHES To TargetScan▶ miR-331-3p:CCCCUGG


A

TAGGTGGAGATGGGGCATGAGGATCCTCCAGGGGAAA  
Depth:5 (RABBIT)  
Ei-value:0.000, Pi-value:0.000  
Er-value:0.000, Pr-value:0.000  
MATCHES To TargetScan▶ miR-331-3p:CCCCUGG


AGC

TAGGTGGAGATGGGGCATGAGGATCCTCCAGGGGAAAAGCTCACTACCACTGGGCAACAACCCTAGGTCAGGAG  
Depth:2 (PIG)  
Ei-value:0.000, Pi-value:0.000  
Er-value:0.000, Pr-value:0.000  
MATCHES To TargetScan▶ miR-140-5p:AGUGGUU▶ miR-142-3p.1:GUAGUGU▶ miR-192-5p/215-5p:UGACCUA▶ miR-199-5p:CCAGUGU▶ miR-296-3p:AGGGUUG▶ miR-331-3p:CCCCUGG


TCACTA

TCACTA  
Depth:5 (RABBIT)  
Ei-value:0.000, Pi-value:0.000  
Er-value:0.000, Pr-value:0.000  
No matches to TargetScan

 17520  


TCACTA  
Depth:5 (RABBIT)  
Ei-value:0.000, Pi-value:0.000  
Er-value:0.000, Pr-value:0.000  
No matches to TargetScan


CCACT

TCACTACCACT  
Depth:4 (DOG)  
Ei-value:0.000, Pi-value:0.000  
Er-value:0.000, Pr-value:0.000  
MATCHES To TargetScan▶ miR-140-5p:AGUGGUU▶ miR-142-3p.1:GUAGUGU


G

TCACTACCACTG  
Depth:3 (COW)  
Ei-value:0.000, Pi-value:0.000  
Er-value:0.000, Pr-value:0.000  
MATCHES To TargetScan▶ miR-140-5p:AGUGGUU▶ miR-142-3p.1:GUAGUGU


G

TAGGTGGAGATGGGGCATGAGGATCCTCCAGGGGAAAAGCTCACTACCACTGGGCAACAACCCTAGGTCAGGAG  
Depth:2 (PIG)  
Ei-value:0.000, Pi-value:0.000  
Er-value:0.000, Pr-value:0.000  
MATCHES To TargetScan▶ miR-140-5p:AGUGGUU▶ miR-142-3p.1:GUAGUGU▶ miR-192-5p/215-5p:UGACCUA▶ miR-199-5p:CCAGUGU▶ miR-296-3p:AGGGUUG▶ miR-331-3p:CCCCUGG


GCAACA

GCAACA  
Depth:6 (MOUSE)  
Ei-value:0.000, Pi-value:0.000  
Er-value:0.000, Pr-value:0.000  
No matches to TargetScan


AC

GCAACAAC  
Depth:5 (RABBIT)  
Ei-value:0.000, Pi-value:0.000  
Er-value:0.000, Pr-value:0.000  
No matches to TargetScan


CCTAGGTCAGGAG

TAGGTGGAGATGGGGCATGAGGATCCTCCAGGGGAAAAGCTCACTACCACTGGGCAACAACCCTAGGTCAGGAG  
Depth:2 (PIG)  
Ei-value:0.000, Pi-value:0.000  
Er-value:0.000, Pr-value:0.000  
MATCHES To TargetScan▶ miR-140-5p:AGUGGUU▶ miR-142-3p.1:GUAGUGU▶ miR-192-5p/215-5p:UGACCUA▶ miR-199-5p:CCAGUGU▶ miR-296-3p:AGGGUUG▶ miR-331-3p:CCCCUGG

--------------

CTTTCCTGG

CTTTCCTGG  
Depth:3 (COW)  
Ei-value:0.000, Pi-value:0.000  
Er-value:0.000, Pr-value:0.000  
MATCHES To TargetScan▶ miR-665:CCAGGAG▶ miR-873-5p.1:CAGGAAC

--

CCAGATAGGAAGAT

CCAGATAGGAAGAT  
Depth:2 (PIG)  
Ei-value:0.000, Pi-value:0.000  
Er-value:0.000, Pr-value:0.000  
MATCHES To TargetScan▶ miR-202-5p:UCCUAUG

-

AAGTCTCAA

AAGTCTCAA  
Depth:2 (PIG)  
Ei-value:0.000, Pi-value:0.000  
Er-value:0.000, Pr-value:0.000  
No matches to TargetScan

-

ACAACCACC

ACAACCACC  
Depth:5 (RABBIT)  
Ei-value:0.000, Pi-value:0.000  
Er-value:0.000, Pr-value:0.000  
No matches to TargetScan


ACAC

ACAACCACCACAC  
Depth:4 (DOG)  
Ei-value:0.000, Pi-value:0.000  
Er-value:0.000, Pr-value:0.000  
No matches to TargetScan

----------------------------- 17640  
 ------------------------------------------------------------------------------------------------------------------------ 17760  
 ------------------------------------------------------------------------------------------------------------------------ 17880  
 -------------------------------------------------------------------------

A

ATTGTTCC  
Depth:2 (PIG)  
Ei-value:0.000, Pi-value:0.000  
Er-value:0.000, Pr-value:0.000  
No matches to TargetScan


TTGTTCC

TTGTTCC  
Depth:4 (DOG)  
Ei-value:0.000, Pi-value:0.000  
Er-value:0.000, Pr-value:0.000  
No matches to TargetScan

---------

TG

TGCCAAATC  
Depth:3 (COW)  
Ei-value:0.000, Pi-value:0.000  
Er-value:0.000, Pr-value:0.000  
MATCHES To TargetScan▶ miR-182-5p:UUGGCAA▶ miR-96-5p/1271-5p:UUGGCAC


CCAAAT

CCAAAT  
Depth:6 (MOUSE)  
Ei-value:0.000, Pi-value:0.000  
Er-value:0.000, Pr-value:0.000  
No matches to TargetScan


C

CCAAATC  
Depth:5 (RABBIT)  
Ei-value:0.000, Pi-value:0.000  
Er-value:0.000, Pr-value:0.000  
No matches to TargetScan

--------------

AAGCAGT

AAGCAGTG  
Depth:2 (PIG)  
Ei-value:0.000, Pi-value:0.000  
Er-value:0.000, Pr-value:0.000  
No matches to TargetScan

 18000  


G

AAGCAGTG  
Depth:2 (PIG)  
Ei-value:0.000, Pi-value:0.000  
Er-value:0.000, Pr-value:0.000  
No matches to TargetScan

-

AGAGAG

AGAGAG  
Depth:2 (PIG)  
Ei-value:0.000, Pi-value:0.010  
Er-value:0.000, Pr-value:0.000  
No matches to TargetScan


CAAGAAA

CAAGAAA  
Depth:5 (RABBIT)  
Ei-value:0.000, Pi-value:0.000  
Er-value:0.000, Pr-value:0.000  
No matches to TargetScan


T

CAAGAAAT  
Depth:3 (COW)  
Ei-value:0.000, Pi-value:0.000  
Er-value:0.000, Pr-value:0.000  
No matches to TargetScan


T

CAAGAAATTTGAACACAC  
Depth:2 (PIG)  
Ei-value:0.000, Pi-value:0.000  
Er-value:0.000, Pr-value:0.000  
No matches to TargetScan


TGAACACAC

TGAACACAC  
Depth:3 (COW)  
Ei-value:0.000, Pi-value:0.000  
Er-value:0.000, Pr-value:0.000  
No matches to TargetScan

-----

G

GAAGATCAACATGCCTG  
Depth:4 (DOG)  
Ei-value:0.000, Pi-value:0.000  
Er-value:0.000, Pr-value:0.000  
No matches to TargetScan


AA

AAGATCAACATGC  
Depth:5 (RABBIT)  
Ei-value:0.000, Pi-value:0.000  
Er-value:0.000, Pr-value:0.000  
No matches to TargetScan


GATCAACATGC

GATCAACATGC  
Depth:6 (MOUSE)  
Ei-value:0.000, Pi-value:0.000  
Er-value:0.000, Pr-value:0.000  
No matches to TargetScan


CTG

GAAGATCAACATGCCTG  
Depth:4 (DOG)  
Ei-value:0.000, Pi-value:0.000  
Er-value:0.000, Pr-value:0.000  
No matches to TargetScan


GC

GAAGATCAACATGCCTGGC  
Depth:2 (PIG)  
Ei-value:0.000, Pi-value:0.000  
Er-value:0.000, Pr-value:0.000  
No matches to TargetScan

----------------------

TGAATGA

TGAATGA  
Depth:2 (PIG)  
Ei-value:0.000, Pi-value:0.010  
Er-value:0.000, Pr-value:0.020  
MATCHES To TargetScan▶ miR-1298-5p:UCAUUCG

--------------------------------

TGTGTAT

TGTGTAT  
Depth:6 (MOUSE)  
Ei-value:0.000, Pi-value:0.000  
Er-value:0.000, Pr-value:0.000  
No matches to TargetScan


TT

TGTGTATTT  
Depth:4 (DOG)  
Ei-value:0.000, Pi-value:0.000  
Er-value:0.000, Pr-value:0.000  
No matches to TargetScan

 18120  


TGTGTATTT  
Depth:4 (DOG)  
Ei-value:0.000, Pi-value:0.000  
Er-value:0.000, Pr-value:0.000  
No matches to TargetScan

------

TTGTC

TTGTCTCTTTCTTTCTT  
Depth:2 (PIG)  
Ei-value:0.000, Pi-value:0.000  
Er-value:0.000, Pr-value:0.000  
MATCHES To TargetScan▶ miR-186-5p:AAAGAAU


TCTTTCTT

TCTTTCTT  
Depth:3 (COW)  
Ei-value:0.000, Pi-value:0.000  
Er-value:0.000, Pr-value:0.000  
No matches to TargetScan


TCTT

TTGTCTCTTTCTTTCTT  
Depth:2 (PIG)  
Ei-value:0.000, Pi-value:0.000  
Er-value:0.000, Pr-value:0.000  
MATCHES To TargetScan▶ miR-186-5p:AAAGAAU

---------------------

TTCTCTA

TTCTCTA  
Depth:2 (PIG)  
Ei-value:0.000, Pi-value:0.010  
Er-value:0.000, Pr-value:0.000  
No matches to TargetScan

---

TG

TGTGTCTTACCCATTTCCATG  
Depth:2 (PIG)  
Ei-value:0.000, Pi-value:0.000  
Er-value:0.000, Pr-value:0.000  
MATCHES To TargetScan▶ miR-203a-3p.1:GAAAUGU▶ miR-208-3p:UAAGACG▶ miR-499a-5p:UAAGACU


TGTCTTA

TGTCTTA  
Depth:4 (DOG)  
Ei-value:0.000, Pi-value:0.000  
Er-value:0.000, Pr-value:0.000  
MATCHES To TargetScan▶ miR-208-3p:UAAGACG▶ miR-499a-5p:UAAGACU


CCCATTTCCATG

TGTCTTACCCATTTCCATG  
Depth:3 (COW)  
Ei-value:0.000, Pi-value:0.000  
Er-value:0.000, Pr-value:0.000  
MATCHES To TargetScan▶ miR-203a-3p.1:GAAAUGU▶ miR-208-3p:UAAGACG▶ miR-499a-5p:UAAGACU

----------------------------------------

TTTTT

TTTTTGT  
Depth:4 (DOG)  
Ei-value:0.000, Pi-value:0.000  
Er-value:0.000, Pr-value:0.000  
No matches to TargetScan

 18240  


GT

TTTTTGT  
Depth:4 (DOG)  
Ei-value:0.000, Pi-value:0.000  
Er-value:0.000, Pr-value:0.000  
No matches to TargetScan

------------

GGTCTGTGTCT

GGTCTGTGTCT  
Depth:2 (PIG)  
Ei-value:0.000, Pi-value:0.000  
Er-value:0.000, Pr-value:0.000  
No matches to TargetScan

-

GTCTTAGA

GTCTTAGA  
Depth:2 (PIG)  
Ei-value:0.000, Pi-value:0.000  
Er-value:0.000, Pr-value:0.000  
MATCHES To TargetScan▶ miR-208-3p:UAAGACG▶ miR-499a-5p:UAAGACU

-------------------

TTT

TTTTTCATTTTGTT  
Depth:2 (PIG)  
Ei-value:0.000, Pi-value:0.000  
Er-value:0.000, Pr-value:0.000  
MATCHES To TargetScan▶ miR-495-3p:AACAAAC


TTCATTTTGTT

TTCATTTTGTT  
Depth:4 (DOG)  
Ei-value:0.000, Pi-value:0.000  
Er-value:0.000, Pr-value:0.000  
MATCHES To TargetScan▶ miR-495-3p:AACAAAC

----------------------------------------------------- 18360  
 ------------------------

CTC

CTCTTTGCTC  
Depth:2 (PIG)  
Ei-value:0.000, Pi-value:0.000  
Er-value:0.000, Pr-value:0.000  
No matches to TargetScan


TTTGCTC

TTTGCTC  
Depth:3 (COW)  
Ei-value:0.000, Pi-value:0.000  
Er-value:0.000, Pr-value:0.000  
No matches to TargetScan

-

TTTCTTGTT

TTTCTTGTT  
Depth:2 (PIG)  
Ei-value:0.000, Pi-value:0.000  
Er-value:0.000, Pr-value:0.000  
No matches to TargetScan

-

TGCCTACCT

TGCCTACCT  
Depth:2 (PIG)  
Ei-value:0.000, Pi-value:0.000  
Er-value:0.000, Pr-value:0.000  
MATCHES To TargetScan▶ miR-196-5p:AGGUAGU

-------

TT

TTTTCTCTTTGTGAA  
Depth:3 (COW)  
Ei-value:0.000, Pi-value:0.000  
Er-value:0.000, Pr-value:0.000  
No matches to TargetScan


TTCTCTTTG

TTCTCTTTG  
Depth:6 (MOUSE)  
Ei-value:0.000, Pi-value:0.000  
Er-value:0.000, Pr-value:0.000  
No matches to TargetScan


TGAA

TTTTCTCTTTGTGAA  
Depth:3 (COW)  
Ei-value:0.000, Pi-value:0.000  
Er-value:0.000, Pr-value:0.000  
No matches to TargetScan

--------------------

TTCCCCTT

TTCCCCTT  
Depth:3 (COW)  
Ei-value:0.000, Pi-value:0.000  
Er-value:0.000, Pr-value:0.000  
No matches to TargetScan


CT

TTCCCCTTCT  
Depth:2 (PIG)  
Ei-value:0.000, Pi-value:0.000  
Er-value:0.000, Pr-value:0.000  
No matches to TargetScan

-

GTTCGTTT

GTTCGTTT  
Depth:2 (PIG)  
Ei-value:0.000, Pi-value:0.000  
Er-value:0.000, Pr-value:0.000  
No matches to TargetScan

---

AT

ATTTCACCT  
Depth:4 (DOG)  
Ei-value:0.000, Pi-value:0.000  
Er-value:0.000, Pr-value:0.000  
MATCHES To TargetScan▶ miR-203a-3p.2:UGAAAUG

 18480  


TTCACCT

ATTTCACCT  
Depth:4 (DOG)  
Ei-value:0.000, Pi-value:0.000  
Er-value:0.000, Pr-value:0.000  
MATCHES To TargetScan▶ miR-203a-3p.2:UGAAAUG

----------------------

TGCTG

TGCTGTTTCTACT  
Depth:3 (COW)  
Ei-value:0.000, Pi-value:0.000  
Er-value:0.000, Pr-value:0.000  
MATCHES To TargetScan▶ miR-411-5p.1:AGUAGAC▶ miR-494-3p:GAAACAU


TTTCTAC

TTTCTAC  
Depth:6 (MOUSE)  
Ei-value:0.000, Pi-value:0.000  
Er-value:0.000, Pr-value:0.000  
No matches to TargetScan


T

TTTCTACT  
Depth:5 (RABBIT)  
Ei-value:0.000, Pi-value:0.000  
Er-value:0.000, Pr-value:0.000  
MATCHES To TargetScan▶ miR-411-5p.1:AGUAGAC

----

ATCTCAC

ATCTCACATTTCTC  
Depth:2 (PIG)  
Ei-value:0.000, Pi-value:0.000  
Er-value:0.000, Pr-value:0.000  
MATCHES To TargetScan▶ miR-203a-3p.1:GAAAUGU


ATTTCTC

ATTTCTC  
Depth:6 (MOUSE)  
Ei-value:0.000, Pi-value:0.000  
Er-value:0.000, Pr-value:0.000  
No matches to TargetScan

-----------------

TGCCTC

TGCCTCTCTTGGGC  
Depth:2 (PIG)  
Ei-value:0.000, Pi-value:0.000  
Er-value:0.000, Pr-value:0.000  
MATCHES To TargetScan▶ miR-335-5p:CAAGAGC


TCTTGGG

TCTTGGG  
Depth:5 (RABBIT)  
Ei-value:0.000, Pi-value:0.000  
Er-value:0.000, Pr-value:0.000  
No matches to TargetScan


C

TCTTGGGC  
Depth:3 (COW)  
Ei-value:0.000, Pi-value:0.000  
Er-value:0.000, Pr-value:0.000  
No matches to TargetScan

----------------------------- 18600  
 -----------------

TTTGTGA

TTTGTGA  
Depth:4 (DOG)  
Ei-value:0.000, Pi-value:0.010  
Er-value:0.000, Pr-value:0.000  
No matches to TargetScan


TTTTC

TTTGTGATTTTC  
Depth:3 (COW)  
Ei-value:0.000, Pi-value:0.000  
Er-value:0.000, Pr-value:0.000  
No matches to TargetScan

----------------

TCTCTGTT

TCTCTGTT  
Depth:4 (DOG)  
Ei-value:0.000, Pi-value:0.000  
Er-value:0.000, Pr-value:0.000  
No matches to TargetScan

--------------------------------

TCACC

TCACCTTTGAGTATTT  
Depth:2 (PIG)  
Ei-value:0.000, Pi-value:0.000  
Er-value:0.000, Pr-value:0.000  
MATCHES To TargetScan▶ miR-18-5p:AAGGUGC▶ miR-200bc-3p/429:AAUACUG▶ miR-371-5p:CUCAAAC


TTTGAGTATTT

TTTGAGTATTT  
Depth:4 (DOG)  
Ei-value:0.000, Pi-value:0.000  
Er-value:0.000, Pr-value:0.000  
MATCHES To TargetScan▶ miR-200bc-3p/429:AAUACUG▶ miR-371-5p:CUCAAAC

--

GCCTCTTC

GCCTCTTC  
Depth:2 (PIG)  
Ei-value:0.000, Pi-value:0.000  
Er-value:0.000, Pr-value:0.000  
No matches to TargetScan

--------- 18720  
 --------

CTTTGATT

CTTTGATT  
Depth:3 (COW)  
Ei-value:0.000, Pi-value:0.000  
Er-value:0.000, Pr-value:0.000  
No matches to TargetScan

------------------------------------

TGTGTGTG

TGTGTGTG  
Depth:4 (DOG)  
Ei-value:0.000, Pi-value:0.000  
Er-value:0.000, Pr-value:0.000  
MATCHES To TargetScan▶ miR-329-3p/362-3p:ACACACC

----------------------

AGGGGCT

AGGGGCTTCCTAACCCCT  
Depth:2 (PIG)  
Ei-value:0.000, Pi-value:0.000  
Er-value:0.000, Pr-value:0.000  
No matches to TargetScan


TCCTAACCCCT

TCCTAACCCCT  
Depth:5 (RABBIT)  
Ei-value:0.000, Pi-value:0.000  
Er-value:0.000, Pr-value:0.000  
No matches to TargetScan

------

TAGGTGCA

TAGGTGCA  
Depth:3 (COW)  
Ei-value:0.000, Pi-value:0.000  
Er-value:0.000, Pr-value:0.000  
No matches to TargetScan

------ 18840  
 ---------------

AAGCATTG

AAGCATTG  
Depth:4 (DOG)  
Ei-value:0.000, Pi-value:0.000  
Er-value:0.000, Pr-value:0.000  
No matches to TargetScan

-------

GTTCC

GTTCCTTATGCCAG  
Depth:2 (PIG)  
Ei-value:0.000, Pi-value:0.000  
Er-value:0.000, Pr-value:0.000  
No matches to TargetScan


TTATGCCA

TTATGCCA  
Depth:5 (RABBIT)  
Ei-value:0.000, Pi-value:0.000  
Er-value:0.000, Pr-value:0.000  
No matches to TargetScan


G

TTATGCCAG  
Depth:4 (DOG)  
Ei-value:0.000, Pi-value:0.000  
Er-value:0.000, Pr-value:0.000  
No matches to TargetScan

------------------------------------

ATGA

ATGATCCAAGACCAA  
Depth:2 (PIG)  
Ei-value:0.000, Pi-value:0.000  
Er-value:0.000, Pr-value:0.000  
MATCHES To TargetScan▶ miR-133a-3p.2/133b:UUGGUCC▶ miR-431-5p:GUCUUGC


TCCAAG

TCCAAG  
Depth:3 (COW)  
Ei-value:0.000, Pi-value:0.000  
Er-value:0.000, Pr-value:0.000  
No matches to TargetScan


ACCAA

ATGATCCAAGACCAA  
Depth:2 (PIG)  
Ei-value:0.000, Pi-value:0.000  
Er-value:0.000, Pr-value:0.000  
MATCHES To TargetScan▶ miR-133a-3p.2/133b:UUGGUCC▶ miR-431-5p:GUCUUGC


T

TAGAAGGCCCAA  
Depth:2 (PIG)  
Ei-value:0.000, Pi-value:0.000  
Er-value:0.000, Pr-value:0.000  
No matches to TargetScan


AGA

AGAAGGCCCAA  
Depth:4 (DOG)  
Ei-value:0.000, Pi-value:0.000  
Er-value:0.000, Pr-value:0.000  
No matches to TargetScan


AGGCCCAA

AGGCCCAA  
Depth:5 (RABBIT)  
Ei-value:0.000, Pi-value:0.000  
Er-value:0.000, Pr-value:0.000  
No matches to TargetScan

-----------

GA

GAGTTGGATGGAAG  
Depth:2 (PIG)  
Ei-value:0.000, Pi-value:0.000  
Er-value:0.000, Pr-value:0.000  
No matches to TargetScan

 18960  


GTTGGATGGAAG

GAGTTGGATGGAAG  
Depth:2 (PIG)  
Ei-value:0.000, Pi-value:0.000  
Er-value:0.000, Pr-value:0.000  
No matches to TargetScan

----

AAGTCT

AAGTCT  
Depth:2 (PIG)  
Ei-value:0.000, Pi-value:0.010  
Er-value:0.000, Pr-value:0.010  
No matches to TargetScan

-----------

AAGGCCAA

AAGGCCAA  
Depth:2 (PIG)  
Ei-value:0.000, Pi-value:0.000  
Er-value:0.000, Pr-value:0.000  
No matches to TargetScan

-

GACCTAAGA

GACCTAAGA  
Depth:2 (PIG)  
Ei-value:0.000, Pi-value:0.000  
Er-value:0.000, Pr-value:0.000  
No matches to TargetScan

----------------------

GAAGGCCC

GAAGGCCC  
Depth:2 (PIG)  
Ei-value:0.000, Pi-value:0.000  
Er-value:0.000, Pr-value:0.000  
No matches to TargetScan

------------

TATC

TATCTCAAGACTAA  
Depth:2 (PIG)  
Ei-value:0.000, Pi-value:0.000  
Er-value:0.000, Pr-value:0.000  
MATCHES To TargetScan▶ miR-431-5p:GUCUUGC


TCAA

TCAAGACTAA  
Depth:4 (DOG)  
Ei-value:0.000, Pi-value:0.000  
Er-value:0.000, Pr-value:0.000  
MATCHES To TargetScan▶ miR-431-5p:GUCUUGC


GACTAA

GACTAA  
Depth:5 (RABBIT)  
Ei-value:0.000, Pi-value:0.000  
Er-value:0.000, Pr-value:0.000  
No matches to TargetScan

------------- 19080  
 ------------------

GAATCTGG

GAATCTGG  
Depth:2 (PIG)  
Ei-value:0.000, Pi-value:0.000  
Er-value:0.000, Pr-value:0.000  
No matches to TargetScan

----

GAT

GATAGAAGC  
Depth:2 (PIG)  
Ei-value:0.000, Pi-value:0.000  
Er-value:0.000, Pr-value:0.000  
No matches to TargetScan


AGAAGC

AGAAGC  
Depth:4 (DOG)  
Ei-value:0.000, Pi-value:0.000  
Er-value:0.000, Pr-value:0.010  
No matches to TargetScan

-----------

GGGAAAT

GGGAAAT  
Depth:2 (PIG)  
Ei-value:0.000, Pi-value:0.000  
Er-value:0.000, Pr-value:0.010  
No matches to TargetScan

--

C

CAAGATGA  
Depth:3 (COW)  
Ei-value:0.000, Pi-value:0.000  
Er-value:0.000, Pr-value:0.000  
No matches to TargetScan


AAGATGA

AAGATGA  
Depth:5 (RABBIT)  
Ei-value:0.000, Pi-value:0.000  
Er-value:0.000, Pr-value:0.000  
No matches to TargetScan

-

AACCCTAAA

AACCCTAAA  
Depth:2 (PIG)  
Ei-value:0.000, Pi-value:0.000  
Er-value:0.000, Pr-value:0.000  
MATCHES To TargetScan▶ miR-296-3p:AGGGUUG

------

CTCT

CTCTTTTCTATTGTT  
Depth:2 (PIG)  
Ei-value:0.000, Pi-value:0.000  
Er-value:0.000, Pr-value:0.000  
No matches to TargetScan


TTTCTATTG

TTTCTATTG  
Depth:3 (COW)  
Ei-value:0.000, Pi-value:0.000  
Er-value:0.000, Pr-value:0.000  
No matches to TargetScan


TT

CTCTTTTCTATTGTT  
Depth:2 (PIG)  
Ei-value:0.000, Pi-value:0.000  
Er-value:0.000, Pr-value:0.000  
No matches to TargetScan

---------------------- 19200  
 --

C

CACTTCTT  
Depth:2 (PIG)  
Ei-value:0.000, Pi-value:0.010  
Er-value:0.000, Pr-value:0.000  
No matches to TargetScan


ACTTCTT

ACTTCTT  
Depth:3 (COW)  
Ei-value:0.000, Pi-value:0.020  
Er-value:0.000, Pr-value:0.000  
No matches to TargetScan

----

TCCTGTT

TCCTGTT  
Depth:2 (PIG)  
Ei-value:0.000, Pi-value:0.020  
Er-value:0.000, Pr-value:0.000  
No matches to TargetScan

-----------------------------------

CTTTTTGATGTT

CTTTTTGATGTT  
Depth:4 (DOG)  
Ei-value:0.000, Pi-value:0.000  
Er-value:0.000, Pr-value:0.000  
No matches to TargetScan


GC

CTTTTTGATGTTGC  
Depth:2 (PIG)  
Ei-value:0.000, Pi-value:0.000  
Er-value:0.000, Pr-value:0.000  
No matches to TargetScan

-

GTTACCTT

GTTACCTT  
Depth:2 (PIG)  
Ei-value:0.000, Pi-value:0.000  
Er-value:0.000, Pr-value:0.010  
No matches to TargetScan

----------------------------

ACAG

ACAGTATTATGCCTGGGCCAGTCTT  
Depth:2 (PIG)  
Ei-value:0.000, Pi-value:0.000  
Er-value:0.000, Pr-value:0.000  
MATCHES To TargetScan▶ miR-193-3p:ACUGGCC▶ miR-200bc-3p/429:AAUACUG▶ miR-328-3p:UGGCCCU▶ miR-369-3p:AUAAUAC▶ miR-655-3p:UAAUACA


TATTATGC

TATTATGC  
Depth:4 (DOG)  
Ei-value:0.000, Pi-value:0.000  
Er-value:0.000, Pr-value:0.000  
MATCHES To TargetScan▶ miR-369-3p:AUAAUAC


C

ACAGTATTATGCCTGGGCCAGTCTT  
Depth:2 (PIG)  
Ei-value:0.000, Pi-value:0.000  
Er-value:0.000, Pr-value:0.000  
MATCHES To TargetScan▶ miR-193-3p:ACUGGCC▶ miR-200bc-3p/429:AAUACUG▶ miR-328-3p:UGGCCCU▶ miR-369-3p:AUAAUAC▶ miR-655-3p:UAAUACA

 19320  


TGGGCCAGTCTT

ACAGTATTATGCCTGGGCCAGTCTT  
Depth:2 (PIG)  
Ei-value:0.000, Pi-value:0.000  
Er-value:0.000, Pr-value:0.000  
MATCHES To TargetScan▶ miR-193-3p:ACUGGCC▶ miR-200bc-3p/429:AAUACUG▶ miR-328-3p:UGGCCCU▶ miR-369-3p:AUAAUAC▶ miR-655-3p:UAAUACA

-------------

TAAACTTC

TAAACTTC  
Depth:3 (COW)  
Ei-value:0.000, Pi-value:0.000  
Er-value:0.000, Pr-value:0.000  
No matches to TargetScan

------------------

CTCCACTTGAGAG

CTCCACTTGAGAG  
Depth:3 (COW)  
Ei-value:0.000, Pi-value:0.000  
Er-value:0.000, Pr-value:0.000  
MATCHES To TargetScan▶ miR-26-5p:UCAAGUA


A

CTCCACTTGAGAGA  
Depth:2 (PIG)  
Ei-value:0.000, Pi-value:0.000  
Er-value:0.000, Pr-value:0.000  
MATCHES To TargetScan▶ miR-26-5p:UCAAGUA

---

ATAGGTGA

ATAGGTGA  
Depth:2 (PIG)  
Ei-value:0.000, Pi-value:0.000  
Er-value:0.000, Pr-value:0.010  
No matches to TargetScan

-----

TATTTCAGT

TATTTCAGT  
Depth:4 (DOG)  
Ei-value:0.000, Pi-value:0.000  
Er-value:0.000, Pr-value:0.000  
MATCHES To TargetScan▶ miR-203a-3p.2:UGAAAUG


CC

TATTTCAGTCC  
Depth:3 (COW)  
Ei-value:0.000, Pi-value:0.000  
Er-value:0.000, Pr-value:0.000  
MATCHES To TargetScan▶ miR-203a-3p.2:UGAAAUG


T

TATTTCAGTCCT  
Depth:2 (PIG)  
Ei-value:0.000, Pi-value:0.000  
Er-value:0.000, Pr-value:0.000  
MATCHES To TargetScan▶ miR-203a-3p.2:UGAAAUG

--------------------------- 19440  


TGAGAAGA

TGAGAAGA  
Depth:2 (PIG)  
Ei-value:0.000, Pi-value:0.000  
Er-value:0.000, Pr-value:0.000  
No matches to TargetScan

---------------------

GGGGAAA

GGGGAAA  
Depth:4 (DOG)  
Ei-value:0.000, Pi-value:0.000  
Er-value:0.000, Pr-value:0.000  
No matches to TargetScan


AAA

GGGGAAAAAA  
Depth:2 (PIG)  
Ei-value:0.000, Pi-value:0.000  
Er-value:0.000, Pr-value:0.000  
No matches to TargetScan

-

GTGCCAGGCT

GTGCCAGGCT  
Depth:2 (PIG)  
Ei-value:0.000, Pi-value:0.000  
Er-value:0.000, Pr-value:0.000  
MATCHES To TargetScan▶ miR-183-5p.2:UGGCACU

-

TCTAGAGAAAA

TCTAGAGAAAA  
Depth:6 (MOUSE)  
Ei-value:0.000, Pi-value:0.000  
Er-value:0.000, Pr-value:0.000  
MATCHES To TargetScan▶ miR-1251-5p:CUCUAGC

--

TGAAGAGATG

TGAAGAGATG  
Depth:5 (RABBIT)  
Ei-value:0.000, Pi-value:0.000  
Er-value:0.000, Pr-value:0.000  
No matches to TargetScan


CTCCA

TGAAGAGATGCTCCA  
Depth:3 (COW)  
Ei-value:0.000, Pi-value:0.000  
Er-value:0.000, Pr-value:0.000  
No matches to TargetScan


GGCCAA

GGCCAATGAGAAGAATTAGACA  
Depth:4 (DOG)  
Ei-value:0.000, Pi-value:0.000  
Er-value:0.000, Pr-value:0.000  
No matches to TargetScan


TGAGAAGAATTAGACA

TGAGAAGAATTAGACA  
Depth:6 (MOUSE)  
Ei-value:0.000, Pi-value:0.000  
Er-value:0.000, Pr-value:0.000  
No matches to TargetScan

-

GAAATACACAGATG

GAAATACACAGATG  
Depth:3 (COW)  
Ei-value:0.000, Pi-value:0.000  
Er-value:0.000, Pr-value:0.000  
No matches to TargetScan

---- 19560  
 ------

C

CTGAGAAG  
Depth:3 (COW)  
Ei-value:0.000, Pi-value:0.000  
Er-value:0.000, Pr-value:0.000  
No matches to TargetScan


TGAGAAG

TGAGAAG  
Depth:4 (DOG)  
Ei-value:0.000, Pi-value:0.000  
Er-value:0.000, Pr-value:0.010  
No matches to TargetScan


CA

CTGAGAAGCA  
Depth:2 (PIG)  
Ei-value:0.000, Pi-value:0.000  
Er-value:0.000, Pr-value:0.000  
No matches to TargetScan

---

GCCA

GCCAGCAACA  
Depth:3 (COW)  
Ei-value:0.000, Pi-value:0.000  
Er-value:0.000, Pr-value:0.000  
No matches to TargetScan


GCAACA

GCAACA  
Depth:6 (MOUSE)  
Ei-value:0.000, Pi-value:0.000  
Er-value:0.000, Pr-value:0.000  
No matches to TargetScan

--------

C

CTTTGAGCTTAGGTGAGCAGGATTC  
Depth:2 (PIG)  
Ei-value:0.000, Pi-value:0.000  
Er-value:0.000, Pr-value:0.000  
MATCHES To TargetScan▶ miR-371-5p:CUCAAAC


TTTGAGCTT

TTTGAGCTT  
Depth:3 (COW)  
Ei-value:0.000, Pi-value:0.000  
Er-value:0.000, Pr-value:0.000  
MATCHES To TargetScan▶ miR-371-5p:CUCAAAC


A

CTTTGAGCTTAGGTGAGCAGGATTC  
Depth:2 (PIG)  
Ei-value:0.000, Pi-value:0.000  
Er-value:0.000, Pr-value:0.000  
MATCHES To TargetScan▶ miR-371-5p:CUCAAAC


GGTGAGC

GGTGAGC  
Depth:4 (DOG)  
Ei-value:0.000, Pi-value:0.000  
Er-value:0.000, Pr-value:0.000  
No matches to TargetScan


AGGAT

GGTGAGCAGGAT  
Depth:3 (COW)  
Ei-value:0.000, Pi-value:0.000  
Er-value:0.000, Pr-value:0.000  
No matches to TargetScan


TC

CTTTGAGCTTAGGTGAGCAGGATTC  
Depth:2 (PIG)  
Ei-value:0.000, Pi-value:0.000  
Er-value:0.000, Pr-value:0.000  
MATCHES To TargetScan▶ miR-371-5p:CUCAAAC

----

GGTTTGGG

GGTTTGGG  
Depth:4 (DOG)  
Ei-value:0.000, Pi-value:0.000  
Er-value:0.000, Pr-value:0.000  
No matches to TargetScan

--

CTAGTGA

CTAGTGATGGTTATG  
Depth:2 (PIG)  
Ei-value:0.000, Pi-value:0.000  
Er-value:0.000, Pr-value:0.000  
No matches to TargetScan


TGGTTA

TGGTTA  
Depth:5 (RABBIT)  
Ei-value:0.000, Pi-value:0.000  
Er-value:0.000, Pr-value:0.000  
No matches to TargetScan


T

TGGTTAT  
Depth:4 (DOG)  
Ei-value:0.000, Pi-value:0.000  
Er-value:0.000, Pr-value:0.000  
No matches to TargetScan


G

TGGTTATG  
Depth:3 (COW)  
Ei-value:0.000, Pi-value:0.000  
Er-value:0.000, Pr-value:0.000  
No matches to TargetScan

----------------

CTGGGACA

CTGGGACA  
Depth:2 (PIG)  
Ei-value:0.000, Pi-value:0.000  
Er-value:0.000, Pr-value:0.000  
No matches to TargetScan

----- 19680  
 ---

GAGGT

GAGGTCCCAAGG  
Depth:2 (PIG)  
Ei-value:0.000, Pi-value:0.000  
Er-value:0.000, Pr-value:0.000  
MATCHES To TargetScan▶ miR-212-5p:CCUUGGC


CCCAAGG

CCCAAGG  
Depth:4 (DOG)  
Ei-value:0.000, Pi-value:0.000  
Er-value:0.000, Pr-value:0.000  
MATCHES To TargetScan▶ miR-212-5p:CCUUGGC

----

AGCC

AGCCTGAACTCCCTGCTCATAGTAGTGGCC  
Depth:2 (PIG)  
Ei-value:0.000, Pi-value:0.000  
Er-value:0.000, Pr-value:0.000  
No matches to TargetScan


TGAACTCCCTGCT

TGAACTCCCTGCT  
Depth:4 (DOG)  
Ei-value:0.000, Pi-value:0.000  
Er-value:0.000, Pr-value:0.000  
No matches to TargetScan


C

TGAACTCCCTGCTCATAGTAGTGGCC  
Depth:3 (COW)  
Ei-value:0.000, Pi-value:0.000  
Er-value:0.000, Pr-value:0.000  
No matches to TargetScan


ATAGTAGTGGCC

ATAGTAGTGGCC  
Depth:4 (DOG)  
Ei-value:0.000, Pi-value:0.000  
Er-value:0.000, Pr-value:0.000  
No matches to TargetScan

-

AATAATTTGG

AATAATTTGG  
Depth:2 (PIG)  
Ei-value:0.000, Pi-value:0.000  
Er-value:0.000, Pr-value:0.000  
No matches to TargetScan

--------------------------

TTTAATAC

TTTAATAC  
Depth:4 (DOG)  
Ei-value:0.000, Pi-value:0.000  
Er-value:0.000, Pr-value:0.000  
MATCHES To TargetScan▶ miR-496.2:GUAUUAC


CCA

TTTAATACCCA  
Depth:2 (PIG)  
Ei-value:0.000, Pi-value:0.000  
Er-value:0.000, Pr-value:0.000  
MATCHES To TargetScan▶ miR-496.2:GUAUUAC

-

CT

CTCTAGGCTTAAAG  
Depth:2 (PIG)  
Ei-value:0.000, Pi-value:0.000  
Er-value:0.000, Pr-value:0.000  
No matches to TargetScan


CT

CTAGGCTTAAAG  
Depth:4 (DOG)  
Ei-value:0.000, Pi-value:0.000  
Er-value:0.000, Pr-value:0.000  
No matches to TargetScan


AGGCTTA

AGGCTTA  
Depth:5 (RABBIT)  
Ei-value:0.000, Pi-value:0.000  
Er-value:0.000, Pr-value:0.000  
No matches to TargetScan


AAG

CTAGGCTTAAAG  
Depth:4 (DOG)  
Ei-value:0.000, Pi-value:0.000  
Er-value:0.000, Pr-value:0.000  
No matches to TargetScan

-------- 19800  
 -------------------

GTTTAAT

GTTTAAT  
Depth:5 (RABBIT)  
Ei-value:0.000, Pi-value:0.000  
Er-value:0.000, Pr-value:0.000  
No matches to TargetScan


ACTTTCCTT

GTTTAATACTTTCCTT  
Depth:2 (PIG)  
Ei-value:0.000, Pi-value:0.000  
Er-value:0.000, Pr-value:0.000  
MATCHES To TargetScan▶ miR-496.2:GUAUUAC

----------------------

GGGAAG

GGGAAG  
Depth:2 (PIG)  
Ei-value:0.000, Pi-value:0.020  
Er-value:0.000, Pr-value:0.020  
No matches to TargetScan

---

ATTTAAATGA

ATTTAAATGA  
Depth:2 (PIG)  
Ei-value:0.000, Pi-value:0.000  
Er-value:0.000, Pr-value:0.000  
No matches to TargetScan

------------------

TGTAAAACA

TGTAAAACA  
Depth:3 (COW)  
Ei-value:0.000, Pi-value:0.000  
Er-value:0.000, Pr-value:0.000  
No matches to TargetScan

----------------- 19920  
 -------------

T

TATTGGCA  
Depth:5 (RABBIT)  
Ei-value:0.000, Pi-value:0.000  
Er-value:0.000, Pr-value:0.000  
No matches to TargetScan


ATTGGCA

ATTGGCA  
Depth:6 (MOUSE)  
Ei-value:0.000, Pi-value:0.000  
Er-value:0.000, Pr-value:0.000  
No matches to TargetScan

------------------------------------

TTGTGAAG

TTGTGAAG  
Depth:6 (MOUSE)  
Ei-value:0.000, Pi-value:0.000  
Er-value:0.000, Pr-value:0.000  
No matches to TargetScan

---

T

TATGTAAATCA  
Depth:3 (COW)  
Ei-value:0.000, Pi-value:0.000  
Er-value:0.000, Pr-value:0.000  
No matches to TargetScan


ATGTAAAT

ATGTAAAT  
Depth:5 (RABBIT)  
Ei-value:0.000, Pi-value:0.000  
Er-value:0.000, Pr-value:0.000  
No matches to TargetScan


CA

TATGTAAATCA  
Depth:3 (COW)  
Ei-value:0.000, Pi-value:0.000  
Er-value:0.000, Pr-value:0.000  
No matches to TargetScan


GGGGTC

TATGTAAATCAGGGGTC  
Depth:2 (PIG)  
Ei-value:0.000, Pi-value:0.000  
Er-value:0.000, Pr-value:0.000  
MATCHES To TargetScan▶ miR-125-5p:CCCUGAG▶ miR-331-3p:CCCCUGG

---------

TTTCTGTAA

TTTCTGTAA  
Depth:2 (PIG)  
Ei-value:0.000, Pi-value:0.000  
Er-value:0.000, Pr-value:0.000  
No matches to TargetScan

----------------- 20040  
 -----

GG

GGGCCATATGGTTTC  
Depth:2 (PIG)  
Ei-value:0.000, Pi-value:0.000  
Er-value:0.000, Pr-value:0.000  
MATCHES To TargetScan▶ miR-328-3p:UGGCCCU


GCCATATGGT

GCCATATGGT  
Depth:3 (COW)  
Ei-value:0.000, Pi-value:0.000  
Er-value:0.000, Pr-value:0.000  
No matches to TargetScan


TTC

GGGCCATATGGTTTC  
Depth:2 (PIG)  
Ei-value:0.000, Pi-value:0.000  
Er-value:0.000, Pr-value:0.000  
MATCHES To TargetScan▶ miR-328-3p:UGGCCCU

------------------------------------------------

CCCCTGATGTA

CCCCTGATGTA  
Depth:2 (PIG)  
Ei-value:0.000, Pi-value:0.000  
Er-value:0.000, Pr-value:0.000  
No matches to TargetScan

----------------

TTCTGAA

TTCTGAA  
Depth:2 (PIG)  
Ei-value:0.000, Pi-value:0.020  
Er-value:0.000, Pr-value:0.020  
No matches to TargetScan

------------

TC

TCTGTGCCTGTCCCTGT  
Depth:2 (PIG)  
Ei-value:0.000, Pi-value:0.000  
Er-value:0.000, Pr-value:0.000  
No matches to TargetScan


TGTG

TGTGCCTGTCCCTGT  
Depth:3 (COW)  
Ei-value:0.000, Pi-value:0.000  
Er-value:0.000, Pr-value:0.000  
No matches to TargetScan

 20160  


C

TGTGCCTGTCCCTGT  
Depth:3 (COW)  
Ei-value:0.000, Pi-value:0.000  
Er-value:0.000, Pr-value:0.000  
No matches to TargetScan


CTGTCCCT

CTGTCCCT  
Depth:4 (DOG)  
Ei-value:0.000, Pi-value:0.000  
Er-value:0.000, Pr-value:0.000  
No matches to TargetScan


GT

TGTGCCTGTCCCTGT  
Depth:3 (COW)  
Ei-value:0.000, Pi-value:0.000  
Er-value:0.000, Pr-value:0.000  
No matches to TargetScan

--

TAGGCACT

TAGGCACT  
Depth:4 (DOG)  
Ei-value:0.000, Pi-value:0.000  
Er-value:0.000, Pr-value:0.000  
No matches to TargetScan


AA

TAGGCACTAA  
Depth:2 (PIG)  
Ei-value:0.000, Pi-value:0.000  
Er-value:0.000, Pr-value:0.000  
No matches to TargetScan

------

AATGATTA

AATGATTA  
Depth:2 (PIG)  
Ei-value:0.000, Pi-value:0.000  
Er-value:0.000, Pr-value:0.000  
MATCHES To TargetScan▶ miR-382-3p:AUCAUUC

---------

ATATCTAGGTGA

ATATCTAGGTGA  
Depth:2 (PIG)  
Ei-value:0.000, Pi-value:0.000  
Er-value:0.000, Pr-value:0.000  
No matches to TargetScan

------------------

AATGTGCTTTGTAAACT

AATGTGCTTTGTAAACT  
Depth:2 (PIG)  
Ei-value:0.000, Pi-value:0.000  
Er-value:0.000, Pr-value:0.000  
MATCHES To TargetScan▶ miR-330-3p:CAAAGCA▶ miR-330-3p.2:AAAGCAC

-

TAAAGCA

TAAAGCA  
Depth:4 (DOG)  
Ei-value:0.000, Pi-value:0.000  
Er-value:0.000, Pr-value:0.000  
No matches to TargetScan


CTT

TAAAGCACTT  
Depth:2 (PIG)  
Ei-value:0.000, Pi-value:0.000  
Er-value:0.000, Pr-value:0.000  
MATCHES To TargetScan▶ miR-302-3p/372-3p/373-3p/520-3p:AAGUGCU▶ miR-302c-3p.2/520-3p:AGUGCUU

---------------- 20280  
 ------------------------------------------------------

TGTGGATACAAA

TGTGGATACAAA  
Depth:2 (PIG)  
Ei-value:0.000, Pi-value:0.000  
Er-value:0.000, Pr-value:0.000  
No matches to TargetScan

--------------------------------

TAT

TATAATGTGCCAGATA  
Depth:3 (COW)  
Ei-value:0.000, Pi-value:0.000  
Er-value:0.000, Pr-value:0.000  
MATCHES To TargetScan▶ miR-183-5p.2:UGGCACU▶ miR-323-3p:ACAUUAC


AATGTGCCAGATA

AATGTGCCAGATA  
Depth:4 (DOG)  
Ei-value:0.000, Pi-value:0.000  
Er-value:0.000, Pr-value:0.000  
MATCHES To TargetScan▶ miR-183-5p.2:UGGCACU

------ 20400  
 --------------------------------

TTCTCAT

TTCTCAT  
Depth:2 (PIG)  
Ei-value:0.000, Pi-value:0.000  
Er-value:0.000, Pr-value:0.010  
No matches to TargetScan

------------------------------

CATA

CATATTAAAGTGCTTTGTA  
Depth:2 (PIG)  
Ei-value:0.000, Pi-value:0.000  
Er-value:0.000, Pr-value:0.000  
MATCHES To TargetScan▶ miR-330-3p:CAAAGCA▶ miR-330-3p.2:AAAGCAC


TTAAAGTG

TTAAAGTG  
Depth:4 (DOG)  
Ei-value:0.000, Pi-value:0.000  
Er-value:0.000, Pr-value:0.000  
No matches to TargetScan


CTTTGTA

TTAAAGTGCTTTGTA  
Depth:3 (COW)  
Ei-value:0.000, Pi-value:0.000  
Er-value:0.000, Pr-value:0.000  
MATCHES To TargetScan▶ miR-330-3p:CAAAGCA▶ miR-330-3p.2:AAAGCAC

-------------------------------- 20520  
 ------------------------------------------------------------------------------------------------------------------------ 20640  
 ------------------------------------------------------------------------------------------------------------------------ 20760  
 -----------

AA

AACTAAAGCA  
Depth:2 (PIG)  
Ei-value:0.000, Pi-value:0.000  
Er-value:0.000, Pr-value:0.000  
No matches to TargetScan


CTAAAGCA

CTAAAGCA  
Depth:4 (DOG)  
Ei-value:0.000, Pi-value:0.000  
Er-value:0.000, Pr-value:0.000  
No matches to TargetScan

------------

CAATGGGCTA

CAATGGGCTA  
Depth:3 (COW)  
Ei-value:0.000, Pi-value:0.000  
Er-value:0.000, Pr-value:0.000  
No matches to TargetScan

-----------

GA

GAATGAATA  
Depth:3 (COW)  
Ei-value:0.000, Pi-value:0.000  
Er-value:0.000, Pr-value:0.000  
MATCHES To TargetScan▶ miR-1298-5p:UCAUUCG


ATGAATA

ATGAATA  
Depth:4 (DOG)  
Ei-value:0.000, Pi-value:0.000  
Er-value:0.000, Pr-value:0.000  
No matches to TargetScan

--------------------------

CCAGCTATT

CCAGCTATT  
Depth:3 (COW)  
Ei-value:0.000, Pi-value:0.000  
Er-value:0.000, Pr-value:0.000  
No matches to TargetScan

------

GGTACTGT

GGTACTGT  
Depth:4 (DOG)  
Ei-value:0.000, Pi-value:0.000  
Er-value:0.000, Pr-value:0.000  
MATCHES To TargetScan▶ miR-101-3p.1:ACAGUAC▶ miR-144-3p:ACAGUAU

-------- 20880  
 ---------------

GTA

GTAATAAGAGG  
Depth:2 (PIG)  
Ei-value:0.000, Pi-value:0.000  
Er-value:0.000, Pr-value:0.000  
No matches to TargetScan


ATAAGAGG

ATAAGAGG  
Depth:4 (DOG)  
Ei-value:0.000, Pi-value:0.000  
Er-value:0.000, Pr-value:0.000  
No matches to TargetScan

--------------------------------------

ATATTGCTTA

ATATTGCTTA  
Depth:2 (PIG)  
Ei-value:0.000, Pi-value:0.000  
Er-value:0.000, Pr-value:0.000  
No matches to TargetScan

-------

CAATC

CAATCAAGACTTTAC  
Depth:2 (PIG)  
Ei-value:0.000, Pi-value:0.000  
Er-value:0.000, Pr-value:0.000  
MATCHES To TargetScan▶ miR-431-5p:GUCUUGC


AAGACTTTAC

AAGACTTTAC  
Depth:3 (COW)  
Ei-value:0.000, Pi-value:0.000  
Er-value:0.000, Pr-value:0.000  
No matches to TargetScan

--

GTGAGGT

GTGAGGT  
Depth:2 (PIG)  
Ei-value:0.000, Pi-value:0.000  
Er-value:0.000, Pr-value:0.000  
No matches to TargetScan

----

T

TTAAATTATTAC  
Depth:2 (PIG)  
Ei-value:0.000, Pi-value:0.000  
Er-value:0.000, Pr-value:0.000  
No matches to TargetScan


TAAATTAT

TAAATTAT  
Depth:4 (DOG)  
Ei-value:0.000, Pi-value:0.010  
Er-value:0.000, Pr-value:0.000  
No matches to TargetScan


TA

TAAATTATTAC  
Depth:3 (COW)  
Ei-value:0.000, Pi-value:0.000  
Er-value:0.000, Pr-value:0.000  
No matches to TargetScan

 21000  


C

TAAATTATTAC  
Depth:3 (COW)  
Ei-value:0.000, Pi-value:0.000  
Er-value:0.000, Pr-value:0.000  
No matches to TargetScan

------------

CC

CCAGGTAAC  
Depth:2 (PIG)  
Ei-value:0.000, Pi-value:0.000  
Er-value:0.000, Pr-value:0.000  
No matches to TargetScan


AGGTAA

AGGTAA  
Depth:3 (COW)  
Ei-value:0.000, Pi-value:0.000  
Er-value:0.000, Pr-value:0.000  
No matches to TargetScan


C

CCAGGTAAC  
Depth:2 (PIG)  
Ei-value:0.000, Pi-value:0.000  
Er-value:0.000, Pr-value:0.000  
No matches to TargetScan

---------------------------

TTTCTAA

TTTCTAA  
Depth:3 (COW)  
Ei-value:0.000, Pi-value:0.000  
Er-value:0.000, Pr-value:0.010  
No matches to TargetScan


A

TTTCTAAA  
Depth:2 (PIG)  
Ei-value:0.000, Pi-value:0.000  
Er-value:0.000, Pr-value:0.000  
No matches to TargetScan

--------------------------------------------

TGA

TGAATAAAACTT  
Depth:2 (PIG)  
Ei-value:0.000, Pi-value:0.000  
Er-value:0.000, Pr-value:0.000  
No matches to TargetScan


ATAAAAC

ATAAAAC  
Depth:4 (DOG)  
Ei-value:0.000, Pi-value:0.010  
Er-value:0.000, Pr-value:0.000  
No matches to TargetScan


TT

TGAATAAAACTT  
Depth:2 (PIG)  
Ei-value:0.000, Pi-value:0.000  
Er-value:0.000, Pr-value:0.000  
No matches to TargetScan

------- 21120  
 ----------

TCTCTAC

TCTCTACAAAATTCTCATTGT  
Depth:2 (PIG)  
Ei-value:0.000, Pi-value:0.000  
Er-value:0.000, Pr-value:0.000  
No matches to TargetScan


AAAATTCTCA

AAAATTCTCA  
Depth:4 (DOG)  
Ei-value:0.000, Pi-value:0.000  
Er-value:0.000, Pr-value:0.000  
No matches to TargetScan


TTGT

TCTCTACAAAATTCTCATTGT  
Depth:2 (PIG)  
Ei-value:0.000, Pi-value:0.000  
Er-value:0.000, Pr-value:0.000  
No matches to TargetScan

------------------------------------

T

TTATACAAAC  
Depth:2 (PIG)  
Ei-value:0.000, Pi-value:0.000  
Er-value:0.000, Pr-value:0.000  
No matches to TargetScan


TATACAAAC

TATACAAAC  
Depth:4 (DOG)  
Ei-value:0.000, Pi-value:0.000  
Er-value:0.000, Pr-value:0.000  
No matches to TargetScan

-

GTTTAAATAC

GTTTAAATAC  
Depth:3 (COW)  
Ei-value:0.000, Pi-value:0.000  
Er-value:0.000, Pr-value:0.000  
No matches to TargetScan

-------------------------

A

ATTGCCTACTATGTGAACTCACTGTTA  
Depth:2 (PIG)  
Ei-value:0.000, Pi-value:0.000  
Er-value:0.000, Pr-value:0.000  
MATCHES To TargetScan▶ miR-132-3p/212-3p:AACAGUC▶ miR-23-3p:UCACAUU▶ miR-376c-3p:ACAUAGA▶ miR-411-5p.2:UAGUAGA


TTGCCT

TTGCCTACTATGTGAACTCACTGTTA  
Depth:3 (COW)  
Ei-value:0.000, Pi-value:0.000  
Er-value:0.000, Pr-value:0.000  
MATCHES To TargetScan▶ miR-132-3p/212-3p:AACAGUC▶ miR-23-3p:UCACAUU▶ miR-376c-3p:ACAUAGA▶ miR-411-5p.2:UAGUAGA

 21240  


ACTAT

TTGCCTACTATGTGAACTCACTGTTA  
Depth:3 (COW)  
Ei-value:0.000, Pi-value:0.000  
Er-value:0.000, Pr-value:0.000  
MATCHES To TargetScan▶ miR-132-3p/212-3p:AACAGUC▶ miR-23-3p:UCACAUU▶ miR-376c-3p:ACAUAGA▶ miR-411-5p.2:UAGUAGA


GTGAACTCA

GTGAACTCA  
Depth:4 (DOG)  
Ei-value:0.000, Pi-value:0.000  
Er-value:0.000, Pr-value:0.000  
No matches to TargetScan


CTGTTA

TTGCCTACTATGTGAACTCACTGTTA  
Depth:3 (COW)  
Ei-value:0.000, Pi-value:0.000  
Er-value:0.000, Pr-value:0.000  
MATCHES To TargetScan▶ miR-132-3p/212-3p:AACAGUC▶ miR-23-3p:UCACAUU▶ miR-376c-3p:ACAUAGA▶ miR-411-5p.2:UAGUAGA

----------------------

ATTTATCAT

ATTTATCAT  
Depth:3 (COW)  
Ei-value:0.000, Pi-value:0.000  
Er-value:0.000, Pr-value:0.000  
No matches to TargetScan

-----------------------------------

TATGTTAGC

TATGTTAGCATTTTGTGAACTCTAA  
Depth:2 (PIG)  
Ei-value:0.000, Pi-value:0.000  
Er-value:0.000, Pr-value:0.000  
No matches to TargetScan


ATTTTGTGAACTCTAA

ATTTTGTGAACTCTAA  
Depth:3 (COW)  
Ei-value:0.000, Pi-value:0.000  
Er-value:0.000, Pr-value:0.000  
No matches to TargetScan

-

GCACCAT

GCACCAT  
Depth:2 (PIG)  
Ei-value:0.000, Pi-value:0.000  
Er-value:0.000, Pr-value:0.000  
No matches to TargetScan

- 21360  
 ------------------------------------

GTACTAG

GTACTAG  
Depth:2 (PIG)  
Ei-value:0.000, Pi-value:0.000  
Er-value:0.000, Pr-value:0.000  
No matches to TargetScan

-----

AAAATTG

AAAATTG  
Depth:3 (COW)  
Ei-value:0.000, Pi-value:0.000  
Er-value:0.000, Pr-value:0.000  
No matches to TargetScan

----

ATAGTTAT

ATAGTTAT  
Depth:2 (PIG)  
Ei-value:0.000, Pi-value:0.000  
Er-value:0.000, Pr-value:0.000  
No matches to TargetScan

----------------------------------------------------

T

TTTGAATA  
Depth:2 (PIG)  
Ei-value:0.000, Pi-value:0.000  
Er-value:0.000, Pr-value:0.010  
No matches to TargetScan

 21480  


TTGAATA

TTTGAATA  
Depth:2 (PIG)  
Ei-value:0.000, Pi-value:0.000  
Er-value:0.000, Pr-value:0.010  
No matches to TargetScan

-----------------

AATGCC

AATGCC  
Depth:2 (PIG)  
Ei-value:0.000, Pi-value:0.000  
Er-value:0.000, Pr-value:0.000  
No matches to TargetScan

-----------------------------------

TGTGCCA

TGTGCCA  
Depth:4 (DOG)  
Ei-value:0.000, Pi-value:0.000  
Er-value:0.000, Pr-value:0.000  
MATCHES To TargetScan▶ miR-183-5p.2:UGGCACU

---------------------

AAGATAA

AAGATAA  
Depth:4 (DOG)  
Ei-value:0.000, Pi-value:0.000  
Er-value:0.000, Pr-value:0.000  
No matches to TargetScan

--------

TTTATTGTGT

TTTATTGTGT  
Depth:2 (PIG)  
Ei-value:0.000, Pi-value:0.000  
Er-value:0.000, Pr-value:0.000  
No matches to TargetScan

-- 21600  
 --

GGT

GGTAGCAGAA  
Depth:2 (PIG)  
Ei-value:0.000, Pi-value:0.000  
Er-value:0.000, Pr-value:0.000  
No matches to TargetScan


AGCAGAA

AGCAGAA  
Depth:3 (COW)  
Ei-value:0.000, Pi-value:0.000  
Er-value:0.000, Pr-value:0.000  
No matches to TargetScan

----

ATGTG

ATGTGTAAAATCAATTT  
Depth:2 (PIG)  
Ei-value:0.000, Pi-value:0.000  
Er-value:0.000, Pr-value:0.000  
No matches to TargetScan


TAAAATCAATTT

TAAAATCAATTT  
Depth:3 (COW)  
Ei-value:0.000, Pi-value:0.000  
Er-value:0.000, Pr-value:0.000  
No matches to TargetScan

--------

TAAACTG

TAAACTG  
Depth:4 (DOG)  
Ei-value:0.000, Pi-value:0.000  
Er-value:0.000, Pr-value:0.000  
No matches to TargetScan

---------

TCTGCTGAATGA

TCTGCTGAATGA  
Depth:3 (COW)  
Ei-value:0.000, Pi-value:0.000  
Er-value:0.000, Pr-value:0.000  
MATCHES To TargetScan▶ miR-1298-5p:UCAUUCG

-

C

CATTGATTA  
Depth:3 (COW)  
Ei-value:0.000, Pi-value:0.000  
Er-value:0.000, Pr-value:0.000  
No matches to TargetScan


ATTGATTA

ATTGATTA  
Depth:4 (DOG)  
Ei-value:0.000, Pi-value:0.000  
Er-value:0.000, Pr-value:0.010  
No matches to TargetScan

---------

TCTTATCC

TCTTATCC  
Depth:2 (PIG)  
Ei-value:0.000, Pi-value:0.000  
Er-value:0.000, Pr-value:0.000  
No matches to TargetScan

--

AGAGATA

AGAGATA  
Depth:4 (DOG)  
Ei-value:0.000, Pi-value:0.000  
Er-value:0.000, Pr-value:0.000  
No matches to TargetScan

--------------- 21720  
 --------------------

TGAACCT

TGAACCT  
Depth:3 (COW)  
Ei-value:0.000, Pi-value:0.000  
Er-value:0.000, Pr-value:0.010  
No matches to TargetScan

-

C

CAACAGAGATCT  
Depth:2 (PIG)  
Ei-value:0.000, Pi-value:0.000  
Er-value:0.000, Pr-value:0.000  
No matches to TargetScan


AA

AACAGAGATCT  
Depth:3 (COW)  
Ei-value:0.000, Pi-value:0.000  
Er-value:0.000, Pr-value:0.000  
No matches to TargetScan


CAGAGATCT

CAGAGATCT  
Depth:4 (DOG)  
Ei-value:0.000, Pi-value:0.000  
Er-value:0.000, Pr-value:0.000  
No matches to TargetScan

--------

A

ATTTACAAAGCCTA  
Depth:2 (PIG)  
Ei-value:0.000, Pi-value:0.000  
Er-value:0.000, Pr-value:0.000  
No matches to TargetScan


TTTACAAAGC

TTTACAAAGC  
Depth:3 (COW)  
Ei-value:0.000, Pi-value:0.000  
Er-value:0.000, Pr-value:0.000  
No matches to TargetScan


CTA

ATTTACAAAGCCTA  
Depth:2 (PIG)  
Ei-value:0.000, Pi-value:0.000  
Er-value:0.000, Pr-value:0.000  
No matches to TargetScan

----

TCTATACA

TCTATACA  
Depth:3 (COW)  
Ei-value:0.000, Pi-value:0.000  
Er-value:0.000, Pr-value:0.000  
No matches to TargetScan

-----

TAGGAAT

TAGGAAT  
Depth:2 (PIG)  
Ei-value:0.000, Pi-value:0.000  
Er-value:0.000, Pr-value:0.000  
No matches to TargetScan

------

TTGGCT

TTGGCT  
Depth:4 (DOG)  
Ei-value:0.000, Pi-value:0.000  
Er-value:0.000, Pr-value:0.000  
No matches to TargetScan

-----------------

TTACT

TTACTTTCT  
Depth:4 (DOG)  
Ei-value:0.000, Pi-value:0.000  
Er-value:0.000, Pr-value:0.010  
No matches to TargetScan

 21840  


TTCT

TTACTTTCT  
Depth:4 (DOG)  
Ei-value:0.000, Pi-value:0.000  
Er-value:0.000, Pr-value:0.010  
No matches to TargetScan

-----------------

ACATGC

ACATGC  
Depth:2 (PIG)  
Ei-value:0.000, Pi-value:0.010  
Er-value:0.000, Pr-value:0.020  
No matches to TargetScan

-

CTAGGATAT

CTAGGATAT  
Depth:3 (COW)  
Ei-value:0.000, Pi-value:0.000  
Er-value:0.000, Pr-value:0.000  
No matches to TargetScan

-

AAAATGA

AAAATGA  
Depth:3 (COW)  
Ei-value:0.000, Pi-value:0.000  
Er-value:0.000, Pr-value:0.000  
No matches to TargetScan


T

AAAATGAT  
Depth:2 (PIG)  
Ei-value:0.000, Pi-value:0.010  
Er-value:0.000, Pr-value:0.000  
MATCHES To TargetScan▶ miR-382-3p:AUCAUUC

----------------------

AGGAAATGA

AGGAAATGA  
Depth:2 (PIG)  
Ei-value:0.000, Pi-value:0.000  
Er-value:0.000, Pr-value:0.000  
No matches to TargetScan

----

ATAGGTGTG

ATAGGTGTG  
Depth:2 (PIG)  
Ei-value:0.000, Pi-value:0.000  
Er-value:0.000, Pr-value:0.000  
No matches to TargetScan

------------------------------ 21960  
 ---------------------------------------------------------------------------------------

ATCCAGACCA

ATCCAGACCA  
Depth:3 (COW)  
Ei-value:0.000, Pi-value:0.000  
Er-value:0.000, Pr-value:0.000  
No matches to TargetScan

-------------------

GCCT

GCCTGATTGA  
Depth:2 (PIG)  
Ei-value:0.000, Pi-value:0.000  
Er-value:0.000, Pr-value:0.000  
No matches to TargetScan

 22080  


GATTGA

GCCTGATTGA  
Depth:2 (PIG)  
Ei-value:0.000, Pi-value:0.000  
Er-value:0.000, Pr-value:0.000  
No matches to TargetScan

------------------------------------------------------

AGATGGA

AGATGGA  
Depth:3 (COW)  
Ei-value:0.000, Pi-value:0.000  
Er-value:0.000, Pr-value:0.000  
No matches to TargetScan

-----------------------------

TAAAG

TAAAGGAGTAAAAAT  
Depth:2 (PIG)  
Ei-value:0.000, Pi-value:0.000  
Er-value:0.000, Pr-value:0.000  
MATCHES To TargetScan▶ miR-483-3p.1:ACUCCUC


GAGTAAAAA

GAGTAAAAA  
Depth:4 (DOG)  
Ei-value:0.000, Pi-value:0.000  
Er-value:0.000, Pr-value:0.000  
No matches to TargetScan


T

TAAAGGAGTAAAAAT  
Depth:2 (PIG)  
Ei-value:0.000, Pi-value:0.000  
Er-value:0.000, Pr-value:0.000  
MATCHES To TargetScan▶ miR-483-3p.1:ACUCCUC

--------- 22200  
 -----------------

ATTTGAT

ATTTGAT  
Depth:4 (DOG)  
Ei-value:0.000, Pi-value:0.010  
Er-value:0.000, Pr-value:0.000  
No matches to TargetScan


AAACA

ATTTGATAAACA  
Depth:2 (PIG)  
Ei-value:0.000, Pi-value:0.000  
Er-value:0.000, Pr-value:0.000  
No matches to TargetScan

--

ATC

ATCTTTTATGT  
Depth:3 (COW)  
Ei-value:0.000, Pi-value:0.000  
Er-value:0.000, Pr-value:0.000  
No matches to TargetScan


TTTTATGT

TTTTATGT  
Depth:4 (DOG)  
Ei-value:0.000, Pi-value:0.000  
Er-value:0.000, Pr-value:0.000  
No matches to TargetScan


GGAATA

ATCTTTTATGTGGAATA  
Depth:2 (PIG)  
Ei-value:0.000, Pi-value:0.000  
Er-value:0.000, Pr-value:0.000  
No matches to TargetScan

----------

GGTCCTGAG

GGTCCTGAG  
Depth:3 (COW)  
Ei-value:0.000, Pi-value:0.000  
Er-value:0.000, Pr-value:0.000  
No matches to TargetScan

------------------------------

AGGGCATTAG

AGGGCATTAG  
Depth:2 (PIG)  
Ei-value:0.000, Pi-value:0.000  
Er-value:0.000, Pr-value:0.000  
MATCHES To TargetScan▶ miR-155-5p:UAAUGCU▶ miR-365-3p:AAUGCCC▶ miR-874-3p:UGCCCUG

------------- 22320  
 ----

GCTGAA

GCTGAA  
Depth:2 (PIG)  
Ei-value:0.000, Pi-value:0.010  
Er-value:0.000, Pr-value:0.000  
No matches to TargetScan

--------------

TTTGATT

TTTGATT  
Depth:2 (PIG)  
Ei-value:0.000, Pi-value:0.020  
Er-value:0.000, Pr-value:0.010  
No matches to TargetScan

-------------------------------------------------------------------

TTGCCTT

TTGCCTT  
Depth:3 (COW)  
Ei-value:0.000, Pi-value:0.000  
Er-value:0.000, Pr-value:0.000  
MATCHES To TargetScan▶ miR-124-3p.1:AAGGCAC

--------------- 22440  
 ----

AAAAAAAGA

AAAAAAAGA  
Depth:2 (PIG)  
Ei-value:0.000, Pi-value:0.000  
Er-value:0.000, Pr-value:0.000  
No matches to TargetScan

----------------------

GACATTTTTCCTAG

GACATTTTTCCTAG  
Depth:2 (PIG)  
Ei-value:0.000, Pi-value:0.000  
Er-value:0.000, Pr-value:0.000  
No matches to TargetScan

------------------------------------------

AAGCCAG

AAGCCAG  
Depth:4 (DOG)  
Ei-value:0.000, Pi-value:0.000  
Er-value:0.000, Pr-value:0.000  
MATCHES To TargetScan▶ miR-149-5p:CUGGCUC▶ miR-3064-5p:CUGGCUG

-----

TG

TGATAAAAG  
Depth:2 (PIG)  
Ei-value:0.000, Pi-value:0.000  
Er-value:0.000, Pr-value:0.000  
No matches to TargetScan


ATAAAAG

ATAAAAG  
Depth:4 (DOG)  
Ei-value:0.000, Pi-value:0.000  
Er-value:0.000, Pr-value:0.000  
No matches to TargetScan

-------- 22560  
 -------

CTTTAATTC

CTTTAATTC  
Depth:3 (COW)  
Ei-value:0.000, Pi-value:0.000  
Er-value:0.000, Pr-value:0.000  
No matches to TargetScan

---

AACATTCTGC

AACATTCTGCTTTTATTA  
Depth:2 (PIG)  
Ei-value:0.000, Pi-value:0.000  
Er-value:0.000, Pr-value:0.000  
MATCHES To TargetScan▶ miR-330-3p.2:AAAGCAC▶ miR-409-3p:AAUGUUG


TTTTATTA

TTTTATTA  
Depth:4 (DOG)  
Ei-value:0.000, Pi-value:0.010  
Er-value:0.000, Pr-value:0.000  
No matches to TargetScan

--

G

GTTAAATGG  
Depth:3 (COW)  
Ei-value:0.000, Pi-value:0.000  
Er-value:0.000, Pr-value:0.000  
No matches to TargetScan


TTAAATGG

TTAAATGG  
Depth:4 (DOG)  
Ei-value:0.000, Pi-value:0.000  
Er-value:0.000, Pr-value:0.000  
No matches to TargetScan


TT

GTTAAATGGTT  
Depth:2 (PIG)  
Ei-value:0.000, Pi-value:0.000  
Er-value:0.000, Pr-value:0.000  
No matches to TargetScan

--------------------

AACAACTAGTT

AACAACTAGTT  
Depth:2 (PIG)  
Ei-value:0.000, Pi-value:0.000  
Er-value:0.000, Pr-value:0.000  
No matches to TargetScan

------------------------------------

CTC

CTCATTGGTCTG  
Depth:2 (PIG)  
Ei-value:0.000, Pi-value:0.000  
Er-value:0.000, Pr-value:0.000  
No matches to TargetScan

 22680  


ATTGGTCTG

CTCATTGGTCTG  
Depth:2 (PIG)  
Ei-value:0.000, Pi-value:0.000  
Er-value:0.000, Pr-value:0.000  
No matches to TargetScan

--------------------------------

TAAAAAAAA

TAAAAAAAA  
Depth:2 (PIG)  
Ei-value:0.000, Pi-value:0.000  
Er-value:0.000, Pr-value:0.000  
No matches to TargetScan

------------------------------------

GTAAAAA

GTAAAAA  
Depth:2 (PIG)  
Ei-value:0.000, Pi-value:0.010  
Er-value:0.000, Pr-value:0.000  
No matches to TargetScan

----------------------

CTGTT

CTGTTCTTAAGT  
Depth:3 (COW)  
Ei-value:0.000, Pi-value:0.000  
Er-value:0.000, Pr-value:0.000  
No matches to TargetScan

 22800  


CTTAAGT

CTGTTCTTAAGT  
Depth:3 (COW)  
Ei-value:0.000, Pi-value:0.000  
Er-value:0.000, Pr-value:0.000  
No matches to TargetScan

---------------------------------------------------------

GGAAACA

GGAAACA  
Depth:2 (PIG)  
Ei-value:0.000, Pi-value:0.000  
Er-value:0.000, Pr-value:0.000  
No matches to TargetScan

---

GAACAAATT

GAACAAATT  
Depth:3 (COW)  
Ei-value:0.000, Pi-value:0.000  
Er-value:0.000, Pr-value:0.000  
MATCHES To TargetScan▶ miR-375:UUGUUCG

---

TAAGAGACTG

TAAGAGACTG  
Depth:2 (PIG)  
Ei-value:0.000, Pi-value:0.000  
Er-value:0.000, Pr-value:0.000  
No matches to TargetScan

-----------

TTAGTTG

TTAGTTG  
Depth:3 (COW)  
Ei-value:0.000, Pi-value:0.000  
Er-value:0.000, Pr-value:0.010  
No matches to TargetScan


A

TTAGTTGA  
Depth:2 (PIG)  
Ei-value:0.000, Pi-value:0.000  
Er-value:0.000, Pr-value:0.000  
No matches to TargetScan

-

AAAC

AAACTTCATTGA  
Depth:3 (COW)  
Ei-value:0.000, Pi-value:0.000  
Er-value:0.000, Pr-value:0.000  
No matches to TargetScan

 22920  


TTCATTGA

AAACTTCATTGA  
Depth:3 (COW)  
Ei-value:0.000, Pi-value:0.000  
Er-value:0.000, Pr-value:0.000  
No matches to TargetScan


G

AAACTTCATTGAG  
Depth:2 (PIG)  
Ei-value:0.000, Pi-value:0.000  
Er-value:0.000, Pr-value:0.000  
No matches to TargetScan

------

TGATAT

TGATAT  
Depth:2 (PIG)  
Ei-value:0.000, Pi-value:0.000  
Er-value:0.000, Pr-value:0.010  
No matches to TargetScan

------------------------------------------------------------

GGAATAAGAGA

GGAATAAGAGA  
Depth:2 (PIG)  
Ei-value:0.000, Pi-value:0.000  
Er-value:0.000, Pr-value:0.000  
No matches to TargetScan

---------------------------- 23040  
 -----

GAAGATGAT

GAAGATGAT  
Depth:2 (PIG)  
Ei-value:0.000, Pi-value:0.000  
Er-value:0.000, Pr-value:0.000  
No matches to TargetScan


GCTAAAT

GCTAAAT  
Depth:2 (PIG)  
Ei-value:0.000, Pi-value:0.020  
Er-value:0.000, Pr-value:0.000  
No matches to TargetScan

---------------

CAAAAG

CAAAAG  
Depth:2 (PIG)  
Ei-value:0.000, Pi-value:0.010  
Er-value:0.000, Pr-value:0.000  
No matches to TargetScan

--------

CTTGGAGATG

CTTGGAGATG  
Depth:2 (PIG)  
Ei-value:0.000, Pi-value:0.000  
Er-value:0.000, Pr-value:0.000  
No matches to TargetScan

-----------------------

AAATGGA

AAATGGA  
Depth:2 (PIG)  
Ei-value:0.000, Pi-value:0.000  
Er-value:0.000, Pr-value:0.010  
No matches to TargetScan

------------------------------ 23160  
 --------------

GTGATACTC

GTGATACTC  
Depth:2 (PIG)  
Ei-value:0.000, Pi-value:0.000  
Er-value:0.000, Pr-value:0.000  
MATCHES To TargetScan▶ miR-496.1:GAGUAUU

----

ACAGAAAACAAAA

ACAGAAAACAAAA  
Depth:4 (DOG)  
Ei-value:0.000, Pi-value:0.000  
Er-value:0.000, Pr-value:0.000  
No matches to TargetScan

-----------------

ACTTGC

ACTTGC  
Depth:2 (PIG)  
Ei-value:0.000, Pi-value:0.020  
Er-value:0.000, Pr-value:0.000  
No matches to TargetScan


TTGG

TTGGCTTGGAAA  
Depth:2 (PIG)  
Ei-value:0.000, Pi-value:0.000  
Er-value:0.000, Pr-value:0.000  
No matches to TargetScan


CTTGGAAA

CTTGGAAA  
Depth:3 (COW)  
Ei-value:0.000, Pi-value:0.000  
Er-value:0.000, Pr-value:0.000  
No matches to TargetScan

-----------------

AGGTTA

AGGTTA  
Depth:4 (DOG)  
Ei-value:0.000, Pi-value:0.000  
Er-value:0.000, Pr-value:0.000  
No matches to TargetScan


CTGTTTATT

AGGTTACTGTTTATT  
Depth:2 (PIG)  
Ei-value:0.000, Pi-value:0.000  
Er-value:0.000, Pr-value:0.000  
MATCHES To TargetScan▶ miR-101-3p.1:ACAGUAC▶ miR-132-3p/212-3p:AACAGUC▶ miR-144-3p:ACAGUAU▶ miR-802:CAGUAAC

------------- 23280  
 -----

TTCATTCT

TTCATTCT  
Depth:4 (DOG)  
Ei-value:0.000, Pi-value:0.000  
Er-value:0.000, Pr-value:0.000  
No matches to TargetScan

-----------------------------------------------------

TTGGCCCC

TTGGCCCCAGAGACATG  
Depth:2 (PIG)  
Ei-value:0.000, Pi-value:0.000  
Er-value:0.000, Pr-value:0.000  
MATCHES To TargetScan▶ miR-326:CUCUGGG


AGAGACA

AGAGACA  
Depth:4 (DOG)  
Ei-value:0.000, Pi-value:0.000  
Er-value:0.000, Pr-value:0.000  
No matches to TargetScan


TG

AGAGACATG  
Depth:3 (COW)  
Ei-value:0.000, Pi-value:0.000  
Er-value:0.000, Pr-value:0.000  
No matches to TargetScan


AAAAAATG

AAAAAATG  
Depth:2 (PIG)  
Ei-value:0.000, Pi-value:0.000  
Er-value:0.000, Pr-value:0.000  
No matches to TargetScan

----------------------------- 23400  
 --------

CCTTTTGG

CCTTTTGG  
Depth:4 (DOG)  
Ei-value:0.000, Pi-value:0.000  
Er-value:0.000, Pr-value:0.000  
No matches to TargetScan


C

CCTTTTGGC  
Depth:3 (COW)  
Ei-value:0.000, Pi-value:0.000  
Er-value:0.000, Pr-value:0.000  
No matches to TargetScan

--

GTTTTCT

GTTTTCT  
Depth:2 (PIG)  
Ei-value:0.000, Pi-value:0.010  
Er-value:0.000, Pr-value:0.010  
No matches to TargetScan

-

GTTAGGGGCA

GTTAGGGGCA  
Depth:2 (PIG)  
Ei-value:0.000, Pi-value:0.000  
Er-value:0.000, Pr-value:0.000  
No matches to TargetScan

-

GGCTTAGT

GGCTTAGT  
Depth:2 (PIG)  
Ei-value:0.000, Pi-value:0.000  
Er-value:0.000, Pr-value:0.000  
No matches to TargetScan

-------

TAAC

TAACATTGTGT  
Depth:2 (PIG)  
Ei-value:0.000, Pi-value:0.000  
Er-value:0.000, Pr-value:0.000  
MATCHES To TargetScan▶ miR-409-3p:AAUGUUG


ATTGTGT

ATTGTGT  
Depth:3 (COW)  
Ei-value:0.000, Pi-value:0.000  
Er-value:0.000, Pr-value:0.010  
No matches to TargetScan

---

TTAATTC

TTAATTC  
Depth:4 (DOG)  
Ei-value:0.000, Pi-value:0.000  
Er-value:0.000, Pr-value:0.000  
No matches to TargetScan

---------------------

ACTCTGGCCACTAC

ACTCTGGCCACTAC  
Depth:4 (DOG)  
Ei-value:0.000, Pi-value:0.000  
Er-value:0.000, Pr-value:0.000  
MATCHES To TargetScan▶ miR-142-3p.1:GUAGUGU

-

ATAAGC

ATAAGC  
Depth:5 (RABBIT)  
Ei-value:0.000, Pi-value:0.010  
Er-value:0.000, Pr-value:0.000  
No matches to TargetScan


AGG

ATAAGCAGG  
Depth:4 (DOG)  
Ei-value:0.000, Pi-value:0.000  
Er-value:0.000, Pr-value:0.000  
No matches to TargetScan

- 23520  
 -----------

GCT

GCTTGCTCCTT  
Depth:2 (PIG)  
Ei-value:0.000, Pi-value:0.000  
Er-value:0.000, Pr-value:0.000  
MATCHES To TargetScan▶ miR-28-5p/708-5p:AGGAGCU


TGCTCCTT

TGCTCCTT  
Depth:3 (COW)  
Ei-value:0.000, Pi-value:0.000  
Er-value:0.000, Pr-value:0.000  
MATCHES To TargetScan▶ miR-28-5p/708-5p:AGGAGCU

-------------

ACTTCA

ACTTCA  
Depth:3 (COW)  
Ei-value:0.000, Pi-value:0.000  
Er-value:0.000, Pr-value:0.000  
No matches to TargetScan

-

TTTTCCTA

TTTTCCTA  
Depth:3 (COW)  
Ei-value:0.000, Pi-value:0.000  
Er-value:0.000, Pr-value:0.000  
No matches to TargetScan


GTCCATCC

TTTTCCTAGTCCATCC  
Depth:2 (PIG)  
Ei-value:0.000, Pi-value:0.000  
Er-value:0.000, Pr-value:0.000  
No matches to TargetScan

---

AT

ATGAAAAATG  
Depth:3 (COW)  
Ei-value:0.000, Pi-value:0.000  
Er-value:0.000, Pr-value:0.000  
No matches to TargetScan


GAAAAATG

GAAAAATG  
Depth:4 (DOG)  
Ei-value:0.000, Pi-value:0.000  
Er-value:0.000, Pr-value:0.000  
No matches to TargetScan

--------------

TGGGCAG

TGGGCAG  
Depth:2 (PIG)  
Ei-value:0.000, Pi-value:0.000  
Er-value:0.000, Pr-value:0.000  
No matches to TargetScan

---------------------------- 23640  
 ----

AGTCTCA

AGTCTCA  
Depth:4 (DOG)  
Ei-value:0.000, Pi-value:0.000  
Er-value:0.000, Pr-value:0.000  
No matches to TargetScan


TTGGTACCA

AGTCTCATTGGTACCA  
Depth:3 (COW)  
Ei-value:0.000, Pi-value:0.000  
Er-value:0.000, Pr-value:0.000  
No matches to TargetScan


GC

AGTCTCATTGGTACCAGC  
Depth:2 (PIG)  
Ei-value:0.000, Pi-value:0.000  
Er-value:0.000, Pr-value:0.000  
MATCHES To TargetScan▶ miR-138-5p:GCUGGUG

----

GGGAAC

GGGAAC  
Depth:2 (PIG)  
Ei-value:0.000, Pi-value:0.000  
Er-value:0.000, Pr-value:0.010  
No matches to TargetScan

-----------

AGCCACAA

AGCCACAA  
Depth:2 (PIG)  
Ei-value:0.000, Pi-value:0.000  
Er-value:0.000, Pr-value:0.000  
No matches to TargetScan

-

TGGTTTTGAA

TGGTTTTGAA  
Depth:4 (DOG)  
Ei-value:0.000, Pi-value:0.000  
Er-value:0.000, Pr-value:0.000  
No matches to TargetScan

---

CATTTAC

CATTTAC  
Depth:2 (PIG)  
Ei-value:0.000, Pi-value:0.000  
Er-value:0.000, Pr-value:0.000  
No matches to TargetScan

---------------

TCAGTTC

TCAGTTC  
Depth:2 (PIG)  
Ei-value:0.000, Pi-value:0.010  
Er-value:0.000, Pr-value:0.000  
No matches to TargetScan

-------------------------- 23760  
 --------

ACAATCC

ACAATCC  
Depth:3 (COW)  
Ei-value:0.000, Pi-value:0.000  
Er-value:0.000, Pr-value:0.000  
MATCHES To TargetScan▶ miR-219-5p:GAUUGUC

---------------------

TGGAGATG

TGGAGATG  
Depth:3 (COW)  
Ei-value:0.000, Pi-value:0.000  
Er-value:0.000, Pr-value:0.000  
No matches to TargetScan

---------------

AGCTTCTC

AGCTTCTC  
Depth:3 (COW)  
Ei-value:0.000, Pi-value:0.000  
Er-value:0.000, Pr-value:0.000  
No matches to TargetScan

--------

C

CTTAGAAAT  
Depth:2 (PIG)  
Ei-value:0.000, Pi-value:0.000  
Er-value:0.000, Pr-value:0.000  
No matches to TargetScan


TTAGAAAT

TTAGAAAT  
Depth:4 (DOG)  
Ei-value:0.000, Pi-value:0.000  
Er-value:0.000, Pr-value:0.000  
No matches to TargetScan

--------------

CATCAAA

CATCAAA  
Depth:4 (DOG)  
Ei-value:0.000, Pi-value:0.000  
Er-value:0.000, Pr-value:0.000  
No matches to TargetScan

--------------- 23880  
 --------------------------------

ATGAAAA

ATGAAAA  
Depth:2 (PIG)  
Ei-value:0.000, Pi-value:0.030  
Er-value:0.000, Pr-value:0.010  
No matches to TargetScan

-------------

CTTAAGTTTTA

CTTAAGTTTTA  
Depth:2 (PIG)  
Ei-value:0.000, Pi-value:0.000  
Er-value:0.000, Pr-value:0.000  
No matches to TargetScan

-----

AATAAAAATTGGAA

AATAAAAATTGGAA  
Depth:2 (PIG)  
Ei-value:0.000, Pi-value:0.000  
Er-value:0.000, Pr-value:0.000  
No matches to TargetScan

------

GGAAAAA

GGAAAAA  
Depth:3 (COW)  
Ei-value:0.000, Pi-value:0.000  
Er-value:0.000, Pr-value:0.000  
No matches to TargetScan

------------------------- 24000  
 --------

TCTAAAT

TCTAAAT  
Depth:2 (PIG)  
Ei-value:0.000, Pi-value:0.000  
Er-value:0.000, Pr-value:0.010  
No matches to TargetScan

-----------

TGGGCTTTG

TGGGCTTTG  
Depth:3 (COW)  
Ei-value:0.000, Pi-value:0.000  
Er-value:0.000, Pr-value:0.000  
MATCHES To TargetScan▶ miR-330-3p:CAAAGCA

----------

TTTTTAAATCACTCA

TTTTTAAATCACTCA  
Depth:4 (DOG)  
Ei-value:0.000, Pi-value:0.000  
Er-value:0.000, Pr-value:0.000  
No matches to TargetScan

-

AGAGGGTGGGA

AGAGGGTGGGA  
Depth:4 (DOG)  
Ei-value:0.000, Pi-value:0.000  
Er-value:0.000, Pr-value:0.000  
No matches to TargetScan

-

AGGAGGAAGAGTGAA

AGGAGGAAGAGTGAA  
Depth:4 (DOG)  
Ei-value:0.000, Pi-value:0.000  
Er-value:0.000, Pr-value:0.000  
MATCHES To TargetScan▶ miR-670-3p:UUCCUCA

-

G

GAAAAGGTCA  
Depth:4 (DOG)  
Ei-value:0.000, Pi-value:0.000  
Er-value:0.000, Pr-value:0.000  
MATCHES To TargetScan▶ miR-192-5p/215-5p:UGACCUA


AAAAGGT

AAAAGGT  
Depth:6 (MOUSE)  
Ei-value:0.000, Pi-value:0.000  
Er-value:0.000, Pr-value:0.000  
No matches to TargetScan


CA

GAAAAGGTCA  
Depth:4 (DOG)  
Ei-value:0.000, Pi-value:0.000  
Er-value:0.000, Pr-value:0.000  
MATCHES To TargetScan▶ miR-192-5p/215-5p:UGACCUA

--------------------- 24120  
 ------------------------------------------------------------------------------------------------------------------------ 24240  
 ---------------------

GGGCAACCTGCCTTTGTTCTG

GGGCAACCTGCCTTTGTTCTG  
Depth:2 (PIG)  
Ei-value:0.000, Pi-value:0.000  
Er-value:0.000, Pr-value:0.000  
MATCHES To TargetScan▶ miR-124-3p.1:AAGGCAC▶ miR-495-3p:AACAAAC

-

A

ATTGGTCTTAA  
Depth:2 (PIG)  
Ei-value:0.000, Pi-value:0.000  
Er-value:0.000, Pr-value:0.000  
MATCHES To TargetScan▶ miR-208-3p:UAAGACG▶ miR-499a-5p:UAAGACU


TTGGTCTTAA

TTGGTCTTAA  
Depth:3 (COW)  
Ei-value:0.000, Pi-value:0.000  
Er-value:0.000, Pr-value:0.000  
MATCHES To TargetScan▶ miR-208-3p:UAAGACG▶ miR-499a-5p:UAAGACU

--------------------------------------------

GTTTAAAT

GTTTAAAT  
Depth:2 (PIG)  
Ei-value:0.000, Pi-value:0.000  
Er-value:0.000, Pr-value:0.000  
No matches to TargetScan

-------------- 24360  
 ------------

AGATGAGGACAAA

AGATGAGGACAAA  
Depth:3 (COW)  
Ei-value:0.000, Pi-value:0.000  
Er-value:0.000, Pr-value:0.000  
No matches to TargetScan

-

TCCTTTGT

TCCTTTGT  
Depth:3 (COW)  
Ei-value:0.000, Pi-value:0.000  
Er-value:0.000, Pr-value:0.000  
No matches to TargetScan

-----------------------------

ACATTTTT

ACATTTTT  
Depth:2 (PIG)  
Ei-value:0.000, Pi-value:0.020  
Er-value:0.000, Pr-value:0.000  
No matches to TargetScan

---------------------

CCTTGTG

CCTTGTG  
Depth:2 (PIG)  
Ei-value:0.000, Pi-value:0.000  
Er-value:0.000, Pr-value:0.020  
No matches to TargetScan

--------------------- 24480  
 ------------------------

TAAAGC

TAAAGC  
Depth:3 (COW)  
Ei-value:0.000, Pi-value:0.000  
Er-value:0.000, Pr-value:0.000  
No matches to TargetScan

------------

CAAAAGAAGAGG

CAAAAGAAGAGG  
Depth:2 (PIG)  
Ei-value:0.000, Pi-value:0.000  
Er-value:0.000, Pr-value:0.000  
No matches to TargetScan

------------------------------

TTCATACA

TTCATACA  
Depth:3 (COW)  
Ei-value:0.000, Pi-value:0.000  
Er-value:0.000, Pr-value:0.000  
No matches to TargetScan

---

TTCAAAGCATC

TTCAAAGCATC  
Depth:3 (COW)  
Ei-value:0.000, Pi-value:0.000  
Er-value:0.000, Pr-value:0.000  
No matches to TargetScan

-------------- 24600  
 ----

GCAAGCCAA

GCAAGCCAA  
Depth:2 (PIG)  
Ei-value:0.000, Pi-value:0.000  
Er-value:0.000, Pr-value:0.000  
No matches to TargetScan

---------------

TGG

TGGAGAAGGAAATAGAT  
Depth:2 (PIG)  
Ei-value:0.000, Pi-value:0.000  
Er-value:0.000, Pr-value:0.000  
No matches to TargetScan


AGAAGGAAATAGA

AGAAGGAAATAGA  
Depth:3 (COW)  
Ei-value:0.000, Pi-value:0.000  
Er-value:0.000, Pr-value:0.000  
No matches to TargetScan


T

TGGAGAAGGAAATAGAT  
Depth:2 (PIG)  
Ei-value:0.000, Pi-value:0.000  
Er-value:0.000, Pr-value:0.000  
No matches to TargetScan

--------------------

TGGAGGGAGC

TGGAGGGAGC  
Depth:3 (COW)  
Ei-value:0.000, Pi-value:0.000  
Er-value:0.000, Pr-value:0.000  
No matches to TargetScan


AA

TGGAGGGAGCAA  
Depth:2 (PIG)  
Ei-value:0.000, Pi-value:0.000  
Er-value:0.000, Pr-value:0.000  
No matches to TargetScan

-

GTTGAAGA

GTTGAAGA  
Depth:2 (PIG)  
Ei-value:0.000, Pi-value:0.000  
Er-value:0.000, Pr-value:0.000  
No matches to TargetScan

-------------------

TAATGTTT

TAATGTTT  
Depth:4 (DOG)  
Ei-value:0.000, Pi-value:0.000  
Er-value:0.000, Pr-value:0.000  
MATCHES To TargetScan▶ miR-323-3p:ACAUUAC▶ miR-543:AACAUUC

------- 24720  
 ----

TAATGTTT

TAATGTTT  
Depth:4 (DOG)  
Ei-value:0.000, Pi-value:0.000  
Er-value:0.000, Pr-value:0.000  
MATCHES To TargetScan▶ miR-323-3p:ACAUUAC▶ miR-543:AACAUUC

---------

TATC

TATCAGCTGGA  
Depth:2 (PIG)  
Ei-value:0.000, Pi-value:0.000  
Er-value:0.000, Pr-value:0.000  
No matches to TargetScan


AGCTGGA

AGCTGGA  
Depth:4 (DOG)  
Ei-value:0.000, Pi-value:0.000  
Er-value:0.000, Pr-value:0.000  
No matches to TargetScan

---------------------------------------

ATTATTGGAAA

ATTATTGGAAA  
Depth:4 (DOG)  
Ei-value:0.000, Pi-value:0.000  
Er-value:0.000, Pr-value:0.000  
No matches to TargetScan


A

ATTATTGGAAAA  
Depth:2 (PIG)  
Ei-value:0.000, Pi-value:0.000  
Er-value:0.000, Pr-value:0.000  
No matches to TargetScan

-

TGGAAAG

TGGAAAG  
Depth:2 (PIG)  
Ei-value:0.000, Pi-value:0.000  
Er-value:0.000, Pr-value:0.010  
No matches to TargetScan

-

AGAAAGTAAC

AGAAAGTAAC  
Depth:4 (DOG)  
Ei-value:0.000, Pi-value:0.000  
Er-value:0.000, Pr-value:0.000  
No matches to TargetScan


TAAAAGCCTTCC

AGAAAGTAACTAAAAGCCTTCCTTTCACAGTTTCTGGCATC  
Depth:2 (PIG)  
Ei-value:0.000, Pi-value:0.000  
Er-value:0.000, Pr-value:0.000  
MATCHES To TargetScan▶ miR-488-3p:UGAAAGG


TTTCAC

TTTCACAGTTTCTGGCATC  
Depth:4 (DOG)  
Ei-value:0.000, Pi-value:0.000  
Er-value:0.000, Pr-value:0.000  
No matches to TargetScan

 24840  


AGTTTCTGGCATC

TTTCACAGTTTCTGGCATC  
Depth:4 (DOG)  
Ei-value:0.000, Pi-value:0.000  
Er-value:0.000, Pr-value:0.000  
No matches to TargetScan

----

ACTAC

ACTACCACTACTGAT  
Depth:2 (PIG)  
Ei-value:0.000, Pi-value:0.000  
Er-value:0.000, Pr-value:0.000  
MATCHES To TargetScan▶ miR-140-5p:AGUGGUU▶ miR-142-3p.1:GUAGUGU▶ miR-199-3p:CAGUAGU


CA

CACTACTGAT  
Depth:3 (COW)  
Ei-value:0.000, Pi-value:0.000  
Er-value:0.000, Pr-value:0.000  
MATCHES To TargetScan▶ miR-142-3p.1:GUAGUGU▶ miR-199-3p:CAGUAGU


CTACTGAT

CTACTGAT  
Depth:4 (DOG)  
Ei-value:0.000, Pi-value:0.000  
Er-value:0.000, Pr-value:0.000  
MATCHES To TargetScan▶ miR-199-3p:CAGUAGU


AAACAAGAATAA

AAACAAGAATAA  
Depth:3 (COW)  
Ei-value:0.000, Pi-value:0.000  
Er-value:0.000, Pr-value:0.000  
MATCHES To TargetScan▶ miR-544a-5p:CUUGUUA


G

AAACAAGAATAAGAGAACAT  
Depth:2 (PIG)  
Ei-value:0.000, Pi-value:0.000  
Er-value:0.000, Pr-value:0.000  
MATCHES To TargetScan▶ miR-544a-5p:CUUGUUA


AGAACAT

AGAACAT  
Depth:4 (DOG)  
Ei-value:0.000, Pi-value:0.000  
Er-value:0.000, Pr-value:0.000  
No matches to TargetScan

---

A

ATCATCTG  
Depth:2 (PIG)  
Ei-value:0.000, Pi-value:0.000  
Er-value:0.000, Pr-value:0.000  
No matches to TargetScan


TCATCTG

TCATCTG  
Depth:4 (DOG)  
Ei-value:0.000, Pi-value:0.010  
Er-value:0.000, Pr-value:0.000  
No matches to TargetScan

----------

CATAAATGAA

CATAAATGAA  
Depth:4 (DOG)  
Ei-value:0.000, Pi-value:0.000  
Er-value:0.000, Pr-value:0.000  
No matches to TargetScan


GTTGTGA

CATAAATGAAGTTGTGA  
Depth:3 (COW)  
Ei-value:0.000, Pi-value:0.000  
Er-value:0.000, Pr-value:0.000  
No matches to TargetScan

--

AAATCT

AAATCT  
Depth:2 (PIG)  
Ei-value:0.000, Pi-value:0.000  
Er-value:0.000, Pr-value:0.000  
No matches to TargetScan

--------------------

TG

TGGCTTC  
Depth:2 (PIG)  
Ei-value:0.000, Pi-value:0.000  
Er-value:0.000, Pr-value:0.000  
No matches to TargetScan

 24960  


GCTTC

TGGCTTC  
Depth:2 (PIG)  
Ei-value:0.000, Pi-value:0.000  
Er-value:0.000, Pr-value:0.000  
No matches to TargetScan

----------------

CTACCTCAAAG

CTACCTCAAAG  
Depth:2 (PIG)  
Ei-value:0.000, Pi-value:0.000  
Er-value:0.000, Pr-value:0.000  
MATCHES To TargetScan▶ let-7-5p/98-5p:GAGGUAG▶ miR-196-5p:AGGUAGU

-------------------------------------------

ACTTG

ACTTGTGAACTGATGTGAAA  
Depth:3 (COW)  
Ei-value:0.000, Pi-value:0.000  
Er-value:0.000, Pr-value:0.000  
MATCHES To TargetScan▶ miR-23-3p:UCACAUU


TGAACTGATGTGAAA

TGAACTGATGTGAAA  
Depth:4 (DOG)  
Ei-value:0.000, Pi-value:0.000  
Er-value:0.000, Pr-value:0.000  
MATCHES To TargetScan▶ miR-23-3p:UCACAUU

---

AGAATCTCT

AGAATCTCT  
Depth:2 (PIG)  
Ei-value:0.000, Pi-value:0.000  
Er-value:0.000, Pr-value:0.000  
No matches to TargetScan

------------- 25080  
 ---

GAAGATTGAAAAAT

GAAGATTGAAAAAT  
Depth:2 (PIG)  
Ei-value:0.000, Pi-value:0.000  
Er-value:0.000, Pr-value:0.000  
No matches to TargetScan

-

TTGTTCA

TTGTTCA  
Depth:3 (COW)  
Ei-value:0.000, Pi-value:0.000  
Er-value:0.000, Pr-value:0.000  
No matches to TargetScan

-------

TG

TGACCACCA  
Depth:2 (PIG)  
Ei-value:0.000, Pi-value:0.000  
Er-value:0.000, Pr-value:0.000  
No matches to TargetScan


ACCACCA

ACCACCA  
Depth:3 (COW)  
Ei-value:0.000, Pi-value:0.000  
Er-value:0.000, Pr-value:0.000  
No matches to TargetScan

----------------------------------------

AATTGAA

AATTGAA  
Depth:2 (PIG)  
Ei-value:0.000, Pi-value:0.020  
Er-value:0.000, Pr-value:0.010  
No matches to TargetScan

-------------------

AAATAAAA

AAATAAAA  
Depth:4 (DOG)  
Ei-value:0.000, Pi-value:0.000  
Er-value:0.000, Pr-value:0.000  
No matches to TargetScan


TA

AAATAAAATA  
Depth:2 (PIG)  
Ei-value:0.000, Pi-value:0.000  
Er-value:0.000, Pr-value:0.000  
No matches to TargetScan

--- 25200  
 --

TTTTGAAAACTT

TTTTGAAAACTT  
Depth:2 (PIG)  
Ei-value:0.000, Pi-value:0.000  
Er-value:0.000, Pr-value:0.000  
No matches to TargetScan

-                                                                                                          25215
```

---

## >COW (14055 bases)

```
 --------------------------------------------------------------------

AGGCAAGA

AGGCAAGA  
Depth:3 (COW)  
Ei-value:0.000, Pi-value:0.000  
Er-value:0.000, Pr-value:0.000  
No matches to TargetScan

-------------------------------------------- 120  


AAACATG

AAACATG  
Depth:4 (DOG)  
Ei-value:0.000, Pi-value:0.000  
Er-value:0.000, Pr-value:0.000  
No matches to TargetScan

--------

CAACAG

CAACAG  
Depth:3 (COW)  
Ei-value:0.000, Pi-value:0.000  
Er-value:0.000, Pr-value:0.000  
No matches to TargetScan

----------------------------

TTCCCATC

TTCCCATC  
Depth:4 (DOG)  
Ei-value:0.000, Pi-value:0.000  
Er-value:0.000, Pr-value:0.000  
No matches to TargetScan

---

CTCTGT

CTCTGT  
Depth:3 (COW)  
Ei-value:0.000, Pi-value:0.000  
Er-value:0.000, Pr-value:0.000  
No matches to TargetScan

------------------------------------------------------ 240  
 ------

C

CTGTTAGTCT  
Depth:4 (DOG)  
Ei-value:0.000, Pi-value:0.000  
Er-value:0.000, Pr-value:0.000  
No matches to TargetScan


TGTTAGTC

TGTTAGTC  
Depth:5 (RABBIT)  
Ei-value:0.000, Pi-value:0.000  
Er-value:0.000, Pr-value:0.000  
No matches to TargetScan


T

CTGTTAGTCT  
Depth:4 (DOG)  
Ei-value:0.000, Pi-value:0.000  
Er-value:0.000, Pr-value:0.000  
No matches to TargetScan

-------------------------------------------------------------------------------------------------------- 360  
 ------------------------------------------------------------------------------------------------------------------------ 480  
 ------------------------------------------------------------------------------------------------------------------------ 600  
 ------------------------------------------------------------------------------------------------------------------------ 720  
 ------------------------------------------------------------------------------------------------------------------------ 840  
 ------------------------------------------------------------------------------------------------------------------------ 960  
 ------------------------------------------------------------------------------------------------------------------------ 1080  
 ------------------------------------------------------------------------------------------------------------------------ 1200  
 ------------------------------------------------------------------------------------------------------------------------ 1320  
 ------------------------------------------------------------------------------------------------------------------------ 1440  
 ------------------------------------------------------------------------------------------------------------------------ 1560  
 ------------------------------------------------------------------------------------------------------------------------ 1680  
 ---------------------------------------

TCATCC

TCATCC  
Depth:4 (DOG)  
Ei-value:0.000, Pi-value:0.020  
Er-value:0.000, Pr-value:0.000  
No matches to TargetScan

--------------------------------------------------------------------------- 1800  
 ------------------------------------------------------------------------------------------------------------------------ 1920  
 ------------------------------------------------------------------------------------------------------------------------ 2040  
 --

GGG

GGGTACTTGGGACTGTTAAT  
Depth:3 (COW)  
Ei-value:0.000, Pi-value:0.000  
Er-value:0.000, Pr-value:0.000  
MATCHES To TargetScan▶ miR-132-3p/212-3p:AACAGUC▶ miR-455-3p.1:CAGUCCA


TACTTGGGACTGTTAAT

TACTTGGGACTGTTAAT  
Depth:4 (DOG)  
Ei-value:0.000, Pi-value:0.000  
Er-value:0.000, Pr-value:0.000  
MATCHES To TargetScan▶ miR-132-3p/212-3p:AACAGUC▶ miR-455-3p.1:CAGUCCA

-----------------------------------------------------------------------------------------

ACTG

ACTGTTAATGTGCT  
Depth:4 (DOG)  
Ei-value:0.000, Pi-value:0.000  
Er-value:0.000, Pr-value:0.000  
MATCHES To TargetScan▶ miR-132-3p/212-3p:AACAGUC▶ miR-323-3p:ACAUUAC


TTAAT

TTAATGTGCT  
Depth:5 (RABBIT)  
Ei-value:0.000, Pi-value:0.000  
Er-value:0.000, Pr-value:0.000  
MATCHES To TargetScan▶ miR-323-3p:ACAUUAC

 2160  


GTGCT

TTAATGTGCT  
Depth:5 (RABBIT)  
Ei-value:0.000, Pi-value:0.000  
Er-value:0.000, Pr-value:0.000  
MATCHES To TargetScan▶ miR-323-3p:ACAUUAC

------------------------------------------------------------------------------------------------------------------- 2280  
 ------------------------------------------------------------------------------------------------------------------------ 2400  
 ------------------------------------------------------------------------------------------------------------------------ 2520  
 ------------------------------------------------------------------------------------------------------------------------ 2640  
 ------------------------------------------------------------------------------------------------------------------------ 2760  
 ------------------------------------------------------------------------------------------------------------------------ 2880  
 ------------------------------------------------------------------------------------------------------------------------ 3000  
 --------------------------------------------------------------------------------

CTTGGGACTC

CTTGGGACTC  
Depth:3 (COW)  
Ei-value:0.000, Pi-value:0.000  
Er-value:0.000, Pr-value:0.000  
No matches to TargetScan


AATGTGCAT

AATGTGCAT  
Depth:6 (MOUSE)  
Ei-value:0.000, Pi-value:0.000  
Er-value:0.000, Pr-value:0.000  
MATCHES To TargetScan▶ miR-501-3p/502-3p:AUGCACC

----------------

CTAAT

CTAATA  
Depth:3 (COW)  
Ei-value:0.000, Pi-value:0.000  
Er-value:0.000, Pr-value:0.000  
No matches to TargetScan

 3120  


A

CTAATA  
Depth:3 (COW)  
Ei-value:0.000, Pi-value:0.000  
Er-value:0.000, Pr-value:0.000  
No matches to TargetScan

----------------------------------------------------------------------------------------------------------------------- 3240  
 -----------

TGCTTCT

TGCTTCT  
Depth:3 (COW)  
Ei-value:0.000, Pi-value:0.000  
Er-value:0.000, Pr-value:0.010  
No matches to TargetScan

------------------------------------------

TATGTTAGA

TATGTTAGA  
Depth:4 (DOG)  
Ei-value:0.000, Pi-value:0.000  
Er-value:0.000, Pr-value:0.000  
No matches to TargetScan

--------------------------------------------------- 3360  
 ---------------

TCTTGG

TCTTGGACTGTTAATGT  
Depth:3 (COW)  
Ei-value:0.000, Pi-value:0.000  
Er-value:0.000, Pr-value:0.000  
MATCHES To TargetScan▶ miR-132-3p/212-3p:AACAGUC▶ miR-323-3p:ACAUUAC▶ miR-455-3p.1:CAGUCCA


ACTGTTAATGT

ACTGTTAATGT  
Depth:4 (DOG)  
Ei-value:0.000, Pi-value:0.000  
Er-value:0.000, Pr-value:0.000  
MATCHES To TargetScan▶ miR-132-3p/212-3p:AACAGUC▶ miR-323-3p:ACAUUAC

----------

ATTTGCT

ATTTGCT  
Depth:4 (DOG)  
Ei-value:0.000, Pi-value:0.000  
Er-value:0.000, Pr-value:0.000  
No matches to TargetScan

------------------

GTAAGGA

GTAAGGA  
Depth:5 (RABBIT)  
Ei-value:0.000, Pi-value:0.000  
Er-value:0.000, Pr-value:0.000  
No matches to TargetScan


CCC

GTAAGGACCC  
Depth:3 (COW)  
Ei-value:0.000, Pi-value:0.000  
Er-value:0.000, Pr-value:0.000  
No matches to TargetScan

------------------------------------------- 3480  
 ---------------------------------

ATCTTAG

ATCTTAG  
Depth:3 (COW)  
Ei-value:0.000, Pi-value:0.000  
Er-value:0.000, Pr-value:0.000  
No matches to TargetScan

--------

TACACATT

TACACATT  
Depth:3 (COW)  
Ei-value:0.000, Pi-value:0.000  
Er-value:0.000, Pr-value:0.000  
No matches to TargetScan

------------------------------------------

ACTTAT

ACTTAT  
Depth:5 (RABBIT)  
Ei-value:0.000, Pi-value:0.000  
Er-value:0.000, Pr-value:0.000  
No matches to TargetScan

---------------- 3600  
 ---------------------------------------

TGTAATT

TGTAATT  
Depth:3 (COW)  
Ei-value:0.000, Pi-value:0.000  
Er-value:0.000, Pr-value:0.000  
No matches to TargetScan

----------

ATGGTC

ATGGTC  
Depth:3 (COW)  
Ei-value:0.000, Pi-value:0.020  
Er-value:0.000, Pr-value:0.000  
No matches to TargetScan

---------------------------------------------------

ATGGGGT

ATGGGGTACT  
Depth:3 (COW)  
Ei-value:0.000, Pi-value:0.000  
Er-value:0.000, Pr-value:0.000  
No matches to TargetScan

 3720  


ACT

ATGGGGTACT  
Depth:3 (COW)  
Ei-value:0.000, Pi-value:0.000  
Er-value:0.000, Pr-value:0.000  
No matches to TargetScan

---

CAC

CACTTAAGGCCCCTTTCTCAA  
Depth:3 (COW)  
Ei-value:0.000, Pi-value:0.000  
Er-value:0.000, Pr-value:0.000  
No matches to TargetScan


TTAAGGCC

TTAAGGCC  
Depth:6 (MOUSE)  
Ei-value:0.000, Pi-value:0.000  
Er-value:0.000, Pr-value:0.000  
No matches to TargetScan


CCTTT

TTAAGGCCCCTTT  
Depth:5 (RABBIT)  
Ei-value:0.000, Pi-value:0.000  
Er-value:0.000, Pr-value:0.000  
No matches to TargetScan


CTCAA

TTAAGGCCCCTTTCTCAA  
Depth:4 (DOG)  
Ei-value:0.000, Pi-value:0.000  
Er-value:0.000, Pr-value:0.000  
No matches to TargetScan

-------

TAATGACAATTACAT

TAATGACAATTACAT  
Depth:3 (COW)  
Ei-value:0.000, Pi-value:0.000  
Er-value:0.000, Pr-value:0.000  
MATCHES To TargetScan▶ miR-411-3p:AUGUAAC

-----------------------------

CTTATATTT

CTTATATTT  
Depth:3 (COW)  
Ei-value:0.000, Pi-value:0.000  
Er-value:0.000, Pr-value:0.000  
MATCHES To TargetScan▶ miR-410-3p:AUAUAAC

---------------

TTTTAATTGACCA

TTTTAATTGACCA  
Depth:3 (COW)  
Ei-value:0.000, Pi-value:0.000  
Er-value:0.000, Pr-value:0.000  
No matches to TargetScan

----- 3840  
 ----------

ACATTAAT

ACATTAAT  
Depth:3 (COW)  
Ei-value:0.000, Pi-value:0.000  
Er-value:0.000, Pr-value:0.000  
No matches to TargetScan

---

CATAATTGCA

CATAATTGCA  
Depth:3 (COW)  
Ei-value:0.000, Pi-value:0.000  
Er-value:0.000, Pr-value:0.000  
No matches to TargetScan

---------------------------------------------------------------

CTAGACAAGGA

CTAGACAAGGA  
Depth:3 (COW)  
Ei-value:0.000, Pi-value:0.000  
Er-value:0.000, Pr-value:0.000  
No matches to TargetScan

--------------- 3960  
 -----------------------------------------------

ACAGTTAATGTG

ACAGTTAATGTG  
Depth:4 (DOG)  
Ei-value:0.000, Pi-value:0.000  
Er-value:0.000, Pr-value:0.000  
MATCHES To TargetScan▶ miR-323-3p:ACAUUAC

------------------------------------------------------------- 4080  
 --------

ATACTGTTT

ATACTGTTT  
Depth:3 (COW)  
Ei-value:0.000, Pi-value:0.000  
Er-value:0.000, Pr-value:0.000  
MATCHES To TargetScan▶ miR-101-3p.1:ACAGUAC▶ miR-132-3p/212-3p:AACAGUC▶ miR-144-3p:ACAGUAU

-------------------------------------------

TTGTCTT

TTGTCTT  
Depth:3 (COW)  
Ei-value:0.000, Pi-value:0.000  
Er-value:0.000, Pr-value:0.010  
No matches to TargetScan

--------------------------------------------

CTCAGCTCT

CTCAGCTCTTGG  
Depth:5 (RABBIT)  
Ei-value:0.000, Pi-value:0.000  
Er-value:0.000, Pr-value:0.000  
MATCHES To TargetScan▶ miR-335-5p:CAAGAGC

 4200  


TGG

CTCAGCTCTTGG  
Depth:5 (RABBIT)  
Ei-value:0.000, Pi-value:0.000  
Er-value:0.000, Pr-value:0.000  
MATCHES To TargetScan▶ miR-335-5p:CAAGAGC


ACA

CTCAGCTCTTGGACA  
Depth:4 (DOG)  
Ei-value:0.000, Pi-value:0.000  
Er-value:0.000, Pr-value:0.000  
MATCHES To TargetScan▶ miR-335-5p:CAAGAGC


ATTAATA

CTCAGCTCTTGGACAATTAATA  
Depth:3 (COW)  
Ei-value:0.000, Pi-value:0.000  
Er-value:0.000, Pr-value:0.000  
MATCHES To TargetScan▶ miR-335-5p:CAAGAGC

-----------------------------------------------------------

GATCAT

GATCAT  
Depth:3 (COW)  
Ei-value:0.000, Pi-value:0.000  
Er-value:0.000, Pr-value:0.000  
No matches to TargetScan

-----------

TAAGGC

TAAGGC  
Depth:3 (COW)  
Ei-value:0.000, Pi-value:0.000  
Er-value:0.000, Pr-value:0.000  
No matches to TargetScan

----------------------

GAA

GAATATTTGCA  
Depth:3 (COW)  
Ei-value:0.000, Pi-value:0.000  
Er-value:0.000, Pr-value:0.000  
No matches to TargetScan

 4320  


TATTTGCA

GAATATTTGCA  
Depth:3 (COW)  
Ei-value:0.000, Pi-value:0.000  
Er-value:0.000, Pr-value:0.000  
No matches to TargetScan

--------------------------------------------------------

ATTACTG

ATTACTG  
Depth:3 (COW)  
Ei-value:0.000, Pi-value:0.010  
Er-value:0.000, Pr-value:0.020  
MATCHES To TargetScan▶ miR-802:CAGUAAC

---

GGGCTGCTGA

GGGCTGCTGA  
Depth:3 (COW)  
Ei-value:0.000, Pi-value:0.000  
Er-value:0.000, Pr-value:0.000  
MATCHES To TargetScan▶ miR-15-5p/16-5p/195-5p/424-5p/497-5p:AGCAGCA▶ miR-503-5p:AGCAGCG

-----

CAAAACTT

CAAAACTT  
Depth:4 (DOG)  
Ei-value:0.000, Pi-value:0.000  
Er-value:0.000, Pr-value:0.000  
No matches to TargetScan

---

CTGGGACTG

CTGGGACTG  
Depth:3 (COW)  
Ei-value:0.000, Pi-value:0.000  
Er-value:0.000, Pr-value:0.000  
MATCHES To TargetScan▶ miR-455-3p.1:CAGUCCA

-------

GCAC

GCACAATG  
Depth:6 (MOUSE)  
Ei-value:0.000, Pi-value:0.000  
Er-value:0.000, Pr-value:0.000  
No matches to TargetScan

 4440  


AATG

GCACAATG  
Depth:6 (MOUSE)  
Ei-value:0.000, Pi-value:0.000  
Er-value:0.000, Pr-value:0.000  
No matches to TargetScan

---------------------

CTCCCTG

CTCCCTG  
Depth:3 (COW)  
Ei-value:0.000, Pi-value:0.000  
Er-value:0.000, Pr-value:0.000  
No matches to TargetScan

----------

GCAAGC

GCAAGC  
Depth:3 (COW)  
Ei-value:0.000, Pi-value:0.000  
Er-value:0.000, Pr-value:0.000  
No matches to TargetScan


A

ACTCCCA  
Depth:4 (DOG)  
Ei-value:0.000, Pi-value:0.000  
Er-value:0.000, Pr-value:0.000  
No matches to TargetScan


CTCCCA

CTCCCA  
Depth:6 (MOUSE)  
Ei-value:0.000, Pi-value:0.000  
Er-value:0.000, Pr-value:0.000  
No matches to TargetScan

----------------------------------------------------------------- 4560  
 ------------------------------------------------------------------------------------------------------------------------ 4680  
 --------------------------------------------------------------------------------

CCCTTTTGCATT

CCCTTTTGCATT  
Depth:4 (DOG)  
Ei-value:0.000, Pi-value:0.000  
Er-value:0.000, Pr-value:0.000  
No matches to TargetScan


G

CCCTTTTGCATTG  
Depth:3 (COW)  
Ei-value:0.000, Pi-value:0.000  
Er-value:0.000, Pr-value:0.000  
No matches to TargetScan

--------------------------- 4800  
 --------------------------------------------------------------------------------------------------------------------

ACTT

ACTTCCTT  
Depth:3 (COW)  
Ei-value:0.000, Pi-value:0.000  
Er-value:0.000, Pr-value:0.000  
No matches to TargetScan

 4920  


CCTT

ACTTCCTT  
Depth:3 (COW)  
Ei-value:0.000, Pi-value:0.000  
Er-value:0.000, Pr-value:0.000  
No matches to TargetScan

-----------------------------------

AGCCCCTTCT

AGCCCCTTCT  
Depth:3 (COW)  
Ei-value:0.000, Pi-value:0.000  
Er-value:0.000, Pr-value:0.000  
No matches to TargetScan

----------

CACAGTA

CACAGTA  
Depth:3 (COW)  
Ei-value:0.000, Pi-value:0.000  
Er-value:0.000, Pr-value:0.000  
No matches to TargetScan

-

TGATTGTC

TGATTGTCCCATTTTT  
Depth:3 (COW)  
Ei-value:0.000, Pi-value:0.000  
Er-value:0.000, Pr-value:0.000  
No matches to TargetScan


CCATTTTT

CCATTTTT  
Depth:4 (DOG)  
Ei-value:0.000, Pi-value:0.000  
Er-value:0.000, Pr-value:0.000  
No matches to TargetScan

--------

CAGCCCA

CAGCCCA  
Depth:4 (DOG)  
Ei-value:0.000, Pi-value:0.000  
Er-value:0.000, Pr-value:0.000  
No matches to TargetScan

---

TCTC

TCTCCCTACCA  
Depth:3 (COW)  
Ei-value:0.000, Pi-value:0.000  
Er-value:0.000, Pr-value:0.000  
No matches to TargetScan


CCTACCA

CCTACCA  
Depth:4 (DOG)  
Ei-value:0.000, Pi-value:0.000  
Er-value:0.000, Pr-value:0.000  
No matches to TargetScan

-------- 5040  
 --------

GTGCAGT

GTGCAGT  
Depth:3 (COW)  
Ei-value:0.000, Pi-value:0.000  
Er-value:0.000, Pr-value:0.000  
MATCHES To TargetScan▶ miR-217:ACUGCAU

----------

AAAAGCAG

AAAAGCAG  
Depth:6 (MOUSE)  
Ei-value:0.000, Pi-value:0.000  
Er-value:0.000, Pr-value:0.000  
No matches to TargetScan

----

GAACTA

GAACTA  
Depth:3 (COW)  
Ei-value:0.000, Pi-value:0.000  
Er-value:0.000, Pr-value:0.000  
No matches to TargetScan

---------------------------------

TTAATGATCC

TTAATGATCC  
Depth:4 (DOG)  
Ei-value:0.000, Pi-value:0.000  
Er-value:0.000, Pr-value:0.000  
MATCHES To TargetScan▶ miR-382-3p:AUCAUUC

--------

ATTATTGT

ATTATTGT  
Depth:3 (COW)  
Ei-value:0.000, Pi-value:0.000  
Er-value:0.000, Pr-value:0.000  
No matches to TargetScan

---

ATTCTGGG

ATTCTGGG  
Depth:4 (DOG)  
Ei-value:0.000, Pi-value:0.000  
Er-value:0.000, Pr-value:0.000  
No matches to TargetScan

------- 5160  
 -----------------------

TG

TGCTTTACT  
Depth:3 (COW)  
Ei-value:0.000, Pi-value:0.000  
Er-value:0.000, Pr-value:0.000  
MATCHES To TargetScan▶ miR-330-3p.2:AAAGCAC


CTTTACT

CTTTACT  
Depth:4 (DOG)  
Ei-value:0.000, Pi-value:0.000  
Er-value:0.000, Pr-value:0.000  
No matches to TargetScan

--

GCAAAAT

GCAAAAT  
Depth:6 (MOUSE)  
Ei-value:0.000, Pi-value:0.000  
Er-value:0.000, Pr-value:0.000  
No matches to TargetScan

----

AAGGCAA

AAGGCAA  
Depth:4 (DOG)  
Ei-value:0.000, Pi-value:0.000  
Er-value:0.000, Pr-value:0.000  
No matches to TargetScan


GTCAGACCCA

AAGGCAAGTCAGACCCA  
Depth:3 (COW)  
Ei-value:0.000, Pi-value:0.000  
Er-value:0.000, Pr-value:0.000  
MATCHES To TargetScan▶ miR-193a-5p:GGGUCUU

-------

TGGATTGC

TGGATTGC  
Depth:4 (DOG)  
Ei-value:0.000, Pi-value:0.000  
Er-value:0.000, Pr-value:0.000  
No matches to TargetScan

------------------------------------------- 5280  
 ------------

GAAGGAAG

GAAGGAAG  
Depth:3 (COW)  
Ei-value:0.000, Pi-value:0.000  
Er-value:0.000, Pr-value:0.000  
No matches to TargetScan

--------------

TGCATTCTTC

TGCATTCTTC  
Depth:5 (RABBIT)  
Ei-value:0.000, Pi-value:0.000  
Er-value:0.000, Pr-value:0.000  
No matches to TargetScan

-------

AGC

AGCAGATTGCCTGG  
Depth:4 (DOG)  
Ei-value:0.000, Pi-value:0.000  
Er-value:0.000, Pr-value:0.000  
No matches to TargetScan


A

AGATTGCCTGG  
Depth:5 (RABBIT)  
Ei-value:0.000, Pi-value:0.000  
Er-value:0.000, Pr-value:0.000  
No matches to TargetScan


GATTGCCTGG

GATTGCCTGG  
Depth:6 (MOUSE)  
Ei-value:0.000, Pi-value:0.000  
Er-value:0.000, Pr-value:0.000  
No matches to TargetScan

-------------------

TTGTATATT

TTGTATATT  
Depth:4 (DOG)  
Ei-value:0.000, Pi-value:0.000  
Er-value:0.000, Pr-value:0.000  
MATCHES To TargetScan▶ miR-381-3p:AUACAAG

------------

TGCCAA

TGCCAA  
Depth:3 (COW)  
Ei-value:0.000, Pi-value:0.000  
Er-value:0.000, Pr-value:0.000  
MATCHES To TargetScan▶ miR-182-5p:UUGGCAA▶ miR-96-5p/1271-5p:UUGGCAC

-

TGCCAGGA

TGCCAGGATACA  
Depth:3 (COW)  
Ei-value:0.000, Pi-value:0.000  
Er-value:0.000, Pr-value:0.000  
No matches to TargetScan

 5400  


TACA

TGCCAGGATACA  
Depth:3 (COW)  
Ei-value:0.000, Pi-value:0.000  
Er-value:0.000, Pr-value:0.000  
No matches to TargetScan

----------------------------------------------

ACATCTGG

ACATCTGG  
Depth:3 (COW)  
Ei-value:0.000, Pi-value:0.000  
Er-value:0.000, Pr-value:0.000  
No matches to TargetScan

----------------

GAT

GATAACCTGGTCATT  
Depth:3 (COW)  
Ei-value:0.000, Pi-value:0.000  
Er-value:0.000, Pr-value:0.000  
MATCHES To TargetScan▶ miR-154-5p:AGGUUAU


AAC

AACCTGGTCATT  
Depth:4 (DOG)  
Ei-value:0.000, Pi-value:0.000  
Er-value:0.000, Pr-value:0.000  
No matches to TargetScan


CTGGTCATT

CTGGTCATT  
Depth:5 (RABBIT)  
Ei-value:0.000, Pi-value:0.000  
Er-value:0.000, Pr-value:0.000  
No matches to TargetScan

----

TTTTGAA

TTTTGAA  
Depth:3 (COW)  
Ei-value:0.000, Pi-value:0.000  
Er-value:0.000, Pr-value:0.010  
No matches to TargetScan

----------

CCATTTAT

CCATTTAT  
Depth:5 (RABBIT)  
Ei-value:0.000, Pi-value:0.000  
Er-value:0.000, Pr-value:0.000  
No matches to TargetScan

-- 5520  
 -----------

TGAC

TGACCAGTGTCTCTCATTT  
Depth:4 (DOG)  
Ei-value:0.000, Pi-value:0.000  
Er-value:0.000, Pr-value:0.000  
No matches to TargetScan


CAGTGTCTCTCATTT

CAGTGTCTCTCATTT  
Depth:5 (RABBIT)  
Ei-value:0.000, Pi-value:0.000  
Er-value:0.000, Pr-value:0.000  
No matches to TargetScan

-----

AGG

AGGGTGGTG  
Depth:4 (DOG)  
Ei-value:0.000, Pi-value:0.000  
Er-value:0.000, Pr-value:0.000  
No matches to TargetScan


GTGGTG

GTGGTG  
Depth:5 (RABBIT)  
Ei-value:0.000, Pi-value:0.000  
Er-value:0.000, Pr-value:0.000  
No matches to TargetScan

-

GTCTGTGGATA

GTCTGTGGATA  
Depth:5 (RABBIT)  
Ei-value:0.000, Pi-value:0.000  
Er-value:0.000, Pr-value:0.000  
MATCHES To TargetScan▶ miR-140-3p.1:CCACAGG


GA

GTCTGTGGATAGA  
Depth:3 (COW)  
Ei-value:0.000, Pi-value:0.000  
Er-value:0.000, Pr-value:0.000  
MATCHES To TargetScan▶ miR-140-3p.1:CCACAGG

---------------

TATTTTA

TATTTTA  
Depth:3 (COW)  
Ei-value:0.000, Pi-value:0.040  
Er-value:0.000, Pr-value:0.020  
No matches to TargetScan

-----------------

TTCTAGA

TTCTAGA  
Depth:4 (DOG)  
Ei-value:0.000, Pi-value:0.000  
Er-value:0.000, Pr-value:0.000  
No matches to TargetScan

---------------- 5640  
 -

AGTATCTTTG

AGTATCTTTG  
Depth:3 (COW)  
Ei-value:0.000, Pi-value:0.000  
Er-value:0.000, Pr-value:0.000  
No matches to TargetScan

-----------------------------------------------

ATTCACTT

ATTCACTT  
Depth:4 (DOG)  
Ei-value:0.000, Pi-value:0.000  
Er-value:0.000, Pr-value:0.000  
No matches to TargetScan

----

GAAAAAC

GAAAAAC  
Depth:4 (DOG)  
Ei-value:0.000, Pi-value:0.000  
Er-value:0.000, Pr-value:0.000  
No matches to TargetScan

----------------------

AATTTCTTCATCTGGAGC

AATTTCTTCATCTGGAGC  
Depth:5 (RABBIT)  
Ei-value:0.000, Pi-value:0.000  
Er-value:0.000, Pr-value:0.000  
No matches to TargetScan

--- 5760  
 ------------

CTTATTT

CTTATTT  
Depth:4 (DOG)  
Ei-value:0.000, Pi-value:0.000  
Er-value:0.000, Pr-value:0.010  
No matches to TargetScan


CAAGAA

CTTATTTCAAGAA  
Depth:3 (COW)  
Ei-value:0.000, Pi-value:0.000  
Er-value:0.000, Pr-value:0.000  
MATCHES To TargetScan▶ miR-203a-3p.2:UGAAAUG

---------------

ATAAAATG

ATAAAATG  
Depth:4 (DOG)  
Ei-value:0.000, Pi-value:0.000  
Er-value:0.000, Pr-value:0.000  
No matches to TargetScan


A

ATAAAATGA  
Depth:3 (COW)  
Ei-value:0.000, Pi-value:0.000  
Er-value:0.000, Pr-value:0.000  
No matches to TargetScan

----------------------------------------------------------------------- 5880  
 -----------------------------------

ACCACACT

ACCACACT  
Depth:3 (COW)  
Ei-value:0.000, Pi-value:0.000  
Er-value:0.000, Pr-value:0.000  
No matches to TargetScan

---

GTGAGG

GTGAGG  
Depth:3 (COW)  
Ei-value:0.000, Pi-value:0.000  
Er-value:0.000, Pr-value:0.000  
No matches to TargetScan

-----------------------------

TTTTATA

TTTTATA  
Depth:3 (COW)  
Ei-value:0.000, Pi-value:0.000  
Er-value:0.000, Pr-value:0.010  
MATCHES To TargetScan▶ miR-340-5p:UAUAAAG

-------

AAAAATAAGCCA

AAAAATAAGCCA  
Depth:5 (RABBIT)  
Ei-value:0.000, Pi-value:0.000  
Er-value:0.000, Pr-value:0.000  
No matches to TargetScan


A

AAAAATAAGCCAA  
Depth:4 (DOG)  
Ei-value:0.000, Pi-value:0.000  
Er-value:0.000, Pr-value:0.000  
No matches to TargetScan

-----------

T

TCTTTTGGATATA  
Depth:3 (COW)  
Ei-value:0.000, Pi-value:0.000  
Er-value:0.000, Pr-value:0.000  
No matches to TargetScan

 6000  


CTTTTGGATATA

TCTTTTGGATATA  
Depth:3 (COW)  
Ei-value:0.000, Pi-value:0.000  
Er-value:0.000, Pr-value:0.000  
No matches to TargetScan

--------------------------

ATGAATAATA

ATGAATAATA  
Depth:4 (DOG)  
Ei-value:0.000, Pi-value:0.000  
Er-value:0.000, Pr-value:0.000  
No matches to TargetScan

-----------

AGTGTACA

AGTGTACA  
Depth:3 (COW)  
Ei-value:0.000, Pi-value:0.000  
Er-value:0.000, Pr-value:0.000  
MATCHES To TargetScan▶ miR-493-5p:UGUACAU

-

GGTGTTT

GGTGTTT  
Depth:3 (COW)  
Ei-value:0.000, Pi-value:0.000  
Er-value:0.000, Pr-value:0.000  
No matches to TargetScan

-------------------

TGGAACTGCT

TGGAACTGCT  
Depth:4 (DOG)  
Ei-value:0.000, Pi-value:0.000  
Er-value:0.000, Pr-value:0.000  
No matches to TargetScan

--------

TAACTA

TAACTA  
Depth:4 (DOG)  
Ei-value:0.000, Pi-value:0.000  
Er-value:0.000, Pr-value:0.000  
No matches to TargetScan

-- 6120  
 --------

CAGCAGTTC

CAGCAGTTC  
Depth:5 (RABBIT)  
Ei-value:0.000, Pi-value:0.000  
Er-value:0.000, Pr-value:0.000  
No matches to TargetScan

-

TTGTAAT

TTGTAAT  
Depth:4 (DOG)  
Ei-value:0.000, Pi-value:0.000  
Er-value:0.000, Pr-value:0.000  
No matches to TargetScan

-

ACTGAAAA

ACTGAAAA  
Depth:5 (RABBIT)  
Ei-value:0.000, Pi-value:0.000  
Er-value:0.000, Pr-value:0.000  
No matches to TargetScan

---------------

GAG

GAGAAGGATGTCAAAAGATCGGC  
Depth:3 (COW)  
Ei-value:0.000, Pi-value:0.000  
Er-value:0.000, Pr-value:0.000  
MATCHES To TargetScan▶ miR-362-5p/500b-5p:AUCCUUG▶ miR-489-3p:UGACAUC


AAGGATG

AAGGATG  
Depth:5 (RABBIT)  
Ei-value:0.000, Pi-value:0.000  
Er-value:0.000, Pr-value:0.000  
MATCHES To TargetScan▶ miR-362-5p/500b-5p:AUCCUUG


TCA

AAGGATGTCAAAAGATC  
Depth:4 (DOG)  
Ei-value:0.000, Pi-value:0.000  
Er-value:0.000, Pr-value:0.000  
MATCHES To TargetScan▶ miR-362-5p/500b-5p:AUCCUUG▶ miR-489-3p:UGACAUC


AAAGATC

AAAGATC  
Depth:6 (MOUSE)  
Ei-value:0.000, Pi-value:0.000  
Er-value:0.000, Pr-value:0.000  
No matches to TargetScan


GGC

GAGAAGGATGTCAAAAGATCGGC  
Depth:3 (COW)  
Ei-value:0.000, Pi-value:0.000  
Er-value:0.000, Pr-value:0.000  
MATCHES To TargetScan▶ miR-362-5p/500b-5p:AUCCUUG▶ miR-489-3p:UGACAUC

-

CAGCTCAGGG

CAGCTCAGGG  
Depth:4 (DOG)  
Ei-value:0.000, Pi-value:0.000  
Er-value:0.000, Pr-value:0.000  
MATCHES To TargetScan▶ miR-125-5p:CCCUGAG

-

GCAGTTTGC

GCAGTTTGC  
Depth:3 (COW)  
Ei-value:0.000, Pi-value:0.000  
Er-value:0.000, Pr-value:0.000  
No matches to TargetScan

-

CTACTAGCTCCT

CTACTAGCTCCT  
Depth:4 (DOG)  
Ei-value:0.000, Pi-value:0.000  
Er-value:0.000, Pr-value:0.000  
MATCHES To TargetScan▶ miR-28-5p/708-5p:AGGAGCU▶ miR-411-5p.2:UAGUAGA

-

GGACAGCTG

GGACAGCTG  
Depth:5 (RABBIT)  
Ei-value:0.000, Pi-value:0.000  
Er-value:0.000, Pr-value:0.000  
No matches to TargetScan


T

GGACAGCTGT  
Depth:4 (DOG)  
Ei-value:0.000, Pi-value:0.000  
Er-value:0.000, Pr-value:0.000  
No matches to TargetScan


A

AAGAAGAGTCTCTGGCTCTTTAGA  
Depth:3 (COW)  
Ei-value:0.000, Pi-value:0.000  
Er-value:0.000, Pr-value:0.000  
No matches to TargetScan


AG

AGAAGAGTCTCTGGCTCTTTA  
Depth:5 (RABBIT)  
Ei-value:0.000, Pi-value:0.000  
Er-value:0.000, Pr-value:0.000  
No matches to TargetScan

 6240  


AAGAGTCTCTGGCTCTTTA

AGAAGAGTCTCTGGCTCTTTA  
Depth:5 (RABBIT)  
Ei-value:0.000, Pi-value:0.000  
Er-value:0.000, Pr-value:0.000  
No matches to TargetScan


GA

AGAAGAGTCTCTGGCTCTTTAGA  
Depth:4 (DOG)  
Ei-value:0.000, Pi-value:0.000  
Er-value:0.000, Pr-value:0.000  
No matches to TargetScan

-----------

ATTCTGAGC

ATTCTGAGC  
Depth:4 (DOG)  
Ei-value:0.000, Pi-value:0.000  
Er-value:0.000, Pr-value:0.000  
No matches to TargetScan

------------------------------------------------------------------------------- 6360  
 ----------------------

GA

GACTGCAA  
Depth:3 (COW)  
Ei-value:0.000, Pi-value:0.000  
Er-value:0.000, Pr-value:0.000  
MATCHES To TargetScan▶ miR-455-3p.2:UGCAGUC


CTGCAA

CTGCAA  
Depth:5 (RABBIT)  
Ei-value:0.000, Pi-value:0.000  
Er-value:0.000, Pr-value:0.000  
No matches to TargetScan

--------------------------------------

TTTGAGAATCTGG

TTTGAGAATCTGG  
Depth:3 (COW)  
Ei-value:0.000, Pi-value:0.000  
Er-value:0.000, Pr-value:0.000  
MATCHES To TargetScan▶ miR-371-5p:CUCAAAC

--

AAGCTCCA

AAGCTCCA  
Depth:3 (COW)  
Ei-value:0.000, Pi-value:0.000  
Er-value:0.000, Pr-value:0.000  
No matches to TargetScan

------------

GGATGG

GGATGG  
Depth:3 (COW)  
Ei-value:0.000, Pi-value:0.000  
Er-value:0.000, Pr-value:0.010  
No matches to TargetScan

----------- 6480  
 -

CTGGAGAAAAAGATCT

CTGGAGAAAAAGATCT  
Depth:3 (COW)  
Ei-value:0.000, Pi-value:0.000  
Er-value:0.000, Pr-value:0.000  
No matches to TargetScan

-------

AAGAATAGGC

AAGAATAGGC  
Depth:5 (RABBIT)  
Ei-value:0.000, Pi-value:0.000  
Er-value:0.000, Pr-value:0.000  
No matches to TargetScan

--------

T

TTACAGTGTTAGTGA  
Depth:3 (COW)  
Ei-value:0.000, Pi-value:0.000  
Er-value:0.000, Pr-value:0.000  
MATCHES To TargetScan▶ miR-141-3p/200a-3p:AACACUG


TACAGTGTTAGTGA

TACAGTGTTAGTGA  
Depth:5 (RABBIT)  
Ei-value:0.000, Pi-value:0.000  
Er-value:0.000, Pr-value:0.000  
MATCHES To TargetScan▶ miR-141-3p/200a-3p:AACACUG

--

CA

CATTCCCTTTGA  
Depth:3 (COW)  
Ei-value:0.000, Pi-value:0.000  
Er-value:0.000, Pr-value:0.000  
MATCHES To TargetScan▶ miR-1-3p/206:GGAAUGU


TTCCCTTTGA

TTCCCTTTGA  
Depth:6 (MOUSE)  
Ei-value:0.000, Pi-value:0.000  
Er-value:0.000, Pr-value:0.000  
No matches to TargetScan

-------

TAGGTGGAGATGGGGCATGAGGATCCTCCAGGGGAA

TAGGTGGAGATGGGGCATGAGGATCCTCCAGGGGAA  
Depth:6 (MOUSE)  
Ei-value:0.000, Pi-value:0.000  
Er-value:0.000, Pr-value:0.000  
MATCHES To TargetScan▶ miR-331-3p:CCCCUGG


A

TAGGTGGAGATGGGGCATGAGGATCCTCCAGGGGAAA  
Depth:5 (RABBIT)  
Ei-value:0.000, Pi-value:0.000  
Er-value:0.000, Pr-value:0.000  
MATCHES To TargetScan▶ miR-331-3p:CCCCUGG

---

TC

TCACTA  
Depth:5 (RABBIT)  
Ei-value:0.000, Pi-value:0.000  
Er-value:0.000, Pr-value:0.000  
No matches to TargetScan

 6600  


ACTA

TCACTA  
Depth:5 (RABBIT)  
Ei-value:0.000, Pi-value:0.000  
Er-value:0.000, Pr-value:0.000  
No matches to TargetScan


CCACT

TCACTACCACT  
Depth:4 (DOG)  
Ei-value:0.000, Pi-value:0.000  
Er-value:0.000, Pr-value:0.000  
MATCHES To TargetScan▶ miR-140-5p:AGUGGUU▶ miR-142-3p.1:GUAGUGU


G

TCACTACCACTG  
Depth:3 (COW)  
Ei-value:0.000, Pi-value:0.000  
Er-value:0.000, Pr-value:0.000  
MATCHES To TargetScan▶ miR-140-5p:AGUGGUU▶ miR-142-3p.1:GUAGUGU

-

GCAACA

GCAACA  
Depth:6 (MOUSE)  
Ei-value:0.000, Pi-value:0.000  
Er-value:0.000, Pr-value:0.000  
No matches to TargetScan


AC

GCAACAAC  
Depth:5 (RABBIT)  
Ei-value:0.000, Pi-value:0.000  
Er-value:0.000, Pr-value:0.000  
No matches to TargetScan

---------------------------

CTTTCCTGG

CTTTCCTGG  
Depth:3 (COW)  
Ei-value:0.000, Pi-value:0.000  
Er-value:0.000, Pr-value:0.000  
MATCHES To TargetScan▶ miR-665:CCAGGAG▶ miR-873-5p.1:CAGGAAC

---------------------------

ACAACCACC

ACAACCACC  
Depth:5 (RABBIT)  
Ei-value:0.000, Pi-value:0.000  
Er-value:0.000, Pr-value:0.000  
No matches to TargetScan


ACAC

ACAACCACCACAC  
Depth:4 (DOG)  
Ei-value:0.000, Pi-value:0.000  
Er-value:0.000, Pr-value:0.000  
No matches to TargetScan

---------------

TTGTTCC

TTGTTCC  
Depth:4 (DOG)  
Ei-value:0.000, Pi-value:0.000  
Er-value:0.000, Pr-value:0.000  
No matches to TargetScan

--- 6720  
 ------

TG

TGCCAAATC  
Depth:3 (COW)  
Ei-value:0.000, Pi-value:0.000  
Er-value:0.000, Pr-value:0.000  
MATCHES To TargetScan▶ miR-182-5p:UUGGCAA▶ miR-96-5p/1271-5p:UUGGCAC


CCAAAT

CCAAAT  
Depth:6 (MOUSE)  
Ei-value:0.000, Pi-value:0.000  
Er-value:0.000, Pr-value:0.000  
No matches to TargetScan


C

CCAAATC  
Depth:5 (RABBIT)  
Ei-value:0.000, Pi-value:0.000  
Er-value:0.000, Pr-value:0.000  
No matches to TargetScan

-----------------------------

CAAGAAA

CAAGAAA  
Depth:5 (RABBIT)  
Ei-value:0.000, Pi-value:0.000  
Er-value:0.000, Pr-value:0.000  
No matches to TargetScan


T

CAAGAAAT  
Depth:3 (COW)  
Ei-value:0.000, Pi-value:0.000  
Er-value:0.000, Pr-value:0.000  
No matches to TargetScan

-

TGAACACAC

TGAACACAC  
Depth:3 (COW)  
Ei-value:0.000, Pi-value:0.000  
Er-value:0.000, Pr-value:0.000  
No matches to TargetScan

----

G

GAAGATCAACATGCCTG  
Depth:4 (DOG)  
Ei-value:0.000, Pi-value:0.000  
Er-value:0.000, Pr-value:0.000  
No matches to TargetScan


AA

AAGATCAACATGC  
Depth:5 (RABBIT)  
Ei-value:0.000, Pi-value:0.000  
Er-value:0.000, Pr-value:0.000  
No matches to TargetScan


GATCAACATGC

GATCAACATGC  
Depth:6 (MOUSE)  
Ei-value:0.000, Pi-value:0.000  
Er-value:0.000, Pr-value:0.000  
No matches to TargetScan


CTG

GAAGATCAACATGCCTG  
Depth:4 (DOG)  
Ei-value:0.000, Pi-value:0.000  
Er-value:0.000, Pr-value:0.000  
No matches to TargetScan

------------------------------------- 6840  
 ---------------------------------------------------------

TGTGTAT

TGTGTAT  
Depth:6 (MOUSE)  
Ei-value:0.000, Pi-value:0.000  
Er-value:0.000, Pr-value:0.000  
No matches to TargetScan


TT

TGTGTATTT  
Depth:4 (DOG)  
Ei-value:0.000, Pi-value:0.000  
Er-value:0.000, Pr-value:0.000  
No matches to TargetScan

-----------------------------------

TCTTTCTT

TCTTTCTT  
Depth:3 (COW)  
Ei-value:0.000, Pi-value:0.000  
Er-value:0.000, Pr-value:0.000  
No matches to TargetScan

----------

T

TGTCTTA  
Depth:4 (DOG)  
Ei-value:0.000, Pi-value:0.000  
Er-value:0.000, Pr-value:0.000  
MATCHES To TargetScan▶ miR-208-3p:UAAGACG▶ miR-499a-5p:UAAGACU

 6960  


GTCTTA

TGTCTTA  
Depth:4 (DOG)  
Ei-value:0.000, Pi-value:0.000  
Er-value:0.000, Pr-value:0.000  
MATCHES To TargetScan▶ miR-208-3p:UAAGACG▶ miR-499a-5p:UAAGACU


CCCATTTCCATG

TGTCTTACCCATTTCCATG  
Depth:3 (COW)  
Ei-value:0.000, Pi-value:0.000  
Er-value:0.000, Pr-value:0.000  
MATCHES To TargetScan▶ miR-203a-3p.1:GAAAUGU▶ miR-208-3p:UAAGACG▶ miR-499a-5p:UAAGACU

---------------------------------------------

TTTTTGT

TTTTTGT  
Depth:4 (DOG)  
Ei-value:0.000, Pi-value:0.000  
Er-value:0.000, Pr-value:0.000  
No matches to TargetScan

------------------------------------------------

TT

TTCATTTTGTT  
Depth:4 (DOG)  
Ei-value:0.000, Pi-value:0.000  
Er-value:0.000, Pr-value:0.000  
MATCHES To TargetScan▶ miR-495-3p:AACAAAC

 7080  


CATTTTGTT

TTCATTTTGTT  
Depth:4 (DOG)  
Ei-value:0.000, Pi-value:0.000  
Er-value:0.000, Pr-value:0.000  
MATCHES To TargetScan▶ miR-495-3p:AACAAAC

---------------------------------------------------------------------------

TTTGCTC

TTTGCTC  
Depth:3 (COW)  
Ei-value:0.000, Pi-value:0.000  
Er-value:0.000, Pr-value:0.000  
No matches to TargetScan

---------------------------

TT

TTTTCTCTTTGTGAA  
Depth:3 (COW)  
Ei-value:0.000, Pi-value:0.000  
Er-value:0.000, Pr-value:0.000  
No matches to TargetScan

 7200  


TTTTCTCTTTGTGAA  
Depth:3 (COW)  
Ei-value:0.000, Pi-value:0.000  
Er-value:0.000, Pr-value:0.000  
No matches to TargetScan


TTCTCTTTG

TTCTCTTTG  
Depth:6 (MOUSE)  
Ei-value:0.000, Pi-value:0.000  
Er-value:0.000, Pr-value:0.000  
No matches to TargetScan


TGAA

TTTTCTCTTTGTGAA  
Depth:3 (COW)  
Ei-value:0.000, Pi-value:0.000  
Er-value:0.000, Pr-value:0.000  
No matches to TargetScan

------------

TTCCCCTT

TTCCCCTT  
Depth:3 (COW)  
Ei-value:0.000, Pi-value:0.000  
Er-value:0.000, Pr-value:0.000  
No matches to TargetScan

-----------

ATTTCACCT

ATTTCACCT  
Depth:4 (DOG)  
Ei-value:0.000, Pi-value:0.000  
Er-value:0.000, Pr-value:0.000  
MATCHES To TargetScan▶ miR-203a-3p.2:UGAAAUG

----------------------

TGCTG

TGCTGTTTCTACT  
Depth:3 (COW)  
Ei-value:0.000, Pi-value:0.000  
Er-value:0.000, Pr-value:0.000  
MATCHES To TargetScan▶ miR-411-5p.1:AGUAGAC▶ miR-494-3p:GAAACAU


TTTCTAC

TTTCTAC  
Depth:6 (MOUSE)  
Ei-value:0.000, Pi-value:0.000  
Er-value:0.000, Pr-value:0.000  
No matches to TargetScan


T

TTTCTACT  
Depth:5 (RABBIT)  
Ei-value:0.000, Pi-value:0.000  
Er-value:0.000, Pr-value:0.000  
MATCHES To TargetScan▶ miR-411-5p.1:AGUAGAC

-------------

ATTTCTC

ATTTCTC  
Depth:6 (MOUSE)  
Ei-value:0.000, Pi-value:0.000  
Er-value:0.000, Pr-value:0.000  
No matches to TargetScan

------------ 7320  
 ------------

TCTTGGG

TCTTGGG  
Depth:5 (RABBIT)  
Ei-value:0.000, Pi-value:0.000  
Er-value:0.000, Pr-value:0.000  
No matches to TargetScan


C

TCTTGGGC  
Depth:3 (COW)  
Ei-value:0.000, Pi-value:0.000  
Er-value:0.000, Pr-value:0.000  
No matches to TargetScan

---------------------------------------------------

TTTGTGA

TTTGTGA  
Depth:4 (DOG)  
Ei-value:0.000, Pi-value:0.010  
Er-value:0.000, Pr-value:0.000  
No matches to TargetScan


TTTTC

TTTGTGATTTTC  
Depth:3 (COW)  
Ei-value:0.000, Pi-value:0.000  
Er-value:0.000, Pr-value:0.000  
No matches to TargetScan

--------------

TCTCTGTT

TCTCTGTT  
Depth:4 (DOG)  
Ei-value:0.000, Pi-value:0.000  
Er-value:0.000, Pr-value:0.000  
No matches to TargetScan

--------------- 7440  
 ----------------------

TTTGAGTATTT

TTTGAGTATTT  
Depth:4 (DOG)  
Ei-value:0.000, Pi-value:0.000  
Er-value:0.000, Pr-value:0.000  
MATCHES To TargetScan▶ miR-200bc-3p/429:AAUACUG▶ miR-371-5p:CUCAAAC

---------------------

CTTTGATT

CTTTGATT  
Depth:3 (COW)  
Ei-value:0.000, Pi-value:0.000  
Er-value:0.000, Pr-value:0.000  
No matches to TargetScan

-----------------------------------------

TGTGTGTG

TGTGTGTG  
Depth:4 (DOG)  
Ei-value:0.000, Pi-value:0.000  
Er-value:0.000, Pr-value:0.000  
MATCHES To TargetScan▶ miR-329-3p/362-3p:ACACACC

--------- 7560  
 -------------------

TCCTAACCCCT

TCCTAACCCCT  
Depth:5 (RABBIT)  
Ei-value:0.000, Pi-value:0.000  
Er-value:0.000, Pr-value:0.000  
No matches to TargetScan

-----

TAGGTGCA

TAGGTGCA  
Depth:3 (COW)  
Ei-value:0.000, Pi-value:0.000  
Er-value:0.000, Pr-value:0.000  
No matches to TargetScan

-------------------

AAGCATTG

AAGCATTG  
Depth:4 (DOG)  
Ei-value:0.000, Pi-value:0.000  
Er-value:0.000, Pr-value:0.000  
No matches to TargetScan

------------

TTATGCCA

TTATGCCA  
Depth:5 (RABBIT)  
Ei-value:0.000, Pi-value:0.000  
Er-value:0.000, Pr-value:0.000  
No matches to TargetScan


G

TTATGCCAG  
Depth:4 (DOG)  
Ei-value:0.000, Pi-value:0.000  
Er-value:0.000, Pr-value:0.000  
No matches to TargetScan

----------------------------- 7680  
 -----

TCCAAG

TCCAAG  
Depth:3 (COW)  
Ei-value:0.000, Pi-value:0.000  
Er-value:0.000, Pr-value:0.000  
No matches to TargetScan

------------------------------------------------------------------------------------------------------------- 7800  
 -----------------------------------------------------

AGA

AGAAGGCCCAA  
Depth:4 (DOG)  
Ei-value:0.000, Pi-value:0.000  
Er-value:0.000, Pr-value:0.000  
No matches to TargetScan


AGGCCCAA

AGGCCCAA  
Depth:5 (RABBIT)  
Ei-value:0.000, Pi-value:0.000  
Er-value:0.000, Pr-value:0.000  
No matches to TargetScan

--------------

TCAA

TCAAGACTAA  
Depth:4 (DOG)  
Ei-value:0.000, Pi-value:0.000  
Er-value:0.000, Pr-value:0.000  
MATCHES To TargetScan▶ miR-431-5p:GUCUUGC


GACTAA

GACTAA  
Depth:5 (RABBIT)  
Ei-value:0.000, Pi-value:0.000  
Er-value:0.000, Pr-value:0.000  
No matches to TargetScan

-------------------------------- 7920  
 --------------

AGAAGC

AGAAGC  
Depth:4 (DOG)  
Ei-value:0.000, Pi-value:0.000  
Er-value:0.000, Pr-value:0.010  
No matches to TargetScan

--------------------

C

CAAGATGA  
Depth:3 (COW)  
Ei-value:0.000, Pi-value:0.000  
Er-value:0.000, Pr-value:0.000  
No matches to TargetScan


AAGATGA

AAGATGA  
Depth:5 (RABBIT)  
Ei-value:0.000, Pi-value:0.000  
Er-value:0.000, Pr-value:0.000  
No matches to TargetScan

-------------------

TTTCTATTG

TTTCTATTG  
Depth:3 (COW)  
Ei-value:0.000, Pi-value:0.000  
Er-value:0.000, Pr-value:0.000  
No matches to TargetScan

-------------------------------------------- 8040  
 -------------------

ACTTCTT

ACTTCTT  
Depth:3 (COW)  
Ei-value:0.000, Pi-value:0.020  
Er-value:0.000, Pr-value:0.000  
No matches to TargetScan

----------

CTTTTTGATGTT

CTTTTTGATGTT  
Depth:4 (DOG)  
Ei-value:0.000, Pi-value:0.000  
Er-value:0.000, Pr-value:0.000  
No matches to TargetScan

---------------------------------------

TATTATGC

TATTATGC  
Depth:4 (DOG)  
Ei-value:0.000, Pi-value:0.000  
Er-value:0.000, Pr-value:0.000  
MATCHES To TargetScan▶ miR-369-3p:AUAAUAC

------------------------- 8160  
 ---------------------------

TAAACTTC

TAAACTTC  
Depth:3 (COW)  
Ei-value:0.000, Pi-value:0.000  
Er-value:0.000, Pr-value:0.000  
No matches to TargetScan

------------------

CTCCACTTGAGAG

CTCCACTTGAGAG  
Depth:3 (COW)  
Ei-value:0.000, Pi-value:0.000  
Er-value:0.000, Pr-value:0.000  
MATCHES To TargetScan▶ miR-26-5p:UCAAGUA

-------------------

TATTTCAGT

TATTTCAGT  
Depth:4 (DOG)  
Ei-value:0.000, Pi-value:0.000  
Er-value:0.000, Pr-value:0.000  
MATCHES To TargetScan▶ miR-203a-3p.2:UGAAAUG


CC

TATTTCAGTCC  
Depth:3 (COW)  
Ei-value:0.000, Pi-value:0.000  
Er-value:0.000, Pr-value:0.000  
MATCHES To TargetScan▶ miR-203a-3p.2:UGAAAUG

------------------------ 8280  
 ------------------------------------

GGGGAAA

GGGGAAA  
Depth:4 (DOG)  
Ei-value:0.000, Pi-value:0.000  
Er-value:0.000, Pr-value:0.000  
No matches to TargetScan

---------------

TCTAGAGAAAA

TCTAGAGAAAA  
Depth:6 (MOUSE)  
Ei-value:0.000, Pi-value:0.000  
Er-value:0.000, Pr-value:0.000  
MATCHES To TargetScan▶ miR-1251-5p:CUCUAGC

--

TGAAGAGATG

TGAAGAGATG  
Depth:5 (RABBIT)  
Ei-value:0.000, Pi-value:0.000  
Er-value:0.000, Pr-value:0.000  
No matches to TargetScan


CTCCA

TGAAGAGATGCTCCA  
Depth:3 (COW)  
Ei-value:0.000, Pi-value:0.000  
Er-value:0.000, Pr-value:0.000  
No matches to TargetScan

--

GGCCAA

GGCCAATGAGAAGAATTAGACA  
Depth:4 (DOG)  
Ei-value:0.000, Pi-value:0.000  
Er-value:0.000, Pr-value:0.000  
No matches to TargetScan


TGAGAAGAATTAGACA

TGAGAAGAATTAGACA  
Depth:6 (MOUSE)  
Ei-value:0.000, Pi-value:0.000  
Er-value:0.000, Pr-value:0.000  
No matches to TargetScan

-

GAAATACAC

GAAATACACAGATG  
Depth:3 (COW)  
Ei-value:0.000, Pi-value:0.000  
Er-value:0.000, Pr-value:0.000  
No matches to TargetScan

 8400  


AGATG

GAAATACACAGATG  
Depth:3 (COW)  
Ei-value:0.000, Pi-value:0.000  
Er-value:0.000, Pr-value:0.000  
No matches to TargetScan

----------

C

CTGAGAAG  
Depth:3 (COW)  
Ei-value:0.000, Pi-value:0.000  
Er-value:0.000, Pr-value:0.000  
No matches to TargetScan


TGAGAAG

TGAGAAG  
Depth:4 (DOG)  
Ei-value:0.000, Pi-value:0.000  
Er-value:0.000, Pr-value:0.010  
No matches to TargetScan

-----

GCCA

GCCAGCAACA  
Depth:3 (COW)  
Ei-value:0.000, Pi-value:0.000  
Er-value:0.000, Pr-value:0.000  
No matches to TargetScan


GCAACA

GCAACA  
Depth:6 (MOUSE)  
Ei-value:0.000, Pi-value:0.000  
Er-value:0.000, Pr-value:0.000  
No matches to TargetScan

---------

TTTGAGCTT

TTTGAGCTT  
Depth:3 (COW)  
Ei-value:0.000, Pi-value:0.000  
Er-value:0.000, Pr-value:0.000  
MATCHES To TargetScan▶ miR-371-5p:CUCAAAC

-

GGTGAGC

GGTGAGC  
Depth:4 (DOG)  
Ei-value:0.000, Pi-value:0.000  
Er-value:0.000, Pr-value:0.000  
No matches to TargetScan


AGGAT

GGTGAGCAGGAT  
Depth:3 (COW)  
Ei-value:0.000, Pi-value:0.000  
Er-value:0.000, Pr-value:0.000  
No matches to TargetScan

------

GGTTTGGG

GGTTTGGG  
Depth:4 (DOG)  
Ei-value:0.000, Pi-value:0.000  
Er-value:0.000, Pr-value:0.000  
No matches to TargetScan

-----------

TGGTTA

TGGTTA  
Depth:5 (RABBIT)  
Ei-value:0.000, Pi-value:0.000  
Er-value:0.000, Pr-value:0.000  
No matches to TargetScan


T

TGGTTAT  
Depth:4 (DOG)  
Ei-value:0.000, Pi-value:0.000  
Er-value:0.000, Pr-value:0.000  
No matches to TargetScan


G

TGGTTATG  
Depth:3 (COW)  
Ei-value:0.000, Pi-value:0.000  
Er-value:0.000, Pr-value:0.000  
No matches to TargetScan

------------------ 8520  
 -------------

CCCAAGG

CCCAAGG  
Depth:4 (DOG)  
Ei-value:0.000, Pi-value:0.000  
Er-value:0.000, Pr-value:0.000  
MATCHES To TargetScan▶ miR-212-5p:CCUUGGC

--------

TGAACTCCCTGCT

TGAACTCCCTGCT  
Depth:4 (DOG)  
Ei-value:0.000, Pi-value:0.000  
Er-value:0.000, Pr-value:0.000  
No matches to TargetScan


C

TGAACTCCCTGCTCATAGTAGTGGCC  
Depth:3 (COW)  
Ei-value:0.000, Pi-value:0.000  
Er-value:0.000, Pr-value:0.000  
No matches to TargetScan


ATAGTAGTGGCC

ATAGTAGTGGCC  
Depth:4 (DOG)  
Ei-value:0.000, Pi-value:0.000  
Er-value:0.000, Pr-value:0.000  
No matches to TargetScan

-------------------------------------

TTTAATAC

TTTAATAC  
Depth:4 (DOG)  
Ei-value:0.000, Pi-value:0.000  
Er-value:0.000, Pr-value:0.000  
MATCHES To TargetScan▶ miR-496.2:GUAUUAC


CT

CTAGGCTTAAAG  
Depth:4 (DOG)  
Ei-value:0.000, Pi-value:0.000  
Er-value:0.000, Pr-value:0.000  
No matches to TargetScan


AGGCTTA

AGGCTTA  
Depth:5 (RABBIT)  
Ei-value:0.000, Pi-value:0.000  
Er-value:0.000, Pr-value:0.000  
No matches to TargetScan


AAG

CTAGGCTTAAAG  
Depth:4 (DOG)  
Ei-value:0.000, Pi-value:0.000  
Er-value:0.000, Pr-value:0.000  
No matches to TargetScan

--------- 8640  
 ------------------

GTTTAAT

GTTTAAT  
Depth:5 (RABBIT)  
Ei-value:0.000, Pi-value:0.000  
Er-value:0.000, Pr-value:0.000  
No matches to TargetScan

--------------------------------------------------------------------

TGTAAAACA

TGTAAAACA  
Depth:3 (COW)  
Ei-value:0.000, Pi-value:0.000  
Er-value:0.000, Pr-value:0.000  
No matches to TargetScan

------------------ 8760  
 ----------------

T

TATTGGCA  
Depth:5 (RABBIT)  
Ei-value:0.000, Pi-value:0.000  
Er-value:0.000, Pr-value:0.000  
No matches to TargetScan


ATTGGCA

ATTGGCA  
Depth:6 (MOUSE)  
Ei-value:0.000, Pi-value:0.000  
Er-value:0.000, Pr-value:0.000  
No matches to TargetScan

------------------------------------

TTGTGAAG

TTGTGAAG  
Depth:6 (MOUSE)  
Ei-value:0.000, Pi-value:0.000  
Er-value:0.000, Pr-value:0.000  
No matches to TargetScan

---

T

TATGTAAATCA  
Depth:3 (COW)  
Ei-value:0.000, Pi-value:0.000  
Er-value:0.000, Pr-value:0.000  
No matches to TargetScan


ATGTAAAT

ATGTAAAT  
Depth:5 (RABBIT)  
Ei-value:0.000, Pi-value:0.000  
Er-value:0.000, Pr-value:0.000  
No matches to TargetScan


CA

TATGTAAATCA  
Depth:3 (COW)  
Ei-value:0.000, Pi-value:0.000  
Er-value:0.000, Pr-value:0.000  
No matches to TargetScan

-------------------------------------- 8880  
 -------------------

GCCATATGGT

GCCATATGGT  
Depth:3 (COW)  
Ei-value:0.000, Pi-value:0.000  
Er-value:0.000, Pr-value:0.000  
No matches to TargetScan

------------------------------------------------------------------------------------------- 9000  
 --------------------

TGTGC

TGTGCCTGTCCCTGT  
Depth:3 (COW)  
Ei-value:0.000, Pi-value:0.000  
Er-value:0.000, Pr-value:0.000  
No matches to TargetScan
[truncated: 111,475 more chars]
