# Supplementary material for: Uncovering deeply conserved motif combinations in rapidly evolving noncoding sequences
Source: Genome Biol. 2021 Jan 11;22:29. doi: 10.1186/s13059-020-02247-1 (PMC7798263; doi:10.1186/s13059-020-02247-1)
Supplement: Supplementary file 4 — Additional file 4. LncLOOM output results for XIST sequences from six mammals. [file 13059_2020_2247_MOESM4_ESM.gz › AdditionalFile4/Html_Files/kmers_in_blocks_level.html]

 MOTIFS IN BLOCKS

# MOTIFS IN BLOCK DIAGRAMS

## Motifs conserved to (and beyond) MOUSE (depth:6)

  

NAVIGATE ▼

▶HUMAN (depth:1)▶PIG (depth:2)▶COW (depth:3)▶DOG (depth:4)▶RABBIT (depth:5)▶MOUSE (depth:6)

  
  
  

## >HUMAN (19280 bases)

```
 ------------------------------------------------------------------------------------------------------------------------ 120  
 ------------------------------------------------------------------------------------------------------------------------ 240  
 ------------------------------------------------------------------------------------------------------------------------ 360  
 ------------------------------------------------------------------------------------------------------------------------ 480  
 ------------------------------------------------------------------------------------------------------------------------ 600  
 ------------------------------------------------------------------------------------------------------------------------ 720  
 ------------------------------------------------------------------------------------------------------------------------ 840  
 ------------------------------------------------------------------------------------------------------------------------ 960  
 ------------------------------------------------------------------------------------------------------------------------ 1080  
 ------------------------------------------------------------------------------------------------------------------------ 1200  
 ------------------------------------------------------------------------------------------------------------------------ 1320  
 ------------------------------------------------------------------------------------------------------------------------ 1440  
 ------------------------------------------------------------------------------------------------------------------------ 1560  
 ------------------------------------------------------------------------------------------------------------------------ 1680  
 ------------------------------------------------------------------------------------------------------------------------ 1800  
 ------------------------------------------------------------------------------------------------------------------------ 1920  
 ------------------------------------------------------------------------------------------------------------------------ 2040  
 ------------------------------------------------------------------------------------------------------------------------ 2160  
 ------------------------------------------------------------------------------------------------------------------------ 2280  
 ------------------------------------------------------------------------------------------------------------------------ 2400  
 ------------------------------------------------------------------------------------------------------------------------ 2520  
 ------------------------------------------------------------------------------------------------------------------------ 2640  
 ------------------------------------------------------------------------------------------------------------------------ 2760  
 ------------------------------------------------------------------------------------------------------------------------ 2880  
 ------------------------------------------------------------------------------------------------------------------------ 3000  
 ------------------------------------------------------------------------------------------------------------------------ 3120  
 ------------------------------------------------------------------------------------------------------------------------ 3240  
 ------------------------------------------------------------------------------------------------------------------------ 3360  
 ------------------------------------------------------------------------------------------------------------------------ 3480  
 ------------------------------------------------------------------------------------------------------------------------ 3600  
 ------------------------------------------------------------------------------------------------------------------------ 3720  
 ------------------------------------------------------------------------------------------------------------------------ 3840  
 ------------------------------------------------------------------------------------------------------------------------ 3960  
 ------------------------------------------------------------------------------------------------------------------------ 4080  
 ------------------------------------------------------------------------------------------------------------------------ 4200  
 ------------------------------------------------------------------------------------------------------------------------ 4320  
 ------------------------------------------------------------------------------------------------------------------------ 4440  
 ------------------------------------------------------------------------------------------------------------------------ 4560  
 ------------------------------------------------------------------------------------------------------------------------ 4680  
 ------------------------------------------------------------------------------------------------------------------------ 4800  
 ------------------------------------------------------------------------------------------------------------------------ 4920  
 -----------------------------------------------------------------------------------------------------------------

AATGTGC

AATGTGCAT  
Depth:6 (MOUSE)  
Ei-value:0.000, Pi-value:0.000  
Er-value:0.000, Pr-value:0.000  
No matches to eCLIP DataMATCHES To TargetScan▶ miR-501-3p/502-3p:AUGCACC

 5040  


AT

AATGTGCAT  
Depth:6 (MOUSE)  
Ei-value:0.000, Pi-value:0.000  
Er-value:0.000, Pr-value:0.000  
No matches to eCLIP DataMATCHES To TargetScan▶ miR-501-3p/502-3p:AUGCACC

---------------------------------------------------------------------------------------------------------------------- 5160  
 ------------------------------------------------------------------------------------------------------------------------ 5280  
 ------------------------------------------------------------------------------------------------------------------------ 5400  
 ------------------------------------------------------------------------------------------------------------------------ 5520  
 ------------------------------------------------------------------------------------------------------------------------ 5640  
 ------------------------------------------------------------------------------------------------------------------------ 5760  
 ------------------------------------------------------------------------------------------------------------------------ 5880  
 ------------------------

TTAAGGCC

TTAAGGCC  
Depth:6 (MOUSE)  
Ei-value:0.000, Pi-value:0.000  
Er-value:0.000, Pr-value:0.000  
eCLIP MATCHES▶HNRNPL (bg=0.64%)No matches to TargetScan

---------------------------------------------------------------------------------------- 6000  
 ------------------------------------------------------------------------------------------------------------------------ 6120  
 ------------------------------------------------------------------------------------------------------------------------ 6240  
 ------------------------------------------------------------------------------------------------------------------------ 6360  
 ------------------------------------------------------------------------------------------------------------------------ 6480  
 ------------------------------------------------------------------------------------------------------------------------ 6600  
 ------------------------------------------------------------------------------------------------------------------------ 6720  
 ------------------------------------------------------------------------------------------------------------------------ 6840  
 ------------------------------------------------------------------------------------------------------------------------ 6960  
 ------------------------------------------------------------------------------------------------------------------------ 7080  
 ------------------------------------------------------------------------------------------------------------------------ 7200  
 ------------------------------------------------------------------------------------------------------------------------ 7320  
 ------------------------------------------------------------------------------------------------------------------------ 7440  
 ------------------------------------------------------------------------------------------------------------------------ 7560  
 ------------------------------------------------------------------------------------------------------------------------ 7680  
 ------------------------------------------------------------------------------------------------------------------------ 7800  
 ------------------------------------------------------------------------------------------------------------------------ 7920  
 ------------------------------------------------------------------------------------------------------------------------ 8040  
 ------------------------------------------------------------------------------------------------------------------------ 8160  
 ------------------------------------------------------------------------------------------------------------------------ 8280  
 ------------------------------------------------------------------------------------------------------------------------ 8400  
 ------------------------------------------------------------------------------------------------------------------------ 8520  
 ------------------------------------------------------------------------------------------------------------------------ 8640  
 ------------------------------------------------------------------------------------------------------------------------ 8760  
 ------------------------------------------------------------------------------------------------------------------------ 8880  
 ------------------------------------------------------------------------------------------------------------------------ 9000  
 ------------------------------------------------------------------------------------------------------------------------ 9120  
 ------------------------------------------------------------------------------------------------------------------------ 9240  
 ------------------------------------------------------------------------------------------------------------------------ 9360  
 ------------------------------------------------------------------------------------------------------------------------ 9480  
 ------------------------------------------------------------------------------------------------------------------------ 9600  
 -----

GCACAATG

GCACAATG  
Depth:6 (MOUSE)  
Ei-value:0.000, Pi-value:0.000  
Er-value:0.000, Pr-value:0.000  
No matches to eCLIP DataNo matches to TargetScan

----------------------------------------------------------------------------------------------------------- 9720  
 --------------------------

CTCCCA

CTCCCA  
Depth:6 (MOUSE)  
Ei-value:0.000, Pi-value:0.000  
Er-value:0.000, Pr-value:0.000  
eCLIP MATCHES▶hnrnpk (bg=12.88%)No matches to TargetScan

---------------------------------------------------------------------------------------- 9840  
 ------------------------------------------------------------------------------------------------------------------------ 9960  
 ------------------------------------------------------------------------------------------------------------------------ 10080  
 ------------------------------------------------------------------------------------------------------------------------ 10200  
 --------

AAAAGCAG

AAAAGCAG  
Depth:6 (MOUSE)  
Ei-value:0.000, Pi-value:0.000  
Er-value:0.000, Pr-value:0.000  
No matches to eCLIP DataNo matches to TargetScan

-------------------------------------------------------------------------------------------------------- 10320  
 -------------------

GCAAAAT

GCAAAAT  
Depth:6 (MOUSE)  
Ei-value:0.000, Pi-value:0.000  
Er-value:0.000, Pr-value:0.000  
No matches to eCLIP DataNo matches to TargetScan

---------------------------------------------------------------------------------------------- 10440  
 ---------------------------------

GATTGCCTGG

GATTGCCTGG  
Depth:6 (MOUSE)  
Ei-value:0.000, Pi-value:0.000  
Er-value:0.000, Pr-value:0.000  
No matches to eCLIP DataNo matches to TargetScan

----------------------------------------------------------------------------- 10560  
 ------------------------------------------------------------------------------------------------------------------------ 10680  
 ------------------------------------------------------------------------------------------------------------------------ 10800  
 ------------------------------------------------------------------------------------------------------------------------ 10920  
 ------------------------------------------------------------------------------------------------------------------------ 11040  
 ------------------------------------------------------------------------------------------------------------------------ 11160  
 ------------------------------------------------------------------------------------------------------------------------ 11280  
 -------

AAAGATC

AAAGATC  
Depth:6 (MOUSE)  
Ei-value:0.000, Pi-value:0.000  
Er-value:0.000, Pr-value:0.000  
eCLIP MATCHES▶SRSF1 (bg=8.47%)▶U2AF2 (bg=1.76%)▶uchl5 (bg=11.16%)No matches to TargetScan

------------------------------------------------------------------------------||-------------------------- 11398  
 --------------------------------------||-------------------------------------------------------------------------------- 11516  
 ---------------------------------------------------------||------------------------------------------------

TTCCCTTTGA

TTCCCTTTGA  
Depth:6 (MOUSE)  
Ei-value:0.000, Pi-value:0.000  
Er-value:0.000, Pr-value:0.000  
eCLIP MATCHES▶ILF3 (bg=3.0%)▶RBM15 (bg=7.27%)▶SRSF7 (bg=2.32%)▶ZNF622 (bg=6.58%)No matches to TargetScan

--- 11634  
 ----

TAGGTGGAGATGGGGCATGAGGATCCTCCAGGGGAA

TAGGTGGAGATGGGGCATGAGGATCCTCCAGGGGAA  
Depth:6 (MOUSE)  
Ei-value:0.000, Pi-value:0.000  
Er-value:0.000, Pr-value:0.000  
eCLIP MATCHES▶ILF3 (bg=3.0%)▶NOLC1 (bg=9.43%)▶RBM15 (bg=7.27%)▶SRSF7 (bg=2.32%)▶ZNF622 (bg=6.58%)MATCHES To TargetScan▶ miR-331-3p:CCCCUGG

-----------------

GCAACA

GCAACA  
Depth:6 (MOUSE)  
Ei-value:0.000, Pi-value:0.000  
Er-value:0.000, Pr-value:0.000  
eCLIP MATCHES▶ILF3 (bg=3.0%)No matches to TargetScan

--------------------------------------------------------- 11754  
 ----------------------------||--------------------

CCAAAT

CCAAAT  
Depth:6 (MOUSE)  
Ei-value:0.000, Pi-value:0.000  
Er-value:0.000, Pr-value:0.000  
eCLIP MATCHES▶GRWD1 (bg=5.13%)▶NOLC1 (bg=9.43%)No matches to TargetScan

---------------------------------------------------------------- 11872  
 -----------------------------

GATCAACATGC

GATCAACATGC  
Depth:6 (MOUSE)  
Ei-value:0.000, Pi-value:0.000  
Er-value:0.000, Pr-value:0.000  
eCLIP MATCHES▶GRWD1 (bg=5.13%)▶NOLC1 (bg=9.43%)▶PTBP1 (bg=3.74%)▶RBM15 (bg=7.27%)▶TRA2A (bg=4.8%)▶uchl5 (bg=11.16%)▶ZNF622 (bg=6.58%)No matches to TargetScan

----------------------------------||

TGTGTAT

TGTGTAT  
Depth:6 (MOUSE)  
Ei-value:0.000, Pi-value:0.000  
Er-value:0.000, Pr-value:0.000  
eCLIP MATCHES▶TARDBP (bg=2.79%)▶ZC3H11A (bg=6.55%)No matches to TargetScan

------------------------------------- 11990  
 ------------------------------------------------------------------------------------------------------------------------ 12110  
 ------------------------------------------------------------------------------------------------------------------------ 12230  
 ------------------------------------

TTCTCTTTG

TTCTCTTTG  
Depth:6 (MOUSE)  
Ei-value:0.000, Pi-value:0.000  
Er-value:0.000, Pr-value:0.000  
eCLIP MATCHES▶MATR3 (bg=2.98%)▶PTBP1 (bg=3.74%)▶SMNDC1 (bg=0.63%)▶TIA1 (bg=4.07%)No matches to TargetScan

--------------------------------------------------------------------------- 12350  
 ---------

TTTCTAC

TTTCTAC  
Depth:6 (MOUSE)  
Ei-value:0.000, Pi-value:0.000  
Er-value:0.000, Pr-value:0.000  
eCLIP MATCHES▶MATR3 (bg=2.98%)▶PTBP1 (bg=3.74%)▶TIA1 (bg=4.07%)No matches to TargetScan

------------

ATTTCTC

ATTTCTC  
Depth:6 (MOUSE)  
Ei-value:0.000, Pi-value:0.000  
Er-value:0.000, Pr-value:0.000  
eCLIP MATCHES▶MATR3 (bg=2.98%)▶PTBP1 (bg=3.74%)▶TIA1 (bg=4.07%)No matches to TargetScan

------------------------------------------------------------------------------------- 12470  
 ------------------------------------------------------------------------------------------------------------------------ 12590  
 ------------------------------------------------------------------------------------------------------------------------ 12710  
 ------------------------------------------------------------------------------------------------------------------------ 12830  
 ------------------------------------------------------------------------------------------------------------------------ 12950  
 ------------------------------------------------------------------------------------------------------------------------ 13070  
 ------------------------------------------------------------------------------------------------------------------------ 13190  
 ------------------------------------------------------------------------------------------------------------------------ 13310  
 ------------------------------------------------------------------------------------------------------------------------ 13430  
 ------------------------------------------------------------------------------------------------------------------------ 13550  
 ------------------------------------------------------------------------------------------------------------------------ 13670  
 ---------------------------

TCTAGAGAAAA

TCTAGAGAAAA  
Depth:6 (MOUSE)  
Ei-value:0.000, Pi-value:0.000  
Er-value:0.000, Pr-value:0.000  
eCLIP MATCHES▶CPSF6 (bg=0.4%)▶LARP4 (bg=4.72%)▶UTP3 (bg=3.66%)▶WDR43 (bg=3.37%)MATCHES To TargetScan▶ miR-1251-5p:CUCUAGC

------------------------

TGAGAAGAATTAGACA

TGAGAAGAATTAGACA  
Depth:6 (MOUSE)  
Ei-value:0.000, Pi-value:0.000  
Er-value:0.000, Pr-value:0.000  
eCLIP MATCHES▶LARP4 (bg=4.72%)▶NOLC1 (bg=9.43%)▶SRSF7 (bg=2.32%)No matches to TargetScan

------------------------------------------ 13790  
 ------------------------------------------------------------------------------------------------------------------------ 13910  
 ------------------------------------------------------------------------------------------------------------------------ 14030  
 --------------------------------------------------------------------------------------------------------

ATTGGCA

ATTGGCA  
Depth:6 (MOUSE)  
Ei-value:0.000, Pi-value:0.000  
Er-value:0.000, Pr-value:0.000  
eCLIP MATCHES▶HNRNPA1 (bg=2.57%)No matches to TargetScan

--------- 14150  
 -------------------------

TTGTGAAG

TTGTGAAG  
Depth:6 (MOUSE)  
Ei-value:0.000, Pi-value:0.000  
Er-value:0.000, Pr-value:0.000  
eCLIP MATCHES▶HNRNPA1 (bg=2.57%)No matches to TargetScan

--------------------------------------------------------------------------------------- 14270  
 ------------------------------------------------------------------------------------------------------------------------ 14390  
 ------------------------------------------------------------------------------------------------------------------------ 14510  
 ------------------------------------------------------------------------------------------------------------------------ 14630  
 ------------------------------------------------------------------------------------------------------------------------ 14750  
 ------------------------------------------------------------------------------------------------------------------------ 14870  
 ------------------------------------------------------------------------------------------------------------------------ 14990  
 ------------------------------------------------------------------------------------------------------------------------ 15110  
 ------------------------------------------------------------------------------------------------------------------------ 15230  
 ------------------------------------------------------------------------------------------------------------------------ 15350  
 ------------------------------------------------------------------------------------------------------------------------ 15470  
 ------------------------------------------------------------------------------------------------------------------------ 15590  
 ------------------------------------------------------------------------------------------------------------------------ 15710  
 ------------------------------------------------------------------------------------------------------------------------ 15830  
 ------------------------------------------------------------------------------------------------------------------------ 15950  
 ------------------------------------------------------------------------------------------------------------------------ 16070  
 ------------------------------------------------------------------------------------------------------------------------ 16190  
 ------------------------------------------------------------------------------------------------------------------------ 16310  
 ------------------------------------------------------------------------------------------------------------------------ 16430  
 ------------------------------------------------------------------------------------------------------------------------ 16550  
 ------------------------------------------------------------------------------------------------------------------------ 16670  
 ------------------------------------------------------------------------------------------------------------------------ 16790  
 ------------------------------------------------------------------------------------------------------------------------ 16910  
 ------------------------------------------------------------------------------------------------------------------------ 17030  
 ------------------------------------------------------------------------------------------------------------------------ 17150  
 ------------------------------------------------------------------------------------------------------------------------ 17270  
 ------------------------------------------------------------------------------------------------------------------------ 17390  
 ------------------------------------------------------------------------------------------------------------------------ 17510  
 ------------------------------------------------------------------------------------------------------------------------ 17630  
 ------------------------------------------------------------------------------------------------------------------------ 17750  
 ------------------------------------------------------------------------------------------------------------------------ 17870  
 ------------------------------------------------------------------------------------------------------------------------ 17990  
 ------------------------------------------------------------------------------------------------------------------------ 18110  
 ------------------------------------------------------------------------------------------------------------------------ 18230  
 ----------------------------------

AAAAGGT

AAAAGGT  
Depth:6 (MOUSE)  
Ei-value:0.000, Pi-value:0.000  
Er-value:0.000, Pr-value:0.000  
eCLIP MATCHES▶ILF3 (bg=3.0%)▶SF3B1 (bg=2.48%)▶ZC3H11A (bg=6.55%)No matches to TargetScan

------------------------------------------------------------------------------- 18350  
 ------------------------------------------------------------------------------------------------------------------------ 18470  
 ------------------------------------------------------------------------------------------------------------------------ 18590  
 ------------------------------------------------------------------------------------------------------------------------ 18710  
 ------------------------------------------------------------------------------------------------------------------------ 18830  
 ------------------------------------------------------------------------------------------------------------------------ 18950  
 ------------------------------------------------------------------------------------------------------------------------ 19070  
 ------------------------------------------------------------------------------------------------------------------------ 19190  
 ------------------------------------------------------------------------------------------                               19280
```

---

## >PIG (25215 bases)

```
 ------------------------------------------------------------------------------------------------------------------------ 120  
 ------------------------------------------------------------------------------------------------------------------------ 240  
 ------------------------------------------------------------------------------------------------------------------------ 360  
 ------------------------------------------------------------------------------------------------------------------------ 480  
 ------------------------------------------------------------------------------------------------------------------------ 600  
 ------------------------------------------------------------------------------------------------------------------------ 720  
 ------------------------------------------------------------------------------------------------------------------------ 840  
 ------------------------------------------------------------------------------------------------------------------------ 960  
 ------------------------------------------------------------------------------------------------------------------------ 1080  
 ------------------------------------------------------------------------------------------------------------------------ 1200  
 ------------------------------------------------------------------------------------------------------------------------ 1320  
 ------------------------------------------------------------------------------------------------------------------------ 1440  
 ------------------------------------------------------------------------------------------------------------------------ 1560  
 ------------------------------------------------------------------------------------------------------------------------ 1680  
 ------------------------------------------------------------------------------------------------------------------------ 1800  
 ------------------------------------------------------------------------------------------------------------------------ 1920  
 ------------------------------------------------------------------------------------------------------------------------ 2040  
 ------------------------------------------------------------------------------------------------------------------------ 2160  
 ------------------------------------------------------------------------------------------------------------------------ 2280  
 ------------------------------------------------------------------------------------------------------------------------ 2400  
 ------------------------------------------------------------------------------------------------------------------------ 2520  
 ------------------------------------------------------------------------------------------------------------------------ 2640  
 ------------------------------------------------------------------------------------------------------------------------ 2760  
 ------------------------------------------------------------------------------------------------------------------------ 2880  
 ------------------------------------------------------------------------------------------------------------------------ 3000  
 ------------------------------------------------------------------------------------------------------------------------ 3120  
 ------------------------------------------------------------------------------------------------------------------------ 3240  
 ------------------------------------------------------------------------------------------------------------------------ 3360  
 ------------------------------------------------------------------------------------------------------------------------ 3480  
 ------------------------------------------------------------------------------------------------------------------------ 3600  
 ------------------------------------------------------------------------------------------------------------------------ 3720  
 ------------------------------------------------------------------------------------------------------------------------ 3840  
 ------------------------------------------------------------------------------------------------------------------------ 3960  
 ------------------------------------------------------------------------------------------------------------------------ 4080  
 ------------------------------------------------------------------------------------------------------------------------ 4200  
 ------------------------------------------------------------------------------------------------------------------------ 4320  
 ------------------------------------------------------------------------------------------------------------------------ 4440  
 ------------------------------------------------------------------------------------------------------------------------ 4560  
 ------------------------------------------------------------------------------------------------------------------------ 4680  
 ------------------------------------------------------------------------------------------------------------------------ 4800  
 ------------------------------------------------------------------------------------------------------------------------ 4920  
 ------------------------------------------------------------------------------------------------------------------------ 5040  
 ------------------------------------------------------------------------------------------------------------------------ 5160  
 ------------------------------------------------------------------------------------------------------------------------ 5280  
 ------------------------------------------------------------------------------------------------------------------------ 5400  
 ------------------------------------------------------------------------------------------------------------------------ 5520  
 ------------------------------------------------------------------------------------------------------------------------ 5640  
 ------------------------------------------------------------------------------------------------------------------------ 5760  
 ------------------------------------------------------------------------------------------------------------------------ 5880  
 ------------------------------------------------------------------------------------------------------------------------ 6000  
 ------------------------------------------------------------------------------------------------------------------------ 6120  
 ------------------------------------------------------------------------------------------------------------------------ 6240  
 ------------------------------------------------------------------------------------------------------------------------ 6360  
 ------------------------------------------------------------------------------------------------------------------------ 6480  
 ------------------------------------------------------------------------------------------------------------------------ 6600  
 ------------------------------------------------------------------------------------------------------------------------ 6720  
 ------------------------------------------------------------------------------------------------------------------------ 6840  
 ------------------------------------------------------------------------------------------------------------------------ 6960  
 ------------------------------------------------------------------------------------------------------------------------ 7080  
 ------------------------------------------------------------------------------------------------------------------------ 7200  
 ------------------------------------------------------------------------------------------------------------------------ 7320  
 ------------------------------------------------------------------------------------------------------------------------ 7440  
 ------------------------------------------------------------------------------------------------------------------------ 7560  
 ------------------------------------------------------------------------------------------------------------------------ 7680  
 ------------------------------------------------------------------------------------------------------------------------ 7800  
 ------------------------------------------------------------------------------------------------------------------------ 7920  
 ------------------------------------------------------------------------------------------------------------------------ 8040  
 ------------------------------------------------------------------------------------------------------------------------ 8160  
 ------------------------------------------------------------------------------------------------------------------------ 8280  
 ------------------------------------------------------------------------------------------------------------------------ 8400  
 ------------------------------------------------------------------------------------------------------------------------ 8520  
 ------------------------------------------------------------------------------------------------------------------------ 8640  
 ------------------------------------------------------------------------------------------------------------------------ 8760  
 ------------------------------------------------------------------------------------------------------------------------ 8880  
 ------------------------------------------------------------------------------------------------------------------------ 9000  
 ------------------------------------------------------------------------------------------------------------------------ 9120  
 ------------------------------------------------------------------------------------------------------------------------ 9240  
 ------------------------------------------------------------------------------------------------------------------------ 9360  
 ------------------------------------------------------------------------------------------------------------------------ 9480  
 ------------------------------------------------------------------------------------------------------------------------ 9600  
 ------------------------------------------------------------------------------------------------------------------------ 9720  
 ------------------------------------------------------------------------------------------------------------------------ 9840  
 ------------------------------------------------------------------------------------------------------------------------ 9960  
 ------------------------------------------------------------------------------------------------------------------------ 10080  
 ------------------------------------------------------------------------------------------------------------------------ 10200  
 ------------------------------------------------------------------------------------------------------------------------ 10320  
 ------------------------------------------------------------------------------------------------------------------------ 10440  
 ------------------------------------------------------------------------------------------------------------------------ 10560  
 ------------------------------------------------------------------------------------------------------------------------ 10680  
 ------------------------------------------------------------------------------------------------------------------------ 10800  
 ------------------------------------------------------------------------------------------------------------------------ 10920  
 ------------------------------------------------------------------------------------------------------------------------ 11040  
 ------------------------------------------------------------------------------------------------------------------------ 11160  
 ------------------------------------------------------------------------------------------------------------------------ 11280  
 ------------------------------------------------------------------------------------------------------------------------ 11400  
 ------------------------------------------------------------------------------------------------------------------------ 11520  
 ------------------------------------------------------------------------------------------------------------------------ 11640  
 ------------------------------------------------------------------------------------------------------------------------ 11760  
 ------------------------------------------------------------------------------------------------------------------------ 11880  
 ------------------------------------------------------------------------------------------------------------------------ 12000  
 ------------------------------------------------------------------------------------------------------------------------ 12120  
 ------------------------------------------------------------------------------------------------------------------------ 12240  
 ------------------------------------------------------------------------------------------------------------------------ 12360  
 ------------------------------------------------------------------------------------------------------------------------ 12480  
 ------------------------------------------------------------------------------------------------------------------------ 12600  
 ------------------------------------------------------------------------------------------------------------------------ 12720  
 ------------------------------------------------------------------------------------------------------------------------ 12840  
 ------------------------------------------------------------------------------------------------------------------------ 12960  
 ------------------------------------------------------------------------------------------------------------------------ 13080  
 ------------------------------------------------------------------------------------------------------------------------ 13200  
 ------------------------------------------------------------------------------------------------------------------------ 13320  
 ------------------------------------------------------------------------------------------------------------------------ 13440  
 ------------------------------------------------------------------------

AATGTGCAT

AATGTGCAT  
Depth:6 (MOUSE)  
Ei-value:0.000, Pi-value:0.000  
Er-value:0.000, Pr-value:0.000  
MATCHES To TargetScan▶ miR-501-3p/502-3p:AUGCACC

--------------------------------------- 13560  
 ------------------------------------------------------------------------------------------------------------------------ 13680  
 ------------------------------------------------------------------------------------------------------------------------ 13800  
 ------------------------------------------------------------------------------------------------------------------------ 13920  
 ------------------------------------------------------------------------------------------------------------------------ 14040  
 ---------------------------------------------------------------------------------------------------------

TTAAGGCC

TTAAGGCC  
Depth:6 (MOUSE)  
Ei-value:0.000, Pi-value:0.000  
Er-value:0.000, Pr-value:0.000  
No matches to TargetScan

------- 14160  
 ------------------------------------------------------------------------------------------------------------------------ 14280  
 ------------------------------------------------------------------------------------------------------------------------ 14400  
 ------------------------------------------------------------------------------------------------------------------------ 14520  
 ------------------------------------------------------------------------------------------------------------------------ 14640  
 ------------------------------------------------------------------------------------------------------------------------ 14760  
 ------------------------------------------------------------------------------------------------------------------------ 14880  
 ------------------------------------------------------------------------------------------------------------------------ 15000  
 ------------------------------------------------------------------------------------------------------------------------ 15120  
 ------------------------------------------------------------------------------------------------------------------------ 15240  
 ---------------------------------------------------------------------------------------------------------

GCACAATG

GCACAATG  
Depth:6 (MOUSE)  
Ei-value:0.000, Pi-value:0.000  
Er-value:0.000, Pr-value:0.000  
No matches to TargetScan

------- 15360  
 ------------------------------------------------------------------------------------------------------------------------ 15480  
 -----

CTCCCA

CTCCCA  
Depth:6 (MOUSE)  
Ei-value:0.000, Pi-value:0.000  
Er-value:0.000, Pr-value:0.000  
No matches to TargetScan

------------------------------------------------------------------------------------------------------------- 15600  
 ------------------------------------------------------------------------------------------------------------------------ 15720  
 ------------------------------------------------------------------------------------------------------------------------ 15840  
 ------------------------------------------------------------------------------------------------------------------------ 15960  
 ---------------------------

AAAAGCAG

AAAAGCAG  
Depth:6 (MOUSE)  
Ei-value:0.000, Pi-value:0.000  
Er-value:0.000, Pr-value:0.000  
No matches to TargetScan

------------------------------------------------------------------------------------- 16080  
 ------------------------------------

GCAAAAT

GCAAAAT  
Depth:6 (MOUSE)  
Ei-value:0.000, Pi-value:0.000  
Er-value:0.000, Pr-value:0.000  
No matches to TargetScan

----------------------------------------------------------------------------- 16200  
 ---------------------------------------------------------------

GATTGCCTGG

GATTGCCTGG  
Depth:6 (MOUSE)  
Ei-value:0.000, Pi-value:0.000  
Er-value:0.000, Pr-value:0.000  
No matches to TargetScan

----------------------------------------------- 16320  
 ------------------------------------------------------------------------------------------------------------------------ 16440  
 ------------------------------------------------------------------------------------------------------------------------ 16560  
 ------------------------------------------------------------------------------------------------------------------------ 16680  
 ------------------------------------------------------------------------------------------------------------------------ 16800  
 ------------------------------------------------------------------------------------------------------------------------ 16920  
 ------------------------------------------------------------------------------------------------------------------------ 17040  
 ------------------------------------------------------------

AAAGATC

AAAGATC  
Depth:6 (MOUSE)  
Ei-value:0.000, Pi-value:0.000  
Er-value:0.000, Pr-value:0.000  
No matches to TargetScan

----------------------------------------------------- 17160  
 ------------------------------------------------------------------------------------------------------------------------ 17280  
 ------------------------------------------------------------------------------------------------------------------------ 17400  
 ---------------------------------------------------------

TTCCCTTTGA

TTCCCTTTGA  
Depth:6 (MOUSE)  
Ei-value:0.000, Pi-value:0.000  
Er-value:0.000, Pr-value:0.000  
No matches to TargetScan

-------

TAGGTGGAGATGGGGCATGAGGATCCTCCAGGGGAA

TAGGTGGAGATGGGGCATGAGGATCCTCCAGGGGAA  
Depth:6 (MOUSE)  
Ei-value:0.000, Pi-value:0.000  
Er-value:0.000, Pr-value:0.000  
MATCHES To TargetScan▶ miR-331-3p:CCCCUGG

---------- 17520  
 -------

GCAACA

GCAACA  
Depth:6 (MOUSE)  
Ei-value:0.000, Pi-value:0.000  
Er-value:0.000, Pr-value:0.000  
No matches to TargetScan

----------------------------------------------------------------------------------------------------------- 17640  
 ------------------------------------------------------------------------------------------------------------------------ 17760  
 ------------------------------------------------------------------------------------------------------------------------ 17880  
 --------------------------------------------------------------------------------------------

CCAAAT

CCAAAT  
Depth:6 (MOUSE)  
Ei-value:0.000, Pi-value:0.000  
Er-value:0.000, Pr-value:0.000  
No matches to TargetScan

---------------------- 18000  
 ----------------------------------

GATCAACATGC

GATCAACATGC  
Depth:6 (MOUSE)  
Ei-value:0.000, Pi-value:0.000  
Er-value:0.000, Pr-value:0.000  
No matches to TargetScan

------------------------------------------------------------------

TGTGTAT

TGTGTAT  
Depth:6 (MOUSE)  
Ei-value:0.000, Pi-value:0.000  
Er-value:0.000, Pr-value:0.000  
No matches to TargetScan

-- 18120  
 ------------------------------------------------------------------------------------------------------------------------ 18240  
 ------------------------------------------------------------------------------------------------------------------------ 18360  
 ---------------------------------------------------------------

TTCTCTTTG

TTCTCTTTG  
Depth:6 (MOUSE)  
Ei-value:0.000, Pi-value:0.000  
Er-value:0.000, Pr-value:0.000  
No matches to TargetScan

------------------------------------------------ 18480  
 ----------------------------------

TTTCTAC

TTTCTAC  
Depth:6 (MOUSE)  
Ei-value:0.000, Pi-value:0.000  
Er-value:0.000, Pr-value:0.000  
No matches to TargetScan

------------

ATTTCTC

ATTTCTC  
Depth:6 (MOUSE)  
Ei-value:0.000, Pi-value:0.000  
Er-value:0.000, Pr-value:0.000  
No matches to TargetScan

------------------------------------------------------------ 18600  
 ------------------------------------------------------------------------------------------------------------------------ 18720  
 ------------------------------------------------------------------------------------------------------------------------ 18840  
 ------------------------------------------------------------------------------------------------------------------------ 18960  
 ------------------------------------------------------------------------------------------------------------------------ 19080  
 ------------------------------------------------------------------------------------------------------------------------ 19200  
 ------------------------------------------------------------------------------------------------------------------------ 19320  
 ------------------------------------------------------------------------------------------------------------------------ 19440  
 ---------------------------------------------------

TCTAGAGAAAA

TCTAGAGAAAA  
Depth:6 (MOUSE)  
Ei-value:0.000, Pi-value:0.000  
Er-value:0.000, Pr-value:0.000  
MATCHES To TargetScan▶ miR-1251-5p:CUCUAGC

-----------------------

TGAGAAGAATTAGACA

TGAGAAGAATTAGACA  
Depth:6 (MOUSE)  
Ei-value:0.000, Pi-value:0.000  
Er-value:0.000, Pr-value:0.000  
No matches to TargetScan

------------------- 19560  
 ------------------------------------------------------------------------------------------------------------------------ 19680  
 ------------------------------------------------------------------------------------------------------------------------ 19800  
 ------------------------------------------------------------------------------------------------------------------------ 19920  
 --------------

ATTGGCA

ATTGGCA  
Depth:6 (MOUSE)  
Ei-value:0.000, Pi-value:0.000  
Er-value:0.000, Pr-value:0.000  
No matches to TargetScan

------------------------------------

TTGTGAAG

TTGTGAAG  
Depth:6 (MOUSE)  
Ei-value:0.000, Pi-value:0.000  
Er-value:0.000, Pr-value:0.000  
No matches to TargetScan

------------------------------------------------------- 20040  
 ------------------------------------------------------------------------------------------------------------------------ 20160  
 ------------------------------------------------------------------------------------------------------------------------ 20280  
 ------------------------------------------------------------------------------------------------------------------------ 20400  
 ------------------------------------------------------------------------------------------------------------------------ 20520  
 ------------------------------------------------------------------------------------------------------------------------ 20640  
 ------------------------------------------------------------------------------------------------------------------------ 20760  
 ------------------------------------------------------------------------------------------------------------------------ 20880  
 ------------------------------------------------------------------------------------------------------------------------ 21000  
 ------------------------------------------------------------------------------------------------------------------------ 21120  
 ------------------------------------------------------------------------------------------------------------------------ 21240  
 ------------------------------------------------------------------------------------------------------------------------ 21360  
 ------------------------------------------------------------------------------------------------------------------------ 21480  
 ------------------------------------------------------------------------------------------------------------------------ 21600  
 ------------------------------------------------------------------------------------------------------------------------ 21720  
 ------------------------------------------------------------------------------------------------------------------------ 21840  
 ------------------------------------------------------------------------------------------------------------------------ 21960  
 ------------------------------------------------------------------------------------------------------------------------ 22080  
 ------------------------------------------------------------------------------------------------------------------------ 22200  
 ------------------------------------------------------------------------------------------------------------------------ 22320  
 ------------------------------------------------------------------------------------------------------------------------ 22440  
 ------------------------------------------------------------------------------------------------------------------------ 22560  
 ------------------------------------------------------------------------------------------------------------------------ 22680  
 ------------------------------------------------------------------------------------------------------------------------ 22800  
 ------------------------------------------------------------------------------------------------------------------------ 22920  
 ------------------------------------------------------------------------------------------------------------------------ 23040  
 ------------------------------------------------------------------------------------------------------------------------ 23160  
 ------------------------------------------------------------------------------------------------------------------------ 23280  
 ------------------------------------------------------------------------------------------------------------------------ 23400  
 ------------------------------------------------------------------------------------------------------------------------ 23520  
 ------------------------------------------------------------------------------------------------------------------------ 23640  
 ------------------------------------------------------------------------------------------------------------------------ 23760  
 ------------------------------------------------------------------------------------------------------------------------ 23880  
 ------------------------------------------------------------------------------------------------------------------------ 24000  
 ------------------------------------------------------------------------------------------

AAAAGGT

AAAAGGT  
Depth:6 (MOUSE)  
Ei-value:0.000, Pi-value:0.000  
Er-value:0.000, Pr-value:0.000  
No matches to TargetScan

----------------------- 24120  
 ------------------------------------------------------------------------------------------------------------------------ 24240  
 ------------------------------------------------------------------------------------------------------------------------ 24360  
 ------------------------------------------------------------------------------------------------------------------------ 24480  
 ------------------------------------------------------------------------------------------------------------------------ 24600  
 ------------------------------------------------------------------------------------------------------------------------ 24720  
 ------------------------------------------------------------------------------------------------------------------------ 24840  
 ------------------------------------------------------------------------------------------------------------------------ 24960  
 ------------------------------------------------------------------------------------------------------------------------ 25080  
 ------------------------------------------------------------------------------------------------------------------------ 25200  
 ---------------                                                                                                          25215
```

---

## >COW (14055 bases)

```
 ------------------------------------------------------------------------------------------------------------------------ 120  
 ------------------------------------------------------------------------------------------------------------------------ 240  
 ------------------------------------------------------------------------------------------------------------------------ 360  
 ------------------------------------------------------------------------------------------------------------------------ 480  
 ------------------------------------------------------------------------------------------------------------------------ 600  
 ------------------------------------------------------------------------------------------------------------------------ 720  
 ------------------------------------------------------------------------------------------------------------------------ 840  
 ------------------------------------------------------------------------------------------------------------------------ 960  
 ------------------------------------------------------------------------------------------------------------------------ 1080  
 ------------------------------------------------------------------------------------------------------------------------ 1200  
 ------------------------------------------------------------------------------------------------------------------------ 1320  
 ------------------------------------------------------------------------------------------------------------------------ 1440  
 ------------------------------------------------------------------------------------------------------------------------ 1560  
 ------------------------------------------------------------------------------------------------------------------------ 1680  
 ------------------------------------------------------------------------------------------------------------------------ 1800  
 ------------------------------------------------------------------------------------------------------------------------ 1920  
 ------------------------------------------------------------------------------------------------------------------------ 2040  
 ------------------------------------------------------------------------------------------------------------------------ 2160  
 ------------------------------------------------------------------------------------------------------------------------ 2280  
 ------------------------------------------------------------------------------------------------------------------------ 2400  
 ------------------------------------------------------------------------------------------------------------------------ 2520  
 ------------------------------------------------------------------------------------------------------------------------ 2640  
 ------------------------------------------------------------------------------------------------------------------------ 2760  
 ------------------------------------------------------------------------------------------------------------------------ 2880  
 ------------------------------------------------------------------------------------------------------------------------ 3000  
 ------------------------------------------------------------------------------------------

AATGTGCAT

AATGTGCAT  
Depth:6 (MOUSE)  
Ei-value:0.000, Pi-value:0.000  
Er-value:0.000, Pr-value:0.000  
MATCHES To TargetScan▶ miR-501-3p/502-3p:AUGCACC

--------------------- 3120  
 ------------------------------------------------------------------------------------------------------------------------ 3240  
 ------------------------------------------------------------------------------------------------------------------------ 3360  
 ------------------------------------------------------------------------------------------------------------------------ 3480  
 ------------------------------------------------------------------------------------------------------------------------ 3600  
 ------------------------------------------------------------------------------------------------------------------------ 3720  
 ---------

TTAAGGCC

TTAAGGCC  
Depth:6 (MOUSE)  
Ei-value:0.000, Pi-value:0.000  
Er-value:0.000, Pr-value:0.000  
No matches to TargetScan

------------------------------------------------------------------------------------------------------- 3840  
 ------------------------------------------------------------------------------------------------------------------------ 3960  
 ------------------------------------------------------------------------------------------------------------------------ 4080  
 ------------------------------------------------------------------------------------------------------------------------ 4200  
 ------------------------------------------------------------------------------------------------------------------------ 4320  
 --------------------------------------------------------------------------------------------------------------------

GCAC

GCACAATG  
Depth:6 (MOUSE)  
Ei-value:0.000, Pi-value:0.000  
Er-value:0.000, Pr-value:0.000  
No matches to TargetScan

 4440  


AATG

GCACAATG  
Depth:6 (MOUSE)  
Ei-value:0.000, Pi-value:0.000  
Er-value:0.000, Pr-value:0.000  
No matches to TargetScan

---------------------------------------------

CTCCCA

CTCCCA  
Depth:6 (MOUSE)  
Ei-value:0.000, Pi-value:0.000  
Er-value:0.000, Pr-value:0.000  
No matches to TargetScan

----------------------------------------------------------------- 4560  
 ------------------------------------------------------------------------------------------------------------------------ 4680  
 ------------------------------------------------------------------------------------------------------------------------ 4800  
 ------------------------------------------------------------------------------------------------------------------------ 4920  
 ------------------------------------------------------------------------------------------------------------------------ 5040  
 -------------------------

AAAAGCAG

AAAAGCAG  
Depth:6 (MOUSE)  
Ei-value:0.000, Pi-value:0.000  
Er-value:0.000, Pr-value:0.000  
No matches to TargetScan

--------------------------------------------------------------------------------------- 5160  
 ----------------------------------

GCAAAAT

GCAAAAT  
Depth:6 (MOUSE)  
Ei-value:0.000, Pi-value:0.000  
Er-value:0.000, Pr-value:0.000  
No matches to TargetScan

------------------------------------------------------------------------------- 5280  
 -------------------------------------------------------

GATTGCCTGG

GATTGCCTGG  
Depth:6 (MOUSE)  
Ei-value:0.000, Pi-value:0.000  
Er-value:0.000, Pr-value:0.000  
No matches to TargetScan

------------------------------------------------------- 5400  
 ------------------------------------------------------------------------------------------------------------------------ 5520  
 ------------------------------------------------------------------------------------------------------------------------ 5640  
 ------------------------------------------------------------------------------------------------------------------------ 5760  
 ------------------------------------------------------------------------------------------------------------------------ 5880  
 ------------------------------------------------------------------------------------------------------------------------ 6000  
 ------------------------------------------------------------------------------------------------------------------------ 6120  
 --------------------------------------------------------------

AAAGATC

AAAGATC  
Depth:6 (MOUSE)  
Ei-value:0.000, Pi-value:0.000  
Er-value:0.000, Pr-value:0.000  
No matches to TargetScan

--------------------------------------------------- 6240  
 ------------------------------------------------------------------------------------------------------------------------ 6360  
 ------------------------------------------------------------------------------------------------------------------------ 6480  
 -------------------------------------------------------------

TTCCCTTTGA

TTCCCTTTGA  
Depth:6 (MOUSE)  
Ei-value:0.000, Pi-value:0.000  
Er-value:0.000, Pr-value:0.000  
No matches to TargetScan

-------

TAGGTGGAGATGGGGCATGAGGATCCTCCAGGGGAA

TAGGTGGAGATGGGGCATGAGGATCCTCCAGGGGAA  
Depth:6 (MOUSE)  
Ei-value:0.000, Pi-value:0.000  
Er-value:0.000, Pr-value:0.000  
MATCHES To TargetScan▶ miR-331-3p:CCCCUGG

------ 6600  
 -----------

GCAACA

GCAACA  
Depth:6 (MOUSE)  
Ei-value:0.000, Pi-value:0.000  
Er-value:0.000, Pr-value:0.000  
No matches to TargetScan

------------------------------------------------------------------------------------------------------- 6720  
 --------

CCAAAT

CCAAAT  
Depth:6 (MOUSE)  
Ei-value:0.000, Pi-value:0.000  
Er-value:0.000, Pr-value:0.000  
No matches to TargetScan

-------------------------------------------------------

GATCAACATGC

GATCAACATGC  
Depth:6 (MOUSE)  
Ei-value:0.000, Pi-value:0.000  
Er-value:0.000, Pr-value:0.000  
No matches to TargetScan

---------------------------------------- 6840  
 ---------------------------------------------------------

TGTGTAT

TGTGTAT  
Depth:6 (MOUSE)  
Ei-value:0.000, Pi-value:0.000  
Er-value:0.000, Pr-value:0.000  
No matches to TargetScan

-------------------------------------------------------- 6960  
 ------------------------------------------------------------------------------------------------------------------------ 7080  
 ------------------------------------------------------------------------------------------------------------------------ 7200  


TTCTCTTTG

TTCTCTTTG  
Depth:6 (MOUSE)  
Ei-value:0.000, Pi-value:0.000  
Er-value:0.000, Pr-value:0.000  
No matches to TargetScan

-----------------------------------------------------------------------

TTTCTAC

TTTCTAC  
Depth:6 (MOUSE)  
Ei-value:0.000, Pi-value:0.000  
Er-value:0.000, Pr-value:0.000  
No matches to TargetScan

--------------

ATTTCTC

ATTTCTC  
Depth:6 (MOUSE)  
Ei-value:0.000, Pi-value:0.000  
Er-value:0.000, Pr-value:0.000  
No matches to TargetScan

------------ 7320  
 ------------------------------------------------------------------------------------------------------------------------ 7440  
 ------------------------------------------------------------------------------------------------------------------------ 7560  
 ------------------------------------------------------------------------------------------------------------------------ 7680  
 ------------------------------------------------------------------------------------------------------------------------ 7800  
 ------------------------------------------------------------------------------------------------------------------------ 7920  
 ------------------------------------------------------------------------------------------------------------------------ 8040  
 ------------------------------------------------------------------------------------------------------------------------ 8160  
 ------------------------------------------------------------------------------------------------------------------------ 8280  
 ----------------------------------------------------------

TCTAGAGAAAA

TCTAGAGAAAA  
Depth:6 (MOUSE)  
Ei-value:0.000, Pi-value:0.000  
Er-value:0.000, Pr-value:0.000  
MATCHES To TargetScan▶ miR-1251-5p:CUCUAGC

-------------------------

TGAGAAGAATTAGACA

TGAGAAGAATTAGACA  
Depth:6 (MOUSE)  
Ei-value:0.000, Pi-value:0.000  
Er-value:0.000, Pr-value:0.000  
No matches to TargetScan

---------- 8400  
 ------------------------------------------------------------------------------------------------------------------------ 8520  
 ------------------------------------------------------------------------------------------------------------------------ 8640  
 ------------------------------------------------------------------------------------------------------------------------ 8760  
 -----------------

ATTGGCA

ATTGGCA  
Depth:6 (MOUSE)  
Ei-value:0.000, Pi-value:0.000  
Er-value:0.000, Pr-value:0.000  
No matches to TargetScan

------------------------------------

TTGTGAAG

TTGTGAAG  
Depth:6 (MOUSE)  
Ei-value:0.000, Pi-value:0.000  
Er-value:0.000, Pr-value:0.000  
No matches to TargetScan

---------------------------------------------------- 8880  
 ------------------------------------------------------------------------------------------------------------------------ 9000  
 ------------------------------------------------------------------------------------------------------------------------ 9120  
 ------------------------------------------------------------------------------------------------------------------------ 9240  
 ------------------------------------------------------------------------------------------------------------------------ 9360  
 ------------------------------------------------------------------------------------------------------------------------ 9480  
 ------------------------------------------------------------------------------------------------------------------------ 9600  
 ------------------------------------------------------------------------------------------------------------------------ 9720  
 ------------------------------------------------------------------------------------------------------------------------ 9840  
 ------------------------------------------------------------------------------------------------------------------------ 9960  
 ------------------------------------------------------------------------------------------------------------------------ 10080  
 ------------------------------------------------------------------------------------------------------------------------ 10200  
 ------------------------------------------------------------------------------------------------------------------------ 10320  
 ------------------------------------------------------------------------------------------------------------------------ 10440  
 ------------------------------------------------------------------------------------------------------------------------ 10560  
 ------------------------------------------------------------------------------------------------------------------------ 10680  
 ------------------------------------------------------------------------------------------------------------------------ 10800  
 ------------------------------------------------------------------------------------------------------------------------ 10920  
 ------------------------------------------------------------------------------------------------------------------------ 11040  
 ------------------------------------------------------------------------------------------------------------------------ 11160  
 ------------------------------------------------------------------------------------------------------------------------ 11280  
 ------------------------------------------------------------------------------------------------------------------------ 11400  
 ------------------------------------------------------------------------------------------------------------------------ 11520  
 ------------------------------------------------------------------------------------------------------------------------ 11640  
 ------------------------------------------------------------------------------------------------------------------------ 11760  
 ------------------------------------------------------------------------------------------------------------------------ 11880  
 ------------------------------------------------------------------------------------------------------------------------ 12000  
 ------------------------------------------------------------------------------------------------------------------------ 12120  
 ------------------------------------------------------------------------------------------------------------------------ 12240  
 ------------------------------------------------------------------------------------------------------------------------ 12360  
 ------------------------------------------------------------------------------------------------------------------------ 12480  
 ------------------------------------------------------------------------------------------------------------------------ 12600  
 ------------------------------------------------------------------------------------------------------------------------ 12720  
 ------------------------------------------------------------------------------------------------------------------------ 12840  
 ------------------------------------------------------------------------------------------------------------------------ 12960  
 ------------------------------------------------------------------------------------------------------------------------ 13080  
 --------------------------------

AAAAGGT

AAAAGGT  
Depth:6 (MOUSE)  
Ei-value:0.000, Pi-value:0.000  
Er-value:0.000, Pr-value:0.000  
No matches to TargetScan

--------------------------------------------------------------------------------- 13200  
 ------------------------------------------------------------------------------------------------------------------------ 13320  
 ------------------------------------------------------------------------------------------------------------------------ 13440  
 ------------------------------------------------------------------------------------------------------------------------ 13560  
 ------------------------------------------------------------------------------------------------------------------------ 13680  
 ------------------------------------------------------------------------------------------------------------------------ 13800  
 ------------------------------------------------------------------------------------------------------------------------ 13920  
 ------------------------------------------------------------------------------------------------------------------------ 14040  
 ---------------                                                                                                          14055
```

---

## >DOG (15827 bases)

```
 ------------------------------------------------------------------------------------------------------------------------ 120  
 ------------------------------------------------------------------------------------------------------------------------ 240  
 ------------------------------------------------------------------------------------------------------------------------ 360  
 ------------------------------------------------------------------------------------------------------------------------ 480  
 ------------------------------------------------------------------------------------------------------------------------ 600  
 ------------------------------------------------------------------------------------------------------------------------ 720  
 ------------------------------------------------------------------------------------------------------------------------ 840  
 ------------------------------------------------------------------------------------------------------------------------ 960  
 ------------------------------------------------------------------------------------------------------------------------ 1080  
 ------------------------------------------------------------------------------------------------------------------------ 1200  
 ------------------------------------------------------------------------------------------------------------------------ 1320  
 ------------------------------------------------------------------------------------------------------------------------ 1440  
 ------------------------------------------------------------------------------------------------------------------------ 1560  
 ------------------------------------------------------------------------------------------------------------------------ 1680  
 ------------------------------------------------------------------------------------------------------------------------ 1800  
 ------------------------------------------------------------------------------------------------------------------------ 1920  
 ------------------------------------------------------------------------------------------------------------------------ 2040  
 ------------------------------------------------------------------------------------------------------------------------ 2160  
 ------------------------------------------------------------------------------------------------------------------------ 2280  
 ------------------------------------------------------------------------------------------------------------------------ 2400  
 ------------------------------------------------------------------------------------------------------------------------ 2520  
 ------------------------------------------------------------------------------------------------------------------------ 2640  
 ------------------------------------------------------------------------------------------------------------------------ 2760  
 ------------------------------------------------------------------------------------------------------------------------ 2880  
 ------------------------------------------------------------------------------------------------------------------------ 3000  
 ------------------------------------------------------------------------------------------------------------------------ 3120  
 ------------------------------------------------------------------------------------------------------------------------ 3240  
 ------------------------------------------------------------------------------------------------------------------------ 3360  
 ------------------------------------------------------------------------------------------------------------------------ 3480  
 ------------------------------------------------------------------------------------------------------------------------ 3600  
 ------------------------------------------------------------------------------------------------------------------------ 3720  
 ----------------------------------------------------------------------------------------------------------------

AATGTGCA

AATGTGCAT  
Depth:6 (MOUSE)  
Ei-value:0.000, Pi-value:0.000  
Er-value:0.000, Pr-value:0.000  
MATCHES To TargetScan▶ miR-501-3p/502-3p:AUGCACC

 3840  


T

AATGTGCAT  
Depth:6 (MOUSE)  
Ei-value:0.000, Pi-value:0.000  
Er-value:0.000, Pr-value:0.000  
MATCHES To TargetScan▶ miR-501-3p/502-3p:AUGCACC

----------------------------------------------------------------------------------------------------------------------- 3960  
 ------------------------------------------------------------------------------------------------------------------------ 4080  
 ------------------------------------------------------------------------------------------------------------------------ 4200  
 ---------------------------------------------------------------------------------------

TTAAGGCC

TTAAGGCC  
Depth:6 (MOUSE)  
Ei-value:0.000, Pi-value:0.000  
Er-value:0.000, Pr-value:0.000  
No matches to TargetScan

------------------------- 4320  
 ------------------------------------------------------------------------------------------------------------------------ 4440  
 ------------------------------------------------------------------------------------------------------------------------ 4560  
 ------------------------------------------------------------------------------------------------------------------------ 4680  
 ------------------------------------------------------------------------------------------------------------------------ 4800  
 ------------------------------------------------------------------------------------------------------------------------ 4920  
 ------------------------------------------------------------------------------------------------------------------------ 5040  
 ------------------------------------------------------------------------------------------------------------------------ 5160  
 ------------------------------------------------------------------------------------------------------------------------ 5280  
 ------------------------------------------------------------------------------------------------------------------------ 5400  
 ------------------------------------------------------------------------------------------------------------------------ 5520  
 ------------------------------------------------------------------------------------------------------------------------ 5640  
 ------------------------------------------------------------------------------------------------------------------------ 5760  
 ------------------------------------------------------------------------------------------------------------------------ 5880  
 --------------------------------------------

GCACAATG

GCACAATG  
Depth:6 (MOUSE)  
Ei-value:0.000, Pi-value:0.000  
Er-value:0.000, Pr-value:0.000  
No matches to TargetScan

--------------------------------------------

CTCCCA

CTCCCA  
Depth:6 (MOUSE)  
Ei-value:0.000, Pi-value:0.000  
Er-value:0.000, Pr-value:0.000  
No matches to TargetScan

------------------ 6000  
 ------------------------------------------------------------------------------------------------------------------------ 6120  
 ------------------------------------------------------------------------------------------------------------------------ 6240  
 ------------------------------------------------------------------------------------------------------------------------ 6360  
 ------------------------------------------------------------------------------------------------------------------------ 6480  
 ---------------------------------------------------

AAAAGCAG

AAAAGCAG  
Depth:6 (MOUSE)  
Ei-value:0.000, Pi-value:0.000  
Er-value:0.000, Pr-value:0.000  
No matches to TargetScan

------------------------------------------------------------- 6600  
 ----------------------------------------------------------

GCAAAAT

GCAAAAT  
Depth:6 (MOUSE)  
Ei-value:0.000, Pi-value:0.000  
Er-value:0.000, Pr-value:0.000  
No matches to TargetScan

------------------------------------------------------- 6720  
 ------------------------------------------------------------------------------

GATTGCCTGG

GATTGCCTGG  
Depth:6 (MOUSE)  
Ei-value:0.000, Pi-value:0.000  
Er-value:0.000, Pr-value:0.000  
No matches to TargetScan

-------------------------------- 6840  
 ------------------------------------------------------------------------------------------------------------------------ 6960  
 ------------------------------------------------------------------------------------------------------------------------ 7080  
 ------------------------------------------------------------------------------------------------------------------------ 7200  
 ------------------------------------------------------------------------------------------------------------------------ 7320  
 ------------------------------------------------------------------------------------------------------------------------ 7440  
 ------------------------------------------------------------------------------------------------------------------------ 7560  
 ------------------------------------------------------------------------------------------------------------------------ 7680  
 ------------------------------------------------------------------------------------------------------------------------ 7800  
 ----------------------------------------------------

AAAGATC

AAAGATC  
Depth:6 (MOUSE)  
Ei-value:0.000, Pi-value:0.000  
Er-value:0.000, Pr-value:0.000  
No matches to TargetScan

------------------------------------------------------------- 7920  
 ------------------------------------------------------------------------------------------------------------------------ 8040  
 -----------------------------------------

TTCCCTTTGA

TTCCCTTTGA  
Depth:6 (MOUSE)  
Ei-value:0.000, Pi-value:0.000  
Er-value:0.000, Pr-value:0.000  
No matches to TargetScan

-------

TAGGTGGAGATGGGGCATGAGGATCCTCCAGGGGAA

TAGGTGGAGATGGGGCATGAGGATCCTCCAGGGGAA  
Depth:6 (MOUSE)  
Ei-value:0.000, Pi-value:0.000  
Er-value:0.000, Pr-value:0.000  
MATCHES To TargetScan▶ miR-331-3p:CCCCUGG

-----------------

GCAACA

GCAACA  
Depth:6 (MOUSE)  
Ei-value:0.000, Pi-value:0.000  
Er-value:0.000, Pr-value:0.000  
No matches to TargetScan

--- 8160  
 ------------------------------------------------------------------------------------------------------------------------ 8280  
 ------------------------------------------------------------------------------------------------------------------------ 8400  
 ------------------------------------------------------------------------------------------------------------------------ 8520  
 --------------

CCAAAT

CCAAAT  
Depth:6 (MOUSE)  
Ei-value:0.000, Pi-value:0.000  
Er-value:0.000, Pr-value:0.000  
No matches to TargetScan

------------------------------------------------------

GATCAACATGC

GATCAACATGC  
Depth:6 (MOUSE)  
Ei-value:0.000, Pi-value:0.000  
Er-value:0.000, Pr-value:0.000  
No matches to TargetScan

----------------------------------

T

TGTGTAT  
Depth:6 (MOUSE)  
Ei-value:0.000, Pi-value:0.000  
Er-value:0.000, Pr-value:0.000  
No matches to TargetScan

 8640  


GTGTAT

TGTGTAT  
Depth:6 (MOUSE)  
Ei-value:0.000, Pi-value:0.000  
Er-value:0.000, Pr-value:0.000  
No matches to TargetScan

------------------------------------------------------------------------------------------------------------------ 8760  
 ----------------------------------------------------------------------------

TTCTCTTTG

TTCTCTTTG  
Depth:6 (MOUSE)  
Ei-value:0.000, Pi-value:0.000  
Er-value:0.000, Pr-value:0.000  
No matches to TargetScan

----------------------------------- 8880  
 ------------------------------------------------------------------------------------------------------------------------ 9000  
 ----------------------------------------

TTTCTAC

TTTCTAC  
Depth:6 (MOUSE)  
Ei-value:0.000, Pi-value:0.000  
Er-value:0.000, Pr-value:0.000  
No matches to TargetScan

------------

ATTTCTC

ATTTCTC  
Depth:6 (MOUSE)  
Ei-value:0.000, Pi-value:0.000  
Er-value:0.000, Pr-value:0.000  
No matches to TargetScan

------------------------------------------------------ 9120  
 ------------------------------------------------------------------------------------------------------------------------ 9240  
 ------------------------------------------------------------------------------------------------------------------------ 9360  
 ------------------------------------------------------------------------------------------------------------------------ 9480  
 ------------------------------------------------------------------------------------------------------------------------ 9600  
 ------------------------------------------------------------------------------------------------------------------------ 9720  
 ------------------------------------------------------------------------------------------------------------------------ 9840  
 ------------------------------------------------------------------------------------------------------------------------ 9960  
 ------------------------------------------------------------------------------------------------------------------------ 10080  
 ------------------------------------------------------------------------------------------------------------------------ 10200  
 ---------------------------------------------------------------------------------------------------

TCTAGAGAAAA

TCTAGAGAAAA  
Depth:6 (MOUSE)  
Ei-value:0.000, Pi-value:0.000  
Er-value:0.000, Pr-value:0.000  
MATCHES To TargetScan▶ miR-1251-5p:CUCUAGC

---------- 10320  
 -------------

TGAGAAGAATTAGACA

TGAGAAGAATTAGACA  
Depth:6 (MOUSE)  
Ei-value:0.000, Pi-value:0.000  
Er-value:0.000, Pr-value:0.000  
No matches to TargetScan

------------------------------------------------------------------------------------------- 10440  
 ------------------------------------------------------------------------------------------------------------------------ 10560  
 ------------------------------------------------------------------------------------------------------------------------ 10680  
 -------------------------------------------------------------

ATTGGCA

ATTGGCA  
Depth:6 (MOUSE)  
Ei-value:0.000, Pi-value:0.000  
Er-value:0.000, Pr-value:0.000  
No matches to TargetScan

-----------------------------------

TTGTGAAG

TTGTGAAG  
Depth:6 (MOUSE)  
Ei-value:0.000, Pi-value:0.000  
Er-value:0.000, Pr-value:0.000  
No matches to TargetScan

--------- 10800  
 ------------------------------------------------------------------------------------------------------------------------ 10920  
 ------------------------------------------------------------------------------------------------------------------------ 11040  
 ------------------------------------------------------------------------------------------------------------------------ 11160  
 ------------------------------------------------------------------------------------------------------------------------ 11280  
 ------------------------------------------------------------------------------------------------------------------------ 11400  
 ------------------------------------------------------------------------------------------------------------------------ 11520  
 ------------------------------------------------------------------------------------------------------------------------ 11640  
 ------------------------------------------------------------------------------------------------------------------------ 11760  
 ------------------------------------------------------------------------------------------------------------------------ 11880  
 ------------------------------------------------------------------------------------------------------------------------ 12000  
 ------------------------------------------------------------------------------------------------------------------------ 12120  
 ------------------------------------------------------------------------------------------------------------------------ 12240  
 ------------------------------------------------------------------------------------------------------------------------ 12360  
 ------------------------------------------------------------------------------------------------------------------------ 12480  
 ------------------------------------------------------------------------------------------------------------------------ 12600  
 ------------------------------------------------------------------------------------------------------------------------ 12720  
 ------------------------------------------------------------------------------------------------------------------------ 12840  
 ------------------------------------------------------------------------------------------------------------------------ 12960  
 ------------------------------------------------------------------------------------------------------------------------ 13080  
 ------------------------------------------------------------------------------------------------------------------------ 13200  
 ------------------------------------------------------------------------------------------------------------------------ 13320  
 ------------------------------------------------------------------------------------------------------------------------ 13440  
 ------------------------------------------------------------------------------------------------------------------------ 13560  
 ------------------------------------------------------------------------------------------------------------------------ 13680  
 ------------------------------------------------------------------------------------------------------------------------ 13800  
 ------------------------------------------------------------------------------------------------------------------------ 13920  
 ------------------------------------------------------------------------------------------------------------------------ 14040  
 ------------------------------------------------------------------------------------------------------------------------ 14160  
 ------------------------------------------------------------------------------------------------------------------------ 14280  
 ------------------------------------------------------------------------------------------------------------------------ 14400  
 ------------------------------------------------------------------------------------------------------------------------ 14520  
 ------------------------------------------------------------------------------------------------------------------------ 14640  
 ------------------------------------------------------------------------------------------------------------------------ 14760  
 ------------------------------------------------------------------------------------------------------------------------ 14880  
 ------------------------------------------------------------------------------------------------------------------------ 15000  
 ----------------------------------

AAAAGGT

AAAAGGT  
Depth:6 (MOUSE)  
Ei-value:0.000, Pi-value:0.000  
Er-value:0.000, Pr-value:0.000  
No matches to TargetScan

------------------------------------------------------------------------------- 15120  
 ------------------------------------------------------------------------------------------------------------------------ 15240  
 ------------------------------------------------------------------------------------------------------------------------ 15360  
 ------------------------------------------------------------------------------------------------------------------------ 15480  
 ------------------------------------------------------------------------------------------------------------------------ 15600  
 ------------------------------------------------------------------------------------------------------------------------ 15720  
 -----------------------------------------------------------------------------------------------------------              15827
```

---

## >RABBIT (10466 bases)

```
 ------------------------------------------------------------------------------------------------------------------------ 120  
 ------------------------------------------------------------------------------------------------------------------------ 240  
 ------------------------------------------------------------------------------------------------------------------------ 360  
 ------------------------------------------------------------------------------------------------------------------------ 480  
 ------------------------------------------------------------------------------------------------------------------------ 600  
 ------------------------------------------------------------------------------------------------------------------------ 720  
 ------------------------------------------------------------------------------------------------------------------------ 840  
 ------------------------------------------------------------------------------------------------------------------------ 960  
 ------------------------------------------------------------------------------------------------------------------------ 1080  
 ------------------------------------------------------------------------------------------------------------------------ 1200  
 ------------------------------------------------------------------------------------------------------------------------ 1320  
 ------------------------------------------------------------------------------------------------------------------------ 1440  
 ------------------------------------------------------------------------------------------------------------------------ 1560  
 ---------------------------------------------------------------------------------------

AATGTGCAT

AATGTGCAT  
Depth:6 (MOUSE)  
Ei-value:0.000, Pi-value:0.000  
Er-value:0.000, Pr-value:0.000  
MATCHES To TargetScan▶ miR-501-3p/502-3p:AUGCACC

------------------------ 1680  
 ------------------------------------------------------------------------------------------------------------------------ 1800  
 ------------------------------------------------------------------------------------------------------------------------ 1920  
 ------------------------------------------------------------------------------------------------------------------------ 2040  
 ------------------------------------------------------------------------------------------------------------------------ 2160  
 ----------------------

TTAAGGCC

TTAAGGCC  
Depth:6 (MOUSE)  
Ei-value:0.000, Pi-value:0.000  
Er-value:0.000, Pr-value:0.000  
No matches to TargetScan

------------------------------------------------------------------------------------------ 2280  
 ------------------------------------------------------------------------------------------------------------------------ 2400  
 ------------------------------------------------------------------------------------------------------------------------ 2520  
 ------------------------------------------------------------------------------------------------------------------------ 2640  
 ------------------------------------------------------------------------------------------------------------------------ 2760  
 ------------------------------------------------------------------------------------------------------------------------ 2880  
 ------------------------------------------------------------------------------------------------------------------------ 3000  
 ------------------------------------------------------------------------------------------------------------------------ 3120  
 ------------------------------------------------------------------------------------------------------------------------ 3240  
 ------------------------------------------------------------------------------------------------------------------------ 3360  
 ------------------------------------------------------------------------------------------------------------------------ 3480  
 ------------------------------------------------------------------------------------------------------------------------ 3600  
 ------------------------------------------------------------------------------------------------------------------------ 3720  
 ------------------------------------------------------------------------------------------------------------------------ 3840  
 ------------------------------------------------------------------------------------------------------------------------ 3960  
 ------------------------------------------------------------------------------------------------------------------------ 4080  
 ------------------------------------------------------------------------------------------------------------------------ 4200  
 ------------------------------------------------------------------------------------------------------------------------ 4320  
 ------------------------------------------------------------------------------------------------------------------------ 4440  
 ------------------------------------------------------------------------------------------------------------------------ 4560  
 ------------------------------------------------------------------------------------------------------------------------ 4680  
 ------------------------------------------------------------------------------------------------------------------------ 4800  
 ------------------------------------------------------------------------------------------------------------------------ 4920  
 ------------------------------------------------------------------------------------------------------------------------ 5040  
 ------------------------------------------------------------------------------------------------------------------------ 5160  
 ------------------------------------------------------------------------------------------------------------------------ 5280  
 ------------------------------------------------------------------------------------------------------------------------ 5400  
 ------------------------------------------------------------------------------------------------------------------------ 5520  
 ---------------------------------------------------------------------------

GCACAATG

GCACAATG  
Depth:6 (MOUSE)  
Ei-value:0.000, Pi-value:0.000  
Er-value:0.000, Pr-value:0.000  
No matches to TargetScan

------------------------------------- 5640  
 --------------

GCACAATG

GCACAATG  
Depth:6 (MOUSE)  
Ei-value:0.000, Pi-value:0.000  
Er-value:0.000, Pr-value:0.000  
No matches to TargetScan

-------------------------------------------------------------------------------------------------- 5760  
 ------------------------------------------------------------------------------------------------------------------------ 5880  
 ------------------------------------------------------------------------------------------------------------------------ 6000  
 ---------------------------------------------------------------------------------------

CTCCCA

CTCCCA  
Depth:6 (MOUSE)  
Ei-value:0.000, Pi-value:0.000  
Er-value:0.000, Pr-value:0.000  
No matches to TargetScan

--------------------------- 6120  
 ------------------------------------------------------------------------------------------------------------------------ 6240  
 -

AAAAGCAG

AAAAGCAG  
Depth:6 (MOUSE)  
Ei-value:0.000, Pi-value:0.000  
Er-value:0.000, Pr-value:0.000  
No matches to TargetScan

--------------------------------------------------------------------------------------------------------------- 6360  
 ------------

GCAAAAT

GCAAAAT  
Depth:6 (MOUSE)  
Ei-value:0.000, Pi-value:0.000  
Er-value:0.000, Pr-value:0.000  
No matches to TargetScan

----------------------------------------------------------------------------------------------------- 6480  
 -------------------

GATTGCCTGG

GATTGCCTGG  
Depth:6 (MOUSE)  
Ei-value:0.000, Pi-value:0.000  
Er-value:0.000, Pr-value:0.000  
No matches to TargetScan

------------------------------------------------------------------------------------------- 6600  
 ------------------------------------------------------------------------------------------------------------------------ 6720  
 ------------------------------------------------------------------------------------------------------------------------ 6840  
 ------------------------------------------------------------------------------------------------------------------------ 6960  
 ------------------------------------------------------------------------------------------------------------------------ 7080  
 ------------------------------------------------------------------------------------------------------------------------ 7200  
 -------------------------------------------------------------------------------------------------------------

AAAGATC

AAAGATC  
Depth:6 (MOUSE)  
Ei-value:0.000, Pi-value:0.000  
Er-value:0.000, Pr-value:0.000  
No matches to TargetScan

---- 7320  
 -------------------------------------------------------------------------||--------------------------------------------- 7438  
 ----------------------------------------------------------------------------------------------||------------------------ 7556  
 -----------------------

TTCCCTTTGA

TTCCCTTTGA  
Depth:6 (MOUSE)  
Ei-value:0.000, Pi-value:0.000  
Er-value:0.000, Pr-value:0.000  
No matches to TargetScan

-------

TAGGTGGAGATGGGGCATGAGGATCCTCCAGGGGAA

TAGGTGGAGATGGGGCATGAGGATCCTCCAGGGGAA  
Depth:6 (MOUSE)  
Ei-value:0.000, Pi-value:0.000  
Er-value:0.000, Pr-value:0.000  
MATCHES To TargetScan▶ miR-331-3p:CCCCUGG

-----------------

GCAACA

GCAACA  
Depth:6 (MOUSE)  
Ei-value:0.000, Pi-value:0.000  
Er-value:0.000, Pr-value:0.000  
No matches to TargetScan

--------------------- 7676  
 ----------------------------------------------------------------||------------------------------------------------------ 7794  
 ------------------------------------------------------------------------------------------------------------------------ 7914  
 -------------------------------------------||-------------------------

CCAAAT

CCAAAT  
Depth:6 (MOUSE)  
Ei-value:0.000, Pi-value:0.000  
Er-value:0.000, Pr-value:0.000  
No matches to TargetScan

-------------------------------------------- 8032  
 ------------------------------------------

GATCAACATGC

GATCAACATGC  
Depth:6 (MOUSE)  
Ei-value:0.000, Pi-value:0.000  
Er-value:0.000, Pr-value:0.000  
No matches to TargetScan

-------------------------------------||

TGTGTAT

TGTGTAT  
Depth:6 (MOUSE)  
Ei-value:0.000, Pi-value:0.000  
Er-value:0.000, Pr-value:0.000  
No matches to TargetScan


TTCTCTTTG

TTCTCTTTG  
Depth:6 (MOUSE)  
Ei-value:0.000, Pi-value:0.000  
Er-value:0.000, Pr-value:0.000  
No matches to TargetScan

------------ 8150  
 ------------------------------------------------------------------------------------------------------------------------ 8270  
 ------------------------------------------------------------------------------------------------

TTTCTAC

TTTCTAC  
Depth:6 (MOUSE)  
Ei-value:0.000, Pi-value:0.000  
Er-value:0.000, Pr-value:0.000  
No matches to TargetScan

----------------- 8390  
 ------------------------------------------------------------------------------------------------------------------------ 8510  
 ---------------

ATTTCTC

ATTTCTC  
Depth:6 (MOUSE)  
Ei-value:0.000, Pi-value:0.000  
Er-value:0.000, Pr-value:0.000  
No matches to TargetScan

-------------------------------------------------------------------------------------------------- 8630  
 ------------------------------------------------------------------------------------------------------------------------ 8750  
 ------------------------------------------------------------------------------------------------------------------------ 8870  
 ------------------------------------------------------------------------------------------------------------------------ 8990  
 ------------------------------------------------------------------------------------------------------------------------ 9110  
 ------------------------------------------------------------------------------------------------------------------------ 9230  
 ------------------------------------------------------------------------------------------------------------------------ 9350  
 ------------------------------------------------------------------------------------------------------------------------ 9470  
 ------------------------------------------------------------------------------------------------------------------------ 9590  
 ------------------------------------------------------------------------------------------------------------------------ 9710  
 -----------------------------------------------------------------------------------------------------------

TCTAGAGAAAA

TCTAGAGAAAA  
Depth:6 (MOUSE)  
Ei-value:0.000, Pi-value:0.000  
Er-value:0.000, Pr-value:0.000  
MATCHES To TargetScan▶ miR-1251-5p:CUCUAGC

-- 9830  
 --------------------

TGAGAAGAATTAGACA

TGAGAAGAATTAGACA  
Depth:6 (MOUSE)  
Ei-value:0.000, Pi-value:0.000  
Er-value:0.000, Pr-value:0.000  
No matches to TargetScan

------------------------------------------------------------------------------------ 9950  
 ------------------------------------------------------------------------------------------------------------------------ 10070  
 ------------------------------------------------------------------------------------------------------------------------ 10190  
 --------------------------------------------------------

ATTGGCA

ATTGGCA  
Depth:6 (MOUSE)  
Ei-value:0.000, Pi-value:0.000  
Er-value:0.000, Pr-value:0.000  
No matches to TargetScan

------------------------------------

TTGTGAAG

TTGTGAAG  
Depth:6 (MOUSE)  
Ei-value:0.000, Pi-value:0.000  
Er-value:0.000, Pr-value:0.000  
No matches to TargetScan

------------- 10310  
 ---------------------------------------------------------------------------------------------

AAAAGGT

AAAAGGT  
Depth:6 (MOUSE)  
Ei-value:0.000, Pi-value:0.000  
Er-value:0.000, Pr-value:0.000  
No matches to TargetScan

-------------------- 10430  
 ------------------------------------                                                                                     10466
```

---

## >MOUSE (17918 bases)

```
 ------------------------------------------------------------------------------------------------------------------------ 120  
 ------------------------------------------------------------------------------------------------------------------------ 240  
 ------------------------------------------------------------------------------------------------------------------------ 360  
 ------------------------------------------------------------------------------------------------------------------------ 480  
 ------------------------------------------------------------------------------------------------------------------------ 600  
 ------------------------------------------------------------------------------------------------------------------------ 720  
 ------------------------------------------------------------------------------------------------------------------------ 840  
 ------------------------------------------------------------------------------------------------------------------------ 960  
 ------------------------------------------------------------------------------------------------------------------------ 1080  
 ------------------------------------------------------------------------------------------------------------------------ 1200  
 ------------------------------------------------------------------------------------------------------------------------ 1320  
 ------------------------------------------------------------------------------------------------------------------------ 1440  
 ------------------------------------------------------------------------------------------------------------------------ 1560  
 ------------------------------------------------------------------------------------------------------------------------ 1680  
 -------------------------------------

AATGTGCAT

AATGTGCAT  
Depth:6 (MOUSE)  
Ei-value:0.000, Pi-value:0.000  
Er-value:0.000, Pr-value:0.000  
MATCHES To TargetScan▶ miR-501-3p/502-3p:AUGCACC

-------------------------------------------------------------------------- 1800  
 ------------------------------------------------------------------------------------------------------------------------ 1920  
 ------------------------------------------------------------------------------------------------------------------------ 2040  
 ------------------------------------------------------------------------------------------------------------------------ 2160  
 ------------------------------------------------------------------------------------------------------------------------ 2280  
 ------------------------------------------------------------------------------------------------------------------------ 2400  
 ------------------------------------------------------------------------------------------------------------------------ 2520  
 ------------------------------------------------------------------------------------------------------------------------ 2640  
 ----------------------------------------------------------------------------------------------------------

TTAAGGCC

TTAAGGCC  
Depth:6 (MOUSE)  
Ei-value:0.000, Pi-value:0.000  
Er-value:0.000, Pr-value:0.000  
No matches to TargetScan

------ 2760  
 ------------------------------------------------------------------------------------------------------------------------ 2880  
 ------------------------------------------------------------------------------------------------------------------------ 3000  
 ------------------------------------------------------------------------------------------------------------------------ 3120  
 ------------------------------------------------------------------------------------------------------------------------ 3240  
 ------------------------------------------------------------------------------------------------------------------------ 3360  
 ------------------------------------------------------------------------------------------------------------------------ 3480  
 ------------------------------------------------------------------------------------------------------------------------ 3600  
 ------------------------------------------------------------------------------------------------------------------------ 3720  
 ------------------------------------------------------------------------------------------------------------------------ 3840  
 ------------------------------------------------------------------------------------------------------------------------ 3960  
 ------------------------------------------------------------------------------------------------------------------------ 4080  
 ------------------------------------------------------------------------------------------------------------------------ 4200  
 ------------------------------------------------------------------------------------------------------------------------ 4320  
 ------------------------------------------------------------------------------------------------------------------------ 4440  
 ------------------------------------------------------------------------------------------------------------------------ 4560  
 ------------------------------------------------------------------------------------------------------------------------ 4680  
 ------------------------------------------------------------------------------------------------------------------------ 4800  
 ------------------------------------------------------------------------------------------------------------------------ 4920  
 ------------------------------------------------------------------------------------------------------------------------ 5040  
 ------------------------------------------------------------------------------------------------------------------------ 5160  
 ------------------------------------------------------------------------------------------------------------------------ 5280  
 ------------------------------------------------------------------------------------------------------------------------ 5400  
 ------------------------------------------------------------------------------------------------------------------------ 5520  
 ------------------------------------------------------------------------------------------------------------------------ 5640  
 ------------------------------------------------------------------------------------------------------------------------ 5760  
 ------------------------------------------------------------------------------------------------------------------------ 5880  
 ------------------------------------------------------------------------------------------------------------------------ 6000  
 ------------------------------------------------------------------------------------------------------------------------ 6120  
 ------------------------------------------------------------------------------------------------------------------------ 6240  
 ------------------------------------------------------------------------------------------------------------------------ 6360  
 ------------------------------------------------------------------------------------------------------------------------ 6480  
 ------------------------------------------------------------------------------------------------------------------------ 6600  
 ------------------------------------------------------------------------------------------------------------------------ 6720  
 ------------------------------------------------------------------------------------------------------------------------ 6840  
 ------------------------------------------------------------------------------------------------------------------------ 6960  
 ------------------------------------------------------------------------------------------------------------------------ 7080  
 ------------------------------------------------------------------------------------------------------------------------ 7200  
 ------------------------------------------------------------------------------------------------------------------------ 7320  
 ------------------------------------------------------------------------------------------------------------------------ 7440  
 ------------------------------------------------------------------------------------------------------------------------ 7560  
 -----------------------------------------------------------

GCACAATG

GCACAATG  
Depth:6 (MOUSE)  
Ei-value:0.000, Pi-value:0.000  
Er-value:0.000, Pr-value:0.000  
No matches to TargetScan

----------------------------------------------------- 7680  
 ------------------------------------------------------------------------------------------------------------------------ 7800  
 ------------------------------------------------------------------------------------------------------------------------ 7920  
 ----------------------------------------------------------------------------------------------------------------

CTCCCA

CTCCCA  
Depth:6 (MOUSE)  
Ei-value:0.000, Pi-value:0.000  
Er-value:0.000, Pr-value:0.000  
No matches to TargetScan

-- 8040  
 --------------------------------------------------------------------------------------------------------

AAAAGCAG

AAAAGCAG  
Depth:6 (MOUSE)  
Ei-value:0.000, Pi-value:0.000  
Er-value:0.000, Pr-value:0.000  
No matches to TargetScan

-------- 8160  
 -------------------------------------------------------------------------------------------------------------------

GCAAA

GCAAAAT  
Depth:6 (MOUSE)  
Ei-value:0.000, Pi-value:0.000  
Er-value:0.000, Pr-value:0.000  
No matches to TargetScan

 8280  


AT

GCAAAAT  
Depth:6 (MOUSE)  
Ei-value:0.000, Pi-value:0.000  
Er-value:0.000, Pr-value:0.000  
No matches to TargetScan

---------------------------------------------------------------------------------------------------------------------- 8400  
 ------------------------------------------------------------------------------------------------------------------------ 8520  
 ------------------------------------------------------------------------------------------------------------------------ 8640  
 ------------------------------------------------------------------------------------------------------------------------ 8760  
 ------------------------------------------------------------------------------------------------------------------------ 8880  
 ------------------------------------------------------------------------------------------------------------------------ 9000  
 ------------------------------------------------------------------------------------------------------------------------ 9120  
 ------------------------------------------------------------------------------------------------------------------------ 9240  
 ------------------------------------------------------------------------------------------------------------------------ 9360  
 ------------------------------------------------------------------------------------------------------------------------ 9480  
 --------------------------------------||--------------------------------------------

GATTGCCTGG

GATTGCCTGG  
Depth:6 (MOUSE)  
Ei-value:0.000, Pi-value:0.000  
Er-value:0.000, Pr-value:0.000  
No matches to TargetScan

-------------------------- 9598  
 -----------||----------------------------------------------------------------------------------------------------------- 9716  
 ---------------------

AAAG||ATC

AAAGATC  
Depth:6 (MOUSE)  
Ei-value:0.000, Pi-value:0.000  
Er-value:0.000, Pr-value:0.000  
No matches to TargetScan

----------------------------------------------

TTCCCTTTGA

TTCCCTTTGA  
Depth:6 (MOUSE)  
Ei-value:0.000, Pi-value:0.000  
Er-value:0.000, Pr-value:0.000  
No matches to TargetScan

-------

TAGGTGGAGATGGGGCATGAGGATCCT

TAGGTGGAGATGGGGCATGAGGATCCTCCAGGGGAA  
Depth:6 (MOUSE)  
Ei-value:0.000, Pi-value:0.000  
Er-value:0.000, Pr-value:0.000  
MATCHES To TargetScan▶ miR-331-3p:CCCCUGG

 9834  


CCAGGGGAA

TAGGTGGAGATGGGGCATGAGGATCCTCCAGGGGAA  
Depth:6 (MOUSE)  
Ei-value:0.000, Pi-value:0.000  
Er-value:0.000, Pr-value:0.000  
MATCHES To TargetScan▶ miR-331-3p:CCCCUGG

-----------------

GCAACA

GCAACA  
Depth:6 (MOUSE)  
Ei-value:0.000, Pi-value:0.000  
Er-value:0.000, Pr-value:0.000  
No matches to TargetScan

--------------------------------------------------------------------------------------|| 9952  
 ------------------------------------------------------------------------------------------------------------------------ 10072  
 ---------------------------||---------

CCAAAT

CCAAAT  
Depth:6 (MOUSE)  
Ei-value:0.000, Pi-value:0.000  
Er-value:0.000, Pr-value:0.000  
No matches to TargetScan

---------------------------------------------------------------------------- 10190  
 --------------------

GATCAACATGC

GATCAACATGC  
Depth:6 (MOUSE)  
Ei-value:0.000, Pi-value:0.000  
Er-value:0.000, Pr-value:0.000  
No matches to TargetScan

---------------------------------||

TGTGTAT

TGTGTAT  
Depth:6 (MOUSE)  
Ei-value:0.000, Pi-value:0.000  
Er-value:0.000, Pr-value:0.000  
No matches to TargetScan

----------------------------------------------- 10308  
 ------------------------------------------------------------------------------------------------------------------------ 10428  
 ------------------------------------------------------------------------------------------------------------------------ 10548  
 ------------------------------------------------------------------------------------------------------------------------ 10668  
 ------------------------------------------------------------------------------------------------------------------------ 10788  
 ------------------------------------------------------------------------------------------------------------------------ 10908  
 ----------------------

TGTGTAT

TGTGTAT  
Depth:6 (MOUSE)  
Ei-value:0.000, Pi-value:0.000  
Er-value:0.000, Pr-value:0.000  
No matches to TargetScan

------------------------------------------------------------------------------------------- 11028  
 ------------------------------------------------------------------------------------------------------------------------ 11148  
 ------------------------------------------------------------------------------------------------------------------------ 11268  
 --------------

TTCTCTTTG

TTCTCTTTG  
Depth:6 (MOUSE)  
Ei-value:0.000, Pi-value:0.000  
Er-value:0.000, Pr-value:0.000  
No matches to TargetScan

------------------------------------------------------------------------------------------------- 11388  
 ------------------------------------------------------------------------------------------------------------------------ 11508  
 ------------------------------------------------------------------------------------------------------------------------ 11628  
 ------------------------------------------------------------------------------------------------------------------------ 11748  
 ------------------------------------------------------------------------------------------------------------------------ 11868  
 ---

TTTCTAC

TTTCTAC  
Depth:6 (MOUSE)  
Ei-value:0.000, Pi-value:0.000  
Er-value:0.000, Pr-value:0.000  
No matches to TargetScan

-------------------------------------------------------------------------------------------------------------- 11988  
 ------------------------------------------------------------------------------------------------------------------------ 12108  
 ------------------------------------------------------------------------------------------------------------------------ 12228  
 -----------------------------------------

ATTTCTC

ATTTCTC  
Depth:6 (MOUSE)  
Ei-value:0.000, Pi-value:0.000  
Er-value:0.000, Pr-value:0.000  
No matches to TargetScan

------------------------------------------------------------------------ 12348  
 ----------------------------------------------------------------------------------------------------------------

TCTAGAGA

TCTAGAGAAAA  
Depth:6 (MOUSE)  
Ei-value:0.000, Pi-value:0.000  
Er-value:0.000, Pr-value:0.000  
MATCHES To TargetScan▶ miR-1251-5p:CUCUAGC

 12468  


AAA

TCTAGAGAAAA  
Depth:6 (MOUSE)  
Ei-value:0.000, Pi-value:0.000  
Er-value:0.000, Pr-value:0.000  
MATCHES To TargetScan▶ miR-1251-5p:CUCUAGC

-----------------------

TGAGAAGAATTAGACA

TGAGAAGAATTAGACA  
Depth:6 (MOUSE)  
Ei-value:0.000, Pi-value:0.000  
Er-value:0.000, Pr-value:0.000  
No matches to TargetScan

------------------------------------------------------------------------------ 12588  
 ------------------------------------------------------------------------------------------------------------------------ 12708  
 ------------------------------------------------------------------------------------------------------------------------ 12828  


ATTGGCA

ATTGGCA  
Depth:6 (MOUSE)  
Ei-value:0.000, Pi-value:0.000  
Er-value:0.000, Pr-value:0.000  
No matches to TargetScan

----------------------------------------------------------------------------------------------------------------- 12948  
 ------------------------------------------------------------------------------------------------------------------------ 13068  
 ------------------------------------------------------------------------------------------------------------------------ 13188  
 ------------------------------------------------------------------------------------------------------------------------ 13308  
 ------------------------------------------------------------------------------------------------------------------------ 13428  
 ------------------------------------------------------------------------------------------------------------------------ 13548  
 ------------------------------------------------------------------------------------------------------------------------ 13668  
 ------------------------------------------------------------------------------------------------------------------------ 13788  
 ------------------------------------------------------------------------------------------------------------------------ 13908  
 ------------------------------------------------------------------------------------------------------------------------ 14028  
 ------------------------------------------------------------------------------------------------------------------------ 14148  
 ------------------------------------------------------------------------------------------------------------------------ 14268  
 ------------------------------------------------------------------------------------------------------------------------ 14388  
 ------------------------------------------------------------------------------------------------------------------------ 14508  
 --------------------------

TTGTGAAG

TTGTGAAG  
Depth:6 (MOUSE)  
Ei-value:0.000, Pi-value:0.000  
Er-value:0.000, Pr-value:0.000  
No matches to TargetScan

-------------------------------------------------------------------------------------- 14628  
 ------------------------------------------------------------------------------------------------------------------------ 14748  
 ------------------------------------------------------------------------------------------------------------------------ 14868  
 --------------

AAAAGGT

AAAAGGT  
Depth:6 (MOUSE)  
Ei-value:0.000, Pi-value:0.000  
Er-value:0.000, Pr-value:0.000  
No matches to TargetScan

--------------------------------------------------------------------------------------------------- 14988  
 ------------------------------------------------------------------------------------------------------------------------ 15108  
 ------------------------------------------------------------------------------------------------------------------------ 15228  
 ------------------------------------------------------------------------------------------------------------------------ 15348  
 ------------------------------------------------------------------------------------------------------------------------ 15468  
 ------------------------------------------------------------------------------------------------------------------------ 15588  
 ------------------------------------------------------------------------------------------------------------------------ 15708  
 ------------------------------------------------------------------------------------------------------------------------ 15828  
 ------------------------------------------------------------------------------------------------------------------------ 15948  
 ------------------------------------------------------------------------------------------------------------------------ 16068  
 ------------------------------------------------------------------------------------------------------------------------ 16188  
 ------------------------------------------------------------------------------------------------------------------------ 16308  
 ------------------------------------------------------------------------------------------------------------------------ 16428  
 ------------------------------------------------------------------------------------------------------------------------ 16548  
 ------------------------------------------------------------------------------------------------------------------------ 16668  
 ------------------------------------------------------------------------------------------------------------------------ 16788  
 ------------------------------------------------------------------------------------------------------------------------ 16908  
 ------------------------------------------------------------------------------------------------------------------------ 17028  
 ------------------------------------------------------------------------------------------------------------------------ 17148  
 ------------------------------------------------------------------------------------------------------------------------ 17268  
 ------------------------------------------------------------------------------------------------------------------------ 17388  
 ------------------------------------------------------------------------------------------------------------------------ 17508  
 ------------------------------------------------------------------------------------------------------------------------ 17628  
 ------------------------------------------------------------------------------------------------------------------------ 17748  
 ------------------------------------------------------------------------------------------------------------------------ 17868  
 --------------------------------------------------                                                                       17918
```

---
