# Supplementary material for: Uncovering deeply conserved motif combinations in rapidly evolving noncoding sequences
Source: Genome Biol. 2021 Jan 11;22:29. doi: 10.1186/s13059-020-02247-1 (PMC7798263; doi:10.1186/s13059-020-02247-1)
Supplement: Supplementary file 4 — Additional file 4. LncLOOM output results for XIST sequences from six mammals. [file 13059_2020_2247_MOESM4_ESM.gz › AdditionalFile4/Html_Files/miRNA_Matches.html]

 miRNA Matches

# Matches to miRNA Families Retrieved from TargetScan

  

\*\*SEED defined as positions 2-7 of mature miRNA sequence  
\*\*MOTIF MATCHES that correspond to the reverse complement of miRNA SEEDS are displayed from 5`-3` relative to lincRNA sequence

  

| Seed Matches to the miRNA miR-186-5p | | | | | |
| --- | --- | --- | --- | --- | --- |
| Seed | Conservation | Species | Matches | | |
|  |  |  | Sequence | Motif | Type |
| AAAGAAU | Conserved | Human (Homo sapiens)  Rhesus (Macaca mulatta)  Mouse (Mus musculus)  Rat (Rattus norvegicus)  Opossum (Monodelphis domestica) | HUMAN | GGTTCTTTCT | 6mer |
|  |  |  | HUMAN | TATTTCTTTAAAAAAA | 7mer-A1 |
|  |  |  | HUMAN | AATTTTTCTTTGGAAT | 6mer |
|  |  |  | HUMAN | TTTGCCCAACGGGGCCGTGGATACCTGCCTTTTAATTCTTTTTT | 7mer-m8 |
|  |  |  | HUMAN | ATTCTTTTGGATATA | 7mer-m8 |
|  |  |  | HUMAN | TTGTCTCTTTCTTTCTT | 6mer |
|  |  |  | PIG | GGTTCTTTCT | 6mer |
|  |  |  | PIG | TATTTCTTTAAAAAAA | 7mer-A1 |
|  |  |  | PIG | AATTTTTCTTTGGAAT | 6mer |
|  |  |  | PIG | TTTGCCCAACGGGGCCGTGGATACCTGCCTTTTAATTCTTTTTT | 7mer-m8 |
|  |  |  | PIG | ATTCTTTTGGATATA | 7mer-m8 |
|  |  |  | PIG | TTGTCTCTTTCTTTCTT | 6mer |

  
  
  
  

| Seed Matches to the miRNA miR-409-3p | | | | | |
| --- | --- | --- | --- | --- | --- |
| Seed | Conservation | Species | Matches | | |
|  |  |  | Sequence | Motif | Type |
| AAUGUUG | Conserved | Human (Homo sapiens)  Rhesus (Macaca mulatta)  Mouse (Mus musculus) | HUMAN | GAACATTTTC | 6mer |
|  |  |  | HUMAN | AACATTCTGCTTTTATTA | 6mer |
|  |  |  | HUMAN | TAACATTGTGT | 6mer |
|  |  |  | PIG | GAACATTTTC | 6mer |
|  |  |  | PIG | AACATTCTGCTTTTATTA | 6mer |
|  |  |  | PIG | TAACATTGTGT | 6mer |

  
  
  
  

| Seed Matches to the miRNA miR-505-3p.1 | | | | | |
| --- | --- | --- | --- | --- | --- |
| Seed | Conservation | Species | Matches | | |
|  |  |  | Sequence | Motif | Type |
| GUCAACA | Conserved | Human (Homo sapiens)  Mouse (Mus musculus) | HUMAN | TTTTTGGTTGAC | 7mer-A1 |
|  |  |  | PIG | TTTTTGGTTGAC | 6mer |

  
  
  
  

| Seed Matches to the miRNA miR-124-3p.1 | | | | | |
| --- | --- | --- | --- | --- | --- |
| Seed | Conservation | Species | Matches | | |
|  |  |  | Sequence | Motif | Type |
| AAGGCAC | Broadly Conserved | Human (Homo sapiens)  Mouse (Mus musculus) | HUMAN | TTTGCCCAACGGGGCCGTGGATACCTGCCTTTTAATTCTTTTTT | 6mer |
|  |  |  | HUMAN | TTGCCTT | 6mer |
|  |  |  | HUMAN | GGGCAACCTGCCTTTGTTCTG | 6mer |
|  |  |  | PIG | TTTGCCCAACGGGGCCGTGGATACCTGCCTTTTAATTCTTTTTT | 6mer |
|  |  |  | PIG | TTGCCTT | 6mer |
|  |  |  | PIG | GGGCAACCTGCCTTTGTTCTG | 6mer |
|  |  |  | COW | TTGCCTT | 6mer |

  
  
  
  

| Seed Matches to the miRNA miR-330-3p.2 | | | | | |
| --- | --- | --- | --- | --- | --- |
| Seed | Conservation | Species | Matches | | |
|  |  |  | Sequence | Motif | Type |
| AAAGCAC | Conserved | Human (Homo sapiens)  Mouse (Mus musculus) | HUMAN | GCCCATCGGGGCCGCGGATACCTGCTTTT | 6mer |
|  |  |  | HUMAN | CTGCTTTTT | 6mer |
|  |  |  | HUMAN | TCGGATACCTGCTTT | 6mer |
|  |  |  | HUMAN | TTTTTCCTTGCCCATCGGGGCCTCGGATACCTGCTTTA | 7mer-A1 |
|  |  |  | HUMAN | TGCTTTGTTAG | 7mer-m8 |
|  |  |  | HUMAN | CTTGCTTTGTTCCCATCCTT | 6mer |
|  |  |  | HUMAN | TTACTGCTTTACT | 7mer-A1 |
|  |  |  | HUMAN | TGCTTTACT | 7mer-A1 |
|  |  |  | HUMAN | AATGTGCTTTGTAAACT | 7mer-m8 |
|  |  |  | HUMAN | CATATTAAAGTGCTTTGTA | 7mer-m8 |
|  |  |  | HUMAN | TTAAAGTGCTTTGTA | 7mer-m8 |
|  |  |  | HUMAN | AACATTCTGCTTTTATTA | 6mer |
|  |  |  | PIG | GCCCATCGGGGCCGCGGATACCTGCTTTT | 6mer |
|  |  |  | PIG | CTGCTTTTT | 6mer |
|  |  |  | PIG | TCGGATACCTGCTTT | 7mer-A1 |
|  |  |  | PIG | TTTTTCCTTGCCCATCGGGGCCTCGGATACCTGCTTTA | 7mer-A1 |
|  |  |  | PIG | TGCTTTGTTAG | 6mer |
|  |  |  | PIG | CTTGCTTTGTTCCCATCCTT | 6mer |
|  |  |  | PIG | TTACTGCTTTACT | 7mer-A1 |
|  |  |  | PIG | TGCTTTACT | 7mer-A1 |
|  |  |  | PIG | AATGTGCTTTGTAAACT | 7mer-m8 |
|  |  |  | PIG | CATATTAAAGTGCTTTGTA | 7mer-m8 |
|  |  |  | PIG | TTAAAGTGCTTTGTA | 7mer-m8 |
|  |  |  | PIG | AACATTCTGCTTTTATTA | 6mer |
|  |  |  | COW | TGCTTTACT | 8mer |
|  |  |  | COW | TTAAAGTGCTTTGTA | 7mer-m8 |

  
  
  
  

| Seed Matches to the miRNA miR-423-5p | | | | | |
| --- | --- | --- | --- | --- | --- |
| Seed | Conservation | Species | Matches | | |
|  |  |  | Sequence | Motif | Type |
| GAGGGGC | Conserved | Human (Homo sapiens)  Rhesus (Macaca mulatta)  Cow (Bos taurus)  Mouse (Mus musculus) | HUMAN | CCCCTCT | 6mer |
|  |  |  | HUMAN | TCCCCTCT | 6mer |
|  |  |  | HUMAN | TCCCCTCC | 6mer |
|  |  |  | PIG | CCCCTCT | 6mer |
|  |  |  | PIG | TCCCCTCT | 6mer |
|  |  |  | PIG | TCCCCTCC | 6mer |

  
  
  
  

| Seed Matches to the miRNA miR-31-5p | | | | | |
| --- | --- | --- | --- | --- | --- |
| Seed | Conservation | Species | Matches | | |
|  |  |  | Sequence | Motif | Type |
| GGCAAGA | Broadly Conserved | Human (Homo sapiens)  Chicken (Gallus gallus)  Mouse (Mus musculus)  Rat (Rattus norvegicus) | HUMAN | TTTTTCCTTGCCCATCGGGGCCTCGGATACCTGCTTTA | 6mer |
|  |  |  | HUMAN | CTTGCCGCA | 7mer-m8 |
|  |  |  | PIG | TTTTTCCTTGCCCATCGGGGCCTCGGATACCTGCTTTA | 6mer |
|  |  |  | PIG | CTTGCCGCA | 6mer |

  
  
  
  

| Seed Matches to the miRNA miR-103-3p/107 | | | | | |
| --- | --- | --- | --- | --- | --- |
| Seed | Conservation | Species | Matches | | |
|  |  |  | Sequence | Motif | Type |
| GCAGCAU | Broadly Conserved | Human (Homo sapiens)  Opossum (Monodelphis domestica) | HUMAN | TTTTGCTGCT | 6mer |
|  |  |  | HUMAN | ATGCTGCACT | 8mer |
|  |  |  | PIG | TTTTGCTGCT | 6mer |
|  |  |  | PIG | ATGCTGCACT | 8mer |

  
  
  
  

| Seed Matches to the miRNA miR-15-5p/16-5p/195-5p/424-5p/497-5p | | | | | |
| --- | --- | --- | --- | --- | --- |
| Seed | Conservation | Species | Matches | | |
|  |  |  | Sequence | Motif | Type |
| AGCAGCA | Broadly Conserved | Human (Homo sapiens)  Rhesus (Macaca mulatta) | HUMAN | TTTTGCTGCT | 7mer-m8 |
|  |  |  | HUMAN | AGGGGCTGCTGAC | 6mer |
|  |  |  | HUMAN | GGGCTGCTGA | 6mer |
|  |  |  | PIG | TTTTGCTGCT | 8mer |
|  |  |  | PIG | AGGGGCTGCTGAC | 6mer |
|  |  |  | PIG | GGGCTGCTGA | 6mer |
|  |  |  | COW | GGGCTGCTGA | 6mer |

  
  
  
  

| Seed Matches to the miRNA miR-503-5p | | | | | |
| --- | --- | --- | --- | --- | --- |
| Seed | Conservation | Species | Matches | | |
|  |  |  | Sequence | Motif | Type |
| AGCAGCG | Conserved | Human (Homo sapiens)  Rhesus (Macaca mulatta)  Mouse (Mus musculus)  Rat (Rattus norvegicus) | HUMAN | TTTTGCTGCT | 6mer |
|  |  |  | HUMAN | AGGGGCTGCTGAC | 6mer |
|  |  |  | HUMAN | GGGCTGCTGA | 6mer |
|  |  |  | PIG | TTTTGCTGCT | 7mer-A1 |
|  |  |  | PIG | AGGGGCTGCTGAC | 6mer |
|  |  |  | PIG | GGGCTGCTGA | 6mer |
|  |  |  | COW | GGGCTGCTGA | 6mer |

  
  
  
  

| Seed Matches to the miRNA miR-342-3p | | | | | |
| --- | --- | --- | --- | --- | --- |
| Seed | Conservation | Species | Matches | | |
|  |  |  | Sequence | Motif | Type |
| CUCACAC | Conserved | Human (Homo sapiens)  Rhesus (Macaca mulatta)  Mouse (Mus musculus)  Rat (Rattus norvegicus) | HUMAN | TGTGTGAGTG | 7mer-m8 |
|  |  |  | PIG | TGTGTGAGTG | 7mer-m8 |

  
  
  
  

| Seed Matches to the miRNA miR-377-3p | | | | | |
| --- | --- | --- | --- | --- | --- |
| Seed | Conservation | Species | Matches | | |
|  |  |  | Sequence | Motif | Type |
| UCACACA | Conserved | Human (Homo sapiens)  Rhesus (Macaca mulatta)  Mouse (Mus musculus) | HUMAN | TGTGTGAGTG | 7mer-m8 |
|  |  |  | PIG | TGTGTGAGTG | 7mer-m8 |

  
  
  
  

| Seed Matches to the miRNA miR-330-3p | | | | | |
| --- | --- | --- | --- | --- | --- |
| Seed | Conservation | Species | Matches | | |
|  |  |  | Sequence | Motif | Type |
| CAAAGCA | Conserved | Human (Homo sapiens)  Rhesus (Macaca mulatta)  Rat (Rattus norvegicus) | HUMAN | GCTTTGG | 6mer |
|  |  |  | HUMAN | TGCTTTGTTAG | 7mer-m8 |
|  |  |  | HUMAN | CTTGCTTTGTTCCCATCCTT | 7mer-m8 |
|  |  |  | HUMAN | AATGTGCTTTGTAAACT | 7mer-m8 |
|  |  |  | HUMAN | CATATTAAAGTGCTTTGTA | 7mer-m8 |
|  |  |  | HUMAN | TTAAAGTGCTTTGTA | 7mer-m8 |
|  |  |  | HUMAN | TGGGCTTTG | 7mer-A1 |
|  |  |  | PIG | GCTTTGG | 6mer |
|  |  |  | PIG | TGCTTTGTTAG | 7mer-m8 |
|  |  |  | PIG | CTTGCTTTGTTCCCATCCTT | 7mer-m8 |
|  |  |  | PIG | AATGTGCTTTGTAAACT | 7mer-m8 |
|  |  |  | PIG | CATATTAAAGTGCTTTGTA | 7mer-m8 |
|  |  |  | PIG | TTAAAGTGCTTTGTA | 7mer-m8 |
|  |  |  | PIG | TGGGCTTTG | 6mer |
|  |  |  | COW | TTAAAGTGCTTTGTA | 7mer-m8 |
|  |  |  | COW | TGGGCTTTG | 6mer |

  
  
  
  

| Seed Matches to the miRNA miR-217 | | | | | |
| --- | --- | --- | --- | --- | --- |
| Seed | Conservation | Species | Matches | | |
|  |  |  | Sequence | Motif | Type |
| ACUGCAU | Broadly Conserved | Human (Homo sapiens)  X. tropicalis (Xenopus tropicalis)  Rhesus (Macaca mulatta)  Chimp (Pan troglodytes)  Dog (Canis lupus familiaris)  Cow (Bos taurus) | HUMAN | TGCAGTTA | 6mer |
|  |  |  | HUMAN | CTCCATTTGCAGTATA | 7mer-A1 |
|  |  |  | HUMAN | AATTGCAGTT | 6mer |
|  |  |  | HUMAN | GTGCAGT | 6mer |
|  |  |  | PIG | TGCAGTTA | 6mer |
|  |  |  | PIG | CTCCATTTGCAGTATA | 7mer-A1 |
|  |  |  | PIG | AATTGCAGTT | 6mer |
|  |  |  | PIG | GTGCAGT | 7mer-A1 |
|  |  |  | COW | GTGCAGT | 7mer-A1 |

  
  
  
  

| Seed Matches to the miRNA miR-670-3p | | | | | |
| --- | --- | --- | --- | --- | --- |
| Seed | Conservation | Species | Matches | | |
|  |  |  | Sequence | Motif | Type |
| UUCCUCA | Conserved | Human (Homo sapiens)  Rhesus (Macaca mulatta)  Mouse (Mus musculus) | HUMAN | GGAGGAAA | 7mer-A1 |
|  |  |  | HUMAN | CAGGCAGAGGAA | 6mer |
|  |  |  | HUMAN | GAAGAGGAAT | 6mer |
|  |  |  | HUMAN | CTTCCTCAAGAGGAACACCTACCCC | 6mer |
|  |  |  | HUMAN | AGGAGGAAGAGTGAA | 6mer |
|  |  |  | PIG | GGAGGAAA | 7mer-A1 |
|  |  |  | PIG | CAGGCAGAGGAA | 7mer-A1 |
|  |  |  | PIG | GAAGAGGAAT | 6mer |
|  |  |  | PIG | CTTCCTCAAGAGGAACACCTACCCC | 6mer |
|  |  |  | PIG | AGGAGGAAGAGTGAA | 6mer |
|  |  |  | COW | AGGAGGAAGAGTGAA | 6mer |
|  |  |  | DOG | AGGAGGAAGAGTGAA | 6mer |

  
  
  
  

| Seed Matches to the miRNA miR-129-3p | | | | | |
| --- | --- | --- | --- | --- | --- |
| Seed | Conservation | Species | Matches | | |
|  |  |  | Sequence | Motif | Type |
| AGCCCUU | Broadly Conserved | Human (Homo sapiens)  Rhesus (Macaca mulatta)  Cow (Bos taurus)  Mouse (Mus musculus)  Rat (Rattus norvegicus) | HUMAN | AGGGCTA | 7mer-A1 |
|  |  |  | PIG | AGGGCTA | 7mer-A1 |

  
  
  
  

| Seed Matches to the miRNA miR-495-3p | | | | | |
| --- | --- | --- | --- | --- | --- |
| Seed | Conservation | Species | Matches | | |
|  |  |  | Sequence | Motif | Type |
| AACAAAC | Conserved | Human (Homo sapiens)  Rhesus (Macaca mulatta)  Mouse (Mus musculus) | HUMAN | TGCTTTGTTAG | 7mer-A1 |
|  |  |  | HUMAN | CTTGCTTTGTTCCCATCCTT | 6mer |
|  |  |  | HUMAN | TTTTTCATTTTGTT | 6mer |
|  |  |  | HUMAN | TTCATTTTGTT | 6mer |
|  |  |  | HUMAN | GGGCAACCTGCCTTTGTTCTG | 6mer |
|  |  |  | PIG | TGCTTTGTTAG | 7mer-A1 |
|  |  |  | PIG | CTTGCTTTGTTCCCATCCTT | 6mer |
|  |  |  | PIG | TTTTTCATTTTGTT | 7mer-A1 |
|  |  |  | PIG | TTCATTTTGTT | 7mer-A1 |
|  |  |  | PIG | GGGCAACCTGCCTTTGTTCTG | 6mer |
|  |  |  | COW | TTCATTTTGTT | 7mer-A1 |
|  |  |  | DOG | TTCATTTTGTT | 6mer |

  
  
  
  

| Seed Matches to the miRNA miR-141-3p/200a-3p | | | | | |
| --- | --- | --- | --- | --- | --- |
| Seed | Conservation | Species | Matches | | |
|  |  |  | Sequence | Motif | Type |
| AACACUG | Broadly Conserved | Human (Homo sapiens)  Mouse (Mus musculus)  Rat (Rattus norvegicus)  Opossum (Monodelphis domestica) | HUMAN | CGCAGTGTTC | 7mer-m8 |
|  |  |  | HUMAN | TGTGGTTTGCTAGTGTT | 6mer |
|  |  |  | HUMAN | TTACAGTGTTAGTGA | 8mer |
|  |  |  | HUMAN | TACAGTGTTAGTGA | 8mer |
|  |  |  | PIG | CGCAGTGTTC | 7mer-m8 |
|  |  |  | PIG | TGTGGTTTGCTAGTGTT | 6mer |
|  |  |  | PIG | TTACAGTGTTAGTGA | 8mer |
|  |  |  | PIG | TACAGTGTTAGTGA | 8mer |
|  |  |  | COW | TTACAGTGTTAGTGA | 8mer |
|  |  |  | COW | TACAGTGTTAGTGA | 8mer |
|  |  |  | DOG | TACAGTGTTAGTGA | 8mer |
|  |  |  | RABBIT | TACAGTGTTAGTGA | 8mer |

  
  
  
  

| Seed Matches to the miRNA miR-299-3p | | | | | |
| --- | --- | --- | --- | --- | --- |
| Seed | Conservation | Species | Matches | | |
|  |  |  | Sequence | Motif | Type |
| AUGUGGG | Conserved | Human (Homo sapiens)  Rhesus (Macaca mulatta)  Mouse (Mus musculus)  Rat (Rattus norvegicus) | HUMAN | CCACAT | 6mer |
|  |  |  | PIG | CCACAT | 6mer |

  
  
  
  

| Seed Matches to the miRNA miR-425-5p | | | | | |
| --- | --- | --- | --- | --- | --- |
| Seed | Conservation | Species | Matches | | |
|  |  |  | Sequence | Motif | Type |
| AUGACAC | Broadly Conserved | Human (Homo sapiens)  X. tropicalis (Xenopus tropicalis)  Rat (Rattus norvegicus)  Opossum (Monodelphis domestica) | HUMAN | TTGTCAT | 7mer-A1 |
|  |  |  | HUMAN | CTCTGTCATTGCT | 6mer |
|  |  |  | HUMAN | TTGTCATA | 7mer-A1 |
|  |  |  | PIG | TTGTCAT | 6mer |
|  |  |  | PIG | CTCTGTCATTGCT | 6mer |
|  |  |  | PIG | TTGTCATA | 7mer-A1 |

  
  
  
  

| Seed Matches to the miRNA miR-542-3p | | | | | |
| --- | --- | --- | --- | --- | --- |
| Seed | Conservation | Species | Matches | | |
|  |  |  | Sequence | Motif | Type |
| GUGACAG | Conserved | Human (Homo sapiens)  Rhesus (Macaca mulatta)  Mouse (Mus musculus)  Rat (Rattus norvegicus) | HUMAN | TGTCACGTGGAC | 6mer |
|  |  |  | PIG | TGTCACGTGGAC | 7mer-m8 |

  
  
  
  

| Seed Matches to the miRNA miR-149-5p | | | | | |
| --- | --- | --- | --- | --- | --- |
| Seed | Conservation | Species | Matches | | |
|  |  |  | Sequence | Motif | Type |
| CUGGCUC | Conserved | Human (Homo sapiens)  Rhesus (Macaca mulatta)  Cow (Bos taurus)  Mouse (Mus musculus)  Rat (Rattus norvegicus) | HUMAN | AGCCAGTCAG | 6mer |
|  |  |  | HUMAN | AAGCCAG | 6mer |
|  |  |  | PIG | AGCCAGTCAG | 6mer |
|  |  |  | PIG | AAGCCAG | 7mer-A1 |
|  |  |  | COW | AAGCCAG | 7mer-A1 |
|  |  |  | DOG | AAGCCAG | 6mer |

  
  
  
  

| Seed Matches to the miRNA miR-193-3p | | | | | |
| --- | --- | --- | --- | --- | --- |
| Seed | Conservation | Species | Matches | | |
|  |  |  | Sequence | Motif | Type |
| ACUGGCC | Broadly Conserved | Human (Homo sapiens)  Chicken (Gallus gallus)  Rhesus (Macaca mulatta)  Cow (Bos taurus)  Mouse (Mus musculus)  Rat (Rattus norvegicus)  Opossum (Monodelphis domestica) | HUMAN | AGCCAGTCAG | 6mer |
|  |  |  | HUMAN | ACAGTATTATGCCTGGGCCAGTCTT | 7mer-m8 |
|  |  |  | PIG | AGCCAGTCAG | 6mer |
|  |  |  | PIG | ACAGTATTATGCCTGGGCCAGTCTT | 7mer-m8 |

  
  
  
  

| Seed Matches to the miRNA miR-3064-5p | | | | | |
| --- | --- | --- | --- | --- | --- |
| Seed | Conservation | Species | Matches | | |
|  |  |  | Sequence | Motif | Type |
| CUGGCUG | Conserved | Human (Homo sapiens) | HUMAN | AGCCAGTCAG | 6mer |
|  |  |  | HUMAN | AAGCCAG | 6mer |
|  |  |  | PIG | AGCCAGTCAG | 7mer-m8 |
|  |  |  | PIG | AAGCCAG | 7mer-A1 |
|  |  |  | COW | AAGCCAG | 7mer-A1 |
|  |  |  | DOG | AAGCCAG | 6mer |

  
  
  
  

| Seed Matches to the miRNA miR-1-3p/206 | | | | | |
| --- | --- | --- | --- | --- | --- |
| Seed | Conservation | Species | Matches | | |
|  |  |  | Sequence | Motif | Type |
| GGAAUGU | Broadly Conserved | Human (Homo sapiens)  Chicken (Gallus gallus)  Rhesus (Macaca mulatta)  Opossum (Monodelphis domestica) | HUMAN | CCATTCCTCTG | 6mer |
|  |  |  | HUMAN | CATTCCCTTTGA | 6mer |
|  |  |  | PIG | CCATTCCTCTG | 6mer |
|  |  |  | PIG | CATTCCCTTTGA | 6mer |
|  |  |  | COW | CATTCCCTTTGA | 6mer |

  
  
  
  

| Seed Matches to the miRNA miR-130-3p/301-3p/454-3p | | | | | |
| --- | --- | --- | --- | --- | --- |
| Seed | Conservation | Species | Matches | | |
|  |  |  | Sequence | Motif | Type |
| AGUGCAA | Broadly Conserved | Human (Homo sapiens)  Chicken (Gallus gallus)  Rhesus (Macaca mulatta) | HUMAN | CTGCACTGT | 6mer |
|  |  |  | HUMAN | ATGCTGCACT | 7mer-A1 |
|  |  |  | PIG | CTGCACTGT | 6mer |
|  |  |  | PIG | ATGCTGCACT | 6mer |

  
  
  
  

| Seed Matches to the miRNA miR-148-3p/152-3p | | | | | |
| --- | --- | --- | --- | --- | --- |
| Seed | Conservation | Species | Matches | | |
|  |  |  | Sequence | Motif | Type |
| CAGUGCA | Broadly Conserved | Human (Homo sapiens)  Rhesus (Macaca mulatta)  Mouse (Mus musculus)  Rat (Rattus norvegicus)  Opossum (Monodelphis domestica) | HUMAN | CTGCACTGT | 7mer-m8 |
|  |  |  | PIG | CTGCACTGT | 7mer-m8 |

  
  
  
  

| Seed Matches to the miRNA miR-133a-3p.1 | | | | | |
| --- | --- | --- | --- | --- | --- |
| Seed | Conservation | Species | Matches | | |
|  |  |  | Sequence | Motif | Type |
| UGGUCCC | Broadly Conserved | Human (Homo sapiens) | HUMAN | CGTGGCAAGGACCAGAATGGATC | 6mer |
|  |  |  | PIG | CGTGGCAAGGACCAGAATGGATC | 6mer |

  
  
  
  

| Seed Matches to the miRNA miR-1224-5p | | | | | |
| --- | --- | --- | --- | --- | --- |
| Seed | Conservation | Species | Matches | | |
|  |  |  | Sequence | Motif | Type |
| UGAGGAC | Conserved | Human (Homo sapiens)  Rhesus (Macaca mulatta)  Chimp (Pan troglodytes)  Mouse (Mus musculus) | HUMAN | CTTCCTCAAGAGGAACACCTACCCC | 7mer-A1 |
|  |  |  | HUMAN | TCTGGAGAAAAAGATCTTCCTCAGAAGAATAGGCTTGTTG | 6mer |
|  |  |  | PIG | CTTCCTCAAGAGGAACACCTACCCC | 7mer-A1 |
|  |  |  | PIG | TCTGGAGAAAAAGATCTTCCTCAGAAGAATAGGCTTGTTG | 6mer |

  
  
  
  

| Seed Matches to the miRNA miR-338-3p | | | | | |
| --- | --- | --- | --- | --- | --- |
| Seed | Conservation | Species | Matches | | |
|  |  |  | Sequence | Motif | Type |
| CCAGCAU | Broadly Conserved | Human (Homo sapiens)  Rhesus (Macaca mulatta)  Mouse (Mus musculus)  Rat (Rattus norvegicus) | HUMAN | TGGCTAATGCTGGGGTCGGATTTTGATTT | 7mer-m8 |
|  |  |  | HUMAN | TTGCTGGG | 6mer |
|  |  |  | PIG | TGGCTAATGCTGGGGTCGGATTTTGATTT | 7mer-m8 |
|  |  |  | PIG | TTGCTGGG | 6mer |

  
  
  
  

| Seed Matches to the miRNA miR-551-3p | | | | | |
| --- | --- | --- | --- | --- | --- |
| Seed | Conservation | Species | Matches | | |
|  |  |  | Sequence | Motif | Type |
| CGACCCA | Broadly Conserved | Human (Homo sapiens)  Chicken (Gallus gallus)  Rhesus (Macaca mulatta)  Mouse (Mus musculus)  Opossum (Monodelphis domestica) | HUMAN | TGGCTAATGCTGGGGTCGGATTTTGATTT | 6mer |
|  |  |  | PIG | TGGCTAATGCTGGGGTCGGATTTTGATTT | 6mer |

  
  
  
  

| Seed Matches to the miRNA miR-489-3p | | | | | |
| --- | --- | --- | --- | --- | --- |
| Seed | Conservation | Species | Matches | | |
|  |  |  | Sequence | Motif | Type |
| UGACAUC | Broadly Conserved | Human (Homo sapiens)  Rhesus (Macaca mulatta)  Rat (Rattus norvegicus) | HUMAN | TTGGATGTCAGTCATA | 7mer-m8 |
|  |  |  | HUMAN | GAGAAGGATGTCAAAAGATCGGC | 8mer |
|  |  |  | HUMAN | AAGGATGTCAAAAGATC | 8mer |
|  |  |  | PIG | TTGGATGTCAGTCATA | 7mer-m8 |
|  |  |  | PIG | GAGAAGGATGTCAAAAGATCGGC | 8mer |
|  |  |  | PIG | AAGGATGTCAAAAGATC | 8mer |
|  |  |  | COW | GAGAAGGATGTCAAAAGATCGGC | 8mer |
|  |  |  | COW | AAGGATGTCAAAAGATC | 8mer |
|  |  |  | DOG | AAGGATGTCAAAAGATC | 8mer |

  
  
  
  

| Seed Matches to the miRNA miR-140-3p.2 | | | | | |
| --- | --- | --- | --- | --- | --- |
| Seed | Conservation | Species | Matches | | |
|  |  |  | Sequence | Motif | Type |
| ACCACAG | Broadly Conserved | Human (Homo sapiens) | HUMAN | TGTGGTTTGCTAGTGTT | 6mer |
|  |  |  | PIG | TGTGGTTTGCTAGTGTT | 6mer |

  
  
  
  

| Seed Matches to the miRNA miR-668-3p | | | | | |
| --- | --- | --- | --- | --- | --- |
| Seed | Conservation | Species | Matches | | |
|  |  |  | Sequence | Motif | Type |
| GUCACUC | Conserved | Human (Homo sapiens)  Mouse (Mus musculus) | HUMAN | CTTAAGTGACTA | 6mer |
|  |  |  | PIG | CTTAAGTGACTA | 6mer |

  
  
  
  

| Seed Matches to the miRNA miR-411-3p | | | | | |
| --- | --- | --- | --- | --- | --- |
| Seed | Conservation | Species | Matches | | |
|  |  |  | Sequence | Motif | Type |
| AUGUAAC | Conserved | Human (Homo sapiens)  Mouse (Mus musculus)  Rat (Rattus norvegicus) | HUMAN | TTACATTTA | 6mer |
|  |  |  | HUMAN | TAATGACAATTACAT | 6mer |
|  |  |  | PIG | TTACATTTA | 7mer-m8 |
|  |  |  | PIG | TAATGACAATTACAT | 6mer |
|  |  |  | COW | TAATGACAATTACAT | 6mer |

  
  
  
  

| Seed Matches to the miRNA miR-224-5p | | | | | |
| --- | --- | --- | --- | --- | --- |
| Seed | Conservation | Species | Matches | | |
|  |  |  | Sequence | Motif | Type |
| AAGUCAC | Conserved | Human (Homo sapiens)  Rhesus (Macaca mulatta)  Mouse (Mus musculus)  Rat (Rattus norvegicus) | HUMAN | CTTTGAAATTGACTTAA | 7mer-A1 |
|  |  |  | PIG | CTTTGAAATTGACTTAA | 7mer-A1 |

  
  
  
  

| Seed Matches to the miRNA miR-205-5p | | | | | |
| --- | --- | --- | --- | --- | --- |
| Seed | Conservation | Species | Matches | | |
|  |  |  | Sequence | Motif | Type |
| CCUUCAU | Broadly Conserved | Human (Homo sapiens)  Mouse (Mus musculus) | HUMAN | TTTGAAGGT | 6mer |
|  |  |  | PIG | TTTGAAGGT | 6mer |

  
  
  
  

| Seed Matches to the miRNA miR-378-3p | | | | | |
| --- | --- | --- | --- | --- | --- |
| Seed | Conservation | Species | Matches | | |
|  |  |  | Sequence | Motif | Type |
| CUGGACU | Conserved | Human (Homo sapiens)  Mouse (Mus musculus)  Rat (Rattus norvegicus) | HUMAN | GTCCAGG | 6mer |
|  |  |  | HUMAN | TTGTCCAGAGTCC | 7mer-A1 |
|  |  |  | PIG | GTCCAGG | 6mer |
|  |  |  | PIG | TTGTCCAGAGTCC | 7mer-A1 |

  
  
  
  

| Seed Matches to the miRNA miR-140-3p.1 | | | | | |
| --- | --- | --- | --- | --- | --- |
| Seed | Conservation | Species | Matches | | |
|  |  |  | Sequence | Motif | Type |
| CCACAGG | Broadly Conserved | Human (Homo sapiens)  Mouse (Mus musculus) | HUMAN | AAACTTGAATTGCTGTGG | 6mer |
|  |  |  | HUMAN | TCTGTGG | 6mer |
|  |  |  | HUMAN | AGGGTGGTGGGTCTGTGGATAGA | 7mer-A1 |
|  |  |  | HUMAN | GTCTGTGGATAGA | 7mer-A1 |
|  |  |  | HUMAN | GTCTGTGGATA | 7mer-A1 |
|  |  |  | PIG | AAACTTGAATTGCTGTGG | 7mer-A1 |
|  |  |  | PIG | TCTGTGG | 7mer-A1 |
|  |  |  | PIG | AGGGTGGTGGGTCTGTGGATAGA | 7mer-A1 |
|  |  |  | PIG | GTCTGTGGATAGA | 7mer-A1 |
|  |  |  | PIG | GTCTGTGGATA | 7mer-A1 |
|  |  |  | COW | GTCTGTGGATAGA | 7mer-A1 |
|  |  |  | COW | GTCTGTGGATA | 7mer-A1 |
|  |  |  | DOG | GTCTGTGGATA | 7mer-A1 |
|  |  |  | RABBIT | GTCTGTGGATA | 7mer-A1 |

  
  
  
  

| Seed Matches to the miRNA miR-26-5p | | | | | |
| --- | --- | --- | --- | --- | --- |
| Seed | Conservation | Species | Matches | | |
|  |  |  | Sequence | Motif | Type |
| UCAAGUA | Broadly Conserved | Human (Homo sapiens)  Chicken (Gallus gallus)  Rhesus (Macaca mulatta)  Mouse (Mus musculus)  Rat (Rattus norvegicus)  Opossum (Monodelphis domestica) | HUMAN | AAACTTGAATTGCTGTGG | 7mer-A1 |
|  |  |  | HUMAN | CTCCACTTGAGAGA | 6mer |
|  |  |  | HUMAN | CTCCACTTGAGAG | 6mer |
|  |  |  | PIG | AAACTTGAATTGCTGTGG | 7mer-A1 |
|  |  |  | PIG | CTCCACTTGAGAGA | 6mer |
|  |  |  | PIG | CTCCACTTGAGAG | 6mer |
|  |  |  | COW | CTCCACTTGAGAG | 6mer |

  
  
  
  

| Seed Matches to the miRNA miR-200bc-3p/429 | | | | | |
| --- | --- | --- | --- | --- | --- |
| Seed | Conservation | Species | Matches | | |
|  |  |  | Sequence | Motif | Type |
| AAUACUG | Broadly Conserved | Human (Homo sapiens)  Rat (Rattus norvegicus)  Opossum (Monodelphis domestica) | HUMAN | TTATTATATTGGAGTATT | 6mer |
|  |  |  | HUMAN | TCACCTTTGAGTATTT | 6mer |
|  |  |  | HUMAN | TTTGAGTATTT | 6mer |
|  |  |  | HUMAN | ACAGTATTATGCCTGGGCCAGTCTT | 8mer |
|  |  |  | PIG | TTATTATATTGGAGTATT | 7mer-A1 |
|  |  |  | PIG | TCACCTTTGAGTATTT | 6mer |
|  |  |  | PIG | TTTGAGTATTT | 6mer |
|  |  |  | PIG | ACAGTATTATGCCTGGGCCAGTCTT | 8mer |
|  |  |  | COW | TTTGAGTATTT | 6mer |
|  |  |  | DOG | TTTGAGTATTT | 6mer |

  
  
  
  

| Seed Matches to the miRNA miR-369-3p | | | | | |
| --- | --- | --- | --- | --- | --- |
| Seed | Conservation | Species | Matches | | |
|  |  |  | Sequence | Motif | Type |
| AUAAUAC | Conserved | Human (Homo sapiens)  Rhesus (Macaca mulatta)  Cow (Bos taurus)  Mouse (Mus musculus)  Rat (Rattus norvegicus) | HUMAN | TTATTATATTGGAGTATT | 7mer-A1 |
|  |  |  | HUMAN | ACAGTATTATGCCTGGGCCAGTCTT | 7mer-m8 |
|  |  |  | HUMAN | TATTATGC | 7mer-m8 |
|  |  |  | PIG | TTATTATATTGGAGTATT | 7mer-A1 |
|  |  |  | PIG | ACAGTATTATGCCTGGGCCAGTCTT | 7mer-m8 |
|  |  |  | PIG | TATTATGC | 7mer-m8 |
|  |  |  | COW | TATTATGC | 6mer |
|  |  |  | DOG | TATTATGC | 6mer |

  
  
  
  

| Seed Matches to the miRNA miR-374-5p | | | | | |
| --- | --- | --- | --- | --- | --- |
| Seed | Conservation | Species | Matches | | |
|  |  |  | Sequence | Motif | Type |
| UAUAAUA | Conserved | Human (Homo sapiens)  Rhesus (Macaca mulatta)  Rat (Rattus norvegicus) | HUMAN | TTATTATATTGGAGTATT | 7mer-m8 |
|  |  |  | PIG | TTATTATATTGGAGTATT | 7mer-m8 |

  
  
  
  

| Seed Matches to the miRNA miR-410-3p | | | | | |
| --- | --- | --- | --- | --- | --- |
| Seed | Conservation | Species | Matches | | |
|  |  |  | Sequence | Motif | Type |
| AUAUAAC | Conserved | Human (Homo sapiens)  Rhesus (Macaca mulatta) | HUMAN | TTATTATATTGGAGTATT | 6mer |
|  |  |  | HUMAN | CTTATATTT | 6mer |
|  |  |  | PIG | TTATTATATTGGAGTATT | 6mer |
|  |  |  | PIG | CTTATATTT | 6mer |
|  |  |  | COW | CTTATATTT | 6mer |

  
  
  
  

| Seed Matches to the miRNA miR-383-5p.1 | | | | | |
| --- | --- | --- | --- | --- | --- |
| Seed | Conservation | Species | Matches | | |
|  |  |  | Sequence | Motif | Type |
| GAUCAGA | Broadly Conserved | Human (Homo sapiens)  Mouse (Mus musculus) | HUMAN | GCATTGCTGATCTT | 6mer |
|  |  |  | HUMAN | ACTGATCATTAGATA | 7mer-A1 |
|  |  |  | PIG | GCATTGCTGATCTT | 6mer |
|  |  |  | PIG | ACTGATCATTAGATA | 7mer-A1 |

  
  
  
  

| Seed Matches to the miRNA miR-383-5p.2 | | | | | |
| --- | --- | --- | --- | --- | --- |
| Seed | Conservation | Species | Matches | | |
|  |  |  | Sequence | Motif | Type |
| AGAUCAG | Broadly Conserved | Human (Homo sapiens)  Mouse (Mus musculus) | HUMAN | GCATTGCTGATCTT | 7mer-m8 |
|  |  |  | PIG | GCATTGCTGATCTT | 7mer-m8 |

  
  
  
  

| Seed Matches to the miRNA miR-219-5p | | | | | |
| --- | --- | --- | --- | --- | --- |
| Seed | Conservation | Species | Matches | | |
|  |  |  | Sequence | Motif | Type |
| GAUUGUC | Broadly Conserved | Human (Homo sapiens)  Chimp (Pan troglodytes)  Dog (Canis lupus familiaris)  Cow (Bos taurus)  Mouse (Mus musculus)  Rat (Rattus norvegicus)  Opossum (Monodelphis domestica) | HUMAN | ACAATCCCATTTG | 6mer |
|  |  |  | HUMAN | ACAATCC | 6mer |
|  |  |  | PIG | ACAATCCCATTTG | 6mer |
|  |  |  | PIG | ACAATCC | 7mer-m8 |
|  |  |  | COW | ACAATCC | 6mer |

  
  
  
  

| Seed Matches to the miRNA miR-101-3p.1 | | | | | |
| --- | --- | --- | --- | --- | --- |
| Seed | Conservation | Species | Matches | | |
|  |  |  | Sequence | Motif | Type |
| ACAGUAC | Broadly Conserved | Human (Homo sapiens)  Mouse (Mus musculus) | HUMAN | TACTGTG | 7mer-m8 |
|  |  |  | HUMAN | ATACTGTTT | 6mer |
|  |  |  | HUMAN | GGTACTGT | 8mer |
|  |  |  | HUMAN | AGGTTACTGTTTATT | 6mer |
|  |  |  | PIG | TACTGTG | 6mer |
|  |  |  | PIG | ATACTGTTT | 6mer |
|  |  |  | PIG | GGTACTGT | 7mer-m8 |
|  |  |  | PIG | AGGTTACTGTTTATT | 6mer |
|  |  |  | COW | ATACTGTTT | 6mer |
|  |  |  | COW | GGTACTGT | 7mer-m8 |
|  |  |  | DOG | GGTACTGT | 7mer-m8 |

  
  
  
  

| Seed Matches to the miRNA miR-128-3p | | | | | |
| --- | --- | --- | --- | --- | --- |
| Seed | Conservation | Species | Matches | | |
|  |  |  | Sequence | Motif | Type |
| CACAGUG | Broadly Conserved | Human (Homo sapiens)  Chicken (Gallus gallus)  Rhesus (Macaca mulatta)  Mouse (Mus musculus)  Rat (Rattus norvegicus)  Opossum (Monodelphis domestica) | HUMAN | TACTGTG | 6mer |
|  |  |  | PIG | TACTGTG | 6mer |

  
  
  
  

| Seed Matches to the miRNA miR-144-3p | | | | | |
| --- | --- | --- | --- | --- | --- |
| Seed | Conservation | Species | Matches | | |
|  |  |  | Sequence | Motif | Type |
| ACAGUAU | Broadly Conserved | Human (Homo sapiens)  Mouse (Mus musculus)  Rat (Rattus norvegicus)  Opossum (Monodelphis domestica) | HUMAN | TACTGTG | 6mer |
|  |  |  | HUMAN | ATACTGTTT | 7mer-m8 |
|  |  |  | HUMAN | GGTACTGT | 7mer-A1 |
|  |  |  | HUMAN | AGGTTACTGTTTATT | 6mer |
|  |  |  | PIG | TACTGTG | 7mer-m8 |
|  |  |  | PIG | ATACTGTTT | 7mer-m8 |
|  |  |  | PIG | GGTACTGT | 6mer |
|  |  |  | PIG | AGGTTACTGTTTATT | 6mer |
|  |  |  | COW | ATACTGTTT | 7mer-m8 |
|  |  |  | COW | GGTACTGT | 6mer |
|  |  |  | DOG | GGTACTGT | 6mer |

  
  
  
  

| Seed Matches to the miRNA miR-193a-5p | | | | | |
| --- | --- | --- | --- | --- | --- |
| Seed | Conservation | Species | Matches | | |
|  |  |  | Sequence | Motif | Type |
| GGGUCUU | Broadly Conserved | Human (Homo sapiens) | HUMAN | AAGACCCAC | 8mer |
|  |  |  | HUMAN | CTCTCAGACCCC | 6mer |
|  |  |  | HUMAN | AAGGCAAGTCAGACCCA | 7mer-A1 |
|  |  |  | PIG | AAGACCCAC | 8mer |
|  |  |  | PIG | CTCTCAGACCCC | 6mer |
|  |  |  | PIG | AAGGCAAGTCAGACCCA | 7mer-A1 |
|  |  |  | COW | AAGGCAAGTCAGACCCA | 7mer-A1 |

  
  
  
  

| Seed Matches to the miRNA miR-155-5p | | | | | |
| --- | --- | --- | --- | --- | --- |
| Seed | Conservation | Species | Matches | | |
|  |  |  | Sequence | Motif | Type |
| UAAUGCU | Broadly Conserved | Human (Homo sapiens)  Mouse (Mus musculus)  Rat (Rattus norvegicus) | HUMAN | ACTACCTACCACCTTGCATTAATAT | 7mer-A1 |
|  |  |  | HUMAN | AGGGCATTAG | 6mer |
|  |  |  | PIG | ACTACCTACCACCTTGCATTAATAT | 7mer-A1 |
|  |  |  | PIG | AGGGCATTAG | 6mer |

  
  
  
  

| Seed Matches to the miRNA miR-18-5p | | | | | |
| --- | --- | --- | --- | --- | --- |
| Seed | Conservation | Species | Matches | | |
|  |  |  | Sequence | Motif | Type |
| AAGGUGC | Broadly Conserved | Human (Homo sapiens)  X. tropicalis (Xenopus tropicalis)  Chicken (Gallus gallus)  Mouse (Mus musculus)  Rat (Rattus norvegicus)  Opossum (Monodelphis domestica) | HUMAN | ACTACCTACCACCTTGCATTAATAT | 6mer |
|  |  |  | HUMAN | CACCTTGGA | 6mer |
|  |  |  | HUMAN | TCACCTTTGAGTATTT | 6mer |
|  |  |  | PIG | ACTACCTACCACCTTGCATTAATAT | 6mer |
|  |  |  | PIG | CACCTTGGA | 6mer |
|  |  |  | PIG | TCACCTTTGAGTATTT | 6mer |

  
  
  
  

| Seed Matches to the miRNA miR-196-5p | | | | | |
| --- | --- | --- | --- | --- | --- |
| Seed | Conservation | Species | Matches | | |
|  |  |  | Sequence | Motif | Type |
| AGGUAGU | Broadly Conserved | Human (Homo sapiens)  Chicken (Gallus gallus)  Rhesus (Macaca mulatta)  Mouse (Mus musculus)  Rat (Rattus norvegicus)  Opossum (Monodelphis domestica) | HUMAN | ACTACCTACCACCTTGCATTAATAT | 8mer |
|  |  |  | HUMAN | TGCCTACCT | 6mer |
|  |  |  | HUMAN | CTACCTCAAAG | 6mer |
|  |  |  | PIG | ACTACCTACCACCTTGCATTAATAT | 8mer |
|  |  |  | PIG | TGCCTACCT | 6mer |
|  |  |  | PIG | CTACCTCAAAG | 6mer |

  
  
  
  

| Seed Matches to the miRNA miR-183-5p.1 | | | | | |
| --- | --- | --- | --- | --- | --- |
| Seed | Conservation | Species | Matches | | |
|  |  |  | Sequence | Motif | Type |
| AUGGCAC | Broadly Conserved | Human (Homo sapiens) | HUMAN | TGCCATT | 6mer |
|  |  |  | PIG | TGCCATT | 6mer |

  
  
  
  

| Seed Matches to the miRNA miR-326 | | | | | |
| --- | --- | --- | --- | --- | --- |
| Seed | Conservation | Species | Matches | | |
|  |  |  | Sequence | Motif | Type |
| CUCUGGG | Conserved | Human (Homo sapiens)  Chimp (Pan troglodytes)  Cow (Bos taurus) | HUMAN | TTGTCCAGAGTCC | 6mer |
|  |  |  | HUMAN | TTGGCCCCAGAGACATG | 8mer |
|  |  |  | PIG | TTGTCCAGAGTCC | 6mer |
|  |  |  | PIG | TTGGCCCCAGAGACATG | 8mer |

  
  
  
  

| Seed Matches to the miRNA miR-331-3p | | | | | |
| --- | --- | --- | --- | --- | --- |
| Seed | Conservation | Species | Matches | | |
|  |  |  | Sequence | Motif | Type |
| CCCCUGG | Conserved | Human (Homo sapiens)  Rhesus (Macaca mulatta)  Cow (Bos taurus)  Mouse (Mus musculus)  Rat (Rattus norvegicus) | HUMAN | CTTTGTATTCCAGCAGGGGACCCTT | 7mer-A1 |
|  |  |  | HUMAN | TAGGTGGAGATGGGGCATGAGGATCCTCCAGGGGAAAAGCTCACTACCACTGGGCAACAACCCTAGGTCAGGAG | 8mer |
|  |  |  | HUMAN | TAGGTGGAGATGGGGCATGAGGATCCTCCAGGGGAAA | 8mer |
|  |  |  | HUMAN | TAGGTGGAGATGGGGCATGAGGATCCTCCAGGGGAA | 8mer |
|  |  |  | HUMAN | TATGTAAATCAGGGGTC | 6mer |
|  |  |  | PIG | CTTTGTATTCCAGCAGGGGACCCTT | 7mer-A1 |
|  |  |  | PIG | TAGGTGGAGATGGGGCATGAGGATCCTCCAGGGGAAAAGCTCACTACCACTGGGCAACAACCCTAGGTCAGGAG | 8mer |
|  |  |  | PIG | TAGGTGGAGATGGGGCATGAGGATCCTCCAGGGGAAA | 8mer |
|  |  |  | PIG | TAGGTGGAGATGGGGCATGAGGATCCTCCAGGGGAA | 8mer |
|  |  |  | PIG | TATGTAAATCAGGGGTC | 6mer |
|  |  |  | COW | TAGGTGGAGATGGGGCATGAGGATCCTCCAGGGGAAA | 8mer |
|  |  |  | COW | TAGGTGGAGATGGGGCATGAGGATCCTCCAGGGGAA | 8mer |
|  |  |  | DOG | TAGGTGGAGATGGGGCATGAGGATCCTCCAGGGGAAA | 8mer |
|  |  |  | DOG | TAGGTGGAGATGGGGCATGAGGATCCTCCAGGGGAA | 8mer |
|  |  |  | RABBIT | TAGGTGGAGATGGGGCATGAGGATCCTCCAGGGGAAA | 8mer |
|  |  |  | RABBIT | TAGGTGGAGATGGGGCATGAGGATCCTCCAGGGGAA | 8mer |
|  |  |  | MOUSE | TAGGTGGAGATGGGGCATGAGGATCCTCCAGGGGAA | 8mer |

  
  
  
  

| Seed Matches to the miRNA miR-381-3p | | | | | |
| --- | --- | --- | --- | --- | --- |
| Seed | Conservation | Species | Matches | | |
|  |  |  | Sequence | Motif | Type |
| AUACAAG | Conserved | Human (Homo sapiens)  Rhesus (Macaca mulatta) | HUMAN | CTTTGTATTCCAGCAGGGGACCCTT | 6mer |
|  |  |  | HUMAN | TTGTATATT | 7mer-A1 |
|  |  |  | PIG | CTTTGTATTCCAGCAGGGGACCCTT | 6mer |
|  |  |  | PIG | TTGTATATT | 8mer |
|  |  |  | COW | TTGTATATT | 8mer |
|  |  |  | DOG | TTGTATATT | 8mer |

  
  
  
  

| Seed Matches to the miRNA miR-25-3p/32-5p/92-3p/363-3p/367-3p | | | | | |
| --- | --- | --- | --- | --- | --- |
| Seed | Conservation | Species | Matches | | |
|  |  |  | Sequence | Motif | Type |
| AUUGCAC | Broadly Conserved | Human (Homo sapiens)  Mouse (Mus musculus) | HUMAN | AAATGCAATT | 6mer |
|  |  |  | HUMAN | GAATATTTGCAATTAT | 6mer |
|  |  |  | PIG | AAATGCAATT | 6mer |
|  |  |  | PIG | GAATATTTGCAATTAT | 6mer |

  
  
  
  

| Seed Matches to the miRNA miR-33-5p | | | | | |
| --- | --- | --- | --- | --- | --- |
| Seed | Conservation | Species | Matches | | |
|  |  |  | Sequence | Motif | Type |
| UGCAUUG | Broadly Conserved | Human (Homo sapiens)  Chicken (Gallus gallus)  Rhesus (Macaca mulatta)  Mouse (Mus musculus)  Rat (Rattus norvegicus)  Opossum (Monodelphis domestica) | HUMAN | AAATGCAATT | 7mer-A1 |
|  |  |  | PIG | AAATGCAATT | 7mer-A1 |

  
  
  
  

| Seed Matches to the miRNA miR-132-3p/212-3p | | | | | |
| --- | --- | --- | --- | --- | --- |
| Seed | Conservation | Species | Matches | | |
|  |  |  | Sequence | Motif | Type |
| AACAGUC | Broadly Conserved | Human (Homo sapiens)  Rhesus (Macaca mulatta)  Mouse (Mus musculus)  Rat (Rattus norvegicus)  Opossum (Monodelphis domestica) | HUMAN | GGGTACTTGGGACTGTTAATG | 8mer |
|  |  |  | HUMAN | GGGTACTTGGGACTGTTAAT | 8mer |
|  |  |  | HUMAN | TACTTGGGACTGTTAAT | 8mer |
|  |  |  | HUMAN | ACTGTTAATGTGCTA | 7mer-A1 |
|  |  |  | HUMAN | ACTGTTAATGTGCT | 7mer-A1 |
|  |  |  | HUMAN | TCTTGGACTGTTAATGTG | 8mer |
|  |  |  | HUMAN | TCTTGGACTGTTAATGT | 8mer |
|  |  |  | HUMAN | ACTGTTAATGT | 8mer |
|  |  |  | HUMAN | ATACTGTTT | 6mer |
|  |  |  | HUMAN | ATTGCCTACTATGTGAACTCACTGTTA | 7mer-A1 |
|  |  |  | HUMAN | TTGCCTACTATGTGAACTCACTGTTA | 7mer-A1 |
|  |  |  | HUMAN | AGGTTACTGTTTATT | 6mer |
|  |  |  | PIG | GGGTACTTGGGACTGTTAATG | 8mer |
|  |  |  | PIG | GGGTACTTGGGACTGTTAAT | 8mer |
|  |  |  | PIG | TACTTGGGACTGTTAAT | 8mer |
|  |  |  | PIG | ACTGTTAATGTGCTA | 8mer |
|  |  |  | PIG | ACTGTTAATGTGCT | 8mer |
|  |  |  | PIG | TCTTGGACTGTTAATGTG | 8mer |
|  |  |  | PIG | TCTTGGACTGTTAATGT | 8mer |
|  |  |  | PIG | ACTGTTAATGT | 8mer |
|  |  |  | PIG | ATACTGTTT | 6mer |
|  |  |  | PIG | ATTGCCTACTATGTGAACTCACTGTTA | 7mer-A1 |
|  |  |  | PIG | TTGCCTACTATGTGAACTCACTGTTA | 7mer-A1 |
|  |  |  | PIG | AGGTTACTGTTTATT | 6mer |
|  |  |  | COW | GGGTACTTGGGACTGTTAAT | 8mer |
|  |  |  | COW | TACTTGGGACTGTTAAT | 8mer |
|  |  |  | COW | ACTGTTAATGTGCT | 8mer |
|  |  |  | COW | TCTTGGACTGTTAATGT | 8mer |
|  |  |  | COW | ACTGTTAATGT | 8mer |
|  |  |  | COW | ATACTGTTT | 6mer |
|  |  |  | COW | TTGCCTACTATGTGAACTCACTGTTA | 7mer-A1 |
|  |  |  | DOG | TACTTGGGACTGTTAAT | 8mer |
|  |  |  | DOG | ACTGTTAATGTGCT | 7mer-A1 |
|  |  |  | DOG | ACTGTTAATGT | 7mer-A1 |

  
  
  
  

| Seed Matches to the miRNA miR-455-3p.1 | | | | | |
| --- | --- | --- | --- | --- | --- |
| Seed | Conservation | Species | Matches | | |
|  |  |  | Sequence | Motif | Type |
| CAGUCCA | Broadly Conserved | Human (Homo sapiens)  Mouse (Mus musculus) | HUMAN | GGGTACTTGGGACTGTTAATG | 6mer |
|  |  |  | HUMAN | GGGTACTTGGGACTGTTAAT | 6mer |
|  |  |  | HUMAN | TACTTGGGACTGTTAAT | 6mer |
|  |  |  | HUMAN | TCTTGGACTGTTAATGTG | 7mer-m8 |
|  |  |  | HUMAN | TCTTGGACTGTTAATGT | 7mer-m8 |
|  |  |  | HUMAN | CTGGGACTG | 6mer |
|  |  |  | PIG | GGGTACTTGGGACTGTTAATG | 6mer |
|  |  |  | PIG | GGGTACTTGGGACTGTTAAT | 6mer |
|  |  |  | PIG | TACTTGGGACTGTTAAT | 6mer |
|  |  |  | PIG | TCTTGGACTGTTAATGTG | 7mer-m8 |
|  |  |  | PIG | TCTTGGACTGTTAATGT | 7mer-m8 |
|  |  |  | PIG | CTGGGACTG | 6mer |
|  |  |  | COW | GGGTACTTGGGACTGTTAAT | 6mer |
|  |  |  | COW | TACTTGGGACTGTTAAT | 6mer |
|  |  |  | COW | TCTTGGACTGTTAATGT | 7mer-m8 |
|  |  |  | COW | CTGGGACTG | 6mer |
|  |  |  | DOG | TACTTGGGACTGTTAAT | 6mer |

  
  
  
  

| Seed Matches to the miRNA miR-323-3p | | | | | |
| --- | --- | --- | --- | --- | --- |
| Seed | Conservation | Species | Matches | | |
|  |  |  | Sequence | Motif | Type |
| ACAUUAC | Conserved | Human (Homo sapiens)  Mouse (Mus musculus)  Rat (Rattus norvegicus) | HUMAN | ACTGTTAATGTGCTA | 6mer |
|  |  |  | HUMAN | ACTGTTAATGTGCT | 6mer |
|  |  |  | HUMAN | TTAATGTGCT | 6mer |
|  |  |  | HUMAN | TAATGTGCAT | 6mer |
|  |  |  | HUMAN | TCTTGGACTGTTAATGTG | 6mer |
|  |  |  | HUMAN | TCTTGGACTGTTAATGT | 6mer |
|  |  |  | HUMAN | ACTGTTAATGT | 6mer |
|  |  |  | HUMAN | TAATGTGT | 6mer |
|  |  |  | HUMAN | TTGGACATTAATGTA | 7mer-A1 |
|  |  |  | HUMAN | ACAGTTAATGTG | 6mer |
|  |  |  | HUMAN | TATAATGTGCCAGATA | 6mer |
|  |  |  | HUMAN | TAATGTTT | 6mer |
|  |  |  | PIG | ACTGTTAATGTGCTA | 6mer |
|  |  |  | PIG | ACTGTTAATGTGCT | 6mer |
|  |  |  | PIG | TTAATGTGCT | 6mer |
|  |  |  | PIG | TAATGTGCAT | 6mer |
|  |  |  | PIG | TCTTGGACTGTTAATGTG | 6mer |
|  |  |  | PIG | TCTTGGACTGTTAATGT | 6mer |
|  |  |  | PIG | ACTGTTAATGT | 6mer |
|  |  |  | PIG | TAATGTGT | 6mer |
|  |  |  | PIG | TTGGACATTAATGTA | 7mer-A1 |
|  |  |  | PIG | ACAGTTAATGTG | 6mer |
|  |  |  | PIG | TATAATGTGCCAGATA | 6mer |
|  |  |  | PIG | TAATGTTT | 6mer |
|  |  |  | PIG | TAATGTTT | 6mer |
|  |  |  | COW | ACTGTTAATGTGCT | 6mer |
|  |  |  | COW | TTAATGTGCT | 6mer |
|  |  |  | COW | TCTTGGACTGTTAATGT | 7mer-A1 |
|  |  |  | COW | ACTGTTAATGT | 7mer-A1 |
|  |  |  | COW | ACAGTTAATGTG | 6mer |
|  |  |  | COW | TATAATGTGCCAGATA | 6mer |
|  |  |  | COW | TAATGTTT | 7mer-m8 |
|  |  |  | COW | TAATGTTT | 6mer |
|  |  |  | DOG | ACTGTTAATGTGCT | 6mer |
|  |  |  | DOG | TTAATGTGCT | 6mer |
|  |  |  | DOG | ACTGTTAATGT | 6mer |
|  |  |  | DOG | ACAGTTAATGTG | 6mer |
|  |  |  | DOG | TAATGTTT | 6mer |
|  |  |  | RABBIT | TTAATGTGCT | 6mer |

  
  
  
  

| Seed Matches to the miRNA miR-501-3p/502-3p | | | | | |
| --- | --- | --- | --- | --- | --- |
| Seed | Conservation | Species | Matches | | |
|  |  |  | Sequence | Motif | Type |
| AUGCACC | Conserved | Human (Homo sapiens) | HUMAN | TAATGTGCAT | 7mer-A1 |
|  |  |  | HUMAN | AATGTGCAT | 7mer-A1 |
|  |  |  | PIG | TAATGTGCAT | 6mer |
|  |  |  | PIG | AATGTGCAT | 6mer |
|  |  |  | COW | AATGTGCAT | 6mer |
|  |  |  | DOG | AATGTGCAT | 7mer-A1 |
|  |  |  | RABBIT | AATGTGCAT | 7mer-A1 |
|  |  |  | MOUSE | AATGTGCAT | 6mer |

  
  
  
  

| Seed Matches to the miRNA miR-142-5p | | | | | |
| --- | --- | --- | --- | --- | --- |
| Seed | Conservation | Species | Matches | | |
|  |  |  | Sequence | Motif | Type |
| AUAAAGU | Broadly Conserved | Human (Homo sapiens)  X. tropicalis (Xenopus tropicalis)  Rhesus (Macaca mulatta)  Cow (Bos taurus)  Mouse (Mus musculus)  Rat (Rattus norvegicus) | HUMAN | CTTTATTGC | 6mer |
|  |  |  | PIG | CTTTATTGC | 7mer-m8 |

  
  
  
  

| Seed Matches to the miRNA miR-203a-3p.1 | | | | | |
| --- | --- | --- | --- | --- | --- |
| Seed | Conservation | Species | Matches | | |
|  |  |  | Sequence | Motif | Type |
| GAAAUGU | Broadly Conserved | Human (Homo sapiens) | HUMAN | CCCATTTCTTG | 6mer |
|  |  |  | HUMAN | TGTGTCTTACCCATTTCCATG | 6mer |
|  |  |  | HUMAN | TGTCTTACCCATTTCCATG | 6mer |
|  |  |  | HUMAN | ATCTCACATTTCTC | 7mer-m8 |
|  |  |  | PIG | CCCATTTCTTG | 6mer |
|  |  |  | PIG | TGTGTCTTACCCATTTCCATG | 6mer |
|  |  |  | PIG | TGTCTTACCCATTTCCATG | 6mer |
|  |  |  | PIG | ATCTCACATTTCTC | 7mer-m8 |
|  |  |  | COW | TGTCTTACCCATTTCCATG | 6mer |

  
  
  
  

| Seed Matches to the miRNA miR-455-3p.2 | | | | | |
| --- | --- | --- | --- | --- | --- |
| Seed | Conservation | Species | Matches | | |
|  |  |  | Sequence | Motif | Type |
| UGCAGUC | Broadly Conserved | Human (Homo sapiens)  Mouse (Mus musculus) | HUMAN | ATAACTGCACATGGCT | 6mer |
|  |  |  | HUMAN | GACTGCAA | 8mer |
|  |  |  | PIG | ATAACTGCACATGGCT | 6mer |
|  |  |  | PIG | GACTGCAA | 8mer |
|  |  |  | COW | GACTGCAA | 8mer |

  
  
  
  

| Seed Matches to the miRNA miR-455-5p | | | | | |
| --- | --- | --- | --- | --- | --- |
| Seed | Conservation | Species | Matches | | |
|  |  |  | Sequence | Motif | Type |
| AUGUGCC | Broadly Conserved | Human (Homo sapiens)  Chicken (Gallus gallus)  Rhesus (Macaca mulatta)  Cow (Bos taurus)  Mouse (Mus musculus)  Rat (Rattus norvegicus)  Opossum (Monodelphis domestica) | HUMAN | ATAACTGCACATGGCT | 6mer |
|  |  |  | PIG | ATAACTGCACATGGCT | 6mer |

  
  
  
  

| Seed Matches to the miRNA miR-142-3p.1 | | | | | |
| --- | --- | --- | --- | --- | --- |
| Seed | Conservation | Species | Matches | | |
|  |  |  | Sequence | Motif | Type |
| GUAGUGU | Broadly Conserved | Human (Homo sapiens)  Mouse (Mus musculus) | HUMAN | CACTACTTT | 6mer |
|  |  |  | HUMAN | TAGGTGGAGATGGGGCATGAGGATCCTCCAGGGGAAAAGCTCACTACCACTGGGCAACAACCCTAGGTCAGGAG | 6mer |
|  |  |  | HUMAN | TCACTACCACTG | 6mer |
|  |  |  | HUMAN | TCACTACCACT | 6mer |
|  |  |  | HUMAN | ACTCTGGCCACTAC | 6mer |
|  |  |  | HUMAN | ACTACCACTACTGAT | 6mer |
|  |  |  | HUMAN | CACTACTGAT | 6mer |
|  |  |  | PIG | CACTACTTT | 7mer-m8 |
|  |  |  | PIG | TAGGTGGAGATGGGGCATGAGGATCCTCCAGGGGAAAAGCTCACTACCACTGGGCAACAACCCTAGGTCAGGAG | 6mer |
|  |  |  | PIG | TCACTACCACTG | 6mer |
|  |  |  | PIG | TCACTACCACT | 6mer |
|  |  |  | PIG | ACTCTGGCCACTAC | 7mer-A1 |
|  |  |  | PIG | ACTACCACTACTGAT | 6mer |
|  |  |  | PIG | CACTACTGAT | 6mer |
|  |  |  | COW | TCACTACCACTG | 6mer |
|  |  |  | COW | TCACTACCACT | 6mer |
|  |  |  | COW | ACTCTGGCCACTAC | 7mer-A1 |
|  |  |  | COW | CACTACTGAT | 7mer-m8 |
|  |  |  | DOG | TCACTACCACT | 6mer |
|  |  |  | DOG | ACTCTGGCCACTAC | 7mer-A1 |

  
  
  
  

| Seed Matches to the miRNA miR-10-5p | | | | | |
| --- | --- | --- | --- | --- | --- |
| Seed | Conservation | Species | Matches | | |
|  |  |  | Sequence | Motif | Type |
| ACCCUGU | Broadly Conserved | Human (Homo sapiens)  Chicken (Gallus gallus)  Rhesus (Macaca mulatta)  Mouse (Mus musculus)  Rat (Rattus norvegicus)  Opossum (Monodelphis domestica) | HUMAN | CAGGGTT | 6mer |
|  |  |  | HUMAN | AGTGTACAGGGTGTTT | 7mer-m8 |
|  |  |  | PIG | CAGGGTT | 6mer |
|  |  |  | PIG | AGTGTACAGGGTGTTT | 7mer-m8 |

  
  
  
  

| Seed Matches to the miRNA miR-504-5p.1 | | | | | |
| --- | --- | --- | --- | --- | --- |
| Seed | Conservation | Species | Matches | | |
|  |  |  | Sequence | Motif | Type |
| ACCCUGG | Conserved | Human (Homo sapiens) | HUMAN | CAGGGTT | 7mer-m8 |
|  |  |  | HUMAN | AGTGTACAGGGTGTTT | 6mer |
|  |  |  | PIG | CAGGGTT | 6mer |
|  |  |  | PIG | AGTGTACAGGGTGTTT | 6mer |

  
  
  
  

| Seed Matches to the miRNA let-7-5p/98-5p | | | | | |
| --- | --- | --- | --- | --- | --- |
| Seed | Conservation | Species | Matches | | |
|  |  |  | Sequence | Motif | Type |
| GAGGUAG | Broadly Conserved | Human (Homo sapiens) | HUMAN | ATACCTC | 6mer |
|  |  |  | HUMAN | CTACCTCAAAG | 8mer |
|  |  |  | PIG | ATACCTC | 7mer-A1 |
|  |  |  | PIG | CTACCTCAAAG | 8mer |

  
  
  
  

| Seed Matches to the miRNA miR-335-5p | | | | | |
| --- | --- | --- | --- | --- | --- |
| Seed | Conservation | Species | Matches | | |
|  |  |  | Sequence | Motif | Type |
| CAAGAGC | Conserved | Human (Homo sapiens)  Rhesus (Macaca mulatta)  Mouse (Mus musculus) | HUMAN | CTCAGCTCTTGGACAATTAATA | 7mer-m8 |
|  |  |  | HUMAN | CTCAGCTCTTGGACA | 7mer-m8 |
|  |  |  | HUMAN | CTCAGCTCTTGG | 7mer-m8 |
|  |  |  | HUMAN | TGCCTCTCTTGGGC | 6mer |
|  |  |  | PIG | CTCAGCTCTTGGACAATTAATA | 7mer-m8 |
|  |  |  | PIG | CTCAGCTCTTGGACA | 7mer-m8 |
|  |  |  | PIG | CTCAGCTCTTGG | 7mer-m8 |
|  |  |  | PIG | TGCCTCTCTTGGGC | 6mer |
|  |  |  | COW | CTCAGCTCTTGGACAATTAATA | 7mer-m8 |
|  |  |  | COW | CTCAGCTCTTGGACA | 7mer-m8 |
|  |  |  | COW | CTCAGCTCTTGG | 7mer-m8 |
|  |  |  | DOG | CTCAGCTCTTGGACA | 7mer-m8 |
|  |  |  | DOG | CTCAGCTCTTGG | 7mer-m8 |
|  |  |  | RABBIT | CTCAGCTCTTGG | 7mer-m8 |

  
  
  
  

| Seed Matches to the miRNA miR-802 | | | | | |
| --- | --- | --- | --- | --- | --- |
| Seed | Conservation | Species | Matches | | |
|  |  |  | Sequence | Motif | Type |
| CAGUAAC | Broadly Conserved | Human (Homo sapiens) | HUMAN | ATTACTG | 6mer |
|  |  |  | HUMAN | TTACTGCTTTACT | 7mer-m8 |
|  |  |  | HUMAN | AGGTTACTGTTTATT | 7mer-m8 |
|  |  |  | PIG | ATTACTG | 7mer-A1 |
|  |  |  | PIG | TTACTGCTTTACT | 6mer |
|  |  |  | PIG | AGGTTACTGTTTATT | 7mer-m8 |
|  |  |  | COW | ATTACTG | 6mer |

  
  
  
  

| Seed Matches to the miRNA miR-496.1 | | | | | |
| --- | --- | --- | --- | --- | --- |
| Seed | Conservation | Species | Matches | | |
|  |  |  | Sequence | Motif | Type |
| GAGUAUU | Conserved | Human (Homo sapiens) | HUMAN | CCATACTCCCA | 6mer |
|  |  |  | HUMAN | GTGATACTC | 6mer |
|  |  |  | PIG | CCATACTCCCA | 6mer |
|  |  |  | PIG | GTGATACTC | 6mer |

  
  
  
  

| Seed Matches to the miRNA miR-140-5p | | | | | |
| --- | --- | --- | --- | --- | --- |
| Seed | Conservation | Species | Matches | | |
|  |  |  | Sequence | Motif | Type |
| AGUGGUU | Broadly Conserved | Human (Homo sapiens)  Rhesus (Macaca mulatta)  Mouse (Mus musculus)  Rat (Rattus norvegicus)  Opossum (Monodelphis domestica) | HUMAN | TCTCCCTACCACTTTG | 6mer |
|  |  |  | HUMAN | TAGGTGGAGATGGGGCATGAGGATCCTCCAGGGGAAAAGCTCACTACCACTGGGCAACAACCCTAGGTCAGGAG | 6mer |
|  |  |  | HUMAN | TCACTACCACTG | 6mer |
|  |  |  | HUMAN | TCACTACCACT | 6mer |
|  |  |  | HUMAN | ACTACCACTACTGAT | 7mer-A1 |
|  |  |  | PIG | TCTCCCTACCACTTTG | 6mer |
|  |  |  | PIG | TAGGTGGAGATGGGGCATGAGGATCCTCCAGGGGAAAAGCTCACTACCACTGGGCAACAACCCTAGGTCAGGAG | 6mer |
|  |  |  | PIG | TCACTACCACTG | 6mer |
|  |  |  | PIG | TCACTACCACT | 6mer |
|  |  |  | PIG | ACTACCACTACTGAT | 7mer-A1 |
|  |  |  | COW | TCACTACCACTG | 6mer |
|  |  |  | COW | TCACTACCACT | 6mer |
|  |  |  | DOG | TCACTACCACT | 7mer-A1 |

  
  
  
  

| Seed Matches to the miRNA miR-17-5p/20-5p/93-5p/106-5p/519-3p | | | | | |
| --- | --- | --- | --- | --- | --- |
| Seed | Conservation | Species | Matches | | |
|  |  |  | Sequence | Motif | Type |
| AAAGUGC | Broadly Conserved | Human (Homo sapiens) | HUMAN | TCTCCCTACCACTTTG | 6mer |
|  |  |  | HUMAN | ATTCACTTTTAGAAAAAC | 6mer |
|  |  |  | PIG | TCTCCCTACCACTTTG | 6mer |
|  |  |  | PIG | ATTCACTTTTAGAAAAAC | 6mer |

  
  
  
  

| Seed Matches to the miRNA miR-382-3p | | | | | |
| --- | --- | --- | --- | --- | --- |
| Seed | Conservation | Species | Matches | | |
|  |  |  | Sequence | Motif | Type |
| AUCAUUC | Conserved | Human (Homo sapiens)  Rhesus (Macaca mulatta)  Mouse (Mus musculus)  Rat (Rattus norvegicus) | HUMAN | TTAATGATCC | 6mer |
|  |  |  | HUMAN | AAATGAT | 7mer-A1 |
|  |  |  | HUMAN | AATGATTA | 6mer |
|  |  |  | HUMAN | AAAATGAT | 6mer |
|  |  |  | PIG | TTAATGATCC | 6mer |
|  |  |  | PIG | AAATGAT | 6mer |
|  |  |  | PIG | AATGATTA | 6mer |
|  |  |  | PIG | AAAATGAT | 7mer-A1 |
|  |  |  | COW | TTAATGATCC | 6mer |
|  |  |  | DOG | TTAATGATCC | 6mer |

  
  
  
  

| Seed Matches to the miRNA miR-182-5p | | | | | |
| --- | --- | --- | --- | --- | --- |
| Seed | Conservation | Species | Matches | | |
|  |  |  | Sequence | Motif | Type |
| UUGGCAA | Broadly Conserved | Human (Homo sapiens)  X. tropicalis (Xenopus tropicalis)  Mouse (Mus musculus)  Opossum (Monodelphis domestica) | HUMAN | TGCCAA | 7mer-A1 |
|  |  |  | HUMAN | TGCCAAATC | 7mer-A1 |
|  |  |  | PIG | TGCCAA | 6mer |
|  |  |  | PIG | TGCCAAATC | 8mer |
|  |  |  | COW | TGCCAA | 6mer |
|  |  |  | COW | TGCCAAATC | 8mer |

  
  
  
  

| Seed Matches to the miRNA miR-96-5p/1271-5p | | | | | |
| --- | --- | --- | --- | --- | --- |
| Seed | Conservation | Species | Matches | | |
|  |  |  | Sequence | Motif | Type |
| UUGGCAC | Broadly Conserved | Human (Homo sapiens) | HUMAN | TGCCAA | 8mer |
|  |  |  | HUMAN | TGCCAAATC | 7mer-A1 |
|  |  |  | PIG | TGCCAA | 6mer |
|  |  |  | PIG | TGCCAAATC | 7mer-A1 |
|  |  |  | COW | TGCCAA | 7mer-m8 |
|  |  |  | COW | TGCCAAATC | 7mer-A1 |

  
  
  
  

| Seed Matches to the miRNA miR-154-5p | | | | | |
| --- | --- | --- | --- | --- | --- |
| Seed | Conservation | Species | Matches | | |
|  |  |  | Sequence | Motif | Type |
| AGGUUAU | Conserved | Human (Homo sapiens)  Rhesus (Macaca mulatta)  Mouse (Mus musculus)  Rat (Rattus norvegicus) | HUMAN | GATAACCTGGTCATT | 7mer-m8 |
|  |  |  | PIG | GATAACCTGGTCATT | 7mer-m8 |
|  |  |  | COW | GATAACCTGGTCATT | 7mer-m8 |

  
  
  
  

| Seed Matches to the miRNA miR-203a-3p.2 | | | | | |
| --- | --- | --- | --- | --- | --- |
| Seed | Conservation | Species | Matches | | |
|  |  |  | Sequence | Motif | Type |
| UGAAAUG | Broadly Conserved | Human (Homo sapiens) | HUMAN | CTTATTTCAAGAA | 7mer-A1 |
|  |  |  | HUMAN | ATTTCACCT | 6mer |
|  |  |  | HUMAN | TATTTCAGTCCT | 6mer |
|  |  |  | HUMAN | TATTTCAGTCC | 6mer |
|  |  |  | HUMAN | TATTTCAGT | 6mer |
|  |  |  | PIG | CTTATTTCAAGAA | 7mer-A1 |
|  |  |  | PIG | ATTTCACCT | 7mer-m8 |
|  |  |  | PIG | TATTTCAGTCCT | 6mer |
|  |  |  | PIG | TATTTCAGTCC | 6mer |
|  |  |  | PIG | TATTTCAGT | 6mer |
|  |  |  | COW | CTTATTTCAAGAA | 7mer-A1 |
|  |  |  | COW | ATTTCACCT | 7mer-m8 |
|  |  |  | COW | TATTTCAGTCC | 6mer |
|  |  |  | COW | TATTTCAGT | 6mer |
|  |  |  | DOG | ATTTCACCT | 7mer-m8 |
|  |  |  | DOG | TATTTCAGT | 6mer |

  
  
  
  

| Seed Matches to the miRNA miR-340-5p | | | | | |
| --- | --- | --- | --- | --- | --- |
| Seed | Conservation | Species | Matches | | |
|  |  |  | Sequence | Motif | Type |
| UAUAAAG | Conserved | Human (Homo sapiens)  Rhesus (Macaca mulatta)  Mouse (Mus musculus)  Rat (Rattus norvegicus) | HUMAN | TTTTATA | 7mer-A1 |
|  |  |  | PIG | TTTTATA | 6mer |
|  |  |  | COW | TTTTATA | 6mer |

  
  
  
  

| Seed Matches to the miRNA miR-339-5p | | | | | |
| --- | --- | --- | --- | --- | --- |
| Seed | Conservation | Species | Matches | | |
|  |  |  | Sequence | Motif | Type |
| CCCUGUC | Conserved | Human (Homo sapiens)  Rhesus (Macaca mulatta)  Mouse (Mus musculus) | HUMAN | AGTGTACAGGGTGTTT | 6mer |
|  |  |  | PIG | AGTGTACAGGGTGTTT | 6mer |

  
  
  
  

| Seed Matches to the miRNA miR-486-5p | | | | | |
| --- | --- | --- | --- | --- | --- |
| Seed | Conservation | Species | Matches | | |
|  |  |  | Sequence | Motif | Type |
| CCUGUAC | Conserved | Human (Homo sapiens)  Rhesus (Macaca mulatta)  Mouse (Mus musculus) | HUMAN | AGTGTACAGGGTGTTT | 7mer-m8 |
|  |  |  | PIG | AGTGTACAGGGTGTTT | 7mer-m8 |

  
  
  
  

| Seed Matches to the miRNA miR-493-5p | | | | | |
| --- | --- | --- | --- | --- | --- |
| Seed | Conservation | Species | Matches | | |
|  |  |  | Sequence | Motif | Type |
| UGUACAU | Conserved | Human (Homo sapiens)  Rhesus (Macaca mulatta)  Mouse (Mus musculus)  Rat (Rattus norvegicus) | HUMAN | AGTGTACAGGGTGTTT | 6mer |
|  |  |  | HUMAN | AGTGTACA | 6mer |
|  |  |  | PIG | AGTGTACAGGGTGTTT | 6mer |
|  |  |  | PIG | AGTGTACA | 6mer |
|  |  |  | COW | AGTGTACA | 7mer-A1 |

  
  
  
  

| Seed Matches to the miRNA miR-362-5p/500b-5p | | | | | |
| --- | --- | --- | --- | --- | --- |
| Seed | Conservation | Species | Matches | | |
|  |  |  | Sequence | Motif | Type |
| AUCCUUG | Conserved | Human (Homo sapiens) | HUMAN | GAGAAGGATGTCAAAAGATCGGC | 6mer |
|  |  |  | HUMAN | AAGGATGTCAAAAGATC | 6mer |
|  |  |  | HUMAN | AAGGATG | 6mer |
|  |  |  | PIG | GAGAAGGATGTCAAAAGATCGGC | 6mer |
|  |  |  | PIG | AAGGATGTCAAAAGATC | 6mer |
|  |  |  | PIG | AAGGATG | 6mer |
|  |  |  | COW | GAGAAGGATGTCAAAAGATCGGC | 6mer |
|  |  |  | COW | AAGGATGTCAAAAGATC | 6mer |
|  |  |  | COW | AAGGATG | 6mer |
|  |  |  | DOG | AAGGATGTCAAAAGATC | 7mer-m8 |
|  |  |  | DOG | AAGGATG | 7mer-m8 |
|  |  |  | RABBIT | AAGGATG | 6mer |

  
  
  
  

| Seed Matches to the miRNA miR-125-5p | | | | | |
| --- | --- | --- | --- | --- | --- |
| Seed | Conservation | Species | Matches | | |
|  |  |  | Sequence | Motif | Type |
| CCCUGAG | Broadly Conserved | Human (Homo sapiens)  Chicken (Gallus gallus)  Opossum (Monodelphis domestica) | HUMAN | CAGCTCAGGG | 8mer |
|  |  |  | HUMAN | TATGTAAATCAGGGGTC | 6mer |
|  |  |  | PIG | CAGCTCAGGG | 7mer-m8 |
|  |  |  | PIG | TATGTAAATCAGGGGTC | 6mer |
|  |  |  | COW | CAGCTCAGGG | 7mer-m8 |
|  |  |  | DOG | CAGCTCAGGG | 7mer-m8 |

  
  
  
  

| Seed Matches to the miRNA miR-28-5p/708-5p | | | | | |
| --- | --- | --- | --- | --- | --- |
| Seed | Conservation | Species | Matches | | |
|  |  |  | Sequence | Motif | Type |
| AGGAGCU | Conserved | Human (Homo sapiens)  Mouse (Mus musculus)  Rat (Rattus norvegicus) | HUMAN | CTACTAGCTCCT | 7mer-m8 |
|  |  |  | HUMAN | GCTTGCTCCTT | 6mer |
|  |  |  | HUMAN | TGCTCCTT | 6mer |
|  |  |  | PIG | CTACTAGCTCCT | 7mer-m8 |
|  |  |  | PIG | GCTTGCTCCTT | 6mer |
|  |  |  | PIG | TGCTCCTT | 6mer |
|  |  |  | COW | CTACTAGCTCCT | 7mer-m8 |
|  |  |  | COW | TGCTCCTT | 6mer |
|  |  |  | DOG | CTACTAGCTCCT | 7mer-m8 |

  
  
  
  

| Seed Matches to the miRNA miR-411-5p.2 | | | | | |
| --- | --- | --- | --- | --- | --- |
| Seed | Conservation | Species | Matches | | |
|  |  |  | Sequence | Motif | Type |
| UAGUAGA | Conserved | Human (Homo sapiens)  Mouse (Mus musculus) | HUMAN | CTACTAGCTCCT | 6mer |
|  |  |  | HUMAN | ATTGCCTACTATGTGAACTCACTGTTA | 6mer |
|  |  |  | HUMAN | TTGCCTACTATGTGAACTCACTGTTA | 6mer |
|  |  |  | PIG | CTACTAGCTCCT | 6mer |
|  |  |  | PIG | ATTGCCTACTATGTGAACTCACTGTTA | 6mer |
|  |  |  | PIG | TTGCCTACTATGTGAACTCACTGTTA | 6mer |
|  |  |  | COW | CTACTAGCTCCT | 6mer |
|  |  |  | COW | TTGCCTACTATGTGAACTCACTGTTA | 6mer |
|  |  |  | DOG | CTACTAGCTCCT | 6mer |

  
  
  
  

| Seed Matches to the miRNA miR-371-5p | | | | | |
| --- | --- | --- | --- | --- | --- |
| Seed | Conservation | Species | Matches | | |
|  |  |  | Sequence | Motif | Type |
| CUCAAAC | Conserved | Human (Homo sapiens)  Rhesus (Macaca mulatta) | HUMAN | TTTGAGAATCTGG | 7mer-A1 |
|  |  |  | HUMAN | TCACCTTTGAGTATTT | 6mer |
|  |  |  | HUMAN | TTTGAGTATTT | 6mer |
|  |  |  | HUMAN | CTTTGAGCTTAGGTGAGCAGGATTC | 6mer |
|  |  |  | HUMAN | TTTGAGCTT | 6mer |
|  |  |  | PIG | TTTGAGAATCTGG | 7mer-A1 |
|  |  |  | PIG | TCACCTTTGAGTATTT | 6mer |
|  |  |  | PIG | TTTGAGTATTT | 6mer |
|  |  |  | PIG | CTTTGAGCTTAGGTGAGCAGGATTC | 6mer |
|  |  |  | PIG | TTTGAGCTT | 6mer |
|  |  |  | COW | TTTGAGAATCTGG | 7mer-A1 |
|  |  |  | COW | TTTGAGTATTT | 6mer |
|  |  |  | COW | TTTGAGCTT | 6mer |
|  |  |  | DOG | TTTGAGTATTT | 6mer |

  
  
  
  

| Seed Matches to the miRNA miR-7-5p | | | | | |
| --- | --- | --- | --- | --- | --- |
| Seed | Conservation | Species | Matches | | |
|  |  |  | Sequence | Motif | Type |
| GGAAGAC | Broadly Conserved | Human (Homo sapiens)  Mouse (Mus musculus)  Rat (Rattus norvegicus) | HUMAN | TCTGGAGAAAAAGATCTTCCTCAGAAGAATAGGCTTGTTG | 6mer |
|  |  |  | PIG | TCTGGAGAAAAAGATCTTCCTCAGAAGAATAGGCTTGTTG | 6mer |

  
  
  
  

| Seed Matches to the miRNA miR-192-5p/215-5p | | | | | |
| --- | --- | --- | --- | --- | --- |
| Seed | Conservation | Species | Matches | | |
|  |  |  | Sequence | Motif | Type |
| UGACCUA | Broadly Conserved | Human (Homo sapiens)  Rhesus (Macaca mulatta)  Mouse (Mus musculus) | HUMAN | TAGGTGGAGATGGGGCATGAGGATCCTCCAGGGGAAAAGCTCACTACCACTGGGCAACAACCCTAGGTCAGGAG | 7mer-m8 |
|  |  |  | HUMAN | GAAAAGGTCA | 7mer-A1 |
|  |  |  | PIG | TAGGTGGAGATGGGGCATGAGGATCCTCCAGGGGAAAAGCTCACTACCACTGGGCAACAACCCTAGGTCAGGAG | 7mer-m8 |
|  |  |  | PIG | GAAAAGGTCA | 6mer |
|  |  |  | COW | GAAAAGGTCA | 7mer-A1 |
|  |  |  | DOG | GAAAAGGTCA | 7mer-A1 |

  
  
  
  

| Seed Matches to the miRNA miR-199-5p | | | | | |
| --- | --- | --- | --- | --- | --- |
| Seed | Conservation | Species | Matches | | |
|  |  |  | Sequence | Motif | Type |
| CCAGUGU | Broadly Conserved | Human (Homo sapiens)  X. tropicalis (Xenopus tropicalis)  Chicken (Gallus gallus)  Rhesus (Macaca mulatta)  Chimp (Pan troglodytes)  Cow (Bos taurus)  Mouse (Mus musculus)  Rat (Rattus norvegicus) | HUMAN | TAGGTGGAGATGGGGCATGAGGATCCTCCAGGGGAAAAGCTCACTACCACTGGGCAACAACCCTAGGTCAGGAG | 6mer |
|  |  |  | PIG | TAGGTGGAGATGGGGCATGAGGATCCTCCAGGGGAAAAGCTCACTACCACTGGGCAACAACCCTAGGTCAGGAG | 6mer |

  
  
  
  

| Seed Matches to the miRNA miR-296-3p | | | | | |
| --- | --- | --- | --- | --- | --- |
| Seed | Conservation | Species | Matches | | |
|  |  |  | Sequence | Motif | Type |
| AGGGUUG | Conserved | Human (Homo sapiens)  Rhesus (Macaca mulatta)  Cow (Bos taurus)  Mouse (Mus musculus)  Rat (Rattus norvegicus) | HUMAN | TAGGTGGAGATGGGGCATGAGGATCCTCCAGGGGAAAAGCTCACTACCACTGGGCAACAACCCTAGGTCAGGAG | 8mer |
|  |  |  | HUMAN | AACCCTAAA | 7mer-A1 |
|  |  |  | PIG | TAGGTGGAGATGGGGCATGAGGATCCTCCAGGGGAAAAGCTCACTACCACTGGGCAACAACCCTAGGTCAGGAG | 8mer |
|  |  |  | PIG | AACCCTAAA | 7mer-A1 |

  
  
  
  

| Seed Matches to the miRNA miR-665 | | | | | |
| --- | --- | --- | --- | --- | --- |
| Seed | Conservation | Species | Matches | | |
|  |  |  | Sequence | Motif | Type |
| CCAGGAG | Conserved | Human (Homo sapiens)  Rhesus (Macaca mulatta)  Chimp (Pan troglodytes)  Dog (Canis lupus familiaris)  Rat (Rattus norvegicus) | HUMAN | CTTTCCTGG | 6mer |
|  |  |  | PIG | CTTTCCTGG | 6mer |
|  |  |  | COW | CTTTCCTGG | 6mer |

  
  
  
  

| Seed Matches to the miRNA miR-873-5p.1 | | | | | |
| --- | --- | --- | --- | --- | --- |
| Seed | Conservation | Species | Matches | | |
|  |  |  | Sequence | Motif | Type |
| CAGGAAC | Conserved | Human (Homo sapiens) | HUMAN | CTTTCCTGG | 6mer |
|  |  |  | PIG | CTTTCCTGG | 6mer |
|  |  |  | COW | CTTTCCTGG | 6mer |

  
  
  
  

| Seed Matches to the miRNA miR-202-5p | | | | | |
| --- | --- | --- | --- | --- | --- |
| Seed | Conservation | Species | Matches | | |
|  |  |  | Sequence | Motif | Type |
| UCCUAUG | Broadly Conserved | Human (Homo sapiens)  Mouse (Mus musculus)  Rat (Rattus norvegicus) | HUMAN | CCAGATAGGAAGAT | 7mer-A1 |
|  |  |  | PIG | CCAGATAGGAAGAT | 7mer-A1 |

  
  
  
  

| Seed Matches to the miRNA miR-1298-5p | | | | | |
| --- | --- | --- | --- | --- | --- |
| Seed | Conservation | Species | Matches | | |
|  |  |  | Sequence | Motif | Type |
| UCAUUCG | Conserved | Human (Homo sapiens)  Rhesus (Macaca mulatta)  Mouse (Mus musculus) | HUMAN | TGAATGA | 7mer-A1 |
|  |  |  | HUMAN | GAATGAATA | 7mer-A1 |
|  |  |  | HUMAN | TCTGCTGAATGA | 6mer |
|  |  |  | PIG | TGAATGA | 6mer |
|  |  |  | PIG | GAATGAATA | 8mer |
|  |  |  | PIG | TCTGCTGAATGA | 7mer-A1 |
|  |  |  | COW | GAATGAATA | 7mer-A1 |
|  |  |  | COW | TCTGCTGAATGA | 7mer-A1 |

  
  
  
  

| Seed Matches to the miRNA miR-208-3p | | | | | |
| --- | --- | --- | --- | --- | --- |
| Seed | Conservation | Species | Matches | | |
|  |  |  | Sequence | Motif | Type |
| UAAGACG | Broadly Conserved | Human (Homo sapiens)  Rhesus (Macaca mulatta)  Mouse (Mus musculus)  Rat (Rattus norvegicus)  Opossum (Monodelphis domestica) | HUMAN | TGTGTCTTACCCATTTCCATG | 6mer |
|  |  |  | HUMAN | TGTCTTACCCATTTCCATG | 6mer |
|  |  |  | HUMAN | TGTCTTA | 6mer |
|  |  |  | HUMAN | GTCTTAGA | 6mer |
|  |  |  | HUMAN | ATTGGTCTTAA | 7mer-A1 |
|  |  |  | HUMAN | TTGGTCTTAA | 7mer-A1 |
|  |  |  | PIG | TGTGTCTTACCCATTTCCATG | 6mer |
|  |  |  | PIG | TGTCTTACCCATTTCCATG | 6mer |
|  |  |  | PIG | TGTCTTA | 6mer |
|  |  |  | PIG | GTCTTAGA | 7mer-m8 |
|  |  |  | PIG | ATTGGTCTTAA | 7mer-A1 |
|  |  |  | PIG | TTGGTCTTAA | 7mer-A1 |
|  |  |  | COW | TGTCTTACCCATTTCCATG | 6mer |
|  |  |  | COW | TGTCTTA | 6mer |
|  |  |  | COW | TTGGTCTTAA | 7mer-A1 |
|  |  |  | DOG | TGTCTTA | 6mer |

  
  
  
  

| Seed Matches to the miRNA miR-499a-5p | | | | | |
| --- | --- | --- | --- | --- | --- |
| Seed | Conservation | Species | Matches | | |
|  |  |  | Sequence | Motif | Type |
| UAAGACU | Broadly Conserved | Human (Homo sapiens) | HUMAN | TGTGTCTTACCCATTTCCATG | 6mer |
|  |  |  | HUMAN | TGTCTTACCCATTTCCATG | 6mer |
|  |  |  | HUMAN | TGTCTTA | 6mer |
|  |  |  | HUMAN | GTCTTAGA | 6mer |
|  |  |  | HUMAN | ATTGGTCTTAA | 7mer-A1 |
|  |  |  | HUMAN | TTGGTCTTAA | 7mer-A1 |
|  |  |  | PIG | TGTGTCTTACCCATTTCCATG | 6mer |
|  |  |  | PIG | TGTCTTACCCATTTCCATG | 6mer |
|  |  |  | PIG | TGTCTTA | 6mer |
|  |  |  | PIG | GTCTTAGA | 6mer |
|  |  |  | PIG | ATTGGTCTTAA | 7mer-A1 |
|  |  |  | PIG | TTGGTCTTAA | 7mer-A1 |
|  |  |  | COW | TGTCTTACCCATTTCCATG | 6mer |
|  |  |  | COW | TGTCTTA | 6mer |
|  |  |  | COW | TTGGTCTTAA | 7mer-A1 |
|  |  |  | DOG | TGTCTTA | 6mer |

  
  
  
  

| Seed Matches to the miRNA miR-411-5p.1 | | | | | |
| --- | --- | --- | --- | --- | --- |
| Seed | Conservation | Species | Matches | | |
|  |  |  | Sequence | Motif | Type |
| AGUAGAC | Conserved | Human (Homo sapiens) | HUMAN | TGCTGTTTCTACT | 6mer |
|  |  |  | HUMAN | TTTCTACT | 6mer |
|  |  |  | PIG | TGCTGTTTCTACT | 6mer |
|  |  |  | PIG | TTTCTACT | 6mer |
|  |  |  | COW | TGCTGTTTCTACT | 6mer |
|  |  |  | COW | TTTCTACT | 6mer |
|  |  |  | DOG | TTTCTACT | 6mer |
|  |  |  | RABBIT | TTTCTACT | 6mer |

  
  
  
  

| Seed Matches to the miRNA miR-494-3p | | | | | |
| --- | --- | --- | --- | --- | --- |
| Seed | Conservation | Species | Matches | | |
|  |  |  | Sequence | Motif | Type |
| GAAACAU | Conserved | Human (Homo sapiens)  Rhesus (Macaca mulatta)  Mouse (Mus musculus)  Rat (Rattus norvegicus) | HUMAN | TGCTGTTTCTACT | 6mer |
|  |  |  | PIG | TGCTGTTTCTACT | 6mer |
|  |  |  | COW | TGCTGTTTCTACT | 6mer |

  
  
  
  

| Seed Matches to the miRNA miR-329-3p/362-3p | | | | | |
| --- | --- | --- | --- | --- | --- |
| Seed | Conservation | Species | Matches | | |
|  |  |  | Sequence | Motif | Type |
| ACACACC | Conserved | Human (Homo sapiens)  Rhesus (Macaca mulatta)  Mouse (Mus musculus)  Rat (Rattus norvegicus) | HUMAN | TGTGTGTG | 6mer |
|  |  |  | PIG | TGTGTGTG | 6mer |
|  |  |  | COW | TGTGTGTG | 6mer |
|  |  |  | DOG | TGTGTGTG | 6mer |

  
  
  
  

| Seed Matches to the miRNA miR-133a-3p.2/133b | | | | | |
| --- | --- | --- | --- | --- | --- |
| Seed | Conservation | Species | Matches | | |
|  |  |  | Sequence | Motif | Type |
| UUGGUCC | Broadly Conserved | Human (Homo sapiens) | HUMAN | ATGATCCAAGACCAA | 7mer-A1 |
|  |  |  | PIG | ATGATCCAAGACCAA | 6mer |

  
  
  
  

| Seed Matches to the miRNA miR-431-5p | | | | | |
| --- | --- | --- | --- | --- | --- |
| Seed | Conservation | Species | Matches | | |
|  |  |  | Sequence | Motif | Type |
| GUCUUGC | Conserved | Human (Homo sapiens)  Mouse (Mus musculus) | HUMAN | ATGATCCAAGACCAA | 6mer |
|  |  |  | HUMAN | TATCTCAAGACTAA | 6mer |
|  |  |  | HUMAN | TCAAGACTAA | 6mer |
|  |  |  | HUMAN | CAATCAAGACTTTAC | 6mer |
|  |  |  | PIG | ATGATCCAAGACCAA | 6mer |
|  |  |  | PIG | TATCTCAAGACTAA | 6mer |
|  |  |  | PIG | TCAAGACTAA | 6mer |
|  |  |  | PIG | CAATCAAGACTTTAC | 6mer |
|  |  |  | COW | TCAAGACTAA | 6mer |
|  |  |  | DOG | TCAAGACTAA | 6mer |

  
  
  
  

| Seed Matches to the miRNA miR-328-3p | | | | | |
| --- | --- | --- | --- | --- | --- |
| Seed | Conservation | Species | Matches | | |
|  |  |  | Sequence | Motif | Type |
| UGGCCCU | Conserved | Human (Homo sapiens)  Mouse (Mus musculus)  Rat (Rattus norvegicus) | HUMAN | ACAGTATTATGCCTGGGCCAGTCTT | 6mer |
|  |  |  | HUMAN | GGGCCATATGGTTTC | 6mer |
|  |  |  | PIG | ACAGTATTATGCCTGGGCCAGTCTT | 6mer |
|  |  |  | PIG | GGGCCATATGGTTTC | 7mer-m8 |

  
  
  
  

| Seed Matches to the miRNA miR-655-3p | | | | | |
| --- | --- | --- | --- | --- | --- |
| Seed | Conservation | Species | Matches | | |
|  |  |  | Sequence | Motif | Type |
| UAAUACA | Conserved | Human (Homo sapiens) | HUMAN | ACAGTATTATGCCTGGGCCAGTCTT | 6mer |
|  |  |  | PIG | ACAGTATTATGCCTGGGCCAGTCTT | 6mer |

  
  
  
  

| Seed Matches to the miRNA miR-183-5p.2 | | | | | |
| --- | --- | --- | --- | --- | --- |
| Seed | Conservation | Species | Matches | | |
|  |  |  | Sequence | Motif | Type |
| UGGCACU | Broadly Conserved | Human (Homo sapiens)  Mouse (Mus musculus) | HUMAN | GTGCCAGGCT | 7mer-m8 |
|  |  |  | HUMAN | TATAATGTGCCAGATA | 6mer |
|  |  |  | HUMAN | AATGTGCCAGATA | 6mer |
|  |  |  | HUMAN | TGTGCCA | 7mer-A1 |
|  |  |  | PIG | GTGCCAGGCT | 6mer |
|  |  |  | PIG | TATAATGTGCCAGATA | 6mer |
|  |  |  | PIG | AATGTGCCAGATA | 6mer |
|  |  |  | PIG | TGTGCCA | 6mer |
|  |  |  | COW | TATAATGTGCCAGATA | 6mer |
|  |  |  | COW | AATGTGCCAGATA | 6mer |
|  |  |  | COW | TGTGCCA | 6mer |
|  |  |  | DOG | AATGTGCCAGATA | 6mer |
|  |  |  | DOG | TGTGCCA | 7mer-A1 |

  
  
  
  

| Seed Matches to the miRNA miR-1251-5p | | | | | |
| --- | --- | --- | --- | --- | --- |
| Seed | Conservation | Species | Matches | | |
|  |  |  | Sequence | Motif | Type |
| CUCUAGC | Conserved | Human (Homo sapiens)  Rhesus (Macaca mulatta)  Mouse (Mus musculus)  Opossum (Monodelphis domestica) | HUMAN | TCTAGAGAAAA | 7mer-A1 |
|  |  |  | PIG | TCTAGAGAAAA | 7mer-A1 |
|  |  |  | COW | TCTAGAGAAAA | 7mer-A1 |
|  |  |  | DOG | TCTAGAGAAAA | 7mer-A1 |
|  |  |  | RABBIT | TCTAGAGAAAA | 7mer-A1 |
|  |  |  | MOUSE | TCTAGAGAAAA | 7mer-A1 |

  
  
  
  

| Seed Matches to the miRNA miR-212-5p | | | | | |
| --- | --- | --- | --- | --- | --- |
| Seed | Conservation | Species | Matches | | |
|  |  |  | Sequence | Motif | Type |
| CCUUGGC | Broadly Conserved | Human (Homo sapiens)  Rhesus (Macaca mulatta)  Mouse (Mus musculus)  Rat (Rattus norvegicus) | HUMAN | GAGGTCCCAAGG | 6mer |
|  |  |  | HUMAN | CCCAAGG | 6mer |
|  |  |  | PIG | GAGGTCCCAAGG | 7mer-A1 |
|  |  |  | PIG | CCCAAGG | 7mer-A1 |
|  |  |  | COW | CCCAAGG | 7mer-A1 |
|  |  |  | DOG | CCCAAGG | 7mer-A1 |

  
  
  
  

| Seed Matches to the miRNA miR-496.2 | | | | | |
| --- | --- | --- | --- | --- | --- |
| Seed | Conservation | Species | Matches | | |
|  |  |  | Sequence | Motif | Type |
| GUAUUAC | Conserved | Human (Homo sapiens) | HUMAN | TTTAATACCCA | 6mer |
|  |  |  | HUMAN | TTTAATAC | 6mer |
|  |  |  | HUMAN | GTTTAATACTTTCCTT | 6mer |
|  |  |  | PIG | TTTAATACCCA | 6mer |
|  |  |  | PIG | TTTAATAC | 6mer |
|  |  |  | PIG | GTTTAATACTTTCCTT | 6mer |
|  |  |  | COW | TTTAATAC | 6mer |
|  |  |  | DOG | TTTAATAC | 6mer |

  
  
  
  

| Seed Matches to the miRNA miR-302-3p/372-3p/373-3p/520-3p | | | | | |
| --- | --- | --- | --- | --- | --- |
| Seed | Conservation | Species | Matches | | |
|  |  |  | Sequence | Motif | Type |
| AAGUGCU | Broadly Conserved | Human (Homo sapiens) | HUMAN | TAAAGCACTT | 7mer-m8 |
|  |  |  | PIG | TAAAGCACTT | 8mer |

  
  
  
  

| Seed Matches to the miRNA miR-302c-3p.2/520-3p | | | | | |
| --- | --- | --- | --- | --- | --- |
| Seed | Conservation | Species | Matches | | |
|  |  |  | Sequence | Motif | Type |
| AGUGCUU | Broadly Conserved | Human (Homo sapiens) | HUMAN | TAAAGCACTT | 7mer-m8 |
|  |  |  | PIG | TAAAGCACTT | 7mer-m8 |

  
  
  
  

| Seed Matches to the miRNA miR-23-3p | | | | | |
| --- | --- | --- | --- | --- | --- |
| Seed | Conservation | Species | Matches | | |
|  |  |  | Sequence | Motif | Type |
| UCACAUU | Broadly Conserved | Human (Homo sapiens)  Chicken (Gallus gallus)  Rhesus (Macaca mulatta)  Cow (Bos taurus)  Mouse (Mus musculus)  Rat (Rattus norvegicus)  Opossum (Monodelphis domestica) | HUMAN | ATTGCCTACTATGTGAACTCACTGTTA | 7mer-A1 |
|  |  |  | HUMAN | TTGCCTACTATGTGAACTCACTGTTA | 7mer-A1 |
|  |  |  | HUMAN | ACTTGTGAACTGATGTGAAA | 7mer-A1 |
|  |  |  | HUMAN | TGAACTGATGTGAAA | 7mer-A1 |
|  |  |  | PIG | ATTGCCTACTATGTGAACTCACTGTTA | 7mer-A1 |
|  |  |  | PIG | TTGCCTACTATGTGAACTCACTGTTA | 7mer-A1 |
|  |  |  | PIG | ACTTGTGAACTGATGTGAAA | 7mer-A1 |
|  |  |  | PIG | TGAACTGATGTGAAA | 7mer-A1 |
|  |  |  | COW | TTGCCTACTATGTGAACTCACTGTTA | 7mer-A1 |
|  |  |  | COW | ACTTGTGAACTGATGTGAAA | 7mer-A1 |
|  |  |  | COW | TGAACTGATGTGAAA | 7mer-A1 |
|  |  |  | DOG | TGAACTGATGTGAAA | 7mer-A1 |

  
  
  
  

| Seed Matches to the miRNA miR-376c-3p | | | | | |
| --- | --- | --- | --- | --- | --- |
| Seed | Conservation | Species | Matches | | |
|  |  |  | Sequence | Motif | Type |
| ACAUAGA | Conserved | Human (Homo sapiens)  Rhesus (Macaca mulatta)  Mouse (Mus musculus) | HUMAN | ATTGCCTACTATGTGAACTCACTGTTA | 6mer |
|  |  |  | HUMAN | TTGCCTACTATGTGAACTCACTGTTA | 6mer |
|  |  |  | PIG | ATTGCCTACTATGTGAACTCACTGTTA | 6mer |
|  |  |  | PIG | TTGCCTACTATGTGAACTCACTGTTA | 6mer |
|  |  |  | COW | TTGCCTACTATGTGAACTCACTGTTA | 6mer |

  
  
  
  

| Seed Matches to the miRNA miR-483-3p.1 | | | | | |
| --- | --- | --- | --- | --- | --- |
| Seed | Conservation | Species | Matches | | |
|  |  |  | Sequence | Motif | Type |
| ACUCCUC | Conserved | Human (Homo sapiens)  Mouse (Mus musculus) | HUMAN | TAAAGGAGTAAAAAT | 7mer-A1 |
|  |  |  | PIG | TAAAGGAGTAAAAAT | 7mer-A1 |

  
  
  
  

| Seed Matches to the miRNA miR-365-3p | | | | | |
| --- | --- | --- | --- | --- | --- |
| Seed | Conservation | Species | Matches | | |
|  |  |  | Sequence | Motif | Type |
| AAUGCCC | Broadly Conserved | Human (Homo sapiens)  Chicken (Gallus gallus)  Rhesus (Macaca mulatta)  Cow (Bos taurus)  Mouse (Mus musculus)  Rat (Rattus norvegicus)  Opossum (Monodelphis domestica) | HUMAN | AGGGCATTAG | 8mer |
|  |  |  | PIG | AGGGCATTAG | 8mer |

  
  
  
  

| Seed Matches to the miRNA miR-874-3p | | | | | |
| --- | --- | --- | --- | --- | --- |
| Seed | Conservation | Species | Matches | | |
|  |  |  | Sequence | Motif | Type |
| UGCCCUG | Conserved | Human (Homo sapiens)  Rhesus (Macaca mulatta)  Mouse (Mus musculus)  Rat (Rattus norvegicus) | HUMAN | AGGGCATTAG | 6mer |
|  |  |  | PIG | AGGGCATTAG | 6mer |

  
  
  
  

| Seed Matches to the miRNA miR-375 | | | | | |
| --- | --- | --- | --- | --- | --- |
| Seed | Conservation | Species | Matches | | |
|  |  |  | Sequence | Motif | Type |
| UUGUUCG | Broadly Conserved | Human (Homo sapiens)  X. tropicalis (Xenopus tropicalis)  Chicken (Gallus gallus)  Rhesus (Macaca mulatta)  Chimp (Pan troglodytes)  Dog (Canis lupus familiaris) | HUMAN | GAACAAATT | 7mer-A1 |
|  |  |  | PIG | GAACAAATT | 7mer-A1 |
|  |  |  | COW | GAACAAATT | 7mer-A1 |

  
  
  
  

| Seed Matches to the miRNA miR-138-5p | | | | | |
| --- | --- | --- | --- | --- | --- |
| Seed | Conservation | Species | Matches | | |
|  |  |  | Sequence | Motif | Type |
| GCUGGUG | Broadly Conserved | Human (Homo sapiens)  Chicken (Gallus gallus)  Rhesus (Macaca mulatta)  Mouse (Mus musculus)  Rat (Rattus norvegicus) | HUMAN | AGTCTCATTGGTACCAGC | 6mer |
|  |  |  | PIG | AGTCTCATTGGTACCAGC | 6mer |

  
  
  
  

| Seed Matches to the miRNA miR-543 | | | | | |
| --- | --- | --- | --- | --- | --- |
| Seed | Conservation | Species | Matches | | |
|  |  |  | Sequence | Motif | Type |
| AACAUUC | Conserved | Human (Homo sapiens)  Chimp (Pan troglodytes)  Dog (Canis lupus familiaris)  Cow (Bos taurus) | HUMAN | TAATGTTT | 6mer |
|  |  |  | PIG | TAATGTTT | 6mer |
|  |  |  | PIG | TAATGTTT | 6mer |
|  |  |  | COW | TAATGTTT | 6mer |
|  |  |  | COW | TAATGTTT | 6mer |
|  |  |  | DOG | TAATGTTT | 6mer |

  
  
  
  

| Seed Matches to the miRNA miR-488-3p | | | | | |
| --- | --- | --- | --- | --- | --- |
| Seed | Conservation | Species | Matches | | |
|  |  |  | Sequence | Motif | Type |
| UGAAAGG | Conserved | Human (Homo sapiens)  Rhesus (Macaca mulatta)  Mouse (Mus musculus)  Rat (Rattus norvegicus) | HUMAN | AGAAAGTAACTAAAAGCCTTCCTTTCACAGTTTCTGGCATC | 7mer-m8 |
|  |  |  | PIG | AGAAAGTAACTAAAAGCCTTCCTTTCACAGTTTCTGGCATC | 7mer-m8 |

  
  
  
  

| Seed Matches to the miRNA miR-199-3p | | | | | |
| --- | --- | --- | --- | --- | --- |
| Seed | Conservation | Species | Matches | | |
|  |  |  | Sequence | Motif | Type |
| CAGUAGU | Broadly Conserved | Human (Homo sapiens)  Rhesus (Macaca mulatta)  Cow (Bos taurus)  Mouse (Mus musculus)  Rat (Rattus norvegicus)  Opossum (Monodelphis domestica) | HUMAN | ACTACCACTACTGAT | 8mer |
|  |  |  | HUMAN | CACTACTGAT | 8mer |
|  |  |  | HUMAN | CTACTGAT | 8mer |
|  |  |  | PIG | ACTACCACTACTGAT | 8mer |
|  |  |  | PIG | CACTACTGAT | 8mer |
|  |  |  | PIG | CTACTGAT | 8mer |
|  |  |  | COW | CACTACTGAT | 8mer |
|  |  |  | COW | CTACTGAT | 8mer |
|  |  |  | DOG | CTACTGAT | 7mer-A1 |

  
  
  
  

| Seed Matches to the miRNA miR-544a-5p | | | | | |
| --- | --- | --- | --- | --- | --- |
| Seed | Conservation | Species | Matches | | |
|  |  |  | Sequence | Motif | Type |
| CUUGUUA | Conserved | Human (Homo sapiens) | HUMAN | AAACAAGAATAAGAGAACAT | 7mer-A1 |
|  |  |  | HUMAN | AAACAAGAATAA | 7mer-A1 |
|  |  |  | PIG | AAACAAGAATAAGAGAACAT | 7mer-A1 |
|  |  |  | PIG | AAACAAGAATAA | 7mer-A1 |
|  |  |  | COW | AAACAAGAATAA | 7mer-A1 |

  
  
  
  
