# Supplementary material for: Uncovering deeply conserved motif combinations in rapidly evolving noncoding sequences
Source: Genome Biol. 2021 Jan 11;22:29. doi: 10.1186/s13059-020-02247-1 (PMC7798263; doi:10.1186/s13059-020-02247-1)
Supplement: Supplementary file 4 — Additional file 4. LncLOOM output results for XIST sequences from six mammals. [file 13059_2020_2247_MOESM4_ESM.gz › AdditionalFile4/Html_Files/kmers_in_seqs.html]

 MOTIFS\_IN\_SEQS\_OVERLAP

# MOTIFS IN SEQUENCES

  

NAVIGATE ▼

▶HUMAN (depth:1)▶PIG (depth:2)▶COW (depth:3)▶DOG (depth:4)▶RABBIT (depth:5)▶MOUSE (depth:6)

  
  
  
  
Coloured by Conservation >>  

## >HUMAN (19280 bases)

```
 CCTTCAGTTCTTAAAGCGCTGCAATTCGCTGCTGCAGCCATATTTCTTACTCTCTCGGGGC

TGGAAGCTT

TGGAAGCTT  
Depth:2 (PIG)  
Ei-value:0.000, Pi-value:0.000  
Er-value:0.000, Pr-value:0.000  
eCLIP MATCHES▶DGCR8 (bg=1.84%)▶HNRNPC (bg=3.65%)▶LSM11 (bg=2.28%)▶NCBP2 (bg=1.49%)▶RBM15 (bg=7.27%)▶RBM22 (bg=4.62%)▶SLTM (bg=2.2%)▶SRSF1 (bg=8.47%)▶uchl5 (bg=11.16%)▶YWHAG (bg=1.87%)No matches to TargetScan

CCTGACTGAA

GATCTCT

GATCTCT  
Depth:2 (PIG)  
Ei-value:0.000, Pi-value:0.010  
Er-value:0.000, Pr-value:0.000  
eCLIP MATCHES▶DGCR8 (bg=1.84%)▶HNRNPC (bg=3.65%)▶LSM11 (bg=2.28%)▶NCBP2 (bg=1.49%)▶RBM15 (bg=7.27%)▶RBM22 (bg=4.62%)▶SLTM (bg=2.2%)▶SRSF1 (bg=8.47%)▶uchl5 (bg=11.16%)▶YWHAG (bg=1.87%)No matches to TargetScan

CTGCACTTGG

GGTTCTTTCT

GGTTCTTTCT  
Depth:2 (PIG)  
Ei-value:0.000, Pi-value:0.000  
Er-value:0.000, Pr-value:0.000  
eCLIP MATCHES▶DGCR8 (bg=1.84%)▶LSM11 (bg=2.28%)▶SRSF1 (bg=8.47%)MATCHES To TargetScan▶ miR-186-5p:AAAGAAU

A

GAACATTTTC

GAACATTTTC  
Depth:2 (PIG)  
Ei-value:0.000, Pi-value:0.000  
Er-value:0.000, Pr-value:0.000  
eCLIP MATCHES▶LSM11 (bg=2.28%)▶RBM15 (bg=7.27%)▶RBM22 (bg=4.62%)▶SRSF1 (bg=8.47%)▶uchl5 (bg=11.16%)MATCHES To TargetScan▶ miR-409-3p:AAUGUUG

TA 120  
 GTCCCCCAACACCCT

TTATGGC

TTATGGC  
Depth:2 (PIG)  
Ei-value:0.000, Pi-value:0.000  
Er-value:0.000, Pr-value:0.010  
eCLIP MATCHES▶HNRNPC (bg=3.65%)▶LSM11 (bg=2.28%)▶RBM15 (bg=7.27%)▶RBM22 (bg=4.62%)▶SRSF1 (bg=8.47%)▶uchl5 (bg=11.16%)No matches to TargetScan

G

TATTTCTTTAAAAAAA

TATTTCTTTAAAAAAA  
Depth:2 (PIG)  
Ei-value:0.000, Pi-value:0.000  
Er-value:0.000, Pr-value:0.000  
eCLIP MATCHES▶HNRNPC (bg=3.65%)▶LSM11 (bg=2.28%)▶RBM15 (bg=7.27%)▶RBM22 (bg=4.62%)▶SRSF1 (bg=8.47%)▶uchl5 (bg=11.16%)MATCHES To TargetScan▶ miR-186-5p:AAAGAAU

TCACCTAAATTC

CATAAAAT

CATAAAAT  
Depth:2 (PIG)  
Ei-value:0.000, Pi-value:0.000  
Er-value:0.000, Pr-value:0.000  
No matches to eCLIP DataNo matches to TargetScan

ATTTTTTTAAATTCTAT

ACTTTCTCCTA

ACTTTCTCCTA  
Depth:2 (PIG)  
Ei-value:0.000, Pi-value:0.000  
Er-value:0.000, Pr-value:0.000  
eCLIP MATCHES▶DGCR8 (bg=1.84%)▶HNRNPC (bg=3.65%)▶LSM11 (bg=2.28%)▶RBFOX2 (bg=4.63%)▶RBM15 (bg=7.27%)▶RBM22 (bg=4.62%)▶SRSF1 (bg=8.47%)▶uchl5 (bg=11.16%)▶YWHAG (bg=1.87%)No matches to TargetScan

GTGTC

TTCTTGACAC

TTCTTGACAC  
Depth:2 (PIG)  
Ei-value:0.000, Pi-value:0.000  
Er-value:0.000, Pr-value:0.000  
eCLIP MATCHES▶DGCR8 (bg=1.84%)▶HNRNPC (bg=3.65%)▶LSM11 (bg=2.28%)▶RBFOX2 (bg=4.63%)▶RBM15 (bg=7.27%)▶RBM22 (bg=4.62%)▶SRSF1 (bg=8.47%)▶uchl5 (bg=11.16%)▶YWHAG (bg=1.87%)No matches to TargetScan

GTCCTCCATATTTTTTTA 240  
 AAGAAAG

TATTTGG

TATTTGG  
Depth:2 (PIG)  
Ei-value:0.000, Pi-value:0.010  
Er-value:0.000, Pr-value:0.000  
No matches to eCLIP DataNo matches to TargetScan

AATATTTTGAGGCAATTTTTAATA

TTTAAGG

TTTAAGG  
Depth:2 (PIG)  
Ei-value:0.000, Pi-value:0.000  
Er-value:0.000, Pr-value:0.010  
eCLIP MATCHES▶HNRNPC (bg=3.65%)No matches to TargetScan


AATTTTTCTTTGGAAT

AATTTTTCTTTGGAAT  
Depth:2 (PIG)  
Ei-value:0.000, Pi-value:0.000  
Er-value:0.000, Pr-value:0.000  
eCLIP MATCHES▶DGCR8 (bg=1.84%)▶HNRNPC (bg=3.65%)▶PUS1 (bg=1.04%)▶RBM15 (bg=7.27%)▶SRSF1 (bg=8.47%)▶uchl5 (bg=11.16%)▶UTP18 (bg=0.72%)MATCHES To TargetScan▶ miR-186-5p:AAAGAAU

CA

TTTTTGGTTGAC

TTTTTGGTTGAC  
Depth:2 (PIG)  
Ei-value:0.000, Pi-value:0.000  
Er-value:0.000, Pr-value:0.000  
eCLIP MATCHES▶DGCR8 (bg=1.84%)▶HNRNPC (bg=3.65%)▶NIPBL (bg=5.39%)▶PUS1 (bg=1.04%)▶RBM15 (bg=7.27%)▶SDAD1 (bg=2.97%)▶SRSF1 (bg=8.47%)▶uchl5 (bg=11.16%)▶UTP18 (bg=0.72%)MATCHES To TargetScan▶ miR-505-3p.1:GUCAACA

ATCTCT

GTTTTTT

GTTTTTT  
Depth:2 (PIG)  
Ei-value:0.000, Pi-value:0.020  
Er-value:0.000, Pr-value:0.020  
eCLIP MATCHES▶DGCR8 (bg=1.84%)▶HNRNPC (bg=3.65%)▶NIPBL (bg=5.39%)▶PUS1 (bg=1.04%)▶RBM15 (bg=7.27%)▶SDAD1 (bg=2.97%)▶SRSF1 (bg=8.47%)▶uchl5 (bg=11.16%)▶UTP18 (bg=0.72%)▶YWHAG (bg=1.87%)No matches to TargetScan

GTGGATCA

GTTTTTT

GTTTTTT  
Depth:2 (PIG)  
Ei-value:0.000, Pi-value:0.020  
Er-value:0.000, Pr-value:0.020  
eCLIP MATCHES▶DGCR8 (bg=1.84%)▶HNRNPC (bg=3.65%)▶NIPBL (bg=5.39%)▶RBM15 (bg=7.27%)▶RBM22 (bg=4.62%)▶SDAD1 (bg=2.97%)▶SRSF1 (bg=8.47%)▶uchl5 (bg=11.16%)▶UTP18 (bg=0.72%)▶YWHAG (bg=1.87%)No matches to TargetScan

ACTCTTCCACTCTCTTT 360  
 TCTATATTT

TGCCCATCGGGGCTG

TGCCCATCGGGGCTG  
Depth:2 (PIG)  
Ei-value:0.000, Pi-value:0.000  
Er-value:0.000, Pr-value:0.000  
eCLIP MATCHES▶DDX51 (bg=1.63%)▶HNRNPC (bg=3.65%)▶NIPBL (bg=5.39%)▶RBM15 (bg=7.27%)▶SDAD1 (bg=2.97%)▶SRSF1 (bg=8.47%)▶U2AF1 (bg=1.17%)▶uchl5 (bg=11.16%)No matches to TargetScan

C

GGATACCTGGTTTTA

GGATACCTGGTTTTA  
Depth:2 (PIG)  
Ei-value:0.000, Pi-value:0.000  
Er-value:0.000, Pr-value:0.000  
eCLIP MATCHES▶DDX51 (bg=1.63%)▶HNRNPC (bg=3.65%)▶NIPBL (bg=5.39%)▶RBM15 (bg=7.27%)▶SDAD1 (bg=2.97%)▶SRSF1 (bg=8.47%)▶U2AF1 (bg=1.17%)▶uchl5 (bg=11.16%)No matches to TargetScan


TTATTTT

TTATTTT  
Depth:2 (PIG)  
Ei-value:0.000, Pi-value:0.010  
Er-value:0.000, Pr-value:0.010  
eCLIP MATCHES▶DDX51 (bg=1.63%)▶HNRNPC (bg=3.65%)▶NIPBL (bg=5.39%)▶RBM15 (bg=7.27%)▶SDAD1 (bg=2.97%)▶SRSF1 (bg=8.47%)▶U2AF1 (bg=1.17%)No matches to TargetScan

TTC

TTTGCCCAACGGGGCCGTGGATACCTGCCTTTTAATTCTTTTTT

TTTGCCCAACGGGGCCGTGGATACCTGCCTTTTAATTCTTTTTT  
Depth:2 (PIG)  
Ei-value:0.000, Pi-value:0.000  
Er-value:0.000, Pr-value:0.000  
eCLIP MATCHES▶DDX51 (bg=1.63%)▶HNRNPC (bg=3.65%)▶NIPBL (bg=5.39%)▶RBM15 (bg=7.27%)▶SDAD1 (bg=2.97%)▶SRSF1 (bg=8.47%)▶U2AF1 (bg=1.17%)▶uchl5 (bg=11.16%)MATCHES To TargetScan▶ miR-124-3p.1:AAGGCAC▶ miR-186-5p:AAAGAAU

ATTC

GCCCATCGGGGCCGCGGATACC

GCCCATCGGGGCCGCGGATACCTGCTTTT  
Depth:2 (PIG)  
Ei-value:0.000, Pi-value:0.000  
Er-value:0.000, Pr-value:0.000  
eCLIP MATCHES▶DDX51 (bg=1.63%)▶HNRNPC (bg=3.65%)▶NIPBL (bg=5.39%)▶RBM15 (bg=7.27%)▶SDAD1 (bg=2.97%)▶SRSF1 (bg=8.47%)▶U2AF1 (bg=1.17%)▶uchl5 (bg=11.16%)MATCHES To TargetScan▶ miR-330-3p.2:AAAGCAC

 480  


TGCTTTT

GCCCATCGGGGCCGCGGATACCTGCTTTT  
Depth:2 (PIG)  
Ei-value:0.000, Pi-value:0.000  
Er-value:0.000, Pr-value:0.000  
eCLIP MATCHES▶DDX51 (bg=1.63%)▶HNRNPC (bg=3.65%)▶NIPBL (bg=5.39%)▶RBM15 (bg=7.27%)▶SDAD1 (bg=2.97%)▶SRSF1 (bg=8.47%)▶U2AF1 (bg=1.17%)▶uchl5 (bg=11.16%)MATCHES To TargetScan▶ miR-330-3p.2:AAAGCAC

T

ATTTTTTTTT

ATTTTTTTTT  
Depth:2 (PIG)  
Ei-value:0.000, Pi-value:0.000  
Er-value:0.000, Pr-value:0.000  
eCLIP MATCHES▶DDX51 (bg=1.63%)▶HNRNPC (bg=3.65%)▶NIPBL (bg=5.39%)▶RBM15 (bg=7.27%)▶SDAD1 (bg=2.97%)▶SRSF1 (bg=8.47%)▶U2AF1 (bg=1.17%)▶uchl5 (bg=11.16%)▶YWHAG (bg=1.87%)No matches to TargetScan


CCTTAGCCCATCGGGG

CCTTAGCCCATCGGGG  
Depth:2 (PIG)  
Ei-value:0.000, Pi-value:0.000  
Er-value:0.000, Pr-value:0.000  
eCLIP MATCHES▶DDX51 (bg=1.63%)▶HNRNPC (bg=3.65%)▶NIPBL (bg=5.39%)▶RBM15 (bg=7.27%)▶SDAD1 (bg=2.97%)▶SRSF1 (bg=8.47%)▶uchl5 (bg=11.16%)▶YWHAG (bg=1.87%)No matches to TargetScan

TA

TCGGATACCTGCTG

TCGGATACCTGCTG  
Depth:2 (PIG)  
Ei-value:0.000, Pi-value:0.000  
Er-value:0.000, Pr-value:0.000  
eCLIP MATCHES▶DDX51 (bg=1.63%)▶HNRNPC (bg=3.65%)▶NIPBL (bg=5.39%)▶RBM15 (bg=7.27%)▶SDAD1 (bg=2.97%)▶SRSF1 (bg=8.47%)▶uchl5 (bg=11.16%)▶YWHAG (bg=1.87%)No matches to TargetScan

ATTCCCTT

CCCCTCT

CCCCTCT  
Depth:2 (PIG)  
Ei-value:0.000, Pi-value:0.000  
Er-value:0.000, Pr-value:0.010  
eCLIP MATCHES▶HNRNPC (bg=3.65%)▶NIPBL (bg=5.39%)▶RBM15 (bg=7.27%)▶SDAD1 (bg=2.97%)▶SRSF1 (bg=8.47%)▶uchl5 (bg=11.16%)MATCHES To TargetScan▶ miR-423-5p:GAGGGGC

G

AACCCC

AACCCC  
Depth:2 (PIG)  
Ei-value:0.000, Pi-value:0.010  
Er-value:0.000, Pr-value:0.010  
eCLIP MATCHES▶HNRNPC (bg=3.65%)▶NIPBL (bg=5.39%)▶RBM15 (bg=7.27%)▶SDAD1 (bg=2.97%)▶SRSF1 (bg=8.47%)▶uchl5 (bg=11.16%)No matches to TargetScan

CAACACTC

TGGCCCATC

TGGCCCATC  
Depth:2 (PIG)  
Ei-value:0.000, Pi-value:0.000  
Er-value:0.000, Pr-value:0.000  
eCLIP MATCHES▶HNRNPC (bg=3.65%)▶NIPBL (bg=5.39%)▶RBM15 (bg=7.27%)▶SDAD1 (bg=2.97%)▶SRSF1 (bg=8.47%)No matches to TargetScan

GGGGTGACGGATAT

CTGCTTTTT

CTGCTTTTT  
Depth:2 (PIG)  
Ei-value:0.000, Pi-value:0.000  
Er-value:0.000, Pr-value:0.000  
eCLIP MATCHES▶CPEB4 (bg=1.89%)▶RBM15 (bg=7.27%)MATCHES To TargetScan▶ miR-330-3p.2:AAAGCAC

AAAAATTT 600  
 TC

TTTTTTTGGCCCATCGGGGC

TTTTTTTGGCCCATCGGGGC  
Depth:2 (PIG)  
Ei-value:0.000, Pi-value:0.000  
Er-value:0.000, Pr-value:0.000  
eCLIP MATCHES▶FASTKD2 (bg=1.99%)▶HNRNPC (bg=3.65%)▶RBM15 (bg=7.27%)▶SRSF1 (bg=8.47%)▶U2AF1 (bg=1.17%)▶YWHAG (bg=1.87%)No matches to TargetScan

T

TCGGATACCTGCTTT

TCGGATACCTGCTTT  
Depth:2 (PIG)  
Ei-value:0.000, Pi-value:0.000  
Er-value:0.000, Pr-value:0.000  
eCLIP MATCHES▶HNRNPC (bg=3.65%)▶RBM15 (bg=7.27%)▶SRSF1 (bg=8.47%)▶U2AF1 (bg=1.17%)MATCHES To TargetScan▶ miR-330-3p.2:AAAGCAC

TTTTTTTTTTA

TTTTTCCTTGCCCATCGGGGCCTCGGATACCTGCTTTA

TTTTTCCTTGCCCATCGGGGCCTCGGATACCTGCTTTA  
Depth:2 (PIG)  
Ei-value:0.000, Pi-value:0.000  
Er-value:0.000, Pr-value:0.000  
eCLIP MATCHES▶AARS (bg=2.18%)▶AKAP1 (bg=0.21%)▶HNRNPC (bg=3.65%)▶NIPBL (bg=5.39%)▶RBM15 (bg=7.27%)▶SDAD1 (bg=2.97%)▶SRSF1 (bg=8.47%)▶uchl5 (bg=11.16%)MATCHES To TargetScan▶ miR-31-5p:GGCAAGA▶ miR-330-3p.2:AAAGCAC

ATTTTTGTTTTTCTG

GCCCATCGGGGCCG

GCCCATCGGGGCCG  
Depth:2 (PIG)  
Ei-value:0.000, Pi-value:0.000  
Er-value:0.000, Pr-value:0.000  
eCLIP MATCHES▶AATF (bg=0.64%)▶HNRNPC (bg=3.65%)▶LSM11 (bg=2.28%)▶NIPBL (bg=5.39%)▶RBM15 (bg=7.27%)▶SDAD1 (bg=2.97%)▶SRSF1 (bg=8.47%)▶U2AF1 (bg=1.17%)No matches to TargetScan

C

GGA

GGATACCTGCTT  
Depth:2 (PIG)  
Ei-value:0.000, Pi-value:0.000  
Er-value:0.000, Pr-value:0.000  
eCLIP MATCHES▶AATF (bg=0.64%)▶HNRNPC (bg=3.65%)▶LSM11 (bg=2.28%)▶NIPBL (bg=5.39%)▶RBM15 (bg=7.27%)▶SRSF1 (bg=8.47%)▶U2AF1 (bg=1.17%)No matches to TargetScan

 720  


TACCTGCTT

GGATACCTGCTT  
Depth:2 (PIG)  
Ei-value:0.000, Pi-value:0.000  
Er-value:0.000, Pr-value:0.000  
eCLIP MATCHES▶AATF (bg=0.64%)▶HNRNPC (bg=3.65%)▶LSM11 (bg=2.28%)▶NIPBL (bg=5.39%)▶RBM15 (bg=7.27%)▶SRSF1 (bg=8.47%)▶U2AF1 (bg=1.17%)No matches to TargetScan

T

GATTTTTTTTTTTCATC

GATTTTTTTTTTTCATC  
Depth:2 (PIG)  
Ei-value:0.000, Pi-value:0.000  
Er-value:0.000, Pr-value:0.000  
eCLIP MATCHES▶HNRNPC (bg=3.65%)▶NIPBL (bg=5.39%)▶RBM15 (bg=7.27%)▶SDAD1 (bg=2.97%)▶SRSF1 (bg=8.47%)▶U2AF1 (bg=1.17%)▶UTP3 (bg=3.66%)No matches to TargetScan

G

CCCATCGG

CCCATCGG  
Depth:2 (PIG)  
Ei-value:0.000, Pi-value:0.000  
Er-value:0.000, Pr-value:0.000  
eCLIP MATCHES▶HNRNPC (bg=3.65%)▶NIPBL (bg=5.39%)▶RBM15 (bg=7.27%)▶SDAD1 (bg=2.97%)▶SRSF1 (bg=8.47%)▶UTP3 (bg=3.66%)▶YWHAG (bg=1.87%)No matches to TargetScan

TGCTTTT

TATGGATG

TATGGATG  
Depth:2 (PIG)  
Ei-value:0.000, Pi-value:0.000  
Er-value:0.000, Pr-value:0.000  
eCLIP MATCHES▶HNRNPC (bg=3.65%)▶NIPBL (bg=5.39%)▶RBM15 (bg=7.27%)▶SDAD1 (bg=2.97%)▶SRSF1 (bg=8.47%)▶uchl5 (bg=11.16%)▶UTP3 (bg=3.66%)▶YWHAG (bg=1.87%)No matches to TargetScan

AAAAAATGTT

GGTTTTGTGG

GGTTTTGTGG  
Depth:2 (PIG)  
Ei-value:0.000, Pi-value:0.000  
Er-value:0.000, Pr-value:0.000  
eCLIP MATCHES▶DGCR8 (bg=1.84%)▶EXOSC5 (bg=5.38%)▶GTF2F1 (bg=0.51%)▶NIPBL (bg=5.39%)▶RBM15 (bg=7.27%)▶SRSF1 (bg=8.47%)▶uchl5 (bg=11.16%)▶YWHAG (bg=1.87%)No matches to TargetScan

GTTGTTGCACTC

TCTGGAAT

TCTGGAAT  
Depth:2 (PIG)  
Ei-value:0.000, Pi-value:0.000  
Er-value:0.000, Pr-value:0.000  
eCLIP MATCHES▶DGCR8 (bg=1.84%)▶EXOSC5 (bg=5.38%)▶GTF2F1 (bg=0.51%)▶NIPBL (bg=5.39%)▶RBM15 (bg=7.27%)▶RBM22 (bg=4.62%)▶SDAD1 (bg=2.97%)▶SRSF1 (bg=8.47%)▶uchl5 (bg=11.16%)▶YWHAG (bg=1.87%)No matches to TargetScan

A

TCTACA

TCTACA  
Depth:2 (PIG)  
Ei-value:0.000, Pi-value:0.000  
Er-value:0.000, Pr-value:0.010  
eCLIP MATCHES▶DGCR8 (bg=1.84%)▶NIPBL (bg=5.39%)▶RBM15 (bg=7.27%)▶RBM22 (bg=4.62%)▶SDAD1 (bg=2.97%)▶SRSF1 (bg=8.47%)▶YWHAG (bg=1.87%)No matches to TargetScan

CTTTT

TTTTGCTGCT

TTTTGCTGCT  
Depth:2 (PIG)  
Ei-value:0.000, Pi-value:0.000  
Er-value:0.000, Pr-value:0.010  
eCLIP MATCHES▶DDX52 (bg=0.46%)▶DGCR8 (bg=1.84%)▶EXOSC5 (bg=5.38%)▶NCBP2 (bg=1.49%)▶NIPBL (bg=5.39%)▶RBM15 (bg=7.27%)▶RBM22 (bg=4.62%)▶SDAD1 (bg=2.97%)▶SRSF1 (bg=8.47%)▶uchl5 (bg=11.16%)▶WDR3 (bg=0.25%)▶YWHAG (bg=1.87%)MATCHES To TargetScan▶ miR-103-3p/107:GCAGCAU▶ miR-15-5p/16-5p/195-5p/424-5p/497-5p:AGCAGCA▶ miR-503-5p:AGCAGCG

GATCA

TT

TTTGGTG  
Depth:2 (PIG)  
Ei-value:0.000, Pi-value:0.000  
Er-value:0.000, Pr-value:0.010  
eCLIP MATCHES▶DDX52 (bg=0.46%)▶DGCR8 (bg=1.84%)▶EXOSC5 (bg=5.38%)▶NCBP2 (bg=1.49%)▶NIPBL (bg=5.39%)▶RBM15 (bg=7.27%)▶RBM22 (bg=4.62%)▶SDAD1 (bg=2.97%)▶SLTM (bg=2.2%)▶SRSF1 (bg=8.47%)▶uchl5 (bg=11.16%)▶UTP3 (bg=3.66%)▶WDR3 (bg=0.25%)▶YWHAG (bg=1.87%)No matches to TargetScan

 840  


TGGTG

TTTGGTG  
Depth:2 (PIG)  
Ei-value:0.000, Pi-value:0.000  
Er-value:0.000, Pr-value:0.010  
eCLIP MATCHES▶DDX52 (bg=0.46%)▶DGCR8 (bg=1.84%)▶EXOSC5 (bg=5.38%)▶NCBP2 (bg=1.49%)▶NIPBL (bg=5.39%)▶RBM15 (bg=7.27%)▶RBM22 (bg=4.62%)▶SDAD1 (bg=2.97%)▶SLTM (bg=2.2%)▶SRSF1 (bg=8.47%)▶uchl5 (bg=11.16%)▶UTP3 (bg=3.66%)▶WDR3 (bg=0.25%)▶YWHAG (bg=1.87%)No matches to TargetScan

G

TGTGTGAGTG

TGTGTGAGTG  
Depth:2 (PIG)  
Ei-value:0.000, Pi-value:0.000  
Er-value:0.000, Pr-value:0.000  
eCLIP MATCHES▶DDX52 (bg=0.46%)▶DGCR8 (bg=1.84%)▶EXOSC5 (bg=5.38%)▶NCBP2 (bg=1.49%)▶NIPBL (bg=5.39%)▶RBM15 (bg=7.27%)▶RBM22 (bg=4.62%)▶SDAD1 (bg=2.97%)▶SLTM (bg=2.2%)▶SRSF1 (bg=8.47%)▶uchl5 (bg=11.16%)▶UTP3 (bg=3.66%)▶WDR3 (bg=0.25%)▶YWHAG (bg=1.87%)MATCHES To TargetScan▶ miR-342-3p:CUCACAC▶ miR-377-3p:UCACACA

TACCTACC

GCTTTGG

GCTTTGG  
Depth:2 (PIG)  
Ei-value:0.000, Pi-value:0.000  
Er-value:0.000, Pr-value:0.010  
eCLIP MATCHES▶DGCR8 (bg=1.84%)▶EXOSC5 (bg=5.38%)▶NCBP2 (bg=1.49%)▶NIPBL (bg=5.39%)▶RBM15 (bg=7.27%)▶RBM22 (bg=4.62%)▶SLTM (bg=2.2%)▶SRSF1 (bg=8.47%)▶uchl5 (bg=11.16%)▶YWHAG (bg=1.87%)MATCHES To TargetScan▶ miR-330-3p:CAAAGCA

CAGAGAATGACTC

TGCAGTTA

TGCAGTTA  
Depth:2 (PIG)  
Ei-value:0.000, Pi-value:0.000  
Er-value:0.000, Pr-value:0.000  
eCLIP MATCHES▶DGCR8 (bg=1.84%)▶NIPBL (bg=5.39%)▶RBM15 (bg=7.27%)▶RBM22 (bg=4.62%)▶SF3B1 (bg=2.48%)▶SLTM (bg=2.2%)▶SRSF1 (bg=8.47%)▶uchl5 (bg=11.16%)MATCHES To TargetScan▶ miR-217:ACUGCAU

AGCTAAGGGCGTGTTCAGATTGT

GGAGGAAA

GGAGGAAA  
Depth:2 (PIG)  
Ei-value:0.000, Pi-value:0.000  
Er-value:0.000, Pr-value:0.000  
eCLIP MATCHES▶DDX51 (bg=1.63%)▶DHX30 (bg=0.14%)▶EXOSC5 (bg=5.38%)▶HNRNPM (bg=4.29%)▶NIPBL (bg=5.39%)▶RBM15 (bg=7.27%)▶SF3B1 (bg=2.48%)▶SLTM (bg=2.2%)▶uchl5 (bg=11.16%)MATCHES To TargetScan▶ miR-670-3p:UUCCUCA

AGTGGCCGCCATTTTAGAC

TTGCCGC

TTGCCGC  
Depth:2 (PIG)  
Ei-value:0.000, Pi-value:0.000  
Er-value:0.000, Pr-value:0.010  
eCLIP MATCHES▶EXOSC5 (bg=5.38%)▶HNRNPM (bg=4.29%)▶NIPBL (bg=5.39%)▶RBM15 (bg=7.27%)▶RBM22 (bg=4.62%)▶uchl5 (bg=11.16%)No matches to TargetScan

ATAA

CTCGGCT

CTCGGCT  
Depth:2 (PIG)  
Ei-value:0.000, Pi-value:0.000  
Er-value:0.000, Pr-value:0.000  
eCLIP MATCHES▶EXOSC5 (bg=5.38%)▶NIPBL (bg=5.39%)▶RBM15 (bg=7.27%)▶RBM22 (bg=4.62%)▶SDAD1 (bg=2.97%)▶uchl5 (bg=11.16%)No matches to TargetScan

 960  


CTCGGCT  
Depth:2 (PIG)  
Ei-value:0.000, Pi-value:0.000  
Er-value:0.000, Pr-value:0.000  
eCLIP MATCHES▶EXOSC5 (bg=5.38%)▶NIPBL (bg=5.39%)▶RBM15 (bg=7.27%)▶RBM22 (bg=4.62%)▶SDAD1 (bg=2.97%)▶uchl5 (bg=11.16%)No matches to TargetScan

T

AGGGCTA

AGGGCTA  
Depth:2 (PIG)  
Ei-value:0.000, Pi-value:0.000  
Er-value:0.000, Pr-value:0.010  
eCLIP MATCHES▶EXOSC5 (bg=5.38%)▶HNRNPA1 (bg=2.57%)▶NIPBL (bg=5.39%)▶RBM15 (bg=7.27%)▶RBM22 (bg=4.62%)▶SDAD1 (bg=2.97%)▶uchl5 (bg=11.16%)MATCHES To TargetScan▶ miR-129-3p:AGCCCUU

GTCGTTTG

TGCTAAGT

TGCTAAGT  
Depth:2 (PIG)  
Ei-value:0.000, Pi-value:0.000  
Er-value:0.000, Pr-value:0.000  
eCLIP MATCHES▶AARS (bg=2.18%)▶EXOSC5 (bg=5.38%)▶HNRNPA1 (bg=2.57%)▶NIPBL (bg=5.39%)▶RBM15 (bg=7.27%)▶RBM22 (bg=4.62%)▶SDAD1 (bg=2.97%)▶uchl5 (bg=11.16%)No matches to TargetScan


TAAACTAGGG

TAAACTAGGGAGGCAAGATG  
Depth:2 (PIG)  
Ei-value:0.000, Pi-value:0.000  
Er-value:0.000, Pr-value:0.000  
eCLIP MATCHES▶AARS (bg=2.18%)▶EXOSC5 (bg=5.38%)▶HNRNPA1 (bg=2.57%)▶NIPBL (bg=5.39%)▶RBM15 (bg=7.27%)▶RBM22 (bg=4.62%)▶SDAD1 (bg=2.97%)▶uchl5 (bg=11.16%)No matches to TargetScan


AGGCAAGA

AGGCAAGA  
Depth:3 (COW)  
Ei-value:0.000, Pi-value:0.000  
Er-value:0.000, Pr-value:0.000  
eCLIP MATCHES▶AARS (bg=2.18%)▶EXOSC5 (bg=5.38%)▶HNRNPA1 (bg=2.57%)▶NIPBL (bg=5.39%)▶RBM15 (bg=7.27%)▶RBM22 (bg=4.62%)▶SDAD1 (bg=2.97%)▶uchl5 (bg=11.16%)No matches to TargetScan


TG

TAAACTAGGGAGGCAAGATG  
Depth:2 (PIG)  
Ei-value:0.000, Pi-value:0.000  
Er-value:0.000, Pr-value:0.000  
eCLIP MATCHES▶AARS (bg=2.18%)▶EXOSC5 (bg=5.38%)▶HNRNPA1 (bg=2.57%)▶NIPBL (bg=5.39%)▶RBM15 (bg=7.27%)▶RBM22 (bg=4.62%)▶SDAD1 (bg=2.97%)▶uchl5 (bg=11.16%)No matches to TargetScan

GATGATAGCAGGT

CAGGCAGAGGAA

CAGGCAGAGGAA  
Depth:2 (PIG)  
Ei-value:0.000, Pi-value:0.000  
Er-value:0.000, Pr-value:0.000  
eCLIP MATCHES▶AARS (bg=2.18%)▶EXOSC5 (bg=5.38%)▶HNRNPA1 (bg=2.57%)▶HNRNPM (bg=4.29%)▶NIPBL (bg=5.39%)▶RBM15 (bg=7.27%)▶RBM22 (bg=4.62%)▶SDAD1 (bg=2.97%)▶uchl5 (bg=11.16%)MATCHES To TargetScan▶ miR-670-3p:UUCCUCA

GTCATG

TGCATTG

TGCATTG  
Depth:2 (PIG)  
Ei-value:0.000, Pi-value:0.010  
Er-value:0.000, Pr-value:0.020  
eCLIP MATCHES▶EXOSC5 (bg=5.38%)▶HNRNPA1 (bg=2.57%)▶HNRNPM (bg=4.29%)▶RBM15 (bg=7.27%)▶RBM22 (bg=4.62%)▶uchl5 (bg=11.16%)No matches to TargetScan

C

ATGAGCTA

ATGAGCTA  
Depth:2 (PIG)  
Ei-value:0.000, Pi-value:0.000  
Er-value:0.000, Pr-value:0.000  
eCLIP MATCHES▶HNRNPA1 (bg=2.57%)▶HNRNPM (bg=4.29%)▶RBM22 (bg=4.62%)▶uchl5 (bg=11.16%)No matches to TargetScan

AACCTATCTGAATGAATT

GATTTGGG

GATTTGGG  
Depth:2 (PIG)  
Ei-value:0.000, Pi-value:0.000  
Er-value:0.000, Pr-value:0.000  
eCLIP MATCHES▶CPEB4 (bg=1.89%)▶EXOSC5 (bg=5.38%)▶GNL3 (bg=0.43%)▶HNRNPM (bg=4.29%)▶TARDBP (bg=2.79%)No matches to TargetScan

G

CT

CTTGTTAGGA  
Depth:2 (PIG)  
Ei-value:0.000, Pi-value:0.000  
Er-value:0.000, Pr-value:0.000  
eCLIP MATCHES▶CPEB4 (bg=1.89%)▶EXOSC5 (bg=5.38%)▶GNL3 (bg=0.43%)▶HNRNPM (bg=4.29%)▶TARDBP (bg=2.79%)▶uchl5 (bg=11.16%)No matches to TargetScan

 1080  


TGTTAGGA

CTTGTTAGGA  
Depth:2 (PIG)  
Ei-value:0.000, Pi-value:0.000  
Er-value:0.000, Pr-value:0.000  
eCLIP MATCHES▶CPEB4 (bg=1.89%)▶EXOSC5 (bg=5.38%)▶GNL3 (bg=0.43%)▶HNRNPM (bg=4.29%)▶TARDBP (bg=2.79%)▶uchl5 (bg=11.16%)No matches to TargetScan

GCTTTGCGTGATTGTTGTATCGGGAGGCAGTAAGAATCATCTTTTATCAGTACAAGGGACTAGTTAAAAATG

GAAGGTT

GAAGGTT  
Depth:2 (PIG)  
Ei-value:0.000, Pi-value:0.000  
Er-value:0.000, Pr-value:0.000  
eCLIP MATCHES▶EXOSC5 (bg=5.38%)▶NPM1 (bg=1.21%)▶RBFOX2 (bg=4.63%)▶RBM15 (bg=7.27%)▶RBM22 (bg=4.62%)▶uchl5 (bg=11.16%)No matches to TargetScan

AGGAAAGACTAAGGTGCAGGGCTT

AAAATGGCG

AAAATGGCGATTTTGAC  
Depth:2 (PIG)  
Ei-value:0.000, Pi-value:0.000  
Er-value:0.000, Pr-value:0.000  
eCLIP MATCHES▶EXOSC5 (bg=5.38%)▶GNL3 (bg=0.43%)▶NPM1 (bg=1.21%)▶RBFOX2 (bg=4.63%)▶RBM15 (bg=7.27%)▶RBM22 (bg=4.62%)▶uchl5 (bg=11.16%)No matches to TargetScan

 1200  


ATTTTGAC

AAAATGGCGATTTTGAC  
Depth:2 (PIG)  
Ei-value:0.000, Pi-value:0.000  
Er-value:0.000, Pr-value:0.000  
eCLIP MATCHES▶EXOSC5 (bg=5.38%)▶GNL3 (bg=0.43%)▶NPM1 (bg=1.21%)▶RBFOX2 (bg=4.63%)▶RBM15 (bg=7.27%)▶RBM22 (bg=4.62%)▶uchl5 (bg=11.16%)No matches to TargetScan

ATTGCG

GCATTGCT

GCATTGCT  
Depth:2 (PIG)  
Ei-value:0.000, Pi-value:0.000  
Er-value:0.000, Pr-value:0.000  
eCLIP MATCHES▶EXOSC5 (bg=5.38%)▶HNRNPM (bg=4.29%)▶RBFOX2 (bg=4.63%)▶RBM15 (bg=7.27%)▶RBM22 (bg=4.62%)▶uchl5 (bg=11.16%)No matches to TargetScan

C

AGCATGGC

AGCATGGC  
Depth:2 (PIG)  
Ei-value:0.000, Pi-value:0.000  
Er-value:0.000, Pr-value:0.010  
eCLIP MATCHES▶EXOSC5 (bg=5.38%)▶HNRNPM (bg=4.29%)▶RBFOX2 (bg=4.63%)▶RBM15 (bg=7.27%)▶RBM22 (bg=4.62%)▶uchl5 (bg=11.16%)No matches to TargetScan

GGGCTG

TGCTTTGTTAG

TGCTTTGTTAG  
Depth:2 (PIG)  
Ei-value:0.000, Pi-value:0.000  
Er-value:0.000, Pr-value:0.000  
eCLIP MATCHES▶EXOSC5 (bg=5.38%)▶HNRNPM (bg=4.29%)▶RBFOX2 (bg=4.63%)▶RBM15 (bg=7.27%)▶RBM22 (bg=4.62%)▶SDAD1 (bg=2.97%)▶uchl5 (bg=11.16%)MATCHES To TargetScan▶ miR-330-3p:CAAAGCA▶ miR-330-3p.2:AAAGCAC▶ miR-495-3p:AACAAAC

GTTGTC

CAAAATGGCGGA

CAAAATGGCGGA  
Depth:2 (PIG)  
Ei-value:0.000, Pi-value:0.000  
Er-value:0.000, Pr-value:0.000  
eCLIP MATCHES▶HNRNPM (bg=4.29%)▶RBFOX2 (bg=4.63%)▶RBM22 (bg=4.62%)▶uchl5 (bg=11.16%)No matches to TargetScan

TCCAGTTCTGT

CGCAGTGTTC

CGCAGTGTTC  
Depth:2 (PIG)  
Ei-value:0.000, Pi-value:0.000  
Er-value:0.000, Pr-value:0.000  
eCLIP MATCHES▶EXOSC5 (bg=5.38%)▶HNRNPM (bg=4.29%)▶RBM22 (bg=4.62%)MATCHES To TargetScan▶ miR-141-3p/200a-3p:AACACUG

A

AGTGGCGGGAAG

AGTGGCGGGAAG  
Depth:2 (PIG)  
Ei-value:0.000, Pi-value:0.000  
Er-value:0.000, Pr-value:0.000  
eCLIP MATCHES▶EXOSC5 (bg=5.38%)▶HNRNPM (bg=4.29%)▶LARP4 (bg=4.72%)▶RBM22 (bg=4.62%)No matches to TargetScan

G

CCACAT

CCACAT  
Depth:2 (PIG)  
Ei-value:0.000, Pi-value:0.030  
Er-value:0.000, Pr-value:0.020  
eCLIP MATCHES▶EXOSC5 (bg=5.38%)▶HNRNPM (bg=4.29%)▶LARP4 (bg=4.72%)▶RBM22 (bg=4.62%)▶SDAD1 (bg=2.97%)MATCHES To TargetScan▶ miR-299-3p:AUGUGGG

CATGATGGGCGAG 1320  
 GCTTTGTTAAGTGGTT

AGCATGG

AGCATGG  
Depth:2 (PIG)  
Ei-value:0.000, Pi-value:0.000  
Er-value:0.000, Pr-value:0.000  
eCLIP MATCHES▶AARS (bg=2.18%)▶EXOSC5 (bg=5.38%)▶HNRNPM (bg=4.29%)▶NIPBL (bg=5.39%)▶RBM15 (bg=7.27%)▶RBM22 (bg=4.62%)▶SDAD1 (bg=2.97%)▶uchl5 (bg=11.16%)No matches to TargetScan

TGGTGGACATGTGCGGTCACACAGG

AAAAGATGGCGGCT

AAAAGATGGCGGCT  
Depth:2 (PIG)  
Ei-value:0.000, Pi-value:0.000  
Er-value:0.000, Pr-value:0.000  
eCLIP MATCHES▶AARS (bg=2.18%)▶EXOSC5 (bg=5.38%)▶HNRNPM (bg=4.29%)▶NIPBL (bg=5.39%)▶RBM15 (bg=7.27%)▶RBM22 (bg=4.62%)▶uchl5 (bg=11.16%)No matches to TargetScan

GAAGGT

CTTGCCGCA

CTTGCCGCA  
Depth:2 (PIG)  
Ei-value:0.000, Pi-value:0.000  
Er-value:0.000, Pr-value:0.000  
eCLIP MATCHES▶AARS (bg=2.18%)▶EXOSC5 (bg=5.38%)▶HNRNPM (bg=4.29%)▶uchl5 (bg=11.16%)MATCHES To TargetScan▶ miR-31-5p:GGCAAGA

GTGT

A

AAAACATGGCGGGCCT  
Depth:2 (PIG)  
Ei-value:0.000, Pi-value:0.000  
Er-value:0.000, Pr-value:0.000  
eCLIP MATCHES▶HNRNPM (bg=4.29%)No matches to TargetScan


AAACATG

AAACATG  
Depth:4 (DOG)  
Ei-value:0.000, Pi-value:0.000  
Er-value:0.000, Pr-value:0.000  
eCLIP MATCHES▶HNRNPM (bg=4.29%)No matches to TargetScan


GCGGGCCT

AAAACATGGCGGGCCT  
Depth:2 (PIG)  
Ei-value:0.000, Pi-value:0.000  
Er-value:0.000, Pr-value:0.000  
eCLIP MATCHES▶HNRNPM (bg=4.29%)No matches to TargetScan

CT

TTGTCTTTGC

TTGTCTTTGC  
Depth:2 (PIG)  
Ei-value:0.000, Pi-value:0.000  
Er-value:0.000, Pr-value:0.000  
eCLIP MATCHES▶HNRNPM (bg=4.29%)No matches to TargetScan

TGTGTGCTTTT 1440  
 CGTGTTGGG

TTTTGCCGCAGGGACAATATGGC

TTTTGCCGCAGGGACAATATGGC  
Depth:2 (PIG)  
Ei-value:0.000, Pi-value:0.000  
Er-value:0.000, Pr-value:0.000  
eCLIP MATCHES▶AKAP8L (bg=2.19%)▶DDX51 (bg=1.63%)▶DDX52 (bg=0.46%)▶EXOSC5 (bg=5.38%)▶GNL3 (bg=0.43%)▶HNRNPM (bg=4.29%)▶HNRNPUL1 (bg=1.16%)▶LARP4 (bg=4.72%)▶METAP2 (bg=0.78%)▶NCBP2 (bg=1.49%)▶RBM22 (bg=4.62%)▶SDAD1 (bg=2.97%)▶SLTM (bg=2.2%)▶uchl5 (bg=11.16%)▶WRN (bg=0.77%)▶XRCC6 (bg=2.91%)No matches to TargetScan

AGGCG

TTGTCAT

TTGTCAT  
Depth:2 (PIG)  
Ei-value:0.000, Pi-value:0.020  
Er-value:0.000, Pr-value:0.040  
eCLIP MATCHES▶DDX51 (bg=1.63%)▶DDX52 (bg=0.46%)▶EXOSC5 (bg=5.38%)▶GNL3 (bg=0.43%)▶HNRNPM (bg=4.29%)▶HNRNPUL1 (bg=1.16%)▶METAP2 (bg=0.78%)▶NCBP2 (bg=1.49%)▶RBM22 (bg=4.62%)▶SLTM (bg=2.2%)▶uchl5 (bg=11.16%)▶WRN (bg=0.77%)▶XRCC6 (bg=2.91%)MATCHES To TargetScan▶ miR-425-5p:AUGACAC

ATGTATATCATGGCTTT

TGTCACGTGGAC

TGTCACGTGGAC  
Depth:2 (PIG)  
Ei-value:0.000, Pi-value:0.000  
Er-value:0.000, Pr-value:0.000  
eCLIP MATCHES▶DDX51 (bg=1.63%)▶EXOSC5 (bg=5.38%)▶HNRNPM (bg=4.29%)MATCHES To TargetScan▶ miR-542-3p:GUGACAG

ATCA

TGGCGGGCT

TGGCGGGCT  
Depth:2 (PIG)  
Ei-value:0.000, Pi-value:0.000  
Er-value:0.000, Pr-value:0.000  
eCLIP MATCHES▶DDX51 (bg=1.63%)▶EXOSC5 (bg=5.38%)▶HNRNPM (bg=4.29%)No matches to TargetScan


TGCCGCATTGTT

TGCCGCATTGTT  
Depth:2 (PIG)  
Ei-value:0.000, Pi-value:0.000  
Er-value:0.000, Pr-value:0.000  
eCLIP MATCHES▶DDX51 (bg=1.63%)▶EXOSC5 (bg=5.38%)▶HNRNPM (bg=4.29%)No matches to TargetScan

A

AAGATGGCGGG

AAGATGGCGGG  
Depth:2 (PIG)  
Ei-value:0.000, Pi-value:0.000  
Er-value:0.000, Pr-value:0.000  
eCLIP MATCHES▶HNRNPM (bg=4.29%)▶RBFOX2 (bg=4.63%)No matches to TargetScan

T

TTTGCCGC

TTTGCCGC  
Depth:2 (PIG)  
Ei-value:0.000, Pi-value:0.000  
Er-value:0.000, Pr-value:0.000  
eCLIP MATCHES▶EXOSC5 (bg=5.38%)▶HNRNPM (bg=4.29%)▶RBFOX2 (bg=4.63%)No matches to TargetScan

C 1560  
 TAGTGCCACGCAGAGCGGGAGAAAAGGTGGGATGGACAGTGC

TGGATTGC

TGGATTGC  
Depth:4 (DOG)  
Ei-value:0.000, Pi-value:0.000  
Er-value:0.000, Pr-value:0.000  
eCLIP MATCHES▶AARS (bg=2.18%)▶EXOSC5 (bg=5.38%)▶HNRNPM (bg=4.29%)▶RBFOX2 (bg=4.63%)▶RBM22 (bg=4.62%)▶SUPV3L1 (bg=1.57%)▶uchl5 (bg=11.16%)No matches to TargetScan

TGCATAACCCAACCAATTAGAAATGGG

GGTGGAATTG

GGTGGAATTG  
Depth:2 (PIG)  
Ei-value:0.000, Pi-value:0.000  
Er-value:0.000, Pr-value:0.000  
eCLIP MATCHES▶EXOSC5 (bg=5.38%)▶NPM1 (bg=1.21%)▶RBM22 (bg=4.62%)▶SDAD1 (bg=2.97%)▶SUPV3L1 (bg=1.57%)▶uchl5 (bg=11.16%)No matches to TargetScan

A

TCACAG

TCACAG  
Depth:2 (PIG)  
Ei-value:0.000, Pi-value:0.010  
Er-value:0.000, Pr-value:0.020  
eCLIP MATCHES▶EXOSC5 (bg=5.38%)▶NPM1 (bg=1.21%)▶RBM22 (bg=4.62%)▶SDAD1 (bg=2.97%)▶uchl5 (bg=11.16%)No matches to TargetScan

CCAATTAGAGCAGAA

GATGGAATTAG

GATGGAATTAG  
Depth:2 (PIG)  
Ei-value:0.000, Pi-value:0.000  
Er-value:0.000, Pr-value:0.000  
eCLIP MATCHES▶EXOSC5 (bg=5.38%)▶NPM1 (bg=1.21%)▶RBM22 (bg=4.62%)▶SDAD1 (bg=2.97%)▶uchl5 (bg=11.16%)No matches to TargetScan

 1680  


GATGGAATTAG  
Depth:2 (PIG)  
Ei-value:0.000, Pi-value:0.000  
Er-value:0.000, Pr-value:0.000  
eCLIP MATCHES▶EXOSC5 (bg=5.38%)▶NPM1 (bg=1.21%)▶RBM22 (bg=4.62%)▶SDAD1 (bg=2.97%)▶uchl5 (bg=11.16%)No matches to TargetScan

ACTGATGACACACTGTCCAGCTACTCAGCGAAGACCTGGGTGAA

TTAGCAT

TTAGCAT  
Depth:2 (PIG)  
Ei-value:0.000, Pi-value:0.020  
Er-value:0.000, Pr-value:0.010  
eCLIP MATCHES▶SLTM (bg=2.2%)No matches to TargetScan

GGCACTTCGCAGCTGTCTTT

AGCCAGTCAG

AGCCAGTCAG  
Depth:2 (PIG)  
Ei-value:0.000, Pi-value:0.000  
Er-value:0.000, Pr-value:0.000  
eCLIP MATCHES▶DDX51 (bg=1.63%)▶HNRNPM (bg=4.29%)▶WRN (bg=0.77%)MATCHES To TargetScan▶ miR-149-5p:CUGGCUC▶ miR-193-3p:ACUGGCC▶ miR-3064-5p:CUGGCUG

GAGAAAGAAGTGGAGG

GGCCACGT

GGCCACGT  
Depth:2 (PIG)  
Ei-value:0.000, Pi-value:0.000  
Er-value:0.000, Pr-value:0.000  
eCLIP MATCHES▶DDX51 (bg=1.63%)▶DROSHA (bg=2.49%)▶EXOSC5 (bg=5.38%)▶HNRNPM (bg=4.29%)▶NCBP2 (bg=1.49%)▶RBM22 (bg=4.62%)▶SLTM (bg=2.2%)▶uchl5 (bg=11.16%)▶UTP3 (bg=3.66%)▶WRN (bg=0.77%)No matches to TargetScan

GTATGT

CTCCCAGTG

CTCCCAGTGGG  
Depth:2 (PIG)  
Ei-value:0.000, Pi-value:0.000  
Er-value:0.000, Pr-value:0.000  
eCLIP MATCHES▶DROSHA (bg=2.49%)▶EXOSC5 (bg=5.38%)▶HNRNPM (bg=4.29%)▶NCBP2 (bg=1.49%)▶RBM22 (bg=4.62%)▶SLTM (bg=2.2%)▶uchl5 (bg=11.16%)▶UTP3 (bg=3.66%)▶WRN (bg=0.77%)No matches to TargetScan

 1800  


GG

CTCCCAGTGGG  
Depth:2 (PIG)  
Ei-value:0.000, Pi-value:0.000  
Er-value:0.000, Pr-value:0.000  
eCLIP MATCHES▶DROSHA (bg=2.49%)▶EXOSC5 (bg=5.38%)▶HNRNPM (bg=4.29%)▶NCBP2 (bg=1.49%)▶RBM22 (bg=4.62%)▶SLTM (bg=2.2%)▶uchl5 (bg=11.16%)▶UTP3 (bg=3.66%)▶WRN (bg=0.77%)No matches to TargetScan

CGGTACACCAGGTGTTTT

CAAGGTCTTT

CAAGGTCTTT  
Depth:2 (PIG)  
Ei-value:0.000, Pi-value:0.000  
Er-value:0.000, Pr-value:0.000  
eCLIP MATCHES▶EXOSC5 (bg=5.38%)No matches to TargetScan

T

CAAGGAC

CAAGGAC  
Depth:2 (PIG)  
Ei-value:0.000, Pi-value:0.000  
Er-value:0.000, Pr-value:0.010  
No matches to eCLIP DataNo matches to TargetScan

ATTTA

GCCTTTCCACCTC

GCCTTTCCACCTC  
Depth:2 (PIG)  
Ei-value:0.000, Pi-value:0.000  
Er-value:0.000, Pr-value:0.000  
eCLIP MATCHES▶EXOSC5 (bg=5.38%)▶PCBP1 (bg=1.07%)▶SDAD1 (bg=2.97%)▶TIA1 (bg=4.07%)▶uchl5 (bg=11.16%)No matches to TargetScan

TG

TCCCCTCT

TCCCCTCT  
Depth:2 (PIG)  
Ei-value:0.000, Pi-value:0.000  
Er-value:0.000, Pr-value:0.000  
eCLIP MATCHES▶EXOSC5 (bg=5.38%)▶PCBP1 (bg=1.07%)▶SDAD1 (bg=2.97%)▶TIA1 (bg=4.07%)▶uchl5 (bg=11.16%)MATCHES To TargetScan▶ miR-423-5p:GAGGGGC

TATTTG

TCCCCTCC

TCCCCTCC  
Depth:2 (PIG)  
Ei-value:0.000, Pi-value:0.000  
Er-value:0.000, Pr-value:0.000  
eCLIP MATCHES▶EXOSC5 (bg=5.38%)▶HNRNPM (bg=4.29%)▶PCBP1 (bg=1.07%)▶SDAD1 (bg=2.97%)▶uchl5 (bg=11.16%)MATCHES To TargetScan▶ miR-423-5p:GAGGGGC

TGTCCAGTGCTGCCTCTTGCAGTGCTGGATATCTGGCTGT 1920  
 GTGGT

CTGAACCTC

CTGAACCTC  
Depth:2 (PIG)  
Ei-value:0.000, Pi-value:0.000  
Er-value:0.000, Pr-value:0.000  
eCLIP MATCHES▶EXOSC5 (bg=5.38%)▶hnrnpk (bg=12.88%)▶PCBP1 (bg=1.07%)No matches to TargetScan

CCT

CCATTCCTCTG

CCATTCCTCTG  
Depth:2 (PIG)  
Ei-value:0.000, Pi-value:0.000  
Er-value:0.000, Pr-value:0.000  
eCLIP MATCHES▶EXOSC5 (bg=5.38%)▶hnrnpk (bg=12.88%)▶PCBP1 (bg=1.07%)MATCHES To TargetScan▶ miR-1-3p/206:GGAAUGU

T

ATTGGTG

ATTGGTG  
Depth:2 (PIG)  
Ei-value:0.000, Pi-value:0.000  
Er-value:0.000, Pr-value:0.010  
eCLIP MATCHES▶EXOSC5 (bg=5.38%)▶hnrnpk (bg=12.88%)No matches to TargetScan

CCTCA

CCTAAGGCTAA

CCTAAGGCTAA  
Depth:2 (PIG)  
Ei-value:0.000, Pi-value:0.000  
Er-value:0.000, Pr-value:0.000  
No matches to eCLIP DataNo matches to TargetScan

GTATA

CCTCCCCC

CCTCCCCC  
Depth:2 (PIG)  
Ei-value:0.000, Pi-value:0.000  
Er-value:0.000, Pr-value:0.000  
No matches to eCLIP DataNo matches to TargetScan

CCCACCCCCCAACCCCCCCAACTCCCCACCCCCACCCCCCACCCCCCACCTCCCC 2040  
 ACCCCCCTACCCCCCTACCCCCCTACCCC

CCTCTG

CCTCTG  
Depth:2 (PIG)  
Ei-value:0.000, Pi-value:0.020  
Er-value:0.000, Pr-value:0.000  
eCLIP MATCHES▶CSTF2T (bg=0.82%)▶DDX51 (bg=1.63%)▶DROSHA (bg=2.49%)▶GTF2F1 (bg=0.51%)▶hnrnpk (bg=12.88%)▶ILF3 (bg=3.0%)▶PCBP1 (bg=1.07%)▶PUM1 (bg=1.56%)No matches to TargetScan

GTCTGCCCTGCA

CTGCACTGT

CTGCACTGT  
Depth:2 (PIG)  
Ei-value:0.000, Pi-value:0.000  
Er-value:0.000, Pr-value:0.000  
eCLIP MATCHES▶CSTF2T (bg=0.82%)▶DGCR8 (bg=1.84%)▶DROSHA (bg=2.49%)▶GTF2F1 (bg=0.51%)▶hnrnpk (bg=12.88%)▶ILF3 (bg=3.0%)▶PCBP1 (bg=1.07%)▶PUM1 (bg=1.56%)▶RBM15 (bg=7.27%)MATCHES To TargetScan▶ miR-130-3p/301-3p/454-3p:AGUGCAA▶ miR-148-3p/152-3p:CAGUGCA

TGCCAT

GGGCAGTGCTCCA

GGGCAGTGCTCCA  
Depth:2 (PIG)  
Ei-value:0.000, Pi-value:0.000  
Er-value:0.000, Pr-value:0.000  
eCLIP MATCHES▶CSTF2T (bg=0.82%)▶DGCR8 (bg=1.84%)▶DROSHA (bg=2.49%)▶GTF2F1 (bg=0.51%)▶hnrnpk (bg=12.88%)▶HNRNPM (bg=4.29%)▶ILF3 (bg=3.0%)▶NCBP2 (bg=1.49%)▶PCBP1 (bg=1.07%)▶PUM1 (bg=1.56%)▶RBM15 (bg=7.27%)No matches to TargetScan

G

GCCTGC

GCCTGC  
Depth:2 (PIG)  
Ei-value:0.000, Pi-value:0.000  
Er-value:0.000, Pr-value:0.000  
eCLIP MATCHES▶CSTF2T (bg=0.82%)▶DGCR8 (bg=1.84%)▶DROSHA (bg=2.49%)▶GTF2F1 (bg=0.51%)▶hnrnpk (bg=12.88%)▶HNRNPM (bg=4.29%)▶HNRNPUL1 (bg=1.16%)▶ILF3 (bg=3.0%)▶NCBP2 (bg=1.49%)▶PCBP1 (bg=1.07%)▶PUM1 (bg=1.56%)▶RBM15 (bg=7.27%)▶SRSF1 (bg=8.47%)No matches to TargetScan

TTGGTGTGGACATGGT

GGTGAG

GGTGAG  
Depth:2 (PIG)  
Ei-value:0.000, Pi-value:0.000  
Er-value:0.000, Pr-value:0.010  
eCLIP MATCHES▶CSTF2T (bg=0.82%)▶DDX52 (bg=0.46%)▶DGCR8 (bg=1.84%)▶DROSHA (bg=2.49%)▶EXOSC5 (bg=5.38%)▶GRWD1 (bg=5.13%)▶GTF2F1 (bg=0.51%)▶hnrnpk (bg=12.88%)▶HNRNPM (bg=4.29%)▶HNRNPUL1 (bg=1.16%)▶ILF3 (bg=3.0%)▶NCBP2 (bg=1.49%)▶PUM1 (bg=1.56%)▶RBM15 (bg=7.27%)▶SF3B1 (bg=2.48%)▶SRSF1 (bg=8.47%)▶SUPV3L1 (bg=1.57%)▶ZNF622 (bg=6.58%)▶ZNF800 (bg=1.92%)No matches to TargetScan

C

CGTGGCAAGGACCAG

CGTGGCAAGGACCAGAATGGATC  
Depth:2 (PIG)  
Ei-value:0.000, Pi-value:0.000  
Er-value:0.000, Pr-value:0.000  
eCLIP MATCHES▶CSTF2T (bg=0.82%)▶DDX52 (bg=0.46%)▶DGCR8 (bg=1.84%)▶DROSHA (bg=2.49%)▶EXOSC5 (bg=5.38%)▶GRWD1 (bg=5.13%)▶GTF2F1 (bg=0.51%)▶hnrnpk (bg=12.88%)▶HNRNPM (bg=4.29%)▶HNRNPUL1 (bg=1.16%)▶ILF3 (bg=3.0%)▶NCBP2 (bg=1.49%)▶PUM1 (bg=1.56%)▶RBM15 (bg=7.27%)▶SF3B1 (bg=2.48%)▶SRSF1 (bg=8.47%)▶SUPV3L1 (bg=1.57%)▶TRA2A (bg=4.8%)▶ZNF622 (bg=6.58%)▶ZNF800 (bg=1.92%)MATCHES To TargetScan▶ miR-133a-3p.1:UGGUCCC

 2160  


AATGGATC

CGTGGCAAGGACCAGAATGGATC  
Depth:2 (PIG)  
Ei-value:0.000, Pi-value:0.000  
Er-value:0.000, Pr-value:0.000  
eCLIP MATCHES▶CSTF2T (bg=0.82%)▶DDX52 (bg=0.46%)▶DGCR8 (bg=1.84%)▶DROSHA (bg=2.49%)▶EXOSC5 (bg=5.38%)▶GRWD1 (bg=5.13%)▶GTF2F1 (bg=0.51%)▶hnrnpk (bg=12.88%)▶HNRNPM (bg=4.29%)▶HNRNPUL1 (bg=1.16%)▶ILF3 (bg=3.0%)▶NCBP2 (bg=1.49%)▶PUM1 (bg=1.56%)▶RBM15 (bg=7.27%)▶SF3B1 (bg=2.48%)▶SRSF1 (bg=8.47%)▶SUPV3L1 (bg=1.57%)▶TRA2A (bg=4.8%)▶ZNF622 (bg=6.58%)▶ZNF800 (bg=1.92%)MATCHES To TargetScan▶ miR-133a-3p.1:UGGUCCC

A

CAGATGATCGTTGGC

CAGATGATCGTTGGCCAACAGGTGGC  
Depth:2 (PIG)  
Ei-value:0.000, Pi-value:0.000  
Er-value:0.000, Pr-value:0.000  
eCLIP MATCHES▶CSTF2T (bg=0.82%)▶GRWD1 (bg=5.13%)▶hnrnpk (bg=12.88%)▶HNRNPM (bg=4.29%)▶ILF3 (bg=3.0%)▶MTPAP (bg=2.21%)▶NCBP2 (bg=1.49%)▶NIPBL (bg=5.39%)▶PUM1 (bg=1.56%)▶RBM15 (bg=7.27%)▶SRSF1 (bg=8.47%)▶TRA2A (bg=4.8%)▶uchl5 (bg=11.16%)▶XRCC6 (bg=2.91%)▶ZNF622 (bg=6.58%)▶ZNF800 (bg=1.92%)No matches to TargetScan


CAACAG

CAACAG  
Depth:3 (COW)  
Ei-value:0.000, Pi-value:0.000  
Er-value:0.000, Pr-value:0.000  
eCLIP MATCHES▶CSTF2T (bg=0.82%)▶GRWD1 (bg=5.13%)▶HNRNPM (bg=4.29%)▶MTPAP (bg=2.21%)▶NCBP2 (bg=1.49%)▶NIPBL (bg=5.39%)▶PUM1 (bg=1.56%)▶RBM15 (bg=7.27%)▶SRSF1 (bg=8.47%)▶TRA2A (bg=4.8%)▶uchl5 (bg=11.16%)▶XRCC6 (bg=2.91%)▶ZNF622 (bg=6.58%)▶ZNF800 (bg=1.92%)No matches to TargetScan


GTGGC

CAGATGATCGTTGGCCAACAGGTGGC  
Depth:2 (PIG)  
Ei-value:0.000, Pi-value:0.000  
Er-value:0.000, Pr-value:0.000  
eCLIP MATCHES▶CSTF2T (bg=0.82%)▶GRWD1 (bg=5.13%)▶hnrnpk (bg=12.88%)▶HNRNPM (bg=4.29%)▶ILF3 (bg=3.0%)▶MTPAP (bg=2.21%)▶NCBP2 (bg=1.49%)▶NIPBL (bg=5.39%)▶PUM1 (bg=1.56%)▶RBM15 (bg=7.27%)▶SRSF1 (bg=8.47%)▶TRA2A (bg=4.8%)▶uchl5 (bg=11.16%)▶XRCC6 (bg=2.91%)▶ZNF622 (bg=6.58%)▶ZNF800 (bg=1.92%)No matches to TargetScan

A

GAAGAGGAAT

GAAGAGGAAT  
Depth:2 (PIG)  
Ei-value:0.000, Pi-value:0.000  
Er-value:0.000, Pr-value:0.000  
eCLIP MATCHES▶CSTF2T (bg=0.82%)▶GRWD1 (bg=5.13%)▶HNRNPM (bg=4.29%)▶MTPAP (bg=2.21%)▶NCBP2 (bg=1.49%)▶NIPBL (bg=5.39%)▶PUM1 (bg=1.56%)▶RBM15 (bg=7.27%)▶SRSF1 (bg=8.47%)▶TRA2A (bg=4.8%)▶uchl5 (bg=11.16%)▶UTP3 (bg=3.66%)▶XRCC6 (bg=2.91%)▶ZNF622 (bg=6.58%)▶ZNF800 (bg=1.92%)MATCHES To TargetScan▶ miR-670-3p:UUCCUCA

TCCTGC

CTTCCTCAAGAGGAACACCTACCCC

CTTCCTCAAGAGGAACACCTACCCC  
Depth:2 (PIG)  
Ei-value:0.000, Pi-value:0.000  
Er-value:0.000, Pr-value:0.000  
eCLIP MATCHES▶CSTF2T (bg=0.82%)▶GRWD1 (bg=5.13%)▶HNRNPM (bg=4.29%)▶MTPAP (bg=2.21%)▶NCBP2 (bg=1.49%)▶NIPBL (bg=5.39%)▶PUM1 (bg=1.56%)▶RBM15 (bg=7.27%)▶SRSF1 (bg=8.47%)▶TRA2A (bg=4.8%)▶uchl5 (bg=11.16%)▶UTP3 (bg=3.66%)▶ZNF622 (bg=6.58%)MATCHES To TargetScan▶ miR-1224-5p:UGAGGAC▶ miR-670-3p:UUCCUCA

T

TGGCTAATGCTGGGGTCGGATTTTGATTT

TGGCTAATGCTGGGGTCGGATTTTGATTT  
Depth:2 (PIG)  
Ei-value:0.000, Pi-value:0.000  
Er-value:0.000, Pr-value:0.000  
eCLIP MATCHES▶GRWD1 (bg=5.13%)▶SRSF1 (bg=8.47%)▶ZNF622 (bg=6.58%)MATCHES To TargetScan▶ miR-338-3p:CCAGCAU▶ miR-551-3p:CGACCCA

ATATTTATCTT

TT

TTGGATGTCAGTCATA  
Depth:2 (PIG)  
Ei-value:0.000, Pi-value:0.000  
Er-value:0.000, Pr-value:0.000  
eCLIP MATCHES▶CPSF6 (bg=0.4%)▶EXOSC5 (bg=5.38%)▶HNRNPUL1 (bg=1.16%)▶KHSRP (bg=0.67%)▶RBM15 (bg=7.27%)▶TIA1 (bg=4.07%)▶XRCC6 (bg=2.91%)MATCHES To TargetScan▶ miR-489-3p:UGACAUC

 2280  


GGATGTCAGTCATA

TTGGATGTCAGTCATA  
Depth:2 (PIG)  
Ei-value:0.000, Pi-value:0.000  
Er-value:0.000, Pr-value:0.000  
eCLIP MATCHES▶CPSF6 (bg=0.4%)▶EXOSC5 (bg=5.38%)▶HNRNPUL1 (bg=1.16%)▶KHSRP (bg=0.67%)▶RBM15 (bg=7.27%)▶TIA1 (bg=4.07%)▶XRCC6 (bg=2.91%)MATCHES To TargetScan▶ miR-489-3p:UGACAUC

CAGTCTGATTT

TGTGGTTTGCTAGTGTT

TGTGGTTTGCTAGTGTT  
Depth:2 (PIG)  
Ei-value:0.000, Pi-value:0.000  
Er-value:0.000, Pr-value:0.000  
eCLIP MATCHES▶CPSF6 (bg=0.4%)▶EXOSC5 (bg=5.38%)▶HNRNPM (bg=4.29%)▶HNRNPUL1 (bg=1.16%)▶KHSRP (bg=0.67%)▶NIPBL (bg=5.39%)▶RBM22 (bg=4.62%)▶TIA1 (bg=4.07%)▶XRCC6 (bg=2.91%)MATCHES To TargetScan▶ miR-140-3p.2:ACCACAG▶ miR-141-3p/200a-3p:AACACUG

TGA

ATTTAAG

ATTTAAG  
Depth:2 (PIG)  
Ei-value:0.000, Pi-value:0.000  
Er-value:0.000, Pr-value:0.000  
eCLIP MATCHES▶EXOSC5 (bg=5.38%)▶KHSRP (bg=0.67%)▶NIPBL (bg=5.39%)▶TIA1 (bg=4.07%)No matches to TargetScan

T

CTTAAGTGACTA

CTTAAGTGACTA  
Depth:2 (PIG)  
Ei-value:0.000, Pi-value:0.000  
Er-value:0.000, Pr-value:0.000  
eCLIP MATCHES▶EXOSC5 (bg=5.38%)▶KHSRP (bg=0.67%)▶NIPBL (bg=5.39%)▶TIA1 (bg=4.07%)MATCHES To TargetScan▶ miR-668-3p:GUCACUC

TTATAGA

AATGTATT

AATGTATT  
Depth:2 (PIG)  
Ei-value:0.000, Pi-value:0.000  
Er-value:0.000, Pr-value:0.000  
eCLIP MATCHES▶KHSRP (bg=0.67%)▶TIA1 (bg=4.07%)No matches to TargetScan

AAGAGGCT

TTATTTGTAGAATTCA

TTATTTGTAGAATTCA  
Depth:2 (PIG)  
Ei-value:0.000, Pi-value:0.000  
Er-value:0.000, Pr-value:0.000  
eCLIP MATCHES▶KHSRP (bg=0.67%)▶TIA1 (bg=4.07%)No matches to TargetScan

CTTTAA

TTACATTTA

TTACATTTA  
Depth:2 (PIG)  
Ei-value:0.000, Pi-value:0.000  
Er-value:0.000, Pr-value:0.000  
eCLIP MATCHES▶KHSRP (bg=0.67%)▶TIA1 (bg=4.07%)MATCHES To TargetScan▶ miR-411-3p:AUGUAAC

A 2400  
 TGAGTTTTTGTTTTGA

GTTCCTT

GTTCCTT  
Depth:2 (PIG)  
Ei-value:0.000, Pi-value:0.010  
Er-value:0.000, Pr-value:0.010  
eCLIP MATCHES▶U2AF2 (bg=1.76%)No matches to TargetScan

A

AAATTCCTTAAAGTTTT

AAATTCCTTAAAGTTTT  
Depth:2 (PIG)  
Ei-value:0.000, Pi-value:0.000  
Er-value:0.000, Pr-value:0.000  
eCLIP MATCHES▶U2AF2 (bg=1.76%)No matches to TargetScan

TAGCTTCTCA

TTACAAAT

TTACAAAT  
Depth:2 (PIG)  
Ei-value:0.000, Pi-value:0.000  
Er-value:0.000, Pr-value:0.000  
eCLIP MATCHES▶U2AF2 (bg=1.76%)No matches to TargetScan

TCCTTAACCTTTTTTTGGCAGTAG

ATAGTCAAAGTCAA

ATAGTCAAAGTCAA  
Depth:2 (PIG)  
Ei-value:0.000, Pi-value:0.000  
Er-value:0.000, Pr-value:0.000  
eCLIP MATCHES▶EXOSC5 (bg=5.38%)▶LSM11 (bg=2.28%)▶SUPV3L1 (bg=1.57%)▶U2AF2 (bg=1.76%)No matches to TargetScan

ATCATTTCTAATGTTTTAAAAAT 2520  
 GTGCTGGTCATTTT

CTTTGAAATTGACTTAA

CTTTGAAATTGACTTAA  
Depth:2 (PIG)  
Ei-value:0.000, Pi-value:0.000  
Er-value:0.000, Pr-value:0.000  
eCLIP MATCHES▶LSM11 (bg=2.28%)▶PUS1 (bg=1.04%)▶SF3B1 (bg=2.48%)MATCHES To TargetScan▶ miR-224-5p:AAGUCAC

CTATTTTCC

TTTGAAG

TTTGAAG  
Depth:2 (PIG)  
Ei-value:0.000, Pi-value:0.010  
Er-value:0.000, Pr-value:0.000  
eCLIP MATCHES▶LSM11 (bg=2.28%)No matches to TargetScan

AGTCTGTAGCACAGAAACAGTAA

AAAATTTAAC

AAAATTTAAC  
Depth:2 (PIG)  
Ei-value:0.000, Pi-value:0.000  
Er-value:0.000, Pr-value:0.000  
eCLIP MATCHES▶LSM11 (bg=2.28%)No matches to TargetScan

TTC

ATGACC

ATGACC  
Depth:2 (PIG)  
Ei-value:0.000, Pi-value:0.000  
Er-value:0.000, Pr-value:0.000  
eCLIP MATCHES▶ILF3 (bg=3.0%)▶LSM11 (bg=2.28%)No matches to TargetScan

TAATGTAAAAAAGAGTG

TTTGAAGGT

TTTGAAGGT  
Depth:2 (PIG)  
Ei-value:0.000, Pi-value:0.000  
Er-value:0.000, Pr-value:0.000  
eCLIP MATCHES▶ILF3 (bg=3.0%)▶LSM11 (bg=2.28%)MATCHES To TargetScan▶ miR-205-5p:CCUUCAU

TTACA 2640  
 CAG

GTCCAGG

GTCCAGG  
Depth:2 (PIG)  
Ei-value:0.000, Pi-value:0.000  
Er-value:0.000, Pr-value:0.000  
eCLIP MATCHES▶ILF3 (bg=3.0%)MATCHES To TargetScan▶ miR-378-3p:CUGGACU

C

CTTGCTTTG

CTTGCTTTGTTCCCATCCTT  
Depth:2 (PIG)  
Ei-value:0.000, Pi-value:0.000  
Er-value:0.000, Pr-value:0.000  
eCLIP MATCHES▶ILF3 (bg=3.0%)MATCHES To TargetScan▶ miR-330-3p:CAAAGCA▶ miR-330-3p.2:AAAGCAC▶ miR-495-3p:AACAAAC


TTCCCATC

TTCCCATC  
Depth:4 (DOG)  
Ei-value:0.000, Pi-value:0.000  
Er-value:0.000, Pr-value:0.000  
eCLIP MATCHES▶ILF3 (bg=3.0%)No matches to TargetScan


CTT

CTTGCTTTGTTCCCATCCTT  
Depth:2 (PIG)  
Ei-value:0.000, Pi-value:0.000  
Er-value:0.000, Pr-value:0.000  
eCLIP MATCHES▶ILF3 (bg=3.0%)MATCHES To TargetScan▶ miR-330-3p:CAAAGCA▶ miR-330-3p.2:AAAGCAC▶ miR-495-3p:AACAAAC

G

ATGCTGCACT

ATGCTGCACT  
Depth:2 (PIG)  
Ei-value:0.000, Pi-value:0.000  
Er-value:0.000, Pr-value:0.000  
No matches to eCLIP DataMATCHES To TargetScan▶ miR-103-3p/107:GCAGCAU▶ miR-130-3p/301-3p/454-3p:AGUGCAA

AATTGACTAATCACCTACTTATCAGACAGG

AAACTTGAATTGCTGTGG

AAACTTGAATTGCTGTGG  
Depth:2 (PIG)  
Ei-value:0.000, Pi-value:0.000  
Er-value:0.000, Pr-value:0.000  
No matches to eCLIP DataMATCHES To TargetScan▶ miR-140-3p.1:CCACAGG▶ miR-26-5p:UCAAGUA

TCTGGTGTCCTCTATTCAGAC

TTATTATAT

TTATTATATTGGAGTATT  
Depth:2 (PIG)  
Ei-value:0.000, Pi-value:0.000  
Er-value:0.000, Pr-value:0.000  
No matches to eCLIP DataMATCHES To TargetScan▶ miR-200bc-3p/429:AAUACUG▶ miR-369-3p:AUAAUAC▶ miR-374-5p:UAUAAUA▶ miR-410-3p:AUAUAAC

 2760  


TGGAGTATT

TTATTATATTGGAGTATT  
Depth:2 (PIG)  
Ei-value:0.000, Pi-value:0.000  
Er-value:0.000, Pr-value:0.000  
No matches to eCLIP DataMATCHES To TargetScan▶ miR-200bc-3p/429:AAUACUG▶ miR-369-3p:AUAAUAC▶ miR-374-5p:UAUAAUA▶ miR-410-3p:AUAUAAC


TCAATTTT

TCAATTTT  
Depth:2 (PIG)  
Ei-value:0.000, Pi-value:0.010  
Er-value:0.000, Pr-value:0.010  
No matches to eCLIP DataNo matches to TargetScan

TCGTTGTATCCTGCCTGCCTAGCATCCAGTTCCTCCCCAGCCCTGC

TCCCAGCAAACCC

TCCCAGCAAACCC  
Depth:2 (PIG)  
Ei-value:0.000, Pi-value:0.000  
Er-value:0.000, Pr-value:0.000  
eCLIP MATCHES▶hnrnpk (bg=12.88%)No matches to TargetScan

CTAGTC

TAGCCCCAGCCC

TAGCCCCAGCCC  
Depth:2 (PIG)  
Ei-value:0.000, Pi-value:0.000  
Er-value:0.000, Pr-value:0.000  
No matches to eCLIP DataNo matches to TargetScan

TACTCCCACCCCGCCCCAG

CCCTGCC

CCCTGCCCCAGCCCCAG  
Depth:2 (PIG)  
Ei-value:0.000, Pi-value:0.000  
Er-value:0.000, Pr-value:0.000  
eCLIP MATCHES▶DROSHA (bg=2.49%)No matches to TargetScan

 2880  


CCAGCCCCAG

CCCTGCCCCAGCCCCAG  
Depth:2 (PIG)  
Ei-value:0.000, Pi-value:0.000  
Er-value:0.000, Pr-value:0.000  
eCLIP MATCHES▶DROSHA (bg=2.49%)No matches to TargetScan

TCCCCTAACCCCCCAGCCCT

AGCCCCAG

AGCCCCAG  
Depth:2 (PIG)  
Ei-value:0.000, Pi-value:0.000  
Er-value:0.000, Pr-value:0.000  
eCLIP MATCHES▶DGCR8 (bg=1.84%)▶DROSHA (bg=2.49%)▶hnrnpk (bg=12.88%)▶SDAD1 (bg=2.97%)▶XRN2 (bg=0.39%)No matches to TargetScan

TC

CCAGTCC

CCAGTCC  
Depth:2 (PIG)  
Ei-value:0.000, Pi-value:0.000  
Er-value:0.000, Pr-value:0.010  
eCLIP MATCHES▶DGCR8 (bg=1.84%)▶DROSHA (bg=2.49%)▶hnrnpk (bg=12.88%)▶SDAD1 (bg=2.97%)▶XRN2 (bg=0.39%)No matches to TargetScan

TAGTTCCTCAGTCCCGCCCAGCTTCTCTCGAAAGTCACTCTAATTTTC

ATTGATT

ATTGATT  
Depth:2 (PIG)  
Ei-value:0.000, Pi-value:0.000  
Er-value:0.000, Pr-value:0.030  
eCLIP MATCHES▶DROSHA (bg=2.49%)▶hnrnpk (bg=12.88%)No matches to TargetScan

CAGTGCTC

AAAATAAGTT

AAAATAAGTT  
Depth:2 (PIG)  
Ei-value:0.000, Pi-value:0.000  
Er-value:0.000, Pr-value:0.000  
eCLIP MATCHES▶DROSHA (bg=2.49%)▶hnrnpk (bg=12.88%)No matches to TargetScan

 3000  


AAAATAAGTT  
Depth:2 (PIG)  
Ei-value:0.000, Pi-value:0.000  
Er-value:0.000, Pr-value:0.000  
eCLIP MATCHES▶DROSHA (bg=2.49%)▶hnrnpk (bg=12.88%)No matches to TargetScan

GTCCATTGCTTATCCTATTAT

ACTGGGATA

ACTGGGATA  
Depth:2 (PIG)  
Ei-value:0.000, Pi-value:0.000  
Er-value:0.000, Pr-value:0.000  
eCLIP MATCHES▶DROSHA (bg=2.49%)▶hnrnpk (bg=12.88%)▶ILF3 (bg=3.0%)No matches to TargetScan

TTCCGTTTACCCTTG

GCATTGCTGATCTT

GCATTGCTGATCTT  
Depth:2 (PIG)  
Ei-value:0.000, Pi-value:0.000  
Er-value:0.000, Pr-value:0.000  
eCLIP MATCHES▶hnrnpk (bg=12.88%)▶ILF3 (bg=3.0%)MATCHES To TargetScan▶ miR-383-5p.1:GAUCAGA▶ miR-383-5p.2:AGAUCAG

CAGTACTGACTCCTTG

ACCATTTTCA

ACCATTTTCA  
Depth:2 (PIG)  
Ei-value:0.000, Pi-value:0.000  
Er-value:0.000, Pr-value:0.000  
eCLIP MATCHES▶hnrnpk (bg=12.88%)▶ILF3 (bg=3.0%)No matches to TargetScan

GTTAATGCAT

ACAATCCCATTTG

ACAATCCCATTTG  
Depth:2 (PIG)  
Ei-value:0.000, Pi-value:0.000  
Er-value:0.000, Pr-value:0.000  
eCLIP MATCHES▶hnrnpk (bg=12.88%)▶HNRNPU (bg=5.92%)▶ILF3 (bg=3.0%)MATCHES To TargetScan▶ miR-219-5p:GAUUGUC

TCTGTGATCTCA 3120  
 GG

ACAAAGAATTT

ACAAAGAATTT  
Depth:2 (PIG)  
Ei-value:0.000, Pi-value:0.000  
Er-value:0.000, Pr-value:0.000  
eCLIP MATCHES▶hnrnpk (bg=12.88%)No matches to TargetScan

CCTTACTCGGTACGTTGAA

GTTAGG

GTTAGG  
Depth:2 (PIG)  
Ei-value:0.000, Pi-value:0.010  
Er-value:0.000, Pr-value:0.010  
No matches to eCLIP DataNo matches to TargetScan

GAATGTCAATTGA

GAGCTT

GAGCTT  
Depth:2 (PIG)  
Ei-value:0.000, Pi-value:0.010  
Er-value:0.000, Pr-value:0.010  
No matches to eCLIP DataNo matches to TargetScan

TC

TATCAGA

TATCAGA  
Depth:2 (PIG)  
Ei-value:0.000, Pi-value:0.000  
Er-value:0.000, Pr-value:0.000  
No matches to eCLIP DataNo matches to TargetScan

GC

ATTATTG

ATTATTG  
Depth:2 (PIG)  
Ei-value:0.000, Pi-value:0.000  
Er-value:0.000, Pr-value:0.010  
No matches to eCLIP DataNo matches to TargetScan

CCCACAATTTGAGTTACTTATCATTTTCTCGATCCCCTGCCCTTA 3240  


AAGGAGAAACCATT

AAGGAGAAACCATT  
Depth:2 (PIG)  
Ei-value:0.000, Pi-value:0.000  
Er-value:0.000, Pr-value:0.000  
eCLIP MATCHES▶EIF3G (bg=0.32%)▶hnrnpk (bg=12.88%)No matches to TargetScan

T

CTCTGT

CTCTGT  
Depth:3 (COW)  
Ei-value:0.000, Pi-value:0.000  
Er-value:0.000, Pr-value:0.000  
eCLIP MATCHES▶EIF3G (bg=0.32%)▶hnrnpk (bg=12.88%)No matches to TargetScan


CATTGCT

CTCTGTCATTGCT  
Depth:2 (PIG)  
Ei-value:0.000, Pi-value:0.000  
Er-value:0.000, Pr-value:0.000  
eCLIP MATCHES▶EIF3G (bg=0.32%)▶hnrnpk (bg=12.88%)MATCHES To TargetScan▶ miR-425-5p:AUGACAC

TCT

GTAGTCA

GTAGTCA  
Depth:2 (PIG)  
Ei-value:0.000, Pi-value:0.000  
Er-value:0.000, Pr-value:0.000  
eCLIP MATCHES▶hnrnpk (bg=12.88%)▶SUPV3L1 (bg=1.57%)No matches to TargetScan

CAGTCCCAATTTTGAGTAGTGATCTTTTCTTGTG

TACTGTG

TACTGTG  
Depth:2 (PIG)  
Ei-value:0.000, Pi-value:0.000  
Er-value:0.000, Pr-value:0.000  
eCLIP MATCHES▶HNRNPU (bg=5.92%)▶WRN (bg=0.77%)MATCHES To TargetScan▶ miR-101-3p.1:ACAGUAC▶ miR-128-3p:CACAGUG▶ miR-144-3p:ACAGUAU

TTGGCCACCTA

AAACTCTTTGCA

AAACTCTTTGCA  
Depth:2 (PIG)  
Ei-value:0.000, Pi-value:0.000  
Er-value:0.000, Pr-value:0.000  
eCLIP MATCHES▶HNRNPU (bg=5.92%)▶WRN (bg=0.77%)No matches to TargetScan

TTGAGTA

AAATTCTAATT

AAATTCTAATTG  
Depth:2 (PIG)  
Ei-value:0.000, Pi-value:0.000  
Er-value:0.000, Pr-value:0.000  
No matches to eCLIP DataNo matches to TargetScan

 3360  


G

AAATTCTAATTG  
Depth:2 (PIG)  
Ei-value:0.000, Pi-value:0.000  
Er-value:0.000, Pr-value:0.000  
No matches to eCLIP DataNo matches to TargetScan

CCA

ATAATCCT

ATAATCCT  
Depth:2 (PIG)  
Ei-value:0.000, Pi-value:0.000  
Er-value:0.000, Pr-value:0.000  
eCLIP MATCHES▶HNRNPU (bg=5.92%)No matches to TargetScan

ACC

CATTGGA

CATTGGA  
Depth:2 (PIG)  
Ei-value:0.000, Pi-value:0.010  
Er-value:0.000, Pr-value:0.010  
eCLIP MATCHES▶HNRNPU (bg=5.92%)No matches to TargetScan

TTAGACAGCACTCTGAACCCCA

TTTGCATTCAGCAG

TTTGCATTCAGCAG  
Depth:2 (PIG)  
Ei-value:0.000, Pi-value:0.000  
Er-value:0.000, Pr-value:0.000  
eCLIP MATCHES▶HNRNPU (bg=5.92%)No matches to TargetScan

GGGGTCGCAGACAACCCGTCTTTTGTTGGACAGTTAAAATGCTCAGTCCCAA

TTGTCATA

TTGTCATA  
Depth:2 (PIG)  
Ei-value:0.000, Pi-value:0.000  
Er-value:0.000, Pr-value:0.000  
eCLIP MATCHES▶HNRNPU (bg=5.92%)MATCHES To TargetScan▶ miR-425-5p:AUGACAC

GC 3480  
 TTTGCCTA

TTAAACAAAGGCA

TTAAACAAAGGCA  
Depth:2 (PIG)  
Ei-value:0.000, Pi-value:0.000  
Er-value:0.000, Pr-value:0.000  
eCLIP MATCHES▶HNRNPU (bg=5.92%)No matches to TargetScan

CCCTACTGCGCTTTTTGCTGTGCTTCTGGAGAATCCTG

CTGTTCTTGGACAATTAAAG

CTGTTCTTGGACAATTAAAG  
Depth:2 (PIG)  
Ei-value:0.000, Pi-value:0.000  
Er-value:0.000, Pr-value:0.000  
eCLIP MATCHES▶HNRNPU (bg=5.92%)No matches to TargetScan

AACAAAGTA

GTAATTG

GTAATTG  
Depth:2 (PIG)  
Ei-value:0.000, Pi-value:0.000  
Er-value:0.000, Pr-value:0.000  
No matches to eCLIP DataNo matches to TargetScan

CTA

ATTGTCTCAC

ATTGTCTCAC  
Depth:2 (PIG)  
Ei-value:0.000, Pi-value:0.000  
Er-value:0.000, Pr-value:0.000  
No matches to eCLIP DataNo matches to TargetScan

C

CATTAATCA

CATTAATCA  
Depth:2 (PIG)  
Ei-value:0.000, Pi-value:0.000  
Er-value:0.000, Pr-value:0.000  
No matches to eCLIP DataNo matches to TargetScan

TG 3600  
 AAGACTACCAGTCGCCCTTGCATTTGCCTTG

AGGCAG

AGGCAG  
Depth:2 (PIG)  
Ei-value:0.000, Pi-value:0.010  
Er-value:0.000, Pr-value:0.020  
No matches to eCLIP DataNo matches to TargetScan

C

GCTGACTA

GCTGACTA  
Depth:2 (PIG)  
Ei-value:0.000, Pi-value:0.000  
Er-value:0.000, Pr-value:0.000  
No matches to eCLIP DataNo matches to TargetScan

CCTGAGATTTAAGAGTTTCTTAA

ATTATTGA

ATTATTGA  
Depth:2 (PIG)  
Ei-value:0.000, Pi-value:0.010  
Er-value:0.000, Pr-value:0.000  
No matches to eCLIP DataNo matches to TargetScan

GTAAAATCCCAATTATCCATAGTTCTGTTAG

TTACAC

TTACAC  
Depth:2 (PIG)  
Ei-value:0.000, Pi-value:0.010  
Er-value:0.000, Pr-value:0.000  
No matches to eCLIP DataNo matches to TargetScan

TATGGC 3720  
 CTTTGCAAACA

TCTTTGCA

TCTTTGCA  
Depth:2 (PIG)  
Ei-value:0.000, Pi-value:0.000  
Er-value:0.000, Pr-value:0.000  
No matches to eCLIP DataNo matches to TargetScan

TAACAGCAGTGGGACTGACTCATTCTTAGAGCCCCTTCCCTT

GGAATATTAATGGATACAAT

GGAATATTAATGGATACAAT  
Depth:2 (PIG)  
Ei-value:0.000, Pi-value:0.000  
Er-value:0.000, Pr-value:0.000  
No matches to eCLIP DataNo matches to TargetScan

AGTAATTAT

TCATGGT

TCATGGT  
Depth:2 (PIG)  
Ei-value:0.000, Pi-value:0.000  
Er-value:0.000, Pr-value:0.020  
No matches to eCLIP DataNo matches to TargetScan

TCTGCGTAACAGAG

AAGACCCAC

AAGACCCAC  
Depth:2 (PIG)  
Ei-value:0.000, Pi-value:0.000  
Er-value:0.000, Pr-value:0.000  
eCLIP MATCHES▶HNRNPUL1 (bg=1.16%)MATCHES To TargetScan▶ miR-193a-5p:GGGUCUU

 3840  


AAGACCCAC  
Depth:2 (PIG)  
Ei-value:0.000, Pi-value:0.000  
Er-value:0.000, Pr-value:0.000  
eCLIP MATCHES▶HNRNPUL1 (bg=1.16%)MATCHES To TargetScan▶ miR-193a-5p:GGGUCUU

TTATGTGTATGCCTTTATCATTGCTCCTAGA

TAGTGTG

TAGTGTG  
Depth:2 (PIG)  
Ei-value:0.000, Pi-value:0.000  
Er-value:0.000, Pr-value:0.000  
No matches to eCLIP DataNo matches to TargetScan

A

ACTACCTACCACCTTGCATTAATAT

ACTACCTACCACCTTGCATTAATAT  
Depth:2 (PIG)  
Ei-value:0.000, Pi-value:0.000  
Er-value:0.000, Pr-value:0.000  
No matches to eCLIP DataMATCHES To TargetScan▶ miR-155-5p:UAAUGCU▶ miR-18-5p:AAGGUGC▶ miR-196-5p:AGGUAGU

GTAAAACACTAATTGCCCATA

GTCCCACT

GTCCCACT  
Depth:2 (PIG)  
Ei-value:0.000, Pi-value:0.000  
Er-value:0.000, Pr-value:0.000  
eCLIP MATCHES▶hnrnpk (bg=12.88%)No matches to TargetScan

CATTAGTCTAGGATGTCCTCTT

TGCCA

TGCCATT  
Depth:2 (PIG)  
Ei-value:0.000, Pi-value:0.010  
Er-value:0.000, Pr-value:0.000  
eCLIP MATCHES▶hnrnpk (bg=12.88%)MATCHES To TargetScan▶ miR-183-5p.1:AUGGCAC

 3960  


TT

TGCCATT  
Depth:2 (PIG)  
Ei-value:0.000, Pi-value:0.010  
Er-value:0.000, Pr-value:0.000  
eCLIP MATCHES▶hnrnpk (bg=12.88%)MATCHES To TargetScan▶ miR-183-5p.1:AUGGCAC

GCTGCT

GAGTTCTGA

GAGTTCTGA  
Depth:2 (PIG)  
Ei-value:0.000, Pi-value:0.000  
Er-value:0.000, Pr-value:0.000  
eCLIP MATCHES▶hnrnpk (bg=12.88%)No matches to TargetScan

CTACCCAAGTTTCC

TTCTCTTAAACA

TTCTCTTAAACA  
Depth:2 (PIG)  
Ei-value:0.000, Pi-value:0.000  
Er-value:0.000, Pr-value:0.000  
No matches to eCLIP DataNo matches to TargetScan

GTTGATA

TGCATAATTGCATATA

TGCATAATTGCATATA  
Depth:2 (PIG)  
Ei-value:0.000, Pi-value:0.000  
Er-value:0.000, Pr-value:0.000  
No matches to eCLIP DataNo matches to TargetScan

TTCATGGTTCTGTGCAATAAAAATGGATTCTCACCCCATCCCACCT

TCTGTGG

TCTGTGG  
Depth:2 (PIG)  
Ei-value:0.000, Pi-value:0.000  
Er-value:0.000, Pr-value:0.010  
eCLIP MATCHES▶HNRNPL (bg=0.64%)▶HNRNPU (bg=5.92%)MATCHES To TargetScan▶ miR-140-3p.1:CCACAGG

G 4080  
 ATGTTGCTAACG

AGTGCA

AGTGCA  
Depth:2 (PIG)  
Ei-value:0.000, Pi-value:0.020  
Er-value:0.000, Pr-value:0.000  
eCLIP MATCHES▶HNRNPU (bg=5.92%)No matches to TargetScan

G

ATTATTCAA

ATTATTCAA  
Depth:2 (PIG)  
Ei-value:0.000, Pi-value:0.000  
Er-value:0.000, Pr-value:0.000  
eCLIP MATCHES▶HNRNPA1 (bg=2.57%)▶HNRNPU (bg=5.92%)No matches to TargetScan

TAACAGCTCTTGA

ACAGTTAAT

ACAGTTAAT  
Depth:2 (PIG)  
Ei-value:0.000, Pi-value:0.000  
Er-value:0.000, Pr-value:0.010  
eCLIP MATCHES▶HNRNPA1 (bg=2.57%)No matches to TargetScan

TT

GCACAGTTGC

GCACAGTTGC  
Depth:2 (PIG)  
Ei-value:0.000, Pi-value:0.000  
Er-value:0.000, Pr-value:0.000  
eCLIP MATCHES▶HNRNPA1 (bg=2.57%)No matches to TargetScan

AA

TTGTCCAGAGTCC

TTGTCCAGAGTCC  
Depth:2 (PIG)  
Ei-value:0.000, Pi-value:0.000  
Er-value:0.000, Pr-value:0.000  
eCLIP MATCHES▶HNRNPA1 (bg=2.57%)MATCHES To TargetScan▶ miR-326:CUCUGGG▶ miR-378-3p:CUGGACU

TGTCCATTAGAAAGGGACTCTGTATCCTATTTGCACGCTACAA 4200  
 T

GTGGGC

GTGGGC  
Depth:2 (PIG)  
Ei-value:0.000, Pi-value:0.000  
Er-value:0.000, Pr-value:0.010  
eCLIP MATCHES▶HNRNPU (bg=5.92%)No matches to TargetScan

TGATCACCCAAGGACTCTTCTTGTGCATTGATGTT

CATAATTG

CATAATTG  
Depth:2 (PIG)  
Ei-value:0.000, Pi-value:0.000  
Er-value:0.000, Pr-value:0.000  
eCLIP MATCHES▶HNRNPU (bg=5.92%)No matches to TargetScan

TATTTGTCCACGATCTTGTGCACTAACCCTTCCACTCC

CTTTGTATTCCAGCAGGGGACCCTT

CTTTGTATTCCAGCAGGGGACCCTT  
Depth:2 (PIG)  
Ei-value:0.000, Pi-value:0.000  
Er-value:0.000, Pr-value:0.000  
eCLIP MATCHES▶hnrnpk (bg=12.88%)▶HNRNPU (bg=5.92%)MATCHES To TargetScan▶ miR-331-3p:CCCCUGG▶ miR-381-3p:AUACAAG

ACTACTC 4320  
 AAGACCTCTGTACTAGGACAGTTTATGTGCACAAT

CCTAATTGATTAGA

CCTAATTGATTAGA  
Depth:2 (PIG)  
Ei-value:0.000, Pi-value:0.000  
Er-value:0.000, Pr-value:0.000  
eCLIP MATCHES▶HNRNPA1 (bg=2.57%)No matches to TargetScan

ACTGAG

TCTTTTAT

TCTTTTAT  
Depth:2 (PIG)  
Ei-value:0.000, Pi-value:0.000  
Er-value:0.000, Pr-value:0.010  
eCLIP MATCHES▶HNRNPA1 (bg=2.57%)▶hnrnpk (bg=12.88%)▶HNRNPU (bg=5.92%)▶UTP3 (bg=3.66%)No matches to TargetScan

ATCAAGGTCCC

TGCATC

TGCATC  
Depth:2 (PIG)  
Ei-value:0.000, Pi-value:0.030  
Er-value:0.000, Pr-value:0.020  
eCLIP MATCHES▶HNRNPA1 (bg=2.57%)▶hnrnpk (bg=12.88%)▶HNRNPU (bg=5.92%)▶UTP3 (bg=3.66%)No matches to TargetScan

ATCTTTGCTTTACATCAAGAGGGTGCTGG

TTACCTA

TTACCTA  
Depth:2 (PIG)  
Ei-value:0.000, Pi-value:0.000  
Er-value:0.000, Pr-value:0.020  
eCLIP MATCHES▶HNRNPU (bg=5.92%)▶UTP3 (bg=3.66%)No matches to TargetScan

ATGC 4440  
 CCCTCCTCCAGAAATTATTGATGTGCA

AAATGCAATT

AAATGCAATT  
Depth:2 (PIG)  
Ei-value:0.000, Pi-value:0.000  
Er-value:0.000, Pr-value:0.000  
eCLIP MATCHES▶AKAP8L (bg=2.19%)MATCHES To TargetScan▶ miR-25-3p/32-5p/92-3p/363-3p/367-3p:AUUGCAC▶ miR-33-5p:UGCAUUG

TCCCTATCTG

C

CTGTTAGTCT  
Depth:4 (DOG)  
Ei-value:0.000, Pi-value:0.000  
Er-value:0.000, Pr-value:0.000  
eCLIP MATCHES▶AKAP8L (bg=2.19%)No matches to TargetScan


TGTTAGTC

TGTTAGTC  
Depth:5 (RABBIT)  
Ei-value:0.000, Pi-value:0.000  
Er-value:0.000, Pr-value:0.000  
eCLIP MATCHES▶AKAP8L (bg=2.19%)No matches to TargetScan


T

CTGTTAGTCT  
Depth:4 (DOG)  
Ei-value:0.000, Pi-value:0.000  
Er-value:0.000, Pr-value:0.000  
eCLIP MATCHES▶AKAP8L (bg=2.19%)No matches to TargetScan

GGGG

TC

TCTCATCCCC  
Depth:2 (PIG)  
Ei-value:0.000, Pi-value:0.000  
Er-value:0.000, Pr-value:0.010  
eCLIP MATCHES▶AKAP8L (bg=2.19%)No matches to TargetScan


TCATCC

TCATCC  
Depth:4 (DOG)  
Ei-value:0.000, Pi-value:0.020  
Er-value:0.000, Pr-value:0.000  
eCLIP MATCHES▶AKAP8L (bg=2.19%)No matches to TargetScan


CC

TCTCATCCCC  
Depth:2 (PIG)  
Ei-value:0.000, Pi-value:0.000  
Er-value:0.000, Pr-value:0.010  
eCLIP MATCHES▶AKAP8L (bg=2.19%)No matches to TargetScan

TCATATT

CCTTTTGT

CCTTTTGT  
Depth:2 (PIG)  
Ei-value:0.000, Pi-value:0.000  
Er-value:0.000, Pr-value:0.010  
eCLIP MATCHES▶AKAP8L (bg=2.19%)No matches to TargetScan

CTTACAGCAGG

GGG

GGGTACTTGGGACTGTTAAT  
Depth:3 (COW)  
Ei-value:0.000, Pi-value:0.000  
Er-value:0.000, Pr-value:0.000  
eCLIP MATCHES▶AKAP8L (bg=2.19%)MATCHES To TargetScan▶ miR-132-3p/212-3p:AACAGUC▶ miR-455-3p.1:CAGUCCA


TACTTGGGACTGTTAAT

TACTTGGGACTGTTAAT  
Depth:4 (DOG)  
Ei-value:0.000, Pi-value:0.000  
Er-value:0.000, Pr-value:0.000  
eCLIP MATCHES▶AKAP8L (bg=2.19%)MATCHES To TargetScan▶ miR-132-3p/212-3p:AACAGUC▶ miR-455-3p.1:CAGUCCA


G

GGGTACTTGGGACTGTTAATG  
Depth:2 (PIG)  
Ei-value:0.000, Pi-value:0.000  
Er-value:0.000, Pr-value:0.000  
eCLIP MATCHES▶AKAP8L (bg=2.19%)MATCHES To TargetScan▶ miR-132-3p/212-3p:AACAGUC▶ miR-455-3p.1:CAGUCCA

CG 4560  
 CATAATTGCAATTATGGTCTTTTCCATTAAATTAAGATCCCAACTGCTCACACCCTCTTAGCATTACAGTAGAGGGTGCTAATCACAAGGACATTTCTTTTGT

ACTG

ACTGTTAATGTGCT  
Depth:4 (DOG)  
Ei-value:0.000, Pi-value:0.000  
Er-value:0.000, Pr-value:0.000  
No matches to eCLIP DataMATCHES To TargetScan▶ miR-132-3p/212-3p:AACAGUC▶ miR-323-3p:ACAUUAC


TTAATGTGCT

TTAATGTGCT  
Depth:5 (RABBIT)  
Ei-value:0.000, Pi-value:0.000  
Er-value:0.000, Pr-value:0.000  
No matches to eCLIP DataMATCHES To TargetScan▶ miR-323-3p:ACAUUAC


A

ACTGTTAATGTGCTA  
Depth:2 (PIG)  
Ei-value:0.000, Pi-value:0.000  
Er-value:0.000, Pr-value:0.000  
No matches to eCLIP DataMATCHES To TargetScan▶ miR-132-3p/212-3p:AACAGUC▶ miR-323-3p:ACAUUAC

CT 4680  
 TGCATTTGTCCCTCTTCCTGTGCACTAAAGACCCCACTCACTTCCCTAGTGTTCAGCAGTGGATGACCTCTAGTCAAGACCTTTGCACTAGGATAGTTAATGTGAACCATGGCAACTGAT 4800  
 CACAACAATGTCTTTCAGATCAGATCCATTTTATCCTCCTTGTTTTACAGCAAGGGATATTAATTACCTATGTTACCTTTCCCTGGGACTATGAATGTGCAAAATTCCAATGTTCATGGT 4920  
 CTCTCCCTTTAAACCTATATTCTACCCCTTTTACATTATAGAAAGGGATGCTGGAAACCCAGAGTCCTTCT

CTTGGGACTC

CTTGGGACTC  
Depth:3 (COW)  
Ei-value:0.000, Pi-value:0.000  
Er-value:0.000, Pr-value:0.000  
No matches to eCLIP DataNo matches to TargetScan


TTAATG

CTTGGGACTCTTAATG  
Depth:2 (PIG)  
Ei-value:0.000, Pi-value:0.000  
Er-value:0.000, Pr-value:0.000  
No matches to eCLIP DataNo matches to TargetScan

TGTATTTCTAATT

ATCCATG

ATCCATG  
Depth:2 (PIG)  
Ei-value:0.000, Pi-value:0.010  
Er-value:0.000, Pr-value:0.000  
No matches to eCLIP DataNo matches to TargetScan

ACTCT

T

TAATGTGCAT  
Depth:2 (PIG)  
Ei-value:0.000, Pi-value:0.000  
Er-value:0.000, Pr-value:0.000  
No matches to eCLIP DataMATCHES To TargetScan▶ miR-323-3p:ACAUUAC▶ miR-501-3p/502-3p:AUGCACC


AATGTGC

AATGTGCAT  
Depth:6 (MOUSE)  
Ei-value:0.000, Pi-value:0.000  
Er-value:0.000, Pr-value:0.000  
No matches to eCLIP DataMATCHES To TargetScan▶ miR-501-3p/502-3p:AUGCACC

 5040  


AT

AATGTGCAT  
Depth:6 (MOUSE)  
Ei-value:0.000, Pi-value:0.000  
Er-value:0.000, Pr-value:0.000  
No matches to eCLIP DataMATCHES To TargetScan▶ miR-501-3p/502-3p:AUGCACC

ATTTTCAATTGCCTAATTGATTTCAATTGTCTAAGACATTTCAAATGTCTAATTGATTAGAACTGAGTCTTTTATATCAAG

CTAATA

CTAATA  
Depth:3 (COW)  
Ei-value:0.000, Pi-value:0.000  
Er-value:0.000, Pr-value:0.000  
No matches to eCLIP DataNo matches to TargetScan

TCTAGCTTTTATATCAAG

CTAATA

CTAATA  
Depth:3 (COW)  
Ei-value:0.000, Pi-value:0.000  
Er-value:0.000, Pr-value:0.000  
No matches to eCLIP DataNo matches to TargetScan

TCTTGAC 5160  
 TTCTCAGCATCATAGAAGGGGGTACTGATTTCCTA

AAGTCTTT

AAGTCTTT  
Depth:2 (PIG)  
Ei-value:0.000, Pi-value:0.000  
Er-value:0.000, Pr-value:0.000  
No matches to eCLIP DataNo matches to TargetScan

CTTGAATTTCTATTA

TGCAAAATT

TGCAAAATT  
Depth:2 (PIG)  
Ei-value:0.000, Pi-value:0.000  
Er-value:0.000, Pr-value:0.000  
eCLIP MATCHES▶SUPV3L1 (bg=1.57%)No matches to TargetScan

GCCCTGAGGCCGGGTGTGGTGGCTCACACCTGTAATCCCAGCACTTTGGGAGG 5280  
 CTGAGGTGGGAAGATCCCTTACTGCCAGGAGTTTGAGACCAGCCTGGCCAACATTAAAAAAAAAAAAAAGTAAGACAATTGCCCTGGAATCCCATCCCCCTCACACCTCCTTGGCAAAGC 5400  
 AGCAGGAGTGCTAACTAGCTAG

TGCTTCT

TGCTTCT  
Depth:3 (COW)  
Ei-value:0.000, Pi-value:0.000  
Er-value:0.000, Pr-value:0.010  
No matches to eCLIP DataNo matches to TargetScan

TCTCTTATACTGCTTAAATGCGCATAATTAGCAGTAGTTGATGTGCCCC

TATGTTAGA

TATGTTAGA  
Depth:4 (DOG)  
Ei-value:0.000, Pi-value:0.000  
Er-value:0.000, Pr-value:0.000  
eCLIP MATCHES▶HNRNPU (bg=5.92%)No matches to TargetScan

G

TAGAATCCC

TAGAATCCC  
Depth:2 (PIG)  
Ei-value:0.000, Pi-value:0.000  
Er-value:0.000, Pr-value:0.000  
eCLIP MATCHES▶HNRNPU (bg=5.92%)No matches to TargetScan

GCTTCCTTGCTCCATTTGCATTA 5520  
 CTGCA

GGAGCTTCT

GGAGCTTCT  
Depth:2 (PIG)  
Ei-value:0.000, Pi-value:0.000  
Er-value:0.000, Pr-value:0.000  
No matches to eCLIP DataNo matches to TargetScan

AACTAGCCTGAATTCACTC

TCTTGG

TCTTGGACTGTTAATGT  
Depth:3 (COW)  
Ei-value:0.000, Pi-value:0.000  
Er-value:0.000, Pr-value:0.000  
No matches to eCLIP DataMATCHES To TargetScan▶ miR-132-3p/212-3p:AACAGUC▶ miR-323-3p:ACAUUAC▶ miR-455-3p.1:CAGUCCA


ACTGTTAATGT

ACTGTTAATGT  
Depth:4 (DOG)  
Ei-value:0.000, Pi-value:0.000  
Er-value:0.000, Pr-value:0.000  
No matches to eCLIP DataMATCHES To TargetScan▶ miR-132-3p/212-3p:AACAGUC▶ miR-323-3p:ACAUUAC


G

TCTTGGACTGTTAATGTG  
Depth:2 (PIG)  
Ei-value:0.000, Pi-value:0.000  
Er-value:0.000, Pr-value:0.000  
No matches to eCLIP DataMATCHES To TargetScan▶ miR-132-3p/212-3p:AACAGUC▶ miR-323-3p:ACAUUAC▶ miR-455-3p.1:CAGUCCA

CATACTTAT

ATTTGCT

ATTTGCT  
Depth:4 (DOG)  
Ei-value:0.000, Pi-value:0.000  
Er-value:0.000, Pr-value:0.000  
No matches to eCLIP DataNo matches to TargetScan

GCTGTACTTTTTTACCAT

GTAAGGA

GTAAGGA  
Depth:5 (RABBIT)  
Ei-value:0.000, Pi-value:0.000  
Er-value:0.000, Pr-value:0.000  
No matches to eCLIP DataNo matches to TargetScan


CCC

GTAAGGACCC  
Depth:3 (COW)  
Ei-value:0.000, Pi-value:0.000  
Er-value:0.000, Pr-value:0.000  
No matches to eCLIP DataNo matches to TargetScan

CACCCACTGTATTTACATCCCAGCT 5640  
 GGAAGTACCTACTA

CTTAAGA

CTTAAGA  
Depth:2 (PIG)  
Ei-value:0.000, Pi-value:0.000  
Er-value:0.000, Pr-value:0.000  
No matches to eCLIP DataNo matches to TargetScan

CCCTTAGACTAGTAAAGTTAGCG

TGCATA

TGCATAATCTTAG  
Depth:2 (PIG)  
Ei-value:0.000, Pi-value:0.000  
Er-value:0.000, Pr-value:0.000  
eCLIP MATCHES▶HNRNPU (bg=5.92%)No matches to TargetScan


ATCTTAG

ATCTTAG  
Depth:3 (COW)  
Ei-value:0.000, Pi-value:0.000  
Er-value:0.000, Pr-value:0.000  
eCLIP MATCHES▶HNRNPU (bg=5.92%)No matches to TargetScan

GTGTTATA

TACACATT

TACACATT  
Depth:3 (COW)  
Ei-value:0.000, Pi-value:0.000  
Er-value:0.000, Pr-value:0.000  
eCLIP MATCHES▶HNRNPU (bg=5.92%)No matches to TargetScan

TTCAGTTGCATACAGTTGTGCCTTTTATC

AGGACTCCT

AGGACTCCT  
Depth:2 (PIG)  
Ei-value:0.000, Pi-value:0.000  
Er-value:0.000, Pr-value:0.000  
eCLIP MATCHES▶HNRNPU (bg=5.92%)No matches to TargetScan

G

T

TACTTAT  
Depth:2 (PIG)  
Ei-value:0.000, Pi-value:0.010  
Er-value:0.000, Pr-value:0.030  
eCLIP MATCHES▶HNRNPU (bg=5.92%)No matches to TargetScan


ACTTAT

ACTTAT  
Depth:5 (RABBIT)  
Ei-value:0.000, Pi-value:0.000  
Er-value:0.000, Pr-value:0.000  
eCLIP MATCHES▶HNRNPU (bg=5.92%)No matches to TargetScan

C 5760  
 AAAGCAGAGAGTGCTAATCAATA

TTAAGC

TTAAGC  
Depth:2 (PIG)  
Ei-value:0.000, Pi-value:0.000  
Er-value:0.000, Pr-value:0.010  
No matches to eCLIP DataNo matches to TargetScan

CCTTCTCTTCGAACTGTAGATGGCA

TGTAATT

TGTAATT  
Depth:3 (COW)  
Ei-value:0.000, Pi-value:0.000  
Er-value:0.000, Pr-value:0.000  
No matches to eCLIP DataNo matches to TargetScan

GCAGTTGTCA

ATGGTC

ATGGTC  
Depth:3 (COW)  
Ei-value:0.000, Pi-value:0.020  
Er-value:0.000, Pr-value:0.000  
No matches to eCLIP DataNo matches to TargetScan


CTT

ATGGTCCTT  
Depth:2 (PIG)  
Ei-value:0.000, Pi-value:0.000  
Er-value:0.000, Pr-value:0.000  
No matches to eCLIP DataNo matches to TargetScan

CAATTAGACTTGGGTTTCTGACCTA

TCACAC

TCACAC  
Depth:2 (PIG)  
Ei-value:0.000, Pi-value:0.010  
Er-value:0.000, Pr-value:0.020  
No matches to eCLIP DataNo matches to TargetScan

CCTCTTTG

C

CTTTATTGC  
Depth:2 (PIG)  
Ei-value:0.000, Pi-value:0.000  
Er-value:0.000, Pr-value:0.000  
eCLIP MATCHES▶HNRNPL (bg=0.64%)MATCHES To TargetScan▶ miR-142-5p:AUAAAGU

 5880  


TTTATTGC

CTTTATTGC  
Depth:2 (PIG)  
Ei-value:0.000, Pi-value:0.000  
Er-value:0.000, Pr-value:0.000  
eCLIP MATCHES▶HNRNPL (bg=0.64%)MATCHES To TargetScan▶ miR-142-5p:AUAAAGU


ATGGGGTACT

ATGGGGTACT  
Depth:3 (COW)  
Ei-value:0.000, Pi-value:0.000  
Er-value:0.000, Pr-value:0.000  
eCLIP MATCHES▶HNRNPL (bg=0.64%)No matches to TargetScan

A

TT

TTCACTTAAGGCCCCTTTCTCAAAC  
Depth:2 (PIG)  
Ei-value:0.000, Pi-value:0.000  
Er-value:0.000, Pr-value:0.000  
eCLIP MATCHES▶HNRNPL (bg=0.64%)No matches to TargetScan


CAC

CACTTAAGGCCCCTTTCTCAA  
Depth:3 (COW)  
Ei-value:0.000, Pi-value:0.000  
Er-value:0.000, Pr-value:0.000  
eCLIP MATCHES▶HNRNPL (bg=0.64%)No matches to TargetScan


TTAAGGCC

TTAAGGCC  
Depth:6 (MOUSE)  
Ei-value:0.000, Pi-value:0.000  
Er-value:0.000, Pr-value:0.000  
eCLIP MATCHES▶HNRNPL (bg=0.64%)No matches to TargetScan


CCTTT

TTAAGGCCCCTTT  
Depth:5 (RABBIT)  
Ei-value:0.000, Pi-value:0.000  
Er-value:0.000, Pr-value:0.000  
eCLIP MATCHES▶HNRNPL (bg=0.64%)No matches to TargetScan


CTCAA

TTAAGGCCCCTTTCTCAA  
Depth:4 (DOG)  
Ei-value:0.000, Pi-value:0.000  
Er-value:0.000, Pr-value:0.000  
eCLIP MATCHES▶HNRNPL (bg=0.64%)No matches to TargetScan


AC

TTCACTTAAGGCCCCTTTCTCAAAC  
Depth:2 (PIG)  
Ei-value:0.000, Pi-value:0.000  
Er-value:0.000, Pr-value:0.000  
eCLIP MATCHES▶HNRNPL (bg=0.64%)No matches to TargetScan

TGTTAATGTGCC

TAATGACAATTACAT

TAATGACAATTACAT  
Depth:3 (COW)  
Ei-value:0.000, Pi-value:0.000  
Er-value:0.000, Pr-value:0.000  
eCLIP MATCHES▶HNRNPL (bg=0.64%)MATCHES To TargetScan▶ miR-411-3p:AUGUAAC

CAGT

ATCCTTCC

ATCCTTCC  
Depth:2 (PIG)  
Ei-value:0.000, Pi-value:0.010  
Er-value:0.000, Pr-value:0.000  
No matches to eCLIP DataNo matches to TargetScan

T

TTTGAAG

TTTGAAG  
Depth:2 (PIG)  
Ei-value:0.000, Pi-value:0.010  
Er-value:0.000, Pr-value:0.000  
eCLIP MATCHES▶HNRNPC (bg=3.65%)No matches to TargetScan

GACAGCATGGTTGGTGACA

CCTAAGG

CCTAAGG  
Depth:2 (PIG)  
Ei-value:0.000, Pi-value:0.010  
Er-value:0.000, Pr-value:0.000  
eCLIP MATCHES▶DDX51 (bg=1.63%)▶HNRNPC (bg=3.65%)No matches to TargetScan

C

CC

CCCATTTCTTG  
Depth:2 (PIG)  
Ei-value:0.000, Pi-value:0.000  
Er-value:0.000, Pr-value:0.000  
eCLIP MATCHES▶DDX51 (bg=1.63%)▶HNRNPC (bg=3.65%)MATCHES To TargetScan▶ miR-203a-3p.1:GAAAUGU

 6000  


CATTTCTTG

CCCATTTCTTG  
Depth:2 (PIG)  
Ei-value:0.000, Pi-value:0.000  
Er-value:0.000, Pr-value:0.000  
eCLIP MATCHES▶DDX51 (bg=1.63%)▶HNRNPC (bg=3.65%)MATCHES To TargetScan▶ miR-203a-3p.1:GAAAUGU

GCCTCCCAATATGTGTGAT

TGTATTTGTC

TGTATTTGTC  
Depth:2 (PIG)  
Ei-value:0.000, Pi-value:0.000  
Er-value:0.000, Pr-value:0.000  
eCLIP MATCHES▶DDX51 (bg=1.63%)No matches to TargetScan

GAGGTTGCTATGCACTAGAGAAGGAAAGTGCTCCCCTCATCCCCACTTTTCC

CTTCCAGCAGGAAGTGCCC

CTTCCAGCAGGAAGTGCCC  
Depth:2 (PIG)  
Ei-value:0.000, Pi-value:0.000  
Er-value:0.000, Pr-value:0.000  
eCLIP MATCHES▶hnrnpk (bg=12.88%)No matches to TargetScan

ACCCCATAAGA 6120  
 CCCTTTTATTTGGAGAGTCTAGGTGCACAATTGTAAGTGA

CCACAAG

CCACAAG  
Depth:2 (PIG)  
Ei-value:0.000, Pi-value:0.000  
Er-value:0.000, Pr-value:0.000  
eCLIP MATCHES▶HNRNPU (bg=5.92%)▶UTP3 (bg=3.66%)No matches to TargetScan

CATGCATCTTGGACATTTATGTGCGTAATCGCACACTGCTCATTCCATGTGAATAAGGTCCTACTCTCCGACC 6240  
 CCTTTTGCAATACAGAAGGGTTGCTGATAACGCAGTCCCCTTTTCTTGGCATGTTGTGTGTGATTATAATCGTCTGGGATCCTATGCACTAGAAAAGGAGGGTCCTCTCCACATACCTCA 6360  
 GTCTCACCTTTCCCTTCCAGCAGGGAGTGCCCACTCCATAAGACTCTCACA

TTTGGACAGTCAAG

TTTGGACAGTCAAG  
Depth:2 (PIG)  
Ei-value:0.000, Pi-value:0.000  
Er-value:0.000, Pr-value:0.000  
eCLIP MATCHES▶hnrnpk (bg=12.88%)No matches to TargetScan

GTGCGTAATTGTTAAGTGAACACAACC

ATGCAC

ATGCAC  
Depth:2 (PIG)  
Ei-value:0.000, Pi-value:0.030  
Er-value:0.000, Pr-value:0.010  
No matches to eCLIP DataNo matches to TargetScan

CTTAGACATGGATTTGCATAAC 6480  
 TACACACAGCTCAACCTATCTGAATAAAATCCTACTCTCAGACCCCTTTTGCAGTACAGCAGGGGTGCTGATCACCAAGGCCCTTTTTCCTGGCCTGGTATGCGTGTGATTATGTTTGTC 6600  
 CCGGTTCCTGTGTATTAGACATGGAAGCCTCCCCTGCCACACTCCACCCCCAATCTTCCTTTCCCTTCCGGCAGGGAGTGCCCTCTCCATAAGACGCTTACGTTTGGACAATCAAGGTGC 6720  
 ACAGTTGTAAGTGACCACAGGCATACACCTTGGACATTAATGTGCATAACCACTTTGCCCATTCCATCTGAATAAGGTCCTACTCTCAGACCCCTTTTGCAGTACAGCAGGGGTGCTGAT 6840  
 CACCAAGGCCCCTTTTCTTGGCCTGTTATGTGCGTGATTATATTTGTCTGGGTTCCTGTGTATTAGACAAGGAAGCCTTCCCCCCGCCCCCACCCCCACTCCCAGTCTTCCTTTCCCTTC 6960  
 CAGCAGGGAGTGCCCCCTCCATAAGATCATTACATTTGGACAATCAAGGTGCACAATTATAAGTGACCACAGCC

ATGCAC

ATGCAC  
Depth:2 (PIG)  
Ei-value:0.000, Pi-value:0.030  
Er-value:0.000, Pr-value:0.010  
eCLIP MATCHES▶hnrnpk (bg=12.88%)▶HNRNPU (bg=5.92%)No matches to TargetScan

CTTGGACATTATTGGACATTAATGTGC

GTAACTG

GTAACTG  
Depth:2 (PIG)  
Ei-value:0.000, Pi-value:0.000  
Er-value:0.000, Pr-value:0.000  
eCLIP MATCHES▶hnrnpk (bg=12.88%)No matches to TargetScan

CACATG 7080  
 GCCCATCCCATCTGAATAAGGTCCTACTCTCAGATGCCCTTTGCAGTACAGCAGGGGTACTGAATCACCAAGGCCCTTTTTCTTGGCCTGTTATGTGTGTGATTATATTTATCCCAGTTT 7200  
 CTGTGTAATAGACATGAAAGCCTCCCCTGCCACACCCCACCTCCAATCTTCCTTTCCCTTCCACCAGGGAGTGTCCACTCCATATACCCTTACATTTGGACAATCAAGGTGCACAATTGT 7320  
 AAGTGAGCATAGGCACT

CACCTTGGA

CACCTTGGA  
Depth:2 (PIG)  
Ei-value:0.000, Pi-value:0.000  
Er-value:0.000, Pr-value:0.000  
eCLIP MATCHES▶hnrnpk (bg=12.88%)▶HNRNPU (bg=5.92%)MATCHES To TargetScan▶ miR-18-5p:AAGGUGC

CATGAATGTGCATAACTGCACATGGCCCATCCCATCTGAATAAGGTCCTACTCTCAGACCCTTTTTGCAGTACAGCAGGGGTGCTGATCACCAA 7440  
 GGCCCCTTTTCCTGGCCTGTTATGTGTGTGATTATATTTGTTCCAGTTCCTGTGTAATAGACATGGAAGCCTCCCCTGCCACACTCCACCCCCAATCTTCCTTTCCCTTCTGGCAGGAAG 7560  
 TACCCGCTCCATAAGACCCTTACATTTGGACAGTCAAGGTGCACAATTGTATGTGACCACAACCATGCACCTTGGACATAAATGTGTGTAACTGCACATGGCCCATCCCATCTGAATAAG 7680  
 GTCCTACTCTCAGACCCCTTTTGCAGTACAGTAGGTGTGCTGATAACCAAGGCCCCTCTTCCTGGCCTGTTAACGTATGTGATTATATTTGTCTGGGTTCCAGTGTATAAGACATGGAAG 7800  
 CCTCCCCTGCCCCACCCCACCCTCAATCTTCCTTTCCCTTCTGGCAGGGAGTGCCAGCTCCATAAGAACCTTACATTTGGACAGTCAAGGTGCACAATTCTAAGTGACCGCAGCCATGCA 7920  
 CCTTGGTCAA

TAATGTGT

TAATGTGT  
Depth:2 (PIG)  
Ei-value:0.000, Pi-value:0.010  
Er-value:0.000, Pr-value:0.000  
No matches to eCLIP DataMATCHES To TargetScan▶ miR-323-3p:ACAUUAC

GTAACTGCACACGGCCTATCTCATCTGAATAAGGCCTTACTCTCAGACCCCTTTTGCAGTACAGCAGGGGTGCTGATAACCAAGGCCCATTTTCCTGGCCTG 8040  
 TTATGTGTGTGATTATATTTGTCCAGGTTTCTGTGTACTAGACAAGGAAGCCTCCTCTGCCCCATCCCATCTACGCATAATCTTTCTTTTCCTCCCAGCAGGGAGTGCTCACTCCATAAG 8160  
 ACCCTTACATTTGGACAATCAAGGTGCACAATTGTAAGTGACCACAACCATGCATCTTGGAAATTTATGTGC

ATAACTGCACATGGCT

ATAACTGCACATGGCT  
Depth:2 (PIG)  
Ei-value:0.000, Pi-value:0.000  
Er-value:0.000, Pr-value:0.000  
No matches to eCLIP DataMATCHES To TargetScan▶ miR-455-3p.2:UGCAGUC▶ miR-455-5p:AUGUGCC

TATCCTATTTGAATAAAGTCCTA

CTCTCAGAC

CTCTCAGACCCC  
Depth:2 (PIG)  
Ei-value:0.000, Pi-value:0.000  
Er-value:0.000, Pr-value:0.000  
eCLIP MATCHES▶SF3B1 (bg=2.48%)MATCHES To TargetScan▶ miR-193a-5p:GGGUCUU

 8280  


CCC

CTCTCAGACCCC  
Depth:2 (PIG)  
Ei-value:0.000, Pi-value:0.000  
Er-value:0.000, Pr-value:0.000  
eCLIP MATCHES▶SF3B1 (bg=2.48%)MATCHES To TargetScan▶ miR-193a-5p:GGGUCUU

CTTTGC

AGTATAGC

AGTATAGC  
Depth:2 (PIG)  
Ei-value:0.000, Pi-value:0.000  
Er-value:0.000, Pr-value:0.000  
eCLIP MATCHES▶SF3B1 (bg=2.48%)No matches to TargetScan

TGGGGTGCTGATCACTGAGG

CCTCTTT

CCTCTTT  
Depth:2 (PIG)  
Ei-value:0.000, Pi-value:0.010  
Er-value:0.000, Pr-value:0.020  
No matches to eCLIP DataNo matches to TargetScan

GCTTGGCTTGTCTATATTCTTGTGTACTAGATAAGGGCACCTTCTCATGG

ACTCCCTTTG

ACTCCCTTTG  
Depth:2 (PIG)  
Ei-value:0.000, Pi-value:0.000  
Er-value:0.000, Pr-value:0.000  
eCLIP MATCHES▶DDX21 (bg=0.25%)No matches to TargetScan

CTTTTCAACAAGGAGT 8400  
 ACC

CACTACTTT

CACTACTTT  
Depth:2 (PIG)  
Ei-value:0.000, Pi-value:0.000  
Er-value:0.000, Pr-value:0.000  
eCLIP MATCHES▶DDX21 (bg=0.25%)MATCHES To TargetScan▶ miR-142-3p.1:GUAGUGU

TTAAGATT

CTTATATTT

CTTATATTT  
Depth:3 (COW)  
Ei-value:0.000, Pi-value:0.000  
Er-value:0.000, Pr-value:0.000  
eCLIP MATCHES▶DDX21 (bg=0.25%)MATCHES To TargetScan▶ miR-410-3p:AUAUAAC

GTC

CAAAGTACATG

CAAAGTACATG  
Depth:2 (PIG)  
Ei-value:0.000, Pi-value:0.000  
Er-value:0.000, Pr-value:0.000  
No matches to eCLIP DataNo matches to TargetScan

G

TTTTAATTGACCA

TTTTAATTGACCA  
Depth:3 (COW)  
Ei-value:0.000, Pi-value:0.000  
Er-value:0.000, Pr-value:0.000  
No matches to eCLIP DataNo matches to TargetScan

CAACAATGTCCC

TTGG

TTGGACATTAATGTA  
Depth:2 (PIG)  
Ei-value:0.000, Pi-value:0.000  
Er-value:0.000, Pr-value:0.000  
No matches to eCLIP DataMATCHES To TargetScan▶ miR-323-3p:ACAUUAC


ACATTAAT

ACATTAAT  
Depth:3 (COW)  
Ei-value:0.000, Pi-value:0.000  
Er-value:0.000, Pr-value:0.000  
No matches to eCLIP DataNo matches to TargetScan


GTA

TTGGACATTAATGTA  
Depth:2 (PIG)  
Ei-value:0.000, Pi-value:0.000  
Er-value:0.000, Pr-value:0.000  
No matches to eCLIP DataMATCHES To TargetScan▶ miR-323-3p:ACAUUAC

TGTAATCACCACATGGTTCATCCTAATTAAACAAAG 8520  
 TTCTACCTTCTCACC

CTCCATTTGCAGTATA

CTCCATTTGCAGTATA  
Depth:2 (PIG)  
Ei-value:0.000, Pi-value:0.000  
Er-value:0.000, Pr-value:0.000  
eCLIP MATCHES▶hnrnpk (bg=12.88%)▶TIA1 (bg=4.07%)MATCHES To TargetScan▶ miR-217:ACUGCAU

C

CAGGGTT

CAGGGTT  
Depth:2 (PIG)  
Ei-value:0.000, Pi-value:0.000  
Er-value:0.000, Pr-value:0.000  
eCLIP MATCHES▶hnrnpk (bg=12.88%)▶TIA1 (bg=4.07%)MATCHES To TargetScan▶ miR-10-5p:ACCCUGU▶ miR-504-5p.1:ACCCUGG

GC

TGACCC

TGACCC  
Depth:2 (PIG)  
Ei-value:0.000, Pi-value:0.030  
Er-value:0.000, Pr-value:0.000  
eCLIP MATCHES▶hnrnpk (bg=12.88%)▶TIA1 (bg=4.07%)No matches to TargetScan

CCTAAGTCCCCTTTTCTTGGCTTGTTGACA

TG

TGCATAATTGCATTT  
Depth:2 (PIG)  
Ei-value:0.000, Pi-value:0.000  
Er-value:0.000, Pr-value:0.000  
eCLIP MATCHES▶hnrnpk (bg=12.88%)▶TIA1 (bg=4.07%)No matches to TargetScan


CATAATTGCA

CATAATTGCA  
Depth:3 (COW)  
Ei-value:0.000, Pi-value:0.000  
Er-value:0.000, Pr-value:0.000  
eCLIP MATCHES▶hnrnpk (bg=12.88%)▶TIA1 (bg=4.07%)No matches to TargetScan


TTT

TGCATAATTGCATTT  
Depth:2 (PIG)  
Ei-value:0.000, Pi-value:0.000  
Er-value:0.000, Pr-value:0.000  
eCLIP MATCHES▶hnrnpk (bg=12.88%)▶TIA1 (bg=4.07%)No matches to TargetScan

ATGTT

GGTTCTTG

GGTTCTTG  
Depth:2 (PIG)  
Ei-value:0.000, Pi-value:0.000  
Er-value:0.000, Pr-value:0.000  
eCLIP MATCHES▶UTP3 (bg=3.66%)No matches to TargetScan

TGCC

CTAGACAAGGA

CTAGACAAGGA  
Depth:3 (COW)  
Ei-value:0.000, Pi-value:0.000  
Er-value:0.000, Pr-value:0.000  
eCLIP MATCHES▶UTP3 (bg=3.66%)No matches to TargetScan

 8640  


CTAGACAAGGA  
Depth:3 (COW)  
Ei-value:0.000, Pi-value:0.000  
Er-value:0.000, Pr-value:0.000  
eCLIP MATCHES▶UTP3 (bg=3.66%)No matches to TargetScan

TGCCCCACCTCTTTTCAATAGTGGGTGCCCACTCCTTATGATCTTTACATTTGA

ACAGTTAATGTG

ACAGTTAATGTG  
Depth:4 (DOG)  
Ei-value:0.000, Pi-value:0.000  
Er-value:0.000, Pr-value:0.000  
eCLIP MATCHES▶HNRNPU (bg=5.92%)MATCHES To TargetScan▶ miR-323-3p:ACAUUAC

AAT

AATTGCAGTT

AATTGCAGTT  
Depth:2 (PIG)  
Ei-value:0.000, Pi-value:0.000  
Er-value:0.000, Pr-value:0.000  
eCLIP MATCHES▶HNRNPU (bg=5.92%)MATCHES To TargetScan▶ miR-217:ACUGCAU

G

TCCACAACCC

TCCACAACCC  
Depth:2 (PIG)  
Ei-value:0.000, Pi-value:0.000  
Er-value:0.000, Pr-value:0.000  
eCLIP MATCHES▶hnrnpk (bg=12.88%)▶HNRNPU (bg=5.92%)No matches to TargetScan

TATCACTTCTAGGACCATT

ATACCTC

ATACCTC  
Depth:2 (PIG)  
Ei-value:0.000, Pi-value:0.000  
Er-value:0.000, Pr-value:0.000  
No matches to eCLIP DataMATCHES To TargetScan▶ let-7-5p/98-5p:GAGGUAG

TTTT 8760  
 GCATTACTGTGGGGT

ATACTGTTT

ATACTGTTT  
Depth:3 (COW)  
Ei-value:0.000, Pi-value:0.000  
Er-value:0.000, Pr-value:0.000  
No matches to eCLIP DataMATCHES To TargetScan▶ miR-101-3p.1:ACAGUAC▶ miR-132-3p/212-3p:AACAGUC▶ miR-144-3p:ACAGUAU

CCCTCCAAGGCCCCTTCTG

GTGGAC

GTGGAC  
Depth:2 (PIG)  
Ei-value:0.000, Pi-value:0.000  
Er-value:0.000, Pr-value:0.010  
No matches to eCLIP DataNo matches to TargetScan

TATCAACATA

TAATTGAAAT

TAATTGAAAT  
Depth:2 (PIG)  
Ei-value:0.000, Pi-value:0.000  
Er-value:0.000, Pr-value:0.000  
No matches to eCLIP DataNo matches to TargetScan

TTTCTT

TTGTCTT

TTGTCTT  
Depth:3 (COW)  
Ei-value:0.000, Pi-value:0.000  
Er-value:0.000, Pr-value:0.010  
No matches to eCLIP DataNo matches to TargetScan

TGTCAGTAGATTAAGGTCATACCCCATCACCTTTCCTT 8880  
 TGTAGTACAACAGGGTGTCCTGATCAACCAAAGTCCTGTTGTTTTGGACTGTTAATATGTGCAATTACATTTGCTCCTGATCTGTGCACTAGATAAGGATCCTACCTACTTTCTTAGTGT 9000  
 TTTTAGCAGGTAGTGCCCACTACTCAAGACTGTCACTTGGAATGTTCATGTGCACAAACTCAATTCTCTAAGCATGTTCCTGTACCACCTTTGCTTTAGAGCAGGGGGATGATATTCACT 9120  
 AAGTGCCCCTTCTTTTGGACTTAATATGCATTAATGCAATTGTCCACCTCTTCTTT

TAGACT

TAGACT  
Depth:2 (PIG)  
Ei-value:0.000, Pi-value:0.000  
Er-value:0.000, Pr-value:0.000  
No matches to eCLIP DataNo matches to TargetScan

AAGAGTTGATCTCCACATATTCCCCTTGCATCAGGGGCATGTTAATTATGAATGAACC 9240  
 CTTTTCTTTTAATATTAATGTCATAATTGTATTTGTGGACCTGTGTAGGAGAAAAAGACCCTATGTTCCTCCCATTACCCTTTGGATTGCTGCTGAGAAGTGT

TAACTA

TAACTA  
Depth:4 (DOG)  
Ei-value:0.000, Pi-value:0.000  
Er-value:0.000, Pr-value:0.000  
No matches to eCLIP DataNo matches to TargetScan

CTCATAAT

CTC

CTCAGCTCTTGG  
Depth:5 (RABBIT)  
Ei-value:0.000, Pi-value:0.000  
Er-value:0.000, Pr-value:0.000  
No matches to eCLIP DataMATCHES To TargetScan▶ miR-335-5p:CAAGAGC

 9360  


AGCTCTTGG

CTCAGCTCTTGG  
Depth:5 (RABBIT)  
Ei-value:0.000, Pi-value:0.000  
Er-value:0.000, Pr-value:0.000  
No matches to eCLIP DataMATCHES To TargetScan▶ miR-335-5p:CAAGAGC


ACA

CTCAGCTCTTGGACA  
Depth:4 (DOG)  
Ei-value:0.000, Pi-value:0.000  
Er-value:0.000, Pr-value:0.000  
No matches to eCLIP DataMATCHES To TargetScan▶ miR-335-5p:CAAGAGC


ATTAATA

CTCAGCTCTTGGACAATTAATA  
Depth:3 (COW)  
Ei-value:0.000, Pi-value:0.000  
Er-value:0.000, Pr-value:0.000  
No matches to eCLIP DataMATCHES To TargetScan▶ miR-335-5p:CAAGAGC

GCATT

AATAACA

AATAACA  
Depth:2 (PIG)  
Ei-value:0.000, Pi-value:0.010  
Er-value:0.000, Pr-value:0.000  
No matches to eCLIP DataNo matches to TargetScan

ATTATCAAGGGC

ACT

ACTGATCATTAGATA  
Depth:2 (PIG)  
Ei-value:0.000, Pi-value:0.000  
Er-value:0.000, Pr-value:0.000  
eCLIP MATCHES▶HNRNPU (bg=5.92%)MATCHES To TargetScan▶ miR-383-5p.1:GAUCAGA


GATCAT

GATCAT  
Depth:3 (COW)  
Ei-value:0.000, Pi-value:0.000  
Er-value:0.000, Pr-value:0.000  
eCLIP MATCHES▶HNRNPU (bg=5.92%)No matches to TargetScan


TAGATA

ACTGATCATTAGATA  
Depth:2 (PIG)  
Ei-value:0.000, Pi-value:0.000  
Er-value:0.000, Pr-value:0.000  
eCLIP MATCHES▶HNRNPU (bg=5.92%)MATCHES To TargetScan▶ miR-383-5p.1:GAUCAGA

AGACTCCTGCTTCCTCGTTGCTTACATCGGGGGTACTGACCC

AC

ACTAAGGCCCC  
Depth:2 (PIG)  
Ei-value:0.000, Pi-value:0.000  
Er-value:0.000, Pr-value:0.000  
No matches to eCLIP DataNo matches to TargetScan


TAAGGC

TAAGGC  
Depth:3 (COW)  
Ei-value:0.000, Pi-value:0.000  
Er-value:0.000, Pr-value:0.000  
No matches to eCLIP DataNo matches to TargetScan


CCC

ACTAAGGCCCC  
Depth:2 (PIG)  
Ei-value:0.000, Pi-value:0.000  
Er-value:0.000, Pr-value:0.000  
No matches to eCLIP DataNo matches to TargetScan

TTGTACTGT 9480  
 TAATGT

GAATATTTGCA

GAATATTTGCA  
Depth:3 (COW)  
Ei-value:0.000, Pi-value:0.000  
Er-value:0.000, Pr-value:0.000  
No matches to eCLIP DataNo matches to TargetScan


ATTAT

GAATATTTGCAATTAT  
Depth:2 (PIG)  
Ei-value:0.000, Pi-value:0.000  
Er-value:0.000, Pr-value:0.000  
No matches to eCLIP DataMATCHES To TargetScan▶ miR-25-3p/32-5p/92-3p/363-3p/367-3p:AUUGCAC

ATATGTCTCCTTCTGGTAGAGTGGGATATTATGCCCTAGTA

TCCCCTT

TCCCCTT  
Depth:2 (PIG)  
Ei-value:0.000, Pi-value:0.000  
Er-value:0.000, Pr-value:0.030  
No matches to eCLIP DataNo matches to TargetScan

TGC

ATTACTG

ATTACTG  
Depth:3 (COW)  
Ei-value:0.000, Pi-value:0.010  
Er-value:0.000, Pr-value:0.020  
No matches to eCLIP DataMATCHES To TargetScan▶ miR-802:CAGUAAC

C

AG

AGGGGCTGCTGAC  
Depth:2 (PIG)  
Ei-value:0.000, Pi-value:0.000  
Er-value:0.000, Pr-value:0.000  
No matches to eCLIP DataMATCHES To TargetScan▶ miR-15-5p/16-5p/195-5p/424-5p/497-5p:AGCAGCA▶ miR-503-5p:AGCAGCG


GGGCTGCTGA

GGGCTGCTGA  
Depth:3 (COW)  
Ei-value:0.000, Pi-value:0.000  
Er-value:0.000, Pr-value:0.000  
No matches to eCLIP DataMATCHES To TargetScan▶ miR-15-5p/16-5p/195-5p/424-5p/497-5p:AGCAGCA▶ miR-503-5p:AGCAGCG


C

AGGGGCTGCTGAC  
Depth:2 (PIG)  
Ei-value:0.000, Pi-value:0.000  
Er-value:0.000, Pr-value:0.000  
No matches to eCLIP DataMATCHES To TargetScan▶ miR-15-5p/16-5p/195-5p/424-5p/497-5p:AGCAGCA▶ miR-503-5p:AGCAGCG

TACT

CAAAACTT

CAAAACTT  
Depth:4 (DOG)  
Ei-value:0.000, Pi-value:0.000  
Er-value:0.000, Pr-value:0.000  
eCLIP MATCHES▶SF3B1 (bg=2.48%)No matches to TargetScan


CT

CAAAACTTCT  
Depth:2 (PIG)  
Ei-value:0.000, Pi-value:0.000  
Er-value:0.000, Pr-value:0.000  
eCLIP MATCHES▶SF3B1 (bg=2.48%)No matches to TargetScan

C

CTGGGACTG

CTGGGACTG  
Depth:3 (COW)  
Ei-value:0.000, Pi-value:0.000  
Er-value:0.000, Pr-value:0.000  
eCLIP MATCHES▶SF3B1 (bg=2.48%)MATCHES To TargetScan▶ miR-455-3p.1:CAGUCCA

TT 9600  
 AATAG

GCACAATG

GCACAATG  
Depth:6 (MOUSE)  
Ei-value:0.000, Pi-value:0.000  
Er-value:0.000, Pr-value:0.000  
No matches to eCLIP DataNo matches to TargetScan

GCAGTTATCAATGGTTTTCTCC

CTCCCTG

CTCCCTG  
Depth:3 (COW)  
Ei-value:0.000, Pi-value:0.000  
Er-value:0.000, Pr-value:0.000  
eCLIP MATCHES▶DDX42 (bg=0.58%)No matches to TargetScan

A

CCTTGTT

CCTTGTT  
Depth:2 (PIG)  
Ei-value:0.000, Pi-value:0.000  
Er-value:0.000, Pr-value:0.040  
eCLIP MATCHES▶DDX42 (bg=0.58%)No matches to TargetScan

AA

GCAAGC

GCAAGC  
Depth:3 (COW)  
Ei-value:0.000, Pi-value:0.000  
Er-value:0.000, Pr-value:0.000  
eCLIP MATCHES▶DDX42 (bg=0.58%)▶hnrnpk (bg=12.88%)No matches to TargetScan


GC

GCAAGCGC  
Depth:2 (PIG)  
Ei-value:0.000, Pi-value:0.000  
Er-value:0.000, Pr-value:0.000  
eCLIP MATCHES▶DDX42 (bg=0.58%)▶hnrnpk (bg=12.88%)No matches to TargetScan

CCCACCCCACCCTTAG

TTTCCCATGG

TTTCCCATGG  
Depth:2 (PIG)  
Ei-value:0.000, Pi-value:0.000  
Er-value:0.000, Pr-value:0.000  
eCLIP MATCHES▶DDX42 (bg=0.58%)▶hnrnpk (bg=12.88%)No matches to TargetScan

C

ATAATAAAGTATAA

ATAATAAAGTATAA  
Depth:2 (PIG)  
Ei-value:0.000, Pi-value:0.000  
Er-value:0.000, Pr-value:0.000  
eCLIP MATCHES▶DDX42 (bg=0.58%)▶KHDRBS1 (bg=1.71%)No matches to TargetScan

GCATTGGAGTATTCCATGC 9720  
 ACTTGTCT

ATCAAACAG

ATCAAACAG  
Depth:2 (PIG)  
Ei-value:0.000, Pi-value:0.000  
Er-value:0.000, Pr-value:0.000  
No matches to eCLIP DataNo matches to TargetScan

TGGT

CCAT

CCATACTCCCA  
Depth:2 (PIG)  
Ei-value:0.000, Pi-value:0.000  
Er-value:0.000, Pr-value:0.000  
eCLIP MATCHES▶hnrnpk (bg=12.88%)MATCHES To TargetScan▶ miR-496.1:GAGUAUU


A

ACTCCCA  
Depth:4 (DOG)  
Ei-value:0.000, Pi-value:0.000  
Er-value:0.000, Pr-value:0.000  
eCLIP MATCHES▶hnrnpk (bg=12.88%)No matches to TargetScan


CTCCCA

CTCCCA  
Depth:6 (MOUSE)  
Ei-value:0.000, Pi-value:0.000  
Er-value:0.000, Pr-value:0.000  
eCLIP MATCHES▶hnrnpk (bg=12.88%)No matches to TargetScan

A

CCCTTTTGCATT

CCCTTTTGCATT  
Depth:4 (DOG)  
Ei-value:0.000, Pi-value:0.000  
Er-value:0.000, Pr-value:0.000  
eCLIP MATCHES▶hnrnpk (bg=12.88%)No matches to TargetScan


G

CCCTTTTGCATTG  
Depth:3 (COW)  
Ei-value:0.000, Pi-value:0.000  
Er-value:0.000, Pr-value:0.000  
eCLIP MATCHES▶hnrnpk (bg=12.88%)No matches to TargetScan

CGCCAGTGTGTAAAATCACAGGTAGCCATGGTGTCATGCTTTATATACGAAGTCTTCCCTCTCTCTGCCCCTTG 9840  
 TGTGCCCTTGGCCCCTTTTTACAGACTATTGCTCACAATCTCAGGTGTCCATATTTGCAGCTATTAGGTAAGATTGTGCTGTCTCCCTCTTCCCTTCCCTCTGCCCTGCCCCTTTTGCCT 9960  
 CT

TTGCTGGG

TTGCTGGG  
Depth:2 (PIG)  
Ei-value:0.000, Pi-value:0.000  
Er-value:0.000, Pr-value:0.010  
eCLIP MATCHES▶hnrnpk (bg=12.88%)MATCHES To TargetScan▶ miR-338-3p:CCAGCAU

TAATGTTGACCAGACAAGG

CCCTTTCT

CCCTTTCT  
Depth:2 (PIG)  
Ei-value:0.000, Pi-value:0.000  
Er-value:0.000, Pr-value:0.000  
eCLIP MATCHES▶hnrnpk (bg=12.88%)No matches to TargetScan

CTTGGACTTAAACAATTCTCAGTTGCACTTTCCTTGGTCCCACCCATTATACATGAACCCCTCT

ACTTCCTT

ACTTCCTT  
Depth:3 (COW)  
Ei-value:0.000, Pi-value:0.000  
Er-value:0.000, Pr-value:0.000  
eCLIP MATCHES▶hnrnpk (bg=12.88%)No matches to TargetScan

TCGCATTGCTT 10080  


CTGAGTA

CTGAGTA  
Depth:2 (PIG)  
Ei-value:0.000, Pi-value:0.010  
Er-value:0.000, Pr-value:0.000  
eCLIP MATCHES▶hnrnpk (bg=12.88%)No matches to TargetScan

TG

CTGACTACCCA

CTGACTACCCA  
Depth:2 (PIG)  
Ei-value:0.000, Pi-value:0.000  
Er-value:0.000, Pr-value:0.000  
eCLIP MATCHES▶hnrnpk (bg=12.88%)No matches to TargetScan

A

AGCCCCTTCT

AGCCCCTTCT  
Depth:3 (COW)  
Ei-value:0.000, Pi-value:0.000  
Er-value:0.000, Pr-value:0.000  
eCLIP MATCHES▶hnrnpk (bg=12.88%)No matches to TargetScan


GTGTTATTAA

AGCCCCTTCTGTGTTATTAA  
Depth:2 (PIG)  
Ei-value:0.000, Pi-value:0.000  
Er-value:0.000, Pr-value:0.000  
eCLIP MATCHES▶hnrnpk (bg=12.88%)No matches to TargetScan

TAAA

CACAGTA

CACAGTA  
Depth:3 (COW)  
Ei-value:0.000, Pi-value:0.000  
Er-value:0.000, Pr-value:0.000  
eCLIP MATCHES▶hnrnpk (bg=12.88%)No matches to TargetScan

C

TGATTGTC

TGATTGTCCCATTTTT  
Depth:3 (COW)  
Ei-value:0.000, Pi-value:0.000  
Er-value:0.000, Pr-value:0.000  
eCLIP MATCHES▶hnrnpk (bg=12.88%)No matches to TargetScan


CCATTTTT

CCATTTTT  
Depth:4 (DOG)  
Ei-value:0.000, Pi-value:0.000  
Er-value:0.000, Pr-value:0.000  
eCLIP MATCHES▶hnrnpk (bg=12.88%)No matches to TargetScan


CAGCCCA

CAGCCCA  
Depth:4 (DOG)  
Ei-value:0.000, Pi-value:0.000  
Er-value:0.000, Pr-value:0.000  
eCLIP MATCHES▶hnrnpk (bg=12.88%)No matches to TargetScan

TCAGTCCAAGA

TCTC

TCTCCCTACCA  
Depth:3 (COW)  
Ei-value:0.000, Pi-value:0.000  
Er-value:0.000, Pr-value:0.000  
eCLIP MATCHES▶hnrnpk (bg=12.88%)No matches to TargetScan


CCTACCA

CCTACCA  
Depth:4 (DOG)  
Ei-value:0.000, Pi-value:0.000  
Er-value:0.000, Pr-value:0.000  
eCLIP MATCHES▶hnrnpk (bg=12.88%)No matches to TargetScan


CTTTG

TCTCCCTACCACTTTG  
Depth:2 (PIG)  
Ei-value:0.000, Pi-value:0.000  
Er-value:0.000, Pr-value:0.000  
eCLIP MATCHES▶hnrnpk (bg=12.88%)MATCHES To TargetScan▶ miR-140-5p:AGUGGUU▶ miR-17-5p/20-5p/93-5p/106-5p/519-3p:AAAGUGC

GTGTGTTG

GTGCAGT

GTGCAGT  
Depth:3 (COW)  
Ei-value:0.000, Pi-value:0.000  
Er-value:0.000, Pr-value:0.000  
eCLIP MATCHES▶hnrnpk (bg=12.88%)MATCHES To TargetScan▶ miR-217:ACUGCAU

G

T

TTGACTA  
Depth:2 (PIG)  
Ei-value:0.000, Pi-value:0.020  
Er-value:0.000, Pr-value:0.000  
No matches to eCLIP DataNo matches to TargetScan

 10200  


TGACTA

TTGACTA  
Depth:2 (PIG)  
Ei-value:0.000, Pi-value:0.020  
Er-value:0.000, Pr-value:0.000  
No matches to eCLIP DataNo matches to TargetScan

TG

AAAAGCAG

AAAAGCAG  
Depth:6 (MOUSE)  
Ei-value:0.000, Pi-value:0.000  
Er-value:0.000, Pr-value:0.000  
No matches to eCLIP DataNo matches to TargetScan

G

CCT

CCTGAACTA  
Depth:2 (PIG)  
Ei-value:0.000, Pi-value:0.000  
Er-value:0.000, Pr-value:0.000  
No matches to eCLIP DataNo matches to TargetScan


GAACTA

GAACTA  
Depth:3 (COW)  
Ei-value:0.000, Pi-value:0.000  
Er-value:0.000, Pr-value:0.000  
No matches to eCLIP DataNo matches to TargetScan

GGTGGATAA

GCCTTCACTC

GCCTTCACTC  
Depth:2 (PIG)  
Ei-value:0.000, Pi-value:0.000  
Er-value:0.000, Pr-value:0.000  
No matches to eCLIP DataNo matches to TargetScan

ATTTTCTTTCATTTA

TTAATGATCC

TTAATGATCC  
Depth:4 (DOG)  
Ei-value:0.000, Pi-value:0.000  
Er-value:0.000, Pr-value:0.000  
No matches to eCLIP DataMATCHES To TargetScan▶ miR-382-3p:AUCAUUC

TAGTTT

CA

CAATTATTGT  
Depth:2 (PIG)  
Ei-value:0.000, Pi-value:0.000  
Er-value:0.000, Pr-value:0.000  
No matches to eCLIP DataNo matches to TargetScan


ATTATTGT

ATTATTGT  
Depth:3 (COW)  
Ei-value:0.000, Pi-value:0.000  
Er-value:0.000, Pr-value:0.000  
No matches to eCLIP DataNo matches to TargetScan

CAG

ATTCTGGG

ATTCTGGG  
Depth:4 (DOG)  
Ei-value:0.000, Pi-value:0.000  
Er-value:0.000, Pr-value:0.000  
No matches to eCLIP DataNo matches to TargetScan


GACA

ATTCTGGGGACA  
Depth:2 (PIG)  
Ei-value:0.000, Pi-value:0.000  
Er-value:0.000, Pr-value:0.000  
No matches to eCLIP DataNo matches to TargetScan

A

GAACCATTC

GAACCATTC  
Depth:2 (PIG)  
Ei-value:0.000, Pi-value:0.000  
Er-value:0.000, Pr-value:0.000  
No matches to eCLIP DataNo matches to TargetScan

TTGCCCACC 10320  
 TGTG

TTAC

TTACTGCTTTACT  
Depth:2 (PIG)  
Ei-value:0.000, Pi-value:0.000  
Er-value:0.000, Pr-value:0.000  
No matches to eCLIP DataMATCHES To TargetScan▶ miR-330-3p.2:AAAGCAC▶ miR-802:CAGUAAC


TG

TGCTTTACT  
Depth:3 (COW)  
Ei-value:0.000, Pi-value:0.000  
Er-value:0.000, Pr-value:0.000  
No matches to eCLIP DataMATCHES To TargetScan▶ miR-330-3p.2:AAAGCAC


CTTTACT

CTTTACT  
Depth:4 (DOG)  
Ei-value:0.000, Pi-value:0.000  
Er-value:0.000, Pr-value:0.000  
No matches to eCLIP DataNo matches to TargetScan

GT

GCAAAAT

GCAAAAT  
Depth:6 (MOUSE)  
Ei-value:0.000, Pi-value:0.000  
Er-value:0.000, Pr-value:0.000  
No matches to eCLIP DataNo matches to TargetScan

ACTG

AAGGCAA

AAGGCAA  
Depth:4 (DOG)  
Ei-value:0.000, Pi-value:0.000  
Er-value:0.000, Pr-value:0.000  
No matches to eCLIP DataNo matches to TargetScan


GTCAGACCCA

AAGGCAAGTCAGACCCA  
Depth:3 (COW)  
Ei-value:0.000, Pi-value:0.000  
Er-value:0.000, Pr-value:0.000  
No matches to eCLIP DataMATCHES To TargetScan▶ miR-193a-5p:GGGUCUU

GGGAGC

TGGATTGC

TGGATTGC  
Depth:4 (DOG)  
Ei-value:0.000, Pi-value:0.000  
Er-value:0.000, Pr-value:0.000  
No matches to eCLIP DataNo matches to TargetScan

CATCCTTTATTTTGTGTTTCCAGTGTACACTATAAAATTG

TCTCCCCAG

TCTCCCCAGGAAGGAAG  
Depth:2 (PIG)  
Ei-value:0.000, Pi-value:0.000  
Er-value:0.000, Pr-value:0.000  
eCLIP MATCHES▶SF3B1 (bg=2.48%)No matches to TargetScan


GAAGGAAG

GAAGGAAG  
Depth:3 (COW)  
Ei-value:0.000, Pi-value:0.000  
Er-value:0.000, Pr-value:0.000  
eCLIP MATCHES▶SF3B1 (bg=2.48%)No matches to TargetScan

GT 10440  
 TGGCACTT

TCTC

TCTCTGCATTCTTC  
Depth:2 (PIG)  
Ei-value:0.000, Pi-value:0.000  
Er-value:0.000, Pr-value:0.000  
eCLIP MATCHES▶SF3B1 (bg=2.48%)No matches to TargetScan


TGCATTCTTC

TGCATTCTTC  
Depth:5 (RABBIT)  
Ei-value:0.000, Pi-value:0.000  
Er-value:0.000, Pr-value:0.000  
eCLIP MATCHES▶SF3B1 (bg=2.48%)No matches to TargetScan

TTTC

CAG

CAGAGCAGATTGCCTGG  
Depth:2 (PIG)  
Ei-value:0.000, Pi-value:0.000  
Er-value:0.000, Pr-value:0.000  
eCLIP MATCHES▶SF3B1 (bg=2.48%)No matches to TargetScan


AGC

AGCAGATTGCCTGG  
Depth:4 (DOG)  
Ei-value:0.000, Pi-value:0.000  
Er-value:0.000, Pr-value:0.000  
eCLIP MATCHES▶SF3B1 (bg=2.48%)No matches to TargetScan


A

AGATTGCCTGG  
Depth:5 (RABBIT)  
Ei-value:0.000, Pi-value:0.000  
Er-value:0.000, Pr-value:0.000  
No matches to eCLIP DataNo matches to TargetScan


GATTGCCTGG

GATTGCCTGG  
Depth:6 (MOUSE)  
Ei-value:0.000, Pi-value:0.000  
Er-value:0.000, Pr-value:0.000  
No matches to eCLIP DataNo matches to TargetScan

T

TAAGAATCTCT

TAAGAATCTCT  
Depth:2 (PIG)  
Ei-value:0.000, Pi-value:0.000  
Er-value:0.000, Pr-value:0.000  
No matches to eCLIP DataNo matches to TargetScan

TGTTGTCCCCT

TTGTATATT

TTGTATATT  
Depth:4 (DOG)  
Ei-value:0.000, Pi-value:0.000  
Er-value:0.000, Pr-value:0.000  
No matches to eCLIP DataMATCHES To TargetScan▶ miR-381-3p:AUACAAG

GTTATTGTAAAG

TGCCAA

TGCCAA  
Depth:3 (COW)  
Ei-value:0.000, Pi-value:0.000  
Er-value:0.000, Pr-value:0.000  
No matches to eCLIP DataMATCHES To TargetScan▶ miR-182-5p:UUGGCAA▶ miR-96-5p/1271-5p:UUGGCAC

A

TGCCAGGATACA

TGCCAGGATACA  
Depth:3 (COW)  
Ei-value:0.000, Pi-value:0.000  
Er-value:0.000, Pr-value:0.000  
No matches to eCLIP DataNo matches to TargetScan

GCCAGAAAAATTGC 10560  
 TTATTATTATTAAAA

AAATTTTTT

AAATTTTTT  
Depth:2 (PIG)  
Ei-value:0.000, Pi-value:0.000  
Er-value:0.000, Pr-value:0.000  
No matches to eCLIP DataNo matches to TargetScan

TAAGAAAG

ACATCTGG

ACATCTGG  
Depth:3 (COW)  
Ei-value:0.000, Pi-value:0.000  
Er-value:0.000, Pr-value:0.000  
No matches to eCLIP DataNo matches to TargetScan

ATTGTAGGGTGGACTC

GAT

GATAACCTGGTCATT  
Depth:3 (COW)  
Ei-value:0.000, Pi-value:0.000  
Er-value:0.000, Pr-value:0.000  
No matches to eCLIP DataMATCHES To TargetScan▶ miR-154-5p:AGGUUAU


AAC

AACCTGGTCATT  
Depth:4 (DOG)  
Ei-value:0.000, Pi-value:0.000  
Er-value:0.000, Pr-value:0.000  
No matches to eCLIP DataNo matches to TargetScan


CTGGTCATT

CTGGTCATT  
Depth:5 (RABBIT)  
Ei-value:0.000, Pi-value:0.000  
Er-value:0.000, Pr-value:0.000  
No matches to eCLIP DataNo matches to TargetScan

ATT

T

TTTTTGAAG  
Depth:2 (PIG)  
Ei-value:0.000, Pi-value:0.000  
Er-value:0.000, Pr-value:0.000  
No matches to eCLIP DataNo matches to TargetScan


TTTTGAA

TTTTGAA  
Depth:3 (COW)  
Ei-value:0.000, Pi-value:0.000  
Er-value:0.000, Pr-value:0.010  
No matches to eCLIP DataNo matches to TargetScan


G

TTTTTGAAG  
Depth:2 (PIG)  
Ei-value:0.000, Pi-value:0.000  
Er-value:0.000, Pr-value:0.000  
No matches to eCLIP DataNo matches to TargetScan

CCAAAATAT

CCATTTAT

CCATTTAT  
Depth:5 (RABBIT)  
Ei-value:0.000, Pi-value:0.000  
Er-value:0.000, Pr-value:0.000  
No matches to eCLIP DataNo matches to TargetScan

ACTATGTACCTGG

TGAC

TGACCAGTGTCTCTCATTT  
Depth:4 (DOG)  
Ei-value:0.000, Pi-value:0.000  
Er-value:0.000, Pr-value:0.000  
eCLIP MATCHES▶SUPV3L1 (bg=1.57%)No matches to TargetScan


CAG

CAGTGTCTCTCATTT  
Depth:5 (RABBIT)  
Ei-value:0.000, Pi-value:0.000  
Er-value:0.000, Pr-value:0.000  
eCLIP MATCHES▶SUPV3L1 (bg=1.57%)No matches to TargetScan

 10680  


TGTCTCTCATTT

CAGTGTCTCTCATTT  
Depth:5 (RABBIT)  
Ei-value:0.000, Pi-value:0.000  
Er-value:0.000, Pr-value:0.000  
eCLIP MATCHES▶SUPV3L1 (bg=1.57%)No matches to TargetScan

TAACTG

AGG

AGGGTGGTG  
Depth:4 (DOG)  
Ei-value:0.000, Pi-value:0.000  
Er-value:0.000, Pr-value:0.000  
eCLIP MATCHES▶SUPV3L1 (bg=1.57%)No matches to TargetScan


GTGGTG

GTGGTG  
Depth:5 (RABBIT)  
Ei-value:0.000, Pi-value:0.000  
Er-value:0.000, Pr-value:0.000  
eCLIP MATCHES▶SUPV3L1 (bg=1.57%)No matches to TargetScan


G

AGGGTGGTGGGTCTGTGGATAGA  
Depth:2 (PIG)  
Ei-value:0.000, Pi-value:0.000  
Er-value:0.000, Pr-value:0.000  
eCLIP MATCHES▶SUPV3L1 (bg=1.57%)MATCHES To TargetScan▶ miR-140-3p.1:CCACAGG


GTCTGTGGATA

GTCTGTGGATA  
Depth:5 (RABBIT)  
Ei-value:0.000, Pi-value:0.000  
Er-value:0.000, Pr-value:0.000  
eCLIP MATCHES▶SUPV3L1 (bg=1.57%)MATCHES To TargetScan▶ miR-140-3p.1:CCACAGG


GA

GTCTGTGGATAGA  
Depth:3 (COW)  
Ei-value:0.000, Pi-value:0.000  
Er-value:0.000, Pr-value:0.000  
eCLIP MATCHES▶SUPV3L1 (bg=1.57%)MATCHES To TargetScan▶ miR-140-3p.1:CCACAGG

ACACTGACTCTTGC

TATTTTA

TATTTTA  
Depth:3 (COW)  
Ei-value:0.000, Pi-value:0.040  
Er-value:0.000, Pr-value:0.020  
eCLIP MATCHES▶SUPV3L1 (bg=1.57%)No matches to TargetScan

ATATCAAAGATA

TTCTAGA

TTCTAGA  
Depth:4 (DOG)  
Ei-value:0.000, Pi-value:0.000  
Er-value:0.000, Pr-value:0.000  
No matches to eCLIP DataNo matches to TargetScan

GTGGAACTCTTAAGACC

AGTATCTTTG

AGTATCTTTG  
Depth:3 (COW)  
Ei-value:0.000, Pi-value:0.000  
Er-value:0.000, Pr-value:0.000  
No matches to eCLIP DataNo matches to TargetScan

TGTGGGCTTTAC 10800  
 CAGC

ATTCACTT

ATTCACTT  
Depth:4 (DOG)  
Ei-value:0.000, Pi-value:0.000  
Er-value:0.000, Pr-value:0.000  
No matches to eCLIP DataNo matches to TargetScan


TTA

ATTCACTTTTAGAAAAAC  
Depth:2 (PIG)  
Ei-value:0.000, Pi-value:0.000  
Er-value:0.000, Pr-value:0.000  
No matches to eCLIP DataMATCHES To TargetScan▶ miR-17-5p/20-5p/93-5p/106-5p/519-3p:AAAGUGC


GAAAAAC

GAAAAAC  
Depth:4 (DOG)  
Ei-value:0.000, Pi-value:0.000  
Er-value:0.000, Pr-value:0.000  
No matches to eCLIP DataNo matches to TargetScan

TACCTAAATTTTA

TAATCCTT

TAATCCTT  
Depth:2 (PIG)  
Ei-value:0.000, Pi-value:0.010  
Er-value:0.000, Pr-value:0.000  
eCLIP MATCHES▶SUPV3L1 (bg=1.57%)No matches to TargetScan

T

AATTTCTTCATCTGGAGC

AATTTCTTCATCTGGAGC  
Depth:5 (RABBIT)  
Ei-value:0.000, Pi-value:0.000  
Er-value:0.000, Pr-value:0.000  
eCLIP MATCHES▶SUPV3L1 (bg=1.57%)▶U2AF2 (bg=1.76%)No matches to TargetScan


A

AATTTCTTCATCTGGAGCA  
Depth:2 (PIG)  
Ei-value:0.000, Pi-value:0.000  
Er-value:0.000, Pr-value:0.000  
eCLIP MATCHES▶SUPV3L1 (bg=1.57%)▶U2AF2 (bg=1.76%)No matches to TargetScan

CCTGCCCCTA

CTTATTT

CTTATTT  
Depth:4 (DOG)  
Ei-value:0.000, Pi-value:0.000  
Er-value:0.000, Pr-value:0.010  
eCLIP MATCHES▶SUPV3L1 (bg=1.57%)▶U2AF2 (bg=1.76%)No matches to TargetScan


CAAGAA

CTTATTTCAAGAA  
Depth:3 (COW)  
Ei-value:0.000, Pi-value:0.000  
Er-value:0.000, Pr-value:0.000  
eCLIP MATCHES▶SUPV3L1 (bg=1.57%)▶U2AF2 (bg=1.76%)MATCHES To TargetScan▶ miR-203a-3p.2:UGAAAUG

GATTGCAGTAAAACGATTAAATGAGGGAACATAT 10920  
 GCAGAGGTGCTTTTAAAAAGCATATGCCACCTTTTTTATTAATTATTAT

ATAAAATG

ATAAAATG  
Depth:4 (DOG)  
Ei-value:0.000, Pi-value:0.000  
Er-value:0.000, Pr-value:0.000  
No matches to eCLIP DataNo matches to TargetScan


A

ATAAAATGA  
Depth:3 (COW)  
Ei-value:0.000, Pi-value:0.000  
Er-value:0.000, Pr-value:0.000  
No matches to eCLIP DataNo matches to TargetScan

AGCATTTAATTATAGTAATAATTTGAAGTAGTTTGAAGT

ACCACACT

ACCACACT  
Depth:3 (COW)  
Ei-value:0.000, Pi-value:0.000  
Er-value:0.000, Pr-value:0.000  
No matches to eCLIP DataNo matches to TargetScan


GA

ACCACACTGA  
Depth:2 (PIG)  
Ei-value:0.000, Pi-value:0.000  
Er-value:0.000, Pr-value:0.000  
No matches to eCLIP DataNo matches to TargetScan

G

GTGAGG

GTGAGG  
Depth:3 (COW)  
Ei-value:0.000, Pi-value:0.000  
Er-value:0.000, Pr-value:0.000  
No matches to eCLIP DataNo matches to TargetScan

ACTTAA 11040  


AAATGAT

AAATGAT  
Depth:2 (PIG)  
Ei-value:0.000, Pi-value:0.020  
Er-value:0.000, Pr-value:0.020  
No matches to eCLIP DataMATCHES To TargetScan▶ miR-382-3p:AUCAUUC

AAGACGAGTTCCCTA

TTTTATA

TTTTATA  
Depth:3 (COW)  
Ei-value:0.000, Pi-value:0.000  
Er-value:0.000, Pr-value:0.010  
No matches to eCLIP DataMATCHES To TargetScan▶ miR-340-5p:UAUAAAG

AG

AAAAATAAGCCA

AAAAATAAGCCA  
Depth:5 (RABBIT)  
Ei-value:0.000, Pi-value:0.000  
Er-value:0.000, Pr-value:0.000  
No matches to eCLIP DataNo matches to TargetScan


A

AAAAATAAGCCAA  
Depth:4 (DOG)  
Ei-value:0.000, Pi-value:0.000  
Er-value:0.000, Pr-value:0.000  
No matches to eCLIP DataNo matches to TargetScan

AATTAAAT

AT

ATTCTTTTGGATATA  
Depth:2 (PIG)  
Ei-value:0.000, Pi-value:0.000  
Er-value:0.000, Pr-value:0.000  
No matches to eCLIP DataMATCHES To TargetScan▶ miR-186-5p:AAAGAAU


TCTTTTGGATATA

TCTTTTGGATATA  
Depth:3 (COW)  
Ei-value:0.000, Pi-value:0.000  
Er-value:0.000, Pr-value:0.000  
No matches to eCLIP DataNo matches to TargetScan

AATTTCAAC

AGTGAGATAGCTGCCT

AGTGAGATAGCTGCCT  
Depth:2 (PIG)  
Ei-value:0.000, Pi-value:0.000  
Er-value:0.000, Pr-value:0.000  
No matches to eCLIP DataNo matches to TargetScan

AGTGGAA

ATGAATAATA

ATGAATAATA  
Depth:4 (DOG)  
Ei-value:0.000, Pi-value:0.000  
Er-value:0.000, Pr-value:0.000  
No matches to eCLIP DataNo matches to TargetScan

TCCCAGCCACT 11160  


AGTGTACA

AGTGTACA  
Depth:3 (COW)  
Ei-value:0.000, Pi-value:0.000  
Er-value:0.000, Pr-value:0.000  
No matches to eCLIP DataMATCHES To TargetScan▶ miR-493-5p:UGUACAU


G

AGTGTACAGGGTGTTT  
Depth:2 (PIG)  
Ei-value:0.000, Pi-value:0.000  
Er-value:0.000, Pr-value:0.000  
No matches to eCLIP DataMATCHES To TargetScan▶ miR-10-5p:ACCCUGU▶ miR-339-5p:CCCUGUC▶ miR-486-5p:CCUGUAC▶ miR-493-5p:UGUACAU▶ miR-504-5p.1:ACCCUGG


GGTGTTT

GGTGTTT  
Depth:3 (COW)  
Ei-value:0.000, Pi-value:0.000  
Er-value:0.000, Pr-value:0.000  
No matches to eCLIP DataNo matches to TargetScan

TGTGGCACAGGATTATGTAATA

TGGAACTGCT

TGGAACTGCT  
Depth:4 (DOG)  
Ei-value:0.000, Pi-value:0.000  
Er-value:0.000, Pr-value:0.000  
No matches to eCLIP DataNo matches to TargetScan

CAAGC

AAA

AAATAACTAGT  
Depth:2 (PIG)  
Ei-value:0.000, Pi-value:0.000  
Er-value:0.000, Pr-value:0.000  
No matches to eCLIP DataNo matches to TargetScan


TAACTA

TAACTA  
Depth:4 (DOG)  
Ei-value:0.000, Pi-value:0.000  
Er-value:0.000, Pr-value:0.000  
No matches to eCLIP DataNo matches to TargetScan


GT

AAATAACTAGT  
Depth:2 (PIG)  
Ei-value:0.000, Pi-value:0.000  
Er-value:0.000, Pr-value:0.000  
No matches to eCLIP DataNo matches to TargetScan

CATCACAA

CAGCAGTTC

CAGCAGTTC  
Depth:5 (RABBIT)  
Ei-value:0.000, Pi-value:0.000  
Er-value:0.000, Pr-value:0.000  
No matches to eCLIP DataNo matches to TargetScan

T

TTGTAAT

TTGTAAT  
Depth:4 (DOG)  
Ei-value:0.000, Pi-value:0.000  
Er-value:0.000, Pr-value:0.000  
No matches to eCLIP DataNo matches to TargetScan

A

ACTGAAAA

ACTGAAAA  
Depth:5 (RABBIT)  
Ei-value:0.000, Pi-value:0.000  
Er-value:0.000, Pr-value:0.000  
No matches to eCLIP DataNo matches to TargetScan

AGAATATTGTTTCTCG

GAG

GAGAAGGATGTCAAAAGATCGGC  
Depth:3 (COW)  
Ei-value:0.000, Pi-value:0.000  
Er-value:0.000, Pr-value:0.000  
eCLIP MATCHES▶SRSF1 (bg=8.47%)▶U2AF2 (bg=1.76%)▶uchl5 (bg=11.16%)MATCHES To TargetScan▶ miR-362-5p/500b-5p:AUCCUUG▶ miR-489-3p:UGACAUC


AAG

AAGGATG  
Depth:5 (RABBIT)  
Ei-value:0.000, Pi-value:0.000  
Er-value:0.000, Pr-value:0.000  
eCLIP MATCHES▶SRSF1 (bg=8.47%)▶U2AF2 (bg=1.76%)▶uchl5 (bg=11.16%)MATCHES To TargetScan▶ miR-362-5p/500b-5p:AUCCUUG

 11280  


GATG

AAGGATG  
Depth:5 (RABBIT)  
Ei-value:0.000, Pi-value:0.000  
Er-value:0.000, Pr-value:0.000  
eCLIP MATCHES▶SRSF1 (bg=8.47%)▶U2AF2 (bg=1.76%)▶uchl5 (bg=11.16%)MATCHES To TargetScan▶ miR-362-5p/500b-5p:AUCCUUG


TCA

AAGGATGTCAAAAGATC  
Depth:4 (DOG)  
Ei-value:0.000, Pi-value:0.000  
Er-value:0.000, Pr-value:0.000  
eCLIP MATCHES▶SRSF1 (bg=8.47%)▶U2AF2 (bg=1.76%)▶uchl5 (bg=11.16%)MATCHES To TargetScan▶ miR-362-5p/500b-5p:AUCCUUG▶ miR-489-3p:UGACAUC


AAAGATC

AAAGATC  
Depth:6 (MOUSE)  
Ei-value:0.000, Pi-value:0.000  
Er-value:0.000, Pr-value:0.000  
eCLIP MATCHES▶SRSF1 (bg=8.47%)▶U2AF2 (bg=1.76%)▶uchl5 (bg=11.16%)No matches to TargetScan


GGC

GAGAAGGATGTCAAAAGATCGGC  
Depth:3 (COW)  
Ei-value:0.000, Pi-value:0.000  
Er-value:0.000, Pr-value:0.000  
eCLIP MATCHES▶SRSF1 (bg=8.47%)▶U2AF2 (bg=1.76%)▶uchl5 (bg=11.16%)MATCHES To TargetScan▶ miR-362-5p/500b-5p:AUCCUUG▶ miR-489-3p:UGACAUC

C

CAGCTCAGGG

CAGCTCAGGG  
Depth:4 (DOG)  
Ei-value:0.000, Pi-value:0.000  
Er-value:0.000, Pr-value:0.000  
eCLIP MATCHES▶SRSF1 (bg=8.47%)▶U2AF2 (bg=1.76%)▶uchl5 (bg=11.16%)MATCHES To TargetScan▶ miR-125-5p:CCCUGAG

A

GCAGTTTGC

GCAGTTTGC  
Depth:3 (COW)  
Ei-value:0.000, Pi-value:0.000  
Er-value:0.000, Pr-value:0.000  
eCLIP MATCHES▶SRSF1 (bg=8.47%)▶U2AF2 (bg=1.76%)▶uchl5 (bg=11.16%)No matches to TargetScan

C

CTACTAGCTCCT

CTACTAGCTCCT  
Depth:4 (DOG)  
Ei-value:0.000, Pi-value:0.000  
Er-value:0.000, Pr-value:0.000  
eCLIP MATCHES▶SRSF1 (bg=8.47%)▶U2AF2 (bg=1.76%)▶uchl5 (bg=11.16%)MATCHES To TargetScan▶ miR-28-5p/708-5p:AGGAGCU▶ miR-411-5p.2:UAGUAGA

C

GGACAGCTG

GGACAGCTG  
Depth:5 (RABBIT)  
Ei-value:0.000, Pi-value:0.000  
Er-value:0.000, Pr-value:0.000  
eCLIP MATCHES▶SRSF1 (bg=8.47%)▶SRSF7 (bg=2.32%)▶U2AF2 (bg=1.76%)▶ZNF622 (bg=6.58%)No matches to TargetScan


T

GGACAGCTGT  
Depth:4 (DOG)  
Ei-value:0.000, Pi-value:0.000  
Er-value:0.000, Pr-value:0.000  
eCLIP MATCHES▶SRSF1 (bg=8.47%)▶SRSF7 (bg=2.32%)▶U2AF2 (bg=1.76%)▶ZNF622 (bg=6.58%)No matches to TargetScan

A

A

AAGAAGAGTCTCTGGCTCTTTAGA  
Depth:3 (COW)  
Ei-value:0.000, Pi-value:0.000  
Er-value:0.000, Pr-value:0.000  
eCLIP MATCHES▶DDX24 (bg=2.97%)▶SRSF1 (bg=8.47%)▶SRSF7 (bg=2.32%)▶U2AF2 (bg=1.76%)▶ZNF622 (bg=6.58%)No matches to TargetScan


AGAAGAGTCTCTGGCTCTTTA

AGAAGAGTCTCTGGCTCTTTA  
Depth:5 (RABBIT)  
Ei-value:0.000, Pi-value:0.000  
Er-value:0.000, Pr-value:0.000  
eCLIP MATCHES▶DDX24 (bg=2.97%)▶SRSF1 (bg=8.47%)▶SRSF7 (bg=2.32%)▶U2AF2 (bg=1.76%)▶ZNF622 (bg=6.58%)No matches to TargetScan


GA

AGAAGAGTCTCTGGCTCTTTAGA  
Depth:4 (DOG)  
Ei-value:0.000, Pi-value:0.000  
Er-value:0.000, Pr-value:0.000  
eCLIP MATCHES▶DDX24 (bg=2.97%)▶SRSF1 (bg=8.47%)▶SRSF7 (bg=2.32%)▶U2AF2 (bg=1.76%)▶ZNF622 (bg=6.58%)No matches to TargetScan

ATACT||GATCCCATTGAAGATACCACGCTGCA 11398  
 TGTGTCCTTAGTAGTCATGTCTCCTTAGGCTCCTCTTG||GAC

ATTCTGAGC

ATTCTGAGC  
Depth:4 (DOG)  
Ei-value:0.000, Pi-value:0.000  
Er-value:0.000, Pr-value:0.000  
eCLIP MATCHES▶DDX24 (bg=2.97%)▶GRWD1 (bg=5.13%)▶MTPAP (bg=2.21%)▶NOLC1 (bg=9.43%)▶SRSF1 (bg=8.47%)▶ZNF622 (bg=6.58%)No matches to TargetScan

ATGTGAGACCTGAG

GA

GACTGCAA  
Depth:3 (COW)  
Ei-value:0.000, Pi-value:0.000  
Er-value:0.000, Pr-value:0.000  
eCLIP MATCHES▶DDX24 (bg=2.97%)▶GRWD1 (bg=5.13%)▶MTPAP (bg=2.21%)▶NOLC1 (bg=9.43%)▶SRSF1 (bg=8.47%)▶UTP3 (bg=3.66%)▶ZNF622 (bg=6.58%)MATCHES To TargetScan▶ miR-455-3p.2:UGCAGUC


CTGCAA

CTGCAA  
Depth:5 (RABBIT)  
Ei-value:0.000, Pi-value:0.000  
Er-value:0.000, Pr-value:0.000  
eCLIP MATCHES▶DDX24 (bg=2.97%)▶GRWD1 (bg=5.13%)▶MTPAP (bg=2.21%)▶NOLC1 (bg=9.43%)▶SRSF1 (bg=8.47%)▶UTP3 (bg=3.66%)▶ZNF622 (bg=6.58%)No matches to TargetScan

ACAGCTATAAGAGGCTCCAAATTAATCATATCTTTCCC

TTTGAGAA

TTTGAGAATCTGG  
Depth:3 (COW)  
Ei-value:0.000, Pi-value:0.000  
Er-value:0.000, Pr-value:0.000  
eCLIP MATCHES▶DDX24 (bg=2.97%)▶GRWD1 (bg=5.13%)▶NOLC1 (bg=9.43%)▶SRSF1 (bg=8.47%)▶uchl5 (bg=11.16%)▶ZNF622 (bg=6.58%)MATCHES To TargetScan▶ miR-371-5p:CUCAAAC

 11516  


TCTGG

TTTGAGAATCTGG  
Depth:3 (COW)  
Ei-value:0.000, Pi-value:0.000  
Er-value:0.000, Pr-value:0.000  
eCLIP MATCHES▶DDX24 (bg=2.97%)▶GRWD1 (bg=5.13%)▶NOLC1 (bg=9.43%)▶SRSF1 (bg=8.47%)▶uchl5 (bg=11.16%)▶ZNF622 (bg=6.58%)MATCHES To TargetScan▶ miR-371-5p:CUCAAAC

CC

AAGCTCCA

AAGCTCCA  
Depth:3 (COW)  
Ei-value:0.000, Pi-value:0.000  
Er-value:0.000, Pr-value:0.000  
eCLIP MATCHES▶DDX24 (bg=2.97%)▶GRWD1 (bg=5.13%)▶NOLC1 (bg=9.43%)▶RBM15 (bg=7.27%)▶SRSF1 (bg=8.47%)▶uchl5 (bg=11.16%)▶ZNF622 (bg=6.58%)No matches to TargetScan

GCT

AATCTA

AATCTA  
Depth:2 (PIG)  
Ei-value:0.000, Pi-value:0.000  
Er-value:0.000, Pr-value:0.000  
eCLIP MATCHES▶DDX24 (bg=2.97%)▶GRWD1 (bg=5.13%)▶NOLC1 (bg=9.43%)▶RBM15 (bg=7.27%)▶SRSF1 (bg=8.47%)▶TARDBP (bg=2.79%)▶uchl5 (bg=11.16%)▶ZNF622 (bg=6.58%)No matches to TargetScan

CTT

GGATGG

GGATGG  
Depth:3 (COW)  
Ei-value:0.000, Pi-value:0.000  
Er-value:0.000, Pr-value:0.010  
eCLIP MATCHES▶DDX24 (bg=2.97%)▶GRWD1 (bg=5.13%)▶NIPBL (bg=5.39%)▶NOLC1 (bg=9.43%)▶RBM15 (bg=7.27%)▶SRSF1 (bg=8.47%)▶TARDBP (bg=2.79%)▶uchl5 (bg=11.16%)▶ZNF622 (bg=6.58%)No matches to TargetScan

GTTGCCAGCTA

T

TCTGGAGAAAAAGATCTTCCTCAGAAGAATAGGCTTGTTG  
Depth:2 (PIG)  
Ei-value:0.000, Pi-value:0.000  
Er-value:0.000, Pr-value:0.000  
eCLIP MATCHES▶DDX24 (bg=2.97%)▶GRWD1 (bg=5.13%)▶NIPBL (bg=5.39%)▶NOLC1 (bg=9.43%)▶SRSF1 (bg=8.47%)▶SRSF7 (bg=2.32%)▶TARDBP (bg=2.79%)▶uchl5 (bg=11.16%)▶ZNF622 (bg=6.58%)MATCHES To TargetScan▶ miR-1224-5p:UGAGGAC▶ miR-7-5p:GGAAGAC


CTGGAGAAAAAG||ATCT

CTGGAGAAAAAGATCT  
Depth:3 (COW)  
Ei-value:0.000, Pi-value:0.000  
Er-value:0.000, Pr-value:0.000  
eCLIP MATCHES▶DDX24 (bg=2.97%)▶GRWD1 (bg=5.13%)▶NIPBL (bg=5.39%)▶NOLC1 (bg=9.43%)▶SRSF1 (bg=8.47%)▶SRSF7 (bg=2.32%)▶TARDBP (bg=2.79%)▶uchl5 (bg=11.16%)▶ZNF622 (bg=6.58%)No matches to TargetScan


TCCTCAG

TCTGGAGAAAAAGATCTTCCTCAGAAGAATAGGCTTGTTG  
Depth:2 (PIG)  
Ei-value:0.000, Pi-value:0.000  
Er-value:0.000, Pr-value:0.000  
eCLIP MATCHES▶DDX24 (bg=2.97%)▶GRWD1 (bg=5.13%)▶NIPBL (bg=5.39%)▶NOLC1 (bg=9.43%)▶SRSF1 (bg=8.47%)▶SRSF7 (bg=2.32%)▶TARDBP (bg=2.79%)▶uchl5 (bg=11.16%)▶ZNF622 (bg=6.58%)MATCHES To TargetScan▶ miR-1224-5p:UGAGGAC▶ miR-7-5p:GGAAGAC


AAGAATAGGC

AAGAATAGGC  
Depth:5 (RABBIT)  
Ei-value:0.000, Pi-value:0.000  
Er-value:0.000, Pr-value:0.000  
eCLIP MATCHES▶NOLC1 (bg=9.43%)▶SRSF7 (bg=2.32%)▶uchl5 (bg=11.16%)No matches to TargetScan


TTGTTG

TCTGGAGAAAAAGATCTTCCTCAGAAGAATAGGCTTGTTG  
Depth:2 (PIG)  
Ei-value:0.000, Pi-value:0.000  
Er-value:0.000, Pr-value:0.000  
eCLIP MATCHES▶DDX24 (bg=2.97%)▶GRWD1 (bg=5.13%)▶NIPBL (bg=5.39%)▶NOLC1 (bg=9.43%)▶SRSF1 (bg=8.47%)▶SRSF7 (bg=2.32%)▶TARDBP (bg=2.79%)▶uchl5 (bg=11.16%)▶ZNF622 (bg=6.58%)MATCHES To TargetScan▶ miR-1224-5p:UGAGGAC▶ miR-7-5p:GGAAGAC

TT

T

TTACAGTGTTAGTGA  
Depth:3 (COW)  
Ei-value:0.000, Pi-value:0.000  
Er-value:0.000, Pr-value:0.000  
eCLIP MATCHES▶ILF3 (bg=3.0%)▶NOLC1 (bg=9.43%)▶RBM15 (bg=7.27%)▶SRSF7 (bg=2.32%)▶ZNF622 (bg=6.58%)MATCHES To TargetScan▶ miR-141-3p/200a-3p:AACACUG


TACAGTGTTAGTGA

TACAGTGTTAGTGA  
Depth:5 (RABBIT)  
Ei-value:0.000, Pi-value:0.000  
Er-value:0.000, Pr-value:0.000  
eCLIP MATCHES▶ILF3 (bg=3.0%)▶NOLC1 (bg=9.43%)▶RBM15 (bg=7.27%)▶SRSF7 (bg=2.32%)▶ZNF622 (bg=6.58%)MATCHES To TargetScan▶ miR-141-3p/200a-3p:AACACUG

TC

CA

CATTCCCTTTGA  
Depth:3 (COW)  
Ei-value:0.000, Pi-value:0.000  
Er-value:0.000, Pr-value:0.000  
eCLIP MATCHES▶ILF3 (bg=3.0%)▶RBM15 (bg=7.27%)▶SRSF7 (bg=2.32%)▶ZNF622 (bg=6.58%)MATCHES To TargetScan▶ miR-1-3p/206:GGAAUGU


TTCCCTTTGA

TTCCCTTTGA  
Depth:6 (MOUSE)  
Ei-value:0.000, Pi-value:0.000  
Er-value:0.000, Pr-value:0.000  
eCLIP MATCHES▶ILF3 (bg=3.0%)▶RBM15 (bg=7.27%)▶SRSF7 (bg=2.32%)▶ZNF622 (bg=6.58%)No matches to TargetScan

CGA 11634  
 TCCC

TAGGTGGAGATGGGGCATGAGGATCCTCCAGGGGAA

TAGGTGGAGATGGGGCATGAGGATCCTCCAGGGGAA  
Depth:6 (MOUSE)  
Ei-value:0.000, Pi-value:0.000  
Er-value:0.000, Pr-value:0.000  
eCLIP MATCHES▶ILF3 (bg=3.0%)▶NOLC1 (bg=9.43%)▶RBM15 (bg=7.27%)▶SRSF7 (bg=2.32%)▶ZNF622 (bg=6.58%)MATCHES To TargetScan▶ miR-331-3p:CCCCUGG


A

TAGGTGGAGATGGGGCATGAGGATCCTCCAGGGGAAA  
Depth:5 (RABBIT)  
Ei-value:0.000, Pi-value:0.000  
Er-value:0.000, Pr-value:0.000  
eCLIP MATCHES▶ILF3 (bg=3.0%)▶NOLC1 (bg=9.43%)▶RBM15 (bg=7.27%)▶SRSF7 (bg=2.32%)▶ZNF622 (bg=6.58%)MATCHES To TargetScan▶ miR-331-3p:CCCCUGG


AGC

TAGGTGGAGATGGGGCATGAGGATCCTCCAGGGGAAAAGCTCACTACCACTGGGCAACAACCCTAGGTCAGGAG  
Depth:2 (PIG)  
Ei-value:0.000, Pi-value:0.000  
Er-value:0.000, Pr-value:0.000  
eCLIP MATCHES▶ILF3 (bg=3.0%)▶NOLC1 (bg=9.43%)▶RBM15 (bg=7.27%)▶SRSF7 (bg=2.32%)▶ZNF622 (bg=6.58%)MATCHES To TargetScan▶ miR-140-5p:AGUGGUU▶ miR-142-3p.1:GUAGUGU▶ miR-192-5p/215-5p:UGACCUA▶ miR-199-5p:CCAGUGU▶ miR-296-3p:AGGGUUG▶ miR-331-3p:CCCCUGG


TCACTA

TCACTA  
Depth:5 (RABBIT)  
Ei-value:0.000, Pi-value:0.000  
Er-value:0.000, Pr-value:0.000  
eCLIP MATCHES▶ILF3 (bg=3.0%)No matches to TargetScan


CCACT

TCACTACCACT  
Depth:4 (DOG)  
Ei-value:0.000, Pi-value:0.000  
Er-value:0.000, Pr-value:0.000  
eCLIP MATCHES▶ILF3 (bg=3.0%)MATCHES To TargetScan▶ miR-140-5p:AGUGGUU▶ miR-142-3p.1:GUAGUGU


G

TCACTACCACTG  
Depth:3 (COW)  
Ei-value:0.000, Pi-value:0.000  
Er-value:0.000, Pr-value:0.000  
eCLIP MATCHES▶ILF3 (bg=3.0%)MATCHES To TargetScan▶ miR-140-5p:AGUGGUU▶ miR-142-3p.1:GUAGUGU


G

TAGGTGGAGATGGGGCATGAGGATCCTCCAGGGGAAAAGCTCACTACCACTGGGCAACAACCCTAGGTCAGGAG  
Depth:2 (PIG)  
Ei-value:0.000, Pi-value:0.000  
Er-value:0.000, Pr-value:0.000  
eCLIP MATCHES▶ILF3 (bg=3.0%)▶NOLC1 (bg=9.43%)▶RBM15 (bg=7.27%)▶SRSF7 (bg=2.32%)▶ZNF622 (bg=6.58%)MATCHES To TargetScan▶ miR-140-5p:AGUGGUU▶ miR-142-3p.1:GUAGUGU▶ miR-192-5p/215-5p:UGACCUA▶ miR-199-5p:CCAGUGU▶ miR-296-3p:AGGGUUG▶ miR-331-3p:CCCCUGG


GCAACA

GCAACA  
Depth:6 (MOUSE)  
Ei-value:0.000, Pi-value:0.000  
Er-value:0.000, Pr-value:0.000  
eCLIP MATCHES▶ILF3 (bg=3.0%)No matches to TargetScan


AC

GCAACAAC  
Depth:5 (RABBIT)  
Ei-value:0.000, Pi-value:0.000  
Er-value:0.000, Pr-value:0.000  
eCLIP MATCHES▶ILF3 (bg=3.0%)No matches to TargetScan


CCTAGGTCAGGAG

TAGGTGGAGATGGGGCATGAGGATCCTCCAGGGGAAAAGCTCACTACCACTGGGCAACAACCCTAGGTCAGGAG  
Depth:2 (PIG)  
Ei-value:0.000, Pi-value:0.000  
Er-value:0.000, Pr-value:0.000  
eCLIP MATCHES▶ILF3 (bg=3.0%)▶NOLC1 (bg=9.43%)▶RBM15 (bg=7.27%)▶SRSF7 (bg=2.32%)▶ZNF622 (bg=6.58%)MATCHES To TargetScan▶ miR-140-5p:AGUGGUU▶ miR-142-3p.1:GUAGUGU▶ miR-192-5p/215-5p:UGACCUA▶ miR-199-5p:CCAGUGU▶ miR-296-3p:AGGGUUG▶ miR-331-3p:CCCCUGG

GTTCTGTCAAGATA

CTTTCCTGG

CTTTCCTGG  
Depth:3 (COW)  
Ei-value:0.000, Pi-value:0.000  
Er-value:0.000, Pr-value:0.000  
eCLIP MATCHES▶ILF3 (bg=3.0%)MATCHES To TargetScan▶ miR-665:CCAGGAG▶ miR-873-5p.1:CAGGAAC

TC

CCAGATAGGAAGAT

CCAGATAGGAAGAT  
Depth:2 (PIG)  
Ei-value:0.000, Pi-value:0.000  
Er-value:0.000, Pr-value:0.000  
eCLIP MATCHES▶ILF3 (bg=3.0%)MATCHES To TargetScan▶ miR-202-5p:UCCUAUG

A

AA

AAGTCTCAA  
Depth:2 (PIG)  
Ei-value:0.000, Pi-value:0.000  
Er-value:0.000, Pr-value:0.000  
No matches to eCLIP DataNo matches to TargetScan

 11754  


GTCTCAA

AAGTCTCAA  
Depth:2 (PIG)  
Ei-value:0.000, Pi-value:0.000  
Er-value:0.000, Pr-value:0.000  
No matches to eCLIP DataNo matches to TargetScan

AA

ACAACCACC

ACAACCACC  
Depth:5 (RABBIT)  
Ei-value:0.000, Pi-value:0.000  
Er-value:0.000, Pr-value:0.000  
eCLIP MATCHES▶PRPF8 (bg=0.26%)No matches to TargetScan


ACAC

ACAACCACCACAC  
Depth:4 (DOG)  
Ei-value:0.000, Pi-value:0.000  
Er-value:0.000, Pr-value:0.000  
eCLIP MATCHES▶PRPF8 (bg=0.26%)No matches to TargetScan

GTCAAG||CTCTTC

A

ATTGTTCC  
Depth:2 (PIG)  
Ei-value:0.000, Pi-value:0.000  
Er-value:0.000, Pr-value:0.000  
eCLIP MATCHES▶GRWD1 (bg=5.13%)▶SF3B4 (bg=0.05%)No matches to TargetScan


TTGTTCC

TTGTTCC  
Depth:4 (DOG)  
Ei-value:0.000, Pi-value:0.000  
Er-value:0.000, Pr-value:0.000  
eCLIP MATCHES▶GRWD1 (bg=5.13%)▶SF3B4 (bg=0.05%)No matches to TargetScan

TATC

TG

TGCCAAATC  
Depth:3 (COW)  
Ei-value:0.000, Pi-value:0.000  
Er-value:0.000, Pr-value:0.000  
eCLIP MATCHES▶GRWD1 (bg=5.13%)▶NOLC1 (bg=9.43%)MATCHES To TargetScan▶ miR-182-5p:UUGGCAA▶ miR-96-5p/1271-5p:UUGGCAC


CCAAAT

CCAAAT  
Depth:6 (MOUSE)  
Ei-value:0.000, Pi-value:0.000  
Er-value:0.000, Pr-value:0.000  
eCLIP MATCHES▶GRWD1 (bg=5.13%)▶NOLC1 (bg=9.43%)No matches to TargetScan


C

CCAAATC  
Depth:5 (RABBIT)  
Ei-value:0.000, Pi-value:0.000  
Er-value:0.000, Pr-value:0.000  
eCLIP MATCHES▶GRWD1 (bg=5.13%)▶NOLC1 (bg=9.43%)No matches to TargetScan

ATTATACTTCCTAC

AAGCAGTG

AAGCAGTG  
Depth:2 (PIG)  
Ei-value:0.000, Pi-value:0.000  
Er-value:0.000, Pr-value:0.000  
eCLIP MATCHES▶GRWD1 (bg=5.13%)▶NOLC1 (bg=9.43%)▶uchl5 (bg=11.16%)No matches to TargetScan

C

AGAGAG

AGAGAG  
Depth:2 (PIG)  
Ei-value:0.000, Pi-value:0.010  
Er-value:0.000, Pr-value:0.000  
eCLIP MATCHES▶GRWD1 (bg=5.13%)▶NOLC1 (bg=9.43%)▶uchl5 (bg=11.16%)▶ZNF622 (bg=6.58%)No matches to TargetScan

CTGAGTCTTCAGCAGGTC

CAAGAAA

CAAGAAA  
Depth:5 (RABBIT)  
Ei-value:0.000, Pi-value:0.000  
Er-value:0.000, Pr-value:0.000  
eCLIP MATCHES▶GRWD1 (bg=5.13%)▶NOLC1 (bg=9.43%)▶uchl5 (bg=11.16%)▶ZNF622 (bg=6.58%)No matches to TargetScan


T

CAAGAAAT  
Depth:3 (COW)  
Ei-value:0.000, Pi-value:0.000  
Er-value:0.000, Pr-value:0.000  
eCLIP MATCHES▶GRWD1 (bg=5.13%)▶NOLC1 (bg=9.43%)▶TRA2A (bg=4.8%)▶uchl5 (bg=11.16%)▶ZNF622 (bg=6.58%)No matches to TargetScan


T

CAAGAAATTTGAACACAC  
Depth:2 (PIG)  
Ei-value:0.000, Pi-value:0.000  
Er-value:0.000, Pr-value:0.000  
eCLIP MATCHES▶GRWD1 (bg=5.13%)▶NOLC1 (bg=9.43%)▶PTBP1 (bg=3.74%)▶RBM15 (bg=7.27%)▶TRA2A (bg=4.8%)▶uchl5 (bg=11.16%)▶ZNF622 (bg=6.58%)No matches to TargetScan


TGAACAC

TGAACACAC  
Depth:3 (COW)  
Ei-value:0.000, Pi-value:0.000  
Er-value:0.000, Pr-value:0.000  
eCLIP MATCHES▶GRWD1 (bg=5.13%)▶NOLC1 (bg=9.43%)▶PTBP1 (bg=3.74%)▶RBM15 (bg=7.27%)▶TRA2A (bg=4.8%)▶uchl5 (bg=11.16%)▶ZNF622 (bg=6.58%)No matches to TargetScan

 11872  


AC

TGAACACAC  
Depth:3 (COW)  
Ei-value:0.000, Pi-value:0.000  
Er-value:0.000, Pr-value:0.000  
eCLIP MATCHES▶GRWD1 (bg=5.13%)▶NOLC1 (bg=9.43%)▶PTBP1 (bg=3.74%)▶RBM15 (bg=7.27%)▶TRA2A (bg=4.8%)▶uchl5 (bg=11.16%)▶ZNF622 (bg=6.58%)No matches to TargetScan

TGAAGGAAGTCAGCCTTCCCACCT

G

GAAGATCAACATGCCTG  
Depth:4 (DOG)  
Ei-value:0.000, Pi-value:0.000  
Er-value:0.000, Pr-value:0.000  
eCLIP MATCHES▶GRWD1 (bg=5.13%)▶NOLC1 (bg=9.43%)▶PTBP1 (bg=3.74%)▶RBM15 (bg=7.27%)▶TRA2A (bg=4.8%)▶uchl5 (bg=11.16%)▶ZNF622 (bg=6.58%)No matches to TargetScan


AA

AAGATCAACATGC  
Depth:5 (RABBIT)  
Ei-value:0.000, Pi-value:0.000  
Er-value:0.000, Pr-value:0.000  
eCLIP MATCHES▶GRWD1 (bg=5.13%)▶NOLC1 (bg=9.43%)▶PTBP1 (bg=3.74%)▶RBM15 (bg=7.27%)▶TRA2A (bg=4.8%)▶uchl5 (bg=11.16%)▶ZNF622 (bg=6.58%)No matches to TargetScan


GATCAACATGC

GATCAACATGC  
Depth:6 (MOUSE)  
Ei-value:0.000, Pi-value:0.000  
Er-value:0.000, Pr-value:0.000  
eCLIP MATCHES▶GRWD1 (bg=5.13%)▶NOLC1 (bg=9.43%)▶PTBP1 (bg=3.74%)▶RBM15 (bg=7.27%)▶TRA2A (bg=4.8%)▶uchl5 (bg=11.16%)▶ZNF622 (bg=6.58%)No matches to TargetScan


CTG

GAAGATCAACATGCCTG  
Depth:4 (DOG)  
Ei-value:0.000, Pi-value:0.000  
Er-value:0.000, Pr-value:0.000  
eCLIP MATCHES▶GRWD1 (bg=5.13%)▶NOLC1 (bg=9.43%)▶PTBP1 (bg=3.74%)▶RBM15 (bg=7.27%)▶TRA2A (bg=4.8%)▶uchl5 (bg=11.16%)▶ZNF622 (bg=6.58%)No matches to TargetScan


GC

GAAGATCAACATGCCTGGC  
Depth:2 (PIG)  
Ei-value:0.000, Pi-value:0.000  
Er-value:0.000, Pr-value:0.000  
eCLIP MATCHES▶GRWD1 (bg=5.13%)▶NOLC1 (bg=9.43%)▶PTBP1 (bg=3.74%)▶RBM15 (bg=7.27%)▶TRA2A (bg=4.8%)▶uchl5 (bg=11.16%)▶ZNF622 (bg=6.58%)No matches to TargetScan

ACTCTAGCACTTGAGGATAGC

TGAATGA

TGAATGA  
Depth:2 (PIG)  
Ei-value:0.000, Pi-value:0.010  
Er-value:0.000, Pr-value:0.020  
eCLIP MATCHES▶AQR (bg=0.33%)▶GRWD1 (bg=5.13%)▶TRA2A (bg=4.8%)MATCHES To TargetScan▶ miR-1298-5p:UCAUUCG

A||

TGTGTAT

TGTGTAT  
Depth:6 (MOUSE)  
Ei-value:0.000, Pi-value:0.000  
Er-value:0.000, Pr-value:0.000  
eCLIP MATCHES▶TARDBP (bg=2.79%)▶ZC3H11A (bg=6.55%)No matches to TargetScan


TT

TGTGTATTT  
Depth:4 (DOG)  
Ei-value:0.000, Pi-value:0.000  
Er-value:0.000, Pr-value:0.000  
eCLIP MATCHES▶TARDBP (bg=2.79%)▶ZC3H11A (bg=6.55%)No matches to TargetScan

CT

TTGTC

TTGTCTCTTTCTTTCTT  
Depth:2 (PIG)  
Ei-value:0.000, Pi-value:0.000  
Er-value:0.000, Pr-value:0.000  
eCLIP MATCHES▶PTBP1 (bg=3.74%)▶TARDBP (bg=2.79%)▶ZC3H11A (bg=6.55%)MATCHES To TargetScan▶ miR-186-5p:AAAGAAU


TCTTTCTT

TCTTTCTT  
Depth:3 (COW)  
Ei-value:0.000, Pi-value:0.000  
Er-value:0.000, Pr-value:0.000  
eCLIP MATCHES▶TARDBP (bg=2.79%)▶ZC3H11A (bg=6.55%)No matches to TargetScan


TCTT

TTGTCTCTTTCTTTCTT  
Depth:2 (PIG)  
Ei-value:0.000, Pi-value:0.000  
Er-value:0.000, Pr-value:0.000  
eCLIP MATCHES▶PTBP1 (bg=3.74%)▶TARDBP (bg=2.79%)▶ZC3H11A (bg=6.55%)MATCHES To TargetScan▶ miR-186-5p:AAAGAAU

GTCTTTGCTCTTTG

TT

TTCTCTA  
Depth:2 (PIG)  
Ei-value:0.000, Pi-value:0.010  
Er-value:0.000, Pr-value:0.000  
eCLIP MATCHES▶MATR3 (bg=2.98%)▶PTBP1 (bg=3.74%)▶TARDBP (bg=2.79%)▶ZC3H11A (bg=6.55%)No matches to TargetScan

 11990  


CTCTA

TTCTCTA  
Depth:2 (PIG)  
Ei-value:0.000, Pi-value:0.010  
Er-value:0.000, Pr-value:0.000  
eCLIP MATCHES▶MATR3 (bg=2.98%)▶PTBP1 (bg=3.74%)▶TARDBP (bg=2.79%)▶ZC3H11A (bg=6.55%)No matches to TargetScan

TCTAAAG

TG

TGTGTCTTACCCATTTCCATG  
Depth:2 (PIG)  
Ei-value:0.000, Pi-value:0.000  
Er-value:0.000, Pr-value:0.000  
eCLIP MATCHES▶MATR3 (bg=2.98%)▶PTBP1 (bg=3.74%)▶TARDBP (bg=2.79%)▶ZC3H11A (bg=6.55%)MATCHES To TargetScan▶ miR-203a-3p.1:GAAAUGU▶ miR-208-3p:UAAGACG▶ miR-499a-5p:UAAGACU


TGTCTTA

TGTCTTA  
Depth:4 (DOG)  
Ei-value:0.000, Pi-value:0.000  
Er-value:0.000, Pr-value:0.000  
eCLIP MATCHES▶MATR3 (bg=2.98%)▶PTBP1 (bg=3.74%)▶TARDBP (bg=2.79%)▶ZC3H11A (bg=6.55%)MATCHES To TargetScan▶ miR-208-3p:UAAGACG▶ miR-499a-5p:UAAGACU


CCCATTTCCATG

TGTCTTACCCATTTCCATG  
Depth:3 (COW)  
Ei-value:0.000, Pi-value:0.000  
Er-value:0.000, Pr-value:0.000  
eCLIP MATCHES▶MATR3 (bg=2.98%)▶PTBP1 (bg=3.74%)▶TARDBP (bg=2.79%)▶ZC3H11A (bg=6.55%)MATCHES To TargetScan▶ miR-203a-3p.1:GAAAUGU▶ miR-208-3p:UAAGACG▶ miR-499a-5p:UAAGACU

TTTCTCTTGCTAATTTCTTTCGTGTGTGCCTTTGCCTCATTTTCTC

TTTTTGT

TTTTTGT  
Depth:4 (DOG)  
Ei-value:0.000, Pi-value:0.000  
Er-value:0.000, Pr-value:0.000  
eCLIP MATCHES▶MATR3 (bg=2.98%)▶PTBP1 (bg=3.74%)▶TARDBP (bg=2.79%)▶TIA1 (bg=4.07%)▶ZC3H11A (bg=6.55%)No matches to TargetScan

TCACAAGAGT

GGTCTGTGTCT

GGTCTGTGTCT  
Depth:2 (PIG)  
Ei-value:0.000, Pi-value:0.000  
Er-value:0.000, Pr-value:0.000  
eCLIP MATCHES▶MATR3 (bg=2.98%)▶PTBP1 (bg=3.74%)▶TIA1 (bg=4.07%)▶ZC3H11A (bg=6.55%)No matches to TargetScan

T

GTCTTAGA

GTCTTAGA  
Depth:2 (PIG)  
Ei-value:0.000, Pi-value:0.000  
Er-value:0.000, Pr-value:0.000  
eCLIP MATCHES▶MATR3 (bg=2.98%)▶PTBP1 (bg=3.74%)▶TIA1 (bg=4.07%)▶ZC3H11A (bg=6.55%)MATCHES To TargetScan▶ miR-208-3p:UAAGACG▶ miR-499a-5p:UAAGACU

CATA 12110  
 TCTCTCA

TTT

TTTTTCATTTTGTT  
Depth:2 (PIG)  
Ei-value:0.000, Pi-value:0.000  
Er-value:0.000, Pr-value:0.000  
No matches to eCLIP DataMATCHES To TargetScan▶ miR-495-3p:AACAAAC


TTCATTTTGTT

TTCATTTTGTT  
Depth:4 (DOG)  
Ei-value:0.000, Pi-value:0.000  
Er-value:0.000, Pr-value:0.000  
No matches to eCLIP DataMATCHES To TargetScan▶ miR-495-3p:AACAAAC

GCTATTT

CTC

CTCTTTGCTC  
Depth:2 (PIG)  
Ei-value:0.000, Pi-value:0.000  
Er-value:0.000, Pr-value:0.000  
eCLIP MATCHES▶MATR3 (bg=2.98%)▶PTBP1 (bg=3.74%)▶TIA1 (bg=4.07%)No matches to TargetScan


TTTGCTC

TTTGCTC  
Depth:3 (COW)  
Ei-value:0.000, Pi-value:0.000  
Er-value:0.000, Pr-value:0.000  
eCLIP MATCHES▶MATR3 (bg=2.98%)▶PTBP1 (bg=3.74%)▶TIA1 (bg=4.07%)No matches to TargetScan

TCCTAGATGTGGCTCTTCTTTCACGCTTTATTTCATGTCTCCTTTTTGGGTCACATGCTGTGTGCTTTTTGTCCT

TTTCTTG

TTTCTTGTT  
Depth:2 (PIG)  
Ei-value:0.000, Pi-value:0.000  
Er-value:0.000, Pr-value:0.000  
eCLIP MATCHES▶MATR3 (bg=2.98%)▶PTBP1 (bg=3.74%)▶SMNDC1 (bg=0.63%)▶TIA1 (bg=4.07%)No matches to TargetScan

 12230  


TT

TTTCTTGTT  
Depth:2 (PIG)  
Ei-value:0.000, Pi-value:0.000  
Er-value:0.000, Pr-value:0.000  
eCLIP MATCHES▶MATR3 (bg=2.98%)▶PTBP1 (bg=3.74%)▶SMNDC1 (bg=0.63%)▶TIA1 (bg=4.07%)No matches to TargetScan

CTGTCTACCTCTCCTTTCTC

TGCCTACCT

TGCCTACCT  
Depth:2 (PIG)  
Ei-value:0.000, Pi-value:0.000  
Er-value:0.000, Pr-value:0.000  
eCLIP MATCHES▶MATR3 (bg=2.98%)▶PTBP1 (bg=3.74%)▶SMNDC1 (bg=0.63%)▶TIA1 (bg=4.07%)MATCHES To TargetScan▶ miR-196-5p:AGGUAGU

CTC

TT

TTTTCTCTTTGTGAA  
Depth:3 (COW)  
Ei-value:0.000, Pi-value:0.000  
Er-value:0.000, Pr-value:0.000  
eCLIP MATCHES▶MATR3 (bg=2.98%)▶PTBP1 (bg=3.74%)▶SMNDC1 (bg=0.63%)▶TIA1 (bg=4.07%)No matches to TargetScan


TTCTCTTTG

TTCTCTTTG  
Depth:6 (MOUSE)  
Ei-value:0.000, Pi-value:0.000  
Er-value:0.000, Pr-value:0.000  
eCLIP MATCHES▶MATR3 (bg=2.98%)▶PTBP1 (bg=3.74%)▶SMNDC1 (bg=0.63%)▶TIA1 (bg=4.07%)No matches to TargetScan


TGAA

TTTTCTCTTTGTGAA  
Depth:3 (COW)  
Ei-value:0.000, Pi-value:0.000  
Er-value:0.000, Pr-value:0.000  
eCLIP MATCHES▶MATR3 (bg=2.98%)▶PTBP1 (bg=3.74%)▶SMNDC1 (bg=0.63%)▶TIA1 (bg=4.07%)No matches to TargetScan

CTGTGATTATTTGTTACCCC

TTCCCCTT

TTCCCCTT  
Depth:3 (COW)  
Ei-value:0.000, Pi-value:0.000  
Er-value:0.000, Pr-value:0.000  
eCLIP MATCHES▶MATR3 (bg=2.98%)▶PTBP1 (bg=3.74%)▶TIA1 (bg=4.07%)No matches to TargetScan


CT

TTCCCCTTCT  
Depth:2 (PIG)  
Ei-value:0.000, Pi-value:0.000  
Er-value:0.000, Pr-value:0.000  
eCLIP MATCHES▶MATR3 (bg=2.98%)▶PTBP1 (bg=3.74%)▶TIA1 (bg=4.07%)No matches to TargetScan

C

GTTCGTTT

GTTCGTTT  
Depth:2 (PIG)  
Ei-value:0.000, Pi-value:0.000  
Er-value:0.000, Pr-value:0.000  
eCLIP MATCHES▶MATR3 (bg=2.98%)▶PTBP1 (bg=3.74%)▶TIA1 (bg=4.07%)No matches to TargetScan

TAA

ATTTCACCT

ATTTCACCT  
Depth:4 (DOG)  
Ei-value:0.000, Pi-value:0.000  
Er-value:0.000, Pr-value:0.000  
eCLIP MATCHES▶TIA1 (bg=4.07%)MATCHES To TargetScan▶ miR-203a-3p.2:UGAAAUG

TTTTTCTGAGTCTGGCCTCC 12350  
 TTTC

TGCTG

TGCTGTTTCTACT  
Depth:3 (COW)  
Ei-value:0.000, Pi-value:0.000  
Er-value:0.000, Pr-value:0.000  
eCLIP MATCHES▶MATR3 (bg=2.98%)▶PTBP1 (bg=3.74%)▶TIA1 (bg=4.07%)MATCHES To TargetScan▶ miR-411-5p.1:AGUAGAC▶ miR-494-3p:GAAACAU


TTTCTAC

TTTCTAC  
Depth:6 (MOUSE)  
Ei-value:0.000, Pi-value:0.000  
Er-value:0.000, Pr-value:0.000  
eCLIP MATCHES▶MATR3 (bg=2.98%)▶PTBP1 (bg=3.74%)▶TIA1 (bg=4.07%)No matches to TargetScan


T

TTTCTACT  
Depth:5 (RABBIT)  
Ei-value:0.000, Pi-value:0.000  
Er-value:0.000, Pr-value:0.000  
eCLIP MATCHES▶MATR3 (bg=2.98%)▶PTBP1 (bg=3.74%)▶TIA1 (bg=4.07%)MATCHES To TargetScan▶ miR-411-5p.1:AGUAGAC

TTTT

ATCTCAC

ATCTCACATTTCTC  
Depth:2 (PIG)  
Ei-value:0.000, Pi-value:0.000  
Er-value:0.000, Pr-value:0.000  
eCLIP MATCHES▶MATR3 (bg=2.98%)▶PTBP1 (bg=3.74%)▶TIA1 (bg=4.07%)MATCHES To TargetScan▶ miR-203a-3p.1:GAAAUGU


ATTTCTC

ATTTCTC  
Depth:6 (MOUSE)  
Ei-value:0.000, Pi-value:0.000  
Er-value:0.000, Pr-value:0.000  
eCLIP MATCHES▶MATR3 (bg=2.98%)▶PTBP1 (bg=3.74%)▶TIA1 (bg=4.07%)No matches to TargetScan

ATTTCTGCATTTCCTTTC

TGCCTC

TGCCTCTCTTGGGC  
Depth:2 (PIG)  
Ei-value:0.000, Pi-value:0.000  
Er-value:0.000, Pr-value:0.000  
eCLIP MATCHES▶MATR3 (bg=2.98%)▶PTBP1 (bg=3.74%)▶SMNDC1 (bg=0.63%)▶TIA1 (bg=4.07%)MATCHES To TargetScan▶ miR-335-5p:CAAGAGC


TCTTGGG

TCTTGGG  
Depth:5 (RABBIT)  
Ei-value:0.000, Pi-value:0.000  
Er-value:0.000, Pr-value:0.000  
eCLIP MATCHES▶MATR3 (bg=2.98%)▶PTBP1 (bg=3.74%)▶SMNDC1 (bg=0.63%)▶TIA1 (bg=4.07%)No matches to TargetScan


C

TCTTGGGC  
Depth:3 (COW)  
Ei-value:0.000, Pi-value:0.000  
Er-value:0.000, Pr-value:0.000  
eCLIP MATCHES▶MATR3 (bg=2.98%)▶PTBP1 (bg=3.74%)▶SMNDC1 (bg=0.63%)▶TIA1 (bg=4.07%)No matches to TargetScan

TATTCTCTCTCTCCTCCCCTGCGTGCCTCAGCATCTCTTGCTG

TTTGTGA

TTTGTGA  
Depth:4 (DOG)  
Ei-value:0.000, Pi-value:0.010  
Er-value:0.000, Pr-value:0.000  
eCLIP MATCHES▶MATR3 (bg=2.98%)▶PTBP1 (bg=3.74%)▶TIA1 (bg=4.07%)No matches to TargetScan


TTT

TTTGTGATTTTC  
Depth:3 (COW)  
Ei-value:0.000, Pi-value:0.000  
Er-value:0.000, Pr-value:0.000  
eCLIP MATCHES▶MATR3 (bg=2.98%)▶PTBP1 (bg=3.74%)▶TIA1 (bg=4.07%)No matches to TargetScan

 12470  


TC

TTTGTGATTTTC  
Depth:3 (COW)  
Ei-value:0.000, Pi-value:0.000  
Er-value:0.000, Pr-value:0.000  
eCLIP MATCHES▶MATR3 (bg=2.98%)▶PTBP1 (bg=3.74%)▶TIA1 (bg=4.07%)No matches to TargetScan

TATTTCAGTATTAA

TCTCTGTT

TCTCTGTT  
Depth:4 (DOG)  
Ei-value:0.000, Pi-value:0.000  
Er-value:0.000, Pr-value:0.000  
eCLIP MATCHES▶MATR3 (bg=2.98%)▶PTBP1 (bg=3.74%)No matches to TargetScan

GGCTTGTATTTGTTCTCTGCTTCTTCCCTTTCTAC

TCACC

TCACCTTTGAGTATTT  
Depth:2 (PIG)  
Ei-value:0.000, Pi-value:0.000  
Er-value:0.000, Pr-value:0.000  
eCLIP MATCHES▶MATR3 (bg=2.98%)▶PTBP1 (bg=3.74%)▶TIA1 (bg=4.07%)MATCHES To TargetScan▶ miR-18-5p:AAGGUGC▶ miR-200bc-3p/429:AAUACUG▶ miR-371-5p:CUCAAAC


TTTGAGTATTT

TTTGAGTATTT  
Depth:4 (DOG)  
Ei-value:0.000, Pi-value:0.000  
Er-value:0.000, Pr-value:0.000  
eCLIP MATCHES▶MATR3 (bg=2.98%)▶PTBP1 (bg=3.74%)▶TIA1 (bg=4.07%)MATCHES To TargetScan▶ miR-200bc-3p/429:AAUACUG▶ miR-371-5p:CUCAAAC

CA

GCCTCTTC

GCCTCTTC  
Depth:2 (PIG)  
Ei-value:0.000, Pi-value:0.000  
Er-value:0.000, Pr-value:0.000  
eCLIP MATCHES▶MATR3 (bg=2.98%)▶PTBP1 (bg=3.74%)▶TIA1 (bg=4.07%)No matches to TargetScan

ATGAATCTATCTCCCTCT

CTTTGATT

CTTTGATT  
Depth:3 (COW)  
Ei-value:0.000, Pi-value:0.000  
Er-value:0.000, Pr-value:0.000  
eCLIP MATCHES▶MATR3 (bg=2.98%)▶PTBP1 (bg=3.74%)▶TIA1 (bg=4.07%)No matches to TargetScan

TCATGTAAT 12590  
 CTCTCCTTAAATATTTCTTTGCATATGTGGGCAAGTGTACG

TGTGTGTG

TGTGTGTG  
Depth:4 (DOG)  
Ei-value:0.000, Pi-value:0.000  
Er-value:0.000, Pr-value:0.000  
eCLIP MATCHES▶AATF (bg=0.64%)▶DDX24 (bg=2.97%)▶NCBP2 (bg=1.49%)▶NOLC1 (bg=9.43%)▶PTBP1 (bg=3.74%)▶SND1 (bg=0.45%)▶SRSF7 (bg=2.32%)▶TARDBP (bg=2.79%)▶WDR43 (bg=3.37%)▶XRCC6 (bg=2.91%)▶ZC3H8 (bg=0.29%)MATCHES To TargetScan▶ miR-329-3p/362-3p:ACACACC

TGTCATGTGTGGCAG

AGGGGCT

AGGGGCTTCCTAACCCCT  
Depth:2 (PIG)  
Ei-value:0.000, Pi-value:0.000  
Er-value:0.000, Pr-value:0.000  
eCLIP MATCHES▶AATF (bg=0.64%)▶DDX24 (bg=2.97%)▶NCBP2 (bg=1.49%)▶NOLC1 (bg=9.43%)▶PTBP1 (bg=3.74%)▶SND1 (bg=0.45%)▶SRSF7 (bg=2.32%)▶TARDBP (bg=2.79%)▶UTP3 (bg=3.66%)▶WDR43 (bg=3.37%)▶XRCC6 (bg=2.91%)▶ZC3H8 (bg=0.29%)No matches to TargetScan


TCCTAACCCCT

TCCTAACCCCT  
Depth:5 (RABBIT)  
Ei-value:0.000, Pi-value:0.000  
Er-value:0.000, Pr-value:0.000  
eCLIP MATCHES▶AATF (bg=0.64%)▶DDX24 (bg=2.97%)▶NCBP2 (bg=1.49%)▶NOLC1 (bg=9.43%)▶PTBP1 (bg=3.74%)▶SND1 (bg=0.45%)▶SRSF7 (bg=2.32%)▶TARDBP (bg=2.79%)▶UTP3 (bg=3.66%)▶WDR43 (bg=3.37%)▶XRCC6 (bg=2.91%)▶ZC3H8 (bg=0.29%)No matches to TargetScan

GCCTGA

TAGGTGCA

TAGGTGCA  
Depth:3 (COW)  
Ei-value:0.000, Pi-value:0.000  
Er-value:0.000, Pr-value:0.000  
eCLIP MATCHES▶DDX24 (bg=2.97%)▶NOLC1 (bg=9.43%)▶SND1 (bg=0.45%)▶SRSF7 (bg=2.32%)▶TARDBP (bg=2.79%)▶UTP3 (bg=3.66%)▶WDR43 (bg=3.37%)▶XRCC6 (bg=2.91%)▶ZC3H8 (bg=0.29%)No matches to TargetScan

GAACGTCGGCTATCAGAGC

AAGCA

AAGCATTG  
Depth:4 (DOG)  
Ei-value:0.000, Pi-value:0.000  
Er-value:0.000, Pr-value:0.000  
eCLIP MATCHES▶DDX24 (bg=2.97%)▶NOLC1 (bg=9.43%)▶NPM1 (bg=1.21%)▶RBFOX2 (bg=4.63%)▶RPS3 (bg=0.76%)▶SRSF1 (bg=8.47%)▶SRSF7 (bg=2.32%)▶TARDBP (bg=2.79%)▶TRA2A (bg=4.8%)▶U2AF2 (bg=1.76%)▶uchl5 (bg=11.16%)▶YWHAG (bg=1.87%)▶ZNF622 (bg=6.58%)No matches to TargetScan

 12710  


TTG

AAGCATTG  
Depth:4 (DOG)  
Ei-value:0.000, Pi-value:0.000  
Er-value:0.000, Pr-value:0.000  
eCLIP MATCHES▶DDX24 (bg=2.97%)▶NOLC1 (bg=9.43%)▶NPM1 (bg=1.21%)▶RBFOX2 (bg=4.63%)▶RPS3 (bg=0.76%)▶SRSF1 (bg=8.47%)▶SRSF7 (bg=2.32%)▶TARDBP (bg=2.79%)▶TRA2A (bg=4.8%)▶U2AF2 (bg=1.76%)▶uchl5 (bg=11.16%)▶YWHAG (bg=1.87%)▶ZNF622 (bg=6.58%)No matches to TargetScan

TGGAGCG

GTTCC

GTTCCTTATGCCAG  
Depth:2 (PIG)  
Ei-value:0.000, Pi-value:0.000  
Er-value:0.000, Pr-value:0.000  
eCLIP MATCHES▶DDX24 (bg=2.97%)▶FASTKD2 (bg=1.99%)▶LARP4 (bg=4.72%)▶NOLC1 (bg=9.43%)▶NPM1 (bg=1.21%)▶RBFOX2 (bg=4.63%)▶RBM15 (bg=7.27%)▶RPS3 (bg=0.76%)▶SRSF1 (bg=8.47%)▶SRSF7 (bg=2.32%)▶TARDBP (bg=2.79%)▶TRA2A (bg=4.8%)▶U2AF2 (bg=1.76%)▶uchl5 (bg=11.16%)▶WDR43 (bg=3.37%)▶YWHAG (bg=1.87%)▶ZC3H11A (bg=6.55%)▶ZNF622 (bg=6.58%)▶ZNF800 (bg=1.92%)No matches to TargetScan


TTATGCCA

TTATGCCA  
Depth:5 (RABBIT)  
Ei-value:0.000, Pi-value:0.000  
Er-value:0.000, Pr-value:0.000  
eCLIP MATCHES▶DDX24 (bg=2.97%)▶FASTKD2 (bg=1.99%)▶LARP4 (bg=4.72%)▶NOLC1 (bg=9.43%)▶NPM1 (bg=1.21%)▶RBFOX2 (bg=4.63%)▶RBM15 (bg=7.27%)▶RPS3 (bg=0.76%)▶SRSF1 (bg=8.47%)▶SRSF7 (bg=2.32%)▶TARDBP (bg=2.79%)▶TRA2A (bg=4.8%)▶U2AF2 (bg=1.76%)▶uchl5 (bg=11.16%)▶WDR43 (bg=3.37%)▶YWHAG (bg=1.87%)▶ZC3H11A (bg=6.55%)▶ZNF622 (bg=6.58%)▶ZNF800 (bg=1.92%)No matches to TargetScan


G

TTATGCCAG  
Depth:4 (DOG)  
Ei-value:0.000, Pi-value:0.000  
Er-value:0.000, Pr-value:0.000  
eCLIP MATCHES▶DDX24 (bg=2.97%)▶FASTKD2 (bg=1.99%)▶LARP4 (bg=4.72%)▶NOLC1 (bg=9.43%)▶NPM1 (bg=1.21%)▶RBFOX2 (bg=4.63%)▶RBM15 (bg=7.27%)▶RPS3 (bg=0.76%)▶SRSF1 (bg=8.47%)▶SRSF7 (bg=2.32%)▶TARDBP (bg=2.79%)▶TRA2A (bg=4.8%)▶U2AF2 (bg=1.76%)▶uchl5 (bg=11.16%)▶WDR43 (bg=3.37%)▶YWHAG (bg=1.87%)▶ZC3H11A (bg=6.55%)▶ZNF622 (bg=6.58%)▶ZNF800 (bg=1.92%)No matches to TargetScan

GCTGCCATGTGAG

ATGA

ATGATCCAAGACCAA  
Depth:2 (PIG)  
Ei-value:0.000, Pi-value:0.000  
Er-value:0.000, Pr-value:0.000  
eCLIP MATCHES▶DDX24 (bg=2.97%)▶FASTKD2 (bg=1.99%)▶LARP4 (bg=4.72%)▶NOLC1 (bg=9.43%)▶NPM1 (bg=1.21%)▶RBFOX2 (bg=4.63%)▶RBM15 (bg=7.27%)▶SRSF1 (bg=8.47%)▶SRSF7 (bg=2.32%)▶TARDBP (bg=2.79%)▶TRA2A (bg=4.8%)▶U2AF2 (bg=1.76%)▶uchl5 (bg=11.16%)▶WDR43 (bg=3.37%)▶YWHAG (bg=1.87%)▶ZC3H11A (bg=6.55%)▶ZNF622 (bg=6.58%)▶ZNF800 (bg=1.92%)MATCHES To TargetScan▶ miR-133a-3p.2/133b:UUGGUCC▶ miR-431-5p:GUCUUGC


TCCAAG

TCCAAG  
Depth:3 (COW)  
Ei-value:0.000, Pi-value:0.000  
Er-value:0.000, Pr-value:0.000  
eCLIP MATCHES▶DDX24 (bg=2.97%)▶FASTKD2 (bg=1.99%)▶LARP4 (bg=4.72%)▶NOLC1 (bg=9.43%)▶NPM1 (bg=1.21%)▶RBFOX2 (bg=4.63%)▶RBM15 (bg=7.27%)▶SRSF1 (bg=8.47%)▶SRSF7 (bg=2.32%)▶TARDBP (bg=2.79%)▶TRA2A (bg=4.8%)▶U2AF2 (bg=1.76%)▶uchl5 (bg=11.16%)▶WDR43 (bg=3.37%)▶YWHAG (bg=1.87%)▶ZC3H11A (bg=6.55%)▶ZNF622 (bg=6.58%)▶ZNF800 (bg=1.92%)No matches to TargetScan


ACCAA

ATGATCCAAGACCAA  
Depth:2 (PIG)  
Ei-value:0.000, Pi-value:0.000  
Er-value:0.000, Pr-value:0.000  
eCLIP MATCHES▶DDX24 (bg=2.97%)▶FASTKD2 (bg=1.99%)▶LARP4 (bg=4.72%)▶NOLC1 (bg=9.43%)▶NPM1 (bg=1.21%)▶RBFOX2 (bg=4.63%)▶RBM15 (bg=7.27%)▶SRSF1 (bg=8.47%)▶SRSF7 (bg=2.32%)▶TARDBP (bg=2.79%)▶TRA2A (bg=4.8%)▶U2AF2 (bg=1.76%)▶uchl5 (bg=11.16%)▶WDR43 (bg=3.37%)▶YWHAG (bg=1.87%)▶ZC3H11A (bg=6.55%)▶ZNF622 (bg=6.58%)▶ZNF800 (bg=1.92%)MATCHES To TargetScan▶ miR-133a-3p.2/133b:UUGGUCC▶ miR-431-5p:GUCUUGC

AACAAGGCCCTAGACTGCAGTAAAACCCAGAACTCAAGTAGGGCAGAAGGTGGAAGGCTCATATGGA

T

TAGAAGGCCCAA  
Depth:2 (PIG)  
Ei-value:0.000, Pi-value:0.000  
Er-value:0.000, Pr-value:0.000  
eCLIP MATCHES▶DDX24 (bg=2.97%)▶LARP4 (bg=4.72%)▶MTPAP (bg=2.21%)▶NOLC1 (bg=9.43%)▶SRSF1 (bg=8.47%)▶SRSF7 (bg=2.32%)▶TRA2A (bg=4.8%)▶uchl5 (bg=11.16%)▶UTP3 (bg=3.66%)▶ZNF622 (bg=6.58%)▶ZNF800 (bg=1.92%)No matches to TargetScan

 12830  


TAGAAGGCCCAA  
Depth:2 (PIG)  
Ei-value:0.000, Pi-value:0.000  
Er-value:0.000, Pr-value:0.000  
eCLIP MATCHES▶DDX24 (bg=2.97%)▶LARP4 (bg=4.72%)▶MTPAP (bg=2.21%)▶NOLC1 (bg=9.43%)▶SRSF1 (bg=8.47%)▶SRSF7 (bg=2.32%)▶TRA2A (bg=4.8%)▶uchl5 (bg=11.16%)▶UTP3 (bg=3.66%)▶ZNF622 (bg=6.58%)▶ZNF800 (bg=1.92%)No matches to TargetScan


AGA

AGAAGGCCCAA  
Depth:4 (DOG)  
Ei-value:0.000, Pi-value:0.000  
Er-value:0.000, Pr-value:0.000  
eCLIP MATCHES▶DDX24 (bg=2.97%)▶LARP4 (bg=4.72%)▶MTPAP (bg=2.21%)▶NOLC1 (bg=9.43%)▶SRSF1 (bg=8.47%)▶SRSF7 (bg=2.32%)▶TRA2A (bg=4.8%)▶uchl5 (bg=11.16%)▶UTP3 (bg=3.66%)▶ZNF622 (bg=6.58%)▶ZNF800 (bg=1.92%)No matches to TargetScan


AGGCCCAA

AGGCCCAA  
Depth:5 (RABBIT)  
Ei-value:0.000, Pi-value:0.000  
Er-value:0.000, Pr-value:0.000  
eCLIP MATCHES▶DDX24 (bg=2.97%)▶LARP4 (bg=4.72%)▶MTPAP (bg=2.21%)▶NOLC1 (bg=9.43%)▶SRSF1 (bg=8.47%)▶SRSF7 (bg=2.32%)▶TRA2A (bg=4.8%)▶uchl5 (bg=11.16%)▶UTP3 (bg=3.66%)▶ZNF622 (bg=6.58%)▶ZNF800 (bg=1.92%)No matches to TargetScan

AGTATAAGACAGATGGTTTGAGACTTGAGACCCGAGGACTAAGATGGAAAGCCCATGTTCCAAGATAGATAGAAGCCTCAGGCCTGAAACCAACAAAAGCCTCAAGAGC 12950  
 CAAGAAAACAGAGGGTGGCCTGAATTGGACCGAAGGCCT

GAGTTGGATGGAAG

GAGTTGGATGGAAG  
Depth:2 (PIG)  
Ei-value:0.000, Pi-value:0.000  
Er-value:0.000, Pr-value:0.000  
eCLIP MATCHES▶AARS (bg=2.18%)▶CPEB4 (bg=1.89%)▶DDX24 (bg=2.97%)▶FASTKD2 (bg=1.99%)▶GRWD1 (bg=5.13%)▶HLTF (bg=0.4%)▶LARP4 (bg=4.72%)▶METAP2 (bg=0.78%)▶MTPAP (bg=2.21%)▶NOLC1 (bg=9.43%)▶PUM1 (bg=1.56%)▶RBFOX2 (bg=4.63%)▶RPS11 (bg=0.63%)▶SAFB (bg=2.69%)▶SLTM (bg=2.2%)▶SRSF1 (bg=8.47%)▶TRA2A (bg=4.8%)▶uchl5 (bg=11.16%)▶XRCC6 (bg=2.91%)▶ZNF622 (bg=6.58%)▶ZNF800 (bg=1.92%)No matches to TargetScan

TCTCAAGGCTTGAGTTAG

AAGTCT

AAGTCT  
Depth:2 (PIG)  
Ei-value:0.000, Pi-value:0.010  
Er-value:0.000, Pr-value:0.010  
eCLIP MATCHES▶CPEB4 (bg=1.89%)▶DDX24 (bg=2.97%)▶DROSHA (bg=2.49%)▶FASTKD2 (bg=1.99%)▶GRWD1 (bg=5.13%)▶HLTF (bg=0.4%)▶LARP4 (bg=4.72%)▶METAP2 (bg=0.78%)▶MTPAP (bg=2.21%)▶NOLC1 (bg=9.43%)▶NPM1 (bg=1.21%)▶PPIL4 (bg=0.52%)▶PUM1 (bg=1.56%)▶RBFOX2 (bg=4.63%)▶RPS11 (bg=0.63%)▶SAFB (bg=2.69%)▶SLTM (bg=2.2%)▶SRSF1 (bg=8.47%)▶TRA2A (bg=4.8%)▶uchl5 (bg=11.16%)▶WDR43 (bg=3.37%)▶XRCC6 (bg=2.91%)▶ZC3H11A (bg=6.55%)▶ZNF622 (bg=6.58%)▶ZNF800 (bg=1.92%)No matches to TargetScan

TAAGACCTGGGACAGGACACATGGAAGGCCTAAGAACTGAGAC 13070  
 TTGTGACAC

AAGGCCAA

AAGGCCAA  
Depth:2 (PIG)  
Ei-value:0.000, Pi-value:0.000  
Er-value:0.000, Pr-value:0.000  
eCLIP MATCHES▶GRWD1 (bg=5.13%)▶MTPAP (bg=2.21%)▶SAFB (bg=2.69%)▶SLTM (bg=2.2%)▶SRSF1 (bg=8.47%)▶TRA2A (bg=4.8%)▶uchl5 (bg=11.16%)▶UTP3 (bg=3.66%)▶WDR43 (bg=3.37%)▶XRCC6 (bg=2.91%)No matches to TargetScan

C

GACCTAAGA

GACCTAAGA  
Depth:2 (PIG)  
Ei-value:0.000, Pi-value:0.000  
Er-value:0.000, Pr-value:0.000  
eCLIP MATCHES▶GRWD1 (bg=5.13%)▶NOLC1 (bg=9.43%)▶SAFB (bg=2.69%)▶SRSF1 (bg=8.47%)▶TRA2A (bg=4.8%)▶UTP3 (bg=3.66%)▶WDR43 (bg=3.37%)▶XRCC6 (bg=2.91%)▶ZNF622 (bg=6.58%)No matches to TargetScan

TTAGCCCAGGGTTGTAGCTGGAAGACCTACAACCCAAGGATG

GAAGGCCC

GAAGGCCC  
Depth:2 (PIG)  
Ei-value:0.000, Pi-value:0.000  
Er-value:0.000, Pr-value:0.000  
eCLIP MATCHES▶RBFOX2 (bg=4.63%)▶SLTM (bg=2.2%)▶SRSF1 (bg=8.47%)▶TRA2A (bg=4.8%)▶ZNF622 (bg=6.58%)No matches to TargetScan

CTGTCACAAAGCCTACCTAGATGGATAGAGGACCCAAGCGAAA 13190  
 AAGG

TATC

TATCTCAAGACTAA  
Depth:2 (PIG)  
Ei-value:0.000, Pi-value:0.000  
Er-value:0.000, Pr-value:0.000  
eCLIP MATCHES▶CPEB4 (bg=1.89%)▶FASTKD2 (bg=1.99%)▶GRWD1 (bg=5.13%)▶LARP4 (bg=4.72%)▶MTPAP (bg=2.21%)▶NOLC1 (bg=9.43%)▶RBFOX2 (bg=4.63%)▶SRSF1 (bg=8.47%)▶TRA2A (bg=4.8%)▶uchl5 (bg=11.16%)▶UTP18 (bg=0.72%)▶UTP3 (bg=3.66%)▶WDR43 (bg=3.37%)▶ZNF622 (bg=6.58%)MATCHES To TargetScan▶ miR-431-5p:GUCUUGC


TCAA

TCAAGACTAA  
Depth:4 (DOG)  
Ei-value:0.000, Pi-value:0.000  
Er-value:0.000, Pr-value:0.000  
eCLIP MATCHES▶CPEB4 (bg=1.89%)▶FASTKD2 (bg=1.99%)▶GRWD1 (bg=5.13%)▶LARP4 (bg=4.72%)▶MTPAP (bg=2.21%)▶NOLC1 (bg=9.43%)▶RBFOX2 (bg=4.63%)▶SRSF1 (bg=8.47%)▶TRA2A (bg=4.8%)▶uchl5 (bg=11.16%)▶UTP18 (bg=0.72%)▶UTP3 (bg=3.66%)▶WDR43 (bg=3.37%)▶ZNF622 (bg=6.58%)MATCHES To TargetScan▶ miR-431-5p:GUCUUGC


GACTAA

GACTAA  
Depth:5 (RABBIT)  
Ei-value:0.000, Pi-value:0.000  
Er-value:0.000, Pr-value:0.000  
eCLIP MATCHES▶CPEB4 (bg=1.89%)▶FASTKD2 (bg=1.99%)▶GRWD1 (bg=5.13%)▶LARP4 (bg=4.72%)▶MTPAP (bg=2.21%)▶NOLC1 (bg=9.43%)▶RBFOX2 (bg=4.63%)▶SRSF1 (bg=8.47%)▶TRA2A (bg=4.8%)▶uchl5 (bg=11.16%)▶UTP18 (bg=0.72%)▶UTP3 (bg=3.66%)▶WDR43 (bg=3.37%)▶ZNF622 (bg=6.58%)No matches to TargetScan

CGGCCG

GAATCTGG

GAATCTGG  
Depth:2 (PIG)  
Ei-value:0.000, Pi-value:0.000  
Er-value:0.000, Pr-value:0.000  
eCLIP MATCHES▶CPEB4 (bg=1.89%)▶FASTKD2 (bg=1.99%)▶GRWD1 (bg=5.13%)▶LARP4 (bg=4.72%)▶MTPAP (bg=2.21%)▶NOLC1 (bg=9.43%)▶PCBP1 (bg=1.07%)▶RBFOX2 (bg=4.63%)▶SRSF1 (bg=8.47%)▶TRA2A (bg=4.8%)▶uchl5 (bg=11.16%)▶UTP18 (bg=0.72%)▶UTP3 (bg=3.66%)▶WDR43 (bg=3.37%)▶ZNF622 (bg=6.58%)No matches to TargetScan

AGGCCCATGACCCAGAACCCAGGAAG

GAT

GATAGAAGC  
Depth:2 (PIG)  
Ei-value:0.000, Pi-value:0.000  
Er-value:0.000, Pr-value:0.000  
eCLIP MATCHES▶CPEB4 (bg=1.89%)▶GRWD1 (bg=5.13%)▶LARP4 (bg=4.72%)▶MTPAP (bg=2.21%)▶NOLC1 (bg=9.43%)▶PCBP1 (bg=1.07%)▶RBFOX2 (bg=4.63%)▶SRSF1 (bg=8.47%)▶TRA2A (bg=4.8%)▶uchl5 (bg=11.16%)▶ZNF622 (bg=6.58%)No matches to TargetScan


AGAAGC

AGAAGC  
Depth:4 (DOG)  
Ei-value:0.000, Pi-value:0.000  
Er-value:0.000, Pr-value:0.010  
eCLIP MATCHES▶CPEB4 (bg=1.89%)▶GRWD1 (bg=5.13%)▶LARP4 (bg=4.72%)▶MTPAP (bg=2.21%)▶NOLC1 (bg=9.43%)▶PCBP1 (bg=1.07%)▶RBFOX2 (bg=4.63%)▶SRSF1 (bg=8.47%)▶TRA2A (bg=4.8%)▶uchl5 (bg=11.16%)▶ZNF622 (bg=6.58%)No matches to TargetScan

TTGAAGACCTG

GGGAAAT

GGGAAAT  
Depth:2 (PIG)  
Ei-value:0.000, Pi-value:0.000  
Er-value:0.000, Pr-value:0.010  
eCLIP MATCHES▶CPEB4 (bg=1.89%)▶FTO (bg=0.32%)▶GRWD1 (bg=5.13%)▶LARP4 (bg=4.72%)▶MTPAP (bg=2.21%)▶SRSF1 (bg=8.47%)▶TRA2A (bg=4.8%)▶uchl5 (bg=11.16%)▶ZNF622 (bg=6.58%)No matches to TargetScan

CC

C

CAAGATGA  
Depth:3 (COW)  
Ei-value:0.000, Pi-value:0.000  
Er-value:0.000, Pr-value:0.000  
eCLIP MATCHES▶CPEB4 (bg=1.89%)▶FTO (bg=0.32%)▶GRWD1 (bg=5.13%)▶LARP4 (bg=4.72%)▶MTPAP (bg=2.21%)▶SRSF1 (bg=8.47%)▶TRA2A (bg=4.8%)▶uchl5 (bg=11.16%)▶ZNF622 (bg=6.58%)No matches to TargetScan


AAGATGA

AAGATGA  
Depth:5 (RABBIT)  
Ei-value:0.000, Pi-value:0.000  
Er-value:0.000, Pr-value:0.000  
eCLIP MATCHES▶CPEB4 (bg=1.89%)▶FTO (bg=0.32%)▶GRWD1 (bg=5.13%)▶LARP4 (bg=4.72%)▶MTPAP (bg=2.21%)▶SRSF1 (bg=8.47%)▶TRA2A (bg=4.8%)▶uchl5 (bg=11.16%)▶ZNF622 (bg=6.58%)No matches to TargetScan

G

AACCCTAAA

AACCCTAAA  
Depth:2 (PIG)  
Ei-value:0.000, Pi-value:0.000  
Er-value:0.000, Pr-value:0.000  
eCLIP MATCHES▶FTO (bg=0.32%)▶GRWD1 (bg=5.13%)▶LARP4 (bg=4.72%)▶MTPAP (bg=2.21%)▶ZNF622 (bg=6.58%)MATCHES To TargetScan▶ miR-296-3p:AGGGUUG

CCCTAC

CTCT

CTCTTTTCTATTGTT  
Depth:2 (PIG)  
Ei-value:0.000, Pi-value:0.000  
Er-value:0.000, Pr-value:0.000  
eCLIP MATCHES▶FTO (bg=0.32%)▶LARP4 (bg=4.72%)No matches to TargetScan


TTTCT

TTTCTATTG  
Depth:3 (COW)  
Ei-value:0.000, Pi-value:0.000  
Er-value:0.000, Pr-value:0.000  
No matches to eCLIP DataNo matches to TargetScan

 13310  


ATTG

TTTCTATTG  
Depth:3 (COW)  
Ei-value:0.000, Pi-value:0.000  
Er-value:0.000, Pr-value:0.000  
No matches to eCLIP DataNo matches to TargetScan


TT

CTCTTTTCTATTGTT  
Depth:2 (PIG)  
Ei-value:0.000, Pi-value:0.000  
Er-value:0.000, Pr-value:0.000  
eCLIP MATCHES▶FTO (bg=0.32%)▶LARP4 (bg=4.72%)No matches to TargetScan

TA

C

CACTTCTT  
Depth:2 (PIG)  
Ei-value:0.000, Pi-value:0.010  
Er-value:0.000, Pr-value:0.000  
eCLIP MATCHES▶NOLC1 (bg=9.43%)No matches to TargetScan


ACTTCTT

ACTTCTT  
Depth:3 (COW)  
Ei-value:0.000, Pi-value:0.020  
Er-value:0.000, Pr-value:0.000  
eCLIP MATCHES▶NOLC1 (bg=9.43%)No matches to TargetScan

ACTCTTAGATATTTCCAGTTC

TCCTGTT

TCCTGTT  
Depth:2 (PIG)  
Ei-value:0.000, Pi-value:0.020  
Er-value:0.000, Pr-value:0.000  
eCLIP MATCHES▶NOLC1 (bg=9.43%)▶TIA1 (bg=4.07%)▶ZC3H11A (bg=6.55%)No matches to TargetScan

TATCTTTAAGCCTGATTCTTTTGAGATGTA

CTTTTTGATGTT

CTTTTTGATGTT  
Depth:4 (DOG)  
Ei-value:0.000, Pi-value:0.000  
Er-value:0.000, Pr-value:0.000  
eCLIP MATCHES▶TIA1 (bg=4.07%)No matches to TargetScan


GC

CTTTTTGATGTTGC  
Depth:2 (PIG)  
Ei-value:0.000, Pi-value:0.000  
Er-value:0.000, Pr-value:0.000  
eCLIP MATCHES▶TIA1 (bg=4.07%)No matches to TargetScan

CG

GTTACCTT

GTTACCTT  
Depth:2 (PIG)  
Ei-value:0.000, Pi-value:0.000  
Er-value:0.000, Pr-value:0.010  
No matches to eCLIP DataNo matches to TargetScan

TAGATTG

ACAG

ACAGTATTATGCCTGGGCCAGTCTT  
Depth:2 (PIG)  
Ei-value:0.000, Pi-value:0.000  
Er-value:0.000, Pr-value:0.000  
No matches to eCLIP DataMATCHES To TargetScan▶ miR-193-3p:ACUGGCC▶ miR-200bc-3p/429:AAUACUG▶ miR-328-3p:UGGCCCU▶ miR-369-3p:AUAAUAC▶ miR-655-3p:UAAUACA


TATTATGC

TATTATGC  
Depth:4 (DOG)  
Ei-value:0.000, Pi-value:0.000  
Er-value:0.000, Pr-value:0.000  
No matches to eCLIP DataMATCHES To TargetScan▶ miR-369-3p:AUAAUAC


CTG

ACAGTATTATGCCTGGGCCAGTCTT  
Depth:2 (PIG)  
Ei-value:0.000, Pi-value:0.000  
Er-value:0.000, Pr-value:0.000  
No matches to eCLIP DataMATCHES To TargetScan▶ miR-193-3p:ACUGGCC▶ miR-200bc-3p/429:AAUACUG▶ miR-328-3p:UGGCCCU▶ miR-369-3p:AUAAUAC▶ miR-655-3p:UAAUACA

 13430  


GGCCAGTCTT

ACAGTATTATGCCTGGGCCAGTCTT  
Depth:2 (PIG)  
Ei-value:0.000, Pi-value:0.000  
Er-value:0.000, Pr-value:0.000  
No matches to eCLIP DataMATCHES To TargetScan▶ miR-193-3p:ACUGGCC▶ miR-200bc-3p/429:AAUACUG▶ miR-328-3p:UGGCCCU▶ miR-369-3p:AUAAUAC▶ miR-655-3p:UAAUACA

GAGCCAGCTTTAAATCACAGCTTTTACCTATTTGTTAGGCTATAGTGTTTTG

TAAACTTC

TAAACTTC  
Depth:3 (COW)  
Ei-value:0.000, Pi-value:0.000  
Er-value:0.000, Pr-value:0.000  
eCLIP MATCHES▶NIPBL (bg=5.39%)▶NOLC1 (bg=9.43%)▶ZC3H11A (bg=6.55%)No matches to TargetScan

TGTTTCTATTCACATCTT

CTCCACTTGAGAG

CTCCACTTGAGAG  
Depth:3 (COW)  
Ei-value:0.000, Pi-value:0.000  
Er-value:0.000, Pr-value:0.000  
eCLIP MATCHES▶NIPBL (bg=5.39%)▶NOLC1 (bg=9.43%)▶ZC3H11A (bg=6.55%)MATCHES To TargetScan▶ miR-26-5p:UCAAGUA


A

CTCCACTTGAGAGA  
Depth:2 (PIG)  
Ei-value:0.000, Pi-value:0.000  
Er-value:0.000, Pr-value:0.000  
eCLIP MATCHES▶NIPBL (bg=5.39%)▶NOLC1 (bg=9.43%)▶ZC3H11A (bg=6.55%)MATCHES To TargetScan▶ miR-26-5p:UCAAGUA

GACACCAAAATCCAGTCA 13550  
 GTATCTAATCTGGCTTTTGTTAACTTCCCTCAGGAGCAGACATTCAT

ATAGGTGA

ATAGGTGA  
Depth:2 (PIG)  
Ei-value:0.000, Pi-value:0.000  
Er-value:0.000, Pr-value:0.010  
eCLIP MATCHES▶NOLC1 (bg=9.43%)▶ZC3H11A (bg=6.55%)No matches to TargetScan

TACTG

TATTTCAGT

TATTTCAGT  
Depth:4 (DOG)  
Ei-value:0.000, Pi-value:0.000  
Er-value:0.000, Pr-value:0.000  
eCLIP MATCHES▶NOLC1 (bg=9.43%)▶ZC3H11A (bg=6.55%)MATCHES To TargetScan▶ miR-203a-3p.2:UGAAAUG


CC

TATTTCAGTCC  
Depth:3 (COW)  
Ei-value:0.000, Pi-value:0.000  
Er-value:0.000, Pr-value:0.000  
eCLIP MATCHES▶NOLC1 (bg=9.43%)▶ZC3H11A (bg=6.55%)MATCHES To TargetScan▶ miR-203a-3p.2:UGAAAUG


T

TATTTCAGTCCT  
Depth:2 (PIG)  
Ei-value:0.000, Pi-value:0.000  
Er-value:0.000, Pr-value:0.000  
eCLIP MATCHES▶NOLC1 (bg=9.43%)▶ZC3H11A (bg=6.55%)MATCHES To TargetScan▶ miR-203a-3p.2:UGAAAUG

TTCTTTTGACCCCAGAAGCCCTAGAC

TGAGAAGA

TGAGAAGA  
Depth:2 (PIG)  
Ei-value:0.000, Pi-value:0.000  
Er-value:0.000, Pr-value:0.000  
eCLIP MATCHES▶LARP4 (bg=4.72%)▶NIPBL (bg=5.39%)▶NOLC1 (bg=9.43%)▶WDR43 (bg=3.37%)▶ZC3H11A (bg=6.55%)No matches to TargetScan

TAAAATGGTCAGGT 13670  
 TGTT

GGGGAAA

GGGGAAA  
Depth:4 (DOG)  
Ei-value:0.000, Pi-value:0.000  
Er-value:0.000, Pr-value:0.000  
eCLIP MATCHES▶CPSF6 (bg=0.4%)▶LARP4 (bg=4.72%)▶WDR43 (bg=3.37%)▶ZC3H11A (bg=6.55%)No matches to TargetScan


AAA

GGGGAAAAAA  
Depth:2 (PIG)  
Ei-value:0.000, Pi-value:0.000  
Er-value:0.000, Pr-value:0.000  
eCLIP MATCHES▶CPSF6 (bg=0.4%)▶LARP4 (bg=4.72%)▶WDR43 (bg=3.37%)▶ZC3H11A (bg=6.55%)No matches to TargetScan

AA

GTGCCAGGCT

GTGCCAGGCT  
Depth:2 (PIG)  
Ei-value:0.000, Pi-value:0.000  
Er-value:0.000, Pr-value:0.000  
eCLIP MATCHES▶CPSF6 (bg=0.4%)▶LARP4 (bg=4.72%)▶WDR43 (bg=3.37%)MATCHES To TargetScan▶ miR-183-5p.2:UGGCACU

C

TCTAGAGAAAA

TCTAGAGAAAA  
Depth:6 (MOUSE)  
Ei-value:0.000, Pi-value:0.000  
Er-value:0.000, Pr-value:0.000  
eCLIP MATCHES▶CPSF6 (bg=0.4%)▶LARP4 (bg=4.72%)▶UTP3 (bg=3.66%)▶WDR43 (bg=3.37%)MATCHES To TargetScan▶ miR-1251-5p:CUCUAGC

ATG

TGAAGAGATG

TGAAGAGATG  
Depth:5 (RABBIT)  
Ei-value:0.000, Pi-value:0.000  
Er-value:0.000, Pr-value:0.000  
eCLIP MATCHES▶CPSF6 (bg=0.4%)▶LARP4 (bg=4.72%)▶SRSF7 (bg=2.32%)▶UTP3 (bg=3.66%)▶WDR43 (bg=3.37%)No matches to TargetScan


CTCCA

TGAAGAGATGCTCCA  
Depth:3 (COW)  
Ei-value:0.000, Pi-value:0.000  
Er-value:0.000, Pr-value:0.000  
eCLIP MATCHES▶CPSF6 (bg=0.4%)▶LARP4 (bg=4.72%)▶SRSF7 (bg=2.32%)▶UTP3 (bg=3.66%)▶WDR43 (bg=3.37%)No matches to TargetScan


GGCCAA

GGCCAATGAGAAGAATTAGACA  
Depth:4 (DOG)  
Ei-value:0.000, Pi-value:0.000  
Er-value:0.000, Pr-value:0.000  
eCLIP MATCHES▶LARP4 (bg=4.72%)▶NOLC1 (bg=9.43%)▶SRSF7 (bg=2.32%)▶UTP3 (bg=3.66%)No matches to TargetScan


TGAGAAGAATTAGACA

TGAGAAGAATTAGACA  
Depth:6 (MOUSE)  
Ei-value:0.000, Pi-value:0.000  
Er-value:0.000, Pr-value:0.000  
eCLIP MATCHES▶LARP4 (bg=4.72%)▶NOLC1 (bg=9.43%)▶SRSF7 (bg=2.32%)No matches to TargetScan

A

GAAATACACAGATG

GAAATACACAGATG  
Depth:3 (COW)  
Ei-value:0.000, Pi-value:0.000  
Er-value:0.000, Pr-value:0.000  
eCLIP MATCHES▶LARP4 (bg=4.72%)▶NOLC1 (bg=9.43%)▶SRSF7 (bg=2.32%)No matches to TargetScan

TGCCAGACTT

C

CTGAGAAG  
Depth:3 (COW)  
Ei-value:0.000, Pi-value:0.000  
Er-value:0.000, Pr-value:0.000  
eCLIP MATCHES▶AARS (bg=2.18%)▶NOLC1 (bg=9.43%)▶PUS1 (bg=1.04%)▶SRSF7 (bg=2.32%)▶ZC3H11A (bg=6.55%)No matches to TargetScan


TGAGAAG

TGAGAAG  
Depth:4 (DOG)  
Ei-value:0.000, Pi-value:0.000  
Er-value:0.000, Pr-value:0.010  
eCLIP MATCHES▶AARS (bg=2.18%)▶NOLC1 (bg=9.43%)▶PUS1 (bg=1.04%)▶SRSF7 (bg=2.32%)▶ZC3H11A (bg=6.55%)No matches to TargetScan


CA

CTGAGAAGCA  
Depth:2 (PIG)  
Ei-value:0.000, Pi-value:0.000  
Er-value:0.000, Pr-value:0.000  
eCLIP MATCHES▶AARS (bg=2.18%)▶NOLC1 (bg=9.43%)▶PUS1 (bg=1.04%)▶SRSF7 (bg=2.32%)▶ZC3H11A (bg=6.55%)No matches to TargetScan

CCT

GCCA

GCCAGCAACA  
Depth:3 (COW)  
Ei-value:0.000, Pi-value:0.000  
Er-value:0.000, Pr-value:0.000  
eCLIP MATCHES▶AARS (bg=2.18%)▶NOLC1 (bg=9.43%)▶PUS1 (bg=1.04%)▶SRSF7 (bg=2.32%)▶ZC3H11A (bg=6.55%)No matches to TargetScan

 13790  


GCCAGCAACA  
Depth:3 (COW)  
Ei-value:0.000, Pi-value:0.000  
Er-value:0.000, Pr-value:0.000  
eCLIP MATCHES▶AARS (bg=2.18%)▶NOLC1 (bg=9.43%)▶PUS1 (bg=1.04%)▶SRSF7 (bg=2.32%)▶ZC3H11A (bg=6.55%)No matches to TargetScan


GCAACA

GCAACA  
Depth:6 (MOUSE)  
Ei-value:0.000, Pi-value:0.000  
Er-value:0.000, Pr-value:0.000  
eCLIP MATCHES▶AARS (bg=2.18%)▶NOLC1 (bg=9.43%)▶PUS1 (bg=1.04%)▶ZC3H11A (bg=6.55%)No matches to TargetScan

GCTTCCTT

C

CTTTGAGCTTAGGTGAGCAGGATTC  
Depth:2 (PIG)  
Ei-value:0.000, Pi-value:0.000  
Er-value:0.000, Pr-value:0.000  
eCLIP MATCHES▶AARS (bg=2.18%)▶AKAP8L (bg=2.19%)▶NOLC1 (bg=9.43%)▶PUS1 (bg=1.04%)▶ZC3H11A (bg=6.55%)MATCHES To TargetScan▶ miR-371-5p:CUCAAAC


TTTGAGCTT

TTTGAGCTT  
Depth:3 (COW)  
Ei-value:0.000, Pi-value:0.000  
Er-value:0.000, Pr-value:0.000  
eCLIP MATCHES▶AARS (bg=2.18%)▶NOLC1 (bg=9.43%)▶PUS1 (bg=1.04%)▶ZC3H11A (bg=6.55%)MATCHES To TargetScan▶ miR-371-5p:CUCAAAC


A

CTTTGAGCTTAGGTGAGCAGGATTC  
Depth:2 (PIG)  
Ei-value:0.000, Pi-value:0.000  
Er-value:0.000, Pr-value:0.000  
eCLIP MATCHES▶AARS (bg=2.18%)▶AKAP8L (bg=2.19%)▶NOLC1 (bg=9.43%)▶PUS1 (bg=1.04%)▶ZC3H11A (bg=6.55%)MATCHES To TargetScan▶ miR-371-5p:CUCAAAC


GGTGAGC

GGTGAGC  
Depth:4 (DOG)  
Ei-value:0.000, Pi-value:0.000  
Er-value:0.000, Pr-value:0.000  
eCLIP MATCHES▶AARS (bg=2.18%)▶NOLC1 (bg=9.43%)▶PUS1 (bg=1.04%)▶ZC3H11A (bg=6.55%)No matches to TargetScan


AGGAT

GGTGAGCAGGAT  
Depth:3 (COW)  
Ei-value:0.000, Pi-value:0.000  
Er-value:0.000, Pr-value:0.000  
eCLIP MATCHES▶AARS (bg=2.18%)▶AKAP8L (bg=2.19%)▶NOLC1 (bg=9.43%)▶PUS1 (bg=1.04%)▶ZC3H11A (bg=6.55%)No matches to TargetScan


TC

CTTTGAGCTTAGGTGAGCAGGATTC  
Depth:2 (PIG)  
Ei-value:0.000, Pi-value:0.000  
Er-value:0.000, Pr-value:0.000  
eCLIP MATCHES▶AARS (bg=2.18%)▶AKAP8L (bg=2.19%)▶NOLC1 (bg=9.43%)▶PUS1 (bg=1.04%)▶ZC3H11A (bg=6.55%)MATCHES To TargetScan▶ miR-371-5p:CUCAAAC

TGG

GGTTTGGG

GGTTTGGG  
Depth:4 (DOG)  
Ei-value:0.000, Pi-value:0.000  
Er-value:0.000, Pr-value:0.000  
eCLIP MATCHES▶AARS (bg=2.18%)▶AKAP8L (bg=2.19%)▶NOLC1 (bg=9.43%)▶PUS1 (bg=1.04%)No matches to TargetScan

ATTT

CTAGTGA

CTAGTGATGGTTATG  
Depth:2 (PIG)  
Ei-value:0.000, Pi-value:0.000  
Er-value:0.000, Pr-value:0.000  
eCLIP MATCHES▶AKAP8L (bg=2.19%)▶NOLC1 (bg=9.43%)▶PUS1 (bg=1.04%)▶SF3B1 (bg=2.48%)No matches to TargetScan


TGGTTA

TGGTTA  
Depth:5 (RABBIT)  
Ei-value:0.000, Pi-value:0.000  
Er-value:0.000, Pr-value:0.000  
eCLIP MATCHES▶AKAP8L (bg=2.19%)▶NOLC1 (bg=9.43%)▶PUS1 (bg=1.04%)▶SF3B1 (bg=2.48%)No matches to TargetScan


T

TGGTTAT  
Depth:4 (DOG)  
Ei-value:0.000, Pi-value:0.000  
Er-value:0.000, Pr-value:0.000  
eCLIP MATCHES▶AKAP8L (bg=2.19%)▶NOLC1 (bg=9.43%)▶PUS1 (bg=1.04%)▶SF3B1 (bg=2.48%)No matches to TargetScan


G

TGGTTATG  
Depth:3 (COW)  
Ei-value:0.000, Pi-value:0.000  
Er-value:0.000, Pr-value:0.000  
eCLIP MATCHES▶AKAP8L (bg=2.19%)▶NOLC1 (bg=9.43%)▶PUS1 (bg=1.04%)▶SF3B1 (bg=2.48%)No matches to TargetScan

GAAAGGGTGACTGTGC

CTGGGACA

CTGGGACA  
Depth:2 (PIG)  
Ei-value:0.000, Pi-value:0.000  
Er-value:0.000, Pr-value:0.000  
eCLIP MATCHES▶AKAP8L (bg=2.19%)▶NOLC1 (bg=9.43%)▶PUS1 (bg=1.04%)▶SF3B1 (bg=2.48%)No matches to TargetScan

AAGC

GAGGT

GAGGTCCCAAGG  
Depth:2 (PIG)  
Ei-value:0.000, Pi-value:0.000  
Er-value:0.000, Pr-value:0.000  
eCLIP MATCHES▶AKAP8L (bg=2.19%)▶PUS1 (bg=1.04%)▶UTP3 (bg=3.66%)MATCHES To TargetScan▶ miR-212-5p:CCUUGGC


CCCAAGG

CCCAAGG  
Depth:4 (DOG)  
Ei-value:0.000, Pi-value:0.000  
Er-value:0.000, Pr-value:0.000  
eCLIP MATCHES▶PUS1 (bg=1.04%)▶UTP3 (bg=3.66%)MATCHES To TargetScan▶ miR-212-5p:CCUUGGC

GGAC

AGCC

AGCCTGAACTCCCTGCTCATAGTAGTGGCC  
Depth:2 (PIG)  
Ei-value:0.000, Pi-value:0.000  
Er-value:0.000, Pr-value:0.000  
eCLIP MATCHES▶UTP3 (bg=3.66%)No matches to TargetScan


TGA

TGAACTCCCTGCT  
Depth:4 (DOG)  
Ei-value:0.000, Pi-value:0.000  
Er-value:0.000, Pr-value:0.000  
eCLIP MATCHES▶UTP3 (bg=3.66%)No matches to TargetScan

 13910  


ACTCCCTGCT

TGAACTCCCTGCT  
Depth:4 (DOG)  
Ei-value:0.000, Pi-value:0.000  
Er-value:0.000, Pr-value:0.000  
eCLIP MATCHES▶UTP3 (bg=3.66%)No matches to TargetScan


C

TGAACTCCCTGCTCATAGTAGTGGCC  
Depth:3 (COW)  
Ei-value:0.000, Pi-value:0.000  
Er-value:0.000, Pr-value:0.000  
eCLIP MATCHES▶UTP3 (bg=3.66%)No matches to TargetScan


ATAGTAGTGGCC

ATAGTAGTGGCC  
Depth:4 (DOG)  
Ei-value:0.000, Pi-value:0.000  
Er-value:0.000, Pr-value:0.000  
No matches to eCLIP DataNo matches to TargetScan

A

AATAATTTGG

AATAATTTGG  
Depth:2 (PIG)  
Ei-value:0.000, Pi-value:0.000  
Er-value:0.000, Pr-value:0.000  
No matches to eCLIP DataNo matches to TargetScan

TGGACTGTGCCAACGCTACTCCTGGG

TTTAATAC

TTTAATAC  
Depth:4 (DOG)  
Ei-value:0.000, Pi-value:0.000  
Er-value:0.000, Pr-value:0.000  
eCLIP MATCHES▶WRN (bg=0.77%)MATCHES To TargetScan▶ miR-496.2:GUAUUAC


CCA

TTTAATACCCA  
Depth:2 (PIG)  
Ei-value:0.000, Pi-value:0.000  
Er-value:0.000, Pr-value:0.000  
eCLIP MATCHES▶WRN (bg=0.77%)MATCHES To TargetScan▶ miR-496.2:GUAUUAC

T

CT

CTCTAGGCTTAAAG  
Depth:2 (PIG)  
Ei-value:0.000, Pi-value:0.000  
Er-value:0.000, Pr-value:0.000  
No matches to eCLIP DataNo matches to TargetScan


CT

CTAGGCTTAAAG  
Depth:4 (DOG)  
Ei-value:0.000, Pi-value:0.000  
Er-value:0.000, Pr-value:0.000  
No matches to eCLIP DataNo matches to TargetScan


AGGCTTA

AGGCTTA  
Depth:5 (RABBIT)  
Ei-value:0.000, Pi-value:0.000  
Er-value:0.000, Pr-value:0.000  
No matches to eCLIP DataNo matches to TargetScan


AAG

CTAGGCTTAAAG  
Depth:4 (DOG)  
Ei-value:0.000, Pi-value:0.000  
Er-value:0.000, Pr-value:0.000  
No matches to eCLIP DataNo matches to TargetScan

ATGAGAGAACCTGGGACTGTTGAGCAT

GTTTAAT

GTTTAAT  
Depth:5 (RABBIT)  
Ei-value:0.000, Pi-value:0.000  
Er-value:0.000, Pr-value:0.000  
No matches to eCLIP DataNo matches to TargetScan

 14030  


GTTTAAT  
Depth:5 (RABBIT)  
Ei-value:0.000, Pi-value:0.000  
Er-value:0.000, Pr-value:0.000  
No matches to eCLIP DataNo matches to TargetScan


ACTTTCCTT

GTTTAATACTTTCCTT  
Depth:2 (PIG)  
Ei-value:0.000, Pi-value:0.000  
Er-value:0.000, Pr-value:0.000  
No matches to eCLIP DataMATCHES To TargetScan▶ miR-496.2:GUAUUAC

GATTTTTTTCTTCCTGTTTATGT

GGGAAG

GGGAAG  
Depth:2 (PIG)  
Ei-value:0.000, Pi-value:0.020  
Er-value:0.000, Pr-value:0.020  
eCLIP MATCHES▶UTP3 (bg=3.66%)No matches to TargetScan

TTG

ATTTAAATGA

ATTTAAATGA  
Depth:2 (PIG)  
Ei-value:0.000, Pi-value:0.000  
Er-value:0.000, Pr-value:0.000  
eCLIP MATCHES▶TARDBP (bg=2.79%)▶UTP3 (bg=3.66%)No matches to TargetScan

CTGATAATGTGTATGAAAGCAC

TGTAAAACA

TGTAAAACA  
Depth:3 (COW)  
Ei-value:0.000, Pi-value:0.000  
Er-value:0.000, Pr-value:0.000  
eCLIP MATCHES▶TARDBP (bg=2.79%)▶WDR43 (bg=3.37%)No matches to TargetScan

TAAGAGAAAAACCAATTAGTG

T

TATTGGCA  
Depth:5 (RABBIT)  
Ei-value:0.000, Pi-value:0.000  
Er-value:0.000, Pr-value:0.000  
eCLIP MATCHES▶HNRNPA1 (bg=2.57%)No matches to TargetScan


ATTGGCA

ATTGGCA  
Depth:6 (MOUSE)  
Ei-value:0.000, Pi-value:0.000  
Er-value:0.000, Pr-value:0.000  
eCLIP MATCHES▶HNRNPA1 (bg=2.57%)No matches to TargetScan

ATCATGCAG 14150  
 TTAACATTTGAAAGTGCAGTGTAAA

TTGTGAAG

TTGTGAAG  
Depth:6 (MOUSE)  
Ei-value:0.000, Pi-value:0.000  
Er-value:0.000, Pr-value:0.000  
eCLIP MATCHES▶HNRNPA1 (bg=2.57%)No matches to TargetScan

CAT

T

TATGTAAATCA  
Depth:3 (COW)  
Ei-value:0.000, Pi-value:0.000  
Er-value:0.000, Pr-value:0.000  
No matches to eCLIP DataNo matches to TargetScan


ATGTAAAT

ATGTAAAT  
Depth:5 (RABBIT)  
Ei-value:0.000, Pi-value:0.000  
Er-value:0.000, Pr-value:0.000  
No matches to eCLIP DataNo matches to TargetScan


CA

TATGTAAATCA  
Depth:3 (COW)  
Ei-value:0.000, Pi-value:0.000  
Er-value:0.000, Pr-value:0.000  
No matches to eCLIP DataNo matches to TargetScan


GGGGTC

TATGTAAATCAGGGGTC  
Depth:2 (PIG)  
Ei-value:0.000, Pi-value:0.000  
Er-value:0.000, Pr-value:0.000  
No matches to eCLIP DataMATCHES To TargetScan▶ miR-125-5p:CCCUGAG▶ miR-331-3p:CCCCUGG

CACAGTT

TTTCTGTAA

TTTCTGTAA  
Depth:2 (PIG)  
Ei-value:0.000, Pi-value:0.000  
Er-value:0.000, Pr-value:0.000  
No matches to eCLIP DataNo matches to TargetScan

GGGGTCAAATCATAAATACTTTAGACTGT

GG

GGGCCATATGGTTTC  
Depth:2 (PIG)  
Ei-value:0.000, Pi-value:0.000  
Er-value:0.000, Pr-value:0.000  
No matches to eCLIP DataMATCHES To TargetScan▶ miR-328-3p:UGGCCCU


GCCATATGGT

GCCATATGGT  
Depth:3 (COW)  
Ei-value:0.000, Pi-value:0.000  
Er-value:0.000, Pr-value:0.000  
No matches to eCLIP DataNo matches to TargetScan


TTC

GGGCCATATGGTTTC  
Depth:2 (PIG)  
Ei-value:0.000, Pi-value:0.000  
Er-value:0.000, Pr-value:0.000  
No matches to eCLIP DataMATCHES To TargetScan▶ miR-328-3p:UGGCCCU

TGTTACA 14270  
 TATTTGTTTTTTAAACAACGTTTTTATAAGGTCAAAATCATTCTTAGTTTTTGAGCCAATTGGATTTGGCCTGCTGTTCATAGCTTACCAC

CCCCTGATGTA

CCCCTGATGTA  
Depth:2 (PIG)  
Ei-value:0.000, Pi-value:0.000  
Er-value:0.000, Pr-value:0.000  
No matches to eCLIP DataNo matches to TargetScan

TTATTTGTTATTCAGAGA 14390  
 AAAT

TTCTGAA

TTCTGAA  
Depth:2 (PIG)  
Ei-value:0.000, Pi-value:0.020  
Er-value:0.000, Pr-value:0.020  
No matches to eCLIP DataNo matches to TargetScan

TACTACTAGTTTCCTTT

TC

TCTGTGCCTGTCCCTGT  
Depth:2 (PIG)  
Ei-value:0.000, Pi-value:0.000  
Er-value:0.000, Pr-value:0.000  
No matches to eCLIP DataNo matches to TargetScan


TGTGC

TGTGCCTGTCCCTGT  
Depth:3 (COW)  
Ei-value:0.000, Pi-value:0.000  
Er-value:0.000, Pr-value:0.000  
No matches to eCLIP DataNo matches to TargetScan


CTGTCCCT

CTGTCCCT  
Depth:4 (DOG)  
Ei-value:0.000, Pi-value:0.000  
Er-value:0.000, Pr-value:0.000  
No matches to eCLIP DataNo matches to TargetScan


GT

TGTGCCTGTCCCTGT  
Depth:3 (COW)  
Ei-value:0.000, Pi-value:0.000  
Er-value:0.000, Pr-value:0.000  
No matches to eCLIP DataNo matches to TargetScan

GC

TAGGCACT

TAGGCACT  
Depth:4 (DOG)  
Ei-value:0.000, Pi-value:0.000  
Er-value:0.000, Pr-value:0.000  
No matches to eCLIP DataNo matches to TargetScan


AA

TAGGCACTAA  
Depth:2 (PIG)  
Ei-value:0.000, Pi-value:0.000  
Er-value:0.000, Pr-value:0.000  
No matches to eCLIP DataNo matches to TargetScan

AAATGC

AATGATTA

AATGATTA  
Depth:2 (PIG)  
Ei-value:0.000, Pi-value:0.000  
Er-value:0.000, Pr-value:0.000  
No matches to eCLIP DataMATCHES To TargetScan▶ miR-382-3p:AUCAUUC

TTG

ATATCTAGGTGA

ATATCTAGGTGA  
Depth:2 (PIG)  
Ei-value:0.000, Pi-value:0.000  
Er-value:0.000, Pr-value:0.000  
eCLIP MATCHES▶HNRNPU (bg=5.92%)No matches to TargetScan

CCTGAAAAAAAATAGTG

AATGTGCTTTGTAAACT

AATGTGCTTTGTAAACT  
Depth:2 (PIG)  
Ei-value:0.000, Pi-value:0.000  
Er-value:0.000, Pr-value:0.000  
eCLIP MATCHES▶HNRNPU (bg=5.92%)MATCHES To TargetScan▶ miR-330-3p:CAAAGCA▶ miR-330-3p.2:AAAGCAC

 14510  


AATGTGCTTTGTAAACT  
Depth:2 (PIG)  
Ei-value:0.000, Pi-value:0.000  
Er-value:0.000, Pr-value:0.000  
eCLIP MATCHES▶HNRNPU (bg=5.92%)MATCHES To TargetScan▶ miR-330-3p:CAAAGCA▶ miR-330-3p.2:AAAGCAC

G

TAAAGCA

TAAAGCA  
Depth:4 (DOG)  
Ei-value:0.000, Pi-value:0.000  
Er-value:0.000, Pr-value:0.000  
eCLIP MATCHES▶LIN28B (bg=0.74%)No matches to TargetScan


CTT

TAAAGCACTT  
Depth:2 (PIG)  
Ei-value:0.000, Pi-value:0.000  
Er-value:0.000, Pr-value:0.000  
eCLIP MATCHES▶LIN28B (bg=0.74%)MATCHES To TargetScan▶ miR-302-3p/372-3p/373-3p/520-3p:AAGUGCU▶ miR-302c-3p.2/520-3p:AGUGCUU

GTATTCTACTGTGATAAGCGT

TGTGGATACAAA

TGTGGATACAAA  
Depth:2 (PIG)  
Ei-value:0.000, Pi-value:0.000  
Er-value:0.000, Pr-value:0.000  
eCLIP MATCHES▶LIN28B (bg=0.74%)▶UTP3 (bg=3.66%)No matches to TargetScan

GAAAGGAGCAAGCATAAAAAAGTGCTCTTTCAAAAGGATATAGTACTATGCAGACACAAGGAATTGTTTGATAAAT 14630  
 GAATAAATTATATGTATATTTGAGGCCAATTTGTGTTTGCTGCTCTGGTAATTTTGAGTAAAAATGCAGTATTCCAGGTATCAGAAACGAAAACACATGGAAACTGCTTTTAAACTTTAA 14750  
 AATATACTGAAAACATAAGGGACTAAGCTTGTTGTGGTCACC

TAT

TATAATGTGCCAGATA  
Depth:3 (COW)  
Ei-value:0.000, Pi-value:0.000  
Er-value:0.000, Pr-value:0.000  
No matches to eCLIP DataMATCHES To TargetScan▶ miR-183-5p.2:UGGCACU▶ miR-323-3p:ACAUUAC


AATGTGCCAGATA

AATGTGCCAGATA  
Depth:4 (DOG)  
Ei-value:0.000, Pi-value:0.000  
Er-value:0.000, Pr-value:0.000  
No matches to eCLIP DataMATCHES To TargetScan▶ miR-183-5p.2:UGGCACU

CCATGCTGGGTGCTAGAGCTACCAAAGGGGGAAAAGTA

TTCTCAT

TTCTCAT  
Depth:2 (PIG)  
Ei-value:0.000, Pi-value:0.000  
Er-value:0.000, Pr-value:0.010  
No matches to eCLIP DataNo matches to TargetScan

AGAACAAAAAATTTCAG 14870  
 AAAGGTG

CATA

CATATTAAAGTGCTTTGTA  
Depth:2 (PIG)  
Ei-value:0.000, Pi-value:0.000  
Er-value:0.000, Pr-value:0.000  
eCLIP MATCHES▶SF3B1 (bg=2.48%)MATCHES To TargetScan▶ miR-330-3p:CAAAGCA▶ miR-330-3p.2:AAAGCAC


TTAAAGTG

TTAAAGTG  
Depth:4 (DOG)  
Ei-value:0.000, Pi-value:0.000  
Er-value:0.000, Pr-value:0.000  
eCLIP MATCHES▶SF3B1 (bg=2.48%)No matches to TargetScan


CTTTGTA

TTAAAGTGCTTTGTA  
Depth:3 (COW)  
Ei-value:0.000, Pi-value:0.000  
Er-value:0.000, Pr-value:0.000  
eCLIP MATCHES▶SF3B1 (bg=2.48%)MATCHES To TargetScan▶ miR-330-3p:CAAAGCA▶ miR-330-3p.2:AAAGCAC


AA

AACTAAAGCA  
Depth:2 (PIG)  
Ei-value:0.000, Pi-value:0.000  
Er-value:0.000, Pr-value:0.000  
eCLIP MATCHES▶SF3B1 (bg=2.48%)No matches to TargetScan


CTAAAGCA

CTAAAGCA  
Depth:4 (DOG)  
Ei-value:0.000, Pi-value:0.000  
Er-value:0.000, Pr-value:0.000  
eCLIP MATCHES▶SF3B1 (bg=2.48%)No matches to TargetScan

TGATACAAATGT

CAATGGGCTA

CAATGGGCTA  
Depth:3 (COW)  
Ei-value:0.000, Pi-value:0.000  
Er-value:0.000, Pr-value:0.000  
No matches to eCLIP DataNo matches to TargetScan

CATATTTATGAATGAATGAATGGAT

GA

GAATGAATA  
Depth:3 (COW)  
Ei-value:0.000, Pi-value:0.000  
Er-value:0.000, Pr-value:0.000  
eCLIP MATCHES▶DROSHA (bg=2.49%)▶TARDBP (bg=2.79%)▶ZC3H11A (bg=6.55%)MATCHES To TargetScan▶ miR-1298-5p:UCAUUCG


ATGAATA

ATGAATA  
Depth:4 (DOG)  
Ei-value:0.000, Pi-value:0.000  
Er-value:0.000, Pr-value:0.000  
eCLIP MATCHES▶DROSHA (bg=2.49%)▶TARDBP (bg=2.79%)▶ZC3H11A (bg=6.55%)No matches to TargetScan

TTAAGTGCCTCTTACATA

CCAGCTATT

CCAGCTATT  
Depth:3 (COW)  
Ei-value:0.000, Pi-value:0.000  
Er-value:0.000, Pr-value:0.000  
eCLIP MATCHES▶AARS (bg=2.18%)▶DROSHA (bg=2.49%)▶ILF3 (bg=3.0%)▶TARDBP (bg=2.79%)▶ZC3H11A (bg=6.55%)No matches to TargetScan

T 14990  
 TG

GGTACTGT

GGTACTGT  
Depth:4 (DOG)  
Ei-value:0.000, Pi-value:0.000  
Er-value:0.000, Pr-value:0.000  
eCLIP MATCHES▶AARS (bg=2.18%)▶DROSHA (bg=2.49%)▶ILF3 (bg=3.0%)▶TARDBP (bg=2.79%)▶ZC3H11A (bg=6.55%)MATCHES To TargetScan▶ miR-101-3p.1:ACAGUAC▶ miR-144-3p:ACAGUAU

AAAATACAAGATTAATTCTCCTAT

GTA

GTAATAAGAGG  
Depth:2 (PIG)  
Ei-value:0.000, Pi-value:0.000  
Er-value:0.000, Pr-value:0.000  
eCLIP MATCHES▶ILF3 (bg=3.0%)No matches to TargetScan


ATAAGAGG

ATAAGAGG  
Depth:4 (DOG)  
Ei-value:0.000, Pi-value:0.000  
Er-value:0.000, Pr-value:0.000  
eCLIP MATCHES▶ILF3 (bg=3.0%)No matches to TargetScan

AAAGTTTATCCTCTATACTATTCAGATGTAAGGAATGAT

ATATTGCTTA

ATATTGCTTA  
Depth:2 (PIG)  
Ei-value:0.000, Pi-value:0.000  
Er-value:0.000, Pr-value:0.000  
No matches to eCLIP DataNo matches to TargetScan

ATTTTAAA

CAATC

CAATCAAGACTTTAC  
Depth:2 (PIG)  
Ei-value:0.000, Pi-value:0.000  
Er-value:0.000, Pr-value:0.000  
No matches to eCLIP DataMATCHES To TargetScan▶ miR-431-5p:GUCUUGC


AAGACTTTAC

AAGACTTTAC  
Depth:3 (COW)  
Ei-value:0.000, Pi-value:0.000  
Er-value:0.000, Pr-value:0.000  
No matches to eCLIP DataNo matches to TargetScan

TG

G

GTGAGGT  
Depth:2 (PIG)  
Ei-value:0.000, Pi-value:0.000  
Er-value:0.000, Pr-value:0.000  
No matches to eCLIP DataNo matches to TargetScan

 15110  


TGAGGT

GTGAGGT  
Depth:2 (PIG)  
Ei-value:0.000, Pi-value:0.000  
Er-value:0.000, Pr-value:0.000  
No matches to eCLIP DataNo matches to TargetScan

TAAG

T

TTAAATTATTAC  
Depth:2 (PIG)  
Ei-value:0.000, Pi-value:0.000  
Er-value:0.000, Pr-value:0.000  
No matches to eCLIP DataNo matches to TargetScan


TAAATTAT

TAAATTAT  
Depth:4 (DOG)  
Ei-value:0.000, Pi-value:0.010  
Er-value:0.000, Pr-value:0.000  
No matches to eCLIP DataNo matches to TargetScan


TAC

TAAATTATTAC  
Depth:3 (COW)  
Ei-value:0.000, Pi-value:0.000  
Er-value:0.000, Pr-value:0.000  
No matches to eCLIP DataNo matches to TargetScan

TGATACATTTTT

CC

CCAGGTAAC  
Depth:2 (PIG)  
Ei-value:0.000, Pi-value:0.000  
Er-value:0.000, Pr-value:0.000  
No matches to eCLIP DataNo matches to TargetScan


AGGTAA

AGGTAA  
Depth:3 (COW)  
Ei-value:0.000, Pi-value:0.000  
Er-value:0.000, Pr-value:0.000  
No matches to eCLIP DataNo matches to TargetScan


C

CCAGGTAAC  
Depth:2 (PIG)  
Ei-value:0.000, Pi-value:0.000  
Er-value:0.000, Pr-value:0.000  
No matches to eCLIP DataNo matches to TargetScan

CAGGAAAGAGCTAGTATGAGGAAATGAAGTAATAGATGTGAGATCCAGACCGAAAGTCACTTAATTCAGCTTGCGAA 15230  
 TGTGC

TTTCTAA

TTTCTAA  
Depth:3 (COW)  
Ei-value:0.000, Pi-value:0.000  
Er-value:0.000, Pr-value:0.010  
No matches to eCLIP DataNo matches to TargetScan


A

TTTCTAAA  
Depth:2 (PIG)  
Ei-value:0.000, Pi-value:0.000  
Er-value:0.000, Pr-value:0.000  
No matches to eCLIP DataNo matches to TargetScan

TTATAAAGCACTTGTAAATGAAAAATTTGATGCTTTCTGTA

TGA

TGAATAAAACTT  
Depth:2 (PIG)  
Ei-value:0.000, Pi-value:0.000  
Er-value:0.000, Pr-value:0.000  
No matches to eCLIP DataNo matches to TargetScan


ATAAAAC

ATAAAAC  
Depth:4 (DOG)  
Ei-value:0.000, Pi-value:0.010  
Er-value:0.000, Pr-value:0.000  
No matches to eCLIP DataNo matches to TargetScan


TT

TGAATAAAACTT  
Depth:2 (PIG)  
Ei-value:0.000, Pi-value:0.000  
Er-value:0.000, Pr-value:0.000  
No matches to eCLIP DataNo matches to TargetScan

TCTGTAAGCTAGGTATTG

TCTCTAC

TCTCTACAAAATTCTCATTGT  
Depth:2 (PIG)  
Ei-value:0.000, Pi-value:0.000  
Er-value:0.000, Pr-value:0.000  
eCLIP MATCHES▶HNRNPU (bg=5.92%)No matches to TargetScan


AAAATTCTCA

AAAATTCTCA  
Depth:4 (DOG)  
Ei-value:0.000, Pi-value:0.000  
Er-value:0.000, Pr-value:0.000  
eCLIP MATCHES▶HNRNPU (bg=5.92%)No matches to TargetScan


TTGT

TCTCTACAAAATTCTCATTGT  
Depth:2 (PIG)  
Ei-value:0.000, Pi-value:0.000  
Er-value:0.000, Pr-value:0.000  
eCLIP MATCHES▶HNRNPU (bg=5.92%)No matches to TargetScan

ATAGTTAAACCACAG 15350  
 TGAGAAGGGTTCTATAAGTAG

T

TTATACAAAC  
Depth:2 (PIG)  
Ei-value:0.000, Pi-value:0.000  
Er-value:0.000, Pr-value:0.000  
No matches to eCLIP DataNo matches to TargetScan


TATACAAAC

TATACAAAC  
Depth:4 (DOG)  
Ei-value:0.000, Pi-value:0.000  
Er-value:0.000, Pr-value:0.000  
No matches to eCLIP DataNo matches to TargetScan

CAAGG

GTTTAAATAC

GTTTAAATAC  
Depth:3 (COW)  
Ei-value:0.000, Pi-value:0.000  
Er-value:0.000, Pr-value:0.000  
No matches to eCLIP DataNo matches to TargetScan

CTGTTAAATAGATCAATTTTG

A

ATTGCCTACTATGTGAACTCACTGTTA  
Depth:2 (PIG)  
Ei-value:0.000, Pi-value:0.000  
Er-value:0.000, Pr-value:0.000  
No matches to eCLIP DataMATCHES To TargetScan▶ miR-132-3p/212-3p:AACAGUC▶ miR-23-3p:UCACAUU▶ miR-376c-3p:ACAUAGA▶ miR-411-5p.2:UAGUAGA


TTGCCTACTAT

TTGCCTACTATGTGAACTCACTGTTA  
Depth:3 (COW)  
Ei-value:0.000, Pi-value:0.000  
Er-value:0.000, Pr-value:0.000  
No matches to eCLIP DataMATCHES To TargetScan▶ miR-132-3p/212-3p:AACAGUC▶ miR-23-3p:UCACAUU▶ miR-376c-3p:ACAUAGA▶ miR-411-5p.2:UAGUAGA


GTGAACTCA

GTGAACTCA  
Depth:4 (DOG)  
Ei-value:0.000, Pi-value:0.000  
Er-value:0.000, Pr-value:0.000  
No matches to eCLIP DataNo matches to TargetScan


CTGTTA

TTGCCTACTATGTGAACTCACTGTTA  
Depth:3 (COW)  
Ei-value:0.000, Pi-value:0.000  
Er-value:0.000, Pr-value:0.000  
No matches to eCLIP DataMATCHES To TargetScan▶ miR-132-3p/212-3p:AACAGUC▶ miR-23-3p:UCACAUU▶ miR-376c-3p:ACAUAGA▶ miR-411-5p.2:UAGUAGA

AAGGCACTGAAA

ATTTATCAT

ATTTATCAT  
Depth:3 (COW)  
Ei-value:0.000, Pi-value:0.000  
Er-value:0.000, Pr-value:0.000  
No matches to eCLIP DataNo matches to TargetScan

ATTTC 15470  
 ATTTAGCCACAGCCAAAAATAAGGCAATACC

TATGTTAGC

TATGTTAGCATTTTGTGAACTCTAA  
Depth:2 (PIG)  
Ei-value:0.000, Pi-value:0.000  
Er-value:0.000, Pr-value:0.000  
No matches to eCLIP DataNo matches to TargetScan


ATTTTGTGAACTCTAA

ATTTTGTGAACTCTAA  
Depth:3 (COW)  
Ei-value:0.000, Pi-value:0.000  
Er-value:0.000, Pr-value:0.000  
No matches to eCLIP DataNo matches to TargetScan

G

GCACCAT

GCACCAT  
Depth:2 (PIG)  
Ei-value:0.000, Pi-value:0.000  
Er-value:0.000, Pr-value:0.000  
No matches to eCLIP DataNo matches to TargetScan

ATAAATGTAACTGTTGATTTTCTCACTTGGTGCTGG

GTACTAG

GTACTAG  
Depth:2 (PIG)  
Ei-value:0.000, Pi-value:0.000  
Er-value:0.000, Pr-value:0.000  
No matches to eCLIP DataNo matches to TargetScan

GTTTAT

AAAATTG

AAAATTG  
Depth:3 (COW)  
Ei-value:0.000, Pi-value:0.000  
Er-value:0.000, Pr-value:0.000  
No matches to eCLIP DataNo matches to TargetScan

 15590  


AAAATTG  
Depth:3 (COW)  
Ei-value:0.000, Pi-value:0.000  
Er-value:0.000, Pr-value:0.000  
No matches to eCLIP DataNo matches to TargetScan

TATG

ATAGTTAT

ATAGTTAT  
Depth:2 (PIG)  
Ei-value:0.000, Pi-value:0.000  
Er-value:0.000, Pr-value:0.000  
No matches to eCLIP DataNo matches to TargetScan

TATATTGTGCAAATAAAGTAGGAAAA

TTTGAATA

TTTGAATA  
Depth:2 (PIG)  
Ei-value:0.000, Pi-value:0.000  
Er-value:0.000, Pr-value:0.010  
eCLIP MATCHES▶HNRNPUL1 (bg=1.16%)No matches to TargetScan

ACAATGATTATCT

TTTGAATA

TTTGAATA  
Depth:2 (PIG)  
Ei-value:0.000, Pi-value:0.000  
Er-value:0.000, Pr-value:0.010  
eCLIP MATCHES▶HNRNPUL1 (bg=1.16%)No matches to TargetScan

CGCATACGCAAGGGATTGGTTGTCTGAAG

AATGCC

AATGCC  
Depth:2 (PIG)  
Ei-value:0.000, Pi-value:0.000  
Er-value:0.000, Pr-value:0.000  
eCLIP MATCHES▶SAFB (bg=2.69%)No matches to TargetScan

ACTATAGTAGTTATCTAT 15710  
 TG

TGTGCCA

TGTGCCA  
Depth:4 (DOG)  
Ei-value:0.000, Pi-value:0.000  
Er-value:0.000, Pr-value:0.000  
No matches to eCLIP DataMATCHES To TargetScan▶ miR-183-5p.2:UGGCACU

ATCTCATTGCTAGGCATTGGGGATGCA

AAGATAA

AAGATAA  
Depth:4 (DOG)  
Ei-value:0.000, Pi-value:0.000  
Er-value:0.000, Pr-value:0.000  
No matches to eCLIP DataNo matches to TargetScan

ACCATC

TTTATTGTGT

TTTATTGTGT  
Depth:2 (PIG)  
Ei-value:0.000, Pi-value:0.000  
Er-value:0.000, Pr-value:0.000  
eCLIP MATCHES▶UTP3 (bg=3.66%)No matches to TargetScan

CTTG

GGT

GGTAGCAGAA  
Depth:2 (PIG)  
Ei-value:0.000, Pi-value:0.000  
Er-value:0.000, Pr-value:0.000  
eCLIP MATCHES▶UTP3 (bg=3.66%)No matches to TargetScan


AGCAGAA

AGCAGAA  
Depth:3 (COW)  
Ei-value:0.000, Pi-value:0.000  
Er-value:0.000, Pr-value:0.000  
eCLIP MATCHES▶UTP3 (bg=3.66%)No matches to TargetScan

GAAAAT

ATGTG

ATGTGTAAAATCAATTT  
Depth:2 (PIG)  
Ei-value:0.000, Pi-value:0.000  
Er-value:0.000, Pr-value:0.000  
eCLIP MATCHES▶UTP3 (bg=3.66%)No matches to TargetScan


TAAAATCAATTT

TAAAATCAATTT  
Depth:3 (COW)  
Ei-value:0.000, Pi-value:0.000  
Er-value:0.000, Pr-value:0.000  
eCLIP MATCHES▶UTP3 (bg=3.66%)No matches to TargetScan

ATAATTTG

TAAACTG

TAAACTG  
Depth:4 (DOG)  
Ei-value:0.000, Pi-value:0.000  
Er-value:0.000, Pr-value:0.000  
eCLIP MATCHES▶HNRNPU (bg=5.92%)No matches to TargetScan

CCACCCATA 15830  
 TATAAGCTATA

TCTGCTGAATGA

TCTGCTGAATGA  
Depth:3 (COW)  
Ei-value:0.000, Pi-value:0.000  
Er-value:0.000, Pr-value:0.000  
No matches to eCLIP DataMATCHES To TargetScan▶ miR-1298-5p:UCAUUCG

T

C

CATTGATTA  
Depth:3 (COW)  
Ei-value:0.000, Pi-value:0.000  
Er-value:0.000, Pr-value:0.000  
No matches to eCLIP DataNo matches to TargetScan


ATTGATTA

ATTGATTA  
Depth:4 (DOG)  
Ei-value:0.000, Pi-value:0.000  
Er-value:0.000, Pr-value:0.010  
No matches to eCLIP DataNo matches to TargetScan

C

TCTTATCC

TCTTATCC  
Depth:2 (PIG)  
Ei-value:0.000, Pi-value:0.000  
Er-value:0.000, Pr-value:0.000  
No matches to eCLIP DataNo matches to TargetScan

TT

AGAGATA

AGAGATA  
Depth:4 (DOG)  
Ei-value:0.000, Pi-value:0.000  
Er-value:0.000, Pr-value:0.000  
No matches to eCLIP DataNo matches to TargetScan

ACAACTGGGGGCACAAACATTTATTATCATTAT

TGAACCT

TGAACCT  
Depth:3 (COW)  
Ei-value:0.000, Pi-value:0.000  
Er-value:0.000, Pr-value:0.010  
eCLIP MATCHES▶HNRNPU (bg=5.92%)No matches to TargetScan

A

C

CAACAGAGATCT  
Depth:2 (PIG)  
Ei-value:0.000, Pi-value:0.000  
Er-value:0.000, Pr-value:0.000  
eCLIP MATCHES▶HNRNPA1 (bg=2.57%)▶HNRNPU (bg=5.92%)No matches to TargetScan


AA

AACAGAGATCT  
Depth:3 (COW)  
Ei-value:0.000, Pi-value:0.000  
Er-value:0.000, Pr-value:0.000  
eCLIP MATCHES▶HNRNPA1 (bg=2.57%)▶HNRNPU (bg=5.92%)No matches to TargetScan


CAGAGATCT

CAGAGATCT  
Depth:4 (DOG)  
Ei-value:0.000, Pi-value:0.000  
Er-value:0.000, Pr-value:0.000  
eCLIP MATCHES▶HNRNPA1 (bg=2.57%)▶HNRNPU (bg=5.92%)No matches to TargetScan

ATGTGTAG

A

ATTTACAAAGCCTA  
Depth:2 (PIG)  
Ei-value:0.000, Pi-value:0.000  
Er-value:0.000, Pr-value:0.000  
eCLIP MATCHES▶HNRNPA1 (bg=2.57%)▶HNRNPU (bg=5.92%)No matches to TargetScan


TTTACAA

TTTACAAAGC  
Depth:3 (COW)  
Ei-value:0.000, Pi-value:0.000  
Er-value:0.000, Pr-value:0.000  
eCLIP MATCHES▶HNRNPA1 (bg=2.57%)▶HNRNPU (bg=5.92%)No matches to TargetScan

 15950  


AGC

TTTACAAAGC  
Depth:3 (COW)  
Ei-value:0.000, Pi-value:0.000  
Er-value:0.000, Pr-value:0.000  
eCLIP MATCHES▶HNRNPA1 (bg=2.57%)▶HNRNPU (bg=5.92%)No matches to TargetScan


CTA

ATTTACAAAGCCTA  
Depth:2 (PIG)  
Ei-value:0.000, Pi-value:0.000  
Er-value:0.000, Pr-value:0.000  
eCLIP MATCHES▶HNRNPA1 (bg=2.57%)▶HNRNPU (bg=5.92%)No matches to TargetScan

CAGT

TCTATACA

TCTATACA  
Depth:3 (COW)  
Ei-value:0.000, Pi-value:0.000  
Er-value:0.000, Pr-value:0.000  
eCLIP MATCHES▶HNRNPA1 (bg=2.57%)▶HNRNPU (bg=5.92%)No matches to TargetScan

GA

TAGGAAT

TAGGAAT  
Depth:2 (PIG)  
Ei-value:0.000, Pi-value:0.000  
Er-value:0.000, Pr-value:0.000  
eCLIP MATCHES▶HNRNPA1 (bg=2.57%)No matches to TargetScan

GAACTA

TTGGCT

TTGGCT  
Depth:4 (DOG)  
Ei-value:0.000, Pi-value:0.000  
Er-value:0.000, Pr-value:0.000  
eCLIP MATCHES▶HNRNPA1 (bg=2.57%)No matches to TargetScan

TACTGAATGGTGA

TTACTTTCT

TTACTTTCT  
Depth:4 (DOG)  
Ei-value:0.000, Pi-value:0.000  
Er-value:0.000, Pr-value:0.010  
eCLIP MATCHES▶UTP3 (bg=3.66%)No matches to TargetScan

GTGGGGCTCGGAACT

ACATGC

ACATGC  
Depth:2 (PIG)  
Ei-value:0.000, Pi-value:0.010  
Er-value:0.000, Pr-value:0.020  
No matches to eCLIP DataNo matches to TargetScan

C

CTAGGATAT

CTAGGATAT  
Depth:3 (COW)  
Ei-value:0.000, Pi-value:0.000  
Er-value:0.000, Pr-value:0.000  
No matches to eCLIP DataNo matches to TargetScan

A

AAAATGA

AAAATGA  
Depth:3 (COW)  
Ei-value:0.000, Pi-value:0.000  
Er-value:0.000, Pr-value:0.000  
No matches to eCLIP DataNo matches to TargetScan


T

AAAATGAT  
Depth:2 (PIG)  
Ei-value:0.000, Pi-value:0.010  
Er-value:0.000, Pr-value:0.000  
No matches to eCLIP DataMATCHES To TargetScan▶ miR-382-3p:AUCAUUC

GTTATCATTATAGAGTGCT 16070  
 CACAGA

AGGAAATGA

AGGAAATGA  
Depth:2 (PIG)  
Ei-value:0.000, Pi-value:0.000  
Er-value:0.000, Pr-value:0.000  
eCLIP MATCHES▶KHDRBS1 (bg=1.71%)No matches to TargetScan

AGTAAT

ATAGGTGTG

ATAGGTGTG  
Depth:2 (PIG)  
Ei-value:0.000, Pi-value:0.000  
Er-value:0.000, Pr-value:0.000  
eCLIP MATCHES▶KHDRBS1 (bg=1.71%)No matches to TargetScan

AG

ATCCAGACCA

ATCCAGACCA  
Depth:3 (COW)  
Ei-value:0.000, Pi-value:0.000  
Er-value:0.000, Pr-value:0.000  
eCLIP MATCHES▶KHDRBS1 (bg=1.71%)No matches to TargetScan

AAAGTCATTTAACAAGTTTATTCAGTGATGAAAACATGGGACAAATGGACTAATATAAGGCAGTGTACTAAGCTGAGT 16190  
 AGAGAGATAAAGTCCTGTCCAGAAGATACATGCTTCCTG

GCCTGATTGA

GCCTGATTGA  
Depth:2 (PIG)  
Ei-value:0.000, Pi-value:0.000  
Er-value:0.000, Pr-value:0.000  
eCLIP MATCHES▶HNRNPA1 (bg=2.57%)No matches to TargetScan

GG

AGATGGA

AGATGGA  
Depth:3 (COW)  
Ei-value:0.000, Pi-value:0.000  
Er-value:0.000, Pr-value:0.000  
No matches to eCLIP DataNo matches to TargetScan

AAATTTTTGCAAAAAACAAGGTGTTGTGGTCTTCCATCCAGTTTCTTAAGTGCTGATGATAA 16310  
 AAGTGAATTAGACCCACCTTGACCTGGCCTACAGAAG

TAAAG

TAAAGGAGTAAAAAT  
Depth:2 (PIG)  
Ei-value:0.000, Pi-value:0.000  
Er-value:0.000, Pr-value:0.000  
No matches to eCLIP DataMATCHES To TargetScan▶ miR-483-3p.1:ACUCCUC


GAGTAAAAA

GAGTAAAAA  
Depth:4 (DOG)  
Ei-value:0.000, Pi-value:0.000  
Er-value:0.000, Pr-value:0.000  
No matches to eCLIP DataNo matches to TargetScan


T

TAAAGGAGTAAAAAT  
Depth:2 (PIG)  
Ei-value:0.000, Pi-value:0.000  
Er-value:0.000, Pr-value:0.000  
No matches to eCLIP DataMATCHES To TargetScan▶ miR-483-3p.1:ACUCCUC

AAATGCCTCAGGCGTGCTTTTTGATTC

ATTTGAT

ATTTGAT  
Depth:4 (DOG)  
Ei-value:0.000, Pi-value:0.010  
Er-value:0.000, Pr-value:0.000  
No matches to eCLIP DataNo matches to TargetScan


AAACA

ATTTGATAAACA  
Depth:2 (PIG)  
Ei-value:0.000, Pi-value:0.000  
Er-value:0.000, Pr-value:0.000  
No matches to eCLIP DataNo matches to TargetScan

AAGC

ATC

ATCTTTTATGT  
Depth:3 (COW)  
Ei-value:0.000, Pi-value:0.000  
Er-value:0.000, Pr-value:0.000  
eCLIP MATCHES▶SAFB (bg=2.69%)No matches to TargetScan


TTTTATGT

TTTTATGT  
Depth:4 (DOG)  
Ei-value:0.000, Pi-value:0.000  
Er-value:0.000, Pr-value:0.000  
eCLIP MATCHES▶SAFB (bg=2.69%)No matches to TargetScan


GGAATA

ATCTTTTATGTGGAATA  
Depth:2 (PIG)  
Ei-value:0.000, Pi-value:0.000  
Er-value:0.000, Pr-value:0.000  
eCLIP MATCHES▶SAFB (bg=2.69%)No matches to TargetScan

TACCATTC 16430  
 TG

GGTCCTGAG

GGTCCTGAG  
Depth:3 (COW)  
Ei-value:0.000, Pi-value:0.000  
Er-value:0.000, Pr-value:0.000  
eCLIP MATCHES▶SAFB (bg=2.69%)No matches to TargetScan

GATAAGAGAGATG

AGGGCATTAG

AGGGCATTAG  
Depth:2 (PIG)  
Ei-value:0.000, Pi-value:0.000  
Er-value:0.000, Pr-value:0.000  
eCLIP MATCHES▶SAFB (bg=2.69%)MATCHES To TargetScan▶ miR-155-5p:UAAUGCU▶ miR-365-3p:AAUGCCC▶ miR-874-3p:UGCCCUG

ATCACTGACA

GCTGAA

GCTGAA  
Depth:2 (PIG)  
Ei-value:0.000, Pi-value:0.010  
Er-value:0.000, Pr-value:0.000  
No matches to eCLIP DataNo matches to TargetScan

GATAGAAGAACATCTTTGG

TTTGATT

TTTGATT  
Depth:2 (PIG)  
Ei-value:0.000, Pi-value:0.020  
Er-value:0.000, Pr-value:0.010  
No matches to eCLIP DataNo matches to TargetScan

GTTTAAATAATATTTCAATGCCTATTCTCTGCAAGGTACTATGT 16550  
 TTCGTAAATTAAATAGGTCTGGCCCAGAAGACCCACTCAA

TTGCCTT

TTGCCTT  
Depth:3 (COW)  
Ei-value:0.000, Pi-value:0.000  
Er-value:0.000, Pr-value:0.000  
eCLIP MATCHES▶KHDRBS1 (bg=1.71%)▶UTP18 (bg=0.72%)MATCHES To TargetScan▶ miR-124-3p.1:AAGGCAC

TGAGATTAAAAAAAAAA

AAAAAAAGA

AAAAAAAGA  
Depth:2 (PIG)  
Ei-value:0.000, Pi-value:0.000  
Er-value:0.000, Pr-value:0.000  
eCLIP MATCHES▶KHDRBS1 (bg=1.71%)No matches to TargetScan

AAGAAAAATGCAAGTTTCTTTCAAAATAAAGA

GACATTTTTCCTAG

GACATTTTTCCTAG  
Depth:2 (PIG)  
Ei-value:0.000, Pi-value:0.000  
Er-value:0.000, Pr-value:0.000  
eCLIP MATCHES▶AARS (bg=2.18%)▶HNRNPU (bg=5.92%)▶SAFB (bg=2.69%)No matches to TargetScan

T 16670  
 TTCAGGAATCCCCCAAATCACTTCCTCATTGGCTTAGTTTA

AAGCCAG

AAGCCAG  
Depth:4 (DOG)  
Ei-value:0.000, Pi-value:0.000  
Er-value:0.000, Pr-value:0.000  
eCLIP MATCHES▶SAFB (bg=2.69%)MATCHES To TargetScan▶ miR-149-5p:CUGGCUC▶ miR-3064-5p:CUGGCUG

GAGAC

TG

TGATAAAAG  
Depth:2 (PIG)  
Ei-value:0.000, Pi-value:0.000  
Er-value:0.000, Pr-value:0.000  
No matches to eCLIP DataNo matches to TargetScan


ATAAAAG

ATAAAAG  
Depth:4 (DOG)  
Ei-value:0.000, Pi-value:0.000  
Er-value:0.000, Pr-value:0.000  
No matches to eCLIP DataNo matches to TargetScan

GGCTCAGGGTTTGTT

CTTTAATTC

CTTTAATTC  
Depth:3 (COW)  
Ei-value:0.000, Pi-value:0.000  
Er-value:0.000, Pr-value:0.000  
No matches to eCLIP DataNo matches to TargetScan

ATTAACTA

AACATTCTGC

AACATTCTGCTTTTATTA  
Depth:2 (PIG)  
Ei-value:0.000, Pi-value:0.000  
Er-value:0.000, Pr-value:0.000  
No matches to eCLIP DataMATCHES To TargetScan▶ miR-330-3p.2:AAAGCAC▶ miR-409-3p:AAUGUUG


TTTTATTA

TTTTATTA  
Depth:4 (DOG)  
Ei-value:0.000, Pi-value:0.010  
Er-value:0.000, Pr-value:0.000  
No matches to eCLIP DataNo matches to TargetScan

CA

G

GTTAAATGG  
Depth:3 (COW)  
Ei-value:0.000, Pi-value:0.000  
Er-value:0.000, Pr-value:0.000  
No matches to eCLIP DataNo matches to TargetScan


TTAAA

TTAAATGG  
Depth:4 (DOG)  
Ei-value:0.000, Pi-value:0.000  
Er-value:0.000, Pr-value:0.000  
No matches to eCLIP DataNo matches to TargetScan

 16790  


TGG

TTAAATGG  
Depth:4 (DOG)  
Ei-value:0.000, Pi-value:0.000  
Er-value:0.000, Pr-value:0.000  
No matches to eCLIP DataNo matches to TargetScan


TT

GTTAAATGGTT  
Depth:2 (PIG)  
Ei-value:0.000, Pi-value:0.000  
Er-value:0.000, Pr-value:0.000  
No matches to eCLIP DataNo matches to TargetScan

CAAGATGT

AACAACTAGTT

AACAACTAGTT  
Depth:2 (PIG)  
Ei-value:0.000, Pi-value:0.000  
Er-value:0.000, Pr-value:0.000  
No matches to eCLIP DataNo matches to TargetScan

TTAAAGGTATTTG

CTCATTGGTCTG

CTCATTGGTCTG  
Depth:2 (PIG)  
Ei-value:0.000, Pi-value:0.000  
Er-value:0.000, Pr-value:0.000  
No matches to eCLIP DataNo matches to TargetScan

GCTTAGAGACAGGAAGACATATGAGCAA

TAAAAAAAA

TAAAAAAAA  
Depth:2 (PIG)  
Ei-value:0.000, Pi-value:0.000  
Er-value:0.000, Pr-value:0.000  
No matches to eCLIP DataNo matches to TargetScan

GATTCTTTTGCATTTACCAATTTA

GTAAAAA

GTAAAAA  
Depth:2 (PIG)  
Ei-value:0.000, Pi-value:0.010  
Er-value:0.000, Pr-value:0.000  
eCLIP MATCHES▶KHDRBS1 (bg=1.71%)No matches to TargetScan

TTT 16910  
 ATTAAAACTGAATAAAGTG

CTGTTCTTAAGT

CTGTTCTTAAGT  
Depth:3 (COW)  
Ei-value:0.000, Pi-value:0.000  
Er-value:0.000, Pr-value:0.000  
eCLIP MATCHES▶KHDRBS1 (bg=1.71%)No matches to TargetScan

GCTTGAAAGACGTAAACCAAAGTGCACTTTATCTCATTTATCTTATGGT

GGAAACA

GGAAACA  
Depth:2 (PIG)  
Ei-value:0.000, Pi-value:0.000  
Er-value:0.000, Pr-value:0.000  
No matches to eCLIP DataNo matches to TargetScan

CAG

GAACAAATT

GAACAAATT  
Depth:3 (COW)  
Ei-value:0.000, Pi-value:0.000  
Er-value:0.000, Pr-value:0.000  
No matches to eCLIP DataMATCHES To TargetScan▶ miR-375:UUGUUCG

CTC

TAAGAGACTG

TAAGAGACTG  
Depth:2 (PIG)  
Ei-value:0.000, Pi-value:0.000  
Er-value:0.000, Pr-value:0.000  
No matches to eCLIP DataNo matches to TargetScan

TGTTTCT

T

TTAGTTG  
Depth:3 (COW)  
Ei-value:0.000, Pi-value:0.000  
Er-value:0.000, Pr-value:0.010  
No matches to eCLIP DataNo matches to TargetScan

 17030  


TAGTTG

TTAGTTG  
Depth:3 (COW)  
Ei-value:0.000, Pi-value:0.000  
Er-value:0.000, Pr-value:0.010  
No matches to eCLIP DataNo matches to TargetScan


A

TTAGTTGA  
Depth:2 (PIG)  
Ei-value:0.000, Pi-value:0.000  
Er-value:0.000, Pr-value:0.000  
No matches to eCLIP DataNo matches to TargetScan

GAAG

AAACTTCATTGA

AAACTTCATTGA  
Depth:3 (COW)  
Ei-value:0.000, Pi-value:0.000  
Er-value:0.000, Pr-value:0.000  
eCLIP MATCHES▶HNRNPA1 (bg=2.57%)No matches to TargetScan


G

AAACTTCATTGAG  
Depth:2 (PIG)  
Ei-value:0.000, Pi-value:0.000  
Er-value:0.000, Pr-value:0.000  
eCLIP MATCHES▶HNRNPA1 (bg=2.57%)No matches to TargetScan

TAGCTG

TGATAT

TGATAT  
Depth:2 (PIG)  
Ei-value:0.000, Pi-value:0.000  
Er-value:0.000, Pr-value:0.010  
eCLIP MATCHES▶HNRNPA1 (bg=2.57%)No matches to TargetScan

GTTCGATACTAAGGAAAAACTAAACAGATCACCTTTGACATGCGTTGTAGAGTG

GGAATAAGAGA

GGAATAAGAGA  
Depth:2 (PIG)  
Ei-value:0.000, Pi-value:0.000  
Er-value:0.000, Pr-value:0.000  
No matches to eCLIP DataNo matches to TargetScan

GGGCTTTTTATTTTTTCGT 17150  
 TCATACGAGTATTGAT

GAAGATGAT

GAAGATGAT  
Depth:2 (PIG)  
Ei-value:0.000, Pi-value:0.000  
Er-value:0.000, Pr-value:0.000  
No matches to eCLIP DataNo matches to TargetScan

ACTAAAT

GCTAAAT

GCTAAAT  
Depth:2 (PIG)  
Ei-value:0.000, Pi-value:0.020  
Er-value:0.000, Pr-value:0.000  
No matches to eCLIP DataNo matches to TargetScan

GAAATATATCTGCTC

CAAAAG

CAAAAG  
Depth:2 (PIG)  
Ei-value:0.000, Pi-value:0.010  
Er-value:0.000, Pr-value:0.000  
No matches to eCLIP DataNo matches to TargetScan

GCATTTATTCTGA

CTTGGAGATG

CTTGGAGATG  
Depth:2 (PIG)  
Ei-value:0.000, Pi-value:0.000  
Er-value:0.000, Pr-value:0.000  
No matches to eCLIP DataNo matches to TargetScan

CAACAAAAACACAA

AAATGGA

AAATGGA  
Depth:2 (PIG)  
Ei-value:0.000, Pi-value:0.000  
Er-value:0.000, Pr-value:0.010  
No matches to eCLIP DataNo matches to TargetScan

ATGAA

GTGATACTC

GTGATACTC  
Depth:2 (PIG)  
Ei-value:0.000, Pi-value:0.000  
Er-value:0.000, Pr-value:0.000  
No matches to eCLIP DataMATCHES To TargetScan▶ miR-496.1:GAGUAUU

TT 17270  
 CATCAAACAGAAGTGACTGTTATCTCAACCATTTTGTTAAATCCTAA

ACAGAAAACAAAA

ACAGAAAACAAAA  
Depth:4 (DOG)  
Ei-value:0.000, Pi-value:0.000  
Er-value:0.000, Pr-value:0.000  
No matches to eCLIP DataNo matches to TargetScan

AAAATCATGACGAAAAGAC

ACTTGC

ACTTGC  
Depth:2 (PIG)  
Ei-value:0.000, Pi-value:0.020  
Er-value:0.000, Pr-value:0.000  
No matches to eCLIP DataNo matches to TargetScan

TTATTAA

TTGG

TTGGCTTGGAAA  
Depth:2 (PIG)  
Ei-value:0.000, Pi-value:0.000  
Er-value:0.000, Pr-value:0.000  
No matches to eCLIP DataNo matches to TargetScan


CTTGGAAA

CTTGGAAA  
Depth:3 (COW)  
Ei-value:0.000, Pi-value:0.000  
Er-value:0.000, Pr-value:0.000  
No matches to eCLIP DataNo matches to TargetScan

GTAGAATATAGGAGAA 17390  


AGGTTA

AGGTTA  
Depth:4 (DOG)  
Ei-value:0.000, Pi-value:0.000  
Er-value:0.000, Pr-value:0.000  
No matches to eCLIP DataNo matches to TargetScan


CTGTTTATT

AGGTTACTGTTTATT  
Depth:2 (PIG)  
Ei-value:0.000, Pi-value:0.000  
Er-value:0.000, Pr-value:0.000  
No matches to eCLIP DataMATCHES To TargetScan▶ miR-101-3p.1:ACAGUAC▶ miR-132-3p/212-3p:AACAGUC▶ miR-144-3p:ACAGUAU▶ miR-802:CAGUAAC

TTTTTTCATGTATTCA

TTCATTCT

TTCATTCT  
Depth:4 (DOG)  
Ei-value:0.000, Pi-value:0.000  
Er-value:0.000, Pr-value:0.000  
No matches to eCLIP DataNo matches to TargetScan

ACAAATATATTCGGGTGCCAATAGGTACTTGGTATAAGGTTT

TTGGCCCC

TTGGCCCCAGAGACATG  
Depth:2 (PIG)  
Ei-value:0.000, Pi-value:0.000  
Er-value:0.000, Pr-value:0.000  
No matches to eCLIP DataMATCHES To TargetScan▶ miR-326:CUCUGGG


AGAGACA

AGAGACA  
Depth:4 (DOG)  
Ei-value:0.000, Pi-value:0.000  
Er-value:0.000, Pr-value:0.000  
No matches to eCLIP DataNo matches to TargetScan


TG

AGAGACATG  
Depth:3 (COW)  
Ei-value:0.000, Pi-value:0.000  
Er-value:0.000, Pr-value:0.000  
No matches to eCLIP DataNo matches to TargetScan

GGA

AAAAAATG

AAAAAATG  
Depth:2 (PIG)  
Ei-value:0.000, Pi-value:0.000  
Er-value:0.000, Pr-value:0.000  
No matches to eCLIP DataNo matches to TargetScan

CATGCCTTCCC 17510  
 AGAGAATGCCTAATACTTT

CCTTTTGG

CCTTTTGG  
Depth:4 (DOG)  
Ei-value:0.000, Pi-value:0.000  
Er-value:0.000, Pr-value:0.000  
No matches to eCLIP DataNo matches to TargetScan


C

CCTTTTGGC  
Depth:3 (COW)  
Ei-value:0.000, Pi-value:0.000  
Er-value:0.000, Pr-value:0.000  
No matches to eCLIP DataNo matches to TargetScan

TT

GTTTTCT

GTTTTCT  
Depth:2 (PIG)  
Ei-value:0.000, Pi-value:0.010  
Er-value:0.000, Pr-value:0.010  
No matches to eCLIP DataNo matches to TargetScan

T

GTTAGGGGCA

GTTAGGGGCA  
Depth:2 (PIG)  
Ei-value:0.000, Pi-value:0.000  
Er-value:0.000, Pr-value:0.000  
No matches to eCLIP DataNo matches to TargetScan

T

GGCTTAGT

GGCTTAGT  
Depth:2 (PIG)  
Ei-value:0.000, Pi-value:0.000  
Er-value:0.000, Pr-value:0.000  
No matches to eCLIP DataNo matches to TargetScan

CCCTAAA

TAAC

TAACATTGTGT  
Depth:2 (PIG)  
Ei-value:0.000, Pi-value:0.000  
Er-value:0.000, Pr-value:0.000  
No matches to eCLIP DataMATCHES To TargetScan▶ miR-409-3p:AAUGUUG


ATTGTGT

ATTGTGT  
Depth:3 (COW)  
Ei-value:0.000, Pi-value:0.000  
Er-value:0.000, Pr-value:0.010  
No matches to eCLIP DataNo matches to TargetScan

GGT

TTAATTC

TTAATTC  
Depth:4 (DOG)  
Ei-value:0.000, Pi-value:0.000  
Er-value:0.000, Pr-value:0.000  
No matches to eCLIP DataNo matches to TargetScan

CTACTCCGTATCTCTTCTACC

ACTCTGGCCACTAC

ACTCTGGCCACTAC  
Depth:4 (DOG)  
Ei-value:0.000, Pi-value:0.000  
Er-value:0.000, Pr-value:0.000  
No matches to eCLIP DataMATCHES To TargetScan▶ miR-142-3p.1:GUAGUGU

 17630  


ACTCTGGCCACTAC  
Depth:4 (DOG)  
Ei-value:0.000, Pi-value:0.000  
Er-value:0.000, Pr-value:0.000  
No matches to eCLIP DataMATCHES To TargetScan▶ miR-142-3p.1:GUAGUGU

G

ATAAGC

ATAAGC  
Depth:5 (RABBIT)  
Ei-value:0.000, Pi-value:0.010  
Er-value:0.000, Pr-value:0.000  
No matches to eCLIP DataNo matches to TargetScan


AGG

ATAAGCAGG  
Depth:4 (DOG)  
Ei-value:0.000, Pi-value:0.000  
Er-value:0.000, Pr-value:0.000  
No matches to eCLIP DataNo matches to TargetScan

TAGCTGGGTTTTGTAGTGA

GCT

GCTTGCTCCTT  
Depth:2 (PIG)  
Ei-value:0.000, Pi-value:0.000  
Er-value:0.000, Pr-value:0.000  
No matches to eCLIP DataMATCHES To TargetScan▶ miR-28-5p/708-5p:AGGAGCU


TGCTCCTT

TGCTCCTT  
Depth:3 (COW)  
Ei-value:0.000, Pi-value:0.000  
Er-value:0.000, Pr-value:0.000  
No matches to eCLIP DataMATCHES To TargetScan▶ miR-28-5p/708-5p:AGGAGCU

AAGTTACAGGAACTCTCCTTATAATAGAC

ACTTCA

ACTTCA  
Depth:3 (COW)  
Ei-value:0.000, Pi-value:0.000  
Er-value:0.000, Pr-value:0.000  
No matches to eCLIP DataNo matches to TargetScan


TTTTCCTA

TTTTCCTA  
Depth:3 (COW)  
Ei-value:0.000, Pi-value:0.000  
Er-value:0.000, Pr-value:0.000  
No matches to eCLIP DataNo matches to TargetScan


GTCCATCC

TTTTCCTAGTCCATCC  
Depth:2 (PIG)  
Ei-value:0.000, Pi-value:0.000  
Er-value:0.000, Pr-value:0.000  
No matches to eCLIP DataNo matches to TargetScan

CTC

AT

ATGAAAAATG  
Depth:3 (COW)  
Ei-value:0.000, Pi-value:0.000  
Er-value:0.000, Pr-value:0.000  
No matches to eCLIP DataNo matches to TargetScan


GAAAAATG

GAAAAATG  
Depth:4 (DOG)  
Ei-value:0.000, Pi-value:0.000  
Er-value:0.000, Pr-value:0.000  
No matches to eCLIP DataNo matches to TargetScan

ACTGACCACTGC

TGGG

TGGGCAG  
Depth:2 (PIG)  
Ei-value:0.000, Pi-value:0.000  
Er-value:0.000, Pr-value:0.000  
No matches to eCLIP DataNo matches to TargetScan

 17750  


CAG

TGGGCAG  
Depth:2 (PIG)  
Ei-value:0.000, Pi-value:0.000  
Er-value:0.000, Pr-value:0.000  
No matches to eCLIP DataNo matches to TargetScan

CAGGAGGGATGATGACCAACTAATTCCCAAACCCC

AGTCTCA

AGTCTCA  
Depth:4 (DOG)  
Ei-value:0.000, Pi-value:0.000  
Er-value:0.000, Pr-value:0.000  
eCLIP MATCHES▶ZC3H11A (bg=6.55%)No matches to TargetScan


TTGGTACCA

AGTCTCATTGGTACCA  
Depth:3 (COW)  
Ei-value:0.000, Pi-value:0.000  
Er-value:0.000, Pr-value:0.000  
eCLIP MATCHES▶NOLC1 (bg=9.43%)▶ZC3H11A (bg=6.55%)No matches to TargetScan


GC

AGTCTCATTGGTACCAGC  
Depth:2 (PIG)  
Ei-value:0.000, Pi-value:0.000  
Er-value:0.000, Pr-value:0.000  
eCLIP MATCHES▶NOLC1 (bg=9.43%)▶RPS3 (bg=0.76%)▶ZC3H11A (bg=6.55%)MATCHES To TargetScan▶ miR-138-5p:GCUGGUG

CTTG

GGGAAC

GGGAAC  
Depth:2 (PIG)  
Ei-value:0.000, Pi-value:0.000  
Er-value:0.000, Pr-value:0.010  
eCLIP MATCHES▶NOLC1 (bg=9.43%)▶RBFOX2 (bg=4.63%)▶RPS3 (bg=0.76%)▶ZC3H11A (bg=6.55%)No matches to TargetScan

CACCTACACTTG

AGCCACAA

AGCCACAA  
Depth:2 (PIG)  
Ei-value:0.000, Pi-value:0.000  
Er-value:0.000, Pr-value:0.000  
eCLIP MATCHES▶RBFOX2 (bg=4.63%)▶RPS3 (bg=0.76%)▶ZC3H11A (bg=6.55%)No matches to TargetScan

T

TGGTTTTGAA

TGGTTTTGAA  
Depth:4 (DOG)  
Ei-value:0.000, Pi-value:0.000  
Er-value:0.000, Pr-value:0.000  
No matches to eCLIP DataNo matches to TargetScan

GTG

CATTTAC

CATTTAC  
Depth:2 (PIG)  
Ei-value:0.000, Pi-value:0.000  
Er-value:0.000, Pr-value:0.000  
No matches to eCLIP DataNo matches to TargetScan

AAGGTTTGTCTAT 17870  
 TT

TCAGTTC

TCAGTTC  
Depth:2 (PIG)  
Ei-value:0.000, Pi-value:0.010  
Er-value:0.000, Pr-value:0.000  
eCLIP MATCHES▶HNRNPL (bg=0.64%)No matches to TargetScan

TTTACTTTTTACATGCTGACACATACATACACTGCCTAAATAGATCTCTTTCAGAA

ACAATCC

ACAATCC  
Depth:3 (COW)  
Ei-value:0.000, Pi-value:0.000  
Er-value:0.000, Pr-value:0.000  
No matches to eCLIP DataMATCHES To TargetScan▶ miR-219-5p:GAUUGUC

TCAGATAACGCATAGCAAAA

TGGAGATG

TGGAGATG  
Depth:3 (COW)  
Ei-value:0.000, Pi-value:0.000  
Er-value:0.000, Pr-value:0.000  
No matches to eCLIP DataNo matches to TargetScan

GAGACATGATTTCTCATGCA 17990  
 AC

AGCTTCTC

AGCTTCTC  
Depth:3 (COW)  
Ei-value:0.000, Pi-value:0.000  
Er-value:0.000, Pr-value:0.000  
No matches to eCLIP DataNo matches to TargetScan

TAATTATAC

C

CTTAGAAAT  
Depth:2 (PIG)  
Ei-value:0.000, Pi-value:0.000  
Er-value:0.000, Pr-value:0.000  
eCLIP MATCHES▶WDR3 (bg=0.25%)No matches to TargetScan


TTAGAAAT

TTAGAAAT  
Depth:4 (DOG)  
Ei-value:0.000, Pi-value:0.000  
Er-value:0.000, Pr-value:0.000  
eCLIP MATCHES▶WDR3 (bg=0.25%)No matches to TargetScan

GTTCTCCTTTTTAT

CATCAAA

CATCAAA  
Depth:4 (DOG)  
Ei-value:0.000, Pi-value:0.000  
Er-value:0.000, Pr-value:0.000  
eCLIP MATCHES▶ZC3H11A (bg=6.55%)No matches to TargetScan

TCTGCTCAAGAAGGGCTTTTTATAGTAGAATAATATCAGTGG

ATGAAAA

ATGAAAA  
Depth:2 (PIG)  
Ei-value:0.000, Pi-value:0.030  
Er-value:0.000, Pr-value:0.010  
eCLIP MATCHES▶ZC3H11A (bg=6.55%)No matches to TargetScan

CAGCTTAACATTTTACCATG

CT

CTTAAGTTTTA  
Depth:2 (PIG)  
Ei-value:0.000, Pi-value:0.000  
Er-value:0.000, Pr-value:0.000  
No matches to eCLIP DataNo matches to TargetScan

 18110  


TAAGTTTTA

CTTAAGTTTTA  
Depth:2 (PIG)  
Ei-value:0.000, Pi-value:0.000  
Er-value:0.000, Pr-value:0.000  
No matches to eCLIP DataNo matches to TargetScan

AGAATAA

AATAAAAATTGGAA

AATAAAAATTGGAA  
Depth:2 (PIG)  
Ei-value:0.000, Pi-value:0.000  
Er-value:0.000, Pr-value:0.000  
No matches to eCLIP DataNo matches to TargetScan

ATAATTGGCCAAAATTGAAA

GGAAAAA

GGAAAAA  
Depth:3 (COW)  
Ei-value:0.000, Pi-value:0.000  
Er-value:0.000, Pr-value:0.000  
No matches to eCLIP DataNo matches to TargetScan

TTTTTTTAAAATTTC

TCTAAAT

TCTAAAT  
Depth:2 (PIG)  
Ei-value:0.000, Pi-value:0.000  
Er-value:0.000, Pr-value:0.010  
eCLIP MATCHES▶PPIL4 (bg=0.52%)No matches to TargetScan

GTAGGCCTGGC

TGGGCTTTG

TGGGCTTTG  
Depth:3 (COW)  
Ei-value:0.000, Pi-value:0.000  
Er-value:0.000, Pr-value:0.000  
eCLIP MATCHES▶NOLC1 (bg=9.43%)▶PPIL4 (bg=0.52%)MATCHES To TargetScan▶ miR-330-3p:CAAAGCA

ACCTTTTCCG

TTTTTAAATCA

TTTTTAAATCACTCA  
Depth:4 (DOG)  
Ei-value:0.000, Pi-value:0.000  
Er-value:0.000, Pr-value:0.000  
eCLIP MATCHES▶ILF3 (bg=3.0%)▶NOLC1 (bg=9.43%)▶PPIL4 (bg=0.52%)No matches to TargetScan

 18230  


CTCA

TTTTTAAATCACTCA  
Depth:4 (DOG)  
Ei-value:0.000, Pi-value:0.000  
Er-value:0.000, Pr-value:0.000  
eCLIP MATCHES▶ILF3 (bg=3.0%)▶NOLC1 (bg=9.43%)▶PPIL4 (bg=0.52%)No matches to TargetScan

C

AGAGGGTGGGA

AGAGGGTGGGA  
Depth:4 (DOG)  
Ei-value:0.000, Pi-value:0.000  
Er-value:0.000, Pr-value:0.000  
eCLIP MATCHES▶ILF3 (bg=3.0%)▶ZC3H11A (bg=6.55%)No matches to TargetScan

C

AGGAGGAAGAGTGAA

AGGAGGAAGAGTGAA  
Depth:4 (DOG)  
Ei-value:0.000, Pi-value:0.000  
Er-value:0.000, Pr-value:0.000  
eCLIP MATCHES▶ILF3 (bg=3.0%)▶ZC3H11A (bg=6.55%)MATCHES To TargetScan▶ miR-670-3p:UUCCUCA

G

G

GAAAAGGTCA  
Depth:4 (DOG)  
Ei-value:0.000, Pi-value:0.000  
Er-value:0.000, Pr-value:0.000  
eCLIP MATCHES▶ILF3 (bg=3.0%)▶SF3B1 (bg=2.48%)▶ZC3H11A (bg=6.55%)MATCHES To TargetScan▶ miR-192-5p/215-5p:UGACCUA


AAAAGGT

AAAAGGT  
Depth:6 (MOUSE)  
Ei-value:0.000, Pi-value:0.000  
Er-value:0.000, Pr-value:0.000  
eCLIP MATCHES▶ILF3 (bg=3.0%)▶SF3B1 (bg=2.48%)▶ZC3H11A (bg=6.55%)No matches to TargetScan


CA

GAAAAGGTCA  
Depth:4 (DOG)  
Ei-value:0.000, Pi-value:0.000  
Er-value:0.000, Pr-value:0.000  
eCLIP MATCHES▶ILF3 (bg=3.0%)▶SF3B1 (bg=2.48%)▶ZC3H11A (bg=6.55%)MATCHES To TargetScan▶ miR-192-5p/215-5p:UGACCUA

AACCTGTTTTAA

GGGCAACCTGCCTTTGTTCTG

GGGCAACCTGCCTTTGTTCTG  
Depth:2 (PIG)  
Ei-value:0.000, Pi-value:0.000  
Er-value:0.000, Pr-value:0.000  
eCLIP MATCHES▶ILF3 (bg=3.0%)▶ZC3H11A (bg=6.55%)MATCHES To TargetScan▶ miR-124-3p.1:AAGGCAC▶ miR-495-3p:AACAAAC

A

A

ATTGGTCTTAA  
Depth:2 (PIG)  
Ei-value:0.000, Pi-value:0.000  
Er-value:0.000, Pr-value:0.000  
eCLIP MATCHES▶ILF3 (bg=3.0%)▶ZC3H11A (bg=6.55%)MATCHES To TargetScan▶ miR-208-3p:UAAGACG▶ miR-499a-5p:UAAGACU


TTGGTCTTAA

TTGGTCTTAA  
Depth:3 (COW)  
Ei-value:0.000, Pi-value:0.000  
Er-value:0.000, Pr-value:0.000  
eCLIP MATCHES▶ILF3 (bg=3.0%)▶ZC3H11A (bg=6.55%)MATCHES To TargetScan▶ miR-208-3p:UAAGACG▶ miR-499a-5p:UAAGACU

GAACATTACCAGCTCCAG

GTTTAAAT

GTTTAAAT  
Depth:2 (PIG)  
Ei-value:0.000, Pi-value:0.000  
Er-value:0.000, Pr-value:0.000  
eCLIP MATCHES▶ILF3 (bg=3.0%)No matches to TargetScan

TGTTCA 18350  
 GTTTCATGCAGTTCCAATAGCTGATCATTGTTG

AGATGAGGACAAA

AGATGAGGACAAA  
Depth:3 (COW)  
Ei-value:0.000, Pi-value:0.000  
Er-value:0.000, Pr-value:0.000  
eCLIP MATCHES▶HNRNPA1 (bg=2.57%)No matches to TargetScan

A

TCCTTTGT

TCCTTTGT  
Depth:3 (COW)  
Ei-value:0.000, Pi-value:0.000  
Er-value:0.000, Pr-value:0.000  
eCLIP MATCHES▶HNRNPA1 (bg=2.57%)No matches to TargetScan

CCTCACTAGTTTGCTTT

ACATTTTT

ACATTTTT  
Depth:2 (PIG)  
Ei-value:0.000, Pi-value:0.020  
Er-value:0.000, Pr-value:0.000  
No matches to eCLIP DataNo matches to TargetScan

GAAAAGTATTATTTTTGTCCAAGTGCTTATCAACTAAA

CC

CCTTGTG  
Depth:2 (PIG)  
Ei-value:0.000, Pi-value:0.000  
Er-value:0.000, Pr-value:0.020  
eCLIP MATCHES▶NOLC1 (bg=9.43%)▶ZC3H11A (bg=6.55%)No matches to TargetScan

 18470  


TTGTG

CCTTGTG  
Depth:2 (PIG)  
Ei-value:0.000, Pi-value:0.000  
Er-value:0.000, Pr-value:0.020  
eCLIP MATCHES▶NOLC1 (bg=9.43%)▶ZC3H11A (bg=6.55%)No matches to TargetScan

TTAGGTAAGAATGGAATTTATTAAGTGAATCAGTGTGACCCTTCTTGTCATAAGATTATCT

TAAAGC

TAAAGC  
Depth:3 (COW)  
Ei-value:0.000, Pi-value:0.000  
Er-value:0.000, Pr-value:0.000  
eCLIP MATCHES▶NOLC1 (bg=9.43%)No matches to TargetScan

TGAAGCCAAAATATGCTT

CAAAAGAAGAGG

CAAAAGAAGAGG  
Depth:2 (PIG)  
Ei-value:0.000, Pi-value:0.000  
Er-value:0.000, Pr-value:0.000  
No matches to eCLIP DataNo matches to TargetScan

ACTTTATTGTTCATTGTA 18590  
 G

TTCATACA

TTCATACA  
Depth:3 (COW)  
Ei-value:0.000, Pi-value:0.000  
Er-value:0.000, Pr-value:0.000  
No matches to eCLIP DataNo matches to TargetScan


TTCAAAGCATC

TTCAAAGCATC  
Depth:3 (COW)  
Ei-value:0.000, Pi-value:0.000  
Er-value:0.000, Pr-value:0.000  
No matches to eCLIP DataNo matches to TargetScan

TGAACTGTAGTTTCTATA

GCAAGCCAA

GCAAGCCAA  
Depth:2 (PIG)  
Ei-value:0.000, Pi-value:0.000  
Er-value:0.000, Pr-value:0.000  
No matches to eCLIP DataNo matches to TargetScan

TTACATCCATAAG

TGG

TGGAGAAGGAAATAGAT  
Depth:2 (PIG)  
Ei-value:0.000, Pi-value:0.000  
Er-value:0.000, Pr-value:0.000  
eCLIP MATCHES▶ZC3H11A (bg=6.55%)No matches to TargetScan


AGAAGGAAATAGA

AGAAGGAAATAGA  
Depth:3 (COW)  
Ei-value:0.000, Pi-value:0.000  
Er-value:0.000, Pr-value:0.000  
eCLIP MATCHES▶ZC3H11A (bg=6.55%)No matches to TargetScan


T

TGGAGAAGGAAATAGAT  
Depth:2 (PIG)  
Ei-value:0.000, Pi-value:0.000  
Er-value:0.000, Pr-value:0.000  
eCLIP MATCHES▶ZC3H11A (bg=6.55%)No matches to TargetScan

AAATGTCAAAGTATGATTGG

TGGAGGGAGC

TGGAGGGAGC  
Depth:3 (COW)  
Ei-value:0.000, Pi-value:0.000  
Er-value:0.000, Pr-value:0.000  
eCLIP MATCHES▶FTO (bg=0.32%)▶LARP4 (bg=4.72%)▶LSM11 (bg=2.28%)▶NOLC1 (bg=9.43%)▶XRCC6 (bg=2.91%)▶ZC3H11A (bg=6.55%)No matches to TargetScan


AA

TGGAGGGAGCAA  
Depth:2 (PIG)  
Ei-value:0.000, Pi-value:0.000  
Er-value:0.000, Pr-value:0.000  
eCLIP MATCHES▶FTO (bg=0.32%)▶LARP4 (bg=4.72%)▶LSM11 (bg=2.28%)▶NOLC1 (bg=9.43%)▶XRCC6 (bg=2.91%)▶ZC3H11A (bg=6.55%)No matches to TargetScan

G

GTTGAAGA

GTTGAAGA  
Depth:2 (PIG)  
Ei-value:0.000, Pi-value:0.000  
Er-value:0.000, Pr-value:0.000  
eCLIP MATCHES▶FTO (bg=0.32%)▶LARP4 (bg=4.72%)▶LSM11 (bg=2.28%)▶NOLC1 (bg=9.43%)▶XRCC6 (bg=2.91%)▶ZC3H11A (bg=6.55%)No matches to TargetScan

TA 18710  
 ATCTGGGGTTGAAATTTTCTAGTTTTCATTCTGTACATTTTTAGTTAGACATCAGATTTGAAATAT

TAATGTTT

TAATGTTT  
Depth:4 (DOG)  
Ei-value:0.000, Pi-value:0.000  
Er-value:0.000, Pr-value:0.000  
eCLIP MATCHES▶CPEB4 (bg=1.89%)▶KHDRBS1 (bg=1.71%)▶LARP4 (bg=4.72%)▶LSM11 (bg=2.28%)▶NOLC1 (bg=9.43%)▶RBFOX2 (bg=4.63%)▶SAFB (bg=2.69%)▶SAFB2 (bg=0.8%)▶WDR43 (bg=3.37%)▶ZC3H11A (bg=6.55%)MATCHES To TargetScan▶ miR-323-3p:ACAUUAC▶ miR-543:AACAUUC

ACCTTTCAATGTGTGG

TATC

TATCAGCTGGA  
Depth:2 (PIG)  
Ei-value:0.000, Pi-value:0.000  
Er-value:0.000, Pr-value:0.000  
eCLIP MATCHES▶CPEB4 (bg=1.89%)▶KHDRBS1 (bg=1.71%)▶LSM11 (bg=2.28%)▶NOLC1 (bg=9.43%)▶RBFOX2 (bg=4.63%)▶SAFB (bg=2.69%)▶SAFB2 (bg=0.8%)▶SF3B1 (bg=2.48%)▶TRA2A (bg=4.8%)▶WDR43 (bg=3.37%)▶ZC3H11A (bg=6.55%)No matches to TargetScan


AGCTGGA

AGCTGGA  
Depth:4 (DOG)  
Ei-value:0.000, Pi-value:0.000  
Er-value:0.000, Pr-value:0.000  
eCLIP MATCHES▶CPEB4 (bg=1.89%)▶KHDRBS1 (bg=1.71%)▶LSM11 (bg=2.28%)▶NOLC1 (bg=9.43%)▶RBFOX2 (bg=4.63%)▶SAFB (bg=2.69%)▶SAFB2 (bg=0.8%)▶SF3B1 (bg=2.48%)▶TRA2A (bg=4.8%)▶WDR43 (bg=3.37%)▶ZC3H11A (bg=6.55%)No matches to TargetScan

CTCAGTAACACCCCTTTCT 18830  
 TCAGCTGGGGATGGGGAATGG

ATTATTGGAAA

ATTATTGGAAA  
Depth:4 (DOG)  
Ei-value:0.000, Pi-value:0.000  
Er-value:0.000, Pr-value:0.000  
eCLIP MATCHES▶FASTKD2 (bg=1.99%)▶FUS (bg=2.21%)▶LARP4 (bg=4.72%)▶NOLC1 (bg=9.43%)▶RBFOX2 (bg=4.63%)▶SAFB (bg=2.69%)▶SAFB2 (bg=0.8%)▶TRA2A (bg=4.8%)▶WDR43 (bg=3.37%)▶ZC3H11A (bg=6.55%)No matches to TargetScan


A

ATTATTGGAAAA  
Depth:2 (PIG)  
Ei-value:0.000, Pi-value:0.000  
Er-value:0.000, Pr-value:0.000  
eCLIP MATCHES▶FASTKD2 (bg=1.99%)▶FUS (bg=2.21%)▶LARP4 (bg=4.72%)▶NOLC1 (bg=9.43%)▶RBFOX2 (bg=4.63%)▶SAFB (bg=2.69%)▶SAFB2 (bg=0.8%)▶TRA2A (bg=4.8%)▶WDR43 (bg=3.37%)▶ZC3H11A (bg=6.55%)No matches to TargetScan


TGGAAAG

TGGAAAG  
Depth:2 (PIG)  
Ei-value:0.000, Pi-value:0.000  
Er-value:0.000, Pr-value:0.010  
eCLIP MATCHES▶FASTKD2 (bg=1.99%)▶FUS (bg=2.21%)▶LARP4 (bg=4.72%)▶NIPBL (bg=5.39%)▶NOLC1 (bg=9.43%)▶RBFOX2 (bg=4.63%)▶SAFB (bg=2.69%)▶SAFB2 (bg=0.8%)▶TRA2A (bg=4.8%)▶WDR43 (bg=3.37%)▶ZC3H11A (bg=6.55%)No matches to TargetScan

A

AGAAAGTAAC

AGAAAGTAAC  
Depth:4 (DOG)  
Ei-value:0.000, Pi-value:0.000  
Er-value:0.000, Pr-value:0.000  
eCLIP MATCHES▶FASTKD2 (bg=1.99%)▶FUS (bg=2.21%)▶LARP4 (bg=4.72%)▶NIPBL (bg=5.39%)▶NOLC1 (bg=9.43%)▶RBFOX2 (bg=4.63%)▶SAFB (bg=2.69%)▶SAFB2 (bg=0.8%)▶TRA2A (bg=4.8%)▶uchl5 (bg=11.16%)▶WDR43 (bg=3.37%)▶ZC3H11A (bg=6.55%)▶ZNF800 (bg=1.92%)No matches to TargetScan


TAAAAGCCTTCC

AGAAAGTAACTAAAAGCCTTCCTTTCACAGTTTCTGGCATC  
Depth:2 (PIG)  
Ei-value:0.000, Pi-value:0.000  
Er-value:0.000, Pr-value:0.000  
eCLIP MATCHES▶FASTKD2 (bg=1.99%)▶FUS (bg=2.21%)▶LARP4 (bg=4.72%)▶NIPBL (bg=5.39%)▶NOLC1 (bg=9.43%)▶RBFOX2 (bg=4.63%)▶SAFB (bg=2.69%)▶SAFB2 (bg=0.8%)▶TRA2A (bg=4.8%)▶uchl5 (bg=11.16%)▶WDR43 (bg=3.37%)▶ZC3H11A (bg=6.55%)▶ZNF800 (bg=1.92%)MATCHES To TargetScan▶ miR-488-3p:UGAAAGG


TTTCACAGTTTCTGGCATC

TTTCACAGTTTCTGGCATC  
Depth:4 (DOG)  
Ei-value:0.000, Pi-value:0.000  
Er-value:0.000, Pr-value:0.000  
eCLIP MATCHES▶FASTKD2 (bg=1.99%)▶FUS (bg=2.21%)▶LARP4 (bg=4.72%)▶NIPBL (bg=5.39%)▶NOLC1 (bg=9.43%)▶RBFOX2 (bg=4.63%)▶SAFB (bg=2.69%)▶SAFB2 (bg=0.8%)▶uchl5 (bg=11.16%)▶WDR43 (bg=3.37%)▶ZC3H11A (bg=6.55%)▶ZNF800 (bg=1.92%)No matches to TargetScan


ACTAC

ACTACCACTACTGAT  
Depth:2 (PIG)  
Ei-value:0.000, Pi-value:0.000  
Er-value:0.000, Pr-value:0.000  
eCLIP MATCHES▶FASTKD2 (bg=1.99%)▶FUS (bg=2.21%)▶LARP4 (bg=4.72%)▶NIPBL (bg=5.39%)▶NOLC1 (bg=9.43%)▶RBFOX2 (bg=4.63%)▶RBM15 (bg=7.27%)▶SAFB (bg=2.69%)▶SAFB2 (bg=0.8%)▶uchl5 (bg=11.16%)▶ZC3H11A (bg=6.55%)▶ZNF800 (bg=1.92%)MATCHES To TargetScan▶ miR-140-5p:AGUGGUU▶ miR-142-3p.1:GUAGUGU▶ miR-199-3p:CAGUAGU


CA

CACTACTGAT  
Depth:3 (COW)  
Ei-value:0.000, Pi-value:0.000  
Er-value:0.000, Pr-value:0.000  
eCLIP MATCHES▶FASTKD2 (bg=1.99%)▶FUS (bg=2.21%)▶LARP4 (bg=4.72%)▶NIPBL (bg=5.39%)▶NOLC1 (bg=9.43%)▶RBFOX2 (bg=4.63%)▶SAFB (bg=2.69%)▶SAFB2 (bg=0.8%)▶uchl5 (bg=11.16%)▶ZC3H11A (bg=6.55%)▶ZNF800 (bg=1.92%)MATCHES To TargetScan▶ miR-142-3p.1:GUAGUGU▶ miR-199-3p:CAGUAGU


CTACTGAT

CTACTGAT  
Depth:4 (DOG)  
Ei-value:0.000, Pi-value:0.000  
Er-value:0.000, Pr-value:0.000  
eCLIP MATCHES▶FASTKD2 (bg=1.99%)▶FUS (bg=2.21%)▶LARP4 (bg=4.72%)▶NIPBL (bg=5.39%)▶NOLC1 (bg=9.43%)▶RBFOX2 (bg=4.63%)▶SAFB (bg=2.69%)▶SAFB2 (bg=0.8%)▶uchl5 (bg=11.16%)▶ZC3H11A (bg=6.55%)▶ZNF800 (bg=1.92%)MATCHES To TargetScan▶ miR-199-3p:CAGUAGU

T

AAACAAGAATAA

AAACAAGAATAA  
Depth:3 (COW)  
Ei-value:0.000, Pi-value:0.000  
Er-value:0.000, Pr-value:0.000  
eCLIP MATCHES▶FASTKD2 (bg=1.99%)▶FUS (bg=2.21%)▶LARP4 (bg=4.72%)▶NIPBL (bg=5.39%)▶NOLC1 (bg=9.43%)▶RBFOX2 (bg=4.63%)▶SAFB2 (bg=0.8%)▶uchl5 (bg=11.16%)▶ZC3H11A (bg=6.55%)MATCHES To TargetScan▶ miR-544a-5p:CUUGUUA


G

AAACAAGAATAAGAGAACAT  
Depth:2 (PIG)  
Ei-value:0.000, Pi-value:0.000  
Er-value:0.000, Pr-value:0.000  
eCLIP MATCHES▶FASTKD2 (bg=1.99%)▶FUS (bg=2.21%)▶LARP4 (bg=4.72%)▶NIPBL (bg=5.39%)▶NOLC1 (bg=9.43%)▶RBFOX2 (bg=4.63%)▶SAFB2 (bg=0.8%)▶uchl5 (bg=11.16%)▶ZC3H11A (bg=6.55%)▶ZNF622 (bg=6.58%)MATCHES To TargetScan▶ miR-544a-5p:CUUGUUA


AGAACAT

AGAACAT  
Depth:4 (DOG)  
Ei-value:0.000, Pi-value:0.000  
Er-value:0.000, Pr-value:0.000  
eCLIP MATCHES▶FASTKD2 (bg=1.99%)▶FUS (bg=2.21%)▶LARP4 (bg=4.72%)▶NIPBL (bg=5.39%)▶NOLC1 (bg=9.43%)▶RBFOX2 (bg=4.63%)▶SAFB2 (bg=0.8%)▶uchl5 (bg=11.16%)▶ZNF622 (bg=6.58%)No matches to TargetScan

TT 18950  
 TATC

A

ATCATCTG  
Depth:2 (PIG)  
Ei-value:0.000, Pi-value:0.000  
Er-value:0.000, Pr-value:0.000  
eCLIP MATCHES▶FUS (bg=2.21%)▶LARP4 (bg=4.72%)▶NOLC1 (bg=9.43%)▶RBFOX2 (bg=4.63%)▶RPS3 (bg=0.76%)▶uchl5 (bg=11.16%)▶ZNF622 (bg=6.58%)No matches to TargetScan


TCATCTG

TCATCTG  
Depth:4 (DOG)  
Ei-value:0.000, Pi-value:0.010  
Er-value:0.000, Pr-value:0.000  
eCLIP MATCHES▶FUS (bg=2.21%)▶LARP4 (bg=4.72%)▶NOLC1 (bg=9.43%)▶RBFOX2 (bg=4.63%)▶RPS3 (bg=0.76%)▶uchl5 (bg=11.16%)▶ZNF622 (bg=6.58%)No matches to TargetScan

CTTTATTCA

CATAAATGAA

CATAAATGAA  
Depth:4 (DOG)  
Ei-value:0.000, Pi-value:0.000  
Er-value:0.000, Pr-value:0.000  
eCLIP MATCHES▶FUS (bg=2.21%)▶NOLC1 (bg=9.43%)▶RPS3 (bg=0.76%)▶uchl5 (bg=11.16%)▶ZNF622 (bg=6.58%)No matches to TargetScan


GTTGTGA

CATAAATGAAGTTGTGA  
Depth:3 (COW)  
Ei-value:0.000, Pi-value:0.000  
Er-value:0.000, Pr-value:0.000  
eCLIP MATCHES▶FUS (bg=2.21%)▶NOLC1 (bg=9.43%)▶RPS3 (bg=0.76%)▶uchl5 (bg=11.16%)▶ZNF622 (bg=6.58%)No matches to TargetScan

TGAAT

AAATCT

AAATCT  
Depth:2 (PIG)  
Ei-value:0.000, Pi-value:0.000  
Er-value:0.000, Pr-value:0.000  
eCLIP MATCHES▶FUS (bg=2.21%)▶NOLC1 (bg=9.43%)▶RBFOX2 (bg=4.63%)▶ZNF622 (bg=6.58%)No matches to TargetScan

GCTTTTATGCAGACACAAGGAATTAAG

TGGCTTC

TGGCTTC  
Depth:2 (PIG)  
Ei-value:0.000, Pi-value:0.000  
Er-value:0.000, Pr-value:0.000  
eCLIP MATCHES▶BUD13 (bg=0.18%)▶FUS (bg=2.21%)▶NOLC1 (bg=9.43%)▶RBFOX2 (bg=4.63%)▶XRCC6 (bg=2.91%)No matches to TargetScan

GTCATTGTCCTT

CTACCTCAAAG

CTACCTCAAAG  
Depth:2 (PIG)  
Ei-value:0.000, Pi-value:0.000  
Er-value:0.000, Pr-value:0.000  
eCLIP MATCHES▶BUD13 (bg=0.18%)▶CPEB4 (bg=1.89%)▶FUS (bg=2.21%)▶NOLC1 (bg=9.43%)▶RBFOX2 (bg=4.63%)▶uchl5 (bg=11.16%)MATCHES To TargetScan▶ let-7-5p/98-5p:GAGGUAG▶ miR-196-5p:AGGUAGU

ATAATTTATTCCAA 19070  
 AAGCTAAGATAAATGGAAGACTCTTGA

ACTTG

ACTTGTGAACTGATGTGAAA  
Depth:3 (COW)  
Ei-value:0.000, Pi-value:0.000  
Er-value:0.000, Pr-value:0.000  
eCLIP MATCHES▶FUS (bg=2.21%)▶NOLC1 (bg=9.43%)▶RBFOX2 (bg=4.63%)▶TRA2A (bg=4.8%)MATCHES To TargetScan▶ miR-23-3p:UCACAUU


TGAACTGATGTGAAA

TGAACTGATGTGAAA  
Depth:4 (DOG)  
Ei-value:0.000, Pi-value:0.000  
Er-value:0.000, Pr-value:0.000  
eCLIP MATCHES▶FUS (bg=2.21%)▶NOLC1 (bg=9.43%)▶RBFOX2 (bg=4.63%)▶TRA2A (bg=4.8%)MATCHES To TargetScan▶ miR-23-3p:UCACAUU

TGC

AGAATCTCT

AGAATCTCT  
Depth:2 (PIG)  
Ei-value:0.000, Pi-value:0.000  
Er-value:0.000, Pr-value:0.000  
eCLIP MATCHES▶FUS (bg=2.21%)▶NOLC1 (bg=9.43%)▶RBFOX2 (bg=4.63%)▶TRA2A (bg=4.8%)▶ZC3H11A (bg=6.55%)▶ZNF622 (bg=6.58%)No matches to TargetScan

TTTGAGTCTTTGCTGTTTG

GAAGATTGAAAAAT

GAAGATTGAAAAAT  
Depth:2 (PIG)  
Ei-value:0.000, Pi-value:0.000  
Er-value:0.000, Pr-value:0.000  
eCLIP MATCHES▶AARS (bg=2.18%)▶CPEB4 (bg=1.89%)▶DROSHA (bg=2.49%)▶FUS (bg=2.21%)▶GRWD1 (bg=5.13%)▶LARP4 (bg=4.72%)▶NOLC1 (bg=9.43%)▶RBFOX2 (bg=4.63%)▶TRA2A (bg=4.8%)▶WDR43 (bg=3.37%)▶XRCC6 (bg=2.91%)▶ZC3H11A (bg=6.55%)▶ZNF622 (bg=6.58%)No matches to TargetScan

A

TTGTTCA

TTGTTCA  
Depth:3 (COW)  
Ei-value:0.000, Pi-value:0.000  
Er-value:0.000, Pr-value:0.000  
eCLIP MATCHES▶AARS (bg=2.18%)▶AATF (bg=0.64%)▶CPEB4 (bg=1.89%)▶DROSHA (bg=2.49%)▶FASTKD2 (bg=1.99%)▶FUS (bg=2.21%)▶GRWD1 (bg=5.13%)▶LARP4 (bg=4.72%)▶LSM11 (bg=2.28%)▶NOLC1 (bg=9.43%)▶RBFOX2 (bg=4.63%)▶TRA2A (bg=4.8%)▶uchl5 (bg=11.16%)▶UTP3 (bg=3.66%)▶WDR43 (bg=3.37%)▶XRCC6 (bg=2.91%)▶ZC3H11A (bg=6.55%)▶ZNF622 (bg=6.58%)No matches to TargetScan

GCATGGG

TG

TGACCACCA  
Depth:2 (PIG)  
Ei-value:0.000, Pi-value:0.000  
Er-value:0.000, Pr-value:0.000  
eCLIP MATCHES▶AARS (bg=2.18%)▶AATF (bg=0.64%)▶AKAP8L (bg=2.19%)▶CPEB4 (bg=1.89%)▶DROSHA (bg=2.49%)▶FASTKD2 (bg=1.99%)▶FUS (bg=2.21%)▶GRWD1 (bg=5.13%)▶KHDRBS1 (bg=1.71%)▶LARP4 (bg=4.72%)▶LSM11 (bg=2.28%)▶NIPBL (bg=5.39%)▶NOLC1 (bg=9.43%)▶RBFOX2 (bg=4.63%)▶RPS3 (bg=0.76%)▶TRA2A (bg=4.8%)▶uchl5 (bg=11.16%)▶UTP3 (bg=3.66%)▶WDR43 (bg=3.37%)▶XRCC6 (bg=2.91%)▶ZC3H11A (bg=6.55%)▶ZNF622 (bg=6.58%)No matches to TargetScan


ACCACCA

ACCACCA  
Depth:3 (COW)  
Ei-value:0.000, Pi-value:0.000  
Er-value:0.000, Pr-value:0.000  
eCLIP MATCHES▶AARS (bg=2.18%)▶AATF (bg=0.64%)▶AKAP8L (bg=2.19%)▶CPEB4 (bg=1.89%)▶DROSHA (bg=2.49%)▶FASTKD2 (bg=1.99%)▶FUS (bg=2.21%)▶GRWD1 (bg=5.13%)▶KHDRBS1 (bg=1.71%)▶LARP4 (bg=4.72%)▶LSM11 (bg=2.28%)▶NIPBL (bg=5.39%)▶NOLC1 (bg=9.43%)▶RBFOX2 (bg=4.63%)▶RPS3 (bg=0.76%)▶TRA2A (bg=4.8%)▶uchl5 (bg=11.16%)▶UTP3 (bg=3.66%)▶WDR43 (bg=3.37%)▶XRCC6 (bg=2.91%)▶ZC3H11A (bg=6.55%)▶ZNF622 (bg=6.58%)No matches to TargetScan

GAAA 19190  
 GTAATCTTAAGCCATCTAGATGTCAC

AATTGAA

AATTGAA  
Depth:2 (PIG)  
Ei-value:0.000, Pi-value:0.020  
Er-value:0.000, Pr-value:0.010  
eCLIP MATCHES▶FASTKD2 (bg=1.99%)▶FUS (bg=2.21%)▶GRWD1 (bg=5.13%)▶LARP4 (bg=4.72%)▶RPS3 (bg=0.76%)▶WDR43 (bg=3.37%)▶ZC3H11A (bg=6.55%)▶ZNF622 (bg=6.58%)No matches to TargetScan

ACAAACTGGGGAGTTGGTTGCTATTGTA

AAATAAAA

AAATAAAA  
Depth:4 (DOG)  
Ei-value:0.000, Pi-value:0.000  
Er-value:0.000, Pr-value:0.000  
eCLIP MATCHES▶WDR43 (bg=3.37%)▶ZC3H11A (bg=6.55%)No matches to TargetScan


TA

AAATAAAATA  
Depth:2 (PIG)  
Ei-value:0.000, Pi-value:0.000  
Er-value:0.000, Pr-value:0.000  
eCLIP MATCHES▶WDR43 (bg=3.37%)▶ZC3H11A (bg=6.55%)No matches to TargetScan

TACTG

TTTTGAAAACTT

TTTTGAAAACTT  
Depth:2 (PIG)  
Ei-value:0.000, Pi-value:0.000  
Er-value:0.000, Pr-value:0.000  
No matches to eCLIP DataNo matches to TargetScan

TG                               19280
```

---

## >PIG (25215 bases)

```
 TATTTCTTCTTTTTCCCGGG

TGGAAGCTT

TGGAAGCTT  
Depth:2 (PIG)  
Ei-value:0.000, Pi-value:0.000  
Er-value:0.000, Pr-value:0.000  
No matches to TargetScan

GCTGGTATTG

GATCTCT

GATCTCT  
Depth:2 (PIG)  
Ei-value:0.000, Pi-value:0.010  
Er-value:0.000, Pr-value:0.000  
No matches to TargetScan

TTGCCCGTGT

GGTTCTTTCT

GGTTCTTTCT  
Depth:2 (PIG)  
Ei-value:0.000, Pi-value:0.000  
Er-value:0.000, Pr-value:0.000  
MATCHES To TargetScan▶ miR-186-5p:AAAGAAU

G

GAACATTTTC

GAACATTTTC  
Depth:2 (PIG)  
Ei-value:0.000, Pi-value:0.000  
Er-value:0.000, Pr-value:0.000  
MATCHES To TargetScan▶ miR-409-3p:AAUGUUG

CAGCCCCCAGCCATGCC

TTATGGC

TTATGGC  
Depth:2 (PIG)  
Ei-value:0.000, Pi-value:0.000  
Er-value:0.000, Pr-value:0.010  
No matches to TargetScan

A

TATTTCTTTAAAAAAA

TATTTCTTTAAAAAAA  
Depth:2 (PIG)  
Ei-value:0.000, Pi-value:0.000  
Er-value:0.000, Pr-value:0.000  
MATCHES To TargetScan▶ miR-186-5p:AAAGAAU

AA 120  
 TCCACCAAAAATT

CATAAAAT

CATAAAAT  
Depth:2 (PIG)  
Ei-value:0.000, Pi-value:0.000  
Er-value:0.000, Pr-value:0.000  
No matches to TargetScan

GTTTTAAAATTTCTAA

ACTTTCTCCTA

ACTTTCTCCTA  
Depth:2 (PIG)  
Ei-value:0.000, Pi-value:0.000  
Er-value:0.000, Pr-value:0.000  
No matches to TargetScan

ATATT

TTCTTGACAC

TTCTTGACAC  
Depth:2 (PIG)  
Ei-value:0.000, Pi-value:0.000  
Er-value:0.000, Pr-value:0.000  
No matches to TargetScan

CTTATCTCTAGTTTACAGT

TATTTGG

TATTTGG  
Depth:2 (PIG)  
Ei-value:0.000, Pi-value:0.010  
Er-value:0.000, Pr-value:0.000  
No matches to TargetScan

GATAT

TTTAAGG

TTTAAGG  
Depth:2 (PIG)  
Ei-value:0.000, Pi-value:0.000  
Er-value:0.000, Pr-value:0.010  
No matches to TargetScan

CAACTTTCTATTTTAAAAT 240  


AATTTTTCTTTGGAAT

AATTTTTCTTTGGAAT  
Depth:2 (PIG)  
Ei-value:0.000, Pi-value:0.000  
Er-value:0.000, Pr-value:0.000  
MATCHES To TargetScan▶ miR-186-5p:AAAGAAU

GT

TTTTTGGTTGAC

TTTTTGGTTGAC  
Depth:2 (PIG)  
Ei-value:0.000, Pi-value:0.000  
Er-value:0.000, Pr-value:0.000  
MATCHES To TargetScan▶ miR-505-3p.1:GUCAACA

TCTTCTG

GTTTTTT

GTTTTTT  
Depth:2 (PIG)  
Ei-value:0.000, Pi-value:0.020  
Er-value:0.000, Pr-value:0.020  
No matches to TargetScan

CGTGGTGTAATTTTCTTTTCCCCCTCCTTTTCTGTGTATTA

TGCCCATCGGGGCTG

TGCCCATCGGGGCTG  
Depth:2 (PIG)  
Ei-value:0.000, Pi-value:0.000  
Er-value:0.000, Pr-value:0.000  
No matches to TargetScan

T

GGATACCTGGTTTTA

GGATACCTGGTTTTA  
Depth:2 (PIG)  
Ei-value:0.000, Pi-value:0.000  
Er-value:0.000, Pr-value:0.000  
No matches to TargetScan

ATAA 360  
 TTGTTAT

TTATTTT

TTATTTT  
Depth:2 (PIG)  
Ei-value:0.000, Pi-value:0.010  
Er-value:0.000, Pr-value:0.010  
No matches to TargetScan


TTTGCCCAACGGGGCCGTGGATACCTGCCTTTTAATTCTTTTTT

TTTGCCCAACGGGGCCGTGGATACCTGCCTTTTAATTCTTTTTT  
Depth:2 (PIG)  
Ei-value:0.000, Pi-value:0.000  
Er-value:0.000, Pr-value:0.000  
MATCHES To TargetScan▶ miR-124-3p.1:AAGGCAC▶ miR-186-5p:AAAGAAU

TAAAGACTTT

GCCCATCGGGGCCGCGGATACCTGCTTTT

GCCCATCGGGGCCGCGGATACCTGCTTTT  
Depth:2 (PIG)  
Ei-value:0.000, Pi-value:0.000  
Er-value:0.000, Pr-value:0.000  
MATCHES To TargetScan▶ miR-330-3p.2:AAAGCAC

A

ATTTTTTTTT

ATTTTTTTTT  
Depth:2 (PIG)  
Ei-value:0.000, Pi-value:0.000  
Er-value:0.000, Pr-value:0.000  
No matches to TargetScan

CC

CCTTAGCCCA

CCTTAGCCCATCGGGG  
Depth:2 (PIG)  
Ei-value:0.000, Pi-value:0.000  
Er-value:0.000, Pr-value:0.000  
No matches to TargetScan

 480  


TCGGGG

CCTTAGCCCATCGGGG  
Depth:2 (PIG)  
Ei-value:0.000, Pi-value:0.000  
Er-value:0.000, Pr-value:0.000  
No matches to TargetScan

CC

TCGGATACCTGCTG

TCGGATACCTGCTG  
Depth:2 (PIG)  
Ei-value:0.000, Pi-value:0.000  
Er-value:0.000, Pr-value:0.000  
No matches to TargetScan

TGTC

CCCCTCT

CCCCTCT  
Depth:2 (PIG)  
Ei-value:0.000, Pi-value:0.000  
Er-value:0.000, Pr-value:0.010  
MATCHES To TargetScan▶ miR-423-5p:GAGGGGC

TTCTCC

AACCCC

AACCCC  
Depth:2 (PIG)  
Ei-value:0.000, Pi-value:0.010  
Er-value:0.000, Pr-value:0.010  
No matches to TargetScan

T

TGGCCCATC

TGGCCCATC  
Depth:2 (PIG)  
Ei-value:0.000, Pi-value:0.000  
Er-value:0.000, Pr-value:0.000  
No matches to TargetScan

AGGGTAATGGATAC

CTGCTTTTT

CTGCTTTTT  
Depth:2 (PIG)  
Ei-value:0.000, Pi-value:0.000  
Er-value:0.000, Pr-value:0.000  
MATCHES To TargetScan▶ miR-330-3p.2:AAAGCAC

TATTTAAAAAAAAAT

TTTTTTTGGCCCATCGGGGC

TTTTTTTGGCCCATCGGGGC  
Depth:2 (PIG)  
Ei-value:0.000, Pi-value:0.000  
Er-value:0.000, Pr-value:0.000  
No matches to TargetScan

C

TCGGAT

TCGGATACCTGCTTT  
Depth:2 (PIG)  
Ei-value:0.000, Pi-value:0.000  
Er-value:0.000, Pr-value:0.000  
MATCHES To TargetScan▶ miR-330-3p.2:AAAGCAC

 600  


ACCTGCTTT

TCGGATACCTGCTTT  
Depth:2 (PIG)  
Ei-value:0.000, Pi-value:0.000  
Er-value:0.000, Pr-value:0.000  
MATCHES To TargetScan▶ miR-330-3p.2:AAAGCAC

AATTTTTT

TTTTTCCTTGCCCATCGGGGCCTCGGATACCTGCTTTA

TTTTTCCTTGCCCATCGGGGCCTCGGATACCTGCTTTA  
Depth:2 (PIG)  
Ei-value:0.000, Pi-value:0.000  
Er-value:0.000, Pr-value:0.000  
MATCHES To TargetScan▶ miR-31-5p:GGCAAGA▶ miR-330-3p.2:AAAGCAC

TTATTTTTTTTTCCTT

GCCCATCGGGGCCG

GCCCATCGGGGCCG  
Depth:2 (PIG)  
Ei-value:0.000, Pi-value:0.000  
Er-value:0.000, Pr-value:0.000  
No matches to TargetScan

T

GGATACCTGCTT

GGATACCTGCTT  
Depth:2 (PIG)  
Ei-value:0.000, Pi-value:0.000  
Er-value:0.000, Pr-value:0.000  
No matches to TargetScan

A

GATTTTTTTTTTTCATC

GATTTTTTTTTTTCATC  
Depth:2 (PIG)  
Ei-value:0.000, Pi-value:0.000  
Er-value:0.000, Pr-value:0.000  
No matches to TargetScan

A

CCC

CCCATCGG  
Depth:2 (PIG)  
Ei-value:0.000, Pi-value:0.000  
Er-value:0.000, Pr-value:0.000  
No matches to TargetScan

 720  


ATCGG

CCCATCGG  
Depth:2 (PIG)  
Ei-value:0.000, Pi-value:0.000  
Er-value:0.000, Pr-value:0.000  
No matches to TargetScan

ACCTTTG

TATGGATG

TATGGATG  
Depth:2 (PIG)  
Ei-value:0.000, Pi-value:0.000  
Er-value:0.000, Pr-value:0.000  
No matches to TargetScan

GAAAAGTGTTG

GGTTTTGTGG

GGTTTTGTGG  
Depth:2 (PIG)  
Ei-value:0.000, Pi-value:0.000  
Er-value:0.000, Pr-value:0.000  
No matches to TargetScan

TTCGTTGTACTG

TCTGGAAT

TCTGGAAT  
Depth:2 (PIG)  
Ei-value:0.000, Pi-value:0.000  
Er-value:0.000, Pr-value:0.000  
No matches to TargetScan

G

TCTACA

TCTACA  
Depth:2 (PIG)  
Ei-value:0.000, Pi-value:0.000  
Er-value:0.000, Pr-value:0.010  
No matches to TargetScan

AAA

TTTTGCTGCT

TTTTGCTGCT  
Depth:2 (PIG)  
Ei-value:0.000, Pi-value:0.000  
Er-value:0.000, Pr-value:0.010  
MATCHES To TargetScan▶ miR-103-3p/107:GCAGCAU▶ miR-15-5p/16-5p/195-5p/424-5p/497-5p:AGCAGCA▶ miR-503-5p:AGCAGCG

AATCG

TTTGGTG

TTTGGTG  
Depth:2 (PIG)  
Ei-value:0.000, Pi-value:0.000  
Er-value:0.000, Pr-value:0.010  
No matches to TargetScan

T

TGTGTGAGTG

TGTGTGAGTG  
Depth:2 (PIG)  
Ei-value:0.000, Pi-value:0.000  
Er-value:0.000, Pr-value:0.000  
MATCHES To TargetScan▶ miR-342-3p:CUCACAC▶ miR-377-3p:UCACACA

GACCTACG

GCTTTGG

GCTTTGG  
Depth:2 (PIG)  
Ei-value:0.000, Pi-value:0.000  
Er-value:0.000, Pr-value:0.010  
MATCHES To TargetScan▶ miR-330-3p:CAAAGCA

T 840  
 TTGGAGATGACTT

TGCAGTTA

TGCAGTTA  
Depth:2 (PIG)  
Ei-value:0.000, Pi-value:0.000  
Er-value:0.000, Pr-value:0.000  
MATCHES To TargetScan▶ miR-217:ACUGCAU

GGCTAGGGGGTTGGTCAGGCTGG

GGAGGAAA

GGAGGAAA  
Depth:2 (PIG)  
Ei-value:0.000, Pi-value:0.000  
Er-value:0.000, Pr-value:0.000  
MATCHES To TargetScan▶ miR-670-3p:UUCCUCA

GATGGCGGCCACTTGAGAT

TTGCCGC

TTGCCGC  
Depth:2 (PIG)  
Ei-value:0.000, Pi-value:0.000  
Er-value:0.000, Pr-value:0.010  
No matches to TargetScan

CCAG

CTCGGCT

CTCGGCT  
Depth:2 (PIG)  
Ei-value:0.000, Pi-value:0.000  
Er-value:0.000, Pr-value:0.000  
No matches to TargetScan

G

AGGGCTA

AGGGCTA  
Depth:2 (PIG)  
Ei-value:0.000, Pi-value:0.000  
Er-value:0.000, Pr-value:0.010  
MATCHES To TargetScan▶ miR-129-3p:AGCCCUU

CTTGTTTA

TGCTAAGT

TGCTAAGT  
Depth:2 (PIG)  
Ei-value:0.000, Pi-value:0.000  
Er-value:0.000, Pr-value:0.000  
No matches to TargetScan

G

TAAACT

TAAACTAGGGAGGCAAGATG  
Depth:2 (PIG)  
Ei-value:0.000, Pi-value:0.000  
Er-value:0.000, Pr-value:0.000  
No matches to TargetScan

 960  


AGGG

TAAACTAGGGAGGCAAGATG  
Depth:2 (PIG)  
Ei-value:0.000, Pi-value:0.000  
Er-value:0.000, Pr-value:0.000  
No matches to TargetScan


AGGCAAGA

AGGCAAGA  
Depth:3 (COW)  
Ei-value:0.000, Pi-value:0.000  
Er-value:0.000, Pr-value:0.000  
No matches to TargetScan


TG

TAAACTAGGGAGGCAAGATG  
Depth:2 (PIG)  
Ei-value:0.000, Pi-value:0.000  
Er-value:0.000, Pr-value:0.000  
No matches to TargetScan

AATAGTGGGA

CAGGCAGAGGAA

CAGGCAGAGGAA  
Depth:2 (PIG)  
Ei-value:0.000, Pi-value:0.000  
Er-value:0.000, Pr-value:0.000  
MATCHES To TargetScan▶ miR-670-3p:UUCCUCA

ATGAATA

TGCATTG

TGCATTG  
Depth:2 (PIG)  
Ei-value:0.000, Pi-value:0.010  
Er-value:0.000, Pr-value:0.020  
No matches to TargetScan

T

ATGAGCTA

ATGAGCTA  
Depth:2 (PIG)  
Ei-value:0.000, Pi-value:0.000  
Er-value:0.000, Pr-value:0.000  
No matches to TargetScan

CGTGTTTTGAATTAGTC

GATTTGGG

GATTTGGG  
Depth:2 (PIG)  
Ei-value:0.000, Pi-value:0.000  
Er-value:0.000, Pr-value:0.000  
No matches to TargetScan

T

CTTGTTAGGA

CTTGTTAGGA  
Depth:2 (PIG)  
Ei-value:0.000, Pi-value:0.000  
Er-value:0.000, Pr-value:0.000  
No matches to TargetScan

CCTTTGCATGGATTGTGGTATCATG 1080  
 AGGTGGAAAAACGGGGTCATCCTGTGTCATATTACAAGAGGCTAATAGAAAATGAGAGGGA

GAAGGTT

GAAGGTT  
Depth:2 (PIG)  
Ei-value:0.000, Pi-value:0.000  
Er-value:0.000, Pr-value:0.000  
No matches to TargetScan

TAGGCGCAGGGTTC

AAAATGGCGATTTTGAC

AAAATGGCGATTTTGAC  
Depth:2 (PIG)  
Ei-value:0.000, Pi-value:0.000  
Er-value:0.000, Pr-value:0.000  
No matches to TargetScan

TTTGCA

GCATTGCT

GCATTGCT  
Depth:2 (PIG)  
Ei-value:0.000, Pi-value:0.000  
Er-value:0.000, Pr-value:0.000  
No matches to TargetScan

T

AGCATG

AGCATGGC  
Depth:2 (PIG)  
Ei-value:0.000, Pi-value:0.000  
Er-value:0.000, Pr-value:0.010  
No matches to TargetScan

 1200  


GC

AGCATGGC  
Depth:2 (PIG)  
Ei-value:0.000, Pi-value:0.000  
Er-value:0.000, Pr-value:0.010  
No matches to TargetScan

TCTC

TGCTTTGTTAG

TGCTTTGTTAG  
Depth:2 (PIG)  
Ei-value:0.000, Pi-value:0.000  
Er-value:0.000, Pr-value:0.000  
MATCHES To TargetScan▶ miR-330-3p:CAAAGCA▶ miR-330-3p.2:AAAGCAC▶ miR-495-3p:AACAAAC

AGTGTT

CAAAATGGCGGA

CAAAATGGCGGA  
Depth:2 (PIG)  
Ei-value:0.000, Pi-value:0.000  
Er-value:0.000, Pr-value:0.000  
No matches to TargetScan

CCCACTTTGC

CGCAGTGTTC

CGCAGTGTTC  
Depth:2 (PIG)  
Ei-value:0.000, Pi-value:0.000  
Er-value:0.000, Pr-value:0.000  
MATCHES To TargetScan▶ miR-141-3p/200a-3p:AACACUG

C

AGTGGCGGGAAG

AGTGGCGGGAAG  
Depth:2 (PIG)  
Ei-value:0.000, Pi-value:0.000  
Er-value:0.000, Pr-value:0.000  
No matches to TargetScan


CCACAT

CCACAT  
Depth:2 (PIG)  
Ei-value:0.000, Pi-value:0.030  
Er-value:0.000, Pr-value:0.020  
MATCHES To TargetScan▶ miR-299-3p:AUGUGGG

TATGGGTGTCTTTGTTCTAGCGTGC

AGCATGG

AGCATGG  
Depth:2 (PIG)  
Ei-value:0.000, Pi-value:0.000  
Er-value:0.000, Pr-value:0.000  
No matches to TargetScan

CGGTGGAAATATTC 1320  
 TGTTACATAGC

AAAAGATGGCGGCT

AAAAGATGGCGGCT  
Depth:2 (PIG)  
Ei-value:0.000, Pi-value:0.000  
Er-value:0.000, Pr-value:0.000  
No matches to TargetScan

CAAGTA

CTTGCCGCA

CTTGCCGCA  
Depth:2 (PIG)  
Ei-value:0.000, Pi-value:0.000  
Er-value:0.000, Pr-value:0.000  
MATCHES To TargetScan▶ miR-31-5p:GGCAAGA

ATCG

A

AAAACATGGCGGGCCT  
Depth:2 (PIG)  
Ei-value:0.000, Pi-value:0.000  
Er-value:0.000, Pr-value:0.000  
No matches to TargetScan


AAACATG

AAACATG  
Depth:4 (DOG)  
Ei-value:0.000, Pi-value:0.000  
Er-value:0.000, Pr-value:0.000  
No matches to TargetScan


GCGGGCCT

AAAACATGGCGGGCCT  
Depth:2 (PIG)  
Ei-value:0.000, Pi-value:0.000  
Er-value:0.000, Pr-value:0.000  
No matches to TargetScan


TTGTCTTTGC

TTGTCTTTGC  
Depth:2 (PIG)  
Ei-value:0.000, Pi-value:0.000  
Er-value:0.000, Pr-value:0.000  
No matches to TargetScan

CGTGTGCATTTCCTGACAAA

TTTTGCCGCAGGGACAATATGGC

TTTTGCCGCAGGGACAATATGGC  
Depth:2 (PIG)  
Ei-value:0.000, Pi-value:0.000  
Er-value:0.000, Pr-value:0.000  
No matches to TargetScan

TGACC

TT

TTGTCAT  
Depth:2 (PIG)  
Ei-value:0.000, Pi-value:0.020  
Er-value:0.000, Pr-value:0.040  
MATCHES To TargetScan▶ miR-425-5p:AUGACAC

 1440  


GTCAT

TTGTCAT  
Depth:2 (PIG)  
Ei-value:0.000, Pi-value:0.020  
Er-value:0.000, Pr-value:0.040  
MATCHES To TargetScan▶ miR-425-5p:AUGACAC

GTGGATAGCATGGCAGTC

TGTCACGTGGAC

TGTCACGTGGAC  
Depth:2 (PIG)  
Ei-value:0.000, Pi-value:0.000  
Er-value:0.000, Pr-value:0.000  
MATCHES To TargetScan▶ miR-542-3p:GUGACAG

GTCATGGCAGGGGTGTTTGACCGTTACATTCT

TGGCGGGCT

TGGCGGGCT  
Depth:2 (PIG)  
Ei-value:0.000, Pi-value:0.000  
Er-value:0.000, Pr-value:0.000  
No matches to TargetScan

TTGCACCAGGAGGGCC

TGCCGCATTGTT

TGCCGCATTGTT  
Depth:2 (PIG)  
Ei-value:0.000, Pi-value:0.000  
Er-value:0.000, Pr-value:0.000  
No matches to TargetScan

C

AAGATGGCGGG

AAGATGGCGGG  
Depth:2 (PIG)  
Ei-value:0.000, Pi-value:0.000  
Er-value:0.000, Pr-value:0.000  
No matches to TargetScan

C

TTT

TTTGCCGC  
Depth:2 (PIG)  
Ei-value:0.000, Pi-value:0.000  
Er-value:0.000, Pr-value:0.000  
No matches to TargetScan

 1560  


GCCGC

TTTGCCGC  
Depth:2 (PIG)  
Ei-value:0.000, Pi-value:0.000  
Er-value:0.000, Pr-value:0.000  
No matches to TargetScan

GAAAAAGTGCAGGAGGGATTGGCAGCGT

TGGATTGC

TGGATTGC  
Depth:4 (DOG)  
Ei-value:0.000, Pi-value:0.000  
Er-value:0.000, Pr-value:0.000  
No matches to TargetScan

CGCCCGACACATCCAATCAGAAAGGGT

GGTGGAATTG

GGTGGAATTG  
Depth:2 (PIG)  
Ei-value:0.000, Pi-value:0.000  
Er-value:0.000, Pr-value:0.000  
No matches to TargetScan

G

TCACAG

TCACAG  
Depth:2 (PIG)  
Ei-value:0.000, Pi-value:0.010  
Er-value:0.000, Pr-value:0.020  
No matches to TargetScan

ACAGTTAGTGGAG

GATGGAATTAG

GATGGAATTAG  
Depth:2 (PIG)  
Ei-value:0.000, Pi-value:0.000  
Er-value:0.000, Pr-value:0.000  
No matches to TargetScan

TCGGAG

TTAGC

TTAGCAT  
Depth:2 (PIG)  
Ei-value:0.000, Pi-value:0.020  
Er-value:0.000, Pr-value:0.010  
No matches to TargetScan

 1680  


AT

TTAGCAT  
Depth:2 (PIG)  
Ei-value:0.000, Pi-value:0.020  
Er-value:0.000, Pr-value:0.010  
No matches to TargetScan

AGCACCTCGCTACCGTCTCTATTC

AGCCAGTCAG

AGCCAGTCAG  
Depth:2 (PIG)  
Ei-value:0.000, Pi-value:0.000  
Er-value:0.000, Pr-value:0.000  
MATCHES To TargetScan▶ miR-149-5p:CUGGCUC▶ miR-193-3p:ACUGGCC▶ miR-3064-5p:CUGGCUG

CACC

GGCCACGT

GGCCACGT  
Depth:2 (PIG)  
Ei-value:0.000, Pi-value:0.000  
Er-value:0.000, Pr-value:0.000  
No matches to TargetScan

TTGTACTA

CTCCCAGTGGG

CTCCCAGTGGG  
Depth:2 (PIG)  
Ei-value:0.000, Pi-value:0.000  
Er-value:0.000, Pr-value:0.000  
No matches to TargetScan

TGGTACC

CAAGGTCTTT

CAAGGTCTTT  
Depth:2 (PIG)  
Ei-value:0.000, Pi-value:0.000  
Er-value:0.000, Pr-value:0.000  
No matches to TargetScan

C

CAAGGAC

CAAGGAC  
Depth:2 (PIG)  
Ei-value:0.000, Pi-value:0.000  
Er-value:0.000, Pr-value:0.010  
No matches to TargetScan

GTTTG

GCCTTTCCACCTC

GCCTTTCCACCTC  
Depth:2 (PIG)  
Ei-value:0.000, Pi-value:0.000  
Er-value:0.000, Pr-value:0.000  
No matches to TargetScan

CC

TCCCCTCT

TCCCCTCT  
Depth:2 (PIG)  
Ei-value:0.000, Pi-value:0.000  
Er-value:0.000, Pr-value:0.000  
MATCHES To TargetScan▶ miR-423-5p:GAGGGGC

 1800  


TCCCCTCT  
Depth:2 (PIG)  
Ei-value:0.000, Pi-value:0.000  
Er-value:0.000, Pr-value:0.000  
MATCHES To TargetScan▶ miR-423-5p:GAGGGGC

CACTGGCTCCC

TCCCCTCC

TCCCCTCC  
Depth:2 (PIG)  
Ei-value:0.000, Pi-value:0.000  
Er-value:0.000, Pr-value:0.000  
MATCHES To TargetScan▶ miR-423-5p:GAGGGGC

AGCATTACCACCTGCTGTGCTGAACTTTAGGCTATATGGG

CTGAACCTC

CTGAACCTC  
Depth:2 (PIG)  
Ei-value:0.000, Pi-value:0.000  
Er-value:0.000, Pr-value:0.000  
No matches to TargetScan

ACA

CCATTCCTCTG

CCATTCCTCTG  
Depth:2 (PIG)  
Ei-value:0.000, Pi-value:0.000  
Er-value:0.000, Pr-value:0.000  
MATCHES To TargetScan▶ miR-1-3p/206:GGAAUGU

C

ATTGGTG

ATTGGTG  
Depth:2 (PIG)  
Ei-value:0.000, Pi-value:0.000  
Er-value:0.000, Pr-value:0.010  
No matches to TargetScan

G

CCTAAGGCTAA

CCTAAGGCTAA  
Depth:2 (PIG)  
Ei-value:0.000, Pi-value:0.000  
Er-value:0.000, Pr-value:0.000  
No matches to TargetScan

CTTACTACCCCTTCCCCC 1920  


CCTCCCCC

CCTCCCCC  
Depth:2 (PIG)  
Ei-value:0.000, Pi-value:0.000  
Er-value:0.000, Pr-value:0.000  
No matches to TargetScan

TCCCCCG

CCTCTG

CCTCTG  
Depth:2 (PIG)  
Ei-value:0.000, Pi-value:0.020  
Er-value:0.000, Pr-value:0.000  
No matches to TargetScan

CTCTT

CTGCACTGT

CTGCACTGT  
Depth:2 (PIG)  
Ei-value:0.000, Pi-value:0.000  
Er-value:0.000, Pr-value:0.000  
MATCHES To TargetScan▶ miR-130-3p/301-3p/454-3p:AGUGCAA▶ miR-148-3p/152-3p:CAGUGCA

GGCCAG

GGGCAGTGCTCCA

GGGCAGTGCTCCA  
Depth:2 (PIG)  
Ei-value:0.000, Pi-value:0.000  
Er-value:0.000, Pr-value:0.000  
No matches to TargetScan

T

GCCTGC

GCCTGC  
Depth:2 (PIG)  
Ei-value:0.000, Pi-value:0.000  
Er-value:0.000, Pr-value:0.000  
No matches to TargetScan

CAAGTGTGAACATGGC

GGTGAG

GGTGAG  
Depth:2 (PIG)  
Ei-value:0.000, Pi-value:0.000  
Er-value:0.000, Pr-value:0.010  
No matches to TargetScan

T

CGTGGCAAGGACCAGAATGGATC

CGTGGCAAGGACCAGAATGGATC  
Depth:2 (PIG)  
Ei-value:0.000, Pi-value:0.000  
Er-value:0.000, Pr-value:0.000  
MATCHES To TargetScan▶ miR-133a-3p.1:UGGUCCC

G

CAGATGATCGTT

CAGATGATCGTTGGCCAACAGGTGGC  
Depth:2 (PIG)  
Ei-value:0.000, Pi-value:0.000  
Er-value:0.000, Pr-value:0.000  
No matches to TargetScan

 2040  


GGC

CAGATGATCGTTGGCCAACAGGTGGC  
Depth:2 (PIG)  
Ei-value:0.000, Pi-value:0.000  
Er-value:0.000, Pr-value:0.000  
No matches to TargetScan


CAACAG

CAACAG  
Depth:3 (COW)  
Ei-value:0.000, Pi-value:0.000  
Er-value:0.000, Pr-value:0.000  
No matches to TargetScan


GTGGC

CAGATGATCGTTGGCCAACAGGTGGC  
Depth:2 (PIG)  
Ei-value:0.000, Pi-value:0.000  
Er-value:0.000, Pr-value:0.000  
No matches to TargetScan

G

GAAGAGGAAT

GAAGAGGAAT  
Depth:2 (PIG)  
Ei-value:0.000, Pi-value:0.000  
Er-value:0.000, Pr-value:0.000  
MATCHES To TargetScan▶ miR-670-3p:UUCCUCA

CCCTGT

CTTCCTCAAGAGGAACACCTACCCC

CTTCCTCAAGAGGAACACCTACCCC  
Depth:2 (PIG)  
Ei-value:0.000, Pi-value:0.000  
Er-value:0.000, Pr-value:0.000  
MATCHES To TargetScan▶ miR-1224-5p:UGAGGAC▶ miR-670-3p:UUCCUCA

G

TGGCTAATGCTGGGGTCGGATTTTGATTT

TGGCTAATGCTGGGGTCGGATTTTGATTT  
Depth:2 (PIG)  
Ei-value:0.000, Pi-value:0.000  
Er-value:0.000, Pr-value:0.000  
MATCHES To TargetScan▶ miR-338-3p:CCAGCAU▶ miR-551-3p:CGACCCA

CTATTTATTTC

TTGGATGTCAGTCATA

TTGGATGTCAGTCATA  
Depth:2 (PIG)  
Ei-value:0.000, Pi-value:0.000  
Er-value:0.000, Pr-value:0.000  
MATCHES To TargetScan▶ miR-489-3p:UGACAUC

TATAGTT 2160  
 TGATTA

TGTGGTTTGCTAGTGTT

TGTGGTTTGCTAGTGTT  
Depth:2 (PIG)  
Ei-value:0.000, Pi-value:0.000  
Er-value:0.000, Pr-value:0.000  
MATCHES To TargetScan▶ miR-140-3p.2:ACCACAG▶ miR-141-3p/200a-3p:AACACUG

CG

ATTTAAG

ATTTAAG  
Depth:2 (PIG)  
Ei-value:0.000, Pi-value:0.000  
Er-value:0.000, Pr-value:0.000  
No matches to TargetScan

C

CTTAAGTGACTA

CTTAAGTGACTA  
Depth:2 (PIG)  
Ei-value:0.000, Pi-value:0.000  
Er-value:0.000, Pr-value:0.000  
MATCHES To TargetScan▶ miR-668-3p:GUCACUC

CTATGGT

AATGTATT

AATGTATT  
Depth:2 (PIG)  
Ei-value:0.000, Pi-value:0.000  
Er-value:0.000, Pr-value:0.000  
No matches to TargetScan

TAGGGACTTTG

TTATTTGTAGAATTCA

TTATTTGTAGAATTCA  
Depth:2 (PIG)  
Ei-value:0.000, Pi-value:0.000  
Er-value:0.000, Pr-value:0.000  
No matches to TargetScan

TTTCAG

TTACATTTA

TTACATTTA  
Depth:2 (PIG)  
Ei-value:0.000, Pi-value:0.000  
Er-value:0.000, Pr-value:0.000  
MATCHES To TargetScan▶ miR-411-3p:AUGUAAC

GTGGGTTTTCATTTTGGG 2280  


GTTCCTT

GTTCCTT  
Depth:2 (PIG)  
Ei-value:0.000, Pi-value:0.010  
Er-value:0.000, Pr-value:0.010  
No matches to TargetScan

TG

AAATTCCTTAAAGTTTT

AAATTCCTTAAAGTTTT  
Depth:2 (PIG)  
Ei-value:0.000, Pi-value:0.000  
Er-value:0.000, Pr-value:0.000  
No matches to TargetScan

CAATTTCTTTTTT

TTACAAAT

TTACAAAT  
Depth:2 (PIG)  
Ei-value:0.000, Pi-value:0.000  
Er-value:0.000, Pr-value:0.000  
No matches to TargetScan

GCTTACATTTCTTTATCTTTAT

ATAGTCAAAGTCAA

ATAGTCAAAGTCAA  
Depth:2 (PIG)  
Ei-value:0.000, Pi-value:0.000  
Er-value:0.000, Pr-value:0.000  
No matches to TargetScan

TAATTTGGCATTTATAAGTTTTTACTACTTTTTTA

CT

CTTTGAAATTGACTTAA  
Depth:2 (PIG)  
Ei-value:0.000, Pi-value:0.000  
Er-value:0.000, Pr-value:0.000  
MATCHES To TargetScan▶ miR-224-5p:AAGUCAC

 2400  


TTGAAATTGACTTAA

CTTTGAAATTGACTTAA  
Depth:2 (PIG)  
Ei-value:0.000, Pi-value:0.000  
Er-value:0.000, Pr-value:0.000  
MATCHES To TargetScan▶ miR-224-5p:AAGUCAC

TGAACTACTTCCTTTTGGATT

TTTGAAG

TTTGAAG  
Depth:2 (PIG)  
Ei-value:0.000, Pi-value:0.010  
Er-value:0.000, Pr-value:0.000  
No matches to TargetScan

TCTTATAGTACAAAAATAGTTAATTT

AAAATTTAAC

AAAATTTAAC  
Depth:2 (PIG)  
Ei-value:0.000, Pi-value:0.000  
Er-value:0.000, Pr-value:0.000  
No matches to TargetScan

ATT

ATGACC

ATGACC  
Depth:2 (PIG)  
Ei-value:0.000, Pi-value:0.000  
Er-value:0.000, Pr-value:0.000  
No matches to TargetScan

AAATAATC

TTTGAAGGT

TTTGAAGGT  
Depth:2 (PIG)  
Ei-value:0.000, Pi-value:0.000  
Er-value:0.000, Pr-value:0.000  
MATCHES To TargetScan▶ miR-205-5p:CCUUCAU

CCTCTTCT

GTCCAGG

GTCCAGG  
Depth:2 (PIG)  
Ei-value:0.000, Pi-value:0.000  
Er-value:0.000, Pr-value:0.000  
MATCHES To TargetScan▶ miR-378-3p:CUGGACU

 2520  


GTCCAGG  
Depth:2 (PIG)  
Ei-value:0.000, Pi-value:0.000  
Er-value:0.000, Pr-value:0.000  
MATCHES To TargetScan▶ miR-378-3p:CUGGACU

T

CTTGCTTTG

CTTGCTTTGTTCCCATCCTT  
Depth:2 (PIG)  
Ei-value:0.000, Pi-value:0.000  
Er-value:0.000, Pr-value:0.000  
MATCHES To TargetScan▶ miR-330-3p:CAAAGCA▶ miR-330-3p.2:AAAGCAC▶ miR-495-3p:AACAAAC


TTCCCATC

TTCCCATC  
Depth:4 (DOG)  
Ei-value:0.000, Pi-value:0.000  
Er-value:0.000, Pr-value:0.000  
No matches to TargetScan


CTT

CTTGCTTTGTTCCCATCCTT  
Depth:2 (PIG)  
Ei-value:0.000, Pi-value:0.000  
Er-value:0.000, Pr-value:0.000  
MATCHES To TargetScan▶ miR-330-3p:CAAAGCA▶ miR-330-3p.2:AAAGCAC▶ miR-495-3p:AACAAAC

A

ATGCTGCACT

ATGCTGCACT  
Depth:2 (PIG)  
Ei-value:0.000, Pi-value:0.000  
Er-value:0.000, Pr-value:0.000  
MATCHES To TargetScan▶ miR-103-3p/107:GCAGCAU▶ miR-130-3p/301-3p/454-3p:AGUGCAA

GATTGAATAATGACCTACCTATCTGCAGA

AAACTTGAATTGCTGTGG

AAACTTGAATTGCTGTGG  
Depth:2 (PIG)  
Ei-value:0.000, Pi-value:0.000  
Er-value:0.000, Pr-value:0.000  
MATCHES To TargetScan▶ miR-140-3p.1:CCACAGG▶ miR-26-5p:UCAAGUA

ACTTCTTCTACTCTAAT

TTATTATATTGGAGTATT

TTATTATATTGGAGTATT  
Depth:2 (PIG)  
Ei-value:0.000, Pi-value:0.000  
Er-value:0.000, Pr-value:0.000  
MATCHES To TargetScan▶ miR-200bc-3p/429:AAUACUG▶ miR-369-3p:AUAAUAC▶ miR-374-5p:UAUAAUA▶ miR-410-3p:AUAUAAC

ATTGCC 2640  
 CATAATT

TCAATTTT

TCAATTTT  
Depth:2 (PIG)  
Ei-value:0.000, Pi-value:0.010  
Er-value:0.000, Pr-value:0.010  
No matches to TargetScan

CTGTGGTGACCTGTCTCCACAGGCCCCAGCTCCTCTCCACACCTCT

TCCCAGCAAACCC

TCCCAGCAAACCC  
Depth:2 (PIG)  
Ei-value:0.000, Pi-value:0.000  
Er-value:0.000, Pr-value:0.000  
No matches to TargetScan

ATATT

TAGCCCCAGCCC

TAGCCCCAGCCC  
Depth:2 (PIG)  
Ei-value:0.000, Pi-value:0.000  
Er-value:0.000, Pr-value:0.000  
No matches to TargetScan

CTGCCCCTGCCCCTGCCCCAGCCCTTGCC 2760  
 CCTGCCCCAGCCCCTGCCCCTGCCCCAGCCCCAGCCCCTGCCCCTGCCCCTGC

CCCTGCCCCAGCCCCAG

CCCTGCCCCAGCCCCAG  
Depth:2 (PIG)  
Ei-value:0.000, Pi-value:0.000  
Er-value:0.000, Pr-value:0.000  
No matches to TargetScan

CCCCAACCA

AGCCCCAG

AGCCCCAG  
Depth:2 (PIG)  
Ei-value:0.000, Pi-value:0.000  
Er-value:0.000, Pr-value:0.000  
No matches to TargetScan

CCCC

AGCCCCAG

AGCCCCAG  
Depth:2 (PIG)  
Ei-value:0.000, Pi-value:0.000  
Er-value:0.000, Pr-value:0.000  
No matches to TargetScan

CCAAGCCCCTGTCCCACTCCT 2880  
 GTTTCTCCCTAAAG

CCAGTCC

CCAGTCC  
Depth:2 (PIG)  
Ei-value:0.000, Pi-value:0.000  
Er-value:0.000, Pr-value:0.010  
No matches to TargetScan

AGTTTGT

ATTGATT

ATTGATT  
Depth:2 (PIG)  
Ei-value:0.000, Pi-value:0.000  
Er-value:0.000, Pr-value:0.030  
No matches to TargetScan

TGATGCTA

AAAATAAGTT

AAAATAAGTT  
Depth:2 (PIG)  
Ei-value:0.000, Pi-value:0.000  
Er-value:0.000, Pr-value:0.000  
No matches to TargetScan

TCAGTTGCTCTTCCATCAG

ACTGGGATA

ACTGGGATA  
Depth:2 (PIG)  
Ei-value:0.000, Pi-value:0.000  
Er-value:0.000, Pr-value:0.000  
No matches to TargetScan

CCTTGTCTGCCTCTA

GCATTGCTGATCTT

GCATTGCTGATCTT  
Depth:2 (PIG)  
Ei-value:0.000, Pi-value:0.000  
Er-value:0.000, Pr-value:0.000  
MATCHES To TargetScan▶ miR-383-5p.1:GAUCAGA▶ miR-383-5p.2:AGAUCAG

GAGTGCTGAC 3000  
 TACCTGAGTC

ACCATTTTCA

ACCATTTTCA  
Depth:2 (PIG)  
Ei-value:0.000, Pi-value:0.000  
Er-value:0.000, Pr-value:0.000  
No matches to TargetScan

ATTAATGTAC

ACAATCCCATTTG

ACAATCCCATTTG  
Depth:2 (PIG)  
Ei-value:0.000, Pi-value:0.000  
Er-value:0.000, Pr-value:0.000  
MATCHES To TargetScan▶ miR-219-5p:GAUUGUC

CCCATTATCTCATGTTAGA

ACAAAGAATTT

ACAAAGAATTT  
Depth:2 (PIG)  
Ei-value:0.000, Pi-value:0.000  
Er-value:0.000, Pr-value:0.000  
No matches to TargetScan

GTGCAATAAT

GTTAGG

GTTAGG  
Depth:2 (PIG)  
Ei-value:0.000, Pi-value:0.010  
Er-value:0.000, Pr-value:0.010  
No matches to TargetScan

AAATTTAATTGT

GAGCTT

GAGCTT  
Depth:2 (PIG)  
Ei-value:0.000, Pi-value:0.010  
Er-value:0.000, Pr-value:0.010  
No matches to TargetScan

CA

TATCAGA

TATCAGA  
Depth:2 (PIG)  
Ei-value:0.000, Pi-value:0.000  
Er-value:0.000, Pr-value:0.000  
No matches to TargetScan

CA

AT

ATTATTG  
Depth:2 (PIG)  
Ei-value:0.000, Pi-value:0.000  
Er-value:0.000, Pr-value:0.010  
No matches to TargetScan

 3120  


TATTG

ATTATTG  
Depth:2 (PIG)  
Ei-value:0.000, Pi-value:0.000  
Er-value:0.000, Pr-value:0.010  
No matches to TargetScan

TCCATAAATCTGCTTACTCATCATCTCAATCTCCTGCCTTTAAGG

AAGGAGAAACCATT

AAGGAGAAACCATT  
Depth:2 (PIG)  
Ei-value:0.000, Pi-value:0.000  
Er-value:0.000, Pr-value:0.000  
No matches to TargetScan

C

CTCTGT

CTCTGT  
Depth:3 (COW)  
Ei-value:0.000, Pi-value:0.000  
Er-value:0.000, Pr-value:0.000  
No matches to TargetScan


CATTGCT

CTCTGTCATTGCT  
Depth:2 (PIG)  
Ei-value:0.000, Pi-value:0.000  
Er-value:0.000, Pr-value:0.000  
MATCHES To TargetScan▶ miR-425-5p:AUGACAC

GCA

GTAGTCA

GTAGTCA  
Depth:2 (PIG)  
Ei-value:0.000, Pi-value:0.000  
Er-value:0.000, Pr-value:0.000  
No matches to TargetScan

AAATCCCAGTTTTCTGGAAAGTGCATATA

TAC

TACTGTG  
Depth:2 (PIG)  
Ei-value:0.000, Pi-value:0.000  
Er-value:0.000, Pr-value:0.000  
MATCHES To TargetScan▶ miR-101-3p.1:ACAGUAC▶ miR-128-3p:CACAGUG▶ miR-144-3p:ACAGUAU

 3240  


TGTG

TACTGTG  
Depth:2 (PIG)  
Ei-value:0.000, Pi-value:0.000  
Er-value:0.000, Pr-value:0.000  
MATCHES To TargetScan▶ miR-101-3p.1:ACAGUAC▶ miR-128-3p:CACAGUG▶ miR-144-3p:ACAGUAU

CTGACCACCT

AAACTCTTTGCA

AAACTCTTTGCA  
Depth:2 (PIG)  
Ei-value:0.000, Pi-value:0.000  
Er-value:0.000, Pr-value:0.000  
No matches to TargetScan

CTCAGTG

AAATTCTAATTG

AAATTCTAATTG  
Depth:2 (PIG)  
Ei-value:0.000, Pi-value:0.000  
Er-value:0.000, Pr-value:0.000  
No matches to TargetScan

TCC

ATAATCCT

ATAATCCT  
Depth:2 (PIG)  
Ei-value:0.000, Pi-value:0.000  
Er-value:0.000, Pr-value:0.000  
No matches to TargetScan

G

CATTGGA

CATTGGA  
Depth:2 (PIG)  
Ei-value:0.000, Pi-value:0.010  
Er-value:0.000, Pr-value:0.010  
No matches to TargetScan

CTAAATCCCAATTTAAAAACCCT

TTTGCATTCAGCAG

TTTGCATTCAGCAG  
Depth:2 (PIG)  
Ei-value:0.000, Pi-value:0.000  
Er-value:0.000, Pr-value:0.000  
No matches to TargetScan

TCCCAG

TTGTCATA

TTGTCATA  
Depth:2 (PIG)  
Ei-value:0.000, Pi-value:0.000  
Er-value:0.000, Pr-value:0.000  
MATCHES To TargetScan▶ miR-425-5p:AUGACAC

CCCTT 3360  
 GTCTG

TTAAACAAAGGCA

TTAAACAAAGGCA  
Depth:2 (PIG)  
Ei-value:0.000, Pi-value:0.000  
Er-value:0.000, Pr-value:0.000  
No matches to TargetScan

TCCTACCATTTTTACCCTCTTGTGAGTCCTC

CTGTTCTTGGACAATTAAAG

CTGTTCTTGGACAATTAAAG  
Depth:2 (PIG)  
Ei-value:0.000, Pi-value:0.000  
Er-value:0.000, Pr-value:0.000  
No matches to TargetScan

TACCAAATT

GTAATTG

GTAATTG  
Depth:2 (PIG)  
Ei-value:0.000, Pi-value:0.000  
Er-value:0.000, Pr-value:0.000  
No matches to TargetScan

TAC

ATTGTCTCAC

ATTGTCTCAC  
Depth:2 (PIG)  
Ei-value:0.000, Pi-value:0.000  
Er-value:0.000, Pr-value:0.000  
No matches to TargetScan

T

CATTAATCA

CATTAATCA  
Depth:2 (PIG)  
Ei-value:0.000, Pi-value:0.000  
Er-value:0.000, Pr-value:0.000  
No matches to TargetScan

ATGACCTTCTGT 3480  
 CCCGTTTGCATTGCTATT

AGGCAG

AGGCAG  
Depth:2 (PIG)  
Ei-value:0.000, Pi-value:0.010  
Er-value:0.000, Pr-value:0.020  
No matches to TargetScan

T

GCTGACTA

GCTGACTA  
Depth:2 (PIG)  
Ei-value:0.000, Pi-value:0.000  
Er-value:0.000, Pr-value:0.000  
No matches to TargetScan

TCTGAGACCATGTTCCTTGAATTATT

ATTATTGA

ATTATTGA  
Depth:2 (PIG)  
Ei-value:0.000, Pi-value:0.010  
Er-value:0.000, Pr-value:0.000  
No matches to TargetScan

CTAGAATACCAATTGTTCA

TTACAC

TTACAC  
Depth:2 (PIG)  
Ei-value:0.000, Pi-value:0.010  
Er-value:0.000, Pr-value:0.000  
No matches to TargetScan

AAATCCTTTGCAGCT

TCTTTGCA

TCTTTGCA  
Depth:2 (PIG)  
Ei-value:0.000, Pi-value:0.000  
Er-value:0.000, Pr-value:0.000  
No matches to TargetScan

CTATA 3600  
 AGGGAAGTGCCCATCACTAAAGTCCTTTACTTC

GGAATATTAATGGATACAAT

GGAATATTAATGGATACAAT  
Depth:2 (PIG)  
Ei-value:0.000, Pi-value:0.000  
Er-value:0.000, Pr-value:0.000  
No matches to TargetScan

CATTACTGG

TCATGGT

TCATGGT  
Depth:2 (PIG)  
Ei-value:0.000, Pi-value:0.000  
Er-value:0.000, Pr-value:0.020  
No matches to TargetScan

CCTGCCTACCAGAT

AAGACCCAC

AAGACCCAC  
Depth:2 (PIG)  
Ei-value:0.000, Pi-value:0.000  
Er-value:0.000, Pr-value:0.000  
MATCHES To TargetScan▶ miR-193a-5p:GGGUCUU

CCATGCTCTTTTCATTGTTCTTAGG

TAG

TAGTGTG  
Depth:2 (PIG)  
Ei-value:0.000, Pi-value:0.000  
Er-value:0.000, Pr-value:0.000  
No matches to TargetScan

 3720  


TGTG

TAGTGTG  
Depth:2 (PIG)  
Ei-value:0.000, Pi-value:0.000  
Er-value:0.000, Pr-value:0.000  
No matches to TargetScan

G

ACTACCTACCACCTTGCATTAATAT

ACTACCTACCACCTTGCATTAATAT  
Depth:2 (PIG)  
Ei-value:0.000, Pi-value:0.000  
Er-value:0.000, Pr-value:0.000  
MATCHES To TargetScan▶ miR-155-5p:UAAUGCU▶ miR-18-5p:AAGGUGC▶ miR-196-5p:AGGUAGU

TTAAAATCCTCATTGTCCATT

GTCCCACT

GTCCCACT  
Depth:2 (PIG)  
Ei-value:0.000, Pi-value:0.000  
Er-value:0.000, Pr-value:0.000  
No matches to TargetScan

GTTAGCCTAGGATATCTCTTACCTCTA

TGCCATT

TGCCATT  
Depth:2 (PIG)  
Ei-value:0.000, Pi-value:0.010  
Er-value:0.000, Pr-value:0.000  
MATCHES To TargetScan▶ miR-183-5p.1:AUGGCAC

TCTTCTAAG

GAGTTCTGA

GAGTTCTGA  
Depth:2 (PIG)  
Ei-value:0.000, Pi-value:0.000  
Er-value:0.000, Pr-value:0.000  
No matches to TargetScan

TTACCCTAA 3840  
 GTCCT

TTCTCTTAAACA

TTCTCTTAAACA  
Depth:2 (PIG)  
Ei-value:0.000, Pi-value:0.000  
Er-value:0.000, Pr-value:0.000  
No matches to TargetScan

ATTTATG

TGCATAATTGCATATA

TGCATAATTGCATATA  
Depth:2 (PIG)  
Ei-value:0.000, Pi-value:0.000  
Er-value:0.000, Pr-value:0.000  
No matches to TargetScan

CCTGTGGTCTCATGCAATTAAAAATTAAATCCTACAC

TCTGTGG

TCTGTGG  
Depth:2 (PIG)  
Ei-value:0.000, Pi-value:0.000  
Er-value:0.000, Pr-value:0.010  
MATCHES To TargetScan▶ miR-140-3p.1:CCACAGG

AATATTGCTAGGT

AGTGCA

AGTGCA  
Depth:2 (PIG)  
Ei-value:0.000, Pi-value:0.020  
Er-value:0.000, Pr-value:0.000  
No matches to TargetScan

A

ATTATTCAA

ATTATTCAA  
Depth:2 (PIG)  
Ei-value:0.000, Pi-value:0.000  
Er-value:0.000, Pr-value:0.000  
No matches to TargetScan

GGACTCA 3960  
 GCTTTGGG

ACAGTTAAT

ACAGTTAAT  
Depth:2 (PIG)  
Ei-value:0.000, Pi-value:0.000  
Er-value:0.000, Pr-value:0.010  
No matches to TargetScan

CC

GCACAGTTGC

GCACAGTTGC  
Depth:2 (PIG)  
Ei-value:0.000, Pi-value:0.000  
Er-value:0.000, Pr-value:0.000  
No matches to TargetScan

GG

TTGTCCAGAGTCC

TTGTCCAGAGTCC  
Depth:2 (PIG)  
Ei-value:0.000, Pi-value:0.000  
Er-value:0.000, Pr-value:0.000  
MATCHES To TargetScan▶ miR-326:CUCUGGG▶ miR-378-3p:CUGGACU

CATCCATTTTA

GTGGGC

GTGGGC  
Depth:2 (PIG)  
Ei-value:0.000, Pi-value:0.000  
Er-value:0.000, Pr-value:0.010  
No matches to TargetScan

CACTGTGTCCCATTTGGGTGCTAGGTGTGTCAGGAGGACCCTTCTGTTGAATTGATAAT 4080  
 GTG

CATAATTG

CATAATTG  
Depth:2 (PIG)  
Ei-value:0.000, Pi-value:0.000  
Er-value:0.000, Pr-value:0.000  
No matches to TargetScan

CATTTGTTCATGTCCTGTGAACTAGTAACATCCACCTG

CTTTGTATTCCAGCAGGGGACCCTT

CTTTGTATTCCAGCAGGGGACCCTT  
Depth:2 (PIG)  
Ei-value:0.000, Pi-value:0.000  
Er-value:0.000, Pr-value:0.000  
MATCHES To TargetScan▶ miR-331-3p:CCCCUGG▶ miR-381-3p:AUACAAG

TTTAACTTAAGAGCGCTGCACTGGGACACTCTACCTGTGCAAA

CCT

CCTAATTGATTAGA  
Depth:2 (PIG)  
Ei-value:0.000, Pi-value:0.000  
Er-value:0.000, Pr-value:0.000  
No matches to TargetScan

 4200  


AATTGATTAGA

CCTAATTGATTAGA  
Depth:2 (PIG)  
Ei-value:0.000, Pi-value:0.000  
Er-value:0.000, Pr-value:0.000  
No matches to TargetScan

CCTGAGCCTTTTGTATTAGGAACCCTGCATTATCTTTACACCTAAAAGGTGCTAATTGCCTAAGGCCATTTCCTGCCCGAACGACTGTGTTTAAACTAGAGTCTCACAC 4320  
 TCAATAACCGCCTTTGCATAAGAGTAGAGGATACTTGTGACAGTTAAGTGGTTTGATTGCAGCTACAGACCCTGCTGTTAGACTAGCATCCATTCTCCTCCTAGATGATTCACATGACCA 4440  
 CAATCCGTACTAGGGACTGTTAATGGGTTTAAATGCAGCTACAGTCCTTTATGTTAATCTAGGATCCCATCCCCAACTCACTCTCTTTTCATTACTGCAGGGCTTACATAGGACTTGTTA 4560  
 ACGACCTTTATTGCACTACTGTCGCTTCTGTTAGTCCAAGACCCCATATCCTCCTAAATCCTGTATTACCGAAAGAATATAAGGGACTGTTAAAGTGCTTAAAGCAGTTACAGTCCCTTA 4680  
 TGTTTATCTAGGACCCATCCTCAACTAACCCCTTTTGCATGACTGCAGAGGGTTCTTGGGACTGTTAATGGGCTTTATTGCAGCTACAGTCCCTTCTATTAATCTGGATTCCTATCCACT 4800  
 CCTACACAATTTGCATTACCACAGACTATACAAGGGACTGTTAATGTGTTTAAATGGAGCTACAGTTCCTTATGTTAAACTAGGATCCCATTTCCACCTAACTCCCTTTTCATTAATGCA 4920  
 GAGCCTACATGGGACTAGTGGGCTTATTTGCACTACTGTCCCT

TCTTTTAT

TCTTTTAT  
Depth:2 (PIG)  
Ei-value:0.000, Pi-value:0.000  
Er-value:0.000, Pr-value:0.010  
No matches to TargetScan

TCTAGGATCCCATCCCTCCTACTGGATTTGCACTACCCTAAAATGTATGAGAGACTGTGGATGTGCTTA 5040  
 AATGTATCTACAGTCCCTTTTGTTAACCTATGATCCCGTTCCCATCTAACACCCTTTGCATGACTGCAGAGGGTACTTGGGATAGTTAATGCGCTCAATTAGAGCTACAGATCCTTCTGT 5160  
 TAGTGGAGATTCCCATGCCCTCCTACTCCATTTGCATTACCAAAGGGAGTACAAGGGACTGTTAATGGGCCTAAATGCAGCTACAGTCCCTATGTTAACCTAGGATCCCATTTCCACCTA 5280  
 ACTCCCTTTTCATTAATACAGAGCCTACATGGGACTTAATGGGCTTTATTGTACTACTGTCCCTTCCATTAATCTAGCATCCCATCCCCACATAATTCCCTTTGCATTACAAGGGAGGAT 5400  
 ACAAGGGACTTTTAATGGGCTTAAATGCAGGTTCAGTCCCTTTCTTATTTTACCTAGGATCCCATTCCCACATAATTCCCTTTGCAAAACCACAGAGTACAAGGTACTGTTACTTACTGT 5520  
 ACTTAAAAGCAGCTATAAGATACTCATGTTGCAACAGAAGAAAGGGTGGGGGAAAAACTGTAATTGCAATGTATACATGTAAGGATAACCTGACCCCCTTGCTGTACAGTGGGAAAAAAA 5640  
 AAAAAAAAAAAAAAGCAGCTATAGTCACTTATGTTTATGTAGAACTCCATTTCCACTAACTCCCTTTTCATGACTGCAGAGGTATCTGGGACTGTTAATGGGCTTTATTGCAGCTACAGT 5760  
 CCCTTCTGTTTGTCTGGATTCCCATCCAGTCCTATACAATTTGCATTACCACAGAATATCTAAGGTAACTAGCTTAATGAGCTTAAATACAGCTACAATCCTTTATGTTAACCTAAAATT 5880  
 CCATCCCCACCTAATTCTTTTAGGACGGCTGCTGAGAGTACATGGGACTGTTAATGGGCTATTTTGCAACTACAGTCCCTTCTGTTAGTCTAGAATCTGTTTCCATCCTACTCCATTTGC 6000  
 ATTACCCTATAGCGTATAAGGGACTGTTAATGTGCTTAAATGGAGCTGTGTAGTCCCTTATCTTAACCTGGTATCACACCCCAACTAATTCTCTTTTCATACTGCAGAGCCTACGTGGAA 6120  
 CTTGTTAATGGACTTAATTGCACTGCTGTCCCGTCTATTAGTCTAGGAGTCCACCCTTCCTACTCAGTTTACATTACCCTAGAGTGTATGAGACTGTTAATGTGGTTAAA

TGCATC

TGCATC  
Depth:2 (PIG)  
Ei-value:0.000, Pi-value:0.030  
Er-value:0.000, Pr-value:0.020  
No matches to TargetScan

TGAC 6240  
 TGTTATGT

TTACCTA

TTACCTA  
Depth:2 (PIG)  
Ei-value:0.000, Pi-value:0.000  
Er-value:0.000, Pr-value:0.020  
No matches to TargetScan

TGATCCCATTCTCACATAATTCCTTTGCAATACCGTAAAAGGTAAAAGGAATGTTACTGTGCTTAAAAGAAGCTATAGTCATTTACATTTATCTAGGACCCTGTT 6360  
 CCCATCTAACTGACTTTCAGTGACACAGGGGGCAGTTGGGACTGTTAATGGGCTTTATTGCAGCTATAGTCCCTTGTGTTAATCTGGAATCACTTTCCTTCCTGCTCAATTTGTATTACA 6480  
 ACTGAAGGGAACTGGGACTGTCAATATGCTTAGGTGCAGCTACAGATCCTCACCTTAACCTAGGATCCCGTTCCCACCTAACTATCTTTTTGTTAATGCAAAGCCTATGTGGGACTTGTT 6600  
 AGTGGGCTTAATCGCACTATTGTCCCTTCTGTTATTTTAGGATCCCCTTTTCCTACTCGATTTGCATTACCTTAAGATGTATGAGACTGGATTTGCTTAAATGCATCTACAGTTCCTATG 6720  
 TTAACCTATGATCCCATTCCCATCTAACACCCTTTGCATGGCTGCAGAGGGAACTTGGGATAGTCAATGGGCTCGATTAGAGCTACAGTCCCTTCTGTTGGTTGAGATTCCCGTTCCCTT 6840  
 ACTCGTTTTGCATTACCAAACGGGGGAAGAAAGGGCTGTTAATATGCTT

AAATGCAATT

AAATGCAATT  
Depth:2 (PIG)  
Ei-value:0.000, Pi-value:0.000  
Er-value:0.000, Pr-value:0.000  
MATCHES To TargetScan▶ miR-25-3p/32-5p/92-3p/363-3p/367-3p:AUUGCAC▶ miR-33-5p:UGCAUUG

ACAGTCCCTTATGCTAACCTGGGATTCATCCCCAAATAACTCCCTTTTCGGGATTGCAGAG 6960  
 GATACTTGGGACTGTTAATGGGCTTAACTGCATCTATAGTCTCTTCTGTAAGTCTAGAATCTGTTCCTTTCCTACTTGATTTGCATTACTACAGAGGATACAAGGGACAGTAAAGAGGCT 7080  
 TAAATATAGCTGCAATCCCTTATGTTAACCTAGGAGCCTGTCCACAGTTATTTCCCTTTTCATTAATGCAGAGCCTACATAGGACTTGTTAATAGCCTTAATTGCTCTACTGACCCCTCC 7200  
 CGTTAGTTTGGGATCCCATCCTCACCTAATTCTCTCTGCATTACCAGGGAGGATACAAGGGACTCTTAATGTGCTTAAATGAAGGTACAGTCCTTTTTTTTAAACCTAGGATTTCATTCC 7320  
 CATGTAACTCCATTTGCACTACCACAGAGGGTACAAGGGGCGAATGAGCCTAAAAGCAGCTACAGTCCCTTATGTTAACCTAAGGATCCTGTCTCCTAGGAACTCCCTTTTCCTGGCTGC 7440  
 AGAGGATACTTGGGACTGTTAGTGAGATTTACTGTAGCTACAGTCTCTTTTATTAGTCTAGATTCCCATTACCATCCTAGATGTTTTGCATTACCACGGACTATACAAGGGACCATTATA 7560  
 GGGCTTAAATGCAGCTCCAGTCCCTTATTAACCTAGAATCTCATCCCCACCTACAGCCTTTTTCATGCTGCAGAGGGTACTTGGGACTGTTGATTGGCTTTATTGCAGCTGCAGTCCCTT 7680  


C

CTGTTAGTCT  
Depth:4 (DOG)  
Ei-value:0.000, Pi-value:0.000  
Er-value:0.000, Pr-value:0.000  
No matches to TargetScan


TGTTAGTC

TGTTAGTC  
Depth:5 (RABBIT)  
Ei-value:0.000, Pi-value:0.000  
Er-value:0.000, Pr-value:0.000  
No matches to TargetScan


T

CTGTTAGTCT  
Depth:4 (DOG)  
Ei-value:0.000, Pi-value:0.000  
Er-value:0.000, Pr-value:0.000  
No matches to TargetScan

AGAATCTGTTTCCATCCTACTCCATTTGCATTACCACAGAGGGGACAAGGGACTGTTAATGTGCTTAAATAGAACCACAGTCCCTTATGTTAAATGAGGATCCCATCCCC 7800  
 AACTAACTCTCTTTTCATTACTGCAGAGCTTAAGTAGGACTTGTTAATAGACTATTGCATTACTGTTCTGTTATTCTATAGGTTCCCATCCCTCATTCTATTTGCATTACCCTAGAGTGT 7920  
 AAAGGGACTGTTAATGAGCTTCTATGGCTCTACAGACCCTTACGTTACCTATGATCTTTTTGCCACTTAACTCCCTTTTCATGCCTATAGAGAGTACTTGGGACTGTTAATGGGCTTTAT 8040  
 TATGGCTACAGTCCCTTCTGTTTGTCCAGAATTTGTTACTTTTCTACTCGACTTGCACAGATGGTACAAGGGACTGTTAATGTGCTTAAATGAAGCTACTGTCTCTTATGTTTAAATAAA 8160  
 ATCCCATTCCCCACCTAAAACTCTTGCATGGCTGTAGAAGGTACTTGGGACAGTTAATGGCTTTAATTAGAGCTACAGTCCCTTCTATTGGTCAATATTCCCATGCCCATACTCCATTTG 8280  
 CATTACCAAAGGGAGTACAAGAGACTGTTGGTATGCTTCAATGCAATTACAGTCTCTTATGTTAACCTACGATCTGATACCCAACTATCTCCCTTTTTATTAATGCAGAAGCTACATGGG 8400  
 ACTTGTTAACGGCTTTAATTGCACTACTGTCCTTCCATTAGTCTAAGATCCCATCCCCAACCTGATTGCCTTTACAGTACCAACCAAGGAAGATACAAGGGACTGTCAGTGTGCTTAAAT 8520  
 GCAATTACAGTCCCTTTTAAATTTTACCCAGGATCCCATTCCCCCTTATCTCCCTTTTCAACACTACAGAGGGTACAATTGACTTACTGTGCTTAAAAGAAGCTATAGTCCCTCATGTTA 8640  
 ACCTAAGGA

TC

TCTCATCCCC  
Depth:2 (PIG)  
Ei-value:0.000, Pi-value:0.000  
Er-value:0.000, Pr-value:0.010  
No matches to TargetScan


TCATCC

TCATCC  
Depth:4 (DOG)  
Ei-value:0.000, Pi-value:0.020  
Er-value:0.000, Pr-value:0.000  
No matches to TargetScan


CC

TCTCATCCCC  
Depth:2 (PIG)  
Ei-value:0.000, Pi-value:0.000  
Er-value:0.000, Pr-value:0.010  
No matches to TargetScan

AACTAACTCTCCTTTCATGACTACAGAGAGTACTTGGGACTGTTAATGGGCTTTATTGTAGCTATAGTC

CCTTTTGT

CCTTTTGT  
Depth:2 (PIG)  
Ei-value:0.000, Pi-value:0.000  
Er-value:0.000, Pr-value:0.010  
No matches to TargetScan

TAGTCTAGACTACCATTTACTACA 8760  
 TGATTTGCATTACCACAGAATATGCAAGGGACTGTTACTGCGCTTAAATGCAGTTACAGACCCTTATGTTAATCTAGAATCCCATCCCCAACTATCTCTCTTCATTAATGCAGAGCCTGC 8880  
 ATAGGACTTATTAACAACCTTTATTGCACTACAGTCTCTTCTGTTAGTCTAGGAGCACATCCCCTCCTAGTTCCCTTTACATTACTAAAGAAGATACAAGGGACTGTTGATGGGCTTAAA 9000  
 TACAACTACTGTCCGTTATGTTAACCTAGGATCCCATCTCCACCTGAATCCGTTTGCATGACTGCAGAGGGACCTTAGGCTTTATTACGGGCGGCTACAGCCCCTTCTGGTAGTTTAGCT 9120  
 TCTGTTGCTGTCCTACTGGATTTGCATTACTACAGATGGTGCAAGAGACTGTTAACGTGTTTATTAAATGGAGCTACAGTCTTTTGGGTTTACTTAGAATCCCTTTTCTCTAACCTAAAA 9240  
 CCCTTTGCATGACTGTAGA

GGG

GGGTACTTGGGACTGTTAAT  
Depth:3 (COW)  
Ei-value:0.000, Pi-value:0.000  
Er-value:0.000, Pr-value:0.000  
MATCHES To TargetScan▶ miR-132-3p/212-3p:AACAGUC▶ miR-455-3p.1:CAGUCCA


TACTTGGGACTGTTAAT

TACTTGGGACTGTTAAT  
Depth:4 (DOG)  
Ei-value:0.000, Pi-value:0.000  
Er-value:0.000, Pr-value:0.000  
MATCHES To TargetScan▶ miR-132-3p/212-3p:AACAGUC▶ miR-455-3p.1:CAGUCCA


G

GGGTACTTGGGACTGTTAATG  
Depth:2 (PIG)  
Ei-value:0.000, Pi-value:0.000  
Er-value:0.000, Pr-value:0.000  
MATCHES To TargetScan▶ miR-132-3p/212-3p:AACAGUC▶ miR-455-3p.1:CAGUCCA

GGCTTAATTAGAGCTATAGTCCCTTTTATTTGTTTAGATTCTCGTGCCTTCCTACTCTATCTGCATTACTAAAGAGACTG 9360  
 TAATGCACTATTTATGTGCTTAAGTGGAGCTCCAGCTCTTGTGTTAACCTAGCATCCTATTTCTACCTAACTCCCTTTTCATAACTGCAGAGCCTATGTGGGACTATTAGTGAACTTTAC 9480  
 TGCAGTTACCTTCTGTTAGTGTAGATTCCCATCCTCTTTTATATGATTTGATAACCACAGACTGTACAATGGACTACCAATGTGCTTAAATGCAGCTACAGTCCTTTATGTTAAGCTAGA 9600  
 ATCCCATTCTCACCTAACTCTTCTCATTGATGCAGAGCCTATATGGGACTTGTTAATGGGCTTCATTGCTCTTCTCTGTCCCTTCTACTAGTCTAGATTCCCAAGCTCTCCTACTCAATT 9720  
 TGCATTACCAAGGGGGGTACAATGG

ACTG

ACTGTTAATGTGCT  
Depth:4 (DOG)  
Ei-value:0.000, Pi-value:0.000  
Er-value:0.000, Pr-value:0.000  
MATCHES To TargetScan▶ miR-132-3p/212-3p:AACAGUC▶ miR-323-3p:ACAUUAC


TTAATGTGCT

TTAATGTGCT  
Depth:5 (RABBIT)  
Ei-value:0.000, Pi-value:0.000  
Er-value:0.000, Pr-value:0.000  
MATCHES To TargetScan▶ miR-323-3p:ACAUUAC


A

ACTGTTAATGTGCTA  
Depth:2 (PIG)  
Ei-value:0.000, Pi-value:0.000  
Er-value:0.000, Pr-value:0.000  
MATCHES To TargetScan▶ miR-132-3p/212-3p:AACAGUC▶ miR-323-3p:ACAUUAC

AAATGCAGCTACAATCTCTTACATTAATCTAGAATCTCATGCCCCAACTAACGCCCTTTGCATGGCTTCAAAGGATA

CTT

CTTGGGACTC  
Depth:3 (COW)  
Ei-value:0.000, Pi-value:0.000  
Er-value:0.000, Pr-value:0.000  
No matches to TargetScan

 9840  


GGGACTC

CTTGGGACTC  
Depth:3 (COW)  
Ei-value:0.000, Pi-value:0.000  
Er-value:0.000, Pr-value:0.000  
No matches to TargetScan


TTAATG

CTTGGGACTCTTAATG  
Depth:2 (PIG)  
Ei-value:0.000, Pi-value:0.000  
Er-value:0.000, Pr-value:0.000  
No matches to TargetScan

GGCTGAAATACAGCTACAGTCTCTTATGATAACCTAGGATCCCATTCTCACCTATCTCCCTTTTCATTGCTGCAGAGCCTGCATGGGACTTGTTAATGGTCTTAATT 9960  
 GCACTGCTGTCCCTTTCGTTAGTCTAGGATTCTATCCCCTCATAAAGAATTACATTACCGTAGAGGTTTCAAGGGAATGGTAATGTGCTAAATGCACATAGTCCCCCTCTTTTTGTAACC 10080  
 TAGGATCCCATTCCACCTAACTCCCTTTGCATTATCACCAACTGTACAAGTGACTGGTAATGGGCTTAAAAGCAGCTATTGTTAACCTAGGATCCCATCCCCATTCAACTTCCTTTGCAT 10200  
 GAGTGCAGAGGACACTTGGGACTGTTAATGAGTTGTATTGCTGCTACAGTCCCTCCTGTTATTCTAGG

ATCCATG

ATCCATG  
Depth:2 (PIG)  
Ei-value:0.000, Pi-value:0.010  
Er-value:0.000, Pr-value:0.000  
No matches to TargetScan

CCTTTTTTGATTTGCATTGCAGGGGAGGGTACTAGGGACTGTTAA 10320  
 TGTACTTAAATAGAGCTACAGTCCTTTATGGTAACCAGGATCTGGATCCCACCTAACTGCCTTGTATTACTTCAGGGCTTACATAGGACTTTTTTAATGGGCTTAATTGCTCTGATTTCC 10440  
 CTTCTTTTAGTCTAGGTTCCCCTCCCTCCTACTCTATTTTCATTACCCCAGAGTGTACAAGGGACTGGTAAGTATGCTTAAATGCAATTATAGTCCCTTCTGTTAGTCTAGGGTCCCATT 10560  
 CCCACCTAATTCCCTTTACATTACCAAAGGAGATACAAGGGACTGTCGTTGTGCTTAAGTGTGGGTACAATCCCCATTTTTTCTTTTTTTAGCCTAGGATCCCTTTCCCACCCAATTGCC 10680  
 TTTGGATGACTGCAGAGAATACTGGGGACTGTTAATGGGCTTTATTGTAGCCATAGTCCTTTTTCTTAGTCTGAGATCCCATCTCCTCCTAAGTGATTAACATTACCCCAGAGTGTATAC 10800  
 AGGACTGGTACTTTGCTAAATGCAATTATAGTCCCTTCTATTAGTCTAGGATCCCATCCCCACCTAAATCCCTTTACATTATCACAGGGGATAGATACAAGGGACTGTTGTGCTTAAATG 10920  
 TAGGTACAGCCCCCCCCCCCCTTTTAACCCTAAGATATCTTTCCCACATAAATGCCTTTGCATGACTGCAGAGGATACTTGGGACTGTTAATGGGCTTTATTGTAGCCACAGTCTCTTTC 11040  
 CATAGTCTGGGATCCCATCCCCTCTTAAATGATTTACATTACTCGCAGAGGGCGCAAGGAACTGCAAAGGTGCTTAAATGCAGGTAGTTGGTTTTTAAACCTAGGATCCCATTCCCAGCC 11160  
 TGACTTGCTTTGAATTACTAAAGAGGCTACAAGGGACTCTTATGTGTTTAAGTGCAGGTACAGTGCCTTTTGTTAACCTCAAAATCCATTCCCACCTAACTCCCTTTTCCTGACTGCAAA 11280  
 GGGTAATTGGGACTGTTAACGGGCTGTACTGCAGCCACATTCCTCTCATAGTCTAGGATCCCATTCCCTCCTATTTGATTTGCATTACCCCAGAGGGTACAAGGGACTTTTAAAGTGCTT 11400  
 AAGTGGGAGTTCCTGTCATGGCACCGCGGAAACGAAACCAACTAAGAACCATGAGGTTTGGGGTTCAATCCCTGGCCTTGCTCAGTGGGTTCAGGATCCAGCGTTGCCCTGGGCTGTGGT 11520  
 GTAGGTCGCAGACGCTGCTCGGATCTGGCATTGCTGTGGCTGTGGTGTAGGCTGGCAGCTGTAGCTCCAATTCGACCCCTAGCCTGGGAACTTCATATGCCACAGGTGTGGCCCTAAAAA 11640  
 AAAAAGACAAAAAAAACCAAAAAAAACAAAAAAACCAAAGTGCTTAAGTGGCACTATAGTCCCTTATGTTATCTAGGACCCCATTCCCATCTAACTTTTCATGACTGCAGAGTGTACTTG 11760  
 GGACTGTAAATGTACTTAACTGCCTATACAGTCTCACCTCTTAGTCCAGATTACATGCCCTCCTACTTGAATTGTATTATAACAGAGGGTACAAGGGACTTTTAATGTGCTTTTTTAAAA 11880  
 GCAGCTAAATTTCCTTACCATAACCTGAAAATCCATTTCCATCTCAAAATCCACTCCCACCTAACTCCCTTTCCATCACAGCAGAGGGTACTTGGGACTGTTAATAGGCTTAATTAAAGC 12000  
 TATTCTCCCTTCTGTTGGTCTAGATTTCCTTTCCCGCCTCTATTTGGATTGTACCTCAGTGTACAATGGACTGTTAACTGTGCTTAAATGCAGGTACAGTCCTTTTTTTAAAACCTAGGA 12120  
 TCCTATTCCCACCTAACTCCCTTTGCTTTACTGCAGAGGGTACTTGGGGCTTGTTAATAGGCTTAGTTGGACTACAGTCCTTTCTGTTAGTCTAGGATCCCATCCCCTCCTACGTGATTT 12240  
 GCATTTCCACAAAGGCTATAAGGGACTGTTAAGGTGCTTATTAAATGCAAATACAGTCCCTTATGTTAACCTGGGATCCCATTCCCACCTATCTCCCTTTGCATGACTGCAAAGGTATTT 12360  
 GAGACCTTTAAAGTAGTTAATGGCTGCTGTAGCCTCTTCCATTATAAGTGTACTTTCCCAAGCACTCCAGCTCCATGTAACCTGGGAGCACATTTGGGGCTGTTAATGTGCTCATTTTTA 12480  
 GCTGTGGTCCCTTTTATAATTCTAGGATCACATCCCTTTCTGCTTCTTTGTGTTACTTGTTGGTATTTGGGACAGAAAAGTACTTAAATGCAGCTGTGATCTAACTTAGGCTCCCAGCCC 12600  
 TCTAACTTTTTACATTACCCCAGTGTATATTTGGGACTGTTAAAGTGCTTAATTGCAGCTGTGGTCCCCTCTGTTAGTCTAATTTCCTAAGCTCACCAATTCCCTTTGTATTACAGCAGA 12720  
 GGGTACTTGGGACTGTTAAAGTGTTTATTTGCAGCTCTGGTCCCTTCTCTTAGTCTGGGGTCCATGCCCCCAGCTTTGTTTGCATTACCACAGAGGGTACTTGGAACTGTGAATGTGCTT 12840  
 AAATTCAGGTGTGGTCCCTTTTAATTTAGGATTCCTCAACTCCTTTTACCAGGGAGGGCATTTGGGACTGTCAAGGTGGTAAGGGCACCTGCGTTCCTTTCTTTCTGTTCATCTAGGATC 12960  
 CCATTCCCTCCTACTCCCTTTGTATTGCATGGGAGGATTTGGGGGGCTCTTAAGGTGCTTAACTGCAGCTGTGGTCCCTCCTATTAGTTTCCAATCCCTTTCTACTCCCTTTGCATTACC 13080  
 ACAGAGGGTACTTGGGACTGTTGTAGTACATCCCATTCTCTCATGCCTGTTAGCATTACAGCACAAGGTCCTGAACACCACGGCCCTTTCTTTTGCACTGTTAATGCACAATTTCATTTG 13200  
 TCCCTCATCTTCTGCAGTATTTATAGACCTCACTCATTTCCCTTGTATTTAGTGGTGAATGCCCTTTACTCAAGACCTGTGTACTAGGATAGTTAAAGTGAGCCATGGTAATTGACCCCA 13320  
 AATAATGTCTTTCACTTAAGTCCCTTGTATTCTTTGTTTTACAAGAAGATGTATTAATTACCTCAGTTACTTCCTTCTGGGAGTGTTTACATGCAAAATTCCAGTGTTCATGGCCCCTTT 13440  
 CTTTAAACTAATACCCCTCCTTTTTTATATTCAGGGGATGTTAGTGACCCAAAGTCCCTCTCTTGGGATTC

T

TAATGTGCAT  
Depth:2 (PIG)  
Ei-value:0.000, Pi-value:0.000  
Er-value:0.000, Pr-value:0.000  
MATCHES To TargetScan▶ miR-323-3p:ACAUUAC▶ miR-501-3p/502-3p:AUGCACC


AATGTGCAT

AATGTGCAT  
Depth:6 (MOUSE)  
Ei-value:0.000, Pi-value:0.000  
Er-value:0.000, Pr-value:0.000  
MATCHES To TargetScan▶ miR-501-3p/502-3p:AUGCACC

GTGCCCTCGTGATAAG

CTAATA

CTAATA  
Depth:3 (COW)  
Ei-value:0.000, Pi-value:0.000  
Er-value:0.000, Pr-value:0.000  
No matches to TargetScan

CGTTGAGTTCAGGGATT 13560  
 ACTGACTACCC

AAGTCTTT

AAGTCTTT  
Depth:2 (PIG)  
Ei-value:0.000, Pi-value:0.000  
Er-value:0.000, Pr-value:0.000  
No matches to TargetScan

TTTTGGTGGGGGTGGGGGACAGTTAATG

TGCAAAATT

TGCAAAATT  
Depth:2 (PIG)  
Ei-value:0.000, Pi-value:0.000  
Er-value:0.000, Pr-value:0.000  
No matches to TargetScan

ATACTAGGATCCCATCCTATCCCACCCCTTTTGCATTTGGGGTGCTGACCAACTGTGGC

TGCTT

TGCTTCT  
Depth:3 (COW)  
Ei-value:0.000, Pi-value:0.000  
Er-value:0.000, Pr-value:0.010  
No matches to TargetScan

 13680  


CT

TGCTTCT  
Depth:3 (COW)  
Ei-value:0.000, Pi-value:0.000  
Er-value:0.000, Pr-value:0.010  
No matches to TargetScan

CCTGTACAGTTTAAATGCACATAATTTCAGTAATCCATGCTCCCT

TATGTTAGA

TATGTTAGA  
Depth:4 (DOG)  
Ei-value:0.000, Pi-value:0.000  
Er-value:0.000, Pr-value:0.000  
No matches to TargetScan

C

TAGAATCCC

TAGAATCCC  
Depth:2 (PIG)  
Ei-value:0.000, Pi-value:0.000  
Er-value:0.000, Pr-value:0.000  
No matches to TargetScan

ATTTTCTTGCCCCATTTACATTACTTTG

GGAGCTTCT

GGAGCTTCT  
Depth:2 (PIG)  
Ei-value:0.000, Pi-value:0.000  
Er-value:0.000, Pr-value:0.000  
No matches to TargetScan

GAATAGCCAAGATTCAT 13800  
 TT

TCTTGG

TCTTGGACTGTTAATGT  
Depth:3 (COW)  
Ei-value:0.000, Pi-value:0.000  
Er-value:0.000, Pr-value:0.000  
MATCHES To TargetScan▶ miR-132-3p/212-3p:AACAGUC▶ miR-323-3p:ACAUUAC▶ miR-455-3p.1:CAGUCCA


ACTGTTAATGT

ACTGTTAATGT  
Depth:4 (DOG)  
Ei-value:0.000, Pi-value:0.000  
Er-value:0.000, Pr-value:0.000  
MATCHES To TargetScan▶ miR-132-3p/212-3p:AACAGUC▶ miR-323-3p:ACAUUAC


G

TCTTGGACTGTTAATGTG  
Depth:2 (PIG)  
Ei-value:0.000, Pi-value:0.000  
Er-value:0.000, Pr-value:0.000  
MATCHES To TargetScan▶ miR-132-3p/212-3p:AACAGUC▶ miR-323-3p:ACAUUAC▶ miR-455-3p.1:CAGUCCA

TATACTGCC

ATTTGCT

ATTTGCT  
Depth:4 (DOG)  
Ei-value:0.000, Pi-value:0.000  
Er-value:0.000, Pr-value:0.000  
No matches to TargetScan

CCTGTACATCAG

GTAAGGA

GTAAGGA  
Depth:5 (RABBIT)  
Ei-value:0.000, Pi-value:0.000  
Er-value:0.000, Pr-value:0.000  
No matches to TargetScan


CCC

GTAAGGACCC  
Depth:3 (COW)  
Ei-value:0.000, Pi-value:0.000  
Er-value:0.000, Pr-value:0.000  
No matches to TargetScan

TCTCCATTCTATTTATATTTCAGCAGGAGGTGCCTACTT

CTTAAGA

CTTAAGA  
Depth:2 (PIG)  
Ei-value:0.000, Pi-value:0.000  
Er-value:0.000, Pr-value:0.000  
No matches to TargetScan

TTTATATACTTCTGCA 13920  
 GTTAATC

TGCATA

TGCATAATCTTAG  
Depth:2 (PIG)  
Ei-value:0.000, Pi-value:0.000  
Er-value:0.000, Pr-value:0.000  
No matches to TargetScan


ATCTTAG

ATCTTAG  
Depth:3 (COW)  
Ei-value:0.000, Pi-value:0.000  
Er-value:0.000, Pr-value:0.000  
No matches to TargetScan

TTGTCCTG

TACACATT

TACACATT  
Depth:3 (COW)  
Ei-value:0.000, Pi-value:0.000  
Er-value:0.000, Pr-value:0.000  
No matches to TargetScan

CTCAATTGTCCACAACTGCTTTTTAGGTT

AGGACTCCT

AGGACTCCT  
Depth:2 (PIG)  
Ei-value:0.000, Pi-value:0.000  
Er-value:0.000, Pr-value:0.000  
No matches to TargetScan

T

T

TACTTAT  
Depth:2 (PIG)  
Ei-value:0.000, Pi-value:0.010  
Er-value:0.000, Pr-value:0.030  
No matches to TargetScan


ACTTAT

ACTTAT  
Depth:5 (RABBIT)  
Ei-value:0.000, Pi-value:0.000  
Er-value:0.000, Pr-value:0.000  
No matches to TargetScan


TTAAGC

TTAAGC  
Depth:2 (PIG)  
Ei-value:0.000, Pi-value:0.000  
Er-value:0.000, Pr-value:0.010  
No matches to TargetScan

ACAGCATACTGACTACCTTAAAGGCCTTGTCT 14040  
 TGGGACTGTTACTATG

TGTAATT

TGTAATT  
Depth:3 (COW)  
Ei-value:0.000, Pi-value:0.000  
Er-value:0.000, Pr-value:0.000  
No matches to TargetScan

ACAATTGTCC

ATGGTC

ATGGTC  
Depth:3 (COW)  
Ei-value:0.000, Pi-value:0.020  
Er-value:0.000, Pr-value:0.000  
No matches to TargetScan


CTT

ATGGTCCTT  
Depth:2 (PIG)  
Ei-value:0.000, Pi-value:0.000  
Er-value:0.000, Pr-value:0.000  
No matches to TargetScan

TGAGTTAACTTGTGTCCCAATTT

TCACAC

TCACAC  
Depth:2 (PIG)  
Ei-value:0.000, Pi-value:0.010  
Er-value:0.000, Pr-value:0.020  
No matches to TargetScan

TCTCTTTA

CTTTATTGC

CTTTATTGC  
Depth:2 (PIG)  
Ei-value:0.000, Pi-value:0.000  
Er-value:0.000, Pr-value:0.000  
MATCHES To TargetScan▶ miR-142-5p:AUAAAGU

A

ATGGGGTACT

ATGGGGTACT  
Depth:3 (COW)  
Ei-value:0.000, Pi-value:0.000  
Er-value:0.000, Pr-value:0.000  
No matches to TargetScan

G

TT

TTCACTTAAGGCCCCTTTCTCAAAC  
Depth:2 (PIG)  
Ei-value:0.000, Pi-value:0.000  
Er-value:0.000, Pr-value:0.000  
No matches to TargetScan


CAC

CACTTAAGGCCCCTTTCTCAA  
Depth:3 (COW)  
Ei-value:0.000, Pi-value:0.000  
Er-value:0.000, Pr-value:0.000  
No matches to TargetScan


TTAAGGCC

TTAAGGCC  
Depth:6 (MOUSE)  
Ei-value:0.000, Pi-value:0.000  
Er-value:0.000, Pr-value:0.000  
No matches to TargetScan


CCTTT

TTAAGGCCCCTTT  
Depth:5 (RABBIT)  
Ei-value:0.000, Pi-value:0.000  
Er-value:0.000, Pr-value:0.000  
No matches to TargetScan


CT

TTAAGGCCCCTTTCTCAA  
Depth:4 (DOG)  
Ei-value:0.000, Pi-value:0.000  
Er-value:0.000, Pr-value:0.000  
No matches to TargetScan

 14160  


CAA

TTAAGGCCCCTTTCTCAA  
Depth:4 (DOG)  
Ei-value:0.000, Pi-value:0.000  
Er-value:0.000, Pr-value:0.000  
No matches to TargetScan


AC

TTCACTTAAGGCCCCTTTCTCAAAC  
Depth:2 (PIG)  
Ei-value:0.000, Pi-value:0.000  
Er-value:0.000, Pr-value:0.000  
No matches to TargetScan

AGTAATATATG

TAATGACAATTACAT

TAATGACAATTACAT  
Depth:3 (COW)  
Ei-value:0.000, Pi-value:0.000  
Er-value:0.000, Pr-value:0.000  
MATCHES To TargetScan▶ miR-411-3p:AUGUAAC

TAGG

ATCCTTCC

ATCCTTCC  
Depth:2 (PIG)  
Ei-value:0.000, Pi-value:0.010  
Er-value:0.000, Pr-value:0.000  
No matches to TargetScan

CCTTCACATTCCC

TTTGAAG

TTTGAAG  
Depth:2 (PIG)  
Ei-value:0.000, Pi-value:0.010  
Er-value:0.000, Pr-value:0.000  
No matches to TargetScan

TACCACAAGGATTCTGATC

CCTAAGG

CCTAAGG  
Depth:2 (PIG)  
Ei-value:0.000, Pi-value:0.010  
Er-value:0.000, Pr-value:0.000  
No matches to TargetScan

T

CCCATTTCTTG

CCCATTTCTTG  
Depth:2 (PIG)  
Ei-value:0.000, Pi-value:0.000  
Er-value:0.000, Pr-value:0.000  
MATCHES To TargetScan▶ miR-203a-3p.1:GAAAUGU

ACCTGTTAATGTACGTGA

T

TGTATTTGTC  
Depth:2 (PIG)  
Ei-value:0.000, Pi-value:0.000  
Er-value:0.000, Pr-value:0.000  
No matches to TargetScan

 14280  


GTATTTGTC

TGTATTTGTC  
Depth:2 (PIG)  
Ei-value:0.000, Pi-value:0.000  
Er-value:0.000, Pr-value:0.000  
No matches to TargetScan

TGGGTTCTTGTGCATTCCTTTTCT

CTTCCAGCAGGAAGTGCCC

CTTCCAGCAGGAAGTGCCC  
Depth:2 (PIG)  
Ei-value:0.000, Pi-value:0.000  
Er-value:0.000, Pr-value:0.000  
No matches to TargetScan

CCT

CCACAAG

CCACAAG  
Depth:2 (PIG)  
Ei-value:0.000, Pi-value:0.000  
Er-value:0.000, Pr-value:0.000  
No matches to TargetScan

ACTGTTAAG

TTTGGACAGTCAAG

TTTGGACAGTCAAG  
Depth:2 (PIG)  
Ei-value:0.000, Pi-value:0.000  
Er-value:0.000, Pr-value:0.000  
No matches to TargetScan


ATGCAC

ATGCAC  
Depth:2 (PIG)  
Ei-value:0.000, Pi-value:0.030  
Er-value:0.000, Pr-value:0.010  
No matches to TargetScan

ATTT

GTAACTG

GTAACTG  
Depth:2 (PIG)  
Ei-value:0.000, Pi-value:0.000  
Er-value:0.000, Pr-value:0.000  
No matches to TargetScan

ACTGCAGCCAGT

CACCTT

CACCTTGGA  
Depth:2 (PIG)  
Ei-value:0.000, Pi-value:0.000  
Er-value:0.000, Pr-value:0.000  
MATCHES To TargetScan▶ miR-18-5p:AAGGUGC

 14400  


GGA

CACCTTGGA  
Depth:2 (PIG)  
Ei-value:0.000, Pi-value:0.000  
Er-value:0.000, Pr-value:0.000  
MATCHES To TargetScan▶ miR-18-5p:AAGGUGC

TGT

TAATGTGT

TAATGTGT  
Depth:2 (PIG)  
Ei-value:0.000, Pi-value:0.010  
Er-value:0.000, Pr-value:0.000  
MATCHES To TargetScan▶ miR-323-3p:ACAUUAC


ATAACTGCACATGGCT

ATAACTGCACATGGCT  
Depth:2 (PIG)  
Ei-value:0.000, Pi-value:0.000  
Er-value:0.000, Pr-value:0.000  
MATCHES To TargetScan▶ miR-455-3p.2:UGCAGUC▶ miR-455-5p:AUGUGCC

CATCCCATATGAATAAGATCCTACC

CTCTCAGACCCC

CTCTCAGACCCC  
Depth:2 (PIG)  
Ei-value:0.000, Pi-value:0.000  
Er-value:0.000, Pr-value:0.000  
MATCHES To TargetScan▶ miR-193a-5p:GGGUCUU

TTCTCT

AGTATAGC

AGTATAGC  
Depth:2 (PIG)  
Ei-value:0.000, Pi-value:0.000  
Er-value:0.000, Pr-value:0.000  
No matches to TargetScan

AAGGGTACTGATTCCTAAGA

CCTCTTT

CCTCTTT  
Depth:2 (PIG)  
Ei-value:0.000, Pi-value:0.010  
Er-value:0.000, Pr-value:0.020  
No matches to TargetScan

CCATGGCTAGTT 14520  
 ATTGTACATAATTTGCTTTTGTACATGTTCCTGTACACTAAATAAGGATGCCCCTCTTCCC

ACTCCCTTTG

ACTCCCTTTG  
Depth:2 (PIG)  
Ei-value:0.000, Pi-value:0.000  
Er-value:0.000, Pr-value:0.000  
No matches to TargetScan

TCTTTTACAGGGAGCGTA

CACTACTTT

CACTACTTT  
Depth:2 (PIG)  
Ei-value:0.000, Pi-value:0.000  
Er-value:0.000, Pr-value:0.000  
MATCHES To TargetScan▶ miR-142-3p.1:GUAGUGU

AAGATC

CTTATATTT

CTTATATTT  
Depth:3 (COW)  
Ei-value:0.000, Pi-value:0.000  
Er-value:0.000, Pr-value:0.000  
MATCHES To TargetScan▶ miR-410-3p:AUAUAAC

ATTTGTA 14640  


CAAAGTACATG

CAAAGTACATG  
Depth:2 (PIG)  
Ei-value:0.000, Pi-value:0.000  
Er-value:0.000, Pr-value:0.000  
No matches to TargetScan

A

TTTTAATTGACCA

TTTTAATTGACCA  
Depth:3 (COW)  
Ei-value:0.000, Pi-value:0.000  
Er-value:0.000, Pr-value:0.000  
No matches to TargetScan

TACCATACCCT

TTGG

TTGGACATTAATGTA  
Depth:2 (PIG)  
Ei-value:0.000, Pi-value:0.000  
Er-value:0.000, Pr-value:0.000  
MATCHES To TargetScan▶ miR-323-3p:ACAUUAC


ACATTAAT

ACATTAAT  
Depth:3 (COW)  
Ei-value:0.000, Pi-value:0.000  
Er-value:0.000, Pr-value:0.000  
No matches to TargetScan


GTA

TTGGACATTAATGTA  
Depth:2 (PIG)  
Ei-value:0.000, Pi-value:0.000  
Er-value:0.000, Pr-value:0.000  
MATCHES To TargetScan▶ miR-323-3p:ACAUUAC

CATAATTACACCTTAATTCATCATATCCTTTTGCT

CTCCATTTGCAGTATA

CTCCATTTGCAGTATA  
Depth:2 (PIG)  
Ei-value:0.000, Pi-value:0.000  
Er-value:0.000, Pr-value:0.000  
MATCHES To TargetScan▶ miR-217:ACUGCAU

T

CAGGGTT

CAGGGTT  
Depth:2 (PIG)  
Ei-value:0.000, Pi-value:0.000  
Er-value:0.000, Pr-value:0.000  
MATCHES To TargetScan▶ miR-10-5p:ACCCUGU▶ miR-504-5p.1:ACCCUGG

TG

TGACCC

TGACCC  
Depth:2 (PIG)  
Ei-value:0.000, Pi-value:0.030  
Er-value:0.000, Pr-value:0.000  
No matches to TargetScan

TA 14760  
 AATCAGCTCTTTCCTTGGCCTATTAATG

TG

TGCATAATTGCATTT  
Depth:2 (PIG)  
Ei-value:0.000, Pi-value:0.000  
Er-value:0.000, Pr-value:0.000  
No matches to TargetScan


CATAATTGCA

CATAATTGCA  
Depth:3 (COW)  
Ei-value:0.000, Pi-value:0.000  
Er-value:0.000, Pr-value:0.000  
No matches to TargetScan


TTT

TGCATAATTGCATTT  
Depth:2 (PIG)  
Ei-value:0.000, Pi-value:0.000  
Er-value:0.000, Pr-value:0.000  
No matches to TargetScan

GTCCA

GGTTCTTG

GGTTCTTG  
Depth:2 (PIG)  
Ei-value:0.000, Pi-value:0.000  
Er-value:0.000, Pr-value:0.000  
No matches to TargetScan

CACA

CTAGACAAGGA

CTAGACAAGGA  
Depth:3 (COW)  
Ei-value:0.000, Pi-value:0.000  
Er-value:0.000, Pr-value:0.000  
No matches to TargetScan

CACCCCCCCACCCCCGCCAACTTCCTATGCCTTCCGGTAAGTAGGTGGT 14880  
 TCCCACTGCTAAGACCTTTATATTTGG

ACAGTTAATGTG

ACAGTTAATGTG  
Depth:4 (DOG)  
Ei-value:0.000, Pi-value:0.000  
Er-value:0.000, Pr-value:0.000  
MATCHES To TargetScan▶ miR-323-3p:ACAUUAC

CAG

AATTGCAGTT

AATTGCAGTT  
Depth:2 (PIG)  
Ei-value:0.000, Pi-value:0.000  
Er-value:0.000, Pr-value:0.000  
MATCHES To TargetScan▶ miR-217:ACUGCAU

T

TCCACAACCC

TCCACAACCC  
Depth:2 (PIG)  
Ei-value:0.000, Pi-value:0.000  
Er-value:0.000, Pr-value:0.000  
No matches to TargetScan

AGTTACTTCCAGGACTGCTGTATCTCCTTTGCA

ATACCTC

ATACCTC  
Depth:2 (PIG)  
Ei-value:0.000, Pi-value:0.000  
Er-value:0.000, Pr-value:0.000  
MATCHES To TargetScan▶ let-7-5p/98-5p:GAGGUAG

AAGGG

ATACTGTTT

ATACTGTTT  
Depth:3 (COW)  
Ei-value:0.000, Pi-value:0.000  
Er-value:0.000, Pr-value:0.000  
MATCHES To TargetScan▶ miR-101-3p.1:ACAGUAC▶ miR-132-3p/212-3p:AACAGUC▶ miR-144-3p:ACAGUAU

TTC 15000  
 CCCAAGATCGTTTCTT

GTGGAC

GTGGAC  
Depth:2 (PIG)  
Ei-value:0.000, Pi-value:0.000  
Er-value:0.000, Pr-value:0.010  
No matches to TargetScan

CGTCAATATATG

TAATTGAAAT

TAATTGAAAT  
Depth:2 (PIG)  
Ei-value:0.000, Pi-value:0.000  
Er-value:0.000, Pr-value:0.000  
No matches to TargetScan

GG

TTGTCTT

TTGTCTT  
Depth:3 (COW)  
Ei-value:0.000, Pi-value:0.000  
Er-value:0.000, Pr-value:0.010  
No matches to TargetScan

CATCCA

TAGACT

TAGACT  
Depth:2 (PIG)  
Ei-value:0.000, Pi-value:0.000  
Er-value:0.000, Pr-value:0.000  
No matches to TargetScan

GAGATAATATCCCCTCAGC

TAACTA

TAACTA  
Depth:4 (DOG)  
Ei-value:0.000, Pi-value:0.000  
Er-value:0.000, Pr-value:0.000  
No matches to TargetScan

TCATGAC

CTCAGCTCTTGG

CTCAGCTCTTGG  
Depth:5 (RABBIT)  
Ei-value:0.000, Pi-value:0.000  
Er-value:0.000, Pr-value:0.000  
MATCHES To TargetScan▶ miR-335-5p:CAAGAGC


ACA

CTCAGCTCTTGGACA  
Depth:4 (DOG)  
Ei-value:0.000, Pi-value:0.000  
Er-value:0.000, Pr-value:0.000  
MATCHES To TargetScan▶ miR-335-5p:CAAGAGC


ATTAATA

CTCAGCTCTTGGACAATTAATA  
Depth:3 (COW)  
Ei-value:0.000, Pi-value:0.000  
Er-value:0.000, Pr-value:0.000  
MATCHES To TargetScan▶ miR-335-5p:CAAGAGC

T 15120  
 TCACC

AATAACA

AATAACA  
Depth:2 (PIG)  
Ei-value:0.000, Pi-value:0.010  
Er-value:0.000, Pr-value:0.000  
No matches to TargetScan

TATCAAAAGT

ACT

ACTGATCATTAGATA  
Depth:2 (PIG)  
Ei-value:0.000, Pi-value:0.000  
Er-value:0.000, Pr-value:0.000  
MATCHES To TargetScan▶ miR-383-5p.1:GAUCAGA


GATCAT

GATCAT  
Depth:3 (COW)  
Ei-value:0.000, Pi-value:0.000  
Er-value:0.000, Pr-value:0.000  
No matches to TargetScan


TAGATA

ACTGATCATTAGATA  
Depth:2 (PIG)  
Ei-value:0.000, Pi-value:0.000  
Er-value:0.000, Pr-value:0.000  
MATCHES To TargetScan▶ miR-383-5p.1:GAUCAGA

GGACCCCTGTTCCCTTACTGTATACATCAGGGATACTG

AC

ACTAAGGCCCC  
Depth:2 (PIG)  
Ei-value:0.000, Pi-value:0.000  
Er-value:0.000, Pr-value:0.000  
No matches to TargetScan


TAAGGC

TAAGGC  
Depth:3 (COW)  
Ei-value:0.000, Pi-value:0.000  
Er-value:0.000, Pr-value:0.000  
No matches to TargetScan


CCC

ACTAAGGCCCC  
Depth:2 (PIG)  
Ei-value:0.000, Pi-value:0.000  
Er-value:0.000, Pr-value:0.000  
No matches to TargetScan

CTTTTTTGACTGTTAATGC

GAATATTTGCA

GAATATTTGCA  
Depth:3 (COW)  
Ei-value:0.000, Pi-value:0.000  
Er-value:0.000, Pr-value:0.000  
No matches to TargetScan


ATTA

GAATATTTGCAATTAT  
Depth:2 (PIG)  
Ei-value:0.000, Pi-value:0.000  
Er-value:0.000, Pr-value:0.000  
MATCHES To TargetScan▶ miR-25-3p/32-5p/92-3p/363-3p/367-3p:AUUGCAC

 15240  


T

GAATATTTGCAATTAT  
Depth:2 (PIG)  
Ei-value:0.000, Pi-value:0.000  
Er-value:0.000, Pr-value:0.000  
MATCHES To TargetScan▶ miR-25-3p/32-5p/92-3p/363-3p/367-3p:AUUGCAC

CTAAT

TCCCCTT

TCCCCTT  
Depth:2 (PIG)  
Ei-value:0.000, Pi-value:0.000  
Er-value:0.000, Pr-value:0.030  
No matches to TargetScan

CTATTAGAGTAGGACATTATTTCCTTGCACCCCATTCGG

ATTACTG

ATTACTG  
Depth:3 (COW)  
Ei-value:0.000, Pi-value:0.010  
Er-value:0.000, Pr-value:0.020  
MATCHES To TargetScan▶ miR-802:CAGUAAC

AA

AG

AGGGGCTGCTGAC  
Depth:2 (PIG)  
Ei-value:0.000, Pi-value:0.000  
Er-value:0.000, Pr-value:0.000  
MATCHES To TargetScan▶ miR-15-5p/16-5p/195-5p/424-5p/497-5p:AGCAGCA▶ miR-503-5p:AGCAGCG


GGGCTGCTGA

GGGCTGCTGA  
Depth:3 (COW)  
Ei-value:0.000, Pi-value:0.000  
Er-value:0.000, Pr-value:0.000  
MATCHES To TargetScan▶ miR-15-5p/16-5p/195-5p/424-5p/497-5p:AGCAGCA▶ miR-503-5p:AGCAGCG


C

AGGGGCTGCTGAC  
Depth:2 (PIG)  
Ei-value:0.000, Pi-value:0.000  
Er-value:0.000, Pr-value:0.000  
MATCHES To TargetScan▶ miR-15-5p/16-5p/195-5p/424-5p/497-5p:AGCAGCA▶ miR-503-5p:AGCAGCG

CACA

CAAAACTT

CAAAACTT  
Depth:4 (DOG)  
Ei-value:0.000, Pi-value:0.000  
Er-value:0.000, Pr-value:0.000  
No matches to TargetScan


CT

CAAAACTTCT  
Depth:2 (PIG)  
Ei-value:0.000, Pi-value:0.000  
Er-value:0.000, Pr-value:0.000  
No matches to TargetScan

A

CTGGGACTG

CTGGGACTG  
Depth:3 (COW)  
Ei-value:0.000, Pi-value:0.000  
Er-value:0.000, Pr-value:0.000  
MATCHES To TargetScan▶ miR-455-3p.1:CAGUCCA

CTGATGA

GCACAATG

GCACAATG  
Depth:6 (MOUSE)  
Ei-value:0.000, Pi-value:0.000  
Er-value:0.000, Pr-value:0.000  
No matches to TargetScan

ACGATGA 15360  
 GAAATGGGTTTTTA

CTCCCTG

CTCCCTG  
Depth:3 (COW)  
Ei-value:0.000, Pi-value:0.000  
Er-value:0.000, Pr-value:0.000  
No matches to TargetScan

G

CCTTGTT

CCTTGTT  
Depth:2 (PIG)  
Ei-value:0.000, Pi-value:0.000  
Er-value:0.000, Pr-value:0.040  
No matches to TargetScan

GG

GCAAGC

GCAAGC  
Depth:3 (COW)  
Ei-value:0.000, Pi-value:0.000  
Er-value:0.000, Pr-value:0.000  
No matches to TargetScan


GC

GCAAGCGC  
Depth:2 (PIG)  
Ei-value:0.000, Pi-value:0.000  
Er-value:0.000, Pr-value:0.000  
No matches to TargetScan

TCCCAGCCCAGCCCCAA

TTTCCCATGG

TTTCCCATGG  
Depth:2 (PIG)  
Ei-value:0.000, Pi-value:0.000  
Er-value:0.000, Pr-value:0.000  
No matches to TargetScan

T

ATAATAAAGTATAA

ATAATAAAGTATAA  
Depth:2 (PIG)  
Ei-value:0.000, Pi-value:0.000  
Er-value:0.000, Pr-value:0.000  
No matches to TargetScan

ATACTGCAGTGTGCCATGAACTTTCC

ATCAAACAG

ATCAAACAG  
Depth:2 (PIG)  
Ei-value:0.000, Pi-value:0.000  
Er-value:0.000, Pr-value:0.000  
No matches to TargetScan

CAGC 15480  


CCAT

CCATACTCCCA  
Depth:2 (PIG)  
Ei-value:0.000, Pi-value:0.000  
Er-value:0.000, Pr-value:0.000  
MATCHES To TargetScan▶ miR-496.1:GAGUAUU


A

ACTCCCA  
Depth:4 (DOG)  
Ei-value:0.000, Pi-value:0.000  
Er-value:0.000, Pr-value:0.000  
No matches to TargetScan


CTCCCA

CTCCCA  
Depth:6 (MOUSE)  
Ei-value:0.000, Pi-value:0.000  
Er-value:0.000, Pr-value:0.000  
No matches to TargetScan

CTCTACTTGCATTGGCTCCAGTGTATCAAATTCCAAATAGCTATGGTCTTGCCCTTATGTTCCCTCTCCATACCCTTTGCCTGCCCTTGGACCTTTCTTATGGACTATT 15600  
 AATGCTCACAATTTTCAGGTGTCCATGTATCCAGATAAGATTGTGCTCCCTTGCCCCTCCTGCCCCTTCCACCCCTGCCCTG

CCCTTTTGCATT

CCCTTTTGCATT  
Depth:4 (DOG)  
Ei-value:0.000, Pi-value:0.000  
Er-value:0.000, Pr-value:0.000  
No matches to TargetScan


G

CCCTTTTGCATTG  
Depth:3 (COW)  
Ei-value:0.000, Pi-value:0.000  
Er-value:0.000, Pr-value:0.000  
No matches to TargetScan


TTGCTGGG

TTGCTGGG  
Depth:2 (PIG)  
Ei-value:0.000, Pi-value:0.000  
Er-value:0.000, Pr-value:0.010  
MATCHES To TargetScan▶ miR-338-3p:CCAGCAU

AAATGTCCACTGGCAAA 15720  
 GCCCTTTTGTTTTTAAGACATTAACAATCCCAGATGTCATTGCTTTGCCCATTTTGAATTGCTGTAATGCCTCAGTTA

CCCTTTCT

CCCTTTCT  
Depth:2 (PIG)  
Ei-value:0.000, Pi-value:0.000  
Er-value:0.000, Pr-value:0.000  
No matches to TargetScan

TTGGTTCCGCCCATCAGACATGGACCCTTCC

ACT

ACTTCCTT  
Depth:3 (COW)  
Ei-value:0.000, Pi-value:0.000  
Er-value:0.000, Pr-value:0.000  
No matches to TargetScan

 15840  


TCCTT

ACTTCCTT  
Depth:3 (COW)  
Ei-value:0.000, Pi-value:0.000  
Er-value:0.000, Pr-value:0.000  
No matches to TargetScan

CTTTGCATTACTC

CTGAGTA

CTGAGTA  
Depth:2 (PIG)  
Ei-value:0.000, Pi-value:0.010  
Er-value:0.000, Pr-value:0.000  
No matches to TargetScan

GTA

CTGACTACCCA

CTGACTACCCA  
Depth:2 (PIG)  
Ei-value:0.000, Pi-value:0.000  
Er-value:0.000, Pr-value:0.000  
No matches to TargetScan

C

AGCCCCTTCT

AGCCCCTTCT  
Depth:3 (COW)  
Ei-value:0.000, Pi-value:0.000  
Er-value:0.000, Pr-value:0.000  
No matches to TargetScan


GTGTTATTAA

AGCCCCTTCTGTGTTATTAA  
Depth:2 (PIG)  
Ei-value:0.000, Pi-value:0.000  
Er-value:0.000, Pr-value:0.000  
No matches to TargetScan


CACAGTA

CACAGTA  
Depth:3 (COW)  
Ei-value:0.000, Pi-value:0.000  
Er-value:0.000, Pr-value:0.000  
No matches to TargetScan

T

TGATTGTC

TGATTGTCCCATTTTT  
Depth:3 (COW)  
Ei-value:0.000, Pi-value:0.000  
Er-value:0.000, Pr-value:0.000  
No matches to TargetScan


CCATTTTT

CCATTTTT  
Depth:4 (DOG)  
Ei-value:0.000, Pi-value:0.000  
Er-value:0.000, Pr-value:0.000  
No matches to TargetScan

T

CAGCCCA

CAGCCCA  
Depth:4 (DOG)  
Ei-value:0.000, Pi-value:0.000  
Er-value:0.000, Pr-value:0.000  
No matches to TargetScan

C

CAGCCCA

CAGCCCA  
Depth:4 (DOG)  
Ei-value:0.000, Pi-value:0.000  
Er-value:0.000, Pr-value:0.000  
No matches to TargetScan

AGG

TCTC

TCTCCCTACCA  
Depth:3 (COW)  
Ei-value:0.000, Pi-value:0.000  
Er-value:0.000, Pr-value:0.000  
No matches to TargetScan


CCTACCA

CCTACCA  
Depth:4 (DOG)  
Ei-value:0.000, Pi-value:0.000  
Er-value:0.000, Pr-value:0.000  
No matches to TargetScan


CTTTG

TCTCCCTACCACTTTG  
Depth:2 (PIG)  
Ei-value:0.000, Pi-value:0.000  
Er-value:0.000, Pr-value:0.000  
MATCHES To TargetScan▶ miR-140-5p:AGUGGUU▶ miR-17-5p/20-5p/93-5p/106-5p/519-3p:AAAGUGC

A 15960  
 TGTTGTATTT

GTGCAGT

GTGCAGT  
Depth:3 (COW)  
Ei-value:0.000, Pi-value:0.000  
Er-value:0.000, Pr-value:0.000  
MATCHES To TargetScan▶ miR-217:ACUGCAU

A

TTGACTA

TTGACTA  
Depth:2 (PIG)  
Ei-value:0.000, Pi-value:0.020  
Er-value:0.000, Pr-value:0.000  
No matches to TargetScan

CC

AAAAGCAG

AAAAGCAG  
Depth:6 (MOUSE)  
Ei-value:0.000, Pi-value:0.000  
Er-value:0.000, Pr-value:0.000  
No matches to TargetScan

A

CCT

CCTGAACTA  
Depth:2 (PIG)  
Ei-value:0.000, Pi-value:0.000  
Er-value:0.000, Pr-value:0.000  
No matches to TargetScan


GAACTA

GAACTA  
Depth:3 (COW)  
Ei-value:0.000, Pi-value:0.000  
Er-value:0.000, Pr-value:0.000  
No matches to TargetScan

TGTGGGTGG

GCCTTCACTC

GCCTTCACTC  
Depth:2 (PIG)  
Ei-value:0.000, Pi-value:0.000  
Er-value:0.000, Pr-value:0.000  
No matches to TargetScan

CTTTTCCTGCATTTG

TTAATGATCC

TTAATGATCC  
Depth:4 (DOG)  
Ei-value:0.000, Pi-value:0.000  
Er-value:0.000, Pr-value:0.000  
MATCHES To TargetScan▶ miR-382-3p:AUCAUUC

CAATTC

CA

CAATTATTGT  
Depth:2 (PIG)  
Ei-value:0.000, Pi-value:0.000  
Er-value:0.000, Pr-value:0.000  
No matches to TargetScan


ATTATTGT

ATTATTGT  
Depth:3 (COW)  
Ei-value:0.000, Pi-value:0.000  
Er-value:0.000, Pr-value:0.000  
No matches to TargetScan

AAC

ATTCTGGG

ATTCTGGG  
Depth:4 (DOG)  
Ei-value:0.000, Pi-value:0.000  
Er-value:0.000, Pr-value:0.000  
No matches to TargetScan


GACA

ATTCTGGGGACA  
Depth:2 (PIG)  
Ei-value:0.000, Pi-value:0.000  
Er-value:0.000, Pr-value:0.000  
No matches to TargetScan

 16080  


ATTCTGGGGACA  
Depth:2 (PIG)  
Ei-value:0.000, Pi-value:0.000  
Er-value:0.000, Pr-value:0.000  
No matches to TargetScan

G

GAACCATTC

GAACCATTC  
Depth:2 (PIG)  
Ei-value:0.000, Pi-value:0.000  
Er-value:0.000, Pr-value:0.000  
No matches to TargetScan

CTGCCCCTCTT

TTAC

TTACTGCTTTACT  
Depth:2 (PIG)  
Ei-value:0.000, Pi-value:0.000  
Er-value:0.000, Pr-value:0.000  
MATCHES To TargetScan▶ miR-330-3p.2:AAAGCAC▶ miR-802:CAGUAAC


TG

TGCTTTACT  
Depth:3 (COW)  
Ei-value:0.000, Pi-value:0.000  
Er-value:0.000, Pr-value:0.000  
MATCHES To TargetScan▶ miR-330-3p.2:AAAGCAC


CTTTACT

CTTTACT  
Depth:4 (DOG)  
Ei-value:0.000, Pi-value:0.000  
Er-value:0.000, Pr-value:0.000  
No matches to TargetScan

AG

GCAAAAT

GCAAAAT  
Depth:6 (MOUSE)  
Ei-value:0.000, Pi-value:0.000  
Er-value:0.000, Pr-value:0.000  
No matches to TargetScan

TTTT

AAGGCAA

AAGGCAA  
Depth:4 (DOG)  
Ei-value:0.000, Pi-value:0.000  
Er-value:0.000, Pr-value:0.000  
No matches to TargetScan


GTCAGACCCA

AAGGCAAGTCAGACCCA  
Depth:3 (COW)  
Ei-value:0.000, Pi-value:0.000  
Er-value:0.000, Pr-value:0.000  
MATCHES To TargetScan▶ miR-193a-5p:GGGUCUU

AGGGAACT

TGGATTGC

TGGATTGC  
Depth:4 (DOG)  
Ei-value:0.000, Pi-value:0.000  
Er-value:0.000, Pr-value:0.000  
No matches to TargetScan

TACCCTGTATTTATTTATTATTTATTATATATAAGTATCA 16200  
 ATTGAAAATTA

TCTCCCCAG

TCTCCCCAGGAAGGAAG  
Depth:2 (PIG)  
Ei-value:0.000, Pi-value:0.000  
Er-value:0.000, Pr-value:0.000  
No matches to TargetScan


GAAGGAAG

GAAGGAAG  
Depth:3 (COW)  
Ei-value:0.000, Pi-value:0.000  
Er-value:0.000, Pr-value:0.000  
No matches to TargetScan

TTTAGCATTG

TCTC

TCTCTGCATTCTTC  
Depth:2 (PIG)  
Ei-value:0.000, Pi-value:0.000  
Er-value:0.000, Pr-value:0.000  
No matches to TargetScan


TGCATTCTTC

TGCATTCTTC  
Depth:5 (RABBIT)  
Ei-value:0.000, Pi-value:0.000  
Er-value:0.000, Pr-value:0.000  
No matches to TargetScan

CTTT

CAG

CAGAGCAGATTGCCTGG  
Depth:2 (PIG)  
Ei-value:0.000, Pi-value:0.000  
Er-value:0.000, Pr-value:0.000  
No matches to TargetScan


AGC

AGCAGATTGCCTGG  
Depth:4 (DOG)  
Ei-value:0.000, Pi-value:0.000  
Er-value:0.000, Pr-value:0.000  
No matches to TargetScan


A

AGATTGCCTGG  
Depth:5 (RABBIT)  
Ei-value:0.000, Pi-value:0.000  
Er-value:0.000, Pr-value:0.000  
No matches to TargetScan


GATTGCCTGG

GATTGCCTGG  
Depth:6 (MOUSE)  
Ei-value:0.000, Pi-value:0.000  
Er-value:0.000, Pr-value:0.000  
No matches to TargetScan

C

TAAGAATCTCT

TAAGAATCTCT  
Depth:2 (PIG)  
Ei-value:0.000, Pi-value:0.000  
Er-value:0.000, Pr-value:0.000  
No matches to TargetScan

CTTGGCCTC

TTGTATATT

TTGTATATT  
Depth:4 (DOG)  
Ei-value:0.000, Pi-value:0.000  
Er-value:0.000, Pr-value:0.000  
MATCHES To TargetScan▶ miR-381-3p:AUACAAG

CCCAACATGTAA

TGCCA

TGCCAA  
Depth:3 (COW)  
Ei-value:0.000, Pi-value:0.000  
Er-value:0.000, Pr-value:0.000  
MATCHES To TargetScan▶ miR-182-5p:UUGGCAA▶ miR-96-5p/1271-5p:UUGGCAC

 16320  


A

TGCCAA  
Depth:3 (COW)  
Ei-value:0.000, Pi-value:0.000  
Er-value:0.000, Pr-value:0.000  
MATCHES To TargetScan▶ miR-182-5p:UUGGCAA▶ miR-96-5p/1271-5p:UUGGCAC

T

TGCCAGGATACA

TGCCAGGATACA  
Depth:3 (COW)  
Ei-value:0.000, Pi-value:0.000  
Er-value:0.000, Pr-value:0.000  
No matches to TargetScan

ACCAAAAAGTTGTTATTTTTT

AAATTTTTT

AAATTTTTT  
Depth:2 (PIG)  
Ei-value:0.000, Pi-value:0.000  
Er-value:0.000, Pr-value:0.000  
No matches to TargetScan

AAAACGT

ACATCTGG

ACATCTGG  
Depth:3 (COW)  
Ei-value:0.000, Pi-value:0.000  
Er-value:0.000, Pr-value:0.000  
No matches to TargetScan

TTTGCAAGGTGGAATT

GAT

GATAACCTGGTCATT  
Depth:3 (COW)  
Ei-value:0.000, Pi-value:0.000  
Er-value:0.000, Pr-value:0.000  
MATCHES To TargetScan▶ miR-154-5p:AGGUUAU


AAC

AACCTGGTCATT  
Depth:4 (DOG)  
Ei-value:0.000, Pi-value:0.000  
Er-value:0.000, Pr-value:0.000  
No matches to TargetScan


CTGGTCATT

CTGGTCATT  
Depth:5 (RABBIT)  
Ei-value:0.000, Pi-value:0.000  
Er-value:0.000, Pr-value:0.000  
No matches to TargetScan

GAA

T

TTTTTGAAG  
Depth:2 (PIG)  
Ei-value:0.000, Pi-value:0.000  
Er-value:0.000, Pr-value:0.000  
No matches to TargetScan


TTTTGAA

TTTTGAA  
Depth:3 (COW)  
Ei-value:0.000, Pi-value:0.000  
Er-value:0.000, Pr-value:0.010  
No matches to TargetScan


G

TTTTTGAAG  
Depth:2 (PIG)  
Ei-value:0.000, Pi-value:0.000  
Er-value:0.000, Pr-value:0.000  
No matches to TargetScan

TCTTAAAAAC

CCATTTAT

CCATTTAT  
Depth:5 (RABBIT)  
Ei-value:0.000, Pi-value:0.000  
Er-value:0.000, Pr-value:0.000  
No matches to TargetScan

 16440  


CCATTTAT  
Depth:5 (RABBIT)  
Ei-value:0.000, Pi-value:0.000  
Er-value:0.000, Pr-value:0.000  
No matches to TargetScan

TCCATGTATCTGA

TGAC

TGACCAGTGTCTCTCATTT  
Depth:4 (DOG)  
Ei-value:0.000, Pi-value:0.000  
Er-value:0.000, Pr-value:0.000  
No matches to TargetScan


CAGTGTCTCTCATTT

CAGTGTCTCTCATTT  
Depth:5 (RABBIT)  
Ei-value:0.000, Pi-value:0.000  
Er-value:0.000, Pr-value:0.000  
No matches to TargetScan

ACTA

AGG

AGGGTGGTG  
Depth:4 (DOG)  
Ei-value:0.000, Pi-value:0.000  
Er-value:0.000, Pr-value:0.000  
No matches to TargetScan


GTGGTG

GTGGTG  
Depth:5 (RABBIT)  
Ei-value:0.000, Pi-value:0.000  
Er-value:0.000, Pr-value:0.000  
No matches to TargetScan


G

AGGGTGGTGGGTCTGTGGATAGA  
Depth:2 (PIG)  
Ei-value:0.000, Pi-value:0.000  
Er-value:0.000, Pr-value:0.000  
MATCHES To TargetScan▶ miR-140-3p.1:CCACAGG


GTCTGTGGATA

GTCTGTGGATA  
Depth:5 (RABBIT)  
Ei-value:0.000, Pi-value:0.000  
Er-value:0.000, Pr-value:0.000  
MATCHES To TargetScan▶ miR-140-3p.1:CCACAGG


GA

GTCTGTGGATAGA  
Depth:3 (COW)  
Ei-value:0.000, Pi-value:0.000  
Er-value:0.000, Pr-value:0.000  
MATCHES To TargetScan▶ miR-140-3p.1:CCACAGG

CGGCTGTGACTTTGA

TATTTTA

TATTTTA  
Depth:3 (COW)  
Ei-value:0.000, Pi-value:0.040  
Er-value:0.000, Pr-value:0.020  
No matches to TargetScan

GTATTACTACCAAAGGAG

TTCTAGA

TTCTAGA  
Depth:4 (DOG)  
Ei-value:0.000, Pi-value:0.000  
Er-value:0.000, Pr-value:0.000  
No matches to TargetScan

ATGGAATTCTTAGG 16560  
 ACA

AGTATCTTTG

AGTATCTTTG  
Depth:3 (COW)  
Ei-value:0.000, Pi-value:0.000  
Er-value:0.000, Pr-value:0.000  
No matches to TargetScan

GGCTCTACCACCATTTTGAAACCATTCCTGTTTTGGCTATACCATT

ATTCACTT

ATTCACTT  
Depth:4 (DOG)  
Ei-value:0.000, Pi-value:0.000  
Er-value:0.000, Pr-value:0.000  
No matches to TargetScan


TTA

ATTCACTTTTAGAAAAAC  
Depth:2 (PIG)  
Ei-value:0.000, Pi-value:0.000  
Er-value:0.000, Pr-value:0.000  
MATCHES To TargetScan▶ miR-17-5p/20-5p/93-5p/106-5p/519-3p:AAAGUGC


GAAAAAC

GAAAAAC  
Depth:4 (DOG)  
Ei-value:0.000, Pi-value:0.000  
Er-value:0.000, Pr-value:0.000  
No matches to TargetScan

AACCTGAACTTCC

TAATCCTT

TAATCCTT  
Depth:2 (PIG)  
Ei-value:0.000, Pi-value:0.010  
Er-value:0.000, Pr-value:0.000  
No matches to TargetScan

A

AATTTCTTCATCTGGAGC

AATTTCTTCATCTGGAGC  
Depth:5 (RABBIT)  
Ei-value:0.000, Pi-value:0.000  
Er-value:0.000, Pr-value:0.000  
No matches to TargetScan


A

AATTTCTTCATCTGGAGCA  
Depth:2 (PIG)  
Ei-value:0.000, Pi-value:0.000  
Er-value:0.000, Pr-value:0.000  
No matches to TargetScan

TC 16680  
 AACCAGCCCCTG

CTTATTT

CTTATTT  
Depth:4 (DOG)  
Ei-value:0.000, Pi-value:0.000  
Er-value:0.000, Pr-value:0.010  
No matches to TargetScan


CAAGAA

CTTATTTCAAGAA  
Depth:3 (COW)  
Ei-value:0.000, Pi-value:0.000  
Er-value:0.000, Pr-value:0.000  
MATCHES To TargetScan▶ miR-203a-3p.2:UGAAAUG

CATTGCTATAAATGG

ATAAAATG

ATAAAATG  
Depth:4 (DOG)  
Ei-value:0.000, Pi-value:0.000  
Er-value:0.000, Pr-value:0.000  
No matches to TargetScan


A

ATAAAATGA  
Depth:3 (COW)  
Ei-value:0.000, Pi-value:0.000  
Er-value:0.000, Pr-value:0.000  
No matches to TargetScan

GAAAACATATCCTGAAGTGCTTTTTGGAAACTGTTGATCACTTTGTTTGATTAATCTGTTAAATAAAATGC 16800  
 GTTACATTAAATTCTTAGCCTAAAGC

ACCACACT

ACCACACT  
Depth:3 (COW)  
Ei-value:0.000, Pi-value:0.000  
Er-value:0.000, Pr-value:0.000  
No matches to TargetScan


GA

ACCACACTGA  
Depth:2 (PIG)  
Ei-value:0.000, Pi-value:0.000  
Er-value:0.000, Pr-value:0.000  
No matches to TargetScan

A

GTGAGG

GTGAGG  
Depth:3 (COW)  
Ei-value:0.000, Pi-value:0.000  
Er-value:0.000, Pr-value:0.000  
No matches to TargetScan

GCTTAG

AAATGAT

AAATGAT  
Depth:2 (PIG)  
Ei-value:0.000, Pi-value:0.020  
Er-value:0.000, Pr-value:0.020  
MATCHES To TargetScan▶ miR-382-3p:AUCAUUC

GGGACCAGTTTTCTG

TTTTATA

TTTTATA  
Depth:3 (COW)  
Ei-value:0.000, Pi-value:0.000  
Er-value:0.000, Pr-value:0.010  
MATCHES To TargetScan▶ miR-340-5p:UAUAAAG

TTAAAAT

AAAAATAAGCCA

AAAAATAAGCCA  
Depth:5 (RABBIT)  
Ei-value:0.000, Pi-value:0.000  
Er-value:0.000, Pr-value:0.000  
No matches to TargetScan


A

AAAAATAAGCCAA  
Depth:4 (DOG)  
Ei-value:0.000, Pi-value:0.000  
Er-value:0.000, Pr-value:0.000  
No matches to TargetScan

GATCTAGTC

AT

ATTCTTTTGGATATA  
Depth:2 (PIG)  
Ei-value:0.000, Pi-value:0.000  
Er-value:0.000, Pr-value:0.000  
MATCHES To TargetScan▶ miR-186-5p:AAAGAAU


TCTTTTGGATA

TCTTTTGGATATA  
Depth:3 (COW)  
Ei-value:0.000, Pi-value:0.000  
Er-value:0.000, Pr-value:0.000  
No matches to TargetScan

 16920  


TA

TCTTTTGGATATA  
Depth:3 (COW)  
Ei-value:0.000, Pi-value:0.000  
Er-value:0.000, Pr-value:0.000  
No matches to TargetScan

GGTTTTCAGG

AGTGAGATAGCTGCCT

AGTGAGATAGCTGCCT  
Depth:2 (PIG)  
Ei-value:0.000, Pi-value:0.000  
Er-value:0.000, Pr-value:0.000  
No matches to TargetScan

GGTTAAG

ATGAATAATA

ATGAATAATA  
Depth:4 (DOG)  
Ei-value:0.000, Pi-value:0.000  
Er-value:0.000, Pr-value:0.000  
No matches to TargetScan

GCCTAGCTTCC

AGTGTACA

AGTGTACA  
Depth:3 (COW)  
Ei-value:0.000, Pi-value:0.000  
Er-value:0.000, Pr-value:0.000  
MATCHES To TargetScan▶ miR-493-5p:UGUACAU


G

AGTGTACAGGGTGTTT  
Depth:2 (PIG)  
Ei-value:0.000, Pi-value:0.000  
Er-value:0.000, Pr-value:0.000  
MATCHES To TargetScan▶ miR-10-5p:ACCCUGU▶ miR-339-5p:CCCUGUC▶ miR-486-5p:CCUGUAC▶ miR-493-5p:UGUACAU▶ miR-504-5p.1:ACCCUGG


GGTGTTT

GGTGTTT  
Depth:3 (COW)  
Ei-value:0.000, Pi-value:0.000  
Er-value:0.000, Pr-value:0.000  
No matches to TargetScan

GATGGCAGAGAAGTATTAATG

TGGAACTGCT

TGGAACTGCT  
Depth:4 (DOG)  
Ei-value:0.000, Pi-value:0.000  
Er-value:0.000, Pr-value:0.000  
No matches to TargetScan

GAAGG

AAA

AAATAACTAGT  
Depth:2 (PIG)  
Ei-value:0.000, Pi-value:0.000  
Er-value:0.000, Pr-value:0.000  
No matches to TargetScan


TAACTA

TAACTA  
Depth:4 (DOG)  
Ei-value:0.000, Pi-value:0.000  
Er-value:0.000, Pr-value:0.000  
No matches to TargetScan


GT

AAATAACTAGT  
Depth:2 (PIG)  
Ei-value:0.000, Pi-value:0.000  
Er-value:0.000, Pr-value:0.000  
No matches to TargetScan

T 17040  
 ATCACTG

CAGCAGTTC

CAGCAGTTC  
Depth:5 (RABBIT)  
Ei-value:0.000, Pi-value:0.000  
Er-value:0.000, Pr-value:0.000  
No matches to TargetScan

C

TTGTAAT

TTGTAAT  
Depth:4 (DOG)  
Ei-value:0.000, Pi-value:0.000  
Er-value:0.000, Pr-value:0.000  
No matches to TargetScan

C

ACTGAAAA

ACTGAAAA  
Depth:5 (RABBIT)  
Ei-value:0.000, Pi-value:0.000  
Er-value:0.000, Pr-value:0.000  
No matches to TargetScan

GGATACTCTTCTCT

GAG

GAGAAGGATGTCAAAAGATCGGC  
Depth:3 (COW)  
Ei-value:0.000, Pi-value:0.000  
Er-value:0.000, Pr-value:0.000  
MATCHES To TargetScan▶ miR-362-5p/500b-5p:AUCCUUG▶ miR-489-3p:UGACAUC


AAGGATG

AAGGATG  
Depth:5 (RABBIT)  
Ei-value:0.000, Pi-value:0.000  
Er-value:0.000, Pr-value:0.000  
MATCHES To TargetScan▶ miR-362-5p/500b-5p:AUCCUUG


TCA

AAGGATGTCAAAAGATC  
Depth:4 (DOG)  
Ei-value:0.000, Pi-value:0.000  
Er-value:0.000, Pr-value:0.000  
MATCHES To TargetScan▶ miR-362-5p/500b-5p:AUCCUUG▶ miR-489-3p:UGACAUC


AAAGATC

AAAGATC  
Depth:6 (MOUSE)  
Ei-value:0.000, Pi-value:0.000  
Er-value:0.000, Pr-value:0.000  
No matches to TargetScan


GGC

GAGAAGGATGTCAAAAGATCGGC  
Depth:3 (COW)  
Ei-value:0.000, Pi-value:0.000  
Er-value:0.000, Pr-value:0.000  
MATCHES To TargetScan▶ miR-362-5p/500b-5p:AUCCUUG▶ miR-489-3p:UGACAUC

T

CAGCTCAGGG

CAGCTCAGGG  
Depth:4 (DOG)  
Ei-value:0.000, Pi-value:0.000  
Er-value:0.000, Pr-value:0.000  
MATCHES To TargetScan▶ miR-125-5p:CCCUGAG

T

GCAGTTTGC

GCAGTTTGC  
Depth:3 (COW)  
Ei-value:0.000, Pi-value:0.000  
Er-value:0.000, Pr-value:0.000  
No matches to TargetScan

A

CTACTAGCTCCT

CTACTAGCTCCT  
Depth:4 (DOG)  
Ei-value:0.000, Pi-value:0.000  
Er-value:0.000, Pr-value:0.000  
MATCHES To TargetScan▶ miR-28-5p/708-5p:AGGAGCU▶ miR-411-5p.2:UAGUAGA

T

GGACAGCTG

GGACAGCTG  
Depth:5 (RABBIT)  
Ei-value:0.000, Pi-value:0.000  
Er-value:0.000, Pr-value:0.000  
No matches to TargetScan


T

GGACAGCTGT  
Depth:4 (DOG)  
Ei-value:0.000, Pi-value:0.000  
Er-value:0.000, Pr-value:0.000  
No matches to TargetScan


A

AAGAAGAGTCTCTGGCTCTTTAGA  
Depth:3 (COW)  
Ei-value:0.000, Pi-value:0.000  
Er-value:0.000, Pr-value:0.000  
No matches to TargetScan


AGAA

AGAAGAGTCTCTGGCTCTTTA  
Depth:5 (RABBIT)  
Ei-value:0.000, Pi-value:0.000  
Er-value:0.000, Pr-value:0.000  
No matches to TargetScan

 17160  


GAGTCTCTGGCTCTTTA

AGAAGAGTCTCTGGCTCTTTA  
Depth:5 (RABBIT)  
Ei-value:0.000, Pi-value:0.000  
Er-value:0.000, Pr-value:0.000  
No matches to TargetScan


GA

AGAAGAGTCTCTGGCTCTTTAGA  
Depth:4 (DOG)  
Ei-value:0.000, Pi-value:0.000  
Er-value:0.000, Pr-value:0.000  
No matches to TargetScan

CTACTGGATGA

ATTCTGAGC

ATTCTGAGC  
Depth:4 (DOG)  
Ei-value:0.000, Pi-value:0.000  
Er-value:0.000, Pr-value:0.000  
No matches to TargetScan

CGGTACCCCCACCTCAAGAGGAAGGATGGATCAATTTTAGGTGAATTGAAGCCTATACTAAACAGCCTCCAAAGGATATTC 17280  
 CAAGCAAGTGAGCCCTGA

GA

GACTGCAA  
Depth:3 (COW)  
Ei-value:0.000, Pi-value:0.000  
Er-value:0.000, Pr-value:0.000  
MATCHES To TargetScan▶ miR-455-3p.2:UGCAGUC


CTGCAA

CTGCAA  
Depth:5 (RABBIT)  
Ei-value:0.000, Pi-value:0.000  
Er-value:0.000, Pr-value:0.000  
No matches to TargetScan

GCAGCTCTAAGAAGTTCCGCATTGATCAGGTCTTCCCT

TTTGAGAATCTGG

TTTGAGAATCTGG  
Depth:3 (COW)  
Ei-value:0.000, Pi-value:0.000  
Er-value:0.000, Pr-value:0.000  
MATCHES To TargetScan▶ miR-371-5p:CUCAAAC

AT

AAGCTCCA

AAGCTCCA  
Depth:3 (COW)  
Ei-value:0.000, Pi-value:0.000  
Er-value:0.000, Pr-value:0.000  
No matches to TargetScan

ACC

AATCTA

AATCTA  
Depth:2 (PIG)  
Ei-value:0.000, Pi-value:0.000  
Er-value:0.000, Pr-value:0.000  
No matches to TargetScan

AAA

GGATGG

GGATGG  
Depth:3 (COW)  
Ei-value:0.000, Pi-value:0.000  
Er-value:0.000, Pr-value:0.010  
No matches to TargetScan

TTTGCAGACTG

T

TCTGGAGAAAAAGATCTTCCTCAGAAGAATAGGCTTGTTG  
Depth:2 (PIG)  
Ei-value:0.000, Pi-value:0.000  
Er-value:0.000, Pr-value:0.000  
MATCHES To TargetScan▶ miR-1224-5p:UGAGGAC▶ miR-7-5p:GGAAGAC


CTG

CTGGAGAAAAAGATCT  
Depth:3 (COW)  
Ei-value:0.000, Pi-value:0.000  
Er-value:0.000, Pr-value:0.000  
No matches to TargetScan

 17400  


GAGAAAAAGATCT

CTGGAGAAAAAGATCT  
Depth:3 (COW)  
Ei-value:0.000, Pi-value:0.000  
Er-value:0.000, Pr-value:0.000  
No matches to TargetScan


TCCTCAG

TCTGGAGAAAAAGATCTTCCTCAGAAGAATAGGCTTGTTG  
Depth:2 (PIG)  
Ei-value:0.000, Pi-value:0.000  
Er-value:0.000, Pr-value:0.000  
MATCHES To TargetScan▶ miR-1224-5p:UGAGGAC▶ miR-7-5p:GGAAGAC


AAGAATAGGC

AAGAATAGGC  
Depth:5 (RABBIT)  
Ei-value:0.000, Pi-value:0.000  
Er-value:0.000, Pr-value:0.000  
No matches to TargetScan


TTGTTG

TCTGGAGAAAAAGATCTTCCTCAGAAGAATAGGCTTGTTG  
Depth:2 (PIG)  
Ei-value:0.000, Pi-value:0.000  
Er-value:0.000, Pr-value:0.000  
MATCHES To TargetScan▶ miR-1224-5p:UGAGGAC▶ miR-7-5p:GGAAGAC

CC

T

TTACAGTGTTAGTGA  
Depth:3 (COW)  
Ei-value:0.000, Pi-value:0.000  
Er-value:0.000, Pr-value:0.000  
MATCHES To TargetScan▶ miR-141-3p/200a-3p:AACACUG


TACAGTGTTAGTGA

TACAGTGTTAGTGA  
Depth:5 (RABBIT)  
Ei-value:0.000, Pi-value:0.000  
Er-value:0.000, Pr-value:0.000  
MATCHES To TargetScan▶ miR-141-3p/200a-3p:AACACUG

CT

CA

CATTCCCTTTGA  
Depth:3 (COW)  
Ei-value:0.000, Pi-value:0.000  
Er-value:0.000, Pr-value:0.000  
MATCHES To TargetScan▶ miR-1-3p/206:GGAAUGU


TTCCCTTTGA

TTCCCTTTGA  
Depth:6 (MOUSE)  
Ei-value:0.000, Pi-value:0.000  
Er-value:0.000, Pr-value:0.000  
No matches to TargetScan

TGATCCT

TAGGTGGAGATGGGGCATGAGGATCCTCCAGGGGAA

TAGGTGGAGATGGGGCATGAGGATCCTCCAGGGGAA  
Depth:6 (MOUSE)  
Ei-value:0.000, Pi-value:0.000  
Er-value:0.000, Pr-value:0.000  
MATCHES To TargetScan▶ miR-331-3p:CCCCUGG


A

TAGGTGGAGATGGGGCATGAGGATCCTCCAGGGGAAA  
Depth:5 (RABBIT)  
Ei-value:0.000, Pi-value:0.000  
Er-value:0.000, Pr-value:0.000  
MATCHES To TargetScan▶ miR-331-3p:CCCCUGG


AGC

TAGGTGGAGATGGGGCATGAGGATCCTCCAGGGGAAAAGCTCACTACCACTGGGCAACAACCCTAGGTCAGGAG  
Depth:2 (PIG)  
Ei-value:0.000, Pi-value:0.000  
Er-value:0.000, Pr-value:0.000  
MATCHES To TargetScan▶ miR-140-5p:AGUGGUU▶ miR-142-3p.1:GUAGUGU▶ miR-192-5p/215-5p:UGACCUA▶ miR-199-5p:CCAGUGU▶ miR-296-3p:AGGGUUG▶ miR-331-3p:CCCCUGG


TCACTA

TCACTA  
Depth:5 (RABBIT)  
Ei-value:0.000, Pi-value:0.000  
Er-value:0.000, Pr-value:0.000  
No matches to TargetScan

 17520  


TCACTA  
Depth:5 (RABBIT)  
Ei-value:0.000, Pi-value:0.000  
Er-value:0.000, Pr-value:0.000  
No matches to TargetScan


CCACT

TCACTACCACT  
Depth:4 (DOG)  
Ei-value:0.000, Pi-value:0.000  
Er-value:0.000, Pr-value:0.000  
MATCHES To TargetScan▶ miR-140-5p:AGUGGUU▶ miR-142-3p.1:GUAGUGU


G

TCACTACCACTG  
Depth:3 (COW)  
Ei-value:0.000, Pi-value:0.000  
Er-value:0.000, Pr-value:0.000  
MATCHES To TargetScan▶ miR-140-5p:AGUGGUU▶ miR-142-3p.1:GUAGUGU


G

TAGGTGGAGATGGGGCATGAGGATCCTCCAGGGGAAAAGCTCACTACCACTGGGCAACAACCCTAGGTCAGGAG  
Depth:2 (PIG)  
Ei-value:0.000, Pi-value:0.000  
Er-value:0.000, Pr-value:0.000  
MATCHES To TargetScan▶ miR-140-5p:AGUGGUU▶ miR-142-3p.1:GUAGUGU▶ miR-192-5p/215-5p:UGACCUA▶ miR-199-5p:CCAGUGU▶ miR-296-3p:AGGGUUG▶ miR-331-3p:CCCCUGG


GCAACA

GCAACA  
Depth:6 (MOUSE)  
Ei-value:0.000, Pi-value:0.000  
Er-value:0.000, Pr-value:0.000  
No matches to TargetScan


AC

GCAACAAC  
Depth:5 (RABBIT)  
Ei-value:0.000, Pi-value:0.000  
Er-value:0.000, Pr-value:0.000  
No matches to TargetScan


CCTAGGTCAGGAG

TAGGTGGAGATGGGGCATGAGGATCCTCCAGGGGAAAAGCTCACTACCACTGGGCAACAACCCTAGGTCAGGAG  
Depth:2 (PIG)  
Ei-value:0.000, Pi-value:0.000  
Er-value:0.000, Pr-value:0.000  
MATCHES To TargetScan▶ miR-140-5p:AGUGGUU▶ miR-142-3p.1:GUAGUGU▶ miR-192-5p/215-5p:UGACCUA▶ miR-199-5p:CCAGUGU▶ miR-296-3p:AGGGUUG▶ miR-331-3p:CCCCUGG

ATTCTACTAAGATT

CTTTCCTGG

CTTTCCTGG  
Depth:3 (COW)  
Ei-value:0.000, Pi-value:0.000  
Er-value:0.000, Pr-value:0.000  
MATCHES To TargetScan▶ miR-665:CCAGGAG▶ miR-873-5p.1:CAGGAAC

GT

CCAGATAGGAAGAT

CCAGATAGGAAGAT  
Depth:2 (PIG)  
Ei-value:0.000, Pi-value:0.000  
Er-value:0.000, Pr-value:0.000  
MATCHES To TargetScan▶ miR-202-5p:UCCUAUG

G

AAGTCTCAA

AAGTCTCAA  
Depth:2 (PIG)  
Ei-value:0.000, Pi-value:0.000  
Er-value:0.000, Pr-value:0.000  
No matches to TargetScan

G

ACAACCACC

ACAACCACC  
Depth:5 (RABBIT)  
Ei-value:0.000, Pi-value:0.000  
Er-value:0.000, Pr-value:0.000  
No matches to TargetScan


ACAC

ACAACCACCACAC  
Depth:4 (DOG)  
Ei-value:0.000, Pi-value:0.000  
Er-value:0.000, Pr-value:0.000  
No matches to TargetScan

ATCTGAGGAAAACAGGGACAACAACTGCC 17640  
 ATATCTTACATTCATCATGACATTCATCATGAGCAGATCATAATTCTGGACCCTTTGATCCCCAAAGCCTCCCTTGAGGCCCTGTTGGAGAACTTCACCAATCATTTATATACTTCAAGA 17760  
 TGCCTTGGGATAACTGGACAAAACAAAAAAACAACCTGTTTGAGAAGATAAAACAGTTGTATCTTTTTATGCTGTGCCTGCTAATTGAAAGAAGCCTCATAACTATGAATGTGAACCTGA 17880  
 CTAGGAATGGAACAAAGTTATCTATCATCAGCCTGGATCTAAAGAATCAGGACCCTGCATACTCTAGCTCCTG

A

ATTGTTCC  
Depth:2 (PIG)  
Ei-value:0.000, Pi-value:0.000  
Er-value:0.000, Pr-value:0.000  
No matches to TargetScan


TTGTTCC

TTGTTCC  
Depth:4 (DOG)  
Ei-value:0.000, Pi-value:0.000  
Er-value:0.000, Pr-value:0.000  
No matches to TargetScan

CTTCATATT

TG

TGCCAAATC  
Depth:3 (COW)  
Ei-value:0.000, Pi-value:0.000  
Er-value:0.000, Pr-value:0.000  
MATCHES To TargetScan▶ miR-182-5p:UUGGCAA▶ miR-96-5p/1271-5p:UUGGCAC


CCAAAT

CCAAAT  
Depth:6 (MOUSE)  
Ei-value:0.000, Pi-value:0.000  
Er-value:0.000, Pr-value:0.000  
No matches to TargetScan


C

CCAAATC  
Depth:5 (RABBIT)  
Ei-value:0.000, Pi-value:0.000  
Er-value:0.000, Pr-value:0.000  
No matches to TargetScan

GTTATCTTTCTAAG

AAGCAGT

AAGCAGTG  
Depth:2 (PIG)  
Ei-value:0.000, Pi-value:0.000  
Er-value:0.000, Pr-value:0.000  
No matches to TargetScan

 18000  


G

AAGCAGTG  
Depth:2 (PIG)  
Ei-value:0.000, Pi-value:0.000  
Er-value:0.000, Pr-value:0.000  
No matches to TargetScan

T

AGAGAG

AGAGAG  
Depth:2 (PIG)  
Ei-value:0.000, Pi-value:0.010  
Er-value:0.000, Pr-value:0.000  
No matches to TargetScan


CAAGAAA

CAAGAAA  
Depth:5 (RABBIT)  
Ei-value:0.000, Pi-value:0.000  
Er-value:0.000, Pr-value:0.000  
No matches to TargetScan


T

CAAGAAAT  
Depth:3 (COW)  
Ei-value:0.000, Pi-value:0.000  
Er-value:0.000, Pr-value:0.000  
No matches to TargetScan


T

CAAGAAATTTGAACACAC  
Depth:2 (PIG)  
Ei-value:0.000, Pi-value:0.000  
Er-value:0.000, Pr-value:0.000  
No matches to TargetScan


TGAACACAC

TGAACACAC  
Depth:3 (COW)  
Ei-value:0.000, Pi-value:0.000  
Er-value:0.000, Pr-value:0.000  
No matches to TargetScan

CAAAG

G

GAAGATCAACATGCCTG  
Depth:4 (DOG)  
Ei-value:0.000, Pi-value:0.000  
Er-value:0.000, Pr-value:0.000  
No matches to TargetScan


AA

AAGATCAACATGC  
Depth:5 (RABBIT)  
Ei-value:0.000, Pi-value:0.000  
Er-value:0.000, Pr-value:0.000  
No matches to TargetScan


GATCAACATGC

GATCAACATGC  
Depth:6 (MOUSE)  
Ei-value:0.000, Pi-value:0.000  
Er-value:0.000, Pr-value:0.000  
No matches to TargetScan


CTG

GAAGATCAACATGCCTG  
Depth:4 (DOG)  
Ei-value:0.000, Pi-value:0.000  
Er-value:0.000, Pr-value:0.000  
No matches to TargetScan


GC

GAAGATCAACATGCCTGGC  
Depth:2 (PIG)  
Ei-value:0.000, Pi-value:0.000  
Er-value:0.000, Pr-value:0.000  
No matches to TargetScan

GGTCTGGCATTTTAAAACAGGA

TGAATGA

TGAATGA  
Depth:2 (PIG)  
Ei-value:0.000, Pi-value:0.010  
Er-value:0.000, Pr-value:0.020  
MATCHES To TargetScan▶ miR-1298-5p:UCAUUCG

TTGTCTGCCTTTGCTTATCTTAAACTCTTAAG

TGTGTAT

TGTGTAT  
Depth:6 (MOUSE)  
Ei-value:0.000, Pi-value:0.000  
Er-value:0.000, Pr-value:0.000  
No matches to TargetScan


TT

TGTGTATTT  
Depth:4 (DOG)  
Ei-value:0.000, Pi-value:0.000  
Er-value:0.000, Pr-value:0.000  
No matches to TargetScan

 18120  


TGTGTATTT  
Depth:4 (DOG)  
Ei-value:0.000, Pi-value:0.000  
Er-value:0.000, Pr-value:0.000  
No matches to TargetScan

GTTTGC

TTGTC

TTGTCTCTTTCTTTCTT  
Depth:2 (PIG)  
Ei-value:0.000, Pi-value:0.000  
Er-value:0.000, Pr-value:0.000  
MATCHES To TargetScan▶ miR-186-5p:AAAGAAU


TCTTTCTT

TCTTTCTT  
Depth:3 (COW)  
Ei-value:0.000, Pi-value:0.000  
Er-value:0.000, Pr-value:0.000  
No matches to TargetScan


TCTT

TTGTCTCTTTCTTTCTT  
Depth:2 (PIG)  
Ei-value:0.000, Pi-value:0.000  
Er-value:0.000, Pr-value:0.000  
MATCHES To TargetScan▶ miR-186-5p:AAAGAAU

CCTTGCGCATCTTTGGTCTTT

TTCTCTA

TTCTCTA  
Depth:2 (PIG)  
Ei-value:0.000, Pi-value:0.010  
Er-value:0.000, Pr-value:0.000  
No matches to TargetScan

AAT

TG

TGTGTCTTACCCATTTCCATG  
Depth:2 (PIG)  
Ei-value:0.000, Pi-value:0.000  
Er-value:0.000, Pr-value:0.000  
MATCHES To TargetScan▶ miR-203a-3p.1:GAAAUGU▶ miR-208-3p:UAAGACG▶ miR-499a-5p:UAAGACU


TGTCTTA

TGTCTTA  
Depth:4 (DOG)  
Ei-value:0.000, Pi-value:0.000  
Er-value:0.000, Pr-value:0.000  
MATCHES To TargetScan▶ miR-208-3p:UAAGACG▶ miR-499a-5p:UAAGACU


CCCATTTCCATG

TGTCTTACCCATTTCCATG  
Depth:3 (COW)  
Ei-value:0.000, Pi-value:0.000  
Er-value:0.000, Pr-value:0.000  
MATCHES To TargetScan▶ miR-203a-3p.1:GAAAUGU▶ miR-208-3p:UAAGACG▶ miR-499a-5p:UAAGACU

ATTCTTTTGCTAGTTTCTTCTCAGTATATCTTTGTCTCCT

TTTTT

TTTTTGT  
Depth:4 (DOG)  
Ei-value:0.000, Pi-value:0.000  
Er-value:0.000, Pr-value:0.000  
No matches to TargetScan

 18240  


GT

TTTTTGT  
Depth:4 (DOG)  
Ei-value:0.000, Pi-value:0.000  
Er-value:0.000, Pr-value:0.000  
No matches to TargetScan

ACCTGAGTGTGC

GGTCTGTGTCT

GGTCTGTGTCT  
Depth:2 (PIG)  
Ei-value:0.000, Pi-value:0.000  
Er-value:0.000, Pr-value:0.000  
No matches to TargetScan

C

GTCTTAGA

GTCTTAGA  
Depth:2 (PIG)  
Ei-value:0.000, Pi-value:0.000  
Er-value:0.000, Pr-value:0.000  
MATCHES To TargetScan▶ miR-208-3p:UAAGACG▶ miR-499a-5p:UAAGACU

TGTCTCTCTCTAGTTTCTT

TTT

TTTTTCATTTTGTT  
Depth:2 (PIG)  
Ei-value:0.000, Pi-value:0.000  
Er-value:0.000, Pr-value:0.000  
MATCHES To TargetScan▶ miR-495-3p:AACAAAC


TTCATTTTGTT

TTCATTTTGTT  
Depth:4 (DOG)  
Ei-value:0.000, Pi-value:0.000  
Er-value:0.000, Pr-value:0.000  
MATCHES To TargetScan▶ miR-495-3p:AACAAAC

ATTGATTCTCCTTGGTCTCCTAGATCTGGCTCTTTCACTGTTGTTCACTTTGT 18360  
 GTCTCTTGAGTCACATACTATGTG

CTC

CTCTTTGCTC  
Depth:2 (PIG)  
Ei-value:0.000, Pi-value:0.000  
Er-value:0.000, Pr-value:0.000  
No matches to TargetScan


TTTGCTC

TTTGCTC  
Depth:3 (COW)  
Ei-value:0.000, Pi-value:0.000  
Er-value:0.000, Pr-value:0.000  
No matches to TargetScan

A

TTTCTTGTT

TTTCTTGTT  
Depth:2 (PIG)  
Ei-value:0.000, Pi-value:0.000  
Er-value:0.000, Pr-value:0.000  
No matches to TargetScan

A

TGCCTACCT

TGCCTACCT  
Depth:2 (PIG)  
Ei-value:0.000, Pi-value:0.000  
Er-value:0.000, Pr-value:0.000  
MATCHES To TargetScan▶ miR-196-5p:AGGUAGU

TTCTTCT

TT

TTTTCTCTTTGTGAA  
Depth:3 (COW)  
Ei-value:0.000, Pi-value:0.000  
Er-value:0.000, Pr-value:0.000  
No matches to TargetScan


TTCTCTTTG

TTCTCTTTG  
Depth:6 (MOUSE)  
Ei-value:0.000, Pi-value:0.000  
Er-value:0.000, Pr-value:0.000  
No matches to TargetScan


TGAA

TTTTCTCTTTGTGAA  
Depth:3 (COW)  
Ei-value:0.000, Pi-value:0.000  
Er-value:0.000, Pr-value:0.000  
No matches to TargetScan

TTCTGATTGTCTGTTATCCA

TTCCCCTT

TTCCCCTT  
Depth:3 (COW)  
Ei-value:0.000, Pi-value:0.000  
Er-value:0.000, Pr-value:0.000  
No matches to TargetScan


CT

TTCCCCTTCT  
Depth:2 (PIG)  
Ei-value:0.000, Pi-value:0.000  
Er-value:0.000, Pr-value:0.000  
No matches to TargetScan

T

GTTCGTTT

GTTCGTTT  
Depth:2 (PIG)  
Ei-value:0.000, Pi-value:0.000  
Er-value:0.000, Pr-value:0.000  
No matches to TargetScan

GAC

AT

ATTTCACCT  
Depth:4 (DOG)  
Ei-value:0.000, Pi-value:0.000  
Er-value:0.000, Pr-value:0.000  
MATCHES To TargetScan▶ miR-203a-3p.2:UGAAAUG

 18480  


TTCACCT

ATTTCACCT  
Depth:4 (DOG)  
Ei-value:0.000, Pi-value:0.000  
Er-value:0.000, Pr-value:0.000  
MATCHES To TargetScan▶ miR-203a-3p.2:UGAAAUG

AGTCTAATACTGGCTACCCTTT

TGCTG

TGCTGTTTCTACT  
Depth:3 (COW)  
Ei-value:0.000, Pi-value:0.000  
Er-value:0.000, Pr-value:0.000  
MATCHES To TargetScan▶ miR-411-5p.1:AGUAGAC▶ miR-494-3p:GAAACAU


TTTCTAC

TTTCTAC  
Depth:6 (MOUSE)  
Ei-value:0.000, Pi-value:0.000  
Er-value:0.000, Pr-value:0.000  
No matches to TargetScan


T

TTTCTACT  
Depth:5 (RABBIT)  
Ei-value:0.000, Pi-value:0.000  
Er-value:0.000, Pr-value:0.000  
MATCHES To TargetScan▶ miR-411-5p.1:AGUAGAC

CCTG

ATCTCAC

ATCTCACATTTCTC  
Depth:2 (PIG)  
Ei-value:0.000, Pi-value:0.000  
Er-value:0.000, Pr-value:0.000  
MATCHES To TargetScan▶ miR-203a-3p.1:GAAAUGU


ATTTCTC

ATTTCTC  
Depth:6 (MOUSE)  
Ei-value:0.000, Pi-value:0.000  
Er-value:0.000, Pr-value:0.000  
No matches to TargetScan

TTTTCACATATTCTTTT

TGCCTC

TGCCTCTCTTGGGC  
Depth:2 (PIG)  
Ei-value:0.000, Pi-value:0.000  
Er-value:0.000, Pr-value:0.000  
MATCHES To TargetScan▶ miR-335-5p:CAAGAGC


TCTTGGG

TCTTGGG  
Depth:5 (RABBIT)  
Ei-value:0.000, Pi-value:0.000  
Er-value:0.000, Pr-value:0.000  
No matches to TargetScan


C

TCTTGGGC  
Depth:3 (COW)  
Ei-value:0.000, Pi-value:0.000  
Er-value:0.000, Pr-value:0.000  
No matches to TargetScan

CATTTTCTCTTTTTCGTGCTTTGTATGCT 18600  
 TCTGTGTCTCTTTGTGC

TTTGTGA

TTTGTGA  
Depth:4 (DOG)  
Ei-value:0.000, Pi-value:0.010  
Er-value:0.000, Pr-value:0.000  
No matches to TargetScan


TTTTC

TTTGTGATTTTC  
Depth:3 (COW)  
Ei-value:0.000, Pi-value:0.000  
Er-value:0.000, Pr-value:0.000  
No matches to TargetScan

CATTTCAACATCCATC

TCTCTGTT

TCTCTGTT  
Depth:4 (DOG)  
Ei-value:0.000, Pi-value:0.000  
Er-value:0.000, Pr-value:0.000  
No matches to TargetScan

CTCTTATTTCTTCTCTACTTTTGACTTCCTAT

TCACC

TCACCTTTGAGTATTT  
Depth:2 (PIG)  
Ei-value:0.000, Pi-value:0.000  
Er-value:0.000, Pr-value:0.000  
MATCHES To TargetScan▶ miR-18-5p:AAGGUGC▶ miR-200bc-3p/429:AAUACUG▶ miR-371-5p:CUCAAAC


TTTGAGTATTT

TTTGAGTATTT  
Depth:4 (DOG)  
Ei-value:0.000, Pi-value:0.000  
Er-value:0.000, Pr-value:0.000  
MATCHES To TargetScan▶ miR-200bc-3p/429:AAUACUG▶ miR-371-5p:CUCAAAC

TG

GCCTCTTC

GCCTCTTC  
Depth:2 (PIG)  
Ei-value:0.000, Pi-value:0.000  
Er-value:0.000, Pr-value:0.000  
No matches to TargetScan

TTGTGTCTA 18720  
 TGTCTCCC

CTTTGATT

CTTTGATT  
Depth:3 (COW)  
Ei-value:0.000, Pi-value:0.000  
Er-value:0.000, Pr-value:0.000  
No matches to TargetScan

ACATGCAATTCTCTCGCCTTGCACATTTTCTATGTA

TGTGTGTG

TGTGTGTG  
Depth:4 (DOG)  
Ei-value:0.000, Pi-value:0.000  
Er-value:0.000, Pr-value:0.000  
MATCHES To TargetScan▶ miR-329-3p/362-3p:ACACACC

GGCTCTTGTGTGTTTGTAACAA

AGGGGCT

AGGGGCTTCCTAACCCCT  
Depth:2 (PIG)  
Ei-value:0.000, Pi-value:0.000  
Er-value:0.000, Pr-value:0.000  
No matches to TargetScan


TCCTAACCCCT

TCCTAACCCCT  
Depth:5 (RABBIT)  
Ei-value:0.000, Pi-value:0.000  
Er-value:0.000, Pr-value:0.000  
No matches to TargetScan

TCTCAG

TAGGTGCA

TAGGTGCA  
Depth:3 (COW)  
Ei-value:0.000, Pi-value:0.000  
Er-value:0.000, Pr-value:0.000  
No matches to TargetScan

AGAGTG 18840  
 TCAGCTTACCAAAAT

AAGCATTG

AAGCATTG  
Depth:4 (DOG)  
Ei-value:0.000, Pi-value:0.000  
Er-value:0.000, Pr-value:0.000  
No matches to TargetScan

CAGAGCT

GTTCC

GTTCCTTATGCCAG  
Depth:2 (PIG)  
Ei-value:0.000, Pi-value:0.000  
Er-value:0.000, Pr-value:0.000  
No matches to TargetScan


TTATGCCA

TTATGCCA  
Depth:5 (RABBIT)  
Ei-value:0.000, Pi-value:0.000  
Er-value:0.000, Pr-value:0.000  
No matches to TargetScan


G

TTATGCCAG  
Depth:4 (DOG)  
Ei-value:0.000, Pi-value:0.000  
Er-value:0.000, Pr-value:0.000  
No matches to TargetScan

ACCATGCTGTGAGATGCTGTGAGATTGTGCTGTGAA

ATGA

ATGATCCAAGACCAA  
Depth:2 (PIG)  
Ei-value:0.000, Pi-value:0.000  
Er-value:0.000, Pr-value:0.000  
MATCHES To TargetScan▶ miR-133a-3p.2/133b:UUGGUCC▶ miR-431-5p:GUCUUGC


TCCAAG

TCCAAG  
Depth:3 (COW)  
Ei-value:0.000, Pi-value:0.000  
Er-value:0.000, Pr-value:0.000  
No matches to TargetScan


ACCAA

ATGATCCAAGACCAA  
Depth:2 (PIG)  
Ei-value:0.000, Pi-value:0.000  
Er-value:0.000, Pr-value:0.000  
MATCHES To TargetScan▶ miR-133a-3p.2/133b:UUGGUCC▶ miR-431-5p:GUCUUGC


T

TAGAAGGCCCAA  
Depth:2 (PIG)  
Ei-value:0.000, Pi-value:0.000  
Er-value:0.000, Pr-value:0.000  
No matches to TargetScan


AGA

AGAAGGCCCAA  
Depth:4 (DOG)  
Ei-value:0.000, Pi-value:0.000  
Er-value:0.000, Pr-value:0.000  
No matches to TargetScan


AGGCCCAA

AGGCCCAA  
Depth:5 (RABBIT)  
Ei-value:0.000, Pi-value:0.000  
Er-value:0.000, Pr-value:0.000  
No matches to TargetScan

GATCTGGACTG

GA

GAGTTGGATGGAAG  
Depth:2 (PIG)  
Ei-value:0.000, Pi-value:0.000  
Er-value:0.000, Pr-value:0.000  
No matches to TargetScan

 18960  


GTTGGATGGAAG

GAGTTGGATGGAAG  
Depth:2 (PIG)  
Ei-value:0.000, Pi-value:0.000  
Er-value:0.000, Pr-value:0.000  
No matches to TargetScan

GCTA

AAGTCT

AAGTCT  
Depth:2 (PIG)  
Ei-value:0.000, Pi-value:0.010  
Er-value:0.000, Pr-value:0.010  
No matches to TargetScan

CAGTGAAACTG

AAGGCCAA

AAGGCCAA  
Depth:2 (PIG)  
Ei-value:0.000, Pi-value:0.000  
Er-value:0.000, Pr-value:0.000  
No matches to TargetScan

A

GACCTAAGA

GACCTAAGA  
Depth:2 (PIG)  
Ei-value:0.000, Pi-value:0.000  
Er-value:0.000, Pr-value:0.000  
No matches to TargetScan

CTCAAGCCTGTGTATATGGACA

GAAGGCCC

GAAGGCCC  
Depth:2 (PIG)  
Ei-value:0.000, Pi-value:0.000  
Er-value:0.000, Pr-value:0.000  
No matches to TargetScan

AAGAGAGACAGA

TATC

TATCTCAAGACTAA  
Depth:2 (PIG)  
Ei-value:0.000, Pi-value:0.000  
Er-value:0.000, Pr-value:0.000  
MATCHES To TargetScan▶ miR-431-5p:GUCUUGC


TCAA

TCAAGACTAA  
Depth:4 (DOG)  
Ei-value:0.000, Pi-value:0.000  
Er-value:0.000, Pr-value:0.000  
MATCHES To TargetScan▶ miR-431-5p:GUCUUGC


GACTAA

GACTAA  
Depth:5 (RABBIT)  
Ei-value:0.000, Pi-value:0.000  
Er-value:0.000, Pr-value:0.000  
No matches to TargetScan

ATTAAGTGGGAAG 19080  
 CTCAAGGTCCATGGCCCA

GAATCTGG

GAATCTGG  
Depth:2 (PIG)  
Ei-value:0.000, Pi-value:0.000  
Er-value:0.000, Pr-value:0.000  
No matches to TargetScan

GAAT

GAT

GATAGAAGC  
Depth:2 (PIG)  
Ei-value:0.000, Pi-value:0.000  
Er-value:0.000, Pr-value:0.000  
No matches to TargetScan


AGAAGC

AGAAGC  
Depth:4 (DOG)  
Ei-value:0.000, Pi-value:0.000  
Er-value:0.000, Pr-value:0.010  
No matches to TargetScan

ACCAAGACCAA

GGGAAAT

GGGAAAT  
Depth:2 (PIG)  
Ei-value:0.000, Pi-value:0.000  
Er-value:0.000, Pr-value:0.010  
No matches to TargetScan

TA

C

CAAGATGA  
Depth:3 (COW)  
Ei-value:0.000, Pi-value:0.000  
Er-value:0.000, Pr-value:0.000  
No matches to TargetScan


AAGATGA

AAGATGA  
Depth:5 (RABBIT)  
Ei-value:0.000, Pi-value:0.000  
Er-value:0.000, Pr-value:0.000  
No matches to TargetScan

T

AACCCTAAA

AACCCTAAA  
Depth:2 (PIG)  
Ei-value:0.000, Pi-value:0.000  
Er-value:0.000, Pr-value:0.000  
MATCHES To TargetScan▶ miR-296-3p:AGGGUUG

TCCCAG

CTCT

CTCTTTTCTATTGTT  
Depth:2 (PIG)  
Ei-value:0.000, Pi-value:0.000  
Er-value:0.000, Pr-value:0.000  
No matches to TargetScan


TTTCTATTG

TTTCTATTG  
Depth:3 (COW)  
Ei-value:0.000, Pi-value:0.000  
Er-value:0.000, Pr-value:0.000  
No matches to TargetScan


TT

CTCTTTTCTATTGTT  
Depth:2 (PIG)  
Ei-value:0.000, Pi-value:0.000  
Er-value:0.000, Pr-value:0.000  
No matches to TargetScan

CTCCTCCCTACTCTTGGATATT 19200  
 TT

C

CACTTCTT  
Depth:2 (PIG)  
Ei-value:0.000, Pi-value:0.010  
Er-value:0.000, Pr-value:0.000  
No matches to TargetScan


ACTTCTT

ACTTCTT  
Depth:3 (COW)  
Ei-value:0.000, Pi-value:0.020  
Er-value:0.000, Pr-value:0.000  
No matches to TargetScan

CCTT

TCCTGTT

TCCTGTT  
Depth:2 (PIG)  
Ei-value:0.000, Pi-value:0.020  
Er-value:0.000, Pr-value:0.000  
No matches to TargetScan

CTTATACCACCATTTAAACCCACTTTTGTGATGTT

CTTTTTGATGTT

CTTTTTGATGTT  
Depth:4 (DOG)  
Ei-value:0.000, Pi-value:0.000  
Er-value:0.000, Pr-value:0.000  
No matches to TargetScan


GC

CTTTTTGATGTTGC  
Depth:2 (PIG)  
Ei-value:0.000, Pi-value:0.000  
Er-value:0.000, Pr-value:0.000  
No matches to TargetScan

T

GTTACCTT

GTTACCTT  
Depth:2 (PIG)  
Ei-value:0.000, Pi-value:0.000  
Er-value:0.000, Pr-value:0.010  
No matches to TargetScan

AAAGTTAAAGAAACATATCTTCAAATCA

ACAG

ACAGTATTATGCCTGGGCCAGTCTT  
Depth:2 (PIG)  
Ei-value:0.000, Pi-value:0.000  
Er-value:0.000, Pr-value:0.000  
MATCHES To TargetScan▶ miR-193-3p:ACUGGCC▶ miR-200bc-3p/429:AAUACUG▶ miR-328-3p:UGGCCCU▶ miR-369-3p:AUAAUAC▶ miR-655-3p:UAAUACA


TATTATGC

TATTATGC  
Depth:4 (DOG)  
Ei-value:0.000, Pi-value:0.000  
Er-value:0.000, Pr-value:0.000  
MATCHES To TargetScan▶ miR-369-3p:AUAAUAC


C

ACAGTATTATGCCTGGGCCAGTCTT  
Depth:2 (PIG)  
Ei-value:0.000, Pi-value:0.000  
Er-value:0.000, Pr-value:0.000  
MATCHES To TargetScan▶ miR-193-3p:ACUGGCC▶ miR-200bc-3p/429:AAUACUG▶ miR-328-3p:UGGCCCU▶ miR-369-3p:AUAAUAC▶ miR-655-3p:UAAUACA

 19320  


TGGGCCAGTCTT

ACAGTATTATGCCTGGGCCAGTCTT  
Depth:2 (PIG)  
Ei-value:0.000, Pi-value:0.000  
Er-value:0.000, Pr-value:0.000  
MATCHES To TargetScan▶ miR-193-3p:ACUGGCC▶ miR-200bc-3p/429:AAUACUG▶ miR-328-3p:UGGCCCU▶ miR-369-3p:AUAAUAC▶ miR-655-3p:UAAUACA

AAACCAGTTTTTT

TAAACTTC

TAAACTTC  
Depth:3 (COW)  
Ei-value:0.000, Pi-value:0.000  
Er-value:0.000, Pr-value:0.000  
No matches to TargetScan

CATTTCTCTTCACCTCCA

CTCCACTTGAGAG

CTCCACTTGAGAG  
Depth:3 (COW)  
Ei-value:0.000, Pi-value:0.000  
Er-value:0.000, Pr-value:0.000  
MATCHES To TargetScan▶ miR-26-5p:UCAAGUA


A

CTCCACTTGAGAGA  
Depth:2 (PIG)  
Ei-value:0.000, Pi-value:0.000  
Er-value:0.000, Pr-value:0.000  
MATCHES To TargetScan▶ miR-26-5p:UCAAGUA

CAC

ATAGGTGA

ATAGGTGA  
Depth:2 (PIG)  
Ei-value:0.000, Pi-value:0.000  
Er-value:0.000, Pr-value:0.010  
No matches to TargetScan

CATTA

TATTTCAGT

TATTTCAGT  
Depth:4 (DOG)  
Ei-value:0.000, Pi-value:0.000  
Er-value:0.000, Pr-value:0.000  
MATCHES To TargetScan▶ miR-203a-3p.2:UGAAAUG


CC

TATTTCAGTCC  
Depth:3 (COW)  
Ei-value:0.000, Pi-value:0.000  
Er-value:0.000, Pr-value:0.000  
MATCHES To TargetScan▶ miR-203a-3p.2:UGAAAUG


T

TATTTCAGTCCT  
Depth:2 (PIG)  
Ei-value:0.000, Pi-value:0.000  
Er-value:0.000, Pr-value:0.000  
MATCHES To TargetScan▶ miR-203a-3p.2:UGAAAUG

CTCTTTTCCTCAGATACTCTAGGCTAA 19440  


TGAGAAGA

TGAGAAGA  
Depth:2 (PIG)  
Ei-value:0.000, Pi-value:0.000  
Er-value:0.000, Pr-value:0.000  
No matches to TargetScan

GGAAAGTATCATGCTGTTGAG

GGGGAAA

GGGGAAA  
Depth:4 (DOG)  
Ei-value:0.000, Pi-value:0.000  
Er-value:0.000, Pr-value:0.000  
No matches to TargetScan


AAA

GGGGAAAAAA  
Depth:2 (PIG)  
Ei-value:0.000, Pi-value:0.000  
Er-value:0.000, Pr-value:0.000  
No matches to TargetScan

T

GTGCCAGGCT

GTGCCAGGCT  
Depth:2 (PIG)  
Ei-value:0.000, Pi-value:0.000  
Er-value:0.000, Pr-value:0.000  
MATCHES To TargetScan▶ miR-183-5p.2:UGGCACU

A

TCTAGAGAAAA

TCTAGAGAAAA  
Depth:6 (MOUSE)  
Ei-value:0.000, Pi-value:0.000  
Er-value:0.000, Pr-value:0.000  
MATCHES To TargetScan▶ miR-1251-5p:CUCUAGC

TA

TGAAGAGATG

TGAAGAGATG  
Depth:5 (RABBIT)  
Ei-value:0.000, Pi-value:0.000  
Er-value:0.000, Pr-value:0.000  
No matches to TargetScan


CTCCA

TGAAGAGATGCTCCA  
Depth:3 (COW)  
Ei-value:0.000, Pi-value:0.000  
Er-value:0.000, Pr-value:0.000  
No matches to TargetScan


GGCCAA

GGCCAATGAGAAGAATTAGACA  
Depth:4 (DOG)  
Ei-value:0.000, Pi-value:0.000  
Er-value:0.000, Pr-value:0.000  
No matches to TargetScan


TGAGAAGAATTAGACA

TGAGAAGAATTAGACA  
Depth:6 (MOUSE)  
Ei-value:0.000, Pi-value:0.000  
Er-value:0.000, Pr-value:0.000  
No matches to TargetScan

G

GAAATACACAGATG

GAAATACACAGATG  
Depth:3 (COW)  
Ei-value:0.000, Pi-value:0.000  
Er-value:0.000, Pr-value:0.000  
No matches to TargetScan

CGCC 19560  
 AGCCTG

C

CTGAGAAG  
Depth:3 (COW)  
Ei-value:0.000, Pi-value:0.000  
Er-value:0.000, Pr-value:0.000  
No matches to TargetScan


TGAGAAG

TGAGAAG  
Depth:4 (DOG)  
Ei-value:0.000, Pi-value:0.000  
Er-value:0.000, Pr-value:0.010  
No matches to TargetScan


CA

CTGAGAAGCA  
Depth:2 (PIG)  
Ei-value:0.000, Pi-value:0.000  
Er-value:0.000, Pr-value:0.000  
No matches to TargetScan

TCA

GCCA

GCCAGCAACA  
Depth:3 (COW)  
Ei-value:0.000, Pi-value:0.000  
Er-value:0.000, Pr-value:0.000  
No matches to TargetScan


GCAACA

GCAACA  
Depth:6 (MOUSE)  
Ei-value:0.000, Pi-value:0.000  
Er-value:0.000, Pr-value:0.000  
No matches to TargetScan

CCTTACTC

C

CTTTGAGCTTAGGTGAGCAGGATTC  
Depth:2 (PIG)  
Ei-value:0.000, Pi-value:0.000  
Er-value:0.000, Pr-value:0.000  
MATCHES To TargetScan▶ miR-371-5p:CUCAAAC


TTTGAGCTT

TTTGAGCTT  
Depth:3 (COW)  
Ei-value:0.000, Pi-value:0.000  
Er-value:0.000, Pr-value:0.000  
MATCHES To TargetScan▶ miR-371-5p:CUCAAAC


A

CTTTGAGCTTAGGTGAGCAGGATTC  
Depth:2 (PIG)  
Ei-value:0.000, Pi-value:0.000  
Er-value:0.000, Pr-value:0.000  
MATCHES To TargetScan▶ miR-371-5p:CUCAAAC


GGTGAGC

GGTGAGC  
Depth:4 (DOG)  
Ei-value:0.000, Pi-value:0.000  
Er-value:0.000, Pr-value:0.000  
No matches to TargetScan


AGGAT

GGTGAGCAGGAT  
Depth:3 (COW)  
Ei-value:0.000, Pi-value:0.000  
Er-value:0.000, Pr-value:0.000  
No matches to TargetScan


TC

CTTTGAGCTTAGGTGAGCAGGATTC  
Depth:2 (PIG)  
Ei-value:0.000, Pi-value:0.000  
Er-value:0.000, Pr-value:0.000  
MATCHES To TargetScan▶ miR-371-5p:CUCAAAC

CTGA

GGTTTGGG

GGTTTGGG  
Depth:4 (DOG)  
Ei-value:0.000, Pi-value:0.000  
Er-value:0.000, Pr-value:0.000  
No matches to TargetScan

TA

CTAGTGA

CTAGTGATGGTTATG  
Depth:2 (PIG)  
Ei-value:0.000, Pi-value:0.000  
Er-value:0.000, Pr-value:0.000  
No matches to TargetScan


TGGTTA

TGGTTA  
Depth:5 (RABBIT)  
Ei-value:0.000, Pi-value:0.000  
Er-value:0.000, Pr-value:0.000  
No matches to TargetScan


T

TGGTTAT  
Depth:4 (DOG)  
Ei-value:0.000, Pi-value:0.000  
Er-value:0.000, Pr-value:0.000  
No matches to TargetScan


G

TGGTTATG  
Depth:3 (COW)  
Ei-value:0.000, Pi-value:0.000  
Er-value:0.000, Pr-value:0.000  
No matches to TargetScan

AAAAAGGAAATTGGGT

CTGGGACA

CTGGGACA  
Depth:2 (PIG)  
Ei-value:0.000, Pi-value:0.000  
Er-value:0.000, Pr-value:0.000  
No matches to TargetScan

GAGTG 19680  
 AGG

GAGGT

GAGGTCCCAAGG  
Depth:2 (PIG)  
Ei-value:0.000, Pi-value:0.000  
Er-value:0.000, Pr-value:0.000  
MATCHES To TargetScan▶ miR-212-5p:CCUUGGC


CCCAAGG

CCCAAGG  
Depth:4 (DOG)  
Ei-value:0.000, Pi-value:0.000  
Er-value:0.000, Pr-value:0.000  
MATCHES To TargetScan▶ miR-212-5p:CCUUGGC

AGAT

AGCC

AGCCTGAACTCCCTGCTCATAGTAGTGGCC  
Depth:2 (PIG)  
Ei-value:0.000, Pi-value:0.000  
Er-value:0.000, Pr-value:0.000  
No matches to TargetScan


TGAACTCCCTGCT

TGAACTCCCTGCT  
Depth:4 (DOG)  
Ei-value:0.000, Pi-value:0.000  
Er-value:0.000, Pr-value:0.000  
No matches to TargetScan


C

TGAACTCCCTGCTCATAGTAGTGGCC  
Depth:3 (COW)  
Ei-value:0.000, Pi-value:0.000  
Er-value:0.000, Pr-value:0.000  
No matches to TargetScan


ATAGTAGTGGCC

ATAGTAGTGGCC  
Depth:4 (DOG)  
Ei-value:0.000, Pi-value:0.000  
Er-value:0.000, Pr-value:0.000  
No matches to TargetScan

T

AATAATTTGG

AATAATTTGG  
Depth:2 (PIG)  
Ei-value:0.000, Pi-value:0.000  
Er-value:0.000, Pr-value:0.000  
No matches to TargetScan

CAAACTGCACTAACCTTGCGCCTGGT

TTTAATAC

TTTAATAC  
Depth:4 (DOG)  
Ei-value:0.000, Pi-value:0.000  
Er-value:0.000, Pr-value:0.000  
MATCHES To TargetScan▶ miR-496.2:GUAUUAC


CCA

TTTAATACCCA  
Depth:2 (PIG)  
Ei-value:0.000, Pi-value:0.000  
Er-value:0.000, Pr-value:0.000  
MATCHES To TargetScan▶ miR-496.2:GUAUUAC

C

CT

CTCTAGGCTTAAAG  
Depth:2 (PIG)  
Ei-value:0.000, Pi-value:0.000  
Er-value:0.000, Pr-value:0.000  
No matches to TargetScan


CT

CTAGGCTTAAAG  
Depth:4 (DOG)  
Ei-value:0.000, Pi-value:0.000  
Er-value:0.000, Pr-value:0.000  
No matches to TargetScan


AGGCTTA

AGGCTTA  
Depth:5 (RABBIT)  
Ei-value:0.000, Pi-value:0.000  
Er-value:0.000, Pr-value:0.000  
No matches to TargetScan


AAG

CTAGGCTTAAAG  
Depth:4 (DOG)  
Ei-value:0.000, Pi-value:0.000  
Er-value:0.000, Pr-value:0.000  
No matches to TargetScan

TTGAAAGA 19800  
 ACTTACGCGTATTTAGCAG

GTTTAAT

GTTTAAT  
Depth:5 (RABBIT)  
Ei-value:0.000, Pi-value:0.000  
Er-value:0.000, Pr-value:0.000  
No matches to TargetScan


ACTTTCCTT

GTTTAATACTTTCCTT  
Depth:2 (PIG)  
Ei-value:0.000, Pi-value:0.000  
Er-value:0.000, Pr-value:0.000  
MATCHES To TargetScan▶ miR-496.2:GUAUUAC

CATTTCTTTCCTCTTACCATGA

GGGAAG

GGGAAG  
Depth:2 (PIG)  
Ei-value:0.000, Pi-value:0.020  
Er-value:0.000, Pr-value:0.020  
No matches to TargetScan

ATA

ATTTAAATGA

ATTTAAATGA  
Depth:2 (PIG)  
Ei-value:0.000, Pi-value:0.000  
Er-value:0.000, Pr-value:0.000  
No matches to TargetScan

AAATATATGTGAAACCTT

TGTAAAACA

TGTAAAACA  
Depth:3 (COW)  
Ei-value:0.000, Pi-value:0.000  
Er-value:0.000, Pr-value:0.000  
No matches to TargetScan

CAAAAAAACAAAGCATT 19920  
 CTCATTAATAACA

T

TATTGGCA  
Depth:5 (RABBIT)  
Ei-value:0.000, Pi-value:0.000  
Er-value:0.000, Pr-value:0.000  
No matches to TargetScan


ATTGGCA

ATTGGCA  
Depth:6 (MOUSE)  
Ei-value:0.000, Pi-value:0.000  
Er-value:0.000, Pr-value:0.000  
No matches to TargetScan

TTTGTGCACAGCAAAGATTTGGAAATGTACTGTCAG

TTGTGAAG

TTGTGAAG  
Depth:6 (MOUSE)  
Ei-value:0.000, Pi-value:0.000  
Er-value:0.000, Pr-value:0.000  
No matches to TargetScan

TAC

T

TATGTAAATCA  
Depth:3 (COW)  
Ei-value:0.000, Pi-value:0.000  
Er-value:0.000, Pr-value:0.000  
No matches to TargetScan


ATGTAAAT

ATGTAAAT  
Depth:5 (RABBIT)  
Ei-value:0.000, Pi-value:0.000  
Er-value:0.000, Pr-value:0.000  
No matches to TargetScan


CA

TATGTAAATCA  
Depth:3 (COW)  
Ei-value:0.000, Pi-value:0.000  
Er-value:0.000, Pr-value:0.000  
No matches to TargetScan


GGGGTC

TATGTAAATCAGGGGTC  
Depth:2 (PIG)  
Ei-value:0.000, Pi-value:0.000  
Er-value:0.000, Pr-value:0.000  
MATCHES To TargetScan▶ miR-125-5p:CCCUGAG▶ miR-331-3p:CCCCUGG

ATCATATGC

TTTCTGTAA

TTTCTGTAA  
Depth:2 (PIG)  
Ei-value:0.000, Pi-value:0.000  
Er-value:0.000, Pr-value:0.000  
No matches to TargetScan

AAGATCAGAAGGTAAAT 20040  
 CTTGA

GG

GGGCCATATGGTTTC  
Depth:2 (PIG)  
Ei-value:0.000, Pi-value:0.000  
Er-value:0.000, Pr-value:0.000  
MATCHES To TargetScan▶ miR-328-3p:UGGCCCU


GCCATATGGT

GCCATATGGT  
Depth:3 (COW)  
Ei-value:0.000, Pi-value:0.000  
Er-value:0.000, Pr-value:0.000  
No matches to TargetScan


TTC

GGGCCATATGGTTTC  
Depth:2 (PIG)  
Ei-value:0.000, Pi-value:0.000  
Er-value:0.000, Pr-value:0.000  
MATCHES To TargetScan▶ miR-328-3p:UGGCCCU

CTGCTTTAAAAAAACAAGTGAATTTGGTCTGTGGGCCATAGTTTATTA

CCCCTGATGTA

CCCCTGATGTA  
Depth:2 (PIG)  
Ei-value:0.000, Pi-value:0.000  
Er-value:0.000, Pr-value:0.000  
No matches to TargetScan

ATTGCCAGGGACTGTG

TTCTGAA

TTCTGAA  
Depth:2 (PIG)  
Ei-value:0.000, Pi-value:0.020  
Er-value:0.000, Pr-value:0.020  
No matches to TargetScan

ATTAATTCCTTC

TC

TCTGTGCCTGTCCCTGT  
Depth:2 (PIG)  
Ei-value:0.000, Pi-value:0.000  
Er-value:0.000, Pr-value:0.000  
No matches to TargetScan


TGTG

TGTGCCTGTCCCTGT  
Depth:3 (COW)  
Ei-value:0.000, Pi-value:0.000  
Er-value:0.000, Pr-value:0.000  
No matches to TargetScan

 20160  


C

TGTGCCTGTCCCTGT  
Depth:3 (COW)  
Ei-value:0.000, Pi-value:0.000  
Er-value:0.000, Pr-value:0.000  
No matches to TargetScan


CTGTCCCT

CTGTCCCT  
Depth:4 (DOG)  
Ei-value:0.000, Pi-value:0.000  
Er-value:0.000, Pr-value:0.000  
No matches to TargetScan


GT

TGTGCCTGTCCCTGT  
Depth:3 (COW)  
Ei-value:0.000, Pi-value:0.000  
Er-value:0.000, Pr-value:0.000  
No matches to TargetScan

AT

TAGGCACT

TAGGCACT  
Depth:4 (DOG)  
Ei-value:0.000, Pi-value:0.000  
Er-value:0.000, Pr-value:0.000  
No matches to TargetScan


AA

TAGGCACTAA  
Depth:2 (PIG)  
Ei-value:0.000, Pi-value:0.000  
Er-value:0.000, Pr-value:0.000  
No matches to TargetScan

GGATGT

AATGATTA

AATGATTA  
Depth:2 (PIG)  
Ei-value:0.000, Pi-value:0.000  
Er-value:0.000, Pr-value:0.000  
MATCHES To TargetScan▶ miR-382-3p:AUCAUUC

CTTATGATC

ATATCTAGGTGA

ATATCTAGGTGA  
Depth:2 (PIG)  
Ei-value:0.000, Pi-value:0.000  
Er-value:0.000, Pr-value:0.000  
No matches to TargetScan

TCCAAAGAAAAGTATGTA

AATGTGCTTTGTAAACT

AATGTGCTTTGTAAACT  
Depth:2 (PIG)  
Ei-value:0.000, Pi-value:0.000  
Er-value:0.000, Pr-value:0.000  
MATCHES To TargetScan▶ miR-330-3p:CAAAGCA▶ miR-330-3p.2:AAAGCAC

A

TAAAGCA

TAAAGCA  
Depth:4 (DOG)  
Ei-value:0.000, Pi-value:0.000  
Er-value:0.000, Pr-value:0.000  
No matches to TargetScan


CTT

TAAAGCACTT  
Depth:2 (PIG)  
Ei-value:0.000, Pi-value:0.000  
Er-value:0.000, Pr-value:0.000  
MATCHES To TargetScan▶ miR-302-3p/372-3p/373-3p/520-3p:AAGUGCU▶ miR-302c-3p.2/520-3p:AGUGCUU

AAAGACTGGTAAATGA 20280  
 ACTCTGATTACATAGTATATACAAGCCAAGCAATGTATATACAGTGTTAGGCAC

TGTGGATACAAA

TGTGGATACAAA  
Depth:2 (PIG)  
Ei-value:0.000, Pi-value:0.000  
Er-value:0.000, Pr-value:0.000  
No matches to TargetScan

AACCTGAGGGATTTAAGATAATTGTGAACACT

TAT

TATAATGTGCCAGATA  
Depth:3 (COW)  
Ei-value:0.000, Pi-value:0.000  
Er-value:0.000, Pr-value:0.000  
MATCHES To TargetScan▶ miR-183-5p.2:UGGCACU▶ miR-323-3p:ACAUUAC


AATGTGCCAGATA

AATGTGCCAGATA  
Depth:4 (DOG)  
Ei-value:0.000, Pi-value:0.000  
Er-value:0.000, Pr-value:0.000  
MATCHES To TargetScan▶ miR-183-5p.2:UGGCACU

ACATTC 20400  
 TAGGCATTACAGATGTCAAAGGAAAAAACAAG

TTCTCAT

TTCTCAT  
Depth:2 (PIG)  
Ei-value:0.000, Pi-value:0.000  
Er-value:0.000, Pr-value:0.010  
No matches to TargetScan

TACAGAATTAAAATTTTTACATAGGAAGTA

CATA

CATATTAAAGTGCTTTGTA  
Depth:2 (PIG)  
Ei-value:0.000, Pi-value:0.000  
Er-value:0.000, Pr-value:0.000  
MATCHES To TargetScan▶ miR-330-3p:CAAAGCA▶ miR-330-3p.2:AAAGCAC


TTAAAGTG

TTAAAGTG  
Depth:4 (DOG)  
Ei-value:0.000, Pi-value:0.000  
Er-value:0.000, Pr-value:0.000  
No matches to TargetScan


CTTTGTA

TTAAAGTGCTTTGTA  
Depth:3 (COW)  
Ei-value:0.000, Pi-value:0.000  
Er-value:0.000, Pr-value:0.000  
MATCHES To TargetScan▶ miR-330-3p:CAAAGCA▶ miR-330-3p.2:AAAGCAC

GGAGTTCCCGTCGTGGCTCAGTGGTTAATGAA 20520  
 CACGACTGGCATCCATGAGGACACAGGTTCAATCCCTGGCCTCGCTCAGTGGGTTAAGGATCTGGTGTTGCCGTGAGCTGTGGTGTAGGTCGTAGATGCGGCTCAGATCCCTCGTTGTTG 20640  
 TGCCTGTGGCGTAGGCCAGCAGTTACAGCTCCAATTCAACCCCTAGCCTGGGAACCTCCATATGCTGCTGGTGCAGCCCTAAAAGACAAAAAGAAAAAAAAAAACAACAAAAAAAAATAA 20760  
 AGTGCTTTGTA

AA

AACTAAAGCA  
Depth:2 (PIG)  
Ei-value:0.000, Pi-value:0.000  
Er-value:0.000, Pr-value:0.000  
No matches to TargetScan


CTAAAGCA

CTAAAGCA  
Depth:4 (DOG)  
Ei-value:0.000, Pi-value:0.000  
Er-value:0.000, Pr-value:0.000  
No matches to TargetScan

CCACATAAGTAC

CAATGGGCTA

CAATGGGCTA  
Depth:3 (COW)  
Ei-value:0.000, Pi-value:0.000  
Er-value:0.000, Pr-value:0.000  
No matches to TargetScan

TATATGTTTAC

GA

GAATGAATA  
Depth:3 (COW)  
Ei-value:0.000, Pi-value:0.000  
Er-value:0.000, Pr-value:0.000  
MATCHES To TargetScan▶ miR-1298-5p:UCAUUCG


ATGAATA

ATGAATA  
Depth:4 (DOG)  
Ei-value:0.000, Pi-value:0.000  
Er-value:0.000, Pr-value:0.000  
No matches to TargetScan

AACAAATATTAATTGCCTTATATGTG

CCAGCTATT

CCAGCTATT  
Depth:3 (COW)  
Ei-value:0.000, Pi-value:0.000  
Er-value:0.000, Pr-value:0.000  
No matches to TargetScan

ATTTCA

GGTACTGT

GGTACTGT  
Depth:4 (DOG)  
Ei-value:0.000, Pi-value:0.000  
Er-value:0.000, Pr-value:0.000  
MATCHES To TargetScan▶ miR-101-3p.1:ACAGUAC▶ miR-144-3p:ACAGUAU

GAAATCTA 20880  
 AGATAATACTCCCAA

GTA

GTAATAAGAGG  
Depth:2 (PIG)  
Ei-value:0.000, Pi-value:0.000  
Er-value:0.000, Pr-value:0.000  
No matches to TargetScan


ATAAGAGG

ATAAGAGG  
Depth:4 (DOG)  
Ei-value:0.000, Pi-value:0.000  
Er-value:0.000, Pr-value:0.000  
No matches to TargetScan

GAGGTTTATGTCTTACACTACAGAGGTATAAGAATTAG

ATATTGCTTA

ATATTGCTTA  
Depth:2 (PIG)  
Ei-value:0.000, Pi-value:0.000  
Er-value:0.000, Pr-value:0.000  
No matches to TargetScan

TTTTAGT

CAATC

CAATCAAGACTTTAC  
Depth:2 (PIG)  
Ei-value:0.000, Pi-value:0.000  
Er-value:0.000, Pr-value:0.000  
MATCHES To TargetScan▶ miR-431-5p:GUCUUGC


AAGACTTTAC

AAGACTTTAC  
Depth:3 (COW)  
Ei-value:0.000, Pi-value:0.000  
Er-value:0.000, Pr-value:0.000  
No matches to TargetScan

CA

GTGAGGT

GTGAGGT  
Depth:2 (PIG)  
Ei-value:0.000, Pi-value:0.000  
Er-value:0.000, Pr-value:0.000  
No matches to TargetScan

CAGA

T

TTAAATTATTAC  
Depth:2 (PIG)  
Ei-value:0.000, Pi-value:0.000  
Er-value:0.000, Pr-value:0.000  
No matches to TargetScan


TAAATTAT

TAAATTAT  
Depth:4 (DOG)  
Ei-value:0.000, Pi-value:0.010  
Er-value:0.000, Pr-value:0.000  
No matches to TargetScan


TA

TAAATTATTAC  
Depth:3 (COW)  
Ei-value:0.000, Pi-value:0.000  
Er-value:0.000, Pr-value:0.000  
No matches to TargetScan

 21000  


C

TAAATTATTAC  
Depth:3 (COW)  
Ei-value:0.000, Pi-value:0.000  
Er-value:0.000, Pr-value:0.000  
No matches to TargetScan

CAGGATGTTTTC

CC

CCAGGTAAC  
Depth:2 (PIG)  
Ei-value:0.000, Pi-value:0.000  
Er-value:0.000, Pr-value:0.000  
No matches to TargetScan


AGGTAA

AGGTAA  
Depth:3 (COW)  
Ei-value:0.000, Pi-value:0.000  
Er-value:0.000, Pr-value:0.000  
No matches to TargetScan


C

CCAGGTAAC  
Depth:2 (PIG)  
Ei-value:0.000, Pi-value:0.000  
Er-value:0.000, Pr-value:0.000  
No matches to TargetScan

AGGAAAATGTAATGAATGAGATTGAGT

TTTCTAA

TTTCTAA  
Depth:3 (COW)  
Ei-value:0.000, Pi-value:0.000  
Er-value:0.000, Pr-value:0.010  
No matches to TargetScan


A

TTTCTAAA  
Depth:2 (PIG)  
Ei-value:0.000, Pi-value:0.000  
Er-value:0.000, Pr-value:0.000  
No matches to TargetScan

CTATAAAATGCTTCATAAACATAAGACATTAGATGTTTTCTATG

TGA

TGAATAAAACTT  
Depth:2 (PIG)  
Ei-value:0.000, Pi-value:0.000  
Er-value:0.000, Pr-value:0.000  
No matches to TargetScan


ATAAAAC

ATAAAAC  
Depth:4 (DOG)  
Ei-value:0.000, Pi-value:0.010  
Er-value:0.000, Pr-value:0.000  
No matches to TargetScan


TT

TGAATAAAACTT  
Depth:2 (PIG)  
Ei-value:0.000, Pi-value:0.000  
Er-value:0.000, Pr-value:0.000  
No matches to TargetScan

ATGTGAG 21120  
 GCAGGCATTA

TCTCTAC

TCTCTACAAAATTCTCATTGT  
Depth:2 (PIG)  
Ei-value:0.000, Pi-value:0.000  
Er-value:0.000, Pr-value:0.000  
No matches to TargetScan


AAAATTCTCA

AAAATTCTCA  
Depth:4 (DOG)  
Ei-value:0.000, Pi-value:0.000  
Er-value:0.000, Pr-value:0.000  
No matches to TargetScan


TTGT

TCTCTACAAAATTCTCATTGT  
Depth:2 (PIG)  
Ei-value:0.000, Pi-value:0.000  
Er-value:0.000, Pr-value:0.000  
No matches to TargetScan

GTGCGTAACCCACTACGAGAAAAATTGTATAAATAT

T

TTATACAAAC  
Depth:2 (PIG)  
Ei-value:0.000, Pi-value:0.000  
Er-value:0.000, Pr-value:0.000  
No matches to TargetScan


TATACAAAC

TATACAAAC  
Depth:4 (DOG)  
Ei-value:0.000, Pi-value:0.000  
Er-value:0.000, Pr-value:0.000  
No matches to TargetScan

T

GTTTAAATAC

GTTTAAATAC  
Depth:3 (COW)  
Ei-value:0.000, Pi-value:0.000  
Er-value:0.000, Pr-value:0.000  
No matches to TargetScan

TTAAGTGAATTAATTATTCATATCA

A

ATTGCCTACTATGTGAACTCACTGTTA  
Depth:2 (PIG)  
Ei-value:0.000, Pi-value:0.000  
Er-value:0.000, Pr-value:0.000  
MATCHES To TargetScan▶ miR-132-3p/212-3p:AACAGUC▶ miR-23-3p:UCACAUU▶ miR-376c-3p:ACAUAGA▶ miR-411-5p.2:UAGUAGA


TTGCCT

TTGCCTACTATGTGAACTCACTGTTA  
Depth:3 (COW)  
Ei-value:0.000, Pi-value:0.000  
Er-value:0.000, Pr-value:0.000  
MATCHES To TargetScan▶ miR-132-3p/212-3p:AACAGUC▶ miR-23-3p:UCACAUU▶ miR-376c-3p:ACAUAGA▶ miR-411-5p.2:UAGUAGA

 21240  


ACTAT

TTGCCTACTATGTGAACTCACTGTTA  
Depth:3 (COW)  
Ei-value:0.000, Pi-value:0.000  
Er-value:0.000, Pr-value:0.000  
MATCHES To TargetScan▶ miR-132-3p/212-3p:AACAGUC▶ miR-23-3p:UCACAUU▶ miR-376c-3p:ACAUAGA▶ miR-411-5p.2:UAGUAGA


GTGAACTCA

GTGAACTCA  
Depth:4 (DOG)  
Ei-value:0.000, Pi-value:0.000  
Er-value:0.000, Pr-value:0.000  
No matches to TargetScan


CTGTTA

TTGCCTACTATGTGAACTCACTGTTA  
Depth:3 (COW)  
Ei-value:0.000, Pi-value:0.000  
Er-value:0.000, Pr-value:0.000  
MATCHES To TargetScan▶ miR-132-3p/212-3p:AACAGUC▶ miR-23-3p:UCACAUU▶ miR-376c-3p:ACAUAGA▶ miR-411-5p.2:UAGUAGA

GAGGCATGAAAGATATGCACAC

ATTTATCAT

ATTTATCAT  
Depth:3 (COW)  
Ei-value:0.000, Pi-value:0.000  
Er-value:0.000, Pr-value:0.000  
No matches to TargetScan

TTTATTGTGCCTGTAAATGAGAATAAAGTAATACA

TATGTTAGC

TATGTTAGCATTTTGTGAACTCTAA  
Depth:2 (PIG)  
Ei-value:0.000, Pi-value:0.000  
Er-value:0.000, Pr-value:0.000  
No matches to TargetScan


ATTTTGTGAACTCTAA

ATTTTGTGAACTCTAA  
Depth:3 (COW)  
Ei-value:0.000, Pi-value:0.000  
Er-value:0.000, Pr-value:0.000  
No matches to TargetScan

A

GCACCAT

GCACCAT  
Depth:2 (PIG)  
Ei-value:0.000, Pi-value:0.000  
Er-value:0.000, Pr-value:0.000  
No matches to TargetScan

G 21360  
 TAAATATAACTATTGATCTTCTTATTTGGTGTGCCT

GTACTAG

GTACTAG  
Depth:2 (PIG)  
Ei-value:0.000, Pi-value:0.000  
Er-value:0.000, Pr-value:0.000  
No matches to TargetScan

TTTAC

AAAATTG

AAAATTG  
Depth:3 (COW)  
Ei-value:0.000, Pi-value:0.000  
Er-value:0.000, Pr-value:0.000  
No matches to TargetScan

CATC

ATAGTTAT

ATAGTTAT  
Depth:2 (PIG)  
Ei-value:0.000, Pi-value:0.000  
Er-value:0.000, Pr-value:0.000  
No matches to TargetScan

ACTATGAAAAAGAGAAAAAAAATTGAGGAAGGATTTTTTTACTCTTGAACAC

T

TTTGAATA  
Depth:2 (PIG)  
Ei-value:0.000, Pi-value:0.000  
Er-value:0.000, Pr-value:0.010  
No matches to TargetScan

 21480  


TTGAATA

TTTGAATA  
Depth:2 (PIG)  
Ei-value:0.000, Pi-value:0.000  
Er-value:0.000, Pr-value:0.010  
No matches to TargetScan

TGATATTTAAAGGATTA

AATGCC

AATGCC  
Depth:2 (PIG)  
Ei-value:0.000, Pi-value:0.000  
Er-value:0.000, Pr-value:0.000  
No matches to TargetScan

TGATGAATGAAAGACTAAATAATAATTTTCTATTA

TGTGCCA

TGTGCCA  
Depth:4 (DOG)  
Ei-value:0.000, Pi-value:0.000  
Er-value:0.000, Pr-value:0.000  
MATCHES To TargetScan▶ miR-183-5p.2:UGGCACU

GTCTCCTGAATCAGGGCTGTG

AAGATAA

AAGATAA  
Depth:4 (DOG)  
Ei-value:0.000, Pi-value:0.000  
Er-value:0.000, Pr-value:0.000  
No matches to TargetScan

TCCATTGT

TTTATTGTGT

TTTATTGTGT  
Depth:2 (PIG)  
Ei-value:0.000, Pi-value:0.000  
Er-value:0.000, Pr-value:0.000  
No matches to TargetScan

TC 21600  
 TA

GGT

GGTAGCAGAA  
Depth:2 (PIG)  
Ei-value:0.000, Pi-value:0.000  
Er-value:0.000, Pr-value:0.000  
No matches to TargetScan


AGCAGAA

AGCAGAA  
Depth:3 (COW)  
Ei-value:0.000, Pi-value:0.000  
Er-value:0.000, Pr-value:0.000  
No matches to TargetScan

TGTA

ATGTG

ATGTGTAAAATCAATTT  
Depth:2 (PIG)  
Ei-value:0.000, Pi-value:0.000  
Er-value:0.000, Pr-value:0.000  
No matches to TargetScan


TAAAATCAATTT

TAAAATCAATTT  
Depth:3 (COW)  
Ei-value:0.000, Pi-value:0.000  
Er-value:0.000, Pr-value:0.000  
No matches to TargetScan

GTAAGTCT

TAAACTG

TAAACTG  
Depth:4 (DOG)  
Ei-value:0.000, Pi-value:0.000  
Er-value:0.000, Pr-value:0.000  
No matches to TargetScan

GCATACATT

TCTGCTGAATGA

TCTGCTGAATGA  
Depth:3 (COW)  
Ei-value:0.000, Pi-value:0.000  
Er-value:0.000, Pr-value:0.000  
MATCHES To TargetScan▶ miR-1298-5p:UCAUUCG

A

C

CATTGATTA  
Depth:3 (COW)  
Ei-value:0.000, Pi-value:0.000  
Er-value:0.000, Pr-value:0.000  
No matches to TargetScan


ATTGATTA

ATTGATTA  
Depth:4 (DOG)  
Ei-value:0.000, Pi-value:0.000  
Er-value:0.000, Pr-value:0.010  
No matches to TargetScan

GTTACTCTT

TCTTATCC

TCTTATCC  
Depth:2 (PIG)  
Ei-value:0.000, Pi-value:0.000  
Er-value:0.000, Pr-value:0.000  
No matches to TargetScan

CC

AGAGATA

AGAGATA  
Depth:4 (DOG)  
Ei-value:0.000, Pi-value:0.000  
Er-value:0.000, Pr-value:0.000  
No matches to TargetScan

GCAAGTGGGGATGCT 21720  
 AAATTCATTGTCATTGTCAC

TGAACCT

TGAACCT  
Depth:3 (COW)  
Ei-value:0.000, Pi-value:0.000  
Er-value:0.000, Pr-value:0.010  
No matches to TargetScan

G

C

CAACAGAGATCT  
Depth:2 (PIG)  
Ei-value:0.000, Pi-value:0.000  
Er-value:0.000, Pr-value:0.000  
No matches to TargetScan


AA

AACAGAGATCT  
Depth:3 (COW)  
Ei-value:0.000, Pi-value:0.000  
Er-value:0.000, Pr-value:0.000  
No matches to TargetScan


CAGAGATCT

CAGAGATCT  
Depth:4 (DOG)  
Ei-value:0.000, Pi-value:0.000  
Er-value:0.000, Pr-value:0.000  
No matches to TargetScan

GTGTGTAA

A

ATTTACAAAGCCTA  
Depth:2 (PIG)  
Ei-value:0.000, Pi-value:0.000  
Er-value:0.000, Pr-value:0.000  
No matches to TargetScan


TTTACAAAGC

TTTACAAAGC  
Depth:3 (COW)  
Ei-value:0.000, Pi-value:0.000  
Er-value:0.000, Pr-value:0.000  
No matches to TargetScan


CTA

ATTTACAAAGCCTA  
Depth:2 (PIG)  
Ei-value:0.000, Pi-value:0.000  
Er-value:0.000, Pr-value:0.000  
No matches to TargetScan

TTGA

TCTATACA

TCTATACA  
Depth:3 (COW)  
Ei-value:0.000, Pi-value:0.000  
Er-value:0.000, Pr-value:0.000  
No matches to TargetScan

AATAT

TAGGAAT

TAGGAAT  
Depth:2 (PIG)  
Ei-value:0.000, Pi-value:0.000  
Er-value:0.000, Pr-value:0.000  
No matches to TargetScan

TAACTG

TTGGCT

TTGGCT  
Depth:4 (DOG)  
Ei-value:0.000, Pi-value:0.000  
Er-value:0.000, Pr-value:0.000  
No matches to TargetScan

CAATGAGTGATTACCAG

TTACT

TTACTTTCT  
Depth:4 (DOG)  
Ei-value:0.000, Pi-value:0.000  
Er-value:0.000, Pr-value:0.010  
No matches to TargetScan

 21840  


TTCT

TTACTTTCT  
Depth:4 (DOG)  
Ei-value:0.000, Pi-value:0.000  
Er-value:0.000, Pr-value:0.010  
No matches to TargetScan

ATGAGGCTCAGAACTGC

ACATGC

ACATGC  
Depth:2 (PIG)  
Ei-value:0.000, Pi-value:0.010  
Er-value:0.000, Pr-value:0.020  
No matches to TargetScan

T

CTAGGATAT

CTAGGATAT  
Depth:3 (COW)  
Ei-value:0.000, Pi-value:0.000  
Er-value:0.000, Pr-value:0.000  
No matches to TargetScan

G

AAAATGA

AAAATGA  
Depth:3 (COW)  
Ei-value:0.000, Pi-value:0.000  
Er-value:0.000, Pr-value:0.000  
No matches to TargetScan


T

AAAATGAT  
Depth:2 (PIG)  
Ei-value:0.000, Pi-value:0.010  
Er-value:0.000, Pr-value:0.000  
MATCHES To TargetScan▶ miR-382-3p:AUCAUUC

ATCCTTATCTGATGTTCCCTGG

AGGAAATGA

AGGAAATGA  
Depth:2 (PIG)  
Ei-value:0.000, Pi-value:0.000  
Er-value:0.000, Pr-value:0.000  
No matches to TargetScan

GGTA

ATAGGTGTG

ATAGGTGTG  
Depth:2 (PIG)  
Ei-value:0.000, Pi-value:0.000  
Er-value:0.000, Pr-value:0.000  
No matches to TargetScan

GGAATCTAGACTGAAAGTTACTTAATCACT 21960  
 GCCAAGCTTATTTGATGGCAAAAACATAGGAAAAATGGAATTGTGCAAGGCAGTATGCTTCTGAATGCTGAGCTAGGTGGAAAGAAA

ATCCAGACCA

ATCCAGACCA  
Depth:3 (COW)  
Ei-value:0.000, Pi-value:0.000  
Er-value:0.000, Pr-value:0.000  
No matches to TargetScan

GAAGGCTACTTGCTCCTTT

GCCT

GCCTGATTGA  
Depth:2 (PIG)  
Ei-value:0.000, Pi-value:0.000  
Er-value:0.000, Pr-value:0.000  
No matches to TargetScan

 22080  


GATTGA

GCCTGATTGA  
Depth:2 (PIG)  
Ei-value:0.000, Pi-value:0.000  
Er-value:0.000, Pr-value:0.000  
No matches to TargetScan

CGAGATGGGATTTTTTTCTTTAAGCAAGATGTTGTGCTTGTTTTTAAATTCTAA

AGATGGA

AGATGGA  
Depth:3 (COW)  
Ei-value:0.000, Pi-value:0.000  
Er-value:0.000, Pr-value:0.000  
No matches to TargetScan

GGTGAATGAGATCCACCCTGACATGGCAT

TAAAG

TAAAGGAGTAAAAAT  
Depth:2 (PIG)  
Ei-value:0.000, Pi-value:0.000  
Er-value:0.000, Pr-value:0.000  
MATCHES To TargetScan▶ miR-483-3p.1:ACUCCUC


GAGTAAAAA

GAGTAAAAA  
Depth:4 (DOG)  
Ei-value:0.000, Pi-value:0.000  
Er-value:0.000, Pr-value:0.000  
No matches to TargetScan


T

TAAAGGAGTAAAAAT  
Depth:2 (PIG)  
Ei-value:0.000, Pi-value:0.000  
Er-value:0.000, Pr-value:0.000  
MATCHES To TargetScan▶ miR-483-3p.1:ACUCCUC

TAATGGCTC 22200  
 AAGTTGCTTTTAGATTA

ATTTGAT

ATTTGAT  
Depth:4 (DOG)  
Ei-value:0.000, Pi-value:0.010  
Er-value:0.000, Pr-value:0.000  
No matches to TargetScan


AAACA

ATTTGATAAACA  
Depth:2 (PIG)  
Ei-value:0.000, Pi-value:0.000  
Er-value:0.000, Pr-value:0.000  
No matches to TargetScan

GT

ATC

ATCTTTTATGT  
Depth:3 (COW)  
Ei-value:0.000, Pi-value:0.000  
Er-value:0.000, Pr-value:0.000  
No matches to TargetScan


TTTTATGT

TTTTATGT  
Depth:4 (DOG)  
Ei-value:0.000, Pi-value:0.000  
Er-value:0.000, Pr-value:0.000  
No matches to TargetScan


GGAATA

ATCTTTTATGTGGAATA  
Depth:2 (PIG)  
Ei-value:0.000, Pi-value:0.000  
Er-value:0.000, Pr-value:0.000  
No matches to TargetScan

CATTGTTCTA

GGTCCTGAG

GGTCCTGAG  
Depth:3 (COW)  
Ei-value:0.000, Pi-value:0.000  
Er-value:0.000, Pr-value:0.000  
No matches to TargetScan

CATAACAGAAATGAAAGGGGCCTAGTCTAA

AGGGCATTAG

AGGGCATTAG  
Depth:2 (PIG)  
Ei-value:0.000, Pi-value:0.000  
Er-value:0.000, Pr-value:0.000  
MATCHES To TargetScan▶ miR-155-5p:UAAUGCU▶ miR-365-3p:AAUGCCC▶ miR-874-3p:UGCCCUG

GTAGATAACAAAT 22320  
 CATG

GCTGAA

GCTGAA  
Depth:2 (PIG)  
Ei-value:0.000, Pi-value:0.010  
Er-value:0.000, Pr-value:0.000  
No matches to TargetScan

AATTCAGAAATGTC

TTTGATT

TTTGATT  
Depth:2 (PIG)  
Ei-value:0.000, Pi-value:0.020  
Er-value:0.000, Pr-value:0.010  
No matches to TargetScan

TCTGGAAATATTTGAGTGCCTCTGGAAGAATTTGTTGATTAGATCTGATCCAAAAAAACCTATTGAC

TTGCCTT

TTGCCTT  
Depth:3 (COW)  
Ei-value:0.000, Pi-value:0.000  
Er-value:0.000, Pr-value:0.000  
MATCHES To TargetScan▶ miR-124-3p.1:AAGGCAC

GGAAATTGAAAAAAT 22440  
 AGGG

AAAAAAAGA

AAAAAAAGA  
Depth:2 (PIG)  
Ei-value:0.000, Pi-value:0.000  
Er-value:0.000, Pr-value:0.000  
No matches to TargetScan

TATGATTCTGTCAATGTAAAAT

GACATTTTTCCTAG

GACATTTTTCCTAG  
Depth:2 (PIG)  
Ei-value:0.000, Pi-value:0.000  
Er-value:0.000, Pr-value:0.000  
No matches to TargetScan

ATCTGGAAAGTCCCAGTGTCACTTGGTCATTGACATAGATTG

AAGCCAG

AAGCCAG  
Depth:4 (DOG)  
Ei-value:0.000, Pi-value:0.000  
Er-value:0.000, Pr-value:0.000  
MATCHES To TargetScan▶ miR-149-5p:CUGGCUC▶ miR-3064-5p:CUGGCUG

AAGAT

TG

TGATAAAAG  
Depth:2 (PIG)  
Ei-value:0.000, Pi-value:0.000  
Er-value:0.000, Pr-value:0.000  
No matches to TargetScan


ATAAAAG

ATAAAAG  
Depth:4 (DOG)  
Ei-value:0.000, Pi-value:0.000  
Er-value:0.000, Pr-value:0.000  
No matches to TargetScan

AGCTCATG 22560  
 GTTTGAC

CTTTAATTC

CTTTAATTC  
Depth:3 (COW)  
Ei-value:0.000, Pi-value:0.000  
Er-value:0.000, Pr-value:0.000  
No matches to TargetScan

TTT

AACATTCTGC

AACATTCTGCTTTTATTA  
Depth:2 (PIG)  
Ei-value:0.000, Pi-value:0.000  
Er-value:0.000, Pr-value:0.000  
MATCHES To TargetScan▶ miR-330-3p.2:AAAGCAC▶ miR-409-3p:AAUGUUG


TTTTATTA

TTTTATTA  
Depth:4 (DOG)  
Ei-value:0.000, Pi-value:0.010  
Er-value:0.000, Pr-value:0.000  
No matches to TargetScan

TG

G

GTTAAATGG  
Depth:3 (COW)  
Ei-value:0.000, Pi-value:0.000  
Er-value:0.000, Pr-value:0.000  
No matches to TargetScan


TTAAATGG

TTAAATGG  
Depth:4 (DOG)  
Ei-value:0.000, Pi-value:0.000  
Er-value:0.000, Pr-value:0.000  
No matches to TargetScan


TT

GTTAAATGGTT  
Depth:2 (PIG)  
Ei-value:0.000, Pi-value:0.000  
Er-value:0.000, Pr-value:0.000  
No matches to TargetScan

TATGATGAAAATCAAGCTGA

AACAACTAGTT

AACAACTAGTT  
Depth:2 (PIG)  
Ei-value:0.000, Pi-value:0.000  
Er-value:0.000, Pr-value:0.000  
No matches to TargetScan

CTAAAAGTGTTTTGTTTTGTTTTTTTTTTTTTTTTA

CTC

CTCATTGGTCTG  
Depth:2 (PIG)  
Ei-value:0.000, Pi-value:0.000  
Er-value:0.000, Pr-value:0.000  
No matches to TargetScan

 22680  


ATTGGTCTG

CTCATTGGTCTG  
Depth:2 (PIG)  
Ei-value:0.000, Pi-value:0.000  
Er-value:0.000, Pr-value:0.000  
No matches to TargetScan

CCAGAGAGTCAGAAAGCCACTTAACTAAAAAC

TAAAAAAAA

TAAAAAAAA  
Depth:2 (PIG)  
Ei-value:0.000, Pi-value:0.000  
Er-value:0.000, Pr-value:0.000  
No matches to TargetScan

AAAAATAATAAAAACTTCCTAGATTTTTCATATTTT

GTAAAAA

GTAAAAA  
Depth:2 (PIG)  
Ei-value:0.000, Pi-value:0.010  
Er-value:0.000, Pr-value:0.000  
No matches to TargetScan

AACTAGTCACTTGAGTGAAGTA

CTGTT

CTGTTCTTAAGT  
Depth:3 (COW)  
Ei-value:0.000, Pi-value:0.000  
Er-value:0.000, Pr-value:0.000  
No matches to TargetScan

 22800  


CTTAAGT

CTGTTCTTAAGT  
Depth:3 (COW)  
Ei-value:0.000, Pi-value:0.000  
Er-value:0.000, Pr-value:0.000  
No matches to TargetScan

ACCAAGAATGAAGATTTTCAAGATCTAGATCAAAATATAATTTTGGTAGCTTATAGA

GGAAACA

GGAAACA  
Depth:2 (PIG)  
Ei-value:0.000, Pi-value:0.000  
Er-value:0.000, Pr-value:0.000  
No matches to TargetScan

TAA

GAACAAATT

GAACAAATT  
Depth:3 (COW)  
Ei-value:0.000, Pi-value:0.000  
Er-value:0.000, Pr-value:0.000  
MATCHES To TargetScan▶ miR-375:UUGUUCG

TAA

TAAGAGACTG

TAAGAGACTG  
Depth:2 (PIG)  
Ei-value:0.000, Pi-value:0.000  
Er-value:0.000, Pr-value:0.000  
No matches to TargetScan

AAGTTCTTTCA

TTAGTTG

TTAGTTG  
Depth:3 (COW)  
Ei-value:0.000, Pi-value:0.000  
Er-value:0.000, Pr-value:0.010  
No matches to TargetScan


A

TTAGTTGA  
Depth:2 (PIG)  
Ei-value:0.000, Pi-value:0.000  
Er-value:0.000, Pr-value:0.000  
No matches to TargetScan

A

AAAC

AAACTTCATTGA  
Depth:3 (COW)  
Ei-value:0.000, Pi-value:0.000  
Er-value:0.000, Pr-value:0.000  
No matches to TargetScan

 22920  


TTCATTGA

AAACTTCATTGA  
Depth:3 (COW)  
Ei-value:0.000, Pi-value:0.000  
Er-value:0.000, Pr-value:0.000  
No matches to TargetScan


G

AAACTTCATTGAG  
Depth:2 (PIG)  
Ei-value:0.000, Pi-value:0.000  
Er-value:0.000, Pr-value:0.000  
No matches to TargetScan

CAGCCA

TGATAT

TGATAT  
Depth:2 (PIG)  
Ei-value:0.000, Pi-value:0.000  
Er-value:0.000, Pr-value:0.010  
No matches to TargetScan

TAAGAAACTGAAATGGAATGGTTAAGTAGACACCTTCTGCTTGGCATGTGTGGTAGCGTA

GGAATAAGAGA

GGAATAAGAGA  
Depth:2 (PIG)  
Ei-value:0.000, Pi-value:0.000  
Er-value:0.000, Pr-value:0.000  
No matches to TargetScan

CCTTTGTGTTTACTCATTCATGTAAACA 23040  
 CTGAA

GAAGATGAT

GAAGATGAT  
Depth:2 (PIG)  
Ei-value:0.000, Pi-value:0.000  
Er-value:0.000, Pr-value:0.000  
No matches to TargetScan


GCTAAAT

GCTAAAT  
Depth:2 (PIG)  
Ei-value:0.000, Pi-value:0.020  
Er-value:0.000, Pr-value:0.000  
No matches to TargetScan

TAAATAGATGTGGCA

CAAAAG

CAAAAG  
Depth:2 (PIG)  
Ei-value:0.000, Pi-value:0.010  
Er-value:0.000, Pr-value:0.000  
No matches to TargetScan

CACTTAGT

CTTGGAGATG

CTTGGAGATG  
Depth:2 (PIG)  
Ei-value:0.000, Pi-value:0.000  
Er-value:0.000, Pr-value:0.000  
No matches to TargetScan

TAGTTAAAAACAACAGTAAGAAG

AAATGGA

AAATGGA  
Depth:2 (PIG)  
Ei-value:0.000, Pi-value:0.000  
Er-value:0.000, Pr-value:0.010  
No matches to TargetScan

GTGAAACTCTTCATGAAACAGAAATGTCTA 23160  
 TTTCAAGGCATTGT

GTGATACTC

GTGATACTC  
Depth:2 (PIG)  
Ei-value:0.000, Pi-value:0.000  
Er-value:0.000, Pr-value:0.000  
MATCHES To TargetScan▶ miR-496.1:GAGUAUU

CTAG

ACAGAAAACAAAA

ACAGAAAACAAAA  
Depth:4 (DOG)  
Ei-value:0.000, Pi-value:0.000  
Er-value:0.000, Pr-value:0.000  
No matches to TargetScan

GATCTTGACTGAAAGAT

ACTTGC

ACTTGC  
Depth:2 (PIG)  
Ei-value:0.000, Pi-value:0.020  
Er-value:0.000, Pr-value:0.000  
No matches to TargetScan


TTGG

TTGGCTTGGAAA  
Depth:2 (PIG)  
Ei-value:0.000, Pi-value:0.000  
Er-value:0.000, Pr-value:0.000  
No matches to TargetScan


CTTGGAAA

CTTGGAAA  
Depth:3 (COW)  
Ei-value:0.000, Pi-value:0.000  
Er-value:0.000, Pr-value:0.000  
No matches to TargetScan

CTGAAATATAAGAGGAG

AGGTTA

AGGTTA  
Depth:4 (DOG)  
Ei-value:0.000, Pi-value:0.000  
Er-value:0.000, Pr-value:0.000  
No matches to TargetScan


CTGTTTATT

AGGTTACTGTTTATT  
Depth:2 (PIG)  
Ei-value:0.000, Pi-value:0.000  
Er-value:0.000, Pr-value:0.000  
MATCHES To TargetScan▶ miR-101-3p.1:ACAGUAC▶ miR-132-3p/212-3p:AACAGUC▶ miR-144-3p:ACAGUAU▶ miR-802:CAGUAAC

CATATTTTCATTT 23280  
 ATTTG

TTCATTCT

TTCATTCT  
Depth:4 (DOG)  
Ei-value:0.000, Pi-value:0.000  
Er-value:0.000, Pr-value:0.000  
No matches to TargetScan

GCCAGTATATTTGAGTGCCTACTGTGTGTAAGCTTTCTTGGCTAGTTTCTTAG

TTGGCCCC

TTGGCCCCAGAGACATG  
Depth:2 (PIG)  
Ei-value:0.000, Pi-value:0.000  
Er-value:0.000, Pr-value:0.000  
MATCHES To TargetScan▶ miR-326:CUCUGGG


AGAGACA

AGAGACA  
Depth:4 (DOG)  
Ei-value:0.000, Pi-value:0.000  
Er-value:0.000, Pr-value:0.000  
No matches to TargetScan


TG

AGAGACATG  
Depth:3 (COW)  
Ei-value:0.000, Pi-value:0.000  
Er-value:0.000, Pr-value:0.000  
No matches to TargetScan


AAAAAATG

AAAAAATG  
Depth:2 (PIG)  
Ei-value:0.000, Pi-value:0.000  
Er-value:0.000, Pr-value:0.000  
No matches to TargetScan

AATGTCTTCCAAGAGAACTTAGAACTTTA 23400  
 AAAACTTG

CCTTTTGG

CCTTTTGG  
Depth:4 (DOG)  
Ei-value:0.000, Pi-value:0.000  
Er-value:0.000, Pr-value:0.000  
No matches to TargetScan


C

CCTTTTGGC  
Depth:3 (COW)  
Ei-value:0.000, Pi-value:0.000  
Er-value:0.000, Pr-value:0.000  
No matches to TargetScan

CA

GTTTTCT

GTTTTCT  
Depth:2 (PIG)  
Ei-value:0.000, Pi-value:0.010  
Er-value:0.000, Pr-value:0.010  
No matches to TargetScan

G

GTTAGGGGCA

GTTAGGGGCA  
Depth:2 (PIG)  
Ei-value:0.000, Pi-value:0.000  
Er-value:0.000, Pr-value:0.000  
No matches to TargetScan

A

GGCTTAGT

GGCTTAGT  
Depth:2 (PIG)  
Ei-value:0.000, Pi-value:0.000  
Er-value:0.000, Pr-value:0.000  
No matches to TargetScan

TCCTCAC

TAAC

TAACATTGTGT  
Depth:2 (PIG)  
Ei-value:0.000, Pi-value:0.000  
Er-value:0.000, Pr-value:0.000  
MATCHES To TargetScan▶ miR-409-3p:AAUGUUG


ATTGTGT

ATTGTGT  
Depth:3 (COW)  
Ei-value:0.000, Pi-value:0.000  
Er-value:0.000, Pr-value:0.010  
No matches to TargetScan

AGC

TTAATTC

TTAATTC  
Depth:4 (DOG)  
Ei-value:0.000, Pi-value:0.000  
Er-value:0.000, Pr-value:0.000  
No matches to TargetScan

TTGCTCCATATTTCCTCTGCT

ACTCTGGCCACTAC

ACTCTGGCCACTAC  
Depth:4 (DOG)  
Ei-value:0.000, Pi-value:0.000  
Er-value:0.000, Pr-value:0.000  
MATCHES To TargetScan▶ miR-142-3p.1:GUAGUGU

A

ATAAGC

ATAAGC  
Depth:5 (RABBIT)  
Ei-value:0.000, Pi-value:0.010  
Er-value:0.000, Pr-value:0.000  
No matches to TargetScan


AGG

ATAAGCAGG  
Depth:4 (DOG)  
Ei-value:0.000, Pi-value:0.000  
Er-value:0.000, Pr-value:0.000  
No matches to TargetScan

G 23520  
 ATTTCTATTGC

GCT

GCTTGCTCCTT  
Depth:2 (PIG)  
Ei-value:0.000, Pi-value:0.000  
Er-value:0.000, Pr-value:0.000  
MATCHES To TargetScan▶ miR-28-5p/708-5p:AGGAGCU


TGCTCCTT

TGCTCCTT  
Depth:3 (COW)  
Ei-value:0.000, Pi-value:0.000  
Er-value:0.000, Pr-value:0.000  
MATCHES To TargetScan▶ miR-28-5p/708-5p:AGGAGCU

CCTTAACAATAGT

ACTTCA

ACTTCA  
Depth:3 (COW)  
Ei-value:0.000, Pi-value:0.000  
Er-value:0.000, Pr-value:0.000  
No matches to TargetScan

T

TTTTCCTA

TTTTCCTA  
Depth:3 (COW)  
Ei-value:0.000, Pi-value:0.000  
Er-value:0.000, Pr-value:0.000  
No matches to TargetScan


GTCCATCC

TTTTCCTAGTCCATCC  
Depth:2 (PIG)  
Ei-value:0.000, Pi-value:0.000  
Er-value:0.000, Pr-value:0.000  
No matches to TargetScan

TTA

AT

ATGAAAAATG  
Depth:3 (COW)  
Ei-value:0.000, Pi-value:0.000  
Er-value:0.000, Pr-value:0.000  
No matches to TargetScan


GAAAAATG

GAAAAATG  
Depth:4 (DOG)  
Ei-value:0.000, Pi-value:0.000  
Er-value:0.000, Pr-value:0.000  
No matches to TargetScan

GCCGTGAACTTTGG

TGGGCAG

TGGGCAG  
Depth:2 (PIG)  
Ei-value:0.000, Pi-value:0.000  
Er-value:0.000, Pr-value:0.000  
No matches to TargetScan

GAGAGATGAGAAACAATTGATTCCCTAA 23640  
 CTCT

AGTCTCA

AGTCTCA  
Depth:4 (DOG)  
Ei-value:0.000, Pi-value:0.000  
Er-value:0.000, Pr-value:0.000  
No matches to TargetScan


TTGGTACCA

AGTCTCATTGGTACCA  
Depth:3 (COW)  
Ei-value:0.000, Pi-value:0.000  
Er-value:0.000, Pr-value:0.000  
No matches to TargetScan


GC

AGTCTCATTGGTACCAGC  
Depth:2 (PIG)  
Ei-value:0.000, Pi-value:0.000  
Er-value:0.000, Pr-value:0.000  
MATCHES To TargetScan▶ miR-138-5p:GCUGGUG

TTTT

GGGAAC

GGGAAC  
Depth:2 (PIG)  
Ei-value:0.000, Pi-value:0.000  
Er-value:0.000, Pr-value:0.010  
No matches to TargetScan

ATTCATATTCA

AGCCACAA

AGCCACAA  
Depth:2 (PIG)  
Ei-value:0.000, Pi-value:0.000  
Er-value:0.000, Pr-value:0.000  
No matches to TargetScan

C

TGGTTTTGAA

TGGTTTTGAA  
Depth:4 (DOG)  
Ei-value:0.000, Pi-value:0.000  
Er-value:0.000, Pr-value:0.000  
No matches to TargetScan

ACA

CATTTAC

CATTTAC  
Depth:2 (PIG)  
Ei-value:0.000, Pi-value:0.000  
Er-value:0.000, Pr-value:0.000  
No matches to TargetScan

CAGTTTTGATGATTC

TCAGTTC

TCAGTTC  
Depth:2 (PIG)  
Ei-value:0.000, Pi-value:0.010  
Er-value:0.000, Pr-value:0.000  
No matches to TargetScan

ACCTTTTATATTATGGCAAATGTATT 23760  
 TTTAAGAG

ACAATCC

ACAATCC  
Depth:3 (COW)  
Ei-value:0.000, Pi-value:0.000  
Er-value:0.000, Pr-value:0.000  
MATCHES To TargetScan▶ miR-219-5p:GAUUGUC

CCCAAAAGTACATAACAAGTT

TGGAGATG

TGGAGATG  
Depth:3 (COW)  
Ei-value:0.000, Pi-value:0.000  
Er-value:0.000, Pr-value:0.000  
No matches to TargetScan

TTTTGGTTTTGCAGT

AGCTTCTC

AGCTTCTC  
Depth:3 (COW)  
Ei-value:0.000, Pi-value:0.000  
Er-value:0.000, Pr-value:0.000  
No matches to TargetScan

ACCTTACA

C

CTTAGAAAT  
Depth:2 (PIG)  
Ei-value:0.000, Pi-value:0.000  
Er-value:0.000, Pr-value:0.000  
No matches to TargetScan


TTAGAAAT

TTAGAAAT  
Depth:4 (DOG)  
Ei-value:0.000, Pi-value:0.000  
Er-value:0.000, Pr-value:0.000  
No matches to TargetScan

CTTTTCCTTCTCAC

CATCAAA

CATCAAA  
Depth:4 (DOG)  
Ei-value:0.000, Pi-value:0.000  
Er-value:0.000, Pr-value:0.000  
No matches to TargetScan

ACCTGCTTAAGAAAG 23880  
 GACTTATTATTTGTAACAGAATAGTGCAGAGA

ATGAAAA

ATGAAAA  
Depth:2 (PIG)  
Ei-value:0.000, Pi-value:0.030  
Er-value:0.000, Pr-value:0.010  
No matches to TargetScan

TAACAATTTTATT

CTTAAGTTTTA

CTTAAGTTTTA  
Depth:2 (PIG)  
Ei-value:0.000, Pi-value:0.000  
Er-value:0.000, Pr-value:0.000  
No matches to TargetScan

GGAAT

AATAAAAATTGGAA

AATAAAAATTGGAA  
Depth:2 (PIG)  
Ei-value:0.000, Pi-value:0.000  
Er-value:0.000, Pr-value:0.000  
No matches to TargetScan

GTAATT

GGAAAAA

GGAAAAA  
Depth:3 (COW)  
Ei-value:0.000, Pi-value:0.000  
Er-value:0.000, Pr-value:0.000  
No matches to TargetScan

ATTGGATAGAAGAAATACCCCCACC 24000  
 AAAAAAAG

TCTAAAT

TCTAAAT  
Depth:2 (PIG)  
Ei-value:0.000, Pi-value:0.000  
Er-value:0.000, Pr-value:0.010  
No matches to TargetScan

TTTGACCCAAA

TGGGCTTTG

TGGGCTTTG  
Depth:3 (COW)  
Ei-value:0.000, Pi-value:0.000  
Er-value:0.000, Pr-value:0.000  
MATCHES To TargetScan▶ miR-330-3p:CAAAGCA

GCCTTTTCTA

TTTTTAAATCACTCA

TTTTTAAATCACTCA  
Depth:4 (DOG)  
Ei-value:0.000, Pi-value:0.000  
Er-value:0.000, Pr-value:0.000  
No matches to TargetScan

G

AGAGGGTGGGA

AGAGGGTGGGA  
Depth:4 (DOG)  
Ei-value:0.000, Pi-value:0.000  
Er-value:0.000, Pr-value:0.000  
No matches to TargetScan

T

AGGAGGAAGAGTGAA

AGGAGGAAGAGTGAA  
Depth:4 (DOG)  
Ei-value:0.000, Pi-value:0.000  
Er-value:0.000, Pr-value:0.000  
MATCHES To TargetScan▶ miR-670-3p:UUCCUCA

A

G

GAAAAGGTCA  
Depth:4 (DOG)  
Ei-value:0.000, Pi-value:0.000  
Er-value:0.000, Pr-value:0.000  
MATCHES To TargetScan▶ miR-192-5p/215-5p:UGACCUA


AAAAGGT

AAAAGGT  
Depth:6 (MOUSE)  
Ei-value:0.000, Pi-value:0.000  
Er-value:0.000, Pr-value:0.000  
No matches to TargetScan


CA

GAAAAGGTCA  
Depth:4 (DOG)  
Ei-value:0.000, Pi-value:0.000  
Er-value:0.000, Pr-value:0.000  
MATCHES To TargetScan▶ miR-192-5p/215-5p:UGACCUA

GACCTAGTTCGATGGGCAACC 24120  
 TGCCTTTGTTCTGGATTGGTCTTAAAAGTATTACTACCTCCAGATCTAACTTGGGGGATTGCATCTTTAGTTTAAATGCAACTCCAGCAGAGAGGGTGGGATAGGAGGAAGAGTGAAAGA 24240  
 AAAGGTCAGACCTAGTTCGAT

GGGCAACCTGCCTTTGTTCTG

GGGCAACCTGCCTTTGTTCTG  
Depth:2 (PIG)  
Ei-value:0.000, Pi-value:0.000  
Er-value:0.000, Pr-value:0.000  
MATCHES To TargetScan▶ miR-124-3p.1:AAGGCAC▶ miR-495-3p:AACAAAC

G

A

ATTGGTCTTAA  
Depth:2 (PIG)  
Ei-value:0.000, Pi-value:0.000  
Er-value:0.000, Pr-value:0.000  
MATCHES To TargetScan▶ miR-208-3p:UAAGACG▶ miR-499a-5p:UAAGACU


TTGGTCTTAA

TTGGTCTTAA  
Depth:3 (COW)  
Ei-value:0.000, Pi-value:0.000  
Er-value:0.000, Pr-value:0.000  
MATCHES To TargetScan▶ miR-208-3p:UAAGACG▶ miR-499a-5p:UAAGACU

AAGTATTACTACCTCCAGATCTAACTTGGGGGATTGCATCTTTA

GTTTAAAT

GTTTAAAT  
Depth:2 (PIG)  
Ei-value:0.000, Pi-value:0.000  
Er-value:0.000, Pr-value:0.000  
No matches to TargetScan

GCAACTCCAGTAAC 24360  
 TGGTCACTATTA

AGATGAGGACAAA

AGATGAGGACAAA  
Depth:3 (COW)  
Ei-value:0.000, Pi-value:0.000  
Er-value:0.000, Pr-value:0.000  
No matches to TargetScan

T

TCCTTTGT

TCCTTTGT  
Depth:3 (COW)  
Ei-value:0.000, Pi-value:0.000  
Er-value:0.000, Pr-value:0.000  
No matches to TargetScan

TCCCCCTCATTTCTATATGTTTTTGAGAA

ACATTTTT

ACATTTTT  
Depth:2 (PIG)  
Ei-value:0.000, Pi-value:0.020  
Er-value:0.000, Pr-value:0.000  
No matches to TargetScan

TGTTAATGGCTGTCAATTAAG

CCTTGTG

CCTTGTG  
Depth:2 (PIG)  
Ei-value:0.000, Pi-value:0.000  
Er-value:0.000, Pr-value:0.020  
No matches to TargetScan

CTAGATGTTATGAAGTTGATT 24480  
 AATTAAATCAATGTAGTCTTAAGC

TAAAGC

TAAAGC  
Depth:3 (COW)  
Ei-value:0.000, Pi-value:0.000  
Er-value:0.000, Pr-value:0.000  
No matches to TargetScan

CGAAATTCATTG

CAAAAGAAGAGG

CAAAAGAAGAGG  
Depth:2 (PIG)  
Ei-value:0.000, Pi-value:0.000  
Er-value:0.000, Pr-value:0.000  
No matches to TargetScan

GCTTTTTTGTTTACTGTAAGTCATACATAT

TTCATACA

TTCATACA  
Depth:3 (COW)  
Ei-value:0.000, Pi-value:0.000  
Er-value:0.000, Pr-value:0.000  
No matches to TargetScan

TAT

TTCAAAGCATC

TTCAAAGCATC  
Depth:3 (COW)  
Ei-value:0.000, Pi-value:0.000  
Er-value:0.000, Pr-value:0.000  
No matches to TargetScan

CAAACTTTAGTTTA 24600  
 CATG

GCAAGCCAA

GCAAGCCAA  
Depth:2 (PIG)  
Ei-value:0.000, Pi-value:0.000  
Er-value:0.000, Pr-value:0.000  
No matches to TargetScan

CAGTAACATCTGTGA

TGG

TGGAGAAGGAAATAGAT  
Depth:2 (PIG)  
Ei-value:0.000, Pi-value:0.000  
Er-value:0.000, Pr-value:0.000  
No matches to TargetScan


AGAAGGAAATAGA

AGAAGGAAATAGA  
Depth:3 (COW)  
Ei-value:0.000, Pi-value:0.000  
Er-value:0.000, Pr-value:0.000  
No matches to TargetScan


T

TGGAGAAGGAAATAGAT  
Depth:2 (PIG)  
Ei-value:0.000, Pi-value:0.000  
Er-value:0.000, Pr-value:0.000  
No matches to TargetScan

GAATGGCGAAGGATAGTTGC

TGGAGGGAGC

TGGAGGGAGC  
Depth:3 (COW)  
Ei-value:0.000, Pi-value:0.000  
Er-value:0.000, Pr-value:0.000  
No matches to TargetScan


AA

TGGAGGGAGCAA  
Depth:2 (PIG)  
Ei-value:0.000, Pi-value:0.000  
Er-value:0.000, Pr-value:0.000  
No matches to TargetScan

A

GTTGAAGA

GTTGAAGA  
Depth:2 (PIG)  
Ei-value:0.000, Pi-value:0.000  
Er-value:0.000, Pr-value:0.000  
No matches to TargetScan

GGATTTTCTGACATGTTAA

TAATGTTT

TAATGTTT  
Depth:4 (DOG)  
Ei-value:0.000, Pi-value:0.000  
Er-value:0.000, Pr-value:0.000  
MATCHES To TargetScan▶ miR-323-3p:ACAUUAC▶ miR-543:AACAUUC

AATAAAA 24720  
 AAAA

TAATGTTT

TAATGTTT  
Depth:4 (DOG)  
Ei-value:0.000, Pi-value:0.000  
Er-value:0.000, Pr-value:0.000  
MATCHES To TargetScan▶ miR-323-3p:ACAUUAC▶ miR-543:AACAUUC

AATATATGA

TATC

TATCAGCTGGA  
Depth:2 (PIG)  
Ei-value:0.000, Pi-value:0.000  
Er-value:0.000, Pr-value:0.000  
No matches to TargetScan


AGCTGGA

AGCTGGA  
Depth:4 (DOG)  
Ei-value:0.000, Pi-value:0.000  
Er-value:0.000, Pr-value:0.000  
No matches to TargetScan

TGCAGTTATTCCTCTTTCTTGAGGTGGGGGTGGGGGTGC

ATTATTGGAAA

ATTATTGGAAA  
Depth:4 (DOG)  
Ei-value:0.000, Pi-value:0.000  
Er-value:0.000, Pr-value:0.000  
No matches to TargetScan


A

ATTATTGGAAAA  
Depth:2 (PIG)  
Ei-value:0.000, Pi-value:0.000  
Er-value:0.000, Pr-value:0.000  
No matches to TargetScan

G

TGGAAAG

TGGAAAG  
Depth:2 (PIG)  
Ei-value:0.000, Pi-value:0.000  
Er-value:0.000, Pr-value:0.010  
No matches to TargetScan

G

AGAAAGTAAC

AGAAAGTAAC  
Depth:4 (DOG)  
Ei-value:0.000, Pi-value:0.000  
Er-value:0.000, Pr-value:0.000  
No matches to TargetScan


TAAAAGCCTTCC

AGAAAGTAACTAAAAGCCTTCCTTTCACAGTTTCTGGCATC  
Depth:2 (PIG)  
Ei-value:0.000, Pi-value:0.000  
Er-value:0.000, Pr-value:0.000  
MATCHES To TargetScan▶ miR-488-3p:UGAAAGG


TTTCAC

TTTCACAGTTTCTGGCATC  
Depth:4 (DOG)  
Ei-value:0.000, Pi-value:0.000  
Er-value:0.000, Pr-value:0.000  
No matches to TargetScan

 24840  


AGTTTCTGGCATC

TTTCACAGTTTCTGGCATC  
Depth:4 (DOG)  
Ei-value:0.000, Pi-value:0.000  
Er-value:0.000, Pr-value:0.000  
No matches to TargetScan

CAAG

ACTAC

ACTACCACTACTGAT  
Depth:2 (PIG)  
Ei-value:0.000, Pi-value:0.000  
Er-value:0.000, Pr-value:0.000  
MATCHES To TargetScan▶ miR-140-5p:AGUGGUU▶ miR-142-3p.1:GUAGUGU▶ miR-199-3p:CAGUAGU


CA

CACTACTGAT  
Depth:3 (COW)  
Ei-value:0.000, Pi-value:0.000  
Er-value:0.000, Pr-value:0.000  
MATCHES To TargetScan▶ miR-142-3p.1:GUAGUGU▶ miR-199-3p:CAGUAGU


CTACTGAT

CTACTGAT  
Depth:4 (DOG)  
Ei-value:0.000, Pi-value:0.000  
Er-value:0.000, Pr-value:0.000  
MATCHES To TargetScan▶ miR-199-3p:CAGUAGU


AAACAAGAATAA

AAACAAGAATAA  
Depth:3 (COW)  
Ei-value:0.000, Pi-value:0.000  
Er-value:0.000, Pr-value:0.000  
MATCHES To TargetScan▶ miR-544a-5p:CUUGUUA


G

AAACAAGAATAAGAGAACAT  
Depth:2 (PIG)  
Ei-value:0.000, Pi-value:0.000  
Er-value:0.000, Pr-value:0.000  
MATCHES To TargetScan▶ miR-544a-5p:CUUGUUA


AGAACAT

AGAACAT  
Depth:4 (DOG)  
Ei-value:0.000, Pi-value:0.000  
Er-value:0.000, Pr-value:0.000  
No matches to TargetScan

GCT

A

ATCATCTG  
Depth:2 (PIG)  
Ei-value:0.000, Pi-value:0.000  
Er-value:0.000, Pr-value:0.000  
No matches to TargetScan


TCATCTG

TCATCTG  
Depth:4 (DOG)  
Ei-value:0.000, Pi-value:0.010  
Er-value:0.000, Pr-value:0.000  
No matches to TargetScan

ATTTTTGTAG

CATAAATGAA

CATAAATGAA  
Depth:4 (DOG)  
Ei-value:0.000, Pi-value:0.000  
Er-value:0.000, Pr-value:0.000  
No matches to TargetScan


GTTGTGA

CATAAATGAAGTTGTGA  
Depth:3 (COW)  
Ei-value:0.000, Pi-value:0.000  
Er-value:0.000, Pr-value:0.000  
No matches to TargetScan

AC

AAATCT

AAATCT  
Depth:2 (PIG)  
Ei-value:0.000, Pi-value:0.000  
Er-value:0.000, Pr-value:0.000  
No matches to TargetScan

TTTTTTTTTTTTAAAGAGAA

TG

TGGCTTC  
Depth:2 (PIG)  
Ei-value:0.000, Pi-value:0.000  
Er-value:0.000, Pr-value:0.000  
No matches to TargetScan

 24960  


GCTTC

TGGCTTC  
Depth:2 (PIG)  
Ei-value:0.000, Pi-value:0.000  
Er-value:0.000, Pr-value:0.000  
No matches to TargetScan

ATCTCTATTCTTTCAC

CTACCTCAAAG

CTACCTCAAAG  
Depth:2 (PIG)  
Ei-value:0.000, Pi-value:0.000  
Er-value:0.000, Pr-value:0.000  
MATCHES To TargetScan▶ let-7-5p/98-5p:GAGGUAG▶ miR-196-5p:AGGUAGU

GGAATCTATGCCAAAATACTCCAGTGAATGCAAGACACACTGG

ACTTG

ACTTGTGAACTGATGTGAAA  
Depth:3 (COW)  
Ei-value:0.000, Pi-value:0.000  
Er-value:0.000, Pr-value:0.000  
MATCHES To TargetScan▶ miR-23-3p:UCACAUU


TGAACTGATGTGAAA

TGAACTGATGTGAAA  
Depth:4 (DOG)  
Ei-value:0.000, Pi-value:0.000  
Er-value:0.000, Pr-value:0.000  
MATCHES To TargetScan▶ miR-23-3p:UCACAUU

CAT

AGAATCTCT

AGAATCTCT  
Depth:2 (PIG)  
Ei-value:0.000, Pi-value:0.000  
Er-value:0.000, Pr-value:0.000  
No matches to TargetScan

GAGCCTTGGTTGT 25080  
 TTT

GAAGATTGAAAAAT

GAAGATTGAAAAAT  
Depth:2 (PIG)  
Ei-value:0.000, Pi-value:0.000  
Er-value:0.000, Pr-value:0.000  
No matches to TargetScan

T

TTGTTCA

TTGTTCA  
Depth:3 (COW)  
Ei-value:0.000, Pi-value:0.000  
Er-value:0.000, Pr-value:0.000  
No matches to TargetScan

ACATGGA

TG

TGACCACCA  
Depth:2 (PIG)  
Ei-value:0.000, Pi-value:0.000  
Er-value:0.000, Pr-value:0.000  
No matches to TargetScan


ACCACCA

ACCACCA  
Depth:3 (COW)  
Ei-value:0.000, Pi-value:0.000  
Er-value:0.000, Pr-value:0.000  
No matches to TargetScan

AAAATCAATGTAAACTTGTCTATGTGCCACAACTGAGACA

AATTGAA

AATTGAA  
Depth:2 (PIG)  
Ei-value:0.000, Pi-value:0.020  
Er-value:0.000, Pr-value:0.010  
No matches to TargetScan

GAGTTTGTTGTTTAATGTC

AAATAAAA

AAATAAAA  
Depth:4 (DOG)  
Ei-value:0.000, Pi-value:0.000  
Er-value:0.000, Pr-value:0.000  
No matches to TargetScan


TA

AAATAAAATA  
Depth:2 (PIG)  
Ei-value:0.000, Pi-value:0.000  
Er-value:0.000, Pr-value:0.000  
No matches to TargetScan

CTG 25200  
 TT

TTTTGAAAACTT

TTTTGAAAACTT  
Depth:2 (PIG)  
Ei-value:0.000, Pi-value:0.000  
Er-value:0.000, Pr-value:0.000  
No matches to TargetScan

A                                                                                                          25215
```

---

## >COW (14055 bases)

```
 GTCATGTCCGACTCCTAGCGACCCCATGGACTGCAGCCCACCAGGCTCCTCCATCCATGAGATTCTCT

AGGCAAGA

AGGCAAGA  
Depth:3 (COW)  
Ei-value:0.000, Pi-value:0.000  
Er-value:0.000, Pr-value:0.000  
No matches to TargetScan

GTACTGGAGTGGGGTGCCATTGCCTTCTCCGACAAGGGACTGTT 120  


AAACATG

AAACATG  
Depth:4 (DOG)  
Ei-value:0.000, Pi-value:0.000  
Er-value:0.000, Pr-value:0.000  
No matches to TargetScan

ATTAAATG

CAACAG

CAACAG  
Depth:3 (COW)  
Ei-value:0.000, Pi-value:0.000  
Er-value:0.000, Pr-value:0.000  
No matches to TargetScan

CAGTTTCTTATGTTAATCTAAGATCCTA

TTCCCATC

TTCCCATC  
Depth:4 (DOG)  
Ei-value:0.000, Pi-value:0.000  
Er-value:0.000, Pr-value:0.000  
No matches to TargetScan

TAA

CTCTGT

CTCTGT  
Depth:3 (COW)  
Ei-value:0.000, Pi-value:0.000  
Er-value:0.000, Pr-value:0.000  
No matches to TargetScan

TTTCATTAATGTAGAGCCTACATGGACTTGTTAATGGGCTTATTTGCACTGCCC 240  
 TTCCTT

C

CTGTTAGTCT  
Depth:4 (DOG)  
Ei-value:0.000, Pi-value:0.000  
Er-value:0.000, Pr-value:0.000  
No matches to TargetScan


TGTTAGTC

TGTTAGTC  
Depth:5 (RABBIT)  
Ei-value:0.000, Pi-value:0.000  
Er-value:0.000, Pr-value:0.000  
No matches to TargetScan


T

CTGTTAGTCT  
Depth:4 (DOG)  
Ei-value:0.000, Pi-value:0.000  
Er-value:0.000, Pr-value:0.000  
No matches to TargetScan

AGATTCCCAGGCCATCCTTGATTTGCATTACCAAAGAGGGTATAATGGACTGTTAATGTGCTAAAATGCTGCTACAGTCCCTTATATTAACCTAGGATCTTATC 360  
 CTCAGCTAAGGCTCTTTGCAGGCTTGCAGAGGATGCTTGGGGCTGTTCATGGCCTTAAATACCACTACATCCCTTATGGTAACCTAGGATCCCATTACCAACTATTAATATCTTCCTTTT 480  
 AAGTACTGCAGAGCCTACATAGAACTTGTTAATGAACTACCATTGTTCCTTTCCTTTGTTTAGATTCCCATCTCCTAAAGATTTACATTACCACGGAGGATACTATGGACTGTTAATGTG 600  
 CCCGTACAGTTCTTTTCCCTCCCCCTCCTTTTTAAAACCCAGGATCCTATTCCCACCTAACTCCCTTTGCATTACCAGTGACTGTACAAGTGCCTGCTAATGCTTTTAAATGCAATTACA 720  
 GTCCCTTATGTTAACCTAGGATCCCACCCCCAACTAAGTCCCTTTGCATGATTGCAGAGGATACTTGGAACTGTTAATGTGATTCACTGCAGCTACATTCCTTTATCTCATCTAGGATCC 840  
 ATTCTCTTCCTTCTTGATTTGCATTATCAGAGAGGATAGAAGGGATGGTTAATGTACTTAAATGGAACTATAGTCCCTTATGATAATCAGGATCCTATTTCTACCTCACTTCCTTTGTAT 960  
 TACTTCAGAGTATACATGGGACTTTTTATGGGCTTAATTGCACTACTGTCCCTTCTTTTAGTCTAGATCCCCATCCTTCCTACTCCATTTGCATTACCCCAAAGTGTACAAGGGACTGTT 1080  
 ATTGTACATAAAGGCAGTTACAGTCCCGTGCTTTAGTCTAAGATTCCCTCTCCCCCTTTAATTCCCTCTACATTACCGAAGGGGATACAAGGGACTGCTAATGTGCTTAAGTGCTGCTTG 1200  
 CAGTCCTTTATGTTACCTAGGATCCCATCCCCACCTAACTCACTTGTCAGAGAGGCTACAAGGGACTGTTAATGTGTTTAAATGCAGGTGTAGTGCCTTAACATCGAAATCCATTCCTGC 1320  
 ATAACTCCCTTTTTCAGGACTGCAGATGGTAATTGAGACTGATAGTGAGCTTTATTGCATTTACAGTCTCTCTTAGTCTAGGATCTCATTCTCTCCTACTAGATTTACATTACCATGACT 1440  
 GTACAAGGGACTTTTAATATGCTTAAATGCAGCTGTAGTCCCTTATGTTAACCTAGGATCCTATTCCCACCTAACTTCCTTTTCATGACTGCAGAGGGTACTTAAGGACTGTTAATGCCT 1560  
 TTACTTGCCTATACAGTCTCATCTGTTGATCTAGATTCCCATGCCCTCCTACTCAATTTGCATTATAAAAGAGGGTACAAGGGACTGTTAACTTGCTTAAATGCGGCTACAGGCCCTTAG 1680  
 ATTAACCTGGGATCCCATTCCCACCTGACTCCATTTACA

TCATCC

TCATCC  
Depth:4 (DOG)  
Ei-value:0.000, Pi-value:0.020  
Er-value:0.000, Pr-value:0.000  
No matches to TargetScan

TAGAGGCGATAAGGGACTTTCATTGTGTTTGAAAGTGAAGTCGCTCAGTTGTGTCCGACTCTTTGCGACCCATGG 1800  
 ACTGTAGTTGGCCAGGCTCCTCCGTCCATGGGGTTTTCCAGGCAAGAGTACTGGACTTGGTTGCCATTTCCTTCTCCAGGGGATCTTCCTGACTCAGGGATCAAACCCGGTCTCCTGCAT 1920  
 TACAGGCAGACTCTTGACCATCTGAGCCACCAGGGAATCTCAATGTGTTTAAATGCAGGTGAAGTCCCTTATGTTAAATTCAAAGTCCTTTCCCACCTAACTGCCTTTTTATCACTGCAG 2040  
 GG

GGG

GGGTACTTGGGACTGTTAAT  
Depth:3 (COW)  
Ei-value:0.000, Pi-value:0.000  
Er-value:0.000, Pr-value:0.000  
MATCHES To TargetScan▶ miR-132-3p/212-3p:AACAGUC▶ miR-455-3p.1:CAGUCCA


TACTTGGGACTGTTAAT

TACTTGGGACTGTTAAT  
Depth:4 (DOG)  
Ei-value:0.000, Pi-value:0.000  
Er-value:0.000, Pr-value:0.000  
MATCHES To TargetScan▶ miR-132-3p/212-3p:AACAGUC▶ miR-455-3p.1:CAGUCCA

GGGACTGTACTTGCTTATCCAATTTCGTTCATTGGTCTAGATTCCCATGCTCTTCTACTTAATTTGCATTATAAAAGAGGGTACAAGGG

ACTG

ACTGTTAATGTGCT  
Depth:4 (DOG)  
Ei-value:0.000, Pi-value:0.000  
Er-value:0.000, Pr-value:0.000  
MATCHES To TargetScan▶ miR-132-3p/212-3p:AACAGUC▶ miR-323-3p:ACAUUAC


TTAAT

TTAATGTGCT  
Depth:5 (RABBIT)  
Ei-value:0.000, Pi-value:0.000  
Er-value:0.000, Pr-value:0.000  
MATCHES To TargetScan▶ miR-323-3p:ACAUUAC

 2160  


GTGCT

TTAATGTGCT  
Depth:5 (RABBIT)  
Ei-value:0.000, Pi-value:0.000  
Er-value:0.000, Pr-value:0.000  
MATCHES To TargetScan▶ miR-323-3p:ACAUUAC

TAAATGCAGCTACAGTCCTAGGATCTCACCCCTCTCTACTCACTTGTGTTACTTGTTGGTATTTGGGACAGAAAGGTGCTTAAATGCGACTGTGGTCCATTTTGTTAACCTAGGA 2280  
 TCCCATGCGCTCTAACTCTTAACATTACCTGGGAGTATACTGGGACTGTAGAACTGCTTAATTGCAACTGTGGTCCCTTCTCTTAGTCTGGAGTCCATGCTCTCCACAATTTTGTTTATA 2400  
 TTACCACAGAAGGTACTTGGAACCTGTGAATGTGTCTAAATGCAGATGTGGTCCCTTTTGTTAGTTTAGGATCCCAGGACTTCCTTTACATTATCTGTGAAGGCATTTGGGACTGTCAAG 2520  
 GTGATAATTGCTGCTGTGTTCCCTTCTGATATCTAGGATCCTGTTCCCTCCTACTTCCACTTCCTTTGCGTTACATGGAAGGGTGCTTAACTGTAGCCATGGTCCCTTCTGTTAGCCTAG 2640  
 TTTGCATTCCCTCTCTACTTCCTTTGCTTACCACAGAAGGTACTGACTATTGTGATGCATCTTATCCTCTCACATTACACCTTAGGGTCCTGAACACTAAGGCCCCTCTTTTTGTGCTTT 2760  
 TTAATGTGCAGTTGCATTTGTTCCTCTTCCTCTGCAGTGTATAAAGACTCCACTTATTTCCCTTGTATTCAGTGATGGATGTCCTTTACTCAAGACCTTTGTACTAGAGTAAACTGAACC 2880  
 GTGACAATTCACCATGAATATATTCTTTCACTTCAGTCCCTTGTATTCTGTTTTATAAAAATATATATTAATTACCTCCATTACTTTTTCTCTGGGAGTATTTACATGCAAAATTCCAGT 3000  
 GTTTAAGGCCCTTCTCTTTAAATTAATATCCCTCCCTTTTTGCATTATATCAGGGGATGTTAGTGACCCAAACTCTTTCT

CTTGGGACTC

CTTGGGACTC  
Depth:3 (COW)  
Ei-value:0.000, Pi-value:0.000  
Er-value:0.000, Pr-value:0.000  
No matches to TargetScan


AATGTGCAT

AATGTGCAT  
Depth:6 (MOUSE)  
Ei-value:0.000, Pi-value:0.000  
Er-value:0.000, Pr-value:0.000  
MATCHES To TargetScan▶ miR-501-3p/502-3p:AUGCACC

GTGCCCTCATGACAAA

CTAAT

CTAATA  
Depth:3 (COW)  
Ei-value:0.000, Pi-value:0.000  
Er-value:0.000, Pr-value:0.000  
No matches to TargetScan

 3120  


A

CTAATA  
Depth:3 (COW)  
Ei-value:0.000, Pi-value:0.000  
Er-value:0.000, Pr-value:0.000  
No matches to TargetScan

TATTGAATTTTTAGTATTAAAACAGGGACTACTGACTAGTTTTTATTTTGGGTAGTTAATGTGCAGAATTACACTAGGAACCCATCCCATTCCACCTTCTTTGCATCAGGGGTCCTAAT 3240  
 CAACTGGCTGC

TGCTTCT

TGCTTCT  
Depth:3 (COW)  
Ei-value:0.000, Pi-value:0.000  
Er-value:0.000, Pr-value:0.010  
No matches to TargetScan

GTACAGTTTAAATGTGCATAATTTCAATAGTCCATGCTCCCT

TATGTTAGA

TATGTTAGA  
Depth:4 (DOG)  
Ei-value:0.000, Pi-value:0.000  
Er-value:0.000, Pr-value:0.000  
No matches to TargetScan

CTAGGATCCCCTATCCTTGCCCCATTTACATTACGGCAGGGGCTTCTGACT 3360  
 AGCTAAGATTCACTG

TCTTGG

TCTTGGACTGTTAATGT  
Depth:3 (COW)  
Ei-value:0.000, Pi-value:0.000  
Er-value:0.000, Pr-value:0.000  
MATCHES To TargetScan▶ miR-132-3p/212-3p:AACAGUC▶ miR-323-3p:ACAUUAC▶ miR-455-3p.1:CAGUCCA


ACTGTTAATGT

ACTGTTAATGT  
Depth:4 (DOG)  
Ei-value:0.000, Pi-value:0.000  
Er-value:0.000, Pr-value:0.000  
MATCHES To TargetScan▶ miR-132-3p/212-3p:AACAGUC▶ miR-323-3p:ACAUUAC

ATGTACTCAC

ATTTGCT

ATTTGCT  
Depth:4 (DOG)  
Ei-value:0.000, Pi-value:0.000  
Er-value:0.000, Pr-value:0.000  
No matches to TargetScan

CCCGTACCTGTACCTCAG

GTAAGGA

GTAAGGA  
Depth:5 (RABBIT)  
Ei-value:0.000, Pi-value:0.000  
Er-value:0.000, Pr-value:0.000  
No matches to TargetScan


CCC

GTAAGGACCC  
Depth:3 (COW)  
Ei-value:0.000, Pi-value:0.000  
Er-value:0.000, Pr-value:0.000  
No matches to TargetScan

TCCCCATTCTATTTACATTTTAGCAGGAGATGCCTACTATTCA 3480  
 AGATCTTACACACTATTGCCGTTAATGTGCACC

ATCTTAG

ATCTTAG  
Depth:3 (COW)  
Ei-value:0.000, Pi-value:0.000  
Er-value:0.000, Pr-value:0.000  
No matches to TargetScan

TTGTCTTG

TACACATT

TACACATT  
Depth:3 (COW)  
Ei-value:0.000, Pi-value:0.000  
Er-value:0.000, Pr-value:0.000  
No matches to TargetScan

CTCAGTTGTCCACAGCTGTGCTTTTTAAATCAGGGCCCTTTC

ACTTAT

ACTTAT  
Depth:5 (RABBIT)  
Ei-value:0.000, Pi-value:0.000  
Er-value:0.000, Pr-value:0.000  
No matches to TargetScan

TGAAGCACAGGTTAAC 3600  
 TTACCACCTTAAAGCCCTTGTTATGGGACTGTGACTATG

TGTAATT

TGTAATT  
Depth:3 (COW)  
Ei-value:0.000, Pi-value:0.000  
Er-value:0.000, Pr-value:0.000  
No matches to TargetScan

ACAATTGTCT

ATGGTC

ATGGTC  
Depth:3 (COW)  
Ei-value:0.000, Pi-value:0.020  
Er-value:0.000, Pr-value:0.000  
No matches to TargetScan

TTCTGAGTTAACTTGTCTCCCTGACTCTTTTCATCCTCTTTGCTTTACTGC

ATGGGGT

ATGGGGTACT  
Depth:3 (COW)  
Ei-value:0.000, Pi-value:0.000  
Er-value:0.000, Pr-value:0.000  
No matches to TargetScan

 3720  


ACT

ATGGGGTACT  
Depth:3 (COW)  
Ei-value:0.000, Pi-value:0.000  
Er-value:0.000, Pr-value:0.000  
No matches to TargetScan

GCC

CAC

CACTTAAGGCCCCTTTCTCAA  
Depth:3 (COW)  
Ei-value:0.000, Pi-value:0.000  
Er-value:0.000, Pr-value:0.000  
No matches to TargetScan


TTAAGGCC

TTAAGGCC  
Depth:6 (MOUSE)  
Ei-value:0.000, Pi-value:0.000  
Er-value:0.000, Pr-value:0.000  
No matches to TargetScan


CCTTT

TTAAGGCCCCTTT  
Depth:5 (RABBIT)  
Ei-value:0.000, Pi-value:0.000  
Er-value:0.000, Pr-value:0.000  
No matches to TargetScan


CTCAA

TTAAGGCCCCTTTCTCAA  
Depth:4 (DOG)  
Ei-value:0.000, Pi-value:0.000  
Er-value:0.000, Pr-value:0.000  
No matches to TargetScan

GTATGTG

TAATGACAATTACAT

TAATGACAATTACAT  
Depth:3 (COW)  
Ei-value:0.000, Pi-value:0.000  
Er-value:0.000, Pr-value:0.000  
MATCHES To TargetScan▶ miR-411-3p:AUGUAAC

TATAGGGGCTTCATACTCCCTTTAAGATT

CTTATATTT

CTTATATTT  
Depth:3 (COW)  
Ei-value:0.000, Pi-value:0.000  
Er-value:0.000, Pr-value:0.000  
MATCHES To TargetScan▶ miR-410-3p:AUAUAAC

ATACAAGGTATATGA

TTTTAATTGACCA

TTTTAATTGACCA  
Depth:3 (COW)  
Ei-value:0.000, Pi-value:0.000  
Er-value:0.000, Pr-value:0.000  
No matches to TargetScan

CACCA 3840  
 TGTCCCTTCA

ACATTAAT

ACATTAAT  
Depth:3 (COW)  
Ei-value:0.000, Pi-value:0.000  
Er-value:0.000, Pr-value:0.000  
No matches to TargetScan

TTA

CATAATTGCA

CATAATTGCA  
Depth:3 (COW)  
Ei-value:0.000, Pi-value:0.000  
Er-value:0.000, Pr-value:0.000  
No matches to TargetScan

ACTTAGTTCGTCTTATCCTGTTGCCCTCCCTTTCCTTAGCTTGTGGATGTGCATTCTTGTGCA

CTAGACAAGGA

CTAGACAAGGA  
Depth:3 (COW)  
Ei-value:0.000, Pi-value:0.000  
Er-value:0.000, Pr-value:0.000  
No matches to TargetScan

CCCTCCCCCTCACTT 3960  
 CCTATGCCTTCCGTAAGTTTGCCCATTGCTAAGACCTTTCCATTTGG

ACAGTTAATGTG

ACAGTTAATGTG  
Depth:4 (DOG)  
Ei-value:0.000, Pi-value:0.000  
Er-value:0.000, Pr-value:0.000  
MATCHES To TargetScan▶ miR-323-3p:ACAUUAC

CAGAGTTGCACTTTCCCATAACCCTATTACTTCTAGGACTATTGTATCTCCTTTGTAATAG 4080  
 TGCAGGGG

ATACTGTTT

ATACTGTTT  
Depth:3 (COW)  
Ei-value:0.000, Pi-value:0.000  
Er-value:0.000, Pr-value:0.000  
MATCHES To TargetScan▶ miR-101-3p.1:ACAGUAC▶ miR-132-3p/212-3p:AACAGUC▶ miR-144-3p:ACAGUAU

CCCCGCCAAGATCCCTTACTGTTAATATACGTAATAGAAATTC

TTGTCTT

TTGTCTT  
Depth:3 (COW)  
Ei-value:0.000, Pi-value:0.000  
Er-value:0.000, Pr-value:0.010  
No matches to TargetScan

TATCAGTGTACTTTGGTAATACTCCCTCAGCCAGCTATCATGAC

CTCAGCTCT

CTCAGCTCTTGG  
Depth:5 (RABBIT)  
Ei-value:0.000, Pi-value:0.000  
Er-value:0.000, Pr-value:0.000  
MATCHES To TargetScan▶ miR-335-5p:CAAGAGC

 4200  


TGG

CTCAGCTCTTGG  
Depth:5 (RABBIT)  
Ei-value:0.000, Pi-value:0.000  
Er-value:0.000, Pr-value:0.000  
MATCHES To TargetScan▶ miR-335-5p:CAAGAGC


ACA

CTCAGCTCTTGGACA  
Depth:4 (DOG)  
Ei-value:0.000, Pi-value:0.000  
Er-value:0.000, Pr-value:0.000  
MATCHES To TargetScan▶ miR-335-5p:CAAGAGC


ATTAATA

CTCAGCTCTTGGACAATTAATA  
Depth:3 (COW)  
Ei-value:0.000, Pi-value:0.000  
Er-value:0.000, Pr-value:0.000  
MATCHES To TargetScan▶ miR-335-5p:CAAGAGC

TGCACCAATAATATATCAAACGTATGATCTTTAGGTAGGATCCCTGTACCCTCACTATG

GATCAT

GATCAT  
Depth:3 (COW)  
Ei-value:0.000, Pi-value:0.000  
Er-value:0.000, Pr-value:0.000  
No matches to TargetScan

GACTGCTGACT

TAAGGC

TAAGGC  
Depth:3 (COW)  
Ei-value:0.000, Pi-value:0.000  
Er-value:0.000, Pr-value:0.000  
No matches to TargetScan

TTCTTTTTTTGACTCTGGGTGT

GAA

GAATATTTGCA  
Depth:3 (COW)  
Ei-value:0.000, Pi-value:0.000  
Er-value:0.000, Pr-value:0.000  
No matches to TargetScan

 4320  


TATTTGCA

GAATATTTGCA  
Depth:3 (COW)  
Ei-value:0.000, Pi-value:0.000  
Er-value:0.000, Pr-value:0.000  
No matches to TargetScan

GTTACCTATACACCCTTCTATTAGAGTAGGACATCATTCCCTCATACCCCCTTTGC

ATTACTG

ATTACTG  
Depth:3 (COW)  
Ei-value:0.000, Pi-value:0.010  
Er-value:0.000, Pr-value:0.020  
MATCHES To TargetScan▶ miR-802:CAGUAAC

CAT

GGGCTGCTGA

GGGCTGCTGA  
Depth:3 (COW)  
Ei-value:0.000, Pi-value:0.000  
Er-value:0.000, Pr-value:0.000  
MATCHES To TargetScan▶ miR-15-5p/16-5p/195-5p/424-5p/497-5p:AGCAGCA▶ miR-503-5p:AGCAGCG

TGACA

CAAAACTT

CAAAACTT  
Depth:4 (DOG)  
Ei-value:0.000, Pi-value:0.000  
Er-value:0.000, Pr-value:0.000  
No matches to TargetScan

TCA

CTGGGACTG

CTGGGACTG  
Depth:3 (COW)  
Ei-value:0.000, Pi-value:0.000  
Er-value:0.000, Pr-value:0.000  
MATCHES To TargetScan▶ miR-455-3p.1:CAGUCCA

CTGATGA

GCAC

GCACAATG  
Depth:6 (MOUSE)  
Ei-value:0.000, Pi-value:0.000  
Er-value:0.000, Pr-value:0.000  
No matches to TargetScan

 4440  


AATG

GCACAATG  
Depth:6 (MOUSE)  
Ei-value:0.000, Pi-value:0.000  
Er-value:0.000, Pr-value:0.000  
No matches to TargetScan

GCAGTTGGCAATGGGTTTTTC

CTCCCTG

CTCCCTG  
Depth:3 (COW)  
Ei-value:0.000, Pi-value:0.000  
Er-value:0.000, Pr-value:0.000  
No matches to TargetScan

GTCTTGTTAG

GCAAGC

GCAAGC  
Depth:3 (COW)  
Ei-value:0.000, Pi-value:0.000  
Er-value:0.000, Pr-value:0.000  
No matches to TargetScan


A

ACTCCCA  
Depth:4 (DOG)  
Ei-value:0.000, Pi-value:0.000  
Er-value:0.000, Pr-value:0.000  
No matches to TargetScan


CTCCCA

CTCCCA  
Depth:6 (MOUSE)  
Ei-value:0.000, Pi-value:0.000  
Er-value:0.000, Pr-value:0.000  
No matches to TargetScan

GCTCCAAATTCTCATGGTATATTAAACTATAAACACTGCACTGTGCTGTGAACTTGTCCATCAAA 4560  
 GGGCGACCCACATTCCCACTCCTTTTGCGTTGGCTCCAGTGTATCAAATTCCAAGTAATCATGGCCTTGCCCTTATCTTCCCTCTACAAACCCCTTGCCTGCCCTTGGACCCTTCTTATG 4680  
 GATTTTTAATGTTCACAATTCCAGGTGTCCATCTCTCCAGATAGGATTATGCTCCCTCACCCCTCCCACCCCTGCCCTGC

CCCTTTTGCATT

CCCTTTTGCATT  
Depth:4 (DOG)  
Ei-value:0.000, Pi-value:0.000  
Er-value:0.000, Pr-value:0.000  
No matches to TargetScan


G

CCCTTTTGCATTG  
Depth:3 (COW)  
Ei-value:0.000, Pi-value:0.000  
Er-value:0.000, Pr-value:0.000  
No matches to TargetScan

TTGCCGGGAAATGTTGACTGAGCAAAA 4800  
 CTCTTTTCTCTTGAATTTACAATGTTAACAGTCCCAGATGTCATTGTTCTGCCCACTTTGAATTTCTATAATTCCCTGTTGCACTTTCCTTGGTCCCACCCATCACGGACTCTTCC

ACTT

ACTTCCTT  
Depth:3 (COW)  
Ei-value:0.000, Pi-value:0.000  
Er-value:0.000, Pr-value:0.000  
No matches to TargetScan

 4920  


CCTT

ACTTCCTT  
Depth:3 (COW)  
Ei-value:0.000, Pi-value:0.000  
Er-value:0.000, Pr-value:0.000  
No matches to TargetScan

CTTTGCATTACTTCTGAATAGTGCTGACCACCCAC

AGCCCCTTCT

AGCCCCTTCT  
Depth:3 (COW)  
Ei-value:0.000, Pi-value:0.000  
Er-value:0.000, Pr-value:0.000  
No matches to TargetScan

TTGTTGTTAA

CACAGTA

CACAGTA  
Depth:3 (COW)  
Ei-value:0.000, Pi-value:0.000  
Er-value:0.000, Pr-value:0.000  
No matches to TargetScan

C

TGATTGTC

TGATTGTCCCATTTTT  
Depth:3 (COW)  
Ei-value:0.000, Pi-value:0.000  
Er-value:0.000, Pr-value:0.000  
No matches to TargetScan


CCATTTTT

CCATTTTT  
Depth:4 (DOG)  
Ei-value:0.000, Pi-value:0.000  
Er-value:0.000, Pr-value:0.000  
No matches to TargetScan

AAGCCCAT

CAGCCCA

CAGCCCA  
Depth:4 (DOG)  
Ei-value:0.000, Pi-value:0.000  
Er-value:0.000, Pr-value:0.000  
No matches to TargetScan

AGA

TCTC

TCTCCCTACCA  
Depth:3 (COW)  
Ei-value:0.000, Pi-value:0.000  
Er-value:0.000, Pr-value:0.000  
No matches to TargetScan


CCTACCA

CCTACCA  
Depth:4 (DOG)  
Ei-value:0.000, Pi-value:0.000  
Er-value:0.000, Pr-value:0.000  
No matches to TargetScan

TTTTGATG 5040  
 TTATATTT

GTGCAGT

GTGCAGT  
Depth:3 (COW)  
Ei-value:0.000, Pi-value:0.000  
Er-value:0.000, Pr-value:0.000  
MATCHES To TargetScan▶ miR-217:ACUGCAU

ATGGACTACC

AAAAGCAG

AAAAGCAG  
Depth:6 (MOUSE)  
Ei-value:0.000, Pi-value:0.000  
Er-value:0.000, Pr-value:0.000  
No matches to TargetScan

GCCA

GAACTA

GAACTA  
Depth:3 (COW)  
Ei-value:0.000, Pi-value:0.000  
Er-value:0.000, Pr-value:0.000  
No matches to TargetScan

GGTGACTGGACCTTACTCCTTTTCCTGCATTTG

TTAATGATCC

TTAATGATCC  
Depth:4 (DOG)  
Ei-value:0.000, Pi-value:0.000  
Er-value:0.000, Pr-value:0.000  
MATCHES To TargetScan▶ miR-382-3p:AUCAUUC

CAGTTCTG

ATTATTGT

ATTATTGT  
Depth:3 (COW)  
Ei-value:0.000, Pi-value:0.000  
Er-value:0.000, Pr-value:0.000  
No matches to TargetScan

CAT

ATTCTGGG

ATTCTGGG  
Depth:4 (DOG)  
Ei-value:0.000, Pi-value:0.000  
Er-value:0.000, Pr-value:0.000  
No matches to TargetScan

AACAGAA 5160  
 CCGTTCCTGATCCCCTCTGTTAG

TG

TGCTTTACT  
Depth:3 (COW)  
Ei-value:0.000, Pi-value:0.000  
Er-value:0.000, Pr-value:0.000  
MATCHES To TargetScan▶ miR-330-3p.2:AAAGCAC


CTTTACT

CTTTACT  
Depth:4 (DOG)  
Ei-value:0.000, Pi-value:0.000  
Er-value:0.000, Pr-value:0.000  
No matches to TargetScan

AG

GCAAAAT

GCAAAAT  
Depth:6 (MOUSE)  
Ei-value:0.000, Pi-value:0.000  
Er-value:0.000, Pr-value:0.000  
No matches to TargetScan

GCTC

AAGGCAA

AAGGCAA  
Depth:4 (DOG)  
Ei-value:0.000, Pi-value:0.000  
Er-value:0.000, Pr-value:0.000  
No matches to TargetScan


GTCAGACCCA

AAGGCAAGTCAGACCCA  
Depth:3 (COW)  
Ei-value:0.000, Pi-value:0.000  
Er-value:0.000, Pr-value:0.000  
MATCHES To TargetScan▶ miR-193a-5p:GGGUCUU

AAGGAAC

TGGATTGC

TGGATTGC  
Depth:4 (DOG)  
Ei-value:0.000, Pi-value:0.000  
Er-value:0.000, Pr-value:0.000  
No matches to TargetScan

TACCCTTTATTTGGGGTTTTCATTATAAATAATCATTTGAAAA 5280  
 TTGACTTCCCAA

GAAGGAAG

GAAGGAAG  
Depth:3 (COW)  
Ei-value:0.000, Pi-value:0.000  
Er-value:0.000, Pr-value:0.000  
No matches to TargetScan

GTTAGCACTATCTG

TGCATTCTTC

TGCATTCTTC  
Depth:5 (RABBIT)  
Ei-value:0.000, Pi-value:0.000  
Er-value:0.000, Pr-value:0.000  
No matches to TargetScan

CTTTCAA

AGC

AGCAGATTGCCTGG  
Depth:4 (DOG)  
Ei-value:0.000, Pi-value:0.000  
Er-value:0.000, Pr-value:0.000  
No matches to TargetScan


A

AGATTGCCTGG  
Depth:5 (RABBIT)  
Ei-value:0.000, Pi-value:0.000  
Er-value:0.000, Pr-value:0.000  
No matches to TargetScan


GATTGCCTGG

GATTGCCTGG  
Depth:6 (MOUSE)  
Ei-value:0.000, Pi-value:0.000  
Er-value:0.000, Pr-value:0.000  
No matches to TargetScan

CTATGTCTCTCCTTTCCTC

TTGTATATT

TTGTATATT  
Depth:4 (DOG)  
Ei-value:0.000, Pi-value:0.000  
Er-value:0.000, Pr-value:0.000  
MATCHES To TargetScan▶ miR-381-3p:AUACAAG

GCCATTGTATAG

TGCCAA

TGCCAA  
Depth:3 (COW)  
Ei-value:0.000, Pi-value:0.000  
Er-value:0.000, Pr-value:0.000  
MATCHES To TargetScan▶ miR-182-5p:UUGGCAA▶ miR-96-5p/1271-5p:UUGGCAC

T

TGCCAGGA

TGCCAGGATACA  
Depth:3 (COW)  
Ei-value:0.000, Pi-value:0.000  
Er-value:0.000, Pr-value:0.000  
No matches to TargetScan

 5400  


TACA

TGCCAGGATACA  
Depth:3 (COW)  
Ei-value:0.000, Pi-value:0.000  
Er-value:0.000, Pr-value:0.000  
No matches to TargetScan

ACCAAAAAGTTTATTTATTTTTTATTTTTTTTATTTTTTAAGAAAG

ACATCTGG

ACATCTGG  
Depth:3 (COW)  
Ei-value:0.000, Pi-value:0.000  
Er-value:0.000, Pr-value:0.000  
No matches to TargetScan

ATTGCAGGGTGGAATT

GAT

GATAACCTGGTCATT  
Depth:3 (COW)  
Ei-value:0.000, Pi-value:0.000  
Er-value:0.000, Pr-value:0.000  
MATCHES To TargetScan▶ miR-154-5p:AGGUUAU


AAC

AACCTGGTCATT  
Depth:4 (DOG)  
Ei-value:0.000, Pi-value:0.000  
Er-value:0.000, Pr-value:0.000  
No matches to TargetScan


CTGGTCATT

CTGGTCATT  
Depth:5 (RABBIT)  
Ei-value:0.000, Pi-value:0.000  
Er-value:0.000, Pr-value:0.000  
No matches to TargetScan

GAAA

TTTTGAA

TTTTGAA  
Depth:3 (COW)  
Ei-value:0.000, Pi-value:0.000  
Er-value:0.000, Pr-value:0.010  
No matches to TargetScan

ATTGGTAAAC

CCATTTAT

CCATTTAT  
Depth:5 (RABBIT)  
Ei-value:0.000, Pi-value:0.000  
Er-value:0.000, Pr-value:0.000  
No matches to TargetScan

AT 5520  
 CATGTACCTGA

TGAC

TGACCAGTGTCTCTCATTT  
Depth:4 (DOG)  
Ei-value:0.000, Pi-value:0.000  
Er-value:0.000, Pr-value:0.000  
No matches to TargetScan


CAGTGTCTCTCATTT

CAGTGTCTCTCATTT  
Depth:5 (RABBIT)  
Ei-value:0.000, Pi-value:0.000  
Er-value:0.000, Pr-value:0.000  
No matches to TargetScan

TACTA

AGG

AGGGTGGTG  
Depth:4 (DOG)  
Ei-value:0.000, Pi-value:0.000  
Er-value:0.000, Pr-value:0.000  
No matches to TargetScan


GTGGTG

GTGGTG  
Depth:5 (RABBIT)  
Ei-value:0.000, Pi-value:0.000  
Er-value:0.000, Pr-value:0.000  
No matches to TargetScan

A

GTCTGTGGATA

GTCTGTGGATA  
Depth:5 (RABBIT)  
Ei-value:0.000, Pi-value:0.000  
Er-value:0.000, Pr-value:0.000  
MATCHES To TargetScan▶ miR-140-3p.1:CCACAGG


GA

GTCTGTGGATAGA  
Depth:3 (COW)  
Ei-value:0.000, Pi-value:0.000  
Er-value:0.000, Pr-value:0.000  
MATCHES To TargetScan▶ miR-140-3p.1:CCACAGG

CCACTGTGACTTTGA

TATTTTA

TATTTTA  
Depth:3 (COW)  
Ei-value:0.000, Pi-value:0.040  
Er-value:0.000, Pr-value:0.020  
No matches to TargetScan

GTATATTACCAAAGGGG

TTCTAGA

TTCTAGA  
Depth:4 (DOG)  
Ei-value:0.000, Pi-value:0.000  
Er-value:0.000, Pr-value:0.000  
No matches to TargetScan

GTGGAACTCTTAAGAC 5640  
 C

AGTATCTTTG

AGTATCTTTG  
Depth:3 (COW)  
Ei-value:0.000, Pi-value:0.000  
Er-value:0.000, Pr-value:0.000  
No matches to TargetScan

GGCTCTACCACCATTTTCAAACCACTCCTTGTTTGAGCTTTACCAGT

ATTCACTT

ATTCACTT  
Depth:4 (DOG)  
Ei-value:0.000, Pi-value:0.000  
Er-value:0.000, Pr-value:0.000  
No matches to TargetScan

CTAG

GAAAAAC

GAAAAAC  
Depth:4 (DOG)  
Ei-value:0.000, Pi-value:0.000  
Er-value:0.000, Pr-value:0.000  
No matches to TargetScan

TATCTAAACTTCCTAATCGTTA

AATTTCTTCATCTGGAGC

AATTTCTTCATCTGGAGC  
Depth:5 (RABBIT)  
Ei-value:0.000, Pi-value:0.000  
Er-value:0.000, Pr-value:0.000  
No matches to TargetScan

TCC 5760  
 AACTAGTCCGTA

CTTATTT

CTTATTT  
Depth:4 (DOG)  
Ei-value:0.000, Pi-value:0.000  
Er-value:0.000, Pr-value:0.010  
No matches to TargetScan


CAAGAA

CTTATTTCAAGAA  
Depth:3 (COW)  
Ei-value:0.000, Pi-value:0.000  
Er-value:0.000, Pr-value:0.000  
MATCHES To TargetScan▶ miR-203a-3p.2:UGAAAUG

GATTGCTGTAAAAGG

ATAAAATG

ATAAAATG  
Depth:4 (DOG)  
Ei-value:0.000, Pi-value:0.000  
Er-value:0.000, Pr-value:0.000  
No matches to TargetScan


A

ATAAAATGA  
Depth:3 (COW)  
Ei-value:0.000, Pi-value:0.000  
Er-value:0.000, Pr-value:0.000  
No matches to TargetScan

GAGAACATGCTGAGGTGCTTTTGAAAACCATAGATCACTTTGTTTGATTAATCTATTACATTTTACGTACC 5880  
 CTGTTTAATTCTGCTATTTTAAAGTTACCTAAAGC

ACCACACT

ACCACACT  
Depth:3 (COW)  
Ei-value:0.000, Pi-value:0.000  
Er-value:0.000, Pr-value:0.000  
No matches to TargetScan

CAA

GTGAGG

GTGAGG  
Depth:3 (COW)  
Ei-value:0.000, Pi-value:0.000  
Er-value:0.000, Pr-value:0.000  
No matches to TargetScan

ACTTAGAAACGATGGAACCAGTTTCCCCA

TTTTATA

TTTTATA  
Depth:3 (COW)  
Ei-value:0.000, Pi-value:0.000  
Er-value:0.000, Pr-value:0.010  
MATCHES To TargetScan▶ miR-340-5p:UAUAAAG

TGAAAAG

AAAAATAAGCCA

AAAAATAAGCCA  
Depth:5 (RABBIT)  
Ei-value:0.000, Pi-value:0.000  
Er-value:0.000, Pr-value:0.000  
No matches to TargetScan


A

AAAAATAAGCCAA  
Depth:4 (DOG)  
Ei-value:0.000, Pi-value:0.000  
Er-value:0.000, Pr-value:0.000  
No matches to TargetScan

GGTCTAATCAG

T

TCTTTTGGATATA  
Depth:3 (COW)  
Ei-value:0.000, Pi-value:0.000  
Er-value:0.000, Pr-value:0.000  
No matches to TargetScan

 6000  


CTTTTGGATATA

TCTTTTGGATATA  
Depth:3 (COW)  
Ei-value:0.000, Pi-value:0.000  
Er-value:0.000, Pr-value:0.000  
No matches to TargetScan

AATTTCAACAGTGAAAATGAATACAA

ATGAATAATA

ATGAATAATA  
Depth:4 (DOG)  
Ei-value:0.000, Pi-value:0.000  
Er-value:0.000, Pr-value:0.000  
No matches to TargetScan

TCTCAGTCTCT

AGTGTACA

AGTGTACA  
Depth:3 (COW)  
Ei-value:0.000, Pi-value:0.000  
Er-value:0.000, Pr-value:0.000  
MATCHES To TargetScan▶ miR-493-5p:UGUACAU

A

GGTGTTT

GGTGTTT  
Depth:3 (COW)  
Ei-value:0.000, Pi-value:0.000  
Er-value:0.000, Pr-value:0.000  
No matches to TargetScan

GGCAGAGAAGTATTTAATA

TGGAACTGCT

TGGAACTGCT  
Depth:4 (DOG)  
Ei-value:0.000, Pi-value:0.000  
Er-value:0.000, Pr-value:0.000  
No matches to TargetScan

GAAGCAAG

TAACTA

TAACTA  
Depth:4 (DOG)  
Ei-value:0.000, Pi-value:0.000  
Er-value:0.000, Pr-value:0.000  
No matches to TargetScan

AT 6120  
 TATCACCA

CAGCAGTTC

CAGCAGTTC  
Depth:5 (RABBIT)  
Ei-value:0.000, Pi-value:0.000  
Er-value:0.000, Pr-value:0.000  
No matches to TargetScan

T

TTGTAAT

TTGTAAT  
Depth:4 (DOG)  
Ei-value:0.000, Pi-value:0.000  
Er-value:0.000, Pr-value:0.000  
No matches to TargetScan

C

ACTGAAAA

ACTGAAAA  
Depth:5 (RABBIT)  
Ei-value:0.000, Pi-value:0.000  
Er-value:0.000, Pr-value:0.000  
No matches to TargetScan

AGGATACTGTTGTCT

GAG

GAGAAGGATGTCAAAAGATCGGC  
Depth:3 (COW)  
Ei-value:0.000, Pi-value:0.000  
Er-value:0.000, Pr-value:0.000  
MATCHES To TargetScan▶ miR-362-5p/500b-5p:AUCCUUG▶ miR-489-3p:UGACAUC


AAGGATG

AAGGATG  
Depth:5 (RABBIT)  
Ei-value:0.000, Pi-value:0.000  
Er-value:0.000, Pr-value:0.000  
MATCHES To TargetScan▶ miR-362-5p/500b-5p:AUCCUUG


TCA

AAGGATGTCAAAAGATC  
Depth:4 (DOG)  
Ei-value:0.000, Pi-value:0.000  
Er-value:0.000, Pr-value:0.000  
MATCHES To TargetScan▶ miR-362-5p/500b-5p:AUCCUUG▶ miR-489-3p:UGACAUC


AAAGATC

AAAGATC  
Depth:6 (MOUSE)  
Ei-value:0.000, Pi-value:0.000  
Er-value:0.000, Pr-value:0.000  
No matches to TargetScan


GGC

GAGAAGGATGTCAAAAGATCGGC  
Depth:3 (COW)  
Ei-value:0.000, Pi-value:0.000  
Er-value:0.000, Pr-value:0.000  
MATCHES To TargetScan▶ miR-362-5p/500b-5p:AUCCUUG▶ miR-489-3p:UGACAUC

C

CAGCTCAGGG

CAGCTCAGGG  
Depth:4 (DOG)  
Ei-value:0.000, Pi-value:0.000  
Er-value:0.000, Pr-value:0.000  
MATCHES To TargetScan▶ miR-125-5p:CCCUGAG

T

GCAGTTTGC

GCAGTTTGC  
Depth:3 (COW)  
Ei-value:0.000, Pi-value:0.000  
Er-value:0.000, Pr-value:0.000  
No matches to TargetScan

A

CTACTAGCTCCT

CTACTAGCTCCT  
Depth:4 (DOG)  
Ei-value:0.000, Pi-value:0.000  
Er-value:0.000, Pr-value:0.000  
MATCHES To TargetScan▶ miR-28-5p/708-5p:AGGAGCU▶ miR-411-5p.2:UAGUAGA

T

GGACAGCTG

GGACAGCTG  
Depth:5 (RABBIT)  
Ei-value:0.000, Pi-value:0.000  
Er-value:0.000, Pr-value:0.000  
No matches to TargetScan


T

GGACAGCTGT  
Depth:4 (DOG)  
Ei-value:0.000, Pi-value:0.000  
Er-value:0.000, Pr-value:0.000  
No matches to TargetScan


A

AAGAAGAGTCTCTGGCTCTTTAGA  
Depth:3 (COW)  
Ei-value:0.000, Pi-value:0.000  
Er-value:0.000, Pr-value:0.000  
No matches to TargetScan


AG

AGAAGAGTCTCTGGCTCTTTA  
Depth:5 (RABBIT)  
Ei-value:0.000, Pi-value:0.000  
Er-value:0.000, Pr-value:0.000  
No matches to TargetScan

 6240  


AAGAGTCTCTGGCTCTTTA

AGAAGAGTCTCTGGCTCTTTA  
Depth:5 (RABBIT)  
Ei-value:0.000, Pi-value:0.000  
Er-value:0.000, Pr-value:0.000  
No matches to TargetScan


GA

AGAAGAGTCTCTGGCTCTTTAGA  
Depth:4 (DOG)  
Ei-value:0.000, Pi-value:0.000  
Er-value:0.000, Pr-value:0.000  
No matches to TargetScan

ATACTGGATGA

ATTCTGAGC

ATTCTGAGC  
Depth:4 (DOG)  
Ei-value:0.000, Pi-value:0.000  
Er-value:0.000, Pr-value:0.000  
No matches to TargetScan

TGGTTCCCCCCCACTCAAGAGGAAGGATGGATCAAGTTTAGGTGGAGTGAAGCCTGCACTGGACAGCATCCAAAGGACA 6360  
 TTCCAAGCATATCAGACCTGAG

GA

GACTGCAA  
Depth:3 (COW)  
Ei-value:0.000, Pi-value:0.000  
Er-value:0.000, Pr-value:0.000  
MATCHES To TargetScan▶ miR-455-3p.2:UGCAGUC


CTGCAA

CTGCAA  
Depth:5 (RABBIT)  
Ei-value:0.000, Pi-value:0.000  
Er-value:0.000, Pr-value:0.000  
No matches to TargetScan

GCAACTTTAAGAAGCTTCATATTCAGCAGGTCTTTCCT

TTTGAGAATCTGG

TTTGAGAATCTGG  
Depth:3 (COW)  
Ei-value:0.000, Pi-value:0.000  
Er-value:0.000, Pr-value:0.000  
MATCHES To TargetScan▶ miR-371-5p:CUCAAAC

AT

AAGCTCCA

AAGCTCCA  
Depth:3 (COW)  
Ei-value:0.000, Pi-value:0.000  
Er-value:0.000, Pr-value:0.000  
No matches to TargetScan

ACCAATCTCATA

GGATGG

GGATGG  
Depth:3 (COW)  
Ei-value:0.000, Pi-value:0.000  
Er-value:0.000, Pr-value:0.010  
No matches to TargetScan

CTTGCAGTTTC 6480  
 C

CTGGAGAAAAAGATCT

CTGGAGAAAAAGATCT  
Depth:3 (COW)  
Ei-value:0.000, Pi-value:0.000  
Er-value:0.000, Pr-value:0.000  
No matches to TargetScan

ACCTCAA

AAGAATAGGC

AAGAATAGGC  
Depth:5 (RABBIT)  
Ei-value:0.000, Pi-value:0.000  
Er-value:0.000, Pr-value:0.000  
No matches to TargetScan

CTGTTGCT

T

TTACAGTGTTAGTGA  
Depth:3 (COW)  
Ei-value:0.000, Pi-value:0.000  
Er-value:0.000, Pr-value:0.000  
MATCHES To TargetScan▶ miR-141-3p/200a-3p:AACACUG


TACAGTGTTAGTGA

TACAGTGTTAGTGA  
Depth:5 (RABBIT)  
Ei-value:0.000, Pi-value:0.000  
Er-value:0.000, Pr-value:0.000  
MATCHES To TargetScan▶ miR-141-3p/200a-3p:AACACUG

CC

CA

CATTCCCTTTGA  
Depth:3 (COW)  
Ei-value:0.000, Pi-value:0.000  
Er-value:0.000, Pr-value:0.000  
MATCHES To TargetScan▶ miR-1-3p/206:GGAAUGU


TTCCCTTTGA

TTCCCTTTGA  
Depth:6 (MOUSE)  
Ei-value:0.000, Pi-value:0.000  
Er-value:0.000, Pr-value:0.000  
No matches to TargetScan

CGATTCC

TAGGTGGAGATGGGGCATGAGGATCCTCCAGGGGAA

TAGGTGGAGATGGGGCATGAGGATCCTCCAGGGGAA  
Depth:6 (MOUSE)  
Ei-value:0.000, Pi-value:0.000  
Er-value:0.000, Pr-value:0.000  
MATCHES To TargetScan▶ miR-331-3p:CCCCUGG


A

TAGGTGGAGATGGGGCATGAGGATCCTCCAGGGGAAA  
Depth:5 (RABBIT)  
Ei-value:0.000, Pi-value:0.000  
Er-value:0.000, Pr-value:0.000  
MATCHES To TargetScan▶ miR-331-3p:CCCCUGG

GAT

TC

TCACTA  
Depth:5 (RABBIT)  
Ei-value:0.000, Pi-value:0.000  
Er-value:0.000, Pr-value:0.000  
No matches to TargetScan

 6600  


ACTA

TCACTA  
Depth:5 (RABBIT)  
Ei-value:0.000, Pi-value:0.000  
Er-value:0.000, Pr-value:0.000  
No matches to TargetScan


CCACT

TCACTACCACT  
Depth:4 (DOG)  
Ei-value:0.000, Pi-value:0.000  
Er-value:0.000, Pr-value:0.000  
MATCHES To TargetScan▶ miR-140-5p:AGUGGUU▶ miR-142-3p.1:GUAGUGU


G

TCACTACCACTG  
Depth:3 (COW)  
Ei-value:0.000, Pi-value:0.000  
Er-value:0.000, Pr-value:0.000  
MATCHES To TargetScan▶ miR-140-5p:AGUGGUU▶ miR-142-3p.1:GUAGUGU

A

GCAACA

GCAACA  
Depth:6 (MOUSE)  
Ei-value:0.000, Pi-value:0.000  
Er-value:0.000, Pr-value:0.000  
No matches to TargetScan


AC

GCAACAAC  
Depth:5 (RABBIT)  
Ei-value:0.000, Pi-value:0.000  
Er-value:0.000, Pr-value:0.000  
No matches to TargetScan

TCTAGGCCAGGAGGTTATACCAAGATT

CTTTCCTGG

CTTTCCTGG  
Depth:3 (COW)  
Ei-value:0.000, Pi-value:0.000  
Er-value:0.000, Pr-value:0.000  
MATCHES To TargetScan▶ miR-665:CCAGGAG▶ miR-873-5p.1:CAGGAAC

GCCCAGTTAAGAAGGTGAAGCCTCAAG

ACAACCACC

ACAACCACC  
Depth:5 (RABBIT)  
Ei-value:0.000, Pi-value:0.000  
Er-value:0.000, Pr-value:0.000  
No matches to TargetScan


ACAC

ACAACCACCACAC  
Depth:4 (DOG)  
Ei-value:0.000, Pi-value:0.000  
Er-value:0.000, Pr-value:0.000  
No matches to TargetScan

ATCCAGAGCTCCTGG

TTGTTCC

TTGTTCC  
Depth:4 (DOG)  
Ei-value:0.000, Pi-value:0.000  
Er-value:0.000, Pr-value:0.000  
No matches to TargetScan

CTT 6720  
 CATATT

TG

TGCCAAATC  
Depth:3 (COW)  
Ei-value:0.000, Pi-value:0.000  
Er-value:0.000, Pr-value:0.000  
MATCHES To TargetScan▶ miR-182-5p:UUGGCAA▶ miR-96-5p/1271-5p:UUGGCAC


CCAAAT

CCAAAT  
Depth:6 (MOUSE)  
Ei-value:0.000, Pi-value:0.000  
Er-value:0.000, Pr-value:0.000  
No matches to TargetScan


C

CCAAATC  
Depth:5 (RABBIT)  
Ei-value:0.000, Pi-value:0.000  
Er-value:0.000, Pr-value:0.000  
No matches to TargetScan

ATTATCTTTCCCTGAAGTAGTGCAAAGAG

CAAGAAA

CAAGAAA  
Depth:5 (RABBIT)  
Ei-value:0.000, Pi-value:0.000  
Er-value:0.000, Pr-value:0.000  
No matches to TargetScan


T

CAAGAAAT  
Depth:3 (COW)  
Ei-value:0.000, Pi-value:0.000  
Er-value:0.000, Pr-value:0.000  
No matches to TargetScan

G

TGAACACAC

TGAACACAC  
Depth:3 (COW)  
Ei-value:0.000, Pi-value:0.000  
Er-value:0.000, Pr-value:0.000  
No matches to TargetScan

CAAG

G

GAAGATCAACATGCCTG  
Depth:4 (DOG)  
Ei-value:0.000, Pi-value:0.000  
Er-value:0.000, Pr-value:0.000  
No matches to TargetScan


AA

AAGATCAACATGC  
Depth:5 (RABBIT)  
Ei-value:0.000, Pi-value:0.000  
Er-value:0.000, Pr-value:0.000  
No matches to TargetScan


GATCAACATGC

GATCAACATGC  
Depth:6 (MOUSE)  
Ei-value:0.000, Pi-value:0.000  
Er-value:0.000, Pr-value:0.000  
No matches to TargetScan


CTG

GAAGATCAACATGCCTG  
Depth:4 (DOG)  
Ei-value:0.000, Pi-value:0.000  
Er-value:0.000, Pr-value:0.000  
No matches to TargetScan

CAATGCTAGCATTTTAGAATAGCAGAATGAATTTGTC 6840  
 TCTTCTGTTTCTTACCCTCTTCCATGTCTGCCTTTGCTTATCTTTTAAACTCATAAG

TGTGTAT

TGTGTAT  
Depth:6 (MOUSE)  
Ei-value:0.000, Pi-value:0.000  
Er-value:0.000, Pr-value:0.000  
No matches to TargetScan


TT

TGTGTATTT  
Depth:4 (DOG)  
Ei-value:0.000, Pi-value:0.000  
Er-value:0.000, Pr-value:0.000  
No matches to TargetScan

GTTTGTTTGTCTGTTTCTTCTTTGAATGTCTTTGG

TCTTTCTT

TCTTTCTT  
Depth:3 (COW)  
Ei-value:0.000, Pi-value:0.000  
Er-value:0.000, Pr-value:0.000  
No matches to TargetScan

GTCTAAAGTA

T

TGTCTTA  
Depth:4 (DOG)  
Ei-value:0.000, Pi-value:0.000  
Er-value:0.000, Pr-value:0.000  
MATCHES To TargetScan▶ miR-208-3p:UAAGACG▶ miR-499a-5p:UAAGACU

 6960  


GTCTTA

TGTCTTA  
Depth:4 (DOG)  
Ei-value:0.000, Pi-value:0.000  
Er-value:0.000, Pr-value:0.000  
MATCHES To TargetScan▶ miR-208-3p:UAAGACG▶ miR-499a-5p:UAAGACU


CCCATTTCCATG

TGTCTTACCCATTTCCATG  
Depth:3 (COW)  
Ei-value:0.000, Pi-value:0.000  
Er-value:0.000, Pr-value:0.000  
MATCHES To TargetScan▶ miR-203a-3p.1:GAAAUGU▶ miR-208-3p:UAAGACG▶ miR-499a-5p:UAAGACU

ATTCTCTTGCTAGTTTCTTCTCTGTATATCTTTGTCTCATTTACT

TTTTTGT

TTTTTGT  
Depth:4 (DOG)  
Ei-value:0.000, Pi-value:0.000  
Er-value:0.000, Pr-value:0.000  
No matches to TargetScan

ACCCAGGAGTGGTTTGTGTCTTGTCTTAAATGTCTCTCTCTAGTTTTC

TT

TTCATTTTGTT  
Depth:4 (DOG)  
Ei-value:0.000, Pi-value:0.000  
Er-value:0.000, Pr-value:0.000  
MATCHES To TargetScan▶ miR-495-3p:AACAAAC

 7080  


CATTTTGTT

TTCATTTTGTT  
Depth:4 (DOG)  
Ei-value:0.000, Pi-value:0.000  
Er-value:0.000, Pr-value:0.000  
MATCHES To TargetScan▶ miR-495-3p:AACAAAC

ACTGATTCTCCTTGCTCTGCTAGATCTAGCTCTTCTTTCACCGTTCTCTGTGAGCCTCTTGAGTTATGTGTGCCT

TTTGCTC

TTTGCTC  
Depth:3 (COW)  
Ei-value:0.000, Pi-value:0.000  
Er-value:0.000, Pr-value:0.000  
No matches to TargetScan

ATTTCTTGCTATGCCTGCCTCTCTTCT

TT

TTTTCTCTTTGTGAA  
Depth:3 (COW)  
Ei-value:0.000, Pi-value:0.000  
Er-value:0.000, Pr-value:0.000  
No matches to TargetScan

 7200  


TTTTCTCTTTGTGAA  
Depth:3 (COW)  
Ei-value:0.000, Pi-value:0.000  
Er-value:0.000, Pr-value:0.000  
No matches to TargetScan


TTCTCTTTG

TTCTCTTTG  
Depth:6 (MOUSE)  
Ei-value:0.000, Pi-value:0.000  
Er-value:0.000, Pr-value:0.000  
No matches to TargetScan


TGAA

TTTTCTCTTTGTGAA  
Depth:3 (COW)  
Ei-value:0.000, Pi-value:0.000  
Er-value:0.000, Pr-value:0.000  
No matches to TargetScan

CTCTGTCACCCG

TTCCCCTT

TTCCCCTT  
Depth:3 (COW)  
Ei-value:0.000, Pi-value:0.000  
Er-value:0.000, Pr-value:0.000  
No matches to TargetScan

GTTGGCTTGAC

ATTTCACCT

ATTTCACCT  
Depth:4 (DOG)  
Ei-value:0.000, Pi-value:0.000  
Er-value:0.000, Pr-value:0.000  
MATCHES To TargetScan▶ miR-203a-3p.2:UGAAAUG

TTTCTGATACTGGCTACCCTTC

TGCTG

TGCTGTTTCTACT  
Depth:3 (COW)  
Ei-value:0.000, Pi-value:0.000  
Er-value:0.000, Pr-value:0.000  
MATCHES To TargetScan▶ miR-411-5p.1:AGUAGAC▶ miR-494-3p:GAAACAU


TTTCTAC

TTTCTAC  
Depth:6 (MOUSE)  
Ei-value:0.000, Pi-value:0.000  
Er-value:0.000, Pr-value:0.000  
No matches to TargetScan


T

TTTCTACT  
Depth:5 (RABBIT)  
Ei-value:0.000, Pi-value:0.000  
Er-value:0.000, Pr-value:0.000  
MATCHES To TargetScan▶ miR-411-5p.1:AGUAGAC

CTTTATCTTGCAT

ATTTCTC

ATTTCTC  
Depth:6 (MOUSE)  
Ei-value:0.000, Pi-value:0.000  
Er-value:0.000, Pr-value:0.000  
No matches to TargetScan

TTTTCTACATAT 7320  
 TCTTTGTGCCTT

TCTTGGG

TCTTGGG  
Depth:5 (RABBIT)  
Ei-value:0.000, Pi-value:0.000  
Er-value:0.000, Pr-value:0.000  
No matches to TargetScan


C

TCTTGGGC  
Depth:3 (COW)  
Ei-value:0.000, Pi-value:0.000  
Er-value:0.000, Pr-value:0.000  
No matches to TargetScan

TATTTTCTCTTTTTTTCCTCATGCTTTGTGTGCCCCAGTGTCTCTTTGTTC

TTTGTGA

TTTGTGA  
Depth:4 (DOG)  
Ei-value:0.000, Pi-value:0.010  
Er-value:0.000, Pr-value:0.000  
No matches to TargetScan


TTTTC

TTTGTGATTTTC  
Depth:3 (COW)  
Ei-value:0.000, Pi-value:0.000  
Er-value:0.000, Pr-value:0.000  
No matches to TargetScan

AATTTCAGCATTCA

TCTCTGTT

TCTCTGTT  
Depth:4 (DOG)  
Ei-value:0.000, Pi-value:0.000  
Er-value:0.000, Pr-value:0.000  
No matches to TargetScan

CTCTTGGTTCTTCTC 7440  
 TCCTTTTGCCTTTCTATTCACT

TTTGAGTATTT

TTTGAGTATTT  
Depth:4 (DOG)  
Ei-value:0.000, Pi-value:0.000  
Er-value:0.000, Pr-value:0.000  
MATCHES To TargetScan▶ miR-200bc-3p/429:AAUACUG▶ miR-371-5p:CUCAAAC

CTTGAGTCTATGTCTCCCCCT

CTTTGATT

CTTTGATT  
Depth:3 (COW)  
Ei-value:0.000, Pi-value:0.000  
Er-value:0.000, Pr-value:0.000  
No matches to TargetScan

TCATGCAATTCTCTCTCCTGGCATATTTTTTCATGCGTGTT

TGTGTGTG

TGTGTGTG  
Depth:4 (DOG)  
Ei-value:0.000, Pi-value:0.000  
Er-value:0.000, Pr-value:0.000  
MATCHES To TargetScan▶ miR-329-3p/362-3p:ACACACC

TGCTCTTTT 7560  
 GTGTGTTTGTAAAGGCGCC

TCCTAACCCCT

TCCTAACCCCT  
Depth:5 (RABBIT)  
Ei-value:0.000, Pi-value:0.000  
Er-value:0.000, Pr-value:0.000  
No matches to TargetScan

TCCAG

TAGGTGCA

TAGGTGCA  
Depth:3 (COW)  
Ei-value:0.000, Pi-value:0.000  
Er-value:0.000, Pr-value:0.000  
No matches to TargetScan

GAGTGTCAGCTATCAAAAT

AAGCATTG

AAGCATTG  
Depth:4 (DOG)  
Ei-value:0.000, Pi-value:0.000  
Er-value:0.000, Pr-value:0.000  
No matches to TargetScan

CAGAGCTGTTCG

TTATGCCA

TTATGCCA  
Depth:5 (RABBIT)  
Ei-value:0.000, Pi-value:0.000  
Er-value:0.000, Pr-value:0.000  
No matches to TargetScan


G

TTATGCCAG  
Depth:4 (DOG)  
Ei-value:0.000, Pi-value:0.000  
Er-value:0.000, Pr-value:0.000  
No matches to TargetScan

GCCGCCCTGTGAGATGATCAAAACCAACA 7680  
 GAAGG

TCCAAG

TCCAAG  
Depth:3 (COW)  
Ei-value:0.000, Pi-value:0.000  
Er-value:0.000, Pr-value:0.000  
No matches to TargetScan

GGTCTAGACTGGAGTTGGATAGAAGACTCAAGTCTCCGTGAGACAGAAGGCCAAAGACCTAGGATGGGATTAAAAAACCTTGTCTTGAAGACCTGTGACCCAAAAGATG 7800  
 GAAGTGCCCTAGCACACACAAAGACCCAGGACTCAAACCTATCTATATAAGAC

AGA

AGAAGGCCCAA  
Depth:4 (DOG)  
Ei-value:0.000, Pi-value:0.000  
Er-value:0.000, Pr-value:0.000  
No matches to TargetScan


AGGCCCAA

AGGCCCAA  
Depth:5 (RABBIT)  
Ei-value:0.000, Pi-value:0.000  
Er-value:0.000, Pr-value:0.000  
No matches to TargetScan

GAGAGACAGATATT

TCAA

TCAAGACTAA  
Depth:4 (DOG)  
Ei-value:0.000, Pi-value:0.000  
Er-value:0.000, Pr-value:0.000  
MATCHES To TargetScan▶ miR-431-5p:GUCUUGC


GACTAA

GACTAA  
Depth:5 (RABBIT)  
Ei-value:0.000, Pi-value:0.000  
Er-value:0.000, Pr-value:0.000  
No matches to TargetScan

ATTAGATGGGAAACTGGAGGTTCGTGACAGAG 7920  
 ATACCAGGAAAGAA

AGAAGC

AGAAGC  
Depth:4 (DOG)  
Ei-value:0.000, Pi-value:0.000  
Er-value:0.000, Pr-value:0.010  
No matches to TargetScan

CCCAGGACCAAAGGAAATTC

C

CAAGATGA  
Depth:3 (COW)  
Ei-value:0.000, Pi-value:0.000  
Er-value:0.000, Pr-value:0.000  
No matches to TargetScan


AAGATGA

AAGATGA  
Depth:5 (RABBIT)  
Ei-value:0.000, Pi-value:0.000  
Er-value:0.000, Pr-value:0.000  
No matches to TargetScan

GACTCTTAAACCCCAGCTG

TTTCTATTG

TTTCTATTG  
Depth:3 (COW)  
Ei-value:0.000, Pi-value:0.000  
Er-value:0.000, Pr-value:0.000  
No matches to TargetScan

CTCTTTTCCCTACTCTTGGACATTTTCAGTTCTCCCTTCCTTCT 8040  
 CATGTCTCCATTTATATCT

ACTTCTT

ACTTCTT  
Depth:3 (COW)  
Ei-value:0.000, Pi-value:0.020  
Er-value:0.000, Pr-value:0.000  
No matches to TargetScan

TTGAGATGTC

CTTTTTGATGTT

CTTTTTGATGTT  
Depth:4 (DOG)  
Ei-value:0.000, Pi-value:0.000  
Er-value:0.000, Pr-value:0.000  
No matches to TargetScan

ACCTTAAAAAAAAAAAAAAACGTATCTTTAGATCAGTAA

TATTATGC

TATTATGC  
Depth:4 (DOG)  
Ei-value:0.000, Pi-value:0.000  
Er-value:0.000, Pr-value:0.000  
MATCHES To TargetScan▶ miR-369-3p:AUAAUAC

TTTGGCCTGTTTTTATTACAGTTTT 8160  
 GAACCATTTATTAAGTTTTTGAAGTTT

TAAACTTC

TAAACTTC  
Depth:3 (COW)  
Ei-value:0.000, Pi-value:0.000  
Er-value:0.000, Pr-value:0.000  
No matches to TargetScan

CATTTCTCTTCACCTCCT

CTCCACTTGAGAG

CTCCACTTGAGAG  
Depth:3 (COW)  
Ei-value:0.000, Pi-value:0.000  
Er-value:0.000, Pr-value:0.000  
MATCHES To TargetScan▶ miR-26-5p:UCAAGUA

GGACACATAGCTGACATTA

TATTTCAGT

TATTTCAGT  
Depth:4 (DOG)  
Ei-value:0.000, Pi-value:0.000  
Er-value:0.000, Pr-value:0.000  
MATCHES To TargetScan▶ miR-203a-3p.2:UGAAAUG


CC

TATTTCAGTCC  
Depth:3 (COW)  
Ei-value:0.000, Pi-value:0.000  
Er-value:0.000, Pr-value:0.000  
MATCHES To TargetScan▶ miR-203a-3p.2:UGAAAUG

CCTCTTTCCTCAGAAGCTCTAGGC 8280  
 TGATGAGAGAAGGAAAGTATCAGGTTCAGTTATTGA

GGGGAAA

GGGGAAA  
Depth:4 (DOG)  
Ei-value:0.000, Pi-value:0.000  
Er-value:0.000, Pr-value:0.000  
No matches to TargetScan

GAAAGTGCCAAGCTA

TCTAGAGAAAA

TCTAGAGAAAA  
Depth:6 (MOUSE)  
Ei-value:0.000, Pi-value:0.000  
Er-value:0.000, Pr-value:0.000  
MATCHES To TargetScan▶ miR-1251-5p:CUCUAGC

TG

TGAAGAGATG

TGAAGAGATG  
Depth:5 (RABBIT)  
Ei-value:0.000, Pi-value:0.000  
Er-value:0.000, Pr-value:0.000  
No matches to TargetScan


CTCCA

TGAAGAGATGCTCCA  
Depth:3 (COW)  
Ei-value:0.000, Pi-value:0.000  
Er-value:0.000, Pr-value:0.000  
No matches to TargetScan

CA

GGCCAA

GGCCAATGAGAAGAATTAGACA  
Depth:4 (DOG)  
Ei-value:0.000, Pi-value:0.000  
Er-value:0.000, Pr-value:0.000  
No matches to TargetScan


TGAGAAGAATTAGACA

TGAGAAGAATTAGACA  
Depth:6 (MOUSE)  
Ei-value:0.000, Pi-value:0.000  
Er-value:0.000, Pr-value:0.000  
No matches to TargetScan

A

GAAATACAC

GAAATACACAGATG  
Depth:3 (COW)  
Ei-value:0.000, Pi-value:0.000  
Er-value:0.000, Pr-value:0.000  
No matches to TargetScan

 8400  


AGATG

GAAATACACAGATG  
Depth:3 (COW)  
Ei-value:0.000, Pi-value:0.000  
Er-value:0.000, Pr-value:0.000  
No matches to TargetScan

TGCCAGTTTG

C

CTGAGAAG  
Depth:3 (COW)  
Ei-value:0.000, Pi-value:0.000  
Er-value:0.000, Pr-value:0.000  
No matches to TargetScan


TGAGAAG

TGAGAAG  
Depth:4 (DOG)  
Ei-value:0.000, Pi-value:0.000  
Er-value:0.000, Pr-value:0.010  
No matches to TargetScan

TGCCA

GCCA

GCCAGCAACA  
Depth:3 (COW)  
Ei-value:0.000, Pi-value:0.000  
Er-value:0.000, Pr-value:0.000  
No matches to TargetScan


GCAACA

GCAACA  
Depth:6 (MOUSE)  
Ei-value:0.000, Pi-value:0.000  
Er-value:0.000, Pr-value:0.000  
No matches to TargetScan

TCTTACTTA

TTTGAGCTT

TTTGAGCTT  
Depth:3 (COW)  
Ei-value:0.000, Pi-value:0.000  
Er-value:0.000, Pr-value:0.000  
MATCHES To TargetScan▶ miR-371-5p:CUCAAAC

G

GGTGAGC

GGTGAGC  
Depth:4 (DOG)  
Ei-value:0.000, Pi-value:0.000  
Er-value:0.000, Pr-value:0.000  
No matches to TargetScan


AGGAT

GGTGAGCAGGAT  
Depth:3 (COW)  
Ei-value:0.000, Pi-value:0.000  
Er-value:0.000, Pr-value:0.000  
No matches to TargetScan

ACCTGA

GGTTTGGG

GGTTTGGG  
Depth:4 (DOG)  
Ei-value:0.000, Pi-value:0.000  
Er-value:0.000, Pr-value:0.000  
No matches to TargetScan

ATTCTTACTGT

TGGTTA

TGGTTA  
Depth:5 (RABBIT)  
Ei-value:0.000, Pi-value:0.000  
Er-value:0.000, Pr-value:0.000  
No matches to TargetScan


T

TGGTTAT  
Depth:4 (DOG)  
Ei-value:0.000, Pi-value:0.000  
Er-value:0.000, Pr-value:0.000  
No matches to TargetScan


G

TGGTTATG  
Depth:3 (COW)  
Ei-value:0.000, Pi-value:0.000  
Er-value:0.000, Pr-value:0.000  
No matches to TargetScan

AAGGAGGATTGGGCCTAA 8520  
 CACATAGTGAGGC

CCCAAGG

CCCAAGG  
Depth:4 (DOG)  
Ei-value:0.000, Pi-value:0.000  
Er-value:0.000, Pr-value:0.000  
MATCHES To TargetScan▶ miR-212-5p:CCUUGGC

AAGGAGCA

TGAACTCCCTGCT

TGAACTCCCTGCT  
Depth:4 (DOG)  
Ei-value:0.000, Pi-value:0.000  
Er-value:0.000, Pr-value:0.000  
No matches to TargetScan


C

TGAACTCCCTGCTCATAGTAGTGGCC  
Depth:3 (COW)  
Ei-value:0.000, Pi-value:0.000  
Er-value:0.000, Pr-value:0.000  
No matches to TargetScan


ATAGTAGTGGCC

ATAGTAGTGGCC  
Depth:4 (DOG)  
Ei-value:0.000, Pi-value:0.000  
Er-value:0.000, Pr-value:0.000  
No matches to TargetScan

TAATAATGTGGTAAGCTGCATAAATTTTATCCCTCGG

TTTAATAC

TTTAATAC  
Depth:4 (DOG)  
Ei-value:0.000, Pi-value:0.000  
Er-value:0.000, Pr-value:0.000  
MATCHES To TargetScan▶ miR-496.2:GUAUUAC


CT

CTAGGCTTAAAG  
Depth:4 (DOG)  
Ei-value:0.000, Pi-value:0.000  
Er-value:0.000, Pr-value:0.000  
No matches to TargetScan


AGGCTTA

AGGCTTA  
Depth:5 (RABBIT)  
Ei-value:0.000, Pi-value:0.000  
Er-value:0.000, Pr-value:0.000  
No matches to TargetScan


AAG

CTAGGCTTAAAG  
Depth:4 (DOG)  
Ei-value:0.000, Pi-value:0.000  
Er-value:0.000, Pr-value:0.000  
No matches to TargetScan

GTGAGAAAA 8640  
 TCTGGGAATATTTAGCAG

GTTTAAT

GTTTAAT  
Depth:5 (RABBIT)  
Ei-value:0.000, Pi-value:0.000  
Er-value:0.000, Pr-value:0.000  
No matches to TargetScan

CCTTTCATTAATTTTTTTCCTCTTACCATAAGGAAAGATAATTTTAGTGATAATATATATGAAAGCAC

TGTAAAACA

TGTAAAACA  
Depth:3 (COW)  
Ei-value:0.000, Pi-value:0.000  
Er-value:0.000, Pr-value:0.000  
No matches to TargetScan

CAGAAAAAAAAGCAAGAC 8760  
 TTTCTCATTAATAATG

T

TATTGGCA  
Depth:5 (RABBIT)  
Ei-value:0.000, Pi-value:0.000  
Er-value:0.000, Pr-value:0.000  
No matches to TargetScan


ATTGGCA

ATTGGCA  
Depth:6 (MOUSE)  
Ei-value:0.000, Pi-value:0.000  
Er-value:0.000, Pr-value:0.000  
No matches to TargetScan

CTCATGCACAGCTAACATTTGAAAATGCGTTGTCAG

TTGTGAAG

TTGTGAAG  
Depth:6 (MOUSE)  
Ei-value:0.000, Pi-value:0.000  
Er-value:0.000, Pr-value:0.000  
No matches to TargetScan

CAT

T

TATGTAAATCA  
Depth:3 (COW)  
Ei-value:0.000, Pi-value:0.000  
Er-value:0.000, Pr-value:0.000  
No matches to TargetScan


ATGTAAAT

ATGTAAAT  
Depth:5 (RABBIT)  
Ei-value:0.000, Pi-value:0.000  
Er-value:0.000, Pr-value:0.000  
No matches to TargetScan


CA

TATGTAAATCA  
Depth:3 (COW)  
Ei-value:0.000, Pi-value:0.000  
Er-value:0.000, Pr-value:0.000  
No matches to TargetScan

AGGGTTATCACACCCTTTTTGTAAAGGATCAGATGGCA 8880  
 ATTATTTTATACTTTGCAA

GCCATATGGT

GCCATATGGT  
Depth:3 (COW)  
Ei-value:0.000, Pi-value:0.000  
Er-value:0.000, Pr-value:0.000  
No matches to TargetScan

CTCTCTCTGCTTTTTTTTATTTTTAAAGTGAATATGGTCTGTGGGCCACAGTTTATCACCCCTGGTGTAATTGCCAGGGACTGTGTGCTGA 9000  
 ATAATATTAATTTCTTTCTT

TGTGC

TGTGCCTGTCCCTGT  
Depth:3 (COW)  
Ei-value:0.000, Pi-value:0.000
[truncated: 111,535 more chars]
