# Supplementary material for: Uncovering deeply conserved motif combinations in rapidly evolving noncoding sequences
Source: Genome Biol. 2021 Jan 11;22:29. doi: 10.1186/s13059-020-02247-1 (PMC7798263; doi:10.1186/s13059-020-02247-1)
Supplement: Supplementary file 4 — Additional file 4. LncLOOM output results for XIST sequences from six mammals. [file 13059_2020_2247_MOESM4_ESM.gz › AdditionalFile4/Html_Files/kmers_in_seqs_level_graded.html]

 MOTIF CONSERVATION

# MOTIF CONSERVATION

## Motifs conserved to (and beyond) MOUSE (depth:6)

  

NAVIGATE ▼

▶HUMAN▶PIG▶COW▶DOG▶RABBIT▶MOUSE

  
  
  
  

## >HUMAN (19280 bases)

```
 CCTTCAGTTCTTAAAGCGCTGCAATTCGCTGCTGCAGCCATATTTCTTACTCTCTCGGGGCTGGAAGCTTCCTGACTGAAGATCTCTCTGCACTTGGGGTTCTTTCTAGAACATTTTCTA 120  
 GTCCCCCAACACCCTTTATGGCGTATTTCTTTAAAAAAATCACCTAAATTCCATAAAATATTTTTTTAAATTCTATACTTTCTCCTAGTGTCTTCTTGACACGTCCTCCATATTTTTTTA 240  
 AAGAAAGTATTTGGAATATTTTGAGGCAATTTTTAATATTTAAGGAATTTTTCTTTGGAATCATTTTTGGTTGACATCTCTGTTTTTTGTGGATCAGTTTTTTACTCTTCCACTCTCTTT 360  
 TCTATATTTTGCCCATCGGGGCTGCGGATACCTGGTTTTATTATTTTTTCTTTGCCCAACGGGGCCGTGGATACCTGCCTTTTAATTCTTTTTTATTCGCCCATCGGGGCCGCGGATACC 480  
 TGCTTTTTATTTTTTTTTCCTTAGCCCATCGGGGTATCGGATACCTGCTGATTCCCTTCCCCTCTGAACCCCCAACACTCTGGCCCATCGGGGTGACGGATATCTGCTTTTTAAAAATTT 600  
 TCTTTTTTTGGCCCATCGGGGCTTCGGATACCTGCTTTTTTTTTTTTTATTTTTCCTTGCCCATCGGGGCCTCGGATACCTGCTTTAATTTTTGTTTTTCTGGCCCATCGGGGCCGCGGA 720  
 TACCTGCTTTGATTTTTTTTTTTCATCGCCCATCGGTGCTTTTTATGGATGAAAAAATGTTGGTTTTGTGGGTTGTTGCACTCTCTGGAATATCTACACTTTTTTTTGCTGCTGATCATT 840  
 TGGTGGTGTGTGAGTGTACCTACCGCTTTGGCAGAGAATGACTCTGCAGTTAAGCTAAGGGCGTGTTCAGATTGTGGAGGAAAAGTGGCCGCCATTTTAGACTTGCCGCATAACTCGGCT 960  
 TAGGGCTAGTCGTTTGTGCTAAGTTAAACTAGGGAGGCAAGATGGATGATAGCAGGTCAGGCAGAGGAAGTCATGTGCATTGCATGAGCTAAACCTATCTGAATGAATTGATTTGGGGCT 1080  
 TGTTAGGAGCTTTGCGTGATTGTTGTATCGGGAGGCAGTAAGAATCATCTTTTATCAGTACAAGGGACTAGTTAAAAATGGAAGGTTAGGAAAGACTAAGGTGCAGGGCTTAAAATGGCG 1200  
 ATTTTGACATTGCGGCATTGCTCAGCATGGCGGGCTGTGCTTTGTTAGGTTGTCCAAAATGGCGGATCCAGTTCTGTCGCAGTGTTCAAGTGGCGGGAAGGCCACATCATGATGGGCGAG 1320  
 GCTTTGTTAAGTGGTTAGCATGGTGGTGGACATGTGCGGTCACACAGGAAAAGATGGCGGCTGAAGGTCTTGCCGCAGTGTAAAACATGGCGGGCCTCTTTGTCTTTGCTGTGTGCTTTT 1440  
 CGTGTTGGGTTTTGCCGCAGGGACAATATGGCAGGCGTTGTCATATGTATATCATGGCTTTTGTCACGTGGACATCATGGCGGGCTTGCCGCATTGTTAAAGATGGCGGGTTTTGCCGCC 1560  
 TAGTGCCACGCAGAGCGGGAGAAAAGGTGGGATGGACAGTGCTGGATTGCTGCATAACCCAACCAATTAGAAATGGGGGTGGAATTGATCACAGCCAATTAGAGCAGAAGATGGAATTAG 1680  
 ACTGATGACACACTGTCCAGCTACTCAGCGAAGACCTGGGTGAATTAGCATGGCACTTCGCAGCTGTCTTTAGCCAGTCAGGAGAAAGAAGTGGAGGGGCCACGTGTATGTCTCCCAGTG 1800  
 GGCGGTACACCAGGTGTTTTCAAGGTCTTTTCAAGGACATTTAGCCTTTCCACCTCTGTCCCCTCTTATTTGTCCCCTCCTGTCCAGTGCTGCCTCTTGCAGTGCTGGATATCTGGCTGT 1920  
 GTGGTCTGAACCTCCCTCCATTCCTCTGTATTGGTGCCTCACCTAAGGCTAAGTATACCTCCCCCCCCACCCCCCAACCCCCCCAACTCCCCACCCCCACCCCCCACCCCCCACCTCCCC 2040  
 ACCCCCCTACCCCCCTACCCCCCTACCCCCCTCTGGTCTGCCCTGCACTGCACTGTTGCCATGGGCAGTGCTCCAGGCCTGCTTGGTGTGGACATGGTGGTGAGCCGTGGCAAGGACCAG 2160  
 AATGGATCACAGATGATCGTTGGCCAACAGGTGGCAGAAGAGGAATTCCTGCCTTCCTCAAGAGGAACACCTACCCCTTGGCTAATGCTGGGGTCGGATTTTGATTTATATTTATCTTTT 2280  
 GGATGTCAGTCATACAGTCTGATTTTGTGGTTTGCTAGTGTTTGAATTTAAGTCTTAAGTGACTATTATAGAAATGTATTAAGAGGCTTTATTTGTAGAATTCACTTTAATTACATTTAA 2400  
 TGAGTTTTTGTTTTGAGTTCCTTAAAATTCCTTAAAGTTTTTAGCTTCTCATTACAAATTCCTTAACCTTTTTTTGGCAGTAGATAGTCAAAGTCAAATCATTTCTAATGTTTTAAAAAT 2520  
 GTGCTGGTCATTTTCTTTGAAATTGACTTAACTATTTTCCTTTGAAGAGTCTGTAGCACAGAAACAGTAAAAAATTTAACTTCATGACCTAATGTAAAAAAGAGTGTTTGAAGGTTTACA 2640  
 CAGGTCCAGGCCTTGCTTTGTTCCCATCCTTGATGCTGCACTAATTGACTAATCACCTACTTATCAGACAGGAAACTTGAATTGCTGTGGTCTGGTGTCCTCTATTCAGACTTATTATAT 2760  
 TGGAGTATTTCAATTTTTCGTTGTATCCTGCCTGCCTAGCATCCAGTTCCTCCCCAGCCCTGCTCCCAGCAAACCCCTAGTCTAGCCCCAGCCCTACTCCCACCCCGCCCCAGCCCTGCC 2880  
 CCAGCCCCAGTCCCCTAACCCCCCAGCCCTAGCCCCAGTCCCAGTCCTAGTTCCTCAGTCCCGCCCAGCTTCTCTCGAAAGTCACTCTAATTTTCATTGATTCAGTGCTCAAAATAAGTT 3000  
 GTCCATTGCTTATCCTATTATACTGGGATATTCCGTTTACCCTTGGCATTGCTGATCTTCAGTACTGACTCCTTGACCATTTTCAGTTAATGCATACAATCCCATTTGTCTGTGATCTCA 3120  
 GGACAAAGAATTTCCTTACTCGGTACGTTGAAGTTAGGGAATGTCAATTGAGAGCTTTCTATCAGAGCATTATTGCCCACAATTTGAGTTACTTATCATTTTCTCGATCCCCTGCCCTTA 3240  
 AAGGAGAAACCATTTCTCTGTCATTGCTTCTGTAGTCACAGTCCCAATTTTGAGTAGTGATCTTTTCTTGTGTACTGTGTTGGCCACCTAAAACTCTTTGCATTGAGTAAAATTCTAATT 3360  
 GCCAATAATCCTACCCATTGGATTAGACAGCACTCTGAACCCCATTTGCATTCAGCAGGGGGTCGCAGACAACCCGTCTTTTGTTGGACAGTTAAAATGCTCAGTCCCAATTGTCATAGC 3480  
 TTTGCCTATTAAACAAAGGCACCCTACTGCGCTTTTTGCTGTGCTTCTGGAGAATCCTGCTGTTCTTGGACAATTAAAGAACAAAGTAGTAATTGCTAATTGTCTCACCCATTAATCATG 3600  
 AAGACTACCAGTCGCCCTTGCATTTGCCTTGAGGCAGCGCTGACTACCTGAGATTTAAGAGTTTCTTAAATTATTGAGTAAAATCCCAATTATCCATAGTTCTGTTAGTTACACTATGGC 3720  
 CTTTGCAAACATCTTTGCATAACAGCAGTGGGACTGACTCATTCTTAGAGCCCCTTCCCTTGGAATATTAATGGATACAATAGTAATTATTCATGGTTCTGCGTAACAGAGAAGACCCAC 3840  
 TTATGTGTATGCCTTTATCATTGCTCCTAGATAGTGTGAACTACCTACCACCTTGCATTAATATGTAAAACACTAATTGCCCATAGTCCCACTCATTAGTCTAGGATGTCCTCTTTGCCA 3960  
 TTGCTGCTGAGTTCTGACTACCCAAGTTTCCTTCTCTTAAACAGTTGATATGCATAATTGCATATATTCATGGTTCTGTGCAATAAAAATGGATTCTCACCCCATCCCACCTTCTGTGGG 4080  
 ATGTTGCTAACGAGTGCAGATTATTCAATAACAGCTCTTGAACAGTTAATTTGCACAGTTGCAATTGTCCAGAGTCCTGTCCATTAGAAAGGGACTCTGTATCCTATTTGCACGCTACAA 4200  
 TGTGGGCTGATCACCCAAGGACTCTTCTTGTGCATTGATGTTCATAATTGTATTTGTCCACGATCTTGTGCACTAACCCTTCCACTCCCTTTGTATTCCAGCAGGGGACCCTTACTACTC 4320  
 AAGACCTCTGTACTAGGACAGTTTATGTGCACAATCCTAATTGATTAGAACTGAGTCTTTTATATCAAGGTCCCTGCATCATCTTTGCTTTACATCAAGAGGGTGCTGGTTACCTAATGC 4440  
 CCCTCCTCCAGAAATTATTGATGTGCAAAATGCAATTTCCCTATCTGCTGTTAGTCTGGGGTCTCATCCCCTCATATTCCTTTTGTCTTACAGCAGGGGGTACTTGGGACTGTTAATGCG 4560  
 CATAATTGCAATTATGGTCTTTTCCATTAAATTAAGATCCCAACTGCTCACACCCTCTTAGCATTACAGTAGAGGGTGCTAATCACAAGGACATTTCTTTTGTACTGTTAATGTGCTACT 4680  
 TGCATTTGTCCCTCTTCCTGTGCACTAAAGACCCCACTCACTTCCCTAGTGTTCAGCAGTGGATGACCTCTAGTCAAGACCTTTGCACTAGGATAGTTAATGTGAACCATGGCAACTGAT 4800  
 CACAACAATGTCTTTCAGATCAGATCCATTTTATCCTCCTTGTTTTACAGCAAGGGATATTAATTACCTATGTTACCTTTCCCTGGGACTATGAATGTGCAAAATTCCAATGTTCATGGT 4920  
 CTCTCCCTTTAAACCTATATTCTACCCCTTTTACATTATAGAAAGGGATGCTGGAAACCCAGAGTCCTTCTCTTGGGACTCTTAATGTGTATTTCTAATTATCCATGACTCTT

AATGTGC

AATGTGCAT  
Depth:6 (MOUSE)  
Ei-value:0.000, Pi-value:0.000  
Er-value:0.000, Pr-value:0.000  
No matches to eCLIP DataMATCHES To TargetScan▶ miR-501-3p/502-3p:AUGCACC

 5040  


AT

AATGTGCAT  
Depth:6 (MOUSE)  
Ei-value:0.000, Pi-value:0.000  
Er-value:0.000, Pr-value:0.000  
No matches to eCLIP DataMATCHES To TargetScan▶ miR-501-3p/502-3p:AUGCACC

ATTTTCAATTGCCTAATTGATTTCAATTGTCTAAGACATTTCAAATGTCTAATTGATTAGAACTGAGTCTTTTATATCAAGCTAATATCTAGCTTTTATATCAAGCTAATATCTTGAC 5160  
 TTCTCAGCATCATAGAAGGGGGTACTGATTTCCTAAAGTCTTTCTTGAATTTCTATTATGCAAAATTGCCCTGAGGCCGGGTGTGGTGGCTCACACCTGTAATCCCAGCACTTTGGGAGG 5280  
 CTGAGGTGGGAAGATCCCTTACTGCCAGGAGTTTGAGACCAGCCTGGCCAACATTAAAAAAAAAAAAAAGTAAGACAATTGCCCTGGAATCCCATCCCCCTCACACCTCCTTGGCAAAGC 5400  
 AGCAGGAGTGCTAACTAGCTAGTGCTTCTTCTCTTATACTGCTTAAATGCGCATAATTAGCAGTAGTTGATGTGCCCCTATGTTAGAGTAGAATCCCGCTTCCTTGCTCCATTTGCATTA 5520  
 CTGCAGGAGCTTCTAACTAGCCTGAATTCACTCTCTTGGACTGTTAATGTGCATACTTATATTTGCTGCTGTACTTTTTTACCATGTAAGGACCCCACCCACTGTATTTACATCCCAGCT 5640  
 GGAAGTACCTACTACTTAAGACCCTTAGACTAGTAAAGTTAGCGTGCATAATCTTAGGTGTTATATACACATTTTCAGTTGCATACAGTTGTGCCTTTTATCAGGACTCCTGTACTTATC 5760  
 AAAGCAGAGAGTGCTAATCAATATTAAGCCCTTCTCTTCGAACTGTAGATGGCATGTAATTGCAGTTGTCAATGGTCCTTCAATTAGACTTGGGTTTCTGACCTATCACACCCTCTTTGC 5880  
 TTTATTGCATGGGGTACTATTCAC

TTAAGGCC

TTAAGGCC  
Depth:6 (MOUSE)  
Ei-value:0.000, Pi-value:0.000  
Er-value:0.000, Pr-value:0.000  
eCLIP MATCHES▶HNRNPL (bg=0.64%)No matches to TargetScan

CCTTTCTCAAACTGTTAATGTGCCTAATGACAATTACATCAGTATCCTTCCTTTTGAAGGACAGCATGGTTGGTGACACCTAAGGCCC 6000  
 CATTTCTTGGCCTCCCAATATGTGTGATTGTATTTGTCGAGGTTGCTATGCACTAGAGAAGGAAAGTGCTCCCCTCATCCCCACTTTTCCCTTCCAGCAGGAAGTGCCCACCCCATAAGA 6120  
 CCCTTTTATTTGGAGAGTCTAGGTGCACAATTGTAAGTGACCACAAGCATGCATCTTGGACATTTATGTGCGTAATCGCACACTGCTCATTCCATGTGAATAAGGTCCTACTCTCCGACC 6240  
 CCTTTTGCAATACAGAAGGGTTGCTGATAACGCAGTCCCCTTTTCTTGGCATGTTGTGTGTGATTATAATCGTCTGGGATCCTATGCACTAGAAAAGGAGGGTCCTCTCCACATACCTCA 6360  
 GTCTCACCTTTCCCTTCCAGCAGGGAGTGCCCACTCCATAAGACTCTCACATTTGGACAGTCAAGGTGCGTAATTGTTAAGTGAACACAACCATGCACCTTAGACATGGATTTGCATAAC 6480  
 TACACACAGCTCAACCTATCTGAATAAAATCCTACTCTCAGACCCCTTTTGCAGTACAGCAGGGGTGCTGATCACCAAGGCCCTTTTTCCTGGCCTGGTATGCGTGTGATTATGTTTGTC 6600  
 CCGGTTCCTGTGTATTAGACATGGAAGCCTCCCCTGCCACACTCCACCCCCAATCTTCCTTTCCCTTCCGGCAGGGAGTGCCCTCTCCATAAGACGCTTACGTTTGGACAATCAAGGTGC 6720  
 ACAGTTGTAAGTGACCACAGGCATACACCTTGGACATTAATGTGCATAACCACTTTGCCCATTCCATCTGAATAAGGTCCTACTCTCAGACCCCTTTTGCAGTACAGCAGGGGTGCTGAT 6840  
 CACCAAGGCCCCTTTTCTTGGCCTGTTATGTGCGTGATTATATTTGTCTGGGTTCCTGTGTATTAGACAAGGAAGCCTTCCCCCCGCCCCCACCCCCACTCCCAGTCTTCCTTTCCCTTC 6960  
 CAGCAGGGAGTGCCCCCTCCATAAGATCATTACATTTGGACAATCAAGGTGCACAATTATAAGTGACCACAGCCATGCACCTTGGACATTATTGGACATTAATGTGCGTAACTGCACATG 7080  
 GCCCATCCCATCTGAATAAGGTCCTACTCTCAGATGCCCTTTGCAGTACAGCAGGGGTACTGAATCACCAAGGCCCTTTTTCTTGGCCTGTTATGTGTGTGATTATATTTATCCCAGTTT 7200  
 CTGTGTAATAGACATGAAAGCCTCCCCTGCCACACCCCACCTCCAATCTTCCTTTCCCTTCCACCAGGGAGTGTCCACTCCATATACCCTTACATTTGGACAATCAAGGTGCACAATTGT 7320  
 AAGTGAGCATAGGCACTCACCTTGGACATGAATGTGCATAACTGCACATGGCCCATCCCATCTGAATAAGGTCCTACTCTCAGACCCTTTTTGCAGTACAGCAGGGGTGCTGATCACCAA 7440  
 GGCCCCTTTTCCTGGCCTGTTATGTGTGTGATTATATTTGTTCCAGTTCCTGTGTAATAGACATGGAAGCCTCCCCTGCCACACTCCACCCCCAATCTTCCTTTCCCTTCTGGCAGGAAG 7560  
 TACCCGCTCCATAAGACCCTTACATTTGGACAGTCAAGGTGCACAATTGTATGTGACCACAACCATGCACCTTGGACATAAATGTGTGTAACTGCACATGGCCCATCCCATCTGAATAAG 7680  
 GTCCTACTCTCAGACCCCTTTTGCAGTACAGTAGGTGTGCTGATAACCAAGGCCCCTCTTCCTGGCCTGTTAACGTATGTGATTATATTTGTCTGGGTTCCAGTGTATAAGACATGGAAG 7800  
 CCTCCCCTGCCCCACCCCACCCTCAATCTTCCTTTCCCTTCTGGCAGGGAGTGCCAGCTCCATAAGAACCTTACATTTGGACAGTCAAGGTGCACAATTCTAAGTGACCGCAGCCATGCA 7920  
 CCTTGGTCAATAATGTGTGTAACTGCACACGGCCTATCTCATCTGAATAAGGCCTTACTCTCAGACCCCTTTTGCAGTACAGCAGGGGTGCTGATAACCAAGGCCCATTTTCCTGGCCTG 8040  
 TTATGTGTGTGATTATATTTGTCCAGGTTTCTGTGTACTAGACAAGGAAGCCTCCTCTGCCCCATCCCATCTACGCATAATCTTTCTTTTCCTCCCAGCAGGGAGTGCTCACTCCATAAG 8160  
 ACCCTTACATTTGGACAATCAAGGTGCACAATTGTAAGTGACCACAACCATGCATCTTGGAAATTTATGTGCATAACTGCACATGGCTTATCCTATTTGAATAAAGTCCTACTCTCAGAC 8280  
 CCCCTTTGCAGTATAGCTGGGGTGCTGATCACTGAGGCCTCTTTGCTTGGCTTGTCTATATTCTTGTGTACTAGATAAGGGCACCTTCTCATGGACTCCCTTTGCTTTTCAACAAGGAGT 8400  
 ACCCACTACTTTTTAAGATTCTTATATTTGTCCAAAGTACATGGTTTTAATTGACCACAACAATGTCCCTTGGACATTAATGTATGTAATCACCACATGGTTCATCCTAATTAAACAAAG 8520  
 TTCTACCTTCTCACCCTCCATTTGCAGTATACCAGGGTTGCTGACCCCCTAAGTCCCCTTTTCTTGGCTTGTTGACATGCATAATTGCATTTATGTTGGTTCTTGTGCCCTAGACAAGGA 8640  
 TGCCCCACCTCTTTTCAATAGTGGGTGCCCACTCCTTATGATCTTTACATTTGAACAGTTAATGTGAATAATTGCAGTTGTCCACAACCCTATCACTTCTAGGACCATTATACCTCTTTT 8760  
 GCATTACTGTGGGGTATACTGTTTCCCTCCAAGGCCCCTTCTGGTGGACTATCAACATATAATTGAAATTTTCTTTTGTCTTTGTCAGTAGATTAAGGTCATACCCCATCACCTTTCCTT 8880  
 TGTAGTACAACAGGGTGTCCTGATCAACCAAAGTCCTGTTGTTTTGGACTGTTAATATGTGCAATTACATTTGCTCCTGATCTGTGCACTAGATAAGGATCCTACCTACTTTCTTAGTGT 9000  
 TTTTAGCAGGTAGTGCCCACTACTCAAGACTGTCACTTGGAATGTTCATGTGCACAAACTCAATTCTCTAAGCATGTTCCTGTACCACCTTTGCTTTAGAGCAGGGGGATGATATTCACT 9120  
 AAGTGCCCCTTCTTTTGGACTTAATATGCATTAATGCAATTGTCCACCTCTTCTTTTAGACTAAGAGTTGATCTCCACATATTCCCCTTGCATCAGGGGCATGTTAATTATGAATGAACC 9240  
 CTTTTCTTTTAATATTAATGTCATAATTGTATTTGTGGACCTGTGTAGGAGAAAAAGACCCTATGTTCCTCCCATTACCCTTTGGATTGCTGCTGAGAAGTGTTAACTACTCATAATCTC 9360  
 AGCTCTTGGACAATTAATAGCATTAATAACAATTATCAAGGGCACTGATCATTAGATAAGACTCCTGCTTCCTCGTTGCTTACATCGGGGGTACTGACCCACTAAGGCCCCTTGTACTGT 9480  
 TAATGTGAATATTTGCAATTATATATGTCTCCTTCTGGTAGAGTGGGATATTATGCCCTAGTATCCCCTTTGCATTACTGCAGGGGCTGCTGACTACTCAAAACTTCTCCTGGGACTGTT 9600  
 AATAG

GCACAATG

GCACAATG  
Depth:6 (MOUSE)  
Ei-value:0.000, Pi-value:0.000  
Er-value:0.000, Pr-value:0.000  
No matches to eCLIP DataNo matches to TargetScan

GCAGTTATCAATGGTTTTCTCCCTCCCTGACCTTGTTAAGCAAGCGCCCCACCCCACCCTTAGTTTCCCATGGCATAATAAAGTATAAGCATTGGAGTATTCCATGC 9720  
 ACTTGTCTATCAAACAGTGGTCCATA

CTCCCA

CTCCCA  
Depth:6 (MOUSE)  
Ei-value:0.000, Pi-value:0.000  
Er-value:0.000, Pr-value:0.000  
eCLIP MATCHES▶hnrnpk (bg=12.88%)No matches to TargetScan

ACCCTTTTGCATTGCGCCAGTGTGTAAAATCACAGGTAGCCATGGTGTCATGCTTTATATACGAAGTCTTCCCTCTCTCTGCCCCTTG 9840  
 TGTGCCCTTGGCCCCTTTTTACAGACTATTGCTCACAATCTCAGGTGTCCATATTTGCAGCTATTAGGTAAGATTGTGCTGTCTCCCTCTTCCCTTCCCTCTGCCCTGCCCCTTTTGCCT 9960  
 CTTTGCTGGGTAATGTTGACCAGACAAGGCCCTTTCTCTTGGACTTAAACAATTCTCAGTTGCACTTTCCTTGGTCCCACCCATTATACATGAACCCCTCTACTTCCTTTCGCATTGCTT 10080  
 CTGAGTATGCTGACTACCCAAAGCCCCTTCTGTGTTATTAATAAACACAGTACTGATTGTCCCATTTTTCAGCCCATCAGTCCAAGATCTCCCTACCACTTTGGTGTGTTGGTGCAGTGT 10200  
 TGACTATG

AAAAGCAG

AAAAGCAG  
Depth:6 (MOUSE)  
Ei-value:0.000, Pi-value:0.000  
Er-value:0.000, Pr-value:0.000  
No matches to eCLIP DataNo matches to TargetScan

GCCTGAACTAGGTGGATAAGCCTTCACTCATTTTCTTTCATTTATTAATGATCCTAGTTTCAATTATTGTCAGATTCTGGGGACAAGAACCATTCTTGCCCACC 10320  
 TGTGTTACTGCTTTACTGT

GCAAAAT

GCAAAAT  
Depth:6 (MOUSE)  
Ei-value:0.000, Pi-value:0.000  
Er-value:0.000, Pr-value:0.000  
No matches to eCLIP DataNo matches to TargetScan

ACTGAAGGCAAGTCAGACCCAGGGAGCTGGATTGCCATCCTTTATTTTGTGTTTCCAGTGTACACTATAAAATTGTCTCCCCAGGAAGGAAGGT 10440  
 TGGCACTTTCTCTGCATTCTTCTTTCCAGAGCA

GATTGCCTGG

GATTGCCTGG  
Depth:6 (MOUSE)  
Ei-value:0.000, Pi-value:0.000  
Er-value:0.000, Pr-value:0.000  
No matches to eCLIP DataNo matches to TargetScan

TTAAGAATCTCTTGTTGTCCCCTTTGTATATTGTTATTGTAAAGTGCCAAATGCCAGGATACAGCCAGAAAAATTGC 10560  
 TTATTATTATTAAAAAAATTTTTTTAAGAAAGACATCTGGATTGTAGGGTGGACTCGATAACCTGGTCATTATTTTTTTGAAGCCAAAATATCCATTTATACTATGTACCTGGTGACCAG 10680  
 TGTCTCTCATTTTAACTGAGGGTGGTGGGTCTGTGGATAGAACACTGACTCTTGCTATTTTAATATCAAAGATATTCTAGAGTGGAACTCTTAAGACCAGTATCTTTGTGTGGGCTTTAC 10800  
 CAGCATTCACTTTTAGAAAAACTACCTAAATTTTATAATCCTTTAATTTCTTCATCTGGAGCACCTGCCCCTACTTATTTCAAGAAGATTGCAGTAAAACGATTAAATGAGGGAACATAT 10920  
 GCAGAGGTGCTTTTAAAAAGCATATGCCACCTTTTTTATTAATTATTATATAAAATGAAGCATTTAATTATAGTAATAATTTGAAGTAGTTTGAAGTACCACACTGAGGTGAGGACTTAA 11040  
 AAATGATAAGACGAGTTCCCTATTTTATAAGAAAAATAAGCCAAAATTAAATATTCTTTTGGATATAAATTTCAACAGTGAGATAGCTGCCTAGTGGAAATGAATAATATCCCAGCCACT 11160  
 AGTGTACAGGGTGTTTTGTGGCACAGGATTATGTAATATGGAACTGCTCAAGCAAATAACTAGTCATCACAACAGCAGTTCTTTGTAATAACTGAAAAAGAATATTGTTTCTCGGAGAAG 11280  
 GATGTCA

AAAGATC

AAAGATC  
Depth:6 (MOUSE)  
Ei-value:0.000, Pi-value:0.000  
Er-value:0.000, Pr-value:0.000  
eCLIP MATCHES▶SRSF1 (bg=8.47%)▶U2AF2 (bg=1.76%)▶uchl5 (bg=11.16%)No matches to TargetScan

GGCCCAGCTCAGGGAGCAGTTTGCCCTACTAGCTCCTCGGACAGCTGTAAAGAAGAGTCTCTGGCTCTTTAGAATACT||GATCCCATTGAAGATACCACGCTGCA 11398  
 TGTGTCCTTAGTAGTCATGTCTCCTTAGGCTCCTCTTG||GACATTCTGAGCATGTGAGACCTGAGGACTGCAAACAGCTATAAGAGGCTCCAAATTAATCATATCTTTCCCTTTGAGAA 11516  
 TCTGGCCAAGCTCCAGCTAATCTACTTGGATGGGTTGCCAGCTATCTGGAGAAAAAG||ATCTTCCTCAGAAGAATAGGCTTGTTGTTTTACAGTGTTAGTGATCCA

TTCCCTTTGA

TTCCCTTTGA  
Depth:6 (MOUSE)  
Ei-value:0.000, Pi-value:0.000  
Er-value:0.000, Pr-value:0.000  
eCLIP MATCHES▶ILF3 (bg=3.0%)▶RBM15 (bg=7.27%)▶SRSF7 (bg=2.32%)▶ZNF622 (bg=6.58%)No matches to TargetScan

CGA 11634  
 TCCC

TAGGTGGAGATGGGGCATGAGGATCCTCCAGGGGAA

TAGGTGGAGATGGGGCATGAGGATCCTCCAGGGGAA  
Depth:6 (MOUSE)  
Ei-value:0.000, Pi-value:0.000  
Er-value:0.000, Pr-value:0.000  
eCLIP MATCHES▶ILF3 (bg=3.0%)▶NOLC1 (bg=9.43%)▶RBM15 (bg=7.27%)▶SRSF7 (bg=2.32%)▶ZNF622 (bg=6.58%)MATCHES To TargetScan▶ miR-331-3p:CCCCUGG

AAGCTCACTACCACTGG

GCAACA

GCAACA  
Depth:6 (MOUSE)  
Ei-value:0.000, Pi-value:0.000  
Er-value:0.000, Pr-value:0.000  
eCLIP MATCHES▶ILF3 (bg=3.0%)No matches to TargetScan

ACCCTAGGTCAGGAGGTTCTGTCAAGATACTTTCCTGGTCCCAGATAGGAAGATAAA 11754  
 GTCTCAAAAACAACCACCACACGTCAAG||CTCTTCATTGTTCCTATCTG

CCAAAT

CCAAAT  
Depth:6 (MOUSE)  
Ei-value:0.000, Pi-value:0.000  
Er-value:0.000, Pr-value:0.000  
eCLIP MATCHES▶GRWD1 (bg=5.13%)▶NOLC1 (bg=9.43%)No matches to TargetScan

CATTATACTTCCTACAAGCAGTGCAGAGAGCTGAGTCTTCAGCAGGTCCAAGAAATTTGAACAC 11872  
 ACTGAAGGAAGTCAGCCTTCCCACCTGAA

GATCAACATGC

GATCAACATGC  
Depth:6 (MOUSE)  
Ei-value:0.000, Pi-value:0.000  
Er-value:0.000, Pr-value:0.000  
eCLIP MATCHES▶GRWD1 (bg=5.13%)▶NOLC1 (bg=9.43%)▶PTBP1 (bg=3.74%)▶RBM15 (bg=7.27%)▶TRA2A (bg=4.8%)▶uchl5 (bg=11.16%)▶ZNF622 (bg=6.58%)No matches to TargetScan

CTGGCACTCTAGCACTTGAGGATAGCTGAATGAA||

TGTGTAT

TGTGTAT  
Depth:6 (MOUSE)  
Ei-value:0.000, Pi-value:0.000  
Er-value:0.000, Pr-value:0.000  
eCLIP MATCHES▶TARDBP (bg=2.79%)▶ZC3H11A (bg=6.55%)No matches to TargetScan

TTCTTTGTCTCTTTCTTTCTTGTCTTTGCTCTTTGTT 11990  
 CTCTATCTAAAGTGTGTCTTACCCATTTCCATGTTTCTCTTGCTAATTTCTTTCGTGTGTGCCTTTGCCTCATTTTCTCTTTTTGTTCACAAGAGTGGTCTGTGTCTTGTCTTAGACATA 12110  
 TCTCTCATTTTTCATTTTGTTGCTATTTCTCTTTGCTCTCCTAGATGTGGCTCTTCTTTCACGCTTTATTTCATGTCTCCTTTTTGGGTCACATGCTGTGTGCTTTTTGTCCTTTTCTTG 12230  
 TTCTGTCTACCTCTCCTTTCTCTGCCTACCTCTCTT

TTCTCTTTG

TTCTCTTTG  
Depth:6 (MOUSE)  
Ei-value:0.000, Pi-value:0.000  
Er-value:0.000, Pr-value:0.000  
eCLIP MATCHES▶MATR3 (bg=2.98%)▶PTBP1 (bg=3.74%)▶SMNDC1 (bg=0.63%)▶TIA1 (bg=4.07%)No matches to TargetScan

TGAACTGTGATTATTTGTTACCCCTTCCCCTTCTCGTTCGTTTTAAATTTCACCTTTTTTCTGAGTCTGGCCTCC 12350  
 TTTCTGCTG

TTTCTAC

TTTCTAC  
Depth:6 (MOUSE)  
Ei-value:0.000, Pi-value:0.000  
Er-value:0.000, Pr-value:0.000  
eCLIP MATCHES▶MATR3 (bg=2.98%)▶PTBP1 (bg=3.74%)▶TIA1 (bg=4.07%)No matches to TargetScan

TTTTTATCTCAC

ATTTCTC

ATTTCTC  
Depth:6 (MOUSE)  
Ei-value:0.000, Pi-value:0.000  
Er-value:0.000, Pr-value:0.000  
eCLIP MATCHES▶MATR3 (bg=2.98%)▶PTBP1 (bg=3.74%)▶TIA1 (bg=4.07%)No matches to TargetScan

ATTTCTGCATTTCCTTTCTGCCTCTCTTGGGCTATTCTCTCTCTCCTCCCCTGCGTGCCTCAGCATCTCTTGCTGTTTGTGATTT 12470  
 TCTATTTCAGTATTAATCTCTGTTGGCTTGTATTTGTTCTCTGCTTCTTCCCTTTCTACTCACCTTTGAGTATTTCAGCCTCTTCATGAATCTATCTCCCTCTCTTTGATTTCATGTAAT 12590  
 CTCTCCTTAAATATTTCTTTGCATATGTGGGCAAGTGTACGTGTGTGTGTGTCATGTGTGGCAGAGGGGCTTCCTAACCCCTGCCTGATAGGTGCAGAACGTCGGCTATCAGAGCAAGCA 12710  
 TTGTGGAGCGGTTCCTTATGCCAGGCTGCCATGTGAGATGATCCAAGACCAAAACAAGGCCCTAGACTGCAGTAAAACCCAGAACTCAAGTAGGGCAGAAGGTGGAAGGCTCATATGGAT 12830  
 AGAAGGCCCAAAGTATAAGACAGATGGTTTGAGACTTGAGACCCGAGGACTAAGATGGAAAGCCCATGTTCCAAGATAGATAGAAGCCTCAGGCCTGAAACCAACAAAAGCCTCAAGAGC 12950  
 CAAGAAAACAGAGGGTGGCCTGAATTGGACCGAAGGCCTGAGTTGGATGGAAGTCTCAAGGCTTGAGTTAGAAGTCTTAAGACCTGGGACAGGACACATGGAAGGCCTAAGAACTGAGAC 13070  
 TTGTGACACAAGGCCAACGACCTAAGATTAGCCCAGGGTTGTAGCTGGAAGACCTACAACCCAAGGATGGAAGGCCCCTGTCACAAAGCCTACCTAGATGGATAGAGGACCCAAGCGAAA 13190  
 AAGGTATCTCAAGACTAACGGCCGGAATCTGGAGGCCCATGACCCAGAACCCAGGAAGGATAGAAGCTTGAAGACCTGGGGAAATCCCAAGATGAGAACCCTAAACCCTACCTCTTTTCT 13310  
 ATTGTTTACACTTCTTACTCTTAGATATTTCCAGTTCTCCTGTTTATCTTTAAGCCTGATTCTTTTGAGATGTACTTTTTGATGTTGCCGGTTACCTTTAGATTGACAGTATTATGCCTG 13430  
 GGCCAGTCTTGAGCCAGCTTTAAATCACAGCTTTTACCTATTTGTTAGGCTATAGTGTTTTGTAAACTTCTGTTTCTATTCACATCTTCTCCACTTGAGAGAGACACCAAAATCCAGTCA 13550  
 GTATCTAATCTGGCTTTTGTTAACTTCCCTCAGGAGCAGACATTCATATAGGTGATACTGTATTTCAGTCCTTTCTTTTGACCCCAGAAGCCCTAGACTGAGAAGATAAAATGGTCAGGT 13670  
 TGTTGGGGAAAAAAAAGTGCCAGGCTC

TCTAGAGAAAA

TCTAGAGAAAA  
Depth:6 (MOUSE)  
Ei-value:0.000, Pi-value:0.000  
Er-value:0.000, Pr-value:0.000  
eCLIP MATCHES▶CPSF6 (bg=0.4%)▶LARP4 (bg=4.72%)▶UTP3 (bg=3.66%)▶WDR43 (bg=3.37%)MATCHES To TargetScan▶ miR-1251-5p:CUCUAGC

ATGTGAAGAGATGCTCCAGGCCAA

TGAGAAGAATTAGACA

TGAGAAGAATTAGACA  
Depth:6 (MOUSE)  
Ei-value:0.000, Pi-value:0.000  
Er-value:0.000, Pr-value:0.000  
eCLIP MATCHES▶LARP4 (bg=4.72%)▶NOLC1 (bg=9.43%)▶SRSF7 (bg=2.32%)No matches to TargetScan

AGAAATACACAGATGTGCCAGACTTCTGAGAAGCACCTGCCA 13790  
 GCAACAGCTTCCTTCTTTGAGCTTAGGTGAGCAGGATTCTGGGGTTTGGGATTTCTAGTGATGGTTATGGAAAGGGTGACTGTGCCTGGGACAAAGCGAGGTCCCAAGGGGACAGCCTGA 13910  
 ACTCCCTGCTCATAGTAGTGGCCAAATAATTTGGTGGACTGTGCCAACGCTACTCCTGGGTTTAATACCCATCTCTAGGCTTAAAGATGAGAGAACCTGGGACTGTTGAGCATGTTTAAT 14030  
 ACTTTCCTTGATTTTTTTCTTCCTGTTTATGTGGGAAGTTGATTTAAATGACTGATAATGTGTATGAAAGCACTGTAAAACATAAGAGAAAAACCAATTAGTGT

ATTGGCA

ATTGGCA  
Depth:6 (MOUSE)  
Ei-value:0.000, Pi-value:0.000  
Er-value:0.000, Pr-value:0.000  
eCLIP MATCHES▶HNRNPA1 (bg=2.57%)No matches to TargetScan

ATCATGCAG 14150  
 TTAACATTTGAAAGTGCAGTGTAAA

TTGTGAAG

TTGTGAAG  
Depth:6 (MOUSE)  
Ei-value:0.000, Pi-value:0.000  
Er-value:0.000, Pr-value:0.000  
eCLIP MATCHES▶HNRNPA1 (bg=2.57%)No matches to TargetScan

CATTATGTAAATCAGGGGTCCACAGTTTTTCTGTAAGGGGTCAAATCATAAATACTTTAGACTGTGGGCCATATGGTTTCTGTTACA 14270  
 TATTTGTTTTTTAAACAACGTTTTTATAAGGTCAAAATCATTCTTAGTTTTTGAGCCAATTGGATTTGGCCTGCTGTTCATAGCTTACCACCCCCTGATGTATTATTTGTTATTCAGAGA 14390  
 AAATTTCTGAATACTACTAGTTTCCTTTTCTGTGCCTGTCCCTGTGCTAGGCACTAAAAATGCAATGATTATTGATATCTAGGTGACCTGAAAAAAAATAGTGAATGTGCTTTGTAAACT 14510  
 GTAAAGCACTTGTATTCTACTGTGATAAGCGTTGTGGATACAAAGAAAGGAGCAAGCATAAAAAAGTGCTCTTTCAAAAGGATATAGTACTATGCAGACACAAGGAATTGTTTGATAAAT 14630  
 GAATAAATTATATGTATATTTGAGGCCAATTTGTGTTTGCTGCTCTGGTAATTTTGAGTAAAAATGCAGTATTCCAGGTATCAGAAACGAAAACACATGGAAACTGCTTTTAAACTTTAA 14750  
 AATATACTGAAAACATAAGGGACTAAGCTTGTTGTGGTCACCTATAATGTGCCAGATACCATGCTGGGTGCTAGAGCTACCAAAGGGGGAAAAGTATTCTCATAGAACAAAAAATTTCAG 14870  
 AAAGGTGCATATTAAAGTGCTTTGTAAACTAAAGCATGATACAAATGTCAATGGGCTACATATTTATGAATGAATGAATGGATGAATGAATATTAAGTGCCTCTTACATACCAGCTATTT 14990  
 TGGGTACTGTAAAATACAAGATTAATTCTCCTATGTAATAAGAGGAAAGTTTATCCTCTATACTATTCAGATGTAAGGAATGATATATTGCTTAATTTTAAACAATCAAGACTTTACTGG 15110  
 TGAGGTTAAGTTAAATTATTACTGATACATTTTTCCAGGTAACCAGGAAAGAGCTAGTATGAGGAAATGAAGTAATAGATGTGAGATCCAGACCGAAAGTCACTTAATTCAGCTTGCGAA 15230  
 TGTGCTTTCTAAATTATAAAGCACTTGTAAATGAAAAATTTGATGCTTTCTGTATGAATAAAACTTTCTGTAAGCTAGGTATTGTCTCTACAAAATTCTCATTGTATAGTTAAACCACAG 15350  
 TGAGAAGGGTTCTATAAGTAGTTATACAAACCAAGGGTTTAAATACCTGTTAAATAGATCAATTTTGATTGCCTACTATGTGAACTCACTGTTAAAGGCACTGAAAATTTATCATATTTC 15470  
 ATTTAGCCACAGCCAAAAATAAGGCAATACCTATGTTAGCATTTTGTGAACTCTAAGGCACCATATAAATGTAACTGTTGATTTTCTCACTTGGTGCTGGGTACTAGGTTTATAAAATTG 15590  
 TATGATAGTTATTATATTGTGCAAATAAAGTAGGAAAATTTGAATAACAATGATTATCTTTTGAATACGCATACGCAAGGGATTGGTTGTCTGAAGAATGCCACTATAGTAGTTATCTAT 15710  
 TGTGTGCCAATCTCATTGCTAGGCATTGGGGATGCAAAGATAAACCATCTTTATTGTGTCTTGGGTAGCAGAAGAAAATATGTGTAAAATCAATTTATAATTTGTAAACTGCCACCCATA 15830  
 TATAAGCTATATCTGCTGAATGATCATTGATTACTCTTATCCTTAGAGATAACAACTGGGGGCACAAACATTTATTATCATTATTGAACCTACAACAGAGATCTATGTGTAGATTTACAA 15950  
 AGCCTACAGTTCTATACAGATAGGAATGAACTATTGGCTTACTGAATGGTGATTACTTTCTGTGGGGCTCGGAACTACATGCCCTAGGATATAAAAATGATGTTATCATTATAGAGTGCT 16070  
 CACAGAAGGAAATGAAGTAATATAGGTGTGAGATCCAGACCAAAAGTCATTTAACAAGTTTATTCAGTGATGAAAACATGGGACAAATGGACTAATATAAGGCAGTGTACTAAGCTGAGT 16190  
 AGAGAGATAAAGTCCTGTCCAGAAGATACATGCTTCCTGGCCTGATTGAGGAGATGGAAAATTTTTGCAAAAAACAAGGTGTTGTGGTCTTCCATCCAGTTTCTTAAGTGCTGATGATAA 16310  
 AAGTGAATTAGACCCACCTTGACCTGGCCTACAGAAGTAAAGGAGTAAAAATAAATGCCTCAGGCGTGCTTTTTGATTCATTTGATAAACAAAGCATCTTTTATGTGGAATATACCATTC 16430  
 TGGGTCCTGAGGATAAGAGAGATGAGGGCATTAGATCACTGACAGCTGAAGATAGAAGAACATCTTTGGTTTGATTGTTTAAATAATATTTCAATGCCTATTCTCTGCAAGGTACTATGT 16550  
 TTCGTAAATTAAATAGGTCTGGCCCAGAAGACCCACTCAATTGCCTTTGAGATTAAAAAAAAAAAAAAAAAGAAAGAAAAATGCAAGTTTCTTTCAAAATAAAGAGACATTTTTCCTAGT 16670  
 TTCAGGAATCCCCCAAATCACTTCCTCATTGGCTTAGTTTAAAGCCAGGAGACTGATAAAAGGGCTCAGGGTTTGTTCTTTAATTCATTAACTAAACATTCTGCTTTTATTACAGTTAAA 16790  
 TGGTTCAAGATGTAACAACTAGTTTTAAAGGTATTTGCTCATTGGTCTGGCTTAGAGACAGGAAGACATATGAGCAATAAAAAAAAGATTCTTTTGCATTTACCAATTTAGTAAAAATTT 16910  
 ATTAAAACTGAATAAAGTGCTGTTCTTAAGTGCTTGAAAGACGTAAACCAAAGTGCACTTTATCTCATTTATCTTATGGTGGAAACACAGGAACAAATTCTCTAAGAGACTGTGTTTCTT 17030  
 TAGTTGAGAAGAAACTTCATTGAGTAGCTGTGATATGTTCGATACTAAGGAAAAACTAAACAGATCACCTTTGACATGCGTTGTAGAGTGGGAATAAGAGAGGGCTTTTTATTTTTTCGT 17150  
 TCATACGAGTATTGATGAAGATGATACTAAATGCTAAATGAAATATATCTGCTCCAAAAGGCATTTATTCTGACTTGGAGATGCAACAAAAACACAAAAATGGAATGAAGTGATACTCTT 17270  
 CATCAAACAGAAGTGACTGTTATCTCAACCATTTTGTTAAATCCTAAACAGAAAACAAAAAAAATCATGACGAAAAGACACTTGCTTATTAATTGGCTTGGAAAGTAGAATATAGGAGAA 17390  
 AGGTTACTGTTTATTTTTTTTCATGTATTCATTCATTCTACAAATATATTCGGGTGCCAATAGGTACTTGGTATAAGGTTTTTGGCCCCAGAGACATGGGAAAAAAATGCATGCCTTCCC 17510  
 AGAGAATGCCTAATACTTTCCTTTTGGCTTGTTTTCTTGTTAGGGGCATGGCTTAGTCCCTAAATAACATTGTGTGGTTTAATTCCTACTCCGTATCTCTTCTACCACTCTGGCCACTAC 17630  
 GATAAGCAGGTAGCTGGGTTTTGTAGTGAGCTTGCTCCTTAAGTTACAGGAACTCTCCTTATAATAGACACTTCATTTTCCTAGTCCATCCCTCATGAAAAATGACTGACCACTGCTGGG 17750  
 CAGCAGGAGGGATGATGACCAACTAATTCCCAAACCCCAGTCTCATTGGTACCAGCCTTGGGGAACCACCTACACTTGAGCCACAATTGGTTTTGAAGTGCATTTACAAGGTTTGTCTAT 17870  
 TTTCAGTTCTTTACTTTTTACATGCTGACACATACATACACTGCCTAAATAGATCTCTTTCAGAAACAATCCTCAGATAACGCATAGCAAAATGGAGATGGAGACATGATTTCTCATGCA 17990  
 ACAGCTTCTCTAATTATACCTTAGAAATGTTCTCCTTTTTATCATCAAATCTGCTCAAGAAGGGCTTTTTATAGTAGAATAATATCAGTGGATGAAAACAGCTTAACATTTTACCATGCT 18110  
 TAAGTTTTAAGAATAAAATAAAAATTGGAAATAATTGGCCAAAATTGAAAGGAAAAATTTTTTTAAAATTTCTCTAAATGTAGGCCTGGCTGGGCTTTGACCTTTTCCGTTTTTAAATCA 18230  
 CTCACAGAGGGTGGGACAGGAGGAAGAGTGAAGG

AAAAGGT

AAAAGGT  
Depth:6 (MOUSE)  
Ei-value:0.000, Pi-value:0.000  
Er-value:0.000, Pr-value:0.000  
eCLIP MATCHES▶ILF3 (bg=3.0%)▶SF3B1 (bg=2.48%)▶ZC3H11A (bg=6.55%)No matches to TargetScan

CAAACCTGTTTTAAGGGCAACCTGCCTTTGTTCTGAATTGGTCTTAAGAACATTACCAGCTCCAGGTTTAAATTGTTCA 18350  
 GTTTCATGCAGTTCCAATAGCTGATCATTGTTGAGATGAGGACAAAATCCTTTGTCCTCACTAGTTTGCTTTACATTTTTGAAAAGTATTATTTTTGTCCAAGTGCTTATCAACTAAACC 18470  
 TTGTGTTAGGTAAGAATGGAATTTATTAAGTGAATCAGTGTGACCCTTCTTGTCATAAGATTATCTTAAAGCTGAAGCCAAAATATGCTTCAAAAGAAGAGGACTTTATTGTTCATTGTA 18590  
 GTTCATACATTCAAAGCATCTGAACTGTAGTTTCTATAGCAAGCCAATTACATCCATAAGTGGAGAAGGAAATAGATAAATGTCAAAGTATGATTGGTGGAGGGAGCAAGGTTGAAGATA 18710  
 ATCTGGGGTTGAAATTTTCTAGTTTTCATTCTGTACATTTTTAGTTAGACATCAGATTTGAAATATTAATGTTTACCTTTCAATGTGTGGTATCAGCTGGACTCAGTAACACCCCTTTCT 18830  
 TCAGCTGGGGATGGGGAATGGATTATTGGAAAATGGAAAGAAGAAAGTAACTAAAAGCCTTCCTTTCACAGTTTCTGGCATCACTACCACTACTGATTAAACAAGAATAAGAGAACATTT 18950  
 TATCATCATCTGCTTTATTCACATAAATGAAGTTGTGATGAATAAATCTGCTTTTATGCAGACACAAGGAATTAAGTGGCTTCGTCATTGTCCTTCTACCTCAAAGATAATTTATTCCAA 19070  
 AAGCTAAGATAAATGGAAGACTCTTGAACTTGTGAACTGATGTGAAATGCAGAATCTCTTTTGAGTCTTTGCTGTTTGGAAGATTGAAAAATATTGTTCAGCATGGGTGACCACCAGAAA 19190  
 GTAATCTTAAGCCATCTAGATGTCACAATTGAAACAAACTGGGGAGTTGGTTGCTATTGTAAAATAAAATATACTGTTTTGAAAACTTTG                               19280
```

|  |  |  |  |  |
| --- | --- | --- | --- | --- |
| | | | | | | | | | |
| 2 |  | 4 |  | 6 |
| Depth of motif conservation (number of species) | | | | |

  
  

---

  

## >PIG (25215 bases)

```
 TATTTCTTCTTTTTCCCGGGTGGAAGCTTGCTGGTATTGGATCTCTTTGCCCGTGTGGTTCTTTCTGGAACATTTTCCAGCCCCCAGCCATGCCTTATGGCATATTTCTTTAAAAAAAAA 120  
 TCCACCAAAAATTCATAAAATGTTTTAAAATTTCTAAACTTTCTCCTAATATTTTCTTGACACCTTATCTCTAGTTTACAGTTATTTGGGATATTTTAAGGCAACTTTCTATTTTAAAAT 240  
 AATTTTTCTTTGGAATGTTTTTTGGTTGACTCTTCTGGTTTTTTCGTGGTGTAATTTTCTTTTCCCCCTCCTTTTCTGTGTATTATGCCCATCGGGGCTGTGGATACCTGGTTTTAATAA 360  
 TTGTTATTTATTTTTTTGCCCAACGGGGCCGTGGATACCTGCCTTTTAATTCTTTTTTTAAAGACTTTGCCCATCGGGGCCGCGGATACCTGCTTTTAATTTTTTTTTCCCCTTAGCCCA 480  
 TCGGGGCCTCGGATACCTGCTGTGTCCCCCTCTTTCTCCAACCCCTTGGCCCATCAGGGTAATGGATACCTGCTTTTTTATTTAAAAAAAAATTTTTTTTGGCCCATCGGGGCCTCGGAT 600  
 ACCTGCTTTAATTTTTTTTTTTCCTTGCCCATCGGGGCCTCGGATACCTGCTTTATTATTTTTTTTTCCTTGCCCATCGGGGCCGTGGATACCTGCTTAGATTTTTTTTTTTCATCACCC 720  
 ATCGGACCTTTGTATGGATGGAAAAGTGTTGGGTTTTGTGGTTCGTTGTACTGTCTGGAATGTCTACAAAATTTTGCTGCTAATCGTTTGGTGTTGTGTGAGTGGACCTACGGCTTTGGT 840  
 TTGGAGATGACTTTGCAGTTAGGCTAGGGGGTTGGTCAGGCTGGGGAGGAAAGATGGCGGCCACTTGAGATTTGCCGCCCAGCTCGGCTGAGGGCTACTTGTTTATGCTAAGTGTAAACT 960  
 AGGGAGGCAAGATGAATAGTGGGACAGGCAGAGGAAATGAATATGCATTGTATGAGCTACGTGTTTTGAATTAGTCGATTTGGGTCTTGTTAGGACCTTTGCATGGATTGTGGTATCATG 1080  
 AGGTGGAAAAACGGGGTCATCCTGTGTCATATTACAAGAGGCTAATAGAAAATGAGAGGGAGAAGGTTTAGGCGCAGGGTTCAAAATGGCGATTTTGACTTTGCAGCATTGCTTAGCATG 1200  
 GCTCTCTGCTTTGTTAGAGTGTTCAAAATGGCGGACCCACTTTGCCGCAGTGTTCCAGTGGCGGGAAGCCACATTATGGGTGTCTTTGTTCTAGCGTGCAGCATGGCGGTGGAAATATTC 1320  
 TGTTACATAGCAAAAGATGGCGGCTCAAGTACTTGCCGCAATCGAAAACATGGCGGGCCTTTGTCTTTGCCGTGTGCATTTCCTGACAAATTTTGCCGCAGGGACAATATGGCTGACCTT 1440  
 GTCATGTGGATAGCATGGCAGTCTGTCACGTGGACGTCATGGCAGGGGTGTTTGACCGTTACATTCTTGGCGGGCTTTGCACCAGGAGGGCCTGCCGCATTGTTCAAGATGGCGGGCTTT 1560  
 GCCGCGAAAAAGTGCAGGAGGGATTGGCAGCGTTGGATTGCCGCCCGACACATCCAATCAGAAAGGGTGGTGGAATTGGTCACAGACAGTTAGTGGAGGATGGAATTAGTCGGAGTTAGC 1680  
 ATAGCACCTCGCTACCGTCTCTATTCAGCCAGTCAGCACCGGCCACGTTTGTACTACTCCCAGTGGGTGGTACCCAAGGTCTTTCCAAGGACGTTTGGCCTTTCCACCTCCCTCCCCTCT 1800  
 CACTGGCTCCCTCCCCTCCAGCATTACCACCTGCTGTGCTGAACTTTAGGCTATATGGGCTGAACCTCACACCATTCCTCTGCATTGGTGGCCTAAGGCTAACTTACTACCCCTTCCCCC 1920  
 CCTCCCCCTCCCCCGCCTCTGCTCTTCTGCACTGTGGCCAGGGGCAGTGCTCCATGCCTGCCAAGTGTGAACATGGCGGTGAGTCGTGGCAAGGACCAGAATGGATCGCAGATGATCGTT 2040  
 GGCCAACAGGTGGCGGAAGAGGAATCCCTGTCTTCCTCAAGAGGAACACCTACCCCGTGGCTAATGCTGGGGTCGGATTTTGATTTCTATTTATTTCTTGGATGTCAGTCATATATAGTT 2160  
 TGATTATGTGGTTTGCTAGTGTTCGATTTAAGCCTTAAGTGACTACTATGGTAATGTATTTAGGGACTTTGTTATTTGTAGAATTCATTTCAGTTACATTTAGTGGGTTTTCATTTTGGG 2280  
 GTTCCTTTGAAATTCCTTAAAGTTTTCAATTTCTTTTTTTTACAAATGCTTACATTTCTTTATCTTTATATAGTCAAAGTCAATAATTTGGCATTTATAAGTTTTTACTACTTTTTTACT 2400  
 TTGAAATTGACTTAATGAACTACTTCCTTTTGGATTTTTGAAGTCTTATAGTACAAAAATAGTTAATTTAAAATTTAACATTATGACCAAATAATCTTTGAAGGTCCTCTTCTGTCCAGG 2520  
 TCTTGCTTTGTTCCCATCCTTAATGCTGCACTGATTGAATAATGACCTACCTATCTGCAGAAAACTTGAATTGCTGTGGACTTCTTCTACTCTAATTTATTATATTGGAGTATTATTGCC 2640  
 CATAATTTCAATTTTCTGTGGTGACCTGTCTCCACAGGCCCCAGCTCCTCTCCACACCTCTTCCCAGCAAACCCATATTTAGCCCCAGCCCCTGCCCCTGCCCCTGCCCCAGCCCTTGCC 2760  
 CCTGCCCCAGCCCCTGCCCCTGCCCCAGCCCCAGCCCCTGCCCCTGCCCCTGCCCCTGCCCCAGCCCCAGCCCCAACCAAGCCCCAGCCCCAGCCCCAGCCAAGCCCCTGTCCCACTCCT 2880  
 GTTTCTCCCTAAAGCCAGTCCAGTTTGTATTGATTTGATGCTAAAAATAAGTTTCAGTTGCTCTTCCATCAGACTGGGATACCTTGTCTGCCTCTAGCATTGCTGATCTTGAGTGCTGAC 3000  
 TACCTGAGTCACCATTTTCAATTAATGTACACAATCCCATTTGCCCATTATCTCATGTTAGAACAAAGAATTTGTGCAATAATGTTAGGAAATTTAATTGTGAGCTTCATATCAGACAAT 3120  
 TATTGTCCATAAATCTGCTTACTCATCATCTCAATCTCCTGCCTTTAAGGAAGGAGAAACCATTCCTCTGTCATTGCTGCAGTAGTCAAAATCCCAGTTTTCTGGAAAGTGCATATATAC 3240  
 TGTGCTGACCACCTAAACTCTTTGCACTCAGTGAAATTCTAATTGTCCATAATCCTGCATTGGACTAAATCCCAATTTAAAAACCCTTTTGCATTCAGCAGTCCCAGTTGTCATACCCTT 3360  
 GTCTGTTAAACAAAGGCATCCTACCATTTTTACCCTCTTGTGAGTCCTCCTGTTCTTGGACAATTAAAGTACCAAATTGTAATTGTACATTGTCTCACTCATTAATCAATGACCTTCTGT 3480  
 CCCGTTTGCATTGCTATTAGGCAGTGCTGACTATCTGAGACCATGTTCCTTGAATTATTATTATTGACTAGAATACCAATTGTTCATTACACAAATCCTTTGCAGCTTCTTTGCACTATA 3600  
 AGGGAAGTGCCCATCACTAAAGTCCTTTACTTCGGAATATTAATGGATACAATCATTACTGGTCATGGTCCTGCCTACCAGATAAGACCCACCCATGCTCTTTTCATTGTTCTTAGGTAG 3720  
 TGTGGACTACCTACCACCTTGCATTAATATTTAAAATCCTCATTGTCCATTGTCCCACTGTTAGCCTAGGATATCTCTTACCTCTATGCCATTTCTTCTAAGGAGTTCTGATTACCCTAA 3840  
 GTCCTTTCTCTTAAACAATTTATGTGCATAATTGCATATACCTGTGGTCTCATGCAATTAAAAATTAAATCCTACACTCTGTGGAATATTGCTAGGTAGTGCAAATTATTCAAGGACTCA 3960  
 GCTTTGGGACAGTTAATCCGCACAGTTGCGGTTGTCCAGAGTCCCATCCATTTTAGTGGGCCACTGTGTCCCATTTGGGTGCTAGGTGTGTCAGGAGGACCCTTCTGTTGAATTGATAAT 4080  
 GTGCATAATTGCATTTGTTCATGTCCTGTGAACTAGTAACATCCACCTGCTTTGTATTCCAGCAGGGGACCCTTTTTAACTTAAGAGCGCTGCACTGGGACACTCTACCTGTGCAAACCT 4200  
 AATTGATTAGACCTGAGCCTTTTGTATTAGGAACCCTGCATTATCTTTACACCTAAAAGGTGCTAATTGCCTAAGGCCATTTCCTGCCCGAACGACTGTGTTTAAACTAGAGTCTCACAC 4320  
 TCAATAACCGCCTTTGCATAAGAGTAGAGGATACTTGTGACAGTTAAGTGGTTTGATTGCAGCTACAGACCCTGCTGTTAGACTAGCATCCATTCTCCTCCTAGATGATTCACATGACCA 4440  
 CAATCCGTACTAGGGACTGTTAATGGGTTTAAATGCAGCTACAGTCCTTTATGTTAATCTAGGATCCCATCCCCAACTCACTCTCTTTTCATTACTGCAGGGCTTACATAGGACTTGTTA 4560  
 ACGACCTTTATTGCACTACTGTCGCTTCTGTTAGTCCAAGACCCCATATCCTCCTAAATCCTGTATTACCGAAAGAATATAAGGGACTGTTAAAGTGCTTAAAGCAGTTACAGTCCCTTA 4680  
 TGTTTATCTAGGACCCATCCTCAACTAACCCCTTTTGCATGACTGCAGAGGGTTCTTGGGACTGTTAATGGGCTTTATTGCAGCTACAGTCCCTTCTATTAATCTGGATTCCTATCCACT 4800  
 CCTACACAATTTGCATTACCACAGACTATACAAGGGACTGTTAATGTGTTTAAATGGAGCTACAGTTCCTTATGTTAAACTAGGATCCCATTTCCACCTAACTCCCTTTTCATTAATGCA 4920  
 GAGCCTACATGGGACTAGTGGGCTTATTTGCACTACTGTCCCTTCTTTTATTCTAGGATCCCATCCCTCCTACTGGATTTGCACTACCCTAAAATGTATGAGAGACTGTGGATGTGCTTA 5040  
 AATGTATCTACAGTCCCTTTTGTTAACCTATGATCCCGTTCCCATCTAACACCCTTTGCATGACTGCAGAGGGTACTTGGGATAGTTAATGCGCTCAATTAGAGCTACAGATCCTTCTGT 5160  
 TAGTGGAGATTCCCATGCCCTCCTACTCCATTTGCATTACCAAAGGGAGTACAAGGGACTGTTAATGGGCCTAAATGCAGCTACAGTCCCTATGTTAACCTAGGATCCCATTTCCACCTA 5280  
 ACTCCCTTTTCATTAATACAGAGCCTACATGGGACTTAATGGGCTTTATTGTACTACTGTCCCTTCCATTAATCTAGCATCCCATCCCCACATAATTCCCTTTGCATTACAAGGGAGGAT 5400  
 ACAAGGGACTTTTAATGGGCTTAAATGCAGGTTCAGTCCCTTTCTTATTTTACCTAGGATCCCATTCCCACATAATTCCCTTTGCAAAACCACAGAGTACAAGGTACTGTTACTTACTGT 5520  
 ACTTAAAAGCAGCTATAAGATACTCATGTTGCAACAGAAGAAAGGGTGGGGGAAAAACTGTAATTGCAATGTATACATGTAAGGATAACCTGACCCCCTTGCTGTACAGTGGGAAAAAAA 5640  
 AAAAAAAAAAAAAAGCAGCTATAGTCACTTATGTTTATGTAGAACTCCATTTCCACTAACTCCCTTTTCATGACTGCAGAGGTATCTGGGACTGTTAATGGGCTTTATTGCAGCTACAGT 5760  
 CCCTTCTGTTTGTCTGGATTCCCATCCAGTCCTATACAATTTGCATTACCACAGAATATCTAAGGTAACTAGCTTAATGAGCTTAAATACAGCTACAATCCTTTATGTTAACCTAAAATT 5880  
 CCATCCCCACCTAATTCTTTTAGGACGGCTGCTGAGAGTACATGGGACTGTTAATGGGCTATTTTGCAACTACAGTCCCTTCTGTTAGTCTAGAATCTGTTTCCATCCTACTCCATTTGC 6000  
 ATTACCCTATAGCGTATAAGGGACTGTTAATGTGCTTAAATGGAGCTGTGTAGTCCCTTATCTTAACCTGGTATCACACCCCAACTAATTCTCTTTTCATACTGCAGAGCCTACGTGGAA 6120  
 CTTGTTAATGGACTTAATTGCACTGCTGTCCCGTCTATTAGTCTAGGAGTCCACCCTTCCTACTCAGTTTACATTACCCTAGAGTGTATGAGACTGTTAATGTGGTTAAATGCATCTGAC 6240  
 TGTTATGTTTACCTATGATCCCATTCTCACATAATTCCTTTGCAATACCGTAAAAGGTAAAAGGAATGTTACTGTGCTTAAAAGAAGCTATAGTCATTTACATTTATCTAGGACCCTGTT 6360  
 CCCATCTAACTGACTTTCAGTGACACAGGGGGCAGTTGGGACTGTTAATGGGCTTTATTGCAGCTATAGTCCCTTGTGTTAATCTGGAATCACTTTCCTTCCTGCTCAATTTGTATTACA 6480  
 ACTGAAGGGAACTGGGACTGTCAATATGCTTAGGTGCAGCTACAGATCCTCACCTTAACCTAGGATCCCGTTCCCACCTAACTATCTTTTTGTTAATGCAAAGCCTATGTGGGACTTGTT 6600  
 AGTGGGCTTAATCGCACTATTGTCCCTTCTGTTATTTTAGGATCCCCTTTTCCTACTCGATTTGCATTACCTTAAGATGTATGAGACTGGATTTGCTTAAATGCATCTACAGTTCCTATG 6720  
 TTAACCTATGATCCCATTCCCATCTAACACCCTTTGCATGGCTGCAGAGGGAACTTGGGATAGTCAATGGGCTCGATTAGAGCTACAGTCCCTTCTGTTGGTTGAGATTCCCGTTCCCTT 6840  
 ACTCGTTTTGCATTACCAAACGGGGGAAGAAAGGGCTGTTAATATGCTTAAATGCAATTACAGTCCCTTATGCTAACCTGGGATTCATCCCCAAATAACTCCCTTTTCGGGATTGCAGAG 6960  
 GATACTTGGGACTGTTAATGGGCTTAACTGCATCTATAGTCTCTTCTGTAAGTCTAGAATCTGTTCCTTTCCTACTTGATTTGCATTACTACAGAGGATACAAGGGACAGTAAAGAGGCT 7080  
 TAAATATAGCTGCAATCCCTTATGTTAACCTAGGAGCCTGTCCACAGTTATTTCCCTTTTCATTAATGCAGAGCCTACATAGGACTTGTTAATAGCCTTAATTGCTCTACTGACCCCTCC 7200  
 CGTTAGTTTGGGATCCCATCCTCACCTAATTCTCTCTGCATTACCAGGGAGGATACAAGGGACTCTTAATGTGCTTAAATGAAGGTACAGTCCTTTTTTTTAAACCTAGGATTTCATTCC 7320  
 CATGTAACTCCATTTGCACTACCACAGAGGGTACAAGGGGCGAATGAGCCTAAAAGCAGCTACAGTCCCTTATGTTAACCTAAGGATCCTGTCTCCTAGGAACTCCCTTTTCCTGGCTGC 7440  
 AGAGGATACTTGGGACTGTTAGTGAGATTTACTGTAGCTACAGTCTCTTTTATTAGTCTAGATTCCCATTACCATCCTAGATGTTTTGCATTACCACGGACTATACAAGGGACCATTATA 7560  
 GGGCTTAAATGCAGCTCCAGTCCCTTATTAACCTAGAATCTCATCCCCACCTACAGCCTTTTTCATGCTGCAGAGGGTACTTGGGACTGTTGATTGGCTTTATTGCAGCTGCAGTCCCTT 7680  
 CTGTTAGTCTAGAATCTGTTTCCATCCTACTCCATTTGCATTACCACAGAGGGGACAAGGGACTGTTAATGTGCTTAAATAGAACCACAGTCCCTTATGTTAAATGAGGATCCCATCCCC 7800  
 AACTAACTCTCTTTTCATTACTGCAGAGCTTAAGTAGGACTTGTTAATAGACTATTGCATTACTGTTCTGTTATTCTATAGGTTCCCATCCCTCATTCTATTTGCATTACCCTAGAGTGT 7920  
 AAAGGGACTGTTAATGAGCTTCTATGGCTCTACAGACCCTTACGTTACCTATGATCTTTTTGCCACTTAACTCCCTTTTCATGCCTATAGAGAGTACTTGGGACTGTTAATGGGCTTTAT 8040  
 TATGGCTACAGTCCCTTCTGTTTGTCCAGAATTTGTTACTTTTCTACTCGACTTGCACAGATGGTACAAGGGACTGTTAATGTGCTTAAATGAAGCTACTGTCTCTTATGTTTAAATAAA 8160  
 ATCCCATTCCCCACCTAAAACTCTTGCATGGCTGTAGAAGGTACTTGGGACAGTTAATGGCTTTAATTAGAGCTACAGTCCCTTCTATTGGTCAATATTCCCATGCCCATACTCCATTTG 8280  
 CATTACCAAAGGGAGTACAAGAGACTGTTGGTATGCTTCAATGCAATTACAGTCTCTTATGTTAACCTACGATCTGATACCCAACTATCTCCCTTTTTATTAATGCAGAAGCTACATGGG 8400  
 ACTTGTTAACGGCTTTAATTGCACTACTGTCCTTCCATTAGTCTAAGATCCCATCCCCAACCTGATTGCCTTTACAGTACCAACCAAGGAAGATACAAGGGACTGTCAGTGTGCTTAAAT 8520  
 GCAATTACAGTCCCTTTTAAATTTTACCCAGGATCCCATTCCCCCTTATCTCCCTTTTCAACACTACAGAGGGTACAATTGACTTACTGTGCTTAAAAGAAGCTATAGTCCCTCATGTTA 8640  
 ACCTAAGGATCTCATCCCCAACTAACTCTCCTTTCATGACTACAGAGAGTACTTGGGACTGTTAATGGGCTTTATTGTAGCTATAGTCCCTTTTGTTAGTCTAGACTACCATTTACTACA 8760  
 TGATTTGCATTACCACAGAATATGCAAGGGACTGTTACTGCGCTTAAATGCAGTTACAGACCCTTATGTTAATCTAGAATCCCATCCCCAACTATCTCTCTTCATTAATGCAGAGCCTGC 8880  
 ATAGGACTTATTAACAACCTTTATTGCACTACAGTCTCTTCTGTTAGTCTAGGAGCACATCCCCTCCTAGTTCCCTTTACATTACTAAAGAAGATACAAGGGACTGTTGATGGGCTTAAA 9000  
 TACAACTACTGTCCGTTATGTTAACCTAGGATCCCATCTCCACCTGAATCCGTTTGCATGACTGCAGAGGGACCTTAGGCTTTATTACGGGCGGCTACAGCCCCTTCTGGTAGTTTAGCT 9120  
 TCTGTTGCTGTCCTACTGGATTTGCATTACTACAGATGGTGCAAGAGACTGTTAACGTGTTTATTAAATGGAGCTACAGTCTTTTGGGTTTACTTAGAATCCCTTTTCTCTAACCTAAAA 9240  
 CCCTTTGCATGACTGTAGAGGGTACTTGGGACTGTTAATGGGCTTAATTAGAGCTATAGTCCCTTTTATTTGTTTAGATTCTCGTGCCTTCCTACTCTATCTGCATTACTAAAGAGACTG 9360  
 TAATGCACTATTTATGTGCTTAAGTGGAGCTCCAGCTCTTGTGTTAACCTAGCATCCTATTTCTACCTAACTCCCTTTTCATAACTGCAGAGCCTATGTGGGACTATTAGTGAACTTTAC 9480  
 TGCAGTTACCTTCTGTTAGTGTAGATTCCCATCCTCTTTTATATGATTTGATAACCACAGACTGTACAATGGACTACCAATGTGCTTAAATGCAGCTACAGTCCTTTATGTTAAGCTAGA 9600  
 ATCCCATTCTCACCTAACTCTTCTCATTGATGCAGAGCCTATATGGGACTTGTTAATGGGCTTCATTGCTCTTCTCTGTCCCTTCTACTAGTCTAGATTCCCAAGCTCTCCTACTCAATT 9720  
 TGCATTACCAAGGGGGGTACAATGGACTGTTAATGTGCTAAAATGCAGCTACAATCTCTTACATTAATCTAGAATCTCATGCCCCAACTAACGCCCTTTGCATGGCTTCAAAGGATACTT 9840  
 GGGACTCTTAATGGGCTGAAATACAGCTACAGTCTCTTATGATAACCTAGGATCCCATTCTCACCTATCTCCCTTTTCATTGCTGCAGAGCCTGCATGGGACTTGTTAATGGTCTTAATT 9960  
 GCACTGCTGTCCCTTTCGTTAGTCTAGGATTCTATCCCCTCATAAAGAATTACATTACCGTAGAGGTTTCAAGGGAATGGTAATGTGCTAAATGCACATAGTCCCCCTCTTTTTGTAACC 10080  
 TAGGATCCCATTCCACCTAACTCCCTTTGCATTATCACCAACTGTACAAGTGACTGGTAATGGGCTTAAAAGCAGCTATTGTTAACCTAGGATCCCATCCCCATTCAACTTCCTTTGCAT 10200  
 GAGTGCAGAGGACACTTGGGACTGTTAATGAGTTGTATTGCTGCTACAGTCCCTCCTGTTATTCTAGGATCCATGCCTTTTTTGATTTGCATTGCAGGGGAGGGTACTAGGGACTGTTAA 10320  
 TGTACTTAAATAGAGCTACAGTCCTTTATGGTAACCAGGATCTGGATCCCACCTAACTGCCTTGTATTACTTCAGGGCTTACATAGGACTTTTTTAATGGGCTTAATTGCTCTGATTTCC 10440  
 CTTCTTTTAGTCTAGGTTCCCCTCCCTCCTACTCTATTTTCATTACCCCAGAGTGTACAAGGGACTGGTAAGTATGCTTAAATGCAATTATAGTCCCTTCTGTTAGTCTAGGGTCCCATT 10560  
 CCCACCTAATTCCCTTTACATTACCAAAGGAGATACAAGGGACTGTCGTTGTGCTTAAGTGTGGGTACAATCCCCATTTTTTCTTTTTTTAGCCTAGGATCCCTTTCCCACCCAATTGCC 10680  
 TTTGGATGACTGCAGAGAATACTGGGGACTGTTAATGGGCTTTATTGTAGCCATAGTCCTTTTTCTTAGTCTGAGATCCCATCTCCTCCTAAGTGATTAACATTACCCCAGAGTGTATAC 10800  
 AGGACTGGTACTTTGCTAAATGCAATTATAGTCCCTTCTATTAGTCTAGGATCCCATCCCCACCTAAATCCCTTTACATTATCACAGGGGATAGATACAAGGGACTGTTGTGCTTAAATG 10920  
 TAGGTACAGCCCCCCCCCCCCTTTTAACCCTAAGATATCTTTCCCACATAAATGCCTTTGCATGACTGCAGAGGATACTTGGGACTGTTAATGGGCTTTATTGTAGCCACAGTCTCTTTC 11040  
 CATAGTCTGGGATCCCATCCCCTCTTAAATGATTTACATTACTCGCAGAGGGCGCAAGGAACTGCAAAGGTGCTTAAATGCAGGTAGTTGGTTTTTAAACCTAGGATCCCATTCCCAGCC 11160  
 TGACTTGCTTTGAATTACTAAAGAGGCTACAAGGGACTCTTATGTGTTTAAGTGCAGGTACAGTGCCTTTTGTTAACCTCAAAATCCATTCCCACCTAACTCCCTTTTCCTGACTGCAAA 11280  
 GGGTAATTGGGACTGTTAACGGGCTGTACTGCAGCCACATTCCTCTCATAGTCTAGGATCCCATTCCCTCCTATTTGATTTGCATTACCCCAGAGGGTACAAGGGACTTTTAAAGTGCTT 11400  
 AAGTGGGAGTTCCTGTCATGGCACCGCGGAAACGAAACCAACTAAGAACCATGAGGTTTGGGGTTCAATCCCTGGCCTTGCTCAGTGGGTTCAGGATCCAGCGTTGCCCTGGGCTGTGGT 11520  
 GTAGGTCGCAGACGCTGCTCGGATCTGGCATTGCTGTGGCTGTGGTGTAGGCTGGCAGCTGTAGCTCCAATTCGACCCCTAGCCTGGGAACTTCATATGCCACAGGTGTGGCCCTAAAAA 11640  
 AAAAAGACAAAAAAAACCAAAAAAAACAAAAAAACCAAAGTGCTTAAGTGGCACTATAGTCCCTTATGTTATCTAGGACCCCATTCCCATCTAACTTTTCATGACTGCAGAGTGTACTTG 11760  
 GGACTGTAAATGTACTTAACTGCCTATACAGTCTCACCTCTTAGTCCAGATTACATGCCCTCCTACTTGAATTGTATTATAACAGAGGGTACAAGGGACTTTTAATGTGCTTTTTTAAAA 11880  
 GCAGCTAAATTTCCTTACCATAACCTGAAAATCCATTTCCATCTCAAAATCCACTCCCACCTAACTCCCTTTCCATCACAGCAGAGGGTACTTGGGACTGTTAATAGGCTTAATTAAAGC 12000  
 TATTCTCCCTTCTGTTGGTCTAGATTTCCTTTCCCGCCTCTATTTGGATTGTACCTCAGTGTACAATGGACTGTTAACTGTGCTTAAATGCAGGTACAGTCCTTTTTTTAAAACCTAGGA 12120  
 TCCTATTCCCACCTAACTCCCTTTGCTTTACTGCAGAGGGTACTTGGGGCTTGTTAATAGGCTTAGTTGGACTACAGTCCTTTCTGTTAGTCTAGGATCCCATCCCCTCCTACGTGATTT 12240  
 GCATTTCCACAAAGGCTATAAGGGACTGTTAAGGTGCTTATTAAATGCAAATACAGTCCCTTATGTTAACCTGGGATCCCATTCCCACCTATCTCCCTTTGCATGACTGCAAAGGTATTT 12360  
 GAGACCTTTAAAGTAGTTAATGGCTGCTGTAGCCTCTTCCATTATAAGTGTACTTTCCCAAGCACTCCAGCTCCATGTAACCTGGGAGCACATTTGGGGCTGTTAATGTGCTCATTTTTA 12480  
 GCTGTGGTCCCTTTTATAATTCTAGGATCACATCCCTTTCTGCTTCTTTGTGTTACTTGTTGGTATTTGGGACAGAAAAGTACTTAAATGCAGCTGTGATCTAACTTAGGCTCCCAGCCC 12600  
 TCTAACTTTTTACATTACCCCAGTGTATATTTGGGACTGTTAAAGTGCTTAATTGCAGCTGTGGTCCCCTCTGTTAGTCTAATTTCCTAAGCTCACCAATTCCCTTTGTATTACAGCAGA 12720  
 GGGTACTTGGGACTGTTAAAGTGTTTATTTGCAGCTCTGGTCCCTTCTCTTAGTCTGGGGTCCATGCCCCCAGCTTTGTTTGCATTACCACAGAGGGTACTTGGAACTGTGAATGTGCTT 12840  
 AAATTCAGGTGTGGTCCCTTTTAATTTAGGATTCCTCAACTCCTTTTACCAGGGAGGGCATTTGGGACTGTCAAGGTGGTAAGGGCACCTGCGTTCCTTTCTTTCTGTTCATCTAGGATC 12960  
 CCATTCCCTCCTACTCCCTTTGTATTGCATGGGAGGATTTGGGGGGCTCTTAAGGTGCTTAACTGCAGCTGTGGTCCCTCCTATTAGTTTCCAATCCCTTTCTACTCCCTTTGCATTACC 13080  
 ACAGAGGGTACTTGGGACTGTTGTAGTACATCCCATTCTCTCATGCCTGTTAGCATTACAGCACAAGGTCCTGAACACCACGGCCCTTTCTTTTGCACTGTTAATGCACAATTTCATTTG 13200  
 TCCCTCATCTTCTGCAGTATTTATAGACCTCACTCATTTCCCTTGTATTTAGTGGTGAATGCCCTTTACTCAAGACCTGTGTACTAGGATAGTTAAAGTGAGCCATGGTAATTGACCCCA 13320  
 AATAATGTCTTTCACTTAAGTCCCTTGTATTCTTTGTTTTACAAGAAGATGTATTAATTACCTCAGTTACTTCCTTCTGGGAGTGTTTACATGCAAAATTCCAGTGTTCATGGCCCCTTT 13440  
 CTTTAAACTAATACCCCTCCTTTTTTATATTCAGGGGATGTTAGTGACCCAAAGTCCCTCTCTTGGGATTCT

AATGTGCAT

AATGTGCAT  
Depth:6 (MOUSE)  
Ei-value:0.000, Pi-value:0.000  
Er-value:0.000, Pr-value:0.000  
MATCHES To TargetScan▶ miR-501-3p/502-3p:AUGCACC

GTGCCCTCGTGATAAGCTAATACGTTGAGTTCAGGGATT 13560  
 ACTGACTACCCAAGTCTTTTTTTGGTGGGGGTGGGGGACAGTTAATGTGCAAAATTATACTAGGATCCCATCCTATCCCACCCCTTTTGCATTTGGGGTGCTGACCAACTGTGGCTGCTT 13680  
 CTCCTGTACAGTTTAAATGCACATAATTTCAGTAATCCATGCTCCCTTATGTTAGACTAGAATCCCATTTTCTTGCCCCATTTACATTACTTTGGGAGCTTCTGAATAGCCAAGATTCAT 13800  
 TTTCTTGGACTGTTAATGTGTATACTGCCATTTGCTCCTGTACATCAGGTAAGGACCCTCTCCATTCTATTTATATTTCAGCAGGAGGTGCCTACTTCTTAAGATTTATATACTTCTGCA 13920  
 GTTAATCTGCATAATCTTAGTTGTCCTGTACACATTCTCAATTGTCCACAACTGCTTTTTAGGTTAGGACTCCTTTACTTATTTAAGCACAGCATACTGACTACCTTAAAGGCCTTGTCT 14040  
 TGGGACTGTTACTATGTGTAATTACAATTGTCCATGGTCCTTTGAGTTAACTTGTGTCCCAATTTTCACACTCTCTTTACTTTATTGCAATGGGGTACTGTTCAC

TTAAGGCC

TTAAGGCC  
Depth:6 (MOUSE)  
Ei-value:0.000, Pi-value:0.000  
Er-value:0.000, Pr-value:0.000  
No matches to TargetScan

CCTTTCT 14160  
 CAAACAGTAATATATGTAATGACAATTACATTAGGATCCTTCCCCTTCACATTCCCTTTGAAGTACCACAAGGATTCTGATCCCTAAGGTCCCATTTCTTGACCTGTTAATGTACGTGAT 14280  
 GTATTTGTCTGGGTTCTTGTGCATTCCTTTTCTCTTCCAGCAGGAAGTGCCCCCTCCACAAGACTGTTAAGTTTGGACAGTCAAGATGCACATTTGTAACTGACTGCAGCCAGTCACCTT 14400  
 GGATGTTAATGTGTATAACTGCACATGGCTCATCCCATATGAATAAGATCCTACCCTCTCAGACCCCTTCTCTAGTATAGCAAGGGTACTGATTCCTAAGACCTCTTTCCATGGCTAGTT 14520  
 ATTGTACATAATTTGCTTTTGTACATGTTCCTGTACACTAAATAAGGATGCCCCTCTTCCCACTCCCTTTGTCTTTTACAGGGAGCGTACACTACTTTAAGATCCTTATATTTATTTGTA 14640  
 CAAAGTACATGATTTTAATTGACCATACCATACCCTTTGGACATTAATGTACATAATTACACCTTAATTCATCATATCCTTTTGCTCTCCATTTGCAGTATATCAGGGTTTGTGACCCTA 14760  
 AATCAGCTCTTTCCTTGGCCTATTAATGTGCATAATTGCATTTGTCCAGGTTCTTGCACACTAGACAAGGACACCCCCCCACCCCCGCCAACTTCCTATGCCTTCCGGTAAGTAGGTGGT 14880  
 TCCCACTGCTAAGACCTTTATATTTGGACAGTTAATGTGCAGAATTGCAGTTTTCCACAACCCAGTTACTTCCAGGACTGCTGTATCTCCTTTGCAATACCTCAAGGGATACTGTTTTTC 15000  
 CCCAAGATCGTTTCTTGTGGACCGTCAATATATGTAATTGAAATGGTTGTCTTCATCCATAGACTGAGATAATATCCCCTCAGCTAACTATCATGACCTCAGCTCTTGGACAATTAATAT 15120  
 TCACCAATAACATATCAAAAGTACTGATCATTAGATAGGACCCCTGTTCCCTTACTGTATACATCAGGGATACTGACTAAGGCCCCCTTTTTTGACTGTTAATGCGAATATTTGCAATTA 15240  
 TCTAATTCCCCTTCTATTAGAGTAGGACATTATTTCCTTGCACCCCATTCGGATTACTGAAAGGGGCTGCTGACCACACAAAACTTCTACTGGGACTGCTGATGA

GCACAATG

GCACAATG  
Depth:6 (MOUSE)  
Ei-value:0.000, Pi-value:0.000  
Er-value:0.000, Pr-value:0.000  
No matches to TargetScan

ACGATGA 15360  
 GAAATGGGTTTTTACTCCCTGGCCTTGTTGGGCAAGCGCTCCCAGCCCAGCCCCAATTTCCCATGGTATAATAAAGTATAAATACTGCAGTGTGCCATGAACTTTCCATCAAACAGCAGC 15480  
 CCATA

CTCCCA

CTCCCA  
Depth:6 (MOUSE)  
Ei-value:0.000, Pi-value:0.000  
Er-value:0.000, Pr-value:0.000  
No matches to TargetScan

CTCTACTTGCATTGGCTCCAGTGTATCAAATTCCAAATAGCTATGGTCTTGCCCTTATGTTCCCTCTCCATACCCTTTGCCTGCCCTTGGACCTTTCTTATGGACTATT 15600  
 AATGCTCACAATTTTCAGGTGTCCATGTATCCAGATAAGATTGTGCTCCCTTGCCCCTCCTGCCCCTTCCACCCCTGCCCTGCCCTTTTGCATTGTTGCTGGGAAATGTCCACTGGCAAA 15720  
 GCCCTTTTGTTTTTAAGACATTAACAATCCCAGATGTCATTGCTTTGCCCATTTTGAATTGCTGTAATGCCTCAGTTACCCTTTCTTTGGTTCCGCCCATCAGACATGGACCCTTCCACT 15840  
 TCCTTCTTTGCATTACTCCTGAGTAGTACTGACTACCCACAGCCCCTTCTGTGTTATTAACACAGTATTGATTGTCCCATTTTTTCAGCCCACCAGCCCAAGGTCTCCCTACCACTTTGA 15960  
 TGTTGTATTTGTGCAGTATTGACTACC

AAAAGCAG

AAAAGCAG  
Depth:6 (MOUSE)  
Ei-value:0.000, Pi-value:0.000  
Er-value:0.000, Pr-value:0.000  
No matches to TargetScan

ACCTGAACTATGTGGGTGGGCCTTCACTCCTTTTCCTGCATTTGTTAATGATCCCAATTCCAATTATTGTAACATTCTGGGGACA 16080  
 GGAACCATTCCTGCCCCTCTTTTACTGCTTTACTAG

GCAAAAT

GCAAAAT  
Depth:6 (MOUSE)  
Ei-value:0.000, Pi-value:0.000  
Er-value:0.000, Pr-value:0.000  
No matches to TargetScan

TTTTAAGGCAAGTCAGACCCAAGGGAACTTGGATTGCTACCCTGTATTTATTTATTATTTATTATATATAAGTATCA 16200  
 ATTGAAAATTATCTCCCCAGGAAGGAAGTTTAGCATTGTCTCTGCATTCTTCCTTTCAGAGCA

GATTGCCTGG

GATTGCCTGG  
Depth:6 (MOUSE)  
Ei-value:0.000, Pi-value:0.000  
Er-value:0.000, Pr-value:0.000  
No matches to TargetScan

CTAAGAATCTCTCTTGGCCTCTTGTATATTCCCAACATGTAATGCCA 16320  
 ATTGCCAGGATACAACCAAAAAGTTGTTATTTTTTAAATTTTTTAAAACGTACATCTGGTTTGCAAGGTGGAATTGATAACCTGGTCATTGAATTTTTGAAGTCTTAAAAACCCATTTAT 16440  
 TCCATGTATCTGATGACCAGTGTCTCTCATTTACTAAGGGTGGTGGGTCTGTGGATAGACGGCTGTGACTTTGATATTTTAGTATTACTACCAAAGGAGTTCTAGAATGGAATTCTTAGG 16560  
 ACAAGTATCTTTGGGCTCTACCACCATTTTGAAACCATTCCTGTTTTGGCTATACCATTATTCACTTTTAGAAAAACAACCTGAACTTCCTAATCCTTAAATTTCTTCATCTGGAGCATC 16680  
 AACCAGCCCCTGCTTATTTCAAGAACATTGCTATAAATGGATAAAATGAGAAAACATATCCTGAAGTGCTTTTTGGAAACTGTTGATCACTTTGTTTGATTAATCTGTTAAATAAAATGC 16800  
 GTTACATTAAATTCTTAGCCTAAAGCACCACACTGAAGTGAGGGCTTAGAAATGATGGGACCAGTTTTCTGTTTTATATTAAAATAAAAATAAGCCAAGATCTAGTCATTCTTTTGGATA 16920  
 TAGGTTTTCAGGAGTGAGATAGCTGCCTGGTTAAGATGAATAATAGCCTAGCTTCCAGTGTACAGGGTGTTTGATGGCAGAGAAGTATTAATGTGGAACTGCTGAAGGAAATAACTAGTT 17040  
 ATCACTGCAGCAGTTCCTTGTAATCACTGAAAAGGATACTCTTCTCTGAGAAGGATGTCA

AAAGATC

AAAGATC  
Depth:6 (MOUSE)  
Ei-value:0.000, Pi-value:0.000  
Er-value:0.000, Pr-value:0.000  
No matches to TargetScan

GGCTCAGCTCAGGGTGCAGTTTGCACTACTAGCTCCTTGGACAGCTGTAAGAA 17160  
 GAGTCTCTGGCTCTTTAGACTACTGGATGAATTCTGAGCCGGTACCCCCACCTCAAGAGGAAGGATGGATCAATTTTAGGTGAATTGAAGCCTATACTAAACAGCCTCCAAAGGATATTC 17280  
 CAAGCAAGTGAGCCCTGAGACTGCAAGCAGCTCTAAGAAGTTCCGCATTGATCAGGTCTTCCCTTTTGAGAATCTGGATAAGCTCCAACCAATCTAAAAGGATGGTTTGCAGACTGTCTG 17400  
 GAGAAAAAGATCTTCCTCAGAAGAATAGGCTTGTTGCCTTACAGTGTTAGTGACTCA

TTCCCTTTGA

TTCCCTTTGA  
Depth:6 (MOUSE)  
Ei-value:0.000, Pi-value:0.000  
Er-value:0.000, Pr-value:0.000  
No matches to TargetScan

TGATCCT

TAGGTGGAGATGGGGCATGAGGATCCTCCAGGGGAA

TAGGTGGAGATGGGGCATGAGGATCCTCCAGGGGAA  
Depth:6 (MOUSE)  
Ei-value:0.000, Pi-value:0.000  
Er-value:0.000, Pr-value:0.000  
MATCHES To TargetScan▶ miR-331-3p:CCCCUGG

AAGCTCACTA 17520  
 CCACTGG

GCAACA

GCAACA  
Depth:6 (MOUSE)  
Ei-value:0.000, Pi-value:0.000  
Er-value:0.000, Pr-value:0.000  
No matches to TargetScan

ACCCTAGGTCAGGAGATTCTACTAAGATTCTTTCCTGGGTCCAGATAGGAAGATGAAGTCTCAAGACAACCACCACACATCTGAGGAAAACAGGGACAACAACTGCC 17640  
 ATATCTTACATTCATCATGACATTCATCATGAGCAGATCATAATTCTGGACCCTTTGATCCCCAAAGCCTCCCTTGAGGCCCTGTTGGAGAACTTCACCAATCATTTATATACTTCAAGA 17760  
 TGCCTTGGGATAACTGGACAAAACAAAAAAACAACCTGTTTGAGAAGATAAAACAGTTGTATCTTTTTATGCTGTGCCTGCTAATTGAAAGAAGCCTCATAACTATGAATGTGAACCTGA 17880  
 CTAGGAATGGAACAAAGTTATCTATCATCAGCCTGGATCTAAAGAATCAGGACCCTGCATACTCTAGCTCCTGATTGTTCCCTTCATATTTG

CCAAAT

CCAAAT  
Depth:6 (MOUSE)  
Ei-value:0.000, Pi-value:0.000  
Er-value:0.000, Pr-value:0.000  
No matches to TargetScan

CGTTATCTTTCTAAGAAGCAGT 18000  
 GTAGAGAGCAAGAAATTTGAACACACCAAAGGAA

GATCAACATGC

GATCAACATGC  
Depth:6 (MOUSE)  
Ei-value:0.000, Pi-value:0.000  
Er-value:0.000, Pr-value:0.000  
No matches to TargetScan

CTGGCGGTCTGGCATTTTAAAACAGGATGAATGATTGTCTGCCTTTGCTTATCTTAAACTCTTAAG

TGTGTAT

TGTGTAT  
Depth:6 (MOUSE)  
Ei-value:0.000, Pi-value:0.000  
Er-value:0.000, Pr-value:0.000  
No matches to TargetScan

TT 18120  
 GTTTGCTTGTCTCTTTCTTTCTTCCTTGCGCATCTTTGGTCTTTTTCTCTAAATTGTGTCTTACCCATTTCCATGATTCTTTTGCTAGTTTCTTCTCAGTATATCTTTGTCTCCTTTTTT 18240  
 GTACCTGAGTGTGCGGTCTGTGTCTCGTCTTAGATGTCTCTCTCTAGTTTCTTTTTTTCATTTTGTTATTGATTCTCCTTGGTCTCCTAGATCTGGCTCTTTCACTGTTGTTCACTTTGT 18360  
 GTCTCTTGAGTCACATACTATGTGCTCTTTGCTCATTTCTTGTTATGCCTACCTTTCTTCTTT

TTCTCTTTG

TTCTCTTTG  
Depth:6 (MOUSE)  
Ei-value:0.000, Pi-value:0.000  
Er-value:0.000, Pr-value:0.000  
No matches to TargetScan

TGAATTCTGATTGTCTGTTATCCATTCCCCTTCTTGTTCGTTTGACAT 18480  
 TTCACCTAGTCTAATACTGGCTACCCTTTTGCTG

TTTCTAC

TTTCTAC  
Depth:6 (MOUSE)  
Ei-value:0.000, Pi-value:0.000  
Er-value:0.000, Pr-value:0.000  
No matches to TargetScan

TCCTGATCTCAC

ATTTCTC

ATTTCTC  
Depth:6 (MOUSE)  
Ei-value:0.000, Pi-value:0.000  
Er-value:0.000, Pr-value:0.000  
No matches to TargetScan

TTTTCACATATTCTTTTTGCCTCTCTTGGGCCATTTTCTCTTTTTCGTGCTTTGTATGCT 18600  
 TCTGTGTCTCTTTGTGCTTTGTGATTTTCCATTTCAACATCCATCTCTCTGTTCTCTTATTTCTTCTCTACTTTTGACTTCCTATTCACCTTTGAGTATTTTGGCCTCTTCTTGTGTCTA 18720  
 TGTCTCCCCTTTGATTACATGCAATTCTCTCGCCTTGCACATTTTCTATGTATGTGTGTGGGCTCTTGTGTGTTTGTAACAAAGGGGCTTCCTAACCCCTTCTCAGTAGGTGCAAGAGTG 18840  
 TCAGCTTACCAAAATAAGCATTGCAGAGCTGTTCCTTATGCCAGACCATGCTGTGAGATGCTGTGAGATTGTGCTGTGAAATGATCCAAGACCAATAGAAGGCCCAAGATCTGGACTGGA 18960  
 GTTGGATGGAAGGCTAAAGTCTCAGTGAAACTGAAGGCCAAAGACCTAAGACTCAAGCCTGTGTATATGGACAGAAGGCCCAAGAGAGACAGATATCTCAAGACTAAATTAAGTGGGAAG 19080  
 CTCAAGGTCCATGGCCCAGAATCTGGGAATGATAGAAGCACCAAGACCAAGGGAAATTACAAGATGATAACCCTAAATCCCAGCTCTTTTCTATTGTTCTCCTCCCTACTCTTGGATATT 19200  
 TTCACTTCTTCCTTTCCTGTTCTTATACCACCATTTAAACCCACTTTTGTGATGTTCTTTTTGATGTTGCTGTTACCTTAAAGTTAAAGAAACATATCTTCAAATCAACAGTATTATGCC 19320  
 TGGGCCAGTCTTAAACCAGTTTTTTTAAACTTCCATTTCTCTTCACCTCCACTCCACTTGAGAGACACATAGGTGACATTATATTTCAGTCCTCTCTTTTCCTCAGATACTCTAGGCTAA 19440  
 TGAGAAGAGGAAAGTATCATGCTGTTGAGGGGGAAAAAATGTGCCAGGCTA

TCTAGAGAAAA

TCTAGAGAAAA  
Depth:6 (MOUSE)  
Ei-value:0.000, Pi-value:0.000  
Er-value:0.000, Pr-value:0.000  
MATCHES To TargetScan▶ miR-1251-5p:CUCUAGC

TATGAAGAGATGCTCCAGGCCAA

TGAGAAGAATTAGACA

TGAGAAGAATTAGACA  
Depth:6 (MOUSE)  
Ei-value:0.000, Pi-value:0.000  
Er-value:0.000, Pr-value:0.000  
No matches to TargetScan

GGAAATACACAGATGCGCC 19560  
 AGCCTGCTGAGAAGCATCAGCCAGCAACACCTTACTCCTTTGAGCTTAGGTGAGCAGGATTCCTGAGGTTTGGGTACTAGTGATGGTTATGAAAAAGGAAATTGGGTCTGGGACAGAGTG 19680  
 AGGGAGGTCCCAAGGAGATAGCCTGAACTCCCTGCTCATAGTAGTGGCCTAATAATTTGGCAAACTGCACTAACCTTGCGCCTGGTTTTAATACCCACCTCTAGGCTTAAAGTTGAAAGA 19800  
 ACTTACGCGTATTTAGCAGGTTTAATACTTTCCTTCATTTCTTTCCTCTTACCATGAGGGAAGATAATTTAAATGAAAATATATGTGAAACCTTTGTAAAACACAAAAAAACAAAGCATT 19920  
 CTCATTAATAACAT

ATTGGCA

ATTGGCA  
Depth:6 (MOUSE)  
Ei-value:0.000, Pi-value:0.000  
Er-value:0.000, Pr-value:0.000  
No matches to TargetScan

TTTGTGCACAGCAAAGATTTGGAAATGTACTGTCAG

TTGTGAAG

TTGTGAAG  
Depth:6 (MOUSE)  
Ei-value:0.000, Pi-value:0.000  
Er-value:0.000, Pr-value:0.000  
No matches to TargetScan

TACTATGTAAATCAGGGGTCATCATATGCTTTCTGTAAAAGATCAGAAGGTAAAT 20040  
 CTTGAGGGCCATATGGTTTCCTGCTTTAAAAAAACAAGTGAATTTGGTCTGTGGGCCATAGTTTATTACCCCTGATGTAATTGCCAGGGACTGTGTTCTGAAATTAATTCCTTCTCTGTG 20160  
 CCTGTCCCTGTATTAGGCACTAAGGATGTAATGATTACTTATGATCATATCTAGGTGATCCAAAGAAAAGTATGTAAATGTGCTTTGTAAACTATAAAGCACTTAAAGACTGGTAAATGA 20280  
 ACTCTGATTACATAGTATATACAAGCCAAGCAATGTATATACAGTGTTAGGCACTGTGGATACAAAAACCTGAGGGATTTAAGATAATTGTGAACACTTATAATGTGCCAGATAACATTC 20400  
 TAGGCATTACAGATGTCAAAGGAAAAAACAAGTTCTCATTACAGAATTAAAATTTTTACATAGGAAGTACATATTAAAGTGCTTTGTAGGAGTTCCCGTCGTGGCTCAGTGGTTAATGAA 20520  
 CACGACTGGCATCCATGAGGACACAGGTTCAATCCCTGGCCTCGCTCAGTGGGTTAAGGATCTGGTGTTGCCGTGAGCTGTGGTGTAGGTCGTAGATGCGGCTCAGATCCCTCGTTGTTG 20640  
 TGCCTGTGGCGTAGGCCAGCAGTTACAGCTCCAATTCAACCCCTAGCCTGGGAACCTCCATATGCTGCTGGTGCAGCCCTAAAAGACAAAAAGAAAAAAAAAAACAACAAAAAAAAATAA 20760  
 AGTGCTTTGTAAACTAAAGCACCACATAAGTACCAATGGGCTATATATGTTTACGAATGAATAAACAAATATTAATTGCCTTATATGTGCCAGCTATTATTTCAGGTACTGTGAAATCTA 20880  
 AGATAATACTCCCAAGTAATAAGAGGGAGGTTTATGTCTTACACTACAGAGGTATAAGAATTAGATATTGCTTATTTTAGTCAATCAAGACTTTACCAGTGAGGTCAGATTAAATTATTA 21000  
 CCAGGATGTTTTCCCAGGTAACAGGAAAATGTAATGAATGAGATTGAGTTTTCTAAACTATAAAATGCTTCATAAACATAAGACATTAGATGTTTTCTATGTGAATAAAACTTATGTGAG 21120  
 GCAGGCATTATCTCTACAAAATTCTCATTGTGTGCGTAACCCACTACGAGAAAAATTGTATAAATATTTATACAAACTGTTTAAATACTTAAGTGAATTAATTATTCATATCAATTGCCT 21240  
 ACTATGTGAACTCACTGTTAGAGGCATGAAAGATATGCACACATTTATCATTTTATTGTGCCTGTAAATGAGAATAAAGTAATACATATGTTAGCATTTTGTGAACTCTAAAGCACCATG 21360  
 TAAATATAACTATTGATCTTCTTATTTGGTGTGCCTGTACTAGTTTACAAAATTGCATCATAGTTATACTATGAAAAAGAGAAAAAAAATTGAGGAAGGATTTTTTTACTCTTGAACACT 21480  
 TTGAATATGATATTTAAAGGATTAAATGCCTGATGAATGAAAGACTAAATAATAATTTTCTATTATGTGCCAGTCTCCTGAATCAGGGCTGTGAAGATAATCCATTGTTTTATTGTGTTC 21600  
 TAGGTAGCAGAATGTAATGTGTAAAATCAATTTGTAAGTCTTAAACTGGCATACATTTCTGCTGAATGAACATTGATTAGTTACTCTTTCTTATCCCCAGAGATAGCAAGTGGGGATGCT 21720  
 AAATTCATTGTCATTGTCACTGAACCTGCAACAGAGATCTGTGTGTAAATTTACAAAGCCTATTGATCTATACAAATATTAGGAATTAACTGTTGGCTCAATGAGTGATTACCAGTTACT 21840  
 TTCTATGAGGCTCAGAACTGCACATGCTCTAGGATATGAAAATGATATCCTTATCTGATGTTCCCTGGAGGAAATGAGGTAATAGGTGTGGGAATCTAGACTGAAAGTTACTTAATCACT 21960  
 GCCAAGCTTATTTGATGGCAAAAACATAGGAAAAATGGAATTGTGCAAGGCAGTATGCTTCTGAATGCTGAGCTAGGTGGAAAGAAAATCCAGACCAGAAGGCTACTTGCTCCTTTGCCT 22080  
 GATTGACGAGATGGGATTTTTTTCTTTAAGCAAGATGTTGTGCTTGTTTTTAAATTCTAAAGATGGAGGTGAATGAGATCCACCCTGACATGGCATTAAAGGAGTAAAAATTAATGGCTC 22200  
 AAGTTGCTTTTAGATTAATTTGATAAACAGTATCTTTTATGTGGAATACATTGTTCTAGGTCCTGAGCATAACAGAAATGAAAGGGGCCTAGTCTAAAGGGCATTAGGTAGATAACAAAT 22320  
 CATGGCTGAAAATTCAGAAATGTCTTTGATTTCTGGAAATATTTGAGTGCCTCTGGAAGAATTTGTTGATTAGATCTGATCCAAAAAAACCTATTGACTTGCCTTGGAAATTGAAAAAAT 22440  
 AGGGAAAAAAAGATATGATTCTGTCAATGTAAAATGACATTTTTCCTAGATCTGGAAAGTCCCAGTGTCACTTGGTCATTGACATAGATTGAAGCCAGAAGATTGATAAAAGAGCTCATG 22560  
 GTTTGACCTTTAATTCTTTAACATTCTGCTTTTATTATGGTTAAATGGTTTATGATGAAAATCAAGCTGAAACAACTAGTTCTAAAAGTGTTTTGTTTTGTTTTTTTTTTTTTTTTACTC 22680  
 ATTGGTCTGCCAGAGAGTCAGAAAGCCACTTAACTAAAAACTAAAAAAAAAAAAATAATAAAAACTTCCTAGATTTTTCATATTTTGTAAAAAAACTAGTCACTTGAGTGAAGTACTGTT 22800  
 CTTAAGTACCAAGAATGAAGATTTTCAAGATCTAGATCAAAATATAATTTTGGTAGCTTATAGAGGAAACATAAGAACAAATTTAATAAGAGACTGAAGTTCTTTCATTAGTTGAAAAAC 22920  
 TTCATTGAGCAGCCATGATATTAAGAAACTGAAATGGAATGGTTAAGTAGACACCTTCTGCTTGGCATGTGTGGTAGCGTAGGAATAAGAGACCTTTGTGTTTACTCATTCATGTAAACA 23040  
 CTGAAGAAGATGATGCTAAATTAAATAGATGTGGCACAAAAGCACTTAGTCTTGGAGATGTAGTTAAAAACAACAGTAAGAAGAAATGGAGTGAAACTCTTCATGAAACAGAAATGTCTA 23160  
 TTTCAAGGCATTGTGTGATACTCCTAGACAGAAAACAAAAGATCTTGACTGAAAGATACTTGCTTGGCTTGGAAACTGAAATATAAGAGGAGAGGTTACTGTTTATTCATATTTTCATTT 23280  
 ATTTGTTCATTCTGCCAGTATATTTGAGTGCCTACTGTGTGTAAGCTTTCTTGGCTAGTTTCTTAGTTGGCCCCAGAGACATGAAAAAATGAATGTCTTCCAAGAGAACTTAGAACTTTA 23400  
 AAAACTTGCCTTTTGGCCAGTTTTCTGGTTAGGGGCAAGGCTTAGTTCCTCACTAACATTGTGTAGCTTAATTCTTGCTCCATATTTCCTCTGCTACTCTGGCCACTACAATAAGCAGGG 23520  
 ATTTCTATTGCGCTTGCTCCTTCCTTAACAATAGTACTTCATTTTTCCTAGTCCATCCTTAATGAAAAATGGCCGTGAACTTTGGTGGGCAGGAGAGATGAGAAACAATTGATTCCCTAA 23640  
 CTCTAGTCTCATTGGTACCAGCTTTTGGGAACATTCATATTCAAGCCACAACTGGTTTTGAAACACATTTACCAGTTTTGATGATTCTCAGTTCACCTTTTATATTATGGCAAATGTATT 23760  
 TTTAAGAGACAATCCCCCAAAAGTACATAACAAGTTTGGAGATGTTTTGGTTTTGCAGTAGCTTCTCACCTTACACTTAGAAATCTTTTCCTTCTCACCATCAAAACCTGCTTAAGAAAG 23880  
 GACTTATTATTTGTAACAGAATAGTGCAGAGAATGAAAATAACAATTTTATTCTTAAGTTTTAGGAATAATAAAAATTGGAAGTAATTGGAAAAAATTGGATAGAAGAAATACCCCCACC 24000  
 AAAAAAAGTCTAAATTTTGACCCAAATGGGCTTTGGCCTTTTCTATTTTTAAATCACTCAGAGAGGGTGGGATAGGAGGAAGAGTGAAAG

AAAAGGT

AAAAGGT  
Depth:6 (MOUSE)  
Ei-value:0.000, Pi-value:0.000  
Er-value:0.000, Pr-value:0.000  
No matches to TargetScan

CAGACCTAGTTCGATGGGCAACC 24120  
 TGCCTTTGTTCTGGATTGGTCTTAAAAGTATTACTACCTCCAGATCTAACTTGGGGGATTGCATCTTTAGTTTAAATGCAACTCCAGCAGAGAGGGTGGGATAGGAGGAAGAGTGAAAGA 24240  
 AAAGGTCAGACCTAGTTCGATGGGCAACCTGCCTTTGTTCTGGATTGGTCTTAAAAGTATTACTACCTCCAGATCTAACTTGGGGGATTGCATCTTTAGTTTAAATGCAACTCCAGTAAC 24360  
 TGGTCACTATTAAGATGAGGACAAATTCCTTTGTTCCCCCTCATTTCTATATGTTTTTGAGAAACATTTTTTGTTAATGGCTGTCAATTAAGCCTTGTGCTAGATGTTATGAAGTTGATT 24480  
 AATTAAATCAATGTAGTCTTAAGCTAAAGCCGAAATTCATTGCAAAAGAAGAGGGCTTTTTTGTTTACTGTAAGTCATACATATTTCATACATATTTCAAAGCATCCAAACTTTAGTTTA 24600  
 CATGGCAAGCCAACAGTAACATCTGTGATGGAGAAGGAAATAGATGAATGGCGAAGGATAGTTGCTGGAGGGAGCAAAGTTGAAGAGGATTTTCTGACATGTTAATAATGTTTAATAAAA 24720  
 AAAATAATGTTTAATATATGATATCAGCTGGATGCAGTTATTCCTCTTTCTTGAGGTGGGGGTGGGGGTGCATTATTGGAAAAGTGGAAAGGAGAAAGTAACTAAAAGCCTTCCTTTCAC 24840  
 AGTTTCTGGCATCCAAGACTACCACTACTGATAAACAAGAATAAGAGAACATGCTATCATCTGATTTTTGTAGCATAAATGAAGTTGTGAACAAATCTTTTTTTTTTTTTAAAGAGAATG 24960  
 GCTTCATCTCTATTCTTTCACCTACCTCAAAGGGAATCTATGCCAAAATACTCCAGTGAATGCAAGACACACTGGACTTGTGAACTGATGTGAAACATAGAATCTCTGAGCCTTGGTTGT 25080  
 TTTGAAGATTGAAAAATTTTGTTCAACATGGATGACCACCAAAAATCAATGTAAACTTGTCTATGTGCCACAACTGAGACAAATTGAAGAGTTTGTTGTTTAATGTCAAATAAAATACTG 25200  
 TTTTTTGAAAACTTA                                                                                                          25215
```

|  |  |  |  |  |
| --- | --- | --- | --- | --- |
| | | | | | | | | | |
| 2 |  | 4 |  | 6 |
| Depth of motif conservation (number of species) | | | | |

  
  

---

  

## >COW (14055 bases)

```
 GTCATGTCCGACTCCTAGCGACCCCATGGACTGCAGCCCACCAGGCTCCTCCATCCATGAGATTCTCTAGGCAAGAGTACTGGAGTGGGGTGCCATTGCCTTCTCCGACAAGGGACTGTT 120  
 AAACATGATTAAATGCAACAGCAGTTTCTTATGTTAATCTAAGATCCTATTCCCATCTAACTCTGTTTTCATTAATGTAGAGCCTACATGGACTTGTTAATGGGCTTATTTGCACTGCCC 240  
 TTCCTTCTGTTAGTCTAGATTCCCAGGCCATCCTTGATTTGCATTACCAAAGAGGGTATAATGGACTGTTAATGTGCTAAAATGCTGCTACAGTCCCTTATATTAACCTAGGATCTTATC 360  
 CTCAGCTAAGGCTCTTTGCAGGCTTGCAGAGGATGCTTGGGGCTGTTCATGGCCTTAAATACCACTACATCCCTTATGGTAACCTAGGATCCCATTACCAACTATTAATATCTTCCTTTT 480  
 AAGTACTGCAGAGCCTACATAGAACTTGTTAATGAACTACCATTGTTCCTTTCCTTTGTTTAGATTCCCATCTCCTAAAGATTTACATTACCACGGAGGATACTATGGACTGTTAATGTG 600  
 CCCGTACAGTTCTTTTCCCTCCCCCTCCTTTTTAAAACCCAGGATCCTATTCCCACCTAACTCCCTTTGCATTACCAGTGACTGTACAAGTGCCTGCTAATGCTTTTAAATGCAATTACA 720  
 GTCCCTTATGTTAACCTAGGATCCCACCCCCAACTAAGTCCCTTTGCATGATTGCAGAGGATACTTGGAACTGTTAATGTGATTCACTGCAGCTACATTCCTTTATCTCATCTAGGATCC 840  
 ATTCTCTTCCTTCTTGATTTGCATTATCAGAGAGGATAGAAGGGATGGTTAATGTACTTAAATGGAACTATAGTCCCTTATGATAATCAGGATCCTATTTCTACCTCACTTCCTTTGTAT 960  
 TACTTCAGAGTATACATGGGACTTTTTATGGGCTTAATTGCACTACTGTCCCTTCTTTTAGTCTAGATCCCCATCCTTCCTACTCCATTTGCATTACCCCAAAGTGTACAAGGGACTGTT 1080  
 ATTGTACATAAAGGCAGTTACAGTCCCGTGCTTTAGTCTAAGATTCCCTCTCCCCCTTTAATTCCCTCTACATTACCGAAGGGGATACAAGGGACTGCTAATGTGCTTAAGTGCTGCTTG 1200  
 CAGTCCTTTATGTTACCTAGGATCCCATCCCCACCTAACTCACTTGTCAGAGAGGCTACAAGGGACTGTTAATGTGTTTAAATGCAGGTGTAGTGCCTTAACATCGAAATCCATTCCTGC 1320  
 ATAACTCCCTTTTTCAGGACTGCAGATGGTAATTGAGACTGATAGTGAGCTTTATTGCATTTACAGTCTCTCTTAGTCTAGGATCTCATTCTCTCCTACTAGATTTACATTACCATGACT 1440  
 GTACAAGGGACTTTTAATATGCTTAAATGCAGCTGTAGTCCCTTATGTTAACCTAGGATCCTATTCCCACCTAACTTCCTTTTCATGACTGCAGAGGGTACTTAAGGACTGTTAATGCCT 1560  
 TTACTTGCCTATACAGTCTCATCTGTTGATCTAGATTCCCATGCCCTCCTACTCAATTTGCATTATAAAAGAGGGTACAAGGGACTGTTAACTTGCTTAAATGCGGCTACAGGCCCTTAG 1680  
 ATTAACCTGGGATCCCATTCCCACCTGACTCCATTTACATCATCCTAGAGGCGATAAGGGACTTTCATTGTGTTTGAAAGTGAAGTCGCTCAGTTGTGTCCGACTCTTTGCGACCCATGG 1800  
 ACTGTAGTTGGCCAGGCTCCTCCGTCCATGGGGTTTTCCAGGCAAGAGTACTGGACTTGGTTGCCATTTCCTTCTCCAGGGGATCTTCCTGACTCAGGGATCAAACCCGGTCTCCTGCAT 1920  
 TACAGGCAGACTCTTGACCATCTGAGCCACCAGGGAATCTCAATGTGTTTAAATGCAGGTGAAGTCCCTTATGTTAAATTCAAAGTCCTTTCCCACCTAACTGCCTTTTTATCACTGCAG 2040  
 GGGGGTACTTGGGACTGTTAATGGGACTGTACTTGCTTATCCAATTTCGTTCATTGGTCTAGATTCCCATGCTCTTCTACTTAATTTGCATTATAAAAGAGGGTACAAGGGACTGTTAAT 2160  
 GTGCTTAAATGCAGCTACAGTCCTAGGATCTCACCCCTCTCTACTCACTTGTGTTACTTGTTGGTATTTGGGACAGAAAGGTGCTTAAATGCGACTGTGGTCCATTTTGTTAACCTAGGA 2280  
 TCCCATGCGCTCTAACTCTTAACATTACCTGGGAGTATACTGGGACTGTAGAACTGCTTAATTGCAACTGTGGTCCCTTCTCTTAGTCTGGAGTCCATGCTCTCCACAATTTTGTTTATA 2400  
 TTACCACAGAAGGTACTTGGAACCTGTGAATGTGTCTAAATGCAGATGTGGTCCCTTTTGTTAGTTTAGGATCCCAGGACTTCCTTTACATTATCTGTGAAGGCATTTGGGACTGTCAAG 2520  
 GTGATAATTGCTGCTGTGTTCCCTTCTGATATCTAGGATCCTGTTCCCTCCTACTTCCACTTCCTTTGCGTTACATGGAAGGGTGCTTAACTGTAGCCATGGTCCCTTCTGTTAGCCTAG 2640  
 TTTGCATTCCCTCTCTACTTCCTTTGCTTACCACAGAAGGTACTGACTATTGTGATGCATCTTATCCTCTCACATTACACCTTAGGGTCCTGAACACTAAGGCCCCTCTTTTTGTGCTTT 2760  
 TTAATGTGCAGTTGCATTTGTTCCTCTTCCTCTGCAGTGTATAAAGACTCCACTTATTTCCCTTGTATTCAGTGATGGATGTCCTTTACTCAAGACCTTTGTACTAGAGTAAACTGAACC 2880  
 GTGACAATTCACCATGAATATATTCTTTCACTTCAGTCCCTTGTATTCTGTTTTATAAAAATATATATTAATTACCTCCATTACTTTTTCTCTGGGAGTATTTACATGCAAAATTCCAGT 3000  
 GTTTAAGGCCCTTCTCTTTAAATTAATATCCCTCCCTTTTTGCATTATATCAGGGGATGTTAGTGACCCAAACTCTTTCTCTTGGGACTC

AATGTGCAT

AATGTGCAT  
Depth:6 (MOUSE)  
Ei-value:0.000, Pi-value:0.000  
Er-value:0.000, Pr-value:0.000  
MATCHES To TargetScan▶ miR-501-3p/502-3p:AUGCACC

GTGCCCTCATGACAAACTAAT 3120  
 ATATTGAATTTTTAGTATTAAAACAGGGACTACTGACTAGTTTTTATTTTGGGTAGTTAATGTGCAGAATTACACTAGGAACCCATCCCATTCCACCTTCTTTGCATCAGGGGTCCTAAT 3240  
 CAACTGGCTGCTGCTTCTGTACAGTTTAAATGTGCATAATTTCAATAGTCCATGCTCCCTTATGTTAGACTAGGATCCCCTATCCTTGCCCCATTTACATTACGGCAGGGGCTTCTGACT 3360  
 AGCTAAGATTCACTGTCTTGGACTGTTAATGTATGTACTCACATTTGCTCCCGTACCTGTACCTCAGGTAAGGACCCTCCCCATTCTATTTACATTTTAGCAGGAGATGCCTACTATTCA 3480  
 AGATCTTACACACTATTGCCGTTAATGTGCACCATCTTAGTTGTCTTGTACACATTCTCAGTTGTCCACAGCTGTGCTTTTTAAATCAGGGCCCTTTCACTTATTGAAGCACAGGTTAAC 3600  
 TTACCACCTTAAAGCCCTTGTTATGGGACTGTGACTATGTGTAATTACAATTGTCTATGGTCTTCTGAGTTAACTTGTCTCCCTGACTCTTTTCATCCTCTTTGCTTTACTGCATGGGGT 3720  
 ACTGCCCAC

TTAAGGCC

TTAAGGCC  
Depth:6 (MOUSE)  
Ei-value:0.000, Pi-value:0.000  
Er-value:0.000, Pr-value:0.000  
No matches to TargetScan

CCTTTCTCAAGTATGTGTAATGACAATTACATTATAGGGGCTTCATACTCCCTTTAAGATTCTTATATTTATACAAGGTATATGATTTTAATTGACCACACCA 3840  
 TGTCCCTTCAACATTAATTTACATAATTGCAACTTAGTTCGTCTTATCCTGTTGCCCTCCCTTTCCTTAGCTTGTGGATGTGCATTCTTGTGCACTAGACAAGGACCCTCCCCCTCACTT 3960  
 CCTATGCCTTCCGTAAGTTTGCCCATTGCTAAGACCTTTCCATTTGGACAGTTAATGTGCAGAGTTGCACTTTCCCATAACCCTATTACTTCTAGGACTATTGTATCTCCTTTGTAATAG 4080  
 TGCAGGGGATACTGTTTCCCCGCCAAGATCCCTTACTGTTAATATACGTAATAGAAATTCTTGTCTTTATCAGTGTACTTTGGTAATACTCCCTCAGCCAGCTATCATGACCTCAGCTCT 4200  
 TGGACAATTAATATGCACCAATAATATATCAAACGTATGATCTTTAGGTAGGATCCCTGTACCCTCACTATGGATCATGACTGCTGACTTAAGGCTTCTTTTTTTGACTCTGGGTGTGAA 4320  
 TATTTGCAGTTACCTATACACCCTTCTATTAGAGTAGGACATCATTCCCTCATACCCCCTTTGCATTACTGCATGGGCTGCTGATGACACAAAACTTTCACTGGGACTGCTGATGA

GCAC

GCACAATG  
Depth:6 (MOUSE)  
Ei-value:0.000, Pi-value:0.000  
Er-value:0.000, Pr-value:0.000  
No matches to TargetScan

 4440  


AATG

GCACAATG  
Depth:6 (MOUSE)  
Ei-value:0.000, Pi-value:0.000  
Er-value:0.000, Pr-value:0.000  
No matches to TargetScan

GCAGTTGGCAATGGGTTTTTCCTCCCTGGTCTTGTTAGGCAAGCA

CTCCCA

CTCCCA  
Depth:6 (MOUSE)  
Ei-value:0.000, Pi-value:0.000  
Er-value:0.000, Pr-value:0.000  
No matches to TargetScan

GCTCCAAATTCTCATGGTATATTAAACTATAAACACTGCACTGTGCTGTGAACTTGTCCATCAAA 4560  
 GGGCGACCCACATTCCCACTCCTTTTGCGTTGGCTCCAGTGTATCAAATTCCAAGTAATCATGGCCTTGCCCTTATCTTCCCTCTACAAACCCCTTGCCTGCCCTTGGACCCTTCTTATG 4680  
 GATTTTTAATGTTCACAATTCCAGGTGTCCATCTCTCCAGATAGGATTATGCTCCCTCACCCCTCCCACCCCTGCCCTGCCCCTTTTGCATTGTTGCCGGGAAATGTTGACTGAGCAAAA 4800  
 CTCTTTTCTCTTGAATTTACAATGTTAACAGTCCCAGATGTCATTGTTCTGCCCACTTTGAATTTCTATAATTCCCTGTTGCACTTTCCTTGGTCCCACCCATCACGGACTCTTCCACTT 4920  
 CCTTCTTTGCATTACTTCTGAATAGTGCTGACCACCCACAGCCCCTTCTTTGTTGTTAACACAGTACTGATTGTCCCATTTTTAAGCCCATCAGCCCAAGATCTCCCTACCATTTTGATG 5040  
 TTATATTTGTGCAGTATGGACTACC

AAAAGCAG

AAAAGCAG  
Depth:6 (MOUSE)  
Ei-value:0.000, Pi-value:0.000  
Er-value:0.000, Pr-value:0.000  
No matches to TargetScan

GCCAGAACTAGGTGACTGGACCTTACTCCTTTTCCTGCATTTGTTAATGATCCCAGTTCTGATTATTGTCATATTCTGGGAACAGAA 5160  
 CCGTTCCTGATCCCCTCTGTTAGTGCTTTACTAG

GCAAAAT

GCAAAAT  
Depth:6 (MOUSE)  
Ei-value:0.000, Pi-value:0.000  
Er-value:0.000, Pr-value:0.000  
No matches to TargetScan

GCTCAAGGCAAGTCAGACCCAAAGGAACTGGATTGCTACCCTTTATTTGGGGTTTTCATTATAAATAATCATTTGAAAA 5280  
 TTGACTTCCCAAGAAGGAAGGTTAGCACTATCTGTGCATTCTTCCTTTCAAAGCA

GATTGCCTGG

GATTGCCTGG  
Depth:6 (MOUSE)  
Ei-value:0.000, Pi-value:0.000  
Er-value:0.000, Pr-value:0.000  
No matches to TargetScan

CTATGTCTCTCCTTTCCTCTTGTATATTGCCATTGTATAGTGCCAATTGCCAGGA 5400  
 TACAACCAAAAAGTTTATTTATTTTTTATTTTTTTTATTTTTTAAGAAAGACATCTGGATTGCAGGGTGGAATTGATAACCTGGTCATTGAAATTTTGAAATTGGTAAACCCATTTATAT 5520  
 CATGTACCTGATGACCAGTGTCTCTCATTTTACTAAGGGTGGTGAGTCTGTGGATAGACCACTGTGACTTTGATATTTTAGTATATTACCAAAGGGGTTCTAGAGTGGAACTCTTAAGAC 5640  
 CAGTATCTTTGGGCTCTACCACCATTTTCAAACCACTCCTTGTTTGAGCTTTACCAGTATTCACTTCTAGGAAAAACTATCTAAACTTCCTAATCGTTAAATTTCTTCATCTGGAGCTCC 5760  
 AACTAGTCCGTACTTATTTCAAGAAGATTGCTGTAAAAGGATAAAATGAGAGAACATGCTGAGGTGCTTTTGAAAACCATAGATCACTTTGTTTGATTAATCTATTACATTTTACGTACC 5880  
 CTGTTTAATTCTGCTATTTTAAAGTTACCTAAAGCACCACACTCAAGTGAGGACTTAGAAACGATGGAACCAGTTTCCCCATTTTATATGAAAAGAAAAATAAGCCAAGGTCTAATCAGT 6000  
 CTTTTGGATATAAATTTCAACAGTGAAAATGAATACAAATGAATAATATCTCAGTCTCTAGTGTACAAGGTGTTTGGCAGAGAAGTATTTAATATGGAACTGCTGAAGCAAGTAACTAAT 6120  
 TATCACCACAGCAGTTCTTTGTAATCACTGAAAAAGGATACTGTTGTCTGAGAAGGATGTCA

AAAGATC

AAAGATC  
Depth:6 (MOUSE)  
Ei-value:0.000, Pi-value:0.000  
Er-value:0.000, Pr-value:0.000  
No matches to TargetScan

GGCCCAGCTCAGGGTGCAGTTTGCACTACTAGCTCCTTGGACAGCTGTAAG 6240  
 AAGAGTCTCTGGCTCTTTAGAATACTGGATGAATTCTGAGCTGGTTCCCCCCCACTCAAGAGGAAGGATGGATCAAGTTTAGGTGGAGTGAAGCCTGCACTGGACAGCATCCAAAGGACA 6360  
 TTCCAAGCATATCAGACCTGAGGACTGCAAGCAACTTTAAGAAGCTTCATATTCAGCAGGTCTTTCCTTTTGAGAATCTGGATAAGCTCCAACCAATCTCATAGGATGGCTTGCAGTTTC 6480  
 CCTGGAGAAAAAGATCTACCTCAAAAGAATAGGCCTGTTGCTTTACAGTGTTAGTGACCCA

TTCCCTTTGA

TTCCCTTTGA  
Depth:6 (MOUSE)  
Ei-value:0.000, Pi-value:0.000  
Er-value:0.000, Pr-value:0.000  
No matches to TargetScan

CGATTCC

TAGGTGGAGATGGGGCATGAGGATCCTCCAGGGGAA

TAGGTGGAGATGGGGCATGAGGATCCTCCAGGGGAA  
Depth:6 (MOUSE)  
Ei-value:0.000, Pi-value:0.000  
Er-value:0.000, Pr-value:0.000  
MATCHES To TargetScan▶ miR-331-3p:CCCCUGG

AGATTC 6600  
 ACTACCACTGA

GCAACA

GCAACA  
Depth:6 (MOUSE)  
Ei-value:0.000, Pi-value:0.000  
Er-value:0.000, Pr-value:0.000  
No matches to TargetScan

ACTCTAGGCCAGGAGGTTATACCAAGATTCTTTCCTGGGCCCAGTTAAGAAGGTGAAGCCTCAAGACAACCACCACACATCCAGAGCTCCTGGTTGTTCCCTT 6720  
 CATATTTG

CCAAAT

CCAAAT  
Depth:6 (MOUSE)  
Ei-value:0.000, Pi-value:0.000  
Er-value:0.000, Pr-value:0.000  
No matches to TargetScan

CATTATCTTTCCCTGAAGTAGTGCAAAGAGCAAGAAATGTGAACACACCAAGGAA

GATCAACATGC

GATCAACATGC  
Depth:6 (MOUSE)  
Ei-value:0.000, Pi-value:0.000  
Er-value:0.000, Pr-value:0.000  
No matches to TargetScan

CTGCAATGCTAGCATTTTAGAATAGCAGAATGAATTTGTC 6840  
 TCTTCTGTTTCTTACCCTCTTCCATGTCTGCCTTTGCTTATCTTTTAAACTCATAAG

TGTGTAT

TGTGTAT  
Depth:6 (MOUSE)  
Ei-value:0.000, Pi-value:0.000  
Er-value:0.000, Pr-value:0.000  
No matches to TargetScan

TTGTTTGTTTGTCTGTTTCTTCTTTGAATGTCTTTGGTCTTTCTTGTCTAAAGTAT 6960  
 GTCTTACCCATTTCCATGATTCTCTTGCTAGTTTCTTCTCTGTATATCTTTGTCTCATTTACTTTTTTGTACCCAGGAGTGGTTTGTGTCTTGTCTTAAATGTCTCTCTCTAGTTTTCTT 7080  
 CATTTTGTTACTGATTCTCCTTGCTCTGCTAGATCTAGCTCTTCTTTCACCGTTCTCTGTGAGCCTCTTGAGTTATGTGTGCCTTTTGCTCATTTCTTGCTATGCCTGCCTCTCTTCTTT 7200  


TTCTCTTTG

TTCTCTTTG  
Depth:6 (MOUSE)  
Ei-value:0.000, Pi-value:0.000  
Er-value:0.000, Pr-value:0.000  
No matches to TargetScan

TGAACTCTGTCACCCGTTCCCCTTGTTGGCTTGACATTTCACCTTTTCTGATACTGGCTACCCTTCTGCTG

TTTCTAC

TTTCTAC  
Depth:6 (MOUSE)  
Ei-value:0.000, Pi-value:0.000  
Er-value:0.000, Pr-value:0.000  
No matches to TargetScan

TCTTTATCTTGCAT

ATTTCTC

ATTTCTC  
Depth:6 (MOUSE)  
Ei-value:0.000, Pi-value:0.000  
Er-value:0.000, Pr-value:0.000  
No matches to TargetScan

TTTTCTACATAT 7320  
 TCTTTGTGCCTTTCTTGGGCTATTTTCTCTTTTTTTCCTCATGCTTTGTGTGCCCCAGTGTCTCTTTGTTCTTTGTGATTTTCAATTTCAGCATTCATCTCTGTTCTCTTGGTTCTTCTC 7440  
 TCCTTTTGCCTTTCTATTCACTTTTGAGTATTTCTTGAGTCTATGTCTCCCCCTCTTTGATTTCATGCAATTCTCTCTCCTGGCATATTTTTTCATGCGTGTTTGTGTGTGTGCTCTTTT 7560  
 GTGTGTTTGTAAAGGCGCCTCCTAACCCCTTCCAGTAGGTGCAGAGTGTCAGCTATCAAAATAAGCATTGCAGAGCTGTTCGTTATGCCAGGCCGCCCTGTGAGATGATCAAAACCAACA 7680  
 GAAGGTCCAAGGGTCTAGACTGGAGTTGGATAGAAGACTCAAGTCTCCGTGAGACAGAAGGCCAAAGACCTAGGATGGGATTAAAAAACCTTGTCTTGAAGACCTGTGACCCAAAAGATG 7800  
 GAAGTGCCCTAGCACACACAAAGACCCAGGACTCAAACCTATCTATATAAGACAGAAGGCCCAAGAGAGACAGATATTTCAAGACTAAATTAGATGGGAAACTGGAGGTTCGTGACAGAG 7920  
 ATACCAGGAAAGAAAGAAGCCCCAGGACCAAAGGAAATTCCAAGATGAGACTCTTAAACCCCAGCTGTTTCTATTGCTCTTTTCCCTACTCTTGGACATTTTCAGTTCTCCCTTCCTTCT 8040  
 CATGTCTCCATTTATATCTACTTCTTTTGAGATGTCCTTTTTGATGTTACCTTAAAAAAAAAAAAAAACGTATCTTTAGATCAGTAATATTATGCTTTGGCCTGTTTTTATTACAGTTTT 8160  
 GAACCATTTATTAAGTTTTTGAAGTTTTAAACTTCCATTTCTCTTCACCTCCTCTCCACTTGAGAGGGACACATAGCTGACATTATATTTCAGTCCCCTCTTTCCTCAGAAGCTCTAGGC 8280  
 TGATGAGAGAAGGAAAGTATCAGGTTCAGTTATTGAGGGGAAAGAAAGTGCCAAGCTA

TCTAGAGAAAA

TCTAGAGAAAA  
Depth:6 (MOUSE)  
Ei-value:0.000, Pi-value:0.000  
Er-value:0.000, Pr-value:0.000  
MATCHES To TargetScan▶ miR-1251-5p:CUCUAGC

TGTGAAGAGATGCTCCACAGGCCAA

TGAGAAGAATTAGACA

TGAGAAGAATTAGACA  
Depth:6 (MOUSE)  
Ei-value:0.000, Pi-value:0.000  
Er-value:0.000, Pr-value:0.000  
No matches to TargetScan

AGAAATACAC 8400  
 AGATGTGCCAGTTTGCTGAGAAGTGCCAGCCAGCAACATCTTACTTATTTGAGCTTGGGTGAGCAGGATACCTGAGGTTTGGGATTCTTACTGTTGGTTATGAAGGAGGATTGGGCCTAA 8520  
 CACATAGTGAGGCCCCAAGGAAGGAGCATGAACTCCCTGCTCATAGTAGTGGCCTAATAATGTGGTAAGCTGCATAAATTTTATCCCTCGGTTTAATACCTAGGCTTAAAGGTGAGAAAA 8640  
 TCTGGGAATATTTAGCAGGTTTAATCCTTTCATTAATTTTTTTCCTCTTACCATAAGGAAAGATAATTTTAGTGATAATATATATGAAAGCACTGTAAAACACAGAAAAAAAAGCAAGAC 8760  
 TTTCTCATTAATAATGT

ATTGGCA

ATTGGCA  
Depth:6 (MOUSE)  
Ei-value:0.000, Pi-value:0.000  
Er-value:0.000, Pr-value:0.000  
No matches to TargetScan

CTCATGCACAGCTAACATTTGAAAATGCGTTGTCAG

TTGTGAAG

TTGTGAAG  
Depth:6 (MOUSE)  
Ei-value:0.000, Pi-value:0.000  
Er-value:0.000, Pr-value:0.000  
No matches to TargetScan

CATTATGTAAATCAAGGGTTATCACACCCTTTTTGTAAAGGATCAGATGGCA 8880  
 ATTATTTTATACTTTGCAAGCCATATGGTCTCTCTCTGCTTTTTTTTATTTTTAAAGTGAATATGGTCTGTGGGCCACAGTTTATCACCCCTGGTGTAATTGCCAGGGACTGTGTGCTGA 9000  
 ATAATATTAATTTCTTTCTTTGTGCCTGTCCCTGTATGCATTAAGGATGCAATGATTACTTATGGTCATATCCAGGTGATATGAAGAAAAATATATGAATGTGACTTATAAACTATTATG 9120  
 CACTTTAAAAGCTTGTCACAATTTTCAGAGTTGATGAATGAACATTGATTACATGGTATATACATTTCTAGGCACTGTATATACAAAGAACAAAAGCGTGAATAAAGGGCTTTCTCAAAA 9240  
 GTATGTAATACTGTACAGATATTAGGAATTAAGTCTATGATAAATGAGTAATTCATGTATTTGTGGCCATTGTGTTTCTGTGGCTCTGGTGATTCTGAGTACAGATGTAGTATTCCAAGT 9360  
 CACAGAAAAGAAAACATTGTTCAGTCGCTAAGTCGTGTCTGACGCTTTGTGACCCCATGAACTGCAGCATGCCAGGCTTCCAAACCCTTAAACTATAAAGCATACTGCAAACACAAGGGA 9480  
 TTTAAGCTATTGTGATTACTTATAATGTGCCAGATAACATCCTGGGCACTACAGATGTCAAAGGAAAAGAATATTCTTATTATAGAATAATTAAAAATTTTAGATAGGCCGTACATTTTA 9600  
 AAGTGCTTTGTACTAAAGCACTGTATAAATACCAATGGGCTATATGTTTATGAATGAATGAATAAGTATTAATTTCCTTATATATGCCAGCTATTGTTTCAGGTACTGTGAAATCTAAGA 9720  
 CAATGCTCCCAAGTGATAAGAGGGATGTTTATGTCCTATACTACTTATATATAAAGAATTAGATATTATTTATTTTAATCAATAAAGACTTTACTGGTGATGTTGAACTAAATTATTACC 9840  
 AGTATATTTCCCAAGGTAATAGGAAAATGTAATGAATGAAATTGAGCTTTCTAACTATAAAATGCTACATAAATATAAAACATTAGGTGTTTTCTCTGTGAACAAAACTTAGGTTGAGAC 9960  
 AGACATTTGTTTGTGTGTGTTAGTCGTTCAATCATGTCTGACTCTTGCGACCCCATGGACTGTAACCCACCAGACTCCTCTCTCTGTGGATTTCTCCACACAAGAAAACTGAAGTGGGTT 10080  
 GTCATTCCCTTCTCCAGGGGATATTCTTGACCCAGAAACTGAACTCAGGTCTCCTGCACTGCAGGCAGATTCTTTACCATCTGAGCCACAGGTTCTCATTGTGTGTTTGACCCACTATGA 10200  
 GAAAGGCAGTATAAAATTCTCAGTATACAAACTGAGAGTTTAAATACTTGGTAAATGAATGGGTCATTAATATTAGTTGCCTACTATGTGAACTCACTGTTATAGGCACTAAAGAGACAC 10320  
 ACAAATTTATCATTTTATTGTGCCTACAGTTGAGAATAGGCAATAACATACGTTAGTATTTTGTGAACTCTAAGAGTACCATATAAGTGTAACTATTGATTTCCTCAGTTGGTATGCCTA 10440  
 ATACTAGGCTTGCAAAATTGTATCATAGCTATTATACCATGAAAAAAGTCAGAAAAAATTTGAGCAAGGATTGTTAATTACTGAACACTTCAAATATGATACATAAAGGACTAAATGTCT 10560  
 GATGAATGCAAGCCTGGATGATAATTATGTGCCAGTCTCCTTGATAAGCATAAGAGATAAGAAGATAATCCATCTTTACTGTGTTCTGGGCAGCAGAAGGTAATATATAAAATCAATTTG 10680  
 TAGGTCTTAAACTGGCATACATATATAAGCCATATCTGCTGAATGAACATTGATTACTTACTCTCTCTTATTCCAAGAGATAGCAACTAGGGATGCAAAAATTCATTGCCATTATTGTTG 10800  
 AACCTGTAACAGAGATCTGTATAAATTTTACAAAGCTTACTGTTCTATACAGATCTAAAGAATTAACTGTTGGCTTAATGAATGGTTACTGATTACTTTCTATCTATAAAGCTCAAAACT 10920  
 GTGTATGCTCTAGGATATAAAAATGACTCTCCTTATCAGATGCTGTAAATAACATGAGTGGGAATCCAGAATAAAAATTACTTAACCATTGCTCAGCTTATTTGATGATAAAAACATGGG 11040  
 CAGAATGGAATTGTGCAAGGCAGTATGCTGAATGCTGAGCTAGGTGGAAAGATAAAATCCAGACCAGAAGGCCACTTGCTCCCTGGCTTGACTAAAGGAATGGAATTTTTTTGTTTCTTT 11160  
 GTTTAAAGAGCAAGATGTTGTCATTCTCCATTGGTCTTTTAAATGCTGAATGCTGAAGATGGAGGTGAATTCAATCCACTCTGGCCTGGCATAGAGAAATTGAAAGAGTAAAAAATGGTT 11280  
 CAAGTTGGCTTTTAGATTAATTTGATGAACAGCATCTTTTATGTAAAATATATCATTCTAGGTCCTGAGCATAAGAGATATGAAATGGGCCTAGTCTAAAGGGCTTTAGATAGATCAAAG 11400  
 ATTATAACTGAAGATCCAAAAACATCTTTGAGTTTTAAGTAAATATTTGAGTTCCCGTTCCCTAGATCCTATTCCTGTTCCTTGAATTCTTGTTCCTATTTTCTTGGTGAAGTCTGGTCC 11520  
 AGAAAACCTATTGACTTGCCTTGGAAATTTAAAAAATAGAAAAACAATATGGTTCTTTCAATGTAAAGTGAAGATATATTTCTTGGCTCTAGAAAGTATCAATGTCATGTGCTCATTGAC 11640  
 CTAGATTGAAGCCAGAAGATTAATAAAAGGCCTCAGAGTTTGTCCTTTAATTCATTAATATTCTGTTTTTATTATAGTTAAATGGGTAAGATGACAATCAAGATTATTATGGCATGGAAT 11760  
 CAGGAAGACAACAAAAAAGATTCTTCCTAGATTTTCCATATTTTGTAAATTATTCACTTGAATGAAGTACTGTTCTTAAGTGCTGAGGAGAATGAAGAGATTTACAAGACCTAGACCAAA 11880  
 ATATACTTTAATTTCACTAGTACATGGAGGACACACAAGAACAAATTTAATAGGAGATTGCAATTCTTTCATTAGTTGGAAAAACTAAAGTAGCTGTGATATTAAGATACTGAGATGGAA 12000  
 TGATTAAACAGATCACTTTCTCTTTGGCATGTTTTGTAGAGAAGGAATAAAAAAGCTTTTCAGTTATTCATACAAGCATTGAAGAAGGTGATGCTAAATGAAATAGATCTGGTAAAAAAG 12120  
 AAATTGTCTTGGAGATGCAGTTAAAAAAAAAAAAAAAACAAGAAATGGAGTGAAACTTCATTGAATGAGTGTCTATTTCAAGGCATTGTGCTAATTCCTAGACAGAAAACAAAAAAGATC 12240  
 TTGACTGAAAGACACTTACTTGACTTGGAAACTGGTATATAAGAGAAGAGGTTATTGTTAATTTTTTAAATTTATTCATTCATTCTACAAGTATGTTTGAGTGCCTATTGTATATAAGCT 12360  
 CCTTAGCTGGGTTCTAGCTAGGTTCTCTAGCTAACCCCCAGAAAGAGACATGAGAAAATGAATGCCTTCCCAGAGAACTTAAAATTTGCCTTTTGGCCTATTTTCTGATCAGGGCTAAAC 12480  
 GGCTTACTTCCTCAATAATATTGTGTGGTTTAATTCCTACTCCATCTTAAACCACTCTGGCCACTACAATAAGCAGGGGTTTTTACTGAACTGCTCCTTAAATTGCAGGATCCTTCCTTA 12600  
 TAATAAAACACTTCAGTTTTCCTAACCCATCCCTCATGAAAAATGGTTGTGATCATAGTGAGCCGAAAGAGGGATGAGAAGCAACTGATTCCCAAACTTCAGTCTCATTGGTACCAACCT 12720  
 TGAAGAACCACTCATACTCAAATCACAATTGGTTTTGAAACACACTTACAGTTTTGTCTATGACAAATATACACTGCCTAAGTCTTTTTTAAAAACAATCCTCTGATATTACTTAGCAAG 12840  
 TTTGGAGATGGAGACATGATTTGTGTTTTGCAACAACAGCTTCTCAACTTACAATTTAGAAATCTTTTCCTTTTCACCATCAAAACCTGCTTAAGATAAGACTTGAAAATAGCCTAACAA 12960  
 TTTTACTATGCTTAAGCCTTAAGAATAATAGAACTTGAAAGTATTTGGCAAATGTTGGAGGGAAAAAAAATCTACATATAGGCCCAAATGGGCTTTGGCCTTTTCTCTTTTTAAATCACT 13080  
 CACAGAGGGTGGGATAGGAGGAAGAGTGAAAG

AAAAGGT

AAAAGGT  
Depth:6 (MOUSE)  
Ei-value:0.000, Pi-value:0.000  
Er-value:0.000, Pr-value:0.000  
No matches to TargetScan

CAAGCCTGATTCTAAAAAGAGTTGGTCTTAAAAGTATTGCTACCTCCAGATCTAACTTGGGGATTGCTGTTCTTAGTGTAA 13200  
 TTCAATTTTAAAAACTGGTCACTGTTAAGATGAGGACAAAGTCCTTTGTCCATCAGTTAAGCCTGGGGTTATATAATTATGGAATTTATTAAATCTATGTGACCTCTCCATCATAAGGTA 13320  
 GTCTTAAAGCTAAAGTCGGTCCACTACGTAGAAAGAGGACTTTATCGTTCACTATAATTCATACACACTTTCAAAGCATCTACACCTTAAAGTTTACATGGCAAGACAACAATACCATCT 13440  
 GTAAAAATAGAAGGAAATAGAAGAATGGCAGGAGATAGTTGCTGGAGGGAGCGAATTTGAATGGGATTTGGGGTAATGTTTGTAGTTTTTGCTTTGTATATTTTTATTTAAGTGTAAGGT 13560  
 GTATCTGATATTAATAATGTTTGGTGTTTAATATGTACTATTAGCTGGACTCAGTAACTCCCCTTTCTCCAGGTAGGGATGAGAGATGCATTATTGGAAAGGAGAAAGTAACAGTCTTCA 13680  
 TTTCACAGTTTCTGGCATCCCAAGGCCACACTACTGATAAACAAGAATAATAGAACATTCTGTCATCTGATTTTTGTGGCATAAATGAAGTTGTGAACAATTTTTTTTTTCTTAAGAAAA 13800  
 AGAAACAGCTTTACATCTACCCTTTTACCTTAAAAGGATCTATGCCAAAATGCTCTGATGAATGGAAGACACACATGGACTTGTGAACTGATGTGAAACACATAATATCTGAGCCTTGGT 13920  
 TGTTGTGAAGACTGAAAAGTCTTGTTCAGTTCGGATAACCACCAAAAAAGCAACCTAAAGCTGTCTATATGTCACAACTGAGACAAATTGGGGAGTTTGTTGTTTAATGTCAAATAAAAA 14040  
 TGTACTGTTTTTAAA                                                                                                          14055
```

|  |  |  |  |  |
| --- | --- | --- | --- | --- |
| | | | | | | | | | |
| 2 |  | 4 |  | 6 |
| Depth of motif conservation (number of species) | | | | |

  
  

---

  

## >DOG (15827 bases)

```
 CCAGCCCCCACCCGTAAAGTTATTCCAATTGGCATTGATTTGGTGCTAAAAATAATTTATCCATTGCTCATCCATAAGACTGAATACCCTCTCTCCCTCTAGCATTGCTGATTTTATGAG 120  
 TTGACTACCTGAGTCCTCATTTTTAATGTACACAATTACATTTGTCCACTATCCCATGTTAGAACAAACAATCCATGTACCTTTGTGTTAATGTTAGAAAGTGTTAGTTGAGAGTTTTAT 240  
 ATCAGACACCCATTGTTCATAATTTTGGTTATTCATCATCTCTCCCCTGCCTTTGAGGAAGGAGAAACCATTCCTCTGTCATTGCTGCAGTAACCCCAGCCCCAGTTTTCTGGGCAGTCT 360  
 TCATATATTGTGCTGATCACCTGAACACTACACTGAGTGAAATTTTAATTGTCCATAATCCCACCTATTGGCTAAGATCACAGTCCTAAACCCTTTTGCTGAATTCAGGAGTGGCTGCAG 480  
 AAAACATGAGTCTTTAAACAGTTAACATGCTCATCCCAGTTGTATCAGCCTTGTTTTGTAAACAAAGGCACCCTACCATGTTTGTCCTGCTTCTGGGATTAAATACCAGATTTAAATTGT 600  
 ACATTATCTTGTCCAGTGATTAGTGACCCTCAGTCTCCTTGGCACTGCTACTAGGAAGTGCTAACTACCTGGGACCATGTTCCTTGATTTATTAATGAGTGTAATCCCAATTGTCCATAT 720  
 CCCTGTTCATTACACAAGTCCTTTGCAGCTTCTTTGCATTATGGCAGAGGTGCTCATCCTCAAAGTCCTTTACTTTGGGATATTAATGAGTACAATCATTATTGTTCATGGTCCTGCCTA 840  
 AAAGATAAGACCCACTCATGCTCTTTTCATTACTCGTAGGTAGTACAGACTACTTACCACTTTGCATTAATATGTAAAATCCTCATTTCCCATGGTTCCACCAGTTAATCTGGGATATAA 960  
 CCTTATCTTTATGCCATAGCTTATGAGTTCTGATTACCCAAGGTCACTTCTCTTAAACAGTTGATGTCCATAATTGCATATATCCATGGTGCTGTGCAATAAAAAACGGAATCCCACCTC 1080  
 ATTCTAACCTCTCTGGGATATTGTTAGGGAGTGCAGATTATTCAGAAACTCAGTTCTTGGACAGTTAATTTACACAGTGGCAGTTGTGCAGAGTCCTGTCCATTTGAAAGGGCCACTGTA 1200  
 TCCCATTTGCATACTATAATGTGGGCTGATCACTCAAGTATCCTCTTGAATTGATAATTTTCAAATTGCATTGGCACACGGTCTGTGAACTAGCCACACCCACTCCCTTTGTAATTTAGC 1320  
 AGGGGACCTTTACTACTCAGGAGCTATACACTTAGGACAGTGTATGTATACACTGTTAATTGATTAGACTGTGTTTTTTGTATCAGGAACCCTGCACTATCTTTACATCTTAAAAGGTGT 1440  
 TAATTACCTAAGGCTCTTATCTTAAAATTGTTCATGTGCAAAATTGTGATCTCCAATGTCTCCTATTGTTAACCTAGAATTCCATTCCCACCTAACTCCTTTTGGATTACTGCAGAGGCT 1560  
 GCTTGGGCCTTTTAAGGTGCTTAAGGCAGTTATGGTCAGTTCTGTTAGTCTATGTTCCCATCCCCTCTTACTTTGCATTACTGCACTGAATACTTGTAAATGTTAATGTTCTTAATTGCA 1680  
 GCTATTATTCTTTCTGTTAGTCTAGGATCCTATCCCCTCCTCCTCATTAAGCATTAGCAGAAAGAGTACATGGGACCGTTAAAGTGCATAAATGCAGCTCTGTCCCTTAAGTTAGTGTGG 1800  
 GATTTCATCCTCTTCAACTCCCTAATGCATTACAGCAGAGTAGTTGGGATCTTTAAGGTCCTCAAAGGCAGATATGGTGCCTTAGGTTAGTATGGGAACCCATCCCCTCCTACTTCCTTT 1920  
 GCATTTCTACAGAGGATACTTGGGAATGTAGCGTGCATAATTGCTGTCAGAGTCCTTTCAGTTTGTCCAGGATCCCAATCCCCTCCCTCTTACTGAGTTTGAATTACACAGAGTACAAGG 2040  
 GACTGTTAATGTGCTTAAATGGCAGTTAAGGTCTCCTCTGATCCCTTCCCTATCTAAATCTCTTTGCATTATTCTCGAGGGTACCTGGGACTGTTGATGTGCATAATTGCAGTTCAGGTC 2160  
 CCTTCTGTTAGATCCTGTCCCAACCTACTCCCTTTGCATTATCATGAAGGGTACAATGGGACTATTAATGAGCTTAATTGCAGCTATAGTTCCTTCTGTTAATCTAGTTTCCCAGGCACT 2280  
 CCTACTTCCTTTGCCTTACAGCAGAGGGTACTTGAGACCTTTAAGGTGCTTAAATGCAGCTGTGGCCTCTTCTGTTAATGTTCCCATGCCCTCTTAACTTTGCATTACTGCAGAGGCTAC 2400  
 TTAGGACTGTTAACGTGCCCACTTGCCACTGTAGTCCCTTCTGTTTGTCTGCGATCCCATCCCCTCCTACAGCATTTGCATTATCACAGAAGGCAAGAGAGACTGTTACTGTACTTAAAG 2520  
 GCCGCTATGGCTTCTTCTCTTGACCTGGGTTCCAATTCCTATCTAACTTCCTTTGCATTACTGCAAAGGATACTTGGGACTGTTAATGTCCTCTAAGGCAGCTATGGTTCCTTACATCAC 2640  
 TGTACGGTTCCATTCTTTATAATTCCCTGTGCATTACTTGAGACCTCTAAGGTGTTTGATTACAGCTATGGTCCCTTCTATTAGTCTAGATTCTCCTATCCTCCAACTCCCTTTGTATTC 2760  
 CCTAGGATTGTTTTTGGGACTGTTAAAAGTGCTTGGTTGCAACTGTGGTCTCTTTTGTTAGTCTGGGGTCCCCTGCCTTCCAACTCCTACACACAGCAGAGGCTACTTGGAACTATTAAT 2880  
 ACGTTTGATTTTAGCTGTGGTCCCTTCCCTAGGGACCCCCTTCCCTTCTATTCACTTTGCATTAGAATGACAGTGTACGTGGAACTGTTAATGTGCTTAAATGTAGCTCTGTTTCTTATG 3000  
 TTAATATAGGATTCCATCCTCTATAGCTCCCTCTGCATTATAGCAGAGGGTACTTGCCACCTTTAAGGTGTTTAAATGTAGCTGTGGTGCCAACTTAGTCTAGGATCCCATTCTCCTTCT 3120  
 ACTCCTTTTATATTACATGGCTGGGCATTTGGGACTGTTACTGTGCTTAAATGCAGTTATGATCCCCTGTGTTAGTGTAGAATTCCATTCTAACATGCTATGCATTACAGCAGAGGGTAC 3240  
 TTGGGACCTTTAAGGTGCTTAAATACAGCTATGGTTTATGGTAAGGGATGCTGAACACCAAGCCCCCCCCCTTTTGTGCATTGTTAAAGTGCATAATTGCATTTGTTCCTTTTTCTCTGC 3360  
 ACTAGATAAGATTTCACTAATTTCCCTTATATTCAGCAGTGGATGCACTTTATTTAAGACCTTTGTACTAGGATAAAGTGAACCATGGTAATTGACCACACTGTGTCTTTCATTTAAGTT 3480  
 CCTTGTACTCTCTTTGTATTAATTACCTCACTTTTTTCTGGAACTATTTGTGTGCAAAATCCTTCTCTTCAAATTAATTTCCCACTCTTTTTATATTACATCAGAGAATGCTGGTATCTC 3600  
 AAAGTTCTTTTCTTGGGATTCTTGATGTGCATATGCCCTCTTGATAAGCTAATATCCTTACTTCTTAGTACTATACCAGAGGGTACTGACCACCTGAAGTCTTTCTGTTGGACAGTTAAT 3720  
 GTGCCCAATTGCACTAGAATCCCAGCCCCATCACACCTTCTCCGTGTCAGGGGTGTTGACCAACTATGACTGCTTTTCCTACACAGTTTATTTTTTTTCCCTACACAGTTTA

AATGTGCA

AATGTGCAT  
Depth:6 (MOUSE)  
Ei-value:0.000, Pi-value:0.000  
Er-value:0.000, Pr-value:0.000  
MATCHES To TargetScan▶ miR-501-3p/502-3p:AUGCACC

 3840  


T

AATGTGCAT  
Depth:6 (MOUSE)  
Ei-value:0.000, Pi-value:0.000  
Er-value:0.000, Pr-value:0.000  
MATCHES To TargetScan▶ miR-501-3p/502-3p:AUGCACC

AATTACGGTAATCCATGCTCCCTTATGTTAGAATAGGATCTCATCTCCTTGCCCCATTTACATTATTGCAGGGGCTTCCGACTAGCCAAGATTCACTCTCTTGAACTGTTAATGTGCAT 3960  
 ACTTACATTTGCTCTTATCTGTGCACCAGGTAAGGATCCTCCCCATACTATTGATATTCCAGCAGGAGGTACCTACTACTCAAGATTTTATACACTAAAACAGTTAATGTGCACAACCTT 4080  
 CCTTGTCTTGTACATATTCAGTTGTCCATAACTGTGCCTTTTAGTTCAGGACTTTGCACTTATTGAAGCAGAGGTGACTGATCACTTTAGAGCCTTTCTCTTGGGACTCTGGATGTGTGT 4200  
 AGTTTCAGTTGTCCACTGTCCTTTGAGTTAACTTGGGTCCCTGACTCTTCACAGCCTCTAAGCTTTACTGCATGGGATACTGTCAAT

TTAAGGCC

TTAAGGCC  
Depth:6 (MOUSE)  
Ei-value:0.000, Pi-value:0.000  
Er-value:0.000, Pr-value:0.000  
No matches to TargetScan

CCTTTCTCAAACAGTTAATGTGCAT 4320  
 GATAACAATTACATTAGGATTCTTCCCCTTTACACTGCCTTTGAAGTATTACAGAGAATTCTGACCCATAAGGTCCCCATTTCTTGGCCTGTTGAGGTGCATGATTGTATTTGTCTGGAT 4440  
 TTCTGTGTACTAGAGAAGAACACCTCTCCATACTCCCTTTATTTAATATTATGCAGGGAGTGGCCCCTCTACAAGGCTGTTACACACTTGAACAGTCAAGGAACACAACTGTAACTGACC 4560  
 ACAGCTATGCACCATGGACATTAATGTGCCTAATTGCACATGGCTCCTTCCATCGAATAAGGTACTACTGTCAGACCCCTTTTATAATAAAGCAAGGATACTGGTCACTAAGGCCTTTTT 4680  
 TCATGGCTTGTGATTAGGCCTAATTGTATTTGTACATGGTCTTGTACACTAAATAAGGACACCCCTCTCCCCACTCCCTTTACCTTTTAGCAGGGAGTGCCCATTACTTTGAGTTCCTTA 4800  
 TATTTGTACAAGGTACATGGTTTTAATTAACCATACCATATCCCTTGAACATTAATGTACATAATTGGAGTTTAGTTCATCCTACCCTGTTGTATGTCTTTGCAATATACCAGGGTTGCT 4920  
 GGCCCCTCACTTTTCCTATTAATGTGCATAATTGTATTTGTCTGGGTTTTTATGCACTAGATGGGGAGACATCCCCACACCCCTACTTCTAAGCCTTCCAGTAGGTGGTGCCCACTGCTA 5040  
 AGACATTTACATTTGGATAGTTAATGTGCAGAGTTTGTAGTTTTCCACAACCTTATCACTTCCAGAACTCATACCTCTTTGCACTGCTGCAGGGAATACTATTTTCTCCCAAAGTTCCTT 5160  
 CTAGCAAACTGCTAACATTCATAATTGAAATTATCTGTTGTCCTACAGCCTCACCTACCCTTTGCAGTACAGCAGAGTGTGTTGATCAACTAAGGTCCTCTTTTGACGTTAATATGTGCC 5280  
 ATTGGATTTTTTCCTGTTTTGTGCACTCAATAAGGACCCTACCTACTTCTTAGCGTGTTCAGCAGGTAGTGTCCACTACTTAAGACTCTTTCACTTGAAAAGTTCATGTGTAAATGTAGT 5400  
 TTGCTAAGAATGCCTTATGCTAGAACCCCTGTACCGTCTGAGTTGGAGTAAGGAGATGCTATTTACCTAATGCCCTTTCTTTTGGACTTTAATGTGTATAATTGCAATTGTCCATCTTTT 5520  
 CTAATGGACTAGGATTGCATCCACTCAAACAACTTTTGCACCAGGGGCATGTTAACCATAAATGAGCTCTTCTCAAGATTATTTATATACATACTTGTATTTGTTTGTGGATCTGTGCAG 5640  
 TGTGAAAGGATGTTCTATGCCCCTACCACTGCCTTTGGATTGTTGCTGAGGAGTGCTGTCATGACCTCAGCTCTTGGACAGTTGATATGCACCACTAGCAATGATGGGACCTATGTACCC 5760  
 CCTTTGTATATTTTAAGGGTACTAGGGCCCCTCTCTTTTGGGCTGTCAGTGGGCGATTATCTATGTTCCCTTCTGTTAGATTTAGGATAATGTTCCCTTAAACTCCCTTTGCATTACTAC 5880  
 AGGGGTTGCTGACCACACAAAACTTCCCCTGGGATTGTTAATGG

GCACAATG

GCACAATG  
Depth:6 (MOUSE)  
Ei-value:0.000, Pi-value:0.000  
Er-value:0.000, Pr-value:0.000  
No matches to TargetScan

CCATTAGCAATGGGTTTTTCCTTCCTGGCCTTATTAGGCAAACA

CTCCCA

CTCCCA  
Depth:6 (MOUSE)  
Ei-value:0.000, Pi-value:0.000  
Er-value:0.000, Pr-value:0.000  
No matches to TargetScan

TCTGCATGGCATAATAGA 6000  
 GTATAAACATTGTAATATGCCGAAAACTTGTCTGTCAAATAGTAACTCATATTACCTCTCCTTTTGTATTGCTCCTATGTATAAAATTCCAAATAAGTCCTGGTCTTCCCTTTATCTTCC 6120  
 CTTTTCCCCACGCTCACGCTTGGACACTTCTTATGGACTATTCTCACAGTTTCAAGTGTTCATGTATTCAGCCATGAAATAAGATTGTCCCCCCCGCCTCCAAACCCCTGCCCCTTTTGC 6240  
 ATTATTGCTGGGGAATGTTTACTGGGTAAGGCCCTTCTCTTGGACTTAAAACATTAACAATCCCTGCTGTCATTGCCTTACCCACTTTATATTGCTGTAATGCCTCAGTTGCACCTTCCT 6360  
 TGGTCCCAGCCATCAGACATGGACCCCTCCATTTTTTTGCATTACTTCTGAGTAGTGCTGACTACCCAAAGTCCCTTCTGTGTTATTAACACAGTGCTGATTATCACGTTTTTCATCTCA 6480  
 TCAGCCCAAGATTTTCCTACCACTTTGATGTTATGTGCAGCATTGACTACC

AAAAGCAG

AAAAGCAG  
Depth:6 (MOUSE)  
Ei-value:0.000, Pi-value:0.000  
Er-value:0.000, Pr-value:0.000  
No matches to TargetScan

ACCTGAATTAGGTGGATGGGCCTTCACTCCTTTTTCTGCATTTGTTAATGATCCTGATTCC 6600  
 AGTTGTCACATTCTGGGACAGGAACTGTCCCTGCCCACCTCTGTTACTACTTTACTGA

GCAAAAT

GCAAAAT  
Depth:6 (MOUSE)  
Ei-value:0.000, Pi-value:0.000  
Er-value:0.000, Pr-value:0.000  
No matches to TargetScan

GCTCAAGGCAAAGTCAGGCCTAGAAAGGTGGATTGCCACTCTTTATTTTGGATTT 6720  
 CCAATATAAGTATCAGTTGAAAATTGTCTACCCAGGAAGAAAGGTTAGCACCTTCTGTGCATTCTTCCTTCCAGAGCA

GATTGCCTGG

GATTGCCTGG  
Depth:6 (MOUSE)  
Ei-value:0.000, Pi-value:0.000  
Er-value:0.000, Pr-value:0.000  
No matches to TargetScan

CCAAGAATCTCTCTTCTCTTGTATATTGCTAT 6840  
 TTTACAGTGCCTGTTGCTAGGTACAATAAAATGCTGTTTGCAAGATGGAATTTTTATTTATTTATTTATTTATTTATTTATTTATTTATTTATTTATTTATTTTTGCAAGATGGAATTGT 6960  
 AAACCTGGTCATTTAATTTTCGAAGTCCAAAAACCCATTTATACCGTGTACCTGATGACCAGTGTCTCTCATTTTACTGAGGGTGGTGGGTCTGTGGATACACATTGTGAATATTCCCAA 7080  
 GAGTTCTAGAATAGAACTCTTAAGACATATATCTTTAGGCTCTACCACCATTTAAAAATCATTATTTGGGCTTTACCACCATTCACTTTTAGAAAAACTACCTAAACTTTCTAATCCTTA 7200  
 AATTTCTTCATCTGGAGCATCAACCTCCCCACCCTACTTATTTAAAGAAGATTGCCGTAAAAGAATGAAATGAGAGAACATATGCTGAGGCACTTTTGAAAACCATAGTTCACCTTGATT 7320  
 AATGTATTACATAAATAAAATGTATCCTGTTTAATTCTGCTAATTTAAAGTAGCTGTAGCGGGATCCCTGGGTGGCGCAGCGGTTTGGCGCCTGCCTTTGGCCCAGGGCGCGATCCTGGA 7440  
 GATCCGGGATCAGATCCTGCGTCAGGCTCCCGGTGCATGGAGCCTGCTTCTCCCTCTGCCTGTGTCTCTGCCTCTCTCTCTCTCACTGTGTGCCTATCATGAATAAATAAAAATTTAAAA 7560  
 AAAAATTAAAGTAGCCGTAGCACTACACTGAAGCAAGGACTTAGAAATGATGGGACCAATTTCCCTGTTATGTATTAAAGGAAAAATAAGCCAAGATCTAATCATTCTTCTGGACATACA 7680  
 TTTCAACAGTGAGATAGCTTCCTGGTAAAATGAATAATATCCTAGCCTCTAGTGTGCAGGGTGTGGCAGAGAAGTGTCTAATATGGAACTGCTGAAGCAAATAACTAGTTATCACAACAG 7800  
 CAGTTCTTTGTAATCACTGAAAAAGAATACTATTCCTCTGACAAGGATGTCA

AAAGATC

AAAGATC  
Depth:6 (MOUSE)  
Ei-value:0.000, Pi-value:0.000  
Er-value:0.000, Pr-value:0.000  
No matches to TargetScan

CGCCCAGCTCAGGGTTGCAGTATGCACTACTAGCTCCTTGGACAGCTGTGAGAAGAGTCTC 7920  
 TGGCTCTTTAGAATACTGGATGAATTCTGAGCTGGTTCTGTACCCCCATTCAAGAGGAAGGATGGATCAATTTTAAGTGGATTGAAGCCTGCAACAGACAGCATCATCCAAAGATGTTCC 8040  
 TCAGAAGAATAGGCTTGTTGCTCTACAGTGTTAGTGACCCG

TTCCCTTTGA

TTCCCTTTGA  
Depth:6 (MOUSE)  
Ei-value:0.000, Pi-value:0.000  
Er-value:0.000, Pr-value:0.000  
No matches to TargetScan

CGATCCC

TAGGTGGAGATGGGGCATGAGGATCCTCCAGGGGAA

TAGGTGGAGATGGGGCATGAGGATCCTCCAGGGGAA  
Depth:6 (MOUSE)  
Ei-value:0.000, Pi-value:0.000  
Er-value:0.000, Pr-value:0.000  
MATCHES To TargetScan▶ miR-331-3p:CCCCUGG

AAGCTCACTACCACTAG

GCAACA

GCAACA  
Depth:6 (MOUSE)  
Ei-value:0.000, Pi-value:0.000  
Er-value:0.000, Pr-value:0.000  
No matches to TargetScan

ACC 8160  
 CTAGGCCAGGAGGTTCTACTACCAAGTTTTTTCCTTGGGCCCAGGGAGGAAGATGAAGTCTCAAAACAACCACCACACATTAGAGGTGGAGAGCATGGGCAGTAACTGCCATGTCTGTGA 8280  
 CATTGATCATGGTCAGATCGTCATTTTGGATCCTTGACCCCAAAGTCTTTCTTGAGGCCCCGTTGGAGAATTTTGACAATCGTTTACATACTTCAAGATACCTTAGGATGTCTTTGGGAT 8400  
 AAAACATGAAACAAGCTGTTAGAGAAGATAAATAGGAGTTATATCATTTCTGCTGTACTTGATGATTGATGGAGCCTGACCAGAAATGGCATAAAGTTTCTATTGTCAGCTCCTGATTGT 8520  
 TCCCTTTTTATCTA

CCAAAT

CCAAAT  
Depth:6 (MOUSE)  
Ei-value:0.000, Pi-value:0.000  
Er-value:0.000, Pr-value:0.000  
No matches to TargetScan

CATTGTCTCCCAAAAGCAGTGCAGAGGGCAAGAAAGTGGAACATATCAAAGGAA

GATCAACATGC

GATCAACATGC  
Depth:6 (MOUSE)  
Ei-value:0.000, Pi-value:0.000  
Er-value:0.000, Pr-value:0.000  
No matches to TargetScan

CTGGCATGCTAGCATTTTAGAACATCAGAATGAA

T

TGTGTAT  
Depth:6 (MOUSE)  
Ei-value:0.000, Pi-value:0.000  
Er-value:0.000, Pr-value:0.000  
No matches to TargetScan

 8640  


GTGTAT

TGTGTAT  
Depth:6 (MOUSE)  
Ei-value:0.000, Pi-value:0.000  
Er-value:0.000, Pr-value:0.000  
No matches to TargetScan

TTCTTTGCTTGTCTATTTCTTTTTTAAATCTCTTTGGTTGTCCTTCTATCTAAAGTGTGTCTTAGCCATTCCTTGATTATTTTGTTAGTTTCGTCTATGTGTATCTTTGTCTCC 8760  
 TTTTGTTTTTTGTTCCTAAGAGTGGTCTGTGCCTTGTCTTAGATGTCTCTCTCTAGTTTTTTCATTTTGTTTCTGA

TTCTCTTTG

TTCTCTTTG  
Depth:6 (MOUSE)  
Ei-value:0.000, Pi-value:0.000  
Er-value:0.000, Pr-value:0.000  
No matches to TargetScan

CTCTCCTAGATCTGGCTCTTCTTTCACTATTCTTC 8880  
 CCTTCGTGTCTCTGTGTCACATGCTGTGTCCTTTTTGTTTATTTCTTGTTCTGCCTATACCTCTCTTCTCTTACTGTTGTGAACTCTGATTGTCTTCCCTTCCTCTTCATGTTGGTTTGA 9000  
 CATTTCACCTTTTCCTCATTCTGGGCTTCCCTTTCTGCTT

TTTCTAC

TTTCTAC  
Depth:6 (MOUSE)  
Ei-value:0.000, Pi-value:0.000  
Er-value:0.000, Pr-value:0.000  
No matches to TargetScan

TCATTATCCCAC

ATTTCTC

ATTTCTC  
Depth:6 (MOUSE)  
Ei-value:0.000, Pi-value:0.000  
Er-value:0.000, Pr-value:0.000  
No matches to TargetScan

TTTTATATGTATTTTTTTCTGCCTCTCTTGGGGGCAGTTTTCTCTCTTTTCTCC 9120  
 TGATCAGTGTTTCTTTGTTCTTTGTGACTTTCCATTTCAGCATTCATCTCTGTTCTCTTCTTTCTTCTCTGCTTTCCCCCTTCTGCTTACCTTTGAGTATTTCAGCCTTTTTATGTGTCT 9240  
 GTGTTTACCCCCTTTGGCTTTATGGAGTCCTCTTGAGTCCTTGCATATTTTGTGCATTTCTATGGTCACGTGTGTGTGTTTGTGACAGAGGGGTTTCCTAACCCCTTCCCAATAGGAACT 9360  
 GAGTGCCAGCTATCAAAATAAGCATTGCAGAGCTGTTCATTATGCCAGGACACCCTATGAGATGATCTAAGAACTATGATGGACAGAAAGTGGAAGGCCCATAATGGATAGAAGGCCCAA 9480  
 CGTAAAATAAAGATGGTCTGAGACTTGTGGCCTGAAGACAAGCATGGAAAACCTGTGTTTCAAGATAGATGGAATCCTCAGGCTTGAGACCATCATGGCTCTGGCTATGATTGAAAATGG 9600  
 TGGCCCTTGTTGGATAGAAGACCTGTCTGAGTTGGATGGAAAGTTCAAATCTTGAGTTAAATAGGAGTTCAAAGACCCAGAGTGGGAGACTGAAGGCTTAAGCATGGGGACTAATGAAAT 9720  
 GTCTGAGACCTAAGACACAAGGTCTAAGTGAGATAAAAACCTAAGATCCAAGGATGGAAGGCCCCAGAGGACTCCTGCCTTTCTAGATGGACAGAAGGCTGAAGAGAAAGATATATCAAG 9840  
 ACTAATCAGGTGGGCAGCTAGAAGCCCATGACACAGAATCCGGGAAGGAAAGAAGCCCCAAGACCAGAGGAAATCCTAAGATGAGAACACAAAACACCAGCTTTTTTCTGTTCCTGTCCT 9960  
 CCCTACTCATGGACATTTTCAGTTCTCCCTTTCCAGTTCTTACATTTTCACATAAACCCACTTGTTGTGAGAAGCCCTTTTTGATGTTGCTATTGCCTTCAGGAGCCATATCTTGAGATT 10080  
 GGCAATATTATGCTTGGGCCAGTCTTAAGCCAATTTTTATCACAGTTTTGAGCCATTTATTAAGTGTTTATTTTTTAAATTTCCATTTCTCTTCATCTCTACTTCTGGAGAGAGACATAT 10200  
 AGGTAACATTGTATTTCAGTTCTCCCTTTTCCTTAGAAGCTCTAGGCTGATAAGCAAAGGCAAGCGTCAGGCTGTTGGGGGAAAAAAAGTGCCAGGCTA

TCTAGAGAAAA

TCTAGAGAAAA  
Depth:6 (MOUSE)  
Ei-value:0.000, Pi-value:0.000  
Er-value:0.000, Pr-value:0.000  
MATCHES To TargetScan▶ miR-1251-5p:CUCUAGC

TGTGAAGAGA 10320  
 TGTTCCAGGCCAA

TGAGAAGAATTAGACA

TGAGAAGAATTAGACA  
Depth:6 (MOUSE)  
Ei-value:0.000, Pi-value:0.000  
Er-value:0.000, Pr-value:0.000  
No matches to TargetScan

AGGAGTACCCAGATATGCCAGCCTGTTGAGAAGCACCAGCCTGCAACACCTTTCTTCTTTGCTCTTGGGTGAGCGGGATTCTTGGGGTTTG 10440  
 GGATTCCTAGTGATGGTTATAAAATTGGGCCTGGGACAGAGTAAGGCCCCAAGGAGACAGTTTGAACTCCCTGCTTATAGTAGTGGCCTAATAATTTGGGAGATTGTGCCAACACTGCTT 10560  
 CTGGGTTTAATACCCACCTCTAGGCTTAAAGGTGAGAGAATTTGGGAAGATTGAGCATGTTTAATCCTTTACTTGATTTTTTTTTCCTACCTTACCATGAGAAGATAATTATAAGTAATT 10680  
 GAGAATATATGTGAAAGCATTGTAACACACAGAAAACACAAGGCATTCTTAGTAATACTAT

ATTGGCA

ATTGGCA  
Depth:6 (MOUSE)  
Ei-value:0.000, Pi-value:0.000  
Er-value:0.000, Pr-value:0.000  
No matches to TargetScan

GCCATGCACAGCTAACATTTAAAGTGCACTGTCAG

TTGTGAAG

TTGTGAAG  
Depth:6 (MOUSE)  
Ei-value:0.000, Pi-value:0.000  
Er-value:0.000, Pr-value:0.000  
No matches to TargetScan

CATTATATA 10800  
 AATCAGGAGTCATCATACCCTTTCCATAAAGGGTCAAATGGTAAATATTTTAAGTTTGTGGTCCAAATGGTCTCAGACATCTTTTTTTTTGTTTTACAACCCTTTAAAAAGGAAGAACTA 10920  
 TTCTTAGCTTGTGAACCAAGTAGATTTGGTCTGGCAGCCATGGTTTTATCACCTGTGATGTAAATACCAGGGACAATGTGCTGAATAATATTTTTTCTGGGTGTCTGTCCCTATGCTAGG 11040  
 CACTAAGGATACAATAATTATTGATGATCACACCTTGGTGACCTGAAGAAAAATATGTGAACGTGCTTTGTAAATTATAAAGCACTTAAAGGTTTATGAGTTTGATTTGATCAACAAATA 11160  
 CTGATTACATAGTAACTCTATTCTACTGTACTAGACACTATAGATTCAAAGATTAAAAGAAGACTGAATAAAGTACTTTGTCAAGTCTGTAATACTATGCAGAATTGTTTGATTAGGAAT 11280  
 TGTTTGATAAACGAATAACTCCTGTATTTGTGGCCATTGTGTTTCTGCTGCTCTGGTAATTTTAAGTAAAAATACAGTATTCCAGGTAGCAGAGAAACAAACACATGGAAACTGCTTTGA 11400  
 AACCATAAGATACACTACAAACACAAGGGATTAAGCTATTGTGAGCACTTAAAATGTGCCAGATACATGCTAGGTGCTACAGATTGTCAAAGGGAGAAAGTGTTATTATGGAATTAAAAA 11520  
 TTTCAGATCATGAAGGGCATATTAAAGTGTTTTAAACTAAAGCACTATATAAATACCAGTGGGTTATATGTTTATTAATCGATGAATATTATCTTTTACGGGCCAGGTACTGTTTGAGTA 11640  
 CTGTGAAATCCAAGATAATGCTCCCATGTAATAAGAGGTAGGTTTATGTCCTAGACTAGTTAGATGTAAAGAATTAGATATTGTTTATTTTAATCAATCAAGGCTTTACTGGGCATATCT 11760  
 AAATTATCACCAGTGTATTTTCCTAGGTGACAAGAAATTAGAATGATTGAGAATGCCCTTGCACTTTCTTTCTTTTTTTTTTTAAGATTTTATTTATTTATTCATGAGAGACCCAGAAAG 11880  
 AGAGAAACAGAGACACAGGCAGAGGGAGAAGCAGGCTCCATGCAGGGAGCCTTATGTGGGACTCAATCCCAGGATTCCAGGATCACGCCCTGGGCTGAAGGCCGACACTAAACCGCTGAG 12000  
 CCACCCAGGTGTCCCCACCCTTGCACTTTCTGGAGTGCTATATAAGTGTAAGATATTAGCTGTTTTCTGTGTGAATAAAACCTTTCTGTGAGATAGGCATTTTCTCTACAAAATTCTCAT 12120  
 TTTGTGCTTGACATCCACTATGATGAAGGTAGTATGAATAGTTATACAAACTGAGGGTTTAGGTACTTGTTGAATTAATGAATATCCTCAGTTGCTTAATAGGTGAACTCATTATTACAG 12240  
 GCACTATAGAGATACACAGACTTAATATTTCATTGTGCTTACAGTTGAAAATAAAACAATATATGTTAGTATTTTGGGAAGTCTACAGCACCGTGTAAGTTTAACTATTTTCTAATTCGG 12360  
 TGCTGAGTAGTAGTATAAATTATATCACAGTCATCATTATGCTATGAAAAAAGTTGAAAATTTTTTAAGGAAGAATTGTTAACTATTGAACACTCTGAATATAATATATAAAGGATTAAA 12480  
 TATCTGATAAATGTAAAACTGGATAATAATTGTCTATTATGTGCCAATCTCCTTGCTAGGCATTGGGAATGTGAAGATAAACCATCTTTATTGTTTCCTGGGTAGCAGCAGATAATGTAT 12600  
 AAAATAAATTTAAAAGTTCTTAAACTGCCATACATATAAGAAATACCTGATGAATTATTTATTATTTATTGATTATTTACCTTCTTTTATCCCCAGAGATAGCGGCTAGGGATGTAAAAA 12720  
 CTCATTATCAGAGATCTGTGTAAATTTATAAAGCCTCGTGTTCTATAAGGAAATAACTTGGCTTAATGAGTGATTACTGGTTACTTTCTTTAAAAGAGCTTATTTAATAAAAACATGGGA 12840  
 AAAATGCCATTGTGCCAGGCAGAATGCTAAATGCTGAGCTCTGTGGAAAGATAAAATCTCAGAAATGTACTTGCTCCCAGGCCTGGCTGAAGAGATGGGAAATATTTTTAAGAAGCAGGG 12960  
 TGTTACAATCCTCTATCAGTCTTTAAAATGTTCAATGCTGAAGGTGGAGGTGAATTATTTCCACCTTGGCCTGGCTTGAGTCATTAAAGGAGTAAAAATAAATCACTAAGGTTTGCTTTT 13080  
 AGATTCATTTGATATATACATCATTTTTTATGTGGAATACATCATTCTAGATCCTAAGAGAATTGAAATGGGCCAAGTTAAGAGCGCATTAGAATAAAGATTATAGATGAAGATAAGAAA 13200  
 ATATCATTGAGTTTTGAGTAAATATTTTAATGCTTATGTTCTGGAAGGTATTATGCTAGATAAATTCGTTGAATGGGTCTGGTCCAGAAAACCCATGGACTTGTCTTGGAGATTAAAAAA 13320  
 ATAGAAAAATAAGGCACATGGTTCTTTTAAAATTAAATGAAGACATTACTCCTAGTTCTAGAGAGTCCCAATGTCACATGCTCATTGACCTAGTTTGAAGCCAGCAGACTGATAAAAGGG 13440  
 CACCTAGGGCTTACCCTTGAATTAATTAATTTACTATTCTGTTTTTATTATAAATGAATGACTTAAGATGAGAATTGAGATAAAACAACTAGTTTTAAAACATTTGCATATTGGCCTGGC 13560  
 TTAGAGTCAGAAAGCACCTTGAGCAATAGATTTTTTTCTAGATTTTCCATATTTTGTAAAAATTTATTGACTGAATAAAGTACTGTTCTGAAATGGTGCAATTGAAGAAATGTAAAGGTC 13680  
 CTTGACCAAAATGTAATTTAATCTCATTAGCTTTTGGTGGAAACATAAGAATAAATTTAATAAGAGAATGCAGTTCTTTCATTAGTTGAAAAAACTTTTTTTTTTTAAAAAACTTTATTG 13800  
 AGCAGCTGTGACATTAAGACACTAAGAATAAATGATCACCTTCTCTTTGGCATGTTTTGTAGAGTAGGAATAAGAGAGGACTTTCTATTCATACAAACATCAAAGAAGGTGATGTTAAAT 13920  
 TGGAATGGATCTGGTATGAAAGGCACTTAATTTATTTTGGAGATTCAGTAAAACAAAACAAAACAAAACAAAAAAAATGGAATGAAATTCTTCCTTAAATGGAAGTGTCTCTTTCAAGGC 14040  
 ATTCTCTTAATTGCTAGACAGAAAACAAAAAAGATCATGACTGAAAGAAAGTTGATTTTTTTTTATTAGCTTAGAGACTAGAATATGAGAGGTTACTGTTCATTTCTTCATTTCTTCATT 14160  
 CTACAGGGTTGAGTGCCTGTTATGTATAAGGTGTTTTGCTATATTCCCAGTTTGCCCCAGAGACAAGAAAAAAATGAATGCTTTCCTAGAGAACATTTAAACTTTCCTTTTGGTTTGTTT 14280  
 TGTGGTAGGGACATGGCTCAGTGCTTAGTACCTTAATAATGTTGTGGTTTTTAATTCCTTTTCCATGTTTCTTTTAGTACTCTGGCCACTACAATAAGCAGGGTTTTCAATTGAGCTAGC 14400  
 TTTTTAAGTATCAGGAACTTTCCTTATAGTAGACACTTTATTTTTACCGACCCATCCCTCAGGAAAAATGGCTGTTTTTGTATTAAATGTTGCCATTAAAATGGCTTTTTTTGTATTAAA 14520  
 TATTAAAGCCACTGCTGGGCAGGAGGAGGGATGACAACTAATTCCCAATCCCCAGTCTCACTGGTAACAGCCTTGAGAAAGCACTCATACTCAGGCCACAATTGGTTTTGAAATGCATTT 14640  
 ACCAGTTTTATCTGCTTTTAATTCTTTACATTTTACATACTGACAAATAAACACTGCCTAAATAGATCTCTTTGAAGAACGATCCTCAGATGGTACATAACATAATGGGAGAGACATGAT 14760  
 TTCTCTTTTGCAACAGCTTTGCAACTTAACCTTTAGAAATCTCACCATCAAAACATGTTTCAGGAAAGGATTTCTTAGAGTGGAATTATGTCAGTGAATAAAAATAGCTTAACAATTTTA 14880  
 CCATGTTTAATTTTTAAGAATAGAAATTGGAAATAATTGGCAAAAATTTGAGGTAAACAACAACAAAAAATTCTCAATCTAGGCCTAACTGGGTTTTGACCTTTTCTATTTTTAAATCAC 15000  
 TCATAGAGGGTGGGATTAGGAGGAAGAGTGAAAG

AAAAGGT

AAAAGGT  
Depth:6 (MOUSE)  
Ei-value:0.000, Pi-value:0.000  
Er-value:0.000, Pr-value:0.000  
No matches to TargetScan

CAAACCAGTCTCTAAGGGCAGCCTGCTTGTGCTTTGGATTGGTTTTAAGAGCATTACTACTTCCAAATCTAACTAGGGG 15120  
 ATTGCCTTTTTTCAGTTTAATGCAATTCCAATAACTAGTCATTGTTGAGATGAAGACACAGTACTTTCTCCCCACTCATTTCTATAAGTTTTTGAGAAACATTTTTGGGCTAAATGGTTA 15240  
 TCAACTAAATACTAATGTTTGGCTTTTAATATGTGGTATTAGCTGGATTCTGTAACACTTGTATTTTCAGGTTGGGGTTGGGGAATGCATTATTGGAAATTGGAAAGGAGAAAGTAACTA 15360  
 AAAGCCTTCCTTTCACAGTTTCTGGCATCCTAAGACTACCCCTACTGATAAGGATAAGAACATGCTGTCATCTGATTTTTATGGCATAAATGAAATTGAACAAAACAGTTTTTTAAAGGA 15480  
 AATTGCTTCGTCTTTACCTTTGTACCTAAAAGGGAATCTATGCCAATACTTCGATAAGTGGGAAGACTTATGACTGGACTTATGACTTGGACTTATGAACTGATGTGAAATGCAGAACCT 15600  
 TTGAGCTTTAAGTGTTTTGAAGATTGAAAAATCTTGCTCAGCATCAGTGACCACAAAAACAATCTAAAGCCATCTACATGTCACAACTGAAACAAATTGGGGAATGTGTTTTCTAATGTC 15720  
 AAATAAAATGTAATGTTTTGAAATTTTTGTATTTTGATGTGACAAGTTCTACCTCCGTGTTCCTAGATACACTAGTAATCTCATTTTTTTCCTACCTTAATCACCCA              15827
```

|  |  |  |  |  |
| --- | --- | --- | --- | --- |
| | | | | | | | | | |
| 2 |  | 4 |  | 6 |
| Depth of motif conservation (number of species) | | | | |

  
  

---

  

## >RABBIT (10466 bases)

```
 CAGCCCCAGCCCCAGCCCAGAGCTCCCCACTCCAAAACCCAGCCCTAACCTCAGTCTTAGCCACTCAGCCAAAGCTTGGCTGCTCCCTAGACCCACTCCAATTTTCATTTGTTGAGCACT 120  
 CAATAAGTTGTCCGTTGCTCATCTTATTTTACTGGCATTGATGATCTTAAATGCTGACCATCTGAGTTGCCATTTTCAGTTAATGCATACAATTCCTTTTGTCCATGGTCCTATGTTAAA 240  
 GACTTCCTGTACTCTGTATGTTAATGTTAAGAAATGTTAACTGAGAACTTCATATGAGGGAATTATTGCCCATAATTCGAGTTACTCATCAACGTCTCAATCCCTTGCCTTTAAGGAAGG 360  
 AGAAACCTTTCCACTGTCATTGCTGCAGTAGTTGAAGTCCCGGTTTTCTGAGAAGTGCTCAGTGGTCTTTTCTCATGTACTGTGTTGACCACCTAAAGCTCTGCATTGAATGAAACTCTA 480  
 ATTTTCCATAATCCTACCCATTGGATTAGATGTTAGTCCAAACCGCATTTGTATTCAGGGGGAGCAAACAACCCAAGTGTTTTGTGGAACAATTAATGTGCTCAATCCCAGGTGTCATAA 600  
 ACTTATCCATTAAACAAAGATACTCTACTGCCCATCTTGCCCTGCATTTGGAAAGTCCTGCTGTTCTTGAGCAGTTAAAGTACAGTAATTGTTCATTGTCTTGCCCATTAATCATGAGGA 720  
 CCTCCAATTCTCTTTGTATTTGCCTTGAGGCAGTGCTAATCACCTGAGACTAGGGTTTTTTCCTCTTCCCAGTAACAAACATCACAATTGTCCATATTCCTGTTCTTTACAAATAAGGGC 840  
 ATTTGCAACATTTTTGCACCACAGCAGAAGTGCTCATTCTCAAAGTCCCTTCTCTTGAAATATGGATGAATACAACTACAGTTGTATATTGTCCTAACAAAAGAAACCCACCTATGCTCT 960  
 TTGCTCCTAGGTTGTGCAACTTGCCAACCACTTTGCATTAATATGTAAAACCTTTCTTTCCCATGGCCACATCCTTTAATGTAGGATGTCCTCTACCTCTATACTATATTGCACTGAGGA 1080  
 GTTGACTACCCGAGATCCCTTCTCTTAAACAGTTGAAATACATTAATTGCATATCATGTGTGTCTTACACAACAAAAATGTATTTGCACCCCATTGTATCTTTTGGGTGTTGTCACTAGG 1200  
 GGGTGCACATTATTCAAGAACCCAGCTCTTGGACAGTTAATTTGCAGTTACAGTTGTCCAGAGTGCTATCCACTGAAGAAGGGCCAATATATCCTGTGTGCATTCTATAATAGTGGGATG 1320  
 ATCACCCAAGGTTACTTTCCTTGAGTTGATGATGCACGTAATTGCATTTGTCTATGATCTTGTGCATTGGCCTCATCCACTCCCTTCATTCTGGGAGACCTTTCCTACTCAAGATCTGCA 1440  
 CTAGGACAGTTGATATGCGCAATTTTAATTTATTAGAATTGAGTCTTTTTAAAAGTCAAAATCCCTGCCACTTTCTTTGCTTTACATCAAGAGGGTGCTTGATTACCTGAGGCCCCTTGT 1560  
 CTTGATATTGCTGATATCTCCTGTTATTCTGGGATCCCATCCCCTCAGGCTCCATTTGCATTGCCTTAGGGGATACTTGGGACTATA

AATGTGCAT

AATGTGCAT  
Depth:6 (MOUSE)  
Ei-value:0.000, Pi-value:0.000  
Er-value:0.000, Pr-value:0.000  
MATCHES To TargetScan▶ miR-501-3p/502-3p:AUGCACC

ATTGTGATACAAAATAACCCTAGG 1680  
 GTCCCATCCCTTCACATCTCCTTAGCATAACAACCAGAATGCTGGCTAGCTATGGCTGCCTTTCCTATACCATTGGAATGCGCACAATTAATAGCAATCTATTCACCCTCAAATTAGAGC 1800  
 AGGATACCATCTTCTTGCCTAATTTGCATTACTGCAGAGGCTAAGATTTACTCTTTTGAATTGTTAATCTTATACTTTCAGTCCTGTATTCCTTACCTGTATACCAGGTAAGGACTCCAC 1920  
 CCACTCTATTTGCATTACAGCAGGAGGTACTTAAAGCCACAGTGCACAATCTTAATTGTTCTCAACACACACTCAGTTGTCCAGAACTGTCTTTTAGATCAGGACTCCTGTACTTATCAA 2040  
 AGCAGAGAATGATAACCGACATAAAACCCTCCTTTTGGGTCTGTAGTTGTATATGATTGCATCTGTCAGTGATCCTTCAAGTTAGACTTGGGACTCTGACTTTTCACACCGTCTTTGCTT 2160  
 TATTGCATGAGACAGTGTTCAC

TTAAGGCC

TTAAGGCC  
Depth:6 (MOUSE)  
Ei-value:0.000, Pi-value:0.000  
Er-value:0.000, Pr-value:0.000  
No matches to TargetScan

CCTTTATTAAACTGTTAATGTAGTTGCATAATGACAACTACATTAGGATTCTTCCCCTTCAAATTCCCAAAAAAAAGTACTATAGGAATG 2280  
 CTGACCACTGATGCTTTAATTTGTGGCCTACTAATATAAGTGATGGTATTTGTCCTTGAGTGCTTATATGCACCAGAGAAGGAAATCCCTCCTCTCCCGTCACCAATTTTACCTTGTCTT 2400  
 CAAGCAGGGATGCTGATTCCATAAAATTCTTATGTTTATATAGAAATGGGACACAGCTGTAAGTGTGCACAGCCAAGCACCTTGGACATTAACGTGTATAACTGCACGTGGCTCATCCCA 2520  
 TTTAAATAAGATCCTACTATGAGGCTCCTTTTGCAGTACAGCAGAGGTGCTGATCACCAAGGCCTATATTGGCCTGTTAATGTGGGTGACTGTATTTATCCAGTTTCTTGTATACTAGAC 2640  
 AAGGAAGCCTCCCTTGCTCACCCCTCCCCCACCTTTCCTTTCCTTTCTAGCACAAATGCCCACTCCATAAATCTTACATTGGGAGAACAAGGTGCACAATTGTAAAGAAGCACAGCCATG 2760  
 CACCTTGGATGTTAACGTGCATAACTTCACGTGGCTCATCCATTTGAATAAGATCCTACTCCTTTTACAATACAGAAGGATGCTGATCACCAAGGCCCCACATGTTGGCCTATTACACGG 2880  
 GTGATTATATTTGTTCAATTTTCTGCTTTCTAAAGATTTACTTATTTATTTGAAAGGCAGAATTACAGAGAGAGAGATCTTCCATCTGCTGGCTCACTCCCTAAAAGGCTGCAACAACCT 3000  
 GGCCTAGGCCAGGCTGAAAGCAGGAGCCAGGAGCTTCCTCTGGGTCTCCCACATTAGTGCAGAGGCCCAGGGACCTGGGACATCTGCTGCTTTGCCAGGCATACCAGCAGGAGTTGGAGG 3120  
 GGAAGTGGAGCAGCCGGGTCTTGAACTGGCACCTATATGGAATGCTGGCACTACAGGCTGTGGCTCTAACCTGCTGTGCCACAGTGCTGGCCCCCAGTTTCCTGCTTTCTCTAGACAAGA 3240  
 AAGCCTCCCCTGCTCCATCGCTAACCCCATCTTCCTCTCCCTTGCAGCAGAGGTGTCCATTCCATATGACCATTACAGTCAAGGTGCACAATTGTAGTGACCACAACCATGTACCGTGGA 3360  
 CATTAATGTACATAAGTGCACAAGGCTCATCCCATTTTGTGGTACAGCAAGAATGCTGATCACTAAGGAACCTATTATTGGCCTGTTAAGTGGTTGATTATATTCGTCCAGTTTCCTGTG 3480  
 TGCCAGAAAAGGCATTTCCCACTGCTCCTCCTGGCCCTCATCTTCCTTTCACTTCCAGTTCTGATGCCTAATCCATAAGACATTACATGGGAACAGTCAAGGTGCACAATTGTAAGTGAC 3600  
 CAAAGCCATGGATCTTGGATATCAACGTGCATAACTAACTACATGCGGCTTGTCCCATTTTGATTAAGCTCCTACTCTCAGAACCCTTTTGAAGTATAGCAGGGGTGCTGAGAAGTAAGG 3720  
 CCTGTAACCTTGGCCTGTTATGTGGGTGATTATATTTGTCTGAGTTCCCATGTATTAGACGAGGAAACCTACTTCCTTCCACCCTCTGCTGCTCCTAGCGTTTTCCTTCCAGAAGAAATG 3840  
 TCCACTCCATAAGATCATTACATGTGGGCAGTCTAGTTTCACAATTATAAATGCCACGACCATGCACCTTGGACATTAATGTTCATAACTGCATGTGGCTCATCCTATTTAAATAAGATC 3960  
 CTACTCTCAGGCCCCATATGCAGTACAGCAGGGGTGCTGATTACCAAGGCCCATATTCTTGGTCTGTTAAATGGGTGATTATAATCTGTGTGCTAGACAAGAAAGTCTTTCTTGCTCCAA 4080  
 CCCCAACCTAATCTTCCTTTCCATATCAGCAAAGATACCTACTCCATAAAACCATTGAATTTGGACAGTGAAGGTGCACAATTTTAAGTGACCACAATGCACCTTGGACATTGATGTGTG 4200  
 TAACTGCGTGTGGCTTGTCCTATTTAGATGAGATCCTACTCAGACCCCTTTTGCAGTACAGCCGGGGTGCTGATCATCAAGGCCCATGTTCTTGGCCTGTTATGTGGGTGACTATATATG 4320  
 AGTTCCCATGTACTTGTTCATATTCCTGTACAGTAGGTAATGCTAAGCCCACCTACTACTTTAATATCTTAATGTTTGTACAAAATAAATGGTTTTTACTTGACCACAACTGGATGCCTT 4440  
 TGACATTAATGTATGTAATCATGACATTGTTCATCCTATTTGACCAAGATCCTATCCTCTTGCTCTCCATTTACAATGTACCAGGGATGCTGACCTCTAAGCTCCCTTTTCTTGGTTTGT 4560  
 TAATGCACATAATATATTTGTCTGGGTTCTTGTGCATTAAACAAGGATAGCCCACCTCTGAGTTCCTATGCCTTTTAATAGGTGGTTTCCACTGACTCAGACCTTTGAATTTGGACAATT 4680  
 AATATGCATAATTGCAGTTGTCCACATCCCCATTACTTCCAAGACCAATGTACCTCCTTTACATTACTGCAGGGGATACTGCCCCCCCCCCCCCAGATCCCATCTAGTGGATCATGAATG 4800  
 TACATAATTGAAATTTTCTTTTGTCTTTGTCAGTAGCATATAAAGTCTTATCCCCTCACTTTTCCTTAGTAGTACAGCAAGATGTACTGCTCAACCAAGGCCCTCTTCGTTTGGCGGGAG 4920  
 TGTTAAGTGCAATTCCATTTTCTCCTGTACCCTTCACTAGATAAGGGTCCCATCAACTTCCTTAGCATTTTCAGCAGGTAGTGCTCACTACAAAAGAATTTTTATTTGGAAAGCTCACAT 5040  
 ACATGAACAGTTTGCTAAGTATGTCCTATACCAGAACTCCTGTAATCTCCCTGCTTTACAGCAGGGGGTGCTATGCACTCAGTGCCTTGTCTTTTGGATTTAATGTGCATTAATGCAATT 5160  
 GCTCCTCTCTTAGGGTATCACTTCCTTATACTCCTTTTTTTTTGAATCAGGGGCATATTAACTATGTATTGGCCCCTTTTCCATGAATATTAATGTACATAATTGGTCTGTGGACCTATG 5280  
 TGGTAGAAAAGAACCCTATGCTCCTACCACTGCCTTGTGATTGCAGCTGAGGAGAGCTAACCATAACCTCAGCTCTTGGGCAATTAATATGCAACAATAACACTTATTGAGAATACGGAT 5400  
 TGTCAGATGGAAACCCTGTACCCTCTTCATTTGTAACAGGGGTGCTGACCAACTGTGACACCTTGAACTATTAACTTGAATATTTGCACTTGGGCTCCCCCTTTCTGTTAGACTGGAGTA 5520  
 ATACACCCTTACATAACCTGTGCATTACTTCAGGGTGTGCTGGCCACTCCAAACTTCTCCTTGGACTGTTCATGG

GCACAATG

GCACAATG  
Depth:6 (MOUSE)  
Ei-value:0.000, Pi-value:0.000  
Er-value:0.000, Pr-value:0.000  
No matches to TargetScan

ACAATCAATGGTTCTTTACCTCTTTGAAACCTCTCCT 5640  
 GGGACTGTTAATAG

GCACAATG

GCACAATG  
Depth:6 (MOUSE)  
Ei-value:0.000, Pi-value:0.000  
Er-value:0.000, Pr-value:0.000  
No matches to TargetScan

GCAATTATCAATGGTTTTTTTCTCTCCCTGGCATTGTTAAACAACACTTTCTTTCCCATGGTGCAATAAATTGTAGTATGCCATGCATTTGTCCATCA 5760  
 AAGAACAACCTATATTTCCATTCCCAGGTAGCCATGGTCTCACCATCTATGCAAGGTTTTCCCTCTCCATTGCCCCTTGTGTGCCCTTGGACCCTTCTCATGGACTATTAATGCTCACAG 5880  
 TCCAGCCATTTGAAAAGATTGTGCTCCTTCTGTCCCATCTGCCCCCACCTGGTCCATTTTGCCTCTTTTGGTATGTAATGTTGACTGAACAAGGCCCTCTCTCTTCGACTTAAAATGTTA 6000  
 ATCCCAAATGCCACTGTTTTACCCACTTTGCATTGCTGTAGTGGCTCAGTTGCACTTTCCTTGGTCCCGCCCATTAGACATGGACCC

CTCCCA

CTCCCA  
Depth:6 (MOUSE)  
Ei-value:0.000, Pi-value:0.000  
Er-value:0.000, Pr-value:0.000  
No matches to TargetScan

CTTACTTTGCATTGCTTCTGAGCACTG 6120  
 CTGACTACCCAAAGCCCTTTCTGTGTTATTAATGAATGAACACAATACTGATTGTCCCACTTTGCAGCCTGTCTTTAAGATCTCTCTACCACTTTGATGTGTTTGTGCAGCATTCATTAA 6240  
 C

AAAAGCAG

AAAAGCAG  
Depth:6 (MOUSE)  
Ei-value:0.000, Pi-value:0.000  
Er-value:0.000, Pr-value:0.000  
No matches to TargetScan

GCCTGGAACTATGTGGATGAACCTTCATTTTTTTCCTGCAATTGCTAATTATTCTGATTTCAATTATTGTCACAATCTGGGTACAGGAGTCATTCCTTCCCAGCTCTATTA 6360  
 TTGCTTTATTGT

GCAAAAT

GCAAAAT  
Depth:6 (MOUSE)  
Ei-value:0.000, Pi-value:0.000  
Er-value:0.000, Pr-value:0.000  
No matches to TargetScan

ATTTACAGCACATCAGGCTCAGTGAGCTGAAATACCATCCTGTATTTTGGGTATTTAACATATAGTGATCAGTTGATTGGAGGAGAGTTGTGATTTTCTTT 6480  
 GCATTCTTCTTCCAGAGAA

GATTGCCTGG

GATTGCCTGG  
Depth:6 (MOUSE)  
Ei-value:0.000, Pi-value:0.000  
Er-value:0.000, Pr-value:0.000  
No matches to TargetScan

TTAAGAATCTTTCTTTTGTGTATTGCTGTGGTGCTGTGCCGACTGCTAGGATACAACCAGAAGCGTTGTTAATTGCTTGTTTTTTAAGAAA 6600  
 GACATCTGGATTACAAGGTGGAATTGATAGGCTGGTCATTAATTTTTTTAAGCTGAAAAGTCCATTTATACTATGTACTTAATGATCAGTGTCTCTCATTTTACCGAGTGTGGTGGGTCT 6720  
 GTGGATAGACCACTGTGACTCTTGTATTATAATTATGGCATTACCAAAAGGGTTCTGGAGTGGAGTGGACCTCTTAAGATCAGTATCTTTGGGCTGTACTACCATTTTAAAATCAATCCT 6840  
 TGTTTGGTTTTTACCACCATTTGCTTTTAGAAAAATGATCTAAATGTTCTAATCCTTCAATTTCTTCATCTGGAGCACCAGCCTCTAATTATTTCAAGAAGATGGCTATAAAAATGATTA 6960  
 AATGAGAGAACATAATGCTGAGGTGCTTCTGAAAACCATAGGTCACCATTTAGTTCTGCTAATAGCTTGAAGCATCACACTGAAGTGAGGACTTAACCATAGAAATGATGGGATCAGTTT 7080  
 CCCCATTTTATAAGAAAAATAAGCCATTACCTCATCATTCTTCTGAACATAAATCTCAGCAGTGGGATAGCTGCCTAGTAAAAAGGAGTAATATCCCGGCCTCTAGTGTACAGTGTCTTA 7200  
 TGCCAAAGGAGTATTTAATGTGGAATTGCTGAAGCACATAGCTAGTCATCACAACAGCAGTTCTTTTTAACCACTGAAAAAGGATACTATGACTCTGAGAAGGATGCCG

AAAGATC

AAAGATC  
Depth:6 (MOUSE)  
Ei-value:0.000, Pi-value:0.000  
Er-value:0.000, Pr-value:0.000  
No matches to TargetScan

AGCC 7320  
 CAGCCCAGGGTGCAGTTTGCACTACTGGCTCCTTGGACAGCTGCAAGAAGAGTCTCTGGCTCTTTAAGATTCT||GGTCATTCCCAACTATGTGAGACCTGAGGACTGAGAGCAGCTGCA 7438  
 AGAGACCTCAAGTTCAGCAGATCTTTACTTTTGAGACTCCAGACCAGTTCCAACTATTCTAAGTGGATGGCTTGCAAGTTGCCTGGAGAAAAAG||ATCTTCCTGGAAGAATAGGCTTGT 7556  
 TGCTTTACAGTGTTAGTGACCCA

TTCCCTTTGA

TTCCCTTTGA  
Depth:6 (MOUSE)  
Ei-value:0.000, Pi-value:0.000  
Er-value:0.000, Pr-value:0.000  
No matches to TargetScan

CGATCCC

TAGGTGGAGATGGGGCATGAGGATCCTCCAGGGGAA

TAGGTGGAGATGGGGCATGAGGATCCTCCAGGGGAA  
Depth:6 (MOUSE)  
Ei-value:0.000, Pi-value:0.000  
Er-value:0.000, Pr-value:0.000  
MATCHES To TargetScan▶ miR-331-3p:CCCCUGG

AAGCTCACTAGCACTGG

GCAACA

GCAACA  
Depth:6 (MOUSE)  
Ei-value:0.000, Pi-value:0.000  
Er-value:0.000, Pr-value:0.000  
No matches to TargetScan

ACCCTAGGTCATGAGGTTCAA 7676  
 CCAAGATACTTCCTTGGGCCCAGATAAGAAGATGAAATCTGAAAGACAACCACCTTGTGTCAAG||GAGAACATGGACAACAACTGCCACATTACAACACCTTGGGATGTCCATGGGATA 7794  
 TAACACACAACAAGCTATTTGAGAAGATAAATAAGAGTTGTAGCTTTTTGCTCTGTGCCTGCTGATTGATGGATGCCACGTAGCTACGAATTAAAACATGGGAAATGACACAGTTTTCTA 7914  
 TCATTGGGCAAGAACTAAAGGATGAGTTCCCTGTGTCCTCTAG||CTCTTGAATGTTCCTTTCATGGCTG

CCAAAT

CCAAAT  
Depth:6 (MOUSE)  
Ei-value:0.000, Pi-value:0.000  
Er-value:0.000, Pr-value:0.000  
No matches to TargetScan

CACCCTTCCCAAAATCAGTGCAAAGAGCTGGGTCTTCCGCAGAA 8032  
 TCAAGAAATTTGAACATGCCAAAAGATGCCTTCCTACCTAAA

GATCAACATGC

GATCAACATGC  
Depth:6 (MOUSE)  
Ei-value:0.000, Pi-value:0.000  
Er-value:0.000, Pr-value:0.000  
No matches to TargetScan

TTGCTGACAATGTAGCACTTCAGAGTAGCAGAATGAA||

TGTGTAT

TGTGTAT  
Depth:6 (MOUSE)  
Ei-value:0.000, Pi-value:0.000  
Er-value:0.000, Pr-value:0.000  
No matches to TargetScan


TTCTCTTTG

TTCTCTTTG  
Depth:6 (MOUSE)  
Ei-value:0.000, Pi-value:0.000  
Er-value:0.000, Pr-value:0.000  
No matches to TargetScan

TCTTTTTTCTTC 8150  
 CTTGTGCGGCTTTGCTCTTCTCTAAAGTGATTGTTATCCATTTCCATGTTTCTCTTGCTAATTTCTTCTATGTGTGCTTTTGCTTCATTTTCTCTTTTTGTCCCTAAGTGTGATCTCTGC 8270  
 CTTGTATTTGTTGTCTCTCTCTAGTTTGTCCACTTTGTTGCTGTTAACTCTTTGCTCTCCTACATCTGGCTCTTCTTTCACTATGTCTTCCTTTTG

TTTCTAC

TTTCTAC  
Depth:6 (MOUSE)  
Ei-value:0.000, Pi-value:0.000  
Er-value:0.000, Pr-value:0.000  
No matches to TargetScan

TCTCTGGGTCATACGCT 8390  
 ATGTGCTTTTTTGCCACTTTCTTCTTCTACCTATCTTTCTTTGTCTCTTTGTGACCCTTCCACTTCGTGTTGGCTTGATGTTTCATGTTTCTCTGATTCTGAGCTCCTTTCTGATGTTTC 8510  
 TCCTCCTTTTCTTGC

ATTTCTC

ATTTCTC  
Depth:6 (MOUSE)  
Ei-value:0.000, Pi-value:0.000  
Er-value:0.000, Pr-value:0.000  
No matches to TargetScan

TTTTCTACCTTCTCTTTTTGCCCCTCTTGGGCTATTTTCTCTCTTTCCTCCTTTGTGTGCCTAAGTGTCTCTTTGCTATTTGTAATTGTCTACCTCAG 8630  
 CATCAATCTCTGCTTATTTGTGTTTCTTCTCTGCTTTTCCCCTCTCTATTAACCTTTGACTCTTTCAGCCTCTATGTTTGCCTCTCTTTGATTTTTATGTAATTTCTCCTTGGATCTGTC 8750  
 TTTGTGTATGTGGGCATGTGTGCATATATGTGCATGTCATGTGTGATTGAGGGGGTTCCTAACCCCTTCCCAATAGGTGCAGAATGTCAGCTATCAAAATGAACAGTGTAGAAGCTGTTT 8870  
 CTTATGCCAAGTTACCTAATGAGATGATCAAGACCAAAACAGCACCCTAAGATCAGAACTGAAGTAAAACCCAAGACCACAAATGGACAGAAGGTGGAAGGTGCATGATGGATAAAAGAC 8990  
 GCAAAGTGAAAGACAGATGGTTTAGACCTTGAACCTTGAGGACTAAGGTTGAAAATACAAGACCCCAAAAAAGATGAGACCCTCAGGCCTGAAACCAAACAGAAACTTTGAAAACTAACA 9110  
 TGTACAGAAAGTGGTCTGAGTTGGACAGAAGGCCAAAGACCCAGGGTGGAGGCTGGAGGCCCAAGAGCTGGAATGGACACATGGGAAGCTTAAAAACTGAGATGTATAATACAAAGCCTA 9230  
 AGACCAAGTTATAGTTTGAAGACCTAAGGCCCATGGATGGAAGGCTGCAGACACAAACAACACCCAGGACTCAAGCCACCCCAGATGGACAGAAGACCCAAGCGAAACAGTGATCTCCTG 9350  
 ACTAAAGGTTGGAAGCTGAAGTTCCCTTAACCAGAGCCCAGGAGGGGAGAAACCCGGGGAAGTCCCAAGATGAGAACCCTAAACCCCAACTCTTTTCTATTGTTGTACCTTCTACTCTTA 9470  
 GATATTTTGAATTTTCTCCTCCCAATTATCATATTGCCATTAAGCCTGCTTCTTTTGTGATGTCCTTCAGAAAGGAATGTGTCTTTAGAGTGGTAATATGCATGGGCCAGTCTTGAGCCA 9590  
 GTTTTTGTTGTAGTTTTTAGCCATTCATTTTTTTCATTTCTCTTCATGTCTATTCCATTTGAGAGAGACAACAAAATTCAATCAATATCTAGTCTGGATTTTGGTATTACACTCAGGAGC 9710  
 AAGCATCTGTATAGGTTATATTGCATTTCTGTCTTCTTTTTTAATCTCAGAAGCCTTGGGCTGATGAGAAGACAAAAGCTGTTGTGGTAAAAAGAAGTGCCAGGCTA

TCTAGAGAAAA

TCTAGAGAAAA  
Depth:6 (MOUSE)  
Ei-value:0.000, Pi-value:0.000  
Er-value:0.000, Pr-value:0.000  
MATCHES To TargetScan▶ miR-1251-5p:CUCUAGC

TG 9830  
 TGAAGAGATGCTCTGGCCAT

TGAGAAGAATTAGACA

TGAGAAGAATTAGACA  
Depth:6 (MOUSE)  
Ei-value:0.000, Pi-value:0.000  
Er-value:0.000, Pr-value:0.000  
No matches to TargetScan

AGAAATAAACAGATCATACCAGCCTTCTGAAAAGCACTAGCCAACAGCACCTTCCTTTGAGCTTATCCAGATTTCTGAGATCTG 9950  
 GAACTTCTAGTAATGGTTACAGATGGGTGATTGCCTGCCCAGAGTGAGGCTTACACTCCCAGCTCATTGTAAAGGCCAGATAATTTGGTGGACCACACAAACCCTATTTCTGAGTTTAAC 10070  
 ATCCACTTGTAGGTTTAAAGATGACAGAACATGAGATGATATAATGGTTTAATCCTTTGCTTGATCACTAATTCTCCCCCTTTTGTGAAGGAAGTTGATGTAAATGATTGATAATGTGTT 10190  
 TTTAAACACTGTAAAACACAAGACAAAAACAAGCAAGGCAACCTCATTAATAGCAT

ATTGGCA

ATTGGCA  
Depth:6 (MOUSE)  
Ei-value:0.000, Pi-value:0.000  
Er-value:0.000, Pr-value:0.000  
No matches to TargetScan

GTCACAGACAATTAACATTTAAAAATACAGTGCCTC

TTGTGAAG

TTGTGAAG  
Depth:6 (MOUSE)  
Ei-value:0.000, Pi-value:0.000  
Er-value:0.000, Pr-value:0.000  
No matches to TargetScan

CATTATGTAAATG 10310  
 CTCATCACACATTTTCTCTGAGGTCAGATCTTAAATATTTAGGTTTTTGGGGCCATAAGCTCTCAGCATGTTTTTTTTTTACACAACCTTTTT

AAAAGGT

AAAAGGT  
Depth:6 (MOUSE)  
Ei-value:0.000, Pi-value:0.000  
Er-value:0.000, Pr-value:0.000  
No matches to TargetScan

AAGAACCATTCCTACCTTGT 10430  
 GAGCCAAGTGGATTTGATCTGCAGTTCAAAGTTTAC                                                                                     10466
```

|  |  |  |  |  |
| --- | --- | --- | --- | --- |
| | | | | | | | | | |
| 2 |  | 4 |  | 6 |
| Depth of motif conservation (number of species) | | | | |

  
  

---

  

## >MOUSE (17918 bases)

```
 CGGCTTGCTCCAGCCATGTTTGCTCGTTTCCCGTGGATGTGCGGTTCTTCCGTGGTTTCTCTCCATCTAAGGAGCTTTGGGGGAACATTTTTAGTTCCCCTACCACCAAGCCTTATGGCT 120  
 TATTTAAGAAAACATATCAAAATTCCACGAGATTTTTGACGTTTTGATATGTTCTGGTAAGATTTTTTTTTTGACATGTCCTCCATACTTTTTGATATTTGTAATATTTTCAGTCAATTT 240  
 TTCATTTTTAAGGAATATTTCTTTGTTGTGCCTTTTGGTTGATACTTGTGTGTGTATGGTGGACTTACCTTTCTTTCATTGTTTATATATTCTTGCCCATCGGGGCCACGGATACCTGTG 360  
 TGTCCTCCCCGCCATTCCATGCCCAACGGGGTTTTGGATACTTACCTGCCTTTTCATTCTTTTTTTTTCTTATTATTTTTTTTTCTAAACTTGCCCATCTGGGCTGTGGATACCTGCTTT 480  
 TATTCTTTTTTTCTTCTCCTTAGCCCATCGGGGCCATGGATACCTGCTTTTTGTAAAAAAAAAAAAAAAAACAAAAAAACCTTTCTCGGTCCATCGGGACCTCGGATACCTGCGTTTAGT 600  
 CTTTTTTTCCCATGCCCAACGGGGCCTCGGATACCTGCTGTTATTATTTTTTTTTCTTTTTCTTTTGCCCATCGGGGCTGTGGATACCTGCTTTAAATTTTTTTTTTCACGGCCCAACGG 720  
 GGCGCTTGGTGGATGGAAATATGGTTTTGTGAGTTATTGCACTACCTGGAATATCTATGCCTCTTATTTGCGTGTACTGTTGCTGCTGATCGTTTGGTGCTGTGTGAGTGAACCTATGGC 840  
 TTAGAAAAACGACTTTGCTCTTAAACTGAGTGGGTGTTCAGGGCGTGGAGAGCCCGCGTCCGCCATTATGGCTTCTGCGTGATACGGCTATTCTCGAGCCAGTTACGCCAAGAATTAGGA 960  
 CACCGAGGAGCACAGCGGACTGGATAAAAGCAACCAATTGCGCTGCGCTAGCTAAAGGCTTTCTTTATATGTGCGGGGTTGCGGGATTCGCCTTGATTTGTGGTAGCATTTGCGGGGTTG 1080  
 TGCTAGCCGGAAGTAGAAAGCCAAGGAGTGCTCGTATTAGTGTGCGGTGTTGCGCGGAAGCCGCAGAGGACTAGGGGATAGGGCTCAGCGTGGGTGTGGGGATTGGGCAGGGTGTGTGTG 1200  
 CATATGGACCCCTGGCGCGGTCCCCCGTGGCTTTAAGGGCTGCTCAGAAGTCTATAAAATGGCGGCTCGGGGGCTCCACCCGAGGCTCGACAGCCCAATCTTTGTTCTGGTGTGTAGCAA 1320  
 TGGATTATAGGACATTTAGGTCGTACAGGAAAAGATGGCGGCTCAAGTTCTTGGTGCGGTATAACGCAAAGGGCTTTGTGTGTCACATGTCAGCTTCATGTCTGAGTTAGCCTGGAGAGG 1440  
 TGGCACATGCTCTTGAATGTGTCTAAGATGGCGGAAGTCATGTGACCTGCCCTCTAGTGGTTTCTTTCAGTGATTTTTTTTTTGGCGGGCTTTAGCTACTTGGCGGGCTTTGCCCGAGGG 1560  
 TACACTTGGTGCATTATGGTAGGGTGTGGTTGGTCCTACCTTGTGCCACTCGAAGCTGAGGCAAGGCTAAGTGGAAGTGTTGGTTGCCACTTGACGTAACTCGTCAGAAATGGGCACAAG 1680  
 TGTGAAAGTGTTGGTGTTTGCTTGACTTCCAGTTAGA

AATGTGCAT

AATGTGCAT  
Depth:6 (MOUSE)  
Ei-value:0.000, Pi-value:0.000  
Er-value:0.000, Pr-value:0.000  
MATCHES To TargetScan▶ miR-501-3p/502-3p:AUGCACC

TATTGCTTGGTGGCCAGGATGGAATTAGACTGTGATGAGTCACTGTCCCATAAGGACGTGAGTTTCGCTTGGTA 1800  
 CTTCACGTGTGTCTTTAGTCATCATTTTTTCGAAGTGCCTGCCCAGGTCGGGAGAGCGCATGCTTGCAATTCTAACACTGAAGTGTTGGATGATGTCGGATCCGATTCGAGAGACCGAGG 1920  
 CTGCGGGTTCTTGGTCGATGTAAATCATTGAAACCTCACCTATTAAAAGAAAGAAAAGTATCTAAGGCCATTTCAAGGACATTTGACTCATCCGCTTGCGTTCATAGTCTCTTACAGTGC 2040  
 TCTATACGTGGCGGTGCAAACTAAAACTCAGCCCGTTCCATTCCTTTGTATTGTTCAGTGGCTAGTCTACTTACACCTTGGCCTCTGATTTAGCCAGCACTGATCTCAAGCGGTTCTCTA 2160  
 AGCCTACTGGGTATAAGTGGTGACTTTGGCCAGAGTCATAGTGGATCACAAATCACTGGTGAAGAGGTAGAATCCTACCTTCTTCCAAAATCTACCCCATGACTATTGCTGGGGTTGCAT 2280  
 TTTGATTTCAATGAATATTTTGGATGCCAACGACACGTCTGATAGTGTGCTTTGCTAGTGTTTGAATTTAAAACCGAAGTGATTGTTTTCAAAATGTATTTACGATTTGCTTACTTGTTG 2400  
 AATTCATTTTAATTACCTTTAGTGAATTGTTACTTTGGAGTCCTTAAAGTTTTCAATAATTTTTTTGGCAGATGATACTCAAATTACTTGGCACTTAAATGTACTTTCTTTCAAACTCAT 2520  
 CCACCGAGCTACTCTTCAAATTTTTAAGTCTTATAACACAGATACTGTTAATGTAAAGTGAACATTATGACTGGATGTCAGGAGTATTTGAGGTTCTATACCAGTTCAGGCTTTGCTTTT 2640  
 GTTGCTATTGTTGATGCTATATTGACTAATGGTTTTACTTGTCAGCAAGAGCCTTGAATTGTAATGCTCTGTGTCCTCTATCAGACTTACTGTTATAATAGTAATA

TTAAGGCC

TTAAGGCC  
Depth:6 (MOUSE)  
Ei-value:0.000, Pi-value:0.000  
Er-value:0.000, Pr-value:0.000  
No matches to TargetScan

TACATT 2760  
 TCAACTTTCTGTGTGTTCTTGCCTTTATGGCATCTAGATTCTCCTCAAGACTCAGCAAATAGTGCTGCTGCTATTGCTGCCCCAGCCCCAGGCCCAGCCCCAGCCCCTGCCCCAGCCCCA 2880  
 GCCCCAGCCCCTGCCCCAGCCCCAGCCCCTGCCCCTGCCCCAGCCCCTGCCCCAGCCCCAGCCCCAGCCCCTACCCCTGCCCCTGCCCCTGCCCCACCCAACCAACCCAATCCAGTCCAG 3000  
 CCCTGCCCCAGCCCAGTCCTAGCCCCAGGCCCAGATACTTTCAGACCTATCCCAAGCCCACTTCTACTTAGAGAAATTCGAATCTTCATTGATTCAGTGCTAAAATGCAGTGTCCATCAC 3120  
 TCAGCCTATAAGACTGAGACAGCCCATCTATACCCCCTCCATACTGACTTCTAGAGTCATGGAATTTCACTTAATGCATAGAATCGTATTGCTAAAATGCAGTGCCCATCACTCAGCCTA 3240  
 TAAGACTGAGATAGCCCATCTATACCCCCTCCATACTGACTTACAGAGTCATGGAGTTTCACTTAATGCATGCAGTCCTATTGCTAAAATGCAGTGCCCATAACTCAGCCTATAAGACTG 3360  
 AGATAGCCCATTTATACCCCATACCCCCTCCATACTGACTTCTAGGGTCATGGAATTTCACTTAATACATAGAATCGTATTGCTAAAATGCAGTGTCCATCACTCAGTCTATAAGACTGA 3480  
 GATATCCCTATGTATACCCCATACTCCCTCCATACTGACTTCCAGAGTCATAGAATTTCACTTTGCATACGGTCCTATTGCTAAAATGCAGTGTCCATCACTCAGTCTATAAGACTGAGA 3600  
 TATCCCTATGTATACCCCATACTCCCTCCATACTGACTTCCAGAGTCATAGAATTTCACTTTGCATACGGTCCTATTGCTAAAATGCAGTGCCCATCACTCAGCCTATAAGACTGAGATA 3720  
 GCCCATCTATACCCCCTCCATACTGACTTCCAGAGTCATGGAATTTCACTTAATGCATGCAGTCCTATTGCTAAAATGCAGTGCCCATCACTCAGCCTATAAGACTGAGATAGCCCATCT 3840  
 ATACCCCATACCCCCTCCATACTGACTTCCAGAGTCATGGAATTTCACTTAATGCATGCAGTCCTATTGCTAAAATGCAGTGCCCATCACTCAGCCTATAAGACTGAGATAGCCCATCTA 3960  
 TACCCACTCCATACTGACTTCCAGAGTCATGGAATTTCACTTAATGCATGCAGTCCTATTGCTAAAATGCAGTGCCCATCACTCAGCCTATAAGACTGAGATAGCCCATCTATACCCACT 4080  
 CCATACTGACTTCCAGAGTCATGGAGTTTCACTTAATGCATGCAGTCCTATTGCTAAAATGCAGTGCCCATAACTCAGCCTATAAGACTGAGATAGCCCATTTATACCCCATACCCCCTC 4200  
 CATACTGACTTCTAGGGTCATGGAATTTCACTTAATGCATAGAATCGTATTGCTAAAATGCAGTGTCCATTACTCAGCCTATAAGACTGAGATATCCCTATGTATACCCCATACCCCCTC 4320  
 CATACTGACTTCCAGAGACATAGAATTTCACTTTGCATACGGTCCTATTGCTAAAATGCAGTGCCCATCACTCAGCCTATAAGACTGAGATATCCCTATCTATACCCTCTACCCCCTCCA 4440  
 TACTGACTTCCAGAGTCATGGAATTTCACATAATGTATAGATTTCTATTGCTAAAATGCAGTGCCCATAACTCAGCCTATAAGACTGAGATAGCCCATCTATACCCCCTCCATACTGAGT 4560  
 TCCAGAGTCATGGAATTTCACTTAATGCATAGAATCGTATTGCTAAAATGCAGTGCCCATCACTCAGCCTATAAGACTGAGCCCATCTATACCCCATACCCCCTCCATACTGACTTCCAG 4680  
 AGTCATGGAATTTCACTTTGCATACAGTCCTACTTTACTTGTCCATGGACAAGTAAACAAAGAACTCTTGTCCTTCATGTTAATCAAGATACACCAATCAAACAAGAGTTTTATATCAGA 4800  
 GACTTGCCATGGAGGTATCATCTCTCAAGTCTCCTTTCCTTTAAGGAAAGAAAACCATTCTGTCATTGCTGTAGTAGTCACAGTCCCAAGTTTCTAAGCAGTGTTCAGTCGTCTTTTCTC 4920  
 ATGTATTACCTTGAGTACTGAATAATTCTGTCAGAAATATTTTGTCCATTGGATTAGACTTTAGCTAGTCCAGCCCTGTGTGCATTTAGCAAAGGGGCAAACACAGGTCTGTTATCAGAC 5040  
 AGTTAAAGTGCTCAGTCCCAATTTTCAAGGCATTGGCCATTAAAGGGGGTAGAATACTATATACTGTTGGCATGCTGTCATGGGTGCTATCGCCCCAGGTCACATCTTTCTAACTGATGG 5160  
 AGATACATTTATTTGCTCATGATATTGTATACTAGTCTCACATGCTTTCTTATTTCAGCCAAAAACCTCTGCACTGGAACATTTTATGTGGATAATCCTGACTAGGAATTGAGTCTTTTC 5280  
 TCAAGGTCCTAATACTACCCTTGCTTTATGTAAAGAGGGTGCTGATTACTTAATGCCTCTTACACAATTGTGCAAAATTGCAGTTGTTCAAGTCCCCTTCTGTTAGTAACCAAGATCCCA 5400  
 TACCCTCATACCCTAATGGGTGACAATCAAGGGTGCCAACCAATGAGACCACTTCTCTGTTCTGGTCTTTCTGCTGTGCTGGGGAATCAAACCTTGAGTCTTGTGTACGCTAGTAAAGCA 5520  
 CTGTCATAGAGCTACAGCCCCACCGTGTGGTGGTTTGAGAGAACAGCCTCTTATGTAGCCTGGGCTGGGCGGGACTTACAGGCATTGCCACCTGTAATGTAAACATATTTGTGCCTGTTG 5640  
 TGTGCACAGCTGCATTTGTCCCTCTTCCTAAGCATTGGATAAAGAAACCAAACTAAGTCAAGTCATTTTGTTGGTAATCAAGAAGACCTTTGATCTGTCCTGTTTTTAACTTCCAGGCTG 5760  
 GCCTGGAACTTAGCATATAACCCAGGCTAGCCTTGAGCTCAGGATCTAGCCTGCGTTTAACAAGTGTTGGCATATCTGGTTCCTACCACTATGCCCTGCATGCAGTCTTTCATATTGTGA 5880  
 ATGTGCATATGTCATTTCACTGTAGTAATCTGCATCTGGTGAAGACTTATTTGTATTGCAGCAGTATTTAAGATCCTTAACATAGTAAATGTGCACAGTGTTAACTCTATTGTACATATT 6000  
 CTCATGTCCACAGTTGTGCCTTTTAGATCAGGACTCCTGTACTTAGCAAAGCAAAGAGGCTCACTAATATAAAGCTTCTTTCATGAGACTATAGATTGAAACGATTCCAATACGGTCAAT 6120  
 GGTCCTTCAAGGTAAGACTTCTGTCTCTGATCATTCATATCCTCTTTGCTTTATGGAATTATGTATGTGCTGTGCACTTGAAACCCCTTCCTCAAACTATTTATGTACATACTGGCAATT 6240  
 TTAGTAGGATCAATTTTACTCTTAACTTTGAAGTACAGAAGTGGTGTTGACCTATAAGGTCCCATTTTGTGGCTTGCTAATAATAATGACTGATTGTAGTAGGCCTTTTCTGTTCACTAC 6360  
 AGAAGGAAACCTGAACAGCGTAAAACTGTAATGGCCATAAACATGTACCTTGCATATTAGTATGCATTTACTGCACACATCTCATTCCATTTGGATACGATCCTACTCTCAAACCCTTTT 6480  
 GCAGTACAGCAAGGGTCACTAATCTTTTGGCTTCTTCATCTTCCTGGACACTGGATAAGGCTGTCCCCTCCTTTCCACTCTTTAATTTCCAGGACTATTACTTTAAAGACTTAATATTTG 6600  
 CATAAAGGATGGGGTTTTTAATTGATAACATGTCCCTTGAACATTAATGTATATAACAGGGACATGATCCATTCATTTTAATAAAAATACTTGGCCAGTTAATGTGTAAAATTACACTTA 6720  
 TCCACAACCTTATTACTTTTCGGACCATTGTATCTCTTGCACTCCTGCAAGGGATACCGTTTATCTCCCAAGGTCCCTGCTAGTGGACCATTAATATACAGTGAATCTTCCTTTGTCTTT 6840  
 GCCAGTAAACAAAGGCCATACTCCTTCGCCTTTCATTTGCACTATATCAGGATATGCTGATCAACAAGGCCGCATTCTTTTGGACTGTTATCATATATTAAATGTATGCGTATGCACTGC 6960  
 CACCTGCTCTGTGCACTTGAAAGGATCCCACTCACTTCCTTAGCACCTTCAGCAGGAAGTGATAATAAGCTCAAGACTTTCATTTGGAAAGTTCACATGTCTAAGCACTTCTCTAAGAAC 7080  
 TACTGTACCCTCTTCTCCGCTTTAAAGCAGAAAGAGGGTTGTACGAAGTGCTCTTCATTTGGACTTAAGTGCATTAATGCAGTTAGTTGTCCATCATTACCTTTGGAGTTGGATTTTACA 7200  
 TCCTTGTACTCTTTTGACACCAGAGGCATATTAATTATTTCTGAGCACTTCTCTTGTCAATATTAATCTGTACCCTTACACATATGACCTGTGCGGCAGCAAAGGTTCTGAAATGCCTAC 7320  
 CTTTTGACTGGGGCTGCTGAGTGGTAGTAACTATTAGTAACCTCAGCATTTGGATGATTACTATGCAAAAATGTCAAGGACCTGTGTGCTCTCTTTGCATACCATCAAGGCTACTGAGTC 7440  
 CCAGAATTAATTGCTAAGTTATGCGTATTTATAACTATGAATGTCTGGAATATTTTGTCCCCTTTACATTATTGCAGAGGTTGCTGAGCCCCCGAAACTACCCGGTACTGTCAATGAGCA 7560  
 CAGGGGCTCTGACGAATGACCTGCTCTCTTCCTTAAACTGATTTTGGGACTCTTAATAG

GCACAATG

GCACAATG  
Depth:6 (MOUSE)  
Ei-value:0.000, Pi-value:0.000  
Er-value:0.000, Pr-value:0.000  
No matches to TargetScan

GCAGTTCTGGATGGTTTATTTTCTACTCCAACTTGAGCAAATCCCCTGCTAGT 7680  
 TTCCCAATGATATAATAAAGTACAGCAGTATGTACACCCAACAATGACCCGGATTTCGACCCTTTTTGCATTGCTTTAATATATACAATCCTAAATAGTCACAATCTCACACTTTATAGT 7800  
 GTTCCTTTTGCCCGGCCTCTAGTTTGTCCATTGACCACTTTTCTGAATCACTAATTCTCACAAACCCATCATTAAGGAAGAGTTTGTGCCCTTTCTCAATTCCATCATGCCATCCCTTTT 7920  
 GCCTCTTTGTTTGAACAGTATTGACTGGGCAAAGCCCTTCTCTTGACTTAAAGTCAACAACACCAGTTTACTCACTTCATATGGCTACAGTGTCTCAGTTGCCTTCTCCTTG

CTCCCA

CTCCCA  
Depth:6 (MOUSE)  
Ei-value:0.000, Pi-value:0.000  
Er-value:0.000, Pr-value:0.000  
No matches to TargetScan

CT 8040  
 GAACAGAGACACCTCGAATTCTTACATTATTCTGGGTAATGTTAATTACCCCAAACACCCTATGTGTCATTAATAAATTTTGGTGTATTTATACACTGAATAGC

AAAAGCAG

AAAAGCAG  
Depth:6 (MOUSE)  
Ei-value:0.000, Pi-value:0.000  
Er-value:0.000, Pr-value:0.000  
No matches to TargetScan

GCCAAAAC 8160  
 TAGGTGGATGAGCCTTCAATCTTTAACTTGCACTTCTAAATTATTCCAATTCCAACTGCTGGCACATTCTAGGGCCAGGAACCATTCTTGCCTACCTTTATTAATGCTTTATTGT

GCAAA

GCAAAAT  
Depth:6 (MOUSE)  
Ei-value:0.000, Pi-value:0.000  
Er-value:0.000, Pr-value:0.000  
No matches to TargetScan

 8280  


AT

GCAAAAT  
Depth:6 (MOUSE)  
Ei-value:0.000, Pi-value:0.000  
Er-value:0.000, Pr-value:0.000  
No matches to TargetScan

ATTGCAGGCAAGTAGCTCAGGGAGTTGGATTGCCACCTTTTACTTGGGGCTTTCCTTTACAGTATGAACTGAAAATTGTCTTCCTGAGAAGGAAGCTTAGCACTTTTCTTTCCGTTCT 8400  
 TCCTCCAGGAAGGAGCCAACTGTCTGCTTAAGAAACTTTAAGCCCGATTTTGTATATTGCTACTGTACAGGACCAACTGCCAGAAAAGTTATTGATAATTTTATTCCTTAAGAAAGGCAT 8520  
 TTGGATTGCAAGGTGGATTGACTGTGAGATCATTAGCTTTTGTGAAGTAAAAATAGCCATTTGTGTCATGTTTCTGAAGACTAAGCAGTGTCTCAGTGTACTGAGGGTGATGAGTCTGTG 8640  
 GAAAGATCAGTGCAACTATTGCAGAATGTTTAAGACAAGTATCTTTGCTTGGTCTTTACTACAAGTTTAACAAAACGAAAAAGTCAATCTTTGTGTGGCCTTTAGTATGATTAACTTTTT 8760  
 GGAAGATGACCTAAGCCTTCTAATCATTATATTTTGTCTGACATTGGTCACCAGTCCTTGCTTATTTTTAAAAGGTGACTGGATGGATTAAATTTGAGAACATGTCAAGTCGCCTTTGAA 8880  
 AATTATATAGGCCATCACATTTAATTAATTCATTCTATCCACCATTAAACTCTGGCAATAATTTGAAGTAGCTTGAAAATTCCTAAAGTGGGAATTTATTTTAGAGATGATAGAACCTGT 9000  
 TTCCCCACTTTACATTTTAAAATATGTCTGCCAGGATCTAATCATTCCTTTAAACGTACACTTCAAAGAGAGATTTTCCTAGTAAGAAAAGAGCTTTCTCTAGTGTGAAGGGTGCTTTGT 9120  
 AGCCGCCGAGTACTTAGGTCTTTTTTGGGAGCTATTGTGTATGAGTGTATGTATGTGTGTGTGTACATGCATGTTGCTGCGCGCAGTCATTCATTCACATGGTGCTCAGACAACAATGGG 9240  
 AGCTGGTTCGTCTATCTTGTGGGTCCTGGAGATCAAAGTGAGATCATCAGGCTTGGCAGCAAGTGCCTTTACCCTCCGCGTGCCATCTTGCCATCCCGCTGCTGAGTGTTTGATATGACA 9360  
 TTGCTGATGAAAATAATCATCACAACAGCAGTTCTCCCAGCATTACTGAGAAATGATACTATTTTTCTGAGGAGGATGTTCAAGTAACTCATCCAGTGCAGGATCCTGCTTGAACTACTG 9480  
 CTCCTCCGTTACATCAGACTCTGGCTGTTTAGACTACA||GGATGAATTTGGAGTCTGTTTTGTGCTCCTGCCTCAAGAAGAAG

GATTGCCTGG

GATTGCCTGG  
Depth:6 (MOUSE)  
Ei-value:0.000, Pi-value:0.000  
Er-value:0.000, Pr-value:0.000  
No matches to TargetScan

ATTTAGAGGAGTGAAGAGTGCTGGAG 9598  
 AGAGCCCAAAG||GGACAAACAATCCCTATGTGAGACTCAAGGACTGCCAGCAGCCTATACAGCTACATTACATCTCAGCAGAACTTCTCTTCAAGTCCTCGCTACTCTGAACAAAAAGC 9716  
 TTACAGGCCACATGGAGAAAA

AAAG||ATC

AAAGATC  
Depth:6 (MOUSE)  
Ei-value:0.000, Pi-value:0.000  
Er-value:0.000, Pr-value:0.000  
No matches to TargetScan

TCCCCCCAGAATTGTGGGCTTGCTGCTTTGCAGTGCTGGCGACCTA

TTCCCTTTGA

TTCCCTTTGA  
Depth:6 (MOUSE)  
Ei-value:0.000, Pi-value:0.000  
Er-value:0.000, Pr-value:0.000  
No matches to TargetScan

CGATCCC

TAGGTGGAGATGGGGCATGAGGATCCT

TAGGTGGAGATGGGGCATGAGGATCCTCCAGGGGAA  
Depth:6 (MOUSE)  
Ei-value:0.000, Pi-value:0.000  
Er-value:0.000, Pr-value:0.000  
MATCHES To TargetScan▶ miR-331-3p:CCCCUGG

 9834  


CCAGGGGAA

TAGGTGGAGATGGGGCATGAGGATCCTCCAGGGGAA  
Depth:6 (MOUSE)  
Ei-value:0.000, Pi-value:0.000  
Er-value:0.000, Pr-value:0.000  
MATCHES To TargetScan▶ miR-331-3p:CCCCUGG

TAGCTCACCACCACTGG

GCAACA

GCAACA  
Depth:6 (MOUSE)  
Ei-value:0.000, Pi-value:0.000  
Er-value:0.000, Pr-value:0.000  
No matches to TargetScan

GGCCTAGCCCAGATTTCAGTGAGACGCTTTCCTGAACCCAGCAAGGAAGACAAAGGCTCAAAGAATGCCACCCTACATCAAAGTAG|| 9952  
 GAGAAAAGCTGCTGCAATAGTGGCACTGACCTTCGAGGAAGCCATTCTGCTCTATTTGGTTCTCTCTCCAGAAGCTAGGAAAGCTTTGCCAGCTGTTTACATACTTCAAGATGCACTGCT 10072  
 ACCCTACTCATGCCATATAATACACAA||TGCCATCTA

CCAAAT

CCAAAT  
Depth:6 (MOUSE)  
Ei-value:0.000, Pi-value:0.000  
Er-value:0.000, Pr-value:0.000  
No matches to TargetScan

ATTACCCTTCCCCAAAGCAGCACAGAAAACTGGGTCTTCAGCGTGATCAAGCAATGTGAACACACAAAAGGAAGGC 10190  
 AGCTTTATAAATGACCCGAG

GATCAACATGC

GATCAACATGC  
Depth:6 (MOUSE)  
Ei-value:0.000, Pi-value:0.000  
Er-value:0.000, Pr-value:0.000  
No matches to TargetScan

CTGACTGCAGCATCTTAAAAGCAATAGAATGAG||

TGTGTAT

TGTGTAT  
Depth:6 (MOUSE)  
Ei-value:0.000, Pi-value:0.000  
Er-value:0.000, Pr-value:0.000  
No matches to TargetScan

TGTGGGTGTGTCTATTTCTTGTTTTATGTATCTATTTTTTCCTTGGT 10308  
 CTGTGTGTCTAATTCTTTGTTACATCTATTTCTTCCTTGCTTTGTGTGTCTATTTCTTCCTTGCTTTGTGTGTCTATTTCTTCCTTGCATTATGTCTAATTCTTTGTTATATCTATTTCT 10428  
 TCCTTGCTTTGTGTCTATTTCTTCCTTGCAGTTGTGTCTAATTCTTTGTTACATCTATTTCTTCCTTGCTTTGTGTGTCTATTTCTTCCTTGCATTGTGTCTAATTCTTTGTTATATCTA 10548  
 TTTCTTCCTTGCTTTGTGTGTCTGTCTTCCTTGCTTTGTGTCTATTTCTTCCTTGCAGTTGTGTCTAATTCTTTGTTACATCTATTTCTTCCTTGCTTTTGTGTGTCTTTCTTTCTTGCT 10668  
 TTTGTGTGTCTATTTCTTCCTTGCAGTTGTGTCTAATTCTTTGTTACATCTATTTCTTCCTTGCTTTTGTGTGTCTATTTCTTCCTTGCATTGTGTCTAATTCTTTGGTATATATATTTC 10788  
 TTCATTGCTTTGTGTGTCTATGTCTCCTTGTGTTGTCTAATTCGTTGTTGCATCTATTTCTTCCTTGCTTTGTGTGTCTATTTCTTCCTTGCTTTGTGTGTCTATGTCTTCCTTGCTTTG 10908  
 TGTGTCTATGTCTTCCTTGTTT

TGTGTAT

TGTGTAT  
Depth:6 (MOUSE)  
Ei-value:0.000, Pi-value:0.000  
Er-value:0.000, Pr-value:0.000  
No matches to TargetScan

CTACTTCTTCCTTGTGTGTCTAATTCTTTGTTACATCTATTTCTTCCTTCCTTTGCATGTCTCCTTCTTTCCTTTGTGTGTCTTTTCTGTC 11028  
 TGCAGTGTGTCTTACCTATTCCCATGTTTCTCCTGCATGTTCTTTCTTGCAGAGCTTTGAGCTTTGTTTCACTTTCTCTGGTGCCTGTGTGGTCTGCTTTGTCTTCACTAGCTATGGCTC 11148  
 TCTGTTTTATCTATCTGGTTGCTATTTCTCTTAGCTTTTCTTTCACTCCTGCCTTTCGTGACTCCCCTTTGGGTCACATGTTGCATGCATCCCTCTCTTTTTCTTGTGCTCACCCCACTT 11268  
 GTTCTTTGTTCAAG

TTCTCTTTG

TTCTCTTTG  
Depth:6 (MOUSE)  
Ei-value:0.000, Pi-value:0.000  
Er-value:0.000, Pr-value:0.000  
No matches to TargetScan

TCAGTCCATTTCAGTTTTCTTTCTGCTGCTTCTATCCTTAGTGAATTCTTGTTTACATTTCTTCCCTGCCTTTCTTGGGCCACTTTCTCTGTTTTCT 11388  
 TTTGTATTTGTGTCTCTTTGCTATTGGTGGATTTCTTATCTCAGCATCATTCTGTTGCTTTGTGTTTGCTTGTGTTTCTATCTTCTACTTTCCTCCTTTCTGTTCACTTTGAGCATTTCA 11508  
 TCTCTTTACAAGTCTGTGTCTCTCTTGTATTCTAAAGTAATCCTTTCTTGGATGTTTCTTTGTATGTACATGTGCGTGTGTGCATGTGTGTTATGTGTGTCATGTGTGAGAGGAGCTTCA 11628  
 TAGCCCCTTCCCAATAGGTCCAGAATGTCACCCGTGGAGCCGTTCCTCACACCAGACTGCCCTGAGAAATAATCTAAGACAAAATACATCATTCCGTCCGGTCAGGATTCAAGTGGCTCT 11748  
 GAAGTGAACGCCCAAGTAGAAGACAGAAGTTTTGCGACTTGAGATTTAAAAGGACCAAAATACACAGATGGCCCGTCTTGAGCTGGCTGGACAGAATGCTGACAACCCAAAGAAGAGGAA 11868  
 CTG

TTTCTAC

TTTCTAC  
Depth:6 (MOUSE)  
Ei-value:0.000, Pi-value:0.000  
Er-value:0.000, Pr-value:0.000  
No matches to TargetScan

AGGACACCTGTGACTTCCAAGAGCGGGGAACTACGTATGTCATAAGACACAAAACCTGAGCTAAGTCCAAGCATAAGACCTAAGGACCCAATCCTATATGGACAGAATAT 11988  
 TTAAGAGATAAAGGCCTATGGCCCAGAACTCTGGAAGGATATTTCTATCCTTCTATCCCCAAGACCAAGAAGGGAAATTCGAAGATGAGACCTGCCCCCCAACCCCAGCATCCCTTTCCA 12108  
 TTTCTTATATTTCTATTTAAGCTGTCTTCACTTGAGATGTAATTTTTCATTGTTGCCATTGCCCATAAAGGAATACGTTTTTAGCTGGATAGTATTGTGCAAGGGTCTGTTTTAAACTGG 12228  
 GTCTTAGCCATTTGTTAAATTGTTGATGTTTTACAACTTCC

ATTTCTC

ATTTCTC  
Depth:6 (MOUSE)  
Ei-value:0.000, Pi-value:0.000  
Er-value:0.000, Pr-value:0.000  
No matches to TargetScan

TTCACATCTGCTCCACTTGAGACGGAACTAAATCCAGCCAGTGTATATAGCCTGACTATTGAAACTTCCCTA 12348  
 GGAATAAGCATGCATACAGATATGCATACTGCCATCCTCCCTACCTCAGAAGCCCTAGGCTGACAAGAAAAGGAAAGCATCAGGTTGTTAGGGGGAAAACAATGTCAGGCTA

TCTAGAGA

TCTAGAGAAAA  
Depth:6 (MOUSE)  
Ei-value:0.000, Pi-value:0.000  
Er-value:0.000, Pr-value:0.000  
MATCHES To TargetScan▶ miR-1251-5p:CUCUAGC

 12468  


AAA

TCTAGAGAAAA  
Depth:6 (MOUSE)  
Ei-value:0.000, Pi-value:0.000  
Er-value:0.000, Pr-value:0.000  
MATCHES To TargetScan▶ miR-1251-5p:CUCUAGC

TATAAAGAGTTGTTCCAGACCAA

TGAGAAGAATTAGACA

TGAGAAGAATTAGACA  
Depth:6 (MOUSE)  
Ei-value:0.000, Pi-value:0.000  
Er-value:0.000, Pr-value:0.000  
No matches to TargetScan

AGCAATATGCAGATGTGCCAACCCTCTGAGAAGCACCAGCCAGTGTCACCTTCTTTCTTTGGGCTTAGGTGAGCAGGG 12588  
 TATGGTTTTCTAATAATGGTTTGGGGACAAAATGAGGTCTGAACTCCCTGCTCATAGTAGTGGCCGAGTAATTTGGTGCATTTCACCAAAGGAACTCCTGGGTCTAATACCTACCTTTAA 12708  
 AATTAATGATGAGAGACTCTAAGGACTACTTAACGGGCTTAATCTTTTTCGTGCCTTCCTCTTCCTCTGTAAGAGGGAAGTTAAATGACACAGGATGAAAAAGTAACATGCTCATAGCAC 12828  


ATTGGCA

ATTGGCA  
Depth:6 (MOUSE)  
Ei-value:0.000, Pi-value:0.000  
Er-value:0.000, Pr-value:0.000  
No matches to TargetScan

ATTATACATGGTTATTATCTGAAAGTGTAGAGCTTTTCCTATAAGGCATCAGACTAAGTACCTGAAGCTTTGTGGGTTCATGGTCTTAGTTGCATATTCCTTAGTTGCAAATC 12948  
 CTTTTCAAAAGGTAAGAAAAAGGCACACTGGTCTATTGCCTGTACTTGATCAAGCCCTGATATGAATGCCAGGGAATGTCTGAGTAACATTAATTTCCTTCCCTGCATATTTTTTGTGCT 13068  
 GAATACTAAGGCTGTGATGCTTCACTGTGGTCACCCCCAGGTAACAAGATATTACCAGGTAACCAGGAAACGTATGAATACGTAAACCATGAAGCCTACTGTAACTTCCAAGTCAGTGCT 13188  
 GAGTATGTATTACATAGTAGCTGAAGTCTACGCCTCTGTGTGCTATAGGCACAAAGATTGCTCTAGGAATAACATGCTTTGTAAAAACAAATATATGAACATAACGGGGCTTGAATGAAT 13308  
 AACAGTCCATATACTTAAGGCCAGTGTGTTTCTTCTGCTTTGGTGAGGCTCAGTAAGTTATATTATACCAGGTAGCAGAAGAGAAAACACATGGAAACTGATTTTAAACTACAAACTAGG 13428  
 TCACTAATGCAGGTGATTGATTACCCTATTCTGATCACCTTCTAATTTCTGAATACCCATGTTCAGCACTGGGAATAACAAAGGGGGACATTACCACAGAACTAGAATTTACAAAAGAAT 13548  
 GCATTAAATAAAGCATTATACAGCTATCAATTGTTCCATGTGTGCAAATGAATGACTACTAACTACCTCTGATGTATCCGATATTGTTTTGGGTACATGAAATATTCATGAGTAACTGCC 13668  
 ATGAAATAAGAATGTTTGCATTCCATACTATTCATAAGGAATGAGCCAATGCTTAATTTAATCAGTCAAAACTTGAGTGATAAGGGCATGTTAATACAAGAACATTTGCCCAGGTCACAT 13788  
 TATGGTTGTGGGTACTTTCTTAACTATAAAGCAGTTCAGTAGTATAAGACAAGACAAATTTTCTATAGAAATAAAGCTGCCTATAAAATAGGCATAGTCTCTACAAAATTTTCATTGTAC 13908  
 TTTTTAGCCCATAATGGGAAGAGTACAGTTAACAAGCTGGGTGTGGTAGCATGTGCTCTGAGCTGAAGCAACAGGACCACTTGAGCCCAGAAATTGGAGGCTAGCCTGGGAAGACCATAA 14028  
 GGTCAATCTCAAACCTGGAGGCTAAATATTGTCTCCCATGTGTATATTCTCTTTCATGGGTACTGGAGAGATACACAGACGTACATTTCAGTGTGTCCACACTTGAGAATAATATGTACG 14148  
 TTGGCATTTTATGAACTCGGAGGTACCATATAAATGTAACAATTCATTTTCTTACTTGGTATCAATTTCCAGGCTTTTAAAATTCTGCCACATTTATTATACTGTGAAAATAAAGTAAAT 14268  
 AAGTAACTGTGAACCACTGAATATATGAAGCATTCAATACTTGATGAGTACATACTGAATGGCAGTCATTTATTACAAAACAGTGCCCTTGCTAGGCACTGGGATGCAAAGAGCATTCTC 14388  
 ATTGTCCTGTGTATCTAAAGAAATTATGCATGAGATTAATTTATAATTTGTAAACTGCCATATATATGTGTATATATGCAATATTTGCCTGGTGTGCAATGACTTTGCTTTTATCCCAGG 14508  
 CATGCACAACAGATCTGTGTGGAGCT

TTGTGAAG

TTGTGAAG  
Depth:6 (MOUSE)  
Ei-value:0.000, Pi-value:0.000  
Er-value:0.000, Pr-value:0.000  
No matches to TargetScan

TCTACAGTTCTATAAAGCCGGGACCTAACTGTTGGCTTTATCAGTGAACAGTGATTACTTTCTAAGTTTCATAATGGCTGAAACTT 14628  
 AATCATAATGCTTATCACCTAACACCACCTAATAATAATTTTACCATGCTATGTGTTGAGCGAACACATAGATTGCTTTCTAGCATTATGTAGCACTTATAGGAGTGAAATCTAGACCAA 14748  
 AACTTCAATTCACTTCAATGAGGAAATGAAAACAGAAAAAAAAAATGGATTTGTGCAAGGCAGTGTGCTAAATGTTACACTGAGTGGACTATGCTGTCTAGGATACTTCCCAGCTGGCTT 14868  
 GACTGAGGAGGTGG

AAAAGGT

AAAAGGT  
Depth:6 (MOUSE)  
Ei-value:0.000, Pi-value:0.000  
Er-value:0.000, Pr-value:0.000  
No matches to TargetScan

TTTATTAATGACAGGAACTTTTTCCATCCAGTTTCTTAAATGTTTGTTGAATGCTGCTGCCAGAGATGAATTACAAACACCTTGCCAGTAAAGGAGTTT 14988  
 TATAGGGCCAGAGTGAGATAATCCCAGAGCATGGGTATCAGGGAACAAAACGGGAAGAGGCCAGAGCATCTGATGGCATGTACTCAGTGTGGCCCAGAACCTCTCGAACTAGATGTACTG 15108  
 GCTGGAGGGACCAAGCATGCAGAACACAACACCTAATGAAACATTGTATATAAAATATGCTAACCTAGGTCCTAAAACTAAAATGTGAGGTGGACCTAGTGTAGATCACTGATCATAGGA 15228  
 GACATGGTCTCATAAAGCCCAGGCTGGTTCTAATTGGTGACTGTCACAGCTTCTCAAGTGCTGAGATTACAGATGTGCTTAACCCATGCCCAGCCTGAAGAATATATCTGATTACTGAGT 15348  
 GAATAATATTTTTAAAGAATTATATATTTTATGTATATGAGTACGCTGTTGCTGTCTTCAGACACACCAGAAGAGGGCACCACATCACATTACAGATGGTTGTGAGCCCCCATGTGGTTG 15468  
 TTGGGATTTGAACTCAGGACCTTCGGAAGAGCAGTCAGACTCTTAACCACTGAGTCATCTCTCCAGCCTTCTGAGTAAATATTTTAACTATAATGGCTGTTTGCGAAACCCAACCAAGGC 15588  
 CAAGATTCCTTCAACATAAACTGGAGACTTCCTAGCTAAGGAAGCTCCAAAAGTCATTTTCTCATTGGCCTAGCTTGAAGCCAGGACAGACTTAAAGTCTGTCCTTTAATTCATTACCCA 15708  
 TTTTCCTTTTCTTACTGTTGAAGTGTTTCAAAGGAGAATCAAGATGAATCGATAATTCTAAACGTATTTGTTCATTGCCTGGCTCAGCGTCATGTGAGCAAGAAGAATATACTATCACAC 15828  
 TCATACTTTTAACTTAAGTGTGATGAAAGTGCAGTTCTAAGTACTAAAATTTCTAAGTACTGAAAAGAACAAAGACATTTAAAGGATGCAACCCAAAGTGTACTTTACCTCAGTAGTTTC 15948  
 TGAGGGGACTGCAGTCACACCTTGAGACTACAGCTCTCACTTTAGCTGGGAAAAACATCAAGGTGTAGAGGAGGCAAGTTAAATAAAAAGTTGCTCCCCTCCTCATGGGCATGCTTGGTA 16068  
 GAGTGGAAATAATAAAAGAGGTTCTCTATTTCCTCGGTTCCACACATTGCAGAAGATGCTACTGGATGCTAAGTGCAACACATTTGTTCCAAAAGGGCACTCAGTGTGACTTACAGATGC 16188  
 CCCGGAAAGCAGAGGGATGCTCTTTATTAAACAGAAATATTAGCTCAAACGTTTTCTAGACTGAAGAACACTTTCCTCATTTCCCACAGTTTGCCTCAGAGGTTGAATACAGGAAGGTTA 16308  
 TTATTCATTCATTTGCTTTATTGGTTCGCCTGTTCTACAAGGATTTGCATGTCTCTTAGGCCTTCACTTGGCTCCTGAGACATGGAAAAAGGAAACATAGACATAGGGAAGTGCTGGATG 16428  
 GGGGGGGGGGGTCTCTTTTCTGGGTAGTGGCACGACTTAGTCCTTAGTCCCCAAGTAATATGCAATGTGAGTCCTCATCCTCATGTCTTCTCCGGCCACTGCAATGAGTGGGAAGCTGGG 16548  
 CTTTGTAGCAAGCCTGACCCTAAAGTTACAGAAGCCCTCCACGCTAAGAAACTCAATTTTCTAGGCCATTTTAGCTATGACTGTGACCACTACTGGTCAGGAGGGATGACAGCCATCTAA 16668  
 GTTCCACAATCTTAGGCTACTTTGCATTATCCTGGGGCAAACAAGCCATTTTTGAGCTGCAGCAGGCTTTGAAATACATTGACCAATTTTGCCTGTGTTCGTTAAACCTTTTACCTTTTT 16788  
 ACATGCTAATGCTCACAGTAATTTAGAAATGTTCTCCTTACTATAATATACTCAAGGTGGCTTGCTATGGTAAAATAATGCCAGTGGATGAAAATAACATTAATGTTTAACATTCTTGCA 16908  
 TAAAATTTAAGAATAATAAAATTGACAACAATCAGAAAACTGGAGGAACGAAAGACCAAATTGAAAGAACTTGAAAAAGATTAAAAATGCCTGTGCTTTGACCCTTTCCATTTTTCTTTC 17028  
 ACTCACAGAGGGTGGGACAGGAGGCCGAGTGAAGGAAAGGGTCCAGCCTGTCTATCTGGAATCTAAGTTGGGACTTTAATGCAGTTCCACAAAATTGGTATTAATTCGCTAAATGTTTCT 17148  
 GAAAATGTATTTTCATCTAAATGGCTATCAGCTAAGCCTTGAGTCAAATGGGAATGAAACAGATTAAGTCAATGTGATCTCTTTATCCAAGTTGCCTTAGAGCTGAAGTCACAATTTGCT 17268  
 GTAAGGAAGCTTATTCATTGTAGCATACGCATACTTTCAAAGTATCTAGACTTTACTTAGTAACCCAATCAGGACATTCAGGCAAAAGAAAAGGAACAGAGAAGATGGAGCCAGGTTGAA 17388  
 GAGGTCTGGGAGTTCAAACAAATTTTTTTCATTTTCATTAAAACTCAATTGGGCATCAAAAGTGTTACTAATATTAGCTTTTAATTAGTGGAAATTGGCTGGATTCAGTAATATCCCTTT 17508  
 GTATGGGTAGGAATGGGCTTACATTTCTGGAATTTGCAAAGGAAAAAATAACTGAAAGCCTTCCTTTCACAGTTACTGCCATCAATATTGCTACCAATTAAGCACATCCTACCATCATCT 17628  
 GCTTTGATCACATAAATGAACTGTGTACCAATCTGTTGTTGAAAGACTGGAGTCATCTTCCCACCAACTGTGAAAAAACACATGGAAAACACCTGGACTTTGTGAACGGATGCGGAATAC 17748  
 AGAACTTCTGTTGACTCTTGGGTGTTTTGAAGACTTGAAAAAAAAAACTGTTGCTTACCAACATGTCACAATGAGTCCGTGTGTGGGTGGGTGGATGGGTGGGTGGGTGGGTGGGTGGGT 17868  
 GGTTGAGTGGGTGGGGTAGTTTGCTGTTAAATAAAATGCTTTGTTTTGAA                                                                       17918
```

|  |  |  |  |  |
| --- | --- | --- | --- | --- |
| | | | | | | | | | |
| 2 |  | 4 |  | 6 |
| Depth of motif conservation (number of species) | | | | |

  
  

---
