# Supplementary material for: Uncovering deeply conserved motif combinations in rapidly evolving noncoding sequences
Source: Genome Biol. 2021 Jan 11;22:29. doi: 10.1186/s13059-020-02247-1 (PMC7798263; doi:10.1186/s13059-020-02247-1)
Supplement: Supplementary file 4 — Additional file 4. LncLOOM output results for XIST sequences from six mammals. [file 13059_2020_2247_MOESM4_ESM.gz › AdditionalFile4/Html_Files/eCLIP_results_BLAT.html]

 eCLIP Matches (BLAT)

# eCLIP Annotation Results (BLAT)

  
  

| Match 1 in HUMAN | | | | | | | |
| --- | --- | --- | --- | --- | --- | --- | --- |
| Motif | Start in Seq (1 Indexed) | End in Seq (1 Indexed) | Strand | Chrm | Exon | Start in Chrm (0 Indexed) | End in Chrm (1 Indexed) |
| TGGAAGCTT | 62 | 70 | - | chrX | 1 | 73072518 | 73072527 |
| eCLIP Fold-Enrichment | Binding Protein | Cell Line | Strand | Chrm | | Start in Chrm (0 Indexed) | End in Chrm (1 Indexed) |
| 2.98904153675756 | DGCR8 (bg=1.84%) | K562 | - | chrX | | 73072486 | 73072537 |
| 3.24734018396388 | HNRNPC (bg=3.65%) | K562 | - | chrX | | 73072505 | 73072537 |
| 2.69045973641287 | LSM11 (bg=2.28%) | K562 | - | chrX | | 73072490 | 73072541 |
| 3.45022359909514 | NCBP2 (bg=1.49%) | K562 | - | chrX | | 73072499 | 73072546 |
| 5.46412526783477 | RBM15 (bg=7.27%) | K562 | - | chrX | | 73072507 | 73072530 |
| 5.06195280312894 | RBM15 (bg=7.27%) | K562 | - | chrX | | 73072514 | 73072525 |
| 2.30684326561529 | RBM22 (bg=4.62%) | K562 | - | chrX | | 73072500 | 73072533 |
| 2.51050054466155 | RBM22 (bg=4.62%) | K562 | - | chrX | | 73072508 | 73072540 |
| 2.43601420622152 | SLTM (bg=2.2%) | K562 | - | chrX | | 73072493 | 73072543 |
| 2.77024707924896 | SLTM (bg=2.2%) | K562 | - | chrX | | 73072497 | 73072542 |
| 4.17519012233491 | SRSF1 (bg=8.47%) | K562 | - | chrX | | 73072482 | 73072541 |
| 4.09450650673659 | SRSF1 (bg=8.47%) | K562 | - | chrX | | 73072488 | 73072540 |
| 3.98616179735591 | uchl5 (bg=11.16%) | K562 | - | chrX | | 73072494 | 73072534 |
| 3.50602603069062 | uchl5 (bg=11.16%) | K562 | - | chrX | | 73072499 | 73072521 |
| 2.19343607734182 | YWHAG (bg=1.87%) | K562 | - | chrX | | 73072493 | 73072543 |

  
  

| Match 2 in HUMAN | | | | | | | |
| --- | --- | --- | --- | --- | --- | --- | --- |
| Motif | Start in Seq (1 Indexed) | End in Seq (1 Indexed) | Strand | Chrm | Exon | Start in Chrm (0 Indexed) | End in Chrm (1 Indexed) |
| GATCTCT | 81 | 87 | - | chrX | 1 | 73072501 | 73072508 |
| eCLIP Fold-Enrichment | Binding Protein | Cell Line | Strand | Chrm | | Start in Chrm (0 Indexed) | End in Chrm (1 Indexed) |
| 2.98904153675756 | DGCR8 (bg=1.84%) | K562 | - | chrX | | 73072486 | 73072537 |
| 3.24734018396388 | HNRNPC (bg=3.65%) | K562 | - | chrX | | 73072505 | 73072537 |
| 2.69045973641287 | LSM11 (bg=2.28%) | K562 | - | chrX | | 73072490 | 73072541 |
| 3.45022359909514 | NCBP2 (bg=1.49%) | K562 | - | chrX | | 73072499 | 73072546 |
| 5.46412526783477 | RBM15 (bg=7.27%) | K562 | - | chrX | | 73072507 | 73072530 |
| 2.30684326561529 | RBM22 (bg=4.62%) | K562 | - | chrX | | 73072500 | 73072533 |
| 2.51050054466155 | RBM22 (bg=4.62%) | K562 | - | chrX | | 73072508 | 73072540 |
| 2.43601420622152 | SLTM (bg=2.2%) | K562 | - | chrX | | 73072493 | 73072543 |
| 2.77024707924896 | SLTM (bg=2.2%) | K562 | - | chrX | | 73072497 | 73072542 |
| 4.17519012233491 | SRSF1 (bg=8.47%) | K562 | - | chrX | | 73072482 | 73072541 |
| 4.09450650673659 | SRSF1 (bg=8.47%) | K562 | - | chrX | | 73072488 | 73072540 |
| 3.98616179735591 | uchl5 (bg=11.16%) | K562 | - | chrX | | 73072494 | 73072534 |
| 3.50602603069062 | uchl5 (bg=11.16%) | K562 | - | chrX | | 73072499 | 73072521 |
| 2.19343607734182 | YWHAG (bg=1.87%) | K562 | - | chrX | | 73072493 | 73072543 |

  
  

| Match 3 in HUMAN | | | | | | | |
| --- | --- | --- | --- | --- | --- | --- | --- |
| Motif | Start in Seq (1 Indexed) | End in Seq (1 Indexed) | Strand | Chrm | Exon | Start in Chrm (0 Indexed) | End in Chrm (1 Indexed) |
| GGTTCTTTCT | 98 | 107 | - | chrX | 1 | 73072481 | 73072491 |
| eCLIP Fold-Enrichment | Binding Protein | Cell Line | Strand | Chrm | | Start in Chrm (0 Indexed) | End in Chrm (1 Indexed) |
| 2.98904153675756 | DGCR8 (bg=1.84%) | K562 | - | chrX | | 73072486 | 73072537 |
| 2.69045973641287 | LSM11 (bg=2.28%) | K562 | - | chrX | | 73072490 | 73072541 |
| 4.17519012233491 | SRSF1 (bg=8.47%) | K562 | - | chrX | | 73072482 | 73072541 |
| 4.09450650673659 | SRSF1 (bg=8.47%) | K562 | - | chrX | | 73072488 | 73072540 |

  
  

| Match 4 in HUMAN | | | | | | | |
| --- | --- | --- | --- | --- | --- | --- | --- |
| Motif | Start in Seq (1 Indexed) | End in Seq (1 Indexed) | Strand | Chrm | Exon | Start in Chrm (0 Indexed) | End in Chrm (1 Indexed) |
| GAACATTTTC | 109 | 118 | - | chrX | 1 | 73072470 | 73072480 |
| eCLIP Fold-Enrichment | Binding Protein | Cell Line | Strand | Chrm | | Start in Chrm (0 Indexed) | End in Chrm (1 Indexed) |
| 2.00116057587698 | LSM11 (bg=2.28%) | K562 | - | chrX | | 73072428 | 73072473 |
| 5.02322743385718 | RBM15 (bg=7.27%) | K562 | - | chrX | | 73072431 | 73072473 |
| 5.15482813931232 | RBM15 (bg=7.27%) | K562 | - | chrX | | 73072455 | 73072473 |
| 2.64269515702642 | RBM22 (bg=4.62%) | K562 | - | chrX | | 73072431 | 73072471 |
| 2.19831880883712 | RBM22 (bg=4.62%) | K562 | - | chrX | | 73072438 | 73072472 |
| 3.85058092385051 | SRSF1 (bg=8.47%) | K562 | - | chrX | | 73072435 | 73072473 |
| 3.94190305989517 | SRSF1 (bg=8.47%) | K562 | - | chrX | | 73072456 | 73072474 |
| 3.90025507912279 | uchl5 (bg=11.16%) | K562 | - | chrX | | 73072462 | 73072474 |
| 3.63530904763559 | uchl5 (bg=11.16%) | K562 | - | chrX | | 73072463 | 73072473 |

  
  

| Match 5 in HUMAN | | | | | | | |
| --- | --- | --- | --- | --- | --- | --- | --- |
| Motif | Start in Seq (1 Indexed) | End in Seq (1 Indexed) | Strand | Chrm | Exon | Start in Chrm (0 Indexed) | End in Chrm (1 Indexed) |
| TTATGGC | 136 | 142 | - | chrX | 1 | 73072446 | 73072453 |
| eCLIP Fold-Enrichment | Binding Protein | Cell Line | Strand | Chrm | | Start in Chrm (0 Indexed) | End in Chrm (1 Indexed) |
| 3.1896246861076 | HNRNPC (bg=3.65%) | K562 | - | chrX | | 73072442 | 73072468 |
| 2.00116057587698 | LSM11 (bg=2.28%) | K562 | - | chrX | | 73072428 | 73072473 |
| 5.02322743385718 | RBM15 (bg=7.27%) | K562 | - | chrX | | 73072431 | 73072473 |
| 5.16175649970273 | RBM15 (bg=7.27%) | K562 | - | chrX | | 73072433 | 73072455 |
| 2.64269515702642 | RBM22 (bg=4.62%) | K562 | - | chrX | | 73072431 | 73072471 |
| 2.19831880883712 | RBM22 (bg=4.62%) | K562 | - | chrX | | 73072438 | 73072472 |
| 3.96912452863019 | SRSF1 (bg=8.47%) | K562 | - | chrX | | 73072435 | 73072456 |
| 3.85058092385051 | SRSF1 (bg=8.47%) | K562 | - | chrX | | 73072435 | 73072473 |
| 3.66567711933822 | uchl5 (bg=11.16%) | K562 | - | chrX | | 73072431 | 73072462 |
| 3.28908803944628 | uchl5 (bg=11.16%) | K562 | - | chrX | | 73072435 | 73072463 |

  
  

| Match 6 in HUMAN | | | | | | | |
| --- | --- | --- | --- | --- | --- | --- | --- |
| Motif | Start in Seq (1 Indexed) | End in Seq (1 Indexed) | Strand | Chrm | Exon | Start in Chrm (0 Indexed) | End in Chrm (1 Indexed) |
| TATTTCTTTAAAAAAA | 144 | 159 | - | chrX | 1 | 73072429 | 73072445 |
| eCLIP Fold-Enrichment | Binding Protein | Cell Line | Strand | Chrm | | Start in Chrm (0 Indexed) | End in Chrm (1 Indexed) |
| 3.1896246861076 | HNRNPC (bg=3.65%) | K562 | - | chrX | | 73072442 | 73072468 |
| 2.00116057587698 | LSM11 (bg=2.28%) | K562 | - | chrX | | 73072428 | 73072473 |
| 5.02322743385718 | RBM15 (bg=7.27%) | K562 | - | chrX | | 73072431 | 73072473 |
| 5.16175649970273 | RBM15 (bg=7.27%) | K562 | - | chrX | | 73072433 | 73072455 |
| 2.64269515702642 | RBM22 (bg=4.62%) | K562 | - | chrX | | 73072431 | 73072471 |
| 2.19831880883712 | RBM22 (bg=4.62%) | K562 | - | chrX | | 73072438 | 73072472 |
| 3.96912452863019 | SRSF1 (bg=8.47%) | K562 | - | chrX | | 73072435 | 73072456 |
| 3.85058092385051 | SRSF1 (bg=8.47%) | K562 | - | chrX | | 73072435 | 73072473 |
| 3.66567711933822 | uchl5 (bg=11.16%) | K562 | - | chrX | | 73072431 | 73072462 |
| 3.28908803944628 | uchl5 (bg=11.16%) | K562 | - | chrX | | 73072435 | 73072463 |

  
  

| Match 7 in HUMAN | | | | | | | |
| --- | --- | --- | --- | --- | --- | --- | --- |
| Motif | Start in Seq (1 Indexed) | End in Seq (1 Indexed) | Strand | Chrm | Exon | Start in Chrm (0 Indexed) | End in Chrm (1 Indexed) |
| ACTTTCTCCTA | 197 | 207 | - | chrX | 1 | 73072381 | 73072392 |
| eCLIP Fold-Enrichment | Binding Protein | Cell Line | Strand | Chrm | | Start in Chrm (0 Indexed) | End in Chrm (1 Indexed) |
| 3.02717666564433 | DGCR8 (bg=1.84%) | K562 | - | chrX | | 73072356 | 73072390 |
| 2.05873061925664 | HNRNPC (bg=3.65%) | K562 | - | chrX | | 73072348 | 73072394 |
| 2.01908248387425 | LSM11 (bg=2.28%) | K562 | - | chrX | | 73072363 | 73072381 |
| 2.69896932817222 | RBFOX2 (bg=4.63%) | K562 | - | chrX | | 73072359 | 73072393 |
| 4.7875136340229 | RBM15 (bg=7.27%) | K562 | - | chrX | | 73072358 | 73072383 |
| 2.71616711369974 | RBM22 (bg=4.62%) | K562 | - | chrX | | 73072358 | 73072402 |
| 2.09943194758566 | RBM22 (bg=4.62%) | K562 | - | chrX | | 73072365 | 73072398 |
| 3.09450650673659 | SRSF1 (bg=8.47%) | K562 | - | chrX | | 73072350 | 73072389 |
| 3.02021546235857 | SRSF1 (bg=8.47%) | K562 | - | chrX | | 73072353 | 73072387 |
| 4.22373174659586 | uchl5 (bg=11.16%) | K562 | - | chrX | | 73072377 | 73072391 |
| 4.09322700360778 | uchl5 (bg=11.16%) | K562 | - | chrX | | 73072379 | 73072391 |
| 2.71471034637028 | YWHAG (bg=1.87%) | K562 | - | chrX | | 73072361 | 73072390 |
| 2.16101459964944 | YWHAG (bg=1.87%) | K562 | - | chrX | | 73072363 | 73072387 |

  
  

| Match 8 in HUMAN | | | | | | | |
| --- | --- | --- | --- | --- | --- | --- | --- |
| Motif | Start in Seq (1 Indexed) | End in Seq (1 Indexed) | Strand | Chrm | Exon | Start in Chrm (0 Indexed) | End in Chrm (1 Indexed) |
| TTCTTGACAC | 213 | 222 | - | chrX | 1 | 73072366 | 73072376 |
| eCLIP Fold-Enrichment | Binding Protein | Cell Line | Strand | Chrm | | Start in Chrm (0 Indexed) | End in Chrm (1 Indexed) |
| 3.02717666564433 | DGCR8 (bg=1.84%) | K562 | - | chrX | | 73072356 | 73072390 |
| 2.05873061925664 | HNRNPC (bg=3.65%) | K562 | - | chrX | | 73072348 | 73072394 |
| 2.01908248387425 | LSM11 (bg=2.28%) | K562 | - | chrX | | 73072363 | 73072381 |
| 2.69896932817222 | RBFOX2 (bg=4.63%) | K562 | - | chrX | | 73072359 | 73072393 |
| 4.79031538957629 | RBM15 (bg=7.27%) | K562 | - | chrX | | 73072355 | 73072374 |
| 4.7875136340229 | RBM15 (bg=7.27%) | K562 | - | chrX | | 73072358 | 73072383 |
| 2.71616711369974 | RBM22 (bg=4.62%) | K562 | - | chrX | | 73072358 | 73072402 |
| 2.09943194758566 | RBM22 (bg=4.62%) | K562 | - | chrX | | 73072365 | 73072398 |
| 3.09450650673659 | SRSF1 (bg=8.47%) | K562 | - | chrX | | 73072350 | 73072389 |
| 3.02021546235857 | SRSF1 (bg=8.47%) | K562 | - | chrX | | 73072353 | 73072387 |
| 4.67913937914478 | uchl5 (bg=11.16%) | K562 | - | chrX | | 73072352 | 73072377 |
| 4.55803333415895 | uchl5 (bg=11.16%) | K562 | - | chrX | | 73072357 | 73072379 |
| 2.71471034637028 | YWHAG (bg=1.87%) | K562 | - | chrX | | 73072361 | 73072390 |
| 2.16101459964944 | YWHAG (bg=1.87%) | K562 | - | chrX | | 73072363 | 73072387 |

  
  

| Match 9 in HUMAN | | | | | | | |
| --- | --- | --- | --- | --- | --- | --- | --- |
| Motif | Start in Seq (1 Indexed) | End in Seq (1 Indexed) | Strand | Chrm | Exon | Start in Chrm (0 Indexed) | End in Chrm (1 Indexed) |
| TTTAAGG | 279 | 285 | - | chrX | 1 | 73072303 | 73072310 |
| eCLIP Fold-Enrichment | Binding Protein | Cell Line | Strand | Chrm | | Start in Chrm (0 Indexed) | End in Chrm (1 Indexed) |
| 2.86031706085464 | HNRNPC (bg=3.65%) | K562 | - | chrX | | 73072250 | 73072307 |
| 2.77495988222398 | HNRNPC (bg=3.65%) | K562 | - | chrX | | 73072251 | 73072306 |

  
  

| Match 10 in HUMAN | | | | | | | |
| --- | --- | --- | --- | --- | --- | --- | --- |
| Motif | Start in Seq (1 Indexed) | End in Seq (1 Indexed) | Strand | Chrm | Exon | Start in Chrm (0 Indexed) | End in Chrm (1 Indexed) |
| AATTTTTCTTTGGAAT | 286 | 301 | - | chrX | 1 | 73072287 | 73072303 |
| eCLIP Fold-Enrichment | Binding Protein | Cell Line | Strand | Chrm | | Start in Chrm (0 Indexed) | End in Chrm (1 Indexed) |
| 2.44468553054425 | DGCR8 (bg=1.84%) | K562 | - | chrX | | 73072253 | 73072289 |
| 2.86031706085464 | HNRNPC (bg=3.65%) | K562 | - | chrX | | 73072250 | 73072307 |
| 2.77495988222398 | HNRNPC (bg=3.65%) | K562 | - | chrX | | 73072251 | 73072306 |
| 3.41063664722927 | PUS1 (bg=1.04%) | K562 | - | chrX | | 73072259 | 73072298 |
| 4.63108447506481 | RBM15 (bg=7.27%) | K562 | - | chrX | | 73072267 | 73072297 |
| 4.46101768442345 | RBM15 (bg=7.27%) | K562 | - | chrX | | 73072284 | 73072294 |
| 3.62502122343538 | SRSF1 (bg=8.47%) | K562 | - | chrX | | 73072268 | 73072292 |
| 3.26579267111581 | SRSF1 (bg=8.47%) | K562 | - | chrX | | 73072269 | 73072288 |
| 2.78613394988336 | uchl5 (bg=11.16%) | K562 | - | chrX | | 73072286 | 73072292 |
| 2.14795110416348 | UTP18 (bg=0.72%) | K562 | - | chrX | | 73072233 | 73072299 |

  
  

| Match 11 in HUMAN | | | | | | | |
| --- | --- | --- | --- | --- | --- | --- | --- |
| Motif | Start in Seq (1 Indexed) | End in Seq (1 Indexed) | Strand | Chrm | Exon | Start in Chrm (0 Indexed) | End in Chrm (1 Indexed) |
| TTTTTGGTTGAC | 304 | 315 | - | chrX | 1 | 73072273 | 73072285 |
| eCLIP Fold-Enrichment | Binding Protein | Cell Line | Strand | Chrm | | Start in Chrm (0 Indexed) | End in Chrm (1 Indexed) |
| 3.20405442772841 | DGCR8 (bg=1.84%) | K562 | - | chrX | | 73072234 | 73072279 |
| 2.44468553054425 | DGCR8 (bg=1.84%) | K562 | - | chrX | | 73072253 | 73072289 |
| 2.86031706085464 | HNRNPC (bg=3.65%) | K562 | - | chrX | | 73072250 | 73072307 |
| 2.77495988222398 | HNRNPC (bg=3.65%) | K562 | - | chrX | | 73072251 | 73072306 |
| 2.72991632861751 | NIPBL (bg=5.39%) | K562 | - | chrX | | 73072199 | 73072286 |
| 2.61281760946063 | NIPBL (bg=5.39%) | K562 | - | chrX | | 73072204 | 73072280 |
| 3.41063664722927 | PUS1 (bg=1.04%) | K562 | - | chrX | | 73072259 | 73072298 |
| 4.81887526998382 | RBM15 (bg=7.27%) | K562 | - | chrX | | 73072267 | 73072284 |
| 4.63108447506481 | RBM15 (bg=7.27%) | K562 | - | chrX | | 73072267 | 73072297 |
| 4.46101768442345 | RBM15 (bg=7.27%) | K562 | - | chrX | | 73072284 | 73072294 |
| 3.37077152591512 | SDAD1 (bg=2.97%) | K562 | - | chrX | | 73072243 | 73072283 |
| 3.62502122343538 | SRSF1 (bg=8.47%) | K562 | - | chrX | | 73072268 | 73072292 |
| 3.26579267111581 | SRSF1 (bg=8.47%) | K562 | - | chrX | | 73072269 | 73072288 |
| 2.50118308170997 | uchl5 (bg=11.16%) | K562 | - | chrX | | 73072249 | 73072286 |
| 2.5267062835303 | uchl5 (bg=11.16%) | K562 | - | chrX | | 73072266 | 73072285 |
| 2.14795110416348 | UTP18 (bg=0.72%) | K562 | - | chrX | | 73072233 | 73072299 |

  
  

| Match 12 in HUMAN | | | | | | | |
| --- | --- | --- | --- | --- | --- | --- | --- |
| Motif | Start in Seq (1 Indexed) | End in Seq (1 Indexed) | Strand | Chrm | Exon | Start in Chrm (0 Indexed) | End in Chrm (1 Indexed) |
| GTTTTTT | 322 | 328 | - | chrX | 1 | 73072260 | 73072267 |
| eCLIP Fold-Enrichment | Binding Protein | Cell Line | Strand | Chrm | | Start in Chrm (0 Indexed) | End in Chrm (1 Indexed) |
| 3.20405442772841 | DGCR8 (bg=1.84%) | K562 | - | chrX | | 73072234 | 73072279 |
| 2.44468553054425 | DGCR8 (bg=1.84%) | K562 | - | chrX | | 73072253 | 73072289 |
| 2.86031706085464 | HNRNPC (bg=3.65%) | K562 | - | chrX | | 73072250 | 73072307 |
| 2.77495988222398 | HNRNPC (bg=3.65%) | K562 | - | chrX | | 73072251 | 73072306 |
| 2.72991632861751 | NIPBL (bg=5.39%) | K562 | - | chrX | | 73072199 | 73072286 |
| 2.61281760946063 | NIPBL (bg=5.39%) | K562 | - | chrX | | 73072204 | 73072280 |
| 3.41063664722927 | PUS1 (bg=1.04%) | K562 | - | chrX | | 73072259 | 73072298 |
| 4.79049749246172 | RBM15 (bg=7.27%) | K562 | - | chrX | | 73072245 | 73072267 |
| 5.03557296208144 | RBM15 (bg=7.27%) | K562 | - | chrX | | 73072246 | 73072267 |
| 4.81887526998382 | RBM15 (bg=7.27%) | K562 | - | chrX | | 73072267 | 73072284 |
| 4.63108447506481 | RBM15 (bg=7.27%) | K562 | - | chrX | | 73072267 | 73072297 |
| 3.37077152591512 | SDAD1 (bg=2.97%) | K562 | - | chrX | | 73072243 | 73072283 |
| 3.54958563711641 | SRSF1 (bg=8.47%) | K562 | - | chrX | | 73072225 | 73072269 |
| 3.84211934510231 | SRSF1 (bg=8.47%) | K562 | - | chrX | | 73072226 | 73072268 |
| 2.346959544723 | uchl5 (bg=11.16%) | K562 | - | chrX | | 73072244 | 73072266 |
| 2.50118308170997 | uchl5 (bg=11.16%) | K562 | - | chrX | | 73072249 | 73072286 |
| 2.5267062835303 | uchl5 (bg=11.16%) | K562 | - | chrX | | 73072266 | 73072285 |
| 2.14795110416348 | UTP18 (bg=0.72%) | K562 | - | chrX | | 73072233 | 73072299 |
| 2.78509967426168 | YWHAG (bg=1.87%) | K562 | - | chrX | | 73072238 | 73072270 |

  
  

| Match 13 in HUMAN | | | | | | | |
| --- | --- | --- | --- | --- | --- | --- | --- |
| Motif | Start in Seq (1 Indexed) | End in Seq (1 Indexed) | Strand | Chrm | Exon | Start in Chrm (0 Indexed) | End in Chrm (1 Indexed) |
| GTTTTTT | 337 | 343 | - | chrX | 1 | 73072245 | 73072252 |
| eCLIP Fold-Enrichment | Binding Protein | Cell Line | Strand | Chrm | | Start in Chrm (0 Indexed) | End in Chrm (1 Indexed) |
| 3.20405442772841 | DGCR8 (bg=1.84%) | K562 | - | chrX | | 73072234 | 73072279 |
| 2.44527956157579 | HNRNPC (bg=3.65%) | K562 | - | chrX | | 73072232 | 73072250 |
| 2.21308099461586 | HNRNPC (bg=3.65%) | K562 | - | chrX | | 73072232 | 73072251 |
| 2.86031706085464 | HNRNPC (bg=3.65%) | K562 | - | chrX | | 73072250 | 73072307 |
| 2.77495988222398 | HNRNPC (bg=3.65%) | K562 | - | chrX | | 73072251 | 73072306 |
| 2.72991632861751 | NIPBL (bg=5.39%) | K562 | - | chrX | | 73072199 | 73072286 |
| 2.61281760946063 | NIPBL (bg=5.39%) | K562 | - | chrX | | 73072204 | 73072280 |
| 4.42622310686476 | RBM15 (bg=7.27%) | K562 | - | chrX | | 73072223 | 73072245 |
| 4.56378465574848 | RBM15 (bg=7.27%) | K562 | - | chrX | | 73072223 | 73072246 |
| 4.79049749246172 | RBM15 (bg=7.27%) | K562 | - | chrX | | 73072245 | 73072267 |
| 5.03557296208144 | RBM15 (bg=7.27%) | K562 | - | chrX | | 73072246 | 73072267 |
| 3.38198580413887 | RBM22 (bg=4.62%) | K562 | - | chrX | | 73072226 | 73072256 |
| 3.37077152591512 | SDAD1 (bg=2.97%) | K562 | - | chrX | | 73072243 | 73072283 |
| 3.54958563711641 | SRSF1 (bg=8.47%) | K562 | - | chrX | | 73072225 | 73072269 |
| 3.84211934510231 | SRSF1 (bg=8.47%) | K562 | - | chrX | | 73072226 | 73072268 |
| 2.346959544723 | uchl5 (bg=11.16%) | K562 | - | chrX | | 73072244 | 73072266 |
| 2.50118308170997 | uchl5 (bg=11.16%) | K562 | - | chrX | | 73072249 | 73072286 |
| 2.14795110416348 | UTP18 (bg=0.72%) | K562 | - | chrX | | 73072233 | 73072299 |
| 2.78509967426168 | YWHAG (bg=1.87%) | K562 | - | chrX | | 73072238 | 73072270 |

  
  

| Match 14 in HUMAN | | | | | | | |
| --- | --- | --- | --- | --- | --- | --- | --- |
| Motif | Start in Seq (1 Indexed) | End in Seq (1 Indexed) | Strand | Chrm | Exon | Start in Chrm (0 Indexed) | End in Chrm (1 Indexed) |
| TGCCCATCGGGGCTG | 370 | 384 | - | chrX | 1 | 73072204 | 73072219 |
| eCLIP Fold-Enrichment | Binding Protein | Cell Line | Strand | Chrm | | Start in Chrm (0 Indexed) | End in Chrm (1 Indexed) |
| 2.6353918967895 | DDX51 (bg=1.63%) | K562 | - | chrX | | 73072188 | 73072226 |
| 2.61084050246287 | HNRNPC (bg=3.65%) | K562 | - | chrX | | 73072183 | 73072232 |
| 2.54645839594316 | HNRNPC (bg=3.65%) | K562 | - | chrX | | 73072184 | 73072221 |
| 2.72991632861751 | NIPBL (bg=5.39%) | K562 | - | chrX | | 73072199 | 73072286 |
| 2.61281760946063 | NIPBL (bg=5.39%) | K562 | - | chrX | | 73072204 | 73072280 |
| 4.62016257624441 | RBM15 (bg=7.27%) | K562 | - | chrX | | 73072186 | 73072223 |
| 5.01889573908096 | RBM15 (bg=7.27%) | K562 | - | chrX | | 73072186 | 73072223 |
| 3.05882751960038 | SDAD1 (bg=2.97%) | K562 | - | chrX | | 73072155 | 73072220 |
| 4.09450650673659 | SRSF1 (bg=8.47%) | K562 | - | chrX | | 73072190 | 73072226 |
| 3.70570483903369 | SRSF1 (bg=8.47%) | K562 | - | chrX | | 73072202 | 73072225 |
| 2.04579580229326 | U2AF1 (bg=1.17%) | K562 | - | chrX | | 73072187 | 73072223 |
| 2.92871152517201 | uchl5 (bg=11.16%) | K562 | - | chrX | | 73072202 | 73072214 |

  
  

| Match 15 in HUMAN | | | | | | | |
| --- | --- | --- | --- | --- | --- | --- | --- |
| Motif | Start in Seq (1 Indexed) | End in Seq (1 Indexed) | Strand | Chrm | Exon | Start in Chrm (0 Indexed) | End in Chrm (1 Indexed) |
| GGATACCTGGTTTTA | 386 | 400 | - | chrX | 1 | 73072188 | 73072203 |
| eCLIP Fold-Enrichment | Binding Protein | Cell Line | Strand | Chrm | | Start in Chrm (0 Indexed) | End in Chrm (1 Indexed) |
| 2.11267917321191 | DDX51 (bg=1.63%) | K562 | - | chrX | | 73072152 | 73072199 |
| 2.6353918967895 | DDX51 (bg=1.63%) | K562 | - | chrX | | 73072188 | 73072226 |
| 2.61084050246287 | HNRNPC (bg=3.65%) | K562 | - | chrX | | 73072183 | 73072232 |
| 2.54645839594316 | HNRNPC (bg=3.65%) | K562 | - | chrX | | 73072184 | 73072221 |
| 2.27890292782896 | NIPBL (bg=5.39%) | K562 | - | chrX | | 73072134 | 73072188 |
| 2.72991632861751 | NIPBL (bg=5.39%) | K562 | - | chrX | | 73072199 | 73072286 |
| 4.62016257624441 | RBM15 (bg=7.27%) | K562 | - | chrX | | 73072186 | 73072223 |
| 5.01889573908096 | RBM15 (bg=7.27%) | K562 | - | chrX | | 73072186 | 73072223 |
| 3.05882751960038 | SDAD1 (bg=2.97%) | K562 | - | chrX | | 73072155 | 73072220 |
| 4.09450650673659 | SRSF1 (bg=8.47%) | K562 | - | chrX | | 73072138 | 73072190 |
| 3.96101188284287 | SRSF1 (bg=8.47%) | K562 | - | chrX | | 73072185 | 73072202 |
| 4.09450650673659 | SRSF1 (bg=8.47%) | K562 | - | chrX | | 73072190 | 73072226 |
| 3.70570483903369 | SRSF1 (bg=8.47%) | K562 | - | chrX | | 73072202 | 73072225 |
| 2.04579580229326 | U2AF1 (bg=1.17%) | K562 | - | chrX | | 73072187 | 73072223 |
| 2.92871152517201 | uchl5 (bg=11.16%) | K562 | - | chrX | | 73072202 | 73072214 |

  
  

| Match 16 in HUMAN | | | | | | | |
| --- | --- | --- | --- | --- | --- | --- | --- |
| Motif | Start in Seq (1 Indexed) | End in Seq (1 Indexed) | Strand | Chrm | Exon | Start in Chrm (0 Indexed) | End in Chrm (1 Indexed) |
| TTATTTT | 401 | 407 | - | chrX | 1 | 73072181 | 73072188 |
| eCLIP Fold-Enrichment | Binding Protein | Cell Line | Strand | Chrm | | Start in Chrm (0 Indexed) | End in Chrm (1 Indexed) |
| 2.11267917321191 | DDX51 (bg=1.63%) | K562 | - | chrX | | 73072152 | 73072199 |
| 2.6353918967895 | DDX51 (bg=1.63%) | K562 | - | chrX | | 73072188 | 73072226 |
| 2.50881956552236 | HNRNPC (bg=3.65%) | K562 | - | chrX | | 73072143 | 73072183 |
| 3.22835708059631 | HNRNPC (bg=3.65%) | K562 | - | chrX | | 73072157 | 73072184 |
| 2.61084050246287 | HNRNPC (bg=3.65%) | K562 | - | chrX | | 73072183 | 73072232 |
| 2.54645839594316 | HNRNPC (bg=3.65%) | K562 | - | chrX | | 73072184 | 73072221 |
| 2.44808072477545 | NIPBL (bg=5.39%) | K562 | - | chrX | | 73072132 | 73072184 |
| 2.27890292782896 | NIPBL (bg=5.39%) | K562 | - | chrX | | 73072134 | 73072188 |
| 4.58433912823059 | RBM15 (bg=7.27%) | K562 | - | chrX | | 73072137 | 73072183 |
| 4.93399583489869 | RBM15 (bg=7.27%) | K562 | - | chrX | | 73072178 | 73072183 |
| 4.65196046713484 | RBM15 (bg=7.27%) | K562 | - | chrX | | 73072183 | 73072186 |
| 4.62016257624441 | RBM15 (bg=7.27%) | K562 | - | chrX | | 73072186 | 73072223 |
| 5.01889573908096 | RBM15 (bg=7.27%) | K562 | - | chrX | | 73072186 | 73072223 |
| 3.05882751960038 | SDAD1 (bg=2.97%) | K562 | - | chrX | | 73072155 | 73072220 |
| 4.15247004583482 | SRSF1 (bg=8.47%) | K562 | - | chrX | | 73072138 | 73072185 |
| 4.09450650673659 | SRSF1 (bg=8.47%) | K562 | - | chrX | | 73072138 | 73072190 |
| 3.96101188284287 | SRSF1 (bg=8.47%) | K562 | - | chrX | | 73072185 | 73072202 |
| 2.04579580229326 | U2AF1 (bg=1.17%) | K562 | - | chrX | | 73072187 | 73072223 |

  
  

| Match 17 in HUMAN | | | | | | | |
| --- | --- | --- | --- | --- | --- | --- | --- |
| Motif | Start in Seq (1 Indexed) | End in Seq (1 Indexed) | Strand | Chrm | Exon | Start in Chrm (0 Indexed) | End in Chrm (1 Indexed) |
| TTTGCCCAACGGGGCCGTGGATACCTGCCTTTTAATTCTTTTTT | 411 | 454 | - | chrX | 1 | 73072134 | 73072178 |
| eCLIP Fold-Enrichment | Binding Protein | Cell Line | Strand | Chrm | | Start in Chrm (0 Indexed) | End in Chrm (1 Indexed) |
| 2.11267917321191 | DDX51 (bg=1.63%) | K562 | - | chrX | | 73072152 | 73072199 |
| 2.89876608383837 | HNRNPC (bg=3.65%) | K562 | - | chrX | | 73072093 | 73072143 |
| 3.44527956157579 | HNRNPC (bg=3.65%) | K562 | - | chrX | | 73072095 | 73072138 |
| 3.31638182839353 | HNRNPC (bg=3.65%) | K562 | - | chrX | | 73072138 | 73072157 |
| 2.50881956552236 | HNRNPC (bg=3.65%) | K562 | - | chrX | | 73072143 | 73072183 |
| 3.22835708059631 | HNRNPC (bg=3.65%) | K562 | - | chrX | | 73072157 | 73072184 |
| 2.44808072477545 | NIPBL (bg=5.39%) | K562 | - | chrX | | 73072132 | 73072184 |
| 2.27890292782896 | NIPBL (bg=5.39%) | K562 | - | chrX | | 73072134 | 73072188 |
| 4.21611566881516 | RBM15 (bg=7.27%) | K562 | - | chrX | | 73072099 | 73072138 |
| 3.97514063719867 | RBM15 (bg=7.27%) | K562 | - | chrX | | 73072100 | 73072137 |
| 4.58433912823059 | RBM15 (bg=7.27%) | K562 | - | chrX | | 73072137 | 73072183 |
| 4.93114229554524 | RBM15 (bg=7.27%) | K562 | - | chrX | | 73072138 | 73072167 |
| 4.87574405688614 | RBM15 (bg=7.27%) | K562 | - | chrX | | 73072167 | 73072178 |
| 4.93399583489869 | RBM15 (bg=7.27%) | K562 | - | chrX | | 73072178 | 73072183 |
| 3.05882751960038 | SDAD1 (bg=2.97%) | K562 | - | chrX | | 73072155 | 73072220 |
| 3.82011457949712 | SRSF1 (bg=8.47%) | K562 | - | chrX | | 73072095 | 73072138 |
| 4.06413285769308 | SRSF1 (bg=8.47%) | K562 | - | chrX | | 73072096 | 73072138 |
| 4.15247004583482 | SRSF1 (bg=8.47%) | K562 | - | chrX | | 73072138 | 73072185 |
| 4.09450650673659 | SRSF1 (bg=8.47%) | K562 | - | chrX | | 73072138 | 73072190 |
| 2.01664945663374 | U2AF1 (bg=1.17%) | K562 | - | chrX | | 73072092 | 73072140 |
| 4.10662931736786 | uchl5 (bg=11.16%) | K562 | - | chrX | | 73072147 | 73072166 |
| 3.69437097119939 | uchl5 (bg=11.16%) | K562 | - | chrX | | 73072147 | 73072179 |
| 3.82871573519529 | uchl5 (bg=11.16%) | K562 | - | chrX | | 73072166 | 73072179 |

  
  

| Match 18 in HUMAN | | | | | | | |
| --- | --- | --- | --- | --- | --- | --- | --- |
| Motif | Start in Seq (1 Indexed) | End in Seq (1 Indexed) | Strand | Chrm | Exon | Start in Chrm (0 Indexed) | End in Chrm (1 Indexed) |
| GCCCATCGGGGCCGCGGATACCTGCTTTT | 459 | 487 | - | chrX | 1 | 73072101 | 73072130 |
| eCLIP Fold-Enrichment | Binding Protein | Cell Line | Strand | Chrm | | Start in Chrm (0 Indexed) | End in Chrm (1 Indexed) |
| 2.1088268477163 | DDX51 (bg=1.63%) | K562 | - | chrX | | 73072056 | 73072102 |
| 2.89876608383837 | HNRNPC (bg=3.65%) | K562 | - | chrX | | 73072093 | 73072143 |
| 3.44527956157579 | HNRNPC (bg=3.65%) | K562 | - | chrX | | 73072095 | 73072138 |
| 2.19158916480843 | NIPBL (bg=5.39%) | K562 | - | chrX | | 73072097 | 73072132 |
| 4.21611566881516 | RBM15 (bg=7.27%) | K562 | - | chrX | | 73072099 | 73072138 |
| 3.97514063719867 | RBM15 (bg=7.27%) | K562 | - | chrX | | 73072100 | 73072137 |
| 3.04884343102775 | SDAD1 (bg=2.97%) | K562 | - | chrX | | 73072115 | 73072124 |
| 3.82011457949712 | SRSF1 (bg=8.47%) | K562 | - | chrX | | 73072095 | 73072138 |
| 4.06413285769308 | SRSF1 (bg=8.47%) | K562 | - | chrX | | 73072096 | 73072138 |
| 2.01664945663374 | U2AF1 (bg=1.17%) | K562 | - | chrX | | 73072092 | 73072140 |
| 3.67121530287965 | uchl5 (bg=11.16%) | K562 | - | chrX | | 73072119 | 73072126 |

  
  

| Match 19 in HUMAN | | | | | | | |
| --- | --- | --- | --- | --- | --- | --- | --- |
| Motif | Start in Seq (1 Indexed) | End in Seq (1 Indexed) | Strand | Chrm | Exon | Start in Chrm (0 Indexed) | End in Chrm (1 Indexed) |
| ATTTTTTTTT | 489 | 498 | - | chrX | 1 | 73072090 | 73072100 |
| eCLIP Fold-Enrichment | Binding Protein | Cell Line | Strand | Chrm | | Start in Chrm (0 Indexed) | End in Chrm (1 Indexed) |
| 2.1088268477163 | DDX51 (bg=1.63%) | K562 | - | chrX | | 73072056 | 73072102 |
| 2.18065951692348 | HNRNPC (bg=3.65%) | K562 | - | chrX | | 73072036 | 73072093 |
| 2.38741306732595 | HNRNPC (bg=3.65%) | K562 | - | chrX | | 73072081 | 73072095 |
| 2.89876608383837 | HNRNPC (bg=3.65%) | K562 | - | chrX | | 73072093 | 73072143 |
| 3.44527956157579 | HNRNPC (bg=3.65%) | K562 | - | chrX | | 73072095 | 73072138 |
| 2.60272853947607 | NIPBL (bg=5.39%) | K562 | - | chrX | | 73072017 | 73072097 |
| 2.19158916480843 | NIPBL (bg=5.39%) | K562 | - | chrX | | 73072097 | 73072132 |
| 3.78072446836026 | RBM15 (bg=7.27%) | K562 | - | chrX | | 73072057 | 73072094 |
| 4.10835256887607 | RBM15 (bg=7.27%) | K562 | - | chrX | | 73072080 | 73072093 |
| 4.21611566881516 | RBM15 (bg=7.27%) | K562 | - | chrX | | 73072099 | 73072138 |
| 3.97514063719867 | RBM15 (bg=7.27%) | K562 | - | chrX | | 73072100 | 73072137 |
| 3.49106575963283 | SDAD1 (bg=2.97%) | K562 | - | chrX | | 73072019 | 73072094 |
| 3.05465750742708 | SDAD1 (bg=2.97%) | K562 | - | chrX | | 73072029 | 73072092 |
| 3.89819577465627 | SRSF1 (bg=8.47%) | K562 | - | chrX | | 73072058 | 73072095 |
| 3.87877781568116 | SRSF1 (bg=8.47%) | K562 | - | chrX | | 73072061 | 73072096 |
| 3.82011457949712 | SRSF1 (bg=8.47%) | K562 | - | chrX | | 73072095 | 73072138 |
| 4.06413285769308 | SRSF1 (bg=8.47%) | K562 | - | chrX | | 73072096 | 73072138 |
| 2.01664945663374 | U2AF1 (bg=1.17%) | K562 | - | chrX | | 73072092 | 73072140 |
| 4.0479057244934 | uchl5 (bg=11.16%) | K562 | - | chrX | | 73072044 | 73072093 |
| 3.62313433368949 | uchl5 (bg=11.16%) | K562 | - | chrX | | 73072056 | 73072092 |
| 2.07728042575499 | YWHAG (bg=1.87%) | K562 | - | chrX | | 73072060 | 73072092 |

  
  

| Match 20 in HUMAN | | | | | | | |
| --- | --- | --- | --- | --- | --- | --- | --- |
| Motif | Start in Seq (1 Indexed) | End in Seq (1 Indexed) | Strand | Chrm | Exon | Start in Chrm (0 Indexed) | End in Chrm (1 Indexed) |
| CCTTAGCCCATCGGGG | 499 | 514 | - | chrX | 1 | 73072074 | 73072090 |
| eCLIP Fold-Enrichment | Binding Protein | Cell Line | Strand | Chrm | | Start in Chrm (0 Indexed) | End in Chrm (1 Indexed) |
| 2.1088268477163 | DDX51 (bg=1.63%) | K562 | - | chrX | | 73072056 | 73072102 |
| 2.18065951692348 | HNRNPC (bg=3.65%) | K562 | - | chrX | | 73072036 | 73072093 |
| 2.44774360152727 | HNRNPC (bg=3.65%) | K562 | - | chrX | | 73072060 | 73072081 |
| 2.38741306732595 | HNRNPC (bg=3.65%) | K562 | - | chrX | | 73072081 | 73072095 |
| 2.60272853947607 | NIPBL (bg=5.39%) | K562 | - | chrX | | 73072017 | 73072097 |
| 3.78072446836026 | RBM15 (bg=7.27%) | K562 | - | chrX | | 73072057 | 73072094 |
| 4.05003597433497 | RBM15 (bg=7.27%) | K562 | - | chrX | | 73072063 | 73072080 |
| 4.10835256887607 | RBM15 (bg=7.27%) | K562 | - | chrX | | 73072080 | 73072093 |
| 3.49106575963283 | SDAD1 (bg=2.97%) | K562 | - | chrX | | 73072019 | 73072094 |
| 3.05465750742708 | SDAD1 (bg=2.97%) | K562 | - | chrX | | 73072029 | 73072092 |
| 3.89819577465627 | SRSF1 (bg=8.47%) | K562 | - | chrX | | 73072058 | 73072095 |
| 3.87877781568116 | SRSF1 (bg=8.47%) | K562 | - | chrX | | 73072061 | 73072096 |
| 4.0479057244934 | uchl5 (bg=11.16%) | K562 | - | chrX | | 73072044 | 73072093 |
| 3.62313433368949 | uchl5 (bg=11.16%) | K562 | - | chrX | | 73072056 | 73072092 |
| 2.07728042575499 | YWHAG (bg=1.87%) | K562 | - | chrX | | 73072060 | 73072092 |

  
  

| Match 21 in HUMAN | | | | | | | |
| --- | --- | --- | --- | --- | --- | --- | --- |
| Motif | Start in Seq (1 Indexed) | End in Seq (1 Indexed) | Strand | Chrm | Exon | Start in Chrm (0 Indexed) | End in Chrm (1 Indexed) |
| TCGGATACCTGCTG | 517 | 530 | - | chrX | 1 | 73072058 | 73072072 |
| eCLIP Fold-Enrichment | Binding Protein | Cell Line | Strand | Chrm | | Start in Chrm (0 Indexed) | End in Chrm (1 Indexed) |
| 2.1088268477163 | DDX51 (bg=1.63%) | K562 | - | chrX | | 73072056 | 73072102 |
| 2.53838896596727 | HNRNPC (bg=3.65%) | K562 | - | chrX | | 73072011 | 73072060 |
| 2.18065951692348 | HNRNPC (bg=3.65%) | K562 | - | chrX | | 73072036 | 73072093 |
| 2.44774360152727 | HNRNPC (bg=3.65%) | K562 | - | chrX | | 73072060 | 73072081 |
| 2.60272853947607 | NIPBL (bg=5.39%) | K562 | - | chrX | | 73072017 | 73072097 |
| 2.18410517328471 | NIPBL (bg=5.39%) | K562 | - | chrX | | 73072041 | 73072070 |
| 3.90034809700424 | RBM15 (bg=7.27%) | K562 | - | chrX | | 73072056 | 73072063 |
| 3.78072446836026 | RBM15 (bg=7.27%) | K562 | - | chrX | | 73072057 | 73072094 |
| 4.05003597433497 | RBM15 (bg=7.27%) | K562 | - | chrX | | 73072063 | 73072080 |
| 3.49106575963283 | SDAD1 (bg=2.97%) | K562 | - | chrX | | 73072019 | 73072094 |
| 3.05465750742708 | SDAD1 (bg=2.97%) | K562 | - | chrX | | 73072029 | 73072092 |
| 3.63137972828005 | SRSF1 (bg=8.47%) | K562 | - | chrX | | 73072028 | 73072058 |
| 3.58754651801671 | SRSF1 (bg=8.47%) | K562 | - | chrX | | 73072030 | 73072061 |
| 3.89819577465627 | SRSF1 (bg=8.47%) | K562 | - | chrX | | 73072058 | 73072095 |
| 3.87877781568116 | SRSF1 (bg=8.47%) | K562 | - | chrX | | 73072061 | 73072096 |
| 4.0479057244934 | uchl5 (bg=11.16%) | K562 | - | chrX | | 73072044 | 73072093 |
| 3.62313433368949 | uchl5 (bg=11.16%) | K562 | - | chrX | | 73072056 | 73072092 |
| 2.07728042575499 | YWHAG (bg=1.87%) | K562 | - | chrX | | 73072060 | 73072092 |

  
  

| Match 22 in HUMAN | | | | | | | |
| --- | --- | --- | --- | --- | --- | --- | --- |
| Motif | Start in Seq (1 Indexed) | End in Seq (1 Indexed) | Strand | Chrm | Exon | Start in Chrm (0 Indexed) | End in Chrm (1 Indexed) |
| CCCCTCT | 539 | 545 | - | chrX | 1 | 73072043 | 73072050 |
| eCLIP Fold-Enrichment | Binding Protein | Cell Line | Strand | Chrm | | Start in Chrm (0 Indexed) | End in Chrm (1 Indexed) |
| 2.53838896596727 | HNRNPC (bg=3.65%) | K562 | - | chrX | | 73072011 | 73072060 |
| 2.18065951692348 | HNRNPC (bg=3.65%) | K562 | - | chrX | | 73072036 | 73072093 |
| 2.60272853947607 | NIPBL (bg=5.39%) | K562 | - | chrX | | 73072017 | 73072097 |
| 2.18410517328471 | NIPBL (bg=5.39%) | K562 | - | chrX | | 73072041 | 73072070 |
| 4.18981446380051 | RBM15 (bg=7.27%) | K562 | - | chrX | | 73072028 | 73072044 |
| 3.87369456168412 | RBM15 (bg=7.27%) | K562 | - | chrX | | 73072029 | 73072046 |
| 3.83379879012066 | RBM15 (bg=7.27%) | K562 | - | chrX | | 73072044 | 73072056 |
| 3.6628472451483 | RBM15 (bg=7.27%) | K562 | - | chrX | | 73072046 | 73072057 |
| 3.49106575963283 | SDAD1 (bg=2.97%) | K562 | - | chrX | | 73072019 | 73072094 |
| 3.05465750742708 | SDAD1 (bg=2.97%) | K562 | - | chrX | | 73072029 | 73072092 |
| 3.63137972828005 | SRSF1 (bg=8.47%) | K562 | - | chrX | | 73072028 | 73072058 |
| 3.58754651801671 | SRSF1 (bg=8.47%) | K562 | - | chrX | | 73072030 | 73072061 |
| 4.16711626449709 | uchl5 (bg=11.16%) | K562 | - | chrX | | 73072038 | 73072044 |
| 4.0479057244934 | uchl5 (bg=11.16%) | K562 | - | chrX | | 73072044 | 73072093 |
| 3.23005256914933 | uchl5 (bg=11.16%) | K562 | - | chrX | | 73072047 | 73072056 |

  
  

| Match 23 in HUMAN | | | | | | | |
| --- | --- | --- | --- | --- | --- | --- | --- |
| Motif | Start in Seq (1 Indexed) | End in Seq (1 Indexed) | Strand | Chrm | Exon | Start in Chrm (0 Indexed) | End in Chrm (1 Indexed) |
| AACCCC | 547 | 552 | - | chrX | 1 | 73072036 | 73072042 |
| eCLIP Fold-Enrichment | Binding Protein | Cell Line | Strand | Chrm | | Start in Chrm (0 Indexed) | End in Chrm (1 Indexed) |
| 2.53838896596727 | HNRNPC (bg=3.65%) | K562 | - | chrX | | 73072011 | 73072060 |
| 3.14269166672446 | HNRNPC (bg=3.65%) | K562 | - | chrX | | 73072016 | 73072036 |
| 2.18065951692348 | HNRNPC (bg=3.65%) | K562 | - | chrX | | 73072036 | 73072093 |
| 2.60272853947607 | NIPBL (bg=5.39%) | K562 | - | chrX | | 73072017 | 73072097 |
| 2.18410517328471 | NIPBL (bg=5.39%) | K562 | - | chrX | | 73072041 | 73072070 |
| 4.18981446380051 | RBM15 (bg=7.27%) | K562 | - | chrX | | 73072028 | 73072044 |
| 3.87369456168412 | RBM15 (bg=7.27%) | K562 | - | chrX | | 73072029 | 73072046 |
| 3.49106575963283 | SDAD1 (bg=2.97%) | K562 | - | chrX | | 73072019 | 73072094 |
| 3.05465750742708 | SDAD1 (bg=2.97%) | K562 | - | chrX | | 73072029 | 73072092 |
| 3.63137972828005 | SRSF1 (bg=8.47%) | K562 | - | chrX | | 73072028 | 73072058 |
| 3.58754651801671 | SRSF1 (bg=8.47%) | K562 | - | chrX | | 73072030 | 73072061 |
| 4.16711626449709 | uchl5 (bg=11.16%) | K562 | - | chrX | | 73072038 | 73072044 |

  
  

| Match 24 in HUMAN | | | | | | | |
| --- | --- | --- | --- | --- | --- | --- | --- |
| Motif | Start in Seq (1 Indexed) | End in Seq (1 Indexed) | Strand | Chrm | Exon | Start in Chrm (0 Indexed) | End in Chrm (1 Indexed) |
| TGGCCCATC | 561 | 569 | - | chrX | 1 | 73072019 | 73072028 |
| eCLIP Fold-Enrichment | Binding Protein | Cell Line | Strand | Chrm | | Start in Chrm (0 Indexed) | End in Chrm (1 Indexed) |
| 2.53838896596727 | HNRNPC (bg=3.65%) | K562 | - | chrX | | 73072011 | 73072060 |
| 3.14269166672446 | HNRNPC (bg=3.65%) | K562 | - | chrX | | 73072016 | 73072036 |
| 2.60272853947607 | NIPBL (bg=5.39%) | K562 | - | chrX | | 73072017 | 73072097 |
| 5.07766489146554 | RBM15 (bg=7.27%) | K562 | - | chrX | | 73071996 | 73072028 |
| 4.42761768893651 | RBM15 (bg=7.27%) | K562 | - | chrX | | 73071999 | 73072029 |
| 4.18981446380051 | RBM15 (bg=7.27%) | K562 | - | chrX | | 73072028 | 73072044 |
| 3.49106575963283 | SDAD1 (bg=2.97%) | K562 | - | chrX | | 73072019 | 73072094 |
| 3.63137972828005 | SRSF1 (bg=8.47%) | K562 | - | chrX | | 73072028 | 73072058 |

  
  

| Match 25 in HUMAN | | | | | | | |
| --- | --- | --- | --- | --- | --- | --- | --- |
| Motif | Start in Seq (1 Indexed) | End in Seq (1 Indexed) | Strand | Chrm | Exon | Start in Chrm (0 Indexed) | End in Chrm (1 Indexed) |
| CTGCTTTTT | 584 | 592 | - | chrX | 1 | 73071996 | 73072005 |
| eCLIP Fold-Enrichment | Binding Protein | Cell Line | Strand | Chrm | | Start in Chrm (0 Indexed) | End in Chrm (1 Indexed) |
| 3.4208144718716 | CPEB4 (bg=1.89%) | K562 | - | chrX | | 73072002 | 73072011 |
| 5.07766489146554 | RBM15 (bg=7.27%) | K562 | - | chrX | | 73071996 | 73072028 |
| 4.42761768893651 | RBM15 (bg=7.27%) | K562 | - | chrX | | 73071999 | 73072029 |

  
  

| Match 26 in HUMAN | | | | | | | |
| --- | --- | --- | --- | --- | --- | --- | --- |
| Motif | Start in Seq (1 Indexed) | End in Seq (1 Indexed) | Strand | Chrm | Exon | Start in Chrm (0 Indexed) | End in Chrm (1 Indexed) |
| TTTTTTTGGCCCATCGGGGC | 603 | 622 | - | chrX | 1 | 73071966 | 73071986 |
| eCLIP Fold-Enrichment | Binding Protein | Cell Line | Strand | Chrm | | Start in Chrm (0 Indexed) | End in Chrm (1 Indexed) |
| 2.47739447798057 | FASTKD2 (bg=1.99%) | K562 | - | chrX | | 73071967 | 73071972 |
| 2.48180543760091 | HNRNPC (bg=3.65%) | K562 | - | chrX | | 73071942 | 73071986 |
| 4.45984916958639 | RBM15 (bg=7.27%) | K562 | - | chrX | | 73071952 | 73071983 |
| 4.54360328904443 | RBM15 (bg=7.27%) | K562 | - | chrX | | 73071953 | 73071971 |
| 4.44117933616868 | RBM15 (bg=7.27%) | K562 | - | chrX | | 73071971 | 73071983 |
| 4.18185385261592 | SRSF1 (bg=8.47%) | K562 | - | chrX | | 73071956 | 73071984 |
| 3.94694931832274 | SRSF1 (bg=8.47%) | K562 | - | chrX | | 73071959 | 73071982 |
| 2.2778182393778 | U2AF1 (bg=1.17%) | K562 | - | chrX | | 73071952 | 73071983 |
| 2.12596615097724 | U2AF1 (bg=1.17%) | K562 | - | chrX | | 73071954 | 73071983 |
| 2.78509967426168 | YWHAG (bg=1.87%) | K562 | - | chrX | | 73071967 | 73071981 |

  
  

| Match 27 in HUMAN | | | | | | | |
| --- | --- | --- | --- | --- | --- | --- | --- |
| Motif | Start in Seq (1 Indexed) | End in Seq (1 Indexed) | Strand | Chrm | Exon | Start in Chrm (0 Indexed) | End in Chrm (1 Indexed) |
| TCGGATACCTGCTTT | 624 | 638 | - | chrX | 1 | 73071950 | 73071965 |
| eCLIP Fold-Enrichment | Binding Protein | Cell Line | Strand | Chrm | | Start in Chrm (0 Indexed) | End in Chrm (1 Indexed) |
| 2.48180543760091 | HNRNPC (bg=3.65%) | K562 | - | chrX | | 73071942 | 73071986 |
| 4.45984916958639 | RBM15 (bg=7.27%) | K562 | - | chrX | | 73071952 | 73071983 |
| 4.54360328904443 | RBM15 (bg=7.27%) | K562 | - | chrX | | 73071953 | 73071971 |
| 4.18185385261592 | SRSF1 (bg=8.47%) | K562 | - | chrX | | 73071956 | 73071984 |
| 3.94694931832274 | SRSF1 (bg=8.47%) | K562 | - | chrX | | 73071959 | 73071982 |
| 2.2778182393778 | U2AF1 (bg=1.17%) | K562 | - | chrX | | 73071952 | 73071983 |
| 2.12596615097724 | U2AF1 (bg=1.17%) | K562 | - | chrX | | 73071954 | 73071983 |

  
  

| Match 28 in HUMAN | | | | | | | |
| --- | --- | --- | --- | --- | --- | --- | --- |
| Motif | Start in Seq (1 Indexed) | End in Seq (1 Indexed) | Strand | Chrm | Exon | Start in Chrm (0 Indexed) | End in Chrm (1 Indexed) |
| TTTTTCCTTGCCCATCGGGGCCTCGGATACCTGCTTTA | 650 | 687 | - | chrX | 1 | 73071901 | 73071939 |
| eCLIP Fold-Enrichment | Binding Protein | Cell Line | Strand | Chrm | | Start in Chrm (0 Indexed) | End in Chrm (1 Indexed) |
| 3.1683745129597 | AARS (bg=2.18%) | K562 | - | chrX | | 73071909 | 73071934 |
| 2.02894297090749 | AKAP1 (bg=0.21%) | K562 | - | chrX | | 73071893 | 73071933 |
| 2.96372152567914 | HNRNPC (bg=3.65%) | K562 | - | chrX | | 73071854 | 73071940 |
| 3.27535456013348 | HNRNPC (bg=3.65%) | K562 | - | chrX | | 73071894 | 73071924 |
| 2.7448398434347 | HNRNPC (bg=3.65%) | K562 | - | chrX | | 73071924 | 73071940 |
| 2.50978668575441 | NIPBL (bg=5.39%) | K562 | - | chrX | | 73071888 | 73071919 |
| 2.01101691916661 | NIPBL (bg=5.39%) | K562 | - | chrX | | 73071919 | 73071935 |
| 4.34789049646578 | RBM15 (bg=7.27%) | K562 | - | chrX | | 73071895 | 73071938 |
| 4.78605336272213 | RBM15 (bg=7.27%) | K562 | - | chrX | | 73071899 | 73071937 |
| 3.82333196132062 | SDAD1 (bg=2.97%) | K562 | - | chrX | | 73071900 | 73071934 |
| 3.52277461936017 | SDAD1 (bg=2.97%) | K562 | - | chrX | | 73071902 | 73071936 |
| 5.20493746572896 | SRSF1 (bg=8.47%) | K562 | - | chrX | | 73071895 | 73071936 |
| 5.0945065067366 | SRSF1 (bg=8.47%) | K562 | - | chrX | | 73071897 | 73071936 |
| 3.53046024900082 | uchl5 (bg=11.16%) | K562 | - | chrX | | 73071903 | 73071936 |
| 3.55996767816711 | uchl5 (bg=11.16%) | K562 | - | chrX | | 73071905 | 73071936 |

  
  

| Match 29 in HUMAN | | | | | | | |
| --- | --- | --- | --- | --- | --- | --- | --- |
| Motif | Start in Seq (1 Indexed) | End in Seq (1 Indexed) | Strand | Chrm | Exon | Start in Chrm (0 Indexed) | End in Chrm (1 Indexed) |
| GCCCATCGGGGCCG | 703 | 716 | - | chrX | 1 | 73071872 | 73071886 |
| eCLIP Fold-Enrichment | Binding Protein | Cell Line | Strand | Chrm | | Start in Chrm (0 Indexed) | End in Chrm (1 Indexed) |
| 2.15487238079379 | AATF (bg=0.64%) | K562 | - | chrX | | 73071859 | 73071891 |
| 2.96372152567914 | HNRNPC (bg=3.65%) | K562 | - | chrX | | 73071854 | 73071940 |
| 2.48979822835444 | HNRNPC (bg=3.65%) | K562 | - | chrX | | 73071855 | 73071894 |
| 2.54959720057303 | LSM11 (bg=2.28%) | K562 | - | chrX | | 73071871 | 73071882 |
| 2.91405518927952 | NIPBL (bg=5.39%) | K562 | - | chrX | | 73071854 | 73071888 |
| 2.9242380465296 | NIPBL (bg=5.39%) | K562 | - | chrX | | 73071864 | 73071874 |
| 3.70922207320167 | RBM15 (bg=7.27%) | K562 | - | chrX | | 73071860 | 73071893 |
| 3.67530413043687 | RBM15 (bg=7.27%) | K562 | - | chrX | | 73071863 | 73071884 |
| 3.28055364530301 | RBM15 (bg=7.27%) | K562 | - | chrX | | 73071884 | 73071895 |
| 3.37077152591512 | SDAD1 (bg=2.97%) | K562 | - | chrX | | 73071874 | 73071880 |
| 5.49444408292394 | SRSF1 (bg=8.47%) | K562 | - | chrX | | 73071850 | 73071895 |
| 5.40839728255854 | SRSF1 (bg=8.47%) | K562 | - | chrX | | 73071854 | 73071897 |
| 2.97825795751889 | U2AF1 (bg=1.17%) | K562 | - | chrX | | 73071855 | 73071892 |
| 2.7238677074059 | U2AF1 (bg=1.17%) | K562 | - | chrX | | 73071855 | 73071895 |

  
  

| Match 30 in HUMAN | | | | | | | |
| --- | --- | --- | --- | --- | --- | --- | --- |
| Motif | Start in Seq (1 Indexed) | End in Seq (1 Indexed) | Strand | Chrm | Exon | Start in Chrm (0 Indexed) | End in Chrm (1 Indexed) |
| GGATACCTGCTT | 718 | 729 | - | chrX | 1 | 73071859 | 73071871 |
| eCLIP Fold-Enrichment | Binding Protein | Cell Line | Strand | Chrm | | Start in Chrm (0 Indexed) | End in Chrm (1 Indexed) |
| 2.15487238079379 | AATF (bg=0.64%) | K562 | - | chrX | | 73071859 | 73071891 |
| 2.96372152567914 | HNRNPC (bg=3.65%) | K562 | - | chrX | | 73071854 | 73071940 |
| 2.48979822835444 | HNRNPC (bg=3.65%) | K562 | - | chrX | | 73071855 | 73071894 |
| 2.54959720057303 | LSM11 (bg=2.28%) | K562 | - | chrX | | 73071871 | 73071882 |
| 2.91405518927952 | NIPBL (bg=5.39%) | K562 | - | chrX | | 73071854 | 73071888 |
| 2.9242380465296 | NIPBL (bg=5.39%) | K562 | - | chrX | | 73071864 | 73071874 |
| 3.70922207320167 | RBM15 (bg=7.27%) | K562 | - | chrX | | 73071860 | 73071893 |
| 3.67530413043687 | RBM15 (bg=7.27%) | K562 | - | chrX | | 73071863 | 73071884 |
| 5.49444408292394 | SRSF1 (bg=8.47%) | K562 | - | chrX | | 73071850 | 73071895 |
| 5.40839728255854 | SRSF1 (bg=8.47%) | K562 | - | chrX | | 73071854 | 73071897 |
| 2.97825795751889 | U2AF1 (bg=1.17%) | K562 | - | chrX | | 73071855 | 73071892 |
| 2.7238677074059 | U2AF1 (bg=1.17%) | K562 | - | chrX | | 73071855 | 73071895 |

  
  

| Match 31 in HUMAN | | | | | | | |
| --- | --- | --- | --- | --- | --- | --- | --- |
| Motif | Start in Seq (1 Indexed) | End in Seq (1 Indexed) | Strand | Chrm | Exon | Start in Chrm (0 Indexed) | End in Chrm (1 Indexed) |
| GATTTTTTTTTTTCATC | 731 | 747 | - | chrX | 1 | 73071841 | 73071858 |
| eCLIP Fold-Enrichment | Binding Protein | Cell Line | Strand | Chrm | | Start in Chrm (0 Indexed) | End in Chrm (1 Indexed) |
| 3.35627455551705 | HNRNPC (bg=3.65%) | K562 | - | chrX | | 73071825 | 73071848 |
| 2.96372152567914 | HNRNPC (bg=3.65%) | K562 | - | chrX | | 73071854 | 73071940 |
| 2.48979822835444 | HNRNPC (bg=3.65%) | K562 | - | chrX | | 73071855 | 73071894 |
| 2.74942448750672 | NIPBL (bg=5.39%) | K562 | - | chrX | | 73071805 | 73071854 |
| 3.11371584539331 | NIPBL (bg=5.39%) | K562 | - | chrX | | 73071808 | 73071855 |
| 2.91405518927952 | NIPBL (bg=5.39%) | K562 | - | chrX | | 73071854 | 73071888 |
| 5.29838283808923 | RBM15 (bg=7.27%) | K562 | - | chrX | | 73071806 | 73071848 |
| 5.81143868114204 | RBM15 (bg=7.27%) | K562 | - | chrX | | 73071807 | 73071848 |
| 4.35582704214764 | SDAD1 (bg=2.97%) | K562 | - | chrX | | 73071822 | 73071841 |
| 4.17812644797272 | SDAD1 (bg=2.97%) | K562 | - | chrX | | 73071822 | 73071845 |
| 4.78256250042185 | SRSF1 (bg=8.47%) | K562 | - | chrX | | 73071807 | 73071854 |
| 4.19113166620393 | SRSF1 (bg=8.47%) | K562 | - | chrX | | 73071835 | 73071850 |
| 5.49444408292394 | SRSF1 (bg=8.47%) | K562 | - | chrX | | 73071850 | 73071895 |
| 5.40839728255854 | SRSF1 (bg=8.47%) | K562 | - | chrX | | 73071854 | 73071897 |
| 2.97825795751889 | U2AF1 (bg=1.17%) | K562 | - | chrX | | 73071855 | 73071892 |
| 2.7238677074059 | U2AF1 (bg=1.17%) | K562 | - | chrX | | 73071855 | 73071895 |
| 2.62413976987541 | UTP3 (bg=3.66%) | K562 | - | chrX | | 73071809 | 73071844 |

  
  

| Match 32 in HUMAN | | | | | | | |
| --- | --- | --- | --- | --- | --- | --- | --- |
| Motif | Start in Seq (1 Indexed) | End in Seq (1 Indexed) | Strand | Chrm | Exon | Start in Chrm (0 Indexed) | End in Chrm (1 Indexed) |
| CCCATCGG | 749 | 756 | - | chrX | 1 | 73071832 | 73071840 |
| eCLIP Fold-Enrichment | Binding Protein | Cell Line | Strand | Chrm | | Start in Chrm (0 Indexed) | End in Chrm (1 Indexed) |
| 3.35627455551705 | HNRNPC (bg=3.65%) | K562 | - | chrX | | 73071825 | 73071848 |
| 2.74942448750672 | NIPBL (bg=5.39%) | K562 | - | chrX | | 73071805 | 73071854 |
| 3.11371584539331 | NIPBL (bg=5.39%) | K562 | - | chrX | | 73071808 | 73071855 |
| 5.29838283808923 | RBM15 (bg=7.27%) | K562 | - | chrX | | 73071806 | 73071848 |
| 5.81143868114204 | RBM15 (bg=7.27%) | K562 | - | chrX | | 73071807 | 73071848 |
| 4.35582704214764 | SDAD1 (bg=2.97%) | K562 | - | chrX | | 73071822 | 73071841 |
| 4.17812644797272 | SDAD1 (bg=2.97%) | K562 | - | chrX | | 73071822 | 73071845 |
| 4.20035019623331 | SRSF1 (bg=8.47%) | K562 | - | chrX | | 73071805 | 73071835 |
| 4.78256250042185 | SRSF1 (bg=8.47%) | K562 | - | chrX | | 73071807 | 73071854 |
| 4.19113166620393 | SRSF1 (bg=8.47%) | K562 | - | chrX | | 73071835 | 73071850 |
| 2.62413976987541 | UTP3 (bg=3.66%) | K562 | - | chrX | | 73071809 | 73071844 |
| 2.16101459964944 | YWHAG (bg=1.87%) | K562 | - | chrX | | 73071817 | 73071834 |

  
  

| Match 33 in HUMAN | | | | | | | |
| --- | --- | --- | --- | --- | --- | --- | --- |
| Motif | Start in Seq (1 Indexed) | End in Seq (1 Indexed) | Strand | Chrm | Exon | Start in Chrm (0 Indexed) | End in Chrm (1 Indexed) |
| TATGGATG | 764 | 771 | - | chrX | 1 | 73071817 | 73071825 |
| eCLIP Fold-Enrichment | Binding Protein | Cell Line | Strand | Chrm | | Start in Chrm (0 Indexed) | End in Chrm (1 Indexed) |
| 3.35627455551705 | HNRNPC (bg=3.65%) | K562 | - | chrX | | 73071825 | 73071848 |
| 2.74942448750672 | NIPBL (bg=5.39%) | K562 | - | chrX | | 73071805 | 73071854 |
| 3.11371584539331 | NIPBL (bg=5.39%) | K562 | - | chrX | | 73071808 | 73071855 |
| 5.29838283808923 | RBM15 (bg=7.27%) | K562 | - | chrX | | 73071806 | 73071848 |
| 5.81143868114204 | RBM15 (bg=7.27%) | K562 | - | chrX | | 73071807 | 73071848 |
| 4.35582704214764 | SDAD1 (bg=2.97%) | K562 | - | chrX | | 73071822 | 73071841 |
| 4.17812644797272 | SDAD1 (bg=2.97%) | K562 | - | chrX | | 73071822 | 73071845 |
| 4.20035019623331 | SRSF1 (bg=8.47%) | K562 | - | chrX | | 73071805 | 73071835 |
| 4.78256250042185 | SRSF1 (bg=8.47%) | K562 | - | chrX | | 73071807 | 73071854 |
| 4.07264131808138 | uchl5 (bg=11.16%) | K562 | - | chrX | | 73071813 | 73071829 |
| 2.62413976987541 | UTP3 (bg=3.66%) | K562 | - | chrX | | 73071809 | 73071844 |
| 2.16101459964944 | YWHAG (bg=1.87%) | K562 | - | chrX | | 73071817 | 73071834 |

  
  

| Match 34 in HUMAN | | | | | | | |
| --- | --- | --- | --- | --- | --- | --- | --- |
| Motif | Start in Seq (1 Indexed) | End in Seq (1 Indexed) | Strand | Chrm | Exon | Start in Chrm (0 Indexed) | End in Chrm (1 Indexed) |
| GGTTTTGTGG | 782 | 791 | - | chrX | 1 | 73071797 | 73071807 |
| eCLIP Fold-Enrichment | Binding Protein | Cell Line | Strand | Chrm | | Start in Chrm (0 Indexed) | End in Chrm (1 Indexed) |
| 2.87284851925303 | DGCR8 (bg=1.84%) | K562 | - | chrX | | 73071771 | 73071804 |
| 4.39050076654841 | EXOSC5 (bg=5.38%) | K562 | - | chrX | | 73071780 | 73071801 |
| 3.82575112896224 | EXOSC5 (bg=5.38%) | K562 | - | chrX | | 73071784 | 73071798 |
| 4.14680604402206 | GTF2F1 (bg=0.51%) | K562 | - | chrX | | 73071784 | 73071797 |
| 2.63950679542869 | NIPBL (bg=5.39%) | K562 | - | chrX | | 73071773 | 73071805 |
| 2.44546300131112 | NIPBL (bg=5.39%) | K562 | - | chrX | | 73071773 | 73071808 |
| 2.74942448750672 | NIPBL (bg=5.39%) | K562 | - | chrX | | 73071805 | 73071854 |
| 5.86960955120714 | RBM15 (bg=7.27%) | K562 | - | chrX | | 73071766 | 73071807 |
| 5.20255113330174 | RBM15 (bg=7.27%) | K562 | - | chrX | | 73071794 | 73071806 |
| 5.29838283808923 | RBM15 (bg=7.27%) | K562 | - | chrX | | 73071806 | 73071848 |
| 5.81143868114204 | RBM15 (bg=7.27%) | K562 | - | chrX | | 73071807 | 73071848 |
| 4.66604849169543 | SRSF1 (bg=8.47%) | K562 | - | chrX | | 73071770 | 73071807 |
| 3.6199749650078 | SRSF1 (bg=8.47%) | K562 | - | chrX | | 73071796 | 73071805 |
| 4.20035019623331 | SRSF1 (bg=8.47%) | K562 | - | chrX | | 73071805 | 73071835 |
| 4.78256250042185 | SRSF1 (bg=8.47%) | K562 | - | chrX | | 73071807 | 73071854 |
| 3.88150359385472 | uchl5 (bg=11.16%) | K562 | - | chrX | | 73071778 | 73071804 |
| 4.05799454211698 | uchl5 (bg=11.16%) | K562 | - | chrX | | 73071781 | 73071805 |
| 2.11521091003631 | YWHAG (bg=1.87%) | K562 | - | chrX | | 73071769 | 73071806 |

  
  

| Match 35 in HUMAN | | | | | | | |
| --- | --- | --- | --- | --- | --- | --- | --- |
| Motif | Start in Seq (1 Indexed) | End in Seq (1 Indexed) | Strand | Chrm | Exon | Start in Chrm (0 Indexed) | End in Chrm (1 Indexed) |
| TCTGGAAT | 804 | 811 | - | chrX | 1 | 73071777 | 73071785 |
| eCLIP Fold-Enrichment | Binding Protein | Cell Line | Strand | Chrm | | Start in Chrm (0 Indexed) | End in Chrm (1 Indexed) |
| 2.87284851925303 | DGCR8 (bg=1.84%) | K562 | - | chrX | | 73071771 | 73071804 |
| 4.39050076654841 | EXOSC5 (bg=5.38%) | K562 | - | chrX | | 73071780 | 73071801 |
| 3.82575112896224 | EXOSC5 (bg=5.38%) | K562 | - | chrX | | 73071784 | 73071798 |
| 4.14680604402206 | GTF2F1 (bg=0.51%) | K562 | - | chrX | | 73071784 | 73071797 |
| 2.63950679542869 | NIPBL (bg=5.39%) | K562 | - | chrX | | 73071773 | 73071805 |
| 2.44546300131112 | NIPBL (bg=5.39%) | K562 | - | chrX | | 73071773 | 73071808 |
| 5.86960955120714 | RBM15 (bg=7.27%) | K562 | - | chrX | | 73071766 | 73071807 |
| 5.43284875272353 | RBM15 (bg=7.27%) | K562 | - | chrX | | 73071769 | 73071794 |
| 2.49948834355768 | RBM22 (bg=4.62%) | K562 | - | chrX | | 73071764 | 73071795 |
| 3.76308894869388 | SDAD1 (bg=2.97%) | K562 | - | chrX | | 73071738 | 73071781 |
| 4.48315827537743 | SRSF1 (bg=8.47%) | K562 | - | chrX | | 73071768 | 73071796 |
| 4.66604849169543 | SRSF1 (bg=8.47%) | K562 | - | chrX | | 73071770 | 73071807 |
| 3.88150359385472 | uchl5 (bg=11.16%) | K562 | - | chrX | | 73071778 | 73071804 |
| 4.05799454211698 | uchl5 (bg=11.16%) | K562 | - | chrX | | 73071781 | 73071805 |
| 2.11521091003631 | YWHAG (bg=1.87%) | K562 | - | chrX | | 73071769 | 73071806 |

  
  

| Match 36 in HUMAN | | | | | | | |
| --- | --- | --- | --- | --- | --- | --- | --- |
| Motif | Start in Seq (1 Indexed) | End in Seq (1 Indexed) | Strand | Chrm | Exon | Start in Chrm (0 Indexed) | End in Chrm (1 Indexed) |
| TCTACA | 813 | 818 | - | chrX | 1 | 73071770 | 73071776 |
| eCLIP Fold-Enrichment | Binding Protein | Cell Line | Strand | Chrm | | Start in Chrm (0 Indexed) | End in Chrm (1 Indexed) |
| 2.61909192700725 | DGCR8 (bg=1.84%) | K562 | - | chrX | | 73071692 | 73071771 |
| 2.87284851925303 | DGCR8 (bg=1.84%) | K562 | - | chrX | | 73071771 | 73071804 |
| 3.5569852882719 | NIPBL (bg=5.39%) | K562 | - | chrX | | 73071691 | 73071773 |
| 2.63950679542869 | NIPBL (bg=5.39%) | K562 | - | chrX | | 73071773 | 73071805 |
| 2.44546300131112 | NIPBL (bg=5.39%) | K562 | - | chrX | | 73071773 | 73071808 |
| 5.86960955120714 | RBM15 (bg=7.27%) | K562 | - | chrX | | 73071766 | 73071807 |
| 5.43284875272353 | RBM15 (bg=7.27%) | K562 | - | chrX | | 73071769 | 73071794 |
| 3.11476360134165 | RBM22 (bg=4.62%) | K562 | - | chrX | | 73071715 | 73071770 |
| 2.49948834355768 | RBM22 (bg=4.62%) | K562 | - | chrX | | 73071764 | 73071795 |
| 3.76308894869388 | SDAD1 (bg=2.97%) | K562 | - | chrX | | 73071738 | 73071781 |
| 4.55790702757684 | SRSF1 (bg=8.47%) | K562 | - | chrX | | 73071753 | 73071770 |
| 4.48315827537743 | SRSF1 (bg=8.47%) | K562 | - | chrX | | 73071768 | 73071796 |
| 4.66604849169543 | SRSF1 (bg=8.47%) | K562 | - | chrX | | 73071770 | 73071807 |
| 2.11521091003631 | YWHAG (bg=1.87%) | K562 | - | chrX | | 73071769 | 73071806 |

  
  

| Match 37 in HUMAN | | | | | | | |
| --- | --- | --- | --- | --- | --- | --- | --- |
| Motif | Start in Seq (1 Indexed) | End in Seq (1 Indexed) | Strand | Chrm | Exon | Start in Chrm (0 Indexed) | End in Chrm (1 Indexed) |
| TTTTGCTGCT | 824 | 833 | - | chrX | 1 | 73071755 | 73071765 |
| eCLIP Fold-Enrichment | Binding Protein | Cell Line | Strand | Chrm | | Start in Chrm (0 Indexed) | End in Chrm (1 Indexed) |
| 2.16330771980541 | DDX52 (bg=0.46%) | K562 | - | chrX | | 73071730 | 73071762 |
| 2.61909192700725 | DGCR8 (bg=1.84%) | K562 | - | chrX | | 73071692 | 73071771 |
| 3.45104230848346 | EXOSC5 (bg=5.38%) | K562 | - | chrX | | 73071711 | 73071762 |
| 3.04232622370337 | EXOSC5 (bg=5.38%) | K562 | - | chrX | | 73071716 | 73071761 |
| 2.2183185368202 | NCBP2 (bg=1.49%) | K562 | - | chrX | | 73071718 | 73071765 |
| 3.5569852882719 | NIPBL (bg=5.39%) | K562 | - | chrX | | 73071691 | 73071773 |
| 2.83865435554392 | NIPBL (bg=5.39%) | K562 | - | chrX | | 73071702 | 73071768 |
| 5.43078192474329 | RBM15 (bg=7.27%) | K562 | - | chrX | | 73071749 | 73071757 |
| 5.30742025115569 | RBM15 (bg=7.27%) | K562 | - | chrX | | 73071750 | 73071757 |
| 5.22151124222315 | RBM15 (bg=7.27%) | K562 | - | chrX | | 73071757 | 73071766 |
| 5.06885094829717 | RBM15 (bg=7.27%) | K562 | - | chrX | | 73071757 | 73071769 |
| 3.39471602164062 | RBM22 (bg=4.62%) | K562 | - | chrX | | 73071686 | 73071764 |
| 3.11476360134165 | RBM22 (bg=4.62%) | K562 | - | chrX | | 73071715 | 73071770 |
| 2.49948834355768 | RBM22 (bg=4.62%) | K562 | - | chrX | | 73071764 | 73071795 |
| 3.50783013559269 | SDAD1 (bg=2.97%) | K562 | - | chrX | | 73071736 | 73071763 |
| 3.76308894869388 | SDAD1 (bg=2.97%) | K562 | - | chrX | | 73071738 | 73071781 |
| 4.55790702757684 | SRSF1 (bg=8.47%) | K562 | - | chrX | | 73071753 | 73071770 |
| 4.26408095047892 | SRSF1 (bg=8.47%) | K562 | - | chrX | | 73071754 | 73071768 |
| 3.8278526211756 | uchl5 (bg=11.16%) | K562 | - | chrX | | 73071701 | 73071764 |
| 3.67638304143123 | uchl5 (bg=11.16%) | K562 | - | chrX | | 73071705 | 73071755 |
| 3.33900423184405 | uchl5 (bg=11.16%) | K562 | - | chrX | | 73071755 | 73071765 |
| 2.27361895378144 | WDR3 (bg=0.25%) | K562 | - | chrX | | 73071726 | 73071758 |
| 2.8846353478126 | YWHAG (bg=1.87%) | K562 | - | chrX | | 73071708 | 73071762 |
| 2.89281848869987 | YWHAG (bg=1.87%) | K562 | - | chrX | | 73071710 | 73071769 |

  
  

| Match 38 in HUMAN | | | | | | | |
| --- | --- | --- | --- | --- | --- | --- | --- |
| Motif | Start in Seq (1 Indexed) | End in Seq (1 Indexed) | Strand | Chrm | Exon | Start in Chrm (0 Indexed) | End in Chrm (1 Indexed) |
| TTTGGTG | 839 | 845 | - | chrX | 1 | 73071743 | 73071750 |
| eCLIP Fold-Enrichment | Binding Protein | Cell Line | Strand | Chrm | | Start in Chrm (0 Indexed) | End in Chrm (1 Indexed) |
| 2.16330771980541 | DDX52 (bg=0.46%) | K562 | - | chrX | | 73071730 | 73071762 |
| 2.61909192700725 | DGCR8 (bg=1.84%) | K562 | - | chrX | | 73071692 | 73071771 |
| 3.45104230848346 | EXOSC5 (bg=5.38%) | K562 | - | chrX | | 73071711 | 73071762 |
| 3.04232622370337 | EXOSC5 (bg=5.38%) | K562 | - | chrX | | 73071716 | 73071761 |
| 2.2183185368202 | NCBP2 (bg=1.49%) | K562 | - | chrX | | 73071718 | 73071765 |
| 3.5569852882719 | NIPBL (bg=5.39%) | K562 | - | chrX | | 73071691 | 73071773 |
| 2.83865435554392 | NIPBL (bg=5.39%) | K562 | - | chrX | | 73071702 | 73071768 |
| 5.000058269403 | RBM15 (bg=7.27%) | K562 | - | chrX | | 73071693 | 73071750 |
| 5.11309438100314 | RBM15 (bg=7.27%) | K562 | - | chrX | | 73071717 | 73071749 |
| 5.43078192474329 | RBM15 (bg=7.27%) | K562 | - | chrX | | 73071749 | 73071757 |
| 5.30742025115569 | RBM15 (bg=7.27%) | K562 | - | chrX | | 73071750 | 73071757 |
| 3.39471602164062 | RBM22 (bg=4.62%) | K562 | - | chrX | | 73071686 | 73071764 |
| 3.11476360134165 | RBM22 (bg=4.62%) | K562 | - | chrX | | 73071715 | 73071770 |
| 3.50783013559269 | SDAD1 (bg=2.97%) | K562 | - | chrX | | 73071736 | 73071763 |
| 3.76308894869388 | SDAD1 (bg=2.97%) | K562 | - | chrX | | 73071738 | 73071781 |
| 2.16037976360809 | SLTM (bg=2.2%) | K562 | - | chrX | | 73071659 | 73071752 |
| 4.36441567973616 | SRSF1 (bg=8.47%) | K562 | - | chrX | | 73071704 | 73071754 |
| 4.49139665930518 | SRSF1 (bg=8.47%) | K562 | - | chrX | | 73071734 | 73071753 |
| 3.8278526211756 | uchl5 (bg=11.16%) | K562 | - | chrX | | 73071701 | 73071764 |
| 3.67638304143123 | uchl5 (bg=11.16%) | K562 | - | chrX | | 73071705 | 73071755 |
| 3.07523579413153 | UTP3 (bg=3.66%) | K562 | - | chrX | | 73071729 | 73071746 |
| 2.27361895378144 | WDR3 (bg=0.25%) | K562 | - | chrX | | 73071726 | 73071758 |
| 2.8846353478126 | YWHAG (bg=1.87%) | K562 | - | chrX | | 73071708 | 73071762 |
| 2.89281848869987 | YWHAG (bg=1.87%) | K562 | - | chrX | | 73071710 | 73071769 |

  
  

| Match 39 in HUMAN | | | | | | | |
| --- | --- | --- | --- | --- | --- | --- | --- |
| Motif | Start in Seq (1 Indexed) | End in Seq (1 Indexed) | Strand | Chrm | Exon | Start in Chrm (0 Indexed) | End in Chrm (1 Indexed) |
| TGTGTGAGTG | 847 | 856 | - | chrX | 1 | 73071732 | 73071742 |
| eCLIP Fold-Enrichment | Binding Protein | Cell Line | Strand | Chrm | | Start in Chrm (0 Indexed) | End in Chrm (1 Indexed) |
| 2.16330771980541 | DDX52 (bg=0.46%) | K562 | - | chrX | | 73071730 | 73071762 |
| 2.61909192700725 | DGCR8 (bg=1.84%) | K562 | - | chrX | | 73071692 | 73071771 |
| 3.45104230848346 | EXOSC5 (bg=5.38%) | K562 | - | chrX | | 73071711 | 73071762 |
| 3.04232622370337 | EXOSC5 (bg=5.38%) | K562 | - | chrX | | 73071716 | 73071761 |
| 2.2183185368202 | NCBP2 (bg=1.49%) | K562 | - | chrX | | 73071718 | 73071765 |
| 3.5569852882719 | NIPBL (bg=5.39%) | K562 | - | chrX | | 73071691 | 73071773 |
| 2.83865435554392 | NIPBL (bg=5.39%) | K562 | - | chrX | | 73071702 | 73071768 |
| 5.000058269403 | RBM15 (bg=7.27%) | K562 | - | chrX | | 73071693 | 73071750 |
| 5.11309438100314 | RBM15 (bg=7.27%) | K562 | - | chrX | | 73071717 | 73071749 |
| 3.39471602164062 | RBM22 (bg=4.62%) | K562 | - | chrX | | 73071686 | 73071764 |
| 3.11476360134165 | RBM22 (bg=4.62%) | K562 | - | chrX | | 73071715 | 73071770 |
| 3.50783013559269 | SDAD1 (bg=2.97%) | K562 | - | chrX | | 73071736 | 73071763 |
| 3.76308894869388 | SDAD1 (bg=2.97%) | K562 | - | chrX | | 73071738 | 73071781 |
| 2.16037976360809 | SLTM (bg=2.2%) | K562 | - | chrX | | 73071659 | 73071752 |
| 3.9018614287942 | SRSF1 (bg=8.47%) | K562 | - | chrX | | 73071678 | 73071734 |
| 4.36441567973616 | SRSF1 (bg=8.47%) | K562 | - | chrX | | 73071704 | 73071754 |
| 4.49139665930518 | SRSF1 (bg=8.47%) | K562 | - | chrX | | 73071734 | 73071753 |
| 3.8278526211756 | uchl5 (bg=11.16%) | K562 | - | chrX | | 73071701 | 73071764 |
| 3.67638304143123 | uchl5 (bg=11.16%) | K562 | - | chrX | | 73071705 | 73071755 |
| 3.07523579413153 | UTP3 (bg=3.66%) | K562 | - | chrX | | 73071729 | 73071746 |
| 2.27361895378144 | WDR3 (bg=0.25%) | K562 | - | chrX | | 73071726 | 73071758 |
| 2.8846353478126 | YWHAG (bg=1.87%) | K562 | - | chrX | | 73071708 | 73071762 |
| 2.89281848869987 | YWHAG (bg=1.87%) | K562 | - | chrX | | 73071710 | 73071769 |

  
  

| Match 40 in HUMAN | | | | | | | |
| --- | --- | --- | --- | --- | --- | --- | --- |
| Motif | Start in Seq (1 Indexed) | End in Seq (1 Indexed) | Strand | Chrm | Exon | Start in Chrm (0 Indexed) | End in Chrm (1 Indexed) |
| GCTTTGG | 865 | 871 | - | chrX | 1 | 73071717 | 73071724 |
| eCLIP Fold-Enrichment | Binding Protein | Cell Line | Strand | Chrm | | Start in Chrm (0 Indexed) | End in Chrm (1 Indexed) |
| 2.61909192700725 | DGCR8 (bg=1.84%) | K562 | - | chrX | | 73071692 | 73071771 |
| 3.45104230848346 | EXOSC5 (bg=5.38%) | K562 | - | chrX | | 73071711 | 73071762 |
| 3.04232622370337 | EXOSC5 (bg=5.38%) | K562 | - | chrX | | 73071716 | 73071761 |
| 2.2183185368202 | NCBP2 (bg=1.49%) | K562 | - | chrX | | 73071718 | 73071765 |
| 3.5569852882719 | NIPBL (bg=5.39%) | K562 | - | chrX | | 73071691 | 73071773 |
| 2.83865435554392 | NIPBL (bg=5.39%) | K562 | - | chrX | | 73071702 | 73071768 |
| 5.000058269403 | RBM15 (bg=7.27%) | K562 | - | chrX | | 73071693 | 73071750 |
| 4.80864004778937 | RBM15 (bg=7.27%) | K562 | - | chrX | | 73071708 | 73071717 |
| 5.11309438100314 | RBM15 (bg=7.27%) | K562 | - | chrX | | 73071717 | 73071749 |
| 3.39471602164062 | RBM22 (bg=4.62%) | K562 | - | chrX | | 73071686 | 73071764 |
| 3.11476360134165 | RBM22 (bg=4.62%) | K562 | - | chrX | | 73071715 | 73071770 |
| 2.16037976360809 | SLTM (bg=2.2%) | K562 | - | chrX | | 73071659 | 73071752 |
| 3.9018614287942 | SRSF1 (bg=8.47%) | K562 | - | chrX | | 73071678 | 73071734 |
| 4.36441567973616 | SRSF1 (bg=8.47%) | K562 | - | chrX | | 73071704 | 73071754 |
| 3.8278526211756 | uchl5 (bg=11.16%) | K562 | - | chrX | | 73071701 | 73071764 |
| 3.67638304143123 | uchl5 (bg=11.16%) | K562 | - | chrX | | 73071705 | 73071755 |
| 2.8846353478126 | YWHAG (bg=1.87%) | K562 | - | chrX | | 73071708 | 73071762 |
| 2.89281848869987 | YWHAG (bg=1.87%) | K562 | - | chrX | | 73071710 | 73071769 |

  
  

| Match 41 in HUMAN | | | | | | | |
| --- | --- | --- | --- | --- | --- | --- | --- |
| Motif | Start in Seq (1 Indexed) | End in Seq (1 Indexed) | Strand | Chrm | Exon | Start in Chrm (0 Indexed) | End in Chrm (1 Indexed) |
| TGCAGTTA | 885 | 892 | - | chrX | 1 | 73071696 | 73071704 |
| eCLIP Fold-Enrichment | Binding Protein | Cell Line | Strand | Chrm | | Start in Chrm (0 Indexed) | End in Chrm (1 Indexed) |
| 2.61909192700725 | DGCR8 (bg=1.84%) | K562 | - | chrX | | 73071692 | 73071771 |
| 3.5569852882719 | NIPBL (bg=5.39%) | K562 | - | chrX | | 73071691 | 73071773 |
| 2.83865435554392 | NIPBL (bg=5.39%) | K562 | - | chrX | | 73071702 | 73071768 |
| 5.000058269403 | RBM15 (bg=7.27%) | K562 | - | chrX | | 73071693 | 73071750 |
| 3.39471602164062 | RBM22 (bg=4.62%) | K562 | - | chrX | | 73071686 | 73071764 |
| 2.62864152108095 | SF3B1 (bg=2.48%) | K562 | - | chrX | | 73071670 | 73071704 |
| 2.16037976360809 | SLTM (bg=2.2%) | K562 | - | chrX | | 73071659 | 73071752 |
| 3.9018614287942 | SRSF1 (bg=8.47%) | K562 | - | chrX | | 73071678 | 73071734 |
| 4.36441567973616 | SRSF1 (bg=8.47%) | K562 | - | chrX | | 73071704 | 73071754 |
| 4.654155249443 | uchl5 (bg=11.16%) | K562 | - | chrX | | 73071655 | 73071701 |
| 3.8278526211756 | uchl5 (bg=11.16%) | K562 | - | chrX | | 73071701 | 73071764 |

  
  

| Match 42 in HUMAN | | | | | | | |
| --- | --- | --- | --- | --- | --- | --- | --- |
| Motif | Start in Seq (1 Indexed) | End in Seq (1 Indexed) | Strand | Chrm | Exon | Start in Chrm (0 Indexed) | End in Chrm (1 Indexed) |
| GGAGGAAA | 916 | 923 | - | chrX | 1 | 73071665 | 73071673 |
| eCLIP Fold-Enrichment | Binding Protein | Cell Line | Strand | Chrm | | Start in Chrm (0 Indexed) | End in Chrm (1 Indexed) |
| 2.10060634091133 | DDX51 (bg=1.63%) | K562 | - | chrX | | 73071648 | 73071683 |
| 2.15980335275215 | DHX30 (bg=0.14%) | K562 | - | chrX | | 73071659 | 73071686 |
| 3.61669840420183 | EXOSC5 (bg=5.38%) | K562 | - | chrX | | 73071625 | 73071674 |
| 3.33020225110458 | HNRNPM (bg=4.29%) | K562 | - | chrX | | 73071643 | 73071674 |
| 2.98971197091769 | HNRNPM (bg=4.29%) | K562 | - | chrX | | 73071646 | 73071673 |
| 3.08901943116788 | NIPBL (bg=5.39%) | K562 | - | chrX | | 73071608 | 73071691 |
| 4.24819379885745 | RBM15 (bg=7.27%) | K562 | - | chrX | | 73071652 | 73071693 |
| 4.17890556463357 | RBM15 (bg=7.27%) | K562 | - | chrX | | 73071654 | 73071688 |
| 2.62864152108095 | SF3B1 (bg=2.48%) | K562 | - | chrX | | 73071670 | 73071704 |
| 2.16037976360809 | SLTM (bg=2.2%) | K562 | - | chrX | | 73071659 | 73071752 |
| 4.654155249443 | uchl5 (bg=11.16%) | K562 | - | chrX | | 73071655 | 73071701 |
| 4.20822620669615 | uchl5 (bg=11.16%) | K562 | - | chrX | | 73071656 | 73071678 |

  
  

| Match 43 in HUMAN | | | | | | | |
| --- | --- | --- | --- | --- | --- | --- | --- |
| Motif | Start in Seq (1 Indexed) | End in Seq (1 Indexed) | Strand | Chrm | Exon | Start in Chrm (0 Indexed) | End in Chrm (1 Indexed) |
| TTGCCGC | 943 | 949 | - | chrX | 1 | 73071639 | 73071646 |
| eCLIP Fold-Enrichment | Binding Protein | Cell Line | Strand | Chrm | | Start in Chrm (0 Indexed) | End in Chrm (1 Indexed) |
| 3.61669840420183 | EXOSC5 (bg=5.38%) | K562 | - | chrX | | 73071625 | 73071674 |
| 3.33020225110458 | HNRNPM (bg=4.29%) | K562 | - | chrX | | 73071643 | 73071674 |
| 2.98971197091769 | HNRNPM (bg=4.29%) | K562 | - | chrX | | 73071646 | 73071673 |
| 3.08901943116788 | NIPBL (bg=5.39%) | K562 | - | chrX | | 73071608 | 73071691 |
| 4.40518029981676 | RBM15 (bg=7.27%) | K562 | - | chrX | | 73071627 | 73071652 |
| 4.25416791428993 | RBM15 (bg=7.27%) | K562 | - | chrX | | 73071632 | 73071654 |
| 3.18035194296922 | RBM22 (bg=4.62%) | K562 | - | chrX | | 73071611 | 73071664 |
| 4.16220165539179 | uchl5 (bg=11.16%) | K562 | - | chrX | | 73071629 | 73071655 |
| 4.02324878193433 | uchl5 (bg=11.16%) | K562 | - | chrX | | 73071632 | 73071656 |

  
  

| Match 44 in HUMAN | | | | | | | |
| --- | --- | --- | --- | --- | --- | --- | --- |
| Motif | Start in Seq (1 Indexed) | End in Seq (1 Indexed) | Strand | Chrm | Exon | Start in Chrm (0 Indexed) | End in Chrm (1 Indexed) |
| CTCGGCT | 954 | 960 | - | chrX | 1 | 73071628 | 73071635 |
| eCLIP Fold-Enrichment | Binding Protein | Cell Line | Strand | Chrm | | Start in Chrm (0 Indexed) | End in Chrm (1 Indexed) |
| 3.61669840420183 | EXOSC5 (bg=5.38%) | K562 | - | chrX | | 73071625 | 73071674 |
| 3.08901943116788 | NIPBL (bg=5.39%) | K562 | - | chrX | | 73071608 | 73071691 |
| 3.92536655704108 | RBM15 (bg=7.27%) | K562 | - | chrX | | 73071621 | 73071632 |
| 4.40518029981676 | RBM15 (bg=7.27%) | K562 | - | chrX | | 73071627 | 73071652 |
| 4.25416791428993 | RBM15 (bg=7.27%) | K562 | - | chrX | | 73071632 | 73071654 |
| 3.18035194296922 | RBM22 (bg=4.62%) | K562 | - | chrX | | 73071611 | 73071664 |
| 2.41807724069347 | SDAD1 (bg=2.97%) | K562 | - | chrX | | 73071561 | 73071629 |
| 3.82157256087648 | uchl5 (bg=11.16%) | K562 | - | chrX | | 73071618 | 73071629 |
| 4.00009354808574 | uchl5 (bg=11.16%) | K562 | - | chrX | | 73071618 | 73071632 |
| 4.16220165539179 | uchl5 (bg=11.16%) | K562 | - | chrX | | 73071629 | 73071655 |
| 4.02324878193433 | uchl5 (bg=11.16%) | K562 | - | chrX | | 73071632 | 73071656 |

  
  

| Match 45 in HUMAN | | | | | | | |
| --- | --- | --- | --- | --- | --- | --- | --- |
| Motif | Start in Seq (1 Indexed) | End in Seq (1 Indexed) | Strand | Chrm | Exon | Start in Chrm (0 Indexed) | End in Chrm (1 Indexed) |
| AGGGCTA | 962 | 968 | - | chrX | 1 | 73071620 | 73071627 |
| eCLIP Fold-Enrichment | Binding Protein | Cell Line | Strand | Chrm | | Start in Chrm (0 Indexed) | End in Chrm (1 Indexed) |
| 4.08903677422076 | EXOSC5 (bg=5.38%) | K562 | - | chrX | | 73071586 | 73071625 |
| 3.61669840420183 | EXOSC5 (bg=5.38%) | K562 | - | chrX | | 73071625 | 73071674 |
| 2.22128093310424 | HNRNPA1 (bg=2.57%) | K562 | - | chrX | | 73071588 | 73071625 |
| 3.08901943116788 | NIPBL (bg=5.39%) | K562 | - | chrX | | 73071608 | 73071691 |
| 4.24819379885745 | RBM15 (bg=7.27%) | K562 | - | chrX | | 73071606 | 73071627 |
| 3.85766169790269 | RBM15 (bg=7.27%) | K562 | - | chrX | | 73071607 | 73071621 |
| 3.92536655704108 | RBM15 (bg=7.27%) | K562 | - | chrX | | 73071621 | 73071632 |
| 4.40518029981676 | RBM15 (bg=7.27%) | K562 | - | chrX | | 73071627 | 73071652 |
| 3.18035194296922 | RBM22 (bg=4.62%) | K562 | - | chrX | | 73071611 | 73071664 |
| 2.41807724069347 | SDAD1 (bg=2.97%) | K562 | - | chrX | | 73071561 | 73071629 |
| 3.82157256087648 | uchl5 (bg=11.16%) | K562 | - | chrX | | 73071618 | 73071629 |
| 4.00009354808574 | uchl5 (bg=11.16%) | K562 | - | chrX | | 73071618 | 73071632 |

  
  

| Match 46 in HUMAN | | | | | | | |
| --- | --- | --- | --- | --- | --- | --- | --- |
| Motif | Start in Seq (1 Indexed) | End in Seq (1 Indexed) | Strand | Chrm | Exon | Start in Chrm (0 Indexed) | End in Chrm (1 Indexed) |
| TGCTAAGT | 977 | 984 | - | chrX | 1 | 73071604 | 73071612 |
| eCLIP Fold-Enrichment | Binding Protein | Cell Line | Strand | Chrm | | Start in Chrm (0 Indexed) | End in Chrm (1 Indexed) |
| 2.58082885122454 | AARS (bg=2.18%) | K562 | - | chrX | | 73071555 | 73071609 |
| 2.16097574481551 | AARS (bg=2.18%) | K562 | - | chrX | | 73071565 | 73071611 |
| 3.76458305582422 | EXOSC5 (bg=5.38%) | K562 | - | chrX | | 73071548 | 73071617 |
| 4.08903677422076 | EXOSC5 (bg=5.38%) | K562 | - | chrX | | 73071586 | 73071625 |
| 2.3369901462874 | HNRNPA1 (bg=2.57%) | K562 | - | chrX | | 73071534 | 73071607 |
| 2.22128093310424 | HNRNPA1 (bg=2.57%) | K562 | - | chrX | | 73071588 | 73071625 |
| 3.54667525266942 | NIPBL (bg=5.39%) | K562 | - | chrX | | 73071559 | 73071617 |
| 3.36002454811026 | NIPBL (bg=5.39%) | K562 | - | chrX | | 73071567 | 73071608 |
| 3.08901943116788 | NIPBL (bg=5.39%) | K562 | - | chrX | | 73071608 | 73071691 |
| 3.99808061996255 | RBM15 (bg=7.27%) | K562 | - | chrX | | 73071548 | 73071606 |
| 3.75440773610566 | RBM15 (bg=7.27%) | K562 | - | chrX | | 73071574 | 73071607 |
| 4.24819379885745 | RBM15 (bg=7.27%) | K562 | - | chrX | | 73071606 | 73071627 |
| 3.85766169790269 | RBM15 (bg=7.27%) | K562 | - | chrX | | 73071607 | 73071621 |
| 3.44338634880302 | RBM22 (bg=4.62%) | K562 | - | chrX | | 73071543 | 73071611 |
| 3.18035194296922 | RBM22 (bg=4.62%) | K562 | - | chrX | | 73071611 | 73071664 |
| 2.41807724069347 | SDAD1 (bg=2.97%) | K562 | - | chrX | | 73071561 | 73071629 |
| 4.35678871106837 | uchl5 (bg=11.16%) | K562 | - | chrX | | 73071597 | 73071606 |
| 4.14653619258535 | uchl5 (bg=11.16%) | K562 | - | chrX | | 73071599 | 73071606 |
| 3.99217237633316 | uchl5 (bg=11.16%) | K562 | - | chrX | | 73071606 | 73071618 |
| 3.9461355519263 | uchl5 (bg=11.16%) | K562 | - | chrX | | 73071606 | 73071618 |

  
  

| Match 47 in HUMAN | | | | | | | |
| --- | --- | --- | --- | --- | --- | --- | --- |
| Motif | Start in Seq (1 Indexed) | End in Seq (1 Indexed) | Strand | Chrm | Exon | Start in Chrm (0 Indexed) | End in Chrm (1 Indexed) |
| TAAACTAGGGAGGCAAGATG | 985 | 1004 | - | chrX | 1 | 73071584 | 73071604 |
| eCLIP Fold-Enrichment | Binding Protein | Cell Line | Strand | Chrm | | Start in Chrm (0 Indexed) | End in Chrm (1 Indexed) |
| 2.58082885122454 | AARS (bg=2.18%) | K562 | - | chrX | | 73071555 | 73071609 |
| 2.16097574481551 | AARS (bg=2.18%) | K562 | - | chrX | | 73071565 | 73071611 |
| 3.97141643420686 | EXOSC5 (bg=5.38%) | K562 | - | chrX | | 73071546 | 73071586 |
| 3.76458305582422 | EXOSC5 (bg=5.38%) | K562 | - | chrX | | 73071548 | 73071617 |
| 4.08903677422076 | EXOSC5 (bg=5.38%) | K562 | - | chrX | | 73071586 | 73071625 |
| 2.49403658339176 | HNRNPA1 (bg=2.57%) | K562 | - | chrX | | 73071532 | 73071588 |
| 2.3369901462874 | HNRNPA1 (bg=2.57%) | K562 | - | chrX | | 73071534 | 73071607 |
| 2.22128093310424 | HNRNPA1 (bg=2.57%) | K562 | - | chrX | | 73071588 | 73071625 |
| 3.54667525266942 | NIPBL (bg=5.39%) | K562 | - | chrX | | 73071559 | 73071617 |
| 3.36002454811026 | NIPBL (bg=5.39%) | K562 | - | chrX | | 73071567 | 73071608 |
| 3.99808061996255 | RBM15 (bg=7.27%) | K562 | - | chrX | | 73071548 | 73071606 |
| 3.75440773610566 | RBM15 (bg=7.27%) | K562 | - | chrX | | 73071574 | 73071607 |
| 3.44338634880302 | RBM22 (bg=4.62%) | K562 | - | chrX | | 73071543 | 73071611 |
| 3.59063623161588 | RBM22 (bg=4.62%) | K562 | - | chrX | | 73071549 | 73071591 |
| 2.41807724069347 | SDAD1 (bg=2.97%) | K562 | - | chrX | | 73071561 | 73071629 |
| 3.65381329754738 | uchl5 (bg=11.16%) | K562 | - | chrX | | 73071541 | 73071588 |
| 3.60110205605084 | uchl5 (bg=11.16%) | K562 | - | chrX | | 73071567 | 73071588 |
| 3.88523288850513 | uchl5 (bg=11.16%) | K562 | - | chrX | | 73071588 | 73071597 |
| 3.80545212708314 | uchl5 (bg=11.16%) | K562 | - | chrX | | 73071588 | 73071599 |
| 4.35678871106837 | uchl5 (bg=11.16%) | K562 | - | chrX | | 73071597 | 73071606 |
| 4.14653619258535 | uchl5 (bg=11.16%) | K562 | - | chrX | | 73071599 | 73071606 |

  
  

| Match 48 in HUMAN | | | | | | | |
| --- | --- | --- | --- | --- | --- | --- | --- |
| Motif | Start in Seq (1 Indexed) | End in Seq (1 Indexed) | Strand | Chrm | Exon | Start in Chrm (0 Indexed) | End in Chrm (1 Indexed) |
| AGGCAAGA | 995 | 1002 | - | chrX | 1 | 73071586 | 73071594 |
| eCLIP Fold-Enrichment | Binding Protein | Cell Line | Strand | Chrm | | Start in Chrm (0 Indexed) | End in Chrm (1 Indexed) |
| 2.58082885122454 | AARS (bg=2.18%) | K562 | - | chrX | | 73071555 | 73071609 |
| 2.16097574481551 | AARS (bg=2.18%) | K562 | - | chrX | | 73071565 | 73071611 |
| 3.97141643420686 | EXOSC5 (bg=5.38%) | K562 | - | chrX | | 73071546 | 73071586 |
| 3.76458305582422 | EXOSC5 (bg=5.38%) | K562 | - | chrX | | 73071548 | 73071617 |
| 4.08903677422076 | EXOSC5 (bg=5.38%) | K562 | - | chrX | | 73071586 | 73071625 |
| 2.49403658339176 | HNRNPA1 (bg=2.57%) | K562 | - | chrX | | 73071532 | 73071588 |
| 2.3369901462874 | HNRNPA1 (bg=2.57%) | K562 | - | chrX | | 73071534 | 73071607 |
| 2.22128093310424 | HNRNPA1 (bg=2.57%) | K562 | - | chrX | | 73071588 | 73071625 |
| 3.54667525266942 | NIPBL (bg=5.39%) | K562 | - | chrX | | 73071559 | 73071617 |
| 3.36002454811026 | NIPBL (bg=5.39%) | K562 | - | chrX | | 73071567 | 73071608 |
| 3.99808061996255 | RBM15 (bg=7.27%) | K562 | - | chrX | | 73071548 | 73071606 |
| 3.75440773610566 | RBM15 (bg=7.27%) | K562 | - | chrX | | 73071574 | 73071607 |
| 3.44338634880302 | RBM22 (bg=4.62%) | K562 | - | chrX | | 73071543 | 73071611 |
| 3.59063623161588 | RBM22 (bg=4.62%) | K562 | - | chrX | | 73071549 | 73071591 |
| 2.41807724069347 | SDAD1 (bg=2.97%) | K562 | - | chrX | | 73071561 | 73071629 |
| 3.65381329754738 | uchl5 (bg=11.16%) | K562 | - | chrX | | 73071541 | 73071588 |
| 3.60110205605084 | uchl5 (bg=11.16%) | K562 | - | chrX | | 73071567 | 73071588 |
| 3.88523288850513 | uchl5 (bg=11.16%) | K562 | - | chrX | | 73071588 | 73071597 |
| 3.80545212708314 | uchl5 (bg=11.16%) | K562 | - | chrX | | 73071588 | 73071599 |

  
  

| Match 49 in HUMAN | | | | | | | |
| --- | --- | --- | --- | --- | --- | --- | --- |
| Motif | Start in Seq (1 Indexed) | End in Seq (1 Indexed) | Strand | Chrm | Exon | Start in Chrm (0 Indexed) | End in Chrm (1 Indexed) |
| CAGGCAGAGGAA | 1018 | 1029 | - | chrX | 1 | 73071559 | 73071571 |
| eCLIP Fold-Enrichment | Binding Protein | Cell Line | Strand | Chrm | | Start in Chrm (0 Indexed) | End in Chrm (1 Indexed) |
| 2.58082885122454 | AARS (bg=2.18%) | K562 | - | chrX | | 73071555 | 73071609 |
| 2.16097574481551 | AARS (bg=2.18%) | K562 | - | chrX | | 73071565 | 73071611 |
| 3.97141643420686 | EXOSC5 (bg=5.38%) | K562 | - | chrX | | 73071546 | 73071586 |
| 3.76458305582422 | EXOSC5 (bg=5.38%) | K562 | - | chrX | | 73071548 | 73071617 |
| 2.49403658339176 | HNRNPA1 (bg=2.57%) | K562 | - | chrX | | 73071532 | 73071588 |
| 2.3369901462874 | HNRNPA1 (bg=2.57%) | K562 | - | chrX | | 73071534 | 73071607 |
| 4.16850088037045 | HNRNPM (bg=4.29%) | K562 | - | chrX | | 73071543 | 73071583 |
| 3.53136724522167 | HNRNPM (bg=4.29%) | K562 | - | chrX | | 73071552 | 73071579 |
| 3.54667525266942 | NIPBL (bg=5.39%) | K562 | - | chrX | | 73071559 | 73071617 |
| 3.36002454811026 | NIPBL (bg=5.39%) | K562 | - | chrX | | 73071567 | 73071608 |
| 3.99808061996255 | RBM15 (bg=7.27%) | K562 | - | chrX | | 73071548 | 73071606 |
| 3.26691104482012 | RBM15 (bg=7.27%) | K562 | - | chrX | | 73071558 | 73071569 |
| 3.4295680457984 | RBM15 (bg=7.27%) | K562 | - | chrX | | 73071569 | 73071574 |
| 3.44338634880302 | RBM22 (bg=4.62%) | K562 | - | chrX | | 73071543 | 73071611 |
| 3.59063623161588 | RBM22 (bg=4.62%) | K562 | - | chrX | | 73071549 | 73071591 |
| 2.41807724069347 | SDAD1 (bg=2.97%) | K562 | - | chrX | | 73071561 | 73071629 |
| 3.65381329754738 | uchl5 (bg=11.16%) | K562 | - | chrX | | 73071541 | 73071588 |
| 3.50299197060099 | uchl5 (bg=11.16%) | K562 | - | chrX | | 73071547 | 73071567 |
| 3.60110205605084 | uchl5 (bg=11.16%) | K562 | - | chrX | | 73071567 | 73071588 |

  
  

| Match 50 in HUMAN | | | | | | | |
| --- | --- | --- | --- | --- | --- | --- | --- |
| Motif | Start in Seq (1 Indexed) | End in Seq (1 Indexed) | Strand | Chrm | Exon | Start in Chrm (0 Indexed) | End in Chrm (1 Indexed) |
| TGCATTG | 1036 | 1042 | - | chrX | 1 | 73071546 | 73071553 |
| eCLIP Fold-Enrichment | Binding Protein | Cell Line | Strand | Chrm | | Start in Chrm (0 Indexed) | End in Chrm (1 Indexed) |
| 3.97141643420686 | EXOSC5 (bg=5.38%) | K562 | - | chrX | | 73071546 | 73071586 |
| 3.76458305582422 | EXOSC5 (bg=5.38%) | K562 | - | chrX | | 73071548 | 73071617 |
| 2.49403658339176 | HNRNPA1 (bg=2.57%) | K562 | - | chrX | | 73071532 | 73071588 |
| 2.3369901462874 | HNRNPA1 (bg=2.57%) | K562 | - | chrX | | 73071534 | 73071607 |
| 4.16850088037045 | HNRNPM (bg=4.29%) | K562 | - | chrX | | 73071543 | 73071583 |
| 3.53136724522167 | HNRNPM (bg=4.29%) | K562 | - | chrX | | 73071552 | 73071579 |
| 3.99808061996255 | RBM15 (bg=7.27%) | K562 | - | chrX | | 73071548 | 73071606 |
| 3.44338634880302 | RBM22 (bg=4.62%) | K562 | - | chrX | | 73071543 | 73071611 |
| 3.59063623161588 | RBM22 (bg=4.62%) | K562 | - | chrX | | 73071549 | 73071591 |
| 3.40665763582746 | uchl5 (bg=11.16%) | K562 | - | chrX | | 73071539 | 73071547 |
| 3.65381329754738 | uchl5 (bg=11.16%) | K562 | - | chrX | | 73071541 | 73071588 |
| 3.50299197060099 | uchl5 (bg=11.16%) | K562 | - | chrX | | 73071547 | 73071567 |

  
  

| Match 51 in HUMAN | | | | | | | |
| --- | --- | --- | --- | --- | --- | --- | --- |
| Motif | Start in Seq (1 Indexed) | End in Seq (1 Indexed) | Strand | Chrm | Exon | Start in Chrm (0 Indexed) | End in Chrm (1 Indexed) |
| ATGAGCTA | 1044 | 1051 | - | chrX | 1 | 73071537 | 73071545 |
| eCLIP Fold-Enrichment | Binding Protein | Cell Line | Strand | Chrm | | Start in Chrm (0 Indexed) | End in Chrm (1 Indexed) |
| 2.49403658339176 | HNRNPA1 (bg=2.57%) | K562 | - | chrX | | 73071532 | 73071588 |
| 2.3369901462874 | HNRNPA1 (bg=2.57%) | K562 | - | chrX | | 73071534 | 73071607 |
| 4.16850088037045 | HNRNPM (bg=4.29%) | K562 | - | chrX | | 73071543 | 73071583 |
| 3.44338634880302 | RBM22 (bg=4.62%) | K562 | - | chrX | | 73071543 | 73071611 |
| 3.40665763582746 | uchl5 (bg=11.16%) | K562 | - | chrX | | 73071539 | 73071547 |
| 3.65381329754738 | uchl5 (bg=11.16%) | K562 | - | chrX | | 73071541 | 73071588 |

  
  

| Match 52 in HUMAN | | | | | | | |
| --- | --- | --- | --- | --- | --- | --- | --- |
| Motif | Start in Seq (1 Indexed) | End in Seq (1 Indexed) | Strand | Chrm | Exon | Start in Chrm (0 Indexed) | End in Chrm (1 Indexed) |
| GATTTGGG | 1070 | 1077 | - | chrX | 1 | 73071511 | 73071519 |
| eCLIP Fold-Enrichment | Binding Protein | Cell Line | Strand | Chrm | | Start in Chrm (0 Indexed) | End in Chrm (1 Indexed) |
| 2.29153145492664 | CPEB4 (bg=1.89%) | K562 | - | chrX | | 73071494 | 73071526 |
| 3.77779327534435 | EXOSC5 (bg=5.38%) | K562 | - | chrX | | 73071459 | 73071511 |
| 2.19274477344022 | GNL3 (bg=0.43%) | K562 | - | chrX | | 73071485 | 73071511 |
| 4.35865640628715 | HNRNPM (bg=4.29%) | K562 | - | chrX | | 73071458 | 73071516 |
| 4.7170595625223 | HNRNPM (bg=4.29%) | K562 | - | chrX | | 73071507 | 73071516 |
| 3.28704369603192 | TARDBP (bg=2.79%) | K562 | - | chrX | | 73071438 | 73071524 |
| 3.26978071129285 | TARDBP (bg=2.79%) | K562 | - | chrX | | 73071444 | 73071524 |

  
  

| Match 53 in HUMAN | | | | | | | |
| --- | --- | --- | --- | --- | --- | --- | --- |
| Motif | Start in Seq (1 Indexed) | End in Seq (1 Indexed) | Strand | Chrm | Exon | Start in Chrm (0 Indexed) | End in Chrm (1 Indexed) |
| CTTGTTAGGA | 1079 | 1088 | - | chrX | 1 | 73071500 | 73071510 |
| eCLIP Fold-Enrichment | Binding Protein | Cell Line | Strand | Chrm | | Start in Chrm (0 Indexed) | End in Chrm (1 Indexed) |
| 2.29153145492664 | CPEB4 (bg=1.89%) | K562 | - | chrX | | 73071494 | 73071526 |
| 3.42195838685101 | EXOSC5 (bg=5.38%) | K562 | - | chrX | | 73071459 | 73071500 |
| 3.77779327534435 | EXOSC5 (bg=5.38%) | K562 | - | chrX | | 73071459 | 73071511 |
| 2.19274477344022 | GNL3 (bg=0.43%) | K562 | - | chrX | | 73071485 | 73071511 |
| 4.35865640628715 | HNRNPM (bg=4.29%) | K562 | - | chrX | | 73071458 | 73071516 |
| 4.13835605227282 | HNRNPM (bg=4.29%) | K562 | - | chrX | | 73071467 | 73071507 |
| 4.7170595625223 | HNRNPM (bg=4.29%) | K562 | - | chrX | | 73071507 | 73071516 |
| 3.28704369603192 | TARDBP (bg=2.79%) | K562 | - | chrX | | 73071438 | 73071524 |
| 3.26978071129285 | TARDBP (bg=2.79%) | K562 | - | chrX | | 73071444 | 73071524 |
| 3.37031045492128 | uchl5 (bg=11.16%) | K562 | - | chrX | | 73071459 | 73071510 |

  
  

| Match 54 in HUMAN | | | | | | | |
| --- | --- | --- | --- | --- | --- | --- | --- |
| Motif | Start in Seq (1 Indexed) | End in Seq (1 Indexed) | Strand | Chrm | Exon | Start in Chrm (0 Indexed) | End in Chrm (1 Indexed) |
| GAAGGTT | 1161 | 1167 | - | chrX | 1 | 73071421 | 73071428 |
| eCLIP Fold-Enrichment | Binding Protein | Cell Line | Strand | Chrm | | Start in Chrm (0 Indexed) | End in Chrm (1 Indexed) |
| 3.01151507661784 | EXOSC5 (bg=5.38%) | K562 | - | chrX | | 73071356 | 73071436 |
| 3.01924495929748 | EXOSC5 (bg=5.38%) | K562 | - | chrX | | 73071390 | 73071437 |
| 2.22270523417746 | NPM1 (bg=1.21%) | K562 | - | chrX | | 73071385 | 73071425 |
| 2.07600951126732 | RBFOX2 (bg=4.63%) | K562 | - | chrX | | 73071321 | 73071463 |
| 2.86468149454535 | RBM15 (bg=7.27%) | K562 | - | chrX | | 73071394 | 73071427 |
| 3.91153518454142 | RBM22 (bg=4.62%) | K562 | - | chrX | | 73071332 | 73071498 |
| 3.74033026027023 | uchl5 (bg=11.16%) | K562 | - | chrX | | 73071404 | 73071437 |
| 3.7577999691769 | uchl5 (bg=11.16%) | K562 | - | chrX | | 73071404 | 73071442 |

  
  

| Match 55 in HUMAN | | | | | | | |
| --- | --- | --- | --- | --- | --- | --- | --- |
| Motif | Start in Seq (1 Indexed) | End in Seq (1 Indexed) | Strand | Chrm | Exon | Start in Chrm (0 Indexed) | End in Chrm (1 Indexed) |
| AAAATGGCGATTTTGAC | 1192 | 1208 | - | chrX | 1 | 73071380 | 73071397 |
| eCLIP Fold-Enrichment | Binding Protein | Cell Line | Strand | Chrm | | Start in Chrm (0 Indexed) | End in Chrm (1 Indexed) |
| 3.99302163865387 | EXOSC5 (bg=5.38%) | K562 | - | chrX | | 73071344 | 73071390 |
| 3.01151507661784 | EXOSC5 (bg=5.38%) | K562 | - | chrX | | 73071356 | 73071436 |
| 3.01924495929748 | EXOSC5 (bg=5.38%) | K562 | - | chrX | | 73071390 | 73071437 |
| 2.32238005385682 | GNL3 (bg=0.43%) | K562 | - | chrX | | 73071379 | 73071395 |
| 2.22270523417746 | NPM1 (bg=1.21%) | K562 | - | chrX | | 73071385 | 73071425 |
| 2.07600951126732 | RBFOX2 (bg=4.63%) | K562 | - | chrX | | 73071321 | 73071463 |
| 3.30665953664248 | RBM15 (bg=7.27%) | K562 | - | chrX | | 73071342 | 73071394 |
| 2.86468149454535 | RBM15 (bg=7.27%) | K562 | - | chrX | | 73071394 | 73071427 |
| 3.91153518454142 | RBM22 (bg=4.62%) | K562 | - | chrX | | 73071332 | 73071498 |
| 3.28578165008746 | RBM22 (bg=4.62%) | K562 | - | chrX | | 73071388 | 73071403 |
| 4.51116933396658 | uchl5 (bg=11.16%) | K562 | - | chrX | | 73071373 | 73071386 |
| 4.42440668779608 | uchl5 (bg=11.16%) | K562 | - | chrX | | 73071375 | 73071387 |
| 4.12912366029855 | uchl5 (bg=11.16%) | K562 | - | chrX | | 73071386 | 73071404 |
| 4.13816489080096 | uchl5 (bg=11.16%) | K562 | - | chrX | | 73071387 | 73071404 |

  
  

| Match 56 in HUMAN | | | | | | | |
| --- | --- | --- | --- | --- | --- | --- | --- |
| Motif | Start in Seq (1 Indexed) | End in Seq (1 Indexed) | Strand | Chrm | Exon | Start in Chrm (0 Indexed) | End in Chrm (1 Indexed) |
| GCATTGCT | 1215 | 1222 | - | chrX | 1 | 73071366 | 73071374 |
| eCLIP Fold-Enrichment | Binding Protein | Cell Line | Strand | Chrm | | Start in Chrm (0 Indexed) | End in Chrm (1 Indexed) |
| 3.99302163865387 | EXOSC5 (bg=5.38%) | K562 | - | chrX | | 73071344 | 73071390 |
| 3.01151507661784 | EXOSC5 (bg=5.38%) | K562 | - | chrX | | 73071356 | 73071436 |
| 5.02280121602849 | HNRNPM (bg=4.29%) | K562 | - | chrX | | 73071318 | 73071377 |
| 4.90499251311537 | HNRNPM (bg=4.29%) | K562 | - | chrX | | 73071321 | 73071372 |
| 2.07600951126732 | RBFOX2 (bg=4.63%) | K562 | - | chrX | | 73071321 | 73071463 |
| 3.30665953664248 | RBM15 (bg=7.27%) | K562 | - | chrX | | 73071342 | 73071394 |
| 3.05759926841144 | RBM15 (bg=7.27%) | K562 | - | chrX | | 73071352 | 73071373 |
| 3.91153518454142 | RBM22 (bg=4.62%) | K562 | - | chrX | | 73071332 | 73071498 |
| 4.53982873878136 | uchl5 (bg=11.16%) | K562 | - | chrX | | 73071329 | 73071373 |
| 4.46959295742905 | uchl5 (bg=11.16%) | K562 | - | chrX | | 73071336 | 73071375 |
| 4.51116933396658 | uchl5 (bg=11.16%) | K562 | - | chrX | | 73071373 | 73071386 |

  
  

| Match 57 in HUMAN | | | | | | | |
| --- | --- | --- | --- | --- | --- | --- | --- |
| Motif | Start in Seq (1 Indexed) | End in Seq (1 Indexed) | Strand | Chrm | Exon | Start in Chrm (0 Indexed) | End in Chrm (1 Indexed) |
| AGCATGGC | 1224 | 1231 | - | chrX | 1 | 73071357 | 73071365 |
| eCLIP Fold-Enrichment | Binding Protein | Cell Line | Strand | Chrm | | Start in Chrm (0 Indexed) | End in Chrm (1 Indexed) |
| 3.99302163865387 | EXOSC5 (bg=5.38%) | K562 | - | chrX | | 73071344 | 73071390 |
| 3.01151507661784 | EXOSC5 (bg=5.38%) | K562 | - | chrX | | 73071356 | 73071436 |
| 5.02280121602849 | HNRNPM (bg=4.29%) | K562 | - | chrX | | 73071318 | 73071377 |
| 4.90499251311537 | HNRNPM (bg=4.29%) | K562 | - | chrX | | 73071321 | 73071372 |
| 2.07600951126732 | RBFOX2 (bg=4.63%) | K562 | - | chrX | | 73071321 | 73071463 |
| 3.30665953664248 | RBM15 (bg=7.27%) | K562 | - | chrX | | 73071342 | 73071394 |
| 3.05759926841144 | RBM15 (bg=7.27%) | K562 | - | chrX | | 73071352 | 73071373 |
| 3.91153518454142 | RBM22 (bg=4.62%) | K562 | - | chrX | | 73071332 | 73071498 |
| 4.53982873878136 | uchl5 (bg=11.16%) | K562 | - | chrX | | 73071329 | 73071373 |
| 4.46959295742905 | uchl5 (bg=11.16%) | K562 | - | chrX | | 73071336 | 73071375 |

  
  

| Match 58 in HUMAN | | | | | | | |
| --- | --- | --- | --- | --- | --- | --- | --- |
| Motif | Start in Seq (1 Indexed) | End in Seq (1 Indexed) | Strand | Chrm | Exon | Start in Chrm (0 Indexed) | End in Chrm (1 Indexed) |
| TGCTTTGTTAG | 1238 | 1248 | - | chrX | 1 | 73071340 | 73071351 |
| eCLIP Fold-Enrichment | Binding Protein | Cell Line | Strand | Chrm | | Start in Chrm (0 Indexed) | End in Chrm (1 Indexed) |
| 3.99302163865387 | EXOSC5 (bg=5.38%) | K562 | - | chrX | | 73071344 | 73071390 |
| 5.02280121602849 | HNRNPM (bg=4.29%) | K562 | - | chrX | | 73071318 | 73071377 |
| 4.90499251311537 | HNRNPM (bg=4.29%) | K562 | - | chrX | | 73071321 | 73071372 |
| 2.07600951126732 | RBFOX2 (bg=4.63%) | K562 | - | chrX | | 73071321 | 73071463 |
| 3.30665953664248 | RBM15 (bg=7.27%) | K562 | - | chrX | | 73071342 | 73071394 |
| 3.91153518454142 | RBM22 (bg=4.62%) | K562 | - | chrX | | 73071332 | 73071498 |
| 3.86397394581797 | SDAD1 (bg=2.97%) | K562 | - | chrX | | 73071343 | 73071350 |
| 4.53982873878136 | uchl5 (bg=11.16%) | K562 | - | chrX | | 73071329 | 73071373 |
| 4.46959295742905 | uchl5 (bg=11.16%) | K562 | - | chrX | | 73071336 | 73071375 |

  
  

| Match 59 in HUMAN | | | | | | | |
| --- | --- | --- | --- | --- | --- | --- | --- |
| Motif | Start in Seq (1 Indexed) | End in Seq (1 Indexed) | Strand | Chrm | Exon | Start in Chrm (0 Indexed) | End in Chrm (1 Indexed) |
| CAAAATGGCGGA | 1255 | 1266 | - | chrX | 1 | 73071322 | 73071334 |
| eCLIP Fold-Enrichment | Binding Protein | Cell Line | Strand | Chrm | | Start in Chrm (0 Indexed) | End in Chrm (1 Indexed) |
| 5.02280121602849 | HNRNPM (bg=4.29%) | K562 | - | chrX | | 73071318 | 73071377 |
| 4.90499251311537 | HNRNPM (bg=4.29%) | K562 | - | chrX | | 73071321 | 73071372 |
| 2.07600951126732 | RBFOX2 (bg=4.63%) | K562 | - | chrX | | 73071321 | 73071463 |
| 3.6009276257895 | RBM22 (bg=4.62%) | K562 | - | chrX | | 73071201 | 73071332 |
| 3.91153518454142 | RBM22 (bg=4.62%) | K562 | - | chrX | | 73071332 | 73071498 |
| 4.53982873878136 | uchl5 (bg=11.16%) | K562 | - | chrX | | 73071329 | 73071373 |

  
  

| Match 60 in HUMAN | | | | | | | |
| --- | --- | --- | --- | --- | --- | --- | --- |
| Motif | Start in Seq (1 Indexed) | End in Seq (1 Indexed) | Strand | Chrm | Exon | Start in Chrm (0 Indexed) | End in Chrm (1 Indexed) |
| CGCAGTGTTC | 1278 | 1287 | - | chrX | 1 | 73071301 | 73071311 |
| eCLIP Fold-Enrichment | Binding Protein | Cell Line | Strand | Chrm | | Start in Chrm (0 Indexed) | End in Chrm (1 Indexed) |
| 3.89348596510295 | EXOSC5 (bg=5.38%) | K562 | - | chrX | | 73071280 | 73071307 |
| 6.31534226869972 | HNRNPM (bg=4.29%) | K562 | - | chrX | | 73071256 | 73071318 |
| 6.32037412570315 | HNRNPM (bg=4.29%) | K562 | - | chrX | | 73071287 | 73071321 |
| 3.6009276257895 | RBM22 (bg=4.62%) | K562 | - | chrX | | 73071201 | 73071332 |

  
  

| Match 61 in HUMAN | | | | | | | |
| --- | --- | --- | --- | --- | --- | --- | --- |
| Motif | Start in Seq (1 Indexed) | End in Seq (1 Indexed) | Strand | Chrm | Exon | Start in Chrm (0 Indexed) | End in Chrm (1 Indexed) |
| AGTGGCGGGAAG | 1289 | 1300 | - | chrX | 1 | 73071288 | 73071300 |
| eCLIP Fold-Enrichment | Binding Protein | Cell Line | Strand | Chrm | | Start in Chrm (0 Indexed) | End in Chrm (1 Indexed) |
| 3.89348596510295 | EXOSC5 (bg=5.38%) | K562 | - | chrX | | 73071280 | 73071307 |
| 6.31534226869972 | HNRNPM (bg=4.29%) | K562 | - | chrX | | 73071256 | 73071318 |
| 6.32037412570315 | HNRNPM (bg=4.29%) | K562 | - | chrX | | 73071287 | 73071321 |
| 2.87768629519631 | LARP4 (bg=4.72%) | K562 | - | chrX | | 73071277 | 73071292 |
| 3.6009276257895 | RBM22 (bg=4.62%) | K562 | - | chrX | | 73071201 | 73071332 |

  
  

| Match 62 in HUMAN | | | | | | | |
| --- | --- | --- | --- | --- | --- | --- | --- |
| Motif | Start in Seq (1 Indexed) | End in Seq (1 Indexed) | Strand | Chrm | Exon | Start in Chrm (0 Indexed) | End in Chrm (1 Indexed) |
| CCACAT | 1302 | 1307 | - | chrX | 1 | 73071281 | 73071287 |
| eCLIP Fold-Enrichment | Binding Protein | Cell Line | Strand | Chrm | | Start in Chrm (0 Indexed) | End in Chrm (1 Indexed) |
| 3.89348596510295 | EXOSC5 (bg=5.38%) | K562 | - | chrX | | 73071280 | 73071307 |
| 6.18473598755512 | HNRNPM (bg=4.29%) | K562 | - | chrX | | 73071256 | 73071287 |
| 6.31534226869972 | HNRNPM (bg=4.29%) | K562 | - | chrX | | 73071256 | 73071318 |
| 6.32037412570315 | HNRNPM (bg=4.29%) | K562 | - | chrX | | 73071287 | 73071321 |
| 2.87768629519631 | LARP4 (bg=4.72%) | K562 | - | chrX | | 73071277 | 73071292 |
| 3.6009276257895 | RBM22 (bg=4.62%) | K562 | - | chrX | | 73071201 | 73071332 |
| 3.48624874333505 | SDAD1 (bg=2.97%) | K562 | - | chrX | | 73071223 | 73071281 |

  
  

| Match 63 in HUMAN | | | | | | | |
| --- | --- | --- | --- | --- | --- | --- | --- |
| Motif | Start in Seq (1 Indexed) | End in Seq (1 Indexed) | Strand | Chrm | Exon | Start in Chrm (0 Indexed) | End in Chrm (1 Indexed) |
| AGCATGG | 1337 | 1343 | - | chrX | 1 | 73071245 | 73071252 |
| eCLIP Fold-Enrichment | Binding Protein | Cell Line | Strand | Chrm | | Start in Chrm (0 Indexed) | End in Chrm (1 Indexed) |
| 3.33669026154036 | AARS (bg=2.18%) | K562 | - | chrX | | 73071196 | 73071247 |
| 3.26531240463024 | AARS (bg=2.18%) | K562 | - | chrX | | 73071203 | 73071256 |
| 4.52944482465756 | EXOSC5 (bg=5.38%) | K562 | - | chrX | | 73071195 | 73071273 |
| 4.072279445805 | EXOSC5 (bg=5.38%) | K562 | - | chrX | | 73071199 | 73071267 |
| 4.99660305552153 | HNRNPM (bg=4.29%) | K562 | - | chrX | | 73071189 | 73071256 |
| 4.86407658159773 | HNRNPM (bg=4.29%) | K562 | - | chrX | | 73071194 | 73071256 |
| 3.26982673913868 | NIPBL (bg=5.39%) | K562 | - | chrX | | 73071210 | 73071263 |
| 4.60897550227588 | RBM15 (bg=7.27%) | K562 | - | chrX | | 73071203 | 73071255 |
| 4.29206452204846 | RBM15 (bg=7.27%) | K562 | - | chrX | | 73071227 | 73071256 |
| 3.6009276257895 | RBM22 (bg=4.62%) | K562 | - | chrX | | 73071201 | 73071332 |
| 3.06868352842052 | RBM22 (bg=4.62%) | K562 | - | chrX | | 73071208 | 73071276 |
| 3.48624874333505 | SDAD1 (bg=2.97%) | K562 | - | chrX | | 73071223 | 73071281 |
| 3.18590204070533 | SDAD1 (bg=2.97%) | K562 | - | chrX | | 73071229 | 73071247 |
| 3.54187091772382 | uchl5 (bg=11.16%) | K562 | - | chrX | | 73071197 | 73071265 |
| 3.52190377519463 | uchl5 (bg=11.16%) | K562 | - | chrX | | 73071215 | 73071259 |

  
  

| Match 64 in HUMAN | | | | | | | |
| --- | --- | --- | --- | --- | --- | --- | --- |
| Motif | Start in Seq (1 Indexed) | End in Seq (1 Indexed) | Strand | Chrm | Exon | Start in Chrm (0 Indexed) | End in Chrm (1 Indexed) |
| AAAAGATGGCGGCT | 1369 | 1382 | - | chrX | 1 | 73071206 | 73071220 |
| eCLIP Fold-Enrichment | Binding Protein | Cell Line | Strand | Chrm | | Start in Chrm (0 Indexed) | End in Chrm (1 Indexed) |
| 3.33669026154036 | AARS (bg=2.18%) | K562 | - | chrX | | 73071196 | 73071247 |
| 3.26531240463024 | AARS (bg=2.18%) | K562 | - | chrX | | 73071203 | 73071256 |
| 4.52944482465756 | EXOSC5 (bg=5.38%) | K562 | - | chrX | | 73071195 | 73071273 |
| 4.072279445805 | EXOSC5 (bg=5.38%) | K562 | - | chrX | | 73071199 | 73071267 |
| 4.99660305552153 | HNRNPM (bg=4.29%) | K562 | - | chrX | | 73071189 | 73071256 |
| 4.86407658159773 | HNRNPM (bg=4.29%) | K562 | - | chrX | | 73071194 | 73071256 |
| 3.26982673913868 | NIPBL (bg=5.39%) | K562 | - | chrX | | 73071210 | 73071263 |
| 4.60897550227588 | RBM15 (bg=7.27%) | K562 | - | chrX | | 73071203 | 73071255 |
| 3.88930535254805 | RBM15 (bg=7.27%) | K562 | - | chrX | | 73071211 | 73071221 |
| 3.6009276257895 | RBM22 (bg=4.62%) | K562 | - | chrX | | 73071201 | 73071332 |
| 3.06868352842052 | RBM22 (bg=4.62%) | K562 | - | chrX | | 73071208 | 73071276 |
| 3.68039693716413 | uchl5 (bg=11.16%) | K562 | - | chrX | | 73071195 | 73071215 |
| 3.54187091772382 | uchl5 (bg=11.16%) | K562 | - | chrX | | 73071197 | 73071265 |
| 3.52190377519463 | uchl5 (bg=11.16%) | K562 | - | chrX | | 73071215 | 73071259 |

  
  

| Match 65 in HUMAN | | | | | | | |
| --- | --- | --- | --- | --- | --- | --- | --- |
| Motif | Start in Seq (1 Indexed) | End in Seq (1 Indexed) | Strand | Chrm | Exon | Start in Chrm (0 Indexed) | End in Chrm (1 Indexed) |
| CTTGCCGCA | 1389 | 1397 | - | chrX | 1 | 73071191 | 73071200 |
| eCLIP Fold-Enrichment | Binding Protein | Cell Line | Strand | Chrm | | Start in Chrm (0 Indexed) | End in Chrm (1 Indexed) |
| 3.33669026154036 | AARS (bg=2.18%) | K562 | - | chrX | | 73071196 | 73071247 |
| 4.52944482465756 | EXOSC5 (bg=5.38%) | K562 | - | chrX | | 73071195 | 73071273 |
| 4.072279445805 | EXOSC5 (bg=5.38%) | K562 | - | chrX | | 73071199 | 73071267 |
| 4.99660305552153 | HNRNPM (bg=4.29%) | K562 | - | chrX | | 73071189 | 73071256 |
| 4.86407658159773 | HNRNPM (bg=4.29%) | K562 | - | chrX | | 73071194 | 73071256 |
| 3.68039693716413 | uchl5 (bg=11.16%) | K562 | - | chrX | | 73071195 | 73071215 |
| 3.54187091772382 | uchl5 (bg=11.16%) | K562 | - | chrX | | 73071197 | 73071265 |

  
  

| Match 66 in HUMAN | | | | | | | |
| --- | --- | --- | --- | --- | --- | --- | --- |
| Motif | Start in Seq (1 Indexed) | End in Seq (1 Indexed) | Strand | Chrm | Exon | Start in Chrm (0 Indexed) | End in Chrm (1 Indexed) |
| AAAACATGGCGGGCCT | 1402 | 1417 | - | chrX | 1 | 73071171 | 73071187 |
| eCLIP Fold-Enrichment | Binding Protein | Cell Line | Strand | Chrm | | Start in Chrm (0 Indexed) | End in Chrm (1 Indexed) |
| 4.61438418144081 | HNRNPM (bg=4.29%) | K562 | - | chrX | | 73071168 | 73071189 |

  
  

| Match 67 in HUMAN | | | | | | | |
| --- | --- | --- | --- | --- | --- | --- | --- |
| Motif | Start in Seq (1 Indexed) | End in Seq (1 Indexed) | Strand | Chrm | Exon | Start in Chrm (0 Indexed) | End in Chrm (1 Indexed) |
| AAACATG | 1403 | 1409 | - | chrX | 1 | 73071179 | 73071186 |
| eCLIP Fold-Enrichment | Binding Protein | Cell Line | Strand | Chrm | | Start in Chrm (0 Indexed) | End in Chrm (1 Indexed) |
| 4.61438418144081 | HNRNPM (bg=4.29%) | K562 | - | chrX | | 73071168 | 73071189 |

  
  

| Match 68 in HUMAN | | | | | | | |
| --- | --- | --- | --- | --- | --- | --- | --- |
| Motif | Start in Seq (1 Indexed) | End in Seq (1 Indexed) | Strand | Chrm | Exon | Start in Chrm (0 Indexed) | End in Chrm (1 Indexed) |
| TTGTCTTTGC | 1420 | 1429 | - | chrX | 1 | 73071159 | 73071169 |
| eCLIP Fold-Enrichment | Binding Protein | Cell Line | Strand | Chrm | | Start in Chrm (0 Indexed) | End in Chrm (1 Indexed) |
| 6.57720189893934 | HNRNPM (bg=4.29%) | K562 | - | chrX | | 73071086 | 73071168 |
| 4.61438418144081 | HNRNPM (bg=4.29%) | K562 | - | chrX | | 73071168 | 73071189 |

  
  

| Match 69 in HUMAN | | | | | | | |
| --- | --- | --- | --- | --- | --- | --- | --- |
| Motif | Start in Seq (1 Indexed) | End in Seq (1 Indexed) | Strand | Chrm | Exon | Start in Chrm (0 Indexed) | End in Chrm (1 Indexed) |
| TTTTGCCGCAGGGACAATATGGC | 1450 | 1472 | - | chrX | 1 | 73071116 | 73071139 |
| eCLIP Fold-Enrichment | Binding Protein | Cell Line | Strand | Chrm | | Start in Chrm (0 Indexed) | End in Chrm (1 Indexed) |
| 2.07159779412732 | AKAP8L (bg=2.19%) | K562 | - | chrX | | 73071129 | 73071149 |
| 2.29435497895444 | DDX51 (bg=1.63%) | K562 | - | chrX | | 73071105 | 73071150 |
| 2.28486969968632 | DDX52 (bg=0.46%) | K562 | - | chrX | | 73071108 | 73071150 |
| 5.0445938087266 | EXOSC5 (bg=5.38%) | K562 | - | chrX | | 73071096 | 73071153 |
| 4.29523641226346 | EXOSC5 (bg=5.38%) | K562 | - | chrX | | 73071100 | 73071148 |
| 2.30127914044672 | GNL3 (bg=0.43%) | K562 | - | chrX | | 73071100 | 73071141 |
| 6.57720189893934 | HNRNPM (bg=4.29%) | K562 | - | chrX | | 73071086 | 73071168 |
| 6.25051879266333 | HNRNPM (bg=4.29%) | K562 | - | chrX | | 73071095 | 73071152 |
| 3.14235437039317 | HNRNPUL1 (bg=1.16%) | K562 | - | chrX | | 73071099 | 73071140 |
| 3.02211634686719 | HNRNPUL1 (bg=1.16%) | K562 | - | chrX | | 73071105 | 73071143 |
| 2.4060652675511 | LARP4 (bg=4.72%) | K562 | - | chrX | | 73071126 | 73071147 |
| 2.32857432563455 | METAP2 (bg=0.78%) | K562 | - | chrX | | 73071106 | 73071147 |
| 2.3831094032366 | NCBP2 (bg=1.49%) | K562 | - | chrX | | 73071111 | 73071153 |
| 2.53816881172174 | RBM22 (bg=4.62%) | K562 | - | chrX | | 73071101 | 73071143 |
| 2.71640484320943 | RBM22 (bg=4.62%) | K562 | - | chrX | | 73071102 | 73071147 |
| 4.77086454142649 | SDAD1 (bg=2.97%) | K562 | - | chrX | | 73071135 | 73071148 |
| 2.21614554223257 | SLTM (bg=2.2%) | K562 | - | chrX | | 73071105 | 73071142 |
| 2.74158249914473 | uchl5 (bg=11.16%) | K562 | - | chrX | | 73071101 | 73071146 |
| 3.04042191266903 | uchl5 (bg=11.16%) | K562 | - | chrX | | 73071101 | 73071147 |
| 3.49471466087889 | WRN (bg=0.77%) | K562 | - | chrX | | 73071099 | 73071145 |
| 2.26130385149565 | XRCC6 (bg=2.91%) | K562 | - | chrX | | 73071110 | 73071148 |
| 2.62267062038674 | XRCC6 (bg=2.91%) | K562 | - | chrX | | 73071112 | 73071152 |

  
  

| Match 70 in HUMAN | | | | | | | |
| --- | --- | --- | --- | --- | --- | --- | --- |
| Motif | Start in Seq (1 Indexed) | End in Seq (1 Indexed) | Strand | Chrm | Exon | Start in Chrm (0 Indexed) | End in Chrm (1 Indexed) |
| TTGTCAT | 1478 | 1484 | - | chrX | 1 | 73071104 | 73071111 |
| eCLIP Fold-Enrichment | Binding Protein | Cell Line | Strand | Chrm | | Start in Chrm (0 Indexed) | End in Chrm (1 Indexed) |
| 2.29435497895444 | DDX51 (bg=1.63%) | K562 | - | chrX | | 73071105 | 73071150 |
| 2.28486969968632 | DDX52 (bg=0.46%) | K562 | - | chrX | | 73071108 | 73071150 |
| 5.0445938087266 | EXOSC5 (bg=5.38%) | K562 | - | chrX | | 73071096 | 73071153 |
| 4.29523641226346 | EXOSC5 (bg=5.38%) | K562 | - | chrX | | 73071100 | 73071148 |
| 2.30127914044672 | GNL3 (bg=0.43%) | K562 | - | chrX | | 73071100 | 73071141 |
| 6.57720189893934 | HNRNPM (bg=4.29%) | K562 | - | chrX | | 73071086 | 73071168 |
| 6.25051879266333 | HNRNPM (bg=4.29%) | K562 | - | chrX | | 73071095 | 73071152 |
| 3.14235437039317 | HNRNPUL1 (bg=1.16%) | K562 | - | chrX | | 73071099 | 73071140 |
| 3.02211634686719 | HNRNPUL1 (bg=1.16%) | K562 | - | chrX | | 73071105 | 73071143 |
| 2.32857432563455 | METAP2 (bg=0.78%) | K562 | - | chrX | | 73071106 | 73071147 |
| 2.3831094032366 | NCBP2 (bg=1.49%) | K562 | - | chrX | | 73071111 | 73071153 |
| 2.53816881172174 | RBM22 (bg=4.62%) | K562 | - | chrX | | 73071101 | 73071143 |
| 2.71640484320943 | RBM22 (bg=4.62%) | K562 | - | chrX | | 73071102 | 73071147 |
| 2.21614554223257 | SLTM (bg=2.2%) | K562 | - | chrX | | 73071105 | 73071142 |
| 2.74158249914473 | uchl5 (bg=11.16%) | K562 | - | chrX | | 73071101 | 73071146 |
| 3.04042191266903 | uchl5 (bg=11.16%) | K562 | - | chrX | | 73071101 | 73071147 |
| 3.49471466087889 | WRN (bg=0.77%) | K562 | - | chrX | | 73071099 | 73071145 |
| 2.26130385149565 | XRCC6 (bg=2.91%) | K562 | - | chrX | | 73071110 | 73071148 |

  
  

| Match 71 in HUMAN | | | | | | | |
| --- | --- | --- | --- | --- | --- | --- | --- |
| Motif | Start in Seq (1 Indexed) | End in Seq (1 Indexed) | Strand | Chrm | Exon | Start in Chrm (0 Indexed) | End in Chrm (1 Indexed) |
| TGTCACGTGGAC | 1502 | 1513 | - | chrX | 1 | 73071075 | 73071087 |
| eCLIP Fold-Enrichment | Binding Protein | Cell Line | Strand | Chrm | | Start in Chrm (0 Indexed) | End in Chrm (1 Indexed) |
| 2.75268303749103 | DDX51 (bg=1.63%) | K562 | - | chrX | | 73071059 | 73071079 |
| 6.35924983256397 | EXOSC5 (bg=5.38%) | K562 | - | chrX | | 73071056 | 73071088 |
| 6.72058358527274 | HNRNPM (bg=4.29%) | K562 | - | chrX | | 73071028 | 73071086 |
| 6.46099252401902 | HNRNPM (bg=4.29%) | K562 | - | chrX | | 73071047 | 73071083 |
| 6.57720189893934 | HNRNPM (bg=4.29%) | K562 | - | chrX | | 73071086 | 73071168 |

  
  

| Match 72 in HUMAN | | | | | | | |
| --- | --- | --- | --- | --- | --- | --- | --- |
| Motif | Start in Seq (1 Indexed) | End in Seq (1 Indexed) | Strand | Chrm | Exon | Start in Chrm (0 Indexed) | End in Chrm (1 Indexed) |
| TGGCGGGCT | 1518 | 1526 | - | chrX | 1 | 73071062 | 73071071 |
| eCLIP Fold-Enrichment | Binding Protein | Cell Line | Strand | Chrm | | Start in Chrm (0 Indexed) | End in Chrm (1 Indexed) |
| 2.75268303749103 | DDX51 (bg=1.63%) | K562 | - | chrX | | 73071059 | 73071079 |
| 6.35924983256397 | EXOSC5 (bg=5.38%) | K562 | - | chrX | | 73071056 | 73071088 |
| 6.72058358527274 | HNRNPM (bg=4.29%) | K562 | - | chrX | | 73071028 | 73071086 |
| 6.46099252401902 | HNRNPM (bg=4.29%) | K562 | - | chrX | | 73071047 | 73071083 |

  
  

| Match 73 in HUMAN | | | | | | | |
| --- | --- | --- | --- | --- | --- | --- | --- |
| Motif | Start in Seq (1 Indexed) | End in Seq (1 Indexed) | Strand | Chrm | Exon | Start in Chrm (0 Indexed) | End in Chrm (1 Indexed) |
| TGCCGCATTGTT | 1527 | 1538 | - | chrX | 1 | 73071050 | 73071062 |
| eCLIP Fold-Enrichment | Binding Protein | Cell Line | Strand | Chrm | | Start in Chrm (0 Indexed) | End in Chrm (1 Indexed) |
| 2.75268303749103 | DDX51 (bg=1.63%) | K562 | - | chrX | | 73071059 | 73071079 |
| 6.35924983256397 | EXOSC5 (bg=5.38%) | K562 | - | chrX | | 73071056 | 73071088 |
| 6.72058358527274 | HNRNPM (bg=4.29%) | K562 | - | chrX | | 73071028 | 73071086 |
| 6.46099252401902 | HNRNPM (bg=4.29%) | K562 | - | chrX | | 73071047 | 73071083 |

  
  

| Match 74 in HUMAN | | | | | | | |
| --- | --- | --- | --- | --- | --- | --- | --- |
| Motif | Start in Seq (1 Indexed) | End in Seq (1 Indexed) | Strand | Chrm | Exon | Start in Chrm (0 Indexed) | End in Chrm (1 Indexed) |
| AAGATGGCGGG | 1540 | 1550 | - | chrX | 1 | 73071038 | 73071049 |
| eCLIP Fold-Enrichment | Binding Protein | Cell Line | Strand | Chrm | | Start in Chrm (0 Indexed) | End in Chrm (1 Indexed) |
| 5.71200895391989 | HNRNPM (bg=4.29%) | K562 | - | chrX | | 73070981 | 73071047 |
| 6.72058358527274 | HNRNPM (bg=4.29%) | K562 | - | chrX | | 73071028 | 73071086 |
| 6.46099252401902 | HNRNPM (bg=4.29%) | K562 | - | chrX | | 73071047 | 73071083 |
| 2.78374787081428 | RBFOX2 (bg=4.63%) | K562 | - | chrX | | 73070993 | 73071042 |

  
  

| Match 75 in HUMAN | | | | | | | |
| --- | --- | --- | --- | --- | --- | --- | --- |
| Motif | Start in Seq (1 Indexed) | End in Seq (1 Indexed) | Strand | Chrm | Exon | Start in Chrm (0 Indexed) | End in Chrm (1 Indexed) |
| TTTGCCGC | 1552 | 1559 | - | chrX | 1 | 73071029 | 73071037 |
| eCLIP Fold-Enrichment | Binding Protein | Cell Line | Strand | Chrm | | Start in Chrm (0 Indexed) | End in Chrm (1 Indexed) |
| 3.82455023435305 | EXOSC5 (bg=5.38%) | K562 | - | chrX | | 73070955 | 73071030 |
| 5.71200895391989 | HNRNPM (bg=4.29%) | K562 | - | chrX | | 73070981 | 73071047 |
| 6.72058358527274 | HNRNPM (bg=4.29%) | K562 | - | chrX | | 73071028 | 73071086 |
| 2.78374787081428 | RBFOX2 (bg=4.63%) | K562 | - | chrX | | 73070993 | 73071042 |

  
  

| Match 76 in HUMAN | | | | | | | |
| --- | --- | --- | --- | --- | --- | --- | --- |
| Motif | Start in Seq (1 Indexed) | End in Seq (1 Indexed) | Strand | Chrm | Exon | Start in Chrm (0 Indexed) | End in Chrm (1 Indexed) |
| TGGATTGC | 1603 | 1610 | - | chrX | 1 | 73070978 | 73070986 |
| eCLIP Fold-Enrichment | Binding Protein | Cell Line | Strand | Chrm | | Start in Chrm (0 Indexed) | End in Chrm (1 Indexed) |
| 2.56747046836952 | AARS (bg=2.18%) | K562 | - | chrX | | 73070952 | 73070985 |
| 3.82455023435305 | EXOSC5 (bg=5.38%) | K562 | - | chrX | | 73070955 | 73071030 |
| 3.47341612826758 | EXOSC5 (bg=5.38%) | K562 | - | chrX | | 73070956 | 73071013 |
| 5.71200895391989 | HNRNPM (bg=4.29%) | K562 | - | chrX | | 73070981 | 73071047 |
| 5.04149451302023 | HNRNPM (bg=4.29%) | K562 | - | chrX | | 73070983 | 73071028 |
| 2.18880128152051 | RBFOX2 (bg=4.63%) | K562 | - | chrX | | 73070956 | 73070993 |
| 3.85842384808186 | RBM22 (bg=4.62%) | K562 | - | chrX | | 73070969 | 73071018 |
| 3.59646377163306 | SUPV3L1 (bg=1.57%) | K562 | - | chrX | | 73070949 | 73071007 |
| 3.76253105057994 | uchl5 (bg=11.16%) | K562 | - | chrX | | 73070953 | 73070985 |
| 3.60113686714351 | uchl5 (bg=11.16%) | K562 | - | chrX | | 73070958 | 73070986 |
| 3.71176110807346 | uchl5 (bg=11.16%) | K562 | - | chrX | | 73070985 | 73071008 |
| 3.60482191395137 | uchl5 (bg=11.16%) | K562 | - | chrX | | 73070986 | 73071003 |

  
  

| Match 77 in HUMAN | | | | | | | |
| --- | --- | --- | --- | --- | --- | --- | --- |
| Motif | Start in Seq (1 Indexed) | End in Seq (1 Indexed) | Strand | Chrm | Exon | Start in Chrm (0 Indexed) | End in Chrm (1 Indexed) |
| GGTGGAATTG | 1638 | 1647 | - | chrX | 1 | 73070941 | 73070951 |
| eCLIP Fold-Enrichment | Binding Protein | Cell Line | Strand | Chrm | | Start in Chrm (0 Indexed) | End in Chrm (1 Indexed) |
| 3.62378782898815 | EXOSC5 (bg=5.38%) | K562 | - | chrX | | 73070891 | 73070955 |
| 3.78684977814584 | EXOSC5 (bg=5.38%) | K562 | - | chrX | | 73070894 | 73070956 |
| 2.6299426436932 | NPM1 (bg=1.21%) | K562 | - | chrX | | 73070911 | 73070966 |
| 3.161792932812 | RBM22 (bg=4.62%) | K562 | - | chrX | | 73070907 | 73070950 |
| 3.35582704214764 | SDAD1 (bg=2.97%) | K562 | - | chrX | | 73070913 | 73070948 |
| 3.10773712008132 | SDAD1 (bg=2.97%) | K562 | - | chrX | | 73070938 | 73070955 |
| 3.59646377163306 | SUPV3L1 (bg=1.57%) | K562 | - | chrX | | 73070949 | 73071007 |
| 3.49582602456025 | uchl5 (bg=11.16%) | K562 | - | chrX | | 73070881 | 73070958 |
| 3.39405759904782 | uchl5 (bg=11.16%) | K562 | - | chrX | | 73070923 | 73070953 |

  
  

| Match 78 in HUMAN | | | | | | | |
| --- | --- | --- | --- | --- | --- | --- | --- |
| Motif | Start in Seq (1 Indexed) | End in Seq (1 Indexed) | Strand | Chrm | Exon | Start in Chrm (0 Indexed) | End in Chrm (1 Indexed) |
| TCACAG | 1649 | 1654 | - | chrX | 1 | 73070934 | 73070940 |
| eCLIP Fold-Enrichment | Binding Protein | Cell Line | Strand | Chrm | | Start in Chrm (0 Indexed) | End in Chrm (1 Indexed) |
| 3.62378782898815 | EXOSC5 (bg=5.38%) | K562 | - | chrX | | 73070891 | 73070955 |
| 3.78684977814584 | EXOSC5 (bg=5.38%) | K562 | - | chrX | | 73070894 | 73070956 |
| 2.6299426436932 | NPM1 (bg=1.21%) | K562 | - | chrX | | 73070911 | 73070966 |
| 3.161792932812 | RBM22 (bg=4.62%) | K562 | - | chrX | | 73070907 | 73070950 |
| 3.35582704214764 | SDAD1 (bg=2.97%) | K562 | - | chrX | | 73070913 | 73070948 |
| 3.10773712008132 | SDAD1 (bg=2.97%) | K562 | - | chrX | | 73070938 | 73070955 |
| 3.49582602456025 | uchl5 (bg=11.16%) | K562 | - | chrX | | 73070881 | 73070958 |
| 3.39405759904782 | uchl5 (bg=11.16%) | K562 | - | chrX | | 73070923 | 73070953 |

  
  

| Match 79 in HUMAN | | | | | | | |
| --- | --- | --- | --- | --- | --- | --- | --- |
| Motif | Start in Seq (1 Indexed) | End in Seq (1 Indexed) | Strand | Chrm | Exon | Start in Chrm (0 Indexed) | End in Chrm (1 Indexed) |
| GATGGAATTAG | 1670 | 1680 | - | chrX | 1 | 73070908 | 73070919 |
| eCLIP Fold-Enrichment | Binding Protein | Cell Line | Strand | Chrm | | Start in Chrm (0 Indexed) | End in Chrm (1 Indexed) |
| 3.62378782898815 | EXOSC5 (bg=5.38%) | K562 | - | chrX | | 73070891 | 73070955 |
| 3.78684977814584 | EXOSC5 (bg=5.38%) | K562 | - | chrX | | 73070894 | 73070956 |
| 2.6299426436932 | NPM1 (bg=1.21%) | K562 | - | chrX | | 73070911 | 73070966 |
| 2.78442326663809 | RBM22 (bg=4.62%) | K562 | - | chrX | | 73070894 | 73070913 |
| 3.161792932812 | RBM22 (bg=4.62%) | K562 | - | chrX | | 73070907 | 73070950 |
| 3.35582704214764 | SDAD1 (bg=2.97%) | K562 | - | chrX | | 73070913 | 73070948 |
| 3.15304949954403 | uchl5 (bg=11.16%) | K562 | - | chrX | | 73070875 | 73070923 |
| 3.49582602456025 | uchl5 (bg=11.16%) | K562 | - | chrX | | 73070881 | 73070958 |

  
  

| Match 80 in HUMAN | | | | | | | |
| --- | --- | --- | --- | --- | --- | --- | --- |
| Motif | Start in Seq (1 Indexed) | End in Seq (1 Indexed) | Strand | Chrm | Exon | Start in Chrm (0 Indexed) | End in Chrm (1 Indexed) |
| TTAGCAT | 1725 | 1731 | - | chrX | 1 | 73070857 | 73070864 |
| eCLIP Fold-Enrichment | Binding Protein | Cell Line | Strand | Chrm | | Start in Chrm (0 Indexed) | End in Chrm (1 Indexed) |
| 2.03111364840005 | SLTM (bg=2.2%) | K562 | - | chrX | | 73070862 | 73070898 |

  
  

| Match 81 in HUMAN | | | | | | | |
| --- | --- | --- | --- | --- | --- | --- | --- |
| Motif | Start in Seq (1 Indexed) | End in Seq (1 Indexed) | Strand | Chrm | Exon | Start in Chrm (0 Indexed) | End in Chrm (1 Indexed) |
| AGCCAGTCAG | 1752 | 1761 | - | chrX | 1 | 73070827 | 73070837 |
| eCLIP Fold-Enrichment | Binding Protein | Cell Line | Strand | Chrm | | Start in Chrm (0 Indexed) | End in Chrm (1 Indexed) |
| 2.21524890685246 | DDX51 (bg=1.63%) | K562 | - | chrX | | 73070808 | 73070853 |
| 5.23351328531316 | HNRNPM (bg=4.29%) | K562 | - | chrX | | 73070769 | 73070827 |
| 5.72682194856537 | HNRNPM (bg=4.29%) | K562 | - | chrX | | 73070769 | 73070834 |
| 2.08028556510081 | WRN (bg=0.77%) | K562 | - | chrX | | 73070789 | 73070828 |

  
  

| Match 82 in HUMAN | | | | | | | |
| --- | --- | --- | --- | --- | --- | --- | --- |
| Motif | Start in Seq (1 Indexed) | End in Seq (1 Indexed) | Strand | Chrm | Exon | Start in Chrm (0 Indexed) | End in Chrm (1 Indexed) |
| GGCCACGT | 1778 | 1785 | - | chrX | 1 | 73070803 | 73070811 |
| eCLIP Fold-Enrichment | Binding Protein | Cell Line | Strand | Chrm | | Start in Chrm (0 Indexed) | End in Chrm (1 Indexed) |
| 2.21524890685246 | DDX51 (bg=1.63%) | K562 | - | chrX | | 73070808 | 73070853 |
| 3.27147249455267 | DROSHA (bg=2.49%) | K562 | - | chrX | | 73070773 | 73070807 |
| 3.98753609957014 | EXOSC5 (bg=5.38%) | K562 | - | chrX | | 73070766 | 73070826 |
| 3.38070575445281 | EXOSC5 (bg=5.38%) | K562 | - | chrX | | 73070794 | 73070822 |
| 5.23351328531316 | HNRNPM (bg=4.29%) | K562 | - | chrX | | 73070769 | 73070827 |
| 5.72682194856537 | HNRNPM (bg=4.29%) | K562 | - | chrX | | 73070769 | 73070834 |
| 2.68780382012142 | NCBP2 (bg=1.49%) | K562 | - | chrX | | 73070784 | 73070811 |
| 2.38893856478064 | RBM22 (bg=4.62%) | K562 | - | chrX | | 73070776 | 73070817 |
| 2.25721249789069 | RBM22 (bg=4.62%) | K562 | - | chrX | | 73070780 | 73070818 |
| 2.19373836055452 | SLTM (bg=2.2%) | K562 | - | chrX | | 73070772 | 73070822 |
| 3.17384546381606 | uchl5 (bg=11.16%) | K562 | - | chrX | | 73070783 | 73070813 |
| 3.08002084844698 | uchl5 (bg=11.16%) | K562 | - | chrX | | 73070794 | 73070807 |
| 2.67304937035635 | UTP3 (bg=3.66%) | K562 | - | chrX | | 73070769 | 73070808 |
| 2.08028556510081 | WRN (bg=0.77%) | K562 | - | chrX | | 73070789 | 73070828 |

  
  

| Match 83 in HUMAN | | | | | | | |
| --- | --- | --- | --- | --- | --- | --- | --- |
| Motif | Start in Seq (1 Indexed) | End in Seq (1 Indexed) | Strand | Chrm | Exon | Start in Chrm (0 Indexed) | End in Chrm (1 Indexed) |
| CTCCCAGTGGG | 1792 | 1802 | - | chrX | 1 | 73070786 | 73070797 |
| eCLIP Fold-Enrichment | Binding Protein | Cell Line | Strand | Chrm | | Start in Chrm (0 Indexed) | End in Chrm (1 Indexed) |
| 3.27147249455267 | DROSHA (bg=2.49%) | K562 | - | chrX | | 73070773 | 73070807 |
| 3.98753609957014 | EXOSC5 (bg=5.38%) | K562 | - | chrX | | 73070766 | 73070826 |
| 3.34842935142905 | EXOSC5 (bg=5.38%) | K562 | - | chrX | | 73070771 | 73070794 |
| 3.38070575445281 | EXOSC5 (bg=5.38%) | K562 | - | chrX | | 73070794 | 73070822 |
| 5.23351328531316 | HNRNPM (bg=4.29%) | K562 | - | chrX | | 73070769 | 73070827 |
| 5.72682194856537 | HNRNPM (bg=4.29%) | K562 | - | chrX | | 73070769 | 73070834 |
| 2.68780382012142 | NCBP2 (bg=1.49%) | K562 | - | chrX | | 73070784 | 73070811 |
| 2.38893856478064 | RBM22 (bg=4.62%) | K562 | - | chrX | | 73070776 | 73070817 |
| 2.25721249789069 | RBM22 (bg=4.62%) | K562 | - | chrX | | 73070780 | 73070818 |
| 2.19373836055452 | SLTM (bg=2.2%) | K562 | - | chrX | | 73070772 | 73070822 |
| 3.17384546381606 | uchl5 (bg=11.16%) | K562 | - | chrX | | 73070783 | 73070813 |
| 3.08002084844698 | uchl5 (bg=11.16%) | K562 | - | chrX | | 73070794 | 73070807 |
| 2.67304937035635 | UTP3 (bg=3.66%) | K562 | - | chrX | | 73070769 | 73070808 |
| 2.08028556510081 | WRN (bg=0.77%) | K562 | - | chrX | | 73070789 | 73070828 |

  
  

| Match 84 in HUMAN | | | | | | | |
| --- | --- | --- | --- | --- | --- | --- | --- |
| Motif | Start in Seq (1 Indexed) | End in Seq (1 Indexed) | Strand | Chrm | Exon | Start in Chrm (0 Indexed) | End in Chrm (1 Indexed) |
| CAAGGTCTTT | 1821 | 1830 | - | chrX | 1 | 73070758 | 73070768 |
| eCLIP Fold-Enrichment | Binding Protein | Cell Line | Strand | Chrm | | Start in Chrm (0 Indexed) | End in Chrm (1 Indexed) |
| 3.98753609957014 | EXOSC5 (bg=5.38%) | K562 | - | chrX | | 73070766 | 73070826 |

  
  

| Match 85 in HUMAN | | | | | | | |
| --- | --- | --- | --- | --- | --- | --- | --- |
| Motif | Start in Seq (1 Indexed) | End in Seq (1 Indexed) | Strand | Chrm | Exon | Start in Chrm (0 Indexed) | End in Chrm (1 Indexed) |
| GCCTTTCCACCTC | 1844 | 1856 | - | chrX | 1 | 73070732 | 73070745 |
| eCLIP Fold-Enrichment | Binding Protein | Cell Line | Strand | Chrm | | Start in Chrm (0 Indexed) | End in Chrm (1 Indexed) |
| 3.52775305087361 | EXOSC5 (bg=5.38%) | K562 | - | chrX | | 73070723 | 73070738 |
| 3.88570861451123 | EXOSC5 (bg=5.38%) | K562 | - | chrX | | 73070727 | 73070748 |
| 2.609377484298 | PCBP1 (bg=1.07%) | K562 | - | chrX | | 73070670 | 73070739 |
| 4.18590204070533 | SDAD1 (bg=2.97%) | K562 | - | chrX | | 73070708 | 73070747 |
| 2.21702702497971 | TIA1 (bg=4.07%) | K562 | - | chrX | | 73070717 | 73070745 |
| 2.85247644471205 | uchl5 (bg=11.16%) | K562 | - | chrX | | 73070727 | 73070737 |

  
  

| Match 86 in HUMAN | | | | | | | |
| --- | --- | --- | --- | --- | --- | --- | --- |
| Motif | Start in Seq (1 Indexed) | End in Seq (1 Indexed) | Strand | Chrm | Exon | Start in Chrm (0 Indexed) | End in Chrm (1 Indexed) |
| TCCCCTCT | 1859 | 1866 | - | chrX | 1 | 73070722 | 73070730 |
| eCLIP Fold-Enrichment | Binding Protein | Cell Line | Strand | Chrm | | Start in Chrm (0 Indexed) | End in Chrm (1 Indexed) |
| 3.43888430237455 | EXOSC5 (bg=5.38%) | K562 | - | chrX | | 73070704 | 73070723 |
| 3.98666147387443 | EXOSC5 (bg=5.38%) | K562 | - | chrX | | 73070704 | 73070727 |
| 3.52775305087361 | EXOSC5 (bg=5.38%) | K562 | - | chrX | | 73070723 | 73070738 |
| 3.88570861451123 | EXOSC5 (bg=5.38%) | K562 | - | chrX | | 73070727 | 73070748 |
| 2.35769332643911 | PCBP1 (bg=1.07%) | K562 | - | chrX | | 73070667 | 73070726 |
| 2.609377484298 | PCBP1 (bg=1.07%) | K562 | - | chrX | | 73070670 | 73070739 |
| 4.18590204070533 | SDAD1 (bg=2.97%) | K562 | - | chrX | | 73070708 | 73070747 |
| 2.21702702497971 | TIA1 (bg=4.07%) | K562 | - | chrX | | 73070717 | 73070745 |
| 3.11013042511195 | uchl5 (bg=11.16%) | K562 | - | chrX | | 73070692 | 73070727 |
| 2.85247644471205 | uchl5 (bg=11.16%) | K562 | - | chrX | | 73070727 | 73070737 |

  
  

| Match 87 in HUMAN | | | | | | | |
| --- | --- | --- | --- | --- | --- | --- | --- |
| Motif | Start in Seq (1 Indexed) | End in Seq (1 Indexed) | Strand | Chrm | Exon | Start in Chrm (0 Indexed) | End in Chrm (1 Indexed) |
| TCCCCTCC | 1873 | 1880 | - | chrX | 1 | 73070708 | 73070716 |
| eCLIP Fold-Enrichment | Binding Protein | Cell Line | Strand | Chrm | | Start in Chrm (0 Indexed) | End in Chrm (1 Indexed) |
| 3.43888430237455 | EXOSC5 (bg=5.38%) | K562 | - | chrX | | 73070704 | 73070723 |
| 3.98666147387443 | EXOSC5 (bg=5.38%) | K562 | - | chrX | | 73070704 | 73070727 |
| 3.15851679179783 | HNRNPM (bg=4.29%) | K562 | - | chrX | | 73070675 | 73070714 |
| 2.35769332643911 | PCBP1 (bg=1.07%) | K562 | - | chrX | | 73070667 | 73070726 |
| 2.609377484298 | PCBP1 (bg=1.07%) | K562 | - | chrX | | 73070670 | 73070739 |
| 4.18590204070533 | SDAD1 (bg=2.97%) | K562 | - | chrX | | 73070708 | 73070747 |
| 3.11013042511195 | uchl5 (bg=11.16%) | K562 | - | chrX | | 73070692 | 73070727 |
| 2.76809530142226 | uchl5 (bg=11.16%) | K562 | - | chrX | | 73070700 | 73070719 |

  
  

| Match 88 in HUMAN | | | | | | | |
| --- | --- | --- | --- | --- | --- | --- | --- |
| Motif | Start in Seq (1 Indexed) | End in Seq (1 Indexed) | Strand | Chrm | Exon | Start in Chrm (0 Indexed) | End in Chrm (1 Indexed) |
| CTGAACCTC | 1926 | 1934 | - | chrX | 1 | 73070654 | 73070663 |
| eCLIP Fold-Enrichment | Binding Protein | Cell Line | Strand | Chrm | | Start in Chrm (0 Indexed) | End in Chrm (1 Indexed) |
| 4.76106363668665 | EXOSC5 (bg=5.38%) | K562 | - | chrX | | 73070639 | 73070677 |
| 4.08539494559526 | EXOSC5 (bg=5.38%) | K562 | - | chrX | | 73070647 | 73070669 |
| 3.29902903439952 | hnrnpk (bg=12.88%) | K562 | - | chrX | | 73070635 | 73070670 |
| 2.64980700914872 | hnrnpk (bg=12.88%) | K562 | - | chrX | | 73070646 | 73070665 |
| 2.36966596810518 | PCBP1 (bg=1.07%) | K562 | - | chrX | | 73070641 | 73070667 |

  
  

| Match 89 in HUMAN | | | | | | | |
| --- | --- | --- | --- | --- | --- | --- | --- |
| Motif | Start in Seq (1 Indexed) | End in Seq (1 Indexed) | Strand | Chrm | Exon | Start in Chrm (0 Indexed) | End in Chrm (1 Indexed) |
| CCATTCCTCTG | 1938 | 1948 | - | chrX | 1 | 73070640 | 73070651 |
| eCLIP Fold-Enrichment | Binding Protein | Cell Line | Strand | Chrm | | Start in Chrm (0 Indexed) | End in Chrm (1 Indexed) |
| 4.76106363668665 | EXOSC5 (bg=5.38%) | K562 | - | chrX | | 73070639 | 73070677 |
| 4.08539494559526 | EXOSC5 (bg=5.38%) | K562 | - | chrX | | 73070647 | 73070669 |
| 3.29902903439952 | hnrnpk (bg=12.88%) | K562 | - | chrX | | 73070635 | 73070670 |
| 2.64980700914872 | hnrnpk (bg=12.88%) | K562 | - | chrX | | 73070646 | 73070665 |
| 2.36966596810518 | PCBP1 (bg=1.07%) | K562 | - | chrX | | 73070641 | 73070667 |

  
  

| Match 90 in HUMAN | | | | | | | |
| --- | --- | --- | --- | --- | --- | --- | --- |
| Motif | Start in Seq (1 Indexed) | End in Seq (1 Indexed) | Strand | Chrm | Exon | Start in Chrm (0 Indexed) | End in Chrm (1 Indexed) |
| ATTGGTG | 1950 | 1956 | - | chrX | 1 | 73070632 | 73070639 |
| eCLIP Fold-Enrichment | Binding Protein | Cell Line | Strand | Chrm | | Start in Chrm (0 Indexed) | End in Chrm (1 Indexed) |
| 4.76106363668665 | EXOSC5 (bg=5.38%) | K562 | - | chrX | | 73070639 | 73070677 |
| 3.29902903439952 | hnrnpk (bg=12.88%) | K562 | - | chrX | | 73070635 | 73070670 |

  
  

| Match 91 in HUMAN | | | | | | | |
| --- | --- | --- | --- | --- | --- | --- | --- |
| Motif | Start in Seq (1 Indexed) | End in Seq (1 Indexed) | Strand | Chrm | Exon | Start in Chrm (0 Indexed) | End in Chrm (1 Indexed) |
| CCTCTG | 2070 | 2075 | - | chrX | 1 | 73070513 | 73070519 |
| eCLIP Fold-Enrichment | Binding Protein | Cell Line | Strand | Chrm | | Start in Chrm (0 Indexed) | End in Chrm (1 Indexed) |
| 2.18243328596613 | CSTF2T (bg=0.82%) | K562 | - | chrX | | 73070466 | 73070524 |
| 2.68695795022469 | CSTF2T (bg=0.82%) | K562 | - | chrX | | 73070493 | 73070529 |
| 2.77867824602397 | DDX51 (bg=1.63%) | K562 | - | chrX | | 73070503 | 73070518 |
| 3.44465990437353 | DROSHA (bg=2.49%) | K562 | - | chrX | | 73070424 | 73070520 |
| 3.29545257184222 | GTF2F1 (bg=0.51%) | K562 | - | chrX | | 73070435 | 73070520 |
| 4.51330500924386 | hnrnpk (bg=12.88%) | K562 | - | chrX | | 73070450 | 73070523 |
| 4.82389718795716 | hnrnpk (bg=12.88%) | K562 | - | chrX | | 73070459 | 73070532 |
| 4.41764603908479 | ILF3 (bg=3.0%) | K562 | - | chrX | | 73070464 | 73070527 |
| 4.04554737148182 | ILF3 (bg=3.0%) | K562 | - | chrX | | 73070466 | 73070524 |
| 4.23216244435525 | PCBP1 (bg=1.07%) | K562 | - | chrX | | 73070454 | 73070522 |
| 5.17572430685181 | PCBP1 (bg=1.07%) | K562 | - | chrX | | 73070461 | 73070519 |
| 2.05975279750985 | PUM1 (bg=1.56%) | K562 | - | chrX | | 73070373 | 73070513 |
| 2.27356544584889 | PUM1 (bg=1.56%) | K562 | - | chrX | | 73070435 | 73070526 |

  
  

| Match 92 in HUMAN | | | | | | | |
| --- | --- | --- | --- | --- | --- | --- | --- |
| Motif | Start in Seq (1 Indexed) | End in Seq (1 Indexed) | Strand | Chrm | Exon | Start in Chrm (0 Indexed) | End in Chrm (1 Indexed) |
| CTGCACTGT | 2088 | 2096 | - | chrX | 1 | 73070492 | 73070501 |
| eCLIP Fold-Enrichment | Binding Protein | Cell Line | Strand | Chrm | | Start in Chrm (0 Indexed) | End in Chrm (1 Indexed) |
| 2.33554847102614 | CSTF2T (bg=0.82%) | K562 | - | chrX | | 73070466 | 73070493 |
| 2.18243328596613 | CSTF2T (bg=0.82%) | K562 | - | chrX | | 73070466 | 73070524 |
| 2.68695795022469 | CSTF2T (bg=0.82%) | K562 | - | chrX | | 73070493 | 73070529 |
| 2.57400403747871 | DGCR8 (bg=1.84%) | K562 | - | chrX | | 73070442 | 73070496 |
| 3.44465990437353 | DROSHA (bg=2.49%) | K562 | - | chrX | | 73070424 | 73070520 |
| 3.87719355544062 | DROSHA (bg=2.49%) | K562 | - | chrX | | 73070467 | 73070512 |
| 3.29545257184222 | GTF2F1 (bg=0.51%) | K562 | - | chrX | | 73070435 | 73070520 |
| 4.51330500924386 | hnrnpk (bg=12.88%) | K562 | - | chrX | | 73070450 | 73070523 |
| 4.82389718795716 | hnrnpk (bg=12.88%) | K562 | - | chrX | | 73070459 | 73070532 |
| 4.41764603908479 | ILF3 (bg=3.0%) | K562 | - | chrX | | 73070464 | 73070527 |
| 4.04554737148182 | ILF3 (bg=3.0%) | K562 | - | chrX | | 73070466 | 73070524 |
| 4.23216244435525 | PCBP1 (bg=1.07%) | K562 | - | chrX | | 73070454 | 73070522 |
| 5.17572430685181 | PCBP1 (bg=1.07%) | K562 | - | chrX | | 73070461 | 73070519 |
| 2.05975279750985 | PUM1 (bg=1.56%) | K562 | - | chrX | | 73070373 | 73070513 |
| 2.27356544584889 | PUM1 (bg=1.56%) | K562 | - | chrX | | 73070435 | 73070526 |
| 3.62401490174002 | RBM15 (bg=7.27%) | K562 | - | chrX | | 73070435 | 73070494 |
| 3.71737035678113 | RBM15 (bg=7.27%) | K562 | - | chrX | | 73070464 | 73070497 |

  
  

| Match 93 in HUMAN | | | | | | | |
| --- | --- | --- | --- | --- | --- | --- | --- |
| Motif | Start in Seq (1 Indexed) | End in Seq (1 Indexed) | Strand | Chrm | Exon | Start in Chrm (0 Indexed) | End in Chrm (1 Indexed) |
| GGGCAGTGCTCCA | 2103 | 2115 | - | chrX | 1 | 73070473 | 73070486 |
| eCLIP Fold-Enrichment | Binding Protein | Cell Line | Strand | Chrm | | Start in Chrm (0 Indexed) | End in Chrm (1 Indexed) |
| 2.33554847102614 | CSTF2T (bg=0.82%) | K562 | - | chrX | | 73070466 | 73070493 |
| 2.18243328596613 | CSTF2T (bg=0.82%) | K562 | - | chrX | | 73070466 | 73070524 |
| 2.57400403747871 | DGCR8 (bg=1.84%) | K562 | - | chrX | | 73070442 | 73070496 |
| 3.44465990437353 | DROSHA (bg=2.49%) | K562 | - | chrX | | 73070424 | 73070520 |
| 3.87719355544062 | DROSHA (bg=2.49%) | K562 | - | chrX | | 73070467 | 73070512 |
| 3.29545257184222 | GTF2F1 (bg=0.51%) | K562 | - | chrX | | 73070435 | 73070520 |
| 4.51330500924386 | hnrnpk (bg=12.88%) | K562 | - | chrX | | 73070450 | 73070523 |
| 4.82389718795716 | hnrnpk (bg=12.88%) | K562 | - | chrX | | 73070459 | 73070532 |
| 5.29853307716357 | HNRNPM (bg=4.29%) | K562 | - | chrX | | 73070412 | 73070488 |
| 5.28779980874279 | HNRNPM (bg=4.29%) | K562 | - | chrX | | 73070412 | 73070490 |
| 4.41764603908479 | ILF3 (bg=3.0%) | K562 | - | chrX | | 73070464 | 73070527 |
| 4.04554737148182 | ILF3 (bg=3.0%) | K562 | - | chrX | | 73070466 | 73070524 |
| 3.89768257606636 | NCBP2 (bg=1.49%) | K562 | - | chrX | | 73070431 | 73070476 |
| 4.23216244435525 | PCBP1 (bg=1.07%) | K562 | - | chrX | | 73070454 | 73070522 |
| 5.17572430685181 | PCBP1 (bg=1.07%) | K562 | - | chrX | | 73070461 | 73070519 |
| 2.05975279750985 | PUM1 (bg=1.56%) | K562 | - | chrX | | 73070373 | 73070513 |
| 2.27356544584889 | PUM1 (bg=1.56%) | K562 | - | chrX | | 73070435 | 73070526 |
| 3.62401490174002 | RBM15 (bg=7.27%) | K562 | - | chrX | | 73070435 | 73070494 |
| 3.71737035678113 | RBM15 (bg=7.27%) | K562 | - | chrX | | 73070464 | 73070497 |

  
  

| Match 94 in HUMAN | | | | | | | |
| --- | --- | --- | --- | --- | --- | --- | --- |
| Motif | Start in Seq (1 Indexed) | End in Seq (1 Indexed) | Strand | Chrm | Exon | Start in Chrm (0 Indexed) | End in Chrm (1 Indexed) |
| GCCTGC | 2117 | 2122 | - | chrX | 1 | 73070466 | 73070472 |
| eCLIP Fold-Enrichment | Binding Protein | Cell Line | Strand | Chrm | | Start in Chrm (0 Indexed) | End in Chrm (1 Indexed) |
| 2.15652667694772 | CSTF2T (bg=0.82%) | K562 | - | chrX | | 73070388 | 73070466 |
| 2.34508362361069 | CSTF2T (bg=0.82%) | K562 | - | chrX | | 73070413 | 73070466 |
| 2.33554847102614 | CSTF2T (bg=0.82%) | K562 | - | chrX | | 73070466 | 73070493 |
| 2.18243328596613 | CSTF2T (bg=0.82%) | K562 | - | chrX | | 73070466 | 73070524 |
| 2.57400403747871 | DGCR8 (bg=1.84%) | K562 | - | chrX | | 73070442 | 73070496 |
| 3.44465990437353 | DROSHA (bg=2.49%) | K562 | - | chrX | | 73070424 | 73070520 |
| 4.27628951085044 | DROSHA (bg=2.49%) | K562 | - | chrX | | 73070426 | 73070467 |
| 3.87719355544062 | DROSHA (bg=2.49%) | K562 | - | chrX | | 73070467 | 73070512 |
| 3.29545257184222 | GTF2F1 (bg=0.51%) | K562 | - | chrX | | 73070435 | 73070520 |
| 4.51330500924386 | hnrnpk (bg=12.88%) | K562 | - | chrX | | 73070450 | 73070523 |
| 4.82389718795716 | hnrnpk (bg=12.88%) | K562 | - | chrX | | 73070459 | 73070532 |
| 5.29853307716357 | HNRNPM (bg=4.29%) | K562 | - | chrX | | 73070412 | 73070488 |
| 5.28779980874279 | HNRNPM (bg=4.29%) | K562 | - | chrX | | 73070412 | 73070490 |
| 2.97242936895086 | HNRNPUL1 (bg=1.16%) | K562 | - | chrX | | 73070426 | 73070467 |
| 2.7331781050193 | ILF3 (bg=3.0%) | K562 | - | chrX | | 73070418 | 73070466 |
| 4.41764603908479 | ILF3 (bg=3.0%) | K562 | - | chrX | | 73070464 | 73070527 |
| 4.04554737148182 | ILF3 (bg=3.0%) | K562 | - | chrX | | 73070466 | 73070524 |
| 3.89768257606636 | NCBP2 (bg=1.49%) | K562 | - | chrX | | 73070431 | 73070476 |
| 4.23216244435525 | PCBP1 (bg=1.07%) | K562 | - | chrX | | 73070454 | 73070522 |
| 5.17572430685181 | PCBP1 (bg=1.07%) | K562 | - | chrX | | 73070461 | 73070519 |
| 2.05975279750985 | PUM1 (bg=1.56%) | K562 | - | chrX | | 73070373 | 73070513 |
| 2.27356544584889 | PUM1 (bg=1.56%) | K562 | - | chrX | | 73070435 | 73070526 |
| 3.62401490174002 | RBM15 (bg=7.27%) | K562 | - | chrX | | 73070435 | 73070494 |
| 3.71737035678113 | RBM15 (bg=7.27%) | K562 | - | chrX | | 73070464 | 73070497 |
| 4.15576462374726 | SRSF1 (bg=8.47%) | K562 | - | chrX | | 73070332 | 73070471 |

  
  

| Match 95 in HUMAN | | | | | | | |
| --- | --- | --- | --- | --- | --- | --- | --- |
| Motif | Start in Seq (1 Indexed) | End in Seq (1 Indexed) | Strand | Chrm | Exon | Start in Chrm (0 Indexed) | End in Chrm (1 Indexed) |
| GGTGAG | 2139 | 2144 | - | chrX | 1 | 73070444 | 73070450 |
| eCLIP Fold-Enrichment | Binding Protein | Cell Line | Strand | Chrm | | Start in Chrm (0 Indexed) | End in Chrm (1 Indexed) |
| 2.15652667694772 | CSTF2T (bg=0.82%) | K562 | - | chrX | | 73070388 | 73070466 |
| 2.34508362361069 | CSTF2T (bg=0.82%) | K562 | - | chrX | | 73070413 | 73070466 |
| 2.31896430892362 | DDX52 (bg=0.46%) | K562 | - | chrX | | 73070439 | 73070454 |
| 2.57400403747871 | DGCR8 (bg=1.84%) | K562 | - | chrX | | 73070442 | 73070496 |
| 3.44465990437353 | DROSHA (bg=2.49%) | K562 | - | chrX | | 73070424 | 73070520 |
| 4.27628951085044 | DROSHA (bg=2.49%) | K562 | - | chrX | | 73070426 | 73070467 |
| 3.16810834521196 | EXOSC5 (bg=5.38%) | K562 | - | chrX | | 73070431 | 73070455 |
| 3.24792179875161 | GRWD1 (bg=5.13%) | K562 | - | chrX | | 73070334 | 73070462 |
| 3.29545257184222 | GTF2F1 (bg=0.51%) | K562 | - | chrX | | 73070435 | 73070520 |
| 5.19412752537253 | hnrnpk (bg=12.88%) | K562 | - | chrX | | 73070417 | 73070450 |
| 5.24544799419468 | hnrnpk (bg=12.88%) | K562 | - | chrX | | 73070418 | 73070459 |
| 4.51330500924386 | hnrnpk (bg=12.88%) | K562 | - | chrX | | 73070450 | 73070523 |
| 5.29853307716357 | HNRNPM (bg=4.29%) | K562 | - | chrX | | 73070412 | 73070488 |
| 5.28779980874279 | HNRNPM (bg=4.29%) | K562 | - | chrX | | 73070412 | 73070490 |
| 2.97242936895086 | HNRNPUL1 (bg=1.16%) | K562 | - | chrX | | 73070426 | 73070467 |
| 3.05854572227728 | ILF3 (bg=3.0%) | K562 | - | chrX | | 73070413 | 73070464 |
| 2.7331781050193 | ILF3 (bg=3.0%) | K562 | - | chrX | | 73070418 | 73070466 |
| 3.89768257606636 | NCBP2 (bg=1.49%) | K562 | - | chrX | | 73070431 | 73070476 |
| 2.05975279750985 | PUM1 (bg=1.56%) | K562 | - | chrX | | 73070373 | 73070513 |
| 2.27356544584889 | PUM1 (bg=1.56%) | K562 | - | chrX | | 73070435 | 73070526 |
| 3.51445694338491 | RBM15 (bg=7.27%) | K562 | - | chrX | | 73070431 | 73070464 |
| 3.62401490174002 | RBM15 (bg=7.27%) | K562 | - | chrX | | 73070435 | 73070494 |
| 2.5639757584274 | SF3B1 (bg=2.48%) | K562 | - | chrX | | 73070435 | 73070454 |
| 4.15576462374726 | SRSF1 (bg=8.47%) | K562 | - | chrX | | 73070332 | 73070471 |
| 3.46890202151809 | SRSF1 (bg=8.47%) | K562 | - | chrX | | 73070338 | 73070449 |
| 4.29438675833687 | SUPV3L1 (bg=1.57%) | K562 | - | chrX | | 73070428 | 73070465 |
| 2.75973128018421 | ZNF622 (bg=6.58%) | K562 | - | chrX | | 73070334 | 73070457 |
| 2.120846863757 | ZNF800 (bg=1.92%) | K562 | - | chrX | | 73070391 | 73070465 |

  
  

| Match 96 in HUMAN | | | | | | | |
| --- | --- | --- | --- | --- | --- | --- | --- |
| Motif | Start in Seq (1 Indexed) | End in Seq (1 Indexed) | Strand | Chrm | Exon | Start in Chrm (0 Indexed) | End in Chrm (1 Indexed) |
| CGTGGCAAGGACCAGAATGGATC | 2146 | 2168 | - | chrX | 1 | 73070420 | 73070443 |
| eCLIP Fold-Enrichment | Binding Protein | Cell Line | Strand | Chrm | | Start in Chrm (0 Indexed) | End in Chrm (1 Indexed) |
| 2.15652667694772 | CSTF2T (bg=0.82%) | K562 | - | chrX | | 73070388 | 73070466 |
| 2.34508362361069 | CSTF2T (bg=0.82%) | K562 | - | chrX | | 73070413 | 73070466 |
| 2.31896430892362 | DDX52 (bg=0.46%) | K562 | - | chrX | | 73070439 | 73070454 |
| 2.57400403747871 | DGCR8 (bg=1.84%) | K562 | - | chrX | | 73070442 | 73070496 |
| 3.44465990437353 | DROSHA (bg=2.49%) | K562 | - | chrX | | 73070424 | 73070520 |
| 4.27628951085044 | DROSHA (bg=2.49%) | K562 | - | chrX | | 73070426 | 73070467 |
| 3.16810834521196 | EXOSC5 (bg=5.38%) | K562 | - | chrX | | 73070431 | 73070455 |
| 2.56098468697931 | GRWD1 (bg=5.13%) | K562 | - | chrX | | 73070324 | 73070437 |
| 3.24792179875161 | GRWD1 (bg=5.13%) | K562 | - | chrX | | 73070334 | 73070462 |
| 3.29545257184222 | GTF2F1 (bg=0.51%) | K562 | - | chrX | | 73070435 | 73070520 |
| 5.19412752537253 | hnrnpk (bg=12.88%) | K562 | - | chrX | | 73070417 | 73070450 |
| 5.24544799419468 | hnrnpk (bg=12.88%) | K562 | - | chrX | | 73070418 | 73070459 |
| 5.29853307716357 | HNRNPM (bg=4.29%) | K562 | - | chrX | | 73070412 | 73070488 |
| 5.28779980874279 | HNRNPM (bg=4.29%) | K562 | - | chrX | | 73070412 | 73070490 |
| 2.97242936895086 | HNRNPUL1 (bg=1.16%) | K562 | - | chrX | | 73070426 | 73070467 |
| 3.05854572227728 | ILF3 (bg=3.0%) | K562 | - | chrX | | 73070413 | 73070464 |
| 2.7331781050193 | ILF3 (bg=3.0%) | K562 | - | chrX | | 73070418 | 73070466 |
| 3.89768257606636 | NCBP2 (bg=1.49%) | K562 | - | chrX | | 73070431 | 73070476 |
| 2.05975279750985 | PUM1 (bg=1.56%) | K562 | - | chrX | | 73070373 | 73070513 |
| 2.27356544584889 | PUM1 (bg=1.56%) | K562 | - | chrX | | 73070435 | 73070526 |
| 3.51445694338491 | RBM15 (bg=7.27%) | K562 | - | chrX | | 73070431 | 73070464 |
| 3.62401490174002 | RBM15 (bg=7.27%) | K562 | - | chrX | | 73070435 | 73070494 |
| 3.85348237562239 | SF3B1 (bg=2.48%) | K562 | - | chrX | | 73070430 | 73070435 |
| 2.5639757584274 | SF3B1 (bg=2.48%) | K562 | - | chrX | | 73070435 | 73070454 |
| 4.15576462374726 | SRSF1 (bg=8.47%) | K562 | - | chrX | | 73070332 | 73070471 |
| 3.46890202151809 | SRSF1 (bg=8.47%) | K562 | - | chrX | | 73070338 | 73070449 |
| 4.29438675833687 | SUPV3L1 (bg=1.57%) | K562 | - | chrX | | 73070428 | 73070465 |
| 2.01026239771938 | TRA2A (bg=4.8%) | K562 | - | chrX | | 73070353 | 73070442 |
| 2.60223895325912 | ZNF622 (bg=6.58%) | K562 | - | chrX | | 73070333 | 73070422 |
| 2.75973128018421 | ZNF622 (bg=6.58%) | K562 | - | chrX | | 73070334 | 73070457 |
| 2.120846863757 | ZNF800 (bg=1.92%) | K562 | - | chrX | | 73070391 | 73070465 |

  
  

| Match 97 in HUMAN | | | | | | | |
| --- | --- | --- | --- | --- | --- | --- | --- |
| Motif | Start in Seq (1 Indexed) | End in Seq (1 Indexed) | Strand | Chrm | Exon | Start in Chrm (0 Indexed) | End in Chrm (1 Indexed) |
| CAGATGATCGTTGGCCAACAGGTGGC | 2170 | 2195 | - | chrX | 1 | 73070393 | 73070419 |
| eCLIP Fold-Enrichment | Binding Protein | Cell Line | Strand | Chrm | | Start in Chrm (0 Indexed) | End in Chrm (1 Indexed) |
| 2.16451137796887 | CSTF2T (bg=0.82%) | K562 | - | chrX | | 73070371 | 73070404 |
| 2.15652667694772 | CSTF2T (bg=0.82%) | K562 | - | chrX | | 73070388 | 73070466 |
| 2.34508362361069 | CSTF2T (bg=0.82%) | K562 | - | chrX | | 73070413 | 73070466 |
| 2.56098468697931 | GRWD1 (bg=5.13%) | K562 | - | chrX | | 73070324 | 73070437 |
| 3.24792179875161 | GRWD1 (bg=5.13%) | K562 | - | chrX | | 73070334 | 73070462 |
| 5.19412752537253 | hnrnpk (bg=12.88%) | K562 | - | chrX | | 73070417 | 73070450 |
| 5.24544799419468 | hnrnpk (bg=12.88%) | K562 | - | chrX | | 73070418 | 73070459 |
| 5.71748408398604 | HNRNPM (bg=4.29%) | K562 | - | chrX | | 73070366 | 73070412 |
| 5.33236667223945 | HNRNPM (bg=4.29%) | K562 | - | chrX | | 73070383 | 73070412 |
| 5.29853307716357 | HNRNPM (bg=4.29%) | K562 | - | chrX | | 73070412 | 73070488 |
| 5.28779980874279 | HNRNPM (bg=4.29%) | K562 | - | chrX | | 73070412 | 73070490 |
| 3.05854572227728 | ILF3 (bg=3.0%) | K562 | - | chrX | | 73070413 | 73070464 |
| 2.7331781050193 | ILF3 (bg=3.0%) | K562 | - | chrX | | 73070418 | 73070466 |
| 3.00529452552861 | MTPAP (bg=2.21%) | K562 | - | chrX | | 73070353 | 73070417 |
| 2.68780382012142 | NCBP2 (bg=1.49%) | K562 | - | chrX | | 73070366 | 73070407 |
| 2.45664273827888 | NIPBL (bg=5.39%) | K562 | - | chrX | | 73070371 | 73070405 |
| 2.05975279750985 | PUM1 (bg=1.56%) | K562 | - | chrX | | 73070373 | 73070513 |
| 3.47263676769028 | RBM15 (bg=7.27%) | K562 | - | chrX | | 73070386 | 73070402 |
| 4.15576462374726 | SRSF1 (bg=8.47%) | K562 | - | chrX | | 73070332 | 73070471 |
| 3.46890202151809 | SRSF1 (bg=8.47%) | K562 | - | chrX | | 73070338 | 73070449 |
| 2.01026239771938 | TRA2A (bg=4.8%) | K562 | - | chrX | | 73070353 | 73070442 |
| 3.29328395746787 | uchl5 (bg=11.16%) | K562 | - | chrX | | 73070361 | 73070409 |
| 3.28363360935417 | uchl5 (bg=11.16%) | K562 | - | chrX | | 73070388 | 73070408 |
| 2.06324321177272 | XRCC6 (bg=2.91%) | K562 | - | chrX | | 73070383 | 73070419 |
| 2.60223895325912 | ZNF622 (bg=6.58%) | K562 | - | chrX | | 73070333 | 73070422 |
| 2.75973128018421 | ZNF622 (bg=6.58%) | K562 | - | chrX | | 73070334 | 73070457 |
| 2.120846863757 | ZNF800 (bg=1.92%) | K562 | - | chrX | | 73070391 | 73070465 |

  
  

| Match 98 in HUMAN | | | | | | | |
| --- | --- | --- | --- | --- | --- | --- | --- |
| Motif | Start in Seq (1 Indexed) | End in Seq (1 Indexed) | Strand | Chrm | Exon | Start in Chrm (0 Indexed) | End in Chrm (1 Indexed) |
| CAACAG | 2185 | 2190 | - | chrX | 1 | 73070398 | 73070404 |
| eCLIP Fold-Enrichment | Binding Protein | Cell Line | Strand | Chrm | | Start in Chrm (0 Indexed) | End in Chrm (1 Indexed) |
| 2.16451137796887 | CSTF2T (bg=0.82%) | K562 | - | chrX | | 73070371 | 73070404 |
| 2.15652667694772 | CSTF2T (bg=0.82%) | K562 | - | chrX | | 73070388 | 73070466 |
| 2.56098468697931 | GRWD1 (bg=5.13%) | K562 | - | chrX | | 73070324 | 73070437 |
| 3.24792179875161 | GRWD1 (bg=5.13%) | K562 | - | chrX | | 73070334 | 73070462 |
| 5.71748408398604 | HNRNPM (bg=4.29%) | K562 | - | chrX | | 73070366 | 73070412 |
| 5.33236667223945 | HNRNPM (bg=4.29%) | K562 | - | chrX | | 73070383 | 73070412 |
| 3.00529452552861 | MTPAP (bg=2.21%) | K562 | - | chrX | | 73070353 | 73070417 |
| 2.68780382012142 | NCBP2 (bg=1.49%) | K562 | - | chrX | | 73070366 | 73070407 |
| 2.45664273827888 | NIPBL (bg=5.39%) | K562 | - | chrX | | 73070371 | 73070405 |
| 2.05975279750985 | PUM1 (bg=1.56%) | K562 | - | chrX | | 73070373 | 73070513 |
| 3.47263676769028 | RBM15 (bg=7.27%) | K562 | - | chrX | | 73070386 | 73070402 |
| 4.15576462374726 | SRSF1 (bg=8.47%) | K562 | - | chrX | | 73070332 | 73070471 |
| 3.46890202151809 | SRSF1 (bg=8.47%) | K562 | - | chrX | | 73070338 | 73070449 |
| 2.01026239771938 | TRA2A (bg=4.8%) | K562 | - | chrX | | 73070353 | 73070442 |
| 3.29328395746787 | uchl5 (bg=11.16%) | K562 | - | chrX | | 73070361 | 73070409 |
| 3.28363360935417 | uchl5 (bg=11.16%) | K562 | - | chrX | | 73070388 | 73070408 |
| 2.06324321177272 | XRCC6 (bg=2.91%) | K562 | - | chrX | | 73070383 | 73070419 |
| 2.60223895325912 | ZNF622 (bg=6.58%) | K562 | - | chrX | | 73070333 | 73070422 |
| 2.75973128018421 | ZNF622 (bg=6.58%) | K562 | - | chrX | | 73070334 | 73070457 |
| 2.120846863757 | ZNF800 (bg=1.92%) | K562 | - | chrX | | 73070391 | 73070465 |

  
  

| Match 99 in HUMAN | | | | | | | |
| --- | --- | --- | --- | --- | --- | --- | --- |
| Motif | Start in Seq (1 Indexed) | End in Seq (1 Indexed) | Strand | Chrm | Exon | Start in Chrm (0 Indexed) | End in Chrm (1 Indexed) |
| GAAGAGGAAT | 2197 | 2206 | - | chrX | 1 | 73070382 | 73070392 |
| eCLIP Fold-Enrichment | Binding Protein | Cell Line | Strand | Chrm | | Start in Chrm (0 Indexed) | End in Chrm (1 Indexed) |
| 2.16451137796887 | CSTF2T (bg=0.82%) | K562 | - | chrX | | 73070371 | 73070404 |
| 2.15652667694772 | CSTF2T (bg=0.82%) | K562 | - | chrX | | 73070388 | 73070466 |
| 2.56098468697931 | GRWD1 (bg=5.13%) | K562 | - | chrX | | 73070324 | 73070437 |
| 3.24792179875161 | GRWD1 (bg=5.13%) | K562 | - | chrX | | 73070334 | 73070462 |
| 5.07124185641428 | HNRNPM (bg=4.29%) | K562 | - | chrX | | 73070364 | 73070383 |
| 5.71748408398604 | HNRNPM (bg=4.29%) | K562 | - | chrX | | 73070366 | 73070412 |
| 5.33236667223945 | HNRNPM (bg=4.29%) | K562 | - | chrX | | 73070383 | 73070412 |
| 3.00529452552861 | MTPAP (bg=2.21%) | K562 | - | chrX | | 73070353 | 73070417 |
| 3.25681206395598 | MTPAP (bg=2.21%) | K562 | - | chrX | | 73070356 | 73070390 |
| 2.68780382012142 | NCBP2 (bg=1.49%) | K562 | - | chrX | | 73070366 | 73070407 |
| 2.45664273827888 | NIPBL (bg=5.39%) | K562 | - | chrX | | 73070371 | 73070405 |
| 2.05975279750985 | PUM1 (bg=1.56%) | K562 | - | chrX | | 73070373 | 73070513 |
| 4.49369838321811 | RBM15 (bg=7.27%) | K562 | - | chrX | | 73070370 | 73070386 |
| 4.48265905249447 | RBM15 (bg=7.27%) | K562 | - | chrX | | 73070371 | 73070388 |
| 3.47263676769028 | RBM15 (bg=7.27%) | K562 | - | chrX | | 73070386 | 73070402 |
| 4.15576462374726 | SRSF1 (bg=8.47%) | K562 | - | chrX | | 73070332 | 73070471 |
| 3.46890202151809 | SRSF1 (bg=8.47%) | K562 | - | chrX | | 73070338 | 73070449 |
| 2.01026239771938 | TRA2A (bg=4.8%) | K562 | - | chrX | | 73070353 | 73070442 |
| 3.22705008098781 | uchl5 (bg=11.16%) | K562 | - | chrX | | 73070353 | 73070388 |
| 3.29328395746787 | uchl5 (bg=11.16%) | K562 | - | chrX | | 73070361 | 73070409 |
| 3.28363360935417 | uchl5 (bg=11.16%) | K562 | - | chrX | | 73070388 | 73070408 |
| 2.50866255245547 | UTP3 (bg=3.66%) | K562 | - | chrX | | 73070376 | 73070384 |
| 2.06324321177272 | XRCC6 (bg=2.91%) | K562 | - | chrX | | 73070383 | 73070419 |
| 2.60223895325912 | ZNF622 (bg=6.58%) | K562 | - | chrX | | 73070333 | 73070422 |
| 2.75973128018421 | ZNF622 (bg=6.58%) | K562 | - | chrX | | 73070334 | 73070457 |
| 2.120846863757 | ZNF800 (bg=1.92%) | K562 | - | chrX | | 73070391 | 73070465 |

  
  

| Match 100 in HUMAN | | | | | | | |
| --- | --- | --- | --- | --- | --- | --- | --- |
| Motif | Start in Seq (1 Indexed) | End in Seq (1 Indexed) | Strand | Chrm | Exon | Start in Chrm (0 Indexed) | End in Chrm (1 Indexed) |
| CTTCCTCAAGAGGAACACCTACCCC | 2213 | 2237 | - | chrX | 1 | 73070351 | 73070376 |
| eCLIP Fold-Enrichment | Binding Protein | Cell Line | Strand | Chrm | | Start in Chrm (0 Indexed) | End in Chrm (1 Indexed) |
| 2.16451137796887 | CSTF2T (bg=0.82%) | K562 | - | chrX | | 73070371 | 73070404 |
| 2.56098468697931 | GRWD1 (bg=5.13%) | K562 | - | chrX | | 73070324 | 73070437 |
| 3.24792179875161 | GRWD1 (bg=5.13%) | K562 | - | chrX | | 73070334 | 73070462 |
| 5.07124185641428 | HNRNPM (bg=4.29%) | K562 | - | chrX | | 73070364 | 73070383 |
| 5.71748408398604 | HNRNPM (bg=4.29%) | K562 | - | chrX | | 73070366 | 73070412 |
| 3.00529452552861 | MTPAP (bg=2.21%) | K562 | - | chrX | | 73070353 | 73070417 |
| 3.25681206395598 | MTPAP (bg=2.21%) | K562 | - | chrX | | 73070356 | 73070390 |
| 2.68780382012142 | NCBP2 (bg=1.49%) | K562 | - | chrX | | 73070366 | 73070407 |
| 2.45664273827888 | NIPBL (bg=5.39%) | K562 | - | chrX | | 73070371 | 73070405 |
| 2.05975279750985 | PUM1 (bg=1.56%) | K562 | - | chrX | | 73070373 | 73070513 |
| 4.49369838321811 | RBM15 (bg=7.27%) | K562 | - | chrX | | 73070370 | 73070386 |
| 4.48265905249447 | RBM15 (bg=7.27%) | K562 | - | chrX | | 73070371 | 73070388 |
| 4.15576462374726 | SRSF1 (bg=8.47%) | K562 | - | chrX | | 73070332 | 73070471 |
| 3.46890202151809 | SRSF1 (bg=8.47%) | K562 | - | chrX | | 73070338 | 73070449 |
| 2.01026239771938 | TRA2A (bg=4.8%) | K562 | - | chrX | | 73070353 | 73070442 |
| 3.22705008098781 | uchl5 (bg=11.16%) | K562 | - | chrX | | 73070353 | 73070388 |
| 3.29328395746787 | uchl5 (bg=11.16%) | K562 | - | chrX | | 73070361 | 73070409 |
| 2.50866255245547 | UTP3 (bg=3.66%) | K562 | - | chrX | | 73070376 | 73070384 |
| 2.60223895325912 | ZNF622 (bg=6.58%) | K562 | - | chrX | | 73070333 | 73070422 |
| 2.75973128018421 | ZNF622 (bg=6.58%) | K562 | - | chrX | | 73070334 | 73070457 |

  
  

| Match 101 in HUMAN | | | | | | | |
| --- | --- | --- | --- | --- | --- | --- | --- |
| Motif | Start in Seq (1 Indexed) | End in Seq (1 Indexed) | Strand | Chrm | Exon | Start in Chrm (0 Indexed) | End in Chrm (1 Indexed) |
| TGGCTAATGCTGGGGTCGGATTTTGATTT | 2239 | 2267 | - | chrX | 1 | 73070321 | 73070350 |
| eCLIP Fold-Enrichment | Binding Protein | Cell Line | Strand | Chrm | | Start in Chrm (0 Indexed) | End in Chrm (1 Indexed) |
| 2.56098468697931 | GRWD1 (bg=5.13%) | K562 | - | chrX | | 73070324 | 73070437 |
| 3.24792179875161 | GRWD1 (bg=5.13%) | K562 | - | chrX | | 73070334 | 73070462 |
| 4.15576462374726 | SRSF1 (bg=8.47%) | K562 | - | chrX | | 73070332 | 73070471 |
| 3.46890202151809 | SRSF1 (bg=8.47%) | K562 | - | chrX | | 73070338 | 73070449 |
| 2.60223895325912 | ZNF622 (bg=6.58%) | K562 | - | chrX | | 73070333 | 73070422 |
| 2.75973128018421 | ZNF622 (bg=6.58%) | K562 | - | chrX | | 73070334 | 73070457 |

  
  

| Match 102 in HUMAN | | | | | | | |
| --- | --- | --- | --- | --- | --- | --- | --- |
| Motif | Start in Seq (1 Indexed) | End in Seq (1 Indexed) | Strand | Chrm | Exon | Start in Chrm (0 Indexed) | End in Chrm (1 Indexed) |
| TTGGATGTCAGTCATA | 2279 | 2294 | - | chrX | 1 | 73070294 | 73070310 |
| eCLIP Fold-Enrichment | Binding Protein | Cell Line | Strand | Chrm | | Start in Chrm (0 Indexed) | End in Chrm (1 Indexed) |
| 3.61970356214025 | CPSF6 (bg=0.4%) | K562 | - | chrX | | 73070267 | 73070308 |
| 3.27379055053629 | EXOSC5 (bg=5.38%) | K562 | - | chrX | | 73070238 | 73070311 |
| 2.6535351723822 | EXOSC5 (bg=5.38%) | K562 | - | chrX | | 73070291 | 73070306 |
| 2.7741888334236 | HNRNPUL1 (bg=1.16%) | K562 | - | chrX | | 73070274 | 73070296 |
| 3.65235226006254 | KHSRP (bg=0.67%) | K562 | - | chrX | | 73070236 | 73070315 |
| 2.78846666503286 | KHSRP (bg=0.67%) | K562 | - | chrX | | 73070262 | 73070315 |
| 2.28828100732762 | RBM15 (bg=7.27%) | K562 | - | chrX | | 73070284 | 73070296 |
| 3.85970502355715 | TIA1 (bg=4.07%) | K562 | - | chrX | | 73070254 | 73070297 |
| 4.32307963832293 | TIA1 (bg=4.07%) | K562 | - | chrX | | 73070255 | 73070309 |
| 3.8691037215594 | TIA1 (bg=4.07%) | K562 | - | chrX | | 73070297 | 73070306 |
| 2.1372437932165 | XRCC6 (bg=2.91%) | K562 | - | chrX | | 73070267 | 73070306 |

  
  

| Match 103 in HUMAN | | | | | | | |
| --- | --- | --- | --- | --- | --- | --- | --- |
| Motif | Start in Seq (1 Indexed) | End in Seq (1 Indexed) | Strand | Chrm | Exon | Start in Chrm (0 Indexed) | End in Chrm (1 Indexed) |
| TGTGGTTTGCTAGTGTT | 2306 | 2322 | - | chrX | 1 | 73070266 | 73070283 |
| eCLIP Fold-Enrichment | Binding Protein | Cell Line | Strand | Chrm | | Start in Chrm (0 Indexed) | End in Chrm (1 Indexed) |
| 3.61970356214025 | CPSF6 (bg=0.4%) | K562 | - | chrX | | 73070267 | 73070308 |
| 3.27379055053629 | EXOSC5 (bg=5.38%) | K562 | - | chrX | | 73070238 | 73070311 |
| 3.46766862707087 | EXOSC5 (bg=5.38%) | K562 | - | chrX | | 73070249 | 73070291 |
| 3.92685194707911 | HNRNPM (bg=4.29%) | K562 | - | chrX | | 73070268 | 73070292 |
| 2.7741888334236 | HNRNPUL1 (bg=1.16%) | K562 | - | chrX | | 73070274 | 73070296 |
| 3.65235226006254 | KHSRP (bg=0.67%) | K562 | - | chrX | | 73070236 | 73070315 |
| 2.78846666503286 | KHSRP (bg=0.67%) | K562 | - | chrX | | 73070262 | 73070315 |
| 2.3767502512271 | NIPBL (bg=5.39%) | K562 | - | chrX | | 73070239 | 73070281 |
| 2.66577877013947 | RBM22 (bg=4.62%) | K562 | - | chrX | | 73070273 | 73070288 |
| 3.85970502355715 | TIA1 (bg=4.07%) | K562 | - | chrX | | 73070254 | 73070297 |
| 4.32307963832293 | TIA1 (bg=4.07%) | K562 | - | chrX | | 73070255 | 73070309 |
| 2.1372437932165 | XRCC6 (bg=2.91%) | K562 | - | chrX | | 73070267 | 73070306 |

  
  

| Match 104 in HUMAN | | | | | | | |
| --- | --- | --- | --- | --- | --- | --- | --- |
| Motif | Start in Seq (1 Indexed) | End in Seq (1 Indexed) | Strand | Chrm | Exon | Start in Chrm (0 Indexed) | End in Chrm (1 Indexed) |
| ATTTAAG | 2326 | 2332 | - | chrX | 1 | 73070256 | 73070263 |
| eCLIP Fold-Enrichment | Binding Protein | Cell Line | Strand | Chrm | | Start in Chrm (0 Indexed) | End in Chrm (1 Indexed) |
| 3.27379055053629 | EXOSC5 (bg=5.38%) | K562 | - | chrX | | 73070238 | 73070311 |
| 3.46766862707087 | EXOSC5 (bg=5.38%) | K562 | - | chrX | | 73070249 | 73070291 |
| 3.74412875931742 | KHSRP (bg=0.67%) | K562 | - | chrX | | 73070236 | 73070262 |
| 3.65235226006254 | KHSRP (bg=0.67%) | K562 | - | chrX | | 73070236 | 73070315 |
| 2.78846666503286 | KHSRP (bg=0.67%) | K562 | - | chrX | | 73070262 | 73070315 |
| 2.3767502512271 | NIPBL (bg=5.39%) | K562 | - | chrX | | 73070239 | 73070281 |
| 3.85970502355715 | TIA1 (bg=4.07%) | K562 | - | chrX | | 73070254 | 73070297 |
| 4.32307963832293 | TIA1 (bg=4.07%) | K562 | - | chrX | | 73070255 | 73070309 |

  
  

| Match 105 in HUMAN | | | | | | | |
| --- | --- | --- | --- | --- | --- | --- | --- |
| Motif | Start in Seq (1 Indexed) | End in Seq (1 Indexed) | Strand | Chrm | Exon | Start in Chrm (0 Indexed) | End in Chrm (1 Indexed) |
| CTTAAGTGACTA | 2334 | 2345 | - | chrX | 1 | 73070243 | 73070255 |
| eCLIP Fold-Enrichment | Binding Protein | Cell Line | Strand | Chrm | | Start in Chrm (0 Indexed) | End in Chrm (1 Indexed) |
| 3.27379055053629 | EXOSC5 (bg=5.38%) | K562 | - | chrX | | 73070238 | 73070311 |
| 3.46766862707087 | EXOSC5 (bg=5.38%) | K562 | - | chrX | | 73070249 | 73070291 |
| 3.74412875931742 | KHSRP (bg=0.67%) | K562 | - | chrX | | 73070236 | 73070262 |
| 3.65235226006254 | KHSRP (bg=0.67%) | K562 | - | chrX | | 73070236 | 73070315 |
| 2.3767502512271 | NIPBL (bg=5.39%) | K562 | - | chrX | | 73070239 | 73070281 |
| 4.31616022438313 | TIA1 (bg=4.07%) | K562 | - | chrX | | 73070238 | 73070255 |
| 3.85970502355715 | TIA1 (bg=4.07%) | K562 | - | chrX | | 73070254 | 73070297 |
| 4.32307963832293 | TIA1 (bg=4.07%) | K562 | - | chrX | | 73070255 | 73070309 |

  
  

| Match 106 in HUMAN | | | | | | | |
| --- | --- | --- | --- | --- | --- | --- | --- |
| Motif | Start in Seq (1 Indexed) | End in Seq (1 Indexed) | Strand | Chrm | Exon | Start in Chrm (0 Indexed) | End in Chrm (1 Indexed) |
| AATGTATT | 2353 | 2360 | - | chrX | 1 | 73070228 | 73070236 |
| eCLIP Fold-Enrichment | Binding Protein | Cell Line | Strand | Chrm | | Start in Chrm (0 Indexed) | End in Chrm (1 Indexed) |
| 4.27596773270562 | KHSRP (bg=0.67%) | K562 | - | chrX | | 73070186 | 73070236 |
| 3.69325037691748 | KHSRP (bg=0.67%) | K562 | - | chrX | | 73070189 | 73070236 |
| 3.74412875931742 | KHSRP (bg=0.67%) | K562 | - | chrX | | 73070236 | 73070262 |
| 3.65235226006254 | KHSRP (bg=0.67%) | K562 | - | chrX | | 73070236 | 73070315 |
| 4.86364801968563 | TIA1 (bg=4.07%) | K562 | - | chrX | | 73070196 | 73070238 |

  
  

| Match 107 in HUMAN | | | | | | | |
| --- | --- | --- | --- | --- | --- | --- | --- |
| Motif | Start in Seq (1 Indexed) | End in Seq (1 Indexed) | Strand | Chrm | Exon | Start in Chrm (0 Indexed) | End in Chrm (1 Indexed) |
| TTATTTGTAGAATTCA | 2369 | 2384 | - | chrX | 1 | 73070204 | 73070220 |
| eCLIP Fold-Enrichment | Binding Protein | Cell Line | Strand | Chrm | | Start in Chrm (0 Indexed) | End in Chrm (1 Indexed) |
| 4.27596773270562 | KHSRP (bg=0.67%) | K562 | - | chrX | | 73070186 | 73070236 |
| 3.69325037691748 | KHSRP (bg=0.67%) | K562 | - | chrX | | 73070189 | 73070236 |
| 4.86364801968563 | TIA1 (bg=4.07%) | K562 | - | chrX | | 73070196 | 73070238 |

  
  

| Match 108 in HUMAN | | | | | | | |
| --- | --- | --- | --- | --- | --- | --- | --- |
| Motif | Start in Seq (1 Indexed) | End in Seq (1 Indexed) | Strand | Chrm | Exon | Start in Chrm (0 Indexed) | End in Chrm (1 Indexed) |
| TTACATTTA | 2391 | 2399 | - | chrX | 1 | 73070189 | 73070198 |
| eCLIP Fold-Enrichment | Binding Protein | Cell Line | Strand | Chrm | | Start in Chrm (0 Indexed) | End in Chrm (1 Indexed) |
| 4.27596773270562 | KHSRP (bg=0.67%) | K562 | - | chrX | | 73070186 | 73070236 |
| 3.69325037691748 | KHSRP (bg=0.67%) | K562 | - | chrX | | 73070189 | 73070236 |
| 4.86364801968563 | TIA1 (bg=4.07%) | K562 | - | chrX | | 73070196 | 73070238 |

  
  

| Match 109 in HUMAN | | | | | | | |
| --- | --- | --- | --- | --- | --- | --- | --- |
| Motif | Start in Seq (1 Indexed) | End in Seq (1 Indexed) | Strand | Chrm | Exon | Start in Chrm (0 Indexed) | End in Chrm (1 Indexed) |
| GTTCCTT | 2417 | 2423 | - | chrX | 1 | 73070165 | 73070172 |
| eCLIP Fold-Enrichment | Binding Protein | Cell Line | Strand | Chrm | | Start in Chrm (0 Indexed) | End in Chrm (1 Indexed) |
| 3.82983842740993 | U2AF2 (bg=1.76%) | K562 | - | chrX | | 73070153 | 73070166 |
| 3.71436120999 | U2AF2 (bg=1.76%) | K562 | - | chrX | | 73070166 | 73070177 |

  
  

| Match 110 in HUMAN | | | | | | | |
| --- | --- | --- | --- | --- | --- | --- | --- |
| Motif | Start in Seq (1 Indexed) | End in Seq (1 Indexed) | Strand | Chrm | Exon | Start in Chrm (0 Indexed) | End in Chrm (1 Indexed) |
| AAATTCCTTAAAGTTTT | 2425 | 2441 | - | chrX | 1 | 73070147 | 73070164 |
| eCLIP Fold-Enrichment | Binding Protein | Cell Line | Strand | Chrm | | Start in Chrm (0 Indexed) | End in Chrm (1 Indexed) |
| 2.82983842740993 | U2AF2 (bg=1.76%) | K562 | - | chrX | | 73070092 | 73070153 |
| 3.82983842740993 | U2AF2 (bg=1.76%) | K562 | - | chrX | | 73070153 | 73070166 |

  
  

| Match 111 in HUMAN | | | | | | | |
| --- | --- | --- | --- | --- | --- | --- | --- |
| Motif | Start in Seq (1 Indexed) | End in Seq (1 Indexed) | Strand | Chrm | Exon | Start in Chrm (0 Indexed) | End in Chrm (1 Indexed) |
| TTACAAAT | 2452 | 2459 | - | chrX | 1 | 73070129 | 73070137 |
| eCLIP Fold-Enrichment | Binding Protein | Cell Line | Strand | Chrm | | Start in Chrm (0 Indexed) | End in Chrm (1 Indexed) |
| 2.82983842740993 | U2AF2 (bg=1.76%) | K562 | - | chrX | | 73070092 | 73070153 |

  
  

| Match 112 in HUMAN | | | | | | | |
| --- | --- | --- | --- | --- | --- | --- | --- |
| Motif | Start in Seq (1 Indexed) | End in Seq (1 Indexed) | Strand | Chrm | Exon | Start in Chrm (0 Indexed) | End in Chrm (1 Indexed) |
| ATAGTCAAAGTCAA | 2484 | 2497 | - | chrX | 1 | 73070091 | 73070105 |
| eCLIP Fold-Enrichment | Binding Protein | Cell Line | Strand | Chrm | | Start in Chrm (0 Indexed) | End in Chrm (1 Indexed) |
| 2.43089151162102 | EXOSC5 (bg=5.38%) | K562 | - | chrX | | 73070087 | 73070116 |
| 2.08718834982839 | EXOSC5 (bg=5.38%) | K562 | - | chrX | | 73070105 | 73070106 |
| 2.39759410712797 | LSM11 (bg=2.28%) | K562 | - | chrX | | 73069956 | 73070095 |
| 4.29438675833687 | SUPV3L1 (bg=1.57%) | K562 | - | chrX | | 73070085 | 73070122 |
| 2.82983842740993 | U2AF2 (bg=1.76%) | K562 | - | chrX | | 73070092 | 73070153 |

  
  

| Match 113 in HUMAN | | | | | | | |
| --- | --- | --- | --- | --- | --- | --- | --- |
| Motif | Start in Seq (1 Indexed) | End in Seq (1 Indexed) | Strand | Chrm | Exon | Start in Chrm (0 Indexed) | End in Chrm (1 Indexed) |
| CTTTGAAATTGACTTAA | 2535 | 2551 | - | chrX | 1 | 73070037 | 73070054 |
| eCLIP Fold-Enrichment | Binding Protein | Cell Line | Strand | Chrm | | Start in Chrm (0 Indexed) | End in Chrm (1 Indexed) |
| 2.39759410712797 | LSM11 (bg=2.28%) | K562 | - | chrX | | 73069956 | 73070095 |
| 3.41063664722927 | PUS1 (bg=1.04%) | K562 | - | chrX | | 73070031 | 73070076 |
| 2.70264210252472 | SF3B1 (bg=2.48%) | K562 | - | chrX | | 73070035 | 73070074 |

  
  

| Match 114 in HUMAN | | | | | | | |
| --- | --- | --- | --- | --- | --- | --- | --- |
| Motif | Start in Seq (1 Indexed) | End in Seq (1 Indexed) | Strand | Chrm | Exon | Start in Chrm (0 Indexed) | End in Chrm (1 Indexed) |
| TTTGAAG | 2561 | 2567 | - | chrX | 1 | 73070021 | 73070028 |
| eCLIP Fold-Enrichment | Binding Protein | Cell Line | Strand | Chrm | | Start in Chrm (0 Indexed) | End in Chrm (1 Indexed) |
| 2.39759410712797 | LSM11 (bg=2.28%) | K562 | - | chrX | | 73069956 | 73070095 |

  
  

| Match 115 in HUMAN | | | | | | | |
| --- | --- | --- | --- | --- | --- | --- | --- |
| Motif | Start in Seq (1 Indexed) | End in Seq (1 Indexed) | Strand | Chrm | Exon | Start in Chrm (0 Indexed) | End in Chrm (1 Indexed) |
| AAAATTTAAC | 2591 | 2600 | - | chrX | 1 | 73069988 | 73069998 |
| eCLIP Fold-Enrichment | Binding Protein | Cell Line | Strand | Chrm | | Start in Chrm (0 Indexed) | End in Chrm (1 Indexed) |
| 2.39759410712797 | LSM11 (bg=2.28%) | K562 | - | chrX | | 73069956 | 73070095 |

  
  

| Match 116 in HUMAN | | | | | | | |
| --- | --- | --- | --- | --- | --- | --- | --- |
| Motif | Start in Seq (1 Indexed) | End in Seq (1 Indexed) | Strand | Chrm | Exon | Start in Chrm (0 Indexed) | End in Chrm (1 Indexed) |
| ATGACC | 2604 | 2609 | - | chrX | 1 | 73069979 | 73069985 |
| eCLIP Fold-Enrichment | Binding Protein | Cell Line | Strand | Chrm | | Start in Chrm (0 Indexed) | End in Chrm (1 Indexed) |
| 2.40029879552089 | ILF3 (bg=3.0%) | K562 | - | chrX | | 73069927 | 73069982 |
| 2.39759410712797 | LSM11 (bg=2.28%) | K562 | - | chrX | | 73069956 | 73070095 |

  
  

| Match 117 in HUMAN | | | | | | | |
| --- | --- | --- | --- | --- | --- | --- | --- |
| Motif | Start in Seq (1 Indexed) | End in Seq (1 Indexed) | Strand | Chrm | Exon | Start in Chrm (0 Indexed) | End in Chrm (1 Indexed) |
| TTTGAAGGT | 2627 | 2635 | - | chrX | 1 | 73069953 | 73069962 |
| eCLIP Fold-Enrichment | Binding Protein | Cell Line | Strand | Chrm | | Start in Chrm (0 Indexed) | End in Chrm (1 Indexed) |
| 2.40029879552089 | ILF3 (bg=3.0%) | K562 | - | chrX | | 73069927 | 73069982 |
| 2.39759410712797 | LSM11 (bg=2.28%) | K562 | - | chrX | | 73069956 | 73070095 |

  
  

| Match 118 in HUMAN | | | | | | | |
| --- | --- | --- | --- | --- | --- | --- | --- |
| Motif | Start in Seq (1 Indexed) | End in Seq (1 Indexed) | Strand | Chrm | Exon | Start in Chrm (0 Indexed) | End in Chrm (1 Indexed) |
| GTCCAGG | 2644 | 2650 | - | chrX | 1 | 73069938 | 73069945 |
| eCLIP Fold-Enrichment | Binding Protein | Cell Line | Strand | Chrm | | Start in Chrm (0 Indexed) | End in Chrm (1 Indexed) |
| 2.40029879552089 | ILF3 (bg=3.0%) | K562 | - | chrX | | 73069927 | 73069982 |

  
  

| Match 119 in HUMAN | | | | | | | |
| --- | --- | --- | --- | --- | --- | --- | --- |
| Motif | Start in Seq (1 Indexed) | End in Seq (1 Indexed) | Strand | Chrm | Exon | Start in Chrm (0 Indexed) | End in Chrm (1 Indexed) |
| CTTGCTTTGTTCCCATCCTT | 2652 | 2671 | - | chrX | 1 | 73069917 | 73069937 |
| eCLIP Fold-Enrichment | Binding Protein | Cell Line | Strand | Chrm | | Start in Chrm (0 Indexed) | End in Chrm (1 Indexed) |
| 2.40029879552089 | ILF3 (bg=3.0%) | K562 | - | chrX | | 73069927 | 73069982 |

  
  

| Match 120 in HUMAN | | | | | | | |
| --- | --- | --- | --- | --- | --- | --- | --- |
| Motif | Start in Seq (1 Indexed) | End in Seq (1 Indexed) | Strand | Chrm | Exon | Start in Chrm (0 Indexed) | End in Chrm (1 Indexed) |
| TTCCCATC | 2661 | 2668 | - | chrX | 1 | 73069920 | 73069928 |
| eCLIP Fold-Enrichment | Binding Protein | Cell Line | Strand | Chrm | | Start in Chrm (0 Indexed) | End in Chrm (1 Indexed) |
| 2.40029879552089 | ILF3 (bg=3.0%) | K562 | - | chrX | | 73069927 | 73069982 |

  
  

| Match 121 in HUMAN | | | | | | | |
| --- | --- | --- | --- | --- | --- | --- | --- |
| Motif | Start in Seq (1 Indexed) | End in Seq (1 Indexed) | Strand | Chrm | Exon | Start in Chrm (0 Indexed) | End in Chrm (1 Indexed) |
| TCCCAGCAAACCC | 2824 | 2836 | - | chrX | 1 | 73069752 | 73069765 |
| eCLIP Fold-Enrichment | Binding Protein | Cell Line | Strand | Chrm | | Start in Chrm (0 Indexed) | End in Chrm (1 Indexed) |
| 4.28392214200931 | hnrnpk (bg=12.88%) | K562 | - | chrX | | 73069751 | 73069777 |

  
  

| Match 122 in HUMAN | | | | | | | |
| --- | --- | --- | --- | --- | --- | --- | --- |
| Motif | Start in Seq (1 Indexed) | End in Seq (1 Indexed) | Strand | Chrm | Exon | Start in Chrm (0 Indexed) | End in Chrm (1 Indexed) |
| CCCTGCCCCAGCCCCAG | 2874 | 2890 | - | chrX | 1 | 73069698 | 73069715 |
| eCLIP Fold-Enrichment | Binding Protein | Cell Line | Strand | Chrm | | Start in Chrm (0 Indexed) | End in Chrm (1 Indexed) |
| 2.31472797803857 | DROSHA (bg=2.49%) | K562 | - | chrX | | 73069566 | 73069701 |
| 2.68485836796035 | DROSHA (bg=2.49%) | K562 | - | chrX | | 73069585 | 73069699 |

  
  

| Match 123 in HUMAN | | | | | | | |
| --- | --- | --- | --- | --- | --- | --- | --- |
| Motif | Start in Seq (1 Indexed) | End in Seq (1 Indexed) | Strand | Chrm | Exon | Start in Chrm (0 Indexed) | End in Chrm (1 Indexed) |
| AGCCCCAG | 2911 | 2918 | - | chrX | 1 | 73069670 | 73069678 |
| eCLIP Fold-Enrichment | Binding Protein | Cell Line | Strand | Chrm | | Start in Chrm (0 Indexed) | End in Chrm (1 Indexed) |
| 2.18168661469995 | DGCR8 (bg=1.84%) | K562 | - | chrX | | 73069631 | 73069679 |
| 2.31472797803857 | DROSHA (bg=2.49%) | K562 | - | chrX | | 73069566 | 73069701 |
| 2.68485836796035 | DROSHA (bg=2.49%) | K562 | - | chrX | | 73069585 | 73069699 |
| 5.32850494813575 | hnrnpk (bg=12.88%) | K562 | - | chrX | | 73069612 | 73069691 |
| 6.62345963649005 | hnrnpk (bg=12.88%) | K562 | - | chrX | | 73069614 | 73069697 |
| 2.07042482328539 | SDAD1 (bg=2.97%) | K562 | - | chrX | | 73069637 | 73069684 |
| 2.01780284141765 | XRN2 (bg=0.39%) | K562 | - | chrX | | 73069619 | 73069694 |

  
  

| Match 124 in HUMAN | | | | | | | |
| --- | --- | --- | --- | --- | --- | --- | --- |
| Motif | Start in Seq (1 Indexed) | End in Seq (1 Indexed) | Strand | Chrm | Exon | Start in Chrm (0 Indexed) | End in Chrm (1 Indexed) |
| CCAGTCC | 2921 | 2927 | - | chrX | 1 | 73069661 | 73069668 |
| eCLIP Fold-Enrichment | Binding Protein | Cell Line | Strand | Chrm | | Start in Chrm (0 Indexed) | End in Chrm (1 Indexed) |
| 2.18168661469995 | DGCR8 (bg=1.84%) | K562 | - | chrX | | 73069631 | 73069679 |
| 2.31472797803857 | DROSHA (bg=2.49%) | K562 | - | chrX | | 73069566 | 73069701 |
| 2.68485836796035 | DROSHA (bg=2.49%) | K562 | - | chrX | | 73069585 | 73069699 |
| 5.32850494813575 | hnrnpk (bg=12.88%) | K562 | - | chrX | | 73069612 | 73069691 |
| 6.62345963649005 | hnrnpk (bg=12.88%) | K562 | - | chrX | | 73069614 | 73069697 |
| 2.07042482328539 | SDAD1 (bg=2.97%) | K562 | - | chrX | | 73069637 | 73069684 |
| 2.01780284141765 | XRN2 (bg=0.39%) | K562 | - | chrX | | 73069619 | 73069694 |

  
  

| Match 125 in HUMAN | | | | | | | |
| --- | --- | --- | --- | --- | --- | --- | --- |
| Motif | Start in Seq (1 Indexed) | End in Seq (1 Indexed) | Strand | Chrm | Exon | Start in Chrm (0 Indexed) | End in Chrm (1 Indexed) |
| ATTGATT | 2976 | 2982 | - | chrX | 1 | 73069606 | 73069613 |
| eCLIP Fold-Enrichment | Binding Protein | Cell Line | Strand | Chrm | | Start in Chrm (0 Indexed) | End in Chrm (1 Indexed) |
| 2.31472797803857 | DROSHA (bg=2.49%) | K562 | - | chrX | | 73069566 | 73069701 |
| 2.68485836796035 | DROSHA (bg=2.49%) | K562 | - | chrX | | 73069585 | 73069699 |
| 4.64332494231534 | hnrnpk (bg=12.88%) | K562 | - | chrX | | 73069589 | 73069614 |
| 4.03007909026689 | hnrnpk (bg=12.88%) | K562 | - | chrX | | 73069592 | 73069612 |
| 5.32850494813575 | hnrnpk (bg=12.88%) | K562 | - | chrX | | 73069612 | 73069691 |

  
  

| Match 126 in HUMAN | | | | | | | |
| --- | --- | --- | --- | --- | --- | --- | --- |
| Motif | Start in Seq (1 Indexed) | End in Seq (1 Indexed) | Strand | Chrm | Exon | Start in Chrm (0 Indexed) | End in Chrm (1 Indexed) |
| AAAATAAGTT | 2991 | 3000 | - | chrX | 1 | 73069588 | 73069598 |
| eCLIP Fold-Enrichment | Binding Protein | Cell Line | Strand | Chrm | | Start in Chrm (0 Indexed) | End in Chrm (1 Indexed) |
| 2.31472797803857 | DROSHA (bg=2.49%) | K562 | - | chrX | | 73069566 | 73069701 |
| 2.68485836796035 | DROSHA (bg=2.49%) | K562 | - | chrX | | 73069585 | 73069699 |
| 4.21479778135449 | hnrnpk (bg=12.88%) | K562 | - | chrX | | 73069563 | 73069589 |
| 3.50962935110046 | hnrnpk (bg=12.88%) | K562 | - | chrX | | 73069567 | 73069592 |
| 4.64332494231534 | hnrnpk (bg=12.88%) | K562 | - | chrX | | 73069589 | 73069614 |
| 4.03007909026689 | hnrnpk (bg=12.88%) | K562 | - | chrX | | 73069592 | 73069612 |

  
  

| Match 127 in HUMAN | | | | | | | |
| --- | --- | --- | --- | --- | --- | --- | --- |
| Motif | Start in Seq (1 Indexed) | End in Seq (1 Indexed) | Strand | Chrm | Exon | Start in Chrm (0 Indexed) | End in Chrm (1 Indexed) |
| ACTGGGATA | 3022 | 3030 | - | chrX | 1 | 73069558 | 73069567 |
| eCLIP Fold-Enrichment | Binding Protein | Cell Line | Strand | Chrm | | Start in Chrm (0 Indexed) | End in Chrm (1 Indexed) |
| 2.31472797803857 | DROSHA (bg=2.49%) | K562 | - | chrX | | 73069566 | 73069701 |
| 3.99376639317643 | hnrnpk (bg=12.88%) | K562 | - | chrX | | 73069468 | 73069563 |
| 4.0588207207998 | hnrnpk (bg=12.88%) | K562 | - | chrX | | 73069505 | 73069567 |
| 4.21479778135449 | hnrnpk (bg=12.88%) | K562 | - | chrX | | 73069563 | 73069589 |
| 3.50962935110046 | hnrnpk (bg=12.88%) | K562 | - | chrX | | 73069567 | 73069592 |
| 2.00798137274213 | ILF3 (bg=3.0%) | K562 | - | chrX | | 73069489 | 73069569 |

  
  

| Match 128 in HUMAN | | | | | | | |
| --- | --- | --- | --- | --- | --- | --- | --- |
| Motif | Start in Seq (1 Indexed) | End in Seq (1 Indexed) | Strand | Chrm | Exon | Start in Chrm (0 Indexed) | End in Chrm (1 Indexed) |
| GCATTGCTGATCTT | 3046 | 3059 | - | chrX | 1 | 73069529 | 73069543 |
| eCLIP Fold-Enrichment | Binding Protein | Cell Line | Strand | Chrm | | Start in Chrm (0 Indexed) | End in Chrm (1 Indexed) |
| 3.99376639317643 | hnrnpk (bg=12.88%) | K562 | - | chrX | | 73069468 | 73069563 |
| 4.0588207207998 | hnrnpk (bg=12.88%) | K562 | - | chrX | | 73069505 | 73069567 |
| 2.00798137274213 | ILF3 (bg=3.0%) | K562 | - | chrX | | 73069489 | 73069569 |

  
  

| Match 129 in HUMAN | | | | | | | |
| --- | --- | --- | --- | --- | --- | --- | --- |
| Motif | Start in Seq (1 Indexed) | End in Seq (1 Indexed) | Strand | Chrm | Exon | Start in Chrm (0 Indexed) | End in Chrm (1 Indexed) |
| ACCATTTTCA | 3076 | 3085 | - | chrX | 1 | 73069503 | 73069513 |
| eCLIP Fold-Enrichment | Binding Protein | Cell Line | Strand | Chrm | | Start in Chrm (0 Indexed) | End in Chrm (1 Indexed) |
| 4.1803217258475 | hnrnpk (bg=12.88%) | K562 | - | chrX | | 73069462 | 73069505 |
| 3.99376639317643 | hnrnpk (bg=12.88%) | K562 | - | chrX | | 73069468 | 73069563 |
| 4.0588207207998 | hnrnpk (bg=12.88%) | K562 | - | chrX | | 73069505 | 73069567 |
| 2.00798137274213 | ILF3 (bg=3.0%) | K562 | - | chrX | | 73069489 | 73069569 |

  
  

| Match 130 in HUMAN | | | | | | | |
| --- | --- | --- | --- | --- | --- | --- | --- |
| Motif | Start in Seq (1 Indexed) | End in Seq (1 Indexed) | Strand | Chrm | Exon | Start in Chrm (0 Indexed) | End in Chrm (1 Indexed) |
| ACAATCCCATTTG | 3096 | 3108 | - | chrX | 1 | 73069480 | 73069493 |
| eCLIP Fold-Enrichment | Binding Protein | Cell Line | Strand | Chrm | | Start in Chrm (0 Indexed) | End in Chrm (1 Indexed) |
| 4.1803217258475 | hnrnpk (bg=12.88%) | K562 | - | chrX | | 73069462 | 73069505 |
| 3.99376639317643 | hnrnpk (bg=12.88%) | K562 | - | chrX | | 73069468 | 73069563 |
| 2.01984275271233 | HNRNPU (bg=5.92%) | K562 | - | chrX | | 73069479 | 73069492 |
| 2.00798137274213 | ILF3 (bg=3.0%) | K562 | - | chrX | | 73069489 | 73069569 |

  
  

| Match 131 in HUMAN | | | | | | | |
| --- | --- | --- | --- | --- | --- | --- | --- |
| Motif | Start in Seq (1 Indexed) | End in Seq (1 Indexed) | Strand | Chrm | Exon | Start in Chrm (0 Indexed) | End in Chrm (1 Indexed) |
| ACAAAGAATTT | 3123 | 3133 | - | chrX | 1 | 73069455 | 73069466 |
| eCLIP Fold-Enrichment | Binding Protein | Cell Line | Strand | Chrm | | Start in Chrm (0 Indexed) | End in Chrm (1 Indexed) |
| 4.1803217258475 | hnrnpk (bg=12.88%) | K562 | - | chrX | | 73069462 | 73069505 |

  
  

| Match 132 in HUMAN | | | | | | | |
| --- | --- | --- | --- | --- | --- | --- | --- |
| Motif | Start in Seq (1 Indexed) | End in Seq (1 Indexed) | Strand | Chrm | Exon | Start in Chrm (0 Indexed) | End in Chrm (1 Indexed) |
| AAGGAGAAACCATT | 3241 | 3254 | - | chrX | 1 | 73069334 | 73069348 |
| eCLIP Fold-Enrichment | Binding Protein | Cell Line | Strand | Chrm | | Start in Chrm (0 Indexed) | End in Chrm (1 Indexed) |
| 2.87437452268477 | EIF3G (bg=0.32%) | K562 | - | chrX | | 73069319 | 73069381 |
| 2.4213298253513 | hnrnpk (bg=12.88%) | K562 | - | chrX | | 73069312 | 73069369 |
| 3.08435765012713 | hnrnpk (bg=12.88%) | K562 | - | chrX | | 73069314 | 73069370 |

  
  

| Match 133 in HUMAN | | | | | | | |
| --- | --- | --- | --- | --- | --- | --- | --- |
| Motif | Start in Seq (1 Indexed) | End in Seq (1 Indexed) | Strand | Chrm | Exon | Start in Chrm (0 Indexed) | End in Chrm (1 Indexed) |
| CTCTGTCATTGCT | 3256 | 3268 | - | chrX | 1 | 73069320 | 73069333 |
| eCLIP Fold-Enrichment | Binding Protein | Cell Line | Strand | Chrm | | Start in Chrm (0 Indexed) | End in Chrm (1 Indexed) |
| 2.87437452268477 | EIF3G (bg=0.32%) | K562 | - | chrX | | 73069319 | 73069381 |
| 2.4213298253513 | hnrnpk (bg=12.88%) | K562 | - | chrX | | 73069312 | 73069369 |
| 3.08435765012713 | hnrnpk (bg=12.88%) | K562 | - | chrX | | 73069314 | 73069370 |

  
  

| Match 134 in HUMAN | | | | | | | |
| --- | --- | --- | --- | --- | --- | --- | --- |
| Motif | Start in Seq (1 Indexed) | End in Seq (1 Indexed) | Strand | Chrm | Exon | Start in Chrm (0 Indexed) | End in Chrm (1 Indexed) |
| CTCTGT | 3256 | 3261 | - | chrX | 1 | 73069327 | 73069333 |
| eCLIP Fold-Enrichment | Binding Protein | Cell Line | Strand | Chrm | | Start in Chrm (0 Indexed) | End in Chrm (1 Indexed) |
| 2.87437452268477 | EIF3G (bg=0.32%) | K562 | - | chrX | | 73069319 | 73069381 |
| 2.4213298253513 | hnrnpk (bg=12.88%) | K562 | - | chrX | | 73069312 | 73069369 |
| 3.08435765012713 | hnrnpk (bg=12.88%) | K562 | - | chrX | | 73069314 | 73069370 |

  
  

| Match 135 in HUMAN | | | | | | | |
| --- | --- | --- | --- | --- | --- | --- | --- |
| Motif | Start in Seq (1 Indexed) | End in Seq (1 Indexed) | Strand | Chrm | Exon | Start in Chrm (0 Indexed) | End in Chrm (1 Indexed) |
| GTAGTCA | 3272 | 3278 | - | chrX | 1 | 73069310 | 73069317 |
| eCLIP Fold-Enrichment | Binding Protein | Cell Line | Strand | Chrm | | Start in Chrm (0 Indexed) | End in Chrm (1 Indexed) |
| 2.4213298253513 | hnrnpk (bg=12.88%) | K562 | - | chrX | | 73069312 | 73069369 |
| 3.08435765012713 | hnrnpk (bg=12.88%) | K562 | - | chrX | | 73069314 | 73069370 |
| 3.09082372357331 | SUPV3L1 (bg=1.57%) | K562 | - | chrX | | 73069309 | 73069315 |

  
  

| Match 136 in HUMAN | | | | | | | |
| --- | --- | --- | --- | --- | --- | --- | --- |
| Motif | Start in Seq (1 Indexed) | End in Seq (1 Indexed) | Strand | Chrm | Exon | Start in Chrm (0 Indexed) | End in Chrm (1 Indexed) |
| TACTGTG | 3313 | 3319 | - | chrX | 1 | 73069269 | 73069276 |
| eCLIP Fold-Enrichment | Binding Protein | Cell Line | Strand | Chrm | | Start in Chrm (0 Indexed) | End in Chrm (1 Indexed) |
| 2.48047711843129 | HNRNPU (bg=5.92%) | K562 | - | chrX | | 73069248 | 73069276 |
| 2.9686458492113 | WRN (bg=0.77%) | K562 | - | chrX | | 73069257 | 73069282 |

  
  

| Match 137 in HUMAN | | | | | | | |
| --- | --- | --- | --- | --- | --- | --- | --- |
| Motif | Start in Seq (1 Indexed) | End in Seq (1 Indexed) | Strand | Chrm | Exon | Start in Chrm (0 Indexed) | End in Chrm (1 Indexed) |
| AAACTCTTTGCA | 3331 | 3342 | - | chrX | 1 | 73069246 | 73069258 |
| eCLIP Fold-Enrichment | Binding Protein | Cell Line | Strand | Chrm | | Start in Chrm (0 Indexed) | End in Chrm (1 Indexed) |
| 2.48047711843129 | HNRNPU (bg=5.92%) | K562 | - | chrX | | 73069248 | 73069276 |
| 2.9686458492113 | WRN (bg=0.77%) | K562 | - | chrX | | 73069257 | 73069282 |

  
  

| Match 138 in HUMAN | | | | | | | |
| --- | --- | --- | --- | --- | --- | --- | --- |
| Motif | Start in Seq (1 Indexed) | End in Seq (1 Indexed) | Strand | Chrm | Exon | Start in Chrm (0 Indexed) | End in Chrm (1 Indexed) |
| ATAATCCT | 3365 | 3372 | - | chrX | 1 | 73069216 | 73069224 |
| eCLIP Fold-Enrichment | Binding Protein | Cell Line | Strand | Chrm | | Start in Chrm (0 Indexed) | End in Chrm (1 Indexed) |
| 2.3131260606528 | HNRNPU (bg=5.92%) | K562 | - | chrX | | 73069182 | 73069224 |

  
  

| Match 139 in HUMAN | | | | | | | |
| --- | --- | --- | --- | --- | --- | --- | --- |
| Motif | Start in Seq (1 Indexed) | End in Seq (1 Indexed) | Strand | Chrm | Exon | Start in Chrm (0 Indexed) | End in Chrm (1 Indexed) |
| CATTGGA | 3376 | 3382 | - | chrX | 1 | 73069206 | 73069213 |
| eCLIP Fold-Enrichment | Binding Protein | Cell Line | Strand | Chrm | | Start in Chrm (0 Indexed) | End in Chrm (1 Indexed) |
| 2.3131260606528 | HNRNPU (bg=5.92%) | K562 | - | chrX | | 73069182 | 73069224 |
| 2.23174356359797 | HNRNPU (bg=5.92%) | K562 | - | chrX | | 73069191 | 73069211 |

  
  

| Match 140 in HUMAN | | | | | | | |
| --- | --- | --- | --- | --- | --- | --- | --- |
| Motif | Start in Seq (1 Indexed) | End in Seq (1 Indexed) | Strand | Chrm | Exon | Start in Chrm (0 Indexed) | End in Chrm (1 Indexed) |
| TTTGCATTCAGCAG | 3405 | 3418 | - | chrX | 1 | 73069170 | 73069184 |
| eCLIP Fold-Enrichment | Binding Protein | Cell Line | Strand | Chrm | | Start in Chrm (0 Indexed) | End in Chrm (1 Indexed) |
| 2.3131260606528 | HNRNPU (bg=5.92%) | K562 | - | chrX | | 73069182 | 73069224 |

  
  

| Match 141 in HUMAN | | | | | | | |
| --- | --- | --- | --- | --- | --- | --- | --- |
| Motif | Start in Seq (1 Indexed) | End in Seq (1 Indexed) | Strand | Chrm | Exon | Start in Chrm (0 Indexed) | End in Chrm (1 Indexed) |
| TTGTCATA | 3471 | 3478 | - | chrX | 1 | 73069110 | 73069118 |
| eCLIP Fold-Enrichment | Binding Protein | Cell Line | Strand | Chrm | | Start in Chrm (0 Indexed) | End in Chrm (1 Indexed) |
| 2.69460192378413 | HNRNPU (bg=5.92%) | K562 | - | chrX | | 73069076 | 73069112 |
| 2.75150431501861 | HNRNPU (bg=5.92%) | K562 | - | chrX | | 73069112 | 73069142 |

  
  

| Match 142 in HUMAN | | | | | | | |
| --- | --- | --- | --- | --- | --- | --- | --- |
| Motif | Start in Seq (1 Indexed) | End in Seq (1 Indexed) | Strand | Chrm | Exon | Start in Chrm (0 Indexed) | End in Chrm (1 Indexed) |
| TTAAACAAAGGCA | 3489 | 3501 | - | chrX | 1 | 73069087 | 73069100 |
| eCLIP Fold-Enrichment | Binding Protein | Cell Line | Strand | Chrm | | Start in Chrm (0 Indexed) | End in Chrm (1 Indexed) |
| 2.01696432637139 | HNRNPU (bg=5.92%) | K562 | - | chrX | | 73069055 | 73069097 |
| 2.69460192378413 | HNRNPU (bg=5.92%) | K562 | - | chrX | | 73069076 | 73069112 |

  
  

| Match 143 in HUMAN | | | | | | | |
| --- | --- | --- | --- | --- | --- | --- | --- |
| Motif | Start in Seq (1 Indexed) | End in Seq (1 Indexed) | Strand | Chrm | Exon | Start in Chrm (0 Indexed) | End in Chrm (1 Indexed) |
| CTGTTCTTGGACAATTAAAG | 3540 | 3559 | - | chrX | 1 | 73069029 | 73069049 |
| eCLIP Fold-Enrichment | Binding Protein | Cell Line | Strand | Chrm | | Start in Chrm (0 Indexed) | End in Chrm (1 Indexed) |
| 2.53551848198925 | HNRNPU (bg=5.92%) | K562 | - | chrX | | 73069041 | 73069076 |

  
  

| Match 144 in HUMAN | | | | | | | |
| --- | --- | --- | --- | --- | --- | --- | --- |
| Motif | Start in Seq (1 Indexed) | End in Seq (1 Indexed) | Strand | Chrm | Exon | Start in Chrm (0 Indexed) | End in Chrm (1 Indexed) |
| AAGACCCAC | 3832 | 3840 | - | chrX | 1 | 73068748 | 73068757 |
| eCLIP Fold-Enrichment | Binding Protein | Cell Line | Strand | Chrm | | Start in Chrm (0 Indexed) | End in Chrm (1 Indexed) |
| 3.2336204520609 | HNRNPUL1 (bg=1.16%) | K562 | - | chrX | | 73068738 | 73068758 |

  
  

| Match 145 in HUMAN | | | | | | | |
| --- | --- | --- | --- | --- | --- | --- | --- |
| Motif | Start in Seq (1 Indexed) | End in Seq (1 Indexed) | Strand | Chrm | Exon | Start in Chrm (0 Indexed) | End in Chrm (1 Indexed) |
| GTCCCACT | 3926 | 3933 | - | chrX | 1 | 73068655 | 73068663 |
| eCLIP Fold-Enrichment | Binding Protein | Cell Line | Strand | Chrm | | Start in Chrm (0 Indexed) | End in Chrm (1 Indexed) |
| 2.31128240373067 | hnrnpk (bg=12.88%) | K562 | - | chrX | | 73068611 | 73068666 |
| 2.8814846795101 | hnrnpk (bg=12.88%) | K562 | - | chrX | | 73068617 | 73068679 |

  
  

| Match 146 in HUMAN | | | | | | | |
| --- | --- | --- | --- | --- | --- | --- | --- |
| Motif | Start in Seq (1 Indexed) | End in Seq (1 Indexed) | Strand | Chrm | Exon | Start in Chrm (0 Indexed) | End in Chrm (1 Indexed) |
| TGCCATT | 3956 | 3962 | - | chrX | 1 | 73068626 | 73068633 |
| eCLIP Fold-Enrichment | Binding Protein | Cell Line | Strand | Chrm | | Start in Chrm (0 Indexed) | End in Chrm (1 Indexed) |
| 2.31128240373067 | hnrnpk (bg=12.88%) | K562 | - | chrX | | 73068611 | 73068666 |
| 2.8814846795101 | hnrnpk (bg=12.88%) | K562 | - | chrX | | 73068617 | 73068679 |

  
  

| Match 147 in HUMAN | | | | | | | |
| --- | --- | --- | --- | --- | --- | --- | --- |
| Motif | Start in Seq (1 Indexed) | End in Seq (1 Indexed) | Strand | Chrm | Exon | Start in Chrm (0 Indexed) | End in Chrm (1 Indexed) |
| GAGTTCTGA | 3969 | 3977 | - | chrX | 1 | 73068611 | 73068620 |
| eCLIP Fold-Enrichment | Binding Protein | Cell Line | Strand | Chrm | | Start in Chrm (0 Indexed) | End in Chrm (1 Indexed) |
| 2.31128240373067 | hnrnpk (bg=12.88%) | K562 | - | chrX | | 73068611 | 73068666 |
| 2.8814846795101 | hnrnpk (bg=12.88%) | K562 | - | chrX | | 73068617 | 73068679 |

  
  

| Match 148 in HUMAN | | | | | | | |
| --- | --- | --- | --- | --- | --- | --- | --- |
| Motif | Start in Seq (1 Indexed) | End in Seq (1 Indexed) | Strand | Chrm | Exon | Start in Chrm (0 Indexed) | End in Chrm (1 Indexed) |
| TCTGTGG | 4073 | 4079 | - | chrX | 1 | 73068509 | 73068516 |
| eCLIP Fold-Enrichment | Binding Protein | Cell Line | Strand | Chrm | | Start in Chrm (0 Indexed) | End in Chrm (1 Indexed) |
| 2.07714140905794 | HNRNPL (bg=0.64%) | K562 | - | chrX | | 73068503 | 73068539 |
| 2.41488877680371 | HNRNPU (bg=5.92%) | K562 | - | chrX | | 73068484 | 73068513 |

  
  

| Match 149 in HUMAN | | | | | | | |
| --- | --- | --- | --- | --- | --- | --- | --- |
| Motif | Start in Seq (1 Indexed) | End in Seq (1 Indexed) | Strand | Chrm | Exon | Start in Chrm (0 Indexed) | End in Chrm (1 Indexed) |
| AGTGCA | 4093 | 4098 | - | chrX | 1 | 73068490 | 73068496 |
| eCLIP Fold-Enrichment | Binding Protein | Cell Line | Strand | Chrm | | Start in Chrm (0 Indexed) | End in Chrm (1 Indexed) |
| 2.41488877680371 | HNRNPU (bg=5.92%) | K562 | - | chrX | | 73068484 | 73068513 |

  
  

| Match 150 in HUMAN | | | | | | | |
| --- | --- | --- | --- | --- | --- | --- | --- |
| Motif | Start in Seq (1 Indexed) | End in Seq (1 Indexed) | Strand | Chrm | Exon | Start in Chrm (0 Indexed) | End in Chrm (1 Indexed) |
| ATTATTCAA | 4100 | 4108 | - | chrX | 1 | 73068480 | 73068489 |
| eCLIP Fold-Enrichment | Binding Protein | Cell Line | Strand | Chrm | | Start in Chrm (0 Indexed) | End in Chrm (1 Indexed) |
| 2.87057100892959 | HNRNPA1 (bg=2.57%) | K562 | - | chrX | | 73068434 | 73068486 |
| 2.41488877680371 | HNRNPU (bg=5.92%) | K562 | - | chrX | | 73068484 | 73068513 |

  
  

| Match 151 in HUMAN | | | | | | | |
| --- | --- | --- | --- | --- | --- | --- | --- |
| Motif | Start in Seq (1 Indexed) | End in Seq (1 Indexed) | Strand | Chrm | Exon | Start in Chrm (0 Indexed) | End in Chrm (1 Indexed) |
| ACAGTTAAT | 4122 | 4130 | - | chrX | 1 | 73068458 | 73068467 |
| eCLIP Fold-Enrichment | Binding Protein | Cell Line | Strand | Chrm | | Start in Chrm (0 Indexed) | End in Chrm (1 Indexed) |
| 2.87057100892959 | HNRNPA1 (bg=2.57%) | K562 | - | chrX | | 73068434 | 73068486 |

  
  

| Match 152 in HUMAN | | | | | | | |
| --- | --- | --- | --- | --- | --- | --- | --- |
| Motif | Start in Seq (1 Indexed) | End in Seq (1 Indexed) | Strand | Chrm | Exon | Start in Chrm (0 Indexed) | End in Chrm (1 Indexed) |
| GCACAGTTGC | 4133 | 4142 | - | chrX | 1 | 73068446 | 73068456 |
| eCLIP Fold-Enrichment | Binding Protein | Cell Line | Strand | Chrm | | Start in Chrm (0 Indexed) | End in Chrm (1 Indexed) |
| 2.87057100892959 | HNRNPA1 (bg=2.57%) | K562 | - | chrX | | 73068434 | 73068486 |

  
  

| Match 153 in HUMAN | | | | | | | |
| --- | --- | --- | --- | --- | --- | --- | --- |
| Motif | Start in Seq (1 Indexed) | End in Seq (1 Indexed) | Strand | Chrm | Exon | Start in Chrm (0 Indexed) | End in Chrm (1 Indexed) |
| TTGTCCAGAGTCC | 4145 | 4157 | - | chrX | 1 | 73068431 | 73068444 |
| eCLIP Fold-Enrichment | Binding Protein | Cell Line | Strand | Chrm | | Start in Chrm (0 Indexed) | End in Chrm (1 Indexed) |
| 2.87057100892959 | HNRNPA1 (bg=2.57%) | K562 | - | chrX | | 73068434 | 73068486 |

  
  

| Match 154 in HUMAN | | | | | | | |
| --- | --- | --- | --- | --- | --- | --- | --- |
| Motif | Start in Seq (1 Indexed) | End in Seq (1 Indexed) | Strand | Chrm | Exon | Start in Chrm (0 Indexed) | End in Chrm (1 Indexed) |
| GTGGGC | 4202 | 4207 | - | chrX | 1 | 73068381 | 73068387 |
| eCLIP Fold-Enrichment | Binding Protein | Cell Line | Strand | Chrm | | Start in Chrm (0 Indexed) | End in Chrm (1 Indexed) |
| 2.0320712187616 | HNRNPU (bg=5.92%) | K562 | - | chrX | | 73068359 | 73068395 |

  
  

| Match 155 in HUMAN | | | | | | | |
| --- | --- | --- | --- | --- | --- | --- | --- |
| Motif | Start in Seq (1 Indexed) | End in Seq (1 Indexed) | Strand | Chrm | Exon | Start in Chrm (0 Indexed) | End in Chrm (1 Indexed) |
| CATAATTG | 4243 | 4250 | - | chrX | 1 | 73068338 | 73068346 |
| eCLIP Fold-Enrichment | Binding Protein | Cell Line | Strand | Chrm | | Start in Chrm (0 Indexed) | End in Chrm (1 Indexed) |
| 3.52778283320964 | HNRNPU (bg=5.92%) | K562 | - | chrX | | 73068301 | 73068338 |

  
  

| Match 156 in HUMAN | | | | | | | |
| --- | --- | --- | --- | --- | --- | --- | --- |
| Motif | Start in Seq (1 Indexed) | End in Seq (1 Indexed) | Strand | Chrm | Exon | Start in Chrm (0 Indexed) | End in Chrm (1 Indexed) |
| CTTTGTATTCCAGCAGGGGACCCTT | 4289 | 4313 | - | chrX | 1 | 73068275 | 73068300 |
| eCLIP Fold-Enrichment | Binding Protein | Cell Line | Strand | Chrm | | Start in Chrm (0 Indexed) | End in Chrm (1 Indexed) |
| 4.8107816881669 | hnrnpk (bg=12.88%) | K562 | - | chrX | | 73068254 | 73068334 |
| 3.87219943048517 | hnrnpk (bg=12.88%) | K562 | - | chrX | | 73068255 | 73068300 |
| 4.89491950698525 | hnrnpk (bg=12.88%) | K562 | - | chrX | | 73068300 | 73068332 |
| 2.77802559615506 | HNRNPU (bg=5.92%) | K562 | - | chrX | | 73068300 | 73068327 |

  
  

| Match 157 in HUMAN | | | | | | | |
| --- | --- | --- | --- | --- | --- | --- | --- |
| Motif | Start in Seq (1 Indexed) | End in Seq (1 Indexed) | Strand | Chrm | Exon | Start in Chrm (0 Indexed) | End in Chrm (1 Indexed) |
| CCTAATTGATTAGA | 4356 | 4369 | - | chrX | 1 | 73068219 | 73068233 |
| eCLIP Fold-Enrichment | Binding Protein | Cell Line | Strand | Chrm | | Start in Chrm (0 Indexed) | End in Chrm (1 Indexed) |
| 2.67988744732048 | HNRNPA1 (bg=2.57%) | K562 | - | chrX | | 73068173 | 73068248 |
| 2.57145539992443 | HNRNPA1 (bg=2.57%) | K562 | - | chrX | | 73068185 | 73068236 |

  
  

| Match 158 in HUMAN | | | | | | | |
| --- | --- | --- | --- | --- | --- | --- | --- |
| Motif | Start in Seq (1 Indexed) | End in Seq (1 Indexed) | Strand | Chrm | Exon | Start in Chrm (0 Indexed) | End in Chrm (1 Indexed) |
| TCTTTTAT | 4376 | 4383 | - | chrX | 1 | 73068205 | 73068213 |
| eCLIP Fold-Enrichment | Binding Protein | Cell Line | Strand | Chrm | | Start in Chrm (0 Indexed) | End in Chrm (1 Indexed) |
| 2.67988744732048 | HNRNPA1 (bg=2.57%) | K562 | - | chrX | | 73068173 | 73068248 |
| 2.57145539992443 | HNRNPA1 (bg=2.57%) | K562 | - | chrX | | 73068185 | 73068236 |
| 2.64980700914872 | hnrnpk (bg=12.88%) | K562 | - | chrX | | 73068182 | 73068205 |
| 3.64504888537939 | HNRNPU (bg=5.92%) | K562 | - | chrX | | 73068179 | 73068217 |
| 2.58980896696129 | UTP3 (bg=3.66%) | K562 | - | chrX | | 73068192 | 73068208 |

  
  

| Match 159 in HUMAN | | | | | | | |
| --- | --- | --- | --- | --- | --- | --- | --- |
| Motif | Start in Seq (1 Indexed) | End in Seq (1 Indexed) | Strand | Chrm | Exon | Start in Chrm (0 Indexed) | End in Chrm (1 Indexed) |
| TGCATC | 4395 | 4400 | - | chrX | 1 | 73068188 | 73068194 |
| eCLIP Fold-Enrichment | Binding Protein | Cell Line | Strand | Chrm | | Start in Chrm (0 Indexed) | End in Chrm (1 Indexed) |
| 2.67988744732048 | HNRNPA1 (bg=2.57%) | K562 | - | chrX | | 73068173 | 73068248 |
| 2.57145539992443 | HNRNPA1 (bg=2.57%) | K562 | - | chrX | | 73068185 | 73068236 |
| 2.64980700914872 | hnrnpk (bg=12.88%) | K562 | - | chrX | | 73068182 | 73068205 |
| 3.64504888537939 | HNRNPU (bg=5.92%) | K562 | - | chrX | | 73068179 | 73068217 |
| 2.71533984904515 | UTP3 (bg=3.66%) | K562 | - | chrX | | 73068188 | 73068192 |
| 2.58980896696129 | UTP3 (bg=3.66%) | K562 | - | chrX | | 73068192 | 73068208 |

  
  

| Match 160 in HUMAN | | | | | | | |
| --- | --- | --- | --- | --- | --- | --- | --- |
| Motif | Start in Seq (1 Indexed) | End in Seq (1 Indexed) | Strand | Chrm | Exon | Start in Chrm (0 Indexed) | End in Chrm (1 Indexed) |
| TTACCTA | 4430 | 4436 | - | chrX | 1 | 73068152 | 73068159 |
| eCLIP Fold-Enrichment | Binding Protein | Cell Line | Strand | Chrm | | Start in Chrm (0 Indexed) | End in Chrm (1 Indexed) |
| 2.16711103704735 | HNRNPU (bg=5.92%) | K562 | - | chrX | | 73068157 | 73068179 |
| 2.33463315268042 | UTP3 (bg=3.66%) | K562 | - | chrX | | 73068157 | 73068182 |

  
  

| Match 161 in HUMAN | | | | | | | |
| --- | --- | --- | --- | --- | --- | --- | --- |
| Motif | Start in Seq (1 Indexed) | End in Seq (1 Indexed) | Strand | Chrm | Exon | Start in Chrm (0 Indexed) | End in Chrm (1 Indexed) |
| AAATGCAATT | 4468 | 4477 | - | chrX | 1 | 73068111 | 73068121 |
| eCLIP Fold-Enrichment | Binding Protein | Cell Line | Strand | Chrm | | Start in Chrm (0 Indexed) | End in Chrm (1 Indexed) |
| 2.26355304026552 | AKAP8L (bg=2.19%) | K562 | - | chrX | | 73068040 | 73068114 |

  
  

| Match 162 in HUMAN | | | | | | | |
| --- | --- | --- | --- | --- | --- | --- | --- |
| Motif | Start in Seq (1 Indexed) | End in Seq (1 Indexed) | Strand | Chrm | Exon | Start in Chrm (0 Indexed) | End in Chrm (1 Indexed) |
| CTGTTAGTCT | 4488 | 4497 | - | chrX | 1 | 73068091 | 73068101 |
| eCLIP Fold-Enrichment | Binding Protein | Cell Line | Strand | Chrm | | Start in Chrm (0 Indexed) | End in Chrm (1 Indexed) |
| 2.26355304026552 | AKAP8L (bg=2.19%) | K562 | - | chrX | | 73068040 | 73068114 |

  
  

| Match 163 in HUMAN | | | | | | | |
| --- | --- | --- | --- | --- | --- | --- | --- |
| Motif | Start in Seq (1 Indexed) | End in Seq (1 Indexed) | Strand | Chrm | Exon | Start in Chrm (0 Indexed) | End in Chrm (1 Indexed) |
| TGTTAGTC | 4489 | 4496 | - | chrX | 1 | 73068092 | 73068100 |
| eCLIP Fold-Enrichment | Binding Protein | Cell Line | Strand | Chrm | | Start in Chrm (0 Indexed) | End in Chrm (1 Indexed) |
| 2.26355304026552 | AKAP8L (bg=2.19%) | K562 | - | chrX | | 73068040 | 73068114 |

  
  

| Match 164 in HUMAN | | | | | | | |
| --- | --- | --- | --- | --- | --- | --- | --- |
| Motif | Start in Seq (1 Indexed) | End in Seq (1 Indexed) | Strand | Chrm | Exon | Start in Chrm (0 Indexed) | End in Chrm (1 Indexed) |
| TCTCATCCCC | 4502 | 4511 | - | chrX | 1 | 73068077 | 73068087 |
| eCLIP Fold-Enrichment | Binding Protein | Cell Line | Strand | Chrm | | Start in Chrm (0 Indexed) | End in Chrm (1 Indexed) |
| 2.26355304026552 | AKAP8L (bg=2.19%) | K562 | - | chrX | | 73068040 | 73068114 |

  
  

| Match 165 in HUMAN | | | | | | | |
| --- | --- | --- | --- | --- | --- | --- | --- |
| Motif | Start in Seq (1 Indexed) | End in Seq (1 Indexed) | Strand | Chrm | Exon | Start in Chrm (0 Indexed) | End in Chrm (1 Indexed) |
| TCATCC | 4504 | 4509 | - | chrX | 1 | 73068079 | 73068085 |
| eCLIP Fold-Enrichment | Binding Protein | Cell Line | Strand | Chrm | | Start in Chrm (0 Indexed) | End in Chrm (1 Indexed) |
| 2.26355304026552 | AKAP8L (bg=2.19%) | K562 | - | chrX | | 73068040 | 73068114 |

  
  

| Match 166 in HUMAN | | | | | | | |
| --- | --- | --- | --- | --- | --- | --- | --- |
| Motif | Start in Seq (1 Indexed) | End in Seq (1 Indexed) | Strand | Chrm | Exon | Start in Chrm (0 Indexed) | End in Chrm (1 Indexed) |
| CCTTTTGT | 4519 | 4526 | - | chrX | 1 | 73068062 | 73068070 |
| eCLIP Fold-Enrichment | Binding Protein | Cell Line | Strand | Chrm | | Start in Chrm (0 Indexed) | End in Chrm (1 Indexed) |
| 2.26355304026552 | AKAP8L (bg=2.19%) | K562 | - | chrX | | 73068040 | 73068114 |

  
  

| Match 167 in HUMAN | | | | | | | |
| --- | --- | --- | --- | --- | --- | --- | --- |
| Motif | Start in Seq (1 Indexed) | End in Seq (1 Indexed) | Strand | Chrm | Exon | Start in Chrm (0 Indexed) | End in Chrm (1 Indexed) |
| GGGTACTTGGGACTGTTAATG | 4538 | 4558 | - | chrX | 1 | 73068030 | 73068051 |
| eCLIP Fold-Enrichment | Binding Protein | Cell Line | Strand | Chrm | | Start in Chrm (0 Indexed) | End in Chrm (1 Indexed) |
| 2.26355304026552 | AKAP8L (bg=2.19%) | K562 | - | chrX | | 73068040 | 73068114 |

  
  

| Match 168 in HUMAN | | | | | | | |
| --- | --- | --- | --- | --- | --- | --- | --- |
| Motif | Start in Seq (1 Indexed) | End in Seq (1 Indexed) | Strand | Chrm | Exon | Start in Chrm (0 Indexed) | End in Chrm (1 Indexed) |
| GGGTACTTGGGACTGTTAAT | 4538 | 4557 | - | chrX | 1 | 73068031 | 73068051 |
| eCLIP Fold-Enrichment | Binding Protein | Cell Line | Strand | Chrm | | Start in Chrm (0 Indexed) | End in Chrm (1 Indexed) |
| 2.26355304026552 | AKAP8L (bg=2.19%) | K562 | - | chrX | | 73068040 | 73068114 |

  
  

| Match 169 in HUMAN | | | | | | | |
| --- | --- | --- | --- | --- | --- | --- | --- |
| Motif | Start in Seq (1 Indexed) | End in Seq (1 Indexed) | Strand | Chrm | Exon | Start in Chrm (0 Indexed) | End in Chrm (1 Indexed) |
| TACTTGGGACTGTTAAT | 4541 | 4557 | - | chrX | 1 | 73068031 | 73068048 |
| eCLIP Fold-Enrichment | Binding Protein | Cell Line | Strand | Chrm | | Start in Chrm (0 Indexed) | End in Chrm (1 Indexed) |
| 2.26355304026552 | AKAP8L (bg=2.19%) | K562 | - | chrX | | 73068040 | 73068114 |

  
  

| Match 170 in HUMAN | | | | | | | |
| --- | --- | --- | --- | --- | --- | --- | --- |
| Motif | Start in Seq (1 Indexed) | End in Seq (1 Indexed) | Strand | Chrm | Exon | Start in Chrm (0 Indexed) | End in Chrm (1 Indexed) |
| TGCAAAATT | 5219 | 5227 | - | chrX | 1 | 73067361 | 73067370 |
| eCLIP Fold-Enrichment | Binding Protein | Cell Line | Strand | Chrm | | Start in Chrm (0 Indexed) | End in Chrm (1 Indexed) |
| 3.89191965515923 | SUPV3L1 (bg=1.57%) | K562 | - | chrX | | 73067328 | 73067372 |

  
  

| Match 171 in HUMAN | | | | | | | |
| --- | --- | --- | --- | --- | --- | --- | --- |
| Motif | Start in Seq (1 Indexed) | End in Seq (1 Indexed) | Strand | Chrm | Exon | Start in Chrm (0 Indexed) | End in Chrm (1 Indexed) |
| TATGTTAGA | 5479 | 5487 | - | chrX | 1 | 73067101 | 73067110 |
| eCLIP Fold-Enrichment | Binding Protein | Cell Line | Strand | Chrm | | Start in Chrm (0 Indexed) | End in Chrm (1 Indexed) |
| 2.66634366374262 | HNRNPU (bg=5.92%) | K562 | - | chrX | | 73067068 | 73067121 |

  
  

| Match 172 in HUMAN | | | | | | | |
| --- | --- | --- | --- | --- | --- | --- | --- |
| Motif | Start in Seq (1 Indexed) | End in Seq (1 Indexed) | Strand | Chrm | Exon | Start in Chrm (0 Indexed) | End in Chrm (1 Indexed) |
| TAGAATCCC | 5489 | 5497 | - | chrX | 1 | 73067091 | 73067100 |
| eCLIP Fold-Enrichment | Binding Protein | Cell Line | Strand | Chrm | | Start in Chrm (0 Indexed) | End in Chrm (1 Indexed) |
| 2.66634366374262 | HNRNPU (bg=5.92%) | K562 | - | chrX | | 73067068 | 73067121 |

  
  

| Match 173 in HUMAN | | | | | | | |
| --- | --- | --- | --- | --- | --- | --- | --- |
| Motif | Start in Seq (1 Indexed) | End in Seq (1 Indexed) | Strand | Chrm | Exon | Start in Chrm (0 Indexed) | End in Chrm (1 Indexed) |
| TGCATAATCTTAG | 5685 | 5697 | - | chrX | 1 | 73066891 | 73066904 |
| eCLIP Fold-Enrichment | Binding Protein | Cell Line | Strand | Chrm | | Start in Chrm (0 Indexed) | End in Chrm (1 Indexed) |
| 2.01527597621862 | HNRNPU (bg=5.92%) | K562 | - | chrX | | 73066857 | 73066917 |

  
  

| Match 174 in HUMAN | | | | | | | |
| --- | --- | --- | --- | --- | --- | --- | --- |
| Motif | Start in Seq (1 Indexed) | End in Seq (1 Indexed) | Strand | Chrm | Exon | Start in Chrm (0 Indexed) | End in Chrm (1 Indexed) |
| ATCTTAG | 5691 | 5697 | - | chrX | 1 | 73066891 | 73066898 |
| eCLIP Fold-Enrichment | Binding Protein | Cell Line | Strand | Chrm | | Start in Chrm (0 Indexed) | End in Chrm (1 Indexed) |
| 2.01527597621862 | HNRNPU (bg=5.92%) | K562 | - | chrX | | 73066857 | 73066917 |

  
  

| Match 175 in HUMAN | | | | | | | |
| --- | --- | --- | --- | --- | --- | --- | --- |
| Motif | Start in Seq (1 Indexed) | End in Seq (1 Indexed) | Strand | Chrm | Exon | Start in Chrm (0 Indexed) | End in Chrm (1 Indexed) |
| TACACATT | 5706 | 5713 | - | chrX | 1 | 73066875 | 73066883 |
| eCLIP Fold-Enrichment | Binding Protein | Cell Line | Strand | Chrm | | Start in Chrm (0 Indexed) | End in Chrm (1 Indexed) |
| 4.45622957218461 | HNRNPU (bg=5.92%) | K562 | - | chrX | | 73066845 | 73066881 |
| 2.01527597621862 | HNRNPU (bg=5.92%) | K562 | - | chrX | | 73066857 | 73066917 |

  
  

| Match 176 in HUMAN | | | | | | | |
| --- | --- | --- | --- | --- | --- | --- | --- |
| Motif | Start in Seq (1 Indexed) | End in Seq (1 Indexed) | Strand | Chrm | Exon | Start in Chrm (0 Indexed) | End in Chrm (1 Indexed) |
| AGGACTCCT | 5743 | 5751 | - | chrX | 1 | 73066837 | 73066846 |
| eCLIP Fold-Enrichment | Binding Protein | Cell Line | Strand | Chrm | | Start in Chrm (0 Indexed) | End in Chrm (1 Indexed) |
| 2.48047711843129 | HNRNPU (bg=5.92%) | K562 | - | chrX | | 73066829 | 73066845 |
| 4.45622957218461 | HNRNPU (bg=5.92%) | K562 | - | chrX | | 73066845 | 73066881 |

  
  

| Match 177 in HUMAN | | | | | | | |
| --- | --- | --- | --- | --- | --- | --- | --- |
| Motif | Start in Seq (1 Indexed) | End in Seq (1 Indexed) | Strand | Chrm | Exon | Start in Chrm (0 Indexed) | End in Chrm (1 Indexed) |
| TACTTAT | 5753 | 5759 | - | chrX | 1 | 73066829 | 73066836 |
| eCLIP Fold-Enrichment | Binding Protein | Cell Line | Strand | Chrm | | Start in Chrm (0 Indexed) | End in Chrm (1 Indexed) |
| 2.48047711843129 | HNRNPU (bg=5.92%) | K562 | - | chrX | | 73066829 | 73066845 |

  
  

| Match 178 in HUMAN | | | | | | | |
| --- | --- | --- | --- | --- | --- | --- | --- |
| Motif | Start in Seq (1 Indexed) | End in Seq (1 Indexed) | Strand | Chrm | Exon | Start in Chrm (0 Indexed) | End in Chrm (1 Indexed) |
| ACTTAT | 5754 | 5759 | - | chrX | 1 | 73066829 | 73066835 |
| eCLIP Fold-Enrichment | Binding Protein | Cell Line | Strand | Chrm | | Start in Chrm (0 Indexed) | End in Chrm (1 Indexed) |
| 2.48047711843129 | HNRNPU (bg=5.92%) | K562 | - | chrX | | 73066829 | 73066845 |

  
  

| Match 179 in HUMAN | | | | | | | |
| --- | --- | --- | --- | --- | --- | --- | --- |
| Motif | Start in Seq (1 Indexed) | End in Seq (1 Indexed) | Strand | Chrm | Exon | Start in Chrm (0 Indexed) | End in Chrm (1 Indexed) |
| CTTTATTGC | 5880 | 5888 | - | chrX | 1 | 73066700 | 73066709 |
| eCLIP Fold-Enrichment | Binding Protein | Cell Line | Strand | Chrm | | Start in Chrm (0 Indexed) | End in Chrm (1 Indexed) |
| 2.64868339401678 | HNRNPL (bg=0.64%) | K562 | - | chrX | | 73066651 | 73066703 |

  
  

| Match 180 in HUMAN | | | | | | | |
| --- | --- | --- | --- | --- | --- | --- | --- |
| Motif | Start in Seq (1 Indexed) | End in Seq (1 Indexed) | Strand | Chrm | Exon | Start in Chrm (0 Indexed) | End in Chrm (1 Indexed) |
| ATGGGGTACT | 5889 | 5898 | - | chrX | 1 | 73066690 | 73066700 |
| eCLIP Fold-Enrichment | Binding Protein | Cell Line | Strand | Chrm | | Start in Chrm (0 Indexed) | End in Chrm (1 Indexed) |
| 2.44803853488201 | HNRNPL (bg=0.64%) | K562 | - | chrX | | 73066636 | 73066697 |
| 2.64868339401678 | HNRNPL (bg=0.64%) | K562 | - | chrX | | 73066651 | 73066703 |

  
  

| Match 181 in HUMAN | | | | | | | |
| --- | --- | --- | --- | --- | --- | --- | --- |
| Motif | Start in Seq (1 Indexed) | End in Seq (1 Indexed) | Strand | Chrm | Exon | Start in Chrm (0 Indexed) | End in Chrm (1 Indexed) |
| TTCACTTAAGGCCCCTTTCTCAAAC | 5900 | 5924 | - | chrX | 1 | 73066664 | 73066689 |
| eCLIP Fold-Enrichment | Binding Protein | Cell Line | Strand | Chrm | | Start in Chrm (0 Indexed) | End in Chrm (1 Indexed) |
| 2.44803853488201 | HNRNPL (bg=0.64%) | K562 | - | chrX | | 73066636 | 73066697 |
| 2.64868339401678 | HNRNPL (bg=0.64%) | K562 | - | chrX | | 73066651 | 73066703 |

  
  

| Match 182 in HUMAN | | | | | | | |
| --- | --- | --- | --- | --- | --- | --- | --- |
| Motif | Start in Seq (1 Indexed) | End in Seq (1 Indexed) | Strand | Chrm | Exon | Start in Chrm (0 Indexed) | End in Chrm (1 Indexed) |
| CACTTAAGGCCCCTTTCTCAA | 5902 | 5922 | - | chrX | 1 | 73066666 | 73066687 |
| eCLIP Fold-Enrichment | Binding Protein | Cell Line | Strand | Chrm | | Start in Chrm (0 Indexed) | End in Chrm (1 Indexed) |
| 2.44803853488201 | HNRNPL (bg=0.64%) | K562 | - | chrX | | 73066636 | 73066697 |
| 2.64868339401678 | HNRNPL (bg=0.64%) | K562 | - | chrX | | 73066651 | 73066703 |

  
  

| Match 183 in HUMAN | | | | | | | |
| --- | --- | --- | --- | --- | --- | --- | --- |
| Motif | Start in Seq (1 Indexed) | End in Seq (1 Indexed) | Strand | Chrm | Exon | Start in Chrm (0 Indexed) | End in Chrm (1 Indexed) |
| TTAAGGCCCCTTTCTCAA | 5905 | 5922 | - | chrX | 1 | 73066666 | 73066684 |
| eCLIP Fold-Enrichment | Binding Protein | Cell Line | Strand | Chrm | | Start in Chrm (0 Indexed) | End in Chrm (1 Indexed) |
| 2.44803853488201 | HNRNPL (bg=0.64%) | K562 | - | chrX | | 73066636 | 73066697 |
| 2.64868339401678 | HNRNPL (bg=0.64%) | K562 | - | chrX | | 73066651 | 73066703 |

  
  

| Match 184 in HUMAN | | | | | | | |
| --- | --- | --- | --- | --- | --- | --- | --- |
| Motif | Start in Seq (1 Indexed) | End in Seq (1 Indexed) | Strand | Chrm | Exon | Start in Chrm (0 Indexed) | End in Chrm (1 Indexed) |
| TTAAGGCCCCTTT | 5905 | 5917 | - | chrX | 1 | 73066671 | 73066684 |
| eCLIP Fold-Enrichment | Binding Protein | Cell Line | Strand | Chrm | | Start in Chrm (0 Indexed) | End in Chrm (1 Indexed) |
| 2.44803853488201 | HNRNPL (bg=0.64%) | K562 | - | chrX | | 73066636 | 73066697 |
| 2.64868339401678 | HNRNPL (bg=0.64%) | K562 | - | chrX | | 73066651 | 73066703 |

  
  

| Match 185 in HUMAN | | | | | | | |
| --- | --- | --- | --- | --- | --- | --- | --- |
| Motif | Start in Seq (1 Indexed) | End in Seq (1 Indexed) | Strand | Chrm | Exon | Start in Chrm (0 Indexed) | End in Chrm (1 Indexed) |
| TTAAGGCC | 5905 | 5912 | - | chrX | 1 | 73066676 | 73066684 |
| eCLIP Fold-Enrichment | Binding Protein | Cell Line | Strand | Chrm | | Start in Chrm (0 Indexed) | End in Chrm (1 Indexed) |
| 2.44803853488201 | HNRNPL (bg=0.64%) | K562 | - | chrX | | 73066636 | 73066697 |
| 2.64868339401678 | HNRNPL (bg=0.64%) | K562 | - | chrX | | 73066651 | 73066703 |

  
  

| Match 186 in HUMAN | | | | | | | |
| --- | --- | --- | --- | --- | --- | --- | --- |
| Motif | Start in Seq (1 Indexed) | End in Seq (1 Indexed) | Strand | Chrm | Exon | Start in Chrm (0 Indexed) | End in Chrm (1 Indexed) |
| TAATGACAATTACAT | 5937 | 5951 | - | chrX | 1 | 73066637 | 73066652 |
| eCLIP Fold-Enrichment | Binding Protein | Cell Line | Strand | Chrm | | Start in Chrm (0 Indexed) | End in Chrm (1 Indexed) |
| 2.44803853488201 | HNRNPL (bg=0.64%) | K562 | - | chrX | | 73066636 | 73066697 |
| 2.64868339401678 | HNRNPL (bg=0.64%) | K562 | - | chrX | | 73066651 | 73066703 |

  
  

| Match 187 in HUMAN | | | | | | | |
| --- | --- | --- | --- | --- | --- | --- | --- |
| Motif | Start in Seq (1 Indexed) | End in Seq (1 Indexed) | Strand | Chrm | Exon | Start in Chrm (0 Indexed) | End in Chrm (1 Indexed) |
| TTTGAAG | 5965 | 5971 | - | chrX | 1 | 73066617 | 73066624 |
| eCLIP Fold-Enrichment | Binding Protein | Cell Line | Strand | Chrm | | Start in Chrm (0 Indexed) | End in Chrm (1 Indexed) |
| 3.1062963025087 | HNRNPC (bg=3.65%) | K562 | - | chrX | | 73066570 | 73066623 |

  
  

| Match 188 in HUMAN | | | | | | | |
| --- | --- | --- | --- | --- | --- | --- | --- |
| Motif | Start in Seq (1 Indexed) | End in Seq (1 Indexed) | Strand | Chrm | Exon | Start in Chrm (0 Indexed) | End in Chrm (1 Indexed) |
| CCTAAGG | 5991 | 5997 | - | chrX | 1 | 73066591 | 73066598 |
| eCLIP Fold-Enrichment | Binding Protein | Cell Line | Strand | Chrm | | Start in Chrm (0 Indexed) | End in Chrm (1 Indexed) |
| 2.12724499311918 | DDX51 (bg=1.63%) | K562 | - | chrX | | 73066559 | 73066593 |
| 3.1062963025087 | HNRNPC (bg=3.65%) | K562 | - | chrX | | 73066570 | 73066623 |

  
  

| Match 189 in HUMAN | | | | | | | |
| --- | --- | --- | --- | --- | --- | --- | --- |
| Motif | Start in Seq (1 Indexed) | End in Seq (1 Indexed) | Strand | Chrm | Exon | Start in Chrm (0 Indexed) | End in Chrm (1 Indexed) |
| CCCATTTCTTG | 5999 | 6009 | - | chrX | 1 | 73066579 | 73066590 |
| eCLIP Fold-Enrichment | Binding Protein | Cell Line | Strand | Chrm | | Start in Chrm (0 Indexed) | End in Chrm (1 Indexed) |
| 2.12724499311918 | DDX51 (bg=1.63%) | K562 | - | chrX | | 73066559 | 73066593 |
| 3.1062963025087 | HNRNPC (bg=3.65%) | K562 | - | chrX | | 73066570 | 73066623 |

  
  

| Match 190 in HUMAN | | | | | | | |
| --- | --- | --- | --- | --- | --- | --- | --- |
| Motif | Start in Seq (1 Indexed) | End in Seq (1 Indexed) | Strand | Chrm | Exon | Start in Chrm (0 Indexed) | End in Chrm (1 Indexed) |
| TGTATTTGTC | 6029 | 6038 | - | chrX | 1 | 73066550 | 73066560 |
| eCLIP Fold-Enrichment | Binding Protein | Cell Line | Strand | Chrm | | Start in Chrm (0 Indexed) | End in Chrm (1 Indexed) |
| 2.12724499311918 | DDX51 (bg=1.63%) | K562 | - | chrX | | 73066559 | 73066593 |

  
  

| Match 191 in HUMAN | | | | | | | |
| --- | --- | --- | --- | --- | --- | --- | --- |
| Motif | Start in Seq (1 Indexed) | End in Seq (1 Indexed) | Strand | Chrm | Exon | Start in Chrm (0 Indexed) | End in Chrm (1 Indexed) |
| CTTCCAGCAGGAAGTGCCC | 6091 | 6109 | - | chrX | 1 | 73066479 | 73066498 |
| eCLIP Fold-Enrichment | Binding Protein | Cell Line | Strand | Chrm | | Start in Chrm (0 Indexed) | End in Chrm (1 Indexed) |
| 3.52135864368257 | hnrnpk (bg=12.88%) | K562 | - | chrX | | 73066438 | 73066514 |
| 4.06784349460507 | hnrnpk (bg=12.88%) | K562 | - | chrX | | 73066443 | 73066512 |

  
  

| Match 192 in HUMAN | | | | | | | |
| --- | --- | --- | --- | --- | --- | --- | --- |
| Motif | Start in Seq (1 Indexed) | End in Seq (1 Indexed) | Strand | Chrm | Exon | Start in Chrm (0 Indexed) | End in Chrm (1 Indexed) |
| CCACAAG | 6161 | 6167 | - | chrX | 1 | 73066421 | 73066428 |
| eCLIP Fold-Enrichment | Binding Protein | Cell Line | Strand | Chrm | | Start in Chrm (0 Indexed) | End in Chrm (1 Indexed) |
| 2.02754301139391 | HNRNPU (bg=5.92%) | K562 | - | chrX | | 73066399 | 73066431 |
| 2.53103036548393 | UTP3 (bg=3.66%) | K562 | - | chrX | | 73066418 | 73066457 |

  
  

| Match 193 in HUMAN | | | | | | | |
| --- | --- | --- | --- | --- | --- | --- | --- |
| Motif | Start in Seq (1 Indexed) | End in Seq (1 Indexed) | Strand | Chrm | Exon | Start in Chrm (0 Indexed) | End in Chrm (1 Indexed) |
| TTTGGACAGTCAAG | 6412 | 6425 | - | chrX | 1 | 73066163 | 73066177 |
| eCLIP Fold-Enrichment | Binding Protein | Cell Line | Strand | Chrm | | Start in Chrm (0 Indexed) | End in Chrm (1 Indexed) |
| 3.57385815591542 | hnrnpk (bg=12.88%) | K562 | - | chrX | | 73066171 | 73066244 |
| 3.88034375810553 | hnrnpk (bg=12.88%) | K562 | - | chrX | | 73066174 | 73066235 |

  
  

| Match 194 in HUMAN | | | | | | | |
| --- | --- | --- | --- | --- | --- | --- | --- |
| Motif | Start in Seq (1 Indexed) | End in Seq (1 Indexed) | Strand | Chrm | Exon | Start in Chrm (0 Indexed) | End in Chrm (1 Indexed) |
| ATGCAC | 7035 | 7040 | - | chrX | 1 | 73065548 | 73065554 |
| eCLIP Fold-Enrichment | Binding Protein | Cell Line | Strand | Chrm | | Start in Chrm (0 Indexed) | End in Chrm (1 Indexed) |
| 3.33786300283398 | hnrnpk (bg=12.88%) | K562 | - | chrX | | 73065536 | 73065573 |
| 3.99682403128505 | hnrnpk (bg=12.88%) | K562 | - | chrX | | 73065546 | 73065575 |
| 2.10850834104433 | HNRNPU (bg=5.92%) | K562 | - | chrX | | 73065529 | 73065572 |
| 2.32641348197707 | HNRNPU (bg=5.92%) | K562 | - | chrX | | 73065530 | 73065575 |

  
  

| Match 195 in HUMAN | | | | | | | |
| --- | --- | --- | --- | --- | --- | --- | --- |
| Motif | Start in Seq (1 Indexed) | End in Seq (1 Indexed) | Strand | Chrm | Exon | Start in Chrm (0 Indexed) | End in Chrm (1 Indexed) |
| GTAACTG | 7068 | 7074 | - | chrX | 1 | 73065514 | 73065521 |
| eCLIP Fold-Enrichment | Binding Protein | Cell Line | Strand | Chrm | | Start in Chrm (0 Indexed) | End in Chrm (1 Indexed) |
| 2.96100869744303 | hnrnpk (bg=12.88%) | K562 | - | chrX | | 73065500 | 73065518 |

  
  

| Match 196 in HUMAN | | | | | | | |
| --- | --- | --- | --- | --- | --- | --- | --- |
| Motif | Start in Seq (1 Indexed) | End in Seq (1 Indexed) | Strand | Chrm | Exon | Start in Chrm (0 Indexed) | End in Chrm (1 Indexed) |
| CACCTTGGA | 7338 | 7346 | - | chrX | 1 | 73065242 | 73065251 |
| eCLIP Fold-Enrichment | Binding Protein | Cell Line | Strand | Chrm | | Start in Chrm (0 Indexed) | End in Chrm (1 Indexed) |
| 5.97173510403608 | hnrnpk (bg=12.88%) | K562 | - | chrX | | 73065240 | 73065276 |
| 6.01327455206564 | hnrnpk (bg=12.88%) | K562 | - | chrX | | 73065247 | 73065269 |
| 2.32975176740229 | HNRNPU (bg=5.92%) | K562 | - | chrX | | 73065230 | 73065272 |
| 2.38094144488037 | HNRNPU (bg=5.92%) | K562 | - | chrX | | 73065238 | 73065275 |

  
  

| Match 197 in HUMAN | | | | | | | |
| --- | --- | --- | --- | --- | --- | --- | --- |
| Motif | Start in Seq (1 Indexed) | End in Seq (1 Indexed) | Strand | Chrm | Exon | Start in Chrm (0 Indexed) | End in Chrm (1 Indexed) |
| CTCTCAGACCCC | 8272 | 8283 | - | chrX | 1 | 73064305 | 73064317 |
| eCLIP Fold-Enrichment | Binding Protein | Cell Line | Strand | Chrm | | Start in Chrm (0 Indexed) | End in Chrm (1 Indexed) |
| 3.62864152108095 | SF3B1 (bg=2.48%) | K562 | - | chrX | | 73064291 | 73064319 |

  
  

| Match 198 in HUMAN | | | | | | | |
| --- | --- | --- | --- | --- | --- | --- | --- |
| Motif | Start in Seq (1 Indexed) | End in Seq (1 Indexed) | Strand | Chrm | Exon | Start in Chrm (0 Indexed) | End in Chrm (1 Indexed) |
| AGTATAGC | 8290 | 8297 | - | chrX | 1 | 73064291 | 73064299 |
| eCLIP Fold-Enrichment | Binding Protein | Cell Line | Strand | Chrm | | Start in Chrm (0 Indexed) | End in Chrm (1 Indexed) |
| 3.16920990244365 | SF3B1 (bg=2.48%) | K562 | - | chrX | | 73064283 | 73064291 |
| 3.62864152108095 | SF3B1 (bg=2.48%) | K562 | - | chrX | | 73064291 | 73064319 |

  
  

| Match 199 in HUMAN | | | | | | | |
| --- | --- | --- | --- | --- | --- | --- | --- |
| Motif | Start in Seq (1 Indexed) | End in Seq (1 Indexed) | Strand | Chrm | Exon | Start in Chrm (0 Indexed) | End in Chrm (1 Indexed) |
| ACTCCCTTTG | 8375 | 8384 | - | chrX | 1 | 73064204 | 73064214 |
| eCLIP Fold-Enrichment | Binding Protein | Cell Line | Strand | Chrm | | Start in Chrm (0 Indexed) | End in Chrm (1 Indexed) |
| 2.76243580572603 | DDX21 (bg=0.25%) | K562 | - | chrX | | 73064163 | 73064211 |

  
  

| Match 200 in HUMAN | | | | | | | |
| --- | --- | --- | --- | --- | --- | --- | --- |
| Motif | Start in Seq (1 Indexed) | End in Seq (1 Indexed) | Strand | Chrm | Exon | Start in Chrm (0 Indexed) | End in Chrm (1 Indexed) |
| CACTACTTT | 8404 | 8412 | - | chrX | 1 | 73064176 | 73064185 |
| eCLIP Fold-Enrichment | Binding Protein | Cell Line | Strand | Chrm | | Start in Chrm (0 Indexed) | End in Chrm (1 Indexed) |
| 2.76243580572603 | DDX21 (bg=0.25%) | K562 | - | chrX | | 73064163 | 73064211 |

  
  

| Match 201 in HUMAN | | | | | | | |
| --- | --- | --- | --- | --- | --- | --- | --- |
| Motif | Start in Seq (1 Indexed) | End in Seq (1 Indexed) | Strand | Chrm | Exon | Start in Chrm (0 Indexed) | End in Chrm (1 Indexed) |
| CTTATATTT | 8421 | 8429 | - | chrX | 1 | 73064159 | 73064168 |
| eCLIP Fold-Enrichment | Binding Protein | Cell Line | Strand | Chrm | | Start in Chrm (0 Indexed) | End in Chrm (1 Indexed) |
| 2.76243580572603 | DDX21 (bg=0.25%) | K562 | - | chrX | | 73064163 | 73064211 |

  
  

| Match 202 in HUMAN | | | | | | | |
| --- | --- | --- | --- | --- | --- | --- | --- |
| Motif | Start in Seq (1 Indexed) | End in Seq (1 Indexed) | Strand | Chrm | Exon | Start in Chrm (0 Indexed) | End in Chrm (1 Indexed) |
| CTCCATTTGCAGTATA | 8536 | 8551 | - | chrX | 1 | 73064037 | 73064053 |
| eCLIP Fold-Enrichment | Binding Protein | Cell Line | Strand | Chrm | | Start in Chrm (0 Indexed) | End in Chrm (1 Indexed) |
| 3.05479084376364 | hnrnpk (bg=12.88%) | K562 | - | chrX | | 73063988 | 73064053 |
| 3.66048549347352 | hnrnpk (bg=12.88%) | K562 | - | chrX | | 73064006 | 73064056 |
| 2.0080379290208 | TIA1 (bg=4.07%) | K562 | - | chrX | | 73063978 | 73064039 |

  
  

| Match 203 in HUMAN | | | | | | | |
| --- | --- | --- | --- | --- | --- | --- | --- |
| Motif | Start in Seq (1 Indexed) | End in Seq (1 Indexed) | Strand | Chrm | Exon | Start in Chrm (0 Indexed) | End in Chrm (1 Indexed) |
| CAGGGTT | 8553 | 8559 | - | chrX | 1 | 73064029 | 73064036 |
| eCLIP Fold-Enrichment | Binding Protein | Cell Line | Strand | Chrm | | Start in Chrm (0 Indexed) | End in Chrm (1 Indexed) |
| 3.05479084376364 | hnrnpk (bg=12.88%) | K562 | - | chrX | | 73063988 | 73064053 |
| 3.66048549347352 | hnrnpk (bg=12.88%) | K562 | - | chrX | | 73064006 | 73064056 |
| 2.0080379290208 | TIA1 (bg=4.07%) | K562 | - | chrX | | 73063978 | 73064039 |

  
  

| Match 204 in HUMAN | | | | | | | |
| --- | --- | --- | --- | --- | --- | --- | --- |
| Motif | Start in Seq (1 Indexed) | End in Seq (1 Indexed) | Strand | Chrm | Exon | Start in Chrm (0 Indexed) | End in Chrm (1 Indexed) |
| TGACCC | 8562 | 8567 | - | chrX | 1 | 73064021 | 73064027 |
| eCLIP Fold-Enrichment | Binding Protein | Cell Line | Strand | Chrm | | Start in Chrm (0 Indexed) | End in Chrm (1 Indexed) |
| 3.05479084376364 | hnrnpk (bg=12.88%) | K562 | - | chrX | | 73063988 | 73064053 |
| 3.66048549347352 | hnrnpk (bg=12.88%) | K562 | - | chrX | | 73064006 | 73064056 |
| 2.0080379290208 | TIA1 (bg=4.07%) | K562 | - | chrX | | 73063978 | 73064039 |

  
  

| Match 205 in HUMAN | | | | | | | |
| --- | --- | --- | --- | --- | --- | --- | --- |
| Motif | Start in Seq (1 Indexed) | End in Seq (1 Indexed) | Strand | Chrm | Exon | Start in Chrm (0 Indexed) | End in Chrm (1 Indexed) |
| TGCATAATTGCATTT | 8598 | 8612 | - | chrX | 1 | 73063976 | 73063991 |
| eCLIP Fold-Enrichment | Binding Protein | Cell Line | Strand | Chrm | | Start in Chrm (0 Indexed) | End in Chrm (1 Indexed) |
| 3.05479084376364 | hnrnpk (bg=12.88%) | K562 | - | chrX | | 73063988 | 73064053 |
| 2.0080379290208 | TIA1 (bg=4.07%) | K562 | - | chrX | | 73063978 | 73064039 |

  
  

| Match 206 in HUMAN | | | | | | | |
| --- | --- | --- | --- | --- | --- | --- | --- |
| Motif | Start in Seq (1 Indexed) | End in Seq (1 Indexed) | Strand | Chrm | Exon | Start in Chrm (0 Indexed) | End in Chrm (1 Indexed) |
| CATAATTGCA | 8600 | 8609 | - | chrX | 1 | 73063979 | 73063989 |
| eCLIP Fold-Enrichment | Binding Protein | Cell Line | Strand | Chrm | | Start in Chrm (0 Indexed) | End in Chrm (1 Indexed) |
| 3.05479084376364 | hnrnpk (bg=12.88%) | K562 | - | chrX | | 73063988 | 73064053 |
| 2.0080379290208 | TIA1 (bg=4.07%) | K562 | - | chrX | | 73063978 | 73064039 |

  
  

| Match 207 in HUMAN | | | | | | | |
| --- | --- | --- | --- | --- | --- | --- | --- |
| Motif | Start in Seq (1 Indexed) | End in Seq (1 Indexed) | Strand | Chrm | Exon | Start in Chrm (0 Indexed) | End in Chrm (1 Indexed) |
| GGTTCTTG | 8618 | 8625 | - | chrX | 1 | 73063963 | 73063971 |
| eCLIP Fold-Enrichment | Binding Protein | Cell Line | Strand | Chrm | | Start in Chrm (0 Indexed) | End in Chrm (1 Indexed) |
| 2.53091527790772 | UTP3 (bg=3.66%) | K562 | - | chrX | | 73063936 | 73063968 |

  
  

| Match 208 in HUMAN | | | | | | | |
| --- | --- | --- | --- | --- | --- | --- | --- |
| Motif | Start in Seq (1 Indexed) | End in Seq (1 Indexed) | Strand | Chrm | Exon | Start in Chrm (0 Indexed) | End in Chrm (1 Indexed) |
| CTAGACAAGGA | 8630 | 8640 | - | chrX | 1 | 73063948 | 73063959 |
| eCLIP Fold-Enrichment | Binding Protein | Cell Line | Strand | Chrm | | Start in Chrm (0 Indexed) | End in Chrm (1 Indexed) |
| 2.53091527790772 | UTP3 (bg=3.66%) | K562 | - | chrX | | 73063936 | 73063968 |

  
  

| Match 209 in HUMAN | | | | | | | |
| --- | --- | --- | --- | --- | --- | --- | --- |
| Motif | Start in Seq (1 Indexed) | End in Seq (1 Indexed) | Strand | Chrm | Exon | Start in Chrm (0 Indexed) | End in Chrm (1 Indexed) |
| ACAGTTAATGTG | 8695 | 8706 | - | chrX | 1 | 73063882 | 73063894 |
| eCLIP Fold-Enrichment | Binding Protein | Cell Line | Strand | Chrm | | Start in Chrm (0 Indexed) | End in Chrm (1 Indexed) |
| 2.35119410148632 | HNRNPU (bg=5.92%) | K562 | - | chrX | | 73063842 | 73063884 |

  
  

| Match 210 in HUMAN | | | | | | | |
| --- | --- | --- | --- | --- | --- | --- | --- |
| Motif | Start in Seq (1 Indexed) | End in Seq (1 Indexed) | Strand | Chrm | Exon | Start in Chrm (0 Indexed) | End in Chrm (1 Indexed) |
| AATTGCAGTT | 8710 | 8719 | - | chrX | 1 | 73063869 | 73063879 |
| eCLIP Fold-Enrichment | Binding Protein | Cell Line | Strand | Chrm | | Start in Chrm (0 Indexed) | End in Chrm (1 Indexed) |
| 2.35119410148632 | HNRNPU (bg=5.92%) | K562 | - | chrX | | 73063842 | 73063884 |

  
  

| Match 211 in HUMAN | | | | | | | |
| --- | --- | --- | --- | --- | --- | --- | --- |
| Motif | Start in Seq (1 Indexed) | End in Seq (1 Indexed) | Strand | Chrm | Exon | Start in Chrm (0 Indexed) | End in Chrm (1 Indexed) |
| TCCACAACCC | 8721 | 8730 | - | chrX | 1 | 73063858 | 73063868 |
| eCLIP Fold-Enrichment | Binding Protein | Cell Line | Strand | Chrm | | Start in Chrm (0 Indexed) | End in Chrm (1 Indexed) |
| 3.73643434670682 | hnrnpk (bg=12.88%) | K562 | - | chrX | | 73063840 | 73063859 |
| 2.35119410148632 | HNRNPU (bg=5.92%) | K562 | - | chrX | | 73063842 | 73063884 |

  
  

| Match 212 in HUMAN | | | | | | | |
| --- | --- | --- | --- | --- | --- | --- | --- |
| Motif | Start in Seq (1 Indexed) | End in Seq (1 Indexed) | Strand | Chrm | Exon | Start in Chrm (0 Indexed) | End in Chrm (1 Indexed) |
| ACTGATCATTAGATA | 9404 | 9418 | - | chrX | 1 | 73063170 | 73063185 |
| eCLIP Fold-Enrichment | Binding Protein | Cell Line | Strand | Chrm | | Start in Chrm (0 Indexed) | End in Chrm (1 Indexed) |
| 2.29605254729386 | HNRNPU (bg=5.92%) | K562 | - | chrX | | 73063178 | 73063196 |

  
  

| Match 213 in HUMAN | | | | | | | |
| --- | --- | --- | --- | --- | --- | --- | --- |
| Motif | Start in Seq (1 Indexed) | End in Seq (1 Indexed) | Strand | Chrm | Exon | Start in Chrm (0 Indexed) | End in Chrm (1 Indexed) |
| GATCAT | 9407 | 9412 | - | chrX | 1 | 73063176 | 73063182 |
| eCLIP Fold-Enrichment | Binding Protein | Cell Line | Strand | Chrm | | Start in Chrm (0 Indexed) | End in Chrm (1 Indexed) |
| 2.29605254729386 | HNRNPU (bg=5.92%) | K562 | - | chrX | | 73063178 | 73063196 |

  
  

| Match 214 in HUMAN | | | | | | | |
| --- | --- | --- | --- | --- | --- | --- | --- |
| Motif | Start in Seq (1 Indexed) | End in Seq (1 Indexed) | Strand | Chrm | Exon | Start in Chrm (0 Indexed) | End in Chrm (1 Indexed) |
| CAAAACTTCT | 9579 | 9588 | - | chrX | 1 | 73063000 | 73063010 |
| eCLIP Fold-Enrichment | Binding Protein | Cell Line | Strand | Chrm | | Start in Chrm (0 Indexed) | End in Chrm (1 Indexed) |
| 3.60836987778586 | SF3B1 (bg=2.48%) | K562 | - | chrX | | 73062995 | 73063005 |

  
  

| Match 215 in HUMAN | | | | | | | |
| --- | --- | --- | --- | --- | --- | --- | --- |
| Motif | Start in Seq (1 Indexed) | End in Seq (1 Indexed) | Strand | Chrm | Exon | Start in Chrm (0 Indexed) | End in Chrm (1 Indexed) |
| CAAAACTT | 9579 | 9586 | - | chrX | 1 | 73063002 | 73063010 |
| eCLIP Fold-Enrichment | Binding Protein | Cell Line | Strand | Chrm | | Start in Chrm (0 Indexed) | End in Chrm (1 Indexed) |
| 3.60836987778586 | SF3B1 (bg=2.48%) | K562 | - | chrX | | 73062995 | 73063005 |

  
  

| Match 216 in HUMAN | | | | | | | |
| --- | --- | --- | --- | --- | --- | --- | --- |
| Motif | Start in Seq (1 Indexed) | End in Seq (1 Indexed) | Strand | Chrm | Exon | Start in Chrm (0 Indexed) | End in Chrm (1 Indexed) |
| CTGGGACTG | 9590 | 9598 | - | chrX | 1 | 73062990 | 73062999 |
| eCLIP Fold-Enrichment | Binding Protein | Cell Line | Strand | Chrm | | Start in Chrm (0 Indexed) | End in Chrm (1 Indexed) |
| 3.60836987778586 | SF3B1 (bg=2.48%) | K562 | - | chrX | | 73062995 | 73063005 |

  
  

| Match 217 in HUMAN | | | | | | | |
| --- | --- | --- | --- | --- | --- | --- | --- |
| Motif | Start in Seq (1 Indexed) | End in Seq (1 Indexed) | Strand | Chrm | Exon | Start in Chrm (0 Indexed) | End in Chrm (1 Indexed) |
| CTCCCTG | 9636 | 9642 | - | chrX | 1 | 73062946 | 73062953 |
| eCLIP Fold-Enrichment | Binding Protein | Cell Line | Strand | Chrm | | Start in Chrm (0 Indexed) | End in Chrm (1 Indexed) |
| 2.63928419163696 | DDX42 (bg=0.58%) | K562 | - | chrX | | 73062891 | 73062949 |
| 2.32065741968885 | DDX42 (bg=0.58%) | K562 | - | chrX | | 73062898 | 73062946 |

  
  

| Match 218 in HUMAN | | | | | | | |
| --- | --- | --- | --- | --- | --- | --- | --- |
| Motif | Start in Seq (1 Indexed) | End in Seq (1 Indexed) | Strand | Chrm | Exon | Start in Chrm (0 Indexed) | End in Chrm (1 Indexed) |
| CCTTGTT | 9644 | 9650 | - | chrX | 1 | 73062938 | 73062945 |
| eCLIP Fold-Enrichment | Binding Protein | Cell Line | Strand | Chrm | | Start in Chrm (0 Indexed) | End in Chrm (1 Indexed) |
| 2.63928419163696 | DDX42 (bg=0.58%) | K562 | - | chrX | | 73062891 | 73062949 |
| 2.32065741968885 | DDX42 (bg=0.58%) | K562 | - | chrX | | 73062898 | 73062946 |

  
  

| Match 219 in HUMAN | | | | | | | |
| --- | --- | --- | --- | --- | --- | --- | --- |
| Motif | Start in Seq (1 Indexed) | End in Seq (1 Indexed) | Strand | Chrm | Exon | Start in Chrm (0 Indexed) | End in Chrm (1 Indexed) |
| GCAAGCGC | 9653 | 9660 | - | chrX | 1 | 73062928 | 73062936 |
| eCLIP Fold-Enrichment | Binding Protein | Cell Line | Strand | Chrm | | Start in Chrm (0 Indexed) | End in Chrm (1 Indexed) |
| 2.63928419163696 | DDX42 (bg=0.58%) | K562 | - | chrX | | 73062891 | 73062949 |
| 2.32065741968885 | DDX42 (bg=0.58%) | K562 | - | chrX | | 73062898 | 73062946 |
| 3.49780391570367 | hnrnpk (bg=12.88%) | K562 | - | chrX | | 73062905 | 73062937 |
| 3.84334955062333 | hnrnpk (bg=12.88%) | K562 | - | chrX | | 73062910 | 73062937 |

  
  

| Match 220 in HUMAN | | | | | | | |
| --- | --- | --- | --- | --- | --- | --- | --- |
| Motif | Start in Seq (1 Indexed) | End in Seq (1 Indexed) | Strand | Chrm | Exon | Start in Chrm (0 Indexed) | End in Chrm (1 Indexed) |
| GCAAGC | 9653 | 9658 | - | chrX | 1 | 73062930 | 73062936 |
| eCLIP Fold-Enrichment | Binding Protein | Cell Line | Strand | Chrm | | Start in Chrm (0 Indexed) | End in Chrm (1 Indexed) |
| 2.63928419163696 | DDX42 (bg=0.58%) | K562 | - | chrX | | 73062891 | 73062949 |
| 2.32065741968885 | DDX42 (bg=0.58%) | K562 | - | chrX | | 73062898 | 73062946 |
| 3.49780391570367 | hnrnpk (bg=12.88%) | K562 | - | chrX | | 73062905 | 73062937 |
| 3.84334955062333 | hnrnpk (bg=12.88%) | K562 | - | chrX | | 73062910 | 73062937 |

  
  

| Match 221 in HUMAN | | | | | | | |
| --- | --- | --- | --- | --- | --- | --- | --- |
| Motif | Start in Seq (1 Indexed) | End in Seq (1 Indexed) | Strand | Chrm | Exon | Start in Chrm (0 Indexed) | End in Chrm (1 Indexed) |
| TTTCCCATGG | 9677 | 9686 | - | chrX | 1 | 73062902 | 73062912 |
| eCLIP Fold-Enrichment | Binding Protein | Cell Line | Strand | Chrm | | Start in Chrm (0 Indexed) | End in Chrm (1 Indexed) |
| 2.63928419163696 | DDX42 (bg=0.58%) | K562 | - | chrX | | 73062891 | 73062949 |
| 2.32065741968885 | DDX42 (bg=0.58%) | K562 | - | chrX | | 73062898 | 73062946 |
| 3.49780391570367 | hnrnpk (bg=12.88%) | K562 | - | chrX | | 73062905 | 73062937 |
| 3.84334955062333 | hnrnpk (bg=12.88%) | K562 | - | chrX | | 73062910 | 73062937 |

  
  

| Match 222 in HUMAN | | | | | | | |
| --- | --- | --- | --- | --- | --- | --- | --- |
| Motif | Start in Seq (1 Indexed) | End in Seq (1 Indexed) | Strand | Chrm | Exon | Start in Chrm (0 Indexed) | End in Chrm (1 Indexed) |
| ATAATAAAGTATAA | 9688 | 9701 | - | chrX | 1 | 73062887 | 73062901 |
| eCLIP Fold-Enrichment | Binding Protein | Cell Line | Strand | Chrm | | Start in Chrm (0 Indexed) | End in Chrm (1 Indexed) |
| 2.63928419163696 | DDX42 (bg=0.58%) | K562 | - | chrX | | 73062891 | 73062949 |
| 2.32065741968885 | DDX42 (bg=0.58%) | K562 | - | chrX | | 73062898 | 73062946 |
| 3.42745106015589 | KHDRBS1 (bg=1.71%) | K562 | - | chrX | | 73062868 | 73062891 |

  
  

| Match 223 in HUMAN | | | | | | | |
| --- | --- | --- | --- | --- | --- | --- | --- |
| Motif | Start in Seq (1 Indexed) | End in Seq (1 Indexed) | Strand | Chrm | Exon | Start in Chrm (0 Indexed) | End in Chrm (1 Indexed) |
| CCATACTCCCA | 9742 | 9752 | - | chrX | 1 | 73062836 | 73062847 |
| eCLIP Fold-Enrichment | Binding Protein | Cell Line | Strand | Chrm | | Start in Chrm (0 Indexed) | End in Chrm (1 Indexed) |
| 3.47988200770641 | hnrnpk (bg=12.88%) | K562 | - | chrX | | 73062802 | 73062843 |
| 4.01784728185547 | hnrnpk (bg=12.88%) | K562 | - | chrX | | 73062816 | 73062850 |

  
  

| Match 224 in HUMAN | | | | | | | |
| --- | --- | --- | --- | --- | --- | --- | --- |
| Motif | Start in Seq (1 Indexed) | End in Seq (1 Indexed) | Strand | Chrm | Exon | Start in Chrm (0 Indexed) | End in Chrm (1 Indexed) |
| ACTCCCA | 9746 | 9752 | - | chrX | 1 | 73062836 | 73062843 |
| eCLIP Fold-Enrichment | Binding Protein | Cell Line | Strand | Chrm | | Start in Chrm (0 Indexed) | End in Chrm (1 Indexed) |
| 3.47988200770641 | hnrnpk (bg=12.88%) | K562 | - | chrX | | 73062802 | 73062843 |
| 4.01784728185547 | hnrnpk (bg=12.88%) | K562 | - | chrX | | 73062816 | 73062850 |

  
  

| Match 225 in HUMAN | | | | | | | |
| --- | --- | --- | --- | --- | --- | --- | --- |
| Motif | Start in Seq (1 Indexed) | End in Seq (1 Indexed) | Strand | Chrm | Exon | Start in Chrm (0 Indexed) | End in Chrm (1 Indexed) |
| CTCCCA | 9747 | 9752 | - | chrX | 1 | 73062836 | 73062842 |
| eCLIP Fold-Enrichment | Binding Protein | Cell Line | Strand | Chrm | | Start in Chrm (0 Indexed) | End in Chrm (1 Indexed) |
| 3.47988200770641 | hnrnpk (bg=12.88%) | K562 | - | chrX | | 73062802 | 73062843 |
| 4.01784728185547 | hnrnpk (bg=12.88%) | K562 | - | chrX | | 73062816 | 73062850 |

  
  

| Match 226 in HUMAN | | | | | | | |
| --- | --- | --- | --- | --- | --- | --- | --- |
| Motif | Start in Seq (1 Indexed) | End in Seq (1 Indexed) | Strand | Chrm | Exon | Start in Chrm (0 Indexed) | End in Chrm (1 Indexed) |
| CCCTTTTGCATTG | 9754 | 9766 | - | chrX | 1 | 73062822 | 73062835 |
| eCLIP Fold-Enrichment | Binding Protein | Cell Line | Strand | Chrm | | Start in Chrm (0 Indexed) | End in Chrm (1 Indexed) |
| 3.47988200770641 | hnrnpk (bg=12.88%) | K562 | - | chrX | | 73062802 | 73062843 |
| 4.01784728185547 | hnrnpk (bg=12.88%) | K562 | - | chrX | | 73062816 | 73062850 |

  
  

| Match 227 in HUMAN | | | | | | | |
| --- | --- | --- | --- | --- | --- | --- | --- |
| Motif | Start in Seq (1 Indexed) | End in Seq (1 Indexed) | Strand | Chrm | Exon | Start in Chrm (0 Indexed) | End in Chrm (1 Indexed) |
| CCCTTTTGCATT | 9754 | 9765 | - | chrX | 1 | 73062823 | 73062835 |
| eCLIP Fold-Enrichment | Binding Protein | Cell Line | Strand | Chrm | | Start in Chrm (0 Indexed) | End in Chrm (1 Indexed) |
| 3.47988200770641 | hnrnpk (bg=12.88%) | K562 | - | chrX | | 73062802 | 73062843 |
| 4.01784728185547 | hnrnpk (bg=12.88%) | K562 | - | chrX | | 73062816 | 73062850 |

  
  

| Match 228 in HUMAN | | | | | | | |
| --- | --- | --- | --- | --- | --- | --- | --- |
| Motif | Start in Seq (1 Indexed) | End in Seq (1 Indexed) | Strand | Chrm | Exon | Start in Chrm (0 Indexed) | End in Chrm (1 Indexed) |
| TTGCTGGG | 9963 | 9970 | - | chrX | 1 | 73062618 | 73062626 |
| eCLIP Fold-Enrichment | Binding Protein | Cell Line | Strand | Chrm | | Start in Chrm (0 Indexed) | End in Chrm (1 Indexed) |
| 4.32139684742798 | hnrnpk (bg=12.88%) | K562 | - | chrX | | 73062595 | 73062671 |
| 3.66970656658643 | hnrnpk (bg=12.88%) | K562 | - | chrX | | 73062599 | 73062699 |

  
  

| Match 229 in HUMAN | | | | | | | |
| --- | --- | --- | --- | --- | --- | --- | --- |
| Motif | Start in Seq (1 Indexed) | End in Seq (1 Indexed) | Strand | Chrm | Exon | Start in Chrm (0 Indexed) | End in Chrm (1 Indexed) |
| CCCTTTCT | 9990 | 9997 | - | chrX | 1 | 73062591 | 73062599 |
| eCLIP Fold-Enrichment | Binding Protein | Cell Line | Strand | Chrm | | Start in Chrm (0 Indexed) | End in Chrm (1 Indexed) |
| 4.55327032177531 | hnrnpk (bg=12.88%) | K562 | - | chrX | | 73062545 | 73062595 |
| 3.95020194248644 | hnrnpk (bg=12.88%) | K562 | - | chrX | | 73062569 | 73062599 |
| 4.32139684742798 | hnrnpk (bg=12.88%) | K562 | - | chrX | | 73062595 | 73062671 |
| 3.66970656658643 | hnrnpk (bg=12.88%) | K562 | - | chrX | | 73062599 | 73062699 |

  
  

| Match 230 in HUMAN | | | | | | | |
| --- | --- | --- | --- | --- | --- | --- | --- |
| Motif | Start in Seq (1 Indexed) | End in Seq (1 Indexed) | Strand | Chrm | Exon | Start in Chrm (0 Indexed) | End in Chrm (1 Indexed) |
| ACTTCCTT | 10062 | 10069 | - | chrX | 1 | 73062519 | 73062527 |
| eCLIP Fold-Enrichment | Binding Protein | Cell Line | Strand | Chrm | | Start in Chrm (0 Indexed) | End in Chrm (1 Indexed) |
| 4.20940278971145 | hnrnpk (bg=12.88%) | K562 | - | chrX | | 73062461 | 73062569 |
| 4.2940109596329 | hnrnpk (bg=12.88%) | K562 | - | chrX | | 73062505 | 73062545 |

  
  

| Match 231 in HUMAN | | | | | | | |
| --- | --- | --- | --- | --- | --- | --- | --- |
| Motif | Start in Seq (1 Indexed) | End in Seq (1 Indexed) | Strand | Chrm | Exon | Start in Chrm (0 Indexed) | End in Chrm (1 Indexed) |
| CTGAGTA | 10081 | 10087 | - | chrX | 1 | 73062501 | 73062508 |
| eCLIP Fold-Enrichment | Binding Protein | Cell Line | Strand | Chrm | | Start in Chrm (0 Indexed) | End in Chrm (1 Indexed) |
| 4.20940278971145 | hnrnpk (bg=12.88%) | K562 | - | chrX | | 73062461 | 73062569 |
| 4.68807132514542 | hnrnpk (bg=12.88%) | K562 | - | chrX | | 73062469 | 73062505 |
| 4.2940109596329 | hnrnpk (bg=12.88%) | K562 | - | chrX | | 73062505 | 73062545 |

  
  

| Match 232 in HUMAN | | | | | | | |
| --- | --- | --- | --- | --- | --- | --- | --- |
| Motif | Start in Seq (1 Indexed) | End in Seq (1 Indexed) | Strand | Chrm | Exon | Start in Chrm (0 Indexed) | End in Chrm (1 Indexed) |
| CTGACTACCCA | 10090 | 10100 | - | chrX | 1 | 73062488 | 73062499 |
| eCLIP Fold-Enrichment | Binding Protein | Cell Line | Strand | Chrm | | Start in Chrm (0 Indexed) | End in Chrm (1 Indexed) |
| 4.20940278971145 | hnrnpk (bg=12.88%) | K562 | - | chrX | | 73062461 | 73062569 |
| 4.68807132514542 | hnrnpk (bg=12.88%) | K562 | - | chrX | | 73062469 | 73062505 |

  
  

| Match 233 in HUMAN | | | | | | | |
| --- | --- | --- | --- | --- | --- | --- | --- |
| Motif | Start in Seq (1 Indexed) | End in Seq (1 Indexed) | Strand | Chrm | Exon | Start in Chrm (0 Indexed) | End in Chrm (1 Indexed) |
| AGCCCCTTCTGTGTTATTAA | 10102 | 10121 | - | chrX | 1 | 73062467 | 73062487 |
| eCLIP Fold-Enrichment | Binding Protein | Cell Line | Strand | Chrm | | Start in Chrm (0 Indexed) | End in Chrm (1 Indexed) |
| 4.20940278971145 | hnrnpk (bg=12.88%) | K562 | - | chrX | | 73062461 | 73062569 |
| 4.68807132514542 | hnrnpk (bg=12.88%) | K562 | - | chrX | | 73062469 | 73062505 |

  
  

| Match 234 in HUMAN | | | | | | | |
| --- | --- | --- | --- | --- | --- | --- | --- |
| Motif | Start in Seq (1 Indexed) | End in Seq (1 Indexed) | Strand | Chrm | Exon | Start in Chrm (0 Indexed) | End in Chrm (1 Indexed) |
| AGCCCCTTCT | 10102 | 10111 | - | chrX | 1 | 73062477 | 73062487 |
| eCLIP Fold-Enrichment | Binding Protein | Cell Line | Strand | Chrm | | Start in Chrm (0 Indexed) | End in Chrm (1 Indexed) |
| 4.20940278971145 | hnrnpk (bg=12.88%) | K562 | - | chrX | | 73062461 | 73062569 |
| 4.68807132514542 | hnrnpk (bg=12.88%) | K562 | - | chrX | | 73062469 | 73062505 |

  
  

| Match 235 in HUMAN | | | | | | | |
| --- | --- | --- | --- | --- | --- | --- | --- |
| Motif | Start in Seq (1 Indexed) | End in Seq (1 Indexed) | Strand | Chrm | Exon | Start in Chrm (0 Indexed) | End in Chrm (1 Indexed) |
| CACAGTA | 10126 | 10132 | - | chrX | 1 | 73062456 | 73062463 |
| eCLIP Fold-Enrichment | Binding Protein | Cell Line | Strand | Chrm | | Start in Chrm (0 Indexed) | End in Chrm (1 Indexed) |
| 3.52427612706486 | hnrnpk (bg=12.88%) | K562 | - | chrX | | 73062429 | 73062461 |
| 4.20940278971145 | hnrnpk (bg=12.88%) | K562 | - | chrX | | 73062461 | 73062569 |

  
  

| Match 236 in HUMAN | | | | | | | |
| --- | --- | --- | --- | --- | --- | --- | --- |
| Motif | Start in Seq (1 Indexed) | End in Seq (1 Indexed) | Strand | Chrm | Exon | Start in Chrm (0 Indexed) | End in Chrm (1 Indexed) |
| TGATTGTCCCATTTTT | 10134 | 10149 | - | chrX | 1 | 73062439 | 73062455 |
| eCLIP Fold-Enrichment | Binding Protein | Cell Line | Strand | Chrm | | Start in Chrm (0 Indexed) | End in Chrm (1 Indexed) |
| 3.72529378910162 | hnrnpk (bg=12.88%) | K562 | - | chrX | | 73062396 | 73062450 |
| 3.52427612706486 | hnrnpk (bg=12.88%) | K562 | - | chrX | | 73062429 | 73062461 |

  
  

| Match 237 in HUMAN | | | | | | | |
| --- | --- | --- | --- | --- | --- | --- | --- |
| Motif | Start in Seq (1 Indexed) | End in Seq (1 Indexed) | Strand | Chrm | Exon | Start in Chrm (0 Indexed) | End in Chrm (1 Indexed) |
| CCATTTTT | 10142 | 10149 | - | chrX | 1 | 73062439 | 73062447 |
| eCLIP Fold-Enrichment | Binding Protein | Cell Line | Strand | Chrm | | Start in Chrm (0 Indexed) | End in Chrm (1 Indexed) |
| 3.72529378910162 | hnrnpk (bg=12.88%) | K562 | - | chrX | | 73062396 | 73062450 |
| 3.52427612706486 | hnrnpk (bg=12.88%) | K562 | - | chrX | | 73062429 | 73062461 |

  
  

| Match 238 in HUMAN | | | | | | | |
| --- | --- | --- | --- | --- | --- | --- | --- |
| Motif | Start in Seq (1 Indexed) | End in Seq (1 Indexed) | Strand | Chrm | Exon | Start in Chrm (0 Indexed) | End in Chrm (1 Indexed) |
| CAGCCCA | 10150 | 10156 | - | chrX | 1 | 73062432 | 73062439 |
| eCLIP Fold-Enrichment | Binding Protein | Cell Line | Strand | Chrm | | Start in Chrm (0 Indexed) | End in Chrm (1 Indexed) |
| 3.72529378910162 | hnrnpk (bg=12.88%) | K562 | - | chrX | | 73062396 | 73062450 |
| 3.52427612706486 | hnrnpk (bg=12.88%) | K562 | - | chrX | | 73062429 | 73062461 |

  
  

| Match 239 in HUMAN | | | | | | | |
| --- | --- | --- | --- | --- | --- | --- | --- |
| Motif | Start in Seq (1 Indexed) | End in Seq (1 Indexed) | Strand | Chrm | Exon | Start in Chrm (0 Indexed) | End in Chrm (1 Indexed) |
| TCTCCCTACCACTTTG | 10168 | 10183 | - | chrX | 1 | 73062405 | 73062421 |
| eCLIP Fold-Enrichment | Binding Protein | Cell Line | Strand | Chrm | | Start in Chrm (0 Indexed) | End in Chrm (1 Indexed) |
| 3.08756458492765 | hnrnpk (bg=12.88%) | K562 | - | chrX | | 73062392 | 73062429 |
| 3.72529378910162 | hnrnpk (bg=12.88%) | K562 | - | chrX | | 73062396 | 73062450 |

  
  

| Match 240 in HUMAN | | | | | | | |
| --- | --- | --- | --- | --- | --- | --- | --- |
| Motif | Start in Seq (1 Indexed) | End in Seq (1 Indexed) | Strand | Chrm | Exon | Start in Chrm (0 Indexed) | End in Chrm (1 Indexed) |
| TCTCCCTACCA | 10168 | 10178 | - | chrX | 1 | 73062410 | 73062421 |
| eCLIP Fold-Enrichment | Binding Protein | Cell Line | Strand | Chrm | | Start in Chrm (0 Indexed) | End in Chrm (1 Indexed) |
| 3.08756458492765 | hnrnpk (bg=12.88%) | K562 | - | chrX | | 73062392 | 73062429 |
| 3.72529378910162 | hnrnpk (bg=12.88%) | K562 | - | chrX | | 73062396 | 73062450 |

  
  

| Match 241 in HUMAN | | | | | | | |
| --- | --- | --- | --- | --- | --- | --- | --- |
| Motif | Start in Seq (1 Indexed) | End in Seq (1 Indexed) | Strand | Chrm | Exon | Start in Chrm (0 Indexed) | End in Chrm (1 Indexed) |
| CCTACCA | 10172 | 10178 | - | chrX | 1 | 73062410 | 73062417 |
| eCLIP Fold-Enrichment | Binding Protein | Cell Line | Strand | Chrm | | Start in Chrm (0 Indexed) | End in Chrm (1 Indexed) |
| 3.08756458492765 | hnrnpk (bg=12.88%) | K562 | - | chrX | | 73062392 | 73062429 |
| 3.72529378910162 | hnrnpk (bg=12.88%) | K562 | - | chrX | | 73062396 | 73062450 |

  
  

| Match 242 in HUMAN | | | | | | | |
| --- | --- | --- | --- | --- | --- | --- | --- |
| Motif | Start in Seq (1 Indexed) | End in Seq (1 Indexed) | Strand | Chrm | Exon | Start in Chrm (0 Indexed) | End in Chrm (1 Indexed) |
| GTGCAGT | 10192 | 10198 | - | chrX | 1 | 73062390 | 73062397 |
| eCLIP Fold-Enrichment | Binding Protein | Cell Line | Strand | Chrm | | Start in Chrm (0 Indexed) | End in Chrm (1 Indexed) |
| 3.08756458492765 | hnrnpk (bg=12.88%) | K562 | - | chrX | | 73062392 | 73062429 |
| 3.72529378910162 | hnrnpk (bg=12.88%) | K562 | - | chrX | | 73062396 | 73062450 |

  
  

| Match 243 in HUMAN | | | | | | | |
| --- | --- | --- | --- | --- | --- | --- | --- |
| Motif | Start in Seq (1 Indexed) | End in Seq (1 Indexed) | Strand | Chrm | Exon | Start in Chrm (0 Indexed) | End in Chrm (1 Indexed) |
| TCTCCCCAGGAAGGAAG | 10422 | 10438 | - | chrX | 1 | 73062150 | 73062167 |
| eCLIP Fold-Enrichment | Binding Protein | Cell Line | Strand | Chrm | | Start in Chrm (0 Indexed) | End in Chrm (1 Indexed) |
| 2.97278130399473 | SF3B1 (bg=2.48%) | K562 | - | chrX | | 73062119 | 73062165 |

  
  

| Match 244 in HUMAN | | | | | | | |
| --- | --- | --- | --- | --- | --- | --- | --- |
| Motif | Start in Seq (1 Indexed) | End in Seq (1 Indexed) | Strand | Chrm | Exon | Start in Chrm (0 Indexed) | End in Chrm (1 Indexed) |
| GAAGGAAG | 10431 | 10438 | - | chrX | 1 | 73062150 | 73062158 |
| eCLIP Fold-Enrichment | Binding Protein | Cell Line | Strand | Chrm | | Start in Chrm (0 Indexed) | End in Chrm (1 Indexed) |
| 2.97278130399473 | SF3B1 (bg=2.48%) | K562 | - | chrX | | 73062119 | 73062165 |

  
  

| Match 245 in HUMAN | | | | | | | |
| --- | --- | --- | --- | --- | --- | --- | --- |
| Motif | Start in Seq (1 Indexed) | End in Seq (1 Indexed) | Strand | Chrm | Exon | Start in Chrm (0 Indexed) | End in Chrm (1 Indexed) |
| TCTCTGCATTCTTC | 10449 | 10462 | - | chrX | 1 | 73062126 | 73062140 |
| eCLIP Fold-Enrichment | Binding Protein | Cell Line | Strand | Chrm | | Start in Chrm (0 Indexed) | End in Chrm (1 Indexed) |
| 2.97278130399473 | SF3B1 (bg=2.48%) | K562 | - | chrX | | 73062119 | 73062165 |

  
  

| Match 246 in HUMAN | | | | | | | |
| --- | --- | --- | --- | --- | --- | --- | --- |
| Motif | Start in Seq (1 Indexed) | End in Seq (1 Indexed) | Strand | Chrm | Exon | Start in Chrm (0 Indexed) | End in Chrm (1 Indexed) |
| TGCATTCTTC | 10453 | 10462 | - | chrX | 1 | 73062126 | 73062136 |
| eCLIP Fold-Enrichment | Binding Protein | Cell Line | Strand | Chrm | | Start in Chrm (0 Indexed) | End in Chrm (1 Indexed) |
| 2.97278130399473 | SF3B1 (bg=2.48%) | K562 | - | chrX | | 73062119 | 73062165 |

  
  

| Match 247 in HUMAN | | | | | | | |
| --- | --- | --- | --- | --- | --- | --- | --- |
| Motif | Start in Seq (1 Indexed) | End in Seq (1 Indexed) | Strand | Chrm | Exon | Start in Chrm (0 Indexed) | End in Chrm (1 Indexed) |
| CAGAGCAGATTGCCTGG | 10467 | 10483 | - | chrX | 1 | 73062105 | 73062122 |
| eCLIP Fold-Enrichment | Binding Protein | Cell Line | Strand | Chrm | | Start in Chrm (0 Indexed) | End in Chrm (1 Indexed) |
| 2.97278130399473 | SF3B1 (bg=2.48%) | K562 | - | chrX | | 73062119 | 73062165 |

  
  

| Match 248 in HUMAN | | | | | | | |
| --- | --- | --- | --- | --- | --- | --- | --- |
| Motif | Start in Seq (1 Indexed) | End in Seq (1 Indexed) | Strand | Chrm | Exon | Start in Chrm (0 Indexed) | End in Chrm (1 Indexed) |
| AGCAGATTGCCTGG | 10470 | 10483 | - | chrX | 1 | 73062105 | 73062119 |
| eCLIP Fold-Enrichment | Binding Protein | Cell Line | Strand | Chrm | | Start in Chrm (0 Indexed) | End in Chrm (1 Indexed) |
| 2.97278130399473 | SF3B1 (bg=2.48%) | K562 | - | chrX | | 73062119 | 73062165 |

  
  

| Match 249 in HUMAN | | | | | | | |
| --- | --- | --- | --- | --- | --- | --- | --- |
| Motif | Start in Seq (1 Indexed) | End in Seq (1 Indexed) | Strand | Chrm | Exon | Start in Chrm (0 Indexed) | End in Chrm (1 Indexed) |
| TGACCAGTGTCTCTCATTT | 10674 | 10692 | - | chrX | 1 | 73061896 | 73061915 |
| eCLIP Fold-Enrichment | Binding Protein | Cell Line | Strand | Chrm | | Start in Chrm (0 Indexed) | End in Chrm (1 Indexed) |
| 2.80703075757272 | SUPV3L1 (bg=1.57%) | K562 | - | chrX | | 73061852 | 73061900 |

  
  

| Match 250 in HUMAN | | | | | | | |
| --- | --- | --- | --- | --- | --- | --- | --- |
| Motif | Start in Seq (1 Indexed) | End in Seq (1 Indexed) | Strand | Chrm | Exon | Start in Chrm (0 Indexed) | End in Chrm (1 Indexed) |
| CAGTGTCTCTCATTT | 10678 | 10692 | - | chrX | 1 | 73061896 | 73061911 |
| eCLIP Fold-Enrichment | Binding Protein | Cell Line | Strand | Chrm | | Start in Chrm (0 Indexed) | End in Chrm (1 Indexed) |
| 2.80703075757272 | SUPV3L1 (bg=1.57%) | K562 | - | chrX | | 73061852 | 73061900 |

  
  

| Match 251 in HUMAN | | | | | | | |
| --- | --- | --- | --- | --- | --- | --- | --- |
| Motif | Start in Seq (1 Indexed) | End in Seq (1 Indexed) | Strand | Chrm | Exon | Start in Chrm (0 Indexed) | End in Chrm (1 Indexed) |
| AGGGTGGTGGGTCTGTGGATAGA | 10699 | 10721 | - | chrX | 1 | 73061867 | 73061890 |
| eCLIP Fold-Enrichment | Binding Protein | Cell Line | Strand | Chrm | | Start in Chrm (0 Indexed) | End in Chrm (1 Indexed) |
| 2.80703075757272 | SUPV3L1 (bg=1.57%) | K562 | - | chrX | | 73061852 | 73061900 |

  
  

| Match 252 in HUMAN | | | | | | | |
| --- | --- | --- | --- | --- | --- | --- | --- |
| Motif | Start in Seq (1 Indexed) | End in Seq (1 Indexed) | Strand | Chrm | Exon | Start in Chrm (0 Indexed) | End in Chrm (1 Indexed) |
| AGGGTGGTG | 10699 | 10707 | - | chrX | 1 | 73061881 | 73061890 |
| eCLIP Fold-Enrichment | Binding Protein | Cell Line | Strand | Chrm | | Start in Chrm (0 Indexed) | End in Chrm (1 Indexed) |
| 2.80703075757272 | SUPV3L1 (bg=1.57%) | K562 | - | chrX | | 73061852 | 73061900 |

  
  

| Match 253 in HUMAN | | | | | | | |
| --- | --- | --- | --- | --- | --- | --- | --- |
| Motif | Start in Seq (1 Indexed) | End in Seq (1 Indexed) | Strand | Chrm | Exon | Start in Chrm (0 Indexed) | End in Chrm (1 Indexed) |
| GTGGTG | 10702 | 10707 | - | chrX | 1 | 73061881 | 73061887 |
| eCLIP Fold-Enrichment | Binding Protein | Cell Line | Strand | Chrm | | Start in Chrm (0 Indexed) | End in Chrm (1 Indexed) |
| 2.80703075757272 | SUPV3L1 (bg=1.57%) | K562 | - | chrX | | 73061852 | 73061900 |

  
  

| Match 254 in HUMAN | | | | | | | |
| --- | --- | --- | --- | --- | --- | --- | --- |
| Motif | Start in Seq (1 Indexed) | End in Seq (1 Indexed) | Strand | Chrm | Exon | Start in Chrm (0 Indexed) | End in Chrm (1 Indexed) |
| GTCTGTGGATAGA | 10709 | 10721 | - | chrX | 1 | 73061867 | 73061880 |
| eCLIP Fold-Enrichment | Binding Protein | Cell Line | Strand | Chrm | | Start in Chrm (0 Indexed) | End in Chrm (1 Indexed) |
| 2.80703075757272 | SUPV3L1 (bg=1.57%) | K562 | - | chrX | | 73061852 | 73061900 |

  
  

| Match 255 in HUMAN | | | | | | | |
| --- | --- | --- | --- | --- | --- | --- | --- |
| Motif | Start in Seq (1 Indexed) | End in Seq (1 Indexed) | Strand | Chrm | Exon | Start in Chrm (0 Indexed) | End in Chrm (1 Indexed) |
| GTCTGTGGATA | 10709 | 10719 | - | chrX | 1 | 73061869 | 73061880 |
| eCLIP Fold-Enrichment | Binding Protein | Cell Line | Strand | Chrm | | Start in Chrm (0 Indexed) | End in Chrm (1 Indexed) |
| 2.80703075757272 | SUPV3L1 (bg=1.57%) | K562 | - | chrX | | 73061852 | 73061900 |

  
  

| Match 256 in HUMAN | | | | | | | |
| --- | --- | --- | --- | --- | --- | --- | --- |
| Motif | Start in Seq (1 Indexed) | End in Seq (1 Indexed) | Strand | Chrm | Exon | Start in Chrm (0 Indexed) | End in Chrm (1 Indexed) |
| TATTTTA | 10736 | 10742 | - | chrX | 1 | 73061846 | 73061853 |
| eCLIP Fold-Enrichment | Binding Protein | Cell Line | Strand | Chrm | | Start in Chrm (0 Indexed) | End in Chrm (1 Indexed) |
| 2.80703075757272 | SUPV3L1 (bg=1.57%) | K562 | - | chrX | | 73061852 | 73061900 |

  
  

| Match 257 in HUMAN | | | | | | | |
| --- | --- | --- | --- | --- | --- | --- | --- |
| Motif | Start in Seq (1 Indexed) | End in Seq (1 Indexed) | Strand | Chrm | Exon | Start in Chrm (0 Indexed) | End in Chrm (1 Indexed) |
| TAATCCTT | 10836 | 10843 | - | chrX | 1 | 73061745 | 73061753 |
| eCLIP Fold-Enrichment | Binding Protein | Cell Line | Strand | Chrm | | Start in Chrm (0 Indexed) | End in Chrm (1 Indexed) |
| 2.57817972433746 | SUPV3L1 (bg=1.57%) | K562 | - | chrX | | 73061677 | 73061749 |

  
  

| Match 258 in HUMAN | | | | | | | |
| --- | --- | --- | --- | --- | --- | --- | --- |
| Motif | Start in Seq (1 Indexed) | End in Seq (1 Indexed) | Strand | Chrm | Exon | Start in Chrm (0 Indexed) | End in Chrm (1 Indexed) |
| AATTTCTTCATCTGGAGCA | 10845 | 10863 | - | chrX | 1 | 73061725 | 73061744 |
| eCLIP Fold-Enrichment | Binding Protein | Cell Line | Strand | Chrm | | Start in Chrm (0 Indexed) | End in Chrm (1 Indexed) |
| 2.57817972433746 | SUPV3L1 (bg=1.57%) | K562 | - | chrX | | 73061677 | 73061749 |
| 2.01204175863068 | U2AF2 (bg=1.76%) | K562 | - | chrX | | 73061696 | 73061739 |

  
  

| Match 259 in HUMAN | | | | | | | |
| --- | --- | --- | --- | --- | --- | --- | --- |
| Motif | Start in Seq (1 Indexed) | End in Seq (1 Indexed) | Strand | Chrm | Exon | Start in Chrm (0 Indexed) | End in Chrm (1 Indexed) |
| AATTTCTTCATCTGGAGC | 10845 | 10862 | - | chrX | 1 | 73061726 | 73061744 |
| eCLIP Fold-Enrichment | Binding Protein | Cell Line | Strand | Chrm | | Start in Chrm (0 Indexed) | End in Chrm (1 Indexed) |
| 2.57817972433746 | SUPV3L1 (bg=1.57%) | K562 | - | chrX | | 73061677 | 73061749 |
| 2.01204175863068 | U2AF2 (bg=1.76%) | K562 | - | chrX | | 73061696 | 73061739 |

  
  

| Match 260 in HUMAN | | | | | | | |
| --- | --- | --- | --- | --- | --- | --- | --- |
| Motif | Start in Seq (1 Indexed) | End in Seq (1 Indexed) | Strand | Chrm | Exon | Start in Chrm (0 Indexed) | End in Chrm (1 Indexed) |
| CTTATTTCAAGAA | 10874 | 10886 | - | chrX | 1 | 73061702 | 73061715 |
| eCLIP Fold-Enrichment | Binding Protein | Cell Line | Strand | Chrm | | Start in Chrm (0 Indexed) | End in Chrm (1 Indexed) |
| 2.57817972433746 | SUPV3L1 (bg=1.57%) | K562 | - | chrX | | 73061677 | 73061749 |
| 2.01204175863068 | U2AF2 (bg=1.76%) | K562 | - | chrX | | 73061696 | 73061739 |

  
  

| Match 261 in HUMAN | | | | | | | |
| --- | --- | --- | --- | --- | --- | --- | --- |
| Motif | Start in Seq (1 Indexed) | End in Seq (1 Indexed) | Strand | Chrm | Exon | Start in Chrm (0 Indexed) | End in Chrm (1 Indexed) |
| CTTATTT | 10874 | 10880 | - | chrX | 1 | 73061708 | 73061715 |
| eCLIP Fold-Enrichment | Binding Protein | Cell Line | Strand | Chrm | | Start in Chrm (0 Indexed) | End in Chrm (1 Indexed) |
| 2.57817972433746 | SUPV3L1 (bg=1.57%) | K562 | - | chrX | | 73061677 | 73061749 |
| 2.01204175863068 | U2AF2 (bg=1.76%) | K562 | - | chrX | | 73061696 | 73061739 |

  
  

| Match 262 in HUMAN | | | | | | | |
| --- | --- | --- | --- | --- | --- | --- | --- |
| Motif | Start in Seq (1 Indexed) | End in Seq (1 Indexed) | Strand | Chrm | Exon | Start in Chrm (0 Indexed) | End in Chrm (1 Indexed) |
| GAGAAGGATGTCAAAAGATCGGC | 11275 | 11297 | - | chrX | 1 | 73061291 | 73061314 |
| eCLIP Fold-Enrichment | Binding Protein | Cell Line | Strand | Chrm | | Start in Chrm (0 Indexed) | End in Chrm (1 Indexed) |
| 3.17519012233491 | SRSF1 (bg=8.47%) | K562 | - | chrX | | 73061217 | 73061316 |
| 3.11722658323668 | SRSF1 (bg=8.47%) | K562 | - | chrX | | 73061235 | 73061317 |
| 2.4513268041562 | U2AF2 (bg=1.76%) | K562 | - | chrX | | 73061239 | 73061323 |
| 2.24594046802796 | U2AF2 (bg=1.76%) | K562 | - | chrX | | 73061246 | 73061323 |
| 2.03767271687932 | uchl5 (bg=11.16%) | K562 | - | chrX | | 73061262 | 73061306 |

  
  

| Match 263 in HUMAN | | | | | | | |
| --- | --- | --- | --- | --- | --- | --- | --- |
| Motif | Start in Seq (1 Indexed) | End in Seq (1 Indexed) | Strand | Chrm | Exon | Start in Chrm (0 Indexed) | End in Chrm (1 Indexed) |
| AAGGATGTCAAAAGATC | 11278 | 11294 | - | chrX | 1 | 73061294 | 73061311 |
| eCLIP Fold-Enrichment | Binding Protein | Cell Line | Strand | Chrm | | Start in Chrm (0 Indexed) | End in Chrm (1 Indexed) |
| 3.17519012233491 | SRSF1 (bg=8.47%) | K562 | - | chrX | | 73061217 | 73061316 |
| 3.11722658323668 | SRSF1 (bg=8.47%) | K562 | - | chrX | | 73061235 | 73061317 |
| 2.4513268041562 | U2AF2 (bg=1.76%) | K562 | - | chrX | | 73061239 | 73061323 |
| 2.24594046802796 | U2AF2 (bg=1.76%) | K562 | - | chrX | | 73061246 | 73061323 |
| 2.03767271687932 | uchl5 (bg=11.16%) | K562 | - | chrX | | 73061262 | 73061306 |

  
  

| Match 264 in HUMAN | | | | | | | |
| --- | --- | --- | --- | --- | --- | --- | --- |
| Motif | Start in Seq (1 Indexed) | End in Seq (1 Indexed) | Strand | Chrm | Exon | Start in Chrm (0 Indexed) | End in Chrm (1 Indexed) |
| AAGGATG | 11278 | 11284 | - | chrX | 1 | 73061304 | 73061311 |
| eCLIP Fold-Enrichment | Binding Protein | Cell Line | Strand | Chrm | | Start in Chrm (0 Indexed) | End in Chrm (1 Indexed) |
| 3.17519012233491 | SRSF1 (bg=8.47%) | K562 | - | chrX | | 73061217 | 73061316 |
| 3.11722658323668 | SRSF1 (bg=8.47%) | K562 | - | chrX | | 73061235 | 73061317 |
| 2.4513268041562 | U2AF2 (bg=1.76%) | K562 | - | chrX | | 73061239 | 73061323 |
| 2.24594046802796 | U2AF2 (bg=1.76%) | K562 | - | chrX | | 73061246 | 73061323 |
| 2.03767271687932 | uchl5 (bg=11.16%) | K562 | - | chrX | | 73061262 | 73061306 |

  
  

| Match 265 in HUMAN | | | | | | | |
| --- | --- | --- | --- | --- | --- | --- | --- |
| Motif | Start in Seq (1 Indexed) | End in Seq (1 Indexed) | Strand | Chrm | Exon | Start in Chrm (0 Indexed) | End in Chrm (1 Indexed) |
| AAAGATC | 11288 | 11294 | - | chrX | 1 | 73061294 | 73061301 |
| eCLIP Fold-Enrichment | Binding Protein | Cell Line | Strand | Chrm | | Start in Chrm (0 Indexed) | End in Chrm (1 Indexed) |
| 3.17519012233491 | SRSF1 (bg=8.47%) | K562 | - | chrX | | 73061217 | 73061316 |
| 3.11722658323668 | SRSF1 (bg=8.47%) | K562 | - | chrX | | 73061235 | 73061317 |
| 2.4513268041562 | U2AF2 (bg=1.76%) | K562 | - | chrX | | 73061239 | 73061323 |
| 2.24594046802796 | U2AF2 (bg=1.76%) | K562 | - | chrX | | 73061246 | 73061323 |
| 2.03767271687932 | uchl5 (bg=11.16%) | K562 | - | chrX | | 73061262 | 73061306 |

  
  

| Match 266 in HUMAN | | | | | | | |
| --- | --- | --- | --- | --- | --- | --- | --- |
| Motif | Start in Seq (1 Indexed) | End in Seq (1 Indexed) | Strand | Chrm | Exon | Start in Chrm (0 Indexed) | End in Chrm (1 Indexed) |
| CAGCTCAGGG | 11299 | 11308 | - | chrX | 1 | 73061280 | 73061290 |
| eCLIP Fold-Enrichment | Binding Protein | Cell Line | Strand | Chrm | | Start in Chrm (0 Indexed) | End in Chrm (1 Indexed) |
| 3.17519012233491 | SRSF1 (bg=8.47%) | K562 | - | chrX | | 73061217 | 73061316 |
| 3.11722658323668 | SRSF1 (bg=8.47%) | K562 | - | chrX | | 73061235 | 73061317 |
| 2.4513268041562 | U2AF2 (bg=1.76%) | K562 | - | chrX | | 73061239 | 73061323 |
| 2.24594046802796 | U2AF2 (bg=1.76%) | K562 | - | chrX | | 73061246 | 73061323 |
| 2.03767271687932 | uchl5 (bg=11.16%) | K562 | - | chrX | | 73061262 | 73061306 |

  
  

| Match 267 in HUMAN | | | | | | | |
| --- | --- | --- | --- | --- | --- | --- | --- |
| Motif | Start in Seq (1 Indexed) | End in Seq (1 Indexed) | Strand | Chrm | Exon | Start in Chrm (0 Indexed) | End in Chrm (1 Indexed) |
| GCAGTTTGC | 11310 | 11318 | - | chrX | 1 | 73061270 | 73061279 |
| eCLIP Fold-Enrichment | Binding Protein | Cell Line | Strand | Chrm | | Start in Chrm (0 Indexed) | End in Chrm (1 Indexed) |
| 3.17519012233491 | SRSF1 (bg=8.47%) | K562 | - | chrX | | 73061217 | 73061316 |
| 3.11722658323668 | SRSF1 (bg=8.47%) | K562 | - | chrX | | 73061235 | 73061317 |
| 2.4513268041562 | U2AF2 (bg=1.76%) | K562 | - | chrX | | 73061239 | 73061323 |
| 2.24594046802796 | U2AF2 (bg=1.76%) | K562 | - | chrX | | 73061246 | 73061323 |
| 2.03767271687932 | uchl5 (bg=11.16%) | K562 | - | chrX | | 73061262 | 73061306 |

  
  

| Match 268 in HUMAN | | | | | | | |
| --- | --- | --- | --- | --- | --- | --- | --- |
| Motif | Start in Seq (1 Indexed) | End in Seq (1 Indexed) | Strand | Chrm | Exon | Start in Chrm (0 Indexed) | End in Chrm (1 Indexed) |
| CTACTAGCTCCT | 11320 | 11331 | - | chrX | 1 | 73061257 | 73061269 |
| eCLIP Fold-Enrichment | Binding Protein | Cell Line | Strand | Chrm | | Start in Chrm (0 Indexed) | End in Chrm (1 Indexed) |
| 3.17519012233491 | SRSF1 (bg=8.47%) | K562 | - | chrX | | 73061217 | 73061316 |
| 3.11722658323668 | SRSF1 (bg=8.47%) | K562 | - | chrX | | 73061235 | 73061317 |
| 2.4513268041562 | U2AF2 (bg=1.76%) | K562 | - | chrX | | 73061239 | 73061323 |
| 2.24594046802796 | U2AF2 (bg=1.76%) | K562 | - | chrX | | 73061246 | 73061323 |
| 2.03767271687932 | uchl5 (bg=11.16%) | K562 | - | chrX | | 73061262 | 73061306 |

  
  

| Match 269 in HUMAN | | | | | | | |
| --- | --- | --- | --- | --- | --- | --- | --- |
| Motif | Start in Seq (1 Indexed) | End in Seq (1 Indexed) | Strand | Chrm | Exon | Start in Chrm (0 Indexed) | End in Chrm (1 Indexed) |
| GGACAGCTGT | 11333 | 11342 | - | chrX | 1 | 73061246 | 73061256 |
| eCLIP Fold-Enrichment | Binding Protein | Cell Line | Strand | Chrm | | Start in Chrm (0 Indexed) | End in Chrm (1 Indexed) |
| 3.17519012233491 | SRSF1 (bg=8.47%) | K562 | - | chrX | | 73061217 | 73061316 |
| 3.11722658323668 | SRSF1 (bg=8.47%) | K562 | - | chrX | | 73061235 | 73061317 |
| 2.15696959332617 | SRSF7 (bg=2.32%) | K562 | - | chrX | | 73061214 | 73061254 |
| 2.4513268041562 | U2AF2 (bg=1.76%) | K562 | - | chrX | | 73061239 | 73061323 |
| 2.24594046802796 | U2AF2 (bg=1.76%) | K562 | - | chrX | | 73061246 | 73061323 |
| 2.11884743567764 | ZNF622 (bg=6.58%) | K562 | - | chrX | | 73061215 | 73061252 |

  
  

| Match 270 in HUMAN | | | | | | | |
| --- | --- | --- | --- | --- | --- | --- | --- |
| Motif | Start in Seq (1 Indexed) | End in Seq (1 Indexed) | Strand | Chrm | Exon | Start in Chrm (0 Indexed) | End in Chrm (1 Indexed) |
| GGACAGCTG | 11333 | 11341 | - | chrX | 1 | 73061247 | 73061256 |
| eCLIP Fold-Enrichment | Binding Protein | Cell Line | Strand | Chrm | | Start in Chrm (0 Indexed) | End in Chrm (1 Indexed) |
| 3.17519012233491 | SRSF1 (bg=8.47%) | K562 | - | chrX | | 73061217 | 73061316 |
| 3.11722658323668 | SRSF1 (bg=8.47%) | K562 | - | chrX | | 73061235 | 73061317 |
| 2.15696959332617 | SRSF7 (bg=2.32%) | K562 | - | chrX | | 73061214 | 73061254 |
| 2.4513268041562 | U2AF2 (bg=1.76%) | K562 | - | chrX | | 73061239 | 73061323 |
| 2.24594046802796 | U2AF2 (bg=1.76%) | K562 | - | chrX | | 73061246 | 73061323 |
| 2.11884743567764 | ZNF622 (bg=6.58%) | K562 | - | chrX | | 73061215 | 73061252 |

  
  

| Match 271 in HUMAN | | | | | | | |
| --- | --- | --- | --- | --- | --- | --- | --- |
| Motif | Start in Seq (1 Indexed) | End in Seq (1 Indexed) | Strand | Chrm | Exon | Start in Chrm (0 Indexed) | End in Chrm (1 Indexed) |
| AAGAAGAGTCTCTGGCTCTTTAGA | 11344 | 11367 | - | chrX | 1 | 73061221 | 73061245 |
| eCLIP Fold-Enrichment | Binding Protein | Cell Line | Strand | Chrm | | Start in Chrm (0 Indexed) | End in Chrm (1 Indexed) |
| 2.53419363565551 | DDX24 (bg=2.97%) | K562 | - | chrX | | 73061212 | 73061244 |
| 3.0945065067366 | SRSF1 (bg=8.47%) | K562 | - | chrX | | 73061216 | 73061235 |
| 3.17519012233491 | SRSF1 (bg=8.47%) | K562 | - | chrX | | 73061217 | 73061316 |
| 3.11722658323668 | SRSF1 (bg=8.47%) | K562 | - | chrX | | 73061235 | 73061317 |
| 2.15696959332617 | SRSF7 (bg=2.32%) | K562 | - | chrX | | 73061214 | 73061254 |
| 2.4513268041562 | U2AF2 (bg=1.76%) | K562 | - | chrX | | 73061239 | 73061323 |
| 2.23080281437738 | ZNF622 (bg=6.58%) | K562 | - | chrX | | 73061215 | 73061236 |
| 2.11884743567764 | ZNF622 (bg=6.58%) | K562 | - | chrX | | 73061215 | 73061252 |

  
  

| Match 272 in HUMAN | | | | | | | |
| --- | --- | --- | --- | --- | --- | --- | --- |
| Motif | Start in Seq (1 Indexed) | End in Seq (1 Indexed) | Strand | Chrm | Exon | Start in Chrm (0 Indexed) | End in Chrm (1 Indexed) |
| AGAAGAGTCTCTGGCTCTTTAGA | 11345 | 11367 | - | chrX | 1 | 73061221 | 73061244 |
| eCLIP Fold-Enrichment | Binding Protein | Cell Line | Strand | Chrm | | Start in Chrm (0 Indexed) | End in Chrm (1 Indexed) |
| 2.53419363565551 | DDX24 (bg=2.97%) | K562 | - | chrX | | 73061212 | 73061244 |
| 3.0945065067366 | SRSF1 (bg=8.47%) | K562 | - | chrX | | 73061216 | 73061235 |
| 3.17519012233491 | SRSF1 (bg=8.47%) | K562 | - | chrX | | 73061217 | 73061316 |
| 3.11722658323668 | SRSF1 (bg=8.47%) | K562 | - | chrX | | 73061235 | 73061317 |
| 2.15696959332617 | SRSF7 (bg=2.32%) | K562 | - | chrX | | 73061214 | 73061254 |
| 2.4513268041562 | U2AF2 (bg=1.76%) | K562 | - | chrX | | 73061239 | 73061323 |
| 2.23080281437738 | ZNF622 (bg=6.58%) | K562 | - | chrX | | 73061215 | 73061236 |
| 2.11884743567764 | ZNF622 (bg=6.58%) | K562 | - | chrX | | 73061215 | 73061252 |

  
  

| Match 273 in HUMAN | | | | | | | |
| --- | --- | --- | --- | --- | --- | --- | --- |
| Motif | Start in Seq (1 Indexed) | End in Seq (1 Indexed) | Strand | Chrm | Exon | Start in Chrm (0 Indexed) | End in Chrm (1 Indexed) |
| AGAAGAGTCTCTGGCTCTTTA | 11345 | 11365 | - | chrX | 1 | 73061223 | 73061244 |
| eCLIP Fold-Enrichment | Binding Protein | Cell Line | Strand | Chrm | | Start in Chrm (0 Indexed) | End in Chrm (1 Indexed) |
| 2.53419363565551 | DDX24 (bg=2.97%) | K562 | - | chrX | | 73061212 | 73061244 |
| 3.0945065067366 | SRSF1 (bg=8.47%) | K562 | - | chrX | | 73061216 | 73061235 |
| 3.17519012233491 | SRSF1 (bg=8.47%) | K562 | - | chrX | | 73061217 | 73061316 |
| 3.11722658323668 | SRSF1 (bg=8.47%) | K562 | - | chrX | | 73061235 | 73061317 |
| 2.15696959332617 | SRSF7 (bg=2.32%) | K562 | - | chrX | | 73061214 | 73061254 |
| 2.4513268041562 | U2AF2 (bg=1.76%) | K562 | - | chrX | | 73061239 | 73061323 |
| 2.23080281437738 | ZNF622 (bg=6.58%) | K562 | - | chrX | | 73061215 | 73061236 |
| 2.11884743567764 | ZNF622 (bg=6.58%) | K562 | - | chrX | | 73061215 | 73061252 |

  
  

| Match 274 in HUMAN | | | | | | | |
| --- | --- | --- | --- | --- | --- | --- | --- |
| Motif | Start in Seq (1 Indexed) | End in Seq (1 Indexed) | Strand | Chrm | Exon | Start in Chrm (0 Indexed) | End in Chrm (1 Indexed) |
| ATTCTGAGC | 11440 | 11448 | - | chrX | 3 | 73053197 | 73053206 |
| eCLIP Fold-Enrichment | Binding Protein | Cell Line | Strand | Chrm | | Start in Chrm (0 Indexed) | End in Chrm (1 Indexed) |
| 2.11915613637667 | DDX24 (bg=2.97%) | K562 | - | chrX | | 73053072 | 73053212 |
| 3.46365048980705 | GRWD1 (bg=5.13%) | K562 | - | chrX | | 73053150 | 73053210 |
| 3.02332790103651 | GRWD1 (bg=5.13%) | K562 | - | chrX | | 73053150 | 73053210 |
| 2.45275350249983 | MTPAP (bg=2.21%) | K562 | - | chrX | | 73053147 | 73053209 |
| 2.0793527402955 | NOLC1 (bg=9.43%) | K562 | - | chrX | | 73053181 | 73053210 |
| 3.79244216101257 | SRSF1 (bg=8.47%) | K562 | - | chrX | | 73053158 | 73053210 |
| 2.86318096063014 | SRSF1 (bg=8.47%) | K562 | - | chrX | | 73053173 | 73053207 |
| 2.3120197180991 | ZNF622 (bg=6.58%) | K562 | - | chrX | | 73053151 | 73053211 |
| 2.54543841669839 | ZNF622 (bg=6.58%) | K562 | - | chrX | | 73053152 | 73053211 |

  
  

| Match 275 in HUMAN | | | | | | | |
| --- | --- | --- | --- | --- | --- | --- | --- |
| Motif | Start in Seq (1 Indexed) | End in Seq (1 Indexed) | Strand | Chrm | Exon | Start in Chrm (0 Indexed) | End in Chrm (1 Indexed) |
| GACTGCAA | 11463 | 11470 | - | chrX | 3 | 73053175 | 73053183 |
| eCLIP Fold-Enrichment | Binding Protein | Cell Line | Strand | Chrm | | Start in Chrm (0 Indexed) | End in Chrm (1 Indexed) |
| 2.11915613637667 | DDX24 (bg=2.97%) | K562 | - | chrX | | 73053072 | 73053212 |
| 3.46365048980705 | GRWD1 (bg=5.13%) | K562 | - | chrX | | 73053150 | 73053210 |
| 3.02332790103651 | GRWD1 (bg=5.13%) | K562 | - | chrX | | 73053150 | 73053210 |
| 2.45275350249983 | MTPAP (bg=2.21%) | K562 | - | chrX | | 73053147 | 73053209 |
| 2.0793527402955 | NOLC1 (bg=9.43%) | K562 | - | chrX | | 73053181 | 73053210 |
| 3.79244216101257 | SRSF1 (bg=8.47%) | K562 | - | chrX | | 73053158 | 73053210 |
| 2.86318096063014 | SRSF1 (bg=8.47%) | K562 | - | chrX | | 73053173 | 73053207 |
| 3.40174734853896 | UTP3 (bg=3.66%) | K562 | - | chrX | | 73053160 | 73053182 |
| 2.3120197180991 | ZNF622 (bg=6.58%) | K562 | - | chrX | | 73053151 | 73053211 |
| 2.54543841669839 | ZNF622 (bg=6.58%) | K562 | - | chrX | | 73053152 | 73053211 |

  
  

| Match 276 in HUMAN | | | | | | | |
| --- | --- | --- | --- | --- | --- | --- | --- |
| Motif | Start in Seq (1 Indexed) | End in Seq (1 Indexed) | Strand | Chrm | Exon | Start in Chrm (0 Indexed) | End in Chrm (1 Indexed) |
| CTGCAA | 11465 | 11470 | - | chrX | 3 | 73053175 | 73053181 |
| eCLIP Fold-Enrichment | Binding Protein | Cell Line | Strand | Chrm | | Start in Chrm (0 Indexed) | End in Chrm (1 Indexed) |
| 2.11915613637667 | DDX24 (bg=2.97%) | K562 | - | chrX | | 73053072 | 73053212 |
| 3.46365048980705 | GRWD1 (bg=5.13%) | K562 | - | chrX | | 73053150 | 73053210 |
| 3.02332790103651 | GRWD1 (bg=5.13%) | K562 | - | chrX | | 73053150 | 73053210 |
| 2.45275350249983 | MTPAP (bg=2.21%) | K562 | - | chrX | | 73053147 | 73053209 |
| 2.0793527402955 | NOLC1 (bg=9.43%) | K562 | - | chrX | | 73053181 | 73053210 |
| 3.79244216101257 | SRSF1 (bg=8.47%) | K562 | - | chrX | | 73053158 | 73053210 |
| 2.86318096063014 | SRSF1 (bg=8.47%) | K562 | - | chrX | | 73053173 | 73053207 |
| 3.40174734853896 | UTP3 (bg=3.66%) | K562 | - | chrX | | 73053160 | 73053182 |
| 2.3120197180991 | ZNF622 (bg=6.58%) | K562 | - | chrX | | 73053151 | 73053211 |
| 2.54543841669839 | ZNF622 (bg=6.58%) | K562 | - | chrX | | 73053152 | 73053211 |

  
  

| Match 277 in HUMAN | | | | | | | |
| --- | --- | --- | --- | --- | --- | --- | --- |
| Motif | Start in Seq (1 Indexed) | End in Seq (1 Indexed) | Strand | Chrm | Exon | Start in Chrm (0 Indexed) | End in Chrm (1 Indexed) |
| TTTGAGAATCTGG | 11509 | 11521 | - | chrX | 3 | 73053124 | 73053137 |
| eCLIP Fold-Enrichment | Binding Protein | Cell Line | Strand | Chrm | | Start in Chrm (0 Indexed) | End in Chrm (1 Indexed) |
| 2.0747697536265 | DDX24 (bg=2.97%) | K562 | - | chrX | | 73053072 | 73053171 |
| 2.11915613637667 | DDX24 (bg=2.97%) | K562 | - | chrX | | 73053072 | 73053212 |
| 2.92308210844434 | GRWD1 (bg=5.13%) | K562 | - | chrX | | 73053070 | 73053150 |
| 2.86354745699974 | GRWD1 (bg=5.13%) | K562 | - | chrX | | 73053071 | 73053150 |
| 2.52833757149769 | NOLC1 (bg=9.43%) | K562 | - | chrX | | 73053072 | 73053151 |
| 2.60721182602904 | NOLC1 (bg=9.43%) | K562 | - | chrX | | 73053082 | 73053137 |
| 2.51305976109129 | SRSF1 (bg=8.47%) | K562 | - | chrX | | 73053073 | 73053137 |
| 2.79220952526715 | uchl5 (bg=11.16%) | K562 | - | chrX | | 73053072 | 73053137 |
| 2.63413085643831 | uchl5 (bg=11.16%) | K562 | - | chrX | | 73053081 | 73053136 |
| 2.51092378651318 | ZNF622 (bg=6.58%) | K562 | - | chrX | | 73053070 | 73053151 |
| 2.3992003460731 | ZNF622 (bg=6.58%) | K562 | - | chrX | | 73053070 | 73053151 |

  
  

| Match 278 in HUMAN | | | | | | | |
| --- | --- | --- | --- | --- | --- | --- | --- |
| Motif | Start in Seq (1 Indexed) | End in Seq (1 Indexed) | Strand | Chrm | Exon | Start in Chrm (0 Indexed) | End in Chrm (1 Indexed) |
| AAGCTCCA | 11524 | 11531 | - | chrX | 3 | 73053114 | 73053122 |
| eCLIP Fold-Enrichment | Binding Protein | Cell Line | Strand | Chrm | | Start in Chrm (0 Indexed) | End in Chrm (1 Indexed) |
| 2.0747697536265 | DDX24 (bg=2.97%) | K562 | - | chrX | | 73053072 | 73053171 |
| 2.11915613637667 | DDX24 (bg=2.97%) | K562 | - | chrX | | 73053072 | 73053212 |
| 2.92308210844434 | GRWD1 (bg=5.13%) | K562 | - | chrX | | 73053070 | 73053150 |
| 2.86354745699974 | GRWD1 (bg=5.13%) | K562 | - | chrX | | 73053071 | 73053150 |
| 2.52833757149769 | NOLC1 (bg=9.43%) | K562 | - | chrX | | 73053072 | 73053151 |
| 2.60721182602904 | NOLC1 (bg=9.43%) | K562 | - | chrX | | 73053082 | 73053137 |
| 2.68634346116051 | RBM15 (bg=7.27%) | K562 | - | chrX | | 73053096 | 73053118 |
| 2.38401312393158 | SRSF1 (bg=8.47%) | K562 | - | chrX | | 73053073 | 73053121 |
| 2.51305976109129 | SRSF1 (bg=8.47%) | K562 | - | chrX | | 73053073 | 73053137 |
| 2.79220952526715 | uchl5 (bg=11.16%) | K562 | - | chrX | | 73053072 | 73053137 |
| 2.63413085643831 | uchl5 (bg=11.16%) | K562 | - | chrX | | 73053081 | 73053136 |
| 2.51092378651318 | ZNF622 (bg=6.58%) | K562 | - | chrX | | 73053070 | 73053151 |
| 2.3992003460731 | ZNF622 (bg=6.58%) | K562 | - | chrX | | 73053070 | 73053151 |

  
  

| Match 279 in HUMAN | | | | | | | |
| --- | --- | --- | --- | --- | --- | --- | --- |
| Motif | Start in Seq (1 Indexed) | End in Seq (1 Indexed) | Strand | Chrm | Exon | Start in Chrm (0 Indexed) | End in Chrm (1 Indexed) |
| AATCTA | 11535 | 11540 | - | chrX | 3 | 73053105 | 73053111 |
| eCLIP Fold-Enrichment | Binding Protein | Cell Line | Strand | Chrm | | Start in Chrm (0 Indexed) | End in Chrm (1 Indexed) |
| 2.0747697536265 | DDX24 (bg=2.97%) | K562 | - | chrX | | 73053072 | 73053171 |
| 2.11915613637667 | DDX24 (bg=2.97%) | K562 | - | chrX | | 73053072 | 73053212 |
| 2.92308210844434 | GRWD1 (bg=5.13%) | K562 | - | chrX | | 73053070 | 73053150 |
| 2.86354745699974 | GRWD1 (bg=5.13%) | K562 | - | chrX | | 73053071 | 73053150 |
| 2.52833757149769 | NOLC1 (bg=9.43%) | K562 | - | chrX | | 73053072 | 73053151 |
| 2.60721182602904 | NOLC1 (bg=9.43%) | K562 | - | chrX | | 73053082 | 73053137 |
| 2.68634346116051 | RBM15 (bg=7.27%) | K562 | - | chrX | | 73053096 | 73053118 |
| 2.38401312393158 | SRSF1 (bg=8.47%) | K562 | - | chrX | | 73053073 | 73053121 |
| 2.51305976109129 | SRSF1 (bg=8.47%) | K562 | - | chrX | | 73053073 | 73053137 |
| 2.17024503774193 | TARDBP (bg=2.79%) | K562 | - | chrX | | 73053071 | 73053112 |
| 2.79220952526715 | uchl5 (bg=11.16%) | K562 | - | chrX | | 73053072 | 73053137 |
| 2.63413085643831 | uchl5 (bg=11.16%) | K562 | - | chrX | | 73053081 | 73053136 |
| 2.51092378651318 | ZNF622 (bg=6.58%) | K562 | - | chrX | | 73053070 | 73053151 |
| 2.3992003460731 | ZNF622 (bg=6.58%) | K562 | - | chrX | | 73053070 | 73053151 |

  
  

| Match 280 in HUMAN | | | | | | | |
| --- | --- | --- | --- | --- | --- | --- | --- |
| Motif | Start in Seq (1 Indexed) | End in Seq (1 Indexed) | Strand | Chrm | Exon | Start in Chrm (0 Indexed) | End in Chrm (1 Indexed) |
| GGATGG | 11544 | 11549 | - | chrX | 3 | 73053096 | 73053102 |
| eCLIP Fold-Enrichment | Binding Protein | Cell Line | Strand | Chrm | | Start in Chrm (0 Indexed) | End in Chrm (1 Indexed) |
| 2.0747697536265 | DDX24 (bg=2.97%) | K562 | - | chrX | | 73053072 | 73053171 |
| 2.11915613637667 | DDX24 (bg=2.97%) | K562 | - | chrX | | 73053072 | 73053212 |
| 2.92308210844434 | GRWD1 (bg=5.13%) | K562 | - | chrX | | 73053070 | 73053150 |
| 2.86354745699974 | GRWD1 (bg=5.13%) | K562 | - | chrX | | 73053071 | 73053150 |
| 2.3767502512271 | NIPBL (bg=5.39%) | K562 | - | chrX | | 73053072 | 73053097 |
| 2.52833757149769 | NOLC1 (bg=9.43%) | K562 | - | chrX | | 73053072 | 73053151 |
| 2.60721182602904 | NOLC1 (bg=9.43%) | K562 | - | chrX | | 73053082 | 73053137 |
| 2.68634346116051 | RBM15 (bg=7.27%) | K562 | - | chrX | | 73053096 | 73053118 |
| 2.38401312393158 | SRSF1 (bg=8.47%) | K562 | - | chrX | | 73053073 | 73053121 |
| 2.51305976109129 | SRSF1 (bg=8.47%) | K562 | - | chrX | | 73053073 | 73053137 |
| 2.17024503774193 | TARDBP (bg=2.79%) | K562 | - | chrX | | 73053071 | 73053112 |
| 2.79220952526715 | uchl5 (bg=11.16%) | K562 | - | chrX | | 73053072 | 73053137 |
| 2.63413085643831 | uchl5 (bg=11.16%) | K562 | - | chrX | | 73053081 | 73053136 |
| 2.51092378651318 | ZNF622 (bg=6.58%) | K562 | - | chrX | | 73053070 | 73053151 |
| 2.3992003460731 | ZNF622 (bg=6.58%) | K562 | - | chrX | | 73053070 | 73053151 |

  
  

| Match 281 in HUMAN | | | | | | | |
| --- | --- | --- | --- | --- | --- | --- | --- |
| Motif | Start in Seq (1 Indexed) | End in Seq (1 Indexed) | Strand | Chrm | Exon | Start in Chrm (0 Indexed) | End in Chrm (1 Indexed) |
| TCTGGAGAAAAAGATCTTCCTCAGAAGAATAGGCTTGTTG | 11561 | 11600 | - | chrX | 4 | 73051082 | 73051109 |
|  |  |  |  |  | 3 | 73053072 | 73053085 |
| eCLIP Fold-Enrichment | Binding Protein | Cell Line | Strand | Chrm | | Start in Chrm (0 Indexed) | End in Chrm (1 Indexed) |
| 2.0747697536265 | DDX24 (bg=2.97%) | K562 | - | chrX | | 73053072 | 73053171 |
| 2.11915613637667 | DDX24 (bg=2.97%) | K562 | - | chrX | | 73053072 | 73053212 |
| 2.92308210844434 | GRWD1 (bg=5.13%) | K562 | - | chrX | | 73053070 | 73053150 |
| 2.86354745699974 | GRWD1 (bg=5.13%) | K562 | - | chrX | | 73053071 | 73053150 |
| 2.3767502512271 | NIPBL (bg=5.39%) | K562 | - | chrX | | 73053072 | 73053097 |
| 2.54169595435271 | NOLC1 (bg=9.43%) | K562 | - | chrX | | 73051064 | 73051110 |
| 2.52833757149769 | NOLC1 (bg=9.43%) | K562 | - | chrX | | 73053072 | 73053151 |
| 2.60721182602904 | NOLC1 (bg=9.43%) | K562 | - | chrX | | 73053082 | 73053137 |
| 2.38401312393158 | SRSF1 (bg=8.47%) | K562 | - | chrX | | 73053073 | 73053121 |
| 2.51305976109129 | SRSF1 (bg=8.47%) | K562 | - | chrX | | 73053073 | 73053137 |
| 2.05145800436287 | SRSF7 (bg=2.32%) | K562 | - | chrX | | 73051035 | 73051110 |
| 2.17024503774193 | TARDBP (bg=2.79%) | K562 | - | chrX | | 73053071 | 73053112 |
| 2.47303204139582 | uchl5 (bg=11.16%) | K562 | - | chrX | | 73051085 | 73051109 |
| 2.49453039185279 | uchl5 (bg=11.16%) | K562 | - | chrX | | 73051087 | 73051106 |
| 2.60556170424154 | uchl5 (bg=11.16%) | K562 | - | chrX | | 73051106 | 73051109 |
| 3.09098853141178 | uchl5 (bg=11.16%) | K562 | - | chrX | | 73053072 | 73053081 |
| 2.79220952526715 | uchl5 (bg=11.16%) | K562 | - | chrX | | 73053072 | 73053137 |
| 2.63413085643831 | uchl5 (bg=11.16%) | K562 | - | chrX | | 73053081 | 73053136 |
| 2.349447310876 | ZNF622 (bg=6.58%) | K562 | - | chrX | | 73051038 | 73051084 |
| 2.74687865829069 | ZNF622 (bg=6.58%) | K562 | - | chrX | | 73051051 | 73051084 |
| 2.51092378651318 | ZNF622 (bg=6.58%) | K562 | - | chrX | | 73053070 | 73053151 |
| 2.3992003460731 | ZNF622 (bg=6.58%) | K562 | - | chrX | | 73053070 | 73053151 |

  
  

| Match 282 in HUMAN | | | | | | | |
| --- | --- | --- | --- | --- | --- | --- | --- |
| Motif | Start in Seq (1 Indexed) | End in Seq (1 Indexed) | Strand | Chrm | Exon | Start in Chrm (0 Indexed) | End in Chrm (1 Indexed) |
| CTGGAGAAAAAGATCT | 11562 | 11577 | - | chrX | 4 | 73051105 | 73051109 |
|  |  |  |  |  | 3 | 73053072 | 73053084 |
| eCLIP Fold-Enrichment | Binding Protein | Cell Line | Strand | Chrm | | Start in Chrm (0 Indexed) | End in Chrm (1 Indexed) |
| 2.0747697536265 | DDX24 (bg=2.97%) | K562 | - | chrX | | 73053072 | 73053171 |
| 2.11915613637667 | DDX24 (bg=2.97%) | K562 | - | chrX | | 73053072 | 73053212 |
| 2.92308210844434 | GRWD1 (bg=5.13%) | K562 | - | chrX | | 73053070 | 73053150 |
| 2.86354745699974 | GRWD1 (bg=5.13%) | K562 | - | chrX | | 73053071 | 73053150 |
| 2.3767502512271 | NIPBL (bg=5.39%) | K562 | - | chrX | | 73053072 | 73053097 |
| 2.54169595435271 | NOLC1 (bg=9.43%) | K562 | - | chrX | | 73051064 | 73051110 |
| 2.52833757149769 | NOLC1 (bg=9.43%) | K562 | - | chrX | | 73053072 | 73053151 |
| 2.60721182602904 | NOLC1 (bg=9.43%) | K562 | - | chrX | | 73053082 | 73053137 |
| 2.38401312393158 | SRSF1 (bg=8.47%) | K562 | - | chrX | | 73053073 | 73053121 |
| 2.51305976109129 | SRSF1 (bg=8.47%) | K562 | - | chrX | | 73053073 | 73053137 |
| 2.05145800436287 | SRSF7 (bg=2.32%) | K562 | - | chrX | | 73051035 | 73051110 |
| 2.17024503774193 | TARDBP (bg=2.79%) | K562 | - | chrX | | 73053071 | 73053112 |
| 2.47303204139582 | uchl5 (bg=11.16%) | K562 | - | chrX | | 73051085 | 73051109 |
| 2.49453039185279 | uchl5 (bg=11.16%) | K562 | - | chrX | | 73051087 | 73051106 |
| 2.60556170424154 | uchl5 (bg=11.16%) | K562 | - | chrX | | 73051106 | 73051109 |
| 3.09098853141178 | uchl5 (bg=11.16%) | K562 | - | chrX | | 73053072 | 73053081 |
| 2.79220952526715 | uchl5 (bg=11.16%) | K562 | - | chrX | | 73053072 | 73053137 |
| 2.63413085643831 | uchl5 (bg=11.16%) | K562 | - | chrX | | 73053081 | 73053136 |
| 2.51092378651318 | ZNF622 (bg=6.58%) | K562 | - | chrX | | 73053070 | 73053151 |
| 2.3992003460731 | ZNF622 (bg=6.58%) | K562 | - | chrX | | 73053070 | 73053151 |

  
  

| Match 283 in HUMAN | | | | | | | |
| --- | --- | --- | --- | --- | --- | --- | --- |
| Motif | Start in Seq (1 Indexed) | End in Seq (1 Indexed) | Strand | Chrm | Exon | Start in Chrm (0 Indexed) | End in Chrm (1 Indexed) |
| AAGAATAGGC | 11585 | 11594 | - | chrX | 4 | 73051088 | 73051098 |
| eCLIP Fold-Enrichment | Binding Protein | Cell Line | Strand | Chrm | | Start in Chrm (0 Indexed) | End in Chrm (1 Indexed) |
| 2.54169595435271 | NOLC1 (bg=9.43%) | K562 | - | chrX | | 73051064 | 73051110 |
| 2.05145800436287 | SRSF7 (bg=2.32%) | K562 | - | chrX | | 73051035 | 73051110 |
| 2.47303204139582 | uchl5 (bg=11.16%) | K562 | - | chrX | | 73051085 | 73051109 |
| 2.49453039185279 | uchl5 (bg=11.16%) | K562 | - | chrX | | 73051087 | 73051106 |

  
  

| Match 284 in HUMAN | | | | | | | |
| --- | --- | --- | --- | --- | --- | --- | --- |
| Motif | Start in Seq (1 Indexed) | End in Seq (1 Indexed) | Strand | Chrm | Exon | Start in Chrm (0 Indexed) | End in Chrm (1 Indexed) |
| TTACAGTGTTAGTGA | 11603 | 11617 | - | chrX | 4 | 73051065 | 73051080 |
| eCLIP Fold-Enrichment | Binding Protein | Cell Line | Strand | Chrm | | Start in Chrm (0 Indexed) | End in Chrm (1 Indexed) |
| 2.34226667414948 | ILF3 (bg=3.0%) | K562 | - | chrX | | 73051043 | 73051068 |
| 2.54169595435271 | NOLC1 (bg=9.43%) | K562 | - | chrX | | 73051064 | 73051110 |
| 2.05230849772113 | RBM15 (bg=7.27%) | K562 | - | chrX | | 73051040 | 73051067 |
| 3.41197398578366 | RBM15 (bg=7.27%) | K562 | - | chrX | | 73051053 | 73051066 |
| 2.87993861818952 | RBM15 (bg=7.27%) | K562 | - | chrX | | 73051066 | 73051079 |
| 2.05145800436287 | SRSF7 (bg=2.32%) | K562 | - | chrX | | 73051035 | 73051110 |
| 2.349447310876 | ZNF622 (bg=6.58%) | K562 | - | chrX | | 73051038 | 73051084 |
| 2.74687865829069 | ZNF622 (bg=6.58%) | K562 | - | chrX | | 73051051 | 73051084 |

  
  

| Match 285 in HUMAN | | | | | | | |
| --- | --- | --- | --- | --- | --- | --- | --- |
| Motif | Start in Seq (1 Indexed) | End in Seq (1 Indexed) | Strand | Chrm | Exon | Start in Chrm (0 Indexed) | End in Chrm (1 Indexed) |
| TACAGTGTTAGTGA | 11604 | 11617 | - | chrX | 4 | 73051065 | 73051079 |
| eCLIP Fold-Enrichment | Binding Protein | Cell Line | Strand | Chrm | | Start in Chrm (0 Indexed) | End in Chrm (1 Indexed) |
| 2.34226667414948 | ILF3 (bg=3.0%) | K562 | - | chrX | | 73051043 | 73051068 |
| 2.54169595435271 | NOLC1 (bg=9.43%) | K562 | - | chrX | | 73051064 | 73051110 |
| 2.05230849772113 | RBM15 (bg=7.27%) | K562 | - | chrX | | 73051040 | 73051067 |
| 3.41197398578366 | RBM15 (bg=7.27%) | K562 | - | chrX | | 73051053 | 73051066 |
| 2.87993861818952 | RBM15 (bg=7.27%) | K562 | - | chrX | | 73051066 | 73051079 |
| 2.05145800436287 | SRSF7 (bg=2.32%) | K562 | - | chrX | | 73051035 | 73051110 |
| 2.349447310876 | ZNF622 (bg=6.58%) | K562 | - | chrX | | 73051038 | 73051084 |
| 2.74687865829069 | ZNF622 (bg=6.58%) | K562 | - | chrX | | 73051051 | 73051084 |

  
  

| Match 286 in HUMAN | | | | | | | |
| --- | --- | --- | --- | --- | --- | --- | --- |
| Motif | Start in Seq (1 Indexed) | End in Seq (1 Indexed) | Strand | Chrm | Exon | Start in Chrm (0 Indexed) | End in Chrm (1 Indexed) |
| CATTCCCTTTGA | 11620 | 11631 | - | chrX | 4 | 73051051 | 73051063 |
| eCLIP Fold-Enrichment | Binding Protein | Cell Line | Strand | Chrm | | Start in Chrm (0 Indexed) | End in Chrm (1 Indexed) |
| 2.34226667414948 | ILF3 (bg=3.0%) | K562 | - | chrX | | 73051043 | 73051068 |
| 3.44930426385966 | RBM15 (bg=7.27%) | K562 | - | chrX | | 73051034 | 73051053 |
| 2.05230849772113 | RBM15 (bg=7.27%) | K562 | - | chrX | | 73051040 | 73051067 |
| 3.41197398578366 | RBM15 (bg=7.27%) | K562 | - | chrX | | 73051053 | 73051066 |
| 2.05145800436287 | SRSF7 (bg=2.32%) | K562 | - | chrX | | 73051035 | 73051110 |
| 2.349447310876 | ZNF622 (bg=6.58%) | K562 | - | chrX | | 73051038 | 73051084 |
| 2.74687865829069 | ZNF622 (bg=6.58%) | K562 | - | chrX | | 73051051 | 73051084 |

  
  

| Match 287 in HUMAN | | | | | | | |
| --- | --- | --- | --- | --- | --- | --- | --- |
| Motif | Start in Seq (1 Indexed) | End in Seq (1 Indexed) | Strand | Chrm | Exon | Start in Chrm (0 Indexed) | End in Chrm (1 Indexed) |
| TTCCCTTTGA | 11622 | 11631 | - | chrX | 4 | 73051051 | 73051061 |
| eCLIP Fold-Enrichment | Binding Protein | Cell Line | Strand | Chrm | | Start in Chrm (0 Indexed) | End in Chrm (1 Indexed) |
| 2.34226667414948 | ILF3 (bg=3.0%) | K562 | - | chrX | | 73051043 | 73051068 |
| 3.44930426385966 | RBM15 (bg=7.27%) | K562 | - | chrX | | 73051034 | 73051053 |
| 2.05230849772113 | RBM15 (bg=7.27%) | K562 | - | chrX | | 73051040 | 73051067 |
| 3.41197398578366 | RBM15 (bg=7.27%) | K562 | - | chrX | | 73051053 | 73051066 |
| 2.05145800436287 | SRSF7 (bg=2.32%) | K562 | - | chrX | | 73051035 | 73051110 |
| 2.349447310876 | ZNF622 (bg=6.58%) | K562 | - | chrX | | 73051038 | 73051084 |
| 2.74687865829069 | ZNF622 (bg=6.58%) | K562 | - | chrX | | 73051051 | 73051084 |

  
  

| Match 288 in HUMAN | | | | | | | |
| --- | --- | --- | --- | --- | --- | --- | --- |
| Motif | Start in Seq (1 Indexed) | End in Seq (1 Indexed) | Strand | Chrm | Exon | Start in Chrm (0 Indexed) | End in Chrm (1 Indexed) |
| TAGGTGGAGATGGGGCATGAGGATCCTCCAGGGGAAAAGCTCACTACCACTGGGCAACAACCCTAGGTCAGGAG | 11639 | 11712 | - | chrX | 4 | 73050970 | 73051044 |
| eCLIP Fold-Enrichment | Binding Protein | Cell Line | Strand | Chrm | | Start in Chrm (0 Indexed) | End in Chrm (1 Indexed) |
| 4.02762546348455 | ILF3 (bg=3.0%) | K562 | - | chrX | | 73050932 | 73050986 |
| 4.76631206279508 | ILF3 (bg=3.0%) | K562 | - | chrX | | 73050939 | 73051043 |
| 4.36642737693631 | ILF3 (bg=3.0%) | K562 | - | chrX | | 73050986 | 73051048 |
| 2.34226667414948 | ILF3 (bg=3.0%) | K562 | - | chrX | | 73051043 | 73051068 |
| 2.23135583374055 | NOLC1 (bg=9.43%) | K562 | - | chrX | | 73051026 | 73051045 |
| 3.44930426385966 | RBM15 (bg=7.27%) | K562 | - | chrX | | 73051034 | 73051053 |
| 2.05230849772113 | RBM15 (bg=7.27%) | K562 | - | chrX | | 73051040 | 73051067 |
| 2.05145800436287 | SRSF7 (bg=2.32%) | K562 | - | chrX | | 73051035 | 73051110 |
| 2.349447310876 | ZNF622 (bg=6.58%) | K562 | - | chrX | | 73051038 | 73051084 |

  
  

| Match 289 in HUMAN | | | | | | | |
| --- | --- | --- | --- | --- | --- | --- | --- |
| Motif | Start in Seq (1 Indexed) | End in Seq (1 Indexed) | Strand | Chrm | Exon | Start in Chrm (0 Indexed) | End in Chrm (1 Indexed) |
| TAGGTGGAGATGGGGCATGAGGATCCTCCAGGGGAAA | 11639 | 11675 | - | chrX | 4 | 73051007 | 73051044 |
| eCLIP Fold-Enrichment | Binding Protein | Cell Line | Strand | Chrm | | Start in Chrm (0 Indexed) | End in Chrm (1 Indexed) |
| 4.76631206279508 | ILF3 (bg=3.0%) | K562 | - | chrX | | 73050939 | 73051043 |
| 4.36642737693631 | ILF3 (bg=3.0%) | K562 | - | chrX | | 73050986 | 73051048 |
| 2.34226667414948 | ILF3 (bg=3.0%) | K562 | - | chrX | | 73051043 | 73051068 |
| 2.23135583374055 | NOLC1 (bg=9.43%) | K562 | - | chrX | | 73051026 | 73051045 |
| 3.44930426385966 | RBM15 (bg=7.27%) | K562 | - | chrX | | 73051034 | 73051053 |
| 2.05230849772113 | RBM15 (bg=7.27%) | K562 | - | chrX | | 73051040 | 73051067 |
| 2.05145800436287 | SRSF7 (bg=2.32%) | K562 | - | chrX | | 73051035 | 73051110 |
| 2.349447310876 | ZNF622 (bg=6.58%) | K562 | - | chrX | | 73051038 | 73051084 |

  
  

| Match 290 in HUMAN | | | | | | | |
| --- | --- | --- | --- | --- | --- | --- | --- |
| Motif | Start in Seq (1 Indexed) | End in Seq (1 Indexed) | Strand | Chrm | Exon | Start in Chrm (0 Indexed) | End in Chrm (1 Indexed) |
| TAGGTGGAGATGGGGCATGAGGATCCTCCAGGGGAA | 11639 | 11674 | - | chrX | 4 | 73051008 | 73051044 |
| eCLIP Fold-Enrichment | Binding Protein | Cell Line | Strand | Chrm | | Start in Chrm (0 Indexed) | End in Chrm (1 Indexed) |
| 4.76631206279508 | ILF3 (bg=3.0%) | K562 | - | chrX | | 73050939 | 73051043 |
| 4.36642737693631 | ILF3 (bg=3.0%) | K562 | - | chrX | | 73050986 | 73051048 |
| 2.34226667414948 | ILF3 (bg=3.0%) | K562 | - | chrX | | 73051043 | 73051068 |
| 2.23135583374055 | NOLC1 (bg=9.43%) | K562 | - | chrX | | 73051026 | 73051045 |
| 3.44930426385966 | RBM15 (bg=7.27%) | K562 | - | chrX | | 73051034 | 73051053 |
| 2.05230849772113 | RBM15 (bg=7.27%) | K562 | - | chrX | | 73051040 | 73051067 |
| 2.05145800436287 | SRSF7 (bg=2.32%) | K562 | - | chrX | | 73051035 | 73051110 |
| 2.349447310876 | ZNF622 (bg=6.58%) | K562 | - | chrX | | 73051038 | 73051084 |

  
  

| Match 291 in HUMAN | | | | | | | |
| --- | --- | --- | --- | --- | --- | --- | --- |
| Motif | Start in Seq (1 Indexed) | End in Seq (1 Indexed) | Strand | Chrm | Exon | Start in Chrm (0 Indexed) | End in Chrm (1 Indexed) |
| TCACTACCACTG | 11679 | 11690 | - | chrX | 4 | 73050992 | 73051004 |
| eCLIP Fold-Enrichment | Binding Protein | Cell Line | Strand | Chrm | | Start in Chrm (0 Indexed) | End in Chrm (1 Indexed) |
| 4.76631206279508 | ILF3 (bg=3.0%) | K562 | - | chrX | | 73050939 | 73051043 |
| 4.36642737693631 | ILF3 (bg=3.0%) | K562 | - | chrX | | 73050986 | 73051048 |

  
  

| Match 292 in HUMAN | | | | | | | |
| --- | --- | --- | --- | --- | --- | --- | --- |
| Motif | Start in Seq (1 Indexed) | End in Seq (1 Indexed) | Strand | Chrm | Exon | Start in Chrm (0 Indexed) | End in Chrm (1 Indexed) |
| TCACTACCACT | 11679 | 11689 | - | chrX | 4 | 73050993 | 73051004 |
| eCLIP Fold-Enrichment | Binding Protein | Cell Line | Strand | Chrm | | Start in Chrm (0 Indexed) | End in Chrm (1 Indexed) |
| 4.76631206279508 | ILF3 (bg=3.0%) | K562 | - | chrX | | 73050939 | 73051043 |
| 4.36642737693631 | ILF3 (bg=3.0%) | K562 | - | chrX | | 73050986 | 73051048 |

  
  

| Match 293 in HUMAN | | | | | | | |
| --- | --- | --- | --- | --- | --- | --- | --- |
| Motif | Start in Seq (1 Indexed) | End in Seq (1 Indexed) | Strand | Chrm | Exon | Start in Chrm (0 Indexed) | End in Chrm (1 Indexed) |
| TCACTA | 11679 | 11684 | - | chrX | 4 | 73050998 | 73051004 |
| eCLIP Fold-Enrichment | Binding Protein | Cell Line | Strand | Chrm | | Start in Chrm (0 Indexed) | End in Chrm (1 Indexed) |
| 4.76631206279508 | ILF3 (bg=3.0%) | K562 | - | chrX | | 73050939 | 73051043 |
| 4.36642737693631 | ILF3 (bg=3.0%) | K562 | - | chrX | | 73050986 | 73051048 |

  
  

| Match 294 in HUMAN | | | | | | | |
| --- | --- | --- | --- | --- | --- | --- | --- |
| Motif | Start in Seq (1 Indexed) | End in Seq (1 Indexed) | Strand | Chrm | Exon | Start in Chrm (0 Indexed) | End in Chrm (1 Indexed) |
| GCAACAAC | 11692 | 11699 | - | chrX | 4 | 73050983 | 73050991 |
| eCLIP Fold-Enrichment | Binding Protein | Cell Line | Strand | Chrm | | Start in Chrm (0 Indexed) | End in Chrm (1 Indexed) |
| 4.02762546348455 | ILF3 (bg=3.0%) | K562 | - | chrX | | 73050932 | 73050986 |
| 4.76631206279508 | ILF3 (bg=3.0%) | K562 | - | chrX | | 73050939 | 73051043 |
| 4.36642737693631 | ILF3 (bg=3.0%) | K562 | - | chrX | | 73050986 | 73051048 |

  
  

| Match 295 in HUMAN | | | | | | | |
| --- | --- | --- | --- | --- | --- | --- | --- |
| Motif | Start in Seq (1 Indexed) | End in Seq (1 Indexed) | Strand | Chrm | Exon | Start in Chrm (0 Indexed) | End in Chrm (1 Indexed) |
| GCAACA | 11692 | 11697 | - | chrX | 4 | 73050985 | 73050991 |
| eCLIP Fold-Enrichment | Binding Protein | Cell Line | Strand | Chrm | | Start in Chrm (0 Indexed) | End in Chrm (1 Indexed) |
| 4.02762546348455 | ILF3 (bg=3.0%) | K562 | - | chrX | | 73050932 | 73050986 |
| 4.76631206279508 | ILF3 (bg=3.0%) | K562 | - | chrX | | 73050939 | 73051043 |
| 4.36642737693631 | ILF3 (bg=3.0%) | K562 | - | chrX | | 73050986 | 73051048 |

  
  

| Match 296 in HUMAN | | | | | | | |
| --- | --- | --- | --- | --- | --- | --- | --- |
| Motif | Start in Seq (1 Indexed) | End in Seq (1 Indexed) | Strand | Chrm | Exon | Start in Chrm (0 Indexed) | End in Chrm (1 Indexed) |
| CTTTCCTGG | 11727 | 11735 | - | chrX | 4 | 73050947 | 73050956 |
| eCLIP Fold-Enrichment | Binding Protein | Cell Line | Strand | Chrm | | Start in Chrm (0 Indexed) | End in Chrm (1 Indexed) |
| 4.02762546348455 | ILF3 (bg=3.0%) | K562 | - | chrX | | 73050932 | 73050986 |
| 4.76631206279508 | ILF3 (bg=3.0%) | K562 | - | chrX | | 73050939 | 73051043 |

  
  

| Match 297 in HUMAN | | | | | | | |
| --- | --- | --- | --- | --- | --- | --- | --- |
| Motif | Start in Seq (1 Indexed) | End in Seq (1 Indexed) | Strand | Chrm | Exon | Start in Chrm (0 Indexed) | End in Chrm (1 Indexed) |
| CCAGATAGGAAGAT | 11738 | 11751 | - | chrX | 4 | 73050931 | 73050945 |
| eCLIP Fold-Enrichment | Binding Protein | Cell Line | Strand | Chrm | | Start in Chrm (0 Indexed) | End in Chrm (1 Indexed) |
| 4.02762546348455 | ILF3 (bg=3.0%) | K562 | - | chrX | | 73050932 | 73050986 |
| 4.76631206279508 | ILF3 (bg=3.0%) | K562 | - | chrX | | 73050939 | 73051043 |

  
  

| Match 298 in HUMAN | | | | | | | |
| --- | --- | --- | --- | --- | --- | --- | --- |
| Motif | Start in Seq (1 Indexed) | End in Seq (1 Indexed) | Strand | Chrm | Exon | Start in Chrm (0 Indexed) | End in Chrm (1 Indexed) |
| ACAACCACCACAC | 11764 | 11776 | - | chrX | 4 | 73050906 | 73050919 |
| eCLIP Fold-Enrichment | Binding Protein | Cell Line | Strand | Chrm | | Start in Chrm (0 Indexed) | End in Chrm (1 Indexed) |
| 2.017422207226 | PRPF8 (bg=0.26%) | K562 | - | chrX | | 73050867 | 73050918 |

  
  

| Match 299 in HUMAN | | | | | | | |
| --- | --- | --- | --- | --- | --- | --- | --- |
| Motif | Start in Seq (1 Indexed) | End in Seq (1 Indexed) | Strand | Chrm | Exon | Start in Chrm (0 Indexed) | End in Chrm (1 Indexed) |
| ACAACCACC | 11764 | 11772 | - | chrX | 4 | 73050910 | 73050919 |
| eCLIP Fold-Enrichment | Binding Protein | Cell Line | Strand | Chrm | | Start in Chrm (0 Indexed) | End in Chrm (1 Indexed) |
| 2.017422207226 | PRPF8 (bg=0.26%) | K562 | - | chrX | | 73050867 | 73050918 |

  
  

| Match 300 in HUMAN | | | | | | | |
| --- | --- | --- | --- | --- | --- | --- | --- |
| Motif | Start in Seq (1 Indexed) | End in Seq (1 Indexed) | Strand | Chrm | Exon | Start in Chrm (0 Indexed) | End in Chrm (1 Indexed) |
| ATTGTTCC | 11789 | 11796 | - | chrX | 5 | 73049052 | 73049060 |
| eCLIP Fold-Enrichment | Binding Protein | Cell Line | Strand | Chrm | | Start in Chrm (0 Indexed) | End in Chrm (1 Indexed) |
| 2.36613398389279 | GRWD1 (bg=5.13%) | K562 | - | chrX | | 73048899 | 73049056 |
| 2.34750786900359 | SF3B4 (bg=0.05%) | K562 | - | chrX | | 73049056 | 73049125 |

  
  

| Match 301 in HUMAN | | | | | | | |
| --- | --- | --- | --- | --- | --- | --- | --- |
| Motif | Start in Seq (1 Indexed) | End in Seq (1 Indexed) | Strand | Chrm | Exon | Start in Chrm (0 Indexed) | End in Chrm (1 Indexed) |
| TTGTTCC | 11790 | 11796 | - | chrX | 5 | 73049052 | 73049059 |
| eCLIP Fold-Enrichment | Binding Protein | Cell Line | Strand | Chrm | | Start in Chrm (0 Indexed) | End in Chrm (1 Indexed) |
| 2.36613398389279 | GRWD1 (bg=5.13%) | K562 | - | chrX | | 73048899 | 73049056 |
| 2.34750786900359 | SF3B4 (bg=0.05%) | K562 | - | chrX | | 73049056 | 73049125 |

  
  

| Match 302 in HUMAN | | | | | | | |
| --- | --- | --- | --- | --- | --- | --- | --- |
| Motif | Start in Seq (1 Indexed) | End in Seq (1 Indexed) | Strand | Chrm | Exon | Start in Chrm (0 Indexed) | End in Chrm (1 Indexed) |
| TGCCAAATC | 11801 | 11809 | - | chrX | 5 | 73049039 | 73049048 |
| eCLIP Fold-Enrichment | Binding Protein | Cell Line | Strand | Chrm | | Start in Chrm (0 Indexed) | End in Chrm (1 Indexed) |
| 2.36613398389279 | GRWD1 (bg=5.13%) | K562 | - | chrX | | 73048899 | 73049056 |
| 2.21199050887362 | NOLC1 (bg=9.43%) | K562 | - | chrX | | 73048935 | 73049049 |

  
  

| Match 303 in HUMAN | | | | | | | |
| --- | --- | --- | --- | --- | --- | --- | --- |
| Motif | Start in Seq (1 Indexed) | End in Seq (1 Indexed) | Strand | Chrm | Exon | Start in Chrm (0 Indexed) | End in Chrm (1 Indexed) |
| CCAAATC | 11803 | 11809 | - | chrX | 5 | 73049039 | 73049046 |
| eCLIP Fold-Enrichment | Binding Protein | Cell Line | Strand | Chrm | | Start in Chrm (0 Indexed) | End in Chrm (1 Indexed) |
| 2.36613398389279 | GRWD1 (bg=5.13%) | K562 | - | chrX | | 73048899 | 73049056 |
| 2.21199050887362 | NOLC1 (bg=9.43%) | K562 | - | chrX | | 73048935 | 73049049 |

  
  

| Match 304 in HUMAN | | | | | | | |
| --- | --- | --- | --- | --- | --- | --- | --- |
| Motif | Start in Seq (1 Indexed) | End in Seq (1 Indexed) | Strand | Chrm | Exon | Start in Chrm (0 Indexed) | End in Chrm (1 Indexed) |
| CCAAAT | 11803 | 11808 | - | chrX | 5 | 73049040 | 73049046 |
| eCLIP Fold-Enrichment | Binding Protein | Cell Line | Strand | Chrm | | Start in Chrm (0 Indexed) | End in Chrm (1 Indexed) |
| 2.36613398389279 | GRWD1 (bg=5.13%) | K562 | - | chrX | | 73048899 | 73049056 |
| 2.21199050887362 | NOLC1 (bg=9.43%) | K562 | - | chrX | | 73048935 | 73049049 |

  
  

| Match 305 in HUMAN | | | | | | | |
| --- | --- | --- | --- | --- | --- | --- | --- |
| Motif | Start in Seq (1 Indexed) | End in Seq (1 Indexed) | Strand | Chrm | Exon | Start in Chrm (0 Indexed) | End in Chrm (1 Indexed) |
| AAGCAGTG | 11824 | 11831 | - | chrX | 5 | 73049017 | 73049025 |
| eCLIP Fold-Enrichment | Binding Protein | Cell Line | Strand | Chrm | | Start in Chrm (0 Indexed) | End in Chrm (1 Indexed) |
| 2.36613398389279 | GRWD1 (bg=5.13%) | K562 | - | chrX | | 73048899 | 73049056 |
| 2.21199050887362 | NOLC1 (bg=9.43%) | K562 | - | chrX | | 73048935 | 73049049 |
| 2.21893076033204 | uchl5 (bg=11.16%) | K562 | - | chrX | | 73048993 | 73049037 |

  
  

| Match 306 in HUMAN | | | | | | | |
| --- | --- | --- | --- | --- | --- | --- | --- |
| Motif | Start in Seq (1 Indexed) | End in Seq (1 Indexed) | Strand | Chrm | Exon | Start in Chrm (0 Indexed) | End in Chrm (1 Indexed) |
| AGAGAG | 11833 | 11838 | - | chrX | 5 | 73049010 | 73049016 |
| eCLIP Fold-Enrichment | Binding Protein | Cell Line | Strand | Chrm | | Start in Chrm (0 Indexed) | End in Chrm (1 Indexed) |
| 2.36613398389279 | GRWD1 (bg=5.13%) | K562 | - | chrX | | 73048899 | 73049056 |
| 2.21199050887362 | NOLC1 (bg=9.43%) | K562 | - | chrX | | 73048935 | 73049049 |
| 2.21893076033204 | uchl5 (bg=11.16%) | K562 | - | chrX | | 73048993 | 73049037 |
| 2.56606534070564 | ZNF622 (bg=6.58%) | K562 | - | chrX | | 73048986 | 73049010 |

  
  

| Match 307 in HUMAN | | | | | | | |
| --- | --- | --- | --- | --- | --- | --- | --- |
| Motif | Start in Seq (1 Indexed) | End in Seq (1 Indexed) | Strand | Chrm | Exon | Start in Chrm (0 Indexed) | End in Chrm (1 Indexed) |
| CAAGAAATTTGAACACAC | 11857 | 11874 | - | chrX | 5 | 73048974 | 73048992 |
| eCLIP Fold-Enrichment | Binding Protein | Cell Line | Strand | Chrm | | Start in Chrm (0 Indexed) | End in Chrm (1 Indexed) |
| 2.36613398389279 | GRWD1 (bg=5.13%) | K562 | - | chrX | | 73048899 | 73049056 |
| 2.21199050887362 | NOLC1 (bg=9.43%) | K562 | - | chrX | | 73048935 | 73049049 |
| 2.38387808129389 | PTBP1 (bg=3.74%) | K562 | - | chrX | | 73048932 | 73048979 |
| 2.03242669966347 | PTBP1 (bg=3.74%) | K562 | - | chrX | | 73048951 | 73048977 |
| 2.69065209477031 | RBM15 (bg=7.27%) | K562 | - | chrX | | 73048937 | 73048978 |
| 2.27665581564309 | TRA2A (bg=4.8%) | K562 | - | chrX | | 73048904 | 73048984 |
| 2.48149362014402 | uchl5 (bg=11.16%) | K562 | - | chrX | | 73048925 | 73048993 |
| 2.08360900104618 | uchl5 (bg=11.16%) | K562 | - | chrX | | 73048927 | 73048985 |
| 2.51092378651318 | ZNF622 (bg=6.58%) | K562 | - | chrX | | 73048915 | 73048986 |
| 2.56606534070564 | ZNF622 (bg=6.58%) | K562 | - | chrX | | 73048986 | 73049010 |

  
  

| Match 308 in HUMAN | | | | | | | |
| --- | --- | --- | --- | --- | --- | --- | --- |
| Motif | Start in Seq (1 Indexed) | End in Seq (1 Indexed) | Strand | Chrm | Exon | Start in Chrm (0 Indexed) | End in Chrm (1 Indexed) |
| CAAGAAAT | 11857 | 11864 | - | chrX | 5 | 73048984 | 73048992 |
| eCLIP Fold-Enrichment | Binding Protein | Cell Line | Strand | Chrm | | Start in Chrm (0 Indexed) | End in Chrm (1 Indexed) |
| 2.36613398389279 | GRWD1 (bg=5.13%) | K562 | - | chrX | | 73048899 | 73049056 |
| 2.21199050887362 | NOLC1 (bg=9.43%) | K562 | - | chrX | | 73048935 | 73049049 |
| 2.27665581564309 | TRA2A (bg=4.8%) | K562 | - | chrX | | 73048904 | 73048984 |
| 2.48149362014402 | uchl5 (bg=11.16%) | K562 | - | chrX | | 73048925 | 73048993 |
| 2.08360900104618 | uchl5 (bg=11.16%) | K562 | - | chrX | | 73048927 | 73048985 |
| 2.51092378651318 | ZNF622 (bg=6.58%) | K562 | - | chrX | | 73048915 | 73048986 |
| 2.56606534070564 | ZNF622 (bg=6.58%) | K562 | - | chrX | | 73048986 | 73049010 |

  
  

| Match 309 in HUMAN | | | | | | | |
| --- | --- | --- | --- | --- | --- | --- | --- |
| Motif | Start in Seq (1 Indexed) | End in Seq (1 Indexed) | Strand | Chrm | Exon | Start in Chrm (0 Indexed) | End in Chrm (1 Indexed) |
| CAAGAAA | 11857 | 11863 | - | chrX | 5 | 73048985 | 73048992 |
| eCLIP Fold-Enrichment | Binding Protein | Cell Line | Strand | Chrm | | Start in Chrm (0 Indexed) | End in Chrm (1 Indexed) |
| 2.36613398389279 | GRWD1 (bg=5.13%) | K562 | - | chrX | | 73048899 | 73049056 |
| 2.21199050887362 | NOLC1 (bg=9.43%) | K562 | - | chrX | | 73048935 | 73049049 |
| 2.48149362014402 | uchl5 (bg=11.16%) | K562 | - | chrX | | 73048925 | 73048993 |
| 2.08360900104618 | uchl5 (bg=11.16%) | K562 | - | chrX | | 73048927 | 73048985 |
| 2.51092378651318 | ZNF622 (bg=6.58%) | K562 | - | chrX | | 73048915 | 73048986 |
| 2.56606534070564 | ZNF622 (bg=6.58%) | K562 | - | chrX | | 73048986 | 73049010 |

  
  

| Match 310 in HUMAN | | | | | | | |
| --- | --- | --- | --- | --- | --- | --- | --- |
| Motif | Start in Seq (1 Indexed) | End in Seq (1 Indexed) | Strand | Chrm | Exon | Start in Chrm (0 Indexed) | End in Chrm (1 Indexed) |
| TGAACACAC | 11866 | 11874 | - | chrX | 5 | 73048974 | 73048983 |
| eCLIP Fold-Enrichment | Binding Protein | Cell Line | Strand | Chrm | | Start in Chrm (0 Indexed) | End in Chrm (1 Indexed) |
| 2.36613398389279 | GRWD1 (bg=5.13%) | K562 | - | chrX | | 73048899 | 73049056 |
| 2.21199050887362 | NOLC1 (bg=9.43%) | K562 | - | chrX | | 73048935 | 73049049 |
| 2.38387808129389 | PTBP1 (bg=3.74%) | K562 | - | chrX | | 73048932 | 73048979 |
| 2.03242669966347 | PTBP1 (bg=3.74%) | K562 | - | chrX | | 73048951 | 73048977 |
| 2.69065209477031 | RBM15 (bg=7.27%) | K562 | - | chrX | | 73048937 | 73048978 |
| 2.27665581564309 | TRA2A (bg=4.8%) | K562 | - | chrX | | 73048904 | 73048984 |
| 2.48149362014402 | uchl5 (bg=11.16%) | K562 | - | chrX | | 73048925 | 73048993 |
| 2.08360900104618 | uchl5 (bg=11.16%) | K562 | - | chrX | | 73048927 | 73048985 |
| 2.51092378651318 | ZNF622 (bg=6.58%) | K562 | - | chrX | | 73048915 | 73048986 |

  
  

| Match 311 in HUMAN | | | | | | | |
| --- | --- | --- | --- | --- | --- | --- | --- |
| Motif | Start in Seq (1 Indexed) | End in Seq (1 Indexed) | Strand | Chrm | Exon | Start in Chrm (0 Indexed) | End in Chrm (1 Indexed) |
| GAAGATCAACATGCCTGGC | 11899 | 11917 | - | chrX | 5 | 73048931 | 73048950 |
| eCLIP Fold-Enrichment | Binding Protein | Cell Line | Strand | Chrm | | Start in Chrm (0 Indexed) | End in Chrm (1 Indexed) |
| 2.36613398389279 | GRWD1 (bg=5.13%) | K562 | - | chrX | | 73048899 | 73049056 |
| 2.21199050887362 | NOLC1 (bg=9.43%) | K562 | - | chrX | | 73048935 | 73049049 |
| 2.38387808129389 | PTBP1 (bg=3.74%) | K562 | - | chrX | | 73048932 | 73048979 |
| 2.69065209477031 | RBM15 (bg=7.27%) | K562 | - | chrX | | 73048937 | 73048978 |
| 2.30078173914957 | RBM15 (bg=7.27%) | K562 | - | chrX | | 73048939 | 73048973 |
| 2.27665581564309 | TRA2A (bg=4.8%) | K562 | - | chrX | | 73048904 | 73048984 |
| 2.48149362014402 | uchl5 (bg=11.16%) | K562 | - | chrX | | 73048925 | 73048993 |
| 2.08360900104618 | uchl5 (bg=11.16%) | K562 | - | chrX | | 73048927 | 73048985 |
| 2.51092378651318 | ZNF622 (bg=6.58%) | K562 | - | chrX | | 73048915 | 73048986 |

  
  

| Match 312 in HUMAN | | | | | | | |
| --- | --- | --- | --- | --- | --- | --- | --- |
| Motif | Start in Seq (1 Indexed) | End in Seq (1 Indexed) | Strand | Chrm | Exon | Start in Chrm (0 Indexed) | End in Chrm (1 Indexed) |
| GAAGATCAACATGCCTG | 11899 | 11915 | - | chrX | 5 | 73048933 | 73048950 |
| eCLIP Fold-Enrichment | Binding Protein | Cell Line | Strand | Chrm | | Start in Chrm (0 Indexed) | End in Chrm (1 Indexed) |
| 2.36613398389279 | GRWD1 (bg=5.13%) | K562 | - | chrX | | 73048899 | 73049056 |
| 2.21199050887362 | NOLC1 (bg=9.43%) | K562 | - | chrX | | 73048935 | 73049049 |
| 2.38387808129389 | PTBP1 (bg=3.74%) | K562 | - | chrX | | 73048932 | 73048979 |
| 2.69065209477031 | RBM15 (bg=7.27%) | K562 | - | chrX | | 73048937 | 73048978 |
| 2.30078173914957 | RBM15 (bg=7.27%) | K562 | - | chrX | | 73048939 | 73048973 |
| 2.27665581564309 | TRA2A (bg=4.8%) | K562 | - | chrX | | 73048904 | 73048984 |
| 2.48149362014402 | uchl5 (bg=11.16%) | K562 | - | chrX | | 73048925 | 73048993 |
| 2.08360900104618 | uchl5 (bg=11.16%) | K562 | - | chrX | | 73048927 | 73048985 |
| 2.51092378651318 | ZNF622 (bg=6.58%) | K562 | - | chrX | | 73048915 | 73048986 |

  
  

| Match 313 in HUMAN | | | | | | | |
| --- | --- | --- | --- | --- | --- | --- | --- |
| Motif | Start in Seq (1 Indexed) | End in Seq (1 Indexed) | Strand | Chrm | Exon | Start in Chrm (0 Indexed) | End in Chrm (1 Indexed) |
| AAGATCAACATGC | 11900 | 11912 | - | chrX | 5 | 73048936 | 73048949 |
| eCLIP Fold-Enrichment | Binding Protein | Cell Line | Strand | Chrm | | Start in Chrm (0 Indexed) | End in Chrm (1 Indexed) |
| 2.36613398389279 | GRWD1 (bg=5.13%) | K562 | - | chrX | | 73048899 | 73049056 |
| 2.21199050887362 | NOLC1 (bg=9.43%) | K562 | - | chrX | | 73048935 | 73049049 |
| 2.38387808129389 | PTBP1 (bg=3.74%) | K562 | - | chrX | | 73048932 | 73048979 |
| 2.69065209477031 | RBM15 (bg=7.27%) | K562 | - | chrX | | 73048937 | 73048978 |
| 2.30078173914957 | RBM15 (bg=7.27%) | K562 | - | chrX | | 73048939 | 73048973 |
| 2.27665581564309 | TRA2A (bg=4.8%) | K562 | - | chrX | | 73048904 | 73048984 |
| 2.48149362014402 | uchl5 (bg=11.16%) | K562 | - | chrX | | 73048925 | 73048993 |
| 2.08360900104618 | uchl5 (bg=11.16%) | K562 | - | chrX | | 73048927 | 73048985 |
| 2.51092378651318 | ZNF622 (bg=6.58%) | K562 | - | chrX | | 73048915 | 73048986 |

  
  

| Match 314 in HUMAN | | | | | | | |
| --- | --- | --- | --- | --- | --- | --- | --- |
| Motif | Start in Seq (1 Indexed) | End in Seq (1 Indexed) | Strand | Chrm | Exon | Start in Chrm (0 Indexed) | End in Chrm (1 Indexed) |
| GATCAACATGC | 11902 | 11912 | - | chrX | 5 | 73048936 | 73048947 |
| eCLIP Fold-Enrichment | Binding Protein | Cell Line | Strand | Chrm | | Start in Chrm (0 Indexed) | End in Chrm (1 Indexed) |
| 2.36613398389279 | GRWD1 (bg=5.13%) | K562 | - | chrX | | 73048899 | 73049056 |
| 2.21199050887362 | NOLC1 (bg=9.43%) | K562 | - | chrX | | 73048935 | 73049049 |
| 2.38387808129389 | PTBP1 (bg=3.74%) | K562 | - | chrX | | 73048932 | 73048979 |
| 2.69065209477031 | RBM15 (bg=7.27%) | K562 | - | chrX | | 73048937 | 73048978 |
| 2.30078173914957 | RBM15 (bg=7.27%) | K562 | - | chrX | | 73048939 | 73048973 |
| 2.27665581564309 | TRA2A (bg=4.8%) | K562 | - | chrX | | 73048904 | 73048984 |
| 2.48149362014402 | uchl5 (bg=11.16%) | K562 | - | chrX | | 73048925 | 73048993 |
| 2.08360900104618 | uchl5 (bg=11.16%) | K562 | - | chrX | | 73048927 | 73048985 |
| 2.51092378651318 | ZNF622 (bg=6.58%) | K562 | - | chrX | | 73048915 | 73048986 |

  
  

| Match 315 in HUMAN | | | | | | | |
| --- | --- | --- | --- | --- | --- | --- | --- |
| Motif | Start in Seq (1 Indexed) | End in Seq (1 Indexed) | Strand | Chrm | Exon | Start in Chrm (0 Indexed) | End in Chrm (1 Indexed) |
| TGAATGA | 11939 | 11945 | - | chrX | 5 | 73048903 | 73048910 |
| eCLIP Fold-Enrichment | Binding Protein | Cell Line | Strand | Chrm | | Start in Chrm (0 Indexed) | End in Chrm (1 Indexed) |
| 5.86513418132423 | AQR (bg=0.33%) | K562 | - | chrX | | 73048842 | 73048903 |
| 6.55204642332421 | AQR (bg=0.33%) | K562 | - | chrX | | 73048850 | 73048905 |
| 2.36613398389279 | GRWD1 (bg=5.13%) | K562 | - | chrX | | 73048899 | 73049056 |
| 2.27665581564309 | TRA2A (bg=4.8%) | K562 | - | chrX | | 73048904 | 73048984 |

  
  

| Match 316 in HUMAN | | | | | | | |
| --- | --- | --- | --- | --- | --- | --- | --- |
| Motif | Start in Seq (1 Indexed) | End in Seq (1 Indexed) | Strand | Chrm | Exon | Start in Chrm (0 Indexed) | End in Chrm (1 Indexed) |
| TGTGTATTT | 11947 | 11955 | - | chrX | 6 | 73047810 | 73047819 |
| eCLIP Fold-Enrichment | Binding Protein | Cell Line | Strand | Chrm | | Start in Chrm (0 Indexed) | End in Chrm (1 Indexed) |
| 2.58278226693842 | TARDBP (bg=2.79%) | K562 | - | chrX | | 73047682 | 73047819 |
| 2.38044667445932 | ZC3H11A (bg=6.55%) | K562 | - | chrX | | 73047693 | 73047813 |

  
  

| Match 317 in HUMAN | | | | | | | |
| --- | --- | --- | --- | --- | --- | --- | --- |
| Motif | Start in Seq (1 Indexed) | End in Seq (1 Indexed) | Strand | Chrm | Exon | Start in Chrm (0 Indexed) | End in Chrm (1 Indexed) |
| TGTGTAT | 11947 | 11953 | - | chrX | 6 | 73047812 | 73047819 |
| eCLIP Fold-Enrichment | Binding Protein | Cell Line | Strand | Chrm | | Start in Chrm (0 Indexed) | End in Chrm (1 Indexed) |
| 2.58278226693842 | TARDBP (bg=2.79%) | K562 | - | chrX | | 73047682 | 73047819 |
| 2.38044667445932 | ZC3H11A (bg=6.55%) | K562 | - | chrX | | 73047693 | 73047813 |

  
  

| Match 318 in HUMAN | | | | | | | |
| --- | --- | --- | --- | --- | --- | --- | --- |
| Motif | Start in Seq (1 Indexed) | End in Seq (1 Indexed) | Strand | Chrm | Exon | Start in Chrm (0 Indexed) | End in Chrm (1 Indexed) |
| TTGTCTCTTTCTTTCTT | 11958 | 11974 | - | chrX | 6 | 73047791 | 73047808 |
| eCLIP Fold-Enrichment | Binding Protein | Cell Line | Strand | Chrm | | Start in Chrm (0 Indexed) | End in Chrm (1 Indexed) |
| 5.26124539015935 | PTBP1 (bg=3.74%) | K562 | - | chrX | | 73047730 | 73047792 |
| 5.37736477433521 | PTBP1 (bg=3.74%) | K562 | - | chrX | | 73047738 | 73047793 |
| 2.58278226693842 | TARDBP (bg=2.79%) | K562 | - | chrX | | 73047682 | 73047819 |
| 2.38044667445932 | ZC3H11A (bg=6.55%) | K562 | - | chrX | | 73047693 | 73047813 |
| 2.39955549740702 | ZC3H11A (bg=6.55%) | K562 | - | chrX | | 73047705 | 73047800 |

  
  

| Match 319 in HUMAN | | | | | | | |
| --- | --- | --- | --- | --- | --- | --- | --- |
| Motif | Start in Seq (1 Indexed) | End in Seq (1 Indexed) | Strand | Chrm | Exon | Start in Chrm (0 Indexed) | End in Chrm (1 Indexed) |
| TCTTTCTT | 11963 | 11970 | - | chrX | 6 | 73047795 | 73047803 |
| eCLIP Fold-Enrichment | Binding Protein | Cell Line | Strand | Chrm | | Start in Chrm (0 Indexed) | End in Chrm (1 Indexed) |
| 2.58278226693842 | TARDBP (bg=2.79%) | K562 | - | chrX | | 73047682 | 73047819 |
| 2.38044667445932 | ZC3H11A (bg=6.55%) | K562 | - | chrX | | 73047693 | 73047813 |
| 2.39955549740702 | ZC3H11A (bg=6.55%) | K562 | - | chrX | | 73047705 | 73047800 |

  
  

| Match 320 in HUMAN | | | | | | | |
| --- | --- | --- | --- | --- | --- | --- | --- |
| Motif | Start in Seq (1 Indexed) | End in Seq (1 Indexed) | Strand | Chrm | Exon | Start in Chrm (0 Indexed) | End in Chrm (1 Indexed) |
| TTCTCTA | 11989 | 11995 | - | chrX | 6 | 73047770 | 73047777 |
| eCLIP Fold-Enrichment | Binding Protein | Cell Line | Strand | Chrm | | Start in Chrm (0 Indexed) | End in Chrm (1 Indexed) |
| 3.06179011142427 | MATR3 (bg=2.98%) | K562 | - | chrX | | 73047743 | 73047779 |
| 2.58636308416758 | MATR3 (bg=2.98%) | K562 | - | chrX | | 73047758 | 73047770 |
| 5.26124539015935 | PTBP1 (bg=3.74%) | K562 | - | chrX | | 73047730 | 73047792 |
| 5.37736477433521 | PTBP1 (bg=3.74%) | K562 | - | chrX | | 73047738 | 73047793 |
| 2.58278226693842 | TARDBP (bg=2.79%) | K562 | - | chrX | | 73047682 | 73047819 |
| 2.38044667445932 | ZC3H11A (bg=6.55%) | K562 | - | chrX | | 73047693 | 73047813 |
| 2.39955549740702 | ZC3H11A (bg=6.55%) | K562 | - | chrX | | 73047705 | 73047800 |

  
  

| Match 321 in HUMAN | | | | | | | |
| --- | --- | --- | --- | --- | --- | --- | --- |
| Motif | Start in Seq (1 Indexed) | End in Seq (1 Indexed) | Strand | Chrm | Exon | Start in Chrm (0 Indexed) | End in Chrm (1 Indexed) |
| TGTGTCTTACCCATTTCCATG | 12003 | 12023 | - | chrX | 6 | 73047742 | 73047763 |
| eCLIP Fold-Enrichment | Binding Protein | Cell Line | Strand | Chrm | | Start in Chrm (0 Indexed) | End in Chrm (1 Indexed) |
| 2.8014298398888 | MATR3 (bg=2.98%) | K562 | - | chrX | | 73047729 | 73047743 |
| 3.06179011142427 | MATR3 (bg=2.98%) | K562 | - | chrX | | 73047743 | 73047779 |
| 2.58636308416758 | MATR3 (bg=2.98%) | K562 | - | chrX | | 73047758 | 73047770 |
| 5.26124539015935 | PTBP1 (bg=3.74%) | K562 | - | chrX | | 73047730 | 73047792 |
| 5.37736477433521 | PTBP1 (bg=3.74%) | K562 | - | chrX | | 73047738 | 73047793 |
| 2.58278226693842 | TARDBP (bg=2.79%) | K562 | - | chrX | | 73047682 | 73047819 |
| 2.38044667445932 | ZC3H11A (bg=6.55%) | K562 | - | chrX | | 73047693 | 73047813 |
| 2.39955549740702 | ZC3H11A (bg=6.55%) | K562 | - | chrX | | 73047705 | 73047800 |

  
  

| Match 322 in HUMAN | | | | | | | |
| --- | --- | --- | --- | --- | --- | --- | --- |
| Motif | Start in Seq (1 Indexed) | End in Seq (1 Indexed) | Strand | Chrm | Exon | Start in Chrm (0 Indexed) | End in Chrm (1 Indexed) |
| TGTCTTACCCATTTCCATG | 12005 | 12023 | - | chrX | 6 | 73047742 | 73047761 |
| eCLIP Fold-Enrichment | Binding Protein | Cell Line | Strand | Chrm | | Start in Chrm (0 Indexed) | End in Chrm (1 Indexed) |
| 2.8014298398888 | MATR3 (bg=2.98%) | K562 | - | chrX | | 73047729 | 73047743 |
| 3.06179011142427 | MATR3 (bg=2.98%) | K562 | - | chrX | | 73047743 | 73047779 |
| 2.58636308416758 | MATR3 (bg=2.98%) | K562 | - | chrX | | 73047758 | 73047770 |
| 5.26124539015935 | PTBP1 (bg=3.74%) | K562 | - | chrX | | 73047730 | 73047792 |
| 5.37736477433521 | PTBP1 (bg=3.74%) | K562 | - | chrX | | 73047738 | 73047793 |
| 2.58278226693842 | TARDBP (bg=2.79%) | K562 | - | chrX | | 73047682 | 73047819 |
| 2.38044667445932 | ZC3H11A (bg=6.55%) | K562 | - | chrX | | 73047693 | 73047813 |
| 2.39955549740702 | ZC3H11A (bg=6.55%) | K562 | - | chrX | | 73047705 | 73047800 |

  
  

| Match 323 in HUMAN | | | | | | | |
| --- | --- | --- | --- | --- | --- | --- | --- |
| Motif | Start in Seq (1 Indexed) | End in Seq (1 Indexed) | Strand | Chrm | Exon | Start in Chrm (0 Indexed) | End in Chrm (1 Indexed) |
| TGTCTTA | 12005 | 12011 | - | chrX | 6 | 73047754 | 73047761 |
| eCLIP Fold-Enrichment | Binding Protein | Cell Line | Strand | Chrm | | Start in Chrm (0 Indexed) | End in Chrm (1 Indexed) |
| 3.06179011142427 | MATR3 (bg=2.98%) | K562 | - | chrX | | 73047743 | 73047779 |
| 2.58636308416758 | MATR3 (bg=2.98%) | K562 | - | chrX | | 73047758 | 73047770 |
| 5.26124539015935 | PTBP1 (bg=3.74%) | K562 | - | chrX | | 73047730 | 73047792 |
| 5.37736477433521 | PTBP1 (bg=3.74%) | K562 | - | chrX | | 73047738 | 73047793 |
| 2.58278226693842 | TARDBP (bg=2.79%) | K562 | - | chrX | | 73047682 | 73047819 |
| 2.38044667445932 | ZC3H11A (bg=6.55%) | K562 | - | chrX | | 73047693 | 73047813 |
| 2.39955549740702 | ZC3H11A (bg=6.55%) | K562 | - | chrX | | 73047705 | 73047800 |

  
  

| Match 324 in HUMAN | | | | | | | |
| --- | --- | --- | --- | --- | --- | --- | --- |
| Motif | Start in Seq (1 Indexed) | End in Seq (1 Indexed) | Strand | Chrm | Exon | Start in Chrm (0 Indexed) | End in Chrm (1 Indexed) |
| TTTTTGT | 12070 | 12076 | - | chrX | 6 | 73047689 | 73047696 |
| eCLIP Fold-Enrichment | Binding Protein | Cell Line | Strand | Chrm | | Start in Chrm (0 Indexed) | End in Chrm (1 Indexed) |
| 5.16132578497519 | MATR3 (bg=2.98%) | K562 | - | chrX | | 73047664 | 73047694 |
| 3.60485942778497 | MATR3 (bg=2.98%) | K562 | - | chrX | | 73047668 | 73047726 |
| 3.78206451502187 | MATR3 (bg=2.98%) | K562 | - | chrX | | 73047694 | 73047729 |
| 6.31354967673777 | PTBP1 (bg=3.74%) | K562 | - | chrX | | 73047652 | 73047695 |
| 5.63605304464966 | PTBP1 (bg=3.74%) | K562 | - | chrX | | 73047655 | 73047695 |
| 5.48438706886963 | PTBP1 (bg=3.74%) | K562 | - | chrX | | 73047695 | 73047727 |
| 5.90129784165881 | PTBP1 (bg=3.74%) | K562 | - | chrX | | 73047695 | 73047738 |
| 2.58278226693842 | TARDBP (bg=2.79%) | K562 | - | chrX | | 73047682 | 73047819 |
| 2.15628888760474 | TIA1 (bg=4.07%) | K562 | - | chrX | | 73047656 | 73047701 |
| 2.82470960220095 | TIA1 (bg=4.07%) | K562 | - | chrX | | 73047656 | 73047722 |
| 2.38044667445932 | ZC3H11A (bg=6.55%) | K562 | - | chrX | | 73047693 | 73047813 |

  
  

| Match 325 in HUMAN | | | | | | | |
| --- | --- | --- | --- | --- | --- | --- | --- |
| Motif | Start in Seq (1 Indexed) | End in Seq (1 Indexed) | Strand | Chrm | Exon | Start in Chrm (0 Indexed) | End in Chrm (1 Indexed) |
| GGTCTGTGTCT | 12087 | 12097 | - | chrX | 6 | 73047668 | 73047679 |
| eCLIP Fold-Enrichment | Binding Protein | Cell Line | Strand | Chrm | | Start in Chrm (0 Indexed) | End in Chrm (1 Indexed) |
| 5.16132578497519 | MATR3 (bg=2.98%) | K562 | - | chrX | | 73047664 | 73047694 |
| 3.60485942778497 | MATR3 (bg=2.98%) | K562 | - | chrX | | 73047668 | 73047726 |
| 6.31354967673777 | PTBP1 (bg=3.74%) | K562 | - | chrX | | 73047652 | 73047695 |
| 5.63605304464966 | PTBP1 (bg=3.74%) | K562 | - | chrX | | 73047655 | 73047695 |
| 2.15628888760474 | TIA1 (bg=4.07%) | K562 | - | chrX | | 73047656 | 73047701 |
| 2.82470960220095 | TIA1 (bg=4.07%) | K562 | - | chrX | | 73047656 | 73047722 |
| 2.15805425312287 | ZC3H11A (bg=6.55%) | K562 | - | chrX | | 73047652 | 73047682 |

  
  

| Match 326 in HUMAN | | | | | | | |
| --- | --- | --- | --- | --- | --- | --- | --- |
| Motif | Start in Seq (1 Indexed) | End in Seq (1 Indexed) | Strand | Chrm | Exon | Start in Chrm (0 Indexed) | End in Chrm (1 Indexed) |
| GTCTTAGA | 12099 | 12106 | - | chrX | 6 | 73047659 | 73047667 |
| eCLIP Fold-Enrichment | Binding Protein | Cell Line | Strand | Chrm | | Start in Chrm (0 Indexed) | End in Chrm (1 Indexed) |
| 5.16132578497519 | MATR3 (bg=2.98%) | K562 | - | chrX | | 73047664 | 73047694 |
| 6.31354967673777 | PTBP1 (bg=3.74%) | K562 | - | chrX | | 73047652 | 73047695 |
| 5.63605304464966 | PTBP1 (bg=3.74%) | K562 | - | chrX | | 73047655 | 73047695 |
| 2.15628888760474 | TIA1 (bg=4.07%) | K562 | - | chrX | | 73047656 | 73047701 |
| 2.82470960220095 | TIA1 (bg=4.07%) | K562 | - | chrX | | 73047656 | 73047722 |
| 2.15805425312287 | ZC3H11A (bg=6.55%) | K562 | - | chrX | | 73047652 | 73047682 |

  
  

| Match 327 in HUMAN | | | | | | | |
| --- | --- | --- | --- | --- | --- | --- | --- |
| Motif | Start in Seq (1 Indexed) | End in Seq (1 Indexed) | Strand | Chrm | Exon | Start in Chrm (0 Indexed) | End in Chrm (1 Indexed) |
| CTCTTTGCTC | 12139 | 12148 | - | chrX | 6 | 73047617 | 73047627 |
| eCLIP Fold-Enrichment | Binding Protein | Cell Line | Strand | Chrm | | Start in Chrm (0 Indexed) | End in Chrm (1 Indexed) |
| 5.10790020474711 | MATR3 (bg=2.98%) | K562 | - | chrX | | 73047577 | 73047631 |
| 5.21195185804515 | MATR3 (bg=2.98%) | K562 | - | chrX | | 73047578 | 73047628 |
| 5.24170967497765 | PTBP1 (bg=3.74%) | K562 | - | chrX | | 73047576 | 73047630 |
| 4.91778413372235 | PTBP1 (bg=3.74%) | K562 | - | chrX | | 73047577 | 73047629 |
| 3.84453919673792 | TIA1 (bg=4.07%) | K562 | - | chrX | | 73047547 | 73047626 |
| 4.55651349125138 | TIA1 (bg=4.07%) | K562 | - | chrX | | 73047571 | 73047627 |

  
  

| Match 328 in HUMAN | | | | | | | |
| --- | --- | --- | --- | --- | --- | --- | --- |
| Motif | Start in Seq (1 Indexed) | End in Seq (1 Indexed) | Strand | Chrm | Exon | Start in Chrm (0 Indexed) | End in Chrm (1 Indexed) |
| TTTGCTC | 12142 | 12148 | - | chrX | 6 | 73047617 | 73047624 |
| eCLIP Fold-Enrichment | Binding Protein | Cell Line | Strand | Chrm | | Start in Chrm (0 Indexed) | End in Chrm (1 Indexed) |
| 5.10790020474711 | MATR3 (bg=2.98%) | K562 | - | chrX | | 73047577 | 73047631 |
| 5.21195185804515 | MATR3 (bg=2.98%) | K562 | - | chrX | | 73047578 | 73047628 |
| 5.24170967497765 | PTBP1 (bg=3.74%) | K562 | - | chrX | | 73047576 | 73047630 |
| 4.91778413372235 | PTBP1 (bg=3.74%) | K562 | - | chrX | | 73047577 | 73047629 |
| 3.84453919673792 | TIA1 (bg=4.07%) | K562 | - | chrX | | 73047547 | 73047626 |
| 4.55651349125138 | TIA1 (bg=4.07%) | K562 | - | chrX | | 73047571 | 73047627 |

  
  

| Match 329 in HUMAN | | | | | | | |
| --- | --- | --- | --- | --- | --- | --- | --- |
| Motif | Start in Seq (1 Indexed) | End in Seq (1 Indexed) | Strand | Chrm | Exon | Start in Chrm (0 Indexed) | End in Chrm (1 Indexed) |
| TTTCTTGTT | 12224 | 12232 | - | chrX | 6 | 73047533 | 73047542 |
| eCLIP Fold-Enrichment | Binding Protein | Cell Line | Strand | Chrm | | Start in Chrm (0 Indexed) | End in Chrm (1 Indexed) |
| 3.58120454732353 | MATR3 (bg=2.98%) | K562 | - | chrX | | 73047476 | 73047552 |
| 3.24860750597503 | MATR3 (bg=2.98%) | K562 | - | chrX | | 73047480 | 73047555 |
| 5.41936694498778 | PTBP1 (bg=3.74%) | K562 | - | chrX | | 73047477 | 73047552 |
| 5.47841297121973 | PTBP1 (bg=3.74%) | K562 | - | chrX | | 73047478 | 73047545 |
| 3.46517572168993 | SMNDC1 (bg=0.63%) | HepG2 | - | chrX | | 73047480 | 73047535 |
| 3.95104533509558 | TIA1 (bg=4.07%) | K562 | - | chrX | | 73047474 | 73047541 |
| 2.79629275188386 | TIA1 (bg=4.07%) | K562 | - | chrX | | 73047474 | 73047547 |
| 3.49713494417245 | TIA1 (bg=4.07%) | K562 | - | chrX | | 73047541 | 73047571 |

  
  

| Match 330 in HUMAN | | | | | | | |
| --- | --- | --- | --- | --- | --- | --- | --- |
| Motif | Start in Seq (1 Indexed) | End in Seq (1 Indexed) | Strand | Chrm | Exon | Start in Chrm (0 Indexed) | End in Chrm (1 Indexed) |
| TGCCTACCT | 12253 | 12261 | - | chrX | 6 | 73047504 | 73047513 |
| eCLIP Fold-Enrichment | Binding Protein | Cell Line | Strand | Chrm | | Start in Chrm (0 Indexed) | End in Chrm (1 Indexed) |
| 3.58120454732353 | MATR3 (bg=2.98%) | K562 | - | chrX | | 73047476 | 73047552 |
| 3.24860750597503 | MATR3 (bg=2.98%) | K562 | - | chrX | | 73047480 | 73047555 |
| 5.41936694498778 | PTBP1 (bg=3.74%) | K562 | - | chrX | | 73047477 | 73047552 |
| 5.47841297121973 | PTBP1 (bg=3.74%) | K562 | - | chrX | | 73047478 | 73047545 |
| 3.46517572168993 | SMNDC1 (bg=0.63%) | HepG2 | - | chrX | | 73047480 | 73047535 |
| 3.95104533509558 | TIA1 (bg=4.07%) | K562 | - | chrX | | 73047474 | 73047541 |
| 2.79629275188386 | TIA1 (bg=4.07%) | K562 | - | chrX | | 73047474 | 73047547 |

  
  

| Match 331 in HUMAN | | | | | | | |
| --- | --- | --- | --- | --- | --- | --- | --- |
| Motif | Start in Seq (1 Indexed) | End in Seq (1 Indexed) | Strand | Chrm | Exon | Start in Chrm (0 Indexed) | End in Chrm (1 Indexed) |
| TTTTCTCTTTGTGAA | 12265 | 12279 | - | chrX | 6 | 73047486 | 73047501 |
| eCLIP Fold-Enrichment | Binding Protein | Cell Line | Strand | Chrm | | Start in Chrm (0 Indexed) | End in Chrm (1 Indexed) |
| 3.58120454732353 | MATR3 (bg=2.98%) | K562 | - | chrX | | 73047476 | 73047552 |
| 3.24860750597503 | MATR3 (bg=2.98%) | K562 | - | chrX | | 73047480 | 73047555 |
| 5.41936694498778 | PTBP1 (bg=3.74%) | K562 | - | chrX | | 73047477 | 73047552 |
| 5.47841297121973 | PTBP1 (bg=3.74%) | K562 | - | chrX | | 73047478 | 73047545 |
| 3.46517572168993 | SMNDC1 (bg=0.63%) | HepG2 | - | chrX | | 73047480 | 73047535 |
| 3.95104533509558 | TIA1 (bg=4.07%) | K562 | - | chrX | | 73047474 | 73047541 |
| 2.79629275188386 | TIA1 (bg=4.07%) | K562 | - | chrX | | 73047474 | 73047547 |

  
  

| Match 332 in HUMAN | | | | | | | |
| --- | --- | --- | --- | --- | --- | --- | --- |
| Motif | Start in Seq (1 Indexed) | End in Seq (1 Indexed) | Strand | Chrm | Exon | Start in Chrm (0 Indexed) | End in Chrm (1 Indexed) |
| TTCTCTTTG | 12267 | 12275 | - | chrX | 6 | 73047490 | 73047499 |
| eCLIP Fold-Enrichment | Binding Protein | Cell Line | Strand | Chrm | | Start in Chrm (0 Indexed) | End in Chrm (1 Indexed) |
| 3.58120454732353 | MATR3 (bg=2.98%) | K562 | - | chrX | | 73047476 | 73047552 |
| 3.24860750597503 | MATR3 (bg=2.98%) | K562 | - | chrX | | 73047480 | 73047555 |
| 5.41936694498778 | PTBP1 (bg=3.74%) | K562 | - | chrX | | 73047477 | 73047552 |
| 5.47841297121973 | PTBP1 (bg=3.74%) | K562 | - | chrX | | 73047478 | 73047545 |
| 3.46517572168993 | SMNDC1 (bg=0.63%) | HepG2 | - | chrX | | 73047480 | 73047535 |
| 3.95104533509558 | TIA1 (bg=4.07%) | K562 | - | chrX | | 73047474 | 73047541 |
| 2.79629275188386 | TIA1 (bg=4.07%) | K562 | - | chrX | | 73047474 | 73047547 |

  
  

| Match 333 in HUMAN | | | | | | | |
| --- | --- | --- | --- | --- | --- | --- | --- |
| Motif | Start in Seq (1 Indexed) | End in Seq (1 Indexed) | Strand | Chrm | Exon | Start in Chrm (0 Indexed) | End in Chrm (1 Indexed) |
| TTCCCCTTCT | 12300 | 12309 | - | chrX | 6 | 73047456 | 73047466 |
| eCLIP Fold-Enrichment | Binding Protein | Cell Line | Strand | Chrm | | Start in Chrm (0 Indexed) | End in Chrm (1 Indexed) |
| 3.77927669765025 | MATR3 (bg=2.98%) | K562 | - | chrX | | 73047449 | 73047476 |
| 3.03426521735205 | MATR3 (bg=2.98%) | K562 | - | chrX | | 73047456 | 73047477 |
| 5.52867409761018 | PTBP1 (bg=3.74%) | K562 | - | chrX | | 73047445 | 73047478 |
| 5.00397025361424 | PTBP1 (bg=3.74%) | K562 | - | chrX | | 73047448 | 73047477 |
| 2.07263711564454 | TIA1 (bg=4.07%) | K562 | - | chrX | | 73047444 | 73047474 |

  
  

| Match 334 in HUMAN | | | | | | | |
| --- | --- | --- | --- | --- | --- | --- | --- |
| Motif | Start in Seq (1 Indexed) | End in Seq (1 Indexed) | Strand | Chrm | Exon | Start in Chrm (0 Indexed) | End in Chrm (1 Indexed) |
| TTCCCCTT | 12300 | 12307 | - | chrX | 6 | 73047458 | 73047466 |
| eCLIP Fold-Enrichment | Binding Protein | Cell Line | Strand | Chrm | | Start in Chrm (0 Indexed) | End in Chrm (1 Indexed) |
| 3.77927669765025 | MATR3 (bg=2.98%) | K562 | - | chrX | | 73047449 | 73047476 |
| 3.03426521735205 | MATR3 (bg=2.98%) | K562 | - | chrX | | 73047456 | 73047477 |
| 5.52867409761018 | PTBP1 (bg=3.74%) | K562 | - | chrX | | 73047445 | 73047478 |
| 5.00397025361424 | PTBP1 (bg=3.74%) | K562 | - | chrX | | 73047448 | 73047477 |
| 2.07263711564454 | TIA1 (bg=4.07%) | K562 | - | chrX | | 73047444 | 73047474 |

  
  

| Match 335 in HUMAN | | | | | | | |
| --- | --- | --- | --- | --- | --- | --- | --- |
| Motif | Start in Seq (1 Indexed) | End in Seq (1 Indexed) | Strand | Chrm | Exon | Start in Chrm (0 Indexed) | End in Chrm (1 Indexed) |
| GTTCGTTT | 12311 | 12318 | - | chrX | 6 | 73047447 | 73047455 |
| eCLIP Fold-Enrichment | Binding Protein | Cell Line | Strand | Chrm | | Start in Chrm (0 Indexed) | End in Chrm (1 Indexed) |
| 3.77927669765025 | MATR3 (bg=2.98%) | K562 | - | chrX | | 73047449 | 73047476 |
| 5.52867409761018 | PTBP1 (bg=3.74%) | K562 | - | chrX | | 73047445 | 73047478 |
| 5.00397025361424 | PTBP1 (bg=3.74%) | K562 | - | chrX | | 73047448 | 73047477 |
| 2.07263711564454 | TIA1 (bg=4.07%) | K562 | - | chrX | | 73047444 | 73047474 |

  
  

| Match 336 in HUMAN | | | | | | | |
| --- | --- | --- | --- | --- | --- | --- | --- |
| Motif | Start in Seq (1 Indexed) | End in Seq (1 Indexed) | Strand | Chrm | Exon | Start in Chrm (0 Indexed) | End in Chrm (1 Indexed) |
| ATTTCACCT | 12322 | 12330 | - | chrX | 6 | 73047435 | 73047444 |
| eCLIP Fold-Enrichment | Binding Protein | Cell Line | Strand | Chrm | | Start in Chrm (0 Indexed) | End in Chrm (1 Indexed) |
| 2.07263711564454 | TIA1 (bg=4.07%) | K562 | - | chrX | | 73047444 | 73047474 |

  
  

| Match 337 in HUMAN | | | | | | | |
| --- | --- | --- | --- | --- | --- | --- | --- |
| Motif | Start in Seq (1 Indexed) | End in Seq (1 Indexed) | Strand | Chrm | Exon | Start in Chrm (0 Indexed) | End in Chrm (1 Indexed) |
| TGCTGTTTCTACT | 12355 | 12367 | - | chrX | 6 | 73047398 | 73047411 |
| eCLIP Fold-Enrichment | Binding Protein | Cell Line | Strand | Chrm | | Start in Chrm (0 Indexed) | End in Chrm (1 Indexed) |
| 4.23715719793483 | MATR3 (bg=2.98%) | K562 | - | chrX | | 73047296 | 73047428 |
| 4.09929278420801 | MATR3 (bg=2.98%) | K562 | - | chrX | | 73047297 | 73047399 |
| 3.45998579839803 | MATR3 (bg=2.98%) | K562 | - | chrX | | 73047399 | 73047434 |
| 4.97396197553977 | PTBP1 (bg=3.74%) | K562 | - | chrX | | 73047392 | 73047420 |
| 5.07271642068919 | PTBP1 (bg=3.74%) | K562 | - | chrX | | 73047395 | 73047431 |
| 3.44309510445956 | TIA1 (bg=4.07%) | K562 | - | chrX | | 73047381 | 73047429 |
| 2.42307542829965 | TIA1 (bg=4.07%) | K562 | - | chrX | | 73047398 | 73047422 |

  
  

| Match 338 in HUMAN | | | | | | | |
| --- | --- | --- | --- | --- | --- | --- | --- |
| Motif | Start in Seq (1 Indexed) | End in Seq (1 Indexed) | Strand | Chrm | Exon | Start in Chrm (0 Indexed) | End in Chrm (1 Indexed) |
| TTTCTACT | 12360 | 12367 | - | chrX | 6 | 73047398 | 73047406 |
| eCLIP Fold-Enrichment | Binding Protein | Cell Line | Strand | Chrm | | Start in Chrm (0 Indexed) | End in Chrm (1 Indexed) |
| 4.23715719793483 | MATR3 (bg=2.98%) | K562 | - | chrX | | 73047296 | 73047428 |
| 4.09929278420801 | MATR3 (bg=2.98%) | K562 | - | chrX | | 73047297 | 73047399 |
| 3.45998579839803 | MATR3 (bg=2.98%) | K562 | - | chrX | | 73047399 | 73047434 |
| 4.97396197553977 | PTBP1 (bg=3.74%) | K562 | - | chrX | | 73047392 | 73047420 |
| 5.07271642068919 | PTBP1 (bg=3.74%) | K562 | - | chrX | | 73047395 | 73047431 |
| 3.44309510445956 | TIA1 (bg=4.07%) | K562 | - | chrX | | 73047381 | 73047429 |
| 2.42307542829965 | TIA1 (bg=4.07%) | K562 | - | chrX | | 73047398 | 73047422 |

  
  

| Match 339 in HUMAN | | | | | | | |
| --- | --- | --- | --- | --- | --- | --- | --- |
| Motif | Start in Seq (1 Indexed) | End in Seq (1 Indexed) | Strand | Chrm | Exon | Start in Chrm (0 Indexed) | End in Chrm (1 Indexed) |
| TTTCTAC | 12360 | 12366 | - | chrX | 6 | 73047399 | 73047406 |
| eCLIP Fold-Enrichment | Binding Protein | Cell Line | Strand | Chrm | | Start in Chrm (0 Indexed) | End in Chrm (1 Indexed) |
| 4.23715719793483 | MATR3 (bg=2.98%) | K562 | - | chrX | | 73047296 | 73047428 |
| 4.09929278420801 | MATR3 (bg=2.98%) | K562 | - | chrX | | 73047297 | 73047399 |
| 3.45998579839803 | MATR3 (bg=2.98%) | K562 | - | chrX | | 73047399 | 73047434 |
| 4.97396197553977 | PTBP1 (bg=3.74%) | K562 | - | chrX | | 73047392 | 73047420 |
| 5.07271642068919 | PTBP1 (bg=3.74%) | K562 | - | chrX | | 73047395 | 73047431 |
| 3.44309510445956 | TIA1 (bg=4.07%) | K562 | - | chrX | | 73047381 | 73047429 |
| 2.42307542829965 | TIA1 (bg=4.07%) | K562 | - | chrX | | 73047398 | 73047422 |

  
  

| Match 340 in HUMAN | | | | | | | |
| --- | --- | --- | --- | --- | --- | --- | --- |
| Motif | Start in Seq (1 Indexed) | End in Seq (1 Indexed) | Strand | Chrm | Exon | Start in Chrm (0 Indexed) | End in Chrm (1 Indexed) |
| ATCTCACATTTCTC | 12372 | 12385 | - | chrX | 6 | 73047380 | 73047394 |
| eCLIP Fold-Enrichment | Binding Protein | Cell Line | Strand | Chrm | | Start in Chrm (0 Indexed) | End in Chrm (1 Indexed) |
| 4.23715719793483 | MATR3 (bg=2.98%) | K562 | - | chrX | | 73047296 | 73047428 |
| 4.09929278420801 | MATR3 (bg=2.98%) | K562 | - | chrX | | 73047297 | 73047399 |
| 5.47785545890179 | PTBP1 (bg=3.74%) | K562 | - | chrX | | 73047352 | 73047385 |
| 5.38584205426305 | PTBP1 (bg=3.74%) | K562 | - | chrX | | 73047353 | 73047392 |
| 5.10771482696771 | PTBP1 (bg=3.74%) | K562 | - | chrX | | 73047385 | 73047395 |
| 4.97396197553977 | PTBP1 (bg=3.74%) | K562 | - | chrX | | 73047392 | 73047420 |
| 3.32341636957169 | TIA1 (bg=4.07%) | K562 | - | chrX | | 73047343 | 73047381 |
| 3.44309510445956 | TIA1 (bg=4.07%) | K562 | - | chrX | | 73047381 | 73047429 |

  
  

| Match 341 in HUMAN | | | | | | | |
| --- | --- | --- | --- | --- | --- | --- | --- |
| Motif | Start in Seq (1 Indexed) | End in Seq (1 Indexed) | Strand | Chrm | Exon | Start in Chrm (0 Indexed) | End in Chrm (1 Indexed) |
| ATTTCTC | 12379 | 12385 | - | chrX | 6 | 73047380 | 73047387 |
| eCLIP Fold-Enrichment | Binding Protein | Cell Line | Strand | Chrm | | Start in Chrm (0 Indexed) | End in Chrm (1 Indexed) |
| 4.23715719793483 | MATR3 (bg=2.98%) | K562 | - | chrX | | 73047296 | 73047428 |
| 4.09929278420801 | MATR3 (bg=2.98%) | K562 | - | chrX | | 73047297 | 73047399 |
| 5.47785545890179 | PTBP1 (bg=3.74%) | K562 | - | chrX | | 73047352 | 73047385 |
| 5.38584205426305 | PTBP1 (bg=3.74%) | K562 | - | chrX | | 73047353 | 73047392 |
| 5.10771482696771 | PTBP1 (bg=3.74%) | K562 | - | chrX | | 73047385 | 73047395 |
| 3.32341636957169 | TIA1 (bg=4.07%) | K562 | - | chrX | | 73047343 | 73047381 |
| 3.44309510445956 | TIA1 (bg=4.07%) | K562 | - | chrX | | 73047381 | 73047429 |

  
  

| Match 342 in HUMAN | | | | | | | |
| --- | --- | --- | --- | --- | --- | --- | --- |
| Motif | Start in Seq (1 Indexed) | End in Seq (1 Indexed) | Strand | Chrm | Exon | Start in Chrm (0 Indexed) | End in Chrm (1 Indexed) |
| TGCCTCTCTTGGGC | 12404 | 12417 | - | chrX | 6 | 73047348 | 73047362 |
| eCLIP Fold-Enrichment | Binding Protein | Cell Line | Strand | Chrm | | Start in Chrm (0 Indexed) | End in Chrm (1 Indexed) |
| 4.23715719793483 | MATR3 (bg=2.98%) | K562 | - | chrX | | 73047296 | 73047428 |
| 4.09929278420801 | MATR3 (bg=2.98%) | K562 | - | chrX | | 73047297 | 73047399 |
| 5.68367188863471 | PTBP1 (bg=3.74%) | K562 | - | chrX | | 73047294 | 73047353 |
| 5.62728896767134 | PTBP1 (bg=3.74%) | K562 | - | chrX | | 73047298 | 73047352 |
| 5.47785545890179 | PTBP1 (bg=3.74%) | K562 | - | chrX | | 73047352 | 73047385 |
| 5.38584205426305 | PTBP1 (bg=3.74%) | K562 | - | chrX | | 73047353 | 73047392 |
| 5.05013822241109 | SMNDC1 (bg=0.63%) | HepG2 | - | chrX | | 73047309 | 73047375 |
| 2.63279401960098 | TIA1 (bg=4.07%) | K562 | - | chrX | | 73047297 | 73047376 |
| 3.32341636957169 | TIA1 (bg=4.07%) | K562 | - | chrX | | 73047343 | 73047381 |

  
  

| Match 343 in HUMAN | | | | | | | |
| --- | --- | --- | --- | --- | --- | --- | --- |
| Motif | Start in Seq (1 Indexed) | End in Seq (1 Indexed) | Strand | Chrm | Exon | Start in Chrm (0 Indexed) | End in Chrm (1 Indexed) |
| TCTTGGGC | 12410 | 12417 | - | chrX | 6 | 73047348 | 73047356 |
| eCLIP Fold-Enrichment | Binding Protein | Cell Line | Strand | Chrm | | Start in Chrm (0 Indexed) | End in Chrm (1 Indexed) |
| 4.23715719793483 | MATR3 (bg=2.98%) | K562 | - | chrX | | 73047296 | 73047428 |
| 4.09929278420801 | MATR3 (bg=2.98%) | K562 | - | chrX | | 73047297 | 73047399 |
| 5.68367188863471 | PTBP1 (bg=3.74%) | K562 | - | chrX | | 73047294 | 73047353 |
| 5.62728896767134 | PTBP1 (bg=3.74%) | K562 | - | chrX | | 73047298 | 73047352 |
| 5.47785545890179 | PTBP1 (bg=3.74%) | K562 | - | chrX | | 73047352 | 73047385 |
| 5.38584205426305 | PTBP1 (bg=3.74%) | K562 | - | chrX | | 73047353 | 73047392 |
| 5.05013822241109 | SMNDC1 (bg=0.63%) | HepG2 | - | chrX | | 73047309 | 73047375 |
| 2.63279401960098 | TIA1 (bg=4.07%) | K562 | - | chrX | | 73047297 | 73047376 |
| 3.32341636957169 | TIA1 (bg=4.07%) | K562 | - | chrX | | 73047343 | 73047381 |

  
  

| Match 344 in HUMAN | | | | | | | |
| --- | --- | --- | --- | --- | --- | --- | --- |
| Motif | Start in Seq (1 Indexed) | End in Seq (1 Indexed) | Strand | Chrm | Exon | Start in Chrm (0 Indexed) | End in Chrm (1 Indexed) |
| TCTTGGG | 12410 | 12416 | - | chrX | 6 | 73047349 | 73047356 |
| eCLIP Fold-Enrichment | Binding Protein | Cell Line | Strand | Chrm | | Start in Chrm (0 Indexed) | End in Chrm (1 Indexed) |
| 4.23715719793483 | MATR3 (bg=2.98%) | K562 | - | chrX | | 73047296 | 73047428 |
| 4.09929278420801 | MATR3 (bg=2.98%) | K562 | - | chrX | | 73047297 | 73047399 |
| 5.68367188863471 | PTBP1 (bg=3.74%) | K562 | - | chrX | | 73047294 | 73047353 |
| 5.62728896767134 | PTBP1 (bg=3.74%) | K562 | - | chrX | | 73047298 | 73047352 |
| 5.47785545890179 | PTBP1 (bg=3.74%) | K562 | - | chrX | | 73047352 | 73047385 |
| 5.38584205426305 | PTBP1 (bg=3.74%) | K562 | - | chrX | | 73047353 | 73047392 |
| 5.05013822241109 | SMNDC1 (bg=0.63%) | HepG2 | - | chrX | | 73047309 | 73047375 |
| 2.63279401960098 | TIA1 (bg=4.07%) | K562 | - | chrX | | 73047297 | 73047376 |
| 3.32341636957169 | TIA1 (bg=4.07%) | K562 | - | chrX | | 73047343 | 73047381 |

  
  

| Match 345 in HUMAN | | | | | | | |
| --- | --- | --- | --- | --- | --- | --- | --- |
| Motif | Start in Seq (1 Indexed) | End in Seq (1 Indexed) | Strand | Chrm | Exon | Start in Chrm (0 Indexed) | End in Chrm (1 Indexed) |
| TTTGTGATTTTC | 12461 | 12472 | - | chrX | 6 | 73047293 | 73047305 |
| eCLIP Fold-Enrichment | Binding Protein | Cell Line | Strand | Chrm | | Start in Chrm (0 Indexed) | End in Chrm (1 Indexed) |
| 4.23715719793483 | MATR3 (bg=2.98%) | K562 | - | chrX | | 73047296 | 73047428 |
| 4.09929278420801 | MATR3 (bg=2.98%) | K562 | - | chrX | | 73047297 | 73047399 |
| 5.68367188863471 | PTBP1 (bg=3.74%) | K562 | - | chrX | | 73047294 | 73047353 |
| 5.62728896767134 | PTBP1 (bg=3.74%) | K562 | - | chrX | | 73047298 | 73047352 |
| 3.56449021197421 | TIA1 (bg=4.07%) | K562 | - | chrX | | 73047296 | 73047343 |
| 2.63279401960098 | TIA1 (bg=4.07%) | K562 | - | chrX | | 73047297 | 73047376 |

  
  

| Match 346 in HUMAN | | | | | | | |
| --- | --- | --- | --- | --- | --- | --- | --- |
| Motif | Start in Seq (1 Indexed) | End in Seq (1 Indexed) | Strand | Chrm | Exon | Start in Chrm (0 Indexed) | End in Chrm (1 Indexed) |
| TTTGTGA | 12461 | 12467 | - | chrX | 6 | 73047298 | 73047305 |
| eCLIP Fold-Enrichment | Binding Protein | Cell Line | Strand | Chrm | | Start in Chrm (0 Indexed) | End in Chrm (1 Indexed) |
| 4.23715719793483 | MATR3 (bg=2.98%) | K562 | - | chrX | | 73047296 | 73047428 |
| 4.09929278420801 | MATR3 (bg=2.98%) | K562 | - | chrX | | 73047297 | 73047399 |
| 5.68367188863471 | PTBP1 (bg=3.74%) | K562 | - | chrX | | 73047294 | 73047353 |
| 5.62728896767134 | PTBP1 (bg=3.74%) | K562 | - | chrX | | 73047298 | 73047352 |
| 3.56449021197421 | TIA1 (bg=4.07%) | K562 | - | chrX | | 73047296 | 73047343 |
| 2.63279401960098 | TIA1 (bg=4.07%) | K562 | - | chrX | | 73047297 | 73047376 |

  
  

| Match 347 in HUMAN | | | | | | | |
| --- | --- | --- | --- | --- | --- | --- | --- |
| Motif | Start in Seq (1 Indexed) | End in Seq (1 Indexed) | Strand | Chrm | Exon | Start in Chrm (0 Indexed) | End in Chrm (1 Indexed) |
| TCTCTGTT | 12487 | 12494 | - | chrX | 6 | 73047271 | 73047279 |
| eCLIP Fold-Enrichment | Binding Protein | Cell Line | Strand | Chrm | | Start in Chrm (0 Indexed) | End in Chrm (1 Indexed) |
| 4.70446811000171 | MATR3 (bg=2.98%) | K562 | - | chrX | | 73047175 | 73047284 |
| 4.3908658898942 | MATR3 (bg=2.98%) | K562 | - | chrX | | 73047185 | 73047281 |
| 5.56734851788503 | PTBP1 (bg=3.74%) | K562 | - | chrX | | 73047229 | 73047275 |
| 5.26188755691528 | PTBP1 (bg=3.74%) | K562 | - | chrX | | 73047265 | 73047278 |

  
  

| Match 348 in HUMAN | | | | | | | |
| --- | --- | --- | --- | --- | --- | --- | --- |
| Motif | Start in Seq (1 Indexed) | End in Seq (1 Indexed) | Strand | Chrm | Exon | Start in Chrm (0 Indexed) | End in Chrm (1 Indexed) |
| TCACCTTTGAGTATTT | 12530 | 12545 | - | chrX | 6 | 73047220 | 73047236 |
| eCLIP Fold-Enrichment | Binding Protein | Cell Line | Strand | Chrm | | Start in Chrm (0 Indexed) | End in Chrm (1 Indexed) |
| 4.70446811000171 | MATR3 (bg=2.98%) | K562 | - | chrX | | 73047175 | 73047284 |
| 4.3908658898942 | MATR3 (bg=2.98%) | K562 | - | chrX | | 73047185 | 73047281 |
| 6.45982693452719 | PTBP1 (bg=3.74%) | K562 | - | chrX | | 73047160 | 73047221 |
| 5.78731420182694 | PTBP1 (bg=3.74%) | K562 | - | chrX | | 73047167 | 73047229 |
| 5.66515527925458 | PTBP1 (bg=3.74%) | K562 | - | chrX | | 73047221 | 73047243 |
| 5.56734851788503 | PTBP1 (bg=3.74%) | K562 | - | chrX | | 73047229 | 73047275 |
| 3.10154980755977 | TIA1 (bg=4.07%) | K562 | - | chrX | | 73047182 | 73047262 |
| 2.42307542829965 | TIA1 (bg=4.07%) | K562 | - | chrX | | 73047200 | 73047266 |

  
  

| Match 349 in HUMAN | | | | | | | |
| --- | --- | --- | --- | --- | --- | --- | --- |
| Motif | Start in Seq (1 Indexed) | End in Seq (1 Indexed) | Strand | Chrm | Exon | Start in Chrm (0 Indexed) | End in Chrm (1 Indexed) |
| TTTGAGTATTT | 12535 | 12545 | - | chrX | 6 | 73047220 | 73047231 |
| eCLIP Fold-Enrichment | Binding Protein | Cell Line | Strand | Chrm | | Start in Chrm (0 Indexed) | End in Chrm (1 Indexed) |
| 4.70446811000171 | MATR3 (bg=2.98%) | K562 | - | chrX | | 73047175 | 73047284 |
| 4.3908658898942 | MATR3 (bg=2.98%) | K562 | - | chrX | | 73047185 | 73047281 |
| 6.45982693452719 | PTBP1 (bg=3.74%) | K562 | - | chrX | | 73047160 | 73047221 |
| 5.78731420182694 | PTBP1 (bg=3.74%) | K562 | - | chrX | | 73047167 | 73047229 |
| 5.66515527925458 | PTBP1 (bg=3.74%) | K562 | - | chrX | | 73047221 | 73047243 |
| 5.56734851788503 | PTBP1 (bg=3.74%) | K562 | - | chrX | | 73047229 | 73047275 |
| 3.10154980755977 | TIA1 (bg=4.07%) | K562 | - | chrX | | 73047182 | 73047262 |
| 2.42307542829965 | TIA1 (bg=4.07%) | K562 | - | chrX | | 73047200 | 73047266 |

  
  

| Match 350 in HUMAN | | | | | | | |
| --- | --- | --- | --- | --- | --- | --- | --- |
| Motif | Start in Seq (1 Indexed) | End in Seq (1 Indexed) | Strand | Chrm | Exon | Start in Chrm (0 Indexed) | End in Chrm (1 Indexed) |
| GCCTCTTC | 12548 | 12555 | - | chrX | 6 | 73047210 | 73047218 |
| eCLIP Fold-Enrichment | Binding Protein | Cell Line | Strand | Chrm | | Start in Chrm (0 Indexed) | End in Chrm (1 Indexed) |
| 4.70446811000171 | MATR3 (bg=2.98%) | K562 | - | chrX | | 73047175 | 73047284 |
| 4.3908658898942 | MATR3 (bg=2.98%) | K562 | - | chrX | | 73047185 | 73047281 |
| 6.45982693452719 | PTBP1 (bg=3.74%) | K562 | - | chrX | | 73047160 | 73047221 |
| 5.78731420182694 | PTBP1 (bg=3.74%) | K562 | - | chrX | | 73047167 | 73047229 |
| 3.10154980755977 | TIA1 (bg=4.07%) | K562 | - | chrX | | 73047182 | 73047262 |
| 2.42307542829965 | TIA1 (bg=4.07%) | K562 | - | chrX | | 73047200 | 73047266 |

  
  

| Match 351 in HUMAN | | | | | | | |
| --- | --- | --- | --- | --- | --- | --- | --- |
| Motif | Start in Seq (1 Indexed) | End in Seq (1 Indexed) | Strand | Chrm | Exon | Start in Chrm (0 Indexed) | End in Chrm (1 Indexed) |
| CTTTGATT | 12574 | 12581 | - | chrX | 6 | 73047184 | 73047192 |
| eCLIP Fold-Enrichment | Binding Protein | Cell Line | Strand | Chrm | | Start in Chrm (0 Indexed) | End in Chrm (1 Indexed) |
| 4.70446811000171 | MATR3 (bg=2.98%) | K562 | - | chrX | | 73047175 | 73047284 |
| 4.3908658898942 | MATR3 (bg=2.98%) | K562 | - | chrX | | 73047185 | 73047281 |
| 6.45982693452719 | PTBP1 (bg=3.74%) | K562 | - | chrX | | 73047160 | 73047221 |
| 5.78731420182694 | PTBP1 (bg=3.74%) | K562 | - | chrX | | 73047167 | 73047229 |
| 3.10154980755977 | TIA1 (bg=4.07%) | K562 | - | chrX | | 73047182 | 73047262 |

  
  

| Match 352 in HUMAN | | | | | | | |
| --- | --- | --- | --- | --- | --- | --- | --- |
| Motif | Start in Seq (1 Indexed) | End in Seq (1 Indexed) | Strand | Chrm | Exon | Start in Chrm (0 Indexed) | End in Chrm (1 Indexed) |
| TGTGTGTG | 12632 | 12639 | - | chrX | 6 | 73047126 | 73047134 |
| eCLIP Fold-Enrichment | Binding Protein | Cell Line | Strand | Chrm | | Start in Chrm (0 Indexed) | End in Chrm (1 Indexed) |
| 2.42033352402212 | AATF (bg=0.64%) | K562 | - | chrX | | 73047088 | 73047142 |
| 2.1598058574823 | DDX24 (bg=2.97%) | K562 | - | chrX | | 73047078 | 73047127 |
| 3.10284131940026 | NCBP2 (bg=1.49%) | K562 | - | chrX | | 73047108 | 73047133 |
| 2.30358118398288 | NOLC1 (bg=9.43%) | K562 | - | chrX | | 73047075 | 73047144 |
| 2.32975953780155 | NOLC1 (bg=9.43%) | K562 | - | chrX | | 73047075 | 73047146 |
| 5.56931608210752 | PTBP1 (bg=3.74%) | K562 | - | chrX | | 73047088 | 73047143 |
| 2.60733612988738 | SND1 (bg=0.45%) | K562 | - | chrX | | 73047069 | 73047153 |
| 2.12823944299506 | SND1 (bg=0.45%) | K562 | - | chrX | | 73047069 | 73047155 |
| 2.35101828622178 | SRSF7 (bg=2.32%) | K562 | - | chrX | | 73047076 | 73047140 |
| 4.93312533065893 | TARDBP (bg=2.79%) | K562 | - | chrX | | 73047066 | 73047148 |
| 4.94783760971273 | TARDBP (bg=2.79%) | K562 | - | chrX | | 73047069 | 73047149 |
| 2.29832154751581 | WDR43 (bg=3.37%) | K562 | - | chrX | | 73047078 | 73047126 |
| 2.27478726891247 | WDR43 (bg=3.37%) | K562 | - | chrX | | 73047084 | 73047126 |
| 2.32487079639227 | XRCC6 (bg=2.91%) | K562 | - | chrX | | 73047079 | 73047142 |
| 2.2027147070379 | ZC3H8 (bg=0.29%) | K562 | - | chrX | | 73047073 | 73047129 |

  
  

| Match 353 in HUMAN | | | | | | | |
| --- | --- | --- | --- | --- | --- | --- | --- |
| Motif | Start in Seq (1 Indexed) | End in Seq (1 Indexed) | Strand | Chrm | Exon | Start in Chrm (0 Indexed) | End in Chrm (1 Indexed) |
| AGGGGCTTCCTAACCCCT | 12655 | 12672 | - | chrX | 6 | 73047093 | 73047111 |
| eCLIP Fold-Enrichment | Binding Protein | Cell Line | Strand | Chrm | | Start in Chrm (0 Indexed) | End in Chrm (1 Indexed) |
| 2.42033352402212 | AATF (bg=0.64%) | K562 | - | chrX | | 73047088 | 73047142 |
| 2.1598058574823 | DDX24 (bg=2.97%) | K562 | - | chrX | | 73047078 | 73047127 |
| 3.0131597934863 | NCBP2 (bg=1.49%) | K562 | - | chrX | | 73047094 | 73047123 |
| 3.10284131940026 | NCBP2 (bg=1.49%) | K562 | - | chrX | | 73047108 | 73047133 |
| 2.30358118398288 | NOLC1 (bg=9.43%) | K562 | - | chrX | | 73047075 | 73047144 |
| 2.32975953780155 | NOLC1 (bg=9.43%) | K562 | - | chrX | | 73047075 | 73047146 |
| 5.56931608210752 | PTBP1 (bg=3.74%) | K562 | - | chrX | | 73047088 | 73047143 |
| 2.60733612988738 | SND1 (bg=0.45%) | K562 | - | chrX | | 73047069 | 73047153 |
| 2.12823944299506 | SND1 (bg=0.45%) | K562 | - | chrX | | 73047069 | 73047155 |
| 2.35101828622178 | SRSF7 (bg=2.32%) | K562 | - | chrX | | 73047076 | 73047140 |
| 4.93312533065893 | TARDBP (bg=2.79%) | K562 | - | chrX | | 73047066 | 73047148 |
| 4.94783760971273 | TARDBP (bg=2.79%) | K562 | - | chrX | | 73047069 | 73047149 |
| 3.79625984442872 | UTP3 (bg=3.66%) | K562 | - | chrX | | 73047089 | 73047121 |
| 2.29832154751581 | WDR43 (bg=3.37%) | K562 | - | chrX | | 73047078 | 73047126 |
| 2.27478726891247 | WDR43 (bg=3.37%) | K562 | - | chrX | | 73047084 | 73047126 |
| 2.32487079639227 | XRCC6 (bg=2.91%) | K562 | - | chrX | | 73047079 | 73047142 |
| 2.2027147070379 | ZC3H8 (bg=0.29%) | K562 | - | chrX | | 73047073 | 73047129 |

  
  

| Match 354 in HUMAN | | | | | | | |
| --- | --- | --- | --- | --- | --- | --- | --- |
| Motif | Start in Seq (1 Indexed) | End in Seq (1 Indexed) | Strand | Chrm | Exon | Start in Chrm (0 Indexed) | End in Chrm (1 Indexed) |
| TCCTAACCCCT | 12662 | 12672 | - | chrX | 6 | 73047093 | 73047104 |
| eCLIP Fold-Enrichment | Binding Protein | Cell Line | Strand | Chrm | | Start in Chrm (0 Indexed) | End in Chrm (1 Indexed) |
| 2.42033352402212 | AATF (bg=0.64%) | K562 | - | chrX | | 73047088 | 73047142 |
| 2.1598058574823 | DDX24 (bg=2.97%) | K562 | - | chrX | | 73047078 | 73047127 |
| 3.0131597934863 | NCBP2 (bg=1.49%) | K562 | - | chrX | | 73047094 | 73047123 |
| 2.30358118398288 | NOLC1 (bg=9.43%) | K562 | - | chrX | | 73047075 | 73047144 |
| 2.32975953780155 | NOLC1 (bg=9.43%) | K562 | - | chrX | | 73047075 | 73047146 |
| 5.56931608210752 | PTBP1 (bg=3.74%) | K562 | - | chrX | | 73047088 | 73047143 |
| 2.60733612988738 | SND1 (bg=0.45%) | K562 | - | chrX | | 73047069 | 73047153 |
| 2.12823944299506 | SND1 (bg=0.45%) | K562 | - | chrX | | 73047069 | 73047155 |
| 2.35101828622178 | SRSF7 (bg=2.32%) | K562 | - | chrX | | 73047076 | 73047140 |
| 4.93312533065893 | TARDBP (bg=2.79%) | K562 | - | chrX | | 73047066 | 73047148 |
| 4.94783760971273 | TARDBP (bg=2.79%) | K562 | - | chrX | | 73047069 | 73047149 |
| 3.79625984442872 | UTP3 (bg=3.66%) | K562 | - | chrX | | 73047089 | 73047121 |
| 2.29832154751581 | WDR43 (bg=3.37%) | K562 | - | chrX | | 73047078 | 73047126 |
| 2.27478726891247 | WDR43 (bg=3.37%) | K562 | - | chrX | | 73047084 | 73047126 |
| 2.32487079639227 | XRCC6 (bg=2.91%) | K562 | - | chrX | | 73047079 | 73047142 |
| 2.2027147070379 | ZC3H8 (bg=0.29%) | K562 | - | chrX | | 73047073 | 73047129 |

  
  

| Match 355 in HUMAN | | | | | | | |
| --- | --- | --- | --- | --- | --- | --- | --- |
| Motif | Start in Seq (1 Indexed) | End in Seq (1 Indexed) | Strand | Chrm | Exon | Start in Chrm (0 Indexed) | End in Chrm (1 Indexed) |
| TAGGTGCA | 12679 | 12686 | - | chrX | 6 | 73047079 | 73047087 |
| eCLIP Fold-Enrichment | Binding Protein | Cell Line | Strand | Chrm | | Start in Chrm (0 Indexed) | End in Chrm (1 Indexed) |
| 2.1598058574823 | DDX24 (bg=2.97%) | K562 | - | chrX | | 73047078 | 73047127 |
| 2.30358118398288 | NOLC1 (bg=9.43%) | K562 | - | chrX | | 73047075 | 73047144 |
| 2.32975953780155 | NOLC1 (bg=9.43%) | K562 | - | chrX | | 73047075 | 73047146 |
| 2.60733612988738 | SND1 (bg=0.45%) | K562 | - | chrX | | 73047069 | 73047153 |
| 2.12823944299506 | SND1 (bg=0.45%) | K562 | - | chrX | | 73047069 | 73047155 |
| 2.35101828622178 | SRSF7 (bg=2.32%) | K562 | - | chrX | | 73047076 | 73047140 |
| 4.93312533065893 | TARDBP (bg=2.79%) | K562 | - | chrX | | 73047066 | 73047148 |
| 4.94783760971273 | TARDBP (bg=2.79%) | K562 | - | chrX | | 73047069 | 73047149 |
| 4.51580838551751 | UTP3 (bg=3.66%) | K562 | - | chrX | | 73047084 | 73047089 |
| 2.29832154751581 | WDR43 (bg=3.37%) | K562 | - | chrX | | 73047078 | 73047126 |
| 2.27478726891247 | WDR43 (bg=3.37%) | K562 | - | chrX | | 73047084 | 73047126 |
| 2.32487079639227 | XRCC6 (bg=2.91%) | K562 | - | chrX | | 73047079 | 73047142 |
| 2.2027147070379 | ZC3H8 (bg=0.29%) | K562 | - | chrX | | 73047073 | 73047129 |

  
  

| Match 356 in HUMAN | | | | | | | |
| --- | --- | --- | --- | --- | --- | --- | --- |
| Motif | Start in Seq (1 Indexed) | End in Seq (1 Indexed) | Strand | Chrm | Exon | Start in Chrm (0 Indexed) | End in Chrm (1 Indexed) |
| AAGCATTG | 12706 | 12713 | - | chrX | 6 | 73047052 | 73047060 |
| eCLIP Fold-Enrichment | Binding Protein | Cell Line | Strand | Chrm | | Start in Chrm (0 Indexed) | End in Chrm (1 Indexed) |
| 2.38580179576284 | DDX24 (bg=2.97%) | K562 | - | chrX | | 73046956 | 73047053 |
| 2.48946443498428 | NOLC1 (bg=9.43%) | K562 | - | chrX | | 73047000 | 73047069 |
| 2.27507721116987 | NOLC1 (bg=9.43%) | K562 | - | chrX | | 73047008 | 73047075 |
| 2.22270523417746 | NPM1 (bg=1.21%) | K562 | - | chrX | | 73047008 | 73047059 |
| 2.21265441538398 | RBFOX2 (bg=4.63%) | K562 | - | chrX | | 73046971 | 73047052 |
| 2.05720325771486 | RPS3 (bg=0.76%) | K562 | - | chrX | | 73047022 | 73047060 |
| 2.16054334637051 | SRSF1 (bg=8.47%) | K562 | - | chrX | | 73046997 | 73047063 |
| 2.15837320827939 | SRSF7 (bg=2.32%) | K562 | - | chrX | | 73046995 | 73047076 |
| 4.21463915710039 | TARDBP (bg=2.79%) | K562 | - | chrX | | 73047011 | 73047066 |
| 4.09984070315778 | TARDBP (bg=2.79%) | K562 | - | chrX | | 73047038 | 73047055 |
| 4.37585296298978 | TARDBP (bg=2.79%) | K562 | - | chrX | | 73047055 | 73047069 |
| 2.50246775752068 | TRA2A (bg=4.8%) | K562 | - | chrX | | 73046987 | 73047066 |
| 2.03628930487736 | U2AF2 (bg=1.76%) | K562 | - | chrX | | 73047011 | 73047063 |
| 3.33964462611812 | uchl5 (bg=11.16%) | K562 | - | chrX | | 73046983 | 73047056 |
| 3.06101347196629 | uchl5 (bg=11.16%) | K562 | - | chrX | | 73046989 | 73047057 |
| 2.16101459964944 | YWHAG (bg=1.87%) | K562 | - | chrX | | 73047010 | 73047052 |
| 2.99876022300722 | ZNF622 (bg=6.58%) | K562 | - | chrX | | 73046994 | 73047071 |
| 2.77941262243908 | ZNF622 (bg=6.58%) | K562 | - | chrX | | 73047011 | 73047071 |

  
  

| Match 357 in HUMAN | | | | | | | |
| --- | --- | --- | --- | --- | --- | --- | --- |
| Motif | Start in Seq (1 Indexed) | End in Seq (1 Indexed) | Strand | Chrm | Exon | Start in Chrm (0 Indexed) | End in Chrm (1 Indexed) |
| GTTCCTTATGCCAG | 12721 | 12734 | - | chrX | 6 | 73047031 | 73047045 |
| eCLIP Fold-Enrichment | Binding Protein | Cell Line | Strand | Chrm | | Start in Chrm (0 Indexed) | End in Chrm (1 Indexed) |
| 2.38580179576284 | DDX24 (bg=2.97%) | K562 | - | chrX | | 73046956 | 73047053 |
| 2.15261145927208 | FASTKD2 (bg=1.99%) | K562 | - | chrX | | 73046998 | 73047050 |
| 2.8684084816083 | LARP4 (bg=4.72%) | K562 | - | chrX | | 73046992 | 73047045 |
| 2.48946443498428 | NOLC1 (bg=9.43%) | K562 | - | chrX | | 73047000 | 73047069 |
| 2.27507721116987 | NOLC1 (bg=9.43%) | K562 | - | chrX | | 73047008 | 73047075 |
| 2.22270523417746 | NPM1 (bg=1.21%) | K562 | - | chrX | | 73047008 | 73047059 |
| 2.21265441538398 | RBFOX2 (bg=4.63%) | K562 | - | chrX | | 73046971 | 73047052 |
| 4.67057614530219 | RBM15 (bg=7.27%) | K562 | - | chrX | | 73047002 | 73047047 |
| 3.61758863258058 | RBM15 (bg=7.27%) | K562 | - | chrX | | 73047025 | 73047038 |
| 2.05720325771486 | RPS3 (bg=0.76%) | K562 | - | chrX | | 73047022 | 73047060 |
| 2.16054334637051 | SRSF1 (bg=8.47%) | K562 | - | chrX | | 73046997 | 73047063 |
| 2.15837320827939 | SRSF7 (bg=2.32%) | K562 | - | chrX | | 73046995 | 73047076 |
| 3.87454839131553 | TARDBP (bg=2.79%) | K562 | - | chrX | | 73047009 | 73047038 |
| 4.21463915710039 | TARDBP (bg=2.79%) | K562 | - | chrX | | 73047011 | 73047066 |
| 4.09984070315778 | TARDBP (bg=2.79%) | K562 | - | chrX | | 73047038 | 73047055 |
| 2.50246775752068 | TRA2A (bg=4.8%) | K562 | - | chrX | | 73046987 | 73047066 |
| 2.25910953324272 | TRA2A (bg=4.8%) | K562 | - | chrX | | 73047008 | 73047050 |
| 2.03628930487736 | U2AF2 (bg=1.76%) | K562 | - | chrX | | 73047011 | 73047063 |
| 3.33964462611812 | uchl5 (bg=11.16%) | K562 | - | chrX | | 73046983 | 73047056 |
| 3.06101347196629 | uchl5 (bg=11.16%) | K562 | - | chrX | | 73046989 | 73047057 |
| 2.49717969024892 | WDR43 (bg=3.37%) | K562 | - | chrX | | 73047004 | 73047034 |
| 2.52071396885226 | WDR43 (bg=3.37%) | K562 | - | chrX | | 73047012 | 73047045 |
| 2.39278225148292 | YWHAG (bg=1.87%) | K562 | - | chrX | | 73047006 | 73047044 |
| 2.16101459964944 | YWHAG (bg=1.87%) | K562 | - | chrX | | 73047010 | 73047052 |
| 2.10467866866754 | ZC3H11A (bg=6.55%) | K562 | - | chrX | | 73047013 | 73047032 |
| 2.10467866866754 | ZC3H11A (bg=6.55%) | K562 | - | chrX | | 73047013 | 73047032 |
| 2.99876022300722 | ZNF622 (bg=6.58%) | K562 | - | chrX | | 73046994 | 73047071 |
| 2.77941262243908 | ZNF622 (bg=6.58%) | K562 | - | chrX | | 73047011 | 73047071 |
| 2.25969830647816 | ZNF800 (bg=1.92%) | K562 | - | chrX | | 73047005 | 73047051 |

  
  

| Match 358 in HUMAN | | | | | | | |
| --- | --- | --- | --- | --- | --- | --- | --- |
| Motif | Start in Seq (1 Indexed) | End in Seq (1 Indexed) | Strand | Chrm | Exon | Start in Chrm (0 Indexed) | End in Chrm (1 Indexed) |
| TTATGCCAG | 12726 | 12734 | - | chrX | 6 | 73047031 | 73047040 |
| eCLIP Fold-Enrichment | Binding Protein | Cell Line | Strand | Chrm | | Start in Chrm (0 Indexed) | End in Chrm (1 Indexed) |
| 2.38580179576284 | DDX24 (bg=2.97%) | K562 | - | chrX | | 73046956 | 73047053 |
| 2.15261145927208 | FASTKD2 (bg=1.99%) | K562 | - | chrX | | 73046998 | 73047050 |
| 2.8684084816083 | LARP4 (bg=4.72%) | K562 | - | chrX | | 73046992 | 73047045 |
| 2.48946443498428 | NOLC1 (bg=9.43%) | K562 | - | chrX | | 73047000 | 73047069 |
| 2.27507721116987 | NOLC1 (bg=9.43%) | K562 | - | chrX | | 73047008 | 73047075 |
| 2.22270523417746 | NPM1 (bg=1.21%) | K562 | - | chrX | | 73047008 | 73047059 |
| 2.21265441538398 | RBFOX2 (bg=4.63%) | K562 | - | chrX | | 73046971 | 73047052 |
| 4.67057614530219 | RBM15 (bg=7.27%) | K562 | - | chrX | | 73047002 | 73047047 |
| 3.61758863258058 | RBM15 (bg=7.27%) | K562 | - | chrX | | 73047025 | 73047038 |
| 2.05720325771486 | RPS3 (bg=0.76%) | K562 | - | chrX | | 73047022 | 73047060 |
| 2.16054334637051 | SRSF1 (bg=8.47%) | K562 | - | chrX | | 73046997 | 73047063 |
| 2.15837320827939 | SRSF7 (bg=2.32%) | K562 | - | chrX | | 73046995 | 73047076 |
| 3.87454839131553 | TARDBP (bg=2.79%) | K562 | - | chrX | | 73047009 | 73047038 |
| 4.21463915710039 | TARDBP (bg=2.79%) | K562 | - | chrX | | 73047011 | 73047066 |
| 4.09984070315778 | TARDBP (bg=2.79%) | K562 | - | chrX | | 73047038 | 73047055 |
| 2.50246775752068 | TRA2A (bg=4.8%) | K562 | - | chrX | | 73046987 | 73047066 |
| 2.25910953324272 | TRA2A (bg=4.8%) | K562 | - | chrX | | 73047008 | 73047050 |
| 2.03628930487736 | U2AF2 (bg=1.76%) | K562 | - | chrX | | 73047011 | 73047063 |
| 3.33964462611812 | uchl5 (bg=11.16%) | K562 | - | chrX | | 73046983 | 73047056 |
| 3.06101347196629 | uchl5 (bg=11.16%) | K562 | - | chrX | | 73046989 | 73047057 |
| 2.49717969024892 | WDR43 (bg=3.37%) | K562 | - | chrX | | 73047004 | 73047034 |
| 2.52071396885226 | WDR43 (bg=3.37%) | K562 | - | chrX | | 73047012 | 73047045 |
| 2.39278225148292 | YWHAG (bg=1.87%) | K562 | - | chrX | | 73047006 | 73047044 |
| 2.16101459964944 | YWHAG (bg=1.87%) | K562 | - | chrX | | 73047010 | 73047052 |
| 2.10467866866754 | ZC3H11A (bg=6.55%) | K562 | - | chrX | | 73047013 | 73047032 |
| 2.10467866866754 | ZC3H11A (bg=6.55%) | K562 | - | chrX | | 73047013 | 73047032 |
| 2.99876022300722 | ZNF622 (bg=6.58%) | K562 | - | chrX | | 73046994 | 73047071 |
| 2.77941262243908 | ZNF622 (bg=6.58%) | K562 | - | chrX | | 73047011 | 73047071 |
| 2.25969830647816 | ZNF800 (bg=1.92%) | K562 | - | chrX | | 73047005 | 73047051 |

  
  

| Match 359 in HUMAN | | | | | | | |
| --- | --- | --- | --- | --- | --- | --- | --- |
| Motif | Start in Seq (1 Indexed) | End in Seq (1 Indexed) | Strand | Chrm | Exon | Start in Chrm (0 Indexed) | End in Chrm (1 Indexed) |
| TTATGCCA | 12726 | 12733 | - | chrX | 6 | 73047032 | 73047040 |
| eCLIP Fold-Enrichment | Binding Protein | Cell Line | Strand | Chrm | | Start in Chrm (0 Indexed) | End in Chrm (1 Indexed) |
| 2.38580179576284 | DDX24 (bg=2.97%) | K562 | - | chrX | | 73046956 | 73047053 |
| 2.15261145927208 | FASTKD2 (bg=1.99%) | K562 | - | chrX | | 73046998 | 73047050 |
| 2.8684084816083 | LARP4 (bg=4.72%) | K562 | - | chrX | | 73046992 | 73047045 |
| 2.48946443498428 | NOLC1 (bg=9.43%) | K562 | - | chrX | | 73047000 | 73047069 |
| 2.27507721116987 | NOLC1 (bg=9.43%) | K562 | - | chrX | | 73047008 | 73047075 |
| 2.22270523417746 | NPM1 (bg=1.21%) | K562 | - | chrX | | 73047008 | 73047059 |
| 2.21265441538398 | RBFOX2 (bg=4.63%) | K562 | - | chrX | | 73046971 | 73047052 |
| 4.67057614530219 | RBM15 (bg=7.27%) | K562 | - | chrX | | 73047002 | 73047047 |
| 3.61758863258058 | RBM15 (bg=7.27%) | K562 | - | chrX | | 73047025 | 73047038 |
| 2.05720325771486 | RPS3 (bg=0.76%) | K562 | - | chrX | | 73047022 | 73047060 |
| 2.16054334637051 | SRSF1 (bg=8.47%) | K562 | - | chrX | | 73046997 | 73047063 |
| 2.15837320827939 | SRSF7 (bg=2.32%) | K562 | - | chrX | | 73046995 | 73047076 |
| 3.87454839131553 | TARDBP (bg=2.79%) | K562 | - | chrX | | 73047009 | 73047038 |
| 4.21463915710039 | TARDBP (bg=2.79%) | K562 | - | chrX | | 73047011 | 73047066 |
| 4.09984070315778 | TARDBP (bg=2.79%) | K562 | - | chrX | | 73047038 | 73047055 |
| 2.50246775752068 | TRA2A (bg=4.8%) | K562 | - | chrX | | 73046987 | 73047066 |
| 2.25910953324272 | TRA2A (bg=4.8%) | K562 | - | chrX | | 73047008 | 73047050 |
| 2.03628930487736 | U2AF2 (bg=1.76%) | K562 | - | chrX | | 73047011 | 73047063 |
| 3.33964462611812 | uchl5 (bg=11.16%) | K562 | - | chrX | | 73046983 | 73047056 |
| 3.06101347196629 | uchl5 (bg=11.16%) | K562 | - | chrX | | 73046989 | 73047057 |
| 2.49717969024892 | WDR43 (bg=3.37%) | K562 | - | chrX | | 73047004 | 73047034 |
| 2.52071396885226 | WDR43 (bg=3.37%) | K562 | - | chrX | | 73047012 | 73047045 |
| 2.39278225148292 | YWHAG (bg=1.87%) | K562 | - | chrX | | 73047006 | 73047044 |
| 2.16101459964944 | YWHAG (bg=1.87%) | K562 | - | chrX | | 73047010 | 73047052 |
| 2.10467866866754 | ZC3H11A (bg=6.55%) | K562 | - | chrX | | 73047013 | 73047032 |
| 2.10467866866754 | ZC3H11A (bg=6.55%) | K562 | - | chrX | | 73047013 | 73047032 |
| 2.99876022300722 | ZNF622 (bg=6.58%) | K562 | - | chrX | | 73046994 | 73047071 |
| 2.77941262243908 | ZNF622 (bg=6.58%) | K562 | - | chrX | | 73047011 | 73047071 |
| 2.25969830647816 | ZNF800 (bg=1.92%) | K562 | - | chrX | | 73047005 | 73047051 |

  
  

| Match 360 in HUMAN | | | | | | | |
| --- | --- | --- | --- | --- | --- | --- | --- |
| Motif | Start in Seq (1 Indexed) | End in Seq (1 Indexed) | Strand | Chrm | Exon | Start in Chrm (0 Indexed) | End in Chrm (1 Indexed) |
| ATGATCCAAGACCAA | 12748 | 12762 | - | chrX | 6 | 73047003 | 73047018 |
| eCLIP Fold-Enrichment | Binding Protein | Cell Line | Strand | Chrm | | Start in Chrm (0 Indexed) | End in Chrm (1 Indexed) |
| 2.38580179576284 | DDX24 (bg=2.97%) | K562 | - | chrX | | 73046956 | 73047053 |
| 2.15261145927208 | FASTKD2 (bg=1.99%) | K562 | - | chrX | | 73046998 | 73047050 |
| 2.8684084816083 | LARP4 (bg=4.72%) | K562 | - | chrX | | 73046992 | 73047045 |
| 2.35421258152609 | NOLC1 (bg=9.43%) | K562 | - | chrX | | 73046861 | 73047008 |
| 2.48946443498428 | NOLC1 (bg=9.43%) | K562 | - | chrX | | 73047000 | 73047069 |
| 2.27507721116987 | NOLC1 (bg=9.43%) | K562 | - | chrX | | 73047008 | 73047075 |
| 2.22270523417746 | NPM1 (bg=1.21%) | K562 | - | chrX | | 73047008 | 73047059 |
| 2.21265441538398 | RBFOX2 (bg=4.63%) | K562 | - | chrX | | 73046971 | 73047052 |
| 4.67057614530219 | RBM15 (bg=7.27%) | K562 | - | chrX | | 73047002 | 73047047 |
| 2.16054334637051 | SRSF1 (bg=8.47%) | K562 | - | chrX | | 73046997 | 73047063 |
| 2.15837320827939 | SRSF7 (bg=2.32%) | K562 | - | chrX | | 73046995 | 73047076 |
| 3.87454839131553 | TARDBP (bg=2.79%) | K562 | - | chrX | | 73047009 | 73047038 |
| 4.21463915710039 | TARDBP (bg=2.79%) | K562 | - | chrX | | 73047011 | 73047066 |
| 2.50246775752068 | TRA2A (bg=4.8%) | K562 | - | chrX | | 73046987 | 73047066 |
| 2.25910953324272 | TRA2A (bg=4.8%) | K562 | - | chrX | | 73047008 | 73047050 |
| 2.03628930487736 | U2AF2 (bg=1.76%) | K562 | - | chrX | | 73047011 | 73047063 |
| 3.33964462611812 | uchl5 (bg=11.16%) | K562 | - | chrX | | 73046983 | 73047056 |
| 3.06101347196629 | uchl5 (bg=11.16%) | K562 | - | chrX | | 73046989 | 73047057 |
| 2.49717969024892 | WDR43 (bg=3.37%) | K562 | - | chrX | | 73047004 | 73047034 |
| 2.52071396885226 | WDR43 (bg=3.37%) | K562 | - | chrX | | 73047012 | 73047045 |
| 2.39278225148292 | YWHAG (bg=1.87%) | K562 | - | chrX | | 73047006 | 73047044 |
| 2.16101459964944 | YWHAG (bg=1.87%) | K562 | - | chrX | | 73047010 | 73047052 |
| 2.10467866866754 | ZC3H11A (bg=6.55%) | K562 | - | chrX | | 73047013 | 73047032 |
| 2.10467866866754 | ZC3H11A (bg=6.55%) | K562 | - | chrX | | 73047013 | 73047032 |
| 2.51847059556652 | ZNF622 (bg=6.58%) | K562 | - | chrX | | 73046952 | 73047011 |
| 2.99876022300722 | ZNF622 (bg=6.58%) | K562 | - | chrX | | 73046994 | 73047071 |
| 2.77941262243908 | ZNF622 (bg=6.58%) | K562 | - | chrX | | 73047011 | 73047071 |
| 2.25969830647816 | ZNF800 (bg=1.92%) | K562 | - | chrX | | 73047005 | 73047051 |

  
  

| Match 361 in HUMAN | | | | | | | |
| --- | --- | --- | --- | --- | --- | --- | --- |
| Motif | Start in Seq (1 Indexed) | End in Seq (1 Indexed) | Strand | Chrm | Exon | Start in Chrm (0 Indexed) | End in Chrm (1 Indexed) |
| TCCAAG | 12752 | 12757 | - | chrX | 6 | 73047008 | 73047014 |
| eCLIP Fold-Enrichment | Binding Protein | Cell Line | Strand | Chrm | | Start in Chrm (0 Indexed) | End in Chrm (1 Indexed) |
| 2.38580179576284 | DDX24 (bg=2.97%) | K562 | - | chrX | | 73046956 | 73047053 |
| 2.15261145927208 | FASTKD2 (bg=1.99%) | K562 | - | chrX | | 73046998 | 73047050 |
| 2.8684084816083 | LARP4 (bg=4.72%) | K562 | - | chrX | | 73046992 | 73047045 |
| 2.35421258152609 | NOLC1 (bg=9.43%) | K562 | - | chrX | | 73046861 | 73047008 |
| 2.48946443498428 | NOLC1 (bg=9.43%) | K562 | - | chrX | | 73047000 | 73047069 |
| 2.27507721116987 | NOLC1 (bg=9.43%) | K562 | - | chrX | | 73047008 | 73047075 |
| 2.22270523417746 | NPM1 (bg=1.21%) | K562 | - | chrX | | 73047008 | 73047059 |
| 2.21265441538398 | RBFOX2 (bg=4.63%) | K562 | - | chrX | | 73046971 | 73047052 |
| 4.67057614530219 | RBM15 (bg=7.27%) | K562 | - | chrX | | 73047002 | 73047047 |
| 2.16054334637051 | SRSF1 (bg=8.47%) | K562 | - | chrX | | 73046997 | 73047063 |
| 2.15837320827939 | SRSF7 (bg=2.32%) | K562 | - | chrX | | 73046995 | 73047076 |
| 3.87454839131553 | TARDBP (bg=2.79%) | K562 | - | chrX | | 73047009 | 73047038 |
| 4.21463915710039 | TARDBP (bg=2.79%) | K562 | - | chrX | | 73047011 | 73047066 |
| 2.50246775752068 | TRA2A (bg=4.8%) | K562 | - | chrX | | 73046987 | 73047066 |
| 2.25910953324272 | TRA2A (bg=4.8%) | K562 | - | chrX | | 73047008 | 73047050 |
| 2.03628930487736 | U2AF2 (bg=1.76%) | K562 | - | chrX | | 73047011 | 73047063 |
| 3.33964462611812 | uchl5 (bg=11.16%) | K562 | - | chrX | | 73046983 | 73047056 |
| 3.06101347196629 | uchl5 (bg=11.16%) | K562 | - | chrX | | 73046989 | 73047057 |
| 2.49717969024892 | WDR43 (bg=3.37%) | K562 | - | chrX | | 73047004 | 73047034 |
| 2.52071396885226 | WDR43 (bg=3.37%) | K562 | - | chrX | | 73047012 | 73047045 |
| 2.39278225148292 | YWHAG (bg=1.87%) | K562 | - | chrX | | 73047006 | 73047044 |
| 2.16101459964944 | YWHAG (bg=1.87%) | K562 | - | chrX | | 73047010 | 73047052 |
| 2.10467866866754 | ZC3H11A (bg=6.55%) | K562 | - | chrX | | 73047013 | 73047032 |
| 2.10467866866754 | ZC3H11A (bg=6.55%) | K562 | - | chrX | | 73047013 | 73047032 |
| 2.51847059556652 | ZNF622 (bg=6.58%) | K562 | - | chrX | | 73046952 | 73047011 |
| 2.99876022300722 | ZNF622 (bg=6.58%) | K562 | - | chrX | | 73046994 | 73047071 |
| 2.77941262243908 | ZNF622 (bg=6.58%) | K562 | - | chrX | | 73047011 | 73047071 |
| 2.25969830647816 | ZNF800 (bg=1.92%) | K562 | - | chrX | | 73047005 | 73047051 |

  
  

| Match 362 in HUMAN | | | | | | | |
| --- | --- | --- | --- | --- | --- | --- | --- |
| Motif | Start in Seq (1 Indexed) | End in Seq (1 Indexed) | Strand | Chrm | Exon | Start in Chrm (0 Indexed) | End in Chrm (1 Indexed) |
| TAGAAGGCCCAA | 12830 | 12841 | - | chrX | 6 | 73046924 | 73046936 |
| eCLIP Fold-Enrichment | Binding Protein | Cell Line | Strand | Chrm | | Start in Chrm (0 Indexed) | End in Chrm (1 Indexed) |
| 2.02009200884756 | DDX24 (bg=2.97%) | K562 | - | chrX | | 73046779 | 73046956 |
| 3.29564127785745 | LARP4 (bg=4.72%) | K562 | - | chrX | | 73046875 | 73046992 |
| 2.28282850105752 | MTPAP (bg=2.21%) | K562 | - | chrX | | 73046929 | 73046990 |
| 2.35421258152609 | NOLC1 (bg=9.43%) | K562 | - | chrX | | 73046861 | 73047008 |
| 2.31689892807304 | SRSF1 (bg=8.47%) | K562 | - | chrX | | 73046839 | 73046965 |
| 2.22548740413792 | SRSF7 (bg=2.32%) | K562 | - | chrX | | 73046883 | 73046932 |
| 2.25468112538583 | TRA2A (bg=4.8%) | K562 | - | chrX | | 73046916 | 73046987 |
| 2.88018408435054 | uchl5 (bg=11.16%) | K562 | - | chrX | | 73046915 | 73046989 |
| 3.1232985808486 | uchl5 (bg=11.16%) | K562 | - | chrX | | 73046917 | 73046983 |
| 3.20910227059656 | UTP3 (bg=3.66%) | K562 | - | chrX | | 73046864 | 73046928 |
| 2.99960542621091 | ZNF622 (bg=6.58%) | K562 | - | chrX | | 73046865 | 73046952 |
| 3.1872135458297 | ZNF622 (bg=6.58%) | K562 | - | chrX | | 73046912 | 73046994 |
| 2.03730588514171 | ZNF800 (bg=1.92%) | K562 | - | chrX | | 73046896 | 73046940 |

  
  

| Match 363 in HUMAN | | | | | | | |
| --- | --- | --- | --- | --- | --- | --- | --- |
| Motif | Start in Seq (1 Indexed) | End in Seq (1 Indexed) | Strand | Chrm | Exon | Start in Chrm (0 Indexed) | End in Chrm (1 Indexed) |
| AGAAGGCCCAA | 12831 | 12841 | - | chrX | 6 | 73046924 | 73046935 |
| eCLIP Fold-Enrichment | Binding Protein | Cell Line | Strand | Chrm | | Start in Chrm (0 Indexed) | End in Chrm (1 Indexed) |
| 2.02009200884756 | DDX24 (bg=2.97%) | K562 | - | chrX | | 73046779 | 73046956 |
| 3.29564127785745 | LARP4 (bg=4.72%) | K562 | - | chrX | | 73046875 | 73046992 |
| 2.28282850105752 | MTPAP (bg=2.21%) | K562 | - | chrX | | 73046929 | 73046990 |
| 2.35421258152609 | NOLC1 (bg=9.43%) | K562 | - | chrX | | 73046861 | 73047008 |
| 2.31689892807304 | SRSF1 (bg=8.47%) | K562 | - | chrX | | 73046839 | 73046965 |
| 2.22548740413792 | SRSF7 (bg=2.32%) | K562 | - | chrX | | 73046883 | 73046932 |
| 2.25468112538583 | TRA2A (bg=4.8%) | K562 | - | chrX | | 73046916 | 73046987 |
| 2.88018408435054 | uchl5 (bg=11.16%) | K562 | - | chrX | | 73046915 | 73046989 |
| 3.1232985808486 | uchl5 (bg=11.16%) | K562 | - | chrX | | 73046917 | 73046983 |
| 3.20910227059656 | UTP3 (bg=3.66%) | K562 | - | chrX | | 73046864 | 73046928 |
| 2.99960542621091 | ZNF622 (bg=6.58%) | K562 | - | chrX | | 73046865 | 73046952 |
| 3.1872135458297 | ZNF622 (bg=6.58%) | K562 | - | chrX | | 73046912 | 73046994 |
| 2.03730588514171 | ZNF800 (bg=1.92%) | K562 | - | chrX | | 73046896 | 73046940 |

  
  

| Match 364 in HUMAN | | | | | | | |
| --- | --- | --- | --- | --- | --- | --- | --- |
| Motif | Start in Seq (1 Indexed) | End in Seq (1 Indexed) | Strand | Chrm | Exon | Start in Chrm (0 Indexed) | End in Chrm (1 Indexed) |
| AGGCCCAA | 12834 | 12841 | - | chrX | 6 | 73046924 | 73046932 |
| eCLIP Fold-Enrichment | Binding Protein | Cell Line | Strand | Chrm | | Start in Chrm (0 Indexed) | End in Chrm (1 Indexed) |
| 2.02009200884756 | DDX24 (bg=2.97%) | K562 | - | chrX | | 73046779 | 73046956 |
| 3.29564127785745 | LARP4 (bg=4.72%) | K562 | - | chrX | | 73046875 | 73046992 |
| 2.28282850105752 | MTPAP (bg=2.21%) | K562 | - | chrX | | 73046929 | 73046990 |
| 2.35421258152609 | NOLC1 (bg=9.43%) | K562 | - | chrX | | 73046861 | 73047008 |
| 2.31689892807304 | SRSF1 (bg=8.47%) | K562 | - | chrX | | 73046839 | 73046965 |
| 2.22548740413792 | SRSF7 (bg=2.32%) | K562 | - | chrX | | 73046883 | 73046932 |
| 2.25468112538583 | TRA2A (bg=4.8%) | K562 | - | chrX | | 73046916 | 73046987 |
| 2.88018408435054 | uchl5 (bg=11.16%) | K562 | - | chrX | | 73046915 | 73046989 |
| 3.1232985808486 | uchl5 (bg=11.16%) | K562 | - | chrX | | 73046917 | 73046983 |
| 3.20910227059656 | UTP3 (bg=3.66%) | K562 | - | chrX | | 73046864 | 73046928 |
| 2.99960542621091 | ZNF622 (bg=6.58%) | K562 | - | chrX | | 73046865 | 73046952 |
| 3.1872135458297 | ZNF622 (bg=6.58%) | K562 | - | chrX | | 73046912 | 73046994 |
| 2.03730588514171 | ZNF800 (bg=1.92%) | K562 | - | chrX | | 73046896 | 73046940 |

  
  

| Match 365 in HUMAN | | | | | | | |
| --- | --- | --- | --- | --- | --- | --- | --- |
| Motif | Start in Seq (1 Indexed) | End in Seq (1 Indexed) | Strand | Chrm | Exon | Start in Chrm (0 Indexed) | End in Chrm (1 Indexed) |
| GAGTTGGATGGAAG | 12990 | 13003 | - | chrX | 6 | 73046762 | 73046776 |
| eCLIP Fold-Enrichment | Binding Protein | Cell Line | Strand | Chrm | | Start in Chrm (0 Indexed) | End in Chrm (1 Indexed) |
| 2.37482539042713 | AARS (bg=2.18%) | K562 | - | chrX | | 73046762 | 73046800 |
| 3.4208144718716 | CPEB4 (bg=1.89%) | K562 | - | chrX | | 73046720 | 73046783 |
| 2.08739736195425 | DDX24 (bg=2.97%) | K562 | - | chrX | | 73046698 | 73046792 |
| 2.0668815852462 | FASTKD2 (bg=1.99%) | K562 | - | chrX | | 73046757 | 73046796 |
| 2.46716266462279 | GRWD1 (bg=5.13%) | K562 | - | chrX | | 73046668 | 73046865 |
| 4.78557858469441 | GRWD1 (bg=5.13%) | K562 | - | chrX | | 73046741 | 73046803 |
| 2.31224310276683 | HLTF (bg=0.4%) | K562 | - | chrX | | 73046740 | 73046806 |
| 2.21935472504878 | HLTF (bg=0.4%) | K562 | - | chrX | | 73046745 | 73046817 |
| 2.59025455132217 | LARP4 (bg=4.72%) | K562 | - | chrX | | 73046707 | 73046783 |
| 3.5347985816733 | LARP4 (bg=4.72%) | K562 | - | chrX | | 73046710 | 73046801 |
| 2.03906770843957 | METAP2 (bg=0.78%) | K562 | - | chrX | | 73046692 | 73046801 |
| 2.70909325575962 | MTPAP (bg=2.21%) | K562 | - | chrX | | 73046746 | 73046800 |
| 2.3577239520332 | NOLC1 (bg=9.43%) | K562 | - | chrX | | 73046709 | 73046787 |
| 2.45207022028861 | PUM1 (bg=1.56%) | K562 | - | chrX | | 73046735 | 73046772 |
| 2.09324826728427 | RBFOX2 (bg=4.63%) | K562 | - | chrX | | 73046698 | 73046793 |
| 2.33961339039485 | RPS11 (bg=0.63%) | K562 | - | chrX | | 73046729 | 73046769 |
| 3.91657339959358 | RPS11 (bg=0.63%) | K562 | - | chrX | | 73046773 | 73046854 |
| 2.04548724060692 | SAFB (bg=2.69%) | K562 | - | chrX | | 73046658 | 73046792 |
| 2.04622054079025 | SLTM (bg=2.2%) | K562 | - | chrX | | 73046758 | 73046800 |
| 3.6199749650078 | SRSF1 (bg=8.47%) | K562 | - | chrX | | 73046682 | 73046796 |
| 3.13403487092323 | SRSF1 (bg=8.47%) | K562 | - | chrX | | 73046712 | 73046777 |
| 3.35421679893674 | TRA2A (bg=4.8%) | K562 | - | chrX | | 73046743 | 73046870 |
| 2.5968049439884 | TRA2A (bg=4.8%) | K562 | - | chrX | | 73046753 | 73046826 |
| 3.4971859031046 | uchl5 (bg=11.16%) | K562 | - | chrX | | 73046740 | 73046819 |
| 3.23561209449123 | uchl5 (bg=11.16%) | K562 | - | chrX | | 73046745 | 73046799 |
| 2.28066917636258 | XRCC6 (bg=2.91%) | K562 | - | chrX | | 73046675 | 73046808 |
| 2.64584031365623 | ZNF622 (bg=6.58%) | K562 | - | chrX | | 73046695 | 73046834 |
| 2.29095810257127 | ZNF622 (bg=6.58%) | K562 | - | chrX | | 73046702 | 73046803 |
| 2.08020487925966 | ZNF800 (bg=1.92%) | K562 | - | chrX | | 73046688 | 73046849 |
| 2.38522918856202 | ZNF800 (bg=1.92%) | K562 | - | chrX | | 73046725 | 73046787 |

  
  

| Match 366 in HUMAN | | | | | | | |
| --- | --- | --- | --- | --- | --- | --- | --- |
| Motif | Start in Seq (1 Indexed) | End in Seq (1 Indexed) | Strand | Chrm | Exon | Start in Chrm (0 Indexed) | End in Chrm (1 Indexed) |
| AAGTCT | 13022 | 13027 | - | chrX | 6 | 73046738 | 73046744 |
| eCLIP Fold-Enrichment | Binding Protein | Cell Line | Strand | Chrm | | Start in Chrm (0 Indexed) | End in Chrm (1 Indexed) |
| 3.4208144718716 | CPEB4 (bg=1.89%) | K562 | - | chrX | | 73046720 | 73046783 |
| 2.08739736195425 | DDX24 (bg=2.97%) | K562 | - | chrX | | 73046698 | 73046792 |
| 2.10731333880168 | DROSHA (bg=2.49%) | K562 | - | chrX | | 73046722 | 73046756 |
| 2.31842035224216 | FASTKD2 (bg=1.99%) | K562 | - | chrX | | 73046692 | 73046748 |
| 2.46716266462279 | GRWD1 (bg=5.13%) | K562 | - | chrX | | 73046668 | 73046865 |
| 3.33811960772319 | GRWD1 (bg=5.13%) | K562 | - | chrX | | 73046679 | 73046741 |
| 4.78557858469441 | GRWD1 (bg=5.13%) | K562 | - | chrX | | 73046741 | 73046803 |
| 2.31224310276683 | HLTF (bg=0.4%) | K562 | - | chrX | | 73046740 | 73046806 |
| 2.59025455132217 | LARP4 (bg=4.72%) | K562 | - | chrX | | 73046707 | 73046783 |
| 3.5347985816733 | LARP4 (bg=4.72%) | K562 | - | chrX | | 73046710 | 73046801 |
| 2.03906770843957 | METAP2 (bg=0.78%) | K562 | - | chrX | | 73046692 | 73046801 |
| 2.86779100177868 | MTPAP (bg=2.21%) | K562 | - | chrX | | 73046682 | 73046746 |
| 2.2243905862636 | MTPAP (bg=2.21%) | K562 | - | chrX | | 73046691 | 73046752 |
| 2.3577239520332 | NOLC1 (bg=9.43%) | K562 | - | chrX | | 73046709 | 73046787 |
| 4.03006015623507 | NPM1 (bg=1.21%) | K562 | - | chrX | | 73046726 | 73046741 |
| 2.15241274859568 | PPIL4 (bg=0.52%) | K562 | - | chrX | | 73046697 | 73046750 |
| 2.45207022028861 | PUM1 (bg=1.56%) | K562 | - | chrX | | 73046735 | 73046772 |
| 2.09324826728427 | RBFOX2 (bg=4.63%) | K562 | - | chrX | | 73046698 | 73046793 |
| 2.33961339039485 | RPS11 (bg=0.63%) | K562 | - | chrX | | 73046729 | 73046769 |
| 2.04548724060692 | SAFB (bg=2.69%) | K562 | - | chrX | | 73046658 | 73046792 |
| 2.55630843993923 | SLTM (bg=2.2%) | K562 | - | chrX | | 73046679 | 73046740 |
| 3.6199749650078 | SRSF1 (bg=8.47%) | K562 | - | chrX | | 73046682 | 73046796 |
| 3.13403487092323 | SRSF1 (bg=8.47%) | K562 | - | chrX | | 73046712 | 73046777 |
| 4.64967268246291 | TRA2A (bg=4.8%) | K562 | - | chrX | | 73046677 | 73046743 |
| 3.68795283204659 | TRA2A (bg=4.8%) | K562 | - | chrX | | 73046688 | 73046748 |
| 3.35421679893674 | TRA2A (bg=4.8%) | K562 | - | chrX | | 73046743 | 73046870 |
| 3.72868691686402 | uchl5 (bg=11.16%) | K562 | - | chrX | | 73046680 | 73046740 |
| 3.16744059184965 | uchl5 (bg=11.16%) | K562 | - | chrX | | 73046692 | 73046745 |
| 3.4971859031046 | uchl5 (bg=11.16%) | K562 | - | chrX | | 73046740 | 73046819 |
| 2.73128095479192 | WDR43 (bg=3.37%) | K562 | - | chrX | | 73046734 | 73046760 |
| 2.28066917636258 | XRCC6 (bg=2.91%) | K562 | - | chrX | | 73046675 | 73046808 |
| 2.42351539635121 | ZC3H11A (bg=6.55%) | K562 | - | chrX | | 73046719 | 73046751 |
| 2.42351539635121 | ZC3H11A (bg=6.55%) | K562 | - | chrX | | 73046719 | 73046751 |
| 2.64584031365623 | ZNF622 (bg=6.58%) | K562 | - | chrX | | 73046695 | 73046834 |
| 2.29095810257127 | ZNF622 (bg=6.58%) | K562 | - | chrX | | 73046702 | 73046803 |
| 2.08020487925966 | ZNF800 (bg=1.92%) | K562 | - | chrX | | 73046688 | 73046849 |
| 2.38522918856202 | ZNF800 (bg=1.92%) | K562 | - | chrX | | 73046725 | 73046787 |

  
  

| Match 367 in HUMAN | | | | | | | |
| --- | --- | --- | --- | --- | --- | --- | --- |
| Motif | Start in Seq (1 Indexed) | End in Seq (1 Indexed) | Strand | Chrm | Exon | Start in Chrm (0 Indexed) | End in Chrm (1 Indexed) |
| AAGGCCAA | 13080 | 13087 | - | chrX | 6 | 73046678 | 73046686 |
| eCLIP Fold-Enrichment | Binding Protein | Cell Line | Strand | Chrm | | Start in Chrm (0 Indexed) | End in Chrm (1 Indexed) |
| 2.46716266462279 | GRWD1 (bg=5.13%) | K562 | - | chrX | | 73046668 | 73046865 |
| 3.33811960772319 | GRWD1 (bg=5.13%) | K562 | - | chrX | | 73046679 | 73046741 |
| 2.86779100177868 | MTPAP (bg=2.21%) | K562 | - | chrX | | 73046682 | 73046746 |
| 2.04548724060692 | SAFB (bg=2.69%) | K562 | - | chrX | | 73046658 | 73046792 |
| 2.55630843993923 | SLTM (bg=2.2%) | K562 | - | chrX | | 73046679 | 73046740 |
| 2.58520954684713 | SRSF1 (bg=8.47%) | K562 | - | chrX | | 73046606 | 73046682 |
| 3.6199749650078 | SRSF1 (bg=8.47%) | K562 | - | chrX | | 73046682 | 73046796 |
| 4.64967268246291 | TRA2A (bg=4.8%) | K562 | - | chrX | | 73046677 | 73046743 |
| 3.72868691686402 | uchl5 (bg=11.16%) | K562 | - | chrX | | 73046680 | 73046740 |
| 3.20910227059656 | UTP3 (bg=3.66%) | K562 | - | chrX | | 73046673 | 73046691 |
| 2.85974976963363 | WDR43 (bg=3.37%) | K562 | - | chrX | | 73046640 | 73046727 |
| 2.28066917636258 | XRCC6 (bg=2.91%) | K562 | - | chrX | | 73046675 | 73046808 |

  
  

| Match 368 in HUMAN | | | | | | | |
| --- | --- | --- | --- | --- | --- | --- | --- |
| Motif | Start in Seq (1 Indexed) | End in Seq (1 Indexed) | Strand | Chrm | Exon | Start in Chrm (0 Indexed) | End in Chrm (1 Indexed) |
| GACCTAAGA | 13089 | 13097 | - | chrX | 6 | 73046668 | 73046677 |
| eCLIP Fold-Enrichment | Binding Protein | Cell Line | Strand | Chrm | | Start in Chrm (0 Indexed) | End in Chrm (1 Indexed) |
| 2.46716266462279 | GRWD1 (bg=5.13%) | K562 | - | chrX | | 73046668 | 73046865 |
| 2.23096680974726 | NOLC1 (bg=9.43%) | K562 | - | chrX | | 73046638 | 73046676 |
| 2.04548724060692 | SAFB (bg=2.69%) | K562 | - | chrX | | 73046658 | 73046792 |
| 2.56843769506901 | SRSF1 (bg=8.47%) | K562 | - | chrX | | 73046583 | 73046670 |
| 2.58520954684713 | SRSF1 (bg=8.47%) | K562 | - | chrX | | 73046606 | 73046682 |
| 3.11717760163589 | TRA2A (bg=4.8%) | K562 | - | chrX | | 73046576 | 73046677 |
| 4.64967268246291 | TRA2A (bg=4.8%) | K562 | - | chrX | | 73046677 | 73046743 |
| 3.20910227059656 | UTP3 (bg=3.66%) | K562 | - | chrX | | 73046673 | 73046691 |
| 2.85974976963363 | WDR43 (bg=3.37%) | K562 | - | chrX | | 73046640 | 73046727 |
| 2.28066917636258 | XRCC6 (bg=2.91%) | K562 | - | chrX | | 73046675 | 73046808 |
| 2.12870462675865 | ZNF622 (bg=6.58%) | K562 | - | chrX | | 73046594 | 73046673 |

  
  

| Match 369 in HUMAN | | | | | | | |
| --- | --- | --- | --- | --- | --- | --- | --- |
| Motif | Start in Seq (1 Indexed) | End in Seq (1 Indexed) | Strand | Chrm | Exon | Start in Chrm (0 Indexed) | End in Chrm (1 Indexed) |
| GAAGGCCC | 13140 | 13147 | - | chrX | 6 | 73046618 | 73046626 |
| eCLIP Fold-Enrichment | Binding Protein | Cell Line | Strand | Chrm | | Start in Chrm (0 Indexed) | End in Chrm (1 Indexed) |
| 2.22503813983981 | RBFOX2 (bg=4.63%) | K562 | - | chrX | | 73046614 | 73046631 |
| 3.03111364840005 | SLTM (bg=2.2%) | K562 | - | chrX | | 73046605 | 73046660 |
| 3.71980717222211 | SLTM (bg=2.2%) | K562 | - | chrX | | 73046626 | 73046647 |
| 2.56843769506901 | SRSF1 (bg=8.47%) | K562 | - | chrX | | 73046583 | 73046670 |
| 2.58520954684713 | SRSF1 (bg=8.47%) | K562 | - | chrX | | 73046606 | 73046682 |
| 3.11717760163589 | TRA2A (bg=4.8%) | K562 | - | chrX | | 73046576 | 73046677 |
| 2.01810143373892 | TRA2A (bg=4.8%) | K562 | - | chrX | | 73046602 | 73046635 |
| 2.02251310090411 | ZNF622 (bg=6.58%) | K562 | - | chrX | | 73046585 | 73046665 |
| 2.12870462675865 | ZNF622 (bg=6.58%) | K562 | - | chrX | | 73046594 | 73046673 |

  
  

| Match 370 in HUMAN | | | | | | | |
| --- | --- | --- | --- | --- | --- | --- | --- |
| Motif | Start in Seq (1 Indexed) | End in Seq (1 Indexed) | Strand | Chrm | Exon | Start in Chrm (0 Indexed) | End in Chrm (1 Indexed) |
| TATCTCAAGACTAA | 13195 | 13208 | - | chrX | 6 | 73046557 | 73046571 |
| eCLIP Fold-Enrichment | Binding Protein | Cell Line | Strand | Chrm | | Start in Chrm (0 Indexed) | End in Chrm (1 Indexed) |
| 2.13657271683066 | CPEB4 (bg=1.89%) | K562 | - | chrX | | 73046480 | 73046580 |
| 2.14404944576866 | FASTKD2 (bg=1.99%) | K562 | - | chrX | | 73046540 | 73046617 |
| 2.71157800325063 | GRWD1 (bg=5.13%) | K562 | - | chrX | | 73046478 | 73046603 |
| 2.91421217122142 | LARP4 (bg=4.72%) | K562 | - | chrX | | 73046462 | 73046582 |
| 3.28282850105752 | MTPAP (bg=2.21%) | K562 | - | chrX | | 73046478 | 73046599 |
| 2.31954781930394 | MTPAP (bg=2.21%) | K562 | - | chrX | | 73046503 | 73046572 |
| 2.859430949105 | NOLC1 (bg=9.43%) | K562 | - | chrX | | 73046514 | 73046588 |
| 2.26697974347128 | NOLC1 (bg=9.43%) | K562 | - | chrX | | 73046522 | 73046574 |
| 2.11400682745107 | RBFOX2 (bg=4.63%) | K562 | - | chrX | | 73046510 | 73046565 |
| 2.22765754222904 | SRSF1 (bg=8.47%) | K562 | - | chrX | | 73046551 | 73046606 |
| 3.01026239771938 | TRA2A (bg=4.8%) | K562 | - | chrX | | 73046480 | 73046576 |
| 2.0505229114313 | TRA2A (bg=4.8%) | K562 | - | chrX | | 73046501 | 73046571 |
| 3.17355112635898 | uchl5 (bg=11.16%) | K562 | - | chrX | | 73046482 | 73046572 |
| 3.65502987513871 | uchl5 (bg=11.16%) | K562 | - | chrX | | 73046491 | 73046575 |
| 2.21834043205488 | UTP18 (bg=0.72%) | K562 | - | chrX | | 73046539 | 73046568 |
| 3.26788087207393 | UTP3 (bg=3.66%) | K562 | - | chrX | | 73046521 | 73046563 |
| 2.29832154751581 | WDR43 (bg=3.37%) | K562 | - | chrX | | 73046525 | 73046557 |
| 2.62275670054319 | ZNF622 (bg=6.58%) | K562 | - | chrX | | 73046479 | 73046594 |
| 2.11592404047088 | ZNF622 (bg=6.58%) | K562 | - | chrX | | 73046493 | 73046585 |

  
  

| Match 371 in HUMAN | | | | | | | |
| --- | --- | --- | --- | --- | --- | --- | --- |
| Motif | Start in Seq (1 Indexed) | End in Seq (1 Indexed) | Strand | Chrm | Exon | Start in Chrm (0 Indexed) | End in Chrm (1 Indexed) |
| TCAAGACTAA | 13199 | 13208 | - | chrX | 6 | 73046557 | 73046567 |
| eCLIP Fold-Enrichment | Binding Protein | Cell Line | Strand | Chrm | | Start in Chrm (0 Indexed) | End in Chrm (1 Indexed) |
| 2.13657271683066 | CPEB4 (bg=1.89%) | K562 | - | chrX | | 73046480 | 73046580 |
| 2.14404944576866 | FASTKD2 (bg=1.99%) | K562 | - | chrX | | 73046540 | 73046617 |
| 2.71157800325063 | GRWD1 (bg=5.13%) | K562 | - | chrX | | 73046478 | 73046603 |
| 2.91421217122142 | LARP4 (bg=4.72%) | K562 | - | chrX | | 73046462 | 73046582 |
| 3.28282850105752 | MTPAP (bg=2.21%) | K562 | - | chrX | | 73046478 | 73046599 |
| 2.31954781930394 | MTPAP (bg=2.21%) | K562 | - | chrX | | 73046503 | 73046572 |
| 2.859430949105 | NOLC1 (bg=9.43%) | K562 | - | chrX | | 73046514 | 73046588 |
| 2.26697974347128 | NOLC1 (bg=9.43%) | K562 | - | chrX | | 73046522 | 73046574 |
| 2.11400682745107 | RBFOX2 (bg=4.63%) | K562 | - | chrX | | 73046510 | 73046565 |
| 2.22765754222904 | SRSF1 (bg=8.47%) | K562 | - | chrX | | 73046551 | 73046606 |
| 3.01026239771938 | TRA2A (bg=4.8%) | K562 | - | chrX | | 73046480 | 73046576 |
| 2.0505229114313 | TRA2A (bg=4.8%) | K562 | - | chrX | | 73046501 | 73046571 |
| 3.17355112635898 | uchl5 (bg=11.16%) | K562 | - | chrX | | 73046482 | 73046572 |
| 3.65502987513871 | uchl5 (bg=11.16%) | K562 | - | chrX | | 73046491 | 73046575 |
| 2.21834043205488 | UTP18 (bg=0.72%) | K562 | - | chrX | | 73046539 | 73046568 |
| 3.26788087207393 | UTP3 (bg=3.66%) | K562 | - | chrX | | 73046521 | 73046563 |
| 2.29832154751581 | WDR43 (bg=3.37%) | K562 | - | chrX | | 73046525 | 73046557 |
| 2.62275670054319 | ZNF622 (bg=6.58%) | K562 | - | chrX | | 73046479 | 73046594 |
| 2.11592404047088 | ZNF622 (bg=6.58%) | K562 | - | chrX | | 73046493 | 73046585 |

  
  

| Match 372 in HUMAN | | | | | | | |
| --- | --- | --- | --- | --- | --- | --- | --- |
| Motif | Start in Seq (1 Indexed) | End in Seq (1 Indexed) | Strand | Chrm | Exon | Start in Chrm (0 Indexed) | End in Chrm (1 Indexed) |
| GACTAA | 13203 | 13208 | - | chrX | 6 | 73046557 | 73046563 |
| eCLIP Fold-Enrichment | Binding Protein | Cell Line | Strand | Chrm | | Start in Chrm (0 Indexed) | End in Chrm (1 Indexed) |
| 2.13657271683066 | CPEB4 (bg=1.89%) | K562 | - | chrX | | 73046480 | 73046580 |
| 2.14404944576866 | FASTKD2 (bg=1.99%) | K562 | - | chrX | | 73046540 | 73046617 |
| 2.71157800325063 | GRWD1 (bg=5.13%) | K562 | - | chrX | | 73046478 | 73046603 |
| 2.91421217122142 | LARP4 (bg=4.72%) | K562 | - | chrX | | 73046462 | 73046582 |
| 3.28282850105752 | MTPAP (bg=2.21%) | K562 | - | chrX | | 73046478 | 73046599 |
| 2.31954781930394 | MTPAP (bg=2.21%) | K562 | - | chrX | | 73046503 | 73046572 |
| 2.859430949105 | NOLC1 (bg=9.43%) | K562 | - | chrX | | 73046514 | 73046588 |
| 2.26697974347128 | NOLC1 (bg=9.43%) | K562 | - | chrX | | 73046522 | 73046574 |
| 2.11400682745107 | RBFOX2 (bg=4.63%) | K562 | - | chrX | | 73046510 | 73046565 |
| 2.22765754222904 | SRSF1 (bg=8.47%) | K562 | - | chrX | | 73046551 | 73046606 |
| 3.01026239771938 | TRA2A (bg=4.8%) | K562 | - | chrX | | 73046480 | 73046576 |
| 2.0505229114313 | TRA2A (bg=4.8%) | K562 | - | chrX | | 73046501 | 73046571 |
| 3.17355112635898 | uchl5 (bg=11.16%) | K562 | - | chrX | | 73046482 | 73046572 |
| 3.65502987513871 | uchl5 (bg=11.16%) | K562 | - | chrX | | 73046491 | 73046575 |
| 2.21834043205488 | UTP18 (bg=0.72%) | K562 | - | chrX | | 73046539 | 73046568 |
| 3.26788087207393 | UTP3 (bg=3.66%) | K562 | - | chrX | | 73046521 | 73046563 |
| 2.29832154751581 | WDR43 (bg=3.37%) | K562 | - | chrX | | 73046525 | 73046557 |
| 2.62275670054319 | ZNF622 (bg=6.58%) | K562 | - | chrX | | 73046479 | 73046594 |
| 2.11592404047088 | ZNF622 (bg=6.58%) | K562 | - | chrX | | 73046493 | 73046585 |

  
  

| Match 373 in HUMAN | | | | | | | |
| --- | --- | --- | --- | --- | --- | --- | --- |
| Motif | Start in Seq (1 Indexed) | End in Seq (1 Indexed) | Strand | Chrm | Exon | Start in Chrm (0 Indexed) | End in Chrm (1 Indexed) |
| GAATCTGG | 13215 | 13222 | - | chrX | 6 | 73046543 | 73046551 |
| eCLIP Fold-Enrichment | Binding Protein | Cell Line | Strand | Chrm | | Start in Chrm (0 Indexed) | End in Chrm (1 Indexed) |
| 2.13657271683066 | CPEB4 (bg=1.89%) | K562 | - | chrX | | 73046480 | 73046580 |
| 2.14404944576866 | FASTKD2 (bg=1.99%) | K562 | - | chrX | | 73046540 | 73046617 |
| 2.71157800325063 | GRWD1 (bg=5.13%) | K562 | - | chrX | | 73046478 | 73046603 |
| 2.91421217122142 | LARP4 (bg=4.72%) | K562 | - | chrX | | 73046462 | 73046582 |
| 3.28282850105752 | MTPAP (bg=2.21%) | K562 | - | chrX | | 73046478 | 73046599 |
| 2.31954781930394 | MTPAP (bg=2.21%) | K562 | - | chrX | | 73046503 | 73046572 |
| 2.859430949105 | NOLC1 (bg=9.43%) | K562 | - | chrX | | 73046514 | 73046588 |
| 2.26697974347128 | NOLC1 (bg=9.43%) | K562 | - | chrX | | 73046522 | 73046574 |
| 2.10287942741028 | PCBP1 (bg=1.07%) | K562 | - | chrX | | 73046510 | 73046550 |
| 2.11400682745107 | RBFOX2 (bg=4.63%) | K562 | - | chrX | | 73046510 | 73046565 |
| 2.54958563711641 | SRSF1 (bg=8.47%) | K562 | - | chrX | | 73046482 | 73046551 |
| 2.22765754222904 | SRSF1 (bg=8.47%) | K562 | - | chrX | | 73046551 | 73046606 |
| 3.01026239771938 | TRA2A (bg=4.8%) | K562 | - | chrX | | 73046480 | 73046576 |
| 2.0505229114313 | TRA2A (bg=4.8%) | K562 | - | chrX | | 73046501 | 73046571 |
| 3.17355112635898 | uchl5 (bg=11.16%) | K562 | - | chrX | | 73046482 | 73046572 |
| 3.65502987513871 | uchl5 (bg=11.16%) | K562 | - | chrX | | 73046491 | 73046575 |
| 2.21834043205488 | UTP18 (bg=0.72%) | K562 | - | chrX | | 73046539 | 73046568 |
| 3.26788087207393 | UTP3 (bg=3.66%) | K562 | - | chrX | | 73046521 | 73046563 |
| 2.29832154751581 | WDR43 (bg=3.37%) | K562 | - | chrX | | 73046525 | 73046557 |
| 2.62275670054319 | ZNF622 (bg=6.58%) | K562 | - | chrX | | 73046479 | 73046594 |
| 2.11592404047088 | ZNF622 (bg=6.58%) | K562 | - | chrX | | 73046493 | 73046585 |

  
  

| Match 374 in HUMAN | | | | | | | |
| --- | --- | --- | --- | --- | --- | --- | --- |
| Motif | Start in Seq (1 Indexed) | End in Seq (1 Indexed) | Strand | Chrm | Exon | Start in Chrm (0 Indexed) | End in Chrm (1 Indexed) |
| GATAGAAGC | 13249 | 13257 | - | chrX | 6 | 73046508 | 73046517 |
| eCLIP Fold-Enrichment | Binding Protein | Cell Line | Strand | Chrm | | Start in Chrm (0 Indexed) | End in Chrm (1 Indexed) |
| 2.13657271683066 | CPEB4 (bg=1.89%) | K562 | - | chrX | | 73046480 | 73046580 |
| 2.71157800325063 | GRWD1 (bg=5.13%) | K562 | - | chrX | | 73046478 | 73046603 |
| 2.91421217122142 | LARP4 (bg=4.72%) | K562 | - | chrX | | 73046462 | 73046582 |
| 3.28282850105752 | MTPAP (bg=2.21%) | K562 | - | chrX | | 73046478 | 73046599 |
| 2.31954781930394 | MTPAP (bg=2.21%) | K562 | - | chrX | | 73046503 | 73046572 |
| 2.859430949105 | NOLC1 (bg=9.43%) | K562 | - | chrX | | 73046514 | 73046588 |
| 2.10287942741028 | PCBP1 (bg=1.07%) | K562 | - | chrX | | 73046510 | 73046550 |
| 2.11400682745107 | RBFOX2 (bg=4.63%) | K562 | - | chrX | | 73046510 | 73046565 |
| 2.54958563711641 | SRSF1 (bg=8.47%) | K562 | - | chrX | | 73046482 | 73046551 |
| 3.01026239771938 | TRA2A (bg=4.8%) | K562 | - | chrX | | 73046480 | 73046576 |
| 2.0505229114313 | TRA2A (bg=4.8%) | K562 | - | chrX | | 73046501 | 73046571 |
| 3.17355112635898 | uchl5 (bg=11.16%) | K562 | - | chrX | | 73046482 | 73046572 |
| 3.65502987513871 | uchl5 (bg=11.16%) | K562 | - | chrX | | 73046491 | 73046575 |
| 2.62275670054319 | ZNF622 (bg=6.58%) | K562 | - | chrX | | 73046479 | 73046594 |
| 2.11592404047088 | ZNF622 (bg=6.58%) | K562 | - | chrX | | 73046493 | 73046585 |

  
  

| Match 375 in HUMAN | | | | | | | |
| --- | --- | --- | --- | --- | --- | --- | --- |
| Motif | Start in Seq (1 Indexed) | End in Seq (1 Indexed) | Strand | Chrm | Exon | Start in Chrm (0 Indexed) | End in Chrm (1 Indexed) |
| AGAAGC | 13252 | 13257 | - | chrX | 6 | 73046508 | 73046514 |
| eCLIP Fold-Enrichment | Binding Protein | Cell Line | Strand | Chrm | | Start in Chrm (0 Indexed) | End in Chrm (1 Indexed) |
| 2.13657271683066 | CPEB4 (bg=1.89%) | K562 | - | chrX | | 73046480 | 73046580 |
| 2.71157800325063 | GRWD1 (bg=5.13%) | K562 | - | chrX | | 73046478 | 73046603 |
| 2.91421217122142 | LARP4 (bg=4.72%) | K562 | - | chrX | | 73046462 | 73046582 |
| 3.28282850105752 | MTPAP (bg=2.21%) | K562 | - | chrX | | 73046478 | 73046599 |
| 2.31954781930394 | MTPAP (bg=2.21%) | K562 | - | chrX | | 73046503 | 73046572 |
| 2.859430949105 | NOLC1 (bg=9.43%) | K562 | - | chrX | | 73046514 | 73046588 |
| 2.10287942741028 | PCBP1 (bg=1.07%) | K562 | - | chrX | | 73046510 | 73046550 |
| 2.11400682745107 | RBFOX2 (bg=4.63%) | K562 | - | chrX | | 73046510 | 73046565 |
| 2.54958563711641 | SRSF1 (bg=8.47%) | K562 | - | chrX | | 73046482 | 73046551 |
| 3.01026239771938 | TRA2A (bg=4.8%) | K562 | - | chrX | | 73046480 | 73046576 |
| 2.0505229114313 | TRA2A (bg=4.8%) | K562 | - | chrX | | 73046501 | 73046571 |
| 3.17355112635898 | uchl5 (bg=11.16%) | K562 | - | chrX | | 73046482 | 73046572 |
| 3.65502987513871 | uchl5 (bg=11.16%) | K562 | - | chrX | | 73046491 | 73046575 |
| 2.62275670054319 | ZNF622 (bg=6.58%) | K562 | - | chrX | | 73046479 | 73046594 |
| 2.11592404047088 | ZNF622 (bg=6.58%) | K562 | - | chrX | | 73046493 | 73046585 |

  
  

| Match 376 in HUMAN | | | | | | | |
| --- | --- | --- | --- | --- | --- | --- | --- |
| Motif | Start in Seq (1 Indexed) | End in Seq (1 Indexed) | Strand | Chrm | Exon | Start in Chrm (0 Indexed) | End in Chrm (1 Indexed) |
| GGGAAAT | 13269 | 13275 | - | chrX | 6 | 73046490 | 73046497 |
| eCLIP Fold-Enrichment | Binding Protein | Cell Line | Strand | Chrm | | Start in Chrm (0 Indexed) | End in Chrm (1 Indexed) |
| 2.13657271683066 | CPEB4 (bg=1.89%) | K562 | - | chrX | | 73046480 | 73046580 |
| 2.49876403216476 | FTO (bg=0.32%) | K562 | - | chrX | | 73046461 | 73046494 |
| 2.71157800325063 | GRWD1 (bg=5.13%) | K562 | - | chrX | | 73046478 | 73046603 |
| 2.91421217122142 | LARP4 (bg=4.72%) | K562 | - | chrX | | 73046462 | 73046582 |
| 3.28282850105752 | MTPAP (bg=2.21%) | K562 | - | chrX | | 73046478 | 73046599 |
| 2.54958563711641 | SRSF1 (bg=8.47%) | K562 | - | chrX | | 73046482 | 73046551 |
| 3.01026239771938 | TRA2A (bg=4.8%) | K562 | - | chrX | | 73046480 | 73046576 |
| 3.17355112635898 | uchl5 (bg=11.16%) | K562 | - | chrX | | 73046482 | 73046572 |
| 3.65502987513871 | uchl5 (bg=11.16%) | K562 | - | chrX | | 73046491 | 73046575 |
| 2.62275670054319 | ZNF622 (bg=6.58%) | K562 | - | chrX | | 73046479 | 73046594 |
| 2.11592404047088 | ZNF622 (bg=6.58%) | K562 | - | chrX | | 73046493 | 73046585 |

  
  

| Match 377 in HUMAN | | | | | | | |
| --- | --- | --- | --- | --- | --- | --- | --- |
| Motif | Start in Seq (1 Indexed) | End in Seq (1 Indexed) | Strand | Chrm | Exon | Start in Chrm (0 Indexed) | End in Chrm (1 Indexed) |
| CAAGATGA | 13278 | 13285 | - | chrX | 6 | 73046480 | 73046488 |
| eCLIP Fold-Enrichment | Binding Protein | Cell Line | Strand | Chrm | | Start in Chrm (0 Indexed) | End in Chrm (1 Indexed) |
| 2.13657271683066 | CPEB4 (bg=1.89%) | K562 | - | chrX | | 73046480 | 73046580 |
| 2.49876403216476 | FTO (bg=0.32%) | K562 | - | chrX | | 73046461 | 73046494 |
| 2.71157800325063 | GRWD1 (bg=5.13%) | K562 | - | chrX | | 73046478 | 73046603 |
| 2.91421217122142 | LARP4 (bg=4.72%) | K562 | - | chrX | | 73046462 | 73046582 |
| 3.28282850105752 | MTPAP (bg=2.21%) | K562 | - | chrX | | 73046478 | 73046599 |
| 2.54958563711641 | SRSF1 (bg=8.47%) | K562 | - | chrX | | 73046482 | 73046551 |
| 3.01026239771938 | TRA2A (bg=4.8%) | K562 | - | chrX | | 73046480 | 73046576 |
| 3.17355112635898 | uchl5 (bg=11.16%) | K562 | - | chrX | | 73046482 | 73046572 |
| 2.62275670054319 | ZNF622 (bg=6.58%) | K562 | - | chrX | | 73046479 | 73046594 |

  
  

| Match 378 in HUMAN | | | | | | | |
| --- | --- | --- | --- | --- | --- | --- | --- |
| Motif | Start in Seq (1 Indexed) | End in Seq (1 Indexed) | Strand | Chrm | Exon | Start in Chrm (0 Indexed) | End in Chrm (1 Indexed) |
| AAGATGA | 13279 | 13285 | - | chrX | 6 | 73046480 | 73046487 |
| eCLIP Fold-Enrichment | Binding Protein | Cell Line | Strand | Chrm | | Start in Chrm (0 Indexed) | End in Chrm (1 Indexed) |
| 2.13657271683066 | CPEB4 (bg=1.89%) | K562 | - | chrX | | 73046480 | 73046580 |
| 2.49876403216476 | FTO (bg=0.32%) | K562 | - | chrX | | 73046461 | 73046494 |
| 2.71157800325063 | GRWD1 (bg=5.13%) | K562 | - | chrX | | 73046478 | 73046603 |
| 2.91421217122142 | LARP4 (bg=4.72%) | K562 | - | chrX | | 73046462 | 73046582 |
| 3.28282850105752 | MTPAP (bg=2.21%) | K562 | - | chrX | | 73046478 | 73046599 |
| 2.54958563711641 | SRSF1 (bg=8.47%) | K562 | - | chrX | | 73046482 | 73046551 |
| 3.01026239771938 | TRA2A (bg=4.8%) | K562 | - | chrX | | 73046480 | 73046576 |
| 3.17355112635898 | uchl5 (bg=11.16%) | K562 | - | chrX | | 73046482 | 73046572 |
| 2.62275670054319 | ZNF622 (bg=6.58%) | K562 | - | chrX | | 73046479 | 73046594 |

  
  

| Match 379 in HUMAN | | | | | | | |
| --- | --- | --- | --- | --- | --- | --- | --- |
| Motif | Start in Seq (1 Indexed) | End in Seq (1 Indexed) | Strand | Chrm | Exon | Start in Chrm (0 Indexed) | End in Chrm (1 Indexed) |
| AACCCTAAA | 13287 | 13295 | - | chrX | 6 | 73046470 | 73046479 |
| eCLIP Fold-Enrichment | Binding Protein | Cell Line | Strand | Chrm | | Start in Chrm (0 Indexed) | End in Chrm (1 Indexed) |
| 2.49876403216476 | FTO (bg=0.32%) | K562 | - | chrX | | 73046461 | 73046494 |
| 2.71157800325063 | GRWD1 (bg=5.13%) | K562 | - | chrX | | 73046478 | 73046603 |
| 2.91421217122142 | LARP4 (bg=4.72%) | K562 | - | chrX | | 73046462 | 73046582 |
| 3.28282850105752 | MTPAP (bg=2.21%) | K562 | - | chrX | | 73046478 | 73046599 |
| 2.62275670054319 | ZNF622 (bg=6.58%) | K562 | - | chrX | | 73046479 | 73046594 |

  
  

| Match 380 in HUMAN | | | | | | | |
| --- | --- | --- | --- | --- | --- | --- | --- |
| Motif | Start in Seq (1 Indexed) | End in Seq (1 Indexed) | Strand | Chrm | Exon | Start in Chrm (0 Indexed) | End in Chrm (1 Indexed) |
| CTCTTTTCTATTGTT | 13302 | 13316 | - | chrX | 6 | 73046449 | 73046464 |
| eCLIP Fold-Enrichment | Binding Protein | Cell Line | Strand | Chrm | | Start in Chrm (0 Indexed) | End in Chrm (1 Indexed) |
| 2.49876403216476 | FTO (bg=0.32%) | K562 | - | chrX | | 73046461 | 73046494 |
| 2.91421217122142 | LARP4 (bg=4.72%) | K562 | - | chrX | | 73046462 | 73046582 |

  
  

| Match 381 in HUMAN | | | | | | | |
| --- | --- | --- | --- | --- | --- | --- | --- |
| Motif | Start in Seq (1 Indexed) | End in Seq (1 Indexed) | Strand | Chrm | Exon | Start in Chrm (0 Indexed) | End in Chrm (1 Indexed) |
| CACTTCTT | 13319 | 13326 | - | chrX | 6 | 73046439 | 73046447 |
| eCLIP Fold-Enrichment | Binding Protein | Cell Line | Strand | Chrm | | Start in Chrm (0 Indexed) | End in Chrm (1 Indexed) |
| 2.13182016018964 | NOLC1 (bg=9.43%) | K562 | - | chrX | | 73046401 | 73046443 |

  
  

| Match 382 in HUMAN | | | | | | | |
| --- | --- | --- | --- | --- | --- | --- | --- |
| Motif | Start in Seq (1 Indexed) | End in Seq (1 Indexed) | Strand | Chrm | Exon | Start in Chrm (0 Indexed) | End in Chrm (1 Indexed) |
| ACTTCTT | 13320 | 13326 | - | chrX | 6 | 73046439 | 73046446 |
| eCLIP Fold-Enrichment | Binding Protein | Cell Line | Strand | Chrm | | Start in Chrm (0 Indexed) | End in Chrm (1 Indexed) |
| 2.13182016018964 | NOLC1 (bg=9.43%) | K562 | - | chrX | | 73046401 | 73046443 |

  
  

| Match 383 in HUMAN | | | | | | | |
| --- | --- | --- | --- | --- | --- | --- | --- |
| Motif | Start in Seq (1 Indexed) | End in Seq (1 Indexed) | Strand | Chrm | Exon | Start in Chrm (0 Indexed) | End in Chrm (1 Indexed) |
| TCCTGTT | 13348 | 13354 | - | chrX | 6 | 73046411 | 73046418 |
| eCLIP Fold-Enrichment | Binding Protein | Cell Line | Strand | Chrm | | Start in Chrm (0 Indexed) | End in Chrm (1 Indexed) |
| 2.13182016018964 | NOLC1 (bg=9.43%) | K562 | - | chrX | | 73046401 | 73046443 |
| 3.9376486011294 | TIA1 (bg=4.07%) | K562 | - | chrX | | 73046370 | 73046437 |
| 4.0341629678299 | TIA1 (bg=4.07%) | K562 | - | chrX | | 73046395 | 73046431 |
| 2.57099667289008 | ZC3H11A (bg=6.55%) | K562 | - | chrX | | 73046400 | 73046435 |
| 2.57099667289008 | ZC3H11A (bg=6.55%) | K562 | - | chrX | | 73046400 | 73046435 |
| 3.6699532916543 | ZC3H11A (bg=6.55%) | K562 | - | chrX | | 73046409 | 73046430 |
| 3.6699532916543 | ZC3H11A (bg=6.55%) | K562 | - | chrX | | 73046409 | 73046430 |

  
  

| Match 384 in HUMAN | | | | | | | |
| --- | --- | --- | --- | --- | --- | --- | --- |
| Motif | Start in Seq (1 Indexed) | End in Seq (1 Indexed) | Strand | Chrm | Exon | Start in Chrm (0 Indexed) | End in Chrm (1 Indexed) |
| CTTTTTGATGTTGC | 13385 | 13398 | - | chrX | 6 | 73046367 | 73046381 |
| eCLIP Fold-Enrichment | Binding Protein | Cell Line | Strand | Chrm | | Start in Chrm (0 Indexed) | End in Chrm (1 Indexed) |
| 3.9376486011294 | TIA1 (bg=4.07%) | K562 | - | chrX | | 73046370 | 73046437 |

  
  

| Match 385 in HUMAN | | | | | | | |
| --- | --- | --- | --- | --- | --- | --- | --- |
| Motif | Start in Seq (1 Indexed) | End in Seq (1 Indexed) | Strand | Chrm | Exon | Start in Chrm (0 Indexed) | End in Chrm (1 Indexed) |
| CTTTTTGATGTT | 13385 | 13396 | - | chrX | 6 | 73046369 | 73046381 |
| eCLIP Fold-Enrichment | Binding Protein | Cell Line | Strand | Chrm | | Start in Chrm (0 Indexed) | End in Chrm (1 Indexed) |
| 3.9376486011294 | TIA1 (bg=4.07%) | K562 | - | chrX | | 73046370 | 73046437 |

  
  

| Match 386 in HUMAN | | | | | | | |
| --- | --- | --- | --- | --- | --- | --- | --- |
| Motif | Start in Seq (1 Indexed) | End in Seq (1 Indexed) | Strand | Chrm | Exon | Start in Chrm (0 Indexed) | End in Chrm (1 Indexed) |
| TAAACTTC | 13493 | 13500 | - | chrX | 6 | 73046265 | 73046273 |
| eCLIP Fold-Enrichment | Binding Protein | Cell Line | Strand | Chrm | | Start in Chrm (0 Indexed) | End in Chrm (1 Indexed) |
| 2.07189566969868 | NIPBL (bg=5.39%) | K562 | - | chrX | | 73046218 | 73046277 |
| 2.6463933330194 | NOLC1 (bg=9.43%) | K562 | - | chrX | | 73046218 | 73046296 |
| 2.35096658585789 | NOLC1 (bg=9.43%) | K562 | - | chrX | | 73046218 | 73046305 |
| 2.70158078270022 | ZC3H11A (bg=6.55%) | K562 | - | chrX | | 73046215 | 73046294 |
| 2.70158078270022 | ZC3H11A (bg=6.55%) | K562 | - | chrX | | 73046215 | 73046294 |
| 2.43291409435346 | ZC3H11A (bg=6.55%) | K562 | - | chrX | | 73046220 | 73046280 |
| 2.43291409435346 | ZC3H11A (bg=6.55%) | K562 | - | chrX | | 73046220 | 73046280 |

  
  

| Match 387 in HUMAN | | | | | | | |
| --- | --- | --- | --- | --- | --- | --- | --- |
| Motif | Start in Seq (1 Indexed) | End in Seq (1 Indexed) | Strand | Chrm | Exon | Start in Chrm (0 Indexed) | End in Chrm (1 Indexed) |
| CTCCACTTGAGAGA | 13519 | 13532 | - | chrX | 6 | 73046233 | 73046247 |
| eCLIP Fold-Enrichment | Binding Protein | Cell Line | Strand | Chrm | | Start in Chrm (0 Indexed) | End in Chrm (1 Indexed) |
| 2.07189566969868 | NIPBL (bg=5.39%) | K562 | - | chrX | | 73046218 | 73046277 |
| 2.6463933330194 | NOLC1 (bg=9.43%) | K562 | - | chrX | | 73046218 | 73046296 |
| 2.35096658585789 | NOLC1 (bg=9.43%) | K562 | - | chrX | | 73046218 | 73046305 |
| 2.70158078270022 | ZC3H11A (bg=6.55%) | K562 | - | chrX | | 73046215 | 73046294 |
| 2.70158078270022 | ZC3H11A (bg=6.55%) | K562 | - | chrX | | 73046215 | 73046294 |
| 2.43291409435346 | ZC3H11A (bg=6.55%) | K562 | - | chrX | | 73046220 | 73046280 |
| 2.43291409435346 | ZC3H11A (bg=6.55%) | K562 | - | chrX | | 73046220 | 73046280 |

  
  

| Match 388 in HUMAN | | | | | | | |
| --- | --- | --- | --- | --- | --- | --- | --- |
| Motif | Start in Seq (1 Indexed) | End in Seq (1 Indexed) | Strand | Chrm | Exon | Start in Chrm (0 Indexed) | End in Chrm (1 Indexed) |
| CTCCACTTGAGAG | 13519 | 13531 | - | chrX | 6 | 73046234 | 73046247 |
| eCLIP Fold-Enrichment | Binding Protein | Cell Line | Strand | Chrm | | Start in Chrm (0 Indexed) | End in Chrm (1 Indexed) |
| 2.07189566969868 | NIPBL (bg=5.39%) | K562 | - | chrX | | 73046218 | 73046277 |
| 2.6463933330194 | NOLC1 (bg=9.43%) | K562 | - | chrX | | 73046218 | 73046296 |
| 2.35096658585789 | NOLC1 (bg=9.43%) | K562 | - | chrX | | 73046218 | 73046305 |
| 2.70158078270022 | ZC3H11A (bg=6.55%) | K562 | - | chrX | | 73046215 | 73046294 |
| 2.70158078270022 | ZC3H11A (bg=6.55%) | K562 | - | chrX | | 73046215 | 73046294 |
| 2.43291409435346 | ZC3H11A (bg=6.55%) | K562 | - | chrX | | 73046220 | 73046280 |
| 2.43291409435346 | ZC3H11A (bg=6.55%) | K562 | - | chrX | | 73046220 | 73046280 |

  
  

| Match 389 in HUMAN | | | | | | | |
| --- | --- | --- | --- | --- | --- | --- | --- |
| Motif | Start in Seq (1 Indexed) | End in Seq (1 Indexed) | Strand | Chrm | Exon | Start in Chrm (0 Indexed) | End in Chrm (1 Indexed) |
| ATAGGTGA | 13598 | 13605 | - | chrX | 6 | 73046160 | 73046168 |
| eCLIP Fold-Enrichment | Binding Protein | Cell Line | Strand | Chrm | | Start in Chrm (0 Indexed) | End in Chrm (1 Indexed) |
| 2.25566725037558 | NOLC1 (bg=9.43%) | K562 | - | chrX | | 73046108 | 73046185 |
| 2.72148359229439 | ZC3H11A (bg=6.55%) | K562 | - | chrX | | 73046149 | 73046194 |
| 2.72148359229439 | ZC3H11A (bg=6.55%) | K562 | - | chrX | | 73046149 | 73046194 |

  
  

| Match 390 in HUMAN | | | | | | | |
| --- | --- | --- | --- | --- | --- | --- | --- |
| Motif | Start in Seq (1 Indexed) | End in Seq (1 Indexed) | Strand | Chrm | Exon | Start in Chrm (0 Indexed) | End in Chrm (1 Indexed) |
| TATTTCAGTCCT | 13611 | 13622 | - | chrX | 6 | 73046143 | 73046155 |
| eCLIP Fold-Enrichment | Binding Protein | Cell Line | Strand | Chrm | | Start in Chrm (0 Indexed) | End in Chrm (1 Indexed) |
| 2.25566725037558 | NOLC1 (bg=9.43%) | K562 | - | chrX | | 73046108 | 73046185 |
| 2.24906857800272 | ZC3H11A (bg=6.55%) | K562 | - | chrX | | 73046083 | 73046158 |
| 2.24906857800272 | ZC3H11A (bg=6.55%) | K562 | - | chrX | | 73046083 | 73046158 |
| 2.72148359229439 | ZC3H11A (bg=6.55%) | K562 | - | chrX | | 73046149 | 73046194 |
| 2.72148359229439 | ZC3H11A (bg=6.55%) | K562 | - | chrX | | 73046149 | 73046194 |

  
  

| Match 391 in HUMAN | | | | | | | |
| --- | --- | --- | --- | --- | --- | --- | --- |
| Motif | Start in Seq (1 Indexed) | End in Seq (1 Indexed) | Strand | Chrm | Exon | Start in Chrm (0 Indexed) | End in Chrm (1 Indexed) |
| TATTTCAGTCC | 13611 | 13621 | - | chrX | 6 | 73046144 | 73046155 |
| eCLIP Fold-Enrichment | Binding Protein | Cell Line | Strand | Chrm | | Start in Chrm (0 Indexed) | End in Chrm (1 Indexed) |
| 2.25566725037558 | NOLC1 (bg=9.43%) | K562 | - | chrX | | 73046108 | 73046185 |
| 2.24906857800272 | ZC3H11A (bg=6.55%) | K562 | - | chrX | | 73046083 | 73046158 |
| 2.24906857800272 | ZC3H11A (bg=6.55%) | K562 | - | chrX | | 73046083 | 73046158 |
| 2.72148359229439 | ZC3H11A (bg=6.55%) | K562 | - | chrX | | 73046149 | 73046194 |
| 2.72148359229439 | ZC3H11A (bg=6.55%) | K562 | - | chrX | | 73046149 | 73046194 |

  
  

| Match 392 in HUMAN | | | | | | | |
| --- | --- | --- | --- | --- | --- | --- | --- |
| Motif | Start in Seq (1 Indexed) | End in Seq (1 Indexed) | Strand | Chrm | Exon | Start in Chrm (0 Indexed) | End in Chrm (1 Indexed) |
| TATTTCAGT | 13611 | 13619 | - | chrX | 6 | 73046146 | 73046155 |
| eCLIP Fold-Enrichment | Binding Protein | Cell Line | Strand | Chrm | | Start in Chrm (0 Indexed) | End in Chrm (1 Indexed) |
| 2.25566725037558 | NOLC1 (bg=9.43%) | K562 | - | chrX | | 73046108 | 73046185 |
| 2.24906857800272 | ZC3H11A (bg=6.55%) | K562 | - | chrX | | 73046083 | 73046158 |
| 2.24906857800272 | ZC3H11A (bg=6.55%) | K562 | - | chrX | | 73046083 | 73046158 |
| 2.72148359229439 | ZC3H11A (bg=6.55%) | K562 | - | chrX | | 73046149 | 73046194 |
| 2.72148359229439 | ZC3H11A (bg=6.55%) | K562 | - | chrX | | 73046149 | 73046194 |

  
  

| Match 393 in HUMAN | | | | | | | |
| --- | --- | --- | --- | --- | --- | --- | --- |
| Motif | Start in Seq (1 Indexed) | End in Seq (1 Indexed) | Strand | Chrm | Exon | Start in Chrm (0 Indexed) | End in Chrm (1 Indexed) |
| TGAGAAGA | 13649 | 13656 | - | chrX | 6 | 73046109 | 73046117 |
| eCLIP Fold-Enrichment | Binding Protein | Cell Line | Strand | Chrm | | Start in Chrm (0 Indexed) | End in Chrm (1 Indexed) |
| 3.11060938402493 | LARP4 (bg=4.72%) | K562 | - | chrX | | 73046010 | 73046119 |
| 2.69867834611447 | NIPBL (bg=5.39%) | K562 | - | chrX | | 73046102 | 73046135 |
| 2.25566725037558 | NOLC1 (bg=9.43%) | K562 | - | chrX | | 73046108 | 73046185 |
| 2.24307840918513 | WDR43 (bg=3.37%) | K562 | - | chrX | | 73046047 | 73046121 |
| 2.24906857800272 | ZC3H11A (bg=6.55%) | K562 | - | chrX | | 73046083 | 73046158 |
| 2.24906857800272 | ZC3H11A (bg=6.55%) | K562 | - | chrX | | 73046083 | 73046158 |

  
  

| Match 394 in HUMAN | | | | | | | |
| --- | --- | --- | --- | --- | --- | --- | --- |
| Motif | Start in Seq (1 Indexed) | End in Seq (1 Indexed) | Strand | Chrm | Exon | Start in Chrm (0 Indexed) | End in Chrm (1 Indexed) |
| GGGGAAAAAA | 13675 | 13684 | - | chrX | 6 | 73046081 | 73046091 |
| eCLIP Fold-Enrichment | Binding Protein | Cell Line | Strand | Chrm | | Start in Chrm (0 Indexed) | End in Chrm (1 Indexed) |
| 3.57925687849069 | CPSF6 (bg=0.4%) | K562 | - | chrX | | 73046054 | 73046090 |
| 3.11060938402493 | LARP4 (bg=4.72%) | K562 | - | chrX | | 73046010 | 73046119 |
| 2.24307840918513 | WDR43 (bg=3.37%) | K562 | - | chrX | | 73046047 | 73046121 |
| 2.24906857800272 | ZC3H11A (bg=6.55%) | K562 | - | chrX | | 73046083 | 73046158 |
| 2.24906857800272 | ZC3H11A (bg=6.55%) | K562 | - | chrX | | 73046083 | 73046158 |

  
  

| Match 395 in HUMAN | | | | | | | |
| --- | --- | --- | --- | --- | --- | --- | --- |
| Motif | Start in Seq (1 Indexed) | End in Seq (1 Indexed) | Strand | Chrm | Exon | Start in Chrm (0 Indexed) | End in Chrm (1 Indexed) |
| GGGGAAA | 13675 | 13681 | - | chrX | 6 | 73046084 | 73046091 |
| eCLIP Fold-Enrichment | Binding Protein | Cell Line | Strand | Chrm | | Start in Chrm (0 Indexed) | End in Chrm (1 Indexed) |
| 3.57925687849069 | CPSF6 (bg=0.4%) | K562 | - | chrX | | 73046054 | 73046090 |
| 3.11060938402493 | LARP4 (bg=4.72%) | K562 | - | chrX | | 73046010 | 73046119 |
| 2.24307840918513 | WDR43 (bg=3.37%) | K562 | - | chrX | | 73046047 | 73046121 |
| 2.24906857800272 | ZC3H11A (bg=6.55%) | K562 | - | chrX | | 73046083 | 73046158 |
| 2.24906857800272 | ZC3H11A (bg=6.55%) | K562 | - | chrX | | 73046083 | 73046158 |

  
  

| Match 396 in HUMAN | | | | | | | |
| --- | --- | --- | --- | --- | --- | --- | --- |
| Motif | Start in Seq (1 Indexed) | End in Seq (1 Indexed) | Strand | Chrm | Exon | Start in Chrm (0 Indexed) | End in Chrm (1 Indexed) |
| GTGCCAGGCT | 13687 | 13696 | - | chrX | 6 | 73046069 | 73046079 |
| eCLIP Fold-Enrichment | Binding Protein | Cell Line | Strand | Chrm | | Start in Chrm (0 Indexed) | End in Chrm (1 Indexed) |
| 3.57925687849069 | CPSF6 (bg=0.4%) | K562 | - | chrX | | 73046054 | 73046090 |
| 3.11060938402493 | LARP4 (bg=4.72%) | K562 | - | chrX | | 73046010 | 73046119 |
| 2.24307840918513 | WDR43 (bg=3.37%) | K562 | - | chrX | | 73046047 | 73046121 |

  
  

| Match 397 in HUMAN | | | | | | | |
| --- | --- | --- | --- | --- | --- | --- | --- |
| Motif | Start in Seq (1 Indexed) | End in Seq (1 Indexed) | Strand | Chrm | Exon | Start in Chrm (0 Indexed) | End in Chrm (1 Indexed) |
| TCTAGAGAAAA | 13698 | 13708 | - | chrX | 6 | 73046057 | 73046068 |
| eCLIP Fold-Enrichment | Binding Protein | Cell Line | Strand | Chrm | | Start in Chrm (0 Indexed) | End in Chrm (1 Indexed) |
| 3.57925687849069 | CPSF6 (bg=0.4%) | K562 | - | chrX | | 73046054 | 73046090 |
| 3.11060938402493 | LARP4 (bg=4.72%) | K562 | - | chrX | | 73046010 | 73046119 |
| 4.67227112715326 | UTP3 (bg=3.66%) | K562 | - | chrX | | 73046041 | 73046058 |
| 2.24307840918513 | WDR43 (bg=3.37%) | K562 | - | chrX | | 73046047 | 73046121 |

  
  

| Match 398 in HUMAN | | | | | | | |
| --- | --- | --- | --- | --- | --- | --- | --- |
| Motif | Start in Seq (1 Indexed) | End in Seq (1 Indexed) | Strand | Chrm | Exon | Start in Chrm (0 Indexed) | End in Chrm (1 Indexed) |
| TGAAGAGATGCTCCAGGCCAATGAGAAGAATTAGACA | 13712 | 13748 | - | chrX | 6 | 73046017 | 73046054 |
| eCLIP Fold-Enrichment | Binding Protein | Cell Line | Strand | Chrm | | Start in Chrm (0 Indexed) | End in Chrm (1 Indexed) |
| 3.57925687849069 | CPSF6 (bg=0.4%) | K562 | - | chrX | | 73046054 | 73046090 |
| 3.11060938402493 | LARP4 (bg=4.72%) | K562 | - | chrX | | 73046010 | 73046119 |
| 2.48017638035465 | NOLC1 (bg=9.43%) | K562 | - | chrX | | 73045880 | 73046017 |
| 2.42000399915997 | SRSF7 (bg=2.32%) | K562 | - | chrX | | 73045978 | 73046052 |
| 4.7597339684036 | UTP3 (bg=3.66%) | K562 | - | chrX | | 73046037 | 73046041 |
| 4.67227112715326 | UTP3 (bg=3.66%) | K562 | - | chrX | | 73046041 | 73046058 |
| 2.24307840918513 | WDR43 (bg=3.37%) | K562 | - | chrX | | 73046047 | 73046121 |

  
  

| Match 399 in HUMAN | | | | | | | |
| --- | --- | --- | --- | --- | --- | --- | --- |
| Motif | Start in Seq (1 Indexed) | End in Seq (1 Indexed) | Strand | Chrm | Exon | Start in Chrm (0 Indexed) | End in Chrm (1 Indexed) |
| TGAAGAGATGCTCCA | 13712 | 13726 | - | chrX | 6 | 73046039 | 73046054 |
| eCLIP Fold-Enrichment | Binding Protein | Cell Line | Strand | Chrm | | Start in Chrm (0 Indexed) | End in Chrm (1 Indexed) |
| 3.57925687849069 | CPSF6 (bg=0.4%) | K562 | - | chrX | | 73046054 | 73046090 |
| 3.11060938402493 | LARP4 (bg=4.72%) | K562 | - | chrX | | 73046010 | 73046119 |
| 2.42000399915997 | SRSF7 (bg=2.32%) | K562 | - | chrX | | 73045978 | 73046052 |
| 4.7597339684036 | UTP3 (bg=3.66%) | K562 | - | chrX | | 73046037 | 73046041 |
| 4.67227112715326 | UTP3 (bg=3.66%) | K562 | - | chrX | | 73046041 | 73046058 |
| 2.24307840918513 | WDR43 (bg=3.37%) | K562 | - | chrX | | 73046047 | 73046121 |

  
  

| Match 400 in HUMAN | | | | | | | |
| --- | --- | --- | --- | --- | --- | --- | --- |
| Motif | Start in Seq (1 Indexed) | End in Seq (1 Indexed) | Strand | Chrm | Exon | Start in Chrm (0 Indexed) | End in Chrm (1 Indexed) |
| TGAAGAGATG | 13712 | 13721 | - | chrX | 6 | 73046044 | 73046054 |
| eCLIP Fold-Enrichment | Binding Protein | Cell Line | Strand | Chrm | | Start in Chrm (0 Indexed) | End in Chrm (1 Indexed) |
[truncated: 124,763 more chars]
