# Supplementary material for: Uncovering deeply conserved motif combinations in rapidly evolving noncoding sequences
Source: Genome Biol. 2021 Jan 11;22:29. doi: 10.1186/s13059-020-02247-1 (PMC7798263; doi:10.1186/s13059-020-02247-1)
Supplement: Supplementary file 4 — Additional file 4. LncLOOM output results for XIST sequences from six mammals. [file 13059_2020_2247_MOESM4_ESM.gz › AdditionalFile4/Html_Files/kmers_in_seqs_layer_conservation.html]

 MOTIF CONSERVATION

# MOTIF CONSERVATION

## Motifs mapped to anchor sequence

  

NAVIGATE ▼

▶PIG▶COW▶DOG▶RABBIT▶MOUSE

  
  
  
  

## >HUMAN TO PIG (19280 bases)

```
 CCTTCAGTTCTTAAAGCGCTGCAATTCGCTGCTGCAGCCATATTTCTTACTCTCTCGGGGC

TGGAAGCTT

TGGAAGCTT  
Depth:2 (PIG)  
Ei-value:0.000, Pi-value:0.000  
Er-value:0.000, Pr-value:0.000  
eCLIP MATCHES▶DGCR8 (bg=1.84%)▶HNRNPC (bg=3.65%)▶LSM11 (bg=2.28%)▶NCBP2 (bg=1.49%)▶RBM15 (bg=7.27%)▶RBM22 (bg=4.62%)▶SLTM (bg=2.2%)▶SRSF1 (bg=8.47%)▶uchl5 (bg=11.16%)▶YWHAG (bg=1.87%)No matches to TargetScan

CCTGACTGAA

GATCTCT

GATCTCT  
Depth:2 (PIG)  
Ei-value:0.000, Pi-value:0.010  
Er-value:0.000, Pr-value:0.000  
eCLIP MATCHES▶DGCR8 (bg=1.84%)▶HNRNPC (bg=3.65%)▶LSM11 (bg=2.28%)▶NCBP2 (bg=1.49%)▶RBM15 (bg=7.27%)▶RBM22 (bg=4.62%)▶SLTM (bg=2.2%)▶SRSF1 (bg=8.47%)▶uchl5 (bg=11.16%)▶YWHAG (bg=1.87%)No matches to TargetScan

CTGCACTTGG

GGTTCTTTCT

GGTTCTTTCT  
Depth:2 (PIG)  
Ei-value:0.000, Pi-value:0.000  
Er-value:0.000, Pr-value:0.000  
eCLIP MATCHES▶DGCR8 (bg=1.84%)▶LSM11 (bg=2.28%)▶SRSF1 (bg=8.47%)MATCHES To TargetScan▶ miR-186-5p:AAAGAAU

A

GAACATTTTC

GAACATTTTC  
Depth:2 (PIG)  
Ei-value:0.000, Pi-value:0.000  
Er-value:0.000, Pr-value:0.000  
eCLIP MATCHES▶LSM11 (bg=2.28%)▶RBM15 (bg=7.27%)▶RBM22 (bg=4.62%)▶SRSF1 (bg=8.47%)▶uchl5 (bg=11.16%)MATCHES To TargetScan▶ miR-409-3p:AAUGUUG

TA 120  
 GTCCCCCAACACCCT

TTATGGC

TTATGGC  
Depth:2 (PIG)  
Ei-value:0.000, Pi-value:0.000  
Er-value:0.000, Pr-value:0.010  
eCLIP MATCHES▶HNRNPC (bg=3.65%)▶LSM11 (bg=2.28%)▶RBM15 (bg=7.27%)▶RBM22 (bg=4.62%)▶SRSF1 (bg=8.47%)▶uchl5 (bg=11.16%)No matches to TargetScan

G

TATTTCTTTAAAAAAA

TATTTCTTTAAAAAAA  
Depth:2 (PIG)  
Ei-value:0.000, Pi-value:0.000  
Er-value:0.000, Pr-value:0.000  
eCLIP MATCHES▶HNRNPC (bg=3.65%)▶LSM11 (bg=2.28%)▶RBM15 (bg=7.27%)▶RBM22 (bg=4.62%)▶SRSF1 (bg=8.47%)▶uchl5 (bg=11.16%)MATCHES To TargetScan▶ miR-186-5p:AAAGAAU

TCACCTAAATTC

CATAAAAT

CATAAAAT  
Depth:2 (PIG)  
Ei-value:0.000, Pi-value:0.000  
Er-value:0.000, Pr-value:0.000  
No matches to eCLIP DataNo matches to TargetScan

ATTTTTTTAAATTCTAT

ACTTTCTCCTA

ACTTTCTCCTA  
Depth:2 (PIG)  
Ei-value:0.000, Pi-value:0.000  
Er-value:0.000, Pr-value:0.000  
eCLIP MATCHES▶DGCR8 (bg=1.84%)▶HNRNPC (bg=3.65%)▶LSM11 (bg=2.28%)▶RBFOX2 (bg=4.63%)▶RBM15 (bg=7.27%)▶RBM22 (bg=4.62%)▶SRSF1 (bg=8.47%)▶uchl5 (bg=11.16%)▶YWHAG (bg=1.87%)No matches to TargetScan

GTGTC

TTCTTGACAC

TTCTTGACAC  
Depth:2 (PIG)  
Ei-value:0.000, Pi-value:0.000  
Er-value:0.000, Pr-value:0.000  
eCLIP MATCHES▶DGCR8 (bg=1.84%)▶HNRNPC (bg=3.65%)▶LSM11 (bg=2.28%)▶RBFOX2 (bg=4.63%)▶RBM15 (bg=7.27%)▶RBM22 (bg=4.62%)▶SRSF1 (bg=8.47%)▶uchl5 (bg=11.16%)▶YWHAG (bg=1.87%)No matches to TargetScan

GTCCTCCATATTTTTTTA 240  
 AAGAAAG

TATTTGG

TATTTGG  
Depth:2 (PIG)  
Ei-value:0.000, Pi-value:0.010  
Er-value:0.000, Pr-value:0.000  
No matches to eCLIP DataNo matches to TargetScan

AATATTTTGAGGCAATTTTTAATA

TTTAAGG

TTTAAGG  
Depth:2 (PIG)  
Ei-value:0.000, Pi-value:0.000  
Er-value:0.000, Pr-value:0.010  
eCLIP MATCHES▶HNRNPC (bg=3.65%)No matches to TargetScan


AATTTTTCTTTGGAAT

AATTTTTCTTTGGAAT  
Depth:2 (PIG)  
Ei-value:0.000, Pi-value:0.000  
Er-value:0.000, Pr-value:0.000  
eCLIP MATCHES▶DGCR8 (bg=1.84%)▶HNRNPC (bg=3.65%)▶PUS1 (bg=1.04%)▶RBM15 (bg=7.27%)▶SRSF1 (bg=8.47%)▶uchl5 (bg=11.16%)▶UTP18 (bg=0.72%)MATCHES To TargetScan▶ miR-186-5p:AAAGAAU

CA

TTTTTGGTTGAC

TTTTTGGTTGAC  
Depth:2 (PIG)  
Ei-value:0.000, Pi-value:0.000  
Er-value:0.000, Pr-value:0.000  
eCLIP MATCHES▶DGCR8 (bg=1.84%)▶HNRNPC (bg=3.65%)▶NIPBL (bg=5.39%)▶PUS1 (bg=1.04%)▶RBM15 (bg=7.27%)▶SDAD1 (bg=2.97%)▶SRSF1 (bg=8.47%)▶uchl5 (bg=11.16%)▶UTP18 (bg=0.72%)MATCHES To TargetScan▶ miR-505-3p.1:GUCAACA

ATCTCT

GTTTTTT

GTTTTTT  
Depth:2 (PIG)  
Ei-value:0.000, Pi-value:0.020  
Er-value:0.000, Pr-value:0.020  
eCLIP MATCHES▶DGCR8 (bg=1.84%)▶HNRNPC (bg=3.65%)▶NIPBL (bg=5.39%)▶PUS1 (bg=1.04%)▶RBM15 (bg=7.27%)▶SDAD1 (bg=2.97%)▶SRSF1 (bg=8.47%)▶uchl5 (bg=11.16%)▶UTP18 (bg=0.72%)▶YWHAG (bg=1.87%)No matches to TargetScan

GTGGATCA

GTTTTTT

GTTTTTT  
Depth:2 (PIG)  
Ei-value:0.000, Pi-value:0.020  
Er-value:0.000, Pr-value:0.020  
eCLIP MATCHES▶DGCR8 (bg=1.84%)▶HNRNPC (bg=3.65%)▶NIPBL (bg=5.39%)▶RBM15 (bg=7.27%)▶RBM22 (bg=4.62%)▶SDAD1 (bg=2.97%)▶SRSF1 (bg=8.47%)▶uchl5 (bg=11.16%)▶UTP18 (bg=0.72%)▶YWHAG (bg=1.87%)No matches to TargetScan

ACTCTTCCACTCTCTTT 360  
 TCTATATTT

TGCCCATCGGGGCTG

TGCCCATCGGGGCTG  
Depth:2 (PIG)  
Ei-value:0.000, Pi-value:0.000  
Er-value:0.000, Pr-value:0.000  
eCLIP MATCHES▶DDX51 (bg=1.63%)▶HNRNPC (bg=3.65%)▶NIPBL (bg=5.39%)▶RBM15 (bg=7.27%)▶SDAD1 (bg=2.97%)▶SRSF1 (bg=8.47%)▶U2AF1 (bg=1.17%)▶uchl5 (bg=11.16%)No matches to TargetScan

C

GGATACCTGGTTTTA

GGATACCTGGTTTTA  
Depth:2 (PIG)  
Ei-value:0.000, Pi-value:0.000  
Er-value:0.000, Pr-value:0.000  
eCLIP MATCHES▶DDX51 (bg=1.63%)▶HNRNPC (bg=3.65%)▶NIPBL (bg=5.39%)▶RBM15 (bg=7.27%)▶SDAD1 (bg=2.97%)▶SRSF1 (bg=8.47%)▶U2AF1 (bg=1.17%)▶uchl5 (bg=11.16%)No matches to TargetScan


TTATTTT

TTATTTT  
Depth:2 (PIG)  
Ei-value:0.000, Pi-value:0.010  
Er-value:0.000, Pr-value:0.010  
eCLIP MATCHES▶DDX51 (bg=1.63%)▶HNRNPC (bg=3.65%)▶NIPBL (bg=5.39%)▶RBM15 (bg=7.27%)▶SDAD1 (bg=2.97%)▶SRSF1 (bg=8.47%)▶U2AF1 (bg=1.17%)No matches to TargetScan

TTC

TTTGCCCAACGGGGCCGTGGATACCTGCCTTTTAATTCTTTTTT

TTTGCCCAACGGGGCCGTGGATACCTGCCTTTTAATTCTTTTTT  
Depth:2 (PIG)  
Ei-value:0.000, Pi-value:0.000  
Er-value:0.000, Pr-value:0.000  
eCLIP MATCHES▶DDX51 (bg=1.63%)▶HNRNPC (bg=3.65%)▶NIPBL (bg=5.39%)▶RBM15 (bg=7.27%)▶SDAD1 (bg=2.97%)▶SRSF1 (bg=8.47%)▶U2AF1 (bg=1.17%)▶uchl5 (bg=11.16%)MATCHES To TargetScan▶ miR-124-3p.1:AAGGCAC▶ miR-186-5p:AAAGAAU

ATTC

GCCCATCGGGGCCGCGGATACC

GCCCATCGGGGCCGCGGATACCTGCTTTT  
Depth:2 (PIG)  
Ei-value:0.000, Pi-value:0.000  
Er-value:0.000, Pr-value:0.000  
eCLIP MATCHES▶DDX51 (bg=1.63%)▶HNRNPC (bg=3.65%)▶NIPBL (bg=5.39%)▶RBM15 (bg=7.27%)▶SDAD1 (bg=2.97%)▶SRSF1 (bg=8.47%)▶U2AF1 (bg=1.17%)▶uchl5 (bg=11.16%)MATCHES To TargetScan▶ miR-330-3p.2:AAAGCAC

 480  


TGCTTTT

GCCCATCGGGGCCGCGGATACCTGCTTTT  
Depth:2 (PIG)  
Ei-value:0.000, Pi-value:0.000  
Er-value:0.000, Pr-value:0.000  
eCLIP MATCHES▶DDX51 (bg=1.63%)▶HNRNPC (bg=3.65%)▶NIPBL (bg=5.39%)▶RBM15 (bg=7.27%)▶SDAD1 (bg=2.97%)▶SRSF1 (bg=8.47%)▶U2AF1 (bg=1.17%)▶uchl5 (bg=11.16%)MATCHES To TargetScan▶ miR-330-3p.2:AAAGCAC

T

ATTTTTTTTT

ATTTTTTTTT  
Depth:2 (PIG)  
Ei-value:0.000, Pi-value:0.000  
Er-value:0.000, Pr-value:0.000  
eCLIP MATCHES▶DDX51 (bg=1.63%)▶HNRNPC (bg=3.65%)▶NIPBL (bg=5.39%)▶RBM15 (bg=7.27%)▶SDAD1 (bg=2.97%)▶SRSF1 (bg=8.47%)▶U2AF1 (bg=1.17%)▶uchl5 (bg=11.16%)▶YWHAG (bg=1.87%)No matches to TargetScan


CCTTAGCCCATCGGGG

CCTTAGCCCATCGGGG  
Depth:2 (PIG)  
Ei-value:0.000, Pi-value:0.000  
Er-value:0.000, Pr-value:0.000  
eCLIP MATCHES▶DDX51 (bg=1.63%)▶HNRNPC (bg=3.65%)▶NIPBL (bg=5.39%)▶RBM15 (bg=7.27%)▶SDAD1 (bg=2.97%)▶SRSF1 (bg=8.47%)▶uchl5 (bg=11.16%)▶YWHAG (bg=1.87%)No matches to TargetScan

TA

TCGGATACCTGCTG

TCGGATACCTGCTG  
Depth:2 (PIG)  
Ei-value:0.000, Pi-value:0.000  
Er-value:0.000, Pr-value:0.000  
eCLIP MATCHES▶DDX51 (bg=1.63%)▶HNRNPC (bg=3.65%)▶NIPBL (bg=5.39%)▶RBM15 (bg=7.27%)▶SDAD1 (bg=2.97%)▶SRSF1 (bg=8.47%)▶uchl5 (bg=11.16%)▶YWHAG (bg=1.87%)No matches to TargetScan

ATTCCCTT

CCCCTCT

CCCCTCT  
Depth:2 (PIG)  
Ei-value:0.000, Pi-value:0.000  
Er-value:0.000, Pr-value:0.010  
eCLIP MATCHES▶HNRNPC (bg=3.65%)▶NIPBL (bg=5.39%)▶RBM15 (bg=7.27%)▶SDAD1 (bg=2.97%)▶SRSF1 (bg=8.47%)▶uchl5 (bg=11.16%)MATCHES To TargetScan▶ miR-423-5p:GAGGGGC

G

AACCCC

AACCCC  
Depth:2 (PIG)  
Ei-value:0.000, Pi-value:0.010  
Er-value:0.000, Pr-value:0.010  
eCLIP MATCHES▶HNRNPC (bg=3.65%)▶NIPBL (bg=5.39%)▶RBM15 (bg=7.27%)▶SDAD1 (bg=2.97%)▶SRSF1 (bg=8.47%)▶uchl5 (bg=11.16%)No matches to TargetScan

CAACACTC

TGGCCCATC

TGGCCCATC  
Depth:2 (PIG)  
Ei-value:0.000, Pi-value:0.000  
Er-value:0.000, Pr-value:0.000  
eCLIP MATCHES▶HNRNPC (bg=3.65%)▶NIPBL (bg=5.39%)▶RBM15 (bg=7.27%)▶SDAD1 (bg=2.97%)▶SRSF1 (bg=8.47%)No matches to TargetScan

GGGGTGACGGATAT

CTGCTTTTT

CTGCTTTTT  
Depth:2 (PIG)  
Ei-value:0.000, Pi-value:0.000  
Er-value:0.000, Pr-value:0.000  
eCLIP MATCHES▶CPEB4 (bg=1.89%)▶RBM15 (bg=7.27%)MATCHES To TargetScan▶ miR-330-3p.2:AAAGCAC

AAAAATTT 600  
 TC

TTTTTTTGGCCCATCGGGGC

TTTTTTTGGCCCATCGGGGC  
Depth:2 (PIG)  
Ei-value:0.000, Pi-value:0.000  
Er-value:0.000, Pr-value:0.000  
eCLIP MATCHES▶FASTKD2 (bg=1.99%)▶HNRNPC (bg=3.65%)▶RBM15 (bg=7.27%)▶SRSF1 (bg=8.47%)▶U2AF1 (bg=1.17%)▶YWHAG (bg=1.87%)No matches to TargetScan

T

TCGGATACCTGCTTT

TCGGATACCTGCTTT  
Depth:2 (PIG)  
Ei-value:0.000, Pi-value:0.000  
Er-value:0.000, Pr-value:0.000  
eCLIP MATCHES▶HNRNPC (bg=3.65%)▶RBM15 (bg=7.27%)▶SRSF1 (bg=8.47%)▶U2AF1 (bg=1.17%)MATCHES To TargetScan▶ miR-330-3p.2:AAAGCAC

TTTTTTTTTTA

TTTTTCCTTGCCCATCGGGGCCTCGGATACCTGCTTTA

TTTTTCCTTGCCCATCGGGGCCTCGGATACCTGCTTTA  
Depth:2 (PIG)  
Ei-value:0.000, Pi-value:0.000  
Er-value:0.000, Pr-value:0.000  
eCLIP MATCHES▶AARS (bg=2.18%)▶AKAP1 (bg=0.21%)▶HNRNPC (bg=3.65%)▶NIPBL (bg=5.39%)▶RBM15 (bg=7.27%)▶SDAD1 (bg=2.97%)▶SRSF1 (bg=8.47%)▶uchl5 (bg=11.16%)MATCHES To TargetScan▶ miR-31-5p:GGCAAGA▶ miR-330-3p.2:AAAGCAC

ATTTTTGTTTTTCTG

GCCCATCGGGGCCG

GCCCATCGGGGCCG  
Depth:2 (PIG)  
Ei-value:0.000, Pi-value:0.000  
Er-value:0.000, Pr-value:0.000  
eCLIP MATCHES▶AATF (bg=0.64%)▶HNRNPC (bg=3.65%)▶LSM11 (bg=2.28%)▶NIPBL (bg=5.39%)▶RBM15 (bg=7.27%)▶SDAD1 (bg=2.97%)▶SRSF1 (bg=8.47%)▶U2AF1 (bg=1.17%)No matches to TargetScan

C

GGA

GGATACCTGCTT  
Depth:2 (PIG)  
Ei-value:0.000, Pi-value:0.000  
Er-value:0.000, Pr-value:0.000  
eCLIP MATCHES▶AATF (bg=0.64%)▶HNRNPC (bg=3.65%)▶LSM11 (bg=2.28%)▶NIPBL (bg=5.39%)▶RBM15 (bg=7.27%)▶SRSF1 (bg=8.47%)▶U2AF1 (bg=1.17%)No matches to TargetScan

 720  


TACCTGCTT

GGATACCTGCTT  
Depth:2 (PIG)  
Ei-value:0.000, Pi-value:0.000  
Er-value:0.000, Pr-value:0.000  
eCLIP MATCHES▶AATF (bg=0.64%)▶HNRNPC (bg=3.65%)▶LSM11 (bg=2.28%)▶NIPBL (bg=5.39%)▶RBM15 (bg=7.27%)▶SRSF1 (bg=8.47%)▶U2AF1 (bg=1.17%)No matches to TargetScan

T

GATTTTTTTTTTTCATC

GATTTTTTTTTTTCATC  
Depth:2 (PIG)  
Ei-value:0.000, Pi-value:0.000  
Er-value:0.000, Pr-value:0.000  
eCLIP MATCHES▶HNRNPC (bg=3.65%)▶NIPBL (bg=5.39%)▶RBM15 (bg=7.27%)▶SDAD1 (bg=2.97%)▶SRSF1 (bg=8.47%)▶U2AF1 (bg=1.17%)▶UTP3 (bg=3.66%)No matches to TargetScan

G

CCCATCGG

CCCATCGG  
Depth:2 (PIG)  
Ei-value:0.000, Pi-value:0.000  
Er-value:0.000, Pr-value:0.000  
eCLIP MATCHES▶HNRNPC (bg=3.65%)▶NIPBL (bg=5.39%)▶RBM15 (bg=7.27%)▶SDAD1 (bg=2.97%)▶SRSF1 (bg=8.47%)▶UTP3 (bg=3.66%)▶YWHAG (bg=1.87%)No matches to TargetScan

TGCTTTT

TATGGATG

TATGGATG  
Depth:2 (PIG)  
Ei-value:0.000, Pi-value:0.000  
Er-value:0.000, Pr-value:0.000  
eCLIP MATCHES▶HNRNPC (bg=3.65%)▶NIPBL (bg=5.39%)▶RBM15 (bg=7.27%)▶SDAD1 (bg=2.97%)▶SRSF1 (bg=8.47%)▶uchl5 (bg=11.16%)▶UTP3 (bg=3.66%)▶YWHAG (bg=1.87%)No matches to TargetScan

AAAAAATGTT

GGTTTTGTGG

GGTTTTGTGG  
Depth:2 (PIG)  
Ei-value:0.000, Pi-value:0.000  
Er-value:0.000, Pr-value:0.000  
eCLIP MATCHES▶DGCR8 (bg=1.84%)▶EXOSC5 (bg=5.38%)▶GTF2F1 (bg=0.51%)▶NIPBL (bg=5.39%)▶RBM15 (bg=7.27%)▶SRSF1 (bg=8.47%)▶uchl5 (bg=11.16%)▶YWHAG (bg=1.87%)No matches to TargetScan

GTTGTTGCACTC

TCTGGAAT

TCTGGAAT  
Depth:2 (PIG)  
Ei-value:0.000, Pi-value:0.000  
Er-value:0.000, Pr-value:0.000  
eCLIP MATCHES▶DGCR8 (bg=1.84%)▶EXOSC5 (bg=5.38%)▶GTF2F1 (bg=0.51%)▶NIPBL (bg=5.39%)▶RBM15 (bg=7.27%)▶RBM22 (bg=4.62%)▶SDAD1 (bg=2.97%)▶SRSF1 (bg=8.47%)▶uchl5 (bg=11.16%)▶YWHAG (bg=1.87%)No matches to TargetScan

A

TCTACA

TCTACA  
Depth:2 (PIG)  
Ei-value:0.000, Pi-value:0.000  
Er-value:0.000, Pr-value:0.010  
eCLIP MATCHES▶DGCR8 (bg=1.84%)▶NIPBL (bg=5.39%)▶RBM15 (bg=7.27%)▶RBM22 (bg=4.62%)▶SDAD1 (bg=2.97%)▶SRSF1 (bg=8.47%)▶YWHAG (bg=1.87%)No matches to TargetScan

CTTTT

TTTTGCTGCT

TTTTGCTGCT  
Depth:2 (PIG)  
Ei-value:0.000, Pi-value:0.000  
Er-value:0.000, Pr-value:0.010  
eCLIP MATCHES▶DDX52 (bg=0.46%)▶DGCR8 (bg=1.84%)▶EXOSC5 (bg=5.38%)▶NCBP2 (bg=1.49%)▶NIPBL (bg=5.39%)▶RBM15 (bg=7.27%)▶RBM22 (bg=4.62%)▶SDAD1 (bg=2.97%)▶SRSF1 (bg=8.47%)▶uchl5 (bg=11.16%)▶WDR3 (bg=0.25%)▶YWHAG (bg=1.87%)MATCHES To TargetScan▶ miR-103-3p/107:GCAGCAU▶ miR-15-5p/16-5p/195-5p/424-5p/497-5p:AGCAGCA▶ miR-503-5p:AGCAGCG

GATCA

TT

TTTGGTG  
Depth:2 (PIG)  
Ei-value:0.000, Pi-value:0.000  
Er-value:0.000, Pr-value:0.010  
eCLIP MATCHES▶DDX52 (bg=0.46%)▶DGCR8 (bg=1.84%)▶EXOSC5 (bg=5.38%)▶NCBP2 (bg=1.49%)▶NIPBL (bg=5.39%)▶RBM15 (bg=7.27%)▶RBM22 (bg=4.62%)▶SDAD1 (bg=2.97%)▶SLTM (bg=2.2%)▶SRSF1 (bg=8.47%)▶uchl5 (bg=11.16%)▶UTP3 (bg=3.66%)▶WDR3 (bg=0.25%)▶YWHAG (bg=1.87%)No matches to TargetScan

 840  


TGGTG

TTTGGTG  
Depth:2 (PIG)  
Ei-value:0.000, Pi-value:0.000  
Er-value:0.000, Pr-value:0.010  
eCLIP MATCHES▶DDX52 (bg=0.46%)▶DGCR8 (bg=1.84%)▶EXOSC5 (bg=5.38%)▶NCBP2 (bg=1.49%)▶NIPBL (bg=5.39%)▶RBM15 (bg=7.27%)▶RBM22 (bg=4.62%)▶SDAD1 (bg=2.97%)▶SLTM (bg=2.2%)▶SRSF1 (bg=8.47%)▶uchl5 (bg=11.16%)▶UTP3 (bg=3.66%)▶WDR3 (bg=0.25%)▶YWHAG (bg=1.87%)No matches to TargetScan

G

TGTGTGAGTG

TGTGTGAGTG  
Depth:2 (PIG)  
Ei-value:0.000, Pi-value:0.000  
Er-value:0.000, Pr-value:0.000  
eCLIP MATCHES▶DDX52 (bg=0.46%)▶DGCR8 (bg=1.84%)▶EXOSC5 (bg=5.38%)▶NCBP2 (bg=1.49%)▶NIPBL (bg=5.39%)▶RBM15 (bg=7.27%)▶RBM22 (bg=4.62%)▶SDAD1 (bg=2.97%)▶SLTM (bg=2.2%)▶SRSF1 (bg=8.47%)▶uchl5 (bg=11.16%)▶UTP3 (bg=3.66%)▶WDR3 (bg=0.25%)▶YWHAG (bg=1.87%)MATCHES To TargetScan▶ miR-342-3p:CUCACAC▶ miR-377-3p:UCACACA

TACCTACC

GCTTTGG

GCTTTGG  
Depth:2 (PIG)  
Ei-value:0.000, Pi-value:0.000  
Er-value:0.000, Pr-value:0.010  
eCLIP MATCHES▶DGCR8 (bg=1.84%)▶EXOSC5 (bg=5.38%)▶NCBP2 (bg=1.49%)▶NIPBL (bg=5.39%)▶RBM15 (bg=7.27%)▶RBM22 (bg=4.62%)▶SLTM (bg=2.2%)▶SRSF1 (bg=8.47%)▶uchl5 (bg=11.16%)▶YWHAG (bg=1.87%)MATCHES To TargetScan▶ miR-330-3p:CAAAGCA

CAGAGAATGACTC

TGCAGTTA

TGCAGTTA  
Depth:2 (PIG)  
Ei-value:0.000, Pi-value:0.000  
Er-value:0.000, Pr-value:0.000  
eCLIP MATCHES▶DGCR8 (bg=1.84%)▶NIPBL (bg=5.39%)▶RBM15 (bg=7.27%)▶RBM22 (bg=4.62%)▶SF3B1 (bg=2.48%)▶SLTM (bg=2.2%)▶SRSF1 (bg=8.47%)▶uchl5 (bg=11.16%)MATCHES To TargetScan▶ miR-217:ACUGCAU

AGCTAAGGGCGTGTTCAGATTGT

GGAGGAAA

GGAGGAAA  
Depth:2 (PIG)  
Ei-value:0.000, Pi-value:0.000  
Er-value:0.000, Pr-value:0.000  
eCLIP MATCHES▶DDX51 (bg=1.63%)▶DHX30 (bg=0.14%)▶EXOSC5 (bg=5.38%)▶HNRNPM (bg=4.29%)▶NIPBL (bg=5.39%)▶RBM15 (bg=7.27%)▶SF3B1 (bg=2.48%)▶SLTM (bg=2.2%)▶uchl5 (bg=11.16%)MATCHES To TargetScan▶ miR-670-3p:UUCCUCA

AGTGGCCGCCATTTTAGAC

TTGCCGC

TTGCCGC  
Depth:2 (PIG)  
Ei-value:0.000, Pi-value:0.000  
Er-value:0.000, Pr-value:0.010  
eCLIP MATCHES▶EXOSC5 (bg=5.38%)▶HNRNPM (bg=4.29%)▶NIPBL (bg=5.39%)▶RBM15 (bg=7.27%)▶RBM22 (bg=4.62%)▶uchl5 (bg=11.16%)No matches to TargetScan

ATAA

CTCGGCT

CTCGGCT  
Depth:2 (PIG)  
Ei-value:0.000, Pi-value:0.000  
Er-value:0.000, Pr-value:0.000  
eCLIP MATCHES▶EXOSC5 (bg=5.38%)▶NIPBL (bg=5.39%)▶RBM15 (bg=7.27%)▶RBM22 (bg=4.62%)▶SDAD1 (bg=2.97%)▶uchl5 (bg=11.16%)No matches to TargetScan

 960  


CTCGGCT  
Depth:2 (PIG)  
Ei-value:0.000, Pi-value:0.000  
Er-value:0.000, Pr-value:0.000  
eCLIP MATCHES▶EXOSC5 (bg=5.38%)▶NIPBL (bg=5.39%)▶RBM15 (bg=7.27%)▶RBM22 (bg=4.62%)▶SDAD1 (bg=2.97%)▶uchl5 (bg=11.16%)No matches to TargetScan

T

AGGGCTA

AGGGCTA  
Depth:2 (PIG)  
Ei-value:0.000, Pi-value:0.000  
Er-value:0.000, Pr-value:0.010  
eCLIP MATCHES▶EXOSC5 (bg=5.38%)▶HNRNPA1 (bg=2.57%)▶NIPBL (bg=5.39%)▶RBM15 (bg=7.27%)▶RBM22 (bg=4.62%)▶SDAD1 (bg=2.97%)▶uchl5 (bg=11.16%)MATCHES To TargetScan▶ miR-129-3p:AGCCCUU

GTCGTTTG

TGCTAAGT

TGCTAAGT  
Depth:2 (PIG)  
Ei-value:0.000, Pi-value:0.000  
Er-value:0.000, Pr-value:0.000  
eCLIP MATCHES▶AARS (bg=2.18%)▶EXOSC5 (bg=5.38%)▶HNRNPA1 (bg=2.57%)▶NIPBL (bg=5.39%)▶RBM15 (bg=7.27%)▶RBM22 (bg=4.62%)▶SDAD1 (bg=2.97%)▶uchl5 (bg=11.16%)No matches to TargetScan


TAAACTAGGG

TAAACTAGGGAGGCAAGATG  
Depth:2 (PIG)  
Ei-value:0.000, Pi-value:0.000  
Er-value:0.000, Pr-value:0.000  
eCLIP MATCHES▶AARS (bg=2.18%)▶EXOSC5 (bg=5.38%)▶HNRNPA1 (bg=2.57%)▶NIPBL (bg=5.39%)▶RBM15 (bg=7.27%)▶RBM22 (bg=4.62%)▶SDAD1 (bg=2.97%)▶uchl5 (bg=11.16%)No matches to TargetScan


AGGCAAGA

AGGCAAGA  
Depth:3 (COW)  
Ei-value:0.000, Pi-value:0.000  
Er-value:0.000, Pr-value:0.000  
eCLIP MATCHES▶AARS (bg=2.18%)▶EXOSC5 (bg=5.38%)▶HNRNPA1 (bg=2.57%)▶NIPBL (bg=5.39%)▶RBM15 (bg=7.27%)▶RBM22 (bg=4.62%)▶SDAD1 (bg=2.97%)▶uchl5 (bg=11.16%)No matches to TargetScan


TG

TAAACTAGGGAGGCAAGATG  
Depth:2 (PIG)  
Ei-value:0.000, Pi-value:0.000  
Er-value:0.000, Pr-value:0.000  
eCLIP MATCHES▶AARS (bg=2.18%)▶EXOSC5 (bg=5.38%)▶HNRNPA1 (bg=2.57%)▶NIPBL (bg=5.39%)▶RBM15 (bg=7.27%)▶RBM22 (bg=4.62%)▶SDAD1 (bg=2.97%)▶uchl5 (bg=11.16%)No matches to TargetScan

GATGATAGCAGGT

CAGGCAGAGGAA

CAGGCAGAGGAA  
Depth:2 (PIG)  
Ei-value:0.000, Pi-value:0.000  
Er-value:0.000, Pr-value:0.000  
eCLIP MATCHES▶AARS (bg=2.18%)▶EXOSC5 (bg=5.38%)▶HNRNPA1 (bg=2.57%)▶HNRNPM (bg=4.29%)▶NIPBL (bg=5.39%)▶RBM15 (bg=7.27%)▶RBM22 (bg=4.62%)▶SDAD1 (bg=2.97%)▶uchl5 (bg=11.16%)MATCHES To TargetScan▶ miR-670-3p:UUCCUCA

GTCATG

TGCATTG

TGCATTG  
Depth:2 (PIG)  
Ei-value:0.000, Pi-value:0.010  
Er-value:0.000, Pr-value:0.020  
eCLIP MATCHES▶EXOSC5 (bg=5.38%)▶HNRNPA1 (bg=2.57%)▶HNRNPM (bg=4.29%)▶RBM15 (bg=7.27%)▶RBM22 (bg=4.62%)▶uchl5 (bg=11.16%)No matches to TargetScan

C

ATGAGCTA

ATGAGCTA  
Depth:2 (PIG)  
Ei-value:0.000, Pi-value:0.000  
Er-value:0.000, Pr-value:0.000  
eCLIP MATCHES▶HNRNPA1 (bg=2.57%)▶HNRNPM (bg=4.29%)▶RBM22 (bg=4.62%)▶uchl5 (bg=11.16%)No matches to TargetScan

AACCTATCTGAATGAATT

GATTTGGG

GATTTGGG  
Depth:2 (PIG)  
Ei-value:0.000, Pi-value:0.000  
Er-value:0.000, Pr-value:0.000  
eCLIP MATCHES▶CPEB4 (bg=1.89%)▶EXOSC5 (bg=5.38%)▶GNL3 (bg=0.43%)▶HNRNPM (bg=4.29%)▶TARDBP (bg=2.79%)No matches to TargetScan

G

CT

CTTGTTAGGA  
Depth:2 (PIG)  
Ei-value:0.000, Pi-value:0.000  
Er-value:0.000, Pr-value:0.000  
eCLIP MATCHES▶CPEB4 (bg=1.89%)▶EXOSC5 (bg=5.38%)▶GNL3 (bg=0.43%)▶HNRNPM (bg=4.29%)▶TARDBP (bg=2.79%)▶uchl5 (bg=11.16%)No matches to TargetScan

 1080  


TGTTAGGA

CTTGTTAGGA  
Depth:2 (PIG)  
Ei-value:0.000, Pi-value:0.000  
Er-value:0.000, Pr-value:0.000  
eCLIP MATCHES▶CPEB4 (bg=1.89%)▶EXOSC5 (bg=5.38%)▶GNL3 (bg=0.43%)▶HNRNPM (bg=4.29%)▶TARDBP (bg=2.79%)▶uchl5 (bg=11.16%)No matches to TargetScan

GCTTTGCGTGATTGTTGTATCGGGAGGCAGTAAGAATCATCTTTTATCAGTACAAGGGACTAGTTAAAAATG

GAAGGTT

GAAGGTT  
Depth:2 (PIG)  
Ei-value:0.000, Pi-value:0.000  
Er-value:0.000, Pr-value:0.000  
eCLIP MATCHES▶EXOSC5 (bg=5.38%)▶NPM1 (bg=1.21%)▶RBFOX2 (bg=4.63%)▶RBM15 (bg=7.27%)▶RBM22 (bg=4.62%)▶uchl5 (bg=11.16%)No matches to TargetScan

AGGAAAGACTAAGGTGCAGGGCTT

AAAATGGCG

AAAATGGCGATTTTGAC  
Depth:2 (PIG)  
Ei-value:0.000, Pi-value:0.000  
Er-value:0.000, Pr-value:0.000  
eCLIP MATCHES▶EXOSC5 (bg=5.38%)▶GNL3 (bg=0.43%)▶NPM1 (bg=1.21%)▶RBFOX2 (bg=4.63%)▶RBM15 (bg=7.27%)▶RBM22 (bg=4.62%)▶uchl5 (bg=11.16%)No matches to TargetScan

 1200  


ATTTTGAC

AAAATGGCGATTTTGAC  
Depth:2 (PIG)  
Ei-value:0.000, Pi-value:0.000  
Er-value:0.000, Pr-value:0.000  
eCLIP MATCHES▶EXOSC5 (bg=5.38%)▶GNL3 (bg=0.43%)▶NPM1 (bg=1.21%)▶RBFOX2 (bg=4.63%)▶RBM15 (bg=7.27%)▶RBM22 (bg=4.62%)▶uchl5 (bg=11.16%)No matches to TargetScan

ATTGCG

GCATTGCT

GCATTGCT  
Depth:2 (PIG)  
Ei-value:0.000, Pi-value:0.000  
Er-value:0.000, Pr-value:0.000  
eCLIP MATCHES▶EXOSC5 (bg=5.38%)▶HNRNPM (bg=4.29%)▶RBFOX2 (bg=4.63%)▶RBM15 (bg=7.27%)▶RBM22 (bg=4.62%)▶uchl5 (bg=11.16%)No matches to TargetScan

C

AGCATGGC

AGCATGGC  
Depth:2 (PIG)  
Ei-value:0.000, Pi-value:0.000  
Er-value:0.000, Pr-value:0.010  
eCLIP MATCHES▶EXOSC5 (bg=5.38%)▶HNRNPM (bg=4.29%)▶RBFOX2 (bg=4.63%)▶RBM15 (bg=7.27%)▶RBM22 (bg=4.62%)▶uchl5 (bg=11.16%)No matches to TargetScan

GGGCTG

TGCTTTGTTAG

TGCTTTGTTAG  
Depth:2 (PIG)  
Ei-value:0.000, Pi-value:0.000  
Er-value:0.000, Pr-value:0.000  
eCLIP MATCHES▶EXOSC5 (bg=5.38%)▶HNRNPM (bg=4.29%)▶RBFOX2 (bg=4.63%)▶RBM15 (bg=7.27%)▶RBM22 (bg=4.62%)▶SDAD1 (bg=2.97%)▶uchl5 (bg=11.16%)MATCHES To TargetScan▶ miR-330-3p:CAAAGCA▶ miR-330-3p.2:AAAGCAC▶ miR-495-3p:AACAAAC

GTTGTC

CAAAATGGCGGA

CAAAATGGCGGA  
Depth:2 (PIG)  
Ei-value:0.000, Pi-value:0.000  
Er-value:0.000, Pr-value:0.000  
eCLIP MATCHES▶HNRNPM (bg=4.29%)▶RBFOX2 (bg=4.63%)▶RBM22 (bg=4.62%)▶uchl5 (bg=11.16%)No matches to TargetScan

TCCAGTTCTGT

CGCAGTGTTC

CGCAGTGTTC  
Depth:2 (PIG)  
Ei-value:0.000, Pi-value:0.000  
Er-value:0.000, Pr-value:0.000  
eCLIP MATCHES▶EXOSC5 (bg=5.38%)▶HNRNPM (bg=4.29%)▶RBM22 (bg=4.62%)MATCHES To TargetScan▶ miR-141-3p/200a-3p:AACACUG

A

AGTGGCGGGAAG

AGTGGCGGGAAG  
Depth:2 (PIG)  
Ei-value:0.000, Pi-value:0.000  
Er-value:0.000, Pr-value:0.000  
eCLIP MATCHES▶EXOSC5 (bg=5.38%)▶HNRNPM (bg=4.29%)▶LARP4 (bg=4.72%)▶RBM22 (bg=4.62%)No matches to TargetScan

G

CCACAT

CCACAT  
Depth:2 (PIG)  
Ei-value:0.000, Pi-value:0.030  
Er-value:0.000, Pr-value:0.020  
eCLIP MATCHES▶EXOSC5 (bg=5.38%)▶HNRNPM (bg=4.29%)▶LARP4 (bg=4.72%)▶RBM22 (bg=4.62%)▶SDAD1 (bg=2.97%)MATCHES To TargetScan▶ miR-299-3p:AUGUGGG

CATGATGGGCGAG 1320  
 GCTTTGTTAAGTGGTT

AGCATGG

AGCATGG  
Depth:2 (PIG)  
Ei-value:0.000, Pi-value:0.000  
Er-value:0.000, Pr-value:0.000  
eCLIP MATCHES▶AARS (bg=2.18%)▶EXOSC5 (bg=5.38%)▶HNRNPM (bg=4.29%)▶NIPBL (bg=5.39%)▶RBM15 (bg=7.27%)▶RBM22 (bg=4.62%)▶SDAD1 (bg=2.97%)▶uchl5 (bg=11.16%)No matches to TargetScan

TGGTGGACATGTGCGGTCACACAGG

AAAAGATGGCGGCT

AAAAGATGGCGGCT  
Depth:2 (PIG)  
Ei-value:0.000, Pi-value:0.000  
Er-value:0.000, Pr-value:0.000  
eCLIP MATCHES▶AARS (bg=2.18%)▶EXOSC5 (bg=5.38%)▶HNRNPM (bg=4.29%)▶NIPBL (bg=5.39%)▶RBM15 (bg=7.27%)▶RBM22 (bg=4.62%)▶uchl5 (bg=11.16%)No matches to TargetScan

GAAGGT

CTTGCCGCA

CTTGCCGCA  
Depth:2 (PIG)  
Ei-value:0.000, Pi-value:0.000  
Er-value:0.000, Pr-value:0.000  
eCLIP MATCHES▶AARS (bg=2.18%)▶EXOSC5 (bg=5.38%)▶HNRNPM (bg=4.29%)▶uchl5 (bg=11.16%)MATCHES To TargetScan▶ miR-31-5p:GGCAAGA

GTGT

A

AAAACATGGCGGGCCT  
Depth:2 (PIG)  
Ei-value:0.000, Pi-value:0.000  
Er-value:0.000, Pr-value:0.000  
eCLIP MATCHES▶HNRNPM (bg=4.29%)No matches to TargetScan


AAACATG

AAACATG  
Depth:4 (DOG)  
Ei-value:0.000, Pi-value:0.000  
Er-value:0.000, Pr-value:0.000  
eCLIP MATCHES▶HNRNPM (bg=4.29%)No matches to TargetScan


GCGGGCCT

AAAACATGGCGGGCCT  
Depth:2 (PIG)  
Ei-value:0.000, Pi-value:0.000  
Er-value:0.000, Pr-value:0.000  
eCLIP MATCHES▶HNRNPM (bg=4.29%)No matches to TargetScan

CT

TTGTCTTTGC

TTGTCTTTGC  
Depth:2 (PIG)  
Ei-value:0.000, Pi-value:0.000  
Er-value:0.000, Pr-value:0.000  
eCLIP MATCHES▶HNRNPM (bg=4.29%)No matches to TargetScan

TGTGTGCTTTT 1440  
 CGTGTTGGG

TTTTGCCGCAGGGACAATATGGC

TTTTGCCGCAGGGACAATATGGC  
Depth:2 (PIG)  
Ei-value:0.000, Pi-value:0.000  
Er-value:0.000, Pr-value:0.000  
eCLIP MATCHES▶AKAP8L (bg=2.19%)▶DDX51 (bg=1.63%)▶DDX52 (bg=0.46%)▶EXOSC5 (bg=5.38%)▶GNL3 (bg=0.43%)▶HNRNPM (bg=4.29%)▶HNRNPUL1 (bg=1.16%)▶LARP4 (bg=4.72%)▶METAP2 (bg=0.78%)▶NCBP2 (bg=1.49%)▶RBM22 (bg=4.62%)▶SDAD1 (bg=2.97%)▶SLTM (bg=2.2%)▶uchl5 (bg=11.16%)▶WRN (bg=0.77%)▶XRCC6 (bg=2.91%)No matches to TargetScan

AGGCG

TTGTCAT

TTGTCAT  
Depth:2 (PIG)  
Ei-value:0.000, Pi-value:0.020  
Er-value:0.000, Pr-value:0.040  
eCLIP MATCHES▶DDX51 (bg=1.63%)▶DDX52 (bg=0.46%)▶EXOSC5 (bg=5.38%)▶GNL3 (bg=0.43%)▶HNRNPM (bg=4.29%)▶HNRNPUL1 (bg=1.16%)▶METAP2 (bg=0.78%)▶NCBP2 (bg=1.49%)▶RBM22 (bg=4.62%)▶SLTM (bg=2.2%)▶uchl5 (bg=11.16%)▶WRN (bg=0.77%)▶XRCC6 (bg=2.91%)MATCHES To TargetScan▶ miR-425-5p:AUGACAC

ATGTATATCATGGCTTT

TGTCACGTGGAC

TGTCACGTGGAC  
Depth:2 (PIG)  
Ei-value:0.000, Pi-value:0.000  
Er-value:0.000, Pr-value:0.000  
eCLIP MATCHES▶DDX51 (bg=1.63%)▶EXOSC5 (bg=5.38%)▶HNRNPM (bg=4.29%)MATCHES To TargetScan▶ miR-542-3p:GUGACAG

ATCA

TGGCGGGCT

TGGCGGGCT  
Depth:2 (PIG)  
Ei-value:0.000, Pi-value:0.000  
Er-value:0.000, Pr-value:0.000  
eCLIP MATCHES▶DDX51 (bg=1.63%)▶EXOSC5 (bg=5.38%)▶HNRNPM (bg=4.29%)No matches to TargetScan


TGCCGCATTGTT

TGCCGCATTGTT  
Depth:2 (PIG)  
Ei-value:0.000, Pi-value:0.000  
Er-value:0.000, Pr-value:0.000  
eCLIP MATCHES▶DDX51 (bg=1.63%)▶EXOSC5 (bg=5.38%)▶HNRNPM (bg=4.29%)No matches to TargetScan

A

AAGATGGCGGG

AAGATGGCGGG  
Depth:2 (PIG)  
Ei-value:0.000, Pi-value:0.000  
Er-value:0.000, Pr-value:0.000  
eCLIP MATCHES▶HNRNPM (bg=4.29%)▶RBFOX2 (bg=4.63%)No matches to TargetScan

T

TTTGCCGC

TTTGCCGC  
Depth:2 (PIG)  
Ei-value:0.000, Pi-value:0.000  
Er-value:0.000, Pr-value:0.000  
eCLIP MATCHES▶EXOSC5 (bg=5.38%)▶HNRNPM (bg=4.29%)▶RBFOX2 (bg=4.63%)No matches to TargetScan

C 1560  
 TAGTGCCACGCAGAGCGGGAGAAAAGGTGGGATGGACAGTGC

TGGATTGC

TGGATTGC  
Depth:4 (DOG)  
Ei-value:0.000, Pi-value:0.000  
Er-value:0.000, Pr-value:0.000  
eCLIP MATCHES▶AARS (bg=2.18%)▶EXOSC5 (bg=5.38%)▶HNRNPM (bg=4.29%)▶RBFOX2 (bg=4.63%)▶RBM22 (bg=4.62%)▶SUPV3L1 (bg=1.57%)▶uchl5 (bg=11.16%)No matches to TargetScan

TGCATAACCCAACCAATTAGAAATGGG

GGTGGAATTG

GGTGGAATTG  
Depth:2 (PIG)  
Ei-value:0.000, Pi-value:0.000  
Er-value:0.000, Pr-value:0.000  
eCLIP MATCHES▶EXOSC5 (bg=5.38%)▶NPM1 (bg=1.21%)▶RBM22 (bg=4.62%)▶SDAD1 (bg=2.97%)▶SUPV3L1 (bg=1.57%)▶uchl5 (bg=11.16%)No matches to TargetScan

A

TCACAG

TCACAG  
Depth:2 (PIG)  
Ei-value:0.000, Pi-value:0.010  
Er-value:0.000, Pr-value:0.020  
eCLIP MATCHES▶EXOSC5 (bg=5.38%)▶NPM1 (bg=1.21%)▶RBM22 (bg=4.62%)▶SDAD1 (bg=2.97%)▶uchl5 (bg=11.16%)No matches to TargetScan

CCAATTAGAGCAGAA

GATGGAATTAG

GATGGAATTAG  
Depth:2 (PIG)  
Ei-value:0.000, Pi-value:0.000  
Er-value:0.000, Pr-value:0.000  
eCLIP MATCHES▶EXOSC5 (bg=5.38%)▶NPM1 (bg=1.21%)▶RBM22 (bg=4.62%)▶SDAD1 (bg=2.97%)▶uchl5 (bg=11.16%)No matches to TargetScan

 1680  


GATGGAATTAG  
Depth:2 (PIG)  
Ei-value:0.000, Pi-value:0.000  
Er-value:0.000, Pr-value:0.000  
eCLIP MATCHES▶EXOSC5 (bg=5.38%)▶NPM1 (bg=1.21%)▶RBM22 (bg=4.62%)▶SDAD1 (bg=2.97%)▶uchl5 (bg=11.16%)No matches to TargetScan

ACTGATGACACACTGTCCAGCTACTCAGCGAAGACCTGGGTGAA

TTAGCAT

TTAGCAT  
Depth:2 (PIG)  
Ei-value:0.000, Pi-value:0.020  
Er-value:0.000, Pr-value:0.010  
eCLIP MATCHES▶SLTM (bg=2.2%)No matches to TargetScan

GGCACTTCGCAGCTGTCTTT

AGCCAGTCAG

AGCCAGTCAG  
Depth:2 (PIG)  
Ei-value:0.000, Pi-value:0.000  
Er-value:0.000, Pr-value:0.000  
eCLIP MATCHES▶DDX51 (bg=1.63%)▶HNRNPM (bg=4.29%)▶WRN (bg=0.77%)MATCHES To TargetScan▶ miR-149-5p:CUGGCUC▶ miR-193-3p:ACUGGCC▶ miR-3064-5p:CUGGCUG

GAGAAAGAAGTGGAGG

GGCCACGT

GGCCACGT  
Depth:2 (PIG)  
Ei-value:0.000, Pi-value:0.000  
Er-value:0.000, Pr-value:0.000  
eCLIP MATCHES▶DDX51 (bg=1.63%)▶DROSHA (bg=2.49%)▶EXOSC5 (bg=5.38%)▶HNRNPM (bg=4.29%)▶NCBP2 (bg=1.49%)▶RBM22 (bg=4.62%)▶SLTM (bg=2.2%)▶uchl5 (bg=11.16%)▶UTP3 (bg=3.66%)▶WRN (bg=0.77%)No matches to TargetScan

GTATGT

CTCCCAGTG

CTCCCAGTGGG  
Depth:2 (PIG)  
Ei-value:0.000, Pi-value:0.000  
Er-value:0.000, Pr-value:0.000  
eCLIP MATCHES▶DROSHA (bg=2.49%)▶EXOSC5 (bg=5.38%)▶HNRNPM (bg=4.29%)▶NCBP2 (bg=1.49%)▶RBM22 (bg=4.62%)▶SLTM (bg=2.2%)▶uchl5 (bg=11.16%)▶UTP3 (bg=3.66%)▶WRN (bg=0.77%)No matches to TargetScan

 1800  


GG

CTCCCAGTGGG  
Depth:2 (PIG)  
Ei-value:0.000, Pi-value:0.000  
Er-value:0.000, Pr-value:0.000  
eCLIP MATCHES▶DROSHA (bg=2.49%)▶EXOSC5 (bg=5.38%)▶HNRNPM (bg=4.29%)▶NCBP2 (bg=1.49%)▶RBM22 (bg=4.62%)▶SLTM (bg=2.2%)▶uchl5 (bg=11.16%)▶UTP3 (bg=3.66%)▶WRN (bg=0.77%)No matches to TargetScan

CGGTACACCAGGTGTTTT

CAAGGTCTTT

CAAGGTCTTT  
Depth:2 (PIG)  
Ei-value:0.000, Pi-value:0.000  
Er-value:0.000, Pr-value:0.000  
eCLIP MATCHES▶EXOSC5 (bg=5.38%)No matches to TargetScan

T

CAAGGAC

CAAGGAC  
Depth:2 (PIG)  
Ei-value:0.000, Pi-value:0.000  
Er-value:0.000, Pr-value:0.010  
No matches to eCLIP DataNo matches to TargetScan

ATTTA

GCCTTTCCACCTC

GCCTTTCCACCTC  
Depth:2 (PIG)  
Ei-value:0.000, Pi-value:0.000  
Er-value:0.000, Pr-value:0.000  
eCLIP MATCHES▶EXOSC5 (bg=5.38%)▶PCBP1 (bg=1.07%)▶SDAD1 (bg=2.97%)▶TIA1 (bg=4.07%)▶uchl5 (bg=11.16%)No matches to TargetScan

TG

TCCCCTCT

TCCCCTCT  
Depth:2 (PIG)  
Ei-value:0.000, Pi-value:0.000  
Er-value:0.000, Pr-value:0.000  
eCLIP MATCHES▶EXOSC5 (bg=5.38%)▶PCBP1 (bg=1.07%)▶SDAD1 (bg=2.97%)▶TIA1 (bg=4.07%)▶uchl5 (bg=11.16%)MATCHES To TargetScan▶ miR-423-5p:GAGGGGC

TATTTG

TCCCCTCC

TCCCCTCC  
Depth:2 (PIG)  
Ei-value:0.000, Pi-value:0.000  
Er-value:0.000, Pr-value:0.000  
eCLIP MATCHES▶EXOSC5 (bg=5.38%)▶HNRNPM (bg=4.29%)▶PCBP1 (bg=1.07%)▶SDAD1 (bg=2.97%)▶uchl5 (bg=11.16%)MATCHES To TargetScan▶ miR-423-5p:GAGGGGC

TGTCCAGTGCTGCCTCTTGCAGTGCTGGATATCTGGCTGT 1920  
 GTGGT

CTGAACCTC

CTGAACCTC  
Depth:2 (PIG)  
Ei-value:0.000, Pi-value:0.000  
Er-value:0.000, Pr-value:0.000  
eCLIP MATCHES▶EXOSC5 (bg=5.38%)▶hnrnpk (bg=12.88%)▶PCBP1 (bg=1.07%)No matches to TargetScan

CCT

CCATTCCTCTG

CCATTCCTCTG  
Depth:2 (PIG)  
Ei-value:0.000, Pi-value:0.000  
Er-value:0.000, Pr-value:0.000  
eCLIP MATCHES▶EXOSC5 (bg=5.38%)▶hnrnpk (bg=12.88%)▶PCBP1 (bg=1.07%)MATCHES To TargetScan▶ miR-1-3p/206:GGAAUGU

T

ATTGGTG

ATTGGTG  
Depth:2 (PIG)  
Ei-value:0.000, Pi-value:0.000  
Er-value:0.000, Pr-value:0.010  
eCLIP MATCHES▶EXOSC5 (bg=5.38%)▶hnrnpk (bg=12.88%)No matches to TargetScan

CCTCA

CCTAAGGCTAA

CCTAAGGCTAA  
Depth:2 (PIG)  
Ei-value:0.000, Pi-value:0.000  
Er-value:0.000, Pr-value:0.000  
No matches to eCLIP DataNo matches to TargetScan

GTATA

CCTCCCCC

CCTCCCCC  
Depth:2 (PIG)  
Ei-value:0.000, Pi-value:0.000  
Er-value:0.000, Pr-value:0.000  
No matches to eCLIP DataNo matches to TargetScan

CCCACCCCCCAACCCCCCCAACTCCCCACCCCCACCCCCCACCCCCCACCTCCCC 2040  
 ACCCCCCTACCCCCCTACCCCCCTACCCC

CCTCTG

CCTCTG  
Depth:2 (PIG)  
Ei-value:0.000, Pi-value:0.020  
Er-value:0.000, Pr-value:0.000  
eCLIP MATCHES▶CSTF2T (bg=0.82%)▶DDX51 (bg=1.63%)▶DROSHA (bg=2.49%)▶GTF2F1 (bg=0.51%)▶hnrnpk (bg=12.88%)▶ILF3 (bg=3.0%)▶PCBP1 (bg=1.07%)▶PUM1 (bg=1.56%)No matches to TargetScan

GTCTGCCCTGCA

CTGCACTGT

CTGCACTGT  
Depth:2 (PIG)  
Ei-value:0.000, Pi-value:0.000  
Er-value:0.000, Pr-value:0.000  
eCLIP MATCHES▶CSTF2T (bg=0.82%)▶DGCR8 (bg=1.84%)▶DROSHA (bg=2.49%)▶GTF2F1 (bg=0.51%)▶hnrnpk (bg=12.88%)▶ILF3 (bg=3.0%)▶PCBP1 (bg=1.07%)▶PUM1 (bg=1.56%)▶RBM15 (bg=7.27%)MATCHES To TargetScan▶ miR-130-3p/301-3p/454-3p:AGUGCAA▶ miR-148-3p/152-3p:CAGUGCA

TGCCAT

GGGCAGTGCTCCA

GGGCAGTGCTCCA  
Depth:2 (PIG)  
Ei-value:0.000, Pi-value:0.000  
Er-value:0.000, Pr-value:0.000  
eCLIP MATCHES▶CSTF2T (bg=0.82%)▶DGCR8 (bg=1.84%)▶DROSHA (bg=2.49%)▶GTF2F1 (bg=0.51%)▶hnrnpk (bg=12.88%)▶HNRNPM (bg=4.29%)▶ILF3 (bg=3.0%)▶NCBP2 (bg=1.49%)▶PCBP1 (bg=1.07%)▶PUM1 (bg=1.56%)▶RBM15 (bg=7.27%)No matches to TargetScan

G

GCCTGC

GCCTGC  
Depth:2 (PIG)  
Ei-value:0.000, Pi-value:0.000  
Er-value:0.000, Pr-value:0.000  
eCLIP MATCHES▶CSTF2T (bg=0.82%)▶DGCR8 (bg=1.84%)▶DROSHA (bg=2.49%)▶GTF2F1 (bg=0.51%)▶hnrnpk (bg=12.88%)▶HNRNPM (bg=4.29%)▶HNRNPUL1 (bg=1.16%)▶ILF3 (bg=3.0%)▶NCBP2 (bg=1.49%)▶PCBP1 (bg=1.07%)▶PUM1 (bg=1.56%)▶RBM15 (bg=7.27%)▶SRSF1 (bg=8.47%)No matches to TargetScan

TTGGTGTGGACATGGT

GGTGAG

GGTGAG  
Depth:2 (PIG)  
Ei-value:0.000, Pi-value:0.000  
Er-value:0.000, Pr-value:0.010  
eCLIP MATCHES▶CSTF2T (bg=0.82%)▶DDX52 (bg=0.46%)▶DGCR8 (bg=1.84%)▶DROSHA (bg=2.49%)▶EXOSC5 (bg=5.38%)▶GRWD1 (bg=5.13%)▶GTF2F1 (bg=0.51%)▶hnrnpk (bg=12.88%)▶HNRNPM (bg=4.29%)▶HNRNPUL1 (bg=1.16%)▶ILF3 (bg=3.0%)▶NCBP2 (bg=1.49%)▶PUM1 (bg=1.56%)▶RBM15 (bg=7.27%)▶SF3B1 (bg=2.48%)▶SRSF1 (bg=8.47%)▶SUPV3L1 (bg=1.57%)▶ZNF622 (bg=6.58%)▶ZNF800 (bg=1.92%)No matches to TargetScan

C

CGTGGCAAGGACCAG

CGTGGCAAGGACCAGAATGGATC  
Depth:2 (PIG)  
Ei-value:0.000, Pi-value:0.000  
Er-value:0.000, Pr-value:0.000  
eCLIP MATCHES▶CSTF2T (bg=0.82%)▶DDX52 (bg=0.46%)▶DGCR8 (bg=1.84%)▶DROSHA (bg=2.49%)▶EXOSC5 (bg=5.38%)▶GRWD1 (bg=5.13%)▶GTF2F1 (bg=0.51%)▶hnrnpk (bg=12.88%)▶HNRNPM (bg=4.29%)▶HNRNPUL1 (bg=1.16%)▶ILF3 (bg=3.0%)▶NCBP2 (bg=1.49%)▶PUM1 (bg=1.56%)▶RBM15 (bg=7.27%)▶SF3B1 (bg=2.48%)▶SRSF1 (bg=8.47%)▶SUPV3L1 (bg=1.57%)▶TRA2A (bg=4.8%)▶ZNF622 (bg=6.58%)▶ZNF800 (bg=1.92%)MATCHES To TargetScan▶ miR-133a-3p.1:UGGUCCC

 2160  


AATGGATC

CGTGGCAAGGACCAGAATGGATC  
Depth:2 (PIG)  
Ei-value:0.000, Pi-value:0.000  
Er-value:0.000, Pr-value:0.000  
eCLIP MATCHES▶CSTF2T (bg=0.82%)▶DDX52 (bg=0.46%)▶DGCR8 (bg=1.84%)▶DROSHA (bg=2.49%)▶EXOSC5 (bg=5.38%)▶GRWD1 (bg=5.13%)▶GTF2F1 (bg=0.51%)▶hnrnpk (bg=12.88%)▶HNRNPM (bg=4.29%)▶HNRNPUL1 (bg=1.16%)▶ILF3 (bg=3.0%)▶NCBP2 (bg=1.49%)▶PUM1 (bg=1.56%)▶RBM15 (bg=7.27%)▶SF3B1 (bg=2.48%)▶SRSF1 (bg=8.47%)▶SUPV3L1 (bg=1.57%)▶TRA2A (bg=4.8%)▶ZNF622 (bg=6.58%)▶ZNF800 (bg=1.92%)MATCHES To TargetScan▶ miR-133a-3p.1:UGGUCCC

A

CAGATGATCGTTGGC

CAGATGATCGTTGGCCAACAGGTGGC  
Depth:2 (PIG)  
Ei-value:0.000, Pi-value:0.000  
Er-value:0.000, Pr-value:0.000  
eCLIP MATCHES▶CSTF2T (bg=0.82%)▶GRWD1 (bg=5.13%)▶hnrnpk (bg=12.88%)▶HNRNPM (bg=4.29%)▶ILF3 (bg=3.0%)▶MTPAP (bg=2.21%)▶NCBP2 (bg=1.49%)▶NIPBL (bg=5.39%)▶PUM1 (bg=1.56%)▶RBM15 (bg=7.27%)▶SRSF1 (bg=8.47%)▶TRA2A (bg=4.8%)▶uchl5 (bg=11.16%)▶XRCC6 (bg=2.91%)▶ZNF622 (bg=6.58%)▶ZNF800 (bg=1.92%)No matches to TargetScan


CAACAG

CAACAG  
Depth:3 (COW)  
Ei-value:0.000, Pi-value:0.000  
Er-value:0.000, Pr-value:0.000  
eCLIP MATCHES▶CSTF2T (bg=0.82%)▶GRWD1 (bg=5.13%)▶HNRNPM (bg=4.29%)▶MTPAP (bg=2.21%)▶NCBP2 (bg=1.49%)▶NIPBL (bg=5.39%)▶PUM1 (bg=1.56%)▶RBM15 (bg=7.27%)▶SRSF1 (bg=8.47%)▶TRA2A (bg=4.8%)▶uchl5 (bg=11.16%)▶XRCC6 (bg=2.91%)▶ZNF622 (bg=6.58%)▶ZNF800 (bg=1.92%)No matches to TargetScan


GTGGC

CAGATGATCGTTGGCCAACAGGTGGC  
Depth:2 (PIG)  
Ei-value:0.000, Pi-value:0.000  
Er-value:0.000, Pr-value:0.000  
eCLIP MATCHES▶CSTF2T (bg=0.82%)▶GRWD1 (bg=5.13%)▶hnrnpk (bg=12.88%)▶HNRNPM (bg=4.29%)▶ILF3 (bg=3.0%)▶MTPAP (bg=2.21%)▶NCBP2 (bg=1.49%)▶NIPBL (bg=5.39%)▶PUM1 (bg=1.56%)▶RBM15 (bg=7.27%)▶SRSF1 (bg=8.47%)▶TRA2A (bg=4.8%)▶uchl5 (bg=11.16%)▶XRCC6 (bg=2.91%)▶ZNF622 (bg=6.58%)▶ZNF800 (bg=1.92%)No matches to TargetScan

A

GAAGAGGAAT

GAAGAGGAAT  
Depth:2 (PIG)  
Ei-value:0.000, Pi-value:0.000  
Er-value:0.000, Pr-value:0.000  
eCLIP MATCHES▶CSTF2T (bg=0.82%)▶GRWD1 (bg=5.13%)▶HNRNPM (bg=4.29%)▶MTPAP (bg=2.21%)▶NCBP2 (bg=1.49%)▶NIPBL (bg=5.39%)▶PUM1 (bg=1.56%)▶RBM15 (bg=7.27%)▶SRSF1 (bg=8.47%)▶TRA2A (bg=4.8%)▶uchl5 (bg=11.16%)▶UTP3 (bg=3.66%)▶XRCC6 (bg=2.91%)▶ZNF622 (bg=6.58%)▶ZNF800 (bg=1.92%)MATCHES To TargetScan▶ miR-670-3p:UUCCUCA

TCCTGC

CTTCCTCAAGAGGAACACCTACCCC

CTTCCTCAAGAGGAACACCTACCCC  
Depth:2 (PIG)  
Ei-value:0.000, Pi-value:0.000  
Er-value:0.000, Pr-value:0.000  
eCLIP MATCHES▶CSTF2T (bg=0.82%)▶GRWD1 (bg=5.13%)▶HNRNPM (bg=4.29%)▶MTPAP (bg=2.21%)▶NCBP2 (bg=1.49%)▶NIPBL (bg=5.39%)▶PUM1 (bg=1.56%)▶RBM15 (bg=7.27%)▶SRSF1 (bg=8.47%)▶TRA2A (bg=4.8%)▶uchl5 (bg=11.16%)▶UTP3 (bg=3.66%)▶ZNF622 (bg=6.58%)MATCHES To TargetScan▶ miR-1224-5p:UGAGGAC▶ miR-670-3p:UUCCUCA

T

TGGCTAATGCTGGGGTCGGATTTTGATTT

TGGCTAATGCTGGGGTCGGATTTTGATTT  
Depth:2 (PIG)  
Ei-value:0.000, Pi-value:0.000  
Er-value:0.000, Pr-value:0.000  
eCLIP MATCHES▶GRWD1 (bg=5.13%)▶SRSF1 (bg=8.47%)▶ZNF622 (bg=6.58%)MATCHES To TargetScan▶ miR-338-3p:CCAGCAU▶ miR-551-3p:CGACCCA

ATATTTATCTT

TT

TTGGATGTCAGTCATA  
Depth:2 (PIG)  
Ei-value:0.000, Pi-value:0.000  
Er-value:0.000, Pr-value:0.000  
eCLIP MATCHES▶CPSF6 (bg=0.4%)▶EXOSC5 (bg=5.38%)▶HNRNPUL1 (bg=1.16%)▶KHSRP (bg=0.67%)▶RBM15 (bg=7.27%)▶TIA1 (bg=4.07%)▶XRCC6 (bg=2.91%)MATCHES To TargetScan▶ miR-489-3p:UGACAUC

 2280  


GGATGTCAGTCATA

TTGGATGTCAGTCATA  
Depth:2 (PIG)  
Ei-value:0.000, Pi-value:0.000  
Er-value:0.000, Pr-value:0.000  
eCLIP MATCHES▶CPSF6 (bg=0.4%)▶EXOSC5 (bg=5.38%)▶HNRNPUL1 (bg=1.16%)▶KHSRP (bg=0.67%)▶RBM15 (bg=7.27%)▶TIA1 (bg=4.07%)▶XRCC6 (bg=2.91%)MATCHES To TargetScan▶ miR-489-3p:UGACAUC

CAGTCTGATTT

TGTGGTTTGCTAGTGTT

TGTGGTTTGCTAGTGTT  
Depth:2 (PIG)  
Ei-value:0.000, Pi-value:0.000  
Er-value:0.000, Pr-value:0.000  
eCLIP MATCHES▶CPSF6 (bg=0.4%)▶EXOSC5 (bg=5.38%)▶HNRNPM (bg=4.29%)▶HNRNPUL1 (bg=1.16%)▶KHSRP (bg=0.67%)▶NIPBL (bg=5.39%)▶RBM22 (bg=4.62%)▶TIA1 (bg=4.07%)▶XRCC6 (bg=2.91%)MATCHES To TargetScan▶ miR-140-3p.2:ACCACAG▶ miR-141-3p/200a-3p:AACACUG

TGA

ATTTAAG

ATTTAAG  
Depth:2 (PIG)  
Ei-value:0.000, Pi-value:0.000  
Er-value:0.000, Pr-value:0.000  
eCLIP MATCHES▶EXOSC5 (bg=5.38%)▶KHSRP (bg=0.67%)▶NIPBL (bg=5.39%)▶TIA1 (bg=4.07%)No matches to TargetScan

T

CTTAAGTGACTA

CTTAAGTGACTA  
Depth:2 (PIG)  
Ei-value:0.000, Pi-value:0.000  
Er-value:0.000, Pr-value:0.000  
eCLIP MATCHES▶EXOSC5 (bg=5.38%)▶KHSRP (bg=0.67%)▶NIPBL (bg=5.39%)▶TIA1 (bg=4.07%)MATCHES To TargetScan▶ miR-668-3p:GUCACUC

TTATAGA

AATGTATT

AATGTATT  
Depth:2 (PIG)  
Ei-value:0.000, Pi-value:0.000  
Er-value:0.000, Pr-value:0.000  
eCLIP MATCHES▶KHSRP (bg=0.67%)▶TIA1 (bg=4.07%)No matches to TargetScan

AAGAGGCT

TTATTTGTAGAATTCA

TTATTTGTAGAATTCA  
Depth:2 (PIG)  
Ei-value:0.000, Pi-value:0.000  
Er-value:0.000, Pr-value:0.000  
eCLIP MATCHES▶KHSRP (bg=0.67%)▶TIA1 (bg=4.07%)No matches to TargetScan

CTTTAA

TTACATTTA

TTACATTTA  
Depth:2 (PIG)  
Ei-value:0.000, Pi-value:0.000  
Er-value:0.000, Pr-value:0.000  
eCLIP MATCHES▶KHSRP (bg=0.67%)▶TIA1 (bg=4.07%)MATCHES To TargetScan▶ miR-411-3p:AUGUAAC

A 2400  
 TGAGTTTTTGTTTTGA

GTTCCTT

GTTCCTT  
Depth:2 (PIG)  
Ei-value:0.000, Pi-value:0.010  
Er-value:0.000, Pr-value:0.010  
eCLIP MATCHES▶U2AF2 (bg=1.76%)No matches to TargetScan

A

AAATTCCTTAAAGTTTT

AAATTCCTTAAAGTTTT  
Depth:2 (PIG)  
Ei-value:0.000, Pi-value:0.000  
Er-value:0.000, Pr-value:0.000  
eCLIP MATCHES▶U2AF2 (bg=1.76%)No matches to TargetScan

TAGCTTCTCA

TTACAAAT

TTACAAAT  
Depth:2 (PIG)  
Ei-value:0.000, Pi-value:0.000  
Er-value:0.000, Pr-value:0.000  
eCLIP MATCHES▶U2AF2 (bg=1.76%)No matches to TargetScan

TCCTTAACCTTTTTTTGGCAGTAG

ATAGTCAAAGTCAA

ATAGTCAAAGTCAA  
Depth:2 (PIG)  
Ei-value:0.000, Pi-value:0.000  
Er-value:0.000, Pr-value:0.000  
eCLIP MATCHES▶EXOSC5 (bg=5.38%)▶LSM11 (bg=2.28%)▶SUPV3L1 (bg=1.57%)▶U2AF2 (bg=1.76%)No matches to TargetScan

ATCATTTCTAATGTTTTAAAAAT 2520  
 GTGCTGGTCATTTT

CTTTGAAATTGACTTAA

CTTTGAAATTGACTTAA  
Depth:2 (PIG)  
Ei-value:0.000, Pi-value:0.000  
Er-value:0.000, Pr-value:0.000  
eCLIP MATCHES▶LSM11 (bg=2.28%)▶PUS1 (bg=1.04%)▶SF3B1 (bg=2.48%)MATCHES To TargetScan▶ miR-224-5p:AAGUCAC

CTATTTTCC

TTTGAAG

TTTGAAG  
Depth:2 (PIG)  
Ei-value:0.000, Pi-value:0.010  
Er-value:0.000, Pr-value:0.000  
eCLIP MATCHES▶LSM11 (bg=2.28%)No matches to TargetScan

AGTCTGTAGCACAGAAACAGTAA

AAAATTTAAC

AAAATTTAAC  
Depth:2 (PIG)  
Ei-value:0.000, Pi-value:0.000  
Er-value:0.000, Pr-value:0.000  
eCLIP MATCHES▶LSM11 (bg=2.28%)No matches to TargetScan

TTC

ATGACC

ATGACC  
Depth:2 (PIG)  
Ei-value:0.000, Pi-value:0.000  
Er-value:0.000, Pr-value:0.000  
eCLIP MATCHES▶ILF3 (bg=3.0%)▶LSM11 (bg=2.28%)No matches to TargetScan

TAATGTAAAAAAGAGTG

TTTGAAGGT

TTTGAAGGT  
Depth:2 (PIG)  
Ei-value:0.000, Pi-value:0.000  
Er-value:0.000, Pr-value:0.000  
eCLIP MATCHES▶ILF3 (bg=3.0%)▶LSM11 (bg=2.28%)MATCHES To TargetScan▶ miR-205-5p:CCUUCAU

TTACA 2640  
 CAG

GTCCAGG

GTCCAGG  
Depth:2 (PIG)  
Ei-value:0.000, Pi-value:0.000  
Er-value:0.000, Pr-value:0.000  
eCLIP MATCHES▶ILF3 (bg=3.0%)MATCHES To TargetScan▶ miR-378-3p:CUGGACU

C

CTTGCTTTG

CTTGCTTTGTTCCCATCCTT  
Depth:2 (PIG)  
Ei-value:0.000, Pi-value:0.000  
Er-value:0.000, Pr-value:0.000  
eCLIP MATCHES▶ILF3 (bg=3.0%)MATCHES To TargetScan▶ miR-330-3p:CAAAGCA▶ miR-330-3p.2:AAAGCAC▶ miR-495-3p:AACAAAC


TTCCCATC

TTCCCATC  
Depth:4 (DOG)  
Ei-value:0.000, Pi-value:0.000  
Er-value:0.000, Pr-value:0.000  
eCLIP MATCHES▶ILF3 (bg=3.0%)No matches to TargetScan


CTT

CTTGCTTTGTTCCCATCCTT  
Depth:2 (PIG)  
Ei-value:0.000, Pi-value:0.000  
Er-value:0.000, Pr-value:0.000  
eCLIP MATCHES▶ILF3 (bg=3.0%)MATCHES To TargetScan▶ miR-330-3p:CAAAGCA▶ miR-330-3p.2:AAAGCAC▶ miR-495-3p:AACAAAC

G

ATGCTGCACT

ATGCTGCACT  
Depth:2 (PIG)  
Ei-value:0.000, Pi-value:0.000  
Er-value:0.000, Pr-value:0.000  
No matches to eCLIP DataMATCHES To TargetScan▶ miR-103-3p/107:GCAGCAU▶ miR-130-3p/301-3p/454-3p:AGUGCAA

AATTGACTAATCACCTACTTATCAGACAGG

AAACTTGAATTGCTGTGG

AAACTTGAATTGCTGTGG  
Depth:2 (PIG)  
Ei-value:0.000, Pi-value:0.000  
Er-value:0.000, Pr-value:0.000  
No matches to eCLIP DataMATCHES To TargetScan▶ miR-140-3p.1:CCACAGG▶ miR-26-5p:UCAAGUA

TCTGGTGTCCTCTATTCAGAC

TTATTATAT

TTATTATATTGGAGTATT  
Depth:2 (PIG)  
Ei-value:0.000, Pi-value:0.000  
Er-value:0.000, Pr-value:0.000  
No matches to eCLIP DataMATCHES To TargetScan▶ miR-200bc-3p/429:AAUACUG▶ miR-369-3p:AUAAUAC▶ miR-374-5p:UAUAAUA▶ miR-410-3p:AUAUAAC

 2760  


TGGAGTATT

TTATTATATTGGAGTATT  
Depth:2 (PIG)  
Ei-value:0.000, Pi-value:0.000  
Er-value:0.000, Pr-value:0.000  
No matches to eCLIP DataMATCHES To TargetScan▶ miR-200bc-3p/429:AAUACUG▶ miR-369-3p:AUAAUAC▶ miR-374-5p:UAUAAUA▶ miR-410-3p:AUAUAAC


TCAATTTT

TCAATTTT  
Depth:2 (PIG)  
Ei-value:0.000, Pi-value:0.010  
Er-value:0.000, Pr-value:0.010  
No matches to eCLIP DataNo matches to TargetScan

TCGTTGTATCCTGCCTGCCTAGCATCCAGTTCCTCCCCAGCCCTGC

TCCCAGCAAACCC

TCCCAGCAAACCC  
Depth:2 (PIG)  
Ei-value:0.000, Pi-value:0.000  
Er-value:0.000, Pr-value:0.000  
eCLIP MATCHES▶hnrnpk (bg=12.88%)No matches to TargetScan

CTAGTC

TAGCCCCAGCCC

TAGCCCCAGCCC  
Depth:2 (PIG)  
Ei-value:0.000, Pi-value:0.000  
Er-value:0.000, Pr-value:0.000  
No matches to eCLIP DataNo matches to TargetScan

TACTCCCACCCCGCCCCAG

CCCTGCC

CCCTGCCCCAGCCCCAG  
Depth:2 (PIG)  
Ei-value:0.000, Pi-value:0.000  
Er-value:0.000, Pr-value:0.000  
eCLIP MATCHES▶DROSHA (bg=2.49%)No matches to TargetScan

 2880  


CCAGCCCCAG

CCCTGCCCCAGCCCCAG  
Depth:2 (PIG)  
Ei-value:0.000, Pi-value:0.000  
Er-value:0.000, Pr-value:0.000  
eCLIP MATCHES▶DROSHA (bg=2.49%)No matches to TargetScan

TCCCCTAACCCCCCAGCCCT

AGCCCCAG

AGCCCCAG  
Depth:2 (PIG)  
Ei-value:0.000, Pi-value:0.000  
Er-value:0.000, Pr-value:0.000  
eCLIP MATCHES▶DGCR8 (bg=1.84%)▶DROSHA (bg=2.49%)▶hnrnpk (bg=12.88%)▶SDAD1 (bg=2.97%)▶XRN2 (bg=0.39%)No matches to TargetScan

TC

CCAGTCC

CCAGTCC  
Depth:2 (PIG)  
Ei-value:0.000, Pi-value:0.000  
Er-value:0.000, Pr-value:0.010  
eCLIP MATCHES▶DGCR8 (bg=1.84%)▶DROSHA (bg=2.49%)▶hnrnpk (bg=12.88%)▶SDAD1 (bg=2.97%)▶XRN2 (bg=0.39%)No matches to TargetScan

TAGTTCCTCAGTCCCGCCCAGCTTCTCTCGAAAGTCACTCTAATTTTC

ATTGATT

ATTGATT  
Depth:2 (PIG)  
Ei-value:0.000, Pi-value:0.000  
Er-value:0.000, Pr-value:0.030  
eCLIP MATCHES▶DROSHA (bg=2.49%)▶hnrnpk (bg=12.88%)No matches to TargetScan

CAGTGCTC

AAAATAAGTT

AAAATAAGTT  
Depth:2 (PIG)  
Ei-value:0.000, Pi-value:0.000  
Er-value:0.000, Pr-value:0.000  
eCLIP MATCHES▶DROSHA (bg=2.49%)▶hnrnpk (bg=12.88%)No matches to TargetScan

 3000  


AAAATAAGTT  
Depth:2 (PIG)  
Ei-value:0.000, Pi-value:0.000  
Er-value:0.000, Pr-value:0.000  
eCLIP MATCHES▶DROSHA (bg=2.49%)▶hnrnpk (bg=12.88%)No matches to TargetScan

GTCCATTGCTTATCCTATTAT

ACTGGGATA

ACTGGGATA  
Depth:2 (PIG)  
Ei-value:0.000, Pi-value:0.000  
Er-value:0.000, Pr-value:0.000  
eCLIP MATCHES▶DROSHA (bg=2.49%)▶hnrnpk (bg=12.88%)▶ILF3 (bg=3.0%)No matches to TargetScan

TTCCGTTTACCCTTG

GCATTGCTGATCTT

GCATTGCTGATCTT  
Depth:2 (PIG)  
Ei-value:0.000, Pi-value:0.000  
Er-value:0.000, Pr-value:0.000  
eCLIP MATCHES▶hnrnpk (bg=12.88%)▶ILF3 (bg=3.0%)MATCHES To TargetScan▶ miR-383-5p.1:GAUCAGA▶ miR-383-5p.2:AGAUCAG

CAGTACTGACTCCTTG

ACCATTTTCA

ACCATTTTCA  
Depth:2 (PIG)  
Ei-value:0.000, Pi-value:0.000  
Er-value:0.000, Pr-value:0.000  
eCLIP MATCHES▶hnrnpk (bg=12.88%)▶ILF3 (bg=3.0%)No matches to TargetScan

GTTAATGCAT

ACAATCCCATTTG

ACAATCCCATTTG  
Depth:2 (PIG)  
Ei-value:0.000, Pi-value:0.000  
Er-value:0.000, Pr-value:0.000  
eCLIP MATCHES▶hnrnpk (bg=12.88%)▶HNRNPU (bg=5.92%)▶ILF3 (bg=3.0%)MATCHES To TargetScan▶ miR-219-5p:GAUUGUC

TCTGTGATCTCA 3120  
 GG

ACAAAGAATTT

ACAAAGAATTT  
Depth:2 (PIG)  
Ei-value:0.000, Pi-value:0.000  
Er-value:0.000, Pr-value:0.000  
eCLIP MATCHES▶hnrnpk (bg=12.88%)No matches to TargetScan

CCTTACTCGGTACGTTGAA

GTTAGG

GTTAGG  
Depth:2 (PIG)  
Ei-value:0.000, Pi-value:0.010  
Er-value:0.000, Pr-value:0.010  
No matches to eCLIP DataNo matches to TargetScan

GAATGTCAATTGA

GAGCTT

GAGCTT  
Depth:2 (PIG)  
Ei-value:0.000, Pi-value:0.010  
Er-value:0.000, Pr-value:0.010  
No matches to eCLIP DataNo matches to TargetScan

TC

TATCAGA

TATCAGA  
Depth:2 (PIG)  
Ei-value:0.000, Pi-value:0.000  
Er-value:0.000, Pr-value:0.000  
No matches to eCLIP DataNo matches to TargetScan

GC

ATTATTG

ATTATTG  
Depth:2 (PIG)  
Ei-value:0.000, Pi-value:0.000  
Er-value:0.000, Pr-value:0.010  
No matches to eCLIP DataNo matches to TargetScan

CCCACAATTTGAGTTACTTATCATTTTCTCGATCCCCTGCCCTTA 3240  


AAGGAGAAACCATT

AAGGAGAAACCATT  
Depth:2 (PIG)  
Ei-value:0.000, Pi-value:0.000  
Er-value:0.000, Pr-value:0.000  
eCLIP MATCHES▶EIF3G (bg=0.32%)▶hnrnpk (bg=12.88%)No matches to TargetScan

T

CTCTGT

CTCTGT  
Depth:3 (COW)  
Ei-value:0.000, Pi-value:0.000  
Er-value:0.000, Pr-value:0.000  
eCLIP MATCHES▶EIF3G (bg=0.32%)▶hnrnpk (bg=12.88%)No matches to TargetScan


CATTGCT

CTCTGTCATTGCT  
Depth:2 (PIG)  
Ei-value:0.000, Pi-value:0.000  
Er-value:0.000, Pr-value:0.000  
eCLIP MATCHES▶EIF3G (bg=0.32%)▶hnrnpk (bg=12.88%)MATCHES To TargetScan▶ miR-425-5p:AUGACAC

TCT

GTAGTCA

GTAGTCA  
Depth:2 (PIG)  
Ei-value:0.000, Pi-value:0.000  
Er-value:0.000, Pr-value:0.000  
eCLIP MATCHES▶hnrnpk (bg=12.88%)▶SUPV3L1 (bg=1.57%)No matches to TargetScan

CAGTCCCAATTTTGAGTAGTGATCTTTTCTTGTG

TACTGTG

TACTGTG  
Depth:2 (PIG)  
Ei-value:0.000, Pi-value:0.000  
Er-value:0.000, Pr-value:0.000  
eCLIP MATCHES▶HNRNPU (bg=5.92%)▶WRN (bg=0.77%)MATCHES To TargetScan▶ miR-101-3p.1:ACAGUAC▶ miR-128-3p:CACAGUG▶ miR-144-3p:ACAGUAU

TTGGCCACCTA

AAACTCTTTGCA

AAACTCTTTGCA  
Depth:2 (PIG)  
Ei-value:0.000, Pi-value:0.000  
Er-value:0.000, Pr-value:0.000  
eCLIP MATCHES▶HNRNPU (bg=5.92%)▶WRN (bg=0.77%)No matches to TargetScan

TTGAGTA

AAATTCTAATT

AAATTCTAATTG  
Depth:2 (PIG)  
Ei-value:0.000, Pi-value:0.000  
Er-value:0.000, Pr-value:0.000  
No matches to eCLIP DataNo matches to TargetScan

 3360  


G

AAATTCTAATTG  
Depth:2 (PIG)  
Ei-value:0.000, Pi-value:0.000  
Er-value:0.000, Pr-value:0.000  
No matches to eCLIP DataNo matches to TargetScan

CCA

ATAATCCT

ATAATCCT  
Depth:2 (PIG)  
Ei-value:0.000, Pi-value:0.000  
Er-value:0.000, Pr-value:0.000  
eCLIP MATCHES▶HNRNPU (bg=5.92%)No matches to TargetScan

ACC

CATTGGA

CATTGGA  
Depth:2 (PIG)  
Ei-value:0.000, Pi-value:0.010  
Er-value:0.000, Pr-value:0.010  
eCLIP MATCHES▶HNRNPU (bg=5.92%)No matches to TargetScan

TTAGACAGCACTCTGAACCCCA

TTTGCATTCAGCAG

TTTGCATTCAGCAG  
Depth:2 (PIG)  
Ei-value:0.000, Pi-value:0.000  
Er-value:0.000, Pr-value:0.000  
eCLIP MATCHES▶HNRNPU (bg=5.92%)No matches to TargetScan

GGGGTCGCAGACAACCCGTCTTTTGTTGGACAGTTAAAATGCTCAGTCCCAA

TTGTCATA

TTGTCATA  
Depth:2 (PIG)  
Ei-value:0.000, Pi-value:0.000  
Er-value:0.000, Pr-value:0.000  
eCLIP MATCHES▶HNRNPU (bg=5.92%)MATCHES To TargetScan▶ miR-425-5p:AUGACAC

GC 3480  
 TTTGCCTA

TTAAACAAAGGCA

TTAAACAAAGGCA  
Depth:2 (PIG)  
Ei-value:0.000, Pi-value:0.000  
Er-value:0.000, Pr-value:0.000  
eCLIP MATCHES▶HNRNPU (bg=5.92%)No matches to TargetScan

CCCTACTGCGCTTTTTGCTGTGCTTCTGGAGAATCCTG

CTGTTCTTGGACAATTAAAG

CTGTTCTTGGACAATTAAAG  
Depth:2 (PIG)  
Ei-value:0.000, Pi-value:0.000  
Er-value:0.000, Pr-value:0.000  
eCLIP MATCHES▶HNRNPU (bg=5.92%)No matches to TargetScan

AACAAAGTA

GTAATTG

GTAATTG  
Depth:2 (PIG)  
Ei-value:0.000, Pi-value:0.000  
Er-value:0.000, Pr-value:0.000  
No matches to eCLIP DataNo matches to TargetScan

CTA

ATTGTCTCAC

ATTGTCTCAC  
Depth:2 (PIG)  
Ei-value:0.000, Pi-value:0.000  
Er-value:0.000, Pr-value:0.000  
No matches to eCLIP DataNo matches to TargetScan

C

CATTAATCA

CATTAATCA  
Depth:2 (PIG)  
Ei-value:0.000, Pi-value:0.000  
Er-value:0.000, Pr-value:0.000  
No matches to eCLIP DataNo matches to TargetScan

TG 3600  
 AAGACTACCAGTCGCCCTTGCATTTGCCTTG

AGGCAG

AGGCAG  
Depth:2 (PIG)  
Ei-value:0.000, Pi-value:0.010  
Er-value:0.000, Pr-value:0.020  
No matches to eCLIP DataNo matches to TargetScan

C

GCTGACTA

GCTGACTA  
Depth:2 (PIG)  
Ei-value:0.000, Pi-value:0.000  
Er-value:0.000, Pr-value:0.000  
No matches to eCLIP DataNo matches to TargetScan

CCTGAGATTTAAGAGTTTCTTAA

ATTATTGA

ATTATTGA  
Depth:2 (PIG)  
Ei-value:0.000, Pi-value:0.010  
Er-value:0.000, Pr-value:0.000  
No matches to eCLIP DataNo matches to TargetScan

GTAAAATCCCAATTATCCATAGTTCTGTTAG

TTACAC

TTACAC  
Depth:2 (PIG)  
Ei-value:0.000, Pi-value:0.010  
Er-value:0.000, Pr-value:0.000  
No matches to eCLIP DataNo matches to TargetScan

TATGGC 3720  
 CTTTGCAAACA

TCTTTGCA

TCTTTGCA  
Depth:2 (PIG)  
Ei-value:0.000, Pi-value:0.000  
Er-value:0.000, Pr-value:0.000  
No matches to eCLIP DataNo matches to TargetScan

TAACAGCAGTGGGACTGACTCATTCTTAGAGCCCCTTCCCTT

GGAATATTAATGGATACAAT

GGAATATTAATGGATACAAT  
Depth:2 (PIG)  
Ei-value:0.000, Pi-value:0.000  
Er-value:0.000, Pr-value:0.000  
No matches to eCLIP DataNo matches to TargetScan

AGTAATTAT

TCATGGT

TCATGGT  
Depth:2 (PIG)  
Ei-value:0.000, Pi-value:0.000  
Er-value:0.000, Pr-value:0.020  
No matches to eCLIP DataNo matches to TargetScan

TCTGCGTAACAGAG

AAGACCCAC

AAGACCCAC  
Depth:2 (PIG)  
Ei-value:0.000, Pi-value:0.000  
Er-value:0.000, Pr-value:0.000  
eCLIP MATCHES▶HNRNPUL1 (bg=1.16%)MATCHES To TargetScan▶ miR-193a-5p:GGGUCUU

 3840  


AAGACCCAC  
Depth:2 (PIG)  
Ei-value:0.000, Pi-value:0.000  
Er-value:0.000, Pr-value:0.000  
eCLIP MATCHES▶HNRNPUL1 (bg=1.16%)MATCHES To TargetScan▶ miR-193a-5p:GGGUCUU

TTATGTGTATGCCTTTATCATTGCTCCTAGA

TAGTGTG

TAGTGTG  
Depth:2 (PIG)  
Ei-value:0.000, Pi-value:0.000  
Er-value:0.000, Pr-value:0.000  
No matches to eCLIP DataNo matches to TargetScan

A

ACTACCTACCACCTTGCATTAATAT

ACTACCTACCACCTTGCATTAATAT  
Depth:2 (PIG)  
Ei-value:0.000, Pi-value:0.000  
Er-value:0.000, Pr-value:0.000  
No matches to eCLIP DataMATCHES To TargetScan▶ miR-155-5p:UAAUGCU▶ miR-18-5p:AAGGUGC▶ miR-196-5p:AGGUAGU

GTAAAACACTAATTGCCCATA

GTCCCACT

GTCCCACT  
Depth:2 (PIG)  
Ei-value:0.000, Pi-value:0.000  
Er-value:0.000, Pr-value:0.000  
eCLIP MATCHES▶hnrnpk (bg=12.88%)No matches to TargetScan

CATTAGTCTAGGATGTCCTCTT

TGCCA

TGCCATT  
Depth:2 (PIG)  
Ei-value:0.000, Pi-value:0.010  
Er-value:0.000, Pr-value:0.000  
eCLIP MATCHES▶hnrnpk (bg=12.88%)MATCHES To TargetScan▶ miR-183-5p.1:AUGGCAC

 3960  


TT

TGCCATT  
Depth:2 (PIG)  
Ei-value:0.000, Pi-value:0.010  
Er-value:0.000, Pr-value:0.000  
eCLIP MATCHES▶hnrnpk (bg=12.88%)MATCHES To TargetScan▶ miR-183-5p.1:AUGGCAC

GCTGCT

GAGTTCTGA

GAGTTCTGA  
Depth:2 (PIG)  
Ei-value:0.000, Pi-value:0.000  
Er-value:0.000, Pr-value:0.000  
eCLIP MATCHES▶hnrnpk (bg=12.88%)No matches to TargetScan

CTACCCAAGTTTCC

TTCTCTTAAACA

TTCTCTTAAACA  
Depth:2 (PIG)  
Ei-value:0.000, Pi-value:0.000  
Er-value:0.000, Pr-value:0.000  
No matches to eCLIP DataNo matches to TargetScan

GTTGATA

TGCATAATTGCATATA

TGCATAATTGCATATA  
Depth:2 (PIG)  
Ei-value:0.000, Pi-value:0.000  
Er-value:0.000, Pr-value:0.000  
No matches to eCLIP DataNo matches to TargetScan

TTCATGGTTCTGTGCAATAAAAATGGATTCTCACCCCATCCCACCT

TCTGTGG

TCTGTGG  
Depth:2 (PIG)  
Ei-value:0.000, Pi-value:0.000  
Er-value:0.000, Pr-value:0.010  
eCLIP MATCHES▶HNRNPL (bg=0.64%)▶HNRNPU (bg=5.92%)MATCHES To TargetScan▶ miR-140-3p.1:CCACAGG

G 4080  
 ATGTTGCTAACG

AGTGCA

AGTGCA  
Depth:2 (PIG)  
Ei-value:0.000, Pi-value:0.020  
Er-value:0.000, Pr-value:0.000  
eCLIP MATCHES▶HNRNPU (bg=5.92%)No matches to TargetScan

G

ATTATTCAA

ATTATTCAA  
Depth:2 (PIG)  
Ei-value:0.000, Pi-value:0.000  
Er-value:0.000, Pr-value:0.000  
eCLIP MATCHES▶HNRNPA1 (bg=2.57%)▶HNRNPU (bg=5.92%)No matches to TargetScan

TAACAGCTCTTGA

ACAGTTAAT

ACAGTTAAT  
Depth:2 (PIG)  
Ei-value:0.000, Pi-value:0.000  
Er-value:0.000, Pr-value:0.010  
eCLIP MATCHES▶HNRNPA1 (bg=2.57%)No matches to TargetScan

TT

GCACAGTTGC

GCACAGTTGC  
Depth:2 (PIG)  
Ei-value:0.000, Pi-value:0.000  
Er-value:0.000, Pr-value:0.000  
eCLIP MATCHES▶HNRNPA1 (bg=2.57%)No matches to TargetScan

AA

TTGTCCAGAGTCC

TTGTCCAGAGTCC  
Depth:2 (PIG)  
Ei-value:0.000, Pi-value:0.000  
Er-value:0.000, Pr-value:0.000  
eCLIP MATCHES▶HNRNPA1 (bg=2.57%)MATCHES To TargetScan▶ miR-326:CUCUGGG▶ miR-378-3p:CUGGACU

TGTCCATTAGAAAGGGACTCTGTATCCTATTTGCACGCTACAA 4200  
 T

GTGGGC

GTGGGC  
Depth:2 (PIG)  
Ei-value:0.000, Pi-value:0.000  
Er-value:0.000, Pr-value:0.010  
eCLIP MATCHES▶HNRNPU (bg=5.92%)No matches to TargetScan

TGATCACCCAAGGACTCTTCTTGTGCATTGATGTT

CATAATTG

CATAATTG  
Depth:2 (PIG)  
Ei-value:0.000, Pi-value:0.000  
Er-value:0.000, Pr-value:0.000  
eCLIP MATCHES▶HNRNPU (bg=5.92%)No matches to TargetScan

TATTTGTCCACGATCTTGTGCACTAACCCTTCCACTCC

CTTTGTATTCCAGCAGGGGACCCTT

CTTTGTATTCCAGCAGGGGACCCTT  
Depth:2 (PIG)  
Ei-value:0.000, Pi-value:0.000  
Er-value:0.000, Pr-value:0.000  
eCLIP MATCHES▶hnrnpk (bg=12.88%)▶HNRNPU (bg=5.92%)MATCHES To TargetScan▶ miR-331-3p:CCCCUGG▶ miR-381-3p:AUACAAG

ACTACTC 4320  
 AAGACCTCTGTACTAGGACAGTTTATGTGCACAAT

CCTAATTGATTAGA

CCTAATTGATTAGA  
Depth:2 (PIG)  
Ei-value:0.000, Pi-value:0.000  
Er-value:0.000, Pr-value:0.000  
eCLIP MATCHES▶HNRNPA1 (bg=2.57%)No matches to TargetScan

ACTGAG

TCTTTTAT

TCTTTTAT  
Depth:2 (PIG)  
Ei-value:0.000, Pi-value:0.000  
Er-value:0.000, Pr-value:0.010  
eCLIP MATCHES▶HNRNPA1 (bg=2.57%)▶hnrnpk (bg=12.88%)▶HNRNPU (bg=5.92%)▶UTP3 (bg=3.66%)No matches to TargetScan

ATCAAGGTCCC

TGCATC

TGCATC  
Depth:2 (PIG)  
Ei-value:0.000, Pi-value:0.030  
Er-value:0.000, Pr-value:0.020  
eCLIP MATCHES▶HNRNPA1 (bg=2.57%)▶hnrnpk (bg=12.88%)▶HNRNPU (bg=5.92%)▶UTP3 (bg=3.66%)No matches to TargetScan

ATCTTTGCTTTACATCAAGAGGGTGCTGG

TTACCTA

TTACCTA  
Depth:2 (PIG)  
Ei-value:0.000, Pi-value:0.000  
Er-value:0.000, Pr-value:0.020  
eCLIP MATCHES▶HNRNPU (bg=5.92%)▶UTP3 (bg=3.66%)No matches to TargetScan

ATGC 4440  
 CCCTCCTCCAGAAATTATTGATGTGCA

AAATGCAATT

AAATGCAATT  
Depth:2 (PIG)  
Ei-value:0.000, Pi-value:0.000  
Er-value:0.000, Pr-value:0.000  
eCLIP MATCHES▶AKAP8L (bg=2.19%)MATCHES To TargetScan▶ miR-25-3p/32-5p/92-3p/363-3p/367-3p:AUUGCAC▶ miR-33-5p:UGCAUUG

TCCCTATCTG

C

CTGTTAGTCT  
Depth:4 (DOG)  
Ei-value:0.000, Pi-value:0.000  
Er-value:0.000, Pr-value:0.000  
eCLIP MATCHES▶AKAP8L (bg=2.19%)No matches to TargetScan


TGTTAGTC

TGTTAGTC  
Depth:5 (RABBIT)  
Ei-value:0.000, Pi-value:0.000  
Er-value:0.000, Pr-value:0.000  
eCLIP MATCHES▶AKAP8L (bg=2.19%)No matches to TargetScan


T

CTGTTAGTCT  
Depth:4 (DOG)  
Ei-value:0.000, Pi-value:0.000  
Er-value:0.000, Pr-value:0.000  
eCLIP MATCHES▶AKAP8L (bg=2.19%)No matches to TargetScan

GGGG

TC

TCTCATCCCC  
Depth:2 (PIG)  
Ei-value:0.000, Pi-value:0.000  
Er-value:0.000, Pr-value:0.010  
eCLIP MATCHES▶AKAP8L (bg=2.19%)No matches to TargetScan


TCATCC

TCATCC  
Depth:4 (DOG)  
Ei-value:0.000, Pi-value:0.020  
Er-value:0.000, Pr-value:0.000  
eCLIP MATCHES▶AKAP8L (bg=2.19%)No matches to TargetScan


CC

TCTCATCCCC  
Depth:2 (PIG)  
Ei-value:0.000, Pi-value:0.000  
Er-value:0.000, Pr-value:0.010  
eCLIP MATCHES▶AKAP8L (bg=2.19%)No matches to TargetScan

TCATATT

CCTTTTGT

CCTTTTGT  
Depth:2 (PIG)  
Ei-value:0.000, Pi-value:0.000  
Er-value:0.000, Pr-value:0.010  
eCLIP MATCHES▶AKAP8L (bg=2.19%)No matches to TargetScan

CTTACAGCAGG

GGG

GGGTACTTGGGACTGTTAAT  
Depth:3 (COW)  
Ei-value:0.000, Pi-value:0.000  
Er-value:0.000, Pr-value:0.000  
eCLIP MATCHES▶AKAP8L (bg=2.19%)MATCHES To TargetScan▶ miR-132-3p/212-3p:AACAGUC▶ miR-455-3p.1:CAGUCCA


TACTTGGGACTGTTAAT

TACTTGGGACTGTTAAT  
Depth:4 (DOG)  
Ei-value:0.000, Pi-value:0.000  
Er-value:0.000, Pr-value:0.000  
eCLIP MATCHES▶AKAP8L (bg=2.19%)MATCHES To TargetScan▶ miR-132-3p/212-3p:AACAGUC▶ miR-455-3p.1:CAGUCCA


G

GGGTACTTGGGACTGTTAATG  
Depth:2 (PIG)  
Ei-value:0.000, Pi-value:0.000  
Er-value:0.000, Pr-value:0.000  
eCLIP MATCHES▶AKAP8L (bg=2.19%)MATCHES To TargetScan▶ miR-132-3p/212-3p:AACAGUC▶ miR-455-3p.1:CAGUCCA

CG 4560  
 CATAATTGCAATTATGGTCTTTTCCATTAAATTAAGATCCCAACTGCTCACACCCTCTTAGCATTACAGTAGAGGGTGCTAATCACAAGGACATTTCTTTTGT

ACTG

ACTGTTAATGTGCT  
Depth:4 (DOG)  
Ei-value:0.000, Pi-value:0.000  
Er-value:0.000, Pr-value:0.000  
No matches to eCLIP DataMATCHES To TargetScan▶ miR-132-3p/212-3p:AACAGUC▶ miR-323-3p:ACAUUAC


TTAATGTGCT

TTAATGTGCT  
Depth:5 (RABBIT)  
Ei-value:0.000, Pi-value:0.000  
Er-value:0.000, Pr-value:0.000  
No matches to eCLIP DataMATCHES To TargetScan▶ miR-323-3p:ACAUUAC


A

ACTGTTAATGTGCTA  
Depth:2 (PIG)  
Ei-value:0.000, Pi-value:0.000  
Er-value:0.000, Pr-value:0.000  
No matches to eCLIP DataMATCHES To TargetScan▶ miR-132-3p/212-3p:AACAGUC▶ miR-323-3p:ACAUUAC

CT 4680  
 TGCATTTGTCCCTCTTCCTGTGCACTAAAGACCCCACTCACTTCCCTAGTGTTCAGCAGTGGATGACCTCTAGTCAAGACCTTTGCACTAGGATAGTTAATGTGAACCATGGCAACTGAT 4800  
 CACAACAATGTCTTTCAGATCAGATCCATTTTATCCTCCTTGTTTTACAGCAAGGGATATTAATTACCTATGTTACCTTTCCCTGGGACTATGAATGTGCAAAATTCCAATGTTCATGGT 4920  
 CTCTCCCTTTAAACCTATATTCTACCCCTTTTACATTATAGAAAGGGATGCTGGAAACCCAGAGTCCTTCT

CTTGGGACTC

CTTGGGACTC  
Depth:3 (COW)  
Ei-value:0.000, Pi-value:0.000  
Er-value:0.000, Pr-value:0.000  
No matches to eCLIP DataNo matches to TargetScan


TTAATG

CTTGGGACTCTTAATG  
Depth:2 (PIG)  
Ei-value:0.000, Pi-value:0.000  
Er-value:0.000, Pr-value:0.000  
No matches to eCLIP DataNo matches to TargetScan

TGTATTTCTAATT

ATCCATG

ATCCATG  
Depth:2 (PIG)  
Ei-value:0.000, Pi-value:0.010  
Er-value:0.000, Pr-value:0.000  
No matches to eCLIP DataNo matches to TargetScan

ACTCT

T

TAATGTGCAT  
Depth:2 (PIG)  
Ei-value:0.000, Pi-value:0.000  
Er-value:0.000, Pr-value:0.000  
No matches to eCLIP DataMATCHES To TargetScan▶ miR-323-3p:ACAUUAC▶ miR-501-3p/502-3p:AUGCACC


AATGTGC

AATGTGCAT  
Depth:6 (MOUSE)  
Ei-value:0.000, Pi-value:0.000  
Er-value:0.000, Pr-value:0.000  
No matches to eCLIP DataMATCHES To TargetScan▶ miR-501-3p/502-3p:AUGCACC

 5040  


AT

AATGTGCAT  
Depth:6 (MOUSE)  
Ei-value:0.000, Pi-value:0.000  
Er-value:0.000, Pr-value:0.000  
No matches to eCLIP DataMATCHES To TargetScan▶ miR-501-3p/502-3p:AUGCACC

ATTTTCAATTGCCTAATTGATTTCAATTGTCTAAGACATTTCAAATGTCTAATTGATTAGAACTGAGTCTTTTATATCAAG

CTAATA

CTAATA  
Depth:3 (COW)  
Ei-value:0.000, Pi-value:0.000  
Er-value:0.000, Pr-value:0.000  
No matches to eCLIP DataNo matches to TargetScan

TCTAGCTTTTATATCAAG

CTAATA

CTAATA  
Depth:3 (COW)  
Ei-value:0.000, Pi-value:0.000  
Er-value:0.000, Pr-value:0.000  
No matches to eCLIP DataNo matches to TargetScan

TCTTGAC 5160  
 TTCTCAGCATCATAGAAGGGGGTACTGATTTCCTA

AAGTCTTT

AAGTCTTT  
Depth:2 (PIG)  
Ei-value:0.000, Pi-value:0.000  
Er-value:0.000, Pr-value:0.000  
No matches to eCLIP DataNo matches to TargetScan

CTTGAATTTCTATTA

TGCAAAATT

TGCAAAATT  
Depth:2 (PIG)  
Ei-value:0.000, Pi-value:0.000  
Er-value:0.000, Pr-value:0.000  
eCLIP MATCHES▶SUPV3L1 (bg=1.57%)No matches to TargetScan

GCCCTGAGGCCGGGTGTGGTGGCTCACACCTGTAATCCCAGCACTTTGGGAGG 5280  
 CTGAGGTGGGAAGATCCCTTACTGCCAGGAGTTTGAGACCAGCCTGGCCAACATTAAAAAAAAAAAAAAGTAAGACAATTGCCCTGGAATCCCATCCCCCTCACACCTCCTTGGCAAAGC 5400  
 AGCAGGAGTGCTAACTAGCTAG

TGCTTCT

TGCTTCT  
Depth:3 (COW)  
Ei-value:0.000, Pi-value:0.000  
Er-value:0.000, Pr-value:0.010  
No matches to eCLIP DataNo matches to TargetScan

TCTCTTATACTGCTTAAATGCGCATAATTAGCAGTAGTTGATGTGCCCC

TATGTTAGA

TATGTTAGA  
Depth:4 (DOG)  
Ei-value:0.000, Pi-value:0.000  
Er-value:0.000, Pr-value:0.000  
eCLIP MATCHES▶HNRNPU (bg=5.92%)No matches to TargetScan

G

TAGAATCCC

TAGAATCCC  
Depth:2 (PIG)  
Ei-value:0.000, Pi-value:0.000  
Er-value:0.000, Pr-value:0.000  
eCLIP MATCHES▶HNRNPU (bg=5.92%)No matches to TargetScan

GCTTCCTTGCTCCATTTGCATTA 5520  
 CTGCA

GGAGCTTCT

GGAGCTTCT  
Depth:2 (PIG)  
Ei-value:0.000, Pi-value:0.000  
Er-value:0.000, Pr-value:0.000  
No matches to eCLIP DataNo matches to TargetScan

AACTAGCCTGAATTCACTC

TCTTGG

TCTTGGACTGTTAATGT  
Depth:3 (COW)  
Ei-value:0.000, Pi-value:0.000  
Er-value:0.000, Pr-value:0.000  
No matches to eCLIP DataMATCHES To TargetScan▶ miR-132-3p/212-3p:AACAGUC▶ miR-323-3p:ACAUUAC▶ miR-455-3p.1:CAGUCCA


ACTGTTAATGT

ACTGTTAATGT  
Depth:4 (DOG)  
Ei-value:0.000, Pi-value:0.000  
Er-value:0.000, Pr-value:0.000  
No matches to eCLIP DataMATCHES To TargetScan▶ miR-132-3p/212-3p:AACAGUC▶ miR-323-3p:ACAUUAC


G

TCTTGGACTGTTAATGTG  
Depth:2 (PIG)  
Ei-value:0.000, Pi-value:0.000  
Er-value:0.000, Pr-value:0.000  
No matches to eCLIP DataMATCHES To TargetScan▶ miR-132-3p/212-3p:AACAGUC▶ miR-323-3p:ACAUUAC▶ miR-455-3p.1:CAGUCCA

CATACTTAT

ATTTGCT

ATTTGCT  
Depth:4 (DOG)  
Ei-value:0.000, Pi-value:0.000  
Er-value:0.000, Pr-value:0.000  
No matches to eCLIP DataNo matches to TargetScan

GCTGTACTTTTTTACCAT

GTAAGGA

GTAAGGA  
Depth:5 (RABBIT)  
Ei-value:0.000, Pi-value:0.000  
Er-value:0.000, Pr-value:0.000  
No matches to eCLIP DataNo matches to TargetScan


CCC

GTAAGGACCC  
Depth:3 (COW)  
Ei-value:0.000, Pi-value:0.000  
Er-value:0.000, Pr-value:0.000  
No matches to eCLIP DataNo matches to TargetScan

CACCCACTGTATTTACATCCCAGCT 5640  
 GGAAGTACCTACTA

CTTAAGA

CTTAAGA  
Depth:2 (PIG)  
Ei-value:0.000, Pi-value:0.000  
Er-value:0.000, Pr-value:0.000  
No matches to eCLIP DataNo matches to TargetScan

CCCTTAGACTAGTAAAGTTAGCG

TGCATA

TGCATAATCTTAG  
Depth:2 (PIG)  
Ei-value:0.000, Pi-value:0.000  
Er-value:0.000, Pr-value:0.000  
eCLIP MATCHES▶HNRNPU (bg=5.92%)No matches to TargetScan


ATCTTAG

ATCTTAG  
Depth:3 (COW)  
Ei-value:0.000, Pi-value:0.000  
Er-value:0.000, Pr-value:0.000  
eCLIP MATCHES▶HNRNPU (bg=5.92%)No matches to TargetScan

GTGTTATA

TACACATT

TACACATT  
Depth:3 (COW)  
Ei-value:0.000, Pi-value:0.000  
Er-value:0.000, Pr-value:0.000  
eCLIP MATCHES▶HNRNPU (bg=5.92%)No matches to TargetScan

TTCAGTTGCATACAGTTGTGCCTTTTATC

AGGACTCCT

AGGACTCCT  
Depth:2 (PIG)  
Ei-value:0.000, Pi-value:0.000  
Er-value:0.000, Pr-value:0.000  
eCLIP MATCHES▶HNRNPU (bg=5.92%)No matches to TargetScan

G

T

TACTTAT  
Depth:2 (PIG)  
Ei-value:0.000, Pi-value:0.010  
Er-value:0.000, Pr-value:0.030  
eCLIP MATCHES▶HNRNPU (bg=5.92%)No matches to TargetScan


ACTTAT

ACTTAT  
Depth:5 (RABBIT)  
Ei-value:0.000, Pi-value:0.000  
Er-value:0.000, Pr-value:0.000  
eCLIP MATCHES▶HNRNPU (bg=5.92%)No matches to TargetScan

C 5760  
 AAAGCAGAGAGTGCTAATCAATA

TTAAGC

TTAAGC  
Depth:2 (PIG)  
Ei-value:0.000, Pi-value:0.000  
Er-value:0.000, Pr-value:0.010  
No matches to eCLIP DataNo matches to TargetScan

CCTTCTCTTCGAACTGTAGATGGCA

TGTAATT

TGTAATT  
Depth:3 (COW)  
Ei-value:0.000, Pi-value:0.000  
Er-value:0.000, Pr-value:0.000  
No matches to eCLIP DataNo matches to TargetScan

GCAGTTGTCA

ATGGTC

ATGGTC  
Depth:3 (COW)  
Ei-value:0.000, Pi-value:0.020  
Er-value:0.000, Pr-value:0.000  
No matches to eCLIP DataNo matches to TargetScan


CTT

ATGGTCCTT  
Depth:2 (PIG)  
Ei-value:0.000, Pi-value:0.000  
Er-value:0.000, Pr-value:0.000  
No matches to eCLIP DataNo matches to TargetScan

CAATTAGACTTGGGTTTCTGACCTA

TCACAC

TCACAC  
Depth:2 (PIG)  
Ei-value:0.000, Pi-value:0.010  
Er-value:0.000, Pr-value:0.020  
No matches to eCLIP DataNo matches to TargetScan

CCTCTTTG

C

CTTTATTGC  
Depth:2 (PIG)  
Ei-value:0.000, Pi-value:0.000  
Er-value:0.000, Pr-value:0.000  
eCLIP MATCHES▶HNRNPL (bg=0.64%)MATCHES To TargetScan▶ miR-142-5p:AUAAAGU

 5880  


TTTATTGC

CTTTATTGC  
Depth:2 (PIG)  
Ei-value:0.000, Pi-value:0.000  
Er-value:0.000, Pr-value:0.000  
eCLIP MATCHES▶HNRNPL (bg=0.64%)MATCHES To TargetScan▶ miR-142-5p:AUAAAGU


ATGGGGTACT

ATGGGGTACT  
Depth:3 (COW)  
Ei-value:0.000, Pi-value:0.000  
Er-value:0.000, Pr-value:0.000  
eCLIP MATCHES▶HNRNPL (bg=0.64%)No matches to TargetScan

A

TT

TTCACTTAAGGCCCCTTTCTCAAAC  
Depth:2 (PIG)  
Ei-value:0.000, Pi-value:0.000  
Er-value:0.000, Pr-value:0.000  
eCLIP MATCHES▶HNRNPL (bg=0.64%)No matches to TargetScan


CAC

CACTTAAGGCCCCTTTCTCAA  
Depth:3 (COW)  
Ei-value:0.000, Pi-value:0.000  
Er-value:0.000, Pr-value:0.000  
eCLIP MATCHES▶HNRNPL (bg=0.64%)No matches to TargetScan


TTAAGGCC

TTAAGGCC  
Depth:6 (MOUSE)  
Ei-value:0.000, Pi-value:0.000  
Er-value:0.000, Pr-value:0.000  
eCLIP MATCHES▶HNRNPL (bg=0.64%)No matches to TargetScan


CCTTT

TTAAGGCCCCTTT  
Depth:5 (RABBIT)  
Ei-value:0.000, Pi-value:0.000  
Er-value:0.000, Pr-value:0.000  
eCLIP MATCHES▶HNRNPL (bg=0.64%)No matches to TargetScan


CTCAA

TTAAGGCCCCTTTCTCAA  
Depth:4 (DOG)  
Ei-value:0.000, Pi-value:0.000  
Er-value:0.000, Pr-value:0.000  
eCLIP MATCHES▶HNRNPL (bg=0.64%)No matches to TargetScan


AC

TTCACTTAAGGCCCCTTTCTCAAAC  
Depth:2 (PIG)  
Ei-value:0.000, Pi-value:0.000  
Er-value:0.000, Pr-value:0.000  
eCLIP MATCHES▶HNRNPL (bg=0.64%)No matches to TargetScan

TGTTAATGTGCC

TAATGACAATTACAT

TAATGACAATTACAT  
Depth:3 (COW)  
Ei-value:0.000, Pi-value:0.000  
Er-value:0.000, Pr-value:0.000  
eCLIP MATCHES▶HNRNPL (bg=0.64%)MATCHES To TargetScan▶ miR-411-3p:AUGUAAC

CAGT

ATCCTTCC

ATCCTTCC  
Depth:2 (PIG)  
Ei-value:0.000, Pi-value:0.010  
Er-value:0.000, Pr-value:0.000  
No matches to eCLIP DataNo matches to TargetScan

T

TTTGAAG

TTTGAAG  
Depth:2 (PIG)  
Ei-value:0.000, Pi-value:0.010  
Er-value:0.000, Pr-value:0.000  
eCLIP MATCHES▶HNRNPC (bg=3.65%)No matches to TargetScan

GACAGCATGGTTGGTGACA

CCTAAGG

CCTAAGG  
Depth:2 (PIG)  
Ei-value:0.000, Pi-value:0.010  
Er-value:0.000, Pr-value:0.000  
eCLIP MATCHES▶DDX51 (bg=1.63%)▶HNRNPC (bg=3.65%)No matches to TargetScan

C

CC

CCCATTTCTTG  
Depth:2 (PIG)  
Ei-value:0.000, Pi-value:0.000  
Er-value:0.000, Pr-value:0.000  
eCLIP MATCHES▶DDX51 (bg=1.63%)▶HNRNPC (bg=3.65%)MATCHES To TargetScan▶ miR-203a-3p.1:GAAAUGU

 6000  


CATTTCTTG

CCCATTTCTTG  
Depth:2 (PIG)  
Ei-value:0.000, Pi-value:0.000  
Er-value:0.000, Pr-value:0.000  
eCLIP MATCHES▶DDX51 (bg=1.63%)▶HNRNPC (bg=3.65%)MATCHES To TargetScan▶ miR-203a-3p.1:GAAAUGU

GCCTCCCAATATGTGTGAT

TGTATTTGTC

TGTATTTGTC  
Depth:2 (PIG)  
Ei-value:0.000, Pi-value:0.000  
Er-value:0.000, Pr-value:0.000  
eCLIP MATCHES▶DDX51 (bg=1.63%)No matches to TargetScan

GAGGTTGCTATGCACTAGAGAAGGAAAGTGCTCCCCTCATCCCCACTTTTCC

CTTCCAGCAGGAAGTGCCC

CTTCCAGCAGGAAGTGCCC  
Depth:2 (PIG)  
Ei-value:0.000, Pi-value:0.000  
Er-value:0.000, Pr-value:0.000  
eCLIP MATCHES▶hnrnpk (bg=12.88%)No matches to TargetScan

ACCCCATAAGA 6120  
 CCCTTTTATTTGGAGAGTCTAGGTGCACAATTGTAAGTGA

CCACAAG

CCACAAG  
Depth:2 (PIG)  
Ei-value:0.000, Pi-value:0.000  
Er-value:0.000, Pr-value:0.000  
eCLIP MATCHES▶HNRNPU (bg=5.92%)▶UTP3 (bg=3.66%)No matches to TargetScan

CATGCATCTTGGACATTTATGTGCGTAATCGCACACTGCTCATTCCATGTGAATAAGGTCCTACTCTCCGACC 6240  
 CCTTTTGCAATACAGAAGGGTTGCTGATAACGCAGTCCCCTTTTCTTGGCATGTTGTGTGTGATTATAATCGTCTGGGATCCTATGCACTAGAAAAGGAGGGTCCTCTCCACATACCTCA 6360  
 GTCTCACCTTTCCCTTCCAGCAGGGAGTGCCCACTCCATAAGACTCTCACA

TTTGGACAGTCAAG

TTTGGACAGTCAAG  
Depth:2 (PIG)  
Ei-value:0.000, Pi-value:0.000  
Er-value:0.000, Pr-value:0.000  
eCLIP MATCHES▶hnrnpk (bg=12.88%)No matches to TargetScan

GTGCGTAATTGTTAAGTGAACACAACC

ATGCAC

ATGCAC  
Depth:2 (PIG)  
Ei-value:0.000, Pi-value:0.030  
Er-value:0.000, Pr-value:0.010  
No matches to eCLIP DataNo matches to TargetScan

CTTAGACATGGATTTGCATAAC 6480  
 TACACACAGCTCAACCTATCTGAATAAAATCCTACTCTCAGACCCCTTTTGCAGTACAGCAGGGGTGCTGATCACCAAGGCCCTTTTTCCTGGCCTGGTATGCGTGTGATTATGTTTGTC 6600  
 CCGGTTCCTGTGTATTAGACATGGAAGCCTCCCCTGCCACACTCCACCCCCAATCTTCCTTTCCCTTCCGGCAGGGAGTGCCCTCTCCATAAGACGCTTACGTTTGGACAATCAAGGTGC 6720  
 ACAGTTGTAAGTGACCACAGGCATACACCTTGGACATTAATGTGCATAACCACTTTGCCCATTCCATCTGAATAAGGTCCTACTCTCAGACCCCTTTTGCAGTACAGCAGGGGTGCTGAT 6840  
 CACCAAGGCCCCTTTTCTTGGCCTGTTATGTGCGTGATTATATTTGTCTGGGTTCCTGTGTATTAGACAAGGAAGCCTTCCCCCCGCCCCCACCCCCACTCCCAGTCTTCCTTTCCCTTC 6960  
 CAGCAGGGAGTGCCCCCTCCATAAGATCATTACATTTGGACAATCAAGGTGCACAATTATAAGTGACCACAGCC

ATGCAC

ATGCAC  
Depth:2 (PIG)  
Ei-value:0.000, Pi-value:0.030  
Er-value:0.000, Pr-value:0.010  
eCLIP MATCHES▶hnrnpk (bg=12.88%)▶HNRNPU (bg=5.92%)No matches to TargetScan

CTTGGACATTATTGGACATTAATGTGC

GTAACTG

GTAACTG  
Depth:2 (PIG)  
Ei-value:0.000, Pi-value:0.000  
Er-value:0.000, Pr-value:0.000  
eCLIP MATCHES▶hnrnpk (bg=12.88%)No matches to TargetScan

CACATG 7080  
 GCCCATCCCATCTGAATAAGGTCCTACTCTCAGATGCCCTTTGCAGTACAGCAGGGGTACTGAATCACCAAGGCCCTTTTTCTTGGCCTGTTATGTGTGTGATTATATTTATCCCAGTTT 7200  
 CTGTGTAATAGACATGAAAGCCTCCCCTGCCACACCCCACCTCCAATCTTCCTTTCCCTTCCACCAGGGAGTGTCCACTCCATATACCCTTACATTTGGACAATCAAGGTGCACAATTGT 7320  
 AAGTGAGCATAGGCACT

CACCTTGGA

CACCTTGGA  
Depth:2 (PIG)  
Ei-value:0.000, Pi-value:0.000  
Er-value:0.000, Pr-value:0.000  
eCLIP MATCHES▶hnrnpk (bg=12.88%)▶HNRNPU (bg=5.92%)MATCHES To TargetScan▶ miR-18-5p:AAGGUGC

CATGAATGTGCATAACTGCACATGGCCCATCCCATCTGAATAAGGTCCTACTCTCAGACCCTTTTTGCAGTACAGCAGGGGTGCTGATCACCAA 7440  
 GGCCCCTTTTCCTGGCCTGTTATGTGTGTGATTATATTTGTTCCAGTTCCTGTGTAATAGACATGGAAGCCTCCCCTGCCACACTCCACCCCCAATCTTCCTTTCCCTTCTGGCAGGAAG 7560  
 TACCCGCTCCATAAGACCCTTACATTTGGACAGTCAAGGTGCACAATTGTATGTGACCACAACCATGCACCTTGGACATAAATGTGTGTAACTGCACATGGCCCATCCCATCTGAATAAG 7680  
 GTCCTACTCTCAGACCCCTTTTGCAGTACAGTAGGTGTGCTGATAACCAAGGCCCCTCTTCCTGGCCTGTTAACGTATGTGATTATATTTGTCTGGGTTCCAGTGTATAAGACATGGAAG 7800  
 CCTCCCCTGCCCCACCCCACCCTCAATCTTCCTTTCCCTTCTGGCAGGGAGTGCCAGCTCCATAAGAACCTTACATTTGGACAGTCAAGGTGCACAATTCTAAGTGACCGCAGCCATGCA 7920  
 CCTTGGTCAA

TAATGTGT

TAATGTGT  
Depth:2 (PIG)  
Ei-value:0.000, Pi-value:0.010  
Er-value:0.000, Pr-value:0.000  
No matches to eCLIP DataMATCHES To TargetScan▶ miR-323-3p:ACAUUAC

GTAACTGCACACGGCCTATCTCATCTGAATAAGGCCTTACTCTCAGACCCCTTTTGCAGTACAGCAGGGGTGCTGATAACCAAGGCCCATTTTCCTGGCCTG 8040  
 TTATGTGTGTGATTATATTTGTCCAGGTTTCTGTGTACTAGACAAGGAAGCCTCCTCTGCCCCATCCCATCTACGCATAATCTTTCTTTTCCTCCCAGCAGGGAGTGCTCACTCCATAAG 8160  
 ACCCTTACATTTGGACAATCAAGGTGCACAATTGTAAGTGACCACAACCATGCATCTTGGAAATTTATGTGC

ATAACTGCACATGGCT

ATAACTGCACATGGCT  
Depth:2 (PIG)  
Ei-value:0.000, Pi-value:0.000  
Er-value:0.000, Pr-value:0.000  
No matches to eCLIP DataMATCHES To TargetScan▶ miR-455-3p.2:UGCAGUC▶ miR-455-5p:AUGUGCC

TATCCTATTTGAATAAAGTCCTA

CTCTCAGAC

CTCTCAGACCCC  
Depth:2 (PIG)  
Ei-value:0.000, Pi-value:0.000  
Er-value:0.000, Pr-value:0.000  
eCLIP MATCHES▶SF3B1 (bg=2.48%)MATCHES To TargetScan▶ miR-193a-5p:GGGUCUU

 8280  


CCC

CTCTCAGACCCC  
Depth:2 (PIG)  
Ei-value:0.000, Pi-value:0.000  
Er-value:0.000, Pr-value:0.000  
eCLIP MATCHES▶SF3B1 (bg=2.48%)MATCHES To TargetScan▶ miR-193a-5p:GGGUCUU

CTTTGC

AGTATAGC

AGTATAGC  
Depth:2 (PIG)  
Ei-value:0.000, Pi-value:0.000  
Er-value:0.000, Pr-value:0.000  
eCLIP MATCHES▶SF3B1 (bg=2.48%)No matches to TargetScan

TGGGGTGCTGATCACTGAGG

CCTCTTT

CCTCTTT  
Depth:2 (PIG)  
Ei-value:0.000, Pi-value:0.010  
Er-value:0.000, Pr-value:0.020  
No matches to eCLIP DataNo matches to TargetScan

GCTTGGCTTGTCTATATTCTTGTGTACTAGATAAGGGCACCTTCTCATGG

ACTCCCTTTG

ACTCCCTTTG  
Depth:2 (PIG)  
Ei-value:0.000, Pi-value:0.000  
Er-value:0.000, Pr-value:0.000  
eCLIP MATCHES▶DDX21 (bg=0.25%)No matches to TargetScan

CTTTTCAACAAGGAGT 8400  
 ACC

CACTACTTT

CACTACTTT  
Depth:2 (PIG)  
Ei-value:0.000, Pi-value:0.000  
Er-value:0.000, Pr-value:0.000  
eCLIP MATCHES▶DDX21 (bg=0.25%)MATCHES To TargetScan▶ miR-142-3p.1:GUAGUGU

TTAAGATT

CTTATATTT

CTTATATTT  
Depth:3 (COW)  
Ei-value:0.000, Pi-value:0.000  
Er-value:0.000, Pr-value:0.000  
eCLIP MATCHES▶DDX21 (bg=0.25%)MATCHES To TargetScan▶ miR-410-3p:AUAUAAC

GTC

CAAAGTACATG

CAAAGTACATG  
Depth:2 (PIG)  
Ei-value:0.000, Pi-value:0.000  
Er-value:0.000, Pr-value:0.000  
No matches to eCLIP DataNo matches to TargetScan

G

TTTTAATTGACCA

TTTTAATTGACCA  
Depth:3 (COW)  
Ei-value:0.000, Pi-value:0.000  
Er-value:0.000, Pr-value:0.000  
No matches to eCLIP DataNo matches to TargetScan

CAACAATGTCCC

TTGG

TTGGACATTAATGTA  
Depth:2 (PIG)  
Ei-value:0.000, Pi-value:0.000  
Er-value:0.000, Pr-value:0.000  
No matches to eCLIP DataMATCHES To TargetScan▶ miR-323-3p:ACAUUAC


ACATTAAT

ACATTAAT  
Depth:3 (COW)  
Ei-value:0.000, Pi-value:0.000  
Er-value:0.000, Pr-value:0.000  
No matches to eCLIP DataNo matches to TargetScan


GTA

TTGGACATTAATGTA  
Depth:2 (PIG)  
Ei-value:0.000, Pi-value:0.000  
Er-value:0.000, Pr-value:0.000  
No matches to eCLIP DataMATCHES To TargetScan▶ miR-323-3p:ACAUUAC

TGTAATCACCACATGGTTCATCCTAATTAAACAAAG 8520  
 TTCTACCTTCTCACC

CTCCATTTGCAGTATA

CTCCATTTGCAGTATA  
Depth:2 (PIG)  
Ei-value:0.000, Pi-value:0.000  
Er-value:0.000, Pr-value:0.000  
eCLIP MATCHES▶hnrnpk (bg=12.88%)▶TIA1 (bg=4.07%)MATCHES To TargetScan▶ miR-217:ACUGCAU

C

CAGGGTT

CAGGGTT  
Depth:2 (PIG)  
Ei-value:0.000, Pi-value:0.000  
Er-value:0.000, Pr-value:0.000  
eCLIP MATCHES▶hnrnpk (bg=12.88%)▶TIA1 (bg=4.07%)MATCHES To TargetScan▶ miR-10-5p:ACCCUGU▶ miR-504-5p.1:ACCCUGG

GC

TGACCC

TGACCC  
Depth:2 (PIG)  
Ei-value:0.000, Pi-value:0.030  
Er-value:0.000, Pr-value:0.000  
eCLIP MATCHES▶hnrnpk (bg=12.88%)▶TIA1 (bg=4.07%)No matches to TargetScan

CCTAAGTCCCCTTTTCTTGGCTTGTTGACA

TG

TGCATAATTGCATTT  
Depth:2 (PIG)  
Ei-value:0.000, Pi-value:0.000  
Er-value:0.000, Pr-value:0.000  
eCLIP MATCHES▶hnrnpk (bg=12.88%)▶TIA1 (bg=4.07%)No matches to TargetScan


CATAATTGCA

CATAATTGCA  
Depth:3 (COW)  
Ei-value:0.000, Pi-value:0.000  
Er-value:0.000, Pr-value:0.000  
eCLIP MATCHES▶hnrnpk (bg=12.88%)▶TIA1 (bg=4.07%)No matches to TargetScan


TTT

TGCATAATTGCATTT  
Depth:2 (PIG)  
Ei-value:0.000, Pi-value:0.000  
Er-value:0.000, Pr-value:0.000  
eCLIP MATCHES▶hnrnpk (bg=12.88%)▶TIA1 (bg=4.07%)No matches to TargetScan

ATGTT

GGTTCTTG

GGTTCTTG  
Depth:2 (PIG)  
Ei-value:0.000, Pi-value:0.000  
Er-value:0.000, Pr-value:0.000  
eCLIP MATCHES▶UTP3 (bg=3.66%)No matches to TargetScan

TGCC

CTAGACAAGGA

CTAGACAAGGA  
Depth:3 (COW)  
Ei-value:0.000, Pi-value:0.000  
Er-value:0.000, Pr-value:0.000  
eCLIP MATCHES▶UTP3 (bg=3.66%)No matches to TargetScan

 8640  


CTAGACAAGGA  
Depth:3 (COW)  
Ei-value:0.000, Pi-value:0.000  
Er-value:0.000, Pr-value:0.000  
eCLIP MATCHES▶UTP3 (bg=3.66%)No matches to TargetScan

TGCCCCACCTCTTTTCAATAGTGGGTGCCCACTCCTTATGATCTTTACATTTGA

ACAGTTAATGTG

ACAGTTAATGTG  
Depth:4 (DOG)  
Ei-value:0.000, Pi-value:0.000  
Er-value:0.000, Pr-value:0.000  
eCLIP MATCHES▶HNRNPU (bg=5.92%)MATCHES To TargetScan▶ miR-323-3p:ACAUUAC

AAT

AATTGCAGTT

AATTGCAGTT  
Depth:2 (PIG)  
Ei-value:0.000, Pi-value:0.000  
Er-value:0.000, Pr-value:0.000  
eCLIP MATCHES▶HNRNPU (bg=5.92%)MATCHES To TargetScan▶ miR-217:ACUGCAU

G

TCCACAACCC

TCCACAACCC  
Depth:2 (PIG)  
Ei-value:0.000, Pi-value:0.000  
Er-value:0.000, Pr-value:0.000  
eCLIP MATCHES▶hnrnpk (bg=12.88%)▶HNRNPU (bg=5.92%)No matches to TargetScan

TATCACTTCTAGGACCATT

ATACCTC

ATACCTC  
Depth:2 (PIG)  
Ei-value:0.000, Pi-value:0.000  
Er-value:0.000, Pr-value:0.000  
No matches to eCLIP DataMATCHES To TargetScan▶ let-7-5p/98-5p:GAGGUAG

TTTT 8760  
 GCATTACTGTGGGGT

ATACTGTTT

ATACTGTTT  
Depth:3 (COW)  
Ei-value:0.000, Pi-value:0.000  
Er-value:0.000, Pr-value:0.000  
No matches to eCLIP DataMATCHES To TargetScan▶ miR-101-3p.1:ACAGUAC▶ miR-132-3p/212-3p:AACAGUC▶ miR-144-3p:ACAGUAU

CCCTCCAAGGCCCCTTCTG

GTGGAC

GTGGAC  
Depth:2 (PIG)  
Ei-value:0.000, Pi-value:0.000  
Er-value:0.000, Pr-value:0.010  
No matches to eCLIP DataNo matches to TargetScan

TATCAACATA

TAATTGAAAT

TAATTGAAAT  
Depth:2 (PIG)  
Ei-value:0.000, Pi-value:0.000  
Er-value:0.000, Pr-value:0.000  
No matches to eCLIP DataNo matches to TargetScan

TTTCTT

TTGTCTT

TTGTCTT  
Depth:3 (COW)  
Ei-value:0.000, Pi-value:0.000  
Er-value:0.000, Pr-value:0.010  
No matches to eCLIP DataNo matches to TargetScan

TGTCAGTAGATTAAGGTCATACCCCATCACCTTTCCTT 8880  
 TGTAGTACAACAGGGTGTCCTGATCAACCAAAGTCCTGTTGTTTTGGACTGTTAATATGTGCAATTACATTTGCTCCTGATCTGTGCACTAGATAAGGATCCTACCTACTTTCTTAGTGT 9000  
 TTTTAGCAGGTAGTGCCCACTACTCAAGACTGTCACTTGGAATGTTCATGTGCACAAACTCAATTCTCTAAGCATGTTCCTGTACCACCTTTGCTTTAGAGCAGGGGGATGATATTCACT 9120  
 AAGTGCCCCTTCTTTTGGACTTAATATGCATTAATGCAATTGTCCACCTCTTCTTT

TAGACT

TAGACT  
Depth:2 (PIG)  
Ei-value:0.000, Pi-value:0.000  
Er-value:0.000, Pr-value:0.000  
No matches to eCLIP DataNo matches to TargetScan

AAGAGTTGATCTCCACATATTCCCCTTGCATCAGGGGCATGTTAATTATGAATGAACC 9240  
 CTTTTCTTTTAATATTAATGTCATAATTGTATTTGTGGACCTGTGTAGGAGAAAAAGACCCTATGTTCCTCCCATTACCCTTTGGATTGCTGCTGAGAAGTGT

TAACTA

TAACTA  
Depth:4 (DOG)  
Ei-value:0.000, Pi-value:0.000  
Er-value:0.000, Pr-value:0.000  
No matches to eCLIP DataNo matches to TargetScan

CTCATAAT

CTC

CTCAGCTCTTGG  
Depth:5 (RABBIT)  
Ei-value:0.000, Pi-value:0.000  
Er-value:0.000, Pr-value:0.000  
No matches to eCLIP DataMATCHES To TargetScan▶ miR-335-5p:CAAGAGC

 9360  


AGCTCTTGG

CTCAGCTCTTGG  
Depth:5 (RABBIT)  
Ei-value:0.000, Pi-value:0.000  
Er-value:0.000, Pr-value:0.000  
No matches to eCLIP DataMATCHES To TargetScan▶ miR-335-5p:CAAGAGC


ACA

CTCAGCTCTTGGACA  
Depth:4 (DOG)  
Ei-value:0.000, Pi-value:0.000  
Er-value:0.000, Pr-value:0.000  
No matches to eCLIP DataMATCHES To TargetScan▶ miR-335-5p:CAAGAGC


ATTAATA

CTCAGCTCTTGGACAATTAATA  
Depth:3 (COW)  
Ei-value:0.000, Pi-value:0.000  
Er-value:0.000, Pr-value:0.000  
No matches to eCLIP DataMATCHES To TargetScan▶ miR-335-5p:CAAGAGC

GCATT

AATAACA

AATAACA  
Depth:2 (PIG)  
Ei-value:0.000, Pi-value:0.010  
Er-value:0.000, Pr-value:0.000  
No matches to eCLIP DataNo matches to TargetScan

ATTATCAAGGGC

ACT

ACTGATCATTAGATA  
Depth:2 (PIG)  
Ei-value:0.000, Pi-value:0.000  
Er-value:0.000, Pr-value:0.000  
eCLIP MATCHES▶HNRNPU (bg=5.92%)MATCHES To TargetScan▶ miR-383-5p.1:GAUCAGA


GATCAT

GATCAT  
Depth:3 (COW)  
Ei-value:0.000, Pi-value:0.000  
Er-value:0.000, Pr-value:0.000  
eCLIP MATCHES▶HNRNPU (bg=5.92%)No matches to TargetScan


TAGATA

ACTGATCATTAGATA  
Depth:2 (PIG)  
Ei-value:0.000, Pi-value:0.000  
Er-value:0.000, Pr-value:0.000  
eCLIP MATCHES▶HNRNPU (bg=5.92%)MATCHES To TargetScan▶ miR-383-5p.1:GAUCAGA

AGACTCCTGCTTCCTCGTTGCTTACATCGGGGGTACTGACCC

AC

ACTAAGGCCCC  
Depth:2 (PIG)  
Ei-value:0.000, Pi-value:0.000  
Er-value:0.000, Pr-value:0.000  
No matches to eCLIP DataNo matches to TargetScan


TAAGGC

TAAGGC  
Depth:3 (COW)  
Ei-value:0.000, Pi-value:0.000  
Er-value:0.000, Pr-value:0.000  
No matches to eCLIP DataNo matches to TargetScan


CCC

ACTAAGGCCCC  
Depth:2 (PIG)  
Ei-value:0.000, Pi-value:0.000  
Er-value:0.000, Pr-value:0.000  
No matches to eCLIP DataNo matches to TargetScan

TTGTACTGT 9480  
 TAATGT

GAATATTTGCA

GAATATTTGCA  
Depth:3 (COW)  
Ei-value:0.000, Pi-value:0.000  
Er-value:0.000, Pr-value:0.000  
No matches to eCLIP DataNo matches to TargetScan


ATTAT

GAATATTTGCAATTAT  
Depth:2 (PIG)  
Ei-value:0.000, Pi-value:0.000  
Er-value:0.000, Pr-value:0.000  
No matches to eCLIP DataMATCHES To TargetScan▶ miR-25-3p/32-5p/92-3p/363-3p/367-3p:AUUGCAC

ATATGTCTCCTTCTGGTAGAGTGGGATATTATGCCCTAGTA

TCCCCTT

TCCCCTT  
Depth:2 (PIG)  
Ei-value:0.000, Pi-value:0.000  
Er-value:0.000, Pr-value:0.030  
No matches to eCLIP DataNo matches to TargetScan

TGC

ATTACTG

ATTACTG  
Depth:3 (COW)  
Ei-value:0.000, Pi-value:0.010  
Er-value:0.000, Pr-value:0.020  
No matches to eCLIP DataMATCHES To TargetScan▶ miR-802:CAGUAAC

C

AG

AGGGGCTGCTGAC  
Depth:2 (PIG)  
Ei-value:0.000, Pi-value:0.000  
Er-value:0.000, Pr-value:0.000  
No matches to eCLIP DataMATCHES To TargetScan▶ miR-15-5p/16-5p/195-5p/424-5p/497-5p:AGCAGCA▶ miR-503-5p:AGCAGCG


GGGCTGCTGA

GGGCTGCTGA  
Depth:3 (COW)  
Ei-value:0.000, Pi-value:0.000  
Er-value:0.000, Pr-value:0.000  
No matches to eCLIP DataMATCHES To TargetScan▶ miR-15-5p/16-5p/195-5p/424-5p/497-5p:AGCAGCA▶ miR-503-5p:AGCAGCG


C

AGGGGCTGCTGAC  
Depth:2 (PIG)  
Ei-value:0.000, Pi-value:0.000  
Er-value:0.000, Pr-value:0.000  
No matches to eCLIP DataMATCHES To TargetScan▶ miR-15-5p/16-5p/195-5p/424-5p/497-5p:AGCAGCA▶ miR-503-5p:AGCAGCG

TACT

CAAAACTT

CAAAACTT  
Depth:4 (DOG)  
Ei-value:0.000, Pi-value:0.000  
Er-value:0.000, Pr-value:0.000  
eCLIP MATCHES▶SF3B1 (bg=2.48%)No matches to TargetScan


CT

CAAAACTTCT  
Depth:2 (PIG)  
Ei-value:0.000, Pi-value:0.000  
Er-value:0.000, Pr-value:0.000  
eCLIP MATCHES▶SF3B1 (bg=2.48%)No matches to TargetScan

C

CTGGGACTG

CTGGGACTG  
Depth:3 (COW)  
Ei-value:0.000, Pi-value:0.000  
Er-value:0.000, Pr-value:0.000  
eCLIP MATCHES▶SF3B1 (bg=2.48%)MATCHES To TargetScan▶ miR-455-3p.1:CAGUCCA

TT 9600  
 AATAG

GCACAATG

GCACAATG  
Depth:6 (MOUSE)  
Ei-value:0.000, Pi-value:0.000  
Er-value:0.000, Pr-value:0.000  
No matches to eCLIP DataNo matches to TargetScan

GCAGTTATCAATGGTTTTCTCC

CTCCCTG

CTCCCTG  
Depth:3 (COW)  
Ei-value:0.000, Pi-value:0.000  
Er-value:0.000, Pr-value:0.000  
eCLIP MATCHES▶DDX42 (bg=0.58%)No matches to TargetScan

A

CCTTGTT

CCTTGTT  
Depth:2 (PIG)  
Ei-value:0.000, Pi-value:0.000  
Er-value:0.000, Pr-value:0.040  
eCLIP MATCHES▶DDX42 (bg=0.58%)No matches to TargetScan

AA

GCAAGC

GCAAGC  
Depth:3 (COW)  
Ei-value:0.000, Pi-value:0.000  
Er-value:0.000, Pr-value:0.000  
eCLIP MATCHES▶DDX42 (bg=0.58%)▶hnrnpk (bg=12.88%)No matches to TargetScan


GC

GCAAGCGC  
Depth:2 (PIG)  
Ei-value:0.000, Pi-value:0.000  
Er-value:0.000, Pr-value:0.000  
eCLIP MATCHES▶DDX42 (bg=0.58%)▶hnrnpk (bg=12.88%)No matches to TargetScan

CCCACCCCACCCTTAG

TTTCCCATGG

TTTCCCATGG  
Depth:2 (PIG)  
Ei-value:0.000, Pi-value:0.000  
Er-value:0.000, Pr-value:0.000  
eCLIP MATCHES▶DDX42 (bg=0.58%)▶hnrnpk (bg=12.88%)No matches to TargetScan

C

ATAATAAAGTATAA

ATAATAAAGTATAA  
Depth:2 (PIG)  
Ei-value:0.000, Pi-value:0.000  
Er-value:0.000, Pr-value:0.000  
eCLIP MATCHES▶DDX42 (bg=0.58%)▶KHDRBS1 (bg=1.71%)No matches to TargetScan

GCATTGGAGTATTCCATGC 9720  
 ACTTGTCT

ATCAAACAG

ATCAAACAG  
Depth:2 (PIG)  
Ei-value:0.000, Pi-value:0.000  
Er-value:0.000, Pr-value:0.000  
No matches to eCLIP DataNo matches to TargetScan

TGGT

CCAT

CCATACTCCCA  
Depth:2 (PIG)  
Ei-value:0.000, Pi-value:0.000  
Er-value:0.000, Pr-value:0.000  
eCLIP MATCHES▶hnrnpk (bg=12.88%)MATCHES To TargetScan▶ miR-496.1:GAGUAUU


A

ACTCCCA  
Depth:4 (DOG)  
Ei-value:0.000, Pi-value:0.000  
Er-value:0.000, Pr-value:0.000  
eCLIP MATCHES▶hnrnpk (bg=12.88%)No matches to TargetScan


CTCCCA

CTCCCA  
Depth:6 (MOUSE)  
Ei-value:0.000, Pi-value:0.000  
Er-value:0.000, Pr-value:0.000  
eCLIP MATCHES▶hnrnpk (bg=12.88%)No matches to TargetScan

A

CCCTTTTGCATT

CCCTTTTGCATT  
Depth:4 (DOG)  
Ei-value:0.000, Pi-value:0.000  
Er-value:0.000, Pr-value:0.000  
eCLIP MATCHES▶hnrnpk (bg=12.88%)No matches to TargetScan


G

CCCTTTTGCATTG  
Depth:3 (COW)  
Ei-value:0.000, Pi-value:0.000  
Er-value:0.000, Pr-value:0.000  
eCLIP MATCHES▶hnrnpk (bg=12.88%)No matches to TargetScan

CGCCAGTGTGTAAAATCACAGGTAGCCATGGTGTCATGCTTTATATACGAAGTCTTCCCTCTCTCTGCCCCTTG 9840  
 TGTGCCCTTGGCCCCTTTTTACAGACTATTGCTCACAATCTCAGGTGTCCATATTTGCAGCTATTAGGTAAGATTGTGCTGTCTCCCTCTTCCCTTCCCTCTGCCCTGCCCCTTTTGCCT 9960  
 CT

TTGCTGGG

TTGCTGGG  
Depth:2 (PIG)  
Ei-value:0.000, Pi-value:0.000  
Er-value:0.000, Pr-value:0.010  
eCLIP MATCHES▶hnrnpk (bg=12.88%)MATCHES To TargetScan▶ miR-338-3p:CCAGCAU

TAATGTTGACCAGACAAGG

CCCTTTCT

CCCTTTCT  
Depth:2 (PIG)  
Ei-value:0.000, Pi-value:0.000  
Er-value:0.000, Pr-value:0.000  
eCLIP MATCHES▶hnrnpk (bg=12.88%)No matches to TargetScan

CTTGGACTTAAACAATTCTCAGTTGCACTTTCCTTGGTCCCACCCATTATACATGAACCCCTCT

ACTTCCTT

ACTTCCTT  
Depth:3 (COW)  
Ei-value:0.000, Pi-value:0.000  
Er-value:0.000, Pr-value:0.000  
eCLIP MATCHES▶hnrnpk (bg=12.88%)No matches to TargetScan

TCGCATTGCTT 10080  


CTGAGTA

CTGAGTA  
Depth:2 (PIG)  
Ei-value:0.000, Pi-value:0.010  
Er-value:0.000, Pr-value:0.000  
eCLIP MATCHES▶hnrnpk (bg=12.88%)No matches to TargetScan

TG

CTGACTACCCA

CTGACTACCCA  
Depth:2 (PIG)  
Ei-value:0.000, Pi-value:0.000  
Er-value:0.000, Pr-value:0.000  
eCLIP MATCHES▶hnrnpk (bg=12.88%)No matches to TargetScan

A

AGCCCCTTCT

AGCCCCTTCT  
Depth:3 (COW)  
Ei-value:0.000, Pi-value:0.000  
Er-value:0.000, Pr-value:0.000  
eCLIP MATCHES▶hnrnpk (bg=12.88%)No matches to TargetScan


GTGTTATTAA

AGCCCCTTCTGTGTTATTAA  
Depth:2 (PIG)  
Ei-value:0.000, Pi-value:0.000  
Er-value:0.000, Pr-value:0.000  
eCLIP MATCHES▶hnrnpk (bg=12.88%)No matches to TargetScan

TAAA

CACAGTA

CACAGTA  
Depth:3 (COW)  
Ei-value:0.000, Pi-value:0.000  
Er-value:0.000, Pr-value:0.000  
eCLIP MATCHES▶hnrnpk (bg=12.88%)No matches to TargetScan

C

TGATTGTC

TGATTGTCCCATTTTT  
Depth:3 (COW)  
Ei-value:0.000, Pi-value:0.000  
Er-value:0.000, Pr-value:0.000  
eCLIP MATCHES▶hnrnpk (bg=12.88%)No matches to TargetScan


CCATTTTT

CCATTTTT  
Depth:4 (DOG)  
Ei-value:0.000, Pi-value:0.000  
Er-value:0.000, Pr-value:0.000  
eCLIP MATCHES▶hnrnpk (bg=12.88%)No matches to TargetScan


CAGCCCA

CAGCCCA  
Depth:4 (DOG)  
Ei-value:0.000, Pi-value:0.000  
Er-value:0.000, Pr-value:0.000  
eCLIP MATCHES▶hnrnpk (bg=12.88%)No matches to TargetScan

TCAGTCCAAGA

TCTC

TCTCCCTACCA  
Depth:3 (COW)  
Ei-value:0.000, Pi-value:0.000  
Er-value:0.000, Pr-value:0.000  
eCLIP MATCHES▶hnrnpk (bg=12.88%)No matches to TargetScan


CCTACCA

CCTACCA  
Depth:4 (DOG)  
Ei-value:0.000, Pi-value:0.000  
Er-value:0.000, Pr-value:0.000  
eCLIP MATCHES▶hnrnpk (bg=12.88%)No matches to TargetScan


CTTTG

TCTCCCTACCACTTTG  
Depth:2 (PIG)  
Ei-value:0.000, Pi-value:0.000  
Er-value:0.000, Pr-value:0.000  
eCLIP MATCHES▶hnrnpk (bg=12.88%)MATCHES To TargetScan▶ miR-140-5p:AGUGGUU▶ miR-17-5p/20-5p/93-5p/106-5p/519-3p:AAAGUGC

GTGTGTTG

GTGCAGT

GTGCAGT  
Depth:3 (COW)  
Ei-value:0.000, Pi-value:0.000  
Er-value:0.000, Pr-value:0.000  
eCLIP MATCHES▶hnrnpk (bg=12.88%)MATCHES To TargetScan▶ miR-217:ACUGCAU

G

T

TTGACTA  
Depth:2 (PIG)  
Ei-value:0.000, Pi-value:0.020  
Er-value:0.000, Pr-value:0.000  
No matches to eCLIP DataNo matches to TargetScan

 10200  


TGACTA

TTGACTA  
Depth:2 (PIG)  
Ei-value:0.000, Pi-value:0.020  
Er-value:0.000, Pr-value:0.000  
No matches to eCLIP DataNo matches to TargetScan

TG

AAAAGCAG

AAAAGCAG  
Depth:6 (MOUSE)  
Ei-value:0.000, Pi-value:0.000  
Er-value:0.000, Pr-value:0.000  
No matches to eCLIP DataNo matches to TargetScan

G

CCT

CCTGAACTA  
Depth:2 (PIG)  
Ei-value:0.000, Pi-value:0.000  
Er-value:0.000, Pr-value:0.000  
No matches to eCLIP DataNo matches to TargetScan


GAACTA

GAACTA  
Depth:3 (COW)  
Ei-value:0.000, Pi-value:0.000  
Er-value:0.000, Pr-value:0.000  
No matches to eCLIP DataNo matches to TargetScan

GGTGGATAA

GCCTTCACTC

GCCTTCACTC  
Depth:2 (PIG)  
Ei-value:0.000, Pi-value:0.000  
Er-value:0.000, Pr-value:0.000  
No matches to eCLIP DataNo matches to TargetScan

ATTTTCTTTCATTTA

TTAATGATCC

TTAATGATCC  
Depth:4 (DOG)  
Ei-value:0.000, Pi-value:0.000  
Er-value:0.000, Pr-value:0.000  
No matches to eCLIP DataMATCHES To TargetScan▶ miR-382-3p:AUCAUUC

TAGTTT

CA

CAATTATTGT  
Depth:2 (PIG)  
Ei-value:0.000, Pi-value:0.000  
Er-value:0.000, Pr-value:0.000  
No matches to eCLIP DataNo matches to TargetScan


ATTATTGT

ATTATTGT  
Depth:3 (COW)  
Ei-value:0.000, Pi-value:0.000  
Er-value:0.000, Pr-value:0.000  
No matches to eCLIP DataNo matches to TargetScan

CAG

ATTCTGGG

ATTCTGGG  
Depth:4 (DOG)  
Ei-value:0.000, Pi-value:0.000  
Er-value:0.000, Pr-value:0.000  
No matches to eCLIP DataNo matches to TargetScan


GACA

ATTCTGGGGACA  
Depth:2 (PIG)  
Ei-value:0.000, Pi-value:0.000  
Er-value:0.000, Pr-value:0.000  
No matches to eCLIP DataNo matches to TargetScan

A

GAACCATTC

GAACCATTC  
Depth:2 (PIG)  
Ei-value:0.000, Pi-value:0.000  
Er-value:0.000, Pr-value:0.000  
No matches to eCLIP DataNo matches to TargetScan

TTGCCCACC 10320  
 TGTG

TTAC

TTACTGCTTTACT  
Depth:2 (PIG)  
Ei-value:0.000, Pi-value:0.000  
Er-value:0.000, Pr-value:0.000  
No matches to eCLIP DataMATCHES To TargetScan▶ miR-330-3p.2:AAAGCAC▶ miR-802:CAGUAAC


TG

TGCTTTACT  
Depth:3 (COW)  
Ei-value:0.000, Pi-value:0.000  
Er-value:0.000, Pr-value:0.000  
No matches to eCLIP DataMATCHES To TargetScan▶ miR-330-3p.2:AAAGCAC


CTTTACT

CTTTACT  
Depth:4 (DOG)  
Ei-value:0.000, Pi-value:0.000  
Er-value:0.000, Pr-value:0.000  
No matches to eCLIP DataNo matches to TargetScan

GT

GCAAAAT

GCAAAAT  
Depth:6 (MOUSE)  
Ei-value:0.000, Pi-value:0.000  
Er-value:0.000, Pr-value:0.000  
No matches to eCLIP DataNo matches to TargetScan

ACTG

AAGGCAA

AAGGCAA  
Depth:4 (DOG)  
Ei-value:0.000, Pi-value:0.000  
Er-value:0.000, Pr-value:0.000  
No matches to eCLIP DataNo matches to TargetScan


GTCAGACCCA

AAGGCAAGTCAGACCCA  
Depth:3 (COW)  
Ei-value:0.000, Pi-value:0.000  
Er-value:0.000, Pr-value:0.000  
No matches to eCLIP DataMATCHES To TargetScan▶ miR-193a-5p:GGGUCUU

GGGAGC

TGGATTGC

TGGATTGC  
Depth:4 (DOG)  
Ei-value:0.000, Pi-value:0.000  
Er-value:0.000, Pr-value:0.000  
No matches to eCLIP DataNo matches to TargetScan

CATCCTTTATTTTGTGTTTCCAGTGTACACTATAAAATTG

TCTCCCCAG

TCTCCCCAGGAAGGAAG  
Depth:2 (PIG)  
Ei-value:0.000, Pi-value:0.000  
Er-value:0.000, Pr-value:0.000  
eCLIP MATCHES▶SF3B1 (bg=2.48%)No matches to TargetScan


GAAGGAAG

GAAGGAAG  
Depth:3 (COW)  
Ei-value:0.000, Pi-value:0.000  
Er-value:0.000, Pr-value:0.000  
eCLIP MATCHES▶SF3B1 (bg=2.48%)No matches to TargetScan

GT 10440  
 TGGCACTT

TCTC

TCTCTGCATTCTTC  
Depth:2 (PIG)  
Ei-value:0.000, Pi-value:0.000  
Er-value:0.000, Pr-value:0.000  
eCLIP MATCHES▶SF3B1 (bg=2.48%)No matches to TargetScan


TGCATTCTTC

TGCATTCTTC  
Depth:5 (RABBIT)  
Ei-value:0.000, Pi-value:0.000  
Er-value:0.000, Pr-value:0.000  
eCLIP MATCHES▶SF3B1 (bg=2.48%)No matches to TargetScan

TTTC

CAG

CAGAGCAGATTGCCTGG  
Depth:2 (PIG)  
Ei-value:0.000, Pi-value:0.000  
Er-value:0.000, Pr-value:0.000  
eCLIP MATCHES▶SF3B1 (bg=2.48%)No matches to TargetScan


AGC

AGCAGATTGCCTGG  
Depth:4 (DOG)  
Ei-value:0.000, Pi-value:0.000  
Er-value:0.000, Pr-value:0.000  
eCLIP MATCHES▶SF3B1 (bg=2.48%)No matches to TargetScan


A

AGATTGCCTGG  
Depth:5 (RABBIT)  
Ei-value:0.000, Pi-value:0.000  
Er-value:0.000, Pr-value:0.000  
No matches to eCLIP DataNo matches to TargetScan


GATTGCCTGG

GATTGCCTGG  
Depth:6 (MOUSE)  
Ei-value:0.000, Pi-value:0.000  
Er-value:0.000, Pr-value:0.000  
No matches to eCLIP DataNo matches to TargetScan

T

TAAGAATCTCT

TAAGAATCTCT  
Depth:2 (PIG)  
Ei-value:0.000, Pi-value:0.000  
Er-value:0.000, Pr-value:0.000  
No matches to eCLIP DataNo matches to TargetScan

TGTTGTCCCCT

TTGTATATT

TTGTATATT  
Depth:4 (DOG)  
Ei-value:0.000, Pi-value:0.000  
Er-value:0.000, Pr-value:0.000  
No matches to eCLIP DataMATCHES To TargetScan▶ miR-381-3p:AUACAAG

GTTATTGTAAAG

TGCCAA

TGCCAA  
Depth:3 (COW)  
Ei-value:0.000, Pi-value:0.000  
Er-value:0.000, Pr-value:0.000  
No matches to eCLIP DataMATCHES To TargetScan▶ miR-182-5p:UUGGCAA▶ miR-96-5p/1271-5p:UUGGCAC

A

TGCCAGGATACA

TGCCAGGATACA  
Depth:3 (COW)  
Ei-value:0.000, Pi-value:0.000  
Er-value:0.000, Pr-value:0.000  
No matches to eCLIP DataNo matches to TargetScan

GCCAGAAAAATTGC 10560  
 TTATTATTATTAAAA

AAATTTTTT

AAATTTTTT  
Depth:2 (PIG)  
Ei-value:0.000, Pi-value:0.000  
Er-value:0.000, Pr-value:0.000  
No matches to eCLIP DataNo matches to TargetScan

TAAGAAAG

ACATCTGG

ACATCTGG  
Depth:3 (COW)  
Ei-value:0.000, Pi-value:0.000  
Er-value:0.000, Pr-value:0.000  
No matches to eCLIP DataNo matches to TargetScan

ATTGTAGGGTGGACTC

GAT

GATAACCTGGTCATT  
Depth:3 (COW)  
Ei-value:0.000, Pi-value:0.000  
Er-value:0.000, Pr-value:0.000  
No matches to eCLIP DataMATCHES To TargetScan▶ miR-154-5p:AGGUUAU


AAC

AACCTGGTCATT  
Depth:4 (DOG)  
Ei-value:0.000, Pi-value:0.000  
Er-value:0.000, Pr-value:0.000  
No matches to eCLIP DataNo matches to TargetScan


CTGGTCATT

CTGGTCATT  
Depth:5 (RABBIT)  
Ei-value:0.000, Pi-value:0.000  
Er-value:0.000, Pr-value:0.000  
No matches to eCLIP DataNo matches to TargetScan

ATT

T

TTTTTGAAG  
Depth:2 (PIG)  
Ei-value:0.000, Pi-value:0.000  
Er-value:0.000, Pr-value:0.000  
No matches to eCLIP DataNo matches to TargetScan


TTTTGAA

TTTTGAA  
Depth:3 (COW)  
Ei-value:0.000, Pi-value:0.000  
Er-value:0.000, Pr-value:0.010  
No matches to eCLIP DataNo matches to TargetScan


G

TTTTTGAAG  
Depth:2 (PIG)  
Ei-value:0.000, Pi-value:0.000  
Er-value:0.000, Pr-value:0.000  
No matches to eCLIP DataNo matches to TargetScan

CCAAAATAT

CCATTTAT

CCATTTAT  
Depth:5 (RABBIT)  
Ei-value:0.000, Pi-value:0.000  
Er-value:0.000, Pr-value:0.000  
No matches to eCLIP DataNo matches to TargetScan

ACTATGTACCTGG

TGAC

TGACCAGTGTCTCTCATTT  
Depth:4 (DOG)  
Ei-value:0.000, Pi-value:0.000  
Er-value:0.000, Pr-value:0.000  
eCLIP MATCHES▶SUPV3L1 (bg=1.57%)No matches to TargetScan


CAG

CAGTGTCTCTCATTT  
Depth:5 (RABBIT)  
Ei-value:0.000, Pi-value:0.000  
Er-value:0.000, Pr-value:0.000  
eCLIP MATCHES▶SUPV3L1 (bg=1.57%)No matches to TargetScan

 10680  


TGTCTCTCATTT

CAGTGTCTCTCATTT  
Depth:5 (RABBIT)  
Ei-value:0.000, Pi-value:0.000  
Er-value:0.000, Pr-value:0.000  
eCLIP MATCHES▶SUPV3L1 (bg=1.57%)No matches to TargetScan

TAACTG

AGG

AGGGTGGTG  
Depth:4 (DOG)  
Ei-value:0.000, Pi-value:0.000  
Er-value:0.000, Pr-value:0.000  
eCLIP MATCHES▶SUPV3L1 (bg=1.57%)No matches to TargetScan


GTGGTG

GTGGTG  
Depth:5 (RABBIT)  
Ei-value:0.000, Pi-value:0.000  
Er-value:0.000, Pr-value:0.000  
eCLIP MATCHES▶SUPV3L1 (bg=1.57%)No matches to TargetScan


G

AGGGTGGTGGGTCTGTGGATAGA  
Depth:2 (PIG)  
Ei-value:0.000, Pi-value:0.000  
Er-value:0.000, Pr-value:0.000  
eCLIP MATCHES▶SUPV3L1 (bg=1.57%)MATCHES To TargetScan▶ miR-140-3p.1:CCACAGG


GTCTGTGGATA

GTCTGTGGATA  
Depth:5 (RABBIT)  
Ei-value:0.000, Pi-value:0.000  
Er-value:0.000, Pr-value:0.000  
eCLIP MATCHES▶SUPV3L1 (bg=1.57%)MATCHES To TargetScan▶ miR-140-3p.1:CCACAGG


GA

GTCTGTGGATAGA  
Depth:3 (COW)  
Ei-value:0.000, Pi-value:0.000  
Er-value:0.000, Pr-value:0.000  
eCLIP MATCHES▶SUPV3L1 (bg=1.57%)MATCHES To TargetScan▶ miR-140-3p.1:CCACAGG

ACACTGACTCTTGC

TATTTTA

TATTTTA  
Depth:3 (COW)  
Ei-value:0.000, Pi-value:0.040  
Er-value:0.000, Pr-value:0.020  
eCLIP MATCHES▶SUPV3L1 (bg=1.57%)No matches to TargetScan

ATATCAAAGATA

TTCTAGA

TTCTAGA  
Depth:4 (DOG)  
Ei-value:0.000, Pi-value:0.000  
Er-value:0.000, Pr-value:0.000  
No matches to eCLIP DataNo matches to TargetScan

GTGGAACTCTTAAGACC

AGTATCTTTG

AGTATCTTTG  
Depth:3 (COW)  
Ei-value:0.000, Pi-value:0.000  
Er-value:0.000, Pr-value:0.000  
No matches to eCLIP DataNo matches to TargetScan

TGTGGGCTTTAC 10800  
 CAGC

ATTCACTT

ATTCACTT  
Depth:4 (DOG)  
Ei-value:0.000, Pi-value:0.000  
Er-value:0.000, Pr-value:0.000  
No matches to eCLIP DataNo matches to TargetScan


TTA

ATTCACTTTTAGAAAAAC  
Depth:2 (PIG)  
Ei-value:0.000, Pi-value:0.000  
Er-value:0.000, Pr-value:0.000  
No matches to eCLIP DataMATCHES To TargetScan▶ miR-17-5p/20-5p/93-5p/106-5p/519-3p:AAAGUGC


GAAAAAC

GAAAAAC  
Depth:4 (DOG)  
Ei-value:0.000, Pi-value:0.000  
Er-value:0.000, Pr-value:0.000  
No matches to eCLIP DataNo matches to TargetScan

TACCTAAATTTTA

TAATCCTT

TAATCCTT  
Depth:2 (PIG)  
Ei-value:0.000, Pi-value:0.010  
Er-value:0.000, Pr-value:0.000  
eCLIP MATCHES▶SUPV3L1 (bg=1.57%)No matches to TargetScan

T

AATTTCTTCATCTGGAGC

AATTTCTTCATCTGGAGC  
Depth:5 (RABBIT)  
Ei-value:0.000, Pi-value:0.000  
Er-value:0.000, Pr-value:0.000  
eCLIP MATCHES▶SUPV3L1 (bg=1.57%)▶U2AF2 (bg=1.76%)No matches to TargetScan


A

AATTTCTTCATCTGGAGCA  
Depth:2 (PIG)  
Ei-value:0.000, Pi-value:0.000  
Er-value:0.000, Pr-value:0.000  
eCLIP MATCHES▶SUPV3L1 (bg=1.57%)▶U2AF2 (bg=1.76%)No matches to TargetScan

CCTGCCCCTA

CTTATTT

CTTATTT  
Depth:4 (DOG)  
Ei-value:0.000, Pi-value:0.000  
Er-value:0.000, Pr-value:0.010  
eCLIP MATCHES▶SUPV3L1 (bg=1.57%)▶U2AF2 (bg=1.76%)No matches to TargetScan


CAAGAA

CTTATTTCAAGAA  
Depth:3 (COW)  
Ei-value:0.000, Pi-value:0.000  
Er-value:0.000, Pr-value:0.000  
eCLIP MATCHES▶SUPV3L1 (bg=1.57%)▶U2AF2 (bg=1.76%)MATCHES To TargetScan▶ miR-203a-3p.2:UGAAAUG

GATTGCAGTAAAACGATTAAATGAGGGAACATAT 10920  
 GCAGAGGTGCTTTTAAAAAGCATATGCCACCTTTTTTATTAATTATTAT

ATAAAATG

ATAAAATG  
Depth:4 (DOG)  
Ei-value:0.000, Pi-value:0.000  
Er-value:0.000, Pr-value:0.000  
No matches to eCLIP DataNo matches to TargetScan


A

ATAAAATGA  
Depth:3 (COW)  
Ei-value:0.000, Pi-value:0.000  
Er-value:0.000, Pr-value:0.000  
No matches to eCLIP DataNo matches to TargetScan

AGCATTTAATTATAGTAATAATTTGAAGTAGTTTGAAGT

ACCACACT

ACCACACT  
Depth:3 (COW)  
Ei-value:0.000, Pi-value:0.000  
Er-value:0.000, Pr-value:0.000  
No matches to eCLIP DataNo matches to TargetScan


GA

ACCACACTGA  
Depth:2 (PIG)  
Ei-value:0.000, Pi-value:0.000  
Er-value:0.000, Pr-value:0.000  
No matches to eCLIP DataNo matches to TargetScan

G

GTGAGG

GTGAGG  
Depth:3 (COW)  
Ei-value:0.000, Pi-value:0.000  
Er-value:0.000, Pr-value:0.000  
No matches to eCLIP DataNo matches to TargetScan

ACTTAA 11040  


AAATGAT

AAATGAT  
Depth:2 (PIG)  
Ei-value:0.000, Pi-value:0.020  
Er-value:0.000, Pr-value:0.020  
No matches to eCLIP DataMATCHES To TargetScan▶ miR-382-3p:AUCAUUC

AAGACGAGTTCCCTA

TTTTATA

TTTTATA  
Depth:3 (COW)  
Ei-value:0.000, Pi-value:0.000  
Er-value:0.000, Pr-value:0.010  
No matches to eCLIP DataMATCHES To TargetScan▶ miR-340-5p:UAUAAAG

AG

AAAAATAAGCCA

AAAAATAAGCCA  
Depth:5 (RABBIT)  
Ei-value:0.000, Pi-value:0.000  
Er-value:0.000, Pr-value:0.000  
No matches to eCLIP DataNo matches to TargetScan


A

AAAAATAAGCCAA  
Depth:4 (DOG)  
Ei-value:0.000, Pi-value:0.000  
Er-value:0.000, Pr-value:0.000  
No matches to eCLIP DataNo matches to TargetScan

AATTAAAT

AT

ATTCTTTTGGATATA  
Depth:2 (PIG)  
Ei-value:0.000, Pi-value:0.000  
Er-value:0.000, Pr-value:0.000  
No matches to eCLIP DataMATCHES To TargetScan▶ miR-186-5p:AAAGAAU


TCTTTTGGATATA

TCTTTTGGATATA  
Depth:3 (COW)  
Ei-value:0.000, Pi-value:0.000  
Er-value:0.000, Pr-value:0.000  
No matches to eCLIP DataNo matches to TargetScan

AATTTCAAC

AGTGAGATAGCTGCCT

AGTGAGATAGCTGCCT  
Depth:2 (PIG)  
Ei-value:0.000, Pi-value:0.000  
Er-value:0.000, Pr-value:0.000  
No matches to eCLIP DataNo matches to TargetScan

AGTGGAA

ATGAATAATA

ATGAATAATA  
Depth:4 (DOG)  
Ei-value:0.000, Pi-value:0.000  
Er-value:0.000, Pr-value:0.000  
No matches to eCLIP DataNo matches to TargetScan

TCCCAGCCACT 11160  


AGTGTACA

AGTGTACA  
Depth:3 (COW)  
Ei-value:0.000, Pi-value:0.000  
Er-value:0.000, Pr-value:0.000  
No matches to eCLIP DataMATCHES To TargetScan▶ miR-493-5p:UGUACAU


G

AGTGTACAGGGTGTTT  
Depth:2 (PIG)  
Ei-value:0.000, Pi-value:0.000  
Er-value:0.000, Pr-value:0.000  
No matches to eCLIP DataMATCHES To TargetScan▶ miR-10-5p:ACCCUGU▶ miR-339-5p:CCCUGUC▶ miR-486-5p:CCUGUAC▶ miR-493-5p:UGUACAU▶ miR-504-5p.1:ACCCUGG


GGTGTTT

GGTGTTT  
Depth:3 (COW)  
Ei-value:0.000, Pi-value:0.000  
Er-value:0.000, Pr-value:0.000  
No matches to eCLIP DataNo matches to TargetScan

TGTGGCACAGGATTATGTAATA

TGGAACTGCT

TGGAACTGCT  
Depth:4 (DOG)  
Ei-value:0.000, Pi-value:0.000  
Er-value:0.000, Pr-value:0.000  
No matches to eCLIP DataNo matches to TargetScan

CAAGC

AAA

AAATAACTAGT  
Depth:2 (PIG)  
Ei-value:0.000, Pi-value:0.000  
Er-value:0.000, Pr-value:0.000  
No matches to eCLIP DataNo matches to TargetScan


TAACTA

TAACTA  
Depth:4 (DOG)  
Ei-value:0.000, Pi-value:0.000  
Er-value:0.000, Pr-value:0.000  
No matches to eCLIP DataNo matches to TargetScan


GT

AAATAACTAGT  
Depth:2 (PIG)  
Ei-value:0.000, Pi-value:0.000  
Er-value:0.000, Pr-value:0.000  
No matches to eCLIP DataNo matches to TargetScan

CATCACAA

CAGCAGTTC

CAGCAGTTC  
Depth:5 (RABBIT)  
Ei-value:0.000, Pi-value:0.000  
Er-value:0.000, Pr-value:0.000  
No matches to eCLIP DataNo matches to TargetScan

T

TTGTAAT

TTGTAAT  
Depth:4 (DOG)  
Ei-value:0.000, Pi-value:0.000  
Er-value:0.000, Pr-value:0.000  
No matches to eCLIP DataNo matches to TargetScan

A

ACTGAAAA

ACTGAAAA  
Depth:5 (RABBIT)  
Ei-value:0.000, Pi-value:0.000  
Er-value:0.000, Pr-value:0.000  
No matches to eCLIP DataNo matches to TargetScan

AGAATATTGTTTCTCG

GAG

GAGAAGGATGTCAAAAGATCGGC  
Depth:3 (COW)  
Ei-value:0.000, Pi-value:0.000  
Er-value:0.000, Pr-value:0.000  
eCLIP MATCHES▶SRSF1 (bg=8.47%)▶U2AF2 (bg=1.76%)▶uchl5 (bg=11.16%)MATCHES To TargetScan▶ miR-362-5p/500b-5p:AUCCUUG▶ miR-489-3p:UGACAUC


AAG

AAGGATG  
Depth:5 (RABBIT)  
Ei-value:0.000, Pi-value:0.000  
Er-value:0.000, Pr-value:0.000  
eCLIP MATCHES▶SRSF1 (bg=8.47%)▶U2AF2 (bg=1.76%)▶uchl5 (bg=11.16%)MATCHES To TargetScan▶ miR-362-5p/500b-5p:AUCCUUG

 11280  


GATG

AAGGATG  
Depth:5 (RABBIT)  
Ei-value:0.000, Pi-value:0.000  
Er-value:0.000, Pr-value:0.000  
eCLIP MATCHES▶SRSF1 (bg=8.47%)▶U2AF2 (bg=1.76%)▶uchl5 (bg=11.16%)MATCHES To TargetScan▶ miR-362-5p/500b-5p:AUCCUUG


TCA

AAGGATGTCAAAAGATC  
Depth:4 (DOG)  
Ei-value:0.000, Pi-value:0.000  
Er-value:0.000, Pr-value:0.000  
eCLIP MATCHES▶SRSF1 (bg=8.47%)▶U2AF2 (bg=1.76%)▶uchl5 (bg=11.16%)MATCHES To TargetScan▶ miR-362-5p/500b-5p:AUCCUUG▶ miR-489-3p:UGACAUC


AAAGATC

AAAGATC  
Depth:6 (MOUSE)  
Ei-value:0.000, Pi-value:0.000  
Er-value:0.000, Pr-value:0.000  
eCLIP MATCHES▶SRSF1 (bg=8.47%)▶U2AF2 (bg=1.76%)▶uchl5 (bg=11.16%)No matches to TargetScan


GGC

GAGAAGGATGTCAAAAGATCGGC  
Depth:3 (COW)  
Ei-value:0.000, Pi-value:0.000  
Er-value:0.000, Pr-value:0.000  
eCLIP MATCHES▶SRSF1 (bg=8.47%)▶U2AF2 (bg=1.76%)▶uchl5 (bg=11.16%)MATCHES To TargetScan▶ miR-362-5p/500b-5p:AUCCUUG▶ miR-489-3p:UGACAUC

C

CAGCTCAGGG

CAGCTCAGGG  
Depth:4 (DOG)  
Ei-value:0.000, Pi-value:0.000  
Er-value:0.000, Pr-value:0.000  
eCLIP MATCHES▶SRSF1 (bg=8.47%)▶U2AF2 (bg=1.76%)▶uchl5 (bg=11.16%)MATCHES To TargetScan▶ miR-125-5p:CCCUGAG

A

GCAGTTTGC

GCAGTTTGC  
Depth:3 (COW)  
Ei-value:0.000, Pi-value:0.000  
Er-value:0.000, Pr-value:0.000  
eCLIP MATCHES▶SRSF1 (bg=8.47%)▶U2AF2 (bg=1.76%)▶uchl5 (bg=11.16%)No matches to TargetScan

C

CTACTAGCTCCT

CTACTAGCTCCT  
Depth:4 (DOG)  
Ei-value:0.000, Pi-value:0.000  
Er-value:0.000, Pr-value:0.000  
eCLIP MATCHES▶SRSF1 (bg=8.47%)▶U2AF2 (bg=1.76%)▶uchl5 (bg=11.16%)MATCHES To TargetScan▶ miR-28-5p/708-5p:AGGAGCU▶ miR-411-5p.2:UAGUAGA

C

GGACAGCTG

GGACAGCTG  
Depth:5 (RABBIT)  
Ei-value:0.000, Pi-value:0.000  
Er-value:0.000, Pr-value:0.000  
eCLIP MATCHES▶SRSF1 (bg=8.47%)▶SRSF7 (bg=2.32%)▶U2AF2 (bg=1.76%)▶ZNF622 (bg=6.58%)No matches to TargetScan


T

GGACAGCTGT  
Depth:4 (DOG)  
Ei-value:0.000, Pi-value:0.000  
Er-value:0.000, Pr-value:0.000  
eCLIP MATCHES▶SRSF1 (bg=8.47%)▶SRSF7 (bg=2.32%)▶U2AF2 (bg=1.76%)▶ZNF622 (bg=6.58%)No matches to TargetScan

A

A

AAGAAGAGTCTCTGGCTCTTTAGA  
Depth:3 (COW)  
Ei-value:0.000, Pi-value:0.000  
Er-value:0.000, Pr-value:0.000  
eCLIP MATCHES▶DDX24 (bg=2.97%)▶SRSF1 (bg=8.47%)▶SRSF7 (bg=2.32%)▶U2AF2 (bg=1.76%)▶ZNF622 (bg=6.58%)No matches to TargetScan


AGAAGAGTCTCTGGCTCTTTA

AGAAGAGTCTCTGGCTCTTTA  
Depth:5 (RABBIT)  
Ei-value:0.000, Pi-value:0.000  
Er-value:0.000, Pr-value:0.000  
eCLIP MATCHES▶DDX24 (bg=2.97%)▶SRSF1 (bg=8.47%)▶SRSF7 (bg=2.32%)▶U2AF2 (bg=1.76%)▶ZNF622 (bg=6.58%)No matches to TargetScan


GA

AGAAGAGTCTCTGGCTCTTTAGA  
Depth:4 (DOG)  
Ei-value:0.000, Pi-value:0.000  
Er-value:0.000, Pr-value:0.000  
eCLIP MATCHES▶DDX24 (bg=2.97%)▶SRSF1 (bg=8.47%)▶SRSF7 (bg=2.32%)▶U2AF2 (bg=1.76%)▶ZNF622 (bg=6.58%)No matches to TargetScan

ATACT||GATCCCATTGAAGATACCACGCTGCA 11398  
 TGTGTCCTTAGTAGTCATGTCTCCTTAGGCTCCTCTTG||GAC

ATTCTGAGC

ATTCTGAGC  
Depth:4 (DOG)  
Ei-value:0.000, Pi-value:0.000  
Er-value:0.000, Pr-value:0.000  
eCLIP MATCHES▶DDX24 (bg=2.97%)▶GRWD1 (bg=5.13%)▶MTPAP (bg=2.21%)▶NOLC1 (bg=9.43%)▶SRSF1 (bg=8.47%)▶ZNF622 (bg=6.58%)No matches to TargetScan

ATGTGAGACCTGAG

GA

GACTGCAA  
Depth:3 (COW)  
Ei-value:0.000, Pi-value:0.000  
Er-value:0.000, Pr-value:0.000  
eCLIP MATCHES▶DDX24 (bg=2.97%)▶GRWD1 (bg=5.13%)▶MTPAP (bg=2.21%)▶NOLC1 (bg=9.43%)▶SRSF1 (bg=8.47%)▶UTP3 (bg=3.66%)▶ZNF622 (bg=6.58%)MATCHES To TargetScan▶ miR-455-3p.2:UGCAGUC


CTGCAA

CTGCAA  
Depth:5 (RABBIT)  
Ei-value:0.000, Pi-value:0.000  
Er-value:0.000, Pr-value:0.000  
eCLIP MATCHES▶DDX24 (bg=2.97%)▶GRWD1 (bg=5.13%)▶MTPAP (bg=2.21%)▶NOLC1 (bg=9.43%)▶SRSF1 (bg=8.47%)▶UTP3 (bg=3.66%)▶ZNF622 (bg=6.58%)No matches to TargetScan

ACAGCTATAAGAGGCTCCAAATTAATCATATCTTTCCC

TTTGAGAA

TTTGAGAATCTGG  
Depth:3 (COW)  
Ei-value:0.000, Pi-value:0.000  
Er-value:0.000, Pr-value:0.000  
eCLIP MATCHES▶DDX24 (bg=2.97%)▶GRWD1 (bg=5.13%)▶NOLC1 (bg=9.43%)▶SRSF1 (bg=8.47%)▶uchl5 (bg=11.16%)▶ZNF622 (bg=6.58%)MATCHES To TargetScan▶ miR-371-5p:CUCAAAC

 11516  


TCTGG

TTTGAGAATCTGG  
Depth:3 (COW)  
Ei-value:0.000, Pi-value:0.000  
Er-value:0.000, Pr-value:0.000  
eCLIP MATCHES▶DDX24 (bg=2.97%)▶GRWD1 (bg=5.13%)▶NOLC1 (bg=9.43%)▶SRSF1 (bg=8.47%)▶uchl5 (bg=11.16%)▶ZNF622 (bg=6.58%)MATCHES To TargetScan▶ miR-371-5p:CUCAAAC

CC

AAGCTCCA

AAGCTCCA  
Depth:3 (COW)  
Ei-value:0.000, Pi-value:0.000  
Er-value:0.000, Pr-value:0.000  
eCLIP MATCHES▶DDX24 (bg=2.97%)▶GRWD1 (bg=5.13%)▶NOLC1 (bg=9.43%)▶RBM15 (bg=7.27%)▶SRSF1 (bg=8.47%)▶uchl5 (bg=11.16%)▶ZNF622 (bg=6.58%)No matches to TargetScan

GCT

AATCTA

AATCTA  
Depth:2 (PIG)  
Ei-value:0.000, Pi-value:0.000  
Er-value:0.000, Pr-value:0.000  
eCLIP MATCHES▶DDX24 (bg=2.97%)▶GRWD1 (bg=5.13%)▶NOLC1 (bg=9.43%)▶RBM15 (bg=7.27%)▶SRSF1 (bg=8.47%)▶TARDBP (bg=2.79%)▶uchl5 (bg=11.16%)▶ZNF622 (bg=6.58%)No matches to TargetScan

CTT

GGATGG

GGATGG  
Depth:3 (COW)  
Ei-value:0.000, Pi-value:0.000  
Er-value:0.000, Pr-value:0.010  
eCLIP MATCHES▶DDX24 (bg=2.97%)▶GRWD1 (bg=5.13%)▶NIPBL (bg=5.39%)▶NOLC1 (bg=9.43%)▶RBM15 (bg=7.27%)▶SRSF1 (bg=8.47%)▶TARDBP (bg=2.79%)▶uchl5 (bg=11.16%)▶ZNF622 (bg=6.58%)No matches to TargetScan

GTTGCCAGCTA

T

TCTGGAGAAAAAGATCTTCCTCAGAAGAATAGGCTTGTTG  
Depth:2 (PIG)  
Ei-value:0.000, Pi-value:0.000  
Er-value:0.000, Pr-value:0.000  
eCLIP MATCHES▶DDX24 (bg=2.97%)▶GRWD1 (bg=5.13%)▶NIPBL (bg=5.39%)▶NOLC1 (bg=9.43%)▶SRSF1 (bg=8.47%)▶SRSF7 (bg=2.32%)▶TARDBP (bg=2.79%)▶uchl5 (bg=11.16%)▶ZNF622 (bg=6.58%)MATCHES To TargetScan▶ miR-1224-5p:UGAGGAC▶ miR-7-5p:GGAAGAC


CTGGAGAAAAAG||ATCT

CTGGAGAAAAAGATCT  
Depth:3 (COW)  
Ei-value:0.000, Pi-value:0.000  
Er-value:0.000, Pr-value:0.000  
eCLIP MATCHES▶DDX24 (bg=2.97%)▶GRWD1 (bg=5.13%)▶NIPBL (bg=5.39%)▶NOLC1 (bg=9.43%)▶SRSF1 (bg=8.47%)▶SRSF7 (bg=2.32%)▶TARDBP (bg=2.79%)▶uchl5 (bg=11.16%)▶ZNF622 (bg=6.58%)No matches to TargetScan


TCCTCAG

TCTGGAGAAAAAGATCTTCCTCAGAAGAATAGGCTTGTTG  
Depth:2 (PIG)  
Ei-value:0.000, Pi-value:0.000  
Er-value:0.000, Pr-value:0.000  
eCLIP MATCHES▶DDX24 (bg=2.97%)▶GRWD1 (bg=5.13%)▶NIPBL (bg=5.39%)▶NOLC1 (bg=9.43%)▶SRSF1 (bg=8.47%)▶SRSF7 (bg=2.32%)▶TARDBP (bg=2.79%)▶uchl5 (bg=11.16%)▶ZNF622 (bg=6.58%)MATCHES To TargetScan▶ miR-1224-5p:UGAGGAC▶ miR-7-5p:GGAAGAC


AAGAATAGGC

AAGAATAGGC  
Depth:5 (RABBIT)  
Ei-value:0.000, Pi-value:0.000  
Er-value:0.000, Pr-value:0.000  
eCLIP MATCHES▶NOLC1 (bg=9.43%)▶SRSF7 (bg=2.32%)▶uchl5 (bg=11.16%)No matches to TargetScan


TTGTTG

TCTGGAGAAAAAGATCTTCCTCAGAAGAATAGGCTTGTTG  
Depth:2 (PIG)  
Ei-value:0.000, Pi-value:0.000  
Er-value:0.000, Pr-value:0.000  
eCLIP MATCHES▶DDX24 (bg=2.97%)▶GRWD1 (bg=5.13%)▶NIPBL (bg=5.39%)▶NOLC1 (bg=9.43%)▶SRSF1 (bg=8.47%)▶SRSF7 (bg=2.32%)▶TARDBP (bg=2.79%)▶uchl5 (bg=11.16%)▶ZNF622 (bg=6.58%)MATCHES To TargetScan▶ miR-1224-5p:UGAGGAC▶ miR-7-5p:GGAAGAC

TT

T

TTACAGTGTTAGTGA  
Depth:3 (COW)  
Ei-value:0.000, Pi-value:0.000  
Er-value:0.000, Pr-value:0.000  
eCLIP MATCHES▶ILF3 (bg=3.0%)▶NOLC1 (bg=9.43%)▶RBM15 (bg=7.27%)▶SRSF7 (bg=2.32%)▶ZNF622 (bg=6.58%)MATCHES To TargetScan▶ miR-141-3p/200a-3p:AACACUG


TACAGTGTTAGTGA

TACAGTGTTAGTGA  
Depth:5 (RABBIT)  
Ei-value:0.000, Pi-value:0.000  
Er-value:0.000, Pr-value:0.000  
eCLIP MATCHES▶ILF3 (bg=3.0%)▶NOLC1 (bg=9.43%)▶RBM15 (bg=7.27%)▶SRSF7 (bg=2.32%)▶ZNF622 (bg=6.58%)MATCHES To TargetScan▶ miR-141-3p/200a-3p:AACACUG

TC

CA

CATTCCCTTTGA  
Depth:3 (COW)  
Ei-value:0.000, Pi-value:0.000  
Er-value:0.000, Pr-value:0.000  
eCLIP MATCHES▶ILF3 (bg=3.0%)▶RBM15 (bg=7.27%)▶SRSF7 (bg=2.32%)▶ZNF622 (bg=6.58%)MATCHES To TargetScan▶ miR-1-3p/206:GGAAUGU


TTCCCTTTGA

TTCCCTTTGA  
Depth:6 (MOUSE)  
Ei-value:0.000, Pi-value:0.000  
Er-value:0.000, Pr-value:0.000  
eCLIP MATCHES▶ILF3 (bg=3.0%)▶RBM15 (bg=7.27%)▶SRSF7 (bg=2.32%)▶ZNF622 (bg=6.58%)No matches to TargetScan

CGA 11634  
 TCCC

TAGGTGGAGATGGGGCATGAGGATCCTCCAGGGGAA

TAGGTGGAGATGGGGCATGAGGATCCTCCAGGGGAA  
Depth:6 (MOUSE)  
Ei-value:0.000, Pi-value:0.000  
Er-value:0.000, Pr-value:0.000  
eCLIP MATCHES▶ILF3 (bg=3.0%)▶NOLC1 (bg=9.43%)▶RBM15 (bg=7.27%)▶SRSF7 (bg=2.32%)▶ZNF622 (bg=6.58%)MATCHES To TargetScan▶ miR-331-3p:CCCCUGG


A

TAGGTGGAGATGGGGCATGAGGATCCTCCAGGGGAAA  
Depth:5 (RABBIT)  
Ei-value:0.000, Pi-value:0.000  
Er-value:0.000, Pr-value:0.000  
eCLIP MATCHES▶ILF3 (bg=3.0%)▶NOLC1 (bg=9.43%)▶RBM15 (bg=7.27%)▶SRSF7 (bg=2.32%)▶ZNF622 (bg=6.58%)MATCHES To TargetScan▶ miR-331-3p:CCCCUGG


AGC

TAGGTGGAGATGGGGCATGAGGATCCTCCAGGGGAAAAGCTCACTACCACTGGGCAACAACCCTAGGTCAGGAG  
Depth:2 (PIG)  
Ei-value:0.000, Pi-value:0.000  
Er-value:0.000, Pr-value:0.000  
eCLIP MATCHES▶ILF3 (bg=3.0%)▶NOLC1 (bg=9.43%)▶RBM15 (bg=7.27%)▶SRSF7 (bg=2.32%)▶ZNF622 (bg=6.58%)MATCHES To TargetScan▶ miR-140-5p:AGUGGUU▶ miR-142-3p.1:GUAGUGU▶ miR-192-5p/215-5p:UGACCUA▶ miR-199-5p:CCAGUGU▶ miR-296-3p:AGGGUUG▶ miR-331-3p:CCCCUGG


TCACTA

TCACTA  
Depth:5 (RABBIT)  
Ei-value:0.000, Pi-value:0.000  
Er-value:0.000, Pr-value:0.000  
eCLIP MATCHES▶ILF3 (bg=3.0%)No matches to TargetScan


CCACT

TCACTACCACT  
Depth:4 (DOG)  
Ei-value:0.000, Pi-value:0.000  
Er-value:0.000, Pr-value:0.000  
eCLIP MATCHES▶ILF3 (bg=3.0%)MATCHES To TargetScan▶ miR-140-5p:AGUGGUU▶ miR-142-3p.1:GUAGUGU


G

TCACTACCACTG  
Depth:3 (COW)  
Ei-value:0.000, Pi-value:0.000  
Er-value:0.000, Pr-value:0.000  
eCLIP MATCHES▶ILF3 (bg=3.0%)MATCHES To TargetScan▶ miR-140-5p:AGUGGUU▶ miR-142-3p.1:GUAGUGU


G

TAGGTGGAGATGGGGCATGAGGATCCTCCAGGGGAAAAGCTCACTACCACTGGGCAACAACCCTAGGTCAGGAG  
Depth:2 (PIG)  
Ei-value:0.000, Pi-value:0.000  
Er-value:0.000, Pr-value:0.000  
eCLIP MATCHES▶ILF3 (bg=3.0%)▶NOLC1 (bg=9.43%)▶RBM15 (bg=7.27%)▶SRSF7 (bg=2.32%)▶ZNF622 (bg=6.58%)MATCHES To TargetScan▶ miR-140-5p:AGUGGUU▶ miR-142-3p.1:GUAGUGU▶ miR-192-5p/215-5p:UGACCUA▶ miR-199-5p:CCAGUGU▶ miR-296-3p:AGGGUUG▶ miR-331-3p:CCCCUGG


GCAACA

GCAACA  
Depth:6 (MOUSE)  
Ei-value:0.000, Pi-value:0.000  
Er-value:0.000, Pr-value:0.000  
eCLIP MATCHES▶ILF3 (bg=3.0%)No matches to TargetScan


AC

GCAACAAC  
Depth:5 (RABBIT)  
Ei-value:0.000, Pi-value:0.000  
Er-value:0.000, Pr-value:0.000  
eCLIP MATCHES▶ILF3 (bg=3.0%)No matches to TargetScan


CCTAGGTCAGGAG

TAGGTGGAGATGGGGCATGAGGATCCTCCAGGGGAAAAGCTCACTACCACTGGGCAACAACCCTAGGTCAGGAG  
Depth:2 (PIG)  
Ei-value:0.000, Pi-value:0.000  
Er-value:0.000, Pr-value:0.000  
eCLIP MATCHES▶ILF3 (bg=3.0%)▶NOLC1 (bg=9.43%)▶RBM15 (bg=7.27%)▶SRSF7 (bg=2.32%)▶ZNF622 (bg=6.58%)MATCHES To TargetScan▶ miR-140-5p:AGUGGUU▶ miR-142-3p.1:GUAGUGU▶ miR-192-5p/215-5p:UGACCUA▶ miR-199-5p:CCAGUGU▶ miR-296-3p:AGGGUUG▶ miR-331-3p:CCCCUGG

GTTCTGTCAAGATA

CTTTCCTGG

CTTTCCTGG  
Depth:3 (COW)  
Ei-value:0.000, Pi-value:0.000  
Er-value:0.000, Pr-value:0.000  
eCLIP MATCHES▶ILF3 (bg=3.0%)MATCHES To TargetScan▶ miR-665:CCAGGAG▶ miR-873-5p.1:CAGGAAC

TC

CCAGATAGGAAGAT

CCAGATAGGAAGAT  
Depth:2 (PIG)  
Ei-value:0.000, Pi-value:0.000  
Er-value:0.000, Pr-value:0.000  
eCLIP MATCHES▶ILF3 (bg=3.0%)MATCHES To TargetScan▶ miR-202-5p:UCCUAUG

A

AA

AAGTCTCAA  
Depth:2 (PIG)  
Ei-value:0.000, Pi-value:0.000  
Er-value:0.000, Pr-value:0.000  
No matches to eCLIP DataNo matches to TargetScan

 11754  


GTCTCAA

AAGTCTCAA  
Depth:2 (PIG)  
Ei-value:0.000, Pi-value:0.000  
Er-value:0.000, Pr-value:0.000  
No matches to eCLIP DataNo matches to TargetScan

AA

ACAACCACC

ACAACCACC  
Depth:5 (RABBIT)  
Ei-value:0.000, Pi-value:0.000  
Er-value:0.000, Pr-value:0.000  
eCLIP MATCHES▶PRPF8 (bg=0.26%)No matches to TargetScan


ACAC

ACAACCACCACAC  
Depth:4 (DOG)  
Ei-value:0.000, Pi-value:0.000  
Er-value:0.000, Pr-value:0.000  
eCLIP MATCHES▶PRPF8 (bg=0.26%)No matches to TargetScan

GTCAAG||CTCTTC

A

ATTGTTCC  
Depth:2 (PIG)  
Ei-value:0.000, Pi-value:0.000  
Er-value:0.000, Pr-value:0.000  
eCLIP MATCHES▶GRWD1 (bg=5.13%)▶SF3B4 (bg=0.05%)No matches to TargetScan


TTGTTCC

TTGTTCC  
Depth:4 (DOG)  
Ei-value:0.000, Pi-value:0.000  
Er-value:0.000, Pr-value:0.000  
eCLIP MATCHES▶GRWD1 (bg=5.13%)▶SF3B4 (bg=0.05%)No matches to TargetScan

TATC

TG

TGCCAAATC  
Depth:3 (COW)  
Ei-value:0.000, Pi-value:0.000  
Er-value:0.000, Pr-value:0.000  
eCLIP MATCHES▶GRWD1 (bg=5.13%)▶NOLC1 (bg=9.43%)MATCHES To TargetScan▶ miR-182-5p:UUGGCAA▶ miR-96-5p/1271-5p:UUGGCAC


CCAAAT

CCAAAT  
Depth:6 (MOUSE)  
Ei-value:0.000, Pi-value:0.000  
Er-value:0.000, Pr-value:0.000  
eCLIP MATCHES▶GRWD1 (bg=5.13%)▶NOLC1 (bg=9.43%)No matches to TargetScan


C

CCAAATC  
Depth:5 (RABBIT)  
Ei-value:0.000, Pi-value:0.000  
Er-value:0.000, Pr-value:0.000  
eCLIP MATCHES▶GRWD1 (bg=5.13%)▶NOLC1 (bg=9.43%)No matches to TargetScan

ATTATACTTCCTAC

AAGCAGTG

AAGCAGTG  
Depth:2 (PIG)  
Ei-value:0.000, Pi-value:0.000  
Er-value:0.000, Pr-value:0.000  
eCLIP MATCHES▶GRWD1 (bg=5.13%)▶NOLC1 (bg=9.43%)▶uchl5 (bg=11.16%)No matches to TargetScan

C

AGAGAG

AGAGAG  
Depth:2 (PIG)  
Ei-value:0.000, Pi-value:0.010  
Er-value:0.000, Pr-value:0.000  
eCLIP MATCHES▶GRWD1 (bg=5.13%)▶NOLC1 (bg=9.43%)▶uchl5 (bg=11.16%)▶ZNF622 (bg=6.58%)No matches to TargetScan

CTGAGTCTTCAGCAGGTC

CAAGAAA

CAAGAAA  
Depth:5 (RABBIT)  
Ei-value:0.000, Pi-value:0.000  
Er-value:0.000, Pr-value:0.000  
eCLIP MATCHES▶GRWD1 (bg=5.13%)▶NOLC1 (bg=9.43%)▶uchl5 (bg=11.16%)▶ZNF622 (bg=6.58%)No matches to TargetScan


T

CAAGAAAT  
Depth:3 (COW)  
Ei-value:0.000, Pi-value:0.000  
Er-value:0.000, Pr-value:0.000  
eCLIP MATCHES▶GRWD1 (bg=5.13%)▶NOLC1 (bg=9.43%)▶TRA2A (bg=4.8%)▶uchl5 (bg=11.16%)▶ZNF622 (bg=6.58%)No matches to TargetScan


T

CAAGAAATTTGAACACAC  
Depth:2 (PIG)  
Ei-value:0.000, Pi-value:0.000  
Er-value:0.000, Pr-value:0.000  
eCLIP MATCHES▶GRWD1 (bg=5.13%)▶NOLC1 (bg=9.43%)▶PTBP1 (bg=3.74%)▶RBM15 (bg=7.27%)▶TRA2A (bg=4.8%)▶uchl5 (bg=11.16%)▶ZNF622 (bg=6.58%)No matches to TargetScan


TGAACAC

TGAACACAC  
Depth:3 (COW)  
Ei-value:0.000, Pi-value:0.000  
Er-value:0.000, Pr-value:0.000  
eCLIP MATCHES▶GRWD1 (bg=5.13%)▶NOLC1 (bg=9.43%)▶PTBP1 (bg=3.74%)▶RBM15 (bg=7.27%)▶TRA2A (bg=4.8%)▶uchl5 (bg=11.16%)▶ZNF622 (bg=6.58%)No matches to TargetScan

 11872  


AC

TGAACACAC  
Depth:3 (COW)  
Ei-value:0.000, Pi-value:0.000  
Er-value:0.000, Pr-value:0.000  
eCLIP MATCHES▶GRWD1 (bg=5.13%)▶NOLC1 (bg=9.43%)▶PTBP1 (bg=3.74%)▶RBM15 (bg=7.27%)▶TRA2A (bg=4.8%)▶uchl5 (bg=11.16%)▶ZNF622 (bg=6.58%)No matches to TargetScan

TGAAGGAAGTCAGCCTTCCCACCT

G

GAAGATCAACATGCCTG  
Depth:4 (DOG)  
Ei-value:0.000, Pi-value:0.000  
Er-value:0.000, Pr-value:0.000  
eCLIP MATCHES▶GRWD1 (bg=5.13%)▶NOLC1 (bg=9.43%)▶PTBP1 (bg=3.74%)▶RBM15 (bg=7.27%)▶TRA2A (bg=4.8%)▶uchl5 (bg=11.16%)▶ZNF622 (bg=6.58%)No matches to TargetScan


AA

AAGATCAACATGC  
Depth:5 (RABBIT)  
Ei-value:0.000, Pi-value:0.000  
Er-value:0.000, Pr-value:0.000  
eCLIP MATCHES▶GRWD1 (bg=5.13%)▶NOLC1 (bg=9.43%)▶PTBP1 (bg=3.74%)▶RBM15 (bg=7.27%)▶TRA2A (bg=4.8%)▶uchl5 (bg=11.16%)▶ZNF622 (bg=6.58%)No matches to TargetScan


GATCAACATGC

GATCAACATGC  
Depth:6 (MOUSE)  
Ei-value:0.000, Pi-value:0.000  
Er-value:0.000, Pr-value:0.000  
eCLIP MATCHES▶GRWD1 (bg=5.13%)▶NOLC1 (bg=9.43%)▶PTBP1 (bg=3.74%)▶RBM15 (bg=7.27%)▶TRA2A (bg=4.8%)▶uchl5 (bg=11.16%)▶ZNF622 (bg=6.58%)No matches to TargetScan


CTG

GAAGATCAACATGCCTG  
Depth:4 (DOG)  
Ei-value:0.000, Pi-value:0.000  
Er-value:0.000, Pr-value:0.000  
eCLIP MATCHES▶GRWD1 (bg=5.13%)▶NOLC1 (bg=9.43%)▶PTBP1 (bg=3.74%)▶RBM15 (bg=7.27%)▶TRA2A (bg=4.8%)▶uchl5 (bg=11.16%)▶ZNF622 (bg=6.58%)No matches to TargetScan


GC

GAAGATCAACATGCCTGGC  
Depth:2 (PIG)  
Ei-value:0.000, Pi-value:0.000  
Er-value:0.000, Pr-value:0.000  
eCLIP MATCHES▶GRWD1 (bg=5.13%)▶NOLC1 (bg=9.43%)▶PTBP1 (bg=3.74%)▶RBM15 (bg=7.27%)▶TRA2A (bg=4.8%)▶uchl5 (bg=11.16%)▶ZNF622 (bg=6.58%)No matches to TargetScan

ACTCTAGCACTTGAGGATAGC

TGAATGA

TGAATGA  
Depth:2 (PIG)  
Ei-value:0.000, Pi-value:0.010  
Er-value:0.000, Pr-value:0.020  
eCLIP MATCHES▶AQR (bg=0.33%)▶GRWD1 (bg=5.13%)▶TRA2A (bg=4.8%)MATCHES To TargetScan▶ miR-1298-5p:UCAUUCG

A||

TGTGTAT

TGTGTAT  
Depth:6 (MOUSE)  
Ei-value:0.000, Pi-value:0.000  
Er-value:0.000, Pr-value:0.000  
eCLIP MATCHES▶TARDBP (bg=2.79%)▶ZC3H11A (bg=6.55%)No matches to TargetScan


TT

TGTGTATTT  
Depth:4 (DOG)  
Ei-value:0.000, Pi-value:0.000  
Er-value:0.000, Pr-value:0.000  
eCLIP MATCHES▶TARDBP (bg=2.79%)▶ZC3H11A (bg=6.55%)No matches to TargetScan

CT

TTGTC

TTGTCTCTTTCTTTCTT  
Depth:2 (PIG)  
Ei-value:0.000, Pi-value:0.000  
Er-value:0.000, Pr-value:0.000  
eCLIP MATCHES▶PTBP1 (bg=3.74%)▶TARDBP (bg=2.79%)▶ZC3H11A (bg=6.55%)MATCHES To TargetScan▶ miR-186-5p:AAAGAAU


TCTTTCTT

TCTTTCTT  
Depth:3 (COW)  
Ei-value:0.000, Pi-value:0.000  
Er-value:0.000, Pr-value:0.000  
eCLIP MATCHES▶TARDBP (bg=2.79%)▶ZC3H11A (bg=6.55%)No matches to TargetScan


TCTT

TTGTCTCTTTCTTTCTT  
Depth:2 (PIG)  
Ei-value:0.000, Pi-value:0.000  
Er-value:0.000, Pr-value:0.000  
eCLIP MATCHES▶PTBP1 (bg=3.74%)▶TARDBP (bg=2.79%)▶ZC3H11A (bg=6.55%)MATCHES To TargetScan▶ miR-186-5p:AAAGAAU

GTCTTTGCTCTTTG

TT

TTCTCTA  
Depth:2 (PIG)  
Ei-value:0.000, Pi-value:0.010  
Er-value:0.000, Pr-value:0.000  
eCLIP MATCHES▶MATR3 (bg=2.98%)▶PTBP1 (bg=3.74%)▶TARDBP (bg=2.79%)▶ZC3H11A (bg=6.55%)No matches to TargetScan

 11990  


CTCTA

TTCTCTA  
Depth:2 (PIG)  
Ei-value:0.000, Pi-value:0.010  
Er-value:0.000, Pr-value:0.000  
eCLIP MATCHES▶MATR3 (bg=2.98%)▶PTBP1 (bg=3.74%)▶TARDBP (bg=2.79%)▶ZC3H11A (bg=6.55%)No matches to TargetScan

TCTAAAG

TG

TGTGTCTTACCCATTTCCATG  
Depth:2 (PIG)  
Ei-value:0.000, Pi-value:0.000  
Er-value:0.000, Pr-value:0.000  
eCLIP MATCHES▶MATR3 (bg=2.98%)▶PTBP1 (bg=3.74%)▶TARDBP (bg=2.79%)▶ZC3H11A (bg=6.55%)MATCHES To TargetScan▶ miR-203a-3p.1:GAAAUGU▶ miR-208-3p:UAAGACG▶ miR-499a-5p:UAAGACU


TGTCTTA

TGTCTTA  
Depth:4 (DOG)  
Ei-value:0.000, Pi-value:0.000  
Er-value:0.000, Pr-value:0.000  
eCLIP MATCHES▶MATR3 (bg=2.98%)▶PTBP1 (bg=3.74%)▶TARDBP (bg=2.79%)▶ZC3H11A (bg=6.55%)MATCHES To TargetScan▶ miR-208-3p:UAAGACG▶ miR-499a-5p:UAAGACU


CCCATTTCCATG

TGTCTTACCCATTTCCATG  
Depth:3 (COW)  
Ei-value:0.000, Pi-value:0.000  
Er-value:0.000, Pr-value:0.000  
eCLIP MATCHES▶MATR3 (bg=2.98%)▶PTBP1 (bg=3.74%)▶TARDBP (bg=2.79%)▶ZC3H11A (bg=6.55%)MATCHES To TargetScan▶ miR-203a-3p.1:GAAAUGU▶ miR-208-3p:UAAGACG▶ miR-499a-5p:UAAGACU

TTTCTCTTGCTAATTTCTTTCGTGTGTGCCTTTGCCTCATTTTCTC

TTTTTGT

TTTTTGT  
Depth:4 (DOG)  
Ei-value:0.000, Pi-value:0.000  
Er-value:0.000, Pr-value:0.000  
eCLIP MATCHES▶MATR3 (bg=2.98%)▶PTBP1 (bg=3.74%)▶TARDBP (bg=2.79%)▶TIA1 (bg=4.07%)▶ZC3H11A (bg=6.55%)No matches to TargetScan

TCACAAGAGT

GGTCTGTGTCT

GGTCTGTGTCT  
Depth:2 (PIG)  
Ei-value:0.000, Pi-value:0.000  
Er-value:0.000, Pr-value:0.000  
eCLIP MATCHES▶MATR3 (bg=2.98%)▶PTBP1 (bg=3.74%)▶TIA1 (bg=4.07%)▶ZC3H11A (bg=6.55%)No matches to TargetScan

T

GTCTTAGA

GTCTTAGA  
Depth:2 (PIG)  
Ei-value:0.000, Pi-value:0.000  
Er-value:0.000, Pr-value:0.000  
eCLIP MATCHES▶MATR3 (bg=2.98%)▶PTBP1 (bg=3.74%)▶TIA1 (bg=4.07%)▶ZC3H11A (bg=6.55%)MATCHES To TargetScan▶ miR-208-3p:UAAGACG▶ miR-499a-5p:UAAGACU

CATA 12110  
 TCTCTCA

TTT

TTTTTCATTTTGTT  
Depth:2 (PIG)  
Ei-value:0.000, Pi-value:0.000  
Er-value:0.000, Pr-value:0.000  
No matches to eCLIP DataMATCHES To TargetScan▶ miR-495-3p:AACAAAC


TTCATTTTGTT

TTCATTTTGTT  
Depth:4 (DOG)  
Ei-value:0.000, Pi-value:0.000  
Er-value:0.000, Pr-value:0.000  
No matches to eCLIP DataMATCHES To TargetScan▶ miR-495-3p:AACAAAC

GCTATTT

CTC

CTCTTTGCTC  
Depth:2 (PIG)  
Ei-value:0.000, Pi-value:0.000  
Er-value:0.000, Pr-value:0.000  
eCLIP MATCHES▶MATR3 (bg=2.98%)▶PTBP1 (bg=3.74%)▶TIA1 (bg=4.07%)No matches to TargetScan


TTTGCTC

TTTGCTC  
Depth:3 (COW)  
Ei-value:0.000, Pi-value:0.000  
Er-value:0.000, Pr-value:0.000  
eCLIP MATCHES▶MATR3 (bg=2.98%)▶PTBP1 (bg=3.74%)▶TIA1 (bg=4.07%)No matches to TargetScan

TCCTAGATGTGGCTCTTCTTTCACGCTTTATTTCATGTCTCCTTTTTGGGTCACATGCTGTGTGCTTTTTGTCCT

TTTCTTG

TTTCTTGTT  
Depth:2 (PIG)  
Ei-value:0.000, Pi-value:0.000  
Er-value:0.000, Pr-value:0.000  
eCLIP MATCHES▶MATR3 (bg=2.98%)▶PTBP1 (bg=3.74%)▶SMNDC1 (bg=0.63%)▶TIA1 (bg=4.07%)No matches to TargetScan

 12230  


TT

TTTCTTGTT  
Depth:2 (PIG)  
Ei-value:0.000, Pi-value:0.000  
Er-value:0.000, Pr-value:0.000  
eCLIP MATCHES▶MATR3 (bg=2.98%)▶PTBP1 (bg=3.74%)▶SMNDC1 (bg=0.63%)▶TIA1 (bg=4.07%)No matches to TargetScan

CTGTCTACCTCTCCTTTCTC

TGCCTACCT

TGCCTACCT  
Depth:2 (PIG)  
Ei-value:0.000, Pi-value:0.000  
Er-value:0.000, Pr-value:0.000  
eCLIP MATCHES▶MATR3 (bg=2.98%)▶PTBP1 (bg=3.74%)▶SMNDC1 (bg=0.63%)▶TIA1 (bg=4.07%)MATCHES To TargetScan▶ miR-196-5p:AGGUAGU

CTC

TT

TTTTCTCTTTGTGAA  
Depth:3 (COW)  
Ei-value:0.000, Pi-value:0.000  
Er-value:0.000, Pr-value:0.000  
eCLIP MATCHES▶MATR3 (bg=2.98%)▶PTBP1 (bg=3.74%)▶SMNDC1 (bg=0.63%)▶TIA1 (bg=4.07%)No matches to TargetScan


TTCTCTTTG

TTCTCTTTG  
Depth:6 (MOUSE)  
Ei-value:0.000, Pi-value:0.000  
Er-value:0.000, Pr-value:0.000  
eCLIP MATCHES▶MATR3 (bg=2.98%)▶PTBP1 (bg=3.74%)▶SMNDC1 (bg=0.63%)▶TIA1 (bg=4.07%)No matches to TargetScan


TGAA

TTTTCTCTTTGTGAA  
Depth:3 (COW)  
Ei-value:0.000, Pi-value:0.000  
Er-value:0.000, Pr-value:0.000  
eCLIP MATCHES▶MATR3 (bg=2.98%)▶PTBP1 (bg=3.74%)▶SMNDC1 (bg=0.63%)▶TIA1 (bg=4.07%)No matches to TargetScan

CTGTGATTATTTGTTACCCC

TTCCCCTT

TTCCCCTT  
Depth:3 (COW)  
Ei-value:0.000, Pi-value:0.000  
Er-value:0.000, Pr-value:0.000  
eCLIP MATCHES▶MATR3 (bg=2.98%)▶PTBP1 (bg=3.74%)▶TIA1 (bg=4.07%)No matches to TargetScan


CT

TTCCCCTTCT  
Depth:2 (PIG)  
Ei-value:0.000, Pi-value:0.000  
Er-value:0.000, Pr-value:0.000  
eCLIP MATCHES▶MATR3 (bg=2.98%)▶PTBP1 (bg=3.74%)▶TIA1 (bg=4.07%)No matches to TargetScan

C

GTTCGTTT

GTTCGTTT  
Depth:2 (PIG)  
Ei-value:0.000, Pi-value:0.000  
Er-value:0.000, Pr-value:0.000  
eCLIP MATCHES▶MATR3 (bg=2.98%)▶PTBP1 (bg=3.74%)▶TIA1 (bg=4.07%)No matches to TargetScan

TAA

ATTTCACCT

ATTTCACCT  
Depth:4 (DOG)  
Ei-value:0.000, Pi-value:0.000  
Er-value:0.000, Pr-value:0.000  
eCLIP MATCHES▶TIA1 (bg=4.07%)MATCHES To TargetScan▶ miR-203a-3p.2:UGAAAUG

TTTTTCTGAGTCTGGCCTCC 12350  
 TTTC

TGCTG

TGCTGTTTCTACT  
Depth:3 (COW)  
Ei-value:0.000, Pi-value:0.000  
Er-value:0.000, Pr-value:0.000  
eCLIP MATCHES▶MATR3 (bg=2.98%)▶PTBP1 (bg=3.74%)▶TIA1 (bg=4.07%)MATCHES To TargetScan▶ miR-411-5p.1:AGUAGAC▶ miR-494-3p:GAAACAU


TTTCTAC

TTTCTAC  
Depth:6 (MOUSE)  
Ei-value:0.000, Pi-value:0.000  
Er-value:0.000, Pr-value:0.000  
eCLIP MATCHES▶MATR3 (bg=2.98%)▶PTBP1 (bg=3.74%)▶TIA1 (bg=4.07%)No matches to TargetScan


T

TTTCTACT  
Depth:5 (RABBIT)  
Ei-value:0.000, Pi-value:0.000  
Er-value:0.000, Pr-value:0.000  
eCLIP MATCHES▶MATR3 (bg=2.98%)▶PTBP1 (bg=3.74%)▶TIA1 (bg=4.07%)MATCHES To TargetScan▶ miR-411-5p.1:AGUAGAC

TTTT

ATCTCAC

ATCTCACATTTCTC  
Depth:2 (PIG)  
Ei-value:0.000, Pi-value:0.000  
Er-value:0.000, Pr-value:0.000  
eCLIP MATCHES▶MATR3 (bg=2.98%)▶PTBP1 (bg=3.74%)▶TIA1 (bg=4.07%)MATCHES To TargetScan▶ miR-203a-3p.1:GAAAUGU


ATTTCTC

ATTTCTC  
Depth:6 (MOUSE)  
Ei-value:0.000, Pi-value:0.000  
Er-value:0.000, Pr-value:0.000  
eCLIP MATCHES▶MATR3 (bg=2.98%)▶PTBP1 (bg=3.74%)▶TIA1 (bg=4.07%)No matches to TargetScan

ATTTCTGCATTTCCTTTC

TGCCTC

TGCCTCTCTTGGGC  
Depth:2 (PIG)  
Ei-value:0.000, Pi-value:0.000  
Er-value:0.000, Pr-value:0.000  
eCLIP MATCHES▶MATR3 (bg=2.98%)▶PTBP1 (bg=3.74%)▶SMNDC1 (bg=0.63%)▶TIA1 (bg=4.07%)MATCHES To TargetScan▶ miR-335-5p:CAAGAGC


TCTTGGG

TCTTGGG  
Depth:5 (RABBIT)  
Ei-value:0.000, Pi-value:0.000  
Er-value:0.000, Pr-value:0.000  
eCLIP MATCHES▶MATR3 (bg=2.98%)▶PTBP1 (bg=3.74%)▶SMNDC1 (bg=0.63%)▶TIA1 (bg=4.07%)No matches to TargetScan


C

TCTTGGGC  
Depth:3 (COW)  
Ei-value:0.000, Pi-value:0.000  
Er-value:0.000, Pr-value:0.000  
eCLIP MATCHES▶MATR3 (bg=2.98%)▶PTBP1 (bg=3.74%)▶SMNDC1 (bg=0.63%)▶TIA1 (bg=4.07%)No matches to TargetScan

TATTCTCTCTCTCCTCCCCTGCGTGCCTCAGCATCTCTTGCTG

TTTGTGA

TTTGTGA  
Depth:4 (DOG)  
Ei-value:0.000, Pi-value:0.010  
Er-value:0.000, Pr-value:0.000  
eCLIP MATCHES▶MATR3 (bg=2.98%)▶PTBP1 (bg=3.74%)▶TIA1 (bg=4.07%)No matches to TargetScan


TTT

TTTGTGATTTTC  
Depth:3 (COW)  
Ei-value:0.000, Pi-value:0.000  
Er-value:0.000, Pr-value:0.000  
eCLIP MATCHES▶MATR3 (bg=2.98%)▶PTBP1 (bg=3.74%)▶TIA1 (bg=4.07%)No matches to TargetScan

 12470  


TC

TTTGTGATTTTC  
Depth:3 (COW)  
Ei-value:0.000, Pi-value:0.000  
Er-value:0.000, Pr-value:0.000  
eCLIP MATCHES▶MATR3 (bg=2.98%)▶PTBP1 (bg=3.74%)▶TIA1 (bg=4.07%)No matches to TargetScan

TATTTCAGTATTAA

TCTCTGTT

TCTCTGTT  
Depth:4 (DOG)  
Ei-value:0.000, Pi-value:0.000  
Er-value:0.000, Pr-value:0.000  
eCLIP MATCHES▶MATR3 (bg=2.98%)▶PTBP1 (bg=3.74%)No matches to TargetScan

GGCTTGTATTTGTTCTCTGCTTCTTCCCTTTCTAC

TCACC

TCACCTTTGAGTATTT  
Depth:2 (PIG)  
Ei-value:0.000, Pi-value:0.000  
Er-value:0.000, Pr-value:0.000  
eCLIP MATCHES▶MATR3 (bg=2.98%)▶PTBP1 (bg=3.74%)▶TIA1 (bg=4.07%)MATCHES To TargetScan▶ miR-18-5p:AAGGUGC▶ miR-200bc-3p/429:AAUACUG▶ miR-371-5p:CUCAAAC


TTTGAGTATTT

TTTGAGTATTT  
Depth:4 (DOG)  
Ei-value:0.000, Pi-value:0.000  
Er-value:0.000, Pr-value:0.000  
eCLIP MATCHES▶MATR3 (bg=2.98%)▶PTBP1 (bg=3.74%)▶TIA1 (bg=4.07%)MATCHES To TargetScan▶ miR-200bc-3p/429:AAUACUG▶ miR-371-5p:CUCAAAC

CA

GCCTCTTC

GCCTCTTC  
Depth:2 (PIG)  
Ei-value:0.000, Pi-value:0.000  
Er-value:0.000, Pr-value:0.000  
eCLIP MATCHES▶MATR3 (bg=2.98%)▶PTBP1 (bg=3.74%)▶TIA1 (bg=4.07%)No matches to TargetScan

ATGAATCTATCTCCCTCT

CTTTGATT

CTTTGATT  
Depth:3 (COW)  
Ei-value:0.000, Pi-value:0.000  
Er-value:0.000, Pr-value:0.000  
eCLIP MATCHES▶MATR3 (bg=2.98%)▶PTBP1 (bg=3.74%)▶TIA1 (bg=4.07%)No matches to TargetScan

TCATGTAAT 12590  
 CTCTCCTTAAATATTTCTTTGCATATGTGGGCAAGTGTACG

TGTGTGTG

TGTGTGTG  
Depth:4 (DOG)  
Ei-value:0.000, Pi-value:0.000  
Er-value:0.000, Pr-value:0.000  
eCLIP MATCHES▶AATF (bg=0.64%)▶DDX24 (bg=2.97%)▶NCBP2 (bg=1.49%)▶NOLC1 (bg=9.43%)▶PTBP1 (bg=3.74%)▶SND1 (bg=0.45%)▶SRSF7 (bg=2.32%)▶TARDBP (bg=2.79%)▶WDR43 (bg=3.37%)▶XRCC6 (bg=2.91%)▶ZC3H8 (bg=0.29%)MATCHES To TargetScan▶ miR-329-3p/362-3p:ACACACC

TGTCATGTGTGGCAG

AGGGGCT

AGGGGCTTCCTAACCCCT  
Depth:2 (PIG)  
Ei-value:0.000, Pi-value:0.000  
Er-value:0.000, Pr-value:0.000  
eCLIP MATCHES▶AATF (bg=0.64%)▶DDX24 (bg=2.97%)▶NCBP2 (bg=1.49%)▶NOLC1 (bg=9.43%)▶PTBP1 (bg=3.74%)▶SND1 (bg=0.45%)▶SRSF7 (bg=2.32%)▶TARDBP (bg=2.79%)▶UTP3 (bg=3.66%)▶WDR43 (bg=3.37%)▶XRCC6 (bg=2.91%)▶ZC3H8 (bg=0.29%)No matches to TargetScan


TCCTAACCCCT

TCCTAACCCCT  
Depth:5 (RABBIT)  
Ei-value:0.000, Pi-value:0.000  
Er-value:0.000, Pr-value:0.000  
eCLIP MATCHES▶AATF (bg=0.64%)▶DDX24 (bg=2.97%)▶NCBP2 (bg=1.49%)▶NOLC1 (bg=9.43%)▶PTBP1 (bg=3.74%)▶SND1 (bg=0.45%)▶SRSF7 (bg=2.32%)▶TARDBP (bg=2.79%)▶UTP3 (bg=3.66%)▶WDR43 (bg=3.37%)▶XRCC6 (bg=2.91%)▶ZC3H8 (bg=0.29%)No matches to TargetScan

GCCTGA

TAGGTGCA

TAGGTGCA  
Depth:3 (COW)  
Ei-value:0.000, Pi-value:0.000  
Er-value:0.000, Pr-value:0.000  
eCLIP MATCHES▶DDX24 (bg=2.97%)▶NOLC1 (bg=9.43%)▶SND1 (bg=0.45%)▶SRSF7 (bg=2.32%)▶TARDBP (bg=2.79%)▶UTP3 (bg=3.66%)▶WDR43 (bg=3.37%)▶XRCC6 (bg=2.91%)▶ZC3H8 (bg=0.29%)No matches to TargetScan

GAACGTCGGCTATCAGAGC

AAGCA

AAGCATTG  
Depth:4 (DOG)  
Ei-value:0.000, Pi-value:0.000  
Er-value:0.000, Pr-value:0.000  
eCLIP MATCHES▶DDX24 (bg=2.97%)▶NOLC1 (bg=9.43%)▶NPM1 (bg=1.21%)▶RBFOX2 (bg=4.63%)▶RPS3 (bg=0.76%)▶SRSF1 (bg=8.47%)▶SRSF7 (bg=2.32%)▶TARDBP (bg=2.79%)▶TRA2A (bg=4.8%)▶U2AF2 (bg=1.76%)▶uchl5 (bg=11.16%)▶YWHAG (bg=1.87%)▶ZNF622 (bg=6.58%)No matches to TargetScan

 12710  


TTG

AAGCATTG  
Depth:4 (DOG)  
Ei-value:0.000, Pi-value:0.000  
Er-value:0.000, Pr-value:0.000  
eCLIP MATCHES▶DDX24 (bg=2.97%)▶NOLC1 (bg=9.43%)▶NPM1 (bg=1.21%)▶RBFOX2 (bg=4.63%)▶RPS3 (bg=0.76%)▶SRSF1 (bg=8.47%)▶SRSF7 (bg=2.32%)▶TARDBP (bg=2.79%)▶TRA2A (bg=4.8%)▶U2AF2 (bg=1.76%)▶uchl5 (bg=11.16%)▶YWHAG (bg=1.87%)▶ZNF622 (bg=6.58%)No matches to TargetScan

TGGAGCG

GTTCC

GTTCCTTATGCCAG  
Depth:2 (PIG)  
Ei-value:0.000, Pi-value:0.000  
Er-value:0.000, Pr-value:0.000  
eCLIP MATCHES▶DDX24 (bg=2.97%)▶FASTKD2 (bg=1.99%)▶LARP4 (bg=4.72%)▶NOLC1 (bg=9.43%)▶NPM1 (bg=1.21%)▶RBFOX2 (bg=4.63%)▶RBM15 (bg=7.27%)▶RPS3 (bg=0.76%)▶SRSF1 (bg=8.47%)▶SRSF7 (bg=2.32%)▶TARDBP (bg=2.79%)▶TRA2A (bg=4.8%)▶U2AF2 (bg=1.76%)▶uchl5 (bg=11.16%)▶WDR43 (bg=3.37%)▶YWHAG (bg=1.87%)▶ZC3H11A (bg=6.55%)▶ZNF622 (bg=6.58%)▶ZNF800 (bg=1.92%)No matches to TargetScan


TTATGCCA

TTATGCCA  
Depth:5 (RABBIT)  
Ei-value:0.000, Pi-value:0.000  
Er-value:0.000, Pr-value:0.000  
eCLIP MATCHES▶DDX24 (bg=2.97%)▶FASTKD2 (bg=1.99%)▶LARP4 (bg=4.72%)▶NOLC1 (bg=9.43%)▶NPM1 (bg=1.21%)▶RBFOX2 (bg=4.63%)▶RBM15 (bg=7.27%)▶RPS3 (bg=0.76%)▶SRSF1 (bg=8.47%)▶SRSF7 (bg=2.32%)▶TARDBP (bg=2.79%)▶TRA2A (bg=4.8%)▶U2AF2 (bg=1.76%)▶uchl5 (bg=11.16%)▶WDR43 (bg=3.37%)▶YWHAG (bg=1.87%)▶ZC3H11A (bg=6.55%)▶ZNF622 (bg=6.58%)▶ZNF800 (bg=1.92%)No matches to TargetScan


G

TTATGCCAG  
Depth:4 (DOG)  
Ei-value:0.000, Pi-value:0.000  
Er-value:0.000, Pr-value:0.000  
eCLIP MATCHES▶DDX24 (bg=2.97%)▶FASTKD2 (bg=1.99%)▶LARP4 (bg=4.72%)▶NOLC1 (bg=9.43%)▶NPM1 (bg=1.21%)▶RBFOX2 (bg=4.63%)▶RBM15 (bg=7.27%)▶RPS3 (bg=0.76%)▶SRSF1 (bg=8.47%)▶SRSF7 (bg=2.32%)▶TARDBP (bg=2.79%)▶TRA2A (bg=4.8%)▶U2AF2 (bg=1.76%)▶uchl5 (bg=11.16%)▶WDR43 (bg=3.37%)▶YWHAG (bg=1.87%)▶ZC3H11A (bg=6.55%)▶ZNF622 (bg=6.58%)▶ZNF800 (bg=1.92%)No matches to TargetScan

GCTGCCATGTGAG

ATGA

ATGATCCAAGACCAA  
Depth:2 (PIG)  
Ei-value:0.000, Pi-value:0.000  
Er-value:0.000, Pr-value:0.000  
eCLIP MATCHES▶DDX24 (bg=2.97%)▶FASTKD2 (bg=1.99%)▶LARP4 (bg=4.72%)▶NOLC1 (bg=9.43%)▶NPM1 (bg=1.21%)▶RBFOX2 (bg=4.63%)▶RBM15 (bg=7.27%)▶SRSF1 (bg=8.47%)▶SRSF7 (bg=2.32%)▶TARDBP (bg=2.79%)▶TRA2A (bg=4.8%)▶U2AF2 (bg=1.76%)▶uchl5 (bg=11.16%)▶WDR43 (bg=3.37%)▶YWHAG (bg=1.87%)▶ZC3H11A (bg=6.55%)▶ZNF622 (bg=6.58%)▶ZNF800 (bg=1.92%)MATCHES To TargetScan▶ miR-133a-3p.2/133b:UUGGUCC▶ miR-431-5p:GUCUUGC


TCCAAG

TCCAAG  
Depth:3 (COW)  
Ei-value:0.000, Pi-value:0.000  
Er-value:0.000, Pr-value:0.000  
eCLIP MATCHES▶DDX24 (bg=2.97%)▶FASTKD2 (bg=1.99%)▶LARP4 (bg=4.72%)▶NOLC1 (bg=9.43%)▶NPM1 (bg=1.21%)▶RBFOX2 (bg=4.63%)▶RBM15 (bg=7.27%)▶SRSF1 (bg=8.47%)▶SRSF7 (bg=2.32%)▶TARDBP (bg=2.79%)▶TRA2A (bg=4.8%)▶U2AF2 (bg=1.76%)▶uchl5 (bg=11.16%)▶WDR43 (bg=3.37%)▶YWHAG (bg=1.87%)▶ZC3H11A (bg=6.55%)▶ZNF622 (bg=6.58%)▶ZNF800 (bg=1.92%)No matches to TargetScan


ACCAA

ATGATCCAAGACCAA  
Depth:2 (PIG)  
Ei-value:0.000, Pi-value:0.000  
Er-value:0.000, Pr-value:0.000  
eCLIP MATCHES▶DDX24 (bg=2.97%)▶FASTKD2 (bg=1.99%)▶LARP4 (bg=4.72%)▶NOLC1 (bg=9.43%)▶NPM1 (bg=1.21%)▶RBFOX2 (bg=4.63%)▶RBM15 (bg=7.27%)▶SRSF1 (bg=8.47%)▶SRSF7 (bg=2.32%)▶TARDBP (bg=2.79%)▶TRA2A (bg=4.8%)▶U2AF2 (bg=1.76%)▶uchl5 (bg=11.16%)▶WDR43 (bg=3.37%)▶YWHAG (bg=1.87%)▶ZC3H11A (bg=6.55%)▶ZNF622 (bg=6.58%)▶ZNF800 (bg=1.92%)MATCHES To TargetScan▶ miR-133a-3p.2/133b:UUGGUCC▶ miR-431-5p:GUCUUGC

AACAAGGCCCTAGACTGCAGTAAAACCCAGAACTCAAGTAGGGCAGAAGGTGGAAGGCTCATATGGA

T

TAGAAGGCCCAA  
Depth:2 (PIG)  
Ei-value:0.000, Pi-value:0.000  
Er-value:0.000, Pr-value:0.000  
eCLIP MATCHES▶DDX24 (bg=2.97%)▶LARP4 (bg=4.72%)▶MTPAP (bg=2.21%)▶NOLC1 (bg=9.43%)▶SRSF1 (bg=8.47%)▶SRSF7 (bg=2.32%)▶TRA2A (bg=4.8%)▶uchl5 (bg=11.16%)▶UTP3 (bg=3.66%)▶ZNF622 (bg=6.58%)▶ZNF800 (bg=1.92%)No matches to TargetScan

 12830  


TAGAAGGCCCAA  
Depth:2 (PIG)  
Ei-value:0.000, Pi-value:0.000  
Er-value:0.000, Pr-value:0.000  
eCLIP MATCHES▶DDX24 (bg=2.97%)▶LARP4 (bg=4.72%)▶MTPAP (bg=2.21%)▶NOLC1 (bg=9.43%)▶SRSF1 (bg=8.47%)▶SRSF7 (bg=2.32%)▶TRA2A (bg=4.8%)▶uchl5 (bg=11.16%)▶UTP3 (bg=3.66%)▶ZNF622 (bg=6.58%)▶ZNF800 (bg=1.92%)No matches to TargetScan


AGA

AGAAGGCCCAA  
Depth:4 (DOG)  
Ei-value:0.000, Pi-value:0.000  
Er-value:0.000, Pr-value:0.000  
eCLIP MATCHES▶DDX24 (bg=2.97%)▶LARP4 (bg=4.72%)▶MTPAP (bg=2.21%)▶NOLC1 (bg=9.43%)▶SRSF1 (bg=8.47%)▶SRSF7 (bg=2.32%)▶TRA2A (bg=4.8%)▶uchl5 (bg=11.16%)▶UTP3 (bg=3.66%)▶ZNF622 (bg=6.58%)▶ZNF800 (bg=1.92%)No matches to TargetScan


AGGCCCAA

AGGCCCAA  
Depth:5 (RABBIT)  
Ei-value:0.000, Pi-value:0.000  
Er-value:0.000, Pr-value:0.000  
eCLIP MATCHES▶DDX24 (bg=2.97%)▶LARP4 (bg=4.72%)▶MTPAP (bg=2.21%)▶NOLC1 (bg=9.43%)▶SRSF1 (bg=8.47%)▶SRSF7 (bg=2.32%)▶TRA2A (bg=4.8%)▶uchl5 (bg=11.16%)▶UTP3 (bg=3.66%)▶ZNF622 (bg=6.58%)▶ZNF800 (bg=1.92%)No matches to TargetScan

AGTATAAGACAGATGGTTTGAGACTTGAGACCCGAGGACTAAGATGGAAAGCCCATGTTCCAAGATAGATAGAAGCCTCAGGCCTGAAACCAACAAAAGCCTCAAGAGC 12950  
 CAAGAAAACAGAGGGTGGCCTGAATTGGACCGAAGGCCT

GAGTTGGATGGAAG

GAGTTGGATGGAAG  
Depth:2 (PIG)  
Ei-value:0.000, Pi-value:0.000  
Er-value:0.000, Pr-value:0.000  
eCLIP MATCHES▶AARS (bg=2.18%)▶CPEB4 (bg=1.89%)▶DDX24 (bg=2.97%)▶FASTKD2 (bg=1.99%)▶GRWD1 (bg=5.13%)▶HLTF (bg=0.4%)▶LARP4 (bg=4.72%)▶METAP2 (bg=0.78%)▶MTPAP (bg=2.21%)▶NOLC1 (bg=9.43%)▶PUM1 (bg=1.56%)▶RBFOX2 (bg=4.63%)▶RPS11 (bg=0.63%)▶SAFB (bg=2.69%)▶SLTM (bg=2.2%)▶SRSF1 (bg=8.47%)▶TRA2A (bg=4.8%)▶uchl5 (bg=11.16%)▶XRCC6 (bg=2.91%)▶ZNF622 (bg=6.58%)▶ZNF800 (bg=1.92%)No matches to TargetScan

TCTCAAGGCTTGAGTTAG

AAGTCT

AAGTCT  
Depth:2 (PIG)  
Ei-value:0.000, Pi-value:0.010  
Er-value:0.000, Pr-value:0.010  
eCLIP MATCHES▶CPEB4 (bg=1.89%)▶DDX24 (bg=2.97%)▶DROSHA (bg=2.49%)▶FASTKD2 (bg=1.99%)▶GRWD1 (bg=5.13%)▶HLTF (bg=0.4%)▶LARP4 (bg=4.72%)▶METAP2 (bg=0.78%)▶MTPAP (bg=2.21%)▶NOLC1 (bg=9.43%)▶NPM1 (bg=1.21%)▶PPIL4 (bg=0.52%)▶PUM1 (bg=1.56%)▶RBFOX2 (bg=4.63%)▶RPS11 (bg=0.63%)▶SAFB (bg=2.69%)▶SLTM (bg=2.2%)▶SRSF1 (bg=8.47%)▶TRA2A (bg=4.8%)▶uchl5 (bg=11.16%)▶WDR43 (bg=3.37%)▶XRCC6 (bg=2.91%)▶ZC3H11A (bg=6.55%)▶ZNF622 (bg=6.58%)▶ZNF800 (bg=1.92%)No matches to TargetScan

TAAGACCTGGGACAGGACACATGGAAGGCCTAAGAACTGAGAC 13070  
 TTGTGACAC

AAGGCCAA

AAGGCCAA  
Depth:2 (PIG)  
Ei-value:0.000, Pi-value:0.000  
Er-value:0.000, Pr-value:0.000  
eCLIP MATCHES▶GRWD1 (bg=5.13%)▶MTPAP (bg=2.21%)▶SAFB (bg=2.69%)▶SLTM (bg=2.2%)▶SRSF1 (bg=8.47%)▶TRA2A (bg=4.8%)▶uchl5 (bg=11.16%)▶UTP3 (bg=3.66%)▶WDR43 (bg=3.37%)▶XRCC6 (bg=2.91%)No matches to TargetScan

C

GACCTAAGA

GACCTAAGA  
Depth:2 (PIG)  
Ei-value:0.000, Pi-value:0.000  
Er-value:0.000, Pr-value:0.000  
eCLIP MATCHES▶GRWD1 (bg=5.13%)▶NOLC1 (bg=9.43%)▶SAFB (bg=2.69%)▶SRSF1 (bg=8.47%)▶TRA2A (bg=4.8%)▶UTP3 (bg=3.66%)▶WDR43 (bg=3.37%)▶XRCC6 (bg=2.91%)▶ZNF622 (bg=6.58%)No matches to TargetScan

TTAGCCCAGGGTTGTAGCTGGAAGACCTACAACCCAAGGATG

GAAGGCCC

GAAGGCCC  
Depth:2 (PIG)  
Ei-value:0.000, Pi-value:0.000  
Er-value:0.000, Pr-value:0.000  
eCLIP MATCHES▶RBFOX2 (bg=4.63%)▶SLTM (bg=2.2%)▶SRSF1 (bg=8.47%)▶TRA2A (bg=4.8%)▶ZNF622 (bg=6.58%)No matches to TargetScan

CTGTCACAAAGCCTACCTAGATGGATAGAGGACCCAAGCGAAA 13190  
 AAGG

TATC

TATCTCAAGACTAA  
Depth:2 (PIG)  
Ei-value:0.000, Pi-value:0.000  
Er-value:0.000, Pr-value:0.000  
eCLIP MATCHES▶CPEB4 (bg=1.89%)▶FASTKD2 (bg=1.99%)▶GRWD1 (bg=5.13%)▶LARP4 (bg=4.72%)▶MTPAP (bg=2.21%)▶NOLC1 (bg=9.43%)▶RBFOX2 (bg=4.63%)▶SRSF1 (bg=8.47%)▶TRA2A (bg=4.8%)▶uchl5 (bg=11.16%)▶UTP18 (bg=0.72%)▶UTP3 (bg=3.66%)▶WDR43 (bg=3.37%)▶ZNF622 (bg=6.58%)MATCHES To TargetScan▶ miR-431-5p:GUCUUGC


TCAA

TCAAGACTAA  
Depth:4 (DOG)  
Ei-value:0.000, Pi-value:0.000  
Er-value:0.000, Pr-value:0.000  
eCLIP MATCHES▶CPEB4 (bg=1.89%)▶FASTKD2 (bg=1.99%)▶GRWD1 (bg=5.13%)▶LARP4 (bg=4.72%)▶MTPAP (bg=2.21%)▶NOLC1 (bg=9.43%)▶RBFOX2 (bg=4.63%)▶SRSF1 (bg=8.47%)▶TRA2A (bg=4.8%)▶uchl5 (bg=11.16%)▶UTP18 (bg=0.72%)▶UTP3 (bg=3.66%)▶WDR43 (bg=3.37%)▶ZNF622 (bg=6.58%)MATCHES To TargetScan▶ miR-431-5p:GUCUUGC


GACTAA

GACTAA  
Depth:5 (RABBIT)  
Ei-value:0.000, Pi-value:0.000  
Er-value:0.000, Pr-value:0.000  
eCLIP MATCHES▶CPEB4 (bg=1.89%)▶FASTKD2 (bg=1.99%)▶GRWD1 (bg=5.13%)▶LARP4 (bg=4.72%)▶MTPAP (bg=2.21%)▶NOLC1 (bg=9.43%)▶RBFOX2 (bg=4.63%)▶SRSF1 (bg=8.47%)▶TRA2A (bg=4.8%)▶uchl5 (bg=11.16%)▶UTP18 (bg=0.72%)▶UTP3 (bg=3.66%)▶WDR43 (bg=3.37%)▶ZNF622 (bg=6.58%)No matches to TargetScan

CGGCCG

GAATCTGG

GAATCTGG  
Depth:2 (PIG)  
Ei-value:0.000, Pi-value:0.000  
Er-value:0.000, Pr-value:0.000  
eCLIP MATCHES▶CPEB4 (bg=1.89%)▶FASTKD2 (bg=1.99%)▶GRWD1 (bg=5.13%)▶LARP4 (bg=4.72%)▶MTPAP (bg=2.21%)▶NOLC1 (bg=9.43%)▶PCBP1 (bg=1.07%)▶RBFOX2 (bg=4.63%)▶SRSF1 (bg=8.47%)▶TRA2A (bg=4.8%)▶uchl5 (bg=11.16%)▶UTP18 (bg=0.72%)▶UTP3 (bg=3.66%)▶WDR43 (bg=3.37%)▶ZNF622 (bg=6.58%)No matches to TargetScan

AGGCCCATGACCCAGAACCCAGGAAG

GAT

GATAGAAGC  
Depth:2 (PIG)  
Ei-value:0.000, Pi-value:0.000  
Er-value:0.000, Pr-value:0.000  
eCLIP MATCHES▶CPEB4 (bg=1.89%)▶GRWD1 (bg=5.13%)▶LARP4 (bg=4.72%)▶MTPAP (bg=2.21%)▶NOLC1 (bg=9.43%)▶PCBP1 (bg=1.07%)▶RBFOX2 (bg=4.63%)▶SRSF1 (bg=8.47%)▶TRA2A (bg=4.8%)▶uchl5 (bg=11.16%)▶ZNF622 (bg=6.58%)No matches to TargetScan


AGAAGC

AGAAGC  
Depth:4 (DOG)  
Ei-value:0.000, Pi-value:0.000  
Er-value:0.000, Pr-value:0.010  
eCLIP MATCHES▶CPEB4 (bg=1.89%)▶GRWD1 (bg=5.13%)▶LARP4 (bg=4.72%)▶MTPAP (bg=2.21%)▶NOLC1 (bg=9.43%)▶PCBP1 (bg=1.07%)▶RBFOX2 (bg=4.63%)▶SRSF1 (bg=8.47%)▶TRA2A (bg=4.8%)▶uchl5 (bg=11.16%)▶ZNF622 (bg=6.58%)No matches to TargetScan

TTGAAGACCTG

GGGAAAT

GGGAAAT  
Depth:2 (PIG)  
Ei-value:0.000, Pi-value:0.000  
Er-value:0.000, Pr-value:0.010  
eCLIP MATCHES▶CPEB4 (bg=1.89%)▶FTO (bg=0.32%)▶GRWD1 (bg=5.13%)▶LARP4 (bg=4.72%)▶MTPAP (bg=2.21%)▶SRSF1 (bg=8.47%)▶TRA2A (bg=4.8%)▶uchl5 (bg=11.16%)▶ZNF622 (bg=6.58%)No matches to TargetScan

CC

C

CAAGATGA  
Depth:3 (COW)  
Ei-value:0.000, Pi-value:0.000  
Er-value:0.000, Pr-value:0.000  
eCLIP MATCHES▶CPEB4 (bg=1.89%)▶FTO (bg=0.32%)▶GRWD1 (bg=5.13%)▶LARP4 (bg=4.72%)▶MTPAP (bg=2.21%)▶SRSF1 (bg=8.47%)▶TRA2A (bg=4.8%)▶uchl5 (bg=11.16%)▶ZNF622 (bg=6.58%)No matches to TargetScan


AAGATGA

AAGATGA  
Depth:5 (RABBIT)  
Ei-value:0.000, Pi-value:0.000  
Er-value:0.000, Pr-value:0.000  
eCLIP MATCHES▶CPEB4 (bg=1.89%)▶FTO (bg=0.32%)▶GRWD1 (bg=5.13%)▶LARP4 (bg=4.72%)▶MTPAP (bg=2.21%)▶SRSF1 (bg=8.47%)▶TRA2A (bg=4.8%)▶uchl5 (bg=11.16%)▶ZNF622 (bg=6.58%)No matches to TargetScan

G

AACCCTAAA

AACCCTAAA  
Depth:2 (PIG)  
Ei-value:0.000, Pi-value:0.000  
Er-value:0.000, Pr-value:0.000  
eCLIP MATCHES▶FTO (bg=0.32%)▶GRWD1 (bg=5.13%)▶LARP4 (bg=4.72%)▶MTPAP (bg=2.21%)▶ZNF622 (bg=6.58%)MATCHES To TargetScan▶ miR-296-3p:AGGGUUG

CCCTAC

CTCT

CTCTTTTCTATTGTT  
Depth:2 (PIG)  
Ei-value:0.000, Pi-value:0.000  
Er-value:0.000, Pr-value:0.000  
eCLIP MATCHES▶FTO (bg=0.32%)▶LARP4 (bg=4.72%)No matches to TargetScan


TTTCT

TTTCTATTG  
Depth:3 (COW)  
Ei-value:0.000, Pi-value:0.000  
Er-value:0.000, Pr-value:0.000  
No matches to eCLIP DataNo matches to TargetScan

 13310  


ATTG

TTTCTATTG  
Depth:3 (COW)  
Ei-value:0.000, Pi-value:0.000  
Er-value:0.000, Pr-value:0.000  
No matches to eCLIP DataNo matches to TargetScan


TT

CTCTTTTCTATTGTT  
Depth:2 (PIG)  
Ei-value:0.000, Pi-value:0.000  
Er-value:0.000, Pr-value:0.000  
eCLIP MATCHES▶FTO (bg=0.32%)▶LARP4 (bg=4.72%)No matches to TargetScan

TA

C

CACTTCTT  
Depth:2 (PIG)  
Ei-value:0.000, Pi-value:0.010  
Er-value:0.000, Pr-value:0.000  
eCLIP MATCHES▶NOLC1 (bg=9.43%)No matches to TargetScan


ACTTCTT

ACTTCTT  
Depth:3 (COW)  
Ei-value:0.000, Pi-value:0.020  
Er-value:0.000, Pr-value:0.000  
eCLIP MATCHES▶NOLC1 (bg=9.43%)No matches to TargetScan

ACTCTTAGATATTTCCAGTTC

TCCTGTT

TCCTGTT  
Depth:2 (PIG)  
Ei-value:0.000, Pi-value:0.020  
Er-value:0.000, Pr-value:0.000  
eCLIP MATCHES▶NOLC1 (bg=9.43%)▶TIA1 (bg=4.07%)▶ZC3H11A (bg=6.55%)No matches to TargetScan

TATCTTTAAGCCTGATTCTTTTGAGATGTA

CTTTTTGATGTT

CTTTTTGATGTT  
Depth:4 (DOG)  
Ei-value:0.000, Pi-value:0.000  
Er-value:0.000, Pr-value:0.000  
eCLIP MATCHES▶TIA1 (bg=4.07%)No matches to TargetScan


GC

CTTTTTGATGTTGC  
Depth:2 (PIG)  
Ei-value:0.000, Pi-value:0.000  
Er-value:0.000, Pr-value:0.000  
eCLIP MATCHES▶TIA1 (bg=4.07%)No matches to TargetScan

CG

GTTACCTT

GTTACCTT  
Depth:2 (PIG)  
Ei-value:0.000, Pi-value:0.000  
Er-value:0.000, Pr-value:0.010  
No matches to eCLIP DataNo matches to TargetScan

TAGATTG

ACAG

ACAGTATTATGCCTGGGCCAGTCTT  
Depth:2 (PIG)  
Ei-value:0.000, Pi-value:0.000  
Er-value:0.000, Pr-value:0.000  
No matches to eCLIP DataMATCHES To TargetScan▶ miR-193-3p:ACUGGCC▶ miR-200bc-3p/429:AAUACUG▶ miR-328-3p:UGGCCCU▶ miR-369-3p:AUAAUAC▶ miR-655-3p:UAAUACA


TATTATGC

TATTATGC  
Depth:4 (DOG)  
Ei-value:0.000, Pi-value:0.000  
Er-value:0.000, Pr-value:0.000  
No matches to eCLIP DataMATCHES To TargetScan▶ miR-369-3p:AUAAUAC


CTG

ACAGTATTATGCCTGGGCCAGTCTT  
Depth:2 (PIG)  
Ei-value:0.000, Pi-value:0.000  
Er-value:0.000, Pr-value:0.000  
No matches to eCLIP DataMATCHES To TargetScan▶ miR-193-3p:ACUGGCC▶ miR-200bc-3p/429:AAUACUG▶ miR-328-3p:UGGCCCU▶ miR-369-3p:AUAAUAC▶ miR-655-3p:UAAUACA

 13430  


GGCCAGTCTT

ACAGTATTATGCCTGGGCCAGTCTT  
Depth:2 (PIG)  
Ei-value:0.000, Pi-value:0.000  
Er-value:0.000, Pr-value:0.000  
No matches to eCLIP DataMATCHES To TargetScan▶ miR-193-3p:ACUGGCC▶ miR-200bc-3p/429:AAUACUG▶ miR-328-3p:UGGCCCU▶ miR-369-3p:AUAAUAC▶ miR-655-3p:UAAUACA

GAGCCAGCTTTAAATCACAGCTTTTACCTATTTGTTAGGCTATAGTGTTTTG

TAAACTTC

TAAACTTC  
Depth:3 (COW)  
Ei-value:0.000, Pi-value:0.000  
Er-value:0.000, Pr-value:0.000  
eCLIP MATCHES▶NIPBL (bg=5.39%)▶NOLC1 (bg=9.43%)▶ZC3H11A (bg=6.55%)No matches to TargetScan

TGTTTCTATTCACATCTT

CTCCACTTGAGAG

CTCCACTTGAGAG  
Depth:3 (COW)  
Ei-value:0.000, Pi-value:0.000  
Er-value:0.000, Pr-value:0.000  
eCLIP MATCHES▶NIPBL (bg=5.39%)▶NOLC1 (bg=9.43%)▶ZC3H11A (bg=6.55%)MATCHES To TargetScan▶ miR-26-5p:UCAAGUA


A

CTCCACTTGAGAGA  
Depth:2 (PIG)  
Ei-value:0.000, Pi-value:0.000  
Er-value:0.000, Pr-value:0.000  
eCLIP MATCHES▶NIPBL (bg=5.39%)▶NOLC1 (bg=9.43%)▶ZC3H11A (bg=6.55%)MATCHES To TargetScan▶ miR-26-5p:UCAAGUA

GACACCAAAATCCAGTCA 13550  
 GTATCTAATCTGGCTTTTGTTAACTTCCCTCAGGAGCAGACATTCAT

ATAGGTGA

ATAGGTGA  
Depth:2 (PIG)  
Ei-value:0.000, Pi-value:0.000  
Er-value:0.000, Pr-value:0.010  
eCLIP MATCHES▶NOLC1 (bg=9.43%)▶ZC3H11A (bg=6.55%)No matches to TargetScan

TACTG

TATTTCAGT

TATTTCAGT  
Depth:4 (DOG)  
Ei-value:0.000, Pi-value:0.000  
Er-value:0.000, Pr-value:0.000  
eCLIP MATCHES▶NOLC1 (bg=9.43%)▶ZC3H11A (bg=6.55%)MATCHES To TargetScan▶ miR-203a-3p.2:UGAAAUG


CC

TATTTCAGTCC  
Depth:3 (COW)  
Ei-value:0.000, Pi-value:0.000  
Er-value:0.000, Pr-value:0.000  
eCLIP MATCHES▶NOLC1 (bg=9.43%)▶ZC3H11A (bg=6.55%)MATCHES To TargetScan▶ miR-203a-3p.2:UGAAAUG


T

TATTTCAGTCCT  
Depth:2 (PIG)  
Ei-value:0.000, Pi-value:0.000  
Er-value:0.000, Pr-value:0.000  
eCLIP MATCHES▶NOLC1 (bg=9.43%)▶ZC3H11A (bg=6.55%)MATCHES To TargetScan▶ miR-203a-3p.2:UGAAAUG

TTCTTTTGACCCCAGAAGCCCTAGAC

TGAGAAGA

TGAGAAGA  
Depth:2 (PIG)  
Ei-value:0.000, Pi-value:0.000  
Er-value:0.000, Pr-value:0.000  
eCLIP MATCHES▶LARP4 (bg=4.72%)▶NIPBL (bg=5.39%)▶NOLC1 (bg=9.43%)▶WDR43 (bg=3.37%)▶ZC3H11A (bg=6.55%)No matches to TargetScan

TAAAATGGTCAGGT 13670  
 TGTT

GGGGAAA

GGGGAAA  
Depth:4 (DOG)  
Ei-value:0.000, Pi-value:0.000  
Er-value:0.000, Pr-value:0.000  
eCLIP MATCHES▶CPSF6 (bg=0.4%)▶LARP4 (bg=4.72%)▶WDR43 (bg=3.37%)▶ZC3H11A (bg=6.55%)No matches to TargetScan


AAA

GGGGAAAAAA  
Depth:2 (PIG)  
Ei-value:0.000, Pi-value:0.000  
Er-value:0.000, Pr-value:0.000  
eCLIP MATCHES▶CPSF6 (bg=0.4%)▶LARP4 (bg=4.72%)▶WDR43 (bg=3.37%)▶ZC3H11A (bg=6.55%)No matches to TargetScan

AA

GTGCCAGGCT

GTGCCAGGCT  
Depth:2 (PIG)  
Ei-value:0.000, Pi-value:0.000  
Er-value:0.000, Pr-value:0.000  
eCLIP MATCHES▶CPSF6 (bg=0.4%)▶LARP4 (bg=4.72%)▶WDR43 (bg=3.37%)MATCHES To TargetScan▶ miR-183-5p.2:UGGCACU

C

TCTAGAGAAAA

TCTAGAGAAAA  
Depth:6 (MOUSE)  
Ei-value:0.000, Pi-value:0.000  
Er-value:0.000, Pr-value:0.000  
eCLIP MATCHES▶CPSF6 (bg=0.4%)▶LARP4 (bg=4.72%)▶UTP3 (bg=3.66%)▶WDR43 (bg=3.37%)MATCHES To TargetScan▶ miR-1251-5p:CUCUAGC

ATG

TGAAGAGATG

TGAAGAGATG  
Depth:5 (RABBIT)  
Ei-value:0.000, Pi-value:0.000  
Er-value:0.000, Pr-value:0.000  
eCLIP MATCHES▶CPSF6 (bg=0.4%)▶LARP4 (bg=4.72%)▶SRSF7 (bg=2.32%)▶UTP3 (bg=3.66%)▶WDR43 (bg=3.37%)No matches to TargetScan


CTCCA

TGAAGAGATGCTCCA  
Depth:3 (COW)  
Ei-value:0.000, Pi-value:0.000  
Er-value:0.000, Pr-value:0.000  
eCLIP MATCHES▶CPSF6 (bg=0.4%)▶LARP4 (bg=4.72%)▶SRSF7 (bg=2.32%)▶UTP3 (bg=3.66%)▶WDR43 (bg=3.37%)No matches to TargetScan


GGCCAA

GGCCAATGAGAAGAATTAGACA  
Depth:4 (DOG)  
Ei-value:0.000, Pi-value:0.000  
Er-value:0.000, Pr-value:0.000  
eCLIP MATCHES▶LARP4 (bg=4.72%)▶NOLC1 (bg=9.43%)▶SRSF7 (bg=2.32%)▶UTP3 (bg=3.66%)No matches to TargetScan


TGAGAAGAATTAGACA

TGAGAAGAATTAGACA  
Depth:6 (MOUSE)  
Ei-value:0.000, Pi-value:0.000  
Er-value:0.000, Pr-value:0.000  
eCLIP MATCHES▶LARP4 (bg=4.72%)▶NOLC1 (bg=9.43%)▶SRSF7 (bg=2.32%)No matches to TargetScan

A

GAAATACACAGATG

GAAATACACAGATG  
Depth:3 (COW)  
Ei-value:0.000, Pi-value:0.000  
Er-value:0.000, Pr-value:0.000  
eCLIP MATCHES▶LARP4 (bg=4.72%)▶NOLC1 (bg=9.43%)▶SRSF7 (bg=2.32%)No matches to TargetScan

TGCCAGACTT

C

CTGAGAAG  
Depth:3 (COW)  
Ei-value:0.000, Pi-value:0.000  
Er-value:0.000, Pr-value:0.000  
eCLIP MATCHES▶AARS (bg=2.18%)▶NOLC1 (bg=9.43%)▶PUS1 (bg=1.04%)▶SRSF7 (bg=2.32%)▶ZC3H11A (bg=6.55%)No matches to TargetScan


TGAGAAG

TGAGAAG  
Depth:4 (DOG)  
Ei-value:0.000, Pi-value:0.000  
Er-value:0.000, Pr-value:0.010  
eCLIP MATCHES▶AARS (bg=2.18%)▶NOLC1 (bg=9.43%)▶PUS1 (bg=1.04%)▶SRSF7 (bg=2.32%)▶ZC3H11A (bg=6.55%)No matches to TargetScan


CA

CTGAGAAGCA  
Depth:2 (PIG)  
Ei-value:0.000, Pi-value:0.000  
Er-value:0.000, Pr-value:0.000  
eCLIP MATCHES▶AARS (bg=2.18%)▶NOLC1 (bg=9.43%)▶PUS1 (bg=1.04%)▶SRSF7 (bg=2.32%)▶ZC3H11A (bg=6.55%)No matches to TargetScan

CCT

GCCA

GCCAGCAACA  
Depth:3 (COW)  
Ei-value:0.000, Pi-value:0.000  
Er-value:0.000, Pr-value:0.000  
eCLIP MATCHES▶AARS (bg=2.18%)▶NOLC1 (bg=9.43%)▶PUS1 (bg=1.04%)▶SRSF7 (bg=2.32%)▶ZC3H11A (bg=6.55%)No matches to TargetScan

 13790  


GCCAGCAACA  
Depth:3 (COW)  
Ei-value:0.000, Pi-value:0.000  
Er-value:0.000, Pr-value:0.000  
eCLIP MATCHES▶AARS (bg=2.18%)▶NOLC1 (bg=9.43%)▶PUS1 (bg=1.04%)▶SRSF7 (bg=2.32%)▶ZC3H11A (bg=6.55%)No matches to TargetScan


GCAACA

GCAACA  
Depth:6 (MOUSE)  
Ei-value:0.000, Pi-value:0.000  
Er-value:0.000, Pr-value:0.000  
eCLIP MATCHES▶AARS (bg=2.18%)▶NOLC1 (bg=9.43%)▶PUS1 (bg=1.04%)▶ZC3H11A (bg=6.55%)No matches to TargetScan

GCTTCCTT

C

CTTTGAGCTTAGGTGAGCAGGATTC  
Depth:2 (PIG)  
Ei-value:0.000, Pi-value:0.000  
Er-value:0.000, Pr-value:0.000  
eCLIP MATCHES▶AARS (bg=2.18%)▶AKAP8L (bg=2.19%)▶NOLC1 (bg=9.43%)▶PUS1 (bg=1.04%)▶ZC3H11A (bg=6.55%)MATCHES To TargetScan▶ miR-371-5p:CUCAAAC


TTTGAGCTT

TTTGAGCTT  
Depth:3 (COW)  
Ei-value:0.000, Pi-value:0.000  
Er-value:0.000, Pr-value:0.000  
eCLIP MATCHES▶AARS (bg=2.18%)▶NOLC1 (bg=9.43%)▶PUS1 (bg=1.04%)▶ZC3H11A (bg=6.55%)MATCHES To TargetScan▶ miR-371-5p:CUCAAAC


A

CTTTGAGCTTAGGTGAGCAGGATTC  
Depth:2 (PIG)  
Ei-value:0.000, Pi-value:0.000  
Er-value:0.000, Pr-value:0.000  
eCLIP MATCHES▶AARS (bg=2.18%)▶AKAP8L (bg=2.19%)▶NOLC1 (bg=9.43%)▶PUS1 (bg=1.04%)▶ZC3H11A (bg=6.55%)MATCHES To TargetScan▶ miR-371-5p:CUCAAAC


GGTGAGC

GGTGAGC  
Depth:4 (DOG)  
Ei-value:0.000, Pi-value:0.000  
Er-value:0.000, Pr-value:0.000  
eCLIP MATCHES▶AARS (bg=2.18%)▶NOLC1 (bg=9.43%)▶PUS1 (bg=1.04%)▶ZC3H11A (bg=6.55%)No matches to TargetScan


AGGAT

GGTGAGCAGGAT  
Depth:3 (COW)  
Ei-value:0.000, Pi-value:0.000  
Er-value:0.000, Pr-value:0.000  
eCLIP MATCHES▶AARS (bg=2.18%)▶AKAP8L (bg=2.19%)▶NOLC1 (bg=9.43%)▶PUS1 (bg=1.04%)▶ZC3H11A (bg=6.55%)No matches to TargetScan


TC

CTTTGAGCTTAGGTGAGCAGGATTC  
Depth:2 (PIG)  
Ei-value:0.000, Pi-value:0.000  
Er-value:0.000, Pr-value:0.000  
eCLIP MATCHES▶AARS (bg=2.18%)▶AKAP8L (bg=2.19%)▶NOLC1 (bg=9.43%)▶PUS1 (bg=1.04%)▶ZC3H11A (bg=6.55%)MATCHES To TargetScan▶ miR-371-5p:CUCAAAC

TGG

GGTTTGGG

GGTTTGGG  
Depth:4 (DOG)  
Ei-value:0.000, Pi-value:0.000  
Er-value:0.000, Pr-value:0.000  
eCLIP MATCHES▶AARS (bg=2.18%)▶AKAP8L (bg=2.19%)▶NOLC1 (bg=9.43%)▶PUS1 (bg=1.04%)No matches to TargetScan

ATTT

CTAGTGA

CTAGTGATGGTTATG  
Depth:2 (PIG)  
Ei-value:0.000, Pi-value:0.000  
Er-value:0.000, Pr-value:0.000  
eCLIP MATCHES▶AKAP8L (bg=2.19%)▶NOLC1 (bg=9.43%)▶PUS1 (bg=1.04%)▶SF3B1 (bg=2.48%)No matches to TargetScan


TGGTTA

TGGTTA  
Depth:5 (RABBIT)  
Ei-value:0.000, Pi-value:0.000  
Er-value:0.000, Pr-value:0.000  
eCLIP MATCHES▶AKAP8L (bg=2.19%)▶NOLC1 (bg=9.43%)▶PUS1 (bg=1.04%)▶SF3B1 (bg=2.48%)No matches to TargetScan


T

TGGTTAT  
Depth:4 (DOG)  
Ei-value:0.000, Pi-value:0.000  
Er-value:0.000, Pr-value:0.000  
eCLIP MATCHES▶AKAP8L (bg=2.19%)▶NOLC1 (bg=9.43%)▶PUS1 (bg=1.04%)▶SF3B1 (bg=2.48%)No matches to TargetScan


G

TGGTTATG  
Depth:3 (COW)  
Ei-value:0.000, Pi-value:0.000  
Er-value:0.000, Pr-value:0.000  
eCLIP MATCHES▶AKAP8L (bg=2.19%)▶NOLC1 (bg=9.43%)▶PUS1 (bg=1.04%)▶SF3B1 (bg=2.48%)No matches to TargetScan

GAAAGGGTGACTGTGC

CTGGGACA

CTGGGACA  
Depth:2 (PIG)  
Ei-value:0.000, Pi-value:0.000  
Er-value:0.000, Pr-value:0.000  
eCLIP MATCHES▶AKAP8L (bg=2.19%)▶NOLC1 (bg=9.43%)▶PUS1 (bg=1.04%)▶SF3B1 (bg=2.48%)No matches to TargetScan

AAGC

GAGGT

GAGGTCCCAAGG  
Depth:2 (PIG)  
Ei-value:0.000, Pi-value:0.000  
Er-value:0.000, Pr-value:0.000  
eCLIP MATCHES▶AKAP8L (bg=2.19%)▶PUS1 (bg=1.04%)▶UTP3 (bg=3.66%)MATCHES To TargetScan▶ miR-212-5p:CCUUGGC


CCCAAGG

CCCAAGG  
Depth:4 (DOG)  
Ei-value:0.000, Pi-value:0.000  
Er-value:0.000, Pr-value:0.000  
eCLIP MATCHES▶PUS1 (bg=1.04%)▶UTP3 (bg=3.66%)MATCHES To TargetScan▶ miR-212-5p:CCUUGGC

GGAC

AGCC

AGCCTGAACTCCCTGCTCATAGTAGTGGCC  
Depth:2 (PIG)  
Ei-value:0.000, Pi-value:0.000  
Er-value:0.000, Pr-value:0.000  
eCLIP MATCHES▶UTP3 (bg=3.66%)No matches to TargetScan


TGA

TGAACTCCCTGCT  
Depth:4 (DOG)  
Ei-value:0.000, Pi-value:0.000  
Er-value:0.000, Pr-value:0.000  
eCLIP MATCHES▶UTP3 (bg=3.66%)No matches to TargetScan

 13910  


ACTCCCTGCT

TGAACTCCCTGCT  
Depth:4 (DOG)  
Ei-value:0.000, Pi-value:0.000  
Er-value:0.000, Pr-value:0.000  
eCLIP MATCHES▶UTP3 (bg=3.66%)No matches to TargetScan


C

TGAACTCCCTGCTCATAGTAGTGGCC  
Depth:3 (COW)  
Ei-value:0.000, Pi-value:0.000  
Er-value:0.000, Pr-value:0.000  
eCLIP MATCHES▶UTP3 (bg=3.66%)No matches to TargetScan


ATAGTAGTGGCC

ATAGTAGTGGCC  
Depth:4 (DOG)  
Ei-value:0.000, Pi-value:0.000  
Er-value:0.000, Pr-value:0.000  
No matches to eCLIP DataNo matches to TargetScan

A

AATAATTTGG

AATAATTTGG  
Depth:2 (PIG)  
Ei-value:0.000, Pi-value:0.000  
Er-value:0.000, Pr-value:0.000  
No matches to eCLIP DataNo matches to TargetScan

TGGACTGTGCCAACGCTACTCCTGGG

TTTAATAC

TTTAATAC  
Depth:4 (DOG)  
Ei-value:0.000, Pi-value:0.000  
Er-value:0.000, Pr-value:0.000  
eCLIP MATCHES▶WRN (bg=0.77%)MATCHES To TargetScan▶ miR-496.2:GUAUUAC


CCA

TTTAATACCCA  
Depth:2 (PIG)  
Ei-value:0.000, Pi-value:0.000  
Er-value:0.000, Pr-value:0.000  
eCLIP MATCHES▶WRN (bg=0.77%)MATCHES To TargetScan▶ miR-496.2:GUAUUAC

T

CT

CTCTAGGCTTAAAG  
Depth:2 (PIG)  
Ei-value:0.000, Pi-value:0.000  
Er-value:0.000, Pr-value:0.000  
No matches to eCLIP DataNo matches to TargetScan


CT

CTAGGCTTAAAG  
Depth:4 (DOG)  
Ei-value:0.000, Pi-value:0.000  
Er-value:0.000, Pr-value:0.000  
No matches to eCLIP DataNo matches to TargetScan


AGGCTTA

AGGCTTA  
Depth:5 (RABBIT)  
Ei-value:0.000, Pi-value:0.000  
Er-value:0.000, Pr-value:0.000  
No matches to eCLIP DataNo matches to TargetScan


AAG

CTAGGCTTAAAG  
Depth:4 (DOG)  
Ei-value:0.000, Pi-value:0.000  
Er-value:0.000, Pr-value:0.000  
No matches to eCLIP DataNo matches to TargetScan

ATGAGAGAACCTGGGACTGTTGAGCAT

GTTTAAT

GTTTAAT  
Depth:5 (RABBIT)  
Ei-value:0.000, Pi-value:0.000  
Er-value:0.000, Pr-value:0.000  
No matches to eCLIP DataNo matches to TargetScan

 14030  


GTTTAAT  
Depth:5 (RABBIT)  
Ei-value:0.000, Pi-value:0.000  
Er-value:0.000, Pr-value:0.000  
No matches to eCLIP DataNo matches to TargetScan


ACTTTCCTT

GTTTAATACTTTCCTT  
Depth:2 (PIG)  
Ei-value:0.000, Pi-value:0.000  
Er-value:0.000, Pr-value:0.000  
No matches to eCLIP DataMATCHES To TargetScan▶ miR-496.2:GUAUUAC

GATTTTTTTCTTCCTGTTTATGT

GGGAAG

GGGAAG  
Depth:2 (PIG)  
Ei-value:0.000, Pi-value:0.020  
Er-value:0.000, Pr-value:0.020  
eCLIP MATCHES▶UTP3 (bg=3.66%)No matches to TargetScan

TTG

ATTTAAATGA

ATTTAAATGA  
Depth:2 (PIG)  
Ei-value:0.000, Pi-value:0.000  
Er-value:0.000, Pr-value:0.000  
eCLIP MATCHES▶TARDBP (bg=2.79%)▶UTP3 (bg=3.66%)No matches to TargetScan

CTGATAATGTGTATGAAAGCAC

TGTAAAACA

TGTAAAACA  
Depth:3 (COW)  
Ei-value:0.000, Pi-value:0.000  
Er-value:0.000, Pr-value:0.000  
eCLIP MATCHES▶TARDBP (bg=2.79%)▶WDR43 (bg=3.37%)No matches to TargetScan

TAAGAGAAAAACCAATTAGTG

T

TATTGGCA  
Depth:5 (RABBIT)  
Ei-value:0.000, Pi-value:0.000  
Er-value:0.000, Pr-value:0.000  
eCLIP MATCHES▶HNRNPA1 (bg=2.57%)No matches to TargetScan


ATTGGCA

ATTGGCA  
Depth:6 (MOUSE)  
Ei-value:0.000, Pi-value:0.000  
Er-value:0.000, Pr-value:0.000  
eCLIP MATCHES▶HNRNPA1 (bg=2.57%)No matches to TargetScan

ATCATGCAG 14150  
 TTAACATTTGAAAGTGCAGTGTAAA

TTGTGAAG

TTGTGAAG  
Depth:6 (MOUSE)  
Ei-value:0.000, Pi-value:0.000  
Er-value:0.000, Pr-value:0.000  
eCLIP MATCHES▶HNRNPA1 (bg=2.57%)No matches to TargetScan

CAT

T

TATGTAAATCA  
Depth:3 (COW)  
Ei-value:0.000, Pi-value:0.000  
Er-value:0.000, Pr-value:0.000  
No matches to eCLIP DataNo matches to TargetScan


ATGTAAAT

ATGTAAAT  
Depth:5 (RABBIT)  
Ei-value:0.000, Pi-value:0.000  
Er-value:0.000, Pr-value:0.000  
No matches to eCLIP DataNo matches to TargetScan


CA

TATGTAAATCA  
Depth:3 (COW)  
Ei-value:0.000, Pi-value:0.000  
Er-value:0.000, Pr-value:0.000  
No matches to eCLIP DataNo matches to TargetScan


GGGGTC

TATGTAAATCAGGGGTC  
Depth:2 (PIG)  
Ei-value:0.000, Pi-value:0.000  
Er-value:0.000, Pr-value:0.000  
No matches to eCLIP DataMATCHES To TargetScan▶ miR-125-5p:CCCUGAG▶ miR-331-3p:CCCCUGG

CACAGTT

TTTCTGTAA

TTTCTGTAA  
Depth:2 (PIG)  
Ei-value:0.000, Pi-value:0.000  
Er-value:0.000, Pr-value:0.000  
No matches to eCLIP DataNo matches to TargetScan

GGGGTCAAATCATAAATACTTTAGACTGT

GG

GGGCCATATGGTTTC  
Depth:2 (PIG)  
Ei-value:0.000, Pi-value:0.000  
Er-value:0.000, Pr-value:0.000  
No matches to eCLIP DataMATCHES To TargetScan▶ miR-328-3p:UGGCCCU


GCCATATGGT

GCCATATGGT  
Depth:3 (COW)  
Ei-value:0.000, Pi-value:0.000  
Er-value:0.000, Pr-value:0.000  
No matches to eCLIP DataNo matches to TargetScan


TTC

GGGCCATATGGTTTC  
Depth:2 (PIG)  
Ei-value:0.000, Pi-value:0.000  
Er-value:0.000, Pr-value:0.000  
No matches to eCLIP DataMATCHES To TargetScan▶ miR-328-3p:UGGCCCU

TGTTACA 14270  
 TATTTGTTTTTTAAACAACGTTTTTATAAGGTCAAAATCATTCTTAGTTTTTGAGCCAATTGGATTTGGCCTGCTGTTCATAGCTTACCAC

CCCCTGATGTA

CCCCTGATGTA  
Depth:2 (PIG)  
Ei-value:0.000, Pi-value:0.000  
Er-value:0.000, Pr-value:0.000  
No matches to eCLIP DataNo matches to TargetScan

TTATTTGTTATTCAGAGA 14390  
 AAAT

TTCTGAA

TTCTGAA  
Depth:2 (PIG)  
Ei-value:0.000, Pi-value:0.020  
Er-value:0.000, Pr-value:0.020  
No matches to eCLIP DataNo matches to TargetScan

TACTACTAGTTTCCTTT

TC

TCTGTGCCTGTCCCTGT  
Depth:2 (PIG)  
Ei-value:0.000, Pi-value:0.000  
Er-value:0.000, Pr-value:0.000  
No matches to eCLIP DataNo matches to TargetScan


TGTGC

TGTGCCTGTCCCTGT  
Depth:3 (COW)  
Ei-value:0.000, Pi-value:0.000  
Er-value:0.000, Pr-value:0.000  
No matches to eCLIP DataNo matches to TargetScan


CTGTCCCT

CTGTCCCT  
Depth:4 (DOG)  
Ei-value:0.000, Pi-value:0.000  
Er-value:0.000, Pr-value:0.000  
No matches to eCLIP DataNo matches to TargetScan


GT

TGTGCCTGTCCCTGT  
Depth:3 (COW)  
Ei-value:0.000, Pi-value:0.000  
Er-value:0.000, Pr-value:0.000  
No matches to eCLIP DataNo matches to TargetScan

GC

TAGGCACT

TAGGCACT  
Depth:4 (DOG)  
Ei-value:0.000, Pi-value:0.000  
Er-value:0.000, Pr-value:0.000  
No matches to eCLIP DataNo matches to TargetScan


AA

TAGGCACTAA  
Depth:2 (PIG)  
Ei-value:0.000, Pi-value:0.000  
Er-value:0.000, Pr-value:0.000  
No matches to eCLIP DataNo matches to TargetScan

AAATGC

AATGATTA

AATGATTA  
Depth:2 (PIG)  
Ei-value:0.000, Pi-value:0.000  
Er-value:0.000, Pr-value:0.000  
No matches to eCLIP DataMATCHES To TargetScan▶ miR-382-3p:AUCAUUC

TTG

ATATCTAGGTGA

ATATCTAGGTGA  
Depth:2 (PIG)  
Ei-value:0.000, Pi-value:0.000  
Er-value:0.000, Pr-value:0.000  
eCLIP MATCHES▶HNRNPU (bg=5.92%)No matches to TargetScan

CCTGAAAAAAAATAGTG

AATGTGCTTTGTAAACT

AATGTGCTTTGTAAACT  
Depth:2 (PIG)  
Ei-value:0.000, Pi-value:0.000  
Er-value:0.000, Pr-value:0.000  
eCLIP MATCHES▶HNRNPU (bg=5.92%)MATCHES To TargetScan▶ miR-330-3p:CAAAGCA▶ miR-330-3p.2:AAAGCAC

 14510  


AATGTGCTTTGTAAACT  
Depth:2 (PIG)  
Ei-value:0.000, Pi-value:0.000  
Er-value:0.000, Pr-value:0.000  
eCLIP MATCHES▶HNRNPU (bg=5.92%)MATCHES To TargetScan▶ miR-330-3p:CAAAGCA▶ miR-330-3p.2:AAAGCAC

G

TAAAGCA

TAAAGCA  
Depth:4 (DOG)  
Ei-value:0.000, Pi-value:0.000  
Er-value:0.000, Pr-value:0.000  
eCLIP MATCHES▶LIN28B (bg=0.74%)No matches to TargetScan


CTT

TAAAGCACTT  
Depth:2 (PIG)  
Ei-value:0.000, Pi-value:0.000  
Er-value:0.000, Pr-value:0.000  
eCLIP MATCHES▶LIN28B (bg=0.74%)MATCHES To TargetScan▶ miR-302-3p/372-3p/373-3p/520-3p:AAGUGCU▶ miR-302c-3p.2/520-3p:AGUGCUU

GTATTCTACTGTGATAAGCGT

TGTGGATACAAA

TGTGGATACAAA  
Depth:2 (PIG)  
Ei-value:0.000, Pi-value:0.000  
Er-value:0.000, Pr-value:0.000  
eCLIP MATCHES▶LIN28B (bg=0.74%)▶UTP3 (bg=3.66%)No matches to TargetScan

GAAAGGAGCAAGCATAAAAAAGTGCTCTTTCAAAAGGATATAGTACTATGCAGACACAAGGAATTGTTTGATAAAT 14630  
 GAATAAATTATATGTATATTTGAGGCCAATTTGTGTTTGCTGCTCTGGTAATTTTGAGTAAAAATGCAGTATTCCAGGTATCAGAAACGAAAACACATGGAAACTGCTTTTAAACTTTAA 14750  
 AATATACTGAAAACATAAGGGACTAAGCTTGTTGTGGTCACC

TAT

TATAATGTGCCAGATA  
Depth:3 (COW)  
Ei-value:0.000, Pi-value:0.000  
Er-value:0.000, Pr-value:0.000  
No matches to eCLIP DataMATCHES To TargetScan▶ miR-183-5p.2:UGGCACU▶ miR-323-3p:ACAUUAC


AATGTGCCAGATA

AATGTGCCAGATA  
Depth:4 (DOG)  
Ei-value:0.000, Pi-value:0.000  
Er-value:0.000, Pr-value:0.000  
No matches to eCLIP DataMATCHES To TargetScan▶ miR-183-5p.2:UGGCACU

CCATGCTGGGTGCTAGAGCTACCAAAGGGGGAAAAGTA

TTCTCAT

TTCTCAT  
Depth:2 (PIG)  
Ei-value:0.000, Pi-value:0.000  
Er-value:0.000, Pr-value:0.010  
No matches to eCLIP DataNo matches to TargetScan

AGAACAAAAAATTTCAG 14870  
 AAAGGTG

CATA

CATATTAAAGTGCTTTGTA  
Depth:2 (PIG)  
Ei-value:0.000, Pi-value:0.000  
Er-value:0.000, Pr-value:0.000  
eCLIP MATCHES▶SF3B1 (bg=2.48%)MATCHES To TargetScan▶ miR-330-3p:CAAAGCA▶ miR-330-3p.2:AAAGCAC


TTAAAGTG

TTAAAGTG  
Depth:4 (DOG)  
Ei-value:0.000, Pi-value:0.000  
Er-value:0.000, Pr-value:0.000  
eCLIP MATCHES▶SF3B1 (bg=2.48%)No matches to TargetScan


CTTTGTA

TTAAAGTGCTTTGTA  
Depth:3 (COW)  
Ei-value:0.000, Pi-value:0.000  
Er-value:0.000, Pr-value:0.000  
eCLIP MATCHES▶SF3B1 (bg=2.48%)MATCHES To TargetScan▶ miR-330-3p:CAAAGCA▶ miR-330-3p.2:AAAGCAC


AA

AACTAAAGCA  
Depth:2 (PIG)  
Ei-value:0.000, Pi-value:0.000  
Er-value:0.000, Pr-value:0.000  
eCLIP MATCHES▶SF3B1 (bg=2.48%)No matches to TargetScan


CTAAAGCA

CTAAAGCA  
Depth:4 (DOG)  
Ei-value:0.000, Pi-value:0.000  
Er-value:0.000, Pr-value:0.000  
eCLIP MATCHES▶SF3B1 (bg=2.48%)No matches to TargetScan

TGATACAAATGT

CAATGGGCTA

CAATGGGCTA  
Depth:3 (COW)  
Ei-value:0.000, Pi-value:0.000  
Er-value:0.000, Pr-value:0.000  
No matches to eCLIP DataNo matches to TargetScan

CATATTTATGAATGAATGAATGGAT

GA

GAATGAATA  
Depth:3 (COW)  
Ei-value:0.000, Pi-value:0.000  
Er-value:0.000, Pr-value:0.000  
eCLIP MATCHES▶DROSHA (bg=2.49%)▶TARDBP (bg=2.79%)▶ZC3H11A (bg=6.55%)MATCHES To TargetScan▶ miR-1298-5p:UCAUUCG


ATGAATA

ATGAATA  
Depth:4 (DOG)  
Ei-value:0.000, Pi-value:0.000  
Er-value:0.000, Pr-value:0.000  
eCLIP MATCHES▶DROSHA (bg=2.49%)▶TARDBP (bg=2.79%)▶ZC3H11A (bg=6.55%)No matches to TargetScan

TTAAGTGCCTCTTACATA

CCAGCTATT

CCAGCTATT  
Depth:3 (COW)  
Ei-value:0.000, Pi-value:0.000  
Er-value:0.000, Pr-value:0.000  
eCLIP MATCHES▶AARS (bg=2.18%)▶DROSHA (bg=2.49%)▶ILF3 (bg=3.0%)▶TARDBP (bg=2.79%)▶ZC3H11A (bg=6.55%)No matches to TargetScan

T 14990  
 TG

GGTACTGT

GGTACTGT  
Depth:4 (DOG)  
Ei-value:0.000, Pi-value:0.000  
Er-value:0.000, Pr-value:0.000  
eCLIP MATCHES▶AARS (bg=2.18%)▶DROSHA (bg=2.49%)▶ILF3 (bg=3.0%)▶TARDBP (bg=2.79%)▶ZC3H11A (bg=6.55%)MATCHES To TargetScan▶ miR-101-3p.1:ACAGUAC▶ miR-144-3p:ACAGUAU

AAAATACAAGATTAATTCTCCTAT

GTA

GTAATAAGAGG  
Depth:2 (PIG)  
Ei-value:0.000, Pi-value:0.000  
Er-value:0.000, Pr-value:0.000  
eCLIP MATCHES▶ILF3 (bg=3.0%)No matches to TargetScan


ATAAGAGG

ATAAGAGG  
Depth:4 (DOG)  
Ei-value:0.000, Pi-value:0.000  
Er-value:0.000, Pr-value:0.000  
eCLIP MATCHES▶ILF3 (bg=3.0%)No matches to TargetScan

AAAGTTTATCCTCTATACTATTCAGATGTAAGGAATGAT

ATATTGCTTA

ATATTGCTTA  
Depth:2 (PIG)  
Ei-value:0.000, Pi-value:0.000  
Er-value:0.000, Pr-value:0.000  
No matches to eCLIP DataNo matches to TargetScan

ATTTTAAA

CAATC

CAATCAAGACTTTAC  
Depth:2 (PIG)  
Ei-value:0.000, Pi-value:0.000  
Er-value:0.000, Pr-value:0.000  
No matches to eCLIP DataMATCHES To TargetScan▶ miR-431-5p:GUCUUGC


AAGACTTTAC

AAGACTTTAC  
Depth:3 (COW)  
Ei-value:0.000, Pi-value:0.000  
Er-value:0.000, Pr-value:0.000  
No matches to eCLIP DataNo matches to TargetScan

TG

G

GTGAGGT  
Depth:2 (PIG)  
Ei-value:0.000, Pi-value:0.000  
Er-value:0.000, Pr-value:0.000  
No matches to eCLIP DataNo matches to TargetScan

 15110  


TGAGGT

GTGAGGT  
Depth:2 (PIG)  
Ei-value:0.000, Pi-value:0.000  
Er-value:0.000, Pr-value:0.000  
No matches to eCLIP DataNo matches to TargetScan

TAAG

T

TTAAATTATTAC  
Depth:2 (PIG)  
Ei-value:0.000, Pi-value:0.000  
Er-value:0.000, Pr-value:0.000  
No matches to eCLIP DataNo matches to TargetScan


TAAATTAT

TAAATTAT  
Depth:4 (DOG)  
Ei-value:0.000, Pi-value:0.010  
Er-value:0.000, Pr-value:0.000  
No matches to eCLIP DataNo matches to TargetScan


TAC

TAAATTATTAC  
Depth:3 (COW)  
Ei-value:0.000, Pi-value:0.000  
Er-value:0.000, Pr-value:0.000  
No matches to eCLIP DataNo matches to TargetScan

TGATACATTTTT

CC

CCAGGTAAC  
Depth:2 (PIG)  
Ei-value:0.000, Pi-value:0.000  
Er-value:0.000, Pr-value:0.000  
No matches to eCLIP DataNo matches to TargetScan


AGGTAA

AGGTAA  
Depth:3 (COW)  
Ei-value:0.000, Pi-value:0.000  
Er-value:0.000, Pr-value:0.000  
No matches to eCLIP DataNo matches to TargetScan


C

CCAGGTAAC  
Depth:2 (PIG)  
Ei-value:0.000, Pi-value:0.000  
Er-value:0.000, Pr-value:0.000  
No matches to eCLIP DataNo matches to TargetScan

CAGGAAAGAGCTAGTATGAGGAAATGAAGTAATAGATGTGAGATCCAGACCGAAAGTCACTTAATTCAGCTTGCGAA 15230  
 TGTGC

TTTCTAA

TTTCTAA  
Depth:3 (COW)  
Ei-value:0.000, Pi-value:0.000  
Er-value:0.000, Pr-value:0.010  
No matches to eCLIP DataNo matches to TargetScan


A

TTTCTAAA  
Depth:2 (PIG)  
Ei-value:0.000, Pi-value:0.000  
Er-value:0.000, Pr-value:0.000  
No matches to eCLIP DataNo matches to TargetScan

TTATAAAGCACTTGTAAATGAAAAATTTGATGCTTTCTGTA

TGA

TGAATAAAACTT  
Depth:2 (PIG)  
Ei-value:0.000, Pi-value:0.000  
Er-value:0.000, Pr-value:0.000  
No matches to eCLIP DataNo matches to TargetScan


ATAAAAC

ATAAAAC  
Depth:4 (DOG)  
Ei-value:0.000, Pi-value:0.010  
Er-value:0.000, Pr-value:0.000  
No matches to eCLIP DataNo matches to TargetScan


TT

TGAATAAAACTT  
Depth:2 (PIG)  
Ei-value:0.000, Pi-value:0.000  
Er-value:0.000, Pr-value:0.000  
No matches to eCLIP DataNo matches to TargetScan

TCTGTAAGCTAGGTATTG

TCTCTAC

TCTCTACAAAATTCTCATTGT  
Depth:2 (PIG)  
Ei-value:0.000, Pi-value:0.000  
Er-value:0.000, Pr-value:0.000  
eCLIP MATCHES▶HNRNPU (bg=5.92%)No matches to TargetScan


AAAATTCTCA

AAAATTCTCA  
Depth:4 (DOG)  
Ei-value:0.000, Pi-value:0.000  
Er-value:0.000, Pr-value:0.000  
eCLIP MATCHES▶HNRNPU (bg=5.92%)No matches to TargetScan


TTGT

TCTCTACAAAATTCTCATTGT  
Depth:2 (PIG)  
Ei-value:0.000, Pi-value:0.000  
Er-value:0.000, Pr-value:0.000  
eCLIP MATCHES▶HNRNPU (bg=5.92%)No matches to TargetScan

ATAGTTAAACCACAG 15350  
 TGAGAAGGGTTCTATAAGTAG

T

TTATACAAAC  
Depth:2 (PIG)  
Ei-value:0.000, Pi-value:0.000  
Er-value:0.000, Pr-value:0.000  
No matches to eCLIP DataNo matches to TargetScan


TATACAAAC

TATACAAAC  
Depth:4 (DOG)  
Ei-value:0.000, Pi-value:0.000  
Er-value:0.000, Pr-value:0.000  
No matches to eCLIP DataNo matches to TargetScan

CAAGG

GTTTAAATAC

GTTTAAATAC  
Depth:3 (COW)  
Ei-value:0.000, Pi-value:0.000  
Er-value:0.000, Pr-value:0.000  
No matches to eCLIP DataNo matches to TargetScan

CTGTTAAATAGATCAATTTTG

A

ATTGCCTACTATGTGAACTCACTGTTA  
Depth:2 (PIG)  
Ei-value:0.000, Pi-value:0.000  
Er-value:0.000, Pr-value:0.000  
No matches to eCLIP DataMATCHES To TargetScan▶ miR-132-3p/212-3p:AACAGUC▶ miR-23-3p:UCACAUU▶ miR-376c-3p:ACAUAGA▶ miR-411-5p.2:UAGUAGA


TTGCCTACTAT

TTGCCTACTATGTGAACTCACTGTTA  
Depth:3 (COW)  
Ei-value:0.000, Pi-value:0.000  
Er-value:0.000, Pr-value:0.000  
No matches to eCLIP DataMATCHES To TargetScan▶ miR-132-3p/212-3p:AACAGUC▶ miR-23-3p:UCACAUU▶ miR-376c-3p:ACAUAGA▶ miR-411-5p.2:UAGUAGA


GTGAACTCA

GTGAACTCA  
Depth:4 (DOG)  
Ei-value:0.000, Pi-value:0.000  
Er-value:0.000, Pr-value:0.000  
No matches to eCLIP DataNo matches to TargetScan


CTGTTA

TTGCCTACTATGTGAACTCACTGTTA  
Depth:3 (COW)  
Ei-value:0.000, Pi-value:0.000  
Er-value:0.000, Pr-value:0.000  
No matches to eCLIP DataMATCHES To TargetScan▶ miR-132-3p/212-3p:AACAGUC▶ miR-23-3p:UCACAUU▶ miR-376c-3p:ACAUAGA▶ miR-411-5p.2:UAGUAGA

AAGGCACTGAAA

ATTTATCAT

ATTTATCAT  
Depth:3 (COW)  
Ei-value:0.000, Pi-value:0.000  
Er-value:0.000, Pr-value:0.000  
No matches to eCLIP DataNo matches to TargetScan

ATTTC 15470  
 ATTTAGCCACAGCCAAAAATAAGGCAATACC

TATGTTAGC

TATGTTAGCATTTTGTGAACTCTAA  
Depth:2 (PIG)  
Ei-value:0.000, Pi-value:0.000  
Er-value:0.000, Pr-value:0.000  
No matches to eCLIP DataNo matches to TargetScan


ATTTTGTGAACTCTAA

ATTTTGTGAACTCTAA  
Depth:3 (COW)  
Ei-value:0.000, Pi-value:0.000  
Er-value:0.000, Pr-value:0.000  
No matches to eCLIP DataNo matches to TargetScan

G

GCACCAT

GCACCAT  
Depth:2 (PIG)  
Ei-value:0.000, Pi-value:0.000  
Er-value:0.000, Pr-value:0.000  
No matches to eCLIP DataNo matches to TargetScan

ATAAATGTAACTGTTGATTTTCTCACTTGGTGCTGG

GTACTAG

GTACTAG  
Depth:2 (PIG)  
Ei-value:0.000, Pi-value:0.000  
Er-value:0.000, Pr-value:0.000  
No matches to eCLIP DataNo matches to TargetScan

GTTTAT

AAAATTG

AAAATTG  
Depth:3 (COW)  
Ei-value:0.000, Pi-value:0.000  
Er-value:0.000, Pr-value:0.000  
No matches to eCLIP DataNo matches to TargetScan

 15590  


AAAATTG  
Depth:3 (COW)  
Ei-value:0.000, Pi-value:0.000  
Er-value:0.000, Pr-value:0.000  
No matches to eCLIP DataNo matches to TargetScan

TATG

ATAGTTAT

ATAGTTAT  
Depth:2 (PIG)  
Ei-value:0.000, Pi-value:0.000  
Er-value:0.000, Pr-value:0.000  
No matches to eCLIP DataNo matches to TargetScan

TATATTGTGCAAATAAAGTAGGAAAA

TTTGAATA

TTTGAATA  
Depth:2 (PIG)  
Ei-value:0.000, Pi-value:0.000  
Er-value:0.000, Pr-value:0.010  
eCLIP MATCHES▶HNRNPUL1 (bg=1.16%)No matches to TargetScan

ACAATGATTATCT

TTTGAATA

TTTGAATA  
Depth:2 (PIG)  
Ei-value:0.000, Pi-value:0.000  
Er-value:0.000, Pr-value:0.010  
eCLIP MATCHES▶HNRNPUL1 (bg=1.16%)No matches to TargetScan

CGCATACGCAAGGGATTGGTTGTCTGAAG

AATGCC

AATGCC  
Depth:2 (PIG)  
Ei-value:0.000, Pi-value:0.000  
Er-value:0.000, Pr-value:0.000  
eCLIP MATCHES▶SAFB (bg=2.69%)No matches to TargetScan

ACTATAGTAGTTATCTAT 15710  
 TG

TGTGCCA

TGTGCCA  
Depth:4 (DOG)  
Ei-value:0.000, Pi-value:0.000  
Er-value:0.000, Pr-value:0.000  
No matches to eCLIP DataMATCHES To TargetScan▶ miR-183-5p.2:UGGCACU

ATCTCATTGCTAGGCATTGGGGATGCA

AAGATAA

AAGATAA  
Depth:4 (DOG)  
Ei-value:0.000, Pi-value:0.000  
Er-value:0.000, Pr-value:0.000  
No matches to eCLIP DataNo matches to TargetScan

ACCATC

TTTATTGTGT

TTTATTGTGT  
Depth:2 (PIG)  
Ei-value:0.000, Pi-value:0.000  
Er-value:0.000, Pr-value:0.000  
eCLIP MATCHES▶UTP3 (bg=3.66%)No matches to TargetScan

CTTG

GGT

GGTAGCAGAA  
Depth:2 (PIG)  
Ei-value:0.000, Pi-value:0.000  
Er-value:0.000, Pr-value:0.000  
eCLIP MATCHES▶UTP3 (bg=3.66%)No matches to TargetScan


AGCAGAA

AGCAGAA  
Depth:3 (COW)  
Ei-value:0.000, Pi-value:0.000  
Er-value:0.000, Pr-value:0.000  
eCLIP MATCHES▶UTP3 (bg=3.66%)No matches to TargetScan

GAAAAT

ATGTG

ATGTGTAAAATCAATTT  
Depth:2 (PIG)  
Ei-value:0.000, Pi-value:0.000  
Er-value:0.000, Pr-value:0.000  
eCLIP MATCHES▶UTP3 (bg=3.66%)No matches to TargetScan


TAAAATCAATTT

TAAAATCAATTT  
Depth:3 (COW)  
Ei-value:0.000, Pi-value:0.000  
Er-value:0.000, Pr-value:0.000  
eCLIP MATCHES▶UTP3 (bg=3.66%)No matches to TargetScan

ATAATTTG

TAAACTG

TAAACTG  
Depth:4 (DOG)  
Ei-value:0.000, Pi-value:0.000  
Er-value:0.000, Pr-value:0.000  
eCLIP MATCHES▶HNRNPU (bg=5.92%)No matches to TargetScan

CCACCCATA 15830  
 TATAAGCTATA

TCTGCTGAATGA

TCTGCTGAATGA  
Depth:3 (COW)  
Ei-value:0.000, Pi-value:0.000  
Er-value:0.000, Pr-value:0.000  
No matches to eCLIP DataMATCHES To TargetScan▶ miR-1298-5p:UCAUUCG

T

C

CATTGATTA  
Depth:3 (COW)  
Ei-value:0.000, Pi-value:0.000  
Er-value:0.000, Pr-value:0.000  
No matches to eCLIP DataNo matches to TargetScan


ATTGATTA

ATTGATTA  
Depth:4 (DOG)  
Ei-value:0.000, Pi-value:0.000  
Er-value:0.000, Pr-value:0.010  
No matches to eCLIP DataNo matches to TargetScan

C

TCTTATCC

TCTTATCC  
Depth:2 (PIG)  
Ei-value:0.000, Pi-value:0.000  
Er-value:0.000, Pr-value:0.000  
No matches to eCLIP DataNo matches to TargetScan

TT

AGAGATA

AGAGATA  
Depth:4 (DOG)  
Ei-value:0.000, Pi-value:0.000  
Er-value:0.000, Pr-value:0.000  
No matches to eCLIP DataNo matches to TargetScan

ACAACTGGGGGCACAAACATTTATTATCATTAT

TGAACCT

TGAACCT  
Depth:3 (COW)  
Ei-value:0.000, Pi-value:0.000  
Er-value:0.000, Pr-value:0.010  
eCLIP MATCHES▶HNRNPU (bg=5.92%)No matches to TargetScan

A

C

CAACAGAGATCT  
Depth:2 (PIG)  
Ei-value:0.000, Pi-value:0.000  
Er-value:0.000, Pr-value:0.000  
eCLIP MATCHES▶HNRNPA1 (bg=2.57%)▶HNRNPU (bg=5.92%)No matches to TargetScan


AA

AACAGAGATCT  
Depth:3 (COW)  
Ei-value:0.000, Pi-value:0.000  
Er-value:0.000, Pr-value:0.000  
eCLIP MATCHES▶HNRNPA1 (bg=2.57%)▶HNRNPU (bg=5.92%)No matches to TargetScan


CAGAGATCT

CAGAGATCT  
Depth:4 (DOG)  
Ei-value:0.000, Pi-value:0.000  
Er-value:0.000, Pr-value:0.000  
eCLIP MATCHES▶HNRNPA1 (bg=2.57%)▶HNRNPU (bg=5.92%)No matches to TargetScan

ATGTGTAG

A

ATTTACAAAGCCTA  
Depth:2 (PIG)  
Ei-value:0.000, Pi-value:0.000  
Er-value:0.000, Pr-value:0.000  
eCLIP MATCHES▶HNRNPA1 (bg=2.57%)▶HNRNPU (bg=5.92%)No matches to TargetScan


TTTACAA

TTTACAAAGC  
Depth:3 (COW)  
Ei-value:0.000, Pi-value:0.000  
Er-value:0.000, Pr-value:0.000  
eCLIP MATCHES▶HNRNPA1 (bg=2.57%)▶HNRNPU (bg=5.92%)No matches to TargetScan

 15950  


AGC

TTTACAAAGC  
Depth:3 (COW)  
Ei-value:0.000, Pi-value:0.000  
Er-value:0.000, Pr-value:0.000  
eCLIP MATCHES▶HNRNPA1 (bg=2.57%)▶HNRNPU (bg=5.92%)No matches to TargetScan


CTA

ATTTACAAAGCCTA  
Depth:2 (PIG)  
Ei-value:0.000, Pi-value:0.000  
Er-value:0.000, Pr-value:0.000  
eCLIP MATCHES▶HNRNPA1 (bg=2.57%)▶HNRNPU (bg=5.92%)No matches to TargetScan

CAGT

TCTATACA

TCTATACA  
Depth:3 (COW)  
Ei-value:0.000, Pi-value:0.000  
Er-value:0.000, Pr-value:0.000  
eCLIP MATCHES▶HNRNPA1 (bg=2.57%)▶HNRNPU (bg=5.92%)No matches to TargetScan

GA

TAGGAAT

TAGGAAT  
Depth:2 (PIG)  
Ei-value:0.000, Pi-value:0.000  
Er-value:0.000, Pr-value:0.000  
eCLIP MATCHES▶HNRNPA1 (bg=2.57%)No matches to TargetScan

GAACTA

TTGGCT

TTGGCT  
Depth:4 (DOG)  
Ei-value:0.000, Pi-value:0.000  
Er-value:0.000, Pr-value:0.000  
eCLIP MATCHES▶HNRNPA1 (bg=2.57%)No matches to TargetScan

TACTGAATGGTGA

TTACTTTCT

TTACTTTCT  
Depth:4 (DOG)  
Ei-value:0.000, Pi-value:0.000  
Er-value:0.000, Pr-value:0.010  
eCLIP MATCHES▶UTP3 (bg=3.66%)No matches to TargetScan

GTGGGGCTCGGAACT

ACATGC

ACATGC  
Depth:2 (PIG)  
Ei-value:0.000, Pi-value:0.010  
Er-value:0.000, Pr-value:0.020  
No matches to eCLIP DataNo matches to TargetScan

C

CTAGGATAT

CTAGGATAT  
Depth:3 (COW)  
Ei-value:0.000, Pi-value:0.000  
Er-value:0.000, Pr-value:0.000  
No matches to eCLIP DataNo matches to TargetScan

A

AAAATGA

AAAATGA  
Depth:3 (COW)  
Ei-value:0.000, Pi-value:0.000  
Er-value:0.000, Pr-value:0.000  
No matches to eCLIP DataNo matches to TargetScan


T

AAAATGAT  
Depth:2 (PIG)  
Ei-value:0.000, Pi-value:0.010  
Er-value:0.000, Pr-value:0.000  
No matches to eCLIP DataMATCHES To TargetScan▶ miR-382-3p:AUCAUUC

GTTATCATTATAGAGTGCT 16070  
 CACAGA

AGGAAATGA

AGGAAATGA  
Depth:2 (PIG)  
Ei-value:0.000, Pi-value:0.000  
Er-value:0.000, Pr-value:0.000  
eCLIP MATCHES▶KHDRBS1 (bg=1.71%)No matches to TargetScan

AGTAAT

ATAGGTGTG

ATAGGTGTG  
Depth:2 (PIG)  
Ei-value:0.000, Pi-value:0.000  
Er-value:0.000, Pr-value:0.000  
eCLIP MATCHES▶KHDRBS1 (bg=1.71%)No matches to TargetScan

AG

ATCCAGACCA

ATCCAGACCA  
Depth:3 (COW)  
Ei-value:0.000, Pi-value:0.000  
Er-value:0.000, Pr-value:0.000  
eCLIP MATCHES▶KHDRBS1 (bg=1.71%)No matches to TargetScan

AAAGTCATTTAACAAGTTTATTCAGTGATGAAAACATGGGACAAATGGACTAATATAAGGCAGTGTACTAAGCTGAGT 16190  
 AGAGAGATAAAGTCCTGTCCAGAAGATACATGCTTCCTG

GCCTGATTGA

GCCTGATTGA  
Depth:2 (PIG)  
Ei-value:0.000, Pi-value:0.000  
Er-value:0.000, Pr-value:0.000  
eCLIP MATCHES▶HNRNPA1 (bg=2.57%)No matches to TargetScan

GG

AGATGGA

AGATGGA  
Depth:3 (COW)  
Ei-value:0.000, Pi-value:0.000  
Er-value:0.000, Pr-value:0.000  
No matches to eCLIP DataNo matches to TargetScan

AAATTTTTGCAAAAAACAAGGTGTTGTGGTCTTCCATCCAGTTTCTTAAGTGCTGATGATAA 16310  
 AAGTGAATTAGACCCACCTTGACCTGGCCTACAGAAG

TAAAG

TAAAGGAGTAAAAAT  
Depth:2 (PIG)  
Ei-value:0.000, Pi-value:0.000  
Er-value:0.000, Pr-value:0.000  
No matches to eCLIP DataMATCHES To TargetScan▶ miR-483-3p.1:ACUCCUC


GAGTAAAAA

GAGTAAAAA  
Depth:4 (DOG)  
Ei-value:0.000, Pi-value:0.000  
Er-value:0.000, Pr-value:0.000  
No matches to eCLIP DataNo matches to TargetScan


T

TAAAGGAGTAAAAAT  
Depth:2 (PIG)  
Ei-value:0.000, Pi-value:0.000  
Er-value:0.000, Pr-value:0.000  
No matches to eCLIP DataMATCHES To TargetScan▶ miR-483-3p.1:ACUCCUC

AAATGCCTCAGGCGTGCTTTTTGATTC

ATTTGAT

ATTTGAT  
Depth:4 (DOG)  
Ei-value:0.000, Pi-value:0.010  
Er-value:0.000, Pr-value:0.000  
No matches to eCLIP DataNo matches to TargetScan


AAACA

ATTTGATAAACA  
Depth:2 (PIG)  
Ei-value:0.000, Pi-value:0.000  
Er-value:0.000, Pr-value:0.000  
No matches to eCLIP DataNo matches to TargetScan

AAGC

ATC

ATCTTTTATGT  
Depth:3 (COW)  
Ei-value:0.000, Pi-value:0.000  
Er-value:0.000, Pr-value:0.000  
eCLIP MATCHES▶SAFB (bg=2.69%)No matches to TargetScan


TTTTATGT

TTTTATGT  
Depth:4 (DOG)  
Ei-value:0.000, Pi-value:0.000  
Er-value:0.000, Pr-value:0.000  
eCLIP MATCHES▶SAFB (bg=2.69%)No matches to TargetScan


GGAATA

ATCTTTTATGTGGAATA  
Depth:2 (PIG)  
Ei-value:0.000, Pi-value:0.000  
Er-value:0.000, Pr-value:0.000  
eCLIP MATCHES▶SAFB (bg=2.69%)No matches to TargetScan

TACCATTC 16430  
 TG

GGTCCTGAG

GGTCCTGAG  
Depth:3 (COW)  
Ei-value:0.000, Pi-value:0.000  
Er-value:0.000, Pr-value:0.000  
eCLIP MATCHES▶SAFB (bg=2.69%)No matches to TargetScan

GATAAGAGAGATG

AGGGCATTAG

AGGGCATTAG  
Depth:2 (PIG)  
Ei-value:0.000, Pi-value:0.000  
Er-value:0.000, Pr-value:0.000  
eCLIP MATCHES▶SAFB (bg=2.69%)MATCHES To TargetScan▶ miR-155-5p:UAAUGCU▶ miR-365-3p:AAUGCCC▶ miR-874-3p:UGCCCUG

ATCACTGACA

GCTGAA

GCTGAA  
Depth:2 (PIG)  
Ei-value:0.000, Pi-value:0.010  
Er-value:0.000, Pr-value:0.000  
No matches to eCLIP DataNo matches to TargetScan

GATAGAAGAACATCTTTGG

TTTGATT

TTTGATT  
Depth:2 (PIG)  
Ei-value:0.000, Pi-value:0.020  
Er-value:0.000, Pr-value:0.010  
No matches to eCLIP DataNo matches to TargetScan

GTTTAAATAATATTTCAATGCCTATTCTCTGCAAGGTACTATGT 16550  
 TTCGTAAATTAAATAGGTCTGGCCCAGAAGACCCACTCAA

TTGCCTT

TTGCCTT  
Depth:3 (COW)  
Ei-value:0.000, Pi-value:0.000  
Er-value:0.000, Pr-value:0.000  
eCLIP MATCHES▶KHDRBS1 (bg=1.71%)▶UTP18 (bg=0.72%)MATCHES To TargetScan▶ miR-124-3p.1:AAGGCAC

TGAGATTAAAAAAAAAA

AAAAAAAGA

AAAAAAAGA  
Depth:2 (PIG)  
Ei-value:0.000, Pi-value:0.000  
Er-value:0.000, Pr-value:0.000  
eCLIP MATCHES▶KHDRBS1 (bg=1.71%)No matches to TargetScan

AAGAAAAATGCAAGTTTCTTTCAAAATAAAGA

GACATTTTTCCTAG

GACATTTTTCCTAG  
Depth:2 (PIG)  
Ei-value:0.000, Pi-value:0.000  
Er-value:0.000, Pr-value:0.000  
eCLIP MATCHES▶AARS (bg=2.18%)▶HNRNPU (bg=5.92%)▶SAFB (bg=2.69%)No matches to TargetScan

T 16670  
 TTCAGGAATCCCCCAAATCACTTCCTCATTGGCTTAGTTTA

AAGCCAG

AAGCCAG  
Depth:4 (DOG)  
Ei-value:0.000, Pi-value:0.000  
Er-value:0.000, Pr-value:0.000  
eCLIP MATCHES▶SAFB (bg=2.69%)MATCHES To TargetScan▶ miR-149-5p:CUGGCUC▶ miR-3064-5p:CUGGCUG

GAGAC

TG

TGATAAAAG  
Depth:2 (PIG)  
Ei-value:0.000, Pi-value:0.000  
Er-value:0.000, Pr-value:0.000  
No matches to eCLIP DataNo matches to TargetScan


ATAAAAG

ATAAAAG  
Depth:4 (DOG)  
Ei-value:0.000, Pi-value:0.000  
Er-value:0.000, Pr-value:0.000  
No matches to eCLIP DataNo matches to TargetScan

GGCTCAGGGTTTGTT

CTTTAATTC

CTTTAATTC  
Depth:3 (COW)  
Ei-value:0.000, Pi-value:0.000  
Er-value:0.000, Pr-value:0.000  
No matches to eCLIP DataNo matches to TargetScan

ATTAACTA

AACATTCTGC

AACATTCTGCTTTTATTA  
Depth:2 (PIG)  
Ei-value:0.000, Pi-value:0.000  
Er-value:0.000, Pr-value:0.000  
No matches to eCLIP DataMATCHES To TargetScan▶ miR-330-3p.2:AAAGCAC▶ miR-409-3p:AAUGUUG


TTTTATTA

TTTTATTA  
Depth:4 (DOG)  
Ei-value:0.000, Pi-value:0.010  
Er-value:0.000, Pr-value:0.000  
No matches to eCLIP DataNo matches to TargetScan

CA

G

GTTAAATGG  
Depth:3 (COW)  
Ei-value:0.000, Pi-value:0.000  
Er-value:0.000, Pr-value:0.000  
No matches to eCLIP DataNo matches to TargetScan


TTAAA

TTAAATGG  
Depth:4 (DOG)  
Ei-value:0.000, Pi-value:0.000  
Er-value:0.000, Pr-value:0.000  
No matches to eCLIP DataNo matches to TargetScan

 16790  


TGG

TTAAATGG  
Depth:4 (DOG)  
Ei-value:0.000, Pi-value:0.000  
Er-value:0.000, Pr-value:0.000  
No matches to eCLIP DataNo matches to TargetScan


TT

GTTAAATGGTT  
Depth:2 (PIG)  
Ei-value:0.000, Pi-value:0.000  
Er-value:0.000, Pr-value:0.000  
No matches to eCLIP DataNo matches to TargetScan

CAAGATGT

AACAACTAGTT

AACAACTAGTT  
Depth:2 (PIG)  
Ei-value:0.000, Pi-value:0.000  
Er-value:0.000, Pr-value:0.000  
No matches to eCLIP DataNo matches to TargetScan

TTAAAGGTATTTG

CTCATTGGTCTG

CTCATTGGTCTG  
Depth:2 (PIG)  
Ei-value:0.000, Pi-value:0.000  
Er-value:0.000, Pr-value:0.000  
No matches to eCLIP DataNo matches to TargetScan

GCTTAGAGACAGGAAGACATATGAGCAA

TAAAAAAAA

TAAAAAAAA  
Depth:2 (PIG)  
Ei-value:0.000, Pi-value:0.000  
Er-value:0.000, Pr-value:0.000  
No matches to eCLIP DataNo matches to TargetScan

GATTCTTTTGCATTTACCAATTTA

GTAAAAA

GTAAAAA  
Depth:2 (PIG)  
Ei-value:0.000, Pi-value:0.010  
Er-value:0.000, Pr-value:0.000  
eCLIP MATCHES▶KHDRBS1 (bg=1.71%)No matches to TargetScan

TTT 16910  
 ATTAAAACTGAATAAAGTG

CTGTTCTTAAGT

CTGTTCTTAAGT  
Depth:3 (COW)  
Ei-value:0.000, Pi-value:0.000  
Er-value:0.000, Pr-value:0.000  
eCLIP MATCHES▶KHDRBS1 (bg=1.71%)No matches to TargetScan

GCTTGAAAGACGTAAACCAAAGTGCACTTTATCTCATTTATCTTATGGT

GGAAACA

GGAAACA  
Depth:2 (PIG)  
Ei-value:0.000, Pi-value:0.000  
Er-value:0.000, Pr-value:0.000  
No matches to eCLIP DataNo matches to TargetScan

CAG

GAACAAATT

GAACAAATT  
Depth:3 (COW)  
Ei-value:0.000, Pi-value:0.000  
Er-value:0.000, Pr-value:0.000  
No matches to eCLIP DataMATCHES To TargetScan▶ miR-375:UUGUUCG

CTC

TAAGAGACTG

TAAGAGACTG  
Depth:2 (PIG)  
Ei-value:0.000, Pi-value:0.000  
Er-value:0.000, Pr-value:0.000  
No matches to eCLIP DataNo matches to TargetScan

TGTTTCT

T

TTAGTTG  
Depth:3 (COW)  
Ei-value:0.000, Pi-value:0.000  
Er-value:0.000, Pr-value:0.010  
No matches to eCLIP DataNo matches to TargetScan

 17030  


TAGTTG

TTAGTTG  
Depth:3 (COW)  
Ei-value:0.000, Pi-value:0.000  
Er-value:0.000, Pr-value:0.010  
No matches to eCLIP DataNo matches to TargetScan


A

TTAGTTGA  
Depth:2 (PIG)  
Ei-value:0.000, Pi-value:0.000  
Er-value:0.000, Pr-value:0.000  
No matches to eCLIP DataNo matches to TargetScan

GAAG

AAACTTCATTGA

AAACTTCATTGA  
Depth:3 (COW)  
Ei-value:0.000, Pi-value:0.000  
Er-value:0.000, Pr-value:0.000  
eCLIP MATCHES▶HNRNPA1 (bg=2.57%)No matches to TargetScan


G

AAACTTCATTGAG  
Depth:2 (PIG)  
Ei-value:0.000, Pi-value:0.000  
Er-value:0.000, Pr-value:0.000  
eCLIP MATCHES▶HNRNPA1 (bg=2.57%)No matches to TargetScan

TAGCTG

TGATAT

TGATAT  
Depth:2 (PIG)  
Ei-value:0.000, Pi-value:0.000  
Er-value:0.000, Pr-value:0.010  
eCLIP MATCHES▶HNRNPA1 (bg=2.57%)No matches to TargetScan

GTTCGATACTAAGGAAAAACTAAACAGATCACCTTTGACATGCGTTGTAGAGTG

GGAATAAGAGA

GGAATAAGAGA  
Depth:2 (PIG)  
Ei-value:0.000, Pi-value:0.000  
Er-value:0.000, Pr-value:0.000  
No matches to eCLIP DataNo matches to TargetScan

GGGCTTTTTATTTTTTCGT 17150  
 TCATACGAGTATTGAT

GAAGATGAT

GAAGATGAT  
Depth:2 (PIG)  
Ei-value:0.000, Pi-value:0.000  
Er-value:0.000, Pr-value:0.000  
No matches to eCLIP DataNo matches to TargetScan

ACTAAAT

GCTAAAT

GCTAAAT  
Depth:2 (PIG)  
Ei-value:0.000, Pi-value:0.020  
Er-value:0.000, Pr-value:0.000  
No matches to eCLIP DataNo matches to TargetScan

GAAATATATCTGCTC

CAAAAG

CAAAAG  
Depth:2 (PIG)  
Ei-value:0.000, Pi-value:0.010  
Er-value:0.000, Pr-value:0.000  
No matches to eCLIP DataNo matches to TargetScan

GCATTTATTCTGA

CTTGGAGATG

CTTGGAGATG  
Depth:2 (PIG)  
Ei-value:0.000, Pi-value:0.000  
Er-value:0.000, Pr-value:0.000  
No matches to eCLIP DataNo matches to TargetScan

CAACAAAAACACAA

AAATGGA

AAATGGA  
Depth:2 (PIG)  
Ei-value:0.000, Pi-value:0.000  
Er-value:0.000, Pr-value:0.010  
No matches to eCLIP DataNo matches to TargetScan

ATGAA

GTGATACTC

GTGATACTC  
Depth:2 (PIG)  
Ei-value:0.000, Pi-value:0.000  
Er-value:0.000, Pr-value:0.000  
No matches to eCLIP DataMATCHES To TargetScan▶ miR-496.1:GAGUAUU

TT 17270  
 CATCAAACAGAAGTGACTGTTATCTCAACCATTTTGTTAAATCCTAA

ACAGAAAACAAAA

ACAGAAAACAAAA  
Depth:4 (DOG)  
Ei-value:0.000, Pi-value:0.000  
Er-value:0.000, Pr-value:0.000  
No matches to eCLIP DataNo matches to TargetScan

AAAATCATGACGAAAAGAC

ACTTGC

ACTTGC  
Depth:2 (PIG)  
Ei-value:0.000, Pi-value:0.020  
Er-value:0.000, Pr-value:0.000  
No matches to eCLIP DataNo matches to TargetScan

TTATTAA

TTGG

TTGGCTTGGAAA  
Depth:2 (PIG)  
Ei-value:0.000, Pi-value:0.000  
Er-value:0.000, Pr-value:0.000  
No matches to eCLIP DataNo matches to TargetScan


CTTGGAAA

CTTGGAAA  
Depth:3 (COW)  
Ei-value:0.000, Pi-value:0.000  
Er-value:0.000, Pr-value:0.000  
No matches to eCLIP DataNo matches to TargetScan

GTAGAATATAGGAGAA 17390  


AGGTTA

AGGTTA  
Depth:4 (DOG)  
Ei-value:0.000, Pi-value:0.000  
Er-value:0.000, Pr-value:0.000  
No matches to eCLIP DataNo matches to TargetScan


CTGTTTATT

AGGTTACTGTTTATT  
Depth:2 (PIG)  
Ei-value:0.000, Pi-value:0.000  
Er-value:0.000, Pr-value:0.000  
No matches to eCLIP DataMATCHES To TargetScan▶ miR-101-3p.1:ACAGUAC▶ miR-132-3p/212-3p:AACAGUC▶ miR-144-3p:ACAGUAU▶ miR-802:CAGUAAC

TTTTTTCATGTATTCA

TTCATTCT

TTCATTCT  
Depth:4 (DOG)  
Ei-value:0.000, Pi-value:0.000  
Er-value:0.000, Pr-value:0.000  
No matches to eCLIP DataNo matches to TargetScan

ACAAATATATTCGGGTGCCAATAGGTACTTGGTATAAGGTTT

TTGGCCCC

TTGGCCCCAGAGACATG  
Depth:2 (PIG)  
Ei-value:0.000, Pi-value:0.000  
Er-value:0.000, Pr-value:0.000  
No matches to eCLIP DataMATCHES To TargetScan▶ miR-326:CUCUGGG


AGAGACA

AGAGACA  
Depth:4 (DOG)  
Ei-value:0.000, Pi-value:0.000  
Er-value:0.000, Pr-value:0.000  
No matches to eCLIP DataNo matches to TargetScan


TG

AGAGACATG  
Depth:3 (COW)  
Ei-value:0.000, Pi-value:0.000  
Er-value:0.000, Pr-value:0.000  
No matches to eCLIP DataNo matches to TargetScan

GGA

AAAAAATG

AAAAAATG  
Depth:2 (PIG)  
Ei-value:0.000, Pi-value:0.000  
Er-value:0.000, Pr-value:0.000  
No matches to eCLIP DataNo matches to TargetScan

CATGCCTTCCC 17510  
 AGAGAATGCCTAATACTTT

CCTTTTGG

CCTTTTGG  
Depth:4 (DOG)  
Ei-value:0.000, Pi-value:0.000  
Er-value:0.000, Pr-value:0.000  
No matches to eCLIP DataNo matches to TargetScan


C

CCTTTTGGC  
Depth:3 (COW)  
Ei-value:0.000, Pi-value:0.000  
Er-value:0.000, Pr-value:0.000  
No matches to eCLIP DataNo matches to TargetScan

TT

GTTTTCT

GTTTTCT  
Depth:2 (PIG)  
Ei-value:0.000, Pi-value:0.010  
Er-value:0.000, Pr-value:0.010  
No matches to eCLIP DataNo matches to TargetScan

T

GTTAGGGGCA

GTTAGGGGCA  
Depth:2 (PIG)  
Ei-value:0.000, Pi-value:0.000  
Er-value:0.000, Pr-value:0.000  
No matches to eCLIP DataNo matches to TargetScan

T

GGCTTAGT

GGCTTAGT  
Depth:2 (PIG)  
Ei-value:0.000, Pi-value:0.000  
Er-value:0.000, Pr-value:0.000  
No matches to eCLIP DataNo matches to TargetScan

CCCTAAA

TAAC

TAACATTGTGT  
Depth:2 (PIG)  
Ei-value:0.000, Pi-value:0.000  
Er-value:0.000, Pr-value:0.000  
No matches to eCLIP DataMATCHES To TargetScan▶ miR-409-3p:AAUGUUG


ATTGTGT

ATTGTGT  
Depth:3 (COW)  
Ei-value:0.000, Pi-value:0.000  
Er-value:0.000, Pr-value:0.010  
No matches to eCLIP DataNo matches to TargetScan

GGT

TTAATTC

TTAATTC  
Depth:4 (DOG)  
Ei-value:0.000, Pi-value:0.000  
Er-value:0.000, Pr-value:0.000  
No matches to eCLIP DataNo matches to TargetScan

CTACTCCGTATCTCTTCTACC

ACTCTGGCCACTAC

ACTCTGGCCACTAC  
Depth:4 (DOG)  
Ei-value:0.000, Pi-value:0.000  
Er-value:0.000, Pr-value:0.000  
No matches to eCLIP DataMATCHES To TargetScan▶ miR-142-3p.1:GUAGUGU

 17630  


ACTCTGGCCACTAC  
Depth:4 (DOG)  
Ei-value:0.000, Pi-value:0.000  
Er-value:0.000, Pr-value:0.000  
No matches to eCLIP DataMATCHES To TargetScan▶ miR-142-3p.1:GUAGUGU

G

ATAAGC

ATAAGC  
Depth:5 (RABBIT)  
Ei-value:0.000, Pi-value:0.010  
Er-value:0.000, Pr-value:0.000  
No matches to eCLIP DataNo matches to TargetScan


AGG

ATAAGCAGG  
Depth:4 (DOG)  
Ei-value:0.000, Pi-value:0.000  
Er-value:0.000, Pr-value:0.000  
No matches to eCLIP DataNo matches to TargetScan

TAGCTGGGTTTTGTAGTGA

GCT

GCTTGCTCCTT  
Depth:2 (PIG)  
Ei-value:0.000, Pi-value:0.000  
Er-value:0.000, Pr-value:0.000  
No matches to eCLIP DataMATCHES To TargetScan▶ miR-28-5p/708-5p:AGGAGCU


TGCTCCTT

TGCTCCTT  
Depth:3 (COW)  
Ei-value:0.000, Pi-value:0.000  
Er-value:0.000, Pr-value:0.000  
No matches to eCLIP DataMATCHES To TargetScan▶ miR-28-5p/708-5p:AGGAGCU

AAGTTACAGGAACTCTCCTTATAATAGAC

ACTTCA

ACTTCA  
Depth:3 (COW)  
Ei-value:0.000, Pi-value:0.000  
Er-value:0.000, Pr-value:0.000  
No matches to eCLIP DataNo matches to TargetScan


TTTTCCTA

TTTTCCTA  
Depth:3 (COW)  
Ei-value:0.000, Pi-value:0.000  
Er-value:0.000, Pr-value:0.000  
No matches to eCLIP DataNo matches to TargetScan


GTCCATCC

TTTTCCTAGTCCATCC  
Depth:2 (PIG)  
Ei-value:0.000, Pi-value:0.000  
Er-value:0.000, Pr-value:0.000  
No matches to eCLIP DataNo matches to TargetScan

CTC

AT

ATGAAAAATG  
Depth:3 (COW)  
Ei-value:0.000, Pi-value:0.000  
Er-value:0.000, Pr-value:0.000  
No matches to eCLIP DataNo matches to TargetScan


GAAAAATG

GAAAAATG  
Depth:4 (DOG)  
Ei-value:0.000, Pi-value:0.000  
Er-value:0.000, Pr-value:0.000  
No matches to eCLIP DataNo matches to TargetScan

ACTGACCACTGC

TGGG

TGGGCAG  
Depth:2 (PIG)  
Ei-value:0.000, Pi-value:0.000  
Er-value:0.000, Pr-value:0.000  
No matches to eCLIP DataNo matches to TargetScan

 17750  


CAG

TGGGCAG  
Depth:2 (PIG)  
Ei-value:0.000, Pi-value:0.000  
Er-value:0.000, Pr-value:0.000  
No matches to eCLIP DataNo matches to TargetScan

CAGGAGGGATGATGACCAACTAATTCCCAAACCCC

AGTCTCA

AGTCTCA  
Depth:4 (DOG)  
Ei-value:0.000, Pi-value:0.000  
Er-value:0.000, Pr-value:0.000  
eCLIP MATCHES▶ZC3H11A (bg=6.55%)No matches to TargetScan


TTGGTACCA

AGTCTCATTGGTACCA  
Depth:3 (COW)  
Ei-value:0.000, Pi-value:0.000  
Er-value:0.000, Pr-value:0.000  
eCLIP MATCHES▶NOLC1 (bg=9.43%)▶ZC3H11A (bg=6.55%)No matches to TargetScan


GC

AGTCTCATTGGTACCAGC  
Depth:2 (PIG)  
Ei-value:0.000, Pi-value:0.000  
Er-value:0.000, Pr-value:0.000  
eCLIP MATCHES▶NOLC1 (bg=9.43%)▶RPS3 (bg=0.76%)▶ZC3H11A (bg=6.55%)MATCHES To TargetScan▶ miR-138-5p:GCUGGUG

CTTG

GGGAAC

GGGAAC  
Depth:2 (PIG)  
Ei-value:0.000, Pi-value:0.000  
Er-value:0.000, Pr-value:0.010  
eCLIP MATCHES▶NOLC1 (bg=9.43%)▶RBFOX2 (bg=4.63%)▶RPS3 (bg=0.76%)▶ZC3H11A (bg=6.55%)No matches to TargetScan

CACCTACACTTG

AGCCACAA

AGCCACAA  
Depth:2 (PIG)  
Ei-value:0.000, Pi-value:0.000  
Er-value:0.000, Pr-value:0.000  
eCLIP MATCHES▶RBFOX2 (bg=4.63%)▶RPS3 (bg=0.76%)▶ZC3H11A (bg=6.55%)No matches to TargetScan

T

TGGTTTTGAA

TGGTTTTGAA  
Depth:4 (DOG)  
Ei-value:0.000, Pi-value:0.000  
Er-value:0.000, Pr-value:0.000  
No matches to eCLIP DataNo matches to TargetScan

GTG

CATTTAC

CATTTAC  
Depth:2 (PIG)  
Ei-value:0.000, Pi-value:0.000  
Er-value:0.000, Pr-value:0.000  
No matches to eCLIP DataNo matches to TargetScan

AAGGTTTGTCTAT 17870  
 TT

TCAGTTC

TCAGTTC  
Depth:2 (PIG)  
Ei-value:0.000, Pi-value:0.010  
Er-value:0.000, Pr-value:0.000  
eCLIP MATCHES▶HNRNPL (bg=0.64%)No matches to TargetScan

TTTACTTTTTACATGCTGACACATACATACACTGCCTAAATAGATCTCTTTCAGAA

ACAATCC

ACAATCC  
Depth:3 (COW)  
Ei-value:0.000, Pi-value:0.000  
Er-value:0.000, Pr-value:0.000  
No matches to eCLIP DataMATCHES To TargetScan▶ miR-219-5p:GAUUGUC

TCAGATAACGCATAGCAAAA

TGGAGATG

TGGAGATG  
Depth:3 (COW)  
Ei-value:0.000, Pi-value:0.000  
Er-value:0.000, Pr-value:0.000  
No matches to eCLIP DataNo matches to TargetScan

GAGACATGATTTCTCATGCA 17990  
 AC

AGCTTCTC

AGCTTCTC  
Depth:3 (COW)  
Ei-value:0.000, Pi-value:0.000  
Er-value:0.000, Pr-value:0.000  
No matches to eCLIP DataNo matches to TargetScan

TAATTATAC

C

CTTAGAAAT  
Depth:2 (PIG)  
Ei-value:0.000, Pi-value:0.000  
Er-value:0.000, Pr-value:0.000  
eCLIP MATCHES▶WDR3 (bg=0.25%)No matches to TargetScan


TTAGAAAT

TTAGAAAT  
Depth:4 (DOG)  
Ei-value:0.000, Pi-value:0.000  
Er-value:0.000, Pr-value:0.000  
eCLIP MATCHES▶WDR3 (bg=0.25%)No matches to TargetScan

GTTCTCCTTTTTAT

CATCAAA

CATCAAA  
Depth:4 (DOG)  
Ei-value:0.000, Pi-value:0.000  
Er-value:0.000, Pr-value:0.000  
eCLIP MATCHES▶ZC3H11A (bg=6.55%)No matches to TargetScan

TCTGCTCAAGAAGGGCTTTTTATAGTAGAATAATATCAGTGG

ATGAAAA

ATGAAAA  
Depth:2 (PIG)  
Ei-value:0.000, Pi-value:0.030  
Er-value:0.000, Pr-value:0.010  
eCLIP MATCHES▶ZC3H11A (bg=6.55%)No matches to TargetScan

CAGCTTAACATTTTACCATG

CT

CTTAAGTTTTA  
Depth:2 (PIG)  
Ei-value:0.000, Pi-value:0.000  
Er-value:0.000, Pr-value:0.000  
No matches to eCLIP DataNo matches to TargetScan

 18110  


TAAGTTTTA

CTTAAGTTTTA  
Depth:2 (PIG)  
Ei-value:0.000, Pi-value:0.000  
Er-value:0.000, Pr-value:0.000  
No matches to eCLIP DataNo matches to TargetScan

AGAATAA

AATAAAAATTGGAA

AATAAAAATTGGAA  
Depth:2 (PIG)  
Ei-value:0.000, Pi-value:0.000  
Er-value:0.000, Pr-value:0.000  
No matches to eCLIP DataNo matches to TargetScan

ATAATTGGCCAAAATTGAAA

GGAAAAA

GGAAAAA  
Depth:3 (COW)  
Ei-value:0.000, Pi-value:0.000  
Er-value:0.000, Pr-value:0.000  
No matches to eCLIP DataNo matches to TargetScan

TTTTTTTAAAATTTC

TCTAAAT

TCTAAAT  
Depth:2 (PIG)  
Ei-value:0.000, Pi-value:0.000  
Er-value:0.000, Pr-value:0.010  
eCLIP MATCHES▶PPIL4 (bg=0.52%)No matches to TargetScan

GTAGGCCTGGC

TGGGCTTTG

TGGGCTTTG  
Depth:3 (COW)  
Ei-value:0.000, Pi-value:0.000  
Er-value:0.000, Pr-value:0.000  
eCLIP MATCHES▶NOLC1 (bg=9.43%)▶PPIL4 (bg=0.52%)MATCHES To TargetScan▶ miR-330-3p:CAAAGCA

ACCTTTTCCG

TTTTTAAATCA

TTTTTAAATCACTCA  
Depth:4 (DOG)  
Ei-value:0.000, Pi-value:0.000  
Er-value:0.000, Pr-value:0.000  
eCLIP MATCHES▶ILF3 (bg=3.0%)▶NOLC1 (bg=9.43%)▶PPIL4 (bg=0.52%)No matches to TargetScan

 18230  


CTCA

TTTTTAAATCACTCA  
Depth:4 (DOG)  
Ei-value:0.000, Pi-value:0.000  
Er-value:0.000, Pr-value:0.000  
eCLIP MATCHES▶ILF3 (bg=3.0%)▶NOLC1 (bg=9.43%)▶PPIL4 (bg=0.52%)No matches to TargetScan

C

AGAGGGTGGGA

AGAGGGTGGGA  
Depth:4 (DOG)  
Ei-value:0.000, Pi-value:0.000  
Er-value:0.000, Pr-value:0.000  
eCLIP MATCHES▶ILF3 (bg=3.0%)▶ZC3H11A (bg=6.55%)No matches to TargetScan

C

AGGAGGAAGAGTGAA

AGGAGGAAGAGTGAA  
Depth:4 (DOG)  
Ei-value:0.000, Pi-value:0.000  
Er-value:0.000, Pr-value:0.000  
eCLIP MATCHES▶ILF3 (bg=3.0%)▶ZC3H11A (bg=6.55%)MATCHES To TargetScan▶ miR-670-3p:UUCCUCA

G

G

GAAAAGGTCA  
Depth:4 (DOG)  
Ei-value:0.000, Pi-value:0.000  
Er-value:0.000, Pr-value:0.000  
eCLIP MATCHES▶ILF3 (bg=3.0%)▶SF3B1 (bg=2.48%)▶ZC3H11A (bg=6.55%)MATCHES To TargetScan▶ miR-192-5p/215-5p:UGACCUA


AAAAGGT

AAAAGGT  
Depth:6 (MOUSE)  
Ei-value:0.000, Pi-value:0.000  
Er-value:0.000, Pr-value:0.000  
eCLIP MATCHES▶ILF3 (bg=3.0%)▶SF3B1 (bg=2.48%)▶ZC3H11A (bg=6.55%)No matches to TargetScan


CA

GAAAAGGTCA  
Depth:4 (DOG)  
Ei-value:0.000, Pi-value:0.000  
Er-value:0.000, Pr-value:0.000  
eCLIP MATCHES▶ILF3 (bg=3.0%)▶SF3B1 (bg=2.48%)▶ZC3H11A (bg=6.55%)MATCHES To TargetScan▶ miR-192-5p/215-5p:UGACCUA

AACCTGTTTTAA

GGGCAACCTGCCTTTGTTCTG

GGGCAACCTGCCTTTGTTCTG  
Depth:2 (PIG)  
Ei-value:0.000, Pi-value:0.000  
Er-value:0.000, Pr-value:0.000  
eCLIP MATCHES▶ILF3 (bg=3.0%)▶ZC3H11A (bg=6.55%)MATCHES To TargetScan▶ miR-124-3p.1:AAGGCAC▶ miR-495-3p:AACAAAC

A

A

ATTGGTCTTAA  
Depth:2 (PIG)  
Ei-value:0.000, Pi-value:0.000  
Er-value:0.000, Pr-value:0.000  
eCLIP MATCHES▶ILF3 (bg=3.0%)▶ZC3H11A (bg=6.55%)MATCHES To TargetScan▶ miR-208-3p:UAAGACG▶ miR-499a-5p:UAAGACU


TTGGTCTTAA

TTGGTCTTAA  
Depth:3 (COW)  
Ei-value:0.000, Pi-value:0.000  
Er-value:0.000, Pr-value:0.000  
eCLIP MATCHES▶ILF3 (bg=3.0%)▶ZC3H11A (bg=6.55%)MATCHES To TargetScan▶ miR-208-3p:UAAGACG▶ miR-499a-5p:UAAGACU

GAACATTACCAGCTCCAG

GTTTAAAT

GTTTAAAT  
Depth:2 (PIG)  
Ei-value:0.000, Pi-value:0.000  
Er-value:0.000, Pr-value:0.000  
eCLIP MATCHES▶ILF3 (bg=3.0%)No matches to TargetScan

TGTTCA 18350  
 GTTTCATGCAGTTCCAATAGCTGATCATTGTTG

AGATGAGGACAAA

AGATGAGGACAAA  
Depth:3 (COW)  
Ei-value:0.000, Pi-value:0.000  
Er-value:0.000, Pr-value:0.000  
eCLIP MATCHES▶HNRNPA1 (bg=2.57%)No matches to TargetScan

A

TCCTTTGT

TCCTTTGT  
Depth:3 (COW)  
Ei-value:0.000, Pi-value:0.000  
Er-value:0.000, Pr-value:0.000  
eCLIP MATCHES▶HNRNPA1 (bg=2.57%)No matches to TargetScan

CCTCACTAGTTTGCTTT

ACATTTTT

ACATTTTT  
Depth:2 (PIG)  
Ei-value:0.000, Pi-value:0.020  
Er-value:0.000, Pr-value:0.000  
No matches to eCLIP DataNo matches to TargetScan

GAAAAGTATTATTTTTGTCCAAGTGCTTATCAACTAAA

CC

CCTTGTG  
Depth:2 (PIG)  
Ei-value:0.000, Pi-value:0.000  
Er-value:0.000, Pr-value:0.020  
eCLIP MATCHES▶NOLC1 (bg=9.43%)▶ZC3H11A (bg=6.55%)No matches to TargetScan

 18470  


TTGTG

CCTTGTG  
Depth:2 (PIG)  
Ei-value:0.000, Pi-value:0.000  
Er-value:0.000, Pr-value:0.020  
eCLIP MATCHES▶NOLC1 (bg=9.43%)▶ZC3H11A (bg=6.55%)No matches to TargetScan

TTAGGTAAGAATGGAATTTATTAAGTGAATCAGTGTGACCCTTCTTGTCATAAGATTATCT

TAAAGC

TAAAGC  
Depth:3 (COW)  
Ei-value:0.000, Pi-value:0.000  
Er-value:0.000, Pr-value:0.000  
eCLIP MATCHES▶NOLC1 (bg=9.43%)No matches to TargetScan

TGAAGCCAAAATATGCTT

CAAAAGAAGAGG

CAAAAGAAGAGG  
Depth:2 (PIG)  
Ei-value:0.000, Pi-value:0.000  
Er-value:0.000, Pr-value:0.000  
No matches to eCLIP DataNo matches to TargetScan

ACTTTATTGTTCATTGTA 18590  
 G

TTCATACA

TTCATACA  
Depth:3 (COW)  
Ei-value:0.000, Pi-value:0.000  
Er-value:0.000, Pr-value:0.000  
No matches to eCLIP DataNo matches to TargetScan


TTCAAAGCATC

TTCAAAGCATC  
Depth:3 (COW)  
Ei-value:0.000, Pi-value:0.000  
Er-value:0.000, Pr-value:0.000  
No matches to eCLIP DataNo matches to TargetScan

TGAACTGTAGTTTCTATA

GCAAGCCAA

GCAAGCCAA  
Depth:2 (PIG)  
Ei-value:0.000, Pi-value:0.000  
Er-value:0.000, Pr-value:0.000  
No matches to eCLIP DataNo matches to TargetScan

TTACATCCATAAG

TGG

TGGAGAAGGAAATAGAT  
Depth:2 (PIG)  
Ei-value:0.000, Pi-value:0.000  
Er-value:0.000, Pr-value:0.000  
eCLIP MATCHES▶ZC3H11A (bg=6.55%)No matches to TargetScan


AGAAGGAAATAGA

AGAAGGAAATAGA  
Depth:3 (COW)  
Ei-value:0.000, Pi-value:0.000  
Er-value:0.000, Pr-value:0.000  
eCLIP MATCHES▶ZC3H11A (bg=6.55%)No matches to TargetScan


T

TGGAGAAGGAAATAGAT  
Depth:2 (PIG)  
Ei-value:0.000, Pi-value:0.000  
Er-value:0.000, Pr-value:0.000  
eCLIP MATCHES▶ZC3H11A (bg=6.55%)No matches to TargetScan

AAATGTCAAAGTATGATTGG

TGGAGGGAGC

TGGAGGGAGC  
Depth:3 (COW)  
Ei-value:0.000, Pi-value:0.000  
Er-value:0.000, Pr-value:0.000  
eCLIP MATCHES▶FTO (bg=0.32%)▶LARP4 (bg=4.72%)▶LSM11 (bg=2.28%)▶NOLC1 (bg=9.43%)▶XRCC6 (bg=2.91%)▶ZC3H11A (bg=6.55%)No matches to TargetScan


AA

TGGAGGGAGCAA  
Depth:2 (PIG)  
Ei-value:0.000, Pi-value:0.000  
Er-value:0.000, Pr-value:0.000  
eCLIP MATCHES▶FTO (bg=0.32%)▶LARP4 (bg=4.72%)▶LSM11 (bg=2.28%)▶NOLC1 (bg=9.43%)▶XRCC6 (bg=2.91%)▶ZC3H11A (bg=6.55%)No matches to TargetScan

G

GTTGAAGA

GTTGAAGA  
Depth:2 (PIG)  
Ei-value:0.000, Pi-value:0.000  
Er-value:0.000, Pr-value:0.000  
eCLIP MATCHES▶FTO (bg=0.32%)▶LARP4 (bg=4.72%)▶LSM11 (bg=2.28%)▶NOLC1 (bg=9.43%)▶XRCC6 (bg=2.91%)▶ZC3H11A (bg=6.55%)No matches to TargetScan

TA 18710  
 ATCTGGGGTTGAAATTTTCTAGTTTTCATTCTGTACATTTTTAGTTAGACATCAGATTTGAAATAT

TAATGTTT

TAATGTTT  
Depth:4 (DOG)  
Ei-value:0.000, Pi-value:0.000  
Er-value:0.000, Pr-value:0.000  
eCLIP MATCHES▶CPEB4 (bg=1.89%)▶KHDRBS1 (bg=1.71%)▶LARP4 (bg=4.72%)▶LSM11 (bg=2.28%)▶NOLC1 (bg=9.43%)▶RBFOX2 (bg=4.63%)▶SAFB (bg=2.69%)▶SAFB2 (bg=0.8%)▶WDR43 (bg=3.37%)▶ZC3H11A (bg=6.55%)MATCHES To TargetScan▶ miR-323-3p:ACAUUAC▶ miR-543:AACAUUC

ACCTTTCAATGTGTGG

TATC

TATCAGCTGGA  
Depth:2 (PIG)  
Ei-value:0.000, Pi-value:0.000  
Er-value:0.000, Pr-value:0.000  
eCLIP MATCHES▶CPEB4 (bg=1.89%)▶KHDRBS1 (bg=1.71%)▶LSM11 (bg=2.28%)▶NOLC1 (bg=9.43%)▶RBFOX2 (bg=4.63%)▶SAFB (bg=2.69%)▶SAFB2 (bg=0.8%)▶SF3B1 (bg=2.48%)▶TRA2A (bg=4.8%)▶WDR43 (bg=3.37%)▶ZC3H11A (bg=6.55%)No matches to TargetScan


AGCTGGA

AGCTGGA  
Depth:4 (DOG)  
Ei-value:0.000, Pi-value:0.000  
Er-value:0.000, Pr-value:0.000  
eCLIP MATCHES▶CPEB4 (bg=1.89%)▶KHDRBS1 (bg=1.71%)▶LSM11 (bg=2.28%)▶NOLC1 (bg=9.43%)▶RBFOX2 (bg=4.63%)▶SAFB (bg=2.69%)▶SAFB2 (bg=0.8%)▶SF3B1 (bg=2.48%)▶TRA2A (bg=4.8%)▶WDR43 (bg=3.37%)▶ZC3H11A (bg=6.55%)No matches to TargetScan

CTCAGTAACACCCCTTTCT 18830  
 TCAGCTGGGGATGGGGAATGG

ATTATTGGAAA

ATTATTGGAAA  
Depth:4 (DOG)  
Ei-value:0.000, Pi-value:0.000  
Er-value:0.000, Pr-value:0.000  
eCLIP MATCHES▶FASTKD2 (bg=1.99%)▶FUS (bg=2.21%)▶LARP4 (bg=4.72%)▶NOLC1 (bg=9.43%)▶RBFOX2 (bg=4.63%)▶SAFB (bg=2.69%)▶SAFB2 (bg=0.8%)▶TRA2A (bg=4.8%)▶WDR43 (bg=3.37%)▶ZC3H11A (bg=6.55%)No matches to TargetScan


A

ATTATTGGAAAA  
Depth:2 (PIG)  
Ei-value:0.000, Pi-value:0.000  
Er-value:0.000, Pr-value:0.000  
eCLIP MATCHES▶FASTKD2 (bg=1.99%)▶FUS (bg=2.21%)▶LARP4 (bg=4.72%)▶NOLC1 (bg=9.43%)▶RBFOX2 (bg=4.63%)▶SAFB (bg=2.69%)▶SAFB2 (bg=0.8%)▶TRA2A (bg=4.8%)▶WDR43 (bg=3.37%)▶ZC3H11A (bg=6.55%)No matches to TargetScan


TGGAAAG

TGGAAAG  
Depth:2 (PIG)  
Ei-value:0.000, Pi-value:0.000  
Er-value:0.000, Pr-value:0.010  
eCLIP MATCHES▶FASTKD2 (bg=1.99%)▶FUS (bg=2.21%)▶LARP4 (bg=4.72%)▶NIPBL (bg=5.39%)▶NOLC1 (bg=9.43%)▶RBFOX2 (bg=4.63%)▶SAFB (bg=2.69%)▶SAFB2 (bg=0.8%)▶TRA2A (bg=4.8%)▶WDR43 (bg=3.37%)▶ZC3H11A (bg=6.55%)No matches to TargetScan

A

AGAAAGTAAC

AGAAAGTAAC  
Depth:4 (DOG)  
Ei-value:0.000, Pi-value:0.000  
Er-value:0.000, Pr-value:0.000  
eCLIP MATCHES▶FASTKD2 (bg=1.99%)▶FUS (bg=2.21%)▶LARP4 (bg=4.72%)▶NIPBL (bg=5.39%)▶NOLC1 (bg=9.43%)▶RBFOX2 (bg=4.63%)▶SAFB (bg=2.69%)▶SAFB2 (bg=0.8%)▶TRA2A (bg=4.8%)▶uchl5 (bg=11.16%)▶WDR43 (bg=3.37%)▶ZC3H11A (bg=6.55%)▶ZNF800 (bg=1.92%)No matches to TargetScan


TAAAAGCCTTCC

AGAAAGTAACTAAAAGCCTTCCTTTCACAGTTTCTGGCATC  
Depth:2 (PIG)  
Ei-value:0.000, Pi-value:0.000  
Er-value:0.000, Pr-value:0.000  
eCLIP MATCHES▶FASTKD2 (bg=1.99%)▶FUS (bg=2.21%)▶LARP4 (bg=4.72%)▶NIPBL (bg=5.39%)▶NOLC1 (bg=9.43%)▶RBFOX2 (bg=4.63%)▶SAFB (bg=2.69%)▶SAFB2 (bg=0.8%)▶TRA2A (bg=4.8%)▶uchl5 (bg=11.16%)▶WDR43 (bg=3.37%)▶ZC3H11A (bg=6.55%)▶ZNF800 (bg=1.92%)MATCHES To TargetScan▶ miR-488-3p:UGAAAGG


TTTCACAGTTTCTGGCATC

TTTCACAGTTTCTGGCATC  
Depth:4 (DOG)  
Ei-value:0.000, Pi-value:0.000  
Er-value:0.000, Pr-value:0.000  
eCLIP MATCHES▶FASTKD2 (bg=1.99%)▶FUS (bg=2.21%)▶LARP4 (bg=4.72%)▶NIPBL (bg=5.39%)▶NOLC1 (bg=9.43%)▶RBFOX2 (bg=4.63%)▶SAFB (bg=2.69%)▶SAFB2 (bg=0.8%)▶uchl5 (bg=11.16%)▶WDR43 (bg=3.37%)▶ZC3H11A (bg=6.55%)▶ZNF800 (bg=1.92%)No matches to TargetScan


ACTAC

ACTACCACTACTGAT  
Depth:2 (PIG)  
Ei-value:0.000, Pi-value:0.000  
Er-value:0.000, Pr-value:0.000  
eCLIP MATCHES▶FASTKD2 (bg=1.99%)▶FUS (bg=2.21%)▶LARP4 (bg=4.72%)▶NIPBL (bg=5.39%)▶NOLC1 (bg=9.43%)▶RBFOX2 (bg=4.63%)▶RBM15 (bg=7.27%)▶SAFB (bg=2.69%)▶SAFB2 (bg=0.8%)▶uchl5 (bg=11.16%)▶ZC3H11A (bg=6.55%)▶ZNF800 (bg=1.92%)MATCHES To TargetScan▶ miR-140-5p:AGUGGUU▶ miR-142-3p.1:GUAGUGU▶ miR-199-3p:CAGUAGU


CA

CACTACTGAT  
Depth:3 (COW)  
Ei-value:0.000, Pi-value:0.000  
Er-value:0.000, Pr-value:0.000  
eCLIP MATCHES▶FASTKD2 (bg=1.99%)▶FUS (bg=2.21%)▶LARP4 (bg=4.72%)▶NIPBL (bg=5.39%)▶NOLC1 (bg=9.43%)▶RBFOX2 (bg=4.63%)▶SAFB (bg=2.69%)▶SAFB2 (bg=0.8%)▶uchl5 (bg=11.16%)▶ZC3H11A (bg=6.55%)▶ZNF800 (bg=1.92%)MATCHES To TargetScan▶ miR-142-3p.1:GUAGUGU▶ miR-199-3p:CAGUAGU


CTACTGAT

CTACTGAT  
Depth:4 (DOG)  
Ei-value:0.000, Pi-value:0.000  
Er-value:0.000, Pr-value:0.000  
eCLIP MATCHES▶FASTKD2 (bg=1.99%)▶FUS (bg=2.21%)▶LARP4 (bg=4.72%)▶NIPBL (bg=5.39%)▶NOLC1 (bg=9.43%)▶RBFOX2 (bg=4.63%)▶SAFB (bg=2.69%)▶SAFB2 (bg=0.8%)▶uchl5 (bg=11.16%)▶ZC3H11A (bg=6.55%)▶ZNF800 (bg=1.92%)MATCHES To TargetScan▶ miR-199-3p:CAGUAGU

T

AAACAAGAATAA

AAACAAGAATAA  
Depth:3 (COW)  
Ei-value:0.000, Pi-value:0.000  
Er-value:0.000, Pr-value:0.000  
eCLIP MATCHES▶FASTKD2 (bg=1.99%)▶FUS (bg=2.21%)▶LARP4 (bg=4.72%)▶NIPBL (bg=5.39%)▶NOLC1 (bg=9.43%)▶RBFOX2 (bg=4.63%)▶SAFB2 (bg=0.8%)▶uchl5 (bg=11.16%)▶ZC3H11A (bg=6.55%)MATCHES To TargetScan▶ miR-544a-5p:CUUGUUA


G

AAACAAGAATAAGAGAACAT  
Depth:2 (PIG)  
Ei-value:0.000, Pi-value:0.000  
Er-value:0.000, Pr-value:0.000  
eCLIP MATCHES▶FASTKD2 (bg=1.99%)▶FUS (bg=2.21%)▶LARP4 (bg=4.72%)▶NIPBL (bg=5.39%)▶NOLC1 (bg=9.43%)▶RBFOX2 (bg=4.63%)▶SAFB2 (bg=0.8%)▶uchl5 (bg=11.16%)▶ZC3H11A (bg=6.55%)▶ZNF622 (bg=6.58%)MATCHES To TargetScan▶ miR-544a-5p:CUUGUUA


AGAACAT

AGAACAT  
Depth:4 (DOG)  
Ei-value:0.000, Pi-value:0.000  
Er-value:0.000, Pr-value:0.000  
eCLIP MATCHES▶FASTKD2 (bg=1.99%)▶FUS (bg=2.21%)▶LARP4 (bg=4.72%)▶NIPBL (bg=5.39%)▶NOLC1 (bg=9.43%)▶RBFOX2 (bg=4.63%)▶SAFB2 (bg=0.8%)▶uchl5 (bg=11.16%)▶ZNF622 (bg=6.58%)No matches to TargetScan

TT 18950  
 TATC

A

ATCATCTG  
Depth:2 (PIG)  
Ei-value:0.000, Pi-value:0.000  
Er-value:0.000, Pr-value:0.000  
eCLIP MATCHES▶FUS (bg=2.21%)▶LARP4 (bg=4.72%)▶NOLC1 (bg=9.43%)▶RBFOX2 (bg=4.63%)▶RPS3 (bg=0.76%)▶uchl5 (bg=11.16%)▶ZNF622 (bg=6.58%)No matches to TargetScan


TCATCTG

TCATCTG  
Depth:4 (DOG)  
Ei-value:0.000, Pi-value:0.010  
Er-value:0.000, Pr-value:0.000  
eCLIP MATCHES▶FUS (bg=2.21%)▶LARP4 (bg=4.72%)▶NOLC1 (bg=9.43%)▶RBFOX2 (bg=4.63%)▶RPS3 (bg=0.76%)▶uchl5 (bg=11.16%)▶ZNF622 (bg=6.58%)No matches to TargetScan

CTTTATTCA

CATAAATGAA

CATAAATGAA  
Depth:4 (DOG)  
Ei-value:0.000, Pi-value:0.000  
Er-value:0.000, Pr-value:0.000  
eCLIP MATCHES▶FUS (bg=2.21%)▶NOLC1 (bg=9.43%)▶RPS3 (bg=0.76%)▶uchl5 (bg=11.16%)▶ZNF622 (bg=6.58%)No matches to TargetScan


GTTGTGA

CATAAATGAAGTTGTGA  
Depth:3 (COW)  
Ei-value:0.000, Pi-value:0.000  
Er-value:0.000, Pr-value:0.000  
eCLIP MATCHES▶FUS (bg=2.21%)▶NOLC1 (bg=9.43%)▶RPS3 (bg=0.76%)▶uchl5 (bg=11.16%)▶ZNF622 (bg=6.58%)No matches to TargetScan

TGAAT

AAATCT

AAATCT  
Depth:2 (PIG)  
Ei-value:0.000, Pi-value:0.000  
Er-value:0.000, Pr-value:0.000  
eCLIP MATCHES▶FUS (bg=2.21%)▶NOLC1 (bg=9.43%)▶RBFOX2 (bg=4.63%)▶ZNF622 (bg=6.58%)No matches to TargetScan

GCTTTTATGCAGACACAAGGAATTAAG

TGGCTTC

TGGCTTC  
Depth:2 (PIG)  
Ei-value:0.000, Pi-value:0.000  
Er-value:0.000, Pr-value:0.000  
eCLIP MATCHES▶BUD13 (bg=0.18%)▶FUS (bg=2.21%)▶NOLC1 (bg=9.43%)▶RBFOX2 (bg=4.63%)▶XRCC6 (bg=2.91%)No matches to TargetScan

GTCATTGTCCTT

CTACCTCAAAG

CTACCTCAAAG  
Depth:2 (PIG)  
Ei-value:0.000, Pi-value:0.000  
Er-value:0.000, Pr-value:0.000  
eCLIP MATCHES▶BUD13 (bg=0.18%)▶CPEB4 (bg=1.89%)▶FUS (bg=2.21%)▶NOLC1 (bg=9.43%)▶RBFOX2 (bg=4.63%)▶uchl5 (bg=11.16%)MATCHES To TargetScan▶ let-7-5p/98-5p:GAGGUAG▶ miR-196-5p:AGGUAGU

ATAATTTATTCCAA 19070  
 AAGCTAAGATAAATGGAAGACTCTTGA

ACTTG

ACTTGTGAACTGATGTGAAA  
Depth:3 (COW)  
Ei-value:0.000, Pi-value:0.000  
Er-value:0.000, Pr-value:0.000  
eCLIP MATCHES▶FUS (bg=2.21%)▶NOLC1 (bg=9.43%)▶RBFOX2 (bg=4.63%)▶TRA2A (bg=4.8%)MATCHES To TargetScan▶ miR-23-3p:UCACAUU


TGAACTGATGTGAAA

TGAACTGATGTGAAA  
Depth:4 (DOG)  
Ei-value:0.000, Pi-value:0.000  
Er-value:0.000, Pr-value:0.000  
eCLIP MATCHES▶FUS (bg=2.21%)▶NOLC1 (bg=9.43%)▶RBFOX2 (bg=4.63%)▶TRA2A (bg=4.8%)MATCHES To TargetScan▶ miR-23-3p:UCACAUU

TGC

AGAATCTCT

AGAATCTCT  
Depth:2 (PIG)  
Ei-value:0.000, Pi-value:0.000  
Er-value:0.000, Pr-value:0.000  
eCLIP MATCHES▶FUS (bg=2.21%)▶NOLC1 (bg=9.43%)▶RBFOX2 (bg=4.63%)▶TRA2A (bg=4.8%)▶ZC3H11A (bg=6.55%)▶ZNF622 (bg=6.58%)No matches to TargetScan

TTTGAGTCTTTGCTGTTTG

GAAGATTGAAAAAT

GAAGATTGAAAAAT  
Depth:2 (PIG)  
Ei-value:0.000, Pi-value:0.000  
Er-value:0.000, Pr-value:0.000  
eCLIP MATCHES▶AARS (bg=2.18%)▶CPEB4 (bg=1.89%)▶DROSHA (bg=2.49%)▶FUS (bg=2.21%)▶GRWD1 (bg=5.13%)▶LARP4 (bg=4.72%)▶NOLC1 (bg=9.43%)▶RBFOX2 (bg=4.63%)▶TRA2A (bg=4.8%)▶WDR43 (bg=3.37%)▶XRCC6 (bg=2.91%)▶ZC3H11A (bg=6.55%)▶ZNF622 (bg=6.58%)No matches to TargetScan

A

TTGTTCA

TTGTTCA  
Depth:3 (COW)  
Ei-value:0.000, Pi-value:0.000  
Er-value:0.000, Pr-value:0.000  
eCLIP MATCHES▶AARS (bg=2.18%)▶AATF (bg=0.64%)▶CPEB4 (bg=1.89%)▶DROSHA (bg=2.49%)▶FASTKD2 (bg=1.99%)▶FUS (bg=2.21%)▶GRWD1 (bg=5.13%)▶LARP4 (bg=4.72%)▶LSM11 (bg=2.28%)▶NOLC1 (bg=9.43%)▶RBFOX2 (bg=4.63%)▶TRA2A (bg=4.8%)▶uchl5 (bg=11.16%)▶UTP3 (bg=3.66%)▶WDR43 (bg=3.37%)▶XRCC6 (bg=2.91%)▶ZC3H11A (bg=6.55%)▶ZNF622 (bg=6.58%)No matches to TargetScan

GCATGGG

TG

TGACCACCA  
Depth:2 (PIG)  
Ei-value:0.000, Pi-value:0.000  
Er-value:0.000, Pr-value:0.000  
eCLIP MATCHES▶AARS (bg=2.18%)▶AATF (bg=0.64%)▶AKAP8L (bg=2.19%)▶CPEB4 (bg=1.89%)▶DROSHA (bg=2.49%)▶FASTKD2 (bg=1.99%)▶FUS (bg=2.21%)▶GRWD1 (bg=5.13%)▶KHDRBS1 (bg=1.71%)▶LARP4 (bg=4.72%)▶LSM11 (bg=2.28%)▶NIPBL (bg=5.39%)▶NOLC1 (bg=9.43%)▶RBFOX2 (bg=4.63%)▶RPS3 (bg=0.76%)▶TRA2A (bg=4.8%)▶uchl5 (bg=11.16%)▶UTP3 (bg=3.66%)▶WDR43 (bg=3.37%)▶XRCC6 (bg=2.91%)▶ZC3H11A (bg=6.55%)▶ZNF622 (bg=6.58%)No matches to TargetScan


ACCACCA

ACCACCA  
Depth:3 (COW)  
Ei-value:0.000, Pi-value:0.000  
Er-value:0.000, Pr-value:0.000  
eCLIP MATCHES▶AARS (bg=2.18%)▶AATF (bg=0.64%)▶AKAP8L (bg=2.19%)▶CPEB4 (bg=1.89%)▶DROSHA (bg=2.49%)▶FASTKD2 (bg=1.99%)▶FUS (bg=2.21%)▶GRWD1 (bg=5.13%)▶KHDRBS1 (bg=1.71%)▶LARP4 (bg=4.72%)▶LSM11 (bg=2.28%)▶NIPBL (bg=5.39%)▶NOLC1 (bg=9.43%)▶RBFOX2 (bg=4.63%)▶RPS3 (bg=0.76%)▶TRA2A (bg=4.8%)▶uchl5 (bg=11.16%)▶UTP3 (bg=3.66%)▶WDR43 (bg=3.37%)▶XRCC6 (bg=2.91%)▶ZC3H11A (bg=6.55%)▶ZNF622 (bg=6.58%)No matches to TargetScan

GAAA 19190  
 GTAATCTTAAGCCATCTAGATGTCAC

AATTGAA

AATTGAA  
Depth:2 (PIG)  
Ei-value:0.000, Pi-value:0.020  
Er-value:0.000, Pr-value:0.010  
eCLIP MATCHES▶FASTKD2 (bg=1.99%)▶FUS (bg=2.21%)▶GRWD1 (bg=5.13%)▶LARP4 (bg=4.72%)▶RPS3 (bg=0.76%)▶WDR43 (bg=3.37%)▶ZC3H11A (bg=6.55%)▶ZNF622 (bg=6.58%)No matches to TargetScan

ACAAACTGGGGAGTTGGTTGCTATTGTA

AAATAAAA

AAATAAAA  
Depth:4 (DOG)  
Ei-value:0.000, Pi-value:0.000  
Er-value:0.000, Pr-value:0.000  
eCLIP MATCHES▶WDR43 (bg=3.37%)▶ZC3H11A (bg=6.55%)No matches to TargetScan


TA

AAATAAAATA  
Depth:2 (PIG)  
Ei-value:0.000, Pi-value:0.000  
Er-value:0.000, Pr-value:0.000  
eCLIP MATCHES▶WDR43 (bg=3.37%)▶ZC3H11A (bg=6.55%)No matches to TargetScan

TACTG

TTTTGAAAACTT

TTTTGAAAACTT  
Depth:2 (PIG)  
Ei-value:0.000, Pi-value:0.000  
Er-value:0.000, Pr-value:0.000  
No matches to eCLIP DataNo matches to TargetScan

TG                               19280
```

|  |  |  |  |  |
| --- | --- | --- | --- | --- |
| | | | | | | | | | |
| 2 |  | 4 |  | 6 |
| Depth of motif conservation (number of species) | | | | |

  
  

---

  

## >HUMAN TO COW (19280 bases)

```
 CCTTCAGTTCTTAAAGCGCTGCAATTCGCTGCTGCAGCCATATTTCTTACTCTCTCGGGGCTGGAAGCTTCCTGACTGAAGATCTCTCTGCACTTGGGGTTCTTTCTAGAACATTTTCTA 120  
 GTCCCCCAACACCCTTTATGGCGTATTTCTTTAAAAAAATCACCTAAATTCCATAAAATATTTTTTTAAATTCTATACTTTCTCCTAGTGTCTTCTTGACACGTCCTCCATATTTTTTTA 240  
 AAGAAAGTATTTGGAATATTTTGAGGCAATTTTTAATATTTAAGGAATTTTTCTTTGGAATCATTTTTGGTTGACATCTCTGTTTTTTGTGGATCAGTTTTTTACTCTTCCACTCTCTTT 360  
 TCTATATTTTGCCCATCGGGGCTGCGGATACCTGGTTTTATTATTTTTTCTTTGCCCAACGGGGCCGTGGATACCTGCCTTTTAATTCTTTTTTATTCGCCCATCGGGGCCGCGGATACC 480  
 TGCTTTTTATTTTTTTTTCCTTAGCCCATCGGGGTATCGGATACCTGCTGATTCCCTTCCCCTCTGAACCCCCAACACTCTGGCCCATCGGGGTGACGGATATCTGCTTTTTAAAAATTT 600  
 TCTTTTTTTGGCCCATCGGGGCTTCGGATACCTGCTTTTTTTTTTTTTATTTTTCCTTGCCCATCGGGGCCTCGGATACCTGCTTTAATTTTTGTTTTTCTGGCCCATCGGGGCCGCGGA 720  
 TACCTGCTTTGATTTTTTTTTTTCATCGCCCATCGGTGCTTTTTATGGATGAAAAAATGTTGGTTTTGTGGGTTGTTGCACTCTCTGGAATATCTACACTTTTTTTTGCTGCTGATCATT 840  
 TGGTGGTGTGTGAGTGTACCTACCGCTTTGGCAGAGAATGACTCTGCAGTTAAGCTAAGGGCGTGTTCAGATTGTGGAGGAAAAGTGGCCGCCATTTTAGACTTGCCGCATAACTCGGCT 960  
 TAGGGCTAGTCGTTTGTGCTAAGTTAAACTAGGG

AGGCAAGA

AGGCAAGA  
Depth:3 (COW)  
Ei-value:0.000, Pi-value:0.000  
Er-value:0.000, Pr-value:0.000  
eCLIP MATCHES▶AARS (bg=2.18%)▶EXOSC5 (bg=5.38%)▶HNRNPA1 (bg=2.57%)▶NIPBL (bg=5.39%)▶RBM15 (bg=7.27%)▶RBM22 (bg=4.62%)▶SDAD1 (bg=2.97%)▶uchl5 (bg=11.16%)No matches to TargetScan

TGGATGATAGCAGGTCAGGCAGAGGAAGTCATGTGCATTGCATGAGCTAAACCTATCTGAATGAATTGATTTGGGGCT 1080  
 TGTTAGGAGCTTTGCGTGATTGTTGTATCGGGAGGCAGTAAGAATCATCTTTTATCAGTACAAGGGACTAGTTAAAAATGGAAGGTTAGGAAAGACTAAGGTGCAGGGCTTAAAATGGCG 1200  
 ATTTTGACATTGCGGCATTGCTCAGCATGGCGGGCTGTGCTTTGTTAGGTTGTCCAAAATGGCGGATCCAGTTCTGTCGCAGTGTTCAAGTGGCGGGAAGGCCACATCATGATGGGCGAG 1320  
 GCTTTGTTAAGTGGTTAGCATGGTGGTGGACATGTGCGGTCACACAGGAAAAGATGGCGGCTGAAGGTCTTGCCGCAGTGTA

AAACATG

AAACATG  
Depth:4 (DOG)  
Ei-value:0.000, Pi-value:0.000  
Er-value:0.000, Pr-value:0.000  
eCLIP MATCHES▶HNRNPM (bg=4.29%)No matches to TargetScan

GCGGGCCTCTTTGTCTTTGCTGTGTGCTTTT 1440  
 CGTGTTGGGTTTTGCCGCAGGGACAATATGGCAGGCGTTGTCATATGTATATCATGGCTTTTGTCACGTGGACATCATGGCGGGCTTGCCGCATTGTTAAAGATGGCGGGTTTTGCCGCC 1560  
 TAGTGCCACGCAGAGCGGGAGAAAAGGTGGGATGGACAGTGCTGGATTGCTGCATAACCCAACCAATTAGAAATGGGGGTGGAATTGATCACAGCCAATTAGAGCAGAAGATGGAATTAG 1680  
 ACTGATGACACACTGTCCAGCTACTCAGCGAAGACCTGGGTGAATTAGCATGGCACTTCGCAGCTGTCTTTAGCCAGTCAGGAGAAAGAAGTGGAGGGGCCACGTGTATGTCTCCCAGTG 1800  
 GGCGGTACACCAGGTGTTTTCAAGGTCTTTTCAAGGACATTTAGCCTTTCCACCTCTGTCCCCTCTTATTTGTCCCCTCCTGTCCAGTGCTGCCTCTTGCAGTGCTGGATATCTGGCTGT 1920  
 GTGGTCTGAACCTCCCTCCATTCCTCTGTATTGGTGCCTCACCTAAGGCTAAGTATACCTCCCCCCCCACCCCCCAACCCCCCCAACTCCCCACCCCCACCCCCCACCCCCCACCTCCCC 2040  
 ACCCCCCTACCCCCCTACCCCCCTACCCCCCTCTGGTCTGCCCTGCACTGCACTGTTGCCATGGGCAGTGCTCCAGGCCTGCTTGGTGTGGACATGGTGGTGAGCCGTGGCAAGGACCAG 2160  
 AATGGATCACAGATGATCGTTGGC

CAACAG

CAACAG  
Depth:3 (COW)  
Ei-value:0.000, Pi-value:0.000  
Er-value:0.000, Pr-value:0.000  
eCLIP MATCHES▶CSTF2T (bg=0.82%)▶GRWD1 (bg=5.13%)▶HNRNPM (bg=4.29%)▶MTPAP (bg=2.21%)▶NCBP2 (bg=1.49%)▶NIPBL (bg=5.39%)▶PUM1 (bg=1.56%)▶RBM15 (bg=7.27%)▶SRSF1 (bg=8.47%)▶TRA2A (bg=4.8%)▶uchl5 (bg=11.16%)▶XRCC6 (bg=2.91%)▶ZNF622 (bg=6.58%)▶ZNF800 (bg=1.92%)No matches to TargetScan

GTGGCAGAAGAGGAATTCCTGCCTTCCTCAAGAGGAACACCTACCCCTTGGCTAATGCTGGGGTCGGATTTTGATTTATATTTATCTTTT 2280  
 GGATGTCAGTCATACAGTCTGATTTTGTGGTTTGCTAGTGTTTGAATTTAAGTCTTAAGTGACTATTATAGAAATGTATTAAGAGGCTTTATTTGTAGAATTCACTTTAATTACATTTAA 2400  
 TGAGTTTTTGTTTTGAGTTCCTTAAAATTCCTTAAAGTTTTTAGCTTCTCATTACAAATTCCTTAACCTTTTTTTGGCAGTAGATAGTCAAAGTCAAATCATTTCTAATGTTTTAAAAAT 2520  
 GTGCTGGTCATTTTCTTTGAAATTGACTTAACTATTTTCCTTTGAAGAGTCTGTAGCACAGAAACAGTAAAAAATTTAACTTCATGACCTAATGTAAAAAAGAGTGTTTGAAGGTTTACA 2640  
 CAGGTCCAGGCCTTGCTTTG

TTCCCATC

TTCCCATC  
Depth:4 (DOG)  
Ei-value:0.000, Pi-value:0.000  
Er-value:0.000, Pr-value:0.000  
eCLIP MATCHES▶ILF3 (bg=3.0%)No matches to TargetScan

CTTGATGCTGCACTAATTGACTAATCACCTACTTATCAGACAGGAAACTTGAATTGCTGTGGTCTGGTGTCCTCTATTCAGACTTATTATAT 2760  
 TGGAGTATTTCAATTTTTCGTTGTATCCTGCCTGCCTAGCATCCAGTTCCTCCCCAGCCCTGCTCCCAGCAAACCCCTAGTCTAGCCCCAGCCCTACTCCCACCCCGCCCCAGCCCTGCC 2880  
 CCAGCCCCAGTCCCCTAACCCCCCAGCCCTAGCCCCAGTCCCAGTCCTAGTTCCTCAGTCCCGCCCAGCTTCTCTCGAAAGTCACTCTAATTTTCATTGATTCAGTGCTCAAAATAAGTT 3000  
 GTCCATTGCTTATCCTATTATACTGGGATATTCCGTTTACCCTTGGCATTGCTGATCTTCAGTACTGACTCCTTGACCATTTTCAGTTAATGCATACAATCCCATTTGTCTGTGATCTCA 3120  
 GGACAAAGAATTTCCTTACTCGGTACGTTGAAGTTAGGGAATGTCAATTGAGAGCTTTCTATCAGAGCATTATTGCCCACAATTTGAGTTACTTATCATTTTCTCGATCCCCTGCCCTTA 3240  
 AAGGAGAAACCATTT

CTCTGT

CTCTGT  
Depth:3 (COW)  
Ei-value:0.000, Pi-value:0.000  
Er-value:0.000, Pr-value:0.000  
eCLIP MATCHES▶EIF3G (bg=0.32%)▶hnrnpk (bg=12.88%)No matches to TargetScan

CATTGCTTCTGTAGTCACAGTCCCAATTTTGAGTAGTGATCTTTTCTTGTGTACTGTGTTGGCCACCTAAAACTCTTTGCATTGAGTAAAATTCTAATT 3360  
 GCCAATAATCCTACCCATTGGATTAGACAGCACTCTGAACCCCATTTGCATTCAGCAGGGGGTCGCAGACAACCCGTCTTTTGTTGGACAGTTAAAATGCTCAGTCCCAATTGTCATAGC 3480  
 TTTGCCTATTAAACAAAGGCACCCTACTGCGCTTTTTGCTGTGCTTCTGGAGAATCCTGCTGTTCTTGGACAATTAAAGAACAAAGTAGTAATTGCTAATTGTCTCACCCATTAATCATG 3600  
 AAGACTACCAGTCGCCCTTGCATTTGCCTTGAGGCAGCGCTGACTACCTGAGATTTAAGAGTTTCTTAAATTATTGAGTAAAATCCCAATTATCCATAGTTCTGTTAGTTACACTATGGC 3720  
 CTTTGCAAACATCTTTGCATAACAGCAGTGGGACTGACTCATTCTTAGAGCCCCTTCCCTTGGAATATTAATGGATACAATAGTAATTATTCATGGTTCTGCGTAACAGAGAAGACCCAC 3840  
 TTATGTGTATGCCTTTATCATTGCTCCTAGATAGTGTGAACTACCTACCACCTTGCATTAATATGTAAAACACTAATTGCCCATAGTCCCACTCATTAGTCTAGGATGTCCTCTTTGCCA 3960  
 TTGCTGCTGAGTTCTGACTACCCAAGTTTCCTTCTCTTAAACAGTTGATATGCATAATTGCATATATTCATGGTTCTGTGCAATAAAAATGGATTCTCACCCCATCCCACCTTCTGTGGG 4080  
 ATGTTGCTAACGAGTGCAGATTATTCAATAACAGCTCTTGAACAGTTAATTTGCACAGTTGCAATTGTCCAGAGTCCTGTCCATTAGAAAGGGACTCTGTATCCTATTTGCACGCTACAA 4200  
 TGTGGGCTGATCACCCAAGGACTCTTCTTGTGCATTGATGTTCATAATTGTATTTGTCCACGATCTTGTGCACTAACCCTTCCACTCCCTTTGTATTCCAGCAGGGGACCCTTACTACTC 4320  
 AAGACCTCTGTACTAGGACAGTTTATGTGCACAATCCTAATTGATTAGAACTGAGTCTTTTATATCAAGGTCCCTGCATCATCTTTGCTTTACATCAAGAGGGTGCTGGTTACCTAATGC 4440  
 CCCTCCTCCAGAAATTATTGATGTGCAAAATGCAATTTCCCTATCTG

C

CTGTTAGTCT  
Depth:4 (DOG)  
Ei-value:0.000, Pi-value:0.000  
Er-value:0.000, Pr-value:0.000  
eCLIP MATCHES▶AKAP8L (bg=2.19%)No matches to TargetScan


TGTTAGTC

TGTTAGTC  
Depth:5 (RABBIT)  
Ei-value:0.000, Pi-value:0.000  
Er-value:0.000, Pr-value:0.000  
eCLIP MATCHES▶AKAP8L (bg=2.19%)No matches to TargetScan


T

CTGTTAGTCT  
Depth:4 (DOG)  
Ei-value:0.000, Pi-value:0.000  
Er-value:0.000, Pr-value:0.000  
eCLIP MATCHES▶AKAP8L (bg=2.19%)No matches to TargetScan

GGGGTC

TCATCC

TCATCC  
Depth:4 (DOG)  
Ei-value:0.000, Pi-value:0.020  
Er-value:0.000, Pr-value:0.000  
eCLIP MATCHES▶AKAP8L (bg=2.19%)No matches to TargetScan

CCTCATATTCCTTTTGTCTTACAGCAGG

GGG

GGGTACTTGGGACTGTTAAT  
Depth:3 (COW)  
Ei-value:0.000, Pi-value:0.000  
Er-value:0.000, Pr-value:0.000  
eCLIP MATCHES▶AKAP8L (bg=2.19%)MATCHES To TargetScan▶ miR-132-3p/212-3p:AACAGUC▶ miR-455-3p.1:CAGUCCA


TACTTGGGACTGTTAAT

TACTTGGGACTGTTAAT  
Depth:4 (DOG)  
Ei-value:0.000, Pi-value:0.000  
Er-value:0.000, Pr-value:0.000  
eCLIP MATCHES▶AKAP8L (bg=2.19%)MATCHES To TargetScan▶ miR-132-3p/212-3p:AACAGUC▶ miR-455-3p.1:CAGUCCA

GCG 4560  
 CATAATTGCAATTATGGTCTTTTCCATTAAATTAAGATCCCAACTGCTCACACCCTCTTAGCATTACAGTAGAGGGTGCTAATCACAAGGACATTTCTTTTGT

ACTG

ACTGTTAATGTGCT  
Depth:4 (DOG)  
Ei-value:0.000, Pi-value:0.000  
Er-value:0.000, Pr-value:0.000  
No matches to eCLIP DataMATCHES To TargetScan▶ miR-132-3p/212-3p:AACAGUC▶ miR-323-3p:ACAUUAC


TTAATGTGCT

TTAATGTGCT  
Depth:5 (RABBIT)  
Ei-value:0.000, Pi-value:0.000  
Er-value:0.000, Pr-value:0.000  
No matches to eCLIP DataMATCHES To TargetScan▶ miR-323-3p:ACAUUAC

ACT 4680  
 TGCATTTGTCCCTCTTCCTGTGCACTAAAGACCCCACTCACTTCCCTAGTGTTCAGCAGTGGATGACCTCTAGTCAAGACCTTTGCACTAGGATAGTTAATGTGAACCATGGCAACTGAT 4800  
 CACAACAATGTCTTTCAGATCAGATCCATTTTATCCTCCTTGTTTTACAGCAAGGGATATTAATTACCTATGTTACCTTTCCCTGGGACTATGAATGTGCAAAATTCCAATGTTCATGGT 4920  
 CTCTCCCTTTAAACCTATATTCTACCCCTTTTACATTATAGAAAGGGATGCTGGAAACCCAGAGTCCTTCT

CTTGGGACTC

CTTGGGACTC  
Depth:3 (COW)  
Ei-value:0.000, Pi-value:0.000  
Er-value:0.000, Pr-value:0.000  
No matches to eCLIP DataNo matches to TargetScan

TTAATGTGTATTTCTAATTATCCATGACTCTT

AATGTGC

AATGTGCAT  
Depth:6 (MOUSE)  
Ei-value:0.000, Pi-value:0.000  
Er-value:0.000, Pr-value:0.000  
No matches to eCLIP DataMATCHES To TargetScan▶ miR-501-3p/502-3p:AUGCACC

 5040  


AT

AATGTGCAT  
Depth:6 (MOUSE)  
Ei-value:0.000, Pi-value:0.000  
Er-value:0.000, Pr-value:0.000  
No matches to eCLIP DataMATCHES To TargetScan▶ miR-501-3p/502-3p:AUGCACC

ATTTTCAATTGCCTAATTGATTTCAATTGTCTAAGACATTTCAAATGTCTAATTGATTAGAACTGAGTCTTTTATATCAAG

CTAATA

CTAATA  
Depth:3 (COW)  
Ei-value:0.000, Pi-value:0.000  
Er-value:0.000, Pr-value:0.000  
No matches to eCLIP DataNo matches to TargetScan

TCTAGCTTTTATATCAAG

CTAATA

CTAATA  
Depth:3 (COW)  
Ei-value:0.000, Pi-value:0.000  
Er-value:0.000, Pr-value:0.000  
No matches to eCLIP DataNo matches to TargetScan

TCTTGAC 5160  
 TTCTCAGCATCATAGAAGGGGGTACTGATTTCCTAAAGTCTTTCTTGAATTTCTATTATGCAAAATTGCCCTGAGGCCGGGTGTGGTGGCTCACACCTGTAATCCCAGCACTTTGGGAGG 5280  
 CTGAGGTGGGAAGATCCCTTACTGCCAGGAGTTTGAGACCAGCCTGGCCAACATTAAAAAAAAAAAAAAGTAAGACAATTGCCCTGGAATCCCATCCCCCTCACACCTCCTTGGCAAAGC 5400  
 AGCAGGAGTGCTAACTAGCTAG

TGCTTCT

TGCTTCT  
Depth:3 (COW)  
Ei-value:0.000, Pi-value:0.000  
Er-value:0.000, Pr-value:0.010  
No matches to eCLIP DataNo matches to TargetScan

TCTCTTATACTGCTTAAATGCGCATAATTAGCAGTAGTTGATGTGCCCC

TATGTTAGA

TATGTTAGA  
Depth:4 (DOG)  
Ei-value:0.000, Pi-value:0.000  
Er-value:0.000, Pr-value:0.000  
eCLIP MATCHES▶HNRNPU (bg=5.92%)No matches to TargetScan

GTAGAATCCCGCTTCCTTGCTCCATTTGCATTA 5520  
 CTGCAGGAGCTTCTAACTAGCCTGAATTCACTC

TCTTGG

TCTTGGACTGTTAATGT  
Depth:3 (COW)  
Ei-value:0.000, Pi-value:0.000  
Er-value:0.000, Pr-value:0.000  
No matches to eCLIP DataMATCHES To TargetScan▶ miR-132-3p/212-3p:AACAGUC▶ miR-323-3p:ACAUUAC▶ miR-455-3p.1:CAGUCCA


ACTGTTAATGT

ACTGTTAATGT  
Depth:4 (DOG)  
Ei-value:0.000, Pi-value:0.000  
Er-value:0.000, Pr-value:0.000  
No matches to eCLIP DataMATCHES To TargetScan▶ miR-132-3p/212-3p:AACAGUC▶ miR-323-3p:ACAUUAC

GCATACTTAT

ATTTGCT

ATTTGCT  
Depth:4 (DOG)  
Ei-value:0.000, Pi-value:0.000  
Er-value:0.000, Pr-value:0.000  
No matches to eCLIP DataNo matches to TargetScan

GCTGTACTTTTTTACCAT

GTAAGGA

GTAAGGA  
Depth:5 (RABBIT)  
Ei-value:0.000, Pi-value:0.000  
Er-value:0.000, Pr-value:0.000  
No matches to eCLIP DataNo matches to TargetScan


CCC

GTAAGGACCC  
Depth:3 (COW)  
Ei-value:0.000, Pi-value:0.000  
Er-value:0.000, Pr-value:0.000  
No matches to eCLIP DataNo matches to TargetScan

CACCCACTGTATTTACATCCCAGCT 5640  
 GGAAGTACCTACTACTTAAGACCCTTAGACTAGTAAAGTTAGCGTGCATA

ATCTTAG

ATCTTAG  
Depth:3 (COW)  
Ei-value:0.000, Pi-value:0.000  
Er-value:0.000, Pr-value:0.000  
eCLIP MATCHES▶HNRNPU (bg=5.92%)No matches to TargetScan

GTGTTATA

TACACATT

TACACATT  
Depth:3 (COW)  
Ei-value:0.000, Pi-value:0.000  
Er-value:0.000, Pr-value:0.000  
eCLIP MATCHES▶HNRNPU (bg=5.92%)No matches to TargetScan

TTCAGTTGCATACAGTTGTGCCTTTTATCAGGACTCCTGT

ACTTAT

ACTTAT  
Depth:5 (RABBIT)  
Ei-value:0.000, Pi-value:0.000  
Er-value:0.000, Pr-value:0.000  
eCLIP MATCHES▶HNRNPU (bg=5.92%)No matches to TargetScan

C 5760  
 AAAGCAGAGAGTGCTAATCAATATTAAGCCCTTCTCTTCGAACTGTAGATGGCA

TGTAATT

TGTAATT  
Depth:3 (COW)  
Ei-value:0.000, Pi-value:0.000  
Er-value:0.000, Pr-value:0.000  
No matches to eCLIP DataNo matches to TargetScan

GCAGTTGTCA

ATGGTC

ATGGTC  
Depth:3 (COW)  
Ei-value:0.000, Pi-value:0.020  
Er-value:0.000, Pr-value:0.000  
No matches to eCLIP DataNo matches to TargetScan

CTTCAATTAGACTTGGGTTTCTGACCTATCACACCCTCTTTGC 5880  
 TTTATTGC

ATGGGGTACT

ATGGGGTACT  
Depth:3 (COW)  
Ei-value:0.000, Pi-value:0.000  
Er-value:0.000, Pr-value:0.000  
eCLIP MATCHES▶HNRNPL (bg=0.64%)No matches to TargetScan

ATT

CAC

CACTTAAGGCCCCTTTCTCAA  
Depth:3 (COW)  
Ei-value:0.000, Pi-value:0.000  
Er-value:0.000, Pr-value:0.000  
eCLIP MATCHES▶HNRNPL (bg=0.64%)No matches to TargetScan


TTAAGGCC

TTAAGGCC  
Depth:6 (MOUSE)  
Ei-value:0.000, Pi-value:0.000  
Er-value:0.000, Pr-value:0.000  
eCLIP MATCHES▶HNRNPL (bg=0.64%)No matches to TargetScan


CCTTT

TTAAGGCCCCTTT  
Depth:5 (RABBIT)  
Ei-value:0.000, Pi-value:0.000  
Er-value:0.000, Pr-value:0.000  
eCLIP MATCHES▶HNRNPL (bg=0.64%)No matches to TargetScan


CTCAA

TTAAGGCCCCTTTCTCAA  
Depth:4 (DOG)  
Ei-value:0.000, Pi-value:0.000  
Er-value:0.000, Pr-value:0.000  
eCLIP MATCHES▶HNRNPL (bg=0.64%)No matches to TargetScan

ACTGTTAATGTGCC

TAATGACAATTACAT

TAATGACAATTACAT  
Depth:3 (COW)  
Ei-value:0.000, Pi-value:0.000  
Er-value:0.000, Pr-value:0.000  
eCLIP MATCHES▶HNRNPL (bg=0.64%)MATCHES To TargetScan▶ miR-411-3p:AUGUAAC

CAGTATCCTTCCTTTTGAAGGACAGCATGGTTGGTGACACCTAAGGCCC 6000  
 CATTTCTTGGCCTCCCAATATGTGTGATTGTATTTGTCGAGGTTGCTATGCACTAGAGAAGGAAAGTGCTCCCCTCATCCCCACTTTTCCCTTCCAGCAGGAAGTGCCCACCCCATAAGA 6120  
 CCCTTTTATTTGGAGAGTCTAGGTGCACAATTGTAAGTGACCACAAGCATGCATCTTGGACATTTATGTGCGTAATCGCACACTGCTCATTCCATGTGAATAAGGTCCTACTCTCCGACC 6240  
 CCTTTTGCAATACAGAAGGGTTGCTGATAACGCAGTCCCCTTTTCTTGGCATGTTGTGTGTGATTATAATCGTCTGGGATCCTATGCACTAGAAAAGGAGGGTCCTCTCCACATACCTCA 6360  
 GTCTCACCTTTCCCTTCCAGCAGGGAGTGCCCACTCCATAAGACTCTCACATTTGGACAGTCAAGGTGCGTAATTGTTAAGTGAACACAACCATGCACCTTAGACATGGATTTGCATAAC 6480  
 TACACACAGCTCAACCTATCTGAATAAAATCCTACTCTCAGACCCCTTTTGCAGTACAGCAGGGGTGCTGATCACCAAGGCCCTTTTTCCTGGCCTGGTATGCGTGTGATTATGTTTGTC 6600  
 CCGGTTCCTGTGTATTAGACATGGAAGCCTCCCCTGCCACACTCCACCCCCAATCTTCCTTTCCCTTCCGGCAGGGAGTGCCCTCTCCATAAGACGCTTACGTTTGGACAATCAAGGTGC 6720  
 ACAGTTGTAAGTGACCACAGGCATACACCTTGGACATTAATGTGCATAACCACTTTGCCCATTCCATCTGAATAAGGTCCTACTCTCAGACCCCTTTTGCAGTACAGCAGGGGTGCTGAT 6840  
 CACCAAGGCCCCTTTTCTTGGCCTGTTATGTGCGTGATTATATTTGTCTGGGTTCCTGTGTATTAGACAAGGAAGCCTTCCCCCCGCCCCCACCCCCACTCCCAGTCTTCCTTTCCCTTC 6960  
 CAGCAGGGAGTGCCCCCTCCATAAGATCATTACATTTGGACAATCAAGGTGCACAATTATAAGTGACCACAGCCATGCACCTTGGACATTATTGGACATTAATGTGCGTAACTGCACATG 7080  
 GCCCATCCCATCTGAATAAGGTCCTACTCTCAGATGCCCTTTGCAGTACAGCAGGGGTACTGAATCACCAAGGCCCTTTTTCTTGGCCTGTTATGTGTGTGATTATATTTATCCCAGTTT 7200  
 CTGTGTAATAGACATGAAAGCCTCCCCTGCCACACCCCACCTCCAATCTTCCTTTCCCTTCCACCAGGGAGTGTCCACTCCATATACCCTTACATTTGGACAATCAAGGTGCACAATTGT 7320  
 AAGTGAGCATAGGCACTCACCTTGGACATGAATGTGCATAACTGCACATGGCCCATCCCATCTGAATAAGGTCCTACTCTCAGACCCTTTTTGCAGTACAGCAGGGGTGCTGATCACCAA 7440  
 GGCCCCTTTTCCTGGCCTGTTATGTGTGTGATTATATTTGTTCCAGTTCCTGTGTAATAGACATGGAAGCCTCCCCTGCCACACTCCACCCCCAATCTTCCTTTCCCTTCTGGCAGGAAG 7560  
 TACCCGCTCCATAAGACCCTTACATTTGGACAGTCAAGGTGCACAATTGTATGTGACCACAACCATGCACCTTGGACATAAATGTGTGTAACTGCACATGGCCCATCCCATCTGAATAAG 7680  
 GTCCTACTCTCAGACCCCTTTTGCAGTACAGTAGGTGTGCTGATAACCAAGGCCCCTCTTCCTGGCCTGTTAACGTATGTGATTATATTTGTCTGGGTTCCAGTGTATAAGACATGGAAG 7800  
 CCTCCCCTGCCCCACCCCACCCTCAATCTTCCTTTCCCTTCTGGCAGGGAGTGCCAGCTCCATAAGAACCTTACATTTGGACAGTCAAGGTGCACAATTCTAAGTGACCGCAGCCATGCA 7920  
 CCTTGGTCAATAATGTGTGTAACTGCACACGGCCTATCTCATCTGAATAAGGCCTTACTCTCAGACCCCTTTTGCAGTACAGCAGGGGTGCTGATAACCAAGGCCCATTTTCCTGGCCTG 8040  
 TTATGTGTGTGATTATATTTGTCCAGGTTTCTGTGTACTAGACAAGGAAGCCTCCTCTGCCCCATCCCATCTACGCATAATCTTTCTTTTCCTCCCAGCAGGGAGTGCTCACTCCATAAG 8160  
 ACCCTTACATTTGGACAATCAAGGTGCACAATTGTAAGTGACCACAACCATGCATCTTGGAAATTTATGTGCATAACTGCACATGGCTTATCCTATTTGAATAAAGTCCTACTCTCAGAC 8280  
 CCCCTTTGCAGTATAGCTGGGGTGCTGATCACTGAGGCCTCTTTGCTTGGCTTGTCTATATTCTTGTGTACTAGATAAGGGCACCTTCTCATGGACTCCCTTTGCTTTTCAACAAGGAGT 8400  
 ACCCACTACTTTTTAAGATT

CTTATATTT

CTTATATTT  
Depth:3 (COW)  
Ei-value:0.000, Pi-value:0.000  
Er-value:0.000, Pr-value:0.000  
eCLIP MATCHES▶DDX21 (bg=0.25%)MATCHES To TargetScan▶ miR-410-3p:AUAUAAC

GTCCAAAGTACATGG

TTTTAATTGACCA

TTTTAATTGACCA  
Depth:3 (COW)  
Ei-value:0.000, Pi-value:0.000  
Er-value:0.000, Pr-value:0.000  
No matches to eCLIP DataNo matches to TargetScan

CAACAATGTCCCTTGG

ACATTAAT

ACATTAAT  
Depth:3 (COW)  
Ei-value:0.000, Pi-value:0.000  
Er-value:0.000, Pr-value:0.000  
No matches to eCLIP DataNo matches to TargetScan

GTATGTAATCACCACATGGTTCATCCTAATTAAACAAAG 8520  
 TTCTACCTTCTCACCCTCCATTTGCAGTATACCAGGGTTGCTGACCCCCTAAGTCCCCTTTTCTTGGCTTGTTGACATG

CATAATTGCA

CATAATTGCA  
Depth:3 (COW)  
Ei-value:0.000, Pi-value:0.000  
Er-value:0.000, Pr-value:0.000  
eCLIP MATCHES▶hnrnpk (bg=12.88%)▶TIA1 (bg=4.07%)No matches to TargetScan

TTTATGTTGGTTCTTGTGCC

CTAGACAAGGA

CTAGACAAGGA  
Depth:3 (COW)  
Ei-value:0.000, Pi-value:0.000  
Er-value:0.000, Pr-value:0.000  
eCLIP MATCHES▶UTP3 (bg=3.66%)No matches to TargetScan

 8640  


CTAGACAAGGA  
Depth:3 (COW)  
Ei-value:0.000, Pi-value:0.000  
Er-value:0.000, Pr-value:0.000  
eCLIP MATCHES▶UTP3 (bg=3.66%)No matches to TargetScan

TGCCCCACCTCTTTTCAATAGTGGGTGCCCACTCCTTATGATCTTTACATTTGA

ACAGTTAATGTG

ACAGTTAATGTG  
Depth:4 (DOG)  
Ei-value:0.000, Pi-value:0.000  
Er-value:0.000, Pr-value:0.000  
eCLIP MATCHES▶HNRNPU (bg=5.92%)MATCHES To TargetScan▶ miR-323-3p:ACAUUAC

AATAATTGCAGTTGTCCACAACCCTATCACTTCTAGGACCATTATACCTCTTTT 8760  
 GCATTACTGTGGGGT

ATACTGTTT

ATACTGTTT  
Depth:3 (COW)  
Ei-value:0.000, Pi-value:0.000  
Er-value:0.000, Pr-value:0.000  
No matches to eCLIP DataMATCHES To TargetScan▶ miR-101-3p.1:ACAGUAC▶ miR-132-3p/212-3p:AACAGUC▶ miR-144-3p:ACAGUAU

CCCTCCAAGGCCCCTTCTGGTGGACTATCAACATATAATTGAAATTTTCTT

TTGTCTT

TTGTCTT  
Depth:3 (COW)  
Ei-value:0.000, Pi-value:0.000  
Er-value:0.000, Pr-value:0.010  
No matches to eCLIP DataNo matches to TargetScan

TGTCAGTAGATTAAGGTCATACCCCATCACCTTTCCTT 8880  
 TGTAGTACAACAGGGTGTCCTGATCAACCAAAGTCCTGTTGTTTTGGACTGTTAATATGTGCAATTACATTTGCTCCTGATCTGTGCACTAGATAAGGATCCTACCTACTTTCTTAGTGT 9000  
 TTTTAGCAGGTAGTGCCCACTACTCAAGACTGTCACTTGGAATGTTCATGTGCACAAACTCAATTCTCTAAGCATGTTCCTGTACCACCTTTGCTTTAGAGCAGGGGGATGATATTCACT 9120  
 AAGTGCCCCTTCTTTTGGACTTAATATGCATTAATGCAATTGTCCACCTCTTCTTTTAGACTAAGAGTTGATCTCCACATATTCCCCTTGCATCAGGGGCATGTTAATTATGAATGAACC 9240  
 CTTTTCTTTTAATATTAATGTCATAATTGTATTTGTGGACCTGTGTAGGAGAAAAAGACCCTATGTTCCTCCCATTACCCTTTGGATTGCTGCTGAGAAGTGTTAACTACTCATAAT

CTC

CTCAGCTCTTGG  
Depth:5 (RABBIT)  
Ei-value:0.000, Pi-value:0.000  
Er-value:0.000, Pr-value:0.000  
No matches to eCLIP DataMATCHES To TargetScan▶ miR-335-5p:CAAGAGC

 9360  


AGCTCTTGG

CTCAGCTCTTGG  
Depth:5 (RABBIT)  
Ei-value:0.000, Pi-value:0.000  
Er-value:0.000, Pr-value:0.000  
No matches to eCLIP DataMATCHES To TargetScan▶ miR-335-5p:CAAGAGC


ACA

CTCAGCTCTTGGACA  
Depth:4 (DOG)  
Ei-value:0.000, Pi-value:0.000  
Er-value:0.000, Pr-value:0.000  
No matches to eCLIP DataMATCHES To TargetScan▶ miR-335-5p:CAAGAGC


ATTAATA

CTCAGCTCTTGGACAATTAATA  
Depth:3 (COW)  
Ei-value:0.000, Pi-value:0.000  
Er-value:0.000, Pr-value:0.000  
No matches to eCLIP DataMATCHES To TargetScan▶ miR-335-5p:CAAGAGC

GCATTAATAACAATTATCAAGGGCACT

GATCAT

GATCAT  
Depth:3 (COW)  
Ei-value:0.000, Pi-value:0.000  
Er-value:0.000, Pr-value:0.000  
eCLIP MATCHES▶HNRNPU (bg=5.92%)No matches to TargetScan

TAGATAAGACTCCTGCTTCCTCGTTGCTTACATCGGGGGTACTGACCCAC

TAAGGC

TAAGGC  
Depth:3 (COW)  
Ei-value:0.000, Pi-value:0.000  
Er-value:0.000, Pr-value:0.000  
No matches to eCLIP DataNo matches to TargetScan

CCCTTGTACTGT 9480  
 TAATGT

GAATATTTGCA

GAATATTTGCA  
Depth:3 (COW)  
Ei-value:0.000, Pi-value:0.000  
Er-value:0.000, Pr-value:0.000  
No matches to eCLIP DataNo matches to TargetScan

ATTATATATGTCTCCTTCTGGTAGAGTGGGATATTATGCCCTAGTATCCCCTTTGC

ATTACTG

ATTACTG  
Depth:3 (COW)  
Ei-value:0.000, Pi-value:0.010  
Er-value:0.000, Pr-value:0.020  
No matches to eCLIP DataMATCHES To TargetScan▶ miR-802:CAGUAAC

CAG

GGGCTGCTGA

GGGCTGCTGA  
Depth:3 (COW)  
Ei-value:0.000, Pi-value:0.000  
Er-value:0.000, Pr-value:0.000  
No matches to eCLIP DataMATCHES To TargetScan▶ miR-15-5p/16-5p/195-5p/424-5p/497-5p:AGCAGCA▶ miR-503-5p:AGCAGCG

CTACT

CAAAACTT

CAAAACTT  
Depth:4 (DOG)  
Ei-value:0.000, Pi-value:0.000  
Er-value:0.000, Pr-value:0.000  
eCLIP MATCHES▶SF3B1 (bg=2.48%)No matches to TargetScan

CTC

CTGGGACTG

CTGGGACTG  
Depth:3 (COW)  
Ei-value:0.000, Pi-value:0.000  
Er-value:0.000, Pr-value:0.000  
eCLIP MATCHES▶SF3B1 (bg=2.48%)MATCHES To TargetScan▶ miR-455-3p.1:CAGUCCA

TT 9600  
 AATAG

GCACAATG

GCACAATG  
Depth:6 (MOUSE)  
Ei-value:0.000, Pi-value:0.000  
Er-value:0.000, Pr-value:0.000  
No matches to eCLIP DataNo matches to TargetScan

GCAGTTATCAATGGTTTTCTCC

CTCCCTG

CTCCCTG  
Depth:3 (COW)  
Ei-value:0.000, Pi-value:0.000  
Er-value:0.000, Pr-value:0.000  
eCLIP MATCHES▶DDX42 (bg=0.58%)No matches to TargetScan

ACCTTGTTAA

GCAAGC

GCAAGC  
Depth:3 (COW)  
Ei-value:0.000, Pi-value:0.000  
Er-value:0.000, Pr-value:0.000  
eCLIP MATCHES▶DDX42 (bg=0.58%)▶hnrnpk (bg=12.88%)No matches to TargetScan

GCCCCACCCCACCCTTAGTTTCCCATGGCATAATAAAGTATAAGCATTGGAGTATTCCATGC 9720  
 ACTTGTCTATCAAACAGTGGTCCAT

A

ACTCCCA  
Depth:4 (DOG)  
Ei-value:0.000, Pi-value:0.000  
Er-value:0.000, Pr-value:0.000  
eCLIP MATCHES▶hnrnpk (bg=12.88%)No matches to TargetScan


CTCCCA

CTCCCA  
Depth:6 (MOUSE)  
Ei-value:0.000, Pi-value:0.000  
Er-value:0.000, Pr-value:0.000  
eCLIP MATCHES▶hnrnpk (bg=12.88%)No matches to TargetScan

A

CCCTTTTGCATT

CCCTTTTGCATT  
Depth:4 (DOG)  
Ei-value:0.000, Pi-value:0.000  
Er-value:0.000, Pr-value:0.000  
eCLIP MATCHES▶hnrnpk (bg=12.88%)No matches to TargetScan


G

CCCTTTTGCATTG  
Depth:3 (COW)  
Ei-value:0.000, Pi-value:0.000  
Er-value:0.000, Pr-value:0.000  
eCLIP MATCHES▶hnrnpk (bg=12.88%)No matches to TargetScan

CGCCAGTGTGTAAAATCACAGGTAGCCATGGTGTCATGCTTTATATACGAAGTCTTCCCTCTCTCTGCCCCTTG 9840  
 TGTGCCCTTGGCCCCTTTTTACAGACTATTGCTCACAATCTCAGGTGTCCATATTTGCAGCTATTAGGTAAGATTGTGCTGTCTCCCTCTTCCCTTCCCTCTGCCCTGCCCCTTTTGCCT 9960  
 CTTTGCTGGGTAATGTTGACCAGACAAGGCCCTTTCTCTTGGACTTAAACAATTCTCAGTTGCACTTTCCTTGGTCCCACCCATTATACATGAACCCCTCT

ACTTCCTT

ACTTCCTT  
Depth:3 (COW)  
Ei-value:0.000, Pi-value:0.000  
Er-value:0.000, Pr-value:0.000  
eCLIP MATCHES▶hnrnpk (bg=12.88%)No matches to TargetScan

TCGCATTGCTT 10080  
 CTGAGTATGCTGACTACCCAA

AGCCCCTTCT

AGCCCCTTCT  
Depth:3 (COW)  
Ei-value:0.000, Pi-value:0.000  
Er-value:0.000, Pr-value:0.000  
eCLIP MATCHES▶hnrnpk (bg=12.88%)No matches to TargetScan

GTGTTATTAATAAA

CACAGTA

CACAGTA  
Depth:3 (COW)  
Ei-value:0.000, Pi-value:0.000  
Er-value:0.000, Pr-value:0.000  
eCLIP MATCHES▶hnrnpk (bg=12.88%)No matches to TargetScan

C

TGATTGTC

TGATTGTCCCATTTTT  
Depth:3 (COW)  
Ei-value:0.000, Pi-value:0.000  
Er-value:0.000, Pr-value:0.000  
eCLIP MATCHES▶hnrnpk (bg=12.88%)No matches to TargetScan


CCATTTTT

CCATTTTT  
Depth:4 (DOG)  
Ei-value:0.000, Pi-value:0.000  
Er-value:0.000, Pr-value:0.000  
eCLIP MATCHES▶hnrnpk (bg=12.88%)No matches to TargetScan


CAGCCCA

CAGCCCA  
Depth:4 (DOG)  
Ei-value:0.000, Pi-value:0.000  
Er-value:0.000, Pr-value:0.000  
eCLIP MATCHES▶hnrnpk (bg=12.88%)No matches to TargetScan

TCAGTCCAAGA

TCTC

TCTCCCTACCA  
Depth:3 (COW)  
Ei-value:0.000, Pi-value:0.000  
Er-value:0.000, Pr-value:0.000  
eCLIP MATCHES▶hnrnpk (bg=12.88%)No matches to TargetScan


CCTACCA

CCTACCA  
Depth:4 (DOG)  
Ei-value:0.000, Pi-value:0.000  
Er-value:0.000, Pr-value:0.000  
eCLIP MATCHES▶hnrnpk (bg=12.88%)No matches to TargetScan

CTTTGGTGTGTTG

GTGCAGT

GTGCAGT  
Depth:3 (COW)  
Ei-value:0.000, Pi-value:0.000  
Er-value:0.000, Pr-value:0.000  
eCLIP MATCHES▶hnrnpk (bg=12.88%)MATCHES To TargetScan▶ miR-217:ACUGCAU

GT 10200  
 TGACTATG

AAAAGCAG

AAAAGCAG  
Depth:6 (MOUSE)  
Ei-value:0.000, Pi-value:0.000  
Er-value:0.000, Pr-value:0.000  
No matches to eCLIP DataNo matches to TargetScan

GCCT

GAACTA

GAACTA  
Depth:3 (COW)  
Ei-value:0.000, Pi-value:0.000  
Er-value:0.000, Pr-value:0.000  
No matches to eCLIP DataNo matches to TargetScan

GGTGGATAAGCCTTCACTCATTTTCTTTCATTTA

TTAATGATCC

TTAATGATCC  
Depth:4 (DOG)  
Ei-value:0.000, Pi-value:0.000  
Er-value:0.000, Pr-value:0.000  
No matches to eCLIP DataMATCHES To TargetScan▶ miR-382-3p:AUCAUUC

TAGTTTCA

ATTATTGT

ATTATTGT  
Depth:3 (COW)  
Ei-value:0.000, Pi-value:0.000  
Er-value:0.000, Pr-value:0.000  
No matches to eCLIP DataNo matches to TargetScan

CAG

ATTCTGGG

ATTCTGGG  
Depth:4 (DOG)  
Ei-value:0.000, Pi-value:0.000  
Er-value:0.000, Pr-value:0.000  
No matches to eCLIP DataNo matches to TargetScan

GACAAGAACCATTCTTGCCCACC 10320  
 TGTGTTAC

TG

TGCTTTACT  
Depth:3 (COW)  
Ei-value:0.000, Pi-value:0.000  
Er-value:0.000, Pr-value:0.000  
No matches to eCLIP DataMATCHES To TargetScan▶ miR-330-3p.2:AAAGCAC


CTTTACT

CTTTACT  
Depth:4 (DOG)  
Ei-value:0.000, Pi-value:0.000  
Er-value:0.000, Pr-value:0.000  
No matches to eCLIP DataNo matches to TargetScan

GT

GCAAAAT

GCAAAAT  
Depth:6 (MOUSE)  
Ei-value:0.000, Pi-value:0.000  
Er-value:0.000, Pr-value:0.000  
No matches to eCLIP DataNo matches to TargetScan

ACTG

AAGGCAA

AAGGCAA  
Depth:4 (DOG)  
Ei-value:0.000, Pi-value:0.000  
Er-value:0.000, Pr-value:0.000  
No matches to eCLIP DataNo matches to TargetScan


GTCAGACCCA

AAGGCAAGTCAGACCCA  
Depth:3 (COW)  
Ei-value:0.000, Pi-value:0.000  
Er-value:0.000, Pr-value:0.000  
No matches to eCLIP DataMATCHES To TargetScan▶ miR-193a-5p:GGGUCUU

GGGAGC

TGGATTGC

TGGATTGC  
Depth:4 (DOG)  
Ei-value:0.000, Pi-value:0.000  
Er-value:0.000, Pr-value:0.000  
No matches to eCLIP DataNo matches to TargetScan

CATCCTTTATTTTGTGTTTCCAGTGTACACTATAAAATTGTCTCCCCAG

GAAGGAAG

GAAGGAAG  
Depth:3 (COW)  
Ei-value:0.000, Pi-value:0.000  
Er-value:0.000, Pr-value:0.000  
eCLIP MATCHES▶SF3B1 (bg=2.48%)No matches to TargetScan

GT 10440  
 TGGCACTTTCTC

TGCATTCTTC

TGCATTCTTC  
Depth:5 (RABBIT)  
Ei-value:0.000, Pi-value:0.000  
Er-value:0.000, Pr-value:0.000  
eCLIP MATCHES▶SF3B1 (bg=2.48%)No matches to TargetScan

TTTCCAG

AGC

AGCAGATTGCCTGG  
Depth:4 (DOG)  
Ei-value:0.000, Pi-value:0.000  
Er-value:0.000, Pr-value:0.000  
eCLIP MATCHES▶SF3B1 (bg=2.48%)No matches to TargetScan


A

AGATTGCCTGG  
Depth:5 (RABBIT)  
Ei-value:0.000, Pi-value:0.000  
Er-value:0.000, Pr-value:0.000  
No matches to eCLIP DataNo matches to TargetScan


GATTGCCTGG

GATTGCCTGG  
Depth:6 (MOUSE)  
Ei-value:0.000, Pi-value:0.000  
Er-value:0.000, Pr-value:0.000  
No matches to eCLIP DataNo matches to TargetScan

TTAAGAATCTCTTGTTGTCCCCT

TTGTATATT

TTGTATATT  
Depth:4 (DOG)  
Ei-value:0.000, Pi-value:0.000  
Er-value:0.000, Pr-value:0.000  
No matches to eCLIP DataMATCHES To TargetScan▶ miR-381-3p:AUACAAG

GTTATTGTAAAG

TGCCAA

TGCCAA  
Depth:3 (COW)  
Ei-value:0.000, Pi-value:0.000  
Er-value:0.000, Pr-value:0.000  
No matches to eCLIP DataMATCHES To TargetScan▶ miR-182-5p:UUGGCAA▶ miR-96-5p/1271-5p:UUGGCAC

A

TGCCAGGATACA

TGCCAGGATACA  
Depth:3 (COW)  
Ei-value:0.000, Pi-value:0.000  
Er-value:0.000, Pr-value:0.000  
No matches to eCLIP DataNo matches to TargetScan

GCCAGAAAAATTGC 10560  
 TTATTATTATTAAAAAAATTTTTTTAAGAAAG

ACATCTGG

ACATCTGG  
Depth:3 (COW)  
Ei-value:0.000, Pi-value:0.000  
Er-value:0.000, Pr-value:0.000  
No matches to eCLIP DataNo matches to TargetScan

ATTGTAGGGTGGACTC

GAT

GATAACCTGGTCATT  
Depth:3 (COW)  
Ei-value:0.000, Pi-value:0.000  
Er-value:0.000, Pr-value:0.000  
No matches to eCLIP DataMATCHES To TargetScan▶ miR-154-5p:AGGUUAU


AAC

AACCTGGTCATT  
Depth:4 (DOG)  
Ei-value:0.000, Pi-value:0.000  
Er-value:0.000, Pr-value:0.000  
No matches to eCLIP DataNo matches to TargetScan


CTGGTCATT

CTGGTCATT  
Depth:5 (RABBIT)  
Ei-value:0.000, Pi-value:0.000  
Er-value:0.000, Pr-value:0.000  
No matches to eCLIP DataNo matches to TargetScan

ATTT

TTTTGAA

TTTTGAA  
Depth:3 (COW)  
Ei-value:0.000, Pi-value:0.000  
Er-value:0.000, Pr-value:0.010  
No matches to eCLIP DataNo matches to TargetScan

GCCAAAATAT

CCATTTAT

CCATTTAT  
Depth:5 (RABBIT)  
Ei-value:0.000, Pi-value:0.000  
Er-value:0.000, Pr-value:0.000  
No matches to eCLIP DataNo matches to TargetScan

ACTATGTACCTGG

TGAC

TGACCAGTGTCTCTCATTT  
Depth:4 (DOG)  
Ei-value:0.000, Pi-value:0.000  
Er-value:0.000, Pr-value:0.000  
eCLIP MATCHES▶SUPV3L1 (bg=1.57%)No matches to TargetScan


CAG

CAGTGTCTCTCATTT  
Depth:5 (RABBIT)  
Ei-value:0.000, Pi-value:0.000  
Er-value:0.000, Pr-value:0.000  
eCLIP MATCHES▶SUPV3L1 (bg=1.57%)No matches to TargetScan

 10680  


TGTCTCTCATTT

CAGTGTCTCTCATTT  
Depth:5 (RABBIT)  
Ei-value:0.000, Pi-value:0.000  
Er-value:0.000, Pr-value:0.000  
eCLIP MATCHES▶SUPV3L1 (bg=1.57%)No matches to TargetScan

TAACTG

AGG

AGGGTGGTG  
Depth:4 (DOG)  
Ei-value:0.000, Pi-value:0.000  
Er-value:0.000, Pr-value:0.000  
eCLIP MATCHES▶SUPV3L1 (bg=1.57%)No matches to TargetScan


GTGGTG

GTGGTG  
Depth:5 (RABBIT)  
Ei-value:0.000, Pi-value:0.000  
Er-value:0.000, Pr-value:0.000  
eCLIP MATCHES▶SUPV3L1 (bg=1.57%)No matches to TargetScan

G

GTCTGTGGATA

GTCTGTGGATA  
Depth:5 (RABBIT)  
Ei-value:0.000, Pi-value:0.000  
Er-value:0.000, Pr-value:0.000  
eCLIP MATCHES▶SUPV3L1 (bg=1.57%)MATCHES To TargetScan▶ miR-140-3p.1:CCACAGG


GA

GTCTGTGGATAGA  
Depth:3 (COW)  
Ei-value:0.000, Pi-value:0.000  
Er-value:0.000, Pr-value:0.000  
eCLIP MATCHES▶SUPV3L1 (bg=1.57%)MATCHES To TargetScan▶ miR-140-3p.1:CCACAGG

ACACTGACTCTTGC

TATTTTA

TATTTTA  
Depth:3 (COW)  
Ei-value:0.000, Pi-value:0.040  
Er-value:0.000, Pr-value:0.020  
eCLIP MATCHES▶SUPV3L1 (bg=1.57%)No matches to TargetScan

ATATCAAAGATA

TTCTAGA

TTCTAGA  
Depth:4 (DOG)  
Ei-value:0.000, Pi-value:0.000  
Er-value:0.000, Pr-value:0.000  
No matches to eCLIP DataNo matches to TargetScan

GTGGAACTCTTAAGACC

AGTATCTTTG

AGTATCTTTG  
Depth:3 (COW)  
Ei-value:0.000, Pi-value:0.000  
Er-value:0.000, Pr-value:0.000  
No matches to eCLIP DataNo matches to TargetScan

TGTGGGCTTTAC 10800  
 CAGC

ATTCACTT

ATTCACTT  
Depth:4 (DOG)  
Ei-value:0.000, Pi-value:0.000  
Er-value:0.000, Pr-value:0.000  
No matches to eCLIP DataNo matches to TargetScan

TTA

GAAAAAC

GAAAAAC  
Depth:4 (DOG)  
Ei-value:0.000, Pi-value:0.000  
Er-value:0.000, Pr-value:0.000  
No matches to eCLIP DataNo matches to TargetScan

TACCTAAATTTTATAATCCTTT

AATTTCTTCATCTGGAGC

AATTTCTTCATCTGGAGC  
Depth:5 (RABBIT)  
Ei-value:0.000, Pi-value:0.000  
Er-value:0.000, Pr-value:0.000  
eCLIP MATCHES▶SUPV3L1 (bg=1.57%)▶U2AF2 (bg=1.76%)No matches to TargetScan

ACCTGCCCCTA

CTTATTT

CTTATTT  
Depth:4 (DOG)  
Ei-value:0.000, Pi-value:0.000  
Er-value:0.000, Pr-value:0.010  
eCLIP MATCHES▶SUPV3L1 (bg=1.57%)▶U2AF2 (bg=1.76%)No matches to TargetScan


CAAGAA

CTTATTTCAAGAA  
Depth:3 (COW)  
Ei-value:0.000, Pi-value:0.000  
Er-value:0.000, Pr-value:0.000  
eCLIP MATCHES▶SUPV3L1 (bg=1.57%)▶U2AF2 (bg=1.76%)MATCHES To TargetScan▶ miR-203a-3p.2:UGAAAUG

GATTGCAGTAAAACGATTAAATGAGGGAACATAT 10920  
 GCAGAGGTGCTTTTAAAAAGCATATGCCACCTTTTTTATTAATTATTAT

ATAAAATG

ATAAAATG  
Depth:4 (DOG)  
Ei-value:0.000, Pi-value:0.000  
Er-value:0.000, Pr-value:0.000  
No matches to eCLIP DataNo matches to TargetScan


A

ATAAAATGA  
Depth:3 (COW)  
Ei-value:0.000, Pi-value:0.000  
Er-value:0.000, Pr-value:0.000  
No matches to eCLIP DataNo matches to TargetScan

AGCATTTAATTATAGTAATAATTTGAAGTAGTTTGAAGT

ACCACACT

ACCACACT  
Depth:3 (COW)  
Ei-value:0.000, Pi-value:0.000  
Er-value:0.000, Pr-value:0.000  
No matches to eCLIP DataNo matches to TargetScan

GAG

GTGAGG

GTGAGG  
Depth:3 (COW)  
Ei-value:0.000, Pi-value:0.000  
Er-value:0.000, Pr-value:0.000  
No matches to eCLIP DataNo matches to TargetScan

ACTTAA 11040  
 AAATGATAAGACGAGTTCCCTA

TTTTATA

TTTTATA  
Depth:3 (COW)  
Ei-value:0.000, Pi-value:0.000  
Er-value:0.000, Pr-value:0.010  
No matches to eCLIP DataMATCHES To TargetScan▶ miR-340-5p:UAUAAAG

AG

AAAAATAAGCCA

AAAAATAAGCCA  
Depth:5 (RABBIT)  
Ei-value:0.000, Pi-value:0.000  
Er-value:0.000, Pr-value:0.000  
No matches to eCLIP DataNo matches to TargetScan


A

AAAAATAAGCCAA  
Depth:4 (DOG)  
Ei-value:0.000, Pi-value:0.000  
Er-value:0.000, Pr-value:0.000  
No matches to eCLIP DataNo matches to TargetScan

AATTAAATAT

TCTTTTGGATATA

TCTTTTGGATATA  
Depth:3 (COW)  
Ei-value:0.000, Pi-value:0.000  
Er-value:0.000, Pr-value:0.000  
No matches to eCLIP DataNo matches to TargetScan

AATTTCAACAGTGAGATAGCTGCCTAGTGGAA

ATGAATAATA

ATGAATAATA  
Depth:4 (DOG)  
Ei-value:0.000, Pi-value:0.000  
Er-value:0.000, Pr-value:0.000  
No matches to eCLIP DataNo matches to TargetScan

TCCCAGCCACT 11160  


AGTGTACA

AGTGTACA  
Depth:3 (COW)  
Ei-value:0.000, Pi-value:0.000  
Er-value:0.000, Pr-value:0.000  
No matches to eCLIP DataMATCHES To TargetScan▶ miR-493-5p:UGUACAU

G

GGTGTTT

GGTGTTT  
Depth:3 (COW)  
Ei-value:0.000, Pi-value:0.000  
Er-value:0.000, Pr-value:0.000  
No matches to eCLIP DataNo matches to TargetScan

TGTGGCACAGGATTATGTAATA

TGGAACTGCT

TGGAACTGCT  
Depth:4 (DOG)  
Ei-value:0.000, Pi-value:0.000  
Er-value:0.000, Pr-value:0.000  
No matches to eCLIP DataNo matches to TargetScan

CAAGCAAA

TAACTA

TAACTA  
Depth:4 (DOG)  
Ei-value:0.000, Pi-value:0.000  
Er-value:0.000, Pr-value:0.000  
No matches to eCLIP DataNo matches to TargetScan

GTCATCACAA

CAGCAGTTC

CAGCAGTTC  
Depth:5 (RABBIT)  
Ei-value:0.000, Pi-value:0.000  
Er-value:0.000, Pr-value:0.000  
No matches to eCLIP DataNo matches to TargetScan

T

TTGTAAT

TTGTAAT  
Depth:4 (DOG)  
Ei-value:0.000, Pi-value:0.000  
Er-value:0.000, Pr-value:0.000  
No matches to eCLIP DataNo matches to TargetScan

A

ACTGAAAA

ACTGAAAA  
Depth:5 (RABBIT)  
Ei-value:0.000, Pi-value:0.000  
Er-value:0.000, Pr-value:0.000  
No matches to eCLIP DataNo matches to TargetScan

AGAATATTGTTTCTCG

GAG

GAGAAGGATGTCAAAAGATCGGC  
Depth:3 (COW)  
Ei-value:0.000, Pi-value:0.000  
Er-value:0.000, Pr-value:0.000  
eCLIP MATCHES▶SRSF1 (bg=8.47%)▶U2AF2 (bg=1.76%)▶uchl5 (bg=11.16%)MATCHES To TargetScan▶ miR-362-5p/500b-5p:AUCCUUG▶ miR-489-3p:UGACAUC


AAG

AAGGATG  
Depth:5 (RABBIT)  
Ei-value:0.000, Pi-value:0.000  
Er-value:0.000, Pr-value:0.000  
eCLIP MATCHES▶SRSF1 (bg=8.47%)▶U2AF2 (bg=1.76%)▶uchl5 (bg=11.16%)MATCHES To TargetScan▶ miR-362-5p/500b-5p:AUCCUUG

 11280  


GATG

AAGGATG  
Depth:5 (RABBIT)  
Ei-value:0.000, Pi-value:0.000  
Er-value:0.000, Pr-value:0.000  
eCLIP MATCHES▶SRSF1 (bg=8.47%)▶U2AF2 (bg=1.76%)▶uchl5 (bg=11.16%)MATCHES To TargetScan▶ miR-362-5p/500b-5p:AUCCUUG


TCA

AAGGATGTCAAAAGATC  
Depth:4 (DOG)  
Ei-value:0.000, Pi-value:0.000  
Er-value:0.000, Pr-value:0.000  
eCLIP MATCHES▶SRSF1 (bg=8.47%)▶U2AF2 (bg=1.76%)▶uchl5 (bg=11.16%)MATCHES To TargetScan▶ miR-362-5p/500b-5p:AUCCUUG▶ miR-489-3p:UGACAUC


AAAGATC

AAAGATC  
Depth:6 (MOUSE)  
Ei-value:0.000, Pi-value:0.000  
Er-value:0.000, Pr-value:0.000  
eCLIP MATCHES▶SRSF1 (bg=8.47%)▶U2AF2 (bg=1.76%)▶uchl5 (bg=11.16%)No matches to TargetScan


GGC

GAGAAGGATGTCAAAAGATCGGC  
Depth:3 (COW)  
Ei-value:0.000, Pi-value:0.000  
Er-value:0.000, Pr-value:0.000  
eCLIP MATCHES▶SRSF1 (bg=8.47%)▶U2AF2 (bg=1.76%)▶uchl5 (bg=11.16%)MATCHES To TargetScan▶ miR-362-5p/500b-5p:AUCCUUG▶ miR-489-3p:UGACAUC

C

CAGCTCAGGG

CAGCTCAGGG  
Depth:4 (DOG)  
Ei-value:0.000, Pi-value:0.000  
Er-value:0.000, Pr-value:0.000  
eCLIP MATCHES▶SRSF1 (bg=8.47%)▶U2AF2 (bg=1.76%)▶uchl5 (bg=11.16%)MATCHES To TargetScan▶ miR-125-5p:CCCUGAG

A

GCAGTTTGC

GCAGTTTGC  
Depth:3 (COW)  
Ei-value:0.000, Pi-value:0.000  
Er-value:0.000, Pr-value:0.000  
eCLIP MATCHES▶SRSF1 (bg=8.47%)▶U2AF2 (bg=1.76%)▶uchl5 (bg=11.16%)No matches to TargetScan

C

CTACTAGCTCCT

CTACTAGCTCCT  
Depth:4 (DOG)  
Ei-value:0.000, Pi-value:0.000  
Er-value:0.000, Pr-value:0.000  
eCLIP MATCHES▶SRSF1 (bg=8.47%)▶U2AF2 (bg=1.76%)▶uchl5 (bg=11.16%)MATCHES To TargetScan▶ miR-28-5p/708-5p:AGGAGCU▶ miR-411-5p.2:UAGUAGA

C

GGACAGCTG

GGACAGCTG  
Depth:5 (RABBIT)  
Ei-value:0.000, Pi-value:0.000  
Er-value:0.000, Pr-value:0.000  
eCLIP MATCHES▶SRSF1 (bg=8.47%)▶SRSF7 (bg=2.32%)▶U2AF2 (bg=1.76%)▶ZNF622 (bg=6.58%)No matches to TargetScan


T

GGACAGCTGT  
Depth:4 (DOG)  
Ei-value:0.000, Pi-value:0.000  
Er-value:0.000, Pr-value:0.000  
eCLIP MATCHES▶SRSF1 (bg=8.47%)▶SRSF7 (bg=2.32%)▶U2AF2 (bg=1.76%)▶ZNF622 (bg=6.58%)No matches to TargetScan

A

A

AAGAAGAGTCTCTGGCTCTTTAGA  
Depth:3 (COW)  
Ei-value:0.000, Pi-value:0.000  
Er-value:0.000, Pr-value:0.000  
eCLIP MATCHES▶DDX24 (bg=2.97%)▶SRSF1 (bg=8.47%)▶SRSF7 (bg=2.32%)▶U2AF2 (bg=1.76%)▶ZNF622 (bg=6.58%)No matches to TargetScan


AGAAGAGTCTCTGGCTCTTTA

AGAAGAGTCTCTGGCTCTTTA  
Depth:5 (RABBIT)  
Ei-value:0.000, Pi-value:0.000  
Er-value:0.000, Pr-value:0.000  
eCLIP MATCHES▶DDX24 (bg=2.97%)▶SRSF1 (bg=8.47%)▶SRSF7 (bg=2.32%)▶U2AF2 (bg=1.76%)▶ZNF622 (bg=6.58%)No matches to TargetScan


GA

AGAAGAGTCTCTGGCTCTTTAGA  
Depth:4 (DOG)  
Ei-value:0.000, Pi-value:0.000  
Er-value:0.000, Pr-value:0.000  
eCLIP MATCHES▶DDX24 (bg=2.97%)▶SRSF1 (bg=8.47%)▶SRSF7 (bg=2.32%)▶U2AF2 (bg=1.76%)▶ZNF622 (bg=6.58%)No matches to TargetScan

ATACT||GATCCCATTGAAGATACCACGCTGCA 11398  
 TGTGTCCTTAGTAGTCATGTCTCCTTAGGCTCCTCTTG||GAC

ATTCTGAGC

ATTCTGAGC  
Depth:4 (DOG)  
Ei-value:0.000, Pi-value:0.000  
Er-value:0.000, Pr-value:0.000  
eCLIP MATCHES▶DDX24 (bg=2.97%)▶GRWD1 (bg=5.13%)▶MTPAP (bg=2.21%)▶NOLC1 (bg=9.43%)▶SRSF1 (bg=8.47%)▶ZNF622 (bg=6.58%)No matches to TargetScan

ATGTGAGACCTGAG

GA

GACTGCAA  
Depth:3 (COW)  
Ei-value:0.000, Pi-value:0.000  
Er-value:0.000, Pr-value:0.000  
eCLIP MATCHES▶DDX24 (bg=2.97%)▶GRWD1 (bg=5.13%)▶MTPAP (bg=2.21%)▶NOLC1 (bg=9.43%)▶SRSF1 (bg=8.47%)▶UTP3 (bg=3.66%)▶ZNF622 (bg=6.58%)MATCHES To TargetScan▶ miR-455-3p.2:UGCAGUC


CTGCAA

CTGCAA  
Depth:5 (RABBIT)  
Ei-value:0.000, Pi-value:0.000  
Er-value:0.000, Pr-value:0.000  
eCLIP MATCHES▶DDX24 (bg=2.97%)▶GRWD1 (bg=5.13%)▶MTPAP (bg=2.21%)▶NOLC1 (bg=9.43%)▶SRSF1 (bg=8.47%)▶UTP3 (bg=3.66%)▶ZNF622 (bg=6.58%)No matches to TargetScan

ACAGCTATAAGAGGCTCCAAATTAATCATATCTTTCCC

TTTGAGAA

TTTGAGAATCTGG  
Depth:3 (COW)  
Ei-value:0.000, Pi-value:0.000  
Er-value:0.000, Pr-value:0.000  
eCLIP MATCHES▶DDX24 (bg=2.97%)▶GRWD1 (bg=5.13%)▶NOLC1 (bg=9.43%)▶SRSF1 (bg=8.47%)▶uchl5 (bg=11.16%)▶ZNF622 (bg=6.58%)MATCHES To TargetScan▶ miR-371-5p:CUCAAAC

 11516  


TCTGG

TTTGAGAATCTGG  
Depth:3 (COW)  
Ei-value:0.000, Pi-value:0.000  
Er-value:0.000, Pr-value:0.000  
eCLIP MATCHES▶DDX24 (bg=2.97%)▶GRWD1 (bg=5.13%)▶NOLC1 (bg=9.43%)▶SRSF1 (bg=8.47%)▶uchl5 (bg=11.16%)▶ZNF622 (bg=6.58%)MATCHES To TargetScan▶ miR-371-5p:CUCAAAC

CC

AAGCTCCA

AAGCTCCA  
Depth:3 (COW)  
Ei-value:0.000, Pi-value:0.000  
Er-value:0.000, Pr-value:0.000  
eCLIP MATCHES▶DDX24 (bg=2.97%)▶GRWD1 (bg=5.13%)▶NOLC1 (bg=9.43%)▶RBM15 (bg=7.27%)▶SRSF1 (bg=8.47%)▶uchl5 (bg=11.16%)▶ZNF622 (bg=6.58%)No matches to TargetScan

GCTAATCTACTT

GGATGG

GGATGG  
Depth:3 (COW)  
Ei-value:0.000, Pi-value:0.000  
Er-value:0.000, Pr-value:0.010  
eCLIP MATCHES▶DDX24 (bg=2.97%)▶GRWD1 (bg=5.13%)▶NIPBL (bg=5.39%)▶NOLC1 (bg=9.43%)▶RBM15 (bg=7.27%)▶SRSF1 (bg=8.47%)▶TARDBP (bg=2.79%)▶uchl5 (bg=11.16%)▶ZNF622 (bg=6.58%)No matches to TargetScan

GTTGCCAGCTAT

CTGGAGAAAAAG||ATCT

CTGGAGAAAAAGATCT  
Depth:3 (COW)  
Ei-value:0.000, Pi-value:0.000  
Er-value:0.000, Pr-value:0.000  
eCLIP MATCHES▶DDX24 (bg=2.97%)▶GRWD1 (bg=5.13%)▶NIPBL (bg=5.39%)▶NOLC1 (bg=9.43%)▶SRSF1 (bg=8.47%)▶SRSF7 (bg=2.32%)▶TARDBP (bg=2.79%)▶uchl5 (bg=11.16%)▶ZNF622 (bg=6.58%)No matches to TargetScan

TCCTCAG

AAGAATAGGC

AAGAATAGGC  
Depth:5 (RABBIT)  
Ei-value:0.000, Pi-value:0.000  
Er-value:0.000, Pr-value:0.000  
eCLIP MATCHES▶NOLC1 (bg=9.43%)▶SRSF7 (bg=2.32%)▶uchl5 (bg=11.16%)No matches to TargetScan

TTGTTGTT

T

TTACAGTGTTAGTGA  
Depth:3 (COW)  
Ei-value:0.000, Pi-value:0.000  
Er-value:0.000, Pr-value:0.000  
eCLIP MATCHES▶ILF3 (bg=3.0%)▶NOLC1 (bg=9.43%)▶RBM15 (bg=7.27%)▶SRSF7 (bg=2.32%)▶ZNF622 (bg=6.58%)MATCHES To TargetScan▶ miR-141-3p/200a-3p:AACACUG


TACAGTGTTAGTGA

TACAGTGTTAGTGA  
Depth:5 (RABBIT)  
Ei-value:0.000, Pi-value:0.000  
Er-value:0.000, Pr-value:0.000  
eCLIP MATCHES▶ILF3 (bg=3.0%)▶NOLC1 (bg=9.43%)▶RBM15 (bg=7.27%)▶SRSF7 (bg=2.32%)▶ZNF622 (bg=6.58%)MATCHES To TargetScan▶ miR-141-3p/200a-3p:AACACUG

TC

CA

CATTCCCTTTGA  
Depth:3 (COW)  
Ei-value:0.000, Pi-value:0.000  
Er-value:0.000, Pr-value:0.000  
eCLIP MATCHES▶ILF3 (bg=3.0%)▶RBM15 (bg=7.27%)▶SRSF7 (bg=2.32%)▶ZNF622 (bg=6.58%)MATCHES To TargetScan▶ miR-1-3p/206:GGAAUGU


TTCCCTTTGA

TTCCCTTTGA  
Depth:6 (MOUSE)  
Ei-value:0.000, Pi-value:0.000  
Er-value:0.000, Pr-value:0.000  
eCLIP MATCHES▶ILF3 (bg=3.0%)▶RBM15 (bg=7.27%)▶SRSF7 (bg=2.32%)▶ZNF622 (bg=6.58%)No matches to TargetScan

CGA 11634  
 TCCC

TAGGTGGAGATGGGGCATGAGGATCCTCCAGGGGAA

TAGGTGGAGATGGGGCATGAGGATCCTCCAGGGGAA  
Depth:6 (MOUSE)  
Ei-value:0.000, Pi-value:0.000  
Er-value:0.000, Pr-value:0.000  
eCLIP MATCHES▶ILF3 (bg=3.0%)▶NOLC1 (bg=9.43%)▶RBM15 (bg=7.27%)▶SRSF7 (bg=2.32%)▶ZNF622 (bg=6.58%)MATCHES To TargetScan▶ miR-331-3p:CCCCUGG


A

TAGGTGGAGATGGGGCATGAGGATCCTCCAGGGGAAA  
Depth:5 (RABBIT)  
Ei-value:0.000, Pi-value:0.000  
Er-value:0.000, Pr-value:0.000  
eCLIP MATCHES▶ILF3 (bg=3.0%)▶NOLC1 (bg=9.43%)▶RBM15 (bg=7.27%)▶SRSF7 (bg=2.32%)▶ZNF622 (bg=6.58%)MATCHES To TargetScan▶ miR-331-3p:CCCCUGG

AGC

TCACTA

TCACTA  
Depth:5 (RABBIT)  
Ei-value:0.000, Pi-value:0.000  
Er-value:0.000, Pr-value:0.000  
eCLIP MATCHES▶ILF3 (bg=3.0%)No matches to TargetScan


CCACT

TCACTACCACT  
Depth:4 (DOG)  
Ei-value:0.000, Pi-value:0.000  
Er-value:0.000, Pr-value:0.000  
eCLIP MATCHES▶ILF3 (bg=3.0%)MATCHES To TargetScan▶ miR-140-5p:AGUGGUU▶ miR-142-3p.1:GUAGUGU


G

TCACTACCACTG  
Depth:3 (COW)  
Ei-value:0.000, Pi-value:0.000  
Er-value:0.000, Pr-value:0.000  
eCLIP MATCHES▶ILF3 (bg=3.0%)MATCHES To TargetScan▶ miR-140-5p:AGUGGUU▶ miR-142-3p.1:GUAGUGU

G

GCAACA

GCAACA  
Depth:6 (MOUSE)  
Ei-value:0.000, Pi-value:0.000  
Er-value:0.000, Pr-value:0.000  
eCLIP MATCHES▶ILF3 (bg=3.0%)No matches to TargetScan


AC

GCAACAAC  
Depth:5 (RABBIT)  
Ei-value:0.000, Pi-value:0.000  
Er-value:0.000, Pr-value:0.000  
eCLIP MATCHES▶ILF3 (bg=3.0%)No matches to TargetScan

CCTAGGTCAGGAGGTTCTGTCAAGATA

CTTTCCTGG

CTTTCCTGG  
Depth:3 (COW)  
Ei-value:0.000, Pi-value:0.000  
Er-value:0.000, Pr-value:0.000  
eCLIP MATCHES▶ILF3 (bg=3.0%)MATCHES To TargetScan▶ miR-665:CCAGGAG▶ miR-873-5p.1:CAGGAAC

TCCCAGATAGGAAGATAAA 11754  
 GTCTCAAAA

ACAACCACC

ACAACCACC  
Depth:5 (RABBIT)  
Ei-value:0.000, Pi-value:0.000  
Er-value:0.000, Pr-value:0.000  
eCLIP MATCHES▶PRPF8 (bg=0.26%)No matches to TargetScan


ACAC

ACAACCACCACAC  
Depth:4 (DOG)  
Ei-value:0.000, Pi-value:0.000  
Er-value:0.000, Pr-value:0.000  
eCLIP MATCHES▶PRPF8 (bg=0.26%)No matches to TargetScan

GTCAAG||CTCTTCA

TTGTTCC

TTGTTCC  
Depth:4 (DOG)  
Ei-value:0.000, Pi-value:0.000  
Er-value:0.000, Pr-value:0.000  
eCLIP MATCHES▶GRWD1 (bg=5.13%)▶SF3B4 (bg=0.05%)No matches to TargetScan

TATC

TG

TGCCAAATC  
Depth:3 (COW)  
Ei-value:0.000, Pi-value:0.000  
Er-value:0.000, Pr-value:0.000  
eCLIP MATCHES▶GRWD1 (bg=5.13%)▶NOLC1 (bg=9.43%)MATCHES To TargetScan▶ miR-182-5p:UUGGCAA▶ miR-96-5p/1271-5p:UUGGCAC


CCAAAT

CCAAAT  
Depth:6 (MOUSE)  
Ei-value:0.000, Pi-value:0.000  
Er-value:0.000, Pr-value:0.000  
eCLIP MATCHES▶GRWD1 (bg=5.13%)▶NOLC1 (bg=9.43%)No matches to TargetScan


C

CCAAATC  
Depth:5 (RABBIT)  
Ei-value:0.000, Pi-value:0.000  
Er-value:0.000, Pr-value:0.000  
eCLIP MATCHES▶GRWD1 (bg=5.13%)▶NOLC1 (bg=9.43%)No matches to TargetScan

ATTATACTTCCTACAAGCAGTGCAGAGAGCTGAGTCTTCAGCAGGTC

CAAGAAA

CAAGAAA  
Depth:5 (RABBIT)  
Ei-value:0.000, Pi-value:0.000  
Er-value:0.000, Pr-value:0.000  
eCLIP MATCHES▶GRWD1 (bg=5.13%)▶NOLC1 (bg=9.43%)▶uchl5 (bg=11.16%)▶ZNF622 (bg=6.58%)No matches to TargetScan


T

CAAGAAAT  
Depth:3 (COW)  
Ei-value:0.000, Pi-value:0.000  
Er-value:0.000, Pr-value:0.000  
eCLIP MATCHES▶GRWD1 (bg=5.13%)▶NOLC1 (bg=9.43%)▶TRA2A (bg=4.8%)▶uchl5 (bg=11.16%)▶ZNF622 (bg=6.58%)No matches to TargetScan

T

TGAACAC

TGAACACAC  
Depth:3 (COW)  
Ei-value:0.000, Pi-value:0.000  
Er-value:0.000, Pr-value:0.000  
eCLIP MATCHES▶GRWD1 (bg=5.13%)▶NOLC1 (bg=9.43%)▶PTBP1 (bg=3.74%)▶RBM15 (bg=7.27%)▶TRA2A (bg=4.8%)▶uchl5 (bg=11.16%)▶ZNF622 (bg=6.58%)No matches to TargetScan

 11872  


AC

TGAACACAC  
Depth:3 (COW)  
Ei-value:0.000, Pi-value:0.000  
Er-value:0.000, Pr-value:0.000  
eCLIP MATCHES▶GRWD1 (bg=5.13%)▶NOLC1 (bg=9.43%)▶PTBP1 (bg=3.74%)▶RBM15 (bg=7.27%)▶TRA2A (bg=4.8%)▶uchl5 (bg=11.16%)▶ZNF622 (bg=6.58%)No matches to TargetScan

TGAAGGAAGTCAGCCTTCCCACCT

G

GAAGATCAACATGCCTG  
Depth:4 (DOG)  
Ei-value:0.000, Pi-value:0.000  
Er-value:0.000, Pr-value:0.000  
eCLIP MATCHES▶GRWD1 (bg=5.13%)▶NOLC1 (bg=9.43%)▶PTBP1 (bg=3.74%)▶RBM15 (bg=7.27%)▶TRA2A (bg=4.8%)▶uchl5 (bg=11.16%)▶ZNF622 (bg=6.58%)No matches to TargetScan


AA

AAGATCAACATGC  
Depth:5 (RABBIT)  
Ei-value:0.000, Pi-value:0.000  
Er-value:0.000, Pr-value:0.000  
eCLIP MATCHES▶GRWD1 (bg=5.13%)▶NOLC1 (bg=9.43%)▶PTBP1 (bg=3.74%)▶RBM15 (bg=7.27%)▶TRA2A (bg=4.8%)▶uchl5 (bg=11.16%)▶ZNF622 (bg=6.58%)No matches to TargetScan


GATCAACATGC

GATCAACATGC  
Depth:6 (MOUSE)  
Ei-value:0.000, Pi-value:0.000  
Er-value:0.000, Pr-value:0.000  
eCLIP MATCHES▶GRWD1 (bg=5.13%)▶NOLC1 (bg=9.43%)▶PTBP1 (bg=3.74%)▶RBM15 (bg=7.27%)▶TRA2A (bg=4.8%)▶uchl5 (bg=11.16%)▶ZNF622 (bg=6.58%)No matches to TargetScan


CTG

GAAGATCAACATGCCTG  
Depth:4 (DOG)  
Ei-value:0.000, Pi-value:0.000  
Er-value:0.000, Pr-value:0.000  
eCLIP MATCHES▶GRWD1 (bg=5.13%)▶NOLC1 (bg=9.43%)▶PTBP1 (bg=3.74%)▶RBM15 (bg=7.27%)▶TRA2A (bg=4.8%)▶uchl5 (bg=11.16%)▶ZNF622 (bg=6.58%)No matches to TargetScan

GCACTCTAGCACTTGAGGATAGCTGAATGAA||

TGTGTAT

TGTGTAT  
Depth:6 (MOUSE)  
Ei-value:0.000, Pi-value:0.000  
Er-value:0.000, Pr-value:0.000  
eCLIP MATCHES▶TARDBP (bg=2.79%)▶ZC3H11A (bg=6.55%)No matches to TargetScan


TT

TGTGTATTT  
Depth:4 (DOG)  
Ei-value:0.000, Pi-value:0.000  
Er-value:0.000, Pr-value:0.000  
eCLIP MATCHES▶TARDBP (bg=2.79%)▶ZC3H11A (bg=6.55%)No matches to TargetScan

CTTTGTC

TCTTTCTT

TCTTTCTT  
Depth:3 (COW)  
Ei-value:0.000, Pi-value:0.000  
Er-value:0.000, Pr-value:0.000  
eCLIP MATCHES▶TARDBP (bg=2.79%)▶ZC3H11A (bg=6.55%)No matches to TargetScan

TCTTGTCTTTGCTCTTTGTT 11990  
 CTCTATCTAAAGTG

TGTCTTA

TGTCTTA  
Depth:4 (DOG)  
Ei-value:0.000, Pi-value:0.000  
Er-value:0.000, Pr-value:0.000  
eCLIP MATCHES▶MATR3 (bg=2.98%)▶PTBP1 (bg=3.74%)▶TARDBP (bg=2.79%)▶ZC3H11A (bg=6.55%)MATCHES To TargetScan▶ miR-208-3p:UAAGACG▶ miR-499a-5p:UAAGACU


CCCATTTCCATG

TGTCTTACCCATTTCCATG  
Depth:3 (COW)  
Ei-value:0.000, Pi-value:0.000  
Er-value:0.000, Pr-value:0.000  
eCLIP MATCHES▶MATR3 (bg=2.98%)▶PTBP1 (bg=3.74%)▶TARDBP (bg=2.79%)▶ZC3H11A (bg=6.55%)MATCHES To TargetScan▶ miR-203a-3p.1:GAAAUGU▶ miR-208-3p:UAAGACG▶ miR-499a-5p:UAAGACU

TTTCTCTTGCTAATTTCTTTCGTGTGTGCCTTTGCCTCATTTTCTC

TTTTTGT

TTTTTGT  
Depth:4 (DOG)  
Ei-value:0.000, Pi-value:0.000  
Er-value:0.000, Pr-value:0.000  
eCLIP MATCHES▶MATR3 (bg=2.98%)▶PTBP1 (bg=3.74%)▶TARDBP (bg=2.79%)▶TIA1 (bg=4.07%)▶ZC3H11A (bg=6.55%)No matches to TargetScan

TCACAAGAGTGGTCTGTGTCTTGTCTTAGACATA 12110  
 TCTCTCATTT

TTCATTTTGTT

TTCATTTTGTT  
Depth:4 (DOG)  
Ei-value:0.000, Pi-value:0.000  
Er-value:0.000, Pr-value:0.000  
No matches to eCLIP DataMATCHES To TargetScan▶ miR-495-3p:AACAAAC

GCTATTTCTC

TTTGCTC

TTTGCTC  
Depth:3 (COW)  
Ei-value:0.000, Pi-value:0.000  
Er-value:0.000, Pr-value:0.000  
eCLIP MATCHES▶MATR3 (bg=2.98%)▶PTBP1 (bg=3.74%)▶TIA1 (bg=4.07%)No matches to TargetScan

TCCTAGATGTGGCTCTTCTTTCACGCTTTATTTCATGTCTCCTTTTTGGGTCACATGCTGTGTGCTTTTTGTCCTTTTCTTG 12230  
 TTCTGTCTACCTCTCCTTTCTCTGCCTACCTCTC

TT

TTTTCTCTTTGTGAA  
Depth:3 (COW)  
Ei-value:0.000, Pi-value:0.000  
Er-value:0.000, Pr-value:0.000  
eCLIP MATCHES▶MATR3 (bg=2.98%)▶PTBP1 (bg=3.74%)▶SMNDC1 (bg=0.63%)▶TIA1 (bg=4.07%)No matches to TargetScan


TTCTCTTTG

TTCTCTTTG  
Depth:6 (MOUSE)  
Ei-value:0.000, Pi-value:0.000  
Er-value:0.000, Pr-value:0.000  
eCLIP MATCHES▶MATR3 (bg=2.98%)▶PTBP1 (bg=3.74%)▶SMNDC1 (bg=0.63%)▶TIA1 (bg=4.07%)No matches to TargetScan


TGAA

TTTTCTCTTTGTGAA  
Depth:3 (COW)  
Ei-value:0.000, Pi-value:0.000  
Er-value:0.000, Pr-value:0.000  
eCLIP MATCHES▶MATR3 (bg=2.98%)▶PTBP1 (bg=3.74%)▶SMNDC1 (bg=0.63%)▶TIA1 (bg=4.07%)No matches to TargetScan

CTGTGATTATTTGTTACCCC

TTCCCCTT

TTCCCCTT  
Depth:3 (COW)  
Ei-value:0.000, Pi-value:0.000  
Er-value:0.000, Pr-value:0.000  
eCLIP MATCHES▶MATR3 (bg=2.98%)▶PTBP1 (bg=3.74%)▶TIA1 (bg=4.07%)No matches to TargetScan

CTCGTTCGTTTTAA

ATTTCACCT

ATTTCACCT  
Depth:4 (DOG)  
Ei-value:0.000, Pi-value:0.000  
Er-value:0.000, Pr-value:0.000  
eCLIP MATCHES▶TIA1 (bg=4.07%)MATCHES To TargetScan▶ miR-203a-3p.2:UGAAAUG

TTTTTCTGAGTCTGGCCTCC 12350  
 TTTC

TGCTG

TGCTGTTTCTACT  
Depth:3 (COW)  
Ei-value:0.000, Pi-value:0.000  
Er-value:0.000, Pr-value:0.000  
eCLIP MATCHES▶MATR3 (bg=2.98%)▶PTBP1 (bg=3.74%)▶TIA1 (bg=4.07%)MATCHES To TargetScan▶ miR-411-5p.1:AGUAGAC▶ miR-494-3p:GAAACAU


TTTCTAC

TTTCTAC  
Depth:6 (MOUSE)  
Ei-value:0.000, Pi-value:0.000  
Er-value:0.000, Pr-value:0.000  
eCLIP MATCHES▶MATR3 (bg=2.98%)▶PTBP1 (bg=3.74%)▶TIA1 (bg=4.07%)No matches to TargetScan


T

TTTCTACT  
Depth:5 (RABBIT)  
Ei-value:0.000, Pi-value:0.000  
Er-value:0.000, Pr-value:0.000  
eCLIP MATCHES▶MATR3 (bg=2.98%)▶PTBP1 (bg=3.74%)▶TIA1 (bg=4.07%)MATCHES To TargetScan▶ miR-411-5p.1:AGUAGAC

TTTTATCTCAC

ATTTCTC

ATTTCTC  
Depth:6 (MOUSE)  
Ei-value:0.000, Pi-value:0.000  
Er-value:0.000, Pr-value:0.000  
eCLIP MATCHES▶MATR3 (bg=2.98%)▶PTBP1 (bg=3.74%)▶TIA1 (bg=4.07%)No matches to TargetScan

ATTTCTGCATTTCCTTTCTGCCTC

TCTTGGG

TCTTGGG  
Depth:5 (RABBIT)  
Ei-value:0.000, Pi-value:0.000  
Er-value:0.000, Pr-value:0.000  
eCLIP MATCHES▶MATR3 (bg=2.98%)▶PTBP1 (bg=3.74%)▶SMNDC1 (bg=0.63%)▶TIA1 (bg=4.07%)No matches to TargetScan


C

TCTTGGGC  
Depth:3 (COW)  
Ei-value:0.000, Pi-value:0.000  
Er-value:0.000, Pr-value:0.000  
eCLIP MATCHES▶MATR3 (bg=2.98%)▶PTBP1 (bg=3.74%)▶SMNDC1 (bg=0.63%)▶TIA1 (bg=4.07%)No matches to TargetScan

TATTCTCTCTCTCCTCCCCTGCGTGCCTCAGCATCTCTTGCTG

TTTGTGA

TTTGTGA  
Depth:4 (DOG)  
Ei-value:0.000, Pi-value:0.010  
Er-value:0.000, Pr-value:0.000  
eCLIP MATCHES▶MATR3 (bg=2.98%)▶PTBP1 (bg=3.74%)▶TIA1 (bg=4.07%)No matches to TargetScan


TTT

TTTGTGATTTTC  
Depth:3 (COW)  
Ei-value:0.000, Pi-value:0.000  
Er-value:0.000, Pr-value:0.000  
eCLIP MATCHES▶MATR3 (bg=2.98%)▶PTBP1 (bg=3.74%)▶TIA1 (bg=4.07%)No matches to TargetScan

 12470  


TC

TTTGTGATTTTC  
Depth:3 (COW)  
Ei-value:0.000, Pi-value:0.000  
Er-value:0.000, Pr-value:0.000  
eCLIP MATCHES▶MATR3 (bg=2.98%)▶PTBP1 (bg=3.74%)▶TIA1 (bg=4.07%)No matches to TargetScan

TATTTCAGTATTAA

TCTCTGTT

TCTCTGTT  
Depth:4 (DOG)  
Ei-value:0.000, Pi-value:0.000  
Er-value:0.000, Pr-value:0.000  
eCLIP MATCHES▶MATR3 (bg=2.98%)▶PTBP1 (bg=3.74%)No matches to TargetScan

GGCTTGTATTTGTTCTCTGCTTCTTCCCTTTCTACTCACC

TTTGAGTATTT

TTTGAGTATTT  
Depth:4 (DOG)  
Ei-value:0.000, Pi-value:0.000  
Er-value:0.000, Pr-value:0.000  
eCLIP MATCHES▶MATR3 (bg=2.98%)▶PTBP1 (bg=3.74%)▶TIA1 (bg=4.07%)MATCHES To TargetScan▶ miR-200bc-3p/429:AAUACUG▶ miR-371-5p:CUCAAAC

CAGCCTCTTCATGAATCTATCTCCCTCT

CTTTGATT

CTTTGATT  
Depth:3 (COW)  
Ei-value:0.000, Pi-value:0.000  
Er-value:0.000, Pr-value:0.000  
eCLIP MATCHES▶MATR3 (bg=2.98%)▶PTBP1 (bg=3.74%)▶TIA1 (bg=4.07%)No matches to TargetScan

TCATGTAAT 12590  
 CTCTCCTTAAATATTTCTTTGCATATGTGGGCAAGTGTACG

TGTGTGTG

TGTGTGTG  
Depth:4 (DOG)  
Ei-value:0.000, Pi-value:0.000  
Er-value:0.000, Pr-value:0.000  
eCLIP MATCHES▶AATF (bg=0.64%)▶DDX24 (bg=2.97%)▶NCBP2 (bg=1.49%)▶NOLC1 (bg=9.43%)▶PTBP1 (bg=3.74%)▶SND1 (bg=0.45%)▶SRSF7 (bg=2.32%)▶TARDBP (bg=2.79%)▶WDR43 (bg=3.37%)▶XRCC6 (bg=2.91%)▶ZC3H8 (bg=0.29%)MATCHES To TargetScan▶ miR-329-3p/362-3p:ACACACC

TGTCATGTGTGGCAGAGGGGCT

TCCTAACCCCT

TCCTAACCCCT  
Depth:5 (RABBIT)  
Ei-value:0.000, Pi-value:0.000  
Er-value:0.000, Pr-value:0.000  
eCLIP MATCHES▶AATF (bg=0.64%)▶DDX24 (bg=2.97%)▶NCBP2 (bg=1.49%)▶NOLC1 (bg=9.43%)▶PTBP1 (bg=3.74%)▶SND1 (bg=0.45%)▶SRSF7 (bg=2.32%)▶TARDBP (bg=2.79%)▶UTP3 (bg=3.66%)▶WDR43 (bg=3.37%)▶XRCC6 (bg=2.91%)▶ZC3H8 (bg=0.29%)No matches to TargetScan

GCCTGA

TAGGTGCA

TAGGTGCA  
Depth:3 (COW)  
Ei-value:0.000, Pi-value:0.000  
Er-value:0.000, Pr-value:0.000  
eCLIP MATCHES▶DDX24 (bg=2.97%)▶NOLC1 (bg=9.43%)▶SND1 (bg=0.45%)▶SRSF7 (bg=2.32%)▶TARDBP (bg=2.79%)▶UTP3 (bg=3.66%)▶WDR43 (bg=3.37%)▶XRCC6 (bg=2.91%)▶ZC3H8 (bg=0.29%)No matches to TargetScan

GAACGTCGGCTATCAGAGC

AAGCA

AAGCATTG  
Depth:4 (DOG)  
Ei-value:0.000, Pi-value:0.000  
Er-value:0.000, Pr-value:0.000  
eCLIP MATCHES▶DDX24 (bg=2.97%)▶NOLC1 (bg=9.43%)▶NPM1 (bg=1.21%)▶RBFOX2 (bg=4.63%)▶RPS3 (bg=0.76%)▶SRSF1 (bg=8.47%)▶SRSF7 (bg=2.32%)▶TARDBP (bg=2.79%)▶TRA2A (bg=4.8%)▶U2AF2 (bg=1.76%)▶uchl5 (bg=11.16%)▶YWHAG (bg=1.87%)▶ZNF622 (bg=6.58%)No matches to TargetScan

 12710  


TTG

AAGCATTG  
Depth:4 (DOG)  
Ei-value:0.000, Pi-value:0.000  
Er-value:0.000, Pr-value:0.000  
eCLIP MATCHES▶DDX24 (bg=2.97%)▶NOLC1 (bg=9.43%)▶NPM1 (bg=1.21%)▶RBFOX2 (bg=4.63%)▶RPS3 (bg=0.76%)▶SRSF1 (bg=8.47%)▶SRSF7 (bg=2.32%)▶TARDBP (bg=2.79%)▶TRA2A (bg=4.8%)▶U2AF2 (bg=1.76%)▶uchl5 (bg=11.16%)▶YWHAG (bg=1.87%)▶ZNF622 (bg=6.58%)No matches to TargetScan

TGGAGCGGTTCC

TTATGCCA

TTATGCCA  
Depth:5 (RABBIT)  
Ei-value:0.000, Pi-value:0.000  
Er-value:0.000, Pr-value:0.000  
eCLIP MATCHES▶DDX24 (bg=2.97%)▶FASTKD2 (bg=1.99%)▶LARP4 (bg=4.72%)▶NOLC1 (bg=9.43%)▶NPM1 (bg=1.21%)▶RBFOX2 (bg=4.63%)▶RBM15 (bg=7.27%)▶RPS3 (bg=0.76%)▶SRSF1 (bg=8.47%)▶SRSF7 (bg=2.32%)▶TARDBP (bg=2.79%)▶TRA2A (bg=4.8%)▶U2AF2 (bg=1.76%)▶uchl5 (bg=11.16%)▶WDR43 (bg=3.37%)▶YWHAG (bg=1.87%)▶ZC3H11A (bg=6.55%)▶ZNF622 (bg=6.58%)▶ZNF800 (bg=1.92%)No matches to TargetScan


G

TTATGCCAG  
Depth:4 (DOG)  
Ei-value:0.000, Pi-value:0.000  
Er-value:0.000, Pr-value:0.000  
eCLIP MATCHES▶DDX24 (bg=2.97%)▶FASTKD2 (bg=1.99%)▶LARP4 (bg=4.72%)▶NOLC1 (bg=9.43%)▶NPM1 (bg=1.21%)▶RBFOX2 (bg=4.63%)▶RBM15 (bg=7.27%)▶RPS3 (bg=0.76%)▶SRSF1 (bg=8.47%)▶SRSF7 (bg=2.32%)▶TARDBP (bg=2.79%)▶TRA2A (bg=4.8%)▶U2AF2 (bg=1.76%)▶uchl5 (bg=11.16%)▶WDR43 (bg=3.37%)▶YWHAG (bg=1.87%)▶ZC3H11A (bg=6.55%)▶ZNF622 (bg=6.58%)▶ZNF800 (bg=1.92%)No matches to TargetScan

GCTGCCATGTGAGATGA

TCCAAG

TCCAAG  
Depth:3 (COW)  
Ei-value:0.000, Pi-value:0.000  
Er-value:0.000, Pr-value:0.000  
eCLIP MATCHES▶DDX24 (bg=2.97%)▶FASTKD2 (bg=1.99%)▶LARP4 (bg=4.72%)▶NOLC1 (bg=9.43%)▶NPM1 (bg=1.21%)▶RBFOX2 (bg=4.63%)▶RBM15 (bg=7.27%)▶SRSF1 (bg=8.47%)▶SRSF7 (bg=2.32%)▶TARDBP (bg=2.79%)▶TRA2A (bg=4.8%)▶U2AF2 (bg=1.76%)▶uchl5 (bg=11.16%)▶WDR43 (bg=3.37%)▶YWHAG (bg=1.87%)▶ZC3H11A (bg=6.55%)▶ZNF622 (bg=6.58%)▶ZNF800 (bg=1.92%)No matches to TargetScan

ACCAAAACAAGGCCCTAGACTGCAGTAAAACCCAGAACTCAAGTAGGGCAGAAGGTGGAAGGCTCATATGGAT 12830  


AGA

AGAAGGCCCAA  
Depth:4 (DOG)  
Ei-value:0.000, Pi-value:0.000  
Er-value:0.000, Pr-value:0.000  
eCLIP MATCHES▶DDX24 (bg=2.97%)▶LARP4 (bg=4.72%)▶MTPAP (bg=2.21%)▶NOLC1 (bg=9.43%)▶SRSF1 (bg=8.47%)▶SRSF7 (bg=2.32%)▶TRA2A (bg=4.8%)▶uchl5 (bg=11.16%)▶UTP3 (bg=3.66%)▶ZNF622 (bg=6.58%)▶ZNF800 (bg=1.92%)No matches to TargetScan


AGGCCCAA

AGGCCCAA  
Depth:5 (RABBIT)  
Ei-value:0.000, Pi-value:0.000  
Er-value:0.000, Pr-value:0.000  
eCLIP MATCHES▶DDX24 (bg=2.97%)▶LARP4 (bg=4.72%)▶MTPAP (bg=2.21%)▶NOLC1 (bg=9.43%)▶SRSF1 (bg=8.47%)▶SRSF7 (bg=2.32%)▶TRA2A (bg=4.8%)▶uchl5 (bg=11.16%)▶UTP3 (bg=3.66%)▶ZNF622 (bg=6.58%)▶ZNF800 (bg=1.92%)No matches to TargetScan

AGTATAAGACAGATGGTTTGAGACTTGAGACCCGAGGACTAAGATGGAAAGCCCATGTTCCAAGATAGATAGAAGCCTCAGGCCTGAAACCAACAAAAGCCTCAAGAGC 12950  
 CAAGAAAACAGAGGGTGGCCTGAATTGGACCGAAGGCCTGAGTTGGATGGAAGTCTCAAGGCTTGAGTTAGAAGTCTTAAGACCTGGGACAGGACACATGGAAGGCCTAAGAACTGAGAC 13070  
 TTGTGACACAAGGCCAACGACCTAAGATTAGCCCAGGGTTGTAGCTGGAAGACCTACAACCCAAGGATGGAAGGCCCCTGTCACAAAGCCTACCTAGATGGATAGAGGACCCAAGCGAAA 13190  
 AAGGTATC

TCAA

TCAAGACTAA  
Depth:4 (DOG)  
Ei-value:0.000, Pi-value:0.000  
Er-value:0.000, Pr-value:0.000  
eCLIP MATCHES▶CPEB4 (bg=1.89%)▶FASTKD2 (bg=1.99%)▶GRWD1 (bg=5.13%)▶LARP4 (bg=4.72%)▶MTPAP (bg=2.21%)▶NOLC1 (bg=9.43%)▶RBFOX2 (bg=4.63%)▶SRSF1 (bg=8.47%)▶TRA2A (bg=4.8%)▶uchl5 (bg=11.16%)▶UTP18 (bg=0.72%)▶UTP3 (bg=3.66%)▶WDR43 (bg=3.37%)▶ZNF622 (bg=6.58%)MATCHES To TargetScan▶ miR-431-5p:GUCUUGC


GACTAA

GACTAA  
Depth:5 (RABBIT)  
Ei-value:0.000, Pi-value:0.000  
Er-value:0.000, Pr-value:0.000  
eCLIP MATCHES▶CPEB4 (bg=1.89%)▶FASTKD2 (bg=1.99%)▶GRWD1 (bg=5.13%)▶LARP4 (bg=4.72%)▶MTPAP (bg=2.21%)▶NOLC1 (bg=9.43%)▶RBFOX2 (bg=4.63%)▶SRSF1 (bg=8.47%)▶TRA2A (bg=4.8%)▶uchl5 (bg=11.16%)▶UTP18 (bg=0.72%)▶UTP3 (bg=3.66%)▶WDR43 (bg=3.37%)▶ZNF622 (bg=6.58%)No matches to TargetScan

CGGCCGGAATCTGGAGGCCCATGACCCAGAACCCAGGAAGGAT

AGAAGC

AGAAGC  
Depth:4 (DOG)  
Ei-value:0.000, Pi-value:0.000  
Er-value:0.000, Pr-value:0.010  
eCLIP MATCHES▶CPEB4 (bg=1.89%)▶GRWD1 (bg=5.13%)▶LARP4 (bg=4.72%)▶MTPAP (bg=2.21%)▶NOLC1 (bg=9.43%)▶PCBP1 (bg=1.07%)▶RBFOX2 (bg=4.63%)▶SRSF1 (bg=8.47%)▶TRA2A (bg=4.8%)▶uchl5 (bg=11.16%)▶ZNF622 (bg=6.58%)No matches to TargetScan

TTGAAGACCTGGGGAAATCC

C

CAAGATGA  
Depth:3 (COW)  
Ei-value:0.000, Pi-value:0.000  
Er-value:0.000, Pr-value:0.000  
eCLIP MATCHES▶CPEB4 (bg=1.89%)▶FTO (bg=0.32%)▶GRWD1 (bg=5.13%)▶LARP4 (bg=4.72%)▶MTPAP (bg=2.21%)▶SRSF1 (bg=8.47%)▶TRA2A (bg=4.8%)▶uchl5 (bg=11.16%)▶ZNF622 (bg=6.58%)No matches to TargetScan


AAGATGA

AAGATGA  
Depth:5 (RABBIT)  
Ei-value:0.000, Pi-value:0.000  
Er-value:0.000, Pr-value:0.000  
eCLIP MATCHES▶CPEB4 (bg=1.89%)▶FTO (bg=0.32%)▶GRWD1 (bg=5.13%)▶LARP4 (bg=4.72%)▶MTPAP (bg=2.21%)▶SRSF1 (bg=8.47%)▶TRA2A (bg=4.8%)▶uchl5 (bg=11.16%)▶ZNF622 (bg=6.58%)No matches to TargetScan

GAACCCTAAACCCTACCTCT

TTTCT

TTTCTATTG  
Depth:3 (COW)  
Ei-value:0.000, Pi-value:0.000  
Er-value:0.000, Pr-value:0.000  
No matches to eCLIP DataNo matches to TargetScan

 13310  


ATTG

TTTCTATTG  
Depth:3 (COW)  
Ei-value:0.000, Pi-value:0.000  
Er-value:0.000, Pr-value:0.000  
No matches to eCLIP DataNo matches to TargetScan

TTTAC

ACTTCTT

ACTTCTT  
Depth:3 (COW)  
Ei-value:0.000, Pi-value:0.020  
Er-value:0.000, Pr-value:0.000  
eCLIP MATCHES▶NOLC1 (bg=9.43%)No matches to TargetScan

ACTCTTAGATATTTCCAGTTCTCCTGTTTATCTTTAAGCCTGATTCTTTTGAGATGTA

CTTTTTGATGTT

CTTTTTGATGTT  
Depth:4 (DOG)  
Ei-value:0.000, Pi-value:0.000  
Er-value:0.000, Pr-value:0.000  
eCLIP MATCHES▶TIA1 (bg=4.07%)No matches to TargetScan

GCCGGTTACCTTTAGATTGACAG

TATTATGC

TATTATGC  
Depth:4 (DOG)  
Ei-value:0.000, Pi-value:0.000  
Er-value:0.000, Pr-value:0.000  
No matches to eCLIP DataMATCHES To TargetScan▶ miR-369-3p:AUAAUAC

CTG 13430  
 GGCCAGTCTTGAGCCAGCTTTAAATCACAGCTTTTACCTATTTGTTAGGCTATAGTGTTTTG

TAAACTTC

TAAACTTC  
Depth:3 (COW)  
Ei-value:0.000, Pi-value:0.000  
Er-value:0.000, Pr-value:0.000  
eCLIP MATCHES▶NIPBL (bg=5.39%)▶NOLC1 (bg=9.43%)▶ZC3H11A (bg=6.55%)No matches to TargetScan

TGTTTCTATTCACATCTT

CTCCACTTGAGAG

CTCCACTTGAGAG  
Depth:3 (COW)  
Ei-value:0.000, Pi-value:0.000  
Er-value:0.000, Pr-value:0.000  
eCLIP MATCHES▶NIPBL (bg=5.39%)▶NOLC1 (bg=9.43%)▶ZC3H11A (bg=6.55%)MATCHES To TargetScan▶ miR-26-5p:UCAAGUA

AGACACCAAAATCCAGTCA 13550  
 GTATCTAATCTGGCTTTTGTTAACTTCCCTCAGGAGCAGACATTCATATAGGTGATACTG

TATTTCAGT

TATTTCAGT  
Depth:4 (DOG)  
Ei-value:0.000, Pi-value:0.000  
Er-value:0.000, Pr-value:0.000  
eCLIP MATCHES▶NOLC1 (bg=9.43%)▶ZC3H11A (bg=6.55%)MATCHES To TargetScan▶ miR-203a-3p.2:UGAAAUG


CC

TATTTCAGTCC  
Depth:3 (COW)  
Ei-value:0.000, Pi-value:0.000  
Er-value:0.000, Pr-value:0.000  
eCLIP MATCHES▶NOLC1 (bg=9.43%)▶ZC3H11A (bg=6.55%)MATCHES To TargetScan▶ miR-203a-3p.2:UGAAAUG

TTTCTTTTGACCCCAGAAGCCCTAGACTGAGAAGATAAAATGGTCAGGT 13670  
 TGTT

GGGGAAA

GGGGAAA  
Depth:4 (DOG)  
Ei-value:0.000, Pi-value:0.000  
Er-value:0.000, Pr-value:0.000  
eCLIP MATCHES▶CPSF6 (bg=0.4%)▶LARP4 (bg=4.72%)▶WDR43 (bg=3.37%)▶ZC3H11A (bg=6.55%)No matches to TargetScan

AAAAAGTGCCAGGCTC

TCTAGAGAAAA

TCTAGAGAAAA  
Depth:6 (MOUSE)  
Ei-value:0.000, Pi-value:0.000  
Er-value:0.000, Pr-value:0.000  
eCLIP MATCHES▶CPSF6 (bg=0.4%)▶LARP4 (bg=4.72%)▶UTP3 (bg=3.66%)▶WDR43 (bg=3.37%)MATCHES To TargetScan▶ miR-1251-5p:CUCUAGC

ATG

TGAAGAGATG

TGAAGAGATG  
Depth:5 (RABBIT)  
Ei-value:0.000, Pi-value:0.000  
Er-value:0.000, Pr-value:0.000  
eCLIP MATCHES▶CPSF6 (bg=0.4%)▶LARP4 (bg=4.72%)▶SRSF7 (bg=2.32%)▶UTP3 (bg=3.66%)▶WDR43 (bg=3.37%)No matches to TargetScan


CTCCA

TGAAGAGATGCTCCA  
Depth:3 (COW)  
Ei-value:0.000, Pi-value:0.000  
Er-value:0.000, Pr-value:0.000  
eCLIP MATCHES▶CPSF6 (bg=0.4%)▶LARP4 (bg=4.72%)▶SRSF7 (bg=2.32%)▶UTP3 (bg=3.66%)▶WDR43 (bg=3.37%)No matches to TargetScan


GGCCAA

GGCCAATGAGAAGAATTAGACA  
Depth:4 (DOG)  
Ei-value:0.000, Pi-value:0.000  
Er-value:0.000, Pr-value:0.000  
eCLIP MATCHES▶LARP4 (bg=4.72%)▶NOLC1 (bg=9.43%)▶SRSF7 (bg=2.32%)▶UTP3 (bg=3.66%)No matches to TargetScan


TGAGAAGAATTAGACA

TGAGAAGAATTAGACA  
Depth:6 (MOUSE)  
Ei-value:0.000, Pi-value:0.000  
Er-value:0.000, Pr-value:0.000  
eCLIP MATCHES▶LARP4 (bg=4.72%)▶NOLC1 (bg=9.43%)▶SRSF7 (bg=2.32%)No matches to TargetScan

A

GAAATACACAGATG

GAAATACACAGATG  
Depth:3 (COW)  
Ei-value:0.000, Pi-value:0.000  
Er-value:0.000, Pr-value:0.000  
eCLIP MATCHES▶LARP4 (bg=4.72%)▶NOLC1 (bg=9.43%)▶SRSF7 (bg=2.32%)No matches to TargetScan

TGCCAGACTT

C

CTGAGAAG  
Depth:3 (COW)  
Ei-value:0.000, Pi-value:0.000  
Er-value:0.000, Pr-value:0.000  
eCLIP MATCHES▶AARS (bg=2.18%)▶NOLC1 (bg=9.43%)▶PUS1 (bg=1.04%)▶SRSF7 (bg=2.32%)▶ZC3H11A (bg=6.55%)No matches to TargetScan


TGAGAAG

TGAGAAG  
Depth:4 (DOG)  
Ei-value:0.000, Pi-value:0.000  
Er-value:0.000, Pr-value:0.010  
eCLIP MATCHES▶AARS (bg=2.18%)▶NOLC1 (bg=9.43%)▶PUS1 (bg=1.04%)▶SRSF7 (bg=2.32%)▶ZC3H11A (bg=6.55%)No matches to TargetScan

CACCT

GCCA

GCCAGCAACA  
Depth:3 (COW)  
Ei-value:0.000, Pi-value:0.000  
Er-value:0.000, Pr-value:0.000  
eCLIP MATCHES▶AARS (bg=2.18%)▶NOLC1 (bg=9.43%)▶PUS1 (bg=1.04%)▶SRSF7 (bg=2.32%)▶ZC3H11A (bg=6.55%)No matches to TargetScan

 13790  


GCCAGCAACA  
Depth:3 (COW)  
Ei-value:0.000, Pi-value:0.000  
Er-value:0.000, Pr-value:0.000  
eCLIP MATCHES▶AARS (bg=2.18%)▶NOLC1 (bg=9.43%)▶PUS1 (bg=1.04%)▶SRSF7 (bg=2.32%)▶ZC3H11A (bg=6.55%)No matches to TargetScan


GCAACA

GCAACA  
Depth:6 (MOUSE)  
Ei-value:0.000, Pi-value:0.000  
Er-value:0.000, Pr-value:0.000  
eCLIP MATCHES▶AARS (bg=2.18%)▶NOLC1 (bg=9.43%)▶PUS1 (bg=1.04%)▶ZC3H11A (bg=6.55%)No matches to TargetScan

GCTTCCTTC

TTTGAGCTT

TTTGAGCTT  
Depth:3 (COW)  
Ei-value:0.000, Pi-value:0.000  
Er-value:0.000, Pr-value:0.000  
eCLIP MATCHES▶AARS (bg=2.18%)▶NOLC1 (bg=9.43%)▶PUS1 (bg=1.04%)▶ZC3H11A (bg=6.55%)MATCHES To TargetScan▶ miR-371-5p:CUCAAAC

A

GGTGAGC

GGTGAGC  
Depth:4 (DOG)  
Ei-value:0.000, Pi-value:0.000  
Er-value:0.000, Pr-value:0.000  
eCLIP MATCHES▶AARS (bg=2.18%)▶NOLC1 (bg=9.43%)▶PUS1 (bg=1.04%)▶ZC3H11A (bg=6.55%)No matches to TargetScan


AGGAT

GGTGAGCAGGAT  
Depth:3 (COW)  
Ei-value:0.000, Pi-value:0.000  
Er-value:0.000, Pr-value:0.000  
eCLIP MATCHES▶AARS (bg=2.18%)▶AKAP8L (bg=2.19%)▶NOLC1 (bg=9.43%)▶PUS1 (bg=1.04%)▶ZC3H11A (bg=6.55%)No matches to TargetScan

TCTGG

GGTTTGGG

GGTTTGGG  
Depth:4 (DOG)  
Ei-value:0.000, Pi-value:0.000  
Er-value:0.000, Pr-value:0.000  
eCLIP MATCHES▶AARS (bg=2.18%)▶AKAP8L (bg=2.19%)▶NOLC1 (bg=9.43%)▶PUS1 (bg=1.04%)No matches to TargetScan

ATTTCTAGTGA

TGGTTA

TGGTTA  
Depth:5 (RABBIT)  
Ei-value:0.000, Pi-value:0.000  
Er-value:0.000, Pr-value:0.000  
eCLIP MATCHES▶AKAP8L (bg=2.19%)▶NOLC1 (bg=9.43%)▶PUS1 (bg=1.04%)▶SF3B1 (bg=2.48%)No matches to TargetScan


T

TGGTTAT  
Depth:4 (DOG)  
Ei-value:0.000, Pi-value:0.000  
Er-value:0.000, Pr-value:0.000  
eCLIP MATCHES▶AKAP8L (bg=2.19%)▶NOLC1 (bg=9.43%)▶PUS1 (bg=1.04%)▶SF3B1 (bg=2.48%)No matches to TargetScan


G

TGGTTATG  
Depth:3 (COW)  
Ei-value:0.000, Pi-value:0.000  
Er-value:0.000, Pr-value:0.000  
eCLIP MATCHES▶AKAP8L (bg=2.19%)▶NOLC1 (bg=9.43%)▶PUS1 (bg=1.04%)▶SF3B1 (bg=2.48%)No matches to TargetScan

GAAAGGGTGACTGTGCCTGGGACAAAGCGAGGT

CCCAAGG

CCCAAGG  
Depth:4 (DOG)  
Ei-value:0.000, Pi-value:0.000  
Er-value:0.000, Pr-value:0.000  
eCLIP MATCHES▶PUS1 (bg=1.04%)▶UTP3 (bg=3.66%)MATCHES To TargetScan▶ miR-212-5p:CCUUGGC

GGACAGCC

TGA

TGAACTCCCTGCT  
Depth:4 (DOG)  
Ei-value:0.000, Pi-value:0.000  
Er-value:0.000, Pr-value:0.000  
eCLIP MATCHES▶UTP3 (bg=3.66%)No matches to TargetScan

 13910  


ACTCCCTGCT

TGAACTCCCTGCT  
Depth:4 (DOG)  
Ei-value:0.000, Pi-value:0.000  
Er-value:0.000, Pr-value:0.000  
eCLIP MATCHES▶UTP3 (bg=3.66%)No matches to TargetScan


C

TGAACTCCCTGCTCATAGTAGTGGCC  
Depth:3 (COW)  
Ei-value:0.000, Pi-value:0.000  
Er-value:0.000, Pr-value:0.000  
eCLIP MATCHES▶UTP3 (bg=3.66%)No matches to TargetScan


ATAGTAGTGGCC

ATAGTAGTGGCC  
Depth:4 (DOG)  
Ei-value:0.000, Pi-value:0.000  
Er-value:0.000, Pr-value:0.000  
No matches to eCLIP DataNo matches to TargetScan

AAATAATTTGGTGGACTGTGCCAACGCTACTCCTGGG

TTTAATAC

TTTAATAC  
Depth:4 (DOG)  
Ei-value:0.000, Pi-value:0.000  
Er-value:0.000, Pr-value:0.000  
eCLIP MATCHES▶WRN (bg=0.77%)MATCHES To TargetScan▶ miR-496.2:GUAUUAC

CCATCT

CT

CTAGGCTTAAAG  
Depth:4 (DOG)  
Ei-value:0.000, Pi-value:0.000  
Er-value:0.000, Pr-value:0.000  
No matches to eCLIP DataNo matches to TargetScan


AGGCTTA

AGGCTTA  
Depth:5 (RABBIT)  
Ei-value:0.000, Pi-value:0.000  
Er-value:0.000, Pr-value:0.000  
No matches to eCLIP DataNo matches to TargetScan


AAG

CTAGGCTTAAAG  
Depth:4 (DOG)  
Ei-value:0.000, Pi-value:0.000  
Er-value:0.000, Pr-value:0.000  
No matches to eCLIP DataNo matches to TargetScan

ATGAGAGAACCTGGGACTGTTGAGCAT

GTTTAAT

GTTTAAT  
Depth:5 (RABBIT)  
Ei-value:0.000, Pi-value:0.000  
Er-value:0.000, Pr-value:0.000  
No matches to eCLIP DataNo matches to TargetScan

 14030  


GTTTAAT  
Depth:5 (RABBIT)  
Ei-value:0.000, Pi-value:0.000  
Er-value:0.000, Pr-value:0.000  
No matches to eCLIP DataNo matches to TargetScan

ACTTTCCTTGATTTTTTTCTTCCTGTTTATGTGGGAAGTTGATTTAAATGACTGATAATGTGTATGAAAGCAC

TGTAAAACA

TGTAAAACA  
Depth:3 (COW)  
Ei-value:0.000, Pi-value:0.000  
Er-value:0.000, Pr-value:0.000  
eCLIP MATCHES▶TARDBP (bg=2.79%)▶WDR43 (bg=3.37%)No matches to TargetScan

TAAGAGAAAAACCAATTAGTG

T

TATTGGCA  
Depth:5 (RABBIT)  
Ei-value:0.000, Pi-value:0.000  
Er-value:0.000, Pr-value:0.000  
eCLIP MATCHES▶HNRNPA1 (bg=2.57%)No matches to TargetScan


ATTGGCA

ATTGGCA  
Depth:6 (MOUSE)  
Ei-value:0.000, Pi-value:0.000  
Er-value:0.000, Pr-value:0.000  
eCLIP MATCHES▶HNRNPA1 (bg=2.57%)No matches to TargetScan

ATCATGCAG 14150  
 TTAACATTTGAAAGTGCAGTGTAAA

TTGTGAAG

TTGTGAAG  
Depth:6 (MOUSE)  
Ei-value:0.000, Pi-value:0.000  
Er-value:0.000, Pr-value:0.000  
eCLIP MATCHES▶HNRNPA1 (bg=2.57%)No matches to TargetScan

CAT

T

TATGTAAATCA  
Depth:3 (COW)  
Ei-value:0.000, Pi-value:0.000  
Er-value:0.000, Pr-value:0.000  
No matches to eCLIP DataNo matches to TargetScan


ATGTAAAT

ATGTAAAT  
Depth:5 (RABBIT)  
Ei-value:0.000, Pi-value:0.000  
Er-value:0.000, Pr-value:0.000  
No matches to eCLIP DataNo matches to TargetScan


CA

TATGTAAATCA  
Depth:3 (COW)  
Ei-value:0.000, Pi-value:0.000  
Er-value:0.000, Pr-value:0.000  
No matches to eCLIP DataNo matches to TargetScan

GGGGTCCACAGTTTTTCTGTAAGGGGTCAAATCATAAATACTTTAGACTGTGG

GCCATATGGT

GCCATATGGT  
Depth:3 (COW)  
Ei-value:0.000, Pi-value:0.000  
Er-value:0.000, Pr-value:0.000  
No matches to eCLIP DataNo matches to TargetScan

TTCTGTTACA 14270  
 TATTTGTTTTTTAAACAACGTTTTTATAAGGTCAAAATCATTCTTAGTTTTTGAGCCAATTGGATTTGGCCTGCTGTTCATAGCTTACCACCCCCTGATGTATTATTTGTTATTCAGAGA 14390  
 AAATTTCTGAATACTACTAGTTTCCTTTTC

TGTGC

TGTGCCTGTCCCTGT  
Depth:3 (COW)  
Ei-value:0.000, Pi-value:0.000  
Er-value:0.000, Pr-value:0.000  
No matches to eCLIP DataNo matches to TargetScan


CTGTCCCT

CTGTCCCT  
Depth:4 (DOG)  
Ei-value:0.000, Pi-value:0.000  
Er-value:0.000, Pr-value:0.000  
No matches to eCLIP DataNo matches to TargetScan


GT

TGTGCCTGTCCCTGT  
Depth:3 (COW)  
Ei-value:0.000, Pi-value:0.000  
Er-value:0.000, Pr-value:0.000  
No matches to eCLIP DataNo matches to TargetScan

GC

TAGGCACT

TAGGCACT  
Depth:4 (DOG)  
Ei-value:0.000, Pi-value:0.000  
Er-value:0.000, Pr-value:0.000  
No matches to eCLIP DataNo matches to TargetScan

AAAAATGCAATGATTATTGATATCTAGGTGACCTGAAAAAAAATAGTGAATGTGCTTTGTAAACT 14510  
 G

TAAAGCA

TAAAGCA  
Depth:4 (DOG)  
Ei-value:0.000, Pi-value:0.000  
Er-value:0.000, Pr-value:0.000  
eCLIP MATCHES▶LIN28B (bg=0.74%)No matches to TargetScan

CTTGTATTCTACTGTGATAAGCGTTGTGGATACAAAGAAAGGAGCAAGCATAAAAAAGTGCTCTTTCAAAAGGATATAGTACTATGCAGACACAAGGAATTGTTTGATAAAT 14630  
 GAATAAATTATATGTATATTTGAGGCCAATTTGTGTTTGCTGCTCTGGTAATTTTGAGTAAAAATGCAGTATTCCAGGTATCAGAAACGAAAACACATGGAAACTGCTTTTAAACTTTAA 14750  
 AATATACTGAAAACATAAGGGACTAAGCTTGTTGTGGTCACC

TAT

TATAATGTGCCAGATA  
Depth:3 (COW)  
Ei-value:0.000, Pi-value:0.000  
Er-value:0.000, Pr-value:0.000  
No matches to eCLIP DataMATCHES To TargetScan▶ miR-183-5p.2:UGGCACU▶ miR-323-3p:ACAUUAC


AATGTGCCAGATA

AATGTGCCAGATA  
Depth:4 (DOG)  
Ei-value:0.000, Pi-value:0.000  
Er-value:0.000, Pr-value:0.000  
No matches to eCLIP DataMATCHES To TargetScan▶ miR-183-5p.2:UGGCACU

CCATGCTGGGTGCTAGAGCTACCAAAGGGGGAAAAGTATTCTCATAGAACAAAAAATTTCAG 14870  
 AAAGGTGCATA

TTAAAGTG

TTAAAGTG  
Depth:4 (DOG)  
Ei-value:0.000, Pi-value:0.000  
Er-value:0.000, Pr-value:0.000  
eCLIP MATCHES▶SF3B1 (bg=2.48%)No matches to TargetScan


CTTTGTA

TTAAAGTGCTTTGTA  
Depth:3 (COW)  
Ei-value:0.000, Pi-value:0.000  
Er-value:0.000, Pr-value:0.000  
eCLIP MATCHES▶SF3B1 (bg=2.48%)MATCHES To TargetScan▶ miR-330-3p:CAAAGCA▶ miR-330-3p.2:AAAGCAC

AA

CTAAAGCA

CTAAAGCA  
Depth:4 (DOG)  
Ei-value:0.000, Pi-value:0.000  
Er-value:0.000, Pr-value:0.000  
eCLIP MATCHES▶SF3B1 (bg=2.48%)No matches to TargetScan

TGATACAAATGT

CAATGGGCTA

CAATGGGCTA  
Depth:3 (COW)  
Ei-value:0.000, Pi-value:0.000  
Er-value:0.000, Pr-value:0.000  
No matches to eCLIP DataNo matches to TargetScan

CATATTTATGAATGAATGAATGGAT

GA

GAATGAATA  
Depth:3 (COW)  
Ei-value:0.000, Pi-value:0.000  
Er-value:0.000, Pr-value:0.000  
eCLIP MATCHES▶DROSHA (bg=2.49%)▶TARDBP (bg=2.79%)▶ZC3H11A (bg=6.55%)MATCHES To TargetScan▶ miR-1298-5p:UCAUUCG


ATGAATA

ATGAATA  
Depth:4 (DOG)  
Ei-value:0.000, Pi-value:0.000  
Er-value:0.000, Pr-value:0.000  
eCLIP MATCHES▶DROSHA (bg=2.49%)▶TARDBP (bg=2.79%)▶ZC3H11A (bg=6.55%)No matches to TargetScan

TTAAGTGCCTCTTACATA

CCAGCTATT

CCAGCTATT  
Depth:3 (COW)  
Ei-value:0.000, Pi-value:0.000  
Er-value:0.000, Pr-value:0.000  
eCLIP MATCHES▶AARS (bg=2.18%)▶DROSHA (bg=2.49%)▶ILF3 (bg=3.0%)▶TARDBP (bg=2.79%)▶ZC3H11A (bg=6.55%)No matches to TargetScan

T 14990  
 TG

GGTACTGT

GGTACTGT  
Depth:4 (DOG)  
Ei-value:0.000, Pi-value:0.000  
Er-value:0.000, Pr-value:0.000  
eCLIP MATCHES▶AARS (bg=2.18%)▶DROSHA (bg=2.49%)▶ILF3 (bg=3.0%)▶TARDBP (bg=2.79%)▶ZC3H11A (bg=6.55%)MATCHES To TargetScan▶ miR-101-3p.1:ACAGUAC▶ miR-144-3p:ACAGUAU

AAAATACAAGATTAATTCTCCTATGTA

ATAAGAGG

ATAAGAGG  
Depth:4 (DOG)  
Ei-value:0.000, Pi-value:0.000  
Er-value:0.000, Pr-value:0.000  
eCLIP MATCHES▶ILF3 (bg=3.0%)No matches to TargetScan

AAAGTTTATCCTCTATACTATTCAGATGTAAGGAATGATATATTGCTTAATTTTAAACAATC

AAGACTTTAC

AAGACTTTAC  
Depth:3 (COW)  
Ei-value:0.000, Pi-value:0.000  
Er-value:0.000, Pr-value:0.000  
No matches to eCLIP DataNo matches to TargetScan

TGG 15110  
 TGAGGTTAAGT

TAAATTAT

TAAATTAT  
Depth:4 (DOG)  
Ei-value:0.000, Pi-value:0.010  
Er-value:0.000, Pr-value:0.000  
No matches to eCLIP DataNo matches to TargetScan


TAC

TAAATTATTAC  
Depth:3 (COW)  
Ei-value:0.000, Pi-value:0.000  
Er-value:0.000, Pr-value:0.000  
No matches to eCLIP DataNo matches to TargetScan

TGATACATTTTTCC

AGGTAA

AGGTAA  
Depth:3 (COW)  
Ei-value:0.000, Pi-value:0.000  
Er-value:0.000, Pr-value:0.000  
No matches to eCLIP DataNo matches to TargetScan

CCAGGAAAGAGCTAGTATGAGGAAATGAAGTAATAGATGTGAGATCCAGACCGAAAGTCACTTAATTCAGCTTGCGAA 15230  
 TGTGC

TTTCTAA

TTTCTAA  
Depth:3 (COW)  
Ei-value:0.000, Pi-value:0.000  
Er-value:0.000, Pr-value:0.010  
No matches to eCLIP DataNo matches to TargetScan

ATTATAAAGCACTTGTAAATGAAAAATTTGATGCTTTCTGTATGA

ATAAAAC

ATAAAAC  
Depth:4 (DOG)  
Ei-value:0.000, Pi-value:0.010  
Er-value:0.000, Pr-value:0.000  
No matches to eCLIP DataNo matches to TargetScan

TTTCTGTAAGCTAGGTATTGTCTCTAC

AAAATTCTCA

AAAATTCTCA  
Depth:4 (DOG)  
Ei-value:0.000, Pi-value:0.000  
Er-value:0.000, Pr-value:0.000  
eCLIP MATCHES▶HNRNPU (bg=5.92%)No matches to TargetScan

TTGTATAGTTAAACCACAG 15350  
 TGAGAAGGGTTCTATAAGTAGT

TATACAAAC

TATACAAAC  
Depth:4 (DOG)  
Ei-value:0.000, Pi-value:0.000  
Er-value:0.000, Pr-value:0.000  
No matches to eCLIP DataNo matches to TargetScan

CAAGG

GTTTAAATAC

GTTTAAATAC  
Depth:3 (COW)  
Ei-value:0.000, Pi-value:0.000  
Er-value:0.000, Pr-value:0.000  
No matches to eCLIP DataNo matches to TargetScan

CTGTTAAATAGATCAATTTTGA

TTGCCTACTAT

TTGCCTACTATGTGAACTCACTGTTA  
Depth:3 (COW)  
Ei-value:0.000, Pi-value:0.000  
Er-value:0.000, Pr-value:0.000  
No matches to eCLIP DataMATCHES To TargetScan▶ miR-132-3p/212-3p:AACAGUC▶ miR-23-3p:UCACAUU▶ miR-376c-3p:ACAUAGA▶ miR-411-5p.2:UAGUAGA


GTGAACTCA

GTGAACTCA  
Depth:4 (DOG)  
Ei-value:0.000, Pi-value:0.000  
Er-value:0.000, Pr-value:0.000  
No matches to eCLIP DataNo matches to TargetScan


CTGTTA

TTGCCTACTATGTGAACTCACTGTTA  
Depth:3 (COW)  
Ei-value:0.000, Pi-value:0.000  
Er-value:0.000, Pr-value:0.000  
No matches to eCLIP DataMATCHES To TargetScan▶ miR-132-3p/212-3p:AACAGUC▶ miR-23-3p:UCACAUU▶ miR-376c-3p:ACAUAGA▶ miR-411-5p.2:UAGUAGA

AAGGCACTGAAA

ATTTATCAT

ATTTATCAT  
Depth:3 (COW)  
Ei-value:0.000, Pi-value:0.000  
Er-value:0.000, Pr-value:0.000  
No matches to eCLIP DataNo matches to TargetScan

ATTTC 15470  
 ATTTAGCCACAGCCAAAAATAAGGCAATACCTATGTTAGC

ATTTTGTGAACTCTAA

ATTTTGTGAACTCTAA  
Depth:3 (COW)  
Ei-value:0.000, Pi-value:0.000  
Er-value:0.000, Pr-value:0.000  
No matches to eCLIP DataNo matches to TargetScan

GGCACCATATAAATGTAACTGTTGATTTTCTCACTTGGTGCTGGGTACTAGGTTTAT

AAAATTG

AAAATTG  
Depth:3 (COW)  
Ei-value:0.000, Pi-value:0.000  
Er-value:0.000, Pr-value:0.000  
No matches to eCLIP DataNo matches to TargetScan

 15590  


AAAATTG  
Depth:3 (COW)  
Ei-value:0.000, Pi-value:0.000  
Er-value:0.000, Pr-value:0.000  
No matches to eCLIP DataNo matches to TargetScan

TATGATAGTTATTATATTGTGCAAATAAAGTAGGAAAATTTGAATAACAATGATTATCTTTTGAATACGCATACGCAAGGGATTGGTTGTCTGAAGAATGCCACTATAGTAGTTATCTAT 15710  
 TG

TGTGCCA

TGTGCCA  
Depth:4 (DOG)  
Ei-value:0.000, Pi-value:0.000  
Er-value:0.000, Pr-value:0.000  
No matches to eCLIP DataMATCHES To TargetScan▶ miR-183-5p.2:UGGCACU

ATCTCATTGCTAGGCATTGGGGATGCA

AAGATAA

AAGATAA  
Depth:4 (DOG)  
Ei-value:0.000, Pi-value:0.000  
Er-value:0.000, Pr-value:0.000  
No matches to eCLIP DataNo matches to TargetScan

ACCATCTTTATTGTGTCTTGGGT

AGCAGAA

AGCAGAA  
Depth:3 (COW)  
Ei-value:0.000, Pi-value:0.000  
Er-value:0.000, Pr-value:0.000  
eCLIP MATCHES▶UTP3 (bg=3.66%)No matches to TargetScan

GAAAATATGTG

TAAAATCAATTT

TAAAATCAATTT  
Depth:3 (COW)  
Ei-value:0.000, Pi-value:0.000  
Er-value:0.000, Pr-value:0.000  
eCLIP MATCHES▶UTP3 (bg=3.66%)No matches to TargetScan

ATAATTTG

TAAACTG

TAAACTG  
Depth:4 (DOG)  
Ei-value:0.000, Pi-value:0.000  
Er-value:0.000, Pr-value:0.000  
eCLIP MATCHES▶HNRNPU (bg=5.92%)No matches to TargetScan

CCACCCATA 15830  
 TATAAGCTATA

TCTGCTGAATGA

TCTGCTGAATGA  
Depth:3 (COW)  
Ei-value:0.000, Pi-value:0.000  
Er-value:0.000, Pr-value:0.000  
No matches to eCLIP DataMATCHES To TargetScan▶ miR-1298-5p:UCAUUCG

T

C

CATTGATTA  
Depth:3 (COW)  
Ei-value:0.000, Pi-value:0.000  
Er-value:0.000, Pr-value:0.000  
No matches to eCLIP DataNo matches to TargetScan


ATTGATTA

ATTGATTA  
Depth:4 (DOG)  
Ei-value:0.000, Pi-value:0.000  
Er-value:0.000, Pr-value:0.010  
No matches to eCLIP DataNo matches to TargetScan

CTCTTATCCTT

AGAGATA

AGAGATA  
Depth:4 (DOG)  
Ei-value:0.000, Pi-value:0.000  
Er-value:0.000, Pr-value:0.000  
No matches to eCLIP DataNo matches to TargetScan

ACAACTGGGGGCACAAACATTTATTATCATTAT

TGAACCT

TGAACCT  
Depth:3 (COW)  
Ei-value:0.000, Pi-value:0.000  
Er-value:0.000, Pr-value:0.010  
eCLIP MATCHES▶HNRNPU (bg=5.92%)No matches to TargetScan

AC

AA

AACAGAGATCT  
Depth:3 (COW)  
Ei-value:0.000, Pi-value:0.000  
Er-value:0.000, Pr-value:0.000  
eCLIP MATCHES▶HNRNPA1 (bg=2.57%)▶HNRNPU (bg=5.92%)No matches to TargetScan


CAGAGATCT

CAGAGATCT  
Depth:4 (DOG)  
Ei-value:0.000, Pi-value:0.000  
Er-value:0.000, Pr-value:0.000  
eCLIP MATCHES▶HNRNPA1 (bg=2.57%)▶HNRNPU (bg=5.92%)No matches to TargetScan

ATGTGTAGA

TTTACAA

TTTACAAAGC  
Depth:3 (COW)  
Ei-value:0.000, Pi-value:0.000  
Er-value:0.000, Pr-value:0.000  
eCLIP MATCHES▶HNRNPA1 (bg=2.57%)▶HNRNPU (bg=5.92%)No matches to TargetScan

 15950  


AGC

TTTACAAAGC  
Depth:3 (COW)  
Ei-value:0.000, Pi-value:0.000  
Er-value:0.000, Pr-value:0.000  
eCLIP MATCHES▶HNRNPA1 (bg=2.57%)▶HNRNPU (bg=5.92%)No matches to TargetScan

CTACAGT

TCTATACA

TCTATACA  
Depth:3 (COW)  
Ei-value:0.000, Pi-value:0.000  
Er-value:0.000, Pr-value:0.000  
eCLIP MATCHES▶HNRNPA1 (bg=2.57%)▶HNRNPU (bg=5.92%)No matches to TargetScan

GATAGGAATGAACTA

TTGGCT

TTGGCT  
Depth:4 (DOG)  
Ei-value:0.000, Pi-value:0.000  
Er-value:0.000, Pr-value:0.000  
eCLIP MATCHES▶HNRNPA1 (bg=2.57%)No matches to TargetScan

TACTGAATGGTGA

TTACTTTCT

TTACTTTCT  
Depth:4 (DOG)  
Ei-value:0.000, Pi-value:0.000  
Er-value:0.000, Pr-value:0.010  
eCLIP MATCHES▶UTP3 (bg=3.66%)No matches to TargetScan

GTGGGGCTCGGAACTACATGCC

CTAGGATAT

CTAGGATAT  
Depth:3 (COW)  
Ei-value:0.000, Pi-value:0.000  
Er-value:0.000, Pr-value:0.000  
No matches to eCLIP DataNo matches to TargetScan

A

AAAATGA

AAAATGA  
Depth:3 (COW)  
Ei-value:0.000, Pi-value:0.000  
Er-value:0.000, Pr-value:0.000  
No matches to eCLIP DataNo matches to TargetScan

TGTTATCATTATAGAGTGCT 16070  
 CACAGAAGGAAATGAAGTAATATAGGTGTGAG

ATCCAGACCA

ATCCAGACCA  
Depth:3 (COW)  
Ei-value:0.000, Pi-value:0.000  
Er-value:0.000, Pr-value:0.000  
eCLIP MATCHES▶KHDRBS1 (bg=1.71%)No matches to TargetScan

AAAGTCATTTAACAAGTTTATTCAGTGATGAAAACATGGGACAAATGGACTAATATAAGGCAGTGTACTAAGCTGAGT 16190  
 AGAGAGATAAAGTCCTGTCCAGAAGATACATGCTTCCTGGCCTGATTGAGG

AGATGGA

AGATGGA  
Depth:3 (COW)  
Ei-value:0.000, Pi-value:0.000  
Er-value:0.000, Pr-value:0.000  
No matches to eCLIP DataNo matches to TargetScan

AAATTTTTGCAAAAAACAAGGTGTTGTGGTCTTCCATCCAGTTTCTTAAGTGCTGATGATAA 16310  
 AAGTGAATTAGACCCACCTTGACCTGGCCTACAGAAGTAAAG

GAGTAAAAA

GAGTAAAAA  
Depth:4 (DOG)  
Ei-value:0.000, Pi-value:0.000  
Er-value:0.000, Pr-value:0.000  
No matches to eCLIP DataNo matches to TargetScan

TAAATGCCTCAGGCGTGCTTTTTGATTC

ATTTGAT

ATTTGAT  
Depth:4 (DOG)  
Ei-value:0.000, Pi-value:0.010  
Er-value:0.000, Pr-value:0.000  
No matches to eCLIP DataNo matches to TargetScan

AAACAAAGC

ATC

ATCTTTTATGT  
Depth:3 (COW)  
Ei-value:0.000, Pi-value:0.000  
Er-value:0.000, Pr-value:0.000  
eCLIP MATCHES▶SAFB (bg=2.69%)No matches to TargetScan


TTTTATGT

TTTTATGT  
Depth:4 (DOG)  
Ei-value:0.000, Pi-value:0.000  
Er-value:0.000, Pr-value:0.000  
eCLIP MATCHES▶SAFB (bg=2.69%)No matches to TargetScan

GGAATATACCATTC 16430  
 TG

GGTCCTGAG

GGTCCTGAG  
Depth:3 (COW)  
Ei-value:0.000, Pi-value:0.000  
Er-value:0.000, Pr-value:0.000  
eCLIP MATCHES▶SAFB (bg=2.69%)No matches to TargetScan

GATAAGAGAGATGAGGGCATTAGATCACTGACAGCTGAAGATAGAAGAACATCTTTGGTTTGATTGTTTAAATAATATTTCAATGCCTATTCTCTGCAAGGTACTATGT 16550  
 TTCGTAAATTAAATAGGTCTGGCCCAGAAGACCCACTCAA

TTGCCTT

TTGCCTT  
Depth:3 (COW)  
Ei-value:0.000, Pi-value:0.000  
Er-value:0.000, Pr-value:0.000  
eCLIP MATCHES▶KHDRBS1 (bg=1.71%)▶UTP18 (bg=0.72%)MATCHES To TargetScan▶ miR-124-3p.1:AAGGCAC

TGAGATTAAAAAAAAAAAAAAAAAGAAAGAAAAATGCAAGTTTCTTTCAAAATAAAGAGACATTTTTCCTAGT 16670  
 TTCAGGAATCCCCCAAATCACTTCCTCATTGGCTTAGTTTA

AAGCCAG

AAGCCAG  
Depth:4 (DOG)  
Ei-value:0.000, Pi-value:0.000  
Er-value:0.000, Pr-value:0.000  
eCLIP MATCHES▶SAFB (bg=2.69%)MATCHES To TargetScan▶ miR-149-5p:CUGGCUC▶ miR-3064-5p:CUGGCUG

GAGACTG

ATAAAAG

ATAAAAG  
Depth:4 (DOG)  
Ei-value:0.000, Pi-value:0.000  
Er-value:0.000, Pr-value:0.000  
No matches to eCLIP DataNo matches to TargetScan

GGCTCAGGGTTTGTT

CTTTAATTC

CTTTAATTC  
Depth:3 (COW)  
Ei-value:0.000, Pi-value:0.000  
Er-value:0.000, Pr-value:0.000  
No matches to eCLIP DataNo matches to TargetScan

ATTAACTAAACATTCTGC

TTTTATTA

TTTTATTA  
Depth:4 (DOG)  
Ei-value:0.000, Pi-value:0.010  
Er-value:0.000, Pr-value:0.000  
No matches to eCLIP DataNo matches to TargetScan

CA

G

GTTAAATGG  
Depth:3 (COW)  
Ei-value:0.000, Pi-value:0.000  
Er-value:0.000, Pr-value:0.000  
No matches to eCLIP DataNo matches to TargetScan


TTAAA

TTAAATGG  
Depth:4 (DOG)  
Ei-value:0.000, Pi-value:0.000  
Er-value:0.000, Pr-value:0.000  
No matches to eCLIP DataNo matches to TargetScan

 16790  


TGG

TTAAATGG  
Depth:4 (DOG)  
Ei-value:0.000, Pi-value:0.000  
Er-value:0.000, Pr-value:0.000  
No matches to eCLIP DataNo matches to TargetScan

TTCAAGATGTAACAACTAGTTTTAAAGGTATTTGCTCATTGGTCTGGCTTAGAGACAGGAAGACATATGAGCAATAAAAAAAAGATTCTTTTGCATTTACCAATTTAGTAAAAATTT 16910  
 ATTAAAACTGAATAAAGTG

CTGTTCTTAAGT

CTGTTCTTAAGT  
Depth:3 (COW)  
Ei-value:0.000, Pi-value:0.000  
Er-value:0.000, Pr-value:0.000  
eCLIP MATCHES▶KHDRBS1 (bg=1.71%)No matches to TargetScan

GCTTGAAAGACGTAAACCAAAGTGCACTTTATCTCATTTATCTTATGGTGGAAACACAG

GAACAAATT

GAACAAATT  
Depth:3 (COW)  
Ei-value:0.000, Pi-value:0.000  
Er-value:0.000, Pr-value:0.000  
No matches to eCLIP DataMATCHES To TargetScan▶ miR-375:UUGUUCG

CTCTAAGAGACTGTGTTTCT

T

TTAGTTG  
Depth:3 (COW)  
Ei-value:0.000, Pi-value:0.000  
Er-value:0.000, Pr-value:0.010  
No matches to eCLIP DataNo matches to TargetScan

 17030  


TAGTTG

TTAGTTG  
Depth:3 (COW)  
Ei-value:0.000, Pi-value:0.000  
Er-value:0.000, Pr-value:0.010  
No matches to eCLIP DataNo matches to TargetScan

AGAAG

AAACTTCATTGA

AAACTTCATTGA  
Depth:3 (COW)  
Ei-value:0.000, Pi-value:0.000  
Er-value:0.000, Pr-value:0.000  
eCLIP MATCHES▶HNRNPA1 (bg=2.57%)No matches to TargetScan

GTAGCTGTGATATGTTCGATACTAAGGAAAAACTAAACAGATCACCTTTGACATGCGTTGTAGAGTGGGAATAAGAGAGGGCTTTTTATTTTTTCGT 17150  
 TCATACGAGTATTGATGAAGATGATACTAAATGCTAAATGAAATATATCTGCTCCAAAAGGCATTTATTCTGACTTGGAGATGCAACAAAAACACAAAAATGGAATGAAGTGATACTCTT 17270  
 CATCAAACAGAAGTGACTGTTATCTCAACCATTTTGTTAAATCCTAA

ACAGAAAACAAAA

ACAGAAAACAAAA  
Depth:4 (DOG)  
Ei-value:0.000, Pi-value:0.000  
Er-value:0.000, Pr-value:0.000  
No matches to eCLIP DataNo matches to TargetScan

AAAATCATGACGAAAAGACACTTGCTTATTAATTGG

CTTGGAAA

CTTGGAAA  
Depth:3 (COW)  
Ei-value:0.000, Pi-value:0.000  
Er-value:0.000, Pr-value:0.000  
No matches to eCLIP DataNo matches to TargetScan

GTAGAATATAGGAGAA 17390  


AGGTTA

AGGTTA  
Depth:4 (DOG)  
Ei-value:0.000, Pi-value:0.000  
Er-value:0.000, Pr-value:0.000  
No matches to eCLIP DataNo matches to TargetScan

CTGTTTATTTTTTTTCATGTATTCA

TTCATTCT

TTCATTCT  
Depth:4 (DOG)  
Ei-value:0.000, Pi-value:0.000  
Er-value:0.000, Pr-value:0.000  
No matches to eCLIP DataNo matches to TargetScan

ACAAATATATTCGGGTGCCAATAGGTACTTGGTATAAGGTTTTTGGCCCC

AGAGACA

AGAGACA  
Depth:4 (DOG)  
Ei-value:0.000, Pi-value:0.000  
Er-value:0.000, Pr-value:0.000  
No matches to eCLIP DataNo matches to TargetScan


TG

AGAGACATG  
Depth:3 (COW)  
Ei-value:0.000, Pi-value:0.000  
Er-value:0.000, Pr-value:0.000  
No matches to eCLIP DataNo matches to TargetScan

GGAAAAAAATGCATGCCTTCCC 17510  
 AGAGAATGCCTAATACTTT

CCTTTTGG

CCTTTTGG  
Depth:4 (DOG)  
Ei-value:0.000, Pi-value:0.000  
Er-value:0.000, Pr-value:0.000  
No matches to eCLIP DataNo matches to TargetScan


C

CCTTTTGGC  
Depth:3 (COW)  
Ei-value:0.000, Pi-value:0.000  
Er-value:0.000, Pr-value:0.000  
No matches to eCLIP DataNo matches to TargetScan

TTGTTTTCTTGTTAGGGGCATGGCTTAGTCCCTAAATAAC

ATTGTGT

ATTGTGT  
Depth:3 (COW)  
Ei-value:0.000, Pi-value:0.000  
Er-value:0.000, Pr-value:0.010  
No matches to eCLIP DataNo matches to TargetScan

GGT

TTAATTC

TTAATTC  
Depth:4 (DOG)  
Ei-value:0.000, Pi-value:0.000  
Er-value:0.000, Pr-value:0.000  
No matches to eCLIP DataNo matches to TargetScan

CTACTCCGTATCTCTTCTACC

ACTCTGGCCACTAC

ACTCTGGCCACTAC  
Depth:4 (DOG)  
Ei-value:0.000, Pi-value:0.000  
Er-value:0.000, Pr-value:0.000  
No matches to eCLIP DataMATCHES To TargetScan▶ miR-142-3p.1:GUAGUGU

 17630  


ACTCTGGCCACTAC  
Depth:4 (DOG)  
Ei-value:0.000, Pi-value:0.000  
Er-value:0.000, Pr-value:0.000  
No matches to eCLIP DataMATCHES To TargetScan▶ miR-142-3p.1:GUAGUGU

G

ATAAGC

ATAAGC  
Depth:5 (RABBIT)  
Ei-value:0.000, Pi-value:0.010  
Er-value:0.000, Pr-value:0.000  
No matches to eCLIP DataNo matches to TargetScan


AGG

ATAAGCAGG  
Depth:4 (DOG)  
Ei-value:0.000, Pi-value:0.000  
Er-value:0.000, Pr-value:0.000  
No matches to eCLIP DataNo matches to TargetScan

TAGCTGGGTTTTGTAGTGAGCT

TGCTCCTT

TGCTCCTT  
Depth:3 (COW)  
Ei-value:0.000, Pi-value:0.000  
Er-value:0.000, Pr-value:0.000  
No matches to eCLIP DataMATCHES To TargetScan▶ miR-28-5p/708-5p:AGGAGCU

AAGTTACAGGAACTCTCCTTATAATAGAC

ACTTCA

ACTTCA  
Depth:3 (COW)  
Ei-value:0.000, Pi-value:0.000  
Er-value:0.000, Pr-value:0.000  
No matches to eCLIP DataNo matches to TargetScan


TTTTCCTA

TTTTCCTA  
Depth:3 (COW)  
Ei-value:0.000, Pi-value:0.000  
Er-value:0.000, Pr-value:0.000  
No matches to eCLIP DataNo matches to TargetScan

GTCCATCCCTC

AT

ATGAAAAATG  
Depth:3 (COW)  
Ei-value:0.000, Pi-value:0.000  
Er-value:0.000, Pr-value:0.000  
No matches to eCLIP DataNo matches to TargetScan


GAAAAATG

GAAAAATG  
Depth:4 (DOG)  
Ei-value:0.000, Pi-value:0.000  
Er-value:0.000, Pr-value:0.000  
No matches to eCLIP DataNo matches to TargetScan

ACTGACCACTGCTGGG 17750  
 CAGCAGGAGGGATGATGACCAACTAATTCCCAAACCCC

AGTCTCA

AGTCTCA  
Depth:4 (DOG)  
Ei-value:0.000, Pi-value:0.000  
Er-value:0.000, Pr-value:0.000  
eCLIP MATCHES▶ZC3H11A (bg=6.55%)No matches to TargetScan


TTGGTACCA

AGTCTCATTGGTACCA  
Depth:3 (COW)  
Ei-value:0.000, Pi-value:0.000  
Er-value:0.000, Pr-value:0.000  
eCLIP MATCHES▶NOLC1 (bg=9.43%)▶ZC3H11A (bg=6.55%)No matches to TargetScan

GCCTTGGGGAACCACCTACACTTGAGCCACAAT

TGGTTTTGAA

TGGTTTTGAA  
Depth:4 (DOG)  
Ei-value:0.000, Pi-value:0.000  
Er-value:0.000, Pr-value:0.000  
No matches to eCLIP DataNo matches to TargetScan

GTGCATTTACAAGGTTTGTCTAT 17870  
 TTTCAGTTCTTTACTTTTTACATGCTGACACATACATACACTGCCTAAATAGATCTCTTTCAGAA

ACAATCC

ACAATCC  
Depth:3 (COW)  
Ei-value:0.000, Pi-value:0.000  
Er-value:0.000, Pr-value:0.000  
No matches to eCLIP DataMATCHES To TargetScan▶ miR-219-5p:GAUUGUC

TCAGATAACGCATAGCAAAA

TGGAGATG

TGGAGATG  
Depth:3 (COW)  
Ei-value:0.000, Pi-value:0.000  
Er-value:0.000, Pr-value:0.000  
No matches to eCLIP DataNo matches to TargetScan

GAGACATGATTTCTCATGCA 17990  
 AC

AGCTTCTC

AGCTTCTC  
Depth:3 (COW)  
Ei-value:0.000, Pi-value:0.000  
Er-value:0.000, Pr-value:0.000  
No matches to eCLIP DataNo matches to TargetScan

TAATTATACC

TTAGAAAT

TTAGAAAT  
Depth:4 (DOG)  
Ei-value:0.000, Pi-value:0.000  
Er-value:0.000, Pr-value:0.000  
eCLIP MATCHES▶WDR3 (bg=0.25%)No matches to TargetScan

GTTCTCCTTTTTAT

CATCAAA

CATCAAA  
Depth:4 (DOG)  
Ei-value:0.000, Pi-value:0.000  
Er-value:0.000, Pr-value:0.000  
eCLIP MATCHES▶ZC3H11A (bg=6.55%)No matches to TargetScan

TCTGCTCAAGAAGGGCTTTTTATAGTAGAATAATATCAGTGGATGAAAACAGCTTAACATTTTACCATGCT 18110  
 TAAGTTTTAAGAATAAAATAAAAATTGGAAATAATTGGCCAAAATTGAAA

GGAAAAA

GGAAAAA  
Depth:3 (COW)  
Ei-value:0.000, Pi-value:0.000  
Er-value:0.000, Pr-value:0.000  
No matches to eCLIP DataNo matches to TargetScan

TTTTTTTAAAATTTCTCTAAATGTAGGCCTGGC

TGGGCTTTG

TGGGCTTTG  
Depth:3 (COW)  
Ei-value:0.000, Pi-value:0.000  
Er-value:0.000, Pr-value:0.000  
eCLIP MATCHES▶NOLC1 (bg=9.43%)▶PPIL4 (bg=0.52%)MATCHES To TargetScan▶ miR-330-3p:CAAAGCA

ACCTTTTCCG

TTTTTAAATCA

TTTTTAAATCACTCA  
Depth:4 (DOG)  
Ei-value:0.000, Pi-value:0.000  
Er-value:0.000, Pr-value:0.000  
eCLIP MATCHES▶ILF3 (bg=3.0%)▶NOLC1 (bg=9.43%)▶PPIL4 (bg=0.52%)No matches to TargetScan

 18230  


CTCA

TTTTTAAATCACTCA  
Depth:4 (DOG)  
Ei-value:0.000, Pi-value:0.000  
Er-value:0.000, Pr-value:0.000  
eCLIP MATCHES▶ILF3 (bg=3.0%)▶NOLC1 (bg=9.43%)▶PPIL4 (bg=0.52%)No matches to TargetScan

C

AGAGGGTGGGA

AGAGGGTGGGA  
Depth:4 (DOG)  
Ei-value:0.000, Pi-value:0.000  
Er-value:0.000, Pr-value:0.000  
eCLIP MATCHES▶ILF3 (bg=3.0%)▶ZC3H11A (bg=6.55%)No matches to TargetScan

C

AGGAGGAAGAGTGAA

AGGAGGAAGAGTGAA  
Depth:4 (DOG)  
Ei-value:0.000, Pi-value:0.000  
Er-value:0.000, Pr-value:0.000  
eCLIP MATCHES▶ILF3 (bg=3.0%)▶ZC3H11A (bg=6.55%)MATCHES To TargetScan▶ miR-670-3p:UUCCUCA

G

G

GAAAAGGTCA  
Depth:4 (DOG)  
Ei-value:0.000, Pi-value:0.000  
Er-value:0.000, Pr-value:0.000  
eCLIP MATCHES▶ILF3 (bg=3.0%)▶SF3B1 (bg=2.48%)▶ZC3H11A (bg=6.55%)MATCHES To TargetScan▶ miR-192-5p/215-5p:UGACCUA


AAAAGGT

AAAAGGT  
Depth:6 (MOUSE)  
Ei-value:0.000, Pi-value:0.000  
Er-value:0.000, Pr-value:0.000  
eCLIP MATCHES▶ILF3 (bg=3.0%)▶SF3B1 (bg=2.48%)▶ZC3H11A (bg=6.55%)No matches to TargetScan


CA

GAAAAGGTCA  
Depth:4 (DOG)  
Ei-value:0.000, Pi-value:0.000  
Er-value:0.000, Pr-value:0.000  
eCLIP MATCHES▶ILF3 (bg=3.0%)▶SF3B1 (bg=2.48%)▶ZC3H11A (bg=6.55%)MATCHES To TargetScan▶ miR-192-5p/215-5p:UGACCUA

AACCTGTTTTAAGGGCAACCTGCCTTTGTTCTGAA

TTGGTCTTAA

TTGGTCTTAA  
Depth:3 (COW)  
Ei-value:0.000, Pi-value:0.000  
Er-value:0.000, Pr-value:0.000  
eCLIP MATCHES▶ILF3 (bg=3.0%)▶ZC3H11A (bg=6.55%)MATCHES To TargetScan▶ miR-208-3p:UAAGACG▶ miR-499a-5p:UAAGACU

GAACATTACCAGCTCCAGGTTTAAATTGTTCA 18350  
 GTTTCATGCAGTTCCAATAGCTGATCATTGTTG

AGATGAGGACAAA

AGATGAGGACAAA  
Depth:3 (COW)  
Ei-value:0.000, Pi-value:0.000  
Er-value:0.000, Pr-value:0.000  
eCLIP MATCHES▶HNRNPA1 (bg=2.57%)No matches to TargetScan

A

TCCTTTGT

TCCTTTGT  
Depth:3 (COW)  
Ei-value:0.000, Pi-value:0.000  
Er-value:0.000, Pr-value:0.000  
eCLIP MATCHES▶HNRNPA1 (bg=2.57%)No matches to TargetScan

CCTCACTAGTTTGCTTTACATTTTTGAAAAGTATTATTTTTGTCCAAGTGCTTATCAACTAAACC 18470  
 TTGTGTTAGGTAAGAATGGAATTTATTAAGTGAATCAGTGTGACCCTTCTTGTCATAAGATTATCT

TAAAGC

TAAAGC  
Depth:3 (COW)  
Ei-value:0.000, Pi-value:0.000  
Er-value:0.000, Pr-value:0.000  
eCLIP MATCHES▶NOLC1 (bg=9.43%)No matches to TargetScan

TGAAGCCAAAATATGCTTCAAAAGAAGAGGACTTTATTGTTCATTGTA 18590  
 G

TTCATACA

TTCATACA  
Depth:3 (COW)  
Ei-value:0.000, Pi-value:0.000  
Er-value:0.000, Pr-value:0.000  
No matches to eCLIP DataNo matches to TargetScan


TTCAAAGCATC

TTCAAAGCATC  
Depth:3 (COW)  
Ei-value:0.000, Pi-value:0.000  
Er-value:0.000, Pr-value:0.000  
No matches to eCLIP DataNo matches to TargetScan

TGAACTGTAGTTTCTATAGCAAGCCAATTACATCCATAAGTGG

AGAAGGAAATAGA

AGAAGGAAATAGA  
Depth:3 (COW)  
Ei-value:0.000, Pi-value:0.000  
Er-value:0.000, Pr-value:0.000  
eCLIP MATCHES▶ZC3H11A (bg=6.55%)No matches to TargetScan

TAAATGTCAAAGTATGATTGG

TGGAGGGAGC

TGGAGGGAGC  
Depth:3 (COW)  
Ei-value:0.000, Pi-value:0.000  
Er-value:0.000, Pr-value:0.000  
eCLIP MATCHES▶FTO (bg=0.32%)▶LARP4 (bg=4.72%)▶LSM11 (bg=2.28%)▶NOLC1 (bg=9.43%)▶XRCC6 (bg=2.91%)▶ZC3H11A (bg=6.55%)No matches to TargetScan

AAGGTTGAAGATA 18710  
 ATCTGGGGTTGAAATTTTCTAGTTTTCATTCTGTACATTTTTAGTTAGACATCAGATTTGAAATAT

TAATGTTT

TAATGTTT  
Depth:4 (DOG)  
Ei-value:0.000, Pi-value:0.000  
Er-value:0.000, Pr-value:0.000  
eCLIP MATCHES▶CPEB4 (bg=1.89%)▶KHDRBS1 (bg=1.71%)▶LARP4 (bg=4.72%)▶LSM11 (bg=2.28%)▶NOLC1 (bg=9.43%)▶RBFOX2 (bg=4.63%)▶SAFB (bg=2.69%)▶SAFB2 (bg=0.8%)▶WDR43 (bg=3.37%)▶ZC3H11A (bg=6.55%)MATCHES To TargetScan▶ miR-323-3p:ACAUUAC▶ miR-543:AACAUUC

ACCTTTCAATGTGTGGTATC

AGCTGGA

AGCTGGA  
Depth:4 (DOG)  
Ei-value:0.000, Pi-value:0.000  
Er-value:0.000, Pr-value:0.000  
eCLIP MATCHES▶CPEB4 (bg=1.89%)▶KHDRBS1 (bg=1.71%)▶LSM11 (bg=2.28%)▶NOLC1 (bg=9.43%)▶RBFOX2 (bg=4.63%)▶SAFB (bg=2.69%)▶SAFB2 (bg=0.8%)▶SF3B1 (bg=2.48%)▶TRA2A (bg=4.8%)▶WDR43 (bg=3.37%)▶ZC3H11A (bg=6.55%)No matches to TargetScan

CTCAGTAACACCCCTTTCT 18830  
 TCAGCTGGGGATGGGGAATGG

ATTATTGGAAA

ATTATTGGAAA  
Depth:4 (DOG)  
Ei-value:0.000, Pi-value:0.000  
Er-value:0.000, Pr-value:0.000  
eCLIP MATCHES▶FASTKD2 (bg=1.99%)▶FUS (bg=2.21%)▶LARP4 (bg=4.72%)▶NOLC1 (bg=9.43%)▶RBFOX2 (bg=4.63%)▶SAFB (bg=2.69%)▶SAFB2 (bg=0.8%)▶TRA2A (bg=4.8%)▶WDR43 (bg=3.37%)▶ZC3H11A (bg=6.55%)No matches to TargetScan

ATGGAAAGA

AGAAAGTAAC

AGAAAGTAAC  
Depth:4 (DOG)  
Ei-value:0.000, Pi-value:0.000  
Er-value:0.000, Pr-value:0.000  
eCLIP MATCHES▶FASTKD2 (bg=1.99%)▶FUS (bg=2.21%)▶LARP4 (bg=4.72%)▶NIPBL (bg=5.39%)▶NOLC1 (bg=9.43%)▶RBFOX2 (bg=4.63%)▶SAFB (bg=2.69%)▶SAFB2 (bg=0.8%)▶TRA2A (bg=4.8%)▶uchl5 (bg=11.16%)▶WDR43 (bg=3.37%)▶ZC3H11A (bg=6.55%)▶ZNF800 (bg=1.92%)No matches to TargetScan

TAAAAGCCTTCC

TTTCACAGTTTCTGGCATC

TTTCACAGTTTCTGGCATC  
Depth:4 (DOG)  
Ei-value:0.000, Pi-value:0.000  
Er-value:0.000, Pr-value:0.000  
eCLIP MATCHES▶FASTKD2 (bg=1.99%)▶FUS (bg=2.21%)▶LARP4 (bg=4.72%)▶NIPBL (bg=5.39%)▶NOLC1 (bg=9.43%)▶RBFOX2 (bg=4.63%)▶SAFB (bg=2.69%)▶SAFB2 (bg=0.8%)▶uchl5 (bg=11.16%)▶WDR43 (bg=3.37%)▶ZC3H11A (bg=6.55%)▶ZNF800 (bg=1.92%)No matches to TargetScan

ACTAC

CA

CACTACTGAT  
Depth:3 (COW)  
Ei-value:0.000, Pi-value:0.000  
Er-value:0.000, Pr-value:0.000  
eCLIP MATCHES▶FASTKD2 (bg=1.99%)▶FUS (bg=2.21%)▶LARP4 (bg=4.72%)▶NIPBL (bg=5.39%)▶NOLC1 (bg=9.43%)▶RBFOX2 (bg=4.63%)▶SAFB (bg=2.69%)▶SAFB2 (bg=0.8%)▶uchl5 (bg=11.16%)▶ZC3H11A (bg=6.55%)▶ZNF800 (bg=1.92%)MATCHES To TargetScan▶ miR-142-3p.1:GUAGUGU▶ miR-199-3p:CAGUAGU


CTACTGAT

CTACTGAT  
Depth:4 (DOG)  
Ei-value:0.000, Pi-value:0.000  
Er-value:0.000, Pr-value:0.000  
eCLIP MATCHES▶FASTKD2 (bg=1.99%)▶FUS (bg=2.21%)▶LARP4 (bg=4.72%)▶NIPBL (bg=5.39%)▶NOLC1 (bg=9.43%)▶RBFOX2 (bg=4.63%)▶SAFB (bg=2.69%)▶SAFB2 (bg=0.8%)▶uchl5 (bg=11.16%)▶ZC3H11A (bg=6.55%)▶ZNF800 (bg=1.92%)MATCHES To TargetScan▶ miR-199-3p:CAGUAGU

T

AAACAAGAATAA

AAACAAGAATAA  
Depth:3 (COW)  
Ei-value:0.000, Pi-value:0.000  
Er-value:0.000, Pr-value:0.000  
eCLIP MATCHES▶FASTKD2 (bg=1.99%)▶FUS (bg=2.21%)▶LARP4 (bg=4.72%)▶NIPBL (bg=5.39%)▶NOLC1 (bg=9.43%)▶RBFOX2 (bg=4.63%)▶SAFB2 (bg=0.8%)▶uchl5 (bg=11.16%)▶ZC3H11A (bg=6.55%)MATCHES To TargetScan▶ miR-544a-5p:CUUGUUA

G

AGAACAT

AGAACAT  
Depth:4 (DOG)  
Ei-value:0.000, Pi-value:0.000  
Er-value:0.000, Pr-value:0.000  
eCLIP MATCHES▶FASTKD2 (bg=1.99%)▶FUS (bg=2.21%)▶LARP4 (bg=4.72%)▶NIPBL (bg=5.39%)▶NOLC1 (bg=9.43%)▶RBFOX2 (bg=4.63%)▶SAFB2 (bg=0.8%)▶uchl5 (bg=11.16%)▶ZNF622 (bg=6.58%)No matches to TargetScan

TT 18950  
 TATCA

TCATCTG

TCATCTG  
Depth:4 (DOG)  
Ei-value:0.000, Pi-value:0.010  
Er-value:0.000, Pr-value:0.000  
eCLIP MATCHES▶FUS (bg=2.21%)▶LARP4 (bg=4.72%)▶NOLC1 (bg=9.43%)▶RBFOX2 (bg=4.63%)▶RPS3 (bg=0.76%)▶uchl5 (bg=11.16%)▶ZNF622 (bg=6.58%)No matches to TargetScan

CTTTATTCA

CATAAATGAA

CATAAATGAA  
Depth:4 (DOG)  
Ei-value:0.000, Pi-value:0.000  
Er-value:0.000, Pr-value:0.000  
eCLIP MATCHES▶FUS (bg=2.21%)▶NOLC1 (bg=9.43%)▶RPS3 (bg=0.76%)▶uchl5 (bg=11.16%)▶ZNF622 (bg=6.58%)No matches to TargetScan


GTTGTGA

CATAAATGAAGTTGTGA  
Depth:3 (COW)  
Ei-value:0.000, Pi-value:0.000  
Er-value:0.000, Pr-value:0.000  
eCLIP MATCHES▶FUS (bg=2.21%)▶NOLC1 (bg=9.43%)▶RPS3 (bg=0.76%)▶uchl5 (bg=11.16%)▶ZNF622 (bg=6.58%)No matches to TargetScan

TGAATAAATCTGCTTTTATGCAGACACAAGGAATTAAGTGGCTTCGTCATTGTCCTTCTACCTCAAAGATAATTTATTCCAA 19070  
 AAGCTAAGATAAATGGAAGACTCTTGA

ACTTG

ACTTGTGAACTGATGTGAAA  
Depth:3 (COW)  
Ei-value:0.000, Pi-value:0.000  
Er-value:0.000, Pr-value:0.000  
eCLIP MATCHES▶FUS (bg=2.21%)▶NOLC1 (bg=9.43%)▶RBFOX2 (bg=4.63%)▶TRA2A (bg=4.8%)MATCHES To TargetScan▶ miR-23-3p:UCACAUU


TGAACTGATGTGAAA

TGAACTGATGTGAAA  
Depth:4 (DOG)  
Ei-value:0.000, Pi-value:0.000  
Er-value:0.000, Pr-value:0.000  
eCLIP MATCHES▶FUS (bg=2.21%)▶NOLC1 (bg=9.43%)▶RBFOX2 (bg=4.63%)▶TRA2A (bg=4.8%)MATCHES To TargetScan▶ miR-23-3p:UCACAUU

TGCAGAATCTCTTTTGAGTCTTTGCTGTTTGGAAGATTGAAAAATA

TTGTTCA

TTGTTCA  
Depth:3 (COW)  
Ei-value:0.000, Pi-value:0.000  
Er-value:0.000, Pr-value:0.000  
eCLIP MATCHES▶AARS (bg=2.18%)▶AATF (bg=0.64%)▶CPEB4 (bg=1.89%)▶DROSHA (bg=2.49%)▶FASTKD2 (bg=1.99%)▶FUS (bg=2.21%)▶GRWD1 (bg=5.13%)▶LARP4 (bg=4.72%)▶LSM11 (bg=2.28%)▶NOLC1 (bg=9.43%)▶RBFOX2 (bg=4.63%)▶TRA2A (bg=4.8%)▶uchl5 (bg=11.16%)▶UTP3 (bg=3.66%)▶WDR43 (bg=3.37%)▶XRCC6 (bg=2.91%)▶ZC3H11A (bg=6.55%)▶ZNF622 (bg=6.58%)No matches to TargetScan

GCATGGGTG

ACCACCA

ACCACCA  
Depth:3 (COW)  
Ei-value:0.000, Pi-value:0.000  
Er-value:0.000, Pr-value:0.000  
eCLIP MATCHES▶AARS (bg=2.18%)▶AATF (bg=0.64%)▶AKAP8L (bg=2.19%)▶CPEB4 (bg=1.89%)▶DROSHA (bg=2.49%)▶FASTKD2 (bg=1.99%)▶FUS (bg=2.21%)▶GRWD1 (bg=5.13%)▶KHDRBS1 (bg=1.71%)▶LARP4 (bg=4.72%)▶LSM11 (bg=2.28%)▶NIPBL (bg=5.39%)▶NOLC1 (bg=9.43%)▶RBFOX2 (bg=4.63%)▶RPS3 (bg=0.76%)▶TRA2A (bg=4.8%)▶uchl5 (bg=11.16%)▶UTP3 (bg=3.66%)▶WDR43 (bg=3.37%)▶XRCC6 (bg=2.91%)▶ZC3H11A (bg=6.55%)▶ZNF622 (bg=6.58%)No matches to TargetScan

GAAA 19190  
 GTAATCTTAAGCCATCTAGATGTCACAATTGAAACAAACTGGGGAGTTGGTTGCTATTGTA

AAATAAAA

AAATAAAA  
Depth:4 (DOG)  
Ei-value:0.000, Pi-value:0.000  
Er-value:0.000, Pr-value:0.000  
eCLIP MATCHES▶WDR43 (bg=3.37%)▶ZC3H11A (bg=6.55%)No matches to TargetScan

TATACTGTTTTGAAAACTTTG                               19280
```

|  |  |  |  |  |
| --- | --- | --- | --- | --- |
| | | | | | | | | | |
| 2 |  | 4 |  | 6 |
| Depth of motif conservation (number of species) | | | | |

  
  

---

  

## >HUMAN TO DOG (19280 bases)

```
 CCTTCAGTTCTTAAAGCGCTGCAATTCGCTGCTGCAGCCATATTTCTTACTCTCTCGGGGCTGGAAGCTTCCTGACTGAAGATCTCTCTGCACTTGGGGTTCTTTCTAGAACATTTTCTA 120  
 GTCCCCCAACACCCTTTATGGCGTATTTCTTTAAAAAAATCACCTAAATTCCATAAAATATTTTTTTAAATTCTATACTTTCTCCTAGTGTCTTCTTGACACGTCCTCCATATTTTTTTA 240  
 AAGAAAGTATTTGGAATATTTTGAGGCAATTTTTAATATTTAAGGAATTTTTCTTTGGAATCATTTTTGGTTGACATCTCTGTTTTTTGTGGATCAGTTTTTTACTCTTCCACTCTCTTT 360  
 TCTATATTTTGCCCATCGGGGCTGCGGATACCTGGTTTTATTATTTTTTCTTTGCCCAACGGGGCCGTGGATACCTGCCTTTTAATTCTTTTTTATTCGCCCATCGGGGCCGCGGATACC 480  
 TGCTTTTTATTTTTTTTTCCTTAGCCCATCGGGGTATCGGATACCTGCTGATTCCCTTCCCCTCTGAACCCCCAACACTCTGGCCCATCGGGGTGACGGATATCTGCTTTTTAAAAATTT 600  
 TCTTTTTTTGGCCCATCGGGGCTTCGGATACCTGCTTTTTTTTTTTTTATTTTTCCTTGCCCATCGGGGCCTCGGATACCTGCTTTAATTTTTGTTTTTCTGGCCCATCGGGGCCGCGGA 720  
 TACCTGCTTTGATTTTTTTTTTTCATCGCCCATCGGTGCTTTTTATGGATGAAAAAATGTTGGTTTTGTGGGTTGTTGCACTCTCTGGAATATCTACACTTTTTTTTGCTGCTGATCATT 840  
 TGGTGGTGTGTGAGTGTACCTACCGCTTTGGCAGAGAATGACTCTGCAGTTAAGCTAAGGGCGTGTTCAGATTGTGGAGGAAAAGTGGCCGCCATTTTAGACTTGCCGCATAACTCGGCT 960  
 TAGGGCTAGTCGTTTGTGCTAAGTTAAACTAGGGAGGCAAGATGGATGATAGCAGGTCAGGCAGAGGAAGTCATGTGCATTGCATGAGCTAAACCTATCTGAATGAATTGATTTGGGGCT 1080  
 TGTTAGGAGCTTTGCGTGATTGTTGTATCGGGAGGCAGTAAGAATCATCTTTTATCAGTACAAGGGACTAGTTAAAAATGGAAGGTTAGGAAAGACTAAGGTGCAGGGCTTAAAATGGCG 1200  
 ATTTTGACATTGCGGCATTGCTCAGCATGGCGGGCTGTGCTTTGTTAGGTTGTCCAAAATGGCGGATCCAGTTCTGTCGCAGTGTTCAAGTGGCGGGAAGGCCACATCATGATGGGCGAG 1320  
 GCTTTGTTAAGTGGTTAGCATGGTGGTGGACATGTGCGGTCACACAGGAAAAGATGGCGGCTGAAGGTCTTGCCGCAGTGTA

AAACATG

AAACATG  
Depth:4 (DOG)  
Ei-value:0.000, Pi-value:0.000  
Er-value:0.000, Pr-value:0.000  
eCLIP MATCHES▶HNRNPM (bg=4.29%)No matches to TargetScan

GCGGGCCTCTTTGTCTTTGCTGTGTGCTTTT 1440  
 CGTGTTGGGTTTTGCCGCAGGGACAATATGGCAGGCGTTGTCATATGTATATCATGGCTTTTGTCACGTGGACATCATGGCGGGCTTGCCGCATTGTTAAAGATGGCGGGTTTTGCCGCC 1560  
 TAGTGCCACGCAGAGCGGGAGAAAAGGTGGGATGGACAGTGCTGGATTGCTGCATAACCCAACCAATTAGAAATGGGGGTGGAATTGATCACAGCCAATTAGAGCAGAAGATGGAATTAG 1680  
 ACTGATGACACACTGTCCAGCTACTCAGCGAAGACCTGGGTGAATTAGCATGGCACTTCGCAGCTGTCTTTAGCCAGTCAGGAGAAAGAAGTGGAGGGGCCACGTGTATGTCTCCCAGTG 1800  
 GGCGGTACACCAGGTGTTTTCAAGGTCTTTTCAAGGACATTTAGCCTTTCCACCTCTGTCCCCTCTTATTTGTCCCCTCCTGTCCAGTGCTGCCTCTTGCAGTGCTGGATATCTGGCTGT 1920  
 GTGGTCTGAACCTCCCTCCATTCCTCTGTATTGGTGCCTCACCTAAGGCTAAGTATACCTCCCCCCCCACCCCCCAACCCCCCCAACTCCCCACCCCCACCCCCCACCCCCCACCTCCCC 2040  
 ACCCCCCTACCCCCCTACCCCCCTACCCCCCTCTGGTCTGCCCTGCACTGCACTGTTGCCATGGGCAGTGCTCCAGGCCTGCTTGGTGTGGACATGGTGGTGAGCCGTGGCAAGGACCAG 2160  
 AATGGATCACAGATGATCGTTGGCCAACAGGTGGCAGAAGAGGAATTCCTGCCTTCCTCAAGAGGAACACCTACCCCTTGGCTAATGCTGGGGTCGGATTTTGATTTATATTTATCTTTT 2280  
 GGATGTCAGTCATACAGTCTGATTTTGTGGTTTGCTAGTGTTTGAATTTAAGTCTTAAGTGACTATTATAGAAATGTATTAAGAGGCTTTATTTGTAGAATTCACTTTAATTACATTTAA 2400  
 TGAGTTTTTGTTTTGAGTTCCTTAAAATTCCTTAAAGTTTTTAGCTTCTCATTACAAATTCCTTAACCTTTTTTTGGCAGTAGATAGTCAAAGTCAAATCATTTCTAATGTTTTAAAAAT 2520  
 GTGCTGGTCATTTTCTTTGAAATTGACTTAACTATTTTCCTTTGAAGAGTCTGTAGCACAGAAACAGTAAAAAATTTAACTTCATGACCTAATGTAAAAAAGAGTGTTTGAAGGTTTACA 2640  
 CAGGTCCAGGCCTTGCTTTG

TTCCCATC

TTCCCATC  
Depth:4 (DOG)  
Ei-value:0.000, Pi-value:0.000  
Er-value:0.000, Pr-value:0.000  
eCLIP MATCHES▶ILF3 (bg=3.0%)No matches to TargetScan

CTTGATGCTGCACTAATTGACTAATCACCTACTTATCAGACAGGAAACTTGAATTGCTGTGGTCTGGTGTCCTCTATTCAGACTTATTATAT 2760  
 TGGAGTATTTCAATTTTTCGTTGTATCCTGCCTGCCTAGCATCCAGTTCCTCCCCAGCCCTGCTCCCAGCAAACCCCTAGTCTAGCCCCAGCCCTACTCCCACCCCGCCCCAGCCCTGCC 2880  
 CCAGCCCCAGTCCCCTAACCCCCCAGCCCTAGCCCCAGTCCCAGTCCTAGTTCCTCAGTCCCGCCCAGCTTCTCTCGAAAGTCACTCTAATTTTCATTGATTCAGTGCTCAAAATAAGTT 3000  
 GTCCATTGCTTATCCTATTATACTGGGATATTCCGTTTACCCTTGGCATTGCTGATCTTCAGTACTGACTCCTTGACCATTTTCAGTTAATGCATACAATCCCATTTGTCTGTGATCTCA 3120  
 GGACAAAGAATTTCCTTACTCGGTACGTTGAAGTTAGGGAATGTCAATTGAGAGCTTTCTATCAGAGCATTATTGCCCACAATTTGAGTTACTTATCATTTTCTCGATCCCCTGCCCTTA 3240  
 AAGGAGAAACCATTTCTCTGTCATTGCTTCTGTAGTCACAGTCCCAATTTTGAGTAGTGATCTTTTCTTGTGTACTGTGTTGGCCACCTAAAACTCTTTGCATTGAGTAAAATTCTAATT 3360  
 GCCAATAATCCTACCCATTGGATTAGACAGCACTCTGAACCCCATTTGCATTCAGCAGGGGGTCGCAGACAACCCGTCTTTTGTTGGACAGTTAAAATGCTCAGTCCCAATTGTCATAGC 3480  
 TTTGCCTATTAAACAAAGGCACCCTACTGCGCTTTTTGCTGTGCTTCTGGAGAATCCTGCTGTTCTTGGACAATTAAAGAACAAAGTAGTAATTGCTAATTGTCTCACCCATTAATCATG 3600  
 AAGACTACCAGTCGCCCTTGCATTTGCCTTGAGGCAGCGCTGACTACCTGAGATTTAAGAGTTTCTTAAATTATTGAGTAAAATCCCAATTATCCATAGTTCTGTTAGTTACACTATGGC 3720  
 CTTTGCAAACATCTTTGCATAACAGCAGTGGGACTGACTCATTCTTAGAGCCCCTTCCCTTGGAATATTAATGGATACAATAGTAATTATTCATGGTTCTGCGTAACAGAGAAGACCCAC 3840  
 TTATGTGTATGCCTTTATCATTGCTCCTAGATAGTGTGAACTACCTACCACCTTGCATTAATATGTAAAACACTAATTGCCCATAGTCCCACTCATTAGTCTAGGATGTCCTCTTTGCCA 3960  
 TTGCTGCTGAGTTCTGACTACCCAAGTTTCCTTCTCTTAAACAGTTGATATGCATAATTGCATATATTCATGGTTCTGTGCAATAAAAATGGATTCTCACCCCATCCCACCTTCTGTGGG 4080  
 ATGTTGCTAACGAGTGCAGATTATTCAATAACAGCTCTTGAACAGTTAATTTGCACAGTTGCAATTGTCCAGAGTCCTGTCCATTAGAAAGGGACTCTGTATCCTATTTGCACGCTACAA 4200  
 TGTGGGCTGATCACCCAAGGACTCTTCTTGTGCATTGATGTTCATAATTGTATTTGTCCACGATCTTGTGCACTAACCCTTCCACTCCCTTTGTATTCCAGCAGGGGACCCTTACTACTC 4320  
 AAGACCTCTGTACTAGGACAGTTTATGTGCACAATCCTAATTGATTAGAACTGAGTCTTTTATATCAAGGTCCCTGCATCATCTTTGCTTTACATCAAGAGGGTGCTGGTTACCTAATGC 4440  
 CCCTCCTCCAGAAATTATTGATGTGCAAAATGCAATTTCCCTATCTG

C

CTGTTAGTCT  
Depth:4 (DOG)  
Ei-value:0.000, Pi-value:0.000  
Er-value:0.000, Pr-value:0.000  
eCLIP MATCHES▶AKAP8L (bg=2.19%)No matches to TargetScan


TGTTAGTC

TGTTAGTC  
Depth:5 (RABBIT)  
Ei-value:0.000, Pi-value:0.000  
Er-value:0.000, Pr-value:0.000  
eCLIP MATCHES▶AKAP8L (bg=2.19%)No matches to TargetScan


T

CTGTTAGTCT  
Depth:4 (DOG)  
Ei-value:0.000, Pi-value:0.000  
Er-value:0.000, Pr-value:0.000  
eCLIP MATCHES▶AKAP8L (bg=2.19%)No matches to TargetScan

GGGGTC

TCATCC

TCATCC  
Depth:4 (DOG)  
Ei-value:0.000, Pi-value:0.020  
Er-value:0.000, Pr-value:0.000  
eCLIP MATCHES▶AKAP8L (bg=2.19%)No matches to TargetScan

CCTCATATTCCTTTTGTCTTACAGCAGGGGG

TACTTGGGACTGTTAAT

TACTTGGGACTGTTAAT  
Depth:4 (DOG)  
Ei-value:0.000, Pi-value:0.000  
Er-value:0.000, Pr-value:0.000  
eCLIP MATCHES▶AKAP8L (bg=2.19%)MATCHES To TargetScan▶ miR-132-3p/212-3p:AACAGUC▶ miR-455-3p.1:CAGUCCA

GCG 4560  
 CATAATTGCAATTATGGTCTTTTCCATTAAATTAAGATCCCAACTGCTCACACCCTCTTAGCATTACAGTAGAGGGTGCTAATCACAAGGACATTTCTTTTGT

ACTG

ACTGTTAATGTGCT  
Depth:4 (DOG)  
Ei-value:0.000, Pi-value:0.000  
Er-value:0.000, Pr-value:0.000  
No matches to eCLIP DataMATCHES To TargetScan▶ miR-132-3p/212-3p:AACAGUC▶ miR-323-3p:ACAUUAC


TTAATGTGCT

TTAATGTGCT  
Depth:5 (RABBIT)  
Ei-value:0.000, Pi-value:0.000  
Er-value:0.000, Pr-value:0.000  
No matches to eCLIP DataMATCHES To TargetScan▶ miR-323-3p:ACAUUAC

ACT 4680  
 TGCATTTGTCCCTCTTCCTGTGCACTAAAGACCCCACTCACTTCCCTAGTGTTCAGCAGTGGATGACCTCTAGTCAAGACCTTTGCACTAGGATAGTTAATGTGAACCATGGCAACTGAT 4800  
 CACAACAATGTCTTTCAGATCAGATCCATTTTATCCTCCTTGTTTTACAGCAAGGGATATTAATTACCTATGTTACCTTTCCCTGGGACTATGAATGTGCAAAATTCCAATGTTCATGGT 4920  
 CTCTCCCTTTAAACCTATATTCTACCCCTTTTACATTATAGAAAGGGATGCTGGAAACCCAGAGTCCTTCTCTTGGGACTCTTAATGTGTATTTCTAATTATCCATGACTCTT

AATGTGC

AATGTGCAT  
Depth:6 (MOUSE)  
Ei-value:0.000, Pi-value:0.000  
Er-value:0.000, Pr-value:0.000  
No matches to eCLIP DataMATCHES To TargetScan▶ miR-501-3p/502-3p:AUGCACC

 5040  


AT

AATGTGCAT  
Depth:6 (MOUSE)  
Ei-value:0.000, Pi-value:0.000  
Er-value:0.000, Pr-value:0.000  
No matches to eCLIP DataMATCHES To TargetScan▶ miR-501-3p/502-3p:AUGCACC

ATTTTCAATTGCCTAATTGATTTCAATTGTCTAAGACATTTCAAATGTCTAATTGATTAGAACTGAGTCTTTTATATCAAGCTAATATCTAGCTTTTATATCAAGCTAATATCTTGAC 5160  
 TTCTCAGCATCATAGAAGGGGGTACTGATTTCCTAAAGTCTTTCTTGAATTTCTATTATGCAAAATTGCCCTGAGGCCGGGTGTGGTGGCTCACACCTGTAATCCCAGCACTTTGGGAGG 5280  
 CTGAGGTGGGAAGATCCCTTACTGCCAGGAGTTTGAGACCAGCCTGGCCAACATTAAAAAAAAAAAAAAGTAAGACAATTGCCCTGGAATCCCATCCCCCTCACACCTCCTTGGCAAAGC 5400  
 AGCAGGAGTGCTAACTAGCTAGTGCTTCTTCTCTTATACTGCTTAAATGCGCATAATTAGCAGTAGTTGATGTGCCCC

TATGTTAGA

TATGTTAGA  
Depth:4 (DOG)  
Ei-value:0.000, Pi-value:0.000  
Er-value:0.000, Pr-value:0.000  
eCLIP MATCHES▶HNRNPU (bg=5.92%)No matches to TargetScan

GTAGAATCCCGCTTCCTTGCTCCATTTGCATTA 5520  
 CTGCAGGAGCTTCTAACTAGCCTGAATTCACTCTCTTGG

ACTGTTAATGT

ACTGTTAATGT  
Depth:4 (DOG)  
Ei-value:0.000, Pi-value:0.000  
Er-value:0.000, Pr-value:0.000  
No matches to eCLIP DataMATCHES To TargetScan▶ miR-132-3p/212-3p:AACAGUC▶ miR-323-3p:ACAUUAC

GCATACTTAT

ATTTGCT

ATTTGCT  
Depth:4 (DOG)  
Ei-value:0.000, Pi-value:0.000  
Er-value:0.000, Pr-value:0.000  
No matches to eCLIP DataNo matches to TargetScan

GCTGTACTTTTTTACCAT

GTAAGGA

GTAAGGA  
Depth:5 (RABBIT)  
Ei-value:0.000, Pi-value:0.000  
Er-value:0.000, Pr-value:0.000  
No matches to eCLIP DataNo matches to TargetScan

CCCCACCCACTGTATTTACATCCCAGCT 5640  
 GGAAGTACCTACTACTTAAGACCCTTAGACTAGTAAAGTTAGCGTGCATAATCTTAGGTGTTATATACACATTTTCAGTTGCATACAGTTGTGCCTTTTATCAGGACTCCTGT

ACTTAT

ACTTAT  
Depth:5 (RABBIT)  
Ei-value:0.000, Pi-value:0.000  
Er-value:0.000, Pr-value:0.000  
eCLIP MATCHES▶HNRNPU (bg=5.92%)No matches to TargetScan

C 5760  
 AAAGCAGAGAGTGCTAATCAATATTAAGCCCTTCTCTTCGAACTGTAGATGGCATGTAATTGCAGTTGTCAATGGTCCTTCAATTAGACTTGGGTTTCTGACCTATCACACCCTCTTTGC 5880  
 TTTATTGCATGGGGTACTATTCAC

TTAAGGCC

TTAAGGCC  
Depth:6 (MOUSE)  
Ei-value:0.000, Pi-value:0.000  
Er-value:0.000, Pr-value:0.000  
eCLIP MATCHES▶HNRNPL (bg=0.64%)No matches to TargetScan


CCTTT

TTAAGGCCCCTTT  
Depth:5 (RABBIT)  
Ei-value:0.000, Pi-value:0.000  
Er-value:0.000, Pr-value:0.000  
eCLIP MATCHES▶HNRNPL (bg=0.64%)No matches to TargetScan


CTCAA

TTAAGGCCCCTTTCTCAA  
Depth:4 (DOG)  
Ei-value:0.000, Pi-value:0.000  
Er-value:0.000, Pr-value:0.000  
eCLIP MATCHES▶HNRNPL (bg=0.64%)No matches to TargetScan

ACTGTTAATGTGCCTAATGACAATTACATCAGTATCCTTCCTTTTGAAGGACAGCATGGTTGGTGACACCTAAGGCCC 6000  
 CATTTCTTGGCCTCCCAATATGTGTGATTGTATTTGTCGAGGTTGCTATGCACTAGAGAAGGAAAGTGCTCCCCTCATCCCCACTTTTCCCTTCCAGCAGGAAGTGCCCACCCCATAAGA 6120  
 CCCTTTTATTTGGAGAGTCTAGGTGCACAATTGTAAGTGACCACAAGCATGCATCTTGGACATTTATGTGCGTAATCGCACACTGCTCATTCCATGTGAATAAGGTCCTACTCTCCGACC 6240  
 CCTTTTGCAATACAGAAGGGTTGCTGATAACGCAGTCCCCTTTTCTTGGCATGTTGTGTGTGATTATAATCGTCTGGGATCCTATGCACTAGAAAAGGAGGGTCCTCTCCACATACCTCA 6360  
 GTCTCACCTTTCCCTTCCAGCAGGGAGTGCCCACTCCATAAGACTCTCACATTTGGACAGTCAAGGTGCGTAATTGTTAAGTGAACACAACCATGCACCTTAGACATGGATTTGCATAAC 6480  
 TACACACAGCTCAACCTATCTGAATAAAATCCTACTCTCAGACCCCTTTTGCAGTACAGCAGGGGTGCTGATCACCAAGGCCCTTTTTCCTGGCCTGGTATGCGTGTGATTATGTTTGTC 6600  
 CCGGTTCCTGTGTATTAGACATGGAAGCCTCCCCTGCCACACTCCACCCCCAATCTTCCTTTCCCTTCCGGCAGGGAGTGCCCTCTCCATAAGACGCTTACGTTTGGACAATCAAGGTGC 6720  
 ACAGTTGTAAGTGACCACAGGCATACACCTTGGACATTAATGTGCATAACCACTTTGCCCATTCCATCTGAATAAGGTCCTACTCTCAGACCCCTTTTGCAGTACAGCAGGGGTGCTGAT 6840  
 CACCAAGGCCCCTTTTCTTGGCCTGTTATGTGCGTGATTATATTTGTCTGGGTTCCTGTGTATTAGACAAGGAAGCCTTCCCCCCGCCCCCACCCCCACTCCCAGTCTTCCTTTCCCTTC 6960  
 CAGCAGGGAGTGCCCCCTCCATAAGATCATTACATTTGGACAATCAAGGTGCACAATTATAAGTGACCACAGCCATGCACCTTGGACATTATTGGACATTAATGTGCGTAACTGCACATG 7080  
 GCCCATCCCATCTGAATAAGGTCCTACTCTCAGATGCCCTTTGCAGTACAGCAGGGGTACTGAATCACCAAGGCCCTTTTTCTTGGCCTGTTATGTGTGTGATTATATTTATCCCAGTTT 7200  
 CTGTGTAATAGACATGAAAGCCTCCCCTGCCACACCCCACCTCCAATCTTCCTTTCCCTTCCACCAGGGAGTGTCCACTCCATATACCCTTACATTTGGACAATCAAGGTGCACAATTGT 7320  
 AAGTGAGCATAGGCACTCACCTTGGACATGAATGTGCATAACTGCACATGGCCCATCCCATCTGAATAAGGTCCTACTCTCAGACCCTTTTTGCAGTACAGCAGGGGTGCTGATCACCAA 7440  
 GGCCCCTTTTCCTGGCCTGTTATGTGTGTGATTATATTTGTTCCAGTTCCTGTGTAATAGACATGGAAGCCTCCCCTGCCACACTCCACCCCCAATCTTCCTTTCCCTTCTGGCAGGAAG 7560  
 TACCCGCTCCATAAGACCCTTACATTTGGACAGTCAAGGTGCACAATTGTATGTGACCACAACCATGCACCTTGGACATAAATGTGTGTAACTGCACATGGCCCATCCCATCTGAATAAG 7680  
 GTCCTACTCTCAGACCCCTTTTGCAGTACAGTAGGTGTGCTGATAACCAAGGCCCCTCTTCCTGGCCTGTTAACGTATGTGATTATATTTGTCTGGGTTCCAGTGTATAAGACATGGAAG 7800  
 CCTCCCCTGCCCCACCCCACCCTCAATCTTCCTTTCCCTTCTGGCAGGGAGTGCCAGCTCCATAAGAACCTTACATTTGGACAGTCAAGGTGCACAATTCTAAGTGACCGCAGCCATGCA 7920  
 CCTTGGTCAATAATGTGTGTAACTGCACACGGCCTATCTCATCTGAATAAGGCCTTACTCTCAGACCCCTTTTGCAGTACAGCAGGGGTGCTGATAACCAAGGCCCATTTTCCTGGCCTG 8040  
 TTATGTGTGTGATTATATTTGTCCAGGTTTCTGTGTACTAGACAAGGAAGCCTCCTCTGCCCCATCCCATCTACGCATAATCTTTCTTTTCCTCCCAGCAGGGAGTGCTCACTCCATAAG 8160  
 ACCCTTACATTTGGACAATCAAGGTGCACAATTGTAAGTGACCACAACCATGCATCTTGGAAATTTATGTGCATAACTGCACATGGCTTATCCTATTTGAATAAAGTCCTACTCTCAGAC 8280  
 CCCCTTTGCAGTATAGCTGGGGTGCTGATCACTGAGGCCTCTTTGCTTGGCTTGTCTATATTCTTGTGTACTAGATAAGGGCACCTTCTCATGGACTCCCTTTGCTTTTCAACAAGGAGT 8400  
 ACCCACTACTTTTTAAGATTCTTATATTTGTCCAAAGTACATGGTTTTAATTGACCACAACAATGTCCCTTGGACATTAATGTATGTAATCACCACATGGTTCATCCTAATTAAACAAAG 8520  
 TTCTACCTTCTCACCCTCCATTTGCAGTATACCAGGGTTGCTGACCCCCTAAGTCCCCTTTTCTTGGCTTGTTGACATGCATAATTGCATTTATGTTGGTTCTTGTGCCCTAGACAAGGA 8640  
 TGCCCCACCTCTTTTCAATAGTGGGTGCCCACTCCTTATGATCTTTACATTTGA

ACAGTTAATGTG

ACAGTTAATGTG  
Depth:4 (DOG)  
Ei-value:0.000, Pi-value:0.000  
Er-value:0.000, Pr-value:0.000  
eCLIP MATCHES▶HNRNPU (bg=5.92%)MATCHES To TargetScan▶ miR-323-3p:ACAUUAC

AATAATTGCAGTTGTCCACAACCCTATCACTTCTAGGACCATTATACCTCTTTT 8760  
 GCATTACTGTGGGGTATACTGTTTCCCTCCAAGGCCCCTTCTGGTGGACTATCAACATATAATTGAAATTTTCTTTTGTCTTTGTCAGTAGATTAAGGTCATACCCCATCACCTTTCCTT 8880  
 TGTAGTACAACAGGGTGTCCTGATCAACCAAAGTCCTGTTGTTTTGGACTGTTAATATGTGCAATTACATTTGCTCCTGATCTGTGCACTAGATAAGGATCCTACCTACTTTCTTAGTGT 9000  
 TTTTAGCAGGTAGTGCCCACTACTCAAGACTGTCACTTGGAATGTTCATGTGCACAAACTCAATTCTCTAAGCATGTTCCTGTACCACCTTTGCTTTAGAGCAGGGGGATGATATTCACT 9120  
 AAGTGCCCCTTCTTTTGGACTTAATATGCATTAATGCAATTGTCCACCTCTTCTTTTAGACTAAGAGTTGATCTCCACATATTCCCCTTGCATCAGGGGCATGTTAATTATGAATGAACC 9240  
 CTTTTCTTTTAATATTAATGTCATAATTGTATTTGTGGACCTGTGTAGGAGAAAAAGACCCTATGTTCCTCCCATTACCCTTTGGATTGCTGCTGAGAAGTGTTAACTACTCATAAT

CTC

CTCAGCTCTTGG  
Depth:5 (RABBIT)  
Ei-value:0.000, Pi-value:0.000  
Er-value:0.000, Pr-value:0.000  
No matches to eCLIP DataMATCHES To TargetScan▶ miR-335-5p:CAAGAGC

 9360  


AGCTCTTGG

CTCAGCTCTTGG  
Depth:5 (RABBIT)  
Ei-value:0.000, Pi-value:0.000  
Er-value:0.000, Pr-value:0.000  
No matches to eCLIP DataMATCHES To TargetScan▶ miR-335-5p:CAAGAGC


ACA

CTCAGCTCTTGGACA  
Depth:4 (DOG)  
Ei-value:0.000, Pi-value:0.000  
Er-value:0.000, Pr-value:0.000  
No matches to eCLIP DataMATCHES To TargetScan▶ miR-335-5p:CAAGAGC

ATTAATAGCATTAATAACAATTATCAAGGGCACTGATCATTAGATAAGACTCCTGCTTCCTCGTTGCTTACATCGGGGGTACTGACCCACTAAGGCCCCTTGTACTGT 9480  
 TAATGTGAATATTTGCAATTATATATGTCTCCTTCTGGTAGAGTGGGATATTATGCCCTAGTATCCCCTTTGCATTACTGCAGGGGCTGCTGACTACT

CAAAACTT

CAAAACTT  
Depth:4 (DOG)  
Ei-value:0.000, Pi-value:0.000  
Er-value:0.000, Pr-value:0.000  
eCLIP MATCHES▶SF3B1 (bg=2.48%)No matches to TargetScan

CTCCTGGGACTGTT 9600  
 AATAG

GCACAATG

GCACAATG  
Depth:6 (MOUSE)  
Ei-value:0.000, Pi-value:0.000  
Er-value:0.000, Pr-value:0.000  
No matches to eCLIP DataNo matches to TargetScan

GCAGTTATCAATGGTTTTCTCCCTCCCTGACCTTGTTAAGCAAGCGCCCCACCCCACCCTTAGTTTCCCATGGCATAATAAAGTATAAGCATTGGAGTATTCCATGC 9720  
 ACTTGTCTATCAAACAGTGGTCCAT

A

ACTCCCA  
Depth:4 (DOG)  
Ei-value:0.000, Pi-value:0.000  
Er-value:0.000, Pr-value:0.000  
eCLIP MATCHES▶hnrnpk (bg=12.88%)No matches to TargetScan


CTCCCA

CTCCCA  
Depth:6 (MOUSE)  
Ei-value:0.000, Pi-value:0.000  
Er-value:0.000, Pr-value:0.000  
eCLIP MATCHES▶hnrnpk (bg=12.88%)No matches to TargetScan

A

CCCTTTTGCATT

CCCTTTTGCATT  
Depth:4 (DOG)  
Ei-value:0.000, Pi-value:0.000  
Er-value:0.000, Pr-value:0.000  
eCLIP MATCHES▶hnrnpk (bg=12.88%)No matches to TargetScan

GCGCCAGTGTGTAAAATCACAGGTAGCCATGGTGTCATGCTTTATATACGAAGTCTTCCCTCTCTCTGCCCCTTG 9840  
 TGTGCCCTTGGCCCCTTTTTACAGACTATTGCTCACAATCTCAGGTGTCCATATTTGCAGCTATTAGGTAAGATTGTGCTGTCTCCCTCTTCCCTTCCCTCTGCCCTGCCCCTTTTGCCT 9960  
 CTTTGCTGGGTAATGTTGACCAGACAAGGCCCTTTCTCTTGGACTTAAACAATTCTCAGTTGCACTTTCCTTGGTCCCACCCATTATACATGAACCCCTCTACTTCCTTTCGCATTGCTT 10080  
 CTGAGTATGCTGACTACCCAAAGCCCCTTCTGTGTTATTAATAAACACAGTACTGATTGTC

CCATTTTT

CCATTTTT  
Depth:4 (DOG)  
Ei-value:0.000, Pi-value:0.000  
Er-value:0.000, Pr-value:0.000  
eCLIP MATCHES▶hnrnpk (bg=12.88%)No matches to TargetScan


CAGCCCA

CAGCCCA  
Depth:4 (DOG)  
Ei-value:0.000, Pi-value:0.000  
Er-value:0.000, Pr-value:0.000  
eCLIP MATCHES▶hnrnpk (bg=12.88%)No matches to TargetScan

TCAGTCCAAGATCTC

CCTACCA

CCTACCA  
Depth:4 (DOG)  
Ei-value:0.000, Pi-value:0.000  
Er-value:0.000, Pr-value:0.000  
eCLIP MATCHES▶hnrnpk (bg=12.88%)No matches to TargetScan

CTTTGGTGTGTTGGTGCAGTGT 10200  
 TGACTATG

AAAAGCAG

AAAAGCAG  
Depth:6 (MOUSE)  
Ei-value:0.000, Pi-value:0.000  
Er-value:0.000, Pr-value:0.000  
No matches to eCLIP DataNo matches to TargetScan

GCCTGAACTAGGTGGATAAGCCTTCACTCATTTTCTTTCATTTA

TTAATGATCC

TTAATGATCC  
Depth:4 (DOG)  
Ei-value:0.000, Pi-value:0.000  
Er-value:0.000, Pr-value:0.000  
No matches to eCLIP DataMATCHES To TargetScan▶ miR-382-3p:AUCAUUC

TAGTTTCAATTATTGTCAG

ATTCTGGG

ATTCTGGG  
Depth:4 (DOG)  
Ei-value:0.000, Pi-value:0.000  
Er-value:0.000, Pr-value:0.000  
No matches to eCLIP DataNo matches to TargetScan

GACAAGAACCATTCTTGCCCACC 10320  
 TGTGTTACTG

CTTTACT

CTTTACT  
Depth:4 (DOG)  
Ei-value:0.000, Pi-value:0.000  
Er-value:0.000, Pr-value:0.000  
No matches to eCLIP DataNo matches to TargetScan

GT

GCAAAAT

GCAAAAT  
Depth:6 (MOUSE)  
Ei-value:0.000, Pi-value:0.000  
Er-value:0.000, Pr-value:0.000  
No matches to eCLIP DataNo matches to TargetScan

ACTG

AAGGCAA

AAGGCAA  
Depth:4 (DOG)  
Ei-value:0.000, Pi-value:0.000  
Er-value:0.000, Pr-value:0.000  
No matches to eCLIP DataNo matches to TargetScan

GTCAGACCCAGGGAGC

TGGATTGC

TGGATTGC  
Depth:4 (DOG)  
Ei-value:0.000, Pi-value:0.000  
Er-value:0.000, Pr-value:0.000  
No matches to eCLIP DataNo matches to TargetScan

CATCCTTTATTTTGTGTTTCCAGTGTACACTATAAAATTGTCTCCCCAGGAAGGAAGGT 10440  
 TGGCACTTTCTC

TGCATTCTTC

TGCATTCTTC  
Depth:5 (RABBIT)  
Ei-value:0.000, Pi-value:0.000  
Er-value:0.000, Pr-value:0.000  
eCLIP MATCHES▶SF3B1 (bg=2.48%)No matches to TargetScan

TTTCCAG

AGC

AGCAGATTGCCTGG  
Depth:4 (DOG)  
Ei-value:0.000, Pi-value:0.000  
Er-value:0.000, Pr-value:0.000  
eCLIP MATCHES▶SF3B1 (bg=2.48%)No matches to TargetScan


A

AGATTGCCTGG  
Depth:5 (RABBIT)  
Ei-value:0.000, Pi-value:0.000  
Er-value:0.000, Pr-value:0.000  
No matches to eCLIP DataNo matches to TargetScan


GATTGCCTGG

GATTGCCTGG  
Depth:6 (MOUSE)  
Ei-value:0.000, Pi-value:0.000  
Er-value:0.000, Pr-value:0.000  
No matches to eCLIP DataNo matches to TargetScan

TTAAGAATCTCTTGTTGTCCCCT

TTGTATATT

TTGTATATT  
Depth:4 (DOG)  
Ei-value:0.000, Pi-value:0.000  
Er-value:0.000, Pr-value:0.000  
No matches to eCLIP DataMATCHES To TargetScan▶ miR-381-3p:AUACAAG

GTTATTGTAAAGTGCCAAATGCCAGGATACAGCCAGAAAAATTGC 10560  
 TTATTATTATTAAAAAAATTTTTTTAAGAAAGACATCTGGATTGTAGGGTGGACTCGAT

AAC

AACCTGGTCATT  
Depth:4 (DOG)  
Ei-value:0.000, Pi-value:0.000  
Er-value:0.000, Pr-value:0.000  
No matches to eCLIP DataNo matches to TargetScan


CTGGTCATT

CTGGTCATT  
Depth:5 (RABBIT)  
Ei-value:0.000, Pi-value:0.000  
Er-value:0.000, Pr-value:0.000  
No matches to eCLIP DataNo matches to TargetScan

ATTTTTTTGAAGCCAAAATAT

CCATTTAT

CCATTTAT  
Depth:5 (RABBIT)  
Ei-value:0.000, Pi-value:0.000  
Er-value:0.000, Pr-value:0.000  
No matches to eCLIP DataNo matches to TargetScan

ACTATGTACCTGG

TGAC

TGACCAGTGTCTCTCATTT  
Depth:4 (DOG)  
Ei-value:0.000, Pi-value:0.000  
Er-value:0.000, Pr-value:0.000  
eCLIP MATCHES▶SUPV3L1 (bg=1.57%)No matches to TargetScan


CAG

CAGTGTCTCTCATTT  
Depth:5 (RABBIT)  
Ei-value:0.000, Pi-value:0.000  
Er-value:0.000, Pr-value:0.000  
eCLIP MATCHES▶SUPV3L1 (bg=1.57%)No matches to TargetScan

 10680  


TGTCTCTCATTT

CAGTGTCTCTCATTT  
Depth:5 (RABBIT)  
Ei-value:0.000, Pi-value:0.000  
Er-value:0.000, Pr-value:0.000  
eCLIP MATCHES▶SUPV3L1 (bg=1.57%)No matches to TargetScan

TAACTG

AGG

AGGGTGGTG  
Depth:4 (DOG)  
Ei-value:0.000, Pi-value:0.000  
Er-value:0.000, Pr-value:0.000  
eCLIP MATCHES▶SUPV3L1 (bg=1.57%)No matches to TargetScan


GTGGTG

GTGGTG  
Depth:5 (RABBIT)  
Ei-value:0.000, Pi-value:0.000  
Er-value:0.000, Pr-value:0.000  
eCLIP MATCHES▶SUPV3L1 (bg=1.57%)No matches to TargetScan

G

GTCTGTGGATA

GTCTGTGGATA  
Depth:5 (RABBIT)  
Ei-value:0.000, Pi-value:0.000  
Er-value:0.000, Pr-value:0.000  
eCLIP MATCHES▶SUPV3L1 (bg=1.57%)MATCHES To TargetScan▶ miR-140-3p.1:CCACAGG

GAACACTGACTCTTGCTATTTTAATATCAAAGATA

TTCTAGA

TTCTAGA  
Depth:4 (DOG)  
Ei-value:0.000, Pi-value:0.000  
Er-value:0.000, Pr-value:0.000  
No matches to eCLIP DataNo matches to TargetScan

GTGGAACTCTTAAGACCAGTATCTTTGTGTGGGCTTTAC 10800  
 CAGC

ATTCACTT

ATTCACTT  
Depth:4 (DOG)  
Ei-value:0.000, Pi-value:0.000  
Er-value:0.000, Pr-value:0.000  
No matches to eCLIP DataNo matches to TargetScan

TTA

GAAAAAC

GAAAAAC  
Depth:4 (DOG)  
Ei-value:0.000, Pi-value:0.000  
Er-value:0.000, Pr-value:0.000  
No matches to eCLIP DataNo matches to TargetScan

TACCTAAATTTTATAATCCTTT

AATTTCTTCATCTGGAGC

AATTTCTTCATCTGGAGC  
Depth:5 (RABBIT)  
Ei-value:0.000, Pi-value:0.000  
Er-value:0.000, Pr-value:0.000  
eCLIP MATCHES▶SUPV3L1 (bg=1.57%)▶U2AF2 (bg=1.76%)No matches to TargetScan

ACCTGCCCCTA

CTTATTT

CTTATTT  
Depth:4 (DOG)  
Ei-value:0.000, Pi-value:0.000  
Er-value:0.000, Pr-value:0.010  
eCLIP MATCHES▶SUPV3L1 (bg=1.57%)▶U2AF2 (bg=1.76%)No matches to TargetScan

CAAGAAGATTGCAGTAAAACGATTAAATGAGGGAACATAT 10920  
 GCAGAGGTGCTTTTAAAAAGCATATGCCACCTTTTTTATTAATTATTAT

ATAAAATG

ATAAAATG  
Depth:4 (DOG)  
Ei-value:0.000, Pi-value:0.000  
Er-value:0.000, Pr-value:0.000  
No matches to eCLIP DataNo matches to TargetScan

AAGCATTTAATTATAGTAATAATTTGAAGTAGTTTGAAGTACCACACTGAGGTGAGGACTTAA 11040  
 AAATGATAAGACGAGTTCCCTATTTTATAAG

AAAAATAAGCCA

AAAAATAAGCCA  
Depth:5 (RABBIT)  
Ei-value:0.000, Pi-value:0.000  
Er-value:0.000, Pr-value:0.000  
No matches to eCLIP DataNo matches to TargetScan


A

AAAAATAAGCCAA  
Depth:4 (DOG)  
Ei-value:0.000, Pi-value:0.000  
Er-value:0.000, Pr-value:0.000  
No matches to eCLIP DataNo matches to TargetScan

AATTAAATATTCTTTTGGATATAAATTTCAACAGTGAGATAGCTGCCTAGTGGAA

ATGAATAATA

ATGAATAATA  
Depth:4 (DOG)  
Ei-value:0.000, Pi-value:0.000  
Er-value:0.000, Pr-value:0.000  
No matches to eCLIP DataNo matches to TargetScan

TCCCAGCCACT 11160  
 AGTGTACAGGGTGTTTTGTGGCACAGGATTATGTAATA

TGGAACTGCT

TGGAACTGCT  
Depth:4 (DOG)  
Ei-value:0.000, Pi-value:0.000  
Er-value:0.000, Pr-value:0.000  
No matches to eCLIP DataNo matches to TargetScan

CAAGCAAA

TAACTA

TAACTA  
Depth:4 (DOG)  
Ei-value:0.000, Pi-value:0.000  
Er-value:0.000, Pr-value:0.000  
No matches to eCLIP DataNo matches to TargetScan

GTCATCACAA

CAGCAGTTC

CAGCAGTTC  
Depth:5 (RABBIT)  
Ei-value:0.000, Pi-value:0.000  
Er-value:0.000, Pr-value:0.000  
No matches to eCLIP DataNo matches to TargetScan

T

TTGTAAT

TTGTAAT  
Depth:4 (DOG)  
Ei-value:0.000, Pi-value:0.000  
Er-value:0.000, Pr-value:0.000  
No matches to eCLIP DataNo matches to TargetScan

A

ACTGAAAA

ACTGAAAA  
Depth:5 (RABBIT)  
Ei-value:0.000, Pi-value:0.000  
Er-value:0.000, Pr-value:0.000  
No matches to eCLIP DataNo matches to TargetScan

AGAATATTGTTTCTCGGAG

AAG

AAGGATG  
Depth:5 (RABBIT)  
Ei-value:0.000, Pi-value:0.000  
Er-value:0.000, Pr-value:0.000  
eCLIP MATCHES▶SRSF1 (bg=8.47%)▶U2AF2 (bg=1.76%)▶uchl5 (bg=11.16%)MATCHES To TargetScan▶ miR-362-5p/500b-5p:AUCCUUG

 11280  


GATG

AAGGATG  
Depth:5 (RABBIT)  
Ei-value:0.000, Pi-value:0.000  
Er-value:0.000, Pr-value:0.000  
eCLIP MATCHES▶SRSF1 (bg=8.47%)▶U2AF2 (bg=1.76%)▶uchl5 (bg=11.16%)MATCHES To TargetScan▶ miR-362-5p/500b-5p:AUCCUUG


TCA

AAGGATGTCAAAAGATC  
Depth:4 (DOG)  
Ei-value:0.000, Pi-value:0.000  
Er-value:0.000, Pr-value:0.000  
eCLIP MATCHES▶SRSF1 (bg=8.47%)▶U2AF2 (bg=1.76%)▶uchl5 (bg=11.16%)MATCHES To TargetScan▶ miR-362-5p/500b-5p:AUCCUUG▶ miR-489-3p:UGACAUC


AAAGATC

AAAGATC  
Depth:6 (MOUSE)  
Ei-value:0.000, Pi-value:0.000  
Er-value:0.000, Pr-value:0.000  
eCLIP MATCHES▶SRSF1 (bg=8.47%)▶U2AF2 (bg=1.76%)▶uchl5 (bg=11.16%)No matches to TargetScan

GGCC

CAGCTCAGGG

CAGCTCAGGG  
Depth:4 (DOG)  
Ei-value:0.000, Pi-value:0.000  
Er-value:0.000, Pr-value:0.000  
eCLIP MATCHES▶SRSF1 (bg=8.47%)▶U2AF2 (bg=1.76%)▶uchl5 (bg=11.16%)MATCHES To TargetScan▶ miR-125-5p:CCCUGAG

AGCAGTTTGCC

CTACTAGCTCCT

CTACTAGCTCCT  
Depth:4 (DOG)  
Ei-value:0.000, Pi-value:0.000  
Er-value:0.000, Pr-value:0.000  
eCLIP MATCHES▶SRSF1 (bg=8.47%)▶U2AF2 (bg=1.76%)▶uchl5 (bg=11.16%)MATCHES To TargetScan▶ miR-28-5p/708-5p:AGGAGCU▶ miR-411-5p.2:UAGUAGA

C

GGACAGCTG

GGACAGCTG  
Depth:5 (RABBIT)  
Ei-value:0.000, Pi-value:0.000  
Er-value:0.000, Pr-value:0.000  
eCLIP MATCHES▶SRSF1 (bg=8.47%)▶SRSF7 (bg=2.32%)▶U2AF2 (bg=1.76%)▶ZNF622 (bg=6.58%)No matches to TargetScan


T

GGACAGCTGT  
Depth:4 (DOG)  
Ei-value:0.000, Pi-value:0.000  
Er-value:0.000, Pr-value:0.000  
eCLIP MATCHES▶SRSF1 (bg=8.47%)▶SRSF7 (bg=2.32%)▶U2AF2 (bg=1.76%)▶ZNF622 (bg=6.58%)No matches to TargetScan

AA

AGAAGAGTCTCTGGCTCTTTA

AGAAGAGTCTCTGGCTCTTTA  
Depth:5 (RABBIT)  
Ei-value:0.000, Pi-value:0.000  
Er-value:0.000, Pr-value:0.000  
eCLIP MATCHES▶DDX24 (bg=2.97%)▶SRSF1 (bg=8.47%)▶SRSF7 (bg=2.32%)▶U2AF2 (bg=1.76%)▶ZNF622 (bg=6.58%)No matches to TargetScan


GA

AGAAGAGTCTCTGGCTCTTTAGA  
Depth:4 (DOG)  
Ei-value:0.000, Pi-value:0.000  
Er-value:0.000, Pr-value:0.000  
eCLIP MATCHES▶DDX24 (bg=2.97%)▶SRSF1 (bg=8.47%)▶SRSF7 (bg=2.32%)▶U2AF2 (bg=1.76%)▶ZNF622 (bg=6.58%)No matches to TargetScan

ATACT||GATCCCATTGAAGATACCACGCTGCA 11398  
 TGTGTCCTTAGTAGTCATGTCTCCTTAGGCTCCTCTTG||GAC

ATTCTGAGC

ATTCTGAGC  
Depth:4 (DOG)  
Ei-value:0.000, Pi-value:0.000  
Er-value:0.000, Pr-value:0.000  
eCLIP MATCHES▶DDX24 (bg=2.97%)▶GRWD1 (bg=5.13%)▶MTPAP (bg=2.21%)▶NOLC1 (bg=9.43%)▶SRSF1 (bg=8.47%)▶ZNF622 (bg=6.58%)No matches to TargetScan

ATGTGAGACCTGAGGA

CTGCAA

CTGCAA  
Depth:5 (RABBIT)  
Ei-value:0.000, Pi-value:0.000  
Er-value:0.000, Pr-value:0.000  
eCLIP MATCHES▶DDX24 (bg=2.97%)▶GRWD1 (bg=5.13%)▶MTPAP (bg=2.21%)▶NOLC1 (bg=9.43%)▶SRSF1 (bg=8.47%)▶UTP3 (bg=3.66%)▶ZNF622 (bg=6.58%)No matches to TargetScan

ACAGCTATAAGAGGCTCCAAATTAATCATATCTTTCCCTTTGAGAA 11516  
 TCTGGCCAAGCTCCAGCTAATCTACTTGGATGGGTTGCCAGCTATCTGGAGAAAAAG||ATCTTCCTCAG

AAGAATAGGC

AAGAATAGGC  
Depth:5 (RABBIT)  
Ei-value:0.000, Pi-value:0.000  
Er-value:0.000, Pr-value:0.000  
eCLIP MATCHES▶NOLC1 (bg=9.43%)▶SRSF7 (bg=2.32%)▶uchl5 (bg=11.16%)No matches to TargetScan

TTGTTGTTT

TACAGTGTTAGTGA

TACAGTGTTAGTGA  
Depth:5 (RABBIT)  
Ei-value:0.000, Pi-value:0.000  
Er-value:0.000, Pr-value:0.000  
eCLIP MATCHES▶ILF3 (bg=3.0%)▶NOLC1 (bg=9.43%)▶RBM15 (bg=7.27%)▶SRSF7 (bg=2.32%)▶ZNF622 (bg=6.58%)MATCHES To TargetScan▶ miR-141-3p/200a-3p:AACACUG

TCCA

TTCCCTTTGA

TTCCCTTTGA  
Depth:6 (MOUSE)  
Ei-value:0.000, Pi-value:0.000  
Er-value:0.000, Pr-value:0.000  
eCLIP MATCHES▶ILF3 (bg=3.0%)▶RBM15 (bg=7.27%)▶SRSF7 (bg=2.32%)▶ZNF622 (bg=6.58%)No matches to TargetScan

CGA 11634  
 TCCC

TAGGTGGAGATGGGGCATGAGGATCCTCCAGGGGAA

TAGGTGGAGATGGGGCATGAGGATCCTCCAGGGGAA  
Depth:6 (MOUSE)  
Ei-value:0.000, Pi-value:0.000  
Er-value:0.000, Pr-value:0.000  
eCLIP MATCHES▶ILF3 (bg=3.0%)▶NOLC1 (bg=9.43%)▶RBM15 (bg=7.27%)▶SRSF7 (bg=2.32%)▶ZNF622 (bg=6.58%)MATCHES To TargetScan▶ miR-331-3p:CCCCUGG


A

TAGGTGGAGATGGGGCATGAGGATCCTCCAGGGGAAA  
Depth:5 (RABBIT)  
Ei-value:0.000, Pi-value:0.000  
Er-value:0.000, Pr-value:0.000  
eCLIP MATCHES▶ILF3 (bg=3.0%)▶NOLC1 (bg=9.43%)▶RBM15 (bg=7.27%)▶SRSF7 (bg=2.32%)▶ZNF622 (bg=6.58%)MATCHES To TargetScan▶ miR-331-3p:CCCCUGG

AGC

TCACTA

TCACTA  
Depth:5 (RABBIT)  
Ei-value:0.000, Pi-value:0.000  
Er-value:0.000, Pr-value:0.000  
eCLIP MATCHES▶ILF3 (bg=3.0%)No matches to TargetScan


CCACT

TCACTACCACT  
Depth:4 (DOG)  
Ei-value:0.000, Pi-value:0.000  
Er-value:0.000, Pr-value:0.000  
eCLIP MATCHES▶ILF3 (bg=3.0%)MATCHES To TargetScan▶ miR-140-5p:AGUGGUU▶ miR-142-3p.1:GUAGUGU

GG

GCAACA

GCAACA  
Depth:6 (MOUSE)  
Ei-value:0.000, Pi-value:0.000  
Er-value:0.000, Pr-value:0.000  
eCLIP MATCHES▶ILF3 (bg=3.0%)No matches to TargetScan


AC

GCAACAAC  
Depth:5 (RABBIT)  
Ei-value:0.000, Pi-value:0.000  
Er-value:0.000, Pr-value:0.000  
eCLIP MATCHES▶ILF3 (bg=3.0%)No matches to TargetScan

CCTAGGTCAGGAGGTTCTGTCAAGATACTTTCCTGGTCCCAGATAGGAAGATAAA 11754  
 GTCTCAAAA

ACAACCACC

ACAACCACC  
Depth:5 (RABBIT)  
Ei-value:0.000, Pi-value:0.000  
Er-value:0.000, Pr-value:0.000  
eCLIP MATCHES▶PRPF8 (bg=0.26%)No matches to TargetScan


ACAC

ACAACCACCACAC  
Depth:4 (DOG)  
Ei-value:0.000, Pi-value:0.000  
Er-value:0.000, Pr-value:0.000  
eCLIP MATCHES▶PRPF8 (bg=0.26%)No matches to TargetScan

GTCAAG||CTCTTCA

TTGTTCC

TTGTTCC  
Depth:4 (DOG)  
Ei-value:0.000, Pi-value:0.000  
Er-value:0.000, Pr-value:0.000  
eCLIP MATCHES▶GRWD1 (bg=5.13%)▶SF3B4 (bg=0.05%)No matches to TargetScan

TATCTG

CCAAAT

CCAAAT  
Depth:6 (MOUSE)  
Ei-value:0.000, Pi-value:0.000  
Er-value:0.000, Pr-value:0.000  
eCLIP MATCHES▶GRWD1 (bg=5.13%)▶NOLC1 (bg=9.43%)No matches to TargetScan


C

CCAAATC  
Depth:5 (RABBIT)  
Ei-value:0.000, Pi-value:0.000  
Er-value:0.000, Pr-value:0.000  
eCLIP MATCHES▶GRWD1 (bg=5.13%)▶NOLC1 (bg=9.43%)No matches to TargetScan

ATTATACTTCCTACAAGCAGTGCAGAGAGCTGAGTCTTCAGCAGGTC

CAAGAAA

CAAGAAA  
Depth:5 (RABBIT)  
Ei-value:0.000, Pi-value:0.000  
Er-value:0.000, Pr-value:0.000  
eCLIP MATCHES▶GRWD1 (bg=5.13%)▶NOLC1 (bg=9.43%)▶uchl5 (bg=11.16%)▶ZNF622 (bg=6.58%)No matches to TargetScan

TTTGAACAC 11872  
 ACTGAAGGAAGTCAGCCTTCCCACCT

G

GAAGATCAACATGCCTG  
Depth:4 (DOG)  
Ei-value:0.000, Pi-value:0.000  
Er-value:0.000, Pr-value:0.000  
eCLIP MATCHES▶GRWD1 (bg=5.13%)▶NOLC1 (bg=9.43%)▶PTBP1 (bg=3.74%)▶RBM15 (bg=7.27%)▶TRA2A (bg=4.8%)▶uchl5 (bg=11.16%)▶ZNF622 (bg=6.58%)No matches to TargetScan


AA

AAGATCAACATGC  
Depth:5 (RABBIT)  
Ei-value:0.000, Pi-value:0.000  
Er-value:0.000, Pr-value:0.000  
eCLIP MATCHES▶GRWD1 (bg=5.13%)▶NOLC1 (bg=9.43%)▶PTBP1 (bg=3.74%)▶RBM15 (bg=7.27%)▶TRA2A (bg=4.8%)▶uchl5 (bg=11.16%)▶ZNF622 (bg=6.58%)No matches to TargetScan


GATCAACATGC

GATCAACATGC  
Depth:6 (MOUSE)  
Ei-value:0.000, Pi-value:0.000  
Er-value:0.000, Pr-value:0.000  
eCLIP MATCHES▶GRWD1 (bg=5.13%)▶NOLC1 (bg=9.43%)▶PTBP1 (bg=3.74%)▶RBM15 (bg=7.27%)▶TRA2A (bg=4.8%)▶uchl5 (bg=11.16%)▶ZNF622 (bg=6.58%)No matches to TargetScan


CTG

GAAGATCAACATGCCTG  
Depth:4 (DOG)  
Ei-value:0.000, Pi-value:0.000  
Er-value:0.000, Pr-value:0.000  
eCLIP MATCHES▶GRWD1 (bg=5.13%)▶NOLC1 (bg=9.43%)▶PTBP1 (bg=3.74%)▶RBM15 (bg=7.27%)▶TRA2A (bg=4.8%)▶uchl5 (bg=11.16%)▶ZNF622 (bg=6.58%)No matches to TargetScan

GCACTCTAGCACTTGAGGATAGCTGAATGAA||

TGTGTAT

TGTGTAT  
Depth:6 (MOUSE)  
Ei-value:0.000, Pi-value:0.000  
Er-value:0.000, Pr-value:0.000  
eCLIP MATCHES▶TARDBP (bg=2.79%)▶ZC3H11A (bg=6.55%)No matches to TargetScan


TT

TGTGTATTT  
Depth:4 (DOG)  
Ei-value:0.000, Pi-value:0.000  
Er-value:0.000, Pr-value:0.000  
eCLIP MATCHES▶TARDBP (bg=2.79%)▶ZC3H11A (bg=6.55%)No matches to TargetScan

CTTTGTCTCTTTCTTTCTTGTCTTTGCTCTTTGTT 11990  
 CTCTATCTAAAGTG

TGTCTTA

TGTCTTA  
Depth:4 (DOG)  
Ei-value:0.000, Pi-value:0.000  
Er-value:0.000, Pr-value:0.000  
eCLIP MATCHES▶MATR3 (bg=2.98%)▶PTBP1 (bg=3.74%)▶TARDBP (bg=2.79%)▶ZC3H11A (bg=6.55%)MATCHES To TargetScan▶ miR-208-3p:UAAGACG▶ miR-499a-5p:UAAGACU

CCCATTTCCATGTTTCTCTTGCTAATTTCTTTCGTGTGTGCCTTTGCCTCATTTTCTC

TTTTTGT

TTTTTGT  
Depth:4 (DOG)  
Ei-value:0.000, Pi-value:0.000  
Er-value:0.000, Pr-value:0.000  
eCLIP MATCHES▶MATR3 (bg=2.98%)▶PTBP1 (bg=3.74%)▶TARDBP (bg=2.79%)▶TIA1 (bg=4.07%)▶ZC3H11A (bg=6.55%)No matches to TargetScan

TCACAAGAGTGGTCTGTGTCTTGTCTTAGACATA 12110  
 TCTCTCATTT

TTCATTTTGTT

TTCATTTTGTT  
Depth:4 (DOG)  
Ei-value:0.000, Pi-value:0.000  
Er-value:0.000, Pr-value:0.000  
No matches to eCLIP DataMATCHES To TargetScan▶ miR-495-3p:AACAAAC

GCTATTTCTCTTTGCTCTCCTAGATGTGGCTCTTCTTTCACGCTTTATTTCATGTCTCCTTTTTGGGTCACATGCTGTGTGCTTTTTGTCCTTTTCTTG 12230  
 TTCTGTCTACCTCTCCTTTCTCTGCCTACCTCTCTT

TTCTCTTTG

TTCTCTTTG  
Depth:6 (MOUSE)  
Ei-value:0.000, Pi-value:0.000  
Er-value:0.000, Pr-value:0.000  
eCLIP MATCHES▶MATR3 (bg=2.98%)▶PTBP1 (bg=3.74%)▶SMNDC1 (bg=0.63%)▶TIA1 (bg=4.07%)No matches to TargetScan

TGAACTGTGATTATTTGTTACCCCTTCCCCTTCTCGTTCGTTTTAA

ATTTCACCT

ATTTCACCT  
Depth:4 (DOG)  
Ei-value:0.000, Pi-value:0.000  
Er-value:0.000, Pr-value:0.000  
eCLIP MATCHES▶TIA1 (bg=4.07%)MATCHES To TargetScan▶ miR-203a-3p.2:UGAAAUG

TTTTTCTGAGTCTGGCCTCC 12350  
 TTTCTGCTG

TTTCTAC

TTTCTAC  
Depth:6 (MOUSE)  
Ei-value:0.000, Pi-value:0.000  
Er-value:0.000, Pr-value:0.000  
eCLIP MATCHES▶MATR3 (bg=2.98%)▶PTBP1 (bg=3.74%)▶TIA1 (bg=4.07%)No matches to TargetScan


T

TTTCTACT  
Depth:5 (RABBIT)  
Ei-value:0.000, Pi-value:0.000  
Er-value:0.000, Pr-value:0.000  
eCLIP MATCHES▶MATR3 (bg=2.98%)▶PTBP1 (bg=3.74%)▶TIA1 (bg=4.07%)MATCHES To TargetScan▶ miR-411-5p.1:AGUAGAC

TTTTATCTCAC

ATTTCTC

ATTTCTC  
Depth:6 (MOUSE)  
Ei-value:0.000, Pi-value:0.000  
Er-value:0.000, Pr-value:0.000  
eCLIP MATCHES▶MATR3 (bg=2.98%)▶PTBP1 (bg=3.74%)▶TIA1 (bg=4.07%)No matches to TargetScan

ATTTCTGCATTTCCTTTCTGCCTC

TCTTGGG

TCTTGGG  
Depth:5 (RABBIT)  
Ei-value:0.000, Pi-value:0.000  
Er-value:0.000, Pr-value:0.000  
eCLIP MATCHES▶MATR3 (bg=2.98%)▶PTBP1 (bg=3.74%)▶SMNDC1 (bg=0.63%)▶TIA1 (bg=4.07%)No matches to TargetScan

CTATTCTCTCTCTCCTCCCCTGCGTGCCTCAGCATCTCTTGCTG

TTTGTGA

TTTGTGA  
Depth:4 (DOG)  
Ei-value:0.000, Pi-value:0.010  
Er-value:0.000, Pr-value:0.000  
eCLIP MATCHES▶MATR3 (bg=2.98%)▶PTBP1 (bg=3.74%)▶TIA1 (bg=4.07%)No matches to TargetScan

TTT 12470  
 TCTATTTCAGTATTAA

TCTCTGTT

TCTCTGTT  
Depth:4 (DOG)  
Ei-value:0.000, Pi-value:0.000  
Er-value:0.000, Pr-value:0.000  
eCLIP MATCHES▶MATR3 (bg=2.98%)▶PTBP1 (bg=3.74%)No matches to TargetScan

GGCTTGTATTTGTTCTCTGCTTCTTCCCTTTCTACTCACC

TTTGAGTATTT

TTTGAGTATTT  
Depth:4 (DOG)  
Ei-value:0.000, Pi-value:0.000  
Er-value:0.000, Pr-value:0.000  
eCLIP MATCHES▶MATR3 (bg=2.98%)▶PTBP1 (bg=3.74%)▶TIA1 (bg=4.07%)MATCHES To TargetScan▶ miR-200bc-3p/429:AAUACUG▶ miR-371-5p:CUCAAAC

CAGCCTCTTCATGAATCTATCTCCCTCTCTTTGATTTCATGTAAT 12590  
 CTCTCCTTAAATATTTCTTTGCATATGTGGGCAAGTGTACG

TGTGTGTG

TGTGTGTG  
Depth:4 (DOG)  
Ei-value:0.000, Pi-value:0.000  
Er-value:0.000, Pr-value:0.000  
eCLIP MATCHES▶AATF (bg=0.64%)▶DDX24 (bg=2.97%)▶NCBP2 (bg=1.49%)▶NOLC1 (bg=9.43%)▶PTBP1 (bg=3.74%)▶SND1 (bg=0.45%)▶SRSF7 (bg=2.32%)▶TARDBP (bg=2.79%)▶WDR43 (bg=3.37%)▶XRCC6 (bg=2.91%)▶ZC3H8 (bg=0.29%)MATCHES To TargetScan▶ miR-329-3p/362-3p:ACACACC

TGTCATGTGTGGCAGAGGGGCT

TCCTAACCCCT

TCCTAACCCCT  
Depth:5 (RABBIT)  
Ei-value:0.000, Pi-value:0.000  
Er-value:0.000, Pr-value:0.000  
eCLIP MATCHES▶AATF (bg=0.64%)▶DDX24 (bg=2.97%)▶NCBP2 (bg=1.49%)▶NOLC1 (bg=9.43%)▶PTBP1 (bg=3.74%)▶SND1 (bg=0.45%)▶SRSF7 (bg=2.32%)▶TARDBP (bg=2.79%)▶UTP3 (bg=3.66%)▶WDR43 (bg=3.37%)▶XRCC6 (bg=2.91%)▶ZC3H8 (bg=0.29%)No matches to TargetScan

GCCTGATAGGTGCAGAACGTCGGCTATCAGAGC

AAGCA

AAGCATTG  
Depth:4 (DOG)  
Ei-value:0.000, Pi-value:0.000  
Er-value:0.000, Pr-value:0.000  
eCLIP MATCHES▶DDX24 (bg=2.97%)▶NOLC1 (bg=9.43%)▶NPM1 (bg=1.21%)▶RBFOX2 (bg=4.63%)▶RPS3 (bg=0.76%)▶SRSF1 (bg=8.47%)▶SRSF7 (bg=2.32%)▶TARDBP (bg=2.79%)▶TRA2A (bg=4.8%)▶U2AF2 (bg=1.76%)▶uchl5 (bg=11.16%)▶YWHAG (bg=1.87%)▶ZNF622 (bg=6.58%)No matches to TargetScan

 12710  


TTG

AAGCATTG  
Depth:4 (DOG)  
Ei-value:0.000, Pi-value:0.000  
Er-value:0.000, Pr-value:0.000  
eCLIP MATCHES▶DDX24 (bg=2.97%)▶NOLC1 (bg=9.43%)▶NPM1 (bg=1.21%)▶RBFOX2 (bg=4.63%)▶RPS3 (bg=0.76%)▶SRSF1 (bg=8.47%)▶SRSF7 (bg=2.32%)▶TARDBP (bg=2.79%)▶TRA2A (bg=4.8%)▶U2AF2 (bg=1.76%)▶uchl5 (bg=11.16%)▶YWHAG (bg=1.87%)▶ZNF622 (bg=6.58%)No matches to TargetScan

TGGAGCGGTTCC

TTATGCCA

TTATGCCA  
Depth:5 (RABBIT)  
Ei-value:0.000, Pi-value:0.000  
Er-value:0.000, Pr-value:0.000  
eCLIP MATCHES▶DDX24 (bg=2.97%)▶FASTKD2 (bg=1.99%)▶LARP4 (bg=4.72%)▶NOLC1 (bg=9.43%)▶NPM1 (bg=1.21%)▶RBFOX2 (bg=4.63%)▶RBM15 (bg=7.27%)▶RPS3 (bg=0.76%)▶SRSF1 (bg=8.47%)▶SRSF7 (bg=2.32%)▶TARDBP (bg=2.79%)▶TRA2A (bg=4.8%)▶U2AF2 (bg=1.76%)▶uchl5 (bg=11.16%)▶WDR43 (bg=3.37%)▶YWHAG (bg=1.87%)▶ZC3H11A (bg=6.55%)▶ZNF622 (bg=6.58%)▶ZNF800 (bg=1.92%)No matches to TargetScan


G

TTATGCCAG  
Depth:4 (DOG)  
Ei-value:0.000, Pi-value:0.000  
Er-value:0.000, Pr-value:0.000  
eCLIP MATCHES▶DDX24 (bg=2.97%)▶FASTKD2 (bg=1.99%)▶LARP4 (bg=4.72%)▶NOLC1 (bg=9.43%)▶NPM1 (bg=1.21%)▶RBFOX2 (bg=4.63%)▶RBM15 (bg=7.27%)▶RPS3 (bg=0.76%)▶SRSF1 (bg=8.47%)▶SRSF7 (bg=2.32%)▶TARDBP (bg=2.79%)▶TRA2A (bg=4.8%)▶U2AF2 (bg=1.76%)▶uchl5 (bg=11.16%)▶WDR43 (bg=3.37%)▶YWHAG (bg=1.87%)▶ZC3H11A (bg=6.55%)▶ZNF622 (bg=6.58%)▶ZNF800 (bg=1.92%)No matches to TargetScan

GCTGCCATGTGAGATGATCCAAGACCAAAACAAGGCCCTAGACTGCAGTAAAACCCAGAACTCAAGTAGGGCAGAAGGTGGAAGGCTCATATGGAT 12830  


AGA

AGAAGGCCCAA  
Depth:4 (DOG)  
Ei-value:0.000, Pi-value:0.000  
Er-value:0.000, Pr-value:0.000  
eCLIP MATCHES▶DDX24 (bg=2.97%)▶LARP4 (bg=4.72%)▶MTPAP (bg=2.21%)▶NOLC1 (bg=9.43%)▶SRSF1 (bg=8.47%)▶SRSF7 (bg=2.32%)▶TRA2A (bg=4.8%)▶uchl5 (bg=11.16%)▶UTP3 (bg=3.66%)▶ZNF622 (bg=6.58%)▶ZNF800 (bg=1.92%)No matches to TargetScan


AGGCCCAA

AGGCCCAA  
Depth:5 (RABBIT)  
Ei-value:0.000, Pi-value:0.000  
Er-value:0.000, Pr-value:0.000  
eCLIP MATCHES▶DDX24 (bg=2.97%)▶LARP4 (bg=4.72%)▶MTPAP (bg=2.21%)▶NOLC1 (bg=9.43%)▶SRSF1 (bg=8.47%)▶SRSF7 (bg=2.32%)▶TRA2A (bg=4.8%)▶uchl5 (bg=11.16%)▶UTP3 (bg=3.66%)▶ZNF622 (bg=6.58%)▶ZNF800 (bg=1.92%)No matches to TargetScan

AGTATAAGACAGATGGTTTGAGACTTGAGACCCGAGGACTAAGATGGAAAGCCCATGTTCCAAGATAGATAGAAGCCTCAGGCCTGAAACCAACAAAAGCCTCAAGAGC 12950  
 CAAGAAAACAGAGGGTGGCCTGAATTGGACCGAAGGCCTGAGTTGGATGGAAGTCTCAAGGCTTGAGTTAGAAGTCTTAAGACCTGGGACAGGACACATGGAAGGCCTAAGAACTGAGAC 13070  
 TTGTGACACAAGGCCAACGACCTAAGATTAGCCCAGGGTTGTAGCTGGAAGACCTACAACCCAAGGATGGAAGGCCCCTGTCACAAAGCCTACCTAGATGGATAGAGGACCCAAGCGAAA 13190  
 AAGGTATC

TCAA

TCAAGACTAA  
Depth:4 (DOG)  
Ei-value:0.000, Pi-value:0.000  
Er-value:0.000, Pr-value:0.000  
eCLIP MATCHES▶CPEB4 (bg=1.89%)▶FASTKD2 (bg=1.99%)▶GRWD1 (bg=5.13%)▶LARP4 (bg=4.72%)▶MTPAP (bg=2.21%)▶NOLC1 (bg=9.43%)▶RBFOX2 (bg=4.63%)▶SRSF1 (bg=8.47%)▶TRA2A (bg=4.8%)▶uchl5 (bg=11.16%)▶UTP18 (bg=0.72%)▶UTP3 (bg=3.66%)▶WDR43 (bg=3.37%)▶ZNF622 (bg=6.58%)MATCHES To TargetScan▶ miR-431-5p:GUCUUGC


GACTAA

GACTAA  
Depth:5 (RABBIT)  
Ei-value:0.000, Pi-value:0.000  
Er-value:0.000, Pr-value:0.000  
eCLIP MATCHES▶CPEB4 (bg=1.89%)▶FASTKD2 (bg=1.99%)▶GRWD1 (bg=5.13%)▶LARP4 (bg=4.72%)▶MTPAP (bg=2.21%)▶NOLC1 (bg=9.43%)▶RBFOX2 (bg=4.63%)▶SRSF1 (bg=8.47%)▶TRA2A (bg=4.8%)▶uchl5 (bg=11.16%)▶UTP18 (bg=0.72%)▶UTP3 (bg=3.66%)▶WDR43 (bg=3.37%)▶ZNF622 (bg=6.58%)No matches to TargetScan

CGGCCGGAATCTGGAGGCCCATGACCCAGAACCCAGGAAGGAT

AGAAGC

AGAAGC  
Depth:4 (DOG)  
Ei-value:0.000, Pi-value:0.000  
Er-value:0.000, Pr-value:0.010  
eCLIP MATCHES▶CPEB4 (bg=1.89%)▶GRWD1 (bg=5.13%)▶LARP4 (bg=4.72%)▶MTPAP (bg=2.21%)▶NOLC1 (bg=9.43%)▶PCBP1 (bg=1.07%)▶RBFOX2 (bg=4.63%)▶SRSF1 (bg=8.47%)▶TRA2A (bg=4.8%)▶uchl5 (bg=11.16%)▶ZNF622 (bg=6.58%)No matches to TargetScan

TTGAAGACCTGGGGAAATCCC

AAGATGA

AAGATGA  
Depth:5 (RABBIT)  
Ei-value:0.000, Pi-value:0.000  
Er-value:0.000, Pr-value:0.000  
eCLIP MATCHES▶CPEB4 (bg=1.89%)▶FTO (bg=0.32%)▶GRWD1 (bg=5.13%)▶LARP4 (bg=4.72%)▶MTPAP (bg=2.21%)▶SRSF1 (bg=8.47%)▶TRA2A (bg=4.8%)▶uchl5 (bg=11.16%)▶ZNF622 (bg=6.58%)No matches to TargetScan

GAACCCTAAACCCTACCTCTTTTCT 13310  
 ATTGTTTACACTTCTTACTCTTAGATATTTCCAGTTCTCCTGTTTATCTTTAAGCCTGATTCTTTTGAGATGTA

CTTTTTGATGTT

CTTTTTGATGTT  
Depth:4 (DOG)  
Ei-value:0.000, Pi-value:0.000  
Er-value:0.000, Pr-value:0.000  
eCLIP MATCHES▶TIA1 (bg=4.07%)No matches to TargetScan

GCCGGTTACCTTTAGATTGACAG

TATTATGC

TATTATGC  
Depth:4 (DOG)  
Ei-value:0.000, Pi-value:0.000  
Er-value:0.000, Pr-value:0.000  
No matches to eCLIP DataMATCHES To TargetScan▶ miR-369-3p:AUAAUAC

CTG 13430  
 GGCCAGTCTTGAGCCAGCTTTAAATCACAGCTTTTACCTATTTGTTAGGCTATAGTGTTTTGTAAACTTCTGTTTCTATTCACATCTTCTCCACTTGAGAGAGACACCAAAATCCAGTCA 13550  
 GTATCTAATCTGGCTTTTGTTAACTTCCCTCAGGAGCAGACATTCATATAGGTGATACTG

TATTTCAGT

TATTTCAGT  
Depth:4 (DOG)  
Ei-value:0.000, Pi-value:0.000  
Er-value:0.000, Pr-value:0.000  
eCLIP MATCHES▶NOLC1 (bg=9.43%)▶ZC3H11A (bg=6.55%)MATCHES To TargetScan▶ miR-203a-3p.2:UGAAAUG

CCTTTCTTTTGACCCCAGAAGCCCTAGACTGAGAAGATAAAATGGTCAGGT 13670  
 TGTT

GGGGAAA

GGGGAAA  
Depth:4 (DOG)  
Ei-value:0.000, Pi-value:0.000  
Er-value:0.000, Pr-value:0.000  
eCLIP MATCHES▶CPSF6 (bg=0.4%)▶LARP4 (bg=4.72%)▶WDR43 (bg=3.37%)▶ZC3H11A (bg=6.55%)No matches to TargetScan

AAAAAGTGCCAGGCTC

TCTAGAGAAAA

TCTAGAGAAAA  
Depth:6 (MOUSE)  
Ei-value:0.000, Pi-value:0.000  
Er-value:0.000, Pr-value:0.000  
eCLIP MATCHES▶CPSF6 (bg=0.4%)▶LARP4 (bg=4.72%)▶UTP3 (bg=3.66%)▶WDR43 (bg=3.37%)MATCHES To TargetScan▶ miR-1251-5p:CUCUAGC

ATG

TGAAGAGATG

TGAAGAGATG  
Depth:5 (RABBIT)  
Ei-value:0.000, Pi-value:0.000  
Er-value:0.000, Pr-value:0.000  
eCLIP MATCHES▶CPSF6 (bg=0.4%)▶LARP4 (bg=4.72%)▶SRSF7 (bg=2.32%)▶UTP3 (bg=3.66%)▶WDR43 (bg=3.37%)No matches to TargetScan

CTCCA

GGCCAA

GGCCAATGAGAAGAATTAGACA  
Depth:4 (DOG)  
Ei-value:0.000, Pi-value:0.000  
Er-value:0.000, Pr-value:0.000  
eCLIP MATCHES▶LARP4 (bg=4.72%)▶NOLC1 (bg=9.43%)▶SRSF7 (bg=2.32%)▶UTP3 (bg=3.66%)No matches to TargetScan


TGAGAAGAATTAGACA

TGAGAAGAATTAGACA  
Depth:6 (MOUSE)  
Ei-value:0.000, Pi-value:0.000  
Er-value:0.000, Pr-value:0.000  
eCLIP MATCHES▶LARP4 (bg=4.72%)▶NOLC1 (bg=9.43%)▶SRSF7 (bg=2.32%)No matches to TargetScan

AGAAATACACAGATGTGCCAGACTTC

TGAGAAG

TGAGAAG  
Depth:4 (DOG)  
Ei-value:0.000, Pi-value:0.000  
Er-value:0.000, Pr-value:0.010  
eCLIP MATCHES▶AARS (bg=2.18%)▶NOLC1 (bg=9.43%)▶PUS1 (bg=1.04%)▶SRSF7 (bg=2.32%)▶ZC3H11A (bg=6.55%)No matches to TargetScan

CACCTGCCA 13790  


GCAACA

GCAACA  
Depth:6 (MOUSE)  
Ei-value:0.000, Pi-value:0.000  
Er-value:0.000, Pr-value:0.000  
eCLIP MATCHES▶AARS (bg=2.18%)▶NOLC1 (bg=9.43%)▶PUS1 (bg=1.04%)▶ZC3H11A (bg=6.55%)No matches to TargetScan

GCTTCCTTCTTTGAGCTTA

GGTGAGC

GGTGAGC  
Depth:4 (DOG)  
Ei-value:0.000, Pi-value:0.000  
Er-value:0.000, Pr-value:0.000  
eCLIP MATCHES▶AARS (bg=2.18%)▶NOLC1 (bg=9.43%)▶PUS1 (bg=1.04%)▶ZC3H11A (bg=6.55%)No matches to TargetScan

AGGATTCTGG

GGTTTGGG

GGTTTGGG  
Depth:4 (DOG)  
Ei-value:0.000, Pi-value:0.000  
Er-value:0.000, Pr-value:0.000  
eCLIP MATCHES▶AARS (bg=2.18%)▶AKAP8L (bg=2.19%)▶NOLC1 (bg=9.43%)▶PUS1 (bg=1.04%)No matches to TargetScan

ATTTCTAGTGA

TGGTTA

TGGTTA  
Depth:5 (RABBIT)  
Ei-value:0.000, Pi-value:0.000  
Er-value:0.000, Pr-value:0.000  
eCLIP MATCHES▶AKAP8L (bg=2.19%)▶NOLC1 (bg=9.43%)▶PUS1 (bg=1.04%)▶SF3B1 (bg=2.48%)No matches to TargetScan


T

TGGTTAT  
Depth:4 (DOG)  
Ei-value:0.000, Pi-value:0.000  
Er-value:0.000, Pr-value:0.000  
eCLIP MATCHES▶AKAP8L (bg=2.19%)▶NOLC1 (bg=9.43%)▶PUS1 (bg=1.04%)▶SF3B1 (bg=2.48%)No matches to TargetScan

GGAAAGGGTGACTGTGCCTGGGACAAAGCGAGGT

CCCAAGG

CCCAAGG  
Depth:4 (DOG)  
Ei-value:0.000, Pi-value:0.000  
Er-value:0.000, Pr-value:0.000  
eCLIP MATCHES▶PUS1 (bg=1.04%)▶UTP3 (bg=3.66%)MATCHES To TargetScan▶ miR-212-5p:CCUUGGC

GGACAGCC

TGA

TGAACTCCCTGCT  
Depth:4 (DOG)  
Ei-value:0.000, Pi-value:0.000  
Er-value:0.000, Pr-value:0.000  
eCLIP MATCHES▶UTP3 (bg=3.66%)No matches to TargetScan

 13910  


ACTCCCTGCT

TGAACTCCCTGCT  
Depth:4 (DOG)  
Ei-value:0.000, Pi-value:0.000  
Er-value:0.000, Pr-value:0.000  
eCLIP MATCHES▶UTP3 (bg=3.66%)No matches to TargetScan

C

ATAGTAGTGGCC

ATAGTAGTGGCC  
Depth:4 (DOG)  
Ei-value:0.000, Pi-value:0.000  
Er-value:0.000, Pr-value:0.000  
No matches to eCLIP DataNo matches to TargetScan

AAATAATTTGGTGGACTGTGCCAACGCTACTCCTGGG

TTTAATAC

TTTAATAC  
Depth:4 (DOG)  
Ei-value:0.000, Pi-value:0.000  
Er-value:0.000, Pr-value:0.000  
eCLIP MATCHES▶WRN (bg=0.77%)MATCHES To TargetScan▶ miR-496.2:GUAUUAC

CCATCT

CT

CTAGGCTTAAAG  
Depth:4 (DOG)  
Ei-value:0.000, Pi-value:0.000  
Er-value:0.000, Pr-value:0.000  
No matches to eCLIP DataNo matches to TargetScan


AGGCTTA

AGGCTTA  
Depth:5 (RABBIT)  
Ei-value:0.000, Pi-value:0.000  
Er-value:0.000, Pr-value:0.000  
No matches to eCLIP DataNo matches to TargetScan


AAG

CTAGGCTTAAAG  
Depth:4 (DOG)  
Ei-value:0.000, Pi-value:0.000  
Er-value:0.000, Pr-value:0.000  
No matches to eCLIP DataNo matches to TargetScan

ATGAGAGAACCTGGGACTGTTGAGCAT

GTTTAAT

GTTTAAT  
Depth:5 (RABBIT)  
Ei-value:0.000, Pi-value:0.000  
Er-value:0.000, Pr-value:0.000  
No matches to eCLIP DataNo matches to TargetScan

 14030  


GTTTAAT  
Depth:5 (RABBIT)  
Ei-value:0.000, Pi-value:0.000  
Er-value:0.000, Pr-value:0.000  
No matches to eCLIP DataNo matches to TargetScan

ACTTTCCTTGATTTTTTTCTTCCTGTTTATGTGGGAAGTTGATTTAAATGACTGATAATGTGTATGAAAGCACTGTAAAACATAAGAGAAAAACCAATTAGTG

T

TATTGGCA  
Depth:5 (RABBIT)  
Ei-value:0.000, Pi-value:0.000  
Er-value:0.000, Pr-value:0.000  
eCLIP MATCHES▶HNRNPA1 (bg=2.57%)No matches to TargetScan


ATTGGCA

ATTGGCA  
Depth:6 (MOUSE)  
Ei-value:0.000, Pi-value:0.000  
Er-value:0.000, Pr-value:0.000  
eCLIP MATCHES▶HNRNPA1 (bg=2.57%)No matches to TargetScan

ATCATGCAG 14150  
 TTAACATTTGAAAGTGCAGTGTAAA

TTGTGAAG

TTGTGAAG  
Depth:6 (MOUSE)  
Ei-value:0.000, Pi-value:0.000  
Er-value:0.000, Pr-value:0.000  
eCLIP MATCHES▶HNRNPA1 (bg=2.57%)No matches to TargetScan

CATT

ATGTAAAT

ATGTAAAT  
Depth:5 (RABBIT)  
Ei-value:0.000, Pi-value:0.000  
Er-value:0.000, Pr-value:0.000  
No matches to eCLIP DataNo matches to TargetScan

CAGGGGTCCACAGTTTTTCTGTAAGGGGTCAAATCATAAATACTTTAGACTGTGGGCCATATGGTTTCTGTTACA 14270  
 TATTTGTTTTTTAAACAACGTTTTTATAAGGTCAAAATCATTCTTAGTTTTTGAGCCAATTGGATTTGGCCTGCTGTTCATAGCTTACCACCCCCTGATGTATTATTTGTTATTCAGAGA 14390  
 AAATTTCTGAATACTACTAGTTTCCTTTTCTGTGC

CTGTCCCT

CTGTCCCT  
Depth:4 (DOG)  
Ei-value:0.000, Pi-value:0.000  
Er-value:0.000, Pr-value:0.000  
No matches to eCLIP DataNo matches to TargetScan

GTGC

TAGGCACT

TAGGCACT  
Depth:4 (DOG)  
Ei-value:0.000, Pi-value:0.000  
Er-value:0.000, Pr-value:0.000  
No matches to eCLIP DataNo matches to TargetScan

AAAAATGCAATGATTATTGATATCTAGGTGACCTGAAAAAAAATAGTGAATGTGCTTTGTAAACT 14510  
 G

TAAAGCA

TAAAGCA  
Depth:4 (DOG)  
Ei-value:0.000, Pi-value:0.000  
Er-value:0.000, Pr-value:0.000  
eCLIP MATCHES▶LIN28B (bg=0.74%)No matches to TargetScan

CTTGTATTCTACTGTGATAAGCGTTGTGGATACAAAGAAAGGAGCAAGCATAAAAAAGTGCTCTTTCAAAAGGATATAGTACTATGCAGACACAAGGAATTGTTTGATAAAT 14630  
 GAATAAATTATATGTATATTTGAGGCCAATTTGTGTTTGCTGCTCTGGTAATTTTGAGTAAAAATGCAGTATTCCAGGTATCAGAAACGAAAACACATGGAAACTGCTTTTAAACTTTAA 14750  
 AATATACTGAAAACATAAGGGACTAAGCTTGTTGTGGTCACCTAT

AATGTGCCAGATA

AATGTGCCAGATA  
Depth:4 (DOG)  
Ei-value:0.000, Pi-value:0.000  
Er-value:0.000, Pr-value:0.000  
No matches to eCLIP DataMATCHES To TargetScan▶ miR-183-5p.2:UGGCACU

CCATGCTGGGTGCTAGAGCTACCAAAGGGGGAAAAGTATTCTCATAGAACAAAAAATTTCAG 14870  
 AAAGGTGCATA

TTAAAGTG

TTAAAGTG  
Depth:4 (DOG)  
Ei-value:0.000, Pi-value:0.000  
Er-value:0.000, Pr-value:0.000  
eCLIP MATCHES▶SF3B1 (bg=2.48%)No matches to TargetScan

CTTTGTAAA

CTAAAGCA

CTAAAGCA  
Depth:4 (DOG)  
Ei-value:0.000, Pi-value:0.000  
Er-value:0.000, Pr-value:0.000  
eCLIP MATCHES▶SF3B1 (bg=2.48%)No matches to TargetScan

TGATACAAATGTCAATGGGCTACATATTTATGAATGAATGAATGGATGA

ATGAATA

ATGAATA  
Depth:4 (DOG)  
Ei-value:0.000, Pi-value:0.000  
Er-value:0.000, Pr-value:0.000  
eCLIP MATCHES▶DROSHA (bg=2.49%)▶TARDBP (bg=2.79%)▶ZC3H11A (bg=6.55%)No matches to TargetScan

TTAAGTGCCTCTTACATACCAGCTATTT 14990  
 TG

GGTACTGT

GGTACTGT  
Depth:4 (DOG)  
Ei-value:0.000, Pi-value:0.000  
Er-value:0.000, Pr-value:0.000  
eCLIP MATCHES▶AARS (bg=2.18%)▶DROSHA (bg=2.49%)▶ILF3 (bg=3.0%)▶TARDBP (bg=2.79%)▶ZC3H11A (bg=6.55%)MATCHES To TargetScan▶ miR-101-3p.1:ACAGUAC▶ miR-144-3p:ACAGUAU

AAAATACAAGATTAATTCTCCTATGTA

ATAAGAGG

ATAAGAGG  
Depth:4 (DOG)  
Ei-value:0.000, Pi-value:0.000  
Er-value:0.000, Pr-value:0.000  
eCLIP MATCHES▶ILF3 (bg=3.0%)No matches to TargetScan

AAAGTTTATCCTCTATACTATTCAGATGTAAGGAATGATATATTGCTTAATTTTAAACAATCAAGACTTTACTGG 15110  
 TGAGGTTAAGT

TAAATTAT

TAAATTAT  
Depth:4 (DOG)  
Ei-value:0.000, Pi-value:0.010  
Er-value:0.000, Pr-value:0.000  
No matches to eCLIP DataNo matches to TargetScan

TACTGATACATTTTTCCAGGTAACCAGGAAAGAGCTAGTATGAGGAAATGAAGTAATAGATGTGAGATCCAGACCGAAAGTCACTTAATTCAGCTTGCGAA 15230  
 TGTGCTTTCTAAATTATAAAGCACTTGTAAATGAAAAATTTGATGCTTTCTGTATGA

ATAAAAC

ATAAAAC  
Depth:4 (DOG)  
Ei-value:0.000, Pi-value:0.010  
Er-value:0.000, Pr-value:0.000  
No matches to eCLIP DataNo matches to TargetScan

TTTCTGTAAGCTAGGTATTGTCTCTAC

AAAATTCTCA

AAAATTCTCA  
Depth:4 (DOG)  
Ei-value:0.000, Pi-value:0.000  
Er-value:0.000, Pr-value:0.000  
eCLIP MATCHES▶HNRNPU (bg=5.92%)No matches to TargetScan

TTGTATAGTTAAACCACAG 15350  
 TGAGAAGGGTTCTATAAGTAGT

TATACAAAC

TATACAAAC  
Depth:4 (DOG)  
Ei-value:0.000, Pi-value:0.000  
Er-value:0.000, Pr-value:0.000  
No matches to eCLIP DataNo matches to TargetScan

CAAGGGTTTAAATACCTGTTAAATAGATCAATTTTGATTGCCTACTAT

GTGAACTCA

GTGAACTCA  
Depth:4 (DOG)  
Ei-value:0.000, Pi-value:0.000  
Er-value:0.000, Pr-value:0.000  
No matches to eCLIP DataNo matches to TargetScan

CTGTTAAAGGCACTGAAAATTTATCATATTTC 15470  
 ATTTAGCCACAGCCAAAAATAAGGCAATACCTATGTTAGCATTTTGTGAACTCTAAGGCACCATATAAATGTAACTGTTGATTTTCTCACTTGGTGCTGGGTACTAGGTTTATAAAATTG 15590  
 TATGATAGTTATTATATTGTGCAAATAAAGTAGGAAAATTTGAATAACAATGATTATCTTTTGAATACGCATACGCAAGGGATTGGTTGTCTGAAGAATGCCACTATAGTAGTTATCTAT 15710  
 TG

TGTGCCA

TGTGCCA  
Depth:4 (DOG)  
Ei-value:0.000, Pi-value:0.000  
Er-value:0.000, Pr-value:0.000  
No matches to eCLIP DataMATCHES To TargetScan▶ miR-183-5p.2:UGGCACU

ATCTCATTGCTAGGCATTGGGGATGCA

AAGATAA

AAGATAA  
Depth:4 (DOG)  
Ei-value:0.000, Pi-value:0.000  
Er-value:0.000, Pr-value:0.000  
No matches to eCLIP DataNo matches to TargetScan

ACCATCTTTATTGTGTCTTGGGTAGCAGAAGAAAATATGTGTAAAATCAATTTATAATTTG

TAAACTG

TAAACTG  
Depth:4 (DOG)  
Ei-value:0.000, Pi-value:0.000  
Er-value:0.000, Pr-value:0.000  
eCLIP MATCHES▶HNRNPU (bg=5.92%)No matches to TargetScan

CCACCCATA 15830  
 TATAAGCTATATCTGCTGAATGATC

ATTGATTA

ATTGATTA  
Depth:4 (DOG)  
Ei-value:0.000, Pi-value:0.000  
Er-value:0.000, Pr-value:0.010  
No matches to eCLIP DataNo matches to TargetScan

CTCTTATCCTT

AGAGATA

AGAGATA  
Depth:4 (DOG)  
Ei-value:0.000, Pi-value:0.000  
Er-value:0.000, Pr-value:0.000  
No matches to eCLIP DataNo matches to TargetScan

ACAACTGGGGGCACAAACATTTATTATCATTATTGAACCTACAA

CAGAGATCT

CAGAGATCT  
Depth:4 (DOG)  
Ei-value:0.000, Pi-value:0.000  
Er-value:0.000, Pr-value:0.000  
eCLIP MATCHES▶HNRNPA1 (bg=2.57%)▶HNRNPU (bg=5.92%)No matches to TargetScan

ATGTGTAGATTTACAA 15950  
 AGCCTACAGTTCTATACAGATAGGAATGAACTA

TTGGCT

TTGGCT  
Depth:4 (DOG)  
Ei-value:0.000, Pi-value:0.000  
Er-value:0.000, Pr-value:0.000  
eCLIP MATCHES▶HNRNPA1 (bg=2.57%)No matches to TargetScan

TACTGAATGGTGA

TTACTTTCT

TTACTTTCT  
Depth:4 (DOG)  
Ei-value:0.000, Pi-value:0.000  
Er-value:0.000, Pr-value:0.010  
eCLIP MATCHES▶UTP3 (bg=3.66%)No matches to TargetScan

GTGGGGCTCGGAACTACATGCCCTAGGATATAAAAATGATGTTATCATTATAGAGTGCT 16070  
 CACAGAAGGAAATGAAGTAATATAGGTGTGAGATCCAGACCAAAAGTCATTTAACAAGTTTATTCAGTGATGAAAACATGGGACAAATGGACTAATATAAGGCAGTGTACTAAGCTGAGT 16190  
 AGAGAGATAAAGTCCTGTCCAGAAGATACATGCTTCCTGGCCTGATTGAGGAGATGGAAAATTTTTGCAAAAAACAAGGTGTTGTGGTCTTCCATCCAGTTTCTTAAGTGCTGATGATAA 16310  
 AAGTGAATTAGACCCACCTTGACCTGGCCTACAGAAGTAAAG

GAGTAAAAA

GAGTAAAAA  
Depth:4 (DOG)  
Ei-value:0.000, Pi-value:0.000  
Er-value:0.000, Pr-value:0.000  
No matches to eCLIP DataNo matches to TargetScan

TAAATGCCTCAGGCGTGCTTTTTGATTC

ATTTGAT

ATTTGAT  
Depth:4 (DOG)  
Ei-value:0.000, Pi-value:0.010  
Er-value:0.000, Pr-value:0.000  
No matches to eCLIP DataNo matches to TargetScan

AAACAAAGCATC

TTTTATGT

TTTTATGT  
Depth:4 (DOG)  
Ei-value:0.000, Pi-value:0.000  
Er-value:0.000, Pr-value:0.000  
eCLIP MATCHES▶SAFB (bg=2.69%)No matches to TargetScan

GGAATATACCATTC 16430  
 TGGGTCCTGAGGATAAGAGAGATGAGGGCATTAGATCACTGACAGCTGAAGATAGAAGAACATCTTTGGTTTGATTGTTTAAATAATATTTCAATGCCTATTCTCTGCAAGGTACTATGT 16550  
 TTCGTAAATTAAATAGGTCTGGCCCAGAAGACCCACTCAATTGCCTTTGAGATTAAAAAAAAAAAAAAAAAGAAAGAAAAATGCAAGTTTCTTTCAAAATAAAGAGACATTTTTCCTAGT 16670  
 TTCAGGAATCCCCCAAATCACTTCCTCATTGGCTTAGTTTA

AAGCCAG

AAGCCAG  
Depth:4 (DOG)  
Ei-value:0.000, Pi-value:0.000  
Er-value:0.000, Pr-value:0.000  
eCLIP MATCHES▶SAFB (bg=2.69%)MATCHES To TargetScan▶ miR-149-5p:CUGGCUC▶ miR-3064-5p:CUGGCUG

GAGACTG

ATAAAAG

ATAAAAG  
Depth:4 (DOG)  
Ei-value:0.000, Pi-value:0.000  
Er-value:0.000, Pr-value:0.000  
No matches to eCLIP DataNo matches to TargetScan

GGCTCAGGGTTTGTTCTTTAATTCATTAACTAAACATTCTGC

TTTTATTA

TTTTATTA  
Depth:4 (DOG)  
Ei-value:0.000, Pi-value:0.010  
Er-value:0.000, Pr-value:0.000  
No matches to eCLIP DataNo matches to TargetScan

CAG

TTAAA

TTAAATGG  
Depth:4 (DOG)  
Ei-value:0.000, Pi-value:0.000  
Er-value:0.000, Pr-value:0.000  
No matches to eCLIP DataNo matches to TargetScan

 16790  


TGG

TTAAATGG  
Depth:4 (DOG)  
Ei-value:0.000, Pi-value:0.000  
Er-value:0.000, Pr-value:0.000  
No matches to eCLIP DataNo matches to TargetScan

TTCAAGATGTAACAACTAGTTTTAAAGGTATTTGCTCATTGGTCTGGCTTAGAGACAGGAAGACATATGAGCAATAAAAAAAAGATTCTTTTGCATTTACCAATTTAGTAAAAATTT 16910  
 ATTAAAACTGAATAAAGTGCTGTTCTTAAGTGCTTGAAAGACGTAAACCAAAGTGCACTTTATCTCATTTATCTTATGGTGGAAACACAGGAACAAATTCTCTAAGAGACTGTGTTTCTT 17030  
 TAGTTGAGAAGAAACTTCATTGAGTAGCTGTGATATGTTCGATACTAAGGAAAAACTAAACAGATCACCTTTGACATGCGTTGTAGAGTGGGAATAAGAGAGGGCTTTTTATTTTTTCGT 17150  
 TCATACGAGTATTGATGAAGATGATACTAAATGCTAAATGAAATATATCTGCTCCAAAAGGCATTTATTCTGACTTGGAGATGCAACAAAAACACAAAAATGGAATGAAGTGATACTCTT 17270  
 CATCAAACAGAAGTGACTGTTATCTCAACCATTTTGTTAAATCCTAA

ACAGAAAACAAAA

ACAGAAAACAAAA  
Depth:4 (DOG)  
Ei-value:0.000, Pi-value:0.000  
Er-value:0.000, Pr-value:0.000  
No matches to eCLIP DataNo matches to TargetScan

AAAATCATGACGAAAAGACACTTGCTTATTAATTGGCTTGGAAAGTAGAATATAGGAGAA 17390  


AGGTTA

AGGTTA  
Depth:4 (DOG)  
Ei-value:0.000, Pi-value:0.000  
Er-value:0.000, Pr-value:0.000  
No matches to eCLIP DataNo matches to TargetScan

CTGTTTATTTTTTTTCATGTATTCA

TTCATTCT

TTCATTCT  
Depth:4 (DOG)  
Ei-value:0.000, Pi-value:0.000  
Er-value:0.000, Pr-value:0.000  
No matches to eCLIP DataNo matches to TargetScan

ACAAATATATTCGGGTGCCAATAGGTACTTGGTATAAGGTTTTTGGCCCC

AGAGACA

AGAGACA  
Depth:4 (DOG)  
Ei-value:0.000, Pi-value:0.000  
Er-value:0.000, Pr-value:0.000  
No matches to eCLIP DataNo matches to TargetScan

TGGGAAAAAAATGCATGCCTTCCC 17510  
 AGAGAATGCCTAATACTTT

CCTTTTGG

CCTTTTGG  
Depth:4 (DOG)  
Ei-value:0.000, Pi-value:0.000  
Er-value:0.000, Pr-value:0.000  
No matches to eCLIP DataNo matches to TargetScan

CTTGTTTTCTTGTTAGGGGCATGGCTTAGTCCCTAAATAACATTGTGTGGT

TTAATTC

TTAATTC  
Depth:4 (DOG)  
Ei-value:0.000, Pi-value:0.000  
Er-value:0.000, Pr-value:0.000  
No matches to eCLIP DataNo matches to TargetScan

CTACTCCGTATCTCTTCTACC

ACTCTGGCCACTAC

ACTCTGGCCACTAC  
Depth:4 (DOG)  
Ei-value:0.000, Pi-value:0.000  
Er-value:0.000, Pr-value:0.000  
No matches to eCLIP DataMATCHES To TargetScan▶ miR-142-3p.1:GUAGUGU

 17630  


ACTCTGGCCACTAC  
Depth:4 (DOG)  
Ei-value:0.000, Pi-value:0.000  
Er-value:0.000, Pr-value:0.000  
No matches to eCLIP DataMATCHES To TargetScan▶ miR-142-3p.1:GUAGUGU

G

ATAAGC

ATAAGC  
Depth:5 (RABBIT)  
Ei-value:0.000, Pi-value:0.010  
Er-value:0.000, Pr-value:0.000  
No matches to eCLIP DataNo matches to TargetScan


AGG

ATAAGCAGG  
Depth:4 (DOG)  
Ei-value:0.000, Pi-value:0.000  
Er-value:0.000, Pr-value:0.000  
No matches to eCLIP DataNo matches to TargetScan

TAGCTGGGTTTTGTAGTGAGCTTGCTCCTTAAGTTACAGGAACTCTCCTTATAATAGACACTTCATTTTCCTAGTCCATCCCTCAT

GAAAAATG

GAAAAATG  
Depth:4 (DOG)  
Ei-value:0.000, Pi-value:0.000  
Er-value:0.000, Pr-value:0.000  
No matches to eCLIP DataNo matches to TargetScan

ACTGACCACTGCTGGG 17750  
 CAGCAGGAGGGATGATGACCAACTAATTCCCAAACCCC

AGTCTCA

AGTCTCA  
Depth:4 (DOG)  
Ei-value:0.000, Pi-value:0.000  
Er-value:0.000, Pr-value:0.000  
eCLIP MATCHES▶ZC3H11A (bg=6.55%)No matches to TargetScan

TTGGTACCAGCCTTGGGGAACCACCTACACTTGAGCCACAAT

TGGTTTTGAA

TGGTTTTGAA  
Depth:4 (DOG)  
Ei-value:0.000, Pi-value:0.000  
Er-value:0.000, Pr-value:0.000  
No matches to eCLIP DataNo matches to TargetScan

GTGCATTTACAAGGTTTGTCTAT 17870  
 TTTCAGTTCTTTACTTTTTACATGCTGACACATACATACACTGCCTAAATAGATCTCTTTCAGAAACAATCCTCAGATAACGCATAGCAAAATGGAGATGGAGACATGATTTCTCATGCA 17990  
 ACAGCTTCTCTAATTATACC

TTAGAAAT

TTAGAAAT  
Depth:4 (DOG)  
Ei-value:0.000, Pi-value:0.000  
Er-value:0.000, Pr-value:0.000  
eCLIP MATCHES▶WDR3 (bg=0.25%)No matches to TargetScan

GTTCTCCTTTTTAT

CATCAAA

CATCAAA  
Depth:4 (DOG)  
Ei-value:0.000, Pi-value:0.000  
Er-value:0.000, Pr-value:0.000  
eCLIP MATCHES▶ZC3H11A (bg=6.55%)No matches to TargetScan

TCTGCTCAAGAAGGGCTTTTTATAGTAGAATAATATCAGTGGATGAAAACAGCTTAACATTTTACCATGCT 18110  
 TAAGTTTTAAGAATAAAATAAAAATTGGAAATAATTGGCCAAAATTGAAAGGAAAAATTTTTTTAAAATTTCTCTAAATGTAGGCCTGGCTGGGCTTTGACCTTTTCCG

TTTTTAAATCA

TTTTTAAATCACTCA  
Depth:4 (DOG)  
Ei-value:0.000, Pi-value:0.000  
Er-value:0.000, Pr-value:0.000  
eCLIP MATCHES▶ILF3 (bg=3.0%)▶NOLC1 (bg=9.43%)▶PPIL4 (bg=0.52%)No matches to TargetScan

 18230  


CTCA

TTTTTAAATCACTCA  
Depth:4 (DOG)  
Ei-value:0.000, Pi-value:0.000  
Er-value:0.000, Pr-value:0.000  
eCLIP MATCHES▶ILF3 (bg=3.0%)▶NOLC1 (bg=9.43%)▶PPIL4 (bg=0.52%)No matches to TargetScan

C

AGAGGGTGGGA

AGAGGGTGGGA  
Depth:4 (DOG)  
Ei-value:0.000, Pi-value:0.000  
Er-value:0.000, Pr-value:0.000  
eCLIP MATCHES▶ILF3 (bg=3.0%)▶ZC3H11A (bg=6.55%)No matches to TargetScan

C

AGGAGGAAGAGTGAA

AGGAGGAAGAGTGAA  
Depth:4 (DOG)  
Ei-value:0.000, Pi-value:0.000  
Er-value:0.000, Pr-value:0.000  
eCLIP MATCHES▶ILF3 (bg=3.0%)▶ZC3H11A (bg=6.55%)MATCHES To TargetScan▶ miR-670-3p:UUCCUCA

G

G

GAAAAGGTCA  
Depth:4 (DOG)  
Ei-value:0.000, Pi-value:0.000  
Er-value:0.000, Pr-value:0.000  
eCLIP MATCHES▶ILF3 (bg=3.0%)▶SF3B1 (bg=2.48%)▶ZC3H11A (bg=6.55%)MATCHES To TargetScan▶ miR-192-5p/215-5p:UGACCUA


AAAAGGT

AAAAGGT  
Depth:6 (MOUSE)  
Ei-value:0.000, Pi-value:0.000  
Er-value:0.000, Pr-value:0.000  
eCLIP MATCHES▶ILF3 (bg=3.0%)▶SF3B1 (bg=2.48%)▶ZC3H11A (bg=6.55%)No matches to TargetScan


CA

GAAAAGGTCA  
Depth:4 (DOG)  
Ei-value:0.000, Pi-value:0.000  
Er-value:0.000, Pr-value:0.000  
eCLIP MATCHES▶ILF3 (bg=3.0%)▶SF3B1 (bg=2.48%)▶ZC3H11A (bg=6.55%)MATCHES To TargetScan▶ miR-192-5p/215-5p:UGACCUA

AACCTGTTTTAAGGGCAACCTGCCTTTGTTCTGAATTGGTCTTAAGAACATTACCAGCTCCAGGTTTAAATTGTTCA 18350  
 GTTTCATGCAGTTCCAATAGCTGATCATTGTTGAGATGAGGACAAAATCCTTTGTCCTCACTAGTTTGCTTTACATTTTTGAAAAGTATTATTTTTGTCCAAGTGCTTATCAACTAAACC 18470  
 TTGTGTTAGGTAAGAATGGAATTTATTAAGTGAATCAGTGTGACCCTTCTTGTCATAAGATTATCTTAAAGCTGAAGCCAAAATATGCTTCAAAAGAAGAGGACTTTATTGTTCATTGTA 18590  
 GTTCATACATTCAAAGCATCTGAACTGTAGTTTCTATAGCAAGCCAATTACATCCATAAGTGGAGAAGGAAATAGATAAATGTCAAAGTATGATTGGTGGAGGGAGCAAGGTTGAAGATA 18710  
 ATCTGGGGTTGAAATTTTCTAGTTTTCATTCTGTACATTTTTAGTTAGACATCAGATTTGAAATAT

TAATGTTT

TAATGTTT  
Depth:4 (DOG)  
Ei-value:0.000, Pi-value:0.000  
Er-value:0.000, Pr-value:0.000  
eCLIP MATCHES▶CPEB4 (bg=1.89%)▶KHDRBS1 (bg=1.71%)▶LARP4 (bg=4.72%)▶LSM11 (bg=2.28%)▶NOLC1 (bg=9.43%)▶RBFOX2 (bg=4.63%)▶SAFB (bg=2.69%)▶SAFB2 (bg=0.8%)▶WDR43 (bg=3.37%)▶ZC3H11A (bg=6.55%)MATCHES To TargetScan▶ miR-323-3p:ACAUUAC▶ miR-543:AACAUUC

ACCTTTCAATGTGTGGTATC

AGCTGGA

AGCTGGA  
Depth:4 (DOG)  
Ei-value:0.000, Pi-value:0.000  
Er-value:0.000, Pr-value:0.000  
eCLIP MATCHES▶CPEB4 (bg=1.89%)▶KHDRBS1 (bg=1.71%)▶LSM11 (bg=2.28%)▶NOLC1 (bg=9.43%)▶RBFOX2 (bg=4.63%)▶SAFB (bg=2.69%)▶SAFB2 (bg=0.8%)▶SF3B1 (bg=2.48%)▶TRA2A (bg=4.8%)▶WDR43 (bg=3.37%)▶ZC3H11A (bg=6.55%)No matches to TargetScan

CTCAGTAACACCCCTTTCT 18830  
 TCAGCTGGGGATGGGGAATGG

ATTATTGGAAA

ATTATTGGAAA  
Depth:4 (DOG)  
Ei-value:0.000, Pi-value:0.000  
Er-value:0.000, Pr-value:0.000  
eCLIP MATCHES▶FASTKD2 (bg=1.99%)▶FUS (bg=2.21%)▶LARP4 (bg=4.72%)▶NOLC1 (bg=9.43%)▶RBFOX2 (bg=4.63%)▶SAFB (bg=2.69%)▶SAFB2 (bg=0.8%)▶TRA2A (bg=4.8%)▶WDR43 (bg=3.37%)▶ZC3H11A (bg=6.55%)No matches to TargetScan

ATGGAAAGA

AGAAAGTAAC

AGAAAGTAAC  
Depth:4 (DOG)  
Ei-value:0.000, Pi-value:0.000  
Er-value:0.000, Pr-value:0.000  
eCLIP MATCHES▶FASTKD2 (bg=1.99%)▶FUS (bg=2.21%)▶LARP4 (bg=4.72%)▶NIPBL (bg=5.39%)▶NOLC1 (bg=9.43%)▶RBFOX2 (bg=4.63%)▶SAFB (bg=2.69%)▶SAFB2 (bg=0.8%)▶TRA2A (bg=4.8%)▶uchl5 (bg=11.16%)▶WDR43 (bg=3.37%)▶ZC3H11A (bg=6.55%)▶ZNF800 (bg=1.92%)No matches to TargetScan

TAAAAGCCTTCC

TTTCACAGTTTCTGGCATC

TTTCACAGTTTCTGGCATC  
Depth:4 (DOG)  
Ei-value:0.000, Pi-value:0.000  
Er-value:0.000, Pr-value:0.000  
eCLIP MATCHES▶FASTKD2 (bg=1.99%)▶FUS (bg=2.21%)▶LARP4 (bg=4.72%)▶NIPBL (bg=5.39%)▶NOLC1 (bg=9.43%)▶RBFOX2 (bg=4.63%)▶SAFB (bg=2.69%)▶SAFB2 (bg=0.8%)▶uchl5 (bg=11.16%)▶WDR43 (bg=3.37%)▶ZC3H11A (bg=6.55%)▶ZNF800 (bg=1.92%)No matches to TargetScan

ACTACCA

CTACTGAT

CTACTGAT  
Depth:4 (DOG)  
Ei-value:0.000, Pi-value:0.000  
Er-value:0.000, Pr-value:0.000  
eCLIP MATCHES▶FASTKD2 (bg=1.99%)▶FUS (bg=2.21%)▶LARP4 (bg=4.72%)▶NIPBL (bg=5.39%)▶NOLC1 (bg=9.43%)▶RBFOX2 (bg=4.63%)▶SAFB (bg=2.69%)▶SAFB2 (bg=0.8%)▶uchl5 (bg=11.16%)▶ZC3H11A (bg=6.55%)▶ZNF800 (bg=1.92%)MATCHES To TargetScan▶ miR-199-3p:CAGUAGU

TAAACAAGAATAAG

AGAACAT

AGAACAT  
Depth:4 (DOG)  
Ei-value:0.000, Pi-value:0.000  
Er-value:0.000, Pr-value:0.000  
eCLIP MATCHES▶FASTKD2 (bg=1.99%)▶FUS (bg=2.21%)▶LARP4 (bg=4.72%)▶NIPBL (bg=5.39%)▶NOLC1 (bg=9.43%)▶RBFOX2 (bg=4.63%)▶SAFB2 (bg=0.8%)▶uchl5 (bg=11.16%)▶ZNF622 (bg=6.58%)No matches to TargetScan

TT 18950  
 TATCA

TCATCTG

TCATCTG  
Depth:4 (DOG)  
Ei-value:0.000, Pi-value:0.010  
Er-value:0.000, Pr-value:0.000  
eCLIP MATCHES▶FUS (bg=2.21%)▶LARP4 (bg=4.72%)▶NOLC1 (bg=9.43%)▶RBFOX2 (bg=4.63%)▶RPS3 (bg=0.76%)▶uchl5 (bg=11.16%)▶ZNF622 (bg=6.58%)No matches to TargetScan

CTTTATTCA

CATAAATGAA

CATAAATGAA  
Depth:4 (DOG)  
Ei-value:0.000, Pi-value:0.000  
Er-value:0.000, Pr-value:0.000  
eCLIP MATCHES▶FUS (bg=2.21%)▶NOLC1 (bg=9.43%)▶RPS3 (bg=0.76%)▶uchl5 (bg=11.16%)▶ZNF622 (bg=6.58%)No matches to TargetScan

GTTGTGATGAATAAATCTGCTTTTATGCAGACACAAGGAATTAAGTGGCTTCGTCATTGTCCTTCTACCTCAAAGATAATTTATTCCAA 19070  
 AAGCTAAGATAAATGGAAGACTCTTGAACTTG

TGAACTGATGTGAAA

TGAACTGATGTGAAA  
Depth:4 (DOG)  
Ei-value:0.000, Pi-value:0.000  
Er-value:0.000, Pr-value:0.000  
eCLIP MATCHES▶FUS (bg=2.21%)▶NOLC1 (bg=9.43%)▶RBFOX2 (bg=4.63%)▶TRA2A (bg=4.8%)MATCHES To TargetScan▶ miR-23-3p:UCACAUU

TGCAGAATCTCTTTTGAGTCTTTGCTGTTTGGAAGATTGAAAAATATTGTTCAGCATGGGTGACCACCAGAAA 19190  
 GTAATCTTAAGCCATCTAGATGTCACAATTGAAACAAACTGGGGAGTTGGTTGCTATTGTA

AAATAAAA

AAATAAAA  
Depth:4 (DOG)  
Ei-value:0.000, Pi-value:0.000  
Er-value:0.000, Pr-value:0.000  
eCLIP MATCHES▶WDR43 (bg=3.37%)▶ZC3H11A (bg=6.55%)No matches to TargetScan

TATACTGTTTTGAAAACTTTG                               19280
```

|  |  |  |  |  |
| --- | --- | --- | --- | --- |
| | | | | | | | | | |
| 2 |  | 4 |  | 6 |
| Depth of motif conservation (number of species) | | | | |

  
  

---

  

## >HUMAN TO RABBIT (19280 bases)

```
 CCTTCAGTTCTTAAAGCGCTGCAATTCGCTGCTGCAGCCATATTTCTTACTCTCTCGGGGCTGGAAGCTTCCTGACTGAAGATCTCTCTGCACTTGGGGTTCTTTCTAGAACATTTTCTA 120  
 GTCCCCCAACACCCTTTATGGCGTATTTCTTTAAAAAAATCACCTAAATTCCATAAAATATTTTTTTAAATTCTATACTTTCTCCTAGTGTCTTCTTGACACGTCCTCCATATTTTTTTA 240  
 AAGAAAGTATTTGGAATATTTTGAGGCAATTTTTAATATTTAAGGAATTTTTCTTTGGAATCATTTTTGGTTGACATCTCTGTTTTTTGTGGATCAGTTTTTTACTCTTCCACTCTCTTT 360  
 TCTATATTTTGCCCATCGGGGCTGCGGATACCTGGTTTTATTATTTTTTCTTTGCCCAACGGGGCCGTGGATACCTGCCTTTTAATTCTTTTTTATTCGCCCATCGGGGCCGCGGATACC 480  
 TGCTTTTTATTTTTTTTTCCTTAGCCCATCGGGGTATCGGATACCTGCTGATTCCCTTCCCCTCTGAACCCCCAACACTCTGGCCCATCGGGGTGACGGATATCTGCTTTTTAAAAATTT 600  
 TCTTTTTTTGGCCCATCGGGGCTTCGGATACCTGCTTTTTTTTTTTTTATTTTTCCTTGCCCATCGGGGCCTCGGATACCTGCTTTAATTTTTGTTTTTCTGGCCCATCGGGGCCGCGGA 720  
 TACCTGCTTTGATTTTTTTTTTTCATCGCCCATCGGTGCTTTTTATGGATGAAAAAATGTTGGTTTTGTGGGTTGTTGCACTCTCTGGAATATCTACACTTTTTTTTGCTGCTGATCATT 840  
 TGGTGGTGTGTGAGTGTACCTACCGCTTTGGCAGAGAATGACTCTGCAGTTAAGCTAAGGGCGTGTTCAGATTGTGGAGGAAAAGTGGCCGCCATTTTAGACTTGCCGCATAACTCGGCT 960  
 TAGGGCTAGTCGTTTGTGCTAAGTTAAACTAGGGAGGCAAGATGGATGATAGCAGGTCAGGCAGAGGAAGTCATGTGCATTGCATGAGCTAAACCTATCTGAATGAATTGATTTGGGGCT 1080  
 TGTTAGGAGCTTTGCGTGATTGTTGTATCGGGAGGCAGTAAGAATCATCTTTTATCAGTACAAGGGACTAGTTAAAAATGGAAGGTTAGGAAAGACTAAGGTGCAGGGCTTAAAATGGCG 1200  
 ATTTTGACATTGCGGCATTGCTCAGCATGGCGGGCTGTGCTTTGTTAGGTTGTCCAAAATGGCGGATCCAGTTCTGTCGCAGTGTTCAAGTGGCGGGAAGGCCACATCATGATGGGCGAG 1320  
 GCTTTGTTAAGTGGTTAGCATGGTGGTGGACATGTGCGGTCACACAGGAAAAGATGGCGGCTGAAGGTCTTGCCGCAGTGTAAAACATGGCGGGCCTCTTTGTCTTTGCTGTGTGCTTTT 1440  
 CGTGTTGGGTTTTGCCGCAGGGACAATATGGCAGGCGTTGTCATATGTATATCATGGCTTTTGTCACGTGGACATCATGGCGGGCTTGCCGCATTGTTAAAGATGGCGGGTTTTGCCGCC 1560  
 TAGTGCCACGCAGAGCGGGAGAAAAGGTGGGATGGACAGTGCTGGATTGCTGCATAACCCAACCAATTAGAAATGGGGGTGGAATTGATCACAGCCAATTAGAGCAGAAGATGGAATTAG 1680  
 ACTGATGACACACTGTCCAGCTACTCAGCGAAGACCTGGGTGAATTAGCATGGCACTTCGCAGCTGTCTTTAGCCAGTCAGGAGAAAGAAGTGGAGGGGCCACGTGTATGTCTCCCAGTG 1800  
 GGCGGTACACCAGGTGTTTTCAAGGTCTTTTCAAGGACATTTAGCCTTTCCACCTCTGTCCCCTCTTATTTGTCCCCTCCTGTCCAGTGCTGCCTCTTGCAGTGCTGGATATCTGGCTGT 1920  
 GTGGTCTGAACCTCCCTCCATTCCTCTGTATTGGTGCCTCACCTAAGGCTAAGTATACCTCCCCCCCCACCCCCCAACCCCCCCAACTCCCCACCCCCACCCCCCACCCCCCACCTCCCC 2040  
 ACCCCCCTACCCCCCTACCCCCCTACCCCCCTCTGGTCTGCCCTGCACTGCACTGTTGCCATGGGCAGTGCTCCAGGCCTGCTTGGTGTGGACATGGTGGTGAGCCGTGGCAAGGACCAG 2160  
 AATGGATCACAGATGATCGTTGGCCAACAGGTGGCAGAAGAGGAATTCCTGCCTTCCTCAAGAGGAACACCTACCCCTTGGCTAATGCTGGGGTCGGATTTTGATTTATATTTATCTTTT 2280  
 GGATGTCAGTCATACAGTCTGATTTTGTGGTTTGCTAGTGTTTGAATTTAAGTCTTAAGTGACTATTATAGAAATGTATTAAGAGGCTTTATTTGTAGAATTCACTTTAATTACATTTAA 2400  
 TGAGTTTTTGTTTTGAGTTCCTTAAAATTCCTTAAAGTTTTTAGCTTCTCATTACAAATTCCTTAACCTTTTTTTGGCAGTAGATAGTCAAAGTCAAATCATTTCTAATGTTTTAAAAAT 2520  
 GTGCTGGTCATTTTCTTTGAAATTGACTTAACTATTTTCCTTTGAAGAGTCTGTAGCACAGAAACAGTAAAAAATTTAACTTCATGACCTAATGTAAAAAAGAGTGTTTGAAGGTTTACA 2640  
 CAGGTCCAGGCCTTGCTTTGTTCCCATCCTTGATGCTGCACTAATTGACTAATCACCTACTTATCAGACAGGAAACTTGAATTGCTGTGGTCTGGTGTCCTCTATTCAGACTTATTATAT 2760  
 TGGAGTATTTCAATTTTTCGTTGTATCCTGCCTGCCTAGCATCCAGTTCCTCCCCAGCCCTGCTCCCAGCAAACCCCTAGTCTAGCCCCAGCCCTACTCCCACCCCGCCCCAGCCCTGCC 2880  
 CCAGCCCCAGTCCCCTAACCCCCCAGCCCTAGCCCCAGTCCCAGTCCTAGTTCCTCAGTCCCGCCCAGCTTCTCTCGAAAGTCACTCTAATTTTCATTGATTCAGTGCTCAAAATAAGTT 3000  
 GTCCATTGCTTATCCTATTATACTGGGATATTCCGTTTACCCTTGGCATTGCTGATCTTCAGTACTGACTCCTTGACCATTTTCAGTTAATGCATACAATCCCATTTGTCTGTGATCTCA 3120  
 GGACAAAGAATTTCCTTACTCGGTACGTTGAAGTTAGGGAATGTCAATTGAGAGCTTTCTATCAGAGCATTATTGCCCACAATTTGAGTTACTTATCATTTTCTCGATCCCCTGCCCTTA 3240  
 AAGGAGAAACCATTTCTCTGTCATTGCTTCTGTAGTCACAGTCCCAATTTTGAGTAGTGATCTTTTCTTGTGTACTGTGTTGGCCACCTAAAACTCTTTGCATTGAGTAAAATTCTAATT 3360  
 GCCAATAATCCTACCCATTGGATTAGACAGCACTCTGAACCCCATTTGCATTCAGCAGGGGGTCGCAGACAACCCGTCTTTTGTTGGACAGTTAAAATGCTCAGTCCCAATTGTCATAGC 3480  
 TTTGCCTATTAAACAAAGGCACCCTACTGCGCTTTTTGCTGTGCTTCTGGAGAATCCTGCTGTTCTTGGACAATTAAAGAACAAAGTAGTAATTGCTAATTGTCTCACCCATTAATCATG 3600  
 AAGACTACCAGTCGCCCTTGCATTTGCCTTGAGGCAGCGCTGACTACCTGAGATTTAAGAGTTTCTTAAATTATTGAGTAAAATCCCAATTATCCATAGTTCTGTTAGTTACACTATGGC 3720  
 CTTTGCAAACATCTTTGCATAACAGCAGTGGGACTGACTCATTCTTAGAGCCCCTTCCCTTGGAATATTAATGGATACAATAGTAATTATTCATGGTTCTGCGTAACAGAGAAGACCCAC 3840  
 TTATGTGTATGCCTTTATCATTGCTCCTAGATAGTGTGAACTACCTACCACCTTGCATTAATATGTAAAACACTAATTGCCCATAGTCCCACTCATTAGTCTAGGATGTCCTCTTTGCCA 3960  
 TTGCTGCTGAGTTCTGACTACCCAAGTTTCCTTCTCTTAAACAGTTGATATGCATAATTGCATATATTCATGGTTCTGTGCAATAAAAATGGATTCTCACCCCATCCCACCTTCTGTGGG 4080  
 ATGTTGCTAACGAGTGCAGATTATTCAATAACAGCTCTTGAACAGTTAATTTGCACAGTTGCAATTGTCCAGAGTCCTGTCCATTAGAAAGGGACTCTGTATCCTATTTGCACGCTACAA 4200  
 TGTGGGCTGATCACCCAAGGACTCTTCTTGTGCATTGATGTTCATAATTGTATTTGTCCACGATCTTGTGCACTAACCCTTCCACTCCCTTTGTATTCCAGCAGGGGACCCTTACTACTC 4320  
 AAGACCTCTGTACTAGGACAGTTTATGTGCACAATCCTAATTGATTAGAACTGAGTCTTTTATATCAAGGTCCCTGCATCATCTTTGCTTTACATCAAGAGGGTGCTGGTTACCTAATGC 4440  
 CCCTCCTCCAGAAATTATTGATGTGCAAAATGCAATTTCCCTATCTGC

TGTTAGTC

TGTTAGTC  
Depth:5 (RABBIT)  
Ei-value:0.000, Pi-value:0.000  
Er-value:0.000, Pr-value:0.000  
eCLIP MATCHES▶AKAP8L (bg=2.19%)No matches to TargetScan

TGGGGTCTCATCCCCTCATATTCCTTTTGTCTTACAGCAGGGGGTACTTGGGACTGTTAATGCG 4560  
 CATAATTGCAATTATGGTCTTTTCCATTAAATTAAGATCCCAACTGCTCACACCCTCTTAGCATTACAGTAGAGGGTGCTAATCACAAGGACATTTCTTTTGTACTG

TTAATGTGCT

TTAATGTGCT  
Depth:5 (RABBIT)  
Ei-value:0.000, Pi-value:0.000  
Er-value:0.000, Pr-value:0.000  
No matches to eCLIP DataMATCHES To TargetScan▶ miR-323-3p:ACAUUAC

ACT 4680  
 TGCATTTGTCCCTCTTCCTGTGCACTAAAGACCCCACTCACTTCCCTAGTGTTCAGCAGTGGATGACCTCTAGTCAAGACCTTTGCACTAGGATAGTTAATGTGAACCATGGCAACTGAT 4800  
 CACAACAATGTCTTTCAGATCAGATCCATTTTATCCTCCTTGTTTTACAGCAAGGGATATTAATTACCTATGTTACCTTTCCCTGGGACTATGAATGTGCAAAATTCCAATGTTCATGGT 4920  
 CTCTCCCTTTAAACCTATATTCTACCCCTTTTACATTATAGAAAGGGATGCTGGAAACCCAGAGTCCTTCTCTTGGGACTCTTAATGTGTATTTCTAATTATCCATGACTCTT

AATGTGC

AATGTGCAT  
Depth:6 (MOUSE)  
Ei-value:0.000, Pi-value:0.000  
Er-value:0.000, Pr-value:0.000  
No matches to eCLIP DataMATCHES To TargetScan▶ miR-501-3p/502-3p:AUGCACC

 5040  


AT

AATGTGCAT  
Depth:6 (MOUSE)  
Ei-value:0.000, Pi-value:0.000  
Er-value:0.000, Pr-value:0.000  
No matches to eCLIP DataMATCHES To TargetScan▶ miR-501-3p/502-3p:AUGCACC

ATTTTCAATTGCCTAATTGATTTCAATTGTCTAAGACATTTCAAATGTCTAATTGATTAGAACTGAGTCTTTTATATCAAGCTAATATCTAGCTTTTATATCAAGCTAATATCTTGAC 5160  
 TTCTCAGCATCATAGAAGGGGGTACTGATTTCCTAAAGTCTTTCTTGAATTTCTATTATGCAAAATTGCCCTGAGGCCGGGTGTGGTGGCTCACACCTGTAATCCCAGCACTTTGGGAGG 5280  
 CTGAGGTGGGAAGATCCCTTACTGCCAGGAGTTTGAGACCAGCCTGGCCAACATTAAAAAAAAAAAAAAGTAAGACAATTGCCCTGGAATCCCATCCCCCTCACACCTCCTTGGCAAAGC 5400  
 AGCAGGAGTGCTAACTAGCTAGTGCTTCTTCTCTTATACTGCTTAAATGCGCATAATTAGCAGTAGTTGATGTGCCCCTATGTTAGAGTAGAATCCCGCTTCCTTGCTCCATTTGCATTA 5520  
 CTGCAGGAGCTTCTAACTAGCCTGAATTCACTCTCTTGGACTGTTAATGTGCATACTTATATTTGCTGCTGTACTTTTTTACCAT

GTAAGGA

GTAAGGA  
Depth:5 (RABBIT)  
Ei-value:0.000, Pi-value:0.000  
Er-value:0.000, Pr-value:0.000  
No matches to eCLIP DataNo matches to TargetScan

CCCCACCCACTGTATTTACATCCCAGCT 5640  
 GGAAGTACCTACTACTTAAGACCCTTAGACTAGTAAAGTTAGCGTGCATAATCTTAGGTGTTATATACACATTTTCAGTTGCATACAGTTGTGCCTTTTATCAGGACTCCTGT

ACTTAT

ACTTAT  
Depth:5 (RABBIT)  
Ei-value:0.000, Pi-value:0.000  
Er-value:0.000, Pr-value:0.000  
eCLIP MATCHES▶HNRNPU (bg=5.92%)No matches to TargetScan

C 5760  
 AAAGCAGAGAGTGCTAATCAATATTAAGCCCTTCTCTTCGAACTGTAGATGGCATGTAATTGCAGTTGTCAATGGTCCTTCAATTAGACTTGGGTTTCTGACCTATCACACCCTCTTTGC 5880  
 TTTATTGCATGGGGTACTATTCAC

TTAAGGCC

TTAAGGCC  
Depth:6 (MOUSE)  
Ei-value:0.000, Pi-value:0.000  
Er-value:0.000, Pr-value:0.000  
eCLIP MATCHES▶HNRNPL (bg=0.64%)No matches to TargetScan


CCTTT

TTAAGGCCCCTTT  
Depth:5 (RABBIT)  
Ei-value:0.000, Pi-value:0.000  
Er-value:0.000, Pr-value:0.000  
eCLIP MATCHES▶HNRNPL (bg=0.64%)No matches to TargetScan

CTCAAACTGTTAATGTGCCTAATGACAATTACATCAGTATCCTTCCTTTTGAAGGACAGCATGGTTGGTGACACCTAAGGCCC 6000  
 CATTTCTTGGCCTCCCAATATGTGTGATTGTATTTGTCGAGGTTGCTATGCACTAGAGAAGGAAAGTGCTCCCCTCATCCCCACTTTTCCCTTCCAGCAGGAAGTGCCCACCCCATAAGA 6120  
 CCCTTTTATTTGGAGAGTCTAGGTGCACAATTGTAAGTGACCACAAGCATGCATCTTGGACATTTATGTGCGTAATCGCACACTGCTCATTCCATGTGAATAAGGTCCTACTCTCCGACC 6240  
 CCTTTTGCAATACAGAAGGGTTGCTGATAACGCAGTCCCCTTTTCTTGGCATGTTGTGTGTGATTATAATCGTCTGGGATCCTATGCACTAGAAAAGGAGGGTCCTCTCCACATACCTCA 6360  
 GTCTCACCTTTCCCTTCCAGCAGGGAGTGCCCACTCCATAAGACTCTCACATTTGGACAGTCAAGGTGCGTAATTGTTAAGTGAACACAACCATGCACCTTAGACATGGATTTGCATAAC 6480  
 TACACACAGCTCAACCTATCTGAATAAAATCCTACTCTCAGACCCCTTTTGCAGTACAGCAGGGGTGCTGATCACCAAGGCCCTTTTTCCTGGCCTGGTATGCGTGTGATTATGTTTGTC 6600  
 CCGGTTCCTGTGTATTAGACATGGAAGCCTCCCCTGCCACACTCCACCCCCAATCTTCCTTTCCCTTCCGGCAGGGAGTGCCCTCTCCATAAGACGCTTACGTTTGGACAATCAAGGTGC 6720  
 ACAGTTGTAAGTGACCACAGGCATACACCTTGGACATTAATGTGCATAACCACTTTGCCCATTCCATCTGAATAAGGTCCTACTCTCAGACCCCTTTTGCAGTACAGCAGGGGTGCTGAT 6840  
 CACCAAGGCCCCTTTTCTTGGCCTGTTATGTGCGTGATTATATTTGTCTGGGTTCCTGTGTATTAGACAAGGAAGCCTTCCCCCCGCCCCCACCCCCACTCCCAGTCTTCCTTTCCCTTC 6960  
 CAGCAGGGAGTGCCCCCTCCATAAGATCATTACATTTGGACAATCAAGGTGCACAATTATAAGTGACCACAGCCATGCACCTTGGACATTATTGGACATTAATGTGCGTAACTGCACATG 7080  
 GCCCATCCCATCTGAATAAGGTCCTACTCTCAGATGCCCTTTGCAGTACAGCAGGGGTACTGAATCACCAAGGCCCTTTTTCTTGGCCTGTTATGTGTGTGATTATATTTATCCCAGTTT 7200  
 CTGTGTAATAGACATGAAAGCCTCCCCTGCCACACCCCACCTCCAATCTTCCTTTCCCTTCCACCAGGGAGTGTCCACTCCATATACCCTTACATTTGGACAATCAAGGTGCACAATTGT 7320  
 AAGTGAGCATAGGCACTCACCTTGGACATGAATGTGCATAACTGCACATGGCCCATCCCATCTGAATAAGGTCCTACTCTCAGACCCTTTTTGCAGTACAGCAGGGGTGCTGATCACCAA 7440  
 GGCCCCTTTTCCTGGCCTGTTATGTGTGTGATTATATTTGTTCCAGTTCCTGTGTAATAGACATGGAAGCCTCCCCTGCCACACTCCACCCCCAATCTTCCTTTCCCTTCTGGCAGGAAG 7560  
 TACCCGCTCCATAAGACCCTTACATTTGGACAGTCAAGGTGCACAATTGTATGTGACCACAACCATGCACCTTGGACATAAATGTGTGTAACTGCACATGGCCCATCCCATCTGAATAAG 7680  
 GTCCTACTCTCAGACCCCTTTTGCAGTACAGTAGGTGTGCTGATAACCAAGGCCCCTCTTCCTGGCCTGTTAACGTATGTGATTATATTTGTCTGGGTTCCAGTGTATAAGACATGGAAG 7800  
 CCTCCCCTGCCCCACCCCACCCTCAATCTTCCTTTCCCTTCTGGCAGGGAGTGCCAGCTCCATAAGAACCTTACATTTGGACAGTCAAGGTGCACAATTCTAAGTGACCGCAGCCATGCA 7920  
 CCTTGGTCAATAATGTGTGTAACTGCACACGGCCTATCTCATCTGAATAAGGCCTTACTCTCAGACCCCTTTTGCAGTACAGCAGGGGTGCTGATAACCAAGGCCCATTTTCCTGGCCTG 8040  
 TTATGTGTGTGATTATATTTGTCCAGGTTTCTGTGTACTAGACAAGGAAGCCTCCTCTGCCCCATCCCATCTACGCATAATCTTTCTTTTCCTCCCAGCAGGGAGTGCTCACTCCATAAG 8160  
 ACCCTTACATTTGGACAATCAAGGTGCACAATTGTAAGTGACCACAACCATGCATCTTGGAAATTTATGTGCATAACTGCACATGGCTTATCCTATTTGAATAAAGTCCTACTCTCAGAC 8280  
 CCCCTTTGCAGTATAGCTGGGGTGCTGATCACTGAGGCCTCTTTGCTTGGCTTGTCTATATTCTTGTGTACTAGATAAGGGCACCTTCTCATGGACTCCCTTTGCTTTTCAACAAGGAGT 8400  
 ACCCACTACTTTTTAAGATTCTTATATTTGTCCAAAGTACATGGTTTTAATTGACCACAACAATGTCCCTTGGACATTAATGTATGTAATCACCACATGGTTCATCCTAATTAAACAAAG 8520  
 TTCTACCTTCTCACCCTCCATTTGCAGTATACCAGGGTTGCTGACCCCCTAAGTCCCCTTTTCTTGGCTTGTTGACATGCATAATTGCATTTATGTTGGTTCTTGTGCCCTAGACAAGGA 8640  
 TGCCCCACCTCTTTTCAATAGTGGGTGCCCACTCCTTATGATCTTTACATTTGAACAGTTAATGTGAATAATTGCAGTTGTCCACAACCCTATCACTTCTAGGACCATTATACCTCTTTT 8760  
 GCATTACTGTGGGGTATACTGTTTCCCTCCAAGGCCCCTTCTGGTGGACTATCAACATATAATTGAAATTTTCTTTTGTCTTTGTCAGTAGATTAAGGTCATACCCCATCACCTTTCCTT 8880  
 TGTAGTACAACAGGGTGTCCTGATCAACCAAAGTCCTGTTGTTTTGGACTGTTAATATGTGCAATTACATTTGCTCCTGATCTGTGCACTAGATAAGGATCCTACCTACTTTCTTAGTGT 9000  
 TTTTAGCAGGTAGTGCCCACTACTCAAGACTGTCACTTGGAATGTTCATGTGCACAAACTCAATTCTCTAAGCATGTTCCTGTACCACCTTTGCTTTAGAGCAGGGGGATGATATTCACT 9120  
 AAGTGCCCCTTCTTTTGGACTTAATATGCATTAATGCAATTGTCCACCTCTTCTTTTAGACTAAGAGTTGATCTCCACATATTCCCCTTGCATCAGGGGCATGTTAATTATGAATGAACC 9240  
 CTTTTCTTTTAATATTAATGTCATAATTGTATTTGTGGACCTGTGTAGGAGAAAAAGACCCTATGTTCCTCCCATTACCCTTTGGATTGCTGCTGAGAAGTGTTAACTACTCATAAT

CTC

CTCAGCTCTTGG  
Depth:5 (RABBIT)  
Ei-value:0.000, Pi-value:0.000  
Er-value:0.000, Pr-value:0.000  
No matches to eCLIP DataMATCHES To TargetScan▶ miR-335-5p:CAAGAGC

 9360  


AGCTCTTGG

CTCAGCTCTTGG  
Depth:5 (RABBIT)  
Ei-value:0.000, Pi-value:0.000  
Er-value:0.000, Pr-value:0.000  
No matches to eCLIP DataMATCHES To TargetScan▶ miR-335-5p:CAAGAGC

ACAATTAATAGCATTAATAACAATTATCAAGGGCACTGATCATTAGATAAGACTCCTGCTTCCTCGTTGCTTACATCGGGGGTACTGACCCACTAAGGCCCCTTGTACTGT 9480  
 TAATGTGAATATTTGCAATTATATATGTCTCCTTCTGGTAGAGTGGGATATTATGCCCTAGTATCCCCTTTGCATTACTGCAGGGGCTGCTGACTACTCAAAACTTCTCCTGGGACTGTT 9600  
 AATAG

GCACAATG

GCACAATG  
Depth:6 (MOUSE)  
Ei-value:0.000, Pi-value:0.000  
Er-value:0.000, Pr-value:0.000  
No matches to eCLIP DataNo matches to TargetScan

GCAGTTATCAATGGTTTTCTCCCTCCCTGACCTTGTTAAGCAAGCGCCCCACCCCACCCTTAGTTTCCCATGGCATAATAAAGTATAAGCATTGGAGTATTCCATGC 9720  
 ACTTGTCTATCAAACAGTGGTCCATA

CTCCCA

CTCCCA  
Depth:6 (MOUSE)  
Ei-value:0.000, Pi-value:0.000  
Er-value:0.000, Pr-value:0.000  
eCLIP MATCHES▶hnrnpk (bg=12.88%)No matches to TargetScan

ACCCTTTTGCATTGCGCCAGTGTGTAAAATCACAGGTAGCCATGGTGTCATGCTTTATATACGAAGTCTTCCCTCTCTCTGCCCCTTG 9840  
 TGTGCCCTTGGCCCCTTTTTACAGACTATTGCTCACAATCTCAGGTGTCCATATTTGCAGCTATTAGGTAAGATTGTGCTGTCTCCCTCTTCCCTTCCCTCTGCCCTGCCCCTTTTGCCT 9960  
 CTTTGCTGGGTAATGTTGACCAGACAAGGCCCTTTCTCTTGGACTTAAACAATTCTCAGTTGCACTTTCCTTGGTCCCACCCATTATACATGAACCCCTCTACTTCCTTTCGCATTGCTT 10080  
 CTGAGTATGCTGACTACCCAAAGCCCCTTCTGTGTTATTAATAAACACAGTACTGATTGTCCCATTTTTCAGCCCATCAGTCCAAGATCTCCCTACCACTTTGGTGTGTTGGTGCAGTGT 10200  
 TGACTATG

AAAAGCAG

AAAAGCAG  
Depth:6 (MOUSE)  
Ei-value:0.000, Pi-value:0.000  
Er-value:0.000, Pr-value:0.000  
No matches to eCLIP DataNo matches to TargetScan

GCCTGAACTAGGTGGATAAGCCTTCACTCATTTTCTTTCATTTATTAATGATCCTAGTTTCAATTATTGTCAGATTCTGGGGACAAGAACCATTCTTGCCCACC 10320  
 TGTGTTACTGCTTTACTGT

GCAAAAT

GCAAAAT  
Depth:6 (MOUSE)  
Ei-value:0.000, Pi-value:0.000  
Er-value:0.000, Pr-value:0.000  
No matches to eCLIP DataNo matches to TargetScan

ACTGAAGGCAAGTCAGACCCAGGGAGCTGGATTGCCATCCTTTATTTTGTGTTTCCAGTGTACACTATAAAATTGTCTCCCCAGGAAGGAAGGT 10440  
 TGGCACTTTCTC

TGCATTCTTC

TGCATTCTTC  
Depth:5 (RABBIT)  
Ei-value:0.000, Pi-value:0.000  
Er-value:0.000, Pr-value:0.000  
eCLIP MATCHES▶SF3B1 (bg=2.48%)No matches to TargetScan

TTTCCAGAGC

A

AGATTGCCTGG  
Depth:5 (RABBIT)  
Ei-value:0.000, Pi-value:0.000  
Er-value:0.000, Pr-value:0.000  
No matches to eCLIP DataNo matches to TargetScan


GATTGCCTGG

GATTGCCTGG  
Depth:6 (MOUSE)  
Ei-value:0.000, Pi-value:0.000  
Er-value:0.000, Pr-value:0.000  
No matches to eCLIP DataNo matches to TargetScan

TTAAGAATCTCTTGTTGTCCCCTTTGTATATTGTTATTGTAAAGTGCCAAATGCCAGGATACAGCCAGAAAAATTGC 10560  
 TTATTATTATTAAAAAAATTTTTTTAAGAAAGACATCTGGATTGTAGGGTGGACTCGATAAC

CTGGTCATT

CTGGTCATT  
Depth:5 (RABBIT)  
Ei-value:0.000, Pi-value:0.000  
Er-value:0.000, Pr-value:0.000  
No matches to eCLIP DataNo matches to TargetScan

ATTTTTTTGAAGCCAAAATAT

CCATTTAT

CCATTTAT  
Depth:5 (RABBIT)  
Ei-value:0.000, Pi-value:0.000  
Er-value:0.000, Pr-value:0.000  
No matches to eCLIP DataNo matches to TargetScan

ACTATGTACCTGGTGAC

CAG

CAGTGTCTCTCATTT  
Depth:5 (RABBIT)  
Ei-value:0.000, Pi-value:0.000  
Er-value:0.000, Pr-value:0.000  
eCLIP MATCHES▶SUPV3L1 (bg=1.57%)No matches to TargetScan

 10680  


TGTCTCTCATTT

CAGTGTCTCTCATTT  
Depth:5 (RABBIT)  
Ei-value:0.000, Pi-value:0.000  
Er-value:0.000, Pr-value:0.000  
eCLIP MATCHES▶SUPV3L1 (bg=1.57%)No matches to TargetScan

TAACTGAGG

GTGGTG

GTGGTG  
Depth:5 (RABBIT)  
Ei-value:0.000, Pi-value:0.000  
Er-value:0.000, Pr-value:0.000  
eCLIP MATCHES▶SUPV3L1 (bg=1.57%)No matches to TargetScan

G

GTCTGTGGATA

GTCTGTGGATA  
Depth:5 (RABBIT)  
Ei-value:0.000, Pi-value:0.000  
Er-value:0.000, Pr-value:0.000  
eCLIP MATCHES▶SUPV3L1 (bg=1.57%)MATCHES To TargetScan▶ miR-140-3p.1:CCACAGG

GAACACTGACTCTTGCTATTTTAATATCAAAGATATTCTAGAGTGGAACTCTTAAGACCAGTATCTTTGTGTGGGCTTTAC 10800  
 CAGCATTCACTTTTAGAAAAACTACCTAAATTTTATAATCCTTT

AATTTCTTCATCTGGAGC

AATTTCTTCATCTGGAGC  
Depth:5 (RABBIT)  
Ei-value:0.000, Pi-value:0.000  
Er-value:0.000, Pr-value:0.000  
eCLIP MATCHES▶SUPV3L1 (bg=1.57%)▶U2AF2 (bg=1.76%)No matches to TargetScan

ACCTGCCCCTACTTATTTCAAGAAGATTGCAGTAAAACGATTAAATGAGGGAACATAT 10920  
 GCAGAGGTGCTTTTAAAAAGCATATGCCACCTTTTTTATTAATTATTATATAAAATGAAGCATTTAATTATAGTAATAATTTGAAGTAGTTTGAAGTACCACACTGAGGTGAGGACTTAA 11040  
 AAATGATAAGACGAGTTCCCTATTTTATAAG

AAAAATAAGCCA

AAAAATAAGCCA  
Depth:5 (RABBIT)  
Ei-value:0.000, Pi-value:0.000  
Er-value:0.000, Pr-value:0.000  
No matches to eCLIP DataNo matches to TargetScan

AAATTAAATATTCTTTTGGATATAAATTTCAACAGTGAGATAGCTGCCTAGTGGAAATGAATAATATCCCAGCCACT 11160  
 AGTGTACAGGGTGTTTTGTGGCACAGGATTATGTAATATGGAACTGCTCAAGCAAATAACTAGTCATCACAA

CAGCAGTTC

CAGCAGTTC  
Depth:5 (RABBIT)  
Ei-value:0.000, Pi-value:0.000  
Er-value:0.000, Pr-value:0.000  
No matches to eCLIP DataNo matches to TargetScan

TTTGTAATA

ACTGAAAA

ACTGAAAA  
Depth:5 (RABBIT)  
Ei-value:0.000, Pi-value:0.000  
Er-value:0.000, Pr-value:0.000  
No matches to eCLIP DataNo matches to TargetScan

AGAATATTGTTTCTCGGAG

AAG

AAGGATG  
Depth:5 (RABBIT)  
Ei-value:0.000, Pi-value:0.000  
Er-value:0.000, Pr-value:0.000  
eCLIP MATCHES▶SRSF1 (bg=8.47%)▶U2AF2 (bg=1.76%)▶uchl5 (bg=11.16%)MATCHES To TargetScan▶ miR-362-5p/500b-5p:AUCCUUG

 11280  


GATG

AAGGATG  
Depth:5 (RABBIT)  
Ei-value:0.000, Pi-value:0.000  
Er-value:0.000, Pr-value:0.000  
eCLIP MATCHES▶SRSF1 (bg=8.47%)▶U2AF2 (bg=1.76%)▶uchl5 (bg=11.16%)MATCHES To TargetScan▶ miR-362-5p/500b-5p:AUCCUUG

TCA

AAAGATC

AAAGATC  
Depth:6 (MOUSE)  
Ei-value:0.000, Pi-value:0.000  
Er-value:0.000, Pr-value:0.000  
eCLIP MATCHES▶SRSF1 (bg=8.47%)▶U2AF2 (bg=1.76%)▶uchl5 (bg=11.16%)No matches to TargetScan

GGCCCAGCTCAGGGAGCAGTTTGCCCTACTAGCTCCTC

GGACAGCTG

GGACAGCTG  
Depth:5 (RABBIT)  
Ei-value:0.000, Pi-value:0.000  
Er-value:0.000, Pr-value:0.000  
eCLIP MATCHES▶SRSF1 (bg=8.47%)▶SRSF7 (bg=2.32%)▶U2AF2 (bg=1.76%)▶ZNF622 (bg=6.58%)No matches to TargetScan

TAA

AGAAGAGTCTCTGGCTCTTTA

AGAAGAGTCTCTGGCTCTTTA  
Depth:5 (RABBIT)  
Ei-value:0.000, Pi-value:0.000  
Er-value:0.000, Pr-value:0.000  
eCLIP MATCHES▶DDX24 (bg=2.97%)▶SRSF1 (bg=8.47%)▶SRSF7 (bg=2.32%)▶U2AF2 (bg=1.76%)▶ZNF622 (bg=6.58%)No matches to TargetScan

GAATACT||GATCCCATTGAAGATACCACGCTGCA 11398  
 TGTGTCCTTAGTAGTCATGTCTCCTTAGGCTCCTCTTG||GACATTCTGAGCATGTGAGACCTGAGGA

CTGCAA

CTGCAA  
Depth:5 (RABBIT)  
Ei-value:0.000, Pi-value:0.000  
Er-value:0.000, Pr-value:0.000  
eCLIP MATCHES▶DDX24 (bg=2.97%)▶GRWD1 (bg=5.13%)▶MTPAP (bg=2.21%)▶NOLC1 (bg=9.43%)▶SRSF1 (bg=8.47%)▶UTP3 (bg=3.66%)▶ZNF622 (bg=6.58%)No matches to TargetScan

ACAGCTATAAGAGGCTCCAAATTAATCATATCTTTCCCTTTGAGAA 11516  
 TCTGGCCAAGCTCCAGCTAATCTACTTGGATGGGTTGCCAGCTATCTGGAGAAAAAG||ATCTTCCTCAG

AAGAATAGGC

AAGAATAGGC  
Depth:5 (RABBIT)  
Ei-value:0.000, Pi-value:0.000  
Er-value:0.000, Pr-value:0.000  
eCLIP MATCHES▶NOLC1 (bg=9.43%)▶SRSF7 (bg=2.32%)▶uchl5 (bg=11.16%)No matches to TargetScan

TTGTTGTTT

TACAGTGTTAGTGA

TACAGTGTTAGTGA  
Depth:5 (RABBIT)  
Ei-value:0.000, Pi-value:0.000  
Er-value:0.000, Pr-value:0.000  
eCLIP MATCHES▶ILF3 (bg=3.0%)▶NOLC1 (bg=9.43%)▶RBM15 (bg=7.27%)▶SRSF7 (bg=2.32%)▶ZNF622 (bg=6.58%)MATCHES To TargetScan▶ miR-141-3p/200a-3p:AACACUG

TCCA

TTCCCTTTGA

TTCCCTTTGA  
Depth:6 (MOUSE)  
Ei-value:0.000, Pi-value:0.000  
Er-value:0.000, Pr-value:0.000  
eCLIP MATCHES▶ILF3 (bg=3.0%)▶RBM15 (bg=7.27%)▶SRSF7 (bg=2.32%)▶ZNF622 (bg=6.58%)No matches to TargetScan

CGA 11634  
 TCCC

TAGGTGGAGATGGGGCATGAGGATCCTCCAGGGGAA

TAGGTGGAGATGGGGCATGAGGATCCTCCAGGGGAA  
Depth:6 (MOUSE)  
Ei-value:0.000, Pi-value:0.000  
Er-value:0.000, Pr-value:0.000  
eCLIP MATCHES▶ILF3 (bg=3.0%)▶NOLC1 (bg=9.43%)▶RBM15 (bg=7.27%)▶SRSF7 (bg=2.32%)▶ZNF622 (bg=6.58%)MATCHES To TargetScan▶ miR-331-3p:CCCCUGG


A

TAGGTGGAGATGGGGCATGAGGATCCTCCAGGGGAAA  
Depth:5 (RABBIT)  
Ei-value:0.000, Pi-value:0.000  
Er-value:0.000, Pr-value:0.000  
eCLIP MATCHES▶ILF3 (bg=3.0%)▶NOLC1 (bg=9.43%)▶RBM15 (bg=7.27%)▶SRSF7 (bg=2.32%)▶ZNF622 (bg=6.58%)MATCHES To TargetScan▶ miR-331-3p:CCCCUGG

AGC

TCACTA

TCACTA  
Depth:5 (RABBIT)  
Ei-value:0.000, Pi-value:0.000  
Er-value:0.000, Pr-value:0.000  
eCLIP MATCHES▶ILF3 (bg=3.0%)No matches to TargetScan

CCACTGG

GCAACA

GCAACA  
Depth:6 (MOUSE)  
Ei-value:0.000, Pi-value:0.000  
Er-value:0.000, Pr-value:0.000  
eCLIP MATCHES▶ILF3 (bg=3.0%)No matches to TargetScan


AC

GCAACAAC  
Depth:5 (RABBIT)  
Ei-value:0.000, Pi-value:0.000  
Er-value:0.000, Pr-value:0.000  
eCLIP MATCHES▶ILF3 (bg=3.0%)No matches to TargetScan

CCTAGGTCAGGAGGTTCTGTCAAGATACTTTCCTGGTCCCAGATAGGAAGATAAA 11754  
 GTCTCAAAA

ACAACCACC

ACAACCACC  
Depth:5 (RABBIT)  
Ei-value:0.000, Pi-value:0.000  
Er-value:0.000, Pr-value:0.000  
eCLIP MATCHES▶PRPF8 (bg=0.26%)No matches to TargetScan

ACACGTCAAG||CTCTTCATTGTTCCTATCTG

CCAAAT

CCAAAT  
Depth:6 (MOUSE)  
Ei-value:0.000, Pi-value:0.000  
Er-value:0.000, Pr-value:0.000  
eCLIP MATCHES▶GRWD1 (bg=5.13%)▶NOLC1 (bg=9.43%)No matches to TargetScan


C

CCAAATC  
Depth:5 (RABBIT)  
Ei-value:0.000, Pi-value:0.000  
Er-value:0.000, Pr-value:0.000  
eCLIP MATCHES▶GRWD1 (bg=5.13%)▶NOLC1 (bg=9.43%)No matches to TargetScan

ATTATACTTCCTACAAGCAGTGCAGAGAGCTGAGTCTTCAGCAGGTC

CAAGAAA

CAAGAAA  
Depth:5 (RABBIT)  
Ei-value:0.000, Pi-value:0.000  
Er-value:0.000, Pr-value:0.000  
eCLIP MATCHES▶GRWD1 (bg=5.13%)▶NOLC1 (bg=9.43%)▶uchl5 (bg=11.16%)▶ZNF622 (bg=6.58%)No matches to TargetScan

TTTGAACAC 11872  
 ACTGAAGGAAGTCAGCCTTCCCACCTG

AA

AAGATCAACATGC  
Depth:5 (RABBIT)  
Ei-value:0.000, Pi-value:0.000  
Er-value:0.000, Pr-value:0.000  
eCLIP MATCHES▶GRWD1 (bg=5.13%)▶NOLC1 (bg=9.43%)▶PTBP1 (bg=3.74%)▶RBM15 (bg=7.27%)▶TRA2A (bg=4.8%)▶uchl5 (bg=11.16%)▶ZNF622 (bg=6.58%)No matches to TargetScan


GATCAACATGC

GATCAACATGC  
Depth:6 (MOUSE)  
Ei-value:0.000, Pi-value:0.000  
Er-value:0.000, Pr-value:0.000  
eCLIP MATCHES▶GRWD1 (bg=5.13%)▶NOLC1 (bg=9.43%)▶PTBP1 (bg=3.74%)▶RBM15 (bg=7.27%)▶TRA2A (bg=4.8%)▶uchl5 (bg=11.16%)▶ZNF622 (bg=6.58%)No matches to TargetScan

CTGGCACTCTAGCACTTGAGGATAGCTGAATGAA||

TGTGTAT

TGTGTAT  
Depth:6 (MOUSE)  
Ei-value:0.000, Pi-value:0.000  
Er-value:0.000, Pr-value:0.000  
eCLIP MATCHES▶TARDBP (bg=2.79%)▶ZC3H11A (bg=6.55%)No matches to TargetScan

TTCTTTGTCTCTTTCTTTCTTGTCTTTGCTCTTTGTT 11990  
 CTCTATCTAAAGTGTGTCTTACCCATTTCCATGTTTCTCTTGCTAATTTCTTTCGTGTGTGCCTTTGCCTCATTTTCTCTTTTTGTTCACAAGAGTGGTCTGTGTCTTGTCTTAGACATA 12110  
 TCTCTCATTTTTCATTTTGTTGCTATTTCTCTTTGCTCTCCTAGATGTGGCTCTTCTTTCACGCTTTATTTCATGTCTCCTTTTTGGGTCACATGCTGTGTGCTTTTTGTCCTTTTCTTG 12230  
 TTCTGTCTACCTCTCCTTTCTCTGCCTACCTCTCTT

TTCTCTTTG

TTCTCTTTG  
Depth:6 (MOUSE)  
Ei-value:0.000, Pi-value:0.000  
Er-value:0.000, Pr-value:0.000  
eCLIP MATCHES▶MATR3 (bg=2.98%)▶PTBP1 (bg=3.74%)▶SMNDC1 (bg=0.63%)▶TIA1 (bg=4.07%)No matches to TargetScan

TGAACTGTGATTATTTGTTACCCCTTCCCCTTCTCGTTCGTTTTAAATTTCACCTTTTTTCTGAGTCTGGCCTCC 12350  
 TTTCTGCTG

TTTCTAC

TTTCTAC  
Depth:6 (MOUSE)  
Ei-value:0.000, Pi-value:0.000  
Er-value:0.000, Pr-value:0.000  
eCLIP MATCHES▶MATR3 (bg=2.98%)▶PTBP1 (bg=3.74%)▶TIA1 (bg=4.07%)No matches to TargetScan


T

TTTCTACT  
Depth:5 (RABBIT)  
Ei-value:0.000, Pi-value:0.000  
Er-value:0.000, Pr-value:0.000  
eCLIP MATCHES▶MATR3 (bg=2.98%)▶PTBP1 (bg=3.74%)▶TIA1 (bg=4.07%)MATCHES To TargetScan▶ miR-411-5p.1:AGUAGAC

TTTTATCTCAC

ATTTCTC

ATTTCTC  
Depth:6 (MOUSE)  
Ei-value:0.000, Pi-value:0.000  
Er-value:0.000, Pr-value:0.000  
eCLIP MATCHES▶MATR3 (bg=2.98%)▶PTBP1 (bg=3.74%)▶TIA1 (bg=4.07%)No matches to TargetScan

ATTTCTGCATTTCCTTTCTGCCTC

TCTTGGG

TCTTGGG  
Depth:5 (RABBIT)  
Ei-value:0.000, Pi-value:0.000  
Er-value:0.000, Pr-value:0.000  
eCLIP MATCHES▶MATR3 (bg=2.98%)▶PTBP1 (bg=3.74%)▶SMNDC1 (bg=0.63%)▶TIA1 (bg=4.07%)No matches to TargetScan

CTATTCTCTCTCTCCTCCCCTGCGTGCCTCAGCATCTCTTGCTGTTTGTGATTT 12470  
 TCTATTTCAGTATTAATCTCTGTTGGCTTGTATTTGTTCTCTGCTTCTTCCCTTTCTACTCACCTTTGAGTATTTCAGCCTCTTCATGAATCTATCTCCCTCTCTTTGATTTCATGTAAT 12590  
 CTCTCCTTAAATATTTCTTTGCATATGTGGGCAAGTGTACGTGTGTGTGTGTCATGTGTGGCAGAGGGGCT

TCCTAACCCCT

TCCTAACCCCT  
Depth:5 (RABBIT)  
Ei-value:0.000, Pi-value:0.000  
Er-value:0.000, Pr-value:0.000  
eCLIP MATCHES▶AATF (bg=0.64%)▶DDX24 (bg=2.97%)▶NCBP2 (bg=1.49%)▶NOLC1 (bg=9.43%)▶PTBP1 (bg=3.74%)▶SND1 (bg=0.45%)▶SRSF7 (bg=2.32%)▶TARDBP (bg=2.79%)▶UTP3 (bg=3.66%)▶WDR43 (bg=3.37%)▶XRCC6 (bg=2.91%)▶ZC3H8 (bg=0.29%)No matches to TargetScan

GCCTGATAGGTGCAGAACGTCGGCTATCAGAGCAAGCA 12710  
 TTGTGGAGCGGTTCC

TTATGCCA

TTATGCCA  
Depth:5 (RABBIT)  
Ei-value:0.000, Pi-value:0.000  
Er-value:0.000, Pr-value:0.000  
eCLIP MATCHES▶DDX24 (bg=2.97%)▶FASTKD2 (bg=1.99%)▶LARP4 (bg=4.72%)▶NOLC1 (bg=9.43%)▶NPM1 (bg=1.21%)▶RBFOX2 (bg=4.63%)▶RBM15 (bg=7.27%)▶RPS3 (bg=0.76%)▶SRSF1 (bg=8.47%)▶SRSF7 (bg=2.32%)▶TARDBP (bg=2.79%)▶TRA2A (bg=4.8%)▶U2AF2 (bg=1.76%)▶uchl5 (bg=11.16%)▶WDR43 (bg=3.37%)▶YWHAG (bg=1.87%)▶ZC3H11A (bg=6.55%)▶ZNF622 (bg=6.58%)▶ZNF800 (bg=1.92%)No matches to TargetScan

GGCTGCCATGTGAGATGATCCAAGACCAAAACAAGGCCCTAGACTGCAGTAAAACCCAGAACTCAAGTAGGGCAGAAGGTGGAAGGCTCATATGGAT 12830  
 AGA

AGGCCCAA

AGGCCCAA  
Depth:5 (RABBIT)  
Ei-value:0.000, Pi-value:0.000  
Er-value:0.000, Pr-value:0.000  
eCLIP MATCHES▶DDX24 (bg=2.97%)▶LARP4 (bg=4.72%)▶MTPAP (bg=2.21%)▶NOLC1 (bg=9.43%)▶SRSF1 (bg=8.47%)▶SRSF7 (bg=2.32%)▶TRA2A (bg=4.8%)▶uchl5 (bg=11.16%)▶UTP3 (bg=3.66%)▶ZNF622 (bg=6.58%)▶ZNF800 (bg=1.92%)No matches to TargetScan

AGTATAAGACAGATGGTTTGAGACTTGAGACCCGAGGACTAAGATGGAAAGCCCATGTTCCAAGATAGATAGAAGCCTCAGGCCTGAAACCAACAAAAGCCTCAAGAGC 12950  
 CAAGAAAACAGAGGGTGGCCTGAATTGGACCGAAGGCCTGAGTTGGATGGAAGTCTCAAGGCTTGAGTTAGAAGTCTTAAGACCTGGGACAGGACACATGGAAGGCCTAAGAACTGAGAC 13070  
 TTGTGACACAAGGCCAACGACCTAAGATTAGCCCAGGGTTGTAGCTGGAAGACCTACAACCCAAGGATGGAAGGCCCCTGTCACAAAGCCTACCTAGATGGATAGAGGACCCAAGCGAAA 13190  
 AAGGTATCTCAA

GACTAA

GACTAA  
Depth:5 (RABBIT)  
Ei-value:0.000, Pi-value:0.000  
Er-value:0.000, Pr-value:0.000  
eCLIP MATCHES▶CPEB4 (bg=1.89%)▶FASTKD2 (bg=1.99%)▶GRWD1 (bg=5.13%)▶LARP4 (bg=4.72%)▶MTPAP (bg=2.21%)▶NOLC1 (bg=9.43%)▶RBFOX2 (bg=4.63%)▶SRSF1 (bg=8.47%)▶TRA2A (bg=4.8%)▶uchl5 (bg=11.16%)▶UTP18 (bg=0.72%)▶UTP3 (bg=3.66%)▶WDR43 (bg=3.37%)▶ZNF622 (bg=6.58%)No matches to TargetScan

CGGCCGGAATCTGGAGGCCCATGACCCAGAACCCAGGAAGGATAGAAGCTTGAAGACCTGGGGAAATCCC

AAGATGA

AAGATGA  
Depth:5 (RABBIT)  
Ei-value:0.000, Pi-value:0.000  
Er-value:0.000, Pr-value:0.000  
eCLIP MATCHES▶CPEB4 (bg=1.89%)▶FTO (bg=0.32%)▶GRWD1 (bg=5.13%)▶LARP4 (bg=4.72%)▶MTPAP (bg=2.21%)▶SRSF1 (bg=8.47%)▶TRA2A (bg=4.8%)▶uchl5 (bg=11.16%)▶ZNF622 (bg=6.58%)No matches to TargetScan

GAACCCTAAACCCTACCTCTTTTCT 13310  
 ATTGTTTACACTTCTTACTCTTAGATATTTCCAGTTCTCCTGTTTATCTTTAAGCCTGATTCTTTTGAGATGTACTTTTTGATGTTGCCGGTTACCTTTAGATTGACAGTATTATGCCTG 13430  
 GGCCAGTCTTGAGCCAGCTTTAAATCACAGCTTTTACCTATTTGTTAGGCTATAGTGTTTTGTAAACTTCTGTTTCTATTCACATCTTCTCCACTTGAGAGAGACACCAAAATCCAGTCA 13550  
 GTATCTAATCTGGCTTTTGTTAACTTCCCTCAGGAGCAGACATTCATATAGGTGATACTGTATTTCAGTCCTTTCTTTTGACCCCAGAAGCCCTAGACTGAGAAGATAAAATGGTCAGGT 13670  
 TGTTGGGGAAAAAAAAGTGCCAGGCTC

TCTAGAGAAAA

TCTAGAGAAAA  
Depth:6 (MOUSE)  
Ei-value:0.000, Pi-value:0.000  
Er-value:0.000, Pr-value:0.000  
eCLIP MATCHES▶CPSF6 (bg=0.4%)▶LARP4 (bg=4.72%)▶UTP3 (bg=3.66%)▶WDR43 (bg=3.37%)MATCHES To TargetScan▶ miR-1251-5p:CUCUAGC

ATG

TGAAGAGATG

TGAAGAGATG  
Depth:5 (RABBIT)  
Ei-value:0.000, Pi-value:0.000  
Er-value:0.000, Pr-value:0.000  
eCLIP MATCHES▶CPSF6 (bg=0.4%)▶LARP4 (bg=4.72%)▶SRSF7 (bg=2.32%)▶UTP3 (bg=3.66%)▶WDR43 (bg=3.37%)No matches to TargetScan

CTCCAGGCCAA

TGAGAAGAATTAGACA

TGAGAAGAATTAGACA  
Depth:6 (MOUSE)  
Ei-value:0.000, Pi-value:0.000  
Er-value:0.000, Pr-value:0.000  
eCLIP MATCHES▶LARP4 (bg=4.72%)▶NOLC1 (bg=9.43%)▶SRSF7 (bg=2.32%)No matches to TargetScan

AGAAATACACAGATGTGCCAGACTTCTGAGAAGCACCTGCCA 13790  
 GCAACAGCTTCCTTCTTTGAGCTTAGGTGAGCAGGATTCTGGGGTTTGGGATTTCTAGTGA

TGGTTA

TGGTTA  
Depth:5 (RABBIT)  
Ei-value:0.000, Pi-value:0.000  
Er-value:0.000, Pr-value:0.000  
eCLIP MATCHES▶AKAP8L (bg=2.19%)▶NOLC1 (bg=9.43%)▶PUS1 (bg=1.04%)▶SF3B1 (bg=2.48%)No matches to TargetScan

TGGAAAGGGTGACTGTGCCTGGGACAAAGCGAGGTCCCAAGGGGACAGCCTGA 13910  
 ACTCCCTGCTCATAGTAGTGGCCAAATAATTTGGTGGACTGTGCCAACGCTACTCCTGGGTTTAATACCCATCTCT

AGGCTTA

AGGCTTA  
Depth:5 (RABBIT)  
Ei-value:0.000, Pi-value:0.000  
Er-value:0.000, Pr-value:0.000  
No matches to eCLIP DataNo matches to TargetScan

AAGATGAGAGAACCTGGGACTGTTGAGCAT

GTTTAAT

GTTTAAT  
Depth:5 (RABBIT)  
Ei-value:0.000, Pi-value:0.000  
Er-value:0.000, Pr-value:0.000  
No matches to eCLIP DataNo matches to TargetScan

 14030  


GTTTAAT  
Depth:5 (RABBIT)  
Ei-value:0.000, Pi-value:0.000  
Er-value:0.000, Pr-value:0.000  
No matches to eCLIP DataNo matches to TargetScan

ACTTTCCTTGATTTTTTTCTTCCTGTTTATGTGGGAAGTTGATTTAAATGACTGATAATGTGTATGAAAGCACTGTAAAACATAAGAGAAAAACCAATTAGTG

T

TATTGGCA  
Depth:5 (RABBIT)  
Ei-value:0.000, Pi-value:0.000  
Er-value:0.000, Pr-value:0.000  
eCLIP MATCHES▶HNRNPA1 (bg=2.57%)No matches to TargetScan


ATTGGCA

ATTGGCA  
Depth:6 (MOUSE)  
Ei-value:0.000, Pi-value:0.000  
Er-value:0.000, Pr-value:0.000  
eCLIP MATCHES▶HNRNPA1 (bg=2.57%)No matches to TargetScan

ATCATGCAG 14150  
 TTAACATTTGAAAGTGCAGTGTAAA

TTGTGAAG

TTGTGAAG  
Depth:6 (MOUSE)  
Ei-value:0.000, Pi-value:0.000  
Er-value:0.000, Pr-value:0.000  
eCLIP MATCHES▶HNRNPA1 (bg=2.57%)No matches to TargetScan

CATT

ATGTAAAT

ATGTAAAT  
Depth:5 (RABBIT)  
Ei-value:0.000, Pi-value:0.000  
Er-value:0.000, Pr-value:0.000  
No matches to eCLIP DataNo matches to TargetScan

CAGGGGTCCACAGTTTTTCTGTAAGGGGTCAAATCATAAATACTTTAGACTGTGGGCCATATGGTTTCTGTTACA 14270  
 TATTTGTTTTTTAAACAACGTTTTTATAAGGTCAAAATCATTCTTAGTTTTTGAGCCAATTGGATTTGGCCTGCTGTTCATAGCTTACCACCCCCTGATGTATTATTTGTTATTCAGAGA 14390  
 AAATTTCTGAATACTACTAGTTTCCTTTTCTGTGCCTGTCCCTGTGCTAGGCACTAAAAATGCAATGATTATTGATATCTAGGTGACCTGAAAAAAAATAGTGAATGTGCTTTGTAAACT 14510  
 GTAAAGCACTTGTATTCTACTGTGATAAGCGTTGTGGATACAAAGAAAGGAGCAAGCATAAAAAAGTGCTCTTTCAAAAGGATATAGTACTATGCAGACACAAGGAATTGTTTGATAAAT 14630  
 GAATAAATTATATGTATATTTGAGGCCAATTTGTGTTTGCTGCTCTGGTAATTTTGAGTAAAAATGCAGTATTCCAGGTATCAGAAACGAAAACACATGGAAACTGCTTTTAAACTTTAA 14750  
 AATATACTGAAAACATAAGGGACTAAGCTTGTTGTGGTCACCTATAATGTGCCAGATACCATGCTGGGTGCTAGAGCTACCAAAGGGGGAAAAGTATTCTCATAGAACAAAAAATTTCAG 14870  
 AAAGGTGCATATTAAAGTGCTTTGTAAACTAAAGCATGATACAAATGTCAATGGGCTACATATTTATGAATGAATGAATGGATGAATGAATATTAAGTGCCTCTTACATACCAGCTATTT 14990  
 TGGGTACTGTAAAATACAAGATTAATTCTCCTATGTAATAAGAGGAAAGTTTATCCTCTATACTATTCAGATGTAAGGAATGATATATTGCTTAATTTTAAACAATCAAGACTTTACTGG 15110  
 TGAGGTTAAGTTAAATTATTACTGATACATTTTTCCAGGTAACCAGGAAAGAGCTAGTATGAGGAAATGAAGTAATAGATGTGAGATCCAGACCGAAAGTCACTTAATTCAGCTTGCGAA 15230  
 TGTGCTTTCTAAATTATAAAGCACTTGTAAATGAAAAATTTGATGCTTTCTGTATGAATAAAACTTTCTGTAAGCTAGGTATTGTCTCTACAAAATTCTCATTGTATAGTTAAACCACAG 15350  
 TGAGAAGGGTTCTATAAGTAGTTATACAAACCAAGGGTTTAAATACCTGTTAAATAGATCAATTTTGATTGCCTACTATGTGAACTCACTGTTAAAGGCACTGAAAATTTATCATATTTC 15470  
 ATTTAGCCACAGCCAAAAATAAGGCAATACCTATGTTAGCATTTTGTGAACTCTAAGGCACCATATAAATGTAACTGTTGATTTTCTCACTTGGTGCTGGGTACTAGGTTTATAAAATTG 15590  
 TATGATAGTTATTATATTGTGCAAATAAAGTAGGAAAATTTGAATAACAATGATTATCTTTTGAATACGCATACGCAAGGGATTGGTTGTCTGAAGAATGCCACTATAGTAGTTATCTAT 15710  
 TGTGTGCCAATCTCATTGCTAGGCATTGGGGATGCAAAGATAAACCATCTTTATTGTGTCTTGGGTAGCAGAAGAAAATATGTGTAAAATCAATTTATAATTTGTAAACTGCCACCCATA 15830  
 TATAAGCTATATCTGCTGAATGATCATTGATTACTCTTATCCTTAGAGATAACAACTGGGGGCACAAACATTTATTATCATTATTGAACCTACAACAGAGATCTATGTGTAGATTTACAA 15950  
 AGCCTACAGTTCTATACAGATAGGAATGAACTATTGGCTTACTGAATGGTGATTACTTTCTGTGGGGCTCGGAACTACATGCCCTAGGATATAAAAATGATGTTATCATTATAGAGTGCT 16070  
 CACAGAAGGAAATGAAGTAATATAGGTGTGAGATCCAGACCAAAAGTCATTTAACAAGTTTATTCAGTGATGAAAACATGGGACAAATGGACTAATATAAGGCAGTGTACTAAGCTGAGT 16190  
 AGAGAGATAAAGTCCTGTCCAGAAGATACATGCTTCCTGGCCTGATTGAGGAGATGGAAAATTTTTGCAAAAAACAAGGTGTTGTGGTCTTCCATCCAGTTTCTTAAGTGCTGATGATAA 16310  
 AAGTGAATTAGACCCACCTTGACCTGGCCTACAGAAGTAAAGGAGTAAAAATAAATGCCTCAGGCGTGCTTTTTGATTCATTTGATAAACAAAGCATCTTTTATGTGGAATATACCATTC 16430  
 TGGGTCCTGAGGATAAGAGAGATGAGGGCATTAGATCACTGACAGCTGAAGATAGAAGAACATCTTTGGTTTGATTGTTTAAATAATATTTCAATGCCTATTCTCTGCAAGGTACTATGT 16550  
 TTCGTAAATTAAATAGGTCTGGCCCAGAAGACCCACTCAATTGCCTTTGAGATTAAAAAAAAAAAAAAAAAGAAAGAAAAATGCAAGTTTCTTTCAAAATAAAGAGACATTTTTCCTAGT 16670  
 TTCAGGAATCCCCCAAATCACTTCCTCATTGGCTTAGTTTAAAGCCAGGAGACTGATAAAAGGGCTCAGGGTTTGTTCTTTAATTCATTAACTAAACATTCTGCTTTTATTACAGTTAAA 16790  
 TGGTTCAAGATGTAACAACTAGTTTTAAAGGTATTTGCTCATTGGTCTGGCTTAGAGACAGGAAGACATATGAGCAATAAAAAAAAGATTCTTTTGCATTTACCAATTTAGTAAAAATTT 16910  
 ATTAAAACTGAATAAAGTGCTGTTCTTAAGTGCTTGAAAGACGTAAACCAAAGTGCACTTTATCTCATTTATCTTATGGTGGAAACACAGGAACAAATTCTCTAAGAGACTGTGTTTCTT 17030  
 TAGTTGAGAAGAAACTTCATTGAGTAGCTGTGATATGTTCGATACTAAGGAAAAACTAAACAGATCACCTTTGACATGCGTTGTAGAGTGGGAATAAGAGAGGGCTTTTTATTTTTTCGT 17150  
 TCATACGAGTATTGATGAAGATGATACTAAATGCTAAATGAAATATATCTGCTCCAAAAGGCATTTATTCTGACTTGGAGATGCAACAAAAACACAAAAATGGAATGAAGTGATACTCTT 17270  
 CATCAAACAGAAGTGACTGTTATCTCAACCATTTTGTTAAATCCTAAACAGAAAACAAAAAAAATCATGACGAAAAGACACTTGCTTATTAATTGGCTTGGAAAGTAGAATATAGGAGAA 17390  
 AGGTTACTGTTTATTTTTTTTCATGTATTCATTCATTCTACAAATATATTCGGGTGCCAATAGGTACTTGGTATAAGGTTTTTGGCCCCAGAGACATGGGAAAAAAATGCATGCCTTCCC 17510  
 AGAGAATGCCTAATACTTTCCTTTTGGCTTGTTTTCTTGTTAGGGGCATGGCTTAGTCCCTAAATAACATTGTGTGGTTTAATTCCTACTCCGTATCTCTTCTACCACTCTGGCCACTAC 17630  
 G

ATAAGC

ATAAGC  
Depth:5 (RABBIT)  
Ei-value:0.000, Pi-value:0.010  
Er-value:0.000, Pr-value:0.000  
No matches to eCLIP DataNo matches to TargetScan

AGGTAGCTGGGTTTTGTAGTGAGCTTGCTCCTTAAGTTACAGGAACTCTCCTTATAATAGACACTTCATTTTCCTAGTCCATCCCTCATGAAAAATGACTGACCACTGCTGGG 17750  
 CAGCAGGAGGGATGATGACCAACTAATTCCCAAACCCCAGTCTCATTGGTACCAGCCTTGGGGAACCACCTACACTTGAGCCACAATTGGTTTTGAAGTGCATTTACAAGGTTTGTCTAT 17870  
 TTTCAGTTCTTTACTTTTTACATGCTGACACATACATACACTGCCTAAATAGATCTCTTTCAGAAACAATCCTCAGATAACGCATAGCAAAATGGAGATGGAGACATGATTTCTCATGCA 17990  
 ACAGCTTCTCTAATTATACCTTAGAAATGTTCTCCTTTTTATCATCAAATCTGCTCAAGAAGGGCTTTTTATAGTAGAATAATATCAGTGGATGAAAACAGCTTAACATTTTACCATGCT 18110  
 TAAGTTTTAAGAATAAAATAAAAATTGGAAATAATTGGCCAAAATTGAAAGGAAAAATTTTTTTAAAATTTCTCTAAATGTAGGCCTGGCTGGGCTTTGACCTTTTCCGTTTTTAAATCA 18230  
 CTCACAGAGGGTGGGACAGGAGGAAGAGTGAAGG

AAAAGGT

AAAAGGT  
Depth:6 (MOUSE)  
Ei-value:0.000, Pi-value:0.000  
Er-value:0.000, Pr-value:0.000  
eCLIP MATCHES▶ILF3 (bg=3.0%)▶SF3B1 (bg=2.48%)▶ZC3H11A (bg=6.55%)No matches to TargetScan

CAAACCTGTTTTAAGGGCAACCTGCCTTTGTTCTGAATTGGTCTTAAGAACATTACCAGCTCCAGGTTTAAATTGTTCA 18350  
 GTTTCATGCAGTTCCAATAGCTGATCATTGTTGAGATGAGGACAAAATCCTTTGTCCTCACTAGTTTGCTTTACATTTTTGAAAAGTATTATTTTTGTCCAAGTGCTTATCAACTAAACC 18470  
 TTGTGTTAGGTAAGAATGGAATTTATTAAGTGAATCAGTGTGACCCTTCTTGTCATAAGATTATCTTAAAGCTGAAGCCAAAATATGCTTCAAAAGAAGAGGACTTTATTGTTCATTGTA 18590  
 GTTCATACATTCAAAGCATCTGAACTGTAGTTTCTATAGCAAGCCAATTACATCCATAAGTGGAGAAGGAAATAGATAAATGTCAAAGTATGATTGGTGGAGGGAGCAAGGTTGAAGATA 18710  
 ATCTGGGGTTGAAATTTTCTAGTTTTCATTCTGTACATTTTTAGTTAGACATCAGATTTGAAATATTAATGTTTACCTTTCAATGTGTGGTATCAGCTGGACTCAGTAACACCCCTTTCT 18830  
 TCAGCTGGGGATGGGGAATGGATTATTGGAAAATGGAAAGAAGAAAGTAACTAAAAGCCTTCCTTTCACAGTTTCTGGCATCACTACCACTACTGATTAAACAAGAATAAGAGAACATTT 18950  
 TATCATCATCTGCTTTATTCACATAAATGAAGTTGTGATGAATAAATCTGCTTTTATGCAGACACAAGGAATTAAGTGGCTTCGTCATTGTCCTTCTACCTCAAAGATAATTTATTCCAA 19070  
 AAGCTAAGATAAATGGAAGACTCTTGAACTTGTGAACTGATGTGAAATGCAGAATCTCTTTTGAGTCTTTGCTGTTTGGAAGATTGAAAAATATTGTTCAGCATGGGTGACCACCAGAAA 19190  
 GTAATCTTAAGCCATCTAGATGTCACAATTGAAACAAACTGGGGAGTTGGTTGCTATTGTAAAATAAAATATACTGTTTTGAAAACTTTG                               19280
```

|  |  |  |  |  |
| --- | --- | --- | --- | --- |
| | | | | | | | | | |
| 2 |  | 4 |  | 6 |
| Depth of motif conservation (number of species) | | | | |

  
  

---

  

## >HUMAN TO MOUSE (19280 bases)

```
 CCTTCAGTTCTTAAAGCGCTGCAATTCGCTGCTGCAGCCATATTTCTTACTCTCTCGGGGCTGGAAGCTTCCTGACTGAAGATCTCTCTGCACTTGGGGTTCTTTCTAGAACATTTTCTA 120  
 GTCCCCCAACACCCTTTATGGCGTATTTCTTTAAAAAAATCACCTAAATTCCATAAAATATTTTTTTAAATTCTATACTTTCTCCTAGTGTCTTCTTGACACGTCCTCCATATTTTTTTA 240  
 AAGAAAGTATTTGGAATATTTTGAGGCAATTTTTAATATTTAAGGAATTTTTCTTTGGAATCATTTTTGGTTGACATCTCTGTTTTTTGTGGATCAGTTTTTTACTCTTCCACTCTCTTT 360  
 TCTATATTTTGCCCATCGGGGCTGCGGATACCTGGTTTTATTATTTTTTCTTTGCCCAACGGGGCCGTGGATACCTGCCTTTTAATTCTTTTTTATTCGCCCATCGGGGCCGCGGATACC 480  
 TGCTTTTTATTTTTTTTTCCTTAGCCCATCGGGGTATCGGATACCTGCTGATTCCCTTCCCCTCTGAACCCCCAACACTCTGGCCCATCGGGGTGACGGATATCTGCTTTTTAAAAATTT 600  
 TCTTTTTTTGGCCCATCGGGGCTTCGGATACCTGCTTTTTTTTTTTTTATTTTTCCTTGCCCATCGGGGCCTCGGATACCTGCTTTAATTTTTGTTTTTCTGGCCCATCGGGGCCGCGGA 720  
 TACCTGCTTTGATTTTTTTTTTTCATCGCCCATCGGTGCTTTTTATGGATGAAAAAATGTTGGTTTTGTGGGTTGTTGCACTCTCTGGAATATCTACACTTTTTTTTGCTGCTGATCATT 840  
 TGGTGGTGTGTGAGTGTACCTACCGCTTTGGCAGAGAATGACTCTGCAGTTAAGCTAAGGGCGTGTTCAGATTGTGGAGGAAAAGTGGCCGCCATTTTAGACTTGCCGCATAACTCGGCT 960  
 TAGGGCTAGTCGTTTGTGCTAAGTTAAACTAGGGAGGCAAGATGGATGATAGCAGGTCAGGCAGAGGAAGTCATGTGCATTGCATGAGCTAAACCTATCTGAATGAATTGATTTGGGGCT 1080  
 TGTTAGGAGCTTTGCGTGATTGTTGTATCGGGAGGCAGTAAGAATCATCTTTTATCAGTACAAGGGACTAGTTAAAAATGGAAGGTTAGGAAAGACTAAGGTGCAGGGCTTAAAATGGCG 1200  
 ATTTTGACATTGCGGCATTGCTCAGCATGGCGGGCTGTGCTTTGTTAGGTTGTCCAAAATGGCGGATCCAGTTCTGTCGCAGTGTTCAAGTGGCGGGAAGGCCACATCATGATGGGCGAG 1320  
 GCTTTGTTAAGTGGTTAGCATGGTGGTGGACATGTGCGGTCACACAGGAAAAGATGGCGGCTGAAGGTCTTGCCGCAGTGTAAAACATGGCGGGCCTCTTTGTCTTTGCTGTGTGCTTTT 1440  
 CGTGTTGGGTTTTGCCGCAGGGACAATATGGCAGGCGTTGTCATATGTATATCATGGCTTTTGTCACGTGGACATCATGGCGGGCTTGCCGCATTGTTAAAGATGGCGGGTTTTGCCGCC 1560  
 TAGTGCCACGCAGAGCGGGAGAAAAGGTGGGATGGACAGTGCTGGATTGCTGCATAACCCAACCAATTAGAAATGGGGGTGGAATTGATCACAGCCAATTAGAGCAGAAGATGGAATTAG 1680  
 ACTGATGACACACTGTCCAGCTACTCAGCGAAGACCTGGGTGAATTAGCATGGCACTTCGCAGCTGTCTTTAGCCAGTCAGGAGAAAGAAGTGGAGGGGCCACGTGTATGTCTCCCAGTG 1800  
 GGCGGTACACCAGGTGTTTTCAAGGTCTTTTCAAGGACATTTAGCCTTTCCACCTCTGTCCCCTCTTATTTGTCCCCTCCTGTCCAGTGCTGCCTCTTGCAGTGCTGGATATCTGGCTGT 1920  
 GTGGTCTGAACCTCCCTCCATTCCTCTGTATTGGTGCCTCACCTAAGGCTAAGTATACCTCCCCCCCCACCCCCCAACCCCCCCAACTCCCCACCCCCACCCCCCACCCCCCACCTCCCC 2040  
 ACCCCCCTACCCCCCTACCCCCCTACCCCCCTCTGGTCTGCCCTGCACTGCACTGTTGCCATGGGCAGTGCTCCAGGCCTGCTTGGTGTGGACATGGTGGTGAGCCGTGGCAAGGACCAG 2160  
 AATGGATCACAGATGATCGTTGGCCAACAGGTGGCAGAAGAGGAATTCCTGCCTTCCTCAAGAGGAACACCTACCCCTTGGCTAATGCTGGGGTCGGATTTTGATTTATATTTATCTTTT 2280  
 GGATGTCAGTCATACAGTCTGATTTTGTGGTTTGCTAGTGTTTGAATTTAAGTCTTAAGTGACTATTATAGAAATGTATTAAGAGGCTTTATTTGTAGAATTCACTTTAATTACATTTAA 2400  
 TGAGTTTTTGTTTTGAGTTCCTTAAAATTCCTTAAAGTTTTTAGCTTCTCATTACAAATTCCTTAACCTTTTTTTGGCAGTAGATAGTCAAAGTCAAATCATTTCTAATGTTTTAAAAAT 2520  
 GTGCTGGTCATTTTCTTTGAAATTGACTTAACTATTTTCCTTTGAAGAGTCTGTAGCACAGAAACAGTAAAAAATTTAACTTCATGACCTAATGTAAAAAAGAGTGTTTGAAGGTTTACA 2640  
 CAGGTCCAGGCCTTGCTTTGTTCCCATCCTTGATGCTGCACTAATTGACTAATCACCTACTTATCAGACAGGAAACTTGAATTGCTGTGGTCTGGTGTCCTCTATTCAGACTTATTATAT 2760  
 TGGAGTATTTCAATTTTTCGTTGTATCCTGCCTGCCTAGCATCCAGTTCCTCCCCAGCCCTGCTCCCAGCAAACCCCTAGTCTAGCCCCAGCCCTACTCCCACCCCGCCCCAGCCCTGCC 2880  
 CCAGCCCCAGTCCCCTAACCCCCCAGCCCTAGCCCCAGTCCCAGTCCTAGTTCCTCAGTCCCGCCCAGCTTCTCTCGAAAGTCACTCTAATTTTCATTGATTCAGTGCTCAAAATAAGTT 3000  
 GTCCATTGCTTATCCTATTATACTGGGATATTCCGTTTACCCTTGGCATTGCTGATCTTCAGTACTGACTCCTTGACCATTTTCAGTTAATGCATACAATCCCATTTGTCTGTGATCTCA 3120
[truncated: 21,837 more chars]
