# Supplementary material for: Uncovering deeply conserved motif combinations in rapidly evolving noncoding sequences
Source: Genome Biol. 2021 Jan 11;22:29. doi: 10.1186/s13059-020-02247-1 (PMC7798263; doi:10.1186/s13059-020-02247-1)
Supplement: Supplementary file 5 — Additional file 5. LncLOOM output results for MALAT1 sequences from 19 vertebrates. [file 13059_2020_2247_MOESM5_ESM.gz › AdditionalFile5/Html_Files/Modules.html]

 MODULES


# MODULES

## start-MOTIF-end--(number of bases in between)--start-MOTIF-end

  

NAVIGATE ▼

▶ZEBRAFISH (depth:19)▶MEDAKA (depth:18)▶NILETILAPIA (depth:16)▶SPOTTEDGAR (depth:14)▶OPOSSUM (depth:13)▶X.TROPICALIS (depth:11)▶SNAKE (depth:10)▶LIZARD (depth:9)▶ALLIGATOR (depth:8)▶TURTLE (depth:7)▶MOUSE (depth:6)▶COW (depth:5)▶PIG (depth:4)▶DOG (depth:3)▶MARMOSET (depth:2)

  
  
  
  
  
  

|  |  |  |  |  |  |  |  |  |  |  |  |  |  |  |  |  |  |
| --- | --- | --- | --- | --- | --- | --- | --- | --- | --- | --- | --- | --- | --- | --- | --- | --- | --- |
| | | | | | | | | | | | | | | | | | | | | | | | | | | | | | | | | | | | |
| 2 |  |  |  |  |  |  |  |  | 11 |  |  |  |  |  |  |  | 19 |
| Depth of motif conservation (number of species) | | | | | | | | | | | | | | | | | |

  
  

# Modules conserved to ZEBRAFISH (Depth: 19)

## Modules in Main Graph (All sequences considered):

```
>HUMAN  
      2505-

TTTGGG

TTTGGG  
Depth:19 (ZEBRAFISH)  
Ei-value:0.000, Pi-value:0.000  
Er-value:0.000, Pr-value:0.000  
eCLIP MATCHES▶ppil4 (bg=43.39%)▶PRPF8 (bg=6.2%)No matches to TargetScan

-2510--(1110)--3621-

TTTTTCAG

TTTTTCAG  
Depth:19 (ZEBRAFISH)  
Ei-value:0.000, Pi-value:0.000  
Er-value:0.000, Pr-value:0.000  
eCLIP MATCHES▶ddx42 (bg=10.33%)▶ppil4 (bg=43.39%)▶safb (bg=40.39%)▶u2af1 (bg=14.02%)▶u2af2 (bg=19.32%)No matches to TargetScan

-3628--(543)--4172-

GATAAG

GATAAG  
Depth:19 (ZEBRAFISH)  
Ei-value:0.000, Pi-value:0.000  
Er-value:0.000, Pr-value:0.000  
eCLIP MATCHES▶cpsf6 (bg=13.45%)▶khsrp (bg=27.4%)▶NIPBL (bg=8.2%)▶ppil4 (bg=43.39%)▶PRPF8 (bg=6.2%)▶rbm15 (bg=11.59%)▶safb (bg=40.39%)▶safb2 (bg=26.89%)▶srsf1 (bg=30.28%)▶srsf7 (bg=22.53%)▶znf622 (bg=18.79%)No matches to TargetScan

-4177--(2833)--7011-

TTTTCTTTT

TTTTCTTTT  
Depth:19 (ZEBRAFISH)  
Ei-value:0.000, Pi-value:0.000  
Er-value:0.000, Pr-value:0.000  
eCLIP MATCHES▶srsf7 (bg=22.53%)MATCHES To TargetScan▶ miR-186-5p:AAAGAAU

-7019--(28)--7048-

CAGGTTTTGCTTT

CAGGTTTTGCTTT  
Depth:19 (ZEBRAFISH)  
Ei-value:0.000, Pi-value:0.000  
Er-value:0.000, Pr-value:0.000  
eCLIP MATCHES▶srsf7 (bg=22.53%)MATCHES To TargetScan▶ miR-330-3p.2:AAAGCAC▶ miR-490-3p:AACCUGG

-7060--(26)--7087-

AAAAAGCAAAA

AAAAAGCAAAA  
Depth:19 (ZEBRAFISH)  
Ei-value:0.000, Pi-value:0.000  
Er-value:0.000, Pr-value:0.000  
No matches to eCLIP DataNo matches to TargetScan

-7097  
  
>MARMOSET  
      2637-

TTTGGG

TTTGGG  
Depth:19 (ZEBRAFISH)  
Ei-value:0.000, Pi-value:0.000  
Er-value:0.000, Pr-value:0.000  
No matches to TargetScan

-2642--(1095)--3738-

TTTTTCAG

TTTTTCAG  
Depth:19 (ZEBRAFISH)  
Ei-value:0.000, Pi-value:0.000  
Er-value:0.000, Pr-value:0.000  
No matches to TargetScan

-3745--(548)--4294-

GATAAG

GATAAG  
Depth:19 (ZEBRAFISH)  
Ei-value:0.000, Pi-value:0.000  
Er-value:0.000, Pr-value:0.000  
No matches to TargetScan

-4299--(2840)--7140-

TTTTCTTTT

TTTTCTTTT  
Depth:19 (ZEBRAFISH)  
Ei-value:0.000, Pi-value:0.000  
Er-value:0.000, Pr-value:0.000  
MATCHES To TargetScan▶ miR-186-5p:AAAGAAU

-7148--(28)--7177-

CAGGTTTTGCTTT

CAGGTTTTGCTTT  
Depth:19 (ZEBRAFISH)  
Ei-value:0.000, Pi-value:0.000  
Er-value:0.000, Pr-value:0.000  
MATCHES To TargetScan▶ miR-330-3p.2:AAAGCAC▶ miR-490-3p:AACCUGG

-7189--(27)--7217-

AAAAAGCAAAA

AAAAAGCAAAA  
Depth:19 (ZEBRAFISH)  
Ei-value:0.000, Pi-value:0.000  
Er-value:0.000, Pr-value:0.000  
No matches to TargetScan

-7227  
  
>DOG  
      2612-

TTTGGG

TTTGGG  
Depth:19 (ZEBRAFISH)  
Ei-value:0.000, Pi-value:0.000  
Er-value:0.000, Pr-value:0.000  
No matches to TargetScan

-2617--(1013)--3631-

TTTTTCAG

TTTTTCAG  
Depth:19 (ZEBRAFISH)  
Ei-value:0.000, Pi-value:0.000  
Er-value:0.000, Pr-value:0.000  
No matches to TargetScan

-3638--(559)--4198-

GATAAG

GATAAG  
Depth:19 (ZEBRAFISH)  
Ei-value:0.000, Pi-value:0.000  
Er-value:0.000, Pr-value:0.000  
No matches to TargetScan

-4203--(2856)--7060-

TTTTCTTTT

TTTTCTTTT  
Depth:19 (ZEBRAFISH)  
Ei-value:0.000, Pi-value:0.000  
Er-value:0.000, Pr-value:0.000  
MATCHES To TargetScan▶ miR-186-5p:AAAGAAU

-7068--(26)--7095-

CAGGTTTTGCTTT

CAGGTTTTGCTTT  
Depth:19 (ZEBRAFISH)  
Ei-value:0.000, Pi-value:0.000  
Er-value:0.000, Pr-value:0.000  
MATCHES To TargetScan▶ miR-330-3p.2:AAAGCAC▶ miR-490-3p:AACCUGG

-7107--(22)--7130-

AAAAAGCAAAA

AAAAAGCAAAA  
Depth:19 (ZEBRAFISH)  
Ei-value:0.000, Pi-value:0.000  
Er-value:0.000, Pr-value:0.000  
No matches to TargetScan

-7140  
  
>PIG  
      2533-

TTTGGG

TTTGGG  
Depth:19 (ZEBRAFISH)  
Ei-value:0.000, Pi-value:0.000  
Er-value:0.000, Pr-value:0.000  
No matches to TargetScan

-2538--(1002)--3541-

TTTTTCAG

TTTTTCAG  
Depth:19 (ZEBRAFISH)  
Ei-value:0.000, Pi-value:0.000  
Er-value:0.000, Pr-value:0.000  
No matches to TargetScan

-3548--(569)--4118-

GATAAG

GATAAG  
Depth:19 (ZEBRAFISH)  
Ei-value:0.000, Pi-value:0.000  
Er-value:0.000, Pr-value:0.000  
No matches to TargetScan

-4123--(2848)--6972-

TTTTCTTTT

TTTTCTTTT  
Depth:19 (ZEBRAFISH)  
Ei-value:0.000, Pi-value:0.000  
Er-value:0.000, Pr-value:0.000  
MATCHES To TargetScan▶ miR-186-5p:AAAGAAU

-6980--(28)--7009-

CAGGTTTTGCTTT

CAGGTTTTGCTTT  
Depth:19 (ZEBRAFISH)  
Ei-value:0.000, Pi-value:0.000  
Er-value:0.000, Pr-value:0.000  
MATCHES To TargetScan▶ miR-330-3p.2:AAAGCAC▶ miR-490-3p:AACCUGG

-7021--(24)--7046-

AAAAAGCAAAA

AAAAAGCAAAA  
Depth:19 (ZEBRAFISH)  
Ei-value:0.000, Pi-value:0.000  
Er-value:0.000, Pr-value:0.000  
No matches to TargetScan

-7056  
  
>COW  
      2417-

TTTGGG

TTTGGG  
Depth:19 (ZEBRAFISH)  
Ei-value:0.000, Pi-value:0.000  
Er-value:0.000, Pr-value:0.000  
No matches to TargetScan

-2422--(1012)--3435-

TTTTTCAG

TTTTTCAG  
Depth:19 (ZEBRAFISH)  
Ei-value:0.000, Pi-value:0.000  
Er-value:0.000, Pr-value:0.000  
No matches to TargetScan

-3442--(555)--3998-

GATAAG

GATAAG  
Depth:19 (ZEBRAFISH)  
Ei-value:0.000, Pi-value:0.000  
Er-value:0.000, Pr-value:0.000  
No matches to TargetScan

-4003--(2899)--6903-

TTTTCTTTT

TTTTCTTTT  
Depth:19 (ZEBRAFISH)  
Ei-value:0.000, Pi-value:0.000  
Er-value:0.000, Pr-value:0.000  
MATCHES To TargetScan▶ miR-186-5p:AAAGAAU

-6911--(30)--6942-

CAGGTTTTGCTTT

CAGGTTTTGCTTT  
Depth:19 (ZEBRAFISH)  
Ei-value:0.000, Pi-value:0.000  
Er-value:0.000, Pr-value:0.000  
MATCHES To TargetScan▶ miR-330-3p.2:AAAGCAC▶ miR-490-3p:AACCUGG

-6954--(23)--6978-

AAAAAGCAAAA

AAAAAGCAAAA  
Depth:19 (ZEBRAFISH)  
Ei-value:0.000, Pi-value:0.000  
Er-value:0.000, Pr-value:0.000  
No matches to TargetScan

-6988  
  
>MOUSE  
      2324-

TTTGGG

TTTGGG  
Depth:19 (ZEBRAFISH)  
Ei-value:0.000, Pi-value:0.000  
Er-value:0.000, Pr-value:0.000  
No matches to TargetScan

-2329--(990)--3320-

TTTTTCAG

TTTTTCAG  
Depth:19 (ZEBRAFISH)  
Ei-value:0.000, Pi-value:0.000  
Er-value:0.000, Pr-value:0.000  
No matches to TargetScan

-3327--(518)--3846-

GATAAG

GATAAG  
Depth:19 (ZEBRAFISH)  
Ei-value:0.000, Pi-value:0.000  
Er-value:0.000, Pr-value:0.000  
No matches to TargetScan

-3851--(2754)--6606-

TTTTCTTTT

TTTTCTTTT  
Depth:19 (ZEBRAFISH)  
Ei-value:0.000, Pi-value:0.000  
Er-value:0.000, Pr-value:0.000  
MATCHES To TargetScan▶ miR-186-5p:AAAGAAU

-6614--(26)--6641-

CAGGTTTTGCTTT

CAGGTTTTGCTTT  
Depth:19 (ZEBRAFISH)  
Ei-value:0.000, Pi-value:0.000  
Er-value:0.000, Pr-value:0.000  
MATCHES To TargetScan▶ miR-330-3p.2:AAAGCAC▶ miR-490-3p:AACCUGG

-6653--(26)--6680-

AAAAAGCAAAA

AAAAAGCAAAA  
Depth:19 (ZEBRAFISH)  
Ei-value:0.000, Pi-value:0.000  
Er-value:0.000, Pr-value:0.000  
No matches to TargetScan

-6690  
  
>TURTLE  
      1271-

TTTGGG

TTTGGG  
Depth:19 (ZEBRAFISH)  
Ei-value:0.000, Pi-value:0.000  
Er-value:0.000, Pr-value:0.000  
No matches to TargetScan

-1276--(1382)--2659-

TTTTTCAG

TTTTTCAG  
Depth:19 (ZEBRAFISH)  
Ei-value:0.000, Pi-value:0.000  
Er-value:0.000, Pr-value:0.000  
No matches to TargetScan

-2666--(648)--3315-

GATAAG

GATAAG  
Depth:19 (ZEBRAFISH)  
Ei-value:0.000, Pi-value:0.000  
Er-value:0.000, Pr-value:0.000  
No matches to TargetScan

-3320--(3412)--6733-

TTTTCTTTT

TTTTCTTTT  
Depth:19 (ZEBRAFISH)  
Ei-value:0.000, Pi-value:0.000  
Er-value:0.000, Pr-value:0.000  
MATCHES To TargetScan▶ miR-186-5p:AAAGAAU

-6741--(23)--6765-

CAGGTTTTGCTTT

CAGGTTTTGCTTT  
Depth:19 (ZEBRAFISH)  
Ei-value:0.000, Pi-value:0.000  
Er-value:0.000, Pr-value:0.000  
MATCHES To TargetScan▶ miR-330-3p.2:AAAGCAC▶ miR-490-3p:AACCUGG

-6777--(21)--6799-

AAAAAGCAAAA

AAAAAGCAAAA  
Depth:19 (ZEBRAFISH)  
Ei-value:0.000, Pi-value:0.000  
Er-value:0.000, Pr-value:0.000  
No matches to TargetScan

-6809  
  
>ALLIGATOR  
      2537-

TTTGGG

TTTGGG  
Depth:19 (ZEBRAFISH)  
Ei-value:0.000, Pi-value:0.000  
Er-value:0.000, Pr-value:0.000  
No matches to TargetScan

-2542--(1322)--3865-

TTTTTCAG

TTTTTCAG  
Depth:19 (ZEBRAFISH)  
Ei-value:0.000, Pi-value:0.000  
Er-value:0.000, Pr-value:0.000  
No matches to TargetScan

-3872--(648)--4521-

GATAAG

GATAAG  
Depth:19 (ZEBRAFISH)  
Ei-value:0.000, Pi-value:0.000  
Er-value:0.000, Pr-value:0.000  
No matches to TargetScan

-4526--(3478)--8005-

TTTTCTTTT

TTTTCTTTT  
Depth:19 (ZEBRAFISH)  
Ei-value:0.000, Pi-value:0.000  
Er-value:0.000, Pr-value:0.000  
MATCHES To TargetScan▶ miR-186-5p:AAAGAAU

-8013--(23)--8037-

CAGGTTTTGCTTT

CAGGTTTTGCTTT  
Depth:19 (ZEBRAFISH)  
Ei-value:0.000, Pi-value:0.000  
Er-value:0.000, Pr-value:0.000  
MATCHES To TargetScan▶ miR-330-3p.2:AAAGCAC▶ miR-490-3p:AACCUGG

-8049--(26)--8076-

AAAAAGCAAAA

AAAAAGCAAAA  
Depth:19 (ZEBRAFISH)  
Ei-value:0.000, Pi-value:0.000  
Er-value:0.000, Pr-value:0.000  
No matches to TargetScan

-8086  
  
>LIZARD  
      2353-

TTTGGG

TTTGGG  
Depth:19 (ZEBRAFISH)  
Ei-value:0.000, Pi-value:0.000  
Er-value:0.000, Pr-value:0.000  
No matches to TargetScan

-2358--(762)--3121-

TTTTTCAG

TTTTTCAG  
Depth:19 (ZEBRAFISH)  
Ei-value:0.000, Pi-value:0.000  
Er-value:0.000, Pr-value:0.000  
No matches to TargetScan

-3128--(588)--3717-

GATAAG

GATAAG  
Depth:19 (ZEBRAFISH)  
Ei-value:0.000, Pi-value:0.000  
Er-value:0.000, Pr-value:0.000  
No matches to TargetScan

-3722--(3176)--6899-

TTTTCTTTT

TTTTCTTTT  
Depth:19 (ZEBRAFISH)  
Ei-value:0.000, Pi-value:0.000  
Er-value:0.000, Pr-value:0.000  
MATCHES To TargetScan▶ miR-186-5p:AAAGAAU

-6907--(24)--6932-

CAGGTTTTGCTTT

CAGGTTTTGCTTT  
Depth:19 (ZEBRAFISH)  
Ei-value:0.000, Pi-value:0.000  
Er-value:0.000, Pr-value:0.000  
MATCHES To TargetScan▶ miR-330-3p.2:AAAGCAC▶ miR-490-3p:AACCUGG

-6944--(21)--6966-

AAAAAGCAAAA

AAAAAGCAAAA  
Depth:19 (ZEBRAFISH)  
Ei-value:0.000, Pi-value:0.000  
Er-value:0.000, Pr-value:0.000  
No matches to TargetScan

-6976  
  
>SNAKE  
      1292-

TTTGGG

TTTGGG  
Depth:19 (ZEBRAFISH)  
Ei-value:0.000, Pi-value:0.000  
Er-value:0.000, Pr-value:0.000  
No matches to TargetScan

-1297--(729)--2027-

TTTGGG

TTTGGG  
Depth:19 (ZEBRAFISH)  
Ei-value:0.000, Pi-value:0.000  
Er-value:0.000, Pr-value:0.000  
No matches to TargetScan

-2032--(1081)--3114-

TTTTTCAG

TTTTTCAG  
Depth:19 (ZEBRAFISH)  
Ei-value:0.000, Pi-value:0.000  
Er-value:0.000, Pr-value:0.000  
No matches to TargetScan

-3121--(558)--3680-

GATAAG

GATAAG  
Depth:19 (ZEBRAFISH)  
Ei-value:0.000, Pi-value:0.000  
Er-value:0.000, Pr-value:0.000  
No matches to TargetScan

-3685--(3037)--6723-

TTTTCTTTT

TTTTCTTTT  
Depth:19 (ZEBRAFISH)  
Ei-value:0.000, Pi-value:0.000  
Er-value:0.000, Pr-value:0.000  
MATCHES To TargetScan▶ miR-186-5p:AAAGAAU

-6731--(25)--6757-

CAGGTTTTGCTTT

CAGGTTTTGCTTT  
Depth:19 (ZEBRAFISH)  
Ei-value:0.000, Pi-value:0.000  
Er-value:0.000, Pr-value:0.000  
MATCHES To TargetScan▶ miR-330-3p.2:AAAGCAC▶ miR-490-3p:AACCUGG

-6769--(29)--6799-

AAAAAGCAAAA

AAAAAGCAAAA  
Depth:19 (ZEBRAFISH)  
Ei-value:0.000, Pi-value:0.000  
Er-value:0.000, Pr-value:0.000  
No matches to TargetScan

-6809  
  
>X.TROPICALIS  
      2299-

TTTGGG

TTTGGG  
Depth:19 (ZEBRAFISH)  
Ei-value:0.000, Pi-value:0.000  
Er-value:0.000, Pr-value:0.000  
No matches to TargetScan

-2304--(2695)--5000-

TTTGGG

TTTGGG  
Depth:19 (ZEBRAFISH)  
Ei-value:0.000, Pi-value:0.000  
Er-value:0.000, Pr-value:0.000  
No matches to TargetScan

-5005--(125)--5131-

TTTGGG

TTTGGG  
Depth:19 (ZEBRAFISH)  
Ei-value:0.000, Pi-value:0.000  
Er-value:0.000, Pr-value:0.000  
No matches to TargetScan

-5136--(3206)--8343-

TTTTTCAG

TTTTTCAG  
Depth:19 (ZEBRAFISH)  
Ei-value:0.000, Pi-value:0.000  
Er-value:0.000, Pr-value:0.000  
No matches to TargetScan

-8350--(1004)--9355-

GATAAG

GATAAG  
Depth:19 (ZEBRAFISH)  
Ei-value:0.000, Pi-value:0.000  
Er-value:0.000, Pr-value:0.000  
No matches to TargetScan

-9360--(2966)--12327-

TTTTCTTTT

TTTTCTTTT  
Depth:19 (ZEBRAFISH)  
Ei-value:0.000, Pi-value:0.000  
Er-value:0.000, Pr-value:0.000  
MATCHES To TargetScan▶ miR-186-5p:AAAGAAU

-12335--(26)--12362-

CAGGTTTTGCTTT

CAGGTTTTGCTTT  
Depth:19 (ZEBRAFISH)  
Ei-value:0.000, Pi-value:0.000  
Er-value:0.000, Pr-value:0.000  
MATCHES To TargetScan▶ miR-330-3p.2:AAAGCAC▶ miR-490-3p:AACCUGG

-12374--(31)--12406-

AAAAAGCAAAA

AAAAAGCAAAA  
Depth:19 (ZEBRAFISH)  
Ei-value:0.000, Pi-value:0.000  
Er-value:0.000, Pr-value:0.000  
No matches to TargetScan

-12416  
  
>SHARK  
      1413-

TTTGGG

TTTGGG  
Depth:19 (ZEBRAFISH)  
Ei-value:0.000, Pi-value:0.000  
Er-value:0.000, Pr-value:0.000  
No matches to TargetScan

-1418--(1015)--2434-

TTTGGG

TTTGGG  
Depth:19 (ZEBRAFISH)  
Ei-value:0.000, Pi-value:0.000  
Er-value:0.000, Pr-value:0.000  
No matches to TargetScan

-2439--(74)--2514-

TTTGGG

TTTGGG  
Depth:19 (ZEBRAFISH)  
Ei-value:0.000, Pi-value:0.000  
Er-value:0.000, Pr-value:0.000  
No matches to TargetScan

-2519--(38)--2558-

TTTTTCAG

TTTTTCAG  
Depth:19 (ZEBRAFISH)  
Ei-value:0.000, Pi-value:0.000  
Er-value:0.000, Pr-value:0.000  
No matches to TargetScan

-2565--(4402)--6968-

GATAAG

GATAAG  
Depth:19 (ZEBRAFISH)  
Ei-value:0.000, Pi-value:0.000  
Er-value:0.000, Pr-value:0.000  
No matches to TargetScan

-6973--(520)--7494-

TTTTCTTTT

TTTTCTTTT  
Depth:19 (ZEBRAFISH)  
Ei-value:0.000, Pi-value:0.000  
Er-value:0.000, Pr-value:0.000  
MATCHES To TargetScan▶ miR-186-5p:AAAGAAU

-7502--(25)--7528-

CAGGTTTTGCTTT

CAGGTTTTGCTTT  
Depth:19 (ZEBRAFISH)  
Ei-value:0.000, Pi-value:0.000  
Er-value:0.000, Pr-value:0.000  
MATCHES To TargetScan▶ miR-330-3p.2:AAAGCAC▶ miR-490-3p:AACCUGG

-7540--(27)--7568-

AAAAAGCAAAA

AAAAAGCAAAA  
Depth:19 (ZEBRAFISH)  
Ei-value:0.000, Pi-value:0.000  
Er-value:0.000, Pr-value:0.000  
No matches to TargetScan

-7578  
  
>OPOSSUM  
      1043-

TTTGGG

TTTGGG  
Depth:19 (ZEBRAFISH)  
Ei-value:0.000, Pi-value:0.000  
Er-value:0.000, Pr-value:0.000  
No matches to TargetScan

-1048--(273)--1322-

TTTGGG

TTTGGG  
Depth:19 (ZEBRAFISH)  
Ei-value:0.000, Pi-value:0.000  
Er-value:0.000, Pr-value:0.000  
No matches to TargetScan

-1327--(111)--1439-

TTTGGG

TTTGGG  
Depth:19 (ZEBRAFISH)  
Ei-value:0.000, Pi-value:0.000  
Er-value:0.000, Pr-value:0.000  
No matches to TargetScan

-1444--(1059)--2504-

TTTTTCAG

TTTTTCAG  
Depth:19 (ZEBRAFISH)  
Ei-value:0.000, Pi-value:0.000  
Er-value:0.000, Pr-value:0.000  
No matches to TargetScan

-2511--(973)--3485-

GATAAG

GATAAG  
Depth:19 (ZEBRAFISH)  
Ei-value:0.000, Pi-value:0.000  
Er-value:0.000, Pr-value:0.000  
No matches to TargetScan

-3490--(2190)--5681-

TTTTCTTTT

TTTTCTTTT  
Depth:19 (ZEBRAFISH)  
Ei-value:0.000, Pi-value:0.000  
Er-value:0.000, Pr-value:0.000  
MATCHES To TargetScan▶ miR-186-5p:AAAGAAU

-5689--(26)--5716-

CAGGTTTTGCTTT

CAGGTTTTGCTTT  
Depth:19 (ZEBRAFISH)  
Ei-value:0.000, Pi-value:0.000  
Er-value:0.000, Pr-value:0.000  
MATCHES To TargetScan▶ miR-330-3p.2:AAAGCAC▶ miR-490-3p:AACCUGG

-5728--(18)--5747-

AAAAAGCAAAA

AAAAAGCAAAA  
Depth:19 (ZEBRAFISH)  
Ei-value:0.000, Pi-value:0.000  
Er-value:0.000, Pr-value:0.000  
No matches to TargetScan

-5757  
  
>SPOTTEDGAR  
      3644-

TTTGGG

TTTGGG  
Depth:19 (ZEBRAFISH)  
Ei-value:0.000, Pi-value:0.000  
Er-value:0.000, Pr-value:0.000  
No matches to TargetScan

-3649--(31)--3681-

TTTTTCAG

TTTTTCAG  
Depth:19 (ZEBRAFISH)  
Ei-value:0.000, Pi-value:0.000  
Er-value:0.000, Pr-value:0.000  
No matches to TargetScan

-3688--(72)--3761-

GATAAG

GATAAG  
Depth:19 (ZEBRAFISH)  
Ei-value:0.000, Pi-value:0.000  
Er-value:0.000, Pr-value:0.000  
No matches to TargetScan

-3766--(274)--4041-

GATAAG

GATAAG  
Depth:19 (ZEBRAFISH)  
Ei-value:0.000, Pi-value:0.000  
Er-value:0.000, Pr-value:0.000  
No matches to TargetScan

-4046--(2564)--6611-

TTTTCTTTT

TTTTCTTTT  
Depth:19 (ZEBRAFISH)  
Ei-value:0.000, Pi-value:0.000  
Er-value:0.000, Pr-value:0.000  
MATCHES To TargetScan▶ miR-186-5p:AAAGAAU

-6619--(295)--6915-

CAGGTTTTGCTTT

CAGGTTTTGCTTT  
Depth:19 (ZEBRAFISH)  
Ei-value:0.000, Pi-value:0.000  
Er-value:0.000, Pr-value:0.000  
MATCHES To TargetScan▶ miR-330-3p.2:AAAGCAC▶ miR-490-3p:AACCUGG

-6927--(28)--6956-

AAAAAGCAAAA

AAAAAGCAAAA  
Depth:19 (ZEBRAFISH)  
Ei-value:0.000, Pi-value:0.000  
Er-value:0.000, Pr-value:0.000  
No matches to TargetScan

-6966  
  
>FUGU  
      1704-

TTTGGG

TTTGGG  
Depth:19 (ZEBRAFISH)  
Ei-value:0.000, Pi-value:0.000  
Er-value:0.000, Pr-value:0.000  
No matches to TargetScan

-1709--(774)--2484-

TTTTTCAG

TTTTTCAG  
Depth:19 (ZEBRAFISH)  
Ei-value:0.000, Pi-value:0.000  
Er-value:0.000, Pr-value:0.000  
No matches to TargetScan

-2491--(316)--2808-

TTTTTCAG

TTTTTCAG  
Depth:19 (ZEBRAFISH)  
Ei-value:0.000, Pi-value:0.000  
Er-value:0.000, Pr-value:0.000  
No matches to TargetScan

-2815--(72)--2888-

GATAAG

GATAAG  
Depth:19 (ZEBRAFISH)  
Ei-value:0.000, Pi-value:0.000  
Er-value:0.000, Pr-value:0.000  
No matches to TargetScan

-2893--(146)--3040-

GATAAG

GATAAG  
Depth:19 (ZEBRAFISH)  
Ei-value:0.000, Pi-value:0.000  
Er-value:0.000, Pr-value:0.000  
No matches to TargetScan

-3045--(1722)--4768-

TTTTCTTTT

TTTTCTTTT  
Depth:19 (ZEBRAFISH)  
Ei-value:0.000, Pi-value:0.000  
Er-value:0.000, Pr-value:0.000  
MATCHES To TargetScan▶ miR-186-5p:AAAGAAU

-4776--(31)--4808-

CAGGTTTTGCTTT

CAGGTTTTGCTTT  
Depth:19 (ZEBRAFISH)  
Ei-value:0.000, Pi-value:0.000  
Er-value:0.000, Pr-value:0.000  
MATCHES To TargetScan▶ miR-330-3p.2:AAAGCAC▶ miR-490-3p:AACCUGG

-4820--(18)--4839-

AAAAAGCAAAA

AAAAAGCAAAA  
Depth:19 (ZEBRAFISH)  
Ei-value:0.000, Pi-value:0.000  
Er-value:0.000, Pr-value:0.000  
No matches to TargetScan

-4849  
  
>NILETILAPIA  
      2795-

TTTGGG

TTTGGG  
Depth:19 (ZEBRAFISH)  
Ei-value:0.000, Pi-value:0.000  
Er-value:0.000, Pr-value:0.000  
No matches to TargetScan

-2800--(581)--3382-

TTTTTCAG

TTTTTCAG  
Depth:19 (ZEBRAFISH)  
Ei-value:0.000, Pi-value:0.000  
Er-value:0.000, Pr-value:0.000  
No matches to TargetScan

-3389--(324)--3714-

TTTTTCAG

TTTTTCAG  
Depth:19 (ZEBRAFISH)  
Ei-value:0.000, Pi-value:0.000  
Er-value:0.000, Pr-value:0.000  
No matches to TargetScan

-3721--(84)--3806-

GATAAG

GATAAG  
Depth:19 (ZEBRAFISH)  
Ei-value:0.000, Pi-value:0.000  
Er-value:0.000, Pr-value:0.000  
No matches to TargetScan

-3811--(2023)--5835-

TTTTCTTTT

TTTTCTTTT  
Depth:19 (ZEBRAFISH)  
Ei-value:0.000, Pi-value:0.000  
Er-value:0.000, Pr-value:0.000  
MATCHES To TargetScan▶ miR-186-5p:AAAGAAU

-5843--(28)--5872-

CAGGTTTTGCTTT

CAGGTTTTGCTTT  
Depth:19 (ZEBRAFISH)  
Ei-value:0.000, Pi-value:0.000  
Er-value:0.000, Pr-value:0.000  
MATCHES To TargetScan▶ miR-330-3p.2:AAAGCAC▶ miR-490-3p:AACCUGG

-5884--(19)--5904-

AAAAAGCAAAA

AAAAAGCAAAA  
Depth:19 (ZEBRAFISH)  
Ei-value:0.000, Pi-value:0.000  
Er-value:0.000, Pr-value:0.000  
No matches to TargetScan

-5914  
  
>STICKLEBACK  
        70-

TTTGGG

TTTGGG  
Depth:19 (ZEBRAFISH)  
Ei-value:0.000, Pi-value:0.000  
Er-value:0.000, Pr-value:0.000  
No matches to TargetScan

-75--(3779)--3855-

TTTGGG

TTTGGG  
Depth:19 (ZEBRAFISH)  
Ei-value:0.000, Pi-value:0.000  
Er-value:0.000, Pr-value:0.000  
No matches to TargetScan

-3860--(31)--3892-

TTTTTCAG

TTTTTCAG  
Depth:19 (ZEBRAFISH)  
Ei-value:0.000, Pi-value:0.000  
Er-value:0.000, Pr-value:0.000  
No matches to TargetScan

-3899--(84)--3984-

GATAAG

GATAAG  
Depth:19 (ZEBRAFISH)  
Ei-value:0.000, Pi-value:0.000  
Er-value:0.000, Pr-value:0.000  
No matches to TargetScan

-3989--(2020)--6010-

TTTTCTTTT

TTTTCTTTT  
Depth:19 (ZEBRAFISH)  
Ei-value:0.000, Pi-value:0.000  
Er-value:0.000, Pr-value:0.000  
MATCHES To TargetScan▶ miR-186-5p:AAAGAAU

-6018--(36)--6055-

CAGGTTTTGCTTT

CAGGTTTTGCTTT  
Depth:19 (ZEBRAFISH)  
Ei-value:0.000, Pi-value:0.000  
Er-value:0.000, Pr-value:0.000  
MATCHES To TargetScan▶ miR-330-3p.2:AAAGCAC▶ miR-490-3p:AACCUGG

-6067--(24)--6092-

AAAAAGCAAAA

AAAAAGCAAAA  
Depth:19 (ZEBRAFISH)  
Ei-value:0.000, Pi-value:0.000  
Er-value:0.000, Pr-value:0.000  
No matches to TargetScan

-6102  
  
>MEDAKA  
      1667-

TTTGGG

TTTGGG  
Depth:19 (ZEBRAFISH)  
Ei-value:0.000, Pi-value:0.000  
Er-value:0.000, Pr-value:0.000  
No matches to TargetScan

-1672--(1439)--3112-

TTTGGG

TTTGGG  
Depth:19 (ZEBRAFISH)  
Ei-value:0.000, Pi-value:0.000  
Er-value:0.000, Pr-value:0.000  
No matches to TargetScan

-3117--(27)--3145-

TTTTTCAG

TTTTTCAG  
Depth:19 (ZEBRAFISH)  
Ei-value:0.000, Pi-value:0.000  
Er-value:0.000, Pr-value:0.000  
No matches to TargetScan

-3152--(80)--3233-

GATAAG

GATAAG  
Depth:19 (ZEBRAFISH)  
Ei-value:0.000, Pi-value:0.000  
Er-value:0.000, Pr-value:0.000  
No matches to TargetScan

-3238--(1778)--5017-

TTTTCTTTT

TTTTCTTTT  
Depth:19 (ZEBRAFISH)  
Ei-value:0.000, Pi-value:0.000  
Er-value:0.000, Pr-value:0.000  
MATCHES To TargetScan▶ miR-186-5p:AAAGAAU

-5025--(26)--5052-

CAGGTTTTGCTTT

CAGGTTTTGCTTT  
Depth:19 (ZEBRAFISH)  
Ei-value:0.000, Pi-value:0.000  
Er-value:0.000, Pr-value:0.000  
MATCHES To TargetScan▶ miR-330-3p.2:AAAGCAC▶ miR-490-3p:AACCUGG

-5064--(21)--5086-

AAAAAGCAAAA

AAAAAGCAAAA  
Depth:19 (ZEBRAFISH)  
Ei-value:0.000, Pi-value:0.000  
Er-value:0.000, Pr-value:0.000  
No matches to TargetScan

-5096  
  
>ZEBRAFISH  
      1977-

TTTGGG

TTTGGG  
Depth:19 (ZEBRAFISH)  
Ei-value:0.000, Pi-value:0.000  
Er-value:0.000, Pr-value:0.000  
No matches to TargetScan

-1982--(1902)--3885-

TTTGGG

TTTGGG  
Depth:19 (ZEBRAFISH)  
Ei-value:0.000, Pi-value:0.000  
Er-value:0.000, Pr-value:0.000  
No matches to TargetScan

-3890--(1)--3892-

TTTGGG

TTTGGG  
Depth:19 (ZEBRAFISH)  
Ei-value:0.000, Pi-value:0.000  
Er-value:0.000, Pr-value:0.000  
No matches to TargetScan

-3897--(913)--4811-

TTTTTCAG

TTTTTCAG  
Depth:19 (ZEBRAFISH)  
Ei-value:0.000, Pi-value:0.000  
Er-value:0.000, Pr-value:0.000  
No matches to TargetScan

-4818--(342)--5161-

TTTTTCAG

TTTTTCAG  
Depth:19 (ZEBRAFISH)  
Ei-value:0.000, Pi-value:0.000  
Er-value:0.000, Pr-value:0.000  
No matches to TargetScan

-5168--(1707)--6876-

GATAAG

GATAAG  
Depth:19 (ZEBRAFISH)  
Ei-value:0.000, Pi-value:0.000  
Er-value:0.000, Pr-value:0.000  
No matches to TargetScan

-6881--(516)--7398-

TTTTCTTTT

TTTTCTTTT  
Depth:19 (ZEBRAFISH)  
Ei-value:0.000, Pi-value:0.000  
Er-value:0.000, Pr-value:0.000  
MATCHES To TargetScan▶ miR-186-5p:AAAGAAU

-7406--(32)--7439-

CAGGTTTTGCTTT

CAGGTTTTGCTTT  
Depth:19 (ZEBRAFISH)  
Ei-value:0.000, Pi-value:0.000  
Er-value:0.000, Pr-value:0.000  
MATCHES To TargetScan▶ miR-330-3p.2:AAAGCAC▶ miR-490-3p:AACCUGG

-7451--(15)--7467-

AAAAAGCAAAA

AAAAAGCAAAA  
Depth:19 (ZEBRAFISH)  
Ei-value:0.000, Pi-value:0.000  
Er-value:0.000, Pr-value:0.000  
No matches to TargetScan

-7477
```

---

# Modules conserved to MEDAKA (Depth: 18)

## Modules in Main Graph (All sequences considered):

```
>HUMAN  
      2505-

TTTGGG

TTTGGG  
Depth:19 (ZEBRAFISH)  
Ei-value:0.000, Pi-value:0.000  
Er-value:0.000, Pr-value:0.000  
eCLIP MATCHES▶ppil4 (bg=43.39%)▶PRPF8 (bg=6.2%)No matches to TargetScan

-2510--(1110)--3621-

TTTTTCAG

TTTTTCAG  
Depth:19 (ZEBRAFISH)  
Ei-value:0.000, Pi-value:0.000  
Er-value:0.000, Pr-value:0.000  
eCLIP MATCHES▶ddx42 (bg=10.33%)▶ppil4 (bg=43.39%)▶safb (bg=40.39%)▶u2af1 (bg=14.02%)▶u2af2 (bg=19.32%)No matches to TargetScan

-3628--(543)--4172-

GATAAG

GATAAG  
Depth:19 (ZEBRAFISH)  
Ei-value:0.000, Pi-value:0.000  
Er-value:0.000, Pr-value:0.000  
eCLIP MATCHES▶cpsf6 (bg=13.45%)▶khsrp (bg=27.4%)▶NIPBL (bg=8.2%)▶ppil4 (bg=43.39%)▶PRPF8 (bg=6.2%)▶rbm15 (bg=11.59%)▶safb (bg=40.39%)▶safb2 (bg=26.89%)▶srsf1 (bg=30.28%)▶srsf7 (bg=22.53%)▶znf622 (bg=18.79%)No matches to TargetScan

-4177--(2833)--7011-

TTTTCTTTT

TTTTCTTTT  
Depth:19 (ZEBRAFISH)  
Ei-value:0.000, Pi-value:0.000  
Er-value:0.000, Pr-value:0.000  
eCLIP MATCHES▶srsf7 (bg=22.53%)MATCHES To TargetScan▶ miR-186-5p:AAAGAAU

-7019--(28)--7048-

CAGGTTTTGCTTT

CAGGTTTTGCTTT  
Depth:19 (ZEBRAFISH)  
Ei-value:0.000, Pi-value:0.000  
Er-value:0.000, Pr-value:0.000  
eCLIP MATCHES▶srsf7 (bg=22.53%)MATCHES To TargetScan▶ miR-330-3p.2:AAAGCAC▶ miR-490-3p:AACCUGG

-7060--(26)--7087-

AAAAAGCAAAA

AAAAAGCAAAA  
Depth:19 (ZEBRAFISH)  
Ei-value:0.000, Pi-value:0.000  
Er-value:0.000, Pr-value:0.000  
No matches to eCLIP DataNo matches to TargetScan


G

AAAAAGCAAAAG  
Depth:18 (MEDAKA)  
Ei-value:0.000, Pi-value:0.000  
Er-value:0.000, Pr-value:0.000  
No matches to eCLIP DataNo matches to TargetScan

-7098--(15)--7114-

ACTCCTG

ACTCCTG  
Depth:18 (MEDAKA)  
Ei-value:0.000, Pi-value:0.000  
Er-value:0.000, Pr-value:0.000  
No matches to eCLIP DataNo matches to TargetScan

-7120  
  
>MARMOSET  
      2637-

TTTGGG

TTTGGG  
Depth:19 (ZEBRAFISH)  
Ei-value:0.000, Pi-value:0.000  
Er-value:0.000, Pr-value:0.000  
No matches to TargetScan

-2642--(1095)--3738-

TTTTTCAG

TTTTTCAG  
Depth:19 (ZEBRAFISH)  
Ei-value:0.000, Pi-value:0.000  
Er-value:0.000, Pr-value:0.000  
No matches to TargetScan

-3745--(548)--4294-

GATAAG

GATAAG  
Depth:19 (ZEBRAFISH)  
Ei-value:0.000, Pi-value:0.000  
Er-value:0.000, Pr-value:0.000  
No matches to TargetScan

-4299--(2840)--7140-

TTTTCTTTT

TTTTCTTTT  
Depth:19 (ZEBRAFISH)  
Ei-value:0.000, Pi-value:0.000  
Er-value:0.000, Pr-value:0.000  
MATCHES To TargetScan▶ miR-186-5p:AAAGAAU

-7148--(28)--7177-

CAGGTTTTGCTTT

CAGGTTTTGCTTT  
Depth:19 (ZEBRAFISH)  
Ei-value:0.000, Pi-value:0.000  
Er-value:0.000, Pr-value:0.000  
MATCHES To TargetScan▶ miR-330-3p.2:AAAGCAC▶ miR-490-3p:AACCUGG

-7189--(27)--7217-

AAAAAGCAAAA

AAAAAGCAAAA  
Depth:19 (ZEBRAFISH)  
Ei-value:0.000, Pi-value:0.000  
Er-value:0.000, Pr-value:0.000  
No matches to TargetScan


G

AAAAAGCAAAAG  
Depth:18 (MEDAKA)  
Ei-value:0.000, Pi-value:0.000  
Er-value:0.000, Pr-value:0.000  
No matches to TargetScan

-7228--(15)--7244-

ACTCCTG

ACTCCTG  
Depth:18 (MEDAKA)  
Ei-value:0.000, Pi-value:0.000  
Er-value:0.000, Pr-value:0.000  
No matches to TargetScan

-7250  
  
>DOG  
      2612-

TTTGGG

TTTGGG  
Depth:19 (ZEBRAFISH)  
Ei-value:0.000, Pi-value:0.000  
Er-value:0.000, Pr-value:0.000  
No matches to TargetScan

-2617--(1013)--3631-

TTTTTCAG

TTTTTCAG  
Depth:19 (ZEBRAFISH)  
Ei-value:0.000, Pi-value:0.000  
Er-value:0.000, Pr-value:0.000  
No matches to TargetScan

-3638--(559)--4198-

GATAAG

GATAAG  
Depth:19 (ZEBRAFISH)  
Ei-value:0.000, Pi-value:0.000  
Er-value:0.000, Pr-value:0.000  
No matches to TargetScan

-4203--(2856)--7060-

TTTTCTTTT

TTTTCTTTT  
Depth:19 (ZEBRAFISH)  
Ei-value:0.000, Pi-value:0.000  
Er-value:0.000, Pr-value:0.000  
MATCHES To TargetScan▶ miR-186-5p:AAAGAAU

-7068--(26)--7095-

CAGGTTTTGCTTT

CAGGTTTTGCTTT  
Depth:19 (ZEBRAFISH)  
Ei-value:0.000, Pi-value:0.000  
Er-value:0.000, Pr-value:0.000  
MATCHES To TargetScan▶ miR-330-3p.2:AAAGCAC▶ miR-490-3p:AACCUGG

-7107--(22)--7130-

AAAAAGCAAAA

AAAAAGCAAAA  
Depth:19 (ZEBRAFISH)  
Ei-value:0.000, Pi-value:0.000  
Er-value:0.000, Pr-value:0.000  
No matches to TargetScan


G

AAAAAGCAAAAG  
Depth:18 (MEDAKA)  
Ei-value:0.000, Pi-value:0.000  
Er-value:0.000, Pr-value:0.000  
No matches to TargetScan

-7141--(15)--7157-

ACTCCTG

ACTCCTG  
Depth:18 (MEDAKA)  
Ei-value:0.000, Pi-value:0.000  
Er-value:0.000, Pr-value:0.000  
No matches to TargetScan

-7163  
  
>PIG  
      2533-

TTTGGG

TTTGGG  
Depth:19 (ZEBRAFISH)  
Ei-value:0.000, Pi-value:0.000  
Er-value:0.000, Pr-value:0.000  
No matches to TargetScan

-2538--(1002)--3541-

TTTTTCAG

TTTTTCAG  
Depth:19 (ZEBRAFISH)  
Ei-value:0.000, Pi-value:0.000  
Er-value:0.000, Pr-value:0.000  
No matches to TargetScan

-3548--(569)--4118-

GATAAG

GATAAG  
Depth:19 (ZEBRAFISH)  
Ei-value:0.000, Pi-value:0.000  
Er-value:0.000, Pr-value:0.000  
No matches to TargetScan

-4123--(2848)--6972-

TTTTCTTTT

TTTTCTTTT  
Depth:19 (ZEBRAFISH)  
Ei-value:0.000, Pi-value:0.000  
Er-value:0.000, Pr-value:0.000  
MATCHES To TargetScan▶ miR-186-5p:AAAGAAU

-6980--(28)--7009-

CAGGTTTTGCTTT

CAGGTTTTGCTTT  
Depth:19 (ZEBRAFISH)  
Ei-value:0.000, Pi-value:0.000  
Er-value:0.000, Pr-value:0.000  
MATCHES To TargetScan▶ miR-330-3p.2:AAAGCAC▶ miR-490-3p:AACCUGG

-7021--(24)--7046-

AAAAAGCAAAA

AAAAAGCAAAA  
Depth:19 (ZEBRAFISH)  
Ei-value:0.000, Pi-value:0.000  
Er-value:0.000, Pr-value:0.000  
No matches to TargetScan


G

AAAAAGCAAAAG  
Depth:18 (MEDAKA)  
Ei-value:0.000, Pi-value:0.000  
Er-value:0.000, Pr-value:0.000  
No matches to TargetScan

-7057--(15)--7073-

ACTCCTG

ACTCCTG  
Depth:18 (MEDAKA)  
Ei-value:0.000, Pi-value:0.000  
Er-value:0.000, Pr-value:0.000  
No matches to TargetScan

-7079  
  
>COW  
      2417-

TTTGGG

TTTGGG  
Depth:19 (ZEBRAFISH)  
Ei-value:0.000, Pi-value:0.000  
Er-value:0.000, Pr-value:0.000  
No matches to TargetScan

-2422--(1012)--3435-

TTTTTCAG

TTTTTCAG  
Depth:19 (ZEBRAFISH)  
Ei-value:0.000, Pi-value:0.000  
Er-value:0.000, Pr-value:0.000  
No matches to TargetScan

-3442--(555)--3998-

GATAAG

GATAAG  
Depth:19 (ZEBRAFISH)  
Ei-value:0.000, Pi-value:0.000  
Er-value:0.000, Pr-value:0.000  
No matches to TargetScan

-4003--(2899)--6903-

TTTTCTTTT

TTTTCTTTT  
Depth:19 (ZEBRAFISH)  
Ei-value:0.000, Pi-value:0.000  
Er-value:0.000, Pr-value:0.000  
MATCHES To TargetScan▶ miR-186-5p:AAAGAAU

-6911--(30)--6942-

CAGGTTTTGCTTT

CAGGTTTTGCTTT  
Depth:19 (ZEBRAFISH)  
Ei-value:0.000, Pi-value:0.000  
Er-value:0.000, Pr-value:0.000  
MATCHES To TargetScan▶ miR-330-3p.2:AAAGCAC▶ miR-490-3p:AACCUGG

-6954--(23)--6978-

AAAAAGCAAAA

AAAAAGCAAAA  
Depth:19 (ZEBRAFISH)  
Ei-value:0.000, Pi-value:0.000  
Er-value:0.000, Pr-value:0.000  
No matches to TargetScan


G

AAAAAGCAAAAG  
Depth:18 (MEDAKA)  
Ei-value:0.000, Pi-value:0.000  
Er-value:0.000, Pr-value:0.000  
No matches to TargetScan

-6989--(15)--7005-

ACTCCTG

ACTCCTG  
Depth:18 (MEDAKA)  
Ei-value:0.000, Pi-value:0.000  
Er-value:0.000, Pr-value:0.000  
No matches to TargetScan

-7011  
  
>MOUSE  
      2324-

TTTGGG

TTTGGG  
Depth:19 (ZEBRAFISH)  
Ei-value:0.000, Pi-value:0.000  
Er-value:0.000, Pr-value:0.000  
No matches to TargetScan

-2329--(990)--3320-

TTTTTCAG

TTTTTCAG  
Depth:19 (ZEBRAFISH)  
Ei-value:0.000, Pi-value:0.000  
Er-value:0.000, Pr-value:0.000  
No matches to TargetScan

-3327--(518)--3846-

GATAAG

GATAAG  
Depth:19 (ZEBRAFISH)  
Ei-value:0.000, Pi-value:0.000  
Er-value:0.000, Pr-value:0.000  
No matches to TargetScan

-3851--(2754)--6606-

TTTTCTTTT

TTTTCTTTT  
Depth:19 (ZEBRAFISH)  
Ei-value:0.000, Pi-value:0.000  
Er-value:0.000, Pr-value:0.000  
MATCHES To TargetScan▶ miR-186-5p:AAAGAAU

-6614--(26)--6641-

CAGGTTTTGCTTT

CAGGTTTTGCTTT  
Depth:19 (ZEBRAFISH)  
Ei-value:0.000, Pi-value:0.000  
Er-value:0.000, Pr-value:0.000  
MATCHES To TargetScan▶ miR-330-3p.2:AAAGCAC▶ miR-490-3p:AACCUGG

-6653--(26)--6680-

AAAAAGCAAAA

AAAAAGCAAAA  
Depth:19 (ZEBRAFISH)  
Ei-value:0.000, Pi-value:0.000  
Er-value:0.000, Pr-value:0.000  
No matches to TargetScan


G

AAAAAGCAAAAG  
Depth:18 (MEDAKA)  
Ei-value:0.000, Pi-value:0.000  
Er-value:0.000, Pr-value:0.000  
No matches to TargetScan

-6691--(15)--6707-

ACTCCTG

ACTCCTG  
Depth:18 (MEDAKA)  
Ei-value:0.000, Pi-value:0.000  
Er-value:0.000, Pr-value:0.000  
No matches to TargetScan

-6713  
  
>TURTLE  
      1271-

TTTGGG

TTTGGG  
Depth:19 (ZEBRAFISH)  
Ei-value:0.000, Pi-value:0.000  
Er-value:0.000, Pr-value:0.000  
No matches to TargetScan

-1276--(1382)--2659-

TTTTTCAG

TTTTTCAG  
Depth:19 (ZEBRAFISH)  
Ei-value:0.000, Pi-value:0.000  
Er-value:0.000, Pr-value:0.000  
No matches to TargetScan

-2666--(648)--3315-

GATAAG

GATAAG  
Depth:19 (ZEBRAFISH)  
Ei-value:0.000, Pi-value:0.000  
Er-value:0.000, Pr-value:0.000  
No matches to TargetScan

-3320--(3412)--6733-

TTTTCTTTT

TTTTCTTTT  
Depth:19 (ZEBRAFISH)  
Ei-value:0.000, Pi-value:0.000  
Er-value:0.000, Pr-value:0.000  
MATCHES To TargetScan▶ miR-186-5p:AAAGAAU

-6741--(23)--6765-

CAGGTTTTGCTTT

CAGGTTTTGCTTT  
Depth:19 (ZEBRAFISH)  
Ei-value:0.000, Pi-value:0.000  
Er-value:0.000, Pr-value:0.000  
MATCHES To TargetScan▶ miR-330-3p.2:AAAGCAC▶ miR-490-3p:AACCUGG

-6777--(21)--6799-

AAAAAGCAAAA

AAAAAGCAAAA  
Depth:19 (ZEBRAFISH)  
Ei-value:0.000, Pi-value:0.000  
Er-value:0.000, Pr-value:0.000  
No matches to TargetScan


G

AAAAAGCAAAAG  
Depth:18 (MEDAKA)  
Ei-value:0.000, Pi-value:0.000  
Er-value:0.000, Pr-value:0.000  
No matches to TargetScan

-6810--(15)--6826-

ACTCCTG

ACTCCTG  
Depth:18 (MEDAKA)  
Ei-value:0.000, Pi-value:0.000  
Er-value:0.000, Pr-value:0.000  
No matches to TargetScan

-6832  
  
>ALLIGATOR  
      2537-

TTTGGG

TTTGGG  
Depth:19 (ZEBRAFISH)  
Ei-value:0.000, Pi-value:0.000  
Er-value:0.000, Pr-value:0.000  
No matches to TargetScan

-2542--(1322)--3865-

TTTTTCAG

TTTTTCAG  
Depth:19 (ZEBRAFISH)  
Ei-value:0.000, Pi-value:0.000  
Er-value:0.000, Pr-value:0.000  
No matches to TargetScan

-3872--(648)--4521-

GATAAG

GATAAG  
Depth:19 (ZEBRAFISH)  
Ei-value:0.000, Pi-value:0.000  
Er-value:0.000, Pr-value:0.000  
No matches to TargetScan

-4526--(3478)--8005-

TTTTCTTTT

TTTTCTTTT  
Depth:19 (ZEBRAFISH)  
Ei-value:0.000, Pi-value:0.000  
Er-value:0.000, Pr-value:0.000  
MATCHES To TargetScan▶ miR-186-5p:AAAGAAU

-8013--(23)--8037-

CAGGTTTTGCTTT

CAGGTTTTGCTTT  
Depth:19 (ZEBRAFISH)  
Ei-value:0.000, Pi-value:0.000  
Er-value:0.000, Pr-value:0.000  
MATCHES To TargetScan▶ miR-330-3p.2:AAAGCAC▶ miR-490-3p:AACCUGG

-8049--(26)--8076-

AAAAAGCAAAA

AAAAAGCAAAA  
Depth:19 (ZEBRAFISH)  
Ei-value:0.000, Pi-value:0.000  
Er-value:0.000, Pr-value:0.000  
No matches to TargetScan


G

AAAAAGCAAAAG  
Depth:18 (MEDAKA)  
Ei-value:0.000, Pi-value:0.000  
Er-value:0.000, Pr-value:0.000  
No matches to TargetScan

-8087--(15)--8103-

ACTCCTG

ACTCCTG  
Depth:18 (MEDAKA)  
Ei-value:0.000, Pi-value:0.000  
Er-value:0.000, Pr-value:0.000  
No matches to TargetScan

-8109  
  
>LIZARD  
      2353-

TTTGGG

TTTGGG  
Depth:19 (ZEBRAFISH)  
Ei-value:0.000, Pi-value:0.000  
Er-value:0.000, Pr-value:0.000  
No matches to TargetScan

-2358--(762)--3121-

TTTTTCAG

TTTTTCAG  
Depth:19 (ZEBRAFISH)  
Ei-value:0.000, Pi-value:0.000  
Er-value:0.000, Pr-value:0.000  
No matches to TargetScan

-3128--(588)--3717-

GATAAG

GATAAG  
Depth:19 (ZEBRAFISH)  
Ei-value:0.000, Pi-value:0.000  
Er-value:0.000, Pr-value:0.000  
No matches to TargetScan

-3722--(3176)--6899-

TTTTCTTTT

TTTTCTTTT  
Depth:19 (ZEBRAFISH)  
Ei-value:0.000, Pi-value:0.000  
Er-value:0.000, Pr-value:0.000  
MATCHES To TargetScan▶ miR-186-5p:AAAGAAU

-6907--(24)--6932-

CAGGTTTTGCTTT

CAGGTTTTGCTTT  
Depth:19 (ZEBRAFISH)  
Ei-value:0.000, Pi-value:0.000  
Er-value:0.000, Pr-value:0.000  
MATCHES To TargetScan▶ miR-330-3p.2:AAAGCAC▶ miR-490-3p:AACCUGG

-6944--(21)--6966-

AAAAAGCAAAA

AAAAAGCAAAA  
Depth:19 (ZEBRAFISH)  
Ei-value:0.000, Pi-value:0.000  
Er-value:0.000, Pr-value:0.000  
No matches to TargetScan


G

AAAAAGCAAAAG  
Depth:18 (MEDAKA)  
Ei-value:0.000, Pi-value:0.000  
Er-value:0.000, Pr-value:0.000  
No matches to TargetScan

-6977--(15)--6993-

ACTCCTG

ACTCCTG  
Depth:18 (MEDAKA)  
Ei-value:0.000, Pi-value:0.000  
Er-value:0.000, Pr-value:0.000  
No matches to TargetScan

-6999  
  
>SNAKE  
      1292-

TTTGGG

TTTGGG  
Depth:19 (ZEBRAFISH)  
Ei-value:0.000, Pi-value:0.000  
Er-value:0.000, Pr-value:0.000  
No matches to TargetScan

-1297--(729)--2027-

TTTGGG

TTTGGG  
Depth:19 (ZEBRAFISH)  
Ei-value:0.000, Pi-value:0.000  
Er-value:0.000, Pr-value:0.000  
No matches to TargetScan

-2032--(1081)--3114-

TTTTTCAG

TTTTTCAG  
Depth:19 (ZEBRAFISH)  
Ei-value:0.000, Pi-value:0.000  
Er-value:0.000, Pr-value:0.000  
No matches to TargetScan

-3121--(558)--3680-

GATAAG

GATAAG  
Depth:19 (ZEBRAFISH)  
Ei-value:0.000, Pi-value:0.000  
Er-value:0.000, Pr-value:0.000  
No matches to TargetScan

-3685--(3037)--6723-

TTTTCTTTT

TTTTCTTTT  
Depth:19 (ZEBRAFISH)  
Ei-value:0.000, Pi-value:0.000  
Er-value:0.000, Pr-value:0.000  
MATCHES To TargetScan▶ miR-186-5p:AAAGAAU

-6731--(25)--6757-

CAGGTTTTGCTTT

CAGGTTTTGCTTT  
Depth:19 (ZEBRAFISH)  
Ei-value:0.000, Pi-value:0.000  
Er-value:0.000, Pr-value:0.000  
MATCHES To TargetScan▶ miR-330-3p.2:AAAGCAC▶ miR-490-3p:AACCUGG

-6769--(29)--6799-

AAAAAGCAAAA

AAAAAGCAAAA  
Depth:19 (ZEBRAFISH)  
Ei-value:0.000, Pi-value:0.000  
Er-value:0.000, Pr-value:0.000  
No matches to TargetScan


G

AAAAAGCAAAAG  
Depth:18 (MEDAKA)  
Ei-value:0.000, Pi-value:0.000  
Er-value:0.000, Pr-value:0.000  
No matches to TargetScan

-6810--(15)--6826-

ACTCCTG

ACTCCTG  
Depth:18 (MEDAKA)  
Ei-value:0.000, Pi-value:0.000  
Er-value:0.000, Pr-value:0.000  
No matches to TargetScan

-6832  
  
>X.TROPICALIS  
      2299-

TTTGGG

TTTGGG  
Depth:19 (ZEBRAFISH)  
Ei-value:0.000, Pi-value:0.000  
Er-value:0.000, Pr-value:0.000  
No matches to TargetScan

-2304--(2695)--5000-

TTTGGG

TTTGGG  
Depth:19 (ZEBRAFISH)  
Ei-value:0.000, Pi-value:0.000  
Er-value:0.000, Pr-value:0.000  
No matches to TargetScan

-5005--(125)--5131-

TTTGGG

TTTGGG  
Depth:19 (ZEBRAFISH)  
Ei-value:0.000, Pi-value:0.000  
Er-value:0.000, Pr-value:0.000  
No matches to TargetScan

-5136--(3206)--8343-

TTTTTCAG

TTTTTCAG  
Depth:19 (ZEBRAFISH)  
Ei-value:0.000, Pi-value:0.000  
Er-value:0.000, Pr-value:0.000  
No matches to TargetScan

-8350--(1004)--9355-

GATAAG

GATAAG  
Depth:19 (ZEBRAFISH)  
Ei-value:0.000, Pi-value:0.000  
Er-value:0.000, Pr-value:0.000  
No matches to TargetScan

-9360--(2966)--12327-

TTTTCTTTT

TTTTCTTTT  
Depth:19 (ZEBRAFISH)  
Ei-value:0.000, Pi-value:0.000  
Er-value:0.000, Pr-value:0.000  
MATCHES To TargetScan▶ miR-186-5p:AAAGAAU

-12335--(26)--12362-

CAGGTTTTGCTTT

CAGGTTTTGCTTT  
Depth:19 (ZEBRAFISH)  
Ei-value:0.000, Pi-value:0.000  
Er-value:0.000, Pr-value:0.000  
MATCHES To TargetScan▶ miR-330-3p.2:AAAGCAC▶ miR-490-3p:AACCUGG

-12374--(31)--12406-

AAAAAGCAAAA

AAAAAGCAAAA  
Depth:19 (ZEBRAFISH)  
Ei-value:0.000, Pi-value:0.000  
Er-value:0.000, Pr-value:0.000  
No matches to TargetScan


G

AAAAAGCAAAAG  
Depth:18 (MEDAKA)  
Ei-value:0.000, Pi-value:0.000  
Er-value:0.000, Pr-value:0.000  
No matches to TargetScan

-12417--(15)--12433-

ACTCCTG

ACTCCTG  
Depth:18 (MEDAKA)  
Ei-value:0.000, Pi-value:0.000  
Er-value:0.000, Pr-value:0.000  
No matches to TargetScan

-12439  
  
>SHARK  
      1413-

TTTGGG

TTTGGG  
Depth:19 (ZEBRAFISH)  
Ei-value:0.000, Pi-value:0.000  
Er-value:0.000, Pr-value:0.000  
No matches to TargetScan

-1418--(1015)--2434-

TTTGGG

TTTGGG  
Depth:19 (ZEBRAFISH)  
Ei-value:0.000, Pi-value:0.000  
Er-value:0.000, Pr-value:0.000  
No matches to TargetScan

-2439--(74)--2514-

TTTGGG

TTTGGG  
Depth:19 (ZEBRAFISH)  
Ei-value:0.000, Pi-value:0.000  
Er-value:0.000, Pr-value:0.000  
No matches to TargetScan

-2519--(38)--2558-

TTTTTCAG

TTTTTCAG  
Depth:19 (ZEBRAFISH)  
Ei-value:0.000, Pi-value:0.000  
Er-value:0.000, Pr-value:0.000  
No matches to TargetScan

-2565--(4402)--6968-

GATAAG

GATAAG  
Depth:19 (ZEBRAFISH)  
Ei-value:0.000, Pi-value:0.000  
Er-value:0.000, Pr-value:0.000  
No matches to TargetScan

-6973--(520)--7494-

TTTTCTTTT

TTTTCTTTT  
Depth:19 (ZEBRAFISH)  
Ei-value:0.000, Pi-value:0.000  
Er-value:0.000, Pr-value:0.000  
MATCHES To TargetScan▶ miR-186-5p:AAAGAAU

-7502--(25)--7528-

CAGGTTTTGCTTT

CAGGTTTTGCTTT  
Depth:19 (ZEBRAFISH)  
Ei-value:0.000, Pi-value:0.000  
Er-value:0.000, Pr-value:0.000  
MATCHES To TargetScan▶ miR-330-3p.2:AAAGCAC▶ miR-490-3p:AACCUGG

-7540--(27)--7568-

AAAAAGCAAAA

AAAAAGCAAAA  
Depth:19 (ZEBRAFISH)  
Ei-value:0.000, Pi-value:0.000  
Er-value:0.000, Pr-value:0.000  
No matches to TargetScan


G

AAAAAGCAAAAG  
Depth:18 (MEDAKA)  
Ei-value:0.000, Pi-value:0.000  
Er-value:0.000, Pr-value:0.000  
No matches to TargetScan

-7579--(15)--7595-

ACTCCTG

ACTCCTG  
Depth:18 (MEDAKA)  
Ei-value:0.000, Pi-value:0.000  
Er-value:0.000, Pr-value:0.000  
No matches to TargetScan

-7601  
  
>OPOSSUM  
      1043-

TTTGGG

TTTGGG  
Depth:19 (ZEBRAFISH)  
Ei-value:0.000, Pi-value:0.000  
Er-value:0.000, Pr-value:0.000  
No matches to TargetScan

-1048--(273)--1322-

TTTGGG

TTTGGG  
Depth:19 (ZEBRAFISH)  
Ei-value:0.000, Pi-value:0.000  
Er-value:0.000, Pr-value:0.000  
No matches to TargetScan

-1327--(111)--1439-

TTTGGG

TTTGGG  
Depth:19 (ZEBRAFISH)  
Ei-value:0.000, Pi-value:0.000  
Er-value:0.000, Pr-value:0.000  
No matches to TargetScan

-1444--(1059)--2504-

TTTTTCAG

TTTTTCAG  
Depth:19 (ZEBRAFISH)  
Ei-value:0.000, Pi-value:0.000  
Er-value:0.000, Pr-value:0.000  
No matches to TargetScan

-2511--(973)--3485-

GATAAG

GATAAG  
Depth:19 (ZEBRAFISH)  
Ei-value:0.000, Pi-value:0.000  
Er-value:0.000, Pr-value:0.000  
No matches to TargetScan

-3490--(2190)--5681-

TTTTCTTTT

TTTTCTTTT  
Depth:19 (ZEBRAFISH)  
Ei-value:0.000, Pi-value:0.000  
Er-value:0.000, Pr-value:0.000  
MATCHES To TargetScan▶ miR-186-5p:AAAGAAU

-5689--(26)--5716-

CAGGTTTTGCTTT

CAGGTTTTGCTTT  
Depth:19 (ZEBRAFISH)  
Ei-value:0.000, Pi-value:0.000  
Er-value:0.000, Pr-value:0.000  
MATCHES To TargetScan▶ miR-330-3p.2:AAAGCAC▶ miR-490-3p:AACCUGG

-5728--(18)--5747-

AAAAAGCAAAA

AAAAAGCAAAA  
Depth:19 (ZEBRAFISH)  
Ei-value:0.000, Pi-value:0.000  
Er-value:0.000, Pr-value:0.000  
No matches to TargetScan


G

AAAAAGCAAAAG  
Depth:18 (MEDAKA)  
Ei-value:0.000, Pi-value:0.000  
Er-value:0.000, Pr-value:0.000  
No matches to TargetScan

-5758--(14)--5773-

ACTCCTG

ACTCCTG  
Depth:18 (MEDAKA)  
Ei-value:0.000, Pi-value:0.000  
Er-value:0.000, Pr-value:0.000  
No matches to TargetScan

-5779  
  
>SPOTTEDGAR  
      3644-

TTTGGG

TTTGGG  
Depth:19 (ZEBRAFISH)  
Ei-value:0.000, Pi-value:0.000  
Er-value:0.000, Pr-value:0.000  
No matches to TargetScan

-3649--(31)--3681-

TTTTTCAG

TTTTTCAG  
Depth:19 (ZEBRAFISH)  
Ei-value:0.000, Pi-value:0.000  
Er-value:0.000, Pr-value:0.000  
No matches to TargetScan

-3688--(72)--3761-

GATAAG

GATAAG  
Depth:19 (ZEBRAFISH)  
Ei-value:0.000, Pi-value:0.000  
Er-value:0.000, Pr-value:0.000  
No matches to TargetScan

-3766--(274)--4041-

GATAAG

GATAAG  
Depth:19 (ZEBRAFISH)  
Ei-value:0.000, Pi-value:0.000  
Er-value:0.000, Pr-value:0.000  
No matches to TargetScan

-4046--(2564)--6611-

TTTTCTTTT

TTTTCTTTT  
Depth:19 (ZEBRAFISH)  
Ei-value:0.000, Pi-value:0.000  
Er-value:0.000, Pr-value:0.000  
MATCHES To TargetScan▶ miR-186-5p:AAAGAAU

-6619--(295)--6915-

CAGGTTTTGCTTT

CAGGTTTTGCTTT  
Depth:19 (ZEBRAFISH)  
Ei-value:0.000, Pi-value:0.000  
Er-value:0.000, Pr-value:0.000  
MATCHES To TargetScan▶ miR-330-3p.2:AAAGCAC▶ miR-490-3p:AACCUGG

-6927--(28)--6956-

AAAAAGCAAAA

AAAAAGCAAAA  
Depth:19 (ZEBRAFISH)  
Ei-value:0.000, Pi-value:0.000  
Er-value:0.000, Pr-value:0.000  
No matches to TargetScan


G

AAAAAGCAAAAG  
Depth:18 (MEDAKA)  
Ei-value:0.000, Pi-value:0.000  
Er-value:0.000, Pr-value:0.000  
No matches to TargetScan

-6967--(14)--6982-

ACTCCTG

ACTCCTG  
Depth:18 (MEDAKA)  
Ei-value:0.000, Pi-value:0.000  
Er-value:0.000, Pr-value:0.000  
No matches to TargetScan

-6988  
  
>FUGU  
      1704-

TTTGGG

TTTGGG  
Depth:19 (ZEBRAFISH)  
Ei-value:0.000, Pi-value:0.000  
Er-value:0.000, Pr-value:0.000  
No matches to TargetScan

-1709--(774)--2484-

TTTTTCAG

TTTTTCAG  
Depth:19 (ZEBRAFISH)  
Ei-value:0.000, Pi-value:0.000  
Er-value:0.000, Pr-value:0.000  
No matches to TargetScan

-2491--(316)--2808-

TTTTTCAG

TTTTTCAG  
Depth:19 (ZEBRAFISH)  
Ei-value:0.000, Pi-value:0.000  
Er-value:0.000, Pr-value:0.000  
No matches to TargetScan

-2815--(72)--2888-

GATAAG

GATAAG  
Depth:19 (ZEBRAFISH)  
Ei-value:0.000, Pi-value:0.000  
Er-value:0.000, Pr-value:0.000  
No matches to TargetScan

-2893--(146)--3040-

GATAAG

GATAAG  
Depth:19 (ZEBRAFISH)  
Ei-value:0.000, Pi-value:0.000  
Er-value:0.000, Pr-value:0.000  
No matches to TargetScan

-3045--(1722)--4768-

TTTTCTTTT

TTTTCTTTT  
Depth:19 (ZEBRAFISH)  
Ei-value:0.000, Pi-value:0.000  
Er-value:0.000, Pr-value:0.000  
MATCHES To TargetScan▶ miR-186-5p:AAAGAAU

-4776--(31)--4808-

CAGGTTTTGCTTT

CAGGTTTTGCTTT  
Depth:19 (ZEBRAFISH)  
Ei-value:0.000, Pi-value:0.000  
Er-value:0.000, Pr-value:0.000  
MATCHES To TargetScan▶ miR-330-3p.2:AAAGCAC▶ miR-490-3p:AACCUGG

-4820--(18)--4839-

AAAAAGCAAAA

AAAAAGCAAAA  
Depth:19 (ZEBRAFISH)  
Ei-value:0.000, Pi-value:0.000  
Er-value:0.000, Pr-value:0.000  
No matches to TargetScan


G

AAAAAGCAAAAG  
Depth:18 (MEDAKA)  
Ei-value:0.000, Pi-value:0.000  
Er-value:0.000, Pr-value:0.000  
No matches to TargetScan

-4850--(14)--4865-

ACTCCTG

ACTCCTG  
Depth:18 (MEDAKA)  
Ei-value:0.000, Pi-value:0.000  
Er-value:0.000, Pr-value:0.000  
No matches to TargetScan

-4871  
  
>NILETILAPIA  
      2795-

TTTGGG

TTTGGG  
Depth:19 (ZEBRAFISH)  
Ei-value:0.000, Pi-value:0.000  
Er-value:0.000, Pr-value:0.000  
No matches to TargetScan

-2800--(581)--3382-

TTTTTCAG

TTTTTCAG  
Depth:19 (ZEBRAFISH)  
Ei-value:0.000, Pi-value:0.000  
Er-value:0.000, Pr-value:0.000  
No matches to TargetScan

-3389--(324)--3714-

TTTTTCAG

TTTTTCAG  
Depth:19 (ZEBRAFISH)  
Ei-value:0.000, Pi-value:0.000  
Er-value:0.000, Pr-value:0.000  
No matches to TargetScan

-3721--(84)--3806-

GATAAG

GATAAG  
Depth:19 (ZEBRAFISH)  
Ei-value:0.000, Pi-value:0.000  
Er-value:0.000, Pr-value:0.000  
No matches to TargetScan

-3811--(2023)--5835-

TTTTCTTTT

TTTTCTTTT  
Depth:19 (ZEBRAFISH)  
Ei-value:0.000, Pi-value:0.000  
Er-value:0.000, Pr-value:0.000  
MATCHES To TargetScan▶ miR-186-5p:AAAGAAU

-5843--(28)--5872-

CAGGTTTTGCTTT

CAGGTTTTGCTTT  
Depth:19 (ZEBRAFISH)  
Ei-value:0.000, Pi-value:0.000  
Er-value:0.000, Pr-value:0.000  
MATCHES To TargetScan▶ miR-330-3p.2:AAAGCAC▶ miR-490-3p:AACCUGG

-5884--(19)--5904-

AAAAAGCAAAA

AAAAAGCAAAA  
Depth:19 (ZEBRAFISH)  
Ei-value:0.000, Pi-value:0.000  
Er-value:0.000, Pr-value:0.000  
No matches to TargetScan


G

AAAAAGCAAAAG  
Depth:18 (MEDAKA)  
Ei-value:0.000, Pi-value:0.000  
Er-value:0.000, Pr-value:0.000  
No matches to TargetScan

-5915--(14)--5930-

ACTCCTG

ACTCCTG  
Depth:18 (MEDAKA)  
Ei-value:0.000, Pi-value:0.000  
Er-value:0.000, Pr-value:0.000  
No matches to TargetScan

-5936  
  
>STICKLEBACK  
        70-

TTTGGG

TTTGGG  
Depth:19 (ZEBRAFISH)  
Ei-value:0.000, Pi-value:0.000  
Er-value:0.000, Pr-value:0.000  
No matches to TargetScan

-75--(3779)--3855-

TTTGGG

TTTGGG  
Depth:19 (ZEBRAFISH)  
Ei-value:0.000, Pi-value:0.000  
Er-value:0.000, Pr-value:0.000  
No matches to TargetScan

-3860--(31)--3892-

TTTTTCAG

TTTTTCAG  
Depth:19 (ZEBRAFISH)  
Ei-value:0.000, Pi-value:0.000  
Er-value:0.000, Pr-value:0.000  
No matches to TargetScan

-3899--(84)--3984-

GATAAG

GATAAG  
Depth:19 (ZEBRAFISH)  
Ei-value:0.000, Pi-value:0.000  
Er-value:0.000, Pr-value:0.000  
No matches to TargetScan

-3989--(2020)--6010-

TTTTCTTTT

TTTTCTTTT  
Depth:19 (ZEBRAFISH)  
Ei-value:0.000, Pi-value:0.000  
Er-value:0.000, Pr-value:0.000  
MATCHES To TargetScan▶ miR-186-5p:AAAGAAU

-6018--(36)--6055-

CAGGTTTTGCTTT

CAGGTTTTGCTTT  
Depth:19 (ZEBRAFISH)  
Ei-value:0.000, Pi-value:0.000  
Er-value:0.000, Pr-value:0.000  
MATCHES To TargetScan▶ miR-330-3p.2:AAAGCAC▶ miR-490-3p:AACCUGG

-6067--(24)--6092-

AAAAAGCAAAA

AAAAAGCAAAA  
Depth:19 (ZEBRAFISH)  
Ei-value:0.000, Pi-value:0.000  
Er-value:0.000, Pr-value:0.000  
No matches to TargetScan


G

AAAAAGCAAAAG  
Depth:18 (MEDAKA)  
Ei-value:0.000, Pi-value:0.000  
Er-value:0.000, Pr-value:0.000  
No matches to TargetScan

-6103--(14)--6118-

ACTCCTG

ACTCCTG  
Depth:18 (MEDAKA)  
Ei-value:0.000, Pi-value:0.000  
Er-value:0.000, Pr-value:0.000  
No matches to TargetScan

-6124  
  
>MEDAKA  
      1667-

TTTGGG

TTTGGG  
Depth:19 (ZEBRAFISH)  
Ei-value:0.000, Pi-value:0.000  
Er-value:0.000, Pr-value:0.000  
No matches to TargetScan

-1672--(1439)--3112-

TTTGGG

TTTGGG  
Depth:19 (ZEBRAFISH)  
Ei-value:0.000, Pi-value:0.000  
Er-value:0.000, Pr-value:0.000  
No matches to TargetScan

-3117--(27)--3145-

TTTTTCAG

TTTTTCAG  
Depth:19 (ZEBRAFISH)  
Ei-value:0.000, Pi-value:0.000  
Er-value:0.000, Pr-value:0.000  
No matches to TargetScan

-3152--(80)--3233-

GATAAG

GATAAG  
Depth:19 (ZEBRAFISH)  
Ei-value:0.000, Pi-value:0.000  
Er-value:0.000, Pr-value:0.000  
No matches to TargetScan

-3238--(1778)--5017-

TTTTCTTTT

TTTTCTTTT  
Depth:19 (ZEBRAFISH)  
Ei-value:0.000, Pi-value:0.000  
Er-value:0.000, Pr-value:0.000  
MATCHES To TargetScan▶ miR-186-5p:AAAGAAU

-5025--(26)--5052-

CAGGTTTTGCTTT

CAGGTTTTGCTTT  
Depth:19 (ZEBRAFISH)  
Ei-value:0.000, Pi-value:0.000  
Er-value:0.000, Pr-value:0.000  
MATCHES To TargetScan▶ miR-330-3p.2:AAAGCAC▶ miR-490-3p:AACCUGG

-5064--(21)--5086-

AAAAAGCAAAA

AAAAAGCAAAA  
Depth:19 (ZEBRAFISH)  
Ei-value:0.000, Pi-value:0.000  
Er-value:0.000, Pr-value:0.000  
No matches to TargetScan


G

AAAAAGCAAAAG  
Depth:18 (MEDAKA)  
Ei-value:0.000, Pi-value:0.000  
Er-value:0.000, Pr-value:0.000  
No matches to TargetScan

-5097--(14)--5112-

ACTCCTG

ACTCCTG  
Depth:18 (MEDAKA)  
Ei-value:0.000, Pi-value:0.000  
Er-value:0.000, Pr-value:0.000  
No matches to TargetScan

-5118
```

---

# Modules conserved to NILETILAPIA (Depth: 16)

## Modules in Main Graph (All sequences considered):

```
>HUMAN  
      2505-

TTTGGG

TTTGGG  
Depth:19 (ZEBRAFISH)  
Ei-value:0.000, Pi-value:0.000  
Er-value:0.000, Pr-value:0.000  
eCLIP MATCHES▶ppil4 (bg=43.39%)▶PRPF8 (bg=6.2%)No matches to TargetScan

-2510--(1110)--3621-

TTTTTCAG

TTTTTCAG  
Depth:19 (ZEBRAFISH)  
Ei-value:0.000, Pi-value:0.000  
Er-value:0.000, Pr-value:0.000  
eCLIP MATCHES▶ddx42 (bg=10.33%)▶ppil4 (bg=43.39%)▶safb (bg=40.39%)▶u2af1 (bg=14.02%)▶u2af2 (bg=19.32%)No matches to TargetScan

-3628--(543)--4172-

GATAAG

GATAAG  
Depth:19 (ZEBRAFISH)  
Ei-value:0.000, Pi-value:0.000  
Er-value:0.000, Pr-value:0.000  
eCLIP MATCHES▶cpsf6 (bg=13.45%)▶khsrp (bg=27.4%)▶NIPBL (bg=8.2%)▶ppil4 (bg=43.39%)▶PRPF8 (bg=6.2%)▶rbm15 (bg=11.59%)▶safb (bg=40.39%)▶safb2 (bg=26.89%)▶srsf1 (bg=30.28%)▶srsf7 (bg=22.53%)▶znf622 (bg=18.79%)No matches to TargetScan

-4177--(2833)--7011-

TTTTCTTTT

TTTTCTTTT  
Depth:19 (ZEBRAFISH)  
Ei-value:0.000, Pi-value:0.000  
Er-value:0.000, Pr-value:0.000  
eCLIP MATCHES▶srsf7 (bg=22.53%)MATCHES To TargetScan▶ miR-186-5p:AAAGAAU

-7019--(28)--7048-

CAGGTTTTGCTTT

CAGGTTTTGCTTT  
Depth:19 (ZEBRAFISH)  
Ei-value:0.000, Pi-value:0.000  
Er-value:0.000, Pr-value:0.000  
eCLIP MATCHES▶srsf7 (bg=22.53%)MATCHES To TargetScan▶ miR-330-3p.2:AAAGCAC▶ miR-490-3p:AACCUGG


T

CAGGTTTTGCTTTT  
Depth:16 (NILETILAPIA)  
Ei-value:0.000, Pi-value:0.000  
Er-value:0.000, Pr-value:0.000  
eCLIP MATCHES▶srsf7 (bg=22.53%)MATCHES To TargetScan▶ miR-330-3p.2:AAAGCAC▶ miR-490-3p:AACCUGG

-7061--(25)--7087-

AAAAAGCAAAA

AAAAAGCAAAA  
Depth:19 (ZEBRAFISH)  
Ei-value:0.000, Pi-value:0.000  
Er-value:0.000, Pr-value:0.000  
No matches to eCLIP DataNo matches to TargetScan


G

AAAAAGCAAAAG  
Depth:18 (MEDAKA)  
Ei-value:0.000, Pi-value:0.000  
Er-value:0.000, Pr-value:0.000  
No matches to eCLIP DataNo matches to TargetScan

-7098--(15)--7114-

ACTCCTG

ACTCCTG  
Depth:18 (MEDAKA)  
Ei-value:0.000, Pi-value:0.000  
Er-value:0.000, Pr-value:0.000  
No matches to eCLIP DataNo matches to TargetScan

-7120  
  
>MARMOSET  
      2637-

TTTGGG

TTTGGG  
Depth:19 (ZEBRAFISH)  
Ei-value:0.000, Pi-value:0.000  
Er-value:0.000, Pr-value:0.000  
No matches to TargetScan

-2642--(1095)--3738-

TTTTTCAG

TTTTTCAG  
Depth:19 (ZEBRAFISH)  
Ei-value:0.000, Pi-value:0.000  
Er-value:0.000, Pr-value:0.000  
No matches to TargetScan

-3745--(548)--4294-

GATAAG

GATAAG  
Depth:19 (ZEBRAFISH)  
Ei-value:0.000, Pi-value:0.000  
Er-value:0.000, Pr-value:0.000  
No matches to TargetScan

-4299--(2840)--7140-

TTTTCTTTT

TTTTCTTTT  
Depth:19 (ZEBRAFISH)  
Ei-value:0.000, Pi-value:0.000  
Er-value:0.000, Pr-value:0.000  
MATCHES To TargetScan▶ miR-186-5p:AAAGAAU

-7148--(28)--7177-

CAGGTTTTGCTTT

CAGGTTTTGCTTT  
Depth:19 (ZEBRAFISH)  
Ei-value:0.000, Pi-value:0.000  
Er-value:0.000, Pr-value:0.000  
MATCHES To TargetScan▶ miR-330-3p.2:AAAGCAC▶ miR-490-3p:AACCUGG


T

CAGGTTTTGCTTTT  
Depth:16 (NILETILAPIA)  
Ei-value:0.000, Pi-value:0.000  
Er-value:0.000, Pr-value:0.000  
MATCHES To TargetScan▶ miR-330-3p.2:AAAGCAC▶ miR-490-3p:AACCUGG

-7190--(26)--7217-

AAAAAGCAAAA

AAAAAGCAAAA  
Depth:19 (ZEBRAFISH)  
Ei-value:0.000, Pi-value:0.000  
Er-value:0.000, Pr-value:0.000  
No matches to TargetScan


G

AAAAAGCAAAAG  
Depth:18 (MEDAKA)  
Ei-value:0.000, Pi-value:0.000  
Er-value:0.000, Pr-value:0.000  
No matches to TargetScan

-7228--(15)--7244-

ACTCCTG

ACTCCTG  
Depth:18 (MEDAKA)  
Ei-value:0.000, Pi-value:0.000  
Er-value:0.000, Pr-value:0.000  
No matches to TargetScan

-7250  
  
>DOG  
      2612-

TTTGGG

TTTGGG  
Depth:19 (ZEBRAFISH)  
Ei-value:0.000, Pi-value:0.000  
Er-value:0.000, Pr-value:0.000  
No matches to TargetScan

-2617--(1013)--3631-

TTTTTCAG

TTTTTCAG  
Depth:19 (ZEBRAFISH)  
Ei-value:0.000, Pi-value:0.000  
Er-value:0.000, Pr-value:0.000  
No matches to TargetScan

-3638--(559)--4198-

GATAAG

GATAAG  
Depth:19 (ZEBRAFISH)  
Ei-value:0.000, Pi-value:0.000  
Er-value:0.000, Pr-value:0.000  
No matches to TargetScan

-4203--(2856)--7060-

TTTTCTTTT

TTTTCTTTT  
Depth:19 (ZEBRAFISH)  
Ei-value:0.000, Pi-value:0.000  
Er-value:0.000, Pr-value:0.000  
MATCHES To TargetScan▶ miR-186-5p:AAAGAAU

-7068--(26)--7095-

CAGGTTTTGCTTT

CAGGTTTTGCTTT  
Depth:19 (ZEBRAFISH)  
Ei-value:0.000, Pi-value:0.000  
Er-value:0.000, Pr-value:0.000  
MATCHES To TargetScan▶ miR-330-3p.2:AAAGCAC▶ miR-490-3p:AACCUGG


T

CAGGTTTTGCTTTT  
Depth:16 (NILETILAPIA)  
Ei-value:0.000, Pi-value:0.000  
Er-value:0.000, Pr-value:0.000  
MATCHES To TargetScan▶ miR-330-3p.2:AAAGCAC▶ miR-490-3p:AACCUGG

-7108--(21)--7130-

AAAAAGCAAAA

AAAAAGCAAAA  
Depth:19 (ZEBRAFISH)  
Ei-value:0.000, Pi-value:0.000  
Er-value:0.000, Pr-value:0.000  
No matches to TargetScan


G

AAAAAGCAAAAG  
Depth:18 (MEDAKA)  
Ei-value:0.000, Pi-value:0.000  
Er-value:0.000, Pr-value:0.000  
No matches to TargetScan

-7141--(15)--7157-

ACTCCTG

ACTCCTG  
Depth:18 (MEDAKA)  
Ei-value:0.000, Pi-value:0.000  
Er-value:0.000, Pr-value:0.000  
No matches to TargetScan

-7163  
  
>PIG  
      2533-

TTTGGG

TTTGGG  
Depth:19 (ZEBRAFISH)  
Ei-value:0.000, Pi-value:0.000  
Er-value:0.000, Pr-value:0.000  
No matches to TargetScan

-2538--(1002)--3541-

TTTTTCAG

TTTTTCAG  
Depth:19 (ZEBRAFISH)  
Ei-value:0.000, Pi-value:0.000  
Er-value:0.000, Pr-value:0.000  
No matches to TargetScan

-3548--(569)--4118-

GATAAG

GATAAG  
Depth:19 (ZEBRAFISH)  
Ei-value:0.000, Pi-value:0.000  
Er-value:0.000, Pr-value:0.000  
No matches to TargetScan

-4123--(2848)--6972-

TTTTCTTTT

TTTTCTTTT  
Depth:19 (ZEBRAFISH)  
Ei-value:0.000, Pi-value:0.000  
Er-value:0.000, Pr-value:0.000  
MATCHES To TargetScan▶ miR-186-5p:AAAGAAU

-6980--(28)--7009-

CAGGTTTTGCTTT

CAGGTTTTGCTTT  
Depth:19 (ZEBRAFISH)  
Ei-value:0.000, Pi-value:0.000  
Er-value:0.000, Pr-value:0.000  
MATCHES To TargetScan▶ miR-330-3p.2:AAAGCAC▶ miR-490-3p:AACCUGG


T

CAGGTTTTGCTTTT  
Depth:16 (NILETILAPIA)  
Ei-value:0.000, Pi-value:0.000  
Er-value:0.000, Pr-value:0.000  
MATCHES To TargetScan▶ miR-330-3p.2:AAAGCAC▶ miR-490-3p:AACCUGG

-7022--(23)--7046-

AAAAAGCAAAA

AAAAAGCAAAA  
Depth:19 (ZEBRAFISH)  
Ei-value:0.000, Pi-value:0.000  
Er-value:0.000, Pr-value:0.000  
No matches to TargetScan


G

AAAAAGCAAAAG  
Depth:18 (MEDAKA)  
Ei-value:0.000, Pi-value:0.000  
Er-value:0.000, Pr-value:0.000  
No matches to TargetScan

-7057--(15)--7073-

ACTCCTG

ACTCCTG  
Depth:18 (MEDAKA)  
Ei-value:0.000, Pi-value:0.000  
Er-value:0.000, Pr-value:0.000  
No matches to TargetScan

-7079  
  
>COW  
      2417-

TTTGGG

TTTGGG  
Depth:19 (ZEBRAFISH)  
Ei-value:0.000, Pi-value:0.000  
Er-value:0.000, Pr-value:0.000  
No matches to TargetScan

-2422--(1012)--3435-

TTTTTCAG

TTTTTCAG  
Depth:19 (ZEBRAFISH)  
Ei-value:0.000, Pi-value:0.000  
Er-value:0.000, Pr-value:0.000  
No matches to TargetScan

-3442--(555)--3998-

GATAAG

GATAAG  
Depth:19 (ZEBRAFISH)  
Ei-value:0.000, Pi-value:0.000  
Er-value:0.000, Pr-value:0.000  
No matches to TargetScan

-4003--(2899)--6903-

TTTTCTTTT

TTTTCTTTT  
Depth:19 (ZEBRAFISH)  
Ei-value:0.000, Pi-value:0.000  
Er-value:0.000, Pr-value:0.000  
MATCHES To TargetScan▶ miR-186-5p:AAAGAAU

-6911--(30)--6942-

CAGGTTTTGCTTT

CAGGTTTTGCTTT  
Depth:19 (ZEBRAFISH)  
Ei-value:0.000, Pi-value:0.000  
Er-value:0.000, Pr-value:0.000  
MATCHES To TargetScan▶ miR-330-3p.2:AAAGCAC▶ miR-490-3p:AACCUGG


T

CAGGTTTTGCTTTT  
Depth:16 (NILETILAPIA)  
Ei-value:0.000, Pi-value:0.000  
Er-value:0.000, Pr-value:0.000  
MATCHES To TargetScan▶ miR-330-3p.2:AAAGCAC▶ miR-490-3p:AACCUGG

-6955--(22)--6978-

AAAAAGCAAAA

AAAAAGCAAAA  
Depth:19 (ZEBRAFISH)  
Ei-value:0.000, Pi-value:0.000  
Er-value:0.000, Pr-value:0.000  
No matches to TargetScan


G

AAAAAGCAAAAG  
Depth:18 (MEDAKA)  
Ei-value:0.000, Pi-value:0.000  
Er-value:0.000, Pr-value:0.000  
No matches to TargetScan

-6989--(15)--7005-

ACTCCTG

ACTCCTG  
Depth:18 (MEDAKA)  
Ei-value:0.000, Pi-value:0.000  
Er-value:0.000, Pr-value:0.000  
No matches to TargetScan

-7011  
  
>MOUSE  
      2324-

TTTGGG

TTTGGG  
Depth:19 (ZEBRAFISH)  
Ei-value:0.000, Pi-value:0.000  
Er-value:0.000, Pr-value:0.000  
No matches to TargetScan

-2329--(990)--3320-

TTTTTCAG

TTTTTCAG  
Depth:19 (ZEBRAFISH)  
Ei-value:0.000, Pi-value:0.000  
Er-value:0.000, Pr-value:0.000  
No matches to TargetScan

-3327--(518)--3846-

GATAAG

GATAAG  
Depth:19 (ZEBRAFISH)  
Ei-value:0.000, Pi-value:0.000  
Er-value:0.000, Pr-value:0.000  
No matches to TargetScan

-3851--(2754)--6606-

TTTTCTTTT

TTTTCTTTT  
Depth:19 (ZEBRAFISH)  
Ei-value:0.000, Pi-value:0.000  
Er-value:0.000, Pr-value:0.000  
MATCHES To TargetScan▶ miR-186-5p:AAAGAAU

-6614--(26)--6641-

CAGGTTTTGCTTT

CAGGTTTTGCTTT  
Depth:19 (ZEBRAFISH)  
Ei-value:0.000, Pi-value:0.000  
Er-value:0.000, Pr-value:0.000  
MATCHES To TargetScan▶ miR-330-3p.2:AAAGCAC▶ miR-490-3p:AACCUGG


T

CAGGTTTTGCTTTT  
Depth:16 (NILETILAPIA)  
Ei-value:0.000, Pi-value:0.000  
Er-value:0.000, Pr-value:0.000  
MATCHES To TargetScan▶ miR-330-3p.2:AAAGCAC▶ miR-490-3p:AACCUGG

-6654--(25)--6680-

AAAAAGCAAAA

AAAAAGCAAAA  
Depth:19 (ZEBRAFISH)  
Ei-value:0.000, Pi-value:0.000  
Er-value:0.000, Pr-value:0.000  
No matches to TargetScan


G

AAAAAGCAAAAG  
Depth:18 (MEDAKA)  
Ei-value:0.000, Pi-value:0.000  
Er-value:0.000, Pr-value:0.000  
No matches to TargetScan

-6691--(15)--6707-

ACTCCTG

ACTCCTG  
Depth:18 (MEDAKA)  
Ei-value:0.000, Pi-value:0.000  
Er-value:0.000, Pr-value:0.000  
No matches to TargetScan

-6713  
  
>TURTLE  
      1271-

TTTGGG

TTTGGG  
Depth:19 (ZEBRAFISH)  
Ei-value:0.000, Pi-value:0.000  
Er-value:0.000, Pr-value:0.000  
No matches to TargetScan

-1276--(1382)--2659-

TTTTTCAG

TTTTTCAG  
Depth:19 (ZEBRAFISH)  
Ei-value:0.000, Pi-value:0.000  
Er-value:0.000, Pr-value:0.000  
No matches to TargetScan

-2666--(648)--3315-

GATAAG

GATAAG  
Depth:19 (ZEBRAFISH)  
Ei-value:0.000, Pi-value:0.000  
Er-value:0.000, Pr-value:0.000  
No matches to TargetScan

-3320--(3412)--6733-

TTTTCTTTT

TTTTCTTTT  
Depth:19 (ZEBRAFISH)  
Ei-value:0.000, Pi-value:0.000  
Er-value:0.000, Pr-value:0.000  
MATCHES To TargetScan▶ miR-186-5p:AAAGAAU

-6741--(23)--6765-

CAGGTTTTGCTTT

CAGGTTTTGCTTT  
Depth:19 (ZEBRAFISH)  
Ei-value:0.000, Pi-value:0.000  
Er-value:0.000, Pr-value:0.000  
MATCHES To TargetScan▶ miR-330-3p.2:AAAGCAC▶ miR-490-3p:AACCUGG


T

CAGGTTTTGCTTTT  
Depth:16 (NILETILAPIA)  
Ei-value:0.000, Pi-value:0.000  
Er-value:0.000, Pr-value:0.000  
MATCHES To TargetScan▶ miR-330-3p.2:AAAGCAC▶ miR-490-3p:AACCUGG

-6778--(20)--6799-

AAAAAGCAAAA

AAAAAGCAAAA  
Depth:19 (ZEBRAFISH)  
Ei-value:0.000, Pi-value:0.000  
Er-value:0.000, Pr-value:0.000  
No matches to TargetScan


G

AAAAAGCAAAAG  
Depth:18 (MEDAKA)  
Ei-value:0.000, Pi-value:0.000  
Er-value:0.000, Pr-value:0.000  
No matches to TargetScan

-6810--(15)--6826-

ACTCCTG

ACTCCTG  
Depth:18 (MEDAKA)  
Ei-value:0.000, Pi-value:0.000  
Er-value:0.000, Pr-value:0.000  
No matches to TargetScan

-6832  
  
>ALLIGATOR  
      2537-

TTTGGG

TTTGGG  
Depth:19 (ZEBRAFISH)  
Ei-value:0.000, Pi-value:0.000  
Er-value:0.000, Pr-value:0.000  
No matches to TargetScan

-2542--(1322)--3865-

TTTTTCAG

TTTTTCAG  
Depth:19 (ZEBRAFISH)  
Ei-value:0.000, Pi-value:0.000  
Er-value:0.000, Pr-value:0.000  
No matches to TargetScan

-3872--(648)--4521-

GATAAG

GATAAG  
Depth:19 (ZEBRAFISH)  
Ei-value:0.000, Pi-value:0.000  
Er-value:0.000, Pr-value:0.000  
No matches to TargetScan

-4526--(3478)--8005-

TTTTCTTTT

TTTTCTTTT  
Depth:19 (ZEBRAFISH)  
Ei-value:0.000, Pi-value:0.000  
Er-value:0.000, Pr-value:0.000  
MATCHES To TargetScan▶ miR-186-5p:AAAGAAU

-8013--(23)--8037-

CAGGTTTTGCTTT

CAGGTTTTGCTTT  
Depth:19 (ZEBRAFISH)  
Ei-value:0.000, Pi-value:0.000  
Er-value:0.000, Pr-value:0.000  
MATCHES To TargetScan▶ miR-330-3p.2:AAAGCAC▶ miR-490-3p:AACCUGG


T

CAGGTTTTGCTTTT  
Depth:16 (NILETILAPIA)  
Ei-value:0.000, Pi-value:0.000  
Er-value:0.000, Pr-value:0.000  
MATCHES To TargetScan▶ miR-330-3p.2:AAAGCAC▶ miR-490-3p:AACCUGG

-8050--(25)--8076-

AAAAAGCAAAA

AAAAAGCAAAA  
Depth:19 (ZEBRAFISH)  
Ei-value:0.000, Pi-value:0.000  
Er-value:0.000, Pr-value:0.000  
No matches to TargetScan


G

AAAAAGCAAAAG  
Depth:18 (MEDAKA)  
Ei-value:0.000, Pi-value:0.000  
Er-value:0.000, Pr-value:0.000  
No matches to TargetScan

-8087--(15)--8103-

ACTCCTG

ACTCCTG  
Depth:18 (MEDAKA)  
Ei-value:0.000, Pi-value:0.000  
Er-value:0.000, Pr-value:0.000  
No matches to TargetScan

-8109  
  
>LIZARD  
      2353-

TTTGGG

TTTGGG  
Depth:19 (ZEBRAFISH)  
Ei-value:0.000, Pi-value:0.000  
Er-value:0.000, Pr-value:0.000  
No matches to TargetScan

-2358--(762)--3121-

TTTTTCAG

TTTTTCAG  
Depth:19 (ZEBRAFISH)  
Ei-value:0.000, Pi-value:0.000  
Er-value:0.000, Pr-value:0.000  
No matches to TargetScan

-3128--(588)--3717-

GATAAG

GATAAG  
Depth:19 (ZEBRAFISH)  
Ei-value:0.000, Pi-value:0.000  
Er-value:0.000, Pr-value:0.000  
No matches to TargetScan

-3722--(3176)--6899-

TTTTCTTTT

TTTTCTTTT  
Depth:19 (ZEBRAFISH)  
Ei-value:0.000, Pi-value:0.000  
Er-value:0.000, Pr-value:0.000  
MATCHES To TargetScan▶ miR-186-5p:AAAGAAU

-6907--(24)--6932-

CAGGTTTTGCTTT

CAGGTTTTGCTTT  
Depth:19 (ZEBRAFISH)  
Ei-value:0.000, Pi-value:0.000  
Er-value:0.000, Pr-value:0.000  
MATCHES To TargetScan▶ miR-330-3p.2:AAAGCAC▶ miR-490-3p:AACCUGG


T

CAGGTTTTGCTTTT  
Depth:16 (NILETILAPIA)  
Ei-value:0.000, Pi-value:0.000  
Er-value:0.000, Pr-value:0.000  
MATCHES To TargetScan▶ miR-330-3p.2:AAAGCAC▶ miR-490-3p:AACCUGG

-6945--(20)--6966-

AAAAAGCAAAA

AAAAAGCAAAA  
Depth:19 (ZEBRAFISH)  
Ei-value:0.000, Pi-value:0.000  
Er-value:0.000, Pr-value:0.000  
No matches to TargetScan


G

AAAAAGCAAAAG  
Depth:18 (MEDAKA)  
Ei-value:0.000, Pi-value:0.000  
Er-value:0.000, Pr-value:0.000  
No matches to TargetScan

-6977--(15)--6993-

ACTCCTG

ACTCCTG  
Depth:18 (MEDAKA)  
Ei-value:0.000, Pi-value:0.000  
Er-value:0.000, Pr-value:0.000  
No matches to TargetScan

-6999  
  
>SNAKE  
      1292-

TTTGGG

TTTGGG  
Depth:19 (ZEBRAFISH)  
Ei-value:0.000, Pi-value:0.000  
Er-value:0.000, Pr-value:0.000  
No matches to TargetScan

-1297--(729)--2027-

TTTGGG

TTTGGG  
Depth:19 (ZEBRAFISH)  
Ei-value:0.000, Pi-value:0.000  
Er-value:0.000, Pr-value:0.000  
No matches to TargetScan

-2032--(1081)--3114-

TTTTTCAG

TTTTTCAG  
Depth:19 (ZEBRAFISH)  
Ei-value:0.000, Pi-value:0.000  
Er-value:0.000, Pr-value:0.000  
No matches to TargetScan

-3121--(558)--3680-

GATAAG

GATAAG  
Depth:19 (ZEBRAFISH)  
Ei-value:0.000, Pi-value:0.000  
Er-value:0.000, Pr-value:0.000  
No matches to TargetScan

-3685--(3037)--6723-

TTTTCTTTT

TTTTCTTTT  
Depth:19 (ZEBRAFISH)  
Ei-value:0.000, Pi-value:0.000  
Er-value:0.000, Pr-value:0.000  
MATCHES To TargetScan▶ miR-186-5p:AAAGAAU

-6731--(25)--6757-

CAGGTTTTGCTTT

CAGGTTTTGCTTT  
Depth:19 (ZEBRAFISH)  
Ei-value:0.000, Pi-value:0.000  
Er-value:0.000, Pr-value:0.000  
MATCHES To TargetScan▶ miR-330-3p.2:AAAGCAC▶ miR-490-3p:AACCUGG


T

CAGGTTTTGCTTTT  
Depth:16 (NILETILAPIA)  
Ei-value:0.000, Pi-value:0.000  
Er-value:0.000, Pr-value:0.000  
MATCHES To TargetScan▶ miR-330-3p.2:AAAGCAC▶ miR-490-3p:AACCUGG

-6770--(28)--6799-

AAAAAGCAAAA

AAAAAGCAAAA  
Depth:19 (ZEBRAFISH)  
Ei-value:0.000, Pi-value:0.000  
Er-value:0.000, Pr-value:0.000  
No matches to TargetScan


G

AAAAAGCAAAAG  
Depth:18 (MEDAKA)  
Ei-value:0.000, Pi-value:0.000  
Er-value:0.000, Pr-value:0.000  
No matches to TargetScan

-6810--(15)--6826-

ACTCCTG

ACTCCTG  
Depth:18 (MEDAKA)  
Ei-value:0.000, Pi-value:0.000  
Er-value:0.000, Pr-value:0.000  
No matches to TargetScan

-6832  
  
>X.TROPICALIS  
      2299-

TTTGGG

TTTGGG  
Depth:19 (ZEBRAFISH)  
Ei-value:0.000, Pi-value:0.000  
Er-value:0.000, Pr-value:0.000  
No matches to TargetScan

-2304--(2695)--5000-

TTTGGG

TTTGGG  
Depth:19 (ZEBRAFISH)  
Ei-value:0.000, Pi-value:0.000  
Er-value:0.000, Pr-value:0.000  
No matches to TargetScan

-5005--(125)--5131-

TTTGGG

TTTGGG  
Depth:19 (ZEBRAFISH)  
Ei-value:0.000, Pi-value:0.000  
Er-value:0.000, Pr-value:0.000  
No matches to TargetScan

-5136--(3206)--8343-

TTTTTCAG

TTTTTCAG  
Depth:19 (ZEBRAFISH)  
Ei-value:0.000, Pi-value:0.000  
Er-value:0.000, Pr-value:0.000  
No matches to TargetScan

-8350--(1004)--9355-

GATAAG

GATAAG  
Depth:19 (ZEBRAFISH)  
Ei-value:0.000, Pi-value:0.000  
Er-value:0.000, Pr-value:0.000  
No matches to TargetScan

-9360--(2966)--12327-

TTTTCTTTT

TTTTCTTTT  
Depth:19 (ZEBRAFISH)  
Ei-value:0.000, Pi-value:0.000  
Er-value:0.000, Pr-value:0.000  
MATCHES To TargetScan▶ miR-186-5p:AAAGAAU

-12335--(26)--12362-

CAGGTTTTGCTTT

CAGGTTTTGCTTT  
Depth:19 (ZEBRAFISH)  
Ei-value:0.000, Pi-value:0.000  
Er-value:0.000, Pr-value:0.000  
MATCHES To TargetScan▶ miR-330-3p.2:AAAGCAC▶ miR-490-3p:AACCUGG


T

CAGGTTTTGCTTTT  
Depth:16 (NILETILAPIA)  
Ei-value:0.000, Pi-value:0.000  
Er-value:0.000, Pr-value:0.000  
MATCHES To TargetScan▶ miR-330-3p.2:AAAGCAC▶ miR-490-3p:AACCUGG

-12375--(30)--12406-

AAAAAGCAAAA

AAAAAGCAAAA  
Depth:19 (ZEBRAFISH)  
Ei-value:0.000, Pi-value:0.000  
Er-value:0.000, Pr-value:0.000  
No matches to TargetScan


G

AAAAAGCAAAAG  
Depth:18 (MEDAKA)  
Ei-value:0.000, Pi-value:0.000  
Er-value:0.000, Pr-value:0.000  
No matches to TargetScan

-12417--(15)--12433-

ACTCCTG

ACTCCTG  
Depth:18 (MEDAKA)  
Ei-value:0.000, Pi-value:0.000  
Er-value:0.000, Pr-value:0.000  
No matches to TargetScan

-12439  
  
>SHARK  
      1413-

TTTGGG

TTTGGG  
Depth:19 (ZEBRAFISH)  
Ei-value:0.000, Pi-value:0.000  
Er-value:0.000, Pr-value:0.000  
No matches to TargetScan

-1418--(1015)--2434-

TTTGGG

TTTGGG  
Depth:19 (ZEBRAFISH)  
Ei-value:0.000, Pi-value:0.000  
Er-value:0.000, Pr-value:0.000  
No matches to TargetScan

-2439--(74)--2514-

TTTGGG

TTTGGG  
Depth:19 (ZEBRAFISH)  
Ei-value:0.000, Pi-value:0.000  
Er-value:0.000, Pr-value:0.000  
No matches to TargetScan

-2519--(38)--2558-

TTTTTCAG

TTTTTCAG  
Depth:19 (ZEBRAFISH)  
Ei-value:0.000, Pi-value:0.000  
Er-value:0.000, Pr-value:0.000  
No matches to TargetScan

-2565--(4402)--6968-

GATAAG

GATAAG  
Depth:19 (ZEBRAFISH)  
Ei-value:0.000, Pi-value:0.000  
Er-value:0.000, Pr-value:0.000  
No matches to TargetScan

-6973--(520)--7494-

TTTTCTTTT

TTTTCTTTT  
Depth:19 (ZEBRAFISH)  
Ei-value:0.000, Pi-value:0.000  
Er-value:0.000, Pr-value:0.000  
MATCHES To TargetScan▶ miR-186-5p:AAAGAAU

-7502--(25)--7528-

CAGGTTTTGCTTT

CAGGTTTTGCTTT  
Depth:19 (ZEBRAFISH)  
Ei-value:0.000, Pi-value:0.000  
Er-value:0.000, Pr-value:0.000  
MATCHES To TargetScan▶ miR-330-3p.2:AAAGCAC▶ miR-490-3p:AACCUGG


T

CAGGTTTTGCTTTT  
Depth:16 (NILETILAPIA)  
Ei-value:0.000, Pi-value:0.000  
Er-value:0.000, Pr-value:0.000  
MATCHES To TargetScan▶ miR-330-3p.2:AAAGCAC▶ miR-490-3p:AACCUGG

-7541--(26)--7568-

AAAAAGCAAAA

AAAAAGCAAAA  
Depth:19 (ZEBRAFISH)  
Ei-value:0.000, Pi-value:0.000  
Er-value:0.000, Pr-value:0.000  
No matches to TargetScan


G

AAAAAGCAAAAG  
Depth:18 (MEDAKA)  
Ei-value:0.000, Pi-value:0.000  
Er-value:0.000, Pr-value:0.000  
No matches to TargetScan

-7579--(15)--7595-

ACTCCTG

ACTCCTG  
Depth:18 (MEDAKA)  
Ei-value:0.000, Pi-value:0.000  
Er-value:0.000, Pr-value:0.000  
No matches to TargetScan

-7601  
  
>OPOSSUM  
      1043-

TTTGGG

TTTGGG  
Depth:19 (ZEBRAFISH)  
Ei-value:0.000, Pi-value:0.000  
Er-value:0.000, Pr-value:0.000  
No matches to TargetScan

-1048--(273)--1322-

TTTGGG

TTTGGG  
Depth:19 (ZEBRAFISH)  
Ei-value:0.000, Pi-value:0.000  
Er-value:0.000, Pr-value:0.000  
No matches to TargetScan

-1327--(111)--1439-

TTTGGG

TTTGGG  
Depth:19 (ZEBRAFISH)  
Ei-value:0.000, Pi-value:0.000  
Er-value:0.000, Pr-value:0.000  
No matches to TargetScan

-1444--(1059)--2504-

TTTTTCAG

TTTTTCAG  
Depth:19 (ZEBRAFISH)  
Ei-value:0.000, Pi-value:0.000  
Er-value:0.000, Pr-value:0.000  
No matches to TargetScan

-2511--(973)--3485-

GATAAG

GATAAG  
Depth:19 (ZEBRAFISH)  
Ei-value:0.000, Pi-value:0.000  
Er-value:0.000, Pr-value:0.000  
No matches to TargetScan

-3490--(2190)--5681-

TTTTCTTTT

TTTTCTTTT  
Depth:19 (ZEBRAFISH)  
Ei-value:0.000, Pi-value:0.000  
Er-value:0.000, Pr-value:0.000  
MATCHES To TargetScan▶ miR-186-5p:AAAGAAU

-5689--(26)--5716-

CAGGTTTTGCTTT

CAGGTTTTGCTTT  
Depth:19 (ZEBRAFISH)  
Ei-value:0.000, Pi-value:0.000  
Er-value:0.000, Pr-value:0.000  
MATCHES To TargetScan▶ miR-330-3p.2:AAAGCAC▶ miR-490-3p:AACCUGG


T

CAGGTTTTGCTTTT  
Depth:16 (NILETILAPIA)  
Ei-value:0.000, Pi-value:0.000  
Er-value:0.000, Pr-value:0.000  
MATCHES To TargetScan▶ miR-330-3p.2:AAAGCAC▶ miR-490-3p:AACCUGG

-5729--(17)--5747-

AAAAAGCAAAA

AAAAAGCAAAA  
Depth:19 (ZEBRAFISH)  
Ei-value:0.000, Pi-value:0.000  
Er-value:0.000, Pr-value:0.000  
No matches to TargetScan


G

AAAAAGCAAAAG  
Depth:18 (MEDAKA)  
Ei-value:0.000, Pi-value:0.000  
Er-value:0.000, Pr-value:0.000  
No matches to TargetScan

-5758--(14)--5773-

ACTCCTG

ACTCCTG  
Depth:18 (MEDAKA)  
Ei-value:0.000, Pi-value:0.000  
Er-value:0.000, Pr-value:0.000  
No matches to TargetScan

-5779  
  
>SPOTTEDGAR  
      3644-

TTTGGG

TTTGGG  
Depth:19 (ZEBRAFISH)  
Ei-value:0.000, Pi-value:0.000  
Er-value:0.000, Pr-value:0.000  
No matches to TargetScan

-3649--(31)--3681-

TTTTTCAG

TTTTTCAG  
Depth:19 (ZEBRAFISH)  
Ei-value:0.000, Pi-value:0.000  
Er-value:0.000, Pr-value:0.000  
No matches to TargetScan

-3688--(72)--3761-

GATAAG

GATAAG  
Depth:19 (ZEBRAFISH)  
Ei-value:0.000, Pi-value:0.000  
Er-value:0.000, Pr-value:0.000  
No matches to TargetScan

-3766--(274)--4041-

GATAAG

GATAAG  
Depth:19 (ZEBRAFISH)  
Ei-value:0.000, Pi-value:0.000  
Er-value:0.000, Pr-value:0.000  
No matches to TargetScan

-4046--(2564)--6611-

TTTTCTTTT

TTTTCTTTT  
Depth:19 (ZEBRAFISH)  
Ei-value:0.000, Pi-value:0.000  
Er-value:0.000, Pr-value:0.000  
MATCHES To TargetScan▶ miR-186-5p:AAAGAAU

-6619--(295)--6915-

CAGGTTTTGCTTT

CAGGTTTTGCTTT  
Depth:19 (ZEBRAFISH)  
Ei-value:0.000, Pi-value:0.000  
Er-value:0.000, Pr-value:0.000  
MATCHES To TargetScan▶ miR-330-3p.2:AAAGCAC▶ miR-490-3p:AACCUGG


T

CAGGTTTTGCTTTT  
Depth:16 (NILETILAPIA)  
Ei-value:0.000, Pi-value:0.000  
Er-value:0.000, Pr-value:0.000  
MATCHES To TargetScan▶ miR-330-3p.2:AAAGCAC▶ miR-490-3p:AACCUGG

-6928--(27)--6956-

AAAAAGCAAAA

AAAAAGCAAAA  
Depth:19 (ZEBRAFISH)  
Ei-value:0.000, Pi-value:0.000  
Er-value:0.000, Pr-value:0.000  
No matches to TargetScan


G

AAAAAGCAAAAG  
Depth:18 (MEDAKA)  
Ei-value:0.000, Pi-value:0.000  
Er-value:0.000, Pr-value:0.000  
No matches to TargetScan

-6967--(14)--6982-

ACTCCTG

ACTCCTG  
Depth:18 (MEDAKA)  
Ei-value:0.000, Pi-value:0.000  
Er-value:0.000, Pr-value:0.000  
No matches to TargetScan

-6988  
  
>FUGU  
      1704-

TTTGGG

TTTGGG  
Depth:19 (ZEBRAFISH)  
Ei-value:0.000, Pi-value:0.000  
Er-value:0.000, Pr-value:0.000  
No matches to TargetScan

-1709--(774)--2484-

TTTTTCAG

TTTTTCAG  
Depth:19 (ZEBRAFISH)  
Ei-value:0.000, Pi-value:0.000  
Er-value:0.000, Pr-value:0.000  
No matches to TargetScan

-2491--(316)--2808-

TTTTTCAG

TTTTTCAG  
Depth:19 (ZEBRAFISH)  
Ei-value:0.000, Pi-value:0.000  
Er-value:0.000, Pr-value:0.000  
No matches to TargetScan

-2815--(72)--2888-

GATAAG

GATAAG  
Depth:19 (ZEBRAFISH)  
Ei-value:0.000, Pi-value:0.000  
Er-value:0.000, Pr-value:0.000  
No matches to TargetScan

-2893--(146)--3040-

GATAAG

GATAAG  
Depth:19 (ZEBRAFISH)  
Ei-value:0.000, Pi-value:0.000  
Er-value:0.000, Pr-value:0.000  
No matches to TargetScan

-3045--(1722)--4768-

TTTTCTTTT

TTTTCTTTT  
Depth:19 (ZEBRAFISH)  
Ei-value:0.000, Pi-value:0.000  
Er-value:0.000, Pr-value:0.000  
MATCHES To TargetScan▶ miR-186-5p:AAAGAAU

-4776--(31)--4808-

CAGGTTTTGCTTT

CAGGTTTTGCTTT  
Depth:19 (ZEBRAFISH)  
Ei-value:0.000, Pi-value:0.000  
Er-value:0.000, Pr-value:0.000  
MATCHES To TargetScan▶ miR-330-3p.2:AAAGCAC▶ miR-490-3p:AACCUGG


T

CAGGTTTTGCTTTT  
Depth:16 (NILETILAPIA)  
Ei-value:0.000, Pi-value:0.000  
Er-value:0.000, Pr-value:0.000  
MATCHES To TargetScan▶ miR-330-3p.2:AAAGCAC▶ miR-490-3p:AACCUGG

-4821--(17)--4839-

AAAAAGCAAAA

AAAAAGCAAAA  
Depth:19 (ZEBRAFISH)  
Ei-value:0.000, Pi-value:0.000  
Er-value:0.000, Pr-value:0.000  
No matches to TargetScan


G

AAAAAGCAAAAG  
Depth:18 (MEDAKA)  
Ei-value:0.000, Pi-value:0.000  
Er-value:0.000, Pr-value:0.000  
No matches to TargetScan

-4850--(14)--4865-

ACTCCTG

ACTCCTG  
Depth:18 (MEDAKA)  
Ei-value:0.000, Pi-value:0.000  
Er-value:0.000, Pr-value:0.000  
No matches to TargetScan

-4871  
  
>NILETILAPIA  
      2795-

TTTGGG

TTTGGG  
Depth:19 (ZEBRAFISH)  
Ei-value:0.000, Pi-value:0.000  
Er-value:0.000, Pr-value:0.000  
No matches to TargetScan

-2800--(581)--3382-

TTTTTCAG

TTTTTCAG  
Depth:19 (ZEBRAFISH)  
Ei-value:0.000, Pi-value:0.000  
Er-value:0.000, Pr-value:0.000  
No matches to TargetScan

-3389--(324)--3714-

TTTTTCAG

TTTTTCAG  
Depth:19 (ZEBRAFISH)  
Ei-value:0.000, Pi-value:0.000  
Er-value:0.000, Pr-value:0.000  
No matches to TargetScan

-3721--(84)--3806-

GATAAG

GATAAG  
Depth:19 (ZEBRAFISH)  
Ei-value:0.000, Pi-value:0.000  
Er-value:0.000, Pr-value:0.000  
No matches to TargetScan

-3811--(2023)--5835-

TTTTCTTTT

TTTTCTTTT  
Depth:19 (ZEBRAFISH)  
Ei-value:0.000, Pi-value:0.000  
Er-value:0.000, Pr-value:0.000  
MATCHES To TargetScan▶ miR-186-5p:AAAGAAU

-5843--(28)--5872-

CAGGTTTTGCTTT

CAGGTTTTGCTTT  
Depth:19 (ZEBRAFISH)  
Ei-value:0.000, Pi-value:0.000  
Er-value:0.000, Pr-value:0.000  
MATCHES To TargetScan▶ miR-330-3p.2:AAAGCAC▶ miR-490-3p:AACCUGG


T

CAGGTTTTGCTTTT  
Depth:16 (NILETILAPIA)  
Ei-value:0.000, Pi-value:0.000  
Er-value:0.000, Pr-value:0.000  
MATCHES To TargetScan▶ miR-330-3p.2:AAAGCAC▶ miR-490-3p:AACCUGG

-5885--(18)--5904-

AAAAAGCAAAA

AAAAAGCAAAA  
Depth:19 (ZEBRAFISH)  
Ei-value:0.000, Pi-value:0.000  
Er-value:0.000, Pr-value:0.000  
No matches to TargetScan


G

AAAAAGCAAAAG  
Depth:18 (MEDAKA)  
Ei-value:0.000, Pi-value:0.000  
Er-value:0.000, Pr-value:0.000  
No matches to TargetScan

-5915--(14)--5930-

ACTCCTG

ACTCCTG  
Depth:18 (MEDAKA)  
Ei-value:0.000, Pi-value:0.000  
Er-value:0.000, Pr-value:0.000  
No matches to TargetScan

-5936
```

---

# Modules conserved to SPOTTEDGAR (Depth: 14)

## Modules in Main Graph (All sequences considered):

```
>HUMAN  
      1794-

TGGTAA

TGGTAA  
Depth:14 (SPOTTEDGAR)  
Ei-value:0.030, Pi-value:0.000  
Er-value:0.010, Pr-value:0.000  
eCLIP MATCHES▶bclaf1 (bg=17.67%)▶DROSHA (bg=1.03%)▶GRWD1 (bg=7.0%)▶hltf (bg=24.28%)▶MTPAP (bg=9.55%)▶NOLC1 (bg=0.67%)▶npm1 (bg=10.22%)▶ppil4 (bg=43.39%)▶rbm15 (bg=11.59%)▶safb (bg=40.39%)▶srsf1 (bg=30.28%)▶TAF15 (bg=9.06%)▶tia1 (bg=23.76%)▶uchl5 (bg=18.56%)▶YWHAG (bg=9.14%)▶ZRANB2 (bg=7.28%)No matches to TargetScan

-1799--(705)--2505-

TTTGGG

TTTGGG  
Depth:19 (ZEBRAFISH)  
Ei-value:0.000, Pi-value:0.000  
Er-value:0.000, Pr-value:0.000  
eCLIP MATCHES▶ppil4 (bg=43.39%)▶PRPF8 (bg=6.2%)No matches to TargetScan

-2510--(1110)--3621-

TTTTTCAG

TTTTTCAG  
Depth:19 (ZEBRAFISH)  
Ei-value:0.000, Pi-value:0.000  
Er-value:0.000, Pr-value:0.000  
eCLIP MATCHES▶ddx42 (bg=10.33%)▶ppil4 (bg=43.39%)▶safb (bg=40.39%)▶u2af1 (bg=14.02%)▶u2af2 (bg=19.32%)No matches to TargetScan

-3628--(543)--4172-

GATAAG

GATAAG  
Depth:19 (ZEBRAFISH)  
Ei-value:0.000, Pi-value:0.000  
Er-value:0.000, Pr-value:0.000  
eCLIP MATCHES▶cpsf6 (bg=13.45%)▶khsrp (bg=27.4%)▶NIPBL (bg=8.2%)▶ppil4 (bg=43.39%)▶PRPF8 (bg=6.2%)▶rbm15 (bg=11.59%)▶safb (bg=40.39%)▶safb2 (bg=26.89%)▶srsf1 (bg=30.28%)▶srsf7 (bg=22.53%)▶znf622 (bg=18.79%)No matches to TargetScan

-4177--(2833)--7011-

TTTTCTTTT

TTTTCTTTT  
Depth:19 (ZEBRAFISH)  
Ei-value:0.000, Pi-value:0.000  
Er-value:0.000, Pr-value:0.000  
eCLIP MATCHES▶srsf7 (bg=22.53%)MATCHES To TargetScan▶ miR-186-5p:AAAGAAU

-7019--(28)--7048-

CAGGTTTTGCTTT

CAGGTTTTGCTTT  
Depth:19 (ZEBRAFISH)  
Ei-value:0.000, Pi-value:0.000  
Er-value:0.000, Pr-value:0.000  
eCLIP MATCHES▶srsf7 (bg=22.53%)MATCHES To TargetScan▶ miR-330-3p.2:AAAGCAC▶ miR-490-3p:AACCUGG


T

CAGGTTTTGCTTTT  
Depth:16 (NILETILAPIA)  
Ei-value:0.000, Pi-value:0.000  
Er-value:0.000, Pr-value:0.000  
eCLIP MATCHES▶srsf7 (bg=22.53%)MATCHES To TargetScan▶ miR-330-3p.2:AAAGCAC▶ miR-490-3p:AACCUGG

-7061--(25)--7087-

AAAAAGCAAAA

AAAAAGCAAAA  
Depth:19 (ZEBRAFISH)  
Ei-value:0.000, Pi-value:0.000  
Er-value:0.000, Pr-value:0.000  
No matches to eCLIP DataNo matches to TargetScan


G

AAAAAGCAAAAG  
Depth:18 (MEDAKA)  
Ei-value:0.000, Pi-value:0.000  
Er-value:0.000, Pr-value:0.000  
No matches to eCLIP DataNo matches to TargetScan

-7098--(15)--7114-

ACTCCTG

ACTCCTG  
Depth:18 (MEDAKA)  
Ei-value:0.000, Pi-value:0.000  
Er-value:0.000, Pr-value:0.000  
No matches to eCLIP DataNo matches to TargetScan


G

ACTCCTGG  
Depth:14 (SPOTTEDGAR)  
Ei-value:0.000, Pi-value:0.000  
Er-value:0.000, Pr-value:0.000  
No matches to eCLIP DataMATCHES To TargetScan▶ miR-665:CCAGGAG

-7121  
  
>MARMOSET  
      1908-

TGGTAA

TGGTAA  
Depth:14 (SPOTTEDGAR)  
Ei-value:0.030, Pi-value:0.000  
Er-value:0.010, Pr-value:0.000  
No matches to TargetScan

-1913--(723)--2637-

TTTGGG

TTTGGG  
Depth:19 (ZEBRAFISH)  
Ei-value:0.000, Pi-value:0.000  
Er-value:0.000, Pr-value:0.000  
No matches to TargetScan

-2642--(1095)--3738-

TTTTTCAG

TTTTTCAG  
Depth:19 (ZEBRAFISH)  
Ei-value:0.000, Pi-value:0.000  
Er-value:0.000, Pr-value:0.000  
No matches to TargetScan

-3745--(548)--4294-

GATAAG

GATAAG  
Depth:19 (ZEBRAFISH)  
Ei-value:0.000, Pi-value:0.000  
Er-value:0.000, Pr-value:0.000  
No matches to TargetScan

-4299--(2840)--7140-

TTTTCTTTT

TTTTCTTTT  
Depth:19 (ZEBRAFISH)  
Ei-value:0.000, Pi-value:0.000  
Er-value:0.000, Pr-value:0.000  
MATCHES To TargetScan▶ miR-186-5p:AAAGAAU

-7148--(28)--7177-

CAGGTTTTGCTTT

CAGGTTTTGCTTT  
Depth:19 (ZEBRAFISH)  
Ei-value:0.000, Pi-value:0.000  
Er-value:0.000, Pr-value:0.000  
MATCHES To TargetScan▶ miR-330-3p.2:AAAGCAC▶ miR-490-3p:AACCUGG


T

CAGGTTTTGCTTTT  
Depth:16 (NILETILAPIA)  
Ei-value:0.000, Pi-value:0.000  
Er-value:0.000, Pr-value:0.000  
MATCHES To TargetScan▶ miR-330-3p.2:AAAGCAC▶ miR-490-3p:AACCUGG

-7190--(26)--7217-

AAAAAGCAAAA

AAAAAGCAAAA  
Depth:19 (ZEBRAFISH)  
Ei-value:0.000, Pi-value:0.000  
Er-value:0.000, Pr-value:0.000  
No matches to TargetScan


G

AAAAAGCAAAAG  
Depth:18 (MEDAKA)  
Ei-value:0.000, Pi-value:0.000  
Er-value:0.000, Pr-value:0.000  
No matches to TargetScan

-7228--(15)--7244-

ACTCCTG

ACTCCTG  
Depth:18 (MEDAKA)  
Ei-value:0.000, Pi-value:0.000  
Er-value:0.000, Pr-value:0.000  
No matches to TargetScan


G

ACTCCTGG  
Depth:14 (SPOTTEDGAR)  
Ei-value:0.000, Pi-value:0.000  
Er-value:0.000, Pr-value:0.000  
MATCHES To TargetScan▶ miR-665:CCAGGAG

-7251  
  
>DOG  
      1928-

TGGTAA

TGGTAA  
Depth:14 (SPOTTEDGAR)  
Ei-value:0.030, Pi-value:0.000  
Er-value:0.010, Pr-value:0.000  
No matches to TargetScan

-1933--(678)--2612-

TTTGGG

TTTGGG  
Depth:19 (ZEBRAFISH)  
Ei-value:0.000, Pi-value:0.000  
Er-value:0.000, Pr-value:0.000  
No matches to TargetScan

-2617--(1013)--3631-

TTTTTCAG

TTTTTCAG  
Depth:19 (ZEBRAFISH)  
Ei-value:0.000, Pi-value:0.000  
Er-value:0.000, Pr-value:0.000  
No matches to TargetScan

-3638--(559)--4198-

GATAAG

GATAAG  
Depth:19 (ZEBRAFISH)  
Ei-value:0.000, Pi-value:0.000  
Er-value:0.000, Pr-value:0.000  
No matches to TargetScan

-4203--(2856)--7060-

TTTTCTTTT

TTTTCTTTT  
Depth:19 (ZEBRAFISH)  
Ei-value:0.000, Pi-value:0.000  
Er-value:0.000, Pr-value:0.000  
MATCHES To TargetScan▶ miR-186-5p:AAAGAAU

-7068--(26)--7095-

CAGGTTTTGCTTT

CAGGTTTTGCTTT  
Depth:19 (ZEBRAFISH)  
Ei-value:0.000, Pi-value:0.000  
Er-value:0.000, Pr-value:0.000  
MATCHES To TargetScan▶ miR-330-3p.2:AAAGCAC▶ miR-490-3p:AACCUGG


T

CAGGTTTTGCTTTT  
Depth:16 (NILETILAPIA)  
Ei-value:0.000, Pi-value:0.000  
Er-value:0.000, Pr-value:0.000  
MATCHES To TargetScan▶ miR-330-3p.2:AAAGCAC▶ miR-490-3p:AACCUGG

-7108--(21)--7130-

AAAAAGCAAAA

AAAAAGCAAAA  
Depth:19 (ZEBRAFISH)  
Ei-value:0.000, Pi-value:0.000  
Er-value:0.000, Pr-value:0.000  
No matches to TargetScan


G

AAAAAGCAAAAG  
Depth:18 (MEDAKA)  
Ei-value:0.000, Pi-value:0.000  
Er-value:0.000, Pr-value:0.000  
No matches to TargetScan

-7141--(15)--7157-

ACTCCTG

ACTCCTG  
Depth:18 (MEDAKA)  
Ei-value:0.000, Pi-value:0.000  
Er-value:0.000, Pr-value:0.000  
No matches to TargetScan


G

ACTCCTGG  
Depth:14 (SPOTTEDGAR)  
Ei-value:0.000, Pi-value:0.000  
Er-value:0.000, Pr-value:0.000  
MATCHES To TargetScan▶ miR-665:CCAGGAG

-7164  
  
>PIG  
      1849-

TGGTAA

TGGTAA  
Depth:14 (SPOTTEDGAR)  
Ei-value:0.030, Pi-value:0.000  
Er-value:0.010, Pr-value:0.000  
No matches to TargetScan

-1854--(678)--2533-

TTTGGG

TTTGGG  
Depth:19 (ZEBRAFISH)  
Ei-value:0.000, Pi-value:0.000  
Er-value:0.000, Pr-value:0.000  
No matches to TargetScan

-2538--(1002)--3541-

TTTTTCAG

TTTTTCAG  
Depth:19 (ZEBRAFISH)  
Ei-value:0.000, Pi-value:0.000  
Er-value:0.000, Pr-value:0.000  
No matches to TargetScan

-3548--(569)--4118-

GATAAG

GATAAG  
Depth:19 (ZEBRAFISH)  
Ei-value:0.000, Pi-value:0.000  
Er-value:0.000, Pr-value:0.000  
No matches to TargetScan

-4123--(2848)--6972-

TTTTCTTTT

TTTTCTTTT  
Depth:19 (ZEBRAFISH)  
Ei-value:0.000, Pi-value:0.000  
Er-value:0.000, Pr-value:0.000  
MATCHES To TargetScan▶ miR-186-5p:AAAGAAU

-6980--(28)--7009-

CAGGTTTTGCTTT

CAGGTTTTGCTTT  
Depth:19 (ZEBRAFISH)  
Ei-value:0.000, Pi-value:0.000  
Er-value:0.000, Pr-value:0.000  
MATCHES To TargetScan▶ miR-330-3p.2:AAAGCAC▶ miR-490-3p:AACCUGG


T

CAGGTTTTGCTTTT  
Depth:16 (NILETILAPIA)  
Ei-value:0.000, Pi-value:0.000  
Er-value:0.000, Pr-value:0.000  
MATCHES To TargetScan▶ miR-330-3p.2:AAAGCAC▶ miR-490-3p:AACCUGG

-7022--(23)--7046-

AAAAAGCAAAA

AAAAAGCAAAA  
Depth:19 (ZEBRAFISH)  
Ei-value:0.000, Pi-value:0.000  
Er-value:0.000, Pr-value:0.000  
No matches to TargetScan


G

AAAAAGCAAAAG  
Depth:18 (MEDAKA)  
Ei-value:0.000, Pi-value:0.000  
Er-value:0.000, Pr-value:0.000  
No matches to TargetScan

-7057--(15)--7073-

ACTCCTG

ACTCCTG  
Depth:18 (MEDAKA)  
Ei-value:0.000, Pi-value:0.000  
Er-value:0.000, Pr-value:0.000  
No matches to TargetScan


G

ACTCCTGG  
Depth:14 (SPOTTEDGAR)  
Ei-value:0.000, Pi-value:0.000  
Er-value:0.000, Pr-value:0.000  
MATCHES To TargetScan▶ miR-665:CCAGGAG

-7080  
  
>COW  
      1716-

TGGTAA

TGGTAA  
Depth:14 (SPOTTEDGAR)  
Ei-value:0.030, Pi-value:0.000  
Er-value:0.010, Pr-value:0.000  
No matches to TargetScan

-1721--(695)--2417-

TTTGGG

TTTGGG  
Depth:19 (ZEBRAFISH)  
Ei-value:0.000, Pi-value:0.000  
Er-value:0.000, Pr-value:0.000  
No matches to TargetScan

-2422--(1012)--3435-

TTTTTCAG

TTTTTCAG  
Depth:19 (ZEBRAFISH)  
Ei-value:0.000, Pi-value:0.000  
Er-value:0.000, Pr-value:0.000  
No matches to TargetScan

-3442--(555)--3998-

GATAAG

GATAAG  
Depth:19 (ZEBRAFISH)  
Ei-value:0.000, Pi-value:0.000  
Er-value:0.000, Pr-value:0.000  
No matches to TargetScan

-4003--(2899)--6903-

TTTTCTTTT

TTTTCTTTT  
Depth:19 (ZEBRAFISH)  
Ei-value:0.000, Pi-value:0.000  
Er-value:0.000, Pr-value:0.000  
MATCHES To TargetScan▶ miR-186-5p:AAAGAAU

-6911--(30)--6942-

CAGGTTTTGCTTT

CAGGTTTTGCTTT  
Depth:19 (ZEBRAFISH)  
Ei-value:0.000, Pi-value:0.000  
Er-value:0.000, Pr-value:0.000  
MATCHES To TargetScan▶ miR-330-3p.2:AAAGCAC▶ miR-490-3p:AACCUGG


T

CAGGTTTTGCTTTT  
Depth:16 (NILETILAPIA)  
Ei-value:0.000, Pi-value:0.000  
Er-value:0.000, Pr-value:0.000  
MATCHES To TargetScan▶ miR-330-3p.2:AAAGCAC▶ miR-490-3p:AACCUGG

-6955--(22)--6978-

AAAAAGCAAAA

AAAAAGCAAAA  
Depth:19 (ZEBRAFISH)  
Ei-value:0.000, Pi-value:0.000  
Er-value:0.000, Pr-value:0.000  
No matches to TargetScan


G

AAAAAGCAAAAG  
Depth:18 (MEDAKA)  
Ei-value:0.000, Pi-value:0.000  
Er-value:0.000, Pr-value:0.000  
No matches to TargetScan

-6989--(15)--7005-

ACTCCTG

ACTCCTG  
Depth:18 (MEDAKA)  
Ei-value:0.000, Pi-value:0.000  
Er-value:0.000, Pr-value:0.000  
No matches to TargetScan


G

ACTCCTGG  
Depth:14 (SPOTTEDGAR)  
Ei-value:0.000, Pi-value:0.000  
Er-value:0.000, Pr-value:0.000  
MATCHES To TargetScan▶ miR-665:CCAGGAG

-7012  
  
>MOUSE  
      1717-

TGGTAA

TGGTAA  
Depth:14 (SPOTTEDGAR)  
Ei-value:0.030, Pi-value:0.000  
Er-value:0.010, Pr-value:0.000  
No matches to TargetScan

-1722--(601)--2324-

TTTGGG

TTTGGG  
Depth:19 (ZEBRAFISH)  
Ei-value:0.000, Pi-value:0.000  
Er-value:0.000, Pr-value:0.000  
No matches to TargetScan

-2329--(990)--3320-

TTTTTCAG

TTTTTCAG  
Depth:19 (ZEBRAFISH)  
Ei-value:0.000, Pi-value:0.000  
Er-value:0.000, Pr-value:0.000  
No matches to TargetScan

-3327--(518)--3846-

GATAAG

GATAAG  
Depth:19 (ZEBRAFISH)  
Ei-value:0.000, Pi-value:0.000  
Er-value:0.000, Pr-value:0.000  
No matches to TargetScan

-3851--(2754)--6606-

TTTTCTTTT

TTTTCTTTT  
Depth:19 (ZEBRAFISH)  
Ei-value:0.000, Pi-value:0.000  
Er-value:0.000, Pr-value:0.000  
MATCHES To TargetScan▶ miR-186-5p:AAAGAAU

-6614--(26)--6641-

CAGGTTTTGCTTT

CAGGTTTTGCTTT  
Depth:19 (ZEBRAFISH)  
Ei-value:0.000, Pi-value:0.000  
Er-value:0.000, Pr-value:0.000  
MATCHES To TargetScan▶ miR-330-3p.2:AAAGCAC▶ miR-490-3p:AACCUGG


T

CAGGTTTTGCTTTT  
Depth:16 (NILETILAPIA)  
Ei-value:0.000, Pi-value:0.000  
Er-value:0.000, Pr-value:0.000  
MATCHES To TargetScan▶ miR-330-3p.2:AAAGCAC▶ miR-490-3p:AACCUGG

-6654--(25)--6680-

AAAAAGCAAAA

AAAAAGCAAAA  
Depth:19 (ZEBRAFISH)  
Ei-value:0.000, Pi-value:0.000  
Er-value:0.000, Pr-value:0.000  
No matches to TargetScan


G

AAAAAGCAAAAG  
Depth:18 (MEDAKA)  
Ei-value:0.000, Pi-value:0.000  
Er-value:0.000, Pr-value:0.000  
No matches to TargetScan

-6691--(15)--6707-

ACTCCTG

ACTCCTG  
Depth:18 (MEDAKA)  
Ei-value:0.000, Pi-value:0.000  
Er-value:0.000, Pr-value:0.000  
No matches to TargetScan


G

ACTCCTGG  
Depth:14 (SPOTTEDGAR)  
Ei-value:0.000, Pi-value:0.000  
Er-value:0.000, Pr-value:0.000  
MATCHES To TargetScan▶ miR-665:CCAGGAG

-6714  
  
>TURTLE  
       361-

TGGTAA

TGGTAA  
Depth:14 (SPOTTEDGAR)  
Ei-value:0.030, Pi-value:0.000  
Er-value:0.010, Pr-value:0.000  
No matches to TargetScan

-366--(904)--1271-

TTTGGG

TTTGGG  
Depth:19 (ZEBRAFISH)  
Ei-value:0.000, Pi-value:0.000  
Er-value:0.000, Pr-value:0.000  
No matches to TargetScan

-1276--(1382)--2659-

TTTTTCAG

TTTTTCAG  
Depth:19 (ZEBRAFISH)  
Ei-value:0.000, Pi-value:0.000  
Er-value:0.000, Pr-value:0.000  
No matches to TargetScan

-2666--(648)--3315-

GATAAG

GATAAG  
Depth:19 (ZEBRAFISH)  
Ei-value:0.000, Pi-value:0.000  
Er-value:0.000, Pr-value:0.000  
No matches to TargetScan

-3320--(3412)--6733-

TTTTCTTTT

TTTTCTTTT  
Depth:19 (ZEBRAFISH)  
Ei-value:0.000, Pi-value:0.000  
Er-value:0.000, Pr-value:0.000  
MATCHES To TargetScan▶ miR-186-5p:AAAGAAU

-6741--(23)--6765-

CAGGTTTTGCTTT

CAGGTTTTGCTTT  
Depth:19 (ZEBRAFISH)  
Ei-value:0.000, Pi-value:0.000  
Er-value:0.000, Pr-value:0.000  
MATCHES To TargetScan▶ miR-330-3p.2:AAAGCAC▶ miR-490-3p:AACCUGG


T

CAGGTTTTGCTTTT  
Depth:16 (NILETILAPIA)  
Ei-value:0.000, Pi-value:0.000  
Er-value:0.000, Pr-value:0.000  
MATCHES To TargetScan▶ miR-330-3p.2:AAAGCAC▶ miR-490-3p:AACCUGG

-6778--(20)--6799-

AAAAAGCAAAA

AAAAAGCAAAA  
Depth:19 (ZEBRAFISH)  
Ei-value:0.000, Pi-value:0.000  
Er-value:0.000, Pr-value:0.000  
No matches to TargetScan


G

AAAAAGCAAAAG  
Depth:18 (MEDAKA)  
Ei-value:0.000, Pi-value:0.000  
Er-value:0.000, Pr-value:0.000  
No matches to TargetScan

-6810--(15)--6826-

ACTCCTG

ACTCCTG  
Depth:18 (MEDAKA)  
Ei-value:0.000, Pi-value:0.000  
Er-value:0.000, Pr-value:0.000  
No matches to TargetScan


G

ACTCCTGG  
Depth:14 (SPOTTEDGAR)  
Ei-value:0.000, Pi-value:0.000  
Er-value:0.000, Pr-value:0.000  
MATCHES To TargetScan▶ miR-665:CCAGGAG

-6833  
  
>ALLIGATOR  
      1688-

TGGTAA

TGGTAA  
Depth:14 (SPOTTEDGAR)  
Ei-value:0.030, Pi-value:0.000  
Er-value:0.010, Pr-value:0.000  
No matches to TargetScan

-1693--(843)--2537-

TTTGGG

TTTGGG  
Depth:19 (ZEBRAFISH)  
Ei-value:0.000, Pi-value:0.000  
Er-value:0.000, Pr-value:0.000  
No matches to TargetScan

-2542--(1322)--3865-

TTTTTCAG

TTTTTCAG  
Depth:19 (ZEBRAFISH)  
Ei-value:0.000, Pi-value:0.000  
Er-value:0.000, Pr-value:0.000  
No matches to TargetScan

-3872--(648)--4521-

GATAAG

GATAAG  
Depth:19 (ZEBRAFISH)  
Ei-value:0.000, Pi-value:0.000  
Er-value:0.000, Pr-value:0.000  
No matches to TargetScan

-4526--(3478)--8005-

TTTTCTTTT

TTTTCTTTT  
Depth:19 (ZEBRAFISH)  
Ei-value:0.000, Pi-value:0.000  
Er-value:0.000, Pr-value:0.000  
MATCHES To TargetScan▶ miR-186-5p:AAAGAAU

-8013--(23)--8037-

CAGGTTTTGCTTT

CAGGTTTTGCTTT  
Depth:19 (ZEBRAFISH)  
Ei-value:0.000, Pi-value:0.000  
Er-value:0.000, Pr-value:0.000  
MATCHES To TargetScan▶ miR-330-3p.2:AAAGCAC▶ miR-490-3p:AACCUGG


T

CAGGTTTTGCTTTT  
Depth:16 (NILETILAPIA)  
Ei-value:0.000, Pi-value:0.000  
Er-value:0.000, Pr-value:0.000  
MATCHES To TargetScan▶ miR-330-3p.2:AAAGCAC▶ miR-490-3p:AACCUGG

-8050--(25)--8076-

AAAAAGCAAAA

AAAAAGCAAAA  
Depth:19 (ZEBRAFISH)  
Ei-value:0.000, Pi-value:0.000  
Er-value:0.000, Pr-value:0.000  
No matches to TargetScan


G

AAAAAGCAAAAG  
Depth:18 (MEDAKA)  
Ei-value:0.000, Pi-value:0.000  
Er-value:0.000, Pr-value:0.000  
No matches to TargetScan

-8087--(15)--8103-

ACTCCTG

ACTCCTG  
Depth:18 (MEDAKA)  
Ei-value:0.000, Pi-value:0.000  
Er-value:0.000, Pr-value:0.000  
No matches to TargetScan


G

ACTCCTGG  
Depth:14 (SPOTTEDGAR)  
Ei-value:0.000, Pi-value:0.000  
Er-value:0.000, Pr-value:0.000  
MATCHES To TargetScan▶ miR-665:CCAGGAG

-8110  
  
>LIZARD  
      1469-

TGGTAA

TGGTAA  
Depth:14 (SPOTTEDGAR)  
Ei-value:0.030, Pi-value:0.000  
Er-value:0.010, Pr-value:0.000  
No matches to TargetScan

-1474--(878)--2353-

TTTGGG

TTTGGG  
Depth:19 (ZEBRAFISH)  
Ei-value:0.000, Pi-value:0.000  
Er-value:0.000, Pr-value:0.000  
No matches to TargetScan

-2358--(762)--3121-

TTTTTCAG

TTTTTCAG  
Depth:19 (ZEBRAFISH)  
Ei-value:0.000, Pi-value:0.000  
Er-value:0.000, Pr-value:0.000  
No matches to TargetScan

-3128--(588)--3717-

GATAAG

GATAAG  
Depth:19 (ZEBRAFISH)  
Ei-value:0.000, Pi-value:0.000  
Er-value:0.000, Pr-value:0.000  
No matches to TargetScan

-3722--(3176)--6899-

TTTTCTTTT

TTTTCTTTT  
Depth:19 (ZEBRAFISH)  
Ei-value:0.000, Pi-value:0.000  
Er-value:0.000, Pr-value:0.000  
MATCHES To TargetScan▶ miR-186-5p:AAAGAAU

-6907--(24)--6932-

CAGGTTTTGCTTT

CAGGTTTTGCTTT  
Depth:19 (ZEBRAFISH)  
Ei-value:0.000, Pi-value:0.000  
Er-value:0.000, Pr-value:0.000  
MATCHES To TargetScan▶ miR-330-3p.2:AAAGCAC▶ miR-490-3p:AACCUGG


T

CAGGTTTTGCTTTT  
Depth:16 (NILETILAPIA)  
Ei-value:0.000, Pi-value:0.000  
Er-value:0.000, Pr-value:0.000  
MATCHES To TargetScan▶ miR-330-3p.2:AAAGCAC▶ miR-490-3p:AACCUGG

-6945--(20)--6966-

AAAAAGCAAAA

AAAAAGCAAAA  
Depth:19 (ZEBRAFISH)  
Ei-value:0.000, Pi-value:0.000  
Er-value:0.000, Pr-value:0.000  
No matches to TargetScan


G

AAAAAGCAAAAG  
Depth:18 (MEDAKA)  
Ei-value:0.000, Pi-value:0.000  
Er-value:0.000, Pr-value:0.000  
No matches to TargetScan

-6977--(15)--6993-

ACTCCTG

ACTCCTG  
Depth:18 (MEDAKA)  
Ei-value:0.000, Pi-value:0.000  
Er-value:0.000, Pr-value:0.000  
No matches to TargetScan


G

ACTCCTGG  
Depth:14 (SPOTTEDGAR)  
Ei-value:0.000, Pi-value:0.000  
Er-value:0.000, Pr-value:0.000  
MATCHES To TargetScan▶ miR-665:CCAGGAG

-7000  
  
>SNAKE  
      1216-

TGGTAA

TGGTAA  
Depth:14 (SPOTTEDGAR)  
Ei-value:0.030, Pi-value:0.000  
Er-value:0.010, Pr-value:0.000  
No matches to TargetScan

-1221--(70)--1292-

TTTGGG

TTTGGG  
Depth:19 (ZEBRAFISH)  
Ei-value:0.000, Pi-value:0.000  
Er-value:0.000, Pr-value:0.000  
No matches to TargetScan

-1297--(729)--2027-

TTTGGG

TTTGGG  
Depth:19 (ZEBRAFISH)  
Ei-value:0.000, Pi-value:0.000  
Er-value:0.000, Pr-value:0.000  
No matches to TargetScan

-2032--(1081)--3114-

TTTTTCAG

TTTTTCAG  
Depth:19 (ZEBRAFISH)  
Ei-value:0.000, Pi-value:0.000  
Er-value:0.000, Pr-value:0.000  
No matches to TargetScan

-3121--(558)--3680-

GATAAG

GATAAG  
Depth:19 (ZEBRAFISH)  
Ei-value:0.000, Pi-value:0.000  
Er-value:0.000, Pr-value:0.000  
No matches to TargetScan

-3685--(3037)--6723-

TTTTCTTTT

TTTTCTTTT  
Depth:19 (ZEBRAFISH)  
Ei-value:0.000, Pi-value:0.000  
Er-value:0.000, Pr-value:0.000  
MATCHES To TargetScan▶ miR-186-5p:AAAGAAU

-6731--(25)--6757-

CAGGTTTTGCTTT

CAGGTTTTGCTTT  
Depth:19 (ZEBRAFISH)  
Ei-value:0.000, Pi-value:0.000  
Er-value:0.000, Pr-value:0.000  
MATCHES To TargetScan▶ miR-330-3p.2:AAAGCAC▶ miR-490-3p:AACCUGG


T

CAGGTTTTGCTTTT  
Depth:16 (NILETILAPIA)  
Ei-value:0.000, Pi-value:0.000  
Er-value:0.000, Pr-value:0.000  
MATCHES To TargetScan▶ miR-330-3p.2:AAAGCAC▶ miR-490-3p:AACCUGG

-6770--(28)--6799-

AAAAAGCAAAA

AAAAAGCAAAA  
Depth:19 (ZEBRAFISH)  
Ei-value:0.000, Pi-value:0.000  
Er-value:0.000, Pr-value:0.000  
No matches to TargetScan


G

AAAAAGCAAAAG  
Depth:18 (MEDAKA)  
Ei-value:0.000, Pi-value:0.000  
Er-value:0.000, Pr-value:0.000  
No matches to TargetScan

-6810--(15)--6826-

ACTCCTG

ACTCCTG  
Depth:18 (MEDAKA)  
Ei-value:0.000, Pi-value:0.000  
Er-value:0.000, Pr-value:0.000  
No matches to TargetScan


G

ACTCCTGG  
Depth:14 (SPOTTEDGAR)  
Ei-value:0.000, Pi-value:0.000  
Er-value:0.000, Pr-value:0.000  
MATCHES To TargetScan▶ miR-665:CCAGGAG

-6833  
  
>X.TROPICALIS  
      1598-

TGGTAA

TGGTAA  
Depth:14 (SPOTTEDGAR)  
Ei-value:0.030, Pi-value:0.000  
Er-value:0.010, Pr-value:0.000  
No matches to TargetScan

-1603--(248)--1852-

TGGTAA

TGGTAA  
Depth:14 (SPOTTEDGAR)  
Ei-value:0.030, Pi-value:0.000  
Er-value:0.010, Pr-value:0.000  
No matches to TargetScan

-1857--(441)--2299-

TTTGGG

TTTGGG  
Depth:19 (ZEBRAFISH)  
Ei-value:0.000, Pi-value:0.000  
Er-value:0.000, Pr-value:0.000  
No matches to TargetScan

-2304--(2695)--5000-

TTTGGG

TTTGGG  
Depth:19 (ZEBRAFISH)  
Ei-value:0.000, Pi-value:0.000  
Er-value:0.000, Pr-value:0.000  
No matches to TargetScan

-5005--(125)--5131-

TTTGGG

TTTGGG  
Depth:19 (ZEBRAFISH)  
Ei-value:0.000, Pi-value:0.000  
Er-value:0.000, Pr-value:0.000  
No matches to TargetScan

-5136--(3206)--8343-

TTTTTCAG

TTTTTCAG  
Depth:19 (ZEBRAFISH)  
Ei-value:0.000, Pi-value:0.000  
Er-value:0.000, Pr-value:0.000  
No matches to TargetScan

-8350--(1004)--9355-

GATAAG

GATAAG  
Depth:19 (ZEBRAFISH)  
Ei-value:0.000, Pi-value:0.000  
Er-value:0.000, Pr-value:0.000  
No matches to TargetScan

-9360--(2966)--12327-

TTTTCTTTT

TTTTCTTTT  
Depth:19 (ZEBRAFISH)  
Ei-value:0.000, Pi-value:0.000  
Er-value:0.000, Pr-value:0.000  
MATCHES To TargetScan▶ miR-186-5p:AAAGAAU

-12335--(26)--12362-

CAGGTTTTGCTTT

CAGGTTTTGCTTT  
Depth:19 (ZEBRAFISH)  
Ei-value:0.000, Pi-value:0.000  
Er-value:0.000, Pr-value:0.000  
MATCHES To TargetScan▶ miR-330-3p.2:AAAGCAC▶ miR-490-3p:AACCUGG


T

CAGGTTTTGCTTTT  
Depth:16 (NILETILAPIA)  
Ei-value:0.000, Pi-value:0.000  
Er-value:0.000, Pr-value:0.000  
MATCHES To TargetScan▶ miR-330-3p.2:AAAGCAC▶ miR-490-3p:AACCUGG

-12375--(30)--12406-

AAAAAGCAAAA

AAAAAGCAAAA  
Depth:19 (ZEBRAFISH)  
Ei-value:0.000, Pi-value:0.000  
Er-value:0.000, Pr-value:0.000  
No matches to TargetScan


G

AAAAAGCAAAAG  
Depth:18 (MEDAKA)  
Ei-value:0.000, Pi-value:0.000  
Er-value:0.000, Pr-value:0.000  
No matches to TargetScan

-12417--(15)--12433-

ACTCCTG

ACTCCTG  
Depth:18 (MEDAKA)  
Ei-value:0.000, Pi-value:0.000  
Er-value:0.000, Pr-value:0.000  
No matches to TargetScan


G

ACTCCTGG  
Depth:14 (SPOTTEDGAR)  
Ei-value:0.000, Pi-value:0.000  
Er-value:0.000, Pr-value:0.000  
MATCHES To TargetScan▶ miR-665:CCAGGAG

-12440  
  
>SHARK  
       389-

TGGTAA

TGGTAA  
Depth:14 (SPOTTEDGAR)  
Ei-value:0.030, Pi-value:0.000  
Er-value:0.010, Pr-value:0.000  
No matches to TargetScan

-394--(939)--1334-

TGGTAA

TGGTAA  
Depth:14 (SPOTTEDGAR)  
Ei-value:0.030, Pi-value:0.000  
Er-value:0.010, Pr-value:0.000  
No matches to TargetScan

-1339--(73)--1413-

TTTGGG

TTTGGG  
Depth:19 (ZEBRAFISH)  
Ei-value:0.000, Pi-value:0.000  
Er-value:0.000, Pr-value:0.000  
No matches to TargetScan

-1418--(1015)--2434-

TTTGGG

TTTGGG  
Depth:19 (ZEBRAFISH)  
Ei-value:0.000, Pi-value:0.000  
Er-value:0.000, Pr-value:0.000  
No matches to TargetScan

-2439--(74)--2514-

TTTGGG

TTTGGG  
Depth:19 (ZEBRAFISH)  
Ei-value:0.000, Pi-value:0.000  
Er-value:0.000, Pr-value:0.000  
No matches to TargetScan

-2519--(38)--2558-

TTTTTCAG

TTTTTCAG  
Depth:19 (ZEBRAFISH)  
Ei-value:0.000, Pi-value:0.000  
Er-value:0.000, Pr-value:0.000  
No matches to TargetScan

-2565--(4402)--6968-

GATAAG

GATAAG  
Depth:19 (ZEBRAFISH)  
Ei-value:0.000, Pi-value:0.000  
Er-value:0.000, Pr-value:0.000  
No matches to TargetScan

-6973--(520)--7494-

TTTTCTTTT

TTTTCTTTT  
Depth:19 (ZEBRAFISH)  
Ei-value:0.000, Pi-value:0.000  
Er-value:0.000, Pr-value:0.000  
MATCHES To TargetScan▶ miR-186-5p:AAAGAAU

-7502--(25)--7528-

CAGGTTTTGCTTT

CAGGTTTTGCTTT  
Depth:19 (ZEBRAFISH)  
Ei-value:0.000, Pi-value:0.000  
Er-value:0.000, Pr-value:0.000  
MATCHES To TargetScan▶ miR-330-3p.2:AAAGCAC▶ miR-490-3p:AACCUGG


T

CAGGTTTTGCTTTT  
Depth:16 (NILETILAPIA)  
Ei-value:0.000, Pi-value:0.000  
Er-value:0.000, Pr-value:0.000  
MATCHES To TargetScan▶ miR-330-3p.2:AAAGCAC▶ miR-490-3p:AACCUGG

-7541--(26)--7568-

AAAAAGCAAAA

AAAAAGCAAAA  
Depth:19 (ZEBRAFISH)  
Ei-value:0.000, Pi-value:0.000  
Er-value:0.000, Pr-value:0.000  
No matches to TargetScan


G

AAAAAGCAAAAG  
Depth:18 (MEDAKA)  
Ei-value:0.000, Pi-value:0.000  
Er-value:0.000, Pr-value:0.000  
No matches to TargetScan

-7579--(15)--7595-

ACTCCTG

ACTCCTG  
Depth:18 (MEDAKA)  
Ei-value:0.000, Pi-value:0.000  
Er-value:0.000, Pr-value:0.000  
No matches to TargetScan


G

ACTCCTGG  
Depth:14 (SPOTTEDGAR)  
Ei-value:0.000, Pi-value:0.000  
Er-value:0.000, Pr-value:0.000  
MATCHES To TargetScan▶ miR-665:CCAGGAG

-7602  
  
>OPOSSUM  
       880-

TGGTAA

TGGTAA  
Depth:14 (SPOTTEDGAR)  
Ei-value:0.030, Pi-value:0.000  
Er-value:0.010, Pr-value:0.000  
No matches to TargetScan

-885--(157)--1043-

TTTGGG

TTTGGG  
Depth:19 (ZEBRAFISH)  
Ei-value:0.000, Pi-value:0.000  
Er-value:0.000, Pr-value:0.000  
No matches to TargetScan

-1048--(273)--1322-

TTTGGG

TTTGGG  
Depth:19 (ZEBRAFISH)  
Ei-value:0.000, Pi-value:0.000  
Er-value:0.000, Pr-value:0.000  
No matches to TargetScan

-1327--(111)--1439-

TTTGGG

TTTGGG  
Depth:19 (ZEBRAFISH)  
Ei-value:0.000, Pi-value:0.000  
Er-value:0.000, Pr-value:0.000  
No matches to TargetScan

-1444--(1059)--2504-

TTTTTCAG

TTTTTCAG  
Depth:19 (ZEBRAFISH)  
Ei-value:0.000, Pi-value:0.000  
Er-value:0.000, Pr-value:0.000  
No matches to TargetScan

-2511--(973)--3485-

GATAAG

GATAAG  
Depth:19 (ZEBRAFISH)  
Ei-value:0.000, Pi-value:0.000  
Er-value:0.000, Pr-value:0.000  
No matches to TargetScan

-3490--(2190)--5681-

TTTTCTTTT

TTTTCTTTT  
Depth:19 (ZEBRAFISH)  
Ei-value:0.000, Pi-value:0.000  
Er-value:0.000, Pr-value:0.000  
MATCHES To TargetScan▶ miR-186-5p:AAAGAAU

-5689--(26)--5716-

CAGGTTTTGCTTT

CAGGTTTTGCTTT  
Depth:19 (ZEBRAFISH)  
Ei-value:0.000, Pi-value:0.000  
Er-value:0.000, Pr-value:0.000  
MATCHES To TargetScan▶ miR-330-3p.2:AAAGCAC▶ miR-490-3p:AACCUGG


T

CAGGTTTTGCTTTT  
Depth:16 (NILETILAPIA)  
Ei-value:0.000, Pi-value:0.000  
Er-value:0.000, Pr-value:0.000  
MATCHES To TargetScan▶ miR-330-3p.2:AAAGCAC▶ miR-490-3p:AACCUGG

-5729--(17)--5747-

AAAAAGCAAAA

AAAAAGCAAAA  
Depth:19 (ZEBRAFISH)  
Ei-value:0.000, Pi-value:0.000  
Er-value:0.000, Pr-value:0.000  
No matches to TargetScan


G

AAAAAGCAAAAG  
Depth:18 (MEDAKA)  
Ei-value:0.000, Pi-value:0.000  
Er-value:0.000, Pr-value:0.000  
No matches to TargetScan

-5758--(14)--5773-

ACTCCTG

ACTCCTG  
Depth:18 (MEDAKA)  
Ei-value:0.000, Pi-value:0.000  
Er-value:0.000, Pr-value:0.000  
No matches to TargetScan


G

ACTCCTGG  
Depth:14 (SPOTTEDGAR)  
Ei-value:0.000, Pi-value:0.000  
Er-value:0.000, Pr-value:0.000  
MATCHES To TargetScan▶ miR-665:CCAGGAG

-5780  
  
>SPOTTEDGAR  
       244-

TGGTAA

TGGTAA  
Depth:14 (SPOTTEDGAR)  
Ei-value:0.030, Pi-value:0.000  
Er-value:0.010, Pr-value:0.000  
No matches to TargetScan

-249--(3394)--3644-

TTTGGG

TTTGGG  
Depth:19 (ZEBRAFISH)  
Ei-value:0.000, Pi-value:0.000  
Er-value:0.000, Pr-value:0.000  
No matches to TargetScan

-3649--(31)--3681-

TTTTTCAG

TTTTTCAG  
Depth:19 (ZEBRAFISH)  
Ei-value:0.000, Pi-value:0.000  
Er-value:0.000, Pr-value:0.000  
No matches to TargetScan

-3688--(72)--3761-

GATAAG

GATAAG  
Depth:19 (ZEBRAFISH)  
Ei-value:0.000, Pi-value:0.000  
Er-value:0.000, Pr-value:0.000  
No matches to TargetScan

-3766--(274)--4041-

GATAAG

GATAAG  
Depth:19 (ZEBRAFISH)  
Ei-value:0.000, Pi-value:0.000  
Er-value:0.000, Pr-value:0.000  
No matches to TargetScan

-4046--(2564)--6611-

TTTTCTTTT

TTTTCTTTT  
Depth:19 (ZEBRAFISH)  
Ei-value:0.000, Pi-value:0.000  
Er-value:0.000, Pr-value:0.000  
MATCHES To TargetScan▶ miR-186-5p:AAAGAAU

-6619--(295)--6915-

CAGGTTTTGCTTT

CAGGTTTTGCTTT  
Depth:19 (ZEBRAFISH)  
Ei-value:0.000, Pi-value:0.000  
Er-value:0.000, Pr-value:0.000  
MATCHES To TargetScan▶ miR-330-3p.2:AAAGCAC▶ miR-490-3p:AACCUGG


T

CAGGTTTTGCTTTT  
Depth:16 (NILETILAPIA)  
Ei-value:0.000, Pi-value:0.000  
Er-value:0.000, Pr-value:0.000  
MATCHES To TargetScan▶ miR-330-3p.2:AAAGCAC▶ miR-490-3p:AACCUGG

-6928--(27)--6956-

AAAAAGCAAAA

AAAAAGCAAAA  
Depth:19 (ZEBRAFISH)  
Ei-value:0.000, Pi-value:0.000  
Er-value:0.000, Pr-value:0.000  
No matches to TargetScan


G

AAAAAGCAAAAG  
Depth:18 (MEDAKA)  
Ei-value:0.000, Pi-value:0.000  
Er-value:0.000, Pr-value:0.000  
No matches to TargetScan

-6967--(14)--6982-

ACTCCTG

ACTCCTG  
Depth:18 (MEDAKA)  
Ei-value:0.000, Pi-value:0.000  
Er-value:0.000, Pr-value:0.000  
No matches to TargetScan


G

ACTCCTGG  
Depth:14 (SPOTTEDGAR)  
Ei-value:0.000, Pi-value:0.000  
Er-value:0.000, Pr-value:0.000  
MATCHES To TargetScan▶ miR-665:CCAGGAG

-6989
```

---

# Modules conserved to OPOSSUM (Depth: 13)

## Modules in Main Graph (All sequences considered):

```
>HUMAN  
      1794-

TGGTAA

TGGTAA  
Depth:14 (SPOTTEDGAR)  
Ei-value:0.030, Pi-value:0.000  
Er-value:0.010, Pr-value:0.000  
eCLIP MATCHES▶bclaf1 (bg=17.67%)▶DROSHA (bg=1.03%)▶GRWD1 (bg=7.0%)▶hltf (bg=24.28%)▶MTPAP (bg=9.55%)▶NOLC1 (bg=0.67%)▶npm1 (bg=10.22%)▶ppil4 (bg=43.39%)▶rbm15 (bg=11.59%)▶safb (bg=40.39%)▶srsf1 (bg=30.28%)▶TAF15 (bg=9.06%)▶tia1 (bg=23.76%)▶uchl5 (bg=18.56%)▶YWHAG (bg=9.14%)▶ZRANB2 (bg=7.28%)No matches to TargetScan

-1799--(705)--2505-

TTTGGG

TTTGGG  
Depth:19 (ZEBRAFISH)  
Ei-value:0.000, Pi-value:0.000  
Er-value:0.000, Pr-value:0.000  
eCLIP MATCHES▶ppil4 (bg=43.39%)▶PRPF8 (bg=6.2%)No matches to TargetScan

-2510--(1110)--3621-

TTTTTCAG

TTTTTCAG  
Depth:19 (ZEBRAFISH)  
Ei-value:0.000, Pi-value:0.000  
Er-value:0.000, Pr-value:0.000  
eCLIP MATCHES▶ddx42 (bg=10.33%)▶ppil4 (bg=43.39%)▶safb (bg=40.39%)▶u2af1 (bg=14.02%)▶u2af2 (bg=19.32%)No matches to TargetScan

-3628--(82)--3711-

AAAGGCAGAA

AAAGGCAGAA  
Depth:13 (OPOSSUM)  
Ei-value:0.000, Pi-value:0.000  
Er-value:0.000, Pr-value:0.000  
eCLIP MATCHES▶hnrnpa1 (bg=18.32%)▶ppil4 (bg=43.39%)No matches to TargetScan

-3720--(451)--4172-

GATAAG

GATAAG  
Depth:19 (ZEBRAFISH)  
Ei-value:0.000, Pi-value:0.000  
Er-value:0.000, Pr-value:0.000  
eCLIP MATCHES▶cpsf6 (bg=13.45%)▶khsrp (bg=27.4%)▶NIPBL (bg=8.2%)▶ppil4 (bg=43.39%)▶PRPF8 (bg=6.2%)▶rbm15 (bg=11.59%)▶safb (bg=40.39%)▶safb2 (bg=26.89%)▶srsf1 (bg=30.28%)▶srsf7 (bg=22.53%)▶znf622 (bg=18.79%)No matches to TargetScan

-4177--(683)--4861-

TAAGGA

TAAGGA  
Depth:13 (OPOSSUM)  
Ei-value:0.000, Pi-value:0.000  
Er-value:0.000, Pr-value:0.000  
eCLIP MATCHES▶cstf2t (bg=12.11%)▶tia1 (bg=23.76%)▶tial1 (bg=15.02%)No matches to TargetScan

-4866--(2144)--7011-

TTTTCTTTT

TTTTCTTTT  
Depth:19 (ZEBRAFISH)  
Ei-value:0.000, Pi-value:0.000  
Er-value:0.000, Pr-value:0.000  
eCLIP MATCHES▶srsf7 (bg=22.53%)MATCHES To TargetScan▶ miR-186-5p:AAAGAAU

-7019--(28)--7048-

CAGGTTTTGCTTT

CAGGTTTTGCTTT  
Depth:19 (ZEBRAFISH)  
Ei-value:0.000, Pi-value:0.000  
Er-value:0.000, Pr-value:0.000  
eCLIP MATCHES▶srsf7 (bg=22.53%)MATCHES To TargetScan▶ miR-330-3p.2:AAAGCAC▶ miR-490-3p:AACCUGG


T

CAGGTTTTGCTTTT  
Depth:16 (NILETILAPIA)  
Ei-value:0.000, Pi-value:0.000  
Er-value:0.000, Pr-value:0.000  
eCLIP MATCHES▶srsf7 (bg=22.53%)MATCHES To TargetScan▶ miR-330-3p.2:AAAGCAC▶ miR-490-3p:AACCUGG

-7061--(24)--7086-

A

AAAAAAGCAAAAG  
Depth:13 (OPOSSUM)  
Ei-value:0.000, Pi-value:0.000  
Er-value:0.000, Pr-value:0.000  
No matches to eCLIP DataNo matches to TargetScan


AAAAAGCAAAA

AAAAAGCAAAA  
Depth:19 (ZEBRAFISH)  
Ei-value:0.000, Pi-value:0.000  
Er-value:0.000, Pr-value:0.000  
No matches to eCLIP DataNo matches to TargetScan


G

AAAAAGCAAAAG  
Depth:18 (MEDAKA)  
Ei-value:0.000, Pi-value:0.000  
Er-value:0.000, Pr-value:0.000  
No matches to eCLIP DataNo matches to TargetScan

-7098--(15)--7114-

ACTCCTG

ACTCCTG  
Depth:18 (MEDAKA)  
Ei-value:0.000, Pi-value:0.000  
Er-value:0.000, Pr-value:0.000  
No matches to eCLIP DataNo matches to TargetScan


G

ACTCCTGG  
Depth:14 (SPOTTEDGAR)  
Ei-value:0.000, Pi-value:0.000  
Er-value:0.000, Pr-value:0.000  
No matches to eCLIP DataMATCHES To TargetScan▶ miR-665:CCAGGAG

-7121--(11)--7133-

GGGTTC

GGGTTC  
Depth:13 (OPOSSUM)  
Ei-value:0.000, Pi-value:0.000  
Er-value:0.000, Pr-value:0.000  
No matches to eCLIP DataNo matches to TargetScan

-7138  
  
>MARMOSET  
      1908-

TGGTAA

TGGTAA  
Depth:14 (SPOTTEDGAR)  
Ei-value:0.030, Pi-value:0.000  
Er-value:0.010, Pr-value:0.000  
No matches to TargetScan

-1913--(723)--2637-

TTTGGG

TTTGGG  
Depth:19 (ZEBRAFISH)  
Ei-value:0.000, Pi-value:0.000  
Er-value:0.000, Pr-value:0.000  
No matches to TargetScan

-2642--(1095)--3738-

TTTTTCAG

TTTTTCAG  
Depth:19 (ZEBRAFISH)  
Ei-value:0.000, Pi-value:0.000  
Er-value:0.000, Pr-value:0.000  
No matches to TargetScan

-3745--(81)--3827-

AAAGGCAGAA

AAAGGCAGAA  
Depth:13 (OPOSSUM)  
Ei-value:0.000, Pi-value:0.000  
Er-value:0.000, Pr-value:0.000  
No matches to TargetScan

-3836--(457)--4294-

GATAAG

GATAAG  
Depth:19 (ZEBRAFISH)  
Ei-value:0.000, Pi-value:0.000  
Er-value:0.000, Pr-value:0.000  
No matches to TargetScan

-4299--(690)--4990-

TAAGGA

TAAGGA  
Depth:13 (OPOSSUM)  
Ei-value:0.000, Pi-value:0.000  
Er-value:0.000, Pr-value:0.000  
No matches to TargetScan

-4995--(2144)--7140-

TTTTCTTTT

TTTTCTTTT  
Depth:19 (ZEBRAFISH)  
Ei-value:0.000, Pi-value:0.000  
Er-value:0.000, Pr-value:0.000  
MATCHES To TargetScan▶ miR-186-5p:AAAGAAU

-7148--(28)--7177-

CAGGTTTTGCTTT

CAGGTTTTGCTTT  
Depth:19 (ZEBRAFISH)  
Ei-value:0.000, Pi-value:0.000  
Er-value:0.000, Pr-value:0.000  
MATCHES To TargetScan▶ miR-330-3p.2:AAAGCAC▶ miR-490-3p:AACCUGG


T

CAGGTTTTGCTTTT  
Depth:16 (NILETILAPIA)  
Ei-value:0.000, Pi-value:0.000  
Er-value:0.000, Pr-value:0.000  
MATCHES To TargetScan▶ miR-330-3p.2:AAAGCAC▶ miR-490-3p:AACCUGG

-7190--(25)--7216-

A

AAAAAAGCAAAAG  
Depth:13 (OPOSSUM)  
Ei-value:0.000, Pi-value:0.000  
Er-value:0.000, Pr-value:0.000  
No matches to TargetScan


AAAAAGCAAAA

AAAAAGCAAAA  
Depth:19 (ZEBRAFISH)  
Ei-value:0.000, Pi-value:0.000  
Er-value:0.000, Pr-value:0.000  
No matches to TargetScan


G

AAAAAGCAAAAG  
Depth:18 (MEDAKA)  
Ei-value:0.000, Pi-value:0.000  
Er-value:0.000, Pr-value:0.000  
No matches to TargetScan

-7228--(15)--7244-

ACTCCTG

ACTCCTG  
Depth:18 (MEDAKA)  
Ei-value:0.000, Pi-value:0.000  
Er-value:0.000, Pr-value:0.000  
No matches to TargetScan


G

ACTCCTGG  
Depth:14 (SPOTTEDGAR)  
Ei-value:0.000, Pi-value:0.000  
Er-value:0.000, Pr-value:0.000  
MATCHES To TargetScan▶ miR-665:CCAGGAG

-7251--(11)--7263-

GGGTTC

GGGTTC  
Depth:13 (OPOSSUM)  
Ei-value:0.000, Pi-value:0.000  
Er-value:0.000, Pr-value:0.000  
No matches to TargetScan

-7268  
  
>DOG  
      1928-

TGGTAA

TGGTAA  
Depth:14 (SPOTTEDGAR)  
Ei-value:0.030, Pi-value:0.000  
Er-value:0.010, Pr-value:0.000  
No matches to TargetScan

-1933--(678)--2612-

TTTGGG

TTTGGG  
Depth:19 (ZEBRAFISH)  
Ei-value:0.000, Pi-value:0.000  
Er-value:0.000, Pr-value:0.000  
No matches to TargetScan

-2617--(1013)--3631-

TTTTTCAG

TTTTTCAG  
Depth:19 (ZEBRAFISH)  
Ei-value:0.000, Pi-value:0.000  
Er-value:0.000, Pr-value:0.000  
No matches to TargetScan

-3638--(79)--3718-

AAAGGCAGAA

AAAGGCAGAA  
Depth:13 (OPOSSUM)  
Ei-value:0.000, Pi-value:0.000  
Er-value:0.000, Pr-value:0.000  
No matches to TargetScan

-3727--(470)--4198-

GATAAG

GATAAG  
Depth:19 (ZEBRAFISH)  
Ei-value:0.000, Pi-value:0.000  
Er-value:0.000, Pr-value:0.000  
No matches to TargetScan

-4203--(676)--4880-

TAAGGA

TAAGGA  
Depth:13 (OPOSSUM)  
Ei-value:0.000, Pi-value:0.000  
Er-value:0.000, Pr-value:0.000  
No matches to TargetScan

-4885--(2174)--7060-

TTTTCTTTT

TTTTCTTTT  
Depth:19 (ZEBRAFISH)  
Ei-value:0.000, Pi-value:0.000  
Er-value:0.000, Pr-value:0.000  
MATCHES To TargetScan▶ miR-186-5p:AAAGAAU

-7068--(26)--7095-

CAGGTTTTGCTTT

CAGGTTTTGCTTT  
Depth:19 (ZEBRAFISH)  
Ei-value:0.000, Pi-value:0.000  
Er-value:0.000, Pr-value:0.000  
MATCHES To TargetScan▶ miR-330-3p.2:AAAGCAC▶ miR-490-3p:AACCUGG


T

CAGGTTTTGCTTTT  
Depth:16 (NILETILAPIA)  
Ei-value:0.000, Pi-value:0.000  
Er-value:0.000, Pr-value:0.000  
MATCHES To TargetScan▶ miR-330-3p.2:AAAGCAC▶ miR-490-3p:AACCUGG

-7108--(20)--7129-

A

AAAAAAGCAAAAG  
Depth:13 (OPOSSUM)  
Ei-value:0.000, Pi-value:0.000  
Er-value:0.000, Pr-value:0.000  
No matches to TargetScan


AAAAAGCAAAA

AAAAAGCAAAA  
Depth:19 (ZEBRAFISH)  
Ei-value:0.000, Pi-value:0.000  
Er-value:0.000, Pr-value:0.000  
No matches to TargetScan


G

AAAAAGCAAAAG  
Depth:18 (MEDAKA)  
Ei-value:0.000, Pi-value:0.000  
Er-value:0.000, Pr-value:0.000  
No matches to TargetScan

-7141--(15)--7157-

ACTCCTG

ACTCCTG  
Depth:18 (MEDAKA)  
Ei-value:0.000, Pi-value:0.000  
Er-value:0.000, Pr-value:0.000  
No matches to TargetScan


G

ACTCCTGG  
Depth:14 (SPOTTEDGAR)  
Ei-value:0.000, Pi-value:0.000  
Er-value:0.000, Pr-value:0.000  
MATCHES To TargetScan▶ miR-665:CCAGGAG

-7164--(12)--7177-

GGGTTC

GGGTTC  
Depth:13 (OPOSSUM)  
Ei-value:0.000, Pi-value:0.000  
Er-value:0.000, Pr-value:0.000  
No matches to TargetScan

-7182  
  
>PIG  
      1849-

TGGTAA

TGGTAA  
Depth:14 (SPOTTEDGAR)  
Ei-value:0.030, Pi-value:0.000  
Er-value:0.010, Pr-value:0.000  
No matches to TargetScan

-1854--(678)--2533-

TTTGGG

TTTGGG  
Depth:19 (ZEBRAFISH)  
Ei-value:0.000, Pi-value:0.000  
Er-value:0.000, Pr-value:0.000  
No matches to TargetScan

-2538--(1002)--3541-

TTTTTCAG

TTTTTCAG  
Depth:19 (ZEBRAFISH)  
Ei-value:0.000, Pi-value:0.000  
Er-value:0.000, Pr-value:0.000  
No matches to TargetScan

-3548--(82)--3631-

AAAGGCAGAA

AAAGGCAGAA  
Depth:13 (OPOSSUM)  
Ei-value:0.000, Pi-value:0.000  
Er-value:0.000, Pr-value:0.000  
No matches to TargetScan

-3640--(477)--4118-

GATAAG

GATAAG  
Depth:19 (ZEBRAFISH)  
Ei-value:0.000, Pi-value:0.000  
Er-value:0.000, Pr-value:0.000  
No matches to TargetScan

-4123--(662)--4786-

TAAGGA

TAAGGA  
Depth:13 (OPOSSUM)  
Ei-value:0.000, Pi-value:0.000  
Er-value:0.000, Pr-value:0.000  
No matches to TargetScan

-4791--(2180)--6972-

TTTTCTTTT

TTTTCTTTT  
Depth:19 (ZEBRAFISH)  
Ei-value:0.000, Pi-value:0.000  
Er-value:0.000, Pr-value:0.000  
MATCHES To TargetScan▶ miR-186-5p:AAAGAAU

-6980--(28)--7009-

CAGGTTTTGCTTT

CAGGTTTTGCTTT  
Depth:19 (ZEBRAFISH)  
Ei-value:0.000, Pi-value:0.000  
Er-value:0.000, Pr-value:0.000  
MATCHES To TargetScan▶ miR-330-3p.2:AAAGCAC▶ miR-490-3p:AACCUGG


T

CAGGTTTTGCTTTT  
Depth:16 (NILETILAPIA)  
Ei-value:0.000, Pi-value:0.000  
Er-value:0.000, Pr-value:0.000  
MATCHES To TargetScan▶ miR-330-3p.2:AAAGCAC▶ miR-490-3p:AACCUGG

-7022--(22)--7045-

A

AAAAAAGCAAAAG  
Depth:13 (OPOSSUM)  
Ei-value:0.000, Pi-value:0.000  
Er-value:0.000, Pr-value:0.000  
No matches to TargetScan


AAAAAGCAAAA

AAAAAGCAAAA  
Depth:19 (ZEBRAFISH)  
Ei-value:0.000, Pi-value:0.000  
Er-value:0.000, Pr-value:0.000  
No matches to TargetScan


G

AAAAAGCAAAAG  
Depth:18 (MEDAKA)  
Ei-value:0.000, Pi-value:0.000  
Er-value:0.000, Pr-value:0.000  
No matches to TargetScan

-7057--(15)--7073-

ACTCCTG

ACTCCTG  
Depth:18 (MEDAKA)  
Ei-value:0.000, Pi-value:0.000  
Er-value:0.000, Pr-value:0.000  
No matches to TargetScan


G

ACTCCTGG  
Depth:14 (SPOTTEDGAR)  
Ei-value:0.000, Pi-value:0.000  
Er-value:0.000, Pr-value:0.000  
MATCHES To TargetScan▶ miR-665:CCAGGAG

-7080--(11)--7092-

GGGTTC

GGGTTC  
Depth:13 (OPOSSUM)  
Ei-value:0.000, Pi-value:0.000  
Er-value:0.000, Pr-value:0.000  
No matches to TargetScan

-7097  
  
>COW  
      1716-

TGGTAA

TGGTAA  
Depth:14 (SPOTTEDGAR)  
Ei-value:0.030, Pi-value:0.000  
Er-value:0.010, Pr-value:0.000  
No matches to TargetScan

-1721--(695)--2417-

TTTGGG

TTTGGG  
Depth:19 (ZEBRAFISH)  
Ei-value:0.000, Pi-value:0.000  
Er-value:0.000, Pr-value:0.000  
No matches to TargetScan

-2422--(1012)--3435-

TTTTTCAG

TTTTTCAG  
Depth:19 (ZEBRAFISH)  
Ei-value:0.000, Pi-value:0.000  
Er-value:0.000, Pr-value:0.000  
No matches to TargetScan

-3442--(82)--3525-

AAAGGCAGAA

AAAGGCAGAA  
Depth:13 (OPOSSUM)  
Ei-value:0.000, Pi-value:0.000  
Er-value:0.000, Pr-value:0.000  
No matches to TargetScan

-3534--(463)--3998-

GATAAG

GATAAG  
Depth:19 (ZEBRAFISH)  
Ei-value:0.000, Pi-value:0.000  
Er-value:0.000, Pr-value:0.000  
No matches to TargetScan

-4003--(653)--4657-

TAAGGA

TAAGGA  
Depth:13 (OPOSSUM)  
Ei-value:0.000, Pi-value:0.000  
Er-value:0.000, Pr-value:0.000  
No matches to TargetScan

-4662--(2240)--6903-

TTTTCTTTT

TTTTCTTTT  
Depth:19 (ZEBRAFISH)  
Ei-value:0.000, Pi-value:0.000  
Er-value:0.000, Pr-value:0.000  
MATCHES To TargetScan▶ miR-186-5p:AAAGAAU

-6911--(30)--6942-

CAGGTTTTGCTTT

CAGGTTTTGCTTT  
Depth:19 (ZEBRAFISH)  
Ei-value:0.000, Pi-value:0.000  
Er-value:0.000, Pr-value:0.000  
MATCHES To TargetScan▶ miR-330-3p.2:AAAGCAC▶ miR-490-3p:AACCUGG


T

CAGGTTTTGCTTTT  
Depth:16 (NILETILAPIA)  
Ei-value:0.000, Pi-value:0.000  
Er-value:0.000, Pr-value:0.000  
MATCHES To TargetScan▶ miR-330-3p.2:AAAGCAC▶ miR-490-3p:AACCUGG

-6955--(21)--6977-

A

AAAAAAGCAAAAG  
Depth:13 (OPOSSUM)  
Ei-value:0.000, Pi-value:0.000  
Er-value:0.000, Pr-value:0.000  
No matches to TargetScan


AAAAAGCAAAA

AAAAAGCAAAA  
Depth:19 (ZEBRAFISH)  
Ei-value:0.000, Pi-value:0.000  
Er-value:0.000, Pr-value:0.000  
No matches to TargetScan


G

AAAAAGCAAAAG  
Depth:18 (MEDAKA)  
Ei-value:0.000, Pi-value:0.000  
Er-value:0.000, Pr-value:0.000  
No matches to TargetScan

-6989--(15)--7005-

ACTCCTG

ACTCCTG  
Depth:18 (MEDAKA)  
Ei-value:0.000, Pi-value:0.000  
Er-value:0.000, Pr-value:0.000  
No matches to TargetScan


G

ACTCCTGG  
Depth:14 (SPOTTEDGAR)  
Ei-value:0.000, Pi-value:0.000  
Er-value:0.000, Pr-value:0.000  
MATCHES To TargetScan▶ miR-665:CCAGGAG

-7012--(11)--7024-

GGGTTC

GGGTTC  
Depth:13 (OPOSSUM)  
Ei-value:0.000, Pi-value:0.000  
Er-value:0.000, Pr-value:0.000  
No matches to TargetScan

-7029  
  
>MOUSE  
      1717-

TGGTAA

TGGTAA  
Depth:14 (SPOTTEDGAR)  
Ei-value:0.030, Pi-value:0.000  
Er-value:0.010, Pr-value:0.000  
No matches to TargetScan

-1722--(601)--2324-

TTTGGG

TTTGGG  
Depth:19 (ZEBRAFISH)  
Ei-value:0.000, Pi-value:0.000  
Er-value:0.000, Pr-value:0.000  
No matches to TargetScan

-2329--(990)--3320-

TTTTTCAG

TTTTTCAG  
Depth:19 (ZEBRAFISH)  
Ei-value:0.000, Pi-value:0.000  
Er-value:0.000, Pr-value:0.000  
No matches to TargetScan

-3327--(85)--3413-

AAAGGCAGAA

AAAGGCAGAA  
Depth:13 (OPOSSUM)  
Ei-value:0.000, Pi-value:0.000  
Er-value:0.000, Pr-value:0.000  
No matches to TargetScan

-3422--(423)--3846-

GATAAG

GATAAG  
Depth:19 (ZEBRAFISH)  
Ei-value:0.000, Pi-value:0.000  
Er-value:0.000, Pr-value:0.000  
No matches to TargetScan

-3851--(673)--4525-

TAAGGA

TAAGGA  
Depth:13 (OPOSSUM)  
Ei-value:0.000, Pi-value:0.000  
Er-value:0.000, Pr-value:0.000  
No matches to TargetScan

-4530--(2075)--6606-

TTTTCTTTT

TTTTCTTTT  
Depth:19 (ZEBRAFISH)  
Ei-value:0.000, Pi-value:0.000  
Er-value:0.000, Pr-value:0.000  
MATCHES To TargetScan▶ miR-186-5p:AAAGAAU

-6614--(26)--6641-

CAGGTTTTGCTTT

CAGGTTTTGCTTT  
Depth:19 (ZEBRAFISH)  
Ei-value:0.000, Pi-value:0.000  
Er-value:0.000, Pr-value:0.000  
MATCHES To TargetScan▶ miR-330-3p.2:AAAGCAC▶ miR-490-3p:AACCUGG


T

CAGGTTTTGCTTTT  
Depth:16 (NILETILAPIA)  
Ei-value:0.000, Pi-value:0.000  
Er-value:0.000, Pr-value:0.000  
MATCHES To TargetScan▶ miR-330-3p.2:AAAGCAC▶ miR-490-3p:AACCUGG

-6654--(24)--6679-

A

AAAAAAGCAAAAG  
Depth:13 (OPOSSUM)  
Ei-value:0.000, Pi-value:0.000  
Er-value:0.000, Pr-value:0.000  
No matches to TargetScan


AAAAAGCAAAA

AAAAAGCAAAA  
Depth:19 (ZEBRAFISH)  
Ei-value:0.000, Pi-value:0.000  
Er-value:0.000, Pr-value:0.000  
No matches to TargetScan


G

AAAAAGCAAAAG  
Depth:18 (MEDAKA)  
Ei-value:0.000, Pi-value:0.000  
Er-value:0.000, Pr-value:0.000  
No matches to TargetScan

-6691--(15)--6707-

ACTCCTG

ACTCCTG  
Depth:18 (MEDAKA)  
Ei-value:0.000, Pi-value:0.000  
Er-value:0.000, Pr-value:0.000  
No matches to TargetScan


G

ACTCCTGG  
Depth:14 (SPOTTEDGAR)  
Ei-value:0.000, Pi-value:0.000  
Er-value:0.000, Pr-value:0.000  
MATCHES To TargetScan▶ miR-665:CCAGGAG

-6714--(11)--6726-

GGGTTC

GGGTTC  
Depth:13 (OPOSSUM)  
Ei-value:0.000, Pi-value:0.000  
Er-value:0.000, Pr-value:0.000  
No matches to TargetScan

-6731  
  
>TURTLE  
       361-

TGGTAA

TGGTAA  
Depth:14 (SPOTTEDGAR)  
Ei-value:0.030, Pi-value:0.000  
Er-value:0.010, Pr-value:0.000  
No matches to TargetScan

-366--(904)--1271-

TTTGGG

TTTGGG  
Depth:19 (ZEBRAFISH)  
Ei-value:0.000, Pi-value:0.000  
Er-value:0.000, Pr-value:0.000  
No matches to TargetScan

-1276--(1382)--2659-

TTTTTCAG

TTTTTCAG  
Depth:19 (ZEBRAFISH)  
Ei-value:0.000, Pi-value:0.000  
Er-value:0.000, Pr-value:0.000  
No matches to TargetScan

-2666--(89)--2756-

AAAGGCAGAA

AAAGGCAGAA  
Depth:13 (OPOSSUM)  
Ei-value:0.000, Pi-value:0.000  
Er-value:0.000, Pr-value:0.000  
No matches to TargetScan

-2765--(549)--3315-

GATAAG

GATAAG  
Depth:19 (ZEBRAFISH)  
Ei-value:0.000, Pi-value:0.000  
Er-value:0.000, Pr-value:0.000  
No matches to TargetScan

-3320--(784)--4105-

TAAGGA

TAAGGA  
Depth:13 (OPOSSUM)  
Ei-value:0.000, Pi-value:0.000  
Er-value:0.000, Pr-value:0.000  
No matches to TargetScan

-4110--(2622)--6733-

TTTTCTTTT

TTTTCTTTT  
Depth:19 (ZEBRAFISH)  
Ei-value:0.000, Pi-value:0.000  
Er-value:0.000, Pr-value:0.000  
MATCHES To TargetScan▶ miR-186-5p:AAAGAAU

-6741--(23)--6765-

CAGGTTTTGCTTT

CAGGTTTTGCTTT  
Depth:19 (ZEBRAFISH)  
Ei-value:0.000, Pi-value:0.000  
Er-value:0.000, Pr-value:0.000  
MATCHES To TargetScan▶ miR-330-3p.2:AAAGCAC▶ miR-490-3p:AACCUGG


T

CAGGTTTTGCTTTT  
Depth:16 (NILETILAPIA)  
Ei-value:0.000, Pi-value:0.000  
Er-value:0.000, Pr-value:0.000  
MATCHES To TargetScan▶ miR-330-3p.2:AAAGCAC▶ miR-490-3p:AACCUGG

-6778--(19)--6798-

A

AAAAAAGCAAAAG  
Depth:13 (OPOSSUM)  
Ei-value:0.000, Pi-value:0.000  
Er-value:0.000, Pr-value:0.000  
No matches to TargetScan


AAAAAGCAAAA

AAAAAGCAAAA  
Depth:19 (ZEBRAFISH)  
Ei-value:0.000, Pi-value:0.000  
Er-value:0.000, Pr-value:0.000  
No matches to TargetScan


G

AAAAAGCAAAAG  
Depth:18 (MEDAKA)  
Ei-value:0.000, Pi-value:0.000  
Er-value:0.000, Pr-value:0.000  
No matches to TargetScan

-6810--(15)--6826-

ACTCCTG

ACTCCTG  
Depth:18 (MEDAKA)  
Ei-value:0.000, Pi-value:0.000  
Er-value:0.000, Pr-value:0.000  
No matches to TargetScan


G

ACTCCTGG  
Depth:14 (SPOTTEDGAR)  
Ei-value:0.000, Pi-value:0.000  
Er-value:0.000, Pr-value:0.000  
MATCHES To TargetScan▶ miR-665:CCAGGAG

-6833--(11)--6845-

GGGTTC

GGGTTC  
Depth:13 (OPOSSUM)  
Ei-value:0.000, Pi-value:0.000  
Er-value:0.000, Pr-value:0.000  
No matches to TargetScan

-6850  
  
>ALLIGATOR  
      1688-

TGGTAA

TGGTAA  
Depth:14 (SPOTTEDGAR)  
Ei-value:0.030, Pi-value:0.000  
Er-value:0.010, Pr-value:0.000  
No matches to TargetScan

-1693--(843)--2537-

TTTGGG

TTTGGG  
Depth:19 (ZEBRAFISH)  
Ei-value:0.000, Pi-value:0.000  
Er-value:0.000, Pr-value:0.000  
No matches to TargetScan

-2542--(1322)--3865-

TTTTTCAG

TTTTTCAG  
Depth:19 (ZEBRAFISH)  
Ei-value:0.000, Pi-value:0.000  
Er-value:0.000, Pr-value:0.000  
No matches to TargetScan

-3872--(92)--3965-

AAAGGCAGAA

AAAGGCAGAA  
Depth:13 (OPOSSUM)  
Ei-value:0.000, Pi-value:0.000  
Er-value:0.000, Pr-value:0.000  
No matches to TargetScan

-3974--(546)--4521-

GATAAG

GATAAG  
Depth:19 (ZEBRAFISH)  
Ei-value:0.000, Pi-value:0.000  
Er-value:0.000, Pr-value:0.000  
No matches to TargetScan

-4526--(805)--5332-

TAAGGA

TAAGGA  
Depth:13 (OPOSSUM)  
Ei-value:0.000, Pi-value:0.000  
Er-value:0.000, Pr-value:0.000  
No matches to TargetScan

-5337--(2667)--8005-

TTTTCTTTT

TTTTCTTTT  
Depth:19 (ZEBRAFISH)  
Ei-value:0.000, Pi-value:0.000  
Er-value:0.000, Pr-value:0.000  
MATCHES To TargetScan▶ miR-186-5p:AAAGAAU

-8013--(23)--8037-

CAGGTTTTGCTTT

CAGGTTTTGCTTT  
Depth:19 (ZEBRAFISH)  
Ei-value:0.000, Pi-value:0.000  
Er-value:0.000, Pr-value:0.000  
MATCHES To TargetScan▶ miR-330-3p.2:AAAGCAC▶ miR-490-3p:AACCUGG


T

CAGGTTTTGCTTTT  
Depth:16 (NILETILAPIA)  
Ei-value:0.000, Pi-value:0.000  
Er-value:0.000, Pr-value:0.000  
MATCHES To TargetScan▶ miR-330-3p.2:AAAGCAC▶ miR-490-3p:AACCUGG

-8050--(24)--8075-

A

AAAAAAGCAAAAG  
Depth:13 (OPOSSUM)  
Ei-value:0.000, Pi-value:0.000  
Er-value:0.000, Pr-value:0.000  
No matches to TargetScan


AAAAAGCAAAA

AAAAAGCAAAA  
Depth:19 (ZEBRAFISH)  
Ei-value:0.000, Pi-value:0.000  
Er-value:0.000, Pr-value:0.000  
No matches to TargetScan


G

AAAAAGCAAAAG  
Depth:18 (MEDAKA)  
Ei-value:0.000, Pi-value:0.000  
Er-value:0.000, Pr-value:0.000  
No matches to TargetScan

-8087--(15)--8103-

ACTCCTG

ACTCCTG  
Depth:18 (MEDAKA)  
Ei-value:0.000, Pi-value:0.000  
Er-value:0.000, Pr-value:0.000  
No matches to TargetScan


G

ACTCCTGG  
Depth:14 (SPOTTEDGAR)  
Ei-value:0.000, Pi-value:0.000  
Er-value:0.000, Pr-value:0.000  
MATCHES To TargetScan▶ miR-665:CCAGGAG

-8110--(11)--8122-

GGGTTC

GGGTTC  
Depth:13 (OPOSSUM)  
Ei-value:0.000, Pi-value:0.000  
Er-value:0.000, Pr-value:0.000  
No matches to TargetScan

-8127  
  
>LIZARD  
      1469-

TGGTAA

TGGTAA  
Depth:14 (SPOTTEDGAR)  
Ei-value:0.030, Pi-value:0.000  
Er-value:0.010, Pr-value:0.000  
No matches to TargetScan

-1474--(878)--2353-

TTTGGG

TTTGGG  
Depth:19 (ZEBRAFISH)  
Ei-value:0.000, Pi-value:0.000  
Er-value:0.000, Pr-value:0.000  
No matches to TargetScan

-2358--(762)--3121-

TTTTTCAG

TTTTTCAG  
Depth:19 (ZEBRAFISH)  
Ei-value:0.000, Pi-value:0.000  
Er-value:0.000, Pr-value:0.000  
No matches to TargetScan

-3128--(90)--3219-

AAAGGCAGAA

AAAGGCAGAA  
Depth:13 (OPOSSUM)  
Ei-value:0.000, Pi-value:0.000  
Er-value:0.000, Pr-value:0.000  
No matches to TargetScan

-3228--(488)--3717-

GATAAG

GATAAG  
Depth:19 (ZEBRAFISH)  
Ei-value:0.000, Pi-value:0.000  
Er-value:0.000, Pr-value:0.000  
No matches to TargetScan

-3722--(701)--4424-

TAAGGA

TAAGGA  
Depth:13 (OPOSSUM)  
Ei-value:0.000, Pi-value:0.000  
Er-value:0.000, Pr-value:0.000  
No matches to TargetScan

-4429--(2469)--6899-

TTTTCTTTT

TTTTCTTTT  
Depth:19 (ZEBRAFISH)  
Ei-value:0.000, Pi-value:0.000  
Er-value:0.000, Pr-value:0.000  
MATCHES To TargetScan▶ miR-186-5p:AAAGAAU

-6907--(24)--6932-

CAGGTTTTGCTTT

CAGGTTTTGCTTT  
Depth:19 (ZEBRAFISH)  
Ei-value:0.000, Pi-value:0.000  
Er-value:0.000, Pr-value:0.000  
MATCHES To TargetScan▶ miR-330-3p.2:AAAGCAC▶ miR-490-3p:AACCUGG


T

CAGGTTTTGCTTTT  
Depth:16 (NILETILAPIA)  
Ei-value:0.000, Pi-value:0.000  
Er-value:0.000, Pr-value:0.000  
MATCHES To TargetScan▶ miR-330-3p.2:AAAGCAC▶ miR-490-3p:AACCUGG

-6945--(19)--6965-

A

AAAAAAGCAAAAG  
Depth:13 (OPOSSUM)  
Ei-value:0.000, Pi-value:0.000  
Er-value:0.000, Pr-value:0.000  
No matches to TargetScan


AAAAAGCAAAA

AAAAAGCAAAA  
Depth:19 (ZEBRAFISH)  
Ei-value:0.000, Pi-value:0.000  
Er-value:0.000, Pr-value:0.000  
No matches to TargetScan


G

AAAAAGCAAAAG  
Depth:18 (MEDAKA)  
Ei-value:0.000, Pi-value:0.000  
Er-value:0.000, Pr-value:0.000  
No matches to TargetScan

-6977--(15)--6993-

ACTCCTG

ACTCCTG  
Depth:18 (MEDAKA)  
Ei-value:0.000, Pi-value:0.000  
Er-value:0.000, Pr-value:0.000  
No matches to TargetScan


G

ACTCCTGG  
Depth:14 (SPOTTEDGAR)  
Ei-value:0.000, Pi-value:0.000  
Er-value:0.000, Pr-value:0.000  
MATCHES To TargetScan▶ miR-665:CCAGGAG

-7000--(11)--7012-

GGGTTC

GGGTTC  
Depth:13 (OPOSSUM)  
Ei-value:0.000, Pi-value:0.000  
Er-value:0.000, Pr-value:0.000  
No matches to TargetScan

-7017  
  
>SNAKE  
      1216-

TGGTAA

TGGTAA  
Depth:14 (SPOTTEDGAR)  
Ei-value:0.030, Pi-value:0.000  
Er-value:0.010, Pr-value:0.000  
No matches to TargetScan

-1221--(70)--1292-

TTTGGG

TTTGGG  
Depth:19 (ZEBRAFISH)  
Ei-value:0.000, Pi-value:0.000  
Er-value:0.000, Pr-value:0.000  
No matches to TargetScan

-1297--(729)--2027-

TTTGGG

TTTGGG  
Depth:19 (ZEBRAFISH)  
Ei-value:0.000, Pi-value:0.000  
Er-value:0.000, Pr-value:0.000  
No matches to TargetScan

-2032--(1081)--3114-

TTTTTCAG

TTTTTCAG  
Depth:19 (ZEBRAFISH)  
Ei-value:0.000, Pi-value:0.000  
Er-value:0.000, Pr-value:0.000  
No matches to TargetScan

-3121--(80)--3202-

AAAGGCAGAA

AAAGGCAGAA  
Depth:13 (OPOSSUM)  
Ei-value:0.000, Pi-value:0.000  
Er-value:0.000, Pr-value:0.000  
No matches to TargetScan

-3211--(468)--3680-

GATAAG

GATAAG  
Depth:19 (ZEBRAFISH)  
Ei-value:0.000, Pi-value:0.000  
Er-value:0.000, Pr-value:0.000  
No matches to TargetScan

-3685--(879)--4565-

TAAGGA

TAAGGA  
Depth:13 (OPOSSUM)  
Ei-value:0.000, Pi-value:0.000  
Er-value:0.000, Pr-value:0.000  
No matches to TargetScan

-4570--(2152)--6723-

TTTTCTTTT

TTTTCTTTT  
Depth:19 (ZEBRAFISH)  
Ei-value:0.000, Pi-value:0.000  
Er-value:0.000, Pr-value:0.000  
MATCHES To TargetScan▶ miR-186-5p:AAAGAAU

-6731--(25)--6757-

CAGGTTTTGCTTT

CAGGTTTTGCTTT  
Depth:19 (ZEBRAFISH)  
Ei-value:0.000, Pi-value:0.000  
Er-value:0.000, Pr-value:0.000  
MATCHES To TargetScan▶ miR-330-3p.2:AAAGCAC▶ miR-490-3p:AACCUGG


T

CAGGTTTTGCTTTT  
Depth:16 (NILETILAPIA)  
Ei-value:0.000, Pi-value:0.000  
Er-value:0.000, Pr-value:0.000  
MATCHES To TargetScan▶ miR-330-3p.2:AAAGCAC▶ miR-490-3p:AACCUGG

-6770--(27)--6798-

A

AAAAAAGCAAAAG  
Depth:13 (OPOSSUM)  
Ei-value:0.000, Pi-value:0.000  
Er-value:0.000, Pr-value:0.000  
No matches to TargetScan


AAAAAGCAAAA

AAAAAGCAAAA  
Depth:19 (ZEBRAFISH)  
Ei-value:0.000, Pi-value:0.000  
Er-value:0.000, Pr-value:0.000  
No matches to TargetScan


G

AAAAAGCAAAAG  
Depth:18 (MEDAKA)  
Ei-value:0.000, Pi-value:0.000  
Er-value:0.000, Pr-value:0.000  
No matches to TargetScan

-6810--(15)--6826-

ACTCCTG

ACTCCTG  
Depth:18 (MEDAKA)  
Ei-value:0.000, Pi-value:0.000  
Er-value:0.000, Pr-value:0.000  
No matches to TargetScan


G

ACTCCTGG  
Depth:14 (SPOTTEDGAR)  
Ei-value:0.000, Pi-value:0.000  
Er-value:0.000, Pr-value:0.000  
MATCHES To TargetScan▶ miR-665:CCAGGAG

-6833--(11)--6845-

GGGTTC

GGGTTC  
Depth:13 (OPOSSUM)  
Ei-value:0.000, Pi-value:0.000  
Er-value:0.000, Pr-value:0.000  
No matches to TargetScan

-6850  
  
>X.TROPICALIS  
      1598-

TGGTAA

TGGTAA  
Depth:14 (SPOTTEDGAR)  
Ei-value:0.030, Pi-value:0.000  
Er-value:0.010, Pr-value:0.000  
No matches to TargetScan

-1603--(248)--1852-

TGGTAA

TGGTAA  
Depth:14 (SPOTTEDGAR)  
Ei-value:0.030, Pi-value:0.000  
Er-value:0.010, Pr-value:0.000  
No matches to TargetScan

-1857--(441)--2299-

TTTGGG

TTTGGG  
Depth:19 (ZEBRAFISH)  
Ei-value:0.000, Pi-value:0.000  
Er-value:0.000, Pr-value:0.000  
No matches to TargetScan

-2304--(2695)--5000-

TTTGGG

TTTGGG  
Depth:19 (ZEBRAFISH)  
Ei-value:0.000, Pi-value:0.000  
Er-value:0.000, Pr-value:0.000  
No matches to TargetScan

-5005--(125)--5131-

TTTGGG

TTTGGG  
Depth:19 (ZEBRAFISH)  
Ei-value:0.000, Pi-value:0.000  
Er-value:0.000, Pr-value:0.000  
No matches to TargetScan

-5136--(3206)--8343-

TTTTTCAG

TTTTTCAG  
Depth:19 (ZEBRAFISH)  
Ei-value:0.000, Pi-value:0.000  
Er-value:0.000, Pr-value:0.000  
No matches to TargetScan

-8350--(704)--9055-

AAAGGCAGAA

AAAGGCAGAA  
Depth:13 (OPOSSUM)  
Ei-value:0.000, Pi-value:0.000  
Er-value:0.000, Pr-value:0.000  
No matches to TargetScan

-9064--(290)--9355-

GATAAG

GATAAG  
Depth:19 (ZEBRAFISH)  
Ei-value:0.000, Pi-value:0.000  
Er-value:0.000, Pr-value:0.000  
No matches to TargetScan

-9360--(2148)--11509-

TAAGGA

TAAGGA  
Depth:13 (OPOSSUM)  
Ei-value:0.000, Pi-value:0.000  
Er-value:0.000, Pr-value:0.000  
No matches to TargetScan

-11514--(812)--12327-

TTTTCTTTT

TTTTCTTTT  
Depth:19 (ZEBRAFISH)  
Ei-value:0.000, Pi-value:0.000  
Er-value:0.000, Pr-value:0.000  
MATCHES To TargetScan▶ miR-186-5p:AAAGAAU

-12335--(26)--12362-

CAGGTTTTGCTTT

CAGGTTTTGCTTT  
Depth:19 (ZEBRAFISH)  
Ei-value:0.000, Pi-value:0.000  
Er-value:0.000, Pr-value:0.000  
MATCHES To TargetScan▶ miR-330-3p.2:AAAGCAC▶ miR-490-3p:AACCUGG


T

CAGGTTTTGCTTTT  
Depth:16 (NILETILAPIA)  
Ei-value:0.000, Pi-value:0.000  
Er-value:0.000, Pr-value:0.000  
MATCHES To TargetScan▶ miR-330-3p.2:AAAGCAC▶ miR-490-3p:AACCUGG

-12375--(29)--12405-

A

AAAAAAGCAAAAG  
Depth:13 (OPOSSUM)  
Ei-value:0.000, Pi-value:0.000  
Er-value:0.000, Pr-value:0.000  
No matches to TargetScan


AAAAAGCAAAA

AAAAAGCAAAA  
Depth:19 (ZEBRAFISH)  
Ei-value:0.000, Pi-value:0.000  
Er-value:0.000, Pr-value:0.000  
No matches to TargetScan


G

AAAAAGCAAAAG  
Depth:18 (MEDAKA)  
Ei-value:0.000, Pi-value:0.000  
Er-value:0.000, Pr-value:0.000  
No matches to TargetScan

-12417--(15)--12433-

ACTCCTG

ACTCCTG  
Depth:18 (MEDAKA)  
Ei-value:0.000, Pi-value:0.000  
Er-value:0.000, Pr-value:0.000  
No matches to TargetScan


G

ACTCCTGG  
Depth:14 (SPOTTEDGAR)  
Ei-value:0.000, Pi-value:0.000  
Er-value:0.000, Pr-value:0.000  
MATCHES To TargetScan▶ miR-665:CCAGGAG

-12440--(11)--12452-

GGGTTC

GGGTTC  
Depth:13 (OPOSSUM)  
Ei-value:0.000, Pi-value:0.000  
Er-value:0.000, Pr-value:0.000  
No matches to TargetScan

-12457  
  
>SHARK  
       389-

TGGTAA

TGGTAA  
Depth:14 (SPOTTEDGAR)  
Ei-value:0.030, Pi-value:0.000  
Er-value:0.010, Pr-value:0.000  
No matches to TargetScan

-394--(939)--1334-

TGGTAA

TGGTAA  
Depth:14 (SPOTTEDGAR)  
Ei-value:0.030, Pi-value:0.000  
Er-value:0.010, Pr-value:0.000  
No matches to TargetScan

-1339--(73)--1413-

TTTGGG

TTTGGG  
Depth:19 (ZEBRAFISH)  
Ei-value:0.000, Pi-value:0.000  
Er-value:0.000, Pr-value:0.000  
No matches to TargetScan

-1418--(1015)--2434-

TTTGGG

TTTGGG  
Depth:19 (ZEBRAFISH)  
Ei-value:0.000, Pi-value:0.000  
Er-value:0.000, Pr-value:0.000  
No matches to TargetScan

-2439--(74)--2514-

TTTGGG

TTTGGG  
Depth:19 (ZEBRAFISH)  
Ei-value:0.000, Pi-value:0.000  
Er-value:0.000, Pr-value:0.000  
No matches to TargetScan

-2519--(38)--2558-

TTTTTCAG

TTTTTCAG  
Depth:19 (ZEBRAFISH)  
Ei-value:0.000, Pi-value:0.000  
Er-value:0.000, Pr-value:0.000  
No matches to TargetScan

-2565--(2051)--4617-

AAAGGCAGAA

AAAGGCAGAA  
Depth:13 (OPOSSUM)  
Ei-value:0.000, Pi-value:0.000  
Er-value:0.000, Pr-value:0.000  
No matches to TargetScan

-4626--(2341)--6968-

GATAAG

GATAAG  
Depth:19 (ZEBRAFISH)  
Ei-value:0.000, Pi-value:0.000  
Er-value:0.000, Pr-value:0.000  
No matches to TargetScan

-6973--(183)--7157-

TAAGGA

TAAGGA  
Depth:13 (OPOSSUM)  
Ei-value:0.000, Pi-value:0.000  
Er-value:0.000, Pr-value:0.000  
No matches to TargetScan

-7162--(331)--7494-

TTTTCTTTT

TTTTCTTTT  
Depth:19 (ZEBRAFISH)  
Ei-value:0.000, Pi-value:0.000  
Er-value:0.000, Pr-value:0.000  
MATCHES To TargetScan▶ miR-186-5p:AAAGAAU

-7502--(25)--7528-

CAGGTTTTGCTTT

CAGGTTTTGCTTT  
Depth:19 (ZEBRAFISH)  
Ei-value:0.000, Pi-value:0.000  
Er-value:0.000, Pr-value:0.000  
MATCHES To TargetScan▶ miR-330-3p.2:AAAGCAC▶ miR-490-3p:AACCUGG


T

CAGGTTTTGCTTTT  
Depth:16 (NILETILAPIA)  
Ei-value:0.000, Pi-value:0.000  
Er-value:0.000, Pr-value:0.000  
MATCHES To TargetScan▶ miR-330-3p.2:AAAGCAC▶ miR-490-3p:AACCUGG

-7541--(25)--7567-

A

AAAAAAGCAAAAG  
Depth:13 (OPOSSUM)  
Ei-value:0.000, Pi-value:0.000  
Er-value:0.000, Pr-value:0.000  
No matches to TargetScan


AAAAAGCAAAA

AAAAAGCAAAA  
Depth:19 (ZEBRAFISH)  
Ei-value:0.000, Pi-value:0.000  
Er-value:0.000, Pr-value:0.000  
No matches to TargetScan


G

AAAAAGCAAAAG  
Depth:18 (MEDAKA)  
Ei-value:0.000, Pi-value:0.000  
Er-value:0.000, Pr-value:0.000  
No matches to TargetScan

-7579--(15)--7595-

ACTCCTG

ACTCCTG  
Depth:18 (MEDAKA)  
Ei-value:0.000, Pi-value:0.000  
Er-value:0.000, Pr-value:0.000  
No matches to TargetScan


G

ACTCCTGG  
Depth:14 (SPOTTEDGAR)  
Ei-value:0.000, Pi-value:0.000  
Er-value:0.000, Pr-value:0.000  
MATCHES To TargetScan▶ miR-665:CCAGGAG

-7602--(11)--7614-

GGGTTC

GGGTTC  
Depth:13 (OPOSSUM)  
Ei-value:0.000, Pi-value:0.000  
Er-value:0.000, Pr-value:0.000  
No matches to TargetScan

-7619  
  
>OPOSSUM  
       880-

TGGTAA

TGGTAA  
Depth:14 (SPOTTEDGAR)  
Ei-value:0.030, Pi-value:0.000  
Er-value:0.010, Pr-value:0.000  
No matches to TargetScan

-885--(157)--1043-

TTTGGG

TTTGGG  
Depth:19 (ZEBRAFISH)  
Ei-value:0.000, Pi-value:0.000  
Er-value:0.000, Pr-value:0.000  
No matches to TargetScan

-1048--(273)--1322-

TTTGGG

TTTGGG  
Depth:19 (ZEBRAFISH)  
Ei-value:0.000, Pi-value:0.000  
Er-value:0.000, Pr-value:0.000  
No matches to TargetScan

-1327--(111)--1439-

TTTGGG

TTTGGG  
Depth:19 (ZEBRAFISH)  
Ei-value:0.000, Pi-value:0.000  
Er-value:0.000, Pr-value:0.000  
No matches to TargetScan

-1444--(1059)--2504-

TTTTTCAG

TTTTTCAG  
Depth:19 (ZEBRAFISH)  
Ei-value:0.000, Pi-value:0.000  
Er-value:0.000, Pr-value:0.000  
No matches to TargetScan

-2511--(470)--2982-

AAAGGCAGAA

AAAGGCAGAA  
Depth:13 (OPOSSUM)  
Ei-value:0.000, Pi-value:0.000  
Er-value:0.000, Pr-value:0.000  
No matches to TargetScan

-2991--(493)--3485-

GATAAG

GATAAG  
Depth:19 (ZEBRAFISH)  
Ei-value:0.000, Pi-value:0.000  
Er-value:0.000, Pr-value:0.000  
No matches to TargetScan

-3490--(362)--3853-

TAAGGA

TAAGGA  
Depth:13 (OPOSSUM)  
Ei-value:0.000, Pi-value:0.000  
Er-value:0.000, Pr-value:0.000  
No matches to TargetScan

-3858--(1822)--5681-

TTTTCTTTT

TTTTCTTTT  
Depth:19 (ZEBRAFISH)  
Ei-value:0.000, Pi-value:0.000  
Er-value:0.000, Pr-value:0.000  
MATCHES To TargetScan▶ miR-186-5p:AAAGAAU

-5689--(26)--5716-

CAGGTTTTGCTTT

CAGGTTTTGCTTT  
Depth:19 (ZEBRAFISH)  
Ei-value:0.000, Pi-value:0.000  
Er-value:0.000, Pr-value:0.000  
MATCHES To TargetScan▶ miR-330-3p.2:AAAGCAC▶ miR-490-3p:AACCUGG


T

CAGGTTTTGCTTTT  
Depth:16 (NILETILAPIA)  
Ei-value:0.000, Pi-value:0.000  
Er-value:0.000, Pr-value:0.000  
MATCHES To TargetScan▶ miR-330-3p.2:AAAGCAC▶ miR-490-3p:AACCUGG

-5729--(16)--5746-

A

AAAAAAGCAAAAG  
Depth:13 (OPOSSUM)  
Ei-value:0.000, Pi-value:0.000  
Er-value:0.000, Pr-value:0.000  
No matches to TargetScan


AAAAAGCAAAA

AAAAAGCAAAA  
Depth:19 (ZEBRAFISH)  
Ei-value:0.000, Pi-value:0.000  
Er-value:0.000, Pr-value:0.000  
No matches to TargetScan


G

AAAAAGCAAAAG  
Depth:18 (MEDAKA)  
Ei-value:0.000, Pi-value:0.000  
Er-value:0.000, Pr-value:0.000  
No matches to TargetScan

-5758--(14)--5773-

ACTCCTG

ACTCCTG  
Depth:18 (MEDAKA)  
Ei-value:0.000, Pi-value:0.000  
Er-value:0.000, Pr-value:0.000  
No matches to TargetScan


G

ACTCCTGG  
Depth:14 (SPOTTEDGAR)  
Ei-value:0.000, Pi-value:0.000  
Er-value:0.000, Pr-value:0.000  
MATCHES To TargetScan▶ miR-665:CCAGGAG

-5780--(10)--5791-

GGGTTC

GGGTTC  
Depth:13 (OPOSSUM)  
Ei-value:0.000, Pi-value:0.000  
Er-value:0.000, Pr-value:0.000  
No matches to TargetScan

-5796
```

---

# Modules conserved to X.TROPICALIS (Depth: 11)

## Modules in Main Graph (All sequences considered):

```
>HUMAN  
      1577-

GGTGAAG

GGTGAAG  
Depth:11 (X.TROPICALIS)  
Ei-value:0.000, Pi-value:0.000  
Er-value:0.000, Pr-value:0.000  
eCLIP MATCHES▶bclaf1 (bg=17.67%)▶EXOSC5 (bg=2.0%)▶fxr2 (bg=10.1%)▶gtf2f1 (bg=10.18%)▶hltf (bg=24.28%)▶MTPAP (bg=9.55%)▶npm1 (bg=10.22%)▶ppil4 (bg=43.39%)▶rbm15 (bg=11.59%)▶rbm22 (bg=12.69%)▶safb (bg=40.39%)▶safb2 (bg=26.89%)▶SMNDC1 (bg=7.08%)▶srsf1 (bg=30.28%)▶SRSF9 (bg=9.67%)▶TAF15 (bg=9.06%)▶tra2a (bg=37.02%)▶TROVE2 (bg=6.96%)▶uchl5 (bg=18.56%)▶znf622 (bg=18.79%)No matches to TargetScan

-1583--(210)--1794-

TGGTAA

TGGTAA  
Depth:14 (SPOTTEDGAR)  
Ei-value:0.030, Pi-value:0.000  
Er-value:0.010, Pr-value:0.000  
eCLIP MATCHES▶bclaf1 (bg=17.67%)▶DROSHA (bg=1.03%)▶GRWD1 (bg=7.0%)▶hltf (bg=24.28%)▶MTPAP (bg=9.55%)▶NOLC1 (bg=0.67%)▶npm1 (bg=10.22%)▶ppil4 (bg=43.39%)▶rbm15 (bg=11.59%)▶safb (bg=40.39%)▶srsf1 (bg=30.28%)▶TAF15 (bg=9.06%)▶tia1 (bg=23.76%)▶uchl5 (bg=18.56%)▶YWHAG (bg=9.14%)▶ZRANB2 (bg=7.28%)No matches to TargetScan

-1799--(705)--2505-

TTTGGG

TTTGGG  
Depth:19 (ZEBRAFISH)  
Ei-value:0.000, Pi-value:0.000  
Er-value:0.000, Pr-value:0.000  
eCLIP MATCHES▶ppil4 (bg=43.39%)▶PRPF8 (bg=6.2%)No matches to TargetScan

-2510--(864)--3375-

GTAAGA

GTAAGA  
Depth:11 (X.TROPICALIS)  
Ei-value:0.000, Pi-value:0.000  
Er-value:0.000, Pr-value:0.000  
eCLIP MATCHES▶HNRNPM (bg=6.37%)No matches to TargetScan

-3380--(240)--3621-

TTTTTCAG

TTTTTCAG  
Depth:19 (ZEBRAFISH)  
Ei-value:0.000, Pi-value:0.000  
Er-value:0.000, Pr-value:0.000  
eCLIP MATCHES▶ddx42 (bg=10.33%)▶ppil4 (bg=43.39%)▶safb (bg=40.39%)▶u2af1 (bg=14.02%)▶u2af2 (bg=19.32%)No matches to TargetScan

-3628--(82)--3711-

AAAGGCAGAA

AAAGGCAGAA  
Depth:13 (OPOSSUM)  
Ei-value:0.000, Pi-value:0.000  
Er-value:0.000, Pr-value:0.000  
eCLIP MATCHES▶hnrnpa1 (bg=18.32%)▶ppil4 (bg=43.39%)No matches to TargetScan


A

AAAGGCAGAAA  
Depth:11 (X.TROPICALIS)  
Ei-value:0.000, Pi-value:0.000  
Er-value:0.000, Pr-value:0.000  
eCLIP MATCHES▶hnrnpa1 (bg=18.32%)▶ppil4 (bg=43.39%)No matches to TargetScan

-3721--(448)--4170-

CA

CAGATAAGT  
Depth:11 (X.TROPICALIS)  
Ei-value:0.000, Pi-value:0.000  
Er-value:0.000, Pr-value:0.000  
eCLIP MATCHES▶cpsf6 (bg=13.45%)▶hltf (bg=24.28%)▶hnrnpa1 (bg=18.32%)▶khsrp (bg=27.4%)▶NIPBL (bg=8.2%)▶ppil4 (bg=43.39%)▶PRPF8 (bg=6.2%)▶rbm15 (bg=11.59%)▶safb (bg=40.39%)▶safb2 (bg=26.89%)▶srsf1 (bg=30.28%)▶srsf7 (bg=22.53%)▶znf622 (bg=18.79%)No matches to TargetScan


GATAAG

GATAAG  
Depth:19 (ZEBRAFISH)  
Ei-value:0.000, Pi-value:0.000  
Er-value:0.000, Pr-value:0.000  
eCLIP MATCHES▶cpsf6 (bg=13.45%)▶khsrp (bg=27.4%)▶NIPBL (bg=8.2%)▶ppil4 (bg=43.39%)▶PRPF8 (bg=6.2%)▶rbm15 (bg=11.59%)▶safb (bg=40.39%)▶safb2 (bg=26.89%)▶srsf1 (bg=30.28%)▶srsf7 (bg=22.53%)▶znf622 (bg=18.79%)No matches to TargetScan


T

CAGATAAGT  
Depth:11 (X.TROPICALIS)  
Ei-value:0.000, Pi-value:0.000  
Er-value:0.000, Pr-value:0.000  
eCLIP MATCHES▶cpsf6 (bg=13.45%)▶hltf (bg=24.28%)▶hnrnpa1 (bg=18.32%)▶khsrp (bg=27.4%)▶NIPBL (bg=8.2%)▶ppil4 (bg=43.39%)▶PRPF8 (bg=6.2%)▶rbm15 (bg=11.59%)▶safb (bg=40.39%)▶safb2 (bg=26.89%)▶srsf1 (bg=30.28%)▶srsf7 (bg=22.53%)▶znf622 (bg=18.79%)No matches to TargetScan

-4178--(18)--4197-

ATTGCAT

ATTGCAT  
Depth:11 (X.TROPICALIS)  
Ei-value:0.000, Pi-value:0.000  
Er-value:0.000, Pr-value:0.000  
eCLIP MATCHES▶cpsf6 (bg=13.45%)▶hltf (bg=24.28%)▶hnrnpa1 (bg=18.32%)▶HNRNPU (bg=9.45%)▶khsrp (bg=27.4%)▶ppil4 (bg=43.39%)▶PRPF8 (bg=6.2%)▶RBFOX2 (bg=3.41%)▶safb (bg=40.39%)▶safb2 (bg=26.89%)▶tia1 (bg=23.76%)▶tial1 (bg=15.02%)No matches to TargetScan

-4203--(657)--4861-

TAAGGA

TAAGGA  
Depth:13 (OPOSSUM)  
Ei-value:0.000, Pi-value:0.000  
Er-value:0.000, Pr-value:0.000  
eCLIP MATCHES▶cstf2t (bg=12.11%)▶tia1 (bg=23.76%)▶tial1 (bg=15.02%)No matches to TargetScan

-4866--(706)--5573-

TTAGGT

TTAGGT  
Depth:11 (X.TROPICALIS)  
Ei-value:0.000, Pi-value:0.000  
Er-value:0.000, Pr-value:0.000  
eCLIP MATCHES▶cstf2t (bg=12.11%)▶HNRNPM (bg=6.37%)▶khsrp (bg=27.4%)▶srsf7 (bg=22.53%)▶TARDBP (bg=5.12%)▶tia1 (bg=23.76%)▶tial1 (bg=15.02%)▶u2af1 (bg=14.02%)▶ZRANB2 (bg=7.28%)No matches to TargetScan

-5578--(39)--5618-

GAAGCC

GAAGCC  
Depth:11 (X.TROPICALIS)  
Ei-value:0.000, Pi-value:0.000  
Er-value:0.000, Pr-value:0.000  
eCLIP MATCHES▶ddx42 (bg=10.33%)▶khsrp (bg=27.4%)▶SF3B4 (bg=4.76%)▶TARDBP (bg=5.12%)▶tia1 (bg=23.76%)▶tial1 (bg=15.02%)▶u2af1 (bg=14.02%)▶u2af2 (bg=19.32%)▶ZRANB2 (bg=7.28%)No matches to TargetScan

-5623--(375)--5999-

AGGTGA

AGGTGA  
Depth:11 (X.TROPICALIS)  
Ei-value:0.000, Pi-value:0.000  
Er-value:0.000, Pr-value:0.010  
eCLIP MATCHES▶GRWD1 (bg=7.0%)▶srsf1 (bg=30.28%)▶srsf7 (bg=22.53%)No matches to TargetScan

-6004--(1006)--7011-

TTTTCTTTT

TTTTCTTTT  
Depth:19 (ZEBRAFISH)  
Ei-value:0.000, Pi-value:0.000  
Er-value:0.000, Pr-value:0.000  
eCLIP MATCHES▶srsf7 (bg=22.53%)MATCHES To TargetScan▶ miR-186-5p:AAAGAAU

-7019--(28)--7048-

CAGGTTTTGCTTT

CAGGTTTTGCTTT  
Depth:19 (ZEBRAFISH)  
Ei-value:0.000, Pi-value:0.000  
Er-value:0.000, Pr-value:0.000  
eCLIP MATCHES▶srsf7 (bg=22.53%)MATCHES To TargetScan▶ miR-330-3p.2:AAAGCAC▶ miR-490-3p:AACCUGG


T

CAGGTTTTGCTTTT  
Depth:16 (NILETILAPIA)  
Ei-value:0.000, Pi-value:0.000  
Er-value:0.000, Pr-value:0.000  
eCLIP MATCHES▶srsf7 (bg=22.53%)MATCHES To TargetScan▶ miR-330-3p.2:AAAGCAC▶ miR-490-3p:AACCUGG

-7061--(24)--7086-

A

AAAAAAGCAAAAG  
Depth:13 (OPOSSUM)  
Ei-value:0.000, Pi-value:0.000  
Er-value:0.000, Pr-value:0.000  
No matches to eCLIP DataNo matches to TargetScan


AAAAAGCAAAA

AAAAAGCAAAA  
Depth:19 (ZEBRAFISH)  
Ei-value:0.000, Pi-value:0.000  
Er-value:0.000, Pr-value:0.000  
No matches to eCLIP DataNo matches to TargetScan


G

AAAAAGCAAAAG  
Depth:18 (MEDAKA)  
Ei-value:0.000, Pi-value:0.000  
Er-value:0.000, Pr-value:0.000  
No matches to eCLIP DataNo matches to TargetScan

-7098--(15)--7114-

ACTCCTG

ACTCCTG  
Depth:18 (MEDAKA)  
Ei-value:0.000, Pi-value:0.000  
Er-value:0.000, Pr-value:0.000  
No matches to eCLIP DataNo matches to TargetScan


G

ACTCCTGG  
Depth:14 (SPOTTEDGAR)  
Ei-value:0.000, Pi-value:0.000  
Er-value:0.000, Pr-value:0.000  
No matches to eCLIP DataMATCHES To TargetScan▶ miR-665:CCAGGAG

-7121--(11)--7133-

GGGTTC

GGGTTC  
Depth:13 (OPOSSUM)  
Ei-value:0.000, Pi-value:0.000  
Er-value:0.000, Pr-value:0.000  
No matches to eCLIP DataNo matches to TargetScan

-7138  
  
>MARMOSET  
      1687-

GGTGAAG

GGTGAAG  
Depth:11 (X.TROPICALIS)  
Ei-value:0.000, Pi-value:0.000  
Er-value:0.000, Pr-value:0.000  
No matches to TargetScan

-1693--(214)--1908-

TGGTAA

TGGTAA  
Depth:14 (SPOTTEDGAR)  
Ei-value:0.030, Pi-value:0.000  
Er-value:0.010, Pr-value:0.000  
No matches to TargetScan

-1913--(723)--2637-

TTTGGG

TTTGGG  
Depth:19 (ZEBRAFISH)  
Ei-value:0.000, Pi-value:0.000  
Er-value:0.000, Pr-value:0.000  
No matches to TargetScan

-2642--(858)--3501-

GTAAGA

GTAAGA  
Depth:11 (X.TROPICALIS)  
Ei-value:0.000, Pi-value:0.000  
Er-value:0.000, Pr-value:0.000  
No matches to TargetScan

-3506--(231)--3738-

TTTTTCAG

TTTTTCAG  
Depth:19 (ZEBRAFISH)  
Ei-value:0.000, Pi-value:0.000  
Er-value:0.000, Pr-value:0.000  
No matches to TargetScan

-3745--(81)--3827-

AAAGGCAGAA

AAAGGCAGAA  
Depth:13 (OPOSSUM)  
Ei-value:0.000, Pi-value:0.000  
Er-value:0.000, Pr-value:0.000  
No matches to TargetScan


A

AAAGGCAGAAA  
Depth:11 (X.TROPICALIS)  
Ei-value:0.000, Pi-value:0.000  
Er-value:0.000, Pr-value:0.000  
No matches to TargetScan

-3837--(454)--4292-

CA

CAGATAAGT  
Depth:11 (X.TROPICALIS)  
Ei-value:0.000, Pi-value:0.000  
Er-value:0.000, Pr-value:0.000  
No matches to TargetScan


GATAAG

GATAAG  
Depth:19 (ZEBRAFISH)  
Ei-value:0.000, Pi-value:0.000  
Er-value:0.000, Pr-value:0.000  
No matches to TargetScan


T

CAGATAAGT  
Depth:11 (X.TROPICALIS)  
Ei-value:0.000, Pi-value:0.000  
Er-value:0.000, Pr-value:0.000  
No matches to TargetScan

-4300--(18)--4319-

ATTGCAT

ATTGCAT  
Depth:11 (X.TROPICALIS)  
Ei-value:0.000, Pi-value:0.000  
Er-value:0.000, Pr-value:0.000  
No matches to TargetScan

-4325--(664)--4990-

TAAGGA

TAAGGA  
Depth:13 (OPOSSUM)  
Ei-value:0.000, Pi-value:0.000  
Er-value:0.000, Pr-value:0.000  
No matches to TargetScan

-4995--(710)--5706-

TTAGGT

TTAGGT  
Depth:11 (X.TROPICALIS)  
Ei-value:0.000, Pi-value:0.000  
Er-value:0.000, Pr-value:0.000  
No matches to TargetScan

-5711--(39)--5751-

GAAGCC

GAAGCC  
Depth:11 (X.TROPICALIS)  
Ei-value:0.000, Pi-value:0.000  
Er-value:0.000, Pr-value:0.000  
No matches to TargetScan

-5756--(381)--6138-

AGGTGA

AGGTGA  
Depth:11 (X.TROPICALIS)  
Ei-value:0.000, Pi-value:0.000  
Er-value:0.000, Pr-value:0.010  
No matches to TargetScan

-6143--(996)--7140-

TTTTCTTTT

TTTTCTTTT  
Depth:19 (ZEBRAFISH)  
Ei-value:0.000, Pi-value:0.000  
Er-value:0.000, Pr-value:0.000  
MATCHES To TargetScan▶ miR-186-5p:AAAGAAU

-7148--(28)--7177-

CAGGTTTTGCTTT

CAGGTTTTGCTTT  
Depth:19 (ZEBRAFISH)  
Ei-value:0.000, Pi-value:0.000  
Er-value:0.000, Pr-value:0.000  
MATCHES To TargetScan▶ miR-330-3p.2:AAAGCAC▶ miR-490-3p:AACCUGG


T

CAGGTTTTGCTTTT  
Depth:16 (NILETILAPIA)  
Ei-value:0.000, Pi-value:0.000  
Er-value:0.000, Pr-value:0.000  
MATCHES To TargetScan▶ miR-330-3p.2:AAAGCAC▶ miR-490-3p:AACCUGG

-7190--(25)--7216-

A

AAAAAAGCAAAAG  
Depth:13 (OPOSSUM)  
Ei-value:0.000, Pi-value:0.000  
Er-value:0.000, Pr-value:0.000  
No matches to TargetScan


AAAAAGCAAAA

AAAAAGCAAAA  
Depth:19 (ZEBRAFISH)  
Ei-value:0.000, Pi-value:0.000  
Er-value:0.000, Pr-value:0.000  
No matches to TargetScan


G

AAAAAGCAAAAG  
Depth:18 (MEDAKA)  
Ei-value:0.000, Pi-value:0.000  
Er-value:0.000, Pr-value:0.000  
No matches to TargetScan

-7228--(15)--7244-

ACTCCTG

ACTCCTG  
Depth:18 (MEDAKA)  
Ei-value:0.000, Pi-value:0.000  
Er-value:0.000, Pr-value:0.000  
No matches to TargetScan


G

ACTCCTGG  
Depth:14 (SPOTTEDGAR)  
Ei-value:0.000, Pi-value:0.000  
Er-value:0.000, Pr-value:0.000  
MATCHES To TargetScan▶ miR-665:CCAGGAG

-7251--(11)--7263-

GGGTTC

GGGTTC  
Depth:13 (OPOSSUM)  
Ei-value:0.000, Pi-value:0.000  
Er-value:0.000, Pr-value:0.000  
No matches to TargetScan

-7268  
  
>DOG  
      1716-

GGTGAAG

GGTGAAG  
Depth:11 (X.TROPICALIS)  
Ei-value:0.000, Pi-value:0.000  
Er-value:0.000, Pr-value:0.000  
No matches to TargetScan

-1722--(205)--1928-

TGGTAA

TGGTAA  
Depth:14 (SPOTTEDGAR)  
Ei-value:0.030, Pi-value:0.000  
Er-value:0.010, Pr-value:0.000  
No matches to TargetScan

-1933--(678)--2612-

TTTGGG

TTTGGG  
Depth:19 (ZEBRAFISH)  
Ei-value:0.000, Pi-value:0.000  
Er-value:0.000, Pr-value:0.000  
No matches to TargetScan

-2617--(815)--3433-

GTAAGA

GTAAGA  
Depth:11 (X.TROPICALIS)  
Ei-value:0.000, Pi-value:0.000  
Er-value:0.000, Pr-value:0.000  
No matches to TargetScan

-3438--(192)--3631-

TTTTTCAG

TTTTTCAG  
Depth:19 (ZEBRAFISH)  
Ei-value:0.000, Pi-value:0.000  
Er-value:0.000, Pr-value:0.000  
No matches to TargetScan

-3638--(79)--3718-

AAAGGCAGAA

AAAGGCAGAA  
Depth:13 (OPOSSUM)  
Ei-value:0.000, Pi-value:0.000  
Er-value:0.000, Pr-value:0.000  
No matches to TargetScan


A

AAAGGCAGAAA  
Depth:11 (X.TROPICALIS)  
Ei-value:0.000, Pi-value:0.000  
Er-value:0.000, Pr-value:0.000  
No matches to TargetScan

-3728--(467)--4196-

CA

CAGATAAGT  
Depth:11 (X.TROPICALIS)  
Ei-value:0.000, Pi-value:0.000  
Er-value:0.000, Pr-value:0.000  
No matches to TargetScan


GATAAG

GATAAG  
Depth:19 (ZEBRAFISH)  
Ei-value:0.000, Pi-value:0.000  
Er-value:0.000, Pr-value:0.000  
No matches to TargetScan


T

CAGATAAGT  
Depth:11 (X.TROPICALIS)  
Ei-value:0.000, Pi-value:0.000  
Er-value:0.000, Pr-value:0.000  
No matches to TargetScan

-4204--(18)--4223-

ATTGCAT

ATTGCAT  
Depth:11 (X.TROPICALIS)  
Ei-value:0.000, Pi-value:0.000  
Er-value:0.000, Pr-value:0.000  
No matches to TargetScan

-4229--(650)--4880-

TAAGGA

TAAGGA  
Depth:13 (OPOSSUM)  
Ei-value:0.000, Pi-value:0.000  
Er-value:0.000, Pr-value:0.000  
No matches to TargetScan

-4885--(704)--5590-

TTAGGT

TTAGGT  
Depth:11 (X.TROPICALIS)  
Ei-value:0.000, Pi-value:0.000  
Er-value:0.000, Pr-value:0.000  
No matches to TargetScan

-5595--(45)--5641-

GAAGCC

GAAGCC  
Depth:11 (X.TROPICALIS)  
Ei-value:0.000, Pi-value:0.000  
Er-value:0.000, Pr-value:0.000  
No matches to TargetScan

-5646--(373)--6020-

AGGTGA

AGGTGA  
Depth:11 (X.TROPICALIS)  
Ei-value:0.000, Pi-value:0.000  
Er-value:0.000, Pr-value:0.010  
No matches to TargetScan

-6025--(1034)--7060-

TTTTCTTTT

TTTTCTTTT  
Depth:19 (ZEBRAFISH)  
Ei-value:0.000, Pi-value:0.000  
Er-value:0.000, Pr-value:0.000  
MATCHES To TargetScan▶ miR-186-5p:AAAGAAU

-7068--(26)--7095-

CAGGTTTTGCTTT

CAGGTTTTGCTTT  
Depth:19 (ZEBRAFISH)  
Ei-value:0.000, Pi-value:0.000  
Er-value:0.000, Pr-value:0.000  
MATCHES To TargetScan▶ miR-330-3p.2:AAAGCAC▶ miR-490-3p:AACCUGG


T

CAGGTTTTGCTTTT  
Depth:16 (NILETILAPIA)  
Ei-value:0.000, Pi-value:0.000  
Er-value:0.000, Pr-value:0.000  
MATCHES To TargetScan▶ miR-330-3p.2:AAAGCAC▶ miR-490-3p:AACCUGG

-7108--(20)--7129-

A

AAAAAAGCAAAAG  
Depth:13 (OPOSSUM)  
Ei-value:0.000, Pi-value:0.000  
Er-value:0.000, Pr-value:0.000  
No matches to TargetScan


AAAAAGCAAAA

AAAAAGCAAAA  
Depth:19 (ZEBRAFISH)  
Ei-value:0.000, Pi-value:0.000  
Er-value:0.000, Pr-value:0.000  
No matches to TargetScan


G

AAAAAGCAAAAG  
Depth:18 (MEDAKA)  
Ei-value:0.000, Pi-value:0.000  
Er-value:0.000, Pr-value:0.000  
No matches to TargetScan

-7141--(15)--7157-

ACTCCTG

ACTCCTG  
Depth:18 (MEDAKA)  
Ei-value:0.000, Pi-value:0.000  
Er-value:0.000, Pr-value:0.000  
No matches to TargetScan


G

ACTCCTGG  
Depth:14 (SPOTTEDGAR)  
Ei-value:0.000, Pi-value:0.000  
Er-value:0.000, Pr-value:0.000  
MATCHES To TargetScan▶ miR-665:CCAGGAG

-7164--(12)--7177-

GGGTTC

GGGTTC  
Depth:13 (OPOSSUM)  
Ei-value:0.000, Pi-value:0.000  
Er-value:0.000, Pr-value:0.000  
No matches to TargetScan

-7182  
  
>PIG  
      1605-

GGTGAAG

GGTGAAG  
Depth:11 (X.TROPICALIS)  
Ei-value:0.000, Pi-value:0.000  
Er-value:0.000, Pr-value:0.000  
No matches to TargetScan

-1611--(237)--1849-

TGGTAA

TGGTAA  
Depth:14 (SPOTTEDGAR)  
Ei-value:0.030, Pi-value:0.000  
Er-value:0.010, Pr-value:0.000  
No matches to TargetScan

-1854--(678)--2533-

TTTGGG

TTTGGG  
Depth:19 (ZEBRAFISH)  
Ei-value:0.000, Pi-value:0.000  
Er-value:0.000, Pr-value:0.000  
No matches to TargetScan

-2538--(802)--3341-

GTAAGA

GTAAGA  
Depth:11 (X.TROPICALIS)  
Ei-value:0.000, Pi-value:0.000  
Er-value:0.000, Pr-value:0.000  
No matches to TargetScan

-3346--(194)--3541-

TTTTTCAG

TTTTTCAG  
Depth:19 (ZEBRAFISH)  
Ei-value:0.000, Pi-value:0.000  
Er-value:0.000, Pr-value:0.000  
No matches to TargetScan

-3548--(82)--3631-

AAAGGCAGAA

AAAGGCAGAA  
Depth:13 (OPOSSUM)  
Ei-value:0.000, Pi-value:0.000  
Er-value:0.000, Pr-value:0.000  
No matches to TargetScan


A

AAAGGCAGAAA  
Depth:11 (X.TROPICALIS)  
Ei-value:0.000, Pi-value:0.000  
Er-value:0.000, Pr-value:0.000  
No matches to TargetScan

-3641--(474)--4116-

CA

CAGATAAGT  
Depth:11 (X.TROPICALIS)  
Ei-value:0.000, Pi-value:0.000  
Er-value:0.000, Pr-value:0.000  
No matches to TargetScan


GATAAG

GATAAG  
Depth:19 (ZEBRAFISH)  
Ei-value:0.000, Pi-value:0.000  
Er-value:0.000, Pr-value:0.000  
No matches to TargetScan


T

CAGATAAGT  
Depth:11 (X.TROPICALIS)  
Ei-value:0.000, Pi-value:0.000  
Er-value:0.000, Pr-value:0.000  
No matches to TargetScan

-4124--(18)--4143-

ATTGCAT

ATTGCAT  
Depth:11 (X.TROPICALIS)  
Ei-value:0.000, Pi-value:0.000  
Er-value:0.000, Pr-value:0.000  
No matches to TargetScan

-4149--(636)--4786-

TAAGGA

TAAGGA  
Depth:13 (OPOSSUM)  
Ei-value:0.000, Pi-value:0.000  
Er-value:0.000, Pr-value:0.000  
No matches to TargetScan

-4791--(713)--5505-

TTAGGT

TTAGGT  
Depth:11 (X.TROPICALIS)  
Ei-value:0.000, Pi-value:0.000  
Er-value:0.000, Pr-value:0.000  
No matches to TargetScan

-5510--(46)--5557-

GAAGCC

GAAGCC  
Depth:11 (X.TROPICALIS)  
Ei-value:0.000, Pi-value:0.000  
Er-value:0.000, Pr-value:0.000  
No matches to TargetScan

-5562--(377)--5940-

AGGTGA

AGGTGA  
Depth:11 (X.TROPICALIS)  
Ei-value:0.000, Pi-value:0.000  
Er-value:0.000, Pr-value:0.010  
No matches to TargetScan

-5945--(1026)--6972-

TTTTCTTTT

TTTTCTTTT  
Depth:19 (ZEBRAFISH)  
Ei-value:0.000, Pi-value:0.000  
Er-value:0.000, Pr-value:0.000  
MATCHES To TargetScan▶ miR-186-5p:AAAGAAU

-6980--(28)--7009-

CAGGTTTTGCTTT

CAGGTTTTGCTTT  
Depth:19 (ZEBRAFISH)  
Ei-value:0.000, Pi-value:0.000  
Er-value:0.000, Pr-value:0.000  
MATCHES To TargetScan▶ miR-330-3p.2:AAAGCAC▶ miR-490-3p:AACCUGG


T

CAGGTTTTGCTTTT  
Depth:16 (NILETILAPIA)  
Ei-value:0.000, Pi-value:0.000  
Er-value:0.000, Pr-value:0.000  
MATCHES To TargetScan▶ miR-330-3p.2:AAAGCAC▶ miR-490-3p:AACCUGG

-7022--(22)--7045-

A

AAAAAAGCAAAAG  
Depth:13 (OPOSSUM)  
Ei-value:0.000, Pi-value:0.000  
Er-value:0.000, Pr-value:0.000  
No matches to TargetScan


AAAAAGCAAAA

AAAAAGCAAAA  
Depth:19 (ZEBRAFISH)  
Ei-value:0.000, Pi-value:0.000  
Er-value:0.000, Pr-value:0.000  
No matches to TargetScan


G

AAAAAGCAAAAG  
Depth:18 (MEDAKA)  
Ei-value:0.000, Pi-value:0.000  
Er-value:0.000, Pr-value:0.000  
No matches to TargetScan

-7057--(15)--7073-

ACTCCTG

ACTCCTG  
Depth:18 (MEDAKA)  
Ei-value:0.000, Pi-value:0.000  
Er-value:0.000, Pr-value:0.000  
No matches to TargetScan


G

ACTCCTGG  
Depth:14 (SPOTTEDGAR)  
Ei-value:0.000, Pi-value:0.000  
Er-value:0.000, Pr-value:0.000  
MATCHES To TargetScan▶ miR-665:CCAGGAG

-7080--(11)--7092-

GGGTTC

GGGTTC  
Depth:13 (OPOSSUM)  
Ei-value:0.000, Pi-value:0.000  
Er-value:0.000, Pr-value:0.000  
No matches to TargetScan

-7097  
  
>COW  
      1484-

GGTGAAG

GGTGAAG  
Depth:11 (X.TROPICALIS)  
Ei-value:0.000, Pi-value:0.000  
Er-value:0.000, Pr-value:0.000  
No matches to TargetScan

-1490--(225)--1716-

TGGTAA

TGGTAA  
Depth:14 (SPOTTEDGAR)  
Ei-value:0.030, Pi-value:0.000  
Er-value:0.010, Pr-value:0.000  
No matches to TargetScan

-1721--(695)--2417-

TTTGGG

TTTGGG  
Depth:19 (ZEBRAFISH)  
Ei-value:0.000, Pi-value:0.000  
Er-value:0.000, Pr-value:0.000  
No matches to TargetScan

-2422--(804)--3227-

GTAAGA

GTAAGA  
Depth:11 (X.TROPICALIS)  
Ei-value:0.000, Pi-value:0.000  
Er-value:0.000, Pr-value:0.000  
No matches to TargetScan

-3232--(202)--3435-

TTTTTCAG

TTTTTCAG  
Depth:19 (ZEBRAFISH)  
Ei-value:0.000, Pi-value:0.000  
Er-value:0.000, Pr-value:0.000  
No matches to TargetScan

-3442--(82)--3525-

AAAGGCAGAA

AAAGGCAGAA  
Depth:13 (OPOSSUM)  
Ei-value:0.000, Pi-value:0.000  
Er-value:0.000, Pr-value:0.000  
No matches to TargetScan


A

AAAGGCAGAAA  
Depth:11 (X.TROPICALIS)  
Ei-value:0.000, Pi-value:0.000  
Er-value:0.000, Pr-value:0.000  
No matches to TargetScan

-3535--(460)--3996-

CA

CAGATAAGT  
Depth:11 (X.TROPICALIS)  
Ei-value:0.000, Pi-value:0.000  
Er-value:0.000, Pr-value:0.000  
No matches to TargetScan


GATAAG

GATAAG  
Depth:19 (ZEBRAFISH)  
Ei-value:0.000, Pi-value:0.000  
Er-value:0.000, Pr-value:0.000  
No matches to TargetScan


T

CAGATAAGT  
Depth:11 (X.TROPICALIS)  
Ei-value:0.000, Pi-value:0.000  
Er-value:0.000, Pr-value:0.000  
No matches to TargetScan

-4004--(18)--4023-

ATTGCAT

ATTGCAT  
Depth:11 (X.TROPICALIS)  
Ei-value:0.000, Pi-value:0.000  
Er-value:0.000, Pr-value:0.000  
No matches to TargetScan

-4029--(627)--4657-

TAAGGA

TAAGGA  
Depth:13 (OPOSSUM)  
Ei-value:0.000, Pi-value:0.000  
Er-value:0.000, Pr-value:0.000  
No matches to TargetScan

-4662--(738)--5401-

TTAGGT

TTAGGT  
Depth:11 (X.TROPICALIS)  
Ei-value:0.000, Pi-value:0.000  
Er-value:0.000, Pr-value:0.000  
No matches to TargetScan

-5406--(45)--5452-

GAAGCC

GAAGCC  
Depth:11 (X.TROPICALIS)  
Ei-value:0.000, Pi-value:0.000  
Er-value:0.000, Pr-value:0.000  
No matches to TargetScan

-5457--(390)--5848-

AGGTGA

AGGTGA  
Depth:11 (X.TROPICALIS)  
Ei-value:0.000, Pi-value:0.000  
Er-value:0.000, Pr-value:0.010  
No matches to TargetScan

-5853--(1049)--6903-

TTTTCTTTT

TTTTCTTTT  
Depth:19 (ZEBRAFISH)  
Ei-value:0.000, Pi-value:0.000  
Er-value:0.000, Pr-value:0.000  
MATCHES To TargetScan▶ miR-186-5p:AAAGAAU

-6911--(30)--6942-

CAGGTTTTGCTTT

CAGGTTTTGCTTT  
Depth:19 (ZEBRAFISH)  
Ei-value:0.000, Pi-value:0.000  
Er-value:0.000, Pr-value:0.000  
MATCHES To TargetScan▶ miR-330-3p.2:AAAGCAC▶ miR-490-3p:AACCUGG


T

CAGGTTTTGCTTTT  
Depth:16 (NILETILAPIA)  
Ei-value:0.000, Pi-value:0.000  
Er-value:0.000, Pr-value:0.000  
MATCHES To TargetScan▶ miR-330-3p.2:AAAGCAC▶ miR-490-3p:AACCUGG

-6955--(21)--6977-

A

AAAAAAGCAAAAG  
Depth:13 (OPOSSUM)  
Ei-value:0.000, Pi-value:0.000  
Er-value:0.000, Pr-value:0.000  
No matches to TargetScan


AAAAAGCAAAA

AAAAAGCAAAA  
Depth:19 (ZEBRAFISH)  
Ei-value:0.000, Pi-value:0.000  
Er-value:0.000, Pr-value:0.000  
No matches to TargetScan


G

AAAAAGCAAAAG  
Depth:18 (MEDAKA)  
Ei-value:0.000, Pi-value:0.000  
Er-value:0.000, Pr-value:0.000  
No matches to TargetScan

-6989--(15)--7005-

ACTCCTG

ACTCCTG  
Depth:18 (MEDAKA)  
Ei-value:0.000, Pi-value:0.000  
Er-value:0.000, Pr-value:0.000  
No matches to TargetScan


G

ACTCCTGG  
Depth:14 (SPOTTEDGAR)  
Ei-value:0.000, Pi-value:0.000  
Er-value:0.000, Pr-value:0.000  
MATCHES To TargetScan▶ miR-665:CCAGGAG

-7012--(11)--7024-

GGGTTC

GGGTTC  
Depth:13 (OPOSSUM)  
Ei-value:0.000, Pi-value:0.000  
Er-value:0.000, Pr-value:0.000  
No matches to TargetScan

-7029  
  
>MOUSE  
      1485-

GGTGAAG

GGTGAAG  
Depth:11 (X.TROPICALIS)  
Ei-value:0.000, Pi-value:0.000  
Er-value:0.000, Pr-value:0.000  
No matches to TargetScan

-1491--(225)--1717-

TGGTAA

TGGTAA  
Depth:14 (SPOTTEDGAR)  
Ei-value:0.030, Pi-value:0.000  
Er-value:0.010, Pr-value:0.000  
No matches to TargetScan

-1722--(601)--2324-

TTTGGG

TTTGGG  
Depth:19 (ZEBRAFISH)  
Ei-value:0.000, Pi-value:0.000  
Er-value:0.000, Pr-value:0.000  
No matches to TargetScan

-2329--(756)--3086-

GTAAGA

GTAAGA  
Depth:11 (X.TROPICALIS)  
Ei-value:0.000, Pi-value:0.000  
Er-value:0.000, Pr-value:0.000  
No matches to TargetScan

-3091--(228)--3320-

TTTTTCAG

TTTTTCAG  
Depth:19 (ZEBRAFISH)  
Ei-value:0.000, Pi-value:0.000  
Er-value:0.000, Pr-value:0.000  
No matches to TargetScan

-3327--(85)--3413-

AAAGGCAGAA

AAAGGCAGAA  
Depth:13 (OPOSSUM)  
Ei-value:0.000, Pi-value:0.000  
Er-value:0.000, Pr-value:0.000  
No matches to TargetScan


A

AAAGGCAGAAA  
Depth:11 (X.TROPICALIS)  
Ei-value:0.000, Pi-value:0.000  
Er-value:0.000, Pr-value:0.000  
No matches to TargetScan

-3423--(420)--3844-

CA

CAGATAAGT  
Depth:11 (X.TROPICALIS)  
Ei-value:0.000, Pi-value:0.000  
Er-value:0.000, Pr-value:0.000  
No matches to TargetScan


GATAAG

GATAAG  
Depth:19 (ZEBRAFISH)  
Ei-value:0.000, Pi-value:0.000  
Er-value:0.000, Pr-value:0.000  
No matches to TargetScan


T

CAGATAAGT  
Depth:11 (X.TROPICALIS)  
Ei-value:0.000, Pi-value:0.000  
Er-value:0.000, Pr-value:0.000  
No matches to TargetScan

-3852--(18)--3871-

ATTGCAT

ATTGCAT  
Depth:11 (X.TROPICALIS)  
Ei-value:0.000, Pi-value:0.000  
Er-value:0.000, Pr-value:0.000  
No matches to TargetScan

-3877--(647)--4525-

TAAGGA

TAAGGA  
Depth:13 (OPOSSUM)  
Ei-value:0.000, Pi-value:0.000  
Er-value:0.000, Pr-value:0.000  
No matches to TargetScan

-4530--(689)--5220-

TTAGGT

TTAGGT  
Depth:11 (X.TROPICALIS)  
Ei-value:0.000, Pi-value:0.000  
Er-value:0.000, Pr-value:0.000  
No matches to TargetScan

-5225--(42)--5268-

GAAGCC

GAAGCC  
Depth:11 (X.TROPICALIS)  
Ei-value:0.000, Pi-value:0.000  
Er-value:0.000, Pr-value:0.000  
No matches to TargetScan

-5273--(393)--5667-

AGGTGA

AGGTGA  
Depth:11 (X.TROPICALIS)  
Ei-value:0.000, Pi-value:0.000  
Er-value:0.000, Pr-value:0.010  
No matches to TargetScan

-5672--(933)--6606-

TTTTCTTTT

TTTTCTTTT  
Depth:19 (ZEBRAFISH)  
Ei-value:0.000, Pi-value:0.000  
Er-value:0.000, Pr-value:0.000  
MATCHES To TargetScan▶ miR-186-5p:AAAGAAU

-6614--(26)--6641-

CAGGTTTTGCTTT

CAGGTTTTGCTTT  
Depth:19 (ZEBRAFISH)  
Ei-value:0.000, Pi-value:0.000  
Er-value:0.000, Pr-value:0.000  
MATCHES To TargetScan▶ miR-330-3p.2:AAAGCAC▶ miR-490-3p:AACCUGG


T

CAGGTTTTGCTTTT  
Depth:16 (NILETILAPIA)  
Ei-value:0.000, Pi-value:0.000  
Er-value:0.000, Pr-value:0.000  
MATCHES To TargetScan▶ miR-330-3p.2:AAAGCAC▶ miR-490-3p:AACCUGG

-6654--(24)--6679-

A

AAAAAAGCAAAAG  
Depth:13 (OPOSSUM)  
Ei-value:0.000, Pi-value:0.000  
Er-value:0.000, Pr-value:0.000  
No matches to TargetScan


AAAAAGCAAAA

AAAAAGCAAAA  
Depth:19 (ZEBRAFISH)  
Ei-value:0.000, Pi-value:0.000  
Er-value:0.000, Pr-value:0.000  
No matches to TargetScan


G

AAAAAGCAAAAG  
Depth:18 (MEDAKA)  
Ei-value:0.000, Pi-value:0.000  
Er-value:0.000, Pr-value:0.000  
No matches to TargetScan

-6691--(15)--6707-

ACTCCTG

ACTCCTG  
Depth:18 (MEDAKA)  
Ei-value:0.000, Pi-value:0.000  
Er-value:0.000, Pr-value:0.000  
No matches to TargetScan


G

ACTCCTGG  
Depth:14 (SPOTTEDGAR)  
Ei-value:0.000, Pi-value:0.000  
Er-value:0.000, Pr-value:0.000  
MATCHES To TargetScan▶ miR-665:CCAGGAG

-6714--(11)--6726-

GGGTTC

GGGTTC  
Depth:13 (OPOSSUM)  
Ei-value:0.000, Pi-value:0.000  
Er-value:0.000, Pr-value:0.000  
No matches to TargetScan

-6731  
  
>TURTLE  
       176-

GGTGAAG

GGTGAAG  
Depth:11 (X.TROPICALIS)  
Ei-value:0.000, Pi-value:0.000  
Er-value:0.000, Pr-value:0.000  
No matches to TargetScan

-182--(178)--361-

TGGTAA

TGGTAA  
Depth:14 (SPOTTEDGAR)  
Ei-value:0.030, Pi-value:0.000  
Er-value:0.010, Pr-value:0.000  
No matches to TargetScan

-366--(904)--1271-

TTTGGG

TTTGGG  
Depth:19 (ZEBRAFISH)  
Ei-value:0.000, Pi-value:0.000  
Er-value:0.000, Pr-value:0.000  
No matches to TargetScan

-1276--(1028)--2305-

GTAAGA

GTAAGA  
Depth:11 (X.TROPICALIS)  
Ei-value:0.000, Pi-value:0.000  
Er-value:0.000, Pr-value:0.000  
No matches to TargetScan

-2310--(348)--2659-

TTTTTCAG

TTTTTCAG  
Depth:19 (ZEBRAFISH)  
Ei-value:0.000, Pi-value:0.000  
Er-value:0.000, Pr-value:0.000  
No matches to TargetScan

-2666--(89)--2756-

AAAGGCAGAA

AAAGGCAGAA  
Depth:13 (OPOSSUM)  
Ei-value:0.000, Pi-value:0.000  
Er-value:0.000, Pr-value:0.000  
No matches to TargetScan


A

AAAGGCAGAAA  
Depth:11 (X.TROPICALIS)  
Ei-value:0.000, Pi-value:0.000  
Er-value:0.000, Pr-value:0.000  
No matches to TargetScan

-2766--(546)--3313-

CA

CAGATAAGT  
Depth:11 (X.TROPICALIS)  
Ei-value:0.000, Pi-value:0.000  
Er-value:0.000, Pr-value:0.000  
No matches to TargetScan


GATAAG

GATAAG  
Depth:19 (ZEBRAFISH)  
Ei-value:0.000, Pi-value:0.000  
Er-value:0.000, Pr-value:0.000  
No matches to TargetScan


T

CAGATAAGT  
Depth:11 (X.TROPICALIS)  
Ei-value:0.000, Pi-value:0.000  
Er-value:0.000, Pr-value:0.000  
No matches to TargetScan

-3321--(21)--3343-

ATTGCAT

ATTGCAT  
Depth:11 (X.TROPICALIS)  
Ei-value:0.000, Pi-value:0.000  
Er-value:0.000, Pr-value:0.000  
No matches to TargetScan

-3349--(755)--4105-

TAAGGA

TAAGGA  
Depth:13 (OPOSSUM)  
Ei-value:0.000, Pi-value:0.000  
Er-value:0.000, Pr-value:0.000  
No matches to TargetScan

-4110--(918)--5029-

TTAGGT

TTAGGT  
Depth:11 (X.TROPICALIS)  
Ei-value:0.000, Pi-value:0.000  
Er-value:0.000, Pr-value:0.000  
No matches to TargetScan

-5034--(59)--5094-

GAAGCC

GAAGCC  
Depth:11 (X.TROPICALIS)  
Ei-value:0.000, Pi-value:0.000  
Er-value:0.000, Pr-value:0.000  
No matches to TargetScan

-5099--(420)--5520-

AGGTGA

AGGTGA  
Depth:11 (X.TROPICALIS)  
Ei-value:0.000, Pi-value:0.000  
Er-value:0.000, Pr-value:0.010  
No matches to TargetScan

-5525--(1207)--6733-

TTTTCTTTT

TTTTCTTTT  
Depth:19 (ZEBRAFISH)  
Ei-value:0.000, Pi-value:0.000  
Er-value:0.000, Pr-value:0.000  
MATCHES To TargetScan▶ miR-186-5p:AAAGAAU

-6741--(23)--6765-

CAGGTTTTGCTTT

CAGGTTTTGCTTT  
Depth:19 (ZEBRAFISH)  
Ei-value:0.000, Pi-value:0.000  
Er-value:0.000, Pr-value:0.000  
MATCHES To TargetScan▶ miR-330-3p.2:AAAGCAC▶ miR-490-3p:AACCUGG


T

CAGGTTTTGCTTTT  
Depth:16 (NILETILAPIA)  
Ei-value:0.000, Pi-value:0.000  
Er-value:0.000, Pr-value:0.000  
MATCHES To TargetScan▶ miR-330-3p.2:AAAGCAC▶ miR-490-3p:AACCUGG

-6778--(19)--6798-

A

AAAAAAGCAAAAG  
Depth:13 (OPOSSUM)  
Ei-value:0.000, Pi-value:0.000  
Er-value:0.000, Pr-value:0.000  
No matches to TargetScan


AAAAAGCAAAA

AAAAAGCAAAA  
Depth:19 (ZEBRAFISH)  
Ei-value:0.000, Pi-value:0.000  
Er-value:0.000, Pr-value:0.000  
No matches to TargetScan


G

AAAAAGCAAAAG  
Depth:18 (MEDAKA)  
Ei-value:0.000, Pi-value:0.000  
Er-value:0.000, Pr-value:0.000  
No matches to TargetScan

-6810--(15)--6826-

ACTCCTG

ACTCCTG  
Depth:18 (MEDAKA)  
Ei-value:0.000, Pi-value:0.000  
Er-value:0.000, Pr-value:0.000  
No matches to TargetScan


G

ACTCCTGG  
Depth:14 (SPOTTEDGAR)  
Ei-value:0.000, Pi-value:0.000  
Er-value:0.000, Pr-value:0.000  
MATCHES To TargetScan▶ miR-665:CCAGGAG

-6833--(11)--6845-

GGGTTC

GGGTTC  
Depth:13 (OPOSSUM)  
Ei-value:0.000, Pi-value:0.000  
Er-value:0.000, Pr-value:0.000  
No matches to TargetScan

-6850  
  
>ALLIGATOR  
      1490-

GGTGAAG

GGTGAAG  
Depth:11 (X.TROPICALIS)  
Ei-value:0.000, Pi-value:0.000  
Er-value:0.000, Pr-value:0.000  
No matches to TargetScan

-1496--(191)--1688-

TGGTAA

TGGTAA  
Depth:14 (SPOTTEDGAR)  
Ei-value:0.030, Pi-value:0.000  
Er-value:0.010, Pr-value:0.000  
No matches to TargetScan

-1693--(843)--2537-

TTTGGG

TTTGGG  
Depth:19 (ZEBRAFISH)  
Ei-value:0.000, Pi-value:0.000  
Er-value:0.000, Pr-value:0.000  
No matches to TargetScan

-2542--(961)--3504-

GTAAGA

GTAAGA  
Depth:11 (X.TROPICALIS)  
Ei-value:0.000, Pi-value:0.000  
Er-value:0.000, Pr-value:0.000  
No matches to TargetScan

-3509--(355)--3865-

TTTTTCAG

TTTTTCAG  
Depth:19 (ZEBRAFISH)  
Ei-value:0.000, Pi-value:0.000  
Er-value:0.000, Pr-value:0.000  
No matches to TargetScan

-3872--(92)--3965-

AAAGGCAGAA

AAAGGCAGAA  
Depth:13 (OPOSSUM)  
Ei-value:0.000, Pi-value:0.000  
Er-value:0.000, Pr-value:0.000  
No matches to TargetScan


A

AAAGGCAGAAA  
Depth:11 (X.TROPICALIS)  
Ei-value:0.000, Pi-value:0.000  
Er-value:0.000, Pr-value:0.000  
No matches to TargetScan

-3975--(543)--4519-

CA

CAGATAAGT  
Depth:11 (X.TROPICALIS)  
Ei-value:0.000, Pi-value:0.000  
Er-value:0.000, Pr-value:0.000  
No matches to TargetScan


GATAAG

GATAAG  
Depth:19 (ZEBRAFISH)  
Ei-value:0.000, Pi-value:0.000  
Er-value:0.000, Pr-value:0.000  
No matches to TargetScan


T

CAGATAAGT  
Depth:11 (X.TROPICALIS)  
Ei-value:0.000, Pi-value:0.000  
Er-value:0.000, Pr-value:0.000  
No matches to TargetScan

-4527--(21)--4549-

ATTGCAT

ATTGCAT  
Depth:11 (X.TROPICALIS)  
Ei-value:0.000, Pi-value:0.000  
Er-value:0.000, Pr-value:0.000  
No matches to TargetScan

-4555--(776)--5332-

TAAGGA

TAAGGA  
Depth:13 (OPOSSUM)  
Ei-value:0.000, Pi-value:0.000  
Er-value:0.000, Pr-value:0.000  
No matches to TargetScan

-5337--(901)--6239-

TTAGGT

TTAGGT  
Depth:11 (X.TROPICALIS)  
Ei-value:0.000, Pi-value:0.000  
Er-value:0.000, Pr-value:0.000  
No matches to TargetScan

-6244--(90)--6335-

GAAGCC

GAAGCC  
Depth:11 (X.TROPICALIS)  
Ei-value:0.000, Pi-value:0.000  
Er-value:0.000, Pr-value:0.000  
No matches to TargetScan

-6340--(446)--6787-

AGGTGA

AGGTGA  
Depth:11 (X.TROPICALIS)  
Ei-value:0.000, Pi-value:0.000  
Er-value:0.000, Pr-value:0.010  
No matches to TargetScan

-6792--(1212)--8005-

TTTTCTTTT

TTTTCTTTT  
Depth:19 (ZEBRAFISH)  
Ei-value:0.000, Pi-value:0.000  
Er-value:0.000, Pr-value:0.000  
MATCHES To TargetScan▶ miR-186-5p:AAAGAAU

-8013--(23)--8037-

CAGGTTTTGCTTT

CAGGTTTTGCTTT  
Depth:19 (ZEBRAFISH)  
Ei-value:0.000, Pi-value:0.000  
Er-value:0.000, Pr-value:0.000  
MATCHES To TargetScan▶ miR-330-3p.2:AAAGCAC▶ miR-490-3p:AACCUGG


T

CAGGTTTTGCTTTT  
Depth:16 (NILETILAPIA)  
Ei-value:0.000, Pi-value:0.000  
Er-value:0.000, Pr-value:0.000  
MATCHES To TargetScan▶ miR-330-3p.2:AAAGCAC▶ miR-490-3p:AACCUGG

-8050--(24)--8075-

A

AAAAAAGCAAAAG  
Depth:13 (OPOSSUM)  
Ei-value:0.000, Pi-value:0.000  
Er-value:0.000, Pr-value:0.000  
No matches to TargetScan


AAAAAGCAAAA

AAAAAGCAAAA  
Depth:19 (ZEBRAFISH)  
Ei-value:0.000, Pi-value:0.000  
Er-value:0.000, Pr-value:0.000  
No matches to TargetScan


G

AAAAAGCAAAAG  
Depth:18 (MEDAKA)  
Ei-value:0.000, Pi-value:0.000  
Er-value:0.000, Pr-value:0.000  
No matches to TargetScan

-8087--(15)--8103-

ACTCCTG

ACTCCTG  
Depth:18 (MEDAKA)  
Ei-value:0.000, Pi-value:0.000  
Er-value:0.000, Pr-value:0.000  
No matches to TargetScan


G

ACTCCTGG  
Depth:14 (SPOTTEDGAR)  
Ei-value:0.000, Pi-value:0.000  
Er-value:0.000, Pr-value:0.000  
MATCHES To TargetScan▶ miR-665:CCAGGAG

-8110--(11)--8122-

GGGTTC

GGGTTC  
Depth:13 (OPOSSUM)  
Ei-value:0.000, Pi-value:0.000  
Er-value:0.000, Pr-value:0.000  
No matches to TargetScan

-8127  
  
>LIZARD  
      1321-

GGTGAAG

GGTGAAG  
Depth:11 (X.TROPICALIS)  
Ei-value:0.000, Pi-value:0.000  
Er-value:0.000, Pr-value:0.000  
No matches to TargetScan

-1327--(141)--1469-

TGGTAA

TGGTAA  
Depth:14 (SPOTTEDGAR)  
Ei-value:0.030, Pi-value:0.000  
Er-value:0.010, Pr-value:0.000  
No matches to TargetScan

-1474--(878)--2353-

TTTGGG

TTTGGG  
Depth:19 (ZEBRAFISH)  
Ei-value:0.000, Pi-value:0.000  
Er-value:0.000, Pr-value:0.000  
No matches to TargetScan

-2358--(508)--2867-

GTAAGA

GTAAGA  
Depth:11 (X.TROPICALIS)  
Ei-value:0.000, Pi-value:0.000  
Er-value:0.000, Pr-value:0.000  
No matches to TargetScan

-2872--(248)--3121-

TTTTTCAG

TTTTTCAG  
Depth:19 (ZEBRAFISH)  
Ei-value:0.000, Pi-value:0.000  
Er-value:0.000, Pr-value:0.000  
No matches to TargetScan

-3128--(90)--3219-

AAAGGCAGAA

AAAGGCAGAA  
Depth:13 (OPOSSUM)  
Ei-value:0.000, Pi-value:0.000  
Er-value:0.000, Pr-value:0.000  
No matches to TargetScan


A

AAAGGCAGAAA  
Depth:11 (X.TROPICALIS)  
Ei-value:0.000, Pi-value:0.000  
Er-value:0.000, Pr-value:0.000  
No matches to TargetScan

-3229--(485)--3715-

CA

CAGATAAGT  
Depth:11 (X.TROPICALIS)  
Ei-value:0.000, Pi-value:0.000  
Er-value:0.000, Pr-value:0.000  
No matches to TargetScan


GATAAG

GATAAG  
Depth:19 (ZEBRAFISH)  
Ei-value:0.000, Pi-value:0.000  
Er-value:0.000, Pr-value:0.000  
No matches to TargetScan


T

CAGATAAGT  
Depth:11 (X.TROPICALIS)  
Ei-value:0.000, Pi-value:0.000  
Er-value:0.000, Pr-value:0.000  
No matches to TargetScan

-3723--(16)--3740-

ATTGCAT

ATTGCAT  
Depth:11 (X.TROPICALIS)  
Ei-value:0.000, Pi-value:0.000  
Er-value:0.000, Pr-value:0.000  
No matches to TargetScan

-3746--(677)--4424-

TAAGGA

TAAGGA  
Depth:13 (OPOSSUM)  
Ei-value:0.000, Pi-value:0.000  
Er-value:0.000, Pr-value:0.000  
No matches to TargetScan

-4429--(899)--5329-

TTAGGT

TTAGGT  
Depth:11 (X.TROPICALIS)  
Ei-value:0.000, Pi-value:0.000  
Er-value:0.000, Pr-value:0.000  
No matches to TargetScan

-5334--(51)--5386-

GAAGCC

GAAGCC  
Depth:11 (X.TROPICALIS)  
Ei-value:0.000, Pi-value:0.000  
Er-value:0.000, Pr-value:0.000  
No matches to TargetScan

-5391--(409)--5801-

AGGTGA

AGGTGA  
Depth:11 (X.TROPICALIS)  
Ei-value:0.000, Pi-value:0.000  
Er-value:0.000, Pr-value:0.010  
No matches to TargetScan

-5806--(1092)--6899-

TTTTCTTTT

TTTTCTTTT  
Depth:19 (ZEBRAFISH)  
Ei-value:0.000, Pi-value:0.000  
Er-value:0.000, Pr-value:0.000  
MATCHES To TargetScan▶ miR-186-5p:AAAGAAU

-6907--(24)--6932-

CAGGTTTTGCTTT

CAGGTTTTGCTTT  
Depth:19 (ZEBRAFISH)  
Ei-value:0.000, Pi-value:0.000  
Er-value:0.000, Pr-value:0.000  
MATCHES To TargetScan▶ miR-330-3p.2:AAAGCAC▶ miR-490-3p:AACCUGG


T

CAGGTTTTGCTTTT  
Depth:16 (NILETILAPIA)  
Ei-value:0.000, Pi-value:0.000  
Er-value:0.000, Pr-value:0.000  
MATCHES To TargetScan▶ miR-330-3p.2:AAAGCAC▶ miR-490-3p:AACCUGG

-6945--(19)--6965-

A

AAAAAAGCAAAAG  
Depth:13 (OPOSSUM)  
Ei-value:0.000, Pi-value:0.000  
Er-value:0.000, Pr-value:0.000  
No matches to TargetScan


AAAAAGCAAAA

AAAAAGCAAAA  
Depth:19 (ZEBRAFISH)  
Ei-value:0.000, Pi-value:0.000  
Er-value:0.000, Pr-value:0.000  
No matches to TargetScan


G

AAAAAGCAAAAG  
Depth:18 (MEDAKA)  
Ei-value:0.000, Pi-value:0.000  
Er-value:0.000, Pr-value:0.000  
No matches to TargetScan

-6977--(15)--6993-

ACTCCTG

ACTCCTG  
Depth:18 (MEDAKA)  
Ei-value:0.000, Pi-value:0.000  
Er-value:0.000, Pr-value:0.000  
No matches to TargetScan


G

ACTCCTGG  
Depth:14 (SPOTTEDGAR)  
Ei-value:0.000, Pi-value:0.000  
Er-value:0.000, Pr-value:0.000  
MATCHES To TargetScan▶ miR-665:CCAGGAG

-7000--(11)--7012-

GGGTTC

GGGTTC  
Depth:13 (OPOSSUM)  
Ei-value:0.000, Pi-value:0.000  
Er-value:0.000, Pr-value:0.000  
No matches to TargetScan

-7017  
  
>SNAKE  
      1061-

GGTGAAG

GGTGAAG  
Depth:11 (X.TROPICALIS)  
Ei-value:0.000, Pi-value:0.000  
Er-value:0.000, Pr-value:0.000  
No matches to TargetScan

-1067--(148)--1216-

TGGTAA

TGGTAA  
Depth:14 (SPOTTEDGAR)  
Ei-value:0.030, Pi-value:0.000  
Er-value:0.010, Pr-value:0.000  
No matches to TargetScan

-1221--(70)--1292-

TTTGGG

TTTGGG  
Depth:19 (ZEBRAFISH)  
Ei-value:0.000, Pi-value:0.000  
Er-value:0.000, Pr-value:0.000  
No matches to TargetScan

-1297--(729)--2027-

TTTGGG

TTTGGG  
Depth:19 (ZEBRAFISH)  
Ei-value:0.000, Pi-value:0.000  
Er-value:0.000, Pr-value:0.000  
No matches to TargetScan

-2032--(840)--2873-

GTAAGA

GTAAGA  
Depth:11 (X.TROPICALIS)  
Ei-value:0.000, Pi-value:0.000  
Er-value:0.000, Pr-value:0.000  
No matches to TargetScan

-2878--(235)--3114-

TTTTTCAG

TTTTTCAG  
Depth:19 (ZEBRAFISH)  
Ei-value:0.000, Pi-value:0.000  
Er-value:0.000, Pr-value:0.000  
No matches to TargetScan

-3121--(80)--3202-

AAAGGCAGAA

AAAGGCAGAA  
Depth:13 (OPOSSUM)  
Ei-value:0.000, Pi-value:0.000  
Er-value:0.000, Pr-value:0.000  
No matches to TargetScan


A

AAAGGCAGAAA  
Depth:11 (X.TROPICALIS)  
Ei-value:0.000, Pi-value:0.000  
Er-value:0.000, Pr-value:0.000  
No matches to TargetScan

-3212--(465)--3678-

CA

CAGATAAGT  
Depth:11 (X.TROPICALIS)  
Ei-value:0.000, Pi-value:0.000  
Er-value:0.000, Pr-value:0.000  
No matches to TargetScan


GATAAG

GATAAG  
Depth:19 (ZEBRAFISH)  
Ei-value:0.000, Pi-value:0.000  
Er-value:0.000, Pr-value:0.000  
No matches to TargetScan


T

CAGATAAGT  
Depth:11 (X.TROPICALIS)  
Ei-value:0.000, Pi-value:0.000  
Er-value:0.000, Pr-value:0.000  
No matches to TargetScan

-3686--(18)--3705-

ATTGCAT

ATTGCAT  
Depth:11 (X.TROPICALIS)  
Ei-value:0.000, Pi-value:0.000  
Er-value:0.000, Pr-value:0.000  
No matches to TargetScan

-3711--(853)--4565-

TAAGGA

TAAGGA  
Depth:13 (OPOSSUM)  
Ei-value:0.000, Pi-value:0.000  
Er-value:0.000, Pr-value:0.000  
No matches to TargetScan

-4570--(637)--5208-

TTAGGT

TTAGGT  
Depth:11 (X.TROPICALIS)  
Ei-value:0.000, Pi-value:0.000  
Er-value:0.000, Pr-value:0.000  
No matches to TargetScan

-5213--(47)--5261-

GAAGCC

GAAGCC  
Depth:11 (X.TROPICALIS)  
Ei-value:0.000, Pi-value:0.000  
Er-value:0.000, Pr-value:0.000  
No matches to TargetScan

-5266--(417)--5684-

AGGTGA

AGGTGA  
Depth:11 (X.TROPICALIS)  
Ei-value:0.000, Pi-value:0.000  
Er-value:0.000, Pr-value:0.010  
No matches to TargetScan

-5689--(1033)--6723-

TTTTCTTTT

TTTTCTTTT  
Depth:19 (ZEBRAFISH)  
Ei-value:0.000, Pi-value:0.000  
Er-value:0.000, Pr-value:0.000  
MATCHES To TargetScan▶ miR-186-5p:AAAGAAU

-6731--(25)--6757-

CAGGTTTTGCTTT

CAGGTTTTGCTTT  
Depth:19 (ZEBRAFISH)  
Ei-value:0.000, Pi-value:0.000  
Er-value:0.000, Pr-value:0.000  
MATCHES To TargetScan▶ miR-330-3p.2:AAAGCAC▶ miR-490-3p:AACCUGG


T

CAGGTTTTGCTTTT  
Depth:16 (NILETILAPIA)  
Ei-value:0.000, Pi-value:0.000  
Er-value:0.000, Pr-value:0.000  
MATCHES To TargetScan▶ miR-330-3p.2:AAAGCAC▶ miR-490-3p:AACCUGG

-6770--(27)--6798-

A

AAAAAAGCAAAAG  
Depth:13 (OPOSSUM)  
Ei-value:0.000, Pi-value:0.000  
Er-value:0.000, Pr-value:0.000  
No matches to TargetScan


AAAAAGCAAAA

AAAAAGCAAAA  
Depth:19 (ZEBRAFISH)  
Ei-value:0.000, Pi-value:0.000  
Er-value:0.000, Pr-value:0.000  
No matches to TargetScan


G

AAAAAGCAAAAG  
Depth:18 (MEDAKA)  
Ei-value:0.000, Pi-value:0.000  
Er-value:0.000, Pr-value:0.000  
No matches to TargetScan

-6810--(15)--6826-

ACTCCTG

ACTCCTG  
Depth:18 (MEDAKA)  
Ei-value:0.000, Pi-value:0.000  
Er-value:0.000, Pr-value:0.000  
No matches to TargetScan


G

ACTCCTGG  
Depth:14 (SPOTTEDGAR)  
Ei-value:0.000, Pi-value:0.000  
Er-value:0.000, Pr-value:0.000  
MATCHES To TargetScan▶ miR-665:CCAGGAG

-6833--(11)--6845-

GGGTTC

GGGTTC  
Depth:13 (OPOSSUM)  
Ei-value:0.000, Pi-value:0.000  
Er-value:0.000, Pr-value:0.000  
No matches to TargetScan

-6850  
  
>X.TROPICALIS  
      1408-

GGTGAAG

GGTGAAG  
Depth:11 (X.TROPICALIS)  
Ei-value:0.000, Pi-value:0.000  
Er-value:0.000, Pr-value:0.000  
No matches to TargetScan

-1414--(183)--1598-

TGGTAA

TGGTAA  
Depth:14 (SPOTTEDGAR)  
Ei-value:0.030, Pi-value:0.000  
Er-value:0.010, Pr-value:0.000  
No matches to TargetScan

-1603--(248)--1852-

TGGTAA

TGGTAA  
Depth:14 (SPOTTEDGAR)  
Ei-value:0.030, Pi-value:0.000  
Er-value:0.010, Pr-value:0.000  
No matches to TargetScan

-1857--(441)--2299-

TTTGGG

TTTGGG  
Depth:19 (ZEBRAFISH)  
Ei-value:0.000, Pi-value:0.000  
Er-value:0.000, Pr-value:0.000  
No matches to TargetScan

-2304--(2695)--5000-

TTTGGG

TTTGGG  
Depth:19 (ZEBRAFISH)  
Ei-value:0.000, Pi-value:0.000  
Er-value:0.000, Pr-value:0.000  
No matches to TargetScan

-5005--(125)--5131-

TTTGGG

TTTGGG  
Depth:19 (ZEBRAFISH)  
Ei-value:0.000, Pi-value:0.000  
Er-value:0.000, Pr-value:0.000  
No matches to TargetScan

-5136--(89)--5226-

GTAAGA

GTAAGA  
Depth:11 (X.TROPICALIS)  
Ei-value:0.000, Pi-value:0.000  
Er-value:0.000, Pr-value:0.000  
No matches to TargetScan

-5231--(1519)--6751-

GTAAGA

GTAAGA  
Depth:11 (X.TROPICALIS)  
Ei-value:0.000, Pi-value:0.000  
Er-value:0.000, Pr-value:0.000  
No matches to TargetScan

-6756--(1586)--8343-

TTTTTCAG

TTTTTCAG  
Depth:19 (ZEBRAFISH)  
Ei-value:0.000, Pi-value:0.000  
Er-value:0.000, Pr-value:0.000  
No matches to TargetScan

-8350--(704)--9055-

AAAGGCAGAA

AAAGGCAGAA  
Depth:13 (OPOSSUM)  
Ei-value:0.000, Pi-value:0.000  
Er-value:0.000, Pr-value:0.000  
No matches to TargetScan


A

AAAGGCAGAAA  
Depth:11 (X.TROPICALIS)  
Ei-value:0.000, Pi-value:0.000  
Er-value:0.000, Pr-value:0.000  
No matches to TargetScan

-9065--(287)--9353-

CA

CAGATAAGT  
Depth:11 (X.TROPICALIS)  
Ei-value:0.000, Pi-value:0.000  
Er-value:0.000, Pr-value:0.000  
No matches to TargetScan


GATAAG

GATAAG  
Depth:19 (ZEBRAFISH)  
Ei-value:0.000, Pi-value:0.000  
Er-value:0.000, Pr-value:0.000  
No matches to TargetScan


T

CAGATAAGT  
Depth:11 (X.TROPICALIS)  
Ei-value:0.000, Pi-value:0.000  
Er-value:0.000, Pr-value:0.000  
No matches to TargetScan

-9361--(1257)--10619-

ATTGCAT

ATTGCAT  
Depth:11 (X.TROPICALIS)  
Ei-value:0.000, Pi-value:0.000  
Er-value:0.000, Pr-value:0.000  
No matches to TargetScan

-10625--(883)--11509-

TAAGGA

TAAGGA  
Depth:13 (OPOSSUM)  
Ei-value:0.000, Pi-value:0.000  
Er-value:0.000, Pr-value:0.000  
No matches to TargetScan

-11514--(470)--11985-

TTAGGT

TTAGGT  
Depth:11 (X.TROPICALIS)  
Ei-value:0.000, Pi-value:0.000  
Er-value:0.000, Pr-value:0.000  
No matches to TargetScan

-11990--(68)--12059-

GAAGCC

GAAGCC  
Depth:11 (X.TROPICALIS)  
Ei-value:0.000, Pi-value:0.000  
Er-value:0.000, Pr-value:0.000  
No matches to TargetScan

-12064--(192)--12257-

AGGTGA

AGGTGA  
Depth:11 (X.TROPICALIS)  
Ei-value:0.000, Pi-value:0.000  
Er-value:0.000, Pr-value:0.010  
No matches to TargetScan

-12262--(64)--12327-

TTTTCTTTT

TTTTCTTTT  
Depth:19 (ZEBRAFISH)  
Ei-value:0.000, Pi-value:0.000  
Er-value:0.000, Pr-value:0.000  
MATCHES To TargetScan▶ miR-186-5p:AAAGAAU

-12335--(26)--12362-

CAGGTTTTGCTTT

CAGGTTTTGCTTT  
Depth:19 (ZEBRAFISH)  
Ei-value:0.000, Pi-value:0.000  
Er-value:0.000, Pr-value:0.000  
MATCHES To TargetScan▶ miR-330-3p.2:AAAGCAC▶ miR-490-3p:AACCUGG


T

CAGGTTTTGCTTTT  
Depth:16 (NILETILAPIA)  
Ei-value:0.000, Pi-value:0.000  
Er-value:0.000, Pr-value:0.000  
MATCHES To TargetScan▶ miR-330-3p.2:AAAGCAC▶ miR-490-3p:AACCUGG

-12375--(29)--12405-

A

AAAAAAGCAAAAG  
Depth:13 (OPOSSUM)  
Ei-value:0.000, Pi-value:0.000  
Er-value:0.000, Pr-value:0.000  
No matches to TargetScan


AAAAAGCAAAA

AAAAAGCAAAA  
Depth:19 (ZEBRAFISH)  
Ei-value:0.000, Pi-value:0.000  
Er-value:0.000, Pr-value:0.000  
No matches to TargetScan


G

AAAAAGCAAAAG  
Depth:18 (MEDAKA)  
Ei-value:0.000, Pi-value:0.000  
Er-value:0.000, Pr-value:0.000  
No matches to TargetScan

-12417--(15)--12433-

ACTCCTG

ACTCCTG  
Depth:18 (MEDAKA)  
Ei-value:0.000, Pi-value:0.000  
Er-value:0.000, Pr-value:0.000  
No matches to TargetScan


G

ACTCCTGG  
Depth:14 (SPOTTEDGAR)  
Ei-value:0.000, Pi-value:0.000  
Er-value:0.000, Pr-value:0.000  
MATCHES To TargetScan▶ miR-665:CCAGGAG

-12440--(11)--12452-

GGGTTC

GGGTTC  
Depth:13 (OPOSSUM)  
Ei-value:0.000, Pi-value:0.000  
Er-value:0.000, Pr-value:0.000  
No matches to TargetScan

-12457
```

---

# Modules conserved to SNAKE (Depth: 10)

## Modules in Main Graph (All sequences considered):

```
>HUMAN  
      1577-

GGTGAAG

GGTGAAG  
Depth:11 (X.TROPICALIS)  
Ei-value:0.000, Pi-value:0.000  
Er-value:0.000, Pr-value:0.000  
eCLIP MATCHES▶bclaf1 (bg=17.67%)▶EXOSC5 (bg=2.0%)▶fxr2 (bg=10.1%)▶gtf2f1 (bg=10.18%)▶hltf (bg=24.28%)▶MTPAP (bg=9.55%)▶npm1 (bg=10.22%)▶ppil4 (bg=43.39%)▶rbm15 (bg=11.59%)▶rbm22 (bg=12.69%)▶safb (bg=40.39%)▶safb2 (bg=26.89%)▶SMNDC1 (bg=7.08%)▶srsf1 (bg=30.28%)▶SRSF9 (bg=9.67%)▶TAF15 (bg=9.06%)▶tra2a (bg=37.02%)▶TROVE2 (bg=6.96%)▶uchl5 (bg=18.56%)▶znf622 (bg=18.79%)No matches to TargetScan

-1583--(210)--1794-

TGGTAA

TGGTAA  
Depth:14 (SPOTTEDGAR)  
Ei-value:0.030, Pi-value:0.000  
Er-value:0.010, Pr-value:0.000  
eCLIP MATCHES▶bclaf1 (bg=17.67%)▶DROSHA (bg=1.03%)▶GRWD1 (bg=7.0%)▶hltf (bg=24.28%)▶MTPAP (bg=9.55%)▶NOLC1 (bg=0.67%)▶npm1 (bg=10.22%)▶ppil4 (bg=43.39%)▶rbm15 (bg=11.59%)▶safb (bg=40.39%)▶srsf1 (bg=30.28%)▶TAF15 (bg=9.06%)▶tia1 (bg=23.76%)▶uchl5 (bg=18.56%)▶YWHAG (bg=9.14%)▶ZRANB2 (bg=7.28%)No matches to TargetScan

-1799--(705)--2505-

TTTGGG

TTTGGG  
Depth:19 (ZEBRAFISH)  
Ei-value:0.000, Pi-value:0.000  
Er-value:0.000, Pr-value:0.000  
eCLIP MATCHES▶ppil4 (bg=43.39%)▶PRPF8 (bg=6.2%)No matches to TargetScan

-2510--(473)--2984-

GGCAAGTAA

GGCAAGTAA  
Depth:10 (SNAKE)  
Ei-value:0.000, Pi-value:0.000  
Er-value:0.000, Pr-value:0.000  
eCLIP MATCHES▶hnrnpa1 (bg=18.32%)▶ppil4 (bg=43.39%)▶safb (bg=40.39%)No matches to TargetScan

-2992--(379)--3372-

CAA

CAAGTAAGA  
Depth:10 (SNAKE)  
Ei-value:0.000, Pi-value:0.000  
Er-value:0.000, Pr-value:0.000  
eCLIP MATCHES▶HNRNPM (bg=6.37%)▶srsf7 (bg=22.53%)No matches to TargetScan


GTAAGA

GTAAGA  
Depth:11 (X.TROPICALIS)  
Ei-value:0.000, Pi-value:0.000  
Er-value:0.000, Pr-value:0.000  
eCLIP MATCHES▶HNRNPM (bg=6.37%)No matches to TargetScan

-3380--(240)--3621-

TTTTTCAG

TTTTTCAG  
Depth:19 (ZEBRAFISH)  
Ei-value:0.000, Pi-value:0.000  
Er-value:0.000, Pr-value:0.000  
eCLIP MATCHES▶ddx42 (bg=10.33%)▶ppil4 (bg=43.39%)▶safb (bg=40.39%)▶u2af1 (bg=14.02%)▶u2af2 (bg=19.32%)No matches to TargetScan


AT

TTTTTCAGAT  
Depth:10 (SNAKE)  
Ei-value:0.000, Pi-value:0.000  
Er-value:0.000, Pr-value:0.000  
eCLIP MATCHES▶ddx42 (bg=10.33%)▶ppil4 (bg=43.39%)▶safb (bg=40.39%)▶u2af1 (bg=14.02%)▶u2af2 (bg=19.32%)No matches to TargetScan

-3630--(80)--3711-

AAAGGCAGAA

AAAGGCAGAA  
Depth:13 (OPOSSUM)  
Ei-value:0.000, Pi-value:0.000  
Er-value:0.000, Pr-value:0.000  
eCLIP MATCHES▶hnrnpa1 (bg=18.32%)▶ppil4 (bg=43.39%)No matches to TargetScan


A

AAAGGCAGAAA  
Depth:11 (X.TROPICALIS)  
Ei-value:0.000, Pi-value:0.000  
Er-value:0.000, Pr-value:0.000  
eCLIP MATCHES▶hnrnpa1 (bg=18.32%)▶ppil4 (bg=43.39%)No matches to TargetScan

-3721--(376)--4098-

ACAGGT

ACAGGT  
Depth:10 (SNAKE)  
Ei-value:0.000, Pi-value:0.000  
Er-value:0.000, Pr-value:0.000  
eCLIP MATCHES▶DGCR8 (bg=2.67%)▶hltf (bg=24.28%)▶khsrp (bg=27.4%)▶LIN28B (bg=1.31%)▶NOLC1 (bg=0.67%)▶ppil4 (bg=43.39%)▶rbm15 (bg=11.59%)▶safb (bg=40.39%)▶safb2 (bg=26.89%)▶srsf1 (bg=30.28%)▶znf622 (bg=18.79%)No matches to TargetScan

-4103--(66)--4170-

CA

CAGATAAGT  
Depth:11 (X.TROPICALIS)  
Ei-value:0.000, Pi-value:0.000  
Er-value:0.000, Pr-value:0.000  
eCLIP MATCHES▶cpsf6 (bg=13.45%)▶hltf (bg=24.28%)▶hnrnpa1 (bg=18.32%)▶khsrp (bg=27.4%)▶NIPBL (bg=8.2%)▶ppil4 (bg=43.39%)▶PRPF8 (bg=6.2%)▶rbm15 (bg=11.59%)▶safb (bg=40.39%)▶safb2 (bg=26.89%)▶srsf1 (bg=30.28%)▶srsf7 (bg=22.53%)▶znf622 (bg=18.79%)No matches to TargetScan


GATAAG

GATAAG  
Depth:19 (ZEBRAFISH)  
Ei-value:0.000, Pi-value:0.000  
Er-value:0.000, Pr-value:0.000  
eCLIP MATCHES▶cpsf6 (bg=13.45%)▶khsrp (bg=27.4%)▶NIPBL (bg=8.2%)▶ppil4 (bg=43.39%)▶PRPF8 (bg=6.2%)▶rbm15 (bg=11.59%)▶safb (bg=40.39%)▶safb2 (bg=26.89%)▶srsf1 (bg=30.28%)▶srsf7 (bg=22.53%)▶znf622 (bg=18.79%)No matches to TargetScan


T

CAGATAAGT  
Depth:11 (X.TROPICALIS)  
Ei-value:0.000, Pi-value:0.000  
Er-value:0.000, Pr-value:0.000  
eCLIP MATCHES▶cpsf6 (bg=13.45%)▶hltf (bg=24.28%)▶hnrnpa1 (bg=18.32%)▶khsrp (bg=27.4%)▶NIPBL (bg=8.2%)▶ppil4 (bg=43.39%)▶PRPF8 (bg=6.2%)▶rbm15 (bg=11.59%)▶safb (bg=40.39%)▶safb2 (bg=26.89%)▶srsf1 (bg=30.28%)▶srsf7 (bg=22.53%)▶znf622 (bg=18.79%)No matches to TargetScan

-4178--(18)--4197-

ATTGCAT

ATTGCAT  
Depth:11 (X.TROPICALIS)  
Ei-value:0.000, Pi-value:0.000  
Er-value:0.000, Pr-value:0.000  
eCLIP MATCHES▶cpsf6 (bg=13.45%)▶hltf (bg=24.28%)▶hnrnpa1 (bg=18.32%)▶HNRNPU (bg=9.45%)▶khsrp (bg=27.4%)▶ppil4 (bg=43.39%)▶PRPF8 (bg=6.2%)▶RBFOX2 (bg=3.41%)▶safb (bg=40.39%)▶safb2 (bg=26.89%)▶tia1 (bg=23.76%)▶tial1 (bg=15.02%)No matches to TargetScan

-4203--(575)--4779-

AGGCAGGA

AGGCAGGA  
Depth:10 (SNAKE)  
Ei-value:0.000, Pi-value:0.000  
Er-value:0.000, Pr-value:0.000  
eCLIP MATCHES▶CSTF2 (bg=7.88%)▶cstf2t (bg=12.11%)▶srsf1 (bg=30.28%)No matches to TargetScan

-4786--(74)--4861-

TAAGGA

TAAGGA  
Depth:13 (OPOSSUM)  
Ei-value:0.000, Pi-value:0.000  
Er-value:0.000, Pr-value:0.000  
eCLIP MATCHES▶cstf2t (bg=12.11%)▶tia1 (bg=23.76%)▶tial1 (bg=15.02%)No matches to TargetScan

-4866--(338)--5205-

TGCAGTG

TGCAGTG  
Depth:10 (SNAKE)  
Ei-value:0.000, Pi-value:0.000  
Er-value:0.000, Pr-value:0.000  
eCLIP MATCHES▶cstf2t (bg=12.11%)▶hnrnpa1 (bg=18.32%)▶HNRNPM (bg=6.37%)▶PRPF8 (bg=6.2%)▶srsf7 (bg=22.53%)▶TARDBP (bg=5.12%)MATCHES To TargetScan▶ miR-217:ACUGCAU

-5211--(142)--5354-

TTGGCAAGTAA

TTGGCAAGTAA  
Depth:10 (SNAKE)  
Ei-value:0.000, Pi-value:0.000  
Er-value:0.000, Pr-value:0.000  
eCLIP MATCHES▶CSTF2 (bg=7.88%)▶cstf2t (bg=12.11%)▶HNRNPC (bg=4.22%)▶khsrp (bg=27.4%)▶ppil4 (bg=43.39%)▶srsf7 (bg=22.53%)▶TARDBP (bg=5.12%)▶tia1 (bg=23.76%)▶u2af1 (bg=14.02%)▶u2af2 (bg=19.32%)No matches to TargetScan

-5364--(208)--5573-

TTAGGT

TTAGGT  
Depth:11 (X.TROPICALIS)  
Ei-value:0.000, Pi-value:0.000  
Er-value:0.000, Pr-value:0.000  
eCLIP MATCHES▶cstf2t (bg=12.11%)▶HNRNPM (bg=6.37%)▶khsrp (bg=27.4%)▶srsf7 (bg=22.53%)▶TARDBP (bg=5.12%)▶tia1 (bg=23.76%)▶tial1 (bg=15.02%)▶u2af1 (bg=14.02%)▶ZRANB2 (bg=7.28%)No matches to TargetScan


AA

TTAGGTAA  
Depth:10 (SNAKE)  
Ei-value:0.000, Pi-value:0.000  
Er-value:0.000, Pr-value:0.000  
eCLIP MATCHES▶cstf2t (bg=12.11%)▶HNRNPM (bg=6.37%)▶khsrp (bg=27.4%)▶srsf7 (bg=22.53%)▶TARDBP (bg=5.12%)▶tia1 (bg=23.76%)▶tial1 (bg=15.02%)▶u2af1 (bg=14.02%)▶ZRANB2 (bg=7.28%)No matches to TargetScan

-5580--(36)--5617-

T

TGAAGCCTTTAGT  
Depth:10 (SNAKE)  
Ei-value:0.000, Pi-value:0.000  
Er-value:0.000, Pr-value:0.000  
eCLIP MATCHES▶ddx42 (bg=10.33%)▶khsrp (bg=27.4%)▶SF3B4 (bg=4.76%)▶SMNDC1 (bg=7.08%)▶TARDBP (bg=5.12%)▶tia1 (bg=23.76%)▶tial1 (bg=15.02%)▶u2af1 (bg=14.02%)▶u2af2 (bg=19.32%)▶ZRANB2 (bg=7.28%)No matches to TargetScan


GAAGCC

GAAGCC  
Depth:11 (X.TROPICALIS)  
Ei-value:0.000, Pi-value:0.000  
Er-value:0.000, Pr-value:0.000  
eCLIP MATCHES▶ddx42 (bg=10.33%)▶khsrp (bg=27.4%)▶SF3B4 (bg=4.76%)▶TARDBP (bg=5.12%)▶tia1 (bg=23.76%)▶tial1 (bg=15.02%)▶u2af1 (bg=14.02%)▶u2af2 (bg=19.32%)▶ZRANB2 (bg=7.28%)No matches to TargetScan


TTTAGT

TGAAGCCTTTAGT  
Depth:10 (SNAKE)  
Ei-value:0.000, Pi-value:0.000  
Er-value:0.000, Pr-value:0.000  
eCLIP MATCHES▶ddx42 (bg=10.33%)▶khsrp (bg=27.4%)▶SF3B4 (bg=4.76%)▶SMNDC1 (bg=7.08%)▶TARDBP (bg=5.12%)▶tia1 (bg=23.76%)▶tial1 (bg=15.02%)▶u2af1 (bg=14.02%)▶u2af2 (bg=19.32%)▶ZRANB2 (bg=7.28%)No matches to TargetScan

-5629--(3)--5633-

TTCCAGAT

TTCCAGAT  
Depth:10 (SNAKE)  
Ei-value:0.000, Pi-value:0.000  
Er-value:0.000, Pr-value:0.000  
eCLIP MATCHES▶ddx42 (bg=10.33%)▶khsrp (bg=27.4%)▶ppil4 (bg=43.39%)▶SF3B4 (bg=4.76%)▶SMNDC1 (bg=7.08%)▶tia1 (bg=23.76%)▶tial1 (bg=15.02%)▶u2af1 (bg=14.02%)▶u2af2 (bg=19.32%)▶ZRANB2 (bg=7.28%)No matches to TargetScan

-5640--(63)--5704-

GGCAAGT

GGCAAGT  
Depth:10 (SNAKE)  
Ei-value:0.000, Pi-value:0.000  
Er-value:0.000, Pr-value:0.000  
eCLIP MATCHES▶bclaf1 (bg=17.67%)▶khsrp (bg=27.4%)▶srsf7 (bg=22.53%)▶u2af2 (bg=19.32%)▶znf622 (bg=18.79%)No matches to TargetScan

-5710--(285)--5996-

AAC

AACAGGTGAA  
Depth:10 (SNAKE)  
Ei-value:0.000, Pi-value:0.000  
Er-value:0.000, Pr-value:0.000  
eCLIP MATCHES▶GRWD1 (bg=7.0%)▶srsf1 (bg=30.28%)▶srsf7 (bg=22.53%)No matches to TargetScan


AGGTGA

AGGTGA  
Depth:11 (X.TROPICALIS)  
Ei-value:0.000, Pi-value:0.000  
Er-value:0.000, Pr-value:0.010  
eCLIP MATCHES▶GRWD1 (bg=7.0%)▶srsf1 (bg=30.28%)▶srsf7 (bg=22.53%)No matches to TargetScan


A

AACAGGTGAA  
Depth:10 (SNAKE)  
Ei-value:0.000, Pi-value:0.000  
Er-value:0.000, Pr-value:0.000  
eCLIP MATCHES▶GRWD1 (bg=7.0%)▶srsf1 (bg=30.28%)▶srsf7 (bg=22.53%)No matches to TargetScan

-6005--(184)--6190-

AAGGCT

AAGGCT  
Depth:10 (SNAKE)  
Ei-value:0.000, Pi-value:0.000  
Er-value:0.000, Pr-value:0.010  
eCLIP MATCHES▶HNRNPU (bg=9.45%)▶tial1 (bg=15.02%)No matches to TargetScan

-6195--(290)--6486-

TTTTCCAG

TTTTCCAG  
Depth:10 (SNAKE)  
Ei-value:0.000, Pi-value:0.000  
Er-value:0.000, Pr-value:0.000  
No matches to eCLIP DataNo matches to TargetScan

-6493--(113)--6607-

TGGATCCT

TGGATCCT  
Depth:10 (SNAKE)  
Ei-value:0.000, Pi-value:0.000  
Er-value:0.000, Pr-value:0.000  
No matches to eCLIP DataNo matches to TargetScan

-6614--(26)--6641-

AAGGTAA

AAGGTAA  
Depth:10 (SNAKE)  
Ei-value:0.000, Pi-value:0.000  
Er-value:0.000, Pr-value:0.000  
eCLIP MATCHES▶PRPF8 (bg=6.2%)No matches to TargetScan

-6647--(246)--6894-

GGGTGGG

GGGTGGG  
Depth:10 (SNAKE)  
Ei-value:0.000, Pi-value:0.000  
Er-value:0.000, Pr-value:0.000  
eCLIP MATCHES▶ddx42 (bg=10.33%)▶khsrp (bg=27.4%)▶QKI (bg=3.74%)▶SF3B4 (bg=4.76%)No matches to TargetScan

-6900--(15)--6916-

TGCTAAGACT

TGCTAAGACT  
Depth:10 (SNAKE)  
Ei-value:0.000, Pi-value:0.000  
Er-value:0.000, Pr-value:0.000  
eCLIP MATCHES▶ddx42 (bg=10.33%)▶khsrp (bg=27.4%)▶QKI (bg=3.74%)▶SF3B4 (bg=4.76%)No matches to TargetScan

-6925--(85)--7011-

TTTTCTTTT

TTTTCTTTT  
Depth:19 (ZEBRAFISH)  
Ei-value:0.000, Pi-value:0.000  
Er-value:0.000, Pr-value:0.000  
eCLIP MATCHES▶srsf7 (bg=22.53%)MATCHES To TargetScan▶ miR-186-5p:AAAGAAU


CCTGAGA

TTTTCTTTTCCTGAGA  
Depth:10 (SNAKE)  
Ei-value:0.000, Pi-value:0.000  
Er-value:0.000, Pr-value:0.000  
eCLIP MATCHES▶srsf7 (bg=22.53%)MATCHES To TargetScan▶ miR-873-5p.1:CAGGAAC▶ miR-186-5p:AAAGAAU

-7026--(18)--7045-

TCT

TCTCAGGTTTTGCTTTT  
Depth:10 (SNAKE)  
Ei-value:0.000, Pi-value:0.000  
Er-value:0.000, Pr-value:0.000  
eCLIP MATCHES▶srsf7 (bg=22.53%)MATCHES To TargetScan▶ miR-330-3p.2:AAAGCAC▶ miR-490-3p:AACCUGG


CAGGTTTTGCTTT

CAGGTTTTGCTTT  
Depth:19 (ZEBRAFISH)  
Ei-value:0.000, Pi-value:0.000  
Er-value:0.000, Pr-value:0.000  
eCLIP MATCHES▶srsf7 (bg=22.53%)MATCHES To TargetScan▶ miR-330-3p.2:AAAGCAC▶ miR-490-3p:AACCUGG


T

CAGGTTTTGCTTTT  
Depth:16 (NILETILAPIA)  
Ei-value:0.000, Pi-value:0.000  
Er-value:0.000, Pr-value:0.000  
eCLIP MATCHES▶srsf7 (bg=22.53%)MATCHES To TargetScan▶ miR-330-3p.2:AAAGCAC▶ miR-490-3p:AACCUGG

-7061--(24)--7086-

A

AAAAAAGCAAAAG  
Depth:13 (OPOSSUM)  
Ei-value:0.000, Pi-value:0.000  
Er-value:0.000, Pr-value:0.000  
No matches to eCLIP DataNo matches to TargetScan


AAAAAGCAAAA

AAAAAGCAAAA  
Depth:19 (ZEBRAFISH)  
Ei-value:0.000, Pi-value:0.000  
Er-value:0.000, Pr-value:0.000  
No matches to eCLIP DataNo matches to TargetScan


G

AAAAAGCAAAAG  
Depth:18 (MEDAKA)  
Ei-value:0.000, Pi-value:0.000  
Er-value:0.000, Pr-value:0.000  
No matches to eCLIP DataNo matches to TargetScan

-7098--(15)--7114-

ACTCCTG

ACTCCTG  
Depth:18 (MEDAKA)  
Ei-value:0.000, Pi-value:0.000  
Er-value:0.000, Pr-value:0.000  
No matches to eCLIP DataNo matches to TargetScan


G

ACTCCTGG  
Depth:14 (SPOTTEDGAR)  
Ei-value:0.000, Pi-value:0.000  
Er-value:0.000, Pr-value:0.000  
No matches to eCLIP DataMATCHES To TargetScan▶ miR-665:CCAGGAG

-7121--(11)--7133-

GGGTTC

GGGTTC  
Depth:13 (OPOSSUM)  
Ei-value:0.000, Pi-value:0.000  
Er-value:0.000, Pr-value:0.000  
No matches to eCLIP DataNo matches to TargetScan

-7138  
  
>MARMOSET  
      1687-

GGTGAAG

GGTGAAG  
Depth:11 (X.TROPICALIS)  
Ei-value:0.000, Pi-value:0.000  
Er-value:0.000, Pr-value:0.000  
No matches to TargetScan

-1693--(214)--1908-

TGGTAA

TGGTAA  
Depth:14 (SPOTTEDGAR)  
Ei-value:0.030, Pi-value:0.000  
Er-value:0.010, Pr-value:0.000  
No matches to TargetScan

-1913--(723)--2637-

TTTGGG

TTTGGG  
Depth:19 (ZEBRAFISH)  
Ei-value:0.000, Pi-value:0.000  
Er-value:0.000, Pr-value:0.000  
No matches to TargetScan

-2642--(472)--3115-

GGCAAGTAA

GGCAAGTAA  
Depth:10 (SNAKE)  
Ei-value:0.000, Pi-value:0.000  
Er-value:0.000, Pr-value:0.000  
No matches to TargetScan

-3123--(374)--3498-

CAA

CAAGTAAGA  
Depth:10 (SNAKE)  
Ei-value:0.000, Pi-value:0.000  
Er-value:0.000, Pr-value:0.000  
No matches to TargetScan


GTAAGA

GTAAGA  
Depth:11 (X.TROPICALIS)  
Ei-value:0.000, Pi-value:0.000  
Er-value:0.000, Pr-value:0.000  
No matches to TargetScan

-3506--(231)--3738-

TTTTTCAG

TTTTTCAG  
Depth:19 (ZEBRAFISH)  
Ei-value:0.000, Pi-value:0.000  
Er-value:0.000, Pr-value:0.000  
No matches to TargetScan


AT

TTTTTCAGAT  
Depth:10 (SNAKE)  
Ei-value:0.000, Pi-value:0.000  
Er-value:0.000, Pr-value:0.000  
No matches to TargetScan

-3747--(79)--3827-

AAAGGCAGAA

AAAGGCAGAA  
Depth:13 (OPOSSUM)  
Ei-value:0.000, Pi-value:0.000  
Er-value:0.000, Pr-value:0.000  
No matches to TargetScan


A

AAAGGCAGAAA  
Depth:11 (X.TROPICALIS)  
Ei-value:0.000, Pi-value:0.000  
Er-value:0.000, Pr-value:0.000  
No matches to TargetScan

-3837--(382)--4220-

ACAGGT

ACAGGT  
Depth:10 (SNAKE)  
Ei-value:0.000, Pi-value:0.000  
Er-value:0.000, Pr-value:0.000  
No matches to TargetScan

-4225--(66)--4292-

CA

CAGATAAGT  
Depth:11 (X.TROPICALIS)  
Ei-value:0.000, Pi-value:0.000  
Er-value:0.000, Pr-value:0.000  
No matches to TargetScan


GATAAG

GATAAG  
Depth:19 (ZEBRAFISH)  
Ei-value:0.000, Pi-value:0.000  
Er-value:0.000, Pr-value:0.000  
No matches to TargetScan


T

CAGATAAGT  
Depth:11 (X.TROPICALIS)  
Ei-value:0.000, Pi-value:0.000  
Er-value:0.000, Pr-value:0.000  
No matches to TargetScan

-4300--(18)--4319-

ATTGCAT

ATTGCAT  
Depth:11 (X.TROPICALIS)  
Ei-value:0.000, Pi-value:0.000  
Er-value:0.000, Pr-value:0.000  
No matches to TargetScan

-4325--(584)--4910-

AGGCAGGA

AGGCAGGA  
Depth:10 (SNAKE)  
Ei-value:0.000, Pi-value:0.000  
Er-value:0.000, Pr-value:0.000  
No matches to TargetScan

-4917--(72)--4990-

TAAGGA

TAAGGA  
Depth:13 (OPOSSUM)  
Ei-value:0.000, Pi-value:0.000  
Er-value:0.000, Pr-value:0.000  
No matches to TargetScan

-4995--(340)--5336-

TGCAGTG

TGCAGTG  
Depth:10 (SNAKE)  
Ei-value:0.000, Pi-value:0.000  
Er-value:0.000, Pr-value:0.000  
MATCHES To TargetScan▶ miR-217:ACUGCAU

-5342--(138)--5481-

TTGGCAAGTAA

TTGGCAAGTAA  
Depth:10 (SNAKE)  
Ei-value:0.000, Pi-value:0.000  
Er-value:0.000, Pr-value:0.000  
No matches to TargetScan

-5491--(214)--5706-

TTAGGT

TTAGGT  
Depth:11 (X.TROPICALIS)  
Ei-value:0.000, Pi-value:0.000  
Er-value:0.000, Pr-value:0.000  
No matches to TargetScan


AA

TTAGGTAA  
Depth:10 (SNAKE)  
Ei-value:0.000, Pi-value:0.000  
Er-value:0.000, Pr-value:0.000  
No matches to TargetScan

-5713--(36)--5750-

T

TGAAGCCTTTAGT  
Depth:10 (SNAKE)  
Ei-value:0.000, Pi-value:0.000  
Er-value:0.000, Pr-value:0.000  
No matches to TargetScan


GAAGCC

GAAGCC  
Depth:11 (X.TROPICALIS)  
Ei-value:0.000, Pi-value:0.000  
Er-value:0.000, Pr-value:0.000  
No matches to TargetScan


TTTAGT

TGAAGCCTTTAGT  
Depth:10 (SNAKE)  
Ei-value:0.000, Pi-value:0.000  
Er-value:0.000, Pr-value:0.000  
No matches to TargetScan

-5762--(3)--5766-

TTCCAGAT

TTCCAGAT  
Depth:10 (SNAKE)  
Ei-value:0.000, Pi-value:0.000  
Er-value:0.000, Pr-value:0.000  
No matches to TargetScan

-5773--(65)--5839-

GGCAAGT

GGCAAGT  
Depth:10 (SNAKE)  
Ei-value:0.000, Pi-value:0.000  
Er-value:0.000, Pr-value:0.000  
No matches to TargetScan

-5845--(289)--6135-

AAC

AACAGGTGAA  
Depth:10 (SNAKE)  
Ei-value:0.000, Pi-value:0.000  
Er-value:0.000, Pr-value:0.000  
No matches to TargetScan


AGGTGA

AGGTGA  
Depth:11 (X.TROPICALIS)  
Ei-value:0.000, Pi-value:0.000  
Er-value:0.000, Pr-value:0.010  
No matches to TargetScan


A

AACAGGTGAA  
Depth:10 (SNAKE)  
Ei-value:0.000, Pi-value:0.000  
Er-value:0.000, Pr-value:0.000  
No matches to TargetScan

-6144--(189)--6334-

AAGGCT

AAGGCT  
Depth:10 (SNAKE)  
Ei-value:0.000, Pi-value:0.000  
Er-value:0.000, Pr-value:0.010  
No matches to TargetScan

-6339--(286)--6626-

TTTTCCAG

TTTTCCAG  
Depth:10 (SNAKE)  
Ei-value:0.000, Pi-value:0.000  
Er-value:0.000, Pr-value:0.000  
No matches to TargetScan

-6633--(113)--6747-

TGGATCCT

TGGATCCT  
Depth:10 (SNAKE)  
Ei-value:0.000, Pi-value:0.000  
Er-value:0.000, Pr-value:0.000  
No matches to TargetScan

-6754--(26)--6781-

AAGGTAA

AAGGTAA  
Depth:10 (SNAKE)  
Ei-value:0.000, Pi-value:0.000  
Er-value:0.000, Pr-value:0.000  
No matches to TargetScan

-6787--(235)--7023-

GGGTGGG

GGGTGGG  
Depth:10 (SNAKE)  
Ei-value:0.000, Pi-value:0.000  
Er-value:0.000, Pr-value:0.000  
No matches to TargetScan

-7029--(15)--7045-

TGCTAAGACT

TGCTAAGACT  
Depth:10 (SNAKE)  
Ei-value:0.000, Pi-value:0.000  
Er-value:0.000, Pr-value:0.000  
No matches to TargetScan

-7054--(85)--7140-

TTTTCTTTT

TTTTCTTTT  
Depth:19 (ZEBRAFISH)  
Ei-value:0.000, Pi-value:0.000  
Er-value:0.000, Pr-value:0.000  
MATCHES To TargetScan▶ miR-186-5p:AAAGAAU


CCTGAGA

TTTTCTTTTCCTGAGA  
Depth:10 (SNAKE)  
Ei-value:0.000, Pi-value:0.000  
Er-value:0.000, Pr-value:0.000  
MATCHES To TargetScan▶ miR-873-5p.1:CAGGAAC▶ miR-186-5p:AAAGAAU

-7155--(18)--7174-

TCT

TCTCAGGTTTTGCTTTT  
Depth:10 (SNAKE)  
Ei-value:0.000, Pi-value:0.000  
Er-value:0.000, Pr-value:0.000  
MATCHES To TargetScan▶ miR-330-3p.2:AAAGCAC▶ miR-490-3p:AACCUGG


CAGGTTTTGCTTT

CAGGTTTTGCTTT  
Depth:19 (ZEBRAFISH)  
Ei-value:0.000, Pi-value:0.000  
Er-value:0.000, Pr-value:0.000  
MATCHES To TargetScan▶ miR-330-3p.2:AAAGCAC▶ miR-490-3p:AACCUGG


T

CAGGTTTTGCTTTT  
Depth:16 (NILETILAPIA)  
Ei-value:0.000, Pi-value:0.000  
Er-value:0.000, Pr-value:0.000  
MATCHES To TargetScan▶ miR-330-3p.2:AAAGCAC▶ miR-490-3p:AACCUGG

-7190--(25)--7216-

A

AAAAAAGCAAAAG  
Depth:13 (OPOSSUM)  
Ei-value:0.000, Pi-value:0.000  
Er-value:0.000, Pr-value:0.000  
No matches to TargetScan


AAAAAGCAAAA

AAAAAGCAAAA  
Depth:19 (ZEBRAFISH)  
Ei-value:0.000, Pi-value:0.000  
Er-value:0.000, Pr-value:0.000  
No matches to TargetScan


G

AAAAAGCAAAAG  
Depth:18 (MEDAKA)  
Ei-value:0.000, Pi-value:0.000  
Er-value:0.000, Pr-value:0.000  
No matches to TargetScan

-7228--(15)--7244-

ACTCCTG

ACTCCTG  
Depth:18 (MEDAKA)  
Ei-value:0.000, Pi-value:0.000  
Er-value:0.000, Pr-value:0.000  
No matches to TargetScan


G

ACTCCTGG  
Depth:14 (SPOTTEDGAR)  
Ei-value:0.000, Pi-value:0.000  
Er-value:0.000, Pr-value:0.000  
MATCHES To TargetScan▶ miR-665:CCAGGAG

-7251--(11)--7263-

GGGTTC

GGGTTC  
Depth:13 (OPOSSUM)  
Ei-value:0.000, Pi-value:0.000  
Er-value:0.000, Pr-value:0.000  
No matches to TargetScan

-7268  
  
>DOG  
      1716-

GGTGAAG

GGTGAAG  
Depth:11 (X.TROPICALIS)  
Ei-value:0.000, Pi-value:0.000  
Er-value:0.000, Pr-value:0.000  
No matches to TargetScan

-1722--(205)--1928-

TGGTAA

TGGTAA  
Depth:14 (SPOTTEDGAR)  
Ei-value:0.030, Pi-value:0.000  
Er-value:0.010, Pr-value:0.000  
No matches to TargetScan

-1933--(678)--2612-

TTTGGG

TTTGGG  
Depth:19 (ZEBRAFISH)  
Ei-value:0.000, Pi-value:0.000  
Er-value:0.000, Pr-value:0.000  
No matches to TargetScan

-2617--(473)--3091-

GGCAAGTAA

GGCAAGTAA  
Depth:10 (SNAKE)  
Ei-value:0.000, Pi-value:0.000  
Er-value:0.000, Pr-value:0.000  
No matches to TargetScan

-3099--(330)--3430-

CAA

CAAGTAAGA  
Depth:10 (SNAKE)  
Ei-value:0.000, Pi-value:0.000  
Er-value:0.000, Pr-value:0.000  
No matches to TargetScan


GTAAGA

GTAAGA  
Depth:11 (X.TROPICALIS)  
Ei-value:0.000, Pi-value:0.000  
Er-value:0.000, Pr-value:0.000  
No matches to TargetScan

-3438--(192)--3631-

TTTTTCAG

TTTTTCAG  
Depth:19 (ZEBRAFISH)  
Ei-value:0.000, Pi-value:0.000  
Er-value:0.000, Pr-value:0.000  
No matches to TargetScan


AT

TTTTTCAGAT  
Depth:10 (SNAKE)  
Ei-value:0.000, Pi-value:0.000  
Er-value:0.000, Pr-value:0.000  
No matches to TargetScan

-3640--(77)--3718-

AAAGGCAGAA

AAAGGCAGAA  
Depth:13 (OPOSSUM)  
Ei-value:0.000, Pi-value:0.000  
Er-value:0.000, Pr-value:0.000  
No matches to TargetScan


A

AAAGGCAGAAA  
Depth:11 (X.TROPICALIS)  
Ei-value:0.000, Pi-value:0.000  
Er-value:0.000, Pr-value:0.000  
No matches to TargetScan

-3728--(395)--4124-

ACAGGT

ACAGGT  
Depth:10 (SNAKE)  
Ei-value:0.000, Pi-value:0.000  
Er-value:0.000, Pr-value:0.000  
No matches to TargetScan

-4129--(66)--4196-

CA

CAGATAAGT  
Depth:11 (X.TROPICALIS)  
Ei-value:0.000, Pi-value:0.000  
Er-value:0.000, Pr-value:0.000  
No matches to TargetScan


GATAAG

GATAAG  
Depth:19 (ZEBRAFISH)  
Ei-value:0.000, Pi-value:0.000  
Er-value:0.000, Pr-value:0.000  
No matches to TargetScan


T

CAGATAAGT  
Depth:11 (X.TROPICALIS)  
Ei-value:0.000, Pi-value:0.000  
Er-value:0.000, Pr-value:0.000  
No matches to TargetScan

-4204--(18)--4223-

ATTGCAT

ATTGCAT  
Depth:11 (X.TROPICALIS)  
Ei-value:0.000, Pi-value:0.000  
Er-value:0.000, Pr-value:0.000  
No matches to TargetScan

-4229--(570)--4800-

AGGCAGGA

AGGCAGGA  
Depth:10 (SNAKE)  
Ei-value:0.000, Pi-value:0.000  
Er-value:0.000, Pr-value:0.000  
No matches to TargetScan

-4807--(72)--4880-

TAAGGA

TAAGGA  
Depth:13 (OPOSSUM)  
Ei-value:0.000, Pi-value:0.000  
Er-value:0.000, Pr-value:0.000  
No matches to TargetScan

-4885--(350)--5236-

TGCAGTG

TGCAGTG  
Depth:10 (SNAKE)  
Ei-value:0.000, Pi-value:0.000  
Er-value:0.000, Pr-value:0.000  
MATCHES To TargetScan▶ miR-217:ACUGCAU

-5242--(132)--5375-

TTGGCAAGTAA

TTGGCAAGTAA  
Depth:10 (SNAKE)  
Ei-value:0.000, Pi-value:0.000  
Er-value:0.000, Pr-value:0.000  
No matches to TargetScan

-5385--(204)--5590-

TTAGGT

TTAGGT  
Depth:11 (X.TROPICALIS)  
Ei-value:0.000, Pi-value:0.000  
Er-value:0.000, Pr-value:0.000  
No matches to TargetScan


AA

TTAGGTAA  
Depth:10 (SNAKE)  
Ei-value:0.000, Pi-value:0.000  
Er-value:0.000, Pr-value:0.000  
No matches to TargetScan

-5597--(42)--5640-

T

TGAAGCCTTTAGT  
Depth:10 (SNAKE)  
Ei-value:0.000, Pi-value:0.000  
Er-value:0.000, Pr-value:0.000  
No matches to TargetScan


GAAGCC

GAAGCC  
Depth:11 (X.TROPICALIS)  
Ei-value:0.000, Pi-value:0.000  
Er-value:0.000, Pr-value:0.000  
No matches to TargetScan


TTTAGT

TGAAGCCTTTAGT  
Depth:10 (SNAKE)  
Ei-value:0.000, Pi-value:0.000  
Er-value:0.000, Pr-value:0.000  
No matches to TargetScan

-5652--(3)--5656-

TTCCAGAT

TTCCAGAT  
Depth:10 (SNAKE)  
Ei-value:0.000, Pi-value:0.000  
Er-value:0.000, Pr-value:0.000  
No matches to TargetScan

-5663--(64)--5728-

GGCAAGT

GGCAAGT  
Depth:10 (SNAKE)  
Ei-value:0.000, Pi-value:0.000  
Er-value:0.000, Pr-value:0.000  
No matches to TargetScan

-5734--(282)--6017-

AAC

AACAGGTGAA  
Depth:10 (SNAKE)  
Ei-value:0.000, Pi-value:0.000  
Er-value:0.000, Pr-value:0.000  
No matches to TargetScan


AGGTGA

AGGTGA  
Depth:11 (X.TROPICALIS)  
Ei-value:0.000, Pi-value:0.000  
Er-value:0.000, Pr-value:0.010  
No matches to TargetScan


A

AACAGGTGAA  
Depth:10 (SNAKE)  
Ei-value:0.000, Pi-value:0.000  
Er-value:0.000, Pr-value:0.000  
No matches to TargetScan

-6026--(190)--6217-

AAGGCT

AAGGCT  
Depth:10 (SNAKE)  
Ei-value:0.000, Pi-value:0.000  
Er-value:0.000, Pr-value:0.010  
No matches to TargetScan

-6222--(328)--6551-

TTTTCCAG

TTTTCCAG  
Depth:10 (SNAKE)  
Ei-value:0.000, Pi-value:0.000  
Er-value:0.000, Pr-value:0.000  
No matches to TargetScan

-6558--(114)--6673-

TGGATCCT

TGGATCCT  
Depth:10 (SNAKE)  
Ei-value:0.000, Pi-value:0.000  
Er-value:0.000, Pr-value:0.000  
No matches to TargetScan

-6680--(26)--6707-

AAGGTAA

AAGGTAA  
Depth:10 (SNAKE)  
Ei-value:0.000, Pi-value:0.000  
Er-value:0.000, Pr-value:0.000  
No matches to TargetScan

-6713--(168)--6882-

GGGTGGG

GGGTGGG  
Depth:10 (SNAKE)  
Ei-value:0.000, Pi-value:0.000  
Er-value:0.000, Pr-value:0.000  
No matches to TargetScan

-6888--(73)--6962-

TGCTAAGACT

TGCTAAGACT  
Depth:10 (SNAKE)  
Ei-value:0.000, Pi-value:0.000  
Er-value:0.000, Pr-value:0.000  
No matches to TargetScan

-6971--(88)--7060-

TTTTCTTTT

TTTTCTTTT  
Depth:19 (ZEBRAFISH)  
Ei-value:0.000, Pi-value:0.000  
Er-value:0.000, Pr-value:0.000  
MATCHES To TargetScan▶ miR-186-5p:AAAGAAU


CCTGAGA

TTTTCTTTTCCTGAGA  
Depth:10 (SNAKE)  
Ei-value:0.000, Pi-value:0.000  
Er-value:0.000, Pr-value:0.000  
MATCHES To TargetScan▶ miR-873-5p.1:CAGGAAC▶ miR-186-5p:AAAGAAU

-7075--(16)--7092-

TCT

TCTCAGGTTTTGCTTTT  
Depth:10 (SNAKE)  
Ei-value:0.000, Pi-value:0.000  
Er-value:0.000, Pr-value:0.000  
MATCHES To TargetScan▶ miR-330-3p.2:AAAGCAC▶ miR-490-3p:AACCUGG


CAGGTTTTGCTTT

CAGGTTTTGCTTT  
Depth:19 (ZEBRAFISH)  
Ei-value:0.000, Pi-value:0.000  
Er-value:0.000, Pr-value:0.000  
MATCHES To TargetScan▶ miR-330-3p.2:AAAGCAC▶ miR-490-3p:AACCUGG


T

CAGGTTTTGCTTTT  
Depth:16 (NILETILAPIA)  
Ei-value:0.000, Pi-value:0.000  
Er-value:0.000, Pr-value:0.000  
MATCHES To TargetScan▶ miR-330-3p.2:AAAGCAC▶ miR-490-3p:AACCUGG

-7108--(20)--7129-

A

AAAAAAGCAAAAG  
Depth:13 (OPOSSUM)  
Ei-value:0.000, Pi-value:0.000  
Er-value:0.000, Pr-value:0.000  
No matches to TargetScan


AAAAAGCAAAA

AAAAAGCAAAA  
Depth:19 (ZEBRAFISH)  
Ei-value:0.000, Pi-value:0.000  
Er-value:0.000, Pr-value:0.000  
No matches to TargetScan


G

AAAAAGCAAAAG  
Depth:18 (MEDAKA)  
Ei-value:0.000, Pi-value:0.000  
Er-value:0.000, Pr-value:0.000  
No matches to TargetScan

-7141--(15)--7157-

ACTCCTG

ACTCCTG  
Depth:18 (MEDAKA)  
Ei-value:0.000, Pi-value:0.000  
Er-value:0.000, Pr-value:0.000  
No matches to TargetScan


G

ACTCCTGG  
Depth:14 (SPOTTEDGAR)  
Ei-value:0.000, Pi-value:0.000  
Er-value:0.000, Pr-value:0.000  
MATCHES To TargetScan▶ miR-665:CCAGGAG

-7164--(12)--7177-

GGGTTC

GGGTTC  
Depth:13 (OPOSSUM)  
Ei-value:0.000, Pi-value:0.000  
Er-value:0.000, Pr-value:0.000  
No matches to TargetScan

-7182  
  
>PIG  
      1605-

GGTGAAG

GGTGAAG  
Depth:11 (X.TROPICALIS)  
Ei-value:0.000, Pi-value:0.000  
Er-value:0.000, Pr-value:0.000  
No matches to TargetScan

-1611--(237)--1849-

TGGTAA

TGGTAA  
Depth:14 (SPOTTEDGAR)  
Ei-value:0.030, Pi-value:0.000  
Er-value:0.010, Pr-value:0.000  
No matches to TargetScan

-1854--(678)--2533-

TTTGGG

TTTGGG  
Depth:19 (ZEBRAFISH)  
Ei-value:0.000, Pi-value:0.000  
Er-value:0.000, Pr-value:0.000  
No matches to TargetScan

-2538--(466)--3005-

GGCAAGTAA

GGCAAGTAA  
Depth:10 (SNAKE)  
Ei-value:0.000, Pi-value:0.000  
Er-value:0.000, Pr-value:0.000  
No matches to TargetScan

-3013--(324)--3338-

CAA

CAAGTAAGA  
Depth:10 (SNAKE)  
Ei-value:0.000, Pi-value:0.000  
Er-value:0.000, Pr-value:0.000  
No matches to TargetScan


GTAAGA

GTAAGA  
Depth:11 (X.TROPICALIS)  
Ei-value:0.000, Pi-value:0.000  
Er-value:0.000, Pr-value:0.000  
No matches to TargetScan

-3346--(194)--3541-

TTTTTCAG

TTTTTCAG  
Depth:19 (ZEBRAFISH)  
Ei-value:0.000, Pi-value:0.000  
Er-value:0.000, Pr-value:0.000  
No matches to TargetScan


AT

TTTTTCAGAT  
Depth:10 (SNAKE)  
Ei-value:0.000, Pi-value:0.000  
Er-value:0.000, Pr-value:0.000  
No matches to TargetScan

-3550--(80)--3631-

AAAGGCAGAA

AAAGGCAGAA  
Depth:13 (OPOSSUM)  
Ei-value:0.000, Pi-value:0.000  
Er-value:0.000, Pr-value:0.000  
No matches to TargetScan


A

AAAGGCAGAAA  
Depth:11 (X.TROPICALIS)  
Ei-value:0.000, Pi-value:0.000  
Er-value:0.000, Pr-value:0.000  
No matches to TargetScan

-3641--(405)--4047-

ACAGGT

ACAGGT  
Depth:10 (SNAKE)  
Ei-value:0.000, Pi-value:0.000  
Er-value:0.000, Pr-value:0.000  
No matches to TargetScan

-4052--(63)--4116-

CA

CAGATAAGT  
Depth:11 (X.TROPICALIS)  
Ei-value:0.000, Pi-value:0.000  
Er-value:0.000, Pr-value:0.000  
No matches to TargetScan


GATAAG

GATAAG  
Depth:19 (ZEBRAFISH)  
Ei-value:0.000, Pi-value:0.000  
Er-value:0.000, Pr-value:0.000  
No matches to TargetScan


T

CAGATAAGT  
Depth:11 (X.TROPICALIS)  
Ei-value:0.000, Pi-value:0.000  
Er-value:0.000, Pr-value:0.000  
No matches to TargetScan

-4124--(18)--4143-

ATTGCAT

ATTGCAT  
Depth:11 (X.TROPICALIS)  
Ei-value:0.000, Pi-value:0.000  
Er-value:0.000, Pr-value:0.000  
No matches to TargetScan

-4149--(549)--4699-

AGGCAGGA

AGGCAGGA  
Depth:10 (SNAKE)  
Ei-value:0.000, Pi-value:0.000  
Er-value:0.000, Pr-value:0.000  
No matches to TargetScan

-4706--(79)--4786-

TAAGGA

TAAGGA  
Depth:13 (OPOSSUM)  
Ei-value:0.000, Pi-value:0.000  
Er-value:0.000, Pr-value:0.000  
No matches to TargetScan

-4791--(349)--5141-

TGCAGTG

TGCAGTG  
Depth:10 (SNAKE)  
Ei-value:0.000, Pi-value:0.000  
Er-value:0.000, Pr-value:0.000  
MATCHES To TargetScan▶ miR-217:ACUGCAU

-5147--(143)--5291-

TTGGCAAGTAA

TTGGCAAGTAA  
Depth:10 (SNAKE)  
Ei-value:0.000, Pi-value:0.000  
Er-value:0.000, Pr-value:0.000  
No matches to TargetScan

-5301--(203)--5505-

TTAGGT

TTAGGT  
Depth:11 (X.TROPICALIS)  
Ei-value:0.000, Pi-value:0.000  
Er-value:0.000, Pr-value:0.000  
No matches to TargetScan


AA

TTAGGTAA  
Depth:10 (SNAKE)  
Ei-value:0.000, Pi-value:0.000  
Er-value:0.000, Pr-value:0.000  
No matches to TargetScan

-5512--(43)--5556-

T

TGAAGCCTTTAGT  
Depth:10 (SNAKE)  
Ei-value:0.000, Pi-value:0.000  
Er-value:0.000, Pr-value:0.000  
No matches to TargetScan


GAAGCC

GAAGCC  
Depth:11 (X.TROPICALIS)  
Ei-value:0.000, Pi-value:0.000  
Er-value:0.000, Pr-value:0.000  
No matches to TargetScan


TTTAGT

TGAAGCCTTTAGT  
Depth:10 (SNAKE)  
Ei-value:0.000, Pi-value:0.000  
Er-value:0.000, Pr-value:0.000  
No matches to TargetScan

-5568--(3)--5572-

TTCCAGAT

TTCCAGAT  
Depth:10 (SNAKE)  
Ei-value:0.000, Pi-value:0.000  
Er-value:0.000, Pr-value:0.000  
No matches to TargetScan

-5579--(65)--5645-

GGCAAGT

GGCAAGT  
Depth:10 (SNAKE)  
Ei-value:0.000, Pi-value:0.000  
Er-value:0.000, Pr-value:0.000  
No matches to TargetScan

-5651--(285)--5937-

AAC

AACAGGTGAA  
Depth:10 (SNAKE)  
Ei-value:0.000, Pi-value:0.000  
Er-value:0.000, Pr-value:0.000  
No matches to TargetScan


AGGTGA

AGGTGA  
Depth:11 (X.TROPICALIS)  
Ei-value:0.000, Pi-value:0.000  
Er-value:0.000, Pr-value:0.010  
No matches to TargetScan


A

AACAGGTGAA  
Depth:10 (SNAKE)  
Ei-value:0.000, Pi-value:0.000  
Er-value:0.000, Pr-value:0.000  
No matches to TargetScan

-5946--(189)--6136-

AAGGCT

AAGGCT  
Depth:10 (SNAKE)  
Ei-value:0.000, Pi-value:0.000  
Er-value:0.000, Pr-value:0.010  
No matches to TargetScan

-6141--(297)--6439-

TTTTCCAG

TTTTCCAG  
Depth:10 (SNAKE)  
Ei-value:0.000, Pi-value:0.000  
Er-value:0.000, Pr-value:0.000  
No matches to TargetScan

-6446--(113)--6560-

TGGATCCT

TGGATCCT  
Depth:10 (SNAKE)  
Ei-value:0.000, Pi-value:0.000  
Er-value:0.000, Pr-value:0.000  
No matches to TargetScan

-6567--(25)--6593-

AAGGTAA

AAGGTAA  
Depth:10 (SNAKE)  
Ei-value:0.000, Pi-value:0.000  
Er-value:0.000, Pr-value:0.000  
No matches to TargetScan

-6599--(247)--6847-

GGGTGGG

GGGTGGG  
Depth:10 (SNAKE)  
Ei-value:0.000, Pi-value:0.000  
Er-value:0.000, Pr-value:0.000  
No matches to TargetScan

-6853--(15)--6869-

TGCTAAGACT

TGCTAAGACT  
Depth:10 (SNAKE)  
Ei-value:0.000, Pi-value:0.000  
Er-value:0.000, Pr-value:0.000  
No matches to TargetScan

-6878--(93)--6972-

TTTTCTTTT

TTTTCTTTT  
Depth:19 (ZEBRAFISH)  
Ei-value:0.000, Pi-value:0.000  
Er-value:0.000, Pr-value:0.000  
MATCHES To TargetScan▶ miR-186-5p:AAAGAAU


CCTGAGA

TTTTCTTTTCCTGAGA  
Depth:10 (SNAKE)  
Ei-value:0.000, Pi-value:0.000  
Er-value:0.000, Pr-value:0.000  
MATCHES To TargetScan▶ miR-873-5p.1:CAGGAAC▶ miR-186-5p:AAAGAAU

-6987--(18)--7006-

TCT

TCTCAGGTTTTGCTTTT  
Depth:10 (SNAKE)  
Ei-value:0.000, Pi-value:0.000  
Er-value:0.000, Pr-value:0.000  
MATCHES To TargetScan▶ miR-330-3p.2:AAAGCAC▶ miR-490-3p:AACCUGG


CAGGTTTTGCTTT

CAGGTTTTGCTTT  
Depth:19 (ZEBRAFISH)  
Ei-value:0.000, Pi-value:0.000  
Er-value:0.000, Pr-value:0.000  
MATCHES To TargetScan▶ miR-330-3p.2:AAAGCAC▶ miR-490-3p:AACCUGG


T

CAGGTTTTGCTTTT  
Depth:16 (NILETILAPIA)  
Ei-value:0.000, Pi-value:0.000  
Er-value:0.000, Pr-value:0.000  
MATCHES To TargetScan▶ miR-330-3p.2:AAAGCAC▶ miR-490-3p:AACCUGG

-7022--(22)--7045-

A

AAAAAAGCAAAAG  
Depth:13 (OPOSSUM)  
Ei-value:0.000, Pi-value:0.000  
Er-value:0.000, Pr-value:0.000  
No matches to TargetScan


AAAAAGCAAAA

AAAAAGCAAAA  
Depth:19 (ZEBRAFISH)  
Ei-value:0.000, Pi-value:0.000  
Er-value:0.000, Pr-value:0.000  
No matches to TargetScan


G

AAAAAGCAAAAG  
Depth:18 (MEDAKA)  
Ei-value:0.000, Pi-value:0.000  
Er-value:0.000, Pr-value:0.000  
No matches to TargetScan

-7057--(15)--7073-

ACTCCTG

ACTCCTG  
Depth:18 (MEDAKA)  
Ei-value:0.000, Pi-value:0.000  
Er-value:0.000, Pr-value:0.000  
No matches to TargetScan


G

ACTCCTGG  
Depth:14 (SPOTTEDGAR)  
Ei-value:0.000, Pi-value:0.000  
Er-value:0.000, Pr-value:0.000  
MATCHES To TargetScan▶ miR-665:CCAGGAG

-7080--(11)--7092-

GGGTTC

GGGTTC  
Depth:13 (OPOSSUM)  
Ei-value:0.000, Pi-value:0.000  
Er-value:0.000, Pr-value:0.000  
No matches to TargetScan

-7097  
  
>COW  
      1484-

GGTGAAG

GGTGAAG  
Depth:11 (X.TROPICALIS)  
Ei-value:0.000, Pi-value:0.000  
Er-value:0.000, Pr-value:0.000  
No matches to TargetScan

-1490--(225)--1716-

TGGTAA

TGGTAA  
Depth:14 (SPOTTEDGAR)  
Ei-value:0.030, Pi-value:0.000  
Er-value:0.010, Pr-value:0.000  
No matches to TargetScan

-1721--(695)--2417-

TTTGGG

TTTGGG  
Depth:19 (ZEBRAFISH)  
Ei-value:0.000, Pi-value:0.000  
Er-value:0.000, Pr-value:0.000  
No matches to TargetScan

-2422--(473)--2896-

GGCAAGTAA

GGCAAGTAA  
Depth:10 (SNAKE)  
Ei-value:0.000, Pi-value:0.000  
Er-value:0.000, Pr-value:0.000  
No matches to TargetScan

-2904--(319)--3224-

CAA

CAAGTAAGA  
Depth:10 (SNAKE)  
Ei-value:0.000, Pi-value:0.000  
Er-value:0.000, Pr-value:0.000  
No matches to TargetScan


GTAAGA

GTAAGA  
Depth:11 (X.TROPICALIS)  
Ei-value:0.000, Pi-value:0.000  
Er-value:0.000, Pr-value:0.000  
No matches to TargetScan

-3232--(202)--3435-

TTTTTCAG

TTTTTCAG  
Depth:19 (ZEBRAFISH)  
Ei-value:0.000, Pi-value:0.000  
Er-value:0.000, Pr-value:0.000  
No matches to TargetScan


AT

TTTTTCAGAT  
Depth:10 (SNAKE)  
Ei-value:0.000, Pi-value:0.000  
Er-value:0.000, Pr-value:0.000  
No matches to TargetScan

-3444--(80)--3525-

AAAGGCAGAA

AAAGGCAGAA  
Depth:13 (OPOSSUM)  
Ei-value:0.000, Pi-value:0.000  
Er-value:0.000, Pr-value:0.000  
No matches to TargetScan


A

AAAGGCAGAAA  
Depth:11 (X.TROPICALIS)  
Ei-value:0.000, Pi-value:0.000  
Er-value:0.000, Pr-value:0.000  
No matches to TargetScan

-3535--(388)--3924-

ACAGGT

ACAGGT  
Depth:10 (SNAKE)  
Ei-value:0.000, Pi-value:0.000  
Er-value:0.000, Pr-value:0.000  
No matches to TargetScan

-3929--(66)--3996-

CA

CAGATAAGT  
Depth:11 (X.TROPICALIS)  
Ei-value:0.000, Pi-value:0.000  
Er-value:0.000, Pr-value:0.000  
No matches to TargetScan


GATAAG

GATAAG  
Depth:19 (ZEBRAFISH)  
Ei-value:0.000, Pi-value:0.000  
Er-value:0.000, Pr-value:0.000  
No matches to TargetScan


T

CAGATAAGT  
Depth:11 (X.TROPICALIS)  
Ei-value:0.000, Pi-value:0.000  
Er-value:0.000, Pr-value:0.000  
No matches to TargetScan

-4004--(18)--4023-

ATTGCAT

ATTGCAT  
Depth:11 (X.TROPICALIS)  
Ei-value:0.000, Pi-value:0.000  
Er-value:0.000, Pr-value:0.000  
No matches to TargetScan

-4029--(547)--4577-

AGGCAGGA

AGGCAGGA  
Depth:10 (SNAKE)  
Ei-value:0.000, Pi-value:0.000  
Er-value:0.000, Pr-value:0.000  
No matches to TargetScan

-4584--(72)--4657-

TAAGGA

TAAGGA  
Depth:13 (OPOSSUM)  
Ei-value:0.000, Pi-value:0.000  
Er-value:0.000, Pr-value:0.000  
No matches to TargetScan

-4662--(361)--5024-

TGCAGTG

TGCAGTG  
Depth:10 (SNAKE)  
Ei-value:0.000, Pi-value:0.000  
Er-value:0.000, Pr-value:0.000  
MATCHES To TargetScan▶ miR-217:ACUGCAU

-5030--(144)--5175-

TTGGCAAGTAA

TTGGCAAGTAA  
Depth:10 (SNAKE)  
Ei-value:0.000, Pi-value:0.000  
Er-value:0.000, Pr-value:0.000  
No matches to TargetScan

-5185--(215)--5401-

TTAGGT

TTAGGT  
Depth:11 (X.TROPICALIS)  
Ei-value:0.000, Pi-value:0.000  
Er-value:0.000, Pr-value:0.000  
No matches to TargetScan


AA

TTAGGTAA  
Depth:10 (SNAKE)  
Ei-value:0.000, Pi-value:0.000  
Er-value:0.000, Pr-value:0.000  
No matches to TargetScan

-5408--(42)--5451-

T

TGAAGCCTTTAGT  
Depth:10 (SNAKE)  
Ei-value:0.000, Pi-value:0.000  
Er-value:0.000, Pr-value:0.000  
No matches to TargetScan


GAAGCC

GAAGCC  
Depth:11 (X.TROPICALIS)  
Ei-value:0.000, Pi-value:0.000  
Er-value:0.000, Pr-value:0.000  
No matches to TargetScan


TTTAGT

TGAAGCCTTTAGT  
Depth:10 (SNAKE)  
Ei-value:0.000, Pi-value:0.000  
Er-value:0.000, Pr-value:0.000  
No matches to TargetScan

-5463--(3)--5467-

TTCCAGAT

TTCCAGAT  
Depth:10 (SNAKE)  
Ei-value:0.000, Pi-value:0.000  
Er-value:0.000, Pr-value:0.000  
No matches to TargetScan

-5474--(64)--5539-

GGCAAGT

GGCAAGT  
Depth:10 (SNAKE)  
Ei-value:0.000, Pi-value:0.000  
Er-value:0.000, Pr-value:0.000  
No matches to TargetScan

-5545--(299)--5845-

AAC

AACAGGTGAA  
Depth:10 (SNAKE)  
Ei-value:0.000, Pi-value:0.000  
Er-value:0.000, Pr-value:0.000  
No matches to TargetScan


AGGTGA

AGGTGA  
Depth:11 (X.TROPICALIS)  
Ei-value:0.000, Pi-value:0.000  
Er-value:0.000, Pr-value:0.010  
No matches to TargetScan


A

AACAGGTGAA  
Depth:10 (SNAKE)  
Ei-value:0.000, Pi-value:0.000  
Er-value:0.000, Pr-value:0.000  
No matches to TargetScan

-5854--(193)--6048-

AAGGCT

AAGGCT  
Depth:10 (SNAKE)  
Ei-value:0.000, Pi-value:0.000  
Er-value:0.000, Pr-value:0.010  
No matches to TargetScan

-6053--(325)--6379-

TTTTCCAG

TTTTCCAG  
Depth:10 (SNAKE)  
Ei-value:0.000, Pi-value:0.000  
Er-value:0.000, Pr-value:0.000  
No matches to TargetScan

-6386--(113)--6500-

TGGATCCT

TGGATCCT  
Depth:10 (SNAKE)  
Ei-value:0.000, Pi-value:0.000  
Er-value:0.000, Pr-value:0.000  
No matches to TargetScan

-6507--(26)--6534-

AAGGTAA

AAGGTAA  
Depth:10 (SNAKE)  
Ei-value:0.000, Pi-value:0.000  
Er-value:0.000, Pr-value:0.000  
No matches to TargetScan

-6540--(184)--6725-

GGGTGGG

GGGTGGG  
Depth:10 (SNAKE)  
Ei-value:0.000, Pi-value:0.000  
Er-value:0.000, Pr-value:0.000  
No matches to TargetScan

-6731--(71)--6803-

TGCTAAGACT

TGCTAAGACT  
Depth:10 (SNAKE)  
Ei-value:0.000, Pi-value:0.000  
Er-value:0.000, Pr-value:0.000  
No matches to TargetScan

-6812--(90)--6903-

TTTTCTTTT

TTTTCTTTT  
Depth:19 (ZEBRAFISH)  
Ei-value:0.000, Pi-value:0.000  
Er-value:0.000, Pr-value:0.000  
MATCHES To TargetScan▶ miR-186-5p:AAAGAAU


CCTGAGA

TTTTCTTTTCCTGAGA  
Depth:10 (SNAKE)  
Ei-value:0.000, Pi-value:0.000  
Er-value:0.000, Pr-value:0.000  
MATCHES To TargetScan▶ miR-873-5p.1:CAGGAAC▶ miR-186-5p:AAAGAAU

-6918--(20)--6939-

TCT

TCTCAGGTTTTGCTTTT  
Depth:10 (SNAKE)  
Ei-value:0.000, Pi-value:0.000  
Er-value:0.000, Pr-value:0.000  
MATCHES To TargetScan▶ miR-330-3p.2:AAAGCAC▶ miR-490-3p:AACCUGG


CAGGTTTTGCTTT

CAGGTTTTGCTTT  
Depth:19 (ZEBRAFISH)  
Ei-value:0.000, Pi-value:0.000  
Er-value:0.000, Pr-value:0.000  
MATCHES To TargetScan▶ miR-330-3p.2:AAAGCAC▶ miR-490-3p:AACCUGG


T

CAGGTTTTGCTTTT  
Depth:16 (NILETILAPIA)  
Ei-value:0.000, Pi-value:0.000  
Er-value:0.000, Pr-value:0.000  
MATCHES To TargetScan▶ miR-330-3p.2:AAAGCAC▶ miR-490-3p:AACCUGG

-6955--(21)--6977-

A

AAAAAAGCAAAAG  
Depth:13 (OPOSSUM)  
Ei-value:0.000, Pi-value:0.000  
Er-value:0.000, Pr-value:0.000  
No matches to TargetScan


AAAAAGCAAAA

AAAAAGCAAAA  
Depth:19 (ZEBRAFISH)  
Ei-value:0.000, Pi-value:0.000  
Er-value:0.000, Pr-value:0.000  
No matches to TargetScan


G

AAAAAGCAAAAG  
Depth:18 (MEDAKA)  
Ei-value:0.000, Pi-value:0.000  
Er-value:0.000, Pr-value:0.000  
No matches to TargetScan

-6989--(15)--7005-

ACTCCTG

ACTCCTG  
Depth:18 (MEDAKA)  
Ei-value:0.000, Pi-value:0.000  
Er-value:0.000, Pr-value:0.000  
No matches to TargetScan


G

ACTCCTGG  
Depth:14 (SPOTTEDGAR)  
Ei-value:0.000, Pi-value:0.000  
Er-value:0.000, Pr-value:0.000  
MATCHES To TargetScan▶ miR-665:CCAGGAG

-7012--(11)--7024-

GGGTTC

GGGTTC  
Depth:13 (OPOSSUM)  
Ei-value:0.000, Pi-value:0.000  
Er-value:0.000, Pr-value:0.000  
No matches to TargetScan

-7029  
  
>MOUSE  
      1485-

GGTGAAG

GGTGAAG  
Depth:11 (X.TROPICALIS)  
Ei-value:0.000, Pi-value:0.000  
Er-value:0.000, Pr-value:0.000  
No matches to TargetScan

-1491--(225)--1717-

TGGTAA

TGGTAA  
Depth:14 (SPOTTEDGAR)  
Ei-value:0.030, Pi-value:0.000  
Er-value:0.010, Pr-value:0.000  
No matches to TargetScan

-1722--(601)--2324-

TTTGGG

TTTGGG  
Depth:19 (ZEBRAFISH)  
Ei-value:0.000, Pi-value:0.000  
Er-value:0.000, Pr-value:0.000  
No matches to TargetScan

-2329--(438)--2768-

GGCAAGTAA

GGCAAGTAA  
Depth:10 (SNAKE)  
Ei-value:0.000, Pi-value:0.000  
Er-value:0.000, Pr-value:0.000  
No matches to TargetScan

-2776--(306)--3083-

CAA

CAAGTAAGA  
Depth:10 (SNAKE)  
Ei-value:0.000, Pi-value:0.000  
Er-value:0.000, Pr-value:0.000  
No matches to TargetScan


GTAAGA

GTAAGA  
Depth:11 (X.TROPICALIS)  
Ei-value:0.000, Pi-value:0.000  
Er-value:0.000, Pr-value:0.000  
No matches to TargetScan

-3091--(228)--3320-

TTTTTCAG

TTTTTCAG  
Depth:19 (ZEBRAFISH)  
Ei-value:0.000, Pi-value:0.000  
Er-value:0.000, Pr-value:0.000  
No matches to TargetScan


AT

TTTTTCAGAT  
Depth:10 (SNAKE)  
Ei-value:0.000, Pi-value:0.000  
Er-value:0.000, Pr-value:0.000  
No matches to TargetScan

-3329--(83)--3413-

AAAGGCAGAA

AAAGGCAGAA  
Depth:13 (OPOSSUM)  
Ei-value:0.000, Pi-value:0.000  
Er-value:0.000, Pr-value:0.000  
No matches to TargetScan


A

AAAGGCAGAAA  
Depth:11 (X.TROPICALIS)  
Ei-value:0.000, Pi-value:0.000  
Er-value:0.000, Pr-value:0.000  
No matches to TargetScan

-3423--(348)--3772-

ACAGGT

ACAGGT  
Depth:10 (SNAKE)  
Ei-value:0.000, Pi-value:0.000  
Er-value:0.000, Pr-value:0.000  
No matches to TargetScan

-3777--(66)--3844-

CA

CAGATAAGT  
Depth:11 (X.TROPICALIS)  
Ei-value:0.000, Pi-value:0.000  
Er-value:0.000, Pr-value:0.000  
No matches to TargetScan


GATAAG

GATAAG  
Depth:19 (ZEBRAFISH)  
Ei-value:0.000, Pi-value:0.000  
Er-value:0.000, Pr-value:0.000  
No matches to TargetScan


T

CAGATAAGT  
Depth:11 (X.TROPICALIS)  
Ei-value:0.000, Pi-value:0.000  
Er-value:0.000, Pr-value:0.000  
No matches to TargetScan

-3852--(18)--3871-

ATTGCAT

ATTGCAT  
Depth:11 (X.TROPICALIS)  
Ei-value:0.000, Pi-value:0.000  
Er-value:0.000, Pr-value:0.000  
No matches to TargetScan

-3877--(568)--4446-

AGGCAGGA

AGGCAGGA  
Depth:10 (SNAKE)  
Ei-value:0.000, Pi-value:0.000  
Er-value:0.000, Pr-value:0.000  
No matches to TargetScan

-4453--(71)--4525-

TAAGGA

TAAGGA  
Depth:13 (OPOSSUM)  
Ei-value:0.000, Pi-value:0.000  
Er-value:0.000, Pr-value:0.000  
No matches to TargetScan

-4530--(345)--4876-

TGCAGTG

TGCAGTG  
Depth:10 (SNAKE)  
Ei-value:0.000, Pi-value:0.000  
Er-value:0.000, Pr-value:0.000  
MATCHES To TargetScan▶ miR-217:ACUGCAU

-4882--(114)--4997-

TTGGCAAGTAA

TTGGCAAGTAA  
Depth:10 (SNAKE)  
Ei-value:0.000, Pi-value:0.000  
Er-value:0.000, Pr-value:0.000  
No matches to TargetScan

-5007--(212)--5220-

TTAGGT

TTAGGT  
Depth:11 (X.TROPICALIS)  
Ei-value:0.000, Pi-value:0.000  
Er-value:0.000, Pr-value:0.000  
No matches to TargetScan


AA

TTAGGTAA  
Depth:10 (SNAKE)  
Ei-value:0.000, Pi-value:0.000  
Er-value:0.000, Pr-value:0.000  
No matches to TargetScan

-5227--(39)--5267-

T

TGAAGCCTTTAGT  
Depth:10 (SNAKE)  
Ei-value:0.000, Pi-value:0.000  
Er-value:0.000, Pr-value:0.000  
No matches to TargetScan


GAAGCC

GAAGCC  
Depth:11 (X.TROPICALIS)  
Ei-value:0.000, Pi-value:0.000  
Er-value:0.000, Pr-value:0.000  
No matches to TargetScan


TTTAGT

TGAAGCCTTTAGT  
Depth:10 (SNAKE)  
Ei-value:0.000, Pi-value:0.000  
Er-value:0.000, Pr-value:0.000  
No matches to TargetScan

-5279--(3)--5283-

TTCCAGAT

TTCCAGAT  
Depth:10 (SNAKE)  
Ei-value:0.000, Pi-value:0.000  
Er-value:0.000, Pr-value:0.000  
No matches to TargetScan

-5290--(61)--5352-

GGCAAGT

GGCAAGT  
Depth:10 (SNAKE)  
Ei-value:0.000, Pi-value:0.000  
Er-value:0.000, Pr-value:0.000  
No matches to TargetScan

-5358--(305)--5664-

AAC

AACAGGTGAA  
Depth:10 (SNAKE)  
Ei-value:0.000, Pi-value:0.000  
Er-value:0.000, Pr-value:0.000  
No matches to TargetScan


AGGTGA

AGGTGA  
Depth:11 (X.TROPICALIS)  
Ei-value:0.000, Pi-value:0.000  
Er-value:0.000, Pr-value:0.010  
No matches to TargetScan


A

AACAGGTGAA  
Depth:10 (SNAKE)  
Ei-value:0.000, Pi-value:0.000  
Er-value:0.000, Pr-value:0.000  
No matches to TargetScan

-5673--(170)--5844-

AAGGCT

AAGGCT  
Depth:10 (SNAKE)  
Ei-value:0.000, Pi-value:0.000  
Er-value:0.000, Pr-value:0.010  
No matches to TargetScan

-5849--(282)--6132-

TTTTCCAG

TTTTCCAG  
Depth:10 (SNAKE)  
Ei-value:0.000, Pi-value:0.000  
Er-value:0.000, Pr-value:0.000  
No matches to TargetScan

-6139--(108)--6248-

TGGATCCT

TGGATCCT  
Depth:10 (SNAKE)  
Ei-value:0.000, Pi-value:0.000  
Er-value:0.000, Pr-value:0.000  
No matches to TargetScan

-6255--(25)--6281-

AAGGTAA

AAGGTAA  
Depth:10 (SNAKE)  
Ei-value:0.000, Pi-value:0.000  
Er-value:0.000, Pr-value:0.000  
No matches to TargetScan

-6287--(157)--6445-

GGGTGGG

GGGTGGG  
Depth:10 (SNAKE)  
Ei-value:0.000, Pi-value:0.000  
Er-value:0.000, Pr-value:0.000  
No matches to TargetScan

-6451--(68)--6520-

TGCTAAGACT

TGCTAAGACT  
Depth:10 (SNAKE)  
Ei-value:0.000, Pi-value:0.000  
Er-value:0.000, Pr-value:0.000  
No matches to TargetScan

-6529--(76)--6606-

TTTTCTTTT

TTTTCTTTT  
Depth:19 (ZEBRAFISH)  
Ei-value:0.000, Pi-value:0.000  
Er-value:0.000, Pr-value:0.000  
MATCHES To TargetScan▶ miR-186-5p:AAAGAAU


CCTGAGA

TTTTCTTTTCCTGAGA  
Depth:10 (SNAKE)  
Ei-value:0.000, Pi-value:0.000  
Er-value:0.000, Pr-value:0.000  
MATCHES To TargetScan▶ miR-873-5p.1:CAGGAAC▶ miR-186-5p:AAAGAAU

-6621--(16)--6638-

TCT

TCTCAGGTTTTGCTTTT  
Depth:10 (SNAKE)  
Ei-value:0.000, Pi-value:0.000  
Er-value:0.000, Pr-value:0.000  
MATCHES To TargetScan▶ miR-330-3p.2:AAAGCAC▶ miR-490-3p:AACCUGG


CAGGTTTTGCTTT

CAGGTTTTGCTTT  
Depth:19 (ZEBRAFISH)  
Ei-value:0.000, Pi-value:0.000  
Er-value:0.000, Pr-value:0.000  
MATCHES To TargetScan▶ miR-330-3p.2:AAAGCAC▶ miR-490-3p:AACCUGG


T

CAGGTTTTGCTTTT  
Depth:16 (NILETILAPIA)  
Ei-value:0.000, Pi-value:0.000  
Er-value:0.000, Pr-value:0.000  
MATCHES To TargetScan▶ miR-330-3p.2:AAAGCAC▶ miR-490-3p:AACCUGG

-6654--(24)--6679-

A

AAAAAAGCAAAAG  
Depth:13 (OPOSSUM)  
Ei-value:0.000, Pi-value:0.000  
Er-value:0.000, Pr-value:0.000  
No matches to TargetScan


AAAAAGCAAAA

AAAAAGCAAAA  
Depth:19 (ZEBRAFISH)  
Ei-value:0.000, Pi-value:0.000  
Er-value:0.000, Pr-value:0.000  
No matches to TargetScan


G

AAAAAGCAAAAG  
Depth:18 (MEDAKA)  
Ei-value:0.000, Pi-value:0.000  
Er-value:0.000, Pr-value:0.000  
No matches to TargetScan

-6691--(15)--6707-

ACTCCTG

ACTCCTG  
Depth:18 (MEDAKA)  
Ei-value:0.000, Pi-value:0.000  
Er-value:0.000, Pr-value:0.000  
No matches to TargetScan


G

ACTCCTGG  
Depth:14 (SPOTTEDGAR)  
Ei-value:0.000, Pi-value:0.000  
Er-value:0.000, Pr-value:0.000  
MATCHES To TargetScan▶ miR-665:CCAGGAG

-6714--(11)--6726-

GGGTTC

GGGTTC  
Depth:13 (OPOSSUM)  
Ei-value:0.000, Pi-value:0.000  
Er-value:0.000, Pr-value:0.000  
No matches to TargetScan

-6731  
  
>TURTLE  
       176-

GGTGAAG

GGTGAAG  
Depth:11 (X.TROPICALIS)  
Ei-value:0.000, Pi-value:0.000  
Er-value:0.000, Pr-value:0.000  
No matches to TargetScan

-182--(178)--361-

TGGTAA

TGGTAA  
Depth:14 (SPOTTEDGAR)  
Ei-value:0.030, Pi-value:0.000  
Er-value:0.010, Pr-value:0.000  
No matches to TargetScan

-366--(904)--1271-

TTTGGG

TTTGGG  
Depth:19 (ZEBRAFISH)  
Ei-value:0.000, Pi-value:0.000  
Er-value:0.000, Pr-value:0.000  
No matches to TargetScan

-1276--(512)--1789-

GGCAAGTAA

GGCAAGTAA  
Depth:10 (SNAKE)  
Ei-value:0.000, Pi-value:0.000  
Er-value:0.000, Pr-value:0.000  
No matches to TargetScan

-1797--(504)--2302-

CAA

CAAGTAAGA  
Depth:10 (SNAKE)  
Ei-value:0.000, Pi-value:0.000  
Er-value:0.000, Pr-value:0.000  
No matches to TargetScan


GTAAGA

GTAAGA  
Depth:11 (X.TROPICALIS)  
Ei-value:0.000, Pi-value:0.000  
Er-value:0.000, Pr-value:0.000  
No matches to TargetScan

-2310--(348)--2659-

TTTTTCAG

TTTTTCAG  
Depth:19 (ZEBRAFISH)  
Ei-value:0.000, Pi-value:0.000  
Er-value:0.000, Pr-value:0.000  
No matches to TargetScan


AT

TTTTTCAGAT  
Depth:10 (SNAKE)  
Ei-value:0.000, Pi-value:0.000  
Er-value:0.000, Pr-value:0.000  
No matches to TargetScan

-2668--(87)--2756-

AAAGGCAGAA

AAAGGCAGAA  
Depth:13 (OPOSSUM)  
Ei-value:0.000, Pi-value:0.000  
Er-value:0.000, Pr-value:0.000  
No matches to TargetScan


A

AAAGGCAGAAA  
Depth:11 (X.TROPICALIS)  
Ei-value:0.000, Pi-value:0.000  
Er-value:0.000, Pr-value:0.000  
No matches to TargetScan

-2766--(468)--3235-

ACAGGT

ACAGGT  
Depth:10 (SNAKE)  
Ei-value:0.000, Pi-value:0.000  
Er-value:0.000, Pr-value:0.000  
No matches to TargetScan

-3240--(72)--3313-

CA

CAGATAAGT  
Depth:11 (X.TROPICALIS)  
Ei-value:0.000, Pi-value:0.000  
Er-value:0.000, Pr-value:0.000  
No matches to TargetScan


GATAAG

GATAAG  
Depth:19 (ZEBRAFISH)  
Ei-value:0.000, Pi-value:0.000  
Er-value:0.000, Pr-value:0.000  
No matches to TargetScan


T

CAGATAAGT  
Depth:11 (X.TROPICALIS)  
Ei-value:0.000, Pi-value:0.000  
Er-value:0.000, Pr-value:0.000  
No matches to TargetScan

-3321--(21)--3343-

ATTGCAT

ATTGCAT  
Depth:11 (X.TROPICALIS)  
Ei-value:0.000, Pi-value:0.000  
Er-value:0.000, Pr-value:0.000  
No matches to TargetScan

-3349--(651)--4001-

AGGCAGGA

AGGCAGGA  
Depth:10 (SNAKE)  
Ei-value:0.000, Pi-value:0.000  
Er-value:0.000, Pr-value:0.000  
No matches to TargetScan

-4008--(96)--4105-

TAAGGA

TAAGGA  
Depth:13 (OPOSSUM)  
Ei-value:0.000, Pi-value:0.000  
Er-value:0.000, Pr-value:0.000  
No matches to TargetScan

-4110--(502)--4613-

TGCAGTG

TGCAGTG  
Depth:10 (SNAKE)  
Ei-value:0.000, Pi-value:0.000  
Er-value:0.000, Pr-value:0.000  
MATCHES To TargetScan▶ miR-217:ACUGCAU

-4619--(164)--4784-

TTGGCAAGTAA

TTGGCAAGTAA  
Depth:10 (SNAKE)  
Ei-value:0.000, Pi-value:0.000  
Er-value:0.000, Pr-value:0.000  
No matches to TargetScan

-4794--(234)--5029-

TTAGGT

TTAGGT  
Depth:11 (X.TROPICALIS)  
Ei-value:0.000, Pi-value:0.000  
Er-value:0.000, Pr-value:0.000  
No matches to TargetScan


AA

TTAGGTAA  
Depth:10 (SNAKE)  
Ei-value:0.000, Pi-value:0.000  
Er-value:0.000, Pr-value:0.000  
No matches to TargetScan

-5036--(56)--5093-

T

TGAAGCCTTTAGT  
Depth:10 (SNAKE)  
Ei-value:0.000, Pi-value:0.000  
Er-value:0.000, Pr-value:0.000  
No matches to TargetScan


GAAGCC

GAAGCC  
Depth:11 (X.TROPICALIS)  
Ei-value:0.000, Pi-value:0.000  
Er-value:0.000, Pr-value:0.000  
No matches to TargetScan


TTTAGT

TGAAGCCTTTAGT  
Depth:10 (SNAKE)  
Ei-value:0.000, Pi-value:0.000  
Er-value:0.000, Pr-value:0.000  
No matches to TargetScan

-5105--(3)--5109-

TTCCAGAT

TTCCAGAT  
Depth:10 (SNAKE)  
Ei-value:0.000, Pi-value:0.000  
Er-value:0.000, Pr-value:0.000  
No matches to TargetScan

-5116--(70)--5187-

GGCAAGT

GGCAAGT  
Depth:10 (SNAKE)  
Ei-value:0.000, Pi-value:0.000  
Er-value:0.000, Pr-value:0.000  
No matches to TargetScan

-5193--(323)--5517-

AAC

AACAGGTGAA  
Depth:10 (SNAKE)  
Ei-value:0.000, Pi-value:0.000  
Er-value:0.000, Pr-value:0.000  
No matches to TargetScan


AGGTGA

AGGTGA  
Depth:11 (X.TROPICALIS)  
Ei-value:0.000, Pi-value:0.000  
Er-value:0.000, Pr-value:0.010  
No matches to TargetScan


A

AACAGGTGAA  
Depth:10 (SNAKE)  
Ei-value:0.000, Pi-value:0.000  
Er-value:0.000, Pr-value:0.000  
No matches to TargetScan

-5526--(232)--5759-

AAGGCT

AAGGCT  
Depth:10 (SNAKE)  
Ei-value:0.000, Pi-value:0.000  
Er-value:0.000, Pr-value:0.010  
No matches to TargetScan

-5764--(384)--6149-

TTTTCCAG

TTTTCCAG  
Depth:10 (SNAKE)  
Ei-value:0.000, Pi-value:0.000  
Er-value:0.000, Pr-value:0.000  
No matches to TargetScan

-6156--(67)--6224-

TGGATCCT

TGGATCCT  
Depth:10 (SNAKE)  
Ei-value:0.000, Pi-value:0.000  
Er-value:0.000, Pr-value:0.000  
No matches to TargetScan

-6231--(25)--6257-

AAGGTAA

AAGGTAA  
Depth:10 (SNAKE)  
Ei-value:0.000, Pi-value:0.000  
Er-value:0.000, Pr-value:0.000  
No matches to TargetScan

-6263--(270)--6534-

GGGTGGG

GGGTGGG  
Depth:10 (SNAKE)  
Ei-value:0.000, Pi-value:0.000  
Er-value:0.000, Pr-value:0.000  
No matches to TargetScan

-6540--(93)--6634-

TGCTAAGACT

TGCTAAGACT  
Depth:10 (SNAKE)  
Ei-value:0.000, Pi-value:0.000  
Er-value:0.000, Pr-value:0.000  
No matches to TargetScan

-6643--(89)--6733-

TTTTCTTTT

TTTTCTTTT  
Depth:19 (ZEBRAFISH)  
Ei-value:0.000, Pi-value:0.000  
Er-value:0.000, Pr-value:0.000  
MATCHES To TargetScan▶ miR-186-5p:AAAGAAU


CCTGAGA

TTTTCTTTTCCTGAGA  
Depth:10 (SNAKE)  
Ei-value:0.000, Pi-value:0.000  
Er-value:0.000, Pr-value:0.000  
MATCHES To TargetScan▶ miR-873-5p.1:CAGGAAC▶ miR-186-5p:AAAGAAU

-6748--(13)--6762-

TCT

TCTCAGGTTTTGCTTTT  
Depth:10 (SNAKE)  
Ei-value:0.000, Pi-value:0.000  
Er-value:0.000, Pr-value:0.000  
MATCHES To TargetScan▶ miR-330-3p.2:AAAGCAC▶ miR-490-3p:AACCUGG


CAGGTTTTGCTTT

CAGGTTTTGCTTT  
Depth:19 (ZEBRAFISH)  
Ei-value:0.000, Pi-value:0.000  
Er-value:0.000, Pr-value:0.000  
MATCHES To TargetScan▶ miR-330-3p.2:AAAGCAC▶ miR-490-3p:AACCUGG


T

CAGGTTTTGCTTTT  
Depth:16 (NILETILAPIA)  
Ei-value:0.000, Pi-value:0.000  
Er-value:0.000, Pr-value:0.000  
MATCHES To TargetScan▶ miR-330-3p.2:AAAGCAC▶ miR-490-3p:AACCUGG

-6778--(19)--6798-

A

AAAAAAGCAAAAG  
Depth:13 (OPOSSUM)  
Ei-value:0.000, Pi-value:0.000  
Er-value:0.000, Pr-value:0.000  
No matches to TargetScan


AAAAAGCAAAA

AAAAAGCAAAA  
Depth:19 (ZEBRAFISH)  
Ei-value:0.000, Pi-value:0.000  
Er-value:0.000, Pr-value:0.000  
No matches to TargetScan


G

AAAAAGCAAAAG  
Depth:18 (MEDAKA)  
Ei-value:0.000, Pi-value:0.000  
Er-value:0.000, Pr-value:0.000  
No matches to TargetScan

-6810--(15)--6826-

ACTCCTG

ACTCCTG  
Depth:18 (MEDAKA)  
Ei-value:0.000, Pi-value:0.000  
Er-value:0.000, Pr-value:0.000  
No matches to TargetScan


G

ACTCCTGG  
Depth:14 (SPOTTEDGAR)  
Ei-value:0.000, Pi-value:0.000  
Er-value:0.000, Pr-value:0.000  
MATCHES To TargetScan▶ miR-665:CCAGGAG

-6833--(11)--6845-

GGGTTC

GGGTTC  
Depth:13 (OPOSSUM)  
Ei-value:0.000, Pi-value:0.000  
Er-value:0.000, Pr-value:0.000  
No matches to TargetScan

-6850  
  
>ALLIGATOR  
      1490-

GGTGAAG

GGTGAAG  
Depth:11 (X.TROPICALIS)  
Ei-value:0.000, Pi-value:0.000  
Er-value:0.000, Pr-value:0.000  
No matches to TargetScan

-1496--(191)--1688-

TGGTAA

TGGTAA  
Depth:14 (SPOTTEDGAR)  
Ei-value:0.030, Pi-value:0.000  
Er-value:0.010, Pr-value:0.000  
No matches to TargetScan

-1693--(843)--2537-

TTTGGG

TTTGGG  
Depth:19 (ZEBRAFISH)  
Ei-value:0.000, Pi-value:0.000  
Er-value:0.000, Pr-value:0.000  
No matches to TargetScan

-2542--(462)--3005-

GGCAAGTAA

GGCAAGTAA  
Depth:10 (SNAKE)  
Ei-value:0.000, Pi-value:0.000  
Er-value:0.000, Pr-value:0.000  
No matches to TargetScan

-3013--(487)--3501-

CAA

CAAGTAAGA  
Depth:10 (SNAKE)  
Ei-value:0.000, Pi-value:0.000  
Er-value:0.000, Pr-value:0.000  
No matches to TargetScan


GTAAGA

GTAAGA  
Depth:11 (X.TROPICALIS)  
Ei-value:0.000, Pi-value:0.000  
Er-value:0.000, Pr-value:0.000  
No matches to TargetScan

-3509--(355)--3865-

TTTTTCAG

TTTTTCAG  
Depth:19 (ZEBRAFISH)  
Ei-value:0.000, Pi-value:0.000  
Er-value:0.000, Pr-value:0.000  
No matches to TargetScan


AT

TTTTTCAGAT  
Depth:10 (SNAKE)  
Ei-value:0.000, Pi-value:0.000  
Er-value:0.000, Pr-value:0.000  
No matches to TargetScan

-3874--(90)--3965-

AAAGGCAGAA

AAAGGCAGAA  
Depth:13 (OPOSSUM)  
Ei-value:0.000, Pi-value:0.000  
Er-value:0.000, Pr-value:0.000  
No matches to TargetScan


A

AAAGGCAGAAA  
Depth:11 (X.TROPICALIS)  
Ei-value:0.000, Pi-value:0.000  
Er-value:0.000, Pr-value:0.000  
No matches to TargetScan

-3975--(463)--4439-

ACAGGT

ACAGGT  
Depth:10 (SNAKE)  
Ei-value:0.000, Pi-value:0.000  
Er-value:0.000, Pr-value:0.000  
No matches to TargetScan

-4444--(74)--4519-

CA

CAGATAAGT  
Depth:11 (X.TROPICALIS)  
Ei-value:0.000, Pi-value:0.000  
Er-value:0.000, Pr-value:0.000  
No matches to TargetScan


GATAAG

GATAAG  
Depth:19 (ZEBRAFISH)  
Ei-value:0.000, Pi-value:0.000  
Er-value:0.000, Pr-value:0.000  
No matches to TargetScan


T

CAGATAAGT  
Depth:11 (X.TROPICALIS)  
Ei-value:0.000, Pi-value:0.000  
Er-value:0.000, Pr-value:0.000  
No matches to TargetScan

-4527--(21)--4549-

ATTGCAT

ATTGCAT  
Depth:11 (X.TROPICALIS)  
Ei-value:0.000, Pi-value:0.000  
Er-value:0.000, Pr-value:0.000  
No matches to TargetScan

-4555--(672)--5228-

AGGCAGGA

AGGCAGGA  
Depth:10 (SNAKE)  
Ei-value:0.000, Pi-value:0.000  
Er-value:0.000, Pr-value:0.000  
No matches to TargetScan

-5235--(96)--5332-

TAAGGA

TAAGGA  
Depth:13 (OPOSSUM)  
Ei-value:0.000, Pi-value:0.000  
Er-value:0.000, Pr-value:0.000  
No matches to TargetScan

-5337--(503)--5841-

TGCAGTG

TGCAGTG  
Depth:10 (SNAKE)  
Ei-value:0.000, Pi-value:0.000  
Er-value:0.000, Pr-value:0.000  
MATCHES To TargetScan▶ miR-217:ACUGCAU

-5847--(148)--5996-

TTGGCAAGTAA

TTGGCAAGTAA  
Depth:10 (SNAKE)  
Ei-value:0.000, Pi-value:0.000  
Er-value:0.000, Pr-value:0.000  
No matches to TargetScan

-6006--(232)--6239-

TTAGGT

TTAGGT  
Depth:11 (X.TROPICALIS)  
Ei-value:0.000, Pi-value:0.000  
Er-value:0.000, Pr-value:0.000  
No matches to TargetScan


AA

TTAGGTAA  
Depth:10 (SNAKE)  
Ei-value:0.000, Pi-value:0.000  
Er-value:0.000, Pr-value:0.000  
No matches to TargetScan

-6246--(87)--6334-

T

TGAAGCCTTTAGT  
Depth:10 (SNAKE)  
Ei-value:0.000, Pi-value:0.000  
Er-value:0.000, Pr-value:0.000  
No matches to TargetScan


GAAGCC

GAAGCC  
Depth:11 (X.TROPICALIS)  
Ei-value:0.000, Pi-value:0.000  
Er-value:0.000, Pr-value:0.000  
No matches to TargetScan


TTTAGT

TGAAGCCTTTAGT  
Depth:10 (SNAKE)  
Ei-value:0.000, Pi-value:0.000  
Er-value:0.000, Pr-value:0.000  
No matches to TargetScan

-6346--(3)--6350-

TTCCAGAT

TTCCAGAT  
Depth:10 (SNAKE)  
Ei-value:0.000, Pi-value:0.000  
Er-value:0.000, Pr-value:0.000  
No matches to TargetScan

-6357--(70)--6428-

GGCAAGT

GGCAAGT  
Depth:10 (SNAKE)  
Ei-value:0.000, Pi-value:0.000  
Er-value:0.000, Pr-value:0.000  
No matches to TargetScan

-6434--(349)--6784-

AAC

AACAGGTGAA  
Depth:10 (SNAKE)  
Ei-value:0.000, Pi-value:0.000  
Er-value:0.000, Pr-value:0.000  
No matches to TargetScan


AGGTGA

AGGTGA  
Depth:11 (X.TROPICALIS)  
Ei-value:0.000, Pi-value:0.000  
Er-value:0.000, Pr-value:0.010  
No matches to TargetScan


A

AACAGGTGAA  
Depth:10 (SNAKE)  
Ei-value:0.000, Pi-value:0.000  
Er-value:0.000, Pr-value:0.000  
No matches to TargetScan

-6793--(260)--7054-

AAGGCT

AAGGCT  
Depth:10 (SNAKE)  
Ei-value:0.000, Pi-value:0.000  
Er-value:0.000, Pr-value:0.010  
No matches to TargetScan

-7059--(359)--7419-

TTTTCCAG

TTTTCCAG  
Depth:10 (SNAKE)  
Ei-value:0.000, Pi-value:0.000  
Er-value:0.000, Pr-value:0.000  
No matches to TargetScan

-7426--(62)--7489-

TGGATCCT

TGGATCCT  
Depth:10 (SNAKE)  
Ei-value:0.000, Pi-value:0.000  
Er-value:0.000, Pr-value:0.000  
No matches to TargetScan

-7496--(23)--7520-

AAGGTAA

AAGGTAA  
Depth:10 (SNAKE)  
Ei-value:0.000, Pi-value:0.000  
Er-value:0.000, Pr-value:0.000  
No matches to TargetScan

-7526--(281)--7808-

GGGTGGG

GGGTGGG  
Depth:10 (SNAKE)  
Ei-value:0.000, Pi-value:0.000  
Er-value:0.000, Pr-value:0.000  
No matches to TargetScan

-7814--(90)--7905-

TGCTAAGACT

TGCTAAGACT  
Depth:10 (SNAKE)  
Ei-value:0.000, Pi-value:0.000  
Er-value:0.000, Pr-value:0.000  
No matches to TargetScan

-7914--(90)--8005-

TTTTCTTTT

TTTTCTTTT  
Depth:19 (ZEBRAFISH)  
Ei-value:0.000, Pi-value:0.000  
Er-value:0.000, Pr-value:0.000  
MATCHES To TargetScan▶ miR-186-5p:AAAGAAU


CCTGAGA

TTTTCTTTTCCTGAGA  
Depth:10 (SNAKE)  
Ei-value:0.000, Pi-value:0.000  
Er-value:0.000, Pr-value:0.000  
MATCHES To TargetScan▶ miR-873-5p.1:CAGGAAC▶ miR-186-5p:AAAGAAU

-8020--(13)--8034-

TCT

TCTCAGGTTTTGCTTTT  
Depth:10 (SNAKE)  
Ei-value:0.000, Pi-value:0.000  
Er-value:0.000, Pr-value:0.000  
MATCHES To TargetScan▶ miR-330-3p.2:AAAGCAC▶ miR-490-3p:AACCUGG


CAGGTTTTGCTTT

CAGGTTTTGCTTT  
Depth:19 (ZEBRAFISH)  
Ei-value:0.000, Pi-value:0.000  
Er-value:0.000, Pr-value:0.000  
MATCHES To TargetScan▶ miR-330-3p.2:AAAGCAC▶ miR-490-3p:AACCUGG


T

CAGGTTTTGCTTTT  
Depth:16 (NILETILAPIA)  
Ei-value:0.000, Pi-value:0.000  
Er-value:0.000, Pr-value:0.000  
MATCHES To TargetScan▶ miR-330-3p.2:AAAGCAC▶ miR-490-3p:AACCUGG

-8050--(24)--8075-

A

AAAAAAGCAAAAG  
Depth:13 (OPOSSUM)  
Ei-value:0.000, Pi-value:0.000  
Er-value:0.000, Pr-value:0.000  
No matches to TargetScan


AAAAAGCAAAA

AAAAAGCAAAA  
Depth:19 (ZEBRAFISH)  
Ei-value:0.000, Pi-value:0.000  
Er-value:0.000, Pr-value:0.000  
No matches to TargetScan


G

AAAAAGCAAAAG  
Depth:18 (MEDAKA)  
Ei-value:0.000, Pi-value:0.000  
Er-value:0.000, Pr-value:0.000  
No matches to TargetScan

-8087--(15)--8103-

ACTCCTG

ACTCCTG  
Depth:18 (MEDAKA)  
Ei-value:0.000, Pi-value:0.000  
Er-value:0.000, Pr-value:0.000  
No matches to TargetScan


G

ACTCCTGG  
Depth:14 (SPOTTEDGAR)  
Ei-value:0.000, Pi-value:0.000  
Er-value:0.000, Pr-value:0.000  
MATCHES To TargetScan▶ miR-665:CCAGGAG

-8110--(11)--8122-

GGGTTC

GGGTTC  
Depth:13 (OPOSSUM)  
Ei-value:0.000, Pi-value:0.000  
Er-value:0.000, Pr-value:0.000  
No matches to TargetScan

-8127  
  
>LIZARD  
      1321-

GGTGAAG

GGTGAAG  
Depth:11 (X.TROPICALIS)  
Ei-value:0.000, Pi-value:0.000  
Er-value:0.000, Pr-value:0.000  
No matches to TargetScan

-1327--(141)--1469-

TGGTAA

TGGTAA  
Depth:14 (SPOTTEDGAR)  
Ei-value:0.030, Pi-value:0.000  
Er-value:0.010, Pr-value:0.000  
No matches to TargetScan

-1474--(878)--2353-

TTTGGG

TTTGGG  
Depth:19 (ZEBRAFISH)  
Ei-value:0.000, Pi-value:0.000  
Er-value:0.000, Pr-value:0.000  
No matches to TargetScan

-2358--(110)--2469-

GGCAAGTAA

GGCAAGTAA  
Depth:10 (SNAKE)  
Ei-value:0.000, Pi-value:0.000  
Er-value:0.000, Pr-value:0.000  
No matches to TargetScan

-2477--(386)--2864-

CAA

CAAGTAAGA  
Depth:10 (SNAKE)  
Ei-value:0.000, Pi-value:0.000  
Er-value:0.000, Pr-value:0.000  
No matches to TargetScan


GTAAGA

GTAAGA  
Depth:11 (X.TROPICALIS)  
Ei-value:0.000, Pi-value:0.000  
Er-value:0.000, Pr-value:0.000  
No matches to TargetScan

-2872--(248)--3121-

TTTTTCAG

TTTTTCAG  
Depth:19 (ZEBRAFISH)  
Ei-value:0.000, Pi-value:0.000  
Er-value:0.000, Pr-value:0.000  
No matches to TargetScan


AT

TTTTTCAGAT  
Depth:10 (SNAKE)  
Ei-value:0.000, Pi-value:0.000  
Er-value:0.000, Pr-value:0.000  
No matches to TargetScan

-3130--(88)--3219-

AAAGGCAGAA

AAAGGCAGAA  
Depth:13 (OPOSSUM)  
Ei-value:0.000, Pi-value:0.000  
Er-value:0.000, Pr-value:0.000  
No matches to TargetScan


A

AAAGGCAGAAA  
Depth:11 (X.TROPICALIS)  
Ei-value:0.000, Pi-value:0.000  
Er-value:0.000, Pr-value:0.000  
No matches to TargetScan

-3229--(410)--3640-

ACAGGT

ACAGGT  
Depth:10 (SNAKE)  
Ei-value:0.000, Pi-value:0.000  
Er-value:0.000, Pr-value:0.000  
No matches to TargetScan

-3645--(69)--3715-

CA

CAGATAAGT  
Depth:11 (X.TROPICALIS)  
Ei-value:0.000, Pi-value:0.000  
Er-value:0.000, Pr-value:0.000  
No matches to TargetScan


GATAAG

GATAAG  
Depth:19 (ZEBRAFISH)  
Ei-value:0.000, Pi-value:0.000  
Er-value:0.000, Pr-value:0.000  
No matches to TargetScan


T

CAGATAAGT  
Depth:11 (X.TROPICALIS)  
Ei-value:0.000, Pi-value:0.000  
Er-value:0.000, Pr-value:0.000  
No matches to TargetScan

-3723--(16)--3740-

ATTGCAT

ATTGCAT  
Depth:11 (X.TROPICALIS)  
Ei-value:0.000, Pi-value:0.000  
Er-value:0.000, Pr-value:0.000  
No matches to TargetScan

-3746--(578)--4325-

AGGCAGGA

AGGCAGGA  
Depth:10 (SNAKE)  
Ei-value:0.000, Pi-value:0.000  
Er-value:0.000, Pr-value:0.000  
No matches to TargetScan

-4332--(91)--4424-

TAAGGA

TAAGGA  
Depth:13 (OPOSSUM)  
Ei-value:0.000, Pi-value:0.000  
Er-value:0.000, Pr-value:0.000  
No matches to TargetScan

-4429--(306)--4736-

TGCAGTG

TGCAGTG  
Depth:10 (SNAKE)  
Ei-value:0.000, Pi-value:0.000  
Er-value:0.000, Pr-value:0.000  
MATCHES To TargetScan▶ miR-217:ACUGCAU

-4742--(392)--5135-

TTGGCAAGTAA

TTGGCAAGTAA  
Depth:10 (SNAKE)  
Ei-value:0.000, Pi-value:0.000  
Er-value:0.000, Pr-value:0.000  
No matches to TargetScan

-5145--(183)--5329-

TTAGGT

TTAGGT  
Depth:11 (X.TROPICALIS)  
Ei-value:0.000, Pi-value:0.000  
Er-value:0.000, Pr-value:0.000  
No matches to TargetScan


AA

TTAGGTAA  
Depth:10 (SNAKE)  
Ei-value:0.000, Pi-value:0.000  
Er-value:0.000, Pr-value:0.000  
No matches to TargetScan

-5336--(48)--5385-

T

TGAAGCCTTTAGT  
Depth:10 (SNAKE)  
Ei-value:0.000, Pi-value:0.000  
Er-value:0.000, Pr-value:0.000  
No matches to TargetScan


GAAGCC

GAAGCC  
Depth:11 (X.TROPICALIS)  
Ei-value:0.000, Pi-value:0.000  
Er-value:0.000, Pr-value:0.000  
No matches to TargetScan


TTTAGT

TGAAGCCTTTAGT  
Depth:10 (SNAKE)  
Ei-value:0.000, Pi-value:0.000  
Er-value:0.000, Pr-value:0.000  
No matches to TargetScan

-5397--(3)--5401-

TTCCAGAT

TTCCAGAT  
Depth:10 (SNAKE)  
Ei-value:0.000, Pi-value:0.000  
Er-value:0.000, Pr-value:0.000  
No matches to TargetScan

-5408--(52)--5461-

GGCAAGT

GGCAAGT  
Depth:10 (SNAKE)  
Ei-value:0.000, Pi-value:0.000  
Er-value:0.000, Pr-value:0.000  
No matches to TargetScan

-5467--(330)--5798-

AAC

AACAGGTGAA  
Depth:10 (SNAKE)  
Ei-value:0.000, Pi-value:0.000  
Er-value:0.000, Pr-value:0.000  
No matches to TargetScan


AGGTGA

AGGTGA  
Depth:11 (X.TROPICALIS)  
Ei-value:0.000, Pi-value:0.000  
Er-value:0.000, Pr-value:0.010  
No matches to TargetScan


A

AACAGGTGAA  
Depth:10 (SNAKE)  
Ei-value:0.000, Pi-value:0.000  
Er-value:0.000, Pr-value:0.000  
No matches to TargetScan

-5807--(39)--5847-

AAGGCT

AAGGCT  
Depth:10 (SNAKE)  
Ei-value:0.000, Pi-value:0.000  
Er-value:0.000, Pr-value:0.010  
No matches to TargetScan

-5852--(501)--6354-

TTTTCCAG

TTTTCCAG  
Depth:10 (SNAKE)  
Ei-value:0.000, Pi-value:0.000  
Er-value:0.000, Pr-value:0.000  
No matches to TargetScan

-6361--(48)--6410-

TGGATCCT

TGGATCCT  
Depth:10 (SNAKE)  
Ei-value:0.000, Pi-value:0.000  
Er-value:0.000, Pr-value:0.000  
No matches to TargetScan

-6417--(18)--6436-

AAGGTAA

AAGGTAA  
Depth:10 (SNAKE)  
Ei-value:0.000, Pi-value:0.000  
Er-value:0.000, Pr-value:0.000  
No matches to TargetScan

-6442--(91)--6534-

AAGGTAA

AAGGTAA  
Depth:10 (SNAKE)  
Ei-value:0.000, Pi-value:0.000  
Er-value:0.000, Pr-value:0.000  
No matches to TargetScan

-6540--(17)--6558-

AAGGTAA

AAGGTAA  
Depth:10 (SNAKE)  
Ei-value:0.000, Pi-value:0.000  
Er-value:0.000, Pr-value:0.000  
No matches to TargetScan

-6564--(146)--6711-

GGGTGGG

GGGTGGG  
Depth:10 (SNAKE)  
Ei-value:0.000, Pi-value:0.000  
Er-value:0.000, Pr-value:0.000  
No matches to TargetScan

-6717--(75)--6793-

TGCTAAGACT

TGCTAAGACT  
Depth:10 (SNAKE)  
Ei-value:0.000, Pi-value:0.000  
Er-value:0.000, Pr-value:0.000  
No matches to TargetScan

-6802--(96)--6899-

TTTTCTTTT

TTTTCTTTT  
Depth:19 (ZEBRAFISH)  
Ei-value:0.000, Pi-value:0.000  
Er-value:0.000, Pr-value:0.000  
MATCHES To TargetScan▶ miR-186-5p:AAAGAAU


CCTGAGA

TTTTCTTTTCCTGAGA  
Depth:10 (SNAKE)  
Ei-value:0.000, Pi-value:0.000  
Er-value:0.000, Pr-value:0.000  
MATCHES To TargetScan▶ miR-873-5p.1:CAGGAAC▶ miR-186-5p:AAAGAAU

-6914--(14)--6929-

TCT

TCTCAGGTTTTGCTTTT  
Depth:10 (SNAKE)  
Ei-value:0.000, Pi-value:0.000  
Er-value:0.000, Pr-value:0.000  
MATCHES To TargetScan▶ miR-330-3p.2:AAAGCAC▶ miR-490-3p:AACCUGG


CAGGTTTTGCTTT

CAGGTTTTGCTTT  
Depth:19 (ZEBRAFISH)  
Ei-value:0.000, Pi-value:0.000  
Er-value:0.000, Pr-value:0.000  
MATCHES To TargetScan▶ miR-330-3p.2:AAAGCAC▶ miR-490-3p:AACCUGG


T

CAGGTTTTGCTTTT  
Depth:16 (NILETILAPIA)  
Ei-value:0.000, Pi-value:0.000  
Er-value:0.000, Pr-value:0.000  
MATCHES To TargetScan▶ miR-330-3p.2:AAAGCAC▶ miR-490-3p:AACCUGG

-6945--(19)--6965-

A

AAAAAAGCAAAAG  
Depth:13 (OPOSSUM)  
Ei-value:0.000, Pi-value:0.000  
Er-value:0.000, Pr-value:0.000  
No matches to TargetScan


AAAAAGCAAAA

AAAAAGCAAAA  
Depth:19 (ZEBRAFISH)  
Ei-value:0.000, Pi-value:0.000  
Er-value:0.000, Pr-value:0.000  
No matches to TargetScan


G

AAAAAGCAAAAG  
Depth:18 (MEDAKA)  
Ei-value:0.000, Pi-value:0.000  
Er-value:0.000, Pr-value:0.000  
No matches to TargetScan

-6977--(15)--6993-

ACTCCTG

ACTCCTG  
Depth:18 (MEDAKA)  
Ei-value:0.000, Pi-value:0.000  
Er-value:0.000, Pr-value:0.000  
No matches to TargetScan


G

ACTCCTGG  
Depth:14 (SPOTTEDGAR)  
Ei-value:0.000, Pi-value:0.000  
Er-value:0.000, Pr-value:0.000  
MATCHES To TargetScan▶ miR-665:CCAGGAG

-7000--(11)--7012-

GGGTTC

GGGTTC  
Depth:13 (OPOSSUM)  
Ei-value:0.000, Pi-value:0.000  
Er-value:0.000, Pr-value:0.000  
No matches to TargetScan

-7017  
  
>SNAKE  
      1061-

GGTGAAG

GGTGAAG  
Depth:11 (X.TROPICALIS)  
Ei-value:0.000, Pi-value:0.000  
Er-value:0.000, Pr-value:0.000  
No matches to TargetScan

-1067--(148)--1216-

TGGTAA

TGGTAA  
Depth:14 (SPOTTEDGAR)  
Ei-value:0.030, Pi-value:0.000  
Er-value:0.010, Pr-value:0.000  
No matches to TargetScan

-1221--(70)--1292-

TTTGGG

TTTGGG  
Depth:19 (ZEBRAFISH)  
Ei-value:0.000, Pi-value:0.000  
Er-value:0.000, Pr-value:0.000  
No matches to TargetScan

-1297--(729)--2027-

TTTGGG

TTTGGG  
Depth:19 (ZEBRAFISH)  
Ei-value:0.000, Pi-value:0.000  
Er-value:0.000, Pr-value:0.000  
No matches to TargetScan

-2032--(414)--2447-

GGCAAGTAA

GGCAAGTAA  
Depth:10 (SNAKE)  
Ei-value:0.000, Pi-value:0.000  
Er-value:0.000, Pr-value:0.000  
No matches to TargetScan

-2455--(414)--2870-

CAA

CAAGTAAGA  
Depth:10 (SNAKE)  
Ei-value:0.000, Pi-value:0.000  
Er-value:0.000, Pr-value:0.000  
No matches to TargetScan


GTAAGA

GTAAGA  
Depth:11 (X.TROPICALIS)  
Ei-value:0.000, Pi-value:0.000  
Er-value:0.000, Pr-value:0.000  
No matches to TargetScan

-2878--(235)--3114-

TTTTTCAG

TTTTTCAG  
Depth:19 (ZEBRAFISH)  
Ei-value:0.000, Pi-value:0.000  
Er-value:0.000, Pr-value:0.000  
No matches to TargetScan


AT

TTTTTCAGAT  
Depth:10 (SNAKE)  
Ei-value:0.000, Pi-value:0.000  
Er-value:0.000, Pr-value:0.000  
No matches to TargetScan

-3123--(78)--3202-

AAAGGCAGAA

AAAGGCAGAA  
Depth:13 (OPOSSUM)  
Ei-value:0.000, Pi-value:0.000  
Er-value:0.000, Pr-value:0.000  
No matches to TargetScan


A

AAAGGCAGAAA  
Depth:11 (X.TROPICALIS)  
Ei-value:0.000, Pi-value:0.000  
Er-value:0.000, Pr-value:0.000  
No matches to TargetScan

-3212--(33)--3246-

ACAGGT

ACAGGT  
Depth:10 (SNAKE)  
Ei-value:0.000, Pi-value:0.000  
Er-value:0.000, Pr-value:0.000  
No matches to TargetScan

-3251--(426)--3678-

CA

CAGATAAGT  
Depth:11 (X.TROPICALIS)  
Ei-value:0.000, Pi-value:0.000  
Er-value:0.000, Pr-value:0.000  
No matches to TargetScan


GATAAG

GATAAG  
Depth:19 (ZEBRAFISH)  
Ei-value:0.000, Pi-value:0.000  
Er-value:0.000, Pr-value:0.000  
No matches to TargetScan


T

CAGATAAGT  
Depth:11 (X.TROPICALIS)  
Ei-value:0.000, Pi-value:0.000  
Er-value:0.000, Pr-value:0.000  
No matches to TargetScan

-3686--(18)--3705-

ATTGCAT

ATTGCAT  
Depth:11 (X.TROPICALIS)  
Ei-value:0.000, Pi-value:0.000  
Er-value:0.000, Pr-value:0.000  
No matches to TargetScan

-3711--(564)--4276-

AGGCAGGA

AGGCAGGA  
Depth:10 (SNAKE)  
Ei-value:0.000, Pi-value:0.000  
Er-value:0.000, Pr-value:0.000  
No matches to TargetScan

-4283--(281)--4565-

TAAGGA

TAAGGA  
Depth:13 (OPOSSUM)  
Ei-value:0.000, Pi-value:0.000  
Er-value:0.000, Pr-value:0.000  
No matches to TargetScan

-4570--(238)--4809-

TGCAGTG

TGCAGTG  
Depth:10 (SNAKE)  
Ei-value:0.000, Pi-value:0.000  
Er-value:0.000, Pr-value:0.000  
MATCHES To TargetScan▶ miR-217:ACUGCAU

-4815--(166)--4982-

TTGGCAAGTAA

TTGGCAAGTAA  
Depth:10 (SNAKE)  
Ei-value:0.000, Pi-value:0.000  
Er-value:0.000, Pr-value:0.000  
No matches to TargetScan

-4992--(215)--5208-

TTAGGT

TTAGGT  
Depth:11 (X.TROPICALIS)  
Ei-value:0.000, Pi-value:0.000  
Er-value:0.000, Pr-value:0.000  
No matches to TargetScan


AA

TTAGGTAA  
Depth:10 (SNAKE)  
Ei-value:0.000, Pi-value:0.000  
Er-value:0.000, Pr-value:0.000  
No matches to TargetScan

-5215--(44)--5260-

T

TGAAGCCTTTAGT  
Depth:10 (SNAKE)  
Ei-value:0.000, Pi-value:0.000  
Er-value:0.000, Pr-value:0.000  
No matches to TargetScan


GAAGCC

GAAGCC  
Depth:11 (X.TROPICALIS)  
Ei-value:0.000, Pi-value:0.000  
Er-value:0.000, Pr-value:0.000  
No matches to TargetScan


TTTAGT

TGAAGCCTTTAGT  
Depth:10 (SNAKE)  
Ei-value:0.000, Pi-value:0.000  
Er-value:0.000, Pr-value:0.000  
No matches to TargetScan

-5272--(4)--5277-

TTCCAGAT

TTCCAGAT  
Depth:10 (SNAKE)  
Ei-value:0.000, Pi-value:0.000  
Er-value:0.000, Pr-value:0.000  
No matches to TargetScan

-5284--(52)--5337-

GGCAAGT

GGCAAGT  
Depth:10 (SNAKE)  
Ei-value:0.000, Pi-value:0.000  
Er-value:0.000, Pr-value:0.000  
No matches to TargetScan

-5343--(337)--5681-

AAC

AACAGGTGAA  
Depth:10 (SNAKE)  
Ei-value:0.000, Pi-value:0.000  
Er-value:0.000, Pr-value:0.000  
No matches to TargetScan


AGGTGA

AGGTGA  
Depth:11 (X.TROPICALIS)  
Ei-value:0.000, Pi-value:0.000  
Er-value:0.000, Pr-value:0.010  
No matches to TargetScan


A

AACAGGTGAA  
Depth:10 (SNAKE)  
Ei-value:0.000, Pi-value:0.000  
Er-value:0.000, Pr-value:0.000  
No matches to TargetScan

-5690--(125)--5816-

AAGGCT

AAGGCT  
Depth:10 (SNAKE)  
Ei-value:0.000, Pi-value:0.000  
Er-value:0.000, Pr-value:0.010  
No matches to TargetScan

-5821--(369)--6191-

TTTTCCAG

TTTTCCAG  
Depth:10 (SNAKE)  
Ei-value:0.000, Pi-value:0.000  
Er-value:0.000, Pr-value:0.000  
No matches to TargetScan

-6198--(73)--6272-

TGGATCCT

TGGATCCT  
Depth:10 (SNAKE)  
Ei-value:0.000, Pi-value:0.000  
Er-value:0.000, Pr-value:0.000  
No matches to TargetScan

-6279--(19)--6299-

AAGGTAA

AAGGTAA  
Depth:10 (SNAKE)  
Ei-value:0.000, Pi-value:0.000  
Er-value:0.000, Pr-value:0.000  
No matches to TargetScan

-6305--(84)--6390-

AAGGTAA

AAGGTAA  
Depth:10 (SNAKE)  
Ei-value:0.000, Pi-value:0.000  
Er-value:0.000, Pr-value:0.000  
No matches to TargetScan

-6396--(225)--6622-

GGGTGGG

GGGTGGG  
Depth:10 (SNAKE)  
Ei-value:0.000, Pi-value:0.000  
Er-value:0.000, Pr-value:0.000  
No matches to TargetScan

-6628--(21)--6650-

TGCTAAGACT

TGCTAAGACT  
Depth:10 (SNAKE)  
Ei-value:0.000, Pi-value:0.000  
Er-value:0.000, Pr-value:0.000  
No matches to TargetScan

-6659--(63)--6723-

TTTTCTTTT

TTTTCTTTT  
Depth:19 (ZEBRAFISH)  
Ei-value:0.000, Pi-value:0.000  
Er-value:0.000, Pr-value:0.000  
MATCHES To TargetScan▶ miR-186-5p:AAAGAAU


CCTGAGA

TTTTCTTTTCCTGAGA  
Depth:10 (SNAKE)  
Ei-value:0.000, Pi-value:0.000  
Er-value:0.000, Pr-value:0.000  
MATCHES To TargetScan▶ miR-873-5p.1:CAGGAAC▶ miR-186-5p:AAAGAAU

-6738--(15)--6754-

TCT

TCTCAGGTTTTGCTTTT  
Depth:10 (SNAKE)  
Ei-value:0.000, Pi-value:0.000  
Er-value:0.000, Pr-value:0.000  
MATCHES To TargetScan▶ miR-330-3p.2:AAAGCAC▶ miR-490-3p:AACCUGG


CAGGTTTTGCTTT

CAGGTTTTGCTTT  
Depth:19 (ZEBRAFISH)  
Ei-value:0.000, Pi-value:0.000  
Er-value:0.000, Pr-value:0.000  
MATCHES To TargetScan▶ miR-330-3p.2:AAAGCAC▶ miR-490-3p:AACCUGG


T

CAGGTTTTGCTTTT  
Depth:16 (NILETILAPIA)  
Ei-value:0.000, Pi-value:0.000  
Er-value:0.000, Pr-value:0.000  
MATCHES To TargetScan▶ miR-330-3p.2:AAAGCAC▶ miR-490-3p:AACCUGG

-6770--(27)--6798-

A

AAAAAAGCAAAAG  
Depth:13 (OPOSSUM)  
Ei-value:0.000, Pi-value:0.000  
Er-value:0.000, Pr-value:0.000  
No matches to TargetScan


AAAAAGCAAAA

AAAAAGCAAAA  
Depth:19 (ZEBRAFISH)  
Ei-value:0.000, Pi-value:0.000  
Er-value:0.000, Pr-value:0.000  
No matches to TargetScan


G

AAAAAGCAAAAG  
Depth:18 (MEDAKA)  
Ei-value:0.000, Pi-value:0.000  
Er-value:0.000, Pr-value:0.000  
No matches to TargetScan

-6810--(15)--6826-

ACTCCTG

ACTCCTG  
Depth:18 (MEDAKA)  
Ei-value:0.000, Pi-value:0.000  
Er-value:0.000, Pr-value:0.000  
No matches to TargetScan


G

ACTCCTGG  
Depth:14 (SPOTTEDGAR)  
Ei-value:0.000, Pi-value:0.000  
Er-value:0.000, Pr-value:0.000  
MATCHES To TargetScan▶ miR-665:CCAGGAG

-6833--(11)--6845-

GGGTTC

GGGTTC  
Depth:13 (OPOSSUM)  
Ei-value:0.000, Pi-value:0.000  
Er-value:0.000, Pr-value:0.000  
No matches to TargetScan

-6850
```

---

# Modules conserved to LIZARD (Depth: 9)

## Modules in Main Graph (All sequences considered):

```
>HUMAN  
      1315-

TTGGACT

TTGGACT  
Depth:9 (LIZARD)  
Ei-value:0.000, Pi-value:0.000  
Er-value:0.000, Pr-value:0.000  
eCLIP MATCHES▶aggf1 (bg=15.15%)▶bclaf1 (bg=17.67%)▶bud13 (bg=12.85%)▶DGCR8 (bg=2.67%)▶hltf (bg=24.28%)▶larp4 (bg=13.51%)▶NIPBL (bg=8.2%)▶npm1 (bg=10.22%)▶ppil4 (bg=43.39%)▶rbm15 (bg=11.59%)▶rbm22 (bg=12.69%)▶safb (bg=40.39%)▶safb2 (bg=26.89%)▶srsf1 (bg=30.28%)▶SUPV3L1 (bg=9.63%)▶tra2a (bg=37.02%)▶uchl5 (bg=18.56%)▶XRCC6 (bg=3.81%)▶YWHAG (bg=9.14%)▶zc3h8 (bg=12.78%)▶znf622 (bg=18.79%)No matches to TargetScan

-1321--(255)--1577-

GGTGAAG

GGTGAAG  
Depth:11 (X.TROPICALIS)  
Ei-value:0.000, Pi-value:0.000  
Er-value:0.000, Pr-value:0.000  
eCLIP MATCHES▶bclaf1 (bg=17.67%)▶EXOSC5 (bg=2.0%)▶fxr2 (bg=10.1%)▶gtf2f1 (bg=10.18%)▶hltf (bg=24.28%)▶MTPAP (bg=9.55%)▶npm1 (bg=10.22%)▶ppil4 (bg=43.39%)▶rbm15 (bg=11.59%)▶rbm22 (bg=12.69%)▶safb (bg=40.39%)▶safb2 (bg=26.89%)▶SMNDC1 (bg=7.08%)▶srsf1 (bg=30.28%)▶SRSF9 (bg=9.67%)▶TAF15 (bg=9.06%)▶tra2a (bg=37.02%)▶TROVE2 (bg=6.96%)▶uchl5 (bg=18.56%)▶znf622 (bg=18.79%)No matches to TargetScan

-1583--(210)--1794-

TGGTAA

TGGTAA  
Depth:14 (SPOTTEDGAR)  
Ei-value:0.030, Pi-value:0.000  
Er-value:0.010, Pr-value:0.000  
eCLIP MATCHES▶bclaf1 (bg=17.67%)▶DROSHA (bg=1.03%)▶GRWD1 (bg=7.0%)▶hltf (bg=24.28%)▶MTPAP (bg=9.55%)▶NOLC1 (bg=0.67%)▶npm1 (bg=10.22%)▶ppil4 (bg=43.39%)▶rbm15 (bg=11.59%)▶safb (bg=40.39%)▶srsf1 (bg=30.28%)▶TAF15 (bg=9.06%)▶tia1 (bg=23.76%)▶uchl5 (bg=18.56%)▶YWHAG (bg=9.14%)▶ZRANB2 (bg=7.28%)No matches to TargetScan

-1799--(705)--2505-

TTTGGG

TTTGGG  
Depth:19 (ZEBRAFISH)  
Ei-value:0.000, Pi-value:0.000  
Er-value:0.000, Pr-value:0.000  
eCLIP MATCHES▶ppil4 (bg=43.39%)▶PRPF8 (bg=6.2%)No matches to TargetScan

-2510--(243)--2754-

GAGCTT

GAGCTT  
Depth:9 (LIZARD)  
Ei-value:0.000, Pi-value:0.000  
Er-value:0.000, Pr-value:0.000  
eCLIP MATCHES▶hnrnpa1 (bg=18.32%)▶ppil4 (bg=43.39%)▶PRPF8 (bg=6.2%)No matches to TargetScan

-2759--(224)--2984-

GGCAAGTAA

GGCAAGTAA  
Depth:10 (SNAKE)  
Ei-value:0.000, Pi-value:0.000  
Er-value:0.000, Pr-value:0.000  
eCLIP MATCHES▶hnrnpa1 (bg=18.32%)▶ppil4 (bg=43.39%)▶safb (bg=40.39%)No matches to TargetScan

-2992--(378)--3371-

T

TCAAGTAAGA  
Depth:9 (LIZARD)  
Ei-value:0.000, Pi-value:0.000  
Er-value:0.000, Pr-value:0.000  
eCLIP MATCHES▶HNRNPM (bg=6.37%)▶srsf7 (bg=22.53%)No matches to TargetScan


CAA

CAAGTAAGA  
Depth:10 (SNAKE)  
Ei-value:0.000, Pi-value:0.000  
Er-value:0.000, Pr-value:0.000  
eCLIP MATCHES▶HNRNPM (bg=6.37%)▶srsf7 (bg=22.53%)No matches to TargetScan


GTAAGA

GTAAGA  
Depth:11 (X.TROPICALIS)  
Ei-value:0.000, Pi-value:0.000  
Er-value:0.000, Pr-value:0.000  
eCLIP MATCHES▶HNRNPM (bg=6.37%)No matches to TargetScan

-3380--(240)--3621-

TTTTTCAG

TTTTTCAG  
Depth:19 (ZEBRAFISH)  
Ei-value:0.000, Pi-value:0.000  
Er-value:0.000, Pr-value:0.000  
eCLIP MATCHES▶ddx42 (bg=10.33%)▶ppil4 (bg=43.39%)▶safb (bg=40.39%)▶u2af1 (bg=14.02%)▶u2af2 (bg=19.32%)No matches to TargetScan


AT

TTTTTCAGAT  
Depth:10 (SNAKE)  
Ei-value:0.000, Pi-value:0.000  
Er-value:0.000, Pr-value:0.000  
eCLIP MATCHES▶ddx42 (bg=10.33%)▶ppil4 (bg=43.39%)▶safb (bg=40.39%)▶u2af1 (bg=14.02%)▶u2af2 (bg=19.32%)No matches to TargetScan

-3630--(72)--3703-

TGATAAGT

TGATAAGT  
Depth:9 (LIZARD)  
Ei-value:0.000, Pi-value:0.000  
Er-value:0.000, Pr-value:0.000  
eCLIP MATCHES▶ppil4 (bg=43.39%)No matches to TargetScan

-3710--(0)--3711-

AAAGGCAGAA

AAAGGCAGAA  
Depth:13 (OPOSSUM)  
Ei-value:0.000, Pi-value:0.000  
Er-value:0.000, Pr-value:0.000  
eCLIP MATCHES▶hnrnpa1 (bg=18.32%)▶ppil4 (bg=43.39%)No matches to TargetScan


A

AAAGGCAGAAA  
Depth:11 (X.TROPICALIS)  
Ei-value:0.000, Pi-value:0.000  
Er-value:0.000, Pr-value:0.000  
eCLIP MATCHES▶hnrnpa1 (bg=18.32%)▶ppil4 (bg=43.39%)No matches to TargetScan


A

AAAGGCAGAAAA  
Depth:9 (LIZARD)  
Ei-value:0.000, Pi-value:0.000  
Er-value:0.000, Pr-value:0.000  
eCLIP MATCHES▶hnrnpa1 (bg=18.32%)▶ppil4 (bg=43.39%)No matches to TargetScan

-3722--(373)--4096-

AG

AGACAGGT  
Depth:9 (LIZARD)  
Ei-value:0.000, Pi-value:0.000  
Er-value:0.000, Pr-value:0.000  
eCLIP MATCHES▶DGCR8 (bg=2.67%)▶hltf (bg=24.28%)▶khsrp (bg=27.4%)▶LIN28B (bg=1.31%)▶NIPBL (bg=8.2%)▶NOLC1 (bg=0.67%)▶ppil4 (bg=43.39%)▶rbm15 (bg=11.59%)▶safb (bg=40.39%)▶safb2 (bg=26.89%)▶srsf1 (bg=30.28%)▶znf622 (bg=18.79%)No matches to TargetScan


ACAGGT

ACAGGT  
Depth:10 (SNAKE)  
Ei-value:0.000, Pi-value:0.000  
Er-value:0.000, Pr-value:0.000  
eCLIP MATCHES▶DGCR8 (bg=2.67%)▶hltf (bg=24.28%)▶khsrp (bg=27.4%)▶LIN28B (bg=1.31%)▶NOLC1 (bg=0.67%)▶ppil4 (bg=43.39%)▶rbm15 (bg=11.59%)▶safb (bg=40.39%)▶safb2 (bg=26.89%)▶srsf1 (bg=30.28%)▶znf622 (bg=18.79%)No matches to TargetScan

-4103--(66)--4170-

CA

CAGATAAGT  
Depth:11 (X.TROPICALIS)  
Ei-value:0.000, Pi-value:0.000  
Er-value:0.000, Pr-value:0.000  
eCLIP MATCHES▶cpsf6 (bg=13.45%)▶hltf (bg=24.28%)▶hnrnpa1 (bg=18.32%)▶khsrp (bg=27.4%)▶NIPBL (bg=8.2%)▶ppil4 (bg=43.39%)▶PRPF8 (bg=6.2%)▶rbm15 (bg=11.59%)▶safb (bg=40.39%)▶safb2 (bg=26.89%)▶srsf1 (bg=30.28%)▶srsf7 (bg=22.53%)▶znf622 (bg=18.79%)No matches to TargetScan


GATAAG

GATAAG  
Depth:19 (ZEBRAFISH)  
Ei-value:0.000, Pi-value:0.000  
Er-value:0.000, Pr-value:0.000  
eCLIP MATCHES▶cpsf6 (bg=13.45%)▶khsrp (bg=27.4%)▶NIPBL (bg=8.2%)▶ppil4 (bg=43.39%)▶PRPF8 (bg=6.2%)▶rbm15 (bg=11.59%)▶safb (bg=40.39%)▶safb2 (bg=26.89%)▶srsf1 (bg=30.28%)▶srsf7 (bg=22.53%)▶znf622 (bg=18.79%)No matches to TargetScan


T

CAGATAAGT  
Depth:11 (X.TROPICALIS)  
Ei-value:0.000, Pi-value:0.000  
Er-value:0.000, Pr-value:0.000  
eCLIP MATCHES▶cpsf6 (bg=13.45%)▶hltf (bg=24.28%)▶hnrnpa1 (bg=18.32%)▶khsrp (bg=27.4%)▶NIPBL (bg=8.2%)▶ppil4 (bg=43.39%)▶PRPF8 (bg=6.2%)▶rbm15 (bg=11.59%)▶safb (bg=40.39%)▶safb2 (bg=26.89%)▶srsf1 (bg=30.28%)▶srsf7 (bg=22.53%)▶znf622 (bg=18.79%)No matches to TargetScan

-4178--(18)--4197-

ATTGCAT

ATTGCAT  
Depth:11 (X.TROPICALIS)  
Ei-value:0.000, Pi-value:0.000  
Er-value:0.000, Pr-value:0.000  
eCLIP MATCHES▶cpsf6 (bg=13.45%)▶hltf (bg=24.28%)▶hnrnpa1 (bg=18.32%)▶HNRNPU (bg=9.45%)▶khsrp (bg=27.4%)▶ppil4 (bg=43.39%)▶PRPF8 (bg=6.2%)▶RBFOX2 (bg=3.41%)▶safb (bg=40.39%)▶safb2 (bg=26.89%)▶tia1 (bg=23.76%)▶tial1 (bg=15.02%)No matches to TargetScan


G

ATTGCATG  
Depth:9 (LIZARD)  
Ei-value:0.000, Pi-value:0.000  
Er-value:0.000, Pr-value:0.000  
eCLIP MATCHES▶cpsf6 (bg=13.45%)▶hltf (bg=24.28%)▶hnrnpa1 (bg=18.32%)▶HNRNPU (bg=9.45%)▶khsrp (bg=27.4%)▶ppil4 (bg=43.39%)▶PRPF8 (bg=6.2%)▶RBFOX2 (bg=3.41%)▶safb (bg=40.39%)▶safb2 (bg=26.89%)▶tia1 (bg=23.76%)▶tial1 (bg=15.02%)No matches to TargetScan

-4204--(419)--4624-

GCTGTC

GCTGTC  
Depth:9 (LIZARD)  
Ei-value:0.000, Pi-value:0.000  
Er-value:0.000, Pr-value:0.000  
eCLIP MATCHES▶cpsf6 (bg=13.45%)▶CSTF2 (bg=7.88%)▶cstf2t (bg=12.11%)▶FMR1 (bg=1.89%)▶khsrp (bg=27.4%)▶zc3h8 (bg=12.78%)No matches to TargetScan

-4629--(149)--4779-

AGGCAGGA

AGGCAGGA  
Depth:10 (SNAKE)  
Ei-value:0.000, Pi-value:0.000  
Er-value:0.000, Pr-value:0.000  
eCLIP MATCHES▶CSTF2 (bg=7.88%)▶cstf2t (bg=12.11%)▶srsf1 (bg=30.28%)No matches to TargetScan

-4786--(74)--4861-

TAAGGA

TAAGGA  
Depth:13 (OPOSSUM)  
Ei-value:0.000, Pi-value:0.000  
Er-value:0.000, Pr-value:0.000  
eCLIP MATCHES▶cstf2t (bg=12.11%)▶tia1 (bg=23.76%)▶tial1 (bg=15.02%)No matches to TargetScan

-4866--(311)--5178-

TTCTGT

TTCTGT  
Depth:9 (LIZARD)  
Ei-value:0.000, Pi-value:0.000  
Er-value:0.000, Pr-value:0.000  
eCLIP MATCHES▶CSTF2 (bg=7.88%)▶cstf2t (bg=12.11%)▶hnrnpa1 (bg=18.32%)▶HNRNPM (bg=6.37%)▶PRPF8 (bg=6.2%)▶srsf1 (bg=30.28%)▶srsf7 (bg=22.53%)▶TARDBP (bg=5.12%)▶tia1 (bg=23.76%)▶u2af1 (bg=14.02%)▶u2af2 (bg=19.32%)No matches to TargetScan

-5183--(21)--5205-

TGCAGTG

TGCAGTG  
Depth:10 (SNAKE)  
Ei-value:0.000, Pi-value:0.000  
Er-value:0.000, Pr-value:0.000  
eCLIP MATCHES▶cstf2t (bg=12.11%)▶hnrnpa1 (bg=18.32%)▶HNRNPM (bg=6.37%)▶PRPF8 (bg=6.2%)▶srsf7 (bg=22.53%)▶TARDBP (bg=5.12%)MATCHES To TargetScan▶ miR-217:ACUGCAU

-5211--(142)--5354-

TTGGCAAGTAA

TTGGCAAGTAA  
Depth:10 (SNAKE)  
Ei-value:0.000, Pi-value:0.000  
Er-value:0.000, Pr-value:0.000  
eCLIP MATCHES▶CSTF2 (bg=7.88%)▶cstf2t (bg=12.11%)▶HNRNPC (bg=4.22%)▶khsrp (bg=27.4%)▶ppil4 (bg=43.39%)▶srsf7 (bg=22.53%)▶TARDBP (bg=5.12%)▶tia1 (bg=23.76%)▶u2af1 (bg=14.02%)▶u2af2 (bg=19.32%)No matches to TargetScan

-5364--(84)--5449-

TGGAGTAGTG

TGGAGTAGTG  
Depth:9 (LIZARD)  
Ei-value:0.000, Pi-value:0.000  
Er-value:0.000, Pr-value:0.000  
eCLIP MATCHES▶CSTF2 (bg=7.88%)▶cstf2t (bg=12.11%)▶DDX21 (bg=1.05%)▶EIF4G2 (bg=2.16%)▶hnrnpa1 (bg=18.32%)▶HNRNPM (bg=6.37%)▶HNRNPU (bg=9.45%)▶IGF2BP1 (bg=1.23%)▶khsrp (bg=27.4%)▶LIN28B (bg=1.31%)▶srsf7 (bg=22.53%)▶TARDBP (bg=5.12%)▶zc3h8 (bg=12.78%)No matches to TargetScan

-5458--(114)--5573-

TTAGGT

TTAGGT  
Depth:11 (X.TROPICALIS)  
Ei-value:0.000, Pi-value:0.000  
Er-value:0.000, Pr-value:0.000  
eCLIP MATCHES▶cstf2t (bg=12.11%)▶HNRNPM (bg=6.37%)▶khsrp (bg=27.4%)▶srsf7 (bg=22.53%)▶TARDBP (bg=5.12%)▶tia1 (bg=23.76%)▶tial1 (bg=15.02%)▶u2af1 (bg=14.02%)▶ZRANB2 (bg=7.28%)No matches to TargetScan


AA

TTAGGTAA  
Depth:10 (SNAKE)  
Ei-value:0.000, Pi-value:0.000  
Er-value:0.000, Pr-value:0.000  
eCLIP MATCHES▶cstf2t (bg=12.11%)▶HNRNPM (bg=6.37%)▶khsrp (bg=27.4%)▶srsf7 (bg=22.53%)▶TARDBP (bg=5.12%)▶tia1 (bg=23.76%)▶tial1 (bg=15.02%)▶u2af1 (bg=14.02%)▶ZRANB2 (bg=7.28%)No matches to TargetScan

-5580--(36)--5617-

T

TGAAGCCTTTAGT  
Depth:10 (SNAKE)  
Ei-value:0.000, Pi-value:0.000  
Er-value:0.000, Pr-value:0.000  
eCLIP MATCHES▶ddx42 (bg=10.33%)▶khsrp (bg=27.4%)▶SF3B4 (bg=4.76%)▶SMNDC1 (bg=7.08%)▶TARDBP (bg=5.12%)▶tia1 (bg=23.76%)▶tial1 (bg=15.02%)▶u2af1 (bg=14.02%)▶u2af2 (bg=19.32%)▶ZRANB2 (bg=7.28%)No matches to TargetScan


GAAGCC

GAAGCC  
Depth:11 (X.TROPICALIS)  
Ei-value:0.000, Pi-value:0.000  
Er-value:0.000, Pr-value:0.000  
eCLIP MATCHES▶ddx42 (bg=10.33%)▶khsrp (bg=27.4%)▶SF3B4 (bg=4.76%)▶TARDBP (bg=5.12%)▶tia1 (bg=23.76%)▶tial1 (bg=15.02%)▶u2af1 (bg=14.02%)▶u2af2 (bg=19.32%)▶ZRANB2 (bg=7.28%)No matches to TargetScan


TTTAGT

TGAAGCCTTTAGT  
Depth:10 (SNAKE)  
Ei-value:0.000, Pi-value:0.000  
Er-value:0.000, Pr-value:0.000  
eCLIP MATCHES▶ddx42 (bg=10.33%)▶khsrp (bg=27.4%)▶SF3B4 (bg=4.76%)▶SMNDC1 (bg=7.08%)▶TARDBP (bg=5.12%)▶tia1 (bg=23.76%)▶tial1 (bg=15.02%)▶u2af1 (bg=14.02%)▶u2af2 (bg=19.32%)▶ZRANB2 (bg=7.28%)No matches to TargetScan

-5629--(3)--5633-

TTCCAGAT

TTCCAGAT  
Depth:10 (SNAKE)  
Ei-value:0.000, Pi-value:0.000  
Er-value:0.000, Pr-value:0.000  
eCLIP MATCHES▶ddx42 (bg=10.33%)▶khsrp (bg=27.4%)▶ppil4 (bg=43.39%)▶SF3B4 (bg=4.76%)▶SMNDC1 (bg=7.08%)▶tia1 (bg=23.76%)▶tial1 (bg=15.02%)▶u2af1 (bg=14.02%)▶u2af2 (bg=19.32%)▶ZRANB2 (bg=7.28%)No matches to TargetScan

-5640--(63)--5704-

GGCAAGT

GGCAAGT  
Depth:10 (SNAKE)  
Ei-value:0.000, Pi-value:0.000  
Er-value:0.000, Pr-value:0.000  
eCLIP MATCHES▶bclaf1 (bg=17.67%)▶khsrp (bg=27.4%)▶srsf7 (bg=22.53%)▶u2af2 (bg=19.32%)▶znf622 (bg=18.79%)No matches to TargetScan

-5710--(285)--5996-

AAC

AACAGGTGAA  
Depth:10 (SNAKE)  
Ei-value:0.000, Pi-value:0.000  
Er-value:0.000, Pr-value:0.000  
eCLIP MATCHES▶GRWD1 (bg=7.0%)▶srsf1 (bg=30.28%)▶srsf7 (bg=22.53%)No matches to TargetScan


AGGTGA

AGGTGA  
Depth:11 (X.TROPICALIS)  
Ei-value:0.000, Pi-value:0.000  
Er-value:0.000, Pr-value:0.010  
eCLIP MATCHES▶GRWD1 (bg=7.0%)▶srsf1 (bg=30.28%)▶srsf7 (bg=22.53%)No matches to TargetScan


A

AACAGGTGAA  
Depth:10 (SNAKE)  
Ei-value:0.000, Pi-value:0.000  
Er-value:0.000, Pr-value:0.000  
eCLIP MATCHES▶GRWD1 (bg=7.0%)▶srsf1 (bg=30.28%)▶srsf7 (bg=22.53%)No matches to TargetScan

-6005--(184)--6190-

AAGGCT

AAGGCT  
Depth:10 (SNAKE)  
Ei-value:0.000, Pi-value:0.000  
Er-value:0.000, Pr-value:0.010  
eCLIP MATCHES▶HNRNPU (bg=9.45%)▶tial1 (bg=15.02%)No matches to TargetScan


T

AAGGCTT  
Depth:9 (LIZARD)  
Ei-value:0.000, Pi-value:0.000  
Er-value:0.000, Pr-value:0.000  
eCLIP MATCHES▶HNRNPU (bg=9.45%)▶tial1 (bg=15.02%)No matches to TargetScan

-6196--(289)--6486-

TTTTCCAG

TTTTCCAG  
Depth:10 (SNAKE)  
Ei-value:0.000, Pi-value:0.000  
Er-value:0.000, Pr-value:0.000  
No matches to eCLIP DataNo matches to TargetScan

-6493--(113)--6607-

TGGATCCT

TGGATCCT  
Depth:10 (SNAKE)  
Ei-value:0.000, Pi-value:0.000  
Er-value:0.000, Pr-value:0.000  
No matches to eCLIP DataNo matches to TargetScan

-6614--(26)--6641-

AAGGTAA

AAGGTAA  
Depth:10 (SNAKE)  
Ei-value:0.000, Pi-value:0.000  
Er-value:0.000, Pr-value:0.000  
eCLIP MATCHES▶PRPF8 (bg=6.2%)No matches to TargetScan

-6647--(246)--6894-

GGGTGGG

GGGTGGG  
Depth:10 (SNAKE)  
Ei-value:0.000, Pi-value:0.000  
Er-value:0.000, Pr-value:0.000  
eCLIP MATCHES▶ddx42 (bg=10.33%)▶khsrp (bg=27.4%)▶QKI (bg=3.74%)▶SF3B4 (bg=4.76%)No matches to TargetScan

-6900--(15)--6916-

TGCTAAGACT

TGCTAAGACT  
Depth:10 (SNAKE)  
Ei-value:0.000, Pi-value:0.000  
Er-value:0.000, Pr-value:0.000  
eCLIP MATCHES▶ddx42 (bg=10.33%)▶khsrp (bg=27.4%)▶QKI (bg=3.74%)▶SF3B4 (bg=4.76%)No matches to TargetScan

-6925--(85)--7011-

TTTTCTTTT

TTTTCTTTT  
Depth:19 (ZEBRAFISH)  
Ei-value:0.000, Pi-value:0.000  
Er-value:0.000, Pr-value:0.000  
eCLIP MATCHES▶srsf7 (bg=22.53%)MATCHES To TargetScan▶ miR-186-5p:AAAGAAU


CCTGAGA

TTTTCTTTTCCTGAGA  
Depth:10 (SNAKE)  
Ei-value:0.000, Pi-value:0.000  
Er-value:0.000, Pr-value:0.000  
eCLIP MATCHES▶srsf7 (bg=22.53%)MATCHES To TargetScan▶ miR-873-5p.1:CAGGAAC▶ miR-186-5p:AAAGAAU

-7026--(18)--7045-

TCT

TCTCAGGTTTTGCTTTT  
Depth:10 (SNAKE)  
Ei-value:0.000, Pi-value:0.000  
Er-value:0.000, Pr-value:0.000  
eCLIP MATCHES▶srsf7 (bg=22.53%)MATCHES To TargetScan▶ miR-330-3p.2:AAAGCAC▶ miR-490-3p:AACCUGG


CAGGTTTTGCTTT

CAGGTTTTGCTTT  
Depth:19 (ZEBRAFISH)  
Ei-value:0.000, Pi-value:0.000  
Er-value:0.000, Pr-value:0.000  
eCLIP MATCHES▶srsf7 (bg=22.53%)MATCHES To TargetScan▶ miR-330-3p.2:AAAGCAC▶ miR-490-3p:AACCUGG


T

CAGGTTTTGCTTTT  
Depth:16 (NILETILAPIA)  
Ei-value:0.000, Pi-value:0.000  
Er-value:0.000, Pr-value:0.000  
eCLIP MATCHES▶srsf7 (bg=22.53%)MATCHES To TargetScan▶ miR-330-3p.2:AAAGCAC▶ miR-490-3p:AACCUGG

-7061--(24)--7086-

A

AAAAAAGCAAAAG  
Depth:13 (OPOSSUM)  
Ei-value:0.000, Pi-value:0.000  
Er-value:0.000, Pr-value:0.000  
No matches to eCLIP DataNo matches to TargetScan


AAAAAGCAAAA

AAAAAGCAAAA  
Depth:19 (ZEBRAFISH)  
Ei-value:0.000, Pi-value:0.000  
Er-value:0.000, Pr-value:0.000  
No matches to eCLIP DataNo matches to TargetScan


G

AAAAAGCAAAAG  
Depth:18 (MEDAKA)  
Ei-value:0.000, Pi-value:0.000  
Er-value:0.000, Pr-value:0.000  
No matches to eCLIP DataNo matches to TargetScan


A

AAAAAAGCAAAAGA  
Depth:9 (LIZARD)  
Ei-value:0.000, Pi-value:0.000  
Er-value:0.000, Pr-value:0.000  
No matches to eCLIP DataNo matches to TargetScan

-7099--(1)--7101-

GCTGGT

GCTGGT  
Depth:9 (LIZARD)  
Ei-value:0.000, Pi-value:0.000  
Er-value:0.000, Pr-value:0.000  
No matches to eCLIP DataNo matches to TargetScan

-7106--(7)--7114-

ACTCCTG

ACTCCTG  
Depth:18 (MEDAKA)  
Ei-value:0.000, Pi-value:0.000  
Er-value:0.000, Pr-value:0.000  
No matches to eCLIP DataNo matches to TargetScan


G

ACTCCTGG  
Depth:14 (SPOTTEDGAR)  
Ei-value:0.000, Pi-value:0.000  
Er-value:0.000, Pr-value:0.000  
No matches to eCLIP DataMATCHES To TargetScan▶ miR-665:CCAGGAG

-7121--(11)--7133-

GGGTTC

GGGTTC  
Depth:13 (OPOSSUM)  
Ei-value:0.000, Pi-value:0.000  
Er-value:0.000, Pr-value:0.000  
No matches to eCLIP DataNo matches to TargetScan


AA

GGGTTCAA  
Depth:9 (LIZARD)  
Ei-value:0.000, Pi-value:0.000  
Er-value:0.000, Pr-value:0.000  
No matches to eCLIP DataNo matches to TargetScan

-7140  
  
>MARMOSET  
      1435-

TTGGACT

TTGGACT  
Depth:9 (LIZARD)  
Ei-value:0.000, Pi-value:0.000  
Er-value:0.000, Pr-value:0.000  
No matches to TargetScan

-1441--(245)--1687-

GGTGAAG

GGTGAAG  
Depth:11 (X.TROPICALIS)  
Ei-value:0.000, Pi-value:0.000  
Er-value:0.000, Pr-value:0.000  
No matches to TargetScan

-1693--(214)--1908-

TGGTAA

TGGTAA  
Depth:14 (SPOTTEDGAR)  
Ei-value:0.030, Pi-value:0.000  
Er-value:0.010, Pr-value:0.000  
No matches to TargetScan

-1913--(723)--2637-

TTTGGG

TTTGGG  
Depth:19 (ZEBRAFISH)  
Ei-value:0.000, Pi-value:0.000  
Er-value:0.000, Pr-value:0.000  
No matches to TargetScan

-2642--(243)--2886-

GAGCTT

GAGCTT  
Depth:9 (LIZARD)  
Ei-value:0.000, Pi-value:0.000  
Er-value:0.000, Pr-value:0.000  
No matches to TargetScan

-2891--(223)--3115-

GGCAAGTAA

GGCAAGTAA  
Depth:10 (SNAKE)  
Ei-value:0.000, Pi-value:0.000  
Er-value:0.000, Pr-value:0.000  
No matches to TargetScan

-3123--(373)--3497-

T

TCAAGTAAGA  
Depth:9 (LIZARD)  
Ei-value:0.000, Pi-value:0.000  
Er-value:0.000, Pr-value:0.000  
No matches to TargetScan


CAA

CAAGTAAGA  
Depth:10 (SNAKE)  
Ei-value:0.000, Pi-value:0.000  
Er-value:0.000, Pr-value:0.000  
No matches to TargetScan


GTAAGA

GTAAGA  
Depth:11 (X.TROPICALIS)  
Ei-value:0.000, Pi-value:0.000  
Er-value:0.000, Pr-value:0.000  
No matches to TargetScan

-3506--(231)--3738-

TTTTTCAG

TTTTTCAG  
Depth:19 (ZEBRAFISH)  
Ei-value:0.000, Pi-value:0.000  
Er-value:0.000, Pr-value:0.000  
No matches to TargetScan


AT

TTTTTCAGAT  
Depth:10 (SNAKE)  
Ei-value:0.000, Pi-value:0.000  
Er-value:0.000, Pr-value:0.000  
No matches to TargetScan

-3747--(71)--3819-

TGATAAGT

TGATAAGT  
Depth:9 (LIZARD)  
Ei-value:0.000, Pi-value:0.000  
Er-value:0.000, Pr-value:0.000  
No matches to TargetScan

-3826--(0)--3827-

AAAGGCAGAA

AAAGGCAGAA  
Depth:13 (OPOSSUM)  
Ei-value:0.000, Pi-value:0.000  
Er-value:0.000, Pr-value:0.000  
No matches to TargetScan


A

AAAGGCAGAAA  
Depth:11 (X.TROPICALIS)  
Ei-value:0.000, Pi-value:0.000  
Er-value:0.000, Pr-value:0.000  
No matches to TargetScan


A

AAAGGCAGAAAA  
Depth:9 (LIZARD)  
Ei-value:0.000, Pi-value:0.000  
Er-value:0.000, Pr-value:0.000  
No matches to TargetScan

-3838--(379)--4218-

AG

AGACAGGT  
Depth:9 (LIZARD)  
Ei-value:0.000, Pi-value:0.000  
Er-value:0.000, Pr-value:0.000  
No matches to TargetScan


ACAGGT

ACAGGT  
Depth:10 (SNAKE)  
Ei-value:0.000, Pi-value:0.000  
Er-value:0.000, Pr-value:0.000  
No matches to TargetScan

-4225--(66)--4292-

CA

CAGATAAGT  
Depth:11 (X.TROPICALIS)  
Ei-value:0.000, Pi-value:0.000  
Er-value:0.000, Pr-value:0.000  
No matches to TargetScan


GATAAG

GATAAG  
Depth:19 (ZEBRAFISH)  
Ei-value:0.000, Pi-value:0.000  
Er-value:0.000, Pr-value:0.000  
No matches to TargetScan


T

CAGATAAGT  
Depth:11 (X.TROPICALIS)  
Ei-value:0.000, Pi-value:0.000  
Er-value:0.000, Pr-value:0.000  
No matches to TargetScan

-4300--(18)--4319-

ATTGCAT

ATTGCAT  
Depth:11 (X.TROPICALIS)  
Ei-value:0.000, Pi-value:0.000  
Er-value:0.000, Pr-value:0.000  
No matches to TargetScan


G

ATTGCATG  
Depth:9 (LIZARD)  
Ei-value:0.000, Pi-value:0.000  
Er-value:0.000, Pr-value:0.000  
No matches to TargetScan

-4326--(430)--4757-

GCTGTC

GCTGTC  
Depth:9 (LIZARD)  
Ei-value:0.000, Pi-value:0.000  
Er-value:0.000, Pr-value:0.000  
No matches to TargetScan

-4762--(147)--4910-

AGGCAGGA

AGGCAGGA  
Depth:10 (SNAKE)  
Ei-value:0.000, Pi-value:0.000  
Er-value:0.000, Pr-value:0.000  
No matches to TargetScan

-4917--(72)--4990-

TAAGGA

TAAGGA  
Depth:13 (OPOSSUM)  
Ei-value:0.000, Pi-value:0.000  
Er-value:0.000, Pr-value:0.000  
No matches to TargetScan

-4995--(310)--5306-

TTCTGT

TTCTGT  
Depth:9 (LIZARD)  
Ei-value:0.000, Pi-value:0.000  
Er-value:0.000, Pr-value:0.000  
No matches to TargetScan

-5311--(24)--5336-

TGCAGTG

TGCAGTG  
Depth:10 (SNAKE)  
Ei-value:0.000, Pi-value:0.000  
Er-value:0.000, Pr-value:0.000  
MATCHES To TargetScan▶ miR-217:ACUGCAU

-5342--(138)--5481-

TTGGCAAGTAA

TTGGCAAGTAA  
Depth:10 (SNAKE)  
Ei-value:0.000, Pi-value:0.000  
Er-value:0.000, Pr-value:0.000  
No matches to TargetScan

-5491--(90)--5582-

TGGAGTAGTG

TGGAGTAGTG  
Depth:9 (LIZARD)  
Ei-value:0.000, Pi-value:0.000  
Er-value:0.000, Pr-value:0.000  
No matches to TargetScan

-5591--(114)--5706-

TTAGGT

TTAGGT  
Depth:11 (X.TROPICALIS)  
Ei-value:0.000, Pi-value:0.000  
Er-value:0.000, Pr-value:0.000  
No matches to TargetScan


AA

TTAGGTAA  
Depth:10 (SNAKE)  
Ei-value:0.000, Pi-value:0.000  
Er-value:0.000, Pr-value:0.000  
No matches to TargetScan

-5713--(36)--5750-

T

TGAAGCCTTTAGT  
Depth:10 (SNAKE)  
Ei-value:0.000, Pi-value:0.000  
Er-value:0.000, Pr-value:0.000  
No matches to TargetScan


GAAGCC

GAAGCC  
Depth:11 (X.TROPICALIS)  
Ei-value:0.000, Pi-value:0.000  
Er-value:0.000, Pr-value:0.000  
No matches to TargetScan


TTTAGT

TGAAGCCTTTAGT  
Depth:10 (SNAKE)  
Ei-value:0.000, Pi-value:0.000  
Er-value:0.000, Pr-value:0.000  
No matches to TargetScan

-5762--(3)--5766-

TTCCAGAT

TTCCAGAT  
Depth:10 (SNAKE)  
Ei-value:0.000, Pi-value:0.000  
Er-value:0.000, Pr-value:0.000  
No matches to TargetScan

-5773--(65)--5839-

GGCAAGT

GGCAAGT  
Depth:10 (SNAKE)  
Ei-value:0.000, Pi-value:0.000  
Er-value:0.000, Pr-value:0.000  
No matches to TargetScan

-5845--(289)--6135-

AAC

AACAGGTGAA  
Depth:10 (SNAKE)  
Ei-value:0.000, Pi-value:0.000  
Er-value:0.000, Pr-value:0.000  
No matches to TargetScan


AGGTGA

AGGTGA  
Depth:11 (X.TROPICALIS)  
Ei-value:0.000, Pi-value:0.000  
Er-value:0.000, Pr-value:0.010  
No matches to TargetScan


A

AACAGGTGAA  
Depth:10 (SNAKE)  
Ei-value:0.000, Pi-value:0.000  
Er-value:0.000, Pr-value:0.000  
No matches to TargetScan

-6144--(189)--6334-

AAGGCT

AAGGCT  
Depth:10 (SNAKE)  
Ei-value:0.000, Pi-value:0.000  
Er-value:0.000, Pr-value:0.010  
No matches to TargetScan


T

AAGGCTT  
Depth:9 (LIZARD)  
Ei-value:0.000, Pi-value:0.000  
Er-value:0.000, Pr-value:0.000  
No matches to TargetScan

-6340--(285)--6626-

TTTTCCAG

TTTTCCAG  
Depth:10 (SNAKE)  
Ei-value:0.000, Pi-value:0.000  
Er-value:0.000, Pr-value:0.000  
No matches to TargetScan

-6633--(113)--6747-

TGGATCCT

TGGATCCT  
Depth:10 (SNAKE)  
Ei-value:0.000, Pi-value:0.000  
Er-value:0.000, Pr-value:0.000  
No matches to TargetScan

-6754--(26)--6781-

AAGGTAA

AAGGTAA  
Depth:10 (SNAKE)  
Ei-value:0.000, Pi-value:0.000  
Er-value:0.000, Pr-value:0.000  
No matches to TargetScan

-6787--(235)--7023-

GGGTGGG

GGGTGGG  
Depth:10 (SNAKE)  
Ei-value:0.000, Pi-value:0.000  
Er-value:0.000, Pr-value:0.000  
No matches to TargetScan

-7029--(15)--7045-

TGCTAAGACT

TGCTAAGACT  
Depth:10 (SNAKE)  
Ei-value:0.000, Pi-value:0.000  
Er-value:0.000, Pr-value:0.000  
No matches to TargetScan

-7054--(85)--7140-

TTTTCTTTT

TTTTCTTTT  
Depth:19 (ZEBRAFISH)  
Ei-value:0.000, Pi-value:0.000  
Er-value:0.000, Pr-value:0.000  
MATCHES To TargetScan▶ miR-186-5p:AAAGAAU


CCTGAGA

TTTTCTTTTCCTGAGA  
Depth:10 (SNAKE)  
Ei-value:0.000, Pi-value:0.000  
Er-value:0.000, Pr-value:0.000  
MATCHES To TargetScan▶ miR-873-5p.1:CAGGAAC▶ miR-186-5p:AAAGAAU

-7155--(18)--7174-

TCT

TCTCAGGTTTTGCTTTT  
Depth:10 (SNAKE)  
Ei-value:0.000, Pi-value:0.000  
Er-value:0.000, Pr-value:0.000  
MATCHES To TargetScan▶ miR-330-3p.2:AAAGCAC▶ miR-490-3p:AACCUGG


CAGGTTTTGCTTT

CAGGTTTTGCTTT  
Depth:19 (ZEBRAFISH)  
Ei-value:0.000, Pi-value:0.000  
Er-value:0.000, Pr-value:0.000  
MATCHES To TargetScan▶ miR-330-3p.2:AAAGCAC▶ miR-490-3p:AACCUGG


T

CAGGTTTTGCTTTT  
Depth:16 (NILETILAPIA)  
Ei-value:0.000, Pi-value:0.000  
Er-value:0.000, Pr-value:0.000  
MATCHES To TargetScan▶ miR-330-3p.2:AAAGCAC▶ miR-490-3p:AACCUGG

-7190--(25)--7216-

A

AAAAAAGCAAAAG  
Depth:13 (OPOSSUM)  
Ei-value:0.000, Pi-value:0.000  
Er-value:0.000, Pr-value:0.000  
No matches to TargetScan


AAAAAGCAAAA

AAAAAGCAAAA  
Depth:19 (ZEBRAFISH)  
Ei-value:0.000, Pi-value:0.000  
Er-value:0.000, Pr-value:0.000  
No matches to TargetScan


G

AAAAAGCAAAAG  
Depth:18 (MEDAKA)  
Ei-value:0.000, Pi-value:0.000  
Er-value:0.000, Pr-value:0.000  
No matches to TargetScan


A

AAAAAAGCAAAAGA  
Depth:9 (LIZARD)  
Ei-value:0.000, Pi-value:0.000  
Er-value:0.000, Pr-value:0.000  
No matches to TargetScan

-7229--(1)--7231-

GCTGGT

GCTGGT  
Depth:9 (LIZARD)  
Ei-value:0.000, Pi-value:0.000  
Er-value:0.000, Pr-value:0.000  
No matches to TargetScan

-7236--(7)--7244-

ACTCCTG

ACTCCTG  
Depth:18 (MEDAKA)  
Ei-value:0.000, Pi-value:0.000  
Er-value:0.000, Pr-value:0.000  
No matches to TargetScan


G

ACTCCTGG  
Depth:14 (SPOTTEDGAR)  
Ei-value:0.000, Pi-value:0.000  
Er-value:0.000, Pr-value:0.000  
MATCHES To TargetScan▶ miR-665:CCAGGAG

-7251--(11)--7263-

GGGTTC

GGGTTC  
Depth:13 (OPOSSUM)  
Ei-value:0.000, Pi-value:0.000  
Er-value:0.000, Pr-value:0.000  
No matches to TargetScan


AA

GGGTTCAA  
Depth:9 (LIZARD)  
Ei-value:0.000, Pi-value:0.000  
Er-value:0.000, Pr-value:0.000  
No matches to TargetScan

-7270  
  
>DOG  
      1450-

TTGGACT

TTGGACT  
Depth:9 (LIZARD)  
Ei-value:0.000, Pi-value:0.000  
Er-value:0.000, Pr-value:0.000  
No matches to TargetScan

-1456--(259)--1716-

GGTGAAG

GGTGAAG  
Depth:11 (X.TROPICALIS)  
Ei-value:0.000, Pi-value:0.000  
Er-value:0.000, Pr-value:0.000  
No matches to TargetScan

-1722--(205)--1928-

TGGTAA

TGGTAA  
Depth:14 (SPOTTEDGAR)  
Ei-value:0.030, Pi-value:0.000  
Er-value:0.010, Pr-value:0.000  
No matches to TargetScan

-1933--(678)--2612-

TTTGGG

TTTGGG  
Depth:19 (ZEBRAFISH)  
Ei-value:0.000, Pi-value:0.000  
Er-value:0.000, Pr-value:0.000  
No matches to TargetScan

-2617--(240)--2858-

GAGCTT

GAGCTT  
Depth:9 (LIZARD)  
Ei-value:0.000, Pi-value:0.000  
Er-value:0.000, Pr-value:0.000  
No matches to TargetScan

-2863--(227)--3091-

GGCAAGTAA

GGCAAGTAA  
Depth:10 (SNAKE)  
Ei-value:0.000, Pi-value:0.000  
Er-value:0.000, Pr-value:0.000  
No matches to TargetScan

-3099--(329)--3429-

T

TCAAGTAAGA  
Depth:9 (LIZARD)  
Ei-value:0.000, Pi-value:0.000  
Er-value:0.000, Pr-value:0.000  
No matches to TargetScan


CAA

CAAGTAAGA  
Depth:10 (SNAKE)  
Ei-value:0.000, Pi-value:0.000  
Er-value:0.000, Pr-value:0.000  
No matches to TargetScan


GTAAGA

GTAAGA  
Depth:11 (X.TROPICALIS)  
Ei-value:0.000, Pi-value:0.000  
Er-value:0.000, Pr-value:0.000  
No matches to TargetScan

-3438--(192)--3631-

TTTTTCAG

TTTTTCAG  
Depth:19 (ZEBRAFISH)  
Ei-value:0.000, Pi-value:0.000  
Er-value:0.000, Pr-value:0.000  
No matches to TargetScan


AT

TTTTTCAGAT  
Depth:10 (SNAKE)  
Ei-value:0.000, Pi-value:0.000  
Er-value:0.000, Pr-value:0.000  
No matches to TargetScan

-3640--(69)--3710-

TGATAAGT

TGATAAGT  
Depth:9 (LIZARD)  
Ei-value:0.000, Pi-value:0.000  
Er-value:0.000, Pr-value:0.000  
No matches to TargetScan

-3717--(0)--3718-

AAAGGCAGAA

AAAGGCAGAA  
Depth:13 (OPOSSUM)  
Ei-value:0.000, Pi-value:0.000  
Er-value:0.000, Pr-value:0.000  
No matches to TargetScan


A

AAAGGCAGAAA  
Depth:11 (X.TROPICALIS)  
Ei-value:0.000, Pi-value:0.000  
Er-value:0.000, Pr-value:0.000  
No matches to TargetScan


A

AAAGGCAGAAAA  
Depth:9 (LIZARD)  
Ei-value:0.000, Pi-value:0.000  
Er-value:0.000, Pr-value:0.000  
No matches to TargetScan

-3729--(392)--4122-

AG

AGACAGGT  
Depth:9 (LIZARD)  
Ei-value:0.000, Pi-value:0.000  
Er-value:0.000, Pr-value:0.000  
No matches to TargetScan


ACAGGT

ACAGGT  
Depth:10 (SNAKE)  
Ei-value:0.000, Pi-value:0.000  
Er-value:0.000, Pr-value:0.000  
No matches to TargetScan

-4129--(66)--4196-

CA

CAGATAAGT  
Depth:11 (X.TROPICALIS)  
Ei-value:0.000, Pi-value:0.000  
Er-value:0.000, Pr-value:0.000  
No matches to TargetScan


GATAAG

GATAAG  
Depth:19 (ZEBRAFISH)  
Ei-value:0.000, Pi-value:0.000  
Er-value:0.000, Pr-value:0.000  
No matches to TargetScan


T

CAGATAAGT  
Depth:11 (X.TROPICALIS)  
Ei-value:0.000, Pi-value:0.000  
Er-value:0.000, Pr-value:0.000  
No matches to TargetScan

-4204--(18)--4223-

ATTGCAT

ATTGCAT  
Depth:11 (X.TROPICALIS)  
Ei-value:0.000, Pi-value:0.000  
Er-value:0.000, Pr-value:0.000  
No matches to TargetScan


G

ATTGCATG  
Depth:9 (LIZARD)  
Ei-value:0.000, Pi-value:0.000  
Er-value:0.000, Pr-value:0.000  
No matches to TargetScan

-4230--(417)--4648-

GCTGTC

GCTGTC  
Depth:9 (LIZARD)  
Ei-value:0.000, Pi-value:0.000  
Er-value:0.000, Pr-value:0.000  
No matches to TargetScan

-4653--(146)--4800-

AGGCAGGA

AGGCAGGA  
Depth:10 (SNAKE)  
Ei-value:0.000, Pi-value:0.000  
Er-value:0.000, Pr-value:0.000  
No matches to TargetScan

-4807--(72)--4880-

TAAGGA

TAAGGA  
Depth:13 (OPOSSUM)  
Ei-value:0.000, Pi-value:0.000  
Er-value:0.000, Pr-value:0.000  
No matches to TargetScan

-4885--(325)--5211-

TTCTGT

TTCTGT  
Depth:9 (LIZARD)  
Ei-value:0.000, Pi-value:0.000  
Er-value:0.000, Pr-value:0.000  
No matches to TargetScan

-5216--(19)--5236-

TGCAGTG

TGCAGTG  
Depth:10 (SNAKE)  
Ei-value:0.000, Pi-value:0.000  
Er-value:0.000, Pr-value:0.000  
MATCHES To TargetScan▶ miR-217:ACUGCAU

-5242--(132)--5375-

TTGGCAAGTAA

TTGGCAAGTAA  
Depth:10 (SNAKE)  
Ei-value:0.000, Pi-value:0.000  
Er-value:0.000, Pr-value:0.000  
No matches to TargetScan

-5385--(83)--5469-

TGGAGTAGTG

TGGAGTAGTG  
Depth:9 (LIZARD)  
Ei-value:0.000, Pi-value:0.000  
Er-value:0.000, Pr-value:0.000  
No matches to TargetScan

-5478--(111)--5590-

TTAGGT

TTAGGT  
Depth:11 (X.TROPICALIS)  
Ei-value:0.000, Pi-value:0.000  
Er-value:0.000, Pr-value:0.000  
No matches to TargetScan


AA

TTAGGTAA  
Depth:10 (SNAKE)  
Ei-value:0.000, Pi-value:0.000  
Er-value:0.000, Pr-value:0.000  
No matches to TargetScan

-5597--(42)--5640-

T

TGAAGCCTTTAGT  
Depth:10 (SNAKE)  
Ei-value:0.000, Pi-value:0.000  
Er-value:0.000, Pr-value:0.000  
No matches to TargetScan


GAAGCC

GAAGCC  
Depth:11 (X.TROPICALIS)  
Ei-value:0.000, Pi-value:0.000  
Er-value:0.000, Pr-value:0.000  
No matches to TargetScan


TTTAGT

TGAAGCCTTTAGT  
Depth:10 (SNAKE)  
Ei-value:0.000, Pi-value:0.000  
Er-value:0.000, Pr-value:0.000  
No matches to TargetScan

-5652--(3)--5656-

TTCCAGAT

TTCCAGAT  
Depth:10 (SNAKE)  
Ei-value:0.000, Pi-value:0.000  
Er-value:0.000, Pr-value:0.000  
No matches to TargetScan

-5663--(64)--5728-

GGCAAGT

GGCAAGT  
Depth:10 (SNAKE)  
Ei-value:0.000, Pi-value:0.000  
Er-value:0.000, Pr-value:0.000  
No matches to TargetScan

-5734--(282)--6017-

AAC

AACAGGTGAA  
Depth:10 (SNAKE)  
Ei-value:0.000, Pi-value:0.000  
Er-value:0.000, Pr-value:0.000  
No matches to TargetScan


AGGTGA

AGGTGA  
Depth:11 (X.TROPICALIS)  
Ei-value:0.000, Pi-value:0.000  
Er-value:0.000, Pr-value:0.010  
No matches to TargetScan


A

AACAGGTGAA  
Depth:10 (SNAKE)  
Ei-value:0.000, Pi-value:0.000  
Er-value:0.000, Pr-value:0.000  
No matches to TargetScan

-6026--(190)--6217-

AAGGCT

AAGGCT  
Depth:10 (SNAKE)  
Ei-value:0.000, Pi-value:0.000  
Er-value:0.000, Pr-value:0.010  
No matches to TargetScan


T

AAGGCTT  
Depth:9 (LIZARD)  
Ei-value:0.000, Pi-value:0.000  
Er-value:0.000, Pr-value:0.000  
No matches to TargetScan

-6223--(327)--6551-

TTTTCCAG

TTTTCCAG  
Depth:10 (SNAKE)  
Ei-value:0.000, Pi-value:0.000  
Er-value:0.000, Pr-value:0.000  
No matches to TargetScan

-6558--(114)--6673-

TGGATCCT

TGGATCCT  
Depth:10 (SNAKE)  
Ei-value:0.000, Pi-value:0.000  
Er-value:0.000, Pr-value:0.000  
No matches to TargetScan

-6680--(26)--6707-

AAGGTAA

AAGGTAA  
Depth:10 (SNAKE)  
Ei-value:0.000, Pi-value:0.000  
Er-value:0.000, Pr-value:0.000  
No matches to TargetScan

-6713--(168)--6882-

GGGTGGG

GGGTGGG  
Depth:10 (SNAKE)  
Ei-value:0.000, Pi-value:0.000  
Er-value:0.000, Pr-value:0.000  
No matches to TargetScan

-6888--(73)--6962-

TGCTAAGACT

TGCTAAGACT  
Depth:10 (SNAKE)  
Ei-value:0.000, Pi-value:0.000  
Er-value:0.000, Pr-value:0.000  
No matches to TargetScan

-6971--(88)--7060-

TTTTCTTTT

TTTTCTTTT  
Depth:19 (ZEBRAFISH)  
Ei-value:0.000, Pi-value:0.000  
Er-value:0.000, Pr-value:0.000  
MATCHES To TargetScan▶ miR-186-5p:AAAGAAU


CCTGAGA

TTTTCTTTTCCTGAGA  
Depth:10 (SNAKE)  
Ei-value:0.000, Pi-value:0.000  
Er-value:0.000, Pr-value:0.000  
MATCHES To TargetScan▶ miR-873-5p.1:CAGGAAC▶ miR-186-5p:AAAGAAU

-7075--(16)--7092-

TCT

TCTCAGGTTTTGCTTTT  
Depth:10 (SNAKE)  
Ei-value:0.000, Pi-value:0.000  
Er-value:0.000, Pr-value:0.000  
MATCHES To TargetScan▶ miR-330-3p.2:AAAGCAC▶ miR-490-3p:AACCUGG


CAGGTTTTGCTTT

CAGGTTTTGCTTT  
Depth:19 (ZEBRAFISH)  
Ei-value:0.000, Pi-value:0.000  
Er-value:0.000, Pr-value:0.000  
MATCHES To TargetScan▶ miR-330-3p.2:AAAGCAC▶ miR-490-3p:AACCUGG


T

CAGGTTTTGCTTTT  
Depth:16 (NILETILAPIA)  
Ei-value:0.000, Pi-value:0.000  
Er-value:0.000, Pr-value:0.000  
MATCHES To TargetScan▶ miR-330-3p.2:AAAGCAC▶ miR-490-3p:AACCUGG

-7108--(20)--7129-

A

AAAAAAGCAAAAG  
Depth:13 (OPOSSUM)  
Ei-value:0.000, Pi-value:0.000  
Er-value:0.000, Pr-value:0.000  
No matches to TargetScan


AAAAAGCAAAA

AAAAAGCAAAA  
Depth:19 (ZEBRAFISH)  
Ei-value:0.000, Pi-value:0.000  
Er-value:0.000, Pr-value:0.000  
No matches to TargetScan


G

AAAAAGCAAAAG  
Depth:18 (MEDAKA)  
Ei-value:0.000, Pi-value:0.000  
Er-value:0.000, Pr-value:0.000  
No matches to TargetScan


A

AAAAAAGCAAAAGA  
Depth:9 (LIZARD)  
Ei-value:0.000, Pi-value:0.000  
Er-value:0.000, Pr-value:0.000  
No matches to TargetScan

-7142--(1)--7144-

GCTGGT

GCTGGT  
Depth:9 (LIZARD)  
Ei-value:0.000, Pi-value:0.000  
Er-value:0.000, Pr-value:0.000  
No matches to TargetScan

-7149--(7)--7157-

ACTCCTG

ACTCCTG  
Depth:18 (MEDAKA)  
Ei-value:0.000, Pi-value:0.000  
Er-value:0.000, Pr-value:0.000  
No matches to TargetScan


G

ACTCCTGG  
Depth:14 (SPOTTEDGAR)  
Ei-value:0.000, Pi-value:0.000  
Er-value:0.000, Pr-value:0.000  
MATCHES To TargetScan▶ miR-665:CCAGGAG

-7164--(12)--7177-

GGGTTC

GGGTTC  
Depth:13 (OPOSSUM)  
Ei-value:0.000, Pi-value:0.000  
Er-value:0.000, Pr-value:0.000  
No matches to TargetScan


AA

GGGTTCAA  
Depth:9 (LIZARD)  
Ei-value:0.000, Pi-value:0.000  
Er-value:0.000, Pr-value:0.000  
No matches to TargetScan

-7184  
  
>PIG  
      1353-

TTGGACT

TTGGACT  
Depth:9 (LIZARD)  
Ei-value:0.000, Pi-value:0.000  
Er-value:0.000, Pr-value:0.000  
No matches to TargetScan

-1359--(245)--1605-

GGTGAAG

GGTGAAG  
Depth:11 (X.TROPICALIS)  
Ei-value:0.000, Pi-value:0.000  
Er-value:0.000, Pr-value:0.000  
No matches to TargetScan

-1611--(237)--1849-

TGGTAA

TGGTAA  
Depth:14 (SPOTTEDGAR)  
Ei-value:0.030, Pi-value:0.000  
Er-value:0.010, Pr-value:0.000  
No matches to TargetScan

-1854--(678)--2533-

TTTGGG

TTTGGG  
Depth:19 (ZEBRAFISH)  
Ei-value:0.000, Pi-value:0.000  
Er-value:0.000, Pr-value:0.000  
No matches to TargetScan

-2538--(236)--2775-

GAGCTT

GAGCTT  
Depth:9 (LIZARD)  
Ei-value:0.000, Pi-value:0.000  
Er-value:0.000, Pr-value:0.000  
No matches to TargetScan

-2780--(224)--3005-

GGCAAGTAA

GGCAAGTAA  
Depth:10 (SNAKE)  
Ei-value:0.000, Pi-value:0.000  
Er-value:0.000, Pr-value:0.000  
No matches to TargetScan

-3013--(323)--3337-

T

TCAAGTAAGA  
Depth:9 (LIZARD)  
Ei-value:0.000, Pi-value:0.000  
Er-value:0.000, Pr-value:0.000  
No matches to TargetScan


CAA

CAAGTAAGA  
Depth:10 (SNAKE)  
Ei-value:0.000, Pi-value:0.000  
Er-value:0.000, Pr-value:0.000  
No matches to TargetScan


GTAAGA

GTAAGA  
Depth:11 (X.TROPICALIS)  
Ei-value:0.000, Pi-value:0.000  
Er-value:0.000, Pr-value:0.000  
No matches to TargetScan

-3346--(194)--3541-

TTTTTCAG

TTTTTCAG  
Depth:19 (ZEBRAFISH)  
Ei-value:0.000, Pi-value:0.000  
Er-value:0.000, Pr-value:0.000  
No matches to TargetScan


AT

TTTTTCAGAT  
Depth:10 (SNAKE)  
Ei-value:0.000, Pi-value:0.000  
Er-value:0.000, Pr-value:0.000  
No matches to TargetScan

-3550--(72)--3623-

TGATAAGT

TGATAAGT  
Depth:9 (LIZARD)  
Ei-value:0.000, Pi-value:0.000  
Er-value:0.000, Pr-value:0.000  
No matches to TargetScan

-3630--(0)--3631-

AAAGGCAGAA

AAAGGCAGAA  
Depth:13 (OPOSSUM)  
Ei-value:0.000, Pi-value:0.000  
Er-value:0.000, Pr-value:0.000  
No matches to TargetScan


A

AAAGGCAGAAA  
Depth:11 (X.TROPICALIS)  
Ei-value:0.000, Pi-value:0.000  
Er-value:0.000, Pr-value:0.000  
No matches to TargetScan


A

AAAGGCAGAAAA  
Depth:9 (LIZARD)  
Ei-value:0.000, Pi-value:0.000  
Er-value:0.000, Pr-value:0.000  
No matches to TargetScan

-3642--(402)--4045-

AG

AGACAGGT  
Depth:9 (LIZARD)  
Ei-value:0.000, Pi-value:0.000  
Er-value:0.000, Pr-value:0.000  
No matches to TargetScan


ACAGGT

ACAGGT  
Depth:10 (SNAKE)  
Ei-value:0.000, Pi-value:0.000  
Er-value:0.000, Pr-value:0.000  
No matches to TargetScan

-4052--(63)--4116-

CA

CAGATAAGT  
Depth:11 (X.TROPICALIS)  
Ei-value:0.000, Pi-value:0.000  
Er-value:0.000, Pr-value:0.000  
No matches to TargetScan


GATAAG

GATAAG  
Depth:19 (ZEBRAFISH)  
Ei-value:0.000, Pi-value:0.000  
Er-value:0.000, Pr-value:0.000  
No matches to TargetScan


T

CAGATAAGT  
Depth:11 (X.TROPICALIS)  
Ei-value:0.000, Pi-value:0.000  
Er-value:0.000, Pr-value:0.000  
No matches to TargetScan

-4124--(18)--4143-

ATTGCAT

ATTGCAT  
Depth:11 (X.TROPICALIS)  
Ei-value:0.000, Pi-value:0.000  
Er-value:0.000, Pr-value:0.000  
No matches to TargetScan


G

ATTGCATG  
Depth:9 (LIZARD)  
Ei-value:0.000, Pi-value:0.000  
Er-value:0.000, Pr-value:0.000  
No matches to TargetScan

-4150--(399)--4550-

GCTGTC

GCTGTC  
Depth:9 (LIZARD)  
Ei-value:0.000, Pi-value:0.000  
Er-value:0.000, Pr-value:0.000  
No matches to TargetScan

-4555--(143)--4699-

AGGCAGGA

AGGCAGGA  
Depth:10 (SNAKE)  
Ei-value:0.000, Pi-value:0.000  
Er-value:0.000, Pr-value:0.000  
No matches to TargetScan

-4706--(79)--4786-

TAAGGA

TAAGGA  
Depth:13 (OPOSSUM)  
Ei-value:0.000, Pi-value:0.000  
Er-value:0.000, Pr-value:0.000  
No matches to TargetScan

-4791--(320)--5112-

TTCTGT

TTCTGT  
Depth:9 (LIZARD)  
Ei-value:0.000, Pi-value:0.000  
Er-value:0.000, Pr-value:0.000  
No matches to TargetScan

-5117--(23)--5141-

TGCAGTG

TGCAGTG  
Depth:10 (SNAKE)  
Ei-value:0.000, Pi-value:0.000  
Er-value:0.000, Pr-value:0.000  
MATCHES To TargetScan▶ miR-217:ACUGCAU

-5147--(143)--5291-

TTGGCAAGTAA

TTGGCAAGTAA  
Depth:10 (SNAKE)  
Ei-value:0.000, Pi-value:0.000  
Er-value:0.000, Pr-value:0.000  
No matches to TargetScan

-5301--(87)--5389-

TGGAGTAGTG

TGGAGTAGTG  
Depth:9 (LIZARD)  
Ei-value:0.000, Pi-value:0.000  
Er-value:0.000, Pr-value:0.000  
No matches to TargetScan

-5398--(106)--5505-

TTAGGT

TTAGGT  
Depth:11 (X.TROPICALIS)  
Ei-value:0.000, Pi-value:0.000  
Er-value:0.000, Pr-value:0.000  
No matches to TargetScan


AA

TTAGGTAA  
Depth:10 (SNAKE)  
Ei-value:0.000, Pi-value:0.000  
Er-value:0.000, Pr-value:0.000  
No matches to TargetScan

-5512--(43)--5556-

T

TGAAGCCTTTAGT  
Depth:10 (SNAKE)  
Ei-value:0.000, Pi-value:0.000  
Er-value:0.000, Pr-value:0.000  
No matches to TargetScan


GAAGCC

GAAGCC  
Depth:11 (X.TROPICALIS)  
Ei-value:0.000, Pi-value:0.000  
Er-value:0.000, Pr-value:0.000  
No matches to TargetScan


TTTAGT

TGAAGCCTTTAGT  
Depth:10 (SNAKE)  
Ei-value:0.000, Pi-value:0.000  
Er-value:0.000, Pr-value:0.000  
No matches to TargetScan

-5568--(3)--5572-

TTCCAGAT

TTCCAGAT  
Depth:10 (SNAKE)  
Ei-value:0.000, Pi-value:0.000  
Er-value:0.000, Pr-value:0.000  
No matches to TargetScan

-5579--(65)--5645-

GGCAAGT

GGCAAGT  
Depth:10 (SNAKE)  
Ei-value:0.000, Pi-value:0.000  
Er-value:0.000, Pr-value:0.000  
No matches to TargetScan

-5651--(285)--5937-

AAC

AACAGGTGAA  
Depth:10 (SNAKE)  
Ei-value:0.000, Pi-value:0.000  
Er-value:0.000, Pr-value:0.000  
No matches to TargetScan


AGGTGA

AGGTGA  
Depth:11 (X.TROPICALIS)  
Ei-value:0.000, Pi-value:0.000  
Er-value:0.000, Pr-value:0.010  
No matches to TargetScan


A

AACAGGTGAA  
Depth:10 (SNAKE)  
Ei-value:0.000, Pi-value:0.000  
Er-value:0.000, Pr-value:0.000  
No matches to TargetScan

-5946--(189)--6136-

AAGGCT

AAGGCT  
Depth:10 (SNAKE)  
Ei-value:0.000, Pi-value:0.000  
Er-value:0.000, Pr-value:0.010  
No matches to TargetScan


T

AAGGCTT  
Depth:9 (LIZARD)  
Ei-value:0.000, Pi-value:0.000  
Er-value:0.000, Pr-value:0.000  
No matches to TargetScan

-6142--(296)--6439-

TTTTCCAG

TTTTCCAG  
Depth:10 (SNAKE)  
Ei-value:0.000, Pi-value:0.000  
Er-value:0.000, Pr-value:0.000  
No matches to TargetScan

-6446--(113)--6560-

TGGATCCT

TGGATCCT  
Depth:10 (SNAKE)  
Ei-value:0.000, Pi-value:0.000  
Er-value:0.000, Pr-value:0.000  
No matches to TargetScan

-6567--(25)--6593-

AAGGTAA

AAGGTAA  
Depth:10 (SNAKE)  
Ei-value:0.000, Pi-value:0.000  
Er-value:0.000, Pr-value:0.000  
No matches to TargetScan

-6599--(247)--6847-

GGGTGGG

GGGTGGG  
Depth:10 (SNAKE)  
Ei-value:0.000, Pi-value:0.000  
Er-value:0.000, Pr-value:0.000  
No matches to TargetScan

-6853--(15)--6869-

TGCTAAGACT

TGCTAAGACT  
Depth:10 (SNAKE)  
Ei-value:0.000, Pi-value:0.000  
Er-value:0.000, Pr-value:0.000  
No matches to TargetScan

-6878--(93)--6972-

TTTTCTTTT

TTTTCTTTT  
Depth:19 (ZEBRAFISH)  
Ei-value:0.000, Pi-value:0.000  
Er-value:0.000, Pr-value:0.000  
MATCHES To TargetScan▶ miR-186-5p:AAAGAAU


CCTGAGA

TTTTCTTTTCCTGAGA  
Depth:10 (SNAKE)  
Ei-value:0.000, Pi-value:0.000  
Er-value:0.000, Pr-value:0.000  
MATCHES To TargetScan▶ miR-873-5p.1:CAGGAAC▶ miR-186-5p:AAAGAAU

-6987--(18)--7006-

TCT

TCTCAGGTTTTGCTTTT  
Depth:10 (SNAKE)  
Ei-value:0.000, Pi-value:0.000  
Er-value:0.000, Pr-value:0.000  
MATCHES To TargetScan▶ miR-330-3p.2:AAAGCAC▶ miR-490-3p:AACCUGG


CAGGTTTTGCTTT

CAGGTTTTGCTTT  
Depth:19 (ZEBRAFISH)  
Ei-value:0.000, Pi-value:0.000  
Er-value:0.000, Pr-value:0.000  
MATCHES To TargetScan▶ miR-330-3p.2:AAAGCAC▶ miR-490-3p:AACCUGG


T

CAGGTTTTGCTTTT  
Depth:16 (NILETILAPIA)  
Ei-value:0.000, Pi-value:0.000  
Er-value:0.000, Pr-value:0.000  
MATCHES To TargetScan▶ miR-330-3p.2:AAAGCAC▶ miR-490-3p:AACCUGG

-7022--(22)--7045-

A

AAAAAAGCAAAAG  
Depth:13 (OPOSSUM)  
Ei-value:0.000, Pi-value:0.000  
Er-value:0.000, Pr-value:0.000  
No matches to TargetScan


AAAAAGCAAAA

AAAAAGCAAAA  
Depth:19 (ZEBRAFISH)  
Ei-value:0.000, Pi-value:0.000  
Er-value:0.000, Pr-value:0.000  
No matches to TargetScan


G

AAAAAGCAAAAG  
Depth:18 (MEDAKA)  
Ei-value:0.000, Pi-value:0.000  
Er-value:0.000, Pr-value:0.000  
No matches to TargetScan


A

AAAAAAGCAAAAGA  
Depth:9 (LIZARD)  
Ei-value:0.000, Pi-value:0.000  
Er-value:0.000, Pr-value:0.000  
No matches to TargetScan

-7058--(1)--7060-

GCTGGT

GCTGGT  
Depth:9 (LIZARD)  
Ei-value:0.000, Pi-value:0.000  
Er-value:0.000, Pr-value:0.000  
No matches to TargetScan

-7065--(7)--7073-

ACTCCTG

ACTCCTG  
Depth:18 (MEDAKA)  
Ei-value:0.000, Pi-value:0.000  
Er-value:0.000, Pr-value:0.000  
No matches to TargetScan


G

ACTCCTGG  
Depth:14 (SPOTTEDGAR)  
Ei-value:0.000, Pi-value:0.000  
Er-value:0.000, Pr-value:0.000  
MATCHES To TargetScan▶ miR-665:CCAGGAG

-7080--(11)--7092-

GGGTTC

GGGTTC  
Depth:13 (OPOSSUM)  
Ei-value:0.000, Pi-value:0.000  
Er-value:0.000, Pr-value:0.000  
No matches to TargetScan


AA

GGGTTCAA  
Depth:9 (LIZARD)  
Ei-value:0.000, Pi-value:0.000  
Er-value:0.000, Pr-value:0.000  
No matches to TargetScan

-7099  
  
>COW  
      1229-

TTGGACT

TTGGACT  
Depth:9 (LIZARD)  
Ei-value:0.000, Pi-value:0.000  
Er-value:0.000, Pr-value:0.000  
No matches to TargetScan

-1235--(248)--1484-

GGTGAAG

GGTGAAG  
Depth:11 (X.TROPICALIS)  
Ei-value:0.000, Pi-value:0.000  
Er-value:0.000, Pr-value:0.000  
No matches to TargetScan

-1490--(225)--1716-

TGGTAA

TGGTAA  
Depth:14 (SPOTTEDGAR)  
Ei-value:0.030, Pi-value:0.000  
Er-value:0.010, Pr-value:0.000  
No matches to TargetScan

-1721--(695)--2417-

TTTGGG

TTTGGG  
Depth:19 (ZEBRAFISH)  
Ei-value:0.000, Pi-value:0.000  
Er-value:0.000, Pr-value:0.000  
No matches to TargetScan

-2422--(239)--2662-

GAGCTT

GAGCTT  
Depth:9 (LIZARD)  
Ei-value:0.000, Pi-value:0.000  
Er-value:0.000, Pr-value:0.000  
No matches to TargetScan

-2667--(228)--2896-

GGCAAGTAA

GGCAAGTAA  
Depth:10 (SNAKE)  
Ei-value:0.000, Pi-value:0.000  
Er-value:0.000, Pr-value:0.000  
No matches to TargetScan

-2904--(318)--3223-

T

TCAAGTAAGA  
Depth:9 (LIZARD)  
Ei-value:0.000, Pi-value:0.000  
Er-value:0.000, Pr-value:0.000  
No matches to TargetScan


CAA

CAAGTAAGA  
Depth:10 (SNAKE)  
Ei-value:0.000, Pi-value:0.000  
Er-value:0.000, Pr-value:0.000  
No matches to TargetScan


GTAAGA

GTAAGA  
Depth:11 (X.TROPICALIS)  
Ei-value:0.000, Pi-value:0.000  
Er-value:0.000, Pr-value:0.000  
No matches to TargetScan

-3232--(202)--3435-

TTTTTCAG

TTTTTCAG  
Depth:19 (ZEBRAFISH)  
Ei-value:0.000, Pi-value:0.000  
Er-value:0.000, Pr-value:0.000  
No matches to TargetScan


AT

TTTTTCAGAT  
Depth:10 (SNAKE)  
Ei-value:0.000, Pi-value:0.000  
Er-value:0.000, Pr-value:0.000  
No matches to TargetScan

-3444--(72)--3517-

TGATAAGT

TGATAAGT  
Depth:9 (LIZARD)  
Ei-value:0.000, Pi-value:0.000  
Er-value:0.000, Pr-value:0.000  
No matches to TargetScan

-3524--(0)--3525-

AAAGGCAGAA

AAAGGCAGAA  
Depth:13 (OPOSSUM)  
Ei-value:0.000, Pi-value:0.000  
Er-value:0.000, Pr-value:0.000  
No matches to TargetScan


A

AAAGGCAGAAA  
Depth:11 (X.TROPICALIS)  
Ei-value:0.000, Pi-value:0.000  
Er-value:0.000, Pr-value:0.000  
No matches to TargetScan


A

AAAGGCAGAAAA  
Depth:9 (LIZARD)  
Ei-value:0.000, Pi-value:0.000  
Er-value:0.000, Pr-value:0.000  
No matches to TargetScan

-3536--(385)--3922-

AG

AGACAGGT  
Depth:9 (LIZARD)  
Ei-value:0.000, Pi-value:0.000  
Er-value:0.000, Pr-value:0.000  
No matches to TargetScan


ACAGGT

ACAGGT  
Depth:10 (SNAKE)  
Ei-value:0.000, Pi-value:0.000  
Er-value:0.000, Pr-value:0.000  
No matches to TargetScan

-3929--(66)--3996-

CA

CAGATAAGT  
Depth:11 (X.TROPICALIS)  
Ei-value:0.000, Pi-value:0.000  
Er-value:0.000, Pr-value:0.000  
No matches to TargetScan


GATAAG

GATAAG  
Depth:19 (ZEBRAFISH)  
Ei-value:0.000, Pi-value:0.000  
Er-value:0.000, Pr-value:0.000  
No matches to TargetScan


T

CAGATAAGT  
Depth:11 (X.TROPICALIS)  
Ei-value:0.000, Pi-value:0.000  
Er-value:0.000, Pr-value:0.000  
No matches to TargetScan

-4004--(18)--4023-

ATTGCAT

ATTGCAT  
Depth:11 (X.TROPICALIS)  
Ei-value:0.000, Pi-value:0.000  
Er-value:0.000, Pr-value:0.000  
No matches to TargetScan


G

ATTGCATG  
Depth:9 (LIZARD)  
Ei-value:0.000, Pi-value:0.000  
Er-value:0.000, Pr-value:0.000  
No matches to TargetScan

-4030--(394)--4425-

GCTGTC

GCTGTC  
Depth:9 (LIZARD)  
Ei-value:0.000, Pi-value:0.000  
Er-value:0.000, Pr-value:0.000  
No matches to TargetScan

-4430--(146)--4577-

AGGCAGGA

AGGCAGGA  
Depth:10 (SNAKE)  
Ei-value:0.000, Pi-value:0.000  
Er-value:0.000, Pr-value:0.000  
No matches to TargetScan

-4584--(72)--4657-

TAAGGA

TAAGGA  
Depth:13 (OPOSSUM)  
Ei-value:0.000, Pi-value:0.000  
Er-value:0.000, Pr-value:0.000  
No matches to TargetScan

-4662--(331)--4994-

TTCTGT

TTCTGT  
Depth:9 (LIZARD)  
Ei-value:0.000, Pi-value:0.000  
Er-value:0.000, Pr-value:0.000  
No matches to TargetScan

-4999--(24)--5024-

TGCAGTG

TGCAGTG  
Depth:10 (SNAKE)  
Ei-value:0.000, Pi-value:0.000  
Er-value:0.000, Pr-value:0.000  
MATCHES To TargetScan▶ miR-217:ACUGCAU

-5030--(144)--5175-

TTGGCAAGTAA

TTGGCAAGTAA  
Depth:10 (SNAKE)  
Ei-value:0.000, Pi-value:0.000  
Er-value:0.000, Pr-value:0.000  
No matches to TargetScan

-5185--(89)--5275-

TGGAGTAGTG

TGGAGTAGTG  
Depth:9 (LIZARD)  
Ei-value:0.000, Pi-value:0.000  
Er-value:0.000, Pr-value:0.000  
No matches to TargetScan

-5284--(116)--5401-

TTAGGT

TTAGGT  
Depth:11 (X.TROPICALIS)  
Ei-value:0.000, Pi-value:0.000  
Er-value:0.000, Pr-value:0.000  
No matches to TargetScan


AA

TTAGGTAA  
Depth:10 (SNAKE)  
Ei-value:0.000, Pi-value:0.000  
Er-value:0.000, Pr-value:0.000  
No matches to TargetScan

-5408--(42)--5451-

T

TGAAGCCTTTAGT  
Depth:10 (SNAKE)  
Ei-value:0.000, Pi-value:0.000  
Er-value:0.000, Pr-value:0.000  
No matches to TargetScan


GAAGCC

GAAGCC  
Depth:11 (X.TROPICALIS)  
Ei-value:0.000, Pi-value:0.000  
Er-value:0.000, Pr-value:0.000  
No matches to TargetScan


TTTAGT

TGAAGCCTTTAGT  
Depth:10 (SNAKE)  
Ei-value:0.000, Pi-value:0.000  
Er-value:0.000, Pr-value:0.000  
No matches to TargetScan

-5463--(3)--5467-

TTCCAGAT

TTCCAGAT  
Depth:10 (SNAKE)  
Ei-value:0.000, Pi-value:0.000  
Er-value:0.000, Pr-value:0.000  
No matches to TargetScan

-5474--(64)--5539-

GGCAAGT

GGCAAGT  
Depth:10 (SNAKE)  
Ei-value:0.000, Pi-value:0.000  
Er-value:0.000, Pr-value:0.000  
No matches to TargetScan

-5545--(299)--5845-

AAC

AACAGGTGAA  
Depth:10 (SNAKE)  
Ei-value:0.000, Pi-value:0.000  
Er-value:0.000, Pr-value:0.000  
No matches to TargetScan


AGGTGA

AGGTGA  
Depth:11 (X.TROPICALIS)  
Ei-value:0.000, Pi-value:0.000  
Er-value:0.000, Pr-value:0.010  
No matches to TargetScan


A

AACAGGTGAA  
Depth:10 (SNAKE)  
Ei-value:0.000, Pi-value:0.000  
Er-value:0.000, Pr-value:0.000  
No matches to TargetScan

-5854--(193)--6048-

AAGGCT

AAGGCT  
Depth:10 (SNAKE)  
Ei-value:0.000, Pi-value:0.000  
Er-value:0.000, Pr-value:0.010  
No matches to TargetScan


T

AAGGCTT  
Depth:9 (LIZARD)  
Ei-value:0.000, Pi-value:0.000  
Er-value:0.000, Pr-value:0.000  
No matches to TargetScan

-6054--(324)--6379-

TTTTCCAG

TTTTCCAG  
Depth:10 (SNAKE)  
Ei-value:0.000, Pi-value:0.000  
Er-value:0.000, Pr-value:0.000  
No matches to TargetScan

-6386--(113)--6500-

TGGATCCT

TGGATCCT  
Depth:10 (SNAKE)  
Ei-value:0.000, Pi-value:0.000  
Er-value:0.000, Pr-value:0.000  
No matches to TargetScan

-6507--(26)--6534-

AAGGTAA

AAGGTAA  
Depth:10 (SNAKE)  
Ei-value:0.000, Pi-value:0.000  
Er-value:0.000, Pr-value:0.000  
No matches to TargetScan

-6540--(184)--6725-

GGGTGGG

GGGTGGG  
Depth:10 (SNAKE)  
Ei-value:0.000, Pi-value:0.000  
Er-value:0.000, Pr-value:0.000  
No matches to TargetScan

-6731--(71)--6803-

TGCTAAGACT

TGCTAAGACT  
Depth:10 (SNAKE)  
Ei-value:0.000, Pi-value:0.000  
Er-value:0.000, Pr-value:0.000  
No matches to TargetScan

-6812--(90)--6903-

TTTTCTTTT

TTTTCTTTT  
Depth:19 (ZEBRAFISH)  
Ei-value:0.000, Pi-value:0.000  
Er-value:0.000, Pr-value:0.000  
MATCHES To TargetScan▶ miR-186-5p:AAAGAAU


CCTGAGA

TTTTCTTTTCCTGAGA  
Depth:10 (SNAKE)  
Ei-value:0.000, Pi-value:0.000  
Er-value:0.000, Pr-value:0.000  
MATCHES To TargetScan▶ miR-873-5p.1:CAGGAAC▶ miR-186-5p:AAAGAAU

-6918--(20)--6939-

TCT

TCTCAGGTTTTGCTTTT  
Depth:10 (SNAKE)  
Ei-value:0.000, Pi-value:0.000  
Er-value:0.000, Pr-value:0.000  
MATCHES To TargetScan▶ miR-330-3p.2:AAAGCAC▶ miR-490-3p:AACCUGG


CAGGTTTTGCTTT

CAGGTTTTGCTTT  
Depth:19 (ZEBRAFISH)  
Ei-value:0.000, Pi-value:0.000  
Er-value:0.000, Pr-value:0.000  
MATCHES To TargetScan▶ miR-330-3p.2:AAAGCAC▶ miR-490-3p:AACCUGG


T

CAGGTTTTGCTTTT  
Depth:16 (NILETILAPIA)  
Ei-value:0.000, Pi-value:0.000  
Er-value:0.000, Pr-value:0.000  
MATCHES To TargetScan▶ miR-330-3p.2:AAAGCAC▶ miR-490-3p:AACCUGG

-6955--(21)--6977-

A

AAAAAAGCAAAAG  
Depth:13 (OPOSSUM)  
Ei-value:0.000, Pi-value:0.000  
Er-value:0.000, Pr-value:0.000  
No matches to TargetScan


AAAAAGCAAAA

AAAAAGCAAAA  
Depth:19 (ZEBRAFISH)  
Ei-value:0.000, Pi-value:0.000  
Er-value:0.000, Pr-value:0.000  
No matches to TargetScan


G

AAAAAGCAAAAG  
Depth:18 (MEDAKA)  
Ei-value:0.000, Pi-value:0.000  
Er-value:0.000, Pr-value:0.000  
No matches to TargetScan


A

AAAAAAGCAAAAGA  
Depth:9 (LIZARD)  
Ei-value:0.000, Pi-value:0.000  
Er-value:0.000, Pr-value:0.000  
No matches to TargetScan

-6990--(1)--6992-

GCTGGT

GCTGGT  
Depth:9 (LIZARD)  
Ei-value:0.000, Pi-value:0.000  
Er-value:0.000, Pr-value:0.000  
No matches to TargetScan

-6997--(7)--7005-

ACTCCTG

ACTCCTG  
Depth:18 (MEDAKA)  
Ei-value:0.000, Pi-value:0.000  
Er-value:0.000, Pr-value:0.000  
No matches to TargetScan


G

ACTCCTGG  
Depth:14 (SPOTTEDGAR)  
Ei-value:0.000, Pi-value:0.000  
Er-value:0.000, Pr-value:0.000  
MATCHES To TargetScan▶ miR-665:CCAGGAG

-7012--(11)--7024-

GGGTTC

GGGTTC  
Depth:13 (OPOSSUM)  
Ei-value:0.000, Pi-value:0.000  
Er-value:0.000, Pr-value:0.000  
No matches to TargetScan


AA

GGGTTCAA  
Depth:9 (LIZARD)  
Ei-value:0.000, Pi-value:0.000  
Er-value:0.000, Pr-value:0.000  
No matches to TargetScan

-7031  
  
>MOUSE  
      1243-

TTGGACT

TTGGACT  
Depth:9 (LIZARD)  
Ei-value:0.000, Pi-value:0.000  
Er-value:0.000, Pr-value:0.000  
No matches to TargetScan

-1249--(235)--1485-

GGTGAAG

GGTGAAG  
Depth:11 (X.TROPICALIS)  
Ei-value:0.000, Pi-value:0.000  
Er-value:0.000, Pr-value:0.000  
No matches to TargetScan

-1491--(225)--1717-

TGGTAA

TGGTAA  
Depth:14 (SPOTTEDGAR)  
Ei-value:0.030, Pi-value:0.000  
Er-value:0.010, Pr-value:0.000  
No matches to TargetScan

-1722--(601)--2324-

TTTGGG

TTTGGG  
Depth:19 (ZEBRAFISH)  
Ei-value:0.000, Pi-value:0.000  
Er-value:0.000, Pr-value:0.000  
No matches to TargetScan

-2329--(231)--2561-

GAGCTT

GAGCTT  
Depth:9 (LIZARD)  
Ei-value:0.000, Pi-value:0.000  
Er-value:0.000, Pr-value:0.000  
No matches to TargetScan

-2566--(201)--2768-

GGCAAGTAA

GGCAAGTAA  
Depth:10 (SNAKE)  
Ei-value:0.000, Pi-value:0.000  
Er-value:0.000, Pr-value:0.000  
No matches to TargetScan

-2776--(305)--3082-

T

TCAAGTAAGA  
Depth:9 (LIZARD)  
Ei-value:0.000, Pi-value:0.000  
Er-value:0.000, Pr-value:0.000  
No matches to TargetScan


CAA

CAAGTAAGA  
Depth:10 (SNAKE)  
Ei-value:0.000, Pi-value:0.000  
Er-value:0.000, Pr-value:0.000  
No matches to TargetScan


GTAAGA

GTAAGA  
Depth:11 (X.TROPICALIS)  
Ei-value:0.000, Pi-value:0.000  
Er-value:0.000, Pr-value:0.000  
No matches to TargetScan

-3091--(228)--3320-

TTTTTCAG

TTTTTCAG  
Depth:19 (ZEBRAFISH)  
Ei-value:0.000, Pi-value:0.000  
Er-value:0.000, Pr-value:0.000  
No matches to TargetScan


AT

TTTTTCAGAT  
Depth:10 (SNAKE)  
Ei-value:0.000, Pi-value:0.000  
Er-value:0.000, Pr-value:0.000  
No matches to TargetScan

-3329--(75)--3405-

TGATAAGT

TGATAAGT  
Depth:9 (LIZARD)  
Ei-value:0.000, Pi-value:0.000  
Er-value:0.000, Pr-value:0.000  
No matches to TargetScan

-3412--(0)--3413-

AAAGGCAGAA

AAAGGCAGAA  
Depth:13 (OPOSSUM)  
Ei-value:0.000, Pi-value:0.000  
Er-value:0.000, Pr-value:0.000  
No matches to TargetScan


A

AAAGGCAGAAA  
Depth:11 (X.TROPICALIS)  
Ei-value:0.000, Pi-value:0.000  
Er-value:0.000, Pr-value:0.000  
No matches to TargetScan


A

AAAGGCAGAAAA  
Depth:9 (LIZARD)  
Ei-value:0.000, Pi-value:0.000  
Er-value:0.000, Pr-value:0.000  
No matches to TargetScan

-3424--(345)--3770-

AG

AGACAGGT  
Depth:9 (LIZARD)  
Ei-value:0.000, Pi-value:0.000  
Er-value:0.000, Pr-value:0.000  
No matches to TargetScan


ACAGGT

ACAGGT  
Depth:10 (SNAKE)  
Ei-value:0.000, Pi-value:0.000  
Er-value:0.000, Pr-value:0.000  
No matches to TargetScan

-3777--(66)--3844-

CA

CAGATAAGT  
Depth:11 (X.TROPICALIS)  
Ei-value:0.000, Pi-value:0.000  
Er-value:0.000, Pr-value:0.000  
No matches to TargetScan


GATAAG

GATAAG  
Depth:19 (ZEBRAFISH)  
Ei-value:0.000, Pi-value:0.000  
Er-value:0.000, Pr-value:0.000  
No matches to TargetScan


T

CAGATAAGT  
Depth:11 (X.TROPICALIS)  
Ei-value:0.000, Pi-value:0.000  
Er-value:0.000, Pr-value:0.000  
No matches to TargetScan

-3852--(18)--3871-

ATTGCAT

ATTGCAT  
Depth:11 (X.TROPICALIS)  
Ei-value:0.000, Pi-value:0.000  
Er-value:0.000, Pr-value:0.000  
No matches to TargetScan


G

ATTGCATG  
Depth:9 (LIZARD)  
Ei-value:0.000, Pi-value:0.000  
Er-value:0.000, Pr-value:0.000  
No matches to TargetScan

-3878--(412)--4291-

GCTGTC

GCTGTC  
Depth:9 (LIZARD)  
Ei-value:0.000, Pi-value:0.000  
Er-value:0.000, Pr-value:0.000  
No matches to TargetScan

-4296--(149)--4446-

AGGCAGGA

AGGCAGGA  
Depth:10 (SNAKE)  
Ei-value:0.000, Pi-value:0.000  
Er-value:0.000, Pr-value:0.000  
No matches to TargetScan

-4453--(71)--4525-

TAAGGA

TAAGGA  
Depth:13 (OPOSSUM)  
Ei-value:0.000, Pi-value:0.000  
Er-value:0.000, Pr-value:0.000  
No matches to TargetScan

-4530--(310)--4841-

TTCTGT

TTCTGT  
Depth:9 (LIZARD)  
Ei-value:0.000, Pi-value:0.000  
Er-value:0.000, Pr-value:0.000  
No matches to TargetScan

-4846--(29)--4876-

TGCAGTG

TGCAGTG  
Depth:10 (SNAKE)  
Ei-value:0.000, Pi-value:0.000  
Er-value:0.000, Pr-value:0.000  
MATCHES To TargetScan▶ miR-217:ACUGCAU

-4882--(114)--4997-

TTGGCAAGTAA

TTGGCAAGTAA  
Depth:10 (SNAKE)  
Ei-value:0.000, Pi-value:0.000  
Er-value:0.000, Pr-value:0.000  
No matches to TargetScan

-5007--(91)--5099-

TGGAGTAGTG

TGGAGTAGTG  
Depth:9 (LIZARD)  
Ei-value:0.000, Pi-value:0.000  
Er-value:0.000, Pr-value:0.000  
No matches to TargetScan

-5108--(111)--5220-

TTAGGT

TTAGGT  
Depth:11 (X.TROPICALIS)  
Ei-value:0.000, Pi-value:0.000  
Er-value:0.000, Pr-value:0.000  
No matches to TargetScan


AA

TTAGGTAA  
Depth:10 (SNAKE)  
Ei-value:0.000, Pi-value:0.000  
Er-value:0.000, Pr-value:0.000  
No matches to TargetScan

-5227--(39)--5267-

T

TGAAGCCTTTAGT  
Depth:10 (SNAKE)  
Ei-value:0.000, Pi-value:0.000  
Er-value:0.000, Pr-value:0.000  
No matches to TargetScan


GAAGCC

GAAGCC  
Depth:11 (X.TROPICALIS)  
Ei-value:0.000, Pi-value:0.000  
Er-value:0.000, Pr-value:0.000  
No matches to TargetScan


TTTAGT

TGAAGCCTTTAGT  
Depth:10 (SNAKE)  
Ei-value:0.000, Pi-value:0.000  
Er-value:0.000, Pr-value:0.000  
No matches to TargetScan

-5279--(3)--5283-

TTCCAGAT

TTCCAGAT  
Depth:10 (SNAKE)  
Ei-value:0.000, Pi-value:0.000  
Er-value:0.000, Pr-value:0.000  
No matches to TargetScan

-5290--(61)--5352-

GGCAAGT

GGCAAGT  
Depth:10 (SNAKE)  
Ei-value:0.000, Pi-value:0.000  
Er-value:0.000, Pr-value:0.000  
No matches to TargetScan

-5358--(305)--5664-

AAC

AACAGGTGAA  
Depth:10 (SNAKE)  
Ei-value:0.000, Pi-value:0.000  
Er-value:0.000, Pr-value:0.000  
No matches to TargetScan


AGGTGA

AGGTGA  
Depth:11 (X.TROPICALIS)  
Ei-value:0.000, Pi-value:0.000  
Er-value:0.000, Pr-value:0.010  
No matches to TargetScan


A

AACAGGTGAA  
Depth:10 (SNAKE)  
Ei-value:0.000, Pi-value:0.000  
Er-value:0.000, Pr-value:0.000  
No matches to TargetScan

-5673--(170)--5844-

AAGGCT

AAGGCT  
Depth:10 (SNAKE)  
Ei-value:0.000, Pi-value:0.000  
Er-value:0.000, Pr-value:0.010  
No matches to TargetScan


T

AAGGCTT  
Depth:9 (LIZARD)  
Ei-value:0.000, Pi-value:0.000  
Er-value:0.000, Pr-value:0.000  
No matches to TargetScan

-5850--(281)--6132-

TTTTCCAG

TTTTCCAG  
Depth:10 (SNAKE)  
Ei-value:0.000, Pi-value:0.000  
Er-value:0.000, Pr-value:0.000  
No matches to TargetScan

-6139--(108)--6248-

TGGATCCT

TGGATCCT  
Depth:10 (SNAKE)  
Ei-value:0.000, Pi-value:0.000  
Er-value:0.000, Pr-value:0.000  
No matches to TargetScan

-6255--(25)--6281-

AAGGTAA

AAGGTAA  
Depth:10 (SNAKE)  
Ei-value:0.000, Pi-value:0.000  
Er-value:0.000, Pr-value:0.000  
No matches to TargetScan

-6287--(157)--6445-

GGGTGGG

GGGTGGG  
Depth:10 (SNAKE)  
Ei-value:0.000, Pi-value:0.000  
Er-value:0.000, Pr-value:0.000  
No matches to TargetScan

-6451--(68)--6520-

TGCTAAGACT

TGCTAAGACT  
Depth:10 (SNAKE)  
Ei-value:0.000, Pi-value:0.000  
Er-value:0.000, Pr-value:0.000  
No matches to TargetScan

-6529--(76)--6606-

TTTTCTTTT

TTTTCTTTT  
Depth:19 (ZEBRAFISH)  
Ei-value:0.000, Pi-value:0.000  
Er-value:0.000, Pr-value:0.000  
MATCHES To TargetScan▶ miR-186-5p:AAAGAAU


CCTGAGA

TTTTCTTTTCCTGAGA  
Depth:10 (SNAKE)  
Ei-value:0.000, Pi-value:0.000  
Er-value:0.000, Pr-value:0.000  
MATCHES To TargetScan▶ miR-873-5p.1:CAGGAAC▶ miR-186-5p:AAAGAAU

-6621--(16)--6638-

TCT

TCTCAGGTTTTGCTTTT  
Depth:10 (SNAKE)  
Ei-value:0.000, Pi-value:0.000  
Er-value:0.000, Pr-value:0.000  
MATCHES To TargetScan▶ miR-330-3p.2:AAAGCAC▶ miR-490-3p:AACCUGG


CAGGTTTTGCTTT

CAGGTTTTGCTTT  
Depth:19 (ZEBRAFISH)  
Ei-value:0.000, Pi-value:0.000  
Er-value:0.000, Pr-value:0.000  
MATCHES To TargetScan▶ miR-330-3p.2:AAAGCAC▶ miR-490-3p:AACCUGG


T

CAGGTTTTGCTTTT  
Depth:16 (NILETILAPIA)  
Ei-value:0.000, Pi-value:0.000  
Er-value:0.000, Pr-value:0.000  
MATCHES To TargetScan▶ miR-330-3p.2:AAAGCAC▶ miR-490-3p:AACCUGG

-6654--(24)--6679-

A

AAAAAAGCAAAAG  
Depth:13 (OPOSSUM)  
Ei-value:0.000, Pi-value:0.000  
Er-value:0.000, Pr-value:0.000  
No matches to TargetScan


AAAAAGCAAAA

AAAAAGCAAAA  
Depth:19 (ZEBRAFISH)  
Ei-value:0.000, Pi-value:0.000  
Er-value:0.000, Pr-value:0.000  
No matches to TargetScan


G

AAAAAGCAAAAG  
Depth:18 (MEDAKA)  
Ei-value:0.000, Pi-value:0.000  
Er-value:0.000, Pr-value:0.000  
No matches to TargetScan


A

AAAAAAGCAAAAGA  
Depth:9 (LIZARD)  
Ei-value:0.000, Pi-value:0.000  
Er-value:0.000, Pr-value:0.000  
No matches to TargetScan

-6692--(1)--6694-

GCTGGT

GCTGGT  
Depth:9 (LIZARD)  
Ei-value:0.000, Pi-value:0.000  
Er-value:0.000, Pr-value:0.000  
No matches to TargetScan

-6699--(7)--6707-

ACTCCTG

ACTCCTG  
Depth:18 (MEDAKA)  
Ei-value:0.000, Pi-value:0.000  
Er-value:0.000, Pr-value:0.000  
No matches to TargetScan


G

ACTCCTGG  
Depth:14 (SPOTTEDGAR)  
Ei-value:0.000, Pi-value:0.000  
Er-value:0.000, Pr-value:0.000  
MATCHES To TargetScan▶ miR-665:CCAGGAG

-6714--(11)--6726-

GGGTTC

GGGTTC  
Depth:13 (OPOSSUM)  
Ei-value:0.000, Pi-value:0.000  
Er-value:0.000, Pr-value:0.000  
No matches to TargetScan


AA

GGGTTCAA  
Depth:9 (LIZARD)  
Ei-value:0.000, Pi-value:0.000  
Er-value:0.000, Pr-value:0.000  
No matches to TargetScan

-6733  
  
>TURTLE  
         7-

TTGGACT

TTGGACT  
Depth:9 (LIZARD)  
Ei-value:0.000, Pi-value:0.000  
Er-value:0.000, Pr-value:0.000  
No matches to TargetScan

-13--(162)--176-

GGTGAAG

GGTGAAG  
Depth:11 (X.TROPICALIS)  
Ei-value:0.000, Pi-value:0.000  
Er-value:0.000, Pr-value:0.000  
No matches to TargetScan

-182--(178)--361-

TGGTAA

TGGTAA  
Depth:14 (SPOTTEDGAR)  
Ei-value:0.030, Pi-value:0.000  
Er-value:0.010, Pr-value:0.000  
No matches to TargetScan

-366--(904)--1271-

TTTGGG

TTTGGG  
Depth:19 (ZEBRAFISH)  
Ei-value:0.000, Pi-value:0.000  
Er-value:0.000, Pr-value:0.000  
No matches to TargetScan

-1276--(276)--1553-

GAGCTT

GAGCTT  
Depth:9 (LIZARD)  
Ei-value:0.000, Pi-value:0.000  
Er-value:0.000, Pr-value:0.000  
No matches to TargetScan

-1558--(230)--1789-

GGCAAGTAA

GGCAAGTAA  
Depth:10 (SNAKE)  
Ei-value:0.000, Pi-value:0.000  
Er-value:0.000, Pr-value:0.000  
No matches to TargetScan

-1797--(503)--2301-

T

TCAAGTAAGA  
Depth:9 (LIZARD)  
Ei-value:0.000, Pi-value:0.000  
Er-value:0.000, Pr-value:0.000  
No matches to TargetScan


CAA

CAAGTAAGA  
Depth:10 (SNAKE)  
Ei-value:0.000, Pi-value:0.000  
Er-value:0.000, Pr-value:0.000  
No matches to TargetScan


GTAAGA

GTAAGA  
Depth:11 (X.TROPICALIS)  
Ei-value:0.000, Pi-value:0.000  
Er-value:0.000, Pr-value:0.000  
No matches to TargetScan

-2310--(348)--2659-

TTTTTCAG

TTTTTCAG  
Depth:19 (ZEBRAFISH)  
Ei-value:0.000, Pi-value:0.000  
Er-value:0.000, Pr-value:0.000  
No matches to TargetScan


AT

TTTTTCAGAT  
Depth:10 (SNAKE)  
Ei-value:0.000, Pi-value:0.000  
Er-value:0.000, Pr-value:0.000  
No matches to TargetScan

-2668--(79)--2748-

TGATAAGT

TGATAAGT  
Depth:9 (LIZARD)  
Ei-value:0.000, Pi-value:0.000  
Er-value:0.000, Pr-value:0.000  
No matches to TargetScan

-2755--(0)--2756-

AAAGGCAGAA

AAAGGCAGAA  
Depth:13 (OPOSSUM)  
Ei-value:0.000, Pi-value:0.000  
Er-value:0.000, Pr-value:0.000  
No matches to TargetScan


A

AAAGGCAGAAA  
Depth:11 (X.TROPICALIS)  
Ei-value:0.000, Pi-value:0.000  
Er-value:0.000, Pr-value:0.000  
No matches to TargetScan


A

AAAGGCAGAAAA  
Depth:9 (LIZARD)  
Ei-value:0.000, Pi-value:0.000  
Er-value:0.000, Pr-value:0.000  
No matches to TargetScan

-2767--(465)--3233-

AG

AGACAGGT  
Depth:9 (LIZARD)  
Ei-value:0.000, Pi-value:0.000  
Er-value:0.000, Pr-value:0.000  
No matches to TargetScan


ACAGGT

ACAGGT  
Depth:10 (SNAKE)  
Ei-value:0.000, Pi-value:0.000  
Er-value:0.000, Pr-value:0.000  
No matches to TargetScan

-3240--(72)--3313-

CA

CAGATAAGT  
Depth:11 (X.TROPICALIS)  
Ei-value:0.000, Pi-value:0.000  
Er-value:0.000, Pr-value:0.000  
No matches to TargetScan


GATAAG

GATAAG  
Depth:19 (ZEBRAFISH)  
Ei-value:0.000, Pi-value:0.000  
Er-value:0.000, Pr-value:0.000  
No matches to TargetScan


T

CAGATAAGT  
Depth:11 (X.TROPICALIS)  
Ei-value:0.000, Pi-value:0.000  
Er-value:0.000, Pr-value:0.000  
No matches to TargetScan

-3321--(21)--3343-

ATTGCAT

ATTGCAT  
Depth:11 (X.TROPICALIS)  
Ei-value:0.000, Pi-value:0.000  
Er-value:0.000, Pr-value:0.000  
No matches to TargetScan


G

ATTGCATG  
Depth:9 (LIZARD)  
Ei-value:0.000, Pi-value:0.000  
Er-value:0.000, Pr-value:0.000  
No matches to TargetScan

-3350--(501)--3852-

GCTGTC

GCTGTC  
Depth:9 (LIZARD)  
Ei-value:0.000, Pi-value:0.000  
Er-value:0.000, Pr-value:0.000  
No matches to TargetScan

-3857--(143)--4001-

AGGCAGGA

AGGCAGGA  
Depth:10 (SNAKE)  
Ei-value:0.000, Pi-value:0.000  
Er-value:0.000, Pr-value:0.000  
No matches to TargetScan

-4008--(96)--4105-

TAAGGA

TAAGGA  
Depth:13 (OPOSSUM)  
Ei-value:0.000, Pi-value:0.000  
Er-value:0.000, Pr-value:0.000  
No matches to TargetScan

-4110--(463)--4574-

TTCTGT

TTCTGT  
Depth:9 (LIZARD)  
Ei-value:0.000, Pi-value:0.000  
Er-value:0.000, Pr-value:0.000  
No matches to TargetScan

-4579--(33)--4613-

TGCAGTG

TGCAGTG  
Depth:10 (SNAKE)  
Ei-value:0.000, Pi-value:0.000  
Er-value:0.000, Pr-value:0.000  
MATCHES To TargetScan▶ miR-217:ACUGCAU

-4619--(164)--4784-

TTGGCAAGTAA

TTGGCAAGTAA  
Depth:10 (SNAKE)  
Ei-value:0.000, Pi-value:0.000  
Er-value:0.000, Pr-value:0.000  
No matches to TargetScan

-4794--(77)--4872-

TGGAGTAGTG

TGGAGTAGTG  
Depth:9 (LIZARD)  
Ei-value:0.000, Pi-value:0.000  
Er-value:0.000, Pr-value:0.000  
No matches to TargetScan

-4881--(147)--5029-

TTAGGT

TTAGGT  
Depth:11 (X.TROPICALIS)  
Ei-value:0.000, Pi-value:0.000  
Er-value:0.000, Pr-value:0.000  
No matches to TargetScan


AA

TTAGGTAA  
Depth:10 (SNAKE)  
Ei-value:0.000, Pi-value:0.000  
Er-value:0.000, Pr-value:0.000  
No matches to TargetScan

-5036--(56)--5093-

T

TGAAGCCTTTAGT  
Depth:10 (SNAKE)  
Ei-value:0.000, Pi-value:0.000  
Er-value:0.000, Pr-value:0.000  
No matches to TargetScan


GAAGCC

GAAGCC  
Depth:11 (X.TROPICALIS)  
Ei-value:0.000, Pi-value:0.000  
Er-value:0.000, Pr-value:0.000  
No matches to TargetScan


TTTAGT

TGAAGCCTTTAGT  
Depth:10 (SNAKE)  
Ei-value:0.000, Pi-value:0.000  
Er-value:0.000, Pr-value:0.000  
No matches to TargetScan

-5105--(3)--5109-

TTCCAGAT

TTCCAGAT  
Depth:10 (SNAKE)  
Ei-value:0.000, Pi-value:0.000  
Er-value:0.000, Pr-value:0.000  
No matches to TargetScan

-5116--(70)--5187-

GGCAAGT

GGCAAGT  
Depth:10 (SNAKE)  
Ei-value:0.000, Pi-value:0.000  
Er-value:0.000, Pr-value:0.000  
No matches to TargetScan

-5193--(323)--5517-

AAC

AACAGGTGAA  
Depth:10 (SNAKE)  
Ei-value:0.000, Pi-value:0.000  
Er-value:0.000, Pr-value:0.000  
No matches to TargetScan


AGGTGA

AGGTGA  
Depth:11 (X.TROPICALIS)  
Ei-value:0.000, Pi-value:0.000  
Er-value:0.000, Pr-value:0.010  
No matches to TargetScan


A

AACAGGTGAA  
Depth:10 (SNAKE)  
Ei-value:0.000, Pi-value:0.000  
Er-value:0.000, Pr-value:0.000  
No matches to TargetScan

-5526--(232)--5759-

AAGGCT

AAGGCT  
Depth:10 (SNAKE)  
Ei-value:0.000, Pi-value:0.000  
Er-value:0.000, Pr-value:0.010  
No matches to TargetScan


T

AAGGCTT  
Depth:9 (LIZARD)  
Ei-value:0.000, Pi-value:0.000  
Er-value:0.000, Pr-value:0.000  
No matches to TargetScan

-5765--(383)--6149-

TTTTCCAG

TTTTCCAG  
Depth:10 (SNAKE)  
Ei-value:0.000, Pi-value:0.000  
Er-value:0.000, Pr-value:0.000  
No matches to TargetScan

-6156--(67)--6224-

TGGATCCT

TGGATCCT  
Depth:10 (SNAKE)  
Ei-value:0.000, Pi-value:0.000  
Er-value:0.000, Pr-value:0.000  
No matches to TargetScan

-6231--(25)--6257-

AAGGTAA

AAGGTAA  
Depth:10 (SNAKE)  
Ei-value:0.000, Pi-value:0.000  
Er-value:0.000, Pr-value:0.000  
No matches to TargetScan

-6263--(270)--6534-

GGGTGGG

GGGTGGG  
Depth:10 (SNAKE)  
Ei-value:0.000, Pi-value:0.000  
Er-value:0.000, Pr-value:0.000  
No matches to TargetScan

-6540--(93)--6634-

TGCTAAGACT

TGCTAAGACT  
Depth:10 (SNAKE)  
Ei-value:0.000, Pi-value:0.000  
Er-value:0.000, Pr-value:0.000  
No matches to TargetScan

-6643--(89)--6733-

TTTTCTTTT

TTTTCTTTT  
Depth:19 (ZEBRAFISH)  
Ei-value:0.000, Pi-value:0.000  
Er-value:0.000, Pr-value:0.000  
MATCHES To TargetScan▶ miR-186-5p:AAAGAAU


CCTGAGA

TTTTCTTTTCCTGAGA  
Depth:10 (SNAKE)  
Ei-value:0.000, Pi-value:0.000  
Er-value:0.000, Pr-value:0.000  
MATCHES To TargetScan▶ miR-873-5p.1:CAGGAAC▶ miR-186-5p:AAAGAAU

-6748--(13)--6762-

TCT

TCTCAGGTTTTGCTTTT  
Depth:10 (SNAKE)  
Ei-value:0.000, Pi-value:0.000  
Er-value:0.000, Pr-value:0.000  
MATCHES To TargetScan▶ miR-330-3p.2:AAAGCAC▶ miR-490-3p:AACCUGG


CAGGTTTTGCTTT

CAGGTTTTGCTTT  
Depth:19 (ZEBRAFISH)  
Ei-value:0.000, Pi-value:0.000  
Er-value:0.000, Pr-value:0.000  
MATCHES To TargetScan▶ miR-330-3p.2:AAAGCAC▶ miR-490-3p:AACCUGG


T

CAGGTTTTGCTTTT  
Depth:16 (NILETILAPIA)  
Ei-value:0.000, Pi-value:0.000  
Er-value:0.000, Pr-value:0.000  
MATCHES To TargetScan▶ miR-330-3p.2:AAAGCAC▶ miR-490-3p:AACCUGG

-6778--(19)--6798-

A

AAAAAAGCAAAAG  
Depth:13 (OPOSSUM)  
Ei-value:0.000, Pi-value:0.000  
Er-value:0.000, Pr-value:0.000  
No matches to TargetScan


AAAAAGCAAAA

AAAAAGCAAAA  
Depth:19 (ZEBRAFISH)  
Ei-value:0.000, Pi-value:0.000  
Er-value:0.000, Pr-value:0.000  
No matches to TargetScan


G

AAAAAGCAAAAG  
Depth:18 (MEDAKA)  
Ei-value:0.000, Pi-value:0.000  
Er-value:0.000, Pr-value:0.000  
No matches to TargetScan


A

AAAAAAGCAAAAGA  
Depth:9 (LIZARD)  
Ei-value:0.000, Pi-value:0.000  
Er-value:0.000, Pr-value:0.000  
No matches to TargetScan

-6811--(1)--6813-

GCTGGT

GCTGGT  
Depth:9 (LIZARD)  
Ei-value:0.000, Pi-value:0.000  
Er-value:0.000, Pr-value:0.000  
No matches to TargetScan

-6818--(7)--6826-

ACTCCTG

ACTCCTG  
Depth:18 (MEDAKA)  
Ei-value:0.000, Pi-value:0.000  
Er-value:0.000, Pr-value:0.000  
No matches to TargetScan


G

ACTCCTGG  
Depth:14 (SPOTTEDGAR)  
Ei-value:0.000, Pi-value:0.000  
Er-value:0.000, Pr-value:0.000  
MATCHES To TargetScan▶ miR-665:CCAGGAG

-6833--(11)--6845-

GGGTTC

GGGTTC  
Depth:13 (OPOSSUM)  
Ei-value:0.000, Pi-value:0.000  
Er-value:0.000, Pr-value:0.000  
No matches to TargetScan


AA

GGGTTCAA  
Depth:9 (LIZARD)  
Ei-value:0.000, Pi-value:0.000  
Er-value:0.000, Pr-value:0.000  
No matches to TargetScan

-6852  
  
>ALLIGATOR  
      1299-

TTGGACT

TTGGACT  
Depth:9 (LIZARD)  
Ei-value:0.000, Pi-value:0.000  
Er-value:0.000, Pr-value:0.000  
No matches to TargetScan

-1305--(184)--1490-

GGTGAAG

GGTGAAG  
Depth:11 (X.TROPICALIS)  
Ei-value:0.000, Pi-value:0.000  
Er-value:0.000, Pr-value:0.000  
No matches to TargetScan

-1496--(191)--1688-

TGGTAA

TGGTAA  
Depth:14 (SPOTTEDGAR)  
Ei-value:0.030, Pi-value:0.000  
Er-value:0.010, Pr-value:0.000  
No matches to TargetScan

-1693--(843)--2537-

TTTGGG

TTTGGG  
Depth:19 (ZEBRAFISH)  
Ei-value:0.000, Pi-value:0.000  
Er-value:0.000, Pr-value:0.000  
No matches to TargetScan

-2542--(216)--2759-

GAGCTT

GAGCTT  
Depth:9 (LIZARD)  
Ei-value:0.000, Pi-value:0.000  
Er-value:0.000, Pr-value:0.000  
No matches to TargetScan

-2764--(48)--2813-

GAGCTT

GAGCTT  
Depth:9 (LIZARD)  
Ei-value:0.000, Pi-value:0.000  
Er-value:0.000, Pr-value:0.000  
No matches to TargetScan

-2818--(186)--3005-

GGCAAGTAA

GGCAAGTAA  
Depth:10 (SNAKE)  
Ei-value:0.000, Pi-value:0.000  
Er-value:0.000, Pr-value:0.000  
No matches to TargetScan

-3013--(486)--3500-

T

TCAAGTAAGA  
Depth:9 (LIZARD)  
Ei-value:0.000, Pi-value:0.000  
Er-value:0.000, Pr-value:0.000  
No matches to TargetScan


CAA

CAAGTAAGA  
Depth:10 (SNAKE)  
Ei-value:0.000, Pi-value:0.000  
Er-value:0.000, Pr-value:0.000  
No matches to TargetScan


GTAAGA

GTAAGA  
Depth:11 (X.TROPICALIS)  
Ei-value:0.000, Pi-value:0.000  
Er-value:0.000, Pr-value:0.000  
No matches to TargetScan

-3509--(355)--3865-

TTTTTCAG

TTTTTCAG  
Depth:19 (ZEBRAFISH)  
Ei-value:0.000, Pi-value:0.000  
Er-value:0.000, Pr-value:0.000  
No matches to TargetScan


AT

TTTTTCAGAT  
Depth:10 (SNAKE)  
Ei-value:0.000, Pi-value:0.000  
Er-value:0.000, Pr-value:0.000  
No matches to TargetScan

-3874--(82)--3957-

TGATAAGT

TGATAAGT  
Depth:9 (LIZARD)  
Ei-value:0.000, Pi-value:0.000  
Er-value:0.000, Pr-value:0.000  
No matches to TargetScan

-3964--(0)--3965-

AAAGGCAGAA

AAAGGCAGAA  
Depth:13 (OPOSSUM)  
Ei-value:0.000, Pi-value:0.000  
Er-value:0.000, Pr-value:0.000  
No matches to TargetScan


A

AAAGGCAGAAA  
Depth:11 (X.TROPICALIS)  
Ei-value:0.000, Pi-value:0.000  
Er-value:0.000, Pr-value:0.000  
No matches to TargetScan


A

AAAGGCAGAAAA  
Depth:9 (LIZARD)  
Ei-value:0.000, Pi-value:0.000  
Er-value:0.000, Pr-value:0.000  
No matches to TargetScan

-3976--(460)--4437-

AG

AGACAGGT  
Depth:9 (LIZARD)  
Ei-value:0.000, Pi-value:0.000  
Er-value:0.000, Pr-value:0.000  
No matches to TargetScan


ACAGGT

ACAGGT  
Depth:10 (SNAKE)  
Ei-value:0.000, Pi-value:0.000  
Er-value:0.000, Pr-value:0.000  
No matches to TargetScan

-4444--(74)--4519-

CA

CAGATAAGT  
Depth:11 (X.TROPICALIS)  
Ei-value:0.000, Pi-value:0.000  
Er-value:0.000, Pr-value:0.000  
No matches to TargetScan


GATAAG

GATAAG  
Depth:19 (ZEBRAFISH)  
Ei-value:0.000, Pi-value:0.000  
Er-value:0.000, Pr-value:0.000  
No matches to TargetScan


T

CAGATAAGT  
Depth:11 (X.TROPICALIS)  
Ei-value:0.000, Pi-value:0.000  
Er-value:0.000, Pr-value:0.000  
No matches to TargetScan

-4527--(21)--4549-

ATTGCAT

ATTGCAT  
Depth:11 (X.TROPICALIS)  
Ei-value:0.000, Pi-value:0.000  
Er-value:0.000, Pr-value:0.000  
No matches to TargetScan


G

ATTGCATG  
Depth:9 (LIZARD)  
Ei-value:0.000, Pi-value:0.000  
Er-value:0.000, Pr-value:0.000  
No matches to TargetScan

-4556--(524)--5081-

GCTGTC

GCTGTC  
Depth:9 (LIZARD)  
Ei-value:0.000, Pi-value:0.000  
Er-value:0.000, Pr-value:0.000  
No matches to TargetScan

-5086--(141)--5228-

AGGCAGGA

AGGCAGGA  
Depth:10 (SNAKE)  
Ei-value:0.000, Pi-value:0.000  
Er-value:0.000, Pr-value:0.000  
No matches to TargetScan

-5235--(96)--5332-

TAAGGA

TAAGGA  
Depth:13 (OPOSSUM)  
Ei-value:0.000, Pi-value:0.000  
Er-value:0.000, Pr-value:0.000  
No matches to TargetScan

-5337--(464)--5802-

TTCTGT

TTCTGT  
Depth:9 (LIZARD)  
Ei-value:0.000, Pi-value:0.000  
Er-value:0.000, Pr-value:0.000  
No matches to TargetScan

-5807--(33)--5841-

TGCAGTG

TGCAGTG  
Depth:10 (SNAKE)  
Ei-value:0.000, Pi-value:0.000  
Er-value:0.000, Pr-value:0.000  
MATCHES To TargetScan▶ miR-217:ACUGCAU

-5847--(148)--5996-

TTGGCAAGTAA

TTGGCAAGTAA  
Depth:10 (SNAKE)  
Ei-value:0.000, Pi-value:0.000  
Er-value:0.000, Pr-value:0.000  
No matches to TargetScan

-6006--(82)--6089-

TGGAGTAGTG

TGGAGTAGTG  
Depth:9 (LIZARD)  
Ei-value:0.000, Pi-value:0.000  
Er-value:0.000, Pr-value:0.000  
No matches to TargetScan

-6098--(140)--6239-

TTAGGT

TTAGGT  
Depth:11 (X.TROPICALIS)  
Ei-value:0.000, Pi-value:0.000  
Er-value:0.000, Pr-value:0.000  
No matches to TargetScan


AA

TTAGGTAA  
Depth:10 (SNAKE)  
Ei-value:0.000, Pi-value:0.000  
Er-value:0.000, Pr-value:0.000  
No matches to TargetScan

-6246--(87)--6334-

T

TGAAGCCTTTAGT  
Depth:10 (SNAKE)  
Ei-value:0.000, Pi-value:0.000  
Er-value:0.000, Pr-value:0.000  
No matches to TargetScan


GAAGCC

GAAGCC  
Depth:11 (X.TROPICALIS)  
Ei-value:0.000, Pi-value:0.000  
Er-value:0.000, Pr-value:0.000  
No matches to TargetScan


TTTAGT

TGAAGCCTTTAGT  
Depth:10 (SNAKE)  
Ei-value:0.000, Pi-value:0.000  
Er-value:0.000, Pr-value:0.000  
No matches to TargetScan

-6346--(3)--6350-

TTCCAGAT

TTCCAGAT  
Depth:10 (SNAKE)  
Ei-value:0.000, Pi-value:0.000  
Er-value:0.000, Pr-value:0.000  
No matches to TargetScan

-6357--(70)--6428-

GGCAAGT

GGCAAGT  
Depth:10 (SNAKE)  
Ei-value:0.000, Pi-value:0.000  
Er-value:0.000, Pr-value:0.000  
No matches to TargetScan

-6434--(349)--6784-

AAC

AACAGGTGAA  
Depth:10 (SNAKE)  
Ei-value:0.000, Pi-value:0.000  
Er-value:0.000, Pr-value:0.000  
No matches to TargetScan


AGGTGA

AGGTGA  
Depth:11 (X.TROPICALIS)  
Ei-value:0.000, Pi-value:0.000  
Er-value:0.000, Pr-value:0.010  
No matches to TargetScan


A

AACAGGTGAA  
Depth:10 (SNAKE)  
Ei-value:0.000, Pi-value:0.000  
Er-value:0.000, Pr-value:0.000  
No matches to TargetScan

-6793--(260)--7054-

AAGGCT

AAGGCT  
Depth:10 (SNAKE)  
Ei-value:0.000, Pi-value:0.000  
Er-value:0.000, Pr-value:0.010  
No matches to TargetScan


T

AAGGCTT  
Depth:9 (LIZARD)  
Ei-value:0.000, Pi-value:0.000  
Er-value:0.000, Pr-value:0.000  
No matches to TargetScan

-7060--(358)--7419-

TTTTCCAG

TTTTCCAG  
Depth:10 (SNAKE)  
Ei-value:0.000, Pi-value:0.000  
Er-value:0.000, Pr-value:0.000  
No matches to TargetScan

-7426--(62)--7489-

TGGATCCT

TGGATCCT  
Depth:10 (SNAKE)  
Ei-value:0.000, Pi-value:0.000  
Er-value:0.000, Pr-value:0.000  
No matches to TargetScan

-7496--(23)--7520-

AAGGTAA

AAGGTAA  
Depth:10 (SNAKE)  
Ei-value:0.000, Pi-value:0.000  
Er-value:0.000, Pr-value:0.000  
No matches to TargetScan

-7526--(281)--7808-

GGGTGGG

GGGTGGG  
Depth:10 (SNAKE)  
Ei-value:0.000, Pi-value:0.000  
Er-value:0.000, Pr-value:0.000  
No matches to TargetScan

-7814--(90)--7905-

TGCTAAGACT

TGCTAAGACT  
Depth:10 (SNAKE)  
Ei-value:0.000, Pi-value:0.000  
Er-value:0.000, Pr-value:0.000  
No matches to TargetScan

-7914--(90)--8005-

TTTTCTTTT

TTTTCTTTT  
Depth:19 (ZEBRAFISH)  
Ei-value:0.000, Pi-value:0.000  
Er-value:0.000, Pr-value:0.000  
MATCHES To TargetScan▶ miR-186-5p:AAAGAAU


CCTGAGA

TTTTCTTTTCCTGAGA  
Depth:10 (SNAKE)  
Ei-value:0.000, Pi-value:0.000  
Er-value:0.000, Pr-value:0.000  
MATCHES To TargetScan▶ miR-873-5p.1:CAGGAAC▶ miR-186-5p:AAAGAAU

-8020--(13)--8034-

TCT

TCTCAGGTTTTGCTTTT  
Depth:10 (SNAKE)  
Ei-value:0.000, Pi-value:0.000  
Er-value:0.000, Pr-value:0.000  
MATCHES To TargetScan▶ miR-330-3p.2:AAAGCAC▶ miR-490-3p:AACCUGG


CAGGTTTTGCTTT

CAGGTTTTGCTTT  
Depth:19 (ZEBRAFISH)  
Ei-value:0.000, Pi-value:0.000  
Er-value:0.000, Pr-value:0.000  
MATCHES To TargetScan▶ miR-330-3p.2:AAAGCAC▶ miR-490-3p:AACCUGG


T

CAGGTTTTGCTTTT  
Depth:16 (NILETILAPIA)  
Ei-value:0.000, Pi-value:0.000  
Er-value:0.000, Pr-value:0.000  
MATCHES To TargetScan▶ miR-330-3p.2:AAAGCAC▶ miR-490-3p:AACCUGG

-8050--(24)--8075-

A

AAAAAAGCAAAAG  
Depth:13 (OPOSSUM)  
Ei-value:0.000, Pi-value:0.000  
Er-value:0.000, Pr-value:0.000  
No matches to TargetScan


AAAAAGCAAAA

AAAAAGCAAAA  
Depth:19 (ZEBRAFISH)  
Ei-value:0.000, Pi-value:0.000  
Er-value:0.000, Pr-value:0.000  
No matches to TargetScan


G

AAAAAGCAAAAG  
Depth:18 (MEDAKA)  
Ei-value:0.000, Pi-value:0.000  
Er-value:0.000, Pr-value:0.000  
No matches to TargetScan


A

AAAAAAGCAAAAGA  
Depth:9 (LIZARD)  
Ei-value:0.000, Pi-value:0.000  
Er-value:0.000, Pr-value:0.000  
No matches to TargetScan

-8088--(1)--8090-

GCTGGT

GCTGGT  
Depth:9 (LIZARD)  
Ei-value:0.000, Pi-value:0.000  
Er-value:0.000, Pr-value:0.000  
No matches to TargetScan

-8095--(7)--8103-

ACTCCTG

ACTCCTG  
Depth:18 (MEDAKA)  
Ei-value:0.000, Pi-value:0.000  
Er-value:0.000, Pr-value:0.000  
No matches to TargetScan


G

ACTCCTGG  
Depth:14 (SPOTTEDGAR)  
Ei-value:0.000, Pi-value:0.000  
Er-value:0.000, Pr-value:0.000  
MATCHES To TargetScan▶ miR-665:CCAGGAG

-8110--(11)--8122-

GGGTTC

GGGTTC  
Depth:13 (OPOSSUM)  
Ei-value:0.000, Pi-value:0.000  
Er-value:0.000, Pr-value:0.000  
No matches to TargetScan


AA

GGGTTCAA  
Depth:9 (LIZARD)  
Ei-value:0.000, Pi-value:0.000  
Er-value:0.000, Pr-value:0.000  
No matches to TargetScan

-8129  
  
>LIZARD  
      1002-

TTGGACT

TTGGACT  
Depth:9 (LIZARD)  
Ei-value:0.000, Pi-value:0.000  
Er-value:0.000, Pr-value:0.000  
No matches to TargetScan

-1008--(312)--1321-

GGTGAAG

GGTGAAG  
Depth:11 (X.TROPICALIS)  
Ei-value:0.000, Pi-value:0.000  
Er-value:0.000, Pr-value:0.000  
No matches to TargetScan

-1327--(141)--1469-

TGGTAA

TGGTAA  
Depth:14 (SPOTTEDGAR)  
Ei-value:0.030, Pi-value:0.000  
Er-value:0.010, Pr-value:0.000  
No matches to TargetScan

-1474--(878)--2353-

TTTGGG

TTTGGG  
Depth:19 (ZEBRAFISH)  
Ei-value:0.000, Pi-value:0.000  
Er-value:0.000, Pr-value:0.000  
No matches to TargetScan

-2358--(91)--2450-

GAGCTT

GAGCTT  
Depth:9 (LIZARD)  
Ei-value:0.000, Pi-value:0.000  
Er-value:0.000, Pr-value:0.000  
No matches to TargetScan

-2455--(13)--2469-

GGCAAGTAA

GGCAAGTAA  
Depth:10 (SNAKE)  
Ei-value:0.000, Pi-value:0.000  
Er-value:0.000, Pr-value:0.000  
No matches to TargetScan

-2477--(385)--2863-

T

TCAAGTAAGA  
Depth:9 (LIZARD)  
Ei-value:0.000, Pi-value:0.000  
Er-value:0.000, Pr-value:0.000  
No matches to TargetScan


CAA

CAAGTAAGA  
Depth:10 (SNAKE)  
Ei-value:0.000, Pi-value:0.000  
Er-value:0.000, Pr-value:0.000  
No matches to TargetScan


GTAAGA

GTAAGA  
Depth:11 (X.TROPICALIS)  
Ei-value:0.000, Pi-value:0.000  
Er-value:0.000, Pr-value:0.000  
No matches to TargetScan

-2872--(248)--3121-

TTTTTCAG

TTTTTCAG  
Depth:19 (ZEBRAFISH)  
Ei-value:0.000, Pi-value:0.000  
Er-value:0.000, Pr-value:0.000  
No matches to TargetScan


AT

TTTTTCAGAT  
Depth:10 (SNAKE)  
Ei-value:0.000, Pi-value:0.000  
Er-value:0.000, Pr-value:0.000  
No matches to TargetScan

-3130--(79)--3210-

TGATAAGT

TGATAAGT  
Depth:9 (LIZARD)  
Ei-value:0.000, Pi-value:0.000  
Er-value:0.000, Pr-value:0.000  
No matches to TargetScan

-3217--(1)--3219-

AAAGGCAGAA

AAAGGCAGAA  
Depth:13 (OPOSSUM)  
Ei-value:0.000, Pi-value:0.000  
Er-value:0.000, Pr-value:0.000  
No matches to TargetScan


A

AAAGGCAGAAA  
Depth:11 (X.TROPICALIS)  
Ei-value:0.000, Pi-value:0.000  
Er-value:0.000, Pr-value:0.000  
No matches to TargetScan


A

AAAGGCAGAAAA  
Depth:9 (LIZARD)  
Ei-value:0.000, Pi-value:0.000  
Er-value:0.000, Pr-value:0.000  
No matches to TargetScan

-3230--(407)--3638-

AG

AGACAGGT  
Depth:9 (LIZARD)  
Ei-value:0.000, Pi-value:0.000  
Er-value:0.000, Pr-value:0.000  
No matches to TargetScan


ACAGGT

ACAGGT  
Depth:10 (SNAKE)  
Ei-value:0.000, Pi-value:0.000  
Er-value:0.000, Pr-value:0.000  
No matches to TargetScan

-3645--(69)--3715-

CA

CAGATAAGT  
Depth:11 (X.TROPICALIS)  
Ei-value:0.000, Pi-value:0.000  
Er-value:0.000, Pr-value:0.000  
No matches to TargetScan


GATAAG

GATAAG  
Depth:19 (ZEBRAFISH)  
Ei-value:0.000, Pi-value:0.000  
Er-value:0.000, Pr-value:0.000  
No matches to TargetScan


T

CAGATAAGT  
Depth:11 (X.TROPICALIS)  
Ei-value:0.000, Pi-value:0.000  
Er-value:0.000, Pr-value:0.000  
No matches to TargetScan

-3723--(16)--3740-

ATTGCAT

ATTGCAT  
Depth:11 (X.TROPICALIS)  
Ei-value:0.000, Pi-value:0.000  
Er-value:0.000, Pr-value:0.000  
No matches to TargetScan


G

ATTGCATG  
Depth:9 (LIZARD)  
Ei-value:0.000, Pi-value:0.000  
Er-value:0.000, Pr-value:0.000  
No matches to TargetScan

-3747--(403)--4151-

GCTGTC

GCTGTC  
Depth:9 (LIZARD)  
Ei-value:0.000, Pi-value:0.000  
Er-value:0.000, Pr-value:0.000  
No matches to TargetScan

-4156--(168)--4325-

AGGCAGGA

AGGCAGGA  
Depth:10 (SNAKE)  
Ei-value:0.000, Pi-value:0.000  
Er-value:0.000, Pr-value:0.000  
No matches to TargetScan

-4332--(91)--4424-

TAAGGA

TAAGGA  
Depth:13 (OPOSSUM)  
Ei-value:0.000, Pi-value:0.000  
Er-value:0.000, Pr-value:0.000  
No matches to TargetScan

-4429--(235)--4665-

TTCTGT

TTCTGT  
Depth:9 (LIZARD)  
Ei-value:0.000, Pi-value:0.000  
Er-value:0.000, Pr-value:0.000  
No matches to TargetScan

-4670--(65)--4736-

TGCAGTG

TGCAGTG  
Depth:10 (SNAKE)  
Ei-value:0.000, Pi-value:0.000  
Er-value:0.000, Pr-value:0.000  
MATCHES To TargetScan▶ miR-217:ACUGCAU

-4742--(392)--5135-

TTGGCAAGTAA

TTGGCAAGTAA  
Depth:10 (SNAKE)  
Ei-value:0.000, Pi-value:0.000  
Er-value:0.000, Pr-value:0.000  
No matches to TargetScan

-5145--(62)--5208-

TGGAGTAGTG

TGGAGTAGTG  
Depth:9 (LIZARD)  
Ei-value:0.000, Pi-value:0.000  
Er-value:0.000, Pr-value:0.000  
No matches to TargetScan

-5217--(111)--5329-

TTAGGT

TTAGGT  
Depth:11 (X.TROPICALIS)  
Ei-value:0.000, Pi-value:0.000  
Er-value:0.000, Pr-value:0.000  
No matches to TargetScan


AA

TTAGGTAA  
Depth:10 (SNAKE)  
Ei-value:0.000, Pi-value:0.000  
Er-value:0.000, Pr-value:0.000  
No matches to TargetScan

-5336--(48)--5385-

T

TGAAGCCTTTAGT  
Depth:10 (SNAKE)  
Ei-value:0.000, Pi-value:0.000  
Er-value:0.000, Pr-value:0.000  
No matches to TargetScan


GAAGCC

GAAGCC  
Depth:11 (X.TROPICALIS)  
Ei-value:0.000, Pi-value:0.000  
Er-value:0.000, Pr-value:0.000  
No matches to TargetScan


TTTAGT

TGAAGCCTTTAGT  
Depth:10 (SNAKE)  
Ei-value:0.000, Pi-value:0.000  
Er-value:0.000, Pr-value:0.000  
No matches to TargetScan

-5397--(3)--5401-

TTCCAGAT

TTCCAGAT  
Depth:10 (SNAKE)  
Ei-value:0.000, Pi-value:0.000  
Er-value:0.000, Pr-value:0.000  
No matches to TargetScan

-5408--(52)--5461-

GGCAAGT

GGCAAGT  
Depth:10 (SNAKE)  
Ei-value:0.000, Pi-value:0.000  
Er-value:0.000, Pr-value:0.000  
No matches to TargetScan

-5467--(330)--5798-

AAC

AACAGGTGAA  
Depth:10 (SNAKE)  
Ei-value:0.000, Pi-value:0.000  
Er-value:0.000, Pr-value:0.000  
No matches to TargetScan


AGGTGA

AGGTGA  
Depth:11 (X.TROPICALIS)  
Ei-value:0.000, Pi-value:0.000  
Er-value:0.000, Pr-value:0.010  
No matches to TargetScan


A

AACAGGTGAA  
Depth:10 (SNAKE)  
Ei-value:0.000, Pi-value:0.000  
Er-value:0.000, Pr-value:0.000  
No matches to TargetScan

-5807--(39)--5847-

AAGGCT

AAGGCT  
Depth:10 (SNAKE)  
Ei-value:0.000, Pi-value:0.000  
Er-value:0.000, Pr-value:0.010  
No matches to TargetScan


T

AAGGCTT  
Depth:9 (LIZARD)  
Ei-value:0.000, Pi-value:0.000  
Er-value:0.000, Pr-value:0.000  
No matches to TargetScan

-5853--(500)--6354-

TTTTCCAG

TTTTCCAG  
Depth:10 (SNAKE)  
Ei-value:0.000, Pi-value:0.000  
Er-value:0.000, Pr-value:0.000  
No matches to TargetScan

-6361--(48)--6410-

TGGATCCT

TGGATCCT  
Depth:10 (SNAKE)  
Ei-value:0.000, Pi-value:0.000  
Er-value:0.000, Pr-value:0.000  
No matches to TargetScan

-6417--(18)--6436-

AAGGTAA

AAGGTAA  
Depth:10 (SNAKE)  
Ei-value:0.000, Pi-value:0.000  
Er-value:0.000, Pr-value:0.000  
No matches to TargetScan

-6442--(91)--6534-

AAGGTAA

AAGGTAA  
Depth:10 (SNAKE)  
Ei-value:0.000, Pi-value:0.000  
Er-value:0.000, Pr-value:0.000  
No matches to TargetScan

-6540--(17)--6558-

AAGGTAA

AAGGTAA  
Depth:10 (SNAKE)  
Ei-value:0.000, Pi-value:0.000  
Er-value:0.000, Pr-value:0.000  
No matches to TargetScan

-6564--(146)--6711-

GGGTGGG

GGGTGGG  
Depth:10 (SNAKE)  
Ei-value:0.000, Pi-value:0.000  
Er-value:0.000, Pr-value:0.000  
No matches to TargetScan

-6717--(75)--6793-

TGCTAAGACT

TGCTAAGACT  
Depth:10 (SNAKE)  
Ei-value:0.000, Pi-value:0.000  
Er-value:0.000, Pr-value:0.000  
No matches to TargetScan

-6802--(96)--6899-

TTTTCTTTT

TTTTCTTTT  
Depth:19 (ZEBRAFISH)  
Ei-value:0.000, Pi-value:0.000  
Er-value:0.000, Pr-value:0.000  
MATCHES To TargetScan▶ miR-186-5p:AAAGAAU


CCTGAGA

TTTTCTTTTCCTGAGA  
Depth:10 (SNAKE)  
Ei-value:0.000, Pi-value:0.000  
Er-value:0.000, Pr-value:0.000  
MATCHES To TargetScan▶ miR-873-5p.1:CAGGAAC▶ miR-186-5p:AAAGAAU

-6914--(14)--6929-

TCT

TCTCAGGTTTTGCTTTT  
Depth:10 (SNAKE)  
Ei-value:0.000, Pi-value:0.000  
Er-value:0.000, Pr-value:0.000  
MATCHES To TargetScan▶ miR-330-3p.2:AAAGCAC▶ miR-490-3p:AACCUGG


CAGGTTTTGCTTT

CAGGTTTTGCTTT  
Depth:19 (ZEBRAFISH)  
Ei-value:0.000, Pi-value:0.000  
Er-value:0.000, Pr-value:0.000  
MATCHES To TargetScan▶ miR-330-3p.2:AAAGCAC▶ miR-490-3p:AACCUGG


T

CAGGTTTTGCTTTT  
Depth:16 (NILETILAPIA)  
Ei-value:0.000, Pi-value:0.000  
Er-value:0.000, Pr-value:0.000  
MATCHES To TargetScan▶ miR-330-3p.2:AAAGCAC▶ miR-490-3p:AACCUGG

-6945--(19)--6965-

A

AAAAAAGCAAAAG  
Depth:13 (OPOSSUM)  
Ei-value:0.000, Pi-value:0.000  
Er-value:0.000, Pr-value:0.000  
No matches to TargetScan


AAAAAGCAAAA

AAAAAGCAAAA  
Depth:19 (ZEBRAFISH)  
Ei-value:0.000, Pi-value:0.000  
Er-value:0.000, Pr-value:0.000  
No matches to TargetScan


G

AAAAAGCAAAAG  
Depth:18 (MEDAKA)  
Ei-value:0.000, Pi-value:0.000  
Er-value:0.000, Pr-value:0.000  
No matches to TargetScan


A

AAAAAAGCAAAAGA  
Depth:9 (LIZARD)  
Ei-value:0.000, Pi-value:0.000  
Er-value:0.000, Pr-value:0.000  
No matches to TargetScan

-6978--(1)--6980-

GCTGGT

GCTGGT  
Depth:9 (LIZARD)  
Ei-value:0.000, Pi-value:0.000  
Er-value:0.000, Pr-value:0.000  
No matches to TargetScan

-6985--(7)--6993-

ACTCCTG

ACTCCTG  
Depth:18 (MEDAKA)  
Ei-value:0.000, Pi-value:0.000  
Er-value:0.000, Pr-value:0.000  
No matches to TargetScan


G

ACTCCTGG  
Depth:14 (SPOTTEDGAR)  
Ei-value:0.000, Pi-value:0.000  
Er-value:0.000, Pr-value:0.000  
MATCHES To TargetScan▶ miR-665:CCAGGAG

-7000--(11)--7012-

GGGTTC

GGGTTC  
Depth:13 (OPOSSUM)  
Ei-value:0.000, Pi-value:0.000  
Er-value:0.000, Pr-value:0.000  
No matches to TargetScan


AA

GGGTTCAA  
Depth:9 (LIZARD)  
Ei-value:0.000, Pi-value:0.000  
Er-value:0.000, Pr-value:0.000  
No matches to TargetScan

-7019
```

---

# Modules conserved to ALLIGATOR (Depth: 8)

## Modules in Main Graph (All sequences considered):

```
>HUMAN  
      1315-

TTGGACT

TTGGACT  
Depth:9 (LIZARD)  
Ei-value:0.000, Pi-value:0.000  
Er-value:0.000, Pr-value:0.000  
eCLIP MATCHES▶aggf1 (bg=15.15%)▶bclaf1 (bg=17.67%)▶bud13 (bg=12.85%)▶DGCR8 (bg=2.67%)▶hltf (bg=24.28%)▶larp4 (bg=13.51%)▶NIPBL (bg=8.2%)▶npm1 (bg=10.22%)▶ppil4 (bg=43.39%)▶rbm15 (bg=11.59%)▶rbm22 (bg=12.69%)▶safb (bg=40.39%)▶safb2 (bg=26.89%)▶srsf1 (bg=30.28%)▶SUPV3L1 (bg=9.63%)▶tra2a (bg=37.02%)▶uchl5 (bg=18.56%)▶XRCC6 (bg=3.81%)▶YWHAG (bg=9.14%)▶zc3h8 (bg=12.78%)▶znf622 (bg=18.79%)No matches to TargetScan

-1321--(30)--1352-

GACTAG

GACTAG  
Depth:8 (ALLIGATOR)  
Ei-value:0.000, Pi-value:0.000  
Er-value:0.000, Pr-value:0.000  
eCLIP MATCHES▶aggf1 (bg=15.15%)▶bclaf1 (bg=17.67%)▶bud13 (bg=12.85%)▶cpsf6 (bg=13.45%)▶gtf2f1 (bg=10.18%)▶hltf (bg=24.28%)▶NIPBL (bg=8.2%)▶npm1 (bg=10.22%)▶ppil4 (bg=43.39%)▶rbm15 (bg=11.59%)▶rbm22 (bg=12.69%)▶safb (bg=40.39%)▶safb2 (bg=26.89%)▶SLBP (bg=6.66%)▶SLTM (bg=7.5%)▶srsf1 (bg=30.28%)▶SRSF9 (bg=9.67%)▶tra2a (bg=37.02%)▶XRCC6 (bg=3.81%)▶YWHAG (bg=9.14%)No matches to TargetScan

-1357--(55)--1413-

AAGACAG

AAGACAG  
Depth:8 (ALLIGATOR)  
Ei-value:0.000, Pi-value:0.000  
Er-value:0.000, Pr-value:0.000  
eCLIP MATCHES▶ppil4 (bg=43.39%)▶safb (bg=40.39%)▶srsf1 (bg=30.28%)▶SRSF9 (bg=9.67%)▶tra2a (bg=37.02%)▶uchl5 (bg=18.56%)▶znf622 (bg=18.79%)No matches to TargetScan

-1419--(157)--1577-

GGTGAAG

GGTGAAG  
Depth:11 (X.TROPICALIS)  
Ei-value:0.000, Pi-value:0.000  
Er-value:0.000, Pr-value:0.000  
eCLIP MATCHES▶bclaf1 (bg=17.67%)▶EXOSC5 (bg=2.0%)▶fxr2 (bg=10.1%)▶gtf2f1 (bg=10.18%)▶hltf (bg=24.28%)▶MTPAP (bg=9.55%)▶npm1 (bg=10.22%)▶ppil4 (bg=43.39%)▶rbm15 (bg=11.59%)▶rbm22 (bg=12.69%)▶safb (bg=40.39%)▶safb2 (bg=26.89%)▶SMNDC1 (bg=7.08%)▶srsf1 (bg=30.28%)▶SRSF9 (bg=9.67%)▶TAF15 (bg=9.06%)▶tra2a (bg=37.02%)▶TROVE2 (bg=6.96%)▶uchl5 (bg=18.56%)▶znf622 (bg=18.79%)No matches to TargetScan

-1583--(37)--1621-

TGGTGA

TGGTGA  
Depth:8 (ALLIGATOR)  
Ei-value:0.000, Pi-value:0.000  
Er-value:0.000, Pr-value:0.000  
eCLIP MATCHES▶aggf1 (bg=15.15%)▶bclaf1 (bg=17.67%)▶EXOSC5 (bg=2.0%)▶fxr2 (bg=10.1%)▶GRWD1 (bg=7.0%)▶gtf2f1 (bg=10.18%)▶hltf (bg=24.28%)▶larp4 (bg=13.51%)▶MTPAP (bg=9.55%)▶npm1 (bg=10.22%)▶ppil4 (bg=43.39%)▶rbm15 (bg=11.59%)▶rbm22 (bg=12.69%)▶safb (bg=40.39%)▶safb2 (bg=26.89%)▶SLTM (bg=7.5%)▶SMNDC1 (bg=7.08%)▶srsf1 (bg=30.28%)▶SRSF9 (bg=9.67%)▶TAF15 (bg=9.06%)▶tra2a (bg=37.02%)▶TROVE2 (bg=6.96%)▶uchl5 (bg=18.56%)▶UTP3 (bg=1.57%)▶YWHAG (bg=9.14%)▶znf622 (bg=18.79%)No matches to TargetScan

-1626--(167)--1794-

TGGTAA

TGGTAA  
Depth:14 (SPOTTEDGAR)  
Ei-value:0.030, Pi-value:0.000  
Er-value:0.010, Pr-value:0.000  
eCLIP MATCHES▶bclaf1 (bg=17.67%)▶DROSHA (bg=1.03%)▶GRWD1 (bg=7.0%)▶hltf (bg=24.28%)▶MTPAP (bg=9.55%)▶NOLC1 (bg=0.67%)▶npm1 (bg=10.22%)▶ppil4 (bg=43.39%)▶rbm15 (bg=11.59%)▶safb (bg=40.39%)▶srsf1 (bg=30.28%)▶TAF15 (bg=9.06%)▶tia1 (bg=23.76%)▶uchl5 (bg=18.56%)▶YWHAG (bg=9.14%)▶ZRANB2 (bg=7.28%)No matches to TargetScan

-1799--(89)--1889-

GTAGGTTT

GTAGGTTT  
Depth:8 (ALLIGATOR)  
Ei-value:0.000, Pi-value:0.000  
Er-value:0.000, Pr-value:0.000  
eCLIP MATCHES▶ddx42 (bg=10.33%)▶khsrp (bg=27.4%)▶ppil4 (bg=43.39%)▶safb (bg=40.39%)▶SF3B4 (bg=4.76%)▶tia1 (bg=23.76%)▶tial1 (bg=15.02%)▶u2af1 (bg=14.02%)▶ZRANB2 (bg=7.28%)No matches to TargetScan

-1896--(424)--2321-

TCAGGATAA

TCAGGATAA  
Depth:8 (ALLIGATOR)  
Ei-value:0.000, Pi-value:0.000  
Er-value:0.000, Pr-value:0.000  
eCLIP MATCHES▶aggf1 (bg=15.15%)▶hnrnpa1 (bg=18.32%)▶HNRNPL (bg=2.63%)No matches to TargetScan

-2329--(175)--2505-

TTTGGG

TTTGGG  
Depth:19 (ZEBRAFISH)  
Ei-value:0.000, Pi-value:0.000  
Er-value:0.000, Pr-value:0.000  
eCLIP MATCHES▶ppil4 (bg=43.39%)▶PRPF8 (bg=6.2%)No matches to TargetScan

-2510--(44)--2555-

TTTCTA

TTTCTA  
Depth:8 (ALLIGATOR)  
Ei-value:0.000, Pi-value:0.000  
Er-value:0.000, Pr-value:0.000  
eCLIP MATCHES▶PCBP2 (bg=4.33%)▶ppil4 (bg=43.39%)▶PRPF8 (bg=6.2%)▶safb (bg=40.39%)No matches to TargetScan

-2560--(16)--2577-

CTATAGT

CTATAGT  
Depth:8 (ALLIGATOR)  
Ei-value:0.000, Pi-value:0.000  
Er-value:0.000, Pr-value:0.000  
eCLIP MATCHES▶ppil4 (bg=43.39%)▶PRPF8 (bg=6.2%)▶safb (bg=40.39%)No matches to TargetScan

-2583--(16)--2600-

GTTAGA

GTTAGA  
Depth:8 (ALLIGATOR)  
Ei-value:0.000, Pi-value:0.000  
Er-value:0.000, Pr-value:0.000  
eCLIP MATCHES▶ppil4 (bg=43.39%)▶safb (bg=40.39%)No matches to TargetScan

-2605--(112)--2718-

TGGTTC

TGGTTC  
Depth:8 (ALLIGATOR)  
Ei-value:0.000, Pi-value:0.000  
Er-value:0.000, Pr-value:0.000  
eCLIP MATCHES▶hnrnpa1 (bg=18.32%)▶ppil4 (bg=43.39%)No matches to TargetScan

-2723--(30)--2754-

GAGCTT

GAGCTT  
Depth:9 (LIZARD)  
Ei-value:0.000, Pi-value:0.000  
Er-value:0.000, Pr-value:0.000  
eCLIP MATCHES▶hnrnpa1 (bg=18.32%)▶ppil4 (bg=43.39%)▶PRPF8 (bg=6.2%)No matches to TargetScan

-2759--(17)--2777-

AAGTTAAGTT

AAGTTAAGTT  
Depth:8 (ALLIGATOR)  
Ei-value:0.000, Pi-value:0.000  
Er-value:0.000, Pr-value:0.000  
eCLIP MATCHES▶hnrnpa1 (bg=18.32%)▶PRPF8 (bg=6.2%)No matches to TargetScan

-2786--(196)--2983-

T

TGGCAAGTAA  
Depth:8 (ALLIGATOR)  
Ei-value:0.000, Pi-value:0.000  
Er-value:0.000, Pr-value:0.000  
eCLIP MATCHES▶hnrnpa1 (bg=18.32%)▶ppil4 (bg=43.39%)▶safb (bg=40.39%)No matches to TargetScan


GGCAAGTAA

GGCAAGTAA  
Depth:10 (SNAKE)  
Ei-value:0.000, Pi-value:0.000  
Er-value:0.000, Pr-value:0.000  
eCLIP MATCHES▶hnrnpa1 (bg=18.32%)▶ppil4 (bg=43.39%)▶safb (bg=40.39%)No matches to TargetScan

-2992--(247)--3240-

GTGTGTGG

GTGTGTGG  
Depth:8 (ALLIGATOR)  
Ei-value:0.000, Pi-value:0.000  
Er-value:0.000, Pr-value:0.000  
eCLIP MATCHES▶HNRNPM (bg=6.37%)▶khsrp (bg=27.4%)▶PRPF8 (bg=6.2%)▶SF3B4 (bg=4.76%)▶SLBP (bg=6.66%)MATCHES To TargetScan▶ miR-329-3p/362-3p:ACACACC

-3247--(20)--3268-

CAGGTG

CAGGTG  
Depth:8 (ALLIGATOR)  
Ei-value:0.000, Pi-value:0.000  
Er-value:0.000, Pr-value:0.000  
eCLIP MATCHES▶HNRNPM (bg=6.37%)▶ppil4 (bg=43.39%)▶PRPF8 (bg=6.2%)▶PUS1 (bg=1.64%)▶safb (bg=40.39%)▶SLBP (bg=6.66%)▶u2af2 (bg=19.32%)No matches to TargetScan

-3273--(52)--3326-

TTAATGA

TTAATGA  
Depth:8 (ALLIGATOR)  
Ei-value:0.000, Pi-value:0.000  
Er-value:0.000, Pr-value:0.000  
eCLIP MATCHES▶ppil4 (bg=43.39%)▶srsf7 (bg=22.53%)No matches to TargetScan

-3332--(38)--3371-

T

TCAAGTAAGA  
Depth:9 (LIZARD)  
Ei-value:0.000, Pi-value:0.000  
Er-value:0.000, Pr-value:0.000  
eCLIP MATCHES▶HNRNPM (bg=6.37%)▶srsf7 (bg=22.53%)No matches to TargetScan


CAA

CAAGTAAGA  
Depth:10 (SNAKE)  
Ei-value:0.000, Pi-value:0.000  
Er-value:0.000, Pr-value:0.000  
eCLIP MATCHES▶HNRNPM (bg=6.37%)▶srsf7 (bg=22.53%)No matches to TargetScan


GTAAGA

GTAAGA  
Depth:11 (X.TROPICALIS)  
Ei-value:0.000, Pi-value:0.000  
Er-value:0.000, Pr-value:0.000  
eCLIP MATCHES▶HNRNPM (bg=6.37%)No matches to TargetScan

-3380--(240)--3621-

TTTTTCAG

TTTTTCAG  
Depth:19 (ZEBRAFISH)  
Ei-value:0.000, Pi-value:0.000  
Er-value:0.000, Pr-value:0.000  
eCLIP MATCHES▶ddx42 (bg=10.33%)▶ppil4 (bg=43.39%)▶safb (bg=40.39%)▶u2af1 (bg=14.02%)▶u2af2 (bg=19.32%)No matches to TargetScan


AT

TTTTTCAGAT  
Depth:10 (SNAKE)  
Ei-value:0.000, Pi-value:0.000  
Er-value:0.000, Pr-value:0.000  
eCLIP MATCHES▶ddx42 (bg=10.33%)▶ppil4 (bg=43.39%)▶safb (bg=40.39%)▶u2af1 (bg=14.02%)▶u2af2 (bg=19.32%)No matches to TargetScan

-3630--(72)--3703-

TGATAAGT

TGATAAGT  
Depth:9 (LIZARD)  
Ei-value:0.000, Pi-value:0.000  
Er-value:0.000, Pr-value:0.000  
eCLIP MATCHES▶ppil4 (bg=43.39%)No matches to TargetScan


AAAGGCAGAA

AAAGGCAGAA  
Depth:13 (OPOSSUM)  
Ei-value:0.000, Pi-value:0.000  
Er-value:0.000, Pr-value:0.000  
eCLIP MATCHES▶hnrnpa1 (bg=18.32%)▶ppil4 (bg=43.39%)No matches to TargetScan


A

AAAGGCAGAAA  
Depth:11 (X.TROPICALIS)  
Ei-value:0.000, Pi-value:0.000  
Er-value:0.000, Pr-value:0.000  
eCLIP MATCHES▶hnrnpa1 (bg=18.32%)▶ppil4 (bg=43.39%)No matches to TargetScan


A

AAAGGCAGAAAA  
Depth:9 (LIZARD)  
Ei-value:0.000, Pi-value:0.000  
Er-value:0.000, Pr-value:0.000  
eCLIP MATCHES▶hnrnpa1 (bg=18.32%)▶ppil4 (bg=43.39%)No matches to TargetScan

-3722--(237)--3960-

CAGAAT

CAGAAT  
Depth:8 (ALLIGATOR)  
Ei-value:0.000, Pi-value:0.000  
Er-value:0.000, Pr-value:0.000  
eCLIP MATCHES▶khdrbs1 (bg=10.41%)▶larp4 (bg=13.51%)▶safb2 (bg=26.89%)▶znf622 (bg=18.79%)No matches to TargetScan

-3965--(76)--4042-

TGGGATC

TGGGATC  
Depth:8 (ALLIGATOR)  
Ei-value:0.000, Pi-value:0.000  
Er-value:0.000, Pr-value:0.000  
eCLIP MATCHES▶bclaf1 (bg=17.67%)▶DGCR8 (bg=2.67%)▶hltf (bg=24.28%)▶khdrbs1 (bg=10.41%)▶khsrp (bg=27.4%)▶larp4 (bg=13.51%)▶NIPBL (bg=8.2%)▶ppil4 (bg=43.39%)▶rbm15 (bg=11.59%)▶safb (bg=40.39%)▶safb2 (bg=26.89%)▶srsf1 (bg=30.28%)▶srsf7 (bg=22.53%)▶SUPV3L1 (bg=9.63%)▶uchl5 (bg=18.56%)No matches to TargetScan

-4048--(47)--4096-

AG

AGACAGGT  
Depth:9 (LIZARD)  
Ei-value:0.000, Pi-value:0.000  
Er-value:0.000, Pr-value:0.000  
eCLIP MATCHES▶DGCR8 (bg=2.67%)▶hltf (bg=24.28%)▶khsrp (bg=27.4%)▶LIN28B (bg=1.31%)▶NIPBL (bg=8.2%)▶NOLC1 (bg=0.67%)▶ppil4 (bg=43.39%)▶rbm15 (bg=11.59%)▶safb (bg=40.39%)▶safb2 (bg=26.89%)▶srsf1 (bg=30.28%)▶znf622 (bg=18.79%)No matches to TargetScan


ACAGGT

ACAGGT  
Depth:10 (SNAKE)  
Ei-value:0.000, Pi-value:0.000  
Er-value:0.000, Pr-value:0.000  
eCLIP MATCHES▶DGCR8 (bg=2.67%)▶hltf (bg=24.28%)▶khsrp (bg=27.4%)▶LIN28B (bg=1.31%)▶NOLC1 (bg=0.67%)▶ppil4 (bg=43.39%)▶rbm15 (bg=11.59%)▶safb (bg=40.39%)▶safb2 (bg=26.89%)▶srsf1 (bg=30.28%)▶znf622 (bg=18.79%)No matches to TargetScan

-4103--(21)--4125-

TTGCTTCA

TTGCTTCA  
Depth:8 (ALLIGATOR)  
Ei-value:0.000, Pi-value:0.000  
Er-value:0.000, Pr-value:0.000  
eCLIP MATCHES▶cpsf6 (bg=13.45%)▶GRWD1 (bg=7.0%)▶hltf (bg=24.28%)▶LIN28B (bg=1.31%)▶NOLC1 (bg=0.67%)▶ppil4 (bg=43.39%)▶rbm15 (bg=11.59%)▶safb (bg=40.39%)▶safb2 (bg=26.89%)▶srsf1 (bg=30.28%)▶srsf7 (bg=22.53%)▶u2af2 (bg=19.32%)▶znf622 (bg=18.79%)No matches to TargetScan

-4132--(37)--4170-

CA

CAGATAAGT  
Depth:11 (X.TROPICALIS)  
Ei-value:0.000, Pi-value:0.000  
Er-value:0.000, Pr-value:0.000  
eCLIP MATCHES▶cpsf6 (bg=13.45%)▶hltf (bg=24.28%)▶hnrnpa1 (bg=18.32%)▶khsrp (bg=27.4%)▶NIPBL (bg=8.2%)▶ppil4 (bg=43.39%)▶PRPF8 (bg=6.2%)▶rbm15 (bg=11.59%)▶safb (bg=40.39%)▶safb2 (bg=26.89%)▶srsf1 (bg=30.28%)▶srsf7 (bg=22.53%)▶znf622 (bg=18.79%)No matches to TargetScan


GATAAG

GATAAG  
Depth:19 (ZEBRAFISH)  
Ei-value:0.000, Pi-value:0.000  
Er-value:0.000, Pr-value:0.000  
eCLIP MATCHES▶cpsf6 (bg=13.45%)▶khsrp (bg=27.4%)▶NIPBL (bg=8.2%)▶ppil4 (bg=43.39%)▶PRPF8 (bg=6.2%)▶rbm15 (bg=11.59%)▶safb (bg=40.39%)▶safb2 (bg=26.89%)▶srsf1 (bg=30.28%)▶srsf7 (bg=22.53%)▶znf622 (bg=18.79%)No matches to TargetScan


T

CAGATAAGT  
Depth:11 (X.TROPICALIS)  
Ei-value:0.000, Pi-value:0.000  
Er-value:0.000, Pr-value:0.000  
eCLIP MATCHES▶cpsf6 (bg=13.45%)▶hltf (bg=24.28%)▶hnrnpa1 (bg=18.32%)▶khsrp (bg=27.4%)▶NIPBL (bg=8.2%)▶ppil4 (bg=43.39%)▶PRPF8 (bg=6.2%)▶rbm15 (bg=11.59%)▶safb (bg=40.39%)▶safb2 (bg=26.89%)▶srsf1 (bg=30.28%)▶srsf7 (bg=22.53%)▶znf622 (bg=18.79%)No matches to TargetScan

-4178--(17)--4196-

T

TATTGCATG  
Depth:8 (ALLIGATOR)  
Ei-value:0.000, Pi-value:0.000  
Er-value:0.000, Pr-value:0.000  
eCLIP MATCHES▶cpsf6 (bg=13.45%)▶hltf (bg=24.28%)▶hnrnpa1 (bg=18.32%)▶HNRNPU (bg=9.45%)▶khsrp (bg=27.4%)▶ppil4 (bg=43.39%)▶PRPF8 (bg=6.2%)▶RBFOX2 (bg=3.41%)▶safb (bg=40.39%)▶safb2 (bg=26.89%)▶tia1 (bg=23.76%)▶tial1 (bg=15.02%)No matches to TargetScan


ATTGCAT

ATTGCAT  
Depth:11 (X.TROPICALIS)  
Ei-value:0.000, Pi-value:0.000  
Er-value:0.000, Pr-value:0.000  
eCLIP MATCHES▶cpsf6 (bg=13.45%)▶hltf (bg=24.28%)▶hnrnpa1 (bg=18.32%)▶HNRNPU (bg=9.45%)▶khsrp (bg=27.4%)▶ppil4 (bg=43.39%)▶PRPF8 (bg=6.2%)▶RBFOX2 (bg=3.41%)▶safb (bg=40.39%)▶safb2 (bg=26.89%)▶tia1 (bg=23.76%)▶tial1 (bg=15.02%)No matches to TargetScan


G

ATTGCATG  
Depth:9 (LIZARD)  
Ei-value:0.000, Pi-value:0.000  
Er-value:0.000, Pr-value:0.000  
eCLIP MATCHES▶cpsf6 (bg=13.45%)▶hltf (bg=24.28%)▶hnrnpa1 (bg=18.32%)▶HNRNPU (bg=9.45%)▶khsrp (bg=27.4%)▶ppil4 (bg=43.39%)▶PRPF8 (bg=6.2%)▶RBFOX2 (bg=3.41%)▶safb (bg=40.39%)▶safb2 (bg=26.89%)▶tia1 (bg=23.76%)▶tial1 (bg=15.02%)No matches to TargetScan

-4204--(118)--4323-

TTAAAAT

TTAAAAT  
Depth:8 (ALLIGATOR)  
Ei-value:0.000, Pi-value:0.000  
Er-value:0.000, Pr-value:0.000  
eCLIP MATCHES▶HNRNPC (bg=4.22%)▶HNRNPU (bg=9.45%)No matches to TargetScan

-4329--(294)--4624-

GCTGTC

GCTGTC  
Depth:9 (LIZARD)  
Ei-value:0.000, Pi-value:0.000  
Er-value:0.000, Pr-value:0.000  
eCLIP MATCHES▶cpsf6 (bg=13.45%)▶CSTF2 (bg=7.88%)▶cstf2t (bg=12.11%)▶FMR1 (bg=1.89%)▶khsrp (bg=27.4%)▶zc3h8 (bg=12.78%)No matches to TargetScan

-4629--(74)--4704-

TTTCAGA

TTTCAGA  
Depth:8 (ALLIGATOR)  
Ei-value:0.000, Pi-value:0.000  
Er-value:0.000, Pr-value:0.000  
eCLIP MATCHES▶CSTF2 (bg=7.88%)▶cstf2t (bg=12.11%)▶FMR1 (bg=1.89%)▶METAP2 (bg=1.11%)▶ppil4 (bg=43.39%)▶u2af2 (bg=19.32%)▶XRN2 (bg=1.09%)▶zc3h8 (bg=12.78%)No matches to TargetScan

-4710--(68)--4779-

AGGCAGGA

AGGCAGGA  
Depth:10 (SNAKE)  
Ei-value:0.000, Pi-value:0.000  
Er-value:0.000, Pr-value:0.000  
eCLIP MATCHES▶CSTF2 (bg=7.88%)▶cstf2t (bg=12.11%)▶srsf1 (bg=30.28%)No matches to TargetScan


AA

AGGCAGGAAA  
Depth:8 (ALLIGATOR)  
Ei-value:0.000, Pi-value:0.000  
Er-value:0.000, Pr-value:0.000  
eCLIP MATCHES▶CSTF2 (bg=7.88%)▶cstf2t (bg=12.11%)▶srsf1 (bg=30.28%)No matches to TargetScan

-4788--(72)--4861-

TAAGGA

TAAGGA  
Depth:13 (OPOSSUM)  
Ei-value:0.000, Pi-value:0.000  
Er-value:0.000, Pr-value:0.000  
eCLIP MATCHES▶cstf2t (bg=12.11%)▶tia1 (bg=23.76%)▶tial1 (bg=15.02%)No matches to TargetScan

-4866--(130)--4997-

TGCATG

TGCATG  
Depth:8 (ALLIGATOR)  
Ei-value:0.000, Pi-value:0.000  
Er-value:0.000, Pr-value:0.000  
eCLIP MATCHES▶CSTF2 (bg=7.88%)▶cstf2t (bg=12.11%)▶SUPV3L1 (bg=9.63%)No matches to TargetScan

-5002--(116)--5119-

TTTCAT

TTTCAT  
Depth:8 (ALLIGATOR)  
Ei-value:0.000, Pi-value:0.000  
Er-value:0.000, Pr-value:0.000  
eCLIP MATCHES▶CSTF2 (bg=7.88%)▶cstf2t (bg=12.11%)▶HNRNPU (bg=9.45%)▶khsrp (bg=27.4%)▶ppil4 (bg=43.39%)▶RBFOX2 (bg=3.41%)▶srsf1 (bg=30.28%)▶u2af1 (bg=14.02%)▶u2af2 (bg=19.32%)▶zc3h8 (bg=12.78%)No matches to TargetScan

-5124--(53)--5178-

TTCTGT

TTCTGT  
Depth:9 (LIZARD)  
Ei-value:0.000, Pi-value:0.000  
Er-value:0.000, Pr-value:0.000  
eCLIP MATCHES▶CSTF2 (bg=7.88%)▶cstf2t (bg=12.11%)▶hnrnpa1 (bg=18.32%)▶HNRNPM (bg=6.37%)▶PRPF8 (bg=6.2%)▶srsf1 (bg=30.28%)▶srsf7 (bg=22.53%)▶TARDBP (bg=5.12%)▶tia1 (bg=23.76%)▶u2af1 (bg=14.02%)▶u2af2 (bg=19.32%)No matches to TargetScan

-5183--(21)--5205-

TGCAGTG

TGCAGTG  
Depth:10 (SNAKE)  
Ei-value:0.000, Pi-value:0.000  
Er-value:0.000, Pr-value:0.000  
eCLIP MATCHES▶cstf2t (bg=12.11%)▶hnrnpa1 (bg=18.32%)▶HNRNPM (bg=6.37%)▶PRPF8 (bg=6.2%)▶srsf7 (bg=22.53%)▶TARDBP (bg=5.12%)MATCHES To TargetScan▶ miR-217:ACUGCAU

-5211--(104)--5316-

AGAATGCAG

AGAATGCAG  
Depth:8 (ALLIGATOR)  
Ei-value:0.000, Pi-value:0.000  
Er-value:0.000, Pr-value:0.000  
eCLIP MATCHES▶CSTF2 (bg=7.88%)▶cstf2t (bg=12.11%)▶DGCR8 (bg=2.67%)▶HNRNPC (bg=4.22%)▶HNRNPU (bg=9.45%)▶khsrp (bg=27.4%)▶ppil4 (bg=43.39%)▶RBFOX2 (bg=3.41%)▶srsf7 (bg=22.53%)▶tia1 (bg=23.76%)▶tial1 (bg=15.02%)▶u2af1 (bg=14.02%)▶u2af2 (bg=19.32%)▶zc3h8 (bg=12.78%)MATCHES To TargetScan▶ miR-33-5p:UGCAUUG

-5324--(29)--5354-

TTGGCAAGTAA

TTGGCAAGTAA  
Depth:10 (SNAKE)  
Ei-value:0.000, Pi-value:0.000  
Er-value:0.000, Pr-value:0.000  
eCLIP MATCHES▶CSTF2 (bg=7.88%)▶cstf2t (bg=12.11%)▶HNRNPC (bg=4.22%)▶khsrp (bg=27.4%)▶ppil4 (bg=43.39%)▶srsf7 (bg=22.53%)▶TARDBP (bg=5.12%)▶tia1 (bg=23.76%)▶u2af1 (bg=14.02%)▶u2af2 (bg=19.32%)No matches to TargetScan

-5364--(84)--5449-

TGGAGTAGTG

TGGAGTAGTG  
Depth:9 (LIZARD)  
Ei-value:0.000, Pi-value:0.000  
Er-value:0.000, Pr-value:0.000  
eCLIP MATCHES▶CSTF2 (bg=7.88%)▶cstf2t (bg=12.11%)▶DDX21 (bg=1.05%)▶EIF4G2 (bg=2.16%)▶hnrnpa1 (bg=18.32%)▶HNRNPM (bg=6.37%)▶HNRNPU (bg=9.45%)▶IGF2BP1 (bg=1.23%)▶khsrp (bg=27.4%)▶LIN28B (bg=1.31%)▶srsf7 (bg=22.53%)▶TARDBP (bg=5.12%)▶zc3h8 (bg=12.78%)No matches to TargetScan

-5458--(114)--5573-

TTAGGT

TTAGGT  
Depth:11 (X.TROPICALIS)  
Ei-value:0.000, Pi-value:0.000  
Er-value:0.000, Pr-value:0.000  
eCLIP MATCHES▶cstf2t (bg=12.11%)▶HNRNPM (bg=6.37%)▶khsrp (bg=27.4%)▶srsf7 (bg=22.53%)▶TARDBP (bg=5.12%)▶tia1 (bg=23.76%)▶tial1 (bg=15.02%)▶u2af1 (bg=14.02%)▶ZRANB2 (bg=7.28%)No matches to TargetScan


AA

TTAGGTAA  
Depth:10 (SNAKE)  
Ei-value:0.000, Pi-value:0.000  
Er-value:0.000, Pr-value:0.000  
eCLIP MATCHES▶cstf2t (bg=12.11%)▶HNRNPM (bg=6.37%)▶khsrp (bg=27.4%)▶srsf7 (bg=22.53%)▶TARDBP (bg=5.12%)▶tia1 (bg=23.76%)▶tial1 (bg=15.02%)▶u2af1 (bg=14.02%)▶ZRANB2 (bg=7.28%)No matches to TargetScan

-5580--(36)--5617-

T

TGAAGCCTTTAGT  
Depth:10 (SNAKE)  
Ei-value:0.000, Pi-value:0.000  
Er-value:0.000, Pr-value:0.000  
eCLIP MATCHES▶ddx42 (bg=10.33%)▶khsrp (bg=27.4%)▶SF3B4 (bg=4.76%)▶SMNDC1 (bg=7.08%)▶TARDBP (bg=5.12%)▶tia1 (bg=23.76%)▶tial1 (bg=15.02%)▶u2af1 (bg=14.02%)▶u2af2 (bg=19.32%)▶ZRANB2 (bg=7.28%)No matches to TargetScan


GAAGCC

GAAGCC  
Depth:11 (X.TROPICALIS)  
Ei-value:0.000, Pi-value:0.000  
Er-value:0.000, Pr-value:0.000  
eCLIP MATCHES▶ddx42 (bg=10.33%)▶khsrp (bg=27.4%)▶SF3B4 (bg=4.76%)▶TARDBP (bg=5.12%)▶tia1 (bg=23.76%)▶tial1 (bg=15.02%)▶u2af1 (bg=14.02%)▶u2af2 (bg=19.32%)▶ZRANB2 (bg=7.28%)No matches to TargetScan


TTTAGT

TGAAGCCTTTAGT  
Depth:10 (SNAKE)  
Ei-value:0.000, Pi-value:0.000  
Er-value:0.000, Pr-value:0.000  
eCLIP MATCHES▶ddx42 (bg=10.33%)▶khsrp (bg=27.4%)▶SF3B4 (bg=4.76%)▶SMNDC1 (bg=7.08%)▶TARDBP (bg=5.12%)▶tia1 (bg=23.76%)▶tial1 (bg=15.02%)▶u2af1 (bg=14.02%)▶u2af2 (bg=19.32%)▶ZRANB2 (bg=7.28%)No matches to TargetScan


CT

TGAAGCCTTTAGTCT  
Depth:8 (ALLIGATOR)  
Ei-value:0.000, Pi-value:0.000  
Er-value:0.000, Pr-value:0.000  
eCLIP MATCHES▶ddx42 (bg=10.33%)▶khsrp (bg=27.4%)▶SF3B4 (bg=4.76%)▶SMNDC1 (bg=7.08%)▶TARDBP (bg=5.12%)▶tia1 (bg=23.76%)▶tial1 (bg=15.02%)▶u2af1 (bg=14.02%)▶u2af2 (bg=19.32%)▶ZRANB2 (bg=7.28%)No matches to TargetScan

-5631--(1)--5633-

TTCCAGAT

TTCCAGAT  
Depth:10 (SNAKE)  
Ei-value:0.000, Pi-value:0.000  
Er-value:0.000, Pr-value:0.000  
eCLIP MATCHES▶ddx42 (bg=10.33%)▶khsrp (bg=27.4%)▶ppil4 (bg=43.39%)▶SF3B4 (bg=4.76%)▶SMNDC1 (bg=7.08%)▶tia1 (bg=23.76%)▶tial1 (bg=15.02%)▶u2af1 (bg=14.02%)▶u2af2 (bg=19.32%)▶ZRANB2 (bg=7.28%)No matches to TargetScan

-5640--(59)--5700-

AACT

AACTGGCAAGTG  
Depth:8 (ALLIGATOR)  
Ei-value:0.000, Pi-value:0.000  
Er-value:0.000, Pr-value:0.000  
eCLIP MATCHES▶bclaf1 (bg=17.67%)▶khsrp (bg=27.4%)▶srsf7 (bg=22.53%)▶u2af2 (bg=19.32%)▶znf622 (bg=18.79%)No matches to TargetScan


GGCAAGT

GGCAAGT  
Depth:10 (SNAKE)  
Ei-value:0.000, Pi-value:0.000  
Er-value:0.000, Pr-value:0.000  
eCLIP MATCHES▶bclaf1 (bg=17.67%)▶khsrp (bg=27.4%)▶srsf7 (bg=22.53%)▶u2af2 (bg=19.32%)▶znf622 (bg=18.79%)No matches to TargetScan


G

AACTGGCAAGTG  
Depth:8 (ALLIGATOR)  
Ei-value:0.000, Pi-value:0.000  
Er-value:0.000, Pr-value:0.000  
eCLIP MATCHES▶bclaf1 (bg=17.67%)▶khsrp (bg=27.4%)▶srsf7 (bg=22.53%)▶u2af2 (bg=19.32%)▶znf622 (bg=18.79%)No matches to TargetScan

-5711--(12)--5724-

CAGTTC

CAGTTC  
Depth:8 (ALLIGATOR)  
Ei-value:0.000, Pi-value:0.000  
Er-value:0.000, Pr-value:0.000  
eCLIP MATCHES▶khsrp (bg=27.4%)▶srsf7 (bg=22.53%)▶tia1 (bg=23.76%)▶tial1 (bg=15.02%)▶u2af2 (bg=19.32%)No matches to TargetScan

-5729--(119)--5849-

AGTAAG

AGTAAG  
Depth:8 (ALLIGATOR)  
Ei-value:0.000, Pi-value:0.000  
Er-value:0.000, Pr-value:0.010  
eCLIP MATCHES▶ddx42 (bg=10.33%)▶u2af1 (bg=14.02%)▶u2af2 (bg=19.32%)▶ZRANB2 (bg=7.28%)No matches to TargetScan

-5854--(66)--5921-

TAGTTCTT

TAGTTCTT  
Depth:8 (ALLIGATOR)  
Ei-value:0.000, Pi-value:0.000  
Er-value:0.000, Pr-value:0.000  
eCLIP MATCHES▶ddx42 (bg=10.33%)▶khsrp (bg=27.4%)▶SF3B4 (bg=4.76%)▶SLBP (bg=6.66%)▶srsf7 (bg=22.53%)▶tia1 (bg=23.76%)▶tial1 (bg=15.02%)▶u2af1 (bg=14.02%)▶u2af2 (bg=19.32%)▶zc3h8 (bg=12.78%)▶ZRANB2 (bg=7.28%)No matches to TargetScan

-5928--(25)--5954-

AAGGAGC

AAGGAGC  
Depth:8 (ALLIGATOR)  
Ei-value:0.000, Pi-value:0.000  
Er-value:0.000, Pr-value:0.000  
eCLIP MATCHES▶GRWD1 (bg=7.0%)▶hnrnpa1 (bg=18.32%)▶ppil4 (bg=43.39%)▶srsf1 (bg=30.28%)▶srsf7 (bg=22.53%)▶tia1 (bg=23.76%)▶tial1 (bg=15.02%)▶u2af1 (bg=14.02%)▶u2af2 (bg=19.32%)▶zc3h8 (bg=12.78%)▶ZRANB2 (bg=7.28%)No matches to TargetScan

-5960--(35)--5996-

AAC

AACAGGTGAA  
Depth:10 (SNAKE)  
Ei-value:0.000, Pi-value:0.000  
Er-value:0.000, Pr-value:0.000  
eCLIP MATCHES▶GRWD1 (bg=7.0%)▶srsf1 (bg=30.28%)▶srsf7 (bg=22.53%)No matches to TargetScan


AGGTGA

AGGTGA  
Depth:11 (X.TROPICALIS)  
Ei-value:0.000, Pi-value:0.000  
Er-value:0.000, Pr-value:0.010  
eCLIP MATCHES▶GRWD1 (bg=7.0%)▶srsf1 (bg=30.28%)▶srsf7 (bg=22.53%)No matches to TargetScan


A

AACAGGTGAA  
Depth:10 (SNAKE)  
Ei-value:0.000, Pi-value:0.000  
Er-value:0.000, Pr-value:0.000  
eCLIP MATCHES▶GRWD1 (bg=7.0%)▶srsf1 (bg=30.28%)▶srsf7 (bg=22.53%)No matches to TargetScan

-6005--(65)--6071-

CAGCTT

CAGCTT  
Depth:8 (ALLIGATOR)  
Ei-value:0.000, Pi-value:0.000  
Er-value:0.000, Pr-value:0.010  
eCLIP MATCHES▶hnrnpa1 (bg=18.32%)▶SLBP (bg=6.66%)▶tia1 (bg=23.76%)▶tial1 (bg=15.02%)▶zc3h8 (bg=12.78%)No matches to TargetScan

-6076--(77)--6154-

TTAAGA

TTAAGA  
Depth:8 (ALLIGATOR)  
Ei-value:0.000, Pi-value:0.010  
Er-value:0.000, Pr-value:0.010  
eCLIP MATCHES▶ddx42 (bg=10.33%)▶HNRNPU (bg=9.45%)▶tial1 (bg=15.02%)No matches to TargetScan

-6159--(30)--6190-

AAGGCT

AAGGCT  
Depth:10 (SNAKE)  
Ei-value:0.000, Pi-value:0.000  
Er-value:0.000, Pr-value:0.010  
eCLIP MATCHES▶HNRNPU (bg=9.45%)▶tial1 (bg=15.02%)No matches to TargetScan


T

AAGGCTT  
Depth:9 (LIZARD)  
Ei-value:0.000, Pi-value:0.000  
Er-value:0.000, Pr-value:0.000  
eCLIP MATCHES▶HNRNPU (bg=9.45%)▶tial1 (bg=15.02%)No matches to TargetScan

-6196--(246)--6443-

AGGGGA

AGGGGA  
Depth:8 (ALLIGATOR)  
Ei-value:0.000, Pi-value:0.000  
Er-value:0.000, Pr-value:0.000  
eCLIP MATCHES▶ddx42 (bg=10.33%)▶DDX51 (bg=0.19%)▶SERBP1 (bg=0.36%)No matches to TargetScan

-6448--(19)--6468-

CAACCA

CAACCA  
Depth:8 (ALLIGATOR)  
Ei-value:0.000, Pi-value:0.000  
Er-value:0.000, Pr-value:0.000  
eCLIP MATCHES▶ddx42 (bg=10.33%)No matches to TargetScan

-6473--(12)--6486-

TTTTCCAG

TTTTCCAG  
Depth:10 (SNAKE)  
Ei-value:0.000, Pi-value:0.000  
Er-value:0.000, Pr-value:0.000  
No matches to eCLIP DataNo matches to TargetScan


AA

TTTTCCAGAA  
Depth:8 (ALLIGATOR)  
Ei-value:0.000, Pi-value:0.000  
Er-value:0.000, Pr-value:0.000  
No matches to eCLIP DataNo matches to TargetScan

-6495--(111)--6607-

TGGATCCT

TGGATCCT  
Depth:10 (SNAKE)  
Ei-value:0.000, Pi-value:0.000  
Er-value:0.000, Pr-value:0.000  
No matches to eCLIP DataNo matches to TargetScan


TG

TGGATCCTTG  
Depth:8 (ALLIGATOR)  
Ei-value:0.000, Pi-value:0.000  
Er-value:0.000, Pr-value:0.000  
No matches to eCLIP DataNo matches to TargetScan

-6616--(24)--6641-

AAGGTAA

AAGGTAA  
Depth:10 (SNAKE)  
Ei-value:0.000, Pi-value:0.000  
Er-value:0.000, Pr-value:0.000  
eCLIP MATCHES▶PRPF8 (bg=6.2%)No matches to TargetScan

-6647--(246)--6894-

GGGTGGG

GGGTGGG  
Depth:10 (SNAKE)  
Ei-value:0.000, Pi-value:0.000  
Er-value:0.000, Pr-value:0.000  
eCLIP MATCHES▶ddx42 (bg=10.33%)▶khsrp (bg=27.4%)▶QKI (bg=3.74%)▶SF3B4 (bg=4.76%)No matches to TargetScan

-6900--(15)--6916-

TGCTAAGACT

TGCTAAGACT  
Depth:10 (SNAKE)  
Ei-value:0.000, Pi-value:0.000  
Er-value:0.000, Pr-value:0.000  
eCLIP MATCHES▶ddx42 (bg=10.33%)▶khsrp (bg=27.4%)▶QKI (bg=3.74%)▶SF3B4 (bg=4.76%)No matches to TargetScan


TTTTCAGGTG

TGCTAAGACTTTTTCAGGTG  
Depth:8 (ALLIGATOR)  
Ei-value:0.000, Pi-value:0.000  
Er-value:0.000, Pr-value:0.000  
eCLIP MATCHES▶ddx42 (bg=10.33%)▶khsrp (bg=27.4%)▶QKI (bg=3.74%)▶SF3B4 (bg=4.76%)▶srsf7 (bg=22.53%)▶tia1 (bg=23.76%)▶u2af2 (bg=19.32%)No matches to TargetScan

-6935--(75)--7011-

TTTTCTTTT

TTTTCTTTT  
Depth:19 (ZEBRAFISH)  
Ei-value:0.000, Pi-value:0.000  
Er-value:0.000, Pr-value:0.000  
eCLIP MATCHES▶srsf7 (bg=22.53%)MATCHES To TargetScan▶ miR-186-5p:AAAGAAU


CCTGAGA

TTTTCTTTTCCTGAGA  
Depth:10 (SNAKE)  
Ei-value:0.000, Pi-value:0.000  
Er-value:0.000, Pr-value:0.000  
eCLIP MATCHES▶srsf7 (bg=22.53%)MATCHES To TargetScan▶ miR-873-5p.1:CAGGAAC▶ miR-186-5p:AAAGAAU

-7026--(18)--7045-

TCT

TCTCAGGTTTTGCTTTT  
Depth:10 (SNAKE)  
Ei-value:0.000, Pi-value:0.000  
Er-value:0.000, Pr-value:0.000  
eCLIP MATCHES▶srsf7 (bg=22.53%)MATCHES To TargetScan▶ miR-330-3p.2:AAAGCAC▶ miR-490-3p:AACCUGG


CAGGTTTTGCTTT

CAGGTTTTGCTTT  
Depth:19 (ZEBRAFISH)  
Ei-value:0.000, Pi-value:0.000  
Er-value:0.000, Pr-value:0.000  
eCLIP MATCHES▶srsf7 (bg=22.53%)MATCHES To TargetScan▶ miR-330-3p.2:AAAGCAC▶ miR-490-3p:AACCUGG


T

CAGGTTTTGCTTTT  
Depth:16 (NILETILAPIA)  
Ei-value:0.000, Pi-value:0.000  
Er-value:0.000, Pr-value:0.000  
eCLIP MATCHES▶srsf7 (bg=22.53%)MATCHES To TargetScan▶ miR-330-3p.2:AAAGCAC▶ miR-490-3p:AACCUGG

-7061--(24)--7086-

A

AAAAAAGCAAAAG  
Depth:13 (OPOSSUM)  
Ei-value:0.000, Pi-value:0.000  
Er-value:0.000, Pr-value:0.000  
No matches to eCLIP DataNo matches to TargetScan


AAAAAGCAAAA

AAAAAGCAAAA  
Depth:19 (ZEBRAFISH)  
Ei-value:0.000, Pi-value:0.000  
Er-value:0.000, Pr-value:0.000  
No matches to eCLIP DataNo matches to TargetScan


G

AAAAAGCAAAAG  
Depth:18 (MEDAKA)  
Ei-value:0.000, Pi-value:0.000  
Er-value:0.000, Pr-value:0.000  
No matches to eCLIP DataNo matches to TargetScan


A

AAAAAAGCAAAAGA  
Depth:9 (LIZARD)  
Ei-value:0.000, Pi-value:0.000  
Er-value:0.000, Pr-value:0.000  
No matches to eCLIP DataNo matches to TargetScan

-7099--(1)--7101-

GCTGGT

GCTGGT  
Depth:9 (LIZARD)  
Ei-value:0.000, Pi-value:0.000  
Er-value:0.000, Pr-value:0.000  
No matches to eCLIP DataNo matches to TargetScan

-7106--(7)--7114-

ACTCCTG

ACTCCTG  
Depth:18 (MEDAKA)  
Ei-value:0.000, Pi-value:0.000  
Er-value:0.000, Pr-value:0.000  
No matches to eCLIP DataNo matches to TargetScan


G

ACTCCTGG  
Depth:14 (SPOTTEDGAR)  
Ei-value:0.000, Pi-value:0.000  
Er-value:0.000, Pr-value:0.000  
No matches to eCLIP DataMATCHES To TargetScan▶ miR-665:CCAGGAG

-7121--(2)--7124-

TCCAGGA

TCCAGGA  
Depth:8 (ALLIGATOR)  
Ei-value:0.000, Pi-value:0.000  
Er-value:0.000, Pr-value:0.000  
No matches to eCLIP DataNo matches to TargetScan

-7130--(2)--7133-

GGGTTC

GGGTTC  
Depth:13 (OPOSSUM)  
Ei-value:0.000, Pi-value:0.000  
Er-value:0.000, Pr-value:0.000  
No matches to eCLIP DataNo matches to TargetScan


AA

GGGTTCAA  
Depth:9 (LIZARD)  
Ei-value:0.000, Pi-value:0.000  
Er-value:0.000, Pr-value:0.000  
No matches to eCLIP DataNo matches to TargetScan

-7140--(12)--7153-

TCTTTGCT

TCTTTGCT  
Depth:8 (ALLIGATOR)  
Ei-value:0.000, Pi-value:0.000  
Er-value:0.000, Pr-value:0.000  
No matches to eCLIP DataNo matches to TargetScan

-7160  
  
>MARMOSET  
      1435-

TTGGACT

TTGGACT  
Depth:9 (LIZARD)  
Ei-value:0.000, Pi-value:0.000  
Er-value:0.000, Pr-value:0.000  
No matches to TargetScan

-1441--(30)--1472-

GACTAG

GACTAG  
Depth:8 (ALLIGATOR)  
Ei-value:0.000, Pi-value:0.000  
Er-value:0.000, Pr-value:0.000  
No matches to TargetScan

-1477--(55)--1533-

AAGACAG

AAGACAG  
Depth:8 (ALLIGATOR)  
Ei-value:0.000, Pi-value:0.000  
Er-value:0.000, Pr-value:0.000  
No matches to TargetScan

-1539--(147)--1687-

GGTGAAG

GGTGAAG  
Depth:11 (X.TROPICALIS)  
Ei-value:0.000, Pi-value:0.000  
Er-value:0.000, Pr-value:0.000  
No matches to TargetScan

-1693--(37)--1731-

TGGTGA

TGGTGA  
Depth:8 (ALLIGATOR)  
Ei-value:0.000, Pi-value:0.000  
Er-value:0.000, Pr-value:0.000  
No matches to TargetScan

-1736--(171)--1908-

TGGTAA

TGGTAA  
Depth:14 (SPOTTEDGAR)  
Ei-value:0.030, Pi-value:0.000  
Er-value:0.010, Pr-value:0.000  
No matches to TargetScan

-1913--(92)--2006-

GTAGGTTT

GTAGGTTT  
Depth:8 (ALLIGATOR)  
Ei-value:0.000, Pi-value:0.000  
Er-value:0.000, Pr-value:0.000  
No matches to TargetScan

-2013--(437)--2451-

TCAGGATAA

TCAGGATAA  
Depth:8 (ALLIGATOR)  
Ei-value:0.000, Pi-value:0.000  
Er-value:0.000, Pr-value:0.000  
No matches to TargetScan

-2459--(177)--2637-

TTTGGG

TTTGGG  
Depth:19 (ZEBRAFISH)  
Ei-value:0.000, Pi-value:0.000  
Er-value:0.000, Pr-value:0.000  
No matches to TargetScan

-2642--(44)--2687-

TTTCTA

TTTCTA  
Depth:8 (ALLIGATOR)  
Ei-value:0.000, Pi-value:0.000  
Er-value:0.000, Pr-value:0.000  
No matches to TargetScan

-2692--(16)--2709-

CTATAGT

CTATAGT  
Depth:8 (ALLIGATOR)  
Ei-value:0.000, Pi-value:0.000  
Er-value:0.000, Pr-value:0.000  
No matches to TargetScan

-2715--(16)--2732-

GTTAGA

GTTAGA  
Depth:8 (ALLIGATOR)  
Ei-value:0.000, Pi-value:0.000  
Er-value:0.000, Pr-value:0.000  
No matches to TargetScan

-2737--(112)--2850-

TGGTTC

TGGTTC  
Depth:8 (ALLIGATOR)  
Ei-value:0.000, Pi-value:0.000  
Er-value:0.000, Pr-value:0.000  
No matches to TargetScan

-2855--(30)--2886-

GAGCTT

GAGCTT  
Depth:9 (LIZARD)  
Ei-value:0.000, Pi-value:0.000  
Er-value:0.000, Pr-value:0.000  
No matches to TargetScan

-2891--(17)--2909-

AAGTTAAGTT

AAGTTAAGTT  
Depth:8 (ALLIGATOR)  
Ei-value:0.000, Pi-value:0.000  
Er-value:0.000, Pr-value:0.000  
No matches to TargetScan

-2918--(195)--3114-

T

TGGCAAGTAA  
Depth:8 (ALLIGATOR)  
Ei-value:0.000, Pi-value:0.000  
Er-value:0.000, Pr-value:0.000  
No matches to TargetScan


GGCAAGTAA

GGCAAGTAA  
Depth:10 (SNAKE)  
Ei-value:0.000, Pi-value:0.000  
Er-value:0.000, Pr-value:0.000  
No matches to TargetScan

-3123--(243)--3367-

GTGTGTGG

GTGTGTGG  
Depth:8 (ALLIGATOR)  
Ei-value:0.000, Pi-value:0.000  
Er-value:0.000, Pr-value:0.000  
MATCHES To TargetScan▶ miR-329-3p/362-3p:ACACACC

-3374--(20)--3395-

CAGGTG

CAGGTG  
Depth:8 (ALLIGATOR)  
Ei-value:0.000, Pi-value:0.000  
Er-value:0.000, Pr-value:0.000  
No matches to TargetScan

-3400--(51)--3452-

TTAATGA

TTAATGA  
Depth:8 (ALLIGATOR)  
Ei-value:0.000, Pi-value:0.000  
Er-value:0.000, Pr-value:0.000  
No matches to TargetScan

-3458--(38)--3497-

T

TCAAGTAAGA  
Depth:9 (LIZARD)  
Ei-value:0.000, Pi-value:0.000  
Er-value:0.000, Pr-value:0.000  
No matches to TargetScan


CAA

CAAGTAAGA  
Depth:10 (SNAKE)  
Ei-value:0.000, Pi-value:0.000  
Er-value:0.000, Pr-value:0.000  
No matches to TargetScan


GTAAGA

GTAAGA  
Depth:11 (X.TROPICALIS)  
Ei-value:0.000, Pi-value:0.000  
Er-value:0.000, Pr-value:0.000  
No matches to TargetScan

-3506--(231)--3738-

TTTTTCAG

TTTTTCAG  
Depth:19 (ZEBRAFISH)  
Ei-value:0.000, Pi-value:0.000  
Er-value:0.000, Pr-value:0.000  
No matches to TargetScan


AT

TTTTTCAGAT  
Depth:10 (SNAKE)  
Ei-value:0.000, Pi-value:0.000  
Er-value:0.000, Pr-value:0.000  
No matches to TargetScan

-3747--(71)--3819-

TGATAAGT

TGATAAGT  
Depth:9 (LIZARD)  
Ei-value:0.000, Pi-value:0.000  
Er-value:0.000, Pr-value:0.000  
No matches to TargetScan


AAAGGCAGAA

AAAGGCAGAA  
Depth:13 (OPOSSUM)  
Ei-value:0.000, Pi-value:0.000  
Er-value:0.000, Pr-value:0.000  
No matches to TargetScan


A

AAAGGCAGAAA  
Depth:11 (X.TROPICALIS)  
Ei-value:0.000, Pi-value:0.000  
Er-value:0.000, Pr-value:0.000  
No matches to TargetScan


A

AAAGGCAGAAAA  
Depth:9 (LIZARD)  
Ei-value:0.000, Pi-value:0.000  
Er-value:0.000, Pr-value:0.000  
No matches to TargetScan

-3838--(243)--4082-

CAGAAT

CAGAAT  
Depth:8 (ALLIGATOR)  
Ei-value:0.000, Pi-value:0.000  
Er-value:0.000, Pr-value:0.000  
No matches to TargetScan

-4087--(76)--4164-

TGGGATC

TGGGATC  
Depth:8 (ALLIGATOR)  
Ei-value:0.000, Pi-value:0.000  
Er-value:0.000, Pr-value:0.000  
No matches to TargetScan

-4170--(47)--4218-

AG

AGACAGGT  
Depth:9 (LIZARD)  
Ei-value:0.000, Pi-value:0.000  
Er-value:0.000, Pr-value:0.000  
No matches to TargetScan


ACAGGT

ACAGGT  
Depth:10 (SNAKE)  
Ei-value:0.000, Pi-value:0.000  
Er-value:0.000, Pr-value:0.000  
No matches to TargetScan

-4225--(21)--4247-

TTGCTTCA

TTGCTTCA  
Depth:8 (ALLIGATOR)  
Ei-value:0.000, Pi-value:0.000  
Er-value:0.000, Pr-value:0.000  
No matches to TargetScan

-4254--(37)--4292-

CA

CAGATAAGT  
Depth:11 (X.TROPICALIS)  
Ei-value:0.000, Pi-value:0.000  
Er-value:0.000, Pr-value:0.000  
No matches to TargetScan


GATAAG

GATAAG  
Depth:19 (ZEBRAFISH)  
Ei-value:0.000, Pi-value:0.000  
Er-value:0.000, Pr-value:0.000  
No matches to TargetScan


T

CAGATAAGT  
Depth:11 (X.TROPICALIS)  
Ei-value:0.000, Pi-value:0.000  
Er-value:0.000, Pr-value:0.000  
No matches to TargetScan

-4300--(17)--4318-

T

TATTGCATG  
Depth:8 (ALLIGATOR)  
Ei-value:0.000, Pi-value:0.000  
Er-value:0.000, Pr-value:0.000  
No matches to TargetScan


ATTGCAT

ATTGCAT  
Depth:11 (X.TROPICALIS)  
Ei-value:0.000, Pi-value:0.000  
Er-value:0.000, Pr-value:0.000  
No matches to TargetScan


G

ATTGCATG  
Depth:9 (LIZARD)  
Ei-value:0.000, Pi-value:0.000  
Er-value:0.000, Pr-value:0.000  
No matches to TargetScan

-4326--(122)--4449-

TTAAAAT

TTAAAAT  
Depth:8 (ALLIGATOR)  
Ei-value:0.000, Pi-value:0.000  
Er-value:0.000, Pr-value:0.000  
No matches to TargetScan

-4455--(301)--4757-

GCTGTC

GCTGTC  
Depth:9 (LIZARD)  
Ei-value:0.000, Pi-value:0.000  
Er-value:0.000, Pr-value:0.000  
No matches to TargetScan

-4762--(69)--4832-

TTTCAGA

TTTCAGA  
Depth:8 (ALLIGATOR)  
Ei-value:0.000, Pi-value:0.000  
Er-value:0.000, Pr-value:0.000  
No matches to TargetScan

-4838--(71)--4910-

AGGCAGGA

AGGCAGGA  
Depth:10 (SNAKE)  
Ei-value:0.000, Pi-value:0.000  
Er-value:0.000, Pr-value:0.000  
No matches to TargetScan


AA

AGGCAGGAAA  
Depth:8 (ALLIGATOR)  
Ei-value:0.000, Pi-value:0.000  
Er-value:0.000, Pr-value:0.000  
No matches to TargetScan

-4919--(70)--4990-

TAAGGA

TAAGGA  
Depth:13 (OPOSSUM)  
Ei-value:0.000, Pi-value:0.000  
Er-value:0.000, Pr-value:0.000  
No matches to TargetScan

-4995--(130)--5126-

TGCATG

TGCATG  
Depth:8 (ALLIGATOR)  
Ei-value:0.000, Pi-value:0.000  
Er-value:0.000, Pr-value:0.000  
No matches to TargetScan

-5131--(115)--5247-

TTTCAT

TTTCAT  
Depth:8 (ALLIGATOR)  
Ei-value:0.000, Pi-value:0.000  
Er-value:0.000, Pr-value:0.000  
No matches to TargetScan

-5252--(53)--5306-

TTCTGT

TTCTGT  
Depth:9 (LIZARD)  
Ei-value:0.000, Pi-value:0.000  
Er-value:0.000, Pr-value:0.000  
No matches to TargetScan

-5311--(24)--5336-

TGCAGTG

TGCAGTG  
Depth:10 (SNAKE)  
Ei-value:0.000, Pi-value:0.000  
Er-value:0.000, Pr-value:0.000  
MATCHES To TargetScan▶ miR-217:ACUGCAU

-5342--(100)--5443-

AGAATGCAG

AGAATGCAG  
Depth:8 (ALLIGATOR)  
Ei-value:0.000, Pi-value:0.000  
Er-value:0.000, Pr-value:0.000  
MATCHES To TargetScan▶ miR-33-5p:UGCAUUG

-5451--(29)--5481-

TTGGCAAGTAA

TTGGCAAGTAA  
Depth:10 (SNAKE)  
Ei-value:0.000, Pi-value:0.000  
Er-value:0.000, Pr-value:0.000  
No matches to TargetScan

-5491--(90)--5582-

TGGAGTAGTG

TGGAGTAGTG  
Depth:9 (LIZARD)  
Ei-value:0.000, Pi-value:0.000  
Er-value:0.000, Pr-value:0.000  
No matches to TargetScan

-5591--(114)--5706-

TTAGGT

TTAGGT  
Depth:11 (X.TROPICALIS)  
Ei-value:0.000, Pi-value:0.000  
Er-value:0.000, Pr-value:0.000  
No matches to TargetScan


AA

TTAGGTAA  
Depth:10 (SNAKE)  
Ei-value:0.000, Pi-value:0.000  
Er-value:0.000, Pr-value:0.000  
No matches to TargetScan

-5713--(36)--5750-

T

TGAAGCCTTTAGT  
Depth:10 (SNAKE)  
Ei-value:0.000, Pi-value:0.000  
Er-value:0.000, Pr-value:0.000  
No matches to TargetScan


GAAGCC

GAAGCC  
Depth:11 (X.TROPICALIS)  
Ei-value:0.000, Pi-value:0.000  
Er-value:0.000, Pr-value:0.000  
No matches to TargetScan


TTTAGT

TGAAGCCTTTAGT  
Depth:10 (SNAKE)  
Ei-value:0.000, Pi-value:0.000  
Er-value:0.000, Pr-value:0.000  
No matches to TargetScan


CT

TGAAGCCTTTAGTCT  
Depth:8 (ALLIGATOR)  
Ei-value:0.000, Pi-value:0.000  
Er-value:0.000, Pr-value:0.000  
No matches to TargetScan

-5764--(1)--5766-

TTCCAGAT

TTCCAGAT  
Depth:10 (SNAKE)  
Ei-value:0.000, Pi-value:0.000  
Er-value:0.000, Pr-value:0.000  
No matches to TargetScan

-5773--(61)--5835-

AACT

AACTGGCAAGTG  
Depth:8 (ALLIGATOR)  
Ei-value:0.000, Pi-value:0.000  
Er-value:0.000, Pr-value:0.000  
No matches to TargetScan


GGCAAGT

GGCAAGT  
Depth:10 (SNAKE)  
Ei-value:0.000, Pi-value:0.000  
Er-value:0.000, Pr-value:0.000  
No matches to TargetScan


G

AACTGGCAAGTG  
Depth:8 (ALLIGATOR)  
Ei-value:0.000, Pi-value:0.000  
Er-value:0.000, Pr-value:0.000  
No matches to TargetScan

-5846--(11)--5858-

CAGTTC

CAGTTC  
Depth:8 (ALLIGATOR)  
Ei-value:0.000, Pi-value:0.000  
Er-value:0.000, Pr-value:0.000  
No matches to TargetScan

-5863--(123)--5987-

AGTAAG

AGTAAG  
Depth:8 (ALLIGATOR)  
Ei-value:0.000, Pi-value:0.000  
Er-value:0.000, Pr-value:0.010  
No matches to TargetScan

-5992--(67)--6060-

TAGTTCTT

TAGTTCTT  
Depth:8 (ALLIGATOR)  
Ei-value:0.000, Pi-value:0.000  
Er-value:0.000, Pr-value:0.000  
No matches to TargetScan

-6067--(25)--6093-

AAGGAGC

AAGGAGC  
Depth:8 (ALLIGATOR)  
Ei-value:0.000, Pi-value:0.000  
Er-value:0.000, Pr-value:0.000  
No matches to TargetScan

-6099--(35)--6135-

AAC

AACAGGTGAA  
Depth:10 (SNAKE)  
Ei-value:0.000, Pi-value:0.000  
Er-value:0.000, Pr-value:0.000  
No matches to TargetScan


AGGTGA

AGGTGA  
Depth:11 (X.TROPICALIS)  
Ei-value:0.000, Pi-value:0.000  
Er-value:0.000, Pr-value:0.010  
No matches to TargetScan


A

AACAGGTGAA  
Depth:10 (SNAKE)  
Ei-value:0.000, Pi-value:0.000  
Er-value:0.000, Pr-value:0.000  
No matches to TargetScan

-6144--(70)--6215-

CAGCTT

CAGCTT  
Depth:8 (ALLIGATOR)  
Ei-value:0.000, Pi-value:0.000  
Er-value:0.000, Pr-value:0.010  
No matches to TargetScan

-6220--(77)--6298-

TTAAGA

TTAAGA  
Depth:8 (ALLIGATOR)  
Ei-value:0.000, Pi-value:0.010  
Er-value:0.000, Pr-value:0.010  
No matches to TargetScan

-6303--(30)--6334-

AAGGCT

AAGGCT  
Depth:10 (SNAKE)  
Ei-value:0.000, Pi-value:0.000  
Er-value:0.000, Pr-value:0.010  
No matches to TargetScan


T

AAGGCTT  
Depth:9 (LIZARD)  
Ei-value:0.000, Pi-value:0.000  
Er-value:0.000, Pr-value:0.000  
No matches to TargetScan

-6340--(242)--6583-

AGGGGA

AGGGGA  
Depth:8 (ALLIGATOR)  
Ei-value:0.000, Pi-value:0.000  
Er-value:0.000, Pr-value:0.000  
No matches to TargetScan

-6588--(19)--6608-

CAACCA

CAACCA  
Depth:8 (ALLIGATOR)  
Ei-value:0.000, Pi-value:0.000  
Er-value:0.000, Pr-value:0.000  
No matches to TargetScan

-6613--(12)--6626-

TTTTCCAG

TTTTCCAG  
Depth:10 (SNAKE)  
Ei-value:0.000, Pi-value:0.000  
Er-value:0.000, Pr-value:0.000  
No matches to TargetScan


AA

TTTTCCAGAA  
Depth:8 (ALLIGATOR)  
Ei-value:0.000, Pi-value:0.000  
Er-value:0.000, Pr-value:0.000  
No matches to TargetScan

-6635--(111)--6747-

TGGATCCT

TGGATCCT  
Depth:10 (SNAKE)  
Ei-value:0.000, Pi-value:0.000  
Er-value:0.000, Pr-value:0.000  
No matches to TargetScan


TG

TGGATCCTTG  
Depth:8 (ALLIGATOR)  
Ei-value:0.000, Pi-value:0.000  
Er-value:0.000, Pr-value:0.000  
No matches to TargetScan

-6756--(24)--6781-

AAGGTAA

AAGGTAA  
Depth:10 (SNAKE)  
Ei-value:0.000, Pi-value:0.000  
Er-value:0.000, Pr-value:0.000  
No matches to TargetScan

-6787--(235)--7023-

GGGTGGG

GGGTGGG  
Depth:10 (SNAKE)  
Ei-value:0.000, Pi-value:0.000  
Er-value:0.000, Pr-value:0.000  
No matches to TargetScan

-7029--(15)--7045-

TGCTAAGACT

TGCTAAGACT  
Depth:10 (SNAKE)  
Ei-value:0.000, Pi-value:0.000  
Er-value:0.000, Pr-value:0.000  
No matches to TargetScan


TTTTCAGGTG

TGCTAAGACTTTTTCAGGTG  
Depth:8 (ALLIGATOR)  
Ei-value:0.000, Pi-value:0.000  
Er-value:0.000, Pr-value:0.000  
No matches to TargetScan

-7064--(75)--7140-

TTTTCTTTT

TTTTCTTTT  
Depth:19 (ZEBRAFISH)  
Ei-value:0.000, Pi-value:0.000  
Er-value:0.000, Pr-value:0.000  
MATCHES To TargetScan▶ miR-186-5p:AAAGAAU


CCTGAGA

TTTTCTTTTCCTGAGA  
Depth:10 (SNAKE)  
Ei-value:0.000, Pi-value:0.000  
Er-value:0.000, Pr-value:0.000  
MATCHES To TargetScan▶ miR-873-5p.1:CAGGAAC▶ miR-186-5p:AAAGAAU

-7155--(18)--7174-

TCT

TCTCAGGTTTTGCTTTT  
Depth:10 (SNAKE)  
Ei-value:0.000, Pi-value:0.000  
Er-value:0.000, Pr-value:0.000  
MATCHES To TargetScan▶ miR-330-3p.2:AAAGCAC▶ miR-490-3p:AACCUGG


CAGGTTTTGCTTT

CAGGTTTTGCTTT  
Depth:19 (ZEBRAFISH)  
Ei-value:0.000, Pi-value:0.000  
Er-value:0.000, Pr-value:0.000  
MATCHES To TargetScan▶ miR-330-3p.2:AAAGCAC▶ miR-490-3p:AACCUGG


T

CAGGTTTTGCTTTT  
Depth:16 (NILETILAPIA)  
Ei-value:0.000, Pi-value:0.000  
Er-value:0.000, Pr-value:0.000  
MATCHES To TargetScan▶ miR-330-3p.2:AAAGCAC▶ miR-490-3p:AACCUGG

-7190--(25)--7216-

A

AAAAAAGCAAAAG  
Depth:13 (OPOSSUM)  
Ei-value:0.000, Pi-value:0.000  
Er-value:0.000, Pr-value:0.000  
No matches to TargetScan


AAAAAGCAAAA

AAAAAGCAAAA  
Depth:19 (ZEBRAFISH)  
Ei-value:0.000, Pi-value:0.000  
Er-value:0.000, Pr-value:0.000  
No matches to TargetScan


G

AAAAAGCAAAAG  
Depth:18 (MEDAKA)  
Ei-value:0.000, Pi-value:0.000  
Er-value:0.000, Pr-value:0.000  
No matches to TargetScan


A

AAAAAAGCAAAAGA  
Depth:9 (LIZARD)  
Ei-value:0.000, Pi-value:0.000  
Er-value:0.000, Pr-value:0.000  
No matches to TargetScan

-7229--(1)--7231-

GCTGGT

GCTGGT  
Depth:9 (LIZARD)  
Ei-value:0.000, Pi-value:0.000  
Er-value:0.000, Pr-value:0.000  
No matches to TargetScan

-7236--(7)--7244-

ACTCCTG

ACTCCTG  
Depth:18 (MEDAKA)  
Ei-value:0.000, Pi-value:0.000  
Er-value:0.000, Pr-value:0.000  
No matches to TargetScan


G

ACTCCTGG  
Depth:14 (SPOTTEDGAR)  
Ei-value:0.000, Pi-value:0.000  
Er-value:0.000, Pr-value:0.000  
MATCHES To TargetScan▶ miR-665:CCAGGAG

-7251--(2)--7254-

TCCAGGA

TCCAGGA  
Depth:8 (ALLIGATOR)  
Ei-value:0.000, Pi-value:0.000  
Er-value:0.000, Pr-value:0.000  
No matches to TargetScan

-7260--(2)--7263-

GGGTTC

GGGTTC  
Depth:13 (OPOSSUM)  
Ei-value:0.000, Pi-value:0.000  
Er-value:0.000, Pr-value:0.000  
No matches to TargetScan


AA

GGGTTCAA  
Depth:9 (LIZARD)  
Ei-value:0.000, Pi-value:0.000  
Er-value:0.000, Pr-value:0.000  
No matches to TargetScan

-7270--(12)--7283-

TCTTTGCT

TCTTTGCT  
Depth:8 (ALLIGATOR)  
Ei-value:0.000, Pi-value:0.000  
Er-value:0.000, Pr-value:0.000  
No matches to TargetScan

-7290  
  
>DOG  
      1450-

TTGGACT

TTGGACT  
Depth:9 (LIZARD)  
Ei-value:0.000, Pi-value:0.000  
Er-value:0.000, Pr-value:0.000  
No matches to TargetScan

-1456--(30)--1487-

GACTAG

GACTAG  
Depth:8 (ALLIGATOR)  
Ei-value:0.000, Pi-value:0.000  
Er-value:0.000, Pr-value:0.000  
No matches to TargetScan

-1492--(55)--1548-

AAGACAG

AAGACAG  
Depth:8 (ALLIGATOR)  
Ei-value:0.000, Pi-value:0.000  
Er-value:0.000, Pr-value:0.000  
No matches to TargetScan

-1554--(161)--1716-

GGTGAAG

GGTGAAG  
Depth:11 (X.TROPICALIS)  
Ei-value:0.000, Pi-value:0.000  
Er-value:0.000, Pr-value:0.000  
No matches to TargetScan

-1722--(38)--1761-

TGGTGA

TGGTGA  
Depth:8 (ALLIGATOR)  
Ei-value:0.000, Pi-value:0.000  
Er-value:0.000, Pr-value:0.000  
No matches to TargetScan

-1766--(161)--1928-

TGGTAA

TGGTAA  
Depth:14 (SPOTTEDGAR)  
Ei-value:0.030, Pi-value:0.000  
Er-value:0.010, Pr-value:0.000  
No matches to TargetScan

-1933--(89)--2023-

GTAGGTTT

GTAGGTTT  
Depth:8 (ALLIGATOR)  
Ei-value:0.000, Pi-value:0.000  
Er-value:0.000, Pr-value:0.000  
No matches to TargetScan

-2030--(403)--2434-

TCAGGATAA

TCAGGATAA  
Depth:8 (ALLIGATOR)  
Ei-value:0.000, Pi-value:0.000  
Er-value:0.000, Pr-value:0.000  
No matches to TargetScan

-2442--(169)--2612-

TTTGGG

TTTGGG  
Depth:19 (ZEBRAFISH)  
Ei-value:0.000, Pi-value:0.000  
Er-value:0.000, Pr-value:0.000  
No matches to TargetScan

-2617--(40)--2658-

TTTCTA

TTTCTA  
Depth:8 (ALLIGATOR)  
Ei-value:0.000, Pi-value:0.000  
Er-value:0.000, Pr-value:0.000  
No matches to TargetScan

-2663--(16)--2680-

CTATAGT

CTATAGT  
Depth:8 (ALLIGATOR)  
Ei-value:0.000, Pi-value:0.000  
Er-value:0.000, Pr-value:0.000  
No matches to TargetScan

-2686--(16)--2703-

GTTAGA

GTTAGA  
Depth:8 (ALLIGATOR)  
Ei-value:0.000, Pi-value:0.000  
Er-value:0.000, Pr-value:0.000  
No matches to TargetScan

-2708--(113)--2822-

TGGTTC

TGGTTC  
Depth:8 (ALLIGATOR)  
Ei-value:0.000, Pi-value:0.000  
Er-value:0.000, Pr-value:0.000  
No matches to TargetScan

-2827--(30)--2858-

GAGCTT

GAGCTT  
Depth:9 (LIZARD)  
Ei-value:0.000, Pi-value:0.000  
Er-value:0.000, Pr-value:0.000  
No matches to TargetScan

-2863--(17)--2881-

AAGTTAAGTT

AAGTTAAGTT  
Depth:8 (ALLIGATOR)  
Ei-value:0.000, Pi-value:0.000  
Er-value:0.000, Pr-value:0.000  
No matches to TargetScan

-2890--(199)--3090-

T

TGGCAAGTAA  
Depth:8 (ALLIGATOR)  
Ei-value:0.000, Pi-value:0.000  
Er-value:0.000, Pr-value:0.000  
No matches to TargetScan


GGCAAGTAA

GGCAAGTAA  
Depth:10 (SNAKE)  
Ei-value:0.000, Pi-value:0.000  
Er-value:0.000, Pr-value:0.000  
No matches to TargetScan

-3099--(198)--3298-

GTGTGTGG

GTGTGTGG  
Depth:8 (ALLIGATOR)  
Ei-value:0.000, Pi-value:0.000  
Er-value:0.000, Pr-value:0.000  
MATCHES To TargetScan▶ miR-329-3p/362-3p:ACACACC

-3305--(20)--3326-

CAGGTG

CAGGTG  
Depth:8 (ALLIGATOR)  
Ei-value:0.000, Pi-value:0.000  
Er-value:0.000, Pr-value:0.000  
No matches to TargetScan

-3331--(51)--3383-

TTAATGA

TTAATGA  
Depth:8 (ALLIGATOR)  
Ei-value:0.000, Pi-value:0.000  
Er-value:0.000, Pr-value:0.000  
No matches to TargetScan

-3389--(39)--3429-

T

TCAAGTAAGA  
Depth:9 (LIZARD)  
Ei-value:0.000, Pi-value:0.000  
Er-value:0.000, Pr-value:0.000  
No matches to TargetScan


CAA

CAAGTAAGA  
Depth:10 (SNAKE)  
Ei-value:0.000, Pi-value:0.000  
Er-value:0.000, Pr-value:0.000  
No matches to TargetScan


GTAAGA

GTAAGA  
Depth:11 (X.TROPICALIS)  
Ei-value:0.000, Pi-value:0.000  
Er-value:0.000, Pr-value:0.000  
No matches to TargetScan

-3438--(192)--3631-

TTTTTCAG

TTTTTCAG  
Depth:19 (ZEBRAFISH)  
Ei-value:0.000, Pi-value:0.000  
Er-value:0.000, Pr-value:0.000  
No matches to TargetScan


AT

TTTTTCAGAT  
Depth:10 (SNAKE)  
Ei-value:0.000, Pi-value:0.000  
Er-value:0.000, Pr-value:0.000  
No matches to TargetScan

-3640--(69)--3710-

TGATAAGT

TGATAAGT  
Depth:9 (LIZARD)  
Ei-value:0.000, Pi-value:0.000  
Er-value:0.000, Pr-value:0.000  
No matches to TargetScan


AAAGGCAGAA

AAAGGCAGAA  
Depth:13 (OPOSSUM)  
Ei-value:0.000, Pi-value:0.000  
Er-value:0.000, Pr-value:0.000  
No matches to TargetScan


A

AAAGGCAGAAA  
Depth:11 (X.TROPICALIS)  
Ei-value:0.000, Pi-value:0.000  
Er-value:0.000, Pr-value:0.000  
No matches to TargetScan


A

AAAGGCAGAAAA  
Depth:9 (LIZARD)  
Ei-value:0.000, Pi-value:0.000  
Er-value:0.000, Pr-value:0.000  
No matches to TargetScan

-3729--(263)--3993-

CAGAAT

CAGAAT  
Depth:8 (ALLIGATOR)  
Ei-value:0.000, Pi-value:0.000  
Er-value:0.000, Pr-value:0.000  
No matches to TargetScan

-3998--(67)--4066-

TGGGATC

TGGGATC  
Depth:8 (ALLIGATOR)  
Ei-value:0.000, Pi-value:0.000  
Er-value:0.000, Pr-value:0.000  
No matches to TargetScan

-4072--(49)--4122-

AG

AGACAGGT  
Depth:9 (LIZARD)  
Ei-value:0.000, Pi-value:0.000  
Er-value:0.000, Pr-value:0.000  
No matches to TargetScan


ACAGGT

ACAGGT  
Depth:10 (SNAKE)  
Ei-value:0.000, Pi-value:0.000  
Er-value:0.000, Pr-value:0.000  
No matches to TargetScan

-4129--(21)--4151-

TTGCTTCA

TTGCTTCA  
Depth:8 (ALLIGATOR)  
Ei-value:0.000, Pi-value:0.000  
Er-value:0.000, Pr-value:0.000  
No matches to TargetScan

-4158--(37)--4196-

CA

CAGATAAGT  
Depth:11 (X.TROPICALIS)  
Ei-value:0.000, Pi-value:0.000  
Er-value:0.000, Pr-value:0.000  
No matches to TargetScan


GATAAG

GATAAG  
Depth:19 (ZEBRAFISH)  
Ei-value:0.000, Pi-value:0.000  
Er-value:0.000, Pr-value:0.000  
No matches to TargetScan


T

CAGATAAGT  
Depth:11 (X.TROPICALIS)  
Ei-value:0.000, Pi-value:0.000  
Er-value:0.000, Pr-value:0.000  
No matches to TargetScan

-4204--(17)--4222-

T

TATTGCATG  
Depth:8 (ALLIGATOR)  
Ei-value:0.000, Pi-value:0.000  
Er-value:0.000, Pr-value:0.000  
No matches to TargetScan


ATTGCAT

ATTGCAT  
Depth:11 (X.TROPICALIS)  
Ei-value:0.000, Pi-value:0.000  
Er-value:0.000, Pr-value:0.000  
No matches to TargetScan


G

ATTGCATG  
Depth:9 (LIZARD)  
Ei-value:0.000, Pi-value:0.000  
Er-value:0.000, Pr-value:0.000  
No matches to TargetScan

-4230--(121)--4352-

TTAAAAT

TTAAAAT  
Depth:8 (ALLIGATOR)  
Ei-value:0.000, Pi-value:0.000  
Er-value:0.000, Pr-value:0.000  
No matches to TargetScan

-4358--(289)--4648-

GCTGTC

GCTGTC  
Depth:9 (LIZARD)  
Ei-value:0.000, Pi-value:0.000  
Er-value:0.000, Pr-value:0.000  
No matches to TargetScan

-4653--(71)--4725-

TTTCAGA

TTTCAGA  
Depth:8 (ALLIGATOR)  
Ei-value:0.000, Pi-value:0.000  
Er-value:0.000, Pr-value:0.000  
No matches to TargetScan

-4731--(68)--4800-

AGGCAGGA

AGGCAGGA  
Depth:10 (SNAKE)  
Ei-value:0.000, Pi-value:0.000  
Er-value:0.000, Pr-value:0.000  
No matches to TargetScan


AA

AGGCAGGAAA  
Depth:8 (ALLIGATOR)  
Ei-value:0.000, Pi-value:0.000  
Er-value:0.000, Pr-value:0.000  
No matches to TargetScan

-4809--(70)--4880-

TAAGGA

TAAGGA  
Depth:13 (OPOSSUM)  
Ei-value:0.000, Pi-value:0.000  
Er-value:0.000, Pr-value:0.000  
No matches to TargetScan

-4885--(134)--5020-

TGCATG

TGCATG  
Depth:8 (ALLIGATOR)  
Ei-value:0.000, Pi-value:0.000  
Er-value:0.000, Pr-value:0.000  
No matches to TargetScan

-5025--(126)--5152-

TTTCAT

TTTCAT  
Depth:8 (ALLIGATOR)  
Ei-value:0.000, Pi-value:0.000  
Er-value:0.000, Pr-value:0.000  
No matches to TargetScan

-5157--(53)--5211-

TTCTGT

TTCTGT  
Depth:9 (LIZARD)  
Ei-value:0.000, Pi-value:0.000  
Er-value:0.000, Pr-value:0.000  
No matches to TargetScan

-5216--(19)--5236-

TGCAGTG

TGCAGTG  
Depth:10 (SNAKE)  
Ei-value:0.000, Pi-value:0.000  
Er-value:0.000, Pr-value:0.000  
MATCHES To TargetScan▶ miR-217:ACUGCAU

-5242--(94)--5337-

AGAATGCAG

AGAATGCAG  
Depth:8 (ALLIGATOR)  
Ei-value:0.000, Pi-value:0.000  
Er-value:0.000, Pr-value:0.000  
MATCHES To TargetScan▶ miR-33-5p:UGCAUUG

-5345--(29)--5375-

TTGGCAAGTAA

TTGGCAAGTAA  
Depth:10 (SNAKE)  
Ei-value:0.000, Pi-value:0.000  
Er-value:0.000, Pr-value:0.000  
No matches to TargetScan

-5385--(83)--5469-

TGGAGTAGTG

TGGAGTAGTG  
Depth:9 (LIZARD)  
Ei-value:0.000, Pi-value:0.000  
Er-value:0.000, Pr-value:0.000  
No matches to TargetScan

-5478--(111)--5590-

TTAGGT

TTAGGT  
Depth:11 (X.TROPICALIS)  
Ei-value:0.000, Pi-value:0.000  
Er-value:0.000, Pr-value:0.000  
No matches to TargetScan


AA

TTAGGTAA  
Depth:10 (SNAKE)  
Ei-value:0.000, Pi-value:0.000  
Er-value:0.000, Pr-value:0.000  
No matches to TargetScan

-5597--(42)--5640-

T

TGAAGCCTTTAGT  
Depth:10 (SNAKE)  
Ei-value:0.000, Pi-value:0.000  
Er-value:0.000, Pr-value:0.000  
No matches to TargetScan


GAAGCC

GAAGCC  
Depth:11 (X.TROPICALIS)  
Ei-value:0.000, Pi-value:0.000  
Er-value:0.000, Pr-value:0.000  
No matches to TargetScan


TTTAGT

TGAAGCCTTTAGT  
Depth:10 (SNAKE)  
Ei-value:0.000, Pi-value:0.000  
Er-value:0.000, Pr-value:0.000  
No matches to TargetScan


CT

TGAAGCCTTTAGTCT  
Depth:8 (ALLIGATOR)  
Ei-value:0.000, Pi-value:0.000  
Er-value:0.000, Pr-value:0.000  
No matches to TargetScan

-5654--(1)--5656-

TTCCAGAT

TTCCAGAT  
Depth:10 (SNAKE)  
Ei-value:0.000, Pi-value:0.000  
Er-value:0.000, Pr-value:0.000  
No matches to TargetScan

-5663--(60)--5724-

AACT

AACTGGCAAGTG  
Depth:8 (ALLIGATOR)  
Ei-value:0.000, Pi-value:0.000  
Er-value:0.000, Pr-value:0.000  
No matches to TargetScan


GGCAAGT

GGCAAGT  
Depth:10 (SNAKE)  
Ei-value:0.000, Pi-value:0.000  
Er-value:0.000, Pr-value:0.000  
No matches to TargetScan


G

AACTGGCAAGTG  
Depth:8 (ALLIGATOR)  
Ei-value:0.000, Pi-value:0.000  
Er-value:0.000, Pr-value:0.000  
No matches to TargetScan

-5735--(12)--5748-

CAGTTC

CAGTTC  
Depth:8 (ALLIGATOR)  
Ei-value:0.000, Pi-value:0.000  
Er-value:0.000, Pr-value:0.000  
No matches to TargetScan

-5753--(118)--5872-

AGTAAG

AGTAAG  
Depth:8 (ALLIGATOR)  
Ei-value:0.000, Pi-value:0.000  
Er-value:0.000, Pr-value:0.010  
No matches to TargetScan

-5877--(64)--5942-

TAGTTCTT

TAGTTCTT  
Depth:8 (ALLIGATOR)  
Ei-value:0.000, Pi-value:0.000  
Er-value:0.000, Pr-value:0.000  
No matches to TargetScan

-5949--(25)--5975-

AAGGAGC

AAGGAGC  
Depth:8 (ALLIGATOR)  
Ei-value:0.000, Pi-value:0.000  
Er-value:0.000, Pr-value:0.000  
No matches to TargetScan

-5981--(35)--6017-

AAC

AACAGGTGAA  
Depth:10 (SNAKE)  
Ei-value:0.000, Pi-value:0.000  
Er-value:0.000, Pr-value:0.000  
No matches to TargetScan


AGGTGA

AGGTGA  
Depth:11 (X.TROPICALIS)  
Ei-value:0.000, Pi-value:0.000  
Er-value:0.000, Pr-value:0.010  
No matches to TargetScan


A

AACAGGTGAA  
Depth:10 (SNAKE)  
Ei-value:0.000, Pi-value:0.000  
Er-value:0.000, Pr-value:0.000  
No matches to TargetScan

-6026--(72)--6099-

CAGCTT

CAGCTT  
Depth:8 (ALLIGATOR)  
Ei-value:0.000, Pi-value:0.000  
Er-value:0.000, Pr-value:0.010  
No matches to TargetScan

-6104--(78)--6183-

TTAAGA

TTAAGA  
Depth:8 (ALLIGATOR)  
Ei-value:0.000, Pi-value:0.010  
Er-value:0.000, Pr-value:0.010  
No matches to TargetScan

-6188--(28)--6217-

AAGGCT

AAGGCT  
Depth:10 (SNAKE)  
Ei-value:0.000, Pi-value:0.000  
Er-value:0.000, Pr-value:0.010  
No matches to TargetScan


T

AAGGCTT  
Depth:9 (LIZARD)  
Ei-value:0.000, Pi-value:0.000  
Er-value:0.000, Pr-value:0.000  
No matches to TargetScan

-6223--(284)--6508-

AGGGGA

AGGGGA  
Depth:8 (ALLIGATOR)  
Ei-value:0.000, Pi-value:0.000  
Er-value:0.000, Pr-value:0.000  
No matches to TargetScan

-6513--(19)--6533-

CAACCA

CAACCA  
Depth:8 (ALLIGATOR)  
Ei-value:0.000, Pi-value:0.000  
Er-value:0.000, Pr-value:0.000  
No matches to TargetScan

-6538--(12)--6551-

TTTTCCAG

TTTTCCAG  
Depth:10 (SNAKE)  
Ei-value:0.000, Pi-value:0.000  
Er-value:0.000, Pr-value:0.000  
No matches to TargetScan


AA

TTTTCCAGAA  
Depth:8 (ALLIGATOR)  
Ei-value:0.000, Pi-value:0.000  
Er-value:0.000, Pr-value:0.000  
No matches to TargetScan

-6560--(112)--6673-

TGGATCCT

TGGATCCT  
Depth:10 (SNAKE)  
Ei-value:0.000, Pi-value:0.000  
Er-value:0.000, Pr-value:0.000  
No matches to TargetScan


TG

TGGATCCTTG  
Depth:8 (ALLIGATOR)  
Ei-value:0.000, Pi-value:0.000  
Er-value:0.000, Pr-value:0.000  
No matches to TargetScan

-6682--(24)--6707-

AAGGTAA

AAGGTAA  
Depth:10 (SNAKE)  
Ei-value:0.000, Pi-value:0.000  
Er-value:0.000, Pr-value:0.000  
No matches to TargetScan

-6713--(168)--6882-

GGGTGGG

GGGTGGG  
Depth:10 (SNAKE)  
Ei-value:0.000, Pi-value:0.000  
Er-value:0.000, Pr-value:0.000  
No matches to TargetScan

-6888--(73)--6962-

TGCTAAGACT

TGCTAAGACT  
Depth:10 (SNAKE)  
Ei-value:0.000, Pi-value:0.000  
Er-value:0.000, Pr-value:0.000  
No matches to TargetScan


TTTTCAGGTG

TGCTAAGACTTTTTCAGGTG  
Depth:8 (ALLIGATOR)  
Ei-value:0.000, Pi-value:0.000  
Er-value:0.000, Pr-value:0.000  
No matches to TargetScan

-6981--(78)--7060-

TTTTCTTTT

TTTTCTTTT  
Depth:19 (ZEBRAFISH)  
Ei-value:0.000, Pi-value:0.000  
Er-value:0.000, Pr-value:0.000  
MATCHES To TargetScan▶ miR-186-5p:AAAGAAU


CCTGAGA

TTTTCTTTTCCTGAGA  
Depth:10 (SNAKE)  
Ei-value:0.000, Pi-value:0.000  
Er-value:0.000, Pr-value:0.000  
MATCHES To TargetScan▶ miR-873-5p.1:CAGGAAC▶ miR-186-5p:AAAGAAU

-7075--(16)--7092-

TCT

TCTCAGGTTTTGCTTTT  
Depth:10 (SNAKE)  
Ei-value:0.000, Pi-value:0.000  
Er-value:0.000, Pr-value:0.000  
MATCHES To TargetScan▶ miR-330-3p.2:AAAGCAC▶ miR-490-3p:AACCUGG


CAGGTTTTGCTTT

CAGGTTTTGCTTT  
Depth:19 (ZEBRAFISH)  
Ei-value:0.000, Pi-value:0.000  
Er-value:0.000, Pr-value:0.000  
MATCHES To TargetScan▶ miR-330-3p.2:AAAGCAC▶ miR-490-3p:AACCUGG


T

CAGGTTTTGCTTTT  
Depth:16 (NILETILAPIA)  
Ei-value:0.000, Pi-value:0.000  
Er-value:0.000, Pr-value:0.000  
MATCHES To TargetScan▶ miR-330-3p.2:AAAGCAC▶ miR-490-3p:AACCUGG

-7108--(20)--7129-

A

AAAAAAGCAAAAG  
Depth:13 (OPOSSUM)  
Ei-value:0.000, Pi-value:0.000  
Er-value:0.000, Pr-value:0.000  
No matches to TargetScan


AAAAAGCAAAA

AAAAAGCAAAA  
Depth:19 (ZEBRAFISH)  
Ei-value:0.000, Pi-value:0.000  
Er-value:0.000, Pr-value:0.000  
No matches to TargetScan


G

AAAAAGCAAAAG  
Depth:18 (MEDAKA)  
Ei-value:0.000, Pi-value:0.000  
Er-value:0.000, Pr-value:0.000  
No matches to TargetScan


A

AAAAAAGCAAAAGA  
Depth:9 (LIZARD)  
Ei-value:0.000, Pi-value:0.000  
Er-value:0.000, Pr-value:0.000  
No matches to TargetScan

-7142--(1)--7144-

GCTGGT

GCTGGT  
Depth:9 (LIZARD)  
Ei-value:0.000, Pi-value:0.000  
Er-value:0.000, Pr-value:0.000  
No matches to TargetScan

-7149--(7)--7157-

ACTCCTG

ACTCCTG  
Depth:18 (MEDAKA)  
Ei-value:0.000, Pi-value:0.000  
Er-value:0.000, Pr-value:0.000  
No matches to TargetScan


G

ACTCCTGG  
Depth:14 (SPOTTEDGAR)  
Ei-value:0.000, Pi-value:0.000  
Er-value:0.000, Pr-value:0.000  
MATCHES To TargetScan▶ miR-665:CCAGGAG

-7164--(3)--7168-

TCCAGGA

TCCAGGA  
Depth:8 (ALLIGATOR)  
Ei-value:0.000, Pi-value:0.000  
Er-value:0.000, Pr-value:0.000  
No matches to TargetScan

-7174--(2)--7177-

GGGTTC

GGGTTC  
Depth:13 (OPOSSUM)  
Ei-value:0.000, Pi-value:0.000  
Er-value:0.000, Pr-value:0.000  
No matches to TargetScan


AA

GGGTTCAA  
Depth:9 (LIZARD)  
Ei-value:0.000, Pi-value:0.000  
Er-value:0.000, Pr-value:0.000  
No matches to TargetScan

-7184--(12)--7197-

TCTTTGCT

TCTTTGCT  
Depth:8 (ALLIGATOR)  
Ei-value:0.000, Pi-value:0.000  
Er-value:0.000, Pr-value:0.000  
No matches to TargetScan

-7204  
  
>PIG  
      1353-

TTGGACT

TTGGACT  
Depth:9 (LIZARD)  
Ei-value:0.000, Pi-value:0.000  
Er-value:0.000, Pr-value:0.000  
No matches to TargetScan

-1359--(30)--1390-

GACTAG

GACTAG  
Depth:8 (ALLIGATOR)  
Ei-value:0.000, Pi-value:0.000  
Er-value:0.000, Pr-value:0.000  
No matches to TargetScan

-1395--(55)--1451-

AAGACAG

AAGACAG  
Depth:8 (ALLIGATOR)  
Ei-value:0.000, Pi-value:0.000  
Er-value:0.000, Pr-value:0.000  
No matches to TargetScan

-1457--(147)--1605-

GGTGAAG

GGTGAAG  
Depth:11 (X.TROPICALIS)  
Ei-value:0.000, Pi-value:0.000  
Er-value:0.000, Pr-value:0.000  
No matches to TargetScan

-1611--(39)--1651-

TGGTGA

TGGTGA  
Depth:8 (ALLIGATOR)  
Ei-value:0.000, Pi-value:0.000  
Er-value:0.000, Pr-value:0.000  
No matches to TargetScan

-1656--(192)--1849-

TGGTAA

TGGTAA  
Depth:14 (SPOTTEDGAR)  
Ei-value:0.030, Pi-value:0.000  
Er-value:0.010, Pr-value:0.000  
No matches to TargetScan

-1854--(99)--1954-

GTAGGTTT

GTAGGTTT  
Depth:8 (ALLIGATOR)  
Ei-value:0.000, Pi-value:0.000  
Er-value:0.000, Pr-value:0.000  
No matches to TargetScan

-1961--(397)--2359-

TCAGGATAA

TCAGGATAA  
Depth:8 (ALLIGATOR)  
Ei-value:0.000, Pi-value:0.000  
Er-value:0.000, Pr-value:0.000  
No matches to TargetScan

-2367--(165)--2533-

TTTGGG

TTTGGG  
Depth:19 (ZEBRAFISH)
[truncated: 2,330,363 more chars]
