# Supplementary material for: Uncovering deeply conserved motif combinations in rapidly evolving noncoding sequences
Source: Genome Biol. 2021 Jan 11;22:29. doi: 10.1186/s13059-020-02247-1 (PMC7798263; doi:10.1186/s13059-020-02247-1)
Supplement: Supplementary file 5 — Additional file 5. LncLOOM output results for MALAT1 sequences from 19 vertebrates. [file 13059_2020_2247_MOESM5_ESM.gz › AdditionalFile5/Html_Files/kmers_in_seqs_level.html]

 MOTIFS\_IN\_SEQS\_OVERLAP

# MOTIFS IN SEQUENCES

## Motifs conserved to (and beyond) ZEBRAFISH (depth:19)

  

NAVIGATE ▼

▶HUMAN (depth:1)▶MARMOSET (depth:2)▶DOG (depth:3)▶PIG (depth:4)▶COW (depth:5)▶MOUSE (depth:6)▶TURTLE (depth:7)▶ALLIGATOR (depth:8)▶LIZARD (depth:9)▶SNAKE (depth:10)▶X.TROPICALIS (depth:11)▶SHARK (depth:12)▶OPOSSUM (depth:13)▶SPOTTEDGAR (depth:14)▶FUGU (depth:15)▶NILETILAPIA (depth:16)▶STICKLEBACK (depth:17)▶MEDAKA (depth:18)▶ZEBRAFISH (depth:19)

  
  
  
  
Coloured by Conservation >>  

## >HUMAN (7504 bases)

```
 ATACGCCTCGCCCGAGCTGTGCGGTAGGCATTGAGGCAGCCAGCGCAGGGGCTTCTGCTGAGGGGGCAGGCGGAGCTTGAGGAAACCGCAGATAAGTTTTTTTCTCTTTGAAAGATAGAG 120  
 ATTAATACAACTACTTAAAAAATATAGTCAATAGGTTACTAAGATATTGCTTAGCGTTAAGTTTTTAACGTAATTTTAATAGCTTAAGATTTTAAGAGAAAATATGAAGACTTAGAAGAG 240  
 TAGCATGAGGAAGGAAAAGATAAAAGGTTTCTAAAACATGACGGAGGTTGAGATGAAGCTTCTTCATGGAGTAAAAAATGTATTTAAAAGAAAATTGAGAGAAAGGACTACAGAGCCCCG 360  
 AATTAATACCAATAGAAGGGCAATGCTTTTAGATTAAAATGAAGGTGACTTAAACAGCTTAAAGTTTAGTTTAAAAGTTGTAGGTGATTAAAATAATTTGAAGGCGATCTTTTAAAAAGA 480  
 GATTAAACCGAAGGTGATTAAAAGACCTTGAAATCCATGACGCAGGGAGAATTGCGTCATTTAAAGCCTAGTTAACGCATTTACTAAACGCAGACGAAAATGGAAAGATTAATTGGGAGT 600  
 GGTAGGATGAAACAATTTGGAGAAGATAGAAGTTTGAAGTGGAAAACTGGAAGACAGAAGTACGGGAAGGCGAAGAAAAGAATAGAGAAGATAGGGAAATTAGAAGATAAAAACATACTT 720  
 TTAGAAGAAAAAAGATAAATTTAAACCTGAAAAGTAGGAAGCAGAAGAAAAAAGACAAGCTAGGAAACAAAAAGCTAAGGGCAAAATGTACAAACTTAGAAGAAAATTGGAAGATAGAAA 840  
 CAAGATAGAAAATGAAAATATTGTCAAGAGTTTCAGATAGAAAATGAAAAACAAGCTAAGACAAGTATTGGAGAAGTATAGAAGATAGAAAAATATAAAGCCAAAAATTGGATAAAATAG 960  
 CACTGAAAAAATGAGGAAATTATTGGTAACCAATTTATTTTAAAAGCCCATCAATTTAATTTCTGGTGGTGCAGAAGTTAGAAGGTAAAGCTTGAGAAGATGAGGGTGTTTACGTAGACC 1080  
 AGAACCAATTTAGAAGAATACTTGAAGCTAGAAGGGGAAGTTGGTTAAAAATCACATCAAAAAGCTACTAAAAGGACTGGTGTAATTTAAAAAAAACTAAGGCAGAAGGCTTTTGGAAGA 1200  
 GTTAGAAGAATTTGGAAGGCCTTAAATATAGTAGCTTAGTTTGAAAAATGTGAAGGACTTTCGTAACGGAAGTAATTCAAGATCAAGAGTAATTACCAACTTAATGTTTTTGCATTGGAC 1320  
 TTTGAGTTAAGATTATTTTTTAAATCCTGAGGACTAGCATTAATTGACAGCTGACCCAGGTGCTACACAGAAGTGGATTCAGTGAATCTAGGAAGACAGCAGCAGACAGGATTCCAGGAA 1440  
 CCAGTGTTTGATGAAGCTAGGACTGAGGAGCAAGCGAGCAAGCAGCAGTTCGTGGTGAAGATAGGAAAAGAGTCCAGGAGCCAGTGCGATTTGGTGAAGGAAGCTAGGAAGAAGGAAGGA 1560  
 GCGCTAACGATTTGGTGGTGAAGCTAGGAAAAAGGATTCCAGGAAGGAGCGAGTGCAATTTGGTGATGAAGGTAGCAGGCGGCTTGGCTTGGCAACCACACGGAGGAGGCGAGCAGGCGT 1680  
 TGTGCGTAGAGGATCCTAGACCAGCATGCCAGTGTGCCAAGGCCACAGGGAAAGCGAGTGGTTGGTAAAAATCCGTGAGGTCGGCAATATGTTGTTTTTCTGGAACTTACTTATGGTAAC 1800  
 CTTTTATTTATTTTCTAATATAATGGGGGAGTTTCGTACTGAGGTGTAAAGGGATTTATATGGGGACGTAGGCCGATTTCCGGGTGTTGTAGGTTTCTCTTTTTCAGGCTTATACTCATG 1920  
 AATCTTGTCTGAAGCTTTTGAGGGCAGACTGCCAAGTCCTGGAGAAATAGTAGATGGCAAGTTTGTGGGTTTTTTTTTTTTACACGAATTTGAGGAAAACCAAATGAATTTGATAGCCAA 2040  
 ATTGAGACAATTTCAGCAAATCTGTAAGCAGTTTGTATGTTTAGTTGGGGTAATGAAGTATTTCAGTTTTGTGAATAGATGACCTGTTTTTACTTCCTCACCCTGAATTCGTTTTGTAAA 2160  
 TGTAGAGTTTGGATGTGTAACTGAGGCGGGGGGGAGTTTTCAGTATTTTTTTTTGTGGGGGTGGGGGCAAAATATGTTTTCAGTTCTTTTTCCCTTAGGTCTGTCTAGAATCCTAAAGGC 2280  
 AAATGACTCAAGGTGTAACAGAAAACAAGAAAATCCAATATCAGGATAATCAGACCACCACAGGTTTACAGTTTATAGAAACTAGAGCAGTTCTCACGTTGAGGTCTGTGGAAGAGATGT 2400  
 CCATTGGAGAAATGGCTGGTAGTTACTCTTTTTTCCCCCCACCCCCTTAATCAGACTTTAAAAGTGCTTAACCCCTTAAACTTGTTATTTTTTACTTGAAGCAT

TTTGGG

TTTGGG  
Depth:19 (ZEBRAFISH)  
Ei-value:0.000, Pi-value:0.000  
Er-value:0.000, Pr-value:0.000  
eCLIP MATCHES▶ppil4 (bg=43.39%)▶PRPF8 (bg=6.2%)No matches to TargetScan

ATGGTCTTAA 2520  
 CAGGGAAGAGAGAGGGTGGGGGAGAAAATGTTTTTTTCTAAGATTTTCCACAGATGCTATAGTACTATTGACAAACTGGGTTAGAGAAGGAGTGTACCGCTGTGCTGTTGGCACGAACAC 2640  
 CTTCAGGGACTGGAGCTGCTTTTATCCTTGGAAGAGTATTCCCAGTTGAAGCTGAAAAGTACAGCACAGTGCAGCTTTGGTTCATATTCAGTCATCTCAGGAGAACTTCAGAAGAGCTTG 2760  
 AGTAGGCCAAATGTTGAAGTTAAGTTTTCCAATAATGTGACTTCTTAAAAGTTTTATTAAAGGGGAGGGGCAAATATTGGCAATTAGTTGGCAGTGGCCTGTTACGGTTGGGATTGGTGG 2880  
 GGTGGGTTTAGGTAATTGTTTAGTTTATGATTGCAGATAAACTCATGCCAGAGAACTTAAAGTCTTAGAATGGAAAAAGTAAAGAAATATCAACTTCCAAGTTGGCAAGTAACTCCCAAT 3000  
 GATTTAGTTTTTTTCCCCCCAGTTTGAATTGGGAAGCTGGGGGAAGTTAAATATGAGCCACTGGGTGTACCAGTGCATTAATTTGGGCAAGGAAAGTGTCATAATTTGATACTGTATCTG 3120  
 TTTTCCTTCAAAGTATAGAGCTTTTGGGGAAGGAAAGTATTGAACTGGGGGTTGGTCTGGCCTACTGGGCTGACATTAACTACAATTATGGGAAATGCAAAAGTTGTTTGGATATGGTAG 3240  
 TGTGTGGTTCTCTTTTGGAATTTTTTTCAGGTGATTTAATAATAATTTAAAACTACTATAGAAACTGCAGAGCAAAGGAAGTGGCTTAATGATCCTGAAGGGATTTCTTCTGATGGTAGC 3360  
 TTTTGTATTATCAAGTAAGATTCTATTTTCAGTTGTGTGTAAGCAAGTTTTTTTTTAGTGTAGGAGAAATACTTTTCCATTGTTTAACTGCAAAACAAGATGTTAAGGTATGCTTCAAAA 3480  
 ATTTTGTAAATTGTTTATTTTAAACTTATCTGTTTGTAAATTGTAACTGATTAAGAATTGTGATAGTTCAGCTTGAATGTCTCTTAGAGGGTGGGCTTTTGTTGATGAGGGAGGGGAAAC 3600  
 TTTTTTTTTTTCTATAGACT

TTTTTCAG

TTTTTCAG  
Depth:19 (ZEBRAFISH)  
Ei-value:0.000, Pi-value:0.000  
Er-value:0.000, Pr-value:0.000  
eCLIP MATCHES▶ddx42 (bg=10.33%)▶ppil4 (bg=43.39%)▶safb (bg=40.39%)▶u2af1 (bg=14.02%)▶u2af2 (bg=19.32%)No matches to TargetScan

ATAACATCTTCTGAGTCATAACCAGCCTGGCAGTATGATGGCCTAGATGCAGAGAAAACAGCTCCTTGGTGAATTGATAAGTAAAGGCAGAA 3720  
 AAGATTATATGTCATACCTCCATTGGGGAATAAGCATAACCCTGAGATTCTTACTACTGATGAGAACATTATCTGCATATGCCAAAAAATTTTAAGCAAATGAAAGCTACCAATTTAAAG 3840  
 TTACGGAATCTACCATTTTAAAGTTAATTGCTTGTCAAGCTATAACCACAAAAATAATGAATTGATGAGAAATACAATGAAGAGGCAATGTCCATCTCAAAATACTGCTTTTACAAAAGC 3960  
 AGAATAAAAGCGAAAAGAAATGAAAATGTTACACTACATTAATCCTGGAATAAAAGAAGCCGAAATAAATGAGAGATGAGTTGGGATCAAGTGGATTGAGGAGGCTGTGCTGTGTGCCAA 4080  
 TGTTTCGTTTGCCTCAGACAGGTATCTCTTCGTTATCAGAAGAGTTGCTTCATTTCATCTGGGAGCAGAAAACAGCAGGCAGCTGTTAACA

GATAAG

GATAAG  
Depth:19 (ZEBRAFISH)  
Ei-value:0.000, Pi-value:0.000  
Er-value:0.000, Pr-value:0.000  
eCLIP MATCHES▶cpsf6 (bg=13.45%)▶khsrp (bg=27.4%)▶NIPBL (bg=8.2%)▶ppil4 (bg=43.39%)▶PRPF8 (bg=6.2%)▶rbm15 (bg=11.59%)▶safb (bg=40.39%)▶safb2 (bg=26.89%)▶srsf1 (bg=30.28%)▶srsf7 (bg=22.53%)▶znf622 (bg=18.79%)No matches to TargetScan

TTTAACTTGCATCTGCAGTATTG 4200  
 CATGTTAGGGATAAGTGCTTATTTTTAAGAGCTGTGGAGTTCTTAAATATCAACCATGGCACTTTCTCCTGACCCCTTCCCTAGGGGATTTCAGGATTGAGAAATTTTTCCATCGAGCCT 4320  
 TTTTAAAATTGTAGGACTTGTTCCTGTGGGCTTCAGTGATGGGATAGTACACTTCACTCAGAGGCATTTGCATCTTTAAATAATTTCTTAAAAGCCTCTAAAGTGATCAGTGCCTTGATG 4440  
 CCAACTAAGGAAATTTGTTTAGCATTGAATCTCTGAAGGCTCTATGAAAGGAATAGCATGATGTGCTGTTAGAATCAGATGTTACTGCTAAAATTTACATGTTGTGATGTAAATTGTGTA 4560  
 GAAAACCATTAAATCATTCAAAATAATAAACTATTTTTATTAGAGAATGTATACTTTTAGAAAGCTGTCTCCTTATTTAAATAAAATAGTGTTTGTCTGTAGTTCAGTGTTGGGGCAATC 4680  
 TTGGGGGGGATTCTTCTCTAATCTTTCAGAAACTTTGTCTGCGAACACTCTTTAATGGACCAGATCAGGATTTGAGCGGAAGAACGAATGTAACTTTAAGGCAGGAAAGACAAATTTTAT 4800  
 TCTTCATAAAGTGATGAGCATATAATAATTCCAGGCACATGGCAATAGAGGCCCTCTAAATAAGGAATAAATAACCTCTTAGACAGGTGGGAGATTATGATCAGAGTAAAAGGTAATTAC 4920  
 ACATTTTATTTCCAGAAAGTCAGGGGTCTATAAATTGACAGTGATTAGAGTAATACTTTTTCACATTTCCAAAGTTTGCATGTTAACTTTAAATGCTTACAATCTTAGAGTGGTAGGCAA 5040  
 TGTTTTACACTATTGACCTTATATAGGGAAGGGAGGGGGTGCCTGTGGGGTTTTAAAGAATTTTCCTTTGCAGAGGCATTTCATCCTTCATGAAGCCATTCAGGATTTTGAATTGCATAT 5160  
 GAGTGCTTGGCTCTTCCTTCTGTTCTAGTGAGTGTATGAGACCTTGCAGTGAGTTTATCAGCATACTCAAAATTTTTTTCCTGGAATTTGGAGGGATGGGAGGAGGGGGTGGGGCTTACT 5280  
 TGTTGTAGCTTTTTTTTTTTTTACAGACTTCACAGAGAATGCAGTTGTCTTGACTTCAGGTCTGTCTGTTCTGTTGGCAAGTAAATGCAGTACTGTTCTGATCCCGCTGCTATTAGAATG 5400  
 CATTGTGAAACGACTGGAGTATGATTAAAAGTTGTGTTCCCCAATGCTTGGAGTAGTGATTGTTGAAGGAAAAAATCCAGCTGAGTGATAAAGGCTGAGTGTTGAGGAAATTTCTGCAGT 5520  
 TTTAAGCAGTCGTATTTGTGATTGAAGCTGAGTACATTTTGCTGGTGTATTTTTAGGTAAAATGCTTTTTGTTCATTTCTGGTGGTGGGAGGGGACTGAAGCCTTTAGTCTTTTCCAGAT 5640  
 GCAACCTTAAAATCAGTGACAAGAAACATTCCAAACAAGCAACAGTCTTCAAGAAATTAAACTGGCAAGTGGAAATGTTTAAACAGTTCAGTGATCTTTAGTGCATTGTTTATGTGTGGG 5760  
 TTTCTCTCTCCCCTCCCTTGGTCTTAATTCTTACATGCAGGAACACTCAGCAGACACACGTATGCGAAGGGCCAGAGAAGCCAGACCCAGTAAGAAAAAATAGCCTATTTACTTTAAATA 5880  
 AACCAAACATTCCATTTTAAATGTGGGGATTGGGAACCACTAGTTCTTTCAGATGGTATTCTTCAGACTATAGAAGGAGCTTCCAGTTGAATTCACCAGTGGACAAAATGAGGAAAACAG 6000  
 GTGAACAAGCTTTTTCTGTATTTACATACAAAGTCAGATCAGTTATGGGACAATAGTATTGAATAGATTTCAGCTTTATGCTGGAGTAACTGGCATGTGAGCAAACTGTGTTGGCGTGGG 6120  
 GGTGGAGGGGTGAGGTGGGCGCTAAGCCTTTTTTTAAGATTTTTCAGGTACCCCTCACTAAAGGCACCGAAGGCTTAAAGTAGGACAACCATGGAGCCTTCCTGTGGCAGGAGAGACAAC 6240  
 AAAGCGCTATTATCCTAAGGTCAAGAGAAGTGTCAGCCTCACCTGATTTTTATTAGTAATGAGGACTTGCCTCAACTCCCTCTTTCTGGAGTGAAGCATCCGAAGGAATGCTTGAAGTAC 6360  
 CCCTGGGCTTCTCTTAACATTTAAGCAAGCTGTTTTTATAGCAGCTCTTAATAATAAAGCCCAAATCTCAAGCGGTGCTTGAAGGGGAGGGAAAGGGGGAAAGCGGGCAACCACTTTTCC 6480  
 CTAGCTTTTCCAGAAGCCTGTTAAAAGCAAGGTCTCCCCACAAGCAACTTCTCTGCCACATCGCCACCCCGTGCCTTTTGATCTAGCACAGACCCTTCACCCCTCACCTCGATGCAGCCA 6600  
 GTAGCTTGGATCCTTGTGGGCATGATCCATAATCGGTTTCAAGGTAACGATGGTGTCGAGGTCTTTGGTGGGTTGAACTATGTTAGAAAAGGCCATTAATTTGCCTGCAAATTGTTAACA 6720  
 GAAGGGTATTAAAACCACAGCTAAGTAGCTCTATTATAATACTTATCCAGTGACTAAAACCAACTTAAACCAGTAAGTGGAGAAATAACATGTTCAAGAACTGTAATGCTGGGTGGGAAC 6840  
 ATGTAACTTGTAGACTGGAGAAGATAGGCATTTGAGTGGCTGAGAGGGCTTTTGGGTGGGAATGCAAAAATTCTCTGCTAAGACTTTTTCAGGTGAACATAACAGACTTGGCCAAGCTAG 6960  
 CATCTTAGCGGAAGCTGATCTCCAATGCTCTTCAGTAGGGTCATGAAGGT

TTTTCTTTT

TTTTCTTTT  
Depth:19 (ZEBRAFISH)  
Ei-value:0.000, Pi-value:0.000  
Er-value:0.000, Pr-value:0.000  
eCLIP MATCHES▶srsf7 (bg=22.53%)MATCHES To TargetScan▶ miR-186-5p:AAAGAAU

CCTGAGAAAACAACACGTATTGTTTTCT

CAGGTTTTGCTTT

CAGGTTTTGCTTT  
Depth:19 (ZEBRAFISH)  
Ei-value:0.000, Pi-value:0.000  
Er-value:0.000, Pr-value:0.000  
eCLIP MATCHES▶srsf7 (bg=22.53%)MATCHES To TargetScan▶ miR-330-3p.2:AAAGCAC▶ miR-490-3p:AACCUGG

TTGGCCTTTTTCTAGCTTAA 7080  
 AAAAAA

AAAAAGCAAAA

AAAAAGCAAAA  
Depth:19 (ZEBRAFISH)  
Ei-value:0.000, Pi-value:0.000  
Er-value:0.000, Pr-value:0.000  
No matches to eCLIP DataNo matches to TargetScan

GATGCTGGTGGTTGGCACTCCTGGTTTCCAGGACGGGGTTCAAATCCCTGCGGCGTCTTTGCTTTGACTACTAATCTGTCTTCAGGACTCTTTCTGTATTTCT 7200  
 CCTTTTCTCTGCAGGTGCTAGTTCTTGGAGTTTTGGGGAGGTGGGAGGTAACAGCACAATATCTTTGAACTATATACATCCTTGATGTATAATTTGTCAGGAGCTTGACTTGATTGTATA 7320  
 TTCATATTTACACGAGAACCTAATATAACTGCCTTGTCTTTTTCAGGTAATAGCCTGCAGCTGGTGTTTTGAGAAGCCCTACTGCTGAAAACTTAACAATTTTGTGTAATAAAAATGGAG 7440  
 AAGCTCTAAATTGTTGTGGTTCTTTTGTGAATAAAAAAATCTTGATTGGGGAAAAAAGATGGGT                                                         7504
```

---

## >MARMOSET (7293 bases)

```
 GAACTATTTTTTTTTGCCTCACAAAGACGGCGGAAGGTGATTGGATTCCGGTGATGTGAGTTGTCCTCCGTCTATAAATACGCCTCGCCGGGGCTCTGGGCAGGCATTGAGGGAGCCAGC 120  
 GCAGGGCAGGAGGAGGGAGGCGCAGCTTGAGGAAACTGCAGATAAGTTTTTCTCTTTAAAATAAGCTAGGCATTAATACAACTGCTTAAATATAAATAGCCAGTAGCTTACTGAGATTGC 240  
 TCAGCATTAAGTTCTTAATTTAATTTTAGTAATTTAAAAAGATGTTTTGATCGTCTGCGCGCTTAGTAGAAAAAAAGTTAAAAAGGTTATAAGAGAAAATAGGAAGATTTAAAAGAGTAG 360  
 CATGAGGAAGGAAGAGAAGGAATAAATGTTTCTGAAACATGACGGAGGTTGAGATGAAGCTGCTTCATGGAGTAGAAAATGTATTTAAAAGAAAATTGAAAAGAAGGACTAGAGTCCTGA 480  
 ATTAATACCTTTAATTAGAAGGGCAGTGCTTTTAGATTATTAAAAAGGTGACTTAAACAGACCTTAAACGTTATTTAAAAAATCGTAGGTGACTAAAATAGTTTGAACGCGATCATTTAA 600  
 AAAGAGATTAAGCCAAAGGTGATTAAAAGACCTTGAAATCCATGACGCATGAAGAATTGCGTCATTTAAAGCCTACTTAGTTTTTACGCGGTTACTAAACGCAGACGAAGATAGGAAAGA 720  
 TTTAATTGGGAGTGGTAGGAGGAAAACAATTTGGAGAAGATTAGAAGTTTGAAGTGGAATACTGGAAGACAGAAGTACAGGAAGGCGAAGAAAAGAATAGAGAAGATAGGGAAATTAGAA 840  
 GATAAAAATACACCTTTTAGAAGACAAAAGGAATCAGAAGTAGGAAGCAGAAGAAAAAAATTAGACAAGCTAGGAAACAAAAAAGAGAAGCTAGAAAACAAGCAACTAAGGGCAAAATGT 960  
 GCAAACTTAGAAGAAAACGAAGATAGAATCAAGCTAGACAATATTGTCAAGAGTTTCAGATAGAAAATGAAAAGCTAAGACAAGTATTGGACAAGCATAGAAGATAGAAAAATTGGATAA 1080  
 AATAGCACAGAAAAAATGAAATTATTGACAACCAATTTAAAAGCCCATCAATTTAATTTCTGATGGTGCAGAAGTTAGAAGGTAAAGTTTCAGAAGATGAGGGTCTTTACGTTATGTAGA 1200  
 CCAGAGCCAATTTAGAAGAATATTTGAAGCTAGAAGGGAAGTCGGTTAAGATTCGCATCAAAAAGCTGCTGAAAGGAATGGAGTAAATTTAAAAACTAAGGCAGAAGGCTTTTGGAAGAG 1320  
 TTAGAAGAATTTGGAAGGCCTTAAAATACGGTAGCTTAGTTTGAAAAATTTAAAGGACTTTTGTAACGGAAGTAATTCAAGATCAAGAGTAATTACCAACTTAATGTTTCCCCATTGGAC 1440  
 TTTGTGCTAACATGATTTTTAAAATCTTGAGGACTAGCGTTAATTGACAGCTGACCCAGGTGCTACACAGAAGTGGATTCAGTGAATCTAGGAAGACAGCAGCAGACAGAATTCCAGGAG 1560  
 CCAGTGTTTGGTGAAGCTAGGACTGAGGAGCAAGCAGCAGTTCGTGGTGAAGATAGGAAGAGTCCAGGAACCAGTGCGATTTGGTGAAGGAAGCTAGGAAGAAGGAAGGAGCGCTAACGA 1680  
 TTTGGTGGTGAAGCTAGGAAAGAGGATTCCAGGAAGGAGCGAGTGCGATTTGGTGATGAAGCTAGCAGGCGGCTTGGCTTGGCAACCACACGGAGGAGTCGAGCAGGCGTTGTGCATAGA 1800  
 TAGAGGATCCTAGACCAGCATGCCAGTATGCCAAGGCTACAGGGAAGGAGAGTGGTTGGTAAAAATCCGTGAGGTCGGCAATATGTTGTTTTTCTGGAACTTGAGTATGGTAATGTTTTA 1920  
 TTTACTTTCCTAATGTAATGGGGGAGTTTCGTACTGAGGTGTAAAAAGGGATTTATATGGGGATGTAGGCCGATTTCCGGGTGTTGTAGGTTTCTCTTTTTCAGGCTTATGCTCATGAAT 2040  
 CTTGTCTGAAGCTTTTGAGGGCAGACTGCCAAGTCCTGGAGGAATAGTAGATGGCAAGTTTGTTTTTGTTTTTGTTTCTTTTTTACCAACTTGCGGAAAATCAAATGAATTTGATAGAGC 2160  
 CAAAATTGAGACAAAATTTCAGCAAATCTGTAAGCAGTTTGTATGTTTAGTTGGGGTAATGCAGTATTTCAATTTTGTGAATAGATGACTTGTTTTTACTTTCTCACCCTGACTTAGTTT 2280  
 TCTAAATGTGGAGTTTGAATGTGTAACGGATGGGGGGGAATTTTGATTGTTTTTTTTTGTGTGTGTGCGGGTGGGGGCAAAATATGTTCTGAGTTCTTTTCCCCTTAGGTCTGTCTAGAA 2400  
 TCCTAAATGCAAATGACTCAAGGTGTAACAGGAAACAAGAAAATCCAGTATCAGGATAATCAGAACACCACAGATTTACAGTTTGTAGAAACTAGAGCAGTTCTCACTTTAAGGTCTGTG 2520  
 GAGGAGCTGTCCATTGGAGAAATGGCTGGTAGTTACTCTTTTTCCCCCCTCCCCCCTTAATTAGATTTTTTAAAGTGCTTAACCCCTTAAACTTGTTACTTTTTCTTTTGAAGTAT

TTTG

TTTGGG  
Depth:19 (ZEBRAFISH)  
Ei-value:0.000, Pi-value:0.000  
Er-value:0.000, Pr-value:0.000  
No matches to TargetScan

 2640  


GG

TTTGGG  
Depth:19 (ZEBRAFISH)  
Ei-value:0.000, Pi-value:0.000  
Er-value:0.000, Pr-value:0.000  
No matches to TargetScan

ATGGTCTTAACAGGGAAGAGAGAGGGTGGGGGAGAAAATGTTTTTTTCTAAGATTTTCCACAGATGCTATAGTACTATTGACAAACTGGGTTAGAGAAGGAGTGTACGGCTGTGCTGT 2760  
 TGGCACGAACACCTTCAGGGACTGGAGCTGCTTTTATACCTGGAAGAGTATTCCCAGTTGAAGCTGAAAAGTACAGCACAGTGCAGCTTTGGTTCATATTCAGTCATCTCAGGAGAACTT 2880  
 CAGAAGAGCTTGAGTAGGCCAAATGTTGAAGTTAAGTTTTCAAATAATGTGACTTCTTAAAAGTTTATTAAAGGGGAGGGGCAAATATTGGCAATTAGTTGGCAGTGGCCTGTTAAGGTT 3000  
 GGGATGGGGGGGAGGGTTTAGGTAATTGTTTAGTTTATGATTGCAGATAAAGTCATGCCAGAGAACTTAAAAGTCTTAGGATGGAAAAGGTAAAGAAATCTCAACTTCCAAGCTGGCAAG 3120  
 TAACTCCCATTCGTTTAGTTTTTGTTTCCCCAATTTTTTAATTGGGAGGCTGGGAGAAGTTAAATAAGAGCCACTGGGTGTACCAGTGCATTGATTTGGGCGAGGAGTGTCGTAATTTGA 3240  
 TACTGTGTCTGTTTTCCCTCAAAGTATAGAGCTTGGGGAAGGAAAGTAATGGACTGGGGGTTGGTCTGGCCCACTGGGCTGACAGTTAACTATTATGGGAAATGCAAAAGTTGTTCGGAT 3360  
 ATGGTAGTGTGTGGTTCTCTTTTGGAATTTTTTTCAGGTGATTTAATATAAATTAAAACTACTATAGGAACTTCAGAGCAAAGGAAGTGGCTTAATGATCCTGAAGGGATTTCTTCTGAT 3480  
 GGTAGCTTTTGTATTATCAAGTAAGATTCTGTTTTCAGTTGTGTGTAAGCAAGTTTTTCTTAGCGTAGGAGAAATATTTTTCCATTGTTCAACTGCAAAGCAAGATGTTAAGGTATGCTT 3600  
 CAAAAACTGTAAATTATGTATTTTAAATGTATCTGTAAGTCATAACTGATTAAGAATTGTGATAGTTCAGCTTGAATGTCTCTTAGAGGGTGGGCTTTTGTTGATGAGGGAGGGGAAACT 3720  
 TTTTTTTTCTGTAGACT

TTTTTCAG

TTTTTCAG  
Depth:19 (ZEBRAFISH)  
Ei-value:0.000, Pi-value:0.000  
Er-value:0.000, Pr-value:0.000  
No matches to TargetScan

ATAACATCTTCTGAGTCATAACCAGCCTGGCAGTGTGATGGCCTAGATGCAGAGAGAACAGCTCCTTGGTGAATGATAAGTAAAGGCAGAAAAGA 3840  
 TTATATGTCATACCTCCATTGGGGAATAAGCATAACCCTGAGATTCTTACTACTGATGAAAACGTTATCTGCATATGCCAAAAAAAAAAAAAATTAAGCAAATGAAAACTACCAATTTAA 3960  
 AGTTACGGAATCTACCATTTTAAAGTTAATTGCTTGTCAAGCTATAACTACAAAAATAATGAATTGATGAGAAATACAATGAAGATCCAATGTCCATCTCAAAATACTGCTTTTACAAAA 4080  
 GCAGAATAAAAAAAGTGAAATGAAATGAAAGTGGTTATACTACATTAATCCTGGAATAAAAGAAGCCGAAATAAGAGATGAGTTGGGATCAAGTGGATTGAGGAGGCTGTGCTGTGTGCC 4200  
 AATCTTTCGTTTGCCTCAGACAGGTATCTCTTCATTATCAGAAGAGTTGCTTCATTTCATCTGGGAGCAGAAAACAGCAGGCAGCTGTTAACA

GATAAG

GATAAG  
Depth:19 (ZEBRAFISH)  
Ei-value:0.000, Pi-value:0.000  
Er-value:0.000, Pr-value:0.000  
No matches to TargetScan

TTTAACTTGCATCTGCAATAT 4320  
 TGCATGTTAGGGATAAGTGTTTATTTTTAAAGAACTGTGGAGTTCTTAAGTATCAACCCATGGCACTTTTTCCTGACCCCTTCCCTAGGGGATTTCAGGATTATGAGAAATTTTCCCATC 4440  
 AAGCCTCTTTAAAATTGTAGGACTTGTTCCTATGGGCTTCTGTGATGGGATAGTACACCTCACTCAGAGGCATTTGCATCTGAAAAATTCCTTAAAAGCCTCTAAAGTGATTAGTGCCGT 4560  
 AAGTAATGCAAGACTAAGGAAATTCGTTTAGCATTGAATCTCTGAAGACTGAAAGGAATAGCATGATGTGCTATTAGAATCAGATGTAAATCTTGCTAAAATTTACATGTTGTGTTGTGA 4680  
 TGTATATTGTGGAGAAAACCATTGAATCAATCAAAATAATAAACTATTTTTATTAGAGAATGTATACTTTTAGAAAGCTGTCTCCTTATTTAAATAAAATACTGTTTGTAGTTTAGTGTT 4800  
 GGGGCAATCTTGGGGGGATTCTTTTCTAATCTTTCAGAAACTTTGTCTGCGAACACACTTTAATGGACAAGATCAGGATTTGAGCGGAAGAACGAATGTAACTTTCTTAAGGCAGGAAAG 4920  
 AAATTTTATTCTTCATAAACTGATGAGCATATAATAATTCCAGGCACATGGCAATAGAGGCCCTCTAAATAAGGAATAAGTAACCTCTTAGACAGGTGGGAGATTATGATCAGAGTAAAA 5040  
 GGTAATTACACATTTTATTTCCAGAAAGTCAGGTCTATAAATTAACAGTAAACTAGAGTAAGTATTTTTTCACATTTCCAGAGTTTGCATGTTAACTTTAAATGCTTACAATCTTAAGAG 5160  
 TATTAGGCAATGTTTTACACTATTGGCCTTTTATAGGCATTGGGGGGGGCCTGTGGGTTTTTAAAGAATTTTCCTTTGCAGAGGCATTTCATCCTTCATGAAGCTGTTCAGGATTTTGAC 5280  
 TTGCATATGAGTGCTTGGCTCTTCCTTCTGTTCTCCTAGTGAGTGTATGAGACCTTGCAGTGAGTTTATCAGCATACTCAAAATTTTTTTCCTGGAATTTGGAGGGATGGGAGGAGGGGG 5400  
 TGGGGCTTACTTGTTTTAGCTTTTTTTTTACAGACTTCACAGAGAATGCAGTTGTCTTGACTTCAGGTCTGTCTGTTCTGTTGGCAAGTAATAAATAATGCAGTACTGTTCTGATCCCGC 5520  
 TGCTATTAGAATGCATTGTGAAACGACTGGAGTATGATTAAAAGTTGTGTTCCCCAATGCTTGGAGTAGTGATTGTTGAAGGAAAAAAATCCAGCTGAGTGATAAAGGCTGAGTGTTGAG 5640  
 GAAATTTCTGCAGTTTTAAGCAGTCGTGTTTGTGACTGAAGCTAAGTACATTTTGCTGTGCATTTTTAGGTAAAATGCTTTTCCTTCATTTCTGCTGGTGGGGGGGCACTGAAGCCTTTA 5760  
 GTCTTTTCCAGATGCAACCTTAAAATCAGTGACAAGAAACAAATACCAAACAAGCAACAATCTTCAAGAAATTTAACTGGCAAGTGGAAATGTTTAACAGTTCAGTGATCTTAGTGCATT 5880  
 GTTTTTGTGTGGGTTTCTCTCTCCCCTCCCTTGGTCTTAATTCTTACATGCAGGAACATTCAGAACAGCAGACATACGTATGAGAAGGGCCAGAGAAGCCAGACCCAGTAAGAAAAAAAT 6000  
 AGCCTATTTACTTTAAACAAACCAAACATTCCATTTTAAATGTGGGGATTGGGAACCACTAGTTCTTTCAGATGGTATTCTTCAGACTATAGAAGGAGCTTCCAGTTGAATTCAACAGTG 6120  
 GACAAAATGAGGACAACAGGTGAACAAGCTTTTTCTGTATTTACATTCAAAGTCAGATCAGTTACGGGACAATAATACAGTATTGAATAGATTTCAGCTTTATGCTGGAGTAATTGGCAT 6240  
 GTGAGCAAGCTGTGTTGGCGTGGGGGTGGAGGGGTGAGGTGGGCGCTAAGCCTTTTCTTAAGATTTTTCAGGTACCCCTCACTAAAGGCACCGAAGGCTTAAAGTAGGACAACCATGGAG 6360  
 CCTTCCTGTGGCAAGAGAGACAACAAAGCGCTATTATCCTAAGGTCAAGAAAAATGGTGTCAGCCTCACCTAATACTTATTAGTAACAAGGACTTGCCTCAACTCTCTTTCTGGAGTGAA 6480  
 GCATCCAAAGGAATGCTTGAAGTACCCTGGGCATCTTTAACATTTAAGCGAGCTGTTTTTATAGCAGCTCTTAATAAAGCCCAAATCTCAAGCGGTGCTTGAAGGGGAGGGAAAGGGGGA 6600  
 AAGCGGGCAACCAGTTTTCCCTAGCTTTTCCAGAAGCCTGTTAAAAGCAAGGTCTCCCCACAAGCAACTTCTCTGCCACATCGCCACCCTGTGCCTTTGGCCTAGCACAGACCCTTCACC 6720  
 CCTCACCTCGATGCAGCCGGTAGCTTTGGATCCTTGTGGGCATGATCCATAATCTGTTTCAAGGTAACAATGGTGCCGAGGTCTTCGGTGGGTTGTACTATACTAGAAAAGGCCATTAAT 6840  
 TTGCCTGCAAATTGTTAACAAAGATTAAGACCACAGCTAAGTACTATATAACGCCCAGTGACTAAAACCAACTTAAACCAGTAAGTGGAGAAATAACATGTTGAAGAGCTGTGATGTTGG 6960  
 GCGGGAACGTGTAACTTGTAGGCTGAAGGATAGGCAATATTAGTGGCTGAGAGAGGACTGCTGGGTGGGAATGCAAAAATTCTCTGCTAAGACTTTTTCAGGTGAACATAACAGACTTGG 7080  
 CCAAGCTAGCATCTTAGCGGAAGCAGATCTCCAATGCTCTTCAGTAGGGTCATGAAGGT

TTTTCTTTT

TTTTCTTTT  
Depth:19 (ZEBRAFISH)  
Ei-value:0.000, Pi-value:0.000  
Er-value:0.000, Pr-value:0.000  
MATCHES To TargetScan▶ miR-186-5p:AAAGAAU

CCTGAGAAAACAACAAGTTTTGTTTTCT

CAGGTTTTGCTTT

CAGGTTTTGCTTT  
Depth:19 (ZEBRAFISH)  
Ei-value:0.000, Pi-value:0.000  
Er-value:0.000, Pr-value:0.000  
MATCHES To TargetScan▶ miR-330-3p.2:AAAGCAC▶ miR-490-3p:AACCUGG

TTGGCCTTTAC 7200  
 CTAGCTTTTTTTAAAA

AAAAAGCAAAA

AAAAAGCAAAA  
Depth:19 (ZEBRAFISH)  
Ei-value:0.000, Pi-value:0.000  
Er-value:0.000, Pr-value:0.000  
No matches to TargetScan

GATGCTGGTAGTTGGTACTCCTGGTTTCCAGGACGGGGTTCAAATCCCTGCGGCATCTTTGCTGGA                            7293
```

---

## >DOG (7477 bases)

```
 TTGTAAAGCTAGGTTCTATTTTTAACCCGGCCATGGAACTATTTTTGCTTCCCCTCACAAAGGCGGCGGAGGGTTATTGAAATCGCGGTGTGAGCTGTCTTCCGTCTATAAATATGCCTC 120  
 TCCCGGGTTCTGCTGCAGGCTTTCAGGCAGCGAGCGCAGAGACGGCTGGTGAGGGAGGAGGGACTGCTTAAGGCGACAGCAGATAAGTTTTTCTTAAAAAAAAAAAAGCTAGGAATTAAA 240  
 GCAATCTTTAGGAAACGTAAATAGGGATAATACTTCTTGAGATATTGCTTAGCATTAAATTCGTAGAGTAGTATTGTAATCTTAAAAAGAGCTTAAAAGGAAAGCAATGAAGATTTTAAA 360  
 GAGTAGCTTGAGAAAGAAAATGCTCTTTGTAGAAAAAGACGGAGGTTAAGAGATGAAGCTTAATGGAGTGGAAAGAAATCTAATTTTAGAAGAATTGTTGAGAGGACTGCAAAGTCCGGG 480  
 ATTCAATAAATCCTTTAAATCTGAGGGCAGCCTTTCAGGTTGTCGTTGAAATGTAAATGACCTAAGTAGAGCCTAAATGTTTTAGACCGGTTGTAGATGGTAAAGATTTAAAAAGTTTGT 600  
 CTTTTAAAAAGCTTAACTTGAAGGTGTTTTAAGAGCCCTTCAAATTCATGGGTTAGTGATTGCGTCATTTGACACCTAGATCTTAAGCTAATCCTTGCTAAAACTTGCAAATATTAATTA 720  
 GTGGTAGGATGAAAATTTGAAGAGATTAGAAAATTGAAGTGGAGAACTCGCAAGACCGAAGTACAGGAAGGTGAAGATTTAAAAGACCAGAAGTAAAGGTGAAGAAAAGACTTAGAGAAG 840  
 ATAGGAAAATTGGAAGAAAAATTACACGCCTTTTAGAAGACAAAAATAATTTAAACCTCGAAGGCAGGAAGCAGAAGAAAAAAATATTTAGAGAAGAACAAGGCAACAAGGTCTCAGATA 960  
 CAAATTGAGAAAGCCAGAAATTGGAAACGAGGTGGAAAATAAGGTCAGGGAAAGTACATCAGGTAGAAAATGAAAAGCTAGGACAACCGCTGAATATAGGCACATAAGATAGACGTCAAA 1080  
 AAAAAGAGAAGTTTAAAGCCAAAAAATTGGAAAAAGTAAAAACAGGAAAACTAAGAATTTTTAAGTAGTTATGGAATCTTTCCAAAAGCCCATGAATTTAATTTCTGGTGCAGAAGCTAG 1200  
 ATTTGAGGGTGATAAAATTACTGTAGACCGAGACCCAGCTTAGCCCAGGAGCTAGAAGGGAAAGATAAAAATCAGATTAACAGCGTTTGCTACCAAAAAGACTTAAGTTACTTAAGTAGA 1320  
 ATATTTCTGGAGTTAAAAATGTGAGGGTCTTTAAATATGGTAGCTTTTAGTGTTCTTGGAGAATTTAAAGAACTTTCATAACTGAAGTAATGCAAGATCAAGAATAACTACCAACTTAAC 1440  
 ATTCTCCCATTGGACTTTGAGCTAAGATGGTTTTTTAAATCCTGAGGACTAGCGTTAATTGGCAGCAGACCCAGGTACTACGCAGAAGTGGATTCAGTGAATCTAGGAAGACAGGAGCGG 1560  
 CAGACAGGATTCCAGGAGCCAGTGTCGGTGAAGCTAGGACTGAGGAGCGAGCTGCAGTTCGTGGTGAAGATAGGAAGGAAGGCGAAGTCCGGGAGCCAGTGCTGTTTGGTGAAGGAAGGA 1680  
 AGCTAGGAAGAAAGAAGGAGCCCTAACGATTTGGAGGTGAAGCTAGGAGAGGATTCCAGAAAGGAGGAGCCAGTGCGATGTGGTGATGAAGCTAGCGATTGGCGACCACGCGGAGGCGGC 1800  
 GAACAGGCGCTGTGGAGGATAGATAGAGGATCCTAGAGCAGCATCCCAGTGTGCGAGGCCCCAGGGAGGACGAGTGGTGTGGTAAAAATACCAGAGATGGGCTAACGTGGTTTTTGTAAC 1920  
 TTCAGTATGGTAACGTTAATTTGCTTTTCTAATGGTAACGGGGGAATTGTGGTACTTAAGCGCTAAGGGATTTATGGGGGTGTAGGCCGATTTCCGGGTGTCGTAGGTTTCTCTTTTTCA 2040  
 GGCTTGTTTTCTCATCTTGTCTGAAGCTTTTGAGGGCAGACTTCCAAGGCCTGGAGGAATGGTAGATGGCAAGTTTTTTTGTTTTGTTTTTAACCAATTTGAGGGAAACCAAATGAATTT 2160  
 GGTAGATCAACCTTGAGACCAAATTCTGGCAAGTTTTGTAAGAAGTTTTTAGCTTGGGGTAATGAAGCCTTTCAGTTTTTTGAATAGATGACCTTATTCTTCCTTCACCCTGCATTCAAG 2280  
 TTTTCTAAATGTGGAGTTTGGCTGTGTATGGGGGGGAGAGTTTTTTGTGGGGATGGGGGCGCAAAAATATGTTTTGAGTTCTTTTCCCCTTAGGTCTGTCTAGAGTCCCAAAGGCAAATG 2400  
 ACTCAAGGTAACAGGAAAAAAGAAAATCCAGTATCAGGATAATCAGAACACCACAGGTTTACAGTTCTAGAAACTAGAGCACGAGCAGCTCTCACTTTTGAGTTCTGTGGCGGAGCTGTC 2520  
 CATTGGAGAAATGGCTGGTAGTTCTTGTTTTTCCATTTATTCATCAGATATTTTAATACATAACCCTTTAAATTCGTTACATTTACTTAAC

TTTGGG

TTTGGG  
Depth:19 (ZEBRAFISH)  
Ei-value:0.000, Pi-value:0.000  
Er-value:0.000, Pr-value:0.000  
No matches to TargetScan

GTGGTCTTAACAGGGAAGAGGGT 2640  
 TGGGGGAGAAATTTTTCTTTCTAAGATTTTCCACAGATGCTATAGTACTATTGACAAACTGGGTTAGAGAAGGATTGTACTGCTATGCTGTTGGCACGAACACCTTCAGGGATTGGAGCT 2760  
 GCTTTTTTCCTTGGAAGAGTATTCCCAGTTGAAGCTGAAATGGTACAGCACAGTGCAGCTTTGGTTCATACTCAGTCTTCTCAGGAGCACTTCAGAAGAGCTTGACTAGGCCAGATGTTG 2880  
 AAGTTAAGTTTTCAAGTAATGTGATCTTTAACTTTTATTAAAGGGGAGGGGCAAATTTTGGCAATTGATAGTTGGCAGTGGCCTGTTAATGGTTGGGATTGGGGAGGGTTGGGGGTTAGG 3000  
 TAATTGTTTAGTTTATGATTTCAGATAACTCATGCCCGAGAACTTAAATACTTAGGATGGAAAAACTAAAGAAATCTCAACTTCCAAGTTGGCAAGTAACTCCCAATCCTCTAGTTTTTG 3120  
 TTTTCCCCCAATTTTTGAATTGGGCAGCTGGGGAAGTTCATGAAGATCCACTGGATTCATAGTGTAAGGCCTGGGGAAGGAAAGTATAAGGACTGGGGGTTGGTCTAGCCCACTGGCTGA 3240  
 CTTAGATATCATCTTGGGAATTTTTTTAAAAATGGCTAAAGTTGTTTGGATATGGTAGTGTGTGGTTCTCTTTTGGAATTTTTTTCAGGTGATTTAATATAACTTAATACTACTATAGGA 3360  
 GCTTCAGAGCAAAGGAAGTGGCTTAATGATCCTGAAGGGATTTCTTCCTGGTGGTAGCTTTTATATTATCAAGTAAGATTCTATTTTCAGTTGTGTATAAGCAAATTTCTTTTCTCTAGT 3480  
 GTAGGAAAAGTATTTTTCCATTAACTGCAAACAAGATGTTAAGGTATGCTTCAAAATTACTGTAAATTGTATATTTTAATCCTTTCTGATAAAAAAGGGGGTGGGGTGTTGTGGATGAGG 3600  
 GCGGGGAAACCTTTTTTTTTTCTGTAGACT

TTTTTCAG

TTTTTCAG  
Depth:19 (ZEBRAFISH)  
Ei-value:0.000, Pi-value:0.000  
Er-value:0.000, Pr-value:0.000  
No matches to TargetScan

ATAACTTCTGAGTCATAACCAGCCTGGCAAATACGATGACCTAGATGGAGAGGGAACAGCATCTTGGTGAATGATAAGTAAA 3720  
 GGCAGAAAAGATTATGTTTCATACCTCCATTGGGGAAAAAAGCATAACCCTGAGATCCTTAACTACTGATGAACGCATTATCTGCATATGCAAAAAAAATTAAGCAAGTGAAACTACCAA 3840  
 TTTAAAGTTAATGGAATCTACCATTTAAAAGTTATGGCCTATCAAGCTATACCCACACCCACAAAAAGAATTGATGAGCTGTAATATAATGAATTGATGAGAAATAACAACGAAGATCAA 3960  
 ATAACCATCTCAAAATACTGCTTTTACAAAAGCAGAATAAAACAAAATGAAAAATGGTAAAATCAACATTAATCCTGGAATAAAAGAAGCTGAAATAATTCAGAGTGGGATCAAATGGAT 4080  
 TGAGAGAAGGCTGTGCTGTTTGCCAATCTTTCGTTTGCCTCAGACAGGTTTCTCTTCATTATCAGAAGAGTTGCTTCATTTCATCTGGGAGCAGAAAACAGCAGGCAGCTGTTAACA

GAT

GATAAG  
Depth:19 (ZEBRAFISH)  
Ei-value:0.000, Pi-value:0.000  
Er-value:0.000, Pr-value:0.000  
No matches to TargetScan

 4200  


AAG

GATAAG  
Depth:19 (ZEBRAFISH)  
Ei-value:0.000, Pi-value:0.000  
Er-value:0.000, Pr-value:0.000  
No matches to TargetScan

TTTAATTTGAATCTGCAGTATTGCATGTTAGGGATAAGTGCTTATTTTTAATAGAACTGTGGAGCTCTTAAGTATTGAACTTGGCTGTGTTTCCTGATCCCTTCCTTAGAGGATTTC 4320  
 AGGATTGTGGGGAATTTTCCCATCTAGCCTTTTAAAATTAGGACTTACCGTGGGCTTCCATGATGGGATAGCACACCTCGTTCGGAGGCAAGTAATTCCTTAAAGGCTTTGAAGAGTAGT 4440  
 AGTGCCTTAATCATAAGGCAATGTTGATAAAACTAAGAAAATTGAATCTCTTAAATTCTACAAAAGGCTAGCATATTGTGCTGTTAAGAATCCGATGTTGTTAATCTTGCTAAAGCTTAA 4560  
 GTTTTGTGTCGTGATGTACGTTGTATAGAAAACATTAAATACTTCAAAATAAAACATTTTAATCTAGAGAATGTATGCTTTTAGAAAGCTGTCTCCTCATTTAAATAAAGTATTGTTTGT 4680  
 TTGTAGTTAGTGTTGGGCAATCTTGGGGGGATTCTTCTCTAATCTTTCAGAAACTTTGTCTGCGAGCACTCTTTAATGGACAAGATCAGGAATTGAGCGGAAGAACGAATTTAACTTTAA 4800  
 GGCAGGAAAAACATTTTATCCTTCGTAAAGTGATGAGCATTTAATAATTCCAGGCACATGGCAATAGAGGCCCTCTAAATAAGGAATAAATACCTCTTAGACAGGTGGGAGATTATGATC 4920  
 AGAATAACAGGTAACTACACCTACACTATTTCCAGAAAGTCAGGGGTCTCTAAATTTGACAGTGGTAGAGTAATAAAATACTGTCAACTTTTCCAGAGTTGCATGTGAACTTTAAATGCT 5040  
 TCTAATCATCTCATCTGATGTTAGCCAGTGTTGACACAACTGGCCTTTTCATATAGGTACTTGGGGGGGGGGCATGTGGGTTTTTAAAGAATTTTCTTTGCAGAGGCTTCATTTCATCCT 5160  
 TCATGAAGCTGTTCAGGATTTTGACTTGCATATAAGCGCTTGGCTCTTCCTTCTGTTCTAGTGAGTGTGAGACCTTGCAGTGTATCAGCATAATATTCAAGTAAACTTTCCCCTGATTTT 5280  
 GGAGGGGAGGAGGGGGAGGGGCTTACTTGTTTTAGCTTTTTTTTTACAGACCACACAGAATGCAGTTGTCTTGACCTCAGGTCTGTCTGTTCTGTTGGCAAGTAATGCAGTACTGTTCTG 5400  
 ATCTGCTGCTATTAGAATGCATTGTGAAACGACTGGAGTATGATTAAAAGTTGTGTTCCCCCAATGCTTGGAGTAGTGATTGTTGAAGGAAATCCAGCTGAGTGATAAAGGCTGAGTGTT 5520  
 GAGGAAATTTCTGCAGCTTTTAAGCATTCGTGTTTGTGATTGAAGCCAAGTACCTTTTGCTGTGCTTCTTTAGGTAAAATGCTTTTTGTTCACTTCTGGTAGGGGGGTGGGGAGGGTACT 5640  
 GAAGCCTTTAGTCTTTTCCAGATGCACCTTAAAATCAGTGACAAGAAATAAATTCCAAACAAGCAACAGCCTTCGAGAAATTTAACTGGCAAGTGGAAACGTTTTATCAGTTCAGTGGTC 5760  
 TTAGTGCATTTTCTTTGTGTGGGTTTCTCTCTCCCCTCCCTTGGTCTTAATTCTTACACACAGGAACATTCAAAACAGCAGACGTATGCGAAGGGCCAGAGAAGCCAGACCAGTAAGGAA 5880  
 AAAAAATAGCTTATTTACTTTAAATAAACAGTCCATTTTAAATGTGGGGATTGGGAACCACTAGTTCTTTCAGATGGTATTCTTCAGACTATAGAAGGAGCTTCCAGTTGAATTCACCAG 6000  
 TGGACAAAATGAGGACAACAGGTGAACACGCTTTTTTCTATGTTTACATACCAAGTCAGATCCGTTATGAATAAGTAATTTAGTGTTAAATAGACTTCCAGCTTTATGCTGGAATAAATA 6120  
 GCATGTGAGCAGCTGTTTGTTGGCCTGGGGGTGGAGGGGTAAGGTGGGCGCTAAGCCTTTTTTTAAGATTTTTCAGGTACCCCTCTAAAGGCACCGAAGGCTTAAGTAGGACAACCATGG 6240  
 AGCCTTCCTGTGGCAAGAGAGACAACAAAGCGCTATTACACTAAGGTCAATAAAAATGGTGTCAGCCTCCTAACCCCAGCTTCTGTTAGAAATGAGGACTTGCCTCAACACTTGGCAGAC 6360  
 ATGCACTGAGGCTCTCTTCGGGGAACGGAGCATCTAAAGGAATGTTTTGAGTACCTTCTGTACCCCTGGGCTTGTCCTGTCCTGACAATTATGCTGCTTTTATAGCAGCTATTTATTTAA 6480  
 AAAGCCCATACCTCAAGTGATGGTTGAAGGGGAGGGAAATGGGGAAAGAGGGCAACCACTTTTCCCTAGCTTTTCCAGAAGCCTGTTAAAAAAGCAAGGCCTCCCCACAAGTGACTACTC 6600  
 TGCCACATCGCCACTCCCTGCCTTTGGCCTAGCGCAGACCCTTCACCCCTCACCTCGATGCTGCCAGTAGCTTGGATCCTTGAGGGCATGACCCCTAATCGGTTTCAAGGTAACAGTGGT 6720  
 GCTGAGGTCTTCTGGTGGGTTGCACTACTAGAAAAGGCCATTAATTTGCCTGCAAATGCTTAAATGTTATTAAAACCACGACTAAATATTATAACACTGTTATCTTGTGGTTAGAACACT 6840  
 TAAGCCAGTGAATGGAGGATAATGTTTGAGACTGTAATGTTGGGTGGGAACATAGACCTTACAAATCAGAGATAGGCATTTTAGTATTGAGGACTGTTGGGGTGGGATTGCAAAAATTCT 6960  
 CTGCTAAGACTTTTTCAGGTGAACATAAAAGAGACTTGGCTAAGCTAGCATCTTAGCTGAAGCAGATACTCCAGTGTTCTTCAGTAGGGTTGTAAAGGT

TTTTCTTTT

TTTTCTTTT  
Depth:19 (ZEBRAFISH)  
Ei-value:0.000, Pi-value:0.000  
Er-value:0.000, Pr-value:0.000  
MATCHES To TargetScan▶ miR-186-5p:AAAGAAU

CCTGAGAAAACA 7080  
 AATTTTTGTTTTCT

CAGGTTTTGCTTT

CAGGTTTTGCTTT  
Depth:19 (ZEBRAFISH)  
Ei-value:0.000, Pi-value:0.000  
Er-value:0.000, Pr-value:0.000  
MATCHES To TargetScan▶ miR-330-3p.2:AAAGCAC▶ miR-490-3p:AACCUGG

TTAGCCTTTTCCTAGCTTAAAA

AAAAAGCAAAA

AAAAAGCAAAA  
Depth:19 (ZEBRAFISH)  
Ei-value:0.000, Pi-value:0.000  
Er-value:0.000, Pr-value:0.000  
No matches to TargetScan

GATGCTGGTGGTTGGCACTCCTGGATTTCCAGGACAGGGTTCAAATCCTTGCAGTGTCTT 7200  
 TGCTTGAACTACGATCTCTTGTCTTCAGGCCTCCTTCCATTTCCTCTTTTTAGATGTTAGGCTTTGGGATTTGGGGGAGATGGAAAAACACTTCGTGGAATTCAGCCAGAAACCTAAATC 7320  
 TGTTTTTATTTATGTGGGAACCTAATGATAACTGCCTTGTCTCTTTAATTTTCAGGTGGTCCCCATGCATCTAGTGTTTGGAGAAGACTTGCTTTGGAAGTCTTTAAAAATGGAAAACGC 7440  
 TTTTCATTGGTGTGATTCTTTTGTGTATAAACCAAAC                                                                                    7477
```

---

## >PIG (7817 bases)

```
 ATTCTATTTTTAACTCCTTCCGAGGAACTATTTTTGCTTCCCTCACAAAGGCGGCGGAGGGTTATTGGATGACGGGGGTGTGAGGTGTCCTTGGGCTATAAATACGCCGCGCGCTGCGGG 120  
 TCGGGAGGCAGGCATTGAGGCAGCGAGAGCAGAGACGGCTGAGAGGCAGGAAACGCTTCTTGAGGAGACCGCAGATAAGTTTTTCTGTTAAGATAGGGATTACTCTTATTCTCTAGGAAT 240  
 TATAAATTAGCGAAAATACTGAGCTGTAGTTTTGAGTTAAATTCGTGAGAGTAGATTTGATAATTAATAAGAAGGTCTTATTTTAGGGGAAAATGGAGACGTCCTTAAAAAATAGTGTGA 360  
 GAAAGGAAGAAAGAAGAAAAAAGATAATATTAGAACATGACGGAGGTTAAGAGCTGAAGCTTCTTCATGGAGTGAGAAAGCTAATTTTAAAAGGAGTTGCCGAGACAGGACTGCAAAGTA 480  
 TGGAATTTTAAAGTCTTAAATTGAAGGGCAGCTTTTTAGATTATTGTTCAAACGTAATGCTATAAATAGAACCTAAACAATACTTTTAAAGTCCAGTCGTAGATGGATTAAAATGTTTTA 600  
 AAAGCCATCTTTAAAGGTCAGTTGAAGGTGATTTAAGAAATGCCTTCAAATTCACGGCGCGATCAAAACTGCGTCATTTGAAGCCTTAAGGCTTTCTTTGGAAGTTGATGGAAGTATTAG 720  
 TGGAGAAAGAAGATTAGAAATTTGAAGTGGAAAACTCGGAAGACAAGTACAGGAAGGTGAAGAAAAGATTAGTGAAGATAGGAAAACTAGAAGATAAAAAATACGGCTTTTAGAAGACAG 840  
 AAAAAAACTAAGCCCAGAAGGTAGGAGGCAGAAGACATCTAGAAAGAGAAGGTTGAAAAGATGTTCGAGTGTTTGAGGTAGAAAATGAGAAACAAGCTGTTGGAGACAAGCATACAAGAT 960  
 AGAGGTCCAAGTAATTGGAAGATAGAAAAGTCCGAAGCCAAAAAATTGGATAAGTACAAGACGCAAAACTAAGAAAAATAATTTTTAATAGTTCAATTTATTTAAAAAGCCCATCAACTT 1080  
 TAATTTGTGGTGGTGCAGAAGTTAGATCTGAGAAGAGGGTTTTAAACTACAGTAGACCAAGACCAACTTAGAAGAATATCTCGAAGCTGTAGAGGGAAGTAAATTTAAAATCACAGCAAA 1200  
 AAGCTACTAAAAAGACTGATGTAAAAATTAAGGTAGACGGAAGAGTTAAAAATTTGAAAGGCCTTAAATCCATACAGTAGTTTAGTTTCTTCTGGAAAACTTAAAGGACGATAAATTCAA 1320  
 GAGCGAGGATAACCAAGTTAACATTTTTTGCATTGGACTTTGAGCTAAGATGAATTTCTAAACCCTGAGGACTAGGCGCTAATTAACAGCTGACCAGGTGCTACACAGAAGTGGATTCAG 1440  
 AGAACCTAGGAAGACAGCAGCAGACAGGATTCCAGGAGCCAGTTTTTGGTGAAACTAGGACTGAGGAGCGAGCGGCAGTTCGTGAAGATAGGAAGGAGGAGCCCGGGAGCCAGTGCGATT 1560  
 TGGTGAAGGAAGCTAGGAAGAAGGAAGGAGCCCTAACGGTTGGAGGTGAAGCTAGGAGAGGATTCCAGGAAGGAGGAAGCCAGTGCGATGTGGTGATGAAGCTAGCGTGCGGCTTGGCGG 1680  
 CGTGGCTTGGCAACCCCGCGGAGGAGGCGAGCAGGCGGTGTGGAGGCTAGATAGAGCGTCCTAGAGCGGCAGCCCAGTGTGCAAGGCCGGCAGGGAGAGCCAGTGGTGTGGTAAAAATCC 1800  
 GTGAGGTCGGCAGTATCGTGGGTTCTGCTTTGTTTCTTCACCTTTTTTTGGTAACAGTACGGTAATCTTTGTTGTATTTCCTAATGGTGATGGGGGAGCTGTGTACTTAAGCATAAAGGG 1920  
 TTTTATGGGGATGTAGGCCGATTTTCGGGTGTTGTAGGTTTCTCTTTTTCAGGCGTATTTTCTCATCTTGTCTGAAGCTTTTGAGGGCAGACTACCAAGGCCCGGAGGAATAGTAGATGG 2040  
 CAAGTGTTTTCGTTTTTAACCAATATGAGGGAAAACAAATGAATTTGATAGAACAACCTTGAGACAGTTTCAGCCAGCTTTGTAAGCAGGTTTTATATTTAGCTTGGTAATGAAGCATTT 2160  
 CCGTTTTGTGAGTAGATGACTTAATTTTCTTTCTCATTCTCTATTCTAAGTTACCTATATATATATAGTTGGGTGTGTAATTCGGGGGGGTAAAGGTTTTGGGGGGGCAAAATATGTTTT 2280  
 GAGTTCTTTTCCCCTTAGGTCTGTCTAGAATTCCAAAGGCAAATGACTCAAGGTAACAGGAAAAAAGAAAATCCAGTATCAGGATAATCAGAACACCACAGGTTTACAGTTTGTAGAAAC 2400  
 TAGAACACGAGCAGCTCTCACTTAAGGTCTGTGATGGAACTCCAGTGGAGAAATGGCTGGTACTTTTTCCACCCCTTAATCAGATTTTTTTTTTAATTAAAGTATGTAACCGGTTATTTT 2520  
 TACTTGAAGTAT

TTTGGG

TTTGGG  
Depth:19 (ZEBRAFISH)  
Ei-value:0.000, Pi-value:0.000  
Er-value:0.000, Pr-value:0.000  
No matches to TargetScan

GTGGTCTTAACAGGGAAGAGGGTGGGGGAGAAAATGTTTTTTTCTAAGACTTTCCACAGATGCTATAGTACTATTGACAAACTGAGTTAGAGAAGGATTGTA 2640  
 CTGCTATGCTGTTGGCACCAAACACCTTCAGGGATTGGAGCTGCTTTTTTCCTTGGAGTATTCCCAGTGAAGCTGAAATATACAGCACAGTGCAGCTTTGGTTCATATTCAGTCATCTCA 2760  
 GGAGAACTTCAGAAGAGCTTGACTAGGCCAAATATTGAAGTTAAGTTTTCAAATAATGGAACTTTTTAAGATGCCTTTTAAAGGGGAGGGGCAAGTTTTGGCAATTAGTTGGCAGTGGCC 2880  
 TGTTTTGGTTGGGGTTGGGTGGGTTGGGTTTAGGTAATTGTTTAGTTTATGATTTCAGATAACTCATGCCAGAGAACTTAAATACTTAGGATGGAAAAACTAAAGAAATCTCACCTCCCA 3000  
 AGTTGGCAAGTAACTCCTGATCCTCTAGTTTCTTGTTTTACCCCAATTTTTGAACTGGACAGCTGGGAACTTCAAAGATTCACTGGATTTAAAGTGTAGGGCTTGGGAAAGGAAAGTGTT 3120  
 GGATTGGGGGTTGGTCTAGCCCACTGGGTTGACCAAGATAAAAACTTGGGGAATTGTTTAAACACTAAAGTTGTTTGGATATGGTAGTGTGTGGTTCTCTTTTGGAATTTTTTTCAGGTG 3240  
 ATTTAATAAAACTTAATACTACTATAGGAGCTTCAGAGCAAAGGAAGTGGCTTAATGATCCTGAAGGGATTTCTTCTGATGGTAGCTTTTATATTATCAAGTAAGATTCTATTTTCAGTT 3360  
 ACATAAGCAAGTTTTTTAGTATAGGAAATTTTTGTATGGCTGCGAGACAAGATGTAAAGGTATGCTTCAAAATTGTTGTAAATTGTGTATTGAATTTGCACCTAAGTATTTGTAAATCAT 3480  
 AATCAATTAAAAGGGGTGGGTTTTTGTTGATGGGGGGGAAACCTTTTTTTTCTGTAGACT

TTTTTCAG

TTTTTCAG  
Depth:19 (ZEBRAFISH)  
Ei-value:0.000, Pi-value:0.000  
Er-value:0.000, Pr-value:0.000  
No matches to TargetScan

ATAACATCTTCTGAGTCATACCCAGCCTGGCAATATGATGGCCTAGATGGAG 3600  
 AGGGGAACAGCTCCTTGGTGAATGATAAGTAAAGGCAGAAAAGATTATATTTCATACCTATCTTCATTGGGCAAAAGCATAACTCTGAAATCCTTACTACTGATGAACTCATGGTCTGCA 3720  
 TATGCAAAAAAAAAATTTAAGCAAATGAAAGCTACCAATTTAAAGTTAATGGAATCTACTCTTAAAGTTATGGCATATCAAGCTGTATCCACAAAAATAATGAATCGATGAGAAATAAAA 3840  
 TAATGAAATGATGAGAAATACCATGAAGATCTAATGCCCACCTCAAAATACTGCTTTTACAAAAGCAGAATAAAAAATGAAATGAAATGAAAATGATAAAGCTACACATTAACCCTGGAA 3960  
 TAAAAGAAGCTGAAATAATTGAAAGGAGTGGGATCAAGTGGATTGAGAGGAAGCTGTGCTGTTTGCCAATCTTTCGTTTGCCTCAGACAGGTTTCTCATTATCAGAAGAGTTGCTTCATA 4080  
 TCATCTGGGAGCAGAAAACAGCAGGCAGCTGTTCACA

GATAAG

GATAAG  
Depth:19 (ZEBRAFISH)  
Ei-value:0.000, Pi-value:0.000  
Er-value:0.000, Pr-value:0.000  
No matches to TargetScan

TTTAACTTGCATCTGCAGTATTGCATGTTAGGGATAAGTGCTATGTTTAATAAGAGCTGTGGAGGTCCTAGGTCCCT 4200  
 TACTCAAGGGATCTCAGGATTTTGAGGAATTTTCCCATCCAGCCTTTTAAAATGAGACAGGCCTTATGCCTGTGGGCTTCCATGATGGGCTAACTCACTTCACTCGAGGCAAGTAATTCC 4320  
 TTAAAGCCTCTTGAGATTAATACCTTAATAATCAAGGATAATGTTGATAAAATAAAAATTTGTTTAGCATTGATTCTTGAAAACCCTATGAAAAGATAGCATAATGTGCTGTTAACCAGA 4440  
 ATCAGATATGTTAACCTTGCTAAAATTTAAATGTTATGTGTATTGTGCAGAAAACCGTAAAATCACTCAAAATAAAATACTTTATTTAGCGAATGTATGCTTTTAGAAAGCTGTCTCCTT 4560  
 ATTTAAATAAAATATTGTTTGTCTGTAGTTAGTGTTGGGCAATCTTGGGGGGATTCTTCTCATCTTTCAGAGACTTTGTCTGCGAACACTCTGTAATGGACAAGATCAGGAATTGAGCTG 4680  
 AAGAACGAATTTAACTTAAGGCAGGAAAGACAAATTTTATCCTTCGTAAAATTGATGACCATTTAATAATTCCAGGCACATGGCCATAGAGACCCTCTAAATAAATAAGGAATAAATACT 4800  
 TATTAGGCAGGTGGGAGATTATGATCAGAAGAACAGGTAACTACACCTACGCATTTTTCAGAAAGTCAGGAGTCGAAATTGACAGTACTGAAGTATAATGCGGTCCACATTTCCAAAGTT 4920  
 TGCATGTTAAATTGAAATGCTTACCATCTCATCTAGTGTTAGCCAATGCTTTACACTTATTAGCCTTTTATATAGACACTTGGTGGGGGGCATGTGGGTTTTTTAAAGAACTTTCCTTTG 5040  
 CAGAGGCTTCGTTTCATCCTTCATGAGGCTGTTCAGGATTTTGACTTGCATATAAACGCTTGGCTCTGCCTTTCTGTCTCCTAGTGGGTGTGTGAGACCTTGCAGTGAGTTTTCAGCATA 5160  
 CTCAAGTAAAAATTTTTTTCCCCCTGATTTTGGAGGGAAGGGAGGAGAGGGTGGGGCTTACTTGTTTTTAGCTTTTTTTTTGCAGACTACACAGAATGCAGTTGTCTTGACCTCAGGTCT 5280  
 GTCTGTTCTGTTGGCAAGTAATAATGCAGTACTGTTCTGACCTGCTGCTATTAGAATGCATTGTGAAACGACTGGAGTATGATTAAAAGTTATGTTTCCCCCAATGCTTGGAGTAGTGAT 5400  
 TGTTGAAGGAAAATCCACTGAGTGATAAAAGGCTGAGTGTTGAGGAAATTTCTGCAGCTTTAAGCATTCATGTTTGTGATTGAAGCAGTCCTTTTTGCTGTGCTTTAGGTAAAATGCTTT 5520  
 TTGTTCACTTCTGGTAGGGGGGGTGGGGAGGGCACTGAAGCCTTTAGTCTTTTCCAGATGCAACTTTAAAATCAGTGACAAGAAATAAGTTCCAAACGAACAACAGACTTCAAGAAGTTT 5640  
 AACTGGCAAGTGGAAACGTTTTAACATCAGTTCAGTGGTCTTAGTGCATTGTGTGTGCAGGTTTCTCTCTTCCCTCCCTTGGTCTCAATTCTTATACACAGGAACATTCAGAACAACAGA 5760  
 TGAAGGGCCAGAGAAGCCAGACCCAGTAAGGAAAAAAAAATAGTTCATTTGCTTTTAATAAACAAACCAAACATTCCATTTAAATGTGGGGATTGGGAACCACTAGTTCTTTCAGATGGT 5880  
 ACTCTTCAGACTAGAAGGAGCTTCCAGTTGAATTCACCAGGGGACAAAATGAGGACAACAGGTGAACAAGCTTTTTCTACGTTTACATACCAAGTCAGATCTGTTCCGGGTAAATTAGTG 6000  
 TCAAATGGCCTTTCATTTCAGCTTTATGCTGGAATAAACAGCATGAGCAAGTTTGTTGGCCTGGGGGTGGAGGGGTGAGGTGGGCGCTAAGCCTTTTTTTAAGATTTTTCAGGTACCCCT 6120  
 CAATAAAGGCACCCGAAGGCTTAAAGTAGGACAACCCTGGCGCCTTCCTGTGGCAAGAGAGACAACAAAGCGCTATTAAACTAAGGTCAATCTAAGTGGTGTCAGCCTCACAACCCCACC 6240  
 TTCGGTTAGACATGAGGACTTGCCTCAACTGCCTCTGGGGAGTGAAGCATCCAAAGGAATGCCTGCGTACCTTGCGTACCCCCGGCCTGTGTAACGCCTCCCTGGGCTGTTTTTAGAGCA 6360  
 GCCCTTGATGAAGCCCAGACCTGAGTGGTGCTTGAAGGGGAGGGAAAGGGGGAAAGCGGGCAACCACTTCTCCCTAGCTTTTCCAGAAACCTGTTAAAAAGCAAGGTCTCCCCACAAGTG 6480  
 ACTTCTCTGCCACATCGTCACCCCCTGCCTTTGGCCTAGCGCAGACCCTTCACCCCTCACCTCGATGCTGTCGGTAGCTTGGATCCTTGTGGGCATGATCCATAAGCAGTTTAAGGTAAC 6600  
 TGGTGCCGAGGTCTTGGGTGGGTTGCACTGTTAGAAAAGACCGTTAATTTGCCTGTAAACGTTTAATGACATTAAGACCACAACTAAAAAGCTCTCAGATGTTTTAAATGTGTAGCATTA 6720  
 TTTCTGTCCAGTGCTGAAAACAAACTTGAACCAGTAAATGGAGAAATAACATGTAAGAACTATACTGTTGGGTGGGAAAGTATAGCAAACTGAAGGAGATAGGCATTTTAGCAGTTGAGG 6840  
 GCTGTTGGGTGGGAATGCAAAAACTCTCTGCTAAGACTTTTTCAGGTGAACATAACAGACTTGGCCAAGCTAGCATCTTAGCTGAAGCAGATTCTCCAGTGCTCTTCAGTAGGGTCTTAA 6960  
 AATTTAAAGGT

TTTTCTTTT

TTTTCTTTT  
Depth:19 (ZEBRAFISH)  
Ei-value:0.000, Pi-value:0.000  
Er-value:0.000, Pr-value:0.000  
MATCHES To TargetScan▶ miR-186-5p:AAAGAAU

CCTGAGAAAACAAAATACTTTGTTTTCT

CAGGTTTTGCTTT

CAGGTTTTGCTTT  
Depth:19 (ZEBRAFISH)  
Ei-value:0.000, Pi-value:0.000  
Er-value:0.000, Pr-value:0.000  
MATCHES To TargetScan▶ miR-330-3p.2:AAAGCAC▶ miR-490-3p:AACCUGG

TTGGCCTTTACCTAGCTTTAAAAA

AAAAAGCAAAA

AAAAAGCAAAA  
Depth:19 (ZEBRAFISH)  
Ei-value:0.000, Pi-value:0.000  
Er-value:0.000, Pr-value:0.000  
No matches to TargetScan

GATGCTGGTGGTTGGCACTCCTGG 7080  
 CTTCCAGGACAGGGTTCAAATCCCTGCGGCGTCTTTGCTTGACTTTTACTTCTGCCTTTCCGTTTTCCTCCTTTTTCTTCAGATGAAGTTAGGGGAAGTGGAAGTAGCTTCAAACTATAC 7200  
 TATACCTCTTGATTGAATTTGTCCTAAACTGGTTTGTGTTTGTATTTTCACAGGAATCTAGTGTAATTACTTTGCCCTTTTAATTTTCAGGTATCTCTCATGCATCTGTTGTTTGGAGCA 7320  
 GTCTCACTATTTTTAAAAATAGATGACATATTAATAAAGGTTGAAAAACTTCATTGGTGTGATTCTTTTGTGCATAAGCTGAACTTCTCTATCCATGAGTAGTCAGAAGATGGGTGTCTT 7440  
 GTGGGGGTTCTTGTTCCTGTTTTAACTAGTTGTCTAAAAAGCACACTGCACTGTCAAGCCACCAAATTTCTAGGGGGGCAGGGGACCCCTCTGGAGGGGGAGGTGGTCTAGTGGGTGAGT 7560  
 GACCCGAAAGACCCAGTAACATACCCACAGAAATAAGAAGGGACACTTTAAAGCAGCAGTTGGCCGTGAGTGTGGCTGGATCTGGAGAGCTGGAGACCACGTGGTCTGGTACGATTCCCA 7680  
 AGCTGTAAAGTGATCTTGTGGTCACCTGTCCTGTGGAATGGCCAGTGTTCCAGCAGATAGACCTTCAATTGCAACTGAAGTGCCCTTTTTAAGTCCTACCCATCTTTTAAGTCCTTGCTT 7800  
 CTCAAGCCCTGTTCCCT                                                                                                        7817
```

---

## >COW (7147 bases)

```
 CTATAAATACTCCGCTGCGGACTGTGCGACAGGCATTGAGGCAGCGAGCGCAGAGATGGCTTTAGGGATGGAGATGCTGCTTGAGGAGCCCGCAGATAAGTTTTTCTATTAAAAAAGATA 120  
 GGCGTTAGTGTATTTCTTTAGGGATTATAAGTAGTGAAAATACGGAGATACTGCTTTGCATTAAGTTCTTAGATAAATTTTAGGGAAAATATGAAGACTTAAAGAGTAGTTGGAGAAAGG 240  
 AAGAGGGAAGAAAAAAGATAATTTTAGAACCTGAGGGAGGTTAAGAGCTGAAATTTTTTCATGGAGTGAAAAATTTAAAAGAATTGATGAGCTAGGACTGCAATGTCTGGATTTTTTTTT 360  
 TAAAGCCTTAACTTGAAGGGCAACCTTTTACCTTAACATTAAAACGTTGGGTTACGTAAATCGAACCTAAACGTTTTAAAAGGTTGAGTTGTAGGTGATTAAAATACTACAAAGGCCATC 480  
 TTAAGATTTAACTGAAGGTGGTTTCAGAAATAATGCCTTCAGATTCACGGCGCAGTAAAAATGGCGTCATTTGAAGCCTGCTTGATACTAAGGCTTTGTTTGGCTAAAGTTGACGGAAAT 600  
 ATTAGGAATGGTAGGAAAGAAAATTGGAAGTTTGAAGTGGAAAATTCGGAAGACAGAAGTACAGGAAGGTGAAGAAAAAACTAGAGAAGATAAGAGATACACGTTTTAGAAGATAAAACC 720  
 TGAGCCTAGTAAGTAGGCAGAAAAAGACAAATCTAGGAGAAAATTGAAAATACGGTCGAGTGCTTAAGATAGAATGAAAAGAAAGCTATTGGACGCAAACATACAAGATAGACGTCCAAA 840  
 AATTTAGATAGTTCAAAATCAATAATGTTGGATAAATTACAGGAACAAAAACTAAGGAAATTTTTATAGCCAATAAGAATTTATTGTAAAAGCCCATCAACTTATTTCTGGTGGTGCAAA 960  
 AGTTAGATCTCAGAAGATGAGGGTTTTTTTTTTTTAAAACTACTGTAGACCAGGACCAACTTAGAAGAAAATCCGGAAACTAGGAGAGTCAATTTAAAATCTCAGCAAAAAGTTACTGAA 1080  
 GGGGACTGAAGTAACTTAAAAGTTAAGAATGTATTTGGGAGAGTTAAAAATCTGAAAGGCCTTAAATGCGGTAGCGTAGTTTCCTTTGGAAAATGTAAAGGACTTTATAAGTGAATTAAA 1200  
 TTGAAGAGCAAGAATAACCATCGAGTTATTGGACTTTGAGCTAAGATGAATGTTTAAATCCTGAGGACTAGGCGCTAAATAACAGCTGACGCAGGTGCTACGCAGAAGTGGATTCAGTGA 1320  
 AGCTAGGAAGACAGGAGCGGCAGACAGGATTCGGAGCCAGTGTTGGTGAAAACTAGGACTGAGGAGCCAGCGGCAGTTCGTGAAGATAGGAAAGAGAAGCCCGGGAGCCAGTGCGACTTG 1440  
 GTGAAGGAAGCTAGGAAGAAGGAGCGAGTCCTAACGATTTGGAGGTGAAGCTAGGAGAGGATTCCAGGAAGGAGGAAGCCAGTGCGGTGTGGTGAAGCTAGCGTGCGGCTTGGCTTGGCA 1560  
 ACCACGCGGAGGCGGCGAGCAGGCGGAGTGGAGGATAGATAGAGGATCCTAGAGCAGCAGACCAGTGTACGAGCCCCGCAGGGAGAGCCAGTGGTGTGGTAAAAATCCGTGAAGTCGGCA 1680  
 CTATCGTGGGTTTTTTTTCCTCCTGTAACTCAGTATGGTAATATCTCTTATTAAATTTTCCTAATAGTAATGGGGGAGTTGTGTACTTAAGCATAAAGGGGATTTTATTACGGGGATGTA 1800  
 GGCCGGTTTTCGGGTGTCGTAGGTTTCTCTTTTTCAGGCCTATTTTCTCATGCATCTTGTCTGGAGCTTTTGAGGGCAGACTTCAAGGCCCGGAGGAATGGTAGATGGCAAGTGTTCTTG 1920  
 TTTTTAACCAATATGAGGGATGTCCTGAATTTGGTAGAACAACTGTGAGACAACTTCAGCAAGCTTTGTAAACAGGTTTTATATTCAGTTTGGGGGTAATGAAGCATTTCAGTTTCGTGA 2040  
 GTAGAGGGCCTACTTTTCTTTCCTCACCCTGTATTTTAAAAGTCTTCTAAATATGTAGTTTCGGTGTATAATTCTGGGCGGGGGGAGTTTTTTTGGGGGGGGAGCAAAATATGTTTTGAG 2160  
 TTCTTTTCCCTTTAGGTCTGTCTAGAATTCCAAAGGCAAATGACTCGAGGTAACAGGCGAAAAGAAAATCCAATATCAGGATAATCAGAACACTGCAGGTTTACAGTTATAGAAACTCTA 2280  
 GCACAAGCAGCTTTCACTTTGAGGTCTGTGGCGCAGCTGTCCATTGGAGTAATGGCTGGTAGTTTATTCTTTTTTTCCCCACCCCTTTATTAAATATTTTTATAAAGTACGTAACTGGTT 2400  
 ATTTTTACTTAAATAT

TTTGGG

TTTGGG  
Depth:19 (ZEBRAFISH)  
Ei-value:0.000, Pi-value:0.000  
Er-value:0.000, Pr-value:0.000  
No matches to TargetScan

GTGGTGTGAACAGGGAAGAGGGTGGGGGAGAAACTTTTTTTTTCTAAGATTTTCCACAGATTCTATAGTACTATTGACAATCTGAGTTAGAGAAGGAT 2520  
 TGTACTGCTACGCTGTTTGGCACCAACACCTTCAGGGATTGGAGCAGCTTTTTTCCTTGGAAGAGTATTCCCAGTGAAGCTGAAATGTACAGCCCAGTGCAGCTTTGGTTCATATTCAGT 2640  
 CATCTCAGGAGAACTTCAGAAGAGCTTGACTAGGCCAAATGTTGAAGTTAAGTTTTCAAATAACGACTTTTAAGATACCTTTATTAAAGGGGAGGGGCAAATTTTGGCGATTTGTAGTTG 2760  
 GCAGTGGCTTGTAATGATTGGGATGGGGGGGGCGTGGGTTTAGGTAATTGTTTAGTTTTTGATTACAGATAAACTCATGCCAGAGAACTTAAATACTTAAGGTGGAAAAACTAAAGAAAT 2880  
 CTCAACTTCCAAGTTGGCAAGTAACTCGAAATCCTCTGGTTCTGTTTTGCCCCAATTTTTGAATTTAACAGATGGGGAAGTTGATGACGATTCACTGGATTTAAAAGAGTAGGGCTTGGG 3000  
 AAAGGAAAATGACTGGGGGTTGGTCTGGCCCTCTGCGCTGACTTAGATAAAAACTTGGGGAATTTTTTTTTAAATGGTAAAGTTCGGATATAGTGTGTGGTTCTCTCTTGGAATATTTTT 3120  
 CAGGTGATTTAATAAAACTTAATACTACTATAGGAGCTTCAGAGCAAAGGAGGTGGCTTAATGATCCTGAAGGGATTTCTTCTGGTGGTAGCTTTTGTATTATCAAGTAAGATTCTTTTT 3240  
 TCGGCTGTATATAAGCAAGATTGTTTGGTTTATTCTTTTCTTTTTTAGCGTAGGAAAAGTTTGTTGTTAAGGTATGCTTCAAAATTATTGTTAATTGTATTTTGAATTTACATCTAAGCC 3360  
 TTGTAAATCATAACTGATAAGAGGGGGTGGGCTTTTGTTGATGAGGGAGGGGAAACCTTTTTTTTTTGTAGACT

TTTTTCAG

TTTTTCAG  
Depth:19 (ZEBRAFISH)  
Ei-value:0.000, Pi-value:0.000  
Er-value:0.000, Pr-value:0.000  
No matches to TargetScan

ATAACATCTTCTGAGTCATAACCAGCCTGGCAATCTGA 3480  
 TGGCCTAGATGGAGAGGGGAACAGCTCCTTGGTGAATGATAAGTAAAGGCAGAAAAGATTGTATTTCATACCTACATTGGGGAAAAAAGCATAACCCTGAGATTTTCTTCTGATGAACAC 3600  
 ATTGTCTGCACATGCAAAAAAAAAAGCAAATGAAAACTACCAATTTAAAGTTAATGGAATCTACTACTATAAAGTTAATGGCTTATCAAGCTGTATCCACAAAAATAATGAATTGATGAA 3720  
 AAATAAAAATAACGATACAATGAAGATCAAAATGTCAATCTCAATACTGCTTTTACAAAAGCAGAATTAAAAAAGCGAAATGAAAATAGCGCTACATATTAATCCTGGAAAAAAAAGAAG 3840  
 CTGAAATAATTGAAATGAGTGGGATCAAGTGAATTGAGAGGAGGAAGAGGCTGTGCTGTTTGCCAATCTTTCGTTTGCCTCAGACAGGTTTTTCTTCATTATCAGAAGAGTTGCTTCATT 3960  
 TCATCTGGGAGCAGAAAACAGCAGGCAGCTGTTAACA

GATAAG

GATAAG  
Depth:19 (ZEBRAFISH)  
Ei-value:0.000, Pi-value:0.000  
Er-value:0.000, Pr-value:0.000  
No matches to TargetScan

TTTAACTTGCATCTGCAGTATTGCATGTTAGGAATAAGTGCTTACCTTTGTGAAGAGCTGTGGAAGTCCTAGGTCCC 4080  
 TTACCTAAGGGATTCCAGGATTGTGAGGCATCGTCCCATCCAGCCTTTTAAAATTAGACAGGACAGCGTTCCTGTGGGCTTTGATGATGGGACAGCACATTTCATTCAAGAGGCAAGTAA 4200  
 TTCCTTAAAGCCTCTTGAGATTAGCAAAGATAATGTTTATAAAACTAAGAACATTTGTTTAGCCTTGAATCTGACAGCCCTATTTAAGGGATAGTATTGTACTGTTAAGAATTCAGATTT 4320  
 TGTTAATCTTGCTAAAATTCAAATGTTACGTGTATTGTGCAGAAAACCATTAAATCATTCAAAATAAAATACTTTTATTTTTAGAGAATGTATGCTTTTAGGAAGCTGTCTCCTTATTTA 4440  
 AATAAAACATTGTTTGTCTGTAGTTAGTGTTGGGGCAATCTGGGGGGGGTTTCTTCTCATCTTTCAGAAACTTTGTCTGCGAACACTCTTTAATGGACAAGATCAGGAAATTGAGCAGAA 4560  
 GAACGAATTTAATTTAAGGCAGGAAAAAATTTTGTCCTTCGTAAAAGTGATGAGCATTTAATAATTCCAGGCACATGGCAATAGAGGCCCTCTAAATAAGGAATAAATACCTCTTAGACA 4680  
 GGTGGGAGATTATGATCAGAAGAACAGGTAACTTAACTCCTACAATGTTTGCAGAAAGTCAGGAGTCTGTCTAGTGGTTAAACTAGAGTAATGGAATGTACTGTCCACATTTCCAGTTTG 4800  
 CATGTTAACTTTGCTTAGTCATCTCATCTGGTGTTAGCCAGTGTTCACACTGTTGGCCTACTTTTTAGATACCTGGTGGGGGGGTGGTTGGTTTTTTTTTTTTTTTTAAAGAATTTTCCT 4920  
 TTGCAGAGGCTTCATTTCATCCTTCGTGAAGCTTTTCAGGATTTTGGCTTTCATGTAAACGCTTGGCTCTGCCTTCTGTTCTCCTAGGGAGTGTTTCAGACCTTGCAGTGAGTTTATCGG 5040  
 CATATTTAAGTATAAACATTTTTCCCCTTGAATTTGGAGGGAAGGGAGGAGAGGGTGGGGCTTACTTGTTTTAGCTTTTTTTTTTACAGACTACACAGAATGCAGTTGTCTTGACCTCAG 5160  
 GTCTGTGTGTTCTCTTGGCAAGTAATACAGTACTGTTCTGATCCGCTGCTATTAGAATGCATTTTGAAACGACTGGAGTATGAGTCAAAGTTGTGTTTCCCCCAATGCTTGGAGTGGAGT 5280  
 AGTGATTGTTAAAAGAAAATCCAGCTGAGTGATAAAAGGCTGAGTGTTGAGGAAATTTCTGCAGCTTTTTAAGCATTCATGTTTGTGATTGGAGCGGAGTCCCTTTTGCTGTGCTTTTTT 5400  
 TTAGGTAAAATGCTTTTTGTTCACTTCTGGTAAGGGGGTGGGGAGGGCACTGAAGCCTTTAGTCTTTTCCAGATGCAACTTTAAAATCAGTGACAAGAAATAATTCCAAACGAGCAGCAC 5520  
 TCTTGAAGAAATTTAACTGGCAAGTGGAAATGTTTACCAACAGTTCAGTGGTCTTAGTGCATTTGTTTTTGTTTGGGTGTCTCTCTCTCCTCCCCTGGTCTTAATTTCATATACAGGAAC 5640  
 ATTCAAAACAACAGACAGACGTATGCGAAAGGCCAGAGAAGCCAGACCCAGTAAGGAAAAATAGCTTACTTACTTTTAAAATAATAAACCAAAAAACACTGCATTTTAAATGTGGGGATT 5760  
 GGGAACCACTAGTTCTTTCAGATGGTATTCTTCAGACTATAGAAGGAGCTTCCAGTTGAATTCACCAGTGGACAAAATGAGGACAACAGGTGAACAAGTTTTTTCTATGTCTACATACCA 5880  
 AGTCAGATCTGTATGAGTAAACAACAATTCAGTGTTAAATGACCTTTCAGCTTTACGCTGGAGTCAACAGCATGAGCAAGTTGTTTGTTGGCCTGGGGGTGGAGGGGTGAGGTGGGCGCT 6000  
 AAGCCTTTTTTTTAAGATTTTTCAGGTACCCCTCACTAAAGGCACCAAAGGCTTAAAGTAGGACAACCATGGAGTCTTCCTGTGGCAAGAGAGACAACAAAGCGCTATTAACTAAGGTCA 6120  
 ATCAAAATGGTGTCGGCCTCACAGCCCCATCTTCTGTTAGAAATGAGGACTTGACTCAACCCCCTTGACAATGTGCATTGAGGCTCTCTGGGGGAGCGAGCATTTAAAGGAATGCTTGAG 6240  
 TACCTTGTATATATATCCCTGTGCTTGTCCTAATATTTAATTTGGCTGTTTTCATAGCAGCTGTTAATGAAGCCTGAACTTCAAGTGATGCTTGAAGGGGAGGGAAAGGGGGAAAGCGGG 6360  
 CAACCACTTTTCCCTAGCTTTTCCAGAAGCCTGTTAAAAAGCAAGGTCTCCCCACAAGTGACTTCTCTGCCACATCGCCACCCTGTGCCTTTGGCCTAGCGCAGACCCTTCACCCCTCAC 6480  
 CTCGATGCTGCTGGTAGCTTGGATCCTTGTGGGCATGATCCATAATCGCTTTTAAGGTAATTGCCAAGGTCTTCCGGTGGGTTGCACTACTAGAAAAGACCATTAATTTACCTGTCAACA 6600  
 GGTAATATTTAGACCACAACTAAAAAGCTCTTAAAGTGTTGTAAATGTGTGGCATTATCCAATGACTTTAAAAAAACATTTCAAACCAGTAAGTGGAGAAATAGCATCTAAGAACTGTCA 6720  
 TATTGGGTGGGAGAGTACATTTATGAACTGAAAAGTGTTTTGGCAGTTGAGAGGGCTGTTGGGTGGGATTGCAAAAATTCTCTGCTAAGACTTTTTCAGGTGAACATAACAGACTTGACC 6840  
 AAGCTAGCACCTTAGCTGAAGCAGATTCTCCAGTGCTCTTCAATAGGGTTATTGATAAAGGT

TTTTCTTTT

TTTTCTTTT  
Depth:19 (ZEBRAFISH)  
Ei-value:0.000, Pi-value:0.000  
Er-value:0.000, Pr-value:0.000  
MATCHES To TargetScan▶ miR-186-5p:AAAGAAU

CCTGAGAAAACAAAATCTTTTTTGTTTTCT

CAGGTTTTGCTTT

CAGGTTTTGCTTT  
Depth:19 (ZEBRAFISH)  
Ei-value:0.000, Pi-value:0.000  
Er-value:0.000, Pr-value:0.000  
MATCHES To TargetScan▶ miR-330-3p.2:AAAGCAC▶ miR-490-3p:AACCUGG

TTTGCC 6960  
 TTTTCTTATCTTAAAAA

AAAAAGCAAAA

AAAAAGCAAAA  
Depth:19 (ZEBRAFISH)  
Ei-value:0.000, Pi-value:0.000  
Er-value:0.000, Pr-value:0.000  
No matches to TargetScan

GATGCTGGTGGTTGGCACTCCTGGTTTCCAGGACGGGGTTCAAATCCCTGTGGCGTCTTTGCTTGACTTTTACTCTCCAGACTCTACTTCCT 7080  
 GTCTCTCAGGTGCTAGGCCTTAGAATTAGGGGAGGTGGAAGATAATAGTGTGAAACTCTGCATTTGT                                                      7147
```

---

## >MOUSE (6982 bases)

```
 AGGCATTCAGGCAGCGAGAGCAGAGCAGCGTAGAGCAGCACAGCTGAGCTCGTGAGGCAGGAGACTCAGCCCGAGGAAATCGCAGATAAGTTTTTAATTAAAAAGATTGAGCAGTAAAAA 120  
 GAATTAGAACTCTAAACTTAAGCTAATAGAGTAGCTTATCGAAATATTACTTAGTCTTAATAATCTAAGAAGATCTTAAGAGATAACATGAAGGCTTATTTAAACAGTTTGAAAAAGGAA 240  
 ATGAGGAGAAAAGTATTTGTACTGTATAATGGAGGCTGACCAGAGCAGTTTAGGAGATTGTAAAGGGAGGTTTTGTGAAGTTCTAAAAGGTTCTAGTTTGAAGGTCGGCCTTGTAGATTA 360  
 AAACGAAGGTTACCTAAATAGAATCTAAGTGGCATTTAAAACAGTAAAGTTGTAGAGAATAGTTTGAAAATGAGGTGTAGTTTTAAAAGATTGAGAAAAGTAGGTTAAGTTGACGGCCGT 480  
 TATAAAAATCCTTCGACTGGCGCATGTACGTTTGAAGGCATGAGTTGGAAACAGGGAAGATGGAAGTGTTAGGCTAGCCGGGCGATGGTGGCGCACGCCTTTAATCCTAGCACTTGGGAG 600  
 GCAGAGGCAGGCGGATTTCTGAGTTCGAGGCCAGCCTGGTCTACAGAGTGAGTTCCAGGACAGCCAGGGCTACACAGAGAAACCCTGTCTTGAAAAAACAAAAAGGTTAGGCTAGTATTT 720  
 GGAGAAAGAAGATTAGAAAATGGAAGTGAAAGACGAAGAAGACATACAGGAAGGTGAAGAAAAAGCTGTTAGAGAAGATAGGAAAATAGAAGACAAAGCATCTTTAGAAGACAGAAAAGG 840  
 TACTTAAAGGCACAGGTAGTAGGAAGCCGAAGAATAGAAGATAGAAAGAAGCAAGATAGAAAAACAAAATGGAAGTTAAGACAACTTTGGATGCCAGCATTCAAGATAGGCAAAGAAGAT 960  
 AAGATTGAGGCCAAAAGGTTGGATAAGATATAAAGTCAGAAGGAAATTATCTTTAAAGCCATAAGTTCAAATTTCTGATGGAGCGAGCAGTTTAGAAGAGTCTTTAGACAGCCACATACA 1080  
 AGATTGAAGCTAGCAATCAAAGCTACTAGGACTGAAGTAAAAAGTTAAGGCAGAATGCCTTTGAAGAGTTAGAAGAATATTAAAAGCCTTAACTTGTAGCTTAATTTTGCTTGATGACAA 1200  
 AAGGACTTTTGATAACAGTTTCAAGATTGTCAGCATTTTGCATTGGACTTGAGCTGAGGTGCTTTTAAAATCCTAACGACTAGCATTGGCAGCTGACCCAGGTCTACACAGAAGTGCATT 1320  
 CAGTGAACTAGGAAGACAGGAGCGGCAGACAGGAGTCCCGAAGCCAGTTTGGTGAAGCTAGGAAGGACTGAGGAGCCAGCAGCAGCAGTGCATGGTGAAGATAGCCCAGGAAAGAGTGCG 1440  
 GTTCGGTGGAGGAAGCTAGGAAGAAGGAGCCATACGGATGTGGTGGTGAAGCTGGGAAAGGGTTCCAGGATGGTGGAGCGAGAGCGAGTTGGTGATGAAGCTAGCTGGCGGCTTGGCTTG 1560  
 TCAACTGCGCGGAGGAGGCGAGCAGGCATTGTGGAGAGGATAGATAGCGGCTCCTAGACCAGCATGCCAGTGTGCAAGAAAGGCTGCAGGGAGAGCATGCGGTGCGGTAACATTCCTTGA 1680  
 GGTCGGCAACATGGTGGTGGTTTTCTGTAACTTGGATGGTAACTTGTTTACTTTGTCTTAATAGTTATGGGGGAGTTGTAGGCTTCTGTGTAAAGAGATATATCTGGGGCTGTATGTAGG 1800  
 CCTTTGCGGGTGTTGTAGGTTTTTCTTTTTCAGGGTTATGTCCTCTTGCATCTTGTCAGAAGCTTTTGAGGGCTGACTGCCAAGGCCCAGAAAGAAGAATGGTAGATGGCAAGTTGTCTT 1920  
 TAACCGCTCAGAGGGGAATGAATGGTAGAGCCAGCACAACCTCCCAGTTTTGTAAGACGTTGTAGTTTGAACAGATGACCTACCACAAGCCTCACTCCTGTGTAGGGGAGGTAATTGGGC 2040  
 AAAGTGCTTTTGGGGGAATGGGGGCAAAATATATTTTGAGTTCTTTTCCCCTTAGGTCTGTCTAGAATCCTAAAGGCAGATGACTCAAGGGAACCAGAAAAAAGGAAATCCACTCTCAGG 2160  
 ATAAGCAGAGCTCGCCAGGTTTACAGTTTGTAGGAAGTAGAGGATGGATGCTAGCTTTCACACTGAGTGTGGAGGAGCTGGCCATGGCGGAATTGCTGGTAGTTTACTCTTTCCCCCTCC 2280  
 CTTAATGAGATTTGTAAAATCCTAAACACTTTTACTTGAAATA

TTTGGG

TTTGGG  
Depth:19 (ZEBRAFISH)  
Ei-value:0.000, Pi-value:0.000  
Er-value:0.000, Pr-value:0.000  
No matches to TargetScan

AGTGGTCTTAACAGGGAGGAGTGGGTGGGGGAAACGTTTTTTTTCTAAGATTTTCCACAGATGCTATAGTT 2400  
 GTGTTGACACACTGGGTTAGAGAAGGCGTGTACTGCTATGCTGTTGGCACGACACCTTCAGGGACTGGAGCTGCCTTTTGTCCTTGGAAGAGTTTTCCCAGTTGCCGCTGAAGTCAGCAC 2520  
 AGTGCGGCTTTGGTTCACAGTCACCTCAGGAGAACCTCAGGAGCTTGGCTAGGCCAGAGGTTGAAGTTAAGTTTTACAGCACCGTGATTTAAAATATTTCATTAAAGGGGAGGGGTAAAA 2640  
 CTTAGTTGGCTGTGGCCTTGTGTTTGGGTGGGTGGGGGTGTTAGGTAATTGTTTAGTTTATGATTTCAGATAATCATACCAGAGAACTTAAATATTTGGAAAAACAGGAAATCTCAGCTT 2760  
 TCAAGTTGGCAAGTAACTCCCAATCCAGTTTTTGCTTCTTTTTTCCTTTTTCTTTTTTTGAGGCGGGCAGCTAAGGAAGGTTGGTTCCTCTGCCGGTCCCTCGAAAGCGTAGGGCTTGGG 2880  
 GGTTGGTCTGGTCCACTGGGATGATGTGATGCTACAGTGGGGACTCTTCTGAAGCTGTTGGATGAATATAGATTGTAGTGTGTGGTTCTCTTTTGAAATTTTTTTCAGGTGACTTAATGT 3000  
 ATCTTAATAACTACTATAGGAACAAAGGAAGTGGCTTTAATGACCCTGAAGGAATTTCTTCTGGTGATAGCTTTTATATTATCAAGTAAGAGATACTATCTCAGTTTTGTATAAGCAAGT 3120  
 CTTTTTCCTAGTGTAGGAGAAATGATTTTCCTTGTGACTAAACAAGATGTAAAGGTATGCTTTTTTTCTTCTTGTGCATTGTATACTTGTGTTTATTTGTAACTTATAATTTAAGAATTA 3240  
 TGATAATTCAGCCTGAATGTCTTTTAGAGGGTGGGCTTTTGTTGATGAGGGAGGGGAAACCTTTTTTTTTCTGTAGACC

TTTTTCAG

TTTTTCAG  
Depth:19 (ZEBRAFISH)  
Ei-value:0.000, Pi-value:0.000  
Er-value:0.000, Pr-value:0.000  
No matches to TargetScan

ATAACACCATCTGAGTCATAACCAGCCTGGCAG 3360  
 TGTGATGACGTAGATGCAGAGGGAGCAGCTCCTTGGTGAATGAGTGATAAGTAAAGGCAGAAAAAATAATGTCATGTCTCCATGGGGAATGAGCATGAGCCAGAGATTGTTCCTACTGAT 3480  
 GAAAAGCTGCATATGCAAAAATTTAAGCAAATGAAAGCAACCAGTATAAAGTTATGGCAATACCTTTAAAAGTTATGGCTTATCTACCAAGCTTTATCCACAAAAGTAAAGAATTGATGA 3600  
 AAAACAGTGAAGATCAAATGTTCATCTCAAAACTGCTTTTACAAAAGCAGAATAGAAATGAAGTGAAAATGCTGCATTAAGCCTGGAGTAAAAAGAAGCTGAGCTTGTTGAGATGAGTGG 3720  
 GATCGAGCGGCTGCGAGGCGGTGCAGTGTGCCAATGTTTCGTTTGCCTCAGACAGGTTTCTCTTCATAAGCAGAAGAGTTGCTTCATTCCATCTCGGAGCAGGAAACAGCAGACTGCTGT 3840  
 TGACA

GATAAG

GATAAG  
Depth:19 (ZEBRAFISH)  
Ei-value:0.000, Pi-value:0.000  
Er-value:0.000, Pr-value:0.000  
No matches to TargetScan

TGTAACTTGGATCTGCAGTATTGCATGTTAGGGATAGATAAGTGCCTTTTTTCTCTTTTTCCAAAAAGACCTGTAGAGCTGTTGAATGTTTGCAGCTGGCCCCTCTTAG 3960  
 GCAGTTCAGAATTTTGAGTAGTTTTCCCATCCAGCCTCTTAAAAATTCCTAAGCCTTGCACCGATGGGCTTTCATGATGGGATAGCTAATAGGCTTTTGCATCGTAAACTTCAACACAAA 4080  
 AGCCTACATGATTAATGCCTACTTTAATTACATTGCTTACAAGATTAAGGAATCTTTATCTTGAAGACCCCATGAAAGGGATCATTATGTGCTGAAAATTAGATGTTCATATTGCTAAAA 4200  
 TTTAAATGTGCTCCAATGTACTTGTGCTTAAAATCATTAAATTATACAAATTAATAAAATACTTCACTAGAGAATGTATGTATTTAGAAGGCTGTCTCCTTATTTAAATAAAGTCTTGTT 4320  
 TGTTGTCTGTAGTTAGTGTGGGCAATTTTGGGGGGATGTTCTTCTCTAATCTTTTCAGAAACTTGACTTCGAACACTTAAGTGGACCAGATCAGGATTTGAGCCAGAAGACCGAAATTAA 4440  
 CTTTAAGGCAGGAAAGACAAATTTTATTCTCCATGCAGTGATGAGCATTTAATAATTGCAGGCCTGGCATAGAGGCCGTCTAACTAAGGACTAAGTACCTTAGGCAGGTGGGAGATGATG 4560  
 GTCAGAGTAAAAGGTAACTACATATTTTGTTTCCAGAAAGTCAGGGGTCTAATTTGACCATGGCTAAACATCTAGGGTAAGACACTTTTCCCCCACATTTCCAAATATGCATGTTGAGTT 4680  
 TAAATGCTTACGATCATCTCATCCACTTTAGCCTTTTGTCACCTCACTTGAGCCACGAGTGGGGTCAGGCATGTGGGTTTAAAGAGTTTTCCTTTGCAGAGCCTCATTTCATCCTTCATG 4800  
 GAGCTGCTCAGGACTTTGCATATAAGCGCTTGCCTCTGTCTTCTGTTCTGCTAGTGAGTGTGTGATGTGAGACCTTGCAGTGAGTTTGTTTTTCCTGGAATGTGGAGGGAGGGGGGGATG 4920  
 GGGCTTACTTGTTCTAGCTTTTTTTTTACAGACCACACAGAATGCAGGTGTCTTGACTTCAGGTCATGTCTGTTCTTTGGCAAGTAATATGTGCAGTACTGTTCCAATCTGCTGCTATTA 5040  
 GAATGCATTGTGACGCGACTGGAGTATGATTAAAGAAAGTTGTGTTTCCCCAAGTGTTTGGAGTAGTGGTTGTTGGAGGAAAAGCCATGAGTAACAGGCTGAGTGTTGAGGAAATGGCTC 5160  
 TCTGCAGCTTTAAGTAACCCGTGTTTGTGATTGGAGCCGAGTCCCTTTGCTGTGCTGCCTTAGGTAAATGTTTTTGTTCATTTCTGGTGAGGGGGGTTGGGAGCACTGAAGCCTTTAGTC 5280  
 TCTTCCAGATTCAACTTAAAATCTGACAAGAAATAAATCAGACAAGCAACATTCTTGAAGAAATTTTAACTGGCAAGTGGAAATGTTTTGAACAGTTCCGTGGTCTTTAGTGCATTATCT 5400  
 TTGTGTAGGTGTTCTCTCTCCCCTCCCTTGGTCTTAATTCTTACATGCAGGAACATTGACAACAGCAGACATCTATCTATTCAAGGGGCCAGAGAATCCAGACCCAGTAAGGAAAAATAG 5520  
 CCCATTTACTTTAAATCGATAAGTGAAGCAGACATGCCATTTTCAGTGTGGGGATTGGGAAGCCCTAGTTCTTTCAGATGTACTTCAGACTGTAGAAGGAGCTTCCAGTTGAATTGAAAT 5640  
 TCACCAGTGGACAAAATGAGGACAACAGGTGAACGAGCCTTTTCTTGTTTAAGATTAGCTACTGGTAATCTAGTGTTGAATCCTCTCCAGCTTCATGCTGGAGCAGCTAGCATGTGATGT 5760  
 AATGTTGGCCTTGGGGTGGAGGGGTGAGGTGGGCGCTAAGCCTTTTTTTAAGATTTTTCAGGTACCCCTCACTAAAGGCACTGAAGGCTTAATGTAGGACAGCGGAGCCTTCCTGTGTGG 5880  
 CAAGAATCAAGCAAGCAGTATTGTATCGAGACCAAAGTGGTATCATGGTCGGTTTTGATTAGCAGTGGGGACTACCCTACCGTAACACCTTGTTGGAATTGAAGCATCCAAAGAAAATAC 6000  
 TTGAGAGGCCCTGGGCTTGTTTTAACATCTGGAAAAAAGGCTGTTTTTATAGCAGCGGTTACCAGCCCAAACCTCAAGTTGTGCTTGCAGGGGAGGGAAAAGGGGGAAAGCGGGCAACCA 6120  
 GTTTCCCCAGCTTTTCCAGAATCCTGTTACAAGGTCTCCCCACAAGTGATTTCTCTGCCACATCGCCACCATGGGCCTTTGGCCTAATCACAGACCCTTCACCCCTCACCTTGATGCAGC 6240  
 CAGTAGCTGGATCCTTGAGGTCACGTTGCATATCGGTTTCAAGGTAACCATGGTGCCAAGGTCCTGTGGGTTGCACCAGAAAAGGCCATCAATTTTCCCCTTGCCTGTAATTTAACATTA 6360  
 AAACCATAGCTAAGATGTTTTATACATAGCACCTATGCAGAGTAAACAAACCAGTATGGGTATAGTATGTTTGATACCAGTGCTGGGTGGGAATGTAGGAAGTCGGATGAAAAGCAAGCC 6480  
 TTTGTAGGAAGTTGTTGGGGTGGGATTGCAAAAATTCTCTGCTAAGACTTTTTCAGGTGGACATAACAGACTTGGCCAAGCTAGCATCTTAGTGGAAGCAGATTCGTCAGTAGGGTTGTA 6600  
 AAGGT

TTTTCTTTT

TTTTCTTTT  
Depth:19 (ZEBRAFISH)  
Ei-value:0.000, Pi-value:0.000  
Er-value:0.000, Pr-value:0.000  
MATCHES To TargetScan▶ miR-186-5p:AAAGAAU

CCTGAGAAAACAACCTTTTGTTTTCT

CAGGTTTTGCTTT

CAGGTTTTGCTTT  
Depth:19 (ZEBRAFISH)  
Ei-value:0.000, Pi-value:0.000  
Er-value:0.000, Pr-value:0.000  
MATCHES To TargetScan▶ miR-330-3p.2:AAAGCAC▶ miR-490-3p:AACCUGG

TTGGCCTTTCCCTAGCTTTAAAAAAA

AAAAAGCAAAA

AAAAAGCAAAA  
Depth:19 (ZEBRAFISH)  
Ei-value:0.000, Pi-value:0.000  
Er-value:0.000, Pr-value:0.000  
No matches to TargetScan

GACGCTGGTGGCTGGCACTCCTGGTTTCCA 6720  
 GGACGGGGTTCAAGTCCCTGCGGTGTCTTTGCTTGACTCTTATATCATGAGGCCATTACATTTTTCTTGGAGGGTTCTAAAGGCTCTGGGTATGGTAGCTGATATCACTGGAACACTCCC 6840  
 CAGCCTCAGTGTTGAACTCTTGATAATTAACTGCATTGTCTTTCAGGTTATGCCCAATTCGTCTTATTACCTCTGAGTCGACACACCTCCTACTATTTATTGAATACTTTGATTTTATGA 6960  
 AATAAAAACTAAATATCTCTCA                                                                                                   6982
```

---

## >TURTLE (7179 bases)

```
 TTTGTGTTGGACTAAGTGGAAAAAATTTGTCTTGGGCTAAAAAGTTAAAAAAAAAAATAGCTGACAAAGGACTAGAATAAAACCAGCAGCCATTTTACATCTGATGCATTGATCAAGGCG 120  
 GAATGTGGATTCAGTGAAGCTAGAAAGACAGGATTTCAAGGAGCCAGTGCAAATTGGTGAAGTTAGAGGGGGAAAAAGAGCCAGTGCAAATTGGTGAAACTAGAGGAAAAAAGAATCACG 240  
 GAAAACAAGATCAAGCAAGAAATTTTTGAAATAATAGATTAGAGGCTTCTAGATCAAGATACCATTGTGAAAGGCAGAAGTGGAAAAATGAGTGAAGACAGCAATAATTTAACCTGTAGT 360  
 TGGTAACCTACTTTCTCCTTGATTTCTTTAAAACACTATTTTCTTTGCTATACTCTTTATTAATAGTAATTTGTAAACGTGCTTATTGTTATGAGAGGGGGTTTGTTTGGAGTGGGTCAA 480  
 GTCGTGGTGTGTCGTAGGTTTTTCTCTTTGCAGGCTTATTTACTCATTGCATCTTGTCTGGAAGCTAATGGGCAGTCTGCCAAGGCCTGGAGAGTGTGGTCATCTTCCCGAGTGTTTAGA 600  
 AAACTGCTGGAATTTTCCTTTCGGCTTTAGGAGTGAGTGGTAGCTAGCGTCTGTATTGCTGTAACCCTACCCAAATTGGGGAAGTCGTGAGCCTGGTGGAGCGACAAGCATCTCTGGTGG 720  
 CAGTGAAGTGTAAGCAAATCACATATAATTTTTTTAAAAAGATTATTGCCCAACAGAACAACAAACTTTGTTTGTTCACGTCTTAAAATATTGTAGGTTATGTATATTATTCCCCATATC 840  
 CTTGGCTGTTCCTAGCAAGAGAAAATAAATGTATTTTTGCCACGCATTCTAGTGGGAAGGGAAAAGGCTTTTGTTAGTCTTGCTCAAAGTGTATTTTTTTTTCCTTGAAGGGAGGGGAAG 960  
 GGGACCAGAAACTTCTTCAGTTTTTTTCCCTAGGTGTGTCTAGATCCTAGAGCCAGACGACTGGAGAGGTGCCAATGTAAAGGATAAAAGAGAACCAGTAATCAGGATAAAACCTCTATT 1080  
 GTATTGATGATGGCAGTGGTTTAGAAATGTGATTGACCTATATTGCAGCTGTAGATATCAATTCAAGTGTGTGGAGAAACTGGCCAGACAGAGGGAAAAGCAAGTTAATTACATTCTATT 1200  
 TTTATAAGTTTGGATTCTTCATAAAGTAATTTACATTTTTTTAATTATCAAACTATAAAGGTACGATTTT

TTTGGG

TTTGGG  
Depth:19 (ZEBRAFISH)  
Ei-value:0.000, Pi-value:0.000  
Er-value:0.000, Pr-value:0.000  
No matches to TargetScan

AGAGGGAAACCTTTTTGTGTATGTTGAAAAGGGTGGTGGTGGGT 1320  
 AGGGATAAAAACAAGCTTGTTTCTAGACTCTTTGCAGATACTATAGTTCCTATTGGCTTGGTTAGAAGTGTGGTTCTATCTACTGTGCTGTTGCGAGAACATTCCCCAGAGTCGGAGCAG 1440  
 CTTTTACCTTGGAAGAAAGGACAGTTTACCTAAAATGGAGTTGGAAAGTTGGATTGGAGCTTGTATTCAGGATTCATGGTCGTTCAGTCATCCTCTAAGGAGAATTTAAGAAGAGCTTGG 1560  
 CTAGTCCAAACTTGAAAGTTAAGTTTTCTCTAGTAAACAAACAAAACAGACAATCTTTGATACAGTATGTGTGTTGTGGGTGGAATTTTAGGTGCAGTTTTATATGGCTTGACATATTTG 1680  
 TGGGGGTGGGGCAACATAATCGTCTATATGTTCTTCAGAGTCTCTCCCTAAACTGCAGAAACATCCATAACTTTAGGACTGAAAAAAAGAATGTTCAGCTTCAATCCTGGCAAGTAATTC 1800  
 TCATAATGTAGCTTTAAATTGTTCTTGGGGAAGTACCCCATTGAGTAATTGGGAATGTTCACACAGGGCCCATATGCATGTATATACTTAAAATGCACACCGTGCTAAAGAGAATAGACA 1920  
 ACGTTAAAACTGTAACCCAGACTAACACAAAGTAGCTTGTTTAATTTTTTTTCCCTGTAGCATAAAACTTACCAAAAATACTCTCTCAGAAGTGGAAAGCTTCCACTAGTCCAAATTAAC 2040  
 ATGGACTTAAGATAAAAAACACACATTCAAATCCTTTCCTAACTATTAGGGTTGTGGTGTCTAGACGCATATTCTGGTGCATATCTGTAATTGTGGGAATCGGTTTGAAACCTTGGATAT 2160  
 AGTGGTGTGTGGCTTTTTCTTTTTTTGGCATTTTTTTTCAGGTGCTTAATGTAACTTAATGCTACTGTAGAGACTTCAGAGAGAAAGAAGTTGCTTAATGACCCTGGTGTGATTCCTTCT 2280  
 GATGGTAGCTTGTGTATTTTTCAAGTAAGATTCTGCTTTCAGTTGTCTGTAACCTAGAAATGTGGGTTTGTTTGAGTGTAGGAATTATGCATTTTTATTTAGATTTTTTTTGTATTAAAA 2400  
 AATGCAACACAGTATGTTACACCATACTTGAAATCCTGTAAAACTCTCAAGTGCACTTACTGTCATAGTAAAAATAAATAAAAAAACGAAAGACCCAAGAGCAGATCAGTTATCAATGTA 2520  
 AACACAGTAATTTTTAAATTAAAACACAGTAATTAAAAAAACAAAAGTAAGAAACATAGTTGTAATGCGTTTAGTTTGAATGTGTCTTTCTTTTTGGGGTGGGGCATGGTGTGGGGAACA 2640  
 ACTTTTGTCTTGTAGACA

TTTTTCAG

TTTTTCAG  
Depth:19 (ZEBRAFISH)  
Ei-value:0.000, Pi-value:0.000  
Er-value:0.000, Pr-value:0.000  
No matches to TargetScan

ATCCGCCATCAGAATCATAGCCCCAACGAGCCCTGAGAAAATATGGCCTCATAAATGCAGAGGGAACAGCTTCTTGGCGAATGATAAGTAAAGG 2760  
 CAGAAAAGATTCTAATATTCCTTACTACTTGATGGAAAAGGATCCCAGATGTTAACAAGTGAAAATCCAATGTGTCTACAAAAAAAAAAAGCCATAACCAATGAAACTGACAAATTTGAA 2880  
 GTAATAAGCATTGACTATCAATTATCTGATCAGAATATTTGCTCTAAGAAATCTCAAAATTATATGCAGTAAAGAGCAAATGGCCTTCTTGAAATACATAATTTCATTAAAGTTGAGTGA 3000  
 ACAACAACTTCCACATCATTGCAATTAAATTGAAAGAAAATACCGGCAGAAAAAATGCATATGCAAATACATATGGCTTCTACCAAACTATTTGGTATGAATATTACCACCTAAATCTTA 3120  
 TGGATTTGAAGAAAGAATATGAATTATTTTGAAACCACAATCTAATTTGGGATCAACTTTCACTACTGTTGGATTTGAGAGGAGGCTTGTGCTGTGTTCCACTGTTTACCTGAGACAGGT 3240  
 TTTCTGCACTATTAATGAGGAGTTTTGCTTCACTTCATCTGGGAGCTGAAAAAAACAAGCAGGCATCTATTACA

GATAAG

GATAAG  
Depth:19 (ZEBRAFISH)  
Ei-value:0.000, Pi-value:0.000  
Er-value:0.000, Pr-value:0.000  
No matches to TargetScan

TTTATCAATTATATCCGCAGATATTGCATGTTAGGGAAAT 3360  
 GTACTTTTTAAATGCTATGAAGCAGTGGAAGCCCTAAACATCTGAACTGGCATTTCTCATTCCCAGCCTTTAGCAGTGTGTGGAATGGGTATGAGGGACTTAACCTACCCCTGTGCCTAT 3480  
 TCAGGGCTGCACTTCCACTTTCTAAATTCCTTTCTCCTCTGTGCACAGGCCACAGTTGGATTGTGCCCATCACCTACAGGCAGATGAGCATAGCAGCAATGCTGTGGTGCTTCCGAACAA 3600  
 ACATGCAATGTATATCCTCCTTTGGAGGCTGAGCCTACTCTGGGCCTACAGTTTGAGAGCCTAAAATGCTATACCAAACAGCACATTATGCTGCCAAAAAACACACCTTATTTTTCTGCT 3720  
 ATTTTGTAGCATTAAAATATATGCAGAAAACATGGGATTAAAAAAAAAAAGCTATAAAGGTACCTGTTAATAAAGTTACTGATGTGCTCTAAGAATAGACAGTTTGTCTTCATTTTTATT 3840  
 AATAAAAATGTGCTGTCAATGTTCTTTGTGTGTGGTTAGCTCTGGGGCAGTGTGGTGGGGGGATTCTTCTTTAATCTTTCAGAATCTTTCTCCAAGAACACCTGATCTTAATTGGACGGG 3960  
 AGCAGGAATTGAACAGAAGACCGAAATTCTTTTTTTATTCAGGCAGGAAAGTCTTTGCAGAATCTTTTCCTTCCCTCCCTCTCCACCCCTCTCTTCATCGAGACAAATGTAGATCAAGGT 4080  
 ACTTGGCAATAGAAGTCCTATAATTAAGGAAAGTATCTGCCCAAGTAATGATTTCTAAAATGCTTTAAACTAGACCAGTGGGGGGCAAAACTTACCAATCAGAGTAAGTTCATAACGGTA 4200  
 AAATTTTGGCAATAAGACTTGCTTTGTCTCCAGAGCTAAAGTCCTGTTAAAATTAAAAAAAAAAAAAAAAAAAAGAACTGACTTATAGTTGGGGGGGAAAACTTTTAACTGCACAATAAT 4320  
 TTGTTAAATTTAACACTAGTCAGTTTGCATATTTAGAAATTTGCATGCTAACAATGCTTGCAACCATTTCCTTCAGTGCTAGTGACGCGACGTCTCATTTTCTACTTCTAATTAACATTA 4440  
 GCGGGGGAAATGGGCAATTTTTTTTTTTAAGTATCTTTCTTTGTAGAAGCTTCATTTCATAATGTATAGACCCTGATCCATCCCTTCATGAAGCTGTTCAGGATGATTTTGCTTGACATG 4560  
 CCTTGGCGCTTTCTTCTGTTCCCCACACCTTGTGTGTGTATGTGAAGGCCCCTGCAGTGAGTTAAACCTCTGTATCTTCAAACATTAACAGCATTTTTTTTTTTTTTTTTTTTTTAATAT 4680  
 CTGGACTGTGGCTGGGGTAGGGTGGGAGGGGCTCACAACTTGTTCTAGCTTTTCTTTTTACAGACTTCGCAGAGAATGCAGTGTCTGAAGCAGGTCTGGTCTGTTGGCAAGTAATGTGCA 4800  
 GTATGCTTGATCTGCTTGGGTAGAGCATTTGAATCGATTGAAATACGTGTCCCAAAGCTTTTGTCCATGTGTGGAGTAGTGATTGTTTGAAAGAAAAAAATACAGCTGAATGACACAAGT 4920  
 GCCTGTGTATTGAAAGGAGCTCTTTACCCTTGGAGTAAAATGCCCCGCAGCTTTAAGCAGTCGGTGTATGTGACTGAGATCCAAGTTCCTTTGGCTGTGCTGCTTTATTTAGGTAACTGC 5040  
 TTTTTGTTGTTGTTCCCTTTCTGAAGTGGGTGGGGGTAGGGGTAGGGGGTACTGAAGCCTTTAGTCTCTTCCAGATACATCTAAAACATCTCATTGACGAGAAATAAAAGCTTCCAAACA 5160  
 AGCAACAATCCTCCAGGAAACTAACTGGCAAGTGACATTTTAAATTCCACCAGTTTAGTGACCATTTTCAGTACCTTGCTTTTGTATGGGTCTCTTCCTTTCTTGGTTTTTTTAACAGTT 5280  
 CTTTTCAAGAAAATACCAAACAAGCCAGTCCCTCATGATGGGTCACAGAAGCCAACCCAGTAAGAATAGTTCTTATTTACTTTAGAACAAAAAATTTAATTCATTTTCATGGAGTTTACT 5400  
 GGGAATTTGGGGGTGGGGGAAGGTGGGGCAACCTAGTTTTAAGATTACTTTTCACAGACCTGTAGAAGGAGCAGCCAGACACAATGAATTGAAAATACCACAGTGGACAAAAAGCAAACA 5520  
 GGTGAATAAACTTTTCTGTATTTACCATAAGATAAAAAGATTCAAAAAAATTTGGAATCTCCTCCCCACCCACAATAAAAAATGGGGAAATAATTAACTTTTATCCAGCTTTATGCTGCA 5640  
 ATTGCACCAATTTAAAATGAGAGACTGTATCTTTGAATCTTTTTAGGGATGTGGGTGGGGGAAAATGGGTTGAGACCTCTTTTGCTTAAGACTTTTGCAGGTGTCCCCATTTAAAGAAAA 5760  
 GGCTTTAAAAAGTAGGAGAACCATGGACCTTTACAGACATCTTGTGACAAAACTGACATGACGACAAAGCAGTTACATCAAGGTCACTCTTAAAATTACATGAATTTATTGAAGGCAAGG 5880  
 GGGTTGCACTTGTAATATAATCTTGCATATACTGAGGATATGTGCCTCAAAGGATGCTCTTTGCATTAACTATATATTGAGCAAGTTAATACATTACTTTTAAAGTAATGTTGAGTACCT 6000  
 TTTGTGTACTTATAGCCATGTTATCACTTCATGTAAACCCCCATTTAAAAACACTAATTCTCAGATCATGGCTTATACTTTAAAATGATGTAAATAGTTAAGGGAGGGGGAGGAGGGGAT 6120  
 AAACCACAACCAGTTTTCGCTAGACTTTTTTTCCAGAAACCTGCTATTCAGTACCTAGTTCAGACTACACACAGCGCTTTTCCTCTTGCTTCAGCTGGTAGCATGGATCCTTGTCACTGG 6240  
 TATGCGGTTTTCTCTTAAGGTAAAATTTTAAAATGTAAGTCTACAAAAAAATTGGGATAATGTACTTTGCCTGGAACTCTCCTAACTTTGGAAGCTAACATCACATTTAAAAAATGTAAT 6360  
 GATGCTTAATTATAAACTATTACTAAAATAGCTTTACCTTCAAATAACACTCAGAATAATATTGACAGGCAAAACCGTCCCAAAACTAAATAGTCAATCTAGACATAACAGATGGCAAAA 6480  
 TACTTAACATTAAAAACCAAAACAACTTAATGGTGCTTTGGAGGAATGTCAGGGGGTGGGTACCCGCCATACTGTCTGTAAATAAACAATTAGAAAGTAAATTTCAACTGTCTTCATTGT 6600  
 GAGGTGGCTTTTGGGAGGGCTCTTGCAACTCTCTGCTAAGACTTTTTCAGGTGTCCTAACAAACTCGAAATAAGTGATTTGTCAGAAGTAGAAGCTTAGATGAAGCAGATTTCTTCAGTG 6720  
 CTCTTTCAAGGT

TTTTCTTTT

TTTTCTTTT  
Depth:19 (ZEBRAFISH)  
Ei-value:0.000, Pi-value:0.000  
Er-value:0.000, Pr-value:0.000  
MATCHES To TargetScan▶ miR-186-5p:AAAGAAU

CCTGAGAGAACAATCTGTTCTCT

CAGGTTTTGCTTT

CAGGTTTTGCTTT  
Depth:19 (ZEBRAFISH)  
Ei-value:0.000, Pi-value:0.000  
Er-value:0.000, Pr-value:0.000  
MATCHES To TargetScan▶ miR-330-3p.2:AAAGCAC▶ miR-490-3p:AACCUGG

TCACCTTTATTATATTAACAA

AAAAAGCAAAA

AAAAAGCAAAA  
Depth:19 (ZEBRAFISH)  
Ei-value:0.000, Pi-value:0.000  
Er-value:0.000, Pr-value:0.000  
No matches to TargetScan

GATGCTGGTGGTTGGCACTCCTGGTTTCCAG 6840  
 GATGGGGTTCAAATCCCTACAGTGTCTTTGCTTTCTTGTAGCATAGAGGTGTAGATACAGACCACTTCAGTGTGGGTTTTCCTAGGTGAGAGCCTAACTTTTTCATGGGGAAATTAGAGC 6960  
 TGTTTGCCTGGACAGACAGTACTTGGGTAATCTTTTGCACTTAACATTTTATCTGTAGGTCACAGTGCAGTTCACAATGGTGGGAGTAACTCTTCACCTTTTACAAAAAGGGAAACTCAG 7080  
 ATGCAGAAAGGACAACTAGCACAGCCAGTCAGTGACAGAGCTGTGACCTGAACCCAGGAGTACTAATTGACAATCCTCTGACTTGGGCAGTGGTTTTCA                      7179
```

---

## >ALLIGATOR (8607 bases)

```
 AGAGCTGCAGGGAGAGCCAGGGAGATGAGACAGCTGGAGGGGGAGGTGAAATAGGGACGGGAAAACAGACCGGTAGCTTTAAAAAGCAGTTTAAATTAGGTTGATAGCATATTGCAAACT 120  
 ATACTAATATTTTAAAGAAAATTTGTAAAGAGGGCTATATTCTATTAAAATATTAATCATAGTTTGATAATTAAAAAGAGACGGCTAACTAGGATTCTCTTGGTTTAAAACCCCTCAGTG 240  
 CTCTTAGAAGAGGAAAAATATGAAGATTTTTAATCTGAAGGGCAATTGGAAGGAAATAGAAGAATTGAAGATTCAGAAATATTTGATAGATTCGGGGGGATGATAAGAGCTTGGCTGAAA 360  
 ATGGTAAATGATGAAAGGAAAATGGGCTTTTTAAAAGCCCCGAAAGGAAAATCCAAGAATGGATTTGGAAGATTAACTTAAGAAACATGAAAATACATAGAATTGAAGATTAAAATACTA 480  
 AATTTGAATTTAAACATTTTAAAAGAAACTGAAAACTAAAAATTTGAAGATAAGAACAAGGTTTAAAAAAAAAAAGAAGATAAATTTAGAAGGCAACCAGAAAAAAATTGAAAATAAATT 600  
 TATAAAAGCAGGAAAAAAAACCAAATTGAAGATGAAATTTTCAACTCAAACATAAAAGCAGGAAAAAAATAAATTGGAAGATGAAATTTTTAACCCAAAACAAGCGCTTTATGCCAGTTT 720  
 GGGGTGGAGGAAAAGATAAGAATGGCTGTTCTAAAAACAGCAAATGCACTGAAGGTCTGCTTCATGCAGAGGGAAAGACAGAGAAAATATTAAAAATAGAGAAAAGATCATAAAGAAGAT 840  
 GATATTTAGGGTCTGTAACTGAAGTTAGACAGCATGAGAAGACTTTCAAGACAGGAGACTTAGAAGAGAGAAGGTTTTGTAATGTTAGAAGGCAGAAGAGAAGGCTTTTAACAAAAGTGA 960  
 AGGCTAGGAGAAGGCAGAAGGCATCTAGAAGAGGATCGGAGAAGCGAGAAGGGGAGTTCAGACTTGTTAAGATGAGCCAAAGCTAGATAAAATAGTAATGGGTCGCTGCCCTGACATGGC 1080  
 CAGCTTAATAGAAATCTAGCGAAAATACAGGAGGAGAAACAAAAAGGACTTGAGGCTTAATCTTTGCAGAAGGATTTAACCTCTTTAATGGCGTGATAGCTCAGTTTGCTCTTTGTAAAA 1200  
 TCCAGCTTACGAATGCCTAGAGTGCTAAACTATTAATTCCGAGGACTTTTGTGAAATATTTCAAGATCAGGAGTAATCTTAAAAGCGGTATCTTTGTGTTGGACTAAGTGGAAAATGTTG 1320  
 TCTTTGAGCTAAAAAGCTTAGAGAAATGTAATGTAGCTGGTAAAGGACTAGACTAAAACCAGCCATTTGACAGCTGATGAGGTGATGAAGGTGGATGCAGTGAAGCTAGGAAGACAGGAT 1440  
 CTCCAGGAGCCAGCTAGGAAAACAGGATCTCCAGAGGCCGGTGCAAATTGGTGAAGCTAGGAAAACAGGATTTCAAGAAGCCAGTACAAATTGGTGAAGCTAGAGGAAAAAAAAAAAAGA 1560  
 ATCATGGAAGGAAGATCAAGCAAGAGAATTTGGAAAGAATAGATTAGAGGCATCTAGATCAAGATACCTTTGTGAAGGCAGACGAGGAAAATTGAGTGAAGACAGCAGTAATGTTAACCT 1680  
 GTCTAGTTGGTAACCTTTCTCCTTGCTTTCTCTTCCACCCTTTTTTTCTGTGTTATGTTCTGTATGAATACCATTGTCAGCGCCTTGTTGTTGTAAGAGGGGGCTTGTTGGGAGGGGGCA 1800  
 AATTGTGGTGTGTAGGTTTTTCTCTTTGCAGGGTTGTTAATTCATATCATCTTATCTGGAAGCTAATGGGCAGTCTGCCAAGGCCTGGAGAGTGTGGTCATCTTCCTGAGTTAGAGCACT 1920  
 GCTGGGATTTTCCTTTCGGCTTTAGGAGTGGTAGCTAGCCTCTGTATTGCTGTCAGTCTGAGCCTGGAGCGCCAAGCACCTCTGGTGGCAGTCAAGTGTGAGCAAACCACACAGGTCTTT 2040  
 TAAAAAAGAGGGATGCTGTTATCCACCACAACCAGCTCGTCCATTCAACCTCACAATATTGTAGGTAATGTGTCCTCTCTCTCTTAGCAAGTGAGAATAAATGTTTGTTGTTTTTTAAAT 2160  
 GTGTTTTGCTGGGAAGGGAAGGTTTTTATTTAGCCTCTATAAATGTACTGTATTTGTTCTTGAAGGGAGGGAGGGGGGCGGAAAACGTCTTCAGTTTTTTTCCCCCCTAGGTATACCTAG 2280  
 AGTCCTAGAGCAAGACGACTGGAGAGGTGACAATTTACAGGAAATGAGACCAGTGATCAGGATAAACCTCACAGTGTGGTGATGGAGGCAGTGGTTAGAAATACATTTGTCCTGTATTGT 2400  
 AGCTGTCAATTGAAATATGTGGAGAAACTATCCAGACAGAGGGACATGCAAGTTAAATGATGCTCTTTTACAAATTTAGACTTTCTAAATGTACAAAGTTGTGTTTTCATATTGTCAAAA 2520  
 TATACAGGTATCTTCT

TTTGGG

TTTGGG  
Depth:19 (ZEBRAFISH)  
Ei-value:0.000, Pi-value:0.000  
Er-value:0.000, Pr-value:0.000  
No matches to TargetScan

GAAAAAAAAACAATTGGTGTATGTCTGTAGGGGAGGGGGAGGGCTGGGCATAAAACAAGCTTGTTTCTAGACTCTTTGCAGATACTATAGTCCCTTTT 2640  
 GGGCTTGGTTAGAAGTGTGGTTCTGCTATGCTGTTGCAAGGACACTGCAGAGGTTGGAGCAGCTTATACCTTGGAAGAAAGGACAGTGTACCTAAAGTGGAGGTGGCAAGTTGGATTGGA 2760  
 GCTTGTATTCAGGATTCATATTATGAGTCACCTAAGGAGGAGGATGTCAGAAGAGCTTGGCTAGTCCAGAAACTTGAAAGTTAAGTTCCCTCTAGTAAACAAAAGACTGTGTTAATGGGG 2880  
 ATGTTAGATGCAGTTGTATGTGGCTGGACATGTCGTGGGGGGTGGGGCGAGGTATTTTTTTATATTTTCAGATTCTACCTAAACAGCAGAAACTTAGGACTGAGAAAGCATCAGCTTCCA 3000  
 CCCTGGCAAGTAATTCTCAGCTTTAAAATCATTCAGAGGCGGTGCCCCTTTAAGGGAAGGTTTTTTTCCAATGCATGTACATCCTGTACAAAGCACCAACTGTGCTAGAAAAAAAAGACT 3120  
 AGGCCAAGCCAAAATCTGGAATTGACGTTAACAAAAAGTAACCATCTAGTATCTTTTTGCTATAGTGCAAATCTTGGCAAAAACCCCAGGTAGGGAGGAAAGCTTCCACTAAAATCATGA 3240  
 GGGACTACAGATGAAATCCAAGTCATTGGGTGAGCGTAGCGGGGTTGTGGTGTGGCAGTAGCTAGTTTGCATACTGTATTGCATATCTGTCACTGGGATTGGATGGAAATCTTTGGATGC 3360  
 GGTGGTGTGTGGCTCTTCTTTGGCATTTTTTGCAGGTGTTTTATGTAACTTAATGCTACTGTAGAGACTTCAGAGAGAAAGAAGGTGCTTAATGACCCTGGTGTGTGATTCCTTCTGATG 3480  
 GTAGCTTGTGTGTTTTTTCTCAAGTAAGATTCTGCTTTCAGTTGTCTGTAAAAGAGAAATACAGGTTGGTCTGAGTGTAGGAATATTTTTTTTATTTACACTTTTTGTATGGAAAAAATG 3600  
 CAGTGAAACATGTTACATCACTTAAAATCTGTTTATATATATATATATATATATATCATTTAAGAACACTTAAAATGTCATAGTAAAACAAAATCAATCTAAAAACCAAAGACTAAATTG 3720  
 ATAAATTTATTAATGTAAACAAATTCGTATTTTTAAGTACAGTAATTAAAAGTAAGAAACATCCTTGTCATGCTTTTAGTTTGAATGTGTCTTTCTTGGGGAGGGGAGGGGCATGGTGTG 3840  
 GGGAACAACTTTGTCTTGTAGACA

TTTTTCAG

TTTTTCAG  
Depth:19 (ZEBRAFISH)  
Ei-value:0.000, Pi-value:0.000  
Er-value:0.000, Pr-value:0.000  
No matches to TargetScan

ATCCACCTTCAGAGTCATAGCCCCAACGAGCCTGGAAAAATATGAAATATGGCGTCATAAATGGAGAGGGCACTTCTTGGCGAATGAT 3960  
 AAGTAAAGGCAGAAAAAAATCTATAATATTCGTTACTACTTGATGGAAAAAGATCCCAGATGTTAACAAGTGAAAATCCAATGTCTCTACGAATGGGGGATAAAAAATAAGCAATGAACC 4080  
 CGACAAATTTGACTATCCAATATCTGATCACAGAATTTGCTCTAAGAAATATCCAAATCCTATACAAGGAAGAGCAAATGTTATTCTTGAAATATCATCTAATTTCACTAAAGCAATGAA 4200  
 AAATGATTTGCCGCAGCATTGCCATTCAATTGGGAGAAAATGCATCAGGAAAAATATACCTATATGTTCTGGCACCTACCCAAATATCCGGTATGAAAATTACCACATAAATCTTACTGG 4320  
 ATTTGAAGAAAGAAATAATGAAATATTGCCAAACAACCATCAAATTTGGGATCAACTGTCACTGCTGCTGGATTTGAGGAGGAGGTGGAGGCTTGTGCTGTCCCATTGTTTACCTCAGAC 4440  
 AGGTTTCTCTGCTCTACTTAATGAGGAGGTTTTTGCTTCATTTCATCTGGGAGCTGACAAACAAGCAGGCACCTATTGCA

GATAAG

GATAAG  
Depth:19 (ZEBRAFISH)  
Ei-value:0.000, Pi-value:0.000  
Er-value:0.000, Pr-value:0.000  
No matches to TargetScan

TTTATCCATTATATCCGCAGATATTGCATGGTAG 4560  
 GAAAGTATGCTTTTTTTTAATTTCCATTTCTTTTCATTGCCTGTAAAGCTCCGGGAGGCCCTTGACATCTGAACTGGCCTTTCCCGGCCCCTGGCTTCAAGCCCCACCCTGTGGAAAGGG 4680  
 ATGATAAAGAAGGAATTGAACCTGCTCAAAGGTTGCTTCCTGCTCCACTCTAAATTCCTATCAGCACAGACTGCACTCAAATTACACCCAGCACTTAAAGGCAGACCAGCCTAGCAGCCA 4800  
 CATGGTGGCTTTTCCAAATGAACACTATATATGATGCATAATCCTCCCTTGGAGGTTGAGCATTCAAATTCTCACGCCCGGGCCTACTCTCCCGGAGCCTGCGACACCCAGCAGACAGCT 4920  
 GTCATTGAAAGCAGCTCCTGCCCTCCTGCTTTTGTTGTTGCATTTCAAATGCCCGCAGAAAACATGGAATTTAAAAAAAAGGCTCTAAAGTCACCTTTAAAATAAAGCTGATTTGACGTG 5040  
 CTGTAAGACTAGAACGCTTGTCTCTTATTAATAAAAATGTGCTGTCAATGTTTCTTGTATGTGGTTAGCTCTGGGGATATCTGGGGGGGATTCTTCTTTCATCTTTCAGAGTCTTTCTCC 5160  
 ATGAACACGTGATCTTTATTGGACAAGAGCAGGAATTGAACAGAAGACTGAAATTATCTTTTTATTCAGGCAGGAAAGTCTTTGCAGACTCTTTTCCTTCCCTCCTTTCCCAACCTTCTC 5280  
 TTCACGGAGACAAATGTAGATCAAGGCACTTGGCAGTAGAGGTCGCATAACTAAGGAGAGTATCTATCTGCCCCAGGAATGATTTCTAAAATGCTATGAGCTAGACCGGTGGGGGGAAAA 5400  
 ACTTACTAACCGGAGTAAGTATCTGACAAAATAAAATTCTGGCTTTGAGGCTTGCTCTGTCTCCAGAGCTCAAGTCCTGCTTCAAATTCAAGCTGACTGATTGCTACAGTCATGGCAAAC 5520  
 TTTTTAACTGCACATCTATTAAAAACCAAAGTGACACACTACTCAGTTTGCATCTTTAGAAATTTGCATGCTAACAATGCTTGCACCCATCTCCTCTAGTGCTAGTGATGTCTCATTTTT 5640  
 CTACTTCCAAGTAACATTTGGGAGGTCCATGGGGGATCAGGGGGCAATTTTTTTTTTGTAGTATCTTTCTTTGCAGAAGCTTCATTTCATAATGTATAGACCCTGATCCATCCCTCCATG 5760  
 AAGCTGTTCAGGATGATTTTGCTTGACATGCTTGCTTTTTCTTCTGTTTCCCACACCTTGTGTGTGTACCTGAAGGCCCCTGCAGTGAGTTAAGTCTCAGTATCTTCAAACATTAACAGC 5880  
 ATTTTTTTTTTAATATTTGGATTGTGGCTGGGGTGGGTGGGGCTCACAATTTGTTCTAGCTTTTTCTTTTTACAGACTTCGCAGAGAATGCAGTGTCTGAAGCAGGTTTGGTCTGTTGGC 6000  
 AAGTAATGTGCAGTGTGCTTGATCTGCTTGTGTAGAGCATTTGAATCGACTTGAGATATGTGTCCCAAAGCCTTTTTTTTCCCATGTGTGGAGTAGTGATTGTTTGAAGGAAAAAAAATC 6120  
 CAGCTGAATGACAAAAAGTGCCTGTGTATTGAAAGGAGCTCTTTTCCCTTCTGAGTAAAAAAAATTCCTGCAGCTTAAGCAGTCGTGTATGTGACCAAGTGCATTTGGCTGTGACGCTTT 6240  
 AGGTAACTGATGCTTTTTTTTTCTGTTGAGTTTTTTTTTTTTCTGTTGTTGATACCGTGTCTAATGGGGGTGGGGGAGAGGAAGGGGGGGTAATGAAGCCTTTAGTCTCTTCCAGATGCA 6360  
 TCTAAACATCTCAGTGGCAAGAAAGTAAAATCTTCTAAACCAGCAACAATCCTTCAGGAAATTAACTGGCAAGTGATGATTTTAAATACCACCAGTTTAGTGACCAGTCTCCGTGCGATG 6480  
 CTTTTGTATGGGTCTCTTCCTTTCTTGGTGTTTCTAACAGTTCAGTTCAAGACAATAACTAAATCAAGCCAATTCCTCACGGTGAGCCACAGAAGCCATCCCAGTAAGAATAGTTCTTAT 6600  
 TTATACTTCAAACAACAAAACCAAACAATTGTTTTAATCGGTTCATGTTCCGTGGCGTTTTTATTGGGAGGGGGTTGGGGGCAACCTAGTGTTAAGATTACTTTTCACAGACCTGTAAAA 6720  
 GGAGCAGCCAGACACACAGTGTTTGAGAATACCACAGTGGACCAAAAAAAATTAAAAAAAATCAACAGGTGAACAAATTTTTCTTTATTTACCTTAAACAAAAAGATTCAAGATCTTAAA 6840  
 AACTGGCATTTCCTCCCCCACCCCACCCACGAAAAAGAAAACAATGGGGGAGTGAGCGCTCTTAATGCATCCCAGCTTCATGCTGCCGCAATGCCTACAGAAATGGAGAGTAGCCGTATC 6960  
 CTTGAATCTTTTTTAGGGAAGTGGGTGGGGGAAAATGGGTTGAGACCTCTTGTGCTTAAGACTCTTGCAGGTGTCCCCATTTTAAAGGCAGACAAGGCTTTACAAACCACGGATCTGCAG 7080  
 CGTGACGACAAAGCAGTTAACGCCAAGGTCACTTGTAATCCTTTGCATTAATGTATTGGAAGAGATGGGGTCACACTGTAATGTTTTATCTACTTTGCTGAGGATCTGTGTCCCAGAGGA 7200  
 AGCCCTTTGCAGTAACTGCGCATCGGGCAGGTTCGTGACATTACTTTCAAAGTAATGTTGTTACGTGCCTTCTGTGTACTTGTAGCCATGACACTAACTTGTGTGAACCCCTGTTTAACT 7320  
 CTTGGATAAAGTCATGGCTTAAGTTTTAAAACAACTGCAAGTACTTTGGGAGGAGGAGGAGGGAAGAGGGGATAAACCACAACCAGTTTTCCCTAGACTTTTCCAGAAACCGGCTATTCA 7440  
 GTATCTAGTTCAGACTAGAGATGGTCCCTCTTGCTTCAGCTGGTAACATGGATCCTTGTCACTGGTGCGGTTTCCTCTTAAGGTAACATATTTTAAACTGTAAAGCTAGAGAAAAACGGG 7560  
 ACAGAGCCGTTTGCCTGGGACTCCTCTGACTTTGGAAGCTAACGTCACAATTTAAAACCAACTCATAATTTAAAACCAATACACTGTGGCAACTGCGCCATGTATAGATTTACTTGGCTT 7680  
 GCAAACAACACTCAACATTTGAAAGGCAAAAAAACCCCAAAACCCTCAAACCCAACAAAACCAAACAGTAAGTAAGTGTAGAGATATCACTGTAAACTCTGTAACATGGTGCTGGTGAGG 7800  
 GCTGTCAGGGTGGGAACCCGCCATACTGCCTGTAAAAGGTGAGGGGGTCGATTTTTCTGTTGTCTCTGGAGCGGCTGGCTCGTGGGAGGGGGCGTGCAACTCTGTGCTAAGACTTTTTCA 7920  
 GGTGGCCTAACAAACTTGCAAATAAGGGAGATTCAGGACTATAGACGCTTAGATGAAGCAGACTTCTTCAGTGCTCTGTCAAGG

TTTTCTTTT

TTTTCTTTT  
Depth:19 (ZEBRAFISH)  
Ei-value:0.000, Pi-value:0.000  
Er-value:0.000, Pr-value:0.000  
MATCHES To TargetScan▶ miR-186-5p:AAAGAAU

CCTGAGAGAACAATCTGTTGTCT

CAGG

CAGGTTTTGCTTT  
Depth:19 (ZEBRAFISH)  
Ei-value:0.000, Pi-value:0.000  
Er-value:0.000, Pr-value:0.000  
MATCHES To TargetScan▶ miR-330-3p.2:AAAGCAC▶ miR-490-3p:AACCUGG

 8040  


TTTTGCTTT

CAGGTTTTGCTTT  
Depth:19 (ZEBRAFISH)  
Ei-value:0.000, Pi-value:0.000  
Er-value:0.000, Pr-value:0.000  
MATCHES To TargetScan▶ miR-330-3p.2:AAAGCAC▶ miR-490-3p:AACCUGG

TCTCCTTGTGCGTACTAACAGCAACA

AAAAAGCAAAA

AAAAAGCAAAA  
Depth:19 (ZEBRAFISH)  
Ei-value:0.000, Pi-value:0.000  
Er-value:0.000, Pr-value:0.000  
No matches to TargetScan

GATGCTGGTGGTTGGCACTCCTGGCCTCCAGGATGGGGTTCAAGTCCCTACAGTGTCTTTGCTTTCTTGGGAGC 8160  
 AGGGAAATATCCACCCTGCCTCCCTAGGGAAGAGACTAACTCTTTCAGGAAAAGAAATAAAGCAGTTCTCCTTGCCCAGGATAAGAGTGAGCTGTAAGATACCGTCCGTTGCAGAGCAGT 8280  
 TGGTAGAGGTGGGAGCTCACTTGCCAGTTAAAACAGCCTTGCAGAGGCAGACGGCCCTGCTTCAGGCCAGCGTCTTGTTAAAAGCAACAGGAATCCTGCCTGCCTTCTCATAAGGTTACT 8400  
 AACACCGGCATAACTAATCGGATGCTGGTAACCCTATCATTTTCCGCAGCCACTGCACGCCTGTTAACCAGATCTGCGGCAAAGCTACGAGTGACTGCAGTCTGTGGGAGAGACAAAATG 8520  
 CAAACGTGTGGTGCTGTGACACCGTAAAAGATTCCAAGTGTTAAATCATGTGGTGAATTAACAGAAATAAAAAATCAGAGTGCAGTA                                  8607
```

---

## >LIZARD (7365 bases)

```
 AGTGAGCCTGTCCCGGAGTATAAAAGCCCGTCCCAGCCCCTCCTTGTTCAGAGAGGCAGGGAGCGCCAGGCAGGAAGGGAGGGTGGGTGGCTGCAGAGCCAAGATAGACTTAAAAGAAAA 120  
 ACTTAAAATATTAAGTCATCTTTTAAAAACTATAACTAATATTTTTTAAAAGTAGAATAAGAAAATCTTATGCTTCTGAAATATTAATTATAGTTTATAACTTAAAACGAGGCATTAAAT 240  
 AAGTAGCTCTTTCAAACAAAAAGAATATTTGAAGAAAAAGAGAAGAATGAAGGCTAATCTTAAAAAACAGGGAGGAAATTAGGTGTTTTCCCGACGGCAGGCTTAAAAAACCAAACAAGG 360  
 CAATTTTTAAGCCAAGCAAAAACTTAAAACTAGAGAAAAAGGCTGAAAAATTAGGGGGAAAAATGAAGAGAGAAAAAAGCAAGATCAATTAATGAAGATAATTAATTTTAGGCTATTGAA 480  
 AAAATTATACTGAAGATTTCAAAGATTATTAAATGAAGATAATTGTAAAGAAATGTTTATATTACAAACTAATTCAATAGGTTTGAATATAAACATGGAAAAGATAGAAAAAATCTGCTT 600  
 CAAGCAGAAGAAAAAAGTTTGGCTTTTAAAAGGCAACTAGAGAAGCAGTTCTTTAAAATACTTAAAAATAGTTGCTGAAGGCAGAAAAACCAAGATAGTTGTTGAAGGCAGAAAAACCAA 720  
 GATAGTTGTTGAAGGCAGAAAAACCAAAGTAGAAGACCAGGCTTAGAAGAGAGAAGGGTAGAAGGCAAGCAACAGTAGAAGAAAGGCAAGTAGAAGAGAGAAGGCAAGGAAGGAAAGTAG 840  
 AAGAGATCAAGGCAGGAAAGAAGAAAAGAAGAAAGGTAGATGTGACCTAGAAGAGAGAAGGAAAGGTAGAAGGCATCATAGAAGAAAAAGAAGAGGAAAAAGGTAGAAGAGGAACCAAAG 960  
 AGAGAGAAAAGGGGGGAGAAAAGCTTTAATTTGTTGTGCTTTTGGACTAACTAGCAAAAAAAGAGGGGATGAAGGACAATGATACTAAATTCCCCAATGCTTGCAGTCTATAGCTTTTTA 1080  
 AGTTGGTGCCAGTGAAGGTGTGGCGTACTTGAGAAAAGTTTGCTTGCTGCAATTTTGTGGGCACTTGCCCCCCCCAAAAAGGACTGTGTTTGGATAGGCTCTTGCTAAGGAGAAGGGAGG 1200  
 TGGTGGGGAAGGAGCTTCCGAAAAACCATACCCGAGGACTCAGATCGTAAACTACGTGGCATGAGGAAAAGGTGGCATTTGTGGCGTCCTGTTGCGGAGATAGAAAGAGAAGCGTTGGAA 1320  
 GGTGAAGGGAGAAAAGAAGGAAGGCAAACCAGCAGATCTCCTGGGAGGAAGAAGCATAGCTATAGAAGGCCGTAGAAAAAGGACCCAGGACCCCTTTGAGGAGGAGGAGGAGGAAGGGAG 1440  
 AAGGAGCGAAGACCTGCTTTGAAGCAGTTGGTAACATCCCATCAGAGGTTTCTGGTCACCTTCTTGTCTGTCCTGGGGTTTCTTTTTCCTTGGGGAGGGCTCTGGGTGTCGCAGGTCTTT 1560  
 TTCCTTGCAGGCAGAAATCGTGGTCTTGTGCTGGGAAGCAGAAAAAAGAGCCATCTTCCTGAGGTTAGAAGTGCTGCTTTTGCCCTCCTGGTCCCCCGAGAGGTAGCTATAGGCCTTATG 1680  
 TGGTTCTAAGGAACGCATGGGTTGAGTCGAGGAGAGATGCAGCCAGTGGACACAAACCAGCAGGAAAAGAGGAGGAAAAAATAAGGGTGAGACTCAAAGGGTGCCCCATTGTAGCCTTTA 1800  
 GAGGGAGATGGGGGGTCCCCAATCTTGTTGAGTTTTTGTTCCTTCAGGTATGTACATGCATAGCCATATTTGTAAGCCAAGGGAATCCTTTTAAGGATGGATCCAATGATAAAAGGCAAA 1920  
 AAAGAGGACAAATGTGTTGAAATCTTCAGCTTTGAGGAAGAAGGGAGCCCTTTGTTGGGTCCTTTTATGGAGCAAAAGGAAGAACAGGAGGGGAACACAAACAAGCCCAACCCAAGAGGA 2040  
 CTTTTCCCCCTTTTAAAATGCTAAGTATACAGTGGGAGAGTGTGATTTAGTGTTGACGGGGAGGGCGGGAGGGTCAACCCCAGCTTCTTTCCAGCCTCTTTGCAGGTACAGTAGCGACCC 2160  
 CTGGTTTAGCAGAGTGGTCCCGCAGGACTATGGCGAGATTCTGGCAAGCGAGGAGAGAGAGCCTAAAATTGGAGATTGTATTTGAGACGTCTCTATTTTCAGCAGAGCTGGCAAGCTAAG 2280  
 TGAAAAGGAGCGAGATCTCATGCGGTTGGTGGTTCCGTGTGTAACGTTGGGTAATGCTGGGGCCGGGTAATG

TTTGGG

TTTGGG  
Depth:19 (ZEBRAFISH)  
Ei-value:0.000, Pi-value:0.000  
Er-value:0.000, Pr-value:0.000  
No matches to TargetScan

CCGGGTGGGCGTTGGCATCAGAATAACACTGCTCTCTGATTC 2400  
 TTTCTCCAGGAAACCCGTAAGGTTAGGCCTGGGGGACAGTAAGAACACAGAGCTTCTAAGCCTGGATGGGCAAGTAATTCCCAACACTGCACCAAAAGGTGTTCAGTACAACTTAGTTCT 2520  
 TAAAAGAAAATGCAAAGGCATCAGAAAAGTCCCTTTCCAGCTGAAATGCTGTTACAAAGTAGGTTTAAGATCAGTTGAGCTGCCACTCCCACACCCCCTCTAAAGGGGAGGAGAAGGCAT 2640  
 GCGGATTTGGGATTTCCAGTGAATGATGGAAAAGGGCTGTTGGGCTTAGGCAAGTGTTAGGGTGGGGTGGAAACATTGTACTTCCGATGCAGTAGCCTGTGGTTTTCTTTTGGCCTTTTT 2760  
 TTCCAGGTACTTAATAGCCTTAAAGGCTTCAGGGAGAAGGAAGGAGCTTATTGAGCCTGGTGGTGTGTGAGTCCTTCTGATGGTAGCTTGTGTAATGTAAATTCAAGTAAGATCCTGCTT 2880  
 TTGATTGTTTCTTCTTGTAAATGAGTCATGGAGCCAAGGAATGACGTATCTGTTGACACCTATCTCTTGTGTAACCGAGTCTGTAACCTACATAAATAACCAGGCAAACTGGTCACAGCA 3000  
 GATGTAAGCCGCGCAACGCTGACCATTAAAAGTGAGCAACCTGTCTTGAATGCACTTAGCTGGAAATGTCTTTTGTCTTGTGTGGGGCGGGTCGTGTGGGGCACAACTCTTGTCTAGACC 3120  


TTTTTCAG

TTTTTCAG  
Depth:19 (ZEBRAFISH)  
Ei-value:0.000, Pi-value:0.000  
Er-value:0.000, Pr-value:0.000  
No matches to TargetScan

ATCCGTCGCCTAAAGGCTGGCCTCTCGAAGTCTGAGAAAAAGTATGGCATCCCATAAGCGGAGGGAGCAGCTCCTTGGGAATGATAAGTAAAAGGCAGAAAATGTCCTATGG 3240  
 CCTCTTGTTTGATGGAAAAAGTACCAGATGTAAAAAGCAATGCAAAAGCCAGGCTCATCCCGGAGTGGGAAAACCCAGCAAATGCCCTAAGCCAGGAACAGGCAAAATTGGAAATATGGA 3360  
 TTGATTGAGGAATTGAGAGAAGTGTTAAAGCAAGGAGAGCAATGCCATTTCTACAGTATGGTCTTTAAGTTGAAAGAATCTTCAGGGAAAAATTCAAAATCAGCAAAGATGCTTTCTACT 3480  
 ACTACCCAAATCTGTGATGTGAATTATTCCTAAATATGCATGGAATGAAGGAGAGAGGAGAGAGAATAATACAAAGTGATAAAATGTTGGATCGGCTGTCTTGCTGGATTGGAGAGGTGG 3600  
 AGGAGGAGGAGGAGGCTTGTTCTGTGTTGTTTGCCTCAGACAGGTGCTTGGCCCTACTAAGGAGCAGAGGTTGGCCTGCCGTCTGGAGCTGTCTAAACCGACAAATATCCGTGACA

GATA

GATAAG  
Depth:19 (ZEBRAFISH)  
Ei-value:0.000, Pi-value:0.000  
Er-value:0.000, Pr-value:0.000  
No matches to TargetScan

 3720  


AG

GATAAG  
Depth:19 (ZEBRAFISH)  
Ei-value:0.000, Pi-value:0.000  
Er-value:0.000, Pr-value:0.000  
No matches to TargetScan

TGACCCACTTCTATGAGATTGCATGTCAGGGAAAGCTGGGAAATGCTGTTTTCAGGGTTGGGTTGACAAGCTTAGGAGCCCCCAGCCAGCCATTGCATCACACACACACACACACACT 3840  
 GCTTATCTTGGGGTGTGCTGGGTTGCCTGGAGCACCCGCCCCCTTGCCAATCTCCCTGGCCTTGGTTGCCTTGTGTCCATTTTATACATCCAGCTCACTTCATGTGAGGCTGAGGAAGAT 3960  
 GCCCAGTGGGCTATGCCCCCCCCCCTTTGGAGCCTGATAAATACTCAGTTACTGATGCTTGTATAATAAAGTTACATAATGGCTTATATACTCCCTTGTGACACACAAAGATGCCACAAA 4080  
 GTTGCAAAGATGTAAGAGAGGAAACACGCCTTAATAATAAAATGACTCACATTTTGAAAGAATAGGAAGTGCTGTCTTAATTATTATTATTATTTTAAATAAAAGTTGTATTTGTGAATG 4200  
 GTCCTGGGGCGTTTTGGCAGGGTGGGGGGCATTCTTCTCGATCTTTCAGGCACTTTCTTCACGAACCACGTGGTCTTTATTGGACAAGAGCAGAAACTGAGCAGAAGATTTGATTCTCTA 4320  
 AATAAGGCAGGAGTGACTTTGCAAAGCCTATTTTCTTCCCTTCCTCATTCCGCTGAGATCCAAGTGTAGACCCAGGCATTTGGCAGCATAGAGGCATTGTAACTAAGGAAAATGATGTGT 4440  
 ATATATACATATATATAACCAAGGGATTTCTAAAATGCTCAAAACTAGACCAGAGGGGGGAAAACACTTTAAGCAGCTTGGGGTAAGTAATAAATATCTTTATGTCTTCCGGCCCAAGAA 4560  
 ATGCTCTCTTCCAGAGCTTGAGCCTCAGTGAATTAAGCAAGGCCAGACCTAAATTGATGGAGGATTTTGTTAGTCTCCTTGTCTGTGTCTGCATTTAACTTGAATTCTGTGCTCACCAAG 4680  
 TCTGCCTATTGAGGACTCCGCATGCCCCTTCCATCTGCTTGCATCCATTTCCTGCTGCAGTGCTTGTGGTTCCATCTCTCCCTAGCTAACATTGGGCTGGGGGTGGAAGTAAGGGGGGCA 4800  
 ACTGTTGAGTGTTTTATTTCAGAGGCTCCATCCCATAATGGTAGCTGCTAGCTCATCTCTTGTGAAGCTGTCCAGAGCTGATTCTCCTTCTGTCACCCCCTCTGTGCCCATGCGCATCCT 4920  
 ACCAAGGCCTACCCAGTGAGTTGCACTTCTATGTATAACAAAAATAGTGATTTTTTTATCTTCTACGTATAACAAAAACTGATTTTTATCTCCTAACGTATAACAAAACGTGATTTTATA 5040  
 TAAGCCAGCTGGGGAGGGGGGAGGGCTGACCTGTTTTCTAGCTCTTCTTGTTTTGCAGACCCGAGATAACACTGCCTGAAGCAGATCTGTTCAGTTGGCAAGTAACATGGTGCCGTGTGC 5160  
 GTAGTCTGCTTGTGCAAAGCATATGATGAATCGGCTTTTCTCTTGTGTGGAGTAGTGATTGTTGGAGGAGAGAAACCCCAATGCCTGTGGTTTGAAAGGAACTCTTGGATTTAAAAACCT 5280  
 TGCTTTGTAAACGGTCCTTGTATGAAAGGGGTGCGTTTGTGTGCCGCTTTAGGTAACCTAAGTTCTCTTGTTCCTCTTTGGCAAAGTGGGGAGGAGGGGGGCACTGAAGCCTTTAGTTTC 5400  
 TTCCAGATGCATCTTGAGATCACATTCACACCAGAAAACAAACCTGAAAGCAGTTCTGCCGGCAAGTTCCCCATCACATTTGATCACCTAGCGACTTTTCTTTAGTGCACTGCTTTTGTG 5520  
 TTGGGGGCGGGGTCTCTCCTTGTCTAGTTTCTTAACAGTTGCTCCAAGAACCACCTGACCTTCCAAACAAAGCCAGCTCTTCACGTTCAGCCACAGAAGTCACCCACCTCCGTAAGCACA 5640  
 GTGGCATCATTCAGGATCTCTTTTTCTCTCCCCACTTCCTTTGGTGTTATACTTGTGGGGGGGAGCCCCTTGTCTTCTCGAGATTGCTTTCCACAGATCAGGAGGGGAGGAGAAAATGCC 5760  
 CGACACTCAAGGAGTTCACACGGCAATGGACAAAAAGAACAGGTGAACCATCTTTTCTCTCTTTCCACACCAAGCGTAAGATGGGAAAGGCTTTTGGGGGGGGAGGTTTAACATTGCCTC 5880  
 CCTGTTCTGCTTCCTCAAATTCAGATCTTTGACCCCAGCTTCATGCTGATCTGTGCCATTCAGACTGTTTTCACTTATTTAACAGGTTGGGTTGAGATCCTTGGCGTCTTCATGGCAAGA 6000  
 GGATATCCCCAGCTATGCTTTATTTAAAGCTCTTTAGCCTCGGAGGCCTCTTTCAATAGAGGAGACCAACAAGCCTTAAGCTCTCTGGCCTCCTTTTCTGGTCAATGGCAATTCAGTTGG 6120  
 AAGCAGAACAAATACTAATTTTACCAAAAAAAAATACAGTATACCCACACAGTGCCCCTTGCAGTAACTCTGCCTAGAGCAGGCAACAATGGCCAATCCATAATAACGAGGCTTATAGCC 6240  
 GTGATGGTCGTAATCTTTGCCTCACGGTCTTCCATTGGCAATGGATAAAAGACCAATGTTGAAAAGGGAGGAGGGCAGGGATTGTGCTGCAACCCGGTTTTTCTTGCTAAGCGTTTTCCA 6360  
 GGAACGTAGCTCAGACCACACAAAGCACTGACTCCATAATACTAAAACCTGGATCCTGCCCGTGCTGCTGTTCTCAAGGTAACACATTTAAAATGCCCCATTTCAAACTAGATTTGGTAA 6480  
 ATGGCTGTAACTTAAGCCTGTAACTCTTCCCTTGGAAACTAAACAGTTGAAGGAAGGTAACTTTAACTTAACTTTTGAAGGTAACCTTAACTTTTGAATTCTCAGAGCTAACCCCCAATA 6600  
 TACTGATAAGTGCTCAGCAGAGATCTCATTTCTAACAAGCCTAATGTTCAATGTTGTCTCTTCAAATAACATGGCTAAGACAGTATATGACTTGTGAGCTGGGGATGCTGGGGTGGGTAC 6720  
 TACTTCACTGACTTAGCCAGCCTTTGTGAATTGTGTGTCAGACGGCTTTTGGGGTGGATGTCTGCAACTGTCTGCTAAGACTCTTTCAGGTCGCAATAAACGAAGGTAGAGAAGTGGAAG 6840  
 CTTTATGGAAGAGATTTCATCCTAATTCCAAAAACAAATAAACAAATAAAATGAAAGG

TTTTCTTTT

TTTTCTTTT  
Depth:19 (ZEBRAFISH)  
Ei-value:0.000, Pi-value:0.000  
Er-value:0.000, Pr-value:0.000  
MATCHES To TargetScan▶ miR-186-5p:AAAGAAU

CCTGAGAGAACAATCTTGTTTTCT

CAGGTTTTGCTTT

CAGGTTTTGCTTT  
Depth:19 (ZEBRAFISH)  
Ei-value:0.000, Pi-value:0.000  
Er-value:0.000, Pr-value:0.000  
MATCHES To TargetScan▶ miR-330-3p.2:AAAGCAC▶ miR-490-3p:AACCUGG

TCCCCTTGTTGCATTG 6960  
 CCCAA

AAAAAGCAAAA

AAAAAGCAAAA  
Depth:19 (ZEBRAFISH)  
Ei-value:0.000, Pi-value:0.000  
Er-value:0.000, Pr-value:0.000  
No matches to TargetScan

GACGCTGGTGGTTGGCACTCCTGGCTTCCGGGACGGGGTTCAAGTCCCTGCGGTGTCCTTGCTTTCCTCAATGCAACTTTGGTGGACTTCTAAGGTCCTGCTGC 7080  
 TGCCAGAGGCATCAACACTAGTTTTGTTTCATACTGTTGTAATTGGTTGCTCTTTTTCTGATTCAAGATTCTATATTAAGAAAAAGACCAGCACCAAGGAACTGCAAAATTTGGGACATA 7200  
 ATGCTCTGAGAGGCCCAAGTTTGTATTGTCATTCTAAGATCACACTGTTACTTTGATGGTTGGAAATAAAGTTTGGAGTTACTTGATCTGCATCTTGTATTGTTCTCTTCATTTATCTTT 7320  
 TCTCCCAGTTTGGGACTAGGGTGCATTGAATAAAAAACCAGGGGG                                                                            7365
```

---

## >SNAKE (6910 bases)

```
 GTTCCGTTGTCAGAGCTGCAGGGAGGCTAGGAGAGAGCGGAGGGAGGTTTTTGCCGGGGAGATAACTAGACTTTAAAAAGGAAACTAAAACAGCCTAAAATCCTTTAAAAGGGATAGCTA 120  
 AGGTTTTTTTTAAAAAAGCAACTAAGGCTTTTCTTCGATTAAAATACTCAGCTACTTAGTGCTTAAAAAGAATAGGCAACCTAGGTAATTCTAAATTAAACCAGAAAATAGAAGAAGATG 240  
 GAATAGAATTGAAGATTTCTTTTAAAATCCGAGGGACAAATTCCTGTAAAAGGGCTTTTATTTAAAAAAAAAACCAAACTCCCTCGAATCTGAAATGAAGGCAAACTGAAAAGATAAGAA 360  
 GGCTTTGAAAGATCAAGAAAATTGAAGATAGAAAACAAGATATAATGGAAAACGGTAGAAAATTAAAAAAAAAAACACCGTTCAACGTTTCAGCTTTAGAAGTTAAAATTAGCCATAGAG 480  
 GAGGTAAAAAAAGAAAAGAGCATGGATGATGCGAAGTCTGCTTCAAGCAGAGCTGACCAAAAGGAAAGAGGAAAAAAAATGGAGGGTTTGTTTCAAAGAAGTGGCAACAAAGGTAACTAT 600  
 AAAAAAAGGTGAGCGCCTTGTTGCTGAAGTAGGAAAAAACAAGACTTGAAGATTTTCTACAGAGGCAAGACTTAGAAGAGAGAATTTAGAAGATGAAGAGAGAAGGAAAAAAAAGACGGG 720  
 TAGGGAAAAAAAGAAGCAAAAATCCAGGCTAAAGAAGTAGAAAATTAGCGAAGAGGAAGGCAAAGGGAACAAAAAGGACTTTAAAAATAGTATAAACAACCCTCTTGCTTGTTTAGTTTC 840  
 GAGCTAAAGCCCTACTGGTGAACCAAAAGCCTTTTCCAAGGGGGCATTGGTTTAAATTTTGCTTGCAAAGCTTTATATTGGTCAGGATGCTATTTAAAAGCAGTTTTGAAGATAGACGCC 960  
 AAAAAAAAAAAGACTTTTTAAAAGCTGTTTTCTCAACTGGCGTGAAAACAAGGAAGATAGGAAGGCGGTAGACCCCCAAGAAAGGAAGCCAAAACCCATTGGTGAAGCAGGGAAGGAAAA 1080  
 GCAGGAAAAGCAAAGTAGAAGAGAGGGCATGGGAAGATCCCTTTAGAGATGCTTCTAGAACAAGCCAAGAATATCCTCTGTTAGGGGACCCATGACGAAGACCCGTAAAAGGCCTCGTGC 1200  
 GTTGCGTATCGTAGTTGGTAACCGCCTCATCTTATTGGCAACGTCTTTTTATTTAGGGCTGCGCTGGTCGTAAATTGGGTTTGTCCGTAAA

TTTGGG

TTTGGG  
Depth:19 (ZEBRAFISH)  
Ei-value:0.000, Pi-value:0.000  
Er-value:0.000, Pr-value:0.000  
No matches to TargetScan

GGTTTATTCTCGGAGCTGCGGGG 1320  
 GGAGGCGGGGCTGTTGGTCTCTCTCTTTACAGGTGTACCGTAATGTCCTTAGTCGGCTGTTTGCTGGGAAGCTATTGGGCGGTCTTCGCAAGCCAACCAAGCCAGCAGTTCCTGTGCTTA 1440  
 AAAAGTAGCCATTGTTTCTGCTTGTTCCCTCTTCTCCCCCCCTTTCCTCCACAAGAAAATAGCTAGAATCCTTGAGGCTGTGTGTAAACCACCCTCCCCAGAATGGGTGAGTTGAAATCC 1560  
 CCTGGTGGGAACGACAGGATTTTGCAGGTAGAGTTACAAAAAGGGTGAAAAATTGTACTTGATTTGTAGCCATTTGCCGGGTGGATAGCTATTCTGGGTGGAGGGGTGGGCATTTGCAAT 1680  
 GTTGTGTTGAGATTTTGTCCCTTCAGGTGTATGTAGAGCCAGAGGAATCCACAAAAAGTCCCCGGTGTGTTTCATAAGGAAAAAAAATGCCAAGGAAGTGACTTGAGGATGGCTTGAATA 1800  
 ACTCCAGTGTAAACAAGCAAAGGAAGGAACATAATGAAACCAGGCGTGGCGATGCAAACTTTTAACTCCTAGGAGGAAATGGCTCCTTGGCATCTGTAGTGGGGGAGGAACTGCGCAGGA 1920  
 CCATAAGTCCCAACCTCTTGGTTGTAACCACCCTTTTTTTATTCCCTACAAGAGGGTTAAGTATATAGATGTTTATAGACAGCAAACATTTCATCTGGGAGTGTGT

TTTGGG

TTTGGG  
Depth:19 (ZEBRAFISH)  
Ei-value:0.000, Pi-value:0.000  
Er-value:0.000, Pr-value:0.000  
No matches to TargetScan

GGGGTGAG 2040  
 GGGTGGGGAGGATAAATGCCAGCTTCTCTCTAGCTTCTCTGCAGGTACTATAGCATCCTCTGGTGGCATGACTTAGAAGTTGCTTGCTGCTCTTATGGCAAAGACCACCACCCTCCCTCT 2160  
 GAGGCTGGACCTCACTTTCCTGGGAAGAAAGGACAGACAGTGCCTAGAATTGGAAGTTGTTGTGGAATTGTGATCCATTTACGCAGCATTTGCAGAAGAGCTGAGAAGTTGAAAGCTAAG 2280  
 TTCTTAAGAGAAAAAAACAACACTTGGTGTTTGATATGAAGTGGAGGGGGATTTTTAATTAACCAGTATTGGGGTGGGGAAGGGGAGAGAATAATTTTCTGTGTTTTTCTTAGATTATTT 2400  
 CTCCAGGATACCCATAATTTTTAGGTCTGGAAAAATAGCCTAGTCTGGCAAGTAATTTTCTGCAGTAGAAATTGAGACAATTGGAGGAGATTGGCCTTTAACGGCAAATGCTTATACATT 2520  
 TCCCGCTAAAAAAGCTGTGTTATGACTAATCTGTAAAAAAATAGATCCAACTCCTACATTTCCTATTAGGGGGAGGAGAGAAGGAAAGCCCCTGAGGAATATACAGATGTGGATTTTTCT 2640  
 GAAAGGAAGGAAGGCTTGGATATTTATAAAATCGGGGCTGCAATGCCCAGGTAGGAAACTGAGAAGTGAAAGAGGAAATCATAATCTTTAGATATAGTAGTTTGTGGTTCTCTTTTGGCA 2760  
 TCTTTTCCAGGTACTTAATATCTCTTAAAAGAGACTTCAGGGAGGAAAGACAACTGAATGATCTGATCCTGTGTGTGTATTCGTTCCGGGGTAGTTTGTATAATTTTTGCAAGTAAGATT 2880  
 CTGCTTTTGATTGTTTCCCTTTTTTTGTAATTCAAATCATTGGTTCTAAGAATGAGTTGCATTAATTCTGTTAAGAGCTTGAAAAAAAAAGAATGATTAGTGATTATTGTAGACTTTTGA 3000  
 GTACCTAATTAATACAACAGTAATCATAACCTCCGTTGACTGCTCATAGCTCATGATGTATCTTGTCTTGTATTGGGGGTGGGGTGGCCTGGGGAACAACTCTTGTCTAGACC

TTTTTCA

TTTTTCAG  
Depth:19 (ZEBRAFISH)  
Ei-value:0.000, Pi-value:0.000  
Er-value:0.000, Pr-value:0.000  
No matches to TargetScan

 3120  


G

TTTTTCAG  
Depth:19 (ZEBRAFISH)  
Ei-value:0.000, Pi-value:0.000  
Er-value:0.000, Pr-value:0.000  
No matches to TargetScan

ATCCGTCCTCAACATTAACCTGCTGAAAAAAAGCCTTGGCATCATAAAAAGCAGAGGGAGCAACATCGTTGCGAATGATAAAAGGCAGAAATACCCTAGATGGAAAAAGGTCCCAGATG 3240  
 TTTTAACAGGTGAAAAAGCCAGAATCTCTGCCAACAGGGGAAAAAAAGATCCACAAAGCTATAAGCAGTGAAGCAGGCAAATTTGAAGTGTATGAAATCAAATGTGAAATTTCTAACCTT 3360  
 ATGGAAATGAGGAGCACAACACATTAAAACCCAGTGCATGAAGGCCTGTGTCTACAAATGCAAATATTAACCAATTAAGAGAATCTTCAGGGGAAACAAAATGGCAAAGGTGCTTTCTCC 3480  
 ACCCAGCTGGCTTCAAAATATTTGATGTGTGAATGATTTCCTAAATCTTAAAATGGAATTGAAGAGAATAATGGAAAAAAAAGCATGGTAGAATTGGATCAATCTTCTTTGGGACTGGAG 3600  
 AGCAGGTGATTTTTGTGTTGGTTGTTTTGCCTCAGCAAAGGTTTGCTTCGTGTTCCGTCTGGGAGCTGAACTGGTAACA

GATAAG

GATAAG  
Depth:19 (ZEBRAFISH)  
Ei-value:0.000, Pi-value:0.000  
Er-value:0.000, Pr-value:0.000  
No matches to TargetScan

TTTGACCATTTCTAAAAGAATTGCATATTGGGGGG 3720  
 AAAAGTGAGTTAAATGTTTATTTTCAGGGCTTTGGGAATGAAATCTTGGAATTTCATAATGCATTATAATATACCCCCTTTCAGGTTTTAAGATGCTTCCTGGGTTGCCTGTGGCCCCCA 3840  
 GATACCTTCTACATGAGCTACTACTCGGTTTATAAAGTCTCTTTTATCATATTCCTTGGCCTCGACTGCCTTGCCTCCTTGTGCTATAGAGGCCACTTCAAGTGGCCCTTAATTGCTGGT 3960  
 CCTTGCACACAAAGGAAGGCATTTTGTGTAGCCCTTGAGTACCTGTATAAAGTCTTCAACATATCAAAATATTTGTACAGTAATGAAAATGTTCAGTGTTTGCCAAAGGGCTAGGAAGTT 4080  
 GTAATCTTTTTCACTGTTCAAATAAAACATGCTGTTTCTGGGTTCATTTTTAATTGTTACTGTGTGAATAGTTGGCATTTGGTGTGGGTGGGTGCGGGGTATTCTTCTTGATCTTTCAGA 4200  
 ATCTTTCTCCTCCGTGAAACACCATCTGATCTCTATTGGACAAGAACAGGAAAATAGAGCTGAAGACTCTAAATCAGGCAGGAGGTTTTCTTTGCACAATGTTTTCCTCTCTTCCCTCCA 4320  
 TCCTTGAGAAAAAGTAGAAAATCCAGGCGCTTGGCTATGCTATATGTAGTCCTCTCCTAAGTAAGTGGTCTTCTCTGGGACGATTTCTAAAATACTTAAAAAACTAGACAAGTTGGGGAA 4440  
 AAATACTTAGAGGTTTTGGGGTAAGTAACAATATTCCTTTAACATATGTATGCTAACTTTTAGGAAGCTCCTTTCTATCCAAGTTTTTATCCTTTGCTCAGCTAAATTGGGTGACCTAAA 4560  
 TTCATAAGGATCAAGTTTGTGCTTTAACAGTATGAATAACACAAGCTTCTATATTTAGAAATCTGCATGCCACTTAAATCTGCTTGCATCAGTCTCTTGTAACACTAGTGACATCTGTTC 4680  
 CAATTCCACCCATGGAAGGGTTTGGGGGGGGGATAGGGGCAACCATTTGTTTGGTGTTATGTTTCAGAGGCTTCTATTTGCGAAGTTCTCCAGGGTGCTATGTACACAACTCTTGCTCCC 4800  
 ACCCTGTATGCAGTGCTTCCTGTGAAGGCTTGGCAGTGAGTTAACTCATGTAATCATGGCACAACTTTTTGGTGGCAGCTTGGTGCTTGTATGGGGGGAGGGGGGGCTCACTGTTTTGTT 4920  
 CCAGTTCTTTTTTTCAGACTCCACCAACAGAAAACACTCTCTGTGATGCCCGTCTGTTCAGTTGGCAAGTAATGTGATGCGCAGTGTGCTTGATCTGCTTGTGTAGAGCATATGAACCCG 5040  
 GCTTTTCTTGGGCAGAGTAATAATACTAGTTGAAAGAGAGAATCCAGTTGATGAGTGACAGAAAAAAAAAATGCCTGTGGTTTGAAAGGAGATTTCTGAGCATCTCATGCAATCCTGCAG 5160  
 TGTAGCCAGTTCTTGTTTGGCTGAGAGACGAGTGGCGGTACTGCTTTTTAGGTAATCTGTGCAACTTTTTCTTCCGGGGGAGGGCTTGGGGGTGGGTACTGAAGCCTTTAGTTTTTTTCC 5280  
 AGATGCACCTTAGGCTCTAATTGACACAAGAAATCACAGCTCCAGGAAGCTTAACTGGCAAGTCACCTTCACATTTCTACCCAATTCCTGACCCGAAGTGGAATGCTTTGTTGTGTGGGT 5400  
 TTCTTCCTGTTGTATTTTCTTAACAGTTTTGATTCAAGAACACACAACACTCCAAAAAAAAACCCCAGCTCCTCATGAGGCGGCACAGAAGCCACTCCAGTAAGAATAGCCTTCCCGAAA 5520  
 ACCCATTTTTTCTCTCTCTCCTTACGTGATGGGATTAATGGGGGGAGCTGTTGGGGGCACCCTTGAAAGGGGGGGGGGGAGTCTAGATTGCTTTTCACAGATCAGTAGAAGGAACCTCCT 5640  
 CCTCCAGACAGGACTCTCACACTGCAGTGGACAGAAATAAAACAGGTGAACATATTTTTCTTTTCCATGCTGTGATATTACAAAGATAGGAAAAAACAGGAGATGTTAACTGATCTCCAT 5760  
 TCTGCCTCTGTAGTTTAACCCAGCTTCATGCTGCTGATTGGAGGGCATCTATCTTAAGGCTGGTTCTGTAAGAATCCCATCCTTTGGGTGCTGAGGTATGAGAACCTGGTGTACTTAAAA 5880  
 GTCTCCGGTATCCTCATCAACAGGTGCTCCTTAAATGGACCCTGTAACAATATTACAACAGAGGCAGATGTTTTAAAATTACAAAAAATAAATAAATGGAATGAGACCGGCTTGTTTGGA 6000  
 ATAATCACCAAACTTGTGCAGAAAAGATGTCTCAGAACCAGCCATCTTGCAGTTAAATGCAACAGGTTGTCCTCCTTTGATTTGCCCCCAAATGCCTAACATTCCTTTAAAACCATGGTA 6120  
 GTAATTGCCAGTCTCGTAGGGTGAAGGAGGGTAGGGGGCTGTACTGCAACCAGGTTTTTTTTGCTAAGAATTTTCCAGGAACAAAGCTGTTCAACATGTAGCTCAAAACACCACAAAAAA 6240  
 ACAAAAACACAGTTGACTTCATGCTAAAACTTGGATCCTGCCCAGGGCTGCTGTTCACAAGGTAATCTGTTAAAAACATATTTGAAAACCTCAGAGCAAATGTTGCCTGTAACTTGTAAA 6360  
 GTTGGAAGCCAACAGCGCCCAGTTCAAGGAAGGTAAATTTTGTTTTTTACACTTTTCCTACGATGTTCATTGTGCCAGGCACAAATCGAGTAAAATCTCTATAAAAGTGTGTTAGCAACA 6480  
 AAGAAAGTAACGAAGAAATGCCGTACCTTTTGAAACCGTAGCAACGAAGCCATTGATGTTTCCTGTCTTTGAGGGACATAATGTGGGTGGTGGTAGGGAGCCTGTGGGCCGACCTAGGAC 6600  
 TTAGGCAGTGTAGGTGGCTATGGGTGGGGAGGGAGGGTGTTTGCAATTCTGCTAAGACTCTTTCAGGTCGTGGTACGTACGTGGTCAGAAGTGGAAGCAAGAAAAAAAAACACAAAAAAG 6720  
 GT

TTTTCTTTT

TTTTCTTTT  
Depth:19 (ZEBRAFISH)  
Ei-value:0.000, Pi-value:0.000  
Er-value:0.000, Pr-value:0.000  
MATCHES To TargetScan▶ miR-186-5p:AAAGAAU

CCTGAGAGAACAAATTGTGTTTTCT

CAGGTTTTGCTTT

CAGGTTTTGCTTT  
Depth:19 (ZEBRAFISH)  
Ei-value:0.000, Pi-value:0.000  
Er-value:0.000, Pr-value:0.000  
MATCHES To TargetScan▶ miR-330-3p.2:AAAGCAC▶ miR-490-3p:AACCUGG

TCACCTTGCTGCATGGCCCAAACCCAAAA

AAAAAGCAAAA

AAAAAGCAAAA  
Depth:19 (ZEBRAFISH)  
Ei-value:0.000, Pi-value:0.000  
Er-value:0.000, Pr-value:0.000  
No matches to TargetScan

GGCATTGGTGGTTGGCACTCCTGGAGTCCAG 6840  
 GACGGGGTTCGAGTCCCTGCGATGCCCTTGCTTTAATCCAAATGCAGTAGGGTGTGTGTTTTGGGGTCCT                                                   6910
```

---

## >X.TROPICALIS (12676 bases)

```
 TATGGGAAGAGCAAAGGACAGGAGAGGCATGCAGACAAGGGAAGGCGTGAGCTCTTTTTTTTTTTTAAATTTAGGCTATAGAATTTCTTTTAACACAGCTACTGGAATTTTAGCTATATT 120  
 CTAGTGGGCTTAAACATTTTTATTTTCTGAAAGTGTAGAGGGAGAATAAAGGCAAACAAGCAAATAGTATTTTATAGATTAGAAGGAACAGTGATCCAGCATCAGGAAAGCAGAGAGGGA 240  
 AAAAAAATCGGCATTACTGAACAACTGTATTTACGCAGCCATGAAATGCATAATAGGCTTTGTGTATTGCCTCACTAGCCTCAAAAAGATTAAAGCTAGGCTCTTTAACAGTAATTTAAT 360  
 GTGGAAGTACGTTCATAGATGATAGCTGAAAACGACCACTGAAAATAACAAAGGGAAATGGCTAGAGCAGATTTCTTTATGAAATGTAAGAGCTAAGGCATACCTGCATCATGCTCAGAA 480  
 AACATAGAAAAGGAGAAAACTGGCAAAGAAAACTGAAGACTAGATTTCTGAAAAGCTAAAAGAAAGTAGTCTATATGTAAAAGGAGAGATTAGAATTCAAAAGCAGACTAGCTTCAGATT 600  
 TCAAAGAAGATATAAAAAGAAAAACAGAAGATAGCTGCCTTGAGAACAATAAATCTTTATTAAACAGCTAACTTACTTATAGTTACCTTGAAAATAGAAGATAATGCAATCATCATATTA 720  
 GAAAATAGAAGAAAGAAGAAATTAGCTGTATTTCAGCAATGTGAGATTTCTAACCCCAGTGAAAACAGAAGTGGCGACAGAAAGACCTGAAGAATAGATGTGTAAATGAAGGAAGAAAAT 840  
 TAAGCAAGGCATTAATATACAATTTTATTCAATCCAGCCCTAAGCTGACAGTACTGATCGAGGTTATGCAGAAAGTTCTATATATAAAAGAACAATCTTGAAGGAAAATTTAAAAAAAAA 960  
 ATAGAAATAACTCTCTAACCAGCAGAAAAAAAAAATCACTAGTTAGTCACGACTAGATTAAGAAGAAGACAGTAAAAGGGTAGTAGGAAGGGTTATTTTCTTTTTAGGAAGAAGCAGGAA 1080  
 GATTTTAGAAGAGGATGAATTAAGAAGCAGGAAGACAAGGAGAAATTGCTTTGAAAGGAAGGACAGGACTGAGATTTTCTGCATAGTGTCTTGCTAGGCAGAATGACTTGTTGGACTCTG 1200  
 GTACTCGTCTTACATTAGCGGTGTAATTATGTGATCACTTGGGAGTTGCGAATCAAAACTTGAGTATATGTACTTCCAGACGGACTCAACTTAAAGCAGTTAGCAATATGCAAGGGGACT 1320  
 GAGTAATTATGGTTGTTGTGCTGGTTTTGAATTATTTGACAACGTGAATGGGAAGCTTAGGACCAAGGCGAAAAATCTGCTGATGTCGGTGAAGTTAGAACGGGCAAGAATCAAGTGGTG 1440  
 GTAGGAAACACAGAAATTAATCAAGTGGCTTCAGTTCAGCTATAGAGCGTGTTGGATGACCCCCAAGGGAGGTAGAATCATCTAGCAATTGATATCCTGGAAGGCAGAGAAAGCAATCTT 1560  
 GTGTTCTAATTGCCTGACCCATCCCATCGTACGTATATGGTAAAAGGGATACAAGTAATTCTTGGGGAGGGAGGGGTTGGGGGGGGGGATGGTCTTTTTCAGCTTTTGTTTTTCAGGACT 1680  
 GTATCCAAGAATTGCTGGTGCAGTGTAAACATCCCCGAACATTTATTACCAGTAGGAGGACTTGGCAAGTTCAAGCCATCTAGCTTTTGGCAAAACCTTCTTCTATACATGGGTAGTTTG 1800  
 TATTTAGTTTGGTCTGTGTAACCTCAGCAGCAGCTGGATCACAGTGCCCTTTGGTAAGAATATTATTTATTTTTTCCAACTAGTTAAAAATACAACTACCCCCATATTATTTCTCTTACC 1920  
 CATTAGTTTAGCTTACTGTCATGGGTTTTTATATGTTCACAGTTGTTACTGAGCCAGTAAGGCATACATCTTTAGGGTAACTGGCGTGGGGTTGGGGGTGCAAATGTTTTTTTATTTAGA 2040  
 TTTTTTGCAGAAGTACATTGTGGTGTATGTATAAATGGCAAGAGTATCTTCTTCTGCTGTGGTTTGCCAGTATCCGGATTCATGCTTTTTCACAGGTAGTTTTTCATTTTTTGCGGGATT 2160  
 GTGTGTATTTGTATGTATATAGATATTTATATAATAACATAAATACGTCACAAAAAGGACATGCTGTTAGCTATTATCCTATAGGAGAGAAATAATACCCAGCATGTTCTGCTTATAAAA 2280  
 AAAATACTGGGTAGGAAT

TTTGGG

TTTGGG  
Depth:19 (ZEBRAFISH)  
Ei-value:0.000, Pi-value:0.000  
Er-value:0.000, Pr-value:0.000  
No matches to TargetScan

TTTAAAAACTTCAAACTAGGAAGTAACTGGGGTTACTCTTATTGTGTGTGTGTATATATAGCAATAAGCATGCCTGTGGGCTCATCTTTGTGCTGG 2400  
 TGTTGCTAAAAACTGATTTATTCAGTTTTAGAACAGGTGTTAATTTAATTAATTGCATTCTAAGTATGCGTATTGCATGTATGTAATGTTTGAATAAAGAATGTGGAGTATGCTGTAATT 2520  
 ATAAGGGTGGGGATTCAAGCTGTTTATGTCTATCCTGTTCAATAAATATGATTAAATTAAGAACCTACATTTACAAACCTAGTTGAAATGTGTCTGTTATATGTTGCTGTGGTGGAGAAT 2640  
 GTTTTGCCTTAGACTCTTTCAGATTCAGTTCGTGAAACTGCACTAAGTTAAAGTCGAACTAACATAACCATCCAAAAGTTTCAAGGTGTTCCTGATGAATGGGTGAAGAGTGATACAGTA 2760  
 TAGCAGTATAAGTCTAAAAGAACAAGGATGTTTCCAAAATTCAAAATATTGAAGCAGGCAAGGTTGCTACTATAGATTAATTGGCTTTTTTTATCCAGAAGTTGTGTTTGTTTTATTTGC 2880  
 AACAAATTCCATTAACACTTTTTCCACCAGCTCAGTAATATTAGGTATGATATTAAATCAAACATATGTAAGTAGAATGTCCTAGTGTGTTGAAAAGATCTGCATTCAAGCAAAAAAGCA 3000  
 GTATCTGAATTTTAAATTATTTTCATTTGCTGATTGCAATGAAAAGTCAGTCTTACAGACTCTCCTATACATCCCAATAAATGAAAAATTCTGTTTGATGATGAACCTAATTCAAACATT 3120  
 ACTCAGTGTTCTCTTATGATTATAAAACAAGCTTTGCTGAAGCTTGCATGGGGATAAATAATCAGGAGAGTGTGGGAGGGTCAGGGAATGTTTTATTGCAGTTTTTATTTTTTAGGATTA 3240  
 TAAAGTATGGCTCCTGGTGCCGTAGCTATTCCTGAGCATAACACAACAGCGGAAAAAGCAAATGTAAGCCAGCTAGGATCTGGGACCAGCGTACCTATATATACATATATCTATACACAG 3360  
 CGTCTGTAATTTCACAGTGTGTTTGTAAAATCAGAAGCAGATTGAACAGCACCCTCTTGTGGGTTTTGGTAAGACTATAACTTTTTGACTTTACAAAAGGGCTAAAACTTTGTACAAAGC 3480  
 TATTTAGTTTTTGTGTTCATTGACTAATTCTAATTTTTGGTAAAGTAATTCAGCAATTGGGATTATTCTACAATATATATTGGGGCCTATGTGGGTTTTTTTTTTTATAAGTAATTGGGA 3600  
 TGGGTTGGGCTGCAAACTTTTTTTGCTAGATTTTTGCAGGAATACACGGTATTGTATATTAAGGAAAGATTATCTTCTGCCGTGGTTTGCAAGAATATGGACTCTGGTACTTTTGCACAG 3720  
 GTAGACTTTTCATATATCATTGGATGGGTTTCTAATATACAGCTTATTCTGAACAACAATGTTGTATCTGTATTGGGGGCTGTTTGTAATGTATCATTTTACAGATTTATAATTACAAAT 3840  
 GGGGAACAGAGAGTAAATCTGTACTTAAATTATTCAGGATTCTCCATACTTTACAAAGAAGGAGATGAACATCACAAAAAACAAAAAAAACATGGAGAAAAGGGGAATAGAAATAAGCAT 3960  
 GCACATGGATATGTTGCCTGTGCTGGTGTTTGTAACAAACATTTTGTAGTCATTATATTACTTAAAGCACCGCTGTTTATTTGTGGTTGCATTATAAATATATACATTGTTTTGGATGTA 4080  
 GAGACATTTATGTCTTGTGTTCAATGAATGTCCTTGGTTTTAAAATCATTATTTTGTAAAGTTGGGAGGGGTGGGTGAGAACATTTTGCCTTAGACTCTTTCAGATTCGTCTTATGAAAT 4200  
 GGCAACAAGCCAGATGCGAATTGCGTTTCCATCCTAAACTTTCAAGGTATCGTTGATGAATGAGTGAACCATAATGCAGAATAGAAACGAAAAGGCAAGGATTGGAAATTTGTGTTTGAC 4320  
 CTCCTGATTATTTGAAAAGAGGCAGCGGCTTTTTATTATGCAGATAAAATCAACACCATAGAAAAATCAGATTTCAGTATCCAGAGGTTCTACTGGAGCAAGAATAAGAGATTTAAGTAT 4440  
 TTACCAGGATTTCAGAGTGTGCAGAAGTGAATCACATACAAGGAAAAGCAAAAATATTACCAATACTACCAATAACCAATATTACATATATTTTCTGCTAGCAATGAAAGACTTGAAAAG 4560  
 ATATAAATTAATATCCAACAAGCATCAAACACCAAATATTTCCTAACTTAAAACTAGCTATGAAAATTGATATCTGAAATGATCAATTAAAATCTACTCCAGGAAAAGATTTCCATGTTG 4680  
 TGTACTTGAAGAATTATTTAGCCCTCACAACAGCGCTTACATTAGAAGAATAAAGTTAGGCTGGATAATAATCAAGTTGAGGAGCCTGCAGGAGAGATCTTGGAGCTAGACGGGCAAGGA 4800  
 TCACGTGGTAGTGGTACAACCAATGCAAAGATCAAGTGGCATCAGTTGAGCTAGAAAACCGTCAAGATCCCAAGGGAGACAGAAACCTTTAGCTGTTTGCGTCCTGGAAGGCAGAGAAAA 4920  
 GAAGCTTGTGTTCTAACTGGCAGACCTATCGTATGTATACAGTAAAATTTAGAATTGGGTGTAGATGGGGGTAGGGATG

TTTGGG

TTTGGG  
Depth:19 (ZEBRAFISH)  
Ei-value:0.000, Pi-value:0.000  
Er-value:0.000, Pr-value:0.000  
No matches to TargetScan

TGTTTTTTTTTTTTTTTTTTTGTAGTTTATTTTTC 5040  
 AGGATTCCGTAAGATATGGCTCCTGGTGCTGTGTATCTCCTGTGCATTTATTACATGTAAGTGAATTTTGTAAATTCAAGCCAGCTAGCA

TTTGGG

TTTGGG  
Depth:19 (ZEBRAFISH)  
Ei-value:0.000, Pi-value:0.000  
Er-value:0.000, Pr-value:0.000  
No matches to TargetScan

AACCCCTCCAGTGTCAATATGTAG 5160  
 ATAGCCGCTAATTTGGTATGGGAAAAATCAGCAACCGATGGATCAGCACCCTTGTGTGGTCTTTGGTAAGAATCACTTTTTTAACATTTCATAAGGGGATAACTGTAGTAAGCCATCCTT 5280  
 TTACTTTATAACCAACTACTGGTTGTATGTAGATAGTACTGTTGTGCAAAAATGAACGGTGGGATTTAAATTAAATGTGTGGTGTAATGAGTTATTTTCTGTAGGGAGGATGGGTTGGAA 5400  
 TGCAAAGTTTTTCTCTAGATTTTTCATATATGCAAATATCTGCGTATATATAAACAATGTCCAGCACCTTATGTGTATACTGGGCAGGAAATGTGGTTCATAAACAGCAAAATAGAAAGT 5520  
 GACAGATATTAGCTTGTCATACAAATATAAGTAAGCTTGCCTGTAGGCTGGCTCTTGTCCTGGTGTTACTATTGACTGGTTTATTAGCTCTAGTTCAAACCTTAATATATTTATGGTTGT 5640  
 AATCTCAATACGTGCATTGTATATATGTAACAATTGCATAAGAAGCGGGTGGGGAAGCAAATGCCTAAGATGTTTTTGACTTTCTGGCTTAATAAACATGATATAAAGAAACTACATTTA 5760  
 GAAAACTTAATTGAAATGTATCTGTTAAGTTGGTGTTGTGGAGAATGTTGTGCTTTAAACTCTTTCAGATTCATCTCATGAAATGGCACCAGGCTAAAATGAGATTTACTTTGAAGTTAC 5880  
 AATCATAAAAGGTATTCCTGATAAATGAGTGAAGAGTGTTACAGAATAGAATTTTAAGTCTGAAATCCAATATTTGACAAGCCAATTGGCATACTTGATGCAGGAAAAGTTGCTACTACA 6000  
 GAAGAATCGACTATACTTATCCAGAGGGTCTCCCAGTATTTTCATCAAATTCTATTACTCTTTATTCCACAAGTTCGATTTGAAAAAGAAATTCATATAAGAATCACTGTGAAGTGCCAA 6120  
 TACAGTTACAAAATAATTCAGTGTATAGAAGTGATCTACACTCAACCAATTAAAAATCTGTAATGGAAAAATTACTTATATAGTGAATTTCAAAGAATATACATTTGCTGGCAGAAATGG 6240  
 AAAAAAAAGTTCTACTTGATCAAAAACATAGCTCTCACATAATGAATTTGATGAACCTTATATTATTATTTGAAAAAAGCATTGTGGAGCTAGGATGGGAAAAAAATCATAATGGTGATA 6360  
 GTGAACCCAAGAAAAATGAAGTGGCTTCAGTTCAACTAGACAGTATATGGAGGACCACAAGGAAAGTTAGAAACTTTGATGTCCCAGAAGAAAAAGCAAGCTTGAGTAATTGCCAATCCC 6480  
 AGAGTATGTATGTTAATGGTATTACAAATAAATATAGTGTGGAGGTGGGGGAGGATGGTGTTTTGCAGTTTTTATTTTTCAGGATTTCACAGTAAAGCTTCTTGTGCCGTAACTATTCCT 6600  
 GAGCATATACTACATGTAAAGTGGAAGAAAGAAACCCAAGCCAAATAGGATCTGGGACCTCCATTCCCTCCATCTTTTTATAGTTAGCTTCTGTAATCTGAAGGTGCCTAAATCAGGAGC 6720  
 AGATGGATCAGCATCCTTTTATGGTCATTGGTAAGAATATATCCCAAAAGAGTAAAAACTTTGTAAAAAGGTTTTTACTTTTTAAGACATTGTATTAGGTAGACTTTTACAAGCGCAGTA 6840  
 CCACTTTTGCACTTCATTATTTAGGTTGAGGGGATTATTCTACAGTATGTATGGGATATTGGGGTACGTTGGTTGATTGGGATGGGTTGGGTTGCAAGCTGTTTTATATATTTTTGCAGA 6960  
 AATGCACAATAATATATATTGTAAGGAATAAGGAAAGATGTACTGTTTTGCAATTTTTGCCCAAGTGGACATTTTATCTTAAACGCAACAATTGTTGTACCTGCATTAGAGGGGTGTCTC 7080  
 TGTGACAAGTATAATTCTACAGATGTATGATTAAAAATAGACATCAAAAAGTTTTTCTATATTTAAGTTATTTACTACTCTCAGTACTTTACAAAGAAGGAGAGTAATGCTTTCTGCAAA 7200  
 TAAAAATGATTTTAATGGGTAGGAAATGTAGATAAATTTAAGCATGCCTGTGGTTATGACCCTTTGTGCTGCTTGCTAATAAGGATAATGTAGTCACATTATTAGTCAAAAGCACCACAA 7320  
 TTCATTTGTGGTTGCATTGAATGTATGTATTGTATGTTTGAGTACTGTACTCGGTACTCTGTGTATGTTCTAACTATAATGGTGAGGATATAAAATAAAAAATATATTTTGGTGTTGGGG 7440  
 GGGGGTTGTGTGAAATATTTCAGTTTAGACTCTTTCAGATTCTATTTGCTAAATGGCAATAAGACAGATGTGAACTGAGTTTGAAGTTACCATCACTTTCGAGGTATCGTTGATGAATGA 7560  
 GTGAGCAGTAATGAAGAATAGAAATAACATAACATTTGAAAGAGCGAGGATTTGAAATTTGCGTCGGACCTCCTGAATATTTGAAAACAGCCAGTGGCTTTATATTATGCAGATAAGATT 7680  
 CATATTATAAAAGAATCCGGATTCAGTATACAGAATTTCTACATATATAAAGAATTCCATTACAGTTTATTGCAAGAGTTCACTTTGACTTACTAACTCAACTTTATAAGAATATTACTG 7800  
 AAGCAAGAATAAGATGTCCAAATTAATCTAAGCATTTACCAGGATTTGAATGGGTAGAAGTAGATCTCAGACAAGAAGCTAAGCATTTGCCAGGATCTGAGTGGGTAGAAGTGAATCTCA 7920  
 AACAAGGAATATCAAGGAAATTCTCAATCCAAAGTATTTTATTACATCAACCTTAAAGACTGAAATTATCAATAATCCACTTGAGAAATGAATTAAAGGTTCTATACTTGAGGTTTAATT 8040  
 TACCCCCCTCAACATTCACAAGCTAATGAAGTTAGGCTGTGCAAGAATCATATAGAAGAGCCTGGAATTGAGATCTTGAAGCTAGAGTGGGCAAGGATGAAGTGATAGTGGCACTCCTGA 8160  
 AGGAAAAATCAAGTGGGTCCAGTTGAGCTAGGAAGCCTTGAAGATCCCAAGGGGAGTTAGAAGCCTCTAGCTGTTGATGTCCTGGAAGGCAGAGAAAGCAAGGTTGTATTGTCAGACCCA 8280  
 TTGTTTTTATATTTAATTTAGATCTGTGGTTTTGGGGATGGGTTTTCTTTTTGTAGTTTTTC

TTTTTCAG

TTTTTCAG  
Depth:19 (ZEBRAFISH)  
Ei-value:0.000, Pi-value:0.000  
Er-value:0.000, Pr-value:0.000  
No matches to TargetScan

GATTGTGTAAAGTAATCCTACTGGAACAGTGTAACTATCCTCAGCATTTA 8400  
 TTACATGTAAGTGGACTAAGTAAATTCAAACCATCTAGCATTTGGGAACCCATCCAGTATGTAGATAGCCTCTCTAACTAGGTATGTAAGAAATCAACAGATGGATCAGCATCTTCTTGC 8520  
 GGTCTTGGGTAAGGCTATGTACCTGCTGTAAAAATATCAATAAAAATGTCTCCTGACTTGTACAAGTGTACCTAACAAGTGAATATGTAGCTGTCTTGACTTTGGATCCATTTGAAATAG 8640  
 ACATTCTCTATTGGAGTTAAGAGTTGCGTATGCTTTGTGATCGTGGCTAATATTGAAGTCCACTGTGTAAAGTAACATTTAAAACATACTACAAATGTATATATTGGGGTGGAAGGGGCT 8760  
 TTCTCTTTTTCAATCTTTCAGAATCGTCCTGTGAAATGGCACCAAACCAATGGGTCTGAGCTTACCCATTCCAGAGTTTCAAGGTATTGCCAAGGAGTGAGAGATGACATGATAAAAGTG 8880  
 AATGAGAGAGAATTTAATTGAAGAAGTGCTCCCTATATTTGCAGTCAGTAGTGATTACAGGATTCTCCACAGATGAATCAGAAAATACAAATATAATCTGCATTGTATTTAACAGACTTC 9000  
 AAAATTAAAATAGTAATTTTACAAGTCTCAACCATTATTGAAATATCAATGCCAAAAGGCAGAAATTATCAACAAAACCCACTCCAAGAAATGAATAAAAATGAAAATCAAGCAGTTCAG 9120  
 TTATGGCTTCAGTTCCCCAACCTTCTGGCATGATATGAAAACAAGAATTAAGTGCACCTCTCTGTGTGGATGAGTTTCCCTTTCGTCTAGCAAATATGAGAGAATTCCTATACCCCAGTG 9240  
 GCTTATCAGGATGAAAGCAAGAGTATGTTTGACCTTCCACAGGCAGACATGGTAAACAGATTAAAGGCCAGTAGACCTTAGCCATCTTGGAGGCTGGAAACAACTATTTTAACA

GATAAG

GATAAG  
Depth:19 (ZEBRAFISH)  
Ei-value:0.000, Pi-value:0.000  
Er-value:0.000, Pr-value:0.000  
No matches to TargetScan

 9360  


GATAAG  
Depth:19 (ZEBRAFISH)  
Ei-value:0.000, Pi-value:0.000  
Er-value:0.000, Pr-value:0.000  
No matches to TargetScan

TGTTATAACAGCATGTGTATTTTTTCCCTTTTGAGAAAAATGTGCCATAAGCAAGAGTGCTTATCTTAATCTGCAACAGCATTCAGGTAGATATAGATATAGAATACATATTAAATTATT 9480  
 ATTTTAAAACACTTCCTCTTGACTTCGGGTACTGGGCTAATGCTTAAATGCATATTCTTGCAATATACATGCTCTATGATCTATACAAATGGTATTTACACTGTATATTTTTGTTTAATT 9600  
 ATTGTGGGGATAATTAGGTTGGAGGGGGGTGCTCTTACCTTGCAGTTTTTCTTTCTTTTCTAATAGGAACCATAAATGTAAGGCTAACAACGATGGAGCTGACTTAATTGGGCAGTGATA 9720  
 GTGAACATTCTACAAGAAAGTACTCTCGCTTAACTCACTATGTTAAATACAAATTGTAGTGCTACTATAAATTTGTGACTAATTATTGTAAGCCTAGTTTGTTCAAGTATTCTTGGTGTT 9840  
 GGGGTTGTGGGGGCTTACCTTGTAGTTTTTTTTCCTTTTCTCTTTACATAGGAGACATAACTGTAAGGCTAACCACGATGGAGCTGAGTTGGGTACATTCTGCAAAGAAATACTCCCGCT 9960  
 TCATTCATTTTGATATACACAAATTGTAGTGCTGCTGTAAATTGGGACTGATTATTGTAAGCATAGTTTGTTTAAATATTCTGGGGTTGAGGGGGCTTACCTTGTAGATCTTTCTTTTTT 10080  
 CTCTTCACATAGGAAGCATTACTTTAAGGCTAACAACGTTGGAGCTGATTTAATTGGGCAAGTGATTGATGCTGATTATTCCACTGACGGATTGGCATTTTAGTTCTGTTCATTATTAAC 10200  
 AAATATTAAACTCACAAAGTAAACTGTAATAATGAACTTTAAATTATTCCCAGAGACATTGGTGAGAAACAGGTTTGCAATTTTCTTTTTCTGAATTTAAATTCAAATATTTGGTATTTC 10320  
 TAAAAATATTAACATTACAAACATAGCATATACAATTCCCAATGAGAACCCTTAAGGGTTTACTCTAAAAAAAGTGTTTATACTCTTTGAATGTGTCTAGCAGTAGCTTAGTCACATCAC 10440  
 ATAGAATGCTGCGGGGAGGGGGGGGGGGGCAACCTTTGTTGTGTGTTTTACAGAGACAACCTACTTTATAAAGTACAGTTACACTCCAGGAGCCAAAGAGGAATGAGTCGTGCAAAAACT 10560  
 TGAGTTTCGTCTTCTGCTTTTCCTGTATGCTTTTGCCTTGTAATACCTGTAAAGTCACATTGCATCTATAACTTCTTTATCGCCGATCAAAAGAAAAGTAGGGAGGCACTCGGGGGATCC 10680  
 GAAACCTTTGTGCTACGTTATATTTTACAGAGCTTTTTTCTCAGTGCAAACTAATTGGATTCTTTAGCAGGTAATGTGCAATGTGTGCATGTCTCCCTTGTAGATCGTCTGCAATGATTG 10800  
 GGAATGTATCTACGTTTGTGTCATGATTGGGCACGAAGTATATTAAATGTGAGCATTAGACCCTAGCTTGTTAATAACCCCTGTGCTGGAAATACCCATGTGATTTATTGTCTAGAATTG 10920  
 AATGTTCTTGGGGACTTTTCTTTTTCAGGGGTTGGAGGGCGAGGGGGAGGAAATTATTCTTTTTTTTTTTTTTTTTTACAGATGCATCTGGCAAAATAGTGAAATGTACAGTATACCTCA 11040  
 AAATGCACTATTGTGCATATTACTGATTCTATTCTCAATCATTTTCTGCCAAGTTCCACTGCATGTTGTTCTATGCGTGCTTTCTTTGTTCTTTCTTCACACTAGTTTGCAAGACTCAAA 11160  
 GAACCTGAATGTGCTGTGAAATCTGCCTCAGTAAGGCTTAGAAAATTTCAGTGGGGATTTGGAGGGGTTCTGTTTCTAGCTTATATCTCACAGATTGGCTAATGGATTTCAAGATCATGG 11280  
 TGGTAGAAGATCAAAGCTCACAATCTAACAGGTCCTGTATGATTGGCAGAGCGTCATAGAAATTCATCTTCTATTATTAACCTGTCTTAAGTGAGGTTGGTGTCTTTATGTTCTTGCTTG 11400  
 TAAAACTGGAATGCTACAACAAATCCATATACCTGACGCATAGAAGAGATAGTTACTTATCCGGCGAAATAGTCTTTATTCAAAATCATAGCTTTACAAACTTTCCCTTAAGGACTATGG 11520  
 CTGTTGCCTTGGTTTGTAATGCCTCCCTCCTTGTGAATGGCTCAATGCCAAATATCCTGAATGCCAAGAGAAGGCTGAATTTTTCCTTTTGCTATCGGGGCTTGTCATTATATTGAAAAT 11640  
 TATCATTCTCTGGGTTGGGGGAGGGAGCTAACCTACAACATATCTTCAGATTTGTCTTTTCAGGTTTGCAATAAAATGGTCCAGCCTAGAAAATTAAGAATAACCTTCTGCTTCTTGTGG 11760  
 GTTCTTAAACTGTTGCTGGATAGCAAAGCCATAATCTTGGTCAGATACTGCACCACAGCAAGGCATCTATATTGAAGGGGGAGTAAAATATCTTGTATCCATACATTATTGAAGTATTTA 11880  
 GATATAGCAAAGCAGTCTCGATTGTGTATACCTCAAAGTTTAGTGGTACGTATGTTCGAGGCCGTCCGCGTTACCTCTTACCTCAACTCTCTTTGAAAATACATTTAGGTCAGTTGTCTT 12000  
 CTTCTTTAAAACCTTTACGTCAATGGTACAATTAATTTGTTCTGTCTGCAGTCTTCTAGAAGCCAAGAACATGCCTGGTTGTGCTACCTAGTCAAGCAAAAATGCCAGCTTCCTTCATAA 12120  
 GAAGCATATCTTTAATTTTTATTAGAGGCAATTTTATTAAAGTCAAATGTAGCCATTTGGCTGTATTCAGAATTCCATATTTGTGCTTCGTTTTGTTATGGGGTAGGCAAACTTAGTGGT 12240  
 CTTTATGACTCTTCCTAGGTGACAGCGCCTATTGAAGCAATAAAAGTACTTAGAAGAAAAAATGCTCTTCCTGTAGTCTCAAAAGG

TTTTCTTTT

TTTTCTTTT  
Depth:19 (ZEBRAFISH)  
Ei-value:0.000, Pi-value:0.000  
Er-value:0.000, Pr-value:0.000  
MATCHES To TargetScan▶ miR-186-5p:AAAGAAU

ACTGGGAGAACATCTTTTTGTTCTT 12360  
 G

CAGGTTTTGCTTT

CAGGTTTTGCTTT  
Depth:19 (ZEBRAFISH)  
Ei-value:0.000, Pi-value:0.000  
Er-value:0.000, Pr-value:0.000  
MATCHES To TargetScan▶ miR-330-3p.2:AAAGCAC▶ miR-490-3p:AACCUGG

TTACCTCTCGAATTACCAAAAAAAAAAAAAA

AAAAAGCAAAA

AAAAAGCAAAA  
Depth:19 (ZEBRAFISH)  
Ei-value:0.000, Pi-value:0.000  
Er-value:0.000, Pr-value:0.000  
No matches to TargetScan

GATACCGGTGGCCGGCACTCCTGGTTTCCAGGATGGGGTTCAAATCCCTGCGGTGTCTTTGCTG 12480  
 AGACTGTACAATCCTCCTGTGCCTTTTTTCCTTATTTTTTGATCTTCTATGTATTTTTAACTTAACGGGTTTAAAACGCAGATAACACAAATTCCATCTATAAAAACAGAGGCTGTTCTT 12600  
 CAGGATGTATCCTAAAATTGCTTACAAATGAATAAGAGTATTTTTTTGTGAAATGCTTTGGCACACTAATGAATAT                                             12676
```

---

## >SHARK (7850 bases)

```
 ACGTCAGTGCTCGCTGCTGCTATAAAAAGCGGCGACGTGAGCCGCAGCGCAGATTGGTGCGGGAGCGAGCGAGGCGGTCGGAGGCGCGGGGGAGGAGGAGCAGCGAGGCGGTCGGAGGCG 120  
 CGGGGGAGGAGGAGCAGCGAGGCGGTCGGAGGCGCGGGGGAGGAGGAGGAGCAGCGAGGCGGGCGGAGGCGCGGGGGAGGAGGAGCAGCGAGGCGGGCGGAGGAGCGAGGCGGGCGAGCG 240  
 AAGCGACAGGGCAGGAGTGCGATGAGGCGATGGGAGCGGGTGGGGGCGTCGGGAGAGGAGGGTTTTTCTTTGGCGGGGGAAGGGGAGCAGTGAGTAGAGGAGAACTGGGTTTAAACGGAG 360  
 GGGGGAGGGCGGTGTTAGGTGTTTATGTTGGTAAAATACAGAGGGAGGGAGCGGTGCGAGTGAGTAGTGAAGGTGTCGAGTCCCCCGAAGATTACTCTCTGTTTAACGGAGCGGACAGGG 480  
 TGGGCGAGTGAGGCCGTGTTACCGCTGAAGGACGGACGGGTTTAGACGGGGAGGGGGGGAGCAAACTCGACGCCGCGTCCGTTGAAGGTTCGAGAATAAAAACGGCTCCGGGGGGCGTGG 600  
 CCTCGATTAGGGGCGGGGCTGCGAGGCGTGTTGACGTGTGCAGCGACGAATCCCCCCCCCCCCCCCGGGGGCCCGCCCAGTCCTGTTGCCAAGGGCGCGGTTTGGTTGATAAATACCTGA 720  
 GTGTTGACTGAGTCACAGTCCGGAGAGGAGAGAGGGTTCACCTCTGGTAGACGGGTTACCCGCCAAGATGGAAGACGGTAGAGGAAAGGACTGAGCTGGGACTTGGGAGCAAAGTTGAGA 840  
 TGATCCCTATCGGGGATTGTGCGGCAGTGCGGCTTTAATGTGTCTGGAATGATGTGTGGGGGACTGCGGTGTGGCTGCGTGTGAAATGTCATGTTTTATGTGAAGGGTAAGAGTTGAAGC 960  
 ATTGACGGAACAAGTTGAAAGGACTGCAAGCGGTTGGTTGGTTGAGGAGGAGAGCAGTGCACGACTGGATCACCATCACAGACGGTATAGCGGCGTTGGACATCGTGAGCAGAGTAAGAG 1080  
 GAAGCAGAGGAAGAAGGGAGCCAAGAGGACGTCAGGAGAAGGGGGTTTGGATAGACAGAAAACAGAATACAGCCCAGATGAGAGTTAGACAGCAGAACTGATGAAGAGAGTACCTGAAAT 1200  
 GAGGCTTTGTGGAAGCAAAGAAGGGCCCGTGATGAGGTAGACAGCGCAGTCCAGATGAAGATGTGGAGGGTTGAAATCTTCTGGACATGTCAGATAGTGAAGAAGAGAAAGGCTGGTTTG 1320  
 TATATTAAGAACATGGTAACTTAAAACCAGGCAACACTATCCTCCGTTACTTTCTGTAATTGAGGGGTTGTAGTAACACTTGTCATTGAGTG

TTTGGG

TTTGGG  
Depth:19 (ZEBRAFISH)  
Ei-value:0.000, Pi-value:0.000  
Er-value:0.000, Pr-value:0.000  
No matches to TargetScan

TGGGCAATGTTTCCAGTTTTAT 1440  
 TTTCCAGGCATATTCTGTACCGGCAGTGCGTACCGTCATGTGAAACGTTTGGCAGCAGGATGAAGCGGCTGTGCAGTGGTTACTGATGTGCCTTTAGTGACGTCTCCATACCCACTGAGA 1560  
 TCCTGTCCATCTGCTTTTGGTTTTTTGTATTTTTTATCAAATTAGGTTGAGATCTACTTTGCTGGATTGTCAGCAAGTCTGCAGCTGGGGGAGACCTTGATGCGAGTATTAAACTGGCAA 1680  
 AGTTTTGAGCAGCTAAGTGACTCTTTCTGACCTGTAATGTGACATATTCTGTGTTTGCAGTGTGAGGAGCATTGGCCACTGAGATATGTGAGCATGGTTTGTAATAATACAGAAGGGAAG 1800  
 TGGGACAGGGTCTGCGTTATAATGCACCGGGGCTTTACGCCCCCTGTGGTGCACCGGGGCTTTACGCCCCCTGTGGTGCACCGGGGCTTTACGCCCCCTGTGGTGCACCGGGGCTTTACG 1920  
 CCCCCTGTGGTGCACCGGGGCTTTACGCCCCCTGTGGTGCACCGGGGCTTTACGCCCCCTGTGGTGCACCGGGGCTTTACGCCCCCTGTGGTGCACCGGGGCTTTACGCCCCCTGTGGTG 2040  
 CACCGGGGCTTTACGCCCCCTGTGGTGCACCGGGGCTTTACGCCCCCTGTGGTGCACCGGGGCTTTACGCCCCCTGTGGTGCACCGGGGCTTTACGCCCCCTGTGGTGCACCGGGGCTTT 2160  
 ACACGCTCGCTGTCGTGCTTTCACGCCCGGGTTGCAATGCCAAGGTTTTGGTGTGCATCTTGTTGAGAATGTTGCAGGTGGTTTTAAGATCACTATAGTGTGAGAGGACAGTCGGTATGT 2280  
 GGACTTTAAGTCTGTAATTCATACATTTGTGTGGAGAACGGCCAATGCACAGGCTTGAGCTGGAAGTTTTGGAATTAGGTTTGGAAGATAGGGAGATGGATGTGTATGGACTTTTAAGCC 2400  
 TCCCAATATAAAGGCTTGGGAAGGGGTGTATTG

TTTGGG

TTTGGG  
Depth:19 (ZEBRAFISH)  
Ei-value:0.000, Pi-value:0.000  
Er-value:0.000, Pr-value:0.000  
No matches to TargetScan

CTCTAAGCCAGGTTTCGGGAGGAACAGTGTATGGTTATCAGGAAAGCAGGGAACAGATTTTAGTTTGACAGAAT

TTTGGG

TTTGGG  
Depth:19 (ZEBRAFISH)  
Ei-value:0.000, Pi-value:0.000  
Er-value:0.000, Pr-value:0.000  
No matches to TargetScan

A 2520  
 GGGATAATGGGTTGGCAAGATGGATTTGTAGTGTTTG

TTTTTCAG

TTTTTCAG  
Depth:19 (ZEBRAFISH)  
Ei-value:0.000, Pi-value:0.000  
Er-value:0.000, Pr-value:0.000  
No matches to TargetScan

GCTTGATACACCATTGGTCAGTTCAATCCCAGGAAAGTAATGCAAGAATGCAATTACCAACCAAGAATTGTTCCA 2640  
 GATCATGAATCTGATGTGTAATGTAGCCTGAATTTATTTGCCTCAAAGCTCTTTATCCTGCTTGAAATGGTTTCATTTGGTATGTATCAGGATACCTGGACTTCTGGTCAGTTAAGGGTG 2760  
 AGCAATCCGTTTCCGATCTGATATAGCCTGAAACCCTCCTAGTAATTTTCTACCTTGTAGTAAATGATGTGATCTTTCCATGAAATAACCTTTATACTGTGCTGCTGCAGTTGACTGATA 2880  
 CAATCCCCCTACTGCGATAGACCATTAGCAAGTTGGTGGGGTAGGAACAAGCAGCTTTTCCTAGACTTTATTTTGCAGATCCTTATTTCCTTTGATGTAAAGATGAAAGGCTTCAAGTAC 3000  
 CCTAACCCCGTTCCTGCAGGATCTTGGAGATTGAAGCGGATCTTGAGTAAAGCAGAAATATTGGATTTTCTTCTACTACTTCATCTTTACTGCAAGCCTCTTGTTTATTTCTCATCTACT 3120  
 CCAGTGCCTTCTTATCTACTAGTACTTCAATTTTGCTAAAAATTGCAGATAAGTTCTTCCAACTTCTTGCCAGTGTTAAGTGTGCTAAATTGAAGAAAATGTAAAATACACTAGCAATTT 3240  
 GTAATTGTTTTGGTGTTGCGAATATTTGCTTTTATGGGAGGGATGGGAACAAAATGGTCTTAGTTTTATTTTGCAGATTGGATCAGTAAGTGCTTGTAAAGGGCTATGTGAAGAATGTGG 3360  
 CATTGCCTGGAAGGTGGAGCTTGCCAGACAAGTGGGCTGAGATCAGCAACATCAGAAGTTAAACATTCCCATGTAATGAGATCGCTTGGGCAAGGAAGTAGACAAATACTTAGGTGGAAT 3480  
 ACGCAGGTGGAAAATTTGCAAATATTGTGGAAATTTAAACTAATCTATAATAATGCTGAAGGCCGCATCTTGACATTTATTAAATTTAGTTTGATTGCTTAATGATACTTGACTTAATTG 3600  
 CCAATATTAAAAAAACAACTGCTTGCTGGATTGGGGGGTGTGGGGGGGGGTTGCTATTAAGACTTTTACAGGCATCCAGACCTCGATTGCCTAATGGAGTAAAAATGCTCATTGGACCAA 3720  
 AAGTGAAGTGGGATTGAGCAGTAATCAGTGTGGAGCAGAGCATCTGAAGTCTTCAGTGTCAAGGTCTCTGTATAATAGGAACAAGTTCTTCTGAGAATGATGACTCGATCATGTTGCTGT 3840  
 ACTGAAGATAAGTCATGAGTAACATTCAATAACTGGAATTCAGTTGCATTTTGTTCATCTAGGCATACTTTAATACAAGTACAATATACATATATATGTGTGAGTGTTCAGGAATGCCAT 3960  
 ACTTTAATACAAATACAGAATATTTCCAGTGAGTGTTCAGGAATACCAAGAACTTGTTTATTATTTTCATGTTTTCTGTGACAGTCACATTGCAATTACATGGCTAAATTTCAGCTGTTG 4080  
 TCACAATCTAAGTAGATTGTGACATCCTGTGAGAAAGTAAAACGTTTAACATACTGTTTTATTACAATTACATTCTGCATAAATAACTAGCAACATTACTACTGTAGCAACATTTTCTGG 4200  
 GGTTAACTCTGGGGAGGGGGAAGCTGTCCCAGGTTTTATTTTCCAGGTTTCTCCCCATCCCCGTTGTCATGATACATACGGCAAGTCTGTCCTTTGTATTGGTGGCTGAAATGGCAAGTA 4320  
 ACTAAAGCGAATAAAGCAGTGAAGTGTTAACGTTGACAGTGGTGCGGAAGGATGCTAATAAAGGCGGAAAAATGCGAAAGGTTGTCTTGTTTTCTGTACTTGAGGTAACGTTTCTGCATG 4440  
 AAATTGTTTAGGATTTGGAGCTTTTTCCTCCAGCAACATTTTCATGTGCTTGTGGCAGTGCTCTGAGTAATAGTGGCATGCTTTATCTGGCGAAGAGGGCAGGGGAAAGCTTGTCAGTTT 4560  
 GTAGTTTAGTTTCCCATGGGAAACTTAACTGTCTGAAGTAACCATTCTGTCACTGGAAAGGCAGAATTCATTCAGATTGTTGAATTTCTATTCATGAATTAGAGACTAGTCTGTGATTCC 4680  
 TGTGCTGATAGATTTGACGGCCGTGTTAGAAACAAACCATCACTTCATCGTTACTTTCTTAACCCACTGAGATAGCTGTTGGGCAGCTACAATGAAAACTGTTTTGAAGAGATTTAATTG 4800  
 TGCAAGCCCCACCTTTAGGCTGCTTAGATGACTTGTATACCTTCCAGAATGATTGAGTTTACCAATGGTAAACTTTACATCCTTGGAAATGATTAAAAGACCCAACAAATTAGAAGTGTT 4920  
 AACATTTTTTTCTTTTTTAAAAAAATAACACTACTGGCTTGTGTGCAAAAGTATCAACATGATTTATATTGTGATGTAACATCCGTAACCTTATCTGGGAATTGGGAGGGAAAATATTTT 5040  
 ATTTAAGACTTTTTCAGGTATTCAAGACTTAGTAGCTGTGATGTAATGTGGAATGTGAAGCTGACGAGTGATTCCCACCAATCGGGCCATAATGGTGTTGAGTCCCATGTTCGACCCTTC 5160  
 TCTAGTTGGATGGATTCAGAGGAACGTCTCCTAATAAGTTGCAGAACGCTGTTTGTTGCCAGTAAAGGTAGTGTGTGATATAGCGACAGAACAGCTGGATGTCTGATGTGGAATATTTCA 5280  
 ACACTGGGGCATACTTTTCTCTCCTCTCCTGCCTCCCATTCTAAAATGTCTTTCAGGTTCATCTAATAGAAATGAATTCGTAGAACGGATGGAGGTGACCTGTTGCAAACTTTTACTCTG 5400  
 CTTTGATAATAAGCCCAGTATTGTATAATTAATATGTTTGCTCACAGTCTTATAGAACATGTACAACTAATTGTAAATGATCAGTTAACTTGCAGCATTGCAATTTTAATGGGATGTTTG 5520  
 GGATGGGAGCAGCACATATTACTTTCTTTGCAGACATGAATACAAACTACCGCATGAAGTGGACACTAAGCGATTACCAGAGGATTAGCTTGTCAAATAAACAGGATCACACAAAGTCTA 5640  
 TTCAGCATCTGGTTCAGGACTTAAAGGCGTAAGAGTAACTCGTTTAACATTGTAACTTTAAACGCAATTCAAAATGCCCTCATTTTATCTTAATTTTGACCCAAATGTAATACATCAGCT 5760  
 GTATCAAACTTCAGTGGAATTAATGAAAGTCTTAAAATGGGAGCATGAAGAATGCAGAAATACTGTTGTCAGTGATGTTATTCTTAGACATGTTACTTAATTCTATTGTAAAATGTGAGG 5880  
 GCATCACTATTTTGAATAGGGTCAAATGTAACCATTAGATGTAGGGATTTTAGATCCCAGTATTCTCTCTCTTATGTGGCTGTATAAAGGGCAGGGAACAGTGTTCTTAGTTTTTCAGGT 6000  
 GTCAAGTCTTCCTCGCTGGCAAATAACCACATGATGCTTCGTCCCTATCCATACTGAACACAGCTTAGAAGGTGCAGTTAACGCTTGAGGAGGTTTAGGGCAGGGGGAACTCCTATGTTC 6120  
 TTGCATCTTTCAGGTACCCCGTCCTTGCTGGCCAATAACTGGAATCTGCCTTCCCTACGCTGGACACCATGTAGTAGGTACAGCATTGGATTCACATTTTTCAGAGGGGTTTAGGGGAGG 6240  
 GAACCCTGTTCTTAATCTTTCAAGTCCTTGCTGATATGTAACTAGAATATACCCTCCCTACGTTGGACACAACTTAACAGGTGCAGCGTTAGATTATAATACAACAATTTACAGGGGTTT 6360  
 AGGGCAGGGGAACTCTGGTGTTCTTAATCTTTCAGGTCCTCGCTGATCAGTAACTAGGATTGCCTTTCCTGCACTGGACCCAACTTAGTAGGTAAAGCTTTGGATTCTAGTACATATTTC 6480  
 TCAAAGGGGTTTAGGGCAAGGGAACTCTGGTGTTCTTAATCTTTCAGGTCCTCGCTGATCAGTAACTAGGATTGCCTTTCCTGCACTGGACCCAACTTAGTAGGTACAGCTTTGGATTCT 6600  
 AGTACATATTTCTCAAAGGGGTTTAGGGCAAGGGAACTCTGGTGTTCTTAATCTTTCAGGTATCCAGTCCTCGATGATGTGTAACTAGAAAATGCCTTCCCTACGCTGGACACAACTTAG 6720  
 CAGGTGCAGAGGGGAACTCTTCTTAATCTTTCAGATCCTCGCTGAACAGTAGCTGGGCTTCGCCTTCCCTACGCTGGACACCACTTAGCAGGTGCTACAAATTTTTCAAGGGGTTTAGGC 6840  
 AGGGGAGGGAACTTCTTAATCTTTCAGGTATCCAGTCCTCGCTGTGAAAGTGACCCACTTCCCTTACCTACGCCGGACATTTTGAACAGGTGCTAAAATAACTAGCATTTACTAATCGTT 6960  
 AAAGAAT

GATAAG

GATAAG  
Depth:19 (ZEBRAFISH)  
Ei-value:0.000, Pi-value:0.000  
Er-value:0.000, Pr-value:0.000  
No matches to TargetScan

GTGGGGGGAGTGGTTGTAGTGTTTTAGGTCCCTCGATCCCTGATGACACCAGTGATAGACGCTGCGTTCAGGACCATTTACACTGCACATTTGTAAGCTTTCTGTAA 7080  
 GATTGTTGTTAGCCAAAGTAAAGTAAACTAGCTTGTGTATCAGTATTAGTCAGTGAGCAAGGCCAACAATCTTAGTTAAGGAAATGCATCTTTCAGAAGTACAGTTATATATTGTCGGGG 7200  
 AGCTAATAACACTGCTTTCATTAACGTCTTGAGGTCTTTCTCTGAACTACAGAGGGTTTTGTTCAGATGCAGTCTCATTGAGTGGTGTATATGACTAAATGTGTCTATGTGAGTGAATGA 7320  
 AATGTGTTTCTGGCCCAACTGCTTTTTGCTCATAAATTGCTTCGGGTGAGGTGTTTTGTTGGAATCTTGAGGTTTTGAAGGTTTTCTGTTGCTGAAGAATACAGTGAGCATCTGATTGGT 7440  
 TGATTTTTTTTTCTGGAAGACTGTACATGCTTTGTTTAGATGATAAAGTTGGC

TTTTCTTTT

TTTTCTTTT  
Depth:19 (ZEBRAFISH)  
Ei-value:0.000, Pi-value:0.000  
Er-value:0.000, Pr-value:0.000  
MATCHES To TargetScan▶ miR-186-5p:AAAGAAU

ACTGGGAGAACAGTTGCTGTTATCT

CAGGTTTTGCTTT

CAGGTTTTGCTTT  
Depth:19 (ZEBRAFISH)  
Ei-value:0.000, Pi-value:0.000  
Er-value:0.000, Pr-value:0.000  
MATCHES To TargetScan▶ miR-330-3p.2:AAAGCAC▶ miR-490-3p:AACCUGG

TGCCTTTATATAATTGTGTA 7560  
 TATAAAA

AAAAAGCAAAA

AAAAAGCAAAA  
Depth:19 (ZEBRAFISH)  
Ei-value:0.000, Pi-value:0.000  
Er-value:0.000, Pr-value:0.000  
No matches to TargetScan

GATGCTGGTGGTTGGCACTCCTGGTTTCCAGGACGGGGTTCAAATCCCTGTGGTATCTCAATGCTTTGTTATTGCTCAGGTGAGGCAGAGGGACTGGAATGG 7680  
 AGTTCCAGAGGGTGGGGGTAGAGTGACAGAAGGTCTGCCCTGCCAATGGTGAAGCAGGCTGGAGCCCACTGAGCGCAAGGAGCGGGCAGCTTGGTAGGGTGTCAGAAGGTCGCTGGCTGC 7800  
 AAAGTTCTGGATGATCTGCAACCTGTAGAGGAAGGAGTTTGGGAGGCCGA                                                                       7850
```

---

## >OPOSSUM (5957 bases)

```
 TGAAGACAAAAATTGTAGCTGCAGAAACCTTAGAAGATAGAAAAAAACATAGGAGCTAAGAAAAGTTAATTCCTCTTGCTAAACTAAAAATTAAACAGGGAGCAATTTTACACTACTTGA 120  
 AGATAAGTGAAGTTTAGAAGAGAAGATAAACAGGTAGAAGAGGAGCATTTTAGAAGTTCCAGGAAGAAAGGGCACTCTGGCAAGATGCTTTTTTTTTAAAGCCAACTAACATGGCTTCTA 240  
 TTCTAGGAAATATGGACTGAGTTTGCTTTATTTTTAAATGATAATTTTTGGAGAGAATTAAGATAGAAGATGCATATTCACTGTTTAAAATAACCCAATTTTGGACTTAAAAAAAAAAAA 360  
 GCAGTACTTGCCAATTGGACTGAGTTATTGGCCAGAGCCTTGCTCTGGATCAGAGTAATTGATTGAGGGAAAGAGTGCCCTTAAGCCAGACTGCGGATGTGAAGCCAGCTAGAAAGGAGA 480  
 AGCAGGAGCGACAGACAGACAGGACCAGGACCAGGACAAGCCCGCGAGGAAGGAAGGAGCCAGTCTGCTGAAGCTAGGAAGGACCCCCCCCCACCCACCCCTGGCAGGAGGATCAGCAGC 600  
 AGCAGGCAGCAGCAGGCAGCGCACGCCCAGGTGTCAGCAGCAGCAGCAGCAGCTTGTAGGCGAGCCAGGACGCTGAGAGCAGAGCAGAGAGAGCCTGGGAGGATGCCCTGGCCGCAGCAG 720  
 GAGCTGATGGTGCACGCGGTGGAGGAAGGAGAGAGGCAGGCAGGAGGGCGCAGCTAGCGCGGTGCGGTGTTGGCTGTTGGTGAAGCAAGCGGGGCCAGTTGTGCCCTTAGAGGCGAAGCG 840  
 GCAGGGCCCTGAGCAAGCAAGCAAGCAAGCGAGCTGTGGTGGTAAATTGGGAAGCTTTGATTTCTTTTTTTGCCTAGTTCTTAATGCCTTTGAATGTGGGAAGGGGGGAGTGCGTGTTCG 960  
 AGTTCGGACGGAGTGCGGTAGTCCTGGTCATGTGAGCAGCAGCAGCAGTTCTCTGGGGGATGTGATGATGTCTGTTAGCCGG

TTTGGG

TTTGGG  
Depth:19 (ZEBRAFISH)  
Ei-value:0.000, Pi-value:0.000  
Er-value:0.000, Pr-value:0.000  
No matches to TargetScan

GGTGTGTGTTGTAGGTTTCTCTTTTGCAGGCT 1080  
 TGTATTTCTCAGTGCATCTGTCTTGTCTGAAGCTTGGGGCAGTCTGCCAAGGCCTGGAGAGTGTTACTGTGTCTGCCTAATTAGGACTCCACCAGGTTGCCTTTCATGCTAAGTGAATGC 1200  
 TAGAGCTAGCCATTATTGCCATGTTTAAAAAGCAAATTGGGGGCCGAGGAGTGAGCCTGAAGAGGGAGAGATGGAGCCCCCCTGCACCTTGTGAGGAAGTAGAAGCAACTGCATTGTAAG 1320  
 C

TTTGGG

TTTGGG  
Depth:19 (ZEBRAFISH)  
Ei-value:0.000, Pi-value:0.000  
Er-value:0.000, Pr-value:0.000  
No matches to TargetScan

CTCGGACACGGCATTCATTTGGAGTGTTTGAATTTTGTGGCTAAACTCTTTCTCCTCTCCCCCTCCCTCCAGTTCTATTTGCGTAAGAAGTAAGAATGTTTTCCTTGAATG

TT

TTTGGG  
Depth:19 (ZEBRAFISH)  
Ei-value:0.000, Pi-value:0.000  
Er-value:0.000, Pr-value:0.000  
No matches to TargetScan

 1440  


TGGG

TTTGGG  
Depth:19 (ZEBRAFISH)  
Ei-value:0.000, Pi-value:0.000  
Er-value:0.000, Pr-value:0.000  
No matches to TargetScan

GGCTCCAAGGGGAGGGCCAAATGGTGTCCAGTTCTTTTCCCTCAGGTGTGTTCTAGAGCTCATGGTCAGATGAACCTCAAGTAACAAAAGAGAAGCCAGTGTCAGGACTCAGGATA 1560  
 AACAAAGCCTGCAGGTTTACATTTGAGGCTTAACTGAGCTCTCCTCAGGGGCCAGGAAGGAGCTGGTCCTGTGGCAGCAGGCCTACGGTAGTCCTACATTTTCCTTGCAGGCTGGGTGGG 1680  
 GGTAGATGCAATGAGTTGTTTCTAAGCCTTTCCCCCCACAGATCACAGATCACTGTAGTCACAATGGTTAGAAAGAAGCCTTGATTGCACTGACATCTCTCCAGCCATGGTTGAGCTGCT 1800  
 TTTTAAACCTGGGAAGAATTGGAAAAATGCAGCTAAGTGTTTGAGAACAGTTGAGTCACCCTCCGAGAGGCTCAGAAGAAAGCATAGGCCCATCTGGCAAGTGAAGGCTTTTAAAGTCCA 1920  
 GTGACCTAAGGAAGTTGGGAGGGCCGAGAAATCCTGCGAGGAGGAGGAAGAGGCGGCCATTTTCAGGAGGCCTAGATGGCGGTATTGTGGTGGGTGGGGGCCTGGGCTAGGTAATTTGTT 2040  
 TATATTTTCAGATAACTCAAAGCCAGAGAGCCTAATAAACTTAGACGGAAAAAAGAGCCATTTTCAGCTTCCAAGTTGGCAAGTAACTCCCCAAATCTGGTAAAAACGTCCATTTTAAAA 2160  
 AAGTTGGCCATTGGGGAAGTAGAACATTAGGACCCTTGGGTCCCTGGAAAATTTTAAATTGTTCAAGGAGAGGAGGAGGAGAGGTAAACAGTTCATAATTGAATCCTTTAAAGTGGCTGG 2280  
 CTGTTCTTTAAAAAAGAGTATCTTAAGGAAAGGAAATTGGTGCAACATCCATCCATCCATCCATCCATCCATCCCTAAAGTGAAAGGTAAAATCTTGGGGTTTTTAAGAGGAGGGCTTTG 2400  
 TCTGTGAAATTGATTTCTGTGTTTTTTTGGCTGTGGTAGTTGGGCGTGAGAAGCGTGTGTGGGTGTGTGTGTGTGTGGATTGGGGGGGTTTTCTCTTGGCATT

TTTTTCAG

TTTTTCAG  
Depth:19 (ZEBRAFISH)  
Ei-value:0.000, Pi-value:0.000  
Er-value:0.000, Pr-value:0.000  
No matches to TargetScan

GTGATTATA 2520  
 TATAAGTAATACACTAGAACTTCAGAGGAAAGGAAGTTGCTAATGATCCAGAGGGATTCCTTCTCATGGTAGCTTTTATATCAAGTAAGACACTGTTTTCAGTTGTGTTAAACAAGTTTC 2640  
 TTCTTAGTGGAGGAATGAGATGCTTTCTTTTTGTGTGACTGCAACAAGATGTTAAGGTATGCTCTTAAGTTCTGTACATTTGCAATTGAAGTAAAGTTTTAAAAATGAATCTAACCAAAG 2760  
 TAATTGATTAAAAAGTAAGATCTTATATACTTTTGTGCTTGAGCTAAGCTCAGACGTGTTGTCTTTATTATGATGTGCGTGGCAGGGCGGCGGGAAGGGGAAATCTTTTTTTTGTCTGTA 2880  
 GACTCTTTGCAGATAACTTCATCTGAGTCATAACCAGCTTGACAAAATGGCGGCCTAAATGGAGAGAGAGGGAACAAGCTCCTTGGTGACAATGATAAGTAAAAGGCAGAAAAGATTATA 3000  
 AATCTAATACCTCATGGGAGGGAAAATACCAAATTCCCTAAATAACCCGAGAATGATGGTATCTACTGGCCAACCACAGAGAATATCCATTATCTGCAAATGCAAAAAGCAAGGAAAAAT 3120  
 GCCAATTATTAAAAAAAAAGCTTTATGGCTTGACAAAAAATGAGCTTATATACCAAAATGTGCCAAAAAAAAGTTATGAACGCTATTTTATTTGCAATGAAGAGAGAAATTTCACCTCGA 3240  
 AATGCTGCTTTTACAAAAGCAGAATGATTAAAAGAGAGAAAAAGGAAAATGCTTATTCGCATACTAAGTGTTCCACGAAGCGGAAATGGTTTGGAGATGAGTGGGAAAAAGGCGTGCGCA 3360  
 GTTTGGATTGAATGAAAGGAGACTATGCTGTCTGTTCCATTTCGTTTACCTCAGACAGGTTCTCTGTTGAGAAGAGTTCATTTCATCTGGGAGCAGAAAACAAGCAGGCTTGGCCTCTCT 3480  
 TACA

GATAAG

GATAAG  
Depth:19 (ZEBRAFISH)  
Ei-value:0.000, Pi-value:0.000  
Er-value:0.000, Pr-value:0.000  
No matches to TargetScan

TTTACTATGCCGTAGCAGTTAACCGATTGCATGTCAGGGCTAATGAGTAAATGCTTTCTTCTTCTGTTAAGACATGCTCAGGGGCTGAGACATCCTGGAGGCAAACGGCC 3600  
 CTTTGTTGAGGGGCTCCTGCAAAAATTAAGTCACTGTTACAGTGCTAGAGAATGATGTCGCTGCACCGCGGAGGAGGCCTTGTCCTCCCCCAGCCCAGCCTTACGTAGTCTAATTAGTTA 3720  
 TTGAATTTTCTCTGCCTTGGATGAGGCCAAGCGAGCCAAAGAGTAACCCGAGATTGGTCTCTGGCTGGATCTAACCAAGTCACTGAGCCTCGCGAGGTCCGTAAAAACCAAAGTTGTTTA 3840  
 AAGTGCCATTTTTAAGGAAGCTCGGATTGTGTTTGCAAGTAAAGTTGAATGCATTGCCTAAGATGAAGCTAGTAAGTAGCGCGATGATGACTAAAGTTTTAGCTGTTGTGGTTGTTGTGG 3960  
 GCAATCTGGGGGGATTCTTCTCTAATCTTTCAGAAACTCACTCCACGAACAAACTTTAATGGACAAGATTCAGGATTGAGCTGAAGACCCGAAATTGACTTTATGCAGGCAGGAAAGACT 4080  
 TAAAAGAAACTAAAAAAGGCCAAATGTAGACCCTTGTGACGAGCAGTTGCTAATTTTGCCAATCAGGCCCTCTATCTAATGAGGAGTAAATCAGCTCTTAGACAGTTGGGACACTTGCTA 4200  
 GGATGAGACAAGCCACAGGTACGGTGTCACGGTGCAGTGTGAGAGGGCTTCTGTGTCCAGAACAGGTCCTTGCGGGACGGCAGCGGCGCCAGAGCCTAGGAGCGCCCCCGACCCCCACTG 4320  
 CTGAGCAGGCTGCGTGCCCAGCGCATGCCGGCCTTGCGGTCACCTCGCGGGTCTTTGCACCCTTCTCGAAGCTGCTCAGGAGCTTGCTGGCGCTGGGCTCTGGCCGCCGCCGCCTCTAGT 4440  
 GTGAGTCCTGCAGGAAGGGAGGCCAGGGCGGGCGGGGCTCACCTTGTAGCTTTCTTCCCGCAGACCCAGAGTGAGTGCGGCCTTGAGCAGGTCTGCTCTGTTGGCACGAGGGCCGCGTGC 4560  
 TCGGAGGAGCGAGTGGGAGCGAGTGTGTGTGTGAGGCTCCCCGGTGACGTGCGGGGGGCGGGGGGCACTGAAGCCTTTAGTCTTTTCCAGATGGAGCCAGGCAGTCAGTGCCAGGAATAA 4680  
 TTGCAAACAAGCGAGCACTGTGGAGGCGCTTCATGGCAAGCGAAACCCAAAGCTGAAGCCGTTGAGTCGTCCCGTCTCGCACCCGTGTCTGTCTCTCTCTTCCCCTCTCCGTGTCTAACT 4800  
 GTCCTGTGCAGGAACACACGAGCCGACTAGCCGCTCACTTTCTTTCAAACCACTTTAAGTCACTCCATATGAGTTCGTGGGGCCGGAGCGGCCTCCGGGCTGCCTTCACGGCCTCTAAAG 4920  
 GGCCTCCAGAATTCACCACAGTGGACAGCCCGACCAGGTGAAGTTTTCTCTTGTTTCCCCAGGAGGCGGCCCGGCTTTAGGCCGGCAGCCTCGCCAGGAGAGGGTGGAGAGGAGGCGCGG 5040  
 TGGGCGCTCAGCCTTCTTTCTTGAGGTTTTTCAGGTTCCCTTCATTAAAGGAGGCGCCTGAGGCTTAGAGTAGCCGCATCCCGGAAGCCTTCTGGTGGCAAGTGACGAGACCCAGAAGTG 5160  
 GGAGCCCCCCCGCCCCCAGCCCAGGAAGCACCGGCACCCGCCTGAGCGCCGCGGCCCCTGCTCCGGGCTCCAAGCCCAGTCGCCGCCCGCATCTTCACGGTGCCCCCGGGAGGCCGGCCA 5280  
 GGCCCGGTGCCGCCTAACGTGGTGCTTCCCGTCCCGTGCCGGGCTCCGCCGCTTTAAAGCGCTTCGGGGAGCCCAGAGCACCACTCCCGAGGGGGGAGGGACGGGGGACACAGCCGCATC 5400  
 CCCCCAGCTTTTCCAGAAGCCTAAACCCAGCCTCTCCCCCCTCCCCCAAGTGACTTCCTGCCACATGGCGGCTAGCCCAGCACCCCCCGCCTCGCCTCACCGGGCTCGGACCCTCGCCGG 5520  
 CGGGCACTTAAGGTAACCAGCAGGCCCCGAGGGGGGAGCCGCACACAGCTAGGGAAGCCAGGGGGGGGGACGCCCAACTCTGTGCTCAGACTTTTGCAGGTGAAGCTCACACACATAGCA 5640  
 GCTTAGCCGAAGATCTTCCTTCCCCACCTTGTCCGAGGGT

TTTTCTTTT

TTTTCTTTT  
Depth:19 (ZEBRAFISH)  
Ei-value:0.000, Pi-value:0.000  
Er-value:0.000, Pr-value:0.000  
MATCHES To TargetScan▶ miR-186-5p:AAAGAAU

CCTGAGGAAACACCCGCTTGTTTCCT

CAGGTTTTGCTTT

CAGGTTTTGCTTT  
Depth:19 (ZEBRAFISH)  
Ei-value:0.000, Pi-value:0.000  
Er-value:0.000, Pr-value:0.000  
MATCHES To TargetScan▶ miR-330-3p.2:AAAGCAC▶ miR-490-3p:AACCUGG

TTCACCTTTAATACTCAA

AAAAAGCAAAA

AAAAAGCAAAA  
Depth:19 (ZEBRAFISH)  
Ei-value:0.000, Pi-value:0.000  
Er-value:0.000, Pr-value:0.000  
No matches to TargetScan

GAT 5760  
 ACTGGTGGTGGCACTCCTGGCTCCAGGACAGGGTTCGAGTCCCTGCAGTGTCTGCGCTTGGCAGCCCCCTCGGACACACAGACCCCCTTCCCCTCCCCCCAGTCCAGCCAAACATTGCTG 5880  
 AGCTTGCAGCGGCTCTTCTAAATAAAATAAAGAAAAAGGAAGAAAAATCCTTCTTGTCTTTGGTCTTTTTAACTGCT                                            5957
```

---

## >SPOTTEDGAR (7306 bases)

```
 GCGTCTTTAGTTTTTTTTAATTCGATGCGTTAGTGTAGATAAAAGTCGAATCATCTGAGCTCGTTTGTTTTATTTATTTTTTTTCTAGAGGGAACAGCGAGGTTTAAATAGGATTGAAGT 120  
 TAGCGAAAAGGCCAGAAACGGGTGACCTTATTTAACTTGTTTTTTAGATTTTTTTTTCAAATAAGGCGACGTAGTTCGTGAACACGGTCGTAAGACGACTCGACACAACGCTCTCTCGGT 240  
 ATTTGGTAAGCCTCGAATCGCTTTTAGTCAATATTAACGTTTTACAGCTGACGTCTCGCCGCGTAGTCGCAGCGAAAACGTCTTAGCTATGTCTCATGCGACGAGCTACTGTAATTTCAA 360  
 GCTCGTCTTTTCGGTGGAAAACATTGTAGCCTATAAAAATGGATGAGGAGGACCATAGTTTTGTGAAGAGGATTGGAGAGCTTGAAAAAAAAATAAAGACGTGGAAATTCAAGATGGCGC 480  
 CGGGGTACCTCCCCGTCAGCAAACGGTCGCCGAAAACAAATCCTTAGAAGAGGAGAATTACTGAAGATTATTTAAGTTGTAGAGAAGAAAATAGGAAGAGGGAACTCTAAAGCGTAAACG 600  
 AAGATCTCAAATCAATCGTAGCTCGATTGACTTAAGCTTGGCACGTTAGATTTAATTCGAAGAGAAAGAAGCGGTAGAAGGGAGGAAAACGAAGCTTTTTTTTAAAAAAAAAATAGAAGC 720  
 TGAAGATTAGAAGGTTTTAGAAGAGAGAAGGGGAAAAAGTGGCCTCGATCGAAGATTTTAATTTAAAGGCGAGGCAGAAAAGGGCTCATTCAAGACCAGGTAGTCTGAAGCAAATGTCGC 840  
 AGAAGAGTGAAAAGCCAGTTGAAGTTGAGCCTGAATTTGAACAGAAGTTGAGCCTTAAAATGAAGATAGCTCTTTTTTTTTCCTGTTCGAAGAAACGAAATTTCCAAAGCACGAAGTTTT 960  
 AATTTTTTTTTTTTAAAGCCTGAAAGCGAGTGTCTAGCGGTGGGAGAGTCACGGTCCGGGGAAACGTGAGCGCAGCGAGGTAGTGACGTCAGCGGAAGGAGCGCAGGGGGGGGCTCGGTG 1080  
 GGCGGAGGGCACCGCCCGGTGGTAGCGCAGGGGAACTGCAGGTCGAAGATGCAGGCTTGTGTCATTCTAGCTAGAGGAATCTGAAGCCAGCAAGCTAAATTTAAACTCAGCTTGGAAGGA 1200  
 AATAGGAAGAGGCGATGGCCCCCTGTCATCAGGAAAGGCTTTCAAATGGACAGTGATGTGCTATTCAATAGCGAAGATGCAGAAGATTTTTCAAGAGGAGACAGGACTGAGTGTGTACCC 1320  
 TTTATGAATTGGGATGGACTTAGTTGGTAGCGGTTTGTATCTGAGGAGGAGGAGGAGCACCAGCGTCAGGAGGAGGTCATGGATCGCAGGAGCCAGGGGCAGGACGAAGAGGAAGGAAGC 1440  
 GAGTCGTGTAGCCGGGCGCGAGGACAGCGAGGGAGGGCGGCGGTAGCGGCAGGGTGGTGACGGCCGGCTGGTGGTCGACGCGGGTAGCGGGAGCGTCCGGTGTCCCAGCAGTCCGGGGGT 1560  
 CGGCGTGCGTAGCTGTGTGAGGGAGGTCCACTCGCTGGAAGAGCTAGGCCACAGAATCCGAGAATTAGTTTTTGTTTTTGTGTTTTTGTGTAGGTGTCACACGTGTTTTTTTTTTTTTTT 1680  
 TTTTTAATTATTTTCAAGCATTTACCACCATTTCAGCCACGCTCGGGATCTGGAACACACTTGCTCGCGTAGTTCTGTTTCTTTCTTTTCCAGGCGAGGTGTAATTTTGAGTTTCTTCAG 1800  
 CCCAGGCCCCAGTGTCGCGCCTCTCGCCTGATGTGGTCTTGTGGTTCCTGGTAGTTCTGCGGCTAATAATCGTAACGCCCCGGTCCGTGGGCTGCCCTGATTCGGAGGAGAGGACTCGTA 1920  
 TCGGACTCCCCCCAGTGATGTAGCGAGCGGCCGGCTCGTCTTTGTGTGTGTGTGCGCTGTGCGACAGTTAACTCCCATAGGCCTCTGCAGTGTCGGATTGGAGAGTCTTCCTCCCCCTCC 2040  
 CCCTTCCTGCAGCAAAGCATAAAAGCTTCCATGGGCTGCCTTCTGAGCACGAAGGAAAGGTAACAACCCGAGGCGAAGGGAGTATCTTCTCTTCGGAGATCCTGTAAGCTCTCCGTGTTA 2160  
 ATGAGGGAGTAACTATTCCCTTTTTGAAGATTATGCTGTTAAGGAGCCGGGGGCGATGTGGGCTTGGGGGTGGTGGGGAAAACCCAGGGTCTTGAGTTTCTTTCTCCACAGGTTCCCCCC 2280  
 TCTAAACTAGGCTCCAAAGAGAATTGATGGCTGAAGAGCTAAAAGGCAATTTGATCCTGGTCTGGCACGTCTGCACAGCTATTCGGTAGTGTGGCAATACTTGAGTTCTATCAGGCAGTG 2400  
 GCCAGTTCTCTGGATGTCTGCTCTTGTAGGCAAGTAGACTATCCTTTTAGCAGTGAATCGGGTTCCTGGAAGTTGGGTTGTAATTATTGGAAGCCCTCGGAGATTGTGATGTTAATATGA 2520  
 AGAGGGGTAGATGACGGCAGGGGTGGTGGTGGTGGGCGGGGGGGTAATCTTAATCTTTTCCCCAGCCTTTTTGCAGCTCTGTATGTAAAGCAGTGTAAAGCCCAGTGGATGCAATAATTT 2640  
 GGATGTCCCAAGCGCATGGTGGGCAAGGGAAGCAACTGAGGGACCCACAGCAGCAAATGACCAGCACAGTCCTGGTGACAGGCAGGCAGGAGCCCACTGGCGTCCTTGTAGAGAGAGGAG 2760  
 TGTGCGATTGTGATGGCCCTGGCACTTGATCGTGTTCAGTCTCTGGGAGTTTCGCGATTGTGAGTGAGGAGCTGTGCACCTGCATCGCCTCTGATCTGGGAGCTGTCCCGGGAGGGGTGG 2880  
 GGCGTCCTCTTACTGTAAAGACTCTTCCAGATGTTGGGTGGAAAAGGCAGCTTGAGGCACCTACAGGCACATCCGCCGTCACTTGGAGAGTGGTTGGATCTTCCATTACAGCTCTTAACA 3000  
 CACAGGCATGTGCGTTTTATCATTTTACACGGGTAATCTGCTGTCTATTAACATTGTACTGTTTTGAGTAGCTTTTGGATACATTGTGGAGTGTAACTCAAGTTTGTGAAATGCAGCATT 3120  
 TGTATGTATTACTCCTTGGGAATGTGGAGGATGTGTGACTGCCACTAGCGTTAGTGGCATCAGTCTTGCAGCGTCTCACGATCTGGGGGTGGGATTGCAGTAATAAACAGGAAGTGGGGG 3240  
 GGGTGTGTGTTCTTGTTCTGCCTTTTTTTCATTCTGCAGTGCCCCAGATCGCTTAAATTCCTGATGCATCAGAAGCAGTCTCCACTAGCCCATGTAATGTCTCGCTTACACAATCCACAT 3360  
 AGTAACGTCCACATACCAAAAATGTTCTCTGCAAAAGAAGGTATTCAAACTGATCTATTATCGAAGTCTTGTTCAATTGCATTTGTAGCTGTGCTGCTTTTTTTCCTGCACTTAGTGCTT 3480  
 GCAGCTGGTTTGCTAGTTCTGTACATCTTTAAAAAAAAACGCCTTGTAAACTTTGAAATGGAGGATTCTCCGTATTGAAATGTAAAGTCTTATTTTACCACGTAATCTTTAAAGAGTAAT 3600  
 AAATACTGTTCTGAATGAGTTTTTATTTTGGTATGCATTGTTT

TTTGGG

TTTGGG  
Depth:19 (ZEBRAFISH)  
Ei-value:0.000, Pi-value:0.000  
Er-value:0.000, Pr-value:0.000  
No matches to TargetScan

GATGGGGCGGGGCCTTTTCTTCTGTTTCCAC

TTTTTCAG

TTTTTCAG  
Depth:19 (ZEBRAFISH)  
Ei-value:0.000, Pi-value:0.000  
Er-value:0.000, Pr-value:0.000  
No matches to TargetScan

GATCTCTGTACTGCTTTCGGCTTTGTAACATA 3720  
 ACGTTGGATGGGCTCGGTAGCAAGTCGAGGTGGAATGAGA

GATAAG

GATAAG  
Depth:19 (ZEBRAFISH)  
Ei-value:0.000, Pi-value:0.000  
Er-value:0.000, Pr-value:0.000  
No matches to TargetScan

TGCTGGGATTTTTGCCTTGGAGAGTGATTTGGAGTAGAGCCCAGTGGGGGCGTCAGACGTGGGCGTGATCTGAA 3840  
 GATCAGTGGAGCTTAGAGCTCTGCTGTAGTTCTGTAAAGATCTGGGAATTCAGTGACTACTGGATATGGAAGCAGAGAGTCTTGGGATTGACGGTGGGGAGTTGTGCGGCAGTTTCTTCG 3960  
 GAAAATTGTATCTTGCAGTGTATGGAAAAGCCCAGCTTCTTCAGTTACAAAGGATTCCTGGAAAAGGCGAGGCCTTCACA

GATAAG

GATAAG  
Depth:19 (ZEBRAFISH)  
Ei-value:0.000, Pi-value:0.000  
Er-value:0.000, Pr-value:0.000  
No matches to TargetScan

TCCCTTTCAGGAGTTTCCTGCCCGTTGTGCTTGA 4080  
 ATCTAGGTAAATGAACGTCGGCGGGGCAGTGGGAAACGTGCTTTGGTAATGTCGTACACCTCTTGGGGTGGGGTATTGTTGTCTTGAGAATGTAGTAGCTGGAGGGGTGGGTGTTTCTCT 4200  
 TTGCATCTTTGCAGAAAAGTCGTTTCAGCTTCACGAAGACCAGACAATCCTCTCAAGTGTGGGGGAATCTCGGCTCTTGACCCCAGGAATCGGCCATCTTGTCTCTCTCCTAACCAGGCA 4320  
 GGCAAAGGATCTTGAGGTGGTAAAATGGGTAGTCTTGGTTTGGGGGGGACTGAACTCGGTTACTGTGCAGCTGTGCAAATGTGCAGCAGCGCCGTCGGTTGTGCAAATGTGCAACTGGAA 4440  
 ATGCCCGGGTGAGGGTGGTTGCGCAACTGTGCAGCGCGCGCTGTGTGATCTGGAAGGGTAACCGAGGAAGCCGCACAACTGTGCAGCTTGTCCAAAACGCATTGCGGCCGCGCAACTGTG 4560  
 CGGCGCGTCTCGACGACATCGGTTGTGCAACTGCGCAGCTGCGGCTTTCTTGTCGAGGGTGGGGTTGTGTCCAGCCTGTTGTCTTTGCAGAGACCCATTTTGAATGAGCAACAAAAGTCC 4680  
 GAACGCAGCCCAGCAAAAAGGTTCCTCGTTCCTGGAGTGTTGAGTAAGGTCAGAACCTTAAAAGCCTTTGATTTTGGGATCCAAGGTCTAAATGCTGGAAAACTCCCAGTAAAAAAAAGA 4800  
 AATTGTGAACATCTTAATTTGAGGTGAGGATTTCAATTTCTTTAGAACACCTGTGGGGAAACTTGCACGCAGCTTAAACTGGCAACTGCATAAAACACTGGCTTGAAGCCTTCAGATGTG 4920  
 ATGCTTTCCTACTTGTCTGTCCCATACGGTAGTGTTGGGTGGAATGTTGTGAGGGCATGTGGAAACTCTTCTTCCAGATTGGCTGTACCCACTTCTAGACTGCGATTGTGTGCAACTCGG 5040  
 GAGCTCTTGGCAAACTGAATGACCTGAATGGAGTCTCGTGGTGTGCCTGTGCTACAGACTGATCCCCCCTCAAGTCCCGTGTGAAGAGGCTTCTCCCTCCTGCTTTTGTGTGTGTGCGCC 5160  
 CCTCTCGTTTCTTCCGGTTTCAAACAGGTGAAGCGAATGCAATGTAGTGAACTTGGGGTCACGAATGTAAAACGGGCTTGGAAGAGGAGTGGAAGTTGCCCTGTTGAGATGGGTCTCGGG 5280  
 GGGTATGGGGGAAACCTTTTCCAGTCTTTCCCTTTCAGATGAACACCATGTGGCTAATAAGCAGCAGCTCCTCCCTGCAAGTTGCTTTGTTGGAAGTTAAGTAACCCTTTCAAGTTTTTC 5400  
 CACTTAACAACACTGATGAGCATGCAGCTTTTAATGCTTACAAAAAGAGCATTTGGTCTTTTTTTGAAGTAGATGTGATGTAAGTGAAGCTGTACTTTCTTTTAAGTTACTCTTGTAACT 5520  
 GGTTACATTTAGGCTCAGGCCTGGCACTGTTGTTAAGGGGGAGGGGGGGGTGGGCACAACAAATCTTGTTTCTTTCCAGATGTTCATCTTTAGTGTACACACACCTGCCCAGCAGTGAGG 5640  
 CACCAGTGAATCTGGCTCCGCCAGCGTAGAGGGGCCCGGCTCTTCTCTCCTGTTTCTCAGTCACTTTAACAGGTCAAACCTCCCCCCCGGATGGAAAATCTTGACTTACTGGCATAAGCG 5760  
 ACTCCTAACTTGGAAAACGGCCACTGGAGCAGCACGTGCTTTTGGGGAGGGGTGTTCGGGTTGCTTTTCAGATGTTTACTCTTCAGTTCGATGACTTTTCCTGGCTCCTACTTAAGCTTG 5880  
 AGGACAAGGCTTTGGAGAGTAAGTTAATCCAACACCAAAACTGATATTACACAGACCTTTCAGAGCCTCAAATGCCCCTAGGGCTAGACCTGGGTGTACTACCTGGTCGAATGTCTTCCA 6000  
 TTAAAATAGGGCATTTTAAGTAGTTTAGTAGGAATACATTAGAGCTAGTTTTGAGGCTCCAGAGTTAAGTGGGGATATCCTGCACACATAAGATAATTTCAGATTAGAAGTGCATTAAAT 6120  
 AAACTCGACTTGGCAGTACAGTAAGCTGAGATCAAATCGGCGAAGCAGTTTTGAGGTTCTAATGGATGGGGTGGGGGGGCTATAAAGTGGTTCTTTAGTTTTTTTTCCCAACAGCTCTAC 6240  
 GTTTTGAGAGGTCACCCAGCAACATACCAACCCTCTTAAAGTGTTTGACCAAGACGCACAGACCCGTGCAGCAACGGGTTCTCCTGGTAAACCATGCAGTTCGCTCTGGTGCGCACGCGC 6360  
 GTGCGGCGCTGATGTTCTCTGTGGGTGCTTGTTCTGTCTCTCTTTAAGAAAAAGAAACACGAAAACGGCGGTCGGAAGACCCGTTGGCCTCTCCAGTCGTAAGACAGTCCTTCGCTTTTT 6480  
 CGTTTCGATTTTTTCAAATACATTTCGGGGGTTCGGTGGGGAATGGGGGACGGCAACCCTTCTCTGTAGTTGTTGCAGGCTCCGTGCGAGGTACGCAGGGACCGTAGGCGAAGCAGCTTT 6600  
 ATCCAAGGGT

TTTTCTTTT

TTTTCTTTT  
Depth:19 (ZEBRAFISH)  
Ei-value:0.000, Pi-value:0.000  
Er-value:0.000, Pr-value:0.000  
MATCHES To TargetScan▶ miR-186-5p:AAAGAAU

CCTGGGGGAACAAGCTCTTGTTCCTCCAGGTTTTGCTTTTTCTCCTTTTAACGAACGCAAAAAAAGCAAAAGGCGCCGGTCGTGGGGCTCCCGGTCTCCGG 6720  
 GACGGGGTTCGACTCCCCGCGGTGTCTCTGCTGCGCCAAGACGCTTCAGCTCTCGGCCTGTTGGTCTCTGTGCCAGAGAGGGTCCTCCGGGCCGAGGGTCGTTCTCCCCCTTGTGTAAAG 6840  
 CTGCAGTGATCGAGTCTCAATGGAGGGTTTTTCTCTTTCCCTGGGGGAACAAAAACCCTGCGTCTCTGTTCCCC

CAGGTTTTGCTTT

CAGGTTTTGCTTT  
Depth:19 (ZEBRAFISH)  
Ei-value:0.000, Pi-value:0.000  
Er-value:0.000, Pr-value:0.000  
MATCHES To TargetScan▶ miR-330-3p.2:AAAGCAC▶ miR-490-3p:AACCUGG

TCTCCCTTTTGATTGGCTTTTTTTTTTT

AAAAA

AAAAAGCAAAA  
Depth:19 (ZEBRAFISH)  
Ei-value:0.000, Pi-value:0.000  
Er-value:0.000, Pr-value:0.000  
No matches to TargetScan

 6960  


GCAAAA

AAAAAGCAAAA  
Depth:19 (ZEBRAFISH)  
Ei-value:0.000, Pi-value:0.000  
Er-value:0.000, Pr-value:0.000  
No matches to TargetScan

GACTCCAGTGGTGGCACTCCTGGTCTCCAGGGTGGGGTCCGACTCCCTGAGGGGTCCTTGCTTTGAATGGGATGATCTGTATCCCGGTTTAAATTGAATTTCATAGGAGTAACG 7080  
 GTCCAATCCCAGCCTGTATGGGTTGACCACCAGTGGGGAATAGAGGTGTGTATGAAACTAGGTCAAACCTAGCCCAAATACCTCCATTCCTCTCGGGGCGGGGGGTGAAATCGCAACTCC 7200  
 AGGATTTCTCAGGATCTGGTTTCATTTGTACTACTGCACTATAAACCAGACAGAACTCCGGCGTTTAGTACTGCGTGCTTGATGTGGAACTTGCTAACCCAATCGA               7306
```

---

## >FUGU (4992 bases)

```
 TAAATACAGACAGTGACGCCATTTCAAGTCAGACCTGCATCGACACGCCACTTTGTGAAGTGGCCTCTCTGCGTCAGGTTAAAAAGAGCTACTCAGCTTCTTTATTTCTTTTTTAAGGTC 120  
 TCAATAGACTATTGTTAGGTGTACGCACTTTTGTCTAGTATTTTCCTTATTTTATTTAAGCATTTAGACTCATTGTTAGGAGGAAGTGACGTTTATGATATGACTCATTGTTCTGGTAGT 240  
 TGATTTGTTCATTCTTTTGAGTGGAGGATTTTTAATTTGACTTCTGTTGGAAGCTAGCCATTTTGTCGAGCCAGGTCTTTATTTTATTTTATTTTTTGGTTATTGCGCGCCTACCCAGCC 360  
 AGCCAAGCTAGAGCCGTTTGTTCGACCCAAATATAGGCGGTAGATTTGTCGAGTGTTTTTTCAAGGTTTTTTTTTTTCTCCCTAAAAATGCAATTCGAAGAAACTCTTAATCCAGATATT 480  
 GTGGTTTTCCTAAACAGTTGGAGCCCTTTCTTAAACAGTAGGCCAAATATGGGCGCTGTGACGTAGTTGCTAGCTGATGTTAGCCATTGGGCTATTTTGAATACTGCCAGTATGGAGAGG 600  
 GCGTGGCTTGGTTTTTAGCCAGTTAAAGTAACAAAAGCTATTTTCTACACAAGAGTAAACTTGTGGCTCTATAAATAGAATAAGCGCAGGCTTTTTTTTATTTTTTTAGATTTTCAGCGG 720  
 AGCTGCAGTTAAATGTTTTTTTTTTTTATTGGGGATTCAAATGGACTGAGTCATGGGGGTGGAAATGGACTTAGTGGTTAGCGGAAGATCTCGGGAGCGGCACGAAAACGGACGGAAGCG 840  
 GAGCGGGACCAGTGCGGAACCAGGCAGTTCAGGACCAGAGGGCGTGGGCGGACCAGTGCAGAACCAGGCAGGACAGGACCAGAGGGCGTGGGCGGACCAGTGCGGTACCAGGCAGAACAG 960  
 GACCAGAGGGCGTGGGAGGACCAGTGCGGTGCCAGGCAGAACAGGACCAGAGGGCGTGGGAGGACCAGTGCGGTGCCAGGCAGGGCAGGACCAGAGGGCATGAAGGGAAGCTGGGCTGAA 1080  
 GAGAGCAGTTGTCCACGTCTGTACGCCAGAGGGAGCCAGAGGTTCAGGACAGTGTAGCAGCATCAAAGGTCACGTCTTCCAGAACAAGCCAGCCAGCGAGTGAGGGGAAATGGGTGCAAG 1200  
 TAGCTTCAGTGTACAGCGAGGCTTTGGTTTGTATATTGGGTATAGGTAACCTTGAAAAATTCTCAGGAATTAGGTTTAAACCGCCATTCCATAAAGTGTTGTGTAGAACAAATTTTCTTG 1320  
 CACAGTTTCTCTTCCCTTTTTCAGGAAATCCTTGTAAAGTGTTTTTTACCGTGATCGCCATCCGCTTGTGAGATGACATGCGTGTCTTGGTCGGTCCACTGTCGTGAATCCCCTGGTATT 1440  
 GTGCTGACAAGGAACGGCTTGTTGGAGTTCCCTGCAGTTCCCTGCGGTCGAAGTAGTTTCCTCAACACGTGGCAGAGGTGTTCCATTGTTCATAGGTCTCCGTAAAGAAGAGAAGTTTTC 1560  
 AGCATCGGGGTGTGAATGGATGCTTTCCCGTGCAACCAGTCGGTGTGTGTAAACTGCAACCCCTCAGAGCTTCAGTACAACTTGGTGCTGCAGATAAGCTACATCAACCTCTTAACTTAT 1680  
 CAGTGAGTTTTTGCATGTGTACA

TTTGGG

TTTGGG  
Depth:19 (ZEBRAFISH)  
Ei-value:0.000, Pi-value:0.000  
Er-value:0.000, Pr-value:0.000  
No matches to TargetScan

TGTAACTATCCAGTACATTAGTTTGCCATCTTTGCATTTTATGACATGTATTGAGCTAGATTTGTGGGAGGGGGGGGGGGGTGGGCATTTA 1800  
 AATGCACCTTTTCTAGTCTTTTCCACAGGCCCGGCCCCCTAAAGCAGGAACGACAGAATCCCATGTAGTTGTATTGAGCTCTCTATTTACTATAGTGCTATGGATCAGCATCATGTACAT 1920  
 CCACAGCTGTGTAAAGAGCCGTAGAAAGGTATTGGTCTGTTGGAAGACTTCAATTCCATGTGTTTGACAGTGTTTATCTGATGGGTAATGGGAGGGGGTTGGGGCTTTAACCAGTCTTCT 2040  
 CTAGACTTTACAGCTCCTTGATACAGGGCAAATGGTTAGGAACATCCAACATACCAGGAACCTTCAGTGGACACTTTCCCCACAGGATAGCAACTACTGAACTAGTGTGCGCTAGCAAGA 2160  
 ACCTCTGTTTTCCTGGGGAGTGTGGTAAAGAGTCTCTTTCCTCGAAGTCATTGAAATTGTATACATCACCTGTGGGGTTGGGAGTGGGCTGTGTATCTGACTCTTGCAGATTAGGTTGTA 2280  
 TGGCCCACTATTGAAAAGCCAACGTTCAAGTCTACGTCGGCAGTTCTTGTGGATGGAATCCTTCACTATTGCGGTATTTAGATGCTCTTTCTGGAAATGGTGTCATTCAATTTTTTAAAA 2400  
 ATGAAATCATTGTACTGTAGCGTTGTACTACTACCGTTGTGGGGGGGGGGGGTTACTCTTGGGGAAAGGCCCTTTCCTTTGCA

TTTTTCAG

TTTTTCAG  
Depth:19 (ZEBRAFISH)  
Ei-value:0.000, Pi-value:0.000  
Er-value:0.000, Pr-value:0.000  
No matches to TargetScan

TGTGGTGTGTCCTGTGTGCTTTCAATACC 2520  
 CAAGACCATCAGGAACTCTAACTCATTGTATGTACAACTTTCCTTGCGCATCAAAGCATCACGTAAGGTCATCTGAAGTGTCACGTTTTTTTAACTTTCATTGTCTGGCTAAATGCCTCA 2640  
 TTGCCATTCCTTTGCAATTAGTTACTATGTCTTGTTTTGACAACACAGTAAGTCAACTATCTGTAAAGGATTTTCTTATTGCATAATAAATGCATAGCTTTGTGTGATTGAATACAGTTG 2760  
 GTATGCATTTGTTCTTTTGGGGTTGTGTGGGGAAGCTTTCTTGTGCT

TTTTTCAG

TTTTTCAG  
Depth:19 (ZEBRAFISH)  
Ei-value:0.000, Pi-value:0.000  
Er-value:0.000, Pr-value:0.000  
No matches to TargetScan

GTGTGAATAAAGCTTGGAGATATGTAGCGCACGGAAGTGACCTGCATTTTTTCAAAAGGGGAGTT 2880  
 AAAGGCA

GATAAG

GATAAG  
Depth:19 (ZEBRAFISH)  
Ei-value:0.000, Pi-value:0.000  
Er-value:0.000, Pr-value:0.000  
No matches to TargetScan

TTGAATCCATTTAATCTTGTTGGAAAGGAGACCCTTGGCCCTGAGGGGAAACATTTGAGCTTGAAATGTGCAATTTAGGTGAACTTCACTTTGAGTTTGATTGGTTG 3000  
 ATTTTAATGGGCCCTAATTGATTGAAACATTCTAGTAAG

GATAAG

GATAAG  
Depth:19 (ZEBRAFISH)  
Ei-value:0.000, Pi-value:0.000  
Er-value:0.000, Pr-value:0.000  
No matches to TargetScan

TGGTCCTGTATTGTAGGGGGTAGGGTCTTTTTCTGCCTGTTTAATCTTTCAGAACAACCCAAAGGAAGAATGCAG 3120  
 TGCAACTTTGACAACACGGAAATCATCTTTTCCTTTGAAATGAACCGGCCCAGGTTGAAACGAGCCCTGTCAGGTTTCTTTTCTCTATGGAACAAGTCCTCAGCTTCTATCCCTTGTGCA 3240  
 AATGTGCAAGTGGCAGGTTATTCAATTTTTGTAACTGCGCAACTGTGTGGTTTCCAAAAATTGGGAGCTGAGCTAAGCTGCGCATGGTGCAGCTTTAATTTGGTCTTGAATGCAACGCAA 3360  
 GTGTGTTGCTGTAGTCGAGTGCATTTCCTTTTTTGGTTGGGTGTATCCCTATACTTTTCTTCACAGAAATCTCTCAGTGCAAGAGTGACTCGCGTACAGGAGGCTCTACCTGGAGCATGT 3480  
 AGTAAGATGTCGGCTTAATCTGTCATTAAAATCTGCAATGTTGTAGTTGACAAAAATGACACTGGATTTGGCTTTAACCTAATTCTCAGACTCGATGCTTTACTAGCATTCTTTTGGCCA 3600  
 TGGGACCCTGTAGCCTTTCATCTTAAACATAAGATGAGTGAAATTTGAATCCTTGTATTGTGTGGTGGGTGTGTTGTGTAGATCACTCTGTTTCAGATCATTTTCGTCCGTCTGCTCCAA 3720  
 TCTCACCCGCCTCTGAATGCTAAGCATCTGTGGAACCTGTCGGCACATGTGTGGCCCCTGAATGAAGTACCAACCCCCCCTGTGCGCTTGAGCTTAAATCCGAACTCCCCGACATTCTTC 3840  
 GCAGAAGGCCTGCTGTCTGTGTTTGTGCCTCTTTTCTTTCCCCCCCTTGGTTTCTCAGGTCAAATTACCTTAAATTGTCTAAATGTATCATCTGGGATGGCCTAACCTGTGATGGAACGC 3960  
 TGAGAATCCAGTCACATTACGATCAAGAGCTGCAAGCTTGTCACACTAATACTGCATCACTTCACTCTCGTGGGTGCGAAGTTAAGTAATTTCGGTTACCATGATTTATTTCGTTAACCA 4080  
 ACCATGGCTTTAATTGTTTTGTAACATACTGCATGCACATCAACTCTGCATAACAGTTAAGTTGACATGATATCCATGTGGTCAATATCACACATTGCAGTATTGGGAATCTGTGGGGTG 4200  
 GGCGGGCGGGCAACCTTCTCTCTTTTCAGTTATCTTGTTCAGGTTAACTTGATACTGAACCAGATGGCTAAACCACTGCAAGGAGTCATGGGACAACTTGGTGTTGCATCAACCTCTAGG 4320  
 GTTCGTTCTCTTTTGCTCGGAGAGTTGACCGTCGTGGTGGTAAATGCTGCTGCACCCGTTGCGGTAAATGACAATCATGACAAATTTTAAAACTTCACGTAAGTGGGACAAATCCTGACA 4440  
 TGATTGATGCGTCATGCTGGTGGCTTTGTGCCGGGGGGGTGGGGGGGGGCCTAAATTGTCTCTCTTTTCACGTTACAGCTGTAAACTTTTGAGTCTTCACTATCCTTCCTGCCAAATTGC 4560  
 GATGGATAAAAGGCTTTGAGCATCACCAGGGATGCTGGTATTGTGGCTGCAGTGCTGACCTAAGAACTTGGAATTCATACAAATACTGTTGTGGGCCATGATGGGGATGGGGAAATGGTT 4680  
 TCCTGCTTTCTAGTTTCAGCTTCGTGACCAAGAGTGAGCAAAAATCCCCTCGCAGTGACGATAATGTAGCGGCCCGGTGTGCCAGGG

TTTTCTTTT

TTTTCTTTT  
Depth:19 (ZEBRAFISH)  
Ei-value:0.000, Pi-value:0.000  
Er-value:0.000, Pr-value:0.000  
MATCHES To TargetScan▶ miR-186-5p:AAAGAAU

CCTGGAGGAACAAATGTCTCGTTT 4800  
 GTTCCTC

CAGGTTTTGCTTT

CAGGTTTTGCTTT  
Depth:19 (ZEBRAFISH)  
Ei-value:0.000, Pi-value:0.000  
Er-value:0.000, Pr-value:0.000  
MATCHES To TargetScan▶ miR-330-3p.2:AAAGCAC▶ miR-490-3p:AACCUGG

TGCCCTTACAGCAACAAC

AAAAAGCAAAA

AAAAAGCAAAA  
Depth:19 (ZEBRAFISH)  
Ei-value:0.000, Pi-value:0.000  
Er-value:0.000, Pr-value:0.000  
No matches to TargetScan

GACCCCGGTGGTGGCACTCCTGACGTCGTCGGGACAGGGTTCGATCCCCTGCGGTGTCTTGCTTTTTAAGT 4920  
 GCTGCAGAGCAAACTTTTATTATGATTTCAGATTAAAAAAATAAATAAAAAATCCTGGTAAAACTTACTGAT                                                 4992
```

---

## >NILETILAPIA (6000 bases)

```
 ATAGCGCTGGTGACGCTATCGCAACTTACACCGCGTTGAAGCTTTTTCGTCTTGCAGTTGGGCGTTGCAGGTTGAGCGTATCTCGGTTTTTTTTCTTCTCCTTTTTCGATCTTTAAAGTT 120  
 CGGGTTTTATTTTCGAACCTATGTAGCCTTGCTCTATCTAGTCTGTTTGTGACAAGTTTAATTTTAGCCTCTTGAGCGTTATTGATCATTATTTTAATGCAAGTACTTTAAATCTCTAGA 240  
 TACCATGTGTCGCTTGTTTTCGGTTTGTTAAACGCTTTTTAGTACTGCTTTATTGCAAATGAGTAAGATTGTAGAAGATGAACACAAATTCTACTGAGTCGAAACGAAGGCCTGATTCCT 360  
 TTTTGTTGTCGTTCTTTTTTTTGTTTTTTTGCCGAGGGCTGTTGAGCTGACGCTAGGCTTAGCCAGCCAAATATATAAATTTATGCCAGTATTGTCAGTTATTCCGCAACAGTTGTTTTG 480  
 TTAGCTGTCATGTTTAGCGTTTGTCTAGCGGTGATCTTGCTTTTTAAAAATATATTCGAAGTAACCATTTTTGGTCATACTAACAGCAGTTGGCTCGAGATAGGAGCTGTAACGTAGAAA 600  
 CGTGTAAACGTTGTCTATTGGCTAATTCAATTTACGTCATGACGTAAGTAGACGGCAGTGGAAAAGGGAGTGGTCAGACTGACGGCCATTATCAACCTTCACGTAGTGTTTTTCCGGAAG 720  
 GTGTAGAAAGCTTTTAGCAACAAAATGGCAGAGGAACAATTTTCATACTTTTGAAATTGATAAGGCAGTGAAGTTAGGTGCCTAATTAGGTTCCTAAAGAGTACTGATTTTGCAATGAGG 840  
 TCTTTCAGATTAGATCACAATCTAACTACAGTTAAGTGACTCTGTTAAATATAAGATTTGTTTTTATTCTGAAGAAACGAAGAATGAAGAAAGAAAATAAGGATGTCCTAGTCGTGATCA 960  
 GACGTAGATTATGAGAAGTGCTAGTAATTGTATTTAAAACGGAGGGTTTATGTGTAAGCTCGGAGGTGCTATCTAACTACGATAGACTAGACCAGCCTGTGTTTAATTTAAAAAAAAAAA 1080  
 AAAAAAAAAAAAATCAGGAACAGATTAAGCAACAACAGCATCAAAGAAGCAATGAAGATGTTTTTAGTTTTTATTTGATAGCGGTTACTTGGATGAGGTCCTTACCTCTGTCTTTTCACT 1200  
 CTTATCCCATTGATCAGGGAAGCATTTTGCAATGCAAAATTTTTATTTTATTTTTTTTAATCCAAGTTTCGCTCAGTTTCCATCAGTCATTAGACTTGAAGTTTCAAGCTATCAATTCTG 1320  
 CATTCAAAGAAAGAAGATAGCATCAGTCAAGTAATGCTGAAGAATGGAAGCTTATCTTAGAGCTCTAGTAGTAGCTCAAGCAATATCAAGTTCAGATTTATGGACTTCTTGAGAAGGAAA 1440  
 AAAAAAAGGTTCATCACTTAGAGACGACAGAAGATTTTTTTTTTGTTTTGATTTTTTTTCTTCGGGGGGAAAATGGACTGAGTTATCACGGGGAGGAATGGACTTAGTTATTAGATGAAG 1560  
 ATCGCAGGACGGCTTGGAGCGAGGTCGCGGGACACGGGGAGGAGCACAGAGGAGCCAGCCGGGCAGGACAGGACCAGAGGGCGTGGGAGGACCAGTGTGGAGCCAGGCAGGACAGGACCA 1680  
 GAGGGCGTGGGAGGACCAGTGTGGAGCCAGGCAGGACAGGACCAGAGGGGCGTGGAGGGGAGGAGCAGAGGGGGCCAAGAGCAGGAAGCATCAGGAGCTGTCGGCCAGTGTACGCCAGAG 1800  
 GGAGCCAGAAACGCAGGGCAGTGTAGAAGCGCAGTGACCAGCCCCGATGCCGATCCAGTGCTGAGAGGGTGGGACGAGGGCGTGCAGTCTTCTAGCGTCACAAGTTTAAAGCGGGGCTTT 1920  
 GGTTTGTATACTGGGTAGAGGTATCTGTGTAACACTCAGTATTTGGTTTTTGTACTTTCCAGAAACATCTGTTGTCGAACAACTTCTCGCACAGCTCTCTCTTCCTCTTTTCAGGCAAGC 2040  
 CGTGTAAAGTGTTTTGTACCGTGGTCACCCCTCCTGTTGTGCGCTGAAATGTGTGGTGGTGTCTGCTGTGATGAATGCCTTGGTAGTGTGCTGACGAGGAGCGTCTTAATGGACTTCTCT 2160  
 ACCCCCCTGCGATCACAAGTAGTTTCCACAAGCGGAGGGAGAGGCAGTCCTTTTGTTGTTCGATAGGTCTTCCCCCAGAGAAGTTTTCTGCATTGGAGTGTGAAAGTGGATGGTCTCCCA 2280  
 TACGGCCAATTGTGTATTCGGCAACCCTTCGGTACTTCAGCTGCACCTCATTGGTACAGATCAGCTACATCTACCTCTTAAACCCAAGCAGTGAGTTAATGCATGGAATGTACATGTGGG 2400  
 TGTAACTATCCTGTACATTAACTCATCATTACATTTCATAAGTATACTGTAATGACTTCACTCAAAAGGGAATTTGCATTGAAATGGTAGTTGATCCTGGTGGGTAAATATTTTTGGCTT 2520  
 AAGGGGAGGGGGGATGGGGGGGTGTTAAAACTTGTTTGGAGTCTTTCTTTTTACAGGCCTGGCCCCCTAACGCGATCATCGACAGAATCCCCCCCTTTCTAGCTGCTTGGTACAAGTTGA 2640  
 GCTCATCCTATATACAGTAGTGATATGGATCGAGATCATGCGCATCCACTGTGCTTAAACGAGATATAGAAAGGTATTTGGTGTTCAGTGACTCAATTAGTTTTCATTTCACTTTTATTT 2760  
 GGAACATGTTGTAAATGGGCTTCCATGAATTTTT

TTTGGG

TTTGGG  
Depth:19 (ZEBRAFISH)  
Ei-value:0.000, Pi-value:0.000  
Er-value:0.000, Pr-value:0.000  
No matches to TargetScan

AGGGGTGGGTTGTGGGGCGTTAATCAGTCTCTTCCTAGACTTCACAGCTCCACATTGCCCAGTGCACATGGCTTGAATAG 2880  
 GAAACATCCAGGCGCAAGCAACAAGATTCAAGTGGACACTTTACAACGCAAGATAGCAACATCAAAAGTAGTCTTCTGATGTAGTGAAGCTCCTTTCCCCAGGGAGAGTGCGGTAATACT 3000  
 TCTCCTTACTAGAAGTCATTGTAATTCTGTACAGGCTTTACGTTGCTTTGTGGGGTTGGGAGGGGGCTGTGTATTTTTTGACTCTTACAGATTAACTTGTAAGGCCCATCCTTCAAAAAG 3120  
 CTACGTCCAAGTCCATGCCTACAGTTAAGGAAGGAGTCCCTCAGGCCTGTGGTATTTTAAATGTTTCTTATGGCAAATGGCTTCAATCTTTTGTGAATTGTATTCATAGTCTTAAAGTTG 3240  
 GCCTTGTTAAAGCAGTTGCAATTGCTGTAATCCCAGTATTGTCTTGTAGCTCTCTACAATGTTACTATAATGCCAGTGTTGTAGTTTTCTGCCATATTGTATTAGGGCAGGGGTGTTGTG 3360  
 GGGAGAGGCTTTTCTTTGCAT

TTTTTCAG

TTTTTCAG  
Depth:19 (ZEBRAFISH)  
Ei-value:0.000, Pi-value:0.000  
Er-value:0.000, Pr-value:0.000  
No matches to TargetScan

TGTGCTGTGTCCTGTGTGCTTTCAATACCCAAGACCATCAGGAACTCCTAACTCATTGTATGTACAAGTTTCCTTGCGCATCGAGCATCAC 3480  
 GTAAGGTGGCTAAATGTGTTCATTTACTTTAACATTCATTATTTGGCTAATTGCCTTCATTGCAATTCCTTTGCAATTAGTTACTTTGCTTTGTATCGCCAATACAATGAGTGAACTATC 3600  
 TGTAAAGGATTTTCTTACTGCATAATAAATGCATAGCTTGTTGTGATTGAATACAGTTTTGGTATGCATTTGTTCTTTTTGGGGGGGCTTGTGTGGGGGAGCTTTTCTTGTGC

TTTTTCA

TTTTTCAG  
Depth:19 (ZEBRAFISH)  
Ei-value:0.000, Pi-value:0.000  
Er-value:0.000, Pr-value:0.000  
No matches to TargetScan

 3720  


G

TTTTTCAG  
Depth:19 (ZEBRAFISH)  
Ei-value:0.000, Pi-value:0.000  
Er-value:0.000, Pr-value:0.000  
No matches to TargetScan

GTATGACTAAAGCTTGGAGCTTTGTAGAGCACGGATGTGACCTGGGCTCGAATGACTTCTATTTCAAAGTGGAAAGTGAAGGCA

GATAAG

GATAAG  
Depth:19 (ZEBRAFISH)  
Ei-value:0.000, Pi-value:0.000  
Er-value:0.000, Pr-value:0.000  
No matches to TargetScan

TTGAATCCATCAAATATTGATGTCGAAAA 3840  
 ACAGAAAATTCTCAGCCCTTTAGGGGTCACTTATCTTGACTGGACTTGCGTGTCCTCATTCTTGAAATGAAAGCAACTTGCTATACTTGACAGTAGAAATAGCACTGCTGTGCTTTCTTA 3960  
 ACAAGTCACTTGTGATAACGGGAAGATTTGATGGACCTTCATTGATTCTGACATTATCTGCAGTAAAGCATTTGGGTAAGGATAACAATAGTCAGTTCTACGATACTGTGGGGAGGGGTA 4080  
 TAGGGGTGTTTCTTTTCTGCCTCTTTGATCTTACAGAACAATCCAAAGGAAGAAAGCAGTGTACCTAGGAGAACACAAGAATCACGCTGCCCTTTGAACTCCACCAACATGCAGTTTGAA 4200  
 CAAAAGGAGTCAGGTTTTTGATGTGTTTACATGGATTAGAGCATTTAGCCCTCAGCTGCTTGCCCTTGTGCAACTGTACAAGTGGCAGGTTGGTCACCTTAGCAACCGCGCAACTGTGCG 4320  
 AAGTTTCAAAATTTAAGCAGCTGAGGAAAGCTGCGCATGGTGCGGCTTAATTTGGTCTGAAATGCAGCGCAAATGTGCTGCCTTAGATTCTTTTGTGCTATGGTTGTGGTTGGGCGTATC 4440  
 CCTACACTTCTCTTCACAGAACTCACAGTGCAAGACTGGTACAAGCACGAGTCAAGTCCTAACCCGGAGCATGTAGTAAGCTGTTGGCTTTACTTGTCAAAACTTCTGCAATGTTGTAGT 4560  
 TGACAAAATGACATCTTGGAACTGGCTTTATGTTAACCTCAAATCATCAGTAACAATGCTTTACTAGCTTATTCCTCTTTGGCCTTGGGGCCCTATTGCTTTGCATCTTAAAACCATAAG 4680  
 ATGAGTAGAACCCGAATCTTTGAGTATTGTGTGGTGGGTGTGTTGTGTAGATCACTCTTTGTTTCAGATCATTCTCGTCTGTCCACTCCGATCTCGCCTGCCTCTGAATGCAAAGCCATC 4800  
 TGTGGAAGCTGTCAGCGATATGAGTGTGGCCCCTGAATGAAGTACCAATCCCCTGTGCGCTTGAGCTTAAATCTGAACCCCTGACGTCCTTCGCAGAAGCCCCCGCTCTGTGTTTGTGTG 4920  
 CCTTTTTCTTTCCCTTTGGTTTCTCAGGTCAAATTACCCCAAATTCTTAATTTTTCATCTGGGATGGCCTAACCTGTGATGGAACACTGAGAATCCAGTCACATTAAGATCAAGAGCTGC 5040  
 AAGCTTGTTTTCGCTAATACTGCATCACTTCAGTCTCGTGGGTGCTAAGTTAAGTAATTTCGGTTCCCTTGATTTTTCTTTAACATACCATGGCTTTAATGTGTAACTCAACATGTTGCA 5160  
 TGCACATCAGCTATGCATAACATTTTATTTGTAAAACAATGTTCATAAAGTCGTTGTCAAACATTGCAGTAGTATTATGGTTAGGAGGGTCTGGGGGGGGCGCAAACACTCTTCTTTCTC 5280  
 CCTTTCAGATTTCTCATTCAGCTTATCTTGCTACTGAACCAGACAGCTTAAAGGTCTGCAGGGAGTCATTGGATCACTTGGTACTGCCAGCCTCTAGGGTTCATTTAATACATTGAAGAG 5400  
 CTGAGCATCAGAGGAAGAATAGCTGTGGTAAATGCTGCATCAGCTGTGGTAAACTACAACCATATTGATAAATTCAATCTCCCATCATATTGGATGAGTCTTGGTACAAGTGTGGGGTTT 5520  
 GGGTGGGGTGGGGGGGGCTTAAATTGTCTGTCTTTTTTCCCATTACAGCCATAAAATTGGCAAGAAAGCCTTCACTGTCAGTTGACCCGCGAGTCCTGCTGAATTGCAGAAGGGAAAATC 5640  
 GCTTTGACAAGGGATTCTGGTATCGTGGCTTTATTATGGCTAAACGGATTTCGTGGAATAATACACCAATACAGTGTCGCTGTGGGGAATTTTGGGGATTGGGGAAACCAGATGTCTCTC 5760  
 TCTGTTCTAGTTTCAGCCTCGTGACCAAGAGAATCCCCGTGTGGTGACAGCATAATTAGCATCCAGGTGTCGGG

TTTTCTTTT

TTTTCTTTT  
Depth:19 (ZEBRAFISH)  
Ei-value:0.000, Pi-value:0.000  
Er-value:0.000, Pr-value:0.000  
MATCHES To TargetScan▶ miR-186-5p:AAAGAAU

CCTGGAAGAACAAATTGTTTTGTTCTTC

CAGGTTTTG

CAGGTTTTGCTTT  
Depth:19 (ZEBRAFISH)  
Ei-value:0.000, Pi-value:0.000  
Er-value:0.000, Pr-value:0.000  
MATCHES To TargetScan▶ miR-330-3p.2:AAAGCAC▶ miR-490-3p:AACCUGG

 5880  


CTTT

CAGGTTTTGCTTT  
Depth:19 (ZEBRAFISH)  
Ei-value:0.000, Pi-value:0.000  
Er-value:0.000, Pr-value:0.000  
MATCHES To TargetScan▶ miR-330-3p.2:AAAGCAC▶ miR-490-3p:AACCUGG

TGCCCTAACTAGCAAAAAC

AAAAAGCAAAA

AAAAAGCAAAA  
Depth:19 (ZEBRAFISH)  
Ei-value:0.000, Pi-value:0.000  
Er-value:0.000, Pr-value:0.000  
No matches to TargetScan

GACTCCGGTGGTGGCACTCCTGACTCTGTCGGGACGGGGTTCAATTCCCTGCGGTGTCTTGCTTTTTTAAATAAGCTTTGTGGGGC 6000  
                                                                                                                          6000
```

---

## >STICKLEBACK (6660 bases)

```
 GTTTGATCCGGTTTTGGACAGTGTTGCAGGCAAGATTCATCCGCGAAGCCGTTACTGTGACGTCAGCTG

TTTGGG

TTTGGG  
Depth:19 (ZEBRAFISH)  
Ei-value:0.000, Pi-value:0.000  
Er-value:0.000, Pr-value:0.000  
No matches to TargetScan

CTGTTAGGTATCATGGGATTTTAACTCTGAATTACTGAACTACTT 120  
 TGCGTATCGTATATTTCCAAATAGGAGTCAGCAGACAACATTAAACAATGAGCATAATAATATCTATGTCTTACCGAGACAATTTAGATGGGATCAGCTATTTCTTTTCAACTACACTAT 240  
 TTATCATTAATATCGCCGTATAGCCTAATTTAGTAATTTGCTTTAATCAACGTTCTGTTCTGAGCTTCAATCTAAGGCCAGAATCAGCTTTTGTAAGCGTTTAAGTTTCAATGTGGGAAC 360  
 TACTTTTTTCTCCGGATGAATATCTTCTCTCGCTAGAGCCGGATCACCCAAACAGTATAAATACCACTATTGACGCCATTCCAACTCAGACAGCATTTTCCACGACTTCGTTTGGTGCCG 480  
 TGCGGGCAGGTTAAACCAATCTTGTTTTCTTTGTTTCATTGTCTGCGAGTCTCACTTGTTATGGGCGAGAATATCTCGGTTTAAGCTATTTTACTTGACTTACTTGATACTTTTTTGTTG 600  
 TCGTGGTGTGATCAGAGCAGTAGGCCCTGTATCGAACGATCAGCTTCAACCCTTTTGAACGTTGATCTATTTTATTTTTTAAGCACAGCCTTTTTGTTTTCTTTTGAAATATGTTTCAGT 720  
 GGGTAGCTCGTCTAATCAACCCTCCATATCATCTCGATTTCGGCGTTAGCTTTTTCTATTTTCTCGACGCGAGTTGACGTTGTATGGTCAACGGCGGGCCACGCGAGCCGAGTTTATGCT 840  
 CACTAACGTTAGTTACGTTGGTAACGTTAGTTTAAAACGTTAGTTACTTTCTAACAGTTTGTTAACTTGTTAACAATATCCCGACTTGTCCTAGAAACATTATGTTCTGGTTCATTTTCA 960  
 ATATAACGACGATAGAGCCATTTCAAGTTGGTGCTTCTAATTTTGGTAGCCATGACGTGGCAGCTTGTAACCGTTAGCTATTGGTTGGTTCGAGCGACTCGTGACGCAACTAGAGGGAGT 1080  
 GGTTAGGGTTTGACCTGCGTCCAGGCTAGCTGTGTCCGCTAGGCTACAATATTGGTGCTAAGAGAAACCTTTTTAATAACGTTAATCGTTTAAACTGTGCGCAATGAAGATGAGATAATG 1200  
 GTCGATCGCGATAGCCCTTACTTAAAATATGAAATATAAAAAGCGACAAATAGTTTCAAGTTGTTCGCATAATATCTAAGAAAGCCAGGGATAACCTTGAAGGGTCGCCGTGTGAAAGGG 1320  
 GAAGAGTTAAGTGGGGAACTTAGCCAACGGAATTTGAGGAACGAGTAGTTGACAAAGAAACCCGGTTGTTTTGGAAAGGATCAGTCACGGCGATCGTATTTGTAGACTCTAAATATGGTG 1440  
 TATGAGGTCCGGGACTTGTGTCTTCGATGTATACCGTCATCTAGGCTGGCTAATTAATTTTTATTCAGTAATTTCTAGTTAATCTTTTATAACGGCCATTTTTCTGGTTTGACGTTATTT 1560  
 CACATTTTATTTCCGTAGACCAGAGAAACGTACCAGTTGTCAAGGTACCAAAGCTCAAATAACCAAAATAGAGCTTTAGTTGTAGCACAATGAAGACGGTGTAGACCTTTTTTTCCTTTT 1680  
 AAGTTATCACCTTTTTTTTTTTTTTTTTTTGGCTGCCTAGAGAAGACAGAAGAGTTTTTTTTTTTTTTTTTCCTGGGGAGGGGAAATGGACTGAGTTGTCAAGGGGAGGAAATGGACTTA 1800  
 GTTAGATGAAGATCGCAGGACCAGAGCGGCGCGGGGCCATGGGAGACGGGAGGACCAGTGTGTGGAGCCAGGCAGGACAGGACCAGAGGGCGTGGGCGGACCACAGTGTTTCCAGGCAGG 1920  
 ACCAGTGGGCGTGGCGGGAGAAGCAGAGGGGCCCGAGAGAGCGTGAGGCAGCTACAGCGGCGAGTGTACACCAGAGGGAGCCAGAGGCGCAGTGCAGGGTAGCAACGCCGCCAACCGCAG 2040  
 CAGAGTGGCCATCGCCGAAGCAGTTGCAGCAGTTCCAGAGCAAGCCAGCCAGTGATCAGAGGGTGGGACGGGGCTCAAGTGTCTTCTTGCTTCTCAAGTTTAAAACGAGGCGGTGGTTTG 2160  
 TATATTGGGCAGCGGTATCTCTCACATTCACAGGCATTTAGTTATTATTCCAGAAGCAATTGTAGAACACAATTTCTCGCACAGATTTCTTCCTCTTTTCAGGCAAGCCGTGTAACGCGT 2280  
 TGTGTACCGTGGTCACCCCTCCCGTTGTGCGCTGACTTGAGTGTGGTGCTGTCTGCTGTGATGAATGCCGTCGTAGTGTGCTGGCGAGAGGAACATCATGTCGGACCTTCTACTCCCCCC 2400  
 TTCCCTGCGATCACAACTAGTTCCCCCCCCAAGCCGGAGGCAGAGGCGCAGTCCCTTTGTCGTCCGATAGGTCCTCCTCAAAGAAGCTTTCAGCATTGGCGTGTGCGAGTGGATGGTCTC 2520  
 CTGTCCGGACGGTTGAGTGTATACTGCAATCTTCAGTACTTCAGCGTCTCATCAGTGGTACAGATCAGCTGCATCTACCTCTTAACCCGAGCAGTGAGTTAATGCATGAAATGTACATGT 2640  
 GGGTGTAACTATCCTGTACATCAACTCCTCGTTTCCCATACTGTTTAGTCCATTTTGCCATCAAAAGGAGTCATGTTTTGATAGTTAATCCTAGATAAAGAAGAACTGGGGTTAGGGGGA 2760  
 GGGGGCATTTAAAACGTGTTTCGAGTTTTCTTTCCACAGGCATGGCCCCCTAAAGCTGATCTTCAAAAGAATCTCCCCCCAATTCTAACCACTTGGTACAAGTTGAGCTCATCTTAAGTT 2880  
 TTATATCGCGGAGGATCGGGATCATTCACACCCGCTTTAAAGAGATGTAGGAAGGTATTTGGTATTGTTCAATACATCCATTAGATTCTTTGGAATGTGTTGTAAACTGGGTTTTATGCA 3000  
 CATAACTTTGGAAGTGGGATGGGGGGGGTTGTGGGGCGTTAATCAGTCTCTTCCCTAGACTTCACAGCTCCATGTTGCCCAACGCAAAATGGCTTGAATAAGAAACCTCCAGGCGCACCC 3120  
 CAACAAGATTCAAGTGGACTTTTTGCCCCGCAGGATAGCAACTTCAAAACTAGTCTCCTGGTGTAGCAGGAAGCTCTGTTTTTCCAGGGAGTGTGGTAACAAGTCTCTTTACTACGAGTC 3240  
 ATTGGAATTCTGTACAGGATTTGCTTTGTTGGGTTGGGAGGGGCTTGTGTATTTGTGACTCTTACAGATTAAGTATTATGGCCTGTCCTTGAAAAATCAACATTCAAGTCCACGTCTACA 3360  
 GTTCTTACGGAAGGAATCCGTCGCGTCCCGGCCTGCGGTATTTGAAGGTTCTTCTGGGGAAATTTCTTGAATCTTAATATGACTTGTCTTCATAGTGCTAGTTGTCCTTATTACGGCAGT 3480  
 ACTGTAATGAAAGTAATCTAGTTCTGTACTGCATTATTGGACGGGTATTGTGGGGAGTGGCTTGTGTCCTTTGCATTTTTCAGTGTGCTTCCTGTGCGCTTTCCAATACCCGAGACCACC 3600  
 AGGAACTCTCCTAACTCATTGTTTGTACAAGTTTCCTTGCGCAGCAAGCATCACGTAAGGTCACTAAACGTGTTCATATTTCTCTTAACATTCATTGTCTGGCTAAATACCCTCCATTGC 3720  
 AATTCCTTTGCAATTAGTTGCTATGCCTTGTATTGACAATACAATGAGTGAACTATCTGTAAAGGATTTTCTTATTGCGTAATAAATGCATAGCTTGTTGTGATTTGAATACACTTTTGG 3840  
 TATGCATTTGTTCT

TTTGGG

TTTGGG  
Depth:19 (ZEBRAFISH)  
Ei-value:0.000, Pi-value:0.000  
Er-value:0.000, Pr-value:0.000  
No matches to TargetScan

GGGGCCTTGTGTGGGGCAGCTTTTCTTGTGC

TTTTTCAG

TTTTTCAG  
Depth:19 (ZEBRAFISH)  
Ei-value:0.000, Pi-value:0.000  
Er-value:0.000, Pr-value:0.000  
No matches to TargetScan

GTATGTCTAAAGCTTGGAGCTTGGTAGAGCACGGATGTGACCAGGGCTTGAATGACATCCT 3960  
 TTTCAAAGTGGAAATTGAAGGCA

GATAAG

GATAAG  
Depth:19 (ZEBRAFISH)  
Ei-value:0.000, Pi-value:0.000  
Er-value:0.000, Pr-value:0.000  
No matches to TargetScan

TGGAATCCATCAGATATTGATGTTGGAAAGCCGAAAATTCTGAACACAAGAAGAGTCTTTATCTTAACTGGGCTCAACTGCCCCTTGTTCT 4080  
 TGACATGAAAGCAACTTGCTATACTTGACTGTAGAAATAGCACTGTTGTGCTTTCTCGACCAGAGTCTTGTGATAACGAGAAGATTTGATGTGCCCTCATTGACTCTAACGTTATCTGCA 4200  
 GTAAAGCATTTGGGTAAGGATAACAATGGTCAGTTCTACGATACTGTGAGGGGGGGTTAGGGGTGTCTCTTGTCTGCCTCTTTGCTCTTACAGAACATACCAAAGGAAGAATGCAGTCTA 4320  
 CCTAGGACCGCACGGGAAACCCTCTGCCCGTTGAAATCAACCAACACGCAGTTTGAACAAACTGAGTCAGGTTTTTTTTCTGTGTAGACTGGAACGATTAGCCCTCAGCTGCTCGTCCTT 4440  
 GCGCAGCTGTGCAAGTGACAGGTTGACTATTCCAAATTACTTGCAGTCGCGCAACTGTGCGACACTTTTCCAAATGTTAAGTAGCTGAGGAAAGCTGCGCATTGTGCAGCTTTAATTTGG 4560  
 TCTGAGATGCAGCGCAACGGTGTTGCTTTAGTGTCGTCTTGTGTTTTGGTTGGGTGTATCCCTACACTTCTCTTCACAGACATCAGGTGCAAGACTGGTTCAAGCACGAGTGAAGTCCTC 4680  
 TAACCTGGAGCATGTAGTAAGATGTTGGCTTACCTTTGTCAAAATAAACTTCTGCAATGTTTTAGTTGACAAACTGACATCTTGGAACTGGCTTTATGTTAACCTCAAATCAACAGTAAC 4800  
 GACAATGCTTTACTAGCATATTCTCTTTGGCCATGGAGCCCTACTACTTTGCATCTTAACCGTAAGATGAGTAGAACCTTGAACCTCTGAGTATTTTGTGGTGGGTGTTGTGTAGATCAC 4920  
 TCTTTGTTTCAGATCATTTTCGTCTGTCCGCTCCGATCTCGCCTGCCTCTGAATGCAAAGCCATCTGTGGGAGCTGTCCGCACATATGAGTGGTCCCTGAATGAAGTACCAACCCCCTGT 5040  
 GCGCTTGAGCTTAATCTGACCCCCCCGACGTCCTTCGCAGAAGCCCCGCTCTCTGTGTTTGTGTGCCCTTTTTTTTTTTTTTTTGTTCTTCCTCCCTCTGGTTTCTCAGGTCGCATTTCC 5160  
 CCCTGAAATTGTCTTAATTTGTCACCTGGGATGGCCTAACCTGTGATGGAGAATTCACTCGCATTAAGATCAAGAGCTGCAAGCTTGTCTCACATTTACTGCATCACTTCTGTCTCATGG 5280  
 GTGCTAAGTTAAGTAAATTTGGTTCTCTTGATTCCTTTTTGTTTTCCTTTTTAACATACCATGGCTTTAATGTGTAACTCGACATGTTGCATGCACATTGGCTCTGCATAACATTTTATC 5400  
 CGTAACACACGACATACTGTTCATGACGTCGTTGTCAAACATTGCAGTAGCATTTGAATCTGGGCGGGGGGAGGGGAGGGGGGCGCGGGCAACAAACATTCTTCTCTCTTCCTTTCAGAT 5520  
 TTCTCGTTCAGCTTAACTTGATTCTGATCCGGGCGACTAAACCTCTGCAGGGAGAGTCGGAGTGACTTGGTACAGCCTCTAGGATGCGTTCTTAATGAATCACTGAATAGCTGTGGTAAA 5640  
 TACTGCTGTTGCGGTCCACAATAACAGCATTGACACAACTTGTCATCGACGAAGAGTGGGTTGTGTGGGGATTCTGCGGGTGGGGGCGGGGCTTAAATTGTCTGTTTTTCTATTGCAGCT 5760  
 GTAAACTTCAGTCTCTGTATGACCCGCAAGTCCAGCTGAATGGCAAAGGGGAAATCCTGTTTGCGTTTCACCAGGGATTGTGGTATCGTGGTTAAAATGATGCAGTGGATTCAGTCTTGT 5880  
 AACGATGCATTTTCGTGGGGAATGCCGGGGATTTGGGGAAACAAGTGTCTTGTCTTCTAGTTTCAGCCTCGTGACCGAGAGAAATCGTCATGTGGTGACAGGAAAATATAGCCGCCAGGT 6000  
 GTCAAGTGG

TTTTCTTTT

TTTTCTTTT  
Depth:19 (ZEBRAFISH)  
Ei-value:0.000, Pi-value:0.000  
Er-value:0.000, Pr-value:0.000  
MATCHES To TargetScan▶ miR-186-5p:AAAGAAU

CCTGGAGGAACAAACCACTTCGGTCTTTTGTTCCTC

CAGGTTTTGCTTT

CAGGTTTTGCTTT  
Depth:19 (ZEBRAFISH)  
Ei-value:0.000, Pi-value:0.000  
Er-value:0.000, Pr-value:0.000  
MATCHES To TargetScan▶ miR-330-3p.2:AAAGCAC▶ miR-490-3p:AACCUGG

CGCCCCTTTAATACAGCAACAACA

AAAAAGCAAAA

AAAAAGCAAAA  
Depth:19 (ZEBRAFISH)  
Ei-value:0.000, Pi-value:0.000  
Er-value:0.000, Pr-value:0.000  
No matches to TargetScan

GACTCCGGTGGTGGCACT 6120  
 CCTGACTCTGTCAGGACGGGGTTCAATCCCCTGCGGTGTCTTGCTTTTATGACTGCTGTTTGGAAAATGTATTTAGCCATTAAAGGCTTCCATTTGAAGTCAATTTAATAATTATTTGAC 6240  
 TTGAATAAATCAACATGACATTTAACTTGTTGAGTTGTACGGCTGAGCTAGCAATCTTACTATTGCACATTTTATCTCCCGAAAACCTATAACGTTTATTAGCCAACTTCGACATGTAGC 6360  
 GTTGGAACTGTGTGCTACATTGTAAAATGGTCCATGTTTTTACTCTTCCACTTTTAACTTCACTTGGTTTAGAAATTTACCCCCATCAAATAGTCTCTAATAGAAATGCTTTAGCGTTGG 6480  
 AGGCTTTGGACAATGAGATGTATCCATAACTCTCTCCTGTGTTTCAGTGTCTGCGAACTAGTGATCTGACAAATGTCCCTTTATCAAACTTGTTTCCTCACGTAGGTTGTAAGACTTGTT 6600  
 AATGCAGTTTGGTCTTATTCTAAGCATCTAATAAATGGAGTGAATCAGTTAACCTTGTCT                                                             6660
```

---

## >MEDAKA (5654 bases)

```
 TTATTGGTCGTTTGGTGACATGATTTTATTCATATAATGTTTATTTAACGGCGCTTATTTCAATTAATATTTTTATTCGAACAATGTTAAGCTTGAAGCGATCTCTTTTGTTTTCTATAG 120  
 TTTGTCGAGTTTGAACGGTTTACTGAACTGTTTTCGCCATTGGGCTGGTGGGCCTTGTCTTGTTGAAGGCATTTATGACGCATTTTTTCCCCCTTGGTTTTTCTGTTGTTGTATTTAGGC 240  
 GACTCGGCAACTTGTAAGTAATTTTTGGTTAGTATTTTTGAAGACTTGACTGCTTATAACCGTTTTTTTCGAGTTACATAATGGACAGGCTGTTAAAGATGGGCTAGGCCGTCCTTCGCC 360  
 AATGTGGCTAGTTAGAAAAAAAAACCTACAAGTAAGATTTTTTTGATATAATTTTTTTTATATATATTTAATTAACAAATGTTCTGCTTTTAAAATTTTTGCTAACCATTAGCTAAGATC 480  
 ACTAGTTAACAATTGACAATACTAGTGTTAACGTAGCATTTTTCATAAAGCTGTGATTGGTTAGCTTGAATAATGCTTCAATGTCAGTGGACGAAAGTCGAAAGGGCGTGGTCAGTTGGC 600  
 TTTTTTTCATTGTACTTCTGTAGCTATGCAGATTTTGATAAAGAAGATAACGACTTTATTTGAAATATTCTTTCTAGAATTAGATTGACTTGAACCGTTGCAAATAAAATCGCCATACAT 720  
 TTGTTAGGCGGTCATTATTTAAAGGAGAAATCTTCTCACTCACAAGAATAGCTGAAGAGGTTCACCGTACCTTTCGTGACGACATATTTTAAGAGCAATTTTACATTTTGTTACAGAAAA 840  
 GATAATGGGTCGGTAATTTTCCCCTTAATTTTACTAATTCTAGTTTTTTAAAGTTTTTTTATTTTTTCATTTAAGATGTGTTGATGAAATAACACTTGAAAGAGGTTTTTTTTTGTCAAA 960  
 CCTAAGATGTGTTAAAGAGGCACATGAAGAAAAATTTTTTTTTCCCAAACAAAGTTTCTACAAGAGTTCAGTTTAGCTCAACAAGTCGAGCCAAAATCGATTAGATTGGACGAAGAAATC 1080  
 TTTTATTTTATTTAATTTTTGGATGAAAATGGACTAAGTCACTCAAGAGTAAAAGGACTCAGATTAGATGATCGTGATTGAAGAATGGACTGACGTTAGATGAAGATCGGGACCAGGGAA 1200  
 GGCGATAGGAAGCGGAAAAGGACCAGAGTGGAGCCAGGCAGGACAAGAGAGCGTGGAGGCGAGAGCAGAGGGGTCCAGGAGGAGGGTCTTCAGGGGAGCTGAGAGGTCCAGGAGGAGGGT 1320  
 CTTCTTCAGGGGAACAGGAGGCGTCATCACGCGATATCGGCATGTGTACACCAGAGGGAGCCAGAGGCACAGGGCAGTGTAGCAGGAATGGTGCTACGAAGTTCCAATGCAAGCCAGCCA 1440  
 ATGCTGAGGGTGGGCGTGGGCAGTCTACTTGCATCACAAGGTTAAAGCGGGCCCTTGGTTTGTATATCGGGTAGAGGTATTTCTGTACACAAGCATTTGGTATTTAAACTTTTCAAAAAT 1560  
 GTTTTGTAGAACAATCCTTCTTGCACAGCTTTCTCTTCCTTTTTTCAGGCAAGCCGTGTAAAACGTTTTGTACCACGGTCACCTCTCCTCTTGCGCGCTGACATGG

TTTGGG

TTTGGG  
Depth:19 (ZEBRAFISH)  
Ei-value:0.000, Pi-value:0.000  
Er-value:0.000, Pr-value:0.000  
No matches to TargetScan

TGATGTCT 1680  
 GCTGTGATGAATGCCTTGGGTAGTGTCCTGGCGTCTGAACCTCATAATTGGACTCCCCTCTTCGATCAGAAGTAGTTTCTCCTCATCTGGAAGGAGTGTCAATCCCTTGTTGTTCGATAG 1800  
 TCTTCCGCTGATGTTCAGCATTGGAGTTTGAGTGGATGGTCCTCTGTGCACCTGGTTGGCGTATATTCAGCACCGCTTCGGTACTTCTGCTGCATCTCAATGGTACAGATCAGCTACATC 1920  
 GACATCTTAACCAAAGCAGTGAGTGATTGCATGAAATGTACGCGTGGGTGTAACTATCCAGTACATTGGCTCGTTGCCTTTGTGGTGTGCAGTAATGACAATTTTAAAGAGATTCTTTAT 2040  
 TTGAAATGGTTTTCCTTTCAATGGGAGGGGAGGTGGGGGGGATTTAAAATGTGTCTGGAGTTTTTCTTTTCACAGGCTTGACTCCCCCCCTCTTAAAGCTCATCATGGAAAAAGTCCCCC 2160  
 GTGCTAACCATTTGGTTGATGTTGACCCCATCTTGCGTACAGTAAAGTTATCGGATTGGAGATCATGCGCATCTACAGTGATTTAAGATGTAGGAAGGTATCTTGTATTCAACAAATCCA 2280  
 AATTTTCTTTGCCATTTCTCGGGGTTTAAACCACACTGGGGGTTTATGGGAGGGCTGGGCTGTGGGGCGTTAATCAGTCTCTTCCCAGACTTCACAGCTCCGTATAGGCTCCTGCCAAAT 2400  
 CGTGTGAATAGGAAACATCCAGGACCAACCAAACAAGATTCAAGTGGACACTTTACCAAGCAGCATAGCAACCTGAAAACTAGTTTCCAGGTGTAGGGAAGCACCCTTTGCCCAAGGAAT 2520  
 ATGGTAACAAGTCTCATTACTAGCAGCGATTATCTTCCTGTTAAAGTTGCTATGTGGGAGGGGATGTGTGCTTCTGACTCTTGCAGATATTTATTTATGGCACATCAGTCAAATCTACGT 2640  
 TCAAGTCTGCATCCACTGTTCAAGAAAGAAACCCCCATTCTCAGGCCTGTGGTATTTAAATGTTCTTCACCAGTTTTTAGTTGTAACTGTATTAGTCCTACATTTTCCCATTTAAGGGTG 2760  
 TAACATTTGCTGTTACAGTGTTGGTGTTGTACTGCTTCGTGTATTGGCAATAGTATTGTGGGGAGGATTTTTTTTTCCCTTTACATTTTTCAGTTTGCTGTGTCCTGTGCGCTTTAATAA 2880  
 CAAGACATTGGAAGATCTTCTGGCTCATTGTATGTACACATTTCCTAGTCCATCGACTATCACGTAAGGTCATAAATTGTTTATCAAAACCTTTTTTGGCCACTTTTTGCACTTAGCTAC 3000  
 CAATTTATTGTACTGTCAAAATTGAGTGATTTAAATTGTAAAGGAATTTTATATTGCATAATAAATGCATAGCTTATGGCGATTCTACAGTTTTGGTATGCATTTGTTCTC

TTTGGG

TTTGGG  
Depth:19 (ZEBRAFISH)  
Ei-value:0.000, Pi-value:0.000  
Er-value:0.000, Pr-value:0.000  
No matches to TargetScan

TTG 3120  
 TATGGGGGGGTACTTTTCTTGTGC

TTTTTCAG

TTTTTCAG  
Depth:19 (ZEBRAFISH)  
Ei-value:0.000, Pi-value:0.000  
Er-value:0.000, Pr-value:0.000  
No matches to TargetScan

GTGTGTAAGTCTTGGGATCTTAGTAGAACATGGAAATGACCCGGGCTTGAAAGACATCCTTCAAAGTGGAATTGAAGGCA

GATAAG

GATAAG  
Depth:19 (ZEBRAFISH)  
Ei-value:0.000, Pi-value:0.000  
Er-value:0.000, Pr-value:0.000  
No matches to TargetScan

TC 3240  
 GATTTCTCCAAAAATTGATGTTTGGACAGCTAGAATCCAGCCGATGTGGGTCACTTACAGTCAGTGGGTGGTGTCTTTAAATCCTTCCTGAAGAAGTCTTTACTAAAACTTAACAGCTGA 3360  
 ATAAGCAAAGTTGTGCGTTTGCAATAATTCTTGATTTGATGGAGCTTCTTGATTTATCTCCAGCTAAGTATTTGGGTAAGGACAAGTCAGTTCTTGTTGGGTGGGAAACTGGACTGGGTT 3480  
 TTTTTTTTTTTTTGTGTGTACTTCTTTGATCCTACAGAACACTAAAAGAAGATTGCAGTGTACATTGGACAAGAACACAACATCATCAGCATGGAGTTTGAACAGACTCAGGTCAACGTT 3600  
 TAAAACTGTTTTGAAACCTAACGAAACAAAGGAATTAGGCCTCAGCTTCTTGCCCTTGTGCAAATGTGCAAGGCTAGGTTAGCCCTTCAAAACTAAATGTGCAACTGTGCATTTCTAAAT 3720  
 TAAAGTAGCTGAGGAAAGCTGTGCATTGTGCAGTTTAATTTGGAATTGCAGTGCAACTGCGCTGCTTTTGAAATGGTACACTTGTCTTGTATGGGCGTGTCCCTACGCTTTTCTTCACAG 3840  
 AATCACCAGTACGAGCCTGGTACAAGCACGGCTGCAGCCCTCTTAACCTGGAGTGTGTAGTAAGATGTTGGCTTTTCAAAAACATCTTAGACCCTGCTATACATTTACCTGAAATCATGC 3960  
 TTTACTAGCAATTTCTTAATCCATGAAGCCCTATTGCTTTGCATCTTAAGATCACTGTTTCGTAGAAATCTTGCATATTGTGTGGTGGGTGTGTTGTGTAGATCCCTCTTTGTTTCAGAT 4080  
 TTTTGTCATCTGTCCACTTCAATCTTGCCTGCGTCTGAATGCAAAGCTGCATCTGGAACCTGTCGGCGTTTGTGTTCATTCCTGAATGAAGTGCCAAACCACTGTGCGCTTGAGCTTGAA 4200  
 TCTGAACCCCTGGTGTCATCTGCAGAAGCCCCGCTGTTTTTGTCTTTTCTATCCCCTTGGTTTCTCAGGTCAAATTGCCAGAAATTTCATCTGGGATCTGATGGAAAACTGAGAACCCTG 4320  
 TCATTTTAAGATCAAGAGCAGCAAGTTTGTTTCTAATACTGCATCACTTCACTCATGGGTGTCAAAGTTAAGTAATTGGTTTCATTTAACATGATTTCTACCATGAAGGATTTTATTTAT 4440  
 AGCATGCAATTCCGTTCTGCCTAATCATGCTTAATGCTCATTAAGTCCTTGTCAAACCGTGTAGCTGTGGGATTTTTTTGGGGGGGGCAACACTATTTTCTTCTTTCCCTTTCAGATAAC 4560  
 TCATTCAGCTTAACTTGCTACTGAGCCAAGCAACATACATCTGCGTTGTCGTGACTTTGGTACACCAGCTTCTAGGCACCATTCTGTAATATGAAACAAGTGATTGGCCGTCTTTGAGGA 4680  
 ATAACAGTTGTGGTAAATGCATCAGCTGTATAATGTATTGACAACTCGTGCTTGTATAGGGAGGGTTTATTGTGGGGTGGGGTGGGGGCTTTAAGTGTCTTCTCTTTTTTTATTGCAGCT 4800  
 TAAGCTTTGCAGGAGAGCTTTCACTGTTTACTTGACGTTGGAAGTCCTGACAGATTGAAAATGGTCATCAGAGACGCTGGTATTGTGGCCCTGTTTATTGAAATCAAGGCATTTATGGAA 4920  
 TTTGTTAACATCTGTTTTGTGGAAATGTCCGGAACTTGGAAATGAACAACTCTTTAGATGAGAGAAAACAGCAGCTAAATGTTTAGAAAGAAAGGG

TTTTCTTTT

TTTTCTTTT  
Depth:19 (ZEBRAFISH)  
Ei-value:0.000, Pi-value:0.000  
Er-value:0.000, Pr-value:0.000  
MATCHES To TargetScan▶ miR-186-5p:AAAGAAU

CCTGGAGGAACAAAT 5040  
 CATTGTTCCTC

CAGGTTTTGCTTT

CAGGTTTTGCTTT  
Depth:19 (ZEBRAFISH)  
Ei-value:0.000, Pi-value:0.000  
Er-value:0.000, Pr-value:0.000  
MATCHES To TargetScan▶ miR-330-3p.2:AAAGCAC▶ miR-490-3p:AACCUGG

TGCCCATAACATAGCTATTAG

AAAAAGCAAAA

AAAAAGCAAAA  
Depth:19 (ZEBRAFISH)  
Ei-value:0.000, Pi-value:0.000  
Er-value:0.000, Pr-value:0.000  
No matches to TargetScan

GACTCCGGTGGTGGCACTCCTGTTTTACTCAGGACGGGGTTCAATTCCCTGCGGTGTCTTGCTT 5160  
 TTTATATGCTATGTGGTGCTCAAAGATTTTTGCAAGAATGGGGATCCCTCCCATGGTGTAACCATGGAACCGGATGTAGACGATAAGCACATGTTATTACCTGCTGTTTAATGAAATACA 5280  
 TGGTTCTTGGACTTGTAATTCAGTTTTTTGTTTTGTCGTGGAGCTGTATTTGCTGTGAAGTTTTTAATATTGAAATAAATGTAAAAAAAAAAAAAAACTTATAAATGAAAACTGGTTTGA 5400  
 ATTTATTGAGTTTCTCAGATGATCACATGAGAACAAACTTTATTTGAAAGGTTAAATTAGAGCCTCTCAACATACCATTCTATATGTATAACATAAGCTGTCAATAAAGTCACTTAAATG 5520  
 GCATAAGGCAGCTCATGTGACAGTATTTCATCCTCCATCCCTATTAGGCAATTACCTGTAACCCTTCTGTCAATTTGAAAGCAGTGAGAAGAAAAGTGGTTAAGATGAGGCAAAGTTAAC 5640  
 CAAGCATTGGTGAT                                                                                                           5654
```

---

## >ZEBRAFISH (7477 bases)

```
 ACTATAAAAGACGAGGCAACAACATTGTGCGTCACGACGGGGTGAGGCGCTATGGAAGGCAGGGAGGCTTCGTTGATCTGGTGAAATGTTCTTATTTTGTTCTGTTTAATTTTTTGATAA 120  
 CTAATAGGCATTTTTATTTACAAACCTTTAAAGTCTTAAAACTCTAGACGTTTTCCGTTGGTTATACAAAGGTTTTCTTTTGAGTAGCAAGCGTGACGAAGATAAAATACAAGCTTACTT 240  
 TTCACTTAAATAGGTAACTTAGAAGTGCCTTTATGAGAAAAAATTCATTTTTTGTTTATCTTAAAAAGACAAAAAACGTCTTATTTAGTCGAACATTTAACGTTTATCTTCAGGTTAATG 360  
 TTTAACTAGCTTACGTTACCGAAAGCCTAAACAAAGCTCGACTGTAAATGGCTAAGCGAACGTTATCCTTTTCACTTAACCTCATTTTAAGCTTATTTTTGTTTCAGCAAGGGTTTTCTG 480  
 AAATTACGTGATTTTATACACTTATTTTTTTGACGCTTGTTTAGTTTGATTCTCGACGCTAGAAAGAATTTACAAAATGGCGGTCGCGACGACGGATTAGATTTCGTTGAAGAAAAGCTG 600  
 TTATGATTTCTCTTTTGTTTTCAAGTCAGAACAACCAATAACAGTTTATATAAACCTATAGAGTTATATTAGACCAACGTTATATACAGACCTACATTTTATTATTTATTTTTCTTTTTA 720  
 CAATACCGATTAGTAACAGCCTTTTATGGTAACAGTAAGGACCCAGGGTATCTACAACCATTTTAACTTGTAAATTTTCAAGGAAAAACTTTTTGTTTCTTTTTTTATCTTCAGACCATA 840  
 ACGAGTAATAGCAGCCTTTTATGGTAGTGAGGTCAAACAGCAAGTCTTTTTTTTCTATCTTGATATTACGATTAGTAAGTTGACCCATTTAGCAAGGATGAACTTAAAAGCAACCAGTTT 960  
 AAATTAGATGGAGGGTAAACGACTACAAGGTTACAGTAGATTTTACAGTAGAAAAAAAAAATTCATTTTTTTGTCATTTAGGTTGACTAGCAAAACCACTAACGACCATTTCAGTACAGC 1080  
 AAGGAAGAGCATATTTAAGCTACAGACTTTCTTAAAAAAATATATATTTTAGTATGCCTGCCATAACTTGAAAATCAATTACAACTTATATTTCATTTAAAAAAAAAAAAAAGTATTTTC 1200  
 TTTTAATATACCGCAAGTCCATTGCAACTTAATTATGATGGCAAACATTTTAAAAATGTATTTCTTTTTATTATAACAGCCATAACAAATGTTACAAGTTTATTATGGTAGTAAACAAAA 1320  
 AAAATCTTTTTAATATGCCAGCTATAATGTAAATGTGTTACAACTTCATTATGAATCCAAATCAGTTTTATTAAAACAAAGCAGCTGCTTTTGTTAAAAAAAAAAAAAAAACAGCAGAGA 1440  
 TTAAATAAACCGATCTACAAGACTAGATTTTTCTAAAATCTGAAAGAACAGTCTAGAACTGCCAATGAGATAGAGGATTAACACAGTCAAGACTGCAGTAGATTTTCTTAAACCAGTTTT 1560  
 GTTTTTACAATTTTAACCTTTTAATGGTCAGGATAAAAAAAAAAAACACTATTACTGAAGATTTCAGTTTTCTTTTTAGTTTGCATACACTTAAGGTATAGTGAGGGCAAACCATCATTG 1680  
 CTATGTTGTAAATCTCATTTTATTTTAAACAGTCATTTATTTATTTTTTTACCCGTTTTATATACAGTAGTACAAATAAATAACTACAATCTAAGGCTACAGTAGATCTTAAAGTTATTT 1800  
 TGCTTGTAAACTATTACACAATAATAACCACTTAAGTAGCAAAAACAGCCTACTGGAGATTTAAAAAATAAAAAAAAAAAGAACTTTTTCTCCAACCCAAAACTTAGCGGCTTATATGAT 1920  
 AGTAAGGGTGAGGTTGTCCACAGTAGAAGTCAACAGTAGATTTTTATTTATTTTCT

TTTGGG

TTTGGG  
Depth:19 (ZEBRAFISH)  
Ei-value:0.000, Pi-value:0.000  
Er-value:0.000, Pr-value:0.000  
No matches to TargetScan

AAAGAAAACTTTTAATTTAGTATTTCAGTCTCTTGACTAATTATAGAGGGTATCAATA 2040  
 ATTAGTCAAGATTACTTTTTTTTTTTTTTTAAACTACAATAGAACAGTTTACGTTACCAAGAATGCAGTATTTACATTTTTATTTGTTTATCTGCAAACCTTATCCAAGAAATGAGGAAC 2160  
 AGCTTCTGATAGCCAGAGTGCCATTTTTAAGAGATTTTATTTTGTTTTTTGCATACCATAACAGAACAATCACCAACGCTTAAAGCCAAAAAAAAAAAGCCAGACAATTTTTTGAAAACT 2280  
 ACAATAGAACAGTTTACGTTAATCAAGAATGCAGTATTTACATTTTTATTTGTTTATCTGCAAACCTTATCCAAGAAATGAAGAACAGCTTCTGATAGCCAGAGTGACATTTATAAGAGA 2400  
 TTTTTATTTTGTTTTTTGCATACCATAACAGAACAATCACCAACGCTTAAAGCCAAAAAAAAAAAAAAAAAGCCAGACAATCAGATGTAAAAGTCTTTTTAAATATTACTTTTGAAAAAA 2520  
 AAAAAAAAAAGCCTGCTCTTGTTGCCTATTAGGAAGAAATTACTGAACATCAAGTGAGTGTTTTTAGTTTGGAAGACAACGATTATAGATGCTGCCTCCTTTTTTTTTCTTTTTTTTTTT 2640  
 TTCTTTTTTTTTTTTGAGAAGAAGAAGAATTGAGTATTTCAAGATTTAAATTTGCAAGCCTAGAAGAATCAATCAACAATGATGCTTTTTTTTTCTACTGAACCACGTAATTGATTGCTG 2760  
 TATGGACTGAGTCATGTTGAGTAACAGCAGTGAAATGGAAGCGTAGGTTGTTTTCATGATCCAAAGATATCAGCAGAAGACGGATGTGCAGAGGACAGGAGAAATGGACTGAGTTTATCC 2880  
 AGTAACAACATAGCTCTTAGATGACGTCGGATGGAAGAAAAAGATCTACAGCAGGGACCAGGGAGAATCAGGACAAGAAAAGATCTACAGCAGGGGCCAGGACCAGGGAGAATCAGGACA 3000  
 AGGTGGCGAGCCAGGCAGAACCAGGGGAGGATCAGAGGGCACAGATCAGGGTGAAGCGCAGCAGTATCCAGAAGGGGGAGCCGGGTCTACAAGGCAGAGTAGCTTCAGATCCACGTTGAC 3120  
 CACCAGGGGGAGCTCAGCTGGTCTAGATCAGAGCAAGCCAAAGATGGGTGTCCCAAAAGCAGCAGGAGAAAGAGGGCTCACAAGCAGAGGGTATCAGGGGTCGTTGCCATAGCACAGTCA 3240  
 TGAGGAGGGCATTTGGTTTGTATGGGTTGAGGTATTCTGTGTAACTTAAGTCATTATTTCAGTTTGTATTCCAGAACCCTTTGCTCGCGAAGTTCTGTTCTTTCCTTTTCAGGCGTGGTG 3360  
 AATGAATGTTTGAAAATCCGATATGGCATCTGTCCCGTTGTTGGCCAAGTCTGATGAATGCCGTTAGTGTGCTGCGGAGACGATCGTCCTTATTCGACCACGCCTCCGCTGAGATTTGGC 3480  
 TATAGGCCCGTGGAAAAGTCGTCCCCCATATCCGCTTAAATCCATTAGGTCTGTCCTGAGAAGCAATCCACGTTCGAAGGGTTTTTCGGAGTTGATGTTCGACTGCGTCCCTACACGACA 3600  
 CCACTACCTCTACTGCAACACCCGCTGCGTCAGTCCAACATATGATGCCAGGGCAGCCGCATATTGCCTGCCGGTAAAATTTTGATGAGTTCATGCATGAATTTTTATTGTAATTTAGAG 3720  
 CAAAAGCTTTGTATATTTAAAAAAAAAAAAAAGAAAAAAAAAAGAAAAGAGAAAAAATATATACAATACGTTTTTGCAAAAGTAAATGCGTTATTGTAAAATTATTAACATACAGATGTT 3840  
 GCAAATGCACTTTCTGAGTAGTTATGTAATGTTATTGCAGTGTT

TTTGGG

TTTGGG  
Depth:19 (ZEBRAFISH)  
Ei-value:0.000, Pi-value:0.000  
Er-value:0.000, Pr-value:0.000  
No matches to TargetScan

G

TTTGGG

TTTGGG  
Depth:19 (ZEBRAFISH)  
Ei-value:0.000, Pi-value:0.000  
Er-value:0.000, Pr-value:0.000  
No matches to TargetScan

AGGGGCAAATTCGTTTCTCGAGTTCTCTTTCCCACAGGCCCTGTTCACATTAGAGGCGATAAA 3960  
 ACCAAAAAGGGATTCCAGGATGTTGTGTGGACCCTTTTAGAGTCAGTTTGACTTCCCTGTAAAGAATTCTTTAAAAGCTTCAAGGAAAGCCTCTGATTTAGGAAGGTACCCCATTGGATT 4080  
 TGTTTTAAAGTCTTGTTTGGAATCATGAACTATACATTTATCAATATTTTCCAGTATTACATGTTGGGATTTGCAATGGGGTGGGAGGGGTGTGGGTCGTTAACCCAAATCTTTTCCTAG 4200  
 ACTTTGCAGCTTGTGAAAGCGGCTTTATTAAGGGTGGCAATGGCTTGAATAGCGCAGTATGGGGAAGGAACCACCAGTGTGTCTGCCAGTGGATGCGTGGCCCTGTTACAAGCCTTCCAT 4320  
 GGCAGCTAGACCAACGTTTACATGGGTAACGCGGCATGGGATCAAACGCTTTCATTTTATTTAACACATCATTATGGCCATTTTCCTATCATTATTGAATTGGTGTTTGGATATTGGTTG 4440  
 TGTATTTGGATATTGTTTATGGGGTTTGTGGATCTGGGGGGGGGGATTGCCTAATTTTGACTCTTACAGATTGAAACAAACCCTTAAAGAACAAAGCTGGTGGTCCAACCAATATCTGCT 4560  
 CCACTTCCCCCACTTGAGCAAAGCTTCTTCGCTAGAGGTCCAGATGCTTTTAACATTTTCTAATGGCAAGTAATTGTGGGTATTCATGTAAATGGCATTCATTTGCTTTTGTCTGTCGTT 4680  
 GTATTGTACTTGAATACAATTTGATGGCTAAGTGTAAGCAGTAACTGTATTAGTTGCTTATTTGTCTTTTTAATTTGTGTATTTTGAAAAGCGGGGATGTGTGTAGGCTTTCTTTTCCTC 4800  
 CCCCTCTGCA

TTTTTCAG

TTTTTCAG  
Depth:19 (ZEBRAFISH)  
Ei-value:0.000, Pi-value:0.000  
Er-value:0.000, Pr-value:0.000  
No matches to TargetScan

TGTGCAGTGTCCCTGAATGCTTTCAACCGAAGATGTCATCCTAACATTGTTTGTAAAGATTTGCTTTCGCATCAAGCAACTTCAGCCTCTTTTCGGTCACGT 4920  
 AAGGTCACTAAGCAAGTCCATTTGTTTCTAACCTTTTCCATTTTTAATAGTCTGTTACAACCTGTAAAATGTGTGGTCGCACACTTATTACAAATTAGTTTTGTAATTTGTATTTAAGTG 5040  
 TACATTCTGTAACTGTTCCCATTTTCAGTTAATAAATGCATAGCCTGTTGTGATTGAAATTTTGGTATGCATTTGTTTCTTGGGGGAATGTGTGGGGCTTTCTTGGGTGTTTCTTTATGC 5160  


TTTTTCAG

TTTTTCAG  
Depth:19 (ZEBRAFISH)  
Ei-value:0.000, Pi-value:0.000  
Er-value:0.000, Pr-value:0.000  
No matches to TargetScan

GTATGAGTGTAAAGCTTGGCGCTTTTTGTAGCTTGGATGCCAGGGCTTGATGTCAACTCTGTGCAAGGTGGAGAGGCAGATCAGTCCAGTCCTACAGCAGTGTGCGAAGGAG 5280  
 GAAAGGCAAATTGTATATACAAAATTTAAGGATATACCGTAAATTTGTAGACTCTTTGGCTTTAAGCCTGTGCTTTTGATATCTGAATCTTGCAGATTTGATGTTTTTACTTGATCATTG 5400  
 GGTGGGTGTAGGGGTCTTGTTGCTTTGCCATTTGGTCTTGCAGAATCTTGCAAAAGCCTTGAAGATTGGCACCTATCAGAAGAAGTTATGACCTGAAATCGCCCGCTTCCACTGAACGCC 5520  
 TCATCAGGCCAGACAGTCGTTCTATAGATTCGTAACCTTTTAAATTTTCTTTAACTGCCCAGCCACTTCTCTGCGCAACTGTGCAGTGACAAATGCTTTACAAATGTATAGCAGCAGTCT 5640  
 TTATTTCAGCTGTCGCACAACTGTGCTTCAGATTCCAAATTTAAAGTGGCTGGGAACGGCTGCGCAACTGTGCAGCTTTATTTGGCATTGGCTCAACAGCCGTACATAAACTGGAAAAGA 5760  
 TGTGTTTGGGGTGGGAAAGGGGATCTGCACTTTTCTCTCTCTTCTGCAGAAATCTCATCAGCCAGCTGTGTGGTGAAAGTTACATGATGTCGCCCTGTGGAGTCTGTAGTTACCAGGTTT 5880  
 CTTGAATCTTTAACTTGATGGTAGAATCCTCATGCAACTTTTTATAAACTTCAGTAACATCAATCGCCAATGCTTCAGAAGCTACACTACCTTTATGCTTCATGCACTTTAAGTTTCTTT 6000  
 TACATCTTAACTCCTCAGTTGAGTCACAGTTAGTATTGCAATCGGTGTGTGGTGGAAGTGTTTGTGTGTAGAAGCTCTTTGTTTCAGATTACCCGTCTGTCCCCCTGCCTTCTCGACTGC 6120  
 ATCCGAATGCAAGGCTGTCTGTGAGAGCAGTTGTCCCCGGTGAATGTCGTCTTCACTTGAATGAATGGCCAATCGAATCCCCCCTTCTGTGCGCTTGAGAGCTTTGTCCAAACCTTGGAA 6240  
 GCCCTTCGCCGAAGTCCCTCTGTGTGTCTGCGCGTCTTTTTTTTTTCTGTTTATCCCCCACCTCCCTCTCCAATTCTTCATTTTCTTAGGTCAATTTCATTTAGGGAGCAAAATTGTCTT 6360  
 GTGTCCGTCTGGGAGAACCCTAACCGCCTGATGAAACCCATCCACTCGCTCTGATCAACCTCATTCGTCATATTTTGCTGCTAATCGTGCATCCCTCTGCTGCTATCTGCTGAGTTAAGT 6480  
 ACATGTAGTTGATCTTAATCTTGTGTCTGGGTAGAAGCATTCACCCTTTTTATAGATATGCATGCACATAACACTTTAAATCAAACCGTATCAGTGTGTGTGTTGGTCGCTTTGGGGAGG 6600  
 GATGGGGCAACACTTTCTCTCTTTTCAGGTACGCTGTTTGTGATTCCTCTAAAAATTAAGGCTGAGCCATAACATGGGAGTGGATGGATGTGCTTGCGTCAAAGCCGTAACTAGTACCTG 6720  
 CATTGGTTTTAAAGTGTTTTTTTTAAAAATTGTGTTGGGATTGGGAAGAGGGAGGGGGGGTTGTGGGCTTGTAAACGTGTTGTTCTTTTCATTGCAGCTATAGACTTGGTAAATCAGTAC 6840  
 TCTGAAGAACTGGGTGTAAAGCGCCGCTACCTGGA

GATAAG

GATAAG  
Depth:19 (ZEBRAFISH)  
Ei-value:0.000, Pi-value:0.000  
Er-value:0.000, Pr-value:0.000  
No matches to TargetScan

TCTATACGTGCACTTTTGCCTCTTAAGAAATTCTGAGAATAGTTCGGGGAGTCTGGGGGGAAACCGAAAACCCTGCGAG 6960  
 TATCTGTCTAGTTACAGGAATGGTGATTTTACAAAGGAGGCTATTTTGTTCTGGCCGTGTTGGTGGAAATCCAAGACCTGGGACCGTTGTGTGGTGGTAAATGACATTTGTATCACATTT 7080  
 GGTTATGAATCTTTGGGGTTGGGTGGGGTGGGGGGGTTCTAAACCCTTTTCTGTGTTTCAGATTTTTGTTTAAATAGGATCACGTCACCTGAATGCAAGTGCAGCCTTGAACAAGACCTA 7200  
 ACTAGTCAGATTTGATCTTCGAAGTAAAGGCGTGTGAATATGCTGCATATTGAAGACAAAATTACTACAGTAATATCGATGGGTTTTGAAGAGTTCGACTGGGGATTTTGGGGGGGAAAC 7320  
 CTGCAATCTTTTGTGTAGTTTCATACTAAAGAAGGACCGTTCGAGACGAGAGTTTTGTAGAGCAGCAGGTTTGAGGT

TTTTCTTTT

TTTTCTTTT  
Depth:19 (ZEBRAFISH)  
Ei-value:0.000, Pi-value:0.000  
Er-value:0.000, Pr-value:0.000  
MATCHES To TargetScan▶ miR-186-5p:AAAGAAU

TTCCTGGGGGAACAATCTTTGGATTGTTCTTT

CA

CAGGTTTTGCTTT  
Depth:19 (ZEBRAFISH)  
Ei-value:0.000, Pi-value:0.000  
Er-value:0.000, Pr-value:0.000  
MATCHES To TargetScan▶ miR-330-3p.2:AAAGCAC▶ miR-490-3p:AACCUGG

 7440  


GGTTTTGCTTT

CAGGTTTTGCTTT  
Depth:19 (ZEBRAFISH)  
Ei-value:0.000, Pi-value:0.000  
Er-value:0.000, Pr-value:0.000  
MATCHES To TargetScan▶ miR-330-3p.2:AAAGCAC▶ miR-490-3p:AACCUGG

TTAACCTCCTAAGAA

AAAAAGCAAAA

AAAAAGCAAAA  
Depth:19 (ZEBRAFISH)  
Ei-value:0.000, Pi-value:0.000  
Er-value:0.000, Pr-value:0.000  
No matches to TargetScan

7477
```

---
