# Supplementary material for: Uncovering deeply conserved motif combinations in rapidly evolving noncoding sequences
Source: Genome Biol. 2021 Jan 11;22:29. doi: 10.1186/s13059-020-02247-1 (PMC7798263; doi:10.1186/s13059-020-02247-1)
Supplement: Supplementary file 5 — Additional file 5. LncLOOM output results for MALAT1 sequences from 19 vertebrates. [file 13059_2020_2247_MOESM5_ESM.gz › AdditionalFile5/Html_Files/kmers_in_blocks.html]

 MOTIFS IN BLOCKS

# MOTIFS IN BLOCK DIAGRAMS

  

NAVIGATE ▼

▶HUMAN (depth:1)▶MARMOSET (depth:2)▶DOG (depth:3)▶PIG (depth:4)▶COW (depth:5)▶MOUSE (depth:6)▶TURTLE (depth:7)▶ALLIGATOR (depth:8)▶LIZARD (depth:9)▶SNAKE (depth:10)▶X.TROPICALIS (depth:11)▶SHARK (depth:12)▶OPOSSUM (depth:13)▶SPOTTEDGAR (depth:14)▶FUGU (depth:15)▶NILETILAPIA (depth:16)▶STICKLEBACK (depth:17)▶MEDAKA (depth:18)▶ZEBRAFISH (depth:19)

  
  
  

## >HUMAN (7504 bases)

```
atacgcctcgcc

atacgcctcgcc  
Depth:2 (MARMOSET)  
Ei-value:0.990, Pi-value:0.000  
Er-value:0.000, Pr-value:0.000  
No matches to eCLIP DataNo matches to TargetScan

-------------

aggcattgagg

aggcattgagg  
Depth:2 (MARMOSET)  
Ei-value:1.000, Pi-value:0.000  
Er-value:0.000, Pr-value:0.000  
eCLIP MATCHES▶NCBP2 (bg=1.99%)▶srsf1 (bg=30.28%)▶tra2a (bg=37.02%)MATCHES To TargetScan▶ miR-532-5p:AUGCCUU▶ miR-365-3p:AAUGCCC

-

agcc

agccagcgcaggg  
Depth:2 (MARMOSET)  
Ei-value:0.680, Pi-value:0.000  
Er-value:0.000, Pr-value:0.000  
eCLIP MATCHES▶NCBP2 (bg=1.99%)▶srsf1 (bg=30.28%)▶tra2a (bg=37.02%)MATCHES To TargetScan▶ miR-149-5p:CUGGCUC▶ miR-3064-5p:CUGGCUG


agcgcag

agcgcag  
Depth:3 (DOG)  
Ei-value:0.350, Pi-value:0.010  
Er-value:0.000, Pr-value:0.000  
eCLIP MATCHES▶NCBP2 (bg=1.99%)▶srsf1 (bg=30.28%)▶tra2a (bg=37.02%)No matches to TargetScan


gg

agccagcgcaggg  
Depth:2 (MARMOSET)  
Ei-value:0.680, Pi-value:0.000  
Er-value:0.000, Pr-value:0.000  
eCLIP MATCHES▶NCBP2 (bg=1.99%)▶srsf1 (bg=30.28%)▶tra2a (bg=37.02%)MATCHES To TargetScan▶ miR-149-5p:CUGGCUC▶ miR-3064-5p:CUGGCUG

-----------------------

agcttgaggaaac

agcttgaggaaac  
Depth:2 (MARMOSET)  
Ei-value:0.680, Pi-value:0.000  
Er-value:0.000, Pr-value:0.000  
eCLIP MATCHES▶hltf (bg=24.28%)▶NCBP2 (bg=1.99%)▶ppil4 (bg=43.39%)▶srsf1 (bg=30.28%)▶tra2a (bg=37.02%)MATCHES To TargetScan▶ miR-670-3p:UUCCUCA

-

GCAGATAAGTTTTT

GCAGATAAGTTTTT  
Depth:6 (MOUSE)  
Ei-value:0.000, Pi-value:0.000  
Er-value:0.000, Pr-value:0.000  
eCLIP MATCHES▶bud13 (bg=12.85%)▶hltf (bg=24.28%)▶NCBP2 (bg=1.99%)▶ppil4 (bg=43.39%)▶srsf1 (bg=30.28%)▶tra2a (bg=37.02%)No matches to TargetScan

------------------- 120  


at

attaatacaact  
Depth:2 (MARMOSET)  
Ei-value:0.990, Pi-value:0.000  
Er-value:0.000, Pr-value:0.000  
eCLIP MATCHES▶bclaf1 (bg=17.67%)▶bud13 (bg=12.85%)▶hltf (bg=24.28%)▶khdrbs1 (bg=10.41%)▶LARP7 (bg=2.17%)▶NCBP2 (bg=1.99%)▶npm1 (bg=10.22%)▶ppil4 (bg=43.39%)▶PUS1 (bg=1.64%)▶srsf1 (bg=30.28%)▶SUPV3L1 (bg=9.63%)▶uchl5 (bg=18.56%)▶YWHAG (bg=9.14%)▶zc3h8 (bg=12.78%)MATCHES To TargetScan▶ miR-496.2:GUAUUAC


taatac

taatac  
Depth:3 (DOG)  
Ei-value:1.000, Pi-value:0.040  
Er-value:0.000, Pr-value:0.000  
eCLIP MATCHES▶khdrbs1 (bg=10.41%)▶LARP7 (bg=2.17%)▶NCBP2 (bg=1.99%)▶npm1 (bg=10.22%)▶ppil4 (bg=43.39%)▶PUS1 (bg=1.64%)▶srsf1 (bg=30.28%)▶SUPV3L1 (bg=9.63%)▶uchl5 (bg=18.56%)▶YWHAG (bg=9.14%)▶zc3h8 (bg=12.78%)MATCHES To TargetScan▶ miR-496.2:GUAUUAC


aact

attaatacaact  
Depth:2 (MARMOSET)  
Ei-value:0.990, Pi-value:0.000  
Er-value:0.000, Pr-value:0.000  
eCLIP MATCHES▶bclaf1 (bg=17.67%)▶bud13 (bg=12.85%)▶hltf (bg=24.28%)▶khdrbs1 (bg=10.41%)▶LARP7 (bg=2.17%)▶NCBP2 (bg=1.99%)▶npm1 (bg=10.22%)▶ppil4 (bg=43.39%)▶PUS1 (bg=1.64%)▶srsf1 (bg=30.28%)▶SUPV3L1 (bg=9.63%)▶uchl5 (bg=18.56%)▶YWHAG (bg=9.14%)▶zc3h8 (bg=12.78%)MATCHES To TargetScan▶ miR-496.2:GUAUUAC

-------

aaatata

aaatata  
Depth:2 (MARMOSET)  
Ei-value:1.000, Pi-value:0.020  
Er-value:0.000, Pr-value:0.000  
eCLIP MATCHES▶bclaf1 (bg=17.67%)▶hltf (bg=24.28%)▶hnrnpa1 (bg=18.32%)▶khdrbs1 (bg=10.41%)▶LARP7 (bg=2.17%)▶NCBP2 (bg=1.99%)▶npm1 (bg=10.22%)▶ppil4 (bg=43.39%)▶PUS1 (bg=1.64%)▶safb (bg=40.39%)▶safb2 (bg=26.89%)▶SUPV3L1 (bg=9.63%)▶uchl5 (bg=18.56%)▶zc3h8 (bg=12.78%)No matches to TargetScan

-------------------

attgct

attgct  
Depth:3 (DOG)  
Ei-value:1.000, Pi-value:0.070  
Er-value:0.000, Pr-value:0.000  
eCLIP MATCHES▶hltf (bg=24.28%)▶hnrnpa1 (bg=18.32%)▶khdrbs1 (bg=10.41%)▶ppil4 (bg=43.39%)▶safb (bg=40.39%)▶safb2 (bg=26.89%)▶zc3h8 (bg=12.78%)No matches to TargetScan

-----

ttaagtt

ttaagtt  
Depth:2 (MARMOSET)  
Ei-value:1.000, Pi-value:0.030  
Er-value:0.000, Pr-value:0.010  
eCLIP MATCHES▶hltf (bg=24.28%)▶hnrnpa1 (bg=18.32%)▶khdrbs1 (bg=10.41%)▶ppil4 (bg=43.39%)▶safb2 (bg=26.89%)▶zc3h8 (bg=12.78%)No matches to TargetScan

-------

taatttta

taatttta  
Depth:2 (MARMOSET)  
Ei-value:1.000, Pi-value:0.000  
Er-value:0.000, Pr-value:0.000  
eCLIP MATCHES▶khdrbs1 (bg=10.41%)▶safb (bg=40.39%)No matches to TargetScan

--------------

taagagaaaata

taagagaaaata  
Depth:2 (MARMOSET)  
Ei-value:0.990, Pi-value:0.000  
Er-value:0.000, Pr-value:0.000  
eCLIP MATCHES▶ppil4 (bg=43.39%)▶safb2 (bg=26.89%)▶tra2a (bg=37.02%)No matches to TargetScan

-----------

AAGAG

AAGAGTAGC  
Depth:3 (DOG)  
Ei-value:0.000, Pi-value:0.000  
Er-value:0.000, Pr-value:0.000  
eCLIP MATCHES▶AQR (bg=4.89%)▶ppil4 (bg=43.39%)▶safb2 (bg=26.89%)▶tra2a (bg=37.02%)No matches to TargetScan

 240  


TAGC

AAGAGTAGC  
Depth:3 (DOG)  
Ei-value:0.000, Pi-value:0.000  
Er-value:0.000, Pr-value:0.000  
eCLIP MATCHES▶AQR (bg=4.89%)▶ppil4 (bg=43.39%)▶safb2 (bg=26.89%)▶tra2a (bg=37.02%)No matches to TargetScan


ATGAGGAAGGAA

AAGAGTAGCATGAGGAAGGAA  
Depth:2 (MARMOSET)  
Ei-value:0.000, Pi-value:0.000  
Er-value:0.000, Pr-value:0.000  
eCLIP MATCHES▶AQR (bg=4.89%)▶ppil4 (bg=43.39%)▶safb (bg=40.39%)▶safb2 (bg=26.89%)▶tra2a (bg=37.02%)MATCHES To TargetScan▶ miR-670-3p:UUCCUCA

----------

gtttct

gtttct  
Depth:2 (MARMOSET)  
Ei-value:1.000, Pi-value:0.020  
Er-value:0.000, Pr-value:0.020  
eCLIP MATCHES▶AQR (bg=4.89%)▶ppil4 (bg=43.39%)▶safb (bg=40.39%)▶safb2 (bg=26.89%)▶srsf1 (bg=30.28%)▶tra2a (bg=37.02%)No matches to TargetScan

-

AAACAT

AAACATGACGGAGGTTGAGATGAAGCT  
Depth:2 (MARMOSET)  
Ei-value:0.000, Pi-value:0.000  
Er-value:0.000, Pr-value:0.000  
eCLIP MATCHES▶AQR (bg=4.89%)▶bclaf1 (bg=17.67%)▶khdrbs1 (bg=10.41%)▶ppil4 (bg=43.39%)▶safb (bg=40.39%)▶safb2 (bg=26.89%)▶srsf1 (bg=30.28%)▶SRSF9 (bg=9.67%)▶tra2a (bg=37.02%)No matches to TargetScan


GACGGAGGTT

GACGGAGGTT  
Depth:3 (DOG)  
Ei-value:0.000, Pi-value:0.000  
Er-value:0.000, Pr-value:0.000  
eCLIP MATCHES▶AQR (bg=4.89%)▶ppil4 (bg=43.39%)▶safb (bg=40.39%)▶safb2 (bg=26.89%)▶srsf1 (bg=30.28%)▶SRSF9 (bg=9.67%)▶tra2a (bg=37.02%)No matches to TargetScan


GAGATGAAGCT

GAGATGAAGCT  
Depth:3 (DOG)  
Ei-value:0.000, Pi-value:0.000  
Er-value:0.000, Pr-value:0.000  
eCLIP MATCHES▶bclaf1 (bg=17.67%)▶khdrbs1 (bg=10.41%)▶ppil4 (bg=43.39%)▶safb (bg=40.39%)▶safb2 (bg=26.89%)▶srsf1 (bg=30.28%)▶SRSF9 (bg=9.67%)▶tra2a (bg=37.02%)No matches to TargetScan

-

cttc

cttcatggagta  
Depth:2 (MARMOSET)  
Ei-value:0.990, Pi-value:0.000  
Er-value:0.000, Pr-value:0.000  
eCLIP MATCHES▶AQR (bg=4.89%)▶bclaf1 (bg=17.67%)▶khdrbs1 (bg=10.41%)▶ppil4 (bg=43.39%)▶safb (bg=40.39%)▶safb2 (bg=26.89%)▶srsf1 (bg=30.28%)▶SRSF9 (bg=9.67%)▶tra2a (bg=37.02%)MATCHES To TargetScan▶ miR-136-5p:CUCCAUU


atggagt

atggagt  
Depth:3 (DOG)  
Ei-value:0.350, Pi-value:0.010  
Er-value:0.000, Pr-value:0.000  
eCLIP MATCHES▶AQR (bg=4.89%)▶bclaf1 (bg=17.67%)▶khdrbs1 (bg=10.41%)▶ppil4 (bg=43.39%)▶safb (bg=40.39%)▶safb2 (bg=26.89%)▶srsf1 (bg=30.28%)▶SRSF9 (bg=9.67%)▶tra2a (bg=37.02%)MATCHES To TargetScan▶ miR-136-5p:CUCCAUU


a

cttcatggagta  
Depth:2 (MARMOSET)  
Ei-value:0.990, Pi-value:0.000  
Er-value:0.000, Pr-value:0.000  
eCLIP MATCHES▶AQR (bg=4.89%)▶bclaf1 (bg=17.67%)▶khdrbs1 (bg=10.41%)▶ppil4 (bg=43.39%)▶safb (bg=40.39%)▶safb2 (bg=26.89%)▶srsf1 (bg=30.28%)▶SRSF9 (bg=9.67%)▶tra2a (bg=37.02%)MATCHES To TargetScan▶ miR-136-5p:CUCCAUU

-

AAAATGT

AAAATGTATTTAAAAGAAAATTGA  
Depth:2 (MARMOSET)  
Ei-value:0.000, Pi-value:0.000  
Er-value:0.000, Pr-value:0.000  
eCLIP MATCHES▶AQR (bg=4.89%)▶bclaf1 (bg=17.67%)▶hltf (bg=24.28%)▶hnrnpa1 (bg=18.32%)▶khdrbs1 (bg=10.41%)▶ppil4 (bg=43.39%)▶safb (bg=40.39%)▶safb2 (bg=26.89%)No matches to TargetScan


ATTTAAAA

ATTTAAAA  
Depth:3 (DOG)  
Ei-value:0.010, Pi-value:0.010  
Er-value:0.000, Pr-value:0.000  
eCLIP MATCHES▶AQR (bg=4.89%)▶hltf (bg=24.28%)▶khdrbs1 (bg=10.41%)▶ppil4 (bg=43.39%)No matches to TargetScan


GAAAATTGA

AAAATGTATTTAAAAGAAAATTGA  
Depth:2 (MARMOSET)  
Ei-value:0.000, Pi-value:0.000  
Er-value:0.000, Pr-value:0.000  
eCLIP MATCHES▶AQR (bg=4.89%)▶bclaf1 (bg=17.67%)▶hltf (bg=24.28%)▶hnrnpa1 (bg=18.32%)▶khdrbs1 (bg=10.41%)▶ppil4 (bg=43.39%)▶safb (bg=40.39%)▶safb2 (bg=26.89%)No matches to TargetScan

----

aaggacta

aaggacta  
Depth:2 (MARMOSET)  
Ei-value:1.000, Pi-value:0.000  
Er-value:0.000, Pr-value:0.000  
eCLIP MATCHES▶bclaf1 (bg=17.67%)▶hltf (bg=24.28%)▶hnrnpa1 (bg=18.32%)▶khdrbs1 (bg=10.41%)▶ppil4 (bg=43.39%)▶safb (bg=40.39%)▶safb2 (bg=26.89%)▶tra2a (bg=37.02%)No matches to TargetScan

---------

g

gaattaatacc  
Depth:2 (MARMOSET)  
Ei-value:1.000, Pi-value:0.000  
Er-value:0.000, Pr-value:0.000  
eCLIP MATCHES▶bclaf1 (bg=17.67%)▶hltf (bg=24.28%)▶hnrnpa1 (bg=18.32%)▶khdrbs1 (bg=10.41%)▶ppil4 (bg=43.39%)▶safb (bg=40.39%)▶safb2 (bg=26.89%)▶tra2a (bg=37.02%)MATCHES To TargetScan▶ miR-496.2:GUAUUAC

 360  


aattaatacc

gaattaatacc  
Depth:2 (MARMOSET)  
Ei-value:1.000, Pi-value:0.000  
Er-value:0.000, Pr-value:0.000  
eCLIP MATCHES▶bclaf1 (bg=17.67%)▶hltf (bg=24.28%)▶hnrnpa1 (bg=18.32%)▶khdrbs1 (bg=10.41%)▶ppil4 (bg=43.39%)▶safb (bg=40.39%)▶safb2 (bg=26.89%)▶tra2a (bg=37.02%)MATCHES To TargetScan▶ miR-496.2:GUAUUAC

--

tagaagggca

tagaagggca  
Depth:2 (MARMOSET)  
Ei-value:1.000, Pi-value:0.000  
Er-value:0.000, Pr-value:0.000  
eCLIP MATCHES▶hltf (bg=24.28%)▶khdrbs1 (bg=10.41%)MATCHES To TargetScan▶ miR-874-3p:UGCCCUG

-

tgcttttagatta

tgcttttagatta  
Depth:2 (MARMOSET)  
Ei-value:0.680, Pi-value:0.000  
Er-value:0.000, Pr-value:0.000  
eCLIP MATCHES▶hltf (bg=24.28%)▶khdrbs1 (bg=10.41%)MATCHES To TargetScan▶ miR-330-3p.2:AAAGCAC

-----

AAGGTGACTTAAACAG

AAGGTGACTTAAACAG  
Depth:2 (MARMOSET)  
Ei-value:0.000, Pi-value:0.000  
Er-value:0.000, Pr-value:0.000  
eCLIP MATCHES▶hltf (bg=24.28%)▶khdrbs1 (bg=10.41%)MATCHES To TargetScan▶ miR-224-5p:AAGUCAC

----------------------

gtaggtga

gtaggtga  
Depth:2 (MARMOSET)  
Ei-value:1.000, Pi-value:0.000  
Er-value:0.000, Pr-value:0.000  
eCLIP MATCHES▶hltf (bg=24.28%)▶hnrnpa1 (bg=18.32%)▶khdrbs1 (bg=10.41%)▶safb (bg=40.39%)▶safb2 (bg=26.89%)No matches to TargetScan

-

taaaata

taaaata  
Depth:2 (MARMOSET)  
Ei-value:1.000, Pi-value:0.040  
Er-value:0.000, Pr-value:0.020  
eCLIP MATCHES▶hltf (bg=24.28%)▶hnrnpa1 (bg=18.32%)▶khdrbs1 (bg=10.41%)▶ppil4 (bg=43.39%)▶safb (bg=40.39%)▶safb2 (bg=26.89%)No matches to TargetScan

--------

gcgatc

gcgatc  
Depth:2 (MARMOSET)  
Ei-value:1.000, Pi-value:0.040  
Er-value:0.000, Pr-value:0.000  
eCLIP MATCHES▶hnrnpa1 (bg=18.32%)▶khdrbs1 (bg=10.41%)▶ppil4 (bg=43.39%)▶safb (bg=40.39%)▶safb2 (bg=26.89%)No matches to TargetScan

-

TTTAAAAAG

TTTAAAAAG  
Depth:3 (DOG)  
Ei-value:0.000, Pi-value:0.000  
Er-value:0.000, Pr-value:0.010  
eCLIP MATCHES▶hnrnpa1 (bg=18.32%)▶khdrbs1 (bg=10.41%)▶ppil4 (bg=43.39%)▶safb (bg=40.39%)▶safb2 (bg=26.89%)No matches to TargetScan


A

TTTAAAAAGAGATTAA  
Depth:2 (MARMOSET)  
Ei-value:0.000, Pi-value:0.000  
Er-value:0.000, Pr-value:0.000  
eCLIP MATCHES▶hnrnpa1 (bg=18.32%)▶khdrbs1 (bg=10.41%)▶ppil4 (bg=43.39%)▶safb (bg=40.39%)▶safb2 (bg=26.89%)MATCHES To TargetScan▶ miR-216a-5p:AAUCUCA▶ miR-216b-5p:AAUCUCU

 480  


GATTAA

TTTAAAAAGAGATTAA  
Depth:2 (MARMOSET)  
Ei-value:0.000, Pi-value:0.000  
Er-value:0.000, Pr-value:0.000  
eCLIP MATCHES▶hnrnpa1 (bg=18.32%)▶khdrbs1 (bg=10.41%)▶ppil4 (bg=43.39%)▶safb (bg=40.39%)▶safb2 (bg=26.89%)MATCHES To TargetScan▶ miR-216a-5p:AAUCUCA▶ miR-216b-5p:AAUCUCU

----

AAGGTGATTAAAAGAC

AAGGTGATTAAAAGACCTTGAAATCCATGACGCA  
Depth:2 (MARMOSET)  
Ei-value:0.000, Pi-value:0.000  
Er-value:0.000, Pr-value:0.000  
eCLIP MATCHES▶hnrnpa1 (bg=18.32%)▶khdrbs1 (bg=10.41%)▶ppil4 (bg=43.39%)▶safb (bg=40.39%)▶safb2 (bg=26.89%)▶TAF15 (bg=9.06%)MATCHES To TargetScan▶ miR-876-5p:GGAUUUC


cttgaa

cttgaa  
Depth:3 (DOG)  
Ei-value:1.000, Pi-value:0.080  
Er-value:0.000, Pr-value:0.000  
eCLIP MATCHES▶khdrbs1 (bg=10.41%)▶safb (bg=40.39%)▶safb2 (bg=26.89%)▶TAF15 (bg=9.06%)No matches to TargetScan


ATCCATGACGCA

AAGGTGATTAAAAGACCTTGAAATCCATGACGCA  
Depth:2 (MARMOSET)  
Ei-value:0.000, Pi-value:0.000  
Er-value:0.000, Pr-value:0.000  
eCLIP MATCHES▶hnrnpa1 (bg=18.32%)▶khdrbs1 (bg=10.41%)▶ppil4 (bg=43.39%)▶safb (bg=40.39%)▶safb2 (bg=26.89%)▶TAF15 (bg=9.06%)MATCHES To TargetScan▶ miR-876-5p:GGAUUUC

---

AGA

AGAATTGCGTCATTTAAAGCCTA  
Depth:2 (MARMOSET)  
Ei-value:0.000, Pi-value:0.000  
Er-value:0.000, Pr-value:0.000  
eCLIP MATCHES▶hltf (bg=24.28%)▶khdrbs1 (bg=10.41%)▶safb (bg=40.39%)▶safb2 (bg=26.89%)▶TAF15 (bg=9.06%)No matches to TargetScan


ATTGCGTCATTT

ATTGCGTCATTT  
Depth:3 (DOG)  
Ei-value:0.000, Pi-value:0.000  
Er-value:0.000, Pr-value:0.000  
eCLIP MATCHES▶hltf (bg=24.28%)▶khdrbs1 (bg=10.41%)▶safb (bg=40.39%)▶safb2 (bg=26.89%)▶TAF15 (bg=9.06%)No matches to TargetScan


AAAGCCTA

AGAATTGCGTCATTTAAAGCCTA  
Depth:2 (MARMOSET)  
Ei-value:0.000, Pi-value:0.000  
Er-value:0.000, Pr-value:0.000  
eCLIP MATCHES▶hltf (bg=24.28%)▶khdrbs1 (bg=10.41%)▶safb (bg=40.39%)▶safb2 (bg=26.89%)▶TAF15 (bg=9.06%)No matches to TargetScan

----------

TTACTAAACGCAGACGAA

TTACTAAACGCAGACGAA  
Depth:2 (MARMOSET)  
Ei-value:0.000, Pi-value:0.000  
Er-value:0.000, Pr-value:0.000  
eCLIP MATCHES▶aggf1 (bg=15.15%)▶khdrbs1 (bg=10.41%)▶larp4 (bg=13.51%)▶safb2 (bg=26.89%)▶srsf1 (bg=30.28%)▶srsf7 (bg=22.53%)▶tra2a (bg=37.02%)MATCHES To TargetScan▶ miR-346:GUCUGCC

---

ggaaaga

ggaaaga  
Depth:2 (MARMOSET)  
Ei-value:1.000, Pi-value:0.010  
Er-value:0.000, Pr-value:0.010  
eCLIP MATCHES▶aggf1 (bg=15.15%)▶bud13 (bg=12.85%)▶cpsf6 (bg=13.45%)▶khdrbs1 (bg=10.41%)▶larp4 (bg=13.51%)▶rbm22 (bg=12.69%)▶safb2 (bg=26.89%)▶srsf1 (bg=30.28%)▶srsf7 (bg=22.53%)▶SRSF9 (bg=9.67%)▶tra2a (bg=37.02%)No matches to TargetScan


TTAATTGGG

TTAATTGGGAGTGGTAGGA  
Depth:2 (MARMOSET)  
Ei-value:0.000, Pi-value:0.000  
Er-value:0.000, Pr-value:0.000  
eCLIP MATCHES▶aggf1 (bg=15.15%)▶bclaf1 (bg=17.67%)▶bud13 (bg=12.85%)▶cpsf6 (bg=13.45%)▶fxr2 (bg=10.1%)▶GPKOW (bg=5.66%)▶gtf2f1 (bg=10.18%)▶khdrbs1 (bg=10.41%)▶larp4 (bg=13.51%)▶LARP7 (bg=2.17%)▶rbm22 (bg=12.69%)▶safb2 (bg=26.89%)▶SMNDC1 (bg=7.08%)▶srsf1 (bg=30.28%)▶srsf7 (bg=22.53%)▶SRSF9 (bg=9.67%)▶TAF15 (bg=9.06%)▶tra2a (bg=37.02%)▶TROVE2 (bg=6.96%)▶uchl5 (bg=18.56%)▶znf622 (bg=18.79%)MATCHES To TargetScan▶ miR-150-5p:CUCCCAA▶ miR-532-3p:CUCCCAC▶ miR-483-3p.2:CACUCCU


AGT

AGTGGTAGGA  
Depth:3 (DOG)  
Ei-value:0.000, Pi-value:0.000  
Er-value:0.000, Pr-value:0.000  
eCLIP MATCHES▶aggf1 (bg=15.15%)▶bclaf1 (bg=17.67%)▶bud13 (bg=12.85%)▶cpsf6 (bg=13.45%)▶fxr2 (bg=10.1%)▶GPKOW (bg=5.66%)▶gtf2f1 (bg=10.18%)▶larp4 (bg=13.51%)▶LARP7 (bg=2.17%)▶rbm22 (bg=12.69%)▶safb2 (bg=26.89%)▶SMNDC1 (bg=7.08%)▶srsf1 (bg=30.28%)▶srsf7 (bg=22.53%)▶SRSF9 (bg=9.67%)▶TAF15 (bg=9.06%)▶tra2a (bg=37.02%)▶TROVE2 (bg=6.96%)▶uchl5 (bg=18.56%)▶znf622 (bg=18.79%)No matches to TargetScan

 600  


GGTAGGA

AGTGGTAGGA  
Depth:3 (DOG)  
Ei-value:0.000, Pi-value:0.000  
Er-value:0.000, Pr-value:0.000  
eCLIP MATCHES▶aggf1 (bg=15.15%)▶bclaf1 (bg=17.67%)▶bud13 (bg=12.85%)▶cpsf6 (bg=13.45%)▶fxr2 (bg=10.1%)▶GPKOW (bg=5.66%)▶gtf2f1 (bg=10.18%)▶larp4 (bg=13.51%)▶LARP7 (bg=2.17%)▶rbm22 (bg=12.69%)▶safb2 (bg=26.89%)▶SMNDC1 (bg=7.08%)▶srsf1 (bg=30.28%)▶srsf7 (bg=22.53%)▶SRSF9 (bg=9.67%)▶TAF15 (bg=9.06%)▶tra2a (bg=37.02%)▶TROVE2 (bg=6.96%)▶uchl5 (bg=18.56%)▶znf622 (bg=18.79%)No matches to TargetScan

--

AAACAATTTGGAGAAGAT

AAACAATTTGGAGAAGAT  
Depth:2 (MARMOSET)  
Ei-value:0.000, Pi-value:0.000  
Er-value:0.000, Pr-value:0.000  
eCLIP MATCHES▶aggf1 (bg=15.15%)▶bclaf1 (bg=17.67%)▶bud13 (bg=12.85%)▶cpsf6 (bg=13.45%)▶fxr2 (bg=10.1%)▶GPKOW (bg=5.66%)▶gtf2f1 (bg=10.18%)▶larp4 (bg=13.51%)▶LARP7 (bg=2.17%)▶rbm22 (bg=12.69%)▶safb (bg=40.39%)▶safb2 (bg=26.89%)▶SMNDC1 (bg=7.08%)▶srsf1 (bg=30.28%)▶srsf7 (bg=22.53%)▶SRSF9 (bg=9.67%)▶TAF15 (bg=9.06%)▶TBRG4 (bg=0.51%)▶tra2a (bg=37.02%)▶TROVE2 (bg=6.96%)▶uchl5 (bg=18.56%)▶znf622 (bg=18.79%)No matches to TargetScan


AGAAGT

AGAAGTTTGAAGTGGAA  
Depth:2 (MARMOSET)  
Ei-value:0.000, Pi-value:0.000  
Er-value:0.000, Pr-value:0.000  
eCLIP MATCHES▶aggf1 (bg=15.15%)▶bclaf1 (bg=17.67%)▶bud13 (bg=12.85%)▶cpsf6 (bg=13.45%)▶fxr2 (bg=10.1%)▶GPKOW (bg=5.66%)▶gtf2f1 (bg=10.18%)▶hltf (bg=24.28%)▶larp4 (bg=13.51%)▶LARP7 (bg=2.17%)▶rbm22 (bg=12.69%)▶safb (bg=40.39%)▶safb2 (bg=26.89%)▶SMNDC1 (bg=7.08%)▶srsf1 (bg=30.28%)▶srsf7 (bg=22.53%)▶SRSF9 (bg=9.67%)▶TAF15 (bg=9.06%)▶TBRG4 (bg=0.51%)▶tra2a (bg=37.02%)▶TROVE2 (bg=6.96%)▶uchl5 (bg=18.56%)▶znf622 (bg=18.79%)No matches to TargetScan


TTGAAGTGGA

TTGAAGTGGA  
Depth:3 (DOG)  
Ei-value:0.000, Pi-value:0.000  
Er-value:0.000, Pr-value:0.000  
eCLIP MATCHES▶aggf1 (bg=15.15%)▶bclaf1 (bg=17.67%)▶bud13 (bg=12.85%)▶cpsf6 (bg=13.45%)▶fxr2 (bg=10.1%)▶GPKOW (bg=5.66%)▶gtf2f1 (bg=10.18%)▶hltf (bg=24.28%)▶larp4 (bg=13.51%)▶LARP7 (bg=2.17%)▶rbm22 (bg=12.69%)▶safb (bg=40.39%)▶safb2 (bg=26.89%)▶SMNDC1 (bg=7.08%)▶srsf1 (bg=30.28%)▶srsf7 (bg=22.53%)▶SRSF9 (bg=9.67%)▶TAF15 (bg=9.06%)▶TBRG4 (bg=0.51%)▶tra2a (bg=37.02%)▶TROVE2 (bg=6.96%)▶uchl5 (bg=18.56%)▶znf622 (bg=18.79%)No matches to TargetScan


A

AGAAGTTTGAAGTGGAA  
Depth:2 (MARMOSET)  
Ei-value:0.000, Pi-value:0.000  
Er-value:0.000, Pr-value:0.000  
eCLIP MATCHES▶aggf1 (bg=15.15%)▶bclaf1 (bg=17.67%)▶bud13 (bg=12.85%)▶cpsf6 (bg=13.45%)▶fxr2 (bg=10.1%)▶GPKOW (bg=5.66%)▶gtf2f1 (bg=10.18%)▶hltf (bg=24.28%)▶larp4 (bg=13.51%)▶LARP7 (bg=2.17%)▶rbm22 (bg=12.69%)▶safb (bg=40.39%)▶safb2 (bg=26.89%)▶SMNDC1 (bg=7.08%)▶srsf1 (bg=30.28%)▶srsf7 (bg=22.53%)▶SRSF9 (bg=9.67%)▶TAF15 (bg=9.06%)▶TBRG4 (bg=0.51%)▶tra2a (bg=37.02%)▶TROVE2 (bg=6.96%)▶uchl5 (bg=18.56%)▶znf622 (bg=18.79%)No matches to TargetScan

-

ACTGGAAGA

ACTGGAAGACAGAAGTAC  
Depth:2 (MARMOSET)  
Ei-value:0.000, Pi-value:0.000  
Er-value:0.000, Pr-value:0.000  
eCLIP MATCHES▶aggf1 (bg=15.15%)▶AQR (bg=4.89%)▶bclaf1 (bg=17.67%)▶bud13 (bg=12.85%)▶cpsf6 (bg=13.45%)▶FASTKD2 (bg=4.54%)▶fxr2 (bg=10.1%)▶GPKOW (bg=5.66%)▶gtf2f1 (bg=10.18%)▶hltf (bg=24.28%)▶larp4 (bg=13.51%)▶LARP7 (bg=2.17%)▶MTPAP (bg=9.55%)▶rbm15 (bg=11.59%)▶rbm22 (bg=12.69%)▶safb2 (bg=26.89%)▶SMNDC1 (bg=7.08%)▶srsf1 (bg=30.28%)▶srsf7 (bg=22.53%)▶SRSF9 (bg=9.67%)▶TAF15 (bg=9.06%)▶TBRG4 (bg=0.51%)▶tra2a (bg=37.02%)▶TROVE2 (bg=6.96%)▶uchl5 (bg=18.56%)▶znf622 (bg=18.79%)MATCHES To TargetScan▶ miR-145-5p:UCCAGUU


CAGAAGTA

CAGAAGTA  
Depth:3 (DOG)  
Ei-value:0.010, Pi-value:0.010  
Er-value:0.000, Pr-value:0.000  
eCLIP MATCHES▶aggf1 (bg=15.15%)▶bclaf1 (bg=17.67%)▶bud13 (bg=12.85%)▶cpsf6 (bg=13.45%)▶FASTKD2 (bg=4.54%)▶fxr2 (bg=10.1%)▶GPKOW (bg=5.66%)▶gtf2f1 (bg=10.18%)▶hltf (bg=24.28%)▶larp4 (bg=13.51%)▶LARP7 (bg=2.17%)▶MTPAP (bg=9.55%)▶rbm15 (bg=11.59%)▶rbm22 (bg=12.69%)▶safb2 (bg=26.89%)▶SMNDC1 (bg=7.08%)▶srsf1 (bg=30.28%)▶srsf7 (bg=22.53%)▶SRSF9 (bg=9.67%)▶TAF15 (bg=9.06%)▶tra2a (bg=37.02%)▶TROVE2 (bg=6.96%)▶uchl5 (bg=18.56%)▶znf622 (bg=18.79%)No matches to TargetScan


C

ACTGGAAGACAGAAGTAC  
Depth:2 (MARMOSET)  
Ei-value:0.000, Pi-value:0.000  
Er-value:0.000, Pr-value:0.000  
eCLIP MATCHES▶aggf1 (bg=15.15%)▶AQR (bg=4.89%)▶bclaf1 (bg=17.67%)▶bud13 (bg=12.85%)▶cpsf6 (bg=13.45%)▶FASTKD2 (bg=4.54%)▶fxr2 (bg=10.1%)▶GPKOW (bg=5.66%)▶gtf2f1 (bg=10.18%)▶hltf (bg=24.28%)▶larp4 (bg=13.51%)▶LARP7 (bg=2.17%)▶MTPAP (bg=9.55%)▶rbm15 (bg=11.59%)▶rbm22 (bg=12.69%)▶safb2 (bg=26.89%)▶SMNDC1 (bg=7.08%)▶srsf1 (bg=30.28%)▶srsf7 (bg=22.53%)▶SRSF9 (bg=9.67%)▶TAF15 (bg=9.06%)▶TBRG4 (bg=0.51%)▶tra2a (bg=37.02%)▶TROVE2 (bg=6.96%)▶uchl5 (bg=18.56%)▶znf622 (bg=18.79%)MATCHES To TargetScan▶ miR-145-5p:UCCAGUU

-

GGAAGGC

GGAAGGCGAAGAAAAGAATAGAGAAGATAGGGAAATTAGAAGATAAAAA  
Depth:2 (MARMOSET)  
Ei-value:0.000, Pi-value:0.000  
Er-value:0.000, Pr-value:0.000  
eCLIP MATCHES▶aggf1 (bg=15.15%)▶AQR (bg=4.89%)▶bclaf1 (bg=17.67%)▶bud13 (bg=12.85%)▶cpsf6 (bg=13.45%)▶FASTKD2 (bg=4.54%)▶fxr2 (bg=10.1%)▶GPKOW (bg=5.66%)▶gtf2f1 (bg=10.18%)▶hltf (bg=24.28%)▶larp4 (bg=13.51%)▶LARP7 (bg=2.17%)▶MTPAP (bg=9.55%)▶rbm15 (bg=11.59%)▶rbm22 (bg=12.69%)▶safb2 (bg=26.89%)▶SMNDC1 (bg=7.08%)▶srsf1 (bg=30.28%)▶srsf7 (bg=22.53%)▶SRSF9 (bg=9.67%)▶TAF15 (bg=9.06%)▶tra2a (bg=37.02%)▶TROVE2 (bg=6.96%)▶uchl5 (bg=18.56%)▶znf622 (bg=18.79%)No matches to TargetScan


GAAGAAAAGA

GAAGAAAAGA  
Depth:3 (DOG)  
Ei-value:0.000, Pi-value:0.000  
Er-value:0.000, Pr-value:0.000  
eCLIP MATCHES▶aggf1 (bg=15.15%)▶AQR (bg=4.89%)▶bclaf1 (bg=17.67%)▶bud13 (bg=12.85%)▶cpsf6 (bg=13.45%)▶FASTKD2 (bg=4.54%)▶fxr2 (bg=10.1%)▶GPKOW (bg=5.66%)▶gtf2f1 (bg=10.18%)▶hltf (bg=24.28%)▶larp4 (bg=13.51%)▶LARP7 (bg=2.17%)▶MTPAP (bg=9.55%)▶rbm15 (bg=11.59%)▶rbm22 (bg=12.69%)▶safb2 (bg=26.89%)▶SMNDC1 (bg=7.08%)▶srsf1 (bg=30.28%)▶srsf7 (bg=22.53%)▶SRSF9 (bg=9.67%)▶TAF15 (bg=9.06%)▶tra2a (bg=37.02%)▶TROVE2 (bg=6.96%)▶uchl5 (bg=18.56%)▶znf622 (bg=18.79%)No matches to TargetScan


A

GGAAGGCGAAGAAAAGAATAGAGAAGATAGGGAAATTAGAAGATAAAAA  
Depth:2 (MARMOSET)  
Ei-value:0.000, Pi-value:0.000  
Er-value:0.000, Pr-value:0.000  
eCLIP MATCHES▶aggf1 (bg=15.15%)▶AQR (bg=4.89%)▶bclaf1 (bg=17.67%)▶bud13 (bg=12.85%)▶cpsf6 (bg=13.45%)▶FASTKD2 (bg=4.54%)▶fxr2 (bg=10.1%)▶GPKOW (bg=5.66%)▶gtf2f1 (bg=10.18%)▶hltf (bg=24.28%)▶larp4 (bg=13.51%)▶LARP7 (bg=2.17%)▶MTPAP (bg=9.55%)▶rbm15 (bg=11.59%)▶rbm22 (bg=12.69%)▶safb2 (bg=26.89%)▶SMNDC1 (bg=7.08%)▶srsf1 (bg=30.28%)▶srsf7 (bg=22.53%)▶SRSF9 (bg=9.67%)▶TAF15 (bg=9.06%)▶tra2a (bg=37.02%)▶TROVE2 (bg=6.96%)▶uchl5 (bg=18.56%)▶znf622 (bg=18.79%)No matches to TargetScan


TAGAG

TAGAGAAGATAGG  
Depth:3 (DOG)  
Ei-value:0.000, Pi-value:0.000  
Er-value:0.000, Pr-value:0.000  
eCLIP MATCHES▶aggf1 (bg=15.15%)▶AQR (bg=4.89%)▶bclaf1 (bg=17.67%)▶bud13 (bg=12.85%)▶cpsf6 (bg=13.45%)▶FASTKD2 (bg=4.54%)▶fxr2 (bg=10.1%)▶GPKOW (bg=5.66%)▶hltf (bg=24.28%)▶larp4 (bg=13.51%)▶LARP7 (bg=2.17%)▶rbm15 (bg=11.59%)▶rbm22 (bg=12.69%)▶safb2 (bg=26.89%)▶SMNDC1 (bg=7.08%)▶srsf1 (bg=30.28%)▶srsf7 (bg=22.53%)▶SRSF9 (bg=9.67%)▶tra2a (bg=37.02%)▶TROVE2 (bg=6.96%)▶uchl5 (bg=18.56%)▶znf622 (bg=18.79%)No matches to TargetScan


AAGATAGG

AAGATAGG  
Depth:5 (COW)  
Ei-value:0.000, Pi-value:0.020  
Er-value:0.000, Pr-value:0.000  
eCLIP MATCHES▶aggf1 (bg=15.15%)▶AQR (bg=4.89%)▶bclaf1 (bg=17.67%)▶bud13 (bg=12.85%)▶cpsf6 (bg=13.45%)▶FASTKD2 (bg=4.54%)▶fxr2 (bg=10.1%)▶GPKOW (bg=5.66%)▶larp4 (bg=13.51%)▶LARP7 (bg=2.17%)▶rbm15 (bg=11.59%)▶rbm22 (bg=12.69%)▶safb2 (bg=26.89%)▶SMNDC1 (bg=7.08%)▶srsf1 (bg=30.28%)▶srsf7 (bg=22.53%)▶SRSF9 (bg=9.67%)▶tra2a (bg=37.02%)▶TROVE2 (bg=6.96%)▶uchl5 (bg=18.56%)▶znf622 (bg=18.79%)No matches to TargetScan


GAAATTAGAAGATAAAAA

GGAAGGCGAAGAAAAGAATAGAGAAGATAGGGAAATTAGAAGATAAAAA  
Depth:2 (MARMOSET)  
Ei-value:0.000, Pi-value:0.000  
Er-value:0.000, Pr-value:0.000  
eCLIP MATCHES▶aggf1 (bg=15.15%)▶AQR (bg=4.89%)▶bclaf1 (bg=17.67%)▶bud13 (bg=12.85%)▶cpsf6 (bg=13.45%)▶FASTKD2 (bg=4.54%)▶fxr2 (bg=10.1%)▶GPKOW (bg=5.66%)▶gtf2f1 (bg=10.18%)▶hltf (bg=24.28%)▶larp4 (bg=13.51%)▶LARP7 (bg=2.17%)▶MTPAP (bg=9.55%)▶rbm15 (bg=11.59%)▶rbm22 (bg=12.69%)▶safb2 (bg=26.89%)▶SMNDC1 (bg=7.08%)▶srsf1 (bg=30.28%)▶srsf7 (bg=22.53%)▶SRSF9 (bg=9.67%)▶TAF15 (bg=9.06%)▶tra2a (bg=37.02%)▶TROVE2 (bg=6.96%)▶uchl5 (bg=18.56%)▶znf622 (bg=18.79%)No matches to TargetScan

----

CTT

CTTTTAGAAGA  
Depth:3 (DOG)  
Ei-value:0.000, Pi-value:0.000  
Er-value:0.000, Pr-value:0.000  
eCLIP MATCHES▶aggf1 (bg=15.15%)▶AQR (bg=4.89%)▶bclaf1 (bg=17.67%)▶bud13 (bg=12.85%)▶cpsf6 (bg=13.45%)▶FASTKD2 (bg=4.54%)▶fxr2 (bg=10.1%)▶GPKOW (bg=5.66%)▶hltf (bg=24.28%)▶larp4 (bg=13.51%)▶rbm15 (bg=11.59%)▶safb2 (bg=26.89%)▶srsf1 (bg=30.28%)▶srsf7 (bg=22.53%)▶tra2a (bg=37.02%)▶TROVE2 (bg=6.96%)No matches to TargetScan

 720  


TTAGAAGA

CTTTTAGAAGA  
Depth:3 (DOG)  
Ei-value:0.000, Pi-value:0.000  
Er-value:0.000, Pr-value:0.000  
eCLIP MATCHES▶aggf1 (bg=15.15%)▶AQR (bg=4.89%)▶bclaf1 (bg=17.67%)▶bud13 (bg=12.85%)▶cpsf6 (bg=13.45%)▶FASTKD2 (bg=4.54%)▶fxr2 (bg=10.1%)▶GPKOW (bg=5.66%)▶hltf (bg=24.28%)▶larp4 (bg=13.51%)▶rbm15 (bg=11.59%)▶safb2 (bg=26.89%)▶srsf1 (bg=30.28%)▶srsf7 (bg=22.53%)▶tra2a (bg=37.02%)▶TROVE2 (bg=6.96%)No matches to TargetScan

-----------------------

AAGT

AAGTAGGAAGCAGAAGAAAAAA  
Depth:2 (MARMOSET)  
Ei-value:0.000, Pi-value:0.000  
Er-value:0.000, Pr-value:0.000  
eCLIP MATCHES▶aggf1 (bg=15.15%)▶AQR (bg=4.89%)▶bclaf1 (bg=17.67%)▶bud13 (bg=12.85%)▶cpsf6 (bg=13.45%)▶FASTKD2 (bg=4.54%)▶FTO (bg=1.11%)▶fxr2 (bg=10.1%)▶GPKOW (bg=5.66%)▶gtf2f1 (bg=10.18%)▶hltf (bg=24.28%)▶larp4 (bg=13.51%)▶MTPAP (bg=9.55%)▶npm1 (bg=10.22%)▶rbm15 (bg=11.59%)▶rbm22 (bg=12.69%)▶safb2 (bg=26.89%)▶SMNDC1 (bg=7.08%)▶srsf1 (bg=30.28%)▶srsf7 (bg=22.53%)▶SRSF9 (bg=9.67%)▶TAF15 (bg=9.06%)▶tra2a (bg=37.02%)▶TROVE2 (bg=6.96%)▶uchl5 (bg=18.56%)▶YWHAG (bg=9.14%)▶zc3h8 (bg=12.78%)▶znf622 (bg=18.79%)No matches to TargetScan


AGGAAGC

AGGAAGCAGAAGAAAAAA  
Depth:3 (DOG)  
Ei-value:0.000, Pi-value:0.000  
Er-value:0.000, Pr-value:0.000  
eCLIP MATCHES▶aggf1 (bg=15.15%)▶AQR (bg=4.89%)▶bclaf1 (bg=17.67%)▶bud13 (bg=12.85%)▶cpsf6 (bg=13.45%)▶FASTKD2 (bg=4.54%)▶FTO (bg=1.11%)▶fxr2 (bg=10.1%)▶GPKOW (bg=5.66%)▶gtf2f1 (bg=10.18%)▶hltf (bg=24.28%)▶larp4 (bg=13.51%)▶MTPAP (bg=9.55%)▶npm1 (bg=10.22%)▶rbm15 (bg=11.59%)▶rbm22 (bg=12.69%)▶safb2 (bg=26.89%)▶SMNDC1 (bg=7.08%)▶srsf1 (bg=30.28%)▶srsf7 (bg=22.53%)▶SRSF9 (bg=9.67%)▶TAF15 (bg=9.06%)▶tra2a (bg=37.02%)▶TROVE2 (bg=6.96%)▶uchl5 (bg=18.56%)▶YWHAG (bg=9.14%)▶zc3h8 (bg=12.78%)▶znf622 (bg=18.79%)No matches to TargetScan


A

AGAAGAAAAAA  
Depth:4 (PIG)  
Ei-value:0.000, Pi-value:0.000  
Er-value:0.000, Pr-value:0.000  
eCLIP MATCHES▶aggf1 (bg=15.15%)▶AQR (bg=4.89%)▶bclaf1 (bg=17.67%)▶bud13 (bg=12.85%)▶cpsf6 (bg=13.45%)▶FASTKD2 (bg=4.54%)▶FTO (bg=1.11%)▶fxr2 (bg=10.1%)▶GPKOW (bg=5.66%)▶gtf2f1 (bg=10.18%)▶hltf (bg=24.28%)▶larp4 (bg=13.51%)▶MTPAP (bg=9.55%)▶npm1 (bg=10.22%)▶rbm22 (bg=12.69%)▶safb2 (bg=26.89%)▶SMNDC1 (bg=7.08%)▶srsf1 (bg=30.28%)▶srsf7 (bg=22.53%)▶SRSF9 (bg=9.67%)▶TAF15 (bg=9.06%)▶tra2a (bg=37.02%)▶TROVE2 (bg=6.96%)▶uchl5 (bg=18.56%)▶YWHAG (bg=9.14%)▶zc3h8 (bg=12.78%)▶znf622 (bg=18.79%)No matches to TargetScan


GAAGAAAAA

GAAGAAAAA  
Depth:6 (MOUSE)  
Ei-value:0.000, Pi-value:0.000  
Er-value:0.000, Pr-value:0.000  
eCLIP MATCHES▶aggf1 (bg=15.15%)▶AQR (bg=4.89%)▶bclaf1 (bg=17.67%)▶bud13 (bg=12.85%)▶cpsf6 (bg=13.45%)▶FASTKD2 (bg=4.54%)▶FTO (bg=1.11%)▶fxr2 (bg=10.1%)▶GPKOW (bg=5.66%)▶gtf2f1 (bg=10.18%)▶hltf (bg=24.28%)▶larp4 (bg=13.51%)▶MTPAP (bg=9.55%)▶npm1 (bg=10.22%)▶rbm22 (bg=12.69%)▶safb2 (bg=26.89%)▶SMNDC1 (bg=7.08%)▶srsf1 (bg=30.28%)▶srsf7 (bg=22.53%)▶SRSF9 (bg=9.67%)▶TAF15 (bg=9.06%)▶tra2a (bg=37.02%)▶TROVE2 (bg=6.96%)▶uchl5 (bg=18.56%)▶YWHAG (bg=9.14%)▶zc3h8 (bg=12.78%)▶znf622 (bg=18.79%)No matches to TargetScan


A

GAAGAAAAAA  
Depth:5 (COW)  
Ei-value:0.000, Pi-value:0.000  
Er-value:0.000, Pr-value:0.000  
eCLIP MATCHES▶aggf1 (bg=15.15%)▶AQR (bg=4.89%)▶bclaf1 (bg=17.67%)▶bud13 (bg=12.85%)▶cpsf6 (bg=13.45%)▶FASTKD2 (bg=4.54%)▶FTO (bg=1.11%)▶fxr2 (bg=10.1%)▶GPKOW (bg=5.66%)▶gtf2f1 (bg=10.18%)▶hltf (bg=24.28%)▶larp4 (bg=13.51%)▶MTPAP (bg=9.55%)▶npm1 (bg=10.22%)▶rbm22 (bg=12.69%)▶safb2 (bg=26.89%)▶SMNDC1 (bg=7.08%)▶srsf1 (bg=30.28%)▶srsf7 (bg=22.53%)▶SRSF9 (bg=9.67%)▶TAF15 (bg=9.06%)▶tra2a (bg=37.02%)▶TROVE2 (bg=6.96%)▶uchl5 (bg=18.56%)▶YWHAG (bg=9.14%)▶zc3h8 (bg=12.78%)▶znf622 (bg=18.79%)No matches to TargetScan


GACAAGCTAGGAAACAAAAA

GACAAGCTAGGAAACAAAAA  
Depth:2 (MARMOSET)  
Ei-value:0.000, Pi-value:0.000  
Er-value:0.000, Pr-value:0.000  
eCLIP MATCHES▶aggf1 (bg=15.15%)▶AQR (bg=4.89%)▶bclaf1 (bg=17.67%)▶bud13 (bg=12.85%)▶cpsf6 (bg=13.45%)▶FASTKD2 (bg=4.54%)▶FTO (bg=1.11%)▶fxr2 (bg=10.1%)▶GPKOW (bg=5.66%)▶gtf2f1 (bg=10.18%)▶hltf (bg=24.28%)▶larp4 (bg=13.51%)▶MTPAP (bg=9.55%)▶npm1 (bg=10.22%)▶rbm22 (bg=12.69%)▶safb2 (bg=26.89%)▶SMNDC1 (bg=7.08%)▶srsf1 (bg=30.28%)▶srsf7 (bg=22.53%)▶SRSF9 (bg=9.67%)▶TAF15 (bg=9.06%)▶tra2a (bg=37.02%)▶TROVE2 (bg=6.96%)▶uchl5 (bg=18.56%)▶YWHAG (bg=9.14%)▶zc3h8 (bg=12.78%)▶znf622 (bg=18.79%)MATCHES To TargetScan▶ miR-129-5p:UUUUUGC

-

CTAAGGGCAAAATGT

CTAAGGGCAAAATGT  
Depth:2 (MARMOSET)  
Ei-value:0.000, Pi-value:0.000  
Er-value:0.000, Pr-value:0.000  
eCLIP MATCHES▶aggf1 (bg=15.15%)▶AQR (bg=4.89%)▶bud13 (bg=12.85%)▶cpsf6 (bg=13.45%)▶FASTKD2 (bg=4.54%)▶FTO (bg=1.11%)▶fxr2 (bg=10.1%)▶GPKOW (bg=5.66%)▶hltf (bg=24.28%)▶larp4 (bg=13.51%)▶npm1 (bg=10.22%)▶rbm22 (bg=12.69%)▶safb2 (bg=26.89%)▶srsf1 (bg=30.28%)▶srsf7 (bg=22.53%)▶SRSF9 (bg=9.67%)▶TAF15 (bg=9.06%)▶tra2a (bg=37.02%)▶TROVE2 (bg=6.96%)▶uchl5 (bg=18.56%)▶zc3h8 (bg=12.78%)▶znf622 (bg=18.79%)MATCHES To TargetScan▶ miR-874-3p:UGCCCUG

-

CAAACTT

CAAACTTAGAAGAAAA  
Depth:2 (MARMOSET)  
Ei-value:0.000, Pi-value:0.000  
Er-value:0.000, Pr-value:0.000  
eCLIP MATCHES▶aggf1 (bg=15.15%)▶AQR (bg=4.89%)▶bclaf1 (bg=17.67%)▶bud13 (bg=12.85%)▶cpsf6 (bg=13.45%)▶FASTKD2 (bg=4.54%)▶fxr2 (bg=10.1%)▶GPKOW (bg=5.66%)▶larp4 (bg=13.51%)▶npm1 (bg=10.22%)▶rbm22 (bg=12.69%)▶safb2 (bg=26.89%)▶srsf1 (bg=30.28%)▶srsf7 (bg=22.53%)▶tra2a (bg=37.02%)▶TROVE2 (bg=6.96%)▶uchl5 (bg=18.56%)▶zc3h8 (bg=12.78%)▶znf622 (bg=18.79%)No matches to TargetScan


agaagaa

agaagaa  
Depth:3 (DOG)  
Ei-value:0.350, Pi-value:0.010  
Er-value:0.000, Pr-value:0.010  
eCLIP MATCHES▶aggf1 (bg=15.15%)▶bclaf1 (bg=17.67%)▶bud13 (bg=12.85%)▶cpsf6 (bg=13.45%)▶FASTKD2 (bg=4.54%)▶fxr2 (bg=10.1%)▶GPKOW (bg=5.66%)▶larp4 (bg=13.51%)▶npm1 (bg=10.22%)▶rbm22 (bg=12.69%)▶safb2 (bg=26.89%)▶srsf1 (bg=30.28%)▶srsf7 (bg=22.53%)▶tra2a (bg=37.02%)▶TROVE2 (bg=6.96%)▶uchl5 (bg=18.56%)▶zc3h8 (bg=12.78%)▶znf622 (bg=18.79%)No matches to TargetScan


AA

CAAACTTAGAAGAAAA  
Depth:2 (MARMOSET)  
Ei-value:0.000, Pi-value:0.000  
Er-value:0.000, Pr-value:0.000  
eCLIP MATCHES▶aggf1 (bg=15.15%)▶AQR (bg=4.89%)▶bclaf1 (bg=17.67%)▶bud13 (bg=12.85%)▶cpsf6 (bg=13.45%)▶FASTKD2 (bg=4.54%)▶fxr2 (bg=10.1%)▶GPKOW (bg=5.66%)▶larp4 (bg=13.51%)▶npm1 (bg=10.22%)▶rbm22 (bg=12.69%)▶safb2 (bg=26.89%)▶srsf1 (bg=30.28%)▶srsf7 (bg=22.53%)▶tra2a (bg=37.02%)▶TROVE2 (bg=6.96%)▶uchl5 (bg=18.56%)▶zc3h8 (bg=12.78%)▶znf622 (bg=18.79%)No matches to TargetScan

---

gaagatagaa

gaagatagaa  
Depth:2 (MARMOSET)  
Ei-value:1.000, Pi-value:0.000  
Er-value:0.000, Pr-value:0.000  
eCLIP MATCHES▶aggf1 (bg=15.15%)▶bclaf1 (bg=17.67%)▶bud13 (bg=12.85%)▶cpsf6 (bg=13.45%)▶FASTKD2 (bg=4.54%)▶fxr2 (bg=10.1%)▶GPKOW (bg=5.66%)▶larp4 (bg=13.51%)▶npm1 (bg=10.22%)▶rbm22 (bg=12.69%)▶safb2 (bg=26.89%)▶SMNDC1 (bg=7.08%)▶srsf1 (bg=30.28%)▶tra2a (bg=37.02%)▶TROVE2 (bg=6.96%)▶uchl5 (bg=18.56%)▶zc3h8 (bg=12.78%)▶znf622 (bg=18.79%)No matches to TargetScan

- 840  
 ----------------

AATATTGTCAAGAGTTTCAGA

AATATTGTCAAGAGTTTCAGATAGAAAATGAAAA  
Depth:2 (MARMOSET)  
Ei-value:0.000, Pi-value:0.000  
Er-value:0.000, Pr-value:0.000  
eCLIP MATCHES▶aggf1 (bg=15.15%)▶bclaf1 (bg=17.67%)▶bud13 (bg=12.85%)▶cpsf6 (bg=13.45%)▶FASTKD2 (bg=4.54%)▶FTO (bg=1.11%)▶fxr2 (bg=10.1%)▶GPKOW (bg=5.66%)▶hltf (bg=24.28%)▶larp4 (bg=13.51%)▶MTPAP (bg=9.55%)▶rbm22 (bg=12.69%)▶safb2 (bg=26.89%)▶srsf1 (bg=30.28%)▶srsf7 (bg=22.53%)▶tra2a (bg=37.02%)▶TROVE2 (bg=6.96%)▶uchl5 (bg=18.56%)▶YWHAG (bg=9.14%)▶zc3h8 (bg=12.78%)▶znf622 (bg=18.79%)▶ZNF800 (bg=3.2%)MATCHES To TargetScan▶ miR-653-5p:UGAAACA


TAGAAAATGA

TAGAAAATGA  
Depth:4 (PIG)  
Ei-value:0.000, Pi-value:0.000  
Er-value:0.000, Pr-value:0.000  
eCLIP MATCHES▶aggf1 (bg=15.15%)▶bclaf1 (bg=17.67%)▶bud13 (bg=12.85%)▶cpsf6 (bg=13.45%)▶FASTKD2 (bg=4.54%)▶FTO (bg=1.11%)▶fxr2 (bg=10.1%)▶GPKOW (bg=5.66%)▶hltf (bg=24.28%)▶larp4 (bg=13.51%)▶MTPAP (bg=9.55%)▶rbm22 (bg=12.69%)▶safb2 (bg=26.89%)▶srsf1 (bg=30.28%)▶srsf7 (bg=22.53%)▶tra2a (bg=37.02%)▶TROVE2 (bg=6.96%)▶uchl5 (bg=18.56%)▶YWHAG (bg=9.14%)▶zc3h8 (bg=12.78%)▶znf622 (bg=18.79%)▶ZNF800 (bg=3.2%)No matches to TargetScan


AAA

TAGAAAATGAAAA  
Depth:3 (DOG)  
Ei-value:0.000, Pi-value:0.000  
Er-value:0.000, Pr-value:0.000  
eCLIP MATCHES▶aggf1 (bg=15.15%)▶bclaf1 (bg=17.67%)▶bud13 (bg=12.85%)▶cpsf6 (bg=13.45%)▶FASTKD2 (bg=4.54%)▶FTO (bg=1.11%)▶fxr2 (bg=10.1%)▶GPKOW (bg=5.66%)▶hltf (bg=24.28%)▶larp4 (bg=13.51%)▶MTPAP (bg=9.55%)▶rbm22 (bg=12.69%)▶safb2 (bg=26.89%)▶srsf1 (bg=30.28%)▶srsf7 (bg=22.53%)▶tra2a (bg=37.02%)▶TROVE2 (bg=6.96%)▶uchl5 (bg=18.56%)▶YWHAG (bg=9.14%)▶zc3h8 (bg=12.78%)▶znf622 (bg=18.79%)▶ZNF800 (bg=3.2%)No matches to TargetScan

----

GCTAAGACAAGTATTGGA

GCTAAGACAAGTATTGGA  
Depth:2 (MARMOSET)  
Ei-value:0.000, Pi-value:0.000  
Er-value:0.000, Pr-value:0.000  
eCLIP MATCHES▶aggf1 (bg=15.15%)▶bclaf1 (bg=17.67%)▶bud13 (bg=12.85%)▶cpsf6 (bg=13.45%)▶FASTKD2 (bg=4.54%)▶FTO (bg=1.11%)▶fxr2 (bg=10.1%)▶GPKOW (bg=5.66%)▶hltf (bg=24.28%)▶larp4 (bg=13.51%)▶MTPAP (bg=9.55%)▶rbm15 (bg=11.59%)▶rbm22 (bg=12.69%)▶safb2 (bg=26.89%)▶srsf1 (bg=30.28%)▶srsf7 (bg=22.53%)▶SUPV3L1 (bg=9.63%)▶tra2a (bg=37.02%)▶TROVE2 (bg=6.96%)▶uchl5 (bg=18.56%)▶YWHAG (bg=9.14%)▶zc3h8 (bg=12.78%)▶znf622 (bg=18.79%)▶ZNF800 (bg=3.2%)MATCHES To TargetScan▶ miR-200bc-3p/429:AAUACUG

-----

atag

atagaagatag  
Depth:2 (MARMOSET)  
Ei-value:1.000, Pi-value:0.000  
Er-value:0.000, Pr-value:0.000  
eCLIP MATCHES▶aggf1 (bg=15.15%)▶bclaf1 (bg=17.67%)▶bud13 (bg=12.85%)▶cpsf6 (bg=13.45%)▶FASTKD2 (bg=4.54%)▶FTO (bg=1.11%)▶fxr2 (bg=10.1%)▶GPKOW (bg=5.66%)▶hltf (bg=24.28%)▶larp4 (bg=13.51%)▶MTPAP (bg=9.55%)▶rbm15 (bg=11.59%)▶rbm22 (bg=12.69%)▶safb2 (bg=26.89%)▶srsf1 (bg=30.28%)▶srsf7 (bg=22.53%)▶SUPV3L1 (bg=9.63%)▶tra2a (bg=37.02%)▶uchl5 (bg=18.56%)▶YWHAG (bg=9.14%)▶zc3h8 (bg=12.78%)▶znf622 (bg=18.79%)▶ZNF800 (bg=3.2%)No matches to TargetScan


AAGATAG

AAGATAG  
Depth:4 (PIG)  
Ei-value:0.000, Pi-value:0.010  
Er-value:0.000, Pr-value:0.000  
eCLIP MATCHES▶aggf1 (bg=15.15%)▶bclaf1 (bg=17.67%)▶bud13 (bg=12.85%)▶cpsf6 (bg=13.45%)▶FASTKD2 (bg=4.54%)▶fxr2 (bg=10.1%)▶GPKOW (bg=5.66%)▶hltf (bg=24.28%)▶larp4 (bg=13.51%)▶MTPAP (bg=9.55%)▶rbm15 (bg=11.59%)▶rbm22 (bg=12.69%)▶safb2 (bg=26.89%)▶srsf1 (bg=30.28%)▶srsf7 (bg=22.53%)▶SUPV3L1 (bg=9.63%)▶tra2a (bg=37.02%)▶uchl5 (bg=18.56%)▶YWHAG (bg=9.14%)▶znf622 (bg=18.79%)▶ZNF800 (bg=3.2%)No matches to TargetScan

--------------

A

AAAAATTGGA  
Depth:4 (PIG)  
Ei-value:0.000, Pi-value:0.000  
Er-value:0.000, Pr-value:0.000  
eCLIP MATCHES▶aggf1 (bg=15.15%)▶bclaf1 (bg=17.67%)▶bud13 (bg=12.85%)▶FASTKD2 (bg=4.54%)▶fxr2 (bg=10.1%)▶GPKOW (bg=5.66%)▶hltf (bg=24.28%)▶larp4 (bg=13.51%)▶MTPAP (bg=9.55%)▶NIPBL (bg=8.2%)▶npm1 (bg=10.22%)▶rbm15 (bg=11.59%)▶safb2 (bg=26.89%)▶srsf7 (bg=22.53%)▶tra2a (bg=37.02%)▶uchl5 (bg=18.56%)▶YWHAG (bg=9.14%)▶znf622 (bg=18.79%)No matches to TargetScan


AAAATTGGA

AAAATTGGA  
Depth:5 (COW)  
Ei-value:0.000, Pi-value:0.000  
Er-value:0.000, Pr-value:0.000  
eCLIP MATCHES▶aggf1 (bg=15.15%)▶bclaf1 (bg=17.67%)▶bud13 (bg=12.85%)▶FASTKD2 (bg=4.54%)▶fxr2 (bg=10.1%)▶GPKOW (bg=5.66%)▶hltf (bg=24.28%)▶larp4 (bg=13.51%)▶MTPAP (bg=9.55%)▶NIPBL (bg=8.2%)▶npm1 (bg=10.22%)▶rbm15 (bg=11.59%)▶safb2 (bg=26.89%)▶srsf7 (bg=22.53%)▶tra2a (bg=37.02%)▶uchl5 (bg=18.56%)▶YWHAG (bg=9.14%)▶znf622 (bg=18.79%)No matches to TargetScan


TAAAATAG

AAAAATTGGATAAAATAGCAC  
Depth:2 (MARMOSET)  
Ei-value:0.000, Pi-value:0.000  
Er-value:0.000, Pr-value:0.000  
eCLIP MATCHES▶aggf1 (bg=15.15%)▶bclaf1 (bg=17.67%)▶bud13 (bg=12.85%)▶FASTKD2 (bg=4.54%)▶fxr2 (bg=10.1%)▶GPKOW (bg=5.66%)▶hltf (bg=24.28%)▶larp4 (bg=13.51%)▶MTPAP (bg=9.55%)▶NIPBL (bg=8.2%)▶npm1 (bg=10.22%)▶rbm15 (bg=11.59%)▶safb2 (bg=26.89%)▶srsf7 (bg=22.53%)▶tra2a (bg=37.02%)▶uchl5 (bg=18.56%)▶YWHAG (bg=9.14%)▶znf622 (bg=18.79%)▶ZNF800 (bg=3.2%)No matches to TargetScan

 960  


CAC

AAAAATTGGATAAAATAGCAC  
Depth:2 (MARMOSET)  
Ei-value:0.000, Pi-value:0.000  
Er-value:0.000, Pr-value:0.000  
eCLIP MATCHES▶aggf1 (bg=15.15%)▶bclaf1 (bg=17.67%)▶bud13 (bg=12.85%)▶FASTKD2 (bg=4.54%)▶fxr2 (bg=10.1%)▶GPKOW (bg=5.66%)▶hltf (bg=24.28%)▶larp4 (bg=13.51%)▶MTPAP (bg=9.55%)▶NIPBL (bg=8.2%)▶npm1 (bg=10.22%)▶rbm15 (bg=11.59%)▶safb2 (bg=26.89%)▶srsf7 (bg=22.53%)▶tra2a (bg=37.02%)▶uchl5 (bg=18.56%)▶YWHAG (bg=9.14%)▶znf622 (bg=18.79%)▶ZNF800 (bg=3.2%)No matches to TargetScan

-

gaaaaaatga

gaaaaaatga  
Depth:2 (MARMOSET)  
Ei-value:1.000, Pi-value:0.000  
Er-value:0.000, Pr-value:0.000  
eCLIP MATCHES▶aggf1 (bg=15.15%)▶bclaf1 (bg=17.67%)▶bud13 (bg=12.85%)▶FASTKD2 (bg=4.54%)▶fxr2 (bg=10.1%)▶GPKOW (bg=5.66%)▶hltf (bg=24.28%)▶larp4 (bg=13.51%)▶MTPAP (bg=9.55%)▶npm1 (bg=10.22%)▶rbm15 (bg=11.59%)▶safb2 (bg=26.89%)▶srsf7 (bg=22.53%)▶tra2a (bg=37.02%)▶uchl5 (bg=18.56%)▶YWHAG (bg=9.14%)▶znf622 (bg=18.79%)▶ZNF800 (bg=3.2%)No matches to TargetScan

---

aattattg

aattattg  
Depth:2 (MARMOSET)  
Ei-value:1.000, Pi-value:0.020  
Er-value:0.000, Pr-value:0.000  
eCLIP MATCHES▶aggf1 (bg=15.15%)▶AQR (bg=4.89%)▶bclaf1 (bg=17.67%)▶bud13 (bg=12.85%)▶FASTKD2 (bg=4.54%)▶fxr2 (bg=10.1%)▶GPKOW (bg=5.66%)▶hltf (bg=24.28%)▶larp4 (bg=13.51%)▶MTPAP (bg=9.55%)▶rbm15 (bg=11.59%)▶safb2 (bg=26.89%)▶tra2a (bg=37.02%)▶uchl5 (bg=18.56%)▶YWHAG (bg=9.14%)▶ZNF800 (bg=3.2%)No matches to TargetScan

--

aaccaa

aaccaa  
Depth:2 (MARMOSET)  
Ei-value:1.000, Pi-value:0.060  
Er-value:0.000, Pr-value:0.030  
eCLIP MATCHES▶aggf1 (bg=15.15%)▶AQR (bg=4.89%)▶bud13 (bg=12.85%)▶FASTKD2 (bg=4.54%)▶fxr2 (bg=10.1%)▶GPKOW (bg=5.66%)▶hltf (bg=24.28%)▶larp4 (bg=13.51%)▶rbm15 (bg=11.59%)▶safb2 (bg=26.89%)▶tra2a (bg=37.02%)▶uchl5 (bg=18.56%)No matches to TargetScan

-----

TTT

TTTAAAAGCCCATCAATTTAATTTCTG  
Depth:2 (MARMOSET)  
Ei-value:0.000, Pi-value:0.000  
Er-value:0.000, Pr-value:0.000  
eCLIP MATCHES▶aggf1 (bg=15.15%)▶AQR (bg=4.89%)▶bclaf1 (bg=17.67%)▶cpsf6 (bg=13.45%)▶DDX24 (bg=0.47%)▶fxr2 (bg=10.1%)▶hltf (bg=24.28%)▶larp4 (bg=13.51%)▶MTPAP (bg=9.55%)▶npm1 (bg=10.22%)▶ppil4 (bg=43.39%)▶safb (bg=40.39%)▶safb2 (bg=26.89%)▶SND1 (bg=0.87%)▶srsf1 (bg=30.28%)▶SUPV3L1 (bg=9.63%)▶TAF15 (bg=9.06%)▶tra2a (bg=37.02%)▶uchl5 (bg=18.56%)▶YBX3 (bg=0.84%)▶znf622 (bg=18.79%)No matches to TargetScan


AAAAGCC

AAAAGCC  
Depth:6 (MOUSE)  
Ei-value:0.000, Pi-value:0.000  
Er-value:0.000, Pr-value:0.000  
eCLIP MATCHES▶aggf1 (bg=15.15%)▶AQR (bg=4.89%)▶bclaf1 (bg=17.67%)▶DDX24 (bg=0.47%)▶fxr2 (bg=10.1%)▶hltf (bg=24.28%)▶larp4 (bg=13.51%)▶MTPAP (bg=9.55%)▶ppil4 (bg=43.39%)▶safb (bg=40.39%)▶safb2 (bg=26.89%)▶SND1 (bg=0.87%)▶srsf1 (bg=30.28%)▶tra2a (bg=37.02%)▶uchl5 (bg=18.56%)▶YBX3 (bg=0.84%)▶znf622 (bg=18.79%)No matches to TargetScan


CAT

AAAAGCCCAT  
Depth:5 (COW)  
Ei-value:0.000, Pi-value:0.000  
Er-value:0.000, Pr-value:0.000  
eCLIP MATCHES▶aggf1 (bg=15.15%)▶AQR (bg=4.89%)▶bclaf1 (bg=17.67%)▶DDX24 (bg=0.47%)▶fxr2 (bg=10.1%)▶hltf (bg=24.28%)▶larp4 (bg=13.51%)▶MTPAP (bg=9.55%)▶npm1 (bg=10.22%)▶ppil4 (bg=43.39%)▶safb (bg=40.39%)▶safb2 (bg=26.89%)▶SND1 (bg=0.87%)▶srsf1 (bg=30.28%)▶tra2a (bg=37.02%)▶uchl5 (bg=18.56%)▶YBX3 (bg=0.84%)▶znf622 (bg=18.79%)No matches to TargetScan


C

TTTAAAAGCCCATCAATTTAATTTCTG  
Depth:2 (MARMOSET)  
Ei-value:0.000, Pi-value:0.000  
Er-value:0.000, Pr-value:0.000  
eCLIP MATCHES▶aggf1 (bg=15.15%)▶AQR (bg=4.89%)▶bclaf1 (bg=17.67%)▶cpsf6 (bg=13.45%)▶DDX24 (bg=0.47%)▶fxr2 (bg=10.1%)▶hltf (bg=24.28%)▶larp4 (bg=13.51%)▶MTPAP (bg=9.55%)▶npm1 (bg=10.22%)▶ppil4 (bg=43.39%)▶safb (bg=40.39%)▶safb2 (bg=26.89%)▶SND1 (bg=0.87%)▶srsf1 (bg=30.28%)▶SUPV3L1 (bg=9.63%)▶TAF15 (bg=9.06%)▶tra2a (bg=37.02%)▶uchl5 (bg=18.56%)▶YBX3 (bg=0.84%)▶znf622 (bg=18.79%)No matches to TargetScan


AA

AATTTAATTTCTG  
Depth:3 (DOG)  
Ei-value:0.000, Pi-value:0.000  
Er-value:0.000, Pr-value:0.000  
eCLIP MATCHES▶aggf1 (bg=15.15%)▶AQR (bg=4.89%)▶bclaf1 (bg=17.67%)▶cpsf6 (bg=13.45%)▶DDX24 (bg=0.47%)▶hltf (bg=24.28%)▶larp4 (bg=13.51%)▶MTPAP (bg=9.55%)▶npm1 (bg=10.22%)▶ppil4 (bg=43.39%)▶safb (bg=40.39%)▶safb2 (bg=26.89%)▶SND1 (bg=0.87%)▶srsf1 (bg=30.28%)▶SUPV3L1 (bg=9.63%)▶TAF15 (bg=9.06%)▶tra2a (bg=37.02%)▶uchl5 (bg=18.56%)▶YBX3 (bg=0.84%)▶znf622 (bg=18.79%)No matches to TargetScan


TTTAATTT

TTTAATTT  
Depth:4 (PIG)  
Ei-value:0.000, Pi-value:0.000  
Er-value:0.000, Pr-value:0.000  
eCLIP MATCHES▶aggf1 (bg=15.15%)▶AQR (bg=4.89%)▶bclaf1 (bg=17.67%)▶cpsf6 (bg=13.45%)▶DDX24 (bg=0.47%)▶hltf (bg=24.28%)▶larp4 (bg=13.51%)▶npm1 (bg=10.22%)▶ppil4 (bg=43.39%)▶safb (bg=40.39%)▶safb2 (bg=26.89%)▶SND1 (bg=0.87%)▶srsf1 (bg=30.28%)▶TAF15 (bg=9.06%)▶tra2a (bg=37.02%)▶uchl5 (bg=18.56%)▶YBX3 (bg=0.84%)▶znf622 (bg=18.79%)No matches to TargetScan


CTG

AATTTAATTTCTG  
Depth:3 (DOG)  
Ei-value:0.000, Pi-value:0.000  
Er-value:0.000, Pr-value:0.000  
eCLIP MATCHES▶aggf1 (bg=15.15%)▶AQR (bg=4.89%)▶bclaf1 (bg=17.67%)▶cpsf6 (bg=13.45%)▶DDX24 (bg=0.47%)▶hltf (bg=24.28%)▶larp4 (bg=13.51%)▶MTPAP (bg=9.55%)▶npm1 (bg=10.22%)▶ppil4 (bg=43.39%)▶safb (bg=40.39%)▶safb2 (bg=26.89%)▶SND1 (bg=0.87%)▶srsf1 (bg=30.28%)▶SUPV3L1 (bg=9.63%)▶TAF15 (bg=9.06%)▶tra2a (bg=37.02%)▶uchl5 (bg=18.56%)▶YBX3 (bg=0.84%)▶znf622 (bg=18.79%)No matches to TargetScan

-

TG

TGGTGCAGAAGTTAGAAGGTAAAG  
Depth:2 (MARMOSET)  
Ei-value:0.000, Pi-value:0.000  
Er-value:0.000, Pr-value:0.000  
eCLIP MATCHES▶aggf1 (bg=15.15%)▶bclaf1 (bg=17.67%)▶cpsf6 (bg=13.45%)▶DDX24 (bg=0.47%)▶fxr2 (bg=10.1%)▶gtf2f1 (bg=10.18%)▶hltf (bg=24.28%)▶larp4 (bg=13.51%)▶npm1 (bg=10.22%)▶ppil4 (bg=43.39%)▶safb (bg=40.39%)▶safb2 (bg=26.89%)▶SND1 (bg=0.87%)▶srsf1 (bg=30.28%)▶srsf7 (bg=22.53%)▶SUPV3L1 (bg=9.63%)▶TAF15 (bg=9.06%)▶tra2a (bg=37.02%)▶uchl5 (bg=18.56%)▶YBX3 (bg=0.84%)▶znf622 (bg=18.79%)No matches to TargetScan


GTGCAGAAG

GTGCAGAAG  
Depth:4 (PIG)  
Ei-value:0.000, Pi-value:0.000  
Er-value:0.000, Pr-value:0.000  
eCLIP MATCHES▶aggf1 (bg=15.15%)▶bclaf1 (bg=17.67%)▶cpsf6 (bg=13.45%)▶DDX24 (bg=0.47%)▶gtf2f1 (bg=10.18%)▶hltf (bg=24.28%)▶larp4 (bg=13.51%)▶npm1 (bg=10.22%)▶ppil4 (bg=43.39%)▶safb (bg=40.39%)▶safb2 (bg=26.89%)▶SND1 (bg=0.87%)▶srsf1 (bg=30.28%)▶srsf7 (bg=22.53%)▶SUPV3L1 (bg=9.63%)▶TAF15 (bg=9.06%)▶tra2a (bg=37.02%)▶uchl5 (bg=18.56%)▶YBX3 (bg=0.84%)▶znf622 (bg=18.79%)No matches to TargetScan


TTAGAAGGTAAAG

TGGTGCAGAAGTTAGAAGGTAAAG  
Depth:2 (MARMOSET)  
Ei-value:0.000, Pi-value:0.000  
Er-value:0.000, Pr-value:0.000  
eCLIP MATCHES▶aggf1 (bg=15.15%)▶bclaf1 (bg=17.67%)▶cpsf6 (bg=13.45%)▶DDX24 (bg=0.47%)▶fxr2 (bg=10.1%)▶gtf2f1 (bg=10.18%)▶hltf (bg=24.28%)▶larp4 (bg=13.51%)▶npm1 (bg=10.22%)▶ppil4 (bg=43.39%)▶safb (bg=40.39%)▶safb2 (bg=26.89%)▶SND1 (bg=0.87%)▶srsf1 (bg=30.28%)▶srsf7 (bg=22.53%)▶SUPV3L1 (bg=9.63%)▶TAF15 (bg=9.06%)▶tra2a (bg=37.02%)▶uchl5 (bg=18.56%)▶YBX3 (bg=0.84%)▶znf622 (bg=18.79%)No matches to TargetScan

----

agaaga

agaagatgagggt  
Depth:2 (MARMOSET)  
Ei-value:0.680, Pi-value:0.000  
Er-value:0.000, Pr-value:0.000  
eCLIP MATCHES▶aggf1 (bg=15.15%)▶bclaf1 (bg=17.67%)▶cpsf6 (bg=13.45%)▶fxr2 (bg=10.1%)▶gtf2f1 (bg=10.18%)▶hltf (bg=24.28%)▶larp4 (bg=13.51%)▶MTPAP (bg=9.55%)▶npm1 (bg=10.22%)▶ppil4 (bg=43.39%)▶rbm22 (bg=12.69%)▶safb (bg=40.39%)▶safb2 (bg=26.89%)▶SND1 (bg=0.87%)▶srsf1 (bg=30.28%)▶srsf7 (bg=22.53%)▶SUPV3L1 (bg=9.63%)▶TAF15 (bg=9.06%)▶tra2a (bg=37.02%)▶uchl5 (bg=18.56%)▶YBX3 (bg=0.84%)▶znf622 (bg=18.79%)No matches to TargetScan


t

tgagggt  
Depth:3 (DOG)  
Ei-value:0.350, Pi-value:0.000  
Er-value:0.000, Pr-value:0.010  
eCLIP MATCHES▶aggf1 (bg=15.15%)▶bclaf1 (bg=17.67%)▶cpsf6 (bg=13.45%)▶fxr2 (bg=10.1%)▶gtf2f1 (bg=10.18%)▶hltf (bg=24.28%)▶larp4 (bg=13.51%)▶MTPAP (bg=9.55%)▶npm1 (bg=10.22%)▶ppil4 (bg=43.39%)▶rbm22 (bg=12.69%)▶safb (bg=40.39%)▶safb2 (bg=26.89%)▶SND1 (bg=0.87%)▶srsf1 (bg=30.28%)▶srsf7 (bg=22.53%)▶SUPV3L1 (bg=9.63%)▶TAF15 (bg=9.06%)▶tra2a (bg=37.02%)▶uchl5 (bg=18.56%)▶znf622 (bg=18.79%)No matches to TargetScan


GAGGGT

GAGGGT  
Depth:5 (COW)  
Ei-value:0.000, Pi-value:0.010  
Er-value:0.000, Pr-value:0.000  
eCLIP MATCHES▶aggf1 (bg=15.15%)▶bclaf1 (bg=17.67%)▶cpsf6 (bg=13.45%)▶fxr2 (bg=10.1%)▶gtf2f1 (bg=10.18%)▶hltf (bg=24.28%)▶larp4 (bg=13.51%)▶MTPAP (bg=9.55%)▶npm1 (bg=10.22%)▶ppil4 (bg=43.39%)▶rbm22 (bg=12.69%)▶safb (bg=40.39%)▶safb2 (bg=26.89%)▶SND1 (bg=0.87%)▶srsf1 (bg=30.28%)▶srsf7 (bg=22.53%)▶SUPV3L1 (bg=9.63%)▶TAF15 (bg=9.06%)▶tra2a (bg=37.02%)▶uchl5 (bg=18.56%)▶znf622 (bg=18.79%)No matches to TargetScan

------

GTAGACC

GTAGACC  
Depth:5 (COW)  
Ei-value:0.000, Pi-value:0.000  
Er-value:0.000, Pr-value:0.000  
eCLIP MATCHES▶aggf1 (bg=15.15%)▶bclaf1 (bg=17.67%)▶cpsf6 (bg=13.45%)▶fxr2 (bg=10.1%)▶gtf2f1 (bg=10.18%)▶hltf (bg=24.28%)▶larp4 (bg=13.51%)▶MTPAP (bg=9.55%)▶npm1 (bg=10.22%)▶ppil4 (bg=43.39%)▶rbm15 (bg=11.59%)▶rbm22 (bg=12.69%)▶safb (bg=40.39%)▶safb2 (bg=26.89%)▶srsf1 (bg=30.28%)▶srsf7 (bg=22.53%)▶SUPV3L1 (bg=9.63%)▶TAF15 (bg=9.06%)▶tra2a (bg=37.02%)▶uchl5 (bg=18.56%)▶znf622 (bg=18.79%)No matches to TargetScan

 1080  


GTAGACC  
Depth:5 (COW)  
Ei-value:0.000, Pi-value:0.000  
Er-value:0.000, Pr-value:0.000  
eCLIP MATCHES▶aggf1 (bg=15.15%)▶bclaf1 (bg=17.67%)▶cpsf6 (bg=13.45%)▶fxr2 (bg=10.1%)▶gtf2f1 (bg=10.18%)▶hltf (bg=24.28%)▶larp4 (bg=13.51%)▶MTPAP (bg=9.55%)▶npm1 (bg=10.22%)▶ppil4 (bg=43.39%)▶rbm15 (bg=11.59%)▶rbm22 (bg=12.69%)▶safb (bg=40.39%)▶safb2 (bg=26.89%)▶srsf1 (bg=30.28%)▶srsf7 (bg=22.53%)▶SUPV3L1 (bg=9.63%)▶TAF15 (bg=9.06%)▶tra2a (bg=37.02%)▶uchl5 (bg=18.56%)▶znf622 (bg=18.79%)No matches to TargetScan


aga

gtagaccaga  
Depth:2 (MARMOSET)  
Ei-value:1.000, Pi-value:0.000  
Er-value:0.000, Pr-value:0.000  
eCLIP MATCHES▶aggf1 (bg=15.15%)▶bclaf1 (bg=17.67%)▶cpsf6 (bg=13.45%)▶fxr2 (bg=10.1%)▶gtf2f1 (bg=10.18%)▶hltf (bg=24.28%)▶larp4 (bg=13.51%)▶MTPAP (bg=9.55%)▶npm1 (bg=10.22%)▶ppil4 (bg=43.39%)▶rbm15 (bg=11.59%)▶rbm22 (bg=12.69%)▶safb (bg=40.39%)▶safb2 (bg=26.89%)▶srsf1 (bg=30.28%)▶srsf7 (bg=22.53%)▶SUPV3L1 (bg=9.63%)▶TAF15 (bg=9.06%)▶tra2a (bg=37.02%)▶uchl5 (bg=18.56%)▶znf622 (bg=18.79%)No matches to TargetScan

-

CCAATTTAGAAGAATA

CCAATTTAGAAGAATA  
Depth:2 (MARMOSET)  
Ei-value:0.000, Pi-value:0.000  
Er-value:0.000, Pr-value:0.000  
eCLIP MATCHES▶aggf1 (bg=15.15%)▶bclaf1 (bg=17.67%)▶cpsf6 (bg=13.45%)▶EIF3H (bg=0.83%)▶fxr2 (bg=10.1%)▶gtf2f1 (bg=10.18%)▶hltf (bg=24.28%)▶larp4 (bg=13.51%)▶MTPAP (bg=9.55%)▶npm1 (bg=10.22%)▶ppil4 (bg=43.39%)▶rbm15 (bg=11.59%)▶rbm22 (bg=12.69%)▶safb (bg=40.39%)▶safb2 (bg=26.89%)▶SLTM (bg=7.5%)▶srsf1 (bg=30.28%)▶srsf7 (bg=22.53%)▶SRSF9 (bg=9.67%)▶SUPV3L1 (bg=9.63%)▶TAF15 (bg=9.06%)▶tra2a (bg=37.02%)▶uchl5 (bg=18.56%)▶YWHAG (bg=9.14%)▶znf622 (bg=18.79%)No matches to TargetScan

-

TTGA

TTGAAGCTAGAAGGG  
Depth:2 (MARMOSET)  
Ei-value:0.000, Pi-value:0.000  
Er-value:0.000, Pr-value:0.000  
eCLIP MATCHES▶aggf1 (bg=15.15%)▶bclaf1 (bg=17.67%)▶bud13 (bg=12.85%)▶cpsf6 (bg=13.45%)▶EIF3H (bg=0.83%)▶fxr2 (bg=10.1%)▶gtf2f1 (bg=10.18%)▶hltf (bg=24.28%)▶larp4 (bg=13.51%)▶MTPAP (bg=9.55%)▶npm1 (bg=10.22%)▶ppil4 (bg=43.39%)▶rbm15 (bg=11.59%)▶rbm22 (bg=12.69%)▶safb (bg=40.39%)▶safb2 (bg=26.89%)▶SLTM (bg=7.5%)▶srsf1 (bg=30.28%)▶srsf7 (bg=22.53%)▶SRSF9 (bg=9.67%)▶SUPV3L1 (bg=9.63%)▶TAF15 (bg=9.06%)▶tra2a (bg=37.02%)▶uchl5 (bg=18.56%)▶XRCC6 (bg=3.81%)▶YWHAG (bg=9.14%)▶znf622 (bg=18.79%)No matches to TargetScan


AGCTAGAAGGG

AGCTAGAAGGG  
Depth:3 (DOG)  
Ei-value:0.000, Pi-value:0.000  
Er-value:0.000, Pr-value:0.000  
eCLIP MATCHES▶aggf1 (bg=15.15%)▶bclaf1 (bg=17.67%)▶bud13 (bg=12.85%)▶cpsf6 (bg=13.45%)▶EIF3H (bg=0.83%)▶fxr2 (bg=10.1%)▶gtf2f1 (bg=10.18%)▶hltf (bg=24.28%)▶larp4 (bg=13.51%)▶MTPAP (bg=9.55%)▶npm1 (bg=10.22%)▶ppil4 (bg=43.39%)▶rbm15 (bg=11.59%)▶rbm22 (bg=12.69%)▶safb (bg=40.39%)▶safb2 (bg=26.89%)▶SLTM (bg=7.5%)▶srsf1 (bg=30.28%)▶srsf7 (bg=22.53%)▶SRSF9 (bg=9.67%)▶SUPV3L1 (bg=9.63%)▶TAF15 (bg=9.06%)▶tra2a (bg=37.02%)▶uchl5 (bg=18.56%)▶XRCC6 (bg=3.81%)▶YWHAG (bg=9.14%)▶znf622 (bg=18.79%)No matches to TargetScan

------

ggttaa

ggttaa  
Depth:2 (MARMOSET)  
Ei-value:1.000, Pi-value:0.110  
Er-value:0.000, Pr-value:0.030  
eCLIP MATCHES▶aggf1 (bg=15.15%)▶bclaf1 (bg=17.67%)▶bud13 (bg=12.85%)▶cpsf6 (bg=13.45%)▶EIF3H (bg=0.83%)▶fxr2 (bg=10.1%)▶gtf2f1 (bg=10.18%)▶hltf (bg=24.28%)▶larp4 (bg=13.51%)▶MTPAP (bg=9.55%)▶NIPBL (bg=8.2%)▶npm1 (bg=10.22%)▶ppil4 (bg=43.39%)▶rbm15 (bg=11.59%)▶rbm22 (bg=12.69%)▶safb (bg=40.39%)▶safb2 (bg=26.89%)▶SLTM (bg=7.5%)▶srsf1 (bg=30.28%)▶srsf7 (bg=22.53%)▶SRSF9 (bg=9.67%)▶SUPV3L1 (bg=9.63%)▶TAF15 (bg=9.06%)▶tra2a (bg=37.02%)▶uchl5 (bg=18.56%)▶XRCC6 (bg=3.81%)▶YWHAG (bg=9.14%)▶znf622 (bg=18.79%)No matches to TargetScan

------

cat

catcaaaaagct  
Depth:2 (MARMOSET)  
Ei-value:0.990, Pi-value:0.000  
Er-value:0.000, Pr-value:0.000  
eCLIP MATCHES▶aggf1 (bg=15.15%)▶bclaf1 (bg=17.67%)▶bud13 (bg=12.85%)▶cpsf6 (bg=13.45%)▶EIF3H (bg=0.83%)▶fxr2 (bg=10.1%)▶gtf2f1 (bg=10.18%)▶hltf (bg=24.28%)▶khdrbs1 (bg=10.41%)▶larp4 (bg=13.51%)▶MTPAP (bg=9.55%)▶NIPBL (bg=8.2%)▶npm1 (bg=10.22%)▶ppil4 (bg=43.39%)▶rbm15 (bg=11.59%)▶rbm22 (bg=12.69%)▶safb (bg=40.39%)▶safb2 (bg=26.89%)▶SLTM (bg=7.5%)▶srsf1 (bg=30.28%)▶srsf7 (bg=22.53%)▶SUPV3L1 (bg=9.63%)▶TAF15 (bg=9.06%)▶tra2a (bg=37.02%)▶uchl5 (bg=18.56%)▶XRCC6 (bg=3.81%)▶YWHAG (bg=9.14%)▶znf622 (bg=18.79%)MATCHES To TargetScan▶ miR-129-5p:UUUUUGC


caaaaag

caaaaag  
Depth:3 (DOG)  
Ei-value:0.350, Pi-value:0.010  
Er-value:0.000, Pr-value:0.000  
eCLIP MATCHES▶aggf1 (bg=15.15%)▶bclaf1 (bg=17.67%)▶bud13 (bg=12.85%)▶cpsf6 (bg=13.45%)▶EIF3H (bg=0.83%)▶fxr2 (bg=10.1%)▶gtf2f1 (bg=10.18%)▶hltf (bg=24.28%)▶khdrbs1 (bg=10.41%)▶larp4 (bg=13.51%)▶MTPAP (bg=9.55%)▶NIPBL (bg=8.2%)▶npm1 (bg=10.22%)▶ppil4 (bg=43.39%)▶rbm15 (bg=11.59%)▶rbm22 (bg=12.69%)▶safb (bg=40.39%)▶safb2 (bg=26.89%)▶SLTM (bg=7.5%)▶srsf1 (bg=30.28%)▶srsf7 (bg=22.53%)▶SUPV3L1 (bg=9.63%)▶TAF15 (bg=9.06%)▶tra2a (bg=37.02%)▶uchl5 (bg=18.56%)▶XRCC6 (bg=3.81%)▶YWHAG (bg=9.14%)▶znf622 (bg=18.79%)MATCHES To TargetScan▶ miR-129-5p:UUUUUGC


ct

catcaaaaagct  
Depth:2 (MARMOSET)  
Ei-value:0.990, Pi-value:0.000  
Er-value:0.000, Pr-value:0.000  
eCLIP MATCHES▶aggf1 (bg=15.15%)▶bclaf1 (bg=17.67%)▶bud13 (bg=12.85%)▶cpsf6 (bg=13.45%)▶EIF3H (bg=0.83%)▶fxr2 (bg=10.1%)▶gtf2f1 (bg=10.18%)▶hltf (bg=24.28%)▶khdrbs1 (bg=10.41%)▶larp4 (bg=13.51%)▶MTPAP (bg=9.55%)▶NIPBL (bg=8.2%)▶npm1 (bg=10.22%)▶ppil4 (bg=43.39%)▶rbm15 (bg=11.59%)▶rbm22 (bg=12.69%)▶safb (bg=40.39%)▶safb2 (bg=26.89%)▶SLTM (bg=7.5%)▶srsf1 (bg=30.28%)▶srsf7 (bg=22.53%)▶SUPV3L1 (bg=9.63%)▶TAF15 (bg=9.06%)▶tra2a (bg=37.02%)▶uchl5 (bg=18.56%)▶XRCC6 (bg=3.81%)▶YWHAG (bg=9.14%)▶znf622 (bg=18.79%)MATCHES To TargetScan▶ miR-129-5p:UUUUUGC

-------------------------

AAAAACTAAGGCAGAAGGCTTTTGGAA

AAAAACTAAGGCAGAAGGCTTTTGGAAGAGTTAGAAGAATTTGGAAGGCCTTAAA  
Depth:2 (MARMOSET)  
Ei-value:0.000, Pi-value:0.000  
Er-value:0.000, Pr-value:0.000  
eCLIP MATCHES▶aggf1 (bg=15.15%)▶AQR (bg=4.89%)▶bclaf1 (bg=17.67%)▶bud13 (bg=12.85%)▶cpsf6 (bg=13.45%)▶FUBP3 (bg=1.53%)▶gtf2f1 (bg=10.18%)▶hltf (bg=24.28%)▶larp4 (bg=13.51%)▶MTPAP (bg=9.55%)▶NIPBL (bg=8.2%)▶ppil4 (bg=43.39%)▶rbm15 (bg=11.59%)▶rbm22 (bg=12.69%)▶safb (bg=40.39%)▶safb2 (bg=26.89%)▶SLTM (bg=7.5%)▶srsf1 (bg=30.28%)▶srsf7 (bg=22.53%)▶SRSF9 (bg=9.67%)▶SUPV3L1 (bg=9.63%)▶tra2a (bg=37.02%)▶uchl5 (bg=18.56%)▶XRCC6 (bg=3.81%)▶YWHAG (bg=9.14%)▶zc3h8 (bg=12.78%)▶znf622 (bg=18.79%)MATCHES To TargetScan▶ miR-124-3p.2/506-3p:UAAGGCA


ga

gagtta  
Depth:3 (DOG)  
Ei-value:1.000, Pi-value:0.070  
Er-value:0.000, Pr-value:0.000  
eCLIP MATCHES▶aggf1 (bg=15.15%)▶AQR (bg=4.89%)▶bclaf1 (bg=17.67%)▶bud13 (bg=12.85%)▶cpsf6 (bg=13.45%)▶FUBP3 (bg=1.53%)▶gtf2f1 (bg=10.18%)▶hltf (bg=24.28%)▶larp4 (bg=13.51%)▶MTPAP (bg=9.55%)▶NIPBL (bg=8.2%)▶ppil4 (bg=43.39%)▶rbm15 (bg=11.59%)▶rbm22 (bg=12.69%)▶safb (bg=40.39%)▶safb2 (bg=26.89%)▶srsf1 (bg=30.28%)▶srsf7 (bg=22.53%)▶SRSF9 (bg=9.67%)▶SUPV3L1 (bg=9.63%)▶tra2a (bg=37.02%)▶uchl5 (bg=18.56%)▶XRCC6 (bg=3.81%)▶YWHAG (bg=9.14%)▶zc3h8 (bg=12.78%)▶znf622 (bg=18.79%)No matches to TargetScan

 1200  


gtta

gagtta  
Depth:3 (DOG)  
Ei-value:1.000, Pi-value:0.070  
Er-value:0.000, Pr-value:0.000  
eCLIP MATCHES▶aggf1 (bg=15.15%)▶AQR (bg=4.89%)▶bclaf1 (bg=17.67%)▶bud13 (bg=12.85%)▶cpsf6 (bg=13.45%)▶FUBP3 (bg=1.53%)▶gtf2f1 (bg=10.18%)▶hltf (bg=24.28%)▶larp4 (bg=13.51%)▶MTPAP (bg=9.55%)▶NIPBL (bg=8.2%)▶ppil4 (bg=43.39%)▶rbm15 (bg=11.59%)▶rbm22 (bg=12.69%)▶safb (bg=40.39%)▶safb2 (bg=26.89%)▶srsf1 (bg=30.28%)▶srsf7 (bg=22.53%)▶SRSF9 (bg=9.67%)▶SUPV3L1 (bg=9.63%)▶tra2a (bg=37.02%)▶uchl5 (bg=18.56%)▶XRCC6 (bg=3.81%)▶YWHAG (bg=9.14%)▶zc3h8 (bg=12.78%)▶znf622 (bg=18.79%)No matches to TargetScan


GAAGAATTTGGAAGGCCTTAAA

AAAAACTAAGGCAGAAGGCTTTTGGAAGAGTTAGAAGAATTTGGAAGGCCTTAAA  
Depth:2 (MARMOSET)  
Ei-value:0.000, Pi-value:0.000  
Er-value:0.000, Pr-value:0.000  
eCLIP MATCHES▶aggf1 (bg=15.15%)▶AQR (bg=4.89%)▶bclaf1 (bg=17.67%)▶bud13 (bg=12.85%)▶cpsf6 (bg=13.45%)▶FUBP3 (bg=1.53%)▶gtf2f1 (bg=10.18%)▶hltf (bg=24.28%)▶larp4 (bg=13.51%)▶MTPAP (bg=9.55%)▶NIPBL (bg=8.2%)▶ppil4 (bg=43.39%)▶rbm15 (bg=11.59%)▶rbm22 (bg=12.69%)▶safb (bg=40.39%)▶safb2 (bg=26.89%)▶SLTM (bg=7.5%)▶srsf1 (bg=30.28%)▶srsf7 (bg=22.53%)▶SRSF9 (bg=9.67%)▶SUPV3L1 (bg=9.63%)▶tra2a (bg=37.02%)▶uchl5 (bg=18.56%)▶XRCC6 (bg=3.81%)▶YWHAG (bg=9.14%)▶zc3h8 (bg=12.78%)▶znf622 (bg=18.79%)MATCHES To TargetScan▶ miR-124-3p.2/506-3p:UAAGGCA

----

gtagctt

gtagctt  
Depth:3 (DOG)  
Ei-value:0.350, Pi-value:0.000  
Er-value:0.000, Pr-value:0.000  
eCLIP MATCHES▶aggf1 (bg=15.15%)▶bclaf1 (bg=17.67%)▶bud13 (bg=12.85%)▶cpsf6 (bg=13.45%)▶hltf (bg=24.28%)▶larp4 (bg=13.51%)▶MTPAP (bg=9.55%)▶NIPBL (bg=8.2%)▶ppil4 (bg=43.39%)▶rbm15 (bg=11.59%)▶rbm22 (bg=12.69%)▶safb (bg=40.39%)▶safb2 (bg=26.89%)▶SLTM (bg=7.5%)▶srsf1 (bg=30.28%)▶srsf7 (bg=22.53%)▶SRSF9 (bg=9.67%)▶SUPV3L1 (bg=9.63%)▶tra2a (bg=37.02%)▶uchl5 (bg=18.56%)▶XRCC6 (bg=3.81%)▶YWHAG (bg=9.14%)▶znf622 (bg=18.79%)No matches to TargetScan


AGTTTGAAAAAT

GTAGCTTAGTTTGAAAAAT  
Depth:2 (MARMOSET)  
Ei-value:0.000, Pi-value:0.000  
Er-value:0.000, Pr-value:0.000  
eCLIP MATCHES▶aggf1 (bg=15.15%)▶bclaf1 (bg=17.67%)▶bud13 (bg=12.85%)▶cpsf6 (bg=13.45%)▶hltf (bg=24.28%)▶hnrnpa1 (bg=18.32%)▶larp4 (bg=13.51%)▶MTPAP (bg=9.55%)▶NIPBL (bg=8.2%)▶ppil4 (bg=43.39%)▶rbm15 (bg=11.59%)▶rbm22 (bg=12.69%)▶safb (bg=40.39%)▶safb2 (bg=26.89%)▶SLTM (bg=7.5%)▶srsf1 (bg=30.28%)▶srsf7 (bg=22.53%)▶SRSF9 (bg=9.67%)▶SUPV3L1 (bg=9.63%)▶tra2a (bg=37.02%)▶uchl5 (bg=18.56%)▶XRCC6 (bg=3.81%)▶YWHAG (bg=9.14%)▶znf622 (bg=18.79%)No matches to TargetScan

---

aaggacttt

aaggacttt  
Depth:2 (MARMOSET)  
Ei-value:1.000, Pi-value:0.000  
Er-value:0.000, Pr-value:0.000  
eCLIP MATCHES▶aggf1 (bg=15.15%)▶bclaf1 (bg=17.67%)▶bud13 (bg=12.85%)▶hltf (bg=24.28%)▶hnrnpa1 (bg=18.32%)▶larp4 (bg=13.51%)▶LARP7 (bg=2.17%)▶MTPAP (bg=9.55%)▶NIPBL (bg=8.2%)▶ppil4 (bg=43.39%)▶rbm15 (bg=11.59%)▶rbm22 (bg=12.69%)▶safb (bg=40.39%)▶safb2 (bg=26.89%)▶srsf1 (bg=30.28%)▶srsf7 (bg=22.53%)▶SRSF9 (bg=9.67%)▶tra2a (bg=37.02%)▶uchl5 (bg=18.56%)▶YWHAG (bg=9.14%)▶znf622 (bg=18.79%)No matches to TargetScan

-

GTAACG

GTAACGGAAGTAATTCAAGATCAAGAGTAATTACCAACTTAATGTTT  
Depth:2 (MARMOSET)  
Ei-value:0.000, Pi-value:0.000  
Er-value:0.000, Pr-value:0.000  
eCLIP MATCHES▶aggf1 (bg=15.15%)▶bclaf1 (bg=17.67%)▶bud13 (bg=12.85%)▶GRWD1 (bg=7.0%)▶hltf (bg=24.28%)▶hnrnpa1 (bg=18.32%)▶larp4 (bg=13.51%)▶LARP7 (bg=2.17%)▶MTPAP (bg=9.55%)▶NIPBL (bg=8.2%)▶ppil4 (bg=43.39%)▶rbm15 (bg=11.59%)▶rbm22 (bg=12.69%)▶safb (bg=40.39%)▶safb2 (bg=26.89%)▶srsf1 (bg=30.28%)▶srsf7 (bg=22.53%)▶SRSF9 (bg=9.67%)▶tra2a (bg=37.02%)▶uchl5 (bg=18.56%)▶XRCC6 (bg=3.81%)▶YWHAG (bg=9.14%)▶znf622 (bg=18.79%)MATCHES To TargetScan▶ miR-382-5p:AAGUUGU▶ miR-323-3p:ACAUUAC▶ miR-543:AACAUUC


GAAGTAAT

GAAGTAAT  
Depth:3 (DOG)  
Ei-value:0.010, Pi-value:0.000  
Er-value:0.000, Pr-value:0.000  
eCLIP MATCHES▶aggf1 (bg=15.15%)▶bclaf1 (bg=17.67%)▶bud13 (bg=12.85%)▶GRWD1 (bg=7.0%)▶hltf (bg=24.28%)▶hnrnpa1 (bg=18.32%)▶larp4 (bg=13.51%)▶LARP7 (bg=2.17%)▶MTPAP (bg=9.55%)▶NIPBL (bg=8.2%)▶ppil4 (bg=43.39%)▶rbm15 (bg=11.59%)▶rbm22 (bg=12.69%)▶safb (bg=40.39%)▶safb2 (bg=26.89%)▶srsf1 (bg=30.28%)▶srsf7 (bg=22.53%)▶SRSF9 (bg=9.67%)▶tra2a (bg=37.02%)▶uchl5 (bg=18.56%)▶YWHAG (bg=9.14%)▶znf622 (bg=18.79%)No matches to TargetScan


T

GTAACGGAAGTAATTCAAGATCAAGAGTAATTACCAACTTAATGTTT  
Depth:2 (MARMOSET)  
Ei-value:0.000, Pi-value:0.000  
Er-value:0.000, Pr-value:0.000  
eCLIP MATCHES▶aggf1 (bg=15.15%)▶bclaf1 (bg=17.67%)▶bud13 (bg=12.85%)▶GRWD1 (bg=7.0%)▶hltf (bg=24.28%)▶hnrnpa1 (bg=18.32%)▶larp4 (bg=13.51%)▶LARP7 (bg=2.17%)▶MTPAP (bg=9.55%)▶NIPBL (bg=8.2%)▶ppil4 (bg=43.39%)▶rbm15 (bg=11.59%)▶rbm22 (bg=12.69%)▶safb (bg=40.39%)▶safb2 (bg=26.89%)▶srsf1 (bg=30.28%)▶srsf7 (bg=22.53%)▶SRSF9 (bg=9.67%)▶tra2a (bg=37.02%)▶uchl5 (bg=18.56%)▶XRCC6 (bg=3.81%)▶YWHAG (bg=9.14%)▶znf622 (bg=18.79%)MATCHES To TargetScan▶ miR-382-5p:AAGUUGU▶ miR-323-3p:ACAUUAC▶ miR-543:AACAUUC


CAAGATCAAGA

CAAGATCAAGA  
Depth:3 (DOG)  
Ei-value:0.000, Pi-value:0.000  
Er-value:0.000, Pr-value:0.000  
eCLIP MATCHES▶aggf1 (bg=15.15%)▶bclaf1 (bg=17.67%)▶bud13 (bg=12.85%)▶GRWD1 (bg=7.0%)▶hltf (bg=24.28%)▶larp4 (bg=13.51%)▶MTPAP (bg=9.55%)▶NIPBL (bg=8.2%)▶ppil4 (bg=43.39%)▶rbm15 (bg=11.59%)▶rbm22 (bg=12.69%)▶safb (bg=40.39%)▶safb2 (bg=26.89%)▶srsf1 (bg=30.28%)▶srsf7 (bg=22.53%)▶SRSF9 (bg=9.67%)▶tra2a (bg=37.02%)▶uchl5 (bg=18.56%)▶XRCC6 (bg=3.81%)▶YWHAG (bg=9.14%)▶znf622 (bg=18.79%)No matches to TargetScan


GTAAT

GTAACGGAAGTAATTCAAGATCAAGAGTAATTACCAACTTAATGTTT  
Depth:2 (MARMOSET)  
Ei-value:0.000, Pi-value:0.000  
Er-value:0.000, Pr-value:0.000  
eCLIP MATCHES▶aggf1 (bg=15.15%)▶bclaf1 (bg=17.67%)▶bud13 (bg=12.85%)▶GRWD1 (bg=7.0%)▶hltf (bg=24.28%)▶hnrnpa1 (bg=18.32%)▶larp4 (bg=13.51%)▶LARP7 (bg=2.17%)▶MTPAP (bg=9.55%)▶NIPBL (bg=8.2%)▶ppil4 (bg=43.39%)▶rbm15 (bg=11.59%)▶rbm22 (bg=12.69%)▶safb (bg=40.39%)▶safb2 (bg=26.89%)▶srsf1 (bg=30.28%)▶srsf7 (bg=22.53%)▶SRSF9 (bg=9.67%)▶tra2a (bg=37.02%)▶uchl5 (bg=18.56%)▶XRCC6 (bg=3.81%)▶YWHAG (bg=9.14%)▶znf622 (bg=18.79%)MATCHES To TargetScan▶ miR-382-5p:AAGUUGU▶ miR-323-3p:ACAUUAC▶ miR-543:AACAUUC


T

TACCAACTTAA  
Depth:3 (DOG)  
Ei-value:0.000, Pi-value:0.000  
Er-value:0.000, Pr-value:0.000  
eCLIP MATCHES▶aggf1 (bg=15.15%)▶bclaf1 (bg=17.67%)▶GRWD1 (bg=7.0%)▶hltf (bg=24.28%)▶larp4 (bg=13.51%)▶MTPAP (bg=9.55%)▶NIPBL (bg=8.2%)▶ppil4 (bg=43.39%)▶rbm15 (bg=11.59%)▶rbm22 (bg=12.69%)▶safb (bg=40.39%)▶safb2 (bg=26.89%)▶srsf1 (bg=30.28%)▶srsf7 (bg=22.53%)▶tra2a (bg=37.02%)▶uchl5 (bg=18.56%)▶XRCC6 (bg=3.81%)▶YWHAG (bg=9.14%)▶znf622 (bg=18.79%)MATCHES To TargetScan▶ miR-382-5p:AAGUUGU


ACCAACTTA

ACCAACTTA  
Depth:5 (COW)  
Ei-value:0.000, Pi-value:0.000  
Er-value:0.000, Pr-value:0.000  
eCLIP MATCHES▶aggf1 (bg=15.15%)▶bclaf1 (bg=17.67%)▶GRWD1 (bg=7.0%)▶hltf (bg=24.28%)▶larp4 (bg=13.51%)▶MTPAP (bg=9.55%)▶NIPBL (bg=8.2%)▶ppil4 (bg=43.39%)▶rbm15 (bg=11.59%)▶rbm22 (bg=12.69%)▶safb (bg=40.39%)▶safb2 (bg=26.89%)▶srsf1 (bg=30.28%)▶srsf7 (bg=22.53%)▶tra2a (bg=37.02%)▶uchl5 (bg=18.56%)▶XRCC6 (bg=3.81%)▶YWHAG (bg=9.14%)▶znf622 (bg=18.79%)MATCHES To TargetScan▶ miR-382-5p:AAGUUGU


A

TACCAACTTAA  
Depth:3 (DOG)  
Ei-value:0.000, Pi-value:0.000  
Er-value:0.000, Pr-value:0.000  
eCLIP MATCHES▶aggf1 (bg=15.15%)▶bclaf1 (bg=17.67%)▶GRWD1 (bg=7.0%)▶hltf (bg=24.28%)▶larp4 (bg=13.51%)▶MTPAP (bg=9.55%)▶NIPBL (bg=8.2%)▶ppil4 (bg=43.39%)▶rbm15 (bg=11.59%)▶rbm22 (bg=12.69%)▶safb (bg=40.39%)▶safb2 (bg=26.89%)▶srsf1 (bg=30.28%)▶srsf7 (bg=22.53%)▶tra2a (bg=37.02%)▶uchl5 (bg=18.56%)▶XRCC6 (bg=3.81%)▶YWHAG (bg=9.14%)▶znf622 (bg=18.79%)MATCHES To TargetScan▶ miR-382-5p:AAGUUGU


TGTTT

GTAACGGAAGTAATTCAAGATCAAGAGTAATTACCAACTTAATGTTT  
Depth:2 (MARMOSET)  
Ei-value:0.000, Pi-value:0.000  
Er-value:0.000, Pr-value:0.000  
eCLIP MATCHES▶aggf1 (bg=15.15%)▶bclaf1 (bg=17.67%)▶bud13 (bg=12.85%)▶GRWD1 (bg=7.0%)▶hltf (bg=24.28%)▶hnrnpa1 (bg=18.32%)▶larp4 (bg=13.51%)▶LARP7 (bg=2.17%)▶MTPAP (bg=9.55%)▶NIPBL (bg=8.2%)▶ppil4 (bg=43.39%)▶rbm15 (bg=11.59%)▶rbm22 (bg=12.69%)▶safb (bg=40.39%)▶safb2 (bg=26.89%)▶srsf1 (bg=30.28%)▶srsf7 (bg=22.53%)▶SRSF9 (bg=9.67%)▶tra2a (bg=37.02%)▶uchl5 (bg=18.56%)▶XRCC6 (bg=3.81%)▶YWHAG (bg=9.14%)▶znf622 (bg=18.79%)MATCHES To TargetScan▶ miR-382-5p:AAGUUGU▶ miR-323-3p:ACAUUAC▶ miR-543:AACAUUC

---

C

CATTGGACTTTG  
Depth:4 (PIG)  
Ei-value:0.000, Pi-value:0.000  
Er-value:0.000, Pr-value:0.000  
eCLIP MATCHES▶aggf1 (bg=15.15%)▶bclaf1 (bg=17.67%)▶bud13 (bg=12.85%)▶cpsf6 (bg=13.45%)▶DGCR8 (bg=2.67%)▶hltf (bg=24.28%)▶larp4 (bg=13.51%)▶NIPBL (bg=8.2%)▶npm1 (bg=10.22%)▶ppil4 (bg=43.39%)▶rbm15 (bg=11.59%)▶rbm22 (bg=12.69%)▶safb (bg=40.39%)▶safb2 (bg=26.89%)▶srsf1 (bg=30.28%)▶SUPV3L1 (bg=9.63%)▶tra2a (bg=37.02%)▶uchl5 (bg=18.56%)▶XRCC6 (bg=3.81%)▶YWHAG (bg=9.14%)▶zc3h8 (bg=12.78%)▶znf622 (bg=18.79%)No matches to TargetScan


A

ATTGGACTT  
Depth:6 (MOUSE)  
Ei-value:0.000, Pi-value:0.000  
Er-value:0.000, Pr-value:0.000  
eCLIP MATCHES▶aggf1 (bg=15.15%)▶bclaf1 (bg=17.67%)▶bud13 (bg=12.85%)▶cpsf6 (bg=13.45%)▶DGCR8 (bg=2.67%)▶hltf (bg=24.28%)▶larp4 (bg=13.51%)▶NIPBL (bg=8.2%)▶npm1 (bg=10.22%)▶ppil4 (bg=43.39%)▶rbm15 (bg=11.59%)▶rbm22 (bg=12.69%)▶safb (bg=40.39%)▶safb2 (bg=26.89%)▶srsf1 (bg=30.28%)▶SUPV3L1 (bg=9.63%)▶tra2a (bg=37.02%)▶uchl5 (bg=18.56%)▶XRCC6 (bg=3.81%)▶YWHAG (bg=9.14%)▶zc3h8 (bg=12.78%)▶znf622 (bg=18.79%)No matches to TargetScan


TTGGAC

TTGGACT  
Depth:9 (LIZARD)  
Ei-value:0.000, Pi-value:0.000  
Er-value:0.000, Pr-value:0.000  
eCLIP MATCHES▶aggf1 (bg=15.15%)▶bclaf1 (bg=17.67%)▶bud13 (bg=12.85%)▶DGCR8 (bg=2.67%)▶hltf (bg=24.28%)▶larp4 (bg=13.51%)▶NIPBL (bg=8.2%)▶npm1 (bg=10.22%)▶ppil4 (bg=43.39%)▶rbm15 (bg=11.59%)▶rbm22 (bg=12.69%)▶safb (bg=40.39%)▶safb2 (bg=26.89%)▶srsf1 (bg=30.28%)▶SUPV3L1 (bg=9.63%)▶tra2a (bg=37.02%)▶uchl5 (bg=18.56%)▶XRCC6 (bg=3.81%)▶YWHAG (bg=9.14%)▶zc3h8 (bg=12.78%)▶znf622 (bg=18.79%)No matches to TargetScan

 1320  


T

TTGGACT  
Depth:9 (LIZARD)  
Ei-value:0.000, Pi-value:0.000  
Er-value:0.000, Pr-value:0.000  
eCLIP MATCHES▶aggf1 (bg=15.15%)▶bclaf1 (bg=17.67%)▶bud13 (bg=12.85%)▶DGCR8 (bg=2.67%)▶hltf (bg=24.28%)▶larp4 (bg=13.51%)▶NIPBL (bg=8.2%)▶npm1 (bg=10.22%)▶ppil4 (bg=43.39%)▶rbm15 (bg=11.59%)▶rbm22 (bg=12.69%)▶safb (bg=40.39%)▶safb2 (bg=26.89%)▶srsf1 (bg=30.28%)▶SUPV3L1 (bg=9.63%)▶tra2a (bg=37.02%)▶uchl5 (bg=18.56%)▶XRCC6 (bg=3.81%)▶YWHAG (bg=9.14%)▶zc3h8 (bg=12.78%)▶znf622 (bg=18.79%)No matches to TargetScan


T

ATTGGACTT  
Depth:6 (MOUSE)  
Ei-value:0.000, Pi-value:0.000  
Er-value:0.000, Pr-value:0.000  
eCLIP MATCHES▶aggf1 (bg=15.15%)▶bclaf1 (bg=17.67%)▶bud13 (bg=12.85%)▶cpsf6 (bg=13.45%)▶DGCR8 (bg=2.67%)▶hltf (bg=24.28%)▶larp4 (bg=13.51%)▶NIPBL (bg=8.2%)▶npm1 (bg=10.22%)▶ppil4 (bg=43.39%)▶rbm15 (bg=11.59%)▶rbm22 (bg=12.69%)▶safb (bg=40.39%)▶safb2 (bg=26.89%)▶srsf1 (bg=30.28%)▶SUPV3L1 (bg=9.63%)▶tra2a (bg=37.02%)▶uchl5 (bg=18.56%)▶XRCC6 (bg=3.81%)▶YWHAG (bg=9.14%)▶zc3h8 (bg=12.78%)▶znf622 (bg=18.79%)No matches to TargetScan


TG

ATTGGACTTTG  
Depth:5 (COW)  
Ei-value:0.000, Pi-value:0.000  
Er-value:0.000, Pr-value:0.000  
eCLIP MATCHES▶aggf1 (bg=15.15%)▶bclaf1 (bg=17.67%)▶bud13 (bg=12.85%)▶cpsf6 (bg=13.45%)▶DGCR8 (bg=2.67%)▶hltf (bg=24.28%)▶larp4 (bg=13.51%)▶NIPBL (bg=8.2%)▶npm1 (bg=10.22%)▶ppil4 (bg=43.39%)▶rbm15 (bg=11.59%)▶rbm22 (bg=12.69%)▶safb (bg=40.39%)▶safb2 (bg=26.89%)▶srsf1 (bg=30.28%)▶SUPV3L1 (bg=9.63%)▶tra2a (bg=37.02%)▶uchl5 (bg=18.56%)▶XRCC6 (bg=3.81%)▶YWHAG (bg=9.14%)▶zc3h8 (bg=12.78%)▶znf622 (bg=18.79%)No matches to TargetScan

------------

TTTTTAAA

TTTTTAAA  
Depth:3 (DOG)  
Ei-value:0.010, Pi-value:0.020  
Er-value:0.000, Pr-value:0.010  
eCLIP MATCHES▶aggf1 (bg=15.15%)▶bclaf1 (bg=17.67%)▶bud13 (bg=12.85%)▶cpsf6 (bg=13.45%)▶gtf2f1 (bg=10.18%)▶hltf (bg=24.28%)▶larp4 (bg=13.51%)▶NIPBL (bg=8.2%)▶npm1 (bg=10.22%)▶ppil4 (bg=43.39%)▶rbm15 (bg=11.59%)▶rbm22 (bg=12.69%)▶safb (bg=40.39%)▶safb2 (bg=26.89%)▶SLBP (bg=6.66%)▶srsf1 (bg=30.28%)▶tra2a (bg=37.02%)▶XRCC6 (bg=3.81%)▶YWHAG (bg=9.14%)▶znf622 (bg=18.79%)No matches to TargetScan

---

TGAG

TGAGGACTAG  
Depth:5 (COW)  
Ei-value:0.000, Pi-value:0.000  
Er-value:0.000, Pr-value:0.000  
eCLIP MATCHES▶aggf1 (bg=15.15%)▶bclaf1 (bg=17.67%)▶bud13 (bg=12.85%)▶cpsf6 (bg=13.45%)▶gtf2f1 (bg=10.18%)▶hltf (bg=24.28%)▶NIPBL (bg=8.2%)▶npm1 (bg=10.22%)▶ppil4 (bg=43.39%)▶rbm15 (bg=11.59%)▶rbm22 (bg=12.69%)▶safb (bg=40.39%)▶safb2 (bg=26.89%)▶SLBP (bg=6.66%)▶SLTM (bg=7.5%)▶srsf1 (bg=30.28%)▶SRSF9 (bg=9.67%)▶tra2a (bg=37.02%)▶XRCC6 (bg=3.81%)▶YWHAG (bg=9.14%)No matches to TargetScan


GACTAG

GACTAG  
Depth:8 (ALLIGATOR)  
Ei-value:0.000, Pi-value:0.000  
Er-value:0.000, Pr-value:0.000  
eCLIP MATCHES▶aggf1 (bg=15.15%)▶bclaf1 (bg=17.67%)▶bud13 (bg=12.85%)▶cpsf6 (bg=13.45%)▶gtf2f1 (bg=10.18%)▶hltf (bg=24.28%)▶NIPBL (bg=8.2%)▶npm1 (bg=10.22%)▶ppil4 (bg=43.39%)▶rbm15 (bg=11.59%)▶rbm22 (bg=12.69%)▶safb (bg=40.39%)▶safb2 (bg=26.89%)▶SLBP (bg=6.66%)▶SLTM (bg=7.5%)▶srsf1 (bg=30.28%)▶SRSF9 (bg=9.67%)▶tra2a (bg=37.02%)▶XRCC6 (bg=3.81%)▶YWHAG (bg=9.14%)No matches to TargetScan


C

TGAGGACTAGC  
Depth:3 (DOG)  
Ei-value:0.000, Pi-value:0.000  
Er-value:0.000, Pr-value:0.000  
eCLIP MATCHES▶aggf1 (bg=15.15%)▶bclaf1 (bg=17.67%)▶bud13 (bg=12.85%)▶cpsf6 (bg=13.45%)▶gtf2f1 (bg=10.18%)▶hltf (bg=24.28%)▶NIPBL (bg=8.2%)▶npm1 (bg=10.22%)▶ppil4 (bg=43.39%)▶rbm15 (bg=11.59%)▶rbm22 (bg=12.69%)▶safb (bg=40.39%)▶safb2 (bg=26.89%)▶SLBP (bg=6.66%)▶SLTM (bg=7.5%)▶srsf1 (bg=30.28%)▶SRSF9 (bg=9.67%)▶tra2a (bg=37.02%)▶XRCC6 (bg=3.81%)▶YWHAG (bg=9.14%)No matches to TargetScan

-

ttaattg

ttaattg  
Depth:3 (DOG)  
Ei-value:0.350, Pi-value:0.030  
Er-value:0.000, Pr-value:0.010  
eCLIP MATCHES▶aggf1 (bg=15.15%)▶bclaf1 (bg=17.67%)▶gtf2f1 (bg=10.18%)▶hltf (bg=24.28%)▶NIPBL (bg=8.2%)▶npm1 (bg=10.22%)▶ppil4 (bg=43.39%)▶rbm22 (bg=12.69%)▶safb (bg=40.39%)▶safb2 (bg=26.89%)▶SLBP (bg=6.66%)▶SLTM (bg=7.5%)▶srsf1 (bg=30.28%)▶SRSF9 (bg=9.67%)▶tra2a (bg=37.02%)▶XRCC6 (bg=3.81%)▶YWHAG (bg=9.14%)No matches to TargetScan


ACAGCT

TTAATTGACAGCTGACCCAGGTGCTACACAGAAGTGGATTCAGTGAATCTAGGAAGACAGCAGCAGACAG  
Depth:2 (MARMOSET)  
Ei-value:0.000, Pi-value:0.000  
Er-value:0.000, Pr-value:0.000  
eCLIP MATCHES▶aggf1 (bg=15.15%)▶bclaf1 (bg=17.67%)▶gtf2f1 (bg=10.18%)▶hltf (bg=24.28%)▶NIPBL (bg=8.2%)▶npm1 (bg=10.22%)▶ppil4 (bg=43.39%)▶rbm22 (bg=12.69%)▶safb (bg=40.39%)▶safb2 (bg=26.89%)▶SLBP (bg=6.66%)▶SLTM (bg=7.5%)▶srsf1 (bg=30.28%)▶SRSF9 (bg=9.67%)▶tra2a (bg=37.02%)▶uchl5 (bg=18.56%)▶XRCC6 (bg=3.81%)▶YWHAG (bg=9.14%)▶znf622 (bg=18.79%)MATCHES To TargetScan▶ miR-29-3p:AGCACCA▶ miR-346:GUCUGCC


GAC

GACCCAGGT  
Depth:3 (DOG)  
Ei-value:0.000, Pi-value:0.000  
Er-value:0.000, Pr-value:0.000  
eCLIP MATCHES▶gtf2f1 (bg=10.18%)▶hltf (bg=24.28%)▶npm1 (bg=10.22%)▶ppil4 (bg=43.39%)▶rbm22 (bg=12.69%)▶safb (bg=40.39%)▶safb2 (bg=26.89%)▶SLTM (bg=7.5%)▶srsf1 (bg=30.28%)▶SRSF9 (bg=9.67%)▶tra2a (bg=37.02%)▶XRCC6 (bg=3.81%)▶YWHAG (bg=9.14%)No matches to TargetScan


CCAGGT

CCAGGT  
Depth:4 (PIG)  
Ei-value:0.010, Pi-value:0.000  
Er-value:0.000, Pr-value:0.000  
eCLIP MATCHES▶gtf2f1 (bg=10.18%)▶hltf (bg=24.28%)▶npm1 (bg=10.22%)▶ppil4 (bg=43.39%)▶rbm22 (bg=12.69%)▶safb (bg=40.39%)▶safb2 (bg=26.89%)▶SLTM (bg=7.5%)▶srsf1 (bg=30.28%)▶SRSF9 (bg=9.67%)▶tra2a (bg=37.02%)▶XRCC6 (bg=3.81%)▶YWHAG (bg=9.14%)No matches to TargetScan


GCTACA

TTAATTGACAGCTGACCCAGGTGCTACACAGAAGTGGATTCAGTGAATCTAGGAAGACAGCAGCAGACAG  
Depth:2 (MARMOSET)  
Ei-value:0.000, Pi-value:0.000  
Er-value:0.000, Pr-value:0.000  
eCLIP MATCHES▶aggf1 (bg=15.15%)▶bclaf1 (bg=17.67%)▶gtf2f1 (bg=10.18%)▶hltf (bg=24.28%)▶NIPBL (bg=8.2%)▶npm1 (bg=10.22%)▶ppil4 (bg=43.39%)▶rbm22 (bg=12.69%)▶safb (bg=40.39%)▶safb2 (bg=26.89%)▶SLBP (bg=6.66%)▶SLTM (bg=7.5%)▶srsf1 (bg=30.28%)▶SRSF9 (bg=9.67%)▶tra2a (bg=37.02%)▶uchl5 (bg=18.56%)▶XRCC6 (bg=3.81%)▶YWHAG (bg=9.14%)▶znf622 (bg=18.79%)MATCHES To TargetScan▶ miR-29-3p:AGCACCA▶ miR-346:GUCUGCC


CAGAAGTG

CAGAAGTG  
Depth:6 (MOUSE)  
Ei-value:0.000, Pi-value:0.000  
Er-value:0.000, Pr-value:0.000  
eCLIP MATCHES▶gtf2f1 (bg=10.18%)▶hltf (bg=24.28%)▶ppil4 (bg=43.39%)▶safb (bg=40.39%)▶SLTM (bg=7.5%)▶srsf1 (bg=30.28%)▶SRSF9 (bg=9.67%)▶tra2a (bg=37.02%)▶XRCC6 (bg=3.81%)No matches to TargetScan


G

CAGAAGTGGATTCAG  
Depth:5 (COW)  
Ei-value:0.000, Pi-value:0.000  
Er-value:0.000, Pr-value:0.000  
eCLIP MATCHES▶gtf2f1 (bg=10.18%)▶hltf (bg=24.28%)▶ppil4 (bg=43.39%)▶safb (bg=40.39%)▶SLTM (bg=7.5%)▶srsf1 (bg=30.28%)▶SRSF9 (bg=9.67%)▶tra2a (bg=37.02%)▶XRCC6 (bg=3.81%)No matches to TargetScan


ATTCAG

ATTCAG  
Depth:7 (TURTLE)  
Ei-value:0.000, Pi-value:0.000  
Er-value:0.000, Pr-value:0.000  
eCLIP MATCHES▶gtf2f1 (bg=10.18%)▶hltf (bg=24.28%)▶ppil4 (bg=43.39%)▶safb (bg=40.39%)▶SLTM (bg=7.5%)▶srsf1 (bg=30.28%)▶SRSF9 (bg=9.67%)▶tra2a (bg=37.02%)▶XRCC6 (bg=3.81%)No matches to TargetScan


TGAAT

CAGAAGTGGATTCAGTGAATCTAGGAAGACAG  
Depth:3 (DOG)  
Ei-value:0.000, Pi-value:0.000  
Er-value:0.000, Pr-value:0.000  
eCLIP MATCHES▶gtf2f1 (bg=10.18%)▶hltf (bg=24.28%)▶ppil4 (bg=43.39%)▶safb (bg=40.39%)▶SLTM (bg=7.5%)▶srsf1 (bg=30.28%)▶SRSF9 (bg=9.67%)▶tra2a (bg=37.02%)▶uchl5 (bg=18.56%)▶XRCC6 (bg=3.81%)▶znf622 (bg=18.79%)No matches to TargetScan


CTAGG

CTAGGAAGACAG  
Depth:6 (MOUSE)  
Ei-value:0.000, Pi-value:0.000  
Er-value:0.000, Pr-value:0.000  
eCLIP MATCHES▶hltf (bg=24.28%)▶ppil4 (bg=43.39%)▶safb (bg=40.39%)▶SLTM (bg=7.5%)▶srsf1 (bg=30.28%)▶SRSF9 (bg=9.67%)▶tra2a (bg=37.02%)▶uchl5 (bg=18.56%)▶znf622 (bg=18.79%)No matches to TargetScan


AAGACAG

AAGACAG  
Depth:8 (ALLIGATOR)  
Ei-value:0.000, Pi-value:0.000  
Er-value:0.000, Pr-value:0.000  
eCLIP MATCHES▶ppil4 (bg=43.39%)▶safb (bg=40.39%)▶srsf1 (bg=30.28%)▶SRSF9 (bg=9.67%)▶tra2a (bg=37.02%)▶uchl5 (bg=18.56%)▶znf622 (bg=18.79%)No matches to TargetScan


CA

TTAATTGACAGCTGACCCAGGTGCTACACAGAAGTGGATTCAGTGAATCTAGGAAGACAGCAGCAGACAG  
Depth:2 (MARMOSET)  
Ei-value:0.000, Pi-value:0.000  
Er-value:0.000, Pr-value:0.000  
eCLIP MATCHES▶aggf1 (bg=15.15%)▶bclaf1 (bg=17.67%)▶gtf2f1 (bg=10.18%)▶hltf (bg=24.28%)▶NIPBL (bg=8.2%)▶npm1 (bg=10.22%)▶ppil4 (bg=43.39%)▶rbm22 (bg=12.69%)▶safb (bg=40.39%)▶safb2 (bg=26.89%)▶SLBP (bg=6.66%)▶SLTM (bg=7.5%)▶srsf1 (bg=30.28%)▶SRSF9 (bg=9.67%)▶tra2a (bg=37.02%)▶uchl5 (bg=18.56%)▶XRCC6 (bg=3.81%)▶YWHAG (bg=9.14%)▶znf622 (bg=18.79%)MATCHES To TargetScan▶ miR-29-3p:AGCACCA▶ miR-346:GUCUGCC


GCAGACAG

GCAGACAG  
Depth:6 (MOUSE)  
Ei-value:0.000, Pi-value:0.000  
Er-value:0.000, Pr-value:0.000  
eCLIP MATCHES▶ppil4 (bg=43.39%)▶safb (bg=40.39%)▶srsf1 (bg=30.28%)▶SRSF9 (bg=9.67%)▶tra2a (bg=37.02%)▶uchl5 (bg=18.56%)▶znf622 (bg=18.79%)MATCHES To TargetScan▶ miR-346:GUCUGCC

-

ATTCCAGGA

ATTCCAGGA  
Depth:4 (PIG)  
Ei-value:0.000, Pi-value:0.000  
Er-value:0.000, Pr-value:0.000  
eCLIP MATCHES▶ppil4 (bg=43.39%)▶safb (bg=40.39%)▶srsf1 (bg=30.28%)▶SRSF9 (bg=9.67%)▶tra2a (bg=37.02%)▶uchl5 (bg=18.56%)▶znf622 (bg=18.79%)No matches to TargetScan

- 1440  


ccagtgt

ccagtgt  
Depth:3 (DOG)  
Ei-value:0.350, Pi-value:0.000  
Er-value:0.000, Pr-value:0.000  
eCLIP MATCHES▶gtf2f1 (bg=10.18%)▶hltf (bg=24.28%)▶ppil4 (bg=43.39%)▶rbm22 (bg=12.69%)▶safb (bg=40.39%)▶safb2 (bg=26.89%)▶SMNDC1 (bg=7.08%)▶srsf1 (bg=30.28%)▶SRSF9 (bg=9.67%)▶tra2a (bg=37.02%)▶uchl5 (bg=18.56%)▶znf622 (bg=18.79%)No matches to TargetScan


ttg

ccagtgtttg  
Depth:2 (MARMOSET)  
Ei-value:1.000, Pi-value:0.000  
Er-value:0.000, Pr-value:0.000  
eCLIP MATCHES▶gtf2f1 (bg=10.18%)▶hltf (bg=24.28%)▶ppil4 (bg=43.39%)▶rbm22 (bg=12.69%)▶safb (bg=40.39%)▶safb2 (bg=26.89%)▶SMNDC1 (bg=7.08%)▶srsf1 (bg=30.28%)▶SRSF9 (bg=9.67%)▶tra2a (bg=37.02%)▶uchl5 (bg=18.56%)▶znf622 (bg=18.79%)MATCHES To TargetScan▶ miR-141-3p/200a-3p:AACACUG

-

TGAAG

TGAAGCTAGGACTGAGGAGC  
Depth:3 (DOG)  
Ei-value:0.000, Pi-value:0.000  
Er-value:0.000, Pr-value:0.000  
eCLIP MATCHES▶gtf2f1 (bg=10.18%)▶hltf (bg=24.28%)▶npm1 (bg=10.22%)▶ppil4 (bg=43.39%)▶rbm22 (bg=12.69%)▶safb (bg=40.39%)▶safb2 (bg=26.89%)▶SMNDC1 (bg=7.08%)▶srsf1 (bg=30.28%)▶SRSF9 (bg=9.67%)▶tra2a (bg=37.02%)▶uchl5 (bg=18.56%)▶znf622 (bg=18.79%)MATCHES To TargetScan▶ miR-455-3p.1:CAGUCCA


CT

CTAGGACTGAGGAGC  
Depth:5 (COW)  
Ei-value:0.000, Pi-value:0.000  
Er-value:0.000, Pr-value:0.000  
eCLIP MATCHES▶gtf2f1 (bg=10.18%)▶hltf (bg=24.28%)▶npm1 (bg=10.22%)▶ppil4 (bg=43.39%)▶rbm22 (bg=12.69%)▶safb (bg=40.39%)▶safb2 (bg=26.89%)▶SMNDC1 (bg=7.08%)▶srsf1 (bg=30.28%)▶SRSF9 (bg=9.67%)▶tra2a (bg=37.02%)▶uchl5 (bg=18.56%)▶znf622 (bg=18.79%)MATCHES To TargetScan▶ miR-455-3p.1:CAGUCCA


AGGACTGAGGAGC

AGGACTGAGGAGC  
Depth:6 (MOUSE)  
Ei-value:0.000, Pi-value:0.000  
Er-value:0.000, Pr-value:0.000  
eCLIP MATCHES▶gtf2f1 (bg=10.18%)▶hltf (bg=24.28%)▶npm1 (bg=10.22%)▶ppil4 (bg=43.39%)▶rbm22 (bg=12.69%)▶safb (bg=40.39%)▶safb2 (bg=26.89%)▶SMNDC1 (bg=7.08%)▶srsf1 (bg=30.28%)▶SRSF9 (bg=9.67%)▶tra2a (bg=37.02%)▶uchl5 (bg=18.56%)▶znf622 (bg=18.79%)MATCHES To TargetScan▶ miR-455-3p.1:CAGUCCA


AA

TGAAGCTAGGACTGAGGAGCAA  
Depth:2 (MARMOSET)  
Ei-value:0.000, Pi-value:0.000  
Er-value:0.000, Pr-value:0.000  
eCLIP MATCHES▶gtf2f1 (bg=10.18%)▶hltf (bg=24.28%)▶npm1 (bg=10.22%)▶ppil4 (bg=43.39%)▶rbm22 (bg=12.69%)▶safb (bg=40.39%)▶safb2 (bg=26.89%)▶SMNDC1 (bg=7.08%)▶srsf1 (bg=30.28%)▶SRSF9 (bg=9.67%)▶tra2a (bg=37.02%)▶uchl5 (bg=18.56%)▶znf622 (bg=18.79%)MATCHES To TargetScan▶ miR-455-3p.1:CAGUCCA

--------

GCA

GCAGCAGTTCGTGGTGAAGATAGGAA  
Depth:2 (MARMOSET)  
Ei-value:0.000, Pi-value:0.000  
Er-value:0.000, Pr-value:0.000  
eCLIP MATCHES▶bclaf1 (bg=17.67%)▶fxr2 (bg=10.1%)▶GRWD1 (bg=7.0%)▶gtf2f1 (bg=10.18%)▶hltf (bg=24.28%)▶MTPAP (bg=9.55%)▶npm1 (bg=10.22%)▶ppil4 (bg=43.39%)▶rbm22 (bg=12.69%)▶safb (bg=40.39%)▶safb2 (bg=26.89%)▶SMNDC1 (bg=7.08%)▶srsf1 (bg=30.28%)▶SRSF9 (bg=9.67%)▶tra2a (bg=37.02%)▶TROVE2 (bg=6.96%)▶uchl5 (bg=18.56%)▶znf622 (bg=18.79%)MATCHES To TargetScan▶ miR-202-5p:UCCUAUG


GCAGTTC

GCAGTTC  
Depth:5 (COW)  
Ei-value:0.000, Pi-value:0.000  
Er-value:0.000, Pr-value:0.000  
eCLIP MATCHES▶gtf2f1 (bg=10.18%)▶hltf (bg=24.28%)▶MTPAP (bg=9.55%)▶npm1 (bg=10.22%)▶ppil4 (bg=43.39%)▶rbm22 (bg=12.69%)▶safb (bg=40.39%)▶safb2 (bg=26.89%)▶SMNDC1 (bg=7.08%)▶srsf1 (bg=30.28%)▶SRSF9 (bg=9.67%)▶tra2a (bg=37.02%)▶uchl5 (bg=18.56%)▶znf622 (bg=18.79%)No matches to TargetScan


GTG

GCAGTTCGTGGTGAAGATAGGAA  
Depth:3 (DOG)  
Ei-value:0.000, Pi-value:0.000  
Er-value:0.000, Pr-value:0.000  
eCLIP MATCHES▶bclaf1 (bg=17.67%)▶fxr2 (bg=10.1%)▶GRWD1 (bg=7.0%)▶gtf2f1 (bg=10.18%)▶hltf (bg=24.28%)▶MTPAP (bg=9.55%)▶npm1 (bg=10.22%)▶ppil4 (bg=43.39%)▶rbm22 (bg=12.69%)▶safb (bg=40.39%)▶safb2 (bg=26.89%)▶SMNDC1 (bg=7.08%)▶srsf1 (bg=30.28%)▶SRSF9 (bg=9.67%)▶tra2a (bg=37.02%)▶TROVE2 (bg=6.96%)▶uchl5 (bg=18.56%)▶znf622 (bg=18.79%)MATCHES To TargetScan▶ miR-202-5p:UCCUAUG


GTGAAGATAG

GTGAAGATAG  
Depth:6 (MOUSE)  
Ei-value:0.000, Pi-value:0.000  
Er-value:0.000, Pr-value:0.000  
eCLIP MATCHES▶bclaf1 (bg=17.67%)▶fxr2 (bg=10.1%)▶gtf2f1 (bg=10.18%)▶MTPAP (bg=9.55%)▶npm1 (bg=10.22%)▶ppil4 (bg=43.39%)▶rbm22 (bg=12.69%)▶safb (bg=40.39%)▶safb2 (bg=26.89%)▶SMNDC1 (bg=7.08%)▶srsf1 (bg=30.28%)▶SRSF9 (bg=9.67%)▶tra2a (bg=37.02%)▶TROVE2 (bg=6.96%)▶uchl5 (bg=18.56%)▶znf622 (bg=18.79%)No matches to TargetScan


GAA

GTGAAGATAGGAA  
Depth:5 (COW)  
Ei-value:0.000, Pi-value:0.000  
Er-value:0.000, Pr-value:0.000  
eCLIP MATCHES▶bclaf1 (bg=17.67%)▶fxr2 (bg=10.1%)▶GRWD1 (bg=7.0%)▶gtf2f1 (bg=10.18%)▶MTPAP (bg=9.55%)▶npm1 (bg=10.22%)▶ppil4 (bg=43.39%)▶rbm22 (bg=12.69%)▶safb (bg=40.39%)▶safb2 (bg=26.89%)▶SMNDC1 (bg=7.08%)▶srsf1 (bg=30.28%)▶SRSF9 (bg=9.67%)▶tra2a (bg=37.02%)▶TROVE2 (bg=6.96%)▶uchl5 (bg=18.56%)▶znf622 (bg=18.79%)MATCHES To TargetScan▶ miR-202-5p:UCCUAUG

--

gagtccagga

gagtccagga  
Depth:2 (MARMOSET)  
Ei-value:1.000, Pi-value:0.000  
Er-value:0.000, Pr-value:0.000  
eCLIP MATCHES▶bclaf1 (bg=17.67%)▶fxr2 (bg=10.1%)▶GRWD1 (bg=7.0%)▶gtf2f1 (bg=10.18%)▶hltf (bg=24.28%)▶MTPAP (bg=9.55%)▶npm1 (bg=10.22%)▶ppil4 (bg=43.39%)▶rbm22 (bg=12.69%)▶safb (bg=40.39%)▶safb2 (bg=26.89%)▶SMNDC1 (bg=7.08%)▶srsf1 (bg=30.28%)▶SRSF9 (bg=9.67%)▶TAF15 (bg=9.06%)▶tra2a (bg=37.02%)▶TROVE2 (bg=6.96%)▶uchl5 (bg=18.56%)▶znf622 (bg=18.79%)MATCHES To TargetScan▶ miR-378-3p:CUGGACU

-

CCAGTGC

CCAGTGC  
Depth:5 (COW)  
Ei-value:0.000, Pi-value:0.000  
Er-value:0.000, Pr-value:0.000  
eCLIP MATCHES▶bclaf1 (bg=17.67%)▶fxr2 (bg=10.1%)▶GRWD1 (bg=7.0%)▶gtf2f1 (bg=10.18%)▶hltf (bg=24.28%)▶MTPAP (bg=9.55%)▶npm1 (bg=10.22%)▶ppil4 (bg=43.39%)▶rbm22 (bg=12.69%)▶safb (bg=40.39%)▶safb2 (bg=26.89%)▶SMNDC1 (bg=7.08%)▶srsf1 (bg=30.28%)▶SRSF9 (bg=9.67%)▶TAF15 (bg=9.06%)▶tra2a (bg=37.02%)▶TROVE2 (bg=6.96%)▶uchl5 (bg=18.56%)▶znf622 (bg=18.79%)No matches to TargetScan


GA

CCAGTGCGATTTGGTGAAGGAAGCTAGGAAGAAGGAAGGAGCGCTAACGATTTGGTGGTGAAGCTAGGAAA  
Depth:2 (MARMOSET)  
Ei-value:0.000, Pi-value:0.000  
Er-value:0.000, Pr-value:0.000  
eCLIP MATCHES▶bclaf1 (bg=17.67%)▶EXOSC5 (bg=2.0%)▶fxr2 (bg=10.1%)▶GRWD1 (bg=7.0%)▶gtf2f1 (bg=10.18%)▶hltf (bg=24.28%)▶MTPAP (bg=9.55%)▶npm1 (bg=10.22%)▶ppil4 (bg=43.39%)▶rbm15 (bg=11.59%)▶rbm22 (bg=12.69%)▶safb (bg=40.39%)▶safb2 (bg=26.89%)▶SLTM (bg=7.5%)▶SMNDC1 (bg=7.08%)▶srsf1 (bg=30.28%)▶SRSF9 (bg=9.67%)▶TAF15 (bg=9.06%)▶tra2a (bg=37.02%)▶TROVE2 (bg=6.96%)▶uchl5 (bg=18.56%)▶znf622 (bg=18.79%)MATCHES To TargetScan▶ miR-205-5p:CCUUCAU


TTTGGT

TTTGGT  
Depth:4 (PIG)  
Ei-value:0.010, Pi-value:0.010  
Er-value:0.000, Pr-value:0.000  
eCLIP MATCHES▶bclaf1 (bg=17.67%)▶fxr2 (bg=10.1%)▶GRWD1 (bg=7.0%)▶gtf2f1 (bg=10.18%)▶hltf (bg=24.28%)▶MTPAP (bg=9.55%)▶npm1 (bg=10.22%)▶ppil4 (bg=43.39%)▶rbm22 (bg=12.69%)▶safb (bg=40.39%)▶safb2 (bg=26.89%)▶SMNDC1 (bg=7.08%)▶srsf1 (bg=30.28%)▶SRSF9 (bg=9.67%)▶TAF15 (bg=9.06%)▶tra2a (bg=37.02%)▶TROVE2 (bg=6.96%)▶uchl5 (bg=18.56%)▶znf622 (bg=18.79%)No matches to TargetScan


GA

GAAGGAAGCTAGGAAGAA  
Depth:5 (COW)  
Ei-value:0.000, Pi-value:0.000  
Er-value:0.000, Pr-value:0.000  
eCLIP MATCHES▶bclaf1 (bg=17.67%)▶fxr2 (bg=10.1%)▶gtf2f1 (bg=10.18%)▶hltf (bg=24.28%)▶MTPAP (bg=9.55%)▶npm1 (bg=10.22%)▶ppil4 (bg=43.39%)▶rbm22 (bg=12.69%)▶safb (bg=40.39%)▶safb2 (bg=26.89%)▶SMNDC1 (bg=7.08%)▶srsf1 (bg=30.28%)▶SRSF9 (bg=9.67%)▶TAF15 (bg=9.06%)▶tra2a (bg=37.02%)▶TROVE2 (bg=6.96%)▶uchl5 (bg=18.56%)▶znf622 (bg=18.79%)No matches to TargetScan


AGGAAGCTAGGAAGAA

AGGAAGCTAGGAAGAA  
Depth:6 (MOUSE)  
Ei-value:0.000, Pi-value:0.000  
Er-value:0.000, Pr-value:0.000  
eCLIP MATCHES▶bclaf1 (bg=17.67%)▶fxr2 (bg=10.1%)▶gtf2f1 (bg=10.18%)▶hltf (bg=24.28%)▶MTPAP (bg=9.55%)▶npm1 (bg=10.22%)▶ppil4 (bg=43.39%)▶rbm22 (bg=12.69%)▶safb (bg=40.39%)▶safb2 (bg=26.89%)▶SMNDC1 (bg=7.08%)▶srsf1 (bg=30.28%)▶SRSF9 (bg=9.67%)▶TAF15 (bg=9.06%)▶tra2a (bg=37.02%)▶TROVE2 (bg=6.96%)▶uchl5 (bg=18.56%)▶znf622 (bg=18.79%)No matches to TargetScan


G

CCAGTGCGATTTGGTGAAGGAAGCTAGGAAGAAGGAAGGAGCGCTAACGATTTGGTGGTGAAGCTAGGAAA  
Depth:2 (MARMOSET)  
Ei-value:0.000, Pi-value:0.000  
Er-value:0.000, Pr-value:0.000  
eCLIP MATCHES▶bclaf1 (bg=17.67%)▶EXOSC5 (bg=2.0%)▶fxr2 (bg=10.1%)▶GRWD1 (bg=7.0%)▶gtf2f1 (bg=10.18%)▶hltf (bg=24.28%)▶MTPAP (bg=9.55%)▶npm1 (bg=10.22%)▶ppil4 (bg=43.39%)▶rbm15 (bg=11.59%)▶rbm22 (bg=12.69%)▶safb (bg=40.39%)▶safb2 (bg=26.89%)▶SLTM (bg=7.5%)▶SMNDC1 (bg=7.08%)▶srsf1 (bg=30.28%)▶SRSF9 (bg=9.67%)▶TAF15 (bg=9.06%)▶tra2a (bg=37.02%)▶TROVE2 (bg=6.96%)▶uchl5 (bg=18.56%)▶znf622 (bg=18.79%)MATCHES To TargetScan▶ miR-205-5p:CCUUCAU


GAAGGA

GAAGGAGC  
Depth:4 (PIG)  
Ei-value:0.000, Pi-value:0.000  
Er-value:0.000, Pr-value:0.000  
eCLIP MATCHES▶bclaf1 (bg=17.67%)▶fxr2 (bg=10.1%)▶gtf2f1 (bg=10.18%)▶hltf (bg=24.28%)▶MTPAP (bg=9.55%)▶npm1 (bg=10.22%)▶ppil4 (bg=43.39%)▶rbm22 (bg=12.69%)▶safb (bg=40.39%)▶safb2 (bg=26.89%)▶SMNDC1 (bg=7.08%)▶srsf1 (bg=30.28%)▶SRSF9 (bg=9.67%)▶TAF15 (bg=9.06%)▶tra2a (bg=37.02%)▶TROVE2 (bg=6.96%)▶uchl5 (bg=18.56%)▶znf622 (bg=18.79%)No matches to TargetScan

 1560  


GC

GAAGGAGC  
Depth:4 (PIG)  
Ei-value:0.000, Pi-value:0.000  
Er-value:0.000, Pr-value:0.000  
eCLIP MATCHES▶bclaf1 (bg=17.67%)▶fxr2 (bg=10.1%)▶gtf2f1 (bg=10.18%)▶hltf (bg=24.28%)▶MTPAP (bg=9.55%)▶npm1 (bg=10.22%)▶ppil4 (bg=43.39%)▶rbm22 (bg=12.69%)▶safb (bg=40.39%)▶safb2 (bg=26.89%)▶SMNDC1 (bg=7.08%)▶srsf1 (bg=30.28%)▶SRSF9 (bg=9.67%)▶TAF15 (bg=9.06%)▶tra2a (bg=37.02%)▶TROVE2 (bg=6.96%)▶uchl5 (bg=18.56%)▶znf622 (bg=18.79%)No matches to TargetScan


G

CCAGTGCGATTTGGTGAAGGAAGCTAGGAAGAAGGAAGGAGCGCTAACGATTTGGTGGTGAAGCTAGGAAA  
Depth:2 (MARMOSET)  
Ei-value:0.000, Pi-value:0.000  
Er-value:0.000, Pr-value:0.000  
eCLIP MATCHES▶bclaf1 (bg=17.67%)▶EXOSC5 (bg=2.0%)▶fxr2 (bg=10.1%)▶GRWD1 (bg=7.0%)▶gtf2f1 (bg=10.18%)▶hltf (bg=24.28%)▶MTPAP (bg=9.55%)▶npm1 (bg=10.22%)▶ppil4 (bg=43.39%)▶rbm15 (bg=11.59%)▶rbm22 (bg=12.69%)▶safb (bg=40.39%)▶safb2 (bg=26.89%)▶SLTM (bg=7.5%)▶SMNDC1 (bg=7.08%)▶srsf1 (bg=30.28%)▶SRSF9 (bg=9.67%)▶TAF15 (bg=9.06%)▶tra2a (bg=37.02%)▶TROVE2 (bg=6.96%)▶uchl5 (bg=18.56%)▶znf622 (bg=18.79%)MATCHES To TargetScan▶ miR-205-5p:CCUUCAU


CTAACG

CTAACG  
Depth:5 (COW)  
Ei-value:0.000, Pi-value:0.000  
Er-value:0.000, Pr-value:0.000  
eCLIP MATCHES▶bclaf1 (bg=17.67%)▶fxr2 (bg=10.1%)▶gtf2f1 (bg=10.18%)▶hltf (bg=24.28%)▶MTPAP (bg=9.55%)▶npm1 (bg=10.22%)▶ppil4 (bg=43.39%)▶rbm22 (bg=12.69%)▶safb (bg=40.39%)▶safb2 (bg=26.89%)▶SMNDC1 (bg=7.08%)▶srsf1 (bg=30.28%)▶SRSF9 (bg=9.67%)▶TAF15 (bg=9.06%)▶tra2a (bg=37.02%)▶TROVE2 (bg=6.96%)▶uchl5 (bg=18.56%)▶znf622 (bg=18.79%)No matches to TargetScan


ATTTGG

CTAACGATTTGG  
Depth:3 (DOG)  
Ei-value:0.000, Pi-value:0.000  
Er-value:0.000, Pr-value:0.000  
eCLIP MATCHES▶bclaf1 (bg=17.67%)▶fxr2 (bg=10.1%)▶gtf2f1 (bg=10.18%)▶hltf (bg=24.28%)▶MTPAP (bg=9.55%)▶npm1 (bg=10.22%)▶ppil4 (bg=43.39%)▶rbm15 (bg=11.59%)▶rbm22 (bg=12.69%)▶safb (bg=40.39%)▶safb2 (bg=26.89%)▶SMNDC1 (bg=7.08%)▶srsf1 (bg=30.28%)▶SRSF9 (bg=9.67%)▶TAF15 (bg=9.06%)▶tra2a (bg=37.02%)▶TROVE2 (bg=6.96%)▶uchl5 (bg=18.56%)▶znf622 (bg=18.79%)No matches to TargetScan


T

CCAGTGCGATTTGGTGAAGGAAGCTAGGAAGAAGGAAGGAGCGCTAACGATTTGGTGGTGAAGCTAGGAAA  
Depth:2 (MARMOSET)  
Ei-value:0.000, Pi-value:0.000  
Er-value:0.000, Pr-value:0.000  
eCLIP MATCHES▶bclaf1 (bg=17.67%)▶EXOSC5 (bg=2.0%)▶fxr2 (bg=10.1%)▶GRWD1 (bg=7.0%)▶gtf2f1 (bg=10.18%)▶hltf (bg=24.28%)▶MTPAP (bg=9.55%)▶npm1 (bg=10.22%)▶ppil4 (bg=43.39%)▶rbm15 (bg=11.59%)▶rbm22 (bg=12.69%)▶safb (bg=40.39%)▶safb2 (bg=26.89%)▶SLTM (bg=7.5%)▶SMNDC1 (bg=7.08%)▶srsf1 (bg=30.28%)▶SRSF9 (bg=9.67%)▶TAF15 (bg=9.06%)▶tra2a (bg=37.02%)▶TROVE2 (bg=6.96%)▶uchl5 (bg=18.56%)▶znf622 (bg=18.79%)MATCHES To TargetScan▶ miR-205-5p:CCUUCAU


GGTGAAG

GGTGAAG  
Depth:11 (X.TROPICALIS)  
Ei-value:0.000, Pi-value:0.000  
Er-value:0.000, Pr-value:0.000  
eCLIP MATCHES▶bclaf1 (bg=17.67%)▶EXOSC5 (bg=2.0%)▶fxr2 (bg=10.1%)▶gtf2f1 (bg=10.18%)▶hltf (bg=24.28%)▶MTPAP (bg=9.55%)▶npm1 (bg=10.22%)▶ppil4 (bg=43.39%)▶rbm15 (bg=11.59%)▶rbm22 (bg=12.69%)▶safb (bg=40.39%)▶safb2 (bg=26.89%)▶SMNDC1 (bg=7.08%)▶srsf1 (bg=30.28%)▶SRSF9 (bg=9.67%)▶TAF15 (bg=9.06%)▶tra2a (bg=37.02%)▶TROVE2 (bg=6.96%)▶uchl5 (bg=18.56%)▶znf622 (bg=18.79%)No matches to TargetScan


CT

GGTGAAGCT  
Depth:6 (MOUSE)  
Ei-value:0.000, Pi-value:0.000  
Er-value:0.000, Pr-value:0.000  
eCLIP MATCHES▶bclaf1 (bg=17.67%)▶EXOSC5 (bg=2.0%)▶fxr2 (bg=10.1%)▶GRWD1 (bg=7.0%)▶gtf2f1 (bg=10.18%)▶hltf (bg=24.28%)▶MTPAP (bg=9.55%)▶npm1 (bg=10.22%)▶ppil4 (bg=43.39%)▶rbm15 (bg=11.59%)▶rbm22 (bg=12.69%)▶safb (bg=40.39%)▶safb2 (bg=26.89%)▶SMNDC1 (bg=7.08%)▶srsf1 (bg=30.28%)▶SRSF9 (bg=9.67%)▶TAF15 (bg=9.06%)▶tra2a (bg=37.02%)▶TROVE2 (bg=6.96%)▶uchl5 (bg=18.56%)▶znf622 (bg=18.79%)No matches to TargetScan


AGGA

GGTGAAGCTAGGA  
Depth:5 (COW)  
Ei-value:0.000, Pi-value:0.000  
Er-value:0.000, Pr-value:0.000  
eCLIP MATCHES▶bclaf1 (bg=17.67%)▶EXOSC5 (bg=2.0%)▶fxr2 (bg=10.1%)▶GRWD1 (bg=7.0%)▶gtf2f1 (bg=10.18%)▶hltf (bg=24.28%)▶MTPAP (bg=9.55%)▶npm1 (bg=10.22%)▶ppil4 (bg=43.39%)▶rbm15 (bg=11.59%)▶rbm22 (bg=12.69%)▶safb (bg=40.39%)▶safb2 (bg=26.89%)▶SMNDC1 (bg=7.08%)▶srsf1 (bg=30.28%)▶SRSF9 (bg=9.67%)▶TAF15 (bg=9.06%)▶tra2a (bg=37.02%)▶TROVE2 (bg=6.96%)▶uchl5 (bg=18.56%)▶znf622 (bg=18.79%)No matches to TargetScan


AA

CCAGTGCGATTTGGTGAAGGAAGCTAGGAAGAAGGAAGGAGCGCTAACGATTTGGTGGTGAAGCTAGGAAA  
Depth:2 (MARMOSET)  
Ei-value:0.000, Pi-value:0.000  
Er-value:0.000, Pr-value:0.000  
eCLIP MATCHES▶bclaf1 (bg=17.67%)▶EXOSC5 (bg=2.0%)▶fxr2 (bg=10.1%)▶GRWD1 (bg=7.0%)▶gtf2f1 (bg=10.18%)▶hltf (bg=24.28%)▶MTPAP (bg=9.55%)▶npm1 (bg=10.22%)▶ppil4 (bg=43.39%)▶rbm15 (bg=11.59%)▶rbm22 (bg=12.69%)▶safb (bg=40.39%)▶safb2 (bg=26.89%)▶SLTM (bg=7.5%)▶SMNDC1 (bg=7.08%)▶srsf1 (bg=30.28%)▶SRSF9 (bg=9.67%)▶TAF15 (bg=9.06%)▶tra2a (bg=37.02%)▶TROVE2 (bg=6.96%)▶uchl5 (bg=18.56%)▶znf622 (bg=18.79%)MATCHES To TargetScan▶ miR-205-5p:CCUUCAU

-

AGGATTCCAG

AGGATTCCAG  
Depth:5 (COW)  
Ei-value:0.000, Pi-value:0.000  
Er-value:0.000, Pr-value:0.000  
eCLIP MATCHES▶bclaf1 (bg=17.67%)▶EXOSC5 (bg=2.0%)▶fxr2 (bg=10.1%)▶GRWD1 (bg=7.0%)▶gtf2f1 (bg=10.18%)▶hltf (bg=24.28%)▶MTPAP (bg=9.55%)▶npm1 (bg=10.22%)▶ppil4 (bg=43.39%)▶rbm15 (bg=11.59%)▶rbm22 (bg=12.69%)▶safb (bg=40.39%)▶safb2 (bg=26.89%)▶SLTM (bg=7.5%)▶SMNDC1 (bg=7.08%)▶srsf1 (bg=30.28%)▶SRSF9 (bg=9.67%)▶TAF15 (bg=9.06%)▶tra2a (bg=37.02%)▶TROVE2 (bg=6.96%)▶uchl5 (bg=18.56%)▶znf622 (bg=18.79%)No matches to TargetScan


GAAGGAGCGAGTGC

AGGATTCCAGGAAGGAGCGAGTGC  
Depth:2 (MARMOSET)  
Ei-value:0.000, Pi-value:0.000  
Er-value:0.000, Pr-value:0.000  
eCLIP MATCHES▶aggf1 (bg=15.15%)▶bclaf1 (bg=17.67%)▶EXOSC5 (bg=2.0%)▶fxr2 (bg=10.1%)▶GRWD1 (bg=7.0%)▶gtf2f1 (bg=10.18%)▶hltf (bg=24.28%)▶larp4 (bg=13.51%)▶MTPAP (bg=9.55%)▶npm1 (bg=10.22%)▶ppil4 (bg=43.39%)▶rbm15 (bg=11.59%)▶rbm22 (bg=12.69%)▶safb (bg=40.39%)▶safb2 (bg=26.89%)▶SLTM (bg=7.5%)▶SMNDC1 (bg=7.08%)▶srsf1 (bg=30.28%)▶SRSF9 (bg=9.67%)▶TAF15 (bg=9.06%)▶tra2a (bg=37.02%)▶TROVE2 (bg=6.96%)▶uchl5 (bg=18.56%)▶YWHAG (bg=9.14%)▶znf622 (bg=18.79%)No matches to TargetScan

-

ATT

ATTTGGTGATGAAG  
Depth:2 (MARMOSET)  
Ei-value:0.020, Pi-value:0.000  
Er-value:0.000, Pr-value:0.000  
eCLIP MATCHES▶aggf1 (bg=15.15%)▶bclaf1 (bg=17.67%)▶EXOSC5 (bg=2.0%)▶fxr2 (bg=10.1%)▶GRWD1 (bg=7.0%)▶gtf2f1 (bg=10.18%)▶hltf (bg=24.28%)▶larp4 (bg=13.51%)▶MTPAP (bg=9.55%)▶npm1 (bg=10.22%)▶ppil4 (bg=43.39%)▶rbm15 (bg=11.59%)▶rbm22 (bg=12.69%)▶safb (bg=40.39%)▶safb2 (bg=26.89%)▶SLTM (bg=7.5%)▶SMNDC1 (bg=7.08%)▶srsf1 (bg=30.28%)▶SRSF9 (bg=9.67%)▶TAF15 (bg=9.06%)▶tra2a (bg=37.02%)▶TROVE2 (bg=6.96%)▶uchl5 (bg=18.56%)▶UTP3 (bg=1.57%)▶YWHAG (bg=9.14%)▶znf622 (bg=18.79%)No matches to TargetScan


TGGTGA

TGGTGA  
Depth:8 (ALLIGATOR)  
Ei-value:0.000, Pi-value:0.000  
Er-value:0.000, Pr-value:0.000  
eCLIP MATCHES▶aggf1 (bg=15.15%)▶bclaf1 (bg=17.67%)▶EXOSC5 (bg=2.0%)▶fxr2 (bg=10.1%)▶GRWD1 (bg=7.0%)▶gtf2f1 (bg=10.18%)▶hltf (bg=24.28%)▶larp4 (bg=13.51%)▶MTPAP (bg=9.55%)▶npm1 (bg=10.22%)▶ppil4 (bg=43.39%)▶rbm15 (bg=11.59%)▶rbm22 (bg=12.69%)▶safb (bg=40.39%)▶safb2 (bg=26.89%)▶SLTM (bg=7.5%)▶SMNDC1 (bg=7.08%)▶srsf1 (bg=30.28%)▶SRSF9 (bg=9.67%)▶TAF15 (bg=9.06%)▶tra2a (bg=37.02%)▶TROVE2 (bg=6.96%)▶uchl5 (bg=18.56%)▶UTP3 (bg=1.57%)▶YWHAG (bg=9.14%)▶znf622 (bg=18.79%)No matches to TargetScan


TGAAG

TGGTGATGAAG  
Depth:4 (PIG)  
Ei-value:0.000, Pi-value:0.000  
Er-value:0.000, Pr-value:0.000  
eCLIP MATCHES▶aggf1 (bg=15.15%)▶bclaf1 (bg=17.67%)▶EXOSC5 (bg=2.0%)▶fxr2 (bg=10.1%)▶GRWD1 (bg=7.0%)▶gtf2f1 (bg=10.18%)▶hltf (bg=24.28%)▶larp4 (bg=13.51%)▶MTPAP (bg=9.55%)▶npm1 (bg=10.22%)▶ppil4 (bg=43.39%)▶rbm15 (bg=11.59%)▶rbm22 (bg=12.69%)▶safb (bg=40.39%)▶safb2 (bg=26.89%)▶SLTM (bg=7.5%)▶SMNDC1 (bg=7.08%)▶srsf1 (bg=30.28%)▶SRSF9 (bg=9.67%)▶TAF15 (bg=9.06%)▶tra2a (bg=37.02%)▶TROVE2 (bg=6.96%)▶uchl5 (bg=18.56%)▶UTP3 (bg=1.57%)▶YWHAG (bg=9.14%)▶znf622 (bg=18.79%)No matches to TargetScan

-

TAGC

TAGCAGGCGGCTTGGCTTGGCAACCACACGGAGGAG  
Depth:2 (MARMOSET)  
Ei-value:0.000, Pi-value:0.000  
Er-value:0.000, Pr-value:0.000  
eCLIP MATCHES▶aggf1 (bg=15.15%)▶bclaf1 (bg=17.67%)▶EXOSC5 (bg=2.0%)▶FUBP3 (bg=1.53%)▶fxr2 (bg=10.1%)▶GRWD1 (bg=7.0%)▶gtf2f1 (bg=10.18%)▶hltf (bg=24.28%)▶khsrp (bg=27.4%)▶larp4 (bg=13.51%)▶MTPAP (bg=9.55%)▶npm1 (bg=10.22%)▶ppil4 (bg=43.39%)▶rbm15 (bg=11.59%)▶rbm22 (bg=12.69%)▶safb (bg=40.39%)▶safb2 (bg=26.89%)▶SLTM (bg=7.5%)▶SMNDC1 (bg=7.08%)▶srsf1 (bg=30.28%)▶SRSF9 (bg=9.67%)▶TAF15 (bg=9.06%)▶tra2a (bg=37.02%)▶TROVE2 (bg=6.96%)▶uchl5 (bg=18.56%)▶UTP3 (bg=1.57%)▶YWHAG (bg=9.14%)▶znf622 (bg=18.79%)▶ZNF800 (bg=3.2%)No matches to TargetScan


a

aggcggc  
Depth:3 (DOG)  
Ei-value:0.350, Pi-value:0.000  
Er-value:0.000, Pr-value:0.000  
eCLIP MATCHES▶aggf1 (bg=15.15%)▶bclaf1 (bg=17.67%)▶EXOSC5 (bg=2.0%)▶FUBP3 (bg=1.53%)▶fxr2 (bg=10.1%)▶GRWD1 (bg=7.0%)▶gtf2f1 (bg=10.18%)▶hltf (bg=24.28%)▶khsrp (bg=27.4%)▶larp4 (bg=13.51%)▶MTPAP (bg=9.55%)▶npm1 (bg=10.22%)▶ppil4 (bg=43.39%)▶rbm15 (bg=11.59%)▶rbm22 (bg=12.69%)▶safb (bg=40.39%)▶safb2 (bg=26.89%)▶SLTM (bg=7.5%)▶SMNDC1 (bg=7.08%)▶srsf1 (bg=30.28%)▶SRSF9 (bg=9.67%)▶TAF15 (bg=9.06%)▶tra2a (bg=37.02%)▶TROVE2 (bg=6.96%)▶uchl5 (bg=18.56%)▶UTP3 (bg=1.57%)▶YWHAG (bg=9.14%)▶znf622 (bg=18.79%)No matches to TargetScan


GGCGGC

GGCGGC  
Depth:6 (MOUSE)  
Ei-value:0.000, Pi-value:0.000  
Er-value:0.000, Pr-value:0.000  
eCLIP MATCHES▶aggf1 (bg=15.15%)▶bclaf1 (bg=17.67%)▶EXOSC5 (bg=2.0%)▶FUBP3 (bg=1.53%)▶fxr2 (bg=10.1%)▶GRWD1 (bg=7.0%)▶gtf2f1 (bg=10.18%)▶hltf (bg=24.28%)▶khsrp (bg=27.4%)▶larp4 (bg=13.51%)▶MTPAP (bg=9.55%)▶npm1 (bg=10.22%)▶ppil4 (bg=43.39%)▶rbm15 (bg=11.59%)▶rbm22 (bg=12.69%)▶safb (bg=40.39%)▶safb2 (bg=26.89%)▶SLTM (bg=7.5%)▶SMNDC1 (bg=7.08%)▶srsf1 (bg=30.28%)▶SRSF9 (bg=9.67%)▶TAF15 (bg=9.06%)▶tra2a (bg=37.02%)▶TROVE2 (bg=6.96%)▶uchl5 (bg=18.56%)▶UTP3 (bg=1.57%)▶YWHAG (bg=9.14%)▶znf622 (bg=18.79%)No matches to TargetScan


TTGGCTTGGCAACCACACGGAGGAG

TAGCAGGCGGCTTGGCTTGGCAACCACACGGAGGAG  
Depth:2 (MARMOSET)  
Ei-value:0.000, Pi-value:0.000  
Er-value:0.000, Pr-value:0.000  
eCLIP MATCHES▶aggf1 (bg=15.15%)▶bclaf1 (bg=17.67%)▶EXOSC5 (bg=2.0%)▶FUBP3 (bg=1.53%)▶fxr2 (bg=10.1%)▶GRWD1 (bg=7.0%)▶gtf2f1 (bg=10.18%)▶hltf (bg=24.28%)▶khsrp (bg=27.4%)▶larp4 (bg=13.51%)▶MTPAP (bg=9.55%)▶npm1 (bg=10.22%)▶ppil4 (bg=43.39%)▶rbm15 (bg=11.59%)▶rbm22 (bg=12.69%)▶safb (bg=40.39%)▶safb2 (bg=26.89%)▶SLTM (bg=7.5%)▶SMNDC1 (bg=7.08%)▶srsf1 (bg=30.28%)▶SRSF9 (bg=9.67%)▶TAF15 (bg=9.06%)▶tra2a (bg=37.02%)▶TROVE2 (bg=6.96%)▶uchl5 (bg=18.56%)▶UTP3 (bg=1.57%)▶YWHAG (bg=9.14%)▶znf622 (bg=18.79%)▶ZNF800 (bg=3.2%)No matches to TargetScan

-

CGAG

CGAGCAGGCGTTGTGC  
Depth:2 (MARMOSET)  
Ei-value:0.000, Pi-value:0.000  
Er-value:0.000, Pr-value:0.000  
eCLIP MATCHES▶aggf1 (bg=15.15%)▶bclaf1 (bg=17.67%)▶EXOSC5 (bg=2.0%)▶FUBP3 (bg=1.53%)▶fxr2 (bg=10.1%)▶GRWD1 (bg=7.0%)▶gtf2f1 (bg=10.18%)▶hltf (bg=24.28%)▶larp4 (bg=13.51%)▶MTPAP (bg=9.55%)▶npm1 (bg=10.22%)▶ppil4 (bg=43.39%)▶rbm15 (bg=11.59%)▶rbm22 (bg=12.69%)▶safb (bg=40.39%)▶safb2 (bg=26.89%)▶SDAD1 (bg=1.11%)▶SLTM (bg=7.5%)▶SMNDC1 (bg=7.08%)▶srsf1 (bg=30.28%)▶srsf7 (bg=22.53%)▶SRSF9 (bg=9.67%)▶TAF15 (bg=9.06%)▶tra2a (bg=37.02%)▶TROVE2 (bg=6.96%)▶uchl5 (bg=18.56%)▶UTP3 (bg=1.57%)▶YWHAG (bg=9.14%)▶znf622 (bg=18.79%)▶ZNF800 (bg=3.2%)No matches to TargetScan


CAGGCG

CAGGCG  
Depth:5 (COW)  
Ei-value:0.000, Pi-value:0.000  
Er-value:0.000, Pr-value:0.000  
eCLIP MATCHES▶aggf1 (bg=15.15%)▶bclaf1 (bg=17.67%)▶EXOSC5 (bg=2.0%)▶FUBP3 (bg=1.53%)▶fxr2 (bg=10.1%)▶GRWD1 (bg=7.0%)▶gtf2f1 (bg=10.18%)▶hltf (bg=24.28%)▶MTPAP (bg=9.55%)▶npm1 (bg=10.22%)▶ppil4 (bg=43.39%)▶rbm15 (bg=11.59%)▶rbm22 (bg=12.69%)▶safb (bg=40.39%)▶safb2 (bg=26.89%)▶SDAD1 (bg=1.11%)▶SLTM (bg=7.5%)▶SMNDC1 (bg=7.08%)▶srsf1 (bg=30.28%)▶srsf7 (bg=22.53%)▶SRSF9 (bg=9.67%)▶TAF15 (bg=9.06%)▶tra2a (bg=37.02%)▶TROVE2 (bg=6.96%)▶uchl5 (bg=18.56%)▶UTP3 (bg=1.57%)▶YWHAG (bg=9.14%)▶znf622 (bg=18.79%)▶ZNF800 (bg=3.2%)No matches to TargetScan


T

CGAGCAGGCGTTGTGC  
Depth:2 (MARMOSET)  
Ei-value:0.000, Pi-value:0.000  
Er-value:0.000, Pr-value:0.000  
eCLIP MATCHES▶aggf1 (bg=15.15%)▶bclaf1 (bg=17.67%)▶EXOSC5 (bg=2.0%)▶FUBP3 (bg=1.53%)▶fxr2 (bg=10.1%)▶GRWD1 (bg=7.0%)▶gtf2f1 (bg=10.18%)▶hltf (bg=24.28%)▶larp4 (bg=13.51%)▶MTPAP (bg=9.55%)▶npm1 (bg=10.22%)▶ppil4 (bg=43.39%)▶rbm15 (bg=11.59%)▶rbm22 (bg=12.69%)▶safb (bg=40.39%)▶safb2 (bg=26.89%)▶SDAD1 (bg=1.11%)▶SLTM (bg=7.5%)▶SMNDC1 (bg=7.08%)▶srsf1 (bg=30.28%)▶srsf7 (bg=22.53%)▶SRSF9 (bg=9.67%)▶TAF15 (bg=9.06%)▶tra2a (bg=37.02%)▶TROVE2 (bg=6.96%)▶uchl5 (bg=18.56%)▶UTP3 (bg=1.57%)▶YWHAG (bg=9.14%)▶znf622 (bg=18.79%)▶ZNF800 (bg=3.2%)No matches to TargetScan

 1680  


TGTGC

CGAGCAGGCGTTGTGC  
Depth:2 (MARMOSET)  
Ei-value:0.000, Pi-value:0.000  
Er-value:0.000, Pr-value:0.000  
eCLIP MATCHES▶aggf1 (bg=15.15%)▶bclaf1 (bg=17.67%)▶EXOSC5 (bg=2.0%)▶FUBP3 (bg=1.53%)▶fxr2 (bg=10.1%)▶GRWD1 (bg=7.0%)▶gtf2f1 (bg=10.18%)▶hltf (bg=24.28%)▶larp4 (bg=13.51%)▶MTPAP (bg=9.55%)▶npm1 (bg=10.22%)▶ppil4 (bg=43.39%)▶rbm15 (bg=11.59%)▶rbm22 (bg=12.69%)▶safb (bg=40.39%)▶safb2 (bg=26.89%)▶SDAD1 (bg=1.11%)▶SLTM (bg=7.5%)▶SMNDC1 (bg=7.08%)▶srsf1 (bg=30.28%)▶srsf7 (bg=22.53%)▶SRSF9 (bg=9.67%)▶TAF15 (bg=9.06%)▶tra2a (bg=37.02%)▶TROVE2 (bg=6.96%)▶uchl5 (bg=18.56%)▶UTP3 (bg=1.57%)▶YWHAG (bg=9.14%)▶znf622 (bg=18.79%)▶ZNF800 (bg=3.2%)No matches to TargetScan

-

TAGAGGA

TAGAGGATCCTAGA  
Depth:3 (DOG)  
Ei-value:0.000, Pi-value:0.000  
Er-value:0.000, Pr-value:0.000  
eCLIP MATCHES▶aggf1 (bg=15.15%)▶bclaf1 (bg=17.67%)▶EXOSC5 (bg=2.0%)▶fxr2 (bg=10.1%)▶GRWD1 (bg=7.0%)▶gtf2f1 (bg=10.18%)▶hltf (bg=24.28%)▶larp4 (bg=13.51%)▶MTPAP (bg=9.55%)▶npm1 (bg=10.22%)▶ppil4 (bg=43.39%)▶rbm15 (bg=11.59%)▶rbm22 (bg=12.69%)▶safb (bg=40.39%)▶safb2 (bg=26.89%)▶SDAD1 (bg=1.11%)▶SLTM (bg=7.5%)▶SMNDC1 (bg=7.08%)▶srsf1 (bg=30.28%)▶srsf7 (bg=22.53%)▶SRSF9 (bg=9.67%)▶TAF15 (bg=9.06%)▶tra2a (bg=37.02%)▶uchl5 (bg=18.56%)▶UTP3 (bg=1.57%)▶znf622 (bg=18.79%)▶ZNF800 (bg=3.2%)No matches to TargetScan


TCCTAGA

TCCTAGA  
Depth:6 (MOUSE)  
Ei-value:0.000, Pi-value:0.000  
Er-value:0.000, Pr-value:0.000  
eCLIP MATCHES▶aggf1 (bg=15.15%)▶bclaf1 (bg=17.67%)▶EXOSC5 (bg=2.0%)▶fxr2 (bg=10.1%)▶GRWD1 (bg=7.0%)▶gtf2f1 (bg=10.18%)▶hltf (bg=24.28%)▶larp4 (bg=13.51%)▶MTPAP (bg=9.55%)▶npm1 (bg=10.22%)▶ppil4 (bg=43.39%)▶rbm15 (bg=11.59%)▶rbm22 (bg=12.69%)▶safb (bg=40.39%)▶safb2 (bg=26.89%)▶SDAD1 (bg=1.11%)▶SLTM (bg=7.5%)▶SMNDC1 (bg=7.08%)▶srsf1 (bg=30.28%)▶srsf7 (bg=22.53%)▶SRSF9 (bg=9.67%)▶TAF15 (bg=9.06%)▶tra2a (bg=37.02%)▶uchl5 (bg=18.56%)▶UTP3 (bg=1.57%)▶znf622 (bg=18.79%)▶ZNF800 (bg=3.2%)No matches to TargetScan


C

TAGAGGATCCTAGACCAGCATGCCAGT  
Depth:2 (MARMOSET)  
Ei-value:0.000, Pi-value:0.000  
Er-value:0.000, Pr-value:0.000  
eCLIP MATCHES▶aggf1 (bg=15.15%)▶bclaf1 (bg=17.67%)▶EXOSC5 (bg=2.0%)▶fxr2 (bg=10.1%)▶GRWD1 (bg=7.0%)▶gtf2f1 (bg=10.18%)▶hltf (bg=24.28%)▶larp4 (bg=13.51%)▶MTPAP (bg=9.55%)▶npm1 (bg=10.22%)▶ppil4 (bg=43.39%)▶rbm15 (bg=11.59%)▶rbm22 (bg=12.69%)▶safb (bg=40.39%)▶safb2 (bg=26.89%)▶SDAD1 (bg=1.11%)▶SLTM (bg=7.5%)▶SMNDC1 (bg=7.08%)▶srsf1 (bg=30.28%)▶srsf7 (bg=22.53%)▶SRSF9 (bg=9.67%)▶TAF15 (bg=9.06%)▶tra2a (bg=37.02%)▶uchl5 (bg=18.56%)▶UTP3 (bg=1.57%)▶znf622 (bg=18.79%)▶ZNF800 (bg=3.2%)MATCHES To TargetScan▶ miR-138-5p:GCUGGUG▶ miR-193-3p:ACUGGCC


cagcat

cagcat  
Depth:3 (DOG)  
Ei-value:1.000, Pi-value:0.020  
Er-value:0.000, Pr-value:0.000  
eCLIP MATCHES▶aggf1 (bg=15.15%)▶bclaf1 (bg=17.67%)▶EXOSC5 (bg=2.0%)▶fxr2 (bg=10.1%)▶GRWD1 (bg=7.0%)▶gtf2f1 (bg=10.18%)▶hltf (bg=24.28%)▶larp4 (bg=13.51%)▶MTPAP (bg=9.55%)▶npm1 (bg=10.22%)▶ppil4 (bg=43.39%)▶rbm15 (bg=11.59%)▶rbm22 (bg=12.69%)▶safb (bg=40.39%)▶safb2 (bg=26.89%)▶SDAD1 (bg=1.11%)▶SLTM (bg=7.5%)▶SMNDC1 (bg=7.08%)▶srsf1 (bg=30.28%)▶srsf7 (bg=22.53%)▶SRSF9 (bg=9.67%)▶TAF15 (bg=9.06%)▶tra2a (bg=37.02%)▶uchl5 (bg=18.56%)▶UTP3 (bg=1.57%)▶znf622 (bg=18.79%)▶ZNF800 (bg=3.2%)No matches to TargetScan


GCCAGT

TAGAGGATCCTAGACCAGCATGCCAGT  
Depth:2 (MARMOSET)  
Ei-value:0.000, Pi-value:0.000  
Er-value:0.000, Pr-value:0.000  
eCLIP MATCHES▶aggf1 (bg=15.15%)▶bclaf1 (bg=17.67%)▶EXOSC5 (bg=2.0%)▶fxr2 (bg=10.1%)▶GRWD1 (bg=7.0%)▶gtf2f1 (bg=10.18%)▶hltf (bg=24.28%)▶larp4 (bg=13.51%)▶MTPAP (bg=9.55%)▶npm1 (bg=10.22%)▶ppil4 (bg=43.39%)▶rbm15 (bg=11.59%)▶rbm22 (bg=12.69%)▶safb (bg=40.39%)▶safb2 (bg=26.89%)▶SDAD1 (bg=1.11%)▶SLTM (bg=7.5%)▶SMNDC1 (bg=7.08%)▶srsf1 (bg=30.28%)▶srsf7 (bg=22.53%)▶SRSF9 (bg=9.67%)▶TAF15 (bg=9.06%)▶tra2a (bg=37.02%)▶uchl5 (bg=18.56%)▶UTP3 (bg=1.57%)▶znf622 (bg=18.79%)▶ZNF800 (bg=3.2%)MATCHES To TargetScan▶ miR-138-5p:GCUGGUG▶ miR-193-3p:ACUGGCC

-

tgccaaggc

tgccaaggc  
Depth:2 (MARMOSET)  
Ei-value:1.000, Pi-value:0.000  
Er-value:0.000, Pr-value:0.000  
eCLIP MATCHES▶aggf1 (bg=15.15%)▶bclaf1 (bg=17.67%)▶EXOSC5 (bg=2.0%)▶fxr2 (bg=10.1%)▶GRWD1 (bg=7.0%)▶gtf2f1 (bg=10.18%)▶hltf (bg=24.28%)▶larp4 (bg=13.51%)▶MTPAP (bg=9.55%)▶npm1 (bg=10.22%)▶ppil4 (bg=43.39%)▶rbm15 (bg=11.59%)▶rbm22 (bg=12.69%)▶safb (bg=40.39%)▶safb2 (bg=26.89%)▶SDAD1 (bg=1.11%)▶SLTM (bg=7.5%)▶SMNDC1 (bg=7.08%)▶srsf1 (bg=30.28%)▶SRSF9 (bg=9.67%)▶TAF15 (bg=9.06%)▶tra2a (bg=37.02%)▶uchl5 (bg=18.56%)▶UTP3 (bg=1.57%)▶YWHAG (bg=9.14%)▶znf622 (bg=18.79%)▶ZNF800 (bg=3.2%)MATCHES To TargetScan▶ miR-212-5p:CCUUGGC▶ miR-182-5p:UUGGCAA▶ miR-96-5p/1271-5p:UUGGCAC

-

a

acagggaa  
Depth:2 (MARMOSET)  
Ei-value:1.000, Pi-value:0.000  
Er-value:0.000, Pr-value:0.000  
eCLIP MATCHES▶bclaf1 (bg=17.67%)▶bud13 (bg=12.85%)▶EXOSC5 (bg=2.0%)▶FUBP3 (bg=1.53%)▶fxr2 (bg=10.1%)▶GRWD1 (bg=7.0%)▶gtf2f1 (bg=10.18%)▶hltf (bg=24.28%)▶MTPAP (bg=9.55%)▶npm1 (bg=10.22%)▶ppil4 (bg=43.39%)▶rbm15 (bg=11.59%)▶rbm22 (bg=12.69%)▶safb (bg=40.39%)▶safb2 (bg=26.89%)▶SDAD1 (bg=1.11%)▶SLTM (bg=7.5%)▶SMNDC1 (bg=7.08%)▶srsf1 (bg=30.28%)▶SRSF9 (bg=9.67%)▶TAF15 (bg=9.06%)▶tra2a (bg=37.02%)▶uchl5 (bg=18.56%)▶UTP3 (bg=1.57%)▶XRCC6 (bg=3.81%)▶YWHAG (bg=9.14%)▶znf622 (bg=18.79%)▶ZNF800 (bg=3.2%)▶ZRANB2 (bg=7.28%)MATCHES To TargetScan▶ miR-339-5p:CCCUGUC


CAGGGA

CAGGGA  
Depth:6 (MOUSE)  
Ei-value:0.000, Pi-value:0.000  
Er-value:0.000, Pr-value:0.000  
eCLIP MATCHES▶bclaf1 (bg=17.67%)▶bud13 (bg=12.85%)▶EXOSC5 (bg=2.0%)▶FUBP3 (bg=1.53%)▶fxr2 (bg=10.1%)▶GRWD1 (bg=7.0%)▶gtf2f1 (bg=10.18%)▶hltf (bg=24.28%)▶MTPAP (bg=9.55%)▶npm1 (bg=10.22%)▶ppil4 (bg=43.39%)▶rbm15 (bg=11.59%)▶rbm22 (bg=12.69%)▶safb (bg=40.39%)▶safb2 (bg=26.89%)▶SDAD1 (bg=1.11%)▶SLTM (bg=7.5%)▶SMNDC1 (bg=7.08%)▶srsf1 (bg=30.28%)▶SRSF9 (bg=9.67%)▶TAF15 (bg=9.06%)▶tra2a (bg=37.02%)▶uchl5 (bg=18.56%)▶UTP3 (bg=1.57%)▶XRCC6 (bg=3.81%)▶YWHAG (bg=9.14%)▶znf622 (bg=18.79%)▶ZNF800 (bg=3.2%)No matches to TargetScan


a

acagggaa  
Depth:2 (MARMOSET)  
Ei-value:1.000, Pi-value:0.000  
Er-value:0.000, Pr-value:0.000  
eCLIP MATCHES▶bclaf1 (bg=17.67%)▶bud13 (bg=12.85%)▶EXOSC5 (bg=2.0%)▶FUBP3 (bg=1.53%)▶fxr2 (bg=10.1%)▶GRWD1 (bg=7.0%)▶gtf2f1 (bg=10.18%)▶hltf (bg=24.28%)▶MTPAP (bg=9.55%)▶npm1 (bg=10.22%)▶ppil4 (bg=43.39%)▶rbm15 (bg=11.59%)▶rbm22 (bg=12.69%)▶safb (bg=40.39%)▶safb2 (bg=26.89%)▶SDAD1 (bg=1.11%)▶SLTM (bg=7.5%)▶SMNDC1 (bg=7.08%)▶srsf1 (bg=30.28%)▶SRSF9 (bg=9.67%)▶TAF15 (bg=9.06%)▶tra2a (bg=37.02%)▶uchl5 (bg=18.56%)▶UTP3 (bg=1.57%)▶XRCC6 (bg=3.81%)▶YWHAG (bg=9.14%)▶znf622 (bg=18.79%)▶ZNF800 (bg=3.2%)▶ZRANB2 (bg=7.28%)MATCHES To TargetScan▶ miR-339-5p:CCCUGUC

---

g

gagtggt  
Depth:3 (DOG)  
Ei-value:0.350, Pi-value:0.010  
Er-value:0.000, Pr-value:0.000  
eCLIP MATCHES▶bclaf1 (bg=17.67%)▶bud13 (bg=12.85%)▶DROSHA (bg=1.03%)▶FUBP3 (bg=1.53%)▶fxr2 (bg=10.1%)▶GRWD1 (bg=7.0%)▶gtf2f1 (bg=10.18%)▶hltf (bg=24.28%)▶larp4 (bg=13.51%)▶MTPAP (bg=9.55%)▶NIPBL (bg=8.2%)▶ppil4 (bg=43.39%)▶rbm15 (bg=11.59%)▶rbm22 (bg=12.69%)▶safb (bg=40.39%)▶safb2 (bg=26.89%)▶SDAD1 (bg=1.11%)▶SLTM (bg=7.5%)▶SMNDC1 (bg=7.08%)▶srsf1 (bg=30.28%)▶SRSF9 (bg=9.67%)▶TAF15 (bg=9.06%)▶tra2a (bg=37.02%)▶uchl5 (bg=18.56%)▶UTP3 (bg=1.57%)▶XRCC6 (bg=3.81%)▶YWHAG (bg=9.14%)▶znf622 (bg=18.79%)▶ZNF800 (bg=3.2%)▶ZRANB2 (bg=7.28%)No matches to TargetScan


AGTGGT

AGTGGT  
Depth:5 (COW)  
Ei-value:0.000, Pi-value:0.020  
Er-value:0.000, Pr-value:0.000  
eCLIP MATCHES▶bclaf1 (bg=17.67%)▶bud13 (bg=12.85%)▶DROSHA (bg=1.03%)▶FUBP3 (bg=1.53%)▶GRWD1 (bg=7.0%)▶gtf2f1 (bg=10.18%)▶hltf (bg=24.28%)▶larp4 (bg=13.51%)▶MTPAP (bg=9.55%)▶NIPBL (bg=8.2%)▶ppil4 (bg=43.39%)▶rbm15 (bg=11.59%)▶rbm22 (bg=12.69%)▶safb (bg=40.39%)▶safb2 (bg=26.89%)▶SDAD1 (bg=1.11%)▶SLTM (bg=7.5%)▶SMNDC1 (bg=7.08%)▶srsf1 (bg=30.28%)▶SRSF9 (bg=9.67%)▶TAF15 (bg=9.06%)▶tra2a (bg=37.02%)▶uchl5 (bg=18.56%)▶UTP3 (bg=1.57%)▶XRCC6 (bg=3.81%)▶YWHAG (bg=9.14%)▶znf622 (bg=18.79%)▶ZNF800 (bg=3.2%)▶ZRANB2 (bg=7.28%)No matches to TargetScan


TGGTAAAAAT

TGGTAAAAAT  
Depth:5 (COW)  
Ei-value:0.000, Pi-value:0.000  
Er-value:0.000, Pr-value:0.000  
eCLIP MATCHES▶bclaf1 (bg=17.67%)▶bud13 (bg=12.85%)▶DROSHA (bg=1.03%)▶FUBP3 (bg=1.53%)▶GRWD1 (bg=7.0%)▶gtf2f1 (bg=10.18%)▶hltf (bg=24.28%)▶larp4 (bg=13.51%)▶MTPAP (bg=9.55%)▶NIPBL (bg=8.2%)▶npm1 (bg=10.22%)▶ppil4 (bg=43.39%)▶rbm15 (bg=11.59%)▶rbm22 (bg=12.69%)▶safb (bg=40.39%)▶safb2 (bg=26.89%)▶SDAD1 (bg=1.11%)▶SLTM (bg=7.5%)▶SMNDC1 (bg=7.08%)▶srsf1 (bg=30.28%)▶SRSF9 (bg=9.67%)▶TAF15 (bg=9.06%)▶tra2a (bg=37.02%)▶uchl5 (bg=18.56%)▶XRCC6 (bg=3.81%)▶YWHAG (bg=9.14%)▶znf622 (bg=18.79%)▶ZNF800 (bg=3.2%)▶ZRANB2 (bg=7.28%)No matches to TargetScan


CCGTGAGGTCGGCAATATGTTGTTTTTCTGGAACTT

GAGTGGTTGGTAAAAATCCGTGAGGTCGGCAATATGTTGTTTTTCTGGAACTT  
Depth:2 (MARMOSET)  
Ei-value:0.000, Pi-value:0.000  
Er-value:0.000, Pr-value:0.000  
eCLIP MATCHES▶bclaf1 (bg=17.67%)▶bud13 (bg=12.85%)▶DROSHA (bg=1.03%)▶FUBP3 (bg=1.53%)▶fxr2 (bg=10.1%)▶GRWD1 (bg=7.0%)▶gtf2f1 (bg=10.18%)▶hltf (bg=24.28%)▶larp4 (bg=13.51%)▶MTPAP (bg=9.55%)▶NIPBL (bg=8.2%)▶NOLC1 (bg=0.67%)▶npm1 (bg=10.22%)▶ppil4 (bg=43.39%)▶rbm15 (bg=11.59%)▶rbm22 (bg=12.69%)▶safb (bg=40.39%)▶safb2 (bg=26.89%)▶SDAD1 (bg=1.11%)▶SLTM (bg=7.5%)▶SMNDC1 (bg=7.08%)▶srsf1 (bg=30.28%)▶SRSF9 (bg=9.67%)▶TAF15 (bg=9.06%)▶tia1 (bg=23.76%)▶tra2a (bg=37.02%)▶uchl5 (bg=18.56%)▶UTP3 (bg=1.57%)▶XRCC6 (bg=3.81%)▶YWHAG (bg=9.14%)▶znf622 (bg=18.79%)▶ZNF800 (bg=3.2%)▶ZRANB2 (bg=7.28%)MATCHES To TargetScan▶ miR-876-5p:GGAUUUC▶ miR-137:UAUUGCU

---

TA

TATGGTAA  
Depth:3 (DOG)  
Ei-value:0.010, Pi-value:0.000  
Er-value:0.000, Pr-value:0.000  
eCLIP MATCHES▶bclaf1 (bg=17.67%)▶DROSHA (bg=1.03%)▶GRWD1 (bg=7.0%)▶hltf (bg=24.28%)▶MTPAP (bg=9.55%)▶NOLC1 (bg=0.67%)▶npm1 (bg=10.22%)▶ppil4 (bg=43.39%)▶rbm15 (bg=11.59%)▶safb (bg=40.39%)▶srsf1 (bg=30.28%)▶TAF15 (bg=9.06%)▶tia1 (bg=23.76%)▶uchl5 (bg=18.56%)▶YWHAG (bg=9.14%)▶ZRANB2 (bg=7.28%)No matches to TargetScan


TGGTAA

TGGTAA  
Depth:14 (SPOTTEDGAR)  
Ei-value:0.030, Pi-value:0.000  
Er-value:0.010, Pr-value:0.000  
eCLIP MATCHES▶bclaf1 (bg=17.67%)▶DROSHA (bg=1.03%)▶GRWD1 (bg=7.0%)▶hltf (bg=24.28%)▶MTPAP (bg=9.55%)▶NOLC1 (bg=0.67%)▶npm1 (bg=10.22%)▶ppil4 (bg=43.39%)▶rbm15 (bg=11.59%)▶safb (bg=40.39%)▶srsf1 (bg=30.28%)▶TAF15 (bg=9.06%)▶tia1 (bg=23.76%)▶uchl5 (bg=18.56%)▶YWHAG (bg=9.14%)▶ZRANB2 (bg=7.28%)No matches to TargetScan

- 1800  
 -

ttttattta

ttttattta  
Depth:2 (MARMOSET)  
Ei-value:1.000, Pi-value:0.000  
Er-value:0.000, Pr-value:0.000  
eCLIP MATCHES▶bclaf1 (bg=17.67%)▶GRWD1 (bg=7.0%)▶hltf (bg=24.28%)▶MTPAP (bg=9.55%)▶npm1 (bg=10.22%)▶rbm15 (bg=11.59%)▶safb (bg=40.39%)▶srsf1 (bg=30.28%)▶tia1 (bg=23.76%)▶uchl5 (bg=18.56%)▶YWHAG (bg=9.14%)No matches to TargetScan

----------

TAATGG

TAATGG  
Depth:5 (COW)  
Ei-value:0.000, Pi-value:0.020  
Er-value:0.000, Pr-value:0.000  
eCLIP MATCHES▶ddx42 (bg=10.33%)▶khsrp (bg=27.4%)▶ppil4 (bg=43.39%)▶safb (bg=40.39%)▶tia1 (bg=23.76%)▶tial1 (bg=15.02%)▶ZRANB2 (bg=7.28%)No matches to TargetScan


GGGAGTTTCGTACTGAGGTGTAAA

TAATGGGGGAGTTTCGTACTGAGGTGTAAA  
Depth:2 (MARMOSET)  
Ei-value:0.000, Pi-value:0.000  
Er-value:0.000, Pr-value:0.000  
eCLIP MATCHES▶ddx42 (bg=10.33%)▶khsrp (bg=27.4%)▶ppil4 (bg=43.39%)▶safb (bg=40.39%)▶tia1 (bg=23.76%)▶tial1 (bg=15.02%)▶ZRANB2 (bg=7.28%)MATCHES To TargetScan▶ miR-1306-5p:CACCUCC


GGGATTTAT

GGGATTTAT  
Depth:3 (DOG)  
Ei-value:0.000, Pi-value:0.020  
Er-value:0.000, Pr-value:0.000  
eCLIP MATCHES▶ddx42 (bg=10.33%)▶khsrp (bg=27.4%)▶ppil4 (bg=43.39%)▶safb (bg=40.39%)▶SF3B4 (bg=4.76%)▶tia1 (bg=23.76%)▶tial1 (bg=15.02%)▶ZRANB2 (bg=7.28%)No matches to TargetScan


ATGGGGA

GGGATTTATATGGGGA  
Depth:2 (MARMOSET)  
Ei-value:0.000, Pi-value:0.000  
Er-value:0.000, Pr-value:0.000  
eCLIP MATCHES▶ddx42 (bg=10.33%)▶khsrp (bg=27.4%)▶ppil4 (bg=43.39%)▶safb (bg=40.39%)▶SF3B4 (bg=4.76%)▶tia1 (bg=23.76%)▶tial1 (bg=15.02%)▶ZRANB2 (bg=7.28%)MATCHES To TargetScan▶ miR-410-3p:AUAUAAC▶ miR-340-5p:UAUAAAG

-

GTAGGCC

GTAGGCC  
Depth:6 (MOUSE)  
Ei-value:0.000, Pi-value:0.000  
Er-value:0.000, Pr-value:0.000  
eCLIP MATCHES▶ddx42 (bg=10.33%)▶khsrp (bg=27.4%)▶ppil4 (bg=43.39%)▶safb (bg=40.39%)▶SF3B4 (bg=4.76%)▶tia1 (bg=23.76%)▶tial1 (bg=15.02%)▶ZRANB2 (bg=7.28%)No matches to TargetScan


G

GTAGGCCG  
Depth:5 (COW)  
Ei-value:0.000, Pi-value:0.000  
Er-value:0.000, Pr-value:0.000  
eCLIP MATCHES▶ddx42 (bg=10.33%)▶khsrp (bg=27.4%)▶ppil4 (bg=43.39%)▶safb (bg=40.39%)▶SF3B4 (bg=4.76%)▶tia1 (bg=23.76%)▶tial1 (bg=15.02%)▶ZRANB2 (bg=7.28%)No matches to TargetScan


ATTT

GTAGGCCGATTT  
Depth:4 (PIG)  
Ei-value:0.000, Pi-value:0.000  
Er-value:0.000, Pr-value:0.000  
eCLIP MATCHES▶ddx42 (bg=10.33%)▶khsrp (bg=27.4%)▶ppil4 (bg=43.39%)▶safb (bg=40.39%)▶SF3B4 (bg=4.76%)▶tia1 (bg=23.76%)▶tial1 (bg=15.02%)▶ZRANB2 (bg=7.28%)No matches to TargetScan


C

GTAGGCCGATTTCCGGGTGT  
Depth:3 (DOG)  
Ei-value:0.000, Pi-value:0.000  
Er-value:0.000, Pr-value:0.000  
eCLIP MATCHES▶ddx42 (bg=10.33%)▶khsrp (bg=27.4%)▶ppil4 (bg=43.39%)▶safb (bg=40.39%)▶SF3B4 (bg=4.76%)▶tia1 (bg=23.76%)▶tial1 (bg=15.02%)▶ZRANB2 (bg=7.28%)No matches to TargetScan


CGGGTGT

CGGGTGT  
Depth:6 (MOUSE)  
Ei-value:0.000, Pi-value:0.000  
Er-value:0.000, Pr-value:0.000  
eCLIP MATCHES▶ddx42 (bg=10.33%)▶khsrp (bg=27.4%)▶ppil4 (bg=43.39%)▶safb (bg=40.39%)▶SF3B4 (bg=4.76%)▶tia1 (bg=23.76%)▶tial1 (bg=15.02%)▶ZRANB2 (bg=7.28%)No matches to TargetScan


T

GTAGGCCGATTTCCGGGTGTTGTAGGTTTCTCTTTTTCAGGCTTAT  
Depth:2 (MARMOSET)  
Ei-value:0.000, Pi-value:0.000  
Er-value:0.000, Pr-value:0.000  
eCLIP MATCHES▶ddx42 (bg=10.33%)▶hltf (bg=24.28%)▶khsrp (bg=27.4%)▶NIPBL (bg=8.2%)▶ppil4 (bg=43.39%)▶safb (bg=40.39%)▶safb2 (bg=26.89%)▶SF3B4 (bg=4.76%)▶srsf1 (bg=30.28%)▶tia1 (bg=23.76%)▶tial1 (bg=15.02%)▶tra2a (bg=37.02%)▶u2af1 (bg=14.02%)▶u2af2 (bg=19.32%)▶ZRANB2 (bg=7.28%)No matches to TargetScan


GTAGGTTT

GTAGGTTT  
Depth:8 (ALLIGATOR)  
Ei-value:0.000, Pi-value:0.000  
Er-value:0.000, Pr-value:0.000  
eCLIP MATCHES▶ddx42 (bg=10.33%)▶khsrp (bg=27.4%)▶ppil4 (bg=43.39%)▶safb (bg=40.39%)▶SF3B4 (bg=4.76%)▶tia1 (bg=23.76%)▶tial1 (bg=15.02%)▶u2af1 (bg=14.02%)▶ZRANB2 (bg=7.28%)No matches to TargetScan


C

GTAGGTTTCTCTTTTTCAGGC  
Depth:5 (COW)  
Ei-value:0.000, Pi-value:0.000  
Er-value:0.000, Pr-value:0.000  
eCLIP MATCHES▶ddx42 (bg=10.33%)▶hltf (bg=24.28%)▶khsrp (bg=27.4%)▶NIPBL (bg=8.2%)▶ppil4 (bg=43.39%)▶safb (bg=40.39%)▶safb2 (bg=26.89%)▶SF3B4 (bg=4.76%)▶srsf1 (bg=30.28%)▶tia1 (bg=23.76%)▶tial1 (bg=15.02%)▶u2af1 (bg=14.02%)▶u2af2 (bg=19.32%)▶ZRANB2 (bg=7.28%)No matches to TargetScan


TCTTTTTCAGG

TCTTTTTCAGG  
Depth:6 (MOUSE)  
Ei-value:0.000, Pi-value:0.000  
Er-value:0.000, Pr-value:0.000  
eCLIP MATCHES▶ddx42 (bg=10.33%)▶hltf (bg=24.28%)▶khsrp (bg=27.4%)▶NIPBL (bg=8.2%)▶ppil4 (bg=43.39%)▶safb (bg=40.39%)▶safb2 (bg=26.89%)▶SF3B4 (bg=4.76%)▶srsf1 (bg=30.28%)▶tia1 (bg=23.76%)▶tial1 (bg=15.02%)▶u2af1 (bg=14.02%)▶u2af2 (bg=19.32%)▶ZRANB2 (bg=7.28%)No matches to TargetScan


C

GTAGGTTTCTCTTTTTCAGGC  
Depth:5 (COW)  
Ei-value:0.000, Pi-value:0.000  
Er-value:0.000, Pr-value:0.000  
eCLIP MATCHES▶ddx42 (bg=10.33%)▶hltf (bg=24.28%)▶khsrp (bg=27.4%)▶NIPBL (bg=8.2%)▶ppil4 (bg=43.39%)▶safb (bg=40.39%)▶safb2 (bg=26.89%)▶SF3B4 (bg=4.76%)▶srsf1 (bg=30.28%)▶tia1 (bg=23.76%)▶tial1 (bg=15.02%)▶u2af1 (bg=14.02%)▶u2af2 (bg=19.32%)▶ZRANB2 (bg=7.28%)No matches to TargetScan


TT

GTAGGTTTCTCTTTTTCAGGCTT  
Depth:3 (DOG)  
Ei-value:0.000, Pi-value:0.000  
Er-value:0.000, Pr-value:0.000  
eCLIP MATCHES▶ddx42 (bg=10.33%)▶hltf (bg=24.28%)▶khsrp (bg=27.4%)▶NIPBL (bg=8.2%)▶ppil4 (bg=43.39%)▶safb (bg=40.39%)▶safb2 (bg=26.89%)▶SF3B4 (bg=4.76%)▶srsf1 (bg=30.28%)▶tia1 (bg=23.76%)▶tial1 (bg=15.02%)▶tra2a (bg=37.02%)▶u2af1 (bg=14.02%)▶u2af2 (bg=19.32%)▶ZRANB2 (bg=7.28%)No matches to TargetScan


AT

GTAGGCCGATTTCCGGGTGTTGTAGGTTTCTCTTTTTCAGGCTTAT  
Depth:2 (MARMOSET)  
Ei-value:0.000, Pi-value:0.000  
Er-value:0.000, Pr-value:0.000  
eCLIP MATCHES▶ddx42 (bg=10.33%)▶hltf (bg=24.28%)▶khsrp (bg=27.4%)▶NIPBL (bg=8.2%)▶ppil4 (bg=43.39%)▶safb (bg=40.39%)▶safb2 (bg=26.89%)▶SF3B4 (bg=4.76%)▶srsf1 (bg=30.28%)▶tia1 (bg=23.76%)▶tial1 (bg=15.02%)▶tra2a (bg=37.02%)▶u2af1 (bg=14.02%)▶u2af2 (bg=19.32%)▶ZRANB2 (bg=7.28%)No matches to TargetScan

-

CTCATG

CTCATGAATCTTGTCTGAAGCTTTTGAGGGCAGACTGCCAAGTCCTGGAG  
Depth:2 (MARMOSET)  
Ei-value:0.000, Pi-value:0.000  
Er-value:0.000, Pr-value:0.000  
eCLIP MATCHES▶BCCIP (bg=0.09%)▶hltf (bg=24.28%)▶khsrp (bg=27.4%)▶NIPBL (bg=8.2%)▶ppil4 (bg=43.39%)▶safb (bg=40.39%)▶safb2 (bg=26.89%)▶srsf1 (bg=30.28%)▶tia1 (bg=23.76%)▶tial1 (bg=15.02%)▶tra2a (bg=37.02%)▶u2af1 (bg=14.02%)▶u2af2 (bg=19.32%)▶ZRANB2 (bg=7.28%)MATCHES To TargetScan▶ miR-433-3p:UCAUGAU▶ miR-665:CCAGGAG▶ miR-371-5p:CUCAAAC▶ miR-34-5p/449-5p:GGCAGUG▶ miR-182-5p:UUGGCAA▶ miR-320:AAAGCUG▶ miR-96-5p/1271-5p:UUGGCAC▶ miR-874-3p:UGCCCUG▶ miR-346:GUCUGCC

 1920  


A

CTCATGAATCTTGTCTGAAGCTTTTGAGGGCAGACTGCCAAGTCCTGGAG  
Depth:2 (MARMOSET)  
Ei-value:0.000, Pi-value:0.000  
Er-value:0.000, Pr-value:0.000  
eCLIP MATCHES▶BCCIP (bg=0.09%)▶hltf (bg=24.28%)▶khsrp (bg=27.4%)▶NIPBL (bg=8.2%)▶ppil4 (bg=43.39%)▶safb (bg=40.39%)▶safb2 (bg=26.89%)▶srsf1 (bg=30.28%)▶tia1 (bg=23.76%)▶tial1 (bg=15.02%)▶tra2a (bg=37.02%)▶u2af1 (bg=14.02%)▶u2af2 (bg=19.32%)▶ZRANB2 (bg=7.28%)MATCHES To TargetScan▶ miR-433-3p:UCAUGAU▶ miR-665:CCAGGAG▶ miR-371-5p:CUCAAAC▶ miR-34-5p/449-5p:GGCAGUG▶ miR-182-5p:UUGGCAA▶ miR-320:AAAGCUG▶ miR-96-5p/1271-5p:UUGGCAC▶ miR-874-3p:UGCCCUG▶ miR-346:GUCUGCC


ATCTTGTC

ATCTTGTC  
Depth:7 (TURTLE)  
Ei-value:0.000, Pi-value:0.000  
Er-value:0.000, Pr-value:0.000  
eCLIP MATCHES▶hltf (bg=24.28%)▶NIPBL (bg=8.2%)▶ppil4 (bg=43.39%)▶safb (bg=40.39%)▶safb2 (bg=26.89%)▶srsf1 (bg=30.28%)▶tia1 (bg=23.76%)▶tial1 (bg=15.02%)▶tra2a (bg=37.02%)▶u2af1 (bg=14.02%)▶u2af2 (bg=19.32%)▶ZRANB2 (bg=7.28%)No matches to TargetScan


TG

ATCTTGTCTG  
Depth:5 (COW)  
Ei-value:0.000, Pi-value:0.000  
Er-value:0.000, Pr-value:0.000  
eCLIP MATCHES▶hltf (bg=24.28%)▶NIPBL (bg=8.2%)▶ppil4 (bg=43.39%)▶safb (bg=40.39%)▶safb2 (bg=26.89%)▶srsf1 (bg=30.28%)▶tia1 (bg=23.76%)▶tial1 (bg=15.02%)▶tra2a (bg=37.02%)▶u2af1 (bg=14.02%)▶u2af2 (bg=19.32%)▶ZRANB2 (bg=7.28%)No matches to TargetScan


A

ATCTTGTCTGAAGCTTTTGAGGGCAGACT  
Depth:4 (PIG)  
Ei-value:0.000, Pi-value:0.000  
Er-value:0.000, Pr-value:0.000  
eCLIP MATCHES▶BCCIP (bg=0.09%)▶hltf (bg=24.28%)▶NIPBL (bg=8.2%)▶ppil4 (bg=43.39%)▶safb (bg=40.39%)▶safb2 (bg=26.89%)▶srsf1 (bg=30.28%)▶tia1 (bg=23.76%)▶tial1 (bg=15.02%)▶tra2a (bg=37.02%)▶u2af1 (bg=14.02%)▶u2af2 (bg=19.32%)▶ZRANB2 (bg=7.28%)MATCHES To TargetScan▶ miR-371-5p:CUCAAAC▶ miR-320:AAAGCUG▶ miR-874-3p:UGCCCUG▶ miR-346:GUCUGCC


AGCTTTTGAGGGC

AGCTTTTGAGGGC  
Depth:6 (MOUSE)  
Ei-value:0.000, Pi-value:0.000  
Er-value:0.000, Pr-value:0.000  
eCLIP MATCHES▶BCCIP (bg=0.09%)▶hltf (bg=24.28%)▶NIPBL (bg=8.2%)▶ppil4 (bg=43.39%)▶safb (bg=40.39%)▶safb2 (bg=26.89%)▶srsf1 (bg=30.28%)▶tia1 (bg=23.76%)▶tial1 (bg=15.02%)▶tra2a (bg=37.02%)▶u2af1 (bg=14.02%)▶u2af2 (bg=19.32%)▶ZRANB2 (bg=7.28%)MATCHES To TargetScan▶ miR-371-5p:CUCAAAC▶ miR-320:AAAGCUG


AGACT

AGCTTTTGAGGGCAGACT  
Depth:5 (COW)  
Ei-value:0.000, Pi-value:0.000  
Er-value:0.000, Pr-value:0.000  
eCLIP MATCHES▶BCCIP (bg=0.09%)▶hltf (bg=24.28%)▶NIPBL (bg=8.2%)▶ppil4 (bg=43.39%)▶safb (bg=40.39%)▶safb2 (bg=26.89%)▶srsf1 (bg=30.28%)▶tia1 (bg=23.76%)▶tial1 (bg=15.02%)▶tra2a (bg=37.02%)▶u2af1 (bg=14.02%)▶u2af2 (bg=19.32%)▶ZRANB2 (bg=7.28%)MATCHES To TargetScan▶ miR-371-5p:CUCAAAC▶ miR-320:AAAGCUG▶ miR-874-3p:UGCCCUG▶ miR-346:GUCUGCC


GCCAAGT

CTCATGAATCTTGTCTGAAGCTTTTGAGGGCAGACTGCCAAGTCCTGGAG  
Depth:2 (MARMOSET)  
Ei-value:0.000, Pi-value:0.000  
Er-value:0.000, Pr-value:0.000  
eCLIP MATCHES▶BCCIP (bg=0.09%)▶hltf (bg=24.28%)▶khsrp (bg=27.4%)▶NIPBL (bg=8.2%)▶ppil4 (bg=43.39%)▶safb (bg=40.39%)▶safb2 (bg=26.89%)▶srsf1 (bg=30.28%)▶tia1 (bg=23.76%)▶tial1 (bg=15.02%)▶tra2a (bg=37.02%)▶u2af1 (bg=14.02%)▶u2af2 (bg=19.32%)▶ZRANB2 (bg=7.28%)MATCHES To TargetScan▶ miR-433-3p:UCAUGAU▶ miR-665:CCAGGAG▶ miR-371-5p:CUCAAAC▶ miR-34-5p/449-5p:GGCAGUG▶ miR-182-5p:UUGGCAA▶ miR-320:AAAGCUG▶ miR-96-5p/1271-5p:UUGGCAC▶ miR-874-3p:UGCCCUG▶ miR-346:GUCUGCC


cctggag

cctggag  
Depth:3 (DOG)  
Ei-value:0.350, Pi-value:0.010  
Er-value:0.000, Pr-value:0.000  
eCLIP MATCHES▶hltf (bg=24.28%)▶ppil4 (bg=43.39%)▶safb2 (bg=26.89%)▶srsf1 (bg=30.28%)▶tra2a (bg=37.02%)▶u2af1 (bg=14.02%)▶u2af2 (bg=19.32%)No matches to TargetScan

-

AATA

AATAGTAGATGGCAAGTTTGT  
Depth:2 (MARMOSET)  
Ei-value:0.000, Pi-value:0.000  
Er-value:0.000, Pr-value:0.000  
eCLIP MATCHES▶srsf1 (bg=30.28%)▶u2af1 (bg=14.02%)▶u2af2 (bg=19.32%)No matches to TargetScan


GTAGATGGCAAGT

GTAGATGGCAAGT  
Depth:6 (MOUSE)  
Ei-value:0.000, Pi-value:0.000  
Er-value:0.000, Pr-value:0.000  
eCLIP MATCHES▶srsf1 (bg=30.28%)No matches to TargetScan


TT

GTAGATGGCAAGTTT  
Depth:3 (DOG)  
Ei-value:0.000, Pi-value:0.000  
Er-value:0.000, Pr-value:0.000  
eCLIP MATCHES▶srsf1 (bg=30.28%)No matches to TargetScan


GT

AATAGTAGATGGCAAGTTTGT  
Depth:2 (MARMOSET)  
Ei-value:0.000, Pi-value:0.000  
Er-value:0.000, Pr-value:0.000  
eCLIP MATCHES▶srsf1 (bg=30.28%)▶u2af1 (bg=14.02%)▶u2af2 (bg=19.32%)No matches to TargetScan

---------

ttttttac

ttttttac  
Depth:2 (MARMOSET)  
Ei-value:1.000, Pi-value:0.000  
Er-value:0.000, Pr-value:0.010  
eCLIP MATCHES▶npm1 (bg=10.22%)▶ppil4 (bg=43.39%)▶safb (bg=40.39%)▶SUPV3L1 (bg=9.63%)▶tial1 (bg=15.02%)▶zc3h8 (bg=12.78%)No matches to TargetScan

-----------------

CAAA

CAAATGAATTTG  
Depth:4 (PIG)  
Ei-value:0.000, Pi-value:0.000  
Er-value:0.000, Pr-value:0.000  
eCLIP MATCHES▶npm1 (bg=10.22%)▶ppil4 (bg=43.39%)▶safb (bg=40.39%)▶safb2 (bg=26.89%)▶SUPV3L1 (bg=9.63%)▶tia1 (bg=23.76%)▶tial1 (bg=15.02%)▶zc3h8 (bg=12.78%)No matches to TargetScan


TGAATTTG

TGAATTTG  
Depth:5 (COW)  
Ei-value:0.000, Pi-value:0.000  
Er-value:0.000, Pr-value:0.000  
eCLIP MATCHES▶npm1 (bg=10.22%)▶ppil4 (bg=43.39%)▶safb (bg=40.39%)▶safb2 (bg=26.89%)▶SUPV3L1 (bg=9.63%)▶tia1 (bg=23.76%)▶tial1 (bg=15.02%)▶zc3h8 (bg=12.78%)No matches to TargetScan


ATAG

CAAATGAATTTGATAG  
Depth:2 (MARMOSET)  
Ei-value:0.000, Pi-value:0.000  
Er-value:0.000, Pr-value:0.000  
eCLIP MATCHES▶npm1 (bg=10.22%)▶ppil4 (bg=43.39%)▶safb (bg=40.39%)▶safb2 (bg=26.89%)▶SUPV3L1 (bg=9.63%)▶tia1 (bg=23.76%)▶tial1 (bg=15.02%)▶zc3h8 (bg=12.78%)No matches to TargetScan

--

aa

aaattgagac  
Depth:2 (MARMOSET)  
Ei-value:1.000, Pi-value:0.000  
Er-value:0.000, Pr-value:0.000  
eCLIP MATCHES▶ppil4 (bg=43.39%)▶safb (bg=40.39%)▶safb2 (bg=26.89%)▶SUPV3L1 (bg=9.63%)▶tia1 (bg=23.76%)▶tial1 (bg=15.02%)▶zc3h8 (bg=12.78%)No matches to TargetScan

 2040  


a

aaattgagac  
Depth:2 (MARMOSET)  
Ei-value:1.000, Pi-value:0.000  
Er-value:0.000, Pr-value:0.000  
eCLIP MATCHES▶ppil4 (bg=43.39%)▶safb (bg=40.39%)▶safb2 (bg=26.89%)▶SUPV3L1 (bg=9.63%)▶tia1 (bg=23.76%)▶tial1 (bg=15.02%)▶zc3h8 (bg=12.78%)No matches to TargetScan


T

TTGAGAC  
Depth:4 (PIG)  
Ei-value:0.000, Pi-value:0.010  
Er-value:0.000, Pr-value:0.000  
eCLIP MATCHES▶ppil4 (bg=43.39%)▶safb (bg=40.39%)▶safb2 (bg=26.89%)▶SUPV3L1 (bg=9.63%)▶zc3h8 (bg=12.78%)No matches to TargetScan


TGAGAC

TGAGAC  
Depth:5 (COW)  
Ei-value:0.000, Pi-value:0.020  
Er-value:0.000, Pr-value:0.000  
eCLIP MATCHES▶ppil4 (bg=43.39%)▶safb (bg=40.39%)▶SUPV3L1 (bg=9.63%)▶zc3h8 (bg=12.78%)No matches to TargetScan


AATTTCAGCAAATCTGTAAGCAGTTTGTATGTTTAG

AATTTCAGCAAATCTGTAAGCAGTTTGTATGTTTAGTTGGGGTAATG  
Depth:2 (MARMOSET)  
Ei-value:0.000, Pi-value:0.000  
Er-value:0.000, Pr-value:0.000  
eCLIP MATCHES▶hnrnpa1 (bg=18.32%)▶ppil4 (bg=43.39%)▶safb (bg=40.39%)▶SUPV3L1 (bg=9.63%)▶zc3h8 (bg=12.78%)MATCHES To TargetScan▶ miR-381-3p:AUACAAG▶ miR-203a-3p.2:UGAAAUG


TTGG

TTGGGGTAATG  
Depth:3 (DOG)  
Ei-value:0.000, Pi-value:0.000  
Er-value:0.000, Pr-value:0.000  
eCLIP MATCHES▶hnrnpa1 (bg=18.32%)▶ppil4 (bg=43.39%)▶SUPV3L1 (bg=9.63%)▶zc3h8 (bg=12.78%)No matches to TargetScan


GGTAAT

GGTAAT  
Depth:6 (MOUSE)  
Ei-value:0.000, Pi-value:0.010  
Er-value:0.000, Pr-value:0.000  
eCLIP MATCHES▶hnrnpa1 (bg=18.32%)▶ppil4 (bg=43.39%)▶SUPV3L1 (bg=9.63%)No matches to TargetScan


G

GGTAATG  
Depth:5 (COW)  
Ei-value:0.000, Pi-value:0.000  
Er-value:0.000, Pr-value:0.000  
eCLIP MATCHES▶hnrnpa1 (bg=18.32%)▶ppil4 (bg=43.39%)▶SUPV3L1 (bg=9.63%)No matches to TargetScan

-

agtatttca

agtatttca  
Depth:2 (MARMOSET)  
Ei-value:1.000, Pi-value:0.000  
Er-value:0.000, Pr-value:0.000  
eCLIP MATCHES▶hnrnpa1 (bg=18.32%)▶ppil4 (bg=43.39%)MATCHES To TargetScan▶ miR-200bc-3p/429:AAUACUG▶ miR-203a-3p.2:UGAAAUG

-

TTTTG

TTTTGTGAATAGATGAC  
Depth:2 (MARMOSET)  
Ei-value:0.000, Pi-value:0.000  
Er-value:0.000, Pr-value:0.000  
eCLIP MATCHES▶hnrnpa1 (bg=18.32%)▶khsrp (bg=27.4%)▶ppil4 (bg=43.39%)▶safb (bg=40.39%)No matches to TargetScan


TGAA

TGAATAGATGAC  
Depth:3 (DOG)  
Ei-value:0.000, Pi-value:0.000  
Er-value:0.000, Pr-value:0.000  
eCLIP MATCHES▶hnrnpa1 (bg=18.32%)▶khsrp (bg=27.4%)▶ppil4 (bg=43.39%)▶safb (bg=40.39%)No matches to TargetScan


TAGATGAC

TAGATGAC  
Depth:4 (PIG)  
Ei-value:0.000, Pi-value:0.000  
Er-value:0.000, Pr-value:0.000  
eCLIP MATCHES▶hnrnpa1 (bg=18.32%)▶khsrp (bg=27.4%)▶ppil4 (bg=43.39%)▶safb (bg=40.39%)No matches to TargetScan

-

tgtttttactt

tgtttttactt  
Depth:2 (MARMOSET)  
Ei-value:1.000, Pi-value:0.000  
Er-value:0.000, Pr-value:0.000  
eCLIP MATCHES▶khsrp (bg=27.4%)▶ppil4 (bg=43.39%)No matches to TargetScan

-

c

ctcaccctga  
Depth:2 (MARMOSET)  
Ei-value:1.000, Pi-value:0.000  
Er-value:0.000, Pr-value:0.000  
eCLIP MATCHES▶khsrp (bg=27.4%)▶ppil4 (bg=43.39%)No matches to TargetScan


TCACCCTG

TCACCCTG  
Depth:3 (DOG)  
Ei-value:0.010, Pi-value:0.000  
Er-value:0.000, Pr-value:0.000  
eCLIP MATCHES▶khsrp (bg=27.4%)▶ppil4 (bg=43.39%)No matches to TargetScan


a

ctcaccctga  
Depth:2 (MARMOSET)  
Ei-value:1.000, Pi-value:0.000  
Er-value:0.000, Pr-value:0.000  
eCLIP MATCHES▶khsrp (bg=27.4%)▶ppil4 (bg=43.39%)No matches to TargetScan

----------

taaa

taaatgt  
Depth:3 (DOG)  
Ei-value:0.350, Pi-value:0.020  
Er-value:0.000, Pr-value:0.000  
eCLIP MATCHES▶khsrp (bg=27.4%)▶ppil4 (bg=43.39%)No matches to TargetScan

 2160  


tgt

taaatgt  
Depth:3 (DOG)  
Ei-value:0.350, Pi-value:0.020  
Er-value:0.000, Pr-value:0.000  
eCLIP MATCHES▶khsrp (bg=27.4%)▶ppil4 (bg=43.39%)No matches to TargetScan

-

gagtttg

gagtttg  
Depth:3 (DOG)  
Ei-value:0.350, Pi-value:0.020  
Er-value:0.000, Pr-value:0.000  
eCLIP MATCHES▶HNRNPC (bg=4.22%)▶khsrp (bg=27.4%)▶ppil4 (bg=43.39%)▶SFPQ (bg=2.48%)No matches to TargetScan

-

atgtgtaac

atgtgtaac  
Depth:2 (MARMOSET)  
Ei-value:1.000, Pi-value:0.000  
Er-value:0.000, Pr-value:0.000  
eCLIP MATCHES▶ddx42 (bg=10.33%)▶HNRNPC (bg=4.22%)▶khsrp (bg=27.4%)▶PUS1 (bg=1.64%)▶SFPQ (bg=2.48%)No matches to TargetScan

------

GGGGGGGA

GGGGGGGA  
Depth:3 (DOG)  
Ei-value:0.010, Pi-value:0.000  
Er-value:0.000, Pr-value:0.000  
eCLIP MATCHES▶bud13 (bg=12.85%)▶ddx42 (bg=10.33%)▶HNRNPC (bg=4.22%)▶ppil4 (bg=43.39%)▶PUS1 (bg=1.64%)▶SFPQ (bg=2.48%)No matches to TargetScan

----------

ttt

tttttttttgtg  
Depth:2 (MARMOSET)  
Ei-value:0.990, Pi-value:0.000  
Er-value:0.000, Pr-value:0.000  
eCLIP MATCHES▶bud13 (bg=12.85%)▶ddx42 (bg=10.33%)▶HNRNPC (bg=4.22%)▶khsrp (bg=27.4%)▶PCBP2 (bg=4.33%)▶ppil4 (bg=43.39%)▶PUS1 (bg=1.64%)▶safb (bg=40.39%)▶safb2 (bg=26.89%)▶SLBP (bg=6.66%)▶TBRG4 (bg=0.51%)▶tia1 (bg=23.76%)▶TROVE2 (bg=6.96%)▶u2af1 (bg=14.02%)▶u2af2 (bg=19.32%)▶zc3h8 (bg=12.78%)▶znf622 (bg=18.79%)No matches to TargetScan


TTTTTTGTG

TTTTTTGTG  
Depth:3 (DOG)  
Ei-value:0.000, Pi-value:0.000  
Er-value:0.000, Pr-value:0.000  
eCLIP MATCHES▶bud13 (bg=12.85%)▶ddx42 (bg=10.33%)▶HNRNPC (bg=4.22%)▶khsrp (bg=27.4%)▶PCBP2 (bg=4.33%)▶ppil4 (bg=43.39%)▶PUS1 (bg=1.64%)▶safb (bg=40.39%)▶safb2 (bg=26.89%)▶SLBP (bg=6.66%)▶TBRG4 (bg=0.51%)▶tia1 (bg=23.76%)▶TROVE2 (bg=6.96%)▶u2af1 (bg=14.02%)▶u2af2 (bg=19.32%)▶zc3h8 (bg=12.78%)▶znf622 (bg=18.79%)No matches to TargetScan

-

GGG

GGGTGGGGGCAAAATATGTT  
Depth:2 (MARMOSET)  
Ei-value:0.000, Pi-value:0.000  
Er-value:0.000, Pr-value:0.000  
eCLIP MATCHES▶AQR (bg=4.89%)▶bud13 (bg=12.85%)▶ddx42 (bg=10.33%)▶HNRNPC (bg=4.22%)▶khsrp (bg=27.4%)▶NIPBL (bg=8.2%)▶PCBP2 (bg=4.33%)▶PPIG (bg=0.25%)▶ppil4 (bg=43.39%)▶PRPF8 (bg=6.2%)▶PUS1 (bg=1.64%)▶safb (bg=40.39%)▶safb2 (bg=26.89%)▶SLBP (bg=6.66%)▶TBRG4 (bg=0.51%)▶tia1 (bg=23.76%)▶TROVE2 (bg=6.96%)▶u2af1 (bg=14.02%)▶u2af2 (bg=19.32%)▶uchl5 (bg=18.56%)▶zc3h8 (bg=12.78%)▶znf622 (bg=18.79%)No matches to TargetScan


t

tgggggc  
Depth:3 (DOG)  
Ei-value:0.350, Pi-value:0.000  
Er-value:0.000, Pr-value:0.000  
eCLIP MATCHES▶bud13 (bg=12.85%)▶ddx42 (bg=10.33%)▶HNRNPC (bg=4.22%)▶khsrp (bg=27.4%)▶PCBP2 (bg=4.33%)▶PPIG (bg=0.25%)▶ppil4 (bg=43.39%)▶PUS1 (bg=1.64%)▶safb (bg=40.39%)▶safb2 (bg=26.89%)▶SLBP (bg=6.66%)▶TBRG4 (bg=0.51%)▶tia1 (bg=23.76%)▶TROVE2 (bg=6.96%)▶u2af1 (bg=14.02%)▶u2af2 (bg=19.32%)▶uchl5 (bg=18.56%)▶zc3h8 (bg=12.78%)▶znf622 (bg=18.79%)No matches to TargetScan


GGGGGC

GGGGGC  
Depth:4 (PIG)  
Ei-value:0.010, Pi-value:0.040  
Er-value:0.000, Pr-value:0.000  
eCLIP MATCHES▶bud13 (bg=12.85%)▶ddx42 (bg=10.33%)▶HNRNPC (bg=4.22%)▶khsrp (bg=27.4%)▶PCBP2 (bg=4.33%)▶PPIG (bg=0.25%)▶ppil4 (bg=43.39%)▶PUS1 (bg=1.64%)▶safb (bg=40.39%)▶safb2 (bg=26.89%)▶SLBP (bg=6.66%)▶TBRG4 (bg=0.51%)▶tia1 (bg=23.76%)▶TROVE2 (bg=6.96%)▶u2af1 (bg=14.02%)▶u2af2 (bg=19.32%)▶uchl5 (bg=18.56%)▶zc3h8 (bg=12.78%)▶znf622 (bg=18.79%)No matches to TargetScan


AAAATAT

AAAATAT  
Depth:7 (TURTLE)  
Ei-value:0.000, Pi-value:0.000  
Er-value:0.000, Pr-value:0.000  
eCLIP MATCHES▶bud13 (bg=12.85%)▶ddx42 (bg=10.33%)▶HNRNPC (bg=4.22%)▶khsrp (bg=27.4%)▶NIPBL (bg=8.2%)▶PCBP2 (bg=4.33%)▶PPIG (bg=0.25%)▶ppil4 (bg=43.39%)▶PRPF8 (bg=6.2%)▶PUS1 (bg=1.64%)▶safb (bg=40.39%)▶safb2 (bg=26.89%)▶SLBP (bg=6.66%)▶tia1 (bg=23.76%)▶TROVE2 (bg=6.96%)▶u2af1 (bg=14.02%)▶u2af2 (bg=19.32%)▶uchl5 (bg=18.56%)▶zc3h8 (bg=12.78%)▶znf622 (bg=18.79%)No matches to TargetScan


GTT

AAAATATGTT  
Depth:5 (COW)  
Ei-value:0.000, Pi-value:0.000  
Er-value:0.000, Pr-value:0.000  
eCLIP MATCHES▶AQR (bg=4.89%)▶bud13 (bg=12.85%)▶ddx42 (bg=10.33%)▶HNRNPC (bg=4.22%)▶khsrp (bg=27.4%)▶NIPBL (bg=8.2%)▶PCBP2 (bg=4.33%)▶PPIG (bg=0.25%)▶ppil4 (bg=43.39%)▶PRPF8 (bg=6.2%)▶PUS1 (bg=1.64%)▶safb (bg=40.39%)▶safb2 (bg=26.89%)▶SLBP (bg=6.66%)▶tia1 (bg=23.76%)▶TROVE2 (bg=6.96%)▶u2af1 (bg=14.02%)▶u2af2 (bg=19.32%)▶uchl5 (bg=18.56%)▶zc3h8 (bg=12.78%)▶znf622 (bg=18.79%)No matches to TargetScan

---

AGTTCTTTT

AGTTCTTTT  
Depth:6 (MOUSE)  
Ei-value:0.000, Pi-value:0.000  
Er-value:0.000, Pr-value:0.000  
eCLIP MATCHES▶AQR (bg=4.89%)▶bud13 (bg=12.85%)▶ddx42 (bg=10.33%)▶DGCR8 (bg=2.67%)▶GNL3 (bg=0.59%)▶HNRNPC (bg=4.22%)▶khsrp (bg=27.4%)▶NIPBL (bg=8.2%)▶npm1 (bg=10.22%)▶PCBP2 (bg=4.33%)▶ppil4 (bg=43.39%)▶PRPF8 (bg=6.2%)▶PUS1 (bg=1.64%)▶safb (bg=40.39%)▶safb2 (bg=26.89%)▶SLBP (bg=6.66%)▶SUPV3L1 (bg=9.63%)▶tia1 (bg=23.76%)▶TROVE2 (bg=6.96%)▶u2af1 (bg=14.02%)▶u2af2 (bg=19.32%)▶zc3h8 (bg=12.78%)MATCHES To TargetScan▶ miR-186-5p:AAAGAAU

-

CCC

CCCTTAGGTCTGTCTAGA  
Depth:4 (PIG)  
Ei-value:0.000, Pi-value:0.000  
Er-value:0.000, Pr-value:0.000  
eCLIP MATCHES▶aggf1 (bg=15.15%)▶AQR (bg=4.89%)▶bud13 (bg=12.85%)▶cpsf6 (bg=13.45%)▶ddx42 (bg=10.33%)▶DGCR8 (bg=2.67%)▶GNL3 (bg=0.59%)▶HNRNPC (bg=4.22%)▶khsrp (bg=27.4%)▶NIPBL (bg=8.2%)▶npm1 (bg=10.22%)▶ppil4 (bg=43.39%)▶PRPF8 (bg=6.2%)▶PUS1 (bg=1.64%)▶safb (bg=40.39%)▶safb2 (bg=26.89%)▶SLBP (bg=6.66%)▶SUPV3L1 (bg=9.63%)▶u2af1 (bg=14.02%)▶u2af2 (bg=19.32%)▶YWHAG (bg=9.14%)▶zc3h8 (bg=12.78%)No matches to TargetScan


TTAGGTC

TTAGGTCTGTCTAGA  
Depth:6 (MOUSE)  
Ei-value:0.000, Pi-value:0.000  
Er-value:0.000, Pr-value:0.000  
eCLIP MATCHES▶aggf1 (bg=15.15%)▶AQR (bg=4.89%)▶cpsf6 (bg=13.45%)▶ddx42 (bg=10.33%)▶DGCR8 (bg=2.67%)▶GNL3 (bg=0.59%)▶HNRNPC (bg=4.22%)▶khsrp (bg=27.4%)▶NIPBL (bg=8.2%)▶npm1 (bg=10.22%)▶ppil4 (bg=43.39%)▶PRPF8 (bg=6.2%)▶PUS1 (bg=1.64%)▶safb (bg=40.39%)▶safb2 (bg=26.89%)▶SLBP (bg=6.66%)▶SUPV3L1 (bg=9.63%)▶u2af1 (bg=14.02%)▶u2af2 (bg=19.32%)▶YWHAG (bg=9.14%)▶zc3h8 (bg=12.78%)No matches to TargetScan


TGTCTAGA

TGTCTAGA  
Depth:7 (TURTLE)  
Ei-value:0.000, Pi-value:0.000  
Er-value:0.000, Pr-value:0.000  
eCLIP MATCHES▶aggf1 (bg=15.15%)▶AQR (bg=4.89%)▶cpsf6 (bg=13.45%)▶ddx42 (bg=10.33%)▶DGCR8 (bg=2.67%)▶GNL3 (bg=0.59%)▶HNRNPC (bg=4.22%)▶khsrp (bg=27.4%)▶NIPBL (bg=8.2%)▶npm1 (bg=10.22%)▶ppil4 (bg=43.39%)▶PRPF8 (bg=6.2%)▶safb (bg=40.39%)▶safb2 (bg=26.89%)▶SLBP (bg=6.66%)▶SUPV3L1 (bg=9.63%)▶u2af1 (bg=14.02%)▶u2af2 (bg=19.32%)▶YWHAG (bg=9.14%)▶zc3h8 (bg=12.78%)No matches to TargetScan


ATCCTAAA

CCCTTAGGTCTGTCTAGAATCCTAAA  
Depth:2 (MARMOSET)  
Ei-value:0.000, Pi-value:0.000  
Er-value:0.000, Pr-value:0.000  
eCLIP MATCHES▶aggf1 (bg=15.15%)▶AQR (bg=4.89%)▶bud13 (bg=12.85%)▶cpsf6 (bg=13.45%)▶ddx42 (bg=10.33%)▶DGCR8 (bg=2.67%)▶GNL3 (bg=0.59%)▶HNRNPC (bg=4.22%)▶khsrp (bg=27.4%)▶NIPBL (bg=8.2%)▶npm1 (bg=10.22%)▶ppil4 (bg=43.39%)▶PRPF8 (bg=6.2%)▶PUS1 (bg=1.64%)▶safb (bg=40.39%)▶safb2 (bg=26.89%)▶SLBP (bg=6.66%)▶SUPV3L1 (bg=9.63%)▶u2af1 (bg=14.02%)▶u2af2 (bg=19.32%)▶YWHAG (bg=9.14%)▶zc3h8 (bg=12.78%)No matches to TargetScan

-

GC

GCAAATGACTC  
Depth:5 (COW)  
Ei-value:0.000, Pi-value:0.000  
Er-value:0.000, Pr-value:0.000  
eCLIP MATCHES▶aggf1 (bg=15.15%)▶AQR (bg=4.89%)▶cpsf6 (bg=13.45%)▶DGCR8 (bg=2.67%)▶GNL3 (bg=0.59%)▶HNRNPC (bg=4.22%)▶khsrp (bg=27.4%)▶npm1 (bg=10.22%)▶ppil4 (bg=43.39%)▶PRPF8 (bg=6.2%)▶safb (bg=40.39%)▶safb2 (bg=26.89%)▶SLBP (bg=6.66%)▶SUPV3L1 (bg=9.63%)▶u2af1 (bg=14.02%)▶u2af2 (bg=19.32%)▶YWHAG (bg=9.14%)No matches to TargetScan

 2280  


AA

GCAAATGACTC  
Depth:5 (COW)  
Ei-value:0.000, Pi-value:0.000  
Er-value:0.000, Pr-value:0.000  
eCLIP MATCHES▶aggf1 (bg=15.15%)▶AQR (bg=4.89%)▶cpsf6 (bg=13.45%)▶DGCR8 (bg=2.67%)▶GNL3 (bg=0.59%)▶HNRNPC (bg=4.22%)▶khsrp (bg=27.4%)▶npm1 (bg=10.22%)▶ppil4 (bg=43.39%)▶PRPF8 (bg=6.2%)▶safb (bg=40.39%)▶safb2 (bg=26.89%)▶SLBP (bg=6.66%)▶SUPV3L1 (bg=9.63%)▶u2af1 (bg=14.02%)▶u2af2 (bg=19.32%)▶YWHAG (bg=9.14%)No matches to TargetScan


ATGACTC

ATGACTC  
Depth:6 (MOUSE)  
Ei-value:0.000, Pi-value:0.000  
Er-value:0.000, Pr-value:0.000  
eCLIP MATCHES▶aggf1 (bg=15.15%)▶AQR (bg=4.89%)▶cpsf6 (bg=13.45%)▶DGCR8 (bg=2.67%)▶GNL3 (bg=0.59%)▶HNRNPC (bg=4.22%)▶khsrp (bg=27.4%)▶npm1 (bg=10.22%)▶ppil4 (bg=43.39%)▶safb (bg=40.39%)▶safb2 (bg=26.89%)▶SLBP (bg=6.66%)▶SUPV3L1 (bg=9.63%)▶u2af1 (bg=14.02%)▶u2af2 (bg=19.32%)▶YWHAG (bg=9.14%)No matches to TargetScan


AAGGT

GCAAATGACTCAAGGT  
Depth:4 (PIG)  
Ei-value:0.000, Pi-value:0.000  
Er-value:0.000, Pr-value:0.000  
eCLIP MATCHES▶aggf1 (bg=15.15%)▶AQR (bg=4.89%)▶cpsf6 (bg=13.45%)▶DGCR8 (bg=2.67%)▶GNL3 (bg=0.59%)▶HNRNPC (bg=4.22%)▶khsrp (bg=27.4%)▶npm1 (bg=10.22%)▶ppil4 (bg=43.39%)▶PRPF8 (bg=6.2%)▶safb (bg=40.39%)▶safb2 (bg=26.89%)▶SLBP (bg=6.66%)▶SUPV3L1 (bg=9.63%)▶u2af1 (bg=14.02%)▶u2af2 (bg=19.32%)▶YWHAG (bg=9.14%)No matches to TargetScan


GTAACAG

GCAAATGACTCAAGGTGTAACAG  
Depth:2 (MARMOSET)  
Ei-value:0.000, Pi-value:0.000  
Er-value:0.000, Pr-value:0.000  
eCLIP MATCHES▶aggf1 (bg=15.15%)▶AQR (bg=4.89%)▶cpsf6 (bg=13.45%)▶DGCR8 (bg=2.67%)▶GNL3 (bg=0.59%)▶HNRNPC (bg=4.22%)▶khsrp (bg=27.4%)▶npm1 (bg=10.22%)▶ppil4 (bg=43.39%)▶PRPF8 (bg=6.2%)▶safb (bg=40.39%)▶safb2 (bg=26.89%)▶SLBP (bg=6.66%)▶SUPV3L1 (bg=9.63%)▶u2af1 (bg=14.02%)▶u2af2 (bg=19.32%)▶YWHAG (bg=9.14%)No matches to TargetScan

-

AAAC

AAACAAGAAAATCCA  
Depth:2 (MARMOSET)  
Ei-value:0.000, Pi-value:0.000  
Er-value:0.000, Pr-value:0.000  
eCLIP MATCHES▶aggf1 (bg=15.15%)▶AQR (bg=4.89%)▶HNRNPL (bg=2.63%)MATCHES To TargetScan▶ miR-876-5p:GGAUUUC▶ miR-544a-5p:CUUGUUA


AAGA

AAGAAAATCCA  
Depth:5 (COW)  
Ei-value:0.000, Pi-value:0.000  
Er-value:0.000, Pr-value:0.000  
eCLIP MATCHES▶aggf1 (bg=15.15%)▶HNRNPL (bg=2.63%)MATCHES To TargetScan▶ miR-876-5p:GGAUUUC


AAATCCA

AAATCCA  
Depth:6 (MOUSE)  
Ei-value:0.000, Pi-value:0.000  
Er-value:0.000, Pr-value:0.000  
eCLIP MATCHES▶aggf1 (bg=15.15%)▶HNRNPL (bg=2.63%)MATCHES To TargetScan▶ miR-876-5p:GGAUUUC

-

TA

TATCAGGATAATCAGA  
Depth:5 (COW)  
Ei-value:0.000, Pi-value:0.000  
Er-value:0.000, Pr-value:0.000  
eCLIP MATCHES▶aggf1 (bg=15.15%)▶hnrnpa1 (bg=18.32%)▶HNRNPL (bg=2.63%)No matches to TargetScan


TCAGGATAA

TCAGGATAA  
Depth:8 (ALLIGATOR)  
Ei-value:0.000, Pi-value:0.000  
Er-value:0.000, Pr-value:0.000  
eCLIP MATCHES▶aggf1 (bg=15.15%)▶hnrnpa1 (bg=18.32%)▶HNRNPL (bg=2.63%)No matches to TargetScan


TCAGA

TATCAGGATAATCAGA  
Depth:5 (COW)  
Ei-value:0.000, Pi-value:0.000  
Er-value:0.000, Pr-value:0.000  
eCLIP MATCHES▶aggf1 (bg=15.15%)▶hnrnpa1 (bg=18.32%)▶HNRNPL (bg=2.63%)No matches to TargetScan

-

CACCACAG

CACCACAG  
Depth:4 (PIG)  
Ei-value:0.000, Pi-value:0.000  
Er-value:0.000, Pr-value:0.000  
eCLIP MATCHES▶aggf1 (bg=15.15%)▶hnrnpa1 (bg=18.32%)▶HNRNPL (bg=2.63%)No matches to TargetScan

-

TTTACAGTT

TTTACAGTT  
Depth:6 (MOUSE)  
Ei-value:0.000, Pi-value:0.000  
Er-value:0.000, Pr-value:0.000  
eCLIP MATCHES▶aggf1 (bg=15.15%)▶hnrnpa1 (bg=18.32%)▶HNRNPL (bg=2.63%)No matches to TargetScan


t

tttacagttt  
Depth:2 (MARMOSET)  
Ei-value:1.000, Pi-value:0.000  
Er-value:0.000, Pr-value:0.000  
eCLIP MATCHES▶aggf1 (bg=15.15%)▶hnrnpa1 (bg=18.32%)▶HNRNPL (bg=2.63%)▶safb (bg=40.39%)No matches to TargetScan

-

TAGAAACT

TAGAAACT  
Depth:5 (COW)  
Ei-value:0.000, Pi-value:0.000  
Er-value:0.000, Pr-value:0.000  
eCLIP MATCHES▶aggf1 (bg=15.15%)▶hnrnpa1 (bg=18.32%)▶HNRNPL (bg=2.63%)▶safb (bg=40.39%)No matches to TargetScan


AGA

TAGAAACTAGA  
Depth:4 (PIG)  
Ei-value:0.000, Pi-value:0.000  
Er-value:0.000, Pr-value:0.000  
eCLIP MATCHES▶aggf1 (bg=15.15%)▶hnrnpa1 (bg=18.32%)▶HNRNPL (bg=2.63%)▶safb (bg=40.39%)No matches to TargetScan


GCA

TAGAAACTAGAGCA  
Depth:3 (DOG)  
Ei-value:0.000, Pi-value:0.000  
Er-value:0.000, Pr-value:0.000  
eCLIP MATCHES▶aggf1 (bg=15.15%)▶hnrnpa1 (bg=18.32%)▶HNRNPL (bg=2.63%)▶safb (bg=40.39%)MATCHES To TargetScan▶ miR-1251-5p:CUCUAGC


GT

TAGAAACTAGAGCAGTTCTCAC  
Depth:2 (MARMOSET)  
Ei-value:0.000, Pi-value:0.000  
Er-value:0.000, Pr-value:0.000  
eCLIP MATCHES▶aggf1 (bg=15.15%)▶hnrnpa1 (bg=18.32%)▶HNRNPL (bg=2.63%)▶safb (bg=40.39%)MATCHES To TargetScan▶ miR-1251-5p:CUCUAGC▶ miR-146-5p:GAGAACU


TCTCAC

TCTCAC  
Depth:4 (PIG)  
Ei-value:0.010, Pi-value:0.010  
Er-value:0.000, Pr-value:0.000  
eCLIP MATCHES▶aggf1 (bg=15.15%)▶HNRNPL (bg=2.63%)▶safb (bg=40.39%)No matches to TargetScan

----

agg

aggtctgtgga  
Depth:2 (MARMOSET)  
Ei-value:1.000, Pi-value:0.000  
Er-value:0.000, Pr-value:0.000  
eCLIP MATCHES▶aggf1 (bg=15.15%)▶HNRNPL (bg=2.63%)▶safb (bg=40.39%)MATCHES To TargetScan▶ miR-140-3p.1:CCACAGG


TCTGTG

TCTGTG  
Depth:5 (COW)  
Ei-value:0.000, Pi-value:0.010  
Er-value:0.000, Pr-value:0.000  
eCLIP MATCHES▶aggf1 (bg=15.15%)▶HNRNPL (bg=2.63%)▶safb (bg=40.39%)No matches to TargetScan


g

tctgtgg  
Depth:3 (DOG)  
Ei-value:0.350, Pi-value:0.000  
Er-value:0.000, Pr-value:0.000  
eCLIP MATCHES▶aggf1 (bg=15.15%)▶HNRNPL (bg=2.63%)▶safb (bg=40.39%)MATCHES To TargetScan▶ miR-140-3p.1:CCACAGG


a

aggtctgtgga  
Depth:2 (MARMOSET)  
Ei-value:1.000, Pi-value:0.000  
Er-value:0.000, Pr-value:0.000  
eCLIP MATCHES▶aggf1 (bg=15.15%)▶HNRNPL (bg=2.63%)▶safb (bg=40.39%)MATCHES To TargetScan▶ miR-140-3p.1:CCACAGG

-----

TGT

TGTCCATTGGAGAAATGGCTGGTAGTT  
Depth:3 (DOG)  
Ei-value:0.000, Pi-value:0.000  
Er-value:0.000, Pr-value:0.000  
eCLIP MATCHES▶aggf1 (bg=15.15%)▶HNRNPL (bg=2.63%)▶safb (bg=40.39%)No matches to TargetScan

 2400  


CCAT

TGTCCATTGGAGAAATGGCTGGTAGTT  
Depth:3 (DOG)  
Ei-value:0.000, Pi-value:0.000  
Er-value:0.000, Pr-value:0.000  
eCLIP MATCHES▶aggf1 (bg=15.15%)▶HNRNPL (bg=2.63%)▶safb (bg=40.39%)No matches to TargetScan


TGGAGA

TGGAGAAATGGCTGGTA  
Depth:4 (PIG)  
Ei-value:0.000, Pi-value:0.000  
Er-value:0.000, Pr-value:0.000  
eCLIP MATCHES▶aggf1 (bg=15.15%)▶HNRNPL (bg=2.63%)▶safb (bg=40.39%)No matches to TargetScan


AATG

AATGGCTGGTA  
Depth:5 (COW)  
Ei-value:0.000, Pi-value:0.000  
Er-value:0.000, Pr-value:0.000  
eCLIP MATCHES▶aggf1 (bg=15.15%)▶HNRNPL (bg=2.63%)▶safb (bg=40.39%)No matches to TargetScan


GCTGGTA

GCTGGTA  
Depth:6 (MOUSE)  
Ei-value:0.000, Pi-value:0.000  
Er-value:0.000, Pr-value:0.000  
eCLIP MATCHES▶aggf1 (bg=15.15%)▶HNRNPL (bg=2.63%)▶safb (bg=40.39%)No matches to TargetScan


GTT

TGTCCATTGGAGAAATGGCTGGTAGTT  
Depth:3 (DOG)  
Ei-value:0.000, Pi-value:0.000  
Er-value:0.000, Pr-value:0.000  
eCLIP MATCHES▶aggf1 (bg=15.15%)▶HNRNPL (bg=2.63%)▶safb (bg=40.39%)No matches to TargetScan


ACTCTTTTT

TGTCCATTGGAGAAATGGCTGGTAGTTACTCTTTTT  
Depth:2 (MARMOSET)  
Ei-value:0.000, Pi-value:0.000  
Er-value:0.000, Pr-value:0.000  
eCLIP MATCHES▶aggf1 (bg=15.15%)▶HNRNPL (bg=2.63%)▶HNRNPU (bg=9.45%)▶ppil4 (bg=43.39%)▶safb (bg=40.39%)No matches to TargetScan

--------

cccccttaat

cccccttaat  
Depth:2 (MARMOSET)  
Ei-value:1.000, Pi-value:0.000  
Er-value:0.000, Pr-value:0.000  
eCLIP MATCHES▶HNRNPC (bg=4.22%)▶HNRNPL (bg=2.63%)▶HNRNPU (bg=9.45%)▶ppil4 (bg=43.39%)No matches to TargetScan

---------

AAAGTG

AAAGTGCTTAACCCCTTAAACTTGTTA  
Depth:2 (MARMOSET)  
Ei-value:0.000, Pi-value:0.000  
Er-value:0.000, Pr-value:0.000  
eCLIP MATCHES▶HNRNPL (bg=2.63%)▶ppil4 (bg=43.39%)▶PRPF8 (bg=6.2%)No matches to TargetScan


cttaac

cttaac  
Depth:3 (DOG)  
Ei-value:1.000, Pi-value:0.040  
Er-value:0.000, Pr-value:0.000  
eCLIP MATCHES▶HNRNPL (bg=2.63%)▶ppil4 (bg=43.39%)No matches to TargetScan


CCCTTAAACTTGTTA

AAAGTGCTTAACCCCTTAAACTTGTTA  
Depth:2 (MARMOSET)  
Ei-value:0.000, Pi-value:0.000  
Er-value:0.000, Pr-value:0.000  
eCLIP MATCHES▶HNRNPL (bg=2.63%)▶ppil4 (bg=43.39%)▶PRPF8 (bg=6.2%)No matches to TargetScan

---------------

AT

ATTTTGGGATGGTCTTAACAGGGAAGAGAGAGGGTGGGGGAGAAAATGTTTTTTTCTAAGATTTTCCACAGATGCTATAGTACTATTGACAAACTGGGTTAGAGAAGGAGTGTAC  
Depth:2 (MARMOSET)  
Ei-value:0.000, Pi-value:0.000  
Er-value:0.000, Pr-value:0.000  
eCLIP MATCHES▶HNRNPL (bg=2.63%)▶PCBP2 (bg=4.33%)▶ppil4 (bg=43.39%)▶PRPF8 (bg=6.2%)▶safb (bg=40.39%)▶safb2 (bg=26.89%)MATCHES To TargetScan▶ miR-499a-5p:UAAGACU▶ miR-208-3p:UAAGACG▶ miR-543:AACAUUC▶ miR-483-3p.2:CACUCCU▶ miR-339-5p:CCCUGUC▶ miR-483-3p.1:ACUCCUC


TTTGGG

TTTGGG  
Depth:19 (ZEBRAFISH)  
Ei-value:0.000, Pi-value:0.000  
Er-value:0.000, Pr-value:0.000  
eCLIP MATCHES▶ppil4 (bg=43.39%)▶PRPF8 (bg=6.2%)No matches to TargetScan


A

ATTTTGGGATGGTCTTAACAGGGAAGAGAGAGGGTGGGGGAGAAAATGTTTTTTTCTAAGATTTTCCACAGATGCTATAGTACTATTGACAAACTGGGTTAGAGAAGGAGTGTAC  
Depth:2 (MARMOSET)  
Ei-value:0.000, Pi-value:0.000  
Er-value:0.000, Pr-value:0.000  
eCLIP MATCHES▶HNRNPL (bg=2.63%)▶PCBP2 (bg=4.33%)▶ppil4 (bg=43.39%)▶PRPF8 (bg=6.2%)▶safb (bg=40.39%)▶safb2 (bg=26.89%)MATCHES To TargetScan▶ miR-499a-5p:UAAGACU▶ miR-208-3p:UAAGACG▶ miR-543:AACAUUC▶ miR-483-3p.2:CACUCCU▶ miR-339-5p:CCCUGUC▶ miR-483-3p.1:ACUCCUC


TGGTCTT

TGGTCTTAACAGGGAAGAG  
Depth:4 (PIG)  
Ei-value:0.000, Pi-value:0.000  
Er-value:0.000, Pr-value:0.000  
eCLIP MATCHES▶PCBP2 (bg=4.33%)▶ppil4 (bg=43.39%)▶PRPF8 (bg=6.2%)MATCHES To TargetScan▶ miR-499a-5p:UAAGACU▶ miR-208-3p:UAAGACG▶ miR-339-5p:CCCUGUC


AA

AACAGGGA  
Depth:6 (MOUSE)  
Ei-value:0.000, Pi-value:0.000  
Er-value:0.000, Pr-value:0.000  
eCLIP MATCHES▶ppil4 (bg=43.39%)▶PRPF8 (bg=6.2%)MATCHES To TargetScan▶ miR-339-5p:CCCUGUC

 2520  


CAGGGA

AACAGGGA  
Depth:6 (MOUSE)  
Ei-value:0.000, Pi-value:0.000  
Er-value:0.000, Pr-value:0.000  
eCLIP MATCHES▶ppil4 (bg=43.39%)▶PRPF8 (bg=6.2%)MATCHES To TargetScan▶ miR-339-5p:CCCUGUC


AGAG

AACAGGGAAGAG  
Depth:5 (COW)  
Ei-value:0.000, Pi-value:0.000  
Er-value:0.000, Pr-value:0.000  
eCLIP MATCHES▶PCBP2 (bg=4.33%)▶ppil4 (bg=43.39%)▶PRPF8 (bg=6.2%)MATCHES To TargetScan▶ miR-339-5p:CCCUGUC


AGAGGG

ATTTTGGGATGGTCTTAACAGGGAAGAGAGAGGGTGGGGGAGAAAATGTTTTTTTCTAAGATTTTCCACAGATGCTATAGTACTATTGACAAACTGGGTTAGAGAAGGAGTGTAC  
Depth:2 (MARMOSET)  
Ei-value:0.000, Pi-value:0.000  
Er-value:0.000, Pr-value:0.000  
eCLIP MATCHES▶HNRNPL (bg=2.63%)▶PCBP2 (bg=4.33%)▶ppil4 (bg=43.39%)▶PRPF8 (bg=6.2%)▶safb (bg=40.39%)▶safb2 (bg=26.89%)MATCHES To TargetScan▶ miR-499a-5p:UAAGACU▶ miR-208-3p:UAAGACG▶ miR-543:AACAUUC▶ miR-483-3p.2:CACUCCU▶ miR-339-5p:CCCUGUC▶ miR-483-3p.1:ACUCCUC


TGGGGGA

TGGGGGA  
Depth:6 (MOUSE)  
Ei-value:0.000, Pi-value:0.000  
Er-value:0.000, Pr-value:0.000  
eCLIP MATCHES▶PCBP2 (bg=4.33%)▶ppil4 (bg=43.39%)▶PRPF8 (bg=6.2%)No matches to TargetScan


GAAA

TGGGGGAGAAA  
Depth:5 (COW)  
Ei-value:0.000, Pi-value:0.000  
Er-value:0.000, Pr-value:0.000  
eCLIP MATCHES▶PCBP2 (bg=4.33%)▶ppil4 (bg=43.39%)▶PRPF8 (bg=6.2%)No matches to TargetScan


ATGTTTT

ATTTTGGGATGGTCTTAACAGGGAAGAGAGAGGGTGGGGGAGAAAATGTTTTTTTCTAAGATTTTCCACAGATGCTATAGTACTATTGACAAACTGGGTTAGAGAAGGAGTGTAC  
Depth:2 (MARMOSET)  
Ei-value:0.000, Pi-value:0.000  
Er-value:0.000, Pr-value:0.000  
eCLIP MATCHES▶HNRNPL (bg=2.63%)▶PCBP2 (bg=4.33%)▶ppil4 (bg=43.39%)▶PRPF8 (bg=6.2%)▶safb (bg=40.39%)▶safb2 (bg=26.89%)MATCHES To TargetScan▶ miR-499a-5p:UAAGACU▶ miR-208-3p:UAAGACG▶ miR-543:AACAUUC▶ miR-483-3p.2:CACUCCU▶ miR-339-5p:CCCUGUC▶ miR-483-3p.1:ACUCCUC


TTTCTA

TTTCTA  
Depth:8 (ALLIGATOR)  
Ei-value:0.000, Pi-value:0.000  
Er-value:0.000, Pr-value:0.000  
eCLIP MATCHES▶PCBP2 (bg=4.33%)▶ppil4 (bg=43.39%)▶PRPF8 (bg=6.2%)▶safb (bg=40.39%)No matches to TargetScan


AGA

TTTCTAAGA  
Depth:6 (MOUSE)  
Ei-value:0.000, Pi-value:0.000  
Er-value:0.000, Pr-value:0.000  
eCLIP MATCHES▶PCBP2 (bg=4.33%)▶ppil4 (bg=43.39%)▶PRPF8 (bg=6.2%)▶safb (bg=40.39%)No matches to TargetScan


T

TTTCTAAGATTTTCCACAGATGCTATAGTACTATTGACAAACTGGGTTAGAGAAGGA  
Depth:3 (DOG)  
Ei-value:0.000, Pi-value:0.000  
Er-value:0.000, Pr-value:0.000  
eCLIP MATCHES▶PCBP2 (bg=4.33%)▶ppil4 (bg=43.39%)▶PRPF8 (bg=6.2%)▶safb (bg=40.39%)▶safb2 (bg=26.89%)No matches to TargetScan


TTTCCACAGAT

TTTCCACAGAT  
Depth:6 (MOUSE)  
Ei-value:0.000, Pi-value:0.000  
Er-value:0.000, Pr-value:0.000  
eCLIP MATCHES▶ppil4 (bg=43.39%)▶PRPF8 (bg=6.2%)▶safb (bg=40.39%)No matches to TargetScan


G

TTTCCACAGATGCTATAGTACTATTGACAAACTG  
Depth:4 (PIG)  
Ei-value:0.000, Pi-value:0.000  
Er-value:0.000, Pr-value:0.000  
eCLIP MATCHES▶ppil4 (bg=43.39%)▶PRPF8 (bg=6.2%)▶safb (bg=40.39%)▶safb2 (bg=26.89%)No matches to TargetScan


CTATAGT

CTATAGT  
Depth:8 (ALLIGATOR)  
Ei-value:0.000, Pi-value:0.000  
Er-value:0.000, Pr-value:0.000  
eCLIP MATCHES▶ppil4 (bg=43.39%)▶PRPF8 (bg=6.2%)▶safb (bg=40.39%)No matches to TargetScan


ACTA

CTATAGTACTATTGACAA  
Depth:5 (COW)  
Ei-value:0.000, Pi-value:0.000  
Er-value:0.000, Pr-value:0.000  
eCLIP MATCHES▶ppil4 (bg=43.39%)▶PRPF8 (bg=6.2%)▶safb (bg=40.39%)▶safb2 (bg=26.89%)No matches to TargetScan


TTGACA

TTGACA  
Depth:6 (MOUSE)  
Ei-value:0.000, Pi-value:0.000  
Er-value:0.000, Pr-value:0.010  
eCLIP MATCHES▶ppil4 (bg=43.39%)▶PRPF8 (bg=6.2%)▶safb (bg=40.39%)▶safb2 (bg=26.89%)No matches to TargetScan


A

CTATAGTACTATTGACAA  
Depth:5 (COW)  
Ei-value:0.000, Pi-value:0.000  
Er-value:0.000, Pr-value:0.000  
eCLIP MATCHES▶ppil4 (bg=43.39%)▶PRPF8 (bg=6.2%)▶safb (bg=40.39%)▶safb2 (bg=26.89%)No matches to TargetScan


ACTG

TTTCCACAGATGCTATAGTACTATTGACAAACTG  
Depth:4 (PIG)  
Ei-value:0.000, Pi-value:0.000  
Er-value:0.000, Pr-value:0.000  
eCLIP MATCHES▶ppil4 (bg=43.39%)▶PRPF8 (bg=6.2%)▶safb (bg=40.39%)▶safb2 (bg=26.89%)No matches to TargetScan


G

TTTCTAAGATTTTCCACAGATGCTATAGTACTATTGACAAACTGGGTTAGAGAAGGA  
Depth:3 (DOG)  
Ei-value:0.000, Pi-value:0.000  
Er-value:0.000, Pr-value:0.000  
eCLIP MATCHES▶PCBP2 (bg=4.33%)▶ppil4 (bg=43.39%)▶PRPF8 (bg=6.2%)▶safb (bg=40.39%)▶safb2 (bg=26.89%)No matches to TargetScan


GTTAGA

GTTAGA  
Depth:8 (ALLIGATOR)  
Ei-value:0.000, Pi-value:0.000  
Er-value:0.000, Pr-value:0.000  
eCLIP MATCHES▶ppil4 (bg=43.39%)▶safb (bg=40.39%)No matches to TargetScan


GAAGG

GTTAGAGAAGG  
Depth:6 (MOUSE)  
Ei-value:0.000, Pi-value:0.000  
Er-value:0.000, Pr-value:0.000  
eCLIP MATCHES▶ppil4 (bg=43.39%)▶safb (bg=40.39%)No matches to TargetScan


A

GTTAGAGAAGGA  
Depth:5 (COW)  
Ei-value:0.000, Pi-value:0.000  
Er-value:0.000, Pr-value:0.000  
eCLIP MATCHES▶ppil4 (bg=43.39%)▶safb (bg=40.39%)No matches to TargetScan


GTGTAC

ATTTTGGGATGGTCTTAACAGGGAAGAGAGAGGGTGGGGGAGAAAATGTTTTTTTCTAAGATTTTCCACAGATGCTATAGTACTATTGACAAACTGGGTTAGAGAAGGAGTGTAC  
Depth:2 (MARMOSET)  
Ei-value:0.000, Pi-value:0.000  
Er-value:0.000, Pr-value:0.000  
eCLIP MATCHES▶HNRNPL (bg=2.63%)▶PCBP2 (bg=4.33%)▶ppil4 (bg=43.39%)▶PRPF8 (bg=6.2%)▶safb (bg=40.39%)▶safb2 (bg=26.89%)MATCHES To TargetScan▶ miR-499a-5p:UAAGACU▶ miR-208-3p:UAAGACG▶ miR-543:AACAUUC▶ miR-483-3p.2:CACUCCU▶ miR-339-5p:CCCUGUC▶ miR-483-3p.1:ACUCCUC

-

GCTG

GCTGTGCTGTTGGCACGAACACCTTCAGGGACTGGAGCTGCTTTTAT  
Depth:2 (MARMOSET)  
Ei-value:0.000, Pi-value:0.000  
Er-value:0.000, Pr-value:0.000  
eCLIP MATCHES▶ppil4 (bg=43.39%)▶safb (bg=40.39%)▶srsf1 (bg=30.28%)MATCHES To TargetScan▶ miR-125-5p:CCCUGAG▶ miR-493-3p:GAAGGUC▶ miR-330-3p.2:AAAGCAC▶ miR-18-5p:AAGGUGC▶ miR-145-5p:UCCAGUU▶ miR-455-3p.1:CAGUCCA▶ miR-15-5p/16-5p/195-5p/424-5p/497-5p:AGCAGCA▶ miR-503-5p:AGCAGCG


TGCTG

TGCTGTTGGCAC  
Depth:4 (PIG)  
Ei-value:0.000, Pi-value:0.000  
Er-value:0.000, Pr-value:0.000  
eCLIP MATCHES▶ppil4 (bg=43.39%)▶safb (bg=40.39%)No matches to TargetScan


TTGGCAC

TTGGCAC  
Depth:6 (MOUSE)  
Ei-value:0.000, Pi-value:0.000  
Er-value:0.000, Pr-value:0.000  
eCLIP MATCHES▶ppil4 (bg=43.39%)▶safb (bg=40.39%)No matches to TargetScan


G

TGCTGTTGGCACGAACACCTTCAGGGA  
Depth:3 (DOG)  
Ei-value:0.000, Pi-value:0.000  
Er-value:0.000, Pr-value:0.000  
eCLIP MATCHES▶ppil4 (bg=43.39%)▶safb (bg=40.39%)▶srsf1 (bg=30.28%)MATCHES To TargetScan▶ miR-125-5p:CCCUGAG▶ miR-493-3p:GAAGGUC▶ miR-18-5p:AAGGUGC


A

AACACCTTCAGGGA  
Depth:5 (COW)  
Ei-value:0.000, Pi-value:0.000  
Er-value:0.000, Pr-value:0.000  
eCLIP MATCHES▶ppil4 (bg=43.39%)▶safb (bg=40.39%)▶srsf1 (bg=30.28%)MATCHES To TargetScan▶ miR-125-5p:CCCUGAG▶ miR-493-3p:GAAGGUC▶ miR-18-5p:AAGGUGC


ACAC

ACACCTTCAGGGA  
Depth:6 (MOUSE)  
Ei-value:0.000, Pi-value:0.000  
Er-value:0.000, Pr-value:0.000  
eCLIP MATCHES▶ppil4 (bg=43.39%)▶safb (bg=40.39%)▶srsf1 (bg=30.28%)MATCHES To TargetScan▶ miR-125-5p:CCCUGAG▶ miR-493-3p:GAAGGUC▶ miR-18-5p:AAGGUGC

 2640  


CTTCAGGGA

ACACCTTCAGGGA  
Depth:6 (MOUSE)  
Ei-value:0.000, Pi-value:0.000  
Er-value:0.000, Pr-value:0.000  
eCLIP MATCHES▶ppil4 (bg=43.39%)▶safb (bg=40.39%)▶srsf1 (bg=30.28%)MATCHES To TargetScan▶ miR-125-5p:CCCUGAG▶ miR-493-3p:GAAGGUC▶ miR-18-5p:AAGGUGC


C

GCTGTGCTGTTGGCACGAACACCTTCAGGGACTGGAGCTGCTTTTAT  
Depth:2 (MARMOSET)  
Ei-value:0.000, Pi-value:0.000  
Er-value:0.000, Pr-value:0.000  
eCLIP MATCHES▶ppil4 (bg=43.39%)▶safb (bg=40.39%)▶srsf1 (bg=30.28%)MATCHES To TargetScan▶ miR-125-5p:CCCUGAG▶ miR-493-3p:GAAGGUC▶ miR-330-3p.2:AAAGCAC▶ miR-18-5p:AAGGUGC▶ miR-145-5p:UCCAGUU▶ miR-455-3p.1:CAGUCCA▶ miR-15-5p/16-5p/195-5p/424-5p/497-5p:AGCAGCA▶ miR-503-5p:AGCAGCG


TGGAGC

TGGAGC  
Depth:6 (MOUSE)  
Ei-value:0.000, Pi-value:0.000  
Er-value:0.000, Pr-value:0.010  
eCLIP MATCHES▶ppil4 (bg=43.39%)▶safb (bg=40.39%)▶srsf1 (bg=30.28%)No matches to TargetScan


TGCTTTT

TGGAGCTGCTTTT  
Depth:4 (PIG)  
Ei-value:0.000, Pi-value:0.000  
Er-value:0.000, Pr-value:0.000  
eCLIP MATCHES▶ppil4 (bg=43.39%)▶safb (bg=40.39%)▶srsf1 (bg=30.28%)MATCHES To TargetScan▶ miR-330-3p.2:AAAGCAC▶ miR-15-5p/16-5p/195-5p/424-5p/497-5p:AGCAGCA▶ miR-503-5p:AGCAGCG


AT

GCTGTGCTGTTGGCACGAACACCTTCAGGGACTGGAGCTGCTTTTAT  
Depth:2 (MARMOSET)  
Ei-value:0.000, Pi-value:0.000  
Er-value:0.000, Pr-value:0.000  
eCLIP MATCHES▶ppil4 (bg=43.39%)▶safb (bg=40.39%)▶srsf1 (bg=30.28%)MATCHES To TargetScan▶ miR-125-5p:CCCUGAG▶ miR-493-3p:GAAGGUC▶ miR-330-3p.2:AAAGCAC▶ miR-18-5p:AAGGUGC▶ miR-145-5p:UCCAGUU▶ miR-455-3p.1:CAGUCCA▶ miR-15-5p/16-5p/195-5p/424-5p/497-5p:AGCAGCA▶ miR-503-5p:AGCAGCG

---

TGGAA

TGGAAGAGTATTCCCAGTTGAAGCTGAAA  
Depth:3 (DOG)  
Ei-value:0.000, Pi-value:0.000  
Er-value:0.000, Pr-value:0.000  
eCLIP MATCHES▶hnrnpa1 (bg=18.32%)▶ppil4 (bg=43.39%)▶safb (bg=40.39%)▶srsf1 (bg=30.28%)MATCHES To TargetScan▶ miR-200bc-3p/429:AAUACUG


GAGTA

GAGTATTCCCAGT  
Depth:5 (COW)  
Ei-value:0.000, Pi-value:0.000  
Er-value:0.000, Pr-value:0.000  
eCLIP MATCHES▶hnrnpa1 (bg=18.32%)▶ppil4 (bg=43.39%)▶safb (bg=40.39%)▶srsf1 (bg=30.28%)MATCHES To TargetScan▶ miR-200bc-3p/429:AAUACUG


TTCCCAGT

TTCCCAGT  
Depth:6 (MOUSE)  
Ei-value:0.000, Pi-value:0.000  
Er-value:0.000, Pr-value:0.000  
eCLIP MATCHES▶hnrnpa1 (bg=18.32%)▶ppil4 (bg=43.39%)▶safb (bg=40.39%)▶srsf1 (bg=30.28%)No matches to TargetScan


T

TGGAAGAGTATTCCCAGTTGAAGCTGAAA  
Depth:3 (DOG)  
Ei-value:0.000, Pi-value:0.000  
Er-value:0.000, Pr-value:0.000  
eCLIP MATCHES▶hnrnpa1 (bg=18.32%)▶ppil4 (bg=43.39%)▶safb (bg=40.39%)▶srsf1 (bg=30.28%)MATCHES To TargetScan▶ miR-200bc-3p/429:AAUACUG


GAA

GAAGCTGAAA  
Depth:5 (COW)  
Ei-value:0.000, Pi-value:0.000  
Er-value:0.000, Pr-value:0.000  
eCLIP MATCHES▶hnrnpa1 (bg=18.32%)▶ppil4 (bg=43.39%)▶srsf1 (bg=30.28%)No matches to TargetScan


GCTGAA

GCTGAA  
Depth:6 (MOUSE)  
Ei-value:0.000, Pi-value:0.020  
Er-value:0.000, Pr-value:0.000  
eCLIP MATCHES▶hnrnpa1 (bg=18.32%)▶ppil4 (bg=43.39%)▶srsf1 (bg=30.28%)No matches to TargetScan


A

GAAGCTGAAA  
Depth:5 (COW)  
Ei-value:0.000, Pi-value:0.000  
Er-value:0.000, Pr-value:0.000  
eCLIP MATCHES▶hnrnpa1 (bg=18.32%)▶ppil4 (bg=43.39%)▶srsf1 (bg=30.28%)No matches to TargetScan


A

TGGAAGAGTATTCCCAGTTGAAGCTGAAAAGTACAGCACAGTGCAGCTTTGGTTCATATTCAGTCATCTCAGGAGAACTTCAGAAGAGCTTGAGTAGGCCAAATGTTGAAGTTAAGTTTTC  
Depth:2 (MARMOSET)  
Ei-value:0.000, Pi-value:0.000  
Er-value:0.000, Pr-value:0.000  
eCLIP MATCHES▶hnrnpa1 (bg=18.32%)▶ppil4 (bg=43.39%)▶PRPF8 (bg=6.2%)▶safb (bg=40.39%)▶srsf1 (bg=30.28%)MATCHES To TargetScan▶ miR-218-5p:UGUGCUU▶ miR-22-3p:AGCUGCC▶ miR-421:UCAACAG▶ miR-505-3p.2:UCAACAC▶ miR-330-3p:CAAAGCA▶ miR-543:AACAUUC▶ miR-320:AAAGCUG▶ miR-200bc-3p/429:AAUACUG▶ miR-143-3p:GAGAUGA


G

GTACAGCACAGTGCAGCTTTGGTTCATA  
Depth:3 (DOG)  
Ei-value:0.000, Pi-value:0.000  
Er-value:0.000, Pr-value:0.000  
eCLIP MATCHES▶hnrnpa1 (bg=18.32%)▶ppil4 (bg=43.39%)MATCHES To TargetScan▶ miR-218-5p:UGUGCUU▶ miR-22-3p:AGCUGCC▶ miR-330-3p:CAAAGCA▶ miR-320:AAAGCUG


TACAGC

TACAGC  
Depth:5 (COW)  
Ei-value:0.000, Pi-value:0.000  
Er-value:0.000, Pr-value:0.000  
eCLIP MATCHES▶hnrnpa1 (bg=18.32%)▶ppil4 (bg=43.39%)No matches to TargetScan


A

TACAGCACAGTGCAGCTTTGGTTCATA  
Depth:4 (PIG)  
Ei-value:0.000, Pi-value:0.000  
Er-value:0.000, Pr-value:0.000  
eCLIP MATCHES▶hnrnpa1 (bg=18.32%)▶ppil4 (bg=43.39%)MATCHES To TargetScan▶ miR-218-5p:UGUGCUU▶ miR-22-3p:AGCUGCC▶ miR-330-3p:CAAAGCA▶ miR-320:AAAGCUG


CAGTGC

CAGTGC  
Depth:6 (MOUSE)  
Ei-value:0.000, Pi-value:0.010  
Er-value:0.000, Pr-value:0.000  
eCLIP MATCHES▶hnrnpa1 (bg=18.32%)▶ppil4 (bg=43.39%)No matches to TargetScan


A

CAGTGCAGCTTTGGTTCATA  
Depth:5 (COW)  
Ei-value:0.000, Pi-value:0.000  
Er-value:0.000, Pr-value:0.000  
eCLIP MATCHES▶hnrnpa1 (bg=18.32%)▶ppil4 (bg=43.39%)MATCHES To TargetScan▶ miR-22-3p:AGCUGCC▶ miR-330-3p:CAAAGCA▶ miR-320:AAAGCUG


GCTT

GCTTTGGTTCA  
Depth:6 (MOUSE)  
Ei-value:0.000, Pi-value:0.000  
Er-value:0.000, Pr-value:0.000  
eCLIP MATCHES▶hnrnpa1 (bg=18.32%)▶ppil4 (bg=43.39%)MATCHES To TargetScan▶ miR-330-3p:CAAAGCA


TGGTTC

TGGTTC  
Depth:8 (ALLIGATOR)  
Ei-value:0.000, Pi-value:0.000  
Er-value:0.000, Pr-value:0.000  
eCLIP MATCHES▶hnrnpa1 (bg=18.32%)▶ppil4 (bg=43.39%)No matches to TargetScan


A

GCTTTGGTTCA  
Depth:6 (MOUSE)  
Ei-value:0.000, Pi-value:0.000  
Er-value:0.000, Pr-value:0.000  
eCLIP MATCHES▶hnrnpa1 (bg=18.32%)▶ppil4 (bg=43.39%)MATCHES To TargetScan▶ miR-330-3p:CAAAGCA


TA

CAGTGCAGCTTTGGTTCATA  
Depth:5 (COW)  
Ei-value:0.000, Pi-value:0.000  
Er-value:0.000, Pr-value:0.000  
eCLIP MATCHES▶hnrnpa1 (bg=18.32%)▶ppil4 (bg=43.39%)MATCHES To TargetScan▶ miR-22-3p:AGCUGCC▶ miR-330-3p:CAAAGCA▶ miR-320:AAAGCUG


T

TGGAAGAGTATTCCCAGTTGAAGCTGAAAAGTACAGCACAGTGCAGCTTTGGTTCATATTCAGTCATCTCAGGAGAACTTCAGAAGAGCTTGAGTAGGCCAAATGTTGAAGTTAAGTTTTC  
Depth:2 (MARMOSET)  
Ei-value:0.000, Pi-value:0.000  
Er-value:0.000, Pr-value:0.000  
eCLIP MATCHES▶hnrnpa1 (bg=18.32%)▶ppil4 (bg=43.39%)▶PRPF8 (bg=6.2%)▶safb (bg=40.39%)▶srsf1 (bg=30.28%)MATCHES To TargetScan▶ miR-218-5p:UGUGCUU▶ miR-22-3p:AGCUGCC▶ miR-421:UCAACAG▶ miR-505-3p.2:UCAACAC▶ miR-330-3p:CAAAGCA▶ miR-543:AACAUUC▶ miR-320:AAAGCUG▶ miR-200bc-3p/429:AAUACUG▶ miR-143-3p:GAGAUGA


TCAGTC

TCAGTC  
Depth:5 (COW)  
Ei-value:0.000, Pi-value:0.010  
Er-value:0.000, Pr-value:0.000  
eCLIP MATCHES▶hnrnpa1 (bg=18.32%)▶ppil4 (bg=43.39%)No matches to TargetScan


A

TGGAAGAGTATTCCCAGTTGAAGCTGAAAAGTACAGCACAGTGCAGCTTTGGTTCATATTCAGTCATCTCAGGAGAACTTCAGAAGAGCTTGAGTAGGCCAAATGTTGAAGTTAAGTTTTC  
Depth:2 (MARMOSET)  
Ei-value:0.000, Pi-value:0.000  
Er-value:0.000, Pr-value:0.000  
eCLIP MATCHES▶hnrnpa1 (bg=18.32%)▶ppil4 (bg=43.39%)▶PRPF8 (bg=6.2%)▶safb (bg=40.39%)▶srsf1 (bg=30.28%)MATCHES To TargetScan▶ miR-218-5p:UGUGCUU▶ miR-22-3p:AGCUGCC▶ miR-421:UCAACAG▶ miR-505-3p.2:UCAACAC▶ miR-330-3p:CAAAGCA▶ miR-543:AACAUUC▶ miR-320:AAAGCUG▶ miR-200bc-3p/429:AAUACUG▶ miR-143-3p:GAGAUGA


T

TCTCAGGAG  
Depth:5 (COW)  
Ei-value:0.000, Pi-value:0.000  
Er-value:0.000, Pr-value:0.000  
eCLIP MATCHES▶hnrnpa1 (bg=18.32%)▶ppil4 (bg=43.39%)▶PRPF8 (bg=6.2%)No matches to TargetScan


CTCAGGAG

CTCAGGAG  
Depth:6 (MOUSE)  
Ei-value:0.000, Pi-value:0.000  
Er-value:0.000, Pr-value:0.000  
eCLIP MATCHES▶hnrnpa1 (bg=18.32%)▶ppil4 (bg=43.39%)▶PRPF8 (bg=6.2%)No matches to TargetScan


A

TGGAAGAGTATTCCCAGTTGAAGCTGAAAAGTACAGCACAGTGCAGCTTTGGTTCATATTCAGTCATCTCAGGAGAACTTCAGAAGAGCTTGAGTAGGCCAAATGTTGAAGTTAAGTTTTC  
Depth:2 (MARMOSET)  
Ei-value:0.000, Pi-value:0.000  
Er-value:0.000, Pr-value:0.000  
eCLIP MATCHES▶hnrnpa1 (bg=18.32%)▶ppil4 (bg=43.39%)▶PRPF8 (bg=6.2%)▶safb (bg=40.39%)▶srsf1 (bg=30.28%)MATCHES To TargetScan▶ miR-218-5p:UGUGCUU▶ miR-22-3p:AGCUGCC▶ miR-421:UCAACAG▶ miR-505-3p.2:UCAACAC▶ miR-330-3p:CAAAGCA▶ miR-543:AACAUUC▶ miR-320:AAAGCUG▶ miR-200bc-3p/429:AAUACUG▶ miR-143-3p:GAGAUGA


ACTTCAGAA

ACTTCAGAAGAGCTTGA  
Depth:5 (COW)  
Ei-value:0.000, Pi-value:0.000  
Er-value:0.000, Pr-value:0.000  
eCLIP MATCHES▶hnrnpa1 (bg=18.32%)▶ppil4 (bg=43.39%)▶PRPF8 (bg=6.2%)No matches to TargetScan


GAGCTT

GAGCTT  
Depth:9 (LIZARD)  
Ei-value:0.000, Pi-value:0.000  
Er-value:0.000, Pr-value:0.000  
eCLIP MATCHES▶hnrnpa1 (bg=18.32%)▶ppil4 (bg=43.39%)▶PRPF8 (bg=6.2%)No matches to TargetScan


G

GAGCTTG  
Depth:7 (TURTLE)  
Ei-value:0.000, Pi-value:0.000  
Er-value:0.000, Pr-value:0.000  
eCLIP MATCHES▶hnrnpa1 (bg=18.32%)▶ppil4 (bg=43.39%)▶PRPF8 (bg=6.2%)No matches to TargetScan

 2760  


GAGCTTG  
Depth:7 (TURTLE)  
Ei-value:0.000, Pi-value:0.000  
Er-value:0.000, Pr-value:0.000  
eCLIP MATCHES▶hnrnpa1 (bg=18.32%)▶ppil4 (bg=43.39%)▶PRPF8 (bg=6.2%)No matches to TargetScan


A

ACTTCAGAAGAGCTTGA  
Depth:5 (COW)  
Ei-value:0.000, Pi-value:0.000  
Er-value:0.000, Pr-value:0.000  
eCLIP MATCHES▶hnrnpa1 (bg=18.32%)▶ppil4 (bg=43.39%)▶PRPF8 (bg=6.2%)No matches to TargetScan


G

TGGAAGAGTATTCCCAGTTGAAGCTGAAAAGTACAGCACAGTGCAGCTTTGGTTCATATTCAGTCATCTCAGGAGAACTTCAGAAGAGCTTGAGTAGGCCAAATGTTGAAGTTAAGTTTTC  
Depth:2 (MARMOSET)  
Ei-value:0.000, Pi-value:0.000  
Er-value:0.000, Pr-value:0.000  
eCLIP MATCHES▶hnrnpa1 (bg=18.32%)▶ppil4 (bg=43.39%)▶PRPF8 (bg=6.2%)▶safb (bg=40.39%)▶srsf1 (bg=30.28%)MATCHES To TargetScan▶ miR-218-5p:UGUGCUU▶ miR-22-3p:AGCUGCC▶ miR-421:UCAACAG▶ miR-505-3p.2:UCAACAC▶ miR-330-3p:CAAAGCA▶ miR-543:AACAUUC▶ miR-320:AAAGCUG▶ miR-200bc-3p/429:AAUACUG▶ miR-143-3p:GAGAUGA


TAGGCCA

TAGGCCA  
Depth:6 (MOUSE)  
Ei-value:0.000, Pi-value:0.000  
Er-value:0.000, Pr-value:0.000  
eCLIP MATCHES▶hnrnpa1 (bg=18.32%)▶ppil4 (bg=43.39%)▶PRPF8 (bg=6.2%)No matches to TargetScan


A

TGGAAGAGTATTCCCAGTTGAAGCTGAAAAGTACAGCACAGTGCAGCTTTGGTTCATATTCAGTCATCTCAGGAGAACTTCAGAAGAGCTTGAGTAGGCCAAATGTTGAAGTTAAGTTTTC  
Depth:2 (MARMOSET)  
Ei-value:0.000, Pi-value:0.000  
Er-value:0.000, Pr-value:0.000  
eCLIP MATCHES▶hnrnpa1 (bg=18.32%)▶ppil4 (bg=43.39%)▶PRPF8 (bg=6.2%)▶safb (bg=40.39%)▶srsf1 (bg=30.28%)MATCHES To TargetScan▶ miR-218-5p:UGUGCUU▶ miR-22-3p:AGCUGCC▶ miR-421:UCAACAG▶ miR-505-3p.2:UCAACAC▶ miR-330-3p:CAAAGCA▶ miR-543:AACAUUC▶ miR-320:AAAGCUG▶ miR-200bc-3p/429:AAUACUG▶ miR-143-3p:GAGAUGA


ATG

ATGTTGAAGTTAAGTTTTC  
Depth:3 (DOG)  
Ei-value:0.000, Pi-value:0.000  
Er-value:0.000, Pr-value:0.000  
eCLIP MATCHES▶hnrnpa1 (bg=18.32%)▶ppil4 (bg=43.39%)▶PRPF8 (bg=6.2%)MATCHES To TargetScan▶ miR-421:UCAACAG▶ miR-505-3p.2:UCAACAC


TTG

TTGAAGTTAAGTTTT  
Depth:6 (MOUSE)  
Ei-value:0.000, Pi-value:0.000  
Er-value:0.000, Pr-value:0.000  
eCLIP MATCHES▶hnrnpa1 (bg=18.32%)▶ppil4 (bg=43.39%)▶PRPF8 (bg=6.2%)No matches to TargetScan


AAGTTAAGTT

AAGTTAAGTT  
Depth:8 (ALLIGATOR)  
Ei-value:0.000, Pi-value:0.000  
Er-value:0.000, Pr-value:0.000  
eCLIP MATCHES▶hnrnpa1 (bg=18.32%)▶PRPF8 (bg=6.2%)No matches to TargetScan


TT

AAGTTAAGTTTT  
Depth:7 (TURTLE)  
Ei-value:0.000, Pi-value:0.000  
Er-value:0.000, Pr-value:0.000  
eCLIP MATCHES▶hnrnpa1 (bg=18.32%)▶PRPF8 (bg=6.2%)No matches to TargetScan


C

TTGAAGTTAAGTTTTC  
Depth:5 (COW)  
Ei-value:0.000, Pi-value:0.000  
Er-value:0.000, Pr-value:0.000  
eCLIP MATCHES▶hnrnpa1 (bg=18.32%)▶ppil4 (bg=43.39%)▶PRPF8 (bg=6.2%)No matches to TargetScan

-

AA

AATAATGTGACTTCTTAAAAG  
Depth:2 (MARMOSET)  
Ei-value:0.000, Pi-value:0.000  
Er-value:0.000, Pr-value:0.000  
eCLIP MATCHES▶hnrnpa1 (bg=18.32%)▶PRPF8 (bg=6.2%)▶rbm22 (bg=12.69%)MATCHES To TargetScan▶ miR-323-3p:ACAUUAC▶ miR-23-3p:UCACAUU▶ miR-224-5p:AAGUCAC


TAATGTGA

TAATGTGA  
Depth:3 (DOG)  
Ei-value:0.010, Pi-value:0.000  
Er-value:0.000, Pr-value:0.000  
eCLIP MATCHES▶hnrnpa1 (bg=18.32%)▶PRPF8 (bg=6.2%)MATCHES To TargetScan▶ miR-323-3p:ACAUUAC▶ miR-23-3p:UCACAUU


CTTCTTAAAAG

AATAATGTGACTTCTTAAAAG  
Depth:2 (MARMOSET)  
Ei-value:0.000, Pi-value:0.000  
Er-value:0.000, Pr-value:0.000  
eCLIP MATCHES▶hnrnpa1 (bg=18.32%)▶PRPF8 (bg=6.2%)▶rbm22 (bg=12.69%)MATCHES To TargetScan▶ miR-323-3p:ACAUUAC▶ miR-23-3p:UCACAUU▶ miR-224-5p:AAGUCAC

-

TTTA

TTTATTAAAGGGGAGGGGCAAAT  
Depth:3 (DOG)  
Ei-value:0.000, Pi-value:0.000  
Er-value:0.000, Pr-value:0.000  
eCLIP MATCHES▶gtf2f1 (bg=10.18%)▶PCBP2 (bg=4.33%)▶PRPF8 (bg=6.2%)▶rbm22 (bg=12.69%)No matches to TargetScan


TTAAAGGGGAGGGG

TTAAAGGGGAGGGG  
Depth:6 (MOUSE)  
Ei-value:0.000, Pi-value:0.000  
Er-value:0.000, Pr-value:0.000  
eCLIP MATCHES▶PCBP2 (bg=4.33%)▶PRPF8 (bg=6.2%)▶rbm22 (bg=12.69%)No matches to TargetScan


CAA

TTAAAGGGGAGGGGCAA  
Depth:5 (COW)  
Ei-value:0.000, Pi-value:0.000  
Er-value:0.000, Pr-value:0.000  
eCLIP MATCHES▶gtf2f1 (bg=10.18%)▶PCBP2 (bg=4.33%)▶PRPF8 (bg=6.2%)▶rbm22 (bg=12.69%)No matches to TargetScan


AT

TTTATTAAAGGGGAGGGGCAAAT  
Depth:3 (DOG)  
Ei-value:0.000, Pi-value:0.000  
Er-value:0.000, Pr-value:0.000  
eCLIP MATCHES▶gtf2f1 (bg=10.18%)▶PCBP2 (bg=4.33%)▶PRPF8 (bg=6.2%)▶rbm22 (bg=12.69%)No matches to TargetScan


A

TTTATTAAAGGGGAGGGGCAAATATTGGCAATTAGTTGGCAGTGGCCTGTTA  
Depth:2 (MARMOSET)  
Ei-value:0.000, Pi-value:0.000  
Er-value:0.000, Pr-value:0.000  
eCLIP MATCHES▶ddx42 (bg=10.33%)▶gtf2f1 (bg=10.18%)▶khsrp (bg=27.4%)▶PCBP2 (bg=4.33%)▶PRPF8 (bg=6.2%)▶rbm22 (bg=12.69%)▶safb (bg=40.39%)▶TAF15 (bg=9.06%)No matches to TargetScan


TTGGCAAT

TTGGCAAT  
Depth:4 (PIG)  
Ei-value:0.000, Pi-value:0.000  
Er-value:0.000, Pr-value:0.000  
eCLIP MATCHES▶ddx42 (bg=10.33%)▶gtf2f1 (bg=10.18%)▶khsrp (bg=27.4%)▶PCBP2 (bg=4.33%)▶rbm22 (bg=12.69%)No matches to TargetScan


TAGTTGGC

TAGTTGGC  
Depth:6 (MOUSE)  
Ei-value:0.000, Pi-value:0.000  
Er-value:0.000, Pr-value:0.000  
eCLIP MATCHES▶ddx42 (bg=10.33%)▶gtf2f1 (bg=10.18%)▶khsrp (bg=27.4%)▶PCBP2 (bg=4.33%)▶rbm22 (bg=12.69%)▶safb (bg=40.39%)No matches to TargetScan


AGTGGC

TAGTTGGCAGTGGC  
Depth:5 (COW)  
Ei-value:0.000, Pi-value:0.000  
Er-value:0.000, Pr-value:0.000  
eCLIP MATCHES▶ddx42 (bg=10.33%)▶gtf2f1 (bg=10.18%)▶khsrp (bg=27.4%)▶PCBP2 (bg=4.33%)▶rbm22 (bg=12.69%)▶safb (bg=40.39%)No matches to TargetScan


CTGTT

TAGTTGGCAGTGGCCTGTT  
Depth:4 (PIG)  
Ei-value:0.000, Pi-value:0.000  
Er-value:0.000, Pr-value:0.000  
eCLIP MATCHES▶ddx42 (bg=10.33%)▶gtf2f1 (bg=10.18%)▶khsrp (bg=27.4%)▶PCBP2 (bg=4.33%)▶rbm22 (bg=12.69%)▶safb (bg=40.39%)▶TAF15 (bg=9.06%)No matches to TargetScan


A

TAGTTGGCAGTGGCCTGTTA  
Depth:3 (DOG)  
Ei-value:0.000, Pi-value:0.000  
Er-value:0.000, Pr-value:0.000  
eCLIP MATCHES▶ddx42 (bg=10.33%)▶gtf2f1 (bg=10.18%)▶khsrp (bg=27.4%)▶PCBP2 (bg=4.33%)▶rbm22 (bg=12.69%)▶safb (bg=40.39%)▶TAF15 (bg=9.06%)No matches to TargetScan

-

GGTTGGG

GGTTGGG  
Depth:4 (PIG)  
Ei-value:0.000, Pi-value:0.000  
Er-value:0.000, Pr-value:0.000  
eCLIP MATCHES▶gtf2f1 (bg=10.18%)▶khsrp (bg=27.4%)▶PCBP2 (bg=4.33%)▶safb (bg=40.39%)▶TAF15 (bg=9.06%)No matches to TargetScan


AT

GGTTGGGAT  
Depth:3 (DOG)  
Ei-value:0.000, Pi-value:0.000  
Er-value:0.000, Pr-value:0.000  
eCLIP MATCHES▶gtf2f1 (bg=10.18%)▶khsrp (bg=27.4%)▶PCBP2 (bg=4.33%)▶safb (bg=40.39%)▶TAF15 (bg=9.06%)No matches to TargetScan

------ 2880  
 ---

GGGT

GGGTTTAGGTAATTGTTTAGTTTATGATTGCAGATAAA  
Depth:2 (MARMOSET)  
Ei-value:0.000, Pi-value:0.000  
Er-value:0.000, Pr-value:0.000  
eCLIP MATCHES▶gtf2f1 (bg=10.18%)▶hnrnpa1 (bg=18.32%)▶khsrp (bg=27.4%)▶PCBP2 (bg=4.33%)▶ppil4 (bg=43.39%)▶safb (bg=40.39%)▶SUPV3L1 (bg=9.63%)▶TAF15 (bg=9.06%)MATCHES To TargetScan▶ miR-154-3p/487-3p:AUCAUAC


TTAGGT

TTAGGT  
Depth:11 (X.TROPICALIS)  
Ei-value:0.000, Pi-value:0.000  
Er-value:0.000, Pr-value:0.000  
eCLIP MATCHES▶gtf2f1 (bg=10.18%)▶khsrp (bg=27.4%)▶PCBP2 (bg=4.33%)▶ppil4 (bg=43.39%)▶safb (bg=40.39%)▶SUPV3L1 (bg=9.63%)▶TAF15 (bg=9.06%)No matches to TargetScan


AATTGTTTAGTTT

TTAGGTAATTGTTTAGTTT  
Depth:6 (MOUSE)  
Ei-value:0.000, Pi-value:0.000  
Er-value:0.000, Pr-value:0.000  
eCLIP MATCHES▶gtf2f1 (bg=10.18%)▶hnrnpa1 (bg=18.32%)▶khsrp (bg=27.4%)▶PCBP2 (bg=4.33%)▶ppil4 (bg=43.39%)▶safb (bg=40.39%)▶SUPV3L1 (bg=9.63%)▶TAF15 (bg=9.06%)No matches to TargetScan


ATGATT

TTAGGTAATTGTTTAGTTTATGATT  
Depth:4 (PIG)  
Ei-value:0.000, Pi-value:0.000  
Er-value:0.000, Pr-value:0.000  
eCLIP MATCHES▶gtf2f1 (bg=10.18%)▶hnrnpa1 (bg=18.32%)▶khsrp (bg=27.4%)▶PCBP2 (bg=4.33%)▶ppil4 (bg=43.39%)▶safb (bg=40.39%)▶SUPV3L1 (bg=9.63%)▶TAF15 (bg=9.06%)MATCHES To TargetScan▶ miR-154-3p/487-3p:AUCAUAC


G

GGGTTTAGGTAATTGTTTAGTTTATGATTGCAGATAAA  
Depth:2 (MARMOSET)  
Ei-value:0.000, Pi-value:0.000  
Er-value:0.000, Pr-value:0.000  
eCLIP MATCHES▶gtf2f1 (bg=10.18%)▶hnrnpa1 (bg=18.32%)▶khsrp (bg=27.4%)▶PCBP2 (bg=4.33%)▶ppil4 (bg=43.39%)▶safb (bg=40.39%)▶SUPV3L1 (bg=9.63%)▶TAF15 (bg=9.06%)MATCHES To TargetScan▶ miR-154-3p/487-3p:AUCAUAC


CAGATAA

CAGATAA  
Depth:6 (MOUSE)  
Ei-value:0.000, Pi-value:0.000  
Er-value:0.000, Pr-value:0.000  
eCLIP MATCHES▶gtf2f1 (bg=10.18%)▶hnrnpa1 (bg=18.32%)▶PCBP2 (bg=4.33%)▶ppil4 (bg=43.39%)▶safb (bg=40.39%)▶SUPV3L1 (bg=9.63%)No matches to TargetScan


A

GGGTTTAGGTAATTGTTTAGTTTATGATTGCAGATAAA  
Depth:2 (MARMOSET)  
Ei-value:0.000, Pi-value:0.000  
Er-value:0.000, Pr-value:0.000  
eCLIP MATCHES▶gtf2f1 (bg=10.18%)▶hnrnpa1 (bg=18.32%)▶khsrp (bg=27.4%)▶PCBP2 (bg=4.33%)▶ppil4 (bg=43.39%)▶safb (bg=40.39%)▶SUPV3L1 (bg=9.63%)▶TAF15 (bg=9.06%)MATCHES To TargetScan▶ miR-154-3p/487-3p:AUCAUAC

-

TCATGCC

TCATGCC  
Depth:5 (COW)  
Ei-value:0.000, Pi-value:0.000  
Er-value:0.000, Pr-value:0.000  
eCLIP MATCHES▶gtf2f1 (bg=10.18%)▶hnrnpa1 (bg=18.32%)▶PCBP2 (bg=4.33%)▶ppil4 (bg=43.39%)▶safb (bg=40.39%)▶safb2 (bg=26.89%)▶SUPV3L1 (bg=9.63%)No matches to TargetScan


A

TCATGCCAGAGAACTTAAA  
Depth:2 (MARMOSET)  
Ei-value:0.000, Pi-value:0.000  
Er-value:0.000, Pr-value:0.000  
eCLIP MATCHES▶gtf2f1 (bg=10.18%)▶hnrnpa1 (bg=18.32%)▶PCBP2 (bg=4.33%)▶ppil4 (bg=43.39%)▶safb (bg=40.39%)▶safb2 (bg=26.89%)▶SUPV3L1 (bg=9.63%)MATCHES To TargetScan▶ miR-326:CUCUGGG


GAGAACTTAAA

GAGAACTTAAA  
Depth:6 (MOUSE)  
Ei-value:0.000, Pi-value:0.000  
Er-value:0.000, Pr-value:0.000  
eCLIP MATCHES▶gtf2f1 (bg=10.18%)▶hnrnpa1 (bg=18.32%)▶PCBP2 (bg=4.33%)▶ppil4 (bg=43.39%)▶safb (bg=40.39%)▶safb2 (bg=26.89%)▶SUPV3L1 (bg=9.63%)No matches to TargetScan


gtcttag

gtcttag  
Depth:2 (MARMOSET)  
Ei-value:1.000, Pi-value:0.020  
Er-value:0.000, Pr-value:0.000  
eCLIP MATCHES▶gtf2f1 (bg=10.18%)▶hnrnpa1 (bg=18.32%)▶PCBP2 (bg=4.33%)▶ppil4 (bg=43.39%)▶safb (bg=40.39%)▶safb2 (bg=26.89%)▶SUPV3L1 (bg=9.63%)MATCHES To TargetScan▶ miR-499a-5p:UAAGACU▶ miR-208-3p:UAAGACG

-

A

ATGGAAAA  
Depth:4 (PIG)  
Ei-value:0.000, Pi-value:0.000  
Er-value:0.000, Pr-value:0.000  
eCLIP MATCHES▶hnrnpa1 (bg=18.32%)▶ppil4 (bg=43.39%)▶safb (bg=40.39%)▶SUPV3L1 (bg=9.63%)No matches to TargetScan


TGGAAAA

TGGAAAA  
Depth:6 (MOUSE)  
Ei-value:0.000, Pi-value:0.000  
Er-value:0.000, Pr-value:0.010  
eCLIP MATCHES▶hnrnpa1 (bg=18.32%)▶ppil4 (bg=43.39%)▶safb (bg=40.39%)▶SUPV3L1 (bg=9.63%)No matches to TargetScan

-

g

gtaaagaaat  
Depth:2 (MARMOSET)  
Ei-value:1.000, Pi-value:0.000  
Er-value:0.000, Pr-value:0.000  
eCLIP MATCHES▶hnrnpa1 (bg=18.32%)▶SUPV3L1 (bg=9.63%)No matches to TargetScan


TAAAGAAAT

TAAAGAAAT  
Depth:5 (COW)  
Ei-value:0.000, Pi-value:0.000  
Er-value:0.000, Pr-value:0.000  
eCLIP MATCHES▶hnrnpa1 (bg=18.32%)▶SUPV3L1 (bg=9.63%)No matches to TargetScan

-

TCAACTTCCAAG

TCAACTTCCAAG  
Depth:3 (DOG)  
Ei-value:0.000, Pi-value:0.000  
Er-value:0.000, Pr-value:0.000  
eCLIP MATCHES▶hnrnpa1 (bg=18.32%)▶ppil4 (bg=43.39%)▶SUPV3L1 (bg=9.63%)MATCHES To TargetScan▶ miR-382-5p:AAGUUGU

-

T

TGGCAAGTAA  
Depth:8 (ALLIGATOR)  
Ei-value:0.000, Pi-value:0.000  
Er-value:0.000, Pr-value:0.000  
eCLIP MATCHES▶hnrnpa1 (bg=18.32%)▶ppil4 (bg=43.39%)▶safb (bg=40.39%)No matches to TargetScan


GGCAAGTAA

GGCAAGTAA  
Depth:10 (SNAKE)  
Ei-value:0.000, Pi-value:0.000  
Er-value:0.000, Pr-value:0.000  
eCLIP MATCHES▶hnrnpa1 (bg=18.32%)▶ppil4 (bg=43.39%)▶safb (bg=40.39%)No matches to TargetScan


CTC

TGGCAAGTAACTC  
Depth:6 (MOUSE)  
Ei-value:0.000, Pi-value:0.000  
Er-value:0.000, Pr-value:0.000  
eCLIP MATCHES▶hnrnpa1 (bg=18.32%)▶ppil4 (bg=43.39%)▶safb (bg=40.39%)No matches to TargetScan


C

TGGCAAGTAACTCC  
Depth:4 (PIG)  
Ei-value:0.000, Pi-value:0.000  
Er-value:0.000, Pr-value:0.000  
eCLIP MATCHES▶hnrnpa1 (bg=18.32%)▶ppil4 (bg=43.39%)▶safb (bg=40.39%)No matches to TargetScan


CA

TGGCAAGTAACTCCCA  
Depth:3 (DOG)  
Ei-value:0.000, Pi-value:0.000  
Er-value:0.000, Pr-value:0.000  
eCLIP MATCHES▶hnrnpa1 (bg=18.32%)▶ppil4 (bg=43.39%)▶safb (bg=40.39%)No matches to TargetScan

-- 3000  
 --

tt

tttagttttt  
Depth:2 (MARMOSET)  
Ei-value:1.000, Pi-value:0.000  
Er-value:0.000, Pr-value:0.000  
eCLIP MATCHES▶HNRNPU (bg=9.45%)▶ppil4 (bg=43.39%)▶safb (bg=40.39%)▶tia1 (bg=23.76%)▶tial1 (bg=15.02%)No matches to TargetScan


tagtttt

tagtttt  
Depth:3 (DOG)  
Ei-value:0.350, Pi-value:0.020  
Er-value:0.000, Pr-value:0.010  
eCLIP MATCHES▶HNRNPU (bg=9.45%)▶ppil4 (bg=43.39%)▶safb (bg=40.39%)▶tia1 (bg=23.76%)▶tial1 (bg=15.02%)No matches to TargetScan


t

tttagttttt  
Depth:2 (MARMOSET)  
Ei-value:1.000, Pi-value:0.000  
Er-value:0.000, Pr-value:0.000  
eCLIP MATCHES▶HNRNPU (bg=9.45%)▶ppil4 (bg=43.39%)▶safb (bg=40.39%)▶tia1 (bg=23.76%)▶tial1 (bg=15.02%)No matches to TargetScan


ttcccc

ttcccc  
Depth:2 (MARMOSET)  
Ei-value:1.000, Pi-value:0.010  
Er-value:0.000, Pr-value:0.000  
eCLIP MATCHES▶HNRNPU (bg=9.45%)▶ppil4 (bg=43.39%)▶safb (bg=40.39%)▶SLBP (bg=6.66%)▶tia1 (bg=23.76%)▶tial1 (bg=15.02%)No matches to TargetScan

--------

aattggga

aattggga  
Depth:2 (MARMOSET)  
Ei-value:1.000, Pi-value:0.000  
Er-value:0.000, Pr-value:0.000  
eCLIP MATCHES▶HNRNPU (bg=9.45%)▶ppil4 (bg=43.39%)▶safb (bg=40.39%)▶SLBP (bg=6.66%)▶SUPV3L1 (bg=9.63%)▶tia1 (bg=23.76%)▶tial1 (bg=15.02%)No matches to TargetScan

-

gctggg

gctggg  
Depth:2 (MARMOSET)  
Ei-value:1.000, Pi-value:0.040  
Er-value:0.000, Pr-value:0.000  
eCLIP MATCHES▶HNRNPU (bg=9.45%)▶ppil4 (bg=43.39%)▶safb (bg=40.39%)▶SLBP (bg=6.66%)▶SUPV3L1 (bg=9.63%)▶tia1 (bg=23.76%)▶tial1 (bg=15.02%)▶zc3h8 (bg=12.78%)No matches to TargetScan

-

gaagttaaata

gaagttaaata  
Depth:2 (MARMOSET)  
Ei-value:1.000, Pi-value:0.000  
Er-value:0.000, Pr-value:0.000  
eCLIP MATCHES▶HNRNPU (bg=9.45%)▶ppil4 (bg=43.39%)▶safb (bg=40.39%)▶SLBP (bg=6.66%)▶SUPV3L1 (bg=9.63%)▶tia1 (bg=23.76%)▶tial1 (bg=15.02%)▶zc3h8 (bg=12.78%)No matches to TargetScan

-

GAGCCACTGGGTGTACCAGTGCATT

GAGCCACTGGGTGTACCAGTGCATT  
Depth:2 (MARMOSET)  
Ei-value:0.000, Pi-value:0.000  
Er-value:0.000, Pr-value:0.000  
eCLIP MATCHES▶DROSHA (bg=1.03%)▶FUS (bg=0.13%)▶HNRNPU (bg=9.45%)▶HNRNPUL1 (bg=1.43%)▶NONO (bg=0.13%)▶ppil4 (bg=43.39%)▶safb (bg=40.39%)▶safb2 (bg=26.89%)▶SFPQ (bg=2.48%)▶SLBP (bg=6.66%)▶SUPV3L1 (bg=9.63%)▶tia1 (bg=23.76%)MATCHES To TargetScan▶ miR-199-5p:CCAGUGU▶ miR-501-3p/502-3p:AUGCACC

-

atttgggc

atttgggc  
Depth:2 (MARMOSET)  
Ei-value:1.000, Pi-value:0.000  
Er-value:0.000, Pr-value:0.000  
eCLIP MATCHES▶DROSHA (bg=1.03%)▶FUS (bg=0.13%)▶HNRNPUL1 (bg=1.43%)▶NONO (bg=0.13%)▶ppil4 (bg=43.39%)▶safb2 (bg=26.89%)▶SFPQ (bg=2.48%)▶SLBP (bg=6.66%)No matches to TargetScan

------

agtgtc

agtgtc  
Depth:2 (MARMOSET)  
Ei-value:1.000, Pi-value:0.050  
Er-value:0.000, Pr-value:0.000  
eCLIP MATCHES▶DROSHA (bg=1.03%)▶HNRNPUL1 (bg=1.43%)▶SFPQ (bg=2.48%)▶SLBP (bg=6.66%)No matches to TargetScan

-

TAATTTGATACTGT

TAATTTGATACTGT  
Depth:2 (MARMOSET)  
Ei-value:0.020, Pi-value:0.000  
Er-value:0.000, Pr-value:0.000  
eCLIP MATCHES▶HNRNPUL1 (bg=1.43%)▶SFPQ (bg=2.48%)▶SLBP (bg=6.66%)MATCHES To TargetScan▶ miR-144-3p:ACAGUAU▶ miR-101-3p.1:ACAGUAC

-

tc

tctgttttcc  
Depth:2 (MARMOSET)  
Ei-value:1.000, Pi-value:0.000  
Er-value:0.000, Pr-value:0.000  
eCLIP MATCHES▶HNRNPUL1 (bg=1.43%)▶khsrp (bg=27.4%)▶SFPQ (bg=2.48%)No matches to TargetScan


TG

TGTTTTCC  
Depth:3 (DOG)  
Ei-value:0.010, Pi-value:0.000  
Er-value:0.000, Pr-value:0.000  
eCLIP MATCHES▶HNRNPUL1 (bg=1.43%)▶khsrp (bg=27.4%)▶SFPQ (bg=2.48%)No matches to TargetScan

 3120  


TTTTCC

TGTTTTCC  
Depth:3 (DOG)  
Ei-value:0.010, Pi-value:0.000  
Er-value:0.000, Pr-value:0.000  
eCLIP MATCHES▶HNRNPUL1 (bg=1.43%)▶khsrp (bg=27.4%)▶SFPQ (bg=2.48%)No matches to TargetScan

-

TCAAAGTATAGAGCTT

TCAAAGTATAGAGCTT  
Depth:2 (MARMOSET)  
Ei-value:0.000, Pi-value:0.000  
Er-value:0.000, Pr-value:0.000  
eCLIP MATCHES▶khsrp (bg=27.4%)▶safb (bg=40.39%)No matches to TargetScan

--

GGGG

GGGGAAGGAAAGTA  
Depth:3 (DOG)  
Ei-value:0.000, Pi-value:0.000  
Er-value:0.000, Pr-value:0.000  
eCLIP MATCHES▶ddx42 (bg=10.33%)▶HNRNPU (bg=9.45%)▶khsrp (bg=27.4%)▶ppil4 (bg=43.39%)▶safb (bg=40.39%)▶tia1 (bg=23.76%)No matches to TargetScan


AAGGAAA

AAGGAAA  
Depth:5 (COW)  
Ei-value:0.000, Pi-value:0.010  
Er-value:0.000, Pr-value:0.000  
eCLIP MATCHES▶ddx42 (bg=10.33%)▶HNRNPU (bg=9.45%)▶khsrp (bg=27.4%)▶ppil4 (bg=43.39%)▶safb (bg=40.39%)▶tia1 (bg=23.76%)No matches to TargetScan


GT

AAGGAAAGT  
Depth:4 (PIG)  
Ei-value:0.000, Pi-value:0.000  
Er-value:0.000, Pr-value:0.000  
eCLIP MATCHES▶ddx42 (bg=10.33%)▶HNRNPU (bg=9.45%)▶khsrp (bg=27.4%)▶ppil4 (bg=43.39%)▶safb (bg=40.39%)▶tia1 (bg=23.76%)No matches to TargetScan


A

GGGGAAGGAAAGTA  
Depth:3 (DOG)  
Ei-value:0.000, Pi-value:0.000  
Er-value:0.000, Pr-value:0.000  
eCLIP MATCHES▶ddx42 (bg=10.33%)▶HNRNPU (bg=9.45%)▶khsrp (bg=27.4%)▶ppil4 (bg=43.39%)▶safb (bg=40.39%)▶tia1 (bg=23.76%)No matches to TargetScan

----

AC

ACTGGGGGTTGGTCT  
Depth:3 (DOG)  
Ei-value:0.000, Pi-value:0.000  
Er-value:0.000, Pr-value:0.000  
eCLIP MATCHES▶ddx42 (bg=10.33%)▶DGCR8 (bg=2.67%)▶HNRNPU (bg=9.45%)▶khsrp (bg=27.4%)▶ppil4 (bg=43.39%)▶safb (bg=40.39%)▶SUPV3L1 (bg=9.63%)No matches to TargetScan


TGGGGGTTGGTCT

TGGGGGTTGGTCT  
Depth:6 (MOUSE)  
Ei-value:0.000, Pi-value:0.000  
Er-value:0.000, Pr-value:0.000  
eCLIP MATCHES▶ddx42 (bg=10.33%)▶DGCR8 (bg=2.67%)▶HNRNPU (bg=9.45%)▶khsrp (bg=27.4%)▶ppil4 (bg=43.39%)▶safb (bg=40.39%)▶SUPV3L1 (bg=9.63%)No matches to TargetScan


GGCC

ACTGGGGGTTGGTCTGGCC  
Depth:2 (MARMOSET)  
Ei-value:0.000, Pi-value:0.000  
Er-value:0.000, Pr-value:0.000  
eCLIP MATCHES▶ddx42 (bg=10.33%)▶DGCR8 (bg=2.67%)▶HNRNPU (bg=9.45%)▶khsrp (bg=27.4%)▶ppil4 (bg=43.39%)▶safb (bg=40.39%)▶SUPV3L1 (bg=9.63%)No matches to TargetScan

-

actg

actgggctgaca  
Depth:2 (MARMOSET)  
Ei-value:0.990, Pi-value:0.000  
Er-value:0.000, Pr-value:0.000  
eCLIP MATCHES▶ddx42 (bg=10.33%)▶DGCR8 (bg=2.67%)▶HNRNPU (bg=9.45%)▶khsrp (bg=27.4%)▶ppil4 (bg=43.39%)▶safb (bg=40.39%)▶SUPV3L1 (bg=9.63%)No matches to TargetScan


ggctgac

ggctgac  
Depth:3 (DOG)  
Ei-value:0.350, Pi-value:0.000  
Er-value:0.000, Pr-value:0.000  
eCLIP MATCHES▶ddx42 (bg=10.33%)▶HNRNPU (bg=9.45%)▶khsrp (bg=27.4%)▶ppil4 (bg=43.39%)▶safb (bg=40.39%)▶SUPV3L1 (bg=9.63%)No matches to TargetScan


a

actgggctgaca  
Depth:2 (MARMOSET)  
Ei-value:0.990, Pi-value:0.000  
Er-value:0.000, Pr-value:0.000  
eCLIP MATCHES▶ddx42 (bg=10.33%)▶DGCR8 (bg=2.67%)▶HNRNPU (bg=9.45%)▶khsrp (bg=27.4%)▶ppil4 (bg=43.39%)▶safb (bg=40.39%)▶SUPV3L1 (bg=9.63%)No matches to TargetScan


ttaact

ttaact  
Depth:2 (MARMOSET)  
Ei-value:1.000, Pi-value:0.050  
Er-value:0.000, Pr-value:0.010  
eCLIP MATCHES▶ddx42 (bg=10.33%)▶HNRNPU (bg=9.45%)▶khsrp (bg=27.4%)▶ppil4 (bg=43.39%)▶safb (bg=40.39%)▶SUPV3L1 (bg=9.63%)No matches to TargetScan

---

ATTA

ATTATGGGAAATGCAAAAGTTGTT  
Depth:2 (MARMOSET)  
Ei-value:0.000, Pi-value:0.000  
Er-value:0.000, Pr-value:0.000  
eCLIP MATCHES▶ddx42 (bg=10.33%)▶HNRNPM (bg=6.37%)▶khsrp (bg=27.4%)▶PRPF8 (bg=6.2%)▶SF3B4 (bg=4.76%)MATCHES To TargetScan▶ miR-33-5p:UGCAUUG


tgggaa

tgggaa  
Depth:3 (DOG)  
Ei-value:1.000, Pi-value:0.070  
Er-value:0.000, Pr-value:0.010  
eCLIP MATCHES▶ddx42 (bg=10.33%)▶khsrp (bg=27.4%)No matches to TargetScan


ATGCA

ATTATGGGAAATGCAAAAGTTGTT  
Depth:2 (MARMOSET)  
Ei-value:0.000, Pi-value:0.000  
Er-value:0.000, Pr-value:0.000  
eCLIP MATCHES▶ddx42 (bg=10.33%)▶HNRNPM (bg=6.37%)▶khsrp (bg=27.4%)▶PRPF8 (bg=6.2%)▶SF3B4 (bg=4.76%)MATCHES To TargetScan▶ miR-33-5p:UGCAUUG


AAAGTTGTT

AAAGTTGTT  
Depth:4 (PIG)  
Ei-value:0.000, Pi-value:0.000  
Er-value:0.000, Pr-value:0.000  
eCLIP MATCHES▶HNRNPM (bg=6.37%)▶khsrp (bg=27.4%)▶PRPF8 (bg=6.2%)▶SF3B4 (bg=4.76%)No matches to TargetScan

-

GGATATGG

GGATATGGTAGTGTGTGGTTCTCTTTTGGAATTTTTTTCAGGTGATTTAATA  
Depth:4 (PIG)  
Ei-value:0.000, Pi-value:0.000  
Er-value:0.000, Pr-value:0.000  
eCLIP MATCHES▶HNRNPM (bg=6.37%)▶khsrp (bg=27.4%)▶ppil4 (bg=43.39%)▶PRPF8 (bg=6.2%)▶PUS1 (bg=1.64%)▶safb (bg=40.39%)▶SF3B4 (bg=4.76%)▶SLBP (bg=6.66%)▶u2af2 (bg=19.32%)MATCHES To TargetScan▶ miR-329-3p/362-3p:ACACACC▶ miR-146-5p:GAGAACU▶ miR-140-3p.2:ACCACAG


TA

TAGTGTGTGGTTCTCT  
Depth:6 (MOUSE)  
Ei-value:0.000, Pi-value:0.000  
Er-value:0.000, Pr-value:0.000  
eCLIP MATCHES▶HNRNPM (bg=6.37%)▶khsrp (bg=27.4%)▶PRPF8 (bg=6.2%)▶SF3B4 (bg=4.76%)▶SLBP (bg=6.66%)MATCHES To TargetScan▶ miR-329-3p/362-3p:ACACACC▶ miR-146-5p:GAGAACU▶ miR-140-3p.2:ACCACAG


G

GTGTGTGG  
Depth:8 (ALLIGATOR)  
Ei-value:0.000, Pi-value:0.000  
Er-value:0.000, Pr-value:0.000  
eCLIP MATCHES▶HNRNPM (bg=6.37%)▶khsrp (bg=27.4%)▶PRPF8 (bg=6.2%)▶SF3B4 (bg=4.76%)▶SLBP (bg=6.66%)MATCHES To TargetScan▶ miR-329-3p/362-3p:ACACACC

 3240  


TGTGTGG

GTGTGTGG  
Depth:8 (ALLIGATOR)  
Ei-value:0.000, Pi-value:0.000  
Er-value:0.000, Pr-value:0.000  
eCLIP MATCHES▶HNRNPM (bg=6.37%)▶khsrp (bg=27.4%)▶PRPF8 (bg=6.2%)▶SF3B4 (bg=4.76%)▶SLBP (bg=6.66%)MATCHES To TargetScan▶ miR-329-3p/362-3p:ACACACC


TTCTCT

TAGTGTGTGGTTCTCT  
Depth:6 (MOUSE)  
Ei-value:0.000, Pi-value:0.000  
Er-value:0.000, Pr-value:0.000  
eCLIP MATCHES▶HNRNPM (bg=6.37%)▶khsrp (bg=27.4%)▶PRPF8 (bg=6.2%)▶SF3B4 (bg=4.76%)▶SLBP (bg=6.66%)MATCHES To TargetScan▶ miR-329-3p/362-3p:ACACACC▶ miR-146-5p:GAGAACU▶ miR-140-3p.2:ACCACAG


T

GGATATGGTAGTGTGTGGTTCTCTTTTGGAATTTTTTTCAGGTGATTTAATA  
Depth:4 (PIG)  
Ei-value:0.000, Pi-value:0.000  
Er-value:0.000, Pr-value:0.000  
eCLIP MATCHES▶HNRNPM (bg=6.37%)▶khsrp (bg=27.4%)▶ppil4 (bg=43.39%)▶PRPF8 (bg=6.2%)▶PUS1 (bg=1.64%)▶safb (bg=40.39%)▶SF3B4 (bg=4.76%)▶SLBP (bg=6.66%)▶u2af2 (bg=19.32%)MATCHES To TargetScan▶ miR-329-3p/362-3p:ACACACC▶ miR-146-5p:GAGAACU▶ miR-140-3p.2:ACCACAG


TTGGAAT

TTGGAAT  
Depth:5 (COW)  
Ei-value:0.000, Pi-value:0.000  
Er-value:0.000, Pr-value:0.000  
eCLIP MATCHES▶HNRNPM (bg=6.37%)▶PRPF8 (bg=6.2%)▶SF3B4 (bg=4.76%)▶SLBP (bg=6.66%)▶u2af2 (bg=19.32%)No matches to TargetScan


T

GGATATGGTAGTGTGTGGTTCTCTTTTGGAATTTTTTTCAGGTGATTTAATA  
Depth:4 (PIG)  
Ei-value:0.000, Pi-value:0.000  
Er-value:0.000, Pr-value:0.000  
eCLIP MATCHES▶HNRNPM (bg=6.37%)▶khsrp (bg=27.4%)▶ppil4 (bg=43.39%)▶PRPF8 (bg=6.2%)▶PUS1 (bg=1.64%)▶safb (bg=40.39%)▶SF3B4 (bg=4.76%)▶SLBP (bg=6.66%)▶u2af2 (bg=19.32%)MATCHES To TargetScan▶ miR-329-3p/362-3p:ACACACC▶ miR-146-5p:GAGAACU▶ miR-140-3p.2:ACCACAG


TTTTT

TTTTTCAGGTG  
Depth:7 (TURTLE)  
Ei-value:0.000, Pi-value:0.000  
Er-value:0.000, Pr-value:0.000  
eCLIP MATCHES▶HNRNPM (bg=6.37%)▶ppil4 (bg=43.39%)▶PRPF8 (bg=6.2%)▶PUS1 (bg=1.64%)▶safb (bg=40.39%)▶SF3B4 (bg=4.76%)▶SLBP (bg=6.66%)▶u2af2 (bg=19.32%)No matches to TargetScan


CAGGTG

CAGGTG  
Depth:8 (ALLIGATOR)  
Ei-value:0.000, Pi-value:0.000  
Er-value:0.000, Pr-value:0.000  
eCLIP MATCHES▶HNRNPM (bg=6.37%)▶ppil4 (bg=43.39%)▶PRPF8 (bg=6.2%)▶PUS1 (bg=1.64%)▶safb (bg=40.39%)▶SLBP (bg=6.66%)▶u2af2 (bg=19.32%)No matches to TargetScan


A

TTTTTCAGGTGA  
Depth:6 (MOUSE)  
Ei-value:0.000, Pi-value:0.000  
Er-value:0.000, Pr-value:0.000  
eCLIP MATCHES▶HNRNPM (bg=6.37%)▶ppil4 (bg=43.39%)▶PRPF8 (bg=6.2%)▶PUS1 (bg=1.64%)▶safb (bg=40.39%)▶SF3B4 (bg=4.76%)▶SLBP (bg=6.66%)▶u2af2 (bg=19.32%)No matches to TargetScan


TTTAATA

TTTTTCAGGTGATTTAATA  
Depth:5 (COW)  
Ei-value:0.000, Pi-value:0.000  
Er-value:0.000, Pr-value:0.000  
eCLIP MATCHES▶HNRNPM (bg=6.37%)▶ppil4 (bg=43.39%)▶PRPF8 (bg=6.2%)▶PUS1 (bg=1.64%)▶safb (bg=40.39%)▶SF3B4 (bg=4.76%)▶SLBP (bg=6.66%)▶u2af2 (bg=19.32%)No matches to TargetScan

-----

TTAAA

TTAAAACTACTATAG  
Depth:2 (MARMOSET)  
Ei-value:0.000, Pi-value:0.000  
Er-value:0.000, Pr-value:0.000  
eCLIP MATCHES▶LARP7 (bg=2.17%)▶ppil4 (bg=43.39%)▶u2af2 (bg=19.32%)MATCHES To TargetScan▶ miR-411-5p.2:UAGUAGA


ACTACTATAG

ACTACTATAG  
Depth:6 (MOUSE)  
Ei-value:0.000, Pi-value:0.000  
Er-value:0.000, Pr-value:0.000  
eCLIP MATCHES▶LARP7 (bg=2.17%)▶ppil4 (bg=43.39%)MATCHES To TargetScan▶ miR-411-5p.2:UAGUAGA

------

CAGAG

CAGAGCAAAGGA  
Depth:5 (COW)  
Ei-value:0.000, Pi-value:0.000  
Er-value:0.000, Pr-value:0.000  
eCLIP MATCHES▶ppil4 (bg=43.39%)▶srsf7 (bg=22.53%)No matches to TargetScan


CAAAGGA

CAAAGGA  
Depth:6 (MOUSE)  
Ei-value:0.000, Pi-value:0.000  
Er-value:0.000, Pr-value:0.000  
eCLIP MATCHES▶ppil4 (bg=43.39%)▶srsf7 (bg=22.53%)No matches to TargetScan


A

CAGAGCAAAGGAAGTGGCTTAATGATCCTGAAGGGATTTCTTC  
Depth:4 (PIG)  
Ei-value:0.000, Pi-value:0.000  
Er-value:0.000, Pr-value:0.000  
eCLIP MATCHES▶HNRNPM (bg=6.37%)▶ppil4 (bg=43.39%)▶srsf7 (bg=22.53%)MATCHES To TargetScan▶ miR-382-3p:AUCAUUC▶ miR-204-5p/211-5p:UCCCUUU▶ miR-188-5p:AUCCCUU▶ miR-205-5p:CCUUCAU


GTGGC

GTGGCTTAATGATCCTGAAGGGATTTCTTC  
Depth:5 (COW)  
Ei-value:0.000, Pi-value:0.000  
Er-value:0.000, Pr-value:0.000  
eCLIP MATCHES▶HNRNPM (bg=6.37%)▶ppil4 (bg=43.39%)▶srsf7 (bg=22.53%)MATCHES To TargetScan▶ miR-382-3p:AUCAUUC▶ miR-204-5p/211-5p:UCCCUUU▶ miR-188-5p:AUCCCUU▶ miR-205-5p:CCUUCAU


TTAATGA

TTAATGA  
Depth:8 (ALLIGATOR)  
Ei-value:0.000, Pi-value:0.000  
Er-value:0.000, Pr-value:0.000  
eCLIP MATCHES▶ppil4 (bg=43.39%)▶srsf7 (bg=22.53%)No matches to TargetScan


T

GTGGCTTAATGATCCTGAAGGGATTTCTTC  
Depth:5 (COW)  
Ei-value:0.000, Pi-value:0.000  
Er-value:0.000, Pr-value:0.000  
eCLIP MATCHES▶HNRNPM (bg=6.37%)▶ppil4 (bg=43.39%)▶srsf7 (bg=22.53%)MATCHES To TargetScan▶ miR-382-3p:AUCAUUC▶ miR-204-5p/211-5p:UCCCUUU▶ miR-188-5p:AUCCCUU▶ miR-205-5p:CCUUCAU


CCTGAAGG

CCTGAAGG  
Depth:6 (MOUSE)  
Ei-value:0.000, Pi-value:0.000  
Er-value:0.000, Pr-value:0.000  
eCLIP MATCHES▶HNRNPM (bg=6.37%)▶ppil4 (bg=43.39%)▶srsf7 (bg=22.53%)MATCHES To TargetScan▶ miR-205-5p:CCUUCAU


G

GTGGCTTAATGATCCTGAAGGGATTTCTTC  
Depth:5 (COW)  
Ei-value:0.000, Pi-value:0.000  
Er-value:0.000, Pr-value:0.000  
eCLIP MATCHES▶HNRNPM (bg=6.37%)▶ppil4 (bg=43.39%)▶srsf7 (bg=22.53%)MATCHES To TargetScan▶ miR-382-3p:AUCAUUC▶ miR-204-5p/211-5p:UCCCUUU▶ miR-188-5p:AUCCCUU▶ miR-205-5p:CCUUCAU


ATTTCTTC

ATTTCTTC  
Depth:6 (MOUSE)  
Ei-value:0.000, Pi-value:0.000  
Er-value:0.000, Pr-value:0.000  
eCLIP MATCHES▶HNRNPM (bg=6.37%)▶srsf7 (bg=22.53%)No matches to TargetScan


TGA

CAGAGCAAAGGAAGTGGCTTAATGATCCTGAAGGGATTTCTTCTGATGGTAGCTTTTGTATTATCAAGTAAGATTCT  
Depth:2 (MARMOSET)  
Ei-value:0.000, Pi-value:0.000  
Er-value:0.000, Pr-value:0.000  
eCLIP MATCHES▶HNRNPM (bg=6.37%)▶ppil4 (bg=43.39%)▶srsf7 (bg=22.53%)MATCHES To TargetScan▶ miR-382-3p:AUCAUUC▶ miR-204-5p/211-5p:UCCCUUU▶ miR-369-3p:AUAAUAC▶ miR-381-3p:AUACAAG▶ miR-188-5p:AUCCCUU▶ miR-320:AAAGCUG▶ miR-655-3p:UAAUACA▶ miR-205-5p:CCUUCAU


TGG

TGGTAGCTTTT  
Depth:5 (COW)  
Ei-value:0.000, Pi-value:0.000  
Er-value:0.000, Pr-value:0.000  
eCLIP MATCHES▶HNRNPM (bg=6.37%)▶srsf7 (bg=22.53%)MATCHES To TargetScan▶ miR-320:AAAGCUG


TAGC

TAGCTTTT  
Depth:6 (MOUSE)  
Ei-value:0.000, Pi-value:0.000  
Er-value:0.000, Pr-value:0.000  
eCLIP MATCHES▶HNRNPM (bg=6.37%)▶srsf7 (bg=22.53%)MATCHES To TargetScan▶ miR-320:AAAGCUG

 3360  


TTTT

TAGCTTTT  
Depth:6 (MOUSE)  
Ei-value:0.000, Pi-value:0.000  
Er-value:0.000, Pr-value:0.000  
eCLIP MATCHES▶HNRNPM (bg=6.37%)▶srsf7 (bg=22.53%)MATCHES To TargetScan▶ miR-320:AAAGCUG


G

CAGAGCAAAGGAAGTGGCTTAATGATCCTGAAGGGATTTCTTCTGATGGTAGCTTTTGTATTATCAAGTAAGATTCT  
Depth:2 (MARMOSET)  
Ei-value:0.000, Pi-value:0.000  
Er-value:0.000, Pr-value:0.000  
eCLIP MATCHES▶HNRNPM (bg=6.37%)▶ppil4 (bg=43.39%)▶srsf7 (bg=22.53%)MATCHES To TargetScan▶ miR-382-3p:AUCAUUC▶ miR-204-5p/211-5p:UCCCUUU▶ miR-369-3p:AUAAUAC▶ miR-381-3p:AUACAAG▶ miR-188-5p:AUCCCUU▶ miR-320:AAAGCUG▶ miR-655-3p:UAAUACA▶ miR-205-5p:CCUUCAU


TATTA

TATTATCAAGTAAGA  
Depth:6 (MOUSE)  
Ei-value:0.000, Pi-value:0.000  
Er-value:0.000, Pr-value:0.000  
eCLIP MATCHES▶HNRNPM (bg=6.37%)▶srsf7 (bg=22.53%)MATCHES To TargetScan▶ miR-369-3p:AUAAUAC


T

TCAAGTAAGA  
Depth:9 (LIZARD)  
Ei-value:0.000, Pi-value:0.000  
Er-value:0.000, Pr-value:0.000  
eCLIP MATCHES▶HNRNPM (bg=6.37%)▶srsf7 (bg=22.53%)No matches to TargetScan


CAA

CAAGTAAGA  
Depth:10 (SNAKE)  
Ei-value:0.000, Pi-value:0.000  
Er-value:0.000, Pr-value:0.000  
eCLIP MATCHES▶HNRNPM (bg=6.37%)▶srsf7 (bg=22.53%)No matches to TargetScan


GTAAGA

GTAAGA  
Depth:11 (X.TROPICALIS)  
Ei-value:0.000, Pi-value:0.000  
Er-value:0.000, Pr-value:0.000  
eCLIP MATCHES▶HNRNPM (bg=6.37%)No matches to TargetScan


TTCT

TATTATCAAGTAAGATTCT  
Depth:5 (COW)  
Ei-value:0.000, Pi-value:0.000  
Er-value:0.000, Pr-value:0.000  
eCLIP MATCHES▶HNRNPM (bg=6.37%)▶srsf7 (bg=22.53%)MATCHES To TargetScan▶ miR-369-3p:AUAAUAC

-

TTTTCAGTT

TTTTCAGTT  
Depth:4 (PIG)  
Ei-value:0.000, Pi-value:0.000  
Er-value:0.000, Pr-value:0.000  
eCLIP MATCHES▶HNRNPM (bg=6.37%)▶ppil4 (bg=43.39%)▶SLBP (bg=6.66%)▶tia1 (bg=23.76%)▶tial1 (bg=15.02%)No matches to TargetScan


GTGT

TTTTCAGTTGTGT  
Depth:3 (DOG)  
Ei-value:0.000, Pi-value:0.000  
Er-value:0.000, Pr-value:0.000  
eCLIP MATCHES▶HNRNPM (bg=6.37%)▶ppil4 (bg=43.39%)▶safb2 (bg=26.89%)▶SLBP (bg=6.66%)▶tia1 (bg=23.76%)▶tial1 (bg=15.02%)No matches to TargetScan


G

TTTTCAGTTGTGTGTAAGCAAGTTTTT  
Depth:2 (MARMOSET)  
Ei-value:0.000, Pi-value:0.000  
Er-value:0.000, Pr-value:0.000  
eCLIP MATCHES▶HNRNPM (bg=6.37%)▶khsrp (bg=27.4%)▶ppil4 (bg=43.39%)▶safb (bg=40.39%)▶safb2 (bg=26.89%)▶SLBP (bg=6.66%)▶SUPV3L1 (bg=9.63%)▶tia1 (bg=23.76%)▶tial1 (bg=15.02%)MATCHES To TargetScan▶ miR-329-3p/362-3p:ACACACC


TAAGCAA

TAAGCAA  
Depth:6 (MOUSE)  
Ei-value:0.000, Pi-value:0.000  
Er-value:0.000, Pr-value:0.000  
eCLIP MATCHES▶HNRNPM (bg=6.37%)▶khsrp (bg=27.4%)▶ppil4 (bg=43.39%)▶safb2 (bg=26.89%)▶SLBP (bg=6.66%)▶tia1 (bg=23.76%)▶tial1 (bg=15.02%)No matches to TargetScan


GTTTTT

TTTTCAGTTGTGTGTAAGCAAGTTTTT  
Depth:2 (MARMOSET)  
Ei-value:0.000, Pi-value:0.000  
Er-value:0.000, Pr-value:0.000  
eCLIP MATCHES▶HNRNPM (bg=6.37%)▶khsrp (bg=27.4%)▶ppil4 (bg=43.39%)▶safb (bg=40.39%)▶safb2 (bg=26.89%)▶SLBP (bg=6.66%)▶SUPV3L1 (bg=9.63%)▶tia1 (bg=23.76%)▶tial1 (bg=15.02%)MATCHES To TargetScan▶ miR-329-3p/362-3p:ACACACC

-------

GTAGGA

GTAGGA  
Depth:7 (TURTLE)  
Ei-value:0.000, Pi-value:0.000  
Er-value:0.000, Pr-value:0.000  
eCLIP MATCHES▶hnrnpa1 (bg=18.32%)▶khsrp (bg=27.4%)▶ppil4 (bg=43.39%)▶safb (bg=40.39%)▶safb2 (bg=26.89%)▶SLBP (bg=6.66%)▶SUPV3L1 (bg=9.63%)▶tia1 (bg=23.76%)▶tial1 (bg=15.02%)▶zc3h8 (bg=12.78%)No matches to TargetScan


gaaata

gtaggagaaata  
Depth:2 (MARMOSET)  
Ei-value:0.990, Pi-value:0.000  
Er-value:0.000, Pr-value:0.000  
eCLIP MATCHES▶hnrnpa1 (bg=18.32%)▶khsrp (bg=27.4%)▶ppil4 (bg=43.39%)▶safb (bg=40.39%)▶safb2 (bg=26.89%)▶SLBP (bg=6.66%)▶SUPV3L1 (bg=9.63%)▶tia1 (bg=23.76%)▶tial1 (bg=15.02%)▶zc3h8 (bg=12.78%)No matches to TargetScan

-

TTTTCCATT

TTTTCCATT  
Depth:3 (DOG)  
Ei-value:0.000, Pi-value:0.000  
Er-value:0.000, Pr-value:0.000  
eCLIP MATCHES▶hnrnpa1 (bg=18.32%)▶khsrp (bg=27.4%)▶ppil4 (bg=43.39%)▶safb (bg=40.39%)▶safb2 (bg=26.89%)▶SLBP (bg=6.66%)▶SUPV3L1 (bg=9.63%)▶tia1 (bg=23.76%)▶tial1 (bg=15.02%)▶zc3h8 (bg=12.78%)No matches to TargetScan


gtt

ttttccattgtt  
Depth:2 (MARMOSET)  
Ei-value:0.990, Pi-value:0.000  
Er-value:0.000, Pr-value:0.000  
eCLIP MATCHES▶hnrnpa1 (bg=18.32%)▶khsrp (bg=27.4%)▶ppil4 (bg=43.39%)▶safb (bg=40.39%)▶safb2 (bg=26.89%)▶SLBP (bg=6.66%)▶SUPV3L1 (bg=9.63%)▶tia1 (bg=23.76%)▶tial1 (bg=15.02%)▶zc3h8 (bg=12.78%)No matches to TargetScan

-

AACTGCAAA

AACTGCAAA  
Depth:3 (DOG)  
Ei-value:0.000, Pi-value:0.000  
Er-value:0.000, Pr-value:0.000  
eCLIP MATCHES▶khsrp (bg=27.4%)▶ppil4 (bg=43.39%)▶safb (bg=40.39%)▶safb2 (bg=26.89%)▶SLBP (bg=6.66%)▶SUPV3L1 (bg=9.63%)▶tia1 (bg=23.76%)▶zc3h8 (bg=12.78%)MATCHES To TargetScan▶ miR-455-3p.2:UGCAGUC

-

CAAGATGT

CAAGATGT  
Depth:4 (PIG)  
Ei-value:0.000, Pi-value:0.000  
Er-value:0.000, Pr-value:0.000  
eCLIP MATCHES▶khsrp (bg=27.4%)▶ppil4 (bg=43.39%)▶safb (bg=40.39%)▶tia1 (bg=23.76%)No matches to TargetScan


T

CAAGATGTTAAGGTATGCTTCAAAA  
Depth:3 (DOG)  
Ei-value:0.000, Pi-value:0.000  
Er-value:0.000, Pr-value:0.000  
eCLIP MATCHES▶cpsf6 (bg=13.45%)▶khsrp (bg=27.4%)▶ppil4 (bg=43.39%)▶safb (bg=40.39%)▶tia1 (bg=23.76%)MATCHES To TargetScan▶ miR-875-5p:AUACCUC


AAGGTATGCTT

AAGGTATGCTT  
Depth:6 (MOUSE)  
Ei-value:0.000, Pi-value:0.000  
Er-value:0.000, Pr-value:0.000  
eCLIP MATCHES▶cpsf6 (bg=13.45%)▶khsrp (bg=27.4%)▶ppil4 (bg=43.39%)MATCHES To TargetScan▶ miR-875-5p:AUACCUC


CAAAA

AAGGTATGCTTCAAAA  
Depth:5 (COW)  
Ei-value:0.000, Pi-value:0.000  
Er-value:0.000, Pr-value:0.000  
eCLIP MATCHES▶cpsf6 (bg=13.45%)▶khsrp (bg=27.4%)▶ppil4 (bg=43.39%)MATCHES To TargetScan▶ miR-875-5p:AUACCUC

 3480  


AAGGTATGCTTCAAAA  
Depth:5 (COW)  
Ei-value:0.000, Pi-value:0.000  
Er-value:0.000, Pr-value:0.000  
eCLIP MATCHES▶cpsf6 (bg=13.45%)▶khsrp (bg=27.4%)▶ppil4 (bg=43.39%)MATCHES To TargetScan▶ miR-875-5p:AUACCUC


A

CAAGATGTTAAGGTATGCTTCAAAAA  
Depth:2 (MARMOSET)  
Ei-value:0.000, Pi-value:0.000  
Er-value:0.000, Pr-value:0.000  
eCLIP MATCHES▶cpsf6 (bg=13.45%)▶khsrp (bg=27.4%)▶ppil4 (bg=43.39%)▶safb (bg=40.39%)▶tia1 (bg=23.76%)MATCHES To TargetScan▶ miR-875-5p:AUACCUC▶ miR-129-5p:UUUUUGC

---

TGTAAAT

TGTAAAT  
Depth:5 (COW)  
Ei-value:0.000, Pi-value:0.010  
Er-value:0.000, Pr-value:0.000  
eCLIP MATCHES▶khsrp (bg=27.4%)No matches to TargetScan


T

TGTAAATT  
Depth:4 (PIG)  
Ei-value:0.000, Pi-value:0.000  
Er-value:0.000, Pr-value:0.000  
eCLIP MATCHES▶khsrp (bg=27.4%)No matches to TargetScan

---

TATTTTAA

TATTTTAA  
Depth:3 (DOG)  
Ei-value:0.010, Pi-value:0.010  
Er-value:0.000, Pr-value:0.000  
eCLIP MATCHES▶CPEB4 (bg=0.72%)▶khsrp (bg=27.4%)▶SFPQ (bg=2.48%)No matches to TargetScan


a

tattttaaa  
Depth:2 (MARMOSET)  
Ei-value:1.000, Pi-value:0.000  
Er-value:0.000, Pr-value:0.000  
eCLIP MATCHES▶CPEB4 (bg=0.72%)▶khsrp (bg=27.4%)▶SFPQ (bg=2.48%)No matches to TargetScan

--

tatctgt

tatctgt  
Depth:2 (MARMOSET)  
Ei-value:1.000, Pi-value:0.010  
Er-value:0.000, Pr-value:0.000  
eCLIP MATCHES▶CPEB4 (bg=0.72%)▶khsrp (bg=27.4%)▶SFPQ (bg=2.48%)No matches to TargetScan

----------

TAACTGATTAAGAATTGTGATAGTTCAGCTTGAATGTCTCTTAGA

TAACTGATTAAGAATTGTGATAGTTCAGCTTGAATGTCTCTTAGAGGGTGGGCTTTTGTTGATGAGGGAGGGGAAACTTTTTTTTT  
Depth:2 (MARMOSET)  
Ei-value:0.000, Pi-value:0.000  
Er-value:0.000, Pr-value:0.000  
eCLIP MATCHES▶CPEB4 (bg=0.72%)▶cpsf6 (bg=13.45%)▶ddx42 (bg=10.33%)▶HNRNPC (bg=4.22%)▶HNRNPM (bg=6.37%)▶khsrp (bg=27.4%)▶SFPQ (bg=2.48%)▶tia1 (bg=23.76%)▶u2af1 (bg=14.02%)▶u2af2 (bg=19.32%)▶ZRANB2 (bg=7.28%)MATCHES To TargetScan▶ miR-421:UCAACAG▶ miR-505-3p.2:UCAACAC▶ miR-181-5p:ACAUUCA▶ miR-495-3p:AACAAAC


GGGTGGG

GGGTGGG  
Depth:10 (SNAKE)  
Ei-value:0.000, Pi-value:0.000  
Er-value:0.000, Pr-value:0.000  
eCLIP MATCHES▶cpsf6 (bg=13.45%)▶HNRNPM (bg=6.37%)▶khsrp (bg=27.4%)▶SFPQ (bg=2.48%)No matches to TargetScan


CTTTTGTT

TAACTGATTAAGAATTGTGATAGTTCAGCTTGAATGTCTCTTAGAGGGTGGGCTTTTGTTGATGAGGGAGGGGAAACTTTTTTTTT  
Depth:2 (MARMOSET)  
Ei-value:0.000, Pi-value:0.000  
Er-value:0.000, Pr-value:0.000  
eCLIP MATCHES▶CPEB4 (bg=0.72%)▶cpsf6 (bg=13.45%)▶ddx42 (bg=10.33%)▶HNRNPC (bg=4.22%)▶HNRNPM (bg=6.37%)▶khsrp (bg=27.4%)▶SFPQ (bg=2.48%)▶tia1 (bg=23.76%)▶u2af1 (bg=14.02%)▶u2af2 (bg=19.32%)▶ZRANB2 (bg=7.28%)MATCHES To TargetScan▶ miR-421:UCAACAG▶ miR-505-3p.2:UCAACAC▶ miR-181-5p:ACAUUCA▶ miR-495-3p:AACAAAC


GATGAGGG

GATGAGGG  
Depth:3 (DOG)  
Ei-value:0.010, Pi-value:0.000  
Er-value:0.000, Pr-value:0.000  
eCLIP MATCHES▶HNRNPC (bg=4.22%)▶HNRNPM (bg=6.37%)▶khsrp (bg=27.4%)▶SFPQ (bg=2.48%)▶u2af1 (bg=14.02%)No matches to TargetScan


A

TAACTGATTAAGAATTGTGATAGTTCAGCTTGAATGTCTCTTAGAGGGTGGGCTTTTGTTGATGAGGGAGGGGAAACTTTTTTTTT  
Depth:2 (MARMOSET)  
Ei-value:0.000, Pi-value:0.000  
Er-value:0.000, Pr-value:0.000  
eCLIP MATCHES▶CPEB4 (bg=0.72%)▶cpsf6 (bg=13.45%)▶ddx42 (bg=10.33%)▶HNRNPC (bg=4.22%)▶HNRNPM (bg=6.37%)▶khsrp (bg=27.4%)▶SFPQ (bg=2.48%)▶tia1 (bg=23.76%)▶u2af1 (bg=14.02%)▶u2af2 (bg=19.32%)▶ZRANB2 (bg=7.28%)MATCHES To TargetScan▶ miR-421:UCAACAG▶ miR-505-3p.2:UCAACAC▶ miR-181-5p:ACAUUCA▶ miR-495-3p:AACAAAC


GGGGAAA

GGGGAAA  
Depth:6 (MOUSE)  
Ei-value:0.000, Pi-value:0.000  
Er-value:0.000, Pr-value:0.000  
eCLIP MATCHES▶HNRNPM (bg=6.37%)▶khsrp (bg=27.4%)▶u2af1 (bg=14.02%)▶u2af2 (bg=19.32%)No matches to TargetScan


C

CTTTTTTTT  
Depth:6 (MOUSE)  
Ei-value:0.000, Pi-value:0.000  
Er-value:0.000, Pr-value:0.000  
eCLIP MATCHES▶ddx42 (bg=10.33%)▶HNRNPM (bg=6.37%)▶khsrp (bg=27.4%)▶u2af1 (bg=14.02%)▶u2af2 (bg=19.32%)No matches to TargetScan

 3600  


TTTTTTTT

CTTTTTTTT  
Depth:6 (MOUSE)  
Ei-value:0.000, Pi-value:0.000  
Er-value:0.000, Pr-value:0.000  
eCLIP MATCHES▶ddx42 (bg=10.33%)▶HNRNPM (bg=6.37%)▶khsrp (bg=27.4%)▶u2af1 (bg=14.02%)▶u2af2 (bg=19.32%)No matches to TargetScan


T

CTTTTTTTTT  
Depth:3 (DOG)  
Ei-value:0.000, Pi-value:0.000  
Er-value:0.000, Pr-value:0.000  
eCLIP MATCHES▶ddx42 (bg=10.33%)▶HNRNPM (bg=6.37%)▶khsrp (bg=27.4%)▶u2af1 (bg=14.02%)▶u2af2 (bg=19.32%)No matches to TargetScan

-----

TAGACT

TAGACTTTTTTCAGATAA  
Depth:5 (COW)  
Ei-value:0.000, Pi-value:0.000  
Er-value:0.000, Pr-value:0.000  
eCLIP MATCHES▶ddx42 (bg=10.33%)▶ppil4 (bg=43.39%)▶safb (bg=40.39%)▶tia1 (bg=23.76%)▶u2af1 (bg=14.02%)▶u2af2 (bg=19.32%)No matches to TargetScan


TTTTTCAG

TTTTTCAG  
Depth:19 (ZEBRAFISH)  
Ei-value:0.000, Pi-value:0.000  
Er-value:0.000, Pr-value:0.000  
eCLIP MATCHES▶ddx42 (bg=10.33%)▶ppil4 (bg=43.39%)▶safb (bg=40.39%)▶u2af1 (bg=14.02%)▶u2af2 (bg=19.32%)No matches to TargetScan


AT

TTTTTCAGAT  
Depth:10 (SNAKE)  
Ei-value:0.000, Pi-value:0.000  
Er-value:0.000, Pr-value:0.000  
eCLIP MATCHES▶ddx42 (bg=10.33%)▶ppil4 (bg=43.39%)▶safb (bg=40.39%)▶u2af1 (bg=14.02%)▶u2af2 (bg=19.32%)No matches to TargetScan


AA

TTTTTCAGATAA  
Depth:6 (MOUSE)  
Ei-value:0.000, Pi-value:0.000  
Er-value:0.000, Pr-value:0.000  
eCLIP MATCHES▶ddx42 (bg=10.33%)▶ppil4 (bg=43.39%)▶safb (bg=40.39%)▶tia1 (bg=23.76%)▶u2af1 (bg=14.02%)▶u2af2 (bg=19.32%)No matches to TargetScan


CAT

TAGACTTTTTTCAGATAACATCTTCTGAGTCATAACCAGCCTGGCAGT  
Depth:2 (MARMOSET)  
Ei-value:0.000, Pi-value:0.000  
Er-value:0.000, Pr-value:0.000  
eCLIP MATCHES▶ddx42 (bg=10.33%)▶ppil4 (bg=43.39%)▶safb (bg=40.39%)▶tia1 (bg=23.76%)▶u2af1 (bg=14.02%)▶u2af2 (bg=19.32%)MATCHES To TargetScan▶ miR-138-5p:GCUGGUG


CT

CTTCTGAGTCATA  
Depth:5 (COW)  
Ei-value:0.000, Pi-value:0.000  
Er-value:0.000, Pr-value:0.000  
eCLIP MATCHES▶ppil4 (bg=43.39%)▶safb (bg=40.39%)▶tia1 (bg=23.76%)▶u2af1 (bg=14.02%)▶u2af2 (bg=19.32%)No matches to TargetScan


TCTGAGTCATA

TCTGAGTCATA  
Depth:6 (MOUSE)  
Ei-value:0.000, Pi-value:0.000  
Er-value:0.000, Pr-value:0.000  
eCLIP MATCHES▶ppil4 (bg=43.39%)▶safb (bg=40.39%)▶tia1 (bg=23.76%)▶u2af1 (bg=14.02%)▶u2af2 (bg=19.32%)No matches to TargetScan


A

CTTCTGAGTCATAACCAGCCTGGCA  
Depth:3 (DOG)  
Ei-value:0.000, Pi-value:0.000  
Er-value:0.000, Pr-value:0.000  
eCLIP MATCHES▶ppil4 (bg=43.39%)▶safb (bg=40.39%)▶tia1 (bg=23.76%)▶u2af1 (bg=14.02%)▶u2af2 (bg=19.32%)MATCHES To TargetScan▶ miR-138-5p:GCUGGUG


CCAGCCTGGCA

CCAGCCTGGCA  
Depth:6 (MOUSE)  
Ei-value:0.000, Pi-value:0.000  
Er-value:0.000, Pr-value:0.000  
eCLIP MATCHES▶ppil4 (bg=43.39%)▶safb (bg=40.39%)▶tia1 (bg=23.76%)▶u2af1 (bg=14.02%)▶u2af2 (bg=19.32%)No matches to TargetScan


GT

TAGACTTTTTTCAGATAACATCTTCTGAGTCATAACCAGCCTGGCAGT  
Depth:2 (MARMOSET)  
Ei-value:0.000, Pi-value:0.000  
Er-value:0.000, Pr-value:0.000  
eCLIP MATCHES▶ddx42 (bg=10.33%)▶ppil4 (bg=43.39%)▶safb (bg=40.39%)▶tia1 (bg=23.76%)▶u2af1 (bg=14.02%)▶u2af2 (bg=19.32%)MATCHES To TargetScan▶ miR-138-5p:GCUGGUG

-

TGATGG

TGATGGCCTAGATGCAGAGA  
Depth:2 (MARMOSET)  
Ei-value:0.000, Pi-value:0.000  
Er-value:0.000, Pr-value:0.000  
eCLIP MATCHES▶ppil4 (bg=43.39%)▶safb (bg=40.39%)▶tia1 (bg=23.76%)▶u2af1 (bg=14.02%)▶u2af2 (bg=19.32%)No matches to TargetScan


CC

CCTAGATG  
Depth:5 (COW)  
Ei-value:0.000, Pi-value:0.000  
Er-value:0.000, Pr-value:0.000  
eCLIP MATCHES▶ppil4 (bg=43.39%)▶safb (bg=40.39%)▶u2af1 (bg=14.02%)▶u2af2 (bg=19.32%)No matches to TargetScan


TAGATG

TAGATG  
Depth:6 (MOUSE)  
Ei-value:0.000, Pi-value:0.000  
Er-value:0.000, Pr-value:0.000  
eCLIP MATCHES▶ppil4 (bg=43.39%)▶safb (bg=40.39%)▶u2af1 (bg=14.02%)▶u2af2 (bg=19.32%)No matches to TargetScan


CAGAGA

TGATGGCCTAGATGCAGAGA  
Depth:2 (MARMOSET)  
Ei-value:0.000, Pi-value:0.000  
Er-value:0.000, Pr-value:0.000  
eCLIP MATCHES▶ppil4 (bg=43.39%)▶safb (bg=40.39%)▶tia1 (bg=23.76%)▶u2af1 (bg=14.02%)▶u2af2 (bg=19.32%)No matches to TargetScan

-

AACAGCTC

AACAGCTCCTTGGTGAA  
Depth:2 (MARMOSET)  
Ei-value:0.000, Pi-value:0.000  
Er-value:0.000, Pr-value:0.000  
eCLIP MATCHES▶ppil4 (bg=43.39%)▶u2af1 (bg=14.02%)▶u2af2 (bg=19.32%)MATCHES To TargetScan▶ miR-28-5p/708-5p:AGGAGCU


CTTGGTGAA

CTTGGTGAA  
Depth:6 (MOUSE)  
Ei-value:0.000, Pi-value:0.000  
Er-value:0.000, Pr-value:0.000  
eCLIP MATCHES▶ppil4 (bg=43.39%)▶u2af2 (bg=19.32%)No matches to TargetScan

-

TGATAAGT

TGATAAGT  
Depth:9 (LIZARD)  
Ei-value:0.000, Pi-value:0.000  
Er-value:0.000, Pr-value:0.000  
eCLIP MATCHES▶ppil4 (bg=43.39%)No matches to TargetScan


AAAGGCAGAA

AAAGGCAGAA  
Depth:13 (OPOSSUM)  
Ei-value:0.000, Pi-value:0.000  
Er-value:0.000, Pr-value:0.000  
eCLIP MATCHES▶hnrnpa1 (bg=18.32%)▶ppil4 (bg=43.39%)No matches to TargetScan

 3720  


AAAGGCAGAA  
Depth:13 (OPOSSUM)  
Ei-value:0.000, Pi-value:0.000  
Er-value:0.000, Pr-value:0.000  
eCLIP MATCHES▶hnrnpa1 (bg=18.32%)▶ppil4 (bg=43.39%)No matches to TargetScan


A

AAAGGCAGAAA  
Depth:11 (X.TROPICALIS)  
Ei-value:0.000, Pi-value:0.000  
Er-value:0.000, Pr-value:0.000  
eCLIP MATCHES▶hnrnpa1 (bg=18.32%)▶ppil4 (bg=43.39%)No matches to TargetScan


A

AAAGGCAGAAAA  
Depth:9 (LIZARD)  
Ei-value:0.000, Pi-value:0.000  
Er-value:0.000, Pr-value:0.000  
eCLIP MATCHES▶hnrnpa1 (bg=18.32%)▶ppil4 (bg=43.39%)No matches to TargetScan


GATT

TGATAAGTAAAGGCAGAAAAGATT  
Depth:5 (COW)  
Ei-value:0.000, Pi-value:0.000  
Er-value:0.000, Pr-value:0.000  
eCLIP MATCHES▶hnrnpa1 (bg=18.32%)▶khsrp (bg=27.4%)▶ppil4 (bg=43.39%)No matches to TargetScan


AT

TGATAAGTAAAGGCAGAAAAGATTAT  
Depth:4 (PIG)  
Ei-value:0.000, Pi-value:0.000  
Er-value:0.000, Pr-value:0.000  
eCLIP MATCHES▶hnrnpa1 (bg=18.32%)▶khsrp (bg=27.4%)▶ppil4 (bg=43.39%)No matches to TargetScan


ATG

TGATAAGTAAAGGCAGAAAAGATTATATGTCATACCTCCATTGGGGAATAAGCATAACCCTGAGATTCTTACTACTGATGA  
Depth:2 (MARMOSET)  
Ei-value:0.000, Pi-value:0.000  
Er-value:0.000, Pr-value:0.000  
eCLIP MATCHES▶hnrnpa1 (bg=18.32%)▶khsrp (bg=27.4%)▶ppil4 (bg=43.39%)▶safb2 (bg=26.89%)MATCHES To TargetScan▶ miR-489-3p:UGACAUC▶ miR-425-5p:AUGACAC▶ miR-296-3p:AGGGUUG▶ miR-410-3p:AUAUAAC▶ miR-216a-5p:AAUCUCA▶ miR-374-5p:UAUAAUA▶ miR-216b-5p:AAUCUCU▶ miR-199-3p:CAGUAGU▶ let-7-5p/98-5p:GAGGUAG


TCATACCT

TCATACCT  
Depth:5 (COW)  
Ei-value:0.000, Pi-value:0.000  
Er-value:0.000, Pr-value:0.000  
eCLIP MATCHES▶hnrnpa1 (bg=18.32%)▶khsrp (bg=27.4%)▶ppil4 (bg=43.39%)No matches to TargetScan


C

TCATACCTCCATTGGGGAA  
Depth:3 (DOG)  
Ei-value:0.000, Pi-value:0.000  
Er-value:0.000, Pr-value:0.000  
eCLIP MATCHES▶hnrnpa1 (bg=18.32%)▶khsrp (bg=27.4%)▶ppil4 (bg=43.39%)▶safb2 (bg=26.89%)MATCHES To TargetScan▶ let-7-5p/98-5p:GAGGUAG


CATTGGG

CATTGGG  
Depth:5 (COW)  
Ei-value:0.000, Pi-value:0.000  
Er-value:0.000, Pr-value:0.000  
eCLIP MATCHES▶hnrnpa1 (bg=18.32%)▶khsrp (bg=27.4%)▶ppil4 (bg=43.39%)▶safb2 (bg=26.89%)No matches to TargetScan


GAA

TCATACCTCCATTGGGGAA  
Depth:3 (DOG)  
Ei-value:0.000, Pi-value:0.000  
Er-value:0.000, Pr-value:0.000  
eCLIP MATCHES▶hnrnpa1 (bg=18.32%)▶khsrp (bg=27.4%)▶ppil4 (bg=43.39%)▶safb2 (bg=26.89%)MATCHES To TargetScan▶ let-7-5p/98-5p:GAGGUAG


T

TGATAAGTAAAGGCAGAAAAGATTATATGTCATACCTCCATTGGGGAATAAGCATAACCCTGAGATTCTTACTACTGATGA  
Depth:2 (MARMOSET)  
Ei-value:0.000, Pi-value:0.000  
Er-value:0.000, Pr-value:0.000  
eCLIP MATCHES▶hnrnpa1 (bg=18.32%)▶khsrp (bg=27.4%)▶ppil4 (bg=43.39%)▶safb2 (bg=26.89%)MATCHES To TargetScan▶ miR-489-3p:UGACAUC▶ miR-425-5p:AUGACAC▶ miR-296-3p:AGGGUUG▶ miR-410-3p:AUAUAAC▶ miR-216a-5p:AAUCUCA▶ miR-374-5p:UAUAAUA▶ miR-216b-5p:AAUCUCU▶ miR-199-3p:CAGUAGU▶ let-7-5p/98-5p:GAGGUAG


AAGCATAAC

AAGCATAAC  
Depth:5 (COW)  
Ei-value:0.000, Pi-value:0.000  
Er-value:0.000, Pr-value:0.000  
eCLIP MATCHES▶hnrnpa1 (bg=18.32%)▶ppil4 (bg=43.39%)No matches to TargetScan


CCTGAGAT

AAGCATAACCCTGAGAT  
Depth:3 (DOG)  
Ei-value:0.000, Pi-value:0.000  
Er-value:0.000, Pr-value:0.000  
eCLIP MATCHES▶hnrnpa1 (bg=18.32%)▶ppil4 (bg=43.39%)MATCHES To TargetScan▶ miR-296-3p:AGGGUUG


TCTT

TGATAAGTAAAGGCAGAAAAGATTATATGTCATACCTCCATTGGGGAATAAGCATAACCCTGAGATTCTTACTACTGATGA  
Depth:2 (MARMOSET)  
Ei-value:0.000, Pi-value:0.000  
Er-value:0.000, Pr-value:0.000  
eCLIP MATCHES▶hnrnpa1 (bg=18.32%)▶khsrp (bg=27.4%)▶ppil4 (bg=43.39%)▶safb2 (bg=26.89%)MATCHES To TargetScan▶ miR-489-3p:UGACAUC▶ miR-425-5p:AUGACAC▶ miR-296-3p:AGGGUUG▶ miR-410-3p:AUAUAAC▶ miR-216a-5p:AAUCUCA▶ miR-374-5p:UAUAAUA▶ miR-216b-5p:AAUCUCU▶ miR-199-3p:CAGUAGU▶ let-7-5p/98-5p:GAGGUAG


ACTA

ACTACTGATGA  
Depth:4 (PIG)  
Ei-value:0.000, Pi-value:0.000  
Er-value:0.000, Pr-value:0.000  
eCLIP MATCHES▶hnrnpa1 (bg=18.32%)▶ppil4 (bg=43.39%)MATCHES To TargetScan▶ miR-199-3p:CAGUAGU


CTGATGA

CTGATGA  
Depth:6 (MOUSE)  
Ei-value:0.000, Pi-value:0.000  
Er-value:0.000, Pr-value:0.000  
eCLIP MATCHES▶hnrnpa1 (bg=18.32%)▶ppil4 (bg=43.39%)No matches to TargetScan

-----

TTA

TTATCTGCATATGC  
Depth:3 (DOG)  
Ei-value:0.000, Pi-value:0.000  
Er-value:0.000, Pr-value:0.000  
eCLIP MATCHES▶ppil4 (bg=43.39%)No matches to TargetScan


TCTGCA

TCTGCA  
Depth:5 (COW)  
Ei-value:0.000, Pi-value:0.000  
Er-value:0.000, Pr-value:0.000  
eCLIP MATCHES▶ppil4 (bg=43.39%)No matches to TargetScan


TATGC

TCTGCATATGC  
Depth:4 (PIG)  
Ei-value:0.000, Pi-value:0.000  
Er-value:0.000, Pr-value:0.000  
eCLIP MATCHES▶ppil4 (bg=43.39%)No matches to TargetScan


CAAAAAA

TTATCTGCATATGCCAAAAAA  
Depth:2 (MARMOSET)  
Ei-value:0.000, Pi-value:0.000  
Er-value:0.000, Pr-value:0.000  
eCLIP MATCHES▶ppil4 (bg=43.39%)MATCHES To TargetScan▶ miR-129-5p:UUUUUGC▶ miR-182-5p:UUGGCAA▶ miR-96-5p/1271-5p:UUGGCAC

--

TTAAGCAA

TTAAGCAA  
Depth:4 (PIG)  
Ei-value:0.000, Pi-value:0.020  
Er-value:0.000, Pr-value:0.000  
eCLIP MATCHES▶safb (bg=40.39%)No matches to TargetScan


ATGAAA

TTAAGCAAATGAAA  
Depth:2 (MARMOSET)  
Ei-value:0.020, Pi-value:0.000  
Er-value:0.000, Pr-value:0.000  
eCLIP MATCHES▶safb (bg=40.39%)No matches to TargetScan

-

CTACCAATT

CTACCAATTTAAAGTTA  
Depth:5 (COW)  
Ei-value:0.000, Pi-value:0.000  
Er-value:0.000, Pr-value:0.000  
eCLIP MATCHES▶HNRNPU (bg=9.45%)▶ppil4 (bg=43.39%)▶safb (bg=40.39%)No matches to TargetScan


TAAAG

TAAAGTTA  
Depth:6 (MOUSE)  
Ei-value:0.000, Pi-value:0.000  
Er-value:0.000, Pr-value:0.000  
eCLIP MATCHES▶HNRNPU (bg=9.45%)▶ppil4 (bg=43.39%)▶safb (bg=40.39%)No matches to TargetScan

 3840  


TTA

TAAAGTTA  
Depth:6 (MOUSE)  
Ei-value:0.000, Pi-value:0.000  
Er-value:0.000, Pr-value:0.000  
eCLIP MATCHES▶HNRNPU (bg=9.45%)▶ppil4 (bg=43.39%)▶safb (bg=40.39%)No matches to TargetScan


C

CTACCAATTTAAAGTTACGGAATCTACCATTTTAAAGTTAATTGCTTGTCAAGCTATAAC  
Depth:2 (MARMOSET)  
Ei-value:0.000, Pi-value:0.000  
Er-value:0.000, Pr-value:0.000  
eCLIP MATCHES▶HNRNPU (bg=9.45%)▶khdrbs1 (bg=10.41%)▶ppil4 (bg=43.39%)▶safb (bg=40.39%)▶safb2 (bg=26.89%)MATCHES To TargetScan▶ miR-379-5p:GGUAGAC


GGAATCTAC

GGAATCTAC  
Depth:5 (COW)  
Ei-value:0.000, Pi-value:0.000  
Er-value:0.000, Pr-value:0.000  
eCLIP MATCHES▶HNRNPU (bg=9.45%)▶ppil4 (bg=43.39%)▶safb (bg=40.39%)▶safb2 (bg=26.89%)No matches to TargetScan


CATTT

GGAATCTACCATTT  
Depth:3 (DOG)  
Ei-value:0.000, Pi-value:0.000  
Er-value:0.000, Pr-value:0.000  
eCLIP MATCHES▶HNRNPU (bg=9.45%)▶khdrbs1 (bg=10.41%)▶ppil4 (bg=43.39%)▶safb (bg=40.39%)▶safb2 (bg=26.89%)MATCHES To TargetScan▶ miR-379-5p:GGUAGAC


T

CTACCAATTTAAAGTTACGGAATCTACCATTTTAAAGTTAATTGCTTGTCAAGCTATAAC  
Depth:2 (MARMOSET)  
Ei-value:0.000, Pi-value:0.000  
Er-value:0.000, Pr-value:0.000  
eCLIP MATCHES▶HNRNPU (bg=9.45%)▶khdrbs1 (bg=10.41%)▶ppil4 (bg=43.39%)▶safb (bg=40.39%)▶safb2 (bg=26.89%)MATCHES To TargetScan▶ miR-379-5p:GGUAGAC


AAAGTTA

AAAGTTA  
Depth:6 (MOUSE)  
Ei-value:0.000, Pi-value:0.000  
Er-value:0.000, Pr-value:0.000  
eCLIP MATCHES▶HNRNPU (bg=9.45%)▶khdrbs1 (bg=10.41%)▶ppil4 (bg=43.39%)▶safb (bg=40.39%)▶safb2 (bg=26.89%)No matches to TargetScan


ATTGCTTG

CTACCAATTTAAAGTTACGGAATCTACCATTTTAAAGTTAATTGCTTGTCAAGCTATAAC  
Depth:2 (MARMOSET)  
Ei-value:0.000, Pi-value:0.000  
Er-value:0.000, Pr-value:0.000  
eCLIP MATCHES▶HNRNPU (bg=9.45%)▶khdrbs1 (bg=10.41%)▶ppil4 (bg=43.39%)▶safb (bg=40.39%)▶safb2 (bg=26.89%)MATCHES To TargetScan▶ miR-379-5p:GGUAGAC


T

TCAAGCT  
Depth:5 (COW)  
Ei-value:0.000, Pi-value:0.000  
Er-value:0.000, Pr-value:0.000  
eCLIP MATCHES▶HNRNPU (bg=9.45%)▶khdrbs1 (bg=10.41%)▶ppil4 (bg=43.39%)▶safb (bg=40.39%)▶safb2 (bg=26.89%)No matches to TargetScan


CAAGCT

CAAGCT  
Depth:6 (MOUSE)  
Ei-value:0.000, Pi-value:0.000  
Er-value:0.000, Pr-value:0.000  
eCLIP MATCHES▶HNRNPU (bg=9.45%)▶khdrbs1 (bg=10.41%)▶ppil4 (bg=43.39%)▶safb (bg=40.39%)▶safb2 (bg=26.89%)No matches to TargetScan


ATA

TCAAGCTATA  
Depth:3 (DOG)  
Ei-value:0.000, Pi-value:0.000  
Er-value:0.000, Pr-value:0.000  
eCLIP MATCHES▶HNRNPU (bg=9.45%)▶khdrbs1 (bg=10.41%)▶ppil4 (bg=43.39%)▶safb (bg=40.39%)▶safb2 (bg=26.89%)No matches to TargetScan


AC

CTACCAATTTAAAGTTACGGAATCTACCATTTTAAAGTTAATTGCTTGTCAAGCTATAAC  
Depth:2 (MARMOSET)  
Ei-value:0.000, Pi-value:0.000  
Er-value:0.000, Pr-value:0.000  
eCLIP MATCHES▶HNRNPU (bg=9.45%)▶khdrbs1 (bg=10.41%)▶ppil4 (bg=43.39%)▶safb (bg=40.39%)▶safb2 (bg=26.89%)MATCHES To TargetScan▶ miR-379-5p:GGUAGAC

-

ACAAAA

ACAAAAATAATGAATTGATGAGAAATACAATGAAGA  
Depth:2 (MARMOSET)  
Ei-value:0.000, Pi-value:0.000  
Er-value:0.000, Pr-value:0.000  
eCLIP MATCHES▶hltf (bg=24.28%)▶HNRNPU (bg=9.45%)▶khdrbs1 (bg=10.41%)▶ppil4 (bg=43.39%)▶safb (bg=40.39%)▶safb2 (bg=26.89%)MATCHES To TargetScan▶ miR-129-5p:UUUUUGC


ATAATGAAT

ATAATGAAT  
Depth:5 (COW)  
Ei-value:0.000, Pi-value:0.000  
Er-value:0.000, Pr-value:0.000  
eCLIP MATCHES▶HNRNPU (bg=9.45%)▶khdrbs1 (bg=10.41%)▶ppil4 (bg=43.39%)▶safb (bg=40.39%)▶safb2 (bg=26.89%)No matches to TargetScan


TGATGA

TGATGA  
Depth:6 (MOUSE)  
Ei-value:0.000, Pi-value:0.030  
Er-value:0.000, Pr-value:0.000  
eCLIP MATCHES▶khdrbs1 (bg=10.41%)▶ppil4 (bg=43.39%)▶safb2 (bg=26.89%)No matches to TargetScan


GAAATA

TGATGAGAAATA  
Depth:4 (PIG)  
Ei-value:0.000, Pi-value:0.000  
Er-value:0.000, Pr-value:0.000  
eCLIP MATCHES▶khdrbs1 (bg=10.41%)▶ppil4 (bg=43.39%)▶safb2 (bg=26.89%)No matches to TargetScan


CAATGAAGA

ACAAAAATAATGAATTGATGAGAAATACAATGAAGA  
Depth:2 (MARMOSET)  
Ei-value:0.000, Pi-value:0.000  
Er-value:0.000, Pr-value:0.000  
eCLIP MATCHES▶hltf (bg=24.28%)▶HNRNPU (bg=9.45%)▶khdrbs1 (bg=10.41%)▶ppil4 (bg=43.39%)▶safb (bg=40.39%)▶safb2 (bg=26.89%)MATCHES To TargetScan▶ miR-129-5p:UUUUUGC

--

CAATGT

CAATGTCCATCTCAAAATACTGCTTTTACAAAAGCAGAATAAAA  
Depth:2 (MARMOSET)  
Ei-value:0.000, Pi-value:0.000  
Er-value:0.000, Pr-value:0.000  
eCLIP MATCHES▶hltf (bg=24.28%)▶khdrbs1 (bg=10.41%)▶larp4 (bg=13.51%)▶ppil4 (bg=43.39%)▶safb2 (bg=26.89%)▶znf622 (bg=18.79%)MATCHES To TargetScan▶ miR-330-3p.2:AAAGCAC▶ miR-143-3p:GAGAUGA


CCAT

CCATCTCAAAATACTGCTTTTACAAAAGCAGAATAAAA  
Depth:3 (DOG)  
Ei-value:0.000, Pi-value:0.000  
Er-value:0.000, Pr-value:0.000  
eCLIP MATCHES▶khdrbs1 (bg=10.41%)▶larp4 (bg=13.51%)▶ppil4 (bg=43.39%)▶safb2 (bg=26.89%)▶znf622 (bg=18.79%)MATCHES To TargetScan▶ miR-330-3p.2:AAAGCAC▶ miR-143-3p:GAGAUGA


CTCAA

CTCAAAATACTGCTTTTACAAAAGCAGAATAAAA  
Depth:4 (PIG)  
Ei-value:0.000, Pi-value:0.000  
Er-value:0.000, Pr-value:0.000  
eCLIP MATCHES▶khdrbs1 (bg=10.41%)▶larp4 (bg=13.51%)▶ppil4 (bg=43.39%)▶safb2 (bg=26.89%)▶znf622 (bg=18.79%)MATCHES To TargetScan▶ miR-330-3p.2:AAAGCAC


AAT

AATACTGCTTTTACAAAAGCAGAAT  
Depth:5 (COW)  
Ei-value:0.000, Pi-value:0.000  
Er-value:0.000, Pr-value:0.000  
eCLIP MATCHES▶khdrbs1 (bg=10.41%)▶larp4 (bg=13.51%)▶ppil4 (bg=43.39%)▶safb2 (bg=26.89%)▶znf622 (bg=18.79%)MATCHES To TargetScan▶ miR-330-3p.2:AAAGCAC


ACTGCTTT

ACTGCTTTTACAAAAGCAGAAT  
Depth:6 (MOUSE)  
Ei-value:0.000, Pi-value:0.000  
Er-value:0.000, Pr-value:0.000  
eCLIP MATCHES▶khdrbs1 (bg=10.41%)▶larp4 (bg=13.51%)▶ppil4 (bg=43.39%)▶safb2 (bg=26.89%)▶znf622 (bg=18.79%)MATCHES To TargetScan▶ miR-330-3p.2:AAAGCAC


TACAAAA

TACAAAA  
Depth:7 (TURTLE)  
Ei-value:0.000, Pi-value:0.000  
Er-value:0.000, Pr-value:0.000  
eCLIP MATCHES▶khdrbs1 (bg=10.41%)▶larp4 (bg=13.51%)▶safb2 (bg=26.89%)▶znf622 (bg=18.79%)No matches to TargetScan


G

ACTGCTTTTACAAAAGCAGAAT  
Depth:6 (MOUSE)  
Ei-value:0.000, Pi-value:0.000  
Er-value:0.000, Pr-value:0.000  
eCLIP MATCHES▶khdrbs1 (bg=10.41%)▶larp4 (bg=13.51%)▶ppil4 (bg=43.39%)▶safb2 (bg=26.89%)▶znf622 (bg=18.79%)MATCHES To TargetScan▶ miR-330-3p.2:AAAGCAC


C

CAGAAT  
Depth:8 (ALLIGATOR)  
Ei-value:0.000, Pi-value:0.000  
Er-value:0.000, Pr-value:0.000  
eCLIP MATCHES▶khdrbs1 (bg=10.41%)▶larp4 (bg=13.51%)▶safb2 (bg=26.89%)▶znf622 (bg=18.79%)No matches to TargetScan

 3960  


AGAAT

CAGAAT  
Depth:8 (ALLIGATOR)  
Ei-value:0.000, Pi-value:0.000  
Er-value:0.000, Pr-value:0.000  
eCLIP MATCHES▶khdrbs1 (bg=10.41%)▶larp4 (bg=13.51%)▶safb2 (bg=26.89%)▶znf622 (bg=18.79%)No matches to TargetScan


AAAA

CTCAAAATACTGCTTTTACAAAAGCAGAATAAAA  
Depth:4 (PIG)  
Ei-value:0.000, Pi-value:0.000  
Er-value:0.000, Pr-value:0.000  
eCLIP MATCHES▶khdrbs1 (bg=10.41%)▶larp4 (bg=13.51%)▶ppil4 (bg=43.39%)▶safb2 (bg=26.89%)▶znf622 (bg=18.79%)MATCHES To TargetScan▶ miR-330-3p.2:AAAGCAC

-------

g

gaaatgaaa  
Depth:2 (MARMOSET)  
Ei-value:1.000, Pi-value:0.000  
Er-value:0.000, Pr-value:0.000  
eCLIP MATCHES▶khdrbs1 (bg=10.41%)▶larp4 (bg=13.51%)▶NIPBL (bg=8.2%)▶znf622 (bg=18.79%)No matches to TargetScan


AAATGAA

AAATGAA  
Depth:6 (MOUSE)  
Ei-value:0.000, Pi-value:0.000  
Er-value:0.000, Pr-value:0.000  
eCLIP MATCHES▶khdrbs1 (bg=10.41%)▶larp4 (bg=13.51%)▶NIPBL (bg=8.2%)▶znf622 (bg=18.79%)No matches to TargetScan


A

AAATGAAA  
Depth:5 (COW)  
Ei-value:0.000, Pi-value:0.000  
Er-value:0.000, Pr-value:0.000  
eCLIP MATCHES▶khdrbs1 (bg=10.41%)▶larp4 (bg=13.51%)▶NIPBL (bg=8.2%)▶znf622 (bg=18.79%)No matches to TargetScan

-------

ACT

ACTACATTAATCCTGGAATAAAAGAAGCCGAAATA  
Depth:2 (MARMOSET)  
Ei-value:0.000, Pi-value:0.000  
Er-value:0.000, Pr-value:0.000  
eCLIP MATCHES▶bclaf1 (bg=17.67%)▶cpsf6 (bg=13.45%)▶khdrbs1 (bg=10.41%)▶khsrp (bg=27.4%)▶larp4 (bg=13.51%)▶NIPBL (bg=8.2%)▶ppil4 (bg=43.39%)▶safb (bg=40.39%)▶safb2 (bg=26.89%)▶srsf1 (bg=30.28%)▶srsf7 (bg=22.53%)▶SUPV3L1 (bg=9.63%)▶uchl5 (bg=18.56%)MATCHES To TargetScan▶ miR-665:CCAGGAG


ACATTAA

ACATTAA  
Depth:4 (PIG)  
Ei-value:0.000, Pi-value:0.010  
Er-value:0.000, Pr-value:0.000  
eCLIP MATCHES▶bclaf1 (bg=17.67%)▶cpsf6 (bg=13.45%)▶khdrbs1 (bg=10.41%)▶khsrp (bg=27.4%)▶larp4 (bg=13.51%)▶NIPBL (bg=8.2%)▶safb2 (bg=26.89%)▶srsf1 (bg=30.28%)▶srsf7 (bg=22.53%)▶uchl5 (bg=18.56%)No matches to TargetScan


T

ACATTAATCCTGGAATAAAAGAAGC  
Depth:3 (DOG)  
Ei-value:0.000, Pi-value:0.000  
Er-value:0.000, Pr-value:0.000  
eCLIP MATCHES▶bclaf1 (bg=17.67%)▶cpsf6 (bg=13.45%)▶khdrbs1 (bg=10.41%)▶khsrp (bg=27.4%)▶larp4 (bg=13.51%)▶NIPBL (bg=8.2%)▶ppil4 (bg=43.39%)▶safb (bg=40.39%)▶safb2 (bg=26.89%)▶srsf1 (bg=30.28%)▶srsf7 (bg=22.53%)▶SUPV3L1 (bg=9.63%)▶uchl5 (bg=18.56%)MATCHES To TargetScan▶ miR-665:CCAGGAG


CCTGGA

CCTGGA  
Depth:6 (MOUSE)  
Ei-value:0.000, Pi-value:0.000  
Er-value:0.000, Pr-value:0.000  
eCLIP MATCHES▶bclaf1 (bg=17.67%)▶cpsf6 (bg=13.45%)▶khdrbs1 (bg=10.41%)▶khsrp (bg=27.4%)▶larp4 (bg=13.51%)▶NIPBL (bg=8.2%)▶ppil4 (bg=43.39%)▶safb (bg=40.39%)▶safb2 (bg=26.89%)▶srsf1 (bg=30.28%)▶srsf7 (bg=22.53%)▶uchl5 (bg=18.56%)No matches to TargetScan


A

CCTGGAA  
Depth:5 (COW)  
Ei-value:0.000, Pi-value:0.000  
Er-value:0.000, Pr-value:0.000  
eCLIP MATCHES▶bclaf1 (bg=17.67%)▶cpsf6 (bg=13.45%)▶khdrbs1 (bg=10.41%)▶khsrp (bg=27.4%)▶larp4 (bg=13.51%)▶NIPBL (bg=8.2%)▶ppil4 (bg=43.39%)▶safb (bg=40.39%)▶safb2 (bg=26.89%)▶srsf1 (bg=30.28%)▶srsf7 (bg=22.53%)▶uchl5 (bg=18.56%)No matches to TargetScan


T

CCTGGAATAAAAGAAGC  
Depth:4 (PIG)  
Ei-value:0.000, Pi-value:0.000  
Er-value:0.000, Pr-value:0.000  
eCLIP MATCHES▶bclaf1 (bg=17.67%)▶cpsf6 (bg=13.45%)▶khdrbs1 (bg=10.41%)▶khsrp (bg=27.4%)▶larp4 (bg=13.51%)▶NIPBL (bg=8.2%)▶ppil4 (bg=43.39%)▶safb (bg=40.39%)▶safb2 (bg=26.89%)▶srsf1 (bg=30.28%)▶srsf7 (bg=22.53%)▶SUPV3L1 (bg=9.63%)▶uchl5 (bg=18.56%)No matches to TargetScan


AAAAGAAGC

AAAAGAAGC  
Depth:6 (MOUSE)  
Ei-value:0.000, Pi-value:0.000  
Er-value:0.000, Pr-value:0.000  
eCLIP MATCHES▶bclaf1 (bg=17.67%)▶cpsf6 (bg=13.45%)▶khdrbs1 (bg=10.41%)▶khsrp (bg=27.4%)▶larp4 (bg=13.51%)▶NIPBL (bg=8.2%)▶ppil4 (bg=43.39%)▶safb (bg=40.39%)▶safb2 (bg=26.89%)▶srsf1 (bg=30.28%)▶srsf7 (bg=22.53%)▶SUPV3L1 (bg=9.63%)▶uchl5 (bg=18.56%)No matches to TargetScan


CGAAATA

ACTACATTAATCCTGGAATAAAAGAAGCCGAAATA  
Depth:2 (MARMOSET)  
Ei-value:0.000, Pi-value:0.000  
Er-value:0.000, Pr-value:0.000  
eCLIP MATCHES▶bclaf1 (bg=17.67%)▶cpsf6 (bg=13.45%)▶khdrbs1 (bg=10.41%)▶khsrp (bg=27.4%)▶larp4 (bg=13.51%)▶NIPBL (bg=8.2%)▶ppil4 (bg=43.39%)▶safb (bg=40.39%)▶safb2 (bg=26.89%)▶srsf1 (bg=30.28%)▶srsf7 (bg=22.53%)▶SUPV3L1 (bg=9.63%)▶uchl5 (bg=18.56%)MATCHES To TargetScan▶ miR-665:CCAGGAG

----

AGAGATGAGT

AGAGATGAGTTGGGATCAAGTGGATTGAGGAGGCTGTGCTGTGTGCCAAT  
Depth:2 (MARMOSET)  
Ei-value:0.000, Pi-value:0.000  
Er-value:0.000, Pr-value:0.000  
eCLIP MATCHES▶bclaf1 (bg=17.67%)▶DGCR8 (bg=2.67%)▶hltf (bg=24.28%)▶khdrbs1 (bg=10.41%)▶khsrp (bg=27.4%)▶larp4 (bg=13.51%)▶LIN28B (bg=1.31%)▶NIPBL (bg=8.2%)▶ppil4 (bg=43.39%)▶rbm15 (bg=11.59%)▶safb (bg=40.39%)▶safb2 (bg=26.89%)▶srsf1 (bg=30.28%)▶srsf7 (bg=22.53%)▶SUPV3L1 (bg=9.63%)▶uchl5 (bg=18.56%)MATCHES To TargetScan▶ miR-182-5p:UUGGCAA▶ miR-183-5p.2:UGGCACU▶ miR-96-5p/1271-5p:UUGGCAC


TGGGATC

TGGGATC  
Depth:8 (ALLIGATOR)  
Ei-value:0.000, Pi-value:0.000  
Er-value:0.000, Pr-value:0.000  
eCLIP MATCHES▶bclaf1 (bg=17.67%)▶DGCR8 (bg=2.67%)▶hltf (bg=24.28%)▶khdrbs1 (bg=10.41%)▶khsrp (bg=27.4%)▶larp4 (bg=13.51%)▶NIPBL (bg=8.2%)▶ppil4 (bg=43.39%)▶rbm15 (bg=11.59%)▶safb (bg=40.39%)▶safb2 (bg=26.89%)▶srsf1 (bg=30.28%)▶srsf7 (bg=22.53%)▶SUPV3L1 (bg=9.63%)▶uchl5 (bg=18.56%)No matches to TargetScan


AA

TGGGATCAA  
Depth:5 (COW)  
Ei-value:0.000, Pi-value:0.000  
Er-value:0.000, Pr-value:0.000  
eCLIP MATCHES▶bclaf1 (bg=17.67%)▶DGCR8 (bg=2.67%)▶hltf (bg=24.28%)▶khdrbs1 (bg=10.41%)▶khsrp (bg=27.4%)▶larp4 (bg=13.51%)▶NIPBL (bg=8.2%)▶ppil4 (bg=43.39%)▶rbm15 (bg=11.59%)▶safb (bg=40.39%)▶safb2 (bg=26.89%)▶srsf1 (bg=30.28%)▶srsf7 (bg=22.53%)▶SUPV3L1 (bg=9.63%)▶uchl5 (bg=18.56%)No matches to TargetScan


G

AGAGATGAGTTGGGATCAAGTGGATTGAGGAGGCTGTGCTGTGTGCCAAT  
Depth:2 (MARMOSET)  
Ei-value:0.000, Pi-value:0.000  
Er-value:0.000, Pr-value:0.000  
eCLIP MATCHES▶bclaf1 (bg=17.67%)▶DGCR8 (bg=2.67%)▶hltf (bg=24.28%)▶khdrbs1 (bg=10.41%)▶khsrp (bg=27.4%)▶larp4 (bg=13.51%)▶LIN28B (bg=1.31%)▶NIPBL (bg=8.2%)▶ppil4 (bg=43.39%)▶rbm15 (bg=11.59%)▶safb (bg=40.39%)▶safb2 (bg=26.89%)▶srsf1 (bg=30.28%)▶srsf7 (bg=22.53%)▶SUPV3L1 (bg=9.63%)▶uchl5 (bg=18.56%)MATCHES To TargetScan▶ miR-182-5p:UUGGCAA▶ miR-183-5p.2:UGGCACU▶ miR-96-5p/1271-5p:UUGGCAC


TGG

TGGATTGAG  
Depth:4 (PIG)  
Ei-value:0.000, Pi-value:0.000  
Er-value:0.000, Pr-value:0.000  
eCLIP MATCHES▶bclaf1 (bg=17.67%)▶DGCR8 (bg=2.67%)▶hltf (bg=24.28%)▶khdrbs1 (bg=10.41%)▶khsrp (bg=27.4%)▶larp4 (bg=13.51%)▶NIPBL (bg=8.2%)▶ppil4 (bg=43.39%)▶rbm15 (bg=11.59%)▶safb (bg=40.39%)▶safb2 (bg=26.89%)▶srsf1 (bg=30.28%)▶srsf7 (bg=22.53%)▶SUPV3L1 (bg=9.63%)▶uchl5 (bg=18.56%)No matches to TargetScan


ATTGAG

ATTGAG  
Depth:5 (COW)  
Ei-value:0.000, Pi-value:0.030  
Er-value:0.000, Pr-value:0.000  
eCLIP MATCHES▶bclaf1 (bg=17.67%)▶DGCR8 (bg=2.67%)▶hltf (bg=24.28%)▶khdrbs1 (bg=10.41%)▶khsrp (bg=27.4%)▶larp4 (bg=13.51%)▶NIPBL (bg=8.2%)▶ppil4 (bg=43.39%)▶rbm15 (bg=11.59%)▶safb (bg=40.39%)▶safb2 (bg=26.89%)▶srsf1 (bg=30.28%)▶srsf7 (bg=22.53%)▶SUPV3L1 (bg=9.63%)▶uchl5 (bg=18.56%)No matches to TargetScan


G

AGAGATGAGTTGGGATCAAGTGGATTGAGGAGGCTGTGCTGTGTGCCAAT  
Depth:2 (MARMOSET)  
Ei-value:0.000, Pi-value:0.000  
Er-value:0.000, Pr-value:0.000  
eCLIP MATCHES▶bclaf1 (bg=17.67%)▶DGCR8 (bg=2.67%)▶hltf (bg=24.28%)▶khdrbs1 (bg=10.41%)▶khsrp (bg=27.4%)▶larp4 (bg=13.51%)▶LIN28B (bg=1.31%)▶NIPBL (bg=8.2%)▶ppil4 (bg=43.39%)▶rbm15 (bg=11.59%)▶safb (bg=40.39%)▶safb2 (bg=26.89%)▶srsf1 (bg=30.28%)▶srsf7 (bg=22.53%)▶SUPV3L1 (bg=9.63%)▶uchl5 (bg=18.56%)MATCHES To TargetScan▶ miR-182-5p:UUGGCAA▶ miR-183-5p.2:UGGCACU▶ miR-96-5p/1271-5p:UUGGCAC


AG

AGGCTGTGCTGT  
Depth:3 (DOG)  
Ei-value:0.000, Pi-value:0.000  
Er-value:0.000, Pr-value:0.000  
eCLIP MATCHES▶bclaf1 (bg=17.67%)▶DGCR8 (bg=2.67%)▶hltf (bg=24.28%)▶khdrbs1 (bg=10.41%)▶khsrp (bg=27.4%)▶larp4 (bg=13.51%)▶NIPBL (bg=8.2%)▶ppil4 (bg=43.39%)▶rbm15 (bg=11.59%)▶safb (bg=40.39%)▶safb2 (bg=26.89%)▶srsf1 (bg=30.28%)▶srsf7 (bg=22.53%)▶SUPV3L1 (bg=9.63%)▶uchl5 (bg=18.56%)No matches to TargetScan


GCTGTGCTGT

GCTGTGCTGT  
Depth:5 (COW)  
Ei-value:0.000, Pi-value:0.000  
Er-value:0.000, Pr-value:0.000  
eCLIP MATCHES▶bclaf1 (bg=17.67%)▶DGCR8 (bg=2.67%)▶hltf (bg=24.28%)▶khdrbs1 (bg=10.41%)▶khsrp (bg=27.4%)▶larp4 (bg=13.51%)▶NIPBL (bg=8.2%)▶ppil4 (bg=43.39%)▶rbm15 (bg=11.59%)▶safb (bg=40.39%)▶safb2 (bg=26.89%)▶srsf1 (bg=30.28%)▶srsf7 (bg=22.53%)▶SUPV3L1 (bg=9.63%)▶uchl5 (bg=18.56%)No matches to TargetScan


G

AGAGATGAGTTGGGATCAAGTGGATTGAGGAGGCTGTGCTGTGTGCCAAT  
Depth:2 (MARMOSET)  
Ei-value:0.000, Pi-value:0.000  
Er-value:0.000, Pr-value:0.000  
eCLIP MATCHES▶bclaf1 (bg=17.67%)▶DGCR8 (bg=2.67%)▶hltf (bg=24.28%)▶khdrbs1 (bg=10.41%)▶khsrp (bg=27.4%)▶larp4 (bg=13.51%)▶LIN28B (bg=1.31%)▶NIPBL (bg=8.2%)▶ppil4 (bg=43.39%)▶rbm15 (bg=11.59%)▶safb (bg=40.39%)▶safb2 (bg=26.89%)▶srsf1 (bg=30.28%)▶srsf7 (bg=22.53%)▶SUPV3L1 (bg=9.63%)▶uchl5 (bg=18.56%)MATCHES To TargetScan▶ miR-182-5p:UUGGCAA▶ miR-183-5p.2:UGGCACU▶ miR-96-5p/1271-5p:UUGGCAC


TGCCAA

TGCCAAT  
Depth:6 (MOUSE)  
Ei-value:0.000, Pi-value:0.000  
Er-value:0.000, Pr-value:0.000  
eCLIP MATCHES▶bclaf1 (bg=17.67%)▶DGCR8 (bg=2.67%)▶hltf (bg=24.28%)▶khdrbs1 (bg=10.41%)▶khsrp (bg=27.4%)▶larp4 (bg=13.51%)▶LIN28B (bg=1.31%)▶NIPBL (bg=8.2%)▶ppil4 (bg=43.39%)▶rbm15 (bg=11.59%)▶safb (bg=40.39%)▶safb2 (bg=26.89%)▶srsf1 (bg=30.28%)▶srsf7 (bg=22.53%)▶SUPV3L1 (bg=9.63%)▶uchl5 (bg=18.56%)MATCHES To TargetScan▶ miR-182-5p:UUGGCAA▶ miR-96-5p/1271-5p:UUGGCAC

 4080  


T

TGCCAAT  
Depth:6 (MOUSE)  
Ei-value:0.000, Pi-value:0.000  
Er-value:0.000, Pr-value:0.000  
eCLIP MATCHES▶bclaf1 (bg=17.67%)▶DGCR8 (bg=2.67%)▶hltf (bg=24.28%)▶khdrbs1 (bg=10.41%)▶khsrp (bg=27.4%)▶larp4 (bg=13.51%)▶LIN28B (bg=1.31%)▶NIPBL (bg=8.2%)▶ppil4 (bg=43.39%)▶rbm15 (bg=11.59%)▶safb (bg=40.39%)▶safb2 (bg=26.89%)▶srsf1 (bg=30.28%)▶srsf7 (bg=22.53%)▶SUPV3L1 (bg=9.63%)▶uchl5 (bg=18.56%)MATCHES To TargetScan▶ miR-182-5p:UUGGCAA▶ miR-96-5p/1271-5p:UUGGCAC

-

TTTCGTTTGCCTC

TTTCGTTTGCCTCAGACAGGT  
Depth:6 (MOUSE)  
Ei-value:0.000, Pi-value:0.000  
Er-value:0.000, Pr-value:0.000  
eCLIP MATCHES▶bclaf1 (bg=17.67%)▶DGCR8 (bg=2.67%)▶hltf (bg=24.28%)▶khdrbs1 (bg=10.41%)▶khsrp (bg=27.4%)▶larp4 (bg=13.51%)▶LIN28B (bg=1.31%)▶NIPBL (bg=8.2%)▶NOLC1 (bg=0.67%)▶ppil4 (bg=43.39%)▶rbm15 (bg=11.59%)▶safb (bg=40.39%)▶safb2 (bg=26.89%)▶srsf1 (bg=30.28%)▶srsf7 (bg=22.53%)▶SUPV3L1 (bg=9.63%)▶uchl5 (bg=18.56%)▶znf622 (bg=18.79%)No matches to TargetScan


AG

AGACAGGT  
Depth:9 (LIZARD)  
Ei-value:0.000, Pi-value:0.000  
Er-value:0.000, Pr-value:0.000  
eCLIP MATCHES▶DGCR8 (bg=2.67%)▶hltf (bg=24.28%)▶khsrp (bg=27.4%)▶LIN28B (bg=1.31%)▶NIPBL (bg=8.2%)▶NOLC1 (bg=0.67%)▶ppil4 (bg=43.39%)▶rbm15 (bg=11.59%)▶safb (bg=40.39%)▶safb2 (bg=26.89%)▶srsf1 (bg=30.28%)▶znf622 (bg=18.79%)No matches to TargetScan


ACAGGT

ACAGGT  
Depth:10 (SNAKE)  
Ei-value:0.000, Pi-value:0.000  
Er-value:0.000, Pr-value:0.000  
eCLIP MATCHES▶DGCR8 (bg=2.67%)▶hltf (bg=24.28%)▶khsrp (bg=27.4%)▶LIN28B (bg=1.31%)▶NOLC1 (bg=0.67%)▶ppil4 (bg=43.39%)▶rbm15 (bg=11.59%)▶safb (bg=40.39%)▶safb2 (bg=26.89%)▶srsf1 (bg=30.28%)▶znf622 (bg=18.79%)No matches to TargetScan


A

TTTCGTTTGCCTCAGACAGGTATCTCTTC  
Depth:2 (MARMOSET)  
Ei-value:0.000, Pi-value:0.000  
Er-value:0.000, Pr-value:0.000  
eCLIP MATCHES▶bclaf1 (bg=17.67%)▶cpsf6 (bg=13.45%)▶DGCR8 (bg=2.67%)▶hltf (bg=24.28%)▶khdrbs1 (bg=10.41%)▶khsrp (bg=27.4%)▶larp4 (bg=13.51%)▶LIN28B (bg=1.31%)▶NIPBL (bg=8.2%)▶NOLC1 (bg=0.67%)▶ppil4 (bg=43.39%)▶rbm15 (bg=11.59%)▶safb (bg=40.39%)▶safb2 (bg=26.89%)▶srsf1 (bg=30.28%)▶srsf7 (bg=22.53%)▶SUPV3L1 (bg=9.63%)▶u2af2 (bg=19.32%)▶uchl5 (bg=18.56%)▶znf622 (bg=18.79%)MATCHES To TargetScan▶ miR-875-5p:AUACCUC


tctcttc

tctcttc  
Depth:3 (DOG)  
Ei-value:0.350, Pi-value:0.000  
Er-value:0.000, Pr-value:0.000  
eCLIP MATCHES▶cpsf6 (bg=13.45%)▶hltf (bg=24.28%)▶LIN28B (bg=1.31%)▶NOLC1 (bg=0.67%)▶ppil4 (bg=43.39%)▶rbm15 (bg=11.59%)▶safb (bg=40.39%)▶safb2 (bg=26.89%)▶srsf1 (bg=30.28%)▶u2af2 (bg=19.32%)▶znf622 (bg=18.79%)No matches to TargetScan

-

TTAT

TTATCAGAAGAGTTGCTTCAT  
Depth:5 (COW)  
Ei-value:0.000, Pi-value:0.000  
Er-value:0.000, Pr-value:0.000  
eCLIP MATCHES▶cpsf6 (bg=13.45%)▶DGCR8 (bg=2.67%)▶GRWD1 (bg=7.0%)▶hltf (bg=24.28%)▶LIN28B (bg=1.31%)▶NOLC1 (bg=0.67%)▶ppil4 (bg=43.39%)▶rbm15 (bg=11.59%)▶safb (bg=40.39%)▶safb2 (bg=26.89%)▶srsf1 (bg=30.28%)▶srsf7 (bg=22.53%)▶u2af2 (bg=19.32%)▶znf622 (bg=18.79%)No matches to TargetScan


CAGAAGAG

CAGAAGAGTTGCTTCAT  
Depth:6 (MOUSE)  
Ei-value:0.000, Pi-value:0.000  
Er-value:0.000, Pr-value:0.000  
eCLIP MATCHES▶cpsf6 (bg=13.45%)▶DGCR8 (bg=2.67%)▶GRWD1 (bg=7.0%)▶hltf (bg=24.28%)▶LIN28B (bg=1.31%)▶NOLC1 (bg=0.67%)▶ppil4 (bg=43.39%)▶rbm15 (bg=11.59%)▶safb (bg=40.39%)▶safb2 (bg=26.89%)▶srsf1 (bg=30.28%)▶srsf7 (bg=22.53%)▶u2af2 (bg=19.32%)▶znf622 (bg=18.79%)No matches to TargetScan


TTGCTTCA

TTGCTTCA  
Depth:8 (ALLIGATOR)  
Ei-value:0.000, Pi-value:0.000  
Er-value:0.000, Pr-value:0.000  
eCLIP MATCHES▶cpsf6 (bg=13.45%)▶GRWD1 (bg=7.0%)▶hltf (bg=24.28%)▶LIN28B (bg=1.31%)▶NOLC1 (bg=0.67%)▶ppil4 (bg=43.39%)▶rbm15 (bg=11.59%)▶safb (bg=40.39%)▶safb2 (bg=26.89%)▶srsf1 (bg=30.28%)▶srsf7 (bg=22.53%)▶u2af2 (bg=19.32%)▶znf622 (bg=18.79%)No matches to TargetScan


T

CAGAAGAGTTGCTTCAT  
Depth:6 (MOUSE)  
Ei-value:0.000, Pi-value:0.000  
Er-value:0.000, Pr-value:0.000  
eCLIP MATCHES▶cpsf6 (bg=13.45%)▶DGCR8 (bg=2.67%)▶GRWD1 (bg=7.0%)▶hltf (bg=24.28%)▶LIN28B (bg=1.31%)▶NOLC1 (bg=0.67%)▶ppil4 (bg=43.39%)▶rbm15 (bg=11.59%)▶safb (bg=40.39%)▶safb2 (bg=26.89%)▶srsf1 (bg=30.28%)▶srsf7 (bg=22.53%)▶u2af2 (bg=19.32%)▶znf622 (bg=18.79%)No matches to TargetScan


T

TTATCAGAAGAGTTGCTTCATTTCATCTGGGAGCAGAAAACAGCAGGCAGCTGTTAACAGATAAGTTTAA  
Depth:3 (DOG)  
Ei-value:0.000, Pi-value:0.000  
Er-value:0.000, Pr-value:0.000  
eCLIP MATCHES▶cpsf6 (bg=13.45%)▶DGCR8 (bg=2.67%)▶GRWD1 (bg=7.0%)▶hltf (bg=24.28%)▶hnrnpa1 (bg=18.32%)▶khsrp (bg=27.4%)▶LIN28B (bg=1.31%)▶NIPBL (bg=8.2%)▶NOLC1 (bg=0.67%)▶ppil4 (bg=43.39%)▶PRPF8 (bg=6.2%)▶rbm15 (bg=11.59%)▶safb (bg=40.39%)▶safb2 (bg=26.89%)▶srsf1 (bg=30.28%)▶srsf7 (bg=22.53%)▶u2af2 (bg=19.32%)▶znf622 (bg=18.79%)MATCHES To TargetScan▶ miR-22-3p:AGCUGCC▶ miR-150-5p:CUCCCAA▶ miR-532-3p:CUCCCAC▶ miR-203a-3p.1:GAAAUGU▶ miR-203a-3p.2:UGAAAUG


TCATCTG

TCATCTGGGAGCAGAAAACAGCAGGCAGCTGTT  
Depth:5 (COW)  
Ei-value:0.000, Pi-value:0.000  
Er-value:0.000, Pr-value:0.000  
eCLIP MATCHES▶cpsf6 (bg=13.45%)▶DGCR8 (bg=2.67%)▶GRWD1 (bg=7.0%)▶hltf (bg=24.28%)▶khsrp (bg=27.4%)▶NIPBL (bg=8.2%)▶ppil4 (bg=43.39%)▶PRPF8 (bg=6.2%)▶rbm15 (bg=11.59%)▶safb (bg=40.39%)▶safb2 (bg=26.89%)▶srsf1 (bg=30.28%)▶srsf7 (bg=22.53%)▶znf622 (bg=18.79%)MATCHES To TargetScan▶ miR-22-3p:AGCUGCC▶ miR-150-5p:CUCCCAA▶ miR-532-3p:CUCCCAC


GGAGCAG

GGAGCAG  
Depth:6 (MOUSE)  
Ei-value:0.000, Pi-value:0.000  
Er-value:0.000, Pr-value:0.000  
eCLIP MATCHES▶cpsf6 (bg=13.45%)▶DGCR8 (bg=2.67%)▶GRWD1 (bg=7.0%)▶hltf (bg=24.28%)▶khsrp (bg=27.4%)▶NIPBL (bg=8.2%)▶ppil4 (bg=43.39%)▶rbm15 (bg=11.59%)▶safb (bg=40.39%)▶safb2 (bg=26.89%)▶srsf1 (bg=30.28%)▶srsf7 (bg=22.53%)▶znf622 (bg=18.79%)No matches to TargetScan


A

TCATCTGGGAGCAGAAAACAGCAGGCAGCTGTT  
Depth:5 (COW)  
Ei-value:0.000, Pi-value:0.000  
Er-value:0.000, Pr-value:0.000  
eCLIP MATCHES▶cpsf6 (bg=13.45%)▶DGCR8 (bg=2.67%)▶GRWD1 (bg=7.0%)▶hltf (bg=24.28%)▶khsrp (bg=27.4%)▶NIPBL (bg=8.2%)▶ppil4 (bg=43.39%)▶PRPF8 (bg=6.2%)▶rbm15 (bg=11.59%)▶safb (bg=40.39%)▶safb2 (bg=26.89%)▶srsf1 (bg=30.28%)▶srsf7 (bg=22.53%)▶znf622 (bg=18.79%)MATCHES To TargetScan▶ miR-22-3p:AGCUGCC▶ miR-150-5p:CUCCCAA▶ miR-532-3p:CUCCCAC


AAACAGCAG

AAACAGCAG  
Depth:6 (MOUSE)  
Ei-value:0.000, Pi-value:0.000  
Er-value:0.000, Pr-value:0.000  
eCLIP MATCHES▶cpsf6 (bg=13.45%)▶DGCR8 (bg=2.67%)▶GRWD1 (bg=7.0%)▶hltf (bg=24.28%)▶khsrp (bg=27.4%)▶NIPBL (bg=8.2%)▶ppil4 (bg=43.39%)▶PRPF8 (bg=6.2%)▶rbm15 (bg=11.59%)▶safb (bg=40.39%)▶safb2 (bg=26.89%)▶srsf1 (bg=30.28%)▶srsf7 (bg=22.53%)▶znf622 (bg=18.79%)No matches to TargetScan


GCAGCTGTT

TCATCTGGGAGCAGAAAACAGCAGGCAGCTGTT  
Depth:5 (COW)  
Ei-value:0.000, Pi-value:0.000  
Er-value:0.000, Pr-value:0.000  
eCLIP MATCHES▶cpsf6 (bg=13.45%)▶DGCR8 (bg=2.67%)▶GRWD1 (bg=7.0%)▶hltf (bg=24.28%)▶khsrp (bg=27.4%)▶NIPBL (bg=8.2%)▶ppil4 (bg=43.39%)▶PRPF8 (bg=6.2%)▶rbm15 (bg=11.59%)▶safb (bg=40.39%)▶safb2 (bg=26.89%)▶srsf1 (bg=30.28%)▶srsf7 (bg=22.53%)▶znf622 (bg=18.79%)MATCHES To TargetScan▶ miR-22-3p:AGCUGCC▶ miR-150-5p:CUCCCAA▶ miR-532-3p:CUCCCAC


A

TTATCAGAAGAGTTGCTTCATTTCATCTGGGAGCAGAAAACAGCAGGCAGCTGTTAACAGATAAGTTTAA  
Depth:3 (DOG)  
Ei-value:0.000, Pi-value:0.000  
Er-value:0.000, Pr-value:0.000  
eCLIP MATCHES▶cpsf6 (bg=13.45%)▶DGCR8 (bg=2.67%)▶GRWD1 (bg=7.0%)▶hltf (bg=24.28%)▶hnrnpa1 (bg=18.32%)▶khsrp (bg=27.4%)▶LIN28B (bg=1.31%)▶NIPBL (bg=8.2%)▶NOLC1 (bg=0.67%)▶ppil4 (bg=43.39%)▶PRPF8 (bg=6.2%)▶rbm15 (bg=11.59%)▶safb (bg=40.39%)▶safb2 (bg=26.89%)▶srsf1 (bg=30.28%)▶srsf7 (bg=22.53%)▶u2af2 (bg=19.32%)▶znf622 (bg=18.79%)MATCHES To TargetScan▶ miR-22-3p:AGCUGCC▶ miR-150-5p:CUCCCAA▶ miR-532-3p:CUCCCAC▶ miR-203a-3p.1:GAAAUGU▶ miR-203a-3p.2:UGAAAUG


A

ACAGATAAGT  
Depth:7 (TURTLE)  
Ei-value:0.000, Pi-value:0.000  
Er-value:0.000, Pr-value:0.000  
eCLIP MATCHES▶cpsf6 (bg=13.45%)▶hltf (bg=24.28%)▶hnrnpa1 (bg=18.32%)▶khsrp (bg=27.4%)▶NIPBL (bg=8.2%)▶ppil4 (bg=43.39%)▶PRPF8 (bg=6.2%)▶rbm15 (bg=11.59%)▶safb (bg=40.39%)▶safb2 (bg=26.89%)▶srsf1 (bg=30.28%)▶srsf7 (bg=22.53%)▶znf622 (bg=18.79%)No matches to TargetScan


CA

CAGATAAGT  
Depth:11 (X.TROPICALIS)  
Ei-value:0.000, Pi-value:0.000  
Er-value:0.000, Pr-value:0.000  
eCLIP MATCHES▶cpsf6 (bg=13.45%)▶hltf (bg=24.28%)▶hnrnpa1 (bg=18.32%)▶khsrp (bg=27.4%)▶NIPBL (bg=8.2%)▶ppil4 (bg=43.39%)▶PRPF8 (bg=6.2%)▶rbm15 (bg=11.59%)▶safb (bg=40.39%)▶safb2 (bg=26.89%)▶srsf1 (bg=30.28%)▶srsf7 (bg=22.53%)▶znf622 (bg=18.79%)No matches to TargetScan


GATAAG

GATAAG  
Depth:19 (ZEBRAFISH)  
Ei-value:0.000, Pi-value:0.000  
Er-value:0.000, Pr-value:0.000  
eCLIP MATCHES▶cpsf6 (bg=13.45%)▶khsrp (bg=27.4%)▶NIPBL (bg=8.2%)▶ppil4 (bg=43.39%)▶PRPF8 (bg=6.2%)▶rbm15 (bg=11.59%)▶safb (bg=40.39%)▶safb2 (bg=26.89%)▶srsf1 (bg=30.28%)▶srsf7 (bg=22.53%)▶znf622 (bg=18.79%)No matches to TargetScan


T

CAGATAAGT  
Depth:11 (X.TROPICALIS)  
Ei-value:0.000, Pi-value:0.000  
Er-value:0.000, Pr-value:0.000  
eCLIP MATCHES▶cpsf6 (bg=13.45%)▶hltf (bg=24.28%)▶hnrnpa1 (bg=18.32%)▶khsrp (bg=27.4%)▶NIPBL (bg=8.2%)▶ppil4 (bg=43.39%)▶PRPF8 (bg=6.2%)▶rbm15 (bg=11.59%)▶safb (bg=40.39%)▶safb2 (bg=26.89%)▶srsf1 (bg=30.28%)▶srsf7 (bg=22.53%)▶znf622 (bg=18.79%)No matches to TargetScan


TTAA

ACAGATAAGTTTAA  
Depth:5 (COW)  
Ei-value:0.000, Pi-value:0.000  
Er-value:0.000, Pr-value:0.000  
eCLIP MATCHES▶cpsf6 (bg=13.45%)▶hltf (bg=24.28%)▶hnrnpa1 (bg=18.32%)▶khsrp (bg=27.4%)▶NIPBL (bg=8.2%)▶ppil4 (bg=43.39%)▶PRPF8 (bg=6.2%)▶rbm15 (bg=11.59%)▶safb (bg=40.39%)▶safb2 (bg=26.89%)▶srsf1 (bg=30.28%)▶srsf7 (bg=22.53%)▶znf622 (bg=18.79%)No matches to TargetScan


CTTGC

TTATCAGAAGAGTTGCTTCATTTCATCTGGGAGCAGAAAACAGCAGGCAGCTGTTAACAGATAAGTTTAACTTGCATCTGCA  
Depth:2 (MARMOSET)  
Ei-value:0.000, Pi-value:0.000  
Er-value:0.000, Pr-value:0.000  
eCLIP MATCHES▶cpsf6 (bg=13.45%)▶DGCR8 (bg=2.67%)▶GRWD1 (bg=7.0%)▶hltf (bg=24.28%)▶hnrnpa1 (bg=18.32%)▶khsrp (bg=27.4%)▶LIN28B (bg=1.31%)▶NIPBL (bg=8.2%)▶NOLC1 (bg=0.67%)▶ppil4 (bg=43.39%)▶PRPF8 (bg=6.2%)▶RBFOX2 (bg=3.41%)▶rbm15 (bg=11.59%)▶safb (bg=40.39%)▶safb2 (bg=26.89%)▶srsf1 (bg=30.28%)▶srsf7 (bg=22.53%)▶tia1 (bg=23.76%)▶tial1 (bg=15.02%)▶u2af2 (bg=19.32%)▶znf622 (bg=18.79%)MATCHES To TargetScan▶ miR-22-3p:AGCUGCC▶ miR-150-5p:CUCCCAA▶ miR-532-3p:CUCCCAC▶ miR-203a-3p.1:GAAAUGU▶ miR-203a-3p.2:UGAAAUG


ATCTGCA

ATCTGCA  
Depth:6 (MOUSE)  
Ei-value:0.000, Pi-value:0.000  
Er-value:0.000, Pr-value:0.000  
eCLIP MATCHES▶cpsf6 (bg=13.45%)▶hltf (bg=24.28%)▶hnrnpa1 (bg=18.32%)▶khsrp (bg=27.4%)▶ppil4 (bg=43.39%)▶PRPF8 (bg=6.2%)▶RBFOX2 (bg=3.41%)▶safb (bg=40.39%)▶tia1 (bg=23.76%)▶tial1 (bg=15.02%)No matches to TargetScan

-

T

TATTGCATG  
Depth:8 (ALLIGATOR)  
Ei-value:0.000, Pi-value:0.000  
Er-value:0.000, Pr-value:0.000  
eCLIP MATCHES▶cpsf6 (bg=13.45%)▶hltf (bg=24.28%)▶hnrnpa1 (bg=18.32%)▶HNRNPU (bg=9.45%)▶khsrp (bg=27.4%)▶ppil4 (bg=43.39%)▶PRPF8 (bg=6.2%)▶RBFOX2 (bg=3.41%)▶safb (bg=40.39%)▶safb2 (bg=26.89%)▶tia1 (bg=23.76%)▶tial1 (bg=15.02%)No matches to TargetScan


ATTG

ATTGCAT  
Depth:11 (X.TROPICALIS)  
Ei-value:0.000, Pi-value:0.000  
Er-value:0.000, Pr-value:0.000  
eCLIP MATCHES▶cpsf6 (bg=13.45%)▶hltf (bg=24.28%)▶hnrnpa1 (bg=18.32%)▶HNRNPU (bg=9.45%)▶khsrp (bg=27.4%)▶ppil4 (bg=43.39%)▶PRPF8 (bg=6.2%)▶RBFOX2 (bg=3.41%)▶safb (bg=40.39%)▶safb2 (bg=26.89%)▶tia1 (bg=23.76%)▶tial1 (bg=15.02%)No matches to TargetScan

 4200  


CAT

ATTGCAT  
Depth:11 (X.TROPICALIS)  
Ei-value:0.000, Pi-value:0.000  
Er-value:0.000, Pr-value:0.000  
eCLIP MATCHES▶cpsf6 (bg=13.45%)▶hltf (bg=24.28%)▶hnrnpa1 (bg=18.32%)▶HNRNPU (bg=9.45%)▶khsrp (bg=27.4%)▶ppil4 (bg=43.39%)▶PRPF8 (bg=6.2%)▶RBFOX2 (bg=3.41%)▶safb (bg=40.39%)▶safb2 (bg=26.89%)▶tia1 (bg=23.76%)▶tial1 (bg=15.02%)No matches to TargetScan


G

ATTGCATG  
Depth:9 (LIZARD)  
Ei-value:0.000, Pi-value:0.000  
Er-value:0.000, Pr-value:0.000  
eCLIP MATCHES▶cpsf6 (bg=13.45%)▶hltf (bg=24.28%)▶hnrnpa1 (bg=18.32%)▶HNRNPU (bg=9.45%)▶khsrp (bg=27.4%)▶ppil4 (bg=43.39%)▶PRPF8 (bg=6.2%)▶RBFOX2 (bg=3.41%)▶safb (bg=40.39%)▶safb2 (bg=26.89%)▶tia1 (bg=23.76%)▶tial1 (bg=15.02%)No matches to TargetScan


TTAGG

TATTGCATGTTAGG  
Depth:7 (TURTLE)  
Ei-value:0.000, Pi-value:0.000  
Er-value:0.000, Pr-value:0.000  
eCLIP MATCHES▶cpsf6 (bg=13.45%)▶hltf (bg=24.28%)▶hnrnpa1 (bg=18.32%)▶HNRNPU (bg=9.45%)▶khsrp (bg=27.4%)▶ppil4 (bg=43.39%)▶PRPF8 (bg=6.2%)▶RBFOX2 (bg=3.41%)▶safb (bg=40.39%)▶safb2 (bg=26.89%)▶tia1 (bg=23.76%)▶tial1 (bg=15.02%)No matches to TargetScan


G

TATTGCATGTTAGGGATAAGTG  
Depth:4 (PIG)  
Ei-value:0.000, Pi-value:0.000  
Er-value:0.000, Pr-value:0.000  
eCLIP MATCHES▶cpsf6 (bg=13.45%)▶hltf (bg=24.28%)▶hnrnpa1 (bg=18.32%)▶HNRNPU (bg=9.45%)▶khsrp (bg=27.4%)▶ppil4 (bg=43.39%)▶PRPF8 (bg=6.2%)▶RBFOX2 (bg=3.41%)▶safb (bg=40.39%)▶safb2 (bg=26.89%)▶tia1 (bg=23.76%)▶tial1 (bg=15.02%)MATCHES To TargetScan▶ miR-188-5p:AUCCCUU


ATAAGTG

ATAAGTG  
Depth:6 (MOUSE)  
Ei-value:0.000, Pi-value:0.000  
Er-value:0.000, Pr-value:0.000  
eCLIP MATCHES▶cpsf6 (bg=13.45%)▶hltf (bg=24.28%)▶hnrnpa1 (bg=18.32%)▶HNRNPU (bg=9.45%)▶khsrp (bg=27.4%)▶ppil4 (bg=43.39%)▶PRPF8 (bg=6.2%)▶RBFOX2 (bg=3.41%)▶safb (bg=40.39%)▶safb2 (bg=26.89%)▶tia1 (bg=23.76%)▶tial1 (bg=15.02%)No matches to TargetScan

-

TTATTTTTAA

TTATTTTTAA  
Depth:3 (DOG)  
Ei-value:0.000, Pi-value:0.000  
Er-value:0.000, Pr-value:0.000  
eCLIP MATCHES▶cpsf6 (bg=13.45%)▶hltf (bg=24.28%)▶hnrnpa1 (bg=18.32%)▶HNRNPU (bg=9.45%)▶khsrp (bg=27.4%)▶ppil4 (bg=43.39%)▶RBFOX2 (bg=3.41%)▶safb (bg=40.39%)▶tia1 (bg=23.76%)▶tial1 (bg=15.02%)No matches to TargetScan

---

CTGTGGA

CTGTGGA  
Depth:5 (COW)  
Ei-value:0.000, Pi-value:0.000  
Er-value:0.000, Pr-value:0.000  
eCLIP MATCHES▶hnrnpa1 (bg=18.32%)▶ppil4 (bg=43.39%)▶RBFOX2 (bg=3.41%)▶safb (bg=40.39%)▶tia1 (bg=23.76%)MATCHES To TargetScan▶ miR-140-3p.1:CCACAGG


G

CTGTGGAG  
Depth:4 (PIG)  
Ei-value:0.000, Pi-value:0.000  
Er-value:0.000, Pr-value:0.000  
eCLIP MATCHES▶hnrnpa1 (bg=18.32%)▶ppil4 (bg=43.39%)▶RBFOX2 (bg=3.41%)▶safb (bg=40.39%)▶tia1 (bg=23.76%)MATCHES To TargetScan▶ miR-140-3p.1:CCACAGG


TTCTTAA

CTGTGGAGTTCTTAA  
Depth:2 (MARMOSET)  
Ei-value:0.000, Pi-value:0.000  
Er-value:0.000, Pr-value:0.000  
eCLIP MATCHES▶hnrnpa1 (bg=18.32%)▶ppil4 (bg=43.39%)▶RBFOX2 (bg=3.41%)▶safb (bg=40.39%)▶tia1 (bg=23.76%)MATCHES To TargetScan▶ miR-140-3p.1:CCACAGG

-

tatcaa

tatcaa  
Depth:2 (MARMOSET)  
Ei-value:1.000, Pi-value:0.110  
Er-value:0.000, Pr-value:0.010  
eCLIP MATCHES▶hnrnpa1 (bg=18.32%)▶RBFOX2 (bg=3.41%)▶tia1 (bg=23.76%)No matches to TargetScan


ccatggcacttt

ccatggcacttt  
Depth:2 (MARMOSET)  
Ei-value:0.990, Pi-value:0.000  
Er-value:0.000, Pr-value:0.000  
No matches to eCLIP DataMATCHES To TargetScan▶ miR-17-5p/20-5p/93-5p/106-5p/519-3p:AAAGUGC▶ miR-302-3p/372-3p/373-3p/520-3p:AAGUGCU

-

TCCTGAC

TCCTGACCCCTTCCCTAGGGGATTTCAGGATT  
Depth:2 (MARMOSET)  
Ei-value:0.000, Pi-value:0.000  
Er-value:0.000, Pr-value:0.000  
eCLIP MATCHES▶ppil4 (bg=43.39%)MATCHES To TargetScan▶ miR-203a-3p.2:UGAAAUG


cccttcc

cccttcc  
Depth:3 (DOG)  
Ei-value:0.350, Pi-value:0.000  
Er-value:0.000, Pr-value:0.000  
eCLIP MATCHES▶ppil4 (bg=43.39%)No matches to TargetScan


CTAGG

TCCTGACCCCTTCCCTAGGGGATTTCAGGATT  
Depth:2 (MARMOSET)  
Ei-value:0.000, Pi-value:0.000  
Er-value:0.000, Pr-value:0.000  
eCLIP MATCHES▶ppil4 (bg=43.39%)MATCHES To TargetScan▶ miR-203a-3p.2:UGAAAUG


GGATT

GGATTTCAGGATT  
Depth:3 (DOG)  
Ei-value:0.000, Pi-value:0.000  
Er-value:0.000, Pr-value:0.000  
No matches to eCLIP DataMATCHES To TargetScan▶ miR-203a-3p.2:UGAAAUG


T

TCAGGATT  
Depth:4 (PIG)  
Ei-value:0.000, Pi-value:0.000  
Er-value:0.000, Pr-value:0.000  
No matches to eCLIP DataNo matches to TargetScan


CAGGATT

CAGGATT  
Depth:5 (COW)  
Ei-value:0.000, Pi-value:0.000  
Er-value:0.000, Pr-value:0.000  
No matches to eCLIP DataNo matches to TargetScan


gagaaatttt

gagaaatttt  
Depth:2 (MARMOSET)  
Ei-value:1.000, Pi-value:0.000  
Er-value:0.000, Pr-value:0.000  
No matches to eCLIP DataNo matches to TargetScan

------------ 4320  
 -

T

TTTAAAAT  
Depth:5 (COW)  
Ei-value:0.000, Pi-value:0.000  
Er-value:0.000, Pr-value:0.000  
eCLIP MATCHES▶HNRNPC (bg=4.22%)▶HNRNPU (bg=9.45%)No matches to TargetScan


TTAAAAT

TTAAAAT  
Depth:8 (ALLIGATOR)  
Ei-value:0.000, Pi-value:0.000  
Er-value:0.000, Pr-value:0.000  
eCLIP MATCHES▶HNRNPC (bg=4.22%)▶HNRNPU (bg=9.45%)No matches to TargetScan


T

TTTAAAATT  
Depth:3 (DOG)  
Ei-value:0.000, Pi-value:0.000  
Er-value:0.000, Pr-value:0.010  
eCLIP MATCHES▶HNRNPC (bg=4.22%)▶HNRNPU (bg=9.45%)No matches to TargetScan


GT

TTTAAAATTGTAGGACTTGTTCCT  
Depth:2 (MARMOSET)  
Ei-value:0.000, Pi-value:0.000  
Er-value:0.000, Pr-value:0.000  
eCLIP MATCHES▶HNRNPC (bg=4.22%)▶HNRNPU (bg=9.45%)No matches to TargetScan


aggactt

aggactt  
Depth:3 (DOG)  
Ei-value:0.350, Pi-value:0.010  
Er-value:0.000, Pr-value:0.000  
No matches to eCLIP DataNo matches to TargetScan


GTTCCT

TTTAAAATTGTAGGACTTGTTCCT  
Depth:2 (MARMOSET)  
Ei-value:0.000, Pi-value:0.000  
Er-value:0.000, Pr-value:0.000  
eCLIP MATCHES▶HNRNPC (bg=4.22%)▶HNRNPU (bg=9.45%)No matches to TargetScan

-

TGGGCTT

TGGGCTT  
Depth:5 (COW)  
Ei-value:0.000, Pi-value:0.000  
Er-value:0.000, Pr-value:0.000  
No matches to eCLIP DataNo matches to TargetScan


C

TGGGCTTC  
Depth:4 (PIG)  
Ei-value:0.000, Pi-value:0.000  
Er-value:0.000, Pr-value:0.000  
No matches to eCLIP DataNo matches to TargetScan

-

G

GTGATGGGATAGTACAC  
Depth:2 (MARMOSET)  
Ei-value:0.000, Pi-value:0.000  
Er-value:0.000, Pr-value:0.000  
eCLIP MATCHES▶ppil4 (bg=43.39%)No matches to TargetScan


TGATGGG

TGATGGG  
Depth:5 (COW)  
Ei-value:0.000, Pi-value:0.010  
Er-value:0.000, Pr-value:0.000  
No matches to eCLIP DataNo matches to TargetScan


ATAG

TGATGGGATAG  
Depth:3 (DOG)  
Ei-value:0.000, Pi-value:0.000  
Er-value:0.000, Pr-value:0.000  
No matches to eCLIP DataNo matches to TargetScan


TACAC

GTGATGGGATAGTACAC  
Depth:2 (MARMOSET)  
Ei-value:0.000, Pi-value:0.000  
Er-value:0.000, Pr-value:0.000  
eCLIP MATCHES▶ppil4 (bg=43.39%)No matches to TargetScan

-

TCACTCA

TCACTCAGAGGCATTTGCATCT  
Depth:2 (MARMOSET)  
Ei-value:0.000, Pi-value:0.000  
Er-value:0.000, Pr-value:0.000  
eCLIP MATCHES▶ppil4 (bg=43.39%)MATCHES To TargetScan▶ miR-532-5p:AUGCCUU▶ miR-365-3p:AAUGCCC


GAGGCA

GAGGCA  
Depth:5 (COW)  
Ei-value:0.000, Pi-value:0.030  
Er-value:0.000, Pr-value:0.000  
No matches to eCLIP DataNo matches to TargetScan


TTTGCATCT

TCACTCAGAGGCATTTGCATCT  
Depth:2 (MARMOSET)  
Ei-value:0.000, Pi-value:0.000  
Er-value:0.000, Pr-value:0.000  
eCLIP MATCHES▶ppil4 (bg=43.39%)MATCHES To TargetScan▶ miR-532-5p:AUGCCUU▶ miR-365-3p:AAUGCCC

-----------

CTTAAAAGCCTCTAAAGTGAT

CTTAAAAGCCTCTAAAGTGAT  
Depth:2 (MARMOSET)  
Ei-value:0.000, Pi-value:0.000  
Er-value:0.000, Pr-value:0.000  
eCLIP MATCHES▶ppil4 (bg=43.39%)MATCHES To TargetScan▶ miR-877-5p:UAGAGGA▶ miR-485-5p:GAGGCUG

-

agtgcc

agtgcc  
Depth:3 (DOG)  
Ei-value:1.000, Pi-value:0.000  
Er-value:0.000, Pr-value:0.020  
eCLIP MATCHES▶ppil4 (bg=43.39%)No matches to TargetScan

------ 4440  
 ---

actaag

actaag  
Depth:3 (DOG)  
Ei-value:1.000, Pi-value:0.040  
Er-value:0.000, Pr-value:0.000  
eCLIP MATCHES▶ppil4 (bg=43.39%)No matches to TargetScan


gaaatt

actaaggaaatt  
Depth:2 (MARMOSET)  
Ei-value:0.990, Pi-value:0.000  
Er-value:0.000, Pr-value:0.000  
eCLIP MATCHES▶ppil4 (bg=43.39%)No matches to TargetScan

-

GTTTAGC

GTTTAGCATTGAATCTCTGAAG  
Depth:2 (MARMOSET)  
Ei-value:0.000, Pi-value:0.000  
Er-value:0.000, Pr-value:0.000  
eCLIP MATCHES▶cpsf6 (bg=13.45%)▶khsrp (bg=27.4%)▶ppil4 (bg=43.39%)No matches to TargetScan


ATTGAATCTCT

ATTGAATCTCT  
Depth:3 (DOG)  
Ei-value:0.000, Pi-value:0.000  
Er-value:0.000, Pr-value:0.000  
eCLIP MATCHES▶cpsf6 (bg=13.45%)▶khsrp (bg=27.4%)▶ppil4 (bg=43.39%)No matches to TargetScan


GAAG

GTTTAGCATTGAATCTCTGAAG  
Depth:2 (MARMOSET)  
Ei-value:0.000, Pi-value:0.000  
Er-value:0.000, Pr-value:0.000  
eCLIP MATCHES▶cpsf6 (bg=13.45%)▶khsrp (bg=27.4%)▶ppil4 (bg=43.39%)No matches to TargetScan

------

TGAAAGGAA

TGAAAGGAATAGCATGATGTGCT  
Depth:2 (MARMOSET)  
Ei-value:0.000, Pi-value:0.000  
Er-value:0.000, Pr-value:0.000  
eCLIP MATCHES▶cpsf6 (bg=13.45%)▶hnrnpa1 (bg=18.32%)▶khsrp (bg=27.4%)▶ppil4 (bg=43.39%)No matches to TargetScan


TAGCAT

TAGCAT  
Depth:4 (PIG)  
Ei-value:0.010, Pi-value:0.030  
Er-value:0.000, Pr-value:0.000  
eCLIP MATCHES▶cpsf6 (bg=13.45%)▶hnrnpa1 (bg=18.32%)▶khsrp (bg=27.4%)▶ppil4 (bg=43.39%)No matches to TargetScan


GATGTGCT

TGAAAGGAATAGCATGATGTGCT  
Depth:2 (MARMOSET)  
Ei-value:0.000, Pi-value:0.000  
Er-value:0.000, Pr-value:0.000  
eCLIP MATCHES▶cpsf6 (bg=13.45%)▶hnrnpa1 (bg=18.32%)▶khsrp (bg=27.4%)▶ppil4 (bg=43.39%)No matches to TargetScan

-

TT

TTAGAATCAGATGT  
Depth:2 (MARMOSET)  
Ei-value:0.020, Pi-value:0.000  
Er-value:0.000, Pr-value:0.000  
eCLIP MATCHES▶cpsf6 (bg=13.45%)▶hnrnpa1 (bg=18.32%)▶khsrp (bg=27.4%)▶ppil4 (bg=43.39%)No matches to TargetScan


AGAATC

AGAATC  
Depth:4 (PIG)  
Ei-value:0.010, Pi-value:0.010  
Er-value:0.000, Pr-value:0.000  
eCLIP MATCHES▶cpsf6 (bg=13.45%)▶hnrnpa1 (bg=18.32%)▶khsrp (bg=27.4%)▶ppil4 (bg=43.39%)No matches to TargetScan


AGATGT

TTAGAATCAGATGT  
Depth:2 (MARMOSET)  
Ei-value:0.020, Pi-value:0.000  
Er-value:0.000, Pr-value:0.000  
eCLIP MATCHES▶cpsf6 (bg=13.45%)▶hnrnpa1 (bg=18.32%)▶khsrp (bg=27.4%)▶ppil4 (bg=43.39%)No matches to TargetScan

---

TGCTAAA

TGCTAAA  
Depth:5 (COW)  
Ei-value:0.000, Pi-value:0.000  
Er-value:0.000, Pr-value:0.000  
eCLIP MATCHES▶cpsf6 (bg=13.45%)▶hnrnpa1 (bg=18.32%)▶khsrp (bg=27.4%)No matches to TargetScan


ATTTACATGTTGTG

TGCTAAAATTTACATGTTGTG  
Depth:2 (MARMOSET)  
Ei-value:0.000, Pi-value:0.000  
Er-value:0.000, Pr-value:0.000  
eCLIP MATCHES▶cpsf6 (bg=13.45%)▶hnrnpa1 (bg=18.32%)▶khsrp (bg=27.4%)MATCHES To TargetScan▶ miR-411-3p:AUGUAAC

------

attgtg

attgtg  
Depth:2 (MARMOSET)  
Ei-value:1.000, Pi-value:0.080  
Er-value:0.000, Pr-value:0.030  
eCLIP MATCHES▶cpsf6 (bg=13.45%)▶HNRNPUL1 (bg=1.43%)▶khsrp (bg=27.4%)No matches to TargetScan

-

A

AGAAAAC  
Depth:5 (COW)  
Ei-value:0.000, Pi-value:0.000  
Er-value:0.000, Pr-value:0.000  
eCLIP MATCHES▶cpsf6 (bg=13.45%)▶HNRNPUL1 (bg=1.43%)▶khsrp (bg=27.4%)No matches to TargetScan

 4560  


GAAAAC

AGAAAAC  
Depth:5 (COW)  
Ei-value:0.000, Pi-value:0.000  
Er-value:0.000, Pr-value:0.000  
eCLIP MATCHES▶cpsf6 (bg=13.45%)▶HNRNPUL1 (bg=1.43%)▶khsrp (bg=27.4%)No matches to TargetScan


catt

agaaaaccatt  
Depth:2 (MARMOSET)  
Ei-value:1.000, Pi-value:0.000  
Er-value:0.000, Pr-value:0.000  
eCLIP MATCHES▶cpsf6 (bg=13.45%)▶HNRNPUL1 (bg=1.43%)▶khsrp (bg=27.4%)No matches to TargetScan

-------

TCAAAATAA

TCAAAATAA  
Depth:5 (COW)  
Ei-value:0.000, Pi-value:0.000  
Er-value:0.000, Pr-value:0.000  
eCLIP MATCHES▶cpsf6 (bg=13.45%)▶khsrp (bg=27.4%)No matches to TargetScan


TAAACTATTTTTAT

TCAAAATAATAAACTATTTTTATTAGAGAATGTATACTTTTAGAAAGCTGTCTCCTTATTTAAATAAAATA  
Depth:2 (MARMOSET)  
Ei-value:0.000, Pi-value:0.000  
Er-value:0.000, Pr-value:0.000  
eCLIP MATCHES▶cpsf6 (bg=13.45%)▶CSTF2 (bg=7.88%)▶cstf2t (bg=12.11%)▶FMR1 (bg=1.89%)▶khsrp (bg=27.4%)▶METAP2 (bg=1.11%)▶XRN2 (bg=1.09%)▶zc3h8 (bg=12.78%)MATCHES To TargetScan▶ miR-181-5p:ACAUUCA


TAGA

TAGAGAATGTAT  
Depth:3 (DOG)  
Ei-value:0.000, Pi-value:0.000  
Er-value:0.000, Pr-value:0.000  
eCLIP MATCHES▶khsrp (bg=27.4%)▶METAP2 (bg=1.11%)▶zc3h8 (bg=12.78%)MATCHES To TargetScan▶ miR-181-5p:ACAUUCA


GAATGTAT

GAATGTAT  
Depth:6 (MOUSE)  
Ei-value:0.000, Pi-value:0.000  
Er-value:0.000, Pr-value:0.000  
eCLIP MATCHES▶khsrp (bg=27.4%)▶METAP2 (bg=1.11%)▶zc3h8 (bg=12.78%)MATCHES To TargetScan▶ miR-181-5p:ACAUUCA


A

TCAAAATAATAAACTATTTTTATTAGAGAATGTATACTTTTAGAAAGCTGTCTCCTTATTTAAATAAAATA  
Depth:2 (MARMOSET)  
Ei-value:0.000, Pi-value:0.000  
Er-value:0.000, Pr-value:0.000  
eCLIP MATCHES▶cpsf6 (bg=13.45%)▶CSTF2 (bg=7.88%)▶cstf2t (bg=12.11%)▶FMR1 (bg=1.89%)▶khsrp (bg=27.4%)▶METAP2 (bg=1.11%)▶XRN2 (bg=1.09%)▶zc3h8 (bg=12.78%)MATCHES To TargetScan▶ miR-181-5p:ACAUUCA


CTTTTAG

CTTTTAG  
Depth:5 (COW)  
Ei-value:0.000, Pi-value:0.010  
Er-value:0.000, Pr-value:0.000  
eCLIP MATCHES▶cpsf6 (bg=13.45%)▶CSTF2 (bg=7.88%)▶cstf2t (bg=12.11%)▶khsrp (bg=27.4%)▶zc3h8 (bg=12.78%)No matches to TargetScan


A

CTTTTAGAAAGCTGTCTCCT  
Depth:4 (PIG)  
Ei-value:0.000, Pi-value:0.000  
Er-value:0.000, Pr-value:0.000  
eCLIP MATCHES▶cpsf6 (bg=13.45%)▶CSTF2 (bg=7.88%)▶cstf2t (bg=12.11%)▶FMR1 (bg=1.89%)▶khsrp (bg=27.4%)▶zc3h8 (bg=12.78%)No matches to TargetScan


AA

AAGCTGTCTCCT  
Depth:5 (COW)  
Ei-value:0.000, Pi-value:0.000  
Er-value:0.000, Pr-value:0.000  
eCLIP MATCHES▶cpsf6 (bg=13.45%)▶CSTF2 (bg=7.88%)▶cstf2t (bg=12.11%)▶FMR1 (bg=1.89%)▶khsrp (bg=27.4%)▶zc3h8 (bg=12.78%)No matches to TargetScan


GCTGTC

GCTGTC  
Depth:9 (LIZARD)  
Ei-value:0.000, Pi-value:0.000  
Er-value:0.000, Pr-value:0.000  
eCLIP MATCHES▶cpsf6 (bg=13.45%)▶CSTF2 (bg=7.88%)▶cstf2t (bg=12.11%)▶FMR1 (bg=1.89%)▶khsrp (bg=27.4%)▶zc3h8 (bg=12.78%)No matches to TargetScan


TCCT

GCTGTCTCCT  
Depth:6 (MOUSE)  
Ei-value:0.000, Pi-value:0.000  
Er-value:0.000, Pr-value:0.000  
eCLIP MATCHES▶cpsf6 (bg=13.45%)▶CSTF2 (bg=7.88%)▶cstf2t (bg=12.11%)▶FMR1 (bg=1.89%)▶khsrp (bg=27.4%)▶zc3h8 (bg=12.78%)No matches to TargetScan


T

TCAAAATAATAAACTATTTTTATTAGAGAATGTATACTTTTAGAAAGCTGTCTCCTTATTTAAATAAAATA  
Depth:2 (MARMOSET)  
Ei-value:0.000, Pi-value:0.000  
Er-value:0.000, Pr-value:0.000  
eCLIP MATCHES▶cpsf6 (bg=13.45%)▶CSTF2 (bg=7.88%)▶cstf2t (bg=12.11%)▶FMR1 (bg=1.89%)▶khsrp (bg=27.4%)▶METAP2 (bg=1.11%)▶XRN2 (bg=1.09%)▶zc3h8 (bg=12.78%)MATCHES To TargetScan▶ miR-181-5p:ACAUUCA


ATTTAAATAAA

ATTTAAATAAA  
Depth:6 (MOUSE)  
Ei-value:0.000, Pi-value:0.000  
Er-value:0.000, Pr-value:0.000  
eCLIP MATCHES▶cpsf6 (bg=13.45%)▶CSTF2 (bg=7.88%)▶cstf2t (bg=12.11%)▶FMR1 (bg=1.89%)▶khsrp (bg=27.4%)▶METAP2 (bg=1.11%)▶XRN2 (bg=1.09%)▶zc3h8 (bg=12.78%)No matches to TargetScan


ATA

TCAAAATAATAAACTATTTTTATTAGAGAATGTATACTTTTAGAAAGCTGTCTCCTTATTTAAATAAAATA  
Depth:2 (MARMOSET)  
Ei-value:0.000, Pi-value:0.000  
Er-value:0.000, Pr-value:0.000  
eCLIP MATCHES▶cpsf6 (bg=13.45%)▶CSTF2 (bg=7.88%)▶cstf2t (bg=12.11%)▶FMR1 (bg=1.89%)▶khsrp (bg=27.4%)▶METAP2 (bg=1.11%)▶XRN2 (bg=1.09%)▶zc3h8 (bg=12.78%)MATCHES To TargetScan▶ miR-181-5p:ACAUUCA

-

TGTTTGT

TGTTTGT  
Depth:6 (MOUSE)  
Ei-value:0.000, Pi-value:0.000  
Er-value:0.000, Pr-value:0.000  
eCLIP MATCHES▶CSTF2 (bg=7.88%)▶cstf2t (bg=12.11%)▶FMR1 (bg=1.89%)▶khsrp (bg=27.4%)▶METAP2 (bg=1.11%)▶XRN2 (bg=1.09%)▶zc3h8 (bg=12.78%)No matches to TargetScan

---------

AGTGTTG

AGTGTTGGGGCAATCTTGGGGGG  
Depth:2 (MARMOSET)  
Ei-value:0.000, Pi-value:0.000  
Er-value:0.000, Pr-value:0.000  
eCLIP MATCHES▶CSTF2 (bg=7.88%)▶cstf2t (bg=12.11%)▶FMR1 (bg=1.89%)▶khsrp (bg=27.4%)▶METAP2 (bg=1.11%)▶XRN2 (bg=1.09%)▶zc3h8 (bg=12.78%)MATCHES To TargetScan▶ miR-141-3p/200a-3p:AACACUG


GGGCAAT

GGGCAAT  
Depth:6 (MOUSE)  
Ei-value:0.000, Pi-value:0.000  
Er-value:0.000, Pr-value:0.000  
eCLIP MATCHES▶CSTF2 (bg=7.88%)▶cstf2t (bg=12.11%)▶FMR1 (bg=1.89%)▶khsrp (bg=27.4%)▶METAP2 (bg=1.11%)▶XRN2 (bg=1.09%)▶zc3h8 (bg=12.78%)No matches to TargetScan


C

GGGCAATCT  
Depth:5 (COW)  
Ei-value:0.000, Pi-value:0.000  
Er-value:0.000, Pr-value:0.000  
eCLIP MATCHES▶CSTF2 (bg=7.88%)▶cstf2t (bg=12.11%)▶FMR1 (bg=1.89%)▶khsrp (bg=27.4%)▶METAP2 (bg=1.11%)▶XRN2 (bg=1.09%)▶zc3h8 (bg=12.78%)No matches to TargetScan

 4680  


T

GGGCAATCT  
Depth:5 (COW)  
Ei-value:0.000, Pi-value:0.000  
Er-value:0.000, Pr-value:0.000  
eCLIP MATCHES▶CSTF2 (bg=7.88%)▶cstf2t (bg=12.11%)▶FMR1 (bg=1.89%)▶khsrp (bg=27.4%)▶METAP2 (bg=1.11%)▶XRN2 (bg=1.09%)▶zc3h8 (bg=12.78%)No matches to TargetScan


TGGGGGG

GGGCAATCTTGGGGGG  
Depth:4 (PIG)  
Ei-value:0.000, Pi-value:0.000  
Er-value:0.000, Pr-value:0.000  
eCLIP MATCHES▶CSTF2 (bg=7.88%)▶cstf2t (bg=12.11%)▶FMR1 (bg=1.89%)▶khsrp (bg=27.4%)▶METAP2 (bg=1.11%)▶XRN2 (bg=1.09%)▶zc3h8 (bg=12.78%)No matches to TargetScan

-

ATTCTT

ATTCTT  
Depth:4 (PIG)  
Ei-value:0.010, Pi-value:0.030  
Er-value:0.000, Pr-value:0.000  
eCLIP MATCHES▶CSTF2 (bg=7.88%)▶cstf2t (bg=12.11%)▶FMR1 (bg=1.89%)▶METAP2 (bg=1.11%)▶XRN2 (bg=1.09%)▶zc3h8 (bg=12.78%)No matches to TargetScan

-

TCTA

TCTAATCTTTCAGAAACTTTGTCTGCGA  
Depth:3 (DOG)  
Ei-value:0.000, Pi-value:0.000  
Er-value:0.000, Pr-value:0.000  
eCLIP MATCHES▶CSTF2 (bg=7.88%)▶cstf2t (bg=12.11%)▶FMR1 (bg=1.89%)▶METAP2 (bg=1.11%)▶ppil4 (bg=43.39%)▶srsf1 (bg=30.28%)▶tia1 (bg=23.76%)▶u2af2 (bg=19.32%)▶XRN2 (bg=1.09%)▶zc3h8 (bg=12.78%)MATCHES To TargetScan▶ miR-488-3p:UGAAAGG


ATC

ATCTTTCAGA  
Depth:5 (COW)  
Ei-value:0.000, Pi-value:0.000  
Er-value:0.000, Pr-value:0.000  
eCLIP MATCHES▶CSTF2 (bg=7.88%)▶cstf2t (bg=12.11%)▶FMR1 (bg=1.89%)▶METAP2 (bg=1.11%)▶ppil4 (bg=43.39%)▶u2af2 (bg=19.32%)▶XRN2 (bg=1.09%)▶zc3h8 (bg=12.78%)MATCHES To TargetScan▶ miR-488-3p:UGAAAGG


TTTCAGA

TTTCAGA  
Depth:8 (ALLIGATOR)  
Ei-value:0.000, Pi-value:0.000  
Er-value:0.000, Pr-value:0.000  
eCLIP MATCHES▶CSTF2 (bg=7.88%)▶cstf2t (bg=12.11%)▶FMR1 (bg=1.89%)▶METAP2 (bg=1.11%)▶ppil4 (bg=43.39%)▶u2af2 (bg=19.32%)▶XRN2 (bg=1.09%)▶zc3h8 (bg=12.78%)No matches to TargetScan


A

TCTAATCTTTCAGAAACTTTGTCTGCGA  
Depth:3 (DOG)  
Ei-value:0.000, Pi-value:0.000  
Er-value:0.000, Pr-value:0.000  
eCLIP MATCHES▶CSTF2 (bg=7.88%)▶cstf2t (bg=12.11%)▶FMR1 (bg=1.89%)▶METAP2 (bg=1.11%)▶ppil4 (bg=43.39%)▶srsf1 (bg=30.28%)▶tia1 (bg=23.76%)▶u2af2 (bg=19.32%)▶XRN2 (bg=1.09%)▶zc3h8 (bg=12.78%)MATCHES To TargetScan▶ miR-488-3p:UGAAAGG


ACTTTGTCTGCGA

ACTTTGTCTGCGA  
Depth:5 (COW)  
Ei-value:0.000, Pi-value:0.000  
Er-value:0.000, Pr-value:0.000  
eCLIP MATCHES▶CSTF2 (bg=7.88%)▶cstf2t (bg=12.11%)▶FMR1 (bg=1.89%)▶METAP2 (bg=1.11%)▶ppil4 (bg=43.39%)▶srsf1 (bg=30.28%)▶tia1 (bg=23.76%)▶u2af2 (bg=19.32%)▶XRN2 (bg=1.09%)▶zc3h8 (bg=12.78%)No matches to TargetScan


ACAC

TCTAATCTTTCAGAAACTTTGTCTGCGAACAC  
Depth:2 (MARMOSET)  
Ei-value:0.000, Pi-value:0.000  
Er-value:0.000, Pr-value:0.000  
eCLIP MATCHES▶CSTF2 (bg=7.88%)▶cstf2t (bg=12.11%)▶FMR1 (bg=1.89%)▶METAP2 (bg=1.11%)▶ppil4 (bg=43.39%)▶srsf1 (bg=30.28%)▶tia1 (bg=23.76%)▶u2af2 (bg=19.32%)▶XRN2 (bg=1.09%)▶zc3h8 (bg=12.78%)MATCHES To TargetScan▶ miR-488-3p:UGAAAGG

-

CTT

CTTTAATGGAC  
Depth:3 (DOG)  
Ei-value:0.000, Pi-value:0.000  
Er-value:0.000, Pr-value:0.000  
eCLIP MATCHES▶CSTF2 (bg=7.88%)▶cstf2t (bg=12.11%)▶ppil4 (bg=43.39%)▶srsf1 (bg=30.28%)▶tia1 (bg=23.76%)▶u2af2 (bg=19.32%)No matches to TargetScan


TAATGGAC

TAATGGAC  
Depth:5 (COW)  
Ei-value:0.000, Pi-value:0.010  
Er-value:0.000, Pr-value:0.000  
eCLIP MATCHES▶CSTF2 (bg=7.88%)▶cstf2t (bg=12.11%)▶ppil4 (bg=43.39%)▶srsf1 (bg=30.28%)▶u2af2 (bg=19.32%)No matches to TargetScan

-

AGATCAGGA

AGATCAGGA  
Depth:6 (MOUSE)  
Ei-value:0.000, Pi-value:0.000  
Er-value:0.000, Pr-value:0.000  
eCLIP MATCHES▶CSTF2 (bg=7.88%)▶cstf2t (bg=12.11%)▶ppil4 (bg=43.39%)▶srsf1 (bg=30.28%)▶u2af2 (bg=19.32%)No matches to TargetScan


T

AGATCAGGATTTGAGCGGAAGAACGAATGTAACTTT  
Depth:2 (MARMOSET)  
Ei-value:0.000, Pi-value:0.000  
Er-value:0.000, Pr-value:0.000  
eCLIP MATCHES▶CSTF2 (bg=7.88%)▶cstf2t (bg=12.11%)▶ppil4 (bg=43.39%)▶srsf1 (bg=30.28%)▶u2af2 (bg=19.32%)MATCHES To TargetScan▶ miR-371-5p:CUCAAAC▶ miR-181-5p:ACAUUCA


TTGAGC

TTGAGC  
Depth:6 (MOUSE)  
Ei-value:0.000, Pi-value:0.010  
Er-value:0.000, Pr-value:0.000  
eCLIP MATCHES▶CSTF2 (bg=7.88%)▶cstf2t (bg=12.11%)▶ppil4 (bg=43.39%)▶srsf1 (bg=30.28%)▶u2af2 (bg=19.32%)No matches to TargetScan


G

TTGAGCGGAAGAACGAAT  
Depth:3 (DOG)  
Ei-value:0.000, Pi-value:0.000  
Er-value:0.000, Pr-value:0.000  
eCLIP MATCHES▶CSTF2 (bg=7.88%)▶cstf2t (bg=12.11%)▶ppil4 (bg=43.39%)▶srsf1 (bg=30.28%)▶u2af2 (bg=19.32%)No matches to TargetScan


GAAGAACGAAT

GAAGAACGAAT  
Depth:5 (COW)  
Ei-value:0.000, Pi-value:0.000  
Er-value:0.000, Pr-value:0.000  
eCLIP MATCHES▶CSTF2 (bg=7.88%)▶cstf2t (bg=12.11%)▶ppil4 (bg=43.39%)▶srsf1 (bg=30.28%)No matches to TargetScan


G

AGATCAGGATTTGAGCGGAAGAACGAATGTAACTTT  
Depth:2 (MARMOSET)  
Ei-value:0.000, Pi-value:0.000  
Er-value:0.000, Pr-value:0.000  
eCLIP MATCHES▶CSTF2 (bg=7.88%)▶cstf2t (bg=12.11%)▶ppil4 (bg=43.39%)▶srsf1 (bg=30.28%)▶u2af2 (bg=19.32%)MATCHES To TargetScan▶ miR-371-5p:CUCAAAC▶ miR-181-5p:ACAUUCA


taactt

taactt  
Depth:4 (PIG)  
Ei-value:0.010, Pi-value:0.060  
Er-value:0.000, Pr-value:0.000  
eCLIP MATCHES▶CSTF2 (bg=7.88%)▶cstf2t (bg=12.11%)▶srsf1 (bg=30.28%)No matches to TargetScan


t

taacttt  
Depth:3 (DOG)  
Ei-value:0.350, Pi-value:0.020  
Er-value:0.000, Pr-value:0.000  
eCLIP MATCHES▶CSTF2 (bg=7.88%)▶cstf2t (bg=12.11%)▶srsf1 (bg=30.28%)No matches to TargetScan


A

AAGGCAGGAAA  
Depth:6 (MOUSE)  
Ei-value:0.000, Pi-value:0.000  
Er-value:0.000, Pr-value:0.000  
eCLIP MATCHES▶CSTF2 (bg=7.88%)▶cstf2t (bg=12.11%)▶srsf1 (bg=30.28%)No matches to TargetScan


AGGCAGGA

AGGCAGGA  
Depth:10 (SNAKE)  
Ei-value:0.000, Pi-value:0.000  
Er-value:0.000, Pr-value:0.000  
eCLIP MATCHES▶CSTF2 (bg=7.88%)▶cstf2t (bg=12.11%)▶srsf1 (bg=30.28%)No matches to TargetScan


AA

AGGCAGGAAA  
Depth:8 (ALLIGATOR)  
Ei-value:0.000, Pi-value:0.000  
Er-value:0.000, Pr-value:0.000  
eCLIP MATCHES▶CSTF2 (bg=7.88%)▶cstf2t (bg=12.11%)▶srsf1 (bg=30.28%)No matches to TargetScan


g

aaggcaggaaag  
Depth:2 (MARMOSET)  
Ei-value:0.990, Pi-value:0.000  
Er-value:0.000, Pr-value:0.000  
eCLIP MATCHES▶CSTF2 (bg=7.88%)▶cstf2t (bg=12.11%)▶srsf1 (bg=30.28%)No matches to TargetScan

--

AA

AAATTTTATTCTTCATAAA  
Depth:2 (MARMOSET)  
Ei-value:0.000, Pi-value:0.000  
Er-value:0.000, Pr-value:0.000  
eCLIP MATCHES▶CSTF2 (bg=7.88%)▶cstf2t (bg=12.11%)▶hltf (bg=24.28%)▶ppil4 (bg=43.39%)▶tial1 (bg=15.02%)No matches to TargetScan


ATTTTAT

ATTTTAT  
Depth:4 (PIG)  
Ei-value:0.000, Pi-value:0.000  
Er-value:0.000, Pr-value:0.000  
eCLIP MATCHES▶CSTF2 (bg=7.88%)▶cstf2t (bg=12.11%)No matches to TargetScan

 4800  


ATTTTAT  
Depth:4 (PIG)  
Ei-value:0.000, Pi-value:0.000  
Er-value:0.000, Pr-value:0.000  
eCLIP MATCHES▶CSTF2 (bg=7.88%)▶cstf2t (bg=12.11%)No matches to TargetScan


TCTTCATAAA

AAATTTTATTCTTCATAAA  
Depth:2 (MARMOSET)  
Ei-value:0.000, Pi-value:0.000  
Er-value:0.000, Pr-value:0.000  
eCLIP MATCHES▶CSTF2 (bg=7.88%)▶cstf2t (bg=12.11%)▶hltf (bg=24.28%)▶ppil4 (bg=43.39%)▶tial1 (bg=15.02%)No matches to TargetScan

-

TGATGA

TGATGA  
Depth:6 (MOUSE)  
Ei-value:0.000, Pi-value:0.030  
Er-value:0.000, Pr-value:0.000  
eCLIP MATCHES▶CSTF2 (bg=7.88%)▶cstf2t (bg=12.11%)▶hltf (bg=24.28%)▶ppil4 (bg=43.39%)▶tia1 (bg=23.76%)▶tial1 (bg=15.02%)No matches to TargetScan


GCAT

TGATGAGCAT  
Depth:3 (DOG)  
Ei-value:0.000, Pi-value:0.000  
Er-value:0.000, Pr-value:0.000  
eCLIP MATCHES▶CSTF2 (bg=7.88%)▶cstf2t (bg=12.11%)▶hltf (bg=24.28%)▶ppil4 (bg=43.39%)▶tia1 (bg=23.76%)▶tial1 (bg=15.02%)No matches to TargetScan


A

TGATGAGCATATAATAATTCCAGGCACATGGCAATAGAGGCCCTCTAAATAAGGAATAA  
Depth:2 (MARMOSET)  
Ei-value:0.000, Pi-value:0.000  
Er-value:0.000, Pr-value:0.000  
eCLIP MATCHES▶CSTF2 (bg=7.88%)▶cstf2t (bg=12.11%)▶hltf (bg=24.28%)▶ppil4 (bg=43.39%)▶tia1 (bg=23.76%)▶tial1 (bg=15.02%)MATCHES To TargetScan▶ miR-877-5p:UAGAGGA▶ miR-455-5p:AUGUGCC▶ miR-137:UAUUGCU


TAATAATT

TAATAATT  
Depth:6 (MOUSE)  
Ei-value:0.000, Pi-value:0.000  
Er-value:0.000, Pr-value:0.000  
eCLIP MATCHES▶cstf2t (bg=12.11%)▶ppil4 (bg=43.39%)▶tia1 (bg=23.76%)▶tial1 (bg=15.02%)No matches to TargetScan


CCAGGCACATGGC

TAATAATTCCAGGCACATGGC  
Depth:5 (COW)  
Ei-value:0.000, Pi-value:0.000  
Er-value:0.000, Pr-value:0.000  
eCLIP MATCHES▶cstf2t (bg=12.11%)▶ppil4 (bg=43.39%)▶tia1 (bg=23.76%)▶tial1 (bg=15.02%)MATCHES To TargetScan▶ miR-455-5p:AUGUGCC


A

TAATAATTCCAGGCACATGGCAATAGAGGCCCTCTAAATAAGGAATAA  
Depth:3 (DOG)  
Ei-value:0.000, Pi-value:0.000  
Er-value:0.000, Pr-value:0.000  
eCLIP MATCHES▶cstf2t (bg=12.11%)▶ppil4 (bg=43.39%)▶tia1 (bg=23.76%)▶tial1 (bg=15.02%)MATCHES To TargetScan▶ miR-877-5p:UAGAGGA▶ miR-455-5p:AUGUGCC▶ miR-137:UAUUGCU


ATAGAG

ATAGAG  
Depth:6 (MOUSE)  
Ei-value:0.000, Pi-value:0.010  
Er-value:0.000, Pr-value:0.000  
eCLIP MATCHES▶cstf2t (bg=12.11%)▶ppil4 (bg=43.39%)▶tia1 (bg=23.76%)▶tial1 (bg=15.02%)No matches to TargetScan


GCCCTC

TAATAATTCCAGGCACATGGCAATAGAGGCCCTCTAAATAAGGAATAA  
Depth:3 (DOG)  
Ei-value:0.000, Pi-value:0.000  
Er-value:0.000, Pr-value:0.000  
eCLIP MATCHES▶cstf2t (bg=12.11%)▶ppil4 (bg=43.39%)▶tia1 (bg=23.76%)▶tial1 (bg=15.02%)MATCHES To TargetScan▶ miR-877-5p:UAGAGGA▶ miR-455-5p:AUGUGCC▶ miR-137:UAUUGCU


TAAA

TAAATAAGGAATAA  
Depth:5 (COW)  
Ei-value:0.000, Pi-value:0.000  
Er-value:0.000, Pr-value:0.000  
eCLIP MATCHES▶cstf2t (bg=12.11%)▶tia1 (bg=23.76%)▶tial1 (bg=15.02%)No matches to TargetScan


TAAGGA

TAAGGA  
Depth:13 (OPOSSUM)  
Ei-value:0.000, Pi-value:0.000  
Er-value:0.000, Pr-value:0.000  
eCLIP MATCHES▶cstf2t (bg=12.11%)▶tia1 (bg=23.76%)▶tial1 (bg=15.02%)No matches to TargetScan


ATAA

TAAATAAGGAATAA  
Depth:5 (COW)  
Ei-value:0.000, Pi-value:0.000  
Er-value:0.000, Pr-value:0.000  
eCLIP MATCHES▶cstf2t (bg=12.11%)▶tia1 (bg=23.76%)▶tial1 (bg=15.02%)No matches to TargetScan

-

TA

TAACCTCTTAGACAGGTGGGAGATTATGATCAGAGTAAAAGGTAATTACACATTTTATTTCCAGAAAGTCAGG  
Depth:2 (MARMOSET)  
Ei-value:0.000, Pi-value:0.000  
Er-value:0.000, Pr-value:0.000  
eCLIP MATCHES▶cstf2t (bg=12.11%)▶SLBP (bg=6.66%)▶tia1 (bg=23.76%)▶zc3h8 (bg=12.78%)MATCHES To TargetScan▶ miR-216a-5p:AAUCUCA▶ miR-150-5p:CUCCCAA▶ miR-532-3p:CUCCCAC▶ miR-216b-5p:AAUCUCU▶ miR-154-3p/487-3p:AUCAUAC▶ miR-154-5p:AGGUUAU


ACCTCTTAGA

ACCTCTTAGACAGGTGGGAGATTATGATCAGA  
Depth:3 (DOG)  
Ei-value:0.000, Pi-value:0.000  
Er-value:0.000, Pr-value:0.000  
No matches to eCLIP DataMATCHES To TargetScan▶ miR-216a-5p:AAUCUCA▶ miR-150-5p:CUCCCAA▶ miR-532-3p:CUCCCAC▶ miR-216b-5p:AAUCUCU▶ miR-154-3p/487-3p:AUCAUAC


CAGGTGGGAGAT

CAGGTGGGAGAT  
Depth:6 (MOUSE)  
Ei-value:0.000, Pi-value:0.000  
Er-value:0.000, Pr-value:0.000  
No matches to eCLIP DataMATCHES To TargetScan▶ miR-150-5p:CUCCCAA▶ miR-532-3p:CUCCCAC


TATGATCAGA

CAGGTGGGAGATTATGATCAGA  
Depth:5 (COW)  
Ei-value:0.000, Pi-value:0.000  
Er-value:0.000, Pr-value:0.000  
No matches to eCLIP DataMATCHES To TargetScan▶ miR-216a-5p:AAUCUCA▶ miR-150-5p:CUCCCAA▶ miR-532-3p:CUCCCAC▶ miR-216b-5p:AAUCUCU▶ miR-154-3p/487-3p:AUCAUAC


GTAAA

TAACCTCTTAGACAGGTGGGAGATTATGATCAGAGTAAAAGGTAATTACACATTTTATTTCCAGAAAGTCAGG  
Depth:2 (MARMOSET)  
Ei-value:0.000, Pi-value:0.000  
Er-value:0.000, Pr-value:0.000  
eCLIP MATCHES▶cstf2t (bg=12.11%)▶SLBP (bg=6.66%)▶tia1 (bg=23.76%)▶zc3h8 (bg=12.78%)MATCHES To TargetScan▶ miR-216a-5p:AAUCUCA▶ miR-150-5p:CUCCCAA▶ miR-532-3p:CUCCCAC▶ miR-216b-5p:AAUCUCU▶ miR-154-3p/487-3p:AUCAUAC▶ miR-154-5p:AGGUUAU


AGGTAA

AGGTAA  
Depth:6 (MOUSE)  
Ei-value:0.000, Pi-value:0.010  
Er-value:0.000, Pr-value:0.000  
No matches to eCLIP DataNo matches to TargetScan


TTAC

TAACCTCTTAGACAGGTGGGAGATTATGATCAGAGTAAAAGGTAATTACACATTTTATTTCCAGAAAGTCAGG  
Depth:2 (MARMOSET)  
Ei-value:0.000, Pi-value:0.000  
Er-value:0.000, Pr-value:0.000  
eCLIP MATCHES▶cstf2t (bg=12.11%)▶SLBP (bg=6.66%)▶tia1 (bg=23.76%)▶zc3h8 (bg=12.78%)MATCHES To TargetScan▶ miR-216a-5p:AAUCUCA▶ miR-150-5p:CUCCCAA▶ miR-532-3p:CUCCCAC▶ miR-216b-5p:AAUCUCU▶ miR-154-3p/487-3p:AUCAUAC▶ miR-154-5p:AGGUUAU

 4920  


ACATTT

TAACCTCTTAGACAGGTGGGAGATTATGATCAGAGTAAAAGGTAATTACACATTTTATTTCCAGAAAGTCAGG  
Depth:2 (MARMOSET)  
Ei-value:0.000, Pi-value:0.000  
Er-value:0.000, Pr-value:0.000  
eCLIP MATCHES▶cstf2t (bg=12.11%)▶SLBP (bg=6.66%)▶tia1 (bg=23.76%)▶zc3h8 (bg=12.78%)MATCHES To TargetScan▶ miR-216a-5p:AAUCUCA▶ miR-150-5p:CUCCCAA▶ miR-532-3p:CUCCCAC▶ miR-216b-5p:AAUCUCU▶ miR-154-3p/487-3p:AUCAUAC▶ miR-154-5p:AGGUUAU


TATTTC

TATTTCCAGAAAGTCAGG  
Depth:3 (DOG)  
Ei-value:0.000, Pi-value:0.000  
Er-value:0.000, Pr-value:0.000  
eCLIP MATCHES▶cstf2t (bg=12.11%)▶SLBP (bg=6.66%)▶tia1 (bg=23.76%)▶zc3h8 (bg=12.78%)No matches to TargetScan


CAGAA

CAGAAAGTCAGG  
Depth:6 (MOUSE)  
Ei-value:0.000, Pi-value:0.000  
Er-value:0.000, Pr-value:0.000  
eCLIP MATCHES▶cstf2t (bg=12.11%)▶SLBP (bg=6.66%)▶tia1 (bg=23.76%)▶zc3h8 (bg=12.78%)No matches to TargetScan


AGTCAG

AGTCAG  
Depth:7 (TURTLE)  
Ei-value:0.000, Pi-value:0.000  
Er-value:0.000, Pr-value:0.000  
eCLIP MATCHES▶cstf2t (bg=12.11%)▶SLBP (bg=6.66%)▶tia1 (bg=23.76%)▶zc3h8 (bg=12.78%)No matches to TargetScan


G

CAGAAAGTCAGG  
Depth:6 (MOUSE)  
Ei-value:0.000, Pi-value:0.000  
Er-value:0.000, Pr-value:0.000  
eCLIP MATCHES▶cstf2t (bg=12.11%)▶SLBP (bg=6.66%)▶tia1 (bg=23.76%)▶zc3h8 (bg=12.78%)No matches to TargetScan

--

tctataaatt

tctataaatt  
Depth:2 (MARMOSET)  
Ei-value:1.000, Pi-value:0.000  
Er-value:0.000, Pr-value:0.000  
eCLIP MATCHES▶cstf2t (bg=12.11%)▶SLBP (bg=6.66%)▶tia1 (bg=23.76%)▶zc3h8 (bg=12.78%)No matches to TargetScan

---------

TAGAGTAA

TAGAGTAA  
Depth:3 (DOG)  
Ei-value:0.010, Pi-value:0.000  
Er-value:0.000, Pr-value:0.000  
eCLIP MATCHES▶cstf2t (bg=12.11%)▶SLBP (bg=6.66%)▶tia1 (bg=23.76%)▶zc3h8 (bg=12.78%)No matches to TargetScan

---

TTTTTCACATTTCCA

TTTTTCACATTTCCA  
Depth:2 (MARMOSET)  
Ei-value:0.000, Pi-value:0.000  
Er-value:0.000, Pr-value:0.000  
eCLIP MATCHES▶CSTF2 (bg=7.88%)▶cstf2t (bg=12.11%)▶ppil4 (bg=43.39%)▶SLBP (bg=6.66%)▶zc3h8 (bg=12.78%)MATCHES To TargetScan▶ miR-203a-3p.1:GAAAUGU

-

AGT

AGTTTGCATGTTAACTTTAAATGCTTACAATCTTA  
Depth:2 (MARMOSET)  
Ei-value:0.000, Pi-value:0.000  
Er-value:0.000, Pr-value:0.000  
eCLIP MATCHES▶CSTF2 (bg=7.88%)▶cstf2t (bg=12.11%)▶khsrp (bg=27.4%)▶RBFOX2 (bg=3.41%)▶SUPV3L1 (bg=9.63%)▶zc3h8 (bg=12.78%)MATCHES To TargetScan▶ miR-219-5p:GAUUGUC


T

TTGCATGT  
Depth:5 (COW)  
Ei-value:0.000, Pi-value:0.000  
Er-value:0.000, Pr-value:0.000  
eCLIP MATCHES▶CSTF2 (bg=7.88%)▶cstf2t (bg=12.11%)▶SUPV3L1 (bg=9.63%)No matches to TargetScan


TGCATG

TGCATG  
Depth:8 (ALLIGATOR)  
Ei-value:0.000, Pi-value:0.000  
Er-value:0.000, Pr-value:0.000  
eCLIP MATCHES▶CSTF2 (bg=7.88%)▶cstf2t (bg=12.11%)▶SUPV3L1 (bg=9.63%)No matches to TargetScan


T

TGCATGT  
Depth:6 (MOUSE)  
Ei-value:0.000, Pi-value:0.000  
Er-value:0.000, Pr-value:0.000  
eCLIP MATCHES▶CSTF2 (bg=7.88%)▶cstf2t (bg=12.11%)▶SUPV3L1 (bg=9.63%)No matches to TargetScan


T

AGTTTGCATGTTAACTTTAAATGCTTACAATCTTA  
Depth:2 (MARMOSET)  
Ei-value:0.000, Pi-value:0.000  
Er-value:0.000, Pr-value:0.000  
eCLIP MATCHES▶CSTF2 (bg=7.88%)▶cstf2t (bg=12.11%)▶khsrp (bg=27.4%)▶RBFOX2 (bg=3.41%)▶SUPV3L1 (bg=9.63%)▶zc3h8 (bg=12.78%)MATCHES To TargetScan▶ miR-219-5p:GAUUGUC


AACTTT

AACTTTAAATGCTT  
Depth:3 (DOG)  
Ei-value:0.000, Pi-value:0.000  
Er-value:0.000, Pr-value:0.000  
eCLIP MATCHES▶CSTF2 (bg=7.88%)▶cstf2t (bg=12.11%)▶khsrp (bg=27.4%)▶RBFOX2 (bg=3.41%)No matches to TargetScan


AAATGCTT

AAATGCTT  
Depth:4 (PIG)  
Ei-value:0.000, Pi-value:0.000  
Er-value:0.000, Pr-value:0.000  
eCLIP MATCHES▶cstf2t (bg=12.11%)▶khsrp (bg=27.4%)▶RBFOX2 (bg=3.41%)No matches to TargetScan


ACAATCTTA

AGTTTGCATGTTAACTTTAAATGCTTACAATCTTA  
Depth:2 (MARMOSET)  
Ei-value:0.000, Pi-value:0.000  
Er-value:0.000, Pr-value:0.000  
eCLIP MATCHES▶CSTF2 (bg=7.88%)▶cstf2t (bg=12.11%)▶khsrp (bg=27.4%)▶RBFOX2 (bg=3.41%)▶SUPV3L1 (bg=9.63%)▶zc3h8 (bg=12.78%)MATCHES To TargetScan▶ miR-219-5p:GAUUGUC

------

TAGGCAA

TAGGCAATGTTTTACACTATTG  
Depth:2 (MARMOSET)  
Ei-value:0.000, Pi-value:0.000  
Er-value:0.000, Pr-value:0.000  
eCLIP MATCHES▶cstf2t (bg=12.11%)▶HNRNPU (bg=9.45%)▶khsrp (bg=27.4%)▶RBFOX2 (bg=3.41%)▶SUPV3L1 (bg=9.63%)▶tial1 (bg=15.02%)MATCHES To TargetScan▶ miR-543:AACAUUC▶ miR-142-3p.2:UAGUGUU

 5040  


TGTTTTACACTATTG

TAGGCAATGTTTTACACTATTG  
Depth:2 (MARMOSET)  
Ei-value:0.000, Pi-value:0.000  
Er-value:0.000, Pr-value:0.000  
eCLIP MATCHES▶cstf2t (bg=12.11%)▶HNRNPU (bg=9.45%)▶khsrp (bg=27.4%)▶RBFOX2 (bg=3.41%)▶SUPV3L1 (bg=9.63%)▶tial1 (bg=15.02%)MATCHES To TargetScan▶ miR-543:AACAUUC▶ miR-142-3p.2:UAGUGUU

------

tatagg

tatagg  
Depth:3 (DOG)  
Ei-value:1.000, Pi-value:0.060  
Er-value:0.000, Pr-value:0.000  
eCLIP MATCHES▶cstf2t (bg=12.11%)▶ddx42 (bg=10.33%)▶HNRNPU (bg=9.45%)▶khsrp (bg=27.4%)▶PCBP2 (bg=4.33%)▶RBFOX2 (bg=3.41%)▶SF3A3 (bg=0.97%)▶SUPV3L1 (bg=9.63%)▶tial1 (bg=15.02%)▶u2af1 (bg=14.02%)▶u2af2 (bg=19.32%)No matches to TargetScan

-------------

gcctgtggg

gcctgtggg  
Depth:2 (MARMOSET)  
Ei-value:1.000, Pi-value:0.000  
Er-value:0.000, Pr-value:0.000  
eCLIP MATCHES▶cstf2t (bg=12.11%)▶ddx42 (bg=10.33%)▶HNRNPU (bg=9.45%)▶PCBP2 (bg=4.33%)▶ppil4 (bg=43.39%)▶RBFOX2 (bg=3.41%)▶SF3A3 (bg=0.97%)▶SUPV3L1 (bg=9.63%)▶tial1 (bg=15.02%)▶u2af1 (bg=14.02%)▶u2af2 (bg=19.32%)MATCHES To TargetScan▶ miR-140-3p.1:CCACAGG

-

T

TTTTAAAGAA  
Depth:5 (COW)  
Ei-value:0.000, Pi-value:0.000  
Er-value:0.000, Pr-value:0.000  
eCLIP MATCHES▶CSTF2 (bg=7.88%)▶cstf2t (bg=12.11%)▶ddx42 (bg=10.33%)▶HNRNPU (bg=9.45%)▶PCBP2 (bg=4.33%)▶RBFOX2 (bg=3.41%)▶SF3A3 (bg=0.97%)▶u2af1 (bg=14.02%)▶u2af2 (bg=19.32%)▶zc3h8 (bg=12.78%)No matches to TargetScan


TTTAAAGA

TTTAAAGA  
Depth:6 (MOUSE)  
Ei-value:0.000, Pi-value:0.000  
Er-value:0.000, Pr-value:0.000  
eCLIP MATCHES▶CSTF2 (bg=7.88%)▶cstf2t (bg=12.11%)▶ddx42 (bg=10.33%)▶HNRNPU (bg=9.45%)▶PCBP2 (bg=4.33%)▶RBFOX2 (bg=3.41%)▶SF3A3 (bg=0.97%)▶u2af1 (bg=14.02%)▶u2af2 (bg=19.32%)▶zc3h8 (bg=12.78%)No matches to TargetScan


A

TTTTAAAGAA  
Depth:5 (COW)  
Ei-value:0.000, Pi-value:0.000  
Er-value:0.000, Pr-value:0.000  
eCLIP MATCHES▶CSTF2 (bg=7.88%)▶cstf2t (bg=12.11%)▶ddx42 (bg=10.33%)▶HNRNPU (bg=9.45%)▶PCBP2 (bg=4.33%)▶RBFOX2 (bg=3.41%)▶SF3A3 (bg=0.97%)▶u2af1 (bg=14.02%)▶u2af2 (bg=19.32%)▶zc3h8 (bg=12.78%)No matches to TargetScan


TTTTC

TTTTAAAGAATTTTC  
Depth:3 (DOG)  
Ei-value:0.000, Pi-value:0.000  
Er-value:0.000, Pr-value:0.000  
eCLIP MATCHES▶CSTF2 (bg=7.88%)▶cstf2t (bg=12.11%)▶ddx42 (bg=10.33%)▶HNRNPU (bg=9.45%)▶PCBP2 (bg=4.33%)▶ppil4 (bg=43.39%)▶RBFOX2 (bg=3.41%)▶SF3A3 (bg=0.97%)▶u2af1 (bg=14.02%)▶u2af2 (bg=19.32%)▶zc3h8 (bg=12.78%)No matches to TargetScan


C

TTTTAAAGAATTTTCCTTTGCAGAGGCATTTCATCCTTCATGAAGC  
Depth:2 (MARMOSET)  
Ei-value:0.000, Pi-value:0.000  
Er-value:0.000, Pr-value:0.000  
eCLIP MATCHES▶CSTF2 (bg=7.88%)▶cstf2t (bg=12.11%)▶ddx42 (bg=10.33%)▶HNRNPU (bg=9.45%)▶khsrp (bg=27.4%)▶PCBP2 (bg=4.33%)▶ppil4 (bg=43.39%)▶RBFOX2 (bg=3.41%)▶SF3A3 (bg=0.97%)▶srsf1 (bg=30.28%)▶u2af1 (bg=14.02%)▶u2af2 (bg=19.32%)▶zc3h8 (bg=12.78%)MATCHES To TargetScan▶ miR-532-5p:AUGCCUU▶ miR-433-3p:UCAUGAU▶ miR-203a-3p.1:GAAAUGU▶ miR-365-3p:AAUGCCC▶ miR-203a-3p.2:UGAAAUG


TTTGCAGAG

TTTGCAGAG  
Depth:6 (MOUSE)  
Ei-value:0.000, Pi-value:0.000  
Er-value:0.000, Pr-value:0.000  
eCLIP MATCHES▶CSTF2 (bg=7.88%)▶cstf2t (bg=12.11%)▶ddx42 (bg=10.33%)▶HNRNPU (bg=9.45%)▶ppil4 (bg=43.39%)▶RBFOX2 (bg=3.41%)▶u2af1 (bg=14.02%)▶u2af2 (bg=19.32%)▶zc3h8 (bg=12.78%)No matches to TargetScan


G

TTTGCAGAGG  
Depth:5 (COW)  
Ei-value:0.000, Pi-value:0.000  
Er-value:0.000, Pr-value:0.000  
eCLIP MATCHES▶CSTF2 (bg=7.88%)▶cstf2t (bg=12.11%)▶ddx42 (bg=10.33%)▶HNRNPU (bg=9.45%)▶khsrp (bg=27.4%)▶ppil4 (bg=43.39%)▶RBFOX2 (bg=3.41%)▶u2af1 (bg=14.02%)▶u2af2 (bg=19.32%)▶zc3h8 (bg=12.78%)No matches to TargetScan


CA

CATTTCATCCTTCATGAAGC  
Depth:3 (DOG)  
Ei-value:0.000, Pi-value:0.000  
Er-value:0.000, Pr-value:0.000  
eCLIP MATCHES▶CSTF2 (bg=7.88%)▶cstf2t (bg=12.11%)▶ddx42 (bg=10.33%)▶HNRNPU (bg=9.45%)▶khsrp (bg=27.4%)▶ppil4 (bg=43.39%)▶RBFOX2 (bg=3.41%)▶srsf1 (bg=30.28%)▶u2af1 (bg=14.02%)▶u2af2 (bg=19.32%)▶zc3h8 (bg=12.78%)MATCHES To TargetScan▶ miR-433-3p:UCAUGAU▶ miR-203a-3p.1:GAAAUGU▶ miR-203a-3p.2:UGAAAUG


TTTCAT

TTTCAT  
Depth:8 (ALLIGATOR)  
Ei-value:0.000, Pi-value:0.000  
Er-value:0.000, Pr-value:0.000  
eCLIP MATCHES▶CSTF2 (bg=7.88%)▶cstf2t (bg=12.11%)▶HNRNPU (bg=9.45%)▶khsrp (bg=27.4%)▶ppil4 (bg=43.39%)▶RBFOX2 (bg=3.41%)▶srsf1 (bg=30.28%)▶u2af1 (bg=14.02%)▶u2af2 (bg=19.32%)▶zc3h8 (bg=12.78%)No matches to TargetScan


CCTTC

TTTCATCCTTC  
Depth:6 (MOUSE)  
Ei-value:0.000, Pi-value:0.000  
Er-value:0.000, Pr-value:0.000  
eCLIP MATCHES▶CSTF2 (bg=7.88%)▶cstf2t (bg=12.11%)▶HNRNPU (bg=9.45%)▶khsrp (bg=27.4%)▶ppil4 (bg=43.39%)▶RBFOX2 (bg=3.41%)▶srsf1 (bg=30.28%)▶u2af1 (bg=14.02%)▶u2af2 (bg=19.32%)▶zc3h8 (bg=12.78%)No matches to TargetScan


ATGA

TTTCATCCTTCATGA  
Depth:4 (PIG)  
Ei-value:0.000, Pi-value:0.000  
Er-value:0.000, Pr-value:0.000  
eCLIP MATCHES▶CSTF2 (bg=7.88%)▶cstf2t (bg=12.11%)▶HNRNPU (bg=9.45%)▶khsrp (bg=27.4%)▶ppil4 (bg=43.39%)▶RBFOX2 (bg=3.41%)▶srsf1 (bg=30.28%)▶u2af1 (bg=14.02%)▶u2af2 (bg=19.32%)▶zc3h8 (bg=12.78%)MATCHES To TargetScan▶ miR-433-3p:UCAUGAU


AGC

CATTTCATCCTTCATGAAGC  
Depth:3 (DOG)  
Ei-value:0.000, Pi-value:0.000  
Er-value:0.000, Pr-value:0.000  
eCLIP MATCHES▶CSTF2 (bg=7.88%)▶cstf2t (bg=12.11%)▶ddx42 (bg=10.33%)▶HNRNPU (bg=9.45%)▶khsrp (bg=27.4%)▶ppil4 (bg=43.39%)▶RBFOX2 (bg=3.41%)▶srsf1 (bg=30.28%)▶u2af1 (bg=14.02%)▶u2af2 (bg=19.32%)▶zc3h8 (bg=12.78%)MATCHES To TargetScan▶ miR-433-3p:UCAUGAU▶ miR-203a-3p.1:GAAAUGU▶ miR-203a-3p.2:UGAAAUG

--

TTCAGGATTTTG

TTCAGGATTTTG  
Depth:5 (COW)  
Ei-value:0.000, Pi-value:0.000  
Er-value:0.000, Pr-value:0.000  
eCLIP MATCHES▶CSTF2 (bg=7.88%)▶cstf2t (bg=12.11%)▶HNRNPU (bg=9.45%)▶khsrp (bg=27.4%)▶ppil4 (bg=43.39%)▶srsf1 (bg=30.28%)▶u2af1 (bg=14.02%)▶u2af2 (bg=19.32%)▶zc3h8 (bg=12.78%)No matches to TargetScan


A

TTCAGGATTTTGA  
Depth:4 (PIG)  
Ei-value:0.000, Pi-value:0.000  
Er-value:0.000, Pr-value:0.000  
eCLIP MATCHES▶CSTF2 (bg=7.88%)▶cstf2t (bg=12.11%)▶HNRNPU (bg=9.45%)▶khsrp (bg=27.4%)▶ppil4 (bg=43.39%)▶srsf1 (bg=30.28%)▶u2af1 (bg=14.02%)▶u2af2 (bg=19.32%)▶zc3h8 (bg=12.78%)No matches to TargetScan

-

TTGCATAT

TTGCATAT  
Depth:4 (PIG)  
Ei-value:0.000, Pi-value:0.010  
Er-value:0.000, Pr-value:0.000  
eCLIP MATCHES▶CSTF2 (bg=7.88%)▶cstf2t (bg=12.11%)▶HNRNPM (bg=6.37%)▶PRPF8 (bg=6.2%)▶TARDBP (bg=5.12%)▶u2af1 (bg=14.02%)▶u2af2 (bg=19.32%)No matches to TargetScan

 5160  


TTGCATAT  
Depth:4 (PIG)  
Ei-value:0.000, Pi-value:0.010  
Er-value:0.000, Pr-value:0.000  
eCLIP MATCHES▶CSTF2 (bg=7.88%)▶cstf2t (bg=12.11%)▶HNRNPM (bg=6.37%)▶PRPF8 (bg=6.2%)▶TARDBP (bg=5.12%)▶u2af1 (bg=14.02%)▶u2af2 (bg=19.32%)No matches to TargetScan


GAGT

TTGCATATGAGTGCTTGGCTCTTCCTTCTGTTCT  
Depth:2 (MARMOSET)  
Ei-value:0.000, Pi-value:0.000  
Er-value:0.000, Pr-value:0.000  
eCLIP MATCHES▶CSTF2 (bg=7.88%)▶cstf2t (bg=12.11%)▶hnrnpa1 (bg=18.32%)▶HNRNPM (bg=6.37%)▶PRPF8 (bg=6.2%)▶srsf1 (bg=30.28%)▶srsf7 (bg=22.53%)▶TARDBP (bg=5.12%)▶tia1 (bg=23.76%)▶u2af1 (bg=14.02%)▶u2af2 (bg=19.32%)MATCHES To TargetScan▶ miR-7-5p:GGAAGAC


GCTTGGCTCT

GCTTGGCTCT  
Depth:5 (COW)  
Ei-value:0.000, Pi-value:0.000  
Er-value:0.000, Pr-value:0.000  
eCLIP MATCHES▶CSTF2 (bg=7.88%)▶cstf2t (bg=12.11%)▶hnrnpa1 (bg=18.32%)▶HNRNPM (bg=6.37%)▶PRPF8 (bg=6.2%)▶srsf1 (bg=30.28%)▶srsf7 (bg=22.53%)▶TARDBP (bg=5.12%)▶tia1 (bg=23.76%)▶u2af1 (bg=14.02%)▶u2af2 (bg=19.32%)No matches to TargetScan


TCC

GCTTGGCTCTTCCTTCTGTTCT  
Depth:3 (DOG)  
Ei-value:0.000, Pi-value:0.000  
Er-value:0.000, Pr-value:0.000  
eCLIP MATCHES▶CSTF2 (bg=7.88%)▶cstf2t (bg=12.11%)▶hnrnpa1 (bg=18.32%)▶HNRNPM (bg=6.37%)▶PRPF8 (bg=6.2%)▶srsf1 (bg=30.28%)▶srsf7 (bg=22.53%)▶TARDBP (bg=5.12%)▶tia1 (bg=23.76%)▶u2af1 (bg=14.02%)▶u2af2 (bg=19.32%)MATCHES To TargetScan▶ miR-7-5p:GGAAGAC


TTCTGT

TTCTGT  
Depth:9 (LIZARD)  
Ei-value:0.000, Pi-value:0.000  
Er-value:0.000, Pr-value:0.000  
eCLIP MATCHES▶CSTF2 (bg=7.88%)▶cstf2t (bg=12.11%)▶hnrnpa1 (bg=18.32%)▶HNRNPM (bg=6.37%)▶PRPF8 (bg=6.2%)▶srsf1 (bg=30.28%)▶srsf7 (bg=22.53%)▶TARDBP (bg=5.12%)▶tia1 (bg=23.76%)▶u2af1 (bg=14.02%)▶u2af2 (bg=19.32%)No matches to TargetScan


TCT

GCTTGGCTCTTCCTTCTGTTCT  
Depth:3 (DOG)  
Ei-value:0.000, Pi-value:0.000  
Er-value:0.000, Pr-value:0.000  
eCLIP MATCHES▶CSTF2 (bg=7.88%)▶cstf2t (bg=12.11%)▶hnrnpa1 (bg=18.32%)▶HNRNPM (bg=6.37%)▶PRPF8 (bg=6.2%)▶srsf1 (bg=30.28%)▶srsf7 (bg=22.53%)▶TARDBP (bg=5.12%)▶tia1 (bg=23.76%)▶u2af1 (bg=14.02%)▶u2af2 (bg=19.32%)MATCHES To TargetScan▶ miR-7-5p:GGAAGAC


AGTGAGTG

AGTGAGTG  
Depth:3 (DOG)  
Ei-value:0.010, Pi-value:0.000  
Er-value:0.000, Pr-value:0.000  
eCLIP MATCHES▶CSTF2 (bg=7.88%)▶cstf2t (bg=12.11%)▶hnrnpa1 (bg=18.32%)▶HNRNPM (bg=6.37%)▶PRPF8 (bg=6.2%)▶srsf1 (bg=30.28%)▶srsf7 (bg=22.53%)▶TARDBP (bg=5.12%)▶tia1 (bg=23.76%)▶u2af1 (bg=14.02%)▶u2af2 (bg=19.32%)No matches to TargetScan


TA

AGTGAGTGTATGAGACCTTGCAGTGAGTTTATCAGCATACTCAAAATTTTTTTCCTGGAATTTGGAGGGATGGGAGGAGGGGGTGGGGCTTACTTGTT  
Depth:2 (MARMOSET)  
Ei-value:0.000, Pi-value:0.000  
Er-value:0.000, Pr-value:0.000  
eCLIP MATCHES▶CSTF2 (bg=7.88%)▶cstf2t (bg=12.11%)▶ddx42 (bg=10.33%)▶FAM120A (bg=0.57%)▶gtf2f1 (bg=10.18%)▶hnrnpa1 (bg=18.32%)▶HNRNPM (bg=6.37%)▶PCBP2 (bg=4.33%)▶PRPF8 (bg=6.2%)▶RBM5 (bg=0.39%)▶SF3A3 (bg=0.97%)▶SF3B4 (bg=4.76%)▶srsf1 (bg=30.28%)▶srsf7 (bg=22.53%)▶TARDBP (bg=5.12%)▶tia1 (bg=23.76%)▶tial1 (bg=15.02%)▶u2af1 (bg=14.02%)▶u2af2 (bg=19.32%)MATCHES To TargetScan▶ miR-665:CCAGGAG▶ miR-217:ACUGCAU▶ miR-150-5p:CUCCCAA▶ miR-532-3p:CUCCCAC▶ miR-496.1:GAGUAUU▶ miR-873-5p.1:CAGGAAC▶ miR-188-5p:AUCCCUU▶ miR-539-3p:UCAUACA


TG

TGAGACCTTGCAGTG  
Depth:4 (PIG)  
Ei-value:0.000, Pi-value:0.000  
Er-value:0.000, Pr-value:0.000  
eCLIP MATCHES▶CSTF2 (bg=7.88%)▶cstf2t (bg=12.11%)▶hnrnpa1 (bg=18.32%)▶HNRNPM (bg=6.37%)▶PRPF8 (bg=6.2%)▶srsf1 (bg=30.28%)▶srsf7 (bg=22.53%)▶TARDBP (bg=5.12%)▶tia1 (bg=23.76%)▶u2af1 (bg=14.02%)▶u2af2 (bg=19.32%)MATCHES To TargetScan▶ miR-217:ACUGCAU


AGACCT

AGACCTTGCAGTG  
Depth:6 (MOUSE)  
Ei-value:0.000, Pi-value:0.000  
Er-value:0.000, Pr-value:0.000  
eCLIP MATCHES▶CSTF2 (bg=7.88%)▶cstf2t (bg=12.11%)▶hnrnpa1 (bg=18.32%)▶HNRNPM (bg=6.37%)▶PRPF8 (bg=6.2%)▶srsf1 (bg=30.28%)▶srsf7 (bg=22.53%)▶TARDBP (bg=5.12%)▶tia1 (bg=23.76%)▶u2af1 (bg=14.02%)▶u2af2 (bg=19.32%)MATCHES To TargetScan▶ miR-217:ACUGCAU


TGCAGTG

TGCAGTG  
Depth:10 (SNAKE)  
Ei-value:0.000, Pi-value:0.000  
Er-value:0.000, Pr-value:0.000  
eCLIP MATCHES▶cstf2t (bg=12.11%)▶hnrnpa1 (bg=18.32%)▶HNRNPM (bg=6.37%)▶PRPF8 (bg=6.2%)▶srsf7 (bg=22.53%)▶TARDBP (bg=5.12%)MATCHES To TargetScan▶ miR-217:ACUGCAU


AGTT

AGTGAGTGTATGAGACCTTGCAGTGAGTTTATCAGCATACTCAAAATTTTTTTCCTGGAATTTGGAGGGATGGGAGGAGGGGGTGGGGCTTACTTGTT  
Depth:2 (MARMOSET)  
Ei-value:0.000, Pi-value:0.000  
Er-value:0.000, Pr-value:0.000  
eCLIP MATCHES▶CSTF2 (bg=7.88%)▶cstf2t (bg=12.11%)▶ddx42 (bg=10.33%)▶FAM120A (bg=0.57%)▶gtf2f1 (bg=10.18%)▶hnrnpa1 (bg=18.32%)▶HNRNPM (bg=6.37%)▶PCBP2 (bg=4.33%)▶PRPF8 (bg=6.2%)▶RBM5 (bg=0.39%)▶SF3A3 (bg=0.97%)▶SF3B4 (bg=4.76%)▶srsf1 (bg=30.28%)▶srsf7 (bg=22.53%)▶TARDBP (bg=5.12%)▶tia1 (bg=23.76%)▶tial1 (bg=15.02%)▶u2af1 (bg=14.02%)▶u2af2 (bg=19.32%)MATCHES To TargetScan▶ miR-665:CCAGGAG▶ miR-217:ACUGCAU▶ miR-150-5p:CUCCCAA▶ miR-532-3p:CUCCCAC▶ miR-496.1:GAGUAUU▶ miR-873-5p.1:CAGGAAC▶ miR-188-5p:AUCCCUU▶ miR-539-3p:UCAUACA


TA

TATCAGCATA  
Depth:3 (DOG)  
Ei-value:0.000, Pi-value:0.000  
Er-value:0.000, Pr-value:0.000  
eCLIP MATCHES▶cstf2t (bg=12.11%)▶hnrnpa1 (bg=18.32%)▶HNRNPM (bg=6.37%)▶PRPF8 (bg=6.2%)▶srsf7 (bg=22.53%)▶TARDBP (bg=5.12%)No matches to TargetScan


TCAGCATA

TCAGCATA  
Depth:4 (PIG)  
Ei-value:0.000, Pi-value:0.000  
Er-value:0.000, Pr-value:0.000  
eCLIP MATCHES▶hnrnpa1 (bg=18.32%)▶PRPF8 (bg=6.2%)▶srsf7 (bg=22.53%)▶TARDBP (bg=5.12%)No matches to TargetScan


CTCAAAATTTTTTTCCTGGAA

AGTGAGTGTATGAGACCTTGCAGTGAGTTTATCAGCATACTCAAAATTTTTTTCCTGGAATTTGGAGGGATGGGAGGAGGGGGTGGGGCTTACTTGTT  
Depth:2 (MARMOSET)  
Ei-value:0.000, Pi-value:0.000  
Er-value:0.000, Pr-value:0.000  
eCLIP MATCHES▶CSTF2 (bg=7.88%)▶cstf2t (bg=12.11%)▶ddx42 (bg=10.33%)▶FAM120A (bg=0.57%)▶gtf2f1 (bg=10.18%)▶hnrnpa1 (bg=18.32%)▶HNRNPM (bg=6.37%)▶PCBP2 (bg=4.33%)▶PRPF8 (bg=6.2%)▶RBM5 (bg=0.39%)▶SF3A3 (bg=0.97%)▶SF3B4 (bg=4.76%)▶srsf1 (bg=30.28%)▶srsf7 (bg=22.53%)▶TARDBP (bg=5.12%)▶tia1 (bg=23.76%)▶tial1 (bg=15.02%)▶u2af1 (bg=14.02%)▶u2af2 (bg=19.32%)MATCHES To TargetScan▶ miR-665:CCAGGAG▶ miR-217:ACUGCAU▶ miR-150-5p:CUCCCAA▶ miR-532-3p:CUCCCAC▶ miR-496.1:GAGUAUU▶ miR-873-5p.1:CAGGAAC▶ miR-188-5p:AUCCCUU▶ miR-539-3p:UCAUACA


TTTGGAG

TTTGGAG  
Depth:5 (COW)  
Ei-value:0.000, Pi-value:0.000  
Er-value:0.000, Pr-value:0.000  
eCLIP MATCHES▶FAM120A (bg=0.57%)▶gtf2f1 (bg=10.18%)▶HNRNPM (bg=6.37%)▶PCBP2 (bg=4.33%)▶RBM5 (bg=0.39%)▶SF3A3 (bg=0.97%)▶SF3B4 (bg=4.76%)▶tia1 (bg=23.76%)▶tial1 (bg=15.02%)▶u2af1 (bg=14.02%)▶u2af2 (bg=19.32%)No matches to TargetScan


GGAT

AGTGAGTGTATGAGACCTTGCAGTGAGTTTATCAGCATACTCAAAATTTTTTTCCTGGAATTTGGAGGGATGGGAGGAGGGGGTGGGGCTTACTTGTT  
Depth:2 (MARMOSET)  
Ei-value:0.000, Pi-value:0.000  
Er-value:0.000, Pr-value:0.000  
eCLIP MATCHES▶CSTF2 (bg=7.88%)▶cstf2t (bg=12.11%)▶ddx42 (bg=10.33%)▶FAM120A (bg=0.57%)▶gtf2f1 (bg=10.18%)▶hnrnpa1 (bg=18.32%)▶HNRNPM (bg=6.37%)▶PCBP2 (bg=4.33%)▶PRPF8 (bg=6.2%)▶RBM5 (bg=0.39%)▶SF3A3 (bg=0.97%)▶SF3B4 (bg=4.76%)▶srsf1 (bg=30.28%)▶srsf7 (bg=22.53%)▶TARDBP (bg=5.12%)▶tia1 (bg=23.76%)▶tial1 (bg=15.02%)▶u2af1 (bg=14.02%)▶u2af2 (bg=19.32%)MATCHES To TargetScan▶ miR-665:CCAGGAG▶ miR-217:ACUGCAU▶ miR-150-5p:CUCCCAA▶ miR-532-3p:CUCCCAC▶ miR-496.1:GAGUAUU▶ miR-873-5p.1:CAGGAAC▶ miR-188-5p:AUCCCUU▶ miR-539-3p:UCAUACA


GGGAGGAG

GGGAGGAG  
Depth:5 (COW)  
Ei-value:0.000, Pi-value:0.000  
Er-value:0.000, Pr-value:0.000  
eCLIP MATCHES▶FAM120A (bg=0.57%)▶gtf2f1 (bg=10.18%)▶HNRNPM (bg=6.37%)▶PCBP2 (bg=4.33%)▶RBM5 (bg=0.39%)▶SF3A3 (bg=0.97%)▶SF3B4 (bg=4.76%)▶tia1 (bg=23.76%)▶tial1 (bg=15.02%)▶u2af1 (bg=14.02%)▶u2af2 (bg=19.32%)No matches to TargetScan


GGGG

GGGAGGAGGGGG  
Depth:3 (DOG)  
Ei-value:0.000, Pi-value:0.000  
Er-value:0.000, Pr-value:0.000  
eCLIP MATCHES▶ddx42 (bg=10.33%)▶FAM120A (bg=0.57%)▶gtf2f1 (bg=10.18%)▶HNRNPM (bg=6.37%)▶PCBP2 (bg=4.33%)▶RBM5 (bg=0.39%)▶SF3A3 (bg=0.97%)▶SF3B4 (bg=4.76%)▶tia1 (bg=23.76%)▶tial1 (bg=15.02%)▶u2af1 (bg=14.02%)▶u2af2 (bg=19.32%)No matches to TargetScan


T

AGTGAGTGTATGAGACCTTGCAGTGAGTTTATCAGCATACTCAAAATTTTTTTCCTGGAATTTGGAGGGATGGGAGGAGGGGGTGGGGCTTACTTGTT  
Depth:2 (MARMOSET)  
Ei-value:0.000, Pi-value:0.000  
Er-value:0.000, Pr-value:0.000  
eCLIP MATCHES▶CSTF2 (bg=7.88%)▶cstf2t (bg=12.11%)▶ddx42 (bg=10.33%)▶FAM120A (bg=0.57%)▶gtf2f1 (bg=10.18%)▶hnrnpa1 (bg=18.32%)▶HNRNPM (bg=6.37%)▶PCBP2 (bg=4.33%)▶PRPF8 (bg=6.2%)▶RBM5 (bg=0.39%)▶SF3A3 (bg=0.97%)▶SF3B4 (bg=4.76%)▶srsf1 (bg=30.28%)▶srsf7 (bg=22.53%)▶TARDBP (bg=5.12%)▶tia1 (bg=23.76%)▶tial1 (bg=15.02%)▶u2af1 (bg=14.02%)▶u2af2 (bg=19.32%)MATCHES To TargetScan▶ miR-665:CCAGGAG▶ miR-217:ACUGCAU▶ miR-150-5p:CUCCCAA▶ miR-532-3p:CUCCCAC▶ miR-496.1:GAGUAUU▶ miR-873-5p.1:CAGGAAC▶ miR-188-5p:AUCCCUU▶ miR-539-3p:UCAUACA


GGGGCTT

GGGGCTTACTTGTT  
Depth:6 (MOUSE)  
Ei-value:0.000, Pi-value:0.000  
Er-value:0.000, Pr-value:0.000  
eCLIP MATCHES▶ddx42 (bg=10.33%)▶FAM120A (bg=0.57%)▶gtf2f1 (bg=10.18%)▶HNRNPM (bg=6.37%)▶PCBP2 (bg=4.33%)▶RBM5 (bg=0.39%)▶SF3A3 (bg=0.97%)▶SF3B4 (bg=4.76%)▶tia1 (bg=23.76%)▶tial1 (bg=15.02%)▶u2af1 (bg=14.02%)▶u2af2 (bg=19.32%)No matches to TargetScan


ACT

ACTTGTT  
Depth:7 (TURTLE)  
Ei-value:0.000, Pi-value:0.000  
Er-value:0.000, Pr-value:0.000  
eCLIP MATCHES▶FAM120A (bg=0.57%)▶gtf2f1 (bg=10.18%)▶HNRNPM (bg=6.37%)▶PCBP2 (bg=4.33%)▶RBM5 (bg=0.39%)▶SF3A3 (bg=0.97%)▶SF3B4 (bg=4.76%)▶tia1 (bg=23.76%)▶u2af1 (bg=14.02%)▶u2af2 (bg=19.32%)No matches to TargetScan

 5280  


TGTT

ACTTGTT  
Depth:7 (TURTLE)  
Ei-value:0.000, Pi-value:0.000  
Er-value:0.000, Pr-value:0.000  
eCLIP MATCHES▶FAM120A (bg=0.57%)▶gtf2f1 (bg=10.18%)▶HNRNPM (bg=6.37%)▶PCBP2 (bg=4.33%)▶RBM5 (bg=0.39%)▶SF3A3 (bg=0.97%)▶SF3B4 (bg=4.76%)▶tia1 (bg=23.76%)▶u2af1 (bg=14.02%)▶u2af2 (bg=19.32%)No matches to TargetScan

---------

TTTTTTTTTACAGAC

TTTTTTTTTACAGAC  
Depth:3 (DOG)  
Ei-value:0.000, Pi-value:0.000  
Er-value:0.000, Pr-value:0.000  
eCLIP MATCHES▶CDC40 (bg=0.09%)▶CSTF2 (bg=7.88%)▶cstf2t (bg=12.11%)▶DGCR8 (bg=2.67%)▶HNRNPC (bg=4.22%)▶HNRNPU (bg=9.45%)▶khsrp (bg=27.4%)▶PCBP2 (bg=4.33%)▶ppil4 (bg=43.39%)▶RBFOX2 (bg=3.41%)▶srsf7 (bg=22.53%)▶SUPV3L1 (bg=9.63%)▶tia1 (bg=23.76%)▶tial1 (bg=15.02%)▶u2af1 (bg=14.02%)▶u2af2 (bg=19.32%)▶zc3h8 (bg=12.78%)No matches to TargetScan


TTCACAG

TTTTTTTTTACAGACTTCACAGAGAATGCAGTTGTCTTGACTTCAGGTCTGTCTGTTCTGTTGGCAAGTAA  
Depth:2 (MARMOSET)  
Ei-value:0.000, Pi-value:0.000  
Er-value:0.000, Pr-value:0.000  
eCLIP MATCHES▶CDC40 (bg=0.09%)▶CSTF2 (bg=7.88%)▶cstf2t (bg=12.11%)▶DGCR8 (bg=2.67%)▶HNRNPC (bg=4.22%)▶HNRNPU (bg=9.45%)▶khsrp (bg=27.4%)▶PCBP2 (bg=4.33%)▶ppil4 (bg=43.39%)▶RBFOX2 (bg=3.41%)▶srsf7 (bg=22.53%)▶SUPV3L1 (bg=9.63%)▶TARDBP (bg=5.12%)▶tia1 (bg=23.76%)▶tial1 (bg=15.02%)▶u2af1 (bg=14.02%)▶u2af2 (bg=19.32%)▶zc3h8 (bg=12.78%)MATCHES To TargetScan▶ miR-217:ACUGCAU▶ miR-33-5p:UGCAUUG▶ miR-224-5p:AAGUCAC


AGAATGCAG

AGAATGCAG  
Depth:8 (ALLIGATOR)  
Ei-value:0.000, Pi-value:0.000  
Er-value:0.000, Pr-value:0.000  
eCLIP MATCHES▶CSTF2 (bg=7.88%)▶cstf2t (bg=12.11%)▶DGCR8 (bg=2.67%)▶HNRNPC (bg=4.22%)▶HNRNPU (bg=9.45%)▶khsrp (bg=27.4%)▶ppil4 (bg=43.39%)▶RBFOX2 (bg=3.41%)▶srsf7 (bg=22.53%)▶tia1 (bg=23.76%)▶tial1 (bg=15.02%)▶u2af1 (bg=14.02%)▶u2af2 (bg=19.32%)▶zc3h8 (bg=12.78%)MATCHES To TargetScan▶ miR-33-5p:UGCAUUG


T

AGAATGCAGTTGTCTTGAC  
Depth:5 (COW)  
Ei-value:0.000, Pi-value:0.000  
Er-value:0.000, Pr-value:0.000  
eCLIP MATCHES▶CSTF2 (bg=7.88%)▶cstf2t (bg=12.11%)▶DGCR8 (bg=2.67%)▶HNRNPC (bg=4.22%)▶HNRNPU (bg=9.45%)▶khsrp (bg=27.4%)▶ppil4 (bg=43.39%)▶RBFOX2 (bg=3.41%)▶srsf7 (bg=22.53%)▶tia1 (bg=23.76%)▶tial1 (bg=15.02%)▶u2af1 (bg=14.02%)▶u2af2 (bg=19.32%)▶zc3h8 (bg=12.78%)MATCHES To TargetScan▶ miR-217:ACUGCAU▶ miR-33-5p:UGCAUUG


TGTCTTGAC

TGTCTTGAC  
Depth:6 (MOUSE)  
Ei-value:0.000, Pi-value:0.000  
Er-value:0.000, Pr-value:0.000  
eCLIP MATCHES▶CSTF2 (bg=7.88%)▶cstf2t (bg=12.11%)▶DGCR8 (bg=2.67%)▶HNRNPC (bg=4.22%)▶HNRNPU (bg=9.45%)▶khsrp (bg=27.4%)▶ppil4 (bg=43.39%)▶RBFOX2 (bg=3.41%)▶srsf7 (bg=22.53%)▶tia1 (bg=23.76%)▶tial1 (bg=15.02%)▶u2af1 (bg=14.02%)▶u2af2 (bg=19.32%)▶zc3h8 (bg=12.78%)No matches to TargetScan


T

TTTTTTTTTACAGACTTCACAGAGAATGCAGTTGTCTTGACTTCAGGTCTGTCTGTTCTGTTGGCAAGTAA  
Depth:2 (MARMOSET)  
Ei-value:0.000, Pi-value:0.000  
Er-value:0.000, Pr-value:0.000  
eCLIP MATCHES▶CDC40 (bg=0.09%)▶CSTF2 (bg=7.88%)▶cstf2t (bg=12.11%)▶DGCR8 (bg=2.67%)▶HNRNPC (bg=4.22%)▶HNRNPU (bg=9.45%)▶khsrp (bg=27.4%)▶PCBP2 (bg=4.33%)▶ppil4 (bg=43.39%)▶RBFOX2 (bg=3.41%)▶srsf7 (bg=22.53%)▶SUPV3L1 (bg=9.63%)▶TARDBP (bg=5.12%)▶tia1 (bg=23.76%)▶tial1 (bg=15.02%)▶u2af1 (bg=14.02%)▶u2af2 (bg=19.32%)▶zc3h8 (bg=12.78%)MATCHES To TargetScan▶ miR-217:ACUGCAU▶ miR-33-5p:UGCAUUG▶ miR-224-5p:AAGUCAC


T

TCAGGTC  
Depth:6 (MOUSE)  
Ei-value:0.000, Pi-value:0.000  
Er-value:0.000, Pr-value:0.000  
eCLIP MATCHES▶CSTF2 (bg=7.88%)▶cstf2t (bg=12.11%)▶DGCR8 (bg=2.67%)▶HNRNPC (bg=4.22%)▶HNRNPU (bg=9.45%)▶khsrp (bg=27.4%)▶ppil4 (bg=43.39%)▶RBFOX2 (bg=3.41%)▶srsf7 (bg=22.53%)▶tia1 (bg=23.76%)▶tial1 (bg=15.02%)▶u2af1 (bg=14.02%)▶u2af2 (bg=19.32%)▶zc3h8 (bg=12.78%)No matches to TargetScan


CAGGTC

CAGGTC  
Depth:7 (TURTLE)  
Ei-value:0.000, Pi-value:0.000  
Er-value:0.000, Pr-value:0.000  
eCLIP MATCHES▶CSTF2 (bg=7.88%)▶cstf2t (bg=12.11%)▶DGCR8 (bg=2.67%)▶HNRNPC (bg=4.22%)▶khsrp (bg=27.4%)▶ppil4 (bg=43.39%)▶RBFOX2 (bg=3.41%)▶srsf7 (bg=22.53%)▶tia1 (bg=23.76%)▶tial1 (bg=15.02%)▶u2af1 (bg=14.02%)▶u2af2 (bg=19.32%)▶zc3h8 (bg=12.78%)No matches to TargetScan


TGT

TCAGGTCTGT  
Depth:5 (COW)  
Ei-value:0.000, Pi-value:0.000  
Er-value:0.000, Pr-value:0.000  
eCLIP MATCHES▶CSTF2 (bg=7.88%)▶cstf2t (bg=12.11%)▶DGCR8 (bg=2.67%)▶HNRNPC (bg=4.22%)▶HNRNPU (bg=9.45%)▶khsrp (bg=27.4%)▶ppil4 (bg=43.39%)▶RBFOX2 (bg=3.41%)▶srsf7 (bg=22.53%)▶TARDBP (bg=5.12%)▶tia1 (bg=23.76%)▶tial1 (bg=15.02%)▶u2af1 (bg=14.02%)▶u2af2 (bg=19.32%)▶zc3h8 (bg=12.78%)No matches to TargetScan


CTGTTCTG

TCAGGTCTGTCTGTTCTGTTGGCAAGTAA  
Depth:4 (PIG)  
Ei-value:0.000, Pi-value:0.000  
Er-value:0.000, Pr-value:0.000  
eCLIP MATCHES▶CSTF2 (bg=7.88%)▶cstf2t (bg=12.11%)▶DGCR8 (bg=2.67%)▶HNRNPC (bg=4.22%)▶HNRNPU (bg=9.45%)▶khsrp (bg=27.4%)▶ppil4 (bg=43.39%)▶RBFOX2 (bg=3.41%)▶srsf7 (bg=22.53%)▶TARDBP (bg=5.12%)▶tia1 (bg=23.76%)▶tial1 (bg=15.02%)▶u2af1 (bg=14.02%)▶u2af2 (bg=19.32%)▶zc3h8 (bg=12.78%)No matches to TargetScan


TTGGCAAGTAA

TTGGCAAGTAA  
Depth:10 (SNAKE)  
Ei-value:0.000, Pi-value:0.000  
Er-value:0.000, Pr-value:0.000  
eCLIP MATCHES▶CSTF2 (bg=7.88%)▶cstf2t (bg=12.11%)▶HNRNPC (bg=4.22%)▶khsrp (bg=27.4%)▶ppil4 (bg=43.39%)▶srsf7 (bg=22.53%)▶TARDBP (bg=5.12%)▶tia1 (bg=23.76%)▶u2af1 (bg=14.02%)▶u2af2 (bg=19.32%)No matches to TargetScan


A

ATGCAGTACTGTTCTGATCCCGCTGCTATTAGAATGCATTGTGAAACGACTGGAGTATGATTAAAAGTTGTGTTCCCCAATGCTTGGAGTAGTGATTGTTGAAGGAAAAAA  
Depth:2 (MARMOSET)  
Ei-value:0.000, Pi-value:0.000  
Er-value:0.000, Pr-value:0.000  
eCLIP MATCHES▶CSTF2 (bg=7.88%)▶cstf2t (bg=12.11%)▶DDX21 (bg=1.05%)▶EIF4G2 (bg=2.16%)▶hnrnpa1 (bg=18.32%)▶HNRNPM (bg=6.37%)▶HNRNPU (bg=9.45%)▶IGF2BP1 (bg=1.23%)▶khsrp (bg=27.4%)▶LIN28B (bg=1.31%)▶srsf7 (bg=22.53%)▶TARDBP (bg=5.12%)▶tia1 (bg=23.76%)▶tial1 (bg=15.02%)▶u2af1 (bg=14.02%)▶u2af2 (bg=19.32%)▶zc3h8 (bg=12.78%)MATCHES To TargetScan▶ miR-421:UCAACAG▶ miR-217:ACUGCAU▶ miR-505-3p.2:UCAACAC▶ miR-132-3p/212-3p:AACAGUC▶ miR-145-5p:UCCAGUU▶ miR-154-3p/487-3p:AUCAUAC▶ miR-33-5p:UGCAUUG▶ miR-205-5p:CCUUCAU▶ miR-15-5p/16-5p/195-5p/424-5p/497-5p:AGCAGCA▶ miR-539-3p:UCAUACA▶ miR-383-5p.1:GAUCAGA▶ miR-503-5p:AGCAGCG▶ miR-144-3p:ACAGUAU▶ miR-101-3p.1:ACAGUAC


TG

TGCAGTACTGTTCTGA  
Depth:4 (PIG)  
Ei-value:0.000, Pi-value:0.000  
Er-value:0.000, Pr-value:0.000  
eCLIP MATCHES▶CSTF2 (bg=7.88%)▶cstf2t (bg=12.11%)▶HNRNPM (bg=6.37%)▶khsrp (bg=27.4%)▶srsf7 (bg=22.53%)▶TARDBP (bg=5.12%)▶tia1 (bg=23.76%)▶tial1 (bg=15.02%)▶u2af1 (bg=14.02%)▶u2af2 (bg=19.32%)MATCHES To TargetScan▶ miR-217:ACUGCAU▶ miR-132-3p/212-3p:AACAGUC▶ miR-144-3p:ACAGUAU▶ miR-101-3p.1:ACAGUAC


CAGTACTGTTC

CAGTACTGTTC  
Depth:6 (MOUSE)  
Ei-value:0.000, Pi-value:0.000  
Er-value:0.000, Pr-value:0.000  
eCLIP MATCHES▶CSTF2 (bg=7.88%)▶cstf2t (bg=12.11%)▶HNRNPM (bg=6.37%)▶khsrp (bg=27.4%)▶srsf7 (bg=22.53%)▶TARDBP (bg=5.12%)▶tia1 (bg=23.76%)▶tial1 (bg=15.02%)▶u2af1 (bg=14.02%)▶u2af2 (bg=19.32%)MATCHES To TargetScan▶ miR-132-3p/212-3p:AACAGUC▶ miR-144-3p:ACAGUAU▶ miR-101-3p.1:ACAGUAC


TGA

CAGTACTGTTCTGA  
Depth:5 (COW)  
Ei-value:0.000, Pi-value:0.000  
Er-value:0.000, Pr-value:0.000  
eCLIP MATCHES▶CSTF2 (bg=7.88%)▶cstf2t (bg=12.11%)▶HNRNPM (bg=6.37%)▶khsrp (bg=27.4%)▶srsf7 (bg=22.53%)▶TARDBP (bg=5.12%)▶tia1 (bg=23.76%)▶tial1 (bg=15.02%)▶u2af1 (bg=14.02%)▶u2af2 (bg=19.32%)MATCHES To TargetScan▶ miR-132-3p/212-3p:AACAGUC▶ miR-144-3p:ACAGUAU▶ miR-101-3p.1:ACAGUAC


TC

TGCAGTACTGTTCTGATC  
Depth:3 (DOG)  
Ei-value:0.000, Pi-value:0.000  
Er-value:0.000, Pr-value:0.000  
eCLIP MATCHES▶CSTF2 (bg=7.88%)▶cstf2t (bg=12.11%)▶HNRNPM (bg=6.37%)▶khsrp (bg=27.4%)▶srsf7 (bg=22.53%)▶TARDBP (bg=5.12%)▶tia1 (bg=23.76%)▶tial1 (bg=15.02%)▶u2af1 (bg=14.02%)▶u2af2 (bg=19.32%)MATCHES To TargetScan▶ miR-217:ACUGCAU▶ miR-132-3p/212-3p:AACAGUC▶ miR-383-5p.1:GAUCAGA▶ miR-144-3p:ACAGUAU▶ miR-101-3p.1:ACAGUAC


CC

ATGCAGTACTGTTCTGATCCCGCTGCTATTAGAATGCATTGTGAAACGACTGGAGTATGATTAAAAGTTGTGTTCCCCAATGCTTGGAGTAGTGATTGTTGAAGGAAAAAA  
Depth:2 (MARMOSET)  
Ei-value:0.000, Pi-value:0.000  
Er-value:0.000, Pr-value:0.000  
eCLIP MATCHES▶CSTF2 (bg=7.88%)▶cstf2t (bg=12.11%)▶DDX21 (bg=1.05%)▶EIF4G2 (bg=2.16%)▶hnrnpa1 (bg=18.32%)▶HNRNPM (bg=6.37%)▶HNRNPU (bg=9.45%)▶IGF2BP1 (bg=1.23%)▶khsrp (bg=27.4%)▶LIN28B (bg=1.31%)▶srsf7 (bg=22.53%)▶TARDBP (bg=5.12%)▶tia1 (bg=23.76%)▶tial1 (bg=15.02%)▶u2af1 (bg=14.02%)▶u2af2 (bg=19.32%)▶zc3h8 (bg=12.78%)MATCHES To TargetScan▶ miR-421:UCAACAG▶ miR-217:ACUGCAU▶ miR-505-3p.2:UCAACAC▶ miR-132-3p/212-3p:AACAGUC▶ miR-145-5p:UCCAGUU▶ miR-154-3p/487-3p:AUCAUAC▶ miR-33-5p:UGCAUUG▶ miR-205-5p:CCUUCAU▶ miR-15-5p/16-5p/195-5p/424-5p/497-5p:AGCAGCA▶ miR-539-3p:UCAUACA▶ miR-383-5p.1:GAUCAGA▶ miR-503-5p:AGCAGCG▶ miR-144-3p:ACAGUAU▶ miR-101-3p.1:ACAGUAC


GCTGCTATTAGAATG

GCTGCTATTAGAATGCATT  
Depth:6 (MOUSE)  
Ei-value:0.000, Pi-value:0.000  
Er-value:0.000, Pr-value:0.000  
eCLIP MATCHES▶CSTF2 (bg=7.88%)▶cstf2t (bg=12.11%)▶HNRNPM (bg=6.37%)▶khsrp (bg=27.4%)▶srsf7 (bg=22.53%)▶TARDBP (bg=5.12%)▶tia1 (bg=23.76%)▶tial1 (bg=15.02%)▶u2af2 (bg=19.32%)MATCHES To TargetScan▶ miR-33-5p:UGCAUUG▶ miR-15-5p/16-5p/195-5p/424-5p/497-5p:AGCAGCA▶ miR-503-5p:AGCAGCG

 5400  


CATT

GCTGCTATTAGAATGCATT  
Depth:6 (MOUSE)  
Ei-value:0.000, Pi-value:0.000  
Er-value:0.000, Pr-value:0.000  
eCLIP MATCHES▶CSTF2 (bg=7.88%)▶cstf2t (bg=12.11%)▶HNRNPM (bg=6.37%)▶khsrp (bg=27.4%)▶srsf7 (bg=22.53%)▶TARDBP (bg=5.12%)▶tia1 (bg=23.76%)▶tial1 (bg=15.02%)▶u2af2 (bg=19.32%)MATCHES To TargetScan▶ miR-33-5p:UGCAUUG▶ miR-15-5p/16-5p/195-5p/424-5p/497-5p:AGCAGCA▶ miR-503-5p:AGCAGCG


G

GCTGCTATTAGAATGCATTGTGAAACGACTGGAGTATGATTAAAAGTT  
Depth:4 (PIG)  
Ei-value:0.000, Pi-value:0.000  
Er-value:0.000, Pr-value:0.000  
eCLIP MATCHES▶CSTF2 (bg=7.88%)▶cstf2t (bg=12.11%)▶EIF4G2 (bg=2.16%)▶hnrnpa1 (bg=18.32%)▶HNRNPM (bg=6.37%)▶khsrp (bg=27.4%)▶LIN28B (bg=1.31%)▶srsf7 (bg=22.53%)▶TARDBP (bg=5.12%)▶tia1 (bg=23.76%)▶tial1 (bg=15.02%)▶u2af2 (bg=19.32%)MATCHES To TargetScan▶ miR-145-5p:UCCAGUU▶ miR-154-3p/487-3p:AUCAUAC▶ miR-33-5p:UGCAUUG▶ miR-15-5p/16-5p/195-5p/424-5p/497-5p:AGCAGCA▶ miR-539-3p:UCAUACA▶ miR-503-5p:AGCAGCG


TGAAA

TGAAACGACTGGAGTATGA  
Depth:5 (COW)  
Ei-value:0.000, Pi-value:0.000  
Er-value:0.000, Pr-value:0.000  
eCLIP MATCHES▶CSTF2 (bg=7.88%)▶cstf2t (bg=12.11%)▶EIF4G2 (bg=2.16%)▶HNRNPM (bg=6.37%)▶khsrp (bg=27.4%)▶LIN28B (bg=1.31%)▶srsf7 (bg=22.53%)▶TARDBP (bg=5.12%)▶u2af2 (bg=19.32%)MATCHES To TargetScan▶ miR-145-5p:UCCAGUU▶ miR-539-3p:UCAUACA


CGACTGG

CGACTGGAGTATGA  
Depth:6 (MOUSE)  
Ei-value:0.000, Pi-value:0.000  
Er-value:0.000, Pr-value:0.000  
eCLIP MATCHES▶CSTF2 (bg=7.88%)▶cstf2t (bg=12.11%)▶EIF4G2 (bg=2.16%)▶HNRNPM (bg=6.37%)▶khsrp (bg=27.4%)▶LIN28B (bg=1.31%)▶srsf7 (bg=22.53%)▶TARDBP (bg=5.12%)▶u2af2 (bg=19.32%)MATCHES To TargetScan▶ miR-145-5p:UCCAGUU▶ miR-539-3p:UCAUACA


AGTATG

AGTATG  
Depth:7 (TURTLE)  
Ei-value:0.000, Pi-value:0.000  
Er-value:0.000, Pr-value:0.000  
eCLIP MATCHES▶CSTF2 (bg=7.88%)▶cstf2t (bg=12.11%)▶EIF4G2 (bg=2.16%)▶HNRNPM (bg=6.37%)▶khsrp (bg=27.4%)▶LIN28B (bg=1.31%)▶srsf7 (bg=22.53%)▶TARDBP (bg=5.12%)No matches to TargetScan


A

CGACTGGAGTATGA  
Depth:6 (MOUSE)  
Ei-value:0.000, Pi-value:0.000  
Er-value:0.000, Pr-value:0.000  
eCLIP MATCHES▶CSTF2 (bg=7.88%)▶cstf2t (bg=12.11%)▶EIF4G2 (bg=2.16%)▶HNRNPM (bg=6.37%)▶khsrp (bg=27.4%)▶LIN28B (bg=1.31%)▶srsf7 (bg=22.53%)▶TARDBP (bg=5.12%)▶u2af2 (bg=19.32%)MATCHES To TargetScan▶ miR-145-5p:UCCAGUU▶ miR-539-3p:UCAUACA


TTAAAAGTT

GCTGCTATTAGAATGCATTGTGAAACGACTGGAGTATGATTAAAAGTT  
Depth:4 (PIG)  
Ei-value:0.000, Pi-value:0.000  
Er-value:0.000, Pr-value:0.000  
eCLIP MATCHES▶CSTF2 (bg=7.88%)▶cstf2t (bg=12.11%)▶EIF4G2 (bg=2.16%)▶hnrnpa1 (bg=18.32%)▶HNRNPM (bg=6.37%)▶khsrp (bg=27.4%)▶LIN28B (bg=1.31%)▶srsf7 (bg=22.53%)▶TARDBP (bg=5.12%)▶tia1 (bg=23.76%)▶tial1 (bg=15.02%)▶u2af2 (bg=19.32%)MATCHES To TargetScan▶ miR-145-5p:UCCAGUU▶ miR-154-3p/487-3p:AUCAUAC▶ miR-33-5p:UGCAUUG▶ miR-15-5p/16-5p/195-5p/424-5p/497-5p:AGCAGCA▶ miR-539-3p:UCAUACA▶ miR-503-5p:AGCAGCG


GTGTT

GCTGCTATTAGAATGCATTGTGAAACGACTGGAGTATGATTAAAAGTTGTGTT  
Depth:3 (DOG)  
Ei-value:0.000, Pi-value:0.000  
Er-value:0.000, Pr-value:0.000  
eCLIP MATCHES▶CSTF2 (bg=7.88%)▶cstf2t (bg=12.11%)▶EIF4G2 (bg=2.16%)▶hnrnpa1 (bg=18.32%)▶HNRNPM (bg=6.37%)▶khsrp (bg=27.4%)▶LIN28B (bg=1.31%)▶srsf7 (bg=22.53%)▶TARDBP (bg=5.12%)▶tia1 (bg=23.76%)▶tial1 (bg=15.02%)▶u2af2 (bg=19.32%)MATCHES To TargetScan▶ miR-145-5p:UCCAGUU▶ miR-154-3p/487-3p:AUCAUAC▶ miR-33-5p:UGCAUUG▶ miR-15-5p/16-5p/195-5p/424-5p/497-5p:AGCAGCA▶ miR-539-3p:UCAUACA▶ miR-503-5p:AGCAGCG


CCCCAA

CCCCAA  
Depth:6 (MOUSE)  
Ei-value:0.000, Pi-value:0.000  
Er-value:0.000, Pr-value:0.000  
eCLIP MATCHES▶cstf2t (bg=12.11%)▶EIF4G2 (bg=2.16%)▶hnrnpa1 (bg=18.32%)▶HNRNPM (bg=6.37%)▶khsrp (bg=27.4%)▶LIN28B (bg=1.31%)▶srsf7 (bg=22.53%)▶TARDBP (bg=5.12%)▶zc3h8 (bg=12.78%)No matches to TargetScan


TGCT

CCCCAATGCT  
Depth:5 (COW)  
Ei-value:0.000, Pi-value:0.000  
Er-value:0.000, Pr-value:0.000  
eCLIP MATCHES▶CSTF2 (bg=7.88%)▶cstf2t (bg=12.11%)▶EIF4G2 (bg=2.16%)▶hnrnpa1 (bg=18.32%)▶HNRNPM (bg=6.37%)▶HNRNPU (bg=9.45%)▶IGF2BP1 (bg=1.23%)▶khsrp (bg=27.4%)▶LIN28B (bg=1.31%)▶srsf7 (bg=22.53%)▶TARDBP (bg=5.12%)▶zc3h8 (bg=12.78%)No matches to TargetScan


TGGAGTAGTG

TGGAGTAGTG  
Depth:9 (LIZARD)  
Ei-value:0.000, Pi-value:0.000  
Er-value:0.000, Pr-value:0.000  
eCLIP MATCHES▶CSTF2 (bg=7.88%)▶cstf2t (bg=12.11%)▶DDX21 (bg=1.05%)▶EIF4G2 (bg=2.16%)▶hnrnpa1 (bg=18.32%)▶HNRNPM (bg=6.37%)▶HNRNPU (bg=9.45%)▶IGF2BP1 (bg=1.23%)▶khsrp (bg=27.4%)▶LIN28B (bg=1.31%)▶srsf7 (bg=22.53%)▶TARDBP (bg=5.12%)▶zc3h8 (bg=12.78%)No matches to TargetScan


ATTGTT

TGGAGTAGTGATTGTT  
Depth:5 (COW)  
Ei-value:0.000, Pi-value:0.000  
Er-value:0.000, Pr-value:0.000  
eCLIP MATCHES▶CSTF2 (bg=7.88%)▶cstf2t (bg=12.11%)▶DDX21 (bg=1.05%)▶EIF4G2 (bg=2.16%)▶hnrnpa1 (bg=18.32%)▶HNRNPM (bg=6.37%)▶HNRNPU (bg=9.45%)▶IGF2BP1 (bg=1.23%)▶khsrp (bg=27.4%)▶LIN28B (bg=1.31%)▶srsf7 (bg=22.53%)▶TARDBP (bg=5.12%)▶zc3h8 (bg=12.78%)No matches to TargetScan


GAAGGAAA

CCCCAATGCTTGGAGTAGTGATTGTTGAAGGAAA  
Depth:4 (PIG)  
Ei-value:0.000, Pi-value:0.000  
Er-value:0.000, Pr-value:0.000  
eCLIP MATCHES▶CSTF2 (bg=7.88%)▶cstf2t (bg=12.11%)▶DDX21 (bg=1.05%)▶EIF4G2 (bg=2.16%)▶hnrnpa1 (bg=18.32%)▶HNRNPM (bg=6.37%)▶HNRNPU (bg=9.45%)▶IGF2BP1 (bg=1.23%)▶khsrp (bg=27.4%)▶LIN28B (bg=1.31%)▶srsf7 (bg=22.53%)▶TARDBP (bg=5.12%)▶zc3h8 (bg=12.78%)MATCHES To TargetScan▶ miR-421:UCAACAG▶ miR-505-3p.2:UCAACAC▶ miR-205-5p:CCUUCAU


AAA

ATGCAGTACTGTTCTGATCCCGCTGCTATTAGAATGCATTGTGAAACGACTGGAGTATGATTAAAAGTTGTGTTCCCCAATGCTTGGAGTAGTGATTGTTGAAGGAAAAAA  
Depth:2 (MARMOSET)  
Ei-value:0.000, Pi-value:0.000  
Er-value:0.000, Pr-value:0.000  
eCLIP MATCHES▶CSTF2 (bg=7.88%)▶cstf2t (bg=12.11%)▶DDX21 (bg=1.05%)▶EIF4G2 (bg=2.16%)▶hnrnpa1 (bg=18.32%)▶HNRNPM (bg=6.37%)▶HNRNPU (bg=9.45%)▶IGF2BP1 (bg=1.23%)▶khsrp (bg=27.4%)▶LIN28B (bg=1.31%)▶srsf7 (bg=22.53%)▶TARDBP (bg=5.12%)▶tia1 (bg=23.76%)▶tial1 (bg=15.02%)▶u2af1 (bg=14.02%)▶u2af2 (bg=19.32%)▶zc3h8 (bg=12.78%)MATCHES To TargetScan▶ miR-421:UCAACAG▶ miR-217:ACUGCAU▶ miR-505-3p.2:UCAACAC▶ miR-132-3p/212-3p:AACAGUC▶ miR-145-5p:UCCAGUU▶ miR-154-3p/487-3p:AUCAUAC▶ miR-33-5p:UGCAUUG▶ miR-205-5p:CCUUCAU▶ miR-15-5p/16-5p/195-5p/424-5p/497-5p:AGCAGCA▶ miR-539-3p:UCAUACA▶ miR-383-5p.1:GAUCAGA▶ miR-503-5p:AGCAGCG▶ miR-144-3p:ACAGUAU▶ miR-101-3p.1:ACAGUAC


TCCAG

TCCAGCTGAGTGATAAAGGCTGAGTGTTGAGGAAATTTCTGCAG  
Depth:3 (DOG)  
Ei-value:0.000, Pi-value:0.000  
Er-value:0.000, Pr-value:0.000  
eCLIP MATCHES▶APOBEC3C (bg=0.77%)▶CSTF2 (bg=7.88%)▶cstf2t (bg=12.11%)▶DDX21 (bg=1.05%)▶EIF4G2 (bg=2.16%)▶hnrnpa1 (bg=18.32%)▶HNRNPM (bg=6.37%)▶IGF2BP1 (bg=1.23%)▶khsrp (bg=27.4%)▶srsf7 (bg=22.53%)▶TARDBP (bg=5.12%)▶zc3h8 (bg=12.78%)MATCHES To TargetScan▶ miR-421:UCAACAG▶ miR-505-3p.2:UCAACAC▶ miR-670-3p:UUCCUCA▶ miR-141-3p/200a-3p:AACACUG


CTGAGTGAT

CTGAGTGAT  
Depth:5 (COW)  
Ei-value:0.000, Pi-value:0.000  
Er-value:0.000, Pr-value:0.000  
eCLIP MATCHES▶APOBEC3C (bg=0.77%)▶CSTF2 (bg=7.88%)▶cstf2t (bg=12.11%)▶DDX21 (bg=1.05%)▶EIF4G2 (bg=2.16%)▶hnrnpa1 (bg=18.32%)▶HNRNPM (bg=6.37%)▶IGF2BP1 (bg=1.23%)▶khsrp (bg=27.4%)▶srsf7 (bg=22.53%)▶TARDBP (bg=5.12%)▶zc3h8 (bg=12.78%)No matches to TargetScan


AA

AAAGGCTGAGTGTTGAGGAAATTTCTGCAG  
Depth:5 (COW)  
Ei-value:0.000, Pi-value:0.000  
Er-value:0.000, Pr-value:0.000  
eCLIP MATCHES▶APOBEC3C (bg=0.77%)▶CSTF2 (bg=7.88%)▶cstf2t (bg=12.11%)▶DDX21 (bg=1.05%)▶EIF4G2 (bg=2.16%)▶hnrnpa1 (bg=18.32%)▶HNRNPM (bg=6.37%)▶IGF2BP1 (bg=1.23%)▶khsrp (bg=27.4%)▶srsf7 (bg=22.53%)▶TARDBP (bg=5.12%)▶zc3h8 (bg=12.78%)MATCHES To TargetScan▶ miR-421:UCAACAG▶ miR-505-3p.2:UCAACAC▶ miR-670-3p:UUCCUCA▶ miR-141-3p/200a-3p:AACACUG


AGGCTGAGTGTTGAGGAAAT

AGGCTGAGTGTTGAGGAAAT  
Depth:6 (MOUSE)  
Ei-value:0.000, Pi-value:0.000  
Er-value:0.000, Pr-value:0.000  
eCLIP MATCHES▶APOBEC3C (bg=0.77%)▶CSTF2 (bg=7.88%)▶cstf2t (bg=12.11%)▶DDX21 (bg=1.05%)▶EIF4G2 (bg=2.16%)▶hnrnpa1 (bg=18.32%)▶HNRNPM (bg=6.37%)▶IGF2BP1 (bg=1.23%)▶khsrp (bg=27.4%)▶srsf7 (bg=22.53%)▶TARDBP (bg=5.12%)▶zc3h8 (bg=12.78%)MATCHES To TargetScan▶ miR-421:UCAACAG▶ miR-505-3p.2:UCAACAC▶ miR-670-3p:UUCCUCA▶ miR-141-3p/200a-3p:AACACUG


T

AAAGGCTGAGTGTTGAGGAAATTTCTGCAG  
Depth:5 (COW)  
Ei-value:0.000, Pi-value:0.000  
Er-value:0.000, Pr-value:0.000  
eCLIP MATCHES▶APOBEC3C (bg=0.77%)▶CSTF2 (bg=7.88%)▶cstf2t (bg=12.11%)▶DDX21 (bg=1.05%)▶EIF4G2 (bg=2.16%)▶hnrnpa1 (bg=18.32%)▶HNRNPM (bg=6.37%)▶IGF2BP1 (bg=1.23%)▶khsrp (bg=27.4%)▶srsf7 (bg=22.53%)▶TARDBP (bg=5.12%)▶zc3h8 (bg=12.78%)MATCHES To TargetScan▶ miR-421:UCAACAG▶ miR-505-3p.2:UCAACAC▶ miR-670-3p:UUCCUCA▶ miR-141-3p/200a-3p:AACACUG


TCTGCAG

TCTGCAG  
Depth:6 (MOUSE)  
Ei-value:0.000, Pi-value:0.000  
Er-value:0.000, Pr-value:0.000  
eCLIP MATCHES▶APOBEC3C (bg=0.77%)▶CSTF2 (bg=7.88%)▶cstf2t (bg=12.11%)▶DDX21 (bg=1.05%)▶EIF4G2 (bg=2.16%)▶hnrnpa1 (bg=18.32%)▶HNRNPM (bg=6.37%)▶IGF2BP1 (bg=1.23%)▶khsrp (bg=27.4%)▶srsf7 (bg=22.53%)▶TARDBP (bg=5.12%)▶zc3h8 (bg=12.78%)No matches to TargetScan


T

TTTTAAGCA  
Depth:3 (DOG)  
Ei-value:0.000, Pi-value:0.000  
Er-value:0.000, Pr-value:0.000  
eCLIP MATCHES▶APOBEC3C (bg=0.77%)▶CSTF2 (bg=7.88%)▶cstf2t (bg=12.11%)▶DDX21 (bg=1.05%)▶EIF4G2 (bg=2.16%)▶hnrnpa1 (bg=18.32%)▶HNRNPM (bg=6.37%)▶IGF2BP1 (bg=1.23%)▶khsrp (bg=27.4%)▶srsf7 (bg=22.53%)▶TARDBP (bg=5.12%)▶zc3h8 (bg=12.78%)No matches to TargetScan

 5520  


TTTTAAGCA  
Depth:3 (DOG)  
Ei-value:0.000, Pi-value:0.000  
Er-value:0.000, Pr-value:0.000  
eCLIP MATCHES▶APOBEC3C (bg=0.77%)▶CSTF2 (bg=7.88%)▶cstf2t (bg=12.11%)▶DDX21 (bg=1.05%)▶EIF4G2 (bg=2.16%)▶hnrnpa1 (bg=18.32%)▶HNRNPM (bg=6.37%)▶IGF2BP1 (bg=1.23%)▶khsrp (bg=27.4%)▶srsf7 (bg=22.53%)▶TARDBP (bg=5.12%)▶zc3h8 (bg=12.78%)No matches to TargetScan


TTTAAG

TTTAAG  
Depth:7 (TURTLE)  
Ei-value:0.000, Pi-value:0.000  
Er-value:0.000, Pr-value:0.000  
eCLIP MATCHES▶APOBEC3C (bg=0.77%)▶CSTF2 (bg=7.88%)▶cstf2t (bg=12.11%)▶DDX21 (bg=1.05%)▶EIF4G2 (bg=2.16%)▶hnrnpa1 (bg=18.32%)▶HNRNPM (bg=6.37%)▶IGF2BP1 (bg=1.23%)▶khsrp (bg=27.4%)▶srsf7 (bg=22.53%)▶TARDBP (bg=5.12%)▶zc3h8 (bg=12.78%)No matches to TargetScan


CA

TTTAAGCA  
Depth:5 (COW)  
Ei-value:0.000, Pi-value:0.000  
Er-value:0.000, Pr-value:0.000  
eCLIP MATCHES▶APOBEC3C (bg=0.77%)▶CSTF2 (bg=7.88%)▶cstf2t (bg=12.11%)▶DDX21 (bg=1.05%)▶EIF4G2 (bg=2.16%)▶hnrnpa1 (bg=18.32%)▶HNRNPM (bg=6.37%)▶IGF2BP1 (bg=1.23%)▶khsrp (bg=27.4%)▶srsf7 (bg=22.53%)▶TARDBP (bg=5.12%)▶zc3h8 (bg=12.78%)No matches to TargetScan


GTCGT

TCCAGCTGAGTGATAAAGGCTGAGTGTTGAGGAAATTTCTGCAGTTTTAAGCAGTCGT  
Depth:2 (MARMOSET)  
Ei-value:0.000, Pi-value:0.000  
Er-value:0.000, Pr-value:0.000  
eCLIP MATCHES▶APOBEC3C (bg=0.77%)▶CSTF2 (bg=7.88%)▶cstf2t (bg=12.11%)▶DDX21 (bg=1.05%)▶EIF4G2 (bg=2.16%)▶hnrnpa1 (bg=18.32%)▶HNRNPM (bg=6.37%)▶HNRNPUL1 (bg=1.43%)▶IGF2BP1 (bg=1.23%)▶khsrp (bg=27.4%)▶srsf7 (bg=22.53%)▶TARDBP (bg=5.12%)▶zc3h8 (bg=12.78%)MATCHES To TargetScan▶ miR-421:UCAACAG▶ miR-217:ACUGCAU▶ miR-505-3p.2:UCAACAC▶ miR-670-3p:UUCCUCA▶ miR-141-3p/200a-3p:AACACUG

-

TTTGTGA

TTTGTGA  
Depth:6 (MOUSE)  
Ei-value:0.000, Pi-value:0.010  
Er-value:0.000, Pr-value:0.000  
eCLIP MATCHES▶APOBEC3C (bg=0.77%)▶cstf2t (bg=12.11%)▶DDX21 (bg=1.05%)▶EIF4G2 (bg=2.16%)▶hnrnpa1 (bg=18.32%)▶HNRNPM (bg=6.37%)▶HNRNPUL1 (bg=1.43%)▶IGF2BP1 (bg=1.23%)▶khsrp (bg=27.4%)▶srsf7 (bg=22.53%)▶TARDBP (bg=5.12%)▶zc3h8 (bg=12.78%)No matches to TargetScan

---------

AGTACA

AGTACATTTTGCTG  
Depth:2 (MARMOSET)  
Ei-value:0.020, Pi-value:0.000  
Er-value:0.000, Pr-value:0.000  
eCLIP MATCHES▶cstf2t (bg=12.11%)▶EIF4G2 (bg=2.16%)▶hnrnpa1 (bg=18.32%)▶HNRNPM (bg=6.37%)▶HNRNPUL1 (bg=1.43%)▶khsrp (bg=27.4%)▶srsf7 (bg=22.53%)▶TARDBP (bg=5.12%)▶tia1 (bg=23.76%)▶tial1 (bg=15.02%)▶u2af1 (bg=14.02%)▶u2af2 (bg=19.32%)▶zc3h8 (bg=12.78%)No matches to TargetScan


T

TTTTGCTG  
Depth:5 (COW)  
Ei-value:0.000, Pi-value:0.000  
Er-value:0.000, Pr-value:0.000  
eCLIP MATCHES▶cstf2t (bg=12.11%)▶EIF4G2 (bg=2.16%)▶hnrnpa1 (bg=18.32%)▶HNRNPM (bg=6.37%)▶HNRNPUL1 (bg=1.43%)▶khsrp (bg=27.4%)▶srsf7 (bg=22.53%)▶TARDBP (bg=5.12%)▶tia1 (bg=23.76%)▶tial1 (bg=15.02%)▶u2af1 (bg=14.02%)▶u2af2 (bg=19.32%)▶zc3h8 (bg=12.78%)No matches to TargetScan


TTTGCTG

TTTGCTG  
Depth:6 (MOUSE)  
Ei-value:0.000, Pi-value:0.000  
Er-value:0.000, Pr-value:0.000  
eCLIP MATCHES▶cstf2t (bg=12.11%)▶EIF4G2 (bg=2.16%)▶hnrnpa1 (bg=18.32%)▶HNRNPM (bg=6.37%)▶HNRNPUL1 (bg=1.43%)▶khsrp (bg=27.4%)▶srsf7 (bg=22.53%)▶TARDBP (bg=5.12%)▶tia1 (bg=23.76%)▶tial1 (bg=15.02%)▶u2af1 (bg=14.02%)▶u2af2 (bg=19.32%)▶zc3h8 (bg=12.78%)No matches to TargetScan

----

ATT

ATTTTTAGGTAAAATGCTTTT  
Depth:2 (MARMOSET)  
Ei-value:0.000, Pi-value:0.000  
Er-value:0.000, Pr-value:0.000  
eCLIP MATCHES▶cstf2t (bg=12.11%)▶EIF4G2 (bg=2.16%)▶hnrnpa1 (bg=18.32%)▶HNRNPM (bg=6.37%)▶HNRNPUL1 (bg=1.43%)▶khsrp (bg=27.4%)▶srsf7 (bg=22.53%)▶TARDBP (bg=5.12%)▶tia1 (bg=23.76%)▶tial1 (bg=15.02%)▶u2af1 (bg=14.02%)▶u2af2 (bg=19.32%)▶ZRANB2 (bg=7.28%)MATCHES To TargetScan▶ miR-330-3p.2:AAAGCAC


T

TTTAGGTAAAATGCTTTT  
Depth:5 (COW)  
Ei-value:0.000, Pi-value:0.000  
Er-value:0.000, Pr-value:0.000  
eCLIP MATCHES▶cstf2t (bg=12.11%)▶EIF4G2 (bg=2.16%)▶hnrnpa1 (bg=18.32%)▶HNRNPM (bg=6.37%)▶HNRNPUL1 (bg=1.43%)▶khsrp (bg=27.4%)▶srsf7 (bg=22.53%)▶TARDBP (bg=5.12%)▶tia1 (bg=23.76%)▶tial1 (bg=15.02%)▶u2af1 (bg=14.02%)▶u2af2 (bg=19.32%)▶ZRANB2 (bg=7.28%)MATCHES To TargetScan▶ miR-330-3p.2:AAAGCAC


TTAGGT

TTAGGT  
Depth:11 (X.TROPICALIS)  
Ei-value:0.000, Pi-value:0.000  
Er-value:0.000, Pr-value:0.000  
eCLIP MATCHES▶cstf2t (bg=12.11%)▶HNRNPM (bg=6.37%)▶khsrp (bg=27.4%)▶srsf7 (bg=22.53%)▶TARDBP (bg=5.12%)▶tia1 (bg=23.76%)▶tial1 (bg=15.02%)▶u2af1 (bg=14.02%)▶ZRANB2 (bg=7.28%)No matches to TargetScan


AA

TTAGGTAA  
Depth:10 (SNAKE)  
Ei-value:0.000, Pi-value:0.000  
Er-value:0.000, Pr-value:0.000  
eCLIP MATCHES▶cstf2t (bg=12.11%)▶HNRNPM (bg=6.37%)▶khsrp (bg=27.4%)▶srsf7 (bg=22.53%)▶TARDBP (bg=5.12%)▶tia1 (bg=23.76%)▶tial1 (bg=15.02%)▶u2af1 (bg=14.02%)▶ZRANB2 (bg=7.28%)No matches to TargetScan


A

TTAGGTAAA  
Depth:6 (MOUSE)  
Ei-value:0.000, Pi-value:0.000  
Er-value:0.000, Pr-value:0.000  
eCLIP MATCHES▶cstf2t (bg=12.11%)▶HNRNPM (bg=6.37%)▶khsrp (bg=27.4%)▶srsf7 (bg=22.53%)▶TARDBP (bg=5.12%)▶tia1 (bg=23.76%)▶tial1 (bg=15.02%)▶u2af1 (bg=14.02%)▶ZRANB2 (bg=7.28%)No matches to TargetScan


ATGCTTTT

TTTAGGTAAAATGCTTTT  
Depth:5 (COW)  
Ei-value:0.000, Pi-value:0.000  
Er-value:0.000, Pr-value:0.000  
eCLIP MATCHES▶cstf2t (bg=12.11%)▶EIF4G2 (bg=2.16%)▶hnrnpa1 (bg=18.32%)▶HNRNPM (bg=6.37%)▶HNRNPUL1 (bg=1.43%)▶khsrp (bg=27.4%)▶srsf7 (bg=22.53%)▶TARDBP (bg=5.12%)▶tia1 (bg=23.76%)▶tial1 (bg=15.02%)▶u2af1 (bg=14.02%)▶u2af2 (bg=19.32%)▶ZRANB2 (bg=7.28%)MATCHES To TargetScan▶ miR-330-3p.2:AAAGCAC

--

ttcatttctg

ttcatttctg  
Depth:2 (MARMOSET)  
Ei-value:1.000, Pi-value:0.000  
Er-value:0.000, Pr-value:0.000  
eCLIP MATCHES▶ddx42 (bg=10.33%)▶khsrp (bg=27.4%)▶SF3B4 (bg=4.76%)▶TARDBP (bg=5.12%)▶tia1 (bg=23.76%)▶tial1 (bg=15.02%)▶u2af1 (bg=14.02%)▶u2af2 (bg=19.32%)▶ZRANB2 (bg=7.28%)MATCHES To TargetScan▶ miR-203a-3p.1:GAAAUGU

-

tggtggg

tggtggg  
Depth:2 (MARMOSET)  
Ei-value:1.000, Pi-value:0.000  
Er-value:0.000, Pr-value:0.000  
eCLIP MATCHES▶ddx42 (bg=10.33%)▶khsrp (bg=27.4%)▶SF3B4 (bg=4.76%)▶TARDBP (bg=5.12%)▶tia1 (bg=23.76%)▶tial1 (bg=15.02%)▶u2af1 (bg=14.02%)▶u2af2 (bg=19.32%)▶ZRANB2 (bg=7.28%)No matches to TargetScan

-----

AC

ACTGAAGCCTTTAGTCT  
Depth:7 (TURTLE)  
Ei-value:0.000, Pi-value:0.000  
Er-value:0.000, Pr-value:0.000  
eCLIP MATCHES▶ddx42 (bg=10.33%)▶khsrp (bg=27.4%)▶SF3B4 (bg=4.76%)▶SMNDC1 (bg=7.08%)▶TARDBP (bg=5.12%)▶tia1 (bg=23.76%)▶tial1 (bg=15.02%)▶u2af1 (bg=14.02%)▶u2af2 (bg=19.32%)▶ZRANB2 (bg=7.28%)No matches to TargetScan


T

TGAAGCCTTTAGT  
Depth:10 (SNAKE)  
Ei-value:0.000, Pi-value:0.000  
Er-value:0.000, Pr-value:0.000  
eCLIP MATCHES▶ddx42 (bg=10.33%)▶khsrp (bg=27.4%)▶SF3B4 (bg=4.76%)▶SMNDC1 (bg=7.08%)▶TARDBP (bg=5.12%)▶tia1 (bg=23.76%)▶tial1 (bg=15.02%)▶u2af1 (bg=14.02%)▶u2af2 (bg=19.32%)▶ZRANB2 (bg=7.28%)No matches to TargetScan


GAAGCC

GAAGCC  
Depth:11 (X.TROPICALIS)  
Ei-value:0.000, Pi-value:0.000  
Er-value:0.000, Pr-value:0.000  
eCLIP MATCHES▶ddx42 (bg=10.33%)▶khsrp (bg=27.4%)▶SF3B4 (bg=4.76%)▶TARDBP (bg=5.12%)▶tia1 (bg=23.76%)▶tial1 (bg=15.02%)▶u2af1 (bg=14.02%)▶u2af2 (bg=19.32%)▶ZRANB2 (bg=7.28%)No matches to TargetScan


TTTAGT

TGAAGCCTTTAGT  
Depth:10 (SNAKE)  
Ei-value:0.000, Pi-value:0.000  
Er-value:0.000, Pr-value:0.000  
eCLIP MATCHES▶ddx42 (bg=10.33%)▶khsrp (bg=27.4%)▶SF3B4 (bg=4.76%)▶SMNDC1 (bg=7.08%)▶TARDBP (bg=5.12%)▶tia1 (bg=23.76%)▶tial1 (bg=15.02%)▶u2af1 (bg=14.02%)▶u2af2 (bg=19.32%)▶ZRANB2 (bg=7.28%)No matches to TargetScan


CT

TGAAGCCTTTAGTCT  
Depth:8 (ALLIGATOR)  
Ei-value:0.000, Pi-value:0.000  
Er-value:0.000, Pr-value:0.000  
eCLIP MATCHES▶ddx42 (bg=10.33%)▶khsrp (bg=27.4%)▶SF3B4 (bg=4.76%)▶SMNDC1 (bg=7.08%)▶TARDBP (bg=5.12%)▶tia1 (bg=23.76%)▶tial1 (bg=15.02%)▶u2af1 (bg=14.02%)▶u2af2 (bg=19.32%)▶ZRANB2 (bg=7.28%)No matches to TargetScan


T

ACTGAAGCCTTTAGTCTTTTCCAGATGCA  
Depth:5 (COW)  
Ei-value:0.000, Pi-value:0.000  
Er-value:0.000, Pr-value:0.000  
eCLIP MATCHES▶ddx42 (bg=10.33%)▶khsrp (bg=27.4%)▶ppil4 (bg=43.39%)▶SF3B4 (bg=4.76%)▶SMNDC1 (bg=7.08%)▶TARDBP (bg=5.12%)▶tia1 (bg=23.76%)▶tial1 (bg=15.02%)▶u2af1 (bg=14.02%)▶u2af2 (bg=19.32%)▶ZRANB2 (bg=7.28%)No matches to TargetScan


TTCCAGAT

TTCCAGAT  
Depth:10 (SNAKE)  
Ei-value:0.000, Pi-value:0.000  
Er-value:0.000, Pr-value:0.000  
eCLIP MATCHES▶ddx42 (bg=10.33%)▶khsrp (bg=27.4%)▶ppil4 (bg=43.39%)▶SF3B4 (bg=4.76%)▶SMNDC1 (bg=7.08%)▶tia1 (bg=23.76%)▶tial1 (bg=15.02%)▶u2af1 (bg=14.02%)▶u2af2 (bg=19.32%)▶ZRANB2 (bg=7.28%)No matches to TargetScan

 5640  


TTCCAGAT  
Depth:10 (SNAKE)  
Ei-value:0.000, Pi-value:0.000  
Er-value:0.000, Pr-value:0.000  
eCLIP MATCHES▶ddx42 (bg=10.33%)▶khsrp (bg=27.4%)▶ppil4 (bg=43.39%)▶SF3B4 (bg=4.76%)▶SMNDC1 (bg=7.08%)▶tia1 (bg=23.76%)▶tial1 (bg=15.02%)▶u2af1 (bg=14.02%)▶u2af2 (bg=19.32%)▶ZRANB2 (bg=7.28%)No matches to TargetScan


GCA

ACTGAAGCCTTTAGTCTTTTCCAGATGCA  
Depth:5 (COW)  
Ei-value:0.000, Pi-value:0.000  
Er-value:0.000, Pr-value:0.000  
eCLIP MATCHES▶ddx42 (bg=10.33%)▶khsrp (bg=27.4%)▶ppil4 (bg=43.39%)▶SF3B4 (bg=4.76%)▶SMNDC1 (bg=7.08%)▶TARDBP (bg=5.12%)▶tia1 (bg=23.76%)▶tial1 (bg=15.02%)▶u2af1 (bg=14.02%)▶u2af2 (bg=19.32%)▶ZRANB2 (bg=7.28%)No matches to TargetScan


A

ACTGAAGCCTTTAGTCTTTTCCAGATGCAACCTTAAAATCAGTGACAAGAAACA  
Depth:2 (MARMOSET)  
Ei-value:0.000, Pi-value:0.000  
Er-value:0.000, Pr-value:0.000  
eCLIP MATCHES▶cpsf6 (bg=13.45%)▶ddx42 (bg=10.33%)▶GRWD1 (bg=7.0%)▶khsrp (bg=27.4%)▶ppil4 (bg=43.39%)▶SF3B4 (bg=4.76%)▶SMNDC1 (bg=7.08%)▶srsf7 (bg=22.53%)▶TARDBP (bg=5.12%)▶tia1 (bg=23.76%)▶tial1 (bg=15.02%)▶u2af1 (bg=14.02%)▶u2af2 (bg=19.32%)▶znf622 (bg=18.79%)▶ZRANB2 (bg=7.28%)MATCHES To TargetScan▶ miR-668-3p:GUCACUC


CC

CCTTAAAATCAGTGACAAGAAA  
Depth:3 (DOG)  
Ei-value:0.000, Pi-value:0.000  
Er-value:0.000, Pr-value:0.000  
eCLIP MATCHES▶cpsf6 (bg=13.45%)▶ddx42 (bg=10.33%)▶GRWD1 (bg=7.0%)▶khsrp (bg=27.4%)▶ppil4 (bg=43.39%)▶SF3B4 (bg=4.76%)▶SMNDC1 (bg=7.08%)▶srsf7 (bg=22.53%)▶tia1 (bg=23.76%)▶u2af1 (bg=14.02%)▶u2af2 (bg=19.32%)▶znf622 (bg=18.79%)▶ZRANB2 (bg=7.28%)MATCHES To TargetScan▶ miR-668-3p:GUCACUC


TTAAAATC

TTAAAATC  
Depth:6 (MOUSE)  
Ei-value:0.000, Pi-value:0.000  
Er-value:0.000, Pr-value:0.000  
eCLIP MATCHES▶ddx42 (bg=10.33%)▶khsrp (bg=27.4%)▶ppil4 (bg=43.39%)▶SMNDC1 (bg=7.08%)▶srsf7 (bg=22.53%)▶tia1 (bg=23.76%)▶u2af1 (bg=14.02%)▶u2af2 (bg=19.32%)▶znf622 (bg=18.79%)▶ZRANB2 (bg=7.28%)No matches to TargetScan


AG

TTAAAATCAGTGACAAGAAA  
Depth:5 (COW)  
Ei-value:0.000, Pi-value:0.000  
Er-value:0.000, Pr-value:0.000  
eCLIP MATCHES▶cpsf6 (bg=13.45%)▶ddx42 (bg=10.33%)▶GRWD1 (bg=7.0%)▶khsrp (bg=27.4%)▶ppil4 (bg=43.39%)▶SMNDC1 (bg=7.08%)▶srsf7 (bg=22.53%)▶tia1 (bg=23.76%)▶u2af1 (bg=14.02%)▶u2af2 (bg=19.32%)▶znf622 (bg=18.79%)▶ZRANB2 (bg=7.28%)MATCHES To TargetScan▶ miR-668-3p:GUCACUC


TGACAAGAAA

TGACAAGAAA  
Depth:6 (MOUSE)  
Ei-value:0.000, Pi-value:0.000  
Er-value:0.000, Pr-value:0.000  
eCLIP MATCHES▶cpsf6 (bg=13.45%)▶GRWD1 (bg=7.0%)▶khsrp (bg=27.4%)▶ppil4 (bg=43.39%)▶SMNDC1 (bg=7.08%)▶srsf7 (bg=22.53%)▶u2af1 (bg=14.02%)▶u2af2 (bg=19.32%)▶znf622 (bg=18.79%)▶ZRANB2 (bg=7.28%)No matches to TargetScan


CA

ACTGAAGCCTTTAGTCTTTTCCAGATGCAACCTTAAAATCAGTGACAAGAAACA  
Depth:2 (MARMOSET)  
Ei-value:0.000, Pi-value:0.000  
Er-value:0.000, Pr-value:0.000  
eCLIP MATCHES▶cpsf6 (bg=13.45%)▶ddx42 (bg=10.33%)▶GRWD1 (bg=7.0%)▶khsrp (bg=27.4%)▶ppil4 (bg=43.39%)▶SF3B4 (bg=4.76%)▶SMNDC1 (bg=7.08%)▶srsf7 (bg=22.53%)▶TARDBP (bg=5.12%)▶tia1 (bg=23.76%)▶tial1 (bg=15.02%)▶u2af1 (bg=14.02%)▶u2af2 (bg=19.32%)▶znf622 (bg=18.79%)▶ZRANB2 (bg=7.28%)MATCHES To TargetScan▶ miR-668-3p:GUCACUC

--

CCAAAC

CCAAAC  
Depth:5 (COW)  
Ei-value:0.000, Pi-value:0.000  
Er-value:0.000, Pr-value:0.000  
eCLIP MATCHES▶cpsf6 (bg=13.45%)▶GRWD1 (bg=7.0%)▶khsrp (bg=27.4%)▶SMNDC1 (bg=7.08%)▶srsf7 (bg=22.53%)▶u2af1 (bg=14.02%)▶u2af2 (bg=19.32%)▶znf622 (bg=18.79%)▶ZRANB2 (bg=7.28%)No matches to TargetScan


AAGCAACA

CCAAACAAGCAACA  
Depth:3 (DOG)  
Ei-value:0.000, Pi-value:0.000  
Er-value:0.000, Pr-value:0.000  
eCLIP MATCHES▶cpsf6 (bg=13.45%)▶GRWD1 (bg=7.0%)▶khsrp (bg=27.4%)▶SMNDC1 (bg=7.08%)▶srsf7 (bg=22.53%)▶u2af1 (bg=14.02%)▶u2af2 (bg=19.32%)▶znf622 (bg=18.79%)▶ZRANB2 (bg=7.28%)MATCHES To TargetScan▶ miR-544a-5p:CUUGUUA

-

tcttca

tcttcaagaaatt  
Depth:2 (MARMOSET)  
Ei-value:0.680, Pi-value:0.000  
Er-value:0.000, Pr-value:0.000  
eCLIP MATCHES▶bclaf1 (bg=17.67%)▶GRWD1 (bg=7.0%)▶khsrp (bg=27.4%)▶srsf7 (bg=22.53%)▶u2af2 (bg=19.32%)▶znf622 (bg=18.79%)No matches to TargetScan


agaaatt

agaaatt  
Depth:3 (DOG)  
Ei-value:0.350, Pi-value:0.030  
Er-value:0.000, Pr-value:0.000  
eCLIP MATCHES▶bclaf1 (bg=17.67%)▶GRWD1 (bg=7.0%)▶khsrp (bg=27.4%)▶srsf7 (bg=22.53%)▶u2af2 (bg=19.32%)▶znf622 (bg=18.79%)No matches to TargetScan

-

AACT

AACTGGCAAGTG  
Depth:8 (ALLIGATOR)  
Ei-value:0.000, Pi-value:0.000  
Er-value:0.000, Pr-value:0.000  
eCLIP MATCHES▶bclaf1 (bg=17.67%)▶khsrp (bg=27.4%)▶srsf7 (bg=22.53%)▶u2af2 (bg=19.32%)▶znf622 (bg=18.79%)No matches to TargetScan


GGCAAGT

GGCAAGT  
Depth:10 (SNAKE)  
Ei-value:0.000, Pi-value:0.000  
Er-value:0.000, Pr-value:0.000  
eCLIP MATCHES▶bclaf1 (bg=17.67%)▶khsrp (bg=27.4%)▶srsf7 (bg=22.53%)▶u2af2 (bg=19.32%)▶znf622 (bg=18.79%)No matches to TargetScan


G

AACTGGCAAGTG  
Depth:8 (ALLIGATOR)  
Ei-value:0.000, Pi-value:0.000  
Er-value:0.000, Pr-value:0.000  
eCLIP MATCHES▶bclaf1 (bg=17.67%)▶khsrp (bg=27.4%)▶srsf7 (bg=22.53%)▶u2af2 (bg=19.32%)▶znf622 (bg=18.79%)No matches to TargetScan


GAAA

AACTGGCAAGTGGAAA  
Depth:6 (MOUSE)  
Ei-value:0.000, Pi-value:0.000  
Er-value:0.000, Pr-value:0.000  
eCLIP MATCHES▶bclaf1 (bg=17.67%)▶khsrp (bg=27.4%)▶srsf7 (bg=22.53%)▶u2af2 (bg=19.32%)▶znf622 (bg=18.79%)No matches to TargetScan


TGTTTAA

AACTGGCAAGTGGAAATGTTTAA  
Depth:2 (MARMOSET)  
Ei-value:0.000, Pi-value:0.000  
Er-value:0.000, Pr-value:0.000  
eCLIP MATCHES▶bclaf1 (bg=17.67%)▶khsrp (bg=27.4%)▶srsf7 (bg=22.53%)▶tia1 (bg=23.76%)▶tial1 (bg=15.02%)▶u2af2 (bg=19.32%)▶znf622 (bg=18.79%)MATCHES To TargetScan▶ miR-543:AACAUUC

-

CAGTTC

CAGTTC  
Depth:8 (ALLIGATOR)  
Ei-value:0.000, Pi-value:0.000  
Er-value:0.000, Pr-value:0.000  
eCLIP MATCHES▶khsrp (bg=27.4%)▶srsf7 (bg=22.53%)▶tia1 (bg=23.76%)▶tial1 (bg=15.02%)▶u2af2 (bg=19.32%)No matches to TargetScan


AGTG

CAGTTCAGTG  
Depth:5 (COW)  
Ei-value:0.000, Pi-value:0.000  
Er-value:0.000, Pr-value:0.000  
eCLIP MATCHES▶hnrnpa1 (bg=18.32%)▶khsrp (bg=27.4%)▶srsf7 (bg=22.53%)▶tia1 (bg=23.76%)▶tial1 (bg=15.02%)▶u2af2 (bg=19.32%)▶ZRANB2 (bg=7.28%)No matches to TargetScan


ATCTT

CAGTTCAGTGATCTT  
Depth:2 (MARMOSET)  
Ei-value:0.000, Pi-value:0.000  
Er-value:0.000, Pr-value:0.000  
eCLIP MATCHES▶hnrnpa1 (bg=18.32%)▶khsrp (bg=27.4%)▶srsf7 (bg=22.53%)▶tia1 (bg=23.76%)▶tial1 (bg=15.02%)▶u2af1 (bg=14.02%)▶u2af2 (bg=19.32%)▶ZRANB2 (bg=7.28%)MATCHES To TargetScan▶ miR-383-5p.2:AGAUCAG

-

AGTGCATT

AGTGCATT  
Depth:6 (MOUSE)  
Ei-value:0.000, Pi-value:0.000  
Er-value:0.000, Pr-value:0.000  
eCLIP MATCHES▶hnrnpa1 (bg=18.32%)▶HNRNPU (bg=9.45%)▶khsrp (bg=27.4%)▶tia1 (bg=23.76%)▶tial1 (bg=15.02%)▶u2af1 (bg=14.02%)▶u2af2 (bg=19.32%)▶ZRANB2 (bg=7.28%)MATCHES To TargetScan▶ miR-501-3p/502-3p:AUGCACC


gttt

agtgcattgttt  
Depth:2 (MARMOSET)  
Ei-value:0.990, Pi-value:0.000  
Er-value:0.000, Pr-value:0.000  
eCLIP MATCHES▶hnrnpa1 (bg=18.32%)▶HNRNPU (bg=9.45%)▶khsrp (bg=27.4%)▶PCBP2 (bg=4.33%)▶SF3B4 (bg=4.76%)▶tia1 (bg=23.76%)▶tial1 (bg=15.02%)▶u2af1 (bg=14.02%)▶u2af2 (bg=19.32%)▶ZRANB2 (bg=7.28%)MATCHES To TargetScan▶ miR-501-3p/502-3p:AUGCACC

-

TGTGTG

TGTGTGGGTTTCTCTCTCCCCTCCCTTGGTCTTAATTCTTACA  
Depth:3 (DOG)  
Ei-value:0.000, Pi-value:0.000  
Er-value:0.000, Pr-value:0.000  
eCLIP MATCHES▶DGCR8 (bg=2.67%)▶HNRNPU (bg=9.45%)▶khsrp (bg=27.4%)▶PCBP2 (bg=4.33%)▶SF3B4 (bg=4.76%)▶tia1 (bg=23.76%)▶tial1 (bg=15.02%)▶u2af1 (bg=14.02%)▶u2af2 (bg=19.32%)▶ZRANB2 (bg=7.28%)MATCHES To TargetScan▶ miR-499a-5p:UAAGACU▶ miR-208-3p:UAAGACG▶ miR-423-5p:GAGGGGC▶ miR-185-5p:GGAGAGA


GG

GGTTTCTCTCT  
Depth:4 (PIG)  
Ei-value:0.000, Pi-value:0.000  
Er-value:0.000, Pr-value:0.000  
eCLIP MATCHES▶khsrp (bg=27.4%)▶PCBP2 (bg=4.33%)▶SF3B4 (bg=4.76%)▶tia1 (bg=23.76%)▶tial1 (bg=15.02%)▶u2af1 (bg=14.02%)▶u2af2 (bg=19.32%)▶ZRANB2 (bg=7.28%)No matches to TargetScan

 5760  


TT

GGTTTCTCTCT  
Depth:4 (PIG)  
Ei-value:0.000, Pi-value:0.000  
Er-value:0.000, Pr-value:0.000  
eCLIP MATCHES▶khsrp (bg=27.4%)▶PCBP2 (bg=4.33%)▶SF3B4 (bg=4.76%)▶tia1 (bg=23.76%)▶tial1 (bg=15.02%)▶u2af1 (bg=14.02%)▶u2af2 (bg=19.32%)▶ZRANB2 (bg=7.28%)No matches to TargetScan


TCTCTCT

TCTCTCT  
Depth:6 (MOUSE)  
Ei-value:0.000, Pi-value:0.000  
Er-value:0.000, Pr-value:0.000  
eCLIP MATCHES▶khsrp (bg=27.4%)▶PCBP2 (bg=4.33%)▶SF3B4 (bg=4.76%)▶tia1 (bg=23.76%)▶tial1 (bg=15.02%)▶u2af1 (bg=14.02%)▶u2af2 (bg=19.32%)▶ZRANB2 (bg=7.28%)No matches to TargetScan


C

TGTGTGGGTTTCTCTCTCCCCTCCCTTGGTCTTAATTCTTACA  
Depth:3 (DOG)  
Ei-value:0.000, Pi-value:0.000  
Er-value:0.000, Pr-value:0.000  
eCLIP MATCHES▶DGCR8 (bg=2.67%)▶HNRNPU (bg=9.45%)▶khsrp (bg=27.4%)▶PCBP2 (bg=4.33%)▶SF3B4 (bg=4.76%)▶tia1 (bg=23.76%)▶tial1 (bg=15.02%)▶u2af1 (bg=14.02%)▶u2af2 (bg=19.32%)▶ZRANB2 (bg=7.28%)MATCHES To TargetScan▶ miR-499a-5p:UAAGACU▶ miR-208-3p:UAAGACG▶ miR-423-5p:GAGGGGC▶ miR-185-5p:GGAGAGA


C

CCCTCCCTTGGTCT  
Depth:4 (PIG)  
Ei-value:0.000, Pi-value:0.000  
Er-value:0.000, Pr-value:0.000  
eCLIP MATCHES▶DGCR8 (bg=2.67%)▶khsrp (bg=27.4%)▶PCBP2 (bg=4.33%)▶SF3B4 (bg=4.76%)▶tia1 (bg=23.76%)▶tial1 (bg=15.02%)▶u2af1 (bg=14.02%)▶u2af2 (bg=19.32%)▶ZRANB2 (bg=7.28%)No matches to TargetScan


CCTCCC

CCTCCC  
Depth:6 (MOUSE)  
Ei-value:0.000, Pi-value:0.000  
Er-value:0.000, Pr-value:0.000  
eCLIP MATCHES▶DGCR8 (bg=2.67%)▶khsrp (bg=27.4%)▶PCBP2 (bg=4.33%)▶SF3B4 (bg=4.76%)▶tia1 (bg=23.76%)▶tial1 (bg=15.02%)▶u2af1 (bg=14.02%)▶u2af2 (bg=19.32%)▶ZRANB2 (bg=7.28%)No matches to TargetScan


T

CCCTCCCTTGGTCT  
Depth:4 (PIG)  
Ei-value:0.000, Pi-value:0.000  
Er-value:0.000, Pr-value:0.000  
eCLIP MATCHES▶DGCR8 (bg=2.67%)▶khsrp (bg=27.4%)▶PCBP2 (bg=4.33%)▶SF3B4 (bg=4.76%)▶tia1 (bg=23.76%)▶tial1 (bg=15.02%)▶u2af1 (bg=14.02%)▶u2af2 (bg=19.32%)▶ZRANB2 (bg=7.28%)No matches to TargetScan


TGGTCT

TGGTCT  
Depth:6 (MOUSE)  
Ei-value:0.000, Pi-value:0.000  
Er-value:0.000, Pr-value:0.000  
eCLIP MATCHES▶DGCR8 (bg=2.67%)▶khsrp (bg=27.4%)▶PCBP2 (bg=4.33%)▶SF3B4 (bg=4.76%)▶tia1 (bg=23.76%)▶tial1 (bg=15.02%)▶u2af1 (bg=14.02%)▶u2af2 (bg=19.32%)▶ZRANB2 (bg=7.28%)No matches to TargetScan


T

TGTGTGGGTTTCTCTCTCCCCTCCCTTGGTCTTAATTCTTACA  
Depth:3 (DOG)  
Ei-value:0.000, Pi-value:0.000  
Er-value:0.000, Pr-value:0.000  
eCLIP MATCHES▶DGCR8 (bg=2.67%)▶HNRNPU (bg=9.45%)▶khsrp (bg=27.4%)▶PCBP2 (bg=4.33%)▶SF3B4 (bg=4.76%)▶tia1 (bg=23.76%)▶tial1 (bg=15.02%)▶u2af1 (bg=14.02%)▶u2af2 (bg=19.32%)▶ZRANB2 (bg=7.28%)MATCHES To TargetScan▶ miR-499a-5p:UAAGACU▶ miR-208-3p:UAAGACG▶ miR-423-5p:GAGGGGC▶ miR-185-5p:GGAGAGA


AATTCTTA

AATTCTTA  
Depth:4 (PIG)  
Ei-value:0.000, Pi-value:0.000  
Er-value:0.000, Pr-value:0.000  
eCLIP MATCHES▶khsrp (bg=27.4%)▶PCBP2 (bg=4.33%)▶SF3B4 (bg=4.76%)▶tia1 (bg=23.76%)▶tial1 (bg=15.02%)▶u2af1 (bg=14.02%)▶u2af2 (bg=19.32%)▶ZRANB2 (bg=7.28%)No matches to TargetScan


CA

TGTGTGGGTTTCTCTCTCCCCTCCCTTGGTCTTAATTCTTACA  
Depth:3 (DOG)  
Ei-value:0.000, Pi-value:0.000  
Er-value:0.000, Pr-value:0.000  
eCLIP MATCHES▶DGCR8 (bg=2.67%)▶HNRNPU (bg=9.45%)▶khsrp (bg=27.4%)▶PCBP2 (bg=4.33%)▶SF3B4 (bg=4.76%)▶tia1 (bg=23.76%)▶tial1 (bg=15.02%)▶u2af1 (bg=14.02%)▶u2af2 (bg=19.32%)▶ZRANB2 (bg=7.28%)MATCHES To TargetScan▶ miR-499a-5p:UAAGACU▶ miR-208-3p:UAAGACG▶ miR-423-5p:GAGGGGC▶ miR-185-5p:GGAGAGA


TG

TGTGTGGGTTTCTCTCTCCCCTCCCTTGGTCTTAATTCTTACATGCAGGAACA  
Depth:2 (MARMOSET)  
Ei-value:0.000, Pi-value:0.000  
Er-value:0.000, Pr-value:0.000  
eCLIP MATCHES▶ddx42 (bg=10.33%)▶DGCR8 (bg=2.67%)▶HNRNPU (bg=9.45%)▶khsrp (bg=27.4%)▶PCBP2 (bg=4.33%)▶SF3B4 (bg=4.76%)▶tia1 (bg=23.76%)▶tial1 (bg=15.02%)▶u2af1 (bg=14.02%)▶u2af2 (bg=19.32%)▶ZRANB2 (bg=7.28%)MATCHES To TargetScan▶ miR-499a-5p:UAAGACU▶ miR-208-3p:UAAGACG▶ miR-423-5p:GAGGGGC▶ miR-185-5p:GGAGAGA▶ miR-411-3p:AUGUAAC


CAGGAACA

CAGGAACA  
Depth:6 (MOUSE)  
Ei-value:0.000, Pi-value:0.000  
Er-value:0.000, Pr-value:0.000  
eCLIP MATCHES▶ddx42 (bg=10.33%)▶khsrp (bg=27.4%)▶tia1 (bg=23.76%)▶tial1 (bg=15.02%)▶u2af1 (bg=14.02%)▶u2af2 (bg=19.32%)▶ZRANB2 (bg=7.28%)No matches to TargetScan

--

CAGCAGAC

CAGCAGAC  
Depth:3 (DOG)  
Ei-value:0.010, Pi-value:0.000  
Er-value:0.000, Pr-value:0.000  
eCLIP MATCHES▶ddx42 (bg=10.33%)▶srsf1 (bg=30.28%)▶tia1 (bg=23.76%)▶u2af1 (bg=14.02%)▶u2af2 (bg=19.32%)▶ZRANB2 (bg=7.28%)MATCHES To TargetScan▶ miR-346:GUCUGCC


a

cagcagaca  
Depth:2 (MARMOSET)  
Ei-value:1.000, Pi-value:0.000  
Er-value:0.000, Pr-value:0.000  
eCLIP MATCHES▶ddx42 (bg=10.33%)▶srsf1 (bg=30.28%)▶tia1 (bg=23.76%)▶u2af1 (bg=14.02%)▶u2af2 (bg=19.32%)▶ZRANB2 (bg=7.28%)MATCHES To TargetScan▶ miR-346:GUCUGCC

-

acgtatg

acgtatg  
Depth:2 (MARMOSET)  
Ei-value:1.000, Pi-value:0.020  
Er-value:0.000, Pr-value:0.000  
eCLIP MATCHES▶ddx42 (bg=10.33%)▶srsf1 (bg=30.28%)▶tia1 (bg=23.76%)▶u2af1 (bg=14.02%)▶u2af2 (bg=19.32%)▶ZRANB2 (bg=7.28%)No matches to TargetScan

-

GAAG

GAAGGGCCAGAGAAGCCAGACC  
Depth:4 (PIG)  
Ei-value:0.000, Pi-value:0.000  
Er-value:0.000, Pr-value:0.000  
eCLIP MATCHES▶ddx42 (bg=10.33%)▶srsf1 (bg=30.28%)▶tia1 (bg=23.76%)▶u2af1 (bg=14.02%)▶u2af2 (bg=19.32%)▶ZRANB2 (bg=7.28%)MATCHES To TargetScan▶ miR-326:CUCUGGG▶ miR-328-3p:UGGCCCU▶ miR-149-5p:CUGGCUC▶ miR-3064-5p:CUGGCUG


GGCCAGAGAA

GGCCAGAGAA  
Depth:6 (MOUSE)  
Ei-value:0.000, Pi-value:0.000  
Er-value:0.000, Pr-value:0.000  
eCLIP MATCHES▶ddx42 (bg=10.33%)▶u2af1 (bg=14.02%)▶u2af2 (bg=19.32%)▶ZRANB2 (bg=7.28%)MATCHES To TargetScan▶ miR-326:CUCUGGG


G

GGCCAGAGAAGCCAGACC  
Depth:5 (COW)  
Ei-value:0.000, Pi-value:0.000  
Er-value:0.000, Pr-value:0.000  
eCLIP MATCHES▶ddx42 (bg=10.33%)▶u2af1 (bg=14.02%)▶u2af2 (bg=19.32%)▶ZRANB2 (bg=7.28%)MATCHES To TargetScan▶ miR-326:CUCUGGG▶ miR-149-5p:CUGGCUC▶ miR-3064-5p:CUGGCUG


CCAGACC

CCAGACC  
Depth:6 (MOUSE)  
Ei-value:0.000, Pi-value:0.000  
Er-value:0.000, Pr-value:0.000  
eCLIP MATCHES▶ddx42 (bg=10.33%)▶u2af1 (bg=14.02%)▶u2af2 (bg=19.32%)▶ZRANB2 (bg=7.28%)No matches to TargetScan


C

GAAGGGCCAGAGAAGCCAGACCCAGTAAG  
Depth:2 (MARMOSET)  
Ei-value:0.000, Pi-value:0.000  
Er-value:0.000, Pr-value:0.000  
eCLIP MATCHES▶ddx42 (bg=10.33%)▶srsf1 (bg=30.28%)▶tia1 (bg=23.76%)▶u2af1 (bg=14.02%)▶u2af2 (bg=19.32%)▶ZRANB2 (bg=7.28%)MATCHES To TargetScan▶ miR-326:CUCUGGG▶ miR-328-3p:UGGCCCU▶ miR-193a-5p:GGGUCUU▶ miR-149-5p:CUGGCUC▶ miR-3064-5p:CUGGCUG


AGTAAG

AGTAAG  
Depth:8 (ALLIGATOR)  
Ei-value:0.000, Pi-value:0.000  
Er-value:0.000, Pr-value:0.010  
eCLIP MATCHES▶ddx42 (bg=10.33%)▶u2af1 (bg=14.02%)▶u2af2 (bg=19.32%)▶ZRANB2 (bg=7.28%)No matches to TargetScan


A

AAAAAATAG  
Depth:4 (PIG)  
Ei-value:0.000, Pi-value:0.000  
Er-value:0.000, Pr-value:0.010  
eCLIP MATCHES▶ddx42 (bg=10.33%)▶tial1 (bg=15.02%)▶u2af1 (bg=14.02%)▶u2af2 (bg=19.32%)No matches to TargetScan


AAAAATAG

AAAAATAG  
Depth:6 (MOUSE)  
Ei-value:0.000, Pi-value:0.000  
Er-value:0.000, Pr-value:0.000  
eCLIP MATCHES▶ddx42 (bg=10.33%)▶tial1 (bg=15.02%)▶u2af1 (bg=14.02%)▶u2af2 (bg=19.32%)No matches to TargetScan


C

AAAAAATAGC  
Depth:3 (DOG)  
Ei-value:0.000, Pi-value:0.000  
Er-value:0.000, Pr-value:0.000  
eCLIP MATCHES▶ddx42 (bg=10.33%)▶tial1 (bg=15.02%)▶u2af1 (bg=14.02%)▶u2af2 (bg=19.32%)No matches to TargetScan


C

AAAAAATAGCCTATTTACTTTAAA  
Depth:2 (MARMOSET)  
Ei-value:0.000, Pi-value:0.000  
Er-value:0.000, Pr-value:0.000  
eCLIP MATCHES▶ddx42 (bg=10.33%)▶tia1 (bg=23.76%)▶tial1 (bg=15.02%)▶u2af1 (bg=14.02%)▶u2af2 (bg=19.32%)No matches to TargetScan


TATTTACTTTAAA

TATTTACTTTAAA  
Depth:3 (DOG)  
Ei-value:0.000, Pi-value:0.000  
Er-value:0.000, Pr-value:0.000  
eCLIP MATCHES▶ddx42 (bg=10.33%)▶tia1 (bg=23.76%)▶tial1 (bg=15.02%)▶u2af2 (bg=19.32%)No matches to TargetScan

-

A

AAACCAAACATTCCATTTTAAATGTGGGGATTGGGAACCACTAGTTCTTTCAGATGGTATTCTTCAGACTATAGAAGGAGCTTCCAGTTGAATTCA  
Depth:2 (MARMOSET)  
Ei-value:0.000, Pi-value:0.000  
Er-value:0.000, Pr-value:0.000  
eCLIP MATCHES▶ddx42 (bg=10.33%)▶GRWD1 (bg=7.0%)▶hnrnpa1 (bg=18.32%)▶khsrp (bg=27.4%)▶ppil4 (bg=43.39%)▶SF3B4 (bg=4.76%)▶SLBP (bg=6.66%)▶srsf1 (bg=30.28%)▶srsf7 (bg=22.53%)▶tia1 (bg=23.76%)▶tial1 (bg=15.02%)▶u2af1 (bg=14.02%)▶u2af2 (bg=19.32%)▶zc3h8 (bg=12.78%)▶znf622 (bg=18.79%)▶ZRANB2 (bg=7.28%)MATCHES To TargetScan▶ miR-140-5p:AGUGGUU▶ miR-488-3p:UGAAAGG▶ miR-409-3p:AAUGUUG▶ miR-1-3p/206:GGAAUGU▶ miR-186-5p:AAAGAAU

 5880  


AACCAAACAT

AAACCAAACATTCCATTTTAAATGTGGGGATTGGGAACCACTAGTTCTTTCAGATGGTATTCTTCAGACTATAGAAGGAGCTTCCAGTTGAATTCA  
Depth:2 (MARMOSET)  
Ei-value:0.000, Pi-value:0.000  
Er-value:0.000, Pr-value:0.000  
eCLIP MATCHES▶ddx42 (bg=10.33%)▶GRWD1 (bg=7.0%)▶hnrnpa1 (bg=18.32%)▶khsrp (bg=27.4%)▶ppil4 (bg=43.39%)▶SF3B4 (bg=4.76%)▶SLBP (bg=6.66%)▶srsf1 (bg=30.28%)▶srsf7 (bg=22.53%)▶tia1 (bg=23.76%)▶tial1 (bg=15.02%)▶u2af1 (bg=14.02%)▶u2af2 (bg=19.32%)▶zc3h8 (bg=12.78%)▶znf622 (bg=18.79%)▶ZRANB2 (bg=7.28%)MATCHES To TargetScan▶ miR-140-5p:AGUGGUU▶ miR-488-3p:UGAAAGG▶ miR-409-3p:AAUGUUG▶ miR-1-3p/206:GGAAUGU▶ miR-186-5p:AAAGAAU


TCCAT

TCCATTTTAAATGTGGGGATTGGGAACCACTAGTTCTTTCAGATGGTATTCTTCAGACTATAGAAGGAGCTTCCAGTTGAATTCA  
Depth:3 (DOG)  
Ei-value:0.000, Pi-value:0.000  
Er-value:0.000, Pr-value:0.000  
eCLIP MATCHES▶ddx42 (bg=10.33%)▶GRWD1 (bg=7.0%)▶hnrnpa1 (bg=18.32%)▶khsrp (bg=27.4%)▶ppil4 (bg=43.39%)▶SF3B4 (bg=4.76%)▶SLBP (bg=6.66%)▶srsf1 (bg=30.28%)▶srsf7 (bg=22.53%)▶tia1 (bg=23.76%)▶tial1 (bg=15.02%)▶u2af1 (bg=14.02%)▶u2af2 (bg=19.32%)▶zc3h8 (bg=12.78%)▶znf622 (bg=18.79%)▶ZRANB2 (bg=7.28%)MATCHES To TargetScan▶ miR-140-5p:AGUGGUU▶ miR-488-3p:UGAAAGG▶ miR-186-5p:AAAGAAU


TTTAAA

TTTAAATGTGGGGATTGGGAACCACTAGTTCTTTCAGATGGTA  
Depth:5 (COW)  
Ei-value:0.000, Pi-value:0.000  
Er-value:0.000, Pr-value:0.000  
eCLIP MATCHES▶ddx42 (bg=10.33%)▶khsrp (bg=27.4%)▶SF3B4 (bg=4.76%)▶SLBP (bg=6.66%)▶srsf1 (bg=30.28%)▶srsf7 (bg=22.53%)▶tia1 (bg=23.76%)▶tial1 (bg=15.02%)▶u2af1 (bg=14.02%)▶u2af2 (bg=19.32%)▶zc3h8 (bg=12.78%)▶ZRANB2 (bg=7.28%)MATCHES To TargetScan▶ miR-140-5p:AGUGGUU▶ miR-488-3p:UGAAAGG▶ miR-186-5p:AAAGAAU


TGTGGGGATTGGGAA

TGTGGGGATTGGGAA  
Depth:6 (MOUSE)  
Ei-value:0.000, Pi-value:0.000  
Er-value:0.000, Pr-value:0.000  
eCLIP MATCHES▶ddx42 (bg=10.33%)▶khsrp (bg=27.4%)▶SF3B4 (bg=4.76%)▶SLBP (bg=6.66%)▶tia1 (bg=23.76%)▶tial1 (bg=15.02%)▶u2af1 (bg=14.02%)▶u2af2 (bg=19.32%)▶zc3h8 (bg=12.78%)No matches to TargetScan


CCA

TTTAAATGTGGGGATTGGGAACCACTAGTTCTTTCAGATGGTA  
Depth:5 (COW)  
Ei-value:0.000, Pi-value:0.000  
Er-value:0.000, Pr-value:0.000  
eCLIP MATCHES▶ddx42 (bg=10.33%)▶khsrp (bg=27.4%)▶SF3B4 (bg=4.76%)▶SLBP (bg=6.66%)▶srsf1 (bg=30.28%)▶srsf7 (bg=22.53%)▶tia1 (bg=23.76%)▶tial1 (bg=15.02%)▶u2af1 (bg=14.02%)▶u2af2 (bg=19.32%)▶zc3h8 (bg=12.78%)▶ZRANB2 (bg=7.28%)MATCHES To TargetScan▶ miR-140-5p:AGUGGUU▶ miR-488-3p:UGAAAGG▶ miR-186-5p:AAAGAAU


C

CTAGTTCTTTCAGATG  
Depth:6 (MOUSE)  
Ei-value:0.000, Pi-value:0.000  
Er-value:0.000, Pr-value:0.000  
eCLIP MATCHES▶ddx42 (bg=10.33%)▶khsrp (bg=27.4%)▶SF3B4 (bg=4.76%)▶SLBP (bg=6.66%)▶srsf7 (bg=22.53%)▶tia1 (bg=23.76%)▶tial1 (bg=15.02%)▶u2af1 (bg=14.02%)▶u2af2 (bg=19.32%)▶zc3h8 (bg=12.78%)▶ZRANB2 (bg=7.28%)MATCHES To TargetScan▶ miR-488-3p:UGAAAGG▶ miR-186-5p:AAAGAAU


TAGTTCTT

TAGTTCTT  
Depth:8 (ALLIGATOR)  
Ei-value:0.000, Pi-value:0.000  
Er-value:0.000, Pr-value:0.000  
eCLIP MATCHES▶ddx42 (bg=10.33%)▶khsrp (bg=27.4%)▶SF3B4 (bg=4.76%)▶SLBP (bg=6.66%)▶srsf7 (bg=22.53%)▶tia1 (bg=23.76%)▶tial1 (bg=15.02%)▶u2af1 (bg=14.02%)▶u2af2 (bg=19.32%)▶zc3h8 (bg=12.78%)▶ZRANB2 (bg=7.28%)No matches to TargetScan


TCAGATG

CTAGTTCTTTCAGATG  
Depth:6 (MOUSE)  
Ei-value:0.000, Pi-value:0.000  
Er-value:0.000, Pr-value:0.000  
eCLIP MATCHES▶ddx42 (bg=10.33%)▶khsrp (bg=27.4%)▶SF3B4 (bg=4.76%)▶SLBP (bg=6.66%)▶srsf7 (bg=22.53%)▶tia1 (bg=23.76%)▶tial1 (bg=15.02%)▶u2af1 (bg=14.02%)▶u2af2 (bg=19.32%)▶zc3h8 (bg=12.78%)▶ZRANB2 (bg=7.28%)MATCHES To TargetScan▶ miR-488-3p:UGAAAGG▶ miR-186-5p:AAAGAAU


GTA

TTTAAATGTGGGGATTGGGAACCACTAGTTCTTTCAGATGGTA  
Depth:5 (COW)  
Ei-value:0.000, Pi-value:0.000  
Er-value:0.000, Pr-value:0.000  
eCLIP MATCHES▶ddx42 (bg=10.33%)▶khsrp (bg=27.4%)▶SF3B4 (bg=4.76%)▶SLBP (bg=6.66%)▶srsf1 (bg=30.28%)▶srsf7 (bg=22.53%)▶tia1 (bg=23.76%)▶tial1 (bg=15.02%)▶u2af1 (bg=14.02%)▶u2af2 (bg=19.32%)▶zc3h8 (bg=12.78%)▶ZRANB2 (bg=7.28%)MATCHES To TargetScan▶ miR-140-5p:AGUGGUU▶ miR-488-3p:UGAAAGG▶ miR-186-5p:AAAGAAU


T

TCCATTTTAAATGTGGGGATTGGGAACCACTAGTTCTTTCAGATGGTATTCTTCAGACTATAGAAGGAGCTTCCAGTTGAATTCA  
Depth:3 (DOG)  
Ei-value:0.000, Pi-value:0.000  
Er-value:0.000, Pr-value:0.000  
eCLIP MATCHES▶ddx42 (bg=10.33%)▶GRWD1 (bg=7.0%)▶hnrnpa1 (bg=18.32%)▶khsrp (bg=27.4%)▶ppil4 (bg=43.39%)▶SF3B4 (bg=4.76%)▶SLBP (bg=6.66%)▶srsf1 (bg=30.28%)▶srsf7 (bg=22.53%)▶tia1 (bg=23.76%)▶tial1 (bg=15.02%)▶u2af1 (bg=14.02%)▶u2af2 (bg=19.32%)▶zc3h8 (bg=12.78%)▶znf622 (bg=18.79%)▶ZRANB2 (bg=7.28%)MATCHES To TargetScan▶ miR-140-5p:AGUGGUU▶ miR-488-3p:UGAAAGG▶ miR-186-5p:AAAGAAU


T

TCTTCAGAC  
Depth:5 (COW)  
Ei-value:0.000, Pi-value:0.000  
Er-value:0.000, Pr-value:0.000  
eCLIP MATCHES▶ddx42 (bg=10.33%)▶GRWD1 (bg=7.0%)▶SLBP (bg=6.66%)▶srsf1 (bg=30.28%)▶srsf7 (bg=22.53%)▶tia1 (bg=23.76%)▶tial1 (bg=15.02%)▶u2af1 (bg=14.02%)▶u2af2 (bg=19.32%)▶zc3h8 (bg=12.78%)▶ZRANB2 (bg=7.28%)No matches to TargetScan


CTTCAGAC

CTTCAGAC  
Depth:6 (MOUSE)  
Ei-value:0.000, Pi-value:0.000  
Er-value:0.000, Pr-value:0.000  
eCLIP MATCHES▶ddx42 (bg=10.33%)▶GRWD1 (bg=7.0%)▶SLBP (bg=6.66%)▶srsf1 (bg=30.28%)▶srsf7 (bg=22.53%)▶tia1 (bg=23.76%)▶tial1 (bg=15.02%)▶u2af1 (bg=14.02%)▶u2af2 (bg=19.32%)▶zc3h8 (bg=12.78%)▶ZRANB2 (bg=7.28%)No matches to TargetScan


TA

TCCATTTTAAATGTGGGGATTGGGAACCACTAGTTCTTTCAGATGGTATTCTTCAGACTATAGAAGGAGCTTCCAGTTGAATTCA  
Depth:3 (DOG)  
Ei-value:0.000, Pi-value:0.000  
Er-value:0.000, Pr-value:0.000  
eCLIP MATCHES▶ddx42 (bg=10.33%)▶GRWD1 (bg=7.0%)▶hnrnpa1 (bg=18.32%)▶khsrp (bg=27.4%)▶ppil4 (bg=43.39%)▶SF3B4 (bg=4.76%)▶SLBP (bg=6.66%)▶srsf1 (bg=30.28%)▶srsf7 (bg=22.53%)▶tia1 (bg=23.76%)▶tial1 (bg=15.02%)▶u2af1 (bg=14.02%)▶u2af2 (bg=19.32%)▶zc3h8 (bg=12.78%)▶znf622 (bg=18.79%)▶ZRANB2 (bg=7.28%)MATCHES To TargetScan▶ miR-140-5p:AGUGGUU▶ miR-488-3p:UGAAAGG▶ miR-186-5p:AAAGAAU


TAG

TAGAAGGAGC  
Depth:7 (TURTLE)  
Ei-value:0.000, Pi-value:0.000  
Er-value:0.000, Pr-value:0.000  
eCLIP MATCHES▶GRWD1 (bg=7.0%)▶hnrnpa1 (bg=18.32%)▶ppil4 (bg=43.39%)▶srsf1 (bg=30.28%)▶srsf7 (bg=22.53%)▶tia1 (bg=23.76%)▶tial1 (bg=15.02%)▶u2af1 (bg=14.02%)▶u2af2 (bg=19.32%)▶zc3h8 (bg=12.78%)▶ZRANB2 (bg=7.28%)No matches to TargetScan


AAGGAGC

AAGGAGC  
Depth:8 (ALLIGATOR)  
Ei-value:0.000, Pi-value:0.000  
Er-value:0.000, Pr-value:0.000  
eCLIP MATCHES▶GRWD1 (bg=7.0%)▶hnrnpa1 (bg=18.32%)▶ppil4 (bg=43.39%)▶srsf1 (bg=30.28%)▶srsf7 (bg=22.53%)▶tia1 (bg=23.76%)▶tial1 (bg=15.02%)▶u2af1 (bg=14.02%)▶u2af2 (bg=19.32%)▶zc3h8 (bg=12.78%)▶ZRANB2 (bg=7.28%)No matches to TargetScan


TTCCAGTTGAATT

TAGAAGGAGCTTCCAGTTGAATT  
Depth:6 (MOUSE)  
Ei-value:0.000, Pi-value:0.000  
Er-value:0.000, Pr-value:0.000  
eCLIP MATCHES▶GRWD1 (bg=7.0%)▶hnrnpa1 (bg=18.32%)▶ppil4 (bg=43.39%)▶srsf1 (bg=30.28%)▶srsf7 (bg=22.53%)▶tia1 (bg=23.76%)▶tial1 (bg=15.02%)▶u2af1 (bg=14.02%)▶u2af2 (bg=19.32%)▶zc3h8 (bg=12.78%)▶znf622 (bg=18.79%)▶ZRANB2 (bg=7.28%)No matches to TargetScan


CA

TAGAAGGAGCTTCCAGTTGAATTCA  
Depth:5 (COW)  
Ei-value:0.000, Pi-value:0.000  
Er-value:0.000, Pr-value:0.000  
eCLIP MATCHES▶GRWD1 (bg=7.0%)▶hnrnpa1 (bg=18.32%)▶ppil4 (bg=43.39%)▶srsf1 (bg=30.28%)▶srsf7 (bg=22.53%)▶tia1 (bg=23.76%)▶tial1 (bg=15.02%)▶u2af1 (bg=14.02%)▶u2af2 (bg=19.32%)▶zc3h8 (bg=12.78%)▶znf622 (bg=18.79%)▶ZRANB2 (bg=7.28%)No matches to TargetScan

-

CAGT

CAGTGGACAAAATGAGGA  
Depth:3 (DOG)  
Ei-value:0.000, Pi-value:0.000  
Er-value:0.000, Pr-value:0.000  
eCLIP MATCHES▶GRWD1 (bg=7.0%)▶hnrnpa1 (bg=18.32%)▶srsf1 (bg=30.28%)▶srsf7 (bg=22.53%)▶u2af1 (bg=14.02%)▶u2af2 (bg=19.32%)▶znf622 (bg=18.79%)▶ZRANB2 (bg=7.28%)No matches to TargetScan


GGACAAAA

GGACAAAA  
Depth:7 (TURTLE)  
Ei-value:0.000, Pi-value:0.000  
Er-value:0.000, Pr-value:0.000  
eCLIP MATCHES▶GRWD1 (bg=7.0%)▶hnrnpa1 (bg=18.32%)▶srsf1 (bg=30.28%)▶srsf7 (bg=22.53%)▶u2af1 (bg=14.02%)▶u2af2 (bg=19.32%)▶znf622 (bg=18.79%)▶ZRANB2 (bg=7.28%)No matches to TargetScan


TGAGGA

GGACAAAATGAGGA  
Depth:6 (MOUSE)  
Ei-value:0.000, Pi-value:0.000  
Er-value:0.000, Pr-value:0.000  
eCLIP MATCHES▶GRWD1 (bg=7.0%)▶hnrnpa1 (bg=18.32%)▶srsf1 (bg=30.28%)▶srsf7 (bg=22.53%)▶u2af1 (bg=14.02%)▶u2af2 (bg=19.32%)▶znf622 (bg=18.79%)▶ZRANB2 (bg=7.28%)No matches to TargetScan

-

AAC

AACAGGTGAA  
Depth:10 (SNAKE)  
Ei-value:0.000, Pi-value:0.000  
Er-value:0.000, Pr-value:0.000  
eCLIP MATCHES▶GRWD1 (bg=7.0%)▶srsf1 (bg=30.28%)▶srsf7 (bg=22.53%)No matches to TargetScan


AG

AGGTGA  
Depth:11 (X.TROPICALIS)  
Ei-value:0.000, Pi-value:0.000  
Er-value:0.000, Pr-value:0.010  
eCLIP MATCHES▶GRWD1 (bg=7.0%)▶srsf1 (bg=30.28%)▶srsf7 (bg=22.53%)No matches to TargetScan

 6000  


GTGA

AGGTGA  
Depth:11 (X.TROPICALIS)  
Ei-value:0.000, Pi-value:0.000  
Er-value:0.000, Pr-value:0.010  
eCLIP MATCHES▶GRWD1 (bg=7.0%)▶srsf1 (bg=30.28%)▶srsf7 (bg=22.53%)No matches to TargetScan


A

AACAGGTGAA  
Depth:10 (SNAKE)  
Ei-value:0.000, Pi-value:0.000  
Er-value:0.000, Pr-value:0.000  
eCLIP MATCHES▶GRWD1 (bg=7.0%)▶srsf1 (bg=30.28%)▶srsf7 (bg=22.53%)No matches to TargetScan


C

AACAGGTGAAC  
Depth:6 (MOUSE)  
Ei-value:0.000, Pi-value:0.000  
Er-value:0.000, Pr-value:0.000  
eCLIP MATCHES▶GRWD1 (bg=7.0%)▶srsf1 (bg=30.28%)▶srsf7 (bg=22.53%)No matches to TargetScan


A

AACAGGTGAACA  
Depth:5 (COW)  
Ei-value:0.000, Pi-value:0.000  
Er-value:0.000, Pr-value:0.000  
eCLIP MATCHES▶GRWD1 (bg=7.0%)▶srsf1 (bg=30.28%)▶srsf7 (bg=22.53%)No matches to TargetScan


A

AACAGGTGAACAAGCTTTTTCTGTATTTACAT  
Depth:2 (MARMOSET)  
Ei-value:0.000, Pi-value:0.000  
Er-value:0.000, Pr-value:0.000  
eCLIP MATCHES▶GRWD1 (bg=7.0%)▶hnrnpa1 (bg=18.32%)▶srsf1 (bg=30.28%)▶srsf7 (bg=22.53%)▶tia1 (bg=23.76%)▶tial1 (bg=15.02%)▶zc3h8 (bg=12.78%)MATCHES To TargetScan▶ miR-320:AAAGCUG▶ miR-544a-5p:CUUGUUA▶ miR-375:UUGUUCG▶ miR-411-3p:AUGUAAC


GCTTTTT

GCTTTTT  
Depth:4 (PIG)  
Ei-value:0.000, Pi-value:0.000  
Er-value:0.000, Pr-value:0.000  
eCLIP MATCHES▶GRWD1 (bg=7.0%)▶srsf1 (bg=30.28%)▶srsf7 (bg=22.53%)▶tial1 (bg=15.02%)No matches to TargetScan


CTGTA

AACAGGTGAACAAGCTTTTTCTGTATTTACAT  
Depth:2 (MARMOSET)  
Ei-value:0.000, Pi-value:0.000  
Er-value:0.000, Pr-value:0.000  
eCLIP MATCHES▶GRWD1 (bg=7.0%)▶hnrnpa1 (bg=18.32%)▶srsf1 (bg=30.28%)▶srsf7 (bg=22.53%)▶tia1 (bg=23.76%)▶tial1 (bg=15.02%)▶zc3h8 (bg=12.78%)MATCHES To TargetScan▶ miR-320:AAAGCUG▶ miR-544a-5p:CUUGUUA▶ miR-375:UUGUUCG▶ miR-411-3p:AUGUAAC


TTTACAT

TTTACAT  
Depth:4 (PIG)  
Ei-value:0.000, Pi-value:0.000  
Er-value:0.000, Pr-value:0.000  
eCLIP MATCHES▶hnrnpa1 (bg=18.32%)▶srsf1 (bg=30.28%)▶tia1 (bg=23.76%)▶tial1 (bg=15.02%)▶zc3h8 (bg=12.78%)MATCHES To TargetScan▶ miR-411-3p:AUGUAAC

-

CA

CAAAGTCAGATCAGTTA  
Depth:2 (MARMOSET)  
Ei-value:0.000, Pi-value:0.000  
Er-value:0.000, Pr-value:0.000  
eCLIP MATCHES▶hnrnpa1 (bg=18.32%)▶tia1 (bg=23.76%)▶tial1 (bg=15.02%)▶zc3h8 (bg=12.78%)No matches to TargetScan


AAGTCAGATC

AAGTCAGATC  
Depth:5 (COW)  
Ei-value:0.000, Pi-value:0.000  
Er-value:0.000, Pr-value:0.000  
eCLIP MATCHES▶hnrnpa1 (bg=18.32%)▶tia1 (bg=23.76%)▶tial1 (bg=15.02%)▶zc3h8 (bg=12.78%)No matches to TargetScan


AGTTA

CAAAGTCAGATCAGTTA  
Depth:2 (MARMOSET)  
Ei-value:0.000, Pi-value:0.000  
Er-value:0.000, Pr-value:0.000  
eCLIP MATCHES▶hnrnpa1 (bg=18.32%)▶tia1 (bg=23.76%)▶tial1 (bg=15.02%)▶zc3h8 (bg=12.78%)No matches to TargetScan

-

gggacaat

gggacaat  
Depth:2 (MARMOSET)  
Ei-value:1.000, Pi-value:0.000  
Er-value:0.000, Pr-value:0.000  
eCLIP MATCHES▶hnrnpa1 (bg=18.32%)▶SLBP (bg=6.66%)▶tia1 (bg=23.76%)▶tial1 (bg=15.02%)▶zc3h8 (bg=12.78%)No matches to TargetScan


AGTAT

AGTATTGAATAGATTTCAGCTTTATGCTGGAGTAA  
Depth:2 (MARMOSET)  
Ei-value:0.000, Pi-value:0.000  
Er-value:0.000, Pr-value:0.000  
eCLIP MATCHES▶hnrnpa1 (bg=18.32%)▶HNRNPU (bg=9.45%)▶SLBP (bg=6.66%)▶tia1 (bg=23.76%)▶tial1 (bg=15.02%)▶zc3h8 (bg=12.78%)MATCHES To TargetScan▶ miR-320:AAAGCUG▶ miR-142-5p:AUAAAGU▶ miR-200bc-3p/429:AAUACUG▶ miR-203a-3p.2:UGAAAUG▶ miR-338-3p:CCAGCAU


tgaata

tgaata  
Depth:3 (DOG)  
Ei-value:1.000, Pi-value:0.040  
Er-value:0.000, Pr-value:0.000  
eCLIP MATCHES▶hnrnpa1 (bg=18.32%)▶SLBP (bg=6.66%)▶tia1 (bg=23.76%)▶tial1 (bg=15.02%)▶zc3h8 (bg=12.78%)No matches to TargetScan


GATTT

AGTATTGAATAGATTTCAGCTTTATGCTGGAGTAA  
Depth:2 (MARMOSET)  
Ei-value:0.000, Pi-value:0.000  
Er-value:0.000, Pr-value:0.000  
eCLIP MATCHES▶hnrnpa1 (bg=18.32%)▶HNRNPU (bg=9.45%)▶SLBP (bg=6.66%)▶tia1 (bg=23.76%)▶tial1 (bg=15.02%)▶zc3h8 (bg=12.78%)MATCHES To TargetScan▶ miR-320:AAAGCUG▶ miR-142-5p:AUAAAGU▶ miR-200bc-3p/429:AAUACUG▶ miR-203a-3p.2:UGAAAUG▶ miR-338-3p:CCAGCAU


CAGCTT

CAGCTT  
Depth:8 (ALLIGATOR)  
Ei-value:0.000, Pi-value:0.000  
Er-value:0.000, Pr-value:0.010  
eCLIP MATCHES▶hnrnpa1 (bg=18.32%)▶SLBP (bg=6.66%)▶tia1 (bg=23.76%)▶tial1 (bg=15.02%)▶zc3h8 (bg=12.78%)No matches to TargetScan


TA

CAGCTTTA  
Depth:5 (COW)  
Ei-value:0.000, Pi-value:0.000  
Er-value:0.000, Pr-value:0.000  
eCLIP MATCHES▶hnrnpa1 (bg=18.32%)▶SLBP (bg=6.66%)▶tia1 (bg=23.76%)▶tial1 (bg=15.02%)▶zc3h8 (bg=12.78%)MATCHES To TargetScan▶ miR-320:AAAGCUG


T

CAGCTTTATGCTGGA  
Depth:4 (PIG)  
Ei-value:0.000, Pi-value:0.000  
Er-value:0.000, Pr-value:0.000  
eCLIP MATCHES▶hnrnpa1 (bg=18.32%)▶SLBP (bg=6.66%)▶tia1 (bg=23.76%)▶tial1 (bg=15.02%)▶zc3h8 (bg=12.78%)MATCHES To TargetScan▶ miR-320:AAAGCUG▶ miR-142-5p:AUAAAGU▶ miR-338-3p:CCAGCAU


GCTGGA

GCTGGA  
Depth:6 (MOUSE)  
Ei-value:0.000, Pi-value:0.000  
Er-value:0.000, Pr-value:0.000  
eCLIP MATCHES▶hnrnpa1 (bg=18.32%)▶SLBP (bg=6.66%)▶tia1 (bg=23.76%)▶tial1 (bg=15.02%)No matches to TargetScan


GTAA

AGTATTGAATAGATTTCAGCTTTATGCTGGAGTAA  
Depth:2 (MARMOSET)  
Ei-value:0.000, Pi-value:0.000  
Er-value:0.000, Pr-value:0.000  
eCLIP MATCHES▶hnrnpa1 (bg=18.32%)▶HNRNPU (bg=9.45%)▶SLBP (bg=6.66%)▶tia1 (bg=23.76%)▶tial1 (bg=15.02%)▶zc3h8 (bg=12.78%)MATCHES To TargetScan▶ miR-320:AAAGCUG▶ miR-142-5p:AUAAAGU▶ miR-200bc-3p/429:AAUACUG▶ miR-203a-3p.2:UGAAAUG▶ miR-338-3p:CCAGCAU

-

TG

TGGCATGTGAGCAA  
Depth:2 (MARMOSET)  
Ei-value:0.020, Pi-value:0.000  
Er-value:0.000, Pr-value:0.000  
eCLIP MATCHES▶ddx42 (bg=10.33%)▶hnrnpa1 (bg=18.32%)▶HNRNPU (bg=9.45%)▶tia1 (bg=23.76%)▶tial1 (bg=15.02%)MATCHES To TargetScan▶ miR-342-3p:CUCACAC▶ miR-23-3p:UCACAUU


GCATG

GCATGTGAGCA  
Depth:3 (DOG)  
Ei-value:0.000, Pi-value:0.000  
Er-value:0.000, Pr-value:0.000  
eCLIP MATCHES▶ddx42 (bg=10.33%)▶hnrnpa1 (bg=18.32%)▶HNRNPU (bg=9.45%)▶tia1 (bg=23.76%)▶tial1 (bg=15.02%)MATCHES To TargetScan▶ miR-342-3p:CUCACAC▶ miR-23-3p:UCACAUU


TGAGCA

TGAGCA  
Depth:5 (COW)  
Ei-value:0.000, Pi-value:0.020  
Er-value:0.000, Pr-value:0.000  
eCLIP MATCHES▶ddx42 (bg=10.33%)▶hnrnpa1 (bg=18.32%)▶tia1 (bg=23.76%)▶tial1 (bg=15.02%)No matches to TargetScan


A

TGGCATGTGAGCAA  
Depth:2 (MARMOSET)  
Ei-value:0.020, Pi-value:0.000  
Er-value:0.000, Pr-value:0.000  
eCLIP MATCHES▶ddx42 (bg=10.33%)▶hnrnpa1 (bg=18.32%)▶HNRNPU (bg=9.45%)▶tia1 (bg=23.76%)▶tial1 (bg=15.02%)MATCHES To TargetScan▶ miR-342-3p:CUCACAC▶ miR-23-3p:UCACAUU

-

CTG

CTGTGTTGGCGTGGGGGTGGAGGGGTGAGGTGGGCGCTAAGCCTTTT  
Depth:2 (MARMOSET)  
Ei-value:0.000, Pi-value:0.000  
Er-value:0.000, Pr-value:0.000  
eCLIP MATCHES▶ddx42 (bg=10.33%)▶hnrnpa1 (bg=18.32%)▶HNRNPU (bg=9.45%)▶tia1 (bg=23.76%)▶tial1 (bg=15.02%)MATCHES To TargetScan▶ miR-1306-5p:CACCUCC


TGTTGGC

TGTTGGC  
Depth:6 (MOUSE)  
Ei-value:0.000, Pi-value:0.000  
Er-value:0.000, Pr-value:0.000  
eCLIP MATCHES▶ddx42 (bg=10.33%)▶hnrnpa1 (bg=18.32%)▶tia1 (bg=23.76%)▶tial1 (bg=15.02%)No matches to TargetScan


G

CTGTGTTGGCGTGGGGGTGGAGGGGTGAGGTGGGCGCTAAGCCTTTT  
Depth:2 (MARMOSET)  
Ei-value:0.000, Pi-value:0.000  
Er-value:0.000, Pr-value:0.000  
eCLIP MATCHES▶ddx42 (bg=10.33%)▶hnrnpa1 (bg=18.32%)▶HNRNPU (bg=9.45%)▶tia1 (bg=23.76%)▶tial1 (bg=15.02%)MATCHES To TargetScan▶ miR-1306-5p:CACCUCC


TG

TGGGGGTGGAGGGGT  
Depth:5 (COW)  
Ei-value:0.000, Pi-value:0.000  
Er-value:0.000, Pr-value:0.000  
eCLIP MATCHES▶ddx42 (bg=10.33%)▶HNRNPU (bg=9.45%)▶tial1 (bg=15.02%)No matches to TargetScan


GG

GGGGTGGAGGGGT  
Depth:6 (MOUSE)  
Ei-value:0.000, Pi-value:0.000  
Er-value:0.000, Pr-value:0.000  
eCLIP MATCHES▶ddx42 (bg=10.33%)▶HNRNPU (bg=9.45%)▶tial1 (bg=15.02%)No matches to TargetScan

 6120  


GGTGGAGGGGT

GGGGTGGAGGGGT  
Depth:6 (MOUSE)  
Ei-value:0.000, Pi-value:0.000  
Er-value:0.000, Pr-value:0.000  
eCLIP MATCHES▶ddx42 (bg=10.33%)▶HNRNPU (bg=9.45%)▶tial1 (bg=15.02%)No matches to TargetScan


G

CTGTGTTGGCGTGGGGGTGGAGGGGTGAGGTGGGCGCTAAGCCTTTT  
Depth:2 (MARMOSET)  
Ei-value:0.000, Pi-value:0.000  
Er-value:0.000, Pr-value:0.000  
eCLIP MATCHES▶ddx42 (bg=10.33%)▶hnrnpa1 (bg=18.32%)▶HNRNPU (bg=9.45%)▶tia1 (bg=23.76%)▶tial1 (bg=15.02%)MATCHES To TargetScan▶ miR-1306-5p:CACCUCC


AGGTGGGCGCTAAGCCTTTT

AGGTGGGCGCTAAGCCTTTT  
Depth:6 (MOUSE)  
Ei-value:0.000, Pi-value:0.000  
Er-value:0.000, Pr-value:0.000  
eCLIP MATCHES▶ddx42 (bg=10.33%)▶HNRNPU (bg=9.45%)▶tial1 (bg=15.02%)No matches to TargetScan

-

TTAAGA

TTAAGA  
Depth:8 (ALLIGATOR)  
Ei-value:0.000, Pi-value:0.010  
Er-value:0.000, Pr-value:0.010  
eCLIP MATCHES▶ddx42 (bg=10.33%)▶HNRNPU (bg=9.45%)▶tial1 (bg=15.02%)No matches to TargetScan


TTTTTCAGGTACCCCTC

TTAAGATTTTTCAGGTACCCCTC  
Depth:6 (MOUSE)  
Ei-value:0.000, Pi-value:0.000  
Er-value:0.000, Pr-value:0.000  
eCLIP MATCHES▶ddx42 (bg=10.33%)▶HNRNPU (bg=9.45%)▶tia1 (bg=23.76%)▶tial1 (bg=15.02%)MATCHES To TargetScan▶ miR-423-5p:GAGGGGC


AC

TTAAGATTTTTCAGGTACCCCTCACTAAAGGCACCGAAGGCTTAAAGTAGGACAACCATGGAGCCTTCCTGTGGCA  
Depth:2 (MARMOSET)  
Ei-value:0.000, Pi-value:0.000  
Er-value:0.000, Pr-value:0.000  
eCLIP MATCHES▶ddx42 (bg=10.33%)▶HNRNPU (bg=9.45%)▶QKI (bg=3.74%)▶srsf1 (bg=30.28%)▶tia1 (bg=23.76%)▶tial1 (bg=15.02%)MATCHES To TargetScan▶ miR-140-3p.1:CCACAGG▶ miR-136-5p:CUCCAUU▶ miR-423-5p:GAGGGGC▶ miR-873-5p.1:CAGGAAC


TAAAGGCAC

TAAAGGCAC  
Depth:6 (MOUSE)  
Ei-value:0.000, Pi-value:0.000  
Er-value:0.000, Pr-value:0.000  
eCLIP MATCHES▶HNRNPU (bg=9.45%)▶tial1 (bg=15.02%)No matches to TargetScan


C

TAAAGGCACC  
Depth:5 (COW)  
Ei-value:0.000, Pi-value:0.000  
Er-value:0.000, Pr-value:0.000  
eCLIP MATCHES▶HNRNPU (bg=9.45%)▶tial1 (bg=15.02%)No matches to TargetScan


G

GAAGGCTT  
Depth:4 (PIG)  
Ei-value:0.000, Pi-value:0.000  
Er-value:0.000, Pr-value:0.000  
eCLIP MATCHES▶HNRNPU (bg=9.45%)▶tial1 (bg=15.02%)No matches to TargetScan


AAGGCT

AAGGCT  
Depth:10 (SNAKE)  
Ei-value:0.000, Pi-value:0.000  
Er-value:0.000, Pr-value:0.010  
eCLIP MATCHES▶HNRNPU (bg=9.45%)▶tial1 (bg=15.02%)No matches to TargetScan


T

AAGGCTT  
Depth:9 (LIZARD)  
Ei-value:0.000, Pi-value:0.000  
Er-value:0.000, Pr-value:0.000  
eCLIP MATCHES▶HNRNPU (bg=9.45%)▶tial1 (bg=15.02%)No matches to TargetScan


A

TTAAGATTTTTCAGGTACCCCTCACTAAAGGCACCGAAGGCTTAAAGTAGGACAACCATGGAGCCTTCCTGTGGCA  
Depth:2 (MARMOSET)  
Ei-value:0.000, Pi-value:0.000  
Er-value:0.000, Pr-value:0.000  
eCLIP MATCHES▶ddx42 (bg=10.33%)▶HNRNPU (bg=9.45%)▶QKI (bg=3.74%)▶srsf1 (bg=30.28%)▶tia1 (bg=23.76%)▶tial1 (bg=15.02%)MATCHES To TargetScan▶ miR-140-3p.1:CCACAGG▶ miR-136-5p:CUCCAUU▶ miR-423-5p:GAGGGGC▶ miR-873-5p.1:CAGGAAC


AA

AAGTAGGACAACC  
Depth:5 (COW)  
Ei-value:0.000, Pi-value:0.000  
Er-value:0.000, Pr-value:0.000  
eCLIP MATCHES▶HNRNPU (bg=9.45%)▶tial1 (bg=15.02%)No matches to TargetScan


GTAGGA

GTAGGA  
Depth:7 (TURTLE)  
Ei-value:0.000, Pi-value:0.000  
Er-value:0.000, Pr-value:0.000  
eCLIP MATCHES▶tial1 (bg=15.02%)No matches to TargetScan


CA

GTAGGACA  
Depth:6 (MOUSE)  
Ei-value:0.000, Pi-value:0.000  
Er-value:0.000, Pr-value:0.000  
eCLIP MATCHES▶tial1 (bg=15.02%)No matches to TargetScan


ACC

AAGTAGGACAACC  
Depth:5 (COW)  
Ei-value:0.000, Pi-value:0.000  
Er-value:0.000, Pr-value:0.000  
eCLIP MATCHES▶HNRNPU (bg=9.45%)▶tial1 (bg=15.02%)No matches to TargetScan


ATGGA

AAGTAGGACAACCATGGAGCCTTCCTGTGGCA  
Depth:3 (DOG)  
Ei-value:0.000, Pi-value:0.000  
Er-value:0.000, Pr-value:0.000  
eCLIP MATCHES▶ddx42 (bg=10.33%)▶HNRNPU (bg=9.45%)▶QKI (bg=3.74%)▶srsf1 (bg=30.28%)▶tial1 (bg=15.02%)MATCHES To TargetScan▶ miR-140-3p.1:CCACAGG▶ miR-136-5p:CUCCAUU▶ miR-873-5p.1:CAGGAAC


GC

GCCTTCCTGTGGCA  
Depth:4 (PIG)  
Ei-value:0.000, Pi-value:0.000  
Er-value:0.000, Pr-value:0.000  
eCLIP MATCHES▶ddx42 (bg=10.33%)▶QKI (bg=3.74%)▶srsf1 (bg=30.28%)▶tial1 (bg=15.02%)MATCHES To TargetScan▶ miR-140-3p.1:CCACAGG▶ miR-873-5p.1:CAGGAAC


CTTCCTGTG

CTTCCTGTG  
Depth:6 (MOUSE)  
Ei-value:0.000, Pi-value:0.000  
Er-value:0.000, Pr-value:0.000  
eCLIP MATCHES▶ddx42 (bg=10.33%)▶QKI (bg=3.74%)▶srsf1 (bg=30.28%)▶tial1 (bg=15.02%)MATCHES To TargetScan▶ miR-873-5p.1:CAGGAAC


GCA

CTTCCTGTGGCA  
Depth:5 (COW)  
Ei-value:0.000, Pi-value:0.000  
Er-value:0.000, Pr-value:0.000  
eCLIP MATCHES▶ddx42 (bg=10.33%)▶QKI (bg=3.74%)▶srsf1 (bg=30.28%)▶tial1 (bg=15.02%)MATCHES To TargetScan▶ miR-140-3p.1:CCACAGG▶ miR-873-5p.1:CAGGAAC

-

GAGAGACAAC

GAGAGACAACAAAGCGCTATTA  
Depth:5 (COW)  
Ei-value:0.000, Pi-value:0.000  
Er-value:0.000, Pr-value:0.000  
eCLIP MATCHES▶ddx42 (bg=10.33%)▶QKI (bg=3.74%)▶srsf1 (bg=30.28%)No matches to TargetScan

 6240  


AAAGCGCTATTA

GAGAGACAACAAAGCGCTATTA  
Depth:5 (COW)  
Ei-value:0.000, Pi-value:0.000  
Er-value:0.000, Pr-value:0.000  
eCLIP MATCHES▶ddx42 (bg=10.33%)▶QKI (bg=3.74%)▶srsf1 (bg=30.28%)No matches to TargetScan


TC

GAGAGACAACAAAGCGCTATTATCCTAAGGTCAAGA  
Depth:2 (MARMOSET)  
Ei-value:0.000, Pi-value:0.000  
Er-value:0.000, Pr-value:0.000  
eCLIP MATCHES▶ddx42 (bg=10.33%)▶HNRNPU (bg=9.45%)▶QKI (bg=3.74%)▶srsf1 (bg=30.28%)MATCHES To TargetScan▶ miR-369-3p:AUAAUAC▶ miR-192-5p/215-5p:UGACCUA


CTAAGGTCAA

CTAAGGTCAA  
Depth:5 (COW)  
Ei-value:0.000, Pi-value:0.000  
Er-value:0.000, Pr-value:0.000  
eCLIP MATCHES▶ddx42 (bg=10.33%)▶HNRNPU (bg=9.45%)▶srsf1 (bg=30.28%)MATCHES To TargetScan▶ miR-192-5p/215-5p:UGACCUA


GA

GAGAGACAACAAAGCGCTATTATCCTAAGGTCAAGA  
Depth:2 (MARMOSET)  
Ei-value:0.000, Pi-value:0.000  
Er-value:0.000, Pr-value:0.000  
eCLIP MATCHES▶ddx42 (bg=10.33%)▶HNRNPU (bg=9.45%)▶QKI (bg=3.74%)▶srsf1 (bg=30.28%)MATCHES To TargetScan▶ miR-369-3p:AUAAUAC▶ miR-192-5p/215-5p:UGACCUA

---

GTGTCAGCCTC

GTGTCAGCCTC  
Depth:4 (PIG)  
Ei-value:0.000, Pi-value:0.000  
Er-value:0.000, Pr-value:0.000  
eCLIP MATCHES▶HNRNPU (bg=9.45%)▶tial1 (bg=15.02%)MATCHES To TargetScan▶ miR-485-5p:GAGGCUG


ACCT

GTGTCAGCCTCACCT  
Depth:2 (MARMOSET)  
Ei-value:0.000, Pi-value:0.000  
Er-value:0.000, Pr-value:0.000  
eCLIP MATCHES▶HNRNPU (bg=9.45%)▶tial1 (bg=15.02%)MATCHES To TargetScan▶ miR-485-5p:GAGGCUG

-----

ttattagtaa

ttattagtaa  
Depth:2 (MARMOSET)  
Ei-value:1.000, Pi-value:0.000  
Er-value:0.000, Pr-value:0.000  
eCLIP MATCHES▶hnrnpa1 (bg=18.32%)▶HNRNPU (bg=9.45%)▶tia1 (bg=23.76%)▶tial1 (bg=15.02%)No matches to TargetScan

--

AGGACTTG

AGGACTTG  
Depth:5 (COW)  
Ei-value:0.000, Pi-value:0.000  
Er-value:0.000, Pr-value:0.000  
eCLIP MATCHES▶hnrnpa1 (bg=18.32%)▶HNRNPU (bg=9.45%)▶tia1 (bg=23.76%)▶tial1 (bg=15.02%)▶XRCC6 (bg=3.81%)No matches to TargetScan


C

AGGACTTGCCTCAAC  
Depth:4 (PIG)  
Ei-value:0.000, Pi-value:0.000  
Er-value:0.000, Pr-value:0.000  
eCLIP MATCHES▶hnrnpa1 (bg=18.32%)▶HNRNPU (bg=9.45%)▶tia1 (bg=23.76%)▶tial1 (bg=15.02%)▶XRCC6 (bg=3.81%)MATCHES To TargetScan▶ miR-31-5p:GGCAAGA


CTCAAC

CTCAAC  
Depth:5 (COW)  
Ei-value:0.000, Pi-value:0.000  
Er-value:0.000, Pr-value:0.000  
eCLIP MATCHES▶HNRNPU (bg=9.45%)▶tia1 (bg=23.76%)▶tial1 (bg=15.02%)▶XRCC6 (bg=3.81%)No matches to TargetScan


T

AGGACTTGCCTCAACT  
Depth:2 (MARMOSET)  
Ei-value:0.000, Pi-value:0.000  
Er-value:0.000, Pr-value:0.000  
eCLIP MATCHES▶hnrnpa1 (bg=18.32%)▶HNRNPU (bg=9.45%)▶tia1 (bg=23.76%)▶tial1 (bg=15.02%)▶XRCC6 (bg=3.81%)▶zc3h8 (bg=12.78%)MATCHES To TargetScan▶ miR-31-5p:GGCAAGA

--

CTCTTTCTGGAGTGA

CTCTTTCTGGAGTGAAGCATCC  
Depth:2 (MARMOSET)  
Ei-value:0.000, Pi-value:0.000  
Er-value:0.000, Pr-value:0.000  
eCLIP MATCHES▶HNRNPU (bg=9.45%)▶NIPBL (bg=8.2%)▶tia1 (bg=23.76%)▶tial1 (bg=15.02%)▶XRCC6 (bg=3.81%)▶zc3h8 (bg=12.78%)MATCHES To TargetScan▶ miR-483-3p.2:CACUCCU


AGCATC

AGCATC  
Depth:4 (PIG)  
Ei-value:0.010, Pi-value:0.000  
Er-value:0.000, Pr-value:0.000  
eCLIP MATCHES▶HNRNPU (bg=9.45%)▶NIPBL (bg=8.2%)▶tia1 (bg=23.76%)▶tial1 (bg=15.02%)▶XRCC6 (bg=3.81%)▶zc3h8 (bg=12.78%)No matches to TargetScan


C

CTCTTTCTGGAGTGAAGCATCC  
Depth:2 (MARMOSET)  
Ei-value:0.000, Pi-value:0.000  
Er-value:0.000, Pr-value:0.000  
eCLIP MATCHES▶HNRNPU (bg=9.45%)▶NIPBL (bg=8.2%)▶tia1 (bg=23.76%)▶tial1 (bg=15.02%)▶XRCC6 (bg=3.81%)▶zc3h8 (bg=12.78%)MATCHES To TargetScan▶ miR-483-3p.2:CACUCCU

-

AAGGAATG

AAGGAATG  
Depth:5 (COW)  
Ei-value:0.000, Pi-value:0.000  
Er-value:0.000, Pr-value:0.000  
eCLIP MATCHES▶tia1 (bg=23.76%)▶XRCC6 (bg=3.81%)No matches to TargetScan


CTTGAA

AAGGAATGCTTGAAGTACCC  
Depth:2 (MARMOSET)  
Ei-value:0.000, Pi-value:0.000  
Er-value:0.000, Pr-value:0.000  
eCLIP MATCHES▶tia1 (bg=23.76%)▶XRCC6 (bg=3.81%)No matches to TargetScan


GTAC

GTACCC  
Depth:4 (PIG)  
Ei-value:0.010, Pi-value:0.000  
Er-value:0.000, Pr-value:0.000  
eCLIP MATCHES▶tia1 (bg=23.76%)No matches to TargetScan

 6360  


CC

GTACCC  
Depth:4 (PIG)  
Ei-value:0.010, Pi-value:0.000  
Er-value:0.000, Pr-value:0.000  
eCLIP MATCHES▶tia1 (bg=23.76%)No matches to TargetScan

-----------

ttaacatttaagc

ttaacatttaagc  
Depth:2 (MARMOSET)  
Ei-value:0.680, Pi-value:0.000  
Er-value:0.000, Pr-value:0.000  
No matches to eCLIP DataMATCHES To TargetScan▶ miR-409-3p:AAUGUUG

-

AGCTGT

AGCTGTTTTTATAGCAGCTCTTAA  
Depth:2 (MARMOSET)  
Ei-value:0.000, Pi-value:0.000  
Er-value:0.000, Pr-value:0.000  
No matches to eCLIP DataMATCHES To TargetScan▶ miR-22-3p:AGCUGCC▶ miR-340-5p:UAUAAAG


TTTTATAGCAGCT

TTTTATAGCAGCT  
Depth:3 (DOG)  
Ei-value:0.000, Pi-value:0.000  
Er-value:0.000, Pr-value:0.000  
No matches to eCLIP DataMATCHES To TargetScan▶ miR-22-3p:AGCUGCC▶ miR-340-5p:UAUAAAG


CTTAA

AGCTGTTTTTATAGCAGCTCTTAA  
Depth:2 (MARMOSET)  
Ei-value:0.000, Pi-value:0.000  
Er-value:0.000, Pr-value:0.000  
No matches to eCLIP DataMATCHES To TargetScan▶ miR-22-3p:AGCUGCC▶ miR-340-5p:UAUAAAG

---

T

TAAAGCCCAAATCTCAAGCGGTGCTTGAAGGGGAGGGAAAGGGGGAAAGCGGGCAACCA  
Depth:2 (MARMOSET)  
Ei-value:0.000, Pi-value:0.000  
Er-value:0.000, Pr-value:0.000  
eCLIP MATCHES▶ddx42 (bg=10.33%)▶DDX51 (bg=0.19%)▶HNRNPU (bg=9.45%)▶SERBP1 (bg=0.36%)MATCHES To TargetScan▶ miR-29-3p:AGCACCA▶ miR-205-5p:CCUUCAU


A

AAAGCCCA  
Depth:3 (DOG)  
Ei-value:0.010, Pi-value:0.000  
Er-value:0.000, Pr-value:0.000  
eCLIP MATCHES▶HNRNPU (bg=9.45%)No matches to TargetScan


AAGCCCA

AAGCCCA  
Depth:4 (PIG)  
Ei-value:0.000, Pi-value:0.000  
Er-value:0.000, Pr-value:0.000  
eCLIP MATCHES▶HNRNPU (bg=9.45%)No matches to TargetScan


AAT

TAAAGCCCAAATCTCAAGCGGTGCTTGAAGGGGAGGGAAAGGGGGAAAGCGGGCAACCA  
Depth:2 (MARMOSET)  
Ei-value:0.000, Pi-value:0.000  
Er-value:0.000, Pr-value:0.000  
eCLIP MATCHES▶ddx42 (bg=10.33%)▶DDX51 (bg=0.19%)▶HNRNPU (bg=9.45%)▶SERBP1 (bg=0.36%)MATCHES To TargetScan▶ miR-29-3p:AGCACCA▶ miR-205-5p:CCUUCAU


ctcaag

ctcaag  
Depth:3 (DOG)  
Ei-value:1.000, Pi-value:0.020  
Er-value:0.000, Pr-value:0.000  
eCLIP MATCHES▶HNRNPU (bg=9.45%)▶SERBP1 (bg=0.36%)No matches to TargetScan


CGGTGC

TAAAGCCCAAATCTCAAGCGGTGCTTGAAGGGGAGGGAAAGGGGGAAAGCGGGCAACCA  
Depth:2 (MARMOSET)  
Ei-value:0.000, Pi-value:0.000  
Er-value:0.000, Pr-value:0.000  
eCLIP MATCHES▶ddx42 (bg=10.33%)▶DDX51 (bg=0.19%)▶HNRNPU (bg=9.45%)▶SERBP1 (bg=0.36%)MATCHES To TargetScan▶ miR-29-3p:AGCACCA▶ miR-205-5p:CCUUCAU


TTGA

TTGAAGGGGAGGGAAA  
Depth:5 (COW)  
Ei-value:0.000, Pi-value:0.000  
Er-value:0.000, Pr-value:0.000  
eCLIP MATCHES▶ddx42 (bg=10.33%)▶DDX51 (bg=0.19%)▶SERBP1 (bg=0.36%)MATCHES To TargetScan▶ miR-205-5p:CCUUCAU


AGGGGA

AGGGGA  
Depth:8 (ALLIGATOR)  
Ei-value:0.000, Pi-value:0.000  
Er-value:0.000, Pr-value:0.000  
eCLIP MATCHES▶ddx42 (bg=10.33%)▶DDX51 (bg=0.19%)▶SERBP1 (bg=0.36%)No matches to TargetScan


GGGAAA

AGGGGAGGGAAA  
Depth:6 (MOUSE)  
Ei-value:0.000, Pi-value:0.000  
Er-value:0.000, Pr-value:0.000  
eCLIP MATCHES▶ddx42 (bg=10.33%)▶DDX51 (bg=0.19%)▶SERBP1 (bg=0.36%)No matches to TargetScan


G

TAAAGCCCAAATCTCAAGCGGTGCTTGAAGGGGAGGGAAAGGGGGAAAGCGGGCAACCA  
Depth:2 (MARMOSET)  
Ei-value:0.000, Pi-value:0.000  
Er-value:0.000, Pr-value:0.000  
eCLIP MATCHES▶ddx42 (bg=10.33%)▶DDX51 (bg=0.19%)▶HNRNPU (bg=9.45%)▶SERBP1 (bg=0.36%)MATCHES To TargetScan▶ miR-29-3p:AGCACCA▶ miR-205-5p:CCUUCAU


GGGGAAAG

GGGGAAAG  
Depth:6 (MOUSE)  
Ei-value:0.000, Pi-value:0.000  
Er-value:0.000, Pr-value:0.000  
eCLIP MATCHES▶ddx42 (bg=10.33%)No matches to TargetScan


C

TAAAGCCCAAATCTCAAGCGGTGCTTGAAGGGGAGGGAAAGGGGGAAAGCGGGCAACCA  
Depth:2 (MARMOSET)  
Ei-value:0.000, Pi-value:0.000  
Er-value:0.000, Pr-value:0.000  
eCLIP MATCHES▶ddx42 (bg=10.33%)▶DDX51 (bg=0.19%)▶HNRNPU (bg=9.45%)▶SERBP1 (bg=0.36%)MATCHES To TargetScan▶ miR-29-3p:AGCACCA▶ miR-205-5p:CCUUCAU


GGG

GGGCAACCA  
Depth:6 (MOUSE)  
Ei-value:0.000, Pi-value:0.000  
Er-value:0.000, Pr-value:0.000  
eCLIP MATCHES▶ddx42 (bg=10.33%)No matches to TargetScan


CAACCA

CAACCA  
Depth:8 (ALLIGATOR)  
Ei-value:0.000, Pi-value:0.000  
Er-value:0.000, Pr-value:0.000  
eCLIP MATCHES▶ddx42 (bg=10.33%)No matches to TargetScan

-

TTT

TTTTCCCTAGCTTTTCCAGAAGCCTGTTAAAA  
Depth:3 (DOG)  
Ei-value:0.000, Pi-value:0.000  
Er-value:0.000, Pr-value:0.000  
eCLIP MATCHES▶ddx42 (bg=10.33%)MATCHES To TargetScan▶ miR-320:AAAGCUG


TCC

TCCCTAGCTTTTCCAGAA  
Depth:5 (COW)  
Ei-value:0.000, Pi-value:0.000  
Er-value:0.000, Pr-value:0.000  
eCLIP MATCHES▶ddx42 (bg=10.33%)MATCHES To TargetScan▶ miR-320:AAAGCUG

 6480  


CT

TCCCTAGCTTTTCCAGAA  
Depth:5 (COW)  
Ei-value:0.000, Pi-value:0.000  
Er-value:0.000, Pr-value:0.000  
eCLIP MATCHES▶ddx42 (bg=10.33%)MATCHES To TargetScan▶ miR-320:AAAGCUG


AGC

AGCTTTTCCAGAA  
Depth:6 (MOUSE)  
Ei-value:0.000, Pi-value:0.000  
Er-value:0.000, Pr-value:0.000  
eCLIP MATCHES▶ddx42 (bg=10.33%)MATCHES To TargetScan▶ miR-320:AAAGCUG


TTTTCCAG

TTTTCCAG  
Depth:10 (SNAKE)  
Ei-value:0.000, Pi-value:0.000  
Er-value:0.000, Pr-value:0.000  
No matches to eCLIP DataNo matches to TargetScan


AA

TTTTCCAGAA  
Depth:8 (ALLIGATOR)  
Ei-value:0.000, Pi-value:0.000  
Er-value:0.000, Pr-value:0.000  
No matches to eCLIP DataNo matches to TargetScan


G

TTTTCCCTAGCTTTTCCAGAAGCCTGTTAAAA  
Depth:3 (DOG)  
Ei-value:0.000, Pi-value:0.000  
Er-value:0.000, Pr-value:0.000  
eCLIP MATCHES▶ddx42 (bg=10.33%)MATCHES To TargetScan▶ miR-320:AAAGCUG


CCTGTTA

CCTGTTA  
Depth:6 (MOUSE)  
Ei-value:0.000, Pi-value:0.000  
Er-value:0.000, Pr-value:0.000  
No matches to eCLIP DataNo matches to TargetScan


AAA

CCTGTTAAAA  
Depth:5 (COW)  
Ei-value:0.000, Pi-value:0.000  
Er-value:0.000, Pr-value:0.000  
No matches to eCLIP DataNo matches to TargetScan


GCAAGG

GCAAGG  
Depth:5 (COW)  
Ei-value:0.000, Pi-value:0.020  
Er-value:0.000, Pr-value:0.010  
No matches to eCLIP DataNo matches to TargetScan


T

TTTTCCCTAGCTTTTCCAGAAGCCTGTTAAAAGCAAGGTCTCCCCACAAGCAACTTCTCTGCCACATCGCCACCC  
Depth:2 (MARMOSET)  
Ei-value:0.000, Pi-value:0.000  
Er-value:0.000, Pr-value:0.000  
eCLIP MATCHES▶ddx42 (bg=10.33%)▶HNRNPU (bg=9.45%)▶tia1 (bg=23.76%)MATCHES To TargetScan▶ miR-382-5p:AAGUUGU▶ miR-299-3p:AUGUGGG▶ miR-320:AAAGCUG▶ miR-491-5p:GUGGGGA


CTCCCCACAAG

CTCCCCACAAG  
Depth:6 (MOUSE)  
Ei-value:0.000, Pi-value:0.000  
Er-value:0.000, Pr-value:0.000  
No matches to eCLIP DataMATCHES To TargetScan▶ miR-491-5p:GUGGGGA


CAACTT

TTTTCCCTAGCTTTTCCAGAAGCCTGTTAAAAGCAAGGTCTCCCCACAAGCAACTTCTCTGCCACATCGCCACCC  
Depth:2 (MARMOSET)  
Ei-value:0.000, Pi-value:0.000  
Er-value:0.000, Pr-value:0.000  
eCLIP MATCHES▶ddx42 (bg=10.33%)▶HNRNPU (bg=9.45%)▶tia1 (bg=23.76%)MATCHES To TargetScan▶ miR-382-5p:AAGUUGU▶ miR-299-3p:AUGUGGG▶ miR-320:AAAGCUG▶ miR-491-5p:GUGGGGA


CTCTGCCACATCG

CTCTGCCACATCG  
Depth:6 (MOUSE)  
Ei-value:0.000, Pi-value:0.000  
Er-value:0.000, Pr-value:0.000  
eCLIP MATCHES▶HNRNPU (bg=9.45%)▶tia1 (bg=23.76%)MATCHES To TargetScan▶ miR-299-3p:AUGUGGG


CCAC

CTCTGCCACATCGCCAC  
Depth:3 (DOG)  
Ei-value:0.000, Pi-value:0.000  
Er-value:0.000, Pr-value:0.000  
eCLIP MATCHES▶HNRNPU (bg=9.45%)▶tia1 (bg=23.76%)MATCHES To TargetScan▶ miR-299-3p:AUGUGGG


CC

TTTTCCCTAGCTTTTCCAGAAGCCTGTTAAAAGCAAGGTCTCCCCACAAGCAACTTCTCTGCCACATCGCCACCC  
Depth:2 (MARMOSET)  
Ei-value:0.000, Pi-value:0.000  
Er-value:0.000, Pr-value:0.000  
eCLIP MATCHES▶ddx42 (bg=10.33%)▶HNRNPU (bg=9.45%)▶tia1 (bg=23.76%)MATCHES To TargetScan▶ miR-382-5p:AAGUUGU▶ miR-299-3p:AUGUGGG▶ miR-320:AAAGCUG▶ miR-491-5p:GUGGGGA

-

g

gtgccttt  
Depth:2 (MARMOSET)  
Ei-value:1.000, Pi-value:0.000  
Er-value:0.000, Pr-value:0.000  
eCLIP MATCHES▶ddx42 (bg=10.33%)▶HNRNPU (bg=9.45%)▶tia1 (bg=23.76%)▶tial1 (bg=15.02%)MATCHES To TargetScan▶ miR-124-3p.1:AAGGCAC


TGCCTTT

TGCCTTT  
Depth:5 (COW)  
Ei-value:0.000, Pi-value:0.000  
Er-value:0.000, Pr-value:0.000  
eCLIP MATCHES▶ddx42 (bg=10.33%)▶HNRNPU (bg=9.45%)▶tia1 (bg=23.76%)▶tial1 (bg=15.02%)MATCHES To TargetScan▶ miR-124-3p.1:AAGGCAC

----

CTAGCA

CTAGCACAGACCCTTCACCCCTCACCTCGATGCAGCC  
Depth:2 (MARMOSET)  
Ei-value:0.000, Pi-value:0.000  
Er-value:0.000, Pr-value:0.000  
eCLIP MATCHES▶ddx42 (bg=10.33%)▶HNRNPU (bg=9.45%)▶tia1 (bg=23.76%)▶tial1 (bg=15.02%)MATCHES To TargetScan▶ miR-218-5p:UGUGCUU▶ miR-423-5p:GAGGGGC▶ miR-193a-5p:GGGUCUU


CAGACCCTTCACCCCTCACCT

CAGACCCTTCACCCCTCACCT  
Depth:6 (MOUSE)  
Ei-value:0.000, Pi-value:0.000  
Er-value:0.000, Pr-value:0.000  
eCLIP MATCHES▶ddx42 (bg=10.33%)▶HNRNPU (bg=9.45%)▶tia1 (bg=23.76%)▶tial1 (bg=15.02%)MATCHES To TargetScan▶ miR-423-5p:GAGGGGC▶ miR-193a-5p:GGGUCUU


CGATGC

CAGACCCTTCACCCCTCACCTCGATGC  
Depth:5 (COW)  
Ei-value:0.000, Pi-value:0.000  
Er-value:0.000, Pr-value:0.000  
eCLIP MATCHES▶ddx42 (bg=10.33%)▶HNRNPU (bg=9.45%)▶tia1 (bg=23.76%)▶tial1 (bg=15.02%)MATCHES To TargetScan▶ miR-423-5p:GAGGGGC▶ miR-193a-5p:GGGUCUU


AGCC

CTAGCACAGACCCTTCACCCCTCACCTCGATGCAGCC  
Depth:2 (MARMOSET)  
Ei-value:0.000, Pi-value:0.000  
Er-value:0.000, Pr-value:0.000  
eCLIP MATCHES▶ddx42 (bg=10.33%)▶HNRNPU (bg=9.45%)▶tia1 (bg=23.76%)▶tial1 (bg=15.02%)MATCHES To TargetScan▶ miR-218-5p:UGUGCUU▶ miR-423-5p:GAGGGGC▶ miR-193a-5p:GGGUCUU

- 6600  
 -----

T

TTGGATCCTTG  
Depth:5 (COW)  
Ei-value:0.000, Pi-value:0.000  
Er-value:0.000, Pr-value:0.000  
No matches to eCLIP DataNo matches to TargetScan


TGGATCCT

TGGATCCT  
Depth:10 (SNAKE)  
Ei-value:0.000, Pi-value:0.000  
Er-value:0.000, Pr-value:0.000  
No matches to eCLIP DataNo matches to TargetScan


TG

TGGATCCTTG  
Depth:8 (ALLIGATOR)  
Ei-value:0.000, Pi-value:0.000  
Er-value:0.000, Pr-value:0.000  
No matches to eCLIP DataNo matches to TargetScan


T

TTGGATCCTTGTGGGCATGATCCATAATC  
Depth:2 (MARMOSET)  
Ei-value:0.000, Pi-value:0.000  
Er-value:0.000, Pr-value:0.000  
No matches to eCLIP DataNo matches to TargetScan


GGGCATGA

GGGCATGA  
Depth:5 (COW)  
Ei-value:0.000, Pi-value:0.000  
Er-value:0.000, Pr-value:0.000  
No matches to eCLIP DataNo matches to TargetScan


TCCATAATC

TTGGATCCTTGTGGGCATGATCCATAATC  
Depth:2 (MARMOSET)  
Ei-value:0.000, Pi-value:0.000  
Er-value:0.000, Pr-value:0.000  
No matches to eCLIP DataNo matches to TargetScan

-

GTTTC

GTTTCAAGGTAAC  
Depth:3 (DOG)  
Ei-value:0.000, Pi-value:0.000  
Er-value:0.000, Pr-value:0.000  
eCLIP MATCHES▶PRPF8 (bg=6.2%)MATCHES To TargetScan▶ miR-653-5p:UGAAACA


AAGGTAA

AAGGTAA  
Depth:10 (SNAKE)  
Ei-value:0.000, Pi-value:0.000  
Er-value:0.000, Pr-value:0.000  
eCLIP MATCHES▶PRPF8 (bg=6.2%)No matches to TargetScan


C

AAGGTAAC  
Depth:4 (PIG)  
Ei-value:0.000, Pi-value:0.000  
Er-value:0.000, Pr-value:0.000  
eCLIP MATCHES▶PRPF8 (bg=6.2%)No matches to TargetScan

-

atggtg

atggtg  
Depth:2 (MARMOSET)  
Ei-value:1.000, Pi-value:0.070  
Er-value:0.000, Pr-value:0.020  
eCLIP MATCHES▶PRPF8 (bg=6.2%)No matches to TargetScan

-

c

cgaggtctt  
Depth:2 (MARMOSET)  
Ei-value:1.000, Pi-value:0.000  
Er-value:0.000, Pr-value:0.000  
eCLIP MATCHES▶PRPF8 (bg=6.2%)No matches to TargetScan


G

GAGGTCTT  
Depth:4 (PIG)  
Ei-value:0.000, Pi-value:0.000  
Er-value:0.000, Pr-value:0.000  
eCLIP MATCHES▶PRPF8 (bg=6.2%)No matches to TargetScan


AGGTCTT

AGGTCTT  
Depth:5 (COW)  
Ei-value:0.000, Pi-value:0.000  
Er-value:0.000, Pr-value:0.000  
eCLIP MATCHES▶PRPF8 (bg=6.2%)No matches to TargetScan

-

G

GGTGGGTTG  
Depth:5 (COW)  
Ei-value:0.000, Pi-value:0.000  
Er-value:0.000, Pr-value:0.000  
eCLIP MATCHES▶PRPF8 (bg=6.2%)No matches to TargetScan


GTGGGTTG

GTGGGTTG  
Depth:6 (MOUSE)  
Ei-value:0.000, Pi-value:0.000  
Er-value:0.000, Pr-value:0.000  
eCLIP MATCHES▶PRPF8 (bg=6.2%)No matches to TargetScan

--------

T

TAGAAAAG  
Depth:5 (COW)  
Ei-value:0.000, Pi-value:0.000  
Er-value:0.000, Pr-value:0.000  
eCLIP MATCHES▶HNRNPU (bg=9.45%)No matches to TargetScan


AGAAAAG

AGAAAAG  
Depth:6 (MOUSE)  
Ei-value:0.000, Pi-value:0.000  
Er-value:0.000, Pr-value:0.000  
eCLIP MATCHES▶HNRNPU (bg=9.45%)No matches to TargetScan


GCCA

TAGAAAAGGCCATTAATTTGCCTGCAAAT  
Depth:3 (DOG)  
Ei-value:0.000, Pi-value:0.000  
Er-value:0.000, Pr-value:0.000  
eCLIP MATCHES▶hnrnpa1 (bg=18.32%)▶HNRNPU (bg=9.45%)No matches to TargetScan


TTAATTT

TTAATTT  
Depth:5 (COW)  
Ei-value:0.000, Pi-value:0.030  
Er-value:0.000, Pr-value:0.000  
eCLIP MATCHES▶hnrnpa1 (bg=18.32%)▶HNRNPU (bg=9.45%)No matches to TargetScan


GCCTG

TTAATTTGCCTG  
Depth:4 (PIG)  
Ei-value:0.000, Pi-value:0.000  
Er-value:0.000, Pr-value:0.000  
eCLIP MATCHES▶hnrnpa1 (bg=18.32%)▶HNRNPU (bg=9.45%)No matches to TargetScan


CAAAT

TAGAAAAGGCCATTAATTTGCCTGCAAAT  
Depth:3 (DOG)  
Ei-value:0.000, Pi-value:0.000  
Er-value:0.000, Pr-value:0.000  
eCLIP MATCHES▶hnrnpa1 (bg=18.32%)▶HNRNPU (bg=9.45%)No matches to TargetScan


TGTTAACA

TAGAAAAGGCCATTAATTTGCCTGCAAATTGTTAACA  
Depth:2 (MARMOSET)  
Ei-value:0.000, Pi-value:0.000  
Er-value:0.000, Pr-value:0.000  
eCLIP MATCHES▶hnrnpa1 (bg=18.32%)▶HNRNPU (bg=9.45%)▶khdrbs1 (bg=10.41%)No matches to TargetScan

 6720  


TAGAAAAGGCCATTAATTTGCCTGCAAATTGTTAACA  
Depth:2 (MARMOSET)  
Ei-value:0.000, Pi-value:0.000  
Er-value:0.000, Pr-value:0.000  
eCLIP MATCHES▶hnrnpa1 (bg=18.32%)▶HNRNPU (bg=9.45%)▶khdrbs1 (bg=10.41%)No matches to TargetScan

-------------

ACCACAGCTAAGTA

ACCACAGCTAAGTA  
Depth:2 (MARMOSET)  
Ei-value:0.020, Pi-value:0.000  
Er-value:0.000, Pr-value:0.000  
eCLIP MATCHES▶hnrnpa1 (bg=18.32%)▶HNRNPU (bg=9.45%)▶khdrbs1 (bg=10.41%)▶QKI (bg=3.74%)No matches to TargetScan

-------------------

CCAGTGAC

CCAGTGACTAAAACCAACTTAAACCAGTAAGTGGAGAAATAACATGTT  
Depth:2 (MARMOSET)  
Ei-value:0.000, Pi-value:0.000  
Er-value:0.000, Pr-value:0.000  
eCLIP MATCHES▶hnrnpa1 (bg=18.32%)▶HNRNPU (bg=9.45%)▶khdrbs1 (bg=10.41%)▶khsrp (bg=27.4%)▶QKI (bg=3.74%)MATCHES To TargetScan▶ miR-382-5p:AAGUUGU▶ miR-299-5p:GGUUUAC▶ miR-668-3p:GUCACUC


TAAAACCA

TAAAACCA  
Depth:3 (DOG)  
Ei-value:0.010, Pi-value:0.000  
Er-value:0.000, Pr-value:0.000  
eCLIP MATCHES▶hnrnpa1 (bg=18.32%)▶HNRNPU (bg=9.45%)▶khdrbs1 (bg=10.41%)▶khsrp (bg=27.4%)▶QKI (bg=3.74%)No matches to TargetScan


ACTTAAACCAGTAAGTGGAGAA

CCAGTGACTAAAACCAACTTAAACCAGTAAGTGGAGAAATAACATGTT  
Depth:2 (MARMOSET)  
Ei-value:0.000, Pi-value:0.000  
Er-value:0.000, Pr-value:0.000  
eCLIP MATCHES▶hnrnpa1 (bg=18.32%)▶HNRNPU (bg=9.45%)▶khdrbs1 (bg=10.41%)▶khsrp (bg=27.4%)▶QKI (bg=3.74%)MATCHES To TargetScan▶ miR-382-5p:AAGUUGU▶ miR-299-5p:GGUUUAC▶ miR-668-3p:GUCACUC


ATAACA

ATAACA  
Depth:4 (PIG)  
Ei-value:0.010, Pi-value:0.010  
Er-value:0.000, Pr-value:0.000  
eCLIP MATCHES▶hnrnpa1 (bg=18.32%)▶khdrbs1 (bg=10.41%)▶khsrp (bg=27.4%)▶QKI (bg=3.74%)No matches to TargetScan


TGTT

CCAGTGACTAAAACCAACTTAAACCAGTAAGTGGAGAAATAACATGTT  
Depth:2 (MARMOSET)  
Ei-value:0.000, Pi-value:0.000  
Er-value:0.000, Pr-value:0.000  
eCLIP MATCHES▶hnrnpa1 (bg=18.32%)▶HNRNPU (bg=9.45%)▶khdrbs1 (bg=10.41%)▶khsrp (bg=27.4%)▶QKI (bg=3.74%)MATCHES To TargetScan▶ miR-382-5p:AAGUUGU▶ miR-299-5p:GGUUUAC▶ miR-668-3p:GUCACUC

-------------------------- 6840  
 -

tgtaacttgtag

tgtaacttgtag  
Depth:2 (MARMOSET)  
Ei-value:0.990, Pi-value:0.000  
Er-value:0.000, Pr-value:0.000  
eCLIP MATCHES▶ddx42 (bg=10.33%)▶khdrbs1 (bg=10.41%)▶khsrp (bg=27.4%)▶QKI (bg=3.74%)No matches to TargetScan

---------

gataggca

gataggca  
Depth:2 (MARMOSET)  
Ei-value:1.000, Pi-value:0.000  
Er-value:0.000, Pr-value:0.000  
eCLIP MATCHES▶ddx42 (bg=10.33%)▶khdrbs1 (bg=10.41%)▶khsrp (bg=27.4%)▶QKI (bg=3.74%)▶SF3B4 (bg=4.76%)No matches to TargetScan

----

agtggctgagag

agtggctgagag  
Depth:2 (MARMOSET)  
Ei-value:0.990, Pi-value:0.000  
Er-value:0.000, Pr-value:0.000  
eCLIP MATCHES▶ddx42 (bg=10.33%)▶khsrp (bg=27.4%)▶QKI (bg=3.74%)▶SF3B4 (bg=4.76%)No matches to TargetScan

------

T

TGGGTGGGA  
Depth:6 (MOUSE)  
Ei-value:0.000, Pi-value:0.000  
Er-value:0.000, Pr-value:0.000  
eCLIP MATCHES▶ddx42 (bg=10.33%)▶khsrp (bg=27.4%)▶QKI (bg=3.74%)▶SF3B4 (bg=4.76%)No matches to TargetScan


GGGTGGG

GGGTGGG  
Depth:10 (SNAKE)  
Ei-value:0.000, Pi-value:0.000  
Er-value:0.000, Pr-value:0.000  
eCLIP MATCHES▶ddx42 (bg=10.33%)▶khsrp (bg=27.4%)▶QKI (bg=3.74%)▶SF3B4 (bg=4.76%)No matches to TargetScan


A

TGGGTGGGA  
Depth:6 (MOUSE)  
Ei-value:0.000, Pi-value:0.000  
Er-value:0.000, Pr-value:0.000  
eCLIP MATCHES▶ddx42 (bg=10.33%)▶khsrp (bg=27.4%)▶QKI (bg=3.74%)▶SF3B4 (bg=4.76%)No matches to TargetScan


A

TGGGTGGGAA  
Depth:4 (PIG)  
Ei-value:0.000, Pi-value:0.000  
Er-value:0.000, Pr-value:0.000  
eCLIP MATCHES▶ddx42 (bg=10.33%)▶khsrp (bg=27.4%)▶QKI (bg=3.74%)▶SF3B4 (bg=4.76%)No matches to TargetScan


TGCAAAAA

TGCAAAAA  
Depth:6 (MOUSE)  
Ei-value:0.000, Pi-value:0.000  
Er-value:0.000, Pr-value:0.000  
eCLIP MATCHES▶ddx42 (bg=10.33%)▶khsrp (bg=27.4%)▶QKI (bg=3.74%)▶SF3B4 (bg=4.76%)MATCHES To TargetScan▶ miR-129-5p:UUUUUGC


T

TGCAAAAATTCTCTGCTAAGACTTTTTCAGGTGAACATAA  
Depth:3 (DOG)  
Ei-value:0.000, Pi-value:0.000  
Er-value:0.000, Pr-value:0.000  
eCLIP MATCHES▶ddx42 (bg=10.33%)▶khsrp (bg=27.4%)▶QKI (bg=3.74%)▶SF3B4 (bg=4.76%)▶srsf7 (bg=22.53%)▶tia1 (bg=23.76%)▶u2af2 (bg=19.32%)MATCHES To TargetScan▶ miR-129-5p:UUUUUGC


TCTC

TCTCTGCTAAGACTTTTTCAGGTG  
Depth:7 (TURTLE)  
Ei-value:0.000, Pi-value:0.000  
Er-value:0.000, Pr-value:0.000  
eCLIP MATCHES▶ddx42 (bg=10.33%)▶khsrp (bg=27.4%)▶QKI (bg=3.74%)▶SF3B4 (bg=4.76%)▶srsf7 (bg=22.53%)▶tia1 (bg=23.76%)▶u2af2 (bg=19.32%)No matches to TargetScan


TGCTAAGACT

TGCTAAGACT  
Depth:10 (SNAKE)  
Ei-value:0.000, Pi-value:0.000  
Er-value:0.000, Pr-value:0.000  
eCLIP MATCHES▶ddx42 (bg=10.33%)▶khsrp (bg=27.4%)▶QKI (bg=3.74%)▶SF3B4 (bg=4.76%)No matches to TargetScan


TTTTCAGGTG

TGCTAAGACTTTTTCAGGTG  
Depth:8 (ALLIGATOR)  
Ei-value:0.000, Pi-value:0.000  
Er-value:0.000, Pr-value:0.000  
eCLIP MATCHES▶ddx42 (bg=10.33%)▶khsrp (bg=27.4%)▶QKI (bg=3.74%)▶SF3B4 (bg=4.76%)▶srsf7 (bg=22.53%)▶tia1 (bg=23.76%)▶u2af2 (bg=19.32%)No matches to TargetScan


A

TCTCTGCTAAGACTTTTTCAGGTGAACATAA  
Depth:5 (COW)  
Ei-value:0.000, Pi-value:0.000  
Er-value:0.000, Pr-value:0.000  
eCLIP MATCHES▶ddx42 (bg=10.33%)▶khsrp (bg=27.4%)▶QKI (bg=3.74%)▶SF3B4 (bg=4.76%)▶srsf7 (bg=22.53%)▶tia1 (bg=23.76%)▶u2af2 (bg=19.32%)No matches to TargetScan


ACATAA

ACATAA  
Depth:6 (MOUSE)  
Ei-value:0.000, Pi-value:0.000  
Er-value:0.000, Pr-value:0.000  
eCLIP MATCHES▶QKI (bg=3.74%)▶srsf7 (bg=22.53%)▶tia1 (bg=23.76%)▶u2af2 (bg=19.32%)No matches to TargetScan


C

TGGGTGGGAATGCAAAAATTCTCTGCTAAGACTTTTTCAGGTGAACATAACAGACTTGGCCAAGCTAGCATCTTAGCGGAAGC  
Depth:2 (MARMOSET)  
Ei-value:0.000, Pi-value:0.000  
Er-value:0.000, Pr-value:0.000  
eCLIP MATCHES▶ddx42 (bg=10.33%)▶khsrp (bg=27.4%)▶QKI (bg=3.74%)▶SF3B4 (bg=4.76%)▶srsf1 (bg=30.28%)▶srsf7 (bg=22.53%)▶tia1 (bg=23.76%)▶u2af2 (bg=19.32%)MATCHES To TargetScan▶ miR-129-5p:UUUUUGC▶ miR-33-5p:UGCAUUG


AGACTTG

AGACTTG  
Depth:6 (MOUSE)  
Ei-value:0.000, Pi-value:0.000  
Er-value:0.000, Pr-value:0.000  
eCLIP MATCHES▶QKI (bg=3.74%)▶srsf7 (bg=22.53%)▶u2af2 (bg=19.32%)No matches to TargetScan


GC

AGACTTGGC  
Depth:4 (PIG)  
Ei-value:0.000, Pi-value:0.000  
Er-value:0.000, Pr-value:0.000  
eCLIP MATCHES▶QKI (bg=3.74%)▶srsf7 (bg=22.53%)▶u2af2 (bg=19.32%)No matches to TargetScan


C

TGGGTGGGAATGCAAAAATTCTCTGCTAAGACTTTTTCAGGTGAACATAACAGACTTGGCCAAGCTAGCATCTTAGCGGAAGC  
Depth:2 (MARMOSET)  
Ei-value:0.000, Pi-value:0.000  
Er-value:0.000, Pr-value:0.000  
eCLIP MATCHES▶ddx42 (bg=10.33%)▶khsrp (bg=27.4%)▶QKI (bg=3.74%)▶SF3B4 (bg=4.76%)▶srsf1 (bg=30.28%)▶srsf7 (bg=22.53%)▶tia1 (bg=23.76%)▶u2af2 (bg=19.32%)MATCHES To TargetScan▶ miR-129-5p:UUUUUGC▶ miR-33-5p:UGCAUUG


AAGCTAG

AAGCTAGCA  
Depth:6 (MOUSE)  
Ei-value:0.000, Pi-value:0.000  
Er-value:0.000, Pr-value:0.000  
eCLIP MATCHES▶QKI (bg=3.74%)▶srsf7 (bg=22.53%)No matches to TargetScan

 6960  


CA

AAGCTAGCA  
Depth:6 (MOUSE)  
Ei-value:0.000, Pi-value:0.000  
Er-value:0.000, Pr-value:0.000  
eCLIP MATCHES▶QKI (bg=3.74%)▶srsf7 (bg=22.53%)No matches to TargetScan


T

AAGCTAGCATCTTAGC  
Depth:4 (PIG)  
Ei-value:0.000, Pi-value:0.000  
Er-value:0.000, Pr-value:0.000  
eCLIP MATCHES▶QKI (bg=3.74%)▶srsf1 (bg=30.28%)▶srsf7 (bg=22.53%)No matches to TargetScan


CTTAGC

CTTAGC  
Depth:5 (COW)  
Ei-value:0.000, Pi-value:0.000  
Er-value:0.000, Pr-value:0.010  
eCLIP MATCHES▶QKI (bg=3.74%)▶srsf1 (bg=30.28%)▶srsf7 (bg=22.53%)No matches to TargetScan


GGAAGC

TGGGTGGGAATGCAAAAATTCTCTGCTAAGACTTTTTCAGGTGAACATAACAGACTTGGCCAAGCTAGCATCTTAGCGGAAGC  
Depth:2 (MARMOSET)  
Ei-value:0.000, Pi-value:0.000  
Er-value:0.000, Pr-value:0.000  
eCLIP MATCHES▶ddx42 (bg=10.33%)▶khsrp (bg=27.4%)▶QKI (bg=3.74%)▶SF3B4 (bg=4.76%)▶srsf1 (bg=30.28%)▶srsf7 (bg=22.53%)▶tia1 (bg=23.76%)▶u2af2 (bg=19.32%)MATCHES To TargetScan▶ miR-129-5p:UUUUUGC▶ miR-33-5p:UGCAUUG

-

GATCTCCAATGC

GATCTCCAATGCTCTTCAGTAGGGTCATGAAGGTTTTTCTTTTCCTGAGAAAACAACA  
Depth:2 (MARMOSET)  
Ei-value:0.000, Pi-value:0.000  
Er-value:0.000, Pr-value:0.000  
eCLIP MATCHES▶srsf1 (bg=30.28%)▶srsf7 (bg=22.53%)MATCHES To TargetScan▶ miR-433-3p:UCAUGAU▶ miR-873-5p.1:CAGGAAC▶ miR-205-5p:CCUUCAU▶ miR-186-5p:AAAGAAU


TCTTCAG

TCTTCAGTAGGGT  
Depth:4 (PIG)  
Ei-value:0.000, Pi-value:0.000  
Er-value:0.000, Pr-value:0.000  
eCLIP MATCHES▶srsf7 (bg=22.53%)No matches to TargetScan


TAGGGT

TAGGGT  
Depth:6 (MOUSE)  
Ei-value:0.000, Pi-value:0.010  
Er-value:0.000, Pr-value:0.000  
eCLIP MATCHES▶srsf7 (bg=22.53%)No matches to TargetScan


CATG

GATCTCCAATGCTCTTCAGTAGGGTCATGAAGGTTTTTCTTTTCCTGAGAAAACAACA  
Depth:2 (MARMOSET)  
Ei-value:0.000, Pi-value:0.000  
Er-value:0.000, Pr-value:0.000  
eCLIP MATCHES▶srsf1 (bg=30.28%)▶srsf7 (bg=22.53%)MATCHES To TargetScan▶ miR-433-3p:UCAUGAU▶ miR-873-5p.1:CAGGAAC▶ miR-205-5p:CCUUCAU▶ miR-186-5p:AAAGAAU


AAGGT

AAGGTTTTTCTTTTCCTGAGA  
Depth:7 (TURTLE)  
Ei-value:0.000, Pi-value:0.000  
Er-value:0.000, Pr-value:0.000  
eCLIP MATCHES▶srsf7 (bg=22.53%)MATCHES To TargetScan▶ miR-873-5p.1:CAGGAAC▶ miR-186-5p:AAAGAAU


TTTTCTTTT

TTTTCTTTT  
Depth:19 (ZEBRAFISH)  
Ei-value:0.000, Pi-value:0.000  
Er-value:0.000, Pr-value:0.000  
eCLIP MATCHES▶srsf7 (bg=22.53%)MATCHES To TargetScan▶ miR-186-5p:AAAGAAU


CCTGAGA

TTTTCTTTTCCTGAGA  
Depth:10 (SNAKE)  
Ei-value:0.000, Pi-value:0.000  
Er-value:0.000, Pr-value:0.000  
eCLIP MATCHES▶srsf7 (bg=22.53%)MATCHES To TargetScan▶ miR-873-5p.1:CAGGAAC▶ miR-186-5p:AAAGAAU


AAACAA

AAGGTTTTTCTTTTCCTGAGAAAACAA  
Depth:6 (MOUSE)  
Ei-value:0.000, Pi-value:0.000  
Er-value:0.000, Pr-value:0.000  
eCLIP MATCHES▶srsf7 (bg=22.53%)MATCHES To TargetScan▶ miR-873-5p.1:CAGGAAC▶ miR-186-5p:AAAGAAU


CA

GATCTCCAATGCTCTTCAGTAGGGTCATGAAGGTTTTTCTTTTCCTGAGAAAACAACA  
Depth:2 (MARMOSET)  
Ei-value:0.000, Pi-value:0.000  
Er-value:0.000, Pr-value:0.000  
eCLIP MATCHES▶srsf1 (bg=30.28%)▶srsf7 (bg=22.53%)MATCHES To TargetScan▶ miR-433-3p:UCAUGAU▶ miR-873-5p.1:CAGGAAC▶ miR-205-5p:CCUUCAU▶ miR-186-5p:AAAGAAU

----

TTGTTT

TTGTTTTCTCAGGTTTTGCTTTTT  
Depth:6 (MOUSE)  
Ei-value:0.000, Pi-value:0.000  
Er-value:0.000, Pr-value:0.000  
eCLIP MATCHES▶srsf7 (bg=22.53%)MATCHES To TargetScan▶ miR-330-3p.2:AAAGCAC▶ miR-490-3p:AACCUGG


TCT

TCTCAGGTTTTGCTTTT  
Depth:10 (SNAKE)  
Ei-value:0.000, Pi-value:0.000  
Er-value:0.000, Pr-value:0.000  
eCLIP MATCHES▶srsf7 (bg=22.53%)MATCHES To TargetScan▶ miR-330-3p.2:AAAGCAC▶ miR-490-3p:AACCUGG


CAGGTTTTGCTTT

CAGGTTTTGCTTT  
Depth:19 (ZEBRAFISH)  
Ei-value:0.000, Pi-value:0.000  
Er-value:0.000, Pr-value:0.000  
eCLIP MATCHES▶srsf7 (bg=22.53%)MATCHES To TargetScan▶ miR-330-3p.2:AAAGCAC▶ miR-490-3p:AACCUGG


T

CAGGTTTTGCTTTT  
Depth:16 (NILETILAPIA)  
Ei-value:0.000, Pi-value:0.000  
Er-value:0.000, Pr-value:0.000  
eCLIP MATCHES▶srsf7 (bg=22.53%)MATCHES To TargetScan▶ miR-330-3p.2:AAAGCAC▶ miR-490-3p:AACCUGG


T

TTGTTTTCTCAGGTTTTGCTTTTT  
Depth:6 (MOUSE)  
Ei-value:0.000, Pi-value:0.000  
Er-value:0.000, Pr-value:0.000  
eCLIP MATCHES▶srsf7 (bg=22.53%)MATCHES To TargetScan▶ miR-330-3p.2:AAAGCAC▶ miR-490-3p:AACCUGG


GGCCTTT

TTGTTTTCTCAGGTTTTGCTTTTTGGCCTTT  
Depth:2 (MARMOSET)  
Ei-value:0.000, Pi-value:0.000  
Er-value:0.000, Pr-value:0.000  
eCLIP MATCHES▶srsf7 (bg=22.53%)MATCHES To TargetScan▶ miR-330-3p.2:AAAGCAC▶ miR-490-3p:AACCUGG

--

CTAGCTT

CTAGCTT  
Depth:4 (PIG)  
Ei-value:0.000, Pi-value:0.000  
Er-value:0.000, Pr-value:0.000  
No matches to eCLIP DataNo matches to TargetScan

-- 7080  
 --

AA

AAAAAAAAAGCAAAAGA  
Depth:6 (MOUSE)  
Ei-value:0.000, Pi-value:0.000  
Er-value:0.000, Pr-value:0.000  
No matches to eCLIP DataNo matches to TargetScan


A

AAAAAAAGCAAAAGA  
Depth:7 (TURTLE)  
Ei-value:0.000, Pi-value:0.000  
Er-value:0.000, Pr-value:0.000  
No matches to eCLIP DataNo matches to TargetScan


A

AAAAAAGCAAAAG  
Depth:13 (OPOSSUM)  
Ei-value:0.000, Pi-value:0.000  
Er-value:0.000, Pr-value:0.000  
No matches to eCLIP DataNo matches to TargetScan


AAAAAGCAAAA

AAAAAGCAAAA  
Depth:19 (ZEBRAFISH)  
Ei-value:0.000, Pi-value:0.000  
Er-value:0.000, Pr-value:0.000  
No matches to eCLIP DataNo matches to TargetScan


G

AAAAAGCAAAAG  
Depth:18 (MEDAKA)  
Ei-value:0.000, Pi-value:0.000  
Er-value:0.000, Pr-value:0.000  
No matches to eCLIP DataNo matches to TargetScan


A

AAAAAAGCAAAAGA  
Depth:9 (LIZARD)  
Ei-value:0.000, Pi-value:0.000  
Er-value:0.000, Pr-value:0.000  
No matches to eCLIP DataNo matches to TargetScan


T

AAAAAAAAAGCAAAAGATGCTGGT  
Depth:5 (COW)  
Ei-value:0.000, Pi-value:0.000  
Er-value:0.000, Pr-value:0.000  
No matches to eCLIP DataMATCHES To TargetScan▶ miR-338-3p:CCAGCAU


GCTGGT

GCTGGT  
Depth:9 (LIZARD)  
Ei-value:0.000, Pi-value:0.000  
Er-value:0.000, Pr-value:0.000  
No matches to eCLIP DataNo matches to TargetScan

-------

ACTCCTG

ACTCCTG  
Depth:18 (MEDAKA)  
Ei-value:0.000, Pi-value:0.000  
Er-value:0.000, Pr-value:0.000  
No matches to eCLIP DataNo matches to TargetScan


G

ACTCCTGG  
Depth:14 (SPOTTEDGAR)  
Ei-value:0.000, Pi-value:0.000  
Er-value:0.000, Pr-value:0.000  
No matches to eCLIP DataMATCHES To TargetScan▶ miR-665:CCAGGAG


T

TTTCCAGGAC  
Depth:3 (DOG)  
Ei-value:0.000, Pi-value:0.000  
Er-value:0.000, Pr-value:0.000  
No matches to eCLIP DataNo matches to TargetScan


T

TTCCAGGA  
Depth:7 (TURTLE)  
Ei-value:0.000, Pi-value:0.000  
Er-value:0.000, Pr-value:0.000  
No matches to eCLIP DataNo matches to TargetScan


TCCAGGA

TCCAGGA  
Depth:8 (ALLIGATOR)  
Ei-value:0.000, Pi-value:0.000  
Er-value:0.000, Pr-value:0.000  
No matches to eCLIP DataNo matches to TargetScan


C

TTCCAGGAC  
Depth:6 (MOUSE)  
Ei-value:0.000, Pi-value:0.000  
Er-value:0.000, Pr-value:0.000  
No matches to eCLIP DataNo matches to TargetScan


G

ACTCCTGGTTTCCAGGACGGGGTTCAAATCCCTGCGGC  
Depth:2 (MARMOSET)  
Ei-value:0.000, Pi-value:0.000  
Er-value:0.000, Pr-value:0.000  
No matches to eCLIP DataMATCHES To TargetScan▶ miR-876-5p:GGAUUUC▶ miR-665:CCAGGAG


GGGTTC

GGGTTC  
Depth:13 (OPOSSUM)  
Ei-value:0.000, Pi-value:0.000  
Er-value:0.000, Pr-value:0.000  
No matches to eCLIP DataNo matches to TargetScan


AA

GGGTTCAA  
Depth:9 (LIZARD)  
Ei-value:0.000, Pi-value:0.000  
Er-value:0.000, Pr-value:0.000  
No matches to eCLIP DataNo matches to TargetScan


ATCC

GGGTTCAAATCC  
Depth:5 (COW)  
Ei-value:0.000, Pi-value:0.000  
Er-value:0.000, Pr-value:0.000  
No matches to eCLIP DataMATCHES To TargetScan▶ miR-876-5p:GGAUUUC


CTGCGGC

ACTCCTGGTTTCCAGGACGGGGTTCAAATCCCTGCGGC  
Depth:2 (MARMOSET)  
Ei-value:0.000, Pi-value:0.000  
Er-value:0.000, Pr-value:0.000  
No matches to eCLIP DataMATCHES To TargetScan▶ miR-876-5p:GGAUUUC▶ miR-665:CCAGGAG

-

TCTTTGCT

TCTTTGCT  
Depth:8 (ALLIGATOR)  
Ei-value:0.000, Pi-value:0.000  
Er-value:0.000, Pr-value:0.000  
No matches to eCLIP DataNo matches to TargetScan

---------------------------------------- 7200  
 ------------------------------------------------------------------------------------------------------------------------ 7320  
 ------------------------------------------------------------------------------------------------------------------------ 7440  
 ----------------------------------------------------------------                                                         7504
```

---

## >MARMOSET (7293 bases)

```
 -----------------------------------------------------------------------------

atacgcctcgcc

atacgcctcgcc  
Depth:2 (MARMOSET)  
Ei-value:0.990, Pi-value:0.000  
Er-value:0.000, Pr-value:0.000  
No matches to TargetScan

------------

aggcattgagg

aggcattgagg  
Depth:2 (MARMOSET)  
Ei-value:1.000, Pi-value:0.000  
Er-value:0.000, Pr-value:0.000  
MATCHES To TargetScan▶ miR-532-5p:AUGCCUU▶ miR-365-3p:AAUGCCC

-

agcc

agccagcgcaggg  
Depth:2 (MARMOSET)  
Ei-value:0.680, Pi-value:0.000  
Er-value:0.000, Pr-value:0.000  
MATCHES To TargetScan▶ miR-149-5p:CUGGCUC▶ miR-3064-5p:CUGGCUG


agc

agcgcag  
Depth:3 (DOG)  
Ei-value:0.350, Pi-value:0.010  
Er-value:0.000, Pr-value:0.000  
No matches to TargetScan

 120  


gcag

agcgcag  
Depth:3 (DOG)  
Ei-value:0.350, Pi-value:0.010  
Er-value:0.000, Pr-value:0.000  
No matches to TargetScan


gg

agccagcgcaggg  
Depth:2 (MARMOSET)  
Ei-value:0.680, Pi-value:0.000  
Er-value:0.000, Pr-value:0.000  
MATCHES To TargetScan▶ miR-149-5p:CUGGCUC▶ miR-3064-5p:CUGGCUG

-----------------

agcttgaggaaac

agcttgaggaaac  
Depth:2 (MARMOSET)  
Ei-value:0.680, Pi-value:0.000  
Er-value:0.000, Pr-value:0.000  
MATCHES To TargetScan▶ miR-670-3p:UUCCUCA

-

GCAGATAAGTTTTT

GCAGATAAGTTTTT  
Depth:6 (MOUSE)  
Ei-value:0.000, Pi-value:0.000  
Er-value:0.000, Pr-value:0.000  
No matches to TargetScan

--------------------

at

attaatacaact  
Depth:2 (MARMOSET)  
Ei-value:0.990, Pi-value:0.000  
Er-value:0.000, Pr-value:0.000  
MATCHES To TargetScan▶ miR-496.2:GUAUUAC


taatac

taatac  
Depth:3 (DOG)  
Ei-value:1.000, Pi-value:0.040  
Er-value:0.000, Pr-value:0.000  
MATCHES To TargetScan▶ miR-496.2:GUAUUAC


aact

attaatacaact  
Depth:2 (MARMOSET)  
Ei-value:0.990, Pi-value:0.000  
Er-value:0.000, Pr-value:0.000  
MATCHES To TargetScan▶ miR-496.2:GUAUUAC

----

aaatata

aaatata  
Depth:2 (MARMOSET)  
Ei-value:1.000, Pi-value:0.020  
Er-value:0.000, Pr-value:0.000  
No matches to TargetScan

---------------------

attgc

attgct  
Depth:3 (DOG)  
Ei-value:1.000, Pi-value:0.070  
Er-value:0.000, Pr-value:0.000  
No matches to TargetScan

 240  


t

attgct  
Depth:3 (DOG)  
Ei-value:1.000, Pi-value:0.070  
Er-value:0.000, Pr-value:0.000  
No matches to TargetScan

-----

ttaagtt

ttaagtt  
Depth:2 (MARMOSET)  
Ei-value:1.000, Pi-value:0.030  
Er-value:0.000, Pr-value:0.010  
No matches to TargetScan

-------

taatttta

taatttta  
Depth:2 (MARMOSET)  
Ei-value:1.000, Pi-value:0.000  
Er-value:0.000, Pr-value:0.000  
No matches to TargetScan

-------------------------------------------------------------

taagagaaaata

taagagaaaata  
Depth:2 (MARMOSET)  
Ei-value:0.990, Pi-value:0.000  
Er-value:0.000, Pr-value:0.000  
No matches to TargetScan

-----------

AAGAGTAG

AAGAGTAGC  
Depth:3 (DOG)  
Ei-value:0.000, Pi-value:0.000  
Er-value:0.000, Pr-value:0.000  
No matches to TargetScan

 360  


C

AAGAGTAGC  
Depth:3 (DOG)  
Ei-value:0.000, Pi-value:0.000  
Er-value:0.000, Pr-value:0.000  
No matches to TargetScan


ATGAGGAAGGAA

AAGAGTAGCATGAGGAAGGAA  
Depth:2 (MARMOSET)  
Ei-value:0.000, Pi-value:0.000  
Er-value:0.000, Pr-value:0.000  
MATCHES To TargetScan▶ miR-670-3p:UUCCUCA

--------------

gtttct

gtttct  
Depth:2 (MARMOSET)  
Ei-value:1.000, Pi-value:0.020  
Er-value:0.000, Pr-value:0.020  
No matches to TargetScan

-

AAACAT

AAACATGACGGAGGTTGAGATGAAGCT  
Depth:2 (MARMOSET)  
Ei-value:0.000, Pi-value:0.000  
Er-value:0.000, Pr-value:0.000  
No matches to TargetScan


GACGGAGGTT

GACGGAGGTT  
Depth:3 (DOG)  
Ei-value:0.000, Pi-value:0.000  
Er-value:0.000, Pr-value:0.000  
No matches to TargetScan


GAGATGAAGCT

GAGATGAAGCT  
Depth:3 (DOG)  
Ei-value:0.000, Pi-value:0.000  
Er-value:0.000, Pr-value:0.000  
No matches to TargetScan

-

cttc

cttcatggagta  
Depth:2 (MARMOSET)  
Ei-value:0.990, Pi-value:0.000  
Er-value:0.000, Pr-value:0.000  
MATCHES To TargetScan▶ miR-136-5p:CUCCAUU


atggagt

atggagt  
Depth:3 (DOG)  
Ei-value:0.350, Pi-value:0.010  
Er-value:0.000, Pr-value:0.000  
MATCHES To TargetScan▶ miR-136-5p:CUCCAUU


a

cttcatggagta  
Depth:2 (MARMOSET)  
Ei-value:0.990, Pi-value:0.000  
Er-value:0.000, Pr-value:0.000  
MATCHES To TargetScan▶ miR-136-5p:CUCCAUU

-

AAAATGT

AAAATGTATTTAAAAGAAAATTGA  
Depth:2 (MARMOSET)  
Ei-value:0.000, Pi-value:0.000  
Er-value:0.000, Pr-value:0.000  
No matches to TargetScan


ATTTAAAA

ATTTAAAA  
Depth:3 (DOG)  
Ei-value:0.010, Pi-value:0.010  
Er-value:0.000, Pr-value:0.000  
No matches to TargetScan


GAAAATTGA

AAAATGTATTTAAAAGAAAATTGA  
Depth:2 (MARMOSET)  
Ei-value:0.000, Pi-value:0.000  
Er-value:0.000, Pr-value:0.000  
No matches to TargetScan

----

aaggacta

aaggacta  
Depth:2 (MARMOSET)  
Ei-value:1.000, Pi-value:0.000  
Er-value:0.000, Pr-value:0.000  
No matches to TargetScan

-------

ga

gaattaatacc  
Depth:2 (MARMOSET)  
Ei-value:1.000, Pi-value:0.000  
Er-value:0.000, Pr-value:0.000  
MATCHES To TargetScan▶ miR-496.2:GUAUUAC

 480  


attaatacc

gaattaatacc  
Depth:2 (MARMOSET)  
Ei-value:1.000, Pi-value:0.000  
Er-value:0.000, Pr-value:0.000  
MATCHES To TargetScan▶ miR-496.2:GUAUUAC

------

tagaagggca

tagaagggca  
Depth:2 (MARMOSET)  
Ei-value:1.000, Pi-value:0.000  
Er-value:0.000, Pr-value:0.000  
MATCHES To TargetScan▶ miR-874-3p:UGCCCUG

-

tgcttttagatta

tgcttttagatta  
Depth:2 (MARMOSET)  
Ei-value:0.680, Pi-value:0.000  
Er-value:0.000, Pr-value:0.000  
MATCHES To TargetScan▶ miR-330-3p.2:AAAGCAC

-----

AAGGTGACTTAAACAG

AAGGTGACTTAAACAG  
Depth:2 (MARMOSET)  
Ei-value:0.000, Pi-value:0.000  
Er-value:0.000, Pr-value:0.000  
MATCHES To TargetScan▶ miR-224-5p:AAGUCAC

------------------------

gtaggtga

gtaggtga  
Depth:2 (MARMOSET)  
Ei-value:1.000, Pi-value:0.000  
Er-value:0.000, Pr-value:0.000  
No matches to TargetScan

-

taaaata

taaaata  
Depth:2 (MARMOSET)  
Ei-value:1.000, Pi-value:0.040  
Er-value:0.000, Pr-value:0.020  
No matches to TargetScan

--------

gcgatc

gcgatc  
Depth:2 (MARMOSET)  
Ei-value:1.000, Pi-value:0.040  
Er-value:0.000, Pr-value:0.000  
No matches to TargetScan

-

TTTAA

TTTAAAAAG  
Depth:3 (DOG)  
Ei-value:0.000, Pi-value:0.000  
Er-value:0.000, Pr-value:0.010  
No matches to TargetScan

 600  


AAAG

TTTAAAAAG  
Depth:3 (DOG)  
Ei-value:0.000, Pi-value:0.000  
Er-value:0.000, Pr-value:0.010  
No matches to TargetScan


AGATTAA

TTTAAAAAGAGATTAA  
Depth:2 (MARMOSET)  
Ei-value:0.000, Pi-value:0.000  
Er-value:0.000, Pr-value:0.000  
MATCHES To TargetScan▶ miR-216a-5p:AAUCUCA▶ miR-216b-5p:AAUCUCU

----

AAGGTGATTAAAAGAC

AAGGTGATTAAAAGACCTTGAAATCCATGACGCA  
Depth:2 (MARMOSET)  
Ei-value:0.000, Pi-value:0.000  
Er-value:0.000, Pr-value:0.000  
MATCHES To TargetScan▶ miR-876-5p:GGAUUUC


cttgaa

cttgaa  
Depth:3 (DOG)  
Ei-value:1.000, Pi-value:0.080  
Er-value:0.000, Pr-value:0.000  
No matches to TargetScan


ATCCATGACGCA

AAGGTGATTAAAAGACCTTGAAATCCATGACGCA  
Depth:2 (MARMOSET)  
Ei-value:0.000, Pi-value:0.000  
Er-value:0.000, Pr-value:0.000  
MATCHES To TargetScan▶ miR-876-5p:GGAUUUC

---

AGA

AGAATTGCGTCATTTAAAGCCTA  
Depth:2 (MARMOSET)  
Ei-value:0.000, Pi-value:0.000  
Er-value:0.000, Pr-value:0.000  
No matches to TargetScan


ATTGCGTCATTT

ATTGCGTCATTT  
Depth:3 (DOG)  
Ei-value:0.000, Pi-value:0.000  
Er-value:0.000, Pr-value:0.000  
No matches to TargetScan


AAAGCCTA

AGAATTGCGTCATTTAAAGCCTA  
Depth:2 (MARMOSET)  
Ei-value:0.000, Pi-value:0.000  
Er-value:0.000, Pr-value:0.000  
No matches to TargetScan

----------------

TTACTAAACGCAGACGAA

TTACTAAACGCAGACGAA  
Depth:2 (MARMOSET)  
Ei-value:0.000, Pi-value:0.000  
Er-value:0.000, Pr-value:0.000  
MATCHES To TargetScan▶ miR-346:GUCUGCC

----

ggaaaga

ggaaaga  
Depth:2 (MARMOSET)  
Ei-value:1.000, Pi-value:0.010  
Er-value:0.000, Pr-value:0.010  
No matches to TargetScan

 720  


ggaaaga  
Depth:2 (MARMOSET)  
Ei-value:1.000, Pi-value:0.010  
Er-value:0.000, Pr-value:0.010  
No matches to TargetScan

-

TTAATTGGG

TTAATTGGGAGTGGTAGGA  
Depth:2 (MARMOSET)  
Ei-value:0.000, Pi-value:0.000  
Er-value:0.000, Pr-value:0.000  
MATCHES To TargetScan▶ miR-150-5p:CUCCCAA▶ miR-532-3p:CUCCCAC▶ miR-483-3p.2:CACUCCU


AGTGGTAGGA

AGTGGTAGGA  
Depth:3 (DOG)  
Ei-value:0.000, Pi-value:0.000  
Er-value:0.000, Pr-value:0.000  
No matches to TargetScan

---

AAACAATTTGGAGAAGAT

AAACAATTTGGAGAAGAT  
Depth:2 (MARMOSET)  
Ei-value:0.000, Pi-value:0.000  
Er-value:0.000, Pr-value:0.000  
No matches to TargetScan

-

AGAAGT

AGAAGTTTGAAGTGGAA  
Depth:2 (MARMOSET)  
Ei-value:0.000, Pi-value:0.000  
Er-value:0.000, Pr-value:0.000  
No matches to TargetScan


TTGAAGTGGA

TTGAAGTGGA  
Depth:3 (DOG)  
Ei-value:0.000, Pi-value:0.000  
Er-value:0.000, Pr-value:0.000  
No matches to TargetScan


A

AGAAGTTTGAAGTGGAA  
Depth:2 (MARMOSET)  
Ei-value:0.000, Pi-value:0.000  
Er-value:0.000, Pr-value:0.000  
No matches to TargetScan

-

ACTGGAAGA

ACTGGAAGACAGAAGTAC  
Depth:2 (MARMOSET)  
Ei-value:0.000, Pi-value:0.000  
Er-value:0.000, Pr-value:0.000  
MATCHES To TargetScan▶ miR-145-5p:UCCAGUU


CAGAAGTA

CAGAAGTA  
Depth:3 (DOG)  
Ei-value:0.010, Pi-value:0.010  
Er-value:0.000, Pr-value:0.000  
No matches to TargetScan


C

ACTGGAAGACAGAAGTAC  
Depth:2 (MARMOSET)  
Ei-value:0.000, Pi-value:0.000  
Er-value:0.000, Pr-value:0.000  
MATCHES To TargetScan▶ miR-145-5p:UCCAGUU

-

GGAAGGC

GGAAGGCGAAGAAAAGAATAGAGAAGATAGGGAAATTAGAAGATAAAAA  
Depth:2 (MARMOSET)  
Ei-value:0.000, Pi-value:0.000  
Er-value:0.000, Pr-value:0.000  
No matches to TargetScan


GAAGAAAAGA

GAAGAAAAGA  
Depth:3 (DOG)  
Ei-value:0.000, Pi-value:0.000  
Er-value:0.000, Pr-value:0.000  
No matches to TargetScan


A

GGAAGGCGAAGAAAAGAATAGAGAAGATAGGGAAATTAGAAGATAAAAA  
Depth:2 (MARMOSET)  
Ei-value:0.000, Pi-value:0.000  
Er-value:0.000, Pr-value:0.000  
No matches to TargetScan


TAGAG

TAGAGAAGATAGG  
Depth:3 (DOG)  
Ei-value:0.000, Pi-value:0.000  
Er-value:0.000, Pr-value:0.000  
No matches to TargetScan


AAGATAGG

AAGATAGG  
Depth:5 (COW)  
Ei-value:0.000, Pi-value:0.020  
Er-value:0.000, Pr-value:0.000  
No matches to TargetScan


GAAATTAGAA

GGAAGGCGAAGAAAAGAATAGAGAAGATAGGGAAATTAGAAGATAAAAA  
Depth:2 (MARMOSET)  
Ei-value:0.000, Pi-value:0.000  
Er-value:0.000, Pr-value:0.000  
No matches to TargetScan

 840  


GATAAAAA

GGAAGGCGAAGAAAAGAATAGAGAAGATAGGGAAATTAGAAGATAAAAA  
Depth:2 (MARMOSET)  
Ei-value:0.000, Pi-value:0.000  
Er-value:0.000, Pr-value:0.000  
No matches to TargetScan

-----

CTTTTAGAAGA

CTTTTAGAAGA  
Depth:3 (DOG)  
Ei-value:0.000, Pi-value:0.000  
Er-value:0.000, Pr-value:0.000  
No matches to TargetScan

-------------

AAGT

AAGTAGGAAGCAGAAGAAAAAA  
Depth:2 (MARMOSET)  
Ei-value:0.000, Pi-value:0.000  
Er-value:0.000, Pr-value:0.000  
No matches to TargetScan


AGGAAGC

AGGAAGCAGAAGAAAAAA  
Depth:3 (DOG)  
Ei-value:0.000, Pi-value:0.000  
Er-value:0.000, Pr-value:0.000  
No matches to TargetScan


A

AGAAGAAAAAA  
Depth:4 (PIG)  
Ei-value:0.000, Pi-value:0.000  
Er-value:0.000, Pr-value:0.000  
No matches to TargetScan


GAAGAAAAA

GAAGAAAAA  
Depth:6 (MOUSE)  
Ei-value:0.000, Pi-value:0.000  
Er-value:0.000, Pr-value:0.000  
No matches to TargetScan


A

GAAGAAAAAA  
Depth:5 (COW)  
Ei-value:0.000, Pi-value:0.000  
Er-value:0.000, Pr-value:0.000  
No matches to TargetScan

----

GACAAGCTAGGAAACAAAAA

GACAAGCTAGGAAACAAAAA  
Depth:2 (MARMOSET)  
Ei-value:0.000, Pi-value:0.000  
Er-value:0.000, Pr-value:0.000  
MATCHES To TargetScan▶ miR-129-5p:UUUUUGC

----------------------

CTAAGGGCAAAATGT

CTAAGGGCAAAATGT  
Depth:2 (MARMOSET)  
Ei-value:0.000, Pi-value:0.000  
Er-value:0.000, Pr-value:0.000  
MATCHES To TargetScan▶ miR-874-3p:UGCCCUG

 960  


CTAAGGGCAAAATGT  
Depth:2 (MARMOSET)  
Ei-value:0.000, Pi-value:0.000  
Er-value:0.000, Pr-value:0.000  
MATCHES To TargetScan▶ miR-874-3p:UGCCCUG

-

CAAACTT

CAAACTTAGAAGAAAA  
Depth:2 (MARMOSET)  
Ei-value:0.000, Pi-value:0.000  
Er-value:0.000, Pr-value:0.000  
No matches to TargetScan


agaagaa

agaagaa  
Depth:3 (DOG)  
Ei-value:0.350, Pi-value:0.010  
Er-value:0.000, Pr-value:0.010  
No matches to TargetScan


AA

CAAACTTAGAAGAAAA  
Depth:2 (MARMOSET)  
Ei-value:0.000, Pi-value:0.000  
Er-value:0.000, Pr-value:0.000  
No matches to TargetScan

-

gaagatagaa

gaagatagaa  
Depth:2 (MARMOSET)  
Ei-value:1.000, Pi-value:0.000  
Er-value:0.000, Pr-value:0.000  
No matches to TargetScan

-----------

AATATTGTCAAGAGTTTCAGA

AATATTGTCAAGAGTTTCAGATAGAAAATGAAAA  
Depth:2 (MARMOSET)  
Ei-value:0.000, Pi-value:0.000  
Er-value:0.000, Pr-value:0.000  
MATCHES To TargetScan▶ miR-653-5p:UGAAACA


TAGAAAATGA

TAGAAAATGA  
Depth:4 (PIG)  
Ei-value:0.000, Pi-value:0.000  
Er-value:0.000, Pr-value:0.000  
No matches to TargetScan


AAA

TAGAAAATGAAAA  
Depth:3 (DOG)  
Ei-value:0.000, Pi-value:0.000  
Er-value:0.000, Pr-value:0.000  
No matches to TargetScan


GCTAAGACAAGTATTGGA

GCTAAGACAAGTATTGGA  
Depth:2 (MARMOSET)  
Ei-value:0.000, Pi-value:0.000  
Er-value:0.000, Pr-value:0.000  
MATCHES To TargetScan▶ miR-200bc-3p/429:AAUACUG

-----

atag

atagaagatag  
Depth:2 (MARMOSET)  
Ei-value:1.000, Pi-value:0.000  
Er-value:0.000, Pr-value:0.000  
No matches to TargetScan


AAGATAG

AAGATAG  
Depth:4 (PIG)  
Ei-value:0.000, Pi-value:0.010  
Er-value:0.000, Pr-value:0.000  
No matches to TargetScan


A

AAAAATTGGA  
Depth:4 (PIG)  
Ei-value:0.000, Pi-value:0.000  
Er-value:0.000, Pr-value:0.000  
No matches to TargetScan


AAAATTGGA

AAAATTGGA  
Depth:5 (COW)  
Ei-value:0.000, Pi-value:0.000  
Er-value:0.000, Pr-value:0.000  
No matches to TargetScan


TAA

AAAAATTGGATAAAATAGCAC  
Depth:2 (MARMOSET)  
Ei-value:0.000, Pi-value:0.000  
Er-value:0.000, Pr-value:0.000  
No matches to TargetScan

 1080  


AATAGCAC

AAAAATTGGATAAAATAGCAC  
Depth:2 (MARMOSET)  
Ei-value:0.000, Pi-value:0.000  
Er-value:0.000, Pr-value:0.000  
No matches to TargetScan

-

gaaaaaatga

gaaaaaatga  
Depth:2 (MARMOSET)  
Ei-value:1.000, Pi-value:0.000  
Er-value:0.000, Pr-value:0.000  
No matches to TargetScan


aattattg

aattattg  
Depth:2 (MARMOSET)  
Ei-value:1.000, Pi-value:0.020  
Er-value:0.000, Pr-value:0.000  
No matches to TargetScan

--

aaccaa

aaccaa  
Depth:2 (MARMOSET)  
Ei-value:1.000, Pi-value:0.060  
Er-value:0.000, Pr-value:0.030  
No matches to TargetScan


TTT

TTTAAAAGCCCATCAATTTAATTTCTG  
Depth:2 (MARMOSET)  
Ei-value:0.000, Pi-value:0.000  
Er-value:0.000, Pr-value:0.000  
No matches to TargetScan


AAAAGCC

AAAAGCC  
Depth:6 (MOUSE)  
Ei-value:0.000, Pi-value:0.000  
Er-value:0.000, Pr-value:0.000  
No matches to TargetScan


CAT

AAAAGCCCAT  
Depth:5 (COW)  
Ei-value:0.000, Pi-value:0.000  
Er-value:0.000, Pr-value:0.000  
No matches to TargetScan


C

TTTAAAAGCCCATCAATTTAATTTCTG  
Depth:2 (MARMOSET)  
Ei-value:0.000, Pi-value:0.000  
Er-value:0.000, Pr-value:0.000  
No matches to TargetScan


AA

AATTTAATTTCTG  
Depth:3 (DOG)  
Ei-value:0.000, Pi-value:0.000  
Er-value:0.000, Pr-value:0.000  
No matches to TargetScan


TTTAATTT

TTTAATTT  
Depth:4 (PIG)  
Ei-value:0.000, Pi-value:0.000  
Er-value:0.000, Pr-value:0.000  
No matches to TargetScan


CTG

AATTTAATTTCTG  
Depth:3 (DOG)  
Ei-value:0.000, Pi-value:0.000  
Er-value:0.000, Pr-value:0.000  
No matches to TargetScan

-

TG

TGGTGCAGAAGTTAGAAGGTAAAG  
Depth:2 (MARMOSET)  
Ei-value:0.000, Pi-value:0.000  
Er-value:0.000, Pr-value:0.000  
No matches to TargetScan


GTGCAGAAG

GTGCAGAAG  
Depth:4 (PIG)  
Ei-value:0.000, Pi-value:0.000  
Er-value:0.000, Pr-value:0.000  
No matches to TargetScan


TTAGAAGGTAAAG

TGGTGCAGAAGTTAGAAGGTAAAG  
Depth:2 (MARMOSET)  
Ei-value:0.000, Pi-value:0.000  
Er-value:0.000, Pr-value:0.000  
No matches to TargetScan

----

agaaga

agaagatgagggt  
Depth:2 (MARMOSET)  
Ei-value:0.680, Pi-value:0.000  
Er-value:0.000, Pr-value:0.000  
No matches to TargetScan


t

tgagggt  
Depth:3 (DOG)  
Ei-value:0.350, Pi-value:0.000  
Er-value:0.000, Pr-value:0.010  
No matches to TargetScan


GAGGGT

GAGGGT  
Depth:5 (COW)  
Ei-value:0.000, Pi-value:0.010  
Er-value:0.000, Pr-value:0.000  
No matches to TargetScan

-----------

GTAGA

GTAGACC  
Depth:5 (COW)  
Ei-value:0.000, Pi-value:0.000  
Er-value:0.000, Pr-value:0.000  
No matches to TargetScan

 1200  


CC

GTAGACC  
Depth:5 (COW)  
Ei-value:0.000, Pi-value:0.000  
Er-value:0.000, Pr-value:0.000  
No matches to TargetScan


aga

gtagaccaga  
Depth:2 (MARMOSET)  
Ei-value:1.000, Pi-value:0.000  
Er-value:0.000, Pr-value:0.000  
No matches to TargetScan

-

CCAATTTAGAAGAATA

CCAATTTAGAAGAATA  
Depth:2 (MARMOSET)  
Ei-value:0.000, Pi-value:0.000  
Er-value:0.000, Pr-value:0.000  
No matches to TargetScan

-

TTGA

TTGAAGCTAGAAGGG  
Depth:2 (MARMOSET)  
Ei-value:0.000, Pi-value:0.000  
Er-value:0.000, Pr-value:0.000  
No matches to TargetScan


AGCTAGAAGGG

AGCTAGAAGGG  
Depth:3 (DOG)  
Ei-value:0.000, Pi-value:0.000  
Er-value:0.000, Pr-value:0.000  
No matches to TargetScan

-----

ggttaa

ggttaa  
Depth:2 (MARMOSET)  
Ei-value:1.000, Pi-value:0.110  
Er-value:0.000, Pr-value:0.030  
No matches to TargetScan

------

cat

catcaaaaagct  
Depth:2 (MARMOSET)  
Ei-value:0.990, Pi-value:0.000  
Er-value:0.000, Pr-value:0.000  
MATCHES To TargetScan▶ miR-129-5p:UUUUUGC


caaaaag

caaaaag  
Depth:3 (DOG)  
Ei-value:0.350, Pi-value:0.010  
Er-value:0.000, Pr-value:0.000  
MATCHES To TargetScan▶ miR-129-5p:UUUUUGC


ct

catcaaaaagct  
Depth:2 (MARMOSET)  
Ei-value:0.990, Pi-value:0.000  
Er-value:0.000, Pr-value:0.000  
MATCHES To TargetScan▶ miR-129-5p:UUUUUGC

-----------------------

AAAAACTAAGGCAGAAGGCTTTTGGAA

AAAAACTAAGGCAGAAGGCTTTTGGAAGAGTTAGAAGAATTTGGAAGGCCTTAAA  
Depth:2 (MARMOSET)  
Ei-value:0.000, Pi-value:0.000  
Er-value:0.000, Pr-value:0.000  
MATCHES To TargetScan▶ miR-124-3p.2/506-3p:UAAGGCA


gag

gagtta  
Depth:3 (DOG)  
Ei-value:1.000, Pi-value:0.070  
Er-value:0.000, Pr-value:0.000  
No matches to TargetScan

 1320  


tta

gagtta  
Depth:3 (DOG)  
Ei-value:1.000, Pi-value:0.070  
Er-value:0.000, Pr-value:0.000  
No matches to TargetScan


GAAGAATTTGGAAGGCCTTAAA

AAAAACTAAGGCAGAAGGCTTTTGGAAGAGTTAGAAGAATTTGGAAGGCCTTAAA  
Depth:2 (MARMOSET)  
Ei-value:0.000, Pi-value:0.000  
Er-value:0.000, Pr-value:0.000  
MATCHES To TargetScan▶ miR-124-3p.2/506-3p:UAAGGCA

-----

gtagctt

gtagctt  
Depth:3 (DOG)  
Ei-value:0.350, Pi-value:0.000  
Er-value:0.000, Pr-value:0.000  
No matches to TargetScan


AGTTTGAAAAAT

GTAGCTTAGTTTGAAAAAT  
Depth:2 (MARMOSET)  
Ei-value:0.000, Pi-value:0.000  
Er-value:0.000, Pr-value:0.000  
No matches to TargetScan

---

aaggacttt

aaggacttt  
Depth:2 (MARMOSET)  
Ei-value:1.000, Pi-value:0.000  
Er-value:0.000, Pr-value:0.000  
No matches to TargetScan

-

GTAACG

GTAACGGAAGTAATTCAAGATCAAGAGTAATTACCAACTTAATGTTT  
Depth:2 (MARMOSET)  
Ei-value:0.000, Pi-value:0.000  
Er-value:0.000, Pr-value:0.000  
MATCHES To TargetScan▶ miR-382-5p:AAGUUGU▶ miR-323-3p:ACAUUAC▶ miR-543:AACAUUC


GAAGTAAT

GAAGTAAT  
Depth:3 (DOG)  
Ei-value:0.010, Pi-value:0.000  
Er-value:0.000, Pr-value:0.000  
No matches to TargetScan


T

GTAACGGAAGTAATTCAAGATCAAGAGTAATTACCAACTTAATGTTT  
Depth:2 (MARMOSET)  
Ei-value:0.000, Pi-value:0.000  
Er-value:0.000, Pr-value:0.000  
MATCHES To TargetScan▶ miR-382-5p:AAGUUGU▶ miR-323-3p:ACAUUAC▶ miR-543:AACAUUC


CAAGATCAAGA

CAAGATCAAGA  
Depth:3 (DOG)  
Ei-value:0.000, Pi-value:0.000  
Er-value:0.000, Pr-value:0.000  
No matches to TargetScan


GTAAT

GTAACGGAAGTAATTCAAGATCAAGAGTAATTACCAACTTAATGTTT  
Depth:2 (MARMOSET)  
Ei-value:0.000, Pi-value:0.000  
Er-value:0.000, Pr-value:0.000  
MATCHES To TargetScan▶ miR-382-5p:AAGUUGU▶ miR-323-3p:ACAUUAC▶ miR-543:AACAUUC


T

TACCAACTTAA  
Depth:3 (DOG)  
Ei-value:0.000, Pi-value:0.000  
Er-value:0.000, Pr-value:0.000  
MATCHES To TargetScan▶ miR-382-5p:AAGUUGU


ACCAACTTA

ACCAACTTA  
Depth:5 (COW)  
Ei-value:0.000, Pi-value:0.000  
Er-value:0.000, Pr-value:0.000  
MATCHES To TargetScan▶ miR-382-5p:AAGUUGU


A

TACCAACTTAA  
Depth:3 (DOG)  
Ei-value:0.000, Pi-value:0.000  
Er-value:0.000, Pr-value:0.000  
MATCHES To TargetScan▶ miR-382-5p:AAGUUGU


TGTTT

GTAACGGAAGTAATTCAAGATCAAGAGTAATTACCAACTTAATGTTT  
Depth:2 (MARMOSET)  
Ei-value:0.000, Pi-value:0.000  
Er-value:0.000, Pr-value:0.000  
MATCHES To TargetScan▶ miR-382-5p:AAGUUGU▶ miR-323-3p:ACAUUAC▶ miR-543:AACAUUC

---

C

CATTGGACTTTG  
Depth:4 (PIG)  
Ei-value:0.000, Pi-value:0.000  
Er-value:0.000, Pr-value:0.000  
No matches to TargetScan


A

ATTGGACTT  
Depth:6 (MOUSE)  
Ei-value:0.000, Pi-value:0.000  
Er-value:0.000, Pr-value:0.000  
No matches to TargetScan


TTGGAC

TTGGACT  
Depth:9 (LIZARD)  
Ei-value:0.000, Pi-value:0.000  
Er-value:0.000, Pr-value:0.000  
No matches to TargetScan

 1440  


T

TTGGACT  
Depth:9 (LIZARD)  
Ei-value:0.000, Pi-value:0.000  
Er-value:0.000, Pr-value:0.000  
No matches to TargetScan


T

ATTGGACTT  
Depth:6 (MOUSE)  
Ei-value:0.000, Pi-value:0.000  
Er-value:0.000, Pr-value:0.000  
No matches to TargetScan


TG

ATTGGACTTTG  
Depth:5 (COW)  
Ei-value:0.000, Pi-value:0.000  
Er-value:0.000, Pr-value:0.000  
No matches to TargetScan

-----------

TTTTTAAA

TTTTTAAA  
Depth:3 (DOG)  
Ei-value:0.010, Pi-value:0.020  
Er-value:0.000, Pr-value:0.010  
No matches to TargetScan

----

TGAG

TGAGGACTAG  
Depth:5 (COW)  
Ei-value:0.000, Pi-value:0.000  
Er-value:0.000, Pr-value:0.000  
No matches to TargetScan


GACTAG

GACTAG  
Depth:8 (ALLIGATOR)  
Ei-value:0.000, Pi-value:0.000  
Er-value:0.000, Pr-value:0.000  
No matches to TargetScan


C

TGAGGACTAGC  
Depth:3 (DOG)  
Ei-value:0.000, Pi-value:0.000  
Er-value:0.000, Pr-value:0.000  
No matches to TargetScan

-

ttaattg

ttaattg  
Depth:3 (DOG)  
Ei-value:0.350, Pi-value:0.030  
Er-value:0.000, Pr-value:0.010  
No matches to TargetScan


ACAGCT

TTAATTGACAGCTGACCCAGGTGCTACACAGAAGTGGATTCAGTGAATCTAGGAAGACAGCAGCAGACAG  
Depth:2 (MARMOSET)  
Ei-value:0.000, Pi-value:0.000  
Er-value:0.000, Pr-value:0.000  
MATCHES To TargetScan▶ miR-29-3p:AGCACCA▶ miR-346:GUCUGCC


GAC

GACCCAGGT  
Depth:3 (DOG)  
Ei-value:0.000, Pi-value:0.000  
Er-value:0.000, Pr-value:0.000  
No matches to TargetScan


CCAGGT

CCAGGT  
Depth:4 (PIG)  
Ei-value:0.010, Pi-value:0.000  
Er-value:0.000, Pr-value:0.000  
No matches to TargetScan


GCTACA

TTAATTGACAGCTGACCCAGGTGCTACACAGAAGTGGATTCAGTGAATCTAGGAAGACAGCAGCAGACAG  
Depth:2 (MARMOSET)  
Ei-value:0.000, Pi-value:0.000  
Er-value:0.000, Pr-value:0.000  
MATCHES To TargetScan▶ miR-29-3p:AGCACCA▶ miR-346:GUCUGCC


CAGAAGTG

CAGAAGTG  
Depth:6 (MOUSE)  
Ei-value:0.000, Pi-value:0.000  
Er-value:0.000, Pr-value:0.000  
No matches to TargetScan


G

CAGAAGTGGATTCAG  
Depth:5 (COW)  
Ei-value:0.000, Pi-value:0.000  
Er-value:0.000, Pr-value:0.000  
No matches to TargetScan


ATTCAG

ATTCAG  
Depth:7 (TURTLE)  
Ei-value:0.000, Pi-value:0.000  
Er-value:0.000, Pr-value:0.000  
No matches to TargetScan


TGAAT

CAGAAGTGGATTCAGTGAATCTAGGAAGACAG  
Depth:3 (DOG)  
Ei-value:0.000, Pi-value:0.000  
Er-value:0.000, Pr-value:0.000  
No matches to TargetScan


CTAGG

CTAGGAAGACAG  
Depth:6 (MOUSE)  
Ei-value:0.000, Pi-value:0.000  
Er-value:0.000, Pr-value:0.000  
No matches to TargetScan


AAGACAG

AAGACAG  
Depth:8 (ALLIGATOR)  
Ei-value:0.000, Pi-value:0.000  
Er-value:0.000, Pr-value:0.000  
No matches to TargetScan


CA

TTAATTGACAGCTGACCCAGGTGCTACACAGAAGTGGATTCAGTGAATCTAGGAAGACAGCAGCAGACAG  
Depth:2 (MARMOSET)  
Ei-value:0.000, Pi-value:0.000  
Er-value:0.000, Pr-value:0.000  
MATCHES To TargetScan▶ miR-29-3p:AGCACCA▶ miR-346:GUCUGCC


GCAGACAG

GCAGACAG  
Depth:6 (MOUSE)  
Ei-value:0.000, Pi-value:0.000  
Er-value:0.000, Pr-value:0.000  
MATCHES To TargetScan▶ miR-346:GUCUGCC

-

ATTCCAGGA

ATTCCAGGA  
Depth:4 (PIG)  
Ei-value:0.000, Pi-value:0.000  
Er-value:0.000, Pr-value:0.000  
No matches to TargetScan

- 1560  


ccagtgt

ccagtgt  
Depth:3 (DOG)  
Ei-value:0.350, Pi-value:0.000  
Er-value:0.000, Pr-value:0.000  
No matches to TargetScan


ttg

ccagtgtttg  
Depth:2 (MARMOSET)  
Ei-value:1.000, Pi-value:0.000  
Er-value:0.000, Pr-value:0.000  
MATCHES To TargetScan▶ miR-141-3p/200a-3p:AACACUG

-

TGAAG

TGAAGCTAGGACTGAGGAGC  
Depth:3 (DOG)  
Ei-value:0.000, Pi-value:0.000  
Er-value:0.000, Pr-value:0.000  
MATCHES To TargetScan▶ miR-455-3p.1:CAGUCCA


CT

CTAGGACTGAGGAGC  
Depth:5 (COW)  
Ei-value:0.000, Pi-value:0.000  
Er-value:0.000, Pr-value:0.000  
MATCHES To TargetScan▶ miR-455-3p.1:CAGUCCA


AGGACTGAGGAGC

AGGACTGAGGAGC  
Depth:6 (MOUSE)  
Ei-value:0.000, Pi-value:0.000  
Er-value:0.000, Pr-value:0.000  
MATCHES To TargetScan▶ miR-455-3p.1:CAGUCCA


AA

TGAAGCTAGGACTGAGGAGCAA  
Depth:2 (MARMOSET)  
Ei-value:0.000, Pi-value:0.000  
Er-value:0.000, Pr-value:0.000  
MATCHES To TargetScan▶ miR-455-3p.1:CAGUCCA


GCA

GCAGCAGTTCGTGGTGAAGATAGGAA  
Depth:2 (MARMOSET)  
Ei-value:0.000, Pi-value:0.000  
Er-value:0.000, Pr-value:0.000  
MATCHES To TargetScan▶ miR-202-5p:UCCUAUG


GCAGTTC

GCAGTTC  
Depth:5 (COW)  
Ei-value:0.000, Pi-value:0.000  
Er-value:0.000, Pr-value:0.000  
No matches to TargetScan


GTG

GCAGTTCGTGGTGAAGATAGGAA  
Depth:3 (DOG)  
Ei-value:0.000, Pi-value:0.000  
Er-value:0.000, Pr-value:0.000  
MATCHES To TargetScan▶ miR-202-5p:UCCUAUG


GTGAAGATAG

GTGAAGATAG  
Depth:6 (MOUSE)  
Ei-value:0.000, Pi-value:0.000  
Er-value:0.000, Pr-value:0.000  
No matches to TargetScan


GAA

GTGAAGATAGGAA  
Depth:5 (COW)  
Ei-value:0.000, Pi-value:0.000  
Er-value:0.000, Pr-value:0.000  
MATCHES To TargetScan▶ miR-202-5p:UCCUAUG


gagtccagga

gagtccagga  
Depth:2 (MARMOSET)  
Ei-value:1.000, Pi-value:0.000  
Er-value:0.000, Pr-value:0.000  
MATCHES To TargetScan▶ miR-378-3p:CUGGACU

-

CCAGTGC

CCAGTGC  
Depth:5 (COW)  
Ei-value:0.000, Pi-value:0.000  
Er-value:0.000, Pr-value:0.000  
No matches to TargetScan


GA

CCAGTGCGATTTGGTGAAGGAAGCTAGGAAGAAGGAAGGAGCGCTAACGATTTGGTGGTGAAGCTAGGAAA  
Depth:2 (MARMOSET)  
Ei-value:0.000, Pi-value:0.000  
Er-value:0.000, Pr-value:0.000  
MATCHES To TargetScan▶ miR-205-5p:CCUUCAU


TTTGGT

TTTGGT  
Depth:4 (PIG)  
Ei-value:0.010, Pi-value:0.010  
Er-value:0.000, Pr-value:0.000  
No matches to TargetScan


GA

GAAGGAAGCTAGGAAGAA  
Depth:5 (COW)  
Ei-value:0.000, Pi-value:0.000  
Er-value:0.000, Pr-value:0.000  
No matches to TargetScan


AGGAAGCTAGGAAGAA

AGGAAGCTAGGAAGAA  
Depth:6 (MOUSE)  
Ei-value:0.000, Pi-value:0.000  
Er-value:0.000, Pr-value:0.000  
No matches to TargetScan


G

CCAGTGCGATTTGGTGAAGGAAGCTAGGAAGAAGGAAGGAGCGCTAACGATTTGGTGGTGAAGCTAGGAAA  
Depth:2 (MARMOSET)  
Ei-value:0.000, Pi-value:0.000  
Er-value:0.000, Pr-value:0.000  
MATCHES To TargetScan▶ miR-205-5p:CCUUCAU


GAAGGAGC

GAAGGAGC  
Depth:4 (PIG)  
Ei-value:0.000, Pi-value:0.000  
Er-value:0.000, Pr-value:0.000  
No matches to TargetScan


G

CCAGTGCGATTTGGTGAAGGAAGCTAGGAAGAAGGAAGGAGCGCTAACGATTTGGTGGTGAAGCTAGGAAA  
Depth:2 (MARMOSET)  
Ei-value:0.000, Pi-value:0.000  
Er-value:0.000, Pr-value:0.000  
MATCHES To TargetScan▶ miR-205-5p:CCUUCAU


CTAACG

CTAACG  
Depth:5 (COW)  
Ei-value:0.000, Pi-value:0.000  
Er-value:0.000, Pr-value:0.000  
No matches to TargetScan


A

CTAACGATTTGG  
Depth:3 (DOG)  
Ei-value:0.000, Pi-value:0.000  
Er-value:0.000, Pr-value:0.000  
No matches to TargetScan

 1680  


TTTGG

CTAACGATTTGG  
Depth:3 (DOG)  
Ei-value:0.000, Pi-value:0.000  
Er-value:0.000, Pr-value:0.000  
No matches to TargetScan


T

CCAGTGCGATTTGGTGAAGGAAGCTAGGAAGAAGGAAGGAGCGCTAACGATTTGGTGGTGAAGCTAGGAAA  
Depth:2 (MARMOSET)  
Ei-value:0.000, Pi-value:0.000  
Er-value:0.000, Pr-value:0.000  
MATCHES To TargetScan▶ miR-205-5p:CCUUCAU


GGTGAAG

GGTGAAG  
Depth:11 (X.TROPICALIS)  
Ei-value:0.000, Pi-value:0.000  
Er-value:0.000, Pr-value:0.000  
No matches to TargetScan


CT

GGTGAAGCT  
Depth:6 (MOUSE)  
Ei-value:0.000, Pi-value:0.000  
Er-value:0.000, Pr-value:0.000  
No matches to TargetScan


AGGA

GGTGAAGCTAGGA  
Depth:5 (COW)  
Ei-value:0.000, Pi-value:0.000  
Er-value:0.000, Pr-value:0.000  
No matches to TargetScan


AA

CCAGTGCGATTTGGTGAAGGAAGCTAGGAAGAAGGAAGGAGCGCTAACGATTTGGTGGTGAAGCTAGGAAA  
Depth:2 (MARMOSET)  
Ei-value:0.000, Pi-value:0.000  
Er-value:0.000, Pr-value:0.000  
MATCHES To TargetScan▶ miR-205-5p:CCUUCAU

-

AGGATTCCAG

AGGATTCCAG  
Depth:5 (COW)  
Ei-value:0.000, Pi-value:0.000  
Er-value:0.000, Pr-value:0.000  
No matches to TargetScan


GAAGGAGCGAGTGC

AGGATTCCAGGAAGGAGCGAGTGC  
Depth:2 (MARMOSET)  
Ei-value:0.000, Pi-value:0.000  
Er-value:0.000, Pr-value:0.000  
No matches to TargetScan

-

ATT

ATTTGGTGATGAAG  
Depth:2 (MARMOSET)  
Ei-value:0.020, Pi-value:0.000  
Er-value:0.000, Pr-value:0.000  
No matches to TargetScan


TGGTGA

TGGTGA  
Depth:8 (ALLIGATOR)  
Ei-value:0.000, Pi-value:0.000  
Er-value:0.000, Pr-value:0.000  
No matches to TargetScan


TGAAG

TGGTGATGAAG  
Depth:4 (PIG)  
Ei-value:0.000, Pi-value:0.000  
Er-value:0.000, Pr-value:0.000  
No matches to TargetScan

-

TAGC

TAGCAGGCGGCTTGGCTTGGCAACCACACGGAGGAG  
Depth:2 (MARMOSET)  
Ei-value:0.000, Pi-value:0.000  
Er-value:0.000, Pr-value:0.000  
No matches to TargetScan


a

aggcggc  
Depth:3 (DOG)  
Ei-value:0.350, Pi-value:0.000  
Er-value:0.000, Pr-value:0.000  
No matches to TargetScan


GGCGGC

GGCGGC  
Depth:6 (MOUSE)  
Ei-value:0.000, Pi-value:0.000  
Er-value:0.000, Pr-value:0.000  
No matches to TargetScan


TTGGCTTGGCAACCACACGGAGGAG

TAGCAGGCGGCTTGGCTTGGCAACCACACGGAGGAG  
Depth:2 (MARMOSET)  
Ei-value:0.000, Pi-value:0.000  
Er-value:0.000, Pr-value:0.000  
No matches to TargetScan

-

CGAG

CGAGCAGGCGTTGTGC  
Depth:2 (MARMOSET)  
Ei-value:0.000, Pi-value:0.000  
Er-value:0.000, Pr-value:0.000  
No matches to TargetScan


CAGGCG

CAGGCG  
Depth:5 (COW)  
Ei-value:0.000, Pi-value:0.000  
Er-value:0.000, Pr-value:0.000  
No matches to TargetScan


TTGTGC

CGAGCAGGCGTTGTGC  
Depth:2 (MARMOSET)  
Ei-value:0.000, Pi-value:0.000  
Er-value:0.000, Pr-value:0.000  
No matches to TargetScan

----- 1800  


TAGAGGA

TAGAGGATCCTAGA  
Depth:3 (DOG)  
Ei-value:0.000, Pi-value:0.000  
Er-value:0.000, Pr-value:0.000  
No matches to TargetScan


TCCTAGA

TCCTAGA  
Depth:6 (MOUSE)  
Ei-value:0.000, Pi-value:0.000  
Er-value:0.000, Pr-value:0.000  
No matches to TargetScan


C

TAGAGGATCCTAGACCAGCATGCCAGT  
Depth:2 (MARMOSET)  
Ei-value:0.000, Pi-value:0.000  
Er-value:0.000, Pr-value:0.000  
MATCHES To TargetScan▶ miR-138-5p:GCUGGUG▶ miR-193-3p:ACUGGCC


cagcat

cagcat  
Depth:3 (DOG)  
Ei-value:1.000, Pi-value:0.020  
Er-value:0.000, Pr-value:0.000  
No matches to TargetScan


GCCAGT

TAGAGGATCCTAGACCAGCATGCCAGT  
Depth:2 (MARMOSET)  
Ei-value:0.000, Pi-value:0.000  
Er-value:0.000, Pr-value:0.000  
MATCHES To TargetScan▶ miR-138-5p:GCUGGUG▶ miR-193-3p:ACUGGCC

-

tgccaaggc

tgccaaggc  
Depth:2 (MARMOSET)  
Ei-value:1.000, Pi-value:0.000  
Er-value:0.000, Pr-value:0.000  
MATCHES To TargetScan▶ miR-212-5p:CCUUGGC▶ miR-182-5p:UUGGCAA▶ miR-96-5p/1271-5p:UUGGCAC

-

a

acagggaa  
Depth:2 (MARMOSET)  
Ei-value:1.000, Pi-value:0.000  
Er-value:0.000, Pr-value:0.000  
MATCHES To TargetScan▶ miR-339-5p:CCCUGUC


CAGGGA

CAGGGA  
Depth:6 (MOUSE)  
Ei-value:0.000, Pi-value:0.000  
Er-value:0.000, Pr-value:0.000  
No matches to TargetScan


a

acagggaa  
Depth:2 (MARMOSET)  
Ei-value:1.000, Pi-value:0.000  
Er-value:0.000, Pr-value:0.000  
MATCHES To TargetScan▶ miR-339-5p:CCCUGUC

---

g

gagtggt  
Depth:3 (DOG)  
Ei-value:0.350, Pi-value:0.010  
Er-value:0.000, Pr-value:0.000  
No matches to TargetScan


AGTGGT

AGTGGT  
Depth:5 (COW)  
Ei-value:0.000, Pi-value:0.020  
Er-value:0.000, Pr-value:0.000  
No matches to TargetScan


TGGTAAAAAT

TGGTAAAAAT  
Depth:5 (COW)  
Ei-value:0.000, Pi-value:0.000  
Er-value:0.000, Pr-value:0.000  
No matches to TargetScan


CCGTGAGGTCGGCAATATGTTGTTTTTCTGGAACTT

GAGTGGTTGGTAAAAATCCGTGAGGTCGGCAATATGTTGTTTTTCTGGAACTT  
Depth:2 (MARMOSET)  
Ei-value:0.000, Pi-value:0.000  
Er-value:0.000, Pr-value:0.000  
MATCHES To TargetScan▶ miR-876-5p:GGAUUUC▶ miR-137:UAUUGCU

---

TA

TATGGTAA  
Depth:3 (DOG)  
Ei-value:0.010, Pi-value:0.000  
Er-value:0.000, Pr-value:0.000  
No matches to TargetScan


TGGTAA

TGGTAA  
Depth:14 (SPOTTEDGAR)  
Ei-value:0.030, Pi-value:0.000  
Er-value:0.010, Pr-value:0.000  
No matches to TargetScan

--

tttta

ttttattta  
Depth:2 (MARMOSET)  
Ei-value:1.000, Pi-value:0.000  
Er-value:0.000, Pr-value:0.000  
No matches to TargetScan

 1920  


ttta

ttttattta  
Depth:2 (MARMOSET)  
Ei-value:1.000, Pi-value:0.000  
Er-value:0.000, Pr-value:0.000  
No matches to TargetScan

-----------

TAATGG

TAATGG  
Depth:5 (COW)  
Ei-value:0.000, Pi-value:0.020  
Er-value:0.000, Pr-value:0.000  
No matches to TargetScan


GGGAGTTTCGTACTGAGGTGTAAA

TAATGGGGGAGTTTCGTACTGAGGTGTAAA  
Depth:2 (MARMOSET)  
Ei-value:0.000, Pi-value:0.000  
Er-value:0.000, Pr-value:0.000  
MATCHES To TargetScan▶ miR-1306-5p:CACCUCC

--

GGGATTTAT

GGGATTTAT  
Depth:3 (DOG)  
Ei-value:0.000, Pi-value:0.020  
Er-value:0.000, Pr-value:0.000  
No matches to TargetScan


ATGGGGA

GGGATTTATATGGGGA  
Depth:2 (MARMOSET)  
Ei-value:0.000, Pi-value:0.000  
Er-value:0.000, Pr-value:0.000  
MATCHES To TargetScan▶ miR-410-3p:AUAUAAC▶ miR-340-5p:UAUAAAG

-

GTAGGCC

GTAGGCC  
Depth:6 (MOUSE)  
Ei-value:0.000, Pi-value:0.000  
Er-value:0.000, Pr-value:0.000  
No matches to TargetScan


G

GTAGGCCG  
Depth:5 (COW)  
Ei-value:0.000, Pi-value:0.000  
Er-value:0.000, Pr-value:0.000  
No matches to TargetScan


ATTT

GTAGGCCGATTT  
Depth:4 (PIG)  
Ei-value:0.000, Pi-value:0.000  
Er-value:0.000, Pr-value:0.000  
No matches to TargetScan


C

GTAGGCCGATTTCCGGGTGT  
Depth:3 (DOG)  
Ei-value:0.000, Pi-value:0.000  
Er-value:0.000, Pr-value:0.000  
No matches to TargetScan


CGGGTGT

CGGGTGT  
Depth:6 (MOUSE)  
Ei-value:0.000, Pi-value:0.000  
Er-value:0.000, Pr-value:0.000  
No matches to TargetScan


T

GTAGGCCGATTTCCGGGTGTTGTAGGTTTCTCTTTTTCAGGCTTAT  
Depth:2 (MARMOSET)  
Ei-value:0.000, Pi-value:0.000  
Er-value:0.000, Pr-value:0.000  
No matches to TargetScan


GTAGGTTT

GTAGGTTT  
Depth:8 (ALLIGATOR)  
Ei-value:0.000, Pi-value:0.000  
Er-value:0.000, Pr-value:0.000  
No matches to TargetScan


C

GTAGGTTTCTCTTTTTCAGGC  
Depth:5 (COW)  
Ei-value:0.000, Pi-value:0.000  
Er-value:0.000, Pr-value:0.000  
No matches to TargetScan


TCTTTTTCAGG

TCTTTTTCAGG  
Depth:6 (MOUSE)  
Ei-value:0.000, Pi-value:0.000  
Er-value:0.000, Pr-value:0.000  
No matches to TargetScan


C

GTAGGTTTCTCTTTTTCAGGC  
Depth:5 (COW)  
Ei-value:0.000, Pi-value:0.000  
Er-value:0.000, Pr-value:0.000  
No matches to TargetScan


TT

GTAGGTTTCTCTTTTTCAGGCTT  
Depth:3 (DOG)  
Ei-value:0.000, Pi-value:0.000  
Er-value:0.000, Pr-value:0.000  
No matches to TargetScan


AT

GTAGGCCGATTTCCGGGTGTTGTAGGTTTCTCTTTTTCAGGCTTAT  
Depth:2 (MARMOSET)  
Ei-value:0.000, Pi-value:0.000  
Er-value:0.000, Pr-value:0.000  
No matches to TargetScan

-

CTCATGA

CTCATGAATCTTGTCTGAAGCTTTTGAGGGCAGACTGCCAAGTCCTGGAG  
Depth:2 (MARMOSET)  
Ei-value:0.000, Pi-value:0.000  
Er-value:0.000, Pr-value:0.000  
MATCHES To TargetScan▶ miR-433-3p:UCAUGAU▶ miR-665:CCAGGAG▶ miR-371-5p:CUCAAAC▶ miR-34-5p/449-5p:GGCAGUG▶ miR-182-5p:UUGGCAA▶ miR-320:AAAGCUG▶ miR-96-5p/1271-5p:UUGGCAC▶ miR-874-3p:UGCCCUG▶ miR-346:GUCUGCC


AT

ATCTTGTC  
Depth:7 (TURTLE)  
Ei-value:0.000, Pi-value:0.000  
Er-value:0.000, Pr-value:0.000  
No matches to TargetScan

 2040  


CTTGTC

ATCTTGTC  
Depth:7 (TURTLE)  
Ei-value:0.000, Pi-value:0.000  
Er-value:0.000, Pr-value:0.000  
No matches to TargetScan


TG

ATCTTGTCTG  
Depth:5 (COW)  
Ei-value:0.000, Pi-value:0.000  
Er-value:0.000, Pr-value:0.000  
No matches to TargetScan


A

ATCTTGTCTGAAGCTTTTGAGGGCAGACT  
Depth:4 (PIG)  
Ei-value:0.000, Pi-value:0.000  
Er-value:0.000, Pr-value:0.000  
MATCHES To TargetScan▶ miR-371-5p:CUCAAAC▶ miR-320:AAAGCUG▶ miR-874-3p:UGCCCUG▶ miR-346:GUCUGCC


AGCTTTTGAGGGC

AGCTTTTGAGGGC  
Depth:6 (MOUSE)  
Ei-value:0.000, Pi-value:0.000  
Er-value:0.000, Pr-value:0.000  
MATCHES To TargetScan▶ miR-371-5p:CUCAAAC▶ miR-320:AAAGCUG


AGACT

AGCTTTTGAGGGCAGACT  
Depth:5 (COW)  
Ei-value:0.000, Pi-value:0.000  
Er-value:0.000, Pr-value:0.000  
MATCHES To TargetScan▶ miR-371-5p:CUCAAAC▶ miR-320:AAAGCUG▶ miR-874-3p:UGCCCUG▶ miR-346:GUCUGCC


GCCAAGT

CTCATGAATCTTGTCTGAAGCTTTTGAGGGCAGACTGCCAAGTCCTGGAG  
Depth:2 (MARMOSET)  
Ei-value:0.000, Pi-value:0.000  
Er-value:0.000, Pr-value:0.000  
MATCHES To TargetScan▶ miR-433-3p:UCAUGAU▶ miR-665:CCAGGAG▶ miR-371-5p:CUCAAAC▶ miR-34-5p/449-5p:GGCAGUG▶ miR-182-5p:UUGGCAA▶ miR-320:AAAGCUG▶ miR-96-5p/1271-5p:UUGGCAC▶ miR-874-3p:UGCCCUG▶ miR-346:GUCUGCC


cctggag

cctggag  
Depth:3 (DOG)  
Ei-value:0.350, Pi-value:0.010  
Er-value:0.000, Pr-value:0.000  
No matches to TargetScan

-

AATA

AATAGTAGATGGCAAGTTTGT  
Depth:2 (MARMOSET)  
Ei-value:0.000, Pi-value:0.000  
Er-value:0.000, Pr-value:0.000  
No matches to TargetScan


GTAGATGGCAAGT

GTAGATGGCAAGT  
Depth:6 (MOUSE)  
Ei-value:0.000, Pi-value:0.000  
Er-value:0.000, Pr-value:0.000  
No matches to TargetScan


TT

GTAGATGGCAAGTTT  
Depth:3 (DOG)  
Ei-value:0.000, Pi-value:0.000  
Er-value:0.000, Pr-value:0.000  
No matches to TargetScan


GT

AATAGTAGATGGCAAGTTTGT  
Depth:2 (MARMOSET)  
Ei-value:0.000, Pi-value:0.000  
Er-value:0.000, Pr-value:0.000  
No matches to TargetScan

---------------

ttttttac

ttttttac  
Depth:2 (MARMOSET)  
Ei-value:1.000, Pi-value:0.000  
Er-value:0.000, Pr-value:0.010  
No matches to TargetScan

---------------

CAAA

CAAATGAATTTG  
Depth:4 (PIG)  
Ei-value:0.000, Pi-value:0.000  
Er-value:0.000, Pr-value:0.000  
No matches to TargetScan


TGAATTTG

TGAATTTG  
Depth:5 (COW)  
Ei-value:0.000, Pi-value:0.000  
Er-value:0.000, Pr-value:0.000  
No matches to TargetScan


ATAG

CAAATGAATTTGATAG  
Depth:2 (MARMOSET)  
Ei-value:0.000, Pi-value:0.000  
Er-value:0.000, Pr-value:0.000  
No matches to TargetScan

--- 2160  
 --

aaa

aaattgagac  
Depth:2 (MARMOSET)  
Ei-value:1.000, Pi-value:0.000  
Er-value:0.000, Pr-value:0.000  
No matches to TargetScan


T

TTGAGAC  
Depth:4 (PIG)  
Ei-value:0.000, Pi-value:0.010  
Er-value:0.000, Pr-value:0.000  
No matches to TargetScan


TGAGAC

TGAGAC  
Depth:5 (COW)  
Ei-value:0.000, Pi-value:0.020  
Er-value:0.000, Pr-value:0.000  
No matches to TargetScan

--

AATTTCAGCAAATCTGTAAGCAGTTTGTATGTTTAG

AATTTCAGCAAATCTGTAAGCAGTTTGTATGTTTAGTTGGGGTAATG  
Depth:2 (MARMOSET)  
Ei-value:0.000, Pi-value:0.000  
Er-value:0.000, Pr-value:0.000  
MATCHES To TargetScan▶ miR-381-3p:AUACAAG▶ miR-203a-3p.2:UGAAAUG


TTGG

TTGGGGTAATG  
Depth:3 (DOG)  
Ei-value:0.000, Pi-value:0.000  
Er-value:0.000, Pr-value:0.000  
No matches to TargetScan


GGTAAT

GGTAAT  
Depth:6 (MOUSE)  
Ei-value:0.000, Pi-value:0.010  
Er-value:0.000, Pr-value:0.000  
No matches to TargetScan


G

GGTAATG  
Depth:5 (COW)  
Ei-value:0.000, Pi-value:0.000
[truncated: 638,327 more chars]
