# Supplementary material for: Uncovering deeply conserved motif combinations in rapidly evolving noncoding sequences
Source: Genome Biol. 2021 Jan 11;22:29. doi: 10.1186/s13059-020-02247-1 (PMC7798263; doi:10.1186/s13059-020-02247-1)
Supplement: Supplementary file 5 — Additional file 5. LncLOOM output results for MALAT1 sequences from 19 vertebrates. [file 13059_2020_2247_MOESM5_ESM.gz › AdditionalFile5/Html_Files/kmers_in_blocks_level.html]

 MOTIFS IN BLOCKS

# MOTIFS IN BLOCK DIAGRAMS

## Motifs conserved to (and beyond) ZEBRAFISH (depth:19)

  

NAVIGATE ▼

▶HUMAN (depth:1)▶MARMOSET (depth:2)▶DOG (depth:3)▶PIG (depth:4)▶COW (depth:5)▶MOUSE (depth:6)▶TURTLE (depth:7)▶ALLIGATOR (depth:8)▶LIZARD (depth:9)▶SNAKE (depth:10)▶X.TROPICALIS (depth:11)▶SHARK (depth:12)▶OPOSSUM (depth:13)▶SPOTTEDGAR (depth:14)▶FUGU (depth:15)▶NILETILAPIA (depth:16)▶STICKLEBACK (depth:17)▶MEDAKA (depth:18)▶ZEBRAFISH (depth:19)

  
  
  

## >HUMAN (7504 bases)

```
 ------------------------------------------------------------------------------------------------------------------------ 120  
 ------------------------------------------------------------------------------------------------------------------------ 240  
 ------------------------------------------------------------------------------------------------------------------------ 360  
 ------------------------------------------------------------------------------------------------------------------------ 480  
 ------------------------------------------------------------------------------------------------------------------------ 600  
 ------------------------------------------------------------------------------------------------------------------------ 720  
 ------------------------------------------------------------------------------------------------------------------------ 840  
 ------------------------------------------------------------------------------------------------------------------------ 960  
 ------------------------------------------------------------------------------------------------------------------------ 1080  
 ------------------------------------------------------------------------------------------------------------------------ 1200  
 ------------------------------------------------------------------------------------------------------------------------ 1320  
 ------------------------------------------------------------------------------------------------------------------------ 1440  
 ------------------------------------------------------------------------------------------------------------------------ 1560  
 ------------------------------------------------------------------------------------------------------------------------ 1680  
 ------------------------------------------------------------------------------------------------------------------------ 1800  
 ------------------------------------------------------------------------------------------------------------------------ 1920  
 ------------------------------------------------------------------------------------------------------------------------ 2040  
 ------------------------------------------------------------------------------------------------------------------------ 2160  
 ------------------------------------------------------------------------------------------------------------------------ 2280  
 ------------------------------------------------------------------------------------------------------------------------ 2400  
 --------------------------------------------------------------------------------------------------------

TTTGGG

TTTGGG  
Depth:19 (ZEBRAFISH)  
Ei-value:0.000, Pi-value:0.000  
Er-value:0.000, Pr-value:0.000  
eCLIP MATCHES▶ppil4 (bg=43.39%)▶PRPF8 (bg=6.2%)No matches to TargetScan

---------- 2520  
 ------------------------------------------------------------------------------------------------------------------------ 2640  
 ------------------------------------------------------------------------------------------------------------------------ 2760  
 ------------------------------------------------------------------------------------------------------------------------ 2880  
 ------------------------------------------------------------------------------------------------------------------------ 3000  
 ------------------------------------------------------------------------------------------------------------------------ 3120  
 ------------------------------------------------------------------------------------------------------------------------ 3240  
 ------------------------------------------------------------------------------------------------------------------------ 3360  
 ------------------------------------------------------------------------------------------------------------------------ 3480  
 ------------------------------------------------------------------------------------------------------------------------ 3600  
 --------------------

TTTTTCAG

TTTTTCAG  
Depth:19 (ZEBRAFISH)  
Ei-value:0.000, Pi-value:0.000  
Er-value:0.000, Pr-value:0.000  
eCLIP MATCHES▶ddx42 (bg=10.33%)▶ppil4 (bg=43.39%)▶safb (bg=40.39%)▶u2af1 (bg=14.02%)▶u2af2 (bg=19.32%)No matches to TargetScan

-------------------------------------------------------------------------------------------- 3720  
 ------------------------------------------------------------------------------------------------------------------------ 3840  
 ------------------------------------------------------------------------------------------------------------------------ 3960  
 ------------------------------------------------------------------------------------------------------------------------ 4080  
 -------------------------------------------------------------------------------------------

GATAAG

GATAAG  
Depth:19 (ZEBRAFISH)  
Ei-value:0.000, Pi-value:0.000  
Er-value:0.000, Pr-value:0.000  
eCLIP MATCHES▶cpsf6 (bg=13.45%)▶khsrp (bg=27.4%)▶NIPBL (bg=8.2%)▶ppil4 (bg=43.39%)▶PRPF8 (bg=6.2%)▶rbm15 (bg=11.59%)▶safb (bg=40.39%)▶safb2 (bg=26.89%)▶srsf1 (bg=30.28%)▶srsf7 (bg=22.53%)▶znf622 (bg=18.79%)No matches to TargetScan

----------------------- 4200  
 ------------------------------------------------------------------------------------------------------------------------ 4320  
 ------------------------------------------------------------------------------------------------------------------------ 4440  
 ------------------------------------------------------------------------------------------------------------------------ 4560  
 ------------------------------------------------------------------------------------------------------------------------ 4680  
 ------------------------------------------------------------------------------------------------------------------------ 4800  
 ------------------------------------------------------------------------------------------------------------------------ 4920  
 ------------------------------------------------------------------------------------------------------------------------ 5040  
 ------------------------------------------------------------------------------------------------------------------------ 5160  
 ------------------------------------------------------------------------------------------------------------------------ 5280  
 ------------------------------------------------------------------------------------------------------------------------ 5400  
 ------------------------------------------------------------------------------------------------------------------------ 5520  
 ------------------------------------------------------------------------------------------------------------------------ 5640  
 ------------------------------------------------------------------------------------------------------------------------ 5760  
 ------------------------------------------------------------------------------------------------------------------------ 5880  
 ------------------------------------------------------------------------------------------------------------------------ 6000  
 ------------------------------------------------------------------------------------------------------------------------ 6120  
 ------------------------------------------------------------------------------------------------------------------------ 6240  
 ------------------------------------------------------------------------------------------------------------------------ 6360  
 ------------------------------------------------------------------------------------------------------------------------ 6480  
 ------------------------------------------------------------------------------------------------------------------------ 6600  
 ------------------------------------------------------------------------------------------------------------------------ 6720  
 ------------------------------------------------------------------------------------------------------------------------ 6840  
 ------------------------------------------------------------------------------------------------------------------------ 6960  
 --------------------------------------------------

TTTTCTTTT

TTTTCTTTT  
Depth:19 (ZEBRAFISH)  
Ei-value:0.000, Pi-value:0.000  
Er-value:0.000, Pr-value:0.000  
eCLIP MATCHES▶srsf7 (bg=22.53%)MATCHES To TargetScan▶ miR-186-5p:AAAGAAU

----------------------------

CAGGTTTTGCTTT

CAGGTTTTGCTTT  
Depth:19 (ZEBRAFISH)  
Ei-value:0.000, Pi-value:0.000  
Er-value:0.000, Pr-value:0.000  
eCLIP MATCHES▶srsf7 (bg=22.53%)MATCHES To TargetScan▶ miR-330-3p.2:AAAGCAC▶ miR-490-3p:AACCUGG

-------------------- 7080  
 ------

AAAAAGCAAAA

AAAAAGCAAAA  
Depth:19 (ZEBRAFISH)  
Ei-value:0.000, Pi-value:0.000  
Er-value:0.000, Pr-value:0.000  
No matches to eCLIP DataNo matches to TargetScan

------------------------------------------------------------------------------------------------------- 7200  
 ------------------------------------------------------------------------------------------------------------------------ 7320  
 ------------------------------------------------------------------------------------------------------------------------ 7440  
 ----------------------------------------------------------------                                                         7504
```

---

## >MARMOSET (7293 bases)

```
 ------------------------------------------------------------------------------------------------------------------------ 120  
 ------------------------------------------------------------------------------------------------------------------------ 240  
 ------------------------------------------------------------------------------------------------------------------------ 360  
 ------------------------------------------------------------------------------------------------------------------------ 480  
 ------------------------------------------------------------------------------------------------------------------------ 600  
 ------------------------------------------------------------------------------------------------------------------------ 720  
 ------------------------------------------------------------------------------------------------------------------------ 840  
 ------------------------------------------------------------------------------------------------------------------------ 960  
 ------------------------------------------------------------------------------------------------------------------------ 1080  
 ------------------------------------------------------------------------------------------------------------------------ 1200  
 ------------------------------------------------------------------------------------------------------------------------ 1320  
 ------------------------------------------------------------------------------------------------------------------------ 1440  
 ------------------------------------------------------------------------------------------------------------------------ 1560  
 ------------------------------------------------------------------------------------------------------------------------ 1680  
 ------------------------------------------------------------------------------------------------------------------------ 1800  
 ------------------------------------------------------------------------------------------------------------------------ 1920  
 ------------------------------------------------------------------------------------------------------------------------ 2040  
 ------------------------------------------------------------------------------------------------------------------------ 2160  
 ------------------------------------------------------------------------------------------------------------------------ 2280  
 ------------------------------------------------------------------------------------------------------------------------ 2400  
 ------------------------------------------------------------------------------------------------------------------------ 2520  
 --------------------------------------------------------------------------------------------------------------------

TTTG

TTTGGG  
Depth:19 (ZEBRAFISH)  
Ei-value:0.000, Pi-value:0.000  
Er-value:0.000, Pr-value:0.000  
No matches to TargetScan

 2640  


GG

TTTGGG  
Depth:19 (ZEBRAFISH)  
Ei-value:0.000, Pi-value:0.000  
Er-value:0.000, Pr-value:0.000  
No matches to TargetScan

---------------------------------------------------------------------------------------------------------------------- 2760  
 ------------------------------------------------------------------------------------------------------------------------ 2880  
 ------------------------------------------------------------------------------------------------------------------------ 3000  
 ------------------------------------------------------------------------------------------------------------------------ 3120  
 ------------------------------------------------------------------------------------------------------------------------ 3240  
 ------------------------------------------------------------------------------------------------------------------------ 3360  
 ------------------------------------------------------------------------------------------------------------------------ 3480  
 ------------------------------------------------------------------------------------------------------------------------ 3600  
 ------------------------------------------------------------------------------------------------------------------------ 3720  
 -----------------

TTTTTCAG

TTTTTCAG  
Depth:19 (ZEBRAFISH)  
Ei-value:0.000, Pi-value:0.000  
Er-value:0.000, Pr-value:0.000  
No matches to TargetScan

----------------------------------------------------------------------------------------------- 3840  
 ------------------------------------------------------------------------------------------------------------------------ 3960  
 ------------------------------------------------------------------------------------------------------------------------ 4080  
 ------------------------------------------------------------------------------------------------------------------------ 4200  
 ---------------------------------------------------------------------------------------------

GATAAG

GATAAG  
Depth:19 (ZEBRAFISH)  
Ei-value:0.000, Pi-value:0.000  
Er-value:0.000, Pr-value:0.000  
No matches to TargetScan

--------------------- 4320  
 ------------------------------------------------------------------------------------------------------------------------ 4440  
 ------------------------------------------------------------------------------------------------------------------------ 4560  
 ------------------------------------------------------------------------------------------------------------------------ 4680  
 ------------------------------------------------------------------------------------------------------------------------ 4800  
 ------------------------------------------------------------------------------------------------------------------------ 4920  
 ------------------------------------------------------------------------------------------------------------------------ 5040  
 ------------------------------------------------------------------------------------------------------------------------ 5160  
 ------------------------------------------------------------------------------------------------------------------------ 5280  
 ------------------------------------------------------------------------------------------------------------------------ 5400  
 ------------------------------------------------------------------------------------------------------------------------ 5520  
 ------------------------------------------------------------------------------------------------------------------------ 5640  
 ------------------------------------------------------------------------------------------------------------------------ 5760  
 ------------------------------------------------------------------------------------------------------------------------ 5880  
 ------------------------------------------------------------------------------------------------------------------------ 6000  
 ------------------------------------------------------------------------------------------------------------------------ 6120  
 ------------------------------------------------------------------------------------------------------------------------ 6240  
 ------------------------------------------------------------------------------------------------------------------------ 6360  
 ------------------------------------------------------------------------------------------------------------------------ 6480  
 ------------------------------------------------------------------------------------------------------------------------ 6600  
 ------------------------------------------------------------------------------------------------------------------------ 6720  
 ------------------------------------------------------------------------------------------------------------------------ 6840  
 ------------------------------------------------------------------------------------------------------------------------ 6960  
 ------------------------------------------------------------------------------------------------------------------------ 7080  
 -----------------------------------------------------------

TTTTCTTTT

TTTTCTTTT  
Depth:19 (ZEBRAFISH)  
Ei-value:0.000, Pi-value:0.000  
Er-value:0.000, Pr-value:0.000  
MATCHES To TargetScan▶ miR-186-5p:AAAGAAU

----------------------------

CAGGTTTTGCTTT

CAGGTTTTGCTTT  
Depth:19 (ZEBRAFISH)  
Ei-value:0.000, Pi-value:0.000  
Er-value:0.000, Pr-value:0.000  
MATCHES To TargetScan▶ miR-330-3p.2:AAAGCAC▶ miR-490-3p:AACCUGG

----------- 7200  
 ----------------

AAAAAGCAAAA

AAAAAGCAAAA  
Depth:19 (ZEBRAFISH)  
Ei-value:0.000, Pi-value:0.000  
Er-value:0.000, Pr-value:0.000  
No matches to TargetScan

------------------------------------------------------------------                            7293
```

---

## >DOG (7477 bases)

```
 ------------------------------------------------------------------------------------------------------------------------ 120  
 ------------------------------------------------------------------------------------------------------------------------ 240  
 ------------------------------------------------------------------------------------------------------------------------ 360  
 ------------------------------------------------------------------------------------------------------------------------ 480  
 ------------------------------------------------------------------------------------------------------------------------ 600  
 ------------------------------------------------------------------------------------------------------------------------ 720  
 ------------------------------------------------------------------------------------------------------------------------ 840  
 ------------------------------------------------------------------------------------------------------------------------ 960  
 ------------------------------------------------------------------------------------------------------------------------ 1080  
 ------------------------------------------------------------------------------------------------------------------------ 1200  
 ------------------------------------------------------------------------------------------------------------------------ 1320  
 ------------------------------------------------------------------------------------------------------------------------ 1440  
 ------------------------------------------------------------------------------------------------------------------------ 1560  
 ------------------------------------------------------------------------------------------------------------------------ 1680  
 ------------------------------------------------------------------------------------------------------------------------ 1800  
 ------------------------------------------------------------------------------------------------------------------------ 1920  
 ------------------------------------------------------------------------------------------------------------------------ 2040  
 ------------------------------------------------------------------------------------------------------------------------ 2160  
 ------------------------------------------------------------------------------------------------------------------------ 2280  
 ------------------------------------------------------------------------------------------------------------------------ 2400  
 ------------------------------------------------------------------------------------------------------------------------ 2520  
 -------------------------------------------------------------------------------------------

TTTGGG

TTTGGG  
Depth:19 (ZEBRAFISH)  
Ei-value:0.000, Pi-value:0.000  
Er-value:0.000, Pr-value:0.000  
No matches to TargetScan

----------------------- 2640  
 ------------------------------------------------------------------------------------------------------------------------ 2760  
 ------------------------------------------------------------------------------------------------------------------------ 2880  
 ------------------------------------------------------------------------------------------------------------------------ 3000  
 ------------------------------------------------------------------------------------------------------------------------ 3120  
 ------------------------------------------------------------------------------------------------------------------------ 3240  
 ------------------------------------------------------------------------------------------------------------------------ 3360  
 ------------------------------------------------------------------------------------------------------------------------ 3480  
 ------------------------------------------------------------------------------------------------------------------------ 3600  
 ------------------------------

TTTTTCAG

TTTTTCAG  
Depth:19 (ZEBRAFISH)  
Ei-value:0.000, Pi-value:0.000  
Er-value:0.000, Pr-value:0.000  
No matches to TargetScan

---------------------------------------------------------------------------------- 3720  
 ------------------------------------------------------------------------------------------------------------------------ 3840  
 ------------------------------------------------------------------------------------------------------------------------ 3960  
 ------------------------------------------------------------------------------------------------------------------------ 4080  
 ---------------------------------------------------------------------------------------------------------------------

GAT

GATAAG  
Depth:19 (ZEBRAFISH)  
Ei-value:0.000, Pi-value:0.000  
Er-value:0.000, Pr-value:0.000  
No matches to TargetScan

 4200  


AAG

GATAAG  
Depth:19 (ZEBRAFISH)  
Ei-value:0.000, Pi-value:0.000  
Er-value:0.000, Pr-value:0.000  
No matches to TargetScan

--------------------------------------------------------------------------------------------------------------------- 4320  
 ------------------------------------------------------------------------------------------------------------------------ 4440  
 ------------------------------------------------------------------------------------------------------------------------ 4560  
 ------------------------------------------------------------------------------------------------------------------------ 4680  
 ------------------------------------------------------------------------------------------------------------------------ 4800  
 ------------------------------------------------------------------------------------------------------------------------ 4920  
 ------------------------------------------------------------------------------------------------------------------------ 5040  
 ------------------------------------------------------------------------------------------------------------------------ 5160  
 ------------------------------------------------------------------------------------------------------------------------ 5280  
 ------------------------------------------------------------------------------------------------------------------------ 5400  
 ------------------------------------------------------------------------------------------------------------------------ 5520  
 ------------------------------------------------------------------------------------------------------------------------ 5640  
 ------------------------------------------------------------------------------------------------------------------------ 5760  
 ------------------------------------------------------------------------------------------------------------------------ 5880  
 ------------------------------------------------------------------------------------------------------------------------ 6000  
 ------------------------------------------------------------------------------------------------------------------------ 6120  
 ------------------------------------------------------------------------------------------------------------------------ 6240  
 ------------------------------------------------------------------------------------------------------------------------ 6360  
 ------------------------------------------------------------------------------------------------------------------------ 6480  
 ------------------------------------------------------------------------------------------------------------------------ 6600  
 ------------------------------------------------------------------------------------------------------------------------ 6720  
 ------------------------------------------------------------------------------------------------------------------------ 6840  
 ------------------------------------------------------------------------------------------------------------------------ 6960  
 ---------------------------------------------------------------------------------------------------

TTTTCTTTT

TTTTCTTTT  
Depth:19 (ZEBRAFISH)  
Ei-value:0.000, Pi-value:0.000  
Er-value:0.000, Pr-value:0.000  
MATCHES To TargetScan▶ miR-186-5p:AAAGAAU

------------ 7080  
 --------------

CAGGTTTTGCTTT

CAGGTTTTGCTTT  
Depth:19 (ZEBRAFISH)  
Ei-value:0.000, Pi-value:0.000  
Er-value:0.000, Pr-value:0.000  
MATCHES To TargetScan▶ miR-330-3p.2:AAAGCAC▶ miR-490-3p:AACCUGG

----------------------

AAAAAGCAAAA

AAAAAGCAAAA  
Depth:19 (ZEBRAFISH)  
Ei-value:0.000, Pi-value:0.000  
Er-value:0.000, Pr-value:0.000  
No matches to TargetScan

------------------------------------------------------------ 7200  
 ------------------------------------------------------------------------------------------------------------------------ 7320  
 ------------------------------------------------------------------------------------------------------------------------ 7440  
 -------------------------------------                                                                                    7477
```

---

## >PIG (7817 bases)

```
 ------------------------------------------------------------------------------------------------------------------------ 120  
 ------------------------------------------------------------------------------------------------------------------------ 240  
 ------------------------------------------------------------------------------------------------------------------------ 360  
 ------------------------------------------------------------------------------------------------------------------------ 480  
 ------------------------------------------------------------------------------------------------------------------------ 600  
 ------------------------------------------------------------------------------------------------------------------------ 720  
 ------------------------------------------------------------------------------------------------------------------------ 840  
 ------------------------------------------------------------------------------------------------------------------------ 960  
 ------------------------------------------------------------------------------------------------------------------------ 1080  
 ------------------------------------------------------------------------------------------------------------------------ 1200  
 ------------------------------------------------------------------------------------------------------------------------ 1320  
 ------------------------------------------------------------------------------------------------------------------------ 1440  
 ------------------------------------------------------------------------------------------------------------------------ 1560  
 ------------------------------------------------------------------------------------------------------------------------ 1680  
 ------------------------------------------------------------------------------------------------------------------------ 1800  
 ------------------------------------------------------------------------------------------------------------------------ 1920  
 ------------------------------------------------------------------------------------------------------------------------ 2040  
 ------------------------------------------------------------------------------------------------------------------------ 2160  
 ------------------------------------------------------------------------------------------------------------------------ 2280  
 ------------------------------------------------------------------------------------------------------------------------ 2400  
 ------------------------------------------------------------------------------------------------------------------------ 2520  
 ------------

TTTGGG

TTTGGG  
Depth:19 (ZEBRAFISH)  
Ei-value:0.000, Pi-value:0.000  
Er-value:0.000, Pr-value:0.000  
No matches to TargetScan

------------------------------------------------------------------------------------------------------ 2640  
 ------------------------------------------------------------------------------------------------------------------------ 2760  
 ------------------------------------------------------------------------------------------------------------------------ 2880  
 ------------------------------------------------------------------------------------------------------------------------ 3000  
 ------------------------------------------------------------------------------------------------------------------------ 3120  
 ------------------------------------------------------------------------------------------------------------------------ 3240  
 ------------------------------------------------------------------------------------------------------------------------ 3360  
 ------------------------------------------------------------------------------------------------------------------------ 3480  
 ------------------------------------------------------------

TTTTTCAG

TTTTTCAG  
Depth:19 (ZEBRAFISH)  
Ei-value:0.000, Pi-value:0.000  
Er-value:0.000, Pr-value:0.000  
No matches to TargetScan

---------------------------------------------------- 3600  
 ------------------------------------------------------------------------------------------------------------------------ 3720  
 ------------------------------------------------------------------------------------------------------------------------ 3840  
 ------------------------------------------------------------------------------------------------------------------------ 3960  
 ------------------------------------------------------------------------------------------------------------------------ 4080  
 -------------------------------------

GATAAG

GATAAG  
Depth:19 (ZEBRAFISH)  
Ei-value:0.000, Pi-value:0.000  
Er-value:0.000, Pr-value:0.000  
No matches to TargetScan

----------------------------------------------------------------------------- 4200  
 ------------------------------------------------------------------------------------------------------------------------ 4320  
 ------------------------------------------------------------------------------------------------------------------------ 4440  
 ------------------------------------------------------------------------------------------------------------------------ 4560  
 ------------------------------------------------------------------------------------------------------------------------ 4680  
 ------------------------------------------------------------------------------------------------------------------------ 4800  
 ------------------------------------------------------------------------------------------------------------------------ 4920  
 ------------------------------------------------------------------------------------------------------------------------ 5040  
 ------------------------------------------------------------------------------------------------------------------------ 5160  
 ------------------------------------------------------------------------------------------------------------------------ 5280  
 ------------------------------------------------------------------------------------------------------------------------ 5400  
 ------------------------------------------------------------------------------------------------------------------------ 5520  
 ------------------------------------------------------------------------------------------------------------------------ 5640  
 ------------------------------------------------------------------------------------------------------------------------ 5760  
 ------------------------------------------------------------------------------------------------------------------------ 5880  
 ------------------------------------------------------------------------------------------------------------------------ 6000  
 ------------------------------------------------------------------------------------------------------------------------ 6120  
 ------------------------------------------------------------------------------------------------------------------------ 6240  
 ------------------------------------------------------------------------------------------------------------------------ 6360  
 ------------------------------------------------------------------------------------------------------------------------ 6480  
 ------------------------------------------------------------------------------------------------------------------------ 6600  
 ------------------------------------------------------------------------------------------------------------------------ 6720  
 ------------------------------------------------------------------------------------------------------------------------ 6840  
 ------------------------------------------------------------------------------------------------------------------------ 6960  
 -----------

TTTTCTTTT

TTTTCTTTT  
Depth:19 (ZEBRAFISH)  
Ei-value:0.000, Pi-value:0.000  
Er-value:0.000, Pr-value:0.000  
MATCHES To TargetScan▶ miR-186-5p:AAAGAAU

----------------------------

CAGGTTTTGCTTT

CAGGTTTTGCTTT  
Depth:19 (ZEBRAFISH)  
Ei-value:0.000, Pi-value:0.000  
Er-value:0.000, Pr-value:0.000  
MATCHES To TargetScan▶ miR-330-3p.2:AAAGCAC▶ miR-490-3p:AACCUGG

------------------------

AAAAAGCAAAA

AAAAAGCAAAA  
Depth:19 (ZEBRAFISH)  
Ei-value:0.000, Pi-value:0.000  
Er-value:0.000, Pr-value:0.000  
No matches to TargetScan

------------------------ 7080  
 ------------------------------------------------------------------------------------------------------------------------ 7200  
 ------------------------------------------------------------------------------------------------------------------------ 7320  
 ------------------------------------------------------------------------------------------------------------------------ 7440  
 ------------------------------------------------------------------------------------------------------------------------ 7560  
 ------------------------------------------------------------------------------------------------------------------------ 7680  
 ------------------------------------------------------------------------------------------------------------------------ 7800  
 -----------------                                                                                                        7817
```

---

## >COW (7147 bases)

```
 ------------------------------------------------------------------------------------------------------------------------ 120  
 ------------------------------------------------------------------------------------------------------------------------ 240  
 ------------------------------------------------------------------------------------------------------------------------ 360  
 ------------------------------------------------------------------------------------------------------------------------ 480  
 ------------------------------------------------------------------------------------------------------------------------ 600  
 ------------------------------------------------------------------------------------------------------------------------ 720  
 ------------------------------------------------------------------------------------------------------------------------ 840  
 ------------------------------------------------------------------------------------------------------------------------ 960  
 ------------------------------------------------------------------------------------------------------------------------ 1080  
 ------------------------------------------------------------------------------------------------------------------------ 1200  
 ------------------------------------------------------------------------------------------------------------------------ 1320  
 ------------------------------------------------------------------------------------------------------------------------ 1440  
 ------------------------------------------------------------------------------------------------------------------------ 1560  
 ------------------------------------------------------------------------------------------------------------------------ 1680  
 ------------------------------------------------------------------------------------------------------------------------ 1800  
 ------------------------------------------------------------------------------------------------------------------------ 1920  
 ------------------------------------------------------------------------------------------------------------------------ 2040  
 ------------------------------------------------------------------------------------------------------------------------ 2160  
 ------------------------------------------------------------------------------------------------------------------------ 2280  
 ------------------------------------------------------------------------------------------------------------------------ 2400  
 ----------------

TTTGGG

TTTGGG  
Depth:19 (ZEBRAFISH)  
Ei-value:0.000, Pi-value:0.000  
Er-value:0.000, Pr-value:0.000  
No matches to TargetScan

-------------------------------------------------------------------------------------------------- 2520  
 ------------------------------------------------------------------------------------------------------------------------ 2640  
 ------------------------------------------------------------------------------------------------------------------------ 2760  
 ------------------------------------------------------------------------------------------------------------------------ 2880  
 ------------------------------------------------------------------------------------------------------------------------ 3000  
 ------------------------------------------------------------------------------------------------------------------------ 3120  
 ------------------------------------------------------------------------------------------------------------------------ 3240  
 ------------------------------------------------------------------------------------------------------------------------ 3360  
 --------------------------------------------------------------------------

TTTTTCAG

TTTTTCAG  
Depth:19 (ZEBRAFISH)  
Ei-value:0.000, Pi-value:0.000  
Er-value:0.000, Pr-value:0.000  
No matches to TargetScan

-------------------------------------- 3480  
 ------------------------------------------------------------------------------------------------------------------------ 3600  
 ------------------------------------------------------------------------------------------------------------------------ 3720  
 ------------------------------------------------------------------------------------------------------------------------ 3840  
 ------------------------------------------------------------------------------------------------------------------------ 3960  
 -------------------------------------

GATAAG

GATAAG  
Depth:19 (ZEBRAFISH)  
Ei-value:0.000, Pi-value:0.000  
Er-value:0.000, Pr-value:0.000  
No matches to TargetScan

----------------------------------------------------------------------------- 4080  
 ------------------------------------------------------------------------------------------------------------------------ 4200  
 ------------------------------------------------------------------------------------------------------------------------ 4320  
 ------------------------------------------------------------------------------------------------------------------------ 4440  
 ------------------------------------------------------------------------------------------------------------------------ 4560  
 ------------------------------------------------------------------------------------------------------------------------ 4680  
 ------------------------------------------------------------------------------------------------------------------------ 4800  
 ------------------------------------------------------------------------------------------------------------------------ 4920  
 ------------------------------------------------------------------------------------------------------------------------ 5040  
 ------------------------------------------------------------------------------------------------------------------------ 5160  
 ------------------------------------------------------------------------------------------------------------------------ 5280  
 ------------------------------------------------------------------------------------------------------------------------ 5400  
 ------------------------------------------------------------------------------------------------------------------------ 5520  
 ------------------------------------------------------------------------------------------------------------------------ 5640  
 ------------------------------------------------------------------------------------------------------------------------ 5760  
 ------------------------------------------------------------------------------------------------------------------------ 5880  
 ------------------------------------------------------------------------------------------------------------------------ 6000  
 ------------------------------------------------------------------------------------------------------------------------ 6120  
 ------------------------------------------------------------------------------------------------------------------------ 6240  
 ------------------------------------------------------------------------------------------------------------------------ 6360  
 ------------------------------------------------------------------------------------------------------------------------ 6480  
 ------------------------------------------------------------------------------------------------------------------------ 6600  
 ------------------------------------------------------------------------------------------------------------------------ 6720  
 ------------------------------------------------------------------------------------------------------------------------ 6840  
 --------------------------------------------------------------

TTTTCTTTT

TTTTCTTTT  
Depth:19 (ZEBRAFISH)  
Ei-value:0.000, Pi-value:0.000  
Er-value:0.000, Pr-value:0.000  
MATCHES To TargetScan▶ miR-186-5p:AAAGAAU

------------------------------

CAGGTTTTGCTTT

CAGGTTTTGCTTT  
Depth:19 (ZEBRAFISH)  
Ei-value:0.000, Pi-value:0.000  
Er-value:0.000, Pr-value:0.000  
MATCHES To TargetScan▶ miR-330-3p.2:AAAGCAC▶ miR-490-3p:AACCUGG

------ 6960  
 -----------------

AAAAAGCAAAA

AAAAAGCAAAA  
Depth:19 (ZEBRAFISH)  
Ei-value:0.000, Pi-value:0.000  
Er-value:0.000, Pr-value:0.000  
No matches to TargetScan

-------------------------------------------------------------------------------------------- 7080  
 -------------------------------------------------------------------                                                      7147
```

---

## >MOUSE (6982 bases)

```
 ------------------------------------------------------------------------------------------------------------------------ 120  
 ------------------------------------------------------------------------------------------------------------------------ 240  
 ------------------------------------------------------------------------------------------------------------------------ 360  
 ------------------------------------------------------------------------------------------------------------------------ 480  
 ------------------------------------------------------------------------------------------------------------------------ 600  
 ------------------------------------------------------------------------------------------------------------------------ 720  
 ------------------------------------------------------------------------------------------------------------------------ 840  
 ------------------------------------------------------------------------------------------------------------------------ 960  
 ------------------------------------------------------------------------------------------------------------------------ 1080  
 ------------------------------------------------------------------------------------------------------------------------ 1200  
 ------------------------------------------------------------------------------------------------------------------------ 1320  
 ------------------------------------------------------------------------------------------------------------------------ 1440  
 ------------------------------------------------------------------------------------------------------------------------ 1560  
 ------------------------------------------------------------------------------------------------------------------------ 1680  
 ------------------------------------------------------------------------------------------------------------------------ 1800  
 ------------------------------------------------------------------------------------------------------------------------ 1920  
 ------------------------------------------------------------------------------------------------------------------------ 2040  
 ------------------------------------------------------------------------------------------------------------------------ 2160  
 ------------------------------------------------------------------------------------------------------------------------ 2280  
 -------------------------------------------

TTTGGG

TTTGGG  
Depth:19 (ZEBRAFISH)  
Ei-value:0.000, Pi-value:0.000  
Er-value:0.000, Pr-value:0.000  
No matches to TargetScan

----------------------------------------------------------------------- 2400  
 ------------------------------------------------------------------------------------------------------------------------ 2520  
 ------------------------------------------------------------------------------------------------------------------------ 2640  
 ------------------------------------------------------------------------------------------------------------------------ 2760  
 ------------------------------------------------------------------------------------------------------------------------ 2880  
 ------------------------------------------------------------------------------------------------------------------------ 3000  
 ------------------------------------------------------------------------------------------------------------------------ 3120  
 ------------------------------------------------------------------------------------------------------------------------ 3240  
 -------------------------------------------------------------------------------

TTTTTCAG

TTTTTCAG  
Depth:19 (ZEBRAFISH)  
Ei-value:0.000, Pi-value:0.000  
Er-value:0.000, Pr-value:0.000  
No matches to TargetScan

--------------------------------- 3360  
 ------------------------------------------------------------------------------------------------------------------------ 3480  
 ------------------------------------------------------------------------------------------------------------------------ 3600  
 ------------------------------------------------------------------------------------------------------------------------ 3720  
 ------------------------------------------------------------------------------------------------------------------------ 3840  
 -----

GATAAG

GATAAG  
Depth:19 (ZEBRAFISH)  
Ei-value:0.000, Pi-value:0.000  
Er-value:0.000, Pr-value:0.000  
No matches to TargetScan

------------------------------------------------------------------------------------------------------------- 3960  
 ------------------------------------------------------------------------------------------------------------------------ 4080  
 ------------------------------------------------------------------------------------------------------------------------ 4200  
 ------------------------------------------------------------------------------------------------------------------------ 4320  
 ------------------------------------------------------------------------------------------------------------------------ 4440  
 ------------------------------------------------------------------------------------------------------------------------ 4560  
 ------------------------------------------------------------------------------------------------------------------------ 4680  
 ------------------------------------------------------------------------------------------------------------------------ 4800  
 ------------------------------------------------------------------------------------------------------------------------ 4920  
 ------------------------------------------------------------------------------------------------------------------------ 5040  
 ------------------------------------------------------------------------------------------------------------------------ 5160  
 ------------------------------------------------------------------------------------------------------------------------ 5280  
 ------------------------------------------------------------------------------------------------------------------------ 5400  
 ------------------------------------------------------------------------------------------------------------------------ 5520  
 ------------------------------------------------------------------------------------------------------------------------ 5640  
 ------------------------------------------------------------------------------------------------------------------------ 5760  
 ------------------------------------------------------------------------------------------------------------------------ 5880  
 ------------------------------------------------------------------------------------------------------------------------ 6000  
 ------------------------------------------------------------------------------------------------------------------------ 6120  
 ------------------------------------------------------------------------------------------------------------------------ 6240  
 ------------------------------------------------------------------------------------------------------------------------ 6360  
 ------------------------------------------------------------------------------------------------------------------------ 6480  
 ------------------------------------------------------------------------------------------------------------------------ 6600  
 -----

TTTTCTTTT

TTTTCTTTT  
Depth:19 (ZEBRAFISH)  
Ei-value:0.000, Pi-value:0.000  
Er-value:0.000, Pr-value:0.000  
MATCHES To TargetScan▶ miR-186-5p:AAAGAAU

--------------------------

CAGGTTTTGCTTT

CAGGTTTTGCTTT  
Depth:19 (ZEBRAFISH)  
Ei-value:0.000, Pi-value:0.000  
Er-value:0.000, Pr-value:0.000  
MATCHES To TargetScan▶ miR-330-3p.2:AAAGCAC▶ miR-490-3p:AACCUGG

--------------------------

AAAAAGCAAAA

AAAAAGCAAAA  
Depth:19 (ZEBRAFISH)  
Ei-value:0.000, Pi-value:0.000  
Er-value:0.000, Pr-value:0.000  
No matches to TargetScan

------------------------------ 6720  
 ------------------------------------------------------------------------------------------------------------------------ 6840  
 ------------------------------------------------------------------------------------------------------------------------ 6960  
 ----------------------                                                                                                   6982
```

---

## >TURTLE (7179 bases)

```
 ------------------------------------------------------------------------------------------------------------------------ 120  
 ------------------------------------------------------------------------------------------------------------------------ 240  
 ------------------------------------------------------------------------------------------------------------------------ 360  
 ------------------------------------------------------------------------------------------------------------------------ 480  
 ------------------------------------------------------------------------------------------------------------------------ 600  
 ------------------------------------------------------------------------------------------------------------------------ 720  
 ------------------------------------------------------------------------------------------------------------------------ 840  
 ------------------------------------------------------------------------------------------------------------------------ 960  
 ------------------------------------------------------------------------------------------------------------------------ 1080  
 ------------------------------------------------------------------------------------------------------------------------ 1200  
 ----------------------------------------------------------------------

TTTGGG

TTTGGG  
Depth:19 (ZEBRAFISH)  
Ei-value:0.000, Pi-value:0.000  
Er-value:0.000, Pr-value:0.000  
No matches to TargetScan

-------------------------------------------- 1320  
 ------------------------------------------------------------------------------------------------------------------------ 1440  
 ------------------------------------------------------------------------------------------------------------------------ 1560  
 ------------------------------------------------------------------------------------------------------------------------ 1680  
 ------------------------------------------------------------------------------------------------------------------------ 1800  
 ------------------------------------------------------------------------------------------------------------------------ 1920  
 ------------------------------------------------------------------------------------------------------------------------ 2040  
 ------------------------------------------------------------------------------------------------------------------------ 2160  
 ------------------------------------------------------------------------------------------------------------------------ 2280  
 ------------------------------------------------------------------------------------------------------------------------ 2400  
 ------------------------------------------------------------------------------------------------------------------------ 2520  
 ------------------------------------------------------------------------------------------------------------------------ 2640  
 ------------------

TTTTTCAG

TTTTTCAG  
Depth:19 (ZEBRAFISH)  
Ei-value:0.000, Pi-value:0.000  
Er-value:0.000, Pr-value:0.000  
No matches to TargetScan

---------------------------------------------------------------------------------------------- 2760  
 ------------------------------------------------------------------------------------------------------------------------ 2880  
 ------------------------------------------------------------------------------------------------------------------------ 3000  
 ------------------------------------------------------------------------------------------------------------------------ 3120  
 ------------------------------------------------------------------------------------------------------------------------ 3240  
 --------------------------------------------------------------------------

GATAAG

GATAAG  
Depth:19 (ZEBRAFISH)  
Ei-value:0.000, Pi-value:0.000  
Er-value:0.000, Pr-value:0.000  
No matches to TargetScan

---------------------------------------- 3360  
 ------------------------------------------------------------------------------------------------------------------------ 3480  
 ------------------------------------------------------------------------------------------------------------------------ 3600  
 ------------------------------------------------------------------------------------------------------------------------ 3720  
 ------------------------------------------------------------------------------------------------------------------------ 3840  
 ------------------------------------------------------------------------------------------------------------------------ 3960  
 ------------------------------------------------------------------------------------------------------------------------ 4080  
 ------------------------------------------------------------------------------------------------------------------------ 4200  
 ------------------------------------------------------------------------------------------------------------------------ 4320  
 ------------------------------------------------------------------------------------------------------------------------ 4440  
 ------------------------------------------------------------------------------------------------------------------------ 4560  
 ------------------------------------------------------------------------------------------------------------------------ 4680  
 ------------------------------------------------------------------------------------------------------------------------ 4800  
 ------------------------------------------------------------------------------------------------------------------------ 4920  
 ------------------------------------------------------------------------------------------------------------------------ 5040  
 ------------------------------------------------------------------------------------------------------------------------ 5160  
 ------------------------------------------------------------------------------------------------------------------------ 5280  
 ------------------------------------------------------------------------------------------------------------------------ 5400  
 ------------------------------------------------------------------------------------------------------------------------ 5520  
 ------------------------------------------------------------------------------------------------------------------------ 5640  
 ------------------------------------------------------------------------------------------------------------------------ 5760  
 ------------------------------------------------------------------------------------------------------------------------ 5880  
 ------------------------------------------------------------------------------------------------------------------------ 6000  
 ------------------------------------------------------------------------------------------------------------------------ 6120  
 ------------------------------------------------------------------------------------------------------------------------ 6240  
 ------------------------------------------------------------------------------------------------------------------------ 6360  
 ------------------------------------------------------------------------------------------------------------------------ 6480  
 ------------------------------------------------------------------------------------------------------------------------ 6600  
 ------------------------------------------------------------------------------------------------------------------------ 6720  
 ------------

TTTTCTTTT

TTTTCTTTT  
Depth:19 (ZEBRAFISH)  
Ei-value:0.000, Pi-value:0.000  
Er-value:0.000, Pr-value:0.000  
MATCHES To TargetScan▶ miR-186-5p:AAAGAAU

-----------------------

CAGGTTTTGCTTT

CAGGTTTTGCTTT  
Depth:19 (ZEBRAFISH)  
Ei-value:0.000, Pi-value:0.000  
Er-value:0.000, Pr-value:0.000  
MATCHES To TargetScan▶ miR-330-3p.2:AAAGCAC▶ miR-490-3p:AACCUGG

---------------------

AAAAAGCAAAA

AAAAAGCAAAA  
Depth:19 (ZEBRAFISH)  
Ei-value:0.000, Pi-value:0.000  
Er-value:0.000, Pr-value:0.000  
No matches to TargetScan

------------------------------- 6840  
 ------------------------------------------------------------------------------------------------------------------------ 6960  
 ------------------------------------------------------------------------------------------------------------------------ 7080  
 ---------------------------------------------------------------------------------------------------                      7179
```

---

## >ALLIGATOR (8607 bases)

```
 ------------------------------------------------------------------------------------------------------------------------ 120  
 ------------------------------------------------------------------------------------------------------------------------ 240  
 ------------------------------------------------------------------------------------------------------------------------ 360  
 ------------------------------------------------------------------------------------------------------------------------ 480  
 ------------------------------------------------------------------------------------------------------------------------ 600  
 ------------------------------------------------------------------------------------------------------------------------ 720  
 ------------------------------------------------------------------------------------------------------------------------ 840  
 ------------------------------------------------------------------------------------------------------------------------ 960  
 ------------------------------------------------------------------------------------------------------------------------ 1080  
 ------------------------------------------------------------------------------------------------------------------------ 1200  
 ------------------------------------------------------------------------------------------------------------------------ 1320  
 ------------------------------------------------------------------------------------------------------------------------ 1440  
 ------------------------------------------------------------------------------------------------------------------------ 1560  
 ------------------------------------------------------------------------------------------------------------------------ 1680  
 ------------------------------------------------------------------------------------------------------------------------ 1800  
 ------------------------------------------------------------------------------------------------------------------------ 1920  
 ------------------------------------------------------------------------------------------------------------------------ 2040  
 ------------------------------------------------------------------------------------------------------------------------ 2160  
 ------------------------------------------------------------------------------------------------------------------------ 2280  
 ------------------------------------------------------------------------------------------------------------------------ 2400  
 ------------------------------------------------------------------------------------------------------------------------ 2520  
 ----------------

TTTGGG

TTTGGG  
Depth:19 (ZEBRAFISH)  
Ei-value:0.000, Pi-value:0.000  
Er-value:0.000, Pr-value:0.000  
No matches to TargetScan

-------------------------------------------------------------------------------------------------- 2640  
 ------------------------------------------------------------------------------------------------------------------------ 2760  
 ------------------------------------------------------------------------------------------------------------------------ 2880  
 ------------------------------------------------------------------------------------------------------------------------ 3000  
 ------------------------------------------------------------------------------------------------------------------------ 3120  
 ------------------------------------------------------------------------------------------------------------------------ 3240  
 ------------------------------------------------------------------------------------------------------------------------ 3360  
 ------------------------------------------------------------------------------------------------------------------------ 3480  
 ------------------------------------------------------------------------------------------------------------------------ 3600  
 ------------------------------------------------------------------------------------------------------------------------ 3720  
 ------------------------------------------------------------------------------------------------------------------------ 3840  
 ------------------------

TTTTTCAG

TTTTTCAG  
Depth:19 (ZEBRAFISH)  
Ei-value:0.000, Pi-value:0.000  
Er-value:0.000, Pr-value:0.000  
No matches to TargetScan

---------------------------------------------------------------------------------------- 3960  
 ------------------------------------------------------------------------------------------------------------------------ 4080  
 ------------------------------------------------------------------------------------------------------------------------ 4200  
 ------------------------------------------------------------------------------------------------------------------------ 4320  
 ------------------------------------------------------------------------------------------------------------------------ 4440  
 --------------------------------------------------------------------------------

GATAAG

GATAAG  
Depth:19 (ZEBRAFISH)  
Ei-value:0.000, Pi-value:0.000  
Er-value:0.000, Pr-value:0.000  
No matches to TargetScan

---------------------------------- 4560  
 ------------------------------------------------------------------------------------------------------------------------ 4680  
 ------------------------------------------------------------------------------------------------------------------------ 4800  
 ------------------------------------------------------------------------------------------------------------------------ 4920  
 ------------------------------------------------------------------------------------------------------------------------ 5040  
 ------------------------------------------------------------------------------------------------------------------------ 5160  
 ------------------------------------------------------------------------------------------------------------------------ 5280  
 ------------------------------------------------------------------------------------------------------------------------ 5400  
 ------------------------------------------------------------------------------------------------------------------------ 5520  
 ------------------------------------------------------------------------------------------------------------------------ 5640  
 ------------------------------------------------------------------------------------------------------------------------ 5760  
 ------------------------------------------------------------------------------------------------------------------------ 5880  
 ------------------------------------------------------------------------------------------------------------------------ 6000  
 ------------------------------------------------------------------------------------------------------------------------ 6120  
 ------------------------------------------------------------------------------------------------------------------------ 6240  
 ------------------------------------------------------------------------------------------------------------------------ 6360  
 ------------------------------------------------------------------------------------------------------------------------ 6480  
 ------------------------------------------------------------------------------------------------------------------------ 6600  
 ------------------------------------------------------------------------------------------------------------------------ 6720  
 ------------------------------------------------------------------------------------------------------------------------ 6840  
 ------------------------------------------------------------------------------------------------------------------------ 6960  
 ------------------------------------------------------------------------------------------------------------------------ 7080  
 ------------------------------------------------------------------------------------------------------------------------ 7200  
 ------------------------------------------------------------------------------------------------------------------------ 7320  
 ------------------------------------------------------------------------------------------------------------------------ 7440  
 ------------------------------------------------------------------------------------------------------------------------ 7560  
 ------------------------------------------------------------------------------------------------------------------------ 7680  
 ------------------------------------------------------------------------------------------------------------------------ 7800  
 ------------------------------------------------------------------------------------------------------------------------ 7920  
 ------------------------------------------------------------------------------------

TTTTCTTTT

TTTTCTTTT  
Depth:19 (ZEBRAFISH)  
Ei-value:0.000, Pi-value:0.000  
Er-value:0.000, Pr-value:0.000  
MATCHES To TargetScan▶ miR-186-5p:AAAGAAU

-----------------------

CAGG

CAGGTTTTGCTTT  
Depth:19 (ZEBRAFISH)  
Ei-value:0.000, Pi-value:0.000  
Er-value:0.000, Pr-value:0.000  
MATCHES To TargetScan▶ miR-330-3p.2:AAAGCAC▶ miR-490-3p:AACCUGG

 8040  


TTTTGCTTT

CAGGTTTTGCTTT  
Depth:19 (ZEBRAFISH)  
Ei-value:0.000, Pi-value:0.000  
Er-value:0.000, Pr-value:0.000  
MATCHES To TargetScan▶ miR-330-3p.2:AAAGCAC▶ miR-490-3p:AACCUGG

--------------------------

AAAAAGCAAAA

AAAAAGCAAAA  
Depth:19 (ZEBRAFISH)  
Ei-value:0.000, Pi-value:0.000  
Er-value:0.000, Pr-value:0.000  
No matches to TargetScan

-------------------------------------------------------------------------- 8160  
 ------------------------------------------------------------------------------------------------------------------------ 8280  
 ------------------------------------------------------------------------------------------------------------------------ 8400  
 ------------------------------------------------------------------------------------------------------------------------ 8520  
 ---------------------------------------------------------------------------------------                                  8607
```

---

## >LIZARD (7365 bases)

```
 ------------------------------------------------------------------------------------------------------------------------ 120  
 ------------------------------------------------------------------------------------------------------------------------ 240  
 ------------------------------------------------------------------------------------------------------------------------ 360  
 ------------------------------------------------------------------------------------------------------------------------ 480  
 ------------------------------------------------------------------------------------------------------------------------ 600  
 ------------------------------------------------------------------------------------------------------------------------ 720  
 ------------------------------------------------------------------------------------------------------------------------ 840  
 ------------------------------------------------------------------------------------------------------------------------ 960  
 ------------------------------------------------------------------------------------------------------------------------ 1080  
 ------------------------------------------------------------------------------------------------------------------------ 1200  
 ------------------------------------------------------------------------------------------------------------------------ 1320  
 ------------------------------------------------------------------------------------------------------------------------ 1440  
 ------------------------------------------------------------------------------------------------------------------------ 1560  
 ------------------------------------------------------------------------------------------------------------------------ 1680  
 ------------------------------------------------------------------------------------------------------------------------ 1800  
 ------------------------------------------------------------------------------------------------------------------------ 1920  
 ------------------------------------------------------------------------------------------------------------------------ 2040  
 ------------------------------------------------------------------------------------------------------------------------ 2160  
 ------------------------------------------------------------------------------------------------------------------------ 2280  
 ------------------------------------------------------------------------

TTTGGG

TTTGGG  
Depth:19 (ZEBRAFISH)  
Ei-value:0.000, Pi-value:0.000  
Er-value:0.000, Pr-value:0.000  
No matches to TargetScan

------------------------------------------ 2400  
 ------------------------------------------------------------------------------------------------------------------------ 2520  
 ------------------------------------------------------------------------------------------------------------------------ 2640  
 ------------------------------------------------------------------------------------------------------------------------ 2760  
 ------------------------------------------------------------------------------------------------------------------------ 2880  
 ------------------------------------------------------------------------------------------------------------------------ 3000  
 ------------------------------------------------------------------------------------------------------------------------ 3120  


TTTTTCAG

TTTTTCAG  
Depth:19 (ZEBRAFISH)  
Ei-value:0.000, Pi-value:0.000  
Er-value:0.000, Pr-value:0.000  
No matches to TargetScan

---------------------------------------------------------------------------------------------------------------- 3240  
 ------------------------------------------------------------------------------------------------------------------------ 3360  
 ------------------------------------------------------------------------------------------------------------------------ 3480  
 ------------------------------------------------------------------------------------------------------------------------ 3600  
 --------------------------------------------------------------------------------------------------------------------

GATA

GATAAG  
Depth:19 (ZEBRAFISH)  
Ei-value:0.000, Pi-value:0.000  
Er-value:0.000, Pr-value:0.000  
No matches to TargetScan

 3720  


AG

GATAAG  
Depth:19 (ZEBRAFISH)  
Ei-value:0.000, Pi-value:0.000  
Er-value:0.000, Pr-value:0.000  
No matches to TargetScan

---------------------------------------------------------------------------------------------------------------------- 3840  
 ------------------------------------------------------------------------------------------------------------------------ 3960  
 ------------------------------------------------------------------------------------------------------------------------ 4080  
 ------------------------------------------------------------------------------------------------------------------------ 4200  
 ------------------------------------------------------------------------------------------------------------------------ 4320  
 ------------------------------------------------------------------------------------------------------------------------ 4440  
 ------------------------------------------------------------------------------------------------------------------------ 4560  
 ------------------------------------------------------------------------------------------------------------------------ 4680  
 ------------------------------------------------------------------------------------------------------------------------ 4800  
 ------------------------------------------------------------------------------------------------------------------------ 4920  
 ------------------------------------------------------------------------------------------------------------------------ 5040  
 ------------------------------------------------------------------------------------------------------------------------ 5160  
 ------------------------------------------------------------------------------------------------------------------------ 5280  
 ------------------------------------------------------------------------------------------------------------------------ 5400  
 ------------------------------------------------------------------------------------------------------------------------ 5520  
 ------------------------------------------------------------------------------------------------------------------------ 5640  
 ------------------------------------------------------------------------------------------------------------------------ 5760  
 ------------------------------------------------------------------------------------------------------------------------ 5880  
 ------------------------------------------------------------------------------------------------------------------------ 6000  
 ------------------------------------------------------------------------------------------------------------------------ 6120  
 ------------------------------------------------------------------------------------------------------------------------ 6240  
 ------------------------------------------------------------------------------------------------------------------------ 6360  
 ------------------------------------------------------------------------------------------------------------------------ 6480  
 ------------------------------------------------------------------------------------------------------------------------ 6600  
 ------------------------------------------------------------------------------------------------------------------------ 6720  
 ------------------------------------------------------------------------------------------------------------------------ 6840  
 ----------------------------------------------------------

TTTTCTTTT

TTTTCTTTT  
Depth:19 (ZEBRAFISH)  
Ei-value:0.000, Pi-value:0.000  
Er-value:0.000, Pr-value:0.000  
MATCHES To TargetScan▶ miR-186-5p:AAAGAAU

------------------------

CAGGTTTTGCTTT

CAGGTTTTGCTTT  
Depth:19 (ZEBRAFISH)  
Ei-value:0.000, Pi-value:0.000  
Er-value:0.000, Pr-value:0.000  
MATCHES To TargetScan▶ miR-330-3p.2:AAAGCAC▶ miR-490-3p:AACCUGG

---------------- 6960  
 -----

AAAAAGCAAAA

AAAAAGCAAAA  
Depth:19 (ZEBRAFISH)  
Ei-value:0.000, Pi-value:0.000  
Er-value:0.000, Pr-value:0.000  
No matches to TargetScan

-------------------------------------------------------------------------------------------------------- 7080  
 ------------------------------------------------------------------------------------------------------------------------ 7200  
 ------------------------------------------------------------------------------------------------------------------------ 7320  
 ---------------------------------------------                                                                            7365
```

---

## >SNAKE (6910 bases)

```
 ------------------------------------------------------------------------------------------------------------------------ 120  
 ------------------------------------------------------------------------------------------------------------------------ 240  
 ------------------------------------------------------------------------------------------------------------------------ 360  
 ------------------------------------------------------------------------------------------------------------------------ 480  
 ------------------------------------------------------------------------------------------------------------------------ 600  
 ------------------------------------------------------------------------------------------------------------------------ 720  
 ------------------------------------------------------------------------------------------------------------------------ 840  
 ------------------------------------------------------------------------------------------------------------------------ 960  
 ------------------------------------------------------------------------------------------------------------------------ 1080  
 ------------------------------------------------------------------------------------------------------------------------ 1200  
 -------------------------------------------------------------------------------------------

TTTGGG

TTTGGG  
Depth:19 (ZEBRAFISH)  
Ei-value:0.000, Pi-value:0.000  
Er-value:0.000, Pr-value:0.000  
No matches to TargetScan

----------------------- 1320  
 ------------------------------------------------------------------------------------------------------------------------ 1440  
 ------------------------------------------------------------------------------------------------------------------------ 1560  
 ------------------------------------------------------------------------------------------------------------------------ 1680  
 ------------------------------------------------------------------------------------------------------------------------ 1800  
 ------------------------------------------------------------------------------------------------------------------------ 1920  
 ----------------------------------------------------------------------------------------------------------

TTTGGG

TTTGGG  
Depth:19 (ZEBRAFISH)  
Ei-value:0.000, Pi-value:0.000  
Er-value:0.000, Pr-value:0.000  
No matches to TargetScan

-------- 2040  
 ------------------------------------------------------------------------------------------------------------------------ 2160  
 ------------------------------------------------------------------------------------------------------------------------ 2280  
 ------------------------------------------------------------------------------------------------------------------------ 2400  
 ------------------------------------------------------------------------------------------------------------------------ 2520  
 ------------------------------------------------------------------------------------------------------------------------ 2640  
 ------------------------------------------------------------------------------------------------------------------------ 2760  
 ------------------------------------------------------------------------------------------------------------------------ 2880  
 ------------------------------------------------------------------------------------------------------------------------ 3000  
 -----------------------------------------------------------------------------------------------------------------

TTTTTCA

TTTTTCAG  
Depth:19 (ZEBRAFISH)  
Ei-value:0.000, Pi-value:0.000  
Er-value:0.000, Pr-value:0.000  
No matches to TargetScan

 3120  


G

TTTTTCAG  
Depth:19 (ZEBRAFISH)  
Ei-value:0.000, Pi-value:0.000  
Er-value:0.000, Pr-value:0.000  
No matches to TargetScan

----------------------------------------------------------------------------------------------------------------------- 3240  
 ------------------------------------------------------------------------------------------------------------------------ 3360  
 ------------------------------------------------------------------------------------------------------------------------ 3480  
 ------------------------------------------------------------------------------------------------------------------------ 3600  
 -------------------------------------------------------------------------------

GATAAG

GATAAG  
Depth:19 (ZEBRAFISH)  
Ei-value:0.000, Pi-value:0.000  
Er-value:0.000, Pr-value:0.000  
No matches to TargetScan

----------------------------------- 3720  
 ------------------------------------------------------------------------------------------------------------------------ 3840  
 ------------------------------------------------------------------------------------------------------------------------ 3960  
 ------------------------------------------------------------------------------------------------------------------------ 4080  
 ------------------------------------------------------------------------------------------------------------------------ 4200  
 ------------------------------------------------------------------------------------------------------------------------ 4320  
 ------------------------------------------------------------------------------------------------------------------------ 4440  
 ------------------------------------------------------------------------------------------------------------------------ 4560  
 ------------------------------------------------------------------------------------------------------------------------ 4680  
 ------------------------------------------------------------------------------------------------------------------------ 4800  
 ------------------------------------------------------------------------------------------------------------------------ 4920  
 ------------------------------------------------------------------------------------------------------------------------ 5040  
 ------------------------------------------------------------------------------------------------------------------------ 5160  
 ------------------------------------------------------------------------------------------------------------------------ 5280  
 ------------------------------------------------------------------------------------------------------------------------ 5400  
 ------------------------------------------------------------------------------------------------------------------------ 5520  
 ------------------------------------------------------------------------------------------------------------------------ 5640  
 ------------------------------------------------------------------------------------------------------------------------ 5760  
 ------------------------------------------------------------------------------------------------------------------------ 5880  
 ------------------------------------------------------------------------------------------------------------------------ 6000  
 ------------------------------------------------------------------------------------------------------------------------ 6120  
 ------------------------------------------------------------------------------------------------------------------------ 6240  
 ------------------------------------------------------------------------------------------------------------------------ 6360  
 ------------------------------------------------------------------------------------------------------------------------ 6480  
 ------------------------------------------------------------------------------------------------------------------------ 6600  
 ------------------------------------------------------------------------------------------------------------------------ 6720  
 --

TTTTCTTTT

TTTTCTTTT  
Depth:19 (ZEBRAFISH)  
Ei-value:0.000, Pi-value:0.000  
Er-value:0.000, Pr-value:0.000  
MATCHES To TargetScan▶ miR-186-5p:AAAGAAU

-------------------------

CAGGTTTTGCTTT

CAGGTTTTGCTTT  
Depth:19 (ZEBRAFISH)  
Ei-value:0.000, Pi-value:0.000  
Er-value:0.000, Pr-value:0.000  
MATCHES To TargetScan▶ miR-330-3p.2:AAAGCAC▶ miR-490-3p:AACCUGG

-----------------------------

AAAAAGCAAAA

AAAAAGCAAAA  
Depth:19 (ZEBRAFISH)  
Ei-value:0.000, Pi-value:0.000  
Er-value:0.000, Pr-value:0.000  
No matches to TargetScan

------------------------------- 6840  
 ----------------------------------------------------------------------                                                   6910
```

---

## >X.TROPICALIS (12676 bases)

```
 ------------------------------------------------------------------------------------------------------------------------ 120  
 ------------------------------------------------------------------------------------------------------------------------ 240  
 ------------------------------------------------------------------------------------------------------------------------ 360  
 ------------------------------------------------------------------------------------------------------------------------ 480  
 ------------------------------------------------------------------------------------------------------------------------ 600  
 ------------------------------------------------------------------------------------------------------------------------ 720  
 ------------------------------------------------------------------------------------------------------------------------ 840  
 ------------------------------------------------------------------------------------------------------------------------ 960  
 ------------------------------------------------------------------------------------------------------------------------ 1080  
 ------------------------------------------------------------------------------------------------------------------------ 1200  
 ------------------------------------------------------------------------------------------------------------------------ 1320  
 ------------------------------------------------------------------------------------------------------------------------ 1440  
 ------------------------------------------------------------------------------------------------------------------------ 1560  
 ------------------------------------------------------------------------------------------------------------------------ 1680  
 ------------------------------------------------------------------------------------------------------------------------ 1800  
 ------------------------------------------------------------------------------------------------------------------------ 1920  
 ------------------------------------------------------------------------------------------------------------------------ 2040  
 ------------------------------------------------------------------------------------------------------------------------ 2160  
 ------------------------------------------------------------------------------------------------------------------------ 2280  
 ------------------

TTTGGG

TTTGGG  
Depth:19 (ZEBRAFISH)  
Ei-value:0.000, Pi-value:0.000  
Er-value:0.000, Pr-value:0.000  
No matches to TargetScan

------------------------------------------------------------------------------------------------ 2400  
 ------------------------------------------------------------------------------------------------------------------------ 2520  
 ------------------------------------------------------------------------------------------------------------------------ 2640  
 ------------------------------------------------------------------------------------------------------------------------ 2760  
 ------------------------------------------------------------------------------------------------------------------------ 2880  
 ------------------------------------------------------------------------------------------------------------------------ 3000  
 ------------------------------------------------------------------------------------------------------------------------ 3120  
 ------------------------------------------------------------------------------------------------------------------------ 3240  
 ------------------------------------------------------------------------------------------------------------------------ 3360  
 ------------------------------------------------------------------------------------------------------------------------ 3480  
 ------------------------------------------------------------------------------------------------------------------------ 3600  
 ------------------------------------------------------------------------------------------------------------------------ 3720  
 ------------------------------------------------------------------------------------------------------------------------ 3840  
 ------------------------------------------------------------------------------------------------------------------------ 3960  
 ------------------------------------------------------------------------------------------------------------------------ 4080  
 ------------------------------------------------------------------------------------------------------------------------ 4200  
 ------------------------------------------------------------------------------------------------------------------------ 4320  
 ------------------------------------------------------------------------------------------------------------------------ 4440  
 ------------------------------------------------------------------------------------------------------------------------ 4560  
 ------------------------------------------------------------------------------------------------------------------------ 4680  
 ------------------------------------------------------------------------------------------------------------------------ 4800  
 ------------------------------------------------------------------------------------------------------------------------ 4920  
 -------------------------------------------------------------------------------

TTTGGG

TTTGGG  
Depth:19 (ZEBRAFISH)  
Ei-value:0.000, Pi-value:0.000  
Er-value:0.000, Pr-value:0.000  
No matches to TargetScan

----------------------------------- 5040  
 ------------------------------------------------------------------------------------------

TTTGGG

TTTGGG  
Depth:19 (ZEBRAFISH)  
Ei-value:0.000, Pi-value:0.000  
Er-value:0.000, Pr-value:0.000  
No matches to TargetScan

------------------------ 5160  
 ------------------------------------------------------------------------------------------------------------------------ 5280  
 ------------------------------------------------------------------------------------------------------------------------ 5400  
 ------------------------------------------------------------------------------------------------------------------------ 5520  
 ------------------------------------------------------------------------------------------------------------------------ 5640  
 ------------------------------------------------------------------------------------------------------------------------ 5760  
 ------------------------------------------------------------------------------------------------------------------------ 5880  
 ------------------------------------------------------------------------------------------------------------------------ 6000  
 ------------------------------------------------------------------------------------------------------------------------ 6120  
 ------------------------------------------------------------------------------------------------------------------------ 6240  
 ------------------------------------------------------------------------------------------------------------------------ 6360  
 ------------------------------------------------------------------------------------------------------------------------ 6480  
 ------------------------------------------------------------------------------------------------------------------------ 6600  
 ------------------------------------------------------------------------------------------------------------------------ 6720  
 ------------------------------------------------------------------------------------------------------------------------ 6840  
 ------------------------------------------------------------------------------------------------------------------------ 6960  
 ------------------------------------------------------------------------------------------------------------------------ 7080  
 ------------------------------------------------------------------------------------------------------------------------ 7200  
 ------------------------------------------------------------------------------------------------------------------------ 7320  
 ------------------------------------------------------------------------------------------------------------------------ 7440  
 ------------------------------------------------------------------------------------------------------------------------ 7560  
 ------------------------------------------------------------------------------------------------------------------------ 7680  
 ------------------------------------------------------------------------------------------------------------------------ 7800  
 ------------------------------------------------------------------------------------------------------------------------ 7920  
 ------------------------------------------------------------------------------------------------------------------------ 8040  
 ------------------------------------------------------------------------------------------------------------------------ 8160  
 ------------------------------------------------------------------------------------------------------------------------ 8280  
 --------------------------------------------------------------

TTTTTCAG

TTTTTCAG  
Depth:19 (ZEBRAFISH)  
Ei-value:0.000, Pi-value:0.000  
Er-value:0.000, Pr-value:0.000  
No matches to TargetScan

-------------------------------------------------- 8400  
 ------------------------------------------------------------------------------------------------------------------------ 8520  
 ------------------------------------------------------------------------------------------------------------------------ 8640  
 ------------------------------------------------------------------------------------------------------------------------ 8760  
 ------------------------------------------------------------------------------------------------------------------------ 8880  
 ------------------------------------------------------------------------------------------------------------------------ 9000  
 ------------------------------------------------------------------------------------------------------------------------ 9120  
 ------------------------------------------------------------------------------------------------------------------------ 9240  
 ------------------------------------------------------------------------------------------------------------------

GATAAG

GATAAG  
Depth:19 (ZEBRAFISH)  
Ei-value:0.000, Pi-value:0.000  
Er-value:0.000, Pr-value:0.000  
No matches to TargetScan

 9360  


GATAAG  
Depth:19 (ZEBRAFISH)  
Ei-value:0.000, Pi-value:0.000  
Er-value:0.000, Pr-value:0.000  
No matches to TargetScan

------------------------------------------------------------------------------------------------------------------------ 9480  
 ------------------------------------------------------------------------------------------------------------------------ 9600  
 ------------------------------------------------------------------------------------------------------------------------ 9720  
 ------------------------------------------------------------------------------------------------------------------------ 9840  
 ------------------------------------------------------------------------------------------------------------------------ 9960  
 ------------------------------------------------------------------------------------------------------------------------ 10080  
 ------------------------------------------------------------------------------------------------------------------------ 10200  
 ------------------------------------------------------------------------------------------------------------------------ 10320  
 ------------------------------------------------------------------------------------------------------------------------ 10440  
 ------------------------------------------------------------------------------------------------------------------------ 10560  
 ------------------------------------------------------------------------------------------------------------------------ 10680  
 ------------------------------------------------------------------------------------------------------------------------ 10800  
 ------------------------------------------------------------------------------------------------------------------------ 10920  
 ------------------------------------------------------------------------------------------------------------------------ 11040  
 ------------------------------------------------------------------------------------------------------------------------ 11160  
 ------------------------------------------------------------------------------------------------------------------------ 11280  
 ------------------------------------------------------------------------------------------------------------------------ 11400  
 ------------------------------------------------------------------------------------------------------------------------ 11520  
 ------------------------------------------------------------------------------------------------------------------------ 11640  
 ------------------------------------------------------------------------------------------------------------------------ 11760  
 ------------------------------------------------------------------------------------------------------------------------ 11880  
 ------------------------------------------------------------------------------------------------------------------------ 12000  
 ------------------------------------------------------------------------------------------------------------------------ 12120  
 ------------------------------------------------------------------------------------------------------------------------ 12240  
 --------------------------------------------------------------------------------------

TTTTCTTTT

TTTTCTTTT  
Depth:19 (ZEBRAFISH)  
Ei-value:0.000, Pi-value:0.000  
Er-value:0.000, Pr-value:0.000  
MATCHES To TargetScan▶ miR-186-5p:AAAGAAU

------------------------- 12360  
 -

CAGGTTTTGCTTT

CAGGTTTTGCTTT  
Depth:19 (ZEBRAFISH)  
Ei-value:0.000, Pi-value:0.000  
Er-value:0.000, Pr-value:0.000  
MATCHES To TargetScan▶ miR-330-3p.2:AAAGCAC▶ miR-490-3p:AACCUGG

-------------------------------

AAAAAGCAAAA

AAAAAGCAAAA  
Depth:19 (ZEBRAFISH)  
Ei-value:0.000, Pi-value:0.000  
Er-value:0.000, Pr-value:0.000  
No matches to TargetScan

---------------------------------------------------------------- 12480  
 ------------------------------------------------------------------------------------------------------------------------ 12600  
 ----------------------------------------------------------------------------                                             12676
```

---

## >SHARK (7850 bases)

```
 ------------------------------------------------------------------------------------------------------------------------ 120  
 ------------------------------------------------------------------------------------------------------------------------ 240  
 ------------------------------------------------------------------------------------------------------------------------ 360  
 ------------------------------------------------------------------------------------------------------------------------ 480  
 ------------------------------------------------------------------------------------------------------------------------ 600  
 ------------------------------------------------------------------------------------------------------------------------ 720  
 ------------------------------------------------------------------------------------------------------------------------ 840  
 ------------------------------------------------------------------------------------------------------------------------ 960  
 ------------------------------------------------------------------------------------------------------------------------ 1080  
 ------------------------------------------------------------------------------------------------------------------------ 1200  
 ------------------------------------------------------------------------------------------------------------------------ 1320  
 --------------------------------------------------------------------------------------------

TTTGGG

TTTGGG  
Depth:19 (ZEBRAFISH)  
Ei-value:0.000, Pi-value:0.000  
Er-value:0.000, Pr-value:0.000  
No matches to TargetScan

---------------------- 1440  
 ------------------------------------------------------------------------------------------------------------------------ 1560  
 ------------------------------------------------------------------------------------------------------------------------ 1680  
 ------------------------------------------------------------------------------------------------------------------------ 1800  
 ------------------------------------------------------------------------------------------------------------------------ 1920  
 ------------------------------------------------------------------------------------------------------------------------ 2040  
 ------------------------------------------------------------------------------------------------------------------------ 2160  
 ------------------------------------------------------------------------------------------------------------------------ 2280  
 ------------------------------------------------------------------------------------------------------------------------ 2400  
 ---------------------------------

TTTGGG

TTTGGG  
Depth:19 (ZEBRAFISH)  
Ei-value:0.000, Pi-value:0.000  
Er-value:0.000, Pr-value:0.000  
No matches to TargetScan

--------------------------------------------------------------------------

TTTGGG

TTTGGG  
Depth:19 (ZEBRAFISH)  
Ei-value:0.000, Pi-value:0.000  
Er-value:0.000, Pr-value:0.000  
No matches to TargetScan

- 2520  
 -------------------------------------

TTTTTCAG

TTTTTCAG  
Depth:19 (ZEBRAFISH)  
Ei-value:0.000, Pi-value:0.000  
Er-value:0.000, Pr-value:0.000  
No matches to TargetScan

--------------------------------------------------------------------------- 2640  
 ------------------------------------------------------------------------------------------------------------------------ 2760  
 ------------------------------------------------------------------------------------------------------------------------ 2880  
 ------------------------------------------------------------------------------------------------------------------------ 3000  
 ------------------------------------------------------------------------------------------------------------------------ 3120  
 ------------------------------------------------------------------------------------------------------------------------ 3240  
 ------------------------------------------------------------------------------------------------------------------------ 3360  
 ------------------------------------------------------------------------------------------------------------------------ 3480  
 ------------------------------------------------------------------------------------------------------------------------ 3600  
 ------------------------------------------------------------------------------------------------------------------------ 3720  
 ------------------------------------------------------------------------------------------------------------------------ 3840  
 ------------------------------------------------------------------------------------------------------------------------ 3960  
 ------------------------------------------------------------------------------------------------------------------------ 4080  
 ------------------------------------------------------------------------------------------------------------------------ 4200  
 ------------------------------------------------------------------------------------------------------------------------ 4320  
 ------------------------------------------------------------------------------------------------------------------------ 4440  
 ------------------------------------------------------------------------------------------------------------------------ 4560  
 ------------------------------------------------------------------------------------------------------------------------ 4680  
 ------------------------------------------------------------------------------------------------------------------------ 4800  
 ------------------------------------------------------------------------------------------------------------------------ 4920  
 ------------------------------------------------------------------------------------------------------------------------ 5040  
 ------------------------------------------------------------------------------------------------------------------------ 5160  
 ------------------------------------------------------------------------------------------------------------------------ 5280  
 ------------------------------------------------------------------------------------------------------------------------ 5400  
 ------------------------------------------------------------------------------------------------------------------------ 5520  
 ------------------------------------------------------------------------------------------------------------------------ 5640  
 ------------------------------------------------------------------------------------------------------------------------ 5760  
 ------------------------------------------------------------------------------------------------------------------------ 5880  
 ------------------------------------------------------------------------------------------------------------------------ 6000  
 ------------------------------------------------------------------------------------------------------------------------ 6120  
 ------------------------------------------------------------------------------------------------------------------------ 6240  
 ------------------------------------------------------------------------------------------------------------------------ 6360  
 ------------------------------------------------------------------------------------------------------------------------ 6480  
 ------------------------------------------------------------------------------------------------------------------------ 6600  
 ------------------------------------------------------------------------------------------------------------------------ 6720  
 ------------------------------------------------------------------------------------------------------------------------ 6840  
 ------------------------------------------------------------------------------------------------------------------------ 6960  
 -------

GATAAG

GATAAG  
Depth:19 (ZEBRAFISH)  
Ei-value:0.000, Pi-value:0.000  
Er-value:0.000, Pr-value:0.000  
No matches to TargetScan

----------------------------------------------------------------------------------------------------------- 7080  
 ------------------------------------------------------------------------------------------------------------------------ 7200  
 ------------------------------------------------------------------------------------------------------------------------ 7320  
 ------------------------------------------------------------------------------------------------------------------------ 7440  
 -----------------------------------------------------

TTTTCTTTT

TTTTCTTTT  
Depth:19 (ZEBRAFISH)  
Ei-value:0.000, Pi-value:0.000  
Er-value:0.000, Pr-value:0.000  
MATCHES To TargetScan▶ miR-186-5p:AAAGAAU

-------------------------

CAGGTTTTGCTTT

CAGGTTTTGCTTT  
Depth:19 (ZEBRAFISH)  
Ei-value:0.000, Pi-value:0.000  
Er-value:0.000, Pr-value:0.000  
MATCHES To TargetScan▶ miR-330-3p.2:AAAGCAC▶ miR-490-3p:AACCUGG

-------------------- 7560  
 -------

AAAAAGCAAAA

AAAAAGCAAAA  
Depth:19 (ZEBRAFISH)  
Ei-value:0.000, Pi-value:0.000  
Er-value:0.000, Pr-value:0.000  
No matches to TargetScan

------------------------------------------------------------------------------------------------------ 7680  
 ------------------------------------------------------------------------------------------------------------------------ 7800  
 --------------------------------------------------                                                                       7850
```

---

## >OPOSSUM (5957 bases)

```
 ------------------------------------------------------------------------------------------------------------------------ 120  
 ------------------------------------------------------------------------------------------------------------------------ 240  
 ------------------------------------------------------------------------------------------------------------------------ 360  
 ------------------------------------------------------------------------------------------------------------------------ 480  
 ------------------------------------------------------------------------------------------------------------------------ 600  
 ------------------------------------------------------------------------------------------------------------------------ 720  
 ------------------------------------------------------------------------------------------------------------------------ 840  
 ------------------------------------------------------------------------------------------------------------------------ 960  
 ----------------------------------------------------------------------------------

TTTGGG

TTTGGG  
Depth:19 (ZEBRAFISH)  
Ei-value:0.000, Pi-value:0.000  
Er-value:0.000, Pr-value:0.000  
No matches to TargetScan

-------------------------------- 1080  
 ------------------------------------------------------------------------------------------------------------------------ 1200  
 ------------------------------------------------------------------------------------------------------------------------ 1320  
 -

TTTGGG

TTTGGG  
Depth:19 (ZEBRAFISH)  
Ei-value:0.000, Pi-value:0.000  
Er-value:0.000, Pr-value:0.000  
No matches to TargetScan

---------------------------------------------------------------------------------------------------------------

TT

TTTGGG  
Depth:19 (ZEBRAFISH)  
Ei-value:0.000, Pi-value:0.000  
Er-value:0.000, Pr-value:0.000  
No matches to TargetScan

 1440  


TGGG

TTTGGG  
Depth:19 (ZEBRAFISH)  
Ei-value:0.000, Pi-value:0.000  
Er-value:0.000, Pr-value:0.000  
No matches to TargetScan

-------------------------------------------------------------------------------------------------------------------- 1560  
 ------------------------------------------------------------------------------------------------------------------------ 1680  
 ------------------------------------------------------------------------------------------------------------------------ 1800  
 ------------------------------------------------------------------------------------------------------------------------ 1920  
 ------------------------------------------------------------------------------------------------------------------------ 2040  
 ------------------------------------------------------------------------------------------------------------------------ 2160  
 ------------------------------------------------------------------------------------------------------------------------ 2280  
 ------------------------------------------------------------------------------------------------------------------------ 2400  
 -------------------------------------------------------------------------------------------------------

TTTTTCAG

TTTTTCAG  
Depth:19 (ZEBRAFISH)  
Ei-value:0.000, Pi-value:0.000  
Er-value:0.000, Pr-value:0.000  
No matches to TargetScan

--------- 2520  
 ------------------------------------------------------------------------------------------------------------------------ 2640  
 ------------------------------------------------------------------------------------------------------------------------ 2760  
 ------------------------------------------------------------------------------------------------------------------------ 2880  
 ------------------------------------------------------------------------------------------------------------------------ 3000  
 ------------------------------------------------------------------------------------------------------------------------ 3120  
 ------------------------------------------------------------------------------------------------------------------------ 3240  
 ------------------------------------------------------------------------------------------------------------------------ 3360  
 ------------------------------------------------------------------------------------------------------------------------ 3480  
 ----

GATAAG

GATAAG  
Depth:19 (ZEBRAFISH)  
Ei-value:0.000, Pi-value:0.000  
Er-value:0.000, Pr-value:0.000  
No matches to TargetScan

-------------------------------------------------------------------------------------------------------------- 3600  
 ------------------------------------------------------------------------------------------------------------------------ 3720  
 ------------------------------------------------------------------------------------------------------------------------ 3840  
 ------------------------------------------------------------------------------------------------------------------------ 3960  
 ------------------------------------------------------------------------------------------------------------------------ 4080  
 ------------------------------------------------------------------------------------------------------------------------ 4200  
 ------------------------------------------------------------------------------------------------------------------------ 4320  
 ------------------------------------------------------------------------------------------------------------------------ 4440  
 ------------------------------------------------------------------------------------------------------------------------ 4560  
 ------------------------------------------------------------------------------------------------------------------------ 4680  
 ------------------------------------------------------------------------------------------------------------------------ 4800  
 ------------------------------------------------------------------------------------------------------------------------ 4920  
 ------------------------------------------------------------------------------------------------------------------------ 5040  
 ------------------------------------------------------------------------------------------------------------------------ 5160  
 ------------------------------------------------------------------------------------------------------------------------ 5280  
 ------------------------------------------------------------------------------------------------------------------------ 5400  
 ------------------------------------------------------------------------------------------------------------------------ 5520  
 ------------------------------------------------------------------------------------------------------------------------ 5640  
 ----------------------------------------

TTTTCTTTT

TTTTCTTTT  
Depth:19 (ZEBRAFISH)  
Ei-value:0.000, Pi-value:0.000  
Er-value:0.000, Pr-value:0.000  
MATCHES To TargetScan▶ miR-186-5p:AAAGAAU

--------------------------

CAGGTTTTGCTTT

CAGGTTTTGCTTT  
Depth:19 (ZEBRAFISH)  
Ei-value:0.000, Pi-value:0.000  
Er-value:0.000, Pr-value:0.000  
MATCHES To TargetScan▶ miR-330-3p.2:AAAGCAC▶ miR-490-3p:AACCUGG

------------------

AAAAAGCAAAA

AAAAAGCAAAA  
Depth:19 (ZEBRAFISH)  
Ei-value:0.000, Pi-value:0.000  
Er-value:0.000, Pr-value:0.000  
No matches to TargetScan

--- 5760  
 ------------------------------------------------------------------------------------------------------------------------ 5880  
 -----------------------------------------------------------------------------                                            5957
```

---

## >SPOTTEDGAR (7306 bases)

```
 ------------------------------------------------------------------------------------------------------------------------ 120  
 ------------------------------------------------------------------------------------------------------------------------ 240  
 ------------------------------------------------------------------------------------------------------------------------ 360  
 ------------------------------------------------------------------------------------------------------------------------ 480  
 ------------------------------------------------------------------------------------------------------------------------ 600  
 ------------------------------------------------------------------------------------------------------------------------ 720  
 ------------------------------------------------------------------------------------------------------------------------ 840  
 ------------------------------------------------------------------------------------------------------------------------ 960  
 ------------------------------------------------------------------------------------------------------------------------ 1080  
 ------------------------------------------------------------------------------------------------------------------------ 1200  
 ------------------------------------------------------------------------------------------------------------------------ 1320  
 ------------------------------------------------------------------------------------------------------------------------ 1440  
 ------------------------------------------------------------------------------------------------------------------------ 1560  
 ------------------------------------------------------------------------------------------------------------------------ 1680  
 ------------------------------------------------------------------------------------------------------------------------ 1800  
 ------------------------------------------------------------------------------------------------------------------------ 1920  
 ------------------------------------------------------------------------------------------------------------------------ 2040  
 ------------------------------------------------------------------------------------------------------------------------ 2160  
 ------------------------------------------------------------------------------------------------------------------------ 2280  
 ------------------------------------------------------------------------------------------------------------------------ 2400  
 ------------------------------------------------------------------------------------------------------------------------ 2520  
 ------------------------------------------------------------------------------------------------------------------------ 2640  
 ------------------------------------------------------------------------------------------------------------------------ 2760  
 ------------------------------------------------------------------------------------------------------------------------ 2880  
 ------------------------------------------------------------------------------------------------------------------------ 3000  
 ------------------------------------------------------------------------------------------------------------------------ 3120  
 ------------------------------------------------------------------------------------------------------------------------ 3240  
 ------------------------------------------------------------------------------------------------------------------------ 3360  
 ------------------------------------------------------------------------------------------------------------------------ 3480  
 ------------------------------------------------------------------------------------------------------------------------ 3600  
 -------------------------------------------

TTTGGG

TTTGGG  
Depth:19 (ZEBRAFISH)  
Ei-value:0.000, Pi-value:0.000  
Er-value:0.000, Pr-value:0.000  
No matches to TargetScan

-------------------------------

TTTTTCAG

TTTTTCAG  
Depth:19 (ZEBRAFISH)  
Ei-value:0.000, Pi-value:0.000  
Er-value:0.000, Pr-value:0.000  
No matches to TargetScan

-------------------------------- 3720  
 ----------------------------------------

GATAAG

GATAAG  
Depth:19 (ZEBRAFISH)  
Ei-value:0.000, Pi-value:0.000  
Er-value:0.000, Pr-value:0.000  
No matches to TargetScan

-------------------------------------------------------------------------- 3840  
 ------------------------------------------------------------------------------------------------------------------------ 3960  
 --------------------------------------------------------------------------------

GATAAG

GATAAG  
Depth:19 (ZEBRAFISH)  
Ei-value:0.000, Pi-value:0.000  
Er-value:0.000, Pr-value:0.000  
No matches to TargetScan

---------------------------------- 4080  
 ------------------------------------------------------------------------------------------------------------------------ 4200  
 ------------------------------------------------------------------------------------------------------------------------ 4320  
 ------------------------------------------------------------------------------------------------------------------------ 4440  
 ------------------------------------------------------------------------------------------------------------------------ 4560  
 ------------------------------------------------------------------------------------------------------------------------ 4680  
 ------------------------------------------------------------------------------------------------------------------------ 4800  
 ------------------------------------------------------------------------------------------------------------------------ 4920  
 ------------------------------------------------------------------------------------------------------------------------ 5040  
 ------------------------------------------------------------------------------------------------------------------------ 5160  
 ------------------------------------------------------------------------------------------------------------------------ 5280  
 ------------------------------------------------------------------------------------------------------------------------ 5400  
 ------------------------------------------------------------------------------------------------------------------------ 5520  
 ------------------------------------------------------------------------------------------------------------------------ 5640  
 ------------------------------------------------------------------------------------------------------------------------ 5760  
 ------------------------------------------------------------------------------------------------------------------------ 5880  
 ------------------------------------------------------------------------------------------------------------------------ 6000  
 ------------------------------------------------------------------------------------------------------------------------ 6120  
 ------------------------------------------------------------------------------------------------------------------------ 6240  
 ------------------------------------------------------------------------------------------------------------------------ 6360  
 ------------------------------------------------------------------------------------------------------------------------ 6480  
 ------------------------------------------------------------------------------------------------------------------------ 6600  
 ----------

TTTTCTTTT

TTTTCTTTT  
Depth:19 (ZEBRAFISH)  
Ei-value:0.000, Pi-value:0.000  
Er-value:0.000, Pr-value:0.000  
MATCHES To TargetScan▶ miR-186-5p:AAAGAAU

----------------------------------------------------------------------------------------------------- 6720  
 ------------------------------------------------------------------------------------------------------------------------ 6840  
 --------------------------------------------------------------------------

CAGGTTTTGCTTT

CAGGTTTTGCTTT  
Depth:19 (ZEBRAFISH)  
Ei-value:0.000, Pi-value:0.000  
Er-value:0.000, Pr-value:0.000  
MATCHES To TargetScan▶ miR-330-3p.2:AAAGCAC▶ miR-490-3p:AACCUGG

----------------------------

AAAAA

AAAAAGCAAAA  
Depth:19 (ZEBRAFISH)  
Ei-value:0.000, Pi-value:0.000  
Er-value:0.000, Pr-value:0.000  
No matches to TargetScan

 6960  


GCAAAA

AAAAAGCAAAA  
Depth:19 (ZEBRAFISH)  
Ei-value:0.000, Pi-value:0.000  
Er-value:0.000, Pr-value:0.000  
No matches to TargetScan

------------------------------------------------------------------------------------------------------------------ 7080  
 ------------------------------------------------------------------------------------------------------------------------ 7200  
 ----------------------------------------------------------------------------------------------------------               7306
```

---

## >FUGU (4992 bases)

```
 ------------------------------------------------------------------------------------------------------------------------ 120  
 ------------------------------------------------------------------------------------------------------------------------ 240  
 ------------------------------------------------------------------------------------------------------------------------ 360  
 ------------------------------------------------------------------------------------------------------------------------ 480  
 ------------------------------------------------------------------------------------------------------------------------ 600  
 ------------------------------------------------------------------------------------------------------------------------ 720  
 ------------------------------------------------------------------------------------------------------------------------ 840  
 ------------------------------------------------------------------------------------------------------------------------ 960  
 ------------------------------------------------------------------------------------------------------------------------ 1080  
 ------------------------------------------------------------------------------------------------------------------------ 1200  
 ------------------------------------------------------------------------------------------------------------------------ 1320  
 ------------------------------------------------------------------------------------------------------------------------ 1440  
 ------------------------------------------------------------------------------------------------------------------------ 1560  
 ------------------------------------------------------------------------------------------------------------------------ 1680  
 -----------------------

TTTGGG

TTTGGG  
Depth:19 (ZEBRAFISH)  
Ei-value:0.000, Pi-value:0.000  
Er-value:0.000, Pr-value:0.000  
No matches to TargetScan

------------------------------------------------------------------------------------------- 1800  
 ------------------------------------------------------------------------------------------------------------------------ 1920  
 ------------------------------------------------------------------------------------------------------------------------ 2040  
 ------------------------------------------------------------------------------------------------------------------------ 2160  
 ------------------------------------------------------------------------------------------------------------------------ 2280  
 ------------------------------------------------------------------------------------------------------------------------ 2400  
 -----------------------------------------------------------------------------------

TTTTTCAG

TTTTTCAG  
Depth:19 (ZEBRAFISH)  
Ei-value:0.000, Pi-value:0.000  
Er-value:0.000, Pr-value:0.000  
No matches to TargetScan

----------------------------- 2520  
 ------------------------------------------------------------------------------------------------------------------------ 2640  
 ------------------------------------------------------------------------------------------------------------------------ 2760  
 -----------------------------------------------

TTTTTCAG

TTTTTCAG  
Depth:19 (ZEBRAFISH)  
Ei-value:0.000, Pi-value:0.000  
Er-value:0.000, Pr-value:0.000  
No matches to TargetScan

----------------------------------------------------------------- 2880  
 -------

GATAAG

GATAAG  
Depth:19 (ZEBRAFISH)  
Ei-value:0.000, Pi-value:0.000  
Er-value:0.000, Pr-value:0.000  
No matches to TargetScan

----------------------------------------------------------------------------------------------------------- 3000  
 ---------------------------------------

GATAAG

GATAAG  
Depth:19 (ZEBRAFISH)  
Ei-value:0.000, Pi-value:0.000  
Er-value:0.000, Pr-value:0.000  
No matches to TargetScan

--------------------------------------------------------------------------- 3120  
 ------------------------------------------------------------------------------------------------------------------------ 3240  
 ------------------------------------------------------------------------------------------------------------------------ 3360  
 ------------------------------------------------------------------------------------------------------------------------ 3480  
 ------------------------------------------------------------------------------------------------------------------------ 3600  
 ------------------------------------------------------------------------------------------------------------------------ 3720  
 ------------------------------------------------------------------------------------------------------------------------ 3840  
 ------------------------------------------------------------------------------------------------------------------------ 3960  
 ------------------------------------------------------------------------------------------------------------------------ 4080  
 ------------------------------------------------------------------------------------------------------------------------ 4200  
 ------------------------------------------------------------------------------------------------------------------------ 4320  
 ------------------------------------------------------------------------------------------------------------------------ 4440  
 ------------------------------------------------------------------------------------------------------------------------ 4560  
 ------------------------------------------------------------------------------------------------------------------------ 4680  
 ---------------------------------------------------------------------------------------

TTTTCTTTT

TTTTCTTTT  
Depth:19 (ZEBRAFISH)  
Ei-value:0.000, Pi-value:0.000  
Er-value:0.000, Pr-value:0.000  
MATCHES To TargetScan▶ miR-186-5p:AAAGAAU

------------------------ 4800  
 -------

CAGGTTTTGCTTT

CAGGTTTTGCTTT  
Depth:19 (ZEBRAFISH)  
Ei-value:0.000, Pi-value:0.000  
Er-value:0.000, Pr-value:0.000  
MATCHES To TargetScan▶ miR-330-3p.2:AAAGCAC▶ miR-490-3p:AACCUGG

------------------

AAAAAGCAAAA

AAAAAGCAAAA  
Depth:19 (ZEBRAFISH)  
Ei-value:0.000, Pi-value:0.000  
Er-value:0.000, Pr-value:0.000  
No matches to TargetScan

----------------------------------------------------------------------- 4920  
 ------------------------------------------------------------------------                                                 4992
```

---

## >NILETILAPIA (6000 bases)

```
 ------------------------------------------------------------------------------------------------------------------------ 120  
 ------------------------------------------------------------------------------------------------------------------------ 240  
 ------------------------------------------------------------------------------------------------------------------------ 360  
 ------------------------------------------------------------------------------------------------------------------------ 480  
 ------------------------------------------------------------------------------------------------------------------------ 600  
 ------------------------------------------------------------------------------------------------------------------------ 720  
 ------------------------------------------------------------------------------------------------------------------------ 840  
 ------------------------------------------------------------------------------------------------------------------------ 960  
 ------------------------------------------------------------------------------------------------------------------------ 1080  
 ------------------------------------------------------------------------------------------------------------------------ 1200  
 ------------------------------------------------------------------------------------------------------------------------ 1320  
 ------------------------------------------------------------------------------------------------------------------------ 1440  
 ------------------------------------------------------------------------------------------------------------------------ 1560  
 ------------------------------------------------------------------------------------------------------------------------ 1680  
 ------------------------------------------------------------------------------------------------------------------------ 1800  
 ------------------------------------------------------------------------------------------------------------------------ 1920  
 ------------------------------------------------------------------------------------------------------------------------ 2040  
 ------------------------------------------------------------------------------------------------------------------------ 2160  
 ------------------------------------------------------------------------------------------------------------------------ 2280  
 ------------------------------------------------------------------------------------------------------------------------ 2400  
 ------------------------------------------------------------------------------------------------------------------------ 2520  
 ------------------------------------------------------------------------------------------------------------------------ 2640  
 ------------------------------------------------------------------------------------------------------------------------ 2760  
 ----------------------------------

TTTGGG

TTTGGG  
Depth:19 (ZEBRAFISH)  
Ei-value:0.000, Pi-value:0.000  
Er-value:0.000, Pr-value:0.000  
No matches to TargetScan

-------------------------------------------------------------------------------- 2880  
 ------------------------------------------------------------------------------------------------------------------------ 3000  
 ------------------------------------------------------------------------------------------------------------------------ 3120  
 ------------------------------------------------------------------------------------------------------------------------ 3240  
 ------------------------------------------------------------------------------------------------------------------------ 3360  
 ---------------------

TTTTTCAG

TTTTTCAG  
Depth:19 (ZEBRAFISH)  
Ei-value:0.000, Pi-value:0.000  
Er-value:0.000, Pr-value:0.000  
No matches to TargetScan

------------------------------------------------------------------------------------------- 3480  
 ------------------------------------------------------------------------------------------------------------------------ 3600  
 -----------------------------------------------------------------------------------------------------------------

TTTTTCA

TTTTTCAG  
Depth:19 (ZEBRAFISH)  
Ei-value:0.000, Pi-value:0.000  
Er-value:0.000, Pr-value:0.000  
No matches to TargetScan

 3720  


G

TTTTTCAG  
Depth:19 (ZEBRAFISH)  
Ei-value:0.000, Pi-value:0.000  
Er-value:0.000, Pr-value:0.000  
No matches to TargetScan

------------------------------------------------------------------------------------

GATAAG

GATAAG  
Depth:19 (ZEBRAFISH)  
Ei-value:0.000, Pi-value:0.000  
Er-value:0.000, Pr-value:0.000  
No matches to TargetScan

----------------------------- 3840  
 ------------------------------------------------------------------------------------------------------------------------ 3960  
 ------------------------------------------------------------------------------------------------------------------------ 4080  
 ------------------------------------------------------------------------------------------------------------------------ 4200  
 ------------------------------------------------------------------------------------------------------------------------ 4320  
 ------------------------------------------------------------------------------------------------------------------------ 4440  
 ------------------------------------------------------------------------------------------------------------------------ 4560  
 ------------------------------------------------------------------------------------------------------------------------ 4680  
 ------------------------------------------------------------------------------------------------------------------------ 4800  
 ------------------------------------------------------------------------------------------------------------------------ 4920  
 ------------------------------------------------------------------------------------------------------------------------ 5040  
 ------------------------------------------------------------------------------------------------------------------------ 5160  
 ------------------------------------------------------------------------------------------------------------------------ 5280  
 ------------------------------------------------------------------------------------------------------------------------ 5400  
 ------------------------------------------------------------------------------------------------------------------------ 5520  
 ------------------------------------------------------------------------------------------------------------------------ 5640  
 ------------------------------------------------------------------------------------------------------------------------ 5760  
 --------------------------------------------------------------------------

TTTTCTTTT

TTTTCTTTT  
Depth:19 (ZEBRAFISH)  
Ei-value:0.000, Pi-value:0.000  
Er-value:0.000, Pr-value:0.000  
MATCHES To TargetScan▶ miR-186-5p:AAAGAAU

----------------------------

CAGGTTTTG

CAGGTTTTGCTTT  
Depth:19 (ZEBRAFISH)  
Ei-value:0.000, Pi-value:0.000  
Er-value:0.000, Pr-value:0.000  
MATCHES To TargetScan▶ miR-330-3p.2:AAAGCAC▶ miR-490-3p:AACCUGG

 5880  


CTTT

CAGGTTTTGCTTT  
Depth:19 (ZEBRAFISH)  
Ei-value:0.000, Pi-value:0.000  
Er-value:0.000, Pr-value:0.000  
MATCHES To TargetScan▶ miR-330-3p.2:AAAGCAC▶ miR-490-3p:AACCUGG

-------------------

AAAAAGCAAAA

AAAAAGCAAAA  
Depth:19 (ZEBRAFISH)  
Ei-value:0.000, Pi-value:0.000  
Er-value:0.000, Pr-value:0.000  
No matches to TargetScan

-------------------------------------------------------------------------------------- 6000  
                                                                                                                          6000
```

---

## >STICKLEBACK (6660 bases)

```
 ---------------------------------------------------------------------

TTTGGG

TTTGGG  
Depth:19 (ZEBRAFISH)  
Ei-value:0.000, Pi-value:0.000  
Er-value:0.000, Pr-value:0.000  
No matches to TargetScan

--------------------------------------------- 120  
 ------------------------------------------------------------------------------------------------------------------------ 240  
 ------------------------------------------------------------------------------------------------------------------------ 360  
 ------------------------------------------------------------------------------------------------------------------------ 480  
 ------------------------------------------------------------------------------------------------------------------------ 600  
 ------------------------------------------------------------------------------------------------------------------------ 720  
 ------------------------------------------------------------------------------------------------------------------------ 840  
 ------------------------------------------------------------------------------------------------------------------------ 960  
 ------------------------------------------------------------------------------------------------------------------------ 1080  
 ------------------------------------------------------------------------------------------------------------------------ 1200  
 ------------------------------------------------------------------------------------------------------------------------ 1320  
 ------------------------------------------------------------------------------------------------------------------------ 1440  
 ------------------------------------------------------------------------------------------------------------------------ 1560  
 ------------------------------------------------------------------------------------------------------------------------ 1680  
 ------------------------------------------------------------------------------------------------------------------------ 1800  
 ------------------------------------------------------------------------------------------------------------------------ 1920  
 ------------------------------------------------------------------------------------------------------------------------ 2040  
 ------------------------------------------------------------------------------------------------------------------------ 2160  
 ------------------------------------------------------------------------------------------------------------------------ 2280  
 ------------------------------------------------------------------------------------------------------------------------ 2400  
 ------------------------------------------------------------------------------------------------------------------------ 2520  
 ------------------------------------------------------------------------------------------------------------------------ 2640  
 ------------------------------------------------------------------------------------------------------------------------ 2760  
 ------------------------------------------------------------------------------------------------------------------------ 2880  
 ------------------------------------------------------------------------------------------------------------------------ 3000  
 ------------------------------------------------------------------------------------------------------------------------ 3120  
 ------------------------------------------------------------------------------------------------------------------------ 3240  
 ------------------------------------------------------------------------------------------------------------------------ 3360  
 ------------------------------------------------------------------------------------------------------------------------ 3480  
 ------------------------------------------------------------------------------------------------------------------------ 3600  
 ------------------------------------------------------------------------------------------------------------------------ 3720  
 ------------------------------------------------------------------------------------------------------------------------ 3840  
 --------------

TTTGGG

TTTGGG  
Depth:19 (ZEBRAFISH)  
Ei-value:0.000, Pi-value:0.000  
Er-value:0.000, Pr-value:0.000  
No matches to TargetScan

-------------------------------

TTTTTCAG

TTTTTCAG  
Depth:19 (ZEBRAFISH)  
Ei-value:0.000, Pi-value:0.000  
Er-value:0.000, Pr-value:0.000  
No matches to TargetScan

------------------------------------------------------------- 3960  
 -----------------------

GATAAG

GATAAG  
Depth:19 (ZEBRAFISH)  
Ei-value:0.000, Pi-value:0.000  
Er-value:0.000, Pr-value:0.000  
No matches to TargetScan

------------------------------------------------------------------------------------------- 4080  
 ------------------------------------------------------------------------------------------------------------------------ 4200  
 ------------------------------------------------------------------------------------------------------------------------ 4320  
 ------------------------------------------------------------------------------------------------------------------------ 4440  
 ------------------------------------------------------------------------------------------------------------------------ 4560  
 ------------------------------------------------------------------------------------------------------------------------ 4680  
 ------------------------------------------------------------------------------------------------------------------------ 4800  
 ------------------------------------------------------------------------------------------------------------------------ 4920  
 ------------------------------------------------------------------------------------------------------------------------ 5040  
 ------------------------------------------------------------------------------------------------------------------------ 5160  
 ------------------------------------------------------------------------------------------------------------------------ 5280  
 ------------------------------------------------------------------------------------------------------------------------ 5400  
 ------------------------------------------------------------------------------------------------------------------------ 5520  
 ------------------------------------------------------------------------------------------------------------------------ 5640  
 ------------------------------------------------------------------------------------------------------------------------ 5760  
 ------------------------------------------------------------------------------------------------------------------------ 5880  
 ------------------------------------------------------------------------------------------------------------------------ 6000  
 ---------

TTTTCTTTT

TTTTCTTTT  
Depth:19 (ZEBRAFISH)  
Ei-value:0.000, Pi-value:0.000  
Er-value:0.000, Pr-value:0.000  
MATCHES To TargetScan▶ miR-186-5p:AAAGAAU

------------------------------------

CAGGTTTTGCTTT

CAGGTTTTGCTTT  
Depth:19 (ZEBRAFISH)  
Ei-value:0.000, Pi-value:0.000  
Er-value:0.000, Pr-value:0.000  
MATCHES To TargetScan▶ miR-330-3p.2:AAAGCAC▶ miR-490-3p:AACCUGG

------------------------

AAAAAGCAAAA

AAAAAGCAAAA  
Depth:19 (ZEBRAFISH)  
Ei-value:0.000, Pi-value:0.000  
Er-value:0.000, Pr-value:0.000  
No matches to TargetScan

------------------ 6120  
 ------------------------------------------------------------------------------------------------------------------------ 6240  
 ------------------------------------------------------------------------------------------------------------------------ 6360  
 ------------------------------------------------------------------------------------------------------------------------ 6480  
 ------------------------------------------------------------------------------------------------------------------------ 6600  
 ------------------------------------------------------------                                                             6660
```

---

## >MEDAKA (5654 bases)

```
 ------------------------------------------------------------------------------------------------------------------------ 120  
 ------------------------------------------------------------------------------------------------------------------------ 240  
 ------------------------------------------------------------------------------------------------------------------------ 360  
 ------------------------------------------------------------------------------------------------------------------------ 480  
 ------------------------------------------------------------------------------------------------------------------------ 600  
 ------------------------------------------------------------------------------------------------------------------------ 720  
 ------------------------------------------------------------------------------------------------------------------------ 840  
 ------------------------------------------------------------------------------------------------------------------------ 960  
 ------------------------------------------------------------------------------------------------------------------------ 1080  
 ------------------------------------------------------------------------------------------------------------------------ 1200  
 ------------------------------------------------------------------------------------------------------------------------ 1320  
 ------------------------------------------------------------------------------------------------------------------------ 1440  
 ------------------------------------------------------------------------------------------------------------------------ 1560  
 ----------------------------------------------------------------------------------------------------------

TTTGGG

TTTGGG  
Depth:19 (ZEBRAFISH)  
Ei-value:0.000, Pi-value:0.000  
Er-value:0.000, Pr-value:0.000  
No matches to TargetScan

-------- 1680  
 ------------------------------------------------------------------------------------------------------------------------ 1800  
 ------------------------------------------------------------------------------------------------------------------------ 1920  
 ------------------------------------------------------------------------------------------------------------------------ 2040  
 ------------------------------------------------------------------------------------------------------------------------ 2160  
 ------------------------------------------------------------------------------------------------------------------------ 2280  
 ------------------------------------------------------------------------------------------------------------------------ 2400  
 ------------------------------------------------------------------------------------------------------------------------ 2520  
 ------------------------------------------------------------------------------------------------------------------------ 2640  
 ------------------------------------------------------------------------------------------------------------------------ 2760  
 ------------------------------------------------------------------------------------------------------------------------ 2880  
 ------------------------------------------------------------------------------------------------------------------------ 3000  
 ---------------------------------------------------------------------------------------------------------------

TTTGGG

TTTGGG  
Depth:19 (ZEBRAFISH)  
Ei-value:0.000, Pi-value:0.000  
Er-value:0.000, Pr-value:0.000  
No matches to TargetScan

--- 3120  
 ------------------------

TTTTTCAG

TTTTTCAG  
Depth:19 (ZEBRAFISH)  
Ei-value:0.000, Pi-value:0.000  
Er-value:0.000, Pr-value:0.000  
No matches to TargetScan

--------------------------------------------------------------------------------

GATAAG

GATAAG  
Depth:19 (ZEBRAFISH)  
Ei-value:0.000, Pi-value:0.000  
Er-value:0.000, Pr-value:0.000  
No matches to TargetScan

-- 3240  
 ------------------------------------------------------------------------------------------------------------------------ 3360  
 ------------------------------------------------------------------------------------------------------------------------ 3480  
 ------------------------------------------------------------------------------------------------------------------------ 3600  
 ------------------------------------------------------------------------------------------------------------------------ 3720  
 ------------------------------------------------------------------------------------------------------------------------ 3840  
 ------------------------------------------------------------------------------------------------------------------------ 3960  
 ------------------------------------------------------------------------------------------------------------------------ 4080  
 ------------------------------------------------------------------------------------------------------------------------ 4200  
 ------------------------------------------------------------------------------------------------------------------------ 4320  
 ------------------------------------------------------------------------------------------------------------------------ 4440  
 ------------------------------------------------------------------------------------------------------------------------ 4560  
 ------------------------------------------------------------------------------------------------------------------------ 4680  
 ------------------------------------------------------------------------------------------------------------------------ 4800  
 ------------------------------------------------------------------------------------------------------------------------ 4920  
 ------------------------------------------------------------------------------------------------

TTTTCTTTT

TTTTCTTTT  
Depth:19 (ZEBRAFISH)  
Ei-value:0.000, Pi-value:0.000  
Er-value:0.000, Pr-value:0.000  
MATCHES To TargetScan▶ miR-186-5p:AAAGAAU

--------------- 5040  
 -----------

CAGGTTTTGCTTT

CAGGTTTTGCTTT  
Depth:19 (ZEBRAFISH)  
Ei-value:0.000, Pi-value:0.000  
Er-value:0.000, Pr-value:0.000  
MATCHES To TargetScan▶ miR-330-3p.2:AAAGCAC▶ miR-490-3p:AACCUGG

---------------------

AAAAAGCAAAA

AAAAAGCAAAA  
Depth:19 (ZEBRAFISH)  
Ei-value:0.000, Pi-value:0.000  
Er-value:0.000, Pr-value:0.000  
No matches to TargetScan

---------------------------------------------------------------- 5160  
 ------------------------------------------------------------------------------------------------------------------------ 5280  
 ------------------------------------------------------------------------------------------------------------------------ 5400  
 ------------------------------------------------------------------------------------------------------------------------ 5520  
 ------------------------------------------------------------------------------------------------------------------------ 5640  
 --------------                                                                                                           5654
```

---

## >ZEBRAFISH (7477 bases)

```
 ------------------------------------------------------------------------------------------------------------------------ 120  
 ------------------------------------------------------------------------------------------------------------------------ 240  
 ------------------------------------------------------------------------------------------------------------------------ 360  
 ------------------------------------------------------------------------------------------------------------------------ 480  
 ------------------------------------------------------------------------------------------------------------------------ 600  
 ------------------------------------------------------------------------------------------------------------------------ 720  
 ------------------------------------------------------------------------------------------------------------------------ 840  
 ------------------------------------------------------------------------------------------------------------------------ 960  
 ------------------------------------------------------------------------------------------------------------------------ 1080  
 ------------------------------------------------------------------------------------------------------------------------ 1200  
 ------------------------------------------------------------------------------------------------------------------------ 1320  
 ------------------------------------------------------------------------------------------------------------------------ 1440  
 ------------------------------------------------------------------------------------------------------------------------ 1560  
 ------------------------------------------------------------------------------------------------------------------------ 1680  
 ------------------------------------------------------------------------------------------------------------------------ 1800  
 ------------------------------------------------------------------------------------------------------------------------ 1920  
 --------------------------------------------------------

TTTGGG

TTTGGG  
Depth:19 (ZEBRAFISH)  
Ei-value:0.000, Pi-value:0.000  
Er-value:0.000, Pr-value:0.000  
No matches to TargetScan

---------------------------------------------------------- 2040  
 ------------------------------------------------------------------------------------------------------------------------ 2160  
 ------------------------------------------------------------------------------------------------------------------------ 2280  
 ------------------------------------------------------------------------------------------------------------------------ 2400  
 ------------------------------------------------------------------------------------------------------------------------ 2520  
 ------------------------------------------------------------------------------------------------------------------------ 2640  
 ------------------------------------------------------------------------------------------------------------------------ 2760  
 ------------------------------------------------------------------------------------------------------------------------ 2880  
 ------------------------------------------------------------------------------------------------------------------------ 3000  
 ------------------------------------------------------------------------------------------------------------------------ 3120  
 ------------------------------------------------------------------------------------------------------------------------ 3240  
 ------------------------------------------------------------------------------------------------------------------------ 3360  
 ------------------------------------------------------------------------------------------------------------------------ 3480  
 ------------------------------------------------------------------------------------------------------------------------ 3600  
 ------------------------------------------------------------------------------------------------------------------------ 3720  
 ------------------------------------------------------------------------------------------------------------------------ 3840  
 --------------------------------------------

TTTGGG

TTTGGG  
Depth:19 (ZEBRAFISH)  
Ei-value:0.000, Pi-value:0.000  
Er-value:0.000, Pr-value:0.000  
No matches to TargetScan

-

TTTGGG

TTTGGG  
Depth:19 (ZEBRAFISH)  
Ei-value:0.000, Pi-value:0.000  
Er-value:0.000, Pr-value:0.000  
No matches to TargetScan

--------------------------------------------------------------- 3960  
 ------------------------------------------------------------------------------------------------------------------------ 4080  
 ------------------------------------------------------------------------------------------------------------------------ 4200  
 ------------------------------------------------------------------------------------------------------------------------ 4320  
 ------------------------------------------------------------------------------------------------------------------------ 4440  
 ------------------------------------------------------------------------------------------------------------------------ 4560  
 ------------------------------------------------------------------------------------------------------------------------ 4680  
 ------------------------------------------------------------------------------------------------------------------------ 4800  
 ----------

TTTTTCAG

TTTTTCAG  
Depth:19 (ZEBRAFISH)  
Ei-value:0.000, Pi-value:0.000  
Er-value:0.000, Pr-value:0.000  
No matches to TargetScan

------------------------------------------------------------------------------------------------------ 4920  
 ------------------------------------------------------------------------------------------------------------------------ 5040  
 ------------------------------------------------------------------------------------------------------------------------ 5160  


TTTTTCAG

TTTTTCAG  
Depth:19 (ZEBRAFISH)  
Ei-value:0.000, Pi-value:0.000  
Er-value:0.000, Pr-value:0.000  
No matches to TargetScan

---------------------------------------------------------------------------------------------------------------- 5280  
 ------------------------------------------------------------------------------------------------------------------------ 5400  
 ------------------------------------------------------------------------------------------------------------------------ 5520  
 ------------------------------------------------------------------------------------------------------------------------ 5640  
 ------------------------------------------------------------------------------------------------------------------------ 5760  
 ------------------------------------------------------------------------------------------------------------------------ 5880  
 ------------------------------------------------------------------------------------------------------------------------ 6000  
 ------------------------------------------------------------------------------------------------------------------------ 6120  
 ------------------------------------------------------------------------------------------------------------------------ 6240  
 ------------------------------------------------------------------------------------------------------------------------ 6360  
 ------------------------------------------------------------------------------------------------------------------------ 6480  
 ------------------------------------------------------------------------------------------------------------------------ 6600  
 ------------------------------------------------------------------------------------------------------------------------ 6720  
 ------------------------------------------------------------------------------------------------------------------------ 6840  
 -----------------------------------

GATAAG

GATAAG  
Depth:19 (ZEBRAFISH)  
Ei-value:0.000, Pi-value:0.000  
Er-value:0.000, Pr-value:0.000  
No matches to TargetScan

------------------------------------------------------------------------------- 6960  
 ------------------------------------------------------------------------------------------------------------------------ 7080  
 ------------------------------------------------------------------------------------------------------------------------ 7200  
 ------------------------------------------------------------------------------------------------------------------------ 7320  
 -----------------------------------------------------------------------------

TTTTCTTTT

TTTTCTTTT  
Depth:19 (ZEBRAFISH)  
Ei-value:0.000, Pi-value:0.000  
Er-value:0.000, Pr-value:0.000  
MATCHES To TargetScan▶ miR-186-5p:AAAGAAU

--------------------------------

CA

CAGGTTTTGCTTT  
Depth:19 (ZEBRAFISH)  
Ei-value:0.000, Pi-value:0.000  
Er-value:0.000, Pr-value:0.000  
MATCHES To TargetScan▶ miR-330-3p.2:AAAGCAC▶ miR-490-3p:AACCUGG

 7440  


GGTTTTGCTTT

CAGGTTTTGCTTT  
Depth:19 (ZEBRAFISH)  
Ei-value:0.000, Pi-value:0.000  
Er-value:0.000, Pr-value:0.000  
MATCHES To TargetScan▶ miR-330-3p.2:AAAGCAC▶ miR-490-3p:AACCUGG

---------------

AAAAAGCAAAA

AAAAAGCAAAA  
Depth:19 (ZEBRAFISH)  
Ei-value:0.000, Pi-value:0.000  
Er-value:0.000, Pr-value:0.000  
No matches to TargetScan

7477
```

---
