# Supplementary material for: Uncovering deeply conserved motif combinations in rapidly evolving noncoding sequences
Source: Genome Biol. 2021 Jan 11;22:29. doi: 10.1186/s13059-020-02247-1 (PMC7798263; doi:10.1186/s13059-020-02247-1)
Supplement: Supplementary file 5 — Additional file 5. LncLOOM output results for MALAT1 sequences from 19 vertebrates. [file 13059_2020_2247_MOESM5_ESM.gz › AdditionalFile5/Html_Files/miRNA_Matches.html]

 miRNA Matches

# Matches to miRNA Families Retrieved from TargetScan

  

\*\*SEED defined as positions 2-7 of mature miRNA sequence  
\*\*MOTIF MATCHES that correspond to the reverse complement of miRNA SEEDS are displayed from 5`-3` relative to lincRNA sequence

  

| Seed Matches to the miRNA miR-411-3p | | | | | |
| --- | --- | --- | --- | --- | --- |
| Seed | Conservation | Species | Matches | | |
|  |  |  | Sequence | Motif | Type |
| AUGUAAC | Conserved | Human (Homo sapiens)  Mouse (Mus musculus)  Rat (Rattus norvegicus) | HUMAN | TGCTAAAATTTACATGTTGTG | 6mer |
|  |  |  | HUMAN | TGTGTGGGTTTCTCTCTCCCCTCCCTTGGTCTTAATTCTTACATGCAGGAACA | 6mer |
|  |  |  | HUMAN | AACAGGTGAACAAGCTTTTTCTGTATTTACAT | 7mer-A1 |
|  |  |  | HUMAN | TTTACAT | 7mer-A1 |
|  |  |  | MARMOSET | TGCTAAAATTTACATGTTGTG | 6mer |
|  |  |  | MARMOSET | TGTGTGGGTTTCTCTCTCCCCTCCCTTGGTCTTAATTCTTACATGCAGGAACA | 6mer |
|  |  |  | MARMOSET | AACAGGTGAACAAGCTTTTTCTGTATTTACAT | 6mer |
|  |  |  | MARMOSET | TTTACAT | 6mer |
|  |  |  | DOG | TTTACAT | 7mer-A1 |
|  |  |  | PIG | TTTACAT | 7mer-A1 |

  
  
  
  

| Seed Matches to the miRNA miR-501-3p/502-3p | | | | | |
| --- | --- | --- | --- | --- | --- |
| Seed | Conservation | Species | Matches | | |
|  |  |  | Sequence | Motif | Type |
| AUGCACC | Conserved | Human (Homo sapiens) | HUMAN | GAGCCACTGGGTGTACCAGTGCATT | 6mer |
|  |  |  | HUMAN | AGTGCATTGTTT | 6mer |
|  |  |  | HUMAN | AGTGCATT | 6mer |
|  |  |  | MARMOSET | GAGCCACTGGGTGTACCAGTGCATT | 6mer |
|  |  |  | MARMOSET | AGTGCATTGTTT | 6mer |
|  |  |  | MARMOSET | AGTGCATT | 6mer |
|  |  |  | DOG | AGTGCATT | 6mer |
|  |  |  | PIG | AGTGCATT | 6mer |
|  |  |  | COW | AGTGCATT | 6mer |
|  |  |  | MOUSE | AGTGCATT | 6mer |

  
  
  
  

| Seed Matches to the miRNA miR-875-5p | | | | | |
| --- | --- | --- | --- | --- | --- |
| Seed | Conservation | Species | Matches | | |
|  |  |  | Sequence | Motif | Type |
| AUACCUC | Conserved | Human (Homo sapiens)  Rhesus (Macaca mulatta)  Mouse (Mus musculus) | HUMAN | CAAGATGTTAAGGTATGCTTCAAAAA | 6mer |
|  |  |  | HUMAN | CAAGATGTTAAGGTATGCTTCAAAA | 6mer |
|  |  |  | HUMAN | AAGGTATGCTTCAAAA | 6mer |
|  |  |  | HUMAN | AAGGTATGCTT | 6mer |
|  |  |  | HUMAN | TTTCGTTTGCCTCAGACAGGTATCTCTTC | 6mer |
|  |  |  | MARMOSET | CAAGATGTTAAGGTATGCTTCAAAAA | 6mer |
|  |  |  | MARMOSET | CAAGATGTTAAGGTATGCTTCAAAA | 6mer |
|  |  |  | MARMOSET | AAGGTATGCTTCAAAA | 6mer |
|  |  |  | MARMOSET | AAGGTATGCTT | 6mer |
|  |  |  | MARMOSET | TTTCGTTTGCCTCAGACAGGTATCTCTTC | 6mer |
|  |  |  | DOG | CAAGATGTTAAGGTATGCTTCAAAA | 6mer |
|  |  |  | DOG | AAGGTATGCTTCAAAA | 6mer |
|  |  |  | DOG | AAGGTATGCTT | 6mer |
|  |  |  | PIG | AAGGTATGCTTCAAAA | 6mer |
|  |  |  | PIG | AAGGTATGCTT | 6mer |
|  |  |  | COW | AAGGTATGCTTCAAAA | 6mer |
|  |  |  | COW | AAGGTATGCTT | 6mer |
|  |  |  | MOUSE | AAGGTATGCTT | 6mer |

  
  
  
  

| Seed Matches to the miRNA miR-323-3p | | | | | |
| --- | --- | --- | --- | --- | --- |
| Seed | Conservation | Species | Matches | | |
|  |  |  | Sequence | Motif | Type |
| ACAUUAC | Conserved | Human (Homo sapiens)  Mouse (Mus musculus)  Rat (Rattus norvegicus) | HUMAN | GTAACGGAAGTAATTCAAGATCAAGAGTAATTACCAACTTAATGTTT | 6mer |
|  |  |  | HUMAN | AATAATGTGACTTCTTAAAAG | 6mer |
|  |  |  | HUMAN | TAATGTGA | 6mer |
|  |  |  | MARMOSET | GTAACGGAAGTAATTCAAGATCAAGAGTAATTACCAACTTAATGTTT | 6mer |
|  |  |  | MARMOSET | AATAATGTGACTTCTTAAAAG | 6mer |
|  |  |  | MARMOSET | TAATGTGA | 6mer |
|  |  |  | DOG | TAATGTGA | 7mer-m8 |

  
  
  
  

| Seed Matches to the miRNA miR-382-5p | | | | | |
| --- | --- | --- | --- | --- | --- |
| Seed | Conservation | Species | Matches | | |
|  |  |  | Sequence | Motif | Type |
| AAGUUGU | Conserved | Human (Homo sapiens)  Rhesus (Macaca mulatta)  Mouse (Mus musculus)  Rat (Rattus norvegicus) | HUMAN | GTAACGGAAGTAATTCAAGATCAAGAGTAATTACCAACTTAATGTTT | 7mer-A1 |
|  |  |  | HUMAN | TACCAACTTAA | 7mer-A1 |
|  |  |  | HUMAN | ACCAACTTA | 7mer-A1 |
|  |  |  | HUMAN | TCAACTTCCAAG | 6mer |
|  |  |  | HUMAN | TTTTCCCTAGCTTTTCCAGAAGCCTGTTAAAAGCAAGGTCTCCCCACAAGCAACTTCTCTGCCACATCGCCACCC | 6mer |
|  |  |  | HUMAN | CCAGTGACTAAAACCAACTTAAACCAGTAAGTGGAGAAATAACATGTT | 7mer-A1 |
|  |  |  | MARMOSET | GTAACGGAAGTAATTCAAGATCAAGAGTAATTACCAACTTAATGTTT | 7mer-A1 |
|  |  |  | MARMOSET | TACCAACTTAA | 7mer-A1 |
|  |  |  | MARMOSET | ACCAACTTA | 7mer-A1 |
|  |  |  | MARMOSET | TCAACTTCCAAG | 6mer |
|  |  |  | MARMOSET | TTTTCCCTAGCTTTTCCAGAAGCCTGTTAAAAGCAAGGTCTCCCCACAAGCAACTTCTCTGCCACATCGCCACCC | 6mer |
|  |  |  | MARMOSET | CCAGTGACTAAAACCAACTTAAACCAGTAAGTGGAGAAATAACATGTT | 7mer-A1 |
|  |  |  | DOG | TACCAACTTAA | 7mer-A1 |
|  |  |  | DOG | ACCAACTTA | 7mer-A1 |
|  |  |  | DOG | TCAACTTCCAAG | 6mer |
|  |  |  | PIG | ACCAACTTA | 7mer-A1 |
|  |  |  | COW | ACCAACTTA | 7mer-A1 |

  
  
  
  

| Seed Matches to the miRNA miR-218-5p | | | | | |
| --- | --- | --- | --- | --- | --- |
| Seed | Conservation | Species | Matches | | |
|  |  |  | Sequence | Motif | Type |
| UGUGCUU | Broadly Conserved | Human (Homo sapiens)  Chicken (Gallus gallus)  Rhesus (Macaca mulatta)  Mouse (Mus musculus)  Rat (Rattus norvegicus)  Opossum (Monodelphis domestica) | HUMAN | TGGAAGAGTATTCCCAGTTGAAGCTGAAAAGTACAGCACAGTGCAGCTTTGGTTCATATTCAGTCATCTCAGGAGAACTTCAGAAGAGCTTGAGTAGGCCAAATGTTGAAGTTAAGTTTTC | 6mer |
|  |  |  | HUMAN | GTACAGCACAGTGCAGCTTTGGTTCATA | 6mer |
|  |  |  | HUMAN | TACAGCACAGTGCAGCTTTGGTTCATA | 6mer |
|  |  |  | HUMAN | CTAGCACAGACCCTTCACCCCTCACCTCGATGCAGCC | 6mer |
|  |  |  | MARMOSET | TGGAAGAGTATTCCCAGTTGAAGCTGAAAAGTACAGCACAGTGCAGCTTTGGTTCATATTCAGTCATCTCAGGAGAACTTCAGAAGAGCTTGAGTAGGCCAAATGTTGAAGTTAAGTTTTC | 6mer |
|  |  |  | MARMOSET | GTACAGCACAGTGCAGCTTTGGTTCATA | 6mer |
|  |  |  | MARMOSET | TACAGCACAGTGCAGCTTTGGTTCATA | 6mer |
|  |  |  | MARMOSET | CTAGCACAGACCCTTCACCCCTCACCTCGATGCAGCC | 6mer |
|  |  |  | DOG | GTACAGCACAGTGCAGCTTTGGTTCATA | 6mer |
|  |  |  | DOG | TACAGCACAGTGCAGCTTTGGTTCATA | 6mer |
|  |  |  | PIG | TACAGCACAGTGCAGCTTTGGTTCATA | 6mer |

  
  
  
  

| Seed Matches to the miRNA miR-505-3p.2 | | | | | |
| --- | --- | --- | --- | --- | --- |
| Seed | Conservation | Species | Matches | | |
|  |  |  | Sequence | Motif | Type |
| UCAACAC | Conserved | Human (Homo sapiens)  Mouse (Mus musculus) | HUMAN | TGGAAGAGTATTCCCAGTTGAAGCTGAAAAGTACAGCACAGTGCAGCTTTGGTTCATATTCAGTCATCTCAGGAGAACTTCAGAAGAGCTTGAGTAGGCCAAATGTTGAAGTTAAGTTTTC | 7mer-A1 |
|  |  |  | HUMAN | ATGTTGAAGTTAAGTTTTC | 7mer-A1 |
|  |  |  | HUMAN | TAACTGATTAAGAATTGTGATAGTTCAGCTTGAATGTCTCTTAGAGGGTGGGCTTTTGTTGATGAGGGAGGGGAAACTTTTTTTTT | 6mer |
|  |  |  | HUMAN | ATGCAGTACTGTTCTGATCCCGCTGCTATTAGAATGCATTGTGAAACGACTGGAGTATGATTAAAAGTTGTGTTCCCCAATGCTTGGAGTAGTGATTGTTGAAGGAAAAAA | 7mer-A1 |
|  |  |  | HUMAN | CCCCAATGCTTGGAGTAGTGATTGTTGAAGGAAA | 7mer-A1 |
|  |  |  | HUMAN | TCCAGCTGAGTGATAAAGGCTGAGTGTTGAGGAAATTTCTGCAGTTTTAAGCAGTCGT | 7mer-m8 |
|  |  |  | HUMAN | TCCAGCTGAGTGATAAAGGCTGAGTGTTGAGGAAATTTCTGCAG | 7mer-m8 |
|  |  |  | HUMAN | AAAGGCTGAGTGTTGAGGAAATTTCTGCAG | 7mer-m8 |
|  |  |  | HUMAN | AGGCTGAGTGTTGAGGAAAT | 7mer-m8 |
|  |  |  | MARMOSET | TGGAAGAGTATTCCCAGTTGAAGCTGAAAAGTACAGCACAGTGCAGCTTTGGTTCATATTCAGTCATCTCAGGAGAACTTCAGAAGAGCTTGAGTAGGCCAAATGTTGAAGTTAAGTTTTC | 7mer-A1 |
|  |  |  | MARMOSET | ATGTTGAAGTTAAGTTTTC | 7mer-A1 |
|  |  |  | MARMOSET | TAACTGATTAAGAATTGTGATAGTTCAGCTTGAATGTCTCTTAGAGGGTGGGCTTTTGTTGATGAGGGAGGGGAAACTTTTTTTTT | 6mer |
|  |  |  | MARMOSET | ATGCAGTACTGTTCTGATCCCGCTGCTATTAGAATGCATTGTGAAACGACTGGAGTATGATTAAAAGTTGTGTTCCCCAATGCTTGGAGTAGTGATTGTTGAAGGAAAAAA | 7mer-A1 |
|  |  |  | MARMOSET | CCCCAATGCTTGGAGTAGTGATTGTTGAAGGAAA | 7mer-A1 |
|  |  |  | MARMOSET | TCCAGCTGAGTGATAAAGGCTGAGTGTTGAGGAAATTTCTGCAGTTTTAAGCAGTCGT | 7mer-m8 |
|  |  |  | MARMOSET | TCCAGCTGAGTGATAAAGGCTGAGTGTTGAGGAAATTTCTGCAG | 7mer-m8 |
|  |  |  | MARMOSET | AAAGGCTGAGTGTTGAGGAAATTTCTGCAG | 7mer-m8 |
|  |  |  | MARMOSET | AGGCTGAGTGTTGAGGAAAT | 7mer-m8 |
|  |  |  | DOG | ATGTTGAAGTTAAGTTTTC | 7mer-A1 |
|  |  |  | DOG | CCCCAATGCTTGGAGTAGTGATTGTTGAAGGAAA | 7mer-A1 |
|  |  |  | DOG | TCCAGCTGAGTGATAAAGGCTGAGTGTTGAGGAAATTTCTGCAG | 7mer-m8 |
|  |  |  | DOG | AAAGGCTGAGTGTTGAGGAAATTTCTGCAG | 7mer-m8 |
|  |  |  | DOG | AGGCTGAGTGTTGAGGAAAT | 7mer-m8 |
|  |  |  | PIG | CCCCAATGCTTGGAGTAGTGATTGTTGAAGGAAA | 7mer-A1 |
|  |  |  | PIG | AAAGGCTGAGTGTTGAGGAAATTTCTGCAG | 7mer-m8 |
|  |  |  | PIG | AGGCTGAGTGTTGAGGAAAT | 7mer-m8 |
|  |  |  | COW | AAAGGCTGAGTGTTGAGGAAATTTCTGCAG | 7mer-m8 |
|  |  |  | COW | AGGCTGAGTGTTGAGGAAAT | 7mer-m8 |
|  |  |  | MOUSE | AGGCTGAGTGTTGAGGAAAT | 7mer-m8 |

  
  
  
  

| Seed Matches to the miRNA miR-421 | | | | | |
| --- | --- | --- | --- | --- | --- |
| Seed | Conservation | Species | Matches | | |
|  |  |  | Sequence | Motif | Type |
| UCAACAG | Conserved | Human (Homo sapiens)  Rhesus (Macaca mulatta)  Chimp (Pan troglodytes)  Dog (Canis lupus familiaris)  Cow (Bos taurus) | HUMAN | TGGAAGAGTATTCCCAGTTGAAGCTGAAAAGTACAGCACAGTGCAGCTTTGGTTCATATTCAGTCATCTCAGGAGAACTTCAGAAGAGCTTGAGTAGGCCAAATGTTGAAGTTAAGTTTTC | 7mer-A1 |
|  |  |  | HUMAN | ATGTTGAAGTTAAGTTTTC | 7mer-A1 |
|  |  |  | HUMAN | TAACTGATTAAGAATTGTGATAGTTCAGCTTGAATGTCTCTTAGAGGGTGGGCTTTTGTTGATGAGGGAGGGGAAACTTTTTTTTT | 6mer |
|  |  |  | HUMAN | ATGCAGTACTGTTCTGATCCCGCTGCTATTAGAATGCATTGTGAAACGACTGGAGTATGATTAAAAGTTGTGTTCCCCAATGCTTGGAGTAGTGATTGTTGAAGGAAAAAA | 7mer-A1 |
|  |  |  | HUMAN | CCCCAATGCTTGGAGTAGTGATTGTTGAAGGAAA | 7mer-A1 |
|  |  |  | HUMAN | TCCAGCTGAGTGATAAAGGCTGAGTGTTGAGGAAATTTCTGCAGTTTTAAGCAGTCGT | 6mer |
|  |  |  | HUMAN | TCCAGCTGAGTGATAAAGGCTGAGTGTTGAGGAAATTTCTGCAG | 6mer |
|  |  |  | HUMAN | AAAGGCTGAGTGTTGAGGAAATTTCTGCAG | 6mer |
|  |  |  | HUMAN | AGGCTGAGTGTTGAGGAAAT | 6mer |
|  |  |  | MARMOSET | TGGAAGAGTATTCCCAGTTGAAGCTGAAAAGTACAGCACAGTGCAGCTTTGGTTCATATTCAGTCATCTCAGGAGAACTTCAGAAGAGCTTGAGTAGGCCAAATGTTGAAGTTAAGTTTTC | 7mer-A1 |
|  |  |  | MARMOSET | ATGTTGAAGTTAAGTTTTC | 7mer-A1 |
|  |  |  | MARMOSET | TAACTGATTAAGAATTGTGATAGTTCAGCTTGAATGTCTCTTAGAGGGTGGGCTTTTGTTGATGAGGGAGGGGAAACTTTTTTTTT | 6mer |
|  |  |  | MARMOSET | ATGCAGTACTGTTCTGATCCCGCTGCTATTAGAATGCATTGTGAAACGACTGGAGTATGATTAAAAGTTGTGTTCCCCAATGCTTGGAGTAGTGATTGTTGAAGGAAAAAA | 7mer-A1 |
|  |  |  | MARMOSET | CCCCAATGCTTGGAGTAGTGATTGTTGAAGGAAA | 7mer-A1 |
|  |  |  | MARMOSET | TCCAGCTGAGTGATAAAGGCTGAGTGTTGAGGAAATTTCTGCAGTTTTAAGCAGTCGT | 6mer |
|  |  |  | MARMOSET | TCCAGCTGAGTGATAAAGGCTGAGTGTTGAGGAAATTTCTGCAG | 6mer |
|  |  |  | MARMOSET | AAAGGCTGAGTGTTGAGGAAATTTCTGCAG | 6mer |
|  |  |  | MARMOSET | AGGCTGAGTGTTGAGGAAAT | 6mer |
|  |  |  | DOG | ATGTTGAAGTTAAGTTTTC | 7mer-A1 |
|  |  |  | DOG | CCCCAATGCTTGGAGTAGTGATTGTTGAAGGAAA | 7mer-A1 |
|  |  |  | DOG | TCCAGCTGAGTGATAAAGGCTGAGTGTTGAGGAAATTTCTGCAG | 6mer |
|  |  |  | DOG | AAAGGCTGAGTGTTGAGGAAATTTCTGCAG | 6mer |
|  |  |  | DOG | AGGCTGAGTGTTGAGGAAAT | 6mer |
|  |  |  | PIG | CCCCAATGCTTGGAGTAGTGATTGTTGAAGGAAA | 7mer-A1 |
|  |  |  | PIG | AAAGGCTGAGTGTTGAGGAAATTTCTGCAG | 6mer |
|  |  |  | PIG | AGGCTGAGTGTTGAGGAAAT | 6mer |
|  |  |  | COW | AAAGGCTGAGTGTTGAGGAAATTTCTGCAG | 6mer |
|  |  |  | COW | AGGCTGAGTGTTGAGGAAAT | 6mer |
|  |  |  | MOUSE | AGGCTGAGTGTTGAGGAAAT | 6mer |

  
  
  
  

| Seed Matches to the miRNA miR-212-5p | | | | | |
| --- | --- | --- | --- | --- | --- |
| Seed | Conservation | Species | Matches | | |
|  |  |  | Sequence | Motif | Type |
| CCUUGGC | Broadly Conserved | Human (Homo sapiens)  Rhesus (Macaca mulatta)  Mouse (Mus musculus)  Rat (Rattus norvegicus) | HUMAN | TGCCAAGGC | 7mer-m8 |
|  |  |  | MARMOSET | TGCCAAGGC | 7mer-m8 |

  
  
  
  

| Seed Matches to the miRNA miR-140-5p | | | | | |
| --- | --- | --- | --- | --- | --- |
| Seed | Conservation | Species | Matches | | |
|  |  |  | Sequence | Motif | Type |
| AGUGGUU | Broadly Conserved | Human (Homo sapiens)  Rhesus (Macaca mulatta)  Mouse (Mus musculus)  Rat (Rattus norvegicus)  Opossum (Monodelphis domestica) | HUMAN | AAACCAAACATTCCATTTTAAATGTGGGGATTGGGAACCACTAGTTCTTTCAGATGGTATTCTTCAGACTATAGAAGGAGCTTCCAGTTGAATTCA | 8mer |
|  |  |  | HUMAN | TCCATTTTAAATGTGGGGATTGGGAACCACTAGTTCTTTCAGATGGTATTCTTCAGACTATAGAAGGAGCTTCCAGTTGAATTCA | 8mer |
|  |  |  | HUMAN | TTTAAATGTGGGGATTGGGAACCACTAGTTCTTTCAGATGGTA | 8mer |
|  |  |  | MARMOSET | AAACCAAACATTCCATTTTAAATGTGGGGATTGGGAACCACTAGTTCTTTCAGATGGTATTCTTCAGACTATAGAAGGAGCTTCCAGTTGAATTCA | 8mer |
|  |  |  | MARMOSET | TCCATTTTAAATGTGGGGATTGGGAACCACTAGTTCTTTCAGATGGTATTCTTCAGACTATAGAAGGAGCTTCCAGTTGAATTCA | 8mer |
|  |  |  | MARMOSET | TTTAAATGTGGGGATTGGGAACCACTAGTTCTTTCAGATGGTA | 8mer |
|  |  |  | DOG | TCCATTTTAAATGTGGGGATTGGGAACCACTAGTTCTTTCAGATGGTATTCTTCAGACTATAGAAGGAGCTTCCAGTTGAATTCA | 8mer |
|  |  |  | DOG | TTTAAATGTGGGGATTGGGAACCACTAGTTCTTTCAGATGGTA | 8mer |
|  |  |  | PIG | TTTAAATGTGGGGATTGGGAACCACTAGTTCTTTCAGATGGTA | 8mer |
|  |  |  | COW | TTTAAATGTGGGGATTGGGAACCACTAGTTCTTTCAGATGGTA | 8mer |

  
  
  
  

| Seed Matches to the miRNA miR-425-5p | | | | | |
| --- | --- | --- | --- | --- | --- |
| Seed | Conservation | Species | Matches | | |
|  |  |  | Sequence | Motif | Type |
| AUGACAC | Broadly Conserved | Human (Homo sapiens)  X. tropicalis (Xenopus tropicalis)  Rat (Rattus norvegicus)  Opossum (Monodelphis domestica) | HUMAN | TGATAAGTAAAGGCAGAAAAGATTATATGTCATACCTCCATTGGGGAATAAGCATAACCCTGAGATTCTTACTACTGATGA | 7mer-A1 |
|  |  |  | MARMOSET | TGATAAGTAAAGGCAGAAAAGATTATATGTCATACCTCCATTGGGGAATAAGCATAACCCTGAGATTCTTACTACTGATGA | 7mer-A1 |

  
  
  
  

| Seed Matches to the miRNA miR-499a-5p | | | | | |
| --- | --- | --- | --- | --- | --- |
| Seed | Conservation | Species | Matches | | |
|  |  |  | Sequence | Motif | Type |
| UAAGACU | Broadly Conserved | Human (Homo sapiens) | HUMAN | ATTTTGGGATGGTCTTAACAGGGAAGAGAGAGGGTGGGGGAGAAAATGTTTTTTTCTAAGATTTTCCACAGATGCTATAGTACTATTGACAAACTGGGTTAGAGAAGGAGTGTAC | 7mer-A1 |
|  |  |  | HUMAN | TGGTCTTAACAGGGAAGAG | 7mer-A1 |
|  |  |  | HUMAN | GTCTTAG | 7mer-m8 |
|  |  |  | HUMAN | TGTGTGGGTTTCTCTCTCCCCTCCCTTGGTCTTAATTCTTACATGCAGGAACA | 7mer-A1 |
|  |  |  | HUMAN | TGTGTGGGTTTCTCTCTCCCCTCCCTTGGTCTTAATTCTTACA | 7mer-A1 |
|  |  |  | MARMOSET | ATTTTGGGATGGTCTTAACAGGGAAGAGAGAGGGTGGGGGAGAAAATGTTTTTTTCTAAGATTTTCCACAGATGCTATAGTACTATTGACAAACTGGGTTAGAGAAGGAGTGTAC | 7mer-A1 |
|  |  |  | MARMOSET | TGGTCTTAACAGGGAAGAG | 7mer-A1 |
|  |  |  | MARMOSET | GTCTTAG | 7mer-m8 |
|  |  |  | MARMOSET | TGTGTGGGTTTCTCTCTCCCCTCCCTTGGTCTTAATTCTTACATGCAGGAACA | 7mer-A1 |
|  |  |  | MARMOSET | TGTGTGGGTTTCTCTCTCCCCTCCCTTGGTCTTAATTCTTACA | 7mer-A1 |
|  |  |  | DOG | TGGTCTTAACAGGGAAGAG | 7mer-A1 |
|  |  |  | DOG | TGTGTGGGTTTCTCTCTCCCCTCCCTTGGTCTTAATTCTTACA | 7mer-A1 |
|  |  |  | PIG | TGGTCTTAACAGGGAAGAG | 7mer-A1 |

  
  
  
  

| Seed Matches to the miRNA miR-22-3p | | | | | |
| --- | --- | --- | --- | --- | --- |
| Seed | Conservation | Species | Matches | | |
|  |  |  | Sequence | Motif | Type |
| AGCUGCC | Broadly Conserved | Human (Homo sapiens)  X. tropicalis (Xenopus tropicalis)  Chicken (Gallus gallus)  Cow (Bos taurus)  Mouse (Mus musculus)  Rat (Rattus norvegicus)  Opossum (Monodelphis domestica) | HUMAN | TGGAAGAGTATTCCCAGTTGAAGCTGAAAAGTACAGCACAGTGCAGCTTTGGTTCATATTCAGTCATCTCAGGAGAACTTCAGAAGAGCTTGAGTAGGCCAAATGTTGAAGTTAAGTTTTC | 6mer |
|  |  |  | HUMAN | GTACAGCACAGTGCAGCTTTGGTTCATA | 6mer |
|  |  |  | HUMAN | TACAGCACAGTGCAGCTTTGGTTCATA | 6mer |
|  |  |  | HUMAN | CAGTGCAGCTTTGGTTCATA | 6mer |
|  |  |  | HUMAN | TTATCAGAAGAGTTGCTTCATTTCATCTGGGAGCAGAAAACAGCAGGCAGCTGTTAACAGATAAGTTTAACTTGCATCTGCA | 7mer-m8 |
|  |  |  | HUMAN | TTATCAGAAGAGTTGCTTCATTTCATCTGGGAGCAGAAAACAGCAGGCAGCTGTTAACAGATAAGTTTAA | 7mer-m8 |
|  |  |  | HUMAN | TCATCTGGGAGCAGAAAACAGCAGGCAGCTGTT | 7mer-m8 |
|  |  |  | HUMAN | AGCTGTTTTTATAGCAGCTCTTAA | 6mer |
|  |  |  | HUMAN | TTTTATAGCAGCT | 6mer |
|  |  |  | MARMOSET | TGGAAGAGTATTCCCAGTTGAAGCTGAAAAGTACAGCACAGTGCAGCTTTGGTTCATATTCAGTCATCTCAGGAGAACTTCAGAAGAGCTTGAGTAGGCCAAATGTTGAAGTTAAGTTTTC | 6mer |
|  |  |  | MARMOSET | GTACAGCACAGTGCAGCTTTGGTTCATA | 6mer |
|  |  |  | MARMOSET | TACAGCACAGTGCAGCTTTGGTTCATA | 6mer |
|  |  |  | MARMOSET | CAGTGCAGCTTTGGTTCATA | 6mer |
|  |  |  | MARMOSET | TTATCAGAAGAGTTGCTTCATTTCATCTGGGAGCAGAAAACAGCAGGCAGCTGTTAACAGATAAGTTTAACTTGCATCTGCA | 7mer-m8 |
|  |  |  | MARMOSET | TTATCAGAAGAGTTGCTTCATTTCATCTGGGAGCAGAAAACAGCAGGCAGCTGTTAACAGATAAGTTTAA | 7mer-m8 |
|  |  |  | MARMOSET | TCATCTGGGAGCAGAAAACAGCAGGCAGCTGTT | 7mer-m8 |
|  |  |  | MARMOSET | AGCTGTTTTTATAGCAGCTCTTAA | 6mer |
|  |  |  | MARMOSET | TTTTATAGCAGCT | 6mer |
|  |  |  | DOG | GTACAGCACAGTGCAGCTTTGGTTCATA | 6mer |
|  |  |  | DOG | TACAGCACAGTGCAGCTTTGGTTCATA | 6mer |
|  |  |  | DOG | CAGTGCAGCTTTGGTTCATA | 6mer |
|  |  |  | DOG | TTATCAGAAGAGTTGCTTCATTTCATCTGGGAGCAGAAAACAGCAGGCAGCTGTTAACAGATAAGTTTAA | 7mer-m8 |
|  |  |  | DOG | TCATCTGGGAGCAGAAAACAGCAGGCAGCTGTT | 7mer-m8 |
|  |  |  | DOG | TTTTATAGCAGCT | 7mer-A1 |
|  |  |  | PIG | TACAGCACAGTGCAGCTTTGGTTCATA | 6mer |
|  |  |  | PIG | CAGTGCAGCTTTGGTTCATA | 6mer |
|  |  |  | PIG | TCATCTGGGAGCAGAAAACAGCAGGCAGCTGTT | 7mer-m8 |
|  |  |  | COW | CAGTGCAGCTTTGGTTCATA | 6mer |
|  |  |  | COW | TCATCTGGGAGCAGAAAACAGCAGGCAGCTGTT | 7mer-m8 |

  
  
  
  

| Seed Matches to the miRNA miR-129-5p | | | | | |
| --- | --- | --- | --- | --- | --- |
| Seed | Conservation | Species | Matches | | |
|  |  |  | Sequence | Motif | Type |
| UUUUUGC | Broadly Conserved | Human (Homo sapiens)  Rhesus (Macaca mulatta)  Cow (Bos taurus)  Mouse (Mus musculus)  Rat (Rattus norvegicus)  Opossum (Monodelphis domestica) | HUMAN | GACAAGCTAGGAAACAAAAA | 6mer |
|  |  |  | HUMAN | CATCAAAAAGCT | 6mer |
|  |  |  | HUMAN | CAAAAAG | 6mer |
|  |  |  | HUMAN | CAAGATGTTAAGGTATGCTTCAAAAA | 6mer |
|  |  |  | HUMAN | TTATCTGCATATGCCAAAAAA | 7mer-A1 |
|  |  |  | HUMAN | ACAAAAATAATGAATTGATGAGAAATACAATGAAGA | 6mer |
|  |  |  | HUMAN | TGGGTGGGAATGCAAAAATTCTCTGCTAAGACTTTTTCAGGTGAACATAACAGACTTGGCCAAGCTAGCATCTTAGCGGAAGC | 7mer-m8 |
|  |  |  | HUMAN | TGCAAAAATTCTCTGCTAAGACTTTTTCAGGTGAACATAA | 7mer-m8 |
|  |  |  | HUMAN | TGCAAAAA | 7mer-m8 |
|  |  |  | MARMOSET | GACAAGCTAGGAAACAAAAA | 7mer-A1 |
|  |  |  | MARMOSET | CATCAAAAAGCT | 6mer |
|  |  |  | MARMOSET | CAAAAAG | 6mer |
|  |  |  | MARMOSET | CAAGATGTTAAGGTATGCTTCAAAAA | 6mer |
|  |  |  | MARMOSET | TTATCTGCATATGCCAAAAAA | 7mer-A1 |
|  |  |  | MARMOSET | ACAAAAATAATGAATTGATGAGAAATACAATGAAGA | 6mer |
|  |  |  | MARMOSET | TGGGTGGGAATGCAAAAATTCTCTGCTAAGACTTTTTCAGGTGAACATAACAGACTTGGCCAAGCTAGCATCTTAGCGGAAGC | 7mer-m8 |
|  |  |  | MARMOSET | TGCAAAAATTCTCTGCTAAGACTTTTTCAGGTGAACATAA | 7mer-m8 |
|  |  |  | MARMOSET | TGCAAAAA | 7mer-m8 |
|  |  |  | DOG | CAAAAAG | 6mer |
|  |  |  | DOG | TGCAAAAATTCTCTGCTAAGACTTTTTCAGGTGAACATAA | 7mer-m8 |
|  |  |  | DOG | TGCAAAAA | 7mer-m8 |
|  |  |  | PIG | TGCAAAAA | 7mer-m8 |
|  |  |  | COW | TGCAAAAA | 7mer-m8 |
|  |  |  | MOUSE | TGCAAAAA | 7mer-m8 |

  
  
  
  

| Seed Matches to the miRNA miR-410-3p | | | | | |
| --- | --- | --- | --- | --- | --- |
| Seed | Conservation | Species | Matches | | |
|  |  |  | Sequence | Motif | Type |
| AUAUAAC | Conserved | Human (Homo sapiens)  Rhesus (Macaca mulatta) | HUMAN | GGGATTTATATGGGGA | 6mer |
|  |  |  | HUMAN | TGATAAGTAAAGGCAGAAAAGATTATATGTCATACCTCCATTGGGGAATAAGCATAACCCTGAGATTCTTACTACTGATGA | 6mer |
|  |  |  | MARMOSET | GGGATTTATATGGGGA | 6mer |
|  |  |  | MARMOSET | TGATAAGTAAAGGCAGAAAAGATTATATGTCATACCTCCATTGGGGAATAAGCATAACCCTGAGATTCTTACTACTGATGA | 6mer |

  
  
  
  

| Seed Matches to the miRNA miR-382-3p | | | | | |
| --- | --- | --- | --- | --- | --- |
| Seed | Conservation | Species | Matches | | |
|  |  |  | Sequence | Motif | Type |
| AUCAUUC | Conserved | Human (Homo sapiens)  Rhesus (Macaca mulatta)  Mouse (Mus musculus)  Rat (Rattus norvegicus) | HUMAN | CAGAGCAAAGGAAGTGGCTTAATGATCCTGAAGGGATTTCTTCTGATGGTAGCTTTTGTATTATCAAGTAAGATTCT | 6mer |
|  |  |  | HUMAN | CAGAGCAAAGGAAGTGGCTTAATGATCCTGAAGGGATTTCTTC | 6mer |
|  |  |  | HUMAN | GTGGCTTAATGATCCTGAAGGGATTTCTTC | 6mer |
|  |  |  | MARMOSET | CAGAGCAAAGGAAGTGGCTTAATGATCCTGAAGGGATTTCTTCTGATGGTAGCTTTTGTATTATCAAGTAAGATTCT | 6mer |
|  |  |  | MARMOSET | CAGAGCAAAGGAAGTGGCTTAATGATCCTGAAGGGATTTCTTC | 6mer |
|  |  |  | MARMOSET | GTGGCTTAATGATCCTGAAGGGATTTCTTC | 6mer |
|  |  |  | DOG | CAGAGCAAAGGAAGTGGCTTAATGATCCTGAAGGGATTTCTTC | 6mer |
|  |  |  | DOG | GTGGCTTAATGATCCTGAAGGGATTTCTTC | 6mer |
|  |  |  | PIG | CAGAGCAAAGGAAGTGGCTTAATGATCCTGAAGGGATTTCTTC | 6mer |
|  |  |  | PIG | GTGGCTTAATGATCCTGAAGGGATTTCTTC | 6mer |
|  |  |  | COW | GTGGCTTAATGATCCTGAAGGGATTTCTTC | 6mer |

  
  
  
  

| Seed Matches to the miRNA miR-338-3p | | | | | |
| --- | --- | --- | --- | --- | --- |
| Seed | Conservation | Species | Matches | | |
|  |  |  | Sequence | Motif | Type |
| CCAGCAU | Broadly Conserved | Human (Homo sapiens)  Rhesus (Macaca mulatta)  Mouse (Mus musculus)  Rat (Rattus norvegicus) | HUMAN | AGTATTGAATAGATTTCAGCTTTATGCTGGAGTAA | 8mer |
|  |  |  | HUMAN | CAGCTTTATGCTGGA | 8mer |
|  |  |  | HUMAN | AAAAAAAAAGCAAAAGATGCTGGT | 7mer-m8 |
|  |  |  | MARMOSET | AGTATTGAATAGATTTCAGCTTTATGCTGGAGTAA | 8mer |
|  |  |  | MARMOSET | CAGCTTTATGCTGGA | 8mer |
|  |  |  | MARMOSET | AAAAAAAAAGCAAAAGATGCTGGT | 7mer-m8 |
|  |  |  | DOG | CAGCTTTATGCTGGA | 8mer |
|  |  |  | DOG | AAAAAAAAAGCAAAAGATGCTGGT | 7mer-m8 |
|  |  |  | PIG | CAGCTTTATGCTGGA | 8mer |
|  |  |  | PIG | AAAAAAAAAGCAAAAGATGCTGGT | 7mer-m8 |
|  |  |  | COW | AAAAAAAAAGCAAAAGATGCTGGT | 7mer-m8 |

  
  
  
  

| Seed Matches to the miRNA miR-138-5p | | | | | |
| --- | --- | --- | --- | --- | --- |
| Seed | Conservation | Species | Matches | | |
|  |  |  | Sequence | Motif | Type |
| GCUGGUG | Broadly Conserved | Human (Homo sapiens)  Chicken (Gallus gallus)  Rhesus (Macaca mulatta)  Mouse (Mus musculus)  Rat (Rattus norvegicus) | HUMAN | TAGAGGATCCTAGACCAGCATGCCAGT | 7mer-A1 |
|  |  |  | HUMAN | TAGACTTTTTTCAGATAACATCTTCTGAGTCATAACCAGCCTGGCAGT | 6mer |
|  |  |  | HUMAN | CTTCTGAGTCATAACCAGCCTGGCA | 6mer |
|  |  |  | MARMOSET | TAGAGGATCCTAGACCAGCATGCCAGT | 7mer-A1 |
|  |  |  | MARMOSET | TAGACTTTTTTCAGATAACATCTTCTGAGTCATAACCAGCCTGGCAGT | 6mer |
|  |  |  | MARMOSET | CTTCTGAGTCATAACCAGCCTGGCA | 6mer |
|  |  |  | DOG | CTTCTGAGTCATAACCAGCCTGGCA | 6mer |

  
  
  
  

| Seed Matches to the miRNA miR-378-3p | | | | | |
| --- | --- | --- | --- | --- | --- |
| Seed | Conservation | Species | Matches | | |
|  |  |  | Sequence | Motif | Type |
| CUGGACU | Conserved | Human (Homo sapiens)  Mouse (Mus musculus)  Rat (Rattus norvegicus) | HUMAN | GAGTCCAGGA | 7mer-m8 |
|  |  |  | MARMOSET | GAGTCCAGGA | 7mer-m8 |

  
  
  
  

| Seed Matches to the miRNA miR-125-5p | | | | | |
| --- | --- | --- | --- | --- | --- |
| Seed | Conservation | Species | Matches | | |
|  |  |  | Sequence | Motif | Type |
| CCCUGAG | Broadly Conserved | Human (Homo sapiens)  Chicken (Gallus gallus)  Opossum (Monodelphis domestica) | HUMAN | GCTGTGCTGTTGGCACGAACACCTTCAGGGACTGGAGCTGCTTTTAT | 7mer-A1 |
|  |  |  | HUMAN | TGCTGTTGGCACGAACACCTTCAGGGA | 7mer-A1 |
|  |  |  | HUMAN | AACACCTTCAGGGA | 7mer-A1 |
|  |  |  | HUMAN | ACACCTTCAGGGA | 7mer-A1 |
|  |  |  | MARMOSET | GCTGTGCTGTTGGCACGAACACCTTCAGGGACTGGAGCTGCTTTTAT | 7mer-A1 |
|  |  |  | MARMOSET | TGCTGTTGGCACGAACACCTTCAGGGA | 7mer-A1 |
|  |  |  | MARMOSET | AACACCTTCAGGGA | 7mer-A1 |
|  |  |  | MARMOSET | ACACCTTCAGGGA | 7mer-A1 |
|  |  |  | DOG | TGCTGTTGGCACGAACACCTTCAGGGA | 7mer-A1 |
|  |  |  | DOG | AACACCTTCAGGGA | 7mer-A1 |
|  |  |  | DOG | ACACCTTCAGGGA | 7mer-A1 |
|  |  |  | PIG | AACACCTTCAGGGA | 7mer-A1 |
|  |  |  | PIG | ACACCTTCAGGGA | 7mer-A1 |
|  |  |  | COW | AACACCTTCAGGGA | 7mer-A1 |
|  |  |  | COW | ACACCTTCAGGGA | 7mer-A1 |
|  |  |  | MOUSE | ACACCTTCAGGGA | 7mer-A1 |

  
  
  
  

| Seed Matches to the miRNA miR-433-3p | | | | | |
| --- | --- | --- | --- | --- | --- |
| Seed | Conservation | Species | Matches | | |
|  |  |  | Sequence | Motif | Type |
| UCAUGAU | Conserved | Human (Homo sapiens)  Rhesus (Macaca mulatta)  Mouse (Mus musculus)  Rat (Rattus norvegicus) | HUMAN | CTCATGAATCTTGTCTGAAGCTTTTGAGGGCAGACTGCCAAGTCCTGGAG | 7mer-A1 |
|  |  |  | HUMAN | TTTTAAAGAATTTTCCTTTGCAGAGGCATTTCATCCTTCATGAAGC | 7mer-A1 |
|  |  |  | HUMAN | CATTTCATCCTTCATGAAGC | 7mer-A1 |
|  |  |  | HUMAN | TTTCATCCTTCATGA | 7mer-A1 |
|  |  |  | HUMAN | GATCTCCAATGCTCTTCAGTAGGGTCATGAAGGTTTTTCTTTTCCTGAGAAAACAACA | 7mer-A1 |
|  |  |  | MARMOSET | CTCATGAATCTTGTCTGAAGCTTTTGAGGGCAGACTGCCAAGTCCTGGAG | 7mer-A1 |
|  |  |  | MARMOSET | TTTTAAAGAATTTTCCTTTGCAGAGGCATTTCATCCTTCATGAAGC | 7mer-A1 |
|  |  |  | MARMOSET | CATTTCATCCTTCATGAAGC | 7mer-A1 |
|  |  |  | MARMOSET | TTTCATCCTTCATGA | 7mer-A1 |
|  |  |  | MARMOSET | GATCTCCAATGCTCTTCAGTAGGGTCATGAAGGTTTTTCTTTTCCTGAGAAAACAACA | 7mer-A1 |
|  |  |  | DOG | CATTTCATCCTTCATGAAGC | 7mer-A1 |
|  |  |  | DOG | TTTCATCCTTCATGA | 7mer-A1 |
|  |  |  | PIG | TTTCATCCTTCATGA | 6mer |

  
  
  
  

| Seed Matches to the miRNA miR-665 | | | | | |
| --- | --- | --- | --- | --- | --- |
| Seed | Conservation | Species | Matches | | |
|  |  |  | Sequence | Motif | Type |
| CCAGGAG | Conserved | Human (Homo sapiens)  Rhesus (Macaca mulatta)  Chimp (Pan troglodytes)  Dog (Canis lupus familiaris)  Rat (Rattus norvegicus) | HUMAN | CTCATGAATCTTGTCTGAAGCTTTTGAGGGCAGACTGCCAAGTCCTGGAG | 7mer-A1 |
|  |  |  | HUMAN | ACTACATTAATCCTGGAATAAAAGAAGCCGAAATA | 7mer-A1 |
|  |  |  | HUMAN | ACATTAATCCTGGAATAAAAGAAGC | 7mer-A1 |
|  |  |  | HUMAN | AGTGAGTGTATGAGACCTTGCAGTGAGTTTATCAGCATACTCAAAATTTTTTTCCTGGAATTTGGAGGGATGGGAGGAGGGGGTGGGGCTTACTTGTT | 7mer-A1 |
|  |  |  | HUMAN | ACTCCTGGTTTCCAGGACGGGGTTCAAATCCCTGCGGC | 7mer-m8 |
|  |  |  | HUMAN | ACTCCTGG | 7mer-m8 |
|  |  |  | MARMOSET | CTCATGAATCTTGTCTGAAGCTTTTGAGGGCAGACTGCCAAGTCCTGGAG | 7mer-A1 |
|  |  |  | MARMOSET | ACTACATTAATCCTGGAATAAAAGAAGCCGAAATA | 7mer-A1 |
|  |  |  | MARMOSET | ACATTAATCCTGGAATAAAAGAAGC | 7mer-A1 |
|  |  |  | MARMOSET | AGTGAGTGTATGAGACCTTGCAGTGAGTTTATCAGCATACTCAAAATTTTTTTCCTGGAATTTGGAGGGATGGGAGGAGGGGGTGGGGCTTACTTGTT | 7mer-A1 |
|  |  |  | MARMOSET | ACTCCTGGTTTCCAGGACGGGGTTCAAATCCCTGCGGC | 7mer-m8 |
|  |  |  | MARMOSET | ACTCCTGG | 7mer-m8 |
|  |  |  | DOG | ACATTAATCCTGGAATAAAAGAAGC | 7mer-A1 |
|  |  |  | DOG | ACTCCTGG | 8mer |
|  |  |  | PIG | ACTCCTGG | 7mer-m8 |
|  |  |  | COW | ACTCCTGG | 7mer-m8 |
|  |  |  | MOUSE | ACTCCTGG | 7mer-m8 |
|  |  |  | TURTLE | ACTCCTGG | 7mer-m8 |
|  |  |  | ALLIGATOR | ACTCCTGG | 7mer-m8 |
|  |  |  | LIZARD | ACTCCTGG | 7mer-m8 |
|  |  |  | SNAKE | ACTCCTGG | 8mer |
|  |  |  | X.TROPICALIS | ACTCCTGG | 7mer-m8 |
|  |  |  | SHARK | ACTCCTGG | 7mer-m8 |
|  |  |  | OPOSSUM | ACTCCTGG | 7mer-m8 |
|  |  |  | SPOTTEDGAR | ACTCCTGG | 7mer-m8 |

  
  
  
  

| Seed Matches to the miRNA miR-216a-5p | | | | | |
| --- | --- | --- | --- | --- | --- |
| Seed | Conservation | Species | Matches | | |
|  |  |  | Sequence | Motif | Type |
| AAUCUCA | Broadly Conserved | Human (Homo sapiens)  Mouse (Mus musculus)  Rat (Rattus norvegicus) | HUMAN | TTTAAAAAGAGATTAA | 7mer-A1 |
|  |  |  | HUMAN | TGATAAGTAAAGGCAGAAAAGATTATATGTCATACCTCCATTGGGGAATAAGCATAACCCTGAGATTCTTACTACTGATGA | 7mer-m8 |
|  |  |  | HUMAN | TAACCTCTTAGACAGGTGGGAGATTATGATCAGAGTAAAAGGTAATTACACATTTTATTTCCAGAAAGTCAGG | 7mer-A1 |
|  |  |  | HUMAN | ACCTCTTAGACAGGTGGGAGATTATGATCAGA | 7mer-A1 |
|  |  |  | HUMAN | CAGGTGGGAGATTATGATCAGA | 7mer-A1 |
|  |  |  | MARMOSET | TTTAAAAAGAGATTAA | 7mer-A1 |
|  |  |  | MARMOSET | TGATAAGTAAAGGCAGAAAAGATTATATGTCATACCTCCATTGGGGAATAAGCATAACCCTGAGATTCTTACTACTGATGA | 7mer-m8 |
|  |  |  | MARMOSET | TAACCTCTTAGACAGGTGGGAGATTATGATCAGAGTAAAAGGTAATTACACATTTTATTTCCAGAAAGTCAGG | 7mer-A1 |
|  |  |  | MARMOSET | ACCTCTTAGACAGGTGGGAGATTATGATCAGA | 7mer-A1 |
|  |  |  | MARMOSET | CAGGTGGGAGATTATGATCAGA | 7mer-A1 |
|  |  |  | DOG | ACCTCTTAGACAGGTGGGAGATTATGATCAGA | 7mer-A1 |
|  |  |  | DOG | CAGGTGGGAGATTATGATCAGA | 7mer-A1 |
|  |  |  | PIG | CAGGTGGGAGATTATGATCAGA | 7mer-A1 |
|  |  |  | COW | CAGGTGGGAGATTATGATCAGA | 7mer-A1 |

  
  
  
  

| Seed Matches to the miRNA miR-488-3p | | | | | |
| --- | --- | --- | --- | --- | --- |
| Seed | Conservation | Species | Matches | | |
|  |  |  | Sequence | Motif | Type |
| UGAAAGG | Conserved | Human (Homo sapiens)  Rhesus (Macaca mulatta)  Mouse (Mus musculus)  Rat (Rattus norvegicus) | HUMAN | TCTAATCTTTCAGAAACTTTGTCTGCGAACAC | 6mer |
|  |  |  | HUMAN | TCTAATCTTTCAGAAACTTTGTCTGCGA | 6mer |
|  |  |  | HUMAN | ATCTTTCAGA | 6mer |
|  |  |  | HUMAN | AAACCAAACATTCCATTTTAAATGTGGGGATTGGGAACCACTAGTTCTTTCAGATGGTATTCTTCAGACTATAGAAGGAGCTTCCAGTTGAATTCA | 6mer |
|  |  |  | HUMAN | TCCATTTTAAATGTGGGGATTGGGAACCACTAGTTCTTTCAGATGGTATTCTTCAGACTATAGAAGGAGCTTCCAGTTGAATTCA | 6mer |
|  |  |  | HUMAN | TTTAAATGTGGGGATTGGGAACCACTAGTTCTTTCAGATGGTA | 6mer |
|  |  |  | HUMAN | CTAGTTCTTTCAGATG | 6mer |
|  |  |  | MARMOSET | TCTAATCTTTCAGAAACTTTGTCTGCGAACAC | 6mer |
|  |  |  | MARMOSET | TCTAATCTTTCAGAAACTTTGTCTGCGA | 6mer |
|  |  |  | MARMOSET | ATCTTTCAGA | 6mer |
|  |  |  | MARMOSET | AAACCAAACATTCCATTTTAAATGTGGGGATTGGGAACCACTAGTTCTTTCAGATGGTATTCTTCAGACTATAGAAGGAGCTTCCAGTTGAATTCA | 6mer |
|  |  |  | MARMOSET | TCCATTTTAAATGTGGGGATTGGGAACCACTAGTTCTTTCAGATGGTATTCTTCAGACTATAGAAGGAGCTTCCAGTTGAATTCA | 6mer |
|  |  |  | MARMOSET | TTTAAATGTGGGGATTGGGAACCACTAGTTCTTTCAGATGGTA | 6mer |
|  |  |  | MARMOSET | CTAGTTCTTTCAGATG | 6mer |
|  |  |  | DOG | TCTAATCTTTCAGAAACTTTGTCTGCGA | 6mer |
|  |  |  | DOG | ATCTTTCAGA | 6mer |
|  |  |  | DOG | TCCATTTTAAATGTGGGGATTGGGAACCACTAGTTCTTTCAGATGGTATTCTTCAGACTATAGAAGGAGCTTCCAGTTGAATTCA | 6mer |
|  |  |  | DOG | TTTAAATGTGGGGATTGGGAACCACTAGTTCTTTCAGATGGTA | 6mer |
|  |  |  | DOG | CTAGTTCTTTCAGATG | 6mer |
|  |  |  | PIG | ATCTTTCAGA | 6mer |
|  |  |  | PIG | TTTAAATGTGGGGATTGGGAACCACTAGTTCTTTCAGATGGTA | 6mer |
|  |  |  | PIG | CTAGTTCTTTCAGATG | 6mer |
|  |  |  | COW | ATCTTTCAGA | 6mer |
|  |  |  | COW | TTTAAATGTGGGGATTGGGAACCACTAGTTCTTTCAGATGGTA | 6mer |
|  |  |  | COW | CTAGTTCTTTCAGATG | 6mer |
|  |  |  | MOUSE | CTAGTTCTTTCAGATG | 6mer |

  
  
  
  

| Seed Matches to the miRNA miR-296-3p | | | | | |
| --- | --- | --- | --- | --- | --- |
| Seed | Conservation | Species | Matches | | |
|  |  |  | Sequence | Motif | Type |
| AGGGUUG | Conserved | Human (Homo sapiens)  Rhesus (Macaca mulatta)  Cow (Bos taurus)  Mouse (Mus musculus)  Rat (Rattus norvegicus) | HUMAN | TGATAAGTAAAGGCAGAAAAGATTATATGTCATACCTCCATTGGGGAATAAGCATAACCCTGAGATTCTTACTACTGATGA | 6mer |
|  |  |  | HUMAN | AAGCATAACCCTGAGAT | 6mer |
|  |  |  | MARMOSET | TGATAAGTAAAGGCAGAAAAGATTATATGTCATACCTCCATTGGGGAATAAGCATAACCCTGAGATTCTTACTACTGATGA | 6mer |
|  |  |  | MARMOSET | AAGCATAACCCTGAGAT | 6mer |
|  |  |  | DOG | AAGCATAACCCTGAGAT | 6mer |

  
  
  
  

| Seed Matches to the miRNA miR-374-5p | | | | | |
| --- | --- | --- | --- | --- | --- |
| Seed | Conservation | Species | Matches | | |
|  |  |  | Sequence | Motif | Type |
| UAUAAUA | Conserved | Human (Homo sapiens)  Rhesus (Macaca mulatta)  Rat (Rattus norvegicus) | HUMAN | TGATAAGTAAAGGCAGAAAAGATTATATGTCATACCTCCATTGGGGAATAAGCATAACCCTGAGATTCTTACTACTGATGA | 6mer |
|  |  |  | MARMOSET | TGATAAGTAAAGGCAGAAAAGATTATATGTCATACCTCCATTGGGGAATAAGCATAACCCTGAGATTCTTACTACTGATGA | 6mer |

  
  
  
  

| Seed Matches to the miRNA miR-204-5p/211-5p | | | | | |
| --- | --- | --- | --- | --- | --- |
| Seed | Conservation | Species | Matches | | |
|  |  |  | Sequence | Motif | Type |
| UCCCUUU | Broadly Conserved | Human (Homo sapiens)  Mouse (Mus musculus)  Rat (Rattus norvegicus) | HUMAN | CAGAGCAAAGGAAGTGGCTTAATGATCCTGAAGGGATTTCTTCTGATGGTAGCTTTTGTATTATCAAGTAAGATTCT | 6mer |
|  |  |  | HUMAN | CAGAGCAAAGGAAGTGGCTTAATGATCCTGAAGGGATTTCTTC | 6mer |
|  |  |  | HUMAN | GTGGCTTAATGATCCTGAAGGGATTTCTTC | 6mer |
|  |  |  | MARMOSET | CAGAGCAAAGGAAGTGGCTTAATGATCCTGAAGGGATTTCTTCTGATGGTAGCTTTTGTATTATCAAGTAAGATTCT | 6mer |
|  |  |  | MARMOSET | CAGAGCAAAGGAAGTGGCTTAATGATCCTGAAGGGATTTCTTC | 6mer |
|  |  |  | MARMOSET | GTGGCTTAATGATCCTGAAGGGATTTCTTC | 6mer |
|  |  |  | DOG | CAGAGCAAAGGAAGTGGCTTAATGATCCTGAAGGGATTTCTTC | 6mer |
|  |  |  | DOG | GTGGCTTAATGATCCTGAAGGGATTTCTTC | 6mer |
|  |  |  | PIG | CAGAGCAAAGGAAGTGGCTTAATGATCCTGAAGGGATTTCTTC | 6mer |
|  |  |  | PIG | GTGGCTTAATGATCCTGAAGGGATTTCTTC | 6mer |
|  |  |  | COW | GTGGCTTAATGATCCTGAAGGGATTTCTTC | 6mer |

  
  
  
  

| Seed Matches to the miRNA miR-493-3p | | | | | |
| --- | --- | --- | --- | --- | --- |
| Seed | Conservation | Species | Matches | | |
|  |  |  | Sequence | Motif | Type |
| GAAGGUC | Conserved | Human (Homo sapiens)  Rhesus (Macaca mulatta)  Mouse (Mus musculus)  Rat (Rattus norvegicus) | HUMAN | GCTGTGCTGTTGGCACGAACACCTTCAGGGACTGGAGCTGCTTTTAT | 7mer-A1 |
|  |  |  | HUMAN | TGCTGTTGGCACGAACACCTTCAGGGA | 7mer-A1 |
|  |  |  | HUMAN | AACACCTTCAGGGA | 7mer-A1 |
|  |  |  | HUMAN | ACACCTTCAGGGA | 7mer-A1 |
|  |  |  | MARMOSET | GCTGTGCTGTTGGCACGAACACCTTCAGGGACTGGAGCTGCTTTTAT | 7mer-A1 |
|  |  |  | MARMOSET | TGCTGTTGGCACGAACACCTTCAGGGA | 7mer-A1 |
|  |  |  | MARMOSET | AACACCTTCAGGGA | 7mer-A1 |
|  |  |  | MARMOSET | ACACCTTCAGGGA | 7mer-A1 |
|  |  |  | DOG | TGCTGTTGGCACGAACACCTTCAGGGA | 7mer-A1 |
|  |  |  | DOG | AACACCTTCAGGGA | 7mer-A1 |
|  |  |  | DOG | ACACCTTCAGGGA | 7mer-A1 |
|  |  |  | PIG | AACACCTTCAGGGA | 7mer-A1 |
|  |  |  | PIG | ACACCTTCAGGGA | 7mer-A1 |
|  |  |  | COW | AACACCTTCAGGGA | 7mer-A1 |
|  |  |  | COW | ACACCTTCAGGGA | 7mer-A1 |
|  |  |  | MOUSE | ACACCTTCAGGGA | 7mer-A1 |

  
  
  
  

| Seed Matches to the miRNA miR-371-5p | | | | | |
| --- | --- | --- | --- | --- | --- |
| Seed | Conservation | Species | Matches | | |
|  |  |  | Sequence | Motif | Type |
| CUCAAAC | Conserved | Human (Homo sapiens)  Rhesus (Macaca mulatta) | HUMAN | CTCATGAATCTTGTCTGAAGCTTTTGAGGGCAGACTGCCAAGTCCTGGAG | 6mer |
|  |  |  | HUMAN | ATCTTGTCTGAAGCTTTTGAGGGCAGACT | 6mer |
|  |  |  | HUMAN | AGCTTTTGAGGGCAGACT | 6mer |
|  |  |  | HUMAN | AGCTTTTGAGGGC | 6mer |
|  |  |  | HUMAN | AGATCAGGATTTGAGCGGAAGAACGAATGTAACTTT | 6mer |
|  |  |  | MARMOSET | CTCATGAATCTTGTCTGAAGCTTTTGAGGGCAGACTGCCAAGTCCTGGAG | 6mer |
|  |  |  | MARMOSET | ATCTTGTCTGAAGCTTTTGAGGGCAGACT | 6mer |
|  |  |  | MARMOSET | AGCTTTTGAGGGCAGACT | 6mer |
|  |  |  | MARMOSET | AGCTTTTGAGGGC | 6mer |
|  |  |  | MARMOSET | AGATCAGGATTTGAGCGGAAGAACGAATGTAACTTT | 6mer |
|  |  |  | DOG | ATCTTGTCTGAAGCTTTTGAGGGCAGACT | 6mer |
|  |  |  | DOG | AGCTTTTGAGGGCAGACT | 6mer |
|  |  |  | DOG | AGCTTTTGAGGGC | 6mer |
|  |  |  | PIG | ATCTTGTCTGAAGCTTTTGAGGGCAGACT | 6mer |
|  |  |  | PIG | AGCTTTTGAGGGCAGACT | 6mer |
|  |  |  | PIG | AGCTTTTGAGGGC | 6mer |
|  |  |  | COW | AGCTTTTGAGGGCAGACT | 6mer |
|  |  |  | COW | AGCTTTTGAGGGC | 6mer |
|  |  |  | MOUSE | AGCTTTTGAGGGC | 6mer |

  
  
  
  

| Seed Matches to the miRNA miR-1306-5p | | | | | |
| --- | --- | --- | --- | --- | --- |
| Seed | Conservation | Species | Matches | | |
|  |  |  | Sequence | Motif | Type |
| CACCUCC | Conserved | Human (Homo sapiens)  Rhesus (Macaca mulatta)  Mouse (Mus musculus)  Rat (Rattus norvegicus) | HUMAN | TAATGGGGGAGTTTCGTACTGAGGTGTAAA | 6mer |
|  |  |  | HUMAN | CTGTGTTGGCGTGGGGGTGGAGGGGTGAGGTGGGCGCTAAGCCTTTT | 6mer |
|  |  |  | MARMOSET | TAATGGGGGAGTTTCGTACTGAGGTGTAAA | 6mer |
|  |  |  | MARMOSET | CTGTGTTGGCGTGGGGGTGGAGGGGTGAGGTGGGCGCTAAGCCTTTT | 6mer |

  
  
  
  

| Seed Matches to the miRNA miR-543 | | | | | |
| --- | --- | --- | --- | --- | --- |
| Seed | Conservation | Species | Matches | | |
|  |  |  | Sequence | Motif | Type |
| AACAUUC | Conserved | Human (Homo sapiens)  Chimp (Pan troglodytes)  Dog (Canis lupus familiaris)  Cow (Bos taurus) | HUMAN | GTAACGGAAGTAATTCAAGATCAAGAGTAATTACCAACTTAATGTTT | 6mer |
|  |  |  | HUMAN | ATTTTGGGATGGTCTTAACAGGGAAGAGAGAGGGTGGGGGAGAAAATGTTTTTTTCTAAGATTTTCCACAGATGCTATAGTACTATTGACAAACTGGGTTAGAGAAGGAGTGTAC | 6mer |
|  |  |  | HUMAN | TGGAAGAGTATTCCCAGTTGAAGCTGAAAAGTACAGCACAGTGCAGCTTTGGTTCATATTCAGTCATCTCAGGAGAACTTCAGAAGAGCTTGAGTAGGCCAAATGTTGAAGTTAAGTTTTC | 6mer |
|  |  |  | HUMAN | TAGGCAATGTTTTACACTATTG | 6mer |
|  |  |  | HUMAN | AACTGGCAAGTGGAAATGTTTAA | 6mer |
|  |  |  | MARMOSET | GTAACGGAAGTAATTCAAGATCAAGAGTAATTACCAACTTAATGTTT | 6mer |
|  |  |  | MARMOSET | ATTTTGGGATGGTCTTAACAGGGAAGAGAGAGGGTGGGGGAGAAAATGTTTTTTTCTAAGATTTTCCACAGATGCTATAGTACTATTGACAAACTGGGTTAGAGAAGGAGTGTAC | 6mer |
|  |  |  | MARMOSET | TGGAAGAGTATTCCCAGTTGAAGCTGAAAAGTACAGCACAGTGCAGCTTTGGTTCATATTCAGTCATCTCAGGAGAACTTCAGAAGAGCTTGAGTAGGCCAAATGTTGAAGTTAAGTTTTC | 6mer |
|  |  |  | MARMOSET | TAGGCAATGTTTTACACTATTG | 6mer |
|  |  |  | MARMOSET | AACTGGCAAGTGGAAATGTTTAA | 6mer |

  
  
  
  

| Seed Matches to the miRNA miR-873-5p.1 | | | | | |
| --- | --- | --- | --- | --- | --- |
| Seed | Conservation | Species | Matches | | |
|  |  |  | Sequence | Motif | Type |
| CAGGAAC | Conserved | Human (Homo sapiens) | HUMAN | AGTGAGTGTATGAGACCTTGCAGTGAGTTTATCAGCATACTCAAAATTTTTTTCCTGGAATTTGGAGGGATGGGAGGAGGGGGTGGGGCTTACTTGTT | 6mer |
|  |  |  | HUMAN | TTAAGATTTTTCAGGTACCCCTCACTAAAGGCACCGAAGGCTTAAAGTAGGACAACCATGGAGCCTTCCTGTGGCA | 6mer |
|  |  |  | HUMAN | AAGTAGGACAACCATGGAGCCTTCCTGTGGCA | 6mer |
|  |  |  | HUMAN | GCCTTCCTGTGGCA | 6mer |
|  |  |  | HUMAN | CTTCCTGTGGCA | 6mer |
|  |  |  | HUMAN | CTTCCTGTG | 6mer |
|  |  |  | HUMAN | GATCTCCAATGCTCTTCAGTAGGGTCATGAAGGTTTTTCTTTTCCTGAGAAAACAACA | 7mer-A1 |
|  |  |  | HUMAN | AAGGTTTTTCTTTTCCTGAGAAAACAA | 7mer-A1 |
|  |  |  | HUMAN | AAGGTTTTTCTTTTCCTGAGA | 7mer-A1 |
|  |  |  | HUMAN | TTTTCTTTTCCTGAGA | 7mer-A1 |
|  |  |  | MARMOSET | AGTGAGTGTATGAGACCTTGCAGTGAGTTTATCAGCATACTCAAAATTTTTTTCCTGGAATTTGGAGGGATGGGAGGAGGGGGTGGGGCTTACTTGTT | 6mer |
|  |  |  | MARMOSET | TTAAGATTTTTCAGGTACCCCTCACTAAAGGCACCGAAGGCTTAAAGTAGGACAACCATGGAGCCTTCCTGTGGCA | 6mer |
|  |  |  | MARMOSET | AAGTAGGACAACCATGGAGCCTTCCTGTGGCA | 6mer |
|  |  |  | MARMOSET | GCCTTCCTGTGGCA | 6mer |
|  |  |  | MARMOSET | CTTCCTGTGGCA | 6mer |
|  |  |  | MARMOSET | CTTCCTGTG | 6mer |
|  |  |  | MARMOSET | GATCTCCAATGCTCTTCAGTAGGGTCATGAAGGTTTTTCTTTTCCTGAGAAAACAACA | 7mer-A1 |
|  |  |  | MARMOSET | AAGGTTTTTCTTTTCCTGAGAAAACAA | 7mer-A1 |
|  |  |  | MARMOSET | AAGGTTTTTCTTTTCCTGAGA | 7mer-A1 |
|  |  |  | MARMOSET | TTTTCTTTTCCTGAGA | 7mer-A1 |
|  |  |  | DOG | AAGTAGGACAACCATGGAGCCTTCCTGTGGCA | 6mer |
|  |  |  | DOG | GCCTTCCTGTGGCA | 6mer |
|  |  |  | DOG | CTTCCTGTGGCA | 6mer |
|  |  |  | DOG | CTTCCTGTG | 6mer |
|  |  |  | DOG | AAGGTTTTTCTTTTCCTGAGAAAACAA | 7mer-A1 |
|  |  |  | DOG | AAGGTTTTTCTTTTCCTGAGA | 7mer-A1 |
|  |  |  | DOG | TTTTCTTTTCCTGAGA | 7mer-A1 |
|  |  |  | PIG | GCCTTCCTGTGGCA | 6mer |
|  |  |  | PIG | CTTCCTGTGGCA | 6mer |
|  |  |  | PIG | CTTCCTGTG | 6mer |
|  |  |  | PIG | AAGGTTTTTCTTTTCCTGAGAAAACAA | 7mer-A1 |
|  |  |  | PIG | AAGGTTTTTCTTTTCCTGAGA | 7mer-A1 |
|  |  |  | PIG | TTTTCTTTTCCTGAGA | 7mer-A1 |
|  |  |  | COW | CTTCCTGTGGCA | 6mer |
|  |  |  | COW | CTTCCTGTG | 6mer |
|  |  |  | COW | AAGGTTTTTCTTTTCCTGAGAAAACAA | 7mer-A1 |
|  |  |  | COW | AAGGTTTTTCTTTTCCTGAGA | 7mer-A1 |
|  |  |  | COW | TTTTCTTTTCCTGAGA | 7mer-A1 |
|  |  |  | MOUSE | CTTCCTGTG | 6mer |
|  |  |  | MOUSE | AAGGTTTTTCTTTTCCTGAGAAAACAA | 7mer-A1 |
|  |  |  | MOUSE | AAGGTTTTTCTTTTCCTGAGA | 7mer-A1 |
|  |  |  | MOUSE | TTTTCTTTTCCTGAGA | 7mer-A1 |
|  |  |  | TURTLE | AAGGTTTTTCTTTTCCTGAGA | 7mer-A1 |
|  |  |  | TURTLE | TTTTCTTTTCCTGAGA | 7mer-A1 |
|  |  |  | ALLIGATOR | TTTTCTTTTCCTGAGA | 7mer-A1 |
|  |  |  | LIZARD | TTTTCTTTTCCTGAGA | 7mer-A1 |
|  |  |  | SNAKE | TTTTCTTTTCCTGAGA | 7mer-A1 |

  
  
  
  

| Seed Matches to the miRNA miR-140-3p.1 | | | | | |
| --- | --- | --- | --- | --- | --- |
| Seed | Conservation | Species | Matches | | |
|  |  |  | Sequence | Motif | Type |
| CCACAGG | Broadly Conserved | Human (Homo sapiens)  Mouse (Mus musculus) | HUMAN | AGGTCTGTGGA | 7mer-A1 |
|  |  |  | HUMAN | TCTGTGG | 7mer-A1 |
|  |  |  | HUMAN | CTGTGGAGTTCTTAA | 7mer-A1 |
|  |  |  | HUMAN | CTGTGGAG | 7mer-A1 |
|  |  |  | HUMAN | CTGTGGA | 7mer-A1 |
|  |  |  | HUMAN | GCCTGTGGG | 7mer-m8 |
|  |  |  | HUMAN | TTAAGATTTTTCAGGTACCCCTCACTAAAGGCACCGAAGGCTTAAAGTAGGACAACCATGGAGCCTTCCTGTGGCA | 7mer-m8 |
|  |  |  | HUMAN | AAGTAGGACAACCATGGAGCCTTCCTGTGGCA | 7mer-m8 |
|  |  |  | HUMAN | GCCTTCCTGTGGCA | 7mer-m8 |
|  |  |  | HUMAN | CTTCCTGTGGCA | 7mer-m8 |
|  |  |  | MARMOSET | AGGTCTGTGGA | 7mer-A1 |
|  |  |  | MARMOSET | TCTGTGG | 7mer-A1 |
|  |  |  | MARMOSET | CTGTGGAGTTCTTAA | 7mer-A1 |
|  |  |  | MARMOSET | CTGTGGAG | 7mer-A1 |
|  |  |  | MARMOSET | CTGTGGA | 7mer-A1 |
|  |  |  | MARMOSET | GCCTGTGGG | 7mer-m8 |
|  |  |  | MARMOSET | TTAAGATTTTTCAGGTACCCCTCACTAAAGGCACCGAAGGCTTAAAGTAGGACAACCATGGAGCCTTCCTGTGGCA | 7mer-m8 |
|  |  |  | MARMOSET | AAGTAGGACAACCATGGAGCCTTCCTGTGGCA | 7mer-m8 |
|  |  |  | MARMOSET | GCCTTCCTGTGGCA | 7mer-m8 |
|  |  |  | MARMOSET | CTTCCTGTGGCA | 7mer-m8 |
|  |  |  | DOG | TCTGTGG | 6mer |
|  |  |  | DOG | CTGTGGAG | 7mer-A1 |
|  |  |  | DOG | CTGTGGA | 7mer-A1 |
|  |  |  | DOG | AAGTAGGACAACCATGGAGCCTTCCTGTGGCA | 7mer-m8 |
|  |  |  | DOG | GCCTTCCTGTGGCA | 7mer-m8 |
|  |  |  | DOG | CTTCCTGTGGCA | 7mer-m8 |
|  |  |  | PIG | CTGTGGAG | 7mer-A1 |
|  |  |  | PIG | CTGTGGA | 7mer-A1 |
|  |  |  | PIG | GCCTTCCTGTGGCA | 7mer-m8 |
|  |  |  | PIG | CTTCCTGTGGCA | 7mer-m8 |
|  |  |  | COW | CTGTGGA | 7mer-A1 |
|  |  |  | COW | CTTCCTGTGGCA | 7mer-m8 |

  
  
  
  

| Seed Matches to the miRNA miR-329-3p/362-3p | | | | | |
| --- | --- | --- | --- | --- | --- |
| Seed | Conservation | Species | Matches | | |
|  |  |  | Sequence | Motif | Type |
| ACACACC | Conserved | Human (Homo sapiens)  Rhesus (Macaca mulatta)  Mouse (Mus musculus)  Rat (Rattus norvegicus) | HUMAN | GGATATGGTAGTGTGTGGTTCTCTTTTGGAATTTTTTTCAGGTGATTTAATA | 6mer |
|  |  |  | HUMAN | TAGTGTGTGGTTCTCT | 6mer |
|  |  |  | HUMAN | GTGTGTGG | 6mer |
|  |  |  | HUMAN | TTTTCAGTTGTGTGTAAGCAAGTTTTT | 7mer-A1 |
|  |  |  | MARMOSET | GGATATGGTAGTGTGTGGTTCTCTTTTGGAATTTTTTTCAGGTGATTTAATA | 6mer |
|  |  |  | MARMOSET | TAGTGTGTGGTTCTCT | 6mer |
|  |  |  | MARMOSET | GTGTGTGG | 6mer |
|  |  |  | MARMOSET | TTTTCAGTTGTGTGTAAGCAAGTTTTT | 7mer-A1 |
|  |  |  | DOG | GGATATGGTAGTGTGTGGTTCTCTTTTGGAATTTTTTTCAGGTGATTTAATA | 6mer |
|  |  |  | DOG | TAGTGTGTGGTTCTCT | 6mer |
|  |  |  | DOG | GTGTGTGG | 6mer |
|  |  |  | PIG | GGATATGGTAGTGTGTGGTTCTCTTTTGGAATTTTTTTCAGGTGATTTAATA | 6mer |
|  |  |  | PIG | TAGTGTGTGGTTCTCT | 6mer |
|  |  |  | PIG | GTGTGTGG | 6mer |
|  |  |  | COW | TAGTGTGTGGTTCTCT | 6mer |
|  |  |  | COW | GTGTGTGG | 6mer |
|  |  |  | MOUSE | TAGTGTGTGGTTCTCT | 6mer |
|  |  |  | MOUSE | GTGTGTGG | 6mer |
|  |  |  | TURTLE | GTGTGTGG | 7mer-m8 |
|  |  |  | ALLIGATOR | GTGTGTGG | 7mer-m8 |

  
  
  
  

| Seed Matches to the miRNA miR-34-5p/449-5p | | | | | |
| --- | --- | --- | --- | --- | --- |
| Seed | Conservation | Species | Matches | | |
|  |  |  | Sequence | Motif | Type |
| GGCAGUG | Broadly Conserved | Human (Homo sapiens)  Chicken (Gallus gallus)  Rhesus (Macaca mulatta)  Mouse (Mus musculus)  Rat (Rattus norvegicus)  Opossum (Monodelphis domestica) | HUMAN | CTCATGAATCTTGTCTGAAGCTTTTGAGGGCAGACTGCCAAGTCCTGGAG | 7mer-A1 |
|  |  |  | MARMOSET | CTCATGAATCTTGTCTGAAGCTTTTGAGGGCAGACTGCCAAGTCCTGGAG | 7mer-A1 |

  
  
  
  

| Seed Matches to the miRNA miR-154-3p/487-3p | | | | | |
| --- | --- | --- | --- | --- | --- |
| Seed | Conservation | Species | Matches | | |
|  |  |  | Sequence | Motif | Type |
| AUCAUAC | Conserved | Human (Homo sapiens) | HUMAN | GGGTTTAGGTAATTGTTTAGTTTATGATTGCAGATAAA | 6mer |
|  |  |  | HUMAN | TTAGGTAATTGTTTAGTTTATGATT | 6mer |
|  |  |  | HUMAN | TAACCTCTTAGACAGGTGGGAGATTATGATCAGAGTAAAAGGTAATTACACATTTTATTTCCAGAAAGTCAGG | 6mer |
|  |  |  | HUMAN | ACCTCTTAGACAGGTGGGAGATTATGATCAGA | 6mer |
|  |  |  | HUMAN | CAGGTGGGAGATTATGATCAGA | 6mer |
|  |  |  | HUMAN | ATGCAGTACTGTTCTGATCCCGCTGCTATTAGAATGCATTGTGAAACGACTGGAGTATGATTAAAAGTTGTGTTCCCCAATGCTTGGAGTAGTGATTGTTGAAGGAAAAAA | 7mer-m8 |
|  |  |  | HUMAN | GCTGCTATTAGAATGCATTGTGAAACGACTGGAGTATGATTAAAAGTTGTGTT | 7mer-m8 |
|  |  |  | HUMAN | GCTGCTATTAGAATGCATTGTGAAACGACTGGAGTATGATTAAAAGTT | 7mer-m8 |
|  |  |  | MARMOSET | GGGTTTAGGTAATTGTTTAGTTTATGATTGCAGATAAA | 6mer |
|  |  |  | MARMOSET | TTAGGTAATTGTTTAGTTTATGATT | 6mer |
|  |  |  | MARMOSET | TAACCTCTTAGACAGGTGGGAGATTATGATCAGAGTAAAAGGTAATTACACATTTTATTTCCAGAAAGTCAGG | 6mer |
|  |  |  | MARMOSET | ACCTCTTAGACAGGTGGGAGATTATGATCAGA | 6mer |
|  |  |  | MARMOSET | CAGGTGGGAGATTATGATCAGA | 6mer |
|  |  |  | MARMOSET | ATGCAGTACTGTTCTGATCCCGCTGCTATTAGAATGCATTGTGAAACGACTGGAGTATGATTAAAAGTTGTGTTCCCCAATGCTTGGAGTAGTGATTGTTGAAGGAAAAAA | 7mer-m8 |
|  |  |  | MARMOSET | GCTGCTATTAGAATGCATTGTGAAACGACTGGAGTATGATTAAAAGTTGTGTT | 7mer-m8 |
|  |  |  | MARMOSET | GCTGCTATTAGAATGCATTGTGAAACGACTGGAGTATGATTAAAAGTT | 7mer-m8 |
|  |  |  | DOG | TTAGGTAATTGTTTAGTTTATGATT | 6mer |
|  |  |  | DOG | ACCTCTTAGACAGGTGGGAGATTATGATCAGA | 6mer |
|  |  |  | DOG | CAGGTGGGAGATTATGATCAGA | 6mer |
|  |  |  | DOG | GCTGCTATTAGAATGCATTGTGAAACGACTGGAGTATGATTAAAAGTTGTGTT | 7mer-m8 |
|  |  |  | DOG | GCTGCTATTAGAATGCATTGTGAAACGACTGGAGTATGATTAAAAGTT | 7mer-m8 |
|  |  |  | PIG | TTAGGTAATTGTTTAGTTTATGATT | 6mer |
|  |  |  | PIG | CAGGTGGGAGATTATGATCAGA | 6mer |
|  |  |  | PIG | GCTGCTATTAGAATGCATTGTGAAACGACTGGAGTATGATTAAAAGTT | 7mer-m8 |
|  |  |  | COW | CAGGTGGGAGATTATGATCAGA | 6mer |

  
  
  
  

| Seed Matches to the miRNA miR-224-5p | | | | | |
| --- | --- | --- | --- | --- | --- |
| Seed | Conservation | Species | Matches | | |
|  |  |  | Sequence | Motif | Type |
| AAGUCAC | Conserved | Human (Homo sapiens)  Rhesus (Macaca mulatta)  Mouse (Mus musculus)  Rat (Rattus norvegicus) | HUMAN | AAGGTGACTTAAACAG | 8mer |
|  |  |  | HUMAN | AATAATGTGACTTCTTAAAAG | 7mer-m8 |
|  |  |  | HUMAN | TTTTTTTTTACAGACTTCACAGAGAATGCAGTTGTCTTGACTTCAGGTCTGTCTGTTCTGTTGGCAAGTAA | 6mer |
|  |  |  | MARMOSET | AAGGTGACTTAAACAG | 8mer |
|  |  |  | MARMOSET | AATAATGTGACTTCTTAAAAG | 7mer-m8 |
|  |  |  | MARMOSET | TTTTTTTTTACAGACTTCACAGAGAATGCAGTTGTCTTGACTTCAGGTCTGTCTGTTCTGTTGGCAAGTAA | 6mer |

  
  
  
  

| Seed Matches to the miRNA miR-670-3p | | | | | |
| --- | --- | --- | --- | --- | --- |
| Seed | Conservation | Species | Matches | | |
|  |  |  | Sequence | Motif | Type |
| UUCCUCA | Conserved | Human (Homo sapiens)  Rhesus (Macaca mulatta)  Mouse (Mus musculus) | HUMAN | AGCTTGAGGAAAC | 8mer |
|  |  |  | HUMAN | AAGAGTAGCATGAGGAAGGAA | 7mer-m8 |
|  |  |  | HUMAN | TCCAGCTGAGTGATAAAGGCTGAGTGTTGAGGAAATTTCTGCAGTTTTAAGCAGTCGT | 8mer |
|  |  |  | HUMAN | TCCAGCTGAGTGATAAAGGCTGAGTGTTGAGGAAATTTCTGCAG | 8mer |
|  |  |  | HUMAN | AAAGGCTGAGTGTTGAGGAAATTTCTGCAG | 8mer |
|  |  |  | HUMAN | AGGCTGAGTGTTGAGGAAAT | 8mer |
|  |  |  | MARMOSET | AGCTTGAGGAAAC | 8mer |
|  |  |  | MARMOSET | AAGAGTAGCATGAGGAAGGAA | 7mer-m8 |
|  |  |  | MARMOSET | TCCAGCTGAGTGATAAAGGCTGAGTGTTGAGGAAATTTCTGCAGTTTTAAGCAGTCGT | 8mer |
|  |  |  | MARMOSET | TCCAGCTGAGTGATAAAGGCTGAGTGTTGAGGAAATTTCTGCAG | 8mer |
|  |  |  | MARMOSET | AAAGGCTGAGTGTTGAGGAAATTTCTGCAG | 8mer |
|  |  |  | MARMOSET | AGGCTGAGTGTTGAGGAAAT | 8mer |
|  |  |  | DOG | TCCAGCTGAGTGATAAAGGCTGAGTGTTGAGGAAATTTCTGCAG | 8mer |
|  |  |  | DOG | AAAGGCTGAGTGTTGAGGAAATTTCTGCAG | 8mer |
|  |  |  | DOG | AGGCTGAGTGTTGAGGAAAT | 8mer |
|  |  |  | PIG | AAAGGCTGAGTGTTGAGGAAATTTCTGCAG | 8mer |
|  |  |  | PIG | AGGCTGAGTGTTGAGGAAAT | 8mer |
|  |  |  | COW | AAAGGCTGAGTGTTGAGGAAATTTCTGCAG | 8mer |
|  |  |  | COW | AGGCTGAGTGTTGAGGAAAT | 8mer |
|  |  |  | MOUSE | AGGCTGAGTGTTGAGGAAAT | 8mer |

  
  
  
  

| Seed Matches to the miRNA miR-411-5p.2 | | | | | |
| --- | --- | --- | --- | --- | --- |
| Seed | Conservation | Species | Matches | | |
|  |  |  | Sequence | Motif | Type |
| UAGUAGA | Conserved | Human (Homo sapiens)  Mouse (Mus musculus) | HUMAN | TTAAAACTACTATAG | 6mer |
|  |  |  | HUMAN | ACTACTATAG | 6mer |
|  |  |  | MARMOSET | TTAAAACTACTATAG | 6mer |
|  |  |  | MARMOSET | ACTACTATAG | 6mer |
|  |  |  | DOG | ACTACTATAG | 6mer |
|  |  |  | PIG | ACTACTATAG | 6mer |
|  |  |  | COW | ACTACTATAG | 6mer |
|  |  |  | MOUSE | ACTACTATAG | 6mer |

  
  
  
  

| Seed Matches to the miRNA miR-532-3p | | | | | |
| --- | --- | --- | --- | --- | --- |
| Seed | Conservation | Species | Matches | | |
|  |  |  | Sequence | Motif | Type |
| CUCCCAC | Conserved | Human (Homo sapiens)  Rhesus (Macaca mulatta)  Mouse (Mus musculus)  Rat (Rattus norvegicus) | HUMAN | TTAATTGGGAGTGGTAGGA | 6mer |
|  |  |  | HUMAN | TTATCAGAAGAGTTGCTTCATTTCATCTGGGAGCAGAAAACAGCAGGCAGCTGTTAACAGATAAGTTTAACTTGCATCTGCA | 6mer |
|  |  |  | HUMAN | TTATCAGAAGAGTTGCTTCATTTCATCTGGGAGCAGAAAACAGCAGGCAGCTGTTAACAGATAAGTTTAA | 6mer |
|  |  |  | HUMAN | TCATCTGGGAGCAGAAAACAGCAGGCAGCTGTT | 6mer |
|  |  |  | HUMAN | TAACCTCTTAGACAGGTGGGAGATTATGATCAGAGTAAAAGGTAATTACACATTTTATTTCCAGAAAGTCAGG | 8mer |
|  |  |  | HUMAN | ACCTCTTAGACAGGTGGGAGATTATGATCAGA | 8mer |
|  |  |  | HUMAN | CAGGTGGGAGATTATGATCAGA | 8mer |
|  |  |  | HUMAN | CAGGTGGGAGAT | 8mer |
|  |  |  | HUMAN | AGTGAGTGTATGAGACCTTGCAGTGAGTTTATCAGCATACTCAAAATTTTTTTCCTGGAATTTGGAGGGATGGGAGGAGGGGGTGGGGCTTACTTGTT | 6mer |
|  |  |  | MARMOSET | TTAATTGGGAGTGGTAGGA | 6mer |
|  |  |  | MARMOSET | TTATCAGAAGAGTTGCTTCATTTCATCTGGGAGCAGAAAACAGCAGGCAGCTGTTAACAGATAAGTTTAACTTGCATCTGCA | 6mer |
|  |  |  | MARMOSET | TTATCAGAAGAGTTGCTTCATTTCATCTGGGAGCAGAAAACAGCAGGCAGCTGTTAACAGATAAGTTTAA | 6mer |
|  |  |  | MARMOSET | TCATCTGGGAGCAGAAAACAGCAGGCAGCTGTT | 6mer |
|  |  |  | MARMOSET | TAACCTCTTAGACAGGTGGGAGATTATGATCAGAGTAAAAGGTAATTACACATTTTATTTCCAGAAAGTCAGG | 8mer |
|  |  |  | MARMOSET | ACCTCTTAGACAGGTGGGAGATTATGATCAGA | 8mer |
|  |  |  | MARMOSET | CAGGTGGGAGATTATGATCAGA | 8mer |
|  |  |  | MARMOSET | CAGGTGGGAGAT | 8mer |
|  |  |  | MARMOSET | AGTGAGTGTATGAGACCTTGCAGTGAGTTTATCAGCATACTCAAAATTTTTTTCCTGGAATTTGGAGGGATGGGAGGAGGGGGTGGGGCTTACTTGTT | 6mer |
|  |  |  | DOG | TTATCAGAAGAGTTGCTTCATTTCATCTGGGAGCAGAAAACAGCAGGCAGCTGTTAACAGATAAGTTTAA | 6mer |
|  |  |  | DOG | TCATCTGGGAGCAGAAAACAGCAGGCAGCTGTT | 6mer |
|  |  |  | DOG | ACCTCTTAGACAGGTGGGAGATTATGATCAGA | 8mer |
|  |  |  | DOG | CAGGTGGGAGATTATGATCAGA | 8mer |
|  |  |  | DOG | CAGGTGGGAGAT | 8mer |
|  |  |  | PIG | TCATCTGGGAGCAGAAAACAGCAGGCAGCTGTT | 6mer |
|  |  |  | PIG | CAGGTGGGAGATTATGATCAGA | 8mer |
|  |  |  | PIG | CAGGTGGGAGAT | 8mer |
|  |  |  | COW | TCATCTGGGAGCAGAAAACAGCAGGCAGCTGTT | 6mer |
|  |  |  | COW | CAGGTGGGAGATTATGATCAGA | 8mer |
|  |  |  | COW | CAGGTGGGAGAT | 8mer |
|  |  |  | MOUSE | CAGGTGGGAGAT | 8mer |

  
  
  
  

| Seed Matches to the miRNA miR-181-5p | | | | | |
| --- | --- | --- | --- | --- | --- |
| Seed | Conservation | Species | Matches | | |
|  |  |  | Sequence | Motif | Type |
| ACAUUCA | Broadly Conserved | Human (Homo sapiens)  X. tropicalis (Xenopus tropicalis)  Chicken (Gallus gallus)  Rhesus (Macaca mulatta)  Chimp (Pan troglodytes)  Mouse (Mus musculus)  Rat (Rattus norvegicus)  Opossum (Monodelphis domestica) | HUMAN | TAACTGATTAAGAATTGTGATAGTTCAGCTTGAATGTCTCTTAGAGGGTGGGCTTTTGTTGATGAGGGAGGGGAAACTTTTTTTTT | 7mer-m8 |
|  |  |  | HUMAN | TCAAAATAATAAACTATTTTTATTAGAGAATGTATACTTTTAGAAAGCTGTCTCCTTATTTAAATAAAATA | 7mer-A1 |
|  |  |  | HUMAN | TAGAGAATGTAT | 7mer-A1 |
|  |  |  | HUMAN | GAATGTAT | 7mer-A1 |
|  |  |  | HUMAN | AGATCAGGATTTGAGCGGAAGAACGAATGTAACTTT | 7mer-A1 |
|  |  |  | MARMOSET | TAACTGATTAAGAATTGTGATAGTTCAGCTTGAATGTCTCTTAGAGGGTGGGCTTTTGTTGATGAGGGAGGGGAAACTTTTTTTTT | 7mer-m8 |
|  |  |  | MARMOSET | TCAAAATAATAAACTATTTTTATTAGAGAATGTATACTTTTAGAAAGCTGTCTCCTTATTTAAATAAAATA | 7mer-A1 |
|  |  |  | MARMOSET | TAGAGAATGTAT | 7mer-A1 |
|  |  |  | MARMOSET | GAATGTAT | 7mer-A1 |
|  |  |  | MARMOSET | AGATCAGGATTTGAGCGGAAGAACGAATGTAACTTT | 7mer-A1 |
|  |  |  | DOG | TAGAGAATGTAT | 7mer-A1 |
|  |  |  | DOG | GAATGTAT | 7mer-A1 |
|  |  |  | PIG | GAATGTAT | 7mer-A1 |
|  |  |  | COW | GAATGTAT | 7mer-A1 |
|  |  |  | MOUSE | GAATGTAT | 7mer-A1 |

  
  
  
  

| Seed Matches to the miRNA miR-136-5p | | | | | |
| --- | --- | --- | --- | --- | --- |
| Seed | Conservation | Species | Matches | | |
|  |  |  | Sequence | Motif | Type |
| CUCCAUU | Conserved | Human (Homo sapiens)  Mouse (Mus musculus)  Rat (Rattus norvegicus) | HUMAN | CTTCATGGAGTA | 6mer |
|  |  |  | HUMAN | ATGGAGT | 6mer |
|  |  |  | HUMAN | TTAAGATTTTTCAGGTACCCCTCACTAAAGGCACCGAAGGCTTAAAGTAGGACAACCATGGAGCCTTCCTGTGGCA | 6mer |
|  |  |  | HUMAN | AAGTAGGACAACCATGGAGCCTTCCTGTGGCA | 6mer |
|  |  |  | MARMOSET | CTTCATGGAGTA | 6mer |
|  |  |  | MARMOSET | ATGGAGT | 6mer |
|  |  |  | MARMOSET | TTAAGATTTTTCAGGTACCCCTCACTAAAGGCACCGAAGGCTTAAAGTAGGACAACCATGGAGCCTTCCTGTGGCA | 6mer |
|  |  |  | MARMOSET | AAGTAGGACAACCATGGAGCCTTCCTGTGGCA | 6mer |
|  |  |  | DOG | ATGGAGT | 7mer-m8 |
|  |  |  | DOG | AAGTAGGACAACCATGGAGCCTTCCTGTGGCA | 6mer |

  
  
  
  

| Seed Matches to the miRNA miR-23-3p | | | | | |
| --- | --- | --- | --- | --- | --- |
| Seed | Conservation | Species | Matches | | |
|  |  |  | Sequence | Motif | Type |
| UCACAUU | Broadly Conserved | Human (Homo sapiens)  Chicken (Gallus gallus)  Rhesus (Macaca mulatta)  Cow (Bos taurus)  Mouse (Mus musculus)  Rat (Rattus norvegicus)  Opossum (Monodelphis domestica) | HUMAN | AATAATGTGACTTCTTAAAAG | 7mer-m8 |
|  |  |  | HUMAN | TAATGTGA | 7mer-m8 |
|  |  |  | HUMAN | TGGCATGTGAGCAA | 6mer |
|  |  |  | HUMAN | GCATGTGAGCA | 6mer |
|  |  |  | MARMOSET | AATAATGTGACTTCTTAAAAG | 7mer-m8 |
|  |  |  | MARMOSET | TAATGTGA | 7mer-m8 |
|  |  |  | MARMOSET | TGGCATGTGAGCAA | 6mer |
|  |  |  | MARMOSET | GCATGTGAGCA | 6mer |
|  |  |  | DOG | TAATGTGA | 7mer-m8 |
|  |  |  | DOG | GCATGTGAGCA | 6mer |

  
  
  
  

| Seed Matches to the miRNA miR-132-3p/212-3p | | | | | |
| --- | --- | --- | --- | --- | --- |
| Seed | Conservation | Species | Matches | | |
|  |  |  | Sequence | Motif | Type |
| AACAGUC | Broadly Conserved | Human (Homo sapiens)  Rhesus (Macaca mulatta)  Mouse (Mus musculus)  Rat (Rattus norvegicus)  Opossum (Monodelphis domestica) | HUMAN | ATGCAGTACTGTTCTGATCCCGCTGCTATTAGAATGCATTGTGAAACGACTGGAGTATGATTAAAAGTTGTGTTCCCCAATGCTTGGAGTAGTGATTGTTGAAGGAAAAAA | 6mer |
|  |  |  | HUMAN | TGCAGTACTGTTCTGATC | 6mer |
|  |  |  | HUMAN | TGCAGTACTGTTCTGA | 6mer |
|  |  |  | HUMAN | CAGTACTGTTCTGA | 6mer |
|  |  |  | HUMAN | CAGTACTGTTC | 6mer |
|  |  |  | MARMOSET | ATGCAGTACTGTTCTGATCCCGCTGCTATTAGAATGCATTGTGAAACGACTGGAGTATGATTAAAAGTTGTGTTCCCCAATGCTTGGAGTAGTGATTGTTGAAGGAAAAAA | 6mer |
|  |  |  | MARMOSET | TGCAGTACTGTTCTGATC | 6mer |
|  |  |  | MARMOSET | TGCAGTACTGTTCTGA | 6mer |
|  |  |  | MARMOSET | CAGTACTGTTCTGA | 6mer |
|  |  |  | MARMOSET | CAGTACTGTTC | 6mer |
|  |  |  | DOG | TGCAGTACTGTTCTGATC | 6mer |
|  |  |  | DOG | TGCAGTACTGTTCTGA | 6mer |
|  |  |  | DOG | CAGTACTGTTCTGA | 6mer |
|  |  |  | DOG | CAGTACTGTTC | 6mer |
|  |  |  | PIG | TGCAGTACTGTTCTGA | 6mer |
|  |  |  | PIG | CAGTACTGTTCTGA | 6mer |
|  |  |  | PIG | CAGTACTGTTC | 6mer |
|  |  |  | COW | CAGTACTGTTCTGA | 6mer |
|  |  |  | COW | CAGTACTGTTC | 6mer |
|  |  |  | MOUSE | CAGTACTGTTC | 6mer |

  
  
  
  

| Seed Matches to the miRNA miR-330-3p | | | | | |
| --- | --- | --- | --- | --- | --- |
| Seed | Conservation | Species | Matches | | |
|  |  |  | Sequence | Motif | Type |
| CAAAGCA | Conserved | Human (Homo sapiens)  Rhesus (Macaca mulatta)  Rat (Rattus norvegicus) | HUMAN | TGGAAGAGTATTCCCAGTTGAAGCTGAAAAGTACAGCACAGTGCAGCTTTGGTTCATATTCAGTCATCTCAGGAGAACTTCAGAAGAGCTTGAGTAGGCCAAATGTTGAAGTTAAGTTTTC | 6mer |
|  |  |  | HUMAN | GTACAGCACAGTGCAGCTTTGGTTCATA | 6mer |
|  |  |  | HUMAN | TACAGCACAGTGCAGCTTTGGTTCATA | 6mer |
|  |  |  | HUMAN | CAGTGCAGCTTTGGTTCATA | 6mer |
|  |  |  | HUMAN | GCTTTGGTTCA | 6mer |
|  |  |  | MARMOSET | TGGAAGAGTATTCCCAGTTGAAGCTGAAAAGTACAGCACAGTGCAGCTTTGGTTCATATTCAGTCATCTCAGGAGAACTTCAGAAGAGCTTGAGTAGGCCAAATGTTGAAGTTAAGTTTTC | 6mer |
|  |  |  | MARMOSET | GTACAGCACAGTGCAGCTTTGGTTCATA | 6mer |
|  |  |  | MARMOSET | TACAGCACAGTGCAGCTTTGGTTCATA | 6mer |
|  |  |  | MARMOSET | CAGTGCAGCTTTGGTTCATA | 6mer |
|  |  |  | MARMOSET | GCTTTGGTTCA | 6mer |
|  |  |  | DOG | GTACAGCACAGTGCAGCTTTGGTTCATA | 6mer |
|  |  |  | DOG | TACAGCACAGTGCAGCTTTGGTTCATA | 6mer |
|  |  |  | DOG | CAGTGCAGCTTTGGTTCATA | 6mer |
|  |  |  | DOG | GCTTTGGTTCA | 6mer |
|  |  |  | PIG | TACAGCACAGTGCAGCTTTGGTTCATA | 6mer |
|  |  |  | PIG | CAGTGCAGCTTTGGTTCATA | 6mer |
|  |  |  | PIG | GCTTTGGTTCA | 6mer |
|  |  |  | COW | CAGTGCAGCTTTGGTTCATA | 6mer |
|  |  |  | COW | GCTTTGGTTCA | 6mer |
|  |  |  | MOUSE | GCTTTGGTTCA | 6mer |

  
  
  
  

| Seed Matches to the miRNA miR-369-3p | | | | | |
| --- | --- | --- | --- | --- | --- |
| Seed | Conservation | Species | Matches | | |
|  |  |  | Sequence | Motif | Type |
| AUAAUAC | Conserved | Human (Homo sapiens)  Rhesus (Macaca mulatta)  Cow (Bos taurus)  Mouse (Mus musculus)  Rat (Rattus norvegicus) | HUMAN | CAGAGCAAAGGAAGTGGCTTAATGATCCTGAAGGGATTTCTTCTGATGGTAGCTTTTGTATTATCAAGTAAGATTCT | 7mer-m8 |
|  |  |  | HUMAN | TATTATCAAGTAAGATTCT | 7mer-m8 |
|  |  |  | HUMAN | TATTATCAAGTAAGA | 7mer-m8 |
|  |  |  | HUMAN | GAGAGACAACAAAGCGCTATTATCCTAAGGTCAAGA | 6mer |
|  |  |  | MARMOSET | CAGAGCAAAGGAAGTGGCTTAATGATCCTGAAGGGATTTCTTCTGATGGTAGCTTTTGTATTATCAAGTAAGATTCT | 7mer-m8 |
|  |  |  | MARMOSET | TATTATCAAGTAAGATTCT | 7mer-m8 |
|  |  |  | MARMOSET | TATTATCAAGTAAGA | 7mer-m8 |
|  |  |  | MARMOSET | GAGAGACAACAAAGCGCTATTATCCTAAGGTCAAGA | 6mer |
|  |  |  | DOG | TATTATCAAGTAAGATTCT | 6mer |
|  |  |  | DOG | TATTATCAAGTAAGA | 6mer |
|  |  |  | PIG | TATTATCAAGTAAGATTCT | 6mer |
|  |  |  | PIG | TATTATCAAGTAAGA | 6mer |
|  |  |  | COW | TATTATCAAGTAAGATTCT | 7mer-m8 |
|  |  |  | COW | TATTATCAAGTAAGA | 7mer-m8 |
|  |  |  | MOUSE | TATTATCAAGTAAGA | 6mer |

  
  
  
  

| Seed Matches to the miRNA miR-1251-5p | | | | | |
| --- | --- | --- | --- | --- | --- |
| Seed | Conservation | Species | Matches | | |
|  |  |  | Sequence | Motif | Type |
| CUCUAGC | Conserved | Human (Homo sapiens)  Rhesus (Macaca mulatta)  Mouse (Mus musculus)  Opossum (Monodelphis domestica) | HUMAN | TAGAAACTAGAGCAGTTCTCAC | 6mer |
|  |  |  | HUMAN | TAGAAACTAGAGCA | 6mer |
|  |  |  | MARMOSET | TAGAAACTAGAGCAGTTCTCAC | 6mer |
|  |  |  | MARMOSET | TAGAAACTAGAGCA | 6mer |
|  |  |  | DOG | TAGAAACTAGAGCA | 6mer |

  
  
  
  

| Seed Matches to the miRNA miR-383-5p.2 | | | | | |
| --- | --- | --- | --- | --- | --- |
| Seed | Conservation | Species | Matches | | |
|  |  |  | Sequence | Motif | Type |
| AGAUCAG | Broadly Conserved | Human (Homo sapiens)  Mouse (Mus musculus) | HUMAN | CAGTTCAGTGATCTT | 6mer |
|  |  |  | MARMOSET | CAGTTCAGTGATCTT | 6mer |

  
  
  
  

| Seed Matches to the miRNA miR-423-5p | | | | | |
| --- | --- | --- | --- | --- | --- |
| Seed | Conservation | Species | Matches | | |
|  |  |  | Sequence | Motif | Type |
| GAGGGGC | Conserved | Human (Homo sapiens)  Rhesus (Macaca mulatta)  Cow (Bos taurus)  Mouse (Mus musculus) | HUMAN | TGTGTGGGTTTCTCTCTCCCCTCCCTTGGTCTTAATTCTTACATGCAGGAACA | 6mer |
|  |  |  | HUMAN | TGTGTGGGTTTCTCTCTCCCCTCCCTTGGTCTTAATTCTTACA | 6mer |
|  |  |  | HUMAN | TTAAGATTTTTCAGGTACCCCTCACTAAAGGCACCGAAGGCTTAAAGTAGGACAACCATGGAGCCTTCCTGTGGCA | 7mer-A1 |
|  |  |  | HUMAN | TTAAGATTTTTCAGGTACCCCTC | 7mer-A1 |
|  |  |  | HUMAN | CTAGCACAGACCCTTCACCCCTCACCTCGATGCAGCC | 7mer-A1 |
|  |  |  | HUMAN | CAGACCCTTCACCCCTCACCTCGATGC | 7mer-A1 |
|  |  |  | HUMAN | CAGACCCTTCACCCCTCACCT | 7mer-A1 |
|  |  |  | MARMOSET | TGTGTGGGTTTCTCTCTCCCCTCCCTTGGTCTTAATTCTTACATGCAGGAACA | 6mer |
|  |  |  | MARMOSET | TGTGTGGGTTTCTCTCTCCCCTCCCTTGGTCTTAATTCTTACA | 6mer |
|  |  |  | MARMOSET | TTAAGATTTTTCAGGTACCCCTCACTAAAGGCACCGAAGGCTTAAAGTAGGACAACCATGGAGCCTTCCTGTGGCA | 7mer-A1 |
|  |  |  | MARMOSET | TTAAGATTTTTCAGGTACCCCTC | 7mer-A1 |
|  |  |  | MARMOSET | CTAGCACAGACCCTTCACCCCTCACCTCGATGCAGCC | 7mer-A1 |
|  |  |  | MARMOSET | CAGACCCTTCACCCCTCACCTCGATGC | 7mer-A1 |
|  |  |  | MARMOSET | CAGACCCTTCACCCCTCACCT | 7mer-A1 |
|  |  |  | DOG | TGTGTGGGTTTCTCTCTCCCCTCCCTTGGTCTTAATTCTTACA | 6mer |
|  |  |  | DOG | TTAAGATTTTTCAGGTACCCCTC | 6mer |
|  |  |  | DOG | CAGACCCTTCACCCCTCACCTCGATGC | 7mer-A1 |
|  |  |  | DOG | CAGACCCTTCACCCCTCACCT | 7mer-A1 |
|  |  |  | PIG | TTAAGATTTTTCAGGTACCCCTC | 7mer-A1 |
|  |  |  | PIG | CAGACCCTTCACCCCTCACCTCGATGC | 7mer-A1 |
|  |  |  | PIG | CAGACCCTTCACCCCTCACCT | 7mer-A1 |
|  |  |  | COW | TTAAGATTTTTCAGGTACCCCTC | 7mer-A1 |
|  |  |  | COW | CAGACCCTTCACCCCTCACCTCGATGC | 7mer-A1 |
|  |  |  | COW | CAGACCCTTCACCCCTCACCT | 7mer-A1 |
|  |  |  | MOUSE | TTAAGATTTTTCAGGTACCCCTC | 7mer-A1 |
|  |  |  | MOUSE | CAGACCCTTCACCCCTCACCT | 7mer-A1 |

  
  
  
  

| Seed Matches to the miRNA miR-877-5p | | | | | |
| --- | --- | --- | --- | --- | --- |
| Seed | Conservation | Species | Matches | | |
|  |  |  | Sequence | Motif | Type |
| UAGAGGA | Conserved | Human (Homo sapiens)  Rhesus (Macaca mulatta)  Mouse (Mus musculus) | HUMAN | CTTAAAAGCCTCTAAAGTGAT | 7mer-A1 |
|  |  |  | HUMAN | TGATGAGCATATAATAATTCCAGGCACATGGCAATAGAGGCCCTCTAAATAAGGAATAA | 7mer-A1 |
|  |  |  | HUMAN | TAATAATTCCAGGCACATGGCAATAGAGGCCCTCTAAATAAGGAATAA | 7mer-A1 |
|  |  |  | MARMOSET | CTTAAAAGCCTCTAAAGTGAT | 7mer-A1 |
|  |  |  | MARMOSET | TGATGAGCATATAATAATTCCAGGCACATGGCAATAGAGGCCCTCTAAATAAGGAATAA | 7mer-A1 |
|  |  |  | MARMOSET | TAATAATTCCAGGCACATGGCAATAGAGGCCCTCTAAATAAGGAATAA | 7mer-A1 |
|  |  |  | DOG | TAATAATTCCAGGCACATGGCAATAGAGGCCCTCTAAATAAGGAATAA | 7mer-A1 |

  
  
  
  

| Seed Matches to the miRNA miR-124-3p.2/506-3p | | | | | |
| --- | --- | --- | --- | --- | --- |
| Seed | Conservation | Species | Matches | | |
|  |  |  | Sequence | Motif | Type |
| UAAGGCA | Broadly Conserved | Human (Homo sapiens) | HUMAN | AAAAACTAAGGCAGAAGGCTTTTGGAAGAGTTAGAAGAATTTGGAAGGCCTTAAA | 7mer-A1 |
|  |  |  | MARMOSET | AAAAACTAAGGCAGAAGGCTTTTGGAAGAGTTAGAAGAATTTGGAAGGCCTTAAA | 7mer-A1 |

  
  
  
  

| Seed Matches to the miRNA miR-31-5p | | | | | |
| --- | --- | --- | --- | --- | --- |
| Seed | Conservation | Species | Matches | | |
|  |  |  | Sequence | Motif | Type |
| GGCAAGA | Broadly Conserved | Human (Homo sapiens)  Chicken (Gallus gallus)  Mouse (Mus musculus)  Rat (Rattus norvegicus) | HUMAN | AGGACTTGCCTCAACT | 6mer |
|  |  |  | HUMAN | AGGACTTGCCTCAAC | 6mer |
|  |  |  | MARMOSET | AGGACTTGCCTCAACT | 6mer |
|  |  |  | MARMOSET | AGGACTTGCCTCAAC | 6mer |
|  |  |  | DOG | AGGACTTGCCTCAAC | 6mer |
|  |  |  | PIG | AGGACTTGCCTCAAC | 6mer |

  
  
  
  

| Seed Matches to the miRNA miR-455-5p | | | | | |
| --- | --- | --- | --- | --- | --- |
| Seed | Conservation | Species | Matches | | |
|  |  |  | Sequence | Motif | Type |
| AUGUGCC | Broadly Conserved | Human (Homo sapiens)  Chicken (Gallus gallus)  Rhesus (Macaca mulatta)  Cow (Bos taurus)  Mouse (Mus musculus)  Rat (Rattus norvegicus)  Opossum (Monodelphis domestica) | HUMAN | TGATGAGCATATAATAATTCCAGGCACATGGCAATAGAGGCCCTCTAAATAAGGAATAA | 7mer-m8 |
|  |  |  | HUMAN | TAATAATTCCAGGCACATGGCAATAGAGGCCCTCTAAATAAGGAATAA | 7mer-m8 |
|  |  |  | HUMAN | TAATAATTCCAGGCACATGGC | 7mer-m8 |
|  |  |  | MARMOSET | TGATGAGCATATAATAATTCCAGGCACATGGCAATAGAGGCCCTCTAAATAAGGAATAA | 7mer-m8 |
|  |  |  | MARMOSET | TAATAATTCCAGGCACATGGCAATAGAGGCCCTCTAAATAAGGAATAA | 7mer-m8 |
|  |  |  | MARMOSET | TAATAATTCCAGGCACATGGC | 7mer-m8 |
|  |  |  | DOG | TAATAATTCCAGGCACATGGCAATAGAGGCCCTCTAAATAAGGAATAA | 7mer-m8 |
|  |  |  | DOG | TAATAATTCCAGGCACATGGC | 7mer-m8 |
|  |  |  | PIG | TAATAATTCCAGGCACATGGC | 7mer-m8 |
|  |  |  | COW | TAATAATTCCAGGCACATGGC | 7mer-m8 |

  
  
  
  

| Seed Matches to the miRNA miR-381-3p | | | | | |
| --- | --- | --- | --- | --- | --- |
| Seed | Conservation | Species | Matches | | |
|  |  |  | Sequence | Motif | Type |
| AUACAAG | Conserved | Human (Homo sapiens)  Rhesus (Macaca mulatta) | HUMAN | AATTTCAGCAAATCTGTAAGCAGTTTGTATGTTTAGTTGGGGTAATG | 6mer |
|  |  |  | HUMAN | CAGAGCAAAGGAAGTGGCTTAATGATCCTGAAGGGATTTCTTCTGATGGTAGCTTTTGTATTATCAAGTAAGATTCT | 6mer |
|  |  |  | MARMOSET | AATTTCAGCAAATCTGTAAGCAGTTTGTATGTTTAGTTGGGGTAATG | 6mer |
|  |  |  | MARMOSET | CAGAGCAAAGGAAGTGGCTTAATGATCCTGAAGGGATTTCTTCTGATGGTAGCTTTTGTATTATCAAGTAAGATTCT | 6mer |

  
  
  
  

| Seed Matches to the miRNA miR-483-3p.2 | | | | | |
| --- | --- | --- | --- | --- | --- |
| Seed | Conservation | Species | Matches | | |
|  |  |  | Sequence | Motif | Type |
| CACUCCU | Conserved | Human (Homo sapiens)  Mouse (Mus musculus) | HUMAN | TTAATTGGGAGTGGTAGGA | 6mer |
|  |  |  | HUMAN | ATTTTGGGATGGTCTTAACAGGGAAGAGAGAGGGTGGGGGAGAAAATGTTTTTTTCTAAGATTTTCCACAGATGCTATAGTACTATTGACAAACTGGGTTAGAGAAGGAGTGTAC | 7mer-m8 |
|  |  |  | HUMAN | CTCTTTCTGGAGTGAAGCATCC | 7mer-A1 |
|  |  |  | MARMOSET | TTAATTGGGAGTGGTAGGA | 6mer |
|  |  |  | MARMOSET | ATTTTGGGATGGTCTTAACAGGGAAGAGAGAGGGTGGGGGAGAAAATGTTTTTTTCTAAGATTTTCCACAGATGCTATAGTACTATTGACAAACTGGGTTAGAGAAGGAGTGTAC | 7mer-m8 |
|  |  |  | MARMOSET | CTCTTTCTGGAGTGAAGCATCC | 7mer-A1 |

  
  
  
  

| Seed Matches to the miRNA miR-182-5p | | | | | |
| --- | --- | --- | --- | --- | --- |
| Seed | Conservation | Species | Matches | | |
|  |  |  | Sequence | Motif | Type |
| UUGGCAA | Broadly Conserved | Human (Homo sapiens)  X. tropicalis (Xenopus tropicalis)  Mouse (Mus musculus)  Opossum (Monodelphis domestica) | HUMAN | TGCCAAGGC | 6mer |
|  |  |  | HUMAN | CTCATGAATCTTGTCTGAAGCTTTTGAGGGCAGACTGCCAAGTCCTGGAG | 6mer |
|  |  |  | HUMAN | TTATCTGCATATGCCAAAAAA | 7mer-A1 |
|  |  |  | HUMAN | AGAGATGAGTTGGGATCAAGTGGATTGAGGAGGCTGTGCTGTGTGCCAAT | 6mer |
|  |  |  | HUMAN | TGCCAAT | 6mer |
|  |  |  | MARMOSET | TGCCAAGGC | 6mer |
|  |  |  | MARMOSET | CTCATGAATCTTGTCTGAAGCTTTTGAGGGCAGACTGCCAAGTCCTGGAG | 6mer |
|  |  |  | MARMOSET | TTATCTGCATATGCCAAAAAA | 7mer-A1 |
|  |  |  | MARMOSET | AGAGATGAGTTGGGATCAAGTGGATTGAGGAGGCTGTGCTGTGTGCCAAT | 6mer |
|  |  |  | MARMOSET | TGCCAAT | 6mer |
|  |  |  | DOG | TGCCAAT | 7mer-m8 |
|  |  |  | PIG | TGCCAAT | 7mer-m8 |
|  |  |  | COW | TGCCAAT | 7mer-m8 |
|  |  |  | MOUSE | TGCCAAT | 6mer |

  
  
  
  

| Seed Matches to the miRNA miR-330-3p.2 | | | | | |
| --- | --- | --- | --- | --- | --- |
| Seed | Conservation | Species | Matches | | |
|  |  |  | Sequence | Motif | Type |
| AAAGCAC | Conserved | Human (Homo sapiens)  Mouse (Mus musculus) | HUMAN | TGCTTTTAGATTA | 6mer |
|  |  |  | HUMAN | GCTGTGCTGTTGGCACGAACACCTTCAGGGACTGGAGCTGCTTTTAT | 6mer |
|  |  |  | HUMAN | TGGAGCTGCTTTT | 6mer |
|  |  |  | HUMAN | CAATGTCCATCTCAAAATACTGCTTTTACAAAAGCAGAATAAAA | 6mer |
|  |  |  | HUMAN | CCATCTCAAAATACTGCTTTTACAAAAGCAGAATAAAA | 6mer |
|  |  |  | HUMAN | CTCAAAATACTGCTTTTACAAAAGCAGAATAAAA | 6mer |
|  |  |  | HUMAN | AATACTGCTTTTACAAAAGCAGAAT | 6mer |
|  |  |  | HUMAN | ACTGCTTTTACAAAAGCAGAAT | 6mer |
|  |  |  | HUMAN | ATTTTTAGGTAAAATGCTTTT | 6mer |
|  |  |  | HUMAN | TTTAGGTAAAATGCTTTT | 6mer |
|  |  |  | HUMAN | TTGTTTTCTCAGGTTTTGCTTTTTGGCCTTT | 6mer |
|  |  |  | HUMAN | TTGTTTTCTCAGGTTTTGCTTTTT | 6mer |
|  |  |  | HUMAN | TCTCAGGTTTTGCTTTT | 6mer |
|  |  |  | HUMAN | CAGGTTTTGCTTTT | 6mer |
|  |  |  | HUMAN | CAGGTTTTGCTTT | 6mer |
|  |  |  | MARMOSET | TGCTTTTAGATTA | 7mer-m8 |
|  |  |  | MARMOSET | GCTGTGCTGTTGGCACGAACACCTTCAGGGACTGGAGCTGCTTTTAT | 6mer |
|  |  |  | MARMOSET | TGGAGCTGCTTTT | 6mer |
|  |  |  | MARMOSET | CAATGTCCATCTCAAAATACTGCTTTTACAAAAGCAGAATAAAA | 6mer |
|  |  |  | MARMOSET | CCATCTCAAAATACTGCTTTTACAAAAGCAGAATAAAA | 6mer |
|  |  |  | MARMOSET | CTCAAAATACTGCTTTTACAAAAGCAGAATAAAA | 6mer |
|  |  |  | MARMOSET | AATACTGCTTTTACAAAAGCAGAAT | 6mer |
|  |  |  | MARMOSET | ACTGCTTTTACAAAAGCAGAAT | 6mer |
|  |  |  | MARMOSET | ATTTTTAGGTAAAATGCTTTT | 6mer |
|  |  |  | MARMOSET | TTTAGGTAAAATGCTTTT | 6mer |
|  |  |  | MARMOSET | TTGTTTTCTCAGGTTTTGCTTTTTGGCCTTT | 6mer |
|  |  |  | MARMOSET | TTGTTTTCTCAGGTTTTGCTTTTT | 6mer |
|  |  |  | MARMOSET | TCTCAGGTTTTGCTTTT | 6mer |
|  |  |  | MARMOSET | CAGGTTTTGCTTTT | 6mer |
|  |  |  | MARMOSET | CAGGTTTTGCTTT | 6mer |
|  |  |  | DOG | TGGAGCTGCTTTT | 6mer |
|  |  |  | DOG | CCATCTCAAAATACTGCTTTTACAAAAGCAGAATAAAA | 6mer |
|  |  |  | DOG | CTCAAAATACTGCTTTTACAAAAGCAGAATAAAA | 6mer |
|  |  |  | DOG | AATACTGCTTTTACAAAAGCAGAAT | 6mer |
|  |  |  | DOG | ACTGCTTTTACAAAAGCAGAAT | 6mer |
|  |  |  | DOG | TTTAGGTAAAATGCTTTT | 6mer |
|  |  |  | DOG | TTGTTTTCTCAGGTTTTGCTTTTT | 6mer |
|  |  |  | DOG | TCTCAGGTTTTGCTTTT | 6mer |
|  |  |  | DOG | CAGGTTTTGCTTTT | 6mer |
|  |  |  | DOG | CAGGTTTTGCTTT | 6mer |
|  |  |  | PIG | TGGAGCTGCTTTT | 6mer |
|  |  |  | PIG | CTCAAAATACTGCTTTTACAAAAGCAGAATAAAA | 6mer |
|  |  |  | PIG | AATACTGCTTTTACAAAAGCAGAAT | 6mer |
|  |  |  | PIG | ACTGCTTTTACAAAAGCAGAAT | 6mer |
|  |  |  | PIG | TTTAGGTAAAATGCTTTT | 6mer |
|  |  |  | PIG | TTGTTTTCTCAGGTTTTGCTTTTT | 6mer |
|  |  |  | PIG | TCTCAGGTTTTGCTTTT | 6mer |
|  |  |  | PIG | CAGGTTTTGCTTTT | 6mer |
|  |  |  | PIG | CAGGTTTTGCTTT | 6mer |
|  |  |  | COW | AATACTGCTTTTACAAAAGCAGAAT | 6mer |
|  |  |  | COW | ACTGCTTTTACAAAAGCAGAAT | 6mer |
|  |  |  | COW | TTTAGGTAAAATGCTTTT | 6mer |
|  |  |  | COW | TTGTTTTCTCAGGTTTTGCTTTTT | 6mer |
|  |  |  | COW | TCTCAGGTTTTGCTTTT | 6mer |
|  |  |  | COW | CAGGTTTTGCTTTT | 6mer |
|  |  |  | COW | CAGGTTTTGCTTT | 6mer |
|  |  |  | MOUSE | ACTGCTTTTACAAAAGCAGAAT | 6mer |
|  |  |  | MOUSE | TTGTTTTCTCAGGTTTTGCTTTTT | 6mer |
|  |  |  | MOUSE | TCTCAGGTTTTGCTTTT | 6mer |
|  |  |  | MOUSE | CAGGTTTTGCTTTT | 6mer |
|  |  |  | MOUSE | CAGGTTTTGCTTT | 6mer |
|  |  |  | TURTLE | TCTCAGGTTTTGCTTTT | 6mer |
|  |  |  | TURTLE | CAGGTTTTGCTTTT | 6mer |
|  |  |  | TURTLE | CAGGTTTTGCTTT | 6mer |
|  |  |  | ALLIGATOR | TCTCAGGTTTTGCTTTT | 6mer |
|  |  |  | ALLIGATOR | CAGGTTTTGCTTTT | 6mer |
|  |  |  | ALLIGATOR | CAGGTTTTGCTTT | 6mer |
|  |  |  | LIZARD | TCTCAGGTTTTGCTTTT | 6mer |
|  |  |  | LIZARD | CAGGTTTTGCTTTT | 6mer |
|  |  |  | LIZARD | CAGGTTTTGCTTT | 6mer |
|  |  |  | SNAKE | TCTCAGGTTTTGCTTTT | 6mer |
|  |  |  | SNAKE | CAGGTTTTGCTTTT | 6mer |
|  |  |  | SNAKE | CAGGTTTTGCTTT | 6mer |
|  |  |  | X.TROPICALIS | CAGGTTTTGCTTTT | 6mer |
|  |  |  | X.TROPICALIS | CAGGTTTTGCTTT | 6mer |
|  |  |  | SHARK | CAGGTTTTGCTTTT | 6mer |
|  |  |  | SHARK | CAGGTTTTGCTTT | 6mer |
|  |  |  | OPOSSUM | CAGGTTTTGCTTTT | 6mer |
|  |  |  | OPOSSUM | CAGGTTTTGCTTT | 6mer |
|  |  |  | SPOTTEDGAR | CAGGTTTTGCTTTT | 6mer |
|  |  |  | SPOTTEDGAR | CAGGTTTTGCTTT | 6mer |
|  |  |  | FUGU | CAGGTTTTGCTTTT | 6mer |
|  |  |  | FUGU | CAGGTTTTGCTTT | 6mer |
|  |  |  | NILETILAPIA | CAGGTTTTGCTTTT | 6mer |
|  |  |  | NILETILAPIA | CAGGTTTTGCTTT | 6mer |
|  |  |  | STICKLEBACK | CAGGTTTTGCTTT | 6mer |
|  |  |  | MEDAKA | CAGGTTTTGCTTT | 6mer |
|  |  |  | ZEBRAFISH | CAGGTTTTGCTTT | 6mer |

  
  
  
  

| Seed Matches to the miRNA miR-18-5p | | | | | |
| --- | --- | --- | --- | --- | --- |
| Seed | Conservation | Species | Matches | | |
|  |  |  | Sequence | Motif | Type |
| AAGGUGC | Broadly Conserved | Human (Homo sapiens)  X. tropicalis (Xenopus tropicalis)  Chicken (Gallus gallus)  Mouse (Mus musculus)  Rat (Rattus norvegicus)  Opossum (Monodelphis domestica) | HUMAN | GCTGTGCTGTTGGCACGAACACCTTCAGGGACTGGAGCTGCTTTTAT | 6mer |
|  |  |  | HUMAN | TGCTGTTGGCACGAACACCTTCAGGGA | 6mer |
|  |  |  | HUMAN | AACACCTTCAGGGA | 6mer |
|  |  |  | HUMAN | ACACCTTCAGGGA | 6mer |
|  |  |  | MARMOSET | GCTGTGCTGTTGGCACGAACACCTTCAGGGACTGGAGCTGCTTTTAT | 6mer |
|  |  |  | MARMOSET | TGCTGTTGGCACGAACACCTTCAGGGA | 6mer |
|  |  |  | MARMOSET | AACACCTTCAGGGA | 6mer |
|  |  |  | MARMOSET | ACACCTTCAGGGA | 6mer |
|  |  |  | DOG | TGCTGTTGGCACGAACACCTTCAGGGA | 6mer |
|  |  |  | DOG | AACACCTTCAGGGA | 6mer |
|  |  |  | DOG | ACACCTTCAGGGA | 6mer |
|  |  |  | PIG | AACACCTTCAGGGA | 6mer |
|  |  |  | PIG | ACACCTTCAGGGA | 6mer |
|  |  |  | COW | AACACCTTCAGGGA | 6mer |
|  |  |  | COW | ACACCTTCAGGGA | 6mer |
|  |  |  | MOUSE | ACACCTTCAGGGA | 6mer |

  
  
  
  

| Seed Matches to the miRNA miR-496.1 | | | | | |
| --- | --- | --- | --- | --- | --- |
| Seed | Conservation | Species | Matches | | |
|  |  |  | Sequence | Motif | Type |
| GAGUAUU | Conserved | Human (Homo sapiens) | HUMAN | AGTGAGTGTATGAGACCTTGCAGTGAGTTTATCAGCATACTCAAAATTTTTTTCCTGGAATTTGGAGGGATGGGAGGAGGGGGTGGGGCTTACTTGTT | 7mer-A1 |
|  |  |  | MARMOSET | AGTGAGTGTATGAGACCTTGCAGTGAGTTTATCAGCATACTCAAAATTTTTTTCCTGGAATTTGGAGGGATGGGAGGAGGGGGTGGGGCTTACTTGTT | 7mer-A1 |

  
  
  
  

| Seed Matches to the miRNA miR-145-5p | | | | | |
| --- | --- | --- | --- | --- | --- |
| Seed | Conservation | Species | Matches | | |
|  |  |  | Sequence | Motif | Type |
| UCCAGUU | Broadly Conserved | Human (Homo sapiens)  Rhesus (Macaca mulatta)  Mouse (Mus musculus)  Rat (Rattus norvegicus)  Opossum (Monodelphis domestica) | HUMAN | ACTGGAAGACAGAAGTAC | 8mer |
|  |  |  | HUMAN | GCTGTGCTGTTGGCACGAACACCTTCAGGGACTGGAGCTGCTTTTAT | 6mer |
|  |  |  | HUMAN | ATGCAGTACTGTTCTGATCCCGCTGCTATTAGAATGCATTGTGAAACGACTGGAGTATGATTAAAAGTTGTGTTCCCCAATGCTTGGAGTAGTGATTGTTGAAGGAAAAAA | 6mer |
|  |  |  | HUMAN | GCTGCTATTAGAATGCATTGTGAAACGACTGGAGTATGATTAAAAGTTGTGTT | 6mer |
|  |  |  | HUMAN | GCTGCTATTAGAATGCATTGTGAAACGACTGGAGTATGATTAAAAGTT | 6mer |
|  |  |  | HUMAN | TGAAACGACTGGAGTATGA | 6mer |
|  |  |  | HUMAN | CGACTGGAGTATGA | 6mer |
|  |  |  | MARMOSET | ACTGGAAGACAGAAGTAC | 7mer-A1 |
|  |  |  | MARMOSET | GCTGTGCTGTTGGCACGAACACCTTCAGGGACTGGAGCTGCTTTTAT | 6mer |
|  |  |  | MARMOSET | ATGCAGTACTGTTCTGATCCCGCTGCTATTAGAATGCATTGTGAAACGACTGGAGTATGATTAAAAGTTGTGTTCCCCAATGCTTGGAGTAGTGATTGTTGAAGGAAAAAA | 6mer |
|  |  |  | MARMOSET | GCTGCTATTAGAATGCATTGTGAAACGACTGGAGTATGATTAAAAGTTGTGTT | 6mer |
|  |  |  | MARMOSET | GCTGCTATTAGAATGCATTGTGAAACGACTGGAGTATGATTAAAAGTT | 6mer |
|  |  |  | MARMOSET | TGAAACGACTGGAGTATGA | 6mer |
|  |  |  | MARMOSET | CGACTGGAGTATGA | 6mer |
|  |  |  | DOG | GCTGCTATTAGAATGCATTGTGAAACGACTGGAGTATGATTAAAAGTTGTGTT | 6mer |
|  |  |  | DOG | GCTGCTATTAGAATGCATTGTGAAACGACTGGAGTATGATTAAAAGTT | 6mer |
|  |  |  | DOG | TGAAACGACTGGAGTATGA | 6mer |
|  |  |  | DOG | CGACTGGAGTATGA | 6mer |
|  |  |  | PIG | GCTGCTATTAGAATGCATTGTGAAACGACTGGAGTATGATTAAAAGTT | 6mer |
|  |  |  | PIG | TGAAACGACTGGAGTATGA | 6mer |
|  |  |  | PIG | CGACTGGAGTATGA | 6mer |
|  |  |  | COW | TGAAACGACTGGAGTATGA | 6mer |
|  |  |  | COW | CGACTGGAGTATGA | 6mer |
|  |  |  | MOUSE | CGACTGGAGTATGA | 6mer |

  
  
  
  

| Seed Matches to the miRNA miR-203a-3p.1 | | | | | |
| --- | --- | --- | --- | --- | --- |
| Seed | Conservation | Species | Matches | | |
|  |  |  | Sequence | Motif | Type |
| GAAAUGU | Broadly Conserved | Human (Homo sapiens) | HUMAN | TTATCAGAAGAGTTGCTTCATTTCATCTGGGAGCAGAAAACAGCAGGCAGCTGTTAACAGATAAGTTTAACTTGCATCTGCA | 7mer-A1 |
|  |  |  | HUMAN | TTATCAGAAGAGTTGCTTCATTTCATCTGGGAGCAGAAAACAGCAGGCAGCTGTTAACAGATAAGTTTAA | 7mer-A1 |
|  |  |  | HUMAN | TTTTTCACATTTCCA | 7mer-m8 |
|  |  |  | HUMAN | TTTTAAAGAATTTTCCTTTGCAGAGGCATTTCATCCTTCATGAAGC | 7mer-A1 |
|  |  |  | HUMAN | CATTTCATCCTTCATGAAGC | 7mer-A1 |
|  |  |  | HUMAN | TTCATTTCTG | 6mer |
|  |  |  | MARMOSET | TTATCAGAAGAGTTGCTTCATTTCATCTGGGAGCAGAAAACAGCAGGCAGCTGTTAACAGATAAGTTTAACTTGCATCTGCA | 7mer-A1 |
|  |  |  | MARMOSET | TTATCAGAAGAGTTGCTTCATTTCATCTGGGAGCAGAAAACAGCAGGCAGCTGTTAACAGATAAGTTTAA | 7mer-A1 |
|  |  |  | MARMOSET | TTTTTCACATTTCCA | 7mer-m8 |
|  |  |  | MARMOSET | TTTTAAAGAATTTTCCTTTGCAGAGGCATTTCATCCTTCATGAAGC | 7mer-A1 |
|  |  |  | MARMOSET | CATTTCATCCTTCATGAAGC | 7mer-A1 |
|  |  |  | MARMOSET | TTCATTTCTG | 6mer |
|  |  |  | DOG | TTATCAGAAGAGTTGCTTCATTTCATCTGGGAGCAGAAAACAGCAGGCAGCTGTTAACAGATAAGTTTAA | 7mer-A1 |
|  |  |  | DOG | CATTTCATCCTTCATGAAGC | 7mer-A1 |

  
  
  
  

| Seed Matches to the miRNA miR-365-3p | | | | | |
| --- | --- | --- | --- | --- | --- |
| Seed | Conservation | Species | Matches | | |
|  |  |  | Sequence | Motif | Type |
| AAUGCCC | Broadly Conserved | Human (Homo sapiens)  Chicken (Gallus gallus)  Rhesus (Macaca mulatta)  Cow (Bos taurus)  Mouse (Mus musculus)  Rat (Rattus norvegicus)  Opossum (Monodelphis domestica) | HUMAN | AGGCATTGAGG | 6mer |
|  |  |  | HUMAN | TCACTCAGAGGCATTTGCATCT | 6mer |
|  |  |  | HUMAN | TTTTAAAGAATTTTCCTTTGCAGAGGCATTTCATCCTTCATGAAGC | 6mer |
|  |  |  | MARMOSET | AGGCATTGAGG | 6mer |
|  |  |  | MARMOSET | TCACTCAGAGGCATTTGCATCT | 6mer |
|  |  |  | MARMOSET | TTTTAAAGAATTTTCCTTTGCAGAGGCATTTCATCCTTCATGAAGC | 6mer |

  
  
  
  

| Seed Matches to the miRNA miR-342-3p | | | | | |
| --- | --- | --- | --- | --- | --- |
| Seed | Conservation | Species | Matches | | |
|  |  |  | Sequence | Motif | Type |
| CUCACAC | Conserved | Human (Homo sapiens)  Rhesus (Macaca mulatta)  Mouse (Mus musculus)  Rat (Rattus norvegicus) | HUMAN | TGGCATGTGAGCAA | 6mer |
|  |  |  | HUMAN | GCATGTGAGCA | 6mer |
|  |  |  | MARMOSET | TGGCATGTGAGCAA | 6mer |
|  |  |  | MARMOSET | GCATGTGAGCA | 6mer |
|  |  |  | DOG | GCATGTGAGCA | 6mer |

  
  
  
  

| Seed Matches to the miRNA miR-146-5p | | | | | |
| --- | --- | --- | --- | --- | --- |
| Seed | Conservation | Species | Matches | | |
|  |  |  | Sequence | Motif | Type |
| GAGAACU | Broadly Conserved | Human (Homo sapiens)  Chicken (Gallus gallus)  Rhesus (Macaca mulatta)  Mouse (Mus musculus)  Rat (Rattus norvegicus)  Opossum (Monodelphis domestica) | HUMAN | TAGAAACTAGAGCAGTTCTCAC | 8mer |
|  |  |  | HUMAN | GGATATGGTAGTGTGTGGTTCTCTTTTGGAATTTTTTTCAGGTGATTTAATA | 6mer |
|  |  |  | HUMAN | TAGTGTGTGGTTCTCT | 6mer |
|  |  |  | MARMOSET | TAGAAACTAGAGCAGTTCTCAC | 8mer |
|  |  |  | MARMOSET | GGATATGGTAGTGTGTGGTTCTCTTTTGGAATTTTTTTCAGGTGATTTAATA | 6mer |
|  |  |  | MARMOSET | TAGTGTGTGGTTCTCT | 6mer |
|  |  |  | DOG | GGATATGGTAGTGTGTGGTTCTCTTTTGGAATTTTTTTCAGGTGATTTAATA | 6mer |
|  |  |  | DOG | TAGTGTGTGGTTCTCT | 6mer |
|  |  |  | PIG | GGATATGGTAGTGTGTGGTTCTCTTTTGGAATTTTTTTCAGGTGATTTAATA | 6mer |
|  |  |  | PIG | TAGTGTGTGGTTCTCT | 6mer |
|  |  |  | COW | TAGTGTGTGGTTCTCT | 6mer |
|  |  |  | MOUSE | TAGTGTGTGGTTCTCT | 6mer |

  
  
  
  

| Seed Matches to the miRNA miR-339-5p | | | | | |
| --- | --- | --- | --- | --- | --- |
| Seed | Conservation | Species | Matches | | |
|  |  |  | Sequence | Motif | Type |
| CCCUGUC | Conserved | Human (Homo sapiens)  Rhesus (Macaca mulatta)  Mouse (Mus musculus) | HUMAN | ACAGGGAA | 7mer-A1 |
|  |  |  | HUMAN | ATTTTGGGATGGTCTTAACAGGGAAGAGAGAGGGTGGGGGAGAAAATGTTTTTTTCTAAGATTTTCCACAGATGCTATAGTACTATTGACAAACTGGGTTAGAGAAGGAGTGTAC | 7mer-A1 |
|  |  |  | HUMAN | TGGTCTTAACAGGGAAGAG | 7mer-A1 |
|  |  |  | HUMAN | AACAGGGAAGAG | 7mer-A1 |
|  |  |  | HUMAN | AACAGGGA | 7mer-A1 |
|  |  |  | MARMOSET | ACAGGGAA | 7mer-A1 |
|  |  |  | MARMOSET | ATTTTGGGATGGTCTTAACAGGGAAGAGAGAGGGTGGGGGAGAAAATGTTTTTTTCTAAGATTTTCCACAGATGCTATAGTACTATTGACAAACTGGGTTAGAGAAGGAGTGTAC | 7mer-A1 |
|  |  |  | MARMOSET | TGGTCTTAACAGGGAAGAG | 7mer-A1 |
|  |  |  | MARMOSET | AACAGGGAAGAG | 7mer-A1 |
|  |  |  | MARMOSET | AACAGGGA | 7mer-A1 |
|  |  |  | DOG | TGGTCTTAACAGGGAAGAG | 7mer-A1 |
|  |  |  | DOG | AACAGGGAAGAG | 7mer-A1 |
|  |  |  | DOG | AACAGGGA | 7mer-A1 |
|  |  |  | PIG | TGGTCTTAACAGGGAAGAG | 7mer-A1 |
|  |  |  | PIG | AACAGGGAAGAG | 7mer-A1 |
|  |  |  | PIG | AACAGGGA | 7mer-A1 |
|  |  |  | COW | AACAGGGAAGAG | 7mer-A1 |
|  |  |  | COW | AACAGGGA | 7mer-A1 |
|  |  |  | MOUSE | AACAGGGA | 7mer-A1 |

  
  
  
  

| Seed Matches to the miRNA miR-489-3p | | | | | |
| --- | --- | --- | --- | --- | --- |
| Seed | Conservation | Species | Matches | | |
|  |  |  | Sequence | Motif | Type |
| UGACAUC | Broadly Conserved | Human (Homo sapiens)  Rhesus (Macaca mulatta)  Rat (Rattus norvegicus) | HUMAN | TGATAAGTAAAGGCAGAAAAGATTATATGTCATACCTCCATTGGGGAATAAGCATAACCCTGAGATTCTTACTACTGATGA | 6mer |
|  |  |  | MARMOSET | TGATAAGTAAAGGCAGAAAAGATTATATGTCATACCTCCATTGGGGAATAAGCATAACCCTGAGATTCTTACTACTGATGA | 6mer |

  
  
  
  

| Seed Matches to the miRNA miR-185-5p | | | | | |
| --- | --- | --- | --- | --- | --- |
| Seed | Conservation | Species | Matches | | |
|  |  |  | Sequence | Motif | Type |
| GGAGAGA | Conserved | Human (Homo sapiens)  Rhesus (Macaca mulatta)  Mouse (Mus musculus)  Rat (Rattus norvegicus) | HUMAN | TGTGTGGGTTTCTCTCTCCCCTCCCTTGGTCTTAATTCTTACATGCAGGAACA | 7mer-m8 |
|  |  |  | HUMAN | TGTGTGGGTTTCTCTCTCCCCTCCCTTGGTCTTAATTCTTACA | 7mer-m8 |
|  |  |  | MARMOSET | TGTGTGGGTTTCTCTCTCCCCTCCCTTGGTCTTAATTCTTACATGCAGGAACA | 7mer-m8 |
|  |  |  | MARMOSET | TGTGTGGGTTTCTCTCTCCCCTCCCTTGGTCTTAATTCTTACA | 7mer-m8 |
|  |  |  | DOG | TGTGTGGGTTTCTCTCTCCCCTCCCTTGGTCTTAATTCTTACA | 7mer-m8 |

  
  
  
  

| Seed Matches to the miRNA miR-137 | | | | | |
| --- | --- | --- | --- | --- | --- |
| Seed | Conservation | Species | Matches | | |
|  |  |  | Sequence | Motif | Type |
| UAUUGCU | Broadly Conserved | Human (Homo sapiens)  Chimp (Pan troglodytes)  Dog (Canis lupus familiaris)  Cow (Bos taurus) | HUMAN | GAGTGGTTGGTAAAAATCCGTGAGGTCGGCAATATGTTGTTTTTCTGGAACTT | 6mer |
|  |  |  | HUMAN | TGATGAGCATATAATAATTCCAGGCACATGGCAATAGAGGCCCTCTAAATAAGGAATAA | 6mer |
|  |  |  | HUMAN | TAATAATTCCAGGCACATGGCAATAGAGGCCCTCTAAATAAGGAATAA | 6mer |
|  |  |  | MARMOSET | GAGTGGTTGGTAAAAATCCGTGAGGTCGGCAATATGTTGTTTTTCTGGAACTT | 6mer |
|  |  |  | MARMOSET | TGATGAGCATATAATAATTCCAGGCACATGGCAATAGAGGCCCTCTAAATAAGGAATAA | 6mer |
|  |  |  | MARMOSET | TAATAATTCCAGGCACATGGCAATAGAGGCCCTCTAAATAAGGAATAA | 6mer |
|  |  |  | DOG | TAATAATTCCAGGCACATGGCAATAGAGGCCCTCTAAATAAGGAATAA | 6mer |

  
  
  
  

| Seed Matches to the miRNA miR-217 | | | | | |
| --- | --- | --- | --- | --- | --- |
| Seed | Conservation | Species | Matches | | |
|  |  |  | Sequence | Motif | Type |
| ACUGCAU | Broadly Conserved | Human (Homo sapiens)  X. tropicalis (Xenopus tropicalis)  Rhesus (Macaca mulatta)  Chimp (Pan troglodytes)  Dog (Canis lupus familiaris)  Cow (Bos taurus) | HUMAN | AGTGAGTGTATGAGACCTTGCAGTGAGTTTATCAGCATACTCAAAATTTTTTTCCTGGAATTTGGAGGGATGGGAGGAGGGGGTGGGGCTTACTTGTT | 6mer |
|  |  |  | HUMAN | TGAGACCTTGCAGTG | 6mer |
|  |  |  | HUMAN | AGACCTTGCAGTG | 6mer |
|  |  |  | HUMAN | TGCAGTG | 6mer |
|  |  |  | HUMAN | TTTTTTTTTACAGACTTCACAGAGAATGCAGTTGTCTTGACTTCAGGTCTGTCTGTTCTGTTGGCAAGTAA | 7mer-m8 |
|  |  |  | HUMAN | AGAATGCAGTTGTCTTGAC | 7mer-m8 |
|  |  |  | HUMAN | ATGCAGTACTGTTCTGATCCCGCTGCTATTAGAATGCATTGTGAAACGACTGGAGTATGATTAAAAGTTGTGTTCCCCAATGCTTGGAGTAGTGATTGTTGAAGGAAAAAA | 8mer |
|  |  |  | HUMAN | TGCAGTACTGTTCTGATC | 8mer |
|  |  |  | HUMAN | TGCAGTACTGTTCTGA | 8mer |
|  |  |  | HUMAN | TCCAGCTGAGTGATAAAGGCTGAGTGTTGAGGAAATTTCTGCAGTTTTAAGCAGTCGT | 6mer |
|  |  |  | MARMOSET | AGTGAGTGTATGAGACCTTGCAGTGAGTTTATCAGCATACTCAAAATTTTTTTCCTGGAATTTGGAGGGATGGGAGGAGGGGGTGGGGCTTACTTGTT | 6mer |
|  |  |  | MARMOSET | TGAGACCTTGCAGTG | 6mer |
|  |  |  | MARMOSET | AGACCTTGCAGTG | 6mer |
|  |  |  | MARMOSET | TGCAGTG | 6mer |
|  |  |  | MARMOSET | TTTTTTTTTACAGACTTCACAGAGAATGCAGTTGTCTTGACTTCAGGTCTGTCTGTTCTGTTGGCAAGTAA | 7mer-m8 |
|  |  |  | MARMOSET | AGAATGCAGTTGTCTTGAC | 7mer-m8 |
|  |  |  | MARMOSET | ATGCAGTACTGTTCTGATCCCGCTGCTATTAGAATGCATTGTGAAACGACTGGAGTATGATTAAAAGTTGTGTTCCCCAATGCTTGGAGTAGTGATTGTTGAAGGAAAAAA | 8mer |
|  |  |  | MARMOSET | TGCAGTACTGTTCTGATC | 8mer |
|  |  |  | MARMOSET | TGCAGTACTGTTCTGA | 8mer |
|  |  |  | MARMOSET | TCCAGCTGAGTGATAAAGGCTGAGTGTTGAGGAAATTTCTGCAGTTTTAAGCAGTCGT | 6mer |
|  |  |  | DOG | TGAGACCTTGCAGTG | 6mer |
|  |  |  | DOG | AGACCTTGCAGTG | 6mer |
|  |  |  | DOG | TGCAGTG | 6mer |
|  |  |  | DOG | AGAATGCAGTTGTCTTGAC | 7mer-m8 |
|  |  |  | DOG | TGCAGTACTGTTCTGATC | 8mer |
|  |  |  | DOG | TGCAGTACTGTTCTGA | 8mer |
|  |  |  | PIG | TGAGACCTTGCAGTG | 6mer |
|  |  |  | PIG | AGACCTTGCAGTG | 6mer |
|  |  |  | PIG | TGCAGTG | 6mer |
|  |  |  | PIG | AGAATGCAGTTGTCTTGAC | 7mer-m8 |
|  |  |  | PIG | TGCAGTACTGTTCTGA | 8mer |
|  |  |  | COW | AGACCTTGCAGTG | 6mer |
|  |  |  | COW | TGCAGTG | 6mer |
|  |  |  | COW | AGAATGCAGTTGTCTTGAC | 7mer-m8 |
|  |  |  | MOUSE | AGACCTTGCAGTG | 6mer |
|  |  |  | MOUSE | TGCAGTG | 6mer |
|  |  |  | TURTLE | TGCAGTG | 6mer |
|  |  |  | ALLIGATOR | TGCAGTG | 6mer |
|  |  |  | LIZARD | TGCAGTG | 6mer |
|  |  |  | SNAKE | TGCAGTG | 7mer-m8 |

  
  
  
  

| Seed Matches to the miRNA miR-183-5p.2 | | | | | |
| --- | --- | --- | --- | --- | --- |
| Seed | Conservation | Species | Matches | | |
|  |  |  | Sequence | Motif | Type |
| UGGCACU | Broadly Conserved | Human (Homo sapiens)  Mouse (Mus musculus) | HUMAN | AGAGATGAGTTGGGATCAAGTGGATTGAGGAGGCTGTGCTGTGTGCCAAT | 7mer-A1 |
|  |  |  | MARMOSET | AGAGATGAGTTGGGATCAAGTGGATTGAGGAGGCTGTGCTGTGTGCCAAT | 7mer-A1 |

  
  
  
  

| Seed Matches to the miRNA miR-216b-5p | | | | | |
| --- | --- | --- | --- | --- | --- |
| Seed | Conservation | Species | Matches | | |
|  |  |  | Sequence | Motif | Type |
| AAUCUCU | Broadly Conserved | Human (Homo sapiens)  Mouse (Mus musculus)  Rat (Rattus norvegicus) | HUMAN | TTTAAAAAGAGATTAA | 8mer |
|  |  |  | HUMAN | TGATAAGTAAAGGCAGAAAAGATTATATGTCATACCTCCATTGGGGAATAAGCATAACCCTGAGATTCTTACTACTGATGA | 6mer |
|  |  |  | HUMAN | TAACCTCTTAGACAGGTGGGAGATTATGATCAGAGTAAAAGGTAATTACACATTTTATTTCCAGAAAGTCAGG | 7mer-A1 |
|  |  |  | HUMAN | ACCTCTTAGACAGGTGGGAGATTATGATCAGA | 7mer-A1 |
|  |  |  | HUMAN | CAGGTGGGAGATTATGATCAGA | 7mer-A1 |
|  |  |  | MARMOSET | TTTAAAAAGAGATTAA | 8mer |
|  |  |  | MARMOSET | TGATAAGTAAAGGCAGAAAAGATTATATGTCATACCTCCATTGGGGAATAAGCATAACCCTGAGATTCTTACTACTGATGA | 6mer |
|  |  |  | MARMOSET | TAACCTCTTAGACAGGTGGGAGATTATGATCAGAGTAAAAGGTAATTACACATTTTATTTCCAGAAAGTCAGG | 7mer-A1 |
|  |  |  | MARMOSET | ACCTCTTAGACAGGTGGGAGATTATGATCAGA | 7mer-A1 |
|  |  |  | MARMOSET | CAGGTGGGAGATTATGATCAGA | 7mer-A1 |
|  |  |  | DOG | ACCTCTTAGACAGGTGGGAGATTATGATCAGA | 7mer-A1 |
|  |  |  | DOG | CAGGTGGGAGATTATGATCAGA | 7mer-A1 |
|  |  |  | PIG | CAGGTGGGAGATTATGATCAGA | 7mer-A1 |
|  |  |  | COW | CAGGTGGGAGATTATGATCAGA | 7mer-A1 |

  
  
  
  

| Seed Matches to the miRNA miR-320 | | | | | |
| --- | --- | --- | --- | --- | --- |
| Seed | Conservation | Species | Matches | | |
|  |  |  | Sequence | Motif | Type |
| AAAGCUG | Conserved | Human (Homo sapiens)  X. tropicalis (Xenopus tropicalis)  Rhesus (Macaca mulatta)  Chimp (Pan troglodytes)  Dog (Canis lupus familiaris) | HUMAN | CTCATGAATCTTGTCTGAAGCTTTTGAGGGCAGACTGCCAAGTCCTGGAG | 6mer |
|  |  |  | HUMAN | ATCTTGTCTGAAGCTTTTGAGGGCAGACT | 6mer |
|  |  |  | HUMAN | AGCTTTTGAGGGCAGACT | 6mer |
|  |  |  | HUMAN | AGCTTTTGAGGGC | 6mer |
|  |  |  | HUMAN | TGGAAGAGTATTCCCAGTTGAAGCTGAAAAGTACAGCACAGTGCAGCTTTGGTTCATATTCAGTCATCTCAGGAGAACTTCAGAAGAGCTTGAGTAGGCCAAATGTTGAAGTTAAGTTTTC | 7mer-m8 |
|  |  |  | HUMAN | GTACAGCACAGTGCAGCTTTGGTTCATA | 7mer-m8 |
|  |  |  | HUMAN | TACAGCACAGTGCAGCTTTGGTTCATA | 7mer-m8 |
|  |  |  | HUMAN | CAGTGCAGCTTTGGTTCATA | 7mer-m8 |
|  |  |  | HUMAN | CAGAGCAAAGGAAGTGGCTTAATGATCCTGAAGGGATTTCTTCTGATGGTAGCTTTTGTATTATCAAGTAAGATTCT | 6mer |
|  |  |  | HUMAN | TGGTAGCTTTT | 6mer |
|  |  |  | HUMAN | TAGCTTTT | 6mer |
|  |  |  | HUMAN | AACAGGTGAACAAGCTTTTTCTGTATTTACAT | 6mer |
|  |  |  | HUMAN | AGTATTGAATAGATTTCAGCTTTATGCTGGAGTAA | 8mer |
|  |  |  | HUMAN | CAGCTTTATGCTGGA | 8mer |
|  |  |  | HUMAN | CAGCTTTA | 8mer |
|  |  |  | HUMAN | TTTTCCCTAGCTTTTCCAGAAGCCTGTTAAAAGCAAGGTCTCCCCACAAGCAACTTCTCTGCCACATCGCCACCC | 6mer |
|  |  |  | HUMAN | TTTTCCCTAGCTTTTCCAGAAGCCTGTTAAAA | 6mer |
|  |  |  | HUMAN | TCCCTAGCTTTTCCAGAA | 6mer |
|  |  |  | HUMAN | AGCTTTTCCAGAA | 6mer |
|  |  |  | MARMOSET | CTCATGAATCTTGTCTGAAGCTTTTGAGGGCAGACTGCCAAGTCCTGGAG | 6mer |
|  |  |  | MARMOSET | ATCTTGTCTGAAGCTTTTGAGGGCAGACT | 6mer |
|  |  |  | MARMOSET | AGCTTTTGAGGGCAGACT | 6mer |
|  |  |  | MARMOSET | AGCTTTTGAGGGC | 6mer |
|  |  |  | MARMOSET | TGGAAGAGTATTCCCAGTTGAAGCTGAAAAGTACAGCACAGTGCAGCTTTGGTTCATATTCAGTCATCTCAGGAGAACTTCAGAAGAGCTTGAGTAGGCCAAATGTTGAAGTTAAGTTTTC | 7mer-m8 |
|  |  |  | MARMOSET | GTACAGCACAGTGCAGCTTTGGTTCATA | 7mer-m8 |
|  |  |  | MARMOSET | TACAGCACAGTGCAGCTTTGGTTCATA | 7mer-m8 |
|  |  |  | MARMOSET | CAGTGCAGCTTTGGTTCATA | 7mer-m8 |
|  |  |  | MARMOSET | CAGAGCAAAGGAAGTGGCTTAATGATCCTGAAGGGATTTCTTCTGATGGTAGCTTTTGTATTATCAAGTAAGATTCT | 6mer |
|  |  |  | MARMOSET | TGGTAGCTTTT | 6mer |
|  |  |  | MARMOSET | TAGCTTTT | 6mer |
|  |  |  | MARMOSET | AACAGGTGAACAAGCTTTTTCTGTATTTACAT | 6mer |
|  |  |  | MARMOSET | AGTATTGAATAGATTTCAGCTTTATGCTGGAGTAA | 8mer |
|  |  |  | MARMOSET | CAGCTTTATGCTGGA | 8mer |
|  |  |  | MARMOSET | CAGCTTTA | 8mer |
|  |  |  | MARMOSET | TTTTCCCTAGCTTTTCCAGAAGCCTGTTAAAAGCAAGGTCTCCCCACAAGCAACTTCTCTGCCACATCGCCACCC | 6mer |
|  |  |  | MARMOSET | TTTTCCCTAGCTTTTCCAGAAGCCTGTTAAAA | 6mer |
|  |  |  | MARMOSET | TCCCTAGCTTTTCCAGAA | 6mer |
|  |  |  | MARMOSET | AGCTTTTCCAGAA | 6mer |
|  |  |  | DOG | ATCTTGTCTGAAGCTTTTGAGGGCAGACT | 6mer |
|  |  |  | DOG | AGCTTTTGAGGGCAGACT | 6mer |
|  |  |  | DOG | AGCTTTTGAGGGC | 6mer |
|  |  |  | DOG | GTACAGCACAGTGCAGCTTTGGTTCATA | 7mer-m8 |
|  |  |  | DOG | TACAGCACAGTGCAGCTTTGGTTCATA | 7mer-m8 |
|  |  |  | DOG | CAGTGCAGCTTTGGTTCATA | 7mer-m8 |
|  |  |  | DOG | TGGTAGCTTTT | 6mer |
|  |  |  | DOG | TAGCTTTT | 6mer |
|  |  |  | DOG | CAGCTTTATGCTGGA | 8mer |
|  |  |  | DOG | CAGCTTTA | 8mer |
|  |  |  | DOG | TTTTCCCTAGCTTTTCCAGAAGCCTGTTAAAA | 6mer |
|  |  |  | DOG | TCCCTAGCTTTTCCAGAA | 6mer |
|  |  |  | DOG | AGCTTTTCCAGAA | 6mer |
|  |  |  | PIG | ATCTTGTCTGAAGCTTTTGAGGGCAGACT | 6mer |
|  |  |  | PIG | AGCTTTTGAGGGCAGACT | 6mer |
|  |  |  | PIG | AGCTTTTGAGGGC | 6mer |
|  |  |  | PIG | TACAGCACAGTGCAGCTTTGGTTCATA | 7mer-m8 |
|  |  |  | PIG | CAGTGCAGCTTTGGTTCATA | 7mer-m8 |
|  |  |  | PIG | TGGTAGCTTTT | 6mer |
|  |  |  | PIG | TAGCTTTT | 6mer |
|  |  |  | PIG | CAGCTTTATGCTGGA | 8mer |
|  |  |  | PIG | CAGCTTTA | 8mer |
|  |  |  | PIG | TCCCTAGCTTTTCCAGAA | 6mer |
|  |  |  | PIG | AGCTTTTCCAGAA | 6mer |
|  |  |  | COW | AGCTTTTGAGGGCAGACT | 6mer |
|  |  |  | COW | AGCTTTTGAGGGC | 6mer |
|  |  |  | COW | CAGTGCAGCTTTGGTTCATA | 7mer-m8 |
|  |  |  | COW | TGGTAGCTTTT | 6mer |
|  |  |  | COW | TAGCTTTT | 6mer |
|  |  |  | COW | CAGCTTTA | 8mer |
|  |  |  | COW | TCCCTAGCTTTTCCAGAA | 6mer |
|  |  |  | COW | AGCTTTTCCAGAA | 6mer |
|  |  |  | MOUSE | AGCTTTTGAGGGC | 6mer |
|  |  |  | MOUSE | TAGCTTTT | 6mer |
|  |  |  | MOUSE | AGCTTTTCCAGAA | 7mer-m8 |

  
  
  
  

| Seed Matches to the miRNA miR-28-5p/708-5p | | | | | |
| --- | --- | --- | --- | --- | --- |
| Seed | Conservation | Species | Matches | | |
|  |  |  | Sequence | Motif | Type |
| AGGAGCU | Conserved | Human (Homo sapiens)  Mouse (Mus musculus)  Rat (Rattus norvegicus) | HUMAN | AACAGCTCCTTGGTGAA | 7mer-m8 |
|  |  |  | MARMOSET | AACAGCTCCTTGGTGAA | 7mer-m8 |

  
  
  
  

| Seed Matches to the miRNA miR-544a-5p | | | | | |
| --- | --- | --- | --- | --- | --- |
| Seed | Conservation | Species | Matches | | |
|  |  |  | Sequence | Motif | Type |
| CUUGUUA | Conserved | Human (Homo sapiens) | HUMAN | AAACAAGAAAATCCA | 7mer-A1 |
|  |  |  | HUMAN | CCAAACAAGCAACA | 6mer |
|  |  |  | HUMAN | AACAGGTGAACAAGCTTTTTCTGTATTTACAT | 6mer |
|  |  |  | MARMOSET | AAACAAGAAAATCCA | 7mer-A1 |
|  |  |  | MARMOSET | CCAAACAAGCAACA | 6mer |
|  |  |  | MARMOSET | AACAGGTGAACAAGCTTTTTCTGTATTTACAT | 6mer |
|  |  |  | DOG | CCAAACAAGCAACA | 6mer |

  
  
  
  

| Seed Matches to the miRNA miR-142-3p.2 | | | | | |
| --- | --- | --- | --- | --- | --- |
| Seed | Conservation | Species | Matches | | |
|  |  |  | Sequence | Motif | Type |
| UAGUGUU | Broadly Conserved | Human (Homo sapiens)  Mouse (Mus musculus) | HUMAN | TAGGCAATGTTTTACACTATTG | 6mer |
|  |  |  | MARMOSET | TAGGCAATGTTTTACACTATTG | 6mer |

  
  
  
  

| Seed Matches to the miRNA miR-496.2 | | | | | |
| --- | --- | --- | --- | --- | --- |
| Seed | Conservation | Species | Matches | | |
|  |  |  | Sequence | Motif | Type |
| GUAUUAC | Conserved | Human (Homo sapiens) | HUMAN | ATTAATACAACT | 7mer-A1 |
|  |  |  | HUMAN | TAATAC | 7mer-A1 |
|  |  |  | HUMAN | GAATTAATACC | 6mer |
|  |  |  | MARMOSET | ATTAATACAACT | 7mer-A1 |
|  |  |  | MARMOSET | TAATAC | 7mer-A1 |
|  |  |  | MARMOSET | GAATTAATACC | 6mer |
|  |  |  | DOG | TAATAC | 6mer |

  
  
  
  

| Seed Matches to the miRNA miR-124-3p.1 | | | | | |
| --- | --- | --- | --- | --- | --- |
| Seed | Conservation | Species | Matches | | |
|  |  |  | Sequence | Motif | Type |
| AAGGCAC | Broadly Conserved | Human (Homo sapiens)  Mouse (Mus musculus) | HUMAN | GTGCCTTT | 7mer-m8 |
|  |  |  | HUMAN | TGCCTTT | 7mer-m8 |
|  |  |  | MARMOSET | GTGCCTTT | 7mer-m8 |
|  |  |  | MARMOSET | TGCCTTT | 7mer-m8 |
|  |  |  | DOG | TGCCTTT | 6mer |
|  |  |  | PIG | TGCCTTT | 6mer |
|  |  |  | COW | TGCCTTT | 7mer-m8 |

  
  
  
  

| Seed Matches to the miRNA miR-455-3p.1 | | | | | |
| --- | --- | --- | --- | --- | --- |
| Seed | Conservation | Species | Matches | | |
|  |  |  | Sequence | Motif | Type |
| CAGUCCA | Broadly Conserved | Human (Homo sapiens)  Mouse (Mus musculus) | HUMAN | TGAAGCTAGGACTGAGGAGCAA | 7mer-A1 |
|  |  |  | HUMAN | TGAAGCTAGGACTGAGGAGC | 7mer-A1 |
|  |  |  | HUMAN | CTAGGACTGAGGAGC | 7mer-A1 |
|  |  |  | HUMAN | AGGACTGAGGAGC | 7mer-A1 |
|  |  |  | HUMAN | GCTGTGCTGTTGGCACGAACACCTTCAGGGACTGGAGCTGCTTTTAT | 6mer |
|  |  |  | MARMOSET | TGAAGCTAGGACTGAGGAGCAA | 7mer-A1 |
|  |  |  | MARMOSET | TGAAGCTAGGACTGAGGAGC | 7mer-A1 |
|  |  |  | MARMOSET | CTAGGACTGAGGAGC | 7mer-A1 |
|  |  |  | MARMOSET | AGGACTGAGGAGC | 7mer-A1 |
|  |  |  | MARMOSET | GCTGTGCTGTTGGCACGAACACCTTCAGGGACTGGAGCTGCTTTTAT | 6mer |
|  |  |  | DOG | TGAAGCTAGGACTGAGGAGC | 7mer-A1 |
|  |  |  | DOG | CTAGGACTGAGGAGC | 7mer-A1 |
|  |  |  | DOG | AGGACTGAGGAGC | 7mer-A1 |
|  |  |  | PIG | CTAGGACTGAGGAGC | 7mer-A1 |
|  |  |  | PIG | AGGACTGAGGAGC | 7mer-A1 |
|  |  |  | COW | CTAGGACTGAGGAGC | 7mer-A1 |
|  |  |  | COW | AGGACTGAGGAGC | 7mer-A1 |
|  |  |  | MOUSE | AGGACTGAGGAGC | 7mer-A1 |

  
  
  
  

| Seed Matches to the miRNA miR-200bc-3p/429 | | | | | |
| --- | --- | --- | --- | --- | --- |
| Seed | Conservation | Species | Matches | | |
|  |  |  | Sequence | Motif | Type |
| AAUACUG | Broadly Conserved | Human (Homo sapiens)  Rat (Rattus norvegicus)  Opossum (Monodelphis domestica) | HUMAN | GCTAAGACAAGTATTGGA | 6mer |
|  |  |  | HUMAN | AGTATTTCA | 6mer |
|  |  |  | HUMAN | TGGAAGAGTATTCCCAGTTGAAGCTGAAAAGTACAGCACAGTGCAGCTTTGGTTCATATTCAGTCATCTCAGGAGAACTTCAGAAGAGCTTGAGTAGGCCAAATGTTGAAGTTAAGTTTTC | 6mer |
|  |  |  | HUMAN | TGGAAGAGTATTCCCAGTTGAAGCTGAAA | 6mer |
|  |  |  | HUMAN | GAGTATTCCCAGT | 6mer |
|  |  |  | HUMAN | AGTATTGAATAGATTTCAGCTTTATGCTGGAGTAA | 6mer |
|  |  |  | MARMOSET | GCTAAGACAAGTATTGGA | 6mer |
|  |  |  | MARMOSET | AGTATTTCA | 7mer-m8 |
|  |  |  | MARMOSET | TGGAAGAGTATTCCCAGTTGAAGCTGAAAAGTACAGCACAGTGCAGCTTTGGTTCATATTCAGTCATCTCAGGAGAACTTCAGAAGAGCTTGAGTAGGCCAAATGTTGAAGTTAAGTTTTC | 6mer |
|  |  |  | MARMOSET | TGGAAGAGTATTCCCAGTTGAAGCTGAAA | 6mer |
|  |  |  | MARMOSET | GAGTATTCCCAGT | 6mer |
|  |  |  | MARMOSET | AGTATTGAATAGATTTCAGCTTTATGCTGGAGTAA | 7mer-m8 |
|  |  |  | DOG | TGGAAGAGTATTCCCAGTTGAAGCTGAAA | 6mer |
|  |  |  | DOG | GAGTATTCCCAGT | 6mer |
|  |  |  | PIG | GAGTATTCCCAGT | 6mer |
|  |  |  | COW | GAGTATTCCCAGT | 6mer |

  
  
  
  

| Seed Matches to the miRNA miR-7-5p | | | | | |
| --- | --- | --- | --- | --- | --- |
| Seed | Conservation | Species | Matches | | |
|  |  |  | Sequence | Motif | Type |
| GGAAGAC | Broadly Conserved | Human (Homo sapiens)  Mouse (Mus musculus)  Rat (Rattus norvegicus) | HUMAN | TTGCATATGAGTGCTTGGCTCTTCCTTCTGTTCT | 6mer |
|  |  |  | HUMAN | GCTTGGCTCTTCCTTCTGTTCT | 6mer |
|  |  |  | MARMOSET | TTGCATATGAGTGCTTGGCTCTTCCTTCTGTTCT | 6mer |
|  |  |  | MARMOSET | GCTTGGCTCTTCCTTCTGTTCT | 6mer |
|  |  |  | DOG | GCTTGGCTCTTCCTTCTGTTCT | 6mer |

  
  
  
  

| Seed Matches to the miRNA miR-199-3p | | | | | |
| --- | --- | --- | --- | --- | --- |
| Seed | Conservation | Species | Matches | | |
|  |  |  | Sequence | Motif | Type |
| CAGUAGU | Broadly Conserved | Human (Homo sapiens)  Rhesus (Macaca mulatta)  Cow (Bos taurus)  Mouse (Mus musculus)  Rat (Rattus norvegicus)  Opossum (Monodelphis domestica) | HUMAN | TGATAAGTAAAGGCAGAAAAGATTATATGTCATACCTCCATTGGGGAATAAGCATAACCCTGAGATTCTTACTACTGATGA | 8mer |
|  |  |  | HUMAN | ACTACTGATGA | 8mer |
|  |  |  | MARMOSET | TGATAAGTAAAGGCAGAAAAGATTATATGTCATACCTCCATTGGGGAATAAGCATAACCCTGAGATTCTTACTACTGATGA | 8mer |
|  |  |  | MARMOSET | ACTACTGATGA | 8mer |
|  |  |  | DOG | ACTACTGATGA | 8mer |
|  |  |  | PIG | ACTACTGATGA | 8mer |

  
  
  
  

| Seed Matches to the miRNA miR-409-3p | | | | | |
| --- | --- | --- | --- | --- | --- |
| Seed | Conservation | Species | Matches | | |
|  |  |  | Sequence | Motif | Type |
| AAUGUUG | Conserved | Human (Homo sapiens)  Rhesus (Macaca mulatta)  Mouse (Mus musculus) | HUMAN | AAACCAAACATTCCATTTTAAATGTGGGGATTGGGAACCACTAGTTCTTTCAGATGGTATTCTTCAGACTATAGAAGGAGCTTCCAGTTGAATTCA | 6mer |
|  |  |  | HUMAN | TTAACATTTAAGC | 6mer |
|  |  |  | MARMOSET | AAACCAAACATTCCATTTTAAATGTGGGGATTGGGAACCACTAGTTCTTTCAGATGGTATTCTTCAGACTATAGAAGGAGCTTCCAGTTGAATTCA | 6mer |
|  |  |  | MARMOSET | TTAACATTTAAGC | 6mer |

  
  
  
  

| Seed Matches to the miRNA miR-340-5p | | | | | |
| --- | --- | --- | --- | --- | --- |
| Seed | Conservation | Species | Matches | | |
|  |  |  | Sequence | Motif | Type |
| UAUAAAG | Conserved | Human (Homo sapiens)  Rhesus (Macaca mulatta)  Mouse (Mus musculus)  Rat (Rattus norvegicus) | HUMAN | GGGATTTATATGGGGA | 6mer |
|  |  |  | HUMAN | AGCTGTTTTTATAGCAGCTCTTAA | 6mer |
|  |  |  | HUMAN | TTTTATAGCAGCT | 6mer |
|  |  |  | MARMOSET | GGGATTTATATGGGGA | 6mer |
|  |  |  | MARMOSET | AGCTGTTTTTATAGCAGCTCTTAA | 6mer |
|  |  |  | MARMOSET | TTTTATAGCAGCT | 6mer |
|  |  |  | DOG | TTTTATAGCAGCT | 6mer |

  
  
  
  

| Seed Matches to the miRNA miR-17-5p/20-5p/93-5p/106-5p/519-3p | | | | | |
| --- | --- | --- | --- | --- | --- |
| Seed | Conservation | Species | Matches | | |
|  |  |  | Sequence | Motif | Type |
| AAAGUGC | Broadly Conserved | Human (Homo sapiens) | HUMAN | CCATGGCACTTT | 7mer-m8 |
|  |  |  | MARMOSET | CCATGGCACTTT | 7mer-m8 |

  
  
  
  

| Seed Matches to the miRNA miR-142-5p | | | | | |
| --- | --- | --- | --- | --- | --- |
| Seed | Conservation | Species | Matches | | |
|  |  |  | Sequence | Motif | Type |
| AUAAAGU | Broadly Conserved | Human (Homo sapiens)  X. tropicalis (Xenopus tropicalis)  Rhesus (Macaca mulatta)  Cow (Bos taurus)  Mouse (Mus musculus)  Rat (Rattus norvegicus) | HUMAN | AGTATTGAATAGATTTCAGCTTTATGCTGGAGTAA | 6mer |
|  |  |  | HUMAN | CAGCTTTATGCTGGA | 6mer |
|  |  |  | MARMOSET | AGTATTGAATAGATTTCAGCTTTATGCTGGAGTAA | 6mer |
|  |  |  | MARMOSET | CAGCTTTATGCTGGA | 6mer |
|  |  |  | DOG | CAGCTTTATGCTGGA | 6mer |
|  |  |  | PIG | CAGCTTTATGCTGGA | 6mer |

  
  
  
  

| Seed Matches to the miRNA miR-1-3p/206 | | | | | |
| --- | --- | --- | --- | --- | --- |
| Seed | Conservation | Species | Matches | | |
|  |  |  | Sequence | Motif | Type |
| GGAAUGU | Broadly Conserved | Human (Homo sapiens)  Chicken (Gallus gallus)  Rhesus (Macaca mulatta)  Opossum (Monodelphis domestica) | HUMAN | AAACCAAACATTCCATTTTAAATGTGGGGATTGGGAACCACTAGTTCTTTCAGATGGTATTCTTCAGACTATAGAAGGAGCTTCCAGTTGAATTCA | 8mer |
|  |  |  | MARMOSET | AAACCAAACATTCCATTTTAAATGTGGGGATTGGGAACCACTAGTTCTTTCAGATGGTATTCTTCAGACTATAGAAGGAGCTTCCAGTTGAATTCA | 8mer |

  
  
  
  

| Seed Matches to the miRNA miR-379-5p | | | | | |
| --- | --- | --- | --- | --- | --- |
| Seed | Conservation | Species | Matches | | |
|  |  |  | Sequence | Motif | Type |
| GGUAGAC | Conserved | Human (Homo sapiens)  Rhesus (Macaca mulatta)  Rat (Rattus norvegicus) | HUMAN | CTACCAATTTAAAGTTACGGAATCTACCATTTTAAAGTTAATTGCTTGTCAAGCTATAAC | 7mer-A1 |
|  |  |  | HUMAN | GGAATCTACCATTT | 7mer-A1 |
|  |  |  | MARMOSET | CTACCAATTTAAAGTTACGGAATCTACCATTTTAAAGTTAATTGCTTGTCAAGCTATAAC | 7mer-A1 |
|  |  |  | MARMOSET | GGAATCTACCATTT | 7mer-A1 |
|  |  |  | DOG | GGAATCTACCATTT | 7mer-A1 |

  
  
  
  

| Seed Matches to the miRNA miR-150-5p | | | | | |
| --- | --- | --- | --- | --- | --- |
| Seed | Conservation | Species | Matches | | |
|  |  |  | Sequence | Motif | Type |
| CUCCCAA | Broadly Conserved | Human (Homo sapiens)  Rhesus (Macaca mulatta)  Mouse (Mus musculus)  Rat (Rattus norvegicus)  Opossum (Monodelphis domestica) | HUMAN | TTAATTGGGAGTGGTAGGA | 7mer-m8 |
|  |  |  | HUMAN | TTATCAGAAGAGTTGCTTCATTTCATCTGGGAGCAGAAAACAGCAGGCAGCTGTTAACAGATAAGTTTAACTTGCATCTGCA | 6mer |
|  |  |  | HUMAN | TTATCAGAAGAGTTGCTTCATTTCATCTGGGAGCAGAAAACAGCAGGCAGCTGTTAACAGATAAGTTTAA | 6mer |
|  |  |  | HUMAN | TCATCTGGGAGCAGAAAACAGCAGGCAGCTGTT | 6mer |
|  |  |  | HUMAN | TAACCTCTTAGACAGGTGGGAGATTATGATCAGAGTAAAAGGTAATTACACATTTTATTTCCAGAAAGTCAGG | 7mer-A1 |
|  |  |  | HUMAN | ACCTCTTAGACAGGTGGGAGATTATGATCAGA | 7mer-A1 |
|  |  |  | HUMAN | CAGGTGGGAGATTATGATCAGA | 7mer-A1 |
|  |  |  | HUMAN | CAGGTGGGAGAT | 7mer-A1 |
|  |  |  | HUMAN | AGTGAGTGTATGAGACCTTGCAGTGAGTTTATCAGCATACTCAAAATTTTTTTCCTGGAATTTGGAGGGATGGGAGGAGGGGGTGGGGCTTACTTGTT | 6mer |
|  |  |  | MARMOSET | TTAATTGGGAGTGGTAGGA | 7mer-m8 |
|  |  |  | MARMOSET | TTATCAGAAGAGTTGCTTCATTTCATCTGGGAGCAGAAAACAGCAGGCAGCTGTTAACAGATAAGTTTAACTTGCATCTGCA | 6mer |
|  |  |  | MARMOSET | TTATCAGAAGAGTTGCTTCATTTCATCTGGGAGCAGAAAACAGCAGGCAGCTGTTAACAGATAAGTTTAA | 6mer |
|  |  |  | MARMOSET | TCATCTGGGAGCAGAAAACAGCAGGCAGCTGTT | 6mer |
|  |  |  | MARMOSET | TAACCTCTTAGACAGGTGGGAGATTATGATCAGAGTAAAAGGTAATTACACATTTTATTTCCAGAAAGTCAGG | 7mer-A1 |
|  |  |  | MARMOSET | ACCTCTTAGACAGGTGGGAGATTATGATCAGA | 7mer-A1 |
|  |  |  | MARMOSET | CAGGTGGGAGATTATGATCAGA | 7mer-A1 |
|  |  |  | MARMOSET | CAGGTGGGAGAT | 7mer-A1 |
|  |  |  | MARMOSET | AGTGAGTGTATGAGACCTTGCAGTGAGTTTATCAGCATACTCAAAATTTTTTTCCTGGAATTTGGAGGGATGGGAGGAGGGGGTGGGGCTTACTTGTT | 6mer |
|  |  |  | DOG | TTATCAGAAGAGTTGCTTCATTTCATCTGGGAGCAGAAAACAGCAGGCAGCTGTTAACAGATAAGTTTAA | 6mer |
|  |  |  | DOG | TCATCTGGGAGCAGAAAACAGCAGGCAGCTGTT | 6mer |
|  |  |  | DOG | ACCTCTTAGACAGGTGGGAGATTATGATCAGA | 7mer-A1 |
|  |  |  | DOG | CAGGTGGGAGATTATGATCAGA | 7mer-A1 |
|  |  |  | DOG | CAGGTGGGAGAT | 7mer-A1 |
|  |  |  | PIG | TCATCTGGGAGCAGAAAACAGCAGGCAGCTGTT | 6mer |
|  |  |  | PIG | CAGGTGGGAGATTATGATCAGA | 7mer-A1 |
|  |  |  | PIG | CAGGTGGGAGAT | 7mer-A1 |
|  |  |  | COW | TCATCTGGGAGCAGAAAACAGCAGGCAGCTGTT | 6mer |
|  |  |  | COW | CAGGTGGGAGATTATGATCAGA | 7mer-A1 |
|  |  |  | COW | CAGGTGGGAGAT | 7mer-A1 |
|  |  |  | MOUSE | CAGGTGGGAGAT | 7mer-A1 |

  
  
  
  

| Seed Matches to the miRNA miR-299-5p | | | | | |
| --- | --- | --- | --- | --- | --- |
| Seed | Conservation | Species | Matches | | |
|  |  |  | Sequence | Motif | Type |
| GGUUUAC | Conserved | Human (Homo sapiens)  Rhesus (Macaca mulatta)  Rat (Rattus norvegicus) | HUMAN | CCAGTGACTAAAACCAACTTAAACCAGTAAGTGGAGAAATAACATGTT | 7mer-A1 |
|  |  |  | MARMOSET | CCAGTGACTAAAACCAACTTAAACCAGTAAGTGGAGAAATAACATGTT | 7mer-A1 |

  
  
  
  

| Seed Matches to the miRNA miR-326 | | | | | |
| --- | --- | --- | --- | --- | --- |
| Seed | Conservation | Species | Matches | | |
|  |  |  | Sequence | Motif | Type |
| CUCUGGG | Conserved | Human (Homo sapiens)  Chimp (Pan troglodytes)  Cow (Bos taurus) | HUMAN | TCATGCCAGAGAACTTAAA | 7mer-A1 |
|  |  |  | HUMAN | GAAGGGCCAGAGAAGCCAGACCCAGTAAG | 7mer-A1 |
|  |  |  | HUMAN | GAAGGGCCAGAGAAGCCAGACC | 7mer-A1 |
|  |  |  | HUMAN | GGCCAGAGAAGCCAGACC | 7mer-A1 |
|  |  |  | HUMAN | GGCCAGAGAA | 7mer-A1 |
|  |  |  | MARMOSET | TCATGCCAGAGAACTTAAA | 7mer-A1 |
|  |  |  | MARMOSET | GAAGGGCCAGAGAAGCCAGACCCAGTAAG | 7mer-A1 |
|  |  |  | MARMOSET | GAAGGGCCAGAGAAGCCAGACC | 7mer-A1 |
|  |  |  | MARMOSET | GGCCAGAGAAGCCAGACC | 7mer-A1 |
|  |  |  | MARMOSET | GGCCAGAGAA | 7mer-A1 |
|  |  |  | DOG | GAAGGGCCAGAGAAGCCAGACC | 7mer-A1 |
|  |  |  | DOG | GGCCAGAGAAGCCAGACC | 7mer-A1 |
|  |  |  | DOG | GGCCAGAGAA | 7mer-A1 |
|  |  |  | PIG | GAAGGGCCAGAGAAGCCAGACC | 7mer-A1 |
|  |  |  | PIG | GGCCAGAGAAGCCAGACC | 7mer-A1 |
|  |  |  | PIG | GGCCAGAGAA | 7mer-A1 |
|  |  |  | COW | GGCCAGAGAAGCCAGACC | 7mer-A1 |
|  |  |  | COW | GGCCAGAGAA | 7mer-A1 |
|  |  |  | MOUSE | GGCCAGAGAA | 7mer-A1 |

  
  
  
  

| Seed Matches to the miRNA miR-653-5p | | | | | |
| --- | --- | --- | --- | --- | --- |
| Seed | Conservation | Species | Matches | | |
|  |  |  | Sequence | Motif | Type |
| UGAAACA | Conserved | Human (Homo sapiens)  Rhesus (Macaca mulatta)  Mouse (Mus musculus)  Rat (Rattus norvegicus) | HUMAN | AATATTGTCAAGAGTTTCAGATAGAAAATGAAAA | 6mer |
|  |  |  | HUMAN | GTTTCAAGGTAAC | 7mer-A1 |
|  |  |  | MARMOSET | AATATTGTCAAGAGTTTCAGATAGAAAATGAAAA | 6mer |
|  |  |  | MARMOSET | GTTTCAAGGTAAC | 8mer |
|  |  |  | DOG | GTTTCAAGGTAAC | 7mer-A1 |

  
  
  
  

| Seed Matches to the miRNA miR-140-3p.2 | | | | | |
| --- | --- | --- | --- | --- | --- |
| Seed | Conservation | Species | Matches | | |
|  |  |  | Sequence | Motif | Type |
| ACCACAG | Broadly Conserved | Human (Homo sapiens) | HUMAN | GGATATGGTAGTGTGTGGTTCTCTTTTGGAATTTTTTTCAGGTGATTTAATA | 6mer |
|  |  |  | HUMAN | TAGTGTGTGGTTCTCT | 6mer |
|  |  |  | MARMOSET | GGATATGGTAGTGTGTGGTTCTCTTTTGGAATTTTTTTCAGGTGATTTAATA | 6mer |
|  |  |  | MARMOSET | TAGTGTGTGGTTCTCT | 6mer |
|  |  |  | DOG | GGATATGGTAGTGTGTGGTTCTCTTTTGGAATTTTTTTCAGGTGATTTAATA | 6mer |
|  |  |  | DOG | TAGTGTGTGGTTCTCT | 6mer |
|  |  |  | PIG | GGATATGGTAGTGTGTGGTTCTCTTTTGGAATTTTTTTCAGGTGATTTAATA | 6mer |
|  |  |  | PIG | TAGTGTGTGGTTCTCT | 6mer |
|  |  |  | COW | TAGTGTGTGGTTCTCT | 6mer |
|  |  |  | MOUSE | TAGTGTGTGGTTCTCT | 6mer |

  
  
  
  

| Seed Matches to the miRNA miR-208-3p | | | | | |
| --- | --- | --- | --- | --- | --- |
| Seed | Conservation | Species | Matches | | |
|  |  |  | Sequence | Motif | Type |
| UAAGACG | Broadly Conserved | Human (Homo sapiens)  Rhesus (Macaca mulatta)  Mouse (Mus musculus)  Rat (Rattus norvegicus)  Opossum (Monodelphis domestica) | HUMAN | ATTTTGGGATGGTCTTAACAGGGAAGAGAGAGGGTGGGGGAGAAAATGTTTTTTTCTAAGATTTTCCACAGATGCTATAGTACTATTGACAAACTGGGTTAGAGAAGGAGTGTAC | 7mer-A1 |
|  |  |  | HUMAN | TGGTCTTAACAGGGAAGAG | 7mer-A1 |
|  |  |  | HUMAN | GTCTTAG | 6mer |
|  |  |  | HUMAN | TGTGTGGGTTTCTCTCTCCCCTCCCTTGGTCTTAATTCTTACATGCAGGAACA | 7mer-A1 |
|  |  |  | HUMAN | TGTGTGGGTTTCTCTCTCCCCTCCCTTGGTCTTAATTCTTACA | 7mer-A1 |
|  |  |  | MARMOSET | ATTTTGGGATGGTCTTAACAGGGAAGAGAGAGGGTGGGGGAGAAAATGTTTTTTTCTAAGATTTTCCACAGATGCTATAGTACTATTGACAAACTGGGTTAGAGAAGGAGTGTAC | 7mer-A1 |
|  |  |  | MARMOSET | TGGTCTTAACAGGGAAGAG | 7mer-A1 |
|  |  |  | MARMOSET | GTCTTAG | 6mer |
|  |  |  | MARMOSET | TGTGTGGGTTTCTCTCTCCCCTCCCTTGGTCTTAATTCTTACATGCAGGAACA | 7mer-A1 |
|  |  |  | MARMOSET | TGTGTGGGTTTCTCTCTCCCCTCCCTTGGTCTTAATTCTTACA | 7mer-A1 |
|  |  |  | DOG | TGGTCTTAACAGGGAAGAG | 7mer-A1 |
|  |  |  | DOG | TGTGTGGGTTTCTCTCTCCCCTCCCTTGGTCTTAATTCTTACA | 7mer-A1 |
|  |  |  | PIG | TGGTCTTAACAGGGAAGAG | 7mer-A1 |

  
  
  
  

| Seed Matches to the miRNA miR-302-3p/372-3p/373-3p/520-3p | | | | | |
| --- | --- | --- | --- | --- | --- |
| Seed | Conservation | Species | Matches | | |
|  |  |  | Sequence | Motif | Type |
| AAGUGCU | Broadly Conserved | Human (Homo sapiens) | HUMAN | CCATGGCACTTT | 6mer |
|  |  |  | MARMOSET | CCATGGCACTTT | 6mer |

  
  
  
  

| Seed Matches to the miRNA miR-96-5p/1271-5p | | | | | |
| --- | --- | --- | --- | --- | --- |
| Seed | Conservation | Species | Matches | | |
|  |  |  | Sequence | Motif | Type |
| UUGGCAC | Broadly Conserved | Human (Homo sapiens) | HUMAN | TGCCAAGGC | 7mer-m8 |
|  |  |  | HUMAN | CTCATGAATCTTGTCTGAAGCTTTTGAGGGCAGACTGCCAAGTCCTGGAG | 6mer |
|  |  |  | HUMAN | TTATCTGCATATGCCAAAAAA | 7mer-A1 |
|  |  |  | HUMAN | AGAGATGAGTTGGGATCAAGTGGATTGAGGAGGCTGTGCTGTGTGCCAAT | 7mer-m8 |
|  |  |  | HUMAN | TGCCAAT | 7mer-m8 |
|  |  |  | MARMOSET | TGCCAAGGC | 6mer |
|  |  |  | MARMOSET | CTCATGAATCTTGTCTGAAGCTTTTGAGGGCAGACTGCCAAGTCCTGGAG | 6mer |
|  |  |  | MARMOSET | TTATCTGCATATGCCAAAAAA | 7mer-A1 |
|  |  |  | MARMOSET | AGAGATGAGTTGGGATCAAGTGGATTGAGGAGGCTGTGCTGTGTGCCAAT | 7mer-m8 |
|  |  |  | MARMOSET | TGCCAAT | 7mer-m8 |
|  |  |  | DOG | TGCCAAT | 6mer |
|  |  |  | PIG | TGCCAAT | 6mer |
|  |  |  | COW | TGCCAAT | 6mer |
|  |  |  | MOUSE | TGCCAAT | 7mer-m8 |

  
  
  
  

| Seed Matches to the miRNA miR-192-5p/215-5p | | | | | |
| --- | --- | --- | --- | --- | --- |
| Seed | Conservation | Species | Matches | | |
|  |  |  | Sequence | Motif | Type |
| UGACCUA | Broadly Conserved | Human (Homo sapiens)  Rhesus (Macaca mulatta)  Mouse (Mus musculus) | HUMAN | GAGAGACAACAAAGCGCTATTATCCTAAGGTCAAGA | 7mer-A1 |
|  |  |  | HUMAN | CTAAGGTCAA | 7mer-A1 |
|  |  |  | MARMOSET | GAGAGACAACAAAGCGCTATTATCCTAAGGTCAAGA | 7mer-A1 |
|  |  |  | MARMOSET | CTAAGGTCAA | 7mer-A1 |
|  |  |  | DOG | CTAAGGTCAA | 7mer-A1 |
|  |  |  | PIG | CTAAGGTCAA | 7mer-A1 |
|  |  |  | COW | CTAAGGTCAA | 7mer-A1 |

  
  
  
  

| Seed Matches to the miRNA miR-346 | | | | | |
| --- | --- | --- | --- | --- | --- |
| Seed | Conservation | Species | Matches | | |
|  |  |  | Sequence | Motif | Type |
| GUCUGCC | Conserved | Human (Homo sapiens)  Rhesus (Macaca mulatta)  Chimp (Pan troglodytes)  Dog (Canis lupus familiaris)  Cow (Bos taurus)  Rat (Rattus norvegicus) | HUMAN | TTACTAAACGCAGACGAA | 6mer |
|  |  |  | HUMAN | TTAATTGACAGCTGACCCAGGTGCTACACAGAAGTGGATTCAGTGAATCTAGGAAGACAGCAGCAGACAG | 7mer-A1 |
|  |  |  | HUMAN | GCAGACAG | 7mer-A1 |
|  |  |  | HUMAN | CTCATGAATCTTGTCTGAAGCTTTTGAGGGCAGACTGCCAAGTCCTGGAG | 7mer-m8 |
|  |  |  | HUMAN | ATCTTGTCTGAAGCTTTTGAGGGCAGACT | 7mer-m8 |
|  |  |  | HUMAN | AGCTTTTGAGGGCAGACT | 7mer-m8 |
|  |  |  | HUMAN | CAGCAGACA | 7mer-A1 |
|  |  |  | HUMAN | CAGCAGAC | 7mer-A1 |
|  |  |  | MARMOSET | TTACTAAACGCAGACGAA | 6mer |
|  |  |  | MARMOSET | TTAATTGACAGCTGACCCAGGTGCTACACAGAAGTGGATTCAGTGAATCTAGGAAGACAGCAGCAGACAG | 7mer-A1 |
|  |  |  | MARMOSET | GCAGACAG | 7mer-A1 |
|  |  |  | MARMOSET | CTCATGAATCTTGTCTGAAGCTTTTGAGGGCAGACTGCCAAGTCCTGGAG | 7mer-m8 |
|  |  |  | MARMOSET | ATCTTGTCTGAAGCTTTTGAGGGCAGACT | 7mer-m8 |
|  |  |  | MARMOSET | AGCTTTTGAGGGCAGACT | 7mer-m8 |
|  |  |  | MARMOSET | CAGCAGACA | 7mer-A1 |
|  |  |  | MARMOSET | CAGCAGAC | 7mer-A1 |
|  |  |  | DOG | GCAGACAG | 8mer |
|  |  |  | DOG | ATCTTGTCTGAAGCTTTTGAGGGCAGACT | 7mer-m8 |
|  |  |  | DOG | AGCTTTTGAGGGCAGACT | 7mer-m8 |
|  |  |  | DOG | CAGCAGAC | 6mer |
|  |  |  | PIG | GCAGACAG | 7mer-A1 |
|  |  |  | PIG | ATCTTGTCTGAAGCTTTTGAGGGCAGACT | 7mer-m8 |
|  |  |  | PIG | AGCTTTTGAGGGCAGACT | 7mer-m8 |
|  |  |  | COW | GCAGACAG | 8mer |
|  |  |  | COW | AGCTTTTGAGGGCAGACT | 7mer-m8 |
|  |  |  | MOUSE | GCAGACAG | 8mer |

  
  
  
  

| Seed Matches to the miRNA miR-33-5p | | | | | |
| --- | --- | --- | --- | --- | --- |
| Seed | Conservation | Species | Matches | | |
|  |  |  | Sequence | Motif | Type |
| UGCAUUG | Broadly Conserved | Human (Homo sapiens)  Chicken (Gallus gallus)  Rhesus (Macaca mulatta)  Mouse (Mus musculus)  Rat (Rattus norvegicus)  Opossum (Monodelphis domestica) | HUMAN | ATTATGGGAAATGCAAAAGTTGTT | 7mer-A1 |
|  |  |  | HUMAN | TTTTTTTTTACAGACTTCACAGAGAATGCAGTTGTCTTGACTTCAGGTCTGTCTGTTCTGTTGGCAAGTAA | 6mer |
|  |  |  | HUMAN | AGAATGCAGTTGTCTTGAC | 6mer |
|  |  |  | HUMAN | AGAATGCAG | 6mer |
|  |  |  | HUMAN | ATGCAGTACTGTTCTGATCCCGCTGCTATTAGAATGCATTGTGAAACGACTGGAGTATGATTAAAAGTTGTGTTCCCCAATGCTTGGAGTAGTGATTGTTGAAGGAAAAAA | 6mer |
|  |  |  | HUMAN | GCTGCTATTAGAATGCATTGTGAAACGACTGGAGTATGATTAAAAGTTGTGTT | 6mer |
|  |  |  | HUMAN | GCTGCTATTAGAATGCATTGTGAAACGACTGGAGTATGATTAAAAGTT | 6mer |
|  |  |  | HUMAN | GCTGCTATTAGAATGCATT | 6mer |
|  |  |  | HUMAN | TGGGTGGGAATGCAAAAATTCTCTGCTAAGACTTTTTCAGGTGAACATAACAGACTTGGCCAAGCTAGCATCTTAGCGGAAGC | 7mer-A1 |
|  |  |  | MARMOSET | ATTATGGGAAATGCAAAAGTTGTT | 7mer-A1 |
|  |  |  | MARMOSET | TTTTTTTTTACAGACTTCACAGAGAATGCAGTTGTCTTGACTTCAGGTCTGTCTGTTCTGTTGGCAAGTAA | 6mer |
|  |  |  | MARMOSET | AGAATGCAGTTGTCTTGAC | 6mer |
|  |  |  | MARMOSET | AGAATGCAG | 6mer |
|  |  |  | MARMOSET | ATGCAGTACTGTTCTGATCCCGCTGCTATTAGAATGCATTGTGAAACGACTGGAGTATGATTAAAAGTTGTGTTCCCCAATGCTTGGAGTAGTGATTGTTGAAGGAAAAAA | 6mer |
|  |  |  | MARMOSET | GCTGCTATTAGAATGCATTGTGAAACGACTGGAGTATGATTAAAAGTTGTGTT | 6mer |
|  |  |  | MARMOSET | GCTGCTATTAGAATGCATTGTGAAACGACTGGAGTATGATTAAAAGTT | 6mer |
|  |  |  | MARMOSET | GCTGCTATTAGAATGCATT | 6mer |
|  |  |  | MARMOSET | TGGGTGGGAATGCAAAAATTCTCTGCTAAGACTTTTTCAGGTGAACATAACAGACTTGGCCAAGCTAGCATCTTAGCGGAAGC | 7mer-A1 |
|  |  |  | DOG | AGAATGCAGTTGTCTTGAC | 6mer |
|  |  |  | DOG | AGAATGCAG | 6mer |
|  |  |  | DOG | GCTGCTATTAGAATGCATTGTGAAACGACTGGAGTATGATTAAAAGTTGTGTT | 6mer |
|  |  |  | DOG | GCTGCTATTAGAATGCATTGTGAAACGACTGGAGTATGATTAAAAGTT | 6mer |
|  |  |  | DOG | GCTGCTATTAGAATGCATT | 6mer |
|  |  |  | PIG | AGAATGCAGTTGTCTTGAC | 6mer |
|  |  |  | PIG | AGAATGCAG | 6mer |
|  |  |  | PIG | GCTGCTATTAGAATGCATTGTGAAACGACTGGAGTATGATTAAAAGTT | 6mer |
|  |  |  | PIG | GCTGCTATTAGAATGCATT | 6mer |
|  |  |  | COW | AGAATGCAGTTGTCTTGAC | 6mer |
|  |  |  | COW | AGAATGCAG | 6mer |
|  |  |  | COW | GCTGCTATTAGAATGCATT | 6mer |
|  |  |  | MOUSE | AGAATGCAG | 6mer |
|  |  |  | MOUSE | GCTGCTATTAGAATGCATT | 6mer |
|  |  |  | TURTLE | AGAATGCAG | 6mer |
|  |  |  | ALLIGATOR | AGAATGCAG | 6mer |

  
  
  
  

| Seed Matches to the miRNA miR-455-3p.2 | | | | | |
| --- | --- | --- | --- | --- | --- |
| Seed | Conservation | Species | Matches | | |
|  |  |  | Sequence | Motif | Type |
| UGCAGUC | Broadly Conserved | Human (Homo sapiens)  Mouse (Mus musculus) | HUMAN | AACTGCAAA | 7mer-A1 |
|  |  |  | MARMOSET | AACTGCAAA | 7mer-A1 |
|  |  |  | DOG | AACTGCAAA | 7mer-A1 |

  
  
  
  

| Seed Matches to the miRNA miR-205-5p | | | | | |
| --- | --- | --- | --- | --- | --- |
| Seed | Conservation | Species | Matches | | |
|  |  |  | Sequence | Motif | Type |
| CCUUCAU | Broadly Conserved | Human (Homo sapiens)  Mouse (Mus musculus) | HUMAN | CCAGTGCGATTTGGTGAAGGAAGCTAGGAAGAAGGAAGGAGCGCTAACGATTTGGTGGTGAAGCTAGGAAA | 7mer-A1 |
|  |  |  | HUMAN | CAGAGCAAAGGAAGTGGCTTAATGATCCTGAAGGGATTTCTTCTGATGGTAGCTTTTGTATTATCAAGTAAGATTCT | 6mer |
|  |  |  | HUMAN | CAGAGCAAAGGAAGTGGCTTAATGATCCTGAAGGGATTTCTTC | 6mer |
|  |  |  | HUMAN | GTGGCTTAATGATCCTGAAGGGATTTCTTC | 6mer |
|  |  |  | HUMAN | CCTGAAGG | 6mer |
|  |  |  | HUMAN | ATGCAGTACTGTTCTGATCCCGCTGCTATTAGAATGCATTGTGAAACGACTGGAGTATGATTAAAAGTTGTGTTCCCCAATGCTTGGAGTAGTGATTGTTGAAGGAAAAAA | 7mer-A1 |
|  |  |  | HUMAN | CCCCAATGCTTGGAGTAGTGATTGTTGAAGGAAA | 7mer-A1 |
|  |  |  | HUMAN | TAAAGCCCAAATCTCAAGCGGTGCTTGAAGGGGAGGGAAAGGGGGAAAGCGGGCAACCA | 6mer |
|  |  |  | HUMAN | TTGAAGGGGAGGGAAA | 6mer |
|  |  |  | HUMAN | GATCTCCAATGCTCTTCAGTAGGGTCATGAAGGTTTTTCTTTTCCTGAGAAAACAACA | 7mer-m8 |
|  |  |  | MARMOSET | CCAGTGCGATTTGGTGAAGGAAGCTAGGAAGAAGGAAGGAGCGCTAACGATTTGGTGGTGAAGCTAGGAAA | 7mer-A1 |
|  |  |  | MARMOSET | CAGAGCAAAGGAAGTGGCTTAATGATCCTGAAGGGATTTCTTCTGATGGTAGCTTTTGTATTATCAAGTAAGATTCT | 6mer |
|  |  |  | MARMOSET | CAGAGCAAAGGAAGTGGCTTAATGATCCTGAAGGGATTTCTTC | 6mer |
|  |  |  | MARMOSET | GTGGCTTAATGATCCTGAAGGGATTTCTTC | 6mer |
|  |  |  | MARMOSET | CCTGAAGG | 6mer |
|  |  |  | MARMOSET | ATGCAGTACTGTTCTGATCCCGCTGCTATTAGAATGCATTGTGAAACGACTGGAGTATGATTAAAAGTTGTGTTCCCCAATGCTTGGAGTAGTGATTGTTGAAGGAAAAAA | 7mer-A1 |
|  |  |  | MARMOSET | CCCCAATGCTTGGAGTAGTGATTGTTGAAGGAAA | 7mer-A1 |
|  |  |  | MARMOSET | TAAAGCCCAAATCTCAAGCGGTGCTTGAAGGGGAGGGAAAGGGGGAAAGCGGGCAACCA | 6mer |
|  |  |  | MARMOSET | TTGAAGGGGAGGGAAA | 6mer |
|  |  |  | MARMOSET | GATCTCCAATGCTCTTCAGTAGGGTCATGAAGGTTTTTCTTTTCCTGAGAAAACAACA | 7mer-m8 |
|  |  |  | DOG | CAGAGCAAAGGAAGTGGCTTAATGATCCTGAAGGGATTTCTTC | 6mer |
|  |  |  | DOG | GTGGCTTAATGATCCTGAAGGGATTTCTTC | 6mer |
|  |  |  | DOG | CCTGAAGG | 6mer |
|  |  |  | DOG | CCCCAATGCTTGGAGTAGTGATTGTTGAAGGAAA | 7mer-A1 |
|  |  |  | DOG | TTGAAGGGGAGGGAAA | 6mer |
|  |  |  | PIG | CAGAGCAAAGGAAGTGGCTTAATGATCCTGAAGGGATTTCTTC | 6mer |
|  |  |  | PIG | GTGGCTTAATGATCCTGAAGGGATTTCTTC | 6mer |
|  |  |  | PIG | CCTGAAGG | 6mer |
|  |  |  | PIG | CCCCAATGCTTGGAGTAGTGATTGTTGAAGGAAA | 7mer-A1 |
|  |  |  | PIG | TTGAAGGGGAGGGAAA | 6mer |
|  |  |  | COW | GTGGCTTAATGATCCTGAAGGGATTTCTTC | 6mer |
|  |  |  | COW | CCTGAAGG | 6mer |
|  |  |  | COW | TTGAAGGGGAGGGAAA | 6mer |
|  |  |  | MOUSE | CCTGAAGG | 7mer-A1 |

  
  
  
  

| Seed Matches to the miRNA miR-203a-3p.2 | | | | | |
| --- | --- | --- | --- | --- | --- |
| Seed | Conservation | Species | Matches | | |
|  |  |  | Sequence | Motif | Type |
| UGAAAUG | Broadly Conserved | Human (Homo sapiens) | HUMAN | AATTTCAGCAAATCTGTAAGCAGTTTGTATGTTTAGTTGGGGTAATG | 6mer |
|  |  |  | HUMAN | AGTATTTCA | 6mer |
|  |  |  | HUMAN | TTATCAGAAGAGTTGCTTCATTTCATCTGGGAGCAGAAAACAGCAGGCAGCTGTTAACAGATAAGTTTAACTTGCATCTGCA | 7mer-m8 |
|  |  |  | HUMAN | TTATCAGAAGAGTTGCTTCATTTCATCTGGGAGCAGAAAACAGCAGGCAGCTGTTAACAGATAAGTTTAA | 7mer-m8 |
|  |  |  | HUMAN | TCCTGACCCCTTCCCTAGGGGATTTCAGGATT | 6mer |
|  |  |  | HUMAN | GGATTTCAGGATT | 6mer |
|  |  |  | HUMAN | TTTTAAAGAATTTTCCTTTGCAGAGGCATTTCATCCTTCATGAAGC | 7mer-m8 |
|  |  |  | HUMAN | CATTTCATCCTTCATGAAGC | 7mer-m8 |
|  |  |  | HUMAN | AGTATTGAATAGATTTCAGCTTTATGCTGGAGTAA | 6mer |
|  |  |  | MARMOSET | AATTTCAGCAAATCTGTAAGCAGTTTGTATGTTTAGTTGGGGTAATG | 6mer |
|  |  |  | MARMOSET | AGTATTTCA | 7mer-A1 |
|  |  |  | MARMOSET | TTATCAGAAGAGTTGCTTCATTTCATCTGGGAGCAGAAAACAGCAGGCAGCTGTTAACAGATAAGTTTAACTTGCATCTGCA | 7mer-m8 |
|  |  |  | MARMOSET | TTATCAGAAGAGTTGCTTCATTTCATCTGGGAGCAGAAAACAGCAGGCAGCTGTTAACAGATAAGTTTAA | 7mer-m8 |
|  |  |  | MARMOSET | TCCTGACCCCTTCCCTAGGGGATTTCAGGATT | 6mer |
|  |  |  | MARMOSET | GGATTTCAGGATT | 6mer |
|  |  |  | MARMOSET | TTTTAAAGAATTTTCCTTTGCAGAGGCATTTCATCCTTCATGAAGC | 7mer-m8 |
|  |  |  | MARMOSET | CATTTCATCCTTCATGAAGC | 7mer-m8 |
|  |  |  | MARMOSET | AGTATTGAATAGATTTCAGCTTTATGCTGGAGTAA | 6mer |
|  |  |  | DOG | TTATCAGAAGAGTTGCTTCATTTCATCTGGGAGCAGAAAACAGCAGGCAGCTGTTAACAGATAAGTTTAA | 7mer-m8 |
|  |  |  | DOG | GGATTTCAGGATT | 6mer |
|  |  |  | DOG | CATTTCATCCTTCATGAAGC | 7mer-m8 |

  
  
  
  

| Seed Matches to the miRNA let-7-5p/98-5p | | | | | |
| --- | --- | --- | --- | --- | --- |
| Seed | Conservation | Species | Matches | | |
|  |  |  | Sequence | Motif | Type |
| GAGGUAG | Broadly Conserved | Human (Homo sapiens) | HUMAN | TGATAAGTAAAGGCAGAAAAGATTATATGTCATACCTCCATTGGGGAATAAGCATAACCCTGAGATTCTTACTACTGATGA | 6mer |
|  |  |  | HUMAN | TCATACCTCCATTGGGGAA | 6mer |
|  |  |  | MARMOSET | TGATAAGTAAAGGCAGAAAAGATTATATGTCATACCTCCATTGGGGAATAAGCATAACCCTGAGATTCTTACTACTGATGA | 6mer |
|  |  |  | MARMOSET | TCATACCTCCATTGGGGAA | 6mer |
|  |  |  | DOG | TCATACCTCCATTGGGGAA | 6mer |

  
  
  
  

| Seed Matches to the miRNA miR-199-5p | | | | | |
| --- | --- | --- | --- | --- | --- |
| Seed | Conservation | Species | Matches | | |
|  |  |  | Sequence | Motif | Type |
| CCAGUGU | Broadly Conserved | Human (Homo sapiens)  X. tropicalis (Xenopus tropicalis)  Chicken (Gallus gallus)  Rhesus (Macaca mulatta)  Chimp (Pan troglodytes)  Cow (Bos taurus)  Mouse (Mus musculus)  Rat (Rattus norvegicus) | HUMAN | GAGCCACTGGGTGTACCAGTGCATT | 6mer |
|  |  |  | MARMOSET | GAGCCACTGGGTGTACCAGTGCATT | 6mer |

  
  
  
  

| Seed Matches to the miRNA miR-495-3p | | | | | |
| --- | --- | --- | --- | --- | --- |
| Seed | Conservation | Species | Matches | | |
|  |  |  | Sequence | Motif | Type |
| AACAAAC | Conserved | Human (Homo sapiens)  Rhesus (Macaca mulatta)  Mouse (Mus musculus) | HUMAN | TAACTGATTAAGAATTGTGATAGTTCAGCTTGAATGTCTCTTAGAGGGTGGGCTTTTGTTGATGAGGGAGGGGAAACTTTTTTTTT | 6mer |
|  |  |  | MARMOSET | TAACTGATTAAGAATTGTGATAGTTCAGCTTGAATGTCTCTTAGAGGGTGGGCTTTTGTTGATGAGGGAGGGGAAACTTTTTTTTT | 6mer |

  
  
  
  

| Seed Matches to the miRNA miR-483-3p.1 | | | | | |
| --- | --- | --- | --- | --- | --- |
| Seed | Conservation | Species | Matches | | |
|  |  |  | Sequence | Motif | Type |
| ACUCCUC | Conserved | Human (Homo sapiens)  Mouse (Mus musculus) | HUMAN | ATTTTGGGATGGTCTTAACAGGGAAGAGAGAGGGTGGGGGAGAAAATGTTTTTTTCTAAGATTTTCCACAGATGCTATAGTACTATTGACAAACTGGGTTAGAGAAGGAGTGTAC | 6mer |
|  |  |  | MARMOSET | ATTTTGGGATGGTCTTAACAGGGAAGAGAGAGGGTGGGGGAGAAAATGTTTTTTTCTAAGATTTTCCACAGATGCTATAGTACTATTGACAAACTGGGTTAGAGAAGGAGTGTAC | 6mer |

  
  
  
  

| Seed Matches to the miRNA miR-15-5p/16-5p/195-5p/424-5p/497-5p | | | | | |
| --- | --- | --- | --- | --- | --- |
| Seed | Conservation | Species | Matches | | |
|  |  |  | Sequence | Motif | Type |
| AGCAGCA | Broadly Conserved | Human (Homo sapiens)  Rhesus (Macaca mulatta) | HUMAN | GCTGTGCTGTTGGCACGAACACCTTCAGGGACTGGAGCTGCTTTTAT | 6mer |
|  |  |  | HUMAN | TGGAGCTGCTTTT | 6mer |
|  |  |  | HUMAN | ATGCAGTACTGTTCTGATCCCGCTGCTATTAGAATGCATTGTGAAACGACTGGAGTATGATTAAAAGTTGTGTTCCCCAATGCTTGGAGTAGTGATTGTTGAAGGAAAAAA | 7mer-A1 |
|  |  |  | HUMAN | GCTGCTATTAGAATGCATTGTGAAACGACTGGAGTATGATTAAAAGTTGTGTT | 7mer-A1 |
|  |  |  | HUMAN | GCTGCTATTAGAATGCATTGTGAAACGACTGGAGTATGATTAAAAGTT | 7mer-A1 |
|  |  |  | HUMAN | GCTGCTATTAGAATGCATT | 7mer-A1 |
|  |  |  | MARMOSET | GCTGTGCTGTTGGCACGAACACCTTCAGGGACTGGAGCTGCTTTTAT | 6mer |
|  |  |  | MARMOSET | TGGAGCTGCTTTT | 6mer |
|  |  |  | MARMOSET | ATGCAGTACTGTTCTGATCCCGCTGCTATTAGAATGCATTGTGAAACGACTGGAGTATGATTAAAAGTTGTGTTCCCCAATGCTTGGAGTAGTGATTGTTGAAGGAAAAAA | 7mer-A1 |
|  |  |  | MARMOSET | GCTGCTATTAGAATGCATTGTGAAACGACTGGAGTATGATTAAAAGTTGTGTT | 7mer-A1 |
|  |  |  | MARMOSET | GCTGCTATTAGAATGCATTGTGAAACGACTGGAGTATGATTAAAAGTT | 7mer-A1 |
|  |  |  | MARMOSET | GCTGCTATTAGAATGCATT | 7mer-A1 |
|  |  |  | DOG | TGGAGCTGCTTTT | 6mer |
|  |  |  | DOG | GCTGCTATTAGAATGCATTGTGAAACGACTGGAGTATGATTAAAAGTTGTGTT | 8mer |
|  |  |  | DOG | GCTGCTATTAGAATGCATTGTGAAACGACTGGAGTATGATTAAAAGTT | 8mer |
|  |  |  | DOG | GCTGCTATTAGAATGCATT | 8mer |
|  |  |  | PIG | TGGAGCTGCTTTT | 6mer |
|  |  |  | PIG | GCTGCTATTAGAATGCATTGTGAAACGACTGGAGTATGATTAAAAGTT | 8mer |
|  |  |  | PIG | GCTGCTATTAGAATGCATT | 8mer |
|  |  |  | COW | GCTGCTATTAGAATGCATT | 7mer-A1 |
|  |  |  | MOUSE | GCTGCTATTAGAATGCATT | 8mer |

  
  
  
  

| Seed Matches to the miRNA miR-29-3p | | | | | |
| --- | --- | --- | --- | --- | --- |
| Seed | Conservation | Species | Matches | | |
|  |  |  | Sequence | Motif | Type |
| AGCACCA | Broadly Conserved | Human (Homo sapiens)  Chicken (Gallus gallus)  Rhesus (Macaca mulatta)  Cow (Bos taurus)  Mouse (Mus musculus)  Rat (Rattus norvegicus)  Opossum (Monodelphis domestica) | HUMAN | TTAATTGACAGCTGACCCAGGTGCTACACAGAAGTGGATTCAGTGAATCTAGGAAGACAGCAGCAGACAG | 7mer-A1 |
|  |  |  | HUMAN | TAAAGCCCAAATCTCAAGCGGTGCTTGAAGGGGAGGGAAAGGGGGAAAGCGGGCAACCA | 6mer |
|  |  |  | MARMOSET | TTAATTGACAGCTGACCCAGGTGCTACACAGAAGTGGATTCAGTGAATCTAGGAAGACAGCAGCAGACAG | 7mer-A1 |
|  |  |  | MARMOSET | TAAAGCCCAAATCTCAAGCGGTGCTTGAAGGGGAGGGAAAGGGGGAAAGCGGGCAACCA | 6mer |

  
  
  
  

| Seed Matches to the miRNA miR-539-3p | | | | | |
| --- | --- | --- | --- | --- | --- |
| Seed | Conservation | Species | Matches | | |
|  |  |  | Sequence | Motif | Type |
| UCAUACA | Conserved | Human (Homo sapiens) | HUMAN | AGTGAGTGTATGAGACCTTGCAGTGAGTTTATCAGCATACTCAAAATTTTTTTCCTGGAATTTGGAGGGATGGGAGGAGGGGGTGGGGCTTACTTGTT | 7mer-m8 |
|  |  |  | HUMAN | ATGCAGTACTGTTCTGATCCCGCTGCTATTAGAATGCATTGTGAAACGACTGGAGTATGATTAAAAGTTGTGTTCCCCAATGCTTGGAGTAGTGATTGTTGAAGGAAAAAA | 6mer |
|  |  |  | HUMAN | GCTGCTATTAGAATGCATTGTGAAACGACTGGAGTATGATTAAAAGTTGTGTT | 6mer |
|  |  |  | HUMAN | GCTGCTATTAGAATGCATTGTGAAACGACTGGAGTATGATTAAAAGTT | 6mer |
|  |  |  | HUMAN | TGAAACGACTGGAGTATGA | 6mer |
|  |  |  | HUMAN | CGACTGGAGTATGA | 6mer |
|  |  |  | MARMOSET | AGTGAGTGTATGAGACCTTGCAGTGAGTTTATCAGCATACTCAAAATTTTTTTCCTGGAATTTGGAGGGATGGGAGGAGGGGGTGGGGCTTACTTGTT | 7mer-m8 |
|  |  |  | MARMOSET | ATGCAGTACTGTTCTGATCCCGCTGCTATTAGAATGCATTGTGAAACGACTGGAGTATGATTAAAAGTTGTGTTCCCCAATGCTTGGAGTAGTGATTGTTGAAGGAAAAAA | 6mer |
|  |  |  | MARMOSET | GCTGCTATTAGAATGCATTGTGAAACGACTGGAGTATGATTAAAAGTTGTGTT | 6mer |
|  |  |  | MARMOSET | GCTGCTATTAGAATGCATTGTGAAACGACTGGAGTATGATTAAAAGTT | 6mer |
|  |  |  | MARMOSET | TGAAACGACTGGAGTATGA | 6mer |
|  |  |  | MARMOSET | CGACTGGAGTATGA | 6mer |
|  |  |  | DOG | GCTGCTATTAGAATGCATTGTGAAACGACTGGAGTATGATTAAAAGTTGTGTT | 6mer |
|  |  |  | DOG | GCTGCTATTAGAATGCATTGTGAAACGACTGGAGTATGATTAAAAGTT | 6mer |
|  |  |  | DOG | TGAAACGACTGGAGTATGA | 6mer |
|  |  |  | DOG | CGACTGGAGTATGA | 6mer |
|  |  |  | PIG | GCTGCTATTAGAATGCATTGTGAAACGACTGGAGTATGATTAAAAGTT | 6mer |
|  |  |  | PIG | TGAAACGACTGGAGTATGA | 6mer |
|  |  |  | PIG | CGACTGGAGTATGA | 6mer |
|  |  |  | COW | TGAAACGACTGGAGTATGA | 6mer |
|  |  |  | COW | CGACTGGAGTATGA | 6mer |
|  |  |  | MOUSE | CGACTGGAGTATGA | 6mer |

  
  
  
  

| Seed Matches to the miRNA miR-143-3p | | | | | |
| --- | --- | --- | --- | --- | --- |
| Seed | Conservation | Species | Matches | | |
|  |  |  | Sequence | Motif | Type |
| GAGAUGA | Broadly Conserved | Human (Homo sapiens)  Rhesus (Macaca mulatta)  Mouse (Mus musculus)  Rat (Rattus norvegicus)  Opossum (Monodelphis domestica) | HUMAN | TGGAAGAGTATTCCCAGTTGAAGCTGAAAAGTACAGCACAGTGCAGCTTTGGTTCATATTCAGTCATCTCAGGAGAACTTCAGAAGAGCTTGAGTAGGCCAAATGTTGAAGTTAAGTTTTC | 8mer |
|  |  |  | HUMAN | CAATGTCCATCTCAAAATACTGCTTTTACAAAAGCAGAATAAAA | 7mer-A1 |
|  |  |  | HUMAN | CCATCTCAAAATACTGCTTTTACAAAAGCAGAATAAAA | 7mer-A1 |
|  |  |  | MARMOSET | TGGAAGAGTATTCCCAGTTGAAGCTGAAAAGTACAGCACAGTGCAGCTTTGGTTCATATTCAGTCATCTCAGGAGAACTTCAGAAGAGCTTGAGTAGGCCAAATGTTGAAGTTAAGTTTTC | 8mer |
|  |  |  | MARMOSET | CAATGTCCATCTCAAAATACTGCTTTTACAAAAGCAGAATAAAA | 7mer-A1 |
|  |  |  | MARMOSET | CCATCTCAAAATACTGCTTTTACAAAAGCAGAATAAAA | 7mer-A1 |
|  |  |  | DOG | CCATCTCAAAATACTGCTTTTACAAAAGCAGAATAAAA | 7mer-A1 |

  
  
  
  

| Seed Matches to the miRNA miR-202-5p | | | | | |
| --- | --- | --- | --- | --- | --- |
| Seed | Conservation | Species | Matches | | |
|  |  |  | Sequence | Motif | Type |
| UCCUAUG | Broadly Conserved | Human (Homo sapiens)  Mouse (Mus musculus)  Rat (Rattus norvegicus) | HUMAN | GCAGCAGTTCGTGGTGAAGATAGGAA | 7mer-A1 |
|  |  |  | HUMAN | GCAGTTCGTGGTGAAGATAGGAA | 7mer-A1 |
|  |  |  | HUMAN | GTGAAGATAGGAA | 7mer-A1 |
|  |  |  | MARMOSET | GCAGCAGTTCGTGGTGAAGATAGGAA | 7mer-A1 |
|  |  |  | MARMOSET | GCAGTTCGTGGTGAAGATAGGAA | 7mer-A1 |
|  |  |  | MARMOSET | GTGAAGATAGGAA | 7mer-A1 |
|  |  |  | DOG | GCAGTTCGTGGTGAAGATAGGAA | 7mer-A1 |
|  |  |  | DOG | GTGAAGATAGGAA | 7mer-A1 |
|  |  |  | PIG | GTGAAGATAGGAA | 7mer-A1 |
|  |  |  | COW | GTGAAGATAGGAA | 7mer-A1 |

  
  
  
  

| Seed Matches to the miRNA miR-193-3p | | | | | |
| --- | --- | --- | --- | --- | --- |
| Seed | Conservation | Species | Matches | | |
|  |  |  | Sequence | Motif | Type |
| ACUGGCC | Broadly Conserved | Human (Homo sapiens)  Chicken (Gallus gallus)  Rhesus (Macaca mulatta)  Cow (Bos taurus)  Mouse (Mus musculus)  Rat (Rattus norvegicus)  Opossum (Monodelphis domestica) | HUMAN | TAGAGGATCCTAGACCAGCATGCCAGT | 6mer |
|  |  |  | MARMOSET | TAGAGGATCCTAGACCAGCATGCCAGT | 7mer-A1 |

  
  
  
  

| Seed Matches to the miRNA miR-383-5p.1 | | | | | |
| --- | --- | --- | --- | --- | --- |
| Seed | Conservation | Species | Matches | | |
|  |  |  | Sequence | Motif | Type |
| GAUCAGA | Broadly Conserved | Human (Homo sapiens)  Mouse (Mus musculus) | HUMAN | ATGCAGTACTGTTCTGATCCCGCTGCTATTAGAATGCATTGTGAAACGACTGGAGTATGATTAAAAGTTGTGTTCCCCAATGCTTGGAGTAGTGATTGTTGAAGGAAAAAA | 7mer-m8 |
|  |  |  | HUMAN | TGCAGTACTGTTCTGATC | 7mer-m8 |
|  |  |  | MARMOSET | ATGCAGTACTGTTCTGATCCCGCTGCTATTAGAATGCATTGTGAAACGACTGGAGTATGATTAAAAGTTGTGTTCCCCAATGCTTGGAGTAGTGATTGTTGAAGGAAAAAA | 7mer-m8 |
|  |  |  | MARMOSET | TGCAGTACTGTTCTGATC | 7mer-m8 |
|  |  |  | DOG | TGCAGTACTGTTCTGATC | 7mer-m8 |

  
  
  
  

| Seed Matches to the miRNA miR-503-5p | | | | | |
| --- | --- | --- | --- | --- | --- |
| Seed | Conservation | Species | Matches | | |
|  |  |  | Sequence | Motif | Type |
| AGCAGCG | Conserved | Human (Homo sapiens)  Rhesus (Macaca mulatta)  Mouse (Mus musculus)  Rat (Rattus norvegicus) | HUMAN | GCTGTGCTGTTGGCACGAACACCTTCAGGGACTGGAGCTGCTTTTAT | 6mer |
|  |  |  | HUMAN | TGGAGCTGCTTTT | 6mer |
|  |  |  | HUMAN | ATGCAGTACTGTTCTGATCCCGCTGCTATTAGAATGCATTGTGAAACGACTGGAGTATGATTAAAAGTTGTGTTCCCCAATGCTTGGAGTAGTGATTGTTGAAGGAAAAAA | 8mer |
|  |  |  | HUMAN | GCTGCTATTAGAATGCATTGTGAAACGACTGGAGTATGATTAAAAGTTGTGTT | 8mer |
|  |  |  | HUMAN | GCTGCTATTAGAATGCATTGTGAAACGACTGGAGTATGATTAAAAGTT | 8mer |
|  |  |  | HUMAN | GCTGCTATTAGAATGCATT | 8mer |
|  |  |  | MARMOSET | GCTGTGCTGTTGGCACGAACACCTTCAGGGACTGGAGCTGCTTTTAT | 6mer |
|  |  |  | MARMOSET | TGGAGCTGCTTTT | 6mer |
|  |  |  | MARMOSET | ATGCAGTACTGTTCTGATCCCGCTGCTATTAGAATGCATTGTGAAACGACTGGAGTATGATTAAAAGTTGTGTTCCCCAATGCTTGGAGTAGTGATTGTTGAAGGAAAAAA | 8mer |
|  |  |  | MARMOSET | GCTGCTATTAGAATGCATTGTGAAACGACTGGAGTATGATTAAAAGTTGTGTT | 8mer |
|  |  |  | MARMOSET | GCTGCTATTAGAATGCATTGTGAAACGACTGGAGTATGATTAAAAGTT | 8mer |
|  |  |  | MARMOSET | GCTGCTATTAGAATGCATT | 8mer |
|  |  |  | DOG | TGGAGCTGCTTTT | 6mer |
|  |  |  | DOG | GCTGCTATTAGAATGCATTGTGAAACGACTGGAGTATGATTAAAAGTTGTGTT | 7mer-A1 |
|  |  |  | DOG | GCTGCTATTAGAATGCATTGTGAAACGACTGGAGTATGATTAAAAGTT | 7mer-A1 |
|  |  |  | DOG | GCTGCTATTAGAATGCATT | 7mer-A1 |
|  |  |  | PIG | TGGAGCTGCTTTT | 6mer |
|  |  |  | PIG | GCTGCTATTAGAATGCATTGTGAAACGACTGGAGTATGATTAAAAGTT | 7mer-A1 |
|  |  |  | PIG | GCTGCTATTAGAATGCATT | 7mer-A1 |
|  |  |  | COW | GCTGCTATTAGAATGCATT | 8mer |
|  |  |  | MOUSE | GCTGCTATTAGAATGCATT | 7mer-A1 |

  
  
  
  

| Seed Matches to the miRNA miR-491-5p | | | | | |
| --- | --- | --- | --- | --- | --- |
| Seed | Conservation | Species | Matches | | |
|  |  |  | Sequence | Motif | Type |
| GUGGGGA | Conserved | Human (Homo sapiens)  Rhesus (Macaca mulatta)  Mouse (Mus musculus) | HUMAN | TTTTCCCTAGCTTTTCCAGAAGCCTGTTAAAAGCAAGGTCTCCCCACAAGCAACTTCTCTGCCACATCGCCACCC | 8mer |
|  |  |  | HUMAN | CTCCCCACAAG | 8mer |
|  |  |  | MARMOSET | TTTTCCCTAGCTTTTCCAGAAGCCTGTTAAAAGCAAGGTCTCCCCACAAGCAACTTCTCTGCCACATCGCCACCC | 8mer |
|  |  |  | MARMOSET | CTCCCCACAAG | 8mer |
|  |  |  | DOG | CTCCCCACAAG | 8mer |
|  |  |  | PIG | CTCCCCACAAG | 8mer |
|  |  |  | COW | CTCCCCACAAG | 8mer |
|  |  |  | MOUSE | CTCCCCACAAG | 8mer |

  
  
  
  

| Seed Matches to the miRNA miR-490-3p | | | | | |
| --- | --- | --- | --- | --- | --- |
| Seed | Conservation | Species | Matches | | |
|  |  |  | Sequence | Motif | Type |
| AACCUGG | Broadly Conserved | Human (Homo sapiens)  Chicken (Gallus gallus)  Rhesus (Macaca mulatta)  Mouse (Mus musculus)  Rat (Rattus norvegicus) | HUMAN | TTGTTTTCTCAGGTTTTGCTTTTTGGCCTTT | 6mer |
|  |  |  | HUMAN | TTGTTTTCTCAGGTTTTGCTTTTT | 6mer |
|  |  |  | HUMAN | TCTCAGGTTTTGCTTTT | 6mer |
|  |  |  | HUMAN | CAGGTTTTGCTTTT | 6mer |
|  |  |  | HUMAN | CAGGTTTTGCTTT | 6mer |
|  |  |  | MARMOSET | TTGTTTTCTCAGGTTTTGCTTTTTGGCCTTT | 6mer |
|  |  |  | MARMOSET | TTGTTTTCTCAGGTTTTGCTTTTT | 6mer |
|  |  |  | MARMOSET | TCTCAGGTTTTGCTTTT | 6mer |
|  |  |  | MARMOSET | CAGGTTTTGCTTTT | 6mer |
|  |  |  | MARMOSET | CAGGTTTTGCTTT | 6mer |
|  |  |  | DOG | TTGTTTTCTCAGGTTTTGCTTTTT | 6mer |
|  |  |  | DOG | TCTCAGGTTTTGCTTTT | 6mer |
|  |  |  | DOG | CAGGTTTTGCTTTT | 6mer |
|  |  |  | DOG | CAGGTTTTGCTTT | 6mer |
|  |  |  | PIG | TTGTTTTCTCAGGTTTTGCTTTTT | 6mer |
|  |  |  | PIG | TCTCAGGTTTTGCTTTT | 6mer |
|  |  |  | PIG | CAGGTTTTGCTTTT | 6mer |
|  |  |  | PIG | CAGGTTTTGCTTT | 6mer |
|  |  |  | COW | TTGTTTTCTCAGGTTTTGCTTTTT | 6mer |
|  |  |  | COW | TCTCAGGTTTTGCTTTT | 6mer |
|  |  |  | COW | CAGGTTTTGCTTTT | 6mer |
|  |  |  | COW | CAGGTTTTGCTTT | 6mer |
|  |  |  | MOUSE | TTGTTTTCTCAGGTTTTGCTTTTT | 6mer |
|  |  |  | MOUSE | TCTCAGGTTTTGCTTTT | 6mer |
|  |  |  | MOUSE | CAGGTTTTGCTTTT | 6mer |
|  |  |  | MOUSE | CAGGTTTTGCTTT | 6mer |
|  |  |  | TURTLE | TCTCAGGTTTTGCTTTT | 6mer |
|  |  |  | TURTLE | CAGGTTTTGCTTTT | 6mer |
|  |  |  | TURTLE | CAGGTTTTGCTTT | 6mer |
|  |  |  | ALLIGATOR | TCTCAGGTTTTGCTTTT | 6mer |
|  |  |  | ALLIGATOR | CAGGTTTTGCTTTT | 6mer |
|  |  |  | ALLIGATOR | CAGGTTTTGCTTT | 6mer |
|  |  |  | LIZARD | TCTCAGGTTTTGCTTTT | 6mer |
|  |  |  | LIZARD | CAGGTTTTGCTTTT | 6mer |
|  |  |  | LIZARD | CAGGTTTTGCTTT | 6mer |
|  |  |  | SNAKE | TCTCAGGTTTTGCTTTT | 6mer |
|  |  |  | SNAKE | CAGGTTTTGCTTTT | 6mer |
|  |  |  | SNAKE | CAGGTTTTGCTTT | 6mer |
|  |  |  | X.TROPICALIS | CAGGTTTTGCTTTT | 6mer |
|  |  |  | X.TROPICALIS | CAGGTTTTGCTTT | 6mer |
|  |  |  | SHARK | CAGGTTTTGCTTTT | 6mer |
|  |  |  | SHARK | CAGGTTTTGCTTT | 6mer |
|  |  |  | OPOSSUM | CAGGTTTTGCTTTT | 6mer |
|  |  |  | OPOSSUM | CAGGTTTTGCTTT | 6mer |
|  |  |  | SPOTTEDGAR | CAGGTTTTGCTTTT | 7mer-m8 |
|  |  |  | SPOTTEDGAR | CAGGTTTTGCTTT | 7mer-m8 |
|  |  |  | FUGU | CAGGTTTTGCTTTT | 7mer-m8 |
|  |  |  | FUGU | CAGGTTTTGCTTT | 7mer-m8 |
|  |  |  | NILETILAPIA | CAGGTTTTGCTTTT | 7mer-m8 |
|  |  |  | NILETILAPIA | CAGGTTTTGCTTT | 7mer-m8 |
|  |  |  | STICKLEBACK | CAGGTTTTGCTTT | 7mer-m8 |
|  |  |  | MEDAKA | CAGGTTTTGCTTT | 7mer-m8 |
|  |  |  | ZEBRAFISH | CAGGTTTTGCTTT | 6mer |

  
  
  
  

| Seed Matches to the miRNA miR-876-5p | | | | | |
| --- | --- | --- | --- | --- | --- |
| Seed | Conservation | Species | Matches | | |
|  |  |  | Sequence | Motif | Type |
| GGAUUUC | Conserved | Human (Homo sapiens)  Rhesus (Macaca mulatta)  Mouse (Mus musculus) | HUMAN | AAGGTGATTAAAAGACCTTGAAATCCATGACGCA | 8mer |
|  |  |  | HUMAN | GAGTGGTTGGTAAAAATCCGTGAGGTCGGCAATATGTTGTTTTTCTGGAACTT | 6mer |
|  |  |  | HUMAN | AAACAAGAAAATCCA | 7mer-A1 |
|  |  |  | HUMAN | AAGAAAATCCA | 7mer-A1 |
|  |  |  | HUMAN | AAATCCA | 7mer-A1 |
|  |  |  | HUMAN | ACTCCTGGTTTCCAGGACGGGGTTCAAATCCCTGCGGC | 6mer |
|  |  |  | HUMAN | GGGTTCAAATCC | 6mer |
|  |  |  | MARMOSET | AAGGTGATTAAAAGACCTTGAAATCCATGACGCA | 8mer |
|  |  |  | MARMOSET | GAGTGGTTGGTAAAAATCCGTGAGGTCGGCAATATGTTGTTTTTCTGGAACTT | 6mer |
|  |  |  | MARMOSET | AAACAAGAAAATCCA | 7mer-A1 |
|  |  |  | MARMOSET | AAGAAAATCCA | 7mer-A1 |
|  |  |  | MARMOSET | AAATCCA | 7mer-A1 |
|  |  |  | MARMOSET | ACTCCTGGTTTCCAGGACGGGGTTCAAATCCCTGCGGC | 6mer |
|  |  |  | MARMOSET | GGGTTCAAATCC | 6mer |
|  |  |  | DOG | AAGAAAATCCA | 7mer-A1 |
|  |  |  | DOG | AAATCCA | 7mer-A1 |
|  |  |  | DOG | GGGTTCAAATCC | 6mer |
|  |  |  | PIG | AAGAAAATCCA | 7mer-A1 |
|  |  |  | PIG | AAATCCA | 7mer-A1 |
|  |  |  | PIG | GGGTTCAAATCC | 6mer |
|  |  |  | COW | AAGAAAATCCA | 7mer-A1 |
|  |  |  | COW | AAATCCA | 7mer-A1 |
|  |  |  | COW | GGGTTCAAATCC | 6mer |
|  |  |  | MOUSE | AAATCCA | 8mer |

  
  
  
  

| Seed Matches to the miRNA miR-188-5p | | | | | |
| --- | --- | --- | --- | --- | --- |
| Seed | Conservation | Species | Matches | | |
|  |  |  | Sequence | Motif | Type |
| AUCCCUU | Conserved | Human (Homo sapiens)  Rhesus (Macaca mulatta)  Mouse (Mus musculus)  Rat (Rattus norvegicus) | HUMAN | CAGAGCAAAGGAAGTGGCTTAATGATCCTGAAGGGATTTCTTCTGATGGTAGCTTTTGTATTATCAAGTAAGATTCT | 7mer-m8 |
|  |  |  | HUMAN | CAGAGCAAAGGAAGTGGCTTAATGATCCTGAAGGGATTTCTTC | 7mer-m8 |
|  |  |  | HUMAN | GTGGCTTAATGATCCTGAAGGGATTTCTTC | 7mer-m8 |
|  |  |  | HUMAN | TATTGCATGTTAGGGATAAGTG | 7mer-A1 |
|  |  |  | HUMAN | AGTGAGTGTATGAGACCTTGCAGTGAGTTTATCAGCATACTCAAAATTTTTTTCCTGGAATTTGGAGGGATGGGAGGAGGGGGTGGGGCTTACTTGTT | 6mer |
|  |  |  | MARMOSET | CAGAGCAAAGGAAGTGGCTTAATGATCCTGAAGGGATTTCTTCTGATGGTAGCTTTTGTATTATCAAGTAAGATTCT | 7mer-m8 |
|  |  |  | MARMOSET | CAGAGCAAAGGAAGTGGCTTAATGATCCTGAAGGGATTTCTTC | 7mer-m8 |
|  |  |  | MARMOSET | GTGGCTTAATGATCCTGAAGGGATTTCTTC | 7mer-m8 |
|  |  |  | MARMOSET | TATTGCATGTTAGGGATAAGTG | 7mer-A1 |
|  |  |  | MARMOSET | AGTGAGTGTATGAGACCTTGCAGTGAGTTTATCAGCATACTCAAAATTTTTTTCCTGGAATTTGGAGGGATGGGAGGAGGGGGTGGGGCTTACTTGTT | 6mer |
|  |  |  | DOG | CAGAGCAAAGGAAGTGGCTTAATGATCCTGAAGGGATTTCTTC | 7mer-m8 |
|  |  |  | DOG | GTGGCTTAATGATCCTGAAGGGATTTCTTC | 7mer-m8 |
|  |  |  | DOG | TATTGCATGTTAGGGATAAGTG | 7mer-A1 |
|  |  |  | PIG | CAGAGCAAAGGAAGTGGCTTAATGATCCTGAAGGGATTTCTTC | 7mer-m8 |
|  |  |  | PIG | GTGGCTTAATGATCCTGAAGGGATTTCTTC | 7mer-m8 |
|  |  |  | PIG | TATTGCATGTTAGGGATAAGTG | 7mer-A1 |
|  |  |  | COW | GTGGCTTAATGATCCTGAAGGGATTTCTTC | 7mer-m8 |

  
  
  
  

| Seed Matches to the miRNA miR-668-3p | | | | | |
| --- | --- | --- | --- | --- | --- |
| Seed | Conservation | Species | Matches | | |
|  |  |  | Sequence | Motif | Type |
| GUCACUC | Conserved | Human (Homo sapiens)  Mouse (Mus musculus) | HUMAN | ACTGAAGCCTTTAGTCTTTTCCAGATGCAACCTTAAAATCAGTGACAAGAAACA | 7mer-A1 |
|  |  |  | HUMAN | CCTTAAAATCAGTGACAAGAAA | 7mer-A1 |
|  |  |  | HUMAN | TTAAAATCAGTGACAAGAAA | 7mer-A1 |
|  |  |  | HUMAN | CCAGTGACTAAAACCAACTTAAACCAGTAAGTGGAGAAATAACATGTT | 6mer |
|  |  |  | MARMOSET | ACTGAAGCCTTTAGTCTTTTCCAGATGCAACCTTAAAATCAGTGACAAGAAACA | 7mer-A1 |
|  |  |  | MARMOSET | CCTTAAAATCAGTGACAAGAAA | 7mer-A1 |
|  |  |  | MARMOSET | TTAAAATCAGTGACAAGAAA | 7mer-A1 |
|  |  |  | MARMOSET | CCAGTGACTAAAACCAACTTAAACCAGTAAGTGGAGAAATAACATGTT | 6mer |
|  |  |  | DOG | CCTTAAAATCAGTGACAAGAAA | 7mer-A1 |
|  |  |  | DOG | TTAAAATCAGTGACAAGAAA | 7mer-A1 |
|  |  |  | PIG | TTAAAATCAGTGACAAGAAA | 7mer-A1 |
|  |  |  | COW | TTAAAATCAGTGACAAGAAA | 7mer-A1 |

  
  
  
  

| Seed Matches to the miRNA miR-655-3p | | | | | |
| --- | --- | --- | --- | --- | --- |
| Seed | Conservation | Species | Matches | | |
|  |  |  | Sequence | Motif | Type |
| UAAUACA | Conserved | Human (Homo sapiens) | HUMAN | CAGAGCAAAGGAAGTGGCTTAATGATCCTGAAGGGATTTCTTCTGATGGTAGCTTTTGTATTATCAAGTAAGATTCT | 7mer-m8 |
|  |  |  | MARMOSET | CAGAGCAAAGGAAGTGGCTTAATGATCCTGAAGGGATTTCTTCTGATGGTAGCTTTTGTATTATCAAGTAAGATTCT | 7mer-m8 |

  
  
  
  

| Seed Matches to the miRNA miR-144-3p | | | | | |
| --- | --- | --- | --- | --- | --- |
| Seed | Conservation | Species | Matches | | |
|  |  |  | Sequence | Motif | Type |
| ACAGUAU | Broadly Conserved | Human (Homo sapiens)  Mouse (Mus musculus)  Rat (Rattus norvegicus)  Opossum (Monodelphis domestica) | HUMAN | TAATTTGATACTGT | 8mer |
|  |  |  | HUMAN | ATGCAGTACTGTTCTGATCCCGCTGCTATTAGAATGCATTGTGAAACGACTGGAGTATGATTAAAAGTTGTGTTCCCCAATGCTTGGAGTAGTGATTGTTGAAGGAAAAAA | 6mer |
|  |  |  | HUMAN | TGCAGTACTGTTCTGATC | 6mer |
|  |  |  | HUMAN | TGCAGTACTGTTCTGA | 6mer |
|  |  |  | HUMAN | CAGTACTGTTCTGA | 6mer |
|  |  |  | HUMAN | CAGTACTGTTC | 6mer |
|  |  |  | MARMOSET | TAATTTGATACTGT | 7mer-m8 |
|  |  |  | MARMOSET | ATGCAGTACTGTTCTGATCCCGCTGCTATTAGAATGCATTGTGAAACGACTGGAGTATGATTAAAAGTTGTGTTCCCCAATGCTTGGAGTAGTGATTGTTGAAGGAAAAAA | 6mer |
|  |  |  | MARMOSET | TGCAGTACTGTTCTGATC | 6mer |
|  |  |  | MARMOSET | TGCAGTACTGTTCTGA | 6mer |
|  |  |  | MARMOSET | CAGTACTGTTCTGA | 6mer |
|  |  |  | MARMOSET | CAGTACTGTTC | 6mer |
|  |  |  | DOG | TGCAGTACTGTTCTGATC | 6mer |
|  |  |  | DOG | TGCAGTACTGTTCTGA | 6mer |
|  |  |  | DOG | CAGTACTGTTCTGA | 6mer |
|  |  |  | DOG | CAGTACTGTTC | 6mer |
|  |  |  | PIG | TGCAGTACTGTTCTGA | 6mer |
|  |  |  | PIG | CAGTACTGTTCTGA | 6mer |
|  |  |  | PIG | CAGTACTGTTC | 6mer |
|  |  |  | COW | CAGTACTGTTCTGA | 6mer |
|  |  |  | COW | CAGTACTGTTC | 6mer |
|  |  |  | MOUSE | CAGTACTGTTC | 6mer |

  
  
  
  

| Seed Matches to the miRNA miR-874-3p | | | | | |
| --- | --- | --- | --- | --- | --- |
| Seed | Conservation | Species | Matches | | |
|  |  |  | Sequence | Motif | Type |
| UGCCCUG | Conserved | Human (Homo sapiens)  Rhesus (Macaca mulatta)  Mouse (Mus musculus)  Rat (Rattus norvegicus) | HUMAN | TAGAAGGGCA | 7mer-A1 |
|  |  |  | HUMAN | CTAAGGGCAAAATGT | 7mer-A1 |
|  |  |  | HUMAN | CTCATGAATCTTGTCTGAAGCTTTTGAGGGCAGACTGCCAAGTCCTGGAG | 6mer |
|  |  |  | HUMAN | ATCTTGTCTGAAGCTTTTGAGGGCAGACT | 6mer |
|  |  |  | HUMAN | AGCTTTTGAGGGCAGACT | 6mer |
|  |  |  | MARMOSET | TAGAAGGGCA | 6mer |
|  |  |  | MARMOSET | CTAAGGGCAAAATGT | 7mer-A1 |
|  |  |  | MARMOSET | CTCATGAATCTTGTCTGAAGCTTTTGAGGGCAGACTGCCAAGTCCTGGAG | 6mer |
|  |  |  | MARMOSET | ATCTTGTCTGAAGCTTTTGAGGGCAGACT | 6mer |
|  |  |  | MARMOSET | AGCTTTTGAGGGCAGACT | 6mer |
|  |  |  | DOG | ATCTTGTCTGAAGCTTTTGAGGGCAGACT | 6mer |
|  |  |  | DOG | AGCTTTTGAGGGCAGACT | 6mer |
|  |  |  | PIG | ATCTTGTCTGAAGCTTTTGAGGGCAGACT | 6mer |
|  |  |  | PIG | AGCTTTTGAGGGCAGACT | 6mer |
|  |  |  | COW | AGCTTTTGAGGGCAGACT | 6mer |

  
  
  
  

| Seed Matches to the miRNA miR-219-5p | | | | | |
| --- | --- | --- | --- | --- | --- |
| Seed | Conservation | Species | Matches | | |
|  |  |  | Sequence | Motif | Type |
| GAUUGUC | Broadly Conserved | Human (Homo sapiens)  Chimp (Pan troglodytes)  Dog (Canis lupus familiaris)  Cow (Bos taurus)  Mouse (Mus musculus)  Rat (Rattus norvegicus)  Opossum (Monodelphis domestica) | HUMAN | AGTTTGCATGTTAACTTTAAATGCTTACAATCTTA | 6mer |
|  |  |  | MARMOSET | AGTTTGCATGTTAACTTTAAATGCTTACAATCTTA | 6mer |

  
  
  
  

| Seed Matches to the miRNA miR-299-3p | | | | | |
| --- | --- | --- | --- | --- | --- |
| Seed | Conservation | Species | Matches | | |
|  |  |  | Sequence | Motif | Type |
| AUGUGGG | Conserved | Human (Homo sapiens)  Rhesus (Macaca mulatta)  Mouse (Mus musculus)  Rat (Rattus norvegicus) | HUMAN | TTTTCCCTAGCTTTTCCAGAAGCCTGTTAAAAGCAAGGTCTCCCCACAAGCAACTTCTCTGCCACATCGCCACCC | 6mer |
|  |  |  | HUMAN | CTCTGCCACATCGCCAC | 6mer |
|  |  |  | HUMAN | CTCTGCCACATCG | 6mer |
|  |  |  | MARMOSET | TTTTCCCTAGCTTTTCCAGAAGCCTGTTAAAAGCAAGGTCTCCCCACAAGCAACTTCTCTGCCACATCGCCACCC | 6mer |
|  |  |  | MARMOSET | CTCTGCCACATCGCCAC | 6mer |
|  |  |  | MARMOSET | CTCTGCCACATCG | 6mer |
|  |  |  | DOG | CTCTGCCACATCGCCAC | 6mer |
|  |  |  | DOG | CTCTGCCACATCG | 6mer |
|  |  |  | PIG | CTCTGCCACATCG | 6mer |
|  |  |  | COW | CTCTGCCACATCG | 6mer |
|  |  |  | MOUSE | CTCTGCCACATCG | 6mer |

  
  
  
  

| Seed Matches to the miRNA miR-101-3p.1 | | | | | |
| --- | --- | --- | --- | --- | --- |
| Seed | Conservation | Species | Matches | | |
|  |  |  | Sequence | Motif | Type |
| ACAGUAC | Broadly Conserved | Human (Homo sapiens)  Mouse (Mus musculus) | HUMAN | TAATTTGATACTGT | 7mer-A1 |
|  |  |  | HUMAN | ATGCAGTACTGTTCTGATCCCGCTGCTATTAGAATGCATTGTGAAACGACTGGAGTATGATTAAAAGTTGTGTTCCCCAATGCTTGGAGTAGTGATTGTTGAAGGAAAAAA | 7mer-m8 |
|  |  |  | HUMAN | TGCAGTACTGTTCTGATC | 7mer-m8 |
|  |  |  | HUMAN | TGCAGTACTGTTCTGA | 7mer-m8 |
|  |  |  | HUMAN | CAGTACTGTTCTGA | 7mer-m8 |
|  |  |  | HUMAN | CAGTACTGTTC | 7mer-m8 |
|  |  |  | MARMOSET | TAATTTGATACTGT | 6mer |
|  |  |  | MARMOSET | ATGCAGTACTGTTCTGATCCCGCTGCTATTAGAATGCATTGTGAAACGACTGGAGTATGATTAAAAGTTGTGTTCCCCAATGCTTGGAGTAGTGATTGTTGAAGGAAAAAA | 7mer-m8 |
|  |  |  | MARMOSET | TGCAGTACTGTTCTGATC | 7mer-m8 |
|  |  |  | MARMOSET | TGCAGTACTGTTCTGA | 7mer-m8 |
|  |  |  | MARMOSET | CAGTACTGTTCTGA | 7mer-m8 |
|  |  |  | MARMOSET | CAGTACTGTTC | 7mer-m8 |
|  |  |  | DOG | TGCAGTACTGTTCTGATC | 7mer-m8 |
|  |  |  | DOG | TGCAGTACTGTTCTGA | 7mer-m8 |
|  |  |  | DOG | CAGTACTGTTCTGA | 7mer-m8 |
|  |  |  | DOG | CAGTACTGTTC | 7mer-m8 |
|  |  |  | PIG | TGCAGTACTGTTCTGA | 7mer-m8 |
|  |  |  | PIG | CAGTACTGTTCTGA | 7mer-m8 |
|  |  |  | PIG | CAGTACTGTTC | 7mer-m8 |
|  |  |  | COW | CAGTACTGTTCTGA | 7mer-m8 |
|  |  |  | COW | CAGTACTGTTC | 7mer-m8 |
|  |  |  | MOUSE | CAGTACTGTTC | 7mer-m8 |

  
  
  
  

| Seed Matches to the miRNA miR-154-5p | | | | | |
| --- | --- | --- | --- | --- | --- |
| Seed | Conservation | Species | Matches | | |
|  |  |  | Sequence | Motif | Type |
| AGGUUAU | Conserved | Human (Homo sapiens)  Rhesus (Macaca mulatta)  Mouse (Mus musculus)  Rat (Rattus norvegicus) | HUMAN | TAACCTCTTAGACAGGTGGGAGATTATGATCAGAGTAAAAGGTAATTACACATTTTATTTCCAGAAAGTCAGG | 7mer-m8 |
|  |  |  | MARMOSET | TAACCTCTTAGACAGGTGGGAGATTATGATCAGAGTAAAAGGTAATTACACATTTTATTTCCAGAAAGTCAGG | 6mer |

  
  
  
  

| Seed Matches to the miRNA miR-3064-5p | | | | | |
| --- | --- | --- | --- | --- | --- |
| Seed | Conservation | Species | Matches | | |
|  |  |  | Sequence | Motif | Type |
| CUGGCUG | Conserved | Human (Homo sapiens) | HUMAN | AGCCAGCGCAGGG | 7mer-m8 |
|  |  |  | HUMAN | GAAGGGCCAGAGAAGCCAGACCCAGTAAG | 7mer-A1 |
|  |  |  | HUMAN | GAAGGGCCAGAGAAGCCAGACC | 7mer-A1 |
|  |  |  | HUMAN | GGCCAGAGAAGCCAGACC | 7mer-A1 |
|  |  |  | MARMOSET | AGCCAGCGCAGGG | 6mer |
|  |  |  | MARMOSET | GAAGGGCCAGAGAAGCCAGACCCAGTAAG | 7mer-A1 |
|  |  |  | MARMOSET | GAAGGGCCAGAGAAGCCAGACC | 7mer-A1 |
|  |  |  | MARMOSET | GGCCAGAGAAGCCAGACC | 7mer-A1 |
|  |  |  | DOG | GAAGGGCCAGAGAAGCCAGACC | 7mer-A1 |
|  |  |  | DOG | GGCCAGAGAAGCCAGACC | 7mer-A1 |
|  |  |  | PIG | GAAGGGCCAGAGAAGCCAGACC | 7mer-A1 |
|  |  |  | PIG | GGCCAGAGAAGCCAGACC | 7mer-A1 |
|  |  |  | COW | GGCCAGAGAAGCCAGACC | 7mer-A1 |

  
  
  
  

| Seed Matches to the miRNA miR-193a-5p | | | | | |
| --- | --- | --- | --- | --- | --- |
| Seed | Conservation | Species | Matches | | |
|  |  |  | Sequence | Motif | Type |
| GGGUCUU | Broadly Conserved | Human (Homo sapiens) | HUMAN | GAAGGGCCAGAGAAGCCAGACCCAGTAAG | 7mer-A1 |
|  |  |  | HUMAN | CTAGCACAGACCCTTCACCCCTCACCTCGATGCAGCC | 6mer |
|  |  |  | HUMAN | CAGACCCTTCACCCCTCACCTCGATGC | 6mer |
|  |  |  | HUMAN | CAGACCCTTCACCCCTCACCT | 6mer |
|  |  |  | MARMOSET | GAAGGGCCAGAGAAGCCAGACCCAGTAAG | 7mer-A1 |
|  |  |  | MARMOSET | CTAGCACAGACCCTTCACCCCTCACCTCGATGCAGCC | 6mer |
|  |  |  | MARMOSET | CAGACCCTTCACCCCTCACCTCGATGC | 6mer |
|  |  |  | MARMOSET | CAGACCCTTCACCCCTCACCT | 6mer |
|  |  |  | DOG | CAGACCCTTCACCCCTCACCTCGATGC | 6mer |
|  |  |  | DOG | CAGACCCTTCACCCCTCACCT | 6mer |
|  |  |  | PIG | CAGACCCTTCACCCCTCACCTCGATGC | 6mer |
|  |  |  | PIG | CAGACCCTTCACCCCTCACCT | 6mer |
|  |  |  | COW | CAGACCCTTCACCCCTCACCTCGATGC | 6mer |
|  |  |  | COW | CAGACCCTTCACCCCTCACCT | 6mer |
|  |  |  | MOUSE | CAGACCCTTCACCCCTCACCT | 6mer |

  
  
  
  

| Seed Matches to the miRNA miR-485-5p | | | | | |
| --- | --- | --- | --- | --- | --- |
| Seed | Conservation | Species | Matches | | |
|  |  |  | Sequence | Motif | Type |
| GAGGCUG | Conserved | Human (Homo sapiens)  Rhesus (Macaca mulatta)  Rat (Rattus norvegicus) | HUMAN | CTTAAAAGCCTCTAAAGTGAT | 6mer |
|  |  |  | HUMAN | GTGTCAGCCTCACCT | 8mer |
|  |  |  | HUMAN | GTGTCAGCCTC | 8mer |
|  |  |  | MARMOSET | CTTAAAAGCCTCTAAAGTGAT | 6mer |
|  |  |  | MARMOSET | GTGTCAGCCTCACCT | 8mer |
|  |  |  | MARMOSET | GTGTCAGCCTC | 8mer |
|  |  |  | DOG | GTGTCAGCCTC | 7mer-m8 |
|  |  |  | PIG | GTGTCAGCCTC | 8mer |

  
  
  
  

| Seed Matches to the miRNA miR-375 | | | | | |
| --- | --- | --- | --- | --- | --- |
| Seed | Conservation | Species | Matches | | |
|  |  |  | Sequence | Motif | Type |
| UUGUUCG | Broadly Conserved | Human (Homo sapiens)  X. tropicalis (Xenopus tropicalis)  Chicken (Gallus gallus)  Rhesus (Macaca mulatta)  Chimp (Pan troglodytes)  Dog (Canis lupus familiaris) | HUMAN | AACAGGTGAACAAGCTTTTTCTGTATTTACAT | 6mer |
|  |  |  | MARMOSET | AACAGGTGAACAAGCTTTTTCTGTATTTACAT | 6mer |

  
  
  
  

| Seed Matches to the miRNA miR-141-3p/200a-3p | | | | | |
| --- | --- | --- | --- | --- | --- |
| Seed | Conservation | Species | Matches | | |
|  |  |  | Sequence | Motif | Type |
| AACACUG | Broadly Conserved | Human (Homo sapiens)  Mouse (Mus musculus)  Rat (Rattus norvegicus)  Opossum (Monodelphis domestica) | HUMAN | CCAGTGTTTG | 7mer-m8 |
|  |  |  | HUMAN | AGTGTTGGGGCAATCTTGGGGGG | 7mer-m8 |
|  |  |  | HUMAN | TCCAGCTGAGTGATAAAGGCTGAGTGTTGAGGAAATTTCTGCAGTTTTAAGCAGTCGT | 6mer |
|  |  |  | HUMAN | TCCAGCTGAGTGATAAAGGCTGAGTGTTGAGGAAATTTCTGCAG | 6mer |
|  |  |  | HUMAN | AAAGGCTGAGTGTTGAGGAAATTTCTGCAG | 6mer |
|  |  |  | HUMAN | AGGCTGAGTGTTGAGGAAAT | 6mer |
|  |  |  | MARMOSET | CCAGTGTTTG | 7mer-m8 |
|  |  |  | MARMOSET | AGTGTTGGGGCAATCTTGGGGGG | 6mer |
|  |  |  | MARMOSET | TCCAGCTGAGTGATAAAGGCTGAGTGTTGAGGAAATTTCTGCAGTTTTAAGCAGTCGT | 6mer |
|  |  |  | MARMOSET | TCCAGCTGAGTGATAAAGGCTGAGTGTTGAGGAAATTTCTGCAG | 6mer |
|  |  |  | MARMOSET | AAAGGCTGAGTGTTGAGGAAATTTCTGCAG | 6mer |
|  |  |  | MARMOSET | AGGCTGAGTGTTGAGGAAAT | 6mer |
|  |  |  | DOG | TCCAGCTGAGTGATAAAGGCTGAGTGTTGAGGAAATTTCTGCAG | 6mer |
|  |  |  | DOG | AAAGGCTGAGTGTTGAGGAAATTTCTGCAG | 6mer |
|  |  |  | DOG | AGGCTGAGTGTTGAGGAAAT | 6mer |
|  |  |  | PIG | AAAGGCTGAGTGTTGAGGAAATTTCTGCAG | 6mer |
|  |  |  | PIG | AGGCTGAGTGTTGAGGAAAT | 6mer |
|  |  |  | COW | AAAGGCTGAGTGTTGAGGAAATTTCTGCAG | 6mer |
|  |  |  | COW | AGGCTGAGTGTTGAGGAAAT | 6mer |
|  |  |  | MOUSE | AGGCTGAGTGTTGAGGAAAT | 6mer |

  
  
  
  

| Seed Matches to the miRNA miR-532-5p | | | | | |
| --- | --- | --- | --- | --- | --- |
| Seed | Conservation | Species | Matches | | |
|  |  |  | Sequence | Motif | Type |
| AUGCCUU | Conserved | Human (Homo sapiens)  Rhesus (Macaca mulatta)  Mouse (Mus musculus) | HUMAN | AGGCATTGAGG | 6mer |
|  |  |  | HUMAN | TCACTCAGAGGCATTTGCATCT | 6mer |
|  |  |  | HUMAN | TTTTAAAGAATTTTCCTTTGCAGAGGCATTTCATCCTTCATGAAGC | 6mer |
|  |  |  | MARMOSET | AGGCATTGAGG | 6mer |
|  |  |  | MARMOSET | TCACTCAGAGGCATTTGCATCT | 6mer |
|  |  |  | MARMOSET | TTTTAAAGAATTTTCCTTTGCAGAGGCATTTCATCCTTCATGAAGC | 6mer |

  
  
  
  

| Seed Matches to the miRNA miR-186-5p | | | | | |
| --- | --- | --- | --- | --- | --- |
| Seed | Conservation | Species | Matches | | |
|  |  |  | Sequence | Motif | Type |
| AAAGAAU | Conserved | Human (Homo sapiens)  Rhesus (Macaca mulatta)  Mouse (Mus musculus)  Rat (Rattus norvegicus)  Opossum (Monodelphis domestica) | HUMAN | AGTTCTTTT | 6mer |
|  |  |  | HUMAN | AAACCAAACATTCCATTTTAAATGTGGGGATTGGGAACCACTAGTTCTTTCAGATGGTATTCTTCAGACTATAGAAGGAGCTTCCAGTTGAATTCA | 6mer |
|  |  |  | HUMAN | TCCATTTTAAATGTGGGGATTGGGAACCACTAGTTCTTTCAGATGGTATTCTTCAGACTATAGAAGGAGCTTCCAGTTGAATTCA | 6mer |
|  |  |  | HUMAN | TTTAAATGTGGGGATTGGGAACCACTAGTTCTTTCAGATGGTA | 6mer |
|  |  |  | HUMAN | CTAGTTCTTTCAGATG | 6mer |
|  |  |  | HUMAN | GATCTCCAATGCTCTTCAGTAGGGTCATGAAGGTTTTTCTTTTCCTGAGAAAACAACA | 6mer |
|  |  |  | HUMAN | AAGGTTTTTCTTTTCCTGAGAAAACAA | 6mer |
|  |  |  | HUMAN | AAGGTTTTTCTTTTCCTGAGA | 6mer |
|  |  |  | HUMAN | TTTTCTTTTCCTGAGA | 6mer |
|  |  |  | HUMAN | TTTTCTTTT | 6mer |
|  |  |  | MARMOSET | AGTTCTTTT | 6mer |
|  |  |  | MARMOSET | AAACCAAACATTCCATTTTAAATGTGGGGATTGGGAACCACTAGTTCTTTCAGATGGTATTCTTCAGACTATAGAAGGAGCTTCCAGTTGAATTCA | 6mer |
|  |  |  | MARMOSET | TCCATTTTAAATGTGGGGATTGGGAACCACTAGTTCTTTCAGATGGTATTCTTCAGACTATAGAAGGAGCTTCCAGTTGAATTCA | 6mer |
|  |  |  | MARMOSET | TTTAAATGTGGGGATTGGGAACCACTAGTTCTTTCAGATGGTA | 6mer |
|  |  |  | MARMOSET | CTAGTTCTTTCAGATG | 6mer |
|  |  |  | MARMOSET | GATCTCCAATGCTCTTCAGTAGGGTCATGAAGGTTTTTCTTTTCCTGAGAAAACAACA | 6mer |
|  |  |  | MARMOSET | AAGGTTTTTCTTTTCCTGAGAAAACAA | 6mer |
|  |  |  | MARMOSET | AAGGTTTTTCTTTTCCTGAGA | 6mer |
|  |  |  | MARMOSET | TTTTCTTTTCCTGAGA | 6mer |
|  |  |  | MARMOSET | TTTTCTTTT | 6mer |
|  |  |  | DOG | AGTTCTTTT | 6mer |
|  |  |  | DOG | TCCATTTTAAATGTGGGGATTGGGAACCACTAGTTCTTTCAGATGGTATTCTTCAGACTATAGAAGGAGCTTCCAGTTGAATTCA | 6mer |
|  |  |  | DOG | TTTAAATGTGGGGATTGGGAACCACTAGTTCTTTCAGATGGTA | 6mer |
|  |  |  | DOG | CTAGTTCTTTCAGATG | 6mer |
|  |  |  | DOG | AAGGTTTTTCTTTTCCTGAGAAAACAA | 6mer |
|  |  |  | DOG | AAGGTTTTTCTTTTCCTGAGA | 6mer |
|  |  |  | DOG | TTTTCTTTTCCTGAGA | 6mer |
|  |  |  | DOG | TTTTCTTTT | 6mer |
|  |  |  | PIG | AGTTCTTTT | 6mer |
|  |  |  | PIG | TTTAAATGTGGGGATTGGGAACCACTAGTTCTTTCAGATGGTA | 6mer |
|  |  |  | PIG | CTAGTTCTTTCAGATG | 6mer |
|  |  |  | PIG | AAGGTTTTTCTTTTCCTGAGAAAACAA | 6mer |
|  |  |  | PIG | AAGGTTTTTCTTTTCCTGAGA | 6mer |
|  |  |  | PIG | TTTTCTTTTCCTGAGA | 6mer |
|  |  |  | PIG | TTTTCTTTT | 6mer |
|  |  |  | COW | AGTTCTTTT | 6mer |
|  |  |  | COW | TTTAAATGTGGGGATTGGGAACCACTAGTTCTTTCAGATGGTA | 6mer |
|  |  |  | COW | CTAGTTCTTTCAGATG | 6mer |
|  |  |  | COW | AAGGTTTTTCTTTTCCTGAGAAAACAA | 6mer |
|  |  |  | COW | AAGGTTTTTCTTTTCCTGAGA | 6mer |
|  |  |  | COW | TTTTCTTTTCCTGAGA | 6mer |
|  |  |  | COW | TTTTCTTTT | 6mer |
|  |  |  | MOUSE | AGTTCTTTT | 6mer |
|  |  |  | MOUSE | CTAGTTCTTTCAGATG | 6mer |
|  |  |  | MOUSE | AAGGTTTTTCTTTTCCTGAGAAAACAA | 6mer |
|  |  |  | MOUSE | AAGGTTTTTCTTTTCCTGAGA | 6mer |
|  |  |  | MOUSE | TTTTCTTTTCCTGAGA | 6mer |
|  |  |  | MOUSE | TTTTCTTTT | 6mer |
|  |  |  | TURTLE | AAGGTTTTTCTTTTCCTGAGA | 6mer |
|  |  |  | TURTLE | TTTTCTTTTCCTGAGA | 6mer |
|  |  |  | TURTLE | TTTTCTTTT | 6mer |
|  |  |  | ALLIGATOR | TTTTCTTTTCCTGAGA | 6mer |
|  |  |  | ALLIGATOR | TTTTCTTTT | 6mer |
|  |  |  | LIZARD | TTTTCTTTTCCTGAGA | 6mer |
|  |  |  | LIZARD | TTTTCTTTT | 6mer |
|  |  |  | SNAKE | TTTTCTTTTCCTGAGA | 6mer |
|  |  |  | SNAKE | TTTTCTTTT | 6mer |
|  |  |  | X.TROPICALIS | TTTTCTTTT | 6mer |
|  |  |  | SHARK | TTTTCTTTT | 6mer |
|  |  |  | OPOSSUM | TTTTCTTTT | 6mer |
|  |  |  | SPOTTEDGAR | TTTTCTTTT | 6mer |
|  |  |  | FUGU | TTTTCTTTT | 6mer |
|  |  |  | NILETILAPIA | TTTTCTTTT | 6mer |
|  |  |  | STICKLEBACK | TTTTCTTTT | 6mer |
|  |  |  | MEDAKA | TTTTCTTTT | 6mer |
|  |  |  | ZEBRAFISH | TTTTCTTTT | 6mer |

  
  
  
  

| Seed Matches to the miRNA miR-328-3p | | | | | |
| --- | --- | --- | --- | --- | --- |
| Seed | Conservation | Species | Matches | | |
|  |  |  | Sequence | Motif | Type |
| UGGCCCU | Conserved | Human (Homo sapiens)  Mouse (Mus musculus)  Rat (Rattus norvegicus) | HUMAN | GAAGGGCCAGAGAAGCCAGACCCAGTAAG | 7mer-m8 |
|  |  |  | HUMAN | GAAGGGCCAGAGAAGCCAGACC | 7mer-m8 |
|  |  |  | MARMOSET | GAAGGGCCAGAGAAGCCAGACCCAGTAAG | 7mer-m8 |
|  |  |  | MARMOSET | GAAGGGCCAGAGAAGCCAGACC | 7mer-m8 |
|  |  |  | DOG | GAAGGGCCAGAGAAGCCAGACC | 7mer-m8 |
|  |  |  | PIG | GAAGGGCCAGAGAAGCCAGACC | 7mer-m8 |

  
  
  
  

| Seed Matches to the miRNA miR-149-5p | | | | | |
| --- | --- | --- | --- | --- | --- |
| Seed | Conservation | Species | Matches | | |
|  |  |  | Sequence | Motif | Type |
| CUGGCUC | Conserved | Human (Homo sapiens)  Rhesus (Macaca mulatta)  Cow (Bos taurus)  Mouse (Mus musculus)  Rat (Rattus norvegicus) | HUMAN | AGCCAGCGCAGGG | 6mer |
|  |  |  | HUMAN | GAAGGGCCAGAGAAGCCAGACCCAGTAAG | 7mer-A1 |
|  |  |  | HUMAN | GAAGGGCCAGAGAAGCCAGACC | 7mer-A1 |
|  |  |  | HUMAN | GGCCAGAGAAGCCAGACC | 7mer-A1 |
|  |  |  | MARMOSET | AGCCAGCGCAGGG | 7mer-m8 |
|  |  |  | MARMOSET | GAAGGGCCAGAGAAGCCAGACCCAGTAAG | 7mer-A1 |
|  |  |  | MARMOSET | GAAGGGCCAGAGAAGCCAGACC | 7mer-A1 |
|  |  |  | MARMOSET | GGCCAGAGAAGCCAGACC | 7mer-A1 |
|  |  |  | DOG | GAAGGGCCAGAGAAGCCAGACC | 7mer-A1 |
|  |  |  | DOG | GGCCAGAGAAGCCAGACC | 7mer-A1 |
|  |  |  | PIG | GAAGGGCCAGAGAAGCCAGACC | 7mer-A1 |
|  |  |  | PIG | GGCCAGAGAAGCCAGACC | 7mer-A1 |
|  |  |  | COW | GGCCAGAGAAGCCAGACC | 7mer-A1 |

  
  
  
  
