# Supplementary material for: Uncovering deeply conserved motif combinations in rapidly evolving noncoding sequences
Source: Genome Biol. 2021 Jan 11;22:29. doi: 10.1186/s13059-020-02247-1 (PMC7798263; doi:10.1186/s13059-020-02247-1)
Supplement: Supplementary file 5 — Additional file 5. LncLOOM output results for MALAT1 sequences from 19 vertebrates. [file 13059_2020_2247_MOESM5_ESM.gz › AdditionalFile5/Html_Files/eCLIP_results_BLAT.html]

 eCLIP Matches (BLAT)

# eCLIP Annotation Results (BLAT)

  
  

| Match 1 in HUMAN | | | | | | | |
| --- | --- | --- | --- | --- | --- | --- | --- |
| Motif | Start in Seq (1 Indexed) | End in Seq (1 Indexed) | Strand | Chrm | Exon | Start in Chrm (0 Indexed) | End in Chrm (1 Indexed) |
| AGGCATTGAGG | 26 | 36 | + | chr11 | 1 | 65266515 | 65266526 |
| eCLIP Fold-Enrichment | Binding Protein | Cell Line | Strand | Chrm | | Start in Chrm (0 Indexed) | End in Chrm (1 Indexed) |
| 3.91236337939 | NCBP2 (bg=1.99%) | HepG2 | + | chr11 | | 65266516 | 65266550 |
| 2.92661855119 | NCBP2 (bg=1.99%) | HepG2 | + | chr11 | | 65266516 | 65266551 |
| 2.62811171836 | srsf1 (bg=30.28%) | K562 | + | chr11 | | 65266523 | 65266561 |
| 2.21671327519 | tra2a (bg=37.02%) | K562 | + | chr11 | | 65266519 | 65266560 |
| 2.14764717961 | tra2a (bg=37.02%) | K562 | + | chr11 | | 65266520 | 65266558 |

  
  

| Match 2 in HUMAN | | | | | | | |
| --- | --- | --- | --- | --- | --- | --- | --- |
| Motif | Start in Seq (1 Indexed) | End in Seq (1 Indexed) | Strand | Chrm | Exon | Start in Chrm (0 Indexed) | End in Chrm (1 Indexed) |
| AGCCAGCGCAGGG | 38 | 50 | + | chr11 | 1 | 65266527 | 65266540 |
| eCLIP Fold-Enrichment | Binding Protein | Cell Line | Strand | Chrm | | Start in Chrm (0 Indexed) | End in Chrm (1 Indexed) |
| 3.91236337939 | NCBP2 (bg=1.99%) | HepG2 | + | chr11 | | 65266516 | 65266550 |
| 2.92661855119 | NCBP2 (bg=1.99%) | HepG2 | + | chr11 | | 65266516 | 65266551 |
| 2.62811171836 | srsf1 (bg=30.28%) | K562 | + | chr11 | | 65266523 | 65266561 |
| 2.21671327519 | tra2a (bg=37.02%) | K562 | + | chr11 | | 65266519 | 65266560 |
| 2.14764717961 | tra2a (bg=37.02%) | K562 | + | chr11 | | 65266520 | 65266558 |

  
  

| Match 3 in HUMAN | | | | | | | |
| --- | --- | --- | --- | --- | --- | --- | --- |
| Motif | Start in Seq (1 Indexed) | End in Seq (1 Indexed) | Strand | Chrm | Exon | Start in Chrm (0 Indexed) | End in Chrm (1 Indexed) |
| AGCGCAG | 42 | 48 | + | chr11 | 1 | 65266531 | 65266538 |
| eCLIP Fold-Enrichment | Binding Protein | Cell Line | Strand | Chrm | | Start in Chrm (0 Indexed) | End in Chrm (1 Indexed) |
| 3.91236337939 | NCBP2 (bg=1.99%) | HepG2 | + | chr11 | | 65266516 | 65266550 |
| 2.92661855119 | NCBP2 (bg=1.99%) | HepG2 | + | chr11 | | 65266516 | 65266551 |
| 2.62811171836 | srsf1 (bg=30.28%) | K562 | + | chr11 | | 65266523 | 65266561 |
| 2.21671327519 | tra2a (bg=37.02%) | K562 | + | chr11 | | 65266519 | 65266560 |
| 2.14764717961 | tra2a (bg=37.02%) | K562 | + | chr11 | | 65266520 | 65266558 |

  
  

| Match 4 in HUMAN | | | | | | | |
| --- | --- | --- | --- | --- | --- | --- | --- |
| Motif | Start in Seq (1 Indexed) | End in Seq (1 Indexed) | Strand | Chrm | Exon | Start in Chrm (0 Indexed) | End in Chrm (1 Indexed) |
| AGCTTGAGGAAAC | 74 | 86 | + | chr11 | 1 | 65266563 | 65266576 |
| eCLIP Fold-Enrichment | Binding Protein | Cell Line | Strand | Chrm | | Start in Chrm (0 Indexed) | End in Chrm (1 Indexed) |
| 2.00306196871 | hltf (bg=24.28%) | K562 | + | chr11 | | 65266561 | 65266578 |
| 3.8936268168 | NCBP2 (bg=1.99%) | HepG2 | + | chr11 | | 65266550 | 65266591 |
| 2.65364342895 | NCBP2 (bg=1.99%) | HepG2 | + | chr11 | | 65266559 | 65266566 |
| 3.13277644654 | NCBP2 (bg=1.99%) | HepG2 | + | chr11 | | 65266566 | 65266596 |
| 2.15224094805 | ppil4 (bg=43.39%) | K562 | + | chr11 | | 65266544 | 65266565 |
| 2.68655473612 | ppil4 (bg=43.39%) | K562 | + | chr11 | | 65266561 | 65266566 |
| 2.82789198724 | ppil4 (bg=43.39%) | K562 | + | chr11 | | 65266565 | 65266571 |
| 2.82020595543 | ppil4 (bg=43.39%) | K562 | + | chr11 | | 65266566 | 65266587 |
| 2.99539158038 | ppil4 (bg=43.39%) | K562 | + | chr11 | | 65266571 | 65266579 |
| 2.9655025604 | srsf1 (bg=30.28%) | K562 | + | chr11 | | 65266561 | 65266565 |
| 2.94901635633 | srsf1 (bg=30.28%) | K562 | + | chr11 | | 65266565 | 65266588 |
| 2.45294609907 | tra2a (bg=37.02%) | K562 | + | chr11 | | 65266560 | 65266571 |
| 2.45369890741 | tra2a (bg=37.02%) | K562 | + | chr11 | | 65266561 | 65266566 |
| 2.57742584277 | tra2a (bg=37.02%) | K562 | + | chr11 | | 65266566 | 65266593 |
| 2.65667502963 | tra2a (bg=37.02%) | K562 | + | chr11 | | 65266571 | 65266575 |
| 2.62353026766 | tra2a (bg=37.02%) | K562 | + | chr11 | | 65266575 | 65266590 |

  
  

| Match 5 in HUMAN | | | | | | | |
| --- | --- | --- | --- | --- | --- | --- | --- |
| Motif | Start in Seq (1 Indexed) | End in Seq (1 Indexed) | Strand | Chrm | Exon | Start in Chrm (0 Indexed) | End in Chrm (1 Indexed) |
| GCAGATAAGTTTTT | 88 | 101 | + | chr11 | 1 | 65266577 | 65266591 |
| eCLIP Fold-Enrichment | Binding Protein | Cell Line | Strand | Chrm | | Start in Chrm (0 Indexed) | End in Chrm (1 Indexed) |
| 2.84434216941 | bud13 (bg=12.85%) | K562 | + | chr11 | | 65266580 | 65266611 |
| 2.00306196871 | hltf (bg=24.28%) | K562 | + | chr11 | | 65266561 | 65266578 |
| 3.8936268168 | NCBP2 (bg=1.99%) | HepG2 | + | chr11 | | 65266550 | 65266591 |
| 3.13277644654 | NCBP2 (bg=1.99%) | HepG2 | + | chr11 | | 65266566 | 65266596 |
| 3.32409435665 | NCBP2 (bg=1.99%) | HepG2 | + | chr11 | | 65266591 | 65266596 |
| 2.82020595543 | ppil4 (bg=43.39%) | K562 | + | chr11 | | 65266566 | 65266587 |
| 2.99539158038 | ppil4 (bg=43.39%) | K562 | + | chr11 | | 65266571 | 65266579 |
| 3.02397499935 | ppil4 (bg=43.39%) | K562 | + | chr11 | | 65266579 | 65266583 |
| 3.01324320196 | ppil4 (bg=43.39%) | K562 | + | chr11 | | 65266583 | 65266589 |
| 2.94901635633 | srsf1 (bg=30.28%) | K562 | + | chr11 | | 65266565 | 65266588 |
| 2.57742584277 | tra2a (bg=37.02%) | K562 | + | chr11 | | 65266566 | 65266593 |
| 2.62353026766 | tra2a (bg=37.02%) | K562 | + | chr11 | | 65266575 | 65266590 |

  
  

| Match 6 in HUMAN | | | | | | | |
| --- | --- | --- | --- | --- | --- | --- | --- |
| Motif | Start in Seq (1 Indexed) | End in Seq (1 Indexed) | Strand | Chrm | Exon | Start in Chrm (0 Indexed) | End in Chrm (1 Indexed) |
| ATTAATACAACT | 121 | 132 | + | chr11 | 1 | 65266610 | 65266622 |
| eCLIP Fold-Enrichment | Binding Protein | Cell Line | Strand | Chrm | | Start in Chrm (0 Indexed) | End in Chrm (1 Indexed) |
| 2.049133087 | bclaf1 (bg=17.67%) | HepG2 | + | chr11 | | 65266619 | 65266649 |
| 2.84434216941 | bud13 (bg=12.85%) | K562 | + | chr11 | | 65266580 | 65266611 |
| 2.24533737097 | hltf (bg=24.28%) | HepG2 | + | chr11 | | 65266619 | 65266640 |
| 2.09886469783 | khdrbs1 (bg=10.41%) | K562 | + | chr11 | | 65266609 | 65266625 |
| 2.70691673492 | khdrbs1 (bg=10.41%) | K562 | + | chr11 | | 65266609 | 65266625 |
| 2.43460939634 | LARP7 (bg=2.17%) | HepG2 | + | chr11 | | 65266604 | 65266633 |
| 2.23867496508 | NCBP2 (bg=1.99%) | HepG2 | + | chr11 | | 65266596 | 65266632 |
| 2.24509918731 | NCBP2 (bg=1.99%) | HepG2 | + | chr11 | | 65266596 | 65266633 |
| 2.36690823786 | npm1 (bg=10.22%) | K562 | + | chr11 | | 65266595 | 65266648 |
| 2.07783160479 | npm1 (bg=10.22%) | K562 | + | chr11 | | 65266596 | 65266631 |
| 3.97530948373 | ppil4 (bg=43.39%) | K562 | + | chr11 | | 65266609 | 65266626 |
| 3.70277058268 | ppil4 (bg=43.39%) | K562 | + | chr11 | | 65266609 | 65266626 |
| 2.23236737598 | PUS1 (bg=1.64%) | K562 | + | chr11 | | 65266600 | 65266612 |
| 2.32420769101 | PUS1 (bg=1.64%) | K562 | + | chr11 | | 65266612 | 65266638 |
| 2.47760847121 | srsf1 (bg=30.28%) | K562 | + | chr11 | | 65266596 | 65266611 |
| 2.15193021607 | srsf1 (bg=30.28%) | K562 | + | chr11 | | 65266611 | 65266627 |
| 3.19862777553 | SUPV3L1 (bg=9.63%) | K562 | + | chr11 | | 65266596 | 65266616 |
| 3.02275944863 | SUPV3L1 (bg=9.63%) | K562 | + | chr11 | | 65266597 | 65266621 |
| 2.63886276014 | SUPV3L1 (bg=9.63%) | K562 | + | chr11 | | 65266616 | 65266645 |
| 2.29045523183 | SUPV3L1 (bg=9.63%) | K562 | + | chr11 | | 65266621 | 65266638 |
| 2.04628401214 | uchl5 (bg=18.56%) | K562 | + | chr11 | | 65266595 | 65266612 |
| 2.2176527122 | uchl5 (bg=18.56%) | K562 | + | chr11 | | 65266612 | 65266625 |
| 2.13873662888 | YWHAG (bg=9.14%) | K562 | + | chr11 | | 65266606 | 65266628 |
| 3.7562856802 | zc3h8 (bg=12.78%) | K562 | + | chr11 | | 65266598 | 65266616 |
| 2.89841323048 | zc3h8 (bg=12.78%) | K562 | + | chr11 | | 65266616 | 65266650 |

  
  

| Match 7 in HUMAN | | | | | | | |
| --- | --- | --- | --- | --- | --- | --- | --- |
| Motif | Start in Seq (1 Indexed) | End in Seq (1 Indexed) | Strand | Chrm | Exon | Start in Chrm (0 Indexed) | End in Chrm (1 Indexed) |
| TAATAC | 123 | 128 | + | chr11 | 1 | 65266612 | 65266618 |
| eCLIP Fold-Enrichment | Binding Protein | Cell Line | Strand | Chrm | | Start in Chrm (0 Indexed) | End in Chrm (1 Indexed) |
| 2.09886469783 | khdrbs1 (bg=10.41%) | K562 | + | chr11 | | 65266609 | 65266625 |
| 2.70691673492 | khdrbs1 (bg=10.41%) | K562 | + | chr11 | | 65266609 | 65266625 |
| 2.43460939634 | LARP7 (bg=2.17%) | HepG2 | + | chr11 | | 65266604 | 65266633 |
| 2.23867496508 | NCBP2 (bg=1.99%) | HepG2 | + | chr11 | | 65266596 | 65266632 |
| 2.24509918731 | NCBP2 (bg=1.99%) | HepG2 | + | chr11 | | 65266596 | 65266633 |
| 2.36690823786 | npm1 (bg=10.22%) | K562 | + | chr11 | | 65266595 | 65266648 |
| 2.07783160479 | npm1 (bg=10.22%) | K562 | + | chr11 | | 65266596 | 65266631 |
| 3.97530948373 | ppil4 (bg=43.39%) | K562 | + | chr11 | | 65266609 | 65266626 |
| 3.70277058268 | ppil4 (bg=43.39%) | K562 | + | chr11 | | 65266609 | 65266626 |
| 2.23236737598 | PUS1 (bg=1.64%) | K562 | + | chr11 | | 65266600 | 65266612 |
| 2.32420769101 | PUS1 (bg=1.64%) | K562 | + | chr11 | | 65266612 | 65266638 |
| 2.15193021607 | srsf1 (bg=30.28%) | K562 | + | chr11 | | 65266611 | 65266627 |
| 3.19862777553 | SUPV3L1 (bg=9.63%) | K562 | + | chr11 | | 65266596 | 65266616 |
| 3.02275944863 | SUPV3L1 (bg=9.63%) | K562 | + | chr11 | | 65266597 | 65266621 |
| 2.63886276014 | SUPV3L1 (bg=9.63%) | K562 | + | chr11 | | 65266616 | 65266645 |
| 2.04628401214 | uchl5 (bg=18.56%) | K562 | + | chr11 | | 65266595 | 65266612 |
| 2.2176527122 | uchl5 (bg=18.56%) | K562 | + | chr11 | | 65266612 | 65266625 |
| 2.13873662888 | YWHAG (bg=9.14%) | K562 | + | chr11 | | 65266606 | 65266628 |
| 3.7562856802 | zc3h8 (bg=12.78%) | K562 | + | chr11 | | 65266598 | 65266616 |
| 2.89841323048 | zc3h8 (bg=12.78%) | K562 | + | chr11 | | 65266616 | 65266650 |

  
  

| Match 8 in HUMAN | | | | | | | |
| --- | --- | --- | --- | --- | --- | --- | --- |
| Motif | Start in Seq (1 Indexed) | End in Seq (1 Indexed) | Strand | Chrm | Exon | Start in Chrm (0 Indexed) | End in Chrm (1 Indexed) |
| AAATATA | 140 | 146 | + | chr11 | 1 | 65266629 | 65266636 |
| eCLIP Fold-Enrichment | Binding Protein | Cell Line | Strand | Chrm | | Start in Chrm (0 Indexed) | End in Chrm (1 Indexed) |
| 2.049133087 | bclaf1 (bg=17.67%) | HepG2 | + | chr11 | | 65266619 | 65266649 |
| 2.24533737097 | hltf (bg=24.28%) | HepG2 | + | chr11 | | 65266619 | 65266640 |
| 2.21744733404 | hnrnpa1 (bg=18.32%) | K562 | + | chr11 | | 65266635 | 65266649 |
| 2.42336347926 | khdrbs1 (bg=10.41%) | K562 | + | chr11 | | 65266625 | 65266631 |
| 2.84796709174 | khdrbs1 (bg=10.41%) | K562 | + | chr11 | | 65266625 | 65266631 |
| 2.75113893058 | khdrbs1 (bg=10.41%) | K562 | + | chr11 | | 65266631 | 65266634 |
| 3.07402540293 | khdrbs1 (bg=10.41%) | K562 | + | chr11 | | 65266631 | 65266634 |
| 2.96920248591 | khdrbs1 (bg=10.41%) | K562 | + | chr11 | | 65266634 | 65266636 |
| 2.75235223853 | khdrbs1 (bg=10.41%) | K562 | + | chr11 | | 65266634 | 65266637 |
| 3.17135946755 | khdrbs1 (bg=10.41%) | K562 | + | chr11 | | 65266636 | 65266643 |
| 2.43460939634 | LARP7 (bg=2.17%) | HepG2 | + | chr11 | | 65266604 | 65266633 |
| 2.23867496508 | NCBP2 (bg=1.99%) | HepG2 | + | chr11 | | 65266596 | 65266632 |
| 2.24509918731 | NCBP2 (bg=1.99%) | HepG2 | + | chr11 | | 65266596 | 65266633 |
| 2.36690823786 | npm1 (bg=10.22%) | K562 | + | chr11 | | 65266595 | 65266648 |
| 2.07783160479 | npm1 (bg=10.22%) | K562 | + | chr11 | | 65266596 | 65266631 |
| 3.69173258376 | ppil4 (bg=43.39%) | K562 | + | chr11 | | 65266626 | 65266631 |
| 3.34001445297 | ppil4 (bg=43.39%) | K562 | + | chr11 | | 65266626 | 65266632 |
| 3.52682116065 | ppil4 (bg=43.39%) | K562 | + | chr11 | | 65266631 | 65266634 |
| 3.09481735877 | ppil4 (bg=43.39%) | K562 | + | chr11 | | 65266632 | 65266634 |
| 3.02707002367 | ppil4 (bg=43.39%) | K562 | + | chr11 | | 65266634 | 65266637 |
| 2.69334343059 | ppil4 (bg=43.39%) | K562 | + | chr11 | | 65266634 | 65266637 |
| 2.32420769101 | PUS1 (bg=1.64%) | K562 | + | chr11 | | 65266612 | 65266638 |
| 2.2850215312 | safb (bg=40.39%) | K562 | + | chr11 | | 65266627 | 65266641 |
| 3.74431746017 | safb2 (bg=26.89%) | K562 | + | chr11 | | 65266634 | 65266641 |
| 2.63886276014 | SUPV3L1 (bg=9.63%) | K562 | + | chr11 | | 65266616 | 65266645 |
| 2.29045523183 | SUPV3L1 (bg=9.63%) | K562 | + | chr11 | | 65266621 | 65266638 |
| 2.17202369482 | uchl5 (bg=18.56%) | K562 | + | chr11 | | 65266625 | 65266646 |
| 2.89841323048 | zc3h8 (bg=12.78%) | K562 | + | chr11 | | 65266616 | 65266650 |

  
  

| Match 9 in HUMAN | | | | | | | |
| --- | --- | --- | --- | --- | --- | --- | --- |
| Motif | Start in Seq (1 Indexed) | End in Seq (1 Indexed) | Strand | Chrm | Exon | Start in Chrm (0 Indexed) | End in Chrm (1 Indexed) |
| ATTGCT | 166 | 171 | + | chr11 | 1 | 65266655 | 65266661 |
| eCLIP Fold-Enrichment | Binding Protein | Cell Line | Strand | Chrm | | Start in Chrm (0 Indexed) | End in Chrm (1 Indexed) |
| 2.1375127524 | hltf (bg=24.28%) | HepG2 | + | chr11 | | 65266642 | 65266667 |
| 2.21572977846 | hnrnpa1 (bg=18.32%) | K562 | + | chr11 | | 65266642 | 65266657 |
| 3.80732110536 | hnrnpa1 (bg=18.32%) | HepG2 | + | chr11 | | 65266646 | 65266675 |
| 2.21246521082 | hnrnpa1 (bg=18.32%) | K562 | + | chr11 | | 65266649 | 65266667 |
| 2.0856709377 | hnrnpa1 (bg=18.32%) | K562 | + | chr11 | | 65266657 | 65266669 |
| 3.19849377483 | hnrnpa1 (bg=18.32%) | HepG2 | + | chr11 | | 65266660 | 65266670 |
| 3.31855429832 | khdrbs1 (bg=10.41%) | K562 | + | chr11 | | 65266651 | 65266656 |
| 3.28027649873 | khdrbs1 (bg=10.41%) | K562 | + | chr11 | | 65266654 | 65266684 |
| 2.96410364015 | khdrbs1 (bg=10.41%) | K562 | + | chr11 | | 65266656 | 65266683 |
| 2.97102519067 | ppil4 (bg=43.39%) | K562 | + | chr11 | | 65266652 | 65266655 |
| 2.49464876162 | ppil4 (bg=43.39%) | K562 | + | chr11 | | 65266654 | 65266666 |
| 2.67605526479 | ppil4 (bg=43.39%) | K562 | + | chr11 | | 65266655 | 65266669 |
| 2.09185298234 | safb (bg=40.39%) | K562 | + | chr11 | | 65266645 | 65266655 |
| 3.13585733193 | safb2 (bg=26.89%) | K562 | + | chr11 | | 65266643 | 65266655 |
| 2.5986871433 | safb2 (bg=26.89%) | K562 | + | chr11 | | 65266655 | 65266671 |
| 2.12012657671 | zc3h8 (bg=12.78%) | K562 | + | chr11 | | 65266650 | 65266668 |

  
  

| Match 10 in HUMAN | | | | | | | |
| --- | --- | --- | --- | --- | --- | --- | --- |
| Motif | Start in Seq (1 Indexed) | End in Seq (1 Indexed) | Strand | Chrm | Exon | Start in Chrm (0 Indexed) | End in Chrm (1 Indexed) |
| TTAAGTT | 177 | 183 | + | chr11 | 1 | 65266666 | 65266673 |
| eCLIP Fold-Enrichment | Binding Protein | Cell Line | Strand | Chrm | | Start in Chrm (0 Indexed) | End in Chrm (1 Indexed) |
| 2.1375127524 | hltf (bg=24.28%) | HepG2 | + | chr11 | | 65266642 | 65266667 |
| 3.80732110536 | hnrnpa1 (bg=18.32%) | HepG2 | + | chr11 | | 65266646 | 65266675 |
| 2.21246521082 | hnrnpa1 (bg=18.32%) | K562 | + | chr11 | | 65266649 | 65266667 |
| 2.0856709377 | hnrnpa1 (bg=18.32%) | K562 | + | chr11 | | 65266657 | 65266669 |
| 3.19849377483 | hnrnpa1 (bg=18.32%) | HepG2 | + | chr11 | | 65266660 | 65266670 |
| 3.28027649873 | khdrbs1 (bg=10.41%) | K562 | + | chr11 | | 65266654 | 65266684 |
| 2.96410364015 | khdrbs1 (bg=10.41%) | K562 | + | chr11 | | 65266656 | 65266683 |
| 2.49464876162 | ppil4 (bg=43.39%) | K562 | + | chr11 | | 65266654 | 65266666 |
| 2.67605526479 | ppil4 (bg=43.39%) | K562 | + | chr11 | | 65266655 | 65266669 |
| 2.5986871433 | safb2 (bg=26.89%) | K562 | + | chr11 | | 65266655 | 65266671 |
| 2.12012657671 | zc3h8 (bg=12.78%) | K562 | + | chr11 | | 65266650 | 65266668 |

  
  

| Match 11 in HUMAN | | | | | | | |
| --- | --- | --- | --- | --- | --- | --- | --- |
| Motif | Start in Seq (1 Indexed) | End in Seq (1 Indexed) | Strand | Chrm | Exon | Start in Chrm (0 Indexed) | End in Chrm (1 Indexed) |
| TAATTTTA | 191 | 198 | + | chr11 | 1 | 65266680 | 65266688 |
| eCLIP Fold-Enrichment | Binding Protein | Cell Line | Strand | Chrm | | Start in Chrm (0 Indexed) | End in Chrm (1 Indexed) |
| 3.28027649873 | khdrbs1 (bg=10.41%) | K562 | + | chr11 | | 65266654 | 65266684 |
| 2.96410364015 | khdrbs1 (bg=10.41%) | K562 | + | chr11 | | 65266656 | 65266683 |
| 2.12050617925 | khdrbs1 (bg=10.41%) | K562 | + | chr11 | | 65266683 | 65266692 |
| 2.11666352287 | khdrbs1 (bg=10.41%) | K562 | + | chr11 | | 65266684 | 65266695 |
| 2.12764358106 | safb (bg=40.39%) | K562 | + | chr11 | | 65266687 | 65266693 |

  
  

| Match 12 in HUMAN | | | | | | | |
| --- | --- | --- | --- | --- | --- | --- | --- |
| Motif | Start in Seq (1 Indexed) | End in Seq (1 Indexed) | Strand | Chrm | Exon | Start in Chrm (0 Indexed) | End in Chrm (1 Indexed) |
| TAAGAGAAAATA | 213 | 224 | + | chr11 | 1 | 65266702 | 65266714 |
| eCLIP Fold-Enrichment | Binding Protein | Cell Line | Strand | Chrm | | Start in Chrm (0 Indexed) | End in Chrm (1 Indexed) |
| 2.77520372994 | ppil4 (bg=43.39%) | K562 | + | chr11 | | 65266706 | 65266728 |
| 2.68374323859 | ppil4 (bg=43.39%) | K562 | + | chr11 | | 65266707 | 65266720 |
| 2.4278639557 | safb2 (bg=26.89%) | K562 | + | chr11 | | 65266704 | 65266730 |
| 3.16477234677 | tra2a (bg=37.02%) | K562 | + | chr11 | | 65266709 | 65266758 |
| 3.52359253208 | tra2a (bg=37.02%) | K562 | + | chr11 | | 65266712 | 65266729 |
| 2.50330606515 | tra2a (bg=37.02%) | HepG2 | + | chr11 | | 65266713 | 65266744 |

  
  

| Match 13 in HUMAN | | | | | | | |
| --- | --- | --- | --- | --- | --- | --- | --- |
| Motif | Start in Seq (1 Indexed) | End in Seq (1 Indexed) | Strand | Chrm | Exon | Start in Chrm (0 Indexed) | End in Chrm (1 Indexed) |
| AAGAGTAGC | 236 | 244 | + | chr11 | 1 | 65266725 | 65266734 |
| eCLIP Fold-Enrichment | Binding Protein | Cell Line | Strand | Chrm | | Start in Chrm (0 Indexed) | End in Chrm (1 Indexed) |
| 4.42685152083 | AQR (bg=4.89%) | K562 | + | chr11 | | 65266734 | 65266774 |
| 2.77520372994 | ppil4 (bg=43.39%) | K562 | + | chr11 | | 65266706 | 65266728 |
| 2.45440199643 | ppil4 (bg=43.39%) | K562 | + | chr11 | | 65266720 | 65266728 |
| 2.77790568443 | ppil4 (bg=43.39%) | K562 | + | chr11 | | 65266728 | 65266734 |
| 2.63078515978 | ppil4 (bg=43.39%) | K562 | + | chr11 | | 65266728 | 65266738 |
| 2.78507470785 | ppil4 (bg=43.39%) | K562 | + | chr11 | | 65266734 | 65266738 |
| 2.4278639557 | safb2 (bg=26.89%) | K562 | + | chr11 | | 65266704 | 65266730 |
| 2.35829589232 | safb2 (bg=26.89%) | K562 | + | chr11 | | 65266730 | 65266735 |
| 3.16477234677 | tra2a (bg=37.02%) | K562 | + | chr11 | | 65266709 | 65266758 |
| 3.52359253208 | tra2a (bg=37.02%) | K562 | + | chr11 | | 65266712 | 65266729 |
| 2.50330606515 | tra2a (bg=37.02%) | HepG2 | + | chr11 | | 65266713 | 65266744 |
| 3.48583280574 | tra2a (bg=37.02%) | K562 | + | chr11 | | 65266729 | 65266737 |

  
  

| Match 14 in HUMAN | | | | | | | |
| --- | --- | --- | --- | --- | --- | --- | --- |
| Motif | Start in Seq (1 Indexed) | End in Seq (1 Indexed) | Strand | Chrm | Exon | Start in Chrm (0 Indexed) | End in Chrm (1 Indexed) |
| AAGAGTAGCATGAGGAAGGAA | 236 | 256 | + | chr11 | 1 | 65266725 | 65266746 |
| eCLIP Fold-Enrichment | Binding Protein | Cell Line | Strand | Chrm | | Start in Chrm (0 Indexed) | End in Chrm (1 Indexed) |
| 4.42685152083 | AQR (bg=4.89%) | K562 | + | chr11 | | 65266734 | 65266774 |
| 2.77520372994 | ppil4 (bg=43.39%) | K562 | + | chr11 | | 65266706 | 65266728 |
| 2.45440199643 | ppil4 (bg=43.39%) | K562 | + | chr11 | | 65266720 | 65266728 |
| 2.77790568443 | ppil4 (bg=43.39%) | K562 | + | chr11 | | 65266728 | 65266734 |
| 2.63078515978 | ppil4 (bg=43.39%) | K562 | + | chr11 | | 65266728 | 65266738 |
| 2.78507470785 | ppil4 (bg=43.39%) | K562 | + | chr11 | | 65266734 | 65266738 |
| 2.77158806897 | ppil4 (bg=43.39%) | K562 | + | chr11 | | 65266738 | 65266742 |
| 2.64152568258 | ppil4 (bg=43.39%) | K562 | + | chr11 | | 65266738 | 65266743 |
| 2.76080595152 | ppil4 (bg=43.39%) | K562 | + | chr11 | | 65266742 | 65266748 |
| 2.63729663088 | ppil4 (bg=43.39%) | K562 | + | chr11 | | 65266743 | 65266750 |
| 2.01765473984 | safb (bg=40.39%) | HepG2 | + | chr11 | | 65266737 | 65266745 |
| 2.4278639557 | safb2 (bg=26.89%) | K562 | + | chr11 | | 65266704 | 65266730 |
| 2.35829589232 | safb2 (bg=26.89%) | K562 | + | chr11 | | 65266730 | 65266735 |
| 2.41790944057 | safb2 (bg=26.89%) | K562 | + | chr11 | | 65266735 | 65266739 |
| 2.51801702683 | safb2 (bg=26.89%) | K562 | + | chr11 | | 65266739 | 65266742 |
| 2.47121625742 | safb2 (bg=26.89%) | K562 | + | chr11 | | 65266742 | 65266748 |
| 3.16477234677 | tra2a (bg=37.02%) | K562 | + | chr11 | | 65266709 | 65266758 |
| 3.52359253208 | tra2a (bg=37.02%) | K562 | + | chr11 | | 65266712 | 65266729 |
| 2.50330606515 | tra2a (bg=37.02%) | HepG2 | + | chr11 | | 65266713 | 65266744 |
| 3.48583280574 | tra2a (bg=37.02%) | K562 | + | chr11 | | 65266729 | 65266737 |
| 3.40223447534 | tra2a (bg=37.02%) | K562 | + | chr11 | | 65266737 | 65266748 |

  
  

| Match 15 in HUMAN | | | | | | | |
| --- | --- | --- | --- | --- | --- | --- | --- |
| Motif | Start in Seq (1 Indexed) | End in Seq (1 Indexed) | Strand | Chrm | Exon | Start in Chrm (0 Indexed) | End in Chrm (1 Indexed) |
| GTTTCT | 267 | 272 | + | chr11 | 1 | 65266756 | 65266762 |
| eCLIP Fold-Enrichment | Binding Protein | Cell Line | Strand | Chrm | | Start in Chrm (0 Indexed) | End in Chrm (1 Indexed) |
| 4.42685152083 | AQR (bg=4.89%) | K562 | + | chr11 | | 65266734 | 65266774 |
| 2.7297572058 | ppil4 (bg=43.39%) | K562 | + | chr11 | | 65266748 | 65266757 |
| 3.01917723876 | ppil4 (bg=43.39%) | K562 | + | chr11 | | 65266757 | 65266771 |
| 3.11599751294 | ppil4 (bg=43.39%) | K562 | + | chr11 | | 65266760 | 65266777 |
| 2.00885032398 | safb (bg=40.39%) | K562 | + | chr11 | | 65266758 | 65266768 |
| 2.00024518858 | safb2 (bg=26.89%) | K562 | + | chr11 | | 65266748 | 65266758 |
| 2.23010902122 | srsf1 (bg=30.28%) | K562 | + | chr11 | | 65266761 | 65266766 |
| 3.16477234677 | tra2a (bg=37.02%) | K562 | + | chr11 | | 65266709 | 65266758 |
| 3.17844708642 | tra2a (bg=37.02%) | K562 | + | chr11 | | 65266748 | 65266758 |
| 2.85211123062 | tra2a (bg=37.02%) | K562 | + | chr11 | | 65266758 | 65266765 |
| 2.93550625109 | tra2a (bg=37.02%) | K562 | + | chr11 | | 65266758 | 65266765 |

  
  

| Match 16 in HUMAN | | | | | | | |
| --- | --- | --- | --- | --- | --- | --- | --- |
| Motif | Start in Seq (1 Indexed) | End in Seq (1 Indexed) | Strand | Chrm | Exon | Start in Chrm (0 Indexed) | End in Chrm (1 Indexed) |
| AAACATGACGGAGGTTGAGATGAAGCT | 274 | 300 | + | chr11 | 1 | 65266763 | 65266790 |
| eCLIP Fold-Enrichment | Binding Protein | Cell Line | Strand | Chrm | | Start in Chrm (0 Indexed) | End in Chrm (1 Indexed) |
| 4.42685152083 | AQR (bg=4.89%) | K562 | + | chr11 | | 65266734 | 65266774 |
| 2.09107640539 | bclaf1 (bg=17.67%) | HepG2 | + | chr11 | | 65266782 | 65266800 |
| 2.29783048949 | bclaf1 (bg=17.67%) | HepG2 | + | chr11 | | 65266784 | 65266801 |
| 2.15532438443 | khdrbs1 (bg=10.41%) | K562 | + | chr11 | | 65266780 | 65266788 |
| 2.11504140639 | khdrbs1 (bg=10.41%) | K562 | + | chr11 | | 65266788 | 65266814 |
| 3.01917723876 | ppil4 (bg=43.39%) | K562 | + | chr11 | | 65266757 | 65266771 |
| 3.11599751294 | ppil4 (bg=43.39%) | K562 | + | chr11 | | 65266760 | 65266777 |
| 3.27964069522 | ppil4 (bg=43.39%) | K562 | + | chr11 | | 65266771 | 65266778 |
| 3.28505138074 | ppil4 (bg=43.39%) | K562 | + | chr11 | | 65266777 | 65266781 |
| 3.05825173902 | ppil4 (bg=43.39%) | K562 | + | chr11 | | 65266778 | 65266788 |
| 3.63868250437 | ppil4 (bg=43.39%) | K562 | + | chr11 | | 65266781 | 65266787 |
| 3.70963618635 | ppil4 (bg=43.39%) | K562 | + | chr11 | | 65266787 | 65266801 |
| 3.44345566929 | ppil4 (bg=43.39%) | K562 | + | chr11 | | 65266788 | 65266800 |
| 2.00885032398 | safb (bg=40.39%) | K562 | + | chr11 | | 65266758 | 65266768 |
| 2.11962455844 | safb (bg=40.39%) | K562 | + | chr11 | | 65266768 | 65266776 |
| 2.0830194471 | safb (bg=40.39%) | K562 | + | chr11 | | 65266775 | 65266784 |
| 2.26566183669 | safb (bg=40.39%) | K562 | + | chr11 | | 65266776 | 65266784 |
| 2.33660812672 | safb (bg=40.39%) | K562 | + | chr11 | | 65266784 | 65266787 |
| 2.1961519738 | safb (bg=40.39%) | K562 | + | chr11 | | 65266784 | 65266788 |
| 2.24517746193 | safb (bg=40.39%) | K562 | + | chr11 | | 65266787 | 65266803 |
| 2.11290543535 | safb (bg=40.39%) | K562 | + | chr11 | | 65266788 | 65266796 |
| 2.04706468361 | safb2 (bg=26.89%) | K562 | + | chr11 | | 65266776 | 65266787 |
| 2.01966696164 | safb2 (bg=26.89%) | K562 | + | chr11 | | 65266787 | 65266804 |
| 2.23010902122 | srsf1 (bg=30.28%) | K562 | + | chr11 | | 65266761 | 65266766 |
| 2.36129842635 | srsf1 (bg=30.28%) | K562 | + | chr11 | | 65266766 | 65266789 |
| 2.61818168318 | srsf1 (bg=30.28%) | K562 | + | chr11 | | 65266789 | 65266801 |
| 2.03111094946 | SRSF9 (bg=9.67%) | HepG2 | + | chr11 | | 65266766 | 65266780 |
| 2.58284547193 | SRSF9 (bg=9.67%) | HepG2 | + | chr11 | | 65266780 | 65266801 |
| 2.85211123062 | tra2a (bg=37.02%) | K562 | + | chr11 | | 65266758 | 65266765 |
| 2.93550625109 | tra2a (bg=37.02%) | K562 | + | chr11 | | 65266758 | 65266765 |
| 2.9049477714 | tra2a (bg=37.02%) | K562 | + | chr11 | | 65266765 | 65266786 |
| 2.86274092709 | tra2a (bg=37.02%) | K562 | + | chr11 | | 65266765 | 65266803 |
| 2.98326647302 | tra2a (bg=37.02%) | K562 | + | chr11 | | 65266786 | 65266802 |

  
  

| Match 17 in HUMAN | | | | | | | |
| --- | --- | --- | --- | --- | --- | --- | --- |
| Motif | Start in Seq (1 Indexed) | End in Seq (1 Indexed) | Strand | Chrm | Exon | Start in Chrm (0 Indexed) | End in Chrm (1 Indexed) |
| GACGGAGGTT | 280 | 289 | + | chr11 | 1 | 65266769 | 65266779 |
| eCLIP Fold-Enrichment | Binding Protein | Cell Line | Strand | Chrm | | Start in Chrm (0 Indexed) | End in Chrm (1 Indexed) |
| 4.42685152083 | AQR (bg=4.89%) | K562 | + | chr11 | | 65266734 | 65266774 |
| 3.01917723876 | ppil4 (bg=43.39%) | K562 | + | chr11 | | 65266757 | 65266771 |
| 3.11599751294 | ppil4 (bg=43.39%) | K562 | + | chr11 | | 65266760 | 65266777 |
| 3.27964069522 | ppil4 (bg=43.39%) | K562 | + | chr11 | | 65266771 | 65266778 |
| 3.28505138074 | ppil4 (bg=43.39%) | K562 | + | chr11 | | 65266777 | 65266781 |
| 3.05825173902 | ppil4 (bg=43.39%) | K562 | + | chr11 | | 65266778 | 65266788 |
| 2.11962455844 | safb (bg=40.39%) | K562 | + | chr11 | | 65266768 | 65266776 |
| 2.0830194471 | safb (bg=40.39%) | K562 | + | chr11 | | 65266775 | 65266784 |
| 2.26566183669 | safb (bg=40.39%) | K562 | + | chr11 | | 65266776 | 65266784 |
| 2.04706468361 | safb2 (bg=26.89%) | K562 | + | chr11 | | 65266776 | 65266787 |
| 2.36129842635 | srsf1 (bg=30.28%) | K562 | + | chr11 | | 65266766 | 65266789 |
| 2.03111094946 | SRSF9 (bg=9.67%) | HepG2 | + | chr11 | | 65266766 | 65266780 |
| 2.9049477714 | tra2a (bg=37.02%) | K562 | + | chr11 | | 65266765 | 65266786 |
| 2.86274092709 | tra2a (bg=37.02%) | K562 | + | chr11 | | 65266765 | 65266803 |

  
  

| Match 18 in HUMAN | | | | | | | |
| --- | --- | --- | --- | --- | --- | --- | --- |
| Motif | Start in Seq (1 Indexed) | End in Seq (1 Indexed) | Strand | Chrm | Exon | Start in Chrm (0 Indexed) | End in Chrm (1 Indexed) |
| GAGATGAAGCT | 290 | 300 | + | chr11 | 1 | 65266779 | 65266790 |
| eCLIP Fold-Enrichment | Binding Protein | Cell Line | Strand | Chrm | | Start in Chrm (0 Indexed) | End in Chrm (1 Indexed) |
| 2.09107640539 | bclaf1 (bg=17.67%) | HepG2 | + | chr11 | | 65266782 | 65266800 |
| 2.29783048949 | bclaf1 (bg=17.67%) | HepG2 | + | chr11 | | 65266784 | 65266801 |
| 2.15532438443 | khdrbs1 (bg=10.41%) | K562 | + | chr11 | | 65266780 | 65266788 |
| 2.11504140639 | khdrbs1 (bg=10.41%) | K562 | + | chr11 | | 65266788 | 65266814 |
| 3.28505138074 | ppil4 (bg=43.39%) | K562 | + | chr11 | | 65266777 | 65266781 |
| 3.05825173902 | ppil4 (bg=43.39%) | K562 | + | chr11 | | 65266778 | 65266788 |
| 3.63868250437 | ppil4 (bg=43.39%) | K562 | + | chr11 | | 65266781 | 65266787 |
| 3.70963618635 | ppil4 (bg=43.39%) | K562 | + | chr11 | | 65266787 | 65266801 |
| 3.44345566929 | ppil4 (bg=43.39%) | K562 | + | chr11 | | 65266788 | 65266800 |
| 2.0830194471 | safb (bg=40.39%) | K562 | + | chr11 | | 65266775 | 65266784 |
| 2.26566183669 | safb (bg=40.39%) | K562 | + | chr11 | | 65266776 | 65266784 |
| 2.33660812672 | safb (bg=40.39%) | K562 | + | chr11 | | 65266784 | 65266787 |
| 2.1961519738 | safb (bg=40.39%) | K562 | + | chr11 | | 65266784 | 65266788 |
| 2.24517746193 | safb (bg=40.39%) | K562 | + | chr11 | | 65266787 | 65266803 |
| 2.11290543535 | safb (bg=40.39%) | K562 | + | chr11 | | 65266788 | 65266796 |
| 2.04706468361 | safb2 (bg=26.89%) | K562 | + | chr11 | | 65266776 | 65266787 |
| 2.01966696164 | safb2 (bg=26.89%) | K562 | + | chr11 | | 65266787 | 65266804 |
| 2.36129842635 | srsf1 (bg=30.28%) | K562 | + | chr11 | | 65266766 | 65266789 |
| 2.61818168318 | srsf1 (bg=30.28%) | K562 | + | chr11 | | 65266789 | 65266801 |
| 2.03111094946 | SRSF9 (bg=9.67%) | HepG2 | + | chr11 | | 65266766 | 65266780 |
| 2.58284547193 | SRSF9 (bg=9.67%) | HepG2 | + | chr11 | | 65266780 | 65266801 |
| 2.9049477714 | tra2a (bg=37.02%) | K562 | + | chr11 | | 65266765 | 65266786 |
| 2.86274092709 | tra2a (bg=37.02%) | K562 | + | chr11 | | 65266765 | 65266803 |
| 2.98326647302 | tra2a (bg=37.02%) | K562 | + | chr11 | | 65266786 | 65266802 |

  
  

| Match 19 in HUMAN | | | | | | | |
| --- | --- | --- | --- | --- | --- | --- | --- |
| Motif | Start in Seq (1 Indexed) | End in Seq (1 Indexed) | Strand | Chrm | Exon | Start in Chrm (0 Indexed) | End in Chrm (1 Indexed) |
| CTTCATGGAGTA | 302 | 313 | + | chr11 | 1 | 65266791 | 65266803 |
| eCLIP Fold-Enrichment | Binding Protein | Cell Line | Strand | Chrm | | Start in Chrm (0 Indexed) | End in Chrm (1 Indexed) |
| 4.07983944555 | AQR (bg=4.89%) | K562 | + | chr11 | | 65266796 | 65266816 |
| 2.09107640539 | bclaf1 (bg=17.67%) | HepG2 | + | chr11 | | 65266782 | 65266800 |
| 2.29783048949 | bclaf1 (bg=17.67%) | HepG2 | + | chr11 | | 65266784 | 65266801 |
| 2.11504140639 | khdrbs1 (bg=10.41%) | K562 | + | chr11 | | 65266788 | 65266814 |
| 3.70963618635 | ppil4 (bg=43.39%) | K562 | + | chr11 | | 65266787 | 65266801 |
| 3.44345566929 | ppil4 (bg=43.39%) | K562 | + | chr11 | | 65266788 | 65266800 |
| 2.24517746193 | safb (bg=40.39%) | K562 | + | chr11 | | 65266787 | 65266803 |
| 2.11290543535 | safb (bg=40.39%) | K562 | + | chr11 | | 65266788 | 65266796 |
| 2.12334407703 | safb (bg=40.39%) | K562 | + | chr11 | | 65266796 | 65266803 |
| 2.01966696164 | safb2 (bg=26.89%) | K562 | + | chr11 | | 65266787 | 65266804 |
| 2.61818168318 | srsf1 (bg=30.28%) | K562 | + | chr11 | | 65266789 | 65266801 |
| 2.58284547193 | SRSF9 (bg=9.67%) | HepG2 | + | chr11 | | 65266780 | 65266801 |
| 2.86274092709 | tra2a (bg=37.02%) | K562 | + | chr11 | | 65266765 | 65266803 |
| 2.98326647302 | tra2a (bg=37.02%) | K562 | + | chr11 | | 65266786 | 65266802 |

  
  

| Match 20 in HUMAN | | | | | | | |
| --- | --- | --- | --- | --- | --- | --- | --- |
| Motif | Start in Seq (1 Indexed) | End in Seq (1 Indexed) | Strand | Chrm | Exon | Start in Chrm (0 Indexed) | End in Chrm (1 Indexed) |
| ATGGAGT | 306 | 312 | + | chr11 | 1 | 65266795 | 65266802 |
| eCLIP Fold-Enrichment | Binding Protein | Cell Line | Strand | Chrm | | Start in Chrm (0 Indexed) | End in Chrm (1 Indexed) |
| 4.07983944555 | AQR (bg=4.89%) | K562 | + | chr11 | | 65266796 | 65266816 |
| 2.09107640539 | bclaf1 (bg=17.67%) | HepG2 | + | chr11 | | 65266782 | 65266800 |
| 2.29783048949 | bclaf1 (bg=17.67%) | HepG2 | + | chr11 | | 65266784 | 65266801 |
| 2.11504140639 | khdrbs1 (bg=10.41%) | K562 | + | chr11 | | 65266788 | 65266814 |
| 3.70963618635 | ppil4 (bg=43.39%) | K562 | + | chr11 | | 65266787 | 65266801 |
| 3.44345566929 | ppil4 (bg=43.39%) | K562 | + | chr11 | | 65266788 | 65266800 |
| 2.24517746193 | safb (bg=40.39%) | K562 | + | chr11 | | 65266787 | 65266803 |
| 2.11290543535 | safb (bg=40.39%) | K562 | + | chr11 | | 65266788 | 65266796 |
| 2.12334407703 | safb (bg=40.39%) | K562 | + | chr11 | | 65266796 | 65266803 |
| 2.01966696164 | safb2 (bg=26.89%) | K562 | + | chr11 | | 65266787 | 65266804 |
| 2.61818168318 | srsf1 (bg=30.28%) | K562 | + | chr11 | | 65266789 | 65266801 |
| 2.58284547193 | SRSF9 (bg=9.67%) | HepG2 | + | chr11 | | 65266780 | 65266801 |
| 2.86274092709 | tra2a (bg=37.02%) | K562 | + | chr11 | | 65266765 | 65266803 |
| 2.98326647302 | tra2a (bg=37.02%) | K562 | + | chr11 | | 65266786 | 65266802 |

  
  

| Match 21 in HUMAN | | | | | | | |
| --- | --- | --- | --- | --- | --- | --- | --- |
| Motif | Start in Seq (1 Indexed) | End in Seq (1 Indexed) | Strand | Chrm | Exon | Start in Chrm (0 Indexed) | End in Chrm (1 Indexed) |
| AAAATGTATTTAAAAGAAAATTGA | 315 | 338 | + | chr11 | 1 | 65266804 | 65266828 |
| eCLIP Fold-Enrichment | Binding Protein | Cell Line | Strand | Chrm | | Start in Chrm (0 Indexed) | End in Chrm (1 Indexed) |
| 4.07983944555 | AQR (bg=4.89%) | K562 | + | chr11 | | 65266796 | 65266816 |
| 2.82235241194 | bclaf1 (bg=17.67%) | HepG2 | + | chr11 | | 65266824 | 65266851 |
| 2.52479532574 | bclaf1 (bg=17.67%) | HepG2 | + | chr11 | | 65266828 | 65266849 |
| 2.16744962632 | hltf (bg=24.28%) | HepG2 | + | chr11 | | 65266816 | 65266862 |
| 2.18225185017 | hltf (bg=24.28%) | HepG2 | + | chr11 | | 65266820 | 65266838 |
| 2.08892180106 | hnrnpa1 (bg=18.32%) | K562 | + | chr11 | | 65266820 | 65266822 |
| 2.18645856851 | hnrnpa1 (bg=18.32%) | HepG2 | + | chr11 | | 65266827 | 65266857 |
| 2.11504140639 | khdrbs1 (bg=10.41%) | K562 | + | chr11 | | 65266788 | 65266814 |
| 2.38694750379 | khdrbs1 (bg=10.41%) | K562 | + | chr11 | | 65266814 | 65266823 |
| 2.76933623342 | khdrbs1 (bg=10.41%) | K562 | + | chr11 | | 65266814 | 65266823 |
| 2.56308535356 | khdrbs1 (bg=10.41%) | K562 | + | chr11 | | 65266823 | 65266830 |
| 2.9571854536 | khdrbs1 (bg=10.41%) | K562 | + | chr11 | | 65266823 | 65266843 |
| 3.75797108653 | ppil4 (bg=43.39%) | K562 | + | chr11 | | 65266818 | 65266823 |
| 3.6235957076 | ppil4 (bg=43.39%) | K562 | + | chr11 | | 65266819 | 65266823 |
| 3.74528798787 | ppil4 (bg=43.39%) | K562 | + | chr11 | | 65266823 | 65266829 |
| 3.91863569175 | ppil4 (bg=43.39%) | K562 | + | chr11 | | 65266823 | 65266832 |
| 2.32381843027 | safb (bg=40.39%) | K562 | + | chr11 | | 65266826 | 65266840 |
| 2.01966696164 | safb2 (bg=26.89%) | K562 | + | chr11 | | 65266787 | 65266804 |
| 2.11001367495 | safb2 (bg=26.89%) | K562 | + | chr11 | | 65266826 | 65266861 |

  
  

| Match 22 in HUMAN | | | | | | | |
| --- | --- | --- | --- | --- | --- | --- | --- |
| Motif | Start in Seq (1 Indexed) | End in Seq (1 Indexed) | Strand | Chrm | Exon | Start in Chrm (0 Indexed) | End in Chrm (1 Indexed) |
| ATTTAAAA | 322 | 329 | + | chr11 | 1 | 65266811 | 65266819 |
| eCLIP Fold-Enrichment | Binding Protein | Cell Line | Strand | Chrm | | Start in Chrm (0 Indexed) | End in Chrm (1 Indexed) |
| 4.07983944555 | AQR (bg=4.89%) | K562 | + | chr11 | | 65266796 | 65266816 |
| 2.16744962632 | hltf (bg=24.28%) | HepG2 | + | chr11 | | 65266816 | 65266862 |
| 2.11504140639 | khdrbs1 (bg=10.41%) | K562 | + | chr11 | | 65266788 | 65266814 |
| 2.38694750379 | khdrbs1 (bg=10.41%) | K562 | + | chr11 | | 65266814 | 65266823 |
| 2.76933623342 | khdrbs1 (bg=10.41%) | K562 | + | chr11 | | 65266814 | 65266823 |
| 3.75797108653 | ppil4 (bg=43.39%) | K562 | + | chr11 | | 65266818 | 65266823 |
| 3.6235957076 | ppil4 (bg=43.39%) | K562 | + | chr11 | | 65266819 | 65266823 |

  
  

| Match 23 in HUMAN | | | | | | | |
| --- | --- | --- | --- | --- | --- | --- | --- |
| Motif | Start in Seq (1 Indexed) | End in Seq (1 Indexed) | Strand | Chrm | Exon | Start in Chrm (0 Indexed) | End in Chrm (1 Indexed) |
| AAGGACTA | 343 | 350 | + | chr11 | 1 | 65266832 | 65266840 |
| eCLIP Fold-Enrichment | Binding Protein | Cell Line | Strand | Chrm | | Start in Chrm (0 Indexed) | End in Chrm (1 Indexed) |
| 2.82235241194 | bclaf1 (bg=17.67%) | HepG2 | + | chr11 | | 65266824 | 65266851 |
| 2.52479532574 | bclaf1 (bg=17.67%) | HepG2 | + | chr11 | | 65266828 | 65266849 |
| 2.16744962632 | hltf (bg=24.28%) | HepG2 | + | chr11 | | 65266816 | 65266862 |
| 2.18225185017 | hltf (bg=24.28%) | HepG2 | + | chr11 | | 65266820 | 65266838 |
| 2.3754848343 | hltf (bg=24.28%) | HepG2 | + | chr11 | | 65266838 | 65266861 |
| 2.18645856851 | hnrnpa1 (bg=18.32%) | HepG2 | + | chr11 | | 65266827 | 65266857 |
| 2.15035881207 | hnrnpa1 (bg=18.32%) | HepG2 | + | chr11 | | 65266829 | 65266857 |
| 2.9571854536 | khdrbs1 (bg=10.41%) | K562 | + | chr11 | | 65266823 | 65266843 |
| 2.53696659262 | khdrbs1 (bg=10.41%) | K562 | + | chr11 | | 65266830 | 65266844 |
| 3.91863569175 | ppil4 (bg=43.39%) | K562 | + | chr11 | | 65266823 | 65266832 |
| 3.65335591205 | ppil4 (bg=43.39%) | K562 | + | chr11 | | 65266829 | 65266858 |
| 3.75389694882 | ppil4 (bg=43.39%) | K562 | + | chr11 | | 65266832 | 65266857 |
| 2.32381843027 | safb (bg=40.39%) | K562 | + | chr11 | | 65266826 | 65266840 |
| 2.37877847111 | safb (bg=40.39%) | K562 | + | chr11 | | 65266840 | 65266853 |
| 2.11001367495 | safb2 (bg=26.89%) | K562 | + | chr11 | | 65266826 | 65266861 |
| 2.94222617522 | tra2a (bg=37.02%) | K562 | + | chr11 | | 65266829 | 65266861 |
| 2.80990597171 | tra2a (bg=37.02%) | K562 | + | chr11 | | 65266832 | 65266854 |

  
  

| Match 24 in HUMAN | | | | | | | |
| --- | --- | --- | --- | --- | --- | --- | --- |
| Motif | Start in Seq (1 Indexed) | End in Seq (1 Indexed) | Strand | Chrm | Exon | Start in Chrm (0 Indexed) | End in Chrm (1 Indexed) |
| GAATTAATACC | 360 | 370 | + | chr11 | 1 | 65266849 | 65266860 |
| eCLIP Fold-Enrichment | Binding Protein | Cell Line | Strand | Chrm | | Start in Chrm (0 Indexed) | End in Chrm (1 Indexed) |
| 2.82235241194 | bclaf1 (bg=17.67%) | HepG2 | + | chr11 | | 65266824 | 65266851 |
| 2.52479532574 | bclaf1 (bg=17.67%) | HepG2 | + | chr11 | | 65266828 | 65266849 |
| 2.16744962632 | hltf (bg=24.28%) | HepG2 | + | chr11 | | 65266816 | 65266862 |
| 2.3754848343 | hltf (bg=24.28%) | HepG2 | + | chr11 | | 65266838 | 65266861 |
| 2.14666613396 | hltf (bg=24.28%) | K562 | + | chr11 | | 65266845 | 65266863 |
| 2.18645856851 | hnrnpa1 (bg=18.32%) | HepG2 | + | chr11 | | 65266827 | 65266857 |
| 2.15035881207 | hnrnpa1 (bg=18.32%) | HepG2 | + | chr11 | | 65266829 | 65266857 |
| 2.99059785486 | khdrbs1 (bg=10.41%) | K562 | + | chr11 | | 65266843 | 65266861 |
| 2.64587410082 | khdrbs1 (bg=10.41%) | K562 | + | chr11 | | 65266844 | 65266879 |
| 3.65335591205 | ppil4 (bg=43.39%) | K562 | + | chr11 | | 65266829 | 65266858 |
| 3.75389694882 | ppil4 (bg=43.39%) | K562 | + | chr11 | | 65266832 | 65266857 |
| 2.37877847111 | safb (bg=40.39%) | K562 | + | chr11 | | 65266840 | 65266853 |
| 2.41873354765 | safb (bg=40.39%) | K562 | + | chr11 | | 65266853 | 65266857 |
| 2.11001367495 | safb2 (bg=26.89%) | K562 | + | chr11 | | 65266826 | 65266861 |
| 2.94222617522 | tra2a (bg=37.02%) | K562 | + | chr11 | | 65266829 | 65266861 |
| 2.80990597171 | tra2a (bg=37.02%) | K562 | + | chr11 | | 65266832 | 65266854 |

  
  

| Match 25 in HUMAN | | | | | | | |
| --- | --- | --- | --- | --- | --- | --- | --- |
| Motif | Start in Seq (1 Indexed) | End in Seq (1 Indexed) | Strand | Chrm | Exon | Start in Chrm (0 Indexed) | End in Chrm (1 Indexed) |
| TAGAAGGGCA | 373 | 382 | + | chr11 | 1 | 65266862 | 65266872 |
| eCLIP Fold-Enrichment | Binding Protein | Cell Line | Strand | Chrm | | Start in Chrm (0 Indexed) | End in Chrm (1 Indexed) |
| 2.16744962632 | hltf (bg=24.28%) | HepG2 | + | chr11 | | 65266816 | 65266862 |
| 2.14666613396 | hltf (bg=24.28%) | K562 | + | chr11 | | 65266845 | 65266863 |
| 2.49092317712 | hltf (bg=24.28%) | K562 | + | chr11 | | 65266863 | 65266872 |
| 2.64587410082 | khdrbs1 (bg=10.41%) | K562 | + | chr11 | | 65266844 | 65266879 |
| 2.2125781755 | khdrbs1 (bg=10.41%) | K562 | + | chr11 | | 65266861 | 65266866 |
| 2.65056392847 | khdrbs1 (bg=10.41%) | K562 | + | chr11 | | 65266866 | 65266880 |

  
  

| Match 26 in HUMAN | | | | | | | |
| --- | --- | --- | --- | --- | --- | --- | --- |
| Motif | Start in Seq (1 Indexed) | End in Seq (1 Indexed) | Strand | Chrm | Exon | Start in Chrm (0 Indexed) | End in Chrm (1 Indexed) |
| TGCTTTTAGATTA | 384 | 396 | + | chr11 | 1 | 65266873 | 65266886 |
| eCLIP Fold-Enrichment | Binding Protein | Cell Line | Strand | Chrm | | Start in Chrm (0 Indexed) | End in Chrm (1 Indexed) |
| 2.66778384963 | hltf (bg=24.28%) | K562 | + | chr11 | | 65266883 | 65266896 |
| 2.64587410082 | khdrbs1 (bg=10.41%) | K562 | + | chr11 | | 65266844 | 65266879 |
| 2.65056392847 | khdrbs1 (bg=10.41%) | K562 | + | chr11 | | 65266866 | 65266880 |
| 3.58389146762 | khdrbs1 (bg=10.41%) | K562 | + | chr11 | | 65266879 | 65266887 |
| 3.56365221863 | khdrbs1 (bg=10.41%) | K562 | + | chr11 | | 65266880 | 65266887 |

  
  

| Match 27 in HUMAN | | | | | | | |
| --- | --- | --- | --- | --- | --- | --- | --- |
| Motif | Start in Seq (1 Indexed) | End in Seq (1 Indexed) | Strand | Chrm | Exon | Start in Chrm (0 Indexed) | End in Chrm (1 Indexed) |
| AAGGTGACTTAAACAG | 402 | 417 | + | chr11 | 1 | 65266891 | 65266907 |
| eCLIP Fold-Enrichment | Binding Protein | Cell Line | Strand | Chrm | | Start in Chrm (0 Indexed) | End in Chrm (1 Indexed) |
| 2.66778384963 | hltf (bg=24.28%) | K562 | + | chr11 | | 65266883 | 65266896 |
| 3.5044275889 | khdrbs1 (bg=10.41%) | K562 | + | chr11 | | 65266887 | 65266893 |
| 3.44929915698 | khdrbs1 (bg=10.41%) | K562 | + | chr11 | | 65266887 | 65266893 |
| 3.43732443965 | khdrbs1 (bg=10.41%) | K562 | + | chr11 | | 65266893 | 65266898 |
| 3.40129045236 | khdrbs1 (bg=10.41%) | K562 | + | chr11 | | 65266893 | 65266904 |
| 3.3631446892 | khdrbs1 (bg=10.41%) | K562 | + | chr11 | | 65266898 | 65266918 |
| 3.3873669124 | khdrbs1 (bg=10.41%) | K562 | + | chr11 | | 65266904 | 65266918 |

  
  

| Match 28 in HUMAN | | | | | | | |
| --- | --- | --- | --- | --- | --- | --- | --- |
| Motif | Start in Seq (1 Indexed) | End in Seq (1 Indexed) | Strand | Chrm | Exon | Start in Chrm (0 Indexed) | End in Chrm (1 Indexed) |
| GTAGGTGA | 440 | 447 | + | chr11 | 1 | 65266929 | 65266937 |
| eCLIP Fold-Enrichment | Binding Protein | Cell Line | Strand | Chrm | | Start in Chrm (0 Indexed) | End in Chrm (1 Indexed) |
| 2.68055760854 | hltf (bg=24.28%) | K562 | + | chr11 | | 65266929 | 65266939 |
| 2.59496899443 | hnrnpa1 (bg=18.32%) | K562 | + | chr11 | | 65266928 | 65266941 |
| 2.54415552305 | hnrnpa1 (bg=18.32%) | K562 | + | chr11 | | 65266929 | 65266938 |
| 3.60615694714 | khdrbs1 (bg=10.41%) | K562 | + | chr11 | | 65266923 | 65266931 |
| 3.53300447411 | khdrbs1 (bg=10.41%) | K562 | + | chr11 | | 65266923 | 65266941 |
| 3.69629275692 | khdrbs1 (bg=10.41%) | K562 | + | chr11 | | 65266931 | 65266941 |
| 2.201118716 | safb (bg=40.39%) | K562 | + | chr11 | | 65266927 | 65266939 |
| 2.26158032272 | safb (bg=40.39%) | HepG2 | + | chr11 | | 65266929 | 65266939 |
| 3.23686374169 | safb2 (bg=26.89%) | K562 | + | chr11 | | 65266928 | 65266940 |
| 2.60862242204 | safb2 (bg=26.89%) | K562 | + | chr11 | | 65266929 | 65266940 |

  
  

| Match 29 in HUMAN | | | | | | | |
| --- | --- | --- | --- | --- | --- | --- | --- |
| Motif | Start in Seq (1 Indexed) | End in Seq (1 Indexed) | Strand | Chrm | Exon | Start in Chrm (0 Indexed) | End in Chrm (1 Indexed) |
| TAAAATA | 449 | 455 | + | chr11 | 1 | 65266938 | 65266945 |
| eCLIP Fold-Enrichment | Binding Protein | Cell Line | Strand | Chrm | | Start in Chrm (0 Indexed) | End in Chrm (1 Indexed) |
| 2.68055760854 | hltf (bg=24.28%) | K562 | + | chr11 | | 65266929 | 65266939 |
| 2.59496899443 | hnrnpa1 (bg=18.32%) | K562 | + | chr11 | | 65266928 | 65266941 |
| 2.54415552305 | hnrnpa1 (bg=18.32%) | K562 | + | chr11 | | 65266929 | 65266938 |
| 2.28304443119 | hnrnpa1 (bg=18.32%) | K562 | + | chr11 | | 65266941 | 65266987 |
| 2.44722669053 | hnrnpa1 (bg=18.32%) | K562 | + | chr11 | | 65266943 | 65266966 |
| 3.53300447411 | khdrbs1 (bg=10.41%) | K562 | + | chr11 | | 65266923 | 65266941 |
| 3.69629275692 | khdrbs1 (bg=10.41%) | K562 | + | chr11 | | 65266931 | 65266941 |
| 3.67650642476 | khdrbs1 (bg=10.41%) | K562 | + | chr11 | | 65266941 | 65266952 |
| 3.88551406013 | khdrbs1 (bg=10.41%) | K562 | + | chr11 | | 65266941 | 65266952 |
| 2.92249038156 | ppil4 (bg=43.39%) | K562 | + | chr11 | | 65266945 | 65266960 |
| 2.201118716 | safb (bg=40.39%) | K562 | + | chr11 | | 65266927 | 65266939 |
| 2.26158032272 | safb (bg=40.39%) | HepG2 | + | chr11 | | 65266929 | 65266939 |
| 2.2736469944 | safb (bg=40.39%) | K562 | + | chr11 | | 65266939 | 65266959 |
| 2.03996426867 | safb (bg=40.39%) | K562 | + | chr11 | | 65266940 | 65266958 |
| 2.25840886609 | safb (bg=40.39%) | HepG2 | + | chr11 | | 65266943 | 65266957 |
| 3.23686374169 | safb2 (bg=26.89%) | K562 | + | chr11 | | 65266928 | 65266940 |
| 2.60862242204 | safb2 (bg=26.89%) | K562 | + | chr11 | | 65266929 | 65266940 |
| 3.11458615775 | safb2 (bg=26.89%) | K562 | + | chr11 | | 65266940 | 65266952 |
| 2.57808454111 | safb2 (bg=26.89%) | K562 | + | chr11 | | 65266940 | 65266959 |

  
  

| Match 30 in HUMAN | | | | | | | |
| --- | --- | --- | --- | --- | --- | --- | --- |
| Motif | Start in Seq (1 Indexed) | End in Seq (1 Indexed) | Strand | Chrm | Exon | Start in Chrm (0 Indexed) | End in Chrm (1 Indexed) |
| GCGATC | 464 | 469 | + | chr11 | 1 | 65266953 | 65266959 |
| eCLIP Fold-Enrichment | Binding Protein | Cell Line | Strand | Chrm | | Start in Chrm (0 Indexed) | End in Chrm (1 Indexed) |
| 2.28304443119 | hnrnpa1 (bg=18.32%) | K562 | + | chr11 | | 65266941 | 65266987 |
| 2.44722669053 | hnrnpa1 (bg=18.32%) | K562 | + | chr11 | | 65266943 | 65266966 |
| 3.55882823635 | khdrbs1 (bg=10.41%) | K562 | + | chr11 | | 65266952 | 65266960 |
| 3.80217638122 | khdrbs1 (bg=10.41%) | K562 | + | chr11 | | 65266952 | 65266960 |
| 2.92249038156 | ppil4 (bg=43.39%) | K562 | + | chr11 | | 65266945 | 65266960 |
| 2.92666362135 | ppil4 (bg=43.39%) | K562 | + | chr11 | | 65266946 | 65266960 |
| 2.2736469944 | safb (bg=40.39%) | K562 | + | chr11 | | 65266939 | 65266959 |
| 2.03996426867 | safb (bg=40.39%) | K562 | + | chr11 | | 65266940 | 65266958 |
| 2.25840886609 | safb (bg=40.39%) | HepG2 | + | chr11 | | 65266943 | 65266957 |
| 2.43539841159 | safb (bg=40.39%) | HepG2 | + | chr11 | | 65266957 | 65266969 |
| 2.16049919833 | safb (bg=40.39%) | K562 | + | chr11 | | 65266958 | 65266966 |
| 2.39803606804 | safb (bg=40.39%) | K562 | + | chr11 | | 65266959 | 65266966 |
| 2.57808454111 | safb2 (bg=26.89%) | K562 | + | chr11 | | 65266940 | 65266959 |
| 3.11893926879 | safb2 (bg=26.89%) | K562 | + | chr11 | | 65266952 | 65266959 |
| 3.00980380118 | safb2 (bg=26.89%) | K562 | + | chr11 | | 65266959 | 65266966 |
| 2.47623528715 | safb2 (bg=26.89%) | K562 | + | chr11 | | 65266959 | 65266966 |

  
  

| Match 31 in HUMAN | | | | | | | |
| --- | --- | --- | --- | --- | --- | --- | --- |
| Motif | Start in Seq (1 Indexed) | End in Seq (1 Indexed) | Strand | Chrm | Exon | Start in Chrm (0 Indexed) | End in Chrm (1 Indexed) |
| TTTAAAAAG | 471 | 479 | + | chr11 | 1 | 65266960 | 65266969 |
| eCLIP Fold-Enrichment | Binding Protein | Cell Line | Strand | Chrm | | Start in Chrm (0 Indexed) | End in Chrm (1 Indexed) |
| 2.28304443119 | hnrnpa1 (bg=18.32%) | K562 | + | chr11 | | 65266941 | 65266987 |
| 2.44722669053 | hnrnpa1 (bg=18.32%) | K562 | + | chr11 | | 65266943 | 65266966 |
| 3.55882823635 | khdrbs1 (bg=10.41%) | K562 | + | chr11 | | 65266952 | 65266960 |
| 3.80217638122 | khdrbs1 (bg=10.41%) | K562 | + | chr11 | | 65266952 | 65266960 |
| 4.00319935211 | khdrbs1 (bg=10.41%) | K562 | + | chr11 | | 65266960 | 65266966 |
| 3.66118105094 | khdrbs1 (bg=10.41%) | K562 | + | chr11 | | 65266960 | 65266967 |
| 4.09761753047 | khdrbs1 (bg=10.41%) | K562 | + | chr11 | | 65266966 | 65266987 |
| 3.88936810201 | khdrbs1 (bg=10.41%) | K562 | + | chr11 | | 65266967 | 65266986 |
| 2.92249038156 | ppil4 (bg=43.39%) | K562 | + | chr11 | | 65266945 | 65266960 |
| 2.92666362135 | ppil4 (bg=43.39%) | K562 | + | chr11 | | 65266946 | 65266960 |
| 2.69338015846 | ppil4 (bg=43.39%) | K562 | + | chr11 | | 65266960 | 65266965 |
| 2.70897096713 | ppil4 (bg=43.39%) | K562 | + | chr11 | | 65266963 | 65266965 |
| 2.43539841159 | safb (bg=40.39%) | HepG2 | + | chr11 | | 65266957 | 65266969 |
| 2.16049919833 | safb (bg=40.39%) | K562 | + | chr11 | | 65266958 | 65266966 |
| 2.39803606804 | safb (bg=40.39%) | K562 | + | chr11 | | 65266959 | 65266966 |
| 2.1123224733 | safb (bg=40.39%) | K562 | + | chr11 | | 65266966 | 65266970 |
| 2.32109462263 | safb (bg=40.39%) | K562 | + | chr11 | | 65266966 | 65266972 |
| 2.56909716447 | safb (bg=40.39%) | HepG2 | + | chr11 | | 65266969 | 65266993 |
| 3.00980380118 | safb2 (bg=26.89%) | K562 | + | chr11 | | 65266959 | 65266966 |
| 2.47623528715 | safb2 (bg=26.89%) | K562 | + | chr11 | | 65266959 | 65266966 |
| 2.77070331056 | safb2 (bg=26.89%) | K562 | + | chr11 | | 65266966 | 65266983 |
| 2.04005052844 | safb2 (bg=26.89%) | K562 | + | chr11 | | 65266966 | 65266992 |

  
  

| Match 32 in HUMAN | | | | | | | |
| --- | --- | --- | --- | --- | --- | --- | --- |
| Motif | Start in Seq (1 Indexed) | End in Seq (1 Indexed) | Strand | Chrm | Exon | Start in Chrm (0 Indexed) | End in Chrm (1 Indexed) |
| TTTAAAAAGAGATTAA | 471 | 486 | + | chr11 | 1 | 65266960 | 65266976 |
| eCLIP Fold-Enrichment | Binding Protein | Cell Line | Strand | Chrm | | Start in Chrm (0 Indexed) | End in Chrm (1 Indexed) |
| 2.28304443119 | hnrnpa1 (bg=18.32%) | K562 | + | chr11 | | 65266941 | 65266987 |
| 2.44722669053 | hnrnpa1 (bg=18.32%) | K562 | + | chr11 | | 65266943 | 65266966 |
| 3.55882823635 | khdrbs1 (bg=10.41%) | K562 | + | chr11 | | 65266952 | 65266960 |
| 3.80217638122 | khdrbs1 (bg=10.41%) | K562 | + | chr11 | | 65266952 | 65266960 |
| 4.00319935211 | khdrbs1 (bg=10.41%) | K562 | + | chr11 | | 65266960 | 65266966 |
| 3.66118105094 | khdrbs1 (bg=10.41%) | K562 | + | chr11 | | 65266960 | 65266967 |
| 4.09761753047 | khdrbs1 (bg=10.41%) | K562 | + | chr11 | | 65266966 | 65266987 |
| 3.88936810201 | khdrbs1 (bg=10.41%) | K562 | + | chr11 | | 65266967 | 65266986 |
| 2.92249038156 | ppil4 (bg=43.39%) | K562 | + | chr11 | | 65266945 | 65266960 |
| 2.92666362135 | ppil4 (bg=43.39%) | K562 | + | chr11 | | 65266946 | 65266960 |
| 2.69338015846 | ppil4 (bg=43.39%) | K562 | + | chr11 | | 65266960 | 65266965 |
| 2.70897096713 | ppil4 (bg=43.39%) | K562 | + | chr11 | | 65266963 | 65266965 |
| 2.62456366737 | ppil4 (bg=43.39%) | K562 | + | chr11 | | 65266972 | 65266979 |
| 2.60932299896 | ppil4 (bg=43.39%) | K562 | + | chr11 | | 65266974 | 65266980 |
| 2.43539841159 | safb (bg=40.39%) | HepG2 | + | chr11 | | 65266957 | 65266969 |
| 2.16049919833 | safb (bg=40.39%) | K562 | + | chr11 | | 65266958 | 65266966 |
| 2.39803606804 | safb (bg=40.39%) | K562 | + | chr11 | | 65266959 | 65266966 |
| 2.1123224733 | safb (bg=40.39%) | K562 | + | chr11 | | 65266966 | 65266970 |
| 2.32109462263 | safb (bg=40.39%) | K562 | + | chr11 | | 65266966 | 65266972 |
| 2.56909716447 | safb (bg=40.39%) | HepG2 | + | chr11 | | 65266969 | 65266993 |
| 2.27453939078 | safb (bg=40.39%) | K562 | + | chr11 | | 65266970 | 65266986 |
| 2.32265878027 | safb (bg=40.39%) | K562 | + | chr11 | | 65266972 | 65266979 |
| 3.00980380118 | safb2 (bg=26.89%) | K562 | + | chr11 | | 65266959 | 65266966 |
| 2.47623528715 | safb2 (bg=26.89%) | K562 | + | chr11 | | 65266959 | 65266966 |
| 2.77070331056 | safb2 (bg=26.89%) | K562 | + | chr11 | | 65266966 | 65266983 |
| 2.04005052844 | safb2 (bg=26.89%) | K562 | + | chr11 | | 65266966 | 65266992 |

  
  

| Match 33 in HUMAN | | | | | | | |
| --- | --- | --- | --- | --- | --- | --- | --- |
| Motif | Start in Seq (1 Indexed) | End in Seq (1 Indexed) | Strand | Chrm | Exon | Start in Chrm (0 Indexed) | End in Chrm (1 Indexed) |
| AAGGTGATTAAAAGACCTTGAAATCCATGACGCA | 491 | 524 | + | chr11 | 1 | 65266980 | 65267014 |
| eCLIP Fold-Enrichment | Binding Protein | Cell Line | Strand | Chrm | | Start in Chrm (0 Indexed) | End in Chrm (1 Indexed) |
| 2.28304443119 | hnrnpa1 (bg=18.32%) | K562 | + | chr11 | | 65266941 | 65266987 |
| 4.09761753047 | khdrbs1 (bg=10.41%) | K562 | + | chr11 | | 65266966 | 65266987 |
| 3.88936810201 | khdrbs1 (bg=10.41%) | K562 | + | chr11 | | 65266967 | 65266986 |
| 3.91030266973 | khdrbs1 (bg=10.41%) | K562 | + | chr11 | | 65266986 | 65266992 |
| 4.29376541793 | khdrbs1 (bg=10.41%) | K562 | + | chr11 | | 65266987 | 65266992 |
| 3.84489851028 | khdrbs1 (bg=10.41%) | K562 | + | chr11 | | 65266992 | 65266996 |
| 4.21394094161 | khdrbs1 (bg=10.41%) | K562 | + | chr11 | | 65266992 | 65266996 |
| 3.77275928705 | khdrbs1 (bg=10.41%) | K562 | + | chr11 | | 65266996 | 65267014 |
| 4.13883781605 | khdrbs1 (bg=10.41%) | K562 | + | chr11 | | 65266996 | 65267014 |
| 3.42353950641 | khdrbs1 (bg=10.41%) | K562 | + | chr11 | | 65267014 | 65267021 |
| 3.83279832001 | khdrbs1 (bg=10.41%) | K562 | + | chr11 | | 65267014 | 65267027 |
| 2.60932299896 | ppil4 (bg=43.39%) | K562 | + | chr11 | | 65266974 | 65266980 |
| 2.56909716447 | safb (bg=40.39%) | HepG2 | + | chr11 | | 65266969 | 65266993 |
| 2.27453939078 | safb (bg=40.39%) | K562 | + | chr11 | | 65266970 | 65266986 |
| 2.47623711239 | safb (bg=40.39%) | K562 | + | chr11 | | 65266979 | 65266986 |
| 2.44775050228 | safb (bg=40.39%) | K562 | + | chr11 | | 65266986 | 65266992 |
| 2.36220004891 | safb (bg=40.39%) | K562 | + | chr11 | | 65266986 | 65266992 |
| 2.36478715813 | safb (bg=40.39%) | K562 | + | chr11 | | 65266992 | 65266995 |
| 2.5094241898 | safb (bg=40.39%) | K562 | + | chr11 | | 65266992 | 65266996 |
| 2.64315918032 | safb (bg=40.39%) | HepG2 | + | chr11 | | 65266993 | 65267006 |
| 2.33569530854 | safb (bg=40.39%) | K562 | + | chr11 | | 65266995 | 65267007 |
| 2.50953065669 | safb (bg=40.39%) | K562 | + | chr11 | | 65266996 | 65267007 |
| 2.66985693727 | safb (bg=40.39%) | HepG2 | + | chr11 | | 65267006 | 65267039 |
| 2.2813551982 | safb (bg=40.39%) | K562 | + | chr11 | | 65267007 | 65267015 |
| 2.36864114916 | safb (bg=40.39%) | K562 | + | chr11 | | 65267007 | 65267021 |
| 2.77070331056 | safb2 (bg=26.89%) | K562 | + | chr11 | | 65266966 | 65266983 |
| 2.04005052844 | safb2 (bg=26.89%) | K562 | + | chr11 | | 65266966 | 65266992 |
| 2.42844177155 | safb2 (bg=26.89%) | K562 | + | chr11 | | 65266983 | 65266986 |
| 2.21795269798 | safb2 (bg=26.89%) | K562 | + | chr11 | | 65266986 | 65266991 |
| 2.09195756958 | safb2 (bg=26.89%) | K562 | + | chr11 | | 65266993 | 65266995 |
| 2.03101971703 | safb2 (bg=26.89%) | K562 | + | chr11 | | 65266995 | 65267002 |
| 2.03882503523 | safb2 (bg=26.89%) | K562 | + | chr11 | | 65267002 | 65267005 |
| 2.1773621434 | TAF15 (bg=9.06%) | HepG2 | + | chr11 | | 65266990 | 65267033 |

  
  

| Match 34 in HUMAN | | | | | | | |
| --- | --- | --- | --- | --- | --- | --- | --- |
| Motif | Start in Seq (1 Indexed) | End in Seq (1 Indexed) | Strand | Chrm | Exon | Start in Chrm (0 Indexed) | End in Chrm (1 Indexed) |
| CTTGAA | 507 | 512 | + | chr11 | 1 | 65266996 | 65267002 |
| eCLIP Fold-Enrichment | Binding Protein | Cell Line | Strand | Chrm | | Start in Chrm (0 Indexed) | End in Chrm (1 Indexed) |
| 3.84489851028 | khdrbs1 (bg=10.41%) | K562 | + | chr11 | | 65266992 | 65266996 |
| 4.21394094161 | khdrbs1 (bg=10.41%) | K562 | + | chr11 | | 65266992 | 65266996 |
| 3.77275928705 | khdrbs1 (bg=10.41%) | K562 | + | chr11 | | 65266996 | 65267014 |
| 4.13883781605 | khdrbs1 (bg=10.41%) | K562 | + | chr11 | | 65266996 | 65267014 |
| 2.5094241898 | safb (bg=40.39%) | K562 | + | chr11 | | 65266992 | 65266996 |
| 2.64315918032 | safb (bg=40.39%) | HepG2 | + | chr11 | | 65266993 | 65267006 |
| 2.33569530854 | safb (bg=40.39%) | K562 | + | chr11 | | 65266995 | 65267007 |
| 2.50953065669 | safb (bg=40.39%) | K562 | + | chr11 | | 65266996 | 65267007 |
| 2.03101971703 | safb2 (bg=26.89%) | K562 | + | chr11 | | 65266995 | 65267002 |
| 2.03882503523 | safb2 (bg=26.89%) | K562 | + | chr11 | | 65267002 | 65267005 |
| 2.1773621434 | TAF15 (bg=9.06%) | HepG2 | + | chr11 | | 65266990 | 65267033 |

  
  

| Match 35 in HUMAN | | | | | | | |
| --- | --- | --- | --- | --- | --- | --- | --- |
| Motif | Start in Seq (1 Indexed) | End in Seq (1 Indexed) | Strand | Chrm | Exon | Start in Chrm (0 Indexed) | End in Chrm (1 Indexed) |
| AGAATTGCGTCATTTAAAGCCTA | 528 | 550 | + | chr11 | 1 | 65267017 | 65267040 |
| eCLIP Fold-Enrichment | Binding Protein | Cell Line | Strand | Chrm | | Start in Chrm (0 Indexed) | End in Chrm (1 Indexed) |
| 2.18674194428 | hltf (bg=24.28%) | HepG2 | + | chr11 | | 65267022 | 65267026 |
| 3.42353950641 | khdrbs1 (bg=10.41%) | K562 | + | chr11 | | 65267014 | 65267021 |
| 3.83279832001 | khdrbs1 (bg=10.41%) | K562 | + | chr11 | | 65267014 | 65267027 |
| 3.30361713296 | khdrbs1 (bg=10.41%) | K562 | + | chr11 | | 65267021 | 65267027 |
| 3.29362379097 | khdrbs1 (bg=10.41%) | K562 | + | chr11 | | 65267027 | 65267034 |
| 3.79571093132 | khdrbs1 (bg=10.41%) | K562 | + | chr11 | | 65267027 | 65267038 |
| 2.84472669248 | khdrbs1 (bg=10.41%) | K562 | + | chr11 | | 65267034 | 65267037 |
| 2.75623100041 | khdrbs1 (bg=10.41%) | K562 | + | chr11 | | 65267037 | 65267040 |
| 3.57030431535 | khdrbs1 (bg=10.41%) | K562 | + | chr11 | | 65267038 | 65267040 |
| 3.62210461283 | khdrbs1 (bg=10.41%) | K562 | + | chr11 | | 65267040 | 65267044 |
| 3.02460446851 | khdrbs1 (bg=10.41%) | K562 | + | chr11 | | 65267040 | 65267049 |
| 2.66985693727 | safb (bg=40.39%) | HepG2 | + | chr11 | | 65267006 | 65267039 |
| 2.36864114916 | safb (bg=40.39%) | K562 | + | chr11 | | 65267007 | 65267021 |
| 2.18518746637 | safb (bg=40.39%) | K562 | + | chr11 | | 65267015 | 65267039 |
| 2.25675660322 | safb (bg=40.39%) | K562 | + | chr11 | | 65267021 | 65267027 |
| 2.27295306268 | safb (bg=40.39%) | K562 | + | chr11 | | 65267027 | 65267030 |
| 2.07982449102 | safb (bg=40.39%) | K562 | + | chr11 | | 65267030 | 65267033 |
| 2.2168775668 | safb (bg=40.39%) | K562 | + | chr11 | | 65267039 | 65267040 |
| 2.09349775638 | safb2 (bg=26.89%) | K562 | + | chr11 | | 65267028 | 65267030 |
| 2.1773621434 | TAF15 (bg=9.06%) | HepG2 | + | chr11 | | 65266990 | 65267033 |

  
  

| Match 36 in HUMAN | | | | | | | |
| --- | --- | --- | --- | --- | --- | --- | --- |
| Motif | Start in Seq (1 Indexed) | End in Seq (1 Indexed) | Strand | Chrm | Exon | Start in Chrm (0 Indexed) | End in Chrm (1 Indexed) |
| ATTGCGTCATTT | 531 | 542 | + | chr11 | 1 | 65267020 | 65267032 |
| eCLIP Fold-Enrichment | Binding Protein | Cell Line | Strand | Chrm | | Start in Chrm (0 Indexed) | End in Chrm (1 Indexed) |
| 2.18674194428 | hltf (bg=24.28%) | HepG2 | + | chr11 | | 65267022 | 65267026 |
| 3.42353950641 | khdrbs1 (bg=10.41%) | K562 | + | chr11 | | 65267014 | 65267021 |
| 3.83279832001 | khdrbs1 (bg=10.41%) | K562 | + | chr11 | | 65267014 | 65267027 |
| 3.30361713296 | khdrbs1 (bg=10.41%) | K562 | + | chr11 | | 65267021 | 65267027 |
| 3.29362379097 | khdrbs1 (bg=10.41%) | K562 | + | chr11 | | 65267027 | 65267034 |
| 3.79571093132 | khdrbs1 (bg=10.41%) | K562 | + | chr11 | | 65267027 | 65267038 |
| 2.66985693727 | safb (bg=40.39%) | HepG2 | + | chr11 | | 65267006 | 65267039 |
| 2.36864114916 | safb (bg=40.39%) | K562 | + | chr11 | | 65267007 | 65267021 |
| 2.18518746637 | safb (bg=40.39%) | K562 | + | chr11 | | 65267015 | 65267039 |
| 2.25675660322 | safb (bg=40.39%) | K562 | + | chr11 | | 65267021 | 65267027 |
| 2.27295306268 | safb (bg=40.39%) | K562 | + | chr11 | | 65267027 | 65267030 |
| 2.07982449102 | safb (bg=40.39%) | K562 | + | chr11 | | 65267030 | 65267033 |
| 2.09349775638 | safb2 (bg=26.89%) | K562 | + | chr11 | | 65267028 | 65267030 |
| 2.1773621434 | TAF15 (bg=9.06%) | HepG2 | + | chr11 | | 65266990 | 65267033 |

  
  

| Match 37 in HUMAN | | | | | | | |
| --- | --- | --- | --- | --- | --- | --- | --- |
| Motif | Start in Seq (1 Indexed) | End in Seq (1 Indexed) | Strand | Chrm | Exon | Start in Chrm (0 Indexed) | End in Chrm (1 Indexed) |
| TTACTAAACGCAGACGAA | 561 | 578 | + | chr11 | 1 | 65267050 | 65267068 |
| eCLIP Fold-Enrichment | Binding Protein | Cell Line | Strand | Chrm | | Start in Chrm (0 Indexed) | End in Chrm (1 Indexed) |
| 2.18096382887 | aggf1 (bg=15.15%) | HepG2 | + | chr11 | | 65267064 | 65267115 |
| 3.13375472572 | khdrbs1 (bg=10.41%) | K562 | + | chr11 | | 65267048 | 65267076 |
| 2.63270630155 | khdrbs1 (bg=10.41%) | K562 | + | chr11 | | 65267049 | 65267076 |
| 2.13314748133 | larp4 (bg=13.51%) | K562 | + | chr11 | | 65267065 | 65267095 |
| 2.19799213818 | safb2 (bg=26.89%) | K562 | + | chr11 | | 65267054 | 65267060 |
| 2.16212967694 | safb2 (bg=26.89%) | K562 | + | chr11 | | 65267060 | 65267075 |
| 2.70447123909 | srsf1 (bg=30.28%) | K562 | + | chr11 | | 65267053 | 65267060 |
| 4.33393746034 | srsf1 (bg=30.28%) | K562 | + | chr11 | | 65267060 | 65267127 |
| 2.16815422907 | srsf7 (bg=22.53%) | K562 | + | chr11 | | 65267068 | 65267115 |
| 3.15565174945 | tra2a (bg=37.02%) | K562 | + | chr11 | | 65267059 | 65267075 |
| 4.40831925427 | tra2a (bg=37.02%) | K562 | + | chr11 | | 65267065 | 65267095 |

  
  

| Match 38 in HUMAN | | | | | | | |
| --- | --- | --- | --- | --- | --- | --- | --- |
| Motif | Start in Seq (1 Indexed) | End in Seq (1 Indexed) | Strand | Chrm | Exon | Start in Chrm (0 Indexed) | End in Chrm (1 Indexed) |
| GGAAAGA | 582 | 588 | + | chr11 | 1 | 65267071 | 65267078 |
| eCLIP Fold-Enrichment | Binding Protein | Cell Line | Strand | Chrm | | Start in Chrm (0 Indexed) | End in Chrm (1 Indexed) |
| 2.18096382887 | aggf1 (bg=15.15%) | HepG2 | + | chr11 | | 65267064 | 65267115 |
| 3.37391870595 | bud13 (bg=12.85%) | HepG2 | + | chr11 | | 65267077 | 65267122 |
| 3.21980668791 | cpsf6 (bg=13.45%) | K562 | + | chr11 | | 65267075 | 65267127 |
| 3.13375472572 | khdrbs1 (bg=10.41%) | K562 | + | chr11 | | 65267048 | 65267076 |
| 2.63270630155 | khdrbs1 (bg=10.41%) | K562 | + | chr11 | | 65267049 | 65267076 |
| 2.18890131612 | khdrbs1 (bg=10.41%) | K562 | + | chr11 | | 65267076 | 65267082 |
| 2.19004819795 | khdrbs1 (bg=10.41%) | K562 | + | chr11 | | 65267076 | 65267083 |
| 2.13314748133 | larp4 (bg=13.51%) | K562 | + | chr11 | | 65267065 | 65267095 |
| 3.29527970554 | rbm22 (bg=12.69%) | HepG2 | + | chr11 | | 65267078 | 65267111 |
| 2.16212967694 | safb2 (bg=26.89%) | K562 | + | chr11 | | 65267060 | 65267075 |
| 2.64601414445 | safb2 (bg=26.89%) | K562 | + | chr11 | | 65267075 | 65267092 |
| 2.47428720786 | safb2 (bg=26.89%) | K562 | + | chr11 | | 65267075 | 65267095 |
| 4.33393746034 | srsf1 (bg=30.28%) | K562 | + | chr11 | | 65267060 | 65267127 |
| 2.16815422907 | srsf7 (bg=22.53%) | K562 | + | chr11 | | 65267068 | 65267115 |
| 4.42807594419 | SRSF9 (bg=9.67%) | HepG2 | + | chr11 | | 65267076 | 65267100 |
| 3.15565174945 | tra2a (bg=37.02%) | K562 | + | chr11 | | 65267059 | 65267075 |
| 4.40831925427 | tra2a (bg=37.02%) | K562 | + | chr11 | | 65267065 | 65267095 |
| 4.62830386505 | tra2a (bg=37.02%) | K562 | + | chr11 | | 65267075 | 65267095 |
| 5.72061034992 | tra2a (bg=37.02%) | HepG2 | + | chr11 | | 65267078 | 65267128 |

  
  

| Match 39 in HUMAN | | | | | | | |
| --- | --- | --- | --- | --- | --- | --- | --- |
| Motif | Start in Seq (1 Indexed) | End in Seq (1 Indexed) | Strand | Chrm | Exon | Start in Chrm (0 Indexed) | End in Chrm (1 Indexed) |
| TTAATTGGGAGTGGTAGGA | 589 | 607 | + | chr11 | 1 | 65267078 | 65267097 |
| eCLIP Fold-Enrichment | Binding Protein | Cell Line | Strand | Chrm | | Start in Chrm (0 Indexed) | End in Chrm (1 Indexed) |
| 2.18096382887 | aggf1 (bg=15.15%) | HepG2 | + | chr11 | | 65267064 | 65267115 |
| 3.5411016405 | bclaf1 (bg=17.67%) | HepG2 | + | chr11 | | 65267082 | 65267153 |
| 3.28361464473 | bclaf1 (bg=17.67%) | HepG2 | + | chr11 | | 65267087 | 65267141 |
| 3.37391870595 | bud13 (bg=12.85%) | HepG2 | + | chr11 | | 65267077 | 65267122 |
| 3.21980668791 | cpsf6 (bg=13.45%) | K562 | + | chr11 | | 65267075 | 65267127 |
| 2.33787314563 | cpsf6 (bg=13.45%) | K562 | + | chr11 | | 65267083 | 65267126 |
| 2.02916249379 | fxr2 (bg=10.1%) | HepG2 | + | chr11 | | 65267091 | 65267220 |
| 2.02833184339 | GPKOW (bg=5.66%) | K562 | + | chr11 | | 65267095 | 65267104 |
| 2.75858085237 | gtf2f1 (bg=10.18%) | HepG2 | + | chr11 | | 65267088 | 65267123 |
| 2.18890131612 | khdrbs1 (bg=10.41%) | K562 | + | chr11 | | 65267076 | 65267082 |
| 2.19004819795 | khdrbs1 (bg=10.41%) | K562 | + | chr11 | | 65267076 | 65267083 |
| 2.13314748133 | larp4 (bg=13.51%) | K562 | + | chr11 | | 65267065 | 65267095 |
| 2.66135062135 | larp4 (bg=13.51%) | K562 | + | chr11 | | 65267095 | 65267103 |
| 3.08038416895 | LARP7 (bg=2.17%) | HepG2 | + | chr11 | | 65267085 | 65267192 |
| 3.29527970554 | rbm22 (bg=12.69%) | HepG2 | + | chr11 | | 65267078 | 65267111 |
| 2.64601414445 | safb2 (bg=26.89%) | K562 | + | chr11 | | 65267075 | 65267092 |
| 2.47428720786 | safb2 (bg=26.89%) | K562 | + | chr11 | | 65267075 | 65267095 |
| 2.64992659019 | safb2 (bg=26.89%) | K562 | + | chr11 | | 65267092 | 65267095 |
| 2.90212224091 | safb2 (bg=26.89%) | K562 | + | chr11 | | 65267095 | 65267113 |
| 2.6997128496 | safb2 (bg=26.89%) | K562 | + | chr11 | | 65267095 | 65267113 |
| 2.80364046129 | SMNDC1 (bg=7.08%) | HepG2 | + | chr11 | | 65267085 | 65267183 |
| 4.33393746034 | srsf1 (bg=30.28%) | K562 | + | chr11 | | 65267060 | 65267127 |
| 4.60708009857 | srsf1 (bg=30.28%) | HepG2 | + | chr11 | | 65267079 | 65267127 |
| 2.9231146063 | srsf1 (bg=30.28%) | K562 | + | chr11 | | 65267079 | 65267127 |
| 2.13981095681 | srsf1 (bg=30.28%) | HepG2 | + | chr11 | | 65267084 | 65267095 |
| 2.95770423866 | srsf1 (bg=30.28%) | HepG2 | + | chr11 | | 65267095 | 65267130 |
| 2.16815422907 | srsf7 (bg=22.53%) | K562 | + | chr11 | | 65267068 | 65267115 |
| 2.41340029978 | srsf7 (bg=22.53%) | HepG2 | + | chr11 | | 65267089 | 65267130 |
| 2.12961147771 | srsf7 (bg=22.53%) | K562 | + | chr11 | | 65267096 | 65267127 |
| 4.42807594419 | SRSF9 (bg=9.67%) | HepG2 | + | chr11 | | 65267076 | 65267100 |
| 2.79577290635 | TAF15 (bg=9.06%) | HepG2 | + | chr11 | | 65267094 | 65267132 |
| 4.40831925427 | tra2a (bg=37.02%) | K562 | + | chr11 | | 65267065 | 65267095 |
| 4.62830386505 | tra2a (bg=37.02%) | K562 | + | chr11 | | 65267075 | 65267095 |
| 5.72061034992 | tra2a (bg=37.02%) | HepG2 | + | chr11 | | 65267078 | 65267128 |
| 4.09386313971 | tra2a (bg=37.02%) | HepG2 | + | chr11 | | 65267079 | 65267209 |
| 4.92476525398 | tra2a (bg=37.02%) | K562 | + | chr11 | | 65267095 | 65267113 |
| 5.38726353353 | tra2a (bg=37.02%) | K562 | + | chr11 | | 65267095 | 65267126 |
| 2.40973791734 | TROVE2 (bg=6.96%) | HepG2 | + | chr11 | | 65267082 | 65267117 |
| 2.39923171119 | uchl5 (bg=18.56%) | K562 | + | chr11 | | 65267079 | 65267096 |
| 2.64679910913 | uchl5 (bg=18.56%) | K562 | + | chr11 | | 65267081 | 65267096 |
| 3.19145597119 | uchl5 (bg=18.56%) | K562 | + | chr11 | | 65267096 | 65267102 |
| 3.06954011215 | uchl5 (bg=18.56%) | K562 | + | chr11 | | 65267096 | 65267112 |
| 2.51736515681 | znf622 (bg=18.79%) | K562 | + | chr11 | | 65267080 | 65267095 |
| 3.17234009228 | znf622 (bg=18.79%) | K562 | + | chr11 | | 65267083 | 65267120 |
| 3.01584022042 | znf622 (bg=18.79%) | K562 | + | chr11 | | 65267095 | 65267118 |

  
  

| Match 40 in HUMAN | | | | | | | |
| --- | --- | --- | --- | --- | --- | --- | --- |
| Motif | Start in Seq (1 Indexed) | End in Seq (1 Indexed) | Strand | Chrm | Exon | Start in Chrm (0 Indexed) | End in Chrm (1 Indexed) |
| AGTGGTAGGA | 598 | 607 | + | chr11 | 1 | 65267087 | 65267097 |
| eCLIP Fold-Enrichment | Binding Protein | Cell Line | Strand | Chrm | | Start in Chrm (0 Indexed) | End in Chrm (1 Indexed) |
| 2.18096382887 | aggf1 (bg=15.15%) | HepG2 | + | chr11 | | 65267064 | 65267115 |
| 3.5411016405 | bclaf1 (bg=17.67%) | HepG2 | + | chr11 | | 65267082 | 65267153 |
| 3.28361464473 | bclaf1 (bg=17.67%) | HepG2 | + | chr11 | | 65267087 | 65267141 |
| 3.37391870595 | bud13 (bg=12.85%) | HepG2 | + | chr11 | | 65267077 | 65267122 |
| 3.21980668791 | cpsf6 (bg=13.45%) | K562 | + | chr11 | | 65267075 | 65267127 |
| 2.33787314563 | cpsf6 (bg=13.45%) | K562 | + | chr11 | | 65267083 | 65267126 |
| 2.02916249379 | fxr2 (bg=10.1%) | HepG2 | + | chr11 | | 65267091 | 65267220 |
| 2.02833184339 | GPKOW (bg=5.66%) | K562 | + | chr11 | | 65267095 | 65267104 |
| 2.75858085237 | gtf2f1 (bg=10.18%) | HepG2 | + | chr11 | | 65267088 | 65267123 |
| 2.13314748133 | larp4 (bg=13.51%) | K562 | + | chr11 | | 65267065 | 65267095 |
| 2.66135062135 | larp4 (bg=13.51%) | K562 | + | chr11 | | 65267095 | 65267103 |
| 3.08038416895 | LARP7 (bg=2.17%) | HepG2 | + | chr11 | | 65267085 | 65267192 |
| 3.29527970554 | rbm22 (bg=12.69%) | HepG2 | + | chr11 | | 65267078 | 65267111 |
| 2.64601414445 | safb2 (bg=26.89%) | K562 | + | chr11 | | 65267075 | 65267092 |
| 2.47428720786 | safb2 (bg=26.89%) | K562 | + | chr11 | | 65267075 | 65267095 |
| 2.64992659019 | safb2 (bg=26.89%) | K562 | + | chr11 | | 65267092 | 65267095 |
| 2.90212224091 | safb2 (bg=26.89%) | K562 | + | chr11 | | 65267095 | 65267113 |
| 2.6997128496 | safb2 (bg=26.89%) | K562 | + | chr11 | | 65267095 | 65267113 |
| 2.80364046129 | SMNDC1 (bg=7.08%) | HepG2 | + | chr11 | | 65267085 | 65267183 |
| 4.33393746034 | srsf1 (bg=30.28%) | K562 | + | chr11 | | 65267060 | 65267127 |
| 4.60708009857 | srsf1 (bg=30.28%) | HepG2 | + | chr11 | | 65267079 | 65267127 |
| 2.9231146063 | srsf1 (bg=30.28%) | K562 | + | chr11 | | 65267079 | 65267127 |
| 2.13981095681 | srsf1 (bg=30.28%) | HepG2 | + | chr11 | | 65267084 | 65267095 |
| 2.95770423866 | srsf1 (bg=30.28%) | HepG2 | + | chr11 | | 65267095 | 65267130 |
| 2.16815422907 | srsf7 (bg=22.53%) | K562 | + | chr11 | | 65267068 | 65267115 |
| 2.41340029978 | srsf7 (bg=22.53%) | HepG2 | + | chr11 | | 65267089 | 65267130 |
| 2.12961147771 | srsf7 (bg=22.53%) | K562 | + | chr11 | | 65267096 | 65267127 |
| 4.42807594419 | SRSF9 (bg=9.67%) | HepG2 | + | chr11 | | 65267076 | 65267100 |
| 2.79577290635 | TAF15 (bg=9.06%) | HepG2 | + | chr11 | | 65267094 | 65267132 |
| 4.40831925427 | tra2a (bg=37.02%) | K562 | + | chr11 | | 65267065 | 65267095 |
| 4.62830386505 | tra2a (bg=37.02%) | K562 | + | chr11 | | 65267075 | 65267095 |
| 5.72061034992 | tra2a (bg=37.02%) | HepG2 | + | chr11 | | 65267078 | 65267128 |
| 4.09386313971 | tra2a (bg=37.02%) | HepG2 | + | chr11 | | 65267079 | 65267209 |
| 4.92476525398 | tra2a (bg=37.02%) | K562 | + | chr11 | | 65267095 | 65267113 |
| 5.38726353353 | tra2a (bg=37.02%) | K562 | + | chr11 | | 65267095 | 65267126 |
| 2.40973791734 | TROVE2 (bg=6.96%) | HepG2 | + | chr11 | | 65267082 | 65267117 |
| 2.39923171119 | uchl5 (bg=18.56%) | K562 | + | chr11 | | 65267079 | 65267096 |
| 2.64679910913 | uchl5 (bg=18.56%) | K562 | + | chr11 | | 65267081 | 65267096 |
| 3.19145597119 | uchl5 (bg=18.56%) | K562 | + | chr11 | | 65267096 | 65267102 |
| 3.06954011215 | uchl5 (bg=18.56%) | K562 | + | chr11 | | 65267096 | 65267112 |
| 2.51736515681 | znf622 (bg=18.79%) | K562 | + | chr11 | | 65267080 | 65267095 |
| 3.17234009228 | znf622 (bg=18.79%) | K562 | + | chr11 | | 65267083 | 65267120 |
| 3.01584022042 | znf622 (bg=18.79%) | K562 | + | chr11 | | 65267095 | 65267118 |

  
  

| Match 41 in HUMAN | | | | | | | |
| --- | --- | --- | --- | --- | --- | --- | --- |
| Motif | Start in Seq (1 Indexed) | End in Seq (1 Indexed) | Strand | Chrm | Exon | Start in Chrm (0 Indexed) | End in Chrm (1 Indexed) |
| AAACAATTTGGAGAAGAT | 610 | 627 | + | chr11 | 1 | 65267099 | 65267117 |
| eCLIP Fold-Enrichment | Binding Protein | Cell Line | Strand | Chrm | | Start in Chrm (0 Indexed) | End in Chrm (1 Indexed) |
| 2.18096382887 | aggf1 (bg=15.15%) | HepG2 | + | chr11 | | 65267064 | 65267115 |
| 2.23623053538 | aggf1 (bg=15.15%) | K562 | + | chr11 | | 65267104 | 65267115 |
| 2.43705025754 | aggf1 (bg=15.15%) | HepG2 | + | chr11 | | 65267115 | 65267121 |
| 2.2000801477 | aggf1 (bg=15.15%) | K562 | + | chr11 | | 65267115 | 65267122 |
| 3.5411016405 | bclaf1 (bg=17.67%) | HepG2 | + | chr11 | | 65267082 | 65267153 |
| 3.28361464473 | bclaf1 (bg=17.67%) | HepG2 | + | chr11 | | 65267087 | 65267141 |
| 3.37391870595 | bud13 (bg=12.85%) | HepG2 | + | chr11 | | 65267077 | 65267122 |
| 3.21980668791 | cpsf6 (bg=13.45%) | K562 | + | chr11 | | 65267075 | 65267127 |
| 2.33787314563 | cpsf6 (bg=13.45%) | K562 | + | chr11 | | 65267083 | 65267126 |
| 2.02916249379 | fxr2 (bg=10.1%) | HepG2 | + | chr11 | | 65267091 | 65267220 |
| 2.02833184339 | GPKOW (bg=5.66%) | K562 | + | chr11 | | 65267095 | 65267104 |
| 2.21138139251 | GPKOW (bg=5.66%) | K562 | + | chr11 | | 65267104 | 65267122 |
| 2.75858085237 | gtf2f1 (bg=10.18%) | HepG2 | + | chr11 | | 65267088 | 65267123 |
| 2.66135062135 | larp4 (bg=13.51%) | K562 | + | chr11 | | 65267095 | 65267103 |
| 2.76660084689 | larp4 (bg=13.51%) | K562 | + | chr11 | | 65267103 | 65267118 |
| 3.08038416895 | LARP7 (bg=2.17%) | HepG2 | + | chr11 | | 65267085 | 65267192 |
| 3.29527970554 | rbm22 (bg=12.69%) | HepG2 | + | chr11 | | 65267078 | 65267111 |
| 3.62398402401 | rbm22 (bg=12.69%) | HepG2 | + | chr11 | | 65267111 | 65267123 |
| 3.66608715451 | safb (bg=40.39%) | HepG2 | + | chr11 | | 65267113 | 65267127 |
| 2.90212224091 | safb2 (bg=26.89%) | K562 | + | chr11 | | 65267095 | 65267113 |
| 2.6997128496 | safb2 (bg=26.89%) | K562 | + | chr11 | | 65267095 | 65267113 |
| 3.05261782162 | safb2 (bg=26.89%) | K562 | + | chr11 | | 65267113 | 65267122 |
| 2.80470063404 | safb2 (bg=26.89%) | K562 | + | chr11 | | 65267113 | 65267122 |
| 2.80364046129 | SMNDC1 (bg=7.08%) | HepG2 | + | chr11 | | 65267085 | 65267183 |
| 4.33393746034 | srsf1 (bg=30.28%) | K562 | + | chr11 | | 65267060 | 65267127 |
| 4.60708009857 | srsf1 (bg=30.28%) | HepG2 | + | chr11 | | 65267079 | 65267127 |
| 2.9231146063 | srsf1 (bg=30.28%) | K562 | + | chr11 | | 65267079 | 65267127 |
| 2.95770423866 | srsf1 (bg=30.28%) | HepG2 | + | chr11 | | 65267095 | 65267130 |
| 2.16815422907 | srsf7 (bg=22.53%) | K562 | + | chr11 | | 65267068 | 65267115 |
| 2.41340029978 | srsf7 (bg=22.53%) | HepG2 | + | chr11 | | 65267089 | 65267130 |
| 2.12961147771 | srsf7 (bg=22.53%) | K562 | + | chr11 | | 65267096 | 65267127 |
| 2.82107362769 | srsf7 (bg=22.53%) | K562 | + | chr11 | | 65267115 | 65267121 |
| 4.42807594419 | SRSF9 (bg=9.67%) | HepG2 | + | chr11 | | 65267076 | 65267100 |
| 4.55775449097 | SRSF9 (bg=9.67%) | HepG2 | + | chr11 | | 65267100 | 65267113 |
| 5.18578571358 | SRSF9 (bg=9.67%) | HepG2 | + | chr11 | | 65267113 | 65267139 |
| 2.79577290635 | TAF15 (bg=9.06%) | HepG2 | + | chr11 | | 65267094 | 65267132 |
| 2.07432138279 | TBRG4 (bg=0.51%) | HepG2 | + | chr11 | | 65267116 | 65267140 |
| 5.72061034992 | tra2a (bg=37.02%) | HepG2 | + | chr11 | | 65267078 | 65267128 |
| 4.09386313971 | tra2a (bg=37.02%) | HepG2 | + | chr11 | | 65267079 | 65267209 |
| 4.92476525398 | tra2a (bg=37.02%) | K562 | + | chr11 | | 65267095 | 65267113 |
| 5.38726353353 | tra2a (bg=37.02%) | K562 | + | chr11 | | 65267095 | 65267126 |
| 5.4440368294 | tra2a (bg=37.02%) | K562 | + | chr11 | | 65267113 | 65267126 |
| 2.40973791734 | TROVE2 (bg=6.96%) | HepG2 | + | chr11 | | 65267082 | 65267117 |
| 2.79695491778 | TROVE2 (bg=6.96%) | HepG2 | + | chr11 | | 65267117 | 65267136 |
| 3.19145597119 | uchl5 (bg=18.56%) | K562 | + | chr11 | | 65267096 | 65267102 |
| 3.06954011215 | uchl5 (bg=18.56%) | K562 | + | chr11 | | 65267096 | 65267112 |
| 3.5115632793 | uchl5 (bg=18.56%) | K562 | + | chr11 | | 65267102 | 65267113 |
| 3.15546914716 | uchl5 (bg=18.56%) | K562 | + | chr11 | | 65267112 | 65267119 |
| 3.72085738799 | uchl5 (bg=18.56%) | K562 | + | chr11 | | 65267113 | 65267130 |
| 3.17234009228 | znf622 (bg=18.79%) | K562 | + | chr11 | | 65267083 | 65267120 |
| 3.01584022042 | znf622 (bg=18.79%) | K562 | + | chr11 | | 65267095 | 65267118 |

  
  

| Match 42 in HUMAN | | | | | | | |
| --- | --- | --- | --- | --- | --- | --- | --- |
| Motif | Start in Seq (1 Indexed) | End in Seq (1 Indexed) | Strand | Chrm | Exon | Start in Chrm (0 Indexed) | End in Chrm (1 Indexed) |
| AGAAGTTTGAAGTGGAA | 628 | 644 | + | chr11 | 1 | 65267117 | 65267134 |
| eCLIP Fold-Enrichment | Binding Protein | Cell Line | Strand | Chrm | | Start in Chrm (0 Indexed) | End in Chrm (1 Indexed) |
| 2.43705025754 | aggf1 (bg=15.15%) | HepG2 | + | chr11 | | 65267115 | 65267121 |
| 2.2000801477 | aggf1 (bg=15.15%) | K562 | + | chr11 | | 65267115 | 65267122 |
| 2.34758444261 | aggf1 (bg=15.15%) | HepG2 | + | chr11 | | 65267121 | 65267127 |
| 2.14620693252 | aggf1 (bg=15.15%) | K562 | + | chr11 | | 65267122 | 65267124 |
| 2.22053929359 | aggf1 (bg=15.15%) | K562 | + | chr11 | | 65267124 | 65267127 |
| 2.29656710912 | aggf1 (bg=15.15%) | K562 | + | chr11 | | 65267127 | 65267129 |
| 2.27645639795 | aggf1 (bg=15.15%) | HepG2 | + | chr11 | | 65267127 | 65267136 |
| 2.40518597064 | aggf1 (bg=15.15%) | K562 | + | chr11 | | 65267129 | 65267136 |
| 3.5411016405 | bclaf1 (bg=17.67%) | HepG2 | + | chr11 | | 65267082 | 65267153 |
| 3.28361464473 | bclaf1 (bg=17.67%) | HepG2 | + | chr11 | | 65267087 | 65267141 |
| 3.37391870595 | bud13 (bg=12.85%) | HepG2 | + | chr11 | | 65267077 | 65267122 |
| 4.1915257905 | bud13 (bg=12.85%) | HepG2 | + | chr11 | | 65267122 | 65267151 |
| 3.21980668791 | cpsf6 (bg=13.45%) | K562 | + | chr11 | | 65267075 | 65267127 |
| 2.33787314563 | cpsf6 (bg=13.45%) | K562 | + | chr11 | | 65267083 | 65267126 |
| 2.4802153632 | cpsf6 (bg=13.45%) | K562 | + | chr11 | | 65267126 | 65267174 |
| 3.74141387901 | cpsf6 (bg=13.45%) | K562 | + | chr11 | | 65267127 | 65267130 |
| 3.62305477164 | cpsf6 (bg=13.45%) | K562 | + | chr11 | | 65267130 | 65267134 |
| 3.28041700197 | cpsf6 (bg=13.45%) | K562 | + | chr11 | | 65267134 | 65267153 |
| 2.02916249379 | fxr2 (bg=10.1%) | HepG2 | + | chr11 | | 65267091 | 65267220 |
| 2.21138139251 | GPKOW (bg=5.66%) | K562 | + | chr11 | | 65267104 | 65267122 |
| 2.29900164833 | GPKOW (bg=5.66%) | K562 | + | chr11 | | 65267118 | 65267128 |
| 2.21321789487 | GPKOW (bg=5.66%) | K562 | + | chr11 | | 65267122 | 65267128 |
| 2.21058221504 | GPKOW (bg=5.66%) | K562 | + | chr11 | | 65267128 | 65267137 |
| 2.26861006162 | GPKOW (bg=5.66%) | K562 | + | chr11 | | 65267128 | 65267139 |
| 2.75858085237 | gtf2f1 (bg=10.18%) | HepG2 | + | chr11 | | 65267088 | 65267123 |
| 3.57434857647 | gtf2f1 (bg=10.18%) | HepG2 | + | chr11 | | 65267123 | 65267170 |
| 2.01675141196 | hltf (bg=24.28%) | HepG2 | + | chr11 | | 65267121 | 65267176 |
| 2.76660084689 | larp4 (bg=13.51%) | K562 | + | chr11 | | 65267103 | 65267118 |
| 2.76328211244 | larp4 (bg=13.51%) | K562 | + | chr11 | | 65267118 | 65267128 |
| 2.68083330217 | larp4 (bg=13.51%) | K562 | + | chr11 | | 65267128 | 65267137 |
| 3.08038416895 | LARP7 (bg=2.17%) | HepG2 | + | chr11 | | 65267085 | 65267192 |
| 3.62398402401 | rbm22 (bg=12.69%) | HepG2 | + | chr11 | | 65267111 | 65267123 |
| 3.58690579335 | rbm22 (bg=12.69%) | HepG2 | + | chr11 | | 65267123 | 65267128 |
| 3.86393167484 | rbm22 (bg=12.69%) | HepG2 | + | chr11 | | 65267128 | 65267135 |
| 3.66608715451 | safb (bg=40.39%) | HepG2 | + | chr11 | | 65267113 | 65267127 |
| 3.05261782162 | safb2 (bg=26.89%) | K562 | + | chr11 | | 65267113 | 65267122 |
| 2.80470063404 | safb2 (bg=26.89%) | K562 | + | chr11 | | 65267113 | 65267122 |
| 3.01756227033 | safb2 (bg=26.89%) | K562 | + | chr11 | | 65267122 | 65267126 |
| 2.73094740516 | safb2 (bg=26.89%) | K562 | + | chr11 | | 65267122 | 65267129 |
| 3.08279137311 | safb2 (bg=26.89%) | K562 | + | chr11 | | 65267126 | 65267129 |
| 3.1839139586 | safb2 (bg=26.89%) | K562 | + | chr11 | | 65267129 | 65267135 |
| 2.73564727959 | safb2 (bg=26.89%) | K562 | + | chr11 | | 65267129 | 65267135 |
| 2.80364046129 | SMNDC1 (bg=7.08%) | HepG2 | + | chr11 | | 65267085 | 65267183 |
| 4.33393746034 | srsf1 (bg=30.28%) | K562 | + | chr11 | | 65267060 | 65267127 |
| 4.60708009857 | srsf1 (bg=30.28%) | HepG2 | + | chr11 | | 65267079 | 65267127 |
| 2.9231146063 | srsf1 (bg=30.28%) | K562 | + | chr11 | | 65267079 | 65267127 |
| 2.95770423866 | srsf1 (bg=30.28%) | HepG2 | + | chr11 | | 65267095 | 65267130 |
| 3.31880599384 | srsf1 (bg=30.28%) | K562 | + | chr11 | | 65267127 | 65267130 |
| 4.8965425265 | srsf1 (bg=30.28%) | K562 | + | chr11 | | 65267127 | 65267130 |
| 5.53728579805 | srsf1 (bg=30.28%) | HepG2 | + | chr11 | | 65267127 | 65267134 |
| 4.85019750077 | srsf1 (bg=30.28%) | K562 | + | chr11 | | 65267130 | 65267134 |
| 3.34548760266 | srsf1 (bg=30.28%) | K562 | + | chr11 | | 65267130 | 65267137 |
| 3.36452843109 | srsf1 (bg=30.28%) | HepG2 | + | chr11 | | 65267130 | 65267189 |
| 4.89138166838 | srsf1 (bg=30.28%) | K562 | + | chr11 | | 65267134 | 65267142 |
| 5.43086995209 | srsf1 (bg=30.28%) | HepG2 | + | chr11 | | 65267134 | 65267173 |
| 2.41340029978 | srsf7 (bg=22.53%) | HepG2 | + | chr11 | | 65267089 | 65267130 |
| 2.12961147771 | srsf7 (bg=22.53%) | K562 | + | chr11 | | 65267096 | 65267127 |
| 2.82107362769 | srsf7 (bg=22.53%) | K562 | + | chr11 | | 65267115 | 65267121 |
| 2.86665972644 | srsf7 (bg=22.53%) | K562 | + | chr11 | | 65267121 | 65267127 |
| 2.95729129319 | srsf7 (bg=22.53%) | K562 | + | chr11 | | 65267127 | 65267129 |
| 2.53880004722 | srsf7 (bg=22.53%) | K562 | + | chr11 | | 65267127 | 65267152 |
| 2.87826528909 | srsf7 (bg=22.53%) | K562 | + | chr11 | | 65267129 | 65267139 |
| 2.76844022786 | srsf7 (bg=22.53%) | HepG2 | + | chr11 | | 65267130 | 65267192 |
| 5.18578571358 | SRSF9 (bg=9.67%) | HepG2 | + | chr11 | | 65267113 | 65267139 |
| 2.79577290635 | TAF15 (bg=9.06%) | HepG2 | + | chr11 | | 65267094 | 65267132 |
| 2.97237624979 | TAF15 (bg=9.06%) | HepG2 | + | chr11 | | 65267132 | 65267168 |
| 2.07432138279 | TBRG4 (bg=0.51%) | HepG2 | + | chr11 | | 65267116 | 65267140 |
| 5.72061034992 | tra2a (bg=37.02%) | HepG2 | + | chr11 | | 65267078 | 65267128 |
| 4.09386313971 | tra2a (bg=37.02%) | HepG2 | + | chr11 | | 65267079 | 65267209 |
| 5.38726353353 | tra2a (bg=37.02%) | K562 | + | chr11 | | 65267095 | 65267126 |
| 5.4440368294 | tra2a (bg=37.02%) | K562 | + | chr11 | | 65267113 | 65267126 |
| 5.48859280628 | tra2a (bg=37.02%) | K562 | + | chr11 | | 65267126 | 65267129 |
| 5.8567375679 | tra2a (bg=37.02%) | K562 | + | chr11 | | 65267126 | 65267134 |
| 6.13987811588 | tra2a (bg=37.02%) | HepG2 | + | chr11 | | 65267128 | 65267135 |
| 6.28000615706 | tra2a (bg=37.02%) | K562 | + | chr11 | | 65267129 | 65267150 |
| 6.43865756135 | tra2a (bg=37.02%) | K562 | + | chr11 | | 65267134 | 65267149 |
| 2.40973791734 | TROVE2 (bg=6.96%) | HepG2 | + | chr11 | | 65267082 | 65267117 |
| 2.79695491778 | TROVE2 (bg=6.96%) | HepG2 | + | chr11 | | 65267117 | 65267136 |
| 3.15546914716 | uchl5 (bg=18.56%) | K562 | + | chr11 | | 65267112 | 65267119 |
| 3.72085738799 | uchl5 (bg=18.56%) | K562 | + | chr11 | | 65267113 | 65267130 |
| 3.33016917937 | uchl5 (bg=18.56%) | K562 | + | chr11 | | 65267119 | 65267129 |
| 3.61248111818 | uchl5 (bg=18.56%) | K562 | + | chr11 | | 65267129 | 65267135 |
| 4.27013885977 | uchl5 (bg=18.56%) | K562 | + | chr11 | | 65267130 | 65267139 |
| 3.17234009228 | znf622 (bg=18.79%) | K562 | + | chr11 | | 65267083 | 65267120 |
| 3.01584022042 | znf622 (bg=18.79%) | K562 | + | chr11 | | 65267095 | 65267118 |
| 3.12864806332 | znf622 (bg=18.79%) | K562 | + | chr11 | | 65267118 | 65267122 |
| 3.59876615253 | znf622 (bg=18.79%) | K562 | + | chr11 | | 65267120 | 65267128 |
| 2.90889792574 | znf622 (bg=18.79%) | K562 | + | chr11 | | 65267122 | 65267127 |
| 3.16214347893 | znf622 (bg=18.79%) | K562 | + | chr11 | | 65267127 | 65267134 |
| 3.68009262286 | znf622 (bg=18.79%) | K562 | + | chr11 | | 65267128 | 65267133 |
| 3.79982377123 | znf622 (bg=18.79%) | K562 | + | chr11 | | 65267133 | 65267140 |
| 3.58211875175 | znf622 (bg=18.79%) | K562 | + | chr11 | | 65267134 | 65267150 |

  
  

| Match 43 in HUMAN | | | | | | | |
| --- | --- | --- | --- | --- | --- | --- | --- |
| Motif | Start in Seq (1 Indexed) | End in Seq (1 Indexed) | Strand | Chrm | Exon | Start in Chrm (0 Indexed) | End in Chrm (1 Indexed) |
| TTGAAGTGGA | 634 | 643 | + | chr11 | 1 | 65267123 | 65267133 |
| eCLIP Fold-Enrichment | Binding Protein | Cell Line | Strand | Chrm | | Start in Chrm (0 Indexed) | End in Chrm (1 Indexed) |
| 2.34758444261 | aggf1 (bg=15.15%) | HepG2 | + | chr11 | | 65267121 | 65267127 |
| 2.14620693252 | aggf1 (bg=15.15%) | K562 | + | chr11 | | 65267122 | 65267124 |
| 2.22053929359 | aggf1 (bg=15.15%) | K562 | + | chr11 | | 65267124 | 65267127 |
| 2.29656710912 | aggf1 (bg=15.15%) | K562 | + | chr11 | | 65267127 | 65267129 |
| 2.27645639795 | aggf1 (bg=15.15%) | HepG2 | + | chr11 | | 65267127 | 65267136 |
| 2.40518597064 | aggf1 (bg=15.15%) | K562 | + | chr11 | | 65267129 | 65267136 |
| 3.5411016405 | bclaf1 (bg=17.67%) | HepG2 | + | chr11 | | 65267082 | 65267153 |
| 3.28361464473 | bclaf1 (bg=17.67%) | HepG2 | + | chr11 | | 65267087 | 65267141 |
| 4.1915257905 | bud13 (bg=12.85%) | HepG2 | + | chr11 | | 65267122 | 65267151 |
| 3.21980668791 | cpsf6 (bg=13.45%) | K562 | + | chr11 | | 65267075 | 65267127 |
| 2.33787314563 | cpsf6 (bg=13.45%) | K562 | + | chr11 | | 65267083 | 65267126 |
| 2.4802153632 | cpsf6 (bg=13.45%) | K562 | + | chr11 | | 65267126 | 65267174 |
| 3.74141387901 | cpsf6 (bg=13.45%) | K562 | + | chr11 | | 65267127 | 65267130 |
| 3.62305477164 | cpsf6 (bg=13.45%) | K562 | + | chr11 | | 65267130 | 65267134 |
| 2.02916249379 | fxr2 (bg=10.1%) | HepG2 | + | chr11 | | 65267091 | 65267220 |
| 2.29900164833 | GPKOW (bg=5.66%) | K562 | + | chr11 | | 65267118 | 65267128 |
| 2.21321789487 | GPKOW (bg=5.66%) | K562 | + | chr11 | | 65267122 | 65267128 |
| 2.21058221504 | GPKOW (bg=5.66%) | K562 | + | chr11 | | 65267128 | 65267137 |
| 2.26861006162 | GPKOW (bg=5.66%) | K562 | + | chr11 | | 65267128 | 65267139 |
| 2.75858085237 | gtf2f1 (bg=10.18%) | HepG2 | + | chr11 | | 65267088 | 65267123 |
| 3.57434857647 | gtf2f1 (bg=10.18%) | HepG2 | + | chr11 | | 65267123 | 65267170 |
| 2.01675141196 | hltf (bg=24.28%) | HepG2 | + | chr11 | | 65267121 | 65267176 |
| 2.76328211244 | larp4 (bg=13.51%) | K562 | + | chr11 | | 65267118 | 65267128 |
| 2.68083330217 | larp4 (bg=13.51%) | K562 | + | chr11 | | 65267128 | 65267137 |
| 3.08038416895 | LARP7 (bg=2.17%) | HepG2 | + | chr11 | | 65267085 | 65267192 |
| 3.62398402401 | rbm22 (bg=12.69%) | HepG2 | + | chr11 | | 65267111 | 65267123 |
| 3.58690579335 | rbm22 (bg=12.69%) | HepG2 | + | chr11 | | 65267123 | 65267128 |
| 3.86393167484 | rbm22 (bg=12.69%) | HepG2 | + | chr11 | | 65267128 | 65267135 |
| 3.66608715451 | safb (bg=40.39%) | HepG2 | + | chr11 | | 65267113 | 65267127 |
| 3.01756227033 | safb2 (bg=26.89%) | K562 | + | chr11 | | 65267122 | 65267126 |
| 2.73094740516 | safb2 (bg=26.89%) | K562 | + | chr11 | | 65267122 | 65267129 |
| 3.08279137311 | safb2 (bg=26.89%) | K562 | + | chr11 | | 65267126 | 65267129 |
| 3.1839139586 | safb2 (bg=26.89%) | K562 | + | chr11 | | 65267129 | 65267135 |
| 2.73564727959 | safb2 (bg=26.89%) | K562 | + | chr11 | | 65267129 | 65267135 |
| 2.80364046129 | SMNDC1 (bg=7.08%) | HepG2 | + | chr11 | | 65267085 | 65267183 |
| 4.33393746034 | srsf1 (bg=30.28%) | K562 | + | chr11 | | 65267060 | 65267127 |
| 4.60708009857 | srsf1 (bg=30.28%) | HepG2 | + | chr11 | | 65267079 | 65267127 |
| 2.9231146063 | srsf1 (bg=30.28%) | K562 | + | chr11 | | 65267079 | 65267127 |
| 2.95770423866 | srsf1 (bg=30.28%) | HepG2 | + | chr11 | | 65267095 | 65267130 |
| 3.31880599384 | srsf1 (bg=30.28%) | K562 | + | chr11 | | 65267127 | 65267130 |
| 4.8965425265 | srsf1 (bg=30.28%) | K562 | + | chr11 | | 65267127 | 65267130 |
| 5.53728579805 | srsf1 (bg=30.28%) | HepG2 | + | chr11 | | 65267127 | 65267134 |
| 4.85019750077 | srsf1 (bg=30.28%) | K562 | + | chr11 | | 65267130 | 65267134 |
| 3.34548760266 | srsf1 (bg=30.28%) | K562 | + | chr11 | | 65267130 | 65267137 |
| 3.36452843109 | srsf1 (bg=30.28%) | HepG2 | + | chr11 | | 65267130 | 65267189 |
| 2.41340029978 | srsf7 (bg=22.53%) | HepG2 | + | chr11 | | 65267089 | 65267130 |
| 2.12961147771 | srsf7 (bg=22.53%) | K562 | + | chr11 | | 65267096 | 65267127 |
| 2.86665972644 | srsf7 (bg=22.53%) | K562 | + | chr11 | | 65267121 | 65267127 |
| 2.95729129319 | srsf7 (bg=22.53%) | K562 | + | chr11 | | 65267127 | 65267129 |
| 2.53880004722 | srsf7 (bg=22.53%) | K562 | + | chr11 | | 65267127 | 65267152 |
| 2.87826528909 | srsf7 (bg=22.53%) | K562 | + | chr11 | | 65267129 | 65267139 |
| 2.76844022786 | srsf7 (bg=22.53%) | HepG2 | + | chr11 | | 65267130 | 65267192 |
| 5.18578571358 | SRSF9 (bg=9.67%) | HepG2 | + | chr11 | | 65267113 | 65267139 |
| 2.79577290635 | TAF15 (bg=9.06%) | HepG2 | + | chr11 | | 65267094 | 65267132 |
| 2.97237624979 | TAF15 (bg=9.06%) | HepG2 | + | chr11 | | 65267132 | 65267168 |
| 2.07432138279 | TBRG4 (bg=0.51%) | HepG2 | + | chr11 | | 65267116 | 65267140 |
| 5.72061034992 | tra2a (bg=37.02%) | HepG2 | + | chr11 | | 65267078 | 65267128 |
| 4.09386313971 | tra2a (bg=37.02%) | HepG2 | + | chr11 | | 65267079 | 65267209 |
| 5.38726353353 | tra2a (bg=37.02%) | K562 | + | chr11 | | 65267095 | 65267126 |
| 5.4440368294 | tra2a (bg=37.02%) | K562 | + | chr11 | | 65267113 | 65267126 |
| 5.48859280628 | tra2a (bg=37.02%) | K562 | + | chr11 | | 65267126 | 65267129 |
| 5.8567375679 | tra2a (bg=37.02%) | K562 | + | chr11 | | 65267126 | 65267134 |
| 6.13987811588 | tra2a (bg=37.02%) | HepG2 | + | chr11 | | 65267128 | 65267135 |
| 6.28000615706 | tra2a (bg=37.02%) | K562 | + | chr11 | | 65267129 | 65267150 |
| 2.79695491778 | TROVE2 (bg=6.96%) | HepG2 | + | chr11 | | 65267117 | 65267136 |
| 3.72085738799 | uchl5 (bg=18.56%) | K562 | + | chr11 | | 65267113 | 65267130 |
| 3.33016917937 | uchl5 (bg=18.56%) | K562 | + | chr11 | | 65267119 | 65267129 |
| 3.61248111818 | uchl5 (bg=18.56%) | K562 | + | chr11 | | 65267129 | 65267135 |
| 4.27013885977 | uchl5 (bg=18.56%) | K562 | + | chr11 | | 65267130 | 65267139 |
| 3.59876615253 | znf622 (bg=18.79%) | K562 | + | chr11 | | 65267120 | 65267128 |
| 2.90889792574 | znf622 (bg=18.79%) | K562 | + | chr11 | | 65267122 | 65267127 |
| 3.16214347893 | znf622 (bg=18.79%) | K562 | + | chr11 | | 65267127 | 65267134 |
| 3.68009262286 | znf622 (bg=18.79%) | K562 | + | chr11 | | 65267128 | 65267133 |
| 3.79982377123 | znf622 (bg=18.79%) | K562 | + | chr11 | | 65267133 | 65267140 |

  
  

| Match 44 in HUMAN | | | | | | | |
| --- | --- | --- | --- | --- | --- | --- | --- |
| Motif | Start in Seq (1 Indexed) | End in Seq (1 Indexed) | Strand | Chrm | Exon | Start in Chrm (0 Indexed) | End in Chrm (1 Indexed) |
| ACTGGAAGACAGAAGTAC | 646 | 663 | + | chr11 | 1 | 65267135 | 65267153 |
| eCLIP Fold-Enrichment | Binding Protein | Cell Line | Strand | Chrm | | Start in Chrm (0 Indexed) | End in Chrm (1 Indexed) |
| 2.27645639795 | aggf1 (bg=15.15%) | HepG2 | + | chr11 | | 65267127 | 65267136 |
| 2.40518597064 | aggf1 (bg=15.15%) | K562 | + | chr11 | | 65267129 | 65267136 |
| 2.47922673988 | aggf1 (bg=15.15%) | K562 | + | chr11 | | 65267136 | 65267141 |
| 2.16804103997 | aggf1 (bg=15.15%) | HepG2 | + | chr11 | | 65267136 | 65267144 |
| 2.58721650992 | aggf1 (bg=15.15%) | K562 | + | chr11 | | 65267141 | 65267145 |
| 2.65283929054 | aggf1 (bg=15.15%) | K562 | + | chr11 | | 65267145 | 65267152 |
| 2.82013080799 | aggf1 (bg=15.15%) | K562 | + | chr11 | | 65267152 | 65267157 |
| 2.14875654764 | AQR (bg=4.89%) | HepG2 | + | chr11 | | 65267153 | 65267163 |
| 3.5411016405 | bclaf1 (bg=17.67%) | HepG2 | + | chr11 | | 65267082 | 65267153 |
| 3.28361464473 | bclaf1 (bg=17.67%) | HepG2 | + | chr11 | | 65267087 | 65267141 |
| 3.40480816275 | bclaf1 (bg=17.67%) | HepG2 | + | chr11 | | 65267141 | 65267150 |
| 3.51718214163 | bclaf1 (bg=17.67%) | HepG2 | + | chr11 | | 65267150 | 65267187 |
| 4.17234158954 | bclaf1 (bg=17.67%) | HepG2 | + | chr11 | | 65267153 | 65267201 |
| 4.1915257905 | bud13 (bg=12.85%) | HepG2 | + | chr11 | | 65267122 | 65267151 |
| 4.61023663539 | bud13 (bg=12.85%) | HepG2 | + | chr11 | | 65267151 | 65267230 |
| 2.4802153632 | cpsf6 (bg=13.45%) | K562 | + | chr11 | | 65267126 | 65267174 |
| 3.28041700197 | cpsf6 (bg=13.45%) | K562 | + | chr11 | | 65267134 | 65267153 |
| 3.27752893622 | cpsf6 (bg=13.45%) | K562 | + | chr11 | | 65267153 | 65267163 |
| 2.06037562139 | FASTKD2 (bg=4.54%) | K562 | + | chr11 | | 65267137 | 65267153 |
| 2.13537833515 | FASTKD2 (bg=4.54%) | K562 | + | chr11 | | 65267153 | 65267173 |
| 2.02916249379 | fxr2 (bg=10.1%) | HepG2 | + | chr11 | | 65267091 | 65267220 |
| 2.21058221504 | GPKOW (bg=5.66%) | K562 | + | chr11 | | 65267128 | 65267137 |
| 2.26861006162 | GPKOW (bg=5.66%) | K562 | + | chr11 | | 65267128 | 65267139 |
| 2.33299625231 | GPKOW (bg=5.66%) | K562 | + | chr11 | | 65267137 | 65267203 |
| 2.14154707557 | GPKOW (bg=5.66%) | K562 | + | chr11 | | 65267139 | 65267153 |
| 2.2345958442 | GPKOW (bg=5.66%) | K562 | + | chr11 | | 65267153 | 65267204 |
| 3.57434857647 | gtf2f1 (bg=10.18%) | HepG2 | + | chr11 | | 65267123 | 65267170 |
| 2.01675141196 | hltf (bg=24.28%) | HepG2 | + | chr11 | | 65267121 | 65267176 |
| 2.68083330217 | larp4 (bg=13.51%) | K562 | + | chr11 | | 65267128 | 65267137 |
| 2.67323940375 | larp4 (bg=13.51%) | K562 | + | chr11 | | 65267137 | 65267152 |
| 2.60579158266 | larp4 (bg=13.51%) | K562 | + | chr11 | | 65267152 | 65267157 |
| 3.08038416895 | LARP7 (bg=2.17%) | HepG2 | + | chr11 | | 65267085 | 65267192 |
| 2.1199614677 | MTPAP (bg=9.55%) | K562 | + | chr11 | | 65267148 | 65267171 |
| 2.04479443816 | rbm15 (bg=11.59%) | HepG2 | + | chr11 | | 65267152 | 65267207 |
| 3.86393167484 | rbm22 (bg=12.69%) | HepG2 | + | chr11 | | 65267128 | 65267135 |
| 3.85817954948 | rbm22 (bg=12.69%) | HepG2 | + | chr11 | | 65267135 | 65267151 |
| 3.91706089456 | rbm22 (bg=12.69%) | HepG2 | + | chr11 | | 65267151 | 65267158 |
| 3.1839139586 | safb2 (bg=26.89%) | K562 | + | chr11 | | 65267129 | 65267135 |
| 2.73564727959 | safb2 (bg=26.89%) | K562 | + | chr11 | | 65267129 | 65267135 |
| 3.11319196482 | safb2 (bg=26.89%) | K562 | + | chr11 | | 65267135 | 65267138 |
| 2.5899878241 | safb2 (bg=26.89%) | K562 | + | chr11 | | 65267135 | 65267138 |
| 3.03612247821 | safb2 (bg=26.89%) | K562 | + | chr11 | | 65267138 | 65267147 |
| 2.5768865381 | safb2 (bg=26.89%) | K562 | + | chr11 | | 65267138 | 65267163 |
| 3.07834158993 | safb2 (bg=26.89%) | K562 | + | chr11 | | 65267147 | 65267155 |
| 2.80364046129 | SMNDC1 (bg=7.08%) | HepG2 | + | chr11 | | 65267085 | 65267183 |
| 3.34548760266 | srsf1 (bg=30.28%) | K562 | + | chr11 | | 65267130 | 65267137 |
| 3.36452843109 | srsf1 (bg=30.28%) | HepG2 | + | chr11 | | 65267130 | 65267189 |
| 4.89138166838 | srsf1 (bg=30.28%) | K562 | + | chr11 | | 65267134 | 65267142 |
| 5.43086995209 | srsf1 (bg=30.28%) | HepG2 | + | chr11 | | 65267134 | 65267173 |
| 3.3663111222 | srsf1 (bg=30.28%) | K562 | + | chr11 | | 65267137 | 65267152 |
| 4.76363071574 | srsf1 (bg=30.28%) | K562 | + | chr11 | | 65267142 | 65267152 |
| 4.74273156389 | srsf1 (bg=30.28%) | K562 | + | chr11 | | 65267152 | 65267157 |
| 3.3103651996 | srsf1 (bg=30.28%) | K562 | + | chr11 | | 65267152 | 65267169 |
| 2.53880004722 | srsf7 (bg=22.53%) | K562 | + | chr11 | | 65267127 | 65267152 |
| 2.87826528909 | srsf7 (bg=22.53%) | K562 | + | chr11 | | 65267129 | 65267139 |
| 2.76844022786 | srsf7 (bg=22.53%) | HepG2 | + | chr11 | | 65267130 | 65267192 |
| 2.70674458956 | srsf7 (bg=22.53%) | K562 | + | chr11 | | 65267139 | 65267154 |
| 2.6467563363 | srsf7 (bg=22.53%) | K562 | + | chr11 | | 65267152 | 65267173 |
| 5.18578571358 | SRSF9 (bg=9.67%) | HepG2 | + | chr11 | | 65267113 | 65267139 |
| 5.39526782119 | SRSF9 (bg=9.67%) | HepG2 | + | chr11 | | 65267139 | 65267181 |
| 2.97237624979 | TAF15 (bg=9.06%) | HepG2 | + | chr11 | | 65267132 | 65267168 |
| 2.07432138279 | TBRG4 (bg=0.51%) | HepG2 | + | chr11 | | 65267116 | 65267140 |
| 4.09386313971 | tra2a (bg=37.02%) | HepG2 | + | chr11 | | 65267079 | 65267209 |
| 6.13987811588 | tra2a (bg=37.02%) | HepG2 | + | chr11 | | 65267128 | 65267135 |
| 6.28000615706 | tra2a (bg=37.02%) | K562 | + | chr11 | | 65267129 | 65267150 |
| 6.43865756135 | tra2a (bg=37.02%) | K562 | + | chr11 | | 65267134 | 65267149 |
| 6.1639007299 | tra2a (bg=37.02%) | HepG2 | + | chr11 | | 65267135 | 65267142 |
| 6.08298073451 | tra2a (bg=37.02%) | HepG2 | + | chr11 | | 65267142 | 65267154 |
| 6.92442043859 | tra2a (bg=37.02%) | K562 | + | chr11 | | 65267149 | 65267154 |
| 6.95988401888 | tra2a (bg=37.02%) | K562 | + | chr11 | | 65267150 | 65267154 |
| 2.79695491778 | TROVE2 (bg=6.96%) | HepG2 | + | chr11 | | 65267117 | 65267136 |
| 2.73705255877 | TROVE2 (bg=6.96%) | HepG2 | + | chr11 | | 65267136 | 65267153 |
| 2.80392272638 | TROVE2 (bg=6.96%) | HepG2 | + | chr11 | | 65267153 | 65267176 |
| 3.61248111818 | uchl5 (bg=18.56%) | K562 | + | chr11 | | 65267129 | 65267135 |
| 4.27013885977 | uchl5 (bg=18.56%) | K562 | + | chr11 | | 65267130 | 65267139 |
| 3.83366777677 | uchl5 (bg=18.56%) | K562 | + | chr11 | | 65267135 | 65267154 |
| 4.2249241798 | uchl5 (bg=18.56%) | K562 | + | chr11 | | 65267139 | 65267154 |
| 3.79982377123 | znf622 (bg=18.79%) | K562 | + | chr11 | | 65267133 | 65267140 |
| 3.58211875175 | znf622 (bg=18.79%) | K562 | + | chr11 | | 65267134 | 65267150 |
| 3.99743927522 | znf622 (bg=18.79%) | K562 | + | chr11 | | 65267140 | 65267148 |
| 3.81752577296 | znf622 (bg=18.79%) | K562 | + | chr11 | | 65267148 | 65267151 |
| 3.59275288435 | znf622 (bg=18.79%) | K562 | + | chr11 | | 65267150 | 65267158 |
| 4.08780546793 | znf622 (bg=18.79%) | K562 | + | chr11 | | 65267151 | 65267157 |

  
  

| Match 45 in HUMAN | | | | | | | |
| --- | --- | --- | --- | --- | --- | --- | --- |
| Motif | Start in Seq (1 Indexed) | End in Seq (1 Indexed) | Strand | Chrm | Exon | Start in Chrm (0 Indexed) | End in Chrm (1 Indexed) |
| CAGAAGTA | 655 | 662 | + | chr11 | 1 | 65267144 | 65267152 |
| eCLIP Fold-Enrichment | Binding Protein | Cell Line | Strand | Chrm | | Start in Chrm (0 Indexed) | End in Chrm (1 Indexed) |
| 2.16804103997 | aggf1 (bg=15.15%) | HepG2 | + | chr11 | | 65267136 | 65267144 |
| 2.58721650992 | aggf1 (bg=15.15%) | K562 | + | chr11 | | 65267141 | 65267145 |
| 2.65283929054 | aggf1 (bg=15.15%) | K562 | + | chr11 | | 65267145 | 65267152 |
| 2.82013080799 | aggf1 (bg=15.15%) | K562 | + | chr11 | | 65267152 | 65267157 |
| 3.5411016405 | bclaf1 (bg=17.67%) | HepG2 | + | chr11 | | 65267082 | 65267153 |
| 3.40480816275 | bclaf1 (bg=17.67%) | HepG2 | + | chr11 | | 65267141 | 65267150 |
| 3.51718214163 | bclaf1 (bg=17.67%) | HepG2 | + | chr11 | | 65267150 | 65267187 |
| 4.1915257905 | bud13 (bg=12.85%) | HepG2 | + | chr11 | | 65267122 | 65267151 |
| 4.61023663539 | bud13 (bg=12.85%) | HepG2 | + | chr11 | | 65267151 | 65267230 |
| 2.4802153632 | cpsf6 (bg=13.45%) | K562 | + | chr11 | | 65267126 | 65267174 |
| 3.28041700197 | cpsf6 (bg=13.45%) | K562 | + | chr11 | | 65267134 | 65267153 |
| 2.06037562139 | FASTKD2 (bg=4.54%) | K562 | + | chr11 | | 65267137 | 65267153 |
| 2.02916249379 | fxr2 (bg=10.1%) | HepG2 | + | chr11 | | 65267091 | 65267220 |
| 2.33299625231 | GPKOW (bg=5.66%) | K562 | + | chr11 | | 65267137 | 65267203 |
| 2.14154707557 | GPKOW (bg=5.66%) | K562 | + | chr11 | | 65267139 | 65267153 |
| 3.57434857647 | gtf2f1 (bg=10.18%) | HepG2 | + | chr11 | | 65267123 | 65267170 |
| 2.01675141196 | hltf (bg=24.28%) | HepG2 | + | chr11 | | 65267121 | 65267176 |
| 2.67323940375 | larp4 (bg=13.51%) | K562 | + | chr11 | | 65267137 | 65267152 |
| 2.60579158266 | larp4 (bg=13.51%) | K562 | + | chr11 | | 65267152 | 65267157 |
| 3.08038416895 | LARP7 (bg=2.17%) | HepG2 | + | chr11 | | 65267085 | 65267192 |
| 2.1199614677 | MTPAP (bg=9.55%) | K562 | + | chr11 | | 65267148 | 65267171 |
| 2.04479443816 | rbm15 (bg=11.59%) | HepG2 | + | chr11 | | 65267152 | 65267207 |
| 3.85817954948 | rbm22 (bg=12.69%) | HepG2 | + | chr11 | | 65267135 | 65267151 |
| 3.91706089456 | rbm22 (bg=12.69%) | HepG2 | + | chr11 | | 65267151 | 65267158 |
| 3.03612247821 | safb2 (bg=26.89%) | K562 | + | chr11 | | 65267138 | 65267147 |
| 2.5768865381 | safb2 (bg=26.89%) | K562 | + | chr11 | | 65267138 | 65267163 |
| 3.07834158993 | safb2 (bg=26.89%) | K562 | + | chr11 | | 65267147 | 65267155 |
| 2.80364046129 | SMNDC1 (bg=7.08%) | HepG2 | + | chr11 | | 65267085 | 65267183 |
| 3.36452843109 | srsf1 (bg=30.28%) | HepG2 | + | chr11 | | 65267130 | 65267189 |
| 5.43086995209 | srsf1 (bg=30.28%) | HepG2 | + | chr11 | | 65267134 | 65267173 |
| 3.3663111222 | srsf1 (bg=30.28%) | K562 | + | chr11 | | 65267137 | 65267152 |
| 4.76363071574 | srsf1 (bg=30.28%) | K562 | + | chr11 | | 65267142 | 65267152 |
| 4.74273156389 | srsf1 (bg=30.28%) | K562 | + | chr11 | | 65267152 | 65267157 |
| 3.3103651996 | srsf1 (bg=30.28%) | K562 | + | chr11 | | 65267152 | 65267169 |
| 2.53880004722 | srsf7 (bg=22.53%) | K562 | + | chr11 | | 65267127 | 65267152 |
| 2.76844022786 | srsf7 (bg=22.53%) | HepG2 | + | chr11 | | 65267130 | 65267192 |
| 2.70674458956 | srsf7 (bg=22.53%) | K562 | + | chr11 | | 65267139 | 65267154 |
| 2.6467563363 | srsf7 (bg=22.53%) | K562 | + | chr11 | | 65267152 | 65267173 |
| 5.39526782119 | SRSF9 (bg=9.67%) | HepG2 | + | chr11 | | 65267139 | 65267181 |
| 2.97237624979 | TAF15 (bg=9.06%) | HepG2 | + | chr11 | | 65267132 | 65267168 |
| 4.09386313971 | tra2a (bg=37.02%) | HepG2 | + | chr11 | | 65267079 | 65267209 |
| 6.28000615706 | tra2a (bg=37.02%) | K562 | + | chr11 | | 65267129 | 65267150 |
| 6.43865756135 | tra2a (bg=37.02%) | K562 | + | chr11 | | 65267134 | 65267149 |
| 6.08298073451 | tra2a (bg=37.02%) | HepG2 | + | chr11 | | 65267142 | 65267154 |
| 6.92442043859 | tra2a (bg=37.02%) | K562 | + | chr11 | | 65267149 | 65267154 |
| 6.95988401888 | tra2a (bg=37.02%) | K562 | + | chr11 | | 65267150 | 65267154 |
| 2.73705255877 | TROVE2 (bg=6.96%) | HepG2 | + | chr11 | | 65267136 | 65267153 |
| 3.83366777677 | uchl5 (bg=18.56%) | K562 | + | chr11 | | 65267135 | 65267154 |
| 4.2249241798 | uchl5 (bg=18.56%) | K562 | + | chr11 | | 65267139 | 65267154 |
| 3.58211875175 | znf622 (bg=18.79%) | K562 | + | chr11 | | 65267134 | 65267150 |
| 3.99743927522 | znf622 (bg=18.79%) | K562 | + | chr11 | | 65267140 | 65267148 |
| 3.81752577296 | znf622 (bg=18.79%) | K562 | + | chr11 | | 65267148 | 65267151 |
| 3.59275288435 | znf622 (bg=18.79%) | K562 | + | chr11 | | 65267150 | 65267158 |
| 4.08780546793 | znf622 (bg=18.79%) | K562 | + | chr11 | | 65267151 | 65267157 |

  
  

| Match 46 in HUMAN | | | | | | | |
| --- | --- | --- | --- | --- | --- | --- | --- |
| Motif | Start in Seq (1 Indexed) | End in Seq (1 Indexed) | Strand | Chrm | Exon | Start in Chrm (0 Indexed) | End in Chrm (1 Indexed) |
| GGAAGGCGAAGAAAAGAATAGAGAAGATAGGGAAATTAGAAGATAAAAA | 665 | 713 | + | chr11 | 1 | 65267154 | 65267203 |
| eCLIP Fold-Enrichment | Binding Protein | Cell Line | Strand | Chrm | | Start in Chrm (0 Indexed) | End in Chrm (1 Indexed) |
| 2.82013080799 | aggf1 (bg=15.15%) | K562 | + | chr11 | | 65267152 | 65267157 |
| 2.66011551871 | aggf1 (bg=15.15%) | K562 | + | chr11 | | 65267157 | 65267162 |
| 2.69298750344 | aggf1 (bg=15.15%) | K562 | + | chr11 | | 65267162 | 65267166 |
| 2.90269997782 | aggf1 (bg=15.15%) | K562 | + | chr11 | | 65267166 | 65267173 |
| 3.26532472149 | aggf1 (bg=15.15%) | K562 | + | chr11 | | 65267173 | 65267180 |
| 3.36580227615 | aggf1 (bg=15.15%) | K562 | + | chr11 | | 65267180 | 65267188 |
| 3.32488965653 | aggf1 (bg=15.15%) | K562 | + | chr11 | | 65267188 | 65267196 |
| 3.5214229532 | aggf1 (bg=15.15%) | K562 | + | chr11 | | 65267196 | 65267205 |
| 2.12706856102 | aggf1 (bg=15.15%) | HepG2 | + | chr11 | | 65267197 | 65267210 |
| 2.14875654764 | AQR (bg=4.89%) | HepG2 | + | chr11 | | 65267153 | 65267163 |
| 2.30620985051 | AQR (bg=4.89%) | HepG2 | + | chr11 | | 65267163 | 65267182 |
| 2.53489162755 | AQR (bg=4.89%) | HepG2 | + | chr11 | | 65267182 | 65267190 |
| 2.37632917397 | AQR (bg=4.89%) | HepG2 | + | chr11 | | 65267190 | 65267208 |
| 3.51718214163 | bclaf1 (bg=17.67%) | HepG2 | + | chr11 | | 65267150 | 65267187 |
| 4.17234158954 | bclaf1 (bg=17.67%) | HepG2 | + | chr11 | | 65267153 | 65267201 |
| 4.61023663539 | bud13 (bg=12.85%) | HepG2 | + | chr11 | | 65267151 | 65267230 |
| 2.00919816575 | bud13 (bg=12.85%) | K562 | + | chr11 | | 65267162 | 65267168 |
| 2.17624704179 | bud13 (bg=12.85%) | K562 | + | chr11 | | 65267168 | 65267172 |
| 2.06544925018 | bud13 (bg=12.85%) | K562 | + | chr11 | | 65267172 | 65267178 |
| 2.05108116008 | bud13 (bg=12.85%) | K562 | + | chr11 | | 65267177 | 65267181 |
| 2.35009579939 | bud13 (bg=12.85%) | K562 | + | chr11 | | 65267178 | 65267181 |
| 2.01855607935 | bud13 (bg=12.85%) | K562 | + | chr11 | | 65267181 | 65267184 |
| 2.14141073614 | bud13 (bg=12.85%) | K562 | + | chr11 | | 65267181 | 65267185 |
| 2.39637598463 | bud13 (bg=12.85%) | K562 | + | chr11 | | 65267184 | 65267189 |
| 2.52249916312 | bud13 (bg=12.85%) | K562 | + | chr11 | | 65267185 | 65267189 |
| 2.54284106858 | bud13 (bg=12.85%) | K562 | + | chr11 | | 65267189 | 65267194 |
| 2.25375571985 | bud13 (bg=12.85%) | K562 | + | chr11 | | 65267189 | 65267207 |
| 2.45565257043 | bud13 (bg=12.85%) | K562 | + | chr11 | | 65267194 | 65267207 |
| 2.4802153632 | cpsf6 (bg=13.45%) | K562 | + | chr11 | | 65267126 | 65267174 |
| 3.27752893622 | cpsf6 (bg=13.45%) | K562 | + | chr11 | | 65267153 | 65267163 |
| 3.18432908594 | cpsf6 (bg=13.45%) | K562 | + | chr11 | | 65267163 | 65267168 |
| 3.2117438941 | cpsf6 (bg=13.45%) | K562 | + | chr11 | | 65267168 | 65267173 |
| 3.22101405695 | cpsf6 (bg=13.45%) | K562 | + | chr11 | | 65267173 | 65267207 |
| 2.34142302678 | cpsf6 (bg=13.45%) | K562 | + | chr11 | | 65267174 | 65267192 |
| 2.67185387121 | cpsf6 (bg=13.45%) | K562 | + | chr11 | | 65267192 | 65267206 |
| 2.13537833515 | FASTKD2 (bg=4.54%) | K562 | + | chr11 | | 65267153 | 65267173 |
| 2.33306712821 | FASTKD2 (bg=4.54%) | K562 | + | chr11 | | 65267173 | 65267207 |
| 2.02916249379 | fxr2 (bg=10.1%) | HepG2 | + | chr11 | | 65267091 | 65267220 |
| 2.33299625231 | GPKOW (bg=5.66%) | K562 | + | chr11 | | 65267137 | 65267203 |
| 2.2345958442 | GPKOW (bg=5.66%) | K562 | + | chr11 | | 65267153 | 65267204 |
| 3.57434857647 | gtf2f1 (bg=10.18%) | HepG2 | + | chr11 | | 65267123 | 65267170 |
| 2.01675141196 | hltf (bg=24.28%) | HepG2 | + | chr11 | | 65267121 | 65267176 |
| 2.60579158266 | larp4 (bg=13.51%) | K562 | + | chr11 | | 65267152 | 65267157 |
| 2.70044902774 | larp4 (bg=13.51%) | K562 | + | chr11 | | 65267157 | 65267168 |
| 2.93798406713 | larp4 (bg=13.51%) | K562 | + | chr11 | | 65267168 | 65267173 |
| 2.98306549147 | larp4 (bg=13.51%) | K562 | + | chr11 | | 65267173 | 65267206 |
| 3.08038416895 | LARP7 (bg=2.17%) | HepG2 | + | chr11 | | 65267085 | 65267192 |
| 2.1199614677 | MTPAP (bg=9.55%) | K562 | + | chr11 | | 65267148 | 65267171 |
| 2.04479443816 | rbm15 (bg=11.59%) | HepG2 | + | chr11 | | 65267152 | 65267207 |
| 3.91706089456 | rbm22 (bg=12.69%) | HepG2 | + | chr11 | | 65267151 | 65267158 |
| 4.14960176189 | rbm22 (bg=12.69%) | HepG2 | + | chr11 | | 65267158 | 65267164 |
| 4.41530863057 | rbm22 (bg=12.69%) | HepG2 | + | chr11 | | 65267164 | 65267201 |
| 2.5768865381 | safb2 (bg=26.89%) | K562 | + | chr11 | | 65267138 | 65267163 |
| 3.07834158993 | safb2 (bg=26.89%) | K562 | + | chr11 | | 65267147 | 65267155 |
| 3.12341563253 | safb2 (bg=26.89%) | K562 | + | chr11 | | 65267155 | 65267168 |
| 2.51218699521 | safb2 (bg=26.89%) | K562 | + | chr11 | | 65267163 | 65267168 |
| 3.22851454004 | safb2 (bg=26.89%) | K562 | + | chr11 | | 65267168 | 65267172 |
| 2.54623196314 | safb2 (bg=26.89%) | K562 | + | chr11 | | 65267168 | 65267172 |
| 2.95151582931 | safb2 (bg=26.89%) | K562 | + | chr11 | | 65267172 | 65267175 |
| 2.29450349779 | safb2 (bg=26.89%) | K562 | + | chr11 | | 65267172 | 65267178 |
| 3.25795321816 | safb2 (bg=26.89%) | K562 | + | chr11 | | 65267175 | 65267178 |
| 3.33000233193 | safb2 (bg=26.89%) | K562 | + | chr11 | | 65267178 | 65267181 |
| 2.61679611215 | safb2 (bg=26.89%) | K562 | + | chr11 | | 65267178 | 65267181 |
| 2.4648847165 | safb2 (bg=26.89%) | K562 | + | chr11 | | 65267181 | 65267184 |
| 3.1934646472 | safb2 (bg=26.89%) | K562 | + | chr11 | | 65267181 | 65267185 |
| 2.68324038165 | safb2 (bg=26.89%) | K562 | + | chr11 | | 65267184 | 65267190 |
| 3.33532797438 | safb2 (bg=26.89%) | K562 | + | chr11 | | 65267185 | 65267189 |
| 2.68375367786 | safb2 (bg=26.89%) | K562 | + | chr11 | | 65267190 | 65267193 |
| 3.47555134425 | safb2 (bg=26.89%) | K562 | + | chr11 | | 65267191 | 65267192 |
| 2.80364046129 | SMNDC1 (bg=7.08%) | HepG2 | + | chr11 | | 65267085 | 65267183 |
| 3.36452843109 | srsf1 (bg=30.28%) | HepG2 | + | chr11 | | 65267130 | 65267189 |
| 5.43086995209 | srsf1 (bg=30.28%) | HepG2 | + | chr11 | | 65267134 | 65267173 |
| 4.74273156389 | srsf1 (bg=30.28%) | K562 | + | chr11 | | 65267152 | 65267157 |
| 3.3103651996 | srsf1 (bg=30.28%) | K562 | + | chr11 | | 65267152 | 65267169 |
| 4.71095324197 | srsf1 (bg=30.28%) | K562 | + | chr11 | | 65267157 | 65267163 |
| 4.76163611423 | srsf1 (bg=30.28%) | K562 | + | chr11 | | 65267163 | 65267168 |
| 4.80001421164 | srsf1 (bg=30.28%) | K562 | + | chr11 | | 65267168 | 65267172 |
| 3.45707658612 | srsf1 (bg=30.28%) | K562 | + | chr11 | | 65267169 | 65267173 |
| 4.66294151906 | srsf1 (bg=30.28%) | K562 | + | chr11 | | 65267172 | 65267178 |
| 5.2322211408 | srsf1 (bg=30.28%) | HepG2 | + | chr11 | | 65267173 | 65267178 |
| 3.4465744617 | srsf1 (bg=30.28%) | K562 | + | chr11 | | 65267173 | 65267178 |
| 4.74757862559 | srsf1 (bg=30.28%) | K562 | + | chr11 | | 65267178 | 65267182 |
| 5.02697301041 | srsf1 (bg=30.28%) | HepG2 | + | chr11 | | 65267178 | 65267191 |
| 3.45752179367 | srsf1 (bg=30.28%) | K562 | + | chr11 | | 65267178 | 65267198 |
| 4.19861786928 | srsf1 (bg=30.28%) | K562 | + | chr11 | | 65267182 | 65267184 |
| 4.36174154544 | srsf1 (bg=30.28%) | K562 | + | chr11 | | 65267184 | 65267190 |
| 4.42959293857 | srsf1 (bg=30.28%) | K562 | + | chr11 | | 65267190 | 65267194 |
| 4.32252022977 | srsf1 (bg=30.28%) | HepG2 | + | chr11 | | 65267191 | 65267194 |
| 4.38936203687 | srsf1 (bg=30.28%) | K562 | + | chr11 | | 65267194 | 65267207 |
| 2.76844022786 | srsf7 (bg=22.53%) | HepG2 | + | chr11 | | 65267130 | 65267192 |
| 2.70674458956 | srsf7 (bg=22.53%) | K562 | + | chr11 | | 65267139 | 65267154 |
| 2.6467563363 | srsf7 (bg=22.53%) | K562 | + | chr11 | | 65267152 | 65267173 |
| 2.62319334591 | srsf7 (bg=22.53%) | K562 | + | chr11 | | 65267154 | 65267174 |
| 2.66286052306 | srsf7 (bg=22.53%) | K562 | + | chr11 | | 65267173 | 65267206 |
| 2.55461100043 | srsf7 (bg=22.53%) | K562 | + | chr11 | | 65267174 | 65267197 |
| 5.39526782119 | SRSF9 (bg=9.67%) | HepG2 | + | chr11 | | 65267139 | 65267181 |
| 5.2733011015 | SRSF9 (bg=9.67%) | HepG2 | + | chr11 | | 65267181 | 65267190 |
| 2.97237624979 | TAF15 (bg=9.06%) | HepG2 | + | chr11 | | 65267132 | 65267168 |
| 4.09386313971 | tra2a (bg=37.02%) | HepG2 | + | chr11 | | 65267079 | 65267209 |
| 6.08298073451 | tra2a (bg=37.02%) | HepG2 | + | chr11 | | 65267142 | 65267154 |
| 6.92442043859 | tra2a (bg=37.02%) | K562 | + | chr11 | | 65267149 | 65267154 |
| 6.95988401888 | tra2a (bg=37.02%) | K562 | + | chr11 | | 65267150 | 65267154 |
| 7.16957558308 | tra2a (bg=37.02%) | K562 | + | chr11 | | 65267154 | 65267157 |
| 7.13408137618 | tra2a (bg=37.02%) | K562 | + | chr11 | | 65267154 | 65267158 |
| 6.04780609921 | tra2a (bg=37.02%) | HepG2 | + | chr11 | | 65267154 | 65267168 |
| 7.15723524745 | tra2a (bg=37.02%) | K562 | + | chr11 | | 65267157 | 65267163 |
| 7.12054260497 | tra2a (bg=37.02%) | K562 | + | chr11 | | 65267158 | 65267168 |
| 7.18075320027 | tra2a (bg=37.02%) | K562 | + | chr11 | | 65267163 | 65267172 |
| 6.17187202049 | tra2a (bg=37.02%) | HepG2 | + | chr11 | | 65267168 | 65267172 |
| 7.23720356262 | tra2a (bg=37.02%) | K562 | + | chr11 | | 65267168 | 65267172 |
| 7.09181012186 | tra2a (bg=37.02%) | K562 | + | chr11 | | 65267172 | 65267175 |
| 7.14169255077 | tra2a (bg=37.02%) | K562 | + | chr11 | | 65267172 | 65267175 |
| 6.24230451441 | tra2a (bg=37.02%) | HepG2 | + | chr11 | | 65267172 | 65267178 |
| 7.38302340368 | tra2a (bg=37.02%) | K562 | + | chr11 | | 65267175 | 65267178 |
| 7.44118894466 | tra2a (bg=37.02%) | K562 | + | chr11 | | 65267175 | 65267178 |
| 7.50679357747 | tra2a (bg=37.02%) | K562 | + | chr11 | | 65267178 | 65267181 |
| 7.54545995788 | tra2a (bg=37.02%) | K562 | + | chr11 | | 65267178 | 65267181 |
| 6.23414480785 | tra2a (bg=37.02%) | HepG2 | + | chr11 | | 65267178 | 65267210 |
| 7.20626805263 | tra2a (bg=37.02%) | K562 | + | chr11 | | 65267181 | 65267184 |
| 7.29605590973 | tra2a (bg=37.02%) | K562 | + | chr11 | | 65267181 | 65267184 |
| 7.24116118183 | tra2a (bg=37.02%) | K562 | + | chr11 | | 65267184 | 65267190 |
| 7.40163267176 | tra2a (bg=37.02%) | K562 | + | chr11 | | 65267184 | 65267190 |
| 6.71133432229 | tra2a (bg=37.02%) | K562 | + | chr11 | | 65267190 | 65267194 |
| 6.97960472107 | tra2a (bg=37.02%) | K562 | + | chr11 | | 65267190 | 65267194 |
| 6.77215935808 | tra2a (bg=37.02%) | K562 | + | chr11 | | 65267194 | 65267197 |
| 6.61612681024 | tra2a (bg=37.02%) | K562 | + | chr11 | | 65267194 | 65267210 |
| 6.64935758673 | tra2a (bg=37.02%) | K562 | + | chr11 | | 65267197 | 65267209 |
| 2.80392272638 | TROVE2 (bg=6.96%) | HepG2 | + | chr11 | | 65267153 | 65267176 |
| 2.97094061895 | TROVE2 (bg=6.96%) | HepG2 | + | chr11 | | 65267176 | 65267209 |
| 3.83366777677 | uchl5 (bg=18.56%) | K562 | + | chr11 | | 65267135 | 65267154 |
| 4.2249241798 | uchl5 (bg=18.56%) | K562 | + | chr11 | | 65267139 | 65267154 |
| 3.93930758704 | uchl5 (bg=18.56%) | K562 | + | chr11 | | 65267154 | 65267163 |
| 4.34485496168 | uchl5 (bg=18.56%) | K562 | + | chr11 | | 65267154 | 65267169 |
| 4.01672965706 | uchl5 (bg=18.56%) | K562 | + | chr11 | | 65267163 | 65267168 |
| 4.10122641452 | uchl5 (bg=18.56%) | K562 | + | chr11 | | 65267168 | 65267173 |
| 4.49787034767 | uchl5 (bg=18.56%) | K562 | + | chr11 | | 65267169 | 65267172 |
| 4.36602033291 | uchl5 (bg=18.56%) | K562 | + | chr11 | | 65267172 | 65267178 |
| 4.13347306076 | uchl5 (bg=18.56%) | K562 | + | chr11 | | 65267173 | 65267178 |
| 3.96566129038 | uchl5 (bg=18.56%) | K562 | + | chr11 | | 65267178 | 65267181 |
| 4.46191697828 | uchl5 (bg=18.56%) | K562 | + | chr11 | | 65267178 | 65267182 |
| 3.67186407461 | uchl5 (bg=18.56%) | K562 | + | chr11 | | 65267181 | 65267185 |
| 4.34374902445 | uchl5 (bg=18.56%) | K562 | + | chr11 | | 65267182 | 65267189 |
| 3.59275288435 | znf622 (bg=18.79%) | K562 | + | chr11 | | 65267150 | 65267158 |
| 4.08780546793 | znf622 (bg=18.79%) | K562 | + | chr11 | | 65267151 | 65267157 |
| 4.34304093066 | znf622 (bg=18.79%) | K562 | + | chr11 | | 65267157 | 65267172 |
| 3.86844063817 | znf622 (bg=18.79%) | K562 | + | chr11 | | 65267158 | 65267163 |
| 3.65169100373 | znf622 (bg=18.79%) | K562 | + | chr11 | | 65267163 | 65267186 |
| 4.20048467175 | znf622 (bg=18.79%) | K562 | + | chr11 | | 65267172 | 65267189 |

  
  

| Match 47 in HUMAN | | | | | | | |
| --- | --- | --- | --- | --- | --- | --- | --- |
| Motif | Start in Seq (1 Indexed) | End in Seq (1 Indexed) | Strand | Chrm | Exon | Start in Chrm (0 Indexed) | End in Chrm (1 Indexed) |
| GAAGAAAAGA | 672 | 681 | + | chr11 | 1 | 65267161 | 65267171 |
| eCLIP Fold-Enrichment | Binding Protein | Cell Line | Strand | Chrm | | Start in Chrm (0 Indexed) | End in Chrm (1 Indexed) |
| 2.66011551871 | aggf1 (bg=15.15%) | K562 | + | chr11 | | 65267157 | 65267162 |
| 2.69298750344 | aggf1 (bg=15.15%) | K562 | + | chr11 | | 65267162 | 65267166 |
| 2.90269997782 | aggf1 (bg=15.15%) | K562 | + | chr11 | | 65267166 | 65267173 |
| 2.14875654764 | AQR (bg=4.89%) | HepG2 | + | chr11 | | 65267153 | 65267163 |
| 2.30620985051 | AQR (bg=4.89%) | HepG2 | + | chr11 | | 65267163 | 65267182 |
| 3.51718214163 | bclaf1 (bg=17.67%) | HepG2 | + | chr11 | | 65267150 | 65267187 |
| 4.17234158954 | bclaf1 (bg=17.67%) | HepG2 | + | chr11 | | 65267153 | 65267201 |
| 4.61023663539 | bud13 (bg=12.85%) | HepG2 | + | chr11 | | 65267151 | 65267230 |
| 2.00919816575 | bud13 (bg=12.85%) | K562 | + | chr11 | | 65267162 | 65267168 |
| 2.17624704179 | bud13 (bg=12.85%) | K562 | + | chr11 | | 65267168 | 65267172 |
| 2.4802153632 | cpsf6 (bg=13.45%) | K562 | + | chr11 | | 65267126 | 65267174 |
| 3.27752893622 | cpsf6 (bg=13.45%) | K562 | + | chr11 | | 65267153 | 65267163 |
| 3.18432908594 | cpsf6 (bg=13.45%) | K562 | + | chr11 | | 65267163 | 65267168 |
| 3.2117438941 | cpsf6 (bg=13.45%) | K562 | + | chr11 | | 65267168 | 65267173 |
| 2.13537833515 | FASTKD2 (bg=4.54%) | K562 | + | chr11 | | 65267153 | 65267173 |
| 2.02916249379 | fxr2 (bg=10.1%) | HepG2 | + | chr11 | | 65267091 | 65267220 |
| 2.33299625231 | GPKOW (bg=5.66%) | K562 | + | chr11 | | 65267137 | 65267203 |
| 2.2345958442 | GPKOW (bg=5.66%) | K562 | + | chr11 | | 65267153 | 65267204 |
| 3.57434857647 | gtf2f1 (bg=10.18%) | HepG2 | + | chr11 | | 65267123 | 65267170 |
| 2.01675141196 | hltf (bg=24.28%) | HepG2 | + | chr11 | | 65267121 | 65267176 |
| 2.70044902774 | larp4 (bg=13.51%) | K562 | + | chr11 | | 65267157 | 65267168 |
| 2.93798406713 | larp4 (bg=13.51%) | K562 | + | chr11 | | 65267168 | 65267173 |
| 3.08038416895 | LARP7 (bg=2.17%) | HepG2 | + | chr11 | | 65267085 | 65267192 |
| 2.1199614677 | MTPAP (bg=9.55%) | K562 | + | chr11 | | 65267148 | 65267171 |
| 2.04479443816 | rbm15 (bg=11.59%) | HepG2 | + | chr11 | | 65267152 | 65267207 |
| 4.14960176189 | rbm22 (bg=12.69%) | HepG2 | + | chr11 | | 65267158 | 65267164 |
| 4.41530863057 | rbm22 (bg=12.69%) | HepG2 | + | chr11 | | 65267164 | 65267201 |
| 2.5768865381 | safb2 (bg=26.89%) | K562 | + | chr11 | | 65267138 | 65267163 |
| 3.12341563253 | safb2 (bg=26.89%) | K562 | + | chr11 | | 65267155 | 65267168 |
| 2.51218699521 | safb2 (bg=26.89%) | K562 | + | chr11 | | 65267163 | 65267168 |
| 3.22851454004 | safb2 (bg=26.89%) | K562 | + | chr11 | | 65267168 | 65267172 |
| 2.54623196314 | safb2 (bg=26.89%) | K562 | + | chr11 | | 65267168 | 65267172 |
| 2.80364046129 | SMNDC1 (bg=7.08%) | HepG2 | + | chr11 | | 65267085 | 65267183 |
| 3.36452843109 | srsf1 (bg=30.28%) | HepG2 | + | chr11 | | 65267130 | 65267189 |
| 5.43086995209 | srsf1 (bg=30.28%) | HepG2 | + | chr11 | | 65267134 | 65267173 |
| 3.3103651996 | srsf1 (bg=30.28%) | K562 | + | chr11 | | 65267152 | 65267169 |
| 4.71095324197 | srsf1 (bg=30.28%) | K562 | + | chr11 | | 65267157 | 65267163 |
| 4.76163611423 | srsf1 (bg=30.28%) | K562 | + | chr11 | | 65267163 | 65267168 |
| 4.80001421164 | srsf1 (bg=30.28%) | K562 | + | chr11 | | 65267168 | 65267172 |
| 3.45707658612 | srsf1 (bg=30.28%) | K562 | + | chr11 | | 65267169 | 65267173 |
| 2.76844022786 | srsf7 (bg=22.53%) | HepG2 | + | chr11 | | 65267130 | 65267192 |
| 2.6467563363 | srsf7 (bg=22.53%) | K562 | + | chr11 | | 65267152 | 65267173 |
| 2.62319334591 | srsf7 (bg=22.53%) | K562 | + | chr11 | | 65267154 | 65267174 |
| 5.39526782119 | SRSF9 (bg=9.67%) | HepG2 | + | chr11 | | 65267139 | 65267181 |
| 2.97237624979 | TAF15 (bg=9.06%) | HepG2 | + | chr11 | | 65267132 | 65267168 |
| 4.09386313971 | tra2a (bg=37.02%) | HepG2 | + | chr11 | | 65267079 | 65267209 |
| 6.04780609921 | tra2a (bg=37.02%) | HepG2 | + | chr11 | | 65267154 | 65267168 |
| 7.15723524745 | tra2a (bg=37.02%) | K562 | + | chr11 | | 65267157 | 65267163 |
| 7.12054260497 | tra2a (bg=37.02%) | K562 | + | chr11 | | 65267158 | 65267168 |
| 7.18075320027 | tra2a (bg=37.02%) | K562 | + | chr11 | | 65267163 | 65267172 |
| 6.17187202049 | tra2a (bg=37.02%) | HepG2 | + | chr11 | | 65267168 | 65267172 |
| 7.23720356262 | tra2a (bg=37.02%) | K562 | + | chr11 | | 65267168 | 65267172 |
| 2.80392272638 | TROVE2 (bg=6.96%) | HepG2 | + | chr11 | | 65267153 | 65267176 |
| 3.93930758704 | uchl5 (bg=18.56%) | K562 | + | chr11 | | 65267154 | 65267163 |
| 4.34485496168 | uchl5 (bg=18.56%) | K562 | + | chr11 | | 65267154 | 65267169 |
| 4.01672965706 | uchl5 (bg=18.56%) | K562 | + | chr11 | | 65267163 | 65267168 |
| 4.10122641452 | uchl5 (bg=18.56%) | K562 | + | chr11 | | 65267168 | 65267173 |
| 4.49787034767 | uchl5 (bg=18.56%) | K562 | + | chr11 | | 65267169 | 65267172 |
| 4.34304093066 | znf622 (bg=18.79%) | K562 | + | chr11 | | 65267157 | 65267172 |
| 3.86844063817 | znf622 (bg=18.79%) | K562 | + | chr11 | | 65267158 | 65267163 |
| 3.65169100373 | znf622 (bg=18.79%) | K562 | + | chr11 | | 65267163 | 65267186 |

  
  

| Match 48 in HUMAN | | | | | | | |
| --- | --- | --- | --- | --- | --- | --- | --- |
| Motif | Start in Seq (1 Indexed) | End in Seq (1 Indexed) | Strand | Chrm | Exon | Start in Chrm (0 Indexed) | End in Chrm (1 Indexed) |
| TAGAGAAGATAGG | 683 | 695 | + | chr11 | 1 | 65267172 | 65267185 |
| eCLIP Fold-Enrichment | Binding Protein | Cell Line | Strand | Chrm | | Start in Chrm (0 Indexed) | End in Chrm (1 Indexed) |
| 2.90269997782 | aggf1 (bg=15.15%) | K562 | + | chr11 | | 65267166 | 65267173 |
| 3.26532472149 | aggf1 (bg=15.15%) | K562 | + | chr11 | | 65267173 | 65267180 |
| 3.36580227615 | aggf1 (bg=15.15%) | K562 | + | chr11 | | 65267180 | 65267188 |
| 2.30620985051 | AQR (bg=4.89%) | HepG2 | + | chr11 | | 65267163 | 65267182 |
| 2.53489162755 | AQR (bg=4.89%) | HepG2 | + | chr11 | | 65267182 | 65267190 |
| 3.51718214163 | bclaf1 (bg=17.67%) | HepG2 | + | chr11 | | 65267150 | 65267187 |
| 4.17234158954 | bclaf1 (bg=17.67%) | HepG2 | + | chr11 | | 65267153 | 65267201 |
| 4.61023663539 | bud13 (bg=12.85%) | HepG2 | + | chr11 | | 65267151 | 65267230 |
| 2.17624704179 | bud13 (bg=12.85%) | K562 | + | chr11 | | 65267168 | 65267172 |
| 2.06544925018 | bud13 (bg=12.85%) | K562 | + | chr11 | | 65267172 | 65267178 |
| 2.05108116008 | bud13 (bg=12.85%) | K562 | + | chr11 | | 65267177 | 65267181 |
| 2.35009579939 | bud13 (bg=12.85%) | K562 | + | chr11 | | 65267178 | 65267181 |
| 2.01855607935 | bud13 (bg=12.85%) | K562 | + | chr11 | | 65267181 | 65267184 |
| 2.14141073614 | bud13 (bg=12.85%) | K562 | + | chr11 | | 65267181 | 65267185 |
| 2.39637598463 | bud13 (bg=12.85%) | K562 | + | chr11 | | 65267184 | 65267189 |
| 2.52249916312 | bud13 (bg=12.85%) | K562 | + | chr11 | | 65267185 | 65267189 |
| 2.4802153632 | cpsf6 (bg=13.45%) | K562 | + | chr11 | | 65267126 | 65267174 |
| 3.2117438941 | cpsf6 (bg=13.45%) | K562 | + | chr11 | | 65267168 | 65267173 |
| 3.22101405695 | cpsf6 (bg=13.45%) | K562 | + | chr11 | | 65267173 | 65267207 |
| 2.34142302678 | cpsf6 (bg=13.45%) | K562 | + | chr11 | | 65267174 | 65267192 |
| 2.13537833515 | FASTKD2 (bg=4.54%) | K562 | + | chr11 | | 65267153 | 65267173 |
| 2.33306712821 | FASTKD2 (bg=4.54%) | K562 | + | chr11 | | 65267173 | 65267207 |
| 2.02916249379 | fxr2 (bg=10.1%) | HepG2 | + | chr11 | | 65267091 | 65267220 |
| 2.33299625231 | GPKOW (bg=5.66%) | K562 | + | chr11 | | 65267137 | 65267203 |
| 2.2345958442 | GPKOW (bg=5.66%) | K562 | + | chr11 | | 65267153 | 65267204 |
| 2.01675141196 | hltf (bg=24.28%) | HepG2 | + | chr11 | | 65267121 | 65267176 |
| 2.93798406713 | larp4 (bg=13.51%) | K562 | + | chr11 | | 65267168 | 65267173 |
| 2.98306549147 | larp4 (bg=13.51%) | K562 | + | chr11 | | 65267173 | 65267206 |
| 3.08038416895 | LARP7 (bg=2.17%) | HepG2 | + | chr11 | | 65267085 | 65267192 |
| 2.04479443816 | rbm15 (bg=11.59%) | HepG2 | + | chr11 | | 65267152 | 65267207 |
| 4.41530863057 | rbm22 (bg=12.69%) | HepG2 | + | chr11 | | 65267164 | 65267201 |
| 3.22851454004 | safb2 (bg=26.89%) | K562 | + | chr11 | | 65267168 | 65267172 |
| 2.54623196314 | safb2 (bg=26.89%) | K562 | + | chr11 | | 65267168 | 65267172 |
| 2.95151582931 | safb2 (bg=26.89%) | K562 | + | chr11 | | 65267172 | 65267175 |
| 2.29450349779 | safb2 (bg=26.89%) | K562 | + | chr11 | | 65267172 | 65267178 |
| 3.25795321816 | safb2 (bg=26.89%) | K562 | + | chr11 | | 65267175 | 65267178 |
| 3.33000233193 | safb2 (bg=26.89%) | K562 | + | chr11 | | 65267178 | 65267181 |
| 2.61679611215 | safb2 (bg=26.89%) | K562 | + | chr11 | | 65267178 | 65267181 |
| 2.4648847165 | safb2 (bg=26.89%) | K562 | + | chr11 | | 65267181 | 65267184 |
| 3.1934646472 | safb2 (bg=26.89%) | K562 | + | chr11 | | 65267181 | 65267185 |
| 2.68324038165 | safb2 (bg=26.89%) | K562 | + | chr11 | | 65267184 | 65267190 |
| 3.33532797438 | safb2 (bg=26.89%) | K562 | + | chr11 | | 65267185 | 65267189 |
| 2.80364046129 | SMNDC1 (bg=7.08%) | HepG2 | + | chr11 | | 65267085 | 65267183 |
| 3.36452843109 | srsf1 (bg=30.28%) | HepG2 | + | chr11 | | 65267130 | 65267189 |
| 5.43086995209 | srsf1 (bg=30.28%) | HepG2 | + | chr11 | | 65267134 | 65267173 |
| 4.80001421164 | srsf1 (bg=30.28%) | K562 | + | chr11 | | 65267168 | 65267172 |
| 3.45707658612 | srsf1 (bg=30.28%) | K562 | + | chr11 | | 65267169 | 65267173 |
| 4.66294151906 | srsf1 (bg=30.28%) | K562 | + | chr11 | | 65267172 | 65267178 |
| 5.2322211408 | srsf1 (bg=30.28%) | HepG2 | + | chr11 | | 65267173 | 65267178 |
| 3.4465744617 | srsf1 (bg=30.28%) | K562 | + | chr11 | | 65267173 | 65267178 |
| 4.74757862559 | srsf1 (bg=30.28%) | K562 | + | chr11 | | 65267178 | 65267182 |
| 5.02697301041 | srsf1 (bg=30.28%) | HepG2 | + | chr11 | | 65267178 | 65267191 |
| 3.45752179367 | srsf1 (bg=30.28%) | K562 | + | chr11 | | 65267178 | 65267198 |
| 4.19861786928 | srsf1 (bg=30.28%) | K562 | + | chr11 | | 65267182 | 65267184 |
| 4.36174154544 | srsf1 (bg=30.28%) | K562 | + | chr11 | | 65267184 | 65267190 |
| 2.76844022786 | srsf7 (bg=22.53%) | HepG2 | + | chr11 | | 65267130 | 65267192 |
| 2.6467563363 | srsf7 (bg=22.53%) | K562 | + | chr11 | | 65267152 | 65267173 |
| 2.62319334591 | srsf7 (bg=22.53%) | K562 | + | chr11 | | 65267154 | 65267174 |
| 2.66286052306 | srsf7 (bg=22.53%) | K562 | + | chr11 | | 65267173 | 65267206 |
| 2.55461100043 | srsf7 (bg=22.53%) | K562 | + | chr11 | | 65267174 | 65267197 |
| 5.39526782119 | SRSF9 (bg=9.67%) | HepG2 | + | chr11 | | 65267139 | 65267181 |
| 5.2733011015 | SRSF9 (bg=9.67%) | HepG2 | + | chr11 | | 65267181 | 65267190 |
| 4.09386313971 | tra2a (bg=37.02%) | HepG2 | + | chr11 | | 65267079 | 65267209 |
| 7.18075320027 | tra2a (bg=37.02%) | K562 | + | chr11 | | 65267163 | 65267172 |
| 6.17187202049 | tra2a (bg=37.02%) | HepG2 | + | chr11 | | 65267168 | 65267172 |
| 7.23720356262 | tra2a (bg=37.02%) | K562 | + | chr11 | | 65267168 | 65267172 |
| 7.09181012186 | tra2a (bg=37.02%) | K562 | + | chr11 | | 65267172 | 65267175 |
| 7.14169255077 | tra2a (bg=37.02%) | K562 | + | chr11 | | 65267172 | 65267175 |
| 6.24230451441 | tra2a (bg=37.02%) | HepG2 | + | chr11 | | 65267172 | 65267178 |
| 7.38302340368 | tra2a (bg=37.02%) | K562 | + | chr11 | | 65267175 | 65267178 |
| 7.44118894466 | tra2a (bg=37.02%) | K562 | + | chr11 | | 65267175 | 65267178 |
| 7.50679357747 | tra2a (bg=37.02%) | K562 | + | chr11 | | 65267178 | 65267181 |
| 7.54545995788 | tra2a (bg=37.02%) | K562 | + | chr11 | | 65267178 | 65267181 |
| 6.23414480785 | tra2a (bg=37.02%) | HepG2 | + | chr11 | | 65267178 | 65267210 |
| 7.20626805263 | tra2a (bg=37.02%) | K562 | + | chr11 | | 65267181 | 65267184 |
| 7.29605590973 | tra2a (bg=37.02%) | K562 | + | chr11 | | 65267181 | 65267184 |
| 7.24116118183 | tra2a (bg=37.02%) | K562 | + | chr11 | | 65267184 | 65267190 |
| 7.40163267176 | tra2a (bg=37.02%) | K562 | + | chr11 | | 65267184 | 65267190 |
| 2.80392272638 | TROVE2 (bg=6.96%) | HepG2 | + | chr11 | | 65267153 | 65267176 |
| 2.97094061895 | TROVE2 (bg=6.96%) | HepG2 | + | chr11 | | 65267176 | 65267209 |
| 4.10122641452 | uchl5 (bg=18.56%) | K562 | + | chr11 | | 65267168 | 65267173 |
| 4.49787034767 | uchl5 (bg=18.56%) | K562 | + | chr11 | | 65267169 | 65267172 |
| 4.36602033291 | uchl5 (bg=18.56%) | K562 | + | chr11 | | 65267172 | 65267178 |
| 4.13347306076 | uchl5 (bg=18.56%) | K562 | + | chr11 | | 65267173 | 65267178 |
| 3.96566129038 | uchl5 (bg=18.56%) | K562 | + | chr11 | | 65267178 | 65267181 |
| 4.46191697828 | uchl5 (bg=18.56%) | K562 | + | chr11 | | 65267178 | 65267182 |
| 3.67186407461 | uchl5 (bg=18.56%) | K562 | + | chr11 | | 65267181 | 65267185 |
| 4.34374902445 | uchl5 (bg=18.56%) | K562 | + | chr11 | | 65267182 | 65267189 |
| 4.34304093066 | znf622 (bg=18.79%) | K562 | + | chr11 | | 65267157 | 65267172 |
| 3.65169100373 | znf622 (bg=18.79%) | K562 | + | chr11 | | 65267163 | 65267186 |
| 4.20048467175 | znf622 (bg=18.79%) | K562 | + | chr11 | | 65267172 | 65267189 |

  
  

| Match 49 in HUMAN | | | | | | | |
| --- | --- | --- | --- | --- | --- | --- | --- |
| Motif | Start in Seq (1 Indexed) | End in Seq (1 Indexed) | Strand | Chrm | Exon | Start in Chrm (0 Indexed) | End in Chrm (1 Indexed) |
| AAGATAGG | 688 | 695 | + | chr11 | 1 | 65267177 | 65267185 |
| eCLIP Fold-Enrichment | Binding Protein | Cell Line | Strand | Chrm | | Start in Chrm (0 Indexed) | End in Chrm (1 Indexed) |
| 3.26532472149 | aggf1 (bg=15.15%) | K562 | + | chr11 | | 65267173 | 65267180 |
| 3.36580227615 | aggf1 (bg=15.15%) | K562 | + | chr11 | | 65267180 | 65267188 |
| 2.30620985051 | AQR (bg=4.89%) | HepG2 | + | chr11 | | 65267163 | 65267182 |
| 2.53489162755 | AQR (bg=4.89%) | HepG2 | + | chr11 | | 65267182 | 65267190 |
| 3.51718214163 | bclaf1 (bg=17.67%) | HepG2 | + | chr11 | | 65267150 | 65267187 |
| 4.17234158954 | bclaf1 (bg=17.67%) | HepG2 | + | chr11 | | 65267153 | 65267201 |
| 4.61023663539 | bud13 (bg=12.85%) | HepG2 | + | chr11 | | 65267151 | 65267230 |
| 2.06544925018 | bud13 (bg=12.85%) | K562 | + | chr11 | | 65267172 | 65267178 |
| 2.05108116008 | bud13 (bg=12.85%) | K562 | + | chr11 | | 65267177 | 65267181 |
| 2.35009579939 | bud13 (bg=12.85%) | K562 | + | chr11 | | 65267178 | 65267181 |
| 2.01855607935 | bud13 (bg=12.85%) | K562 | + | chr11 | | 65267181 | 65267184 |
| 2.14141073614 | bud13 (bg=12.85%) | K562 | + | chr11 | | 65267181 | 65267185 |
| 2.39637598463 | bud13 (bg=12.85%) | K562 | + | chr11 | | 65267184 | 65267189 |
| 2.52249916312 | bud13 (bg=12.85%) | K562 | + | chr11 | | 65267185 | 65267189 |
| 3.22101405695 | cpsf6 (bg=13.45%) | K562 | + | chr11 | | 65267173 | 65267207 |
| 2.34142302678 | cpsf6 (bg=13.45%) | K562 | + | chr11 | | 65267174 | 65267192 |
| 2.33306712821 | FASTKD2 (bg=4.54%) | K562 | + | chr11 | | 65267173 | 65267207 |
| 2.02916249379 | fxr2 (bg=10.1%) | HepG2 | + | chr11 | | 65267091 | 65267220 |
| 2.33299625231 | GPKOW (bg=5.66%) | K562 | + | chr11 | | 65267137 | 65267203 |
| 2.2345958442 | GPKOW (bg=5.66%) | K562 | + | chr11 | | 65267153 | 65267204 |
| 2.98306549147 | larp4 (bg=13.51%) | K562 | + | chr11 | | 65267173 | 65267206 |
| 3.08038416895 | LARP7 (bg=2.17%) | HepG2 | + | chr11 | | 65267085 | 65267192 |
| 2.04479443816 | rbm15 (bg=11.59%) | HepG2 | + | chr11 | | 65267152 | 65267207 |
| 4.41530863057 | rbm22 (bg=12.69%) | HepG2 | + | chr11 | | 65267164 | 65267201 |
| 2.29450349779 | safb2 (bg=26.89%) | K562 | + | chr11 | | 65267172 | 65267178 |
| 3.25795321816 | safb2 (bg=26.89%) | K562 | + | chr11 | | 65267175 | 65267178 |
| 3.33000233193 | safb2 (bg=26.89%) | K562 | + | chr11 | | 65267178 | 65267181 |
| 2.61679611215 | safb2 (bg=26.89%) | K562 | + | chr11 | | 65267178 | 65267181 |
| 2.4648847165 | safb2 (bg=26.89%) | K562 | + | chr11 | | 65267181 | 65267184 |
| 3.1934646472 | safb2 (bg=26.89%) | K562 | + | chr11 | | 65267181 | 65267185 |
| 2.68324038165 | safb2 (bg=26.89%) | K562 | + | chr11 | | 65267184 | 65267190 |
| 3.33532797438 | safb2 (bg=26.89%) | K562 | + | chr11 | | 65267185 | 65267189 |
| 2.80364046129 | SMNDC1 (bg=7.08%) | HepG2 | + | chr11 | | 65267085 | 65267183 |
| 3.36452843109 | srsf1 (bg=30.28%) | HepG2 | + | chr11 | | 65267130 | 65267189 |
| 4.66294151906 | srsf1 (bg=30.28%) | K562 | + | chr11 | | 65267172 | 65267178 |
| 5.2322211408 | srsf1 (bg=30.28%) | HepG2 | + | chr11 | | 65267173 | 65267178 |
| 3.4465744617 | srsf1 (bg=30.28%) | K562 | + | chr11 | | 65267173 | 65267178 |
| 4.74757862559 | srsf1 (bg=30.28%) | K562 | + | chr11 | | 65267178 | 65267182 |
| 5.02697301041 | srsf1 (bg=30.28%) | HepG2 | + | chr11 | | 65267178 | 65267191 |
| 3.45752179367 | srsf1 (bg=30.28%) | K562 | + | chr11 | | 65267178 | 65267198 |
| 4.19861786928 | srsf1 (bg=30.28%) | K562 | + | chr11 | | 65267182 | 65267184 |
| 4.36174154544 | srsf1 (bg=30.28%) | K562 | + | chr11 | | 65267184 | 65267190 |
| 2.76844022786 | srsf7 (bg=22.53%) | HepG2 | + | chr11 | | 65267130 | 65267192 |
| 2.66286052306 | srsf7 (bg=22.53%) | K562 | + | chr11 | | 65267173 | 65267206 |
| 2.55461100043 | srsf7 (bg=22.53%) | K562 | + | chr11 | | 65267174 | 65267197 |
| 5.39526782119 | SRSF9 (bg=9.67%) | HepG2 | + | chr11 | | 65267139 | 65267181 |
| 5.2733011015 | SRSF9 (bg=9.67%) | HepG2 | + | chr11 | | 65267181 | 65267190 |
| 4.09386313971 | tra2a (bg=37.02%) | HepG2 | + | chr11 | | 65267079 | 65267209 |
| 6.24230451441 | tra2a (bg=37.02%) | HepG2 | + | chr11 | | 65267172 | 65267178 |
| 7.38302340368 | tra2a (bg=37.02%) | K562 | + | chr11 | | 65267175 | 65267178 |
| 7.44118894466 | tra2a (bg=37.02%) | K562 | + | chr11 | | 65267175 | 65267178 |
| 7.50679357747 | tra2a (bg=37.02%) | K562 | + | chr11 | | 65267178 | 65267181 |
| 7.54545995788 | tra2a (bg=37.02%) | K562 | + | chr11 | | 65267178 | 65267181 |
| 6.23414480785 | tra2a (bg=37.02%) | HepG2 | + | chr11 | | 65267178 | 65267210 |
| 7.20626805263 | tra2a (bg=37.02%) | K562 | + | chr11 | | 65267181 | 65267184 |
| 7.29605590973 | tra2a (bg=37.02%) | K562 | + | chr11 | | 65267181 | 65267184 |
| 7.24116118183 | tra2a (bg=37.02%) | K562 | + | chr11 | | 65267184 | 65267190 |
| 7.40163267176 | tra2a (bg=37.02%) | K562 | + | chr11 | | 65267184 | 65267190 |
| 2.97094061895 | TROVE2 (bg=6.96%) | HepG2 | + | chr11 | | 65267176 | 65267209 |
| 4.36602033291 | uchl5 (bg=18.56%) | K562 | + | chr11 | | 65267172 | 65267178 |
| 4.13347306076 | uchl5 (bg=18.56%) | K562 | + | chr11 | | 65267173 | 65267178 |
| 3.96566129038 | uchl5 (bg=18.56%) | K562 | + | chr11 | | 65267178 | 65267181 |
| 4.46191697828 | uchl5 (bg=18.56%) | K562 | + | chr11 | | 65267178 | 65267182 |
| 3.67186407461 | uchl5 (bg=18.56%) | K562 | + | chr11 | | 65267181 | 65267185 |
| 4.34374902445 | uchl5 (bg=18.56%) | K562 | + | chr11 | | 65267182 | 65267189 |
| 3.65169100373 | znf622 (bg=18.79%) | K562 | + | chr11 | | 65267163 | 65267186 |
| 4.20048467175 | znf622 (bg=18.79%) | K562 | + | chr11 | | 65267172 | 65267189 |

  
  

| Match 50 in HUMAN | | | | | | | |
| --- | --- | --- | --- | --- | --- | --- | --- |
| Motif | Start in Seq (1 Indexed) | End in Seq (1 Indexed) | Strand | Chrm | Exon | Start in Chrm (0 Indexed) | End in Chrm (1 Indexed) |
| CTTTTAGAAGA | 718 | 728 | + | chr11 | 1 | 65267207 | 65267218 |
| eCLIP Fold-Enrichment | Binding Protein | Cell Line | Strand | Chrm | | Start in Chrm (0 Indexed) | End in Chrm (1 Indexed) |
| 2.12706856102 | aggf1 (bg=15.15%) | HepG2 | + | chr11 | | 65267197 | 65267210 |
| 3.47575766334 | aggf1 (bg=15.15%) | K562 | + | chr11 | | 65267205 | 65267209 |
| 2.08934368515 | aggf1 (bg=15.15%) | K562 | + | chr11 | | 65267206 | 65267226 |
| 3.56853526525 | aggf1 (bg=15.15%) | K562 | + | chr11 | | 65267209 | 65267228 |
| 2.37632917397 | AQR (bg=4.89%) | HepG2 | + | chr11 | | 65267190 | 65267208 |
| 2.141970248 | AQR (bg=4.89%) | HepG2 | + | chr11 | | 65267208 | 65267226 |
| 3.84475679966 | bclaf1 (bg=17.67%) | HepG2 | + | chr11 | | 65267210 | 65267219 |
| 4.61023663539 | bud13 (bg=12.85%) | HepG2 | + | chr11 | | 65267151 | 65267230 |
| 2.25375571985 | bud13 (bg=12.85%) | K562 | + | chr11 | | 65267189 | 65267207 |
| 2.45565257043 | bud13 (bg=12.85%) | K562 | + | chr11 | | 65267194 | 65267207 |
| 2.2673193533 | bud13 (bg=12.85%) | K562 | + | chr11 | | 65267207 | 65267215 |
| 2.07920238637 | bud13 (bg=12.85%) | K562 | + | chr11 | | 65267207 | 65267227 |
| 2.23272899972 | bud13 (bg=12.85%) | K562 | + | chr11 | | 65267215 | 65267227 |
| 3.22101405695 | cpsf6 (bg=13.45%) | K562 | + | chr11 | | 65267173 | 65267207 |
| 3.88695021926 | cpsf6 (bg=13.45%) | K562 | + | chr11 | | 65267207 | 65267226 |
| 3.14485405434 | cpsf6 (bg=13.45%) | K562 | + | chr11 | | 65267210 | 65267222 |
| 2.33306712821 | FASTKD2 (bg=4.54%) | K562 | + | chr11 | | 65267173 | 65267207 |
| 2.06186485275 | FASTKD2 (bg=4.54%) | K562 | + | chr11 | | 65267207 | 65267224 |
| 2.02916249379 | fxr2 (bg=10.1%) | HepG2 | + | chr11 | | 65267091 | 65267220 |
| 2.17620601683 | GPKOW (bg=5.66%) | K562 | + | chr11 | | 65267210 | 65267223 |
| 2.36104404804 | hltf (bg=24.28%) | HepG2 | + | chr11 | | 65267213 | 65267222 |
| 2.80567272626 | larp4 (bg=13.51%) | K562 | + | chr11 | | 65267206 | 65267226 |
| 2.04479443816 | rbm15 (bg=11.59%) | HepG2 | + | chr11 | | 65267152 | 65267207 |
| 2.24957900364 | rbm15 (bg=11.59%) | HepG2 | + | chr11 | | 65267207 | 65267223 |
| 2.90099774702 | safb2 (bg=26.89%) | K562 | + | chr11 | | 65267214 | 65267218 |
| 3.61048855536 | safb2 (bg=26.89%) | K562 | + | chr11 | | 65267214 | 65267219 |
| 4.38936203687 | srsf1 (bg=30.28%) | K562 | + | chr11 | | 65267194 | 65267207 |
| 4.08306306204 | srsf1 (bg=30.28%) | K562 | + | chr11 | | 65267207 | 65267226 |
| 2.76108277301 | srsf1 (bg=30.28%) | K562 | + | chr11 | | 65267213 | 65267220 |
| 2.58262419998 | srsf7 (bg=22.53%) | K562 | + | chr11 | | 65267206 | 65267225 |
| 2.43618566948 | srsf7 (bg=22.53%) | K562 | + | chr11 | | 65267215 | 65267221 |
| 4.09386313971 | tra2a (bg=37.02%) | HepG2 | + | chr11 | | 65267079 | 65267209 |
| 6.23414480785 | tra2a (bg=37.02%) | HepG2 | + | chr11 | | 65267178 | 65267210 |
| 6.61612681024 | tra2a (bg=37.02%) | K562 | + | chr11 | | 65267194 | 65267210 |
| 6.64935758673 | tra2a (bg=37.02%) | K562 | + | chr11 | | 65267197 | 65267209 |
| 6.57051825247 | tra2a (bg=37.02%) | K562 | + | chr11 | | 65267209 | 65267214 |
| 4.5007510397 | tra2a (bg=37.02%) | HepG2 | + | chr11 | | 65267209 | 65267223 |
| 6.17831641953 | tra2a (bg=37.02%) | HepG2 | + | chr11 | | 65267210 | 65267226 |
| 5.92570975349 | tra2a (bg=37.02%) | K562 | + | chr11 | | 65267210 | 65267228 |
| 6.25405630552 | tra2a (bg=37.02%) | K562 | + | chr11 | | 65267214 | 65267222 |
| 2.97094061895 | TROVE2 (bg=6.96%) | HepG2 | + | chr11 | | 65267176 | 65267209 |
| 2.15209005805 | TROVE2 (bg=6.96%) | HepG2 | + | chr11 | | 65267209 | 65267224 |

  
  

| Match 51 in HUMAN | | | | | | | |
| --- | --- | --- | --- | --- | --- | --- | --- |
| Motif | Start in Seq (1 Indexed) | End in Seq (1 Indexed) | Strand | Chrm | Exon | Start in Chrm (0 Indexed) | End in Chrm (1 Indexed) |
| AAGTAGGAAGCAGAAGAAAAAA | 752 | 773 | + | chr11 | 1 | 65267241 | 65267263 |
| eCLIP Fold-Enrichment | Binding Protein | Cell Line | Strand | Chrm | | Start in Chrm (0 Indexed) | End in Chrm (1 Indexed) |
| 3.68053087987 | aggf1 (bg=15.15%) | K562 | + | chr11 | | 65267228 | 65267244 |
| 3.499097184 | aggf1 (bg=15.15%) | K562 | + | chr11 | | 65267244 | 65267262 |
| 2.93658338586 | aggf1 (bg=15.15%) | K562 | + | chr11 | | 65267262 | 65267279 |
| 2.16450355093 | AQR (bg=4.89%) | HepG2 | + | chr11 | | 65267226 | 65267247 |
| 2.19054907827 | AQR (bg=4.89%) | HepG2 | + | chr11 | | 65267247 | 65267259 |
| 2.24068715925 | AQR (bg=4.89%) | HepG2 | + | chr11 | | 65267259 | 65267265 |
| 3.73367396152 | bclaf1 (bg=17.67%) | HepG2 | + | chr11 | | 65267228 | 65267258 |
| 3.8754802553 | bclaf1 (bg=17.67%) | HepG2 | + | chr11 | | 65267229 | 65267265 |
| 3.66041502544 | bclaf1 (bg=17.67%) | HepG2 | + | chr11 | | 65267258 | 65267280 |
| 2.39416939709 | bud13 (bg=12.85%) | K562 | + | chr11 | | 65267227 | 65267255 |
| 2.50334978883 | bud13 (bg=12.85%) | HepG2 | + | chr11 | | 65267230 | 65267263 |
| 2.03899903424 | bud13 (bg=12.85%) | K562 | + | chr11 | | 65267242 | 65267246 |
| 2.0157307384 | bud13 (bg=12.85%) | K562 | + | chr11 | | 65267246 | 65267260 |
| 2.4605118231 | bud13 (bg=12.85%) | K562 | + | chr11 | | 65267255 | 65267260 |
| 2.14665244889 | bud13 (bg=12.85%) | K562 | + | chr11 | | 65267260 | 65267269 |
| 2.46161609591 | bud13 (bg=12.85%) | K562 | + | chr11 | | 65267260 | 65267273 |
| 2.1314756175 | bud13 (bg=12.85%) | HepG2 | + | chr11 | | 65267263 | 65267268 |
| 3.50462703769 | cpsf6 (bg=13.45%) | K562 | + | chr11 | | 65267226 | 65267259 |
| 2.59319606945 | cpsf6 (bg=13.45%) | K562 | + | chr11 | | 65267229 | 65267300 |
| 3.11501566237 | cpsf6 (bg=13.45%) | K562 | + | chr11 | | 65267259 | 65267309 |
| 2.23396057651 | FASTKD2 (bg=4.54%) | K562 | + | chr11 | | 65267229 | 65267258 |
| 2.36366240454 | FASTKD2 (bg=4.54%) | K562 | + | chr11 | | 65267258 | 65267285 |
| 2.01160631692 | FTO (bg=1.11%) | K562 | + | chr11 | | 65267241 | 65267258 |
| 2.09718281337 | FTO (bg=1.11%) | K562 | + | chr11 | | 65267258 | 65267288 |
| 2.57398657312 | fxr2 (bg=10.1%) | HepG2 | + | chr11 | | 65267220 | 65267312 |
| 2.49434921061 | GPKOW (bg=5.66%) | K562 | + | chr11 | | 65267228 | 65267259 |
| 2.24466015604 | GPKOW (bg=5.66%) | K562 | + | chr11 | | 65267229 | 65267261 |
| 2.64679580475 | GPKOW (bg=5.66%) | K562 | + | chr11 | | 65267259 | 65267284 |
| 2.32188827677 | GPKOW (bg=5.66%) | K562 | + | chr11 | | 65267261 | 65267282 |
| 3.24731030221 | gtf2f1 (bg=10.18%) | HepG2 | + | chr11 | | 65267240 | 65267263 |
| 2.28331455438 | hltf (bg=24.28%) | HepG2 | + | chr11 | | 65267229 | 65267258 |
| 2.6104407171 | hltf (bg=24.28%) | HepG2 | + | chr11 | | 65267230 | 65267254 |
| 2.36660595829 | hltf (bg=24.28%) | HepG2 | + | chr11 | | 65267254 | 65267263 |
| 2.19748890429 | hltf (bg=24.28%) | HepG2 | + | chr11 | | 65267258 | 65267287 |
| 2.17726025994 | hltf (bg=24.28%) | HepG2 | + | chr11 | | 65267263 | 65267276 |
| 3.05863680037 | larp4 (bg=13.51%) | K562 | + | chr11 | | 65267226 | 65267258 |
| 3.2047969553 | larp4 (bg=13.51%) | K562 | + | chr11 | | 65267258 | 65267285 |
| 2.74952812016 | MTPAP (bg=9.55%) | K562 | + | chr11 | | 65267231 | 65267246 |
| 2.44202709591 | MTPAP (bg=9.55%) | K562 | + | chr11 | | 65267246 | 65267283 |
| 2.69482851663 | npm1 (bg=10.22%) | K562 | + | chr11 | | 65267234 | 65267291 |
| 2.25382847665 | rbm15 (bg=11.59%) | HepG2 | + | chr11 | | 65267223 | 65267245 |
| 4.02379254966 | rbm22 (bg=12.69%) | HepG2 | + | chr11 | | 65267232 | 65267262 |
| 3.53109896683 | rbm22 (bg=12.69%) | HepG2 | + | chr11 | | 65267262 | 65267278 |
| 4.29511466172 | safb2 (bg=26.89%) | K562 | + | chr11 | | 65267230 | 65267242 |
| 3.71049711616 | safb2 (bg=26.89%) | K562 | + | chr11 | | 65267234 | 65267242 |
| 4.51171714259 | safb2 (bg=26.89%) | K562 | + | chr11 | | 65267242 | 65267246 |
| 4.06937885038 | safb2 (bg=26.89%) | K562 | + | chr11 | | 65267242 | 65267255 |
| 4.59722196544 | safb2 (bg=26.89%) | K562 | + | chr11 | | 65267246 | 65267255 |
| 4.61560138861 | safb2 (bg=26.89%) | K562 | + | chr11 | | 65267255 | 65267260 |
| 4.13058716957 | safb2 (bg=26.89%) | K562 | + | chr11 | | 65267255 | 65267260 |
| 4.10591641106 | safb2 (bg=26.89%) | K562 | + | chr11 | | 65267260 | 65267272 |
| 3.69823660491 | safb2 (bg=26.89%) | K562 | + | chr11 | | 65267260 | 65267272 |
| 2.40640424838 | SMNDC1 (bg=7.08%) | HepG2 | + | chr11 | | 65267240 | 65267274 |
| 2.50504261378 | srsf1 (bg=30.28%) | K562 | + | chr11 | | 65267233 | 65267259 |
| 4.0889512714 | srsf1 (bg=30.28%) | K562 | + | chr11 | | 65267234 | 65267241 |
| 3.86435690026 | srsf1 (bg=30.28%) | K562 | + | chr11 | | 65267241 | 65267256 |
| 3.70220438365 | srsf1 (bg=30.28%) | K562 | + | chr11 | | 65267256 | 65267261 |
| 2.10659957872 | srsf1 (bg=30.28%) | K562 | + | chr11 | | 65267259 | 65267290 |
| 3.5127897199 | srsf1 (bg=30.28%) | K562 | + | chr11 | | 65267261 | 65267295 |
| 2.61932318704 | srsf7 (bg=22.53%) | K562 | + | chr11 | | 65267230 | 65267261 |
| 2.49714339714 | srsf7 (bg=22.53%) | K562 | + | chr11 | | 65267234 | 65267241 |
| 2.42529829883 | srsf7 (bg=22.53%) | K562 | + | chr11 | | 65267241 | 65267260 |
| 2.54440329227 | srsf7 (bg=22.53%) | K562 | + | chr11 | | 65267260 | 65267275 |
| 2.65552932803 | srsf7 (bg=22.53%) | K562 | + | chr11 | | 65267261 | 65267294 |
| 3.74116628913 | SRSF9 (bg=9.67%) | HepG2 | + | chr11 | | 65267257 | 65267287 |
| 2.01684847706 | TAF15 (bg=9.06%) | K562 | + | chr11 | | 65267241 | 65267256 |
| 2.6418112258 | TAF15 (bg=9.06%) | HepG2 | + | chr11 | | 65267248 | 65267287 |
| 3.69259126683 | tra2a (bg=37.02%) | HepG2 | + | chr11 | | 65267223 | 65267307 |
| 5.64737493311 | tra2a (bg=37.02%) | HepG2 | + | chr11 | | 65267226 | 65267259 |
| 6.16658979576 | tra2a (bg=37.02%) | K562 | + | chr11 | | 65267234 | 65267242 |
| 6.46316759763 | tra2a (bg=37.02%) | K562 | + | chr11 | | 65267234 | 65267243 |
| 6.21370452531 | tra2a (bg=37.02%) | K562 | + | chr11 | | 65267242 | 65267244 |
| 6.42971360979 | tra2a (bg=37.02%) | K562 | + | chr11 | | 65267243 | 65267254 |
| 6.13939441827 | tra2a (bg=37.02%) | K562 | + | chr11 | | 65267244 | 65267246 |
| 6.00625981405 | tra2a (bg=37.02%) | K562 | + | chr11 | | 65267246 | 65267255 |
| 6.25479603795 | tra2a (bg=37.02%) | K562 | + | chr11 | | 65267254 | 65267260 |
| 6.00318291258 | tra2a (bg=37.02%) | K562 | + | chr11 | | 65267255 | 65267259 |
| 5.86842560883 | tra2a (bg=37.02%) | K562 | + | chr11 | | 65267259 | 65267272 |
| 5.27142582393 | tra2a (bg=37.02%) | HepG2 | + | chr11 | | 65267259 | 65267297 |
| 6.0711542442 | tra2a (bg=37.02%) | K562 | + | chr11 | | 65267260 | 65267268 |
| 2.17543462991 | TROVE2 (bg=6.96%) | HepG2 | + | chr11 | | 65267229 | 65267258 |
| 2.26542048653 | TROVE2 (bg=6.96%) | HepG2 | + | chr11 | | 65267258 | 65267300 |
| 3.12901017566 | uchl5 (bg=18.56%) | K562 | + | chr11 | | 65267231 | 65267243 |
| 2.5414590362 | uchl5 (bg=18.56%) | K562 | + | chr11 | | 65267231 | 65267257 |
| 2.96308808814 | uchl5 (bg=18.56%) | K562 | + | chr11 | | 65267243 | 65267259 |
| 2.60778636936 | uchl5 (bg=18.56%) | K562 | + | chr11 | | 65267257 | 65267283 |
| 3.04104302397 | uchl5 (bg=18.56%) | K562 | + | chr11 | | 65267259 | 65267269 |
| 2.23408450546 | YWHAG (bg=9.14%) | K562 | + | chr11 | | 65267234 | 65267278 |
| 2.07223161097 | zc3h8 (bg=12.78%) | K562 | + | chr11 | | 65267242 | 65267280 |
| 2.29797932099 | zc3h8 (bg=12.78%) | K562 | + | chr11 | | 65267245 | 65267274 |
| 3.49453099038 | znf622 (bg=18.79%) | K562 | + | chr11 | | 65267236 | 65267256 |
| 3.59337289376 | znf622 (bg=18.79%) | K562 | + | chr11 | | 65267256 | 65267273 |

  
  

| Match 52 in HUMAN | | | | | | | |
| --- | --- | --- | --- | --- | --- | --- | --- |
| Motif | Start in Seq (1 Indexed) | End in Seq (1 Indexed) | Strand | Chrm | Exon | Start in Chrm (0 Indexed) | End in Chrm (1 Indexed) |
| AGGAAGCAGAAGAAAAAA | 756 | 773 | + | chr11 | 1 | 65267245 | 65267263 |
| eCLIP Fold-Enrichment | Binding Protein | Cell Line | Strand | Chrm | | Start in Chrm (0 Indexed) | End in Chrm (1 Indexed) |
| 3.499097184 | aggf1 (bg=15.15%) | K562 | + | chr11 | | 65267244 | 65267262 |
| 2.93658338586 | aggf1 (bg=15.15%) | K562 | + | chr11 | | 65267262 | 65267279 |
| 2.16450355093 | AQR (bg=4.89%) | HepG2 | + | chr11 | | 65267226 | 65267247 |
| 2.19054907827 | AQR (bg=4.89%) | HepG2 | + | chr11 | | 65267247 | 65267259 |
| 2.24068715925 | AQR (bg=4.89%) | HepG2 | + | chr11 | | 65267259 | 65267265 |
| 3.73367396152 | bclaf1 (bg=17.67%) | HepG2 | + | chr11 | | 65267228 | 65267258 |
| 3.8754802553 | bclaf1 (bg=17.67%) | HepG2 | + | chr11 | | 65267229 | 65267265 |
| 3.66041502544 | bclaf1 (bg=17.67%) | HepG2 | + | chr11 | | 65267258 | 65267280 |
| 2.39416939709 | bud13 (bg=12.85%) | K562 | + | chr11 | | 65267227 | 65267255 |
| 2.50334978883 | bud13 (bg=12.85%) | HepG2 | + | chr11 | | 65267230 | 65267263 |
| 2.03899903424 | bud13 (bg=12.85%) | K562 | + | chr11 | | 65267242 | 65267246 |
| 2.0157307384 | bud13 (bg=12.85%) | K562 | + | chr11 | | 65267246 | 65267260 |
| 2.4605118231 | bud13 (bg=12.85%) | K562 | + | chr11 | | 65267255 | 65267260 |
| 2.14665244889 | bud13 (bg=12.85%) | K562 | + | chr11 | | 65267260 | 65267269 |
| 2.46161609591 | bud13 (bg=12.85%) | K562 | + | chr11 | | 65267260 | 65267273 |
| 2.1314756175 | bud13 (bg=12.85%) | HepG2 | + | chr11 | | 65267263 | 65267268 |
| 3.50462703769 | cpsf6 (bg=13.45%) | K562 | + | chr11 | | 65267226 | 65267259 |
| 2.59319606945 | cpsf6 (bg=13.45%) | K562 | + | chr11 | | 65267229 | 65267300 |
| 3.11501566237 | cpsf6 (bg=13.45%) | K562 | + | chr11 | | 65267259 | 65267309 |
| 2.23396057651 | FASTKD2 (bg=4.54%) | K562 | + | chr11 | | 65267229 | 65267258 |
| 2.36366240454 | FASTKD2 (bg=4.54%) | K562 | + | chr11 | | 65267258 | 65267285 |
| 2.01160631692 | FTO (bg=1.11%) | K562 | + | chr11 | | 65267241 | 65267258 |
| 2.09718281337 | FTO (bg=1.11%) | K562 | + | chr11 | | 65267258 | 65267288 |
| 2.57398657312 | fxr2 (bg=10.1%) | HepG2 | + | chr11 | | 65267220 | 65267312 |
| 2.49434921061 | GPKOW (bg=5.66%) | K562 | + | chr11 | | 65267228 | 65267259 |
| 2.24466015604 | GPKOW (bg=5.66%) | K562 | + | chr11 | | 65267229 | 65267261 |
| 2.64679580475 | GPKOW (bg=5.66%) | K562 | + | chr11 | | 65267259 | 65267284 |
| 2.32188827677 | GPKOW (bg=5.66%) | K562 | + | chr11 | | 65267261 | 65267282 |
| 3.24731030221 | gtf2f1 (bg=10.18%) | HepG2 | + | chr11 | | 65267240 | 65267263 |
| 2.28331455438 | hltf (bg=24.28%) | HepG2 | + | chr11 | | 65267229 | 65267258 |
| 2.6104407171 | hltf (bg=24.28%) | HepG2 | + | chr11 | | 65267230 | 65267254 |
| 2.36660595829 | hltf (bg=24.28%) | HepG2 | + | chr11 | | 65267254 | 65267263 |
| 2.19748890429 | hltf (bg=24.28%) | HepG2 | + | chr11 | | 65267258 | 65267287 |
| 2.17726025994 | hltf (bg=24.28%) | HepG2 | + | chr11 | | 65267263 | 65267276 |
| 3.05863680037 | larp4 (bg=13.51%) | K562 | + | chr11 | | 65267226 | 65267258 |
| 3.2047969553 | larp4 (bg=13.51%) | K562 | + | chr11 | | 65267258 | 65267285 |
| 2.74952812016 | MTPAP (bg=9.55%) | K562 | + | chr11 | | 65267231 | 65267246 |
| 2.44202709591 | MTPAP (bg=9.55%) | K562 | + | chr11 | | 65267246 | 65267283 |
| 2.69482851663 | npm1 (bg=10.22%) | K562 | + | chr11 | | 65267234 | 65267291 |
| 2.25382847665 | rbm15 (bg=11.59%) | HepG2 | + | chr11 | | 65267223 | 65267245 |
| 4.02379254966 | rbm22 (bg=12.69%) | HepG2 | + | chr11 | | 65267232 | 65267262 |
| 3.53109896683 | rbm22 (bg=12.69%) | HepG2 | + | chr11 | | 65267262 | 65267278 |
| 4.51171714259 | safb2 (bg=26.89%) | K562 | + | chr11 | | 65267242 | 65267246 |
| 4.06937885038 | safb2 (bg=26.89%) | K562 | + | chr11 | | 65267242 | 65267255 |
| 4.59722196544 | safb2 (bg=26.89%) | K562 | + | chr11 | | 65267246 | 65267255 |
| 4.61560138861 | safb2 (bg=26.89%) | K562 | + | chr11 | | 65267255 | 65267260 |
| 4.13058716957 | safb2 (bg=26.89%) | K562 | + | chr11 | | 65267255 | 65267260 |
| 4.10591641106 | safb2 (bg=26.89%) | K562 | + | chr11 | | 65267260 | 65267272 |
| 3.69823660491 | safb2 (bg=26.89%) | K562 | + | chr11 | | 65267260 | 65267272 |
| 2.40640424838 | SMNDC1 (bg=7.08%) | HepG2 | + | chr11 | | 65267240 | 65267274 |
| 2.50504261378 | srsf1 (bg=30.28%) | K562 | + | chr11 | | 65267233 | 65267259 |
| 3.86435690026 | srsf1 (bg=30.28%) | K562 | + | chr11 | | 65267241 | 65267256 |
| 3.70220438365 | srsf1 (bg=30.28%) | K562 | + | chr11 | | 65267256 | 65267261 |
| 2.10659957872 | srsf1 (bg=30.28%) | K562 | + | chr11 | | 65267259 | 65267290 |
| 3.5127897199 | srsf1 (bg=30.28%) | K562 | + | chr11 | | 65267261 | 65267295 |
| 2.61932318704 | srsf7 (bg=22.53%) | K562 | + | chr11 | | 65267230 | 65267261 |
| 2.42529829883 | srsf7 (bg=22.53%) | K562 | + | chr11 | | 65267241 | 65267260 |
| 2.54440329227 | srsf7 (bg=22.53%) | K562 | + | chr11 | | 65267260 | 65267275 |
| 2.65552932803 | srsf7 (bg=22.53%) | K562 | + | chr11 | | 65267261 | 65267294 |
| 3.74116628913 | SRSF9 (bg=9.67%) | HepG2 | + | chr11 | | 65267257 | 65267287 |
| 2.01684847706 | TAF15 (bg=9.06%) | K562 | + | chr11 | | 65267241 | 65267256 |
| 2.6418112258 | TAF15 (bg=9.06%) | HepG2 | + | chr11 | | 65267248 | 65267287 |
| 3.69259126683 | tra2a (bg=37.02%) | HepG2 | + | chr11 | | 65267223 | 65267307 |
| 5.64737493311 | tra2a (bg=37.02%) | HepG2 | + | chr11 | | 65267226 | 65267259 |
| 6.42971360979 | tra2a (bg=37.02%) | K562 | + | chr11 | | 65267243 | 65267254 |
| 6.13939441827 | tra2a (bg=37.02%) | K562 | + | chr11 | | 65267244 | 65267246 |
| 6.00625981405 | tra2a (bg=37.02%) | K562 | + | chr11 | | 65267246 | 65267255 |
| 6.25479603795 | tra2a (bg=37.02%) | K562 | + | chr11 | | 65267254 | 65267260 |
| 6.00318291258 | tra2a (bg=37.02%) | K562 | + | chr11 | | 65267255 | 65267259 |
| 5.86842560883 | tra2a (bg=37.02%) | K562 | + | chr11 | | 65267259 | 65267272 |
| 5.27142582393 | tra2a (bg=37.02%) | HepG2 | + | chr11 | | 65267259 | 65267297 |
| 6.0711542442 | tra2a (bg=37.02%) | K562 | + | chr11 | | 65267260 | 65267268 |
| 2.17543462991 | TROVE2 (bg=6.96%) | HepG2 | + | chr11 | | 65267229 | 65267258 |
| 2.26542048653 | TROVE2 (bg=6.96%) | HepG2 | + | chr11 | | 65267258 | 65267300 |
| 2.5414590362 | uchl5 (bg=18.56%) | K562 | + | chr11 | | 65267231 | 65267257 |
| 2.96308808814 | uchl5 (bg=18.56%) | K562 | + | chr11 | | 65267243 | 65267259 |
| 2.60778636936 | uchl5 (bg=18.56%) | K562 | + | chr11 | | 65267257 | 65267283 |
| 3.04104302397 | uchl5 (bg=18.56%) | K562 | + | chr11 | | 65267259 | 65267269 |
| 2.23408450546 | YWHAG (bg=9.14%) | K562 | + | chr11 | | 65267234 | 65267278 |
| 2.07223161097 | zc3h8 (bg=12.78%) | K562 | + | chr11 | | 65267242 | 65267280 |
| 2.29797932099 | zc3h8 (bg=12.78%) | K562 | + | chr11 | | 65267245 | 65267274 |
| 3.49453099038 | znf622 (bg=18.79%) | K562 | + | chr11 | | 65267236 | 65267256 |
| 3.59337289376 | znf622 (bg=18.79%) | K562 | + | chr11 | | 65267256 | 65267273 |

  
  

| Match 53 in HUMAN | | | | | | | |
| --- | --- | --- | --- | --- | --- | --- | --- |
| Motif | Start in Seq (1 Indexed) | End in Seq (1 Indexed) | Strand | Chrm | Exon | Start in Chrm (0 Indexed) | End in Chrm (1 Indexed) |
| AGAAGAAAAAA | 763 | 773 | + | chr11 | 1 | 65267252 | 65267263 |
| eCLIP Fold-Enrichment | Binding Protein | Cell Line | Strand | Chrm | | Start in Chrm (0 Indexed) | End in Chrm (1 Indexed) |
| 3.499097184 | aggf1 (bg=15.15%) | K562 | + | chr11 | | 65267244 | 65267262 |
| 2.93658338586 | aggf1 (bg=15.15%) | K562 | + | chr11 | | 65267262 | 65267279 |
| 2.19054907827 | AQR (bg=4.89%) | HepG2 | + | chr11 | | 65267247 | 65267259 |
| 2.24068715925 | AQR (bg=4.89%) | HepG2 | + | chr11 | | 65267259 | 65267265 |
| 3.73367396152 | bclaf1 (bg=17.67%) | HepG2 | + | chr11 | | 65267228 | 65267258 |
| 3.8754802553 | bclaf1 (bg=17.67%) | HepG2 | + | chr11 | | 65267229 | 65267265 |
| 3.66041502544 | bclaf1 (bg=17.67%) | HepG2 | + | chr11 | | 65267258 | 65267280 |
| 2.39416939709 | bud13 (bg=12.85%) | K562 | + | chr11 | | 65267227 | 65267255 |
| 2.50334978883 | bud13 (bg=12.85%) | HepG2 | + | chr11 | | 65267230 | 65267263 |
| 2.0157307384 | bud13 (bg=12.85%) | K562 | + | chr11 | | 65267246 | 65267260 |
| 2.4605118231 | bud13 (bg=12.85%) | K562 | + | chr11 | | 65267255 | 65267260 |
| 2.14665244889 | bud13 (bg=12.85%) | K562 | + | chr11 | | 65267260 | 65267269 |
| 2.46161609591 | bud13 (bg=12.85%) | K562 | + | chr11 | | 65267260 | 65267273 |
| 2.1314756175 | bud13 (bg=12.85%) | HepG2 | + | chr11 | | 65267263 | 65267268 |
| 3.50462703769 | cpsf6 (bg=13.45%) | K562 | + | chr11 | | 65267226 | 65267259 |
| 2.59319606945 | cpsf6 (bg=13.45%) | K562 | + | chr11 | | 65267229 | 65267300 |
| 3.11501566237 | cpsf6 (bg=13.45%) | K562 | + | chr11 | | 65267259 | 65267309 |
| 2.23396057651 | FASTKD2 (bg=4.54%) | K562 | + | chr11 | | 65267229 | 65267258 |
| 2.36366240454 | FASTKD2 (bg=4.54%) | K562 | + | chr11 | | 65267258 | 65267285 |
| 2.01160631692 | FTO (bg=1.11%) | K562 | + | chr11 | | 65267241 | 65267258 |
| 2.09718281337 | FTO (bg=1.11%) | K562 | + | chr11 | | 65267258 | 65267288 |
| 2.57398657312 | fxr2 (bg=10.1%) | HepG2 | + | chr11 | | 65267220 | 65267312 |
| 2.49434921061 | GPKOW (bg=5.66%) | K562 | + | chr11 | | 65267228 | 65267259 |
| 2.24466015604 | GPKOW (bg=5.66%) | K562 | + | chr11 | | 65267229 | 65267261 |
| 2.64679580475 | GPKOW (bg=5.66%) | K562 | + | chr11 | | 65267259 | 65267284 |
| 2.32188827677 | GPKOW (bg=5.66%) | K562 | + | chr11 | | 65267261 | 65267282 |
| 3.24731030221 | gtf2f1 (bg=10.18%) | HepG2 | + | chr11 | | 65267240 | 65267263 |
| 2.28331455438 | hltf (bg=24.28%) | HepG2 | + | chr11 | | 65267229 | 65267258 |
| 2.6104407171 | hltf (bg=24.28%) | HepG2 | + | chr11 | | 65267230 | 65267254 |
| 2.36660595829 | hltf (bg=24.28%) | HepG2 | + | chr11 | | 65267254 | 65267263 |
| 2.19748890429 | hltf (bg=24.28%) | HepG2 | + | chr11 | | 65267258 | 65267287 |
| 2.17726025994 | hltf (bg=24.28%) | HepG2 | + | chr11 | | 65267263 | 65267276 |
| 3.05863680037 | larp4 (bg=13.51%) | K562 | + | chr11 | | 65267226 | 65267258 |
| 3.2047969553 | larp4 (bg=13.51%) | K562 | + | chr11 | | 65267258 | 65267285 |
| 2.44202709591 | MTPAP (bg=9.55%) | K562 | + | chr11 | | 65267246 | 65267283 |
| 2.69482851663 | npm1 (bg=10.22%) | K562 | + | chr11 | | 65267234 | 65267291 |
| 4.02379254966 | rbm22 (bg=12.69%) | HepG2 | + | chr11 | | 65267232 | 65267262 |
| 3.53109896683 | rbm22 (bg=12.69%) | HepG2 | + | chr11 | | 65267262 | 65267278 |
| 4.06937885038 | safb2 (bg=26.89%) | K562 | + | chr11 | | 65267242 | 65267255 |
| 4.59722196544 | safb2 (bg=26.89%) | K562 | + | chr11 | | 65267246 | 65267255 |
| 4.61560138861 | safb2 (bg=26.89%) | K562 | + | chr11 | | 65267255 | 65267260 |
| 4.13058716957 | safb2 (bg=26.89%) | K562 | + | chr11 | | 65267255 | 65267260 |
| 4.10591641106 | safb2 (bg=26.89%) | K562 | + | chr11 | | 65267260 | 65267272 |
| 3.69823660491 | safb2 (bg=26.89%) | K562 | + | chr11 | | 65267260 | 65267272 |
| 2.40640424838 | SMNDC1 (bg=7.08%) | HepG2 | + | chr11 | | 65267240 | 65267274 |
| 2.50504261378 | srsf1 (bg=30.28%) | K562 | + | chr11 | | 65267233 | 65267259 |
| 3.86435690026 | srsf1 (bg=30.28%) | K562 | + | chr11 | | 65267241 | 65267256 |
| 3.70220438365 | srsf1 (bg=30.28%) | K562 | + | chr11 | | 65267256 | 65267261 |
| 2.10659957872 | srsf1 (bg=30.28%) | K562 | + | chr11 | | 65267259 | 65267290 |
| 3.5127897199 | srsf1 (bg=30.28%) | K562 | + | chr11 | | 65267261 | 65267295 |
| 2.61932318704 | srsf7 (bg=22.53%) | K562 | + | chr11 | | 65267230 | 65267261 |
| 2.42529829883 | srsf7 (bg=22.53%) | K562 | + | chr11 | | 65267241 | 65267260 |
| 2.54440329227 | srsf7 (bg=22.53%) | K562 | + | chr11 | | 65267260 | 65267275 |
| 2.65552932803 | srsf7 (bg=22.53%) | K562 | + | chr11 | | 65267261 | 65267294 |
| 3.74116628913 | SRSF9 (bg=9.67%) | HepG2 | + | chr11 | | 65267257 | 65267287 |
| 2.01684847706 | TAF15 (bg=9.06%) | K562 | + | chr11 | | 65267241 | 65267256 |
| 2.6418112258 | TAF15 (bg=9.06%) | HepG2 | + | chr11 | | 65267248 | 65267287 |
| 3.69259126683 | tra2a (bg=37.02%) | HepG2 | + | chr11 | | 65267223 | 65267307 |
| 5.64737493311 | tra2a (bg=37.02%) | HepG2 | + | chr11 | | 65267226 | 65267259 |
| 6.42971360979 | tra2a (bg=37.02%) | K562 | + | chr11 | | 65267243 | 65267254 |
| 6.00625981405 | tra2a (bg=37.02%) | K562 | + | chr11 | | 65267246 | 65267255 |
| 6.25479603795 | tra2a (bg=37.02%) | K562 | + | chr11 | | 65267254 | 65267260 |
| 6.00318291258 | tra2a (bg=37.02%) | K562 | + | chr11 | | 65267255 | 65267259 |
| 5.86842560883 | tra2a (bg=37.02%) | K562 | + | chr11 | | 65267259 | 65267272 |
| 5.27142582393 | tra2a (bg=37.02%) | HepG2 | + | chr11 | | 65267259 | 65267297 |
| 6.0711542442 | tra2a (bg=37.02%) | K562 | + | chr11 | | 65267260 | 65267268 |
| 2.17543462991 | TROVE2 (bg=6.96%) | HepG2 | + | chr11 | | 65267229 | 65267258 |
| 2.26542048653 | TROVE2 (bg=6.96%) | HepG2 | + | chr11 | | 65267258 | 65267300 |
| 2.5414590362 | uchl5 (bg=18.56%) | K562 | + | chr11 | | 65267231 | 65267257 |
| 2.96308808814 | uchl5 (bg=18.56%) | K562 | + | chr11 | | 65267243 | 65267259 |
| 2.60778636936 | uchl5 (bg=18.56%) | K562 | + | chr11 | | 65267257 | 65267283 |
| 3.04104302397 | uchl5 (bg=18.56%) | K562 | + | chr11 | | 65267259 | 65267269 |
| 2.23408450546 | YWHAG (bg=9.14%) | K562 | + | chr11 | | 65267234 | 65267278 |
| 2.07223161097 | zc3h8 (bg=12.78%) | K562 | + | chr11 | | 65267242 | 65267280 |
| 2.29797932099 | zc3h8 (bg=12.78%) | K562 | + | chr11 | | 65267245 | 65267274 |
| 3.49453099038 | znf622 (bg=18.79%) | K562 | + | chr11 | | 65267236 | 65267256 |
| 3.59337289376 | znf622 (bg=18.79%) | K562 | + | chr11 | | 65267256 | 65267273 |

  
  

| Match 54 in HUMAN | | | | | | | |
| --- | --- | --- | --- | --- | --- | --- | --- |
| Motif | Start in Seq (1 Indexed) | End in Seq (1 Indexed) | Strand | Chrm | Exon | Start in Chrm (0 Indexed) | End in Chrm (1 Indexed) |
| GAAGAAAAA | 764 | 772 | + | chr11 | 1 | 65267253 | 65267262 |
| eCLIP Fold-Enrichment | Binding Protein | Cell Line | Strand | Chrm | | Start in Chrm (0 Indexed) | End in Chrm (1 Indexed) |
| 3.499097184 | aggf1 (bg=15.15%) | K562 | + | chr11 | | 65267244 | 65267262 |
| 2.93658338586 | aggf1 (bg=15.15%) | K562 | + | chr11 | | 65267262 | 65267279 |
| 2.19054907827 | AQR (bg=4.89%) | HepG2 | + | chr11 | | 65267247 | 65267259 |
| 2.24068715925 | AQR (bg=4.89%) | HepG2 | + | chr11 | | 65267259 | 65267265 |
| 3.73367396152 | bclaf1 (bg=17.67%) | HepG2 | + | chr11 | | 65267228 | 65267258 |
| 3.8754802553 | bclaf1 (bg=17.67%) | HepG2 | + | chr11 | | 65267229 | 65267265 |
| 3.66041502544 | bclaf1 (bg=17.67%) | HepG2 | + | chr11 | | 65267258 | 65267280 |
| 2.39416939709 | bud13 (bg=12.85%) | K562 | + | chr11 | | 65267227 | 65267255 |
| 2.50334978883 | bud13 (bg=12.85%) | HepG2 | + | chr11 | | 65267230 | 65267263 |
| 2.0157307384 | bud13 (bg=12.85%) | K562 | + | chr11 | | 65267246 | 65267260 |
| 2.4605118231 | bud13 (bg=12.85%) | K562 | + | chr11 | | 65267255 | 65267260 |
| 2.14665244889 | bud13 (bg=12.85%) | K562 | + | chr11 | | 65267260 | 65267269 |
| 2.46161609591 | bud13 (bg=12.85%) | K562 | + | chr11 | | 65267260 | 65267273 |
| 3.50462703769 | cpsf6 (bg=13.45%) | K562 | + | chr11 | | 65267226 | 65267259 |
| 2.59319606945 | cpsf6 (bg=13.45%) | K562 | + | chr11 | | 65267229 | 65267300 |
| 3.11501566237 | cpsf6 (bg=13.45%) | K562 | + | chr11 | | 65267259 | 65267309 |
| 2.23396057651 | FASTKD2 (bg=4.54%) | K562 | + | chr11 | | 65267229 | 65267258 |
| 2.36366240454 | FASTKD2 (bg=4.54%) | K562 | + | chr11 | | 65267258 | 65267285 |
| 2.01160631692 | FTO (bg=1.11%) | K562 | + | chr11 | | 65267241 | 65267258 |
| 2.09718281337 | FTO (bg=1.11%) | K562 | + | chr11 | | 65267258 | 65267288 |
| 2.57398657312 | fxr2 (bg=10.1%) | HepG2 | + | chr11 | | 65267220 | 65267312 |
| 2.49434921061 | GPKOW (bg=5.66%) | K562 | + | chr11 | | 65267228 | 65267259 |
| 2.24466015604 | GPKOW (bg=5.66%) | K562 | + | chr11 | | 65267229 | 65267261 |
| 2.64679580475 | GPKOW (bg=5.66%) | K562 | + | chr11 | | 65267259 | 65267284 |
| 2.32188827677 | GPKOW (bg=5.66%) | K562 | + | chr11 | | 65267261 | 65267282 |
| 3.24731030221 | gtf2f1 (bg=10.18%) | HepG2 | + | chr11 | | 65267240 | 65267263 |
| 2.28331455438 | hltf (bg=24.28%) | HepG2 | + | chr11 | | 65267229 | 65267258 |
| 2.6104407171 | hltf (bg=24.28%) | HepG2 | + | chr11 | | 65267230 | 65267254 |
| 2.36660595829 | hltf (bg=24.28%) | HepG2 | + | chr11 | | 65267254 | 65267263 |
| 2.19748890429 | hltf (bg=24.28%) | HepG2 | + | chr11 | | 65267258 | 65267287 |
| 3.05863680037 | larp4 (bg=13.51%) | K562 | + | chr11 | | 65267226 | 65267258 |
| 3.2047969553 | larp4 (bg=13.51%) | K562 | + | chr11 | | 65267258 | 65267285 |
| 2.44202709591 | MTPAP (bg=9.55%) | K562 | + | chr11 | | 65267246 | 65267283 |
| 2.69482851663 | npm1 (bg=10.22%) | K562 | + | chr11 | | 65267234 | 65267291 |
| 4.02379254966 | rbm22 (bg=12.69%) | HepG2 | + | chr11 | | 65267232 | 65267262 |
| 3.53109896683 | rbm22 (bg=12.69%) | HepG2 | + | chr11 | | 65267262 | 65267278 |
| 4.06937885038 | safb2 (bg=26.89%) | K562 | + | chr11 | | 65267242 | 65267255 |
| 4.59722196544 | safb2 (bg=26.89%) | K562 | + | chr11 | | 65267246 | 65267255 |
| 4.61560138861 | safb2 (bg=26.89%) | K562 | + | chr11 | | 65267255 | 65267260 |
| 4.13058716957 | safb2 (bg=26.89%) | K562 | + | chr11 | | 65267255 | 65267260 |
| 4.10591641106 | safb2 (bg=26.89%) | K562 | + | chr11 | | 65267260 | 65267272 |
| 3.69823660491 | safb2 (bg=26.89%) | K562 | + | chr11 | | 65267260 | 65267272 |
| 2.40640424838 | SMNDC1 (bg=7.08%) | HepG2 | + | chr11 | | 65267240 | 65267274 |
| 2.50504261378 | srsf1 (bg=30.28%) | K562 | + | chr11 | | 65267233 | 65267259 |
| 3.86435690026 | srsf1 (bg=30.28%) | K562 | + | chr11 | | 65267241 | 65267256 |
| 3.70220438365 | srsf1 (bg=30.28%) | K562 | + | chr11 | | 65267256 | 65267261 |
| 2.10659957872 | srsf1 (bg=30.28%) | K562 | + | chr11 | | 65267259 | 65267290 |
| 3.5127897199 | srsf1 (bg=30.28%) | K562 | + | chr11 | | 65267261 | 65267295 |
| 2.61932318704 | srsf7 (bg=22.53%) | K562 | + | chr11 | | 65267230 | 65267261 |
| 2.42529829883 | srsf7 (bg=22.53%) | K562 | + | chr11 | | 65267241 | 65267260 |
| 2.54440329227 | srsf7 (bg=22.53%) | K562 | + | chr11 | | 65267260 | 65267275 |
| 2.65552932803 | srsf7 (bg=22.53%) | K562 | + | chr11 | | 65267261 | 65267294 |
| 3.74116628913 | SRSF9 (bg=9.67%) | HepG2 | + | chr11 | | 65267257 | 65267287 |
| 2.01684847706 | TAF15 (bg=9.06%) | K562 | + | chr11 | | 65267241 | 65267256 |
| 2.6418112258 | TAF15 (bg=9.06%) | HepG2 | + | chr11 | | 65267248 | 65267287 |
| 3.69259126683 | tra2a (bg=37.02%) | HepG2 | + | chr11 | | 65267223 | 65267307 |
| 5.64737493311 | tra2a (bg=37.02%) | HepG2 | + | chr11 | | 65267226 | 65267259 |
| 6.42971360979 | tra2a (bg=37.02%) | K562 | + | chr11 | | 65267243 | 65267254 |
| 6.00625981405 | tra2a (bg=37.02%) | K562 | + | chr11 | | 65267246 | 65267255 |
| 6.25479603795 | tra2a (bg=37.02%) | K562 | + | chr11 | | 65267254 | 65267260 |
| 6.00318291258 | tra2a (bg=37.02%) | K562 | + | chr11 | | 65267255 | 65267259 |
| 5.86842560883 | tra2a (bg=37.02%) | K562 | + | chr11 | | 65267259 | 65267272 |
| 5.27142582393 | tra2a (bg=37.02%) | HepG2 | + | chr11 | | 65267259 | 65267297 |
| 6.0711542442 | tra2a (bg=37.02%) | K562 | + | chr11 | | 65267260 | 65267268 |
| 2.17543462991 | TROVE2 (bg=6.96%) | HepG2 | + | chr11 | | 65267229 | 65267258 |
| 2.26542048653 | TROVE2 (bg=6.96%) | HepG2 | + | chr11 | | 65267258 | 65267300 |
| 2.5414590362 | uchl5 (bg=18.56%) | K562 | + | chr11 | | 65267231 | 65267257 |
| 2.96308808814 | uchl5 (bg=18.56%) | K562 | + | chr11 | | 65267243 | 65267259 |
| 2.60778636936 | uchl5 (bg=18.56%) | K562 | + | chr11 | | 65267257 | 65267283 |
| 3.04104302397 | uchl5 (bg=18.56%) | K562 | + | chr11 | | 65267259 | 65267269 |
| 2.23408450546 | YWHAG (bg=9.14%) | K562 | + | chr11 | | 65267234 | 65267278 |
| 2.07223161097 | zc3h8 (bg=12.78%) | K562 | + | chr11 | | 65267242 | 65267280 |
| 2.29797932099 | zc3h8 (bg=12.78%) | K562 | + | chr11 | | 65267245 | 65267274 |
| 3.49453099038 | znf622 (bg=18.79%) | K562 | + | chr11 | | 65267236 | 65267256 |
| 3.59337289376 | znf622 (bg=18.79%) | K562 | + | chr11 | | 65267256 | 65267273 |

  
  

| Match 55 in HUMAN | | | | | | | |
| --- | --- | --- | --- | --- | --- | --- | --- |
| Motif | Start in Seq (1 Indexed) | End in Seq (1 Indexed) | Strand | Chrm | Exon | Start in Chrm (0 Indexed) | End in Chrm (1 Indexed) |
| GAAGAAAAAA | 764 | 773 | + | chr11 | 1 | 65267253 | 65267263 |
| eCLIP Fold-Enrichment | Binding Protein | Cell Line | Strand | Chrm | | Start in Chrm (0 Indexed) | End in Chrm (1 Indexed) |
| 3.499097184 | aggf1 (bg=15.15%) | K562 | + | chr11 | | 65267244 | 65267262 |
| 2.93658338586 | aggf1 (bg=15.15%) | K562 | + | chr11 | | 65267262 | 65267279 |
| 2.19054907827 | AQR (bg=4.89%) | HepG2 | + | chr11 | | 65267247 | 65267259 |
| 2.24068715925 | AQR (bg=4.89%) | HepG2 | + | chr11 | | 65267259 | 65267265 |
| 3.73367396152 | bclaf1 (bg=17.67%) | HepG2 | + | chr11 | | 65267228 | 65267258 |
| 3.8754802553 | bclaf1 (bg=17.67%) | HepG2 | + | chr11 | | 65267229 | 65267265 |
| 3.66041502544 | bclaf1 (bg=17.67%) | HepG2 | + | chr11 | | 65267258 | 65267280 |
| 2.39416939709 | bud13 (bg=12.85%) | K562 | + | chr11 | | 65267227 | 65267255 |
| 2.50334978883 | bud13 (bg=12.85%) | HepG2 | + | chr11 | | 65267230 | 65267263 |
| 2.0157307384 | bud13 (bg=12.85%) | K562 | + | chr11 | | 65267246 | 65267260 |
| 2.4605118231 | bud13 (bg=12.85%) | K562 | + | chr11 | | 65267255 | 65267260 |
| 2.14665244889 | bud13 (bg=12.85%) | K562 | + | chr11 | | 65267260 | 65267269 |
| 2.46161609591 | bud13 (bg=12.85%) | K562 | + | chr11 | | 65267260 | 65267273 |
| 2.1314756175 | bud13 (bg=12.85%) | HepG2 | + | chr11 | | 65267263 | 65267268 |
| 3.50462703769 | cpsf6 (bg=13.45%) | K562 | + | chr11 | | 65267226 | 65267259 |
| 2.59319606945 | cpsf6 (bg=13.45%) | K562 | + | chr11 | | 65267229 | 65267300 |
| 3.11501566237 | cpsf6 (bg=13.45%) | K562 | + | chr11 | | 65267259 | 65267309 |
| 2.23396057651 | FASTKD2 (bg=4.54%) | K562 | + | chr11 | | 65267229 | 65267258 |
| 2.36366240454 | FASTKD2 (bg=4.54%) | K562 | + | chr11 | | 65267258 | 65267285 |
| 2.01160631692 | FTO (bg=1.11%) | K562 | + | chr11 | | 65267241 | 65267258 |
| 2.09718281337 | FTO (bg=1.11%) | K562 | + | chr11 | | 65267258 | 65267288 |
| 2.57398657312 | fxr2 (bg=10.1%) | HepG2 | + | chr11 | | 65267220 | 65267312 |
| 2.49434921061 | GPKOW (bg=5.66%) | K562 | + | chr11 | | 65267228 | 65267259 |
| 2.24466015604 | GPKOW (bg=5.66%) | K562 | + | chr11 | | 65267229 | 65267261 |
| 2.64679580475 | GPKOW (bg=5.66%) | K562 | + | chr11 | | 65267259 | 65267284 |
| 2.32188827677 | GPKOW (bg=5.66%) | K562 | + | chr11 | | 65267261 | 65267282 |
| 3.24731030221 | gtf2f1 (bg=10.18%) | HepG2 | + | chr11 | | 65267240 | 65267263 |
| 2.28331455438 | hltf (bg=24.28%) | HepG2 | + | chr11 | | 65267229 | 65267258 |
| 2.6104407171 | hltf (bg=24.28%) | HepG2 | + | chr11 | | 65267230 | 65267254 |
| 2.36660595829 | hltf (bg=24.28%) | HepG2 | + | chr11 | | 65267254 | 65267263 |
| 2.19748890429 | hltf (bg=24.28%) | HepG2 | + | chr11 | | 65267258 | 65267287 |
| 2.17726025994 | hltf (bg=24.28%) | HepG2 | + | chr11 | | 65267263 | 65267276 |
| 3.05863680037 | larp4 (bg=13.51%) | K562 | + | chr11 | | 65267226 | 65267258 |
| 3.2047969553 | larp4 (bg=13.51%) | K562 | + | chr11 | | 65267258 | 65267285 |
| 2.44202709591 | MTPAP (bg=9.55%) | K562 | + | chr11 | | 65267246 | 65267283 |
| 2.69482851663 | npm1 (bg=10.22%) | K562 | + | chr11 | | 65267234 | 65267291 |
| 4.02379254966 | rbm22 (bg=12.69%) | HepG2 | + | chr11 | | 65267232 | 65267262 |
| 3.53109896683 | rbm22 (bg=12.69%) | HepG2 | + | chr11 | | 65267262 | 65267278 |
| 4.06937885038 | safb2 (bg=26.89%) | K562 | + | chr11 | | 65267242 | 65267255 |
| 4.59722196544 | safb2 (bg=26.89%) | K562 | + | chr11 | | 65267246 | 65267255 |
| 4.61560138861 | safb2 (bg=26.89%) | K562 | + | chr11 | | 65267255 | 65267260 |
| 4.13058716957 | safb2 (bg=26.89%) | K562 | + | chr11 | | 65267255 | 65267260 |
| 4.10591641106 | safb2 (bg=26.89%) | K562 | + | chr11 | | 65267260 | 65267272 |
| 3.69823660491 | safb2 (bg=26.89%) | K562 | + | chr11 | | 65267260 | 65267272 |
| 2.40640424838 | SMNDC1 (bg=7.08%) | HepG2 | + | chr11 | | 65267240 | 65267274 |
| 2.50504261378 | srsf1 (bg=30.28%) | K562 | + | chr11 | | 65267233 | 65267259 |
| 3.86435690026 | srsf1 (bg=30.28%) | K562 | + | chr11 | | 65267241 | 65267256 |
| 3.70220438365 | srsf1 (bg=30.28%) | K562 | + | chr11 | | 65267256 | 65267261 |
| 2.10659957872 | srsf1 (bg=30.28%) | K562 | + | chr11 | | 65267259 | 65267290 |
| 3.5127897199 | srsf1 (bg=30.28%) | K562 | + | chr11 | | 65267261 | 65267295 |
| 2.61932318704 | srsf7 (bg=22.53%) | K562 | + | chr11 | | 65267230 | 65267261 |
| 2.42529829883 | srsf7 (bg=22.53%) | K562 | + | chr11 | | 65267241 | 65267260 |
| 2.54440329227 | srsf7 (bg=22.53%) | K562 | + | chr11 | | 65267260 | 65267275 |
| 2.65552932803 | srsf7 (bg=22.53%) | K562 | + | chr11 | | 65267261 | 65267294 |
| 3.74116628913 | SRSF9 (bg=9.67%) | HepG2 | + | chr11 | | 65267257 | 65267287 |
| 2.01684847706 | TAF15 (bg=9.06%) | K562 | + | chr11 | | 65267241 | 65267256 |
| 2.6418112258 | TAF15 (bg=9.06%) | HepG2 | + | chr11 | | 65267248 | 65267287 |
| 3.69259126683 | tra2a (bg=37.02%) | HepG2 | + | chr11 | | 65267223 | 65267307 |
| 5.64737493311 | tra2a (bg=37.02%) | HepG2 | + | chr11 | | 65267226 | 65267259 |
| 6.42971360979 | tra2a (bg=37.02%) | K562 | + | chr11 | | 65267243 | 65267254 |
| 6.00625981405 | tra2a (bg=37.02%) | K562 | + | chr11 | | 65267246 | 65267255 |
| 6.25479603795 | tra2a (bg=37.02%) | K562 | + | chr11 | | 65267254 | 65267260 |
| 6.00318291258 | tra2a (bg=37.02%) | K562 | + | chr11 | | 65267255 | 65267259 |
| 5.86842560883 | tra2a (bg=37.02%) | K562 | + | chr11 | | 65267259 | 65267272 |
| 5.27142582393 | tra2a (bg=37.02%) | HepG2 | + | chr11 | | 65267259 | 65267297 |
| 6.0711542442 | tra2a (bg=37.02%) | K562 | + | chr11 | | 65267260 | 65267268 |
| 2.17543462991 | TROVE2 (bg=6.96%) | HepG2 | + | chr11 | | 65267229 | 65267258 |
| 2.26542048653 | TROVE2 (bg=6.96%) | HepG2 | + | chr11 | | 65267258 | 65267300 |
| 2.5414590362 | uchl5 (bg=18.56%) | K562 | + | chr11 | | 65267231 | 65267257 |
| 2.96308808814 | uchl5 (bg=18.56%) | K562 | + | chr11 | | 65267243 | 65267259 |
| 2.60778636936 | uchl5 (bg=18.56%) | K562 | + | chr11 | | 65267257 | 65267283 |
| 3.04104302397 | uchl5 (bg=18.56%) | K562 | + | chr11 | | 65267259 | 65267269 |
| 2.23408450546 | YWHAG (bg=9.14%) | K562 | + | chr11 | | 65267234 | 65267278 |
| 2.07223161097 | zc3h8 (bg=12.78%) | K562 | + | chr11 | | 65267242 | 65267280 |
| 2.29797932099 | zc3h8 (bg=12.78%) | K562 | + | chr11 | | 65267245 | 65267274 |
| 3.49453099038 | znf622 (bg=18.79%) | K562 | + | chr11 | | 65267236 | 65267256 |
| 3.59337289376 | znf622 (bg=18.79%) | K562 | + | chr11 | | 65267256 | 65267273 |

  
  

| Match 56 in HUMAN | | | | | | | |
| --- | --- | --- | --- | --- | --- | --- | --- |
| Motif | Start in Seq (1 Indexed) | End in Seq (1 Indexed) | Strand | Chrm | Exon | Start in Chrm (0 Indexed) | End in Chrm (1 Indexed) |
| GACAAGCTAGGAAACAAAAA | 774 | 793 | + | chr11 | 1 | 65267263 | 65267283 |
| eCLIP Fold-Enrichment | Binding Protein | Cell Line | Strand | Chrm | | Start in Chrm (0 Indexed) | End in Chrm (1 Indexed) |
| 2.93658338586 | aggf1 (bg=15.15%) | K562 | + | chr11 | | 65267262 | 65267279 |
| 2.55982784822 | aggf1 (bg=15.15%) | K562 | + | chr11 | | 65267279 | 65267293 |
| 2.24068715925 | AQR (bg=4.89%) | HepG2 | + | chr11 | | 65267259 | 65267265 |
| 3.8754802553 | bclaf1 (bg=17.67%) | HepG2 | + | chr11 | | 65267229 | 65267265 |
| 3.66041502544 | bclaf1 (bg=17.67%) | HepG2 | + | chr11 | | 65267258 | 65267280 |
| 3.64792925706 | bclaf1 (bg=17.67%) | HepG2 | + | chr11 | | 65267265 | 65267277 |
| 2.50334978883 | bud13 (bg=12.85%) | HepG2 | + | chr11 | | 65267230 | 65267263 |
| 2.14665244889 | bud13 (bg=12.85%) | K562 | + | chr11 | | 65267260 | 65267269 |
| 2.46161609591 | bud13 (bg=12.85%) | K562 | + | chr11 | | 65267260 | 65267273 |
| 2.1314756175 | bud13 (bg=12.85%) | HepG2 | + | chr11 | | 65267263 | 65267268 |
| 2.14325278528 | bud13 (bg=12.85%) | HepG2 | + | chr11 | | 65267268 | 65267279 |
| 2.1293899032 | bud13 (bg=12.85%) | K562 | + | chr11 | | 65267269 | 65267273 |
| 2.19718575781 | bud13 (bg=12.85%) | K562 | + | chr11 | | 65267273 | 65267282 |
| 2.44859901643 | bud13 (bg=12.85%) | K562 | + | chr11 | | 65267273 | 65267282 |
| 2.02892384958 | bud13 (bg=12.85%) | HepG2 | + | chr11 | | 65267279 | 65267301 |
| 2.38456693896 | bud13 (bg=12.85%) | K562 | + | chr11 | | 65267282 | 65267288 |
| 2.16781963158 | bud13 (bg=12.85%) | K562 | + | chr11 | | 65267282 | 65267296 |
| 2.59319606945 | cpsf6 (bg=13.45%) | K562 | + | chr11 | | 65267229 | 65267300 |
| 3.11501566237 | cpsf6 (bg=13.45%) | K562 | + | chr11 | | 65267259 | 65267309 |
| 2.36366240454 | FASTKD2 (bg=4.54%) | K562 | + | chr11 | | 65267258 | 65267285 |
| 2.09718281337 | FTO (bg=1.11%) | K562 | + | chr11 | | 65267258 | 65267288 |
| 2.57398657312 | fxr2 (bg=10.1%) | HepG2 | + | chr11 | | 65267220 | 65267312 |
| 2.64679580475 | GPKOW (bg=5.66%) | K562 | + | chr11 | | 65267259 | 65267284 |
| 2.32188827677 | GPKOW (bg=5.66%) | K562 | + | chr11 | | 65267261 | 65267282 |
| 3.24731030221 | gtf2f1 (bg=10.18%) | HepG2 | + | chr11 | | 65267240 | 65267263 |
| 2.89736783115 | gtf2f1 (bg=10.18%) | HepG2 | + | chr11 | | 65267268 | 65267273 |
| 2.36660595829 | hltf (bg=24.28%) | HepG2 | + | chr11 | | 65267254 | 65267263 |
| 2.19748890429 | hltf (bg=24.28%) | HepG2 | + | chr11 | | 65267258 | 65267287 |
| 2.17726025994 | hltf (bg=24.28%) | HepG2 | + | chr11 | | 65267263 | 65267276 |
| 3.2047969553 | larp4 (bg=13.51%) | K562 | + | chr11 | | 65267258 | 65267285 |
| 2.44202709591 | MTPAP (bg=9.55%) | K562 | + | chr11 | | 65267246 | 65267283 |
| 2.69482851663 | npm1 (bg=10.22%) | K562 | + | chr11 | | 65267234 | 65267291 |
| 3.53109896683 | rbm22 (bg=12.69%) | HepG2 | + | chr11 | | 65267262 | 65267278 |
| 3.12757545556 | rbm22 (bg=12.69%) | HepG2 | + | chr11 | | 65267278 | 65267295 |
| 4.10591641106 | safb2 (bg=26.89%) | K562 | + | chr11 | | 65267260 | 65267272 |
| 3.69823660491 | safb2 (bg=26.89%) | K562 | + | chr11 | | 65267260 | 65267272 |
| 3.58102040102 | safb2 (bg=26.89%) | K562 | + | chr11 | | 65267272 | 65267276 |
| 3.90773326342 | safb2 (bg=26.89%) | K562 | + | chr11 | | 65267272 | 65267277 |
| 3.49889476531 | safb2 (bg=26.89%) | K562 | + | chr11 | | 65267276 | 65267289 |
| 3.80424626264 | safb2 (bg=26.89%) | K562 | + | chr11 | | 65267277 | 65267283 |
| 3.75223533261 | safb2 (bg=26.89%) | K562 | + | chr11 | | 65267283 | 65267296 |
| 2.40640424838 | SMNDC1 (bg=7.08%) | HepG2 | + | chr11 | | 65267240 | 65267274 |
| 2.10659957872 | srsf1 (bg=30.28%) | K562 | + | chr11 | | 65267259 | 65267290 |
| 3.5127897199 | srsf1 (bg=30.28%) | K562 | + | chr11 | | 65267261 | 65267295 |
| 2.54440329227 | srsf7 (bg=22.53%) | K562 | + | chr11 | | 65267260 | 65267275 |
| 2.65552932803 | srsf7 (bg=22.53%) | K562 | + | chr11 | | 65267261 | 65267294 |
| 2.40738822197 | srsf7 (bg=22.53%) | K562 | + | chr11 | | 65267275 | 65267295 |
| 3.74116628913 | SRSF9 (bg=9.67%) | HepG2 | + | chr11 | | 65267257 | 65267287 |
| 2.6418112258 | TAF15 (bg=9.06%) | HepG2 | + | chr11 | | 65267248 | 65267287 |
| 3.69259126683 | tra2a (bg=37.02%) | HepG2 | + | chr11 | | 65267223 | 65267307 |
| 5.86842560883 | tra2a (bg=37.02%) | K562 | + | chr11 | | 65267259 | 65267272 |
| 5.27142582393 | tra2a (bg=37.02%) | HepG2 | + | chr11 | | 65267259 | 65267297 |
| 6.0711542442 | tra2a (bg=37.02%) | K562 | + | chr11 | | 65267260 | 65267268 |
| 6.13227025368 | tra2a (bg=37.02%) | K562 | + | chr11 | | 65267268 | 65267272 |
| 5.85084183271 | tra2a (bg=37.02%) | K562 | + | chr11 | | 65267272 | 65267276 |
| 6.09031119725 | tra2a (bg=37.02%) | K562 | + | chr11 | | 65267272 | 65267276 |
| 5.77782577583 | tra2a (bg=37.02%) | K562 | + | chr11 | | 65267276 | 65267282 |
| 5.971936535 | tra2a (bg=37.02%) | K562 | + | chr11 | | 65267276 | 65267283 |
| 5.72170082665 | tra2a (bg=37.02%) | K562 | + | chr11 | | 65267282 | 65267284 |
| 5.86913879461 | tra2a (bg=37.02%) | K562 | + | chr11 | | 65267283 | 65267288 |
| 2.26542048653 | TROVE2 (bg=6.96%) | HepG2 | + | chr11 | | 65267258 | 65267300 |
| 2.60778636936 | uchl5 (bg=18.56%) | K562 | + | chr11 | | 65267257 | 65267283 |
| 3.04104302397 | uchl5 (bg=18.56%) | K562 | + | chr11 | | 65267259 | 65267269 |
| 3.00570230224 | uchl5 (bg=18.56%) | K562 | + | chr11 | | 65267269 | 65267273 |
| 3.01261278278 | uchl5 (bg=18.56%) | K562 | + | chr11 | | 65267273 | 65267277 |
| 2.90049378543 | uchl5 (bg=18.56%) | K562 | + | chr11 | | 65267277 | 65267283 |
| 2.62413381028 | uchl5 (bg=18.56%) | K562 | + | chr11 | | 65267283 | 65267294 |
| 2.97174354054 | uchl5 (bg=18.56%) | K562 | + | chr11 | | 65267283 | 65267296 |
| 2.23408450546 | YWHAG (bg=9.14%) | K562 | + | chr11 | | 65267234 | 65267278 |
| 2.07223161097 | zc3h8 (bg=12.78%) | K562 | + | chr11 | | 65267242 | 65267280 |
| 2.29797932099 | zc3h8 (bg=12.78%) | K562 | + | chr11 | | 65267245 | 65267274 |
| 3.59337289376 | znf622 (bg=18.79%) | K562 | + | chr11 | | 65267256 | 65267273 |
| 3.01047127463 | znf622 (bg=18.79%) | K562 | + | chr11 | | 65267273 | 65267274 |
| 3.56901368813 | znf622 (bg=18.79%) | K562 | + | chr11 | | 65267273 | 65267278 |

  
  

| Match 57 in HUMAN | | | | | | | |
| --- | --- | --- | --- | --- | --- | --- | --- |
| Motif | Start in Seq (1 Indexed) | End in Seq (1 Indexed) | Strand | Chrm | Exon | Start in Chrm (0 Indexed) | End in Chrm (1 Indexed) |
| CTAAGGGCAAAATGT | 795 | 809 | + | chr11 | 1 | 65267284 | 65267299 |
| eCLIP Fold-Enrichment | Binding Protein | Cell Line | Strand | Chrm | | Start in Chrm (0 Indexed) | End in Chrm (1 Indexed) |
| 2.55982784822 | aggf1 (bg=15.15%) | K562 | + | chr11 | | 65267279 | 65267293 |
| 2.82691715282 | aggf1 (bg=15.15%) | K562 | + | chr11 | | 65267293 | 65267297 |
| 2.70247301349 | aggf1 (bg=15.15%) | K562 | + | chr11 | | 65267297 | 65267304 |
| 2.09972729171 | AQR (bg=4.89%) | HepG2 | + | chr11 | | 65267295 | 65267304 |
| 2.02892384958 | bud13 (bg=12.85%) | HepG2 | + | chr11 | | 65267279 | 65267301 |
| 2.38456693896 | bud13 (bg=12.85%) | K562 | + | chr11 | | 65267282 | 65267288 |
| 2.16781963158 | bud13 (bg=12.85%) | K562 | + | chr11 | | 65267282 | 65267296 |
| 2.38142472286 | bud13 (bg=12.85%) | K562 | + | chr11 | | 65267288 | 65267295 |
| 2.33767033289 | bud13 (bg=12.85%) | K562 | + | chr11 | | 65267295 | 65267303 |
| 2.07609413608 | bud13 (bg=12.85%) | K562 | + | chr11 | | 65267296 | 65267305 |
| 2.59319606945 | cpsf6 (bg=13.45%) | K562 | + | chr11 | | 65267229 | 65267300 |
| 3.11501566237 | cpsf6 (bg=13.45%) | K562 | + | chr11 | | 65267259 | 65267309 |
| 2.36366240454 | FASTKD2 (bg=4.54%) | K562 | + | chr11 | | 65267258 | 65267285 |
| 2.05694749564 | FASTKD2 (bg=4.54%) | K562 | + | chr11 | | 65267285 | 65267297 |
| 2.37839712805 | FASTKD2 (bg=4.54%) | K562 | + | chr11 | | 65267297 | 65267334 |
| 2.09718281337 | FTO (bg=1.11%) | K562 | + | chr11 | | 65267258 | 65267288 |
| 2.57398657312 | fxr2 (bg=10.1%) | HepG2 | + | chr11 | | 65267220 | 65267312 |
| 2.64679580475 | GPKOW (bg=5.66%) | K562 | + | chr11 | | 65267259 | 65267284 |
| 2.33834822591 | GPKOW (bg=5.66%) | K562 | + | chr11 | | 65267284 | 65267294 |
| 2.31184814754 | GPKOW (bg=5.66%) | K562 | + | chr11 | | 65267294 | 65267334 |
| 2.19748890429 | hltf (bg=24.28%) | HepG2 | + | chr11 | | 65267258 | 65267287 |
| 3.2047969553 | larp4 (bg=13.51%) | K562 | + | chr11 | | 65267258 | 65267285 |
| 3.01481611216 | larp4 (bg=13.51%) | K562 | + | chr11 | | 65267285 | 65267295 |
| 3.08990219548 | larp4 (bg=13.51%) | K562 | + | chr11 | | 65267295 | 65267302 |
| 2.69482851663 | npm1 (bg=10.22%) | K562 | + | chr11 | | 65267234 | 65267291 |
| 3.12757545556 | rbm22 (bg=12.69%) | HepG2 | + | chr11 | | 65267278 | 65267295 |
| 3.49889476531 | safb2 (bg=26.89%) | K562 | + | chr11 | | 65267276 | 65267289 |
| 3.75223533261 | safb2 (bg=26.89%) | K562 | + | chr11 | | 65267283 | 65267296 |
| 3.45522779332 | safb2 (bg=26.89%) | K562 | + | chr11 | | 65267289 | 65267296 |
| 3.41691145478 | safb2 (bg=26.89%) | K562 | + | chr11 | | 65267296 | 65267299 |
| 3.06292282699 | safb2 (bg=26.89%) | K562 | + | chr11 | | 65267296 | 65267303 |
| 3.2636682736 | safb2 (bg=26.89%) | K562 | + | chr11 | | 65267299 | 65267305 |
| 2.10659957872 | srsf1 (bg=30.28%) | K562 | + | chr11 | | 65267259 | 65267290 |
| 3.5127897199 | srsf1 (bg=30.28%) | K562 | + | chr11 | | 65267261 | 65267295 |
| 3.21271752221 | srsf1 (bg=30.28%) | K562 | + | chr11 | | 65267295 | 65267303 |
| 2.65552932803 | srsf7 (bg=22.53%) | K562 | + | chr11 | | 65267261 | 65267294 |
| 2.40738822197 | srsf7 (bg=22.53%) | K562 | + | chr11 | | 65267275 | 65267295 |
| 2.24562721315 | srsf7 (bg=22.53%) | K562 | + | chr11 | | 65267295 | 65267311 |
| 2.02119678918 | srsf7 (bg=22.53%) | K562 | + | chr11 | | 65267297 | 65267305 |
| 3.74116628913 | SRSF9 (bg=9.67%) | HepG2 | + | chr11 | | 65267257 | 65267287 |
| 2.6418112258 | TAF15 (bg=9.06%) | HepG2 | + | chr11 | | 65267248 | 65267287 |
| 3.69259126683 | tra2a (bg=37.02%) | HepG2 | + | chr11 | | 65267223 | 65267307 |
| 5.27142582393 | tra2a (bg=37.02%) | HepG2 | + | chr11 | | 65267259 | 65267297 |
| 5.72170082665 | tra2a (bg=37.02%) | K562 | + | chr11 | | 65267282 | 65267284 |
| 5.86913879461 | tra2a (bg=37.02%) | K562 | + | chr11 | | 65267283 | 65267288 |
| 5.79476026409 | tra2a (bg=37.02%) | K562 | + | chr11 | | 65267284 | 65267292 |
| 5.95666073111 | tra2a (bg=37.02%) | K562 | + | chr11 | | 65267288 | 65267296 |
| 5.79797180714 | tra2a (bg=37.02%) | K562 | + | chr11 | | 65267292 | 65267295 |
| 5.92150440733 | tra2a (bg=37.02%) | K562 | + | chr11 | | 65267295 | 65267305 |
| 6.01274058107 | tra2a (bg=37.02%) | K562 | + | chr11 | | 65267296 | 65267303 |
| 5.11570444224 | tra2a (bg=37.02%) | HepG2 | + | chr11 | | 65267297 | 65267346 |
| 2.26542048653 | TROVE2 (bg=6.96%) | HepG2 | + | chr11 | | 65267258 | 65267300 |
| 2.62413381028 | uchl5 (bg=18.56%) | K562 | + | chr11 | | 65267283 | 65267294 |
| 2.97174354054 | uchl5 (bg=18.56%) | K562 | + | chr11 | | 65267283 | 65267296 |
| 2.7482946764 | uchl5 (bg=18.56%) | K562 | + | chr11 | | 65267294 | 65267304 |
| 3.01002213944 | uchl5 (bg=18.56%) | K562 | + | chr11 | | 65267296 | 65267304 |
| 3.30069882235 | zc3h8 (bg=12.78%) | K562 | + | chr11 | | 65267293 | 65267375 |
| 3.762500165 | znf622 (bg=18.79%) | K562 | + | chr11 | | 65267286 | 65267296 |
| 3.73840643239 | znf622 (bg=18.79%) | K562 | + | chr11 | | 65267296 | 65267310 |

  
  

| Match 58 in HUMAN | | | | | | | |
| --- | --- | --- | --- | --- | --- | --- | --- |
| Motif | Start in Seq (1 Indexed) | End in Seq (1 Indexed) | Strand | Chrm | Exon | Start in Chrm (0 Indexed) | End in Chrm (1 Indexed) |
| CAAACTTAGAAGAAAA | 811 | 826 | + | chr11 | 1 | 65267300 | 65267316 |
| eCLIP Fold-Enrichment | Binding Protein | Cell Line | Strand | Chrm | | Start in Chrm (0 Indexed) | End in Chrm (1 Indexed) |
| 2.70247301349 | aggf1 (bg=15.15%) | K562 | + | chr11 | | 65267297 | 65267304 |
| 2.79541675164 | aggf1 (bg=15.15%) | K562 | + | chr11 | | 65267304 | 65267315 |
| 2.90401242941 | aggf1 (bg=15.15%) | K562 | + | chr11 | | 65267315 | 65267321 |
| 2.09972729171 | AQR (bg=4.89%) | HepG2 | + | chr11 | | 65267295 | 65267304 |
| 4.05275760056 | bclaf1 (bg=17.67%) | HepG2 | + | chr11 | | 65267314 | 65267334 |
| 2.02892384958 | bud13 (bg=12.85%) | HepG2 | + | chr11 | | 65267279 | 65267301 |
| 2.33767033289 | bud13 (bg=12.85%) | K562 | + | chr11 | | 65267295 | 65267303 |
| 2.07609413608 | bud13 (bg=12.85%) | K562 | + | chr11 | | 65267296 | 65267305 |
| 2.2767024783 | bud13 (bg=12.85%) | HepG2 | + | chr11 | | 65267301 | 65267340 |
| 2.3266908775 | bud13 (bg=12.85%) | K562 | + | chr11 | | 65267303 | 65267310 |
| 2.56126742833 | bud13 (bg=12.85%) | K562 | + | chr11 | | 65267310 | 65267315 |
| 2.57359456436 | bud13 (bg=12.85%) | K562 | + | chr11 | | 65267315 | 65267320 |
| 2.23878990817 | bud13 (bg=12.85%) | K562 | + | chr11 | | 65267315 | 65267321 |
| 2.59319606945 | cpsf6 (bg=13.45%) | K562 | + | chr11 | | 65267229 | 65267300 |
| 3.11501566237 | cpsf6 (bg=13.45%) | K562 | + | chr11 | | 65267259 | 65267309 |
| 2.84209598348 | cpsf6 (bg=13.45%) | K562 | + | chr11 | | 65267309 | 65267321 |
| 2.37839712805 | FASTKD2 (bg=4.54%) | K562 | + | chr11 | | 65267297 | 65267334 |
| 2.57398657312 | fxr2 (bg=10.1%) | HepG2 | + | chr11 | | 65267220 | 65267312 |
| 2.40986981466 | fxr2 (bg=10.1%) | HepG2 | + | chr11 | | 65267312 | 65267382 |
| 2.31184814754 | GPKOW (bg=5.66%) | K562 | + | chr11 | | 65267294 | 65267334 |
| 3.08990219548 | larp4 (bg=13.51%) | K562 | + | chr11 | | 65267295 | 65267302 |
| 3.18013485638 | larp4 (bg=13.51%) | K562 | + | chr11 | | 65267302 | 65267332 |
| 2.67686369108 | npm1 (bg=10.22%) | K562 | + | chr11 | | 65267305 | 65267319 |
| 3.21274283883 | rbm22 (bg=12.69%) | HepG2 | + | chr11 | | 65267308 | 65267339 |
| 3.06292282699 | safb2 (bg=26.89%) | K562 | + | chr11 | | 65267296 | 65267303 |
| 3.2636682736 | safb2 (bg=26.89%) | K562 | + | chr11 | | 65267299 | 65267305 |
| 2.99955786537 | safb2 (bg=26.89%) | K562 | + | chr11 | | 65267303 | 65267306 |
| 3.13830042237 | safb2 (bg=26.89%) | K562 | + | chr11 | | 65267305 | 65267315 |
| 3.06487518912 | safb2 (bg=26.89%) | K562 | + | chr11 | | 65267306 | 65267309 |
| 3.11679208823 | safb2 (bg=26.89%) | K562 | + | chr11 | | 65267309 | 65267315 |
| 3.67705411581 | safb2 (bg=26.89%) | K562 | + | chr11 | | 65267315 | 65267318 |
| 3.43804478165 | safb2 (bg=26.89%) | K562 | + | chr11 | | 65267315 | 65267320 |
| 3.21271752221 | srsf1 (bg=30.28%) | K562 | + | chr11 | | 65267295 | 65267303 |
| 3.07585088081 | srsf1 (bg=30.28%) | K562 | + | chr11 | | 65267303 | 65267314 |
| 3.216342229 | srsf1 (bg=30.28%) | K562 | + | chr11 | | 65267314 | 65267333 |
| 2.24562721315 | srsf7 (bg=22.53%) | K562 | + | chr11 | | 65267295 | 65267311 |
| 2.02119678918 | srsf7 (bg=22.53%) | K562 | + | chr11 | | 65267297 | 65267305 |
| 3.69259126683 | tra2a (bg=37.02%) | HepG2 | + | chr11 | | 65267223 | 65267307 |
| 5.92150440733 | tra2a (bg=37.02%) | K562 | + | chr11 | | 65267295 | 65267305 |
| 6.01274058107 | tra2a (bg=37.02%) | K562 | + | chr11 | | 65267296 | 65267303 |
| 5.11570444224 | tra2a (bg=37.02%) | HepG2 | + | chr11 | | 65267297 | 65267346 |
| 6.02417438261 | tra2a (bg=37.02%) | K562 | + | chr11 | | 65267303 | 65267306 |
| 5.57356769763 | tra2a (bg=37.02%) | K562 | + | chr11 | | 65267305 | 65267315 |
| 6.171235777 | tra2a (bg=37.02%) | K562 | + | chr11 | | 65267306 | 65267310 |
| 3.18387175151 | tra2a (bg=37.02%) | HepG2 | + | chr11 | | 65267307 | 65267344 |
| 6.51415207461 | tra2a (bg=37.02%) | K562 | + | chr11 | | 65267310 | 65267315 |
| 6.02769306106 | tra2a (bg=37.02%) | K562 | + | chr11 | | 65267315 | 65267318 |
| 6.50891324887 | tra2a (bg=37.02%) | K562 | + | chr11 | | 65267315 | 65267319 |
| 2.26542048653 | TROVE2 (bg=6.96%) | HepG2 | + | chr11 | | 65267258 | 65267300 |
| 2.29034283918 | TROVE2 (bg=6.96%) | HepG2 | + | chr11 | | 65267303 | 65267353 |
| 2.7482946764 | uchl5 (bg=18.56%) | K562 | + | chr11 | | 65267294 | 65267304 |
| 3.01002213944 | uchl5 (bg=18.56%) | K562 | + | chr11 | | 65267296 | 65267304 |
| 2.68630611452 | uchl5 (bg=18.56%) | K562 | + | chr11 | | 65267304 | 65267313 |
| 2.96978146094 | uchl5 (bg=18.56%) | K562 | + | chr11 | | 65267308 | 65267332 |
| 2.77186925664 | uchl5 (bg=18.56%) | K562 | + | chr11 | | 65267313 | 65267331 |
| 3.30069882235 | zc3h8 (bg=12.78%) | K562 | + | chr11 | | 65267293 | 65267375 |
| 2.63080835914 | zc3h8 (bg=12.78%) | K562 | + | chr11 | | 65267310 | 65267355 |
| 3.73840643239 | znf622 (bg=18.79%) | K562 | + | chr11 | | 65267296 | 65267310 |
| 3.04179877167 | znf622 (bg=18.79%) | K562 | + | chr11 | | 65267301 | 65267303 |
| 3.85748635353 | znf622 (bg=18.79%) | K562 | + | chr11 | | 65267310 | 65267345 |

  
  

| Match 59 in HUMAN | | | | | | | |
| --- | --- | --- | --- | --- | --- | --- | --- |
| Motif | Start in Seq (1 Indexed) | End in Seq (1 Indexed) | Strand | Chrm | Exon | Start in Chrm (0 Indexed) | End in Chrm (1 Indexed) |
| AGAAGAA | 818 | 824 | + | chr11 | 1 | 65267307 | 65267314 |
| eCLIP Fold-Enrichment | Binding Protein | Cell Line | Strand | Chrm | | Start in Chrm (0 Indexed) | End in Chrm (1 Indexed) |
| 2.79541675164 | aggf1 (bg=15.15%) | K562 | + | chr11 | | 65267304 | 65267315 |
| 4.05275760056 | bclaf1 (bg=17.67%) | HepG2 | + | chr11 | | 65267314 | 65267334 |
| 2.2767024783 | bud13 (bg=12.85%) | HepG2 | + | chr11 | | 65267301 | 65267340 |
| 2.3266908775 | bud13 (bg=12.85%) | K562 | + | chr11 | | 65267303 | 65267310 |
| 2.56126742833 | bud13 (bg=12.85%) | K562 | + | chr11 | | 65267310 | 65267315 |
| 3.11501566237 | cpsf6 (bg=13.45%) | K562 | + | chr11 | | 65267259 | 65267309 |
| 2.84209598348 | cpsf6 (bg=13.45%) | K562 | + | chr11 | | 65267309 | 65267321 |
| 2.37839712805 | FASTKD2 (bg=4.54%) | K562 | + | chr11 | | 65267297 | 65267334 |
| 2.57398657312 | fxr2 (bg=10.1%) | HepG2 | + | chr11 | | 65267220 | 65267312 |
| 2.40986981466 | fxr2 (bg=10.1%) | HepG2 | + | chr11 | | 65267312 | 65267382 |
| 2.31184814754 | GPKOW (bg=5.66%) | K562 | + | chr11 | | 65267294 | 65267334 |
| 3.18013485638 | larp4 (bg=13.51%) | K562 | + | chr11 | | 65267302 | 65267332 |
| 2.67686369108 | npm1 (bg=10.22%) | K562 | + | chr11 | | 65267305 | 65267319 |
| 3.21274283883 | rbm22 (bg=12.69%) | HepG2 | + | chr11 | | 65267308 | 65267339 |
| 3.13830042237 | safb2 (bg=26.89%) | K562 | + | chr11 | | 65267305 | 65267315 |
| 3.06487518912 | safb2 (bg=26.89%) | K562 | + | chr11 | | 65267306 | 65267309 |
| 3.11679208823 | safb2 (bg=26.89%) | K562 | + | chr11 | | 65267309 | 65267315 |
| 3.07585088081 | srsf1 (bg=30.28%) | K562 | + | chr11 | | 65267303 | 65267314 |
| 3.216342229 | srsf1 (bg=30.28%) | K562 | + | chr11 | | 65267314 | 65267333 |
| 2.24562721315 | srsf7 (bg=22.53%) | K562 | + | chr11 | | 65267295 | 65267311 |
| 3.69259126683 | tra2a (bg=37.02%) | HepG2 | + | chr11 | | 65267223 | 65267307 |
| 5.11570444224 | tra2a (bg=37.02%) | HepG2 | + | chr11 | | 65267297 | 65267346 |
| 5.57356769763 | tra2a (bg=37.02%) | K562 | + | chr11 | | 65267305 | 65267315 |
| 6.171235777 | tra2a (bg=37.02%) | K562 | + | chr11 | | 65267306 | 65267310 |
| 3.18387175151 | tra2a (bg=37.02%) | HepG2 | + | chr11 | | 65267307 | 65267344 |
| 6.51415207461 | tra2a (bg=37.02%) | K562 | + | chr11 | | 65267310 | 65267315 |
| 2.29034283918 | TROVE2 (bg=6.96%) | HepG2 | + | chr11 | | 65267303 | 65267353 |
| 2.68630611452 | uchl5 (bg=18.56%) | K562 | + | chr11 | | 65267304 | 65267313 |
| 2.96978146094 | uchl5 (bg=18.56%) | K562 | + | chr11 | | 65267308 | 65267332 |
| 2.77186925664 | uchl5 (bg=18.56%) | K562 | + | chr11 | | 65267313 | 65267331 |
| 3.30069882235 | zc3h8 (bg=12.78%) | K562 | + | chr11 | | 65267293 | 65267375 |
| 2.63080835914 | zc3h8 (bg=12.78%) | K562 | + | chr11 | | 65267310 | 65267355 |
| 3.73840643239 | znf622 (bg=18.79%) | K562 | + | chr11 | | 65267296 | 65267310 |
| 3.85748635353 | znf622 (bg=18.79%) | K562 | + | chr11 | | 65267310 | 65267345 |

  
  

| Match 60 in HUMAN | | | | | | | |
| --- | --- | --- | --- | --- | --- | --- | --- |
| Motif | Start in Seq (1 Indexed) | End in Seq (1 Indexed) | Strand | Chrm | Exon | Start in Chrm (0 Indexed) | End in Chrm (1 Indexed) |
| GAAGATAGAA | 830 | 839 | + | chr11 | 1 | 65267319 | 65267329 |
| eCLIP Fold-Enrichment | Binding Protein | Cell Line | Strand | Chrm | | Start in Chrm (0 Indexed) | End in Chrm (1 Indexed) |
| 2.90401242941 | aggf1 (bg=15.15%) | K562 | + | chr11 | | 65267315 | 65267321 |
| 2.94081467469 | aggf1 (bg=15.15%) | K562 | + | chr11 | | 65267321 | 65267323 |
| 2.85076883376 | aggf1 (bg=15.15%) | K562 | + | chr11 | | 65267323 | 65267328 |
| 2.91936431482 | aggf1 (bg=15.15%) | K562 | + | chr11 | | 65267328 | 65267332 |
| 4.05275760056 | bclaf1 (bg=17.67%) | HepG2 | + | chr11 | | 65267314 | 65267334 |
| 3.83554362149 | bclaf1 (bg=17.67%) | HepG2 | + | chr11 | | 65267321 | 65267340 |
| 2.2767024783 | bud13 (bg=12.85%) | HepG2 | + | chr11 | | 65267301 | 65267340 |
| 2.57359456436 | bud13 (bg=12.85%) | K562 | + | chr11 | | 65267315 | 65267320 |
| 2.23878990817 | bud13 (bg=12.85%) | K562 | + | chr11 | | 65267315 | 65267321 |
| 2.50286648581 | bud13 (bg=12.85%) | K562 | + | chr11 | | 65267320 | 65267331 |
| 2.25996242145 | bud13 (bg=12.85%) | K562 | + | chr11 | | 65267321 | 65267332 |
| 2.84209598348 | cpsf6 (bg=13.45%) | K562 | + | chr11 | | 65267309 | 65267321 |
| 3.18789784948 | cpsf6 (bg=13.45%) | K562 | + | chr11 | | 65267321 | 65267347 |
| 2.67672268793 | cpsf6 (bg=13.45%) | K562 | + | chr11 | | 65267322 | 65267332 |
| 2.37839712805 | FASTKD2 (bg=4.54%) | K562 | + | chr11 | | 65267297 | 65267334 |
| 2.40986981466 | fxr2 (bg=10.1%) | HepG2 | + | chr11 | | 65267312 | 65267382 |
| 2.31184814754 | GPKOW (bg=5.66%) | K562 | + | chr11 | | 65267294 | 65267334 |
| 3.18013485638 | larp4 (bg=13.51%) | K562 | + | chr11 | | 65267302 | 65267332 |
| 2.67686369108 | npm1 (bg=10.22%) | K562 | + | chr11 | | 65267305 | 65267319 |
| 3.21274283883 | rbm22 (bg=12.69%) | HepG2 | + | chr11 | | 65267308 | 65267339 |
| 3.43804478165 | safb2 (bg=26.89%) | K562 | + | chr11 | | 65267315 | 65267320 |
| 3.68305593864 | safb2 (bg=26.89%) | K562 | + | chr11 | | 65267318 | 65267334 |
| 3.40424619541 | safb2 (bg=26.89%) | K562 | + | chr11 | | 65267320 | 65267331 |
| 2.2978797916 | SMNDC1 (bg=7.08%) | HepG2 | + | chr11 | | 65267318 | 65267329 |
| 3.216342229 | srsf1 (bg=30.28%) | K562 | + | chr11 | | 65267314 | 65267333 |
| 5.11570444224 | tra2a (bg=37.02%) | HepG2 | + | chr11 | | 65267297 | 65267346 |
| 3.18387175151 | tra2a (bg=37.02%) | HepG2 | + | chr11 | | 65267307 | 65267344 |
| 6.50891324887 | tra2a (bg=37.02%) | K562 | + | chr11 | | 65267315 | 65267319 |
| 6.03161939527 | tra2a (bg=37.02%) | K562 | + | chr11 | | 65267318 | 65267321 |
| 6.50001092059 | tra2a (bg=37.02%) | K562 | + | chr11 | | 65267319 | 65267322 |
| 6.08000346901 | tra2a (bg=37.02%) | K562 | + | chr11 | | 65267321 | 65267330 |
| 6.55329009651 | tra2a (bg=37.02%) | K562 | + | chr11 | | 65267322 | 65267331 |
| 2.29034283918 | TROVE2 (bg=6.96%) | HepG2 | + | chr11 | | 65267303 | 65267353 |
| 2.96978146094 | uchl5 (bg=18.56%) | K562 | + | chr11 | | 65267308 | 65267332 |
| 2.77186925664 | uchl5 (bg=18.56%) | K562 | + | chr11 | | 65267313 | 65267331 |
| 3.30069882235 | zc3h8 (bg=12.78%) | K562 | + | chr11 | | 65267293 | 65267375 |
| 2.63080835914 | zc3h8 (bg=12.78%) | K562 | + | chr11 | | 65267310 | 65267355 |
| 3.85748635353 | znf622 (bg=18.79%) | K562 | + | chr11 | | 65267310 | 65267345 |
| 3.13338768229 | znf622 (bg=18.79%) | K562 | + | chr11 | | 65267317 | 65267326 |
| 3.41893492202 | znf622 (bg=18.79%) | K562 | + | chr11 | | 65267326 | 65267343 |

  
  

| Match 61 in HUMAN | | | | | | | |
| --- | --- | --- | --- | --- | --- | --- | --- |
| Motif | Start in Seq (1 Indexed) | End in Seq (1 Indexed) | Strand | Chrm | Exon | Start in Chrm (0 Indexed) | End in Chrm (1 Indexed) |
| AATATTGTCAAGAGTTTCAGATAGAAAATGAAAA | 857 | 890 | + | chr11 | 1 | 65267346 | 65267380 |
| eCLIP Fold-Enrichment | Binding Protein | Cell Line | Strand | Chrm | | Start in Chrm (0 Indexed) | End in Chrm (1 Indexed) |
| 2.85962022459 | aggf1 (bg=15.15%) | K562 | + | chr11 | | 65267341 | 65267347 |
| 2.8524368474 | aggf1 (bg=15.15%) | K562 | + | chr11 | | 65267347 | 65267351 |
| 2.70346078766 | aggf1 (bg=15.15%) | K562 | + | chr11 | | 65267351 | 65267358 |
| 2.92053704921 | aggf1 (bg=15.15%) | K562 | + | chr11 | | 65267358 | 65267372 |
| 2.86684108234 | aggf1 (bg=15.15%) | K562 | + | chr11 | | 65267372 | 65267377 |
| 2.28888345661 | aggf1 (bg=15.15%) | K562 | + | chr11 | | 65267377 | 65267380 |
| 2.77676040026 | aggf1 (bg=15.15%) | K562 | + | chr11 | | 65267380 | 65267402 |
| 3.81809577438 | bclaf1 (bg=17.67%) | HepG2 | + | chr11 | | 65267346 | 65267379 |
| 3.67472341861 | bclaf1 (bg=17.67%) | HepG2 | + | chr11 | | 65267349 | 65267363 |
| 3.59518465363 | bclaf1 (bg=17.67%) | HepG2 | + | chr11 | | 65267363 | 65267396 |
| 4.06206977484 | bclaf1 (bg=17.67%) | HepG2 | + | chr11 | | 65267379 | 65267473 |
| 2.3580264557 | bud13 (bg=12.85%) | HepG2 | + | chr11 | | 65267340 | 65267368 |
| 2.40675489243 | bud13 (bg=12.85%) | K562 | + | chr11 | | 65267343 | 65267349 |
| 2.12824456302 | bud13 (bg=12.85%) | K562 | + | chr11 | | 65267345 | 65267349 |
| 2.29861082849 | bud13 (bg=12.85%) | K562 | + | chr11 | | 65267349 | 65267354 |
| 2.20877657674 | bud13 (bg=12.85%) | K562 | + | chr11 | | 65267354 | 65267361 |
| 2.23009275381 | bud13 (bg=12.85%) | K562 | + | chr11 | | 65267361 | 65267365 |
| 2.04220585012 | bud13 (bg=12.85%) | K562 | + | chr11 | | 65267361 | 65267367 |
| 2.33829612357 | bud13 (bg=12.85%) | K562 | + | chr11 | | 65267365 | 65267386 |
| 2.06524170519 | bud13 (bg=12.85%) | K562 | + | chr11 | | 65267367 | 65267370 |
| 2.4861404988 | bud13 (bg=12.85%) | HepG2 | + | chr11 | | 65267368 | 65267379 |
| 2.12921930212 | bud13 (bg=12.85%) | K562 | + | chr11 | | 65267370 | 65267380 |
| 2.08811123109 | bud13 (bg=12.85%) | HepG2 | + | chr11 | | 65267379 | 65267427 |
| 2.18754999924 | bud13 (bg=12.85%) | K562 | + | chr11 | | 65267380 | 65267391 |
| 3.18789784948 | cpsf6 (bg=13.45%) | K562 | + | chr11 | | 65267321 | 65267347 |
| 3.20338309698 | cpsf6 (bg=13.45%) | K562 | + | chr11 | | 65267347 | 65267368 |
| 3.31989387324 | cpsf6 (bg=13.45%) | K562 | + | chr11 | | 65267368 | 65267400 |
| 2.79418062892 | cpsf6 (bg=13.45%) | K562 | + | chr11 | | 65267376 | 65267394 |
| 2.40003222883 | FASTKD2 (bg=4.54%) | K562 | + | chr11 | | 65267334 | 65267347 |
| 2.27958431553 | FASTKD2 (bg=4.54%) | K562 | + | chr11 | | 65267347 | 65267360 |
| 2.19781245507 | FASTKD2 (bg=4.54%) | K562 | + | chr11 | | 65267360 | 65267370 |
| 2.37885701838 | FASTKD2 (bg=4.54%) | K562 | + | chr11 | | 65267370 | 65267395 |
| 2.07350414972 | FTO (bg=1.11%) | K562 | + | chr11 | | 65267372 | 65267398 |
| 2.40986981466 | fxr2 (bg=10.1%) | HepG2 | + | chr11 | | 65267312 | 65267382 |
| 2.13428335456 | GPKOW (bg=5.66%) | K562 | + | chr11 | | 65267334 | 65267346 |
| 2.20778635876 | GPKOW (bg=5.66%) | K562 | + | chr11 | | 65267346 | 65267361 |
| 2.23384332245 | GPKOW (bg=5.66%) | K562 | + | chr11 | | 65267361 | 65267369 |
| 2.42752002977 | GPKOW (bg=5.66%) | K562 | + | chr11 | | 65267369 | 65267395 |
| 2.72786357884 | hltf (bg=24.28%) | HepG2 | + | chr11 | | 65267353 | 65267358 |
| 2.51314384172 | hltf (bg=24.28%) | HepG2 | + | chr11 | | 65267354 | 65267363 |
| 2.80034528569 | hltf (bg=24.28%) | HepG2 | + | chr11 | | 65267358 | 65267380 |
| 2.58481942532 | hltf (bg=24.28%) | HepG2 | + | chr11 | | 65267363 | 65267396 |
| 2.60282753299 | hltf (bg=24.28%) | HepG2 | + | chr11 | | 65267380 | 65267404 |
| 3.23379251125 | larp4 (bg=13.51%) | K562 | + | chr11 | | 65267332 | 65267346 |
| 3.31263840358 | larp4 (bg=13.51%) | K562 | + | chr11 | | 65267346 | 65267356 |
| 3.22248816552 | larp4 (bg=13.51%) | K562 | + | chr11 | | 65267356 | 65267361 |
| 3.25042077123 | larp4 (bg=13.51%) | K562 | + | chr11 | | 65267361 | 65267369 |
| 3.42942926062 | larp4 (bg=13.51%) | K562 | + | chr11 | | 65267369 | 65267395 |
| 2.89288198274 | MTPAP (bg=9.55%) | K562 | + | chr11 | | 65267362 | 65267391 |
| 2.08228935078 | MTPAP (bg=9.55%) | K562 | + | chr11 | | 65267379 | 65267388 |
| 3.06906398801 | rbm22 (bg=12.69%) | HepG2 | + | chr11 | | 65267349 | 65267366 |
| 2.93822329599 | rbm22 (bg=12.69%) | HepG2 | + | chr11 | | 65267366 | 65267377 |
| 3.50174224203 | safb2 (bg=26.89%) | K562 | + | chr11 | | 65267342 | 65267349 |
| 3.08168784122 | safb2 (bg=26.89%) | K562 | + | chr11 | | 65267344 | 65267349 |
| 3.76721919829 | safb2 (bg=26.89%) | K562 | + | chr11 | | 65267349 | 65267354 |
| 3.44114402031 | safb2 (bg=26.89%) | K562 | + | chr11 | | 65267349 | 65267354 |
| 3.83453486643 | safb2 (bg=26.89%) | K562 | + | chr11 | | 65267354 | 65267358 |
| 3.43189117354 | safb2 (bg=26.89%) | K562 | + | chr11 | | 65267354 | 65267367 |
| 3.97000382237 | safb2 (bg=26.89%) | K562 | + | chr11 | | 65267358 | 65267364 |
| 4.06468622739 | safb2 (bg=26.89%) | K562 | + | chr11 | | 65267364 | 65267367 |
| 3.91759862435 | safb2 (bg=26.89%) | K562 | + | chr11 | | 65267367 | 65267371 |
| 3.50589340732 | safb2 (bg=26.89%) | K562 | + | chr11 | | 65267367 | 65267371 |
| 4.10303888825 | safb2 (bg=26.89%) | K562 | + | chr11 | | 65267371 | 65267380 |
| 3.61057254536 | safb2 (bg=26.89%) | K562 | + | chr11 | | 65267371 | 65267387 |
| 4.05602456061 | safb2 (bg=26.89%) | K562 | + | chr11 | | 65267380 | 65267385 |
| 2.91121247028 | srsf1 (bg=30.28%) | K562 | + | chr11 | | 65267376 | 65267396 |
| 2.00830221244 | srsf7 (bg=22.53%) | K562 | + | chr11 | | 65267346 | 65267360 |
| 2.20202843131 | srsf7 (bg=22.53%) | K562 | + | chr11 | | 65267347 | 65267361 |
| 2.43721328919 | srsf7 (bg=22.53%) | K562 | + | chr11 | | 65267360 | 65267395 |
| 2.2447879601 | srsf7 (bg=22.53%) | K562 | + | chr11 | | 65267361 | 65267369 |
| 2.53092737624 | srsf7 (bg=22.53%) | K562 | + | chr11 | | 65267369 | 65267391 |
| 5.11570444224 | tra2a (bg=37.02%) | HepG2 | + | chr11 | | 65267297 | 65267346 |
| 5.90308450352 | tra2a (bg=37.02%) | K562 | + | chr11 | | 65267330 | 65267349 |
| 6.33537699387 | tra2a (bg=37.02%) | K562 | + | chr11 | | 65267331 | 65267347 |
| 3.11945502301 | tra2a (bg=37.02%) | HepG2 | + | chr11 | | 65267344 | 65267366 |
| 5.31849407758 | tra2a (bg=37.02%) | HepG2 | + | chr11 | | 65267346 | 65267366 |
| 6.32677343192 | tra2a (bg=37.02%) | K562 | + | chr11 | | 65267347 | 65267350 |
| 5.77711470254 | tra2a (bg=37.02%) | K562 | + | chr11 | | 65267349 | 65267354 |
| 6.01753877583 | tra2a (bg=37.02%) | K562 | + | chr11 | | 65267350 | 65267354 |
| 5.78425872283 | tra2a (bg=37.02%) | K562 | + | chr11 | | 65267354 | 65267358 |
| 5.98666042924 | tra2a (bg=37.02%) | K562 | + | chr11 | | 65267354 | 65267363 |
| 5.93727726241 | tra2a (bg=37.02%) | K562 | + | chr11 | | 65267358 | 65267364 |
| 6.05588881633 | tra2a (bg=37.02%) | K562 | + | chr11 | | 65267363 | 65267367 |
| 5.90933348887 | tra2a (bg=37.02%) | K562 | + | chr11 | | 65267364 | 65267367 |
| 3.66803803247 | tra2a (bg=37.02%) | HepG2 | + | chr11 | | 65267366 | 65267392 |
| 5.72894430737 | tra2a (bg=37.02%) | HepG2 | + | chr11 | | 65267366 | 65267472 |
| 5.83690865839 | tra2a (bg=37.02%) | K562 | + | chr11 | | 65267367 | 65267371 |
| 6.08614682116 | tra2a (bg=37.02%) | K562 | + | chr11 | | 65267367 | 65267372 |
| 6.06034168471 | tra2a (bg=37.02%) | K562 | + | chr11 | | 65267371 | 65267380 |
| 6.24504527661 | tra2a (bg=37.02%) | K562 | + | chr11 | | 65267372 | 65267386 |
| 5.970457729 | tra2a (bg=37.02%) | K562 | + | chr11 | | 65267380 | 65267384 |
| 2.29034283918 | TROVE2 (bg=6.96%) | HepG2 | + | chr11 | | 65267303 | 65267353 |
| 2.07516244617 | TROVE2 (bg=6.96%) | HepG2 | + | chr11 | | 65267353 | 65267396 |
| 2.64678436697 | uchl5 (bg=18.56%) | K562 | + | chr11 | | 65267331 | 65267346 |
| 3.09472147661 | uchl5 (bg=18.56%) | K562 | + | chr11 | | 65267332 | 65267348 |
| 2.86339184507 | uchl5 (bg=18.56%) | K562 | + | chr11 | | 65267346 | 65267354 |
| 3.20352588614 | uchl5 (bg=18.56%) | K562 | + | chr11 | | 65267348 | 65267354 |
| 2.86829016266 | uchl5 (bg=18.56%) | K562 | + | chr11 | | 65267354 | 65267361 |
| 3.17912644447 | uchl5 (bg=18.56%) | K562 | + | chr11 | | 65267354 | 65267364 |
| 2.79553264789 | uchl5 (bg=18.56%) | K562 | + | chr11 | | 65267361 | 65267370 |
| 3.2252004879 | uchl5 (bg=18.56%) | K562 | + | chr11 | | 65267364 | 65267380 |
| 2.45084252983 | uchl5 (bg=18.56%) | HepG2 | + | chr11 | | 65267367 | 65267376 |
| 3.03943634241 | uchl5 (bg=18.56%) | K562 | + | chr11 | | 65267370 | 65267395 |
| 3.28306109775 | uchl5 (bg=18.56%) | K562 | + | chr11 | | 65267380 | 65267386 |
| 2.0004648287 | YWHAG (bg=9.14%) | K562 | + | chr11 | | 65267367 | 65267384 |
| 3.30069882235 | zc3h8 (bg=12.78%) | K562 | + | chr11 | | 65267293 | 65267375 |
| 2.63080835914 | zc3h8 (bg=12.78%) | K562 | + | chr11 | | 65267310 | 65267355 |
| 3.82659289093 | znf622 (bg=18.79%) | K562 | + | chr11 | | 65267343 | 65267359 |
| 4.05093500969 | znf622 (bg=18.79%) | K562 | + | chr11 | | 65267348 | 65267359 |
| 4.22522178211 | znf622 (bg=18.79%) | K562 | + | chr11 | | 65267359 | 65267369 |
| 3.81053729551 | znf622 (bg=18.79%) | K562 | + | chr11 | | 65267359 | 65267371 |
| 4.40530054561 | znf622 (bg=18.79%) | K562 | + | chr11 | | 65267369 | 65267395 |
| 3.71411816167 | znf622 (bg=18.79%) | K562 | + | chr11 | | 65267371 | 65267378 |
| 3.39396764017 | znf622 (bg=18.79%) | K562 | + | chr11 | | 65267378 | 65267406 |
| 2.02605380232 | ZNF800 (bg=3.2%) | HepG2 | + | chr11 | | 65267350 | 65267430 |

  
  

| Match 62 in HUMAN | | | | | | | |
| --- | --- | --- | --- | --- | --- | --- | --- |
| Motif | Start in Seq (1 Indexed) | End in Seq (1 Indexed) | Strand | Chrm | Exon | Start in Chrm (0 Indexed) | End in Chrm (1 Indexed) |
| TAGAAAATGAAAA | 878 | 890 | + | chr11 | 1 | 65267367 | 65267380 |
| eCLIP Fold-Enrichment | Binding Protein | Cell Line | Strand | Chrm | | Start in Chrm (0 Indexed) | End in Chrm (1 Indexed) |
| 2.92053704921 | aggf1 (bg=15.15%) | K562 | + | chr11 | | 65267358 | 65267372 |
| 2.86684108234 | aggf1 (bg=15.15%) | K562 | + | chr11 | | 65267372 | 65267377 |
| 2.28888345661 | aggf1 (bg=15.15%) | K562 | + | chr11 | | 65267377 | 65267380 |
| 2.77676040026 | aggf1 (bg=15.15%) | K562 | + | chr11 | | 65267380 | 65267402 |
| 3.81809577438 | bclaf1 (bg=17.67%) | HepG2 | + | chr11 | | 65267346 | 65267379 |
| 3.59518465363 | bclaf1 (bg=17.67%) | HepG2 | + | chr11 | | 65267363 | 65267396 |
| 4.06206977484 | bclaf1 (bg=17.67%) | HepG2 | + | chr11 | | 65267379 | 65267473 |
| 2.3580264557 | bud13 (bg=12.85%) | HepG2 | + | chr11 | | 65267340 | 65267368 |
| 2.04220585012 | bud13 (bg=12.85%) | K562 | + | chr11 | | 65267361 | 65267367 |
| 2.33829612357 | bud13 (bg=12.85%) | K562 | + | chr11 | | 65267365 | 65267386 |
| 2.06524170519 | bud13 (bg=12.85%) | K562 | + | chr11 | | 65267367 | 65267370 |
| 2.4861404988 | bud13 (bg=12.85%) | HepG2 | + | chr11 | | 65267368 | 65267379 |
| 2.12921930212 | bud13 (bg=12.85%) | K562 | + | chr11 | | 65267370 | 65267380 |
| 2.08811123109 | bud13 (bg=12.85%) | HepG2 | + | chr11 | | 65267379 | 65267427 |
| 2.18754999924 | bud13 (bg=12.85%) | K562 | + | chr11 | | 65267380 | 65267391 |
| 3.20338309698 | cpsf6 (bg=13.45%) | K562 | + | chr11 | | 65267347 | 65267368 |
| 3.31989387324 | cpsf6 (bg=13.45%) | K562 | + | chr11 | | 65267368 | 65267400 |
| 2.79418062892 | cpsf6 (bg=13.45%) | K562 | + | chr11 | | 65267376 | 65267394 |
| 2.19781245507 | FASTKD2 (bg=4.54%) | K562 | + | chr11 | | 65267360 | 65267370 |
| 2.37885701838 | FASTKD2 (bg=4.54%) | K562 | + | chr11 | | 65267370 | 65267395 |
| 2.07350414972 | FTO (bg=1.11%) | K562 | + | chr11 | | 65267372 | 65267398 |
| 2.40986981466 | fxr2 (bg=10.1%) | HepG2 | + | chr11 | | 65267312 | 65267382 |
| 2.23384332245 | GPKOW (bg=5.66%) | K562 | + | chr11 | | 65267361 | 65267369 |
| 2.42752002977 | GPKOW (bg=5.66%) | K562 | + | chr11 | | 65267369 | 65267395 |
| 2.80034528569 | hltf (bg=24.28%) | HepG2 | + | chr11 | | 65267358 | 65267380 |
| 2.58481942532 | hltf (bg=24.28%) | HepG2 | + | chr11 | | 65267363 | 65267396 |
| 2.60282753299 | hltf (bg=24.28%) | HepG2 | + | chr11 | | 65267380 | 65267404 |
| 3.25042077123 | larp4 (bg=13.51%) | K562 | + | chr11 | | 65267361 | 65267369 |
| 3.42942926062 | larp4 (bg=13.51%) | K562 | + | chr11 | | 65267369 | 65267395 |
| 2.89288198274 | MTPAP (bg=9.55%) | K562 | + | chr11 | | 65267362 | 65267391 |
| 2.08228935078 | MTPAP (bg=9.55%) | K562 | + | chr11 | | 65267379 | 65267388 |
| 2.93822329599 | rbm22 (bg=12.69%) | HepG2 | + | chr11 | | 65267366 | 65267377 |
| 3.43189117354 | safb2 (bg=26.89%) | K562 | + | chr11 | | 65267354 | 65267367 |
| 4.06468622739 | safb2 (bg=26.89%) | K562 | + | chr11 | | 65267364 | 65267367 |
| 3.91759862435 | safb2 (bg=26.89%) | K562 | + | chr11 | | 65267367 | 65267371 |
| 3.50589340732 | safb2 (bg=26.89%) | K562 | + | chr11 | | 65267367 | 65267371 |
| 4.10303888825 | safb2 (bg=26.89%) | K562 | + | chr11 | | 65267371 | 65267380 |
| 3.61057254536 | safb2 (bg=26.89%) | K562 | + | chr11 | | 65267371 | 65267387 |
| 4.05602456061 | safb2 (bg=26.89%) | K562 | + | chr11 | | 65267380 | 65267385 |
| 2.91121247028 | srsf1 (bg=30.28%) | K562 | + | chr11 | | 65267376 | 65267396 |
| 2.43721328919 | srsf7 (bg=22.53%) | K562 | + | chr11 | | 65267360 | 65267395 |
| 2.2447879601 | srsf7 (bg=22.53%) | K562 | + | chr11 | | 65267361 | 65267369 |
| 2.53092737624 | srsf7 (bg=22.53%) | K562 | + | chr11 | | 65267369 | 65267391 |
| 6.05588881633 | tra2a (bg=37.02%) | K562 | + | chr11 | | 65267363 | 65267367 |
| 5.90933348887 | tra2a (bg=37.02%) | K562 | + | chr11 | | 65267364 | 65267367 |
| 3.66803803247 | tra2a (bg=37.02%) | HepG2 | + | chr11 | | 65267366 | 65267392 |
| 5.72894430737 | tra2a (bg=37.02%) | HepG2 | + | chr11 | | 65267366 | 65267472 |
| 5.83690865839 | tra2a (bg=37.02%) | K562 | + | chr11 | | 65267367 | 65267371 |
| 6.08614682116 | tra2a (bg=37.02%) | K562 | + | chr11 | | 65267367 | 65267372 |
| 6.06034168471 | tra2a (bg=37.02%) | K562 | + | chr11 | | 65267371 | 65267380 |
| 6.24504527661 | tra2a (bg=37.02%) | K562 | + | chr11 | | 65267372 | 65267386 |
| 5.970457729 | tra2a (bg=37.02%) | K562 | + | chr11 | | 65267380 | 65267384 |
| 2.07516244617 | TROVE2 (bg=6.96%) | HepG2 | + | chr11 | | 65267353 | 65267396 |
| 2.79553264789 | uchl5 (bg=18.56%) | K562 | + | chr11 | | 65267361 | 65267370 |
| 3.2252004879 | uchl5 (bg=18.56%) | K562 | + | chr11 | | 65267364 | 65267380 |
| 2.45084252983 | uchl5 (bg=18.56%) | HepG2 | + | chr11 | | 65267367 | 65267376 |
| 3.03943634241 | uchl5 (bg=18.56%) | K562 | + | chr11 | | 65267370 | 65267395 |
| 3.28306109775 | uchl5 (bg=18.56%) | K562 | + | chr11 | | 65267380 | 65267386 |
| 2.0004648287 | YWHAG (bg=9.14%) | K562 | + | chr11 | | 65267367 | 65267384 |
| 3.30069882235 | zc3h8 (bg=12.78%) | K562 | + | chr11 | | 65267293 | 65267375 |
| 4.22522178211 | znf622 (bg=18.79%) | K562 | + | chr11 | | 65267359 | 65267369 |
| 3.81053729551 | znf622 (bg=18.79%) | K562 | + | chr11 | | 65267359 | 65267371 |
| 4.40530054561 | znf622 (bg=18.79%) | K562 | + | chr11 | | 65267369 | 65267395 |
| 3.71411816167 | znf622 (bg=18.79%) | K562 | + | chr11 | | 65267371 | 65267378 |
| 3.39396764017 | znf622 (bg=18.79%) | K562 | + | chr11 | | 65267378 | 65267406 |
| 2.02605380232 | ZNF800 (bg=3.2%) | HepG2 | + | chr11 | | 65267350 | 65267430 |

  
  

| Match 63 in HUMAN | | | | | | | |
| --- | --- | --- | --- | --- | --- | --- | --- |
| Motif | Start in Seq (1 Indexed) | End in Seq (1 Indexed) | Strand | Chrm | Exon | Start in Chrm (0 Indexed) | End in Chrm (1 Indexed) |
| TAGAAAATGA | 878 | 887 | + | chr11 | 1 | 65267367 | 65267377 |
| eCLIP Fold-Enrichment | Binding Protein | Cell Line | Strand | Chrm | | Start in Chrm (0 Indexed) | End in Chrm (1 Indexed) |
| 2.92053704921 | aggf1 (bg=15.15%) | K562 | + | chr11 | | 65267358 | 65267372 |
| 2.86684108234 | aggf1 (bg=15.15%) | K562 | + | chr11 | | 65267372 | 65267377 |
| 2.28888345661 | aggf1 (bg=15.15%) | K562 | + | chr11 | | 65267377 | 65267380 |
| 3.81809577438 | bclaf1 (bg=17.67%) | HepG2 | + | chr11 | | 65267346 | 65267379 |
| 3.59518465363 | bclaf1 (bg=17.67%) | HepG2 | + | chr11 | | 65267363 | 65267396 |
| 2.3580264557 | bud13 (bg=12.85%) | HepG2 | + | chr11 | | 65267340 | 65267368 |
| 2.04220585012 | bud13 (bg=12.85%) | K562 | + | chr11 | | 65267361 | 65267367 |
| 2.33829612357 | bud13 (bg=12.85%) | K562 | + | chr11 | | 65267365 | 65267386 |
| 2.06524170519 | bud13 (bg=12.85%) | K562 | + | chr11 | | 65267367 | 65267370 |
| 2.4861404988 | bud13 (bg=12.85%) | HepG2 | + | chr11 | | 65267368 | 65267379 |
| 2.12921930212 | bud13 (bg=12.85%) | K562 | + | chr11 | | 65267370 | 65267380 |
| 3.20338309698 | cpsf6 (bg=13.45%) | K562 | + | chr11 | | 65267347 | 65267368 |
| 3.31989387324 | cpsf6 (bg=13.45%) | K562 | + | chr11 | | 65267368 | 65267400 |
| 2.79418062892 | cpsf6 (bg=13.45%) | K562 | + | chr11 | | 65267376 | 65267394 |
| 2.19781245507 | FASTKD2 (bg=4.54%) | K562 | + | chr11 | | 65267360 | 65267370 |
| 2.37885701838 | FASTKD2 (bg=4.54%) | K562 | + | chr11 | | 65267370 | 65267395 |
| 2.07350414972 | FTO (bg=1.11%) | K562 | + | chr11 | | 65267372 | 65267398 |
| 2.40986981466 | fxr2 (bg=10.1%) | HepG2 | + | chr11 | | 65267312 | 65267382 |
| 2.23384332245 | GPKOW (bg=5.66%) | K562 | + | chr11 | | 65267361 | 65267369 |
| 2.42752002977 | GPKOW (bg=5.66%) | K562 | + | chr11 | | 65267369 | 65267395 |
| 2.80034528569 | hltf (bg=24.28%) | HepG2 | + | chr11 | | 65267358 | 65267380 |
| 2.58481942532 | hltf (bg=24.28%) | HepG2 | + | chr11 | | 65267363 | 65267396 |
| 3.25042077123 | larp4 (bg=13.51%) | K562 | + | chr11 | | 65267361 | 65267369 |
| 3.42942926062 | larp4 (bg=13.51%) | K562 | + | chr11 | | 65267369 | 65267395 |
| 2.89288198274 | MTPAP (bg=9.55%) | K562 | + | chr11 | | 65267362 | 65267391 |
| 2.93822329599 | rbm22 (bg=12.69%) | HepG2 | + | chr11 | | 65267366 | 65267377 |
| 3.43189117354 | safb2 (bg=26.89%) | K562 | + | chr11 | | 65267354 | 65267367 |
| 4.06468622739 | safb2 (bg=26.89%) | K562 | + | chr11 | | 65267364 | 65267367 |
| 3.91759862435 | safb2 (bg=26.89%) | K562 | + | chr11 | | 65267367 | 65267371 |
| 3.50589340732 | safb2 (bg=26.89%) | K562 | + | chr11 | | 65267367 | 65267371 |
| 4.10303888825 | safb2 (bg=26.89%) | K562 | + | chr11 | | 65267371 | 65267380 |
| 3.61057254536 | safb2 (bg=26.89%) | K562 | + | chr11 | | 65267371 | 65267387 |
| 2.91121247028 | srsf1 (bg=30.28%) | K562 | + | chr11 | | 65267376 | 65267396 |
| 2.43721328919 | srsf7 (bg=22.53%) | K562 | + | chr11 | | 65267360 | 65267395 |
| 2.2447879601 | srsf7 (bg=22.53%) | K562 | + | chr11 | | 65267361 | 65267369 |
| 2.53092737624 | srsf7 (bg=22.53%) | K562 | + | chr11 | | 65267369 | 65267391 |
| 6.05588881633 | tra2a (bg=37.02%) | K562 | + | chr11 | | 65267363 | 65267367 |
| 5.90933348887 | tra2a (bg=37.02%) | K562 | + | chr11 | | 65267364 | 65267367 |
| 3.66803803247 | tra2a (bg=37.02%) | HepG2 | + | chr11 | | 65267366 | 65267392 |
| 5.72894430737 | tra2a (bg=37.02%) | HepG2 | + | chr11 | | 65267366 | 65267472 |
| 5.83690865839 | tra2a (bg=37.02%) | K562 | + | chr11 | | 65267367 | 65267371 |
| 6.08614682116 | tra2a (bg=37.02%) | K562 | + | chr11 | | 65267367 | 65267372 |
| 6.06034168471 | tra2a (bg=37.02%) | K562 | + | chr11 | | 65267371 | 65267380 |
| 6.24504527661 | tra2a (bg=37.02%) | K562 | + | chr11 | | 65267372 | 65267386 |
| 2.07516244617 | TROVE2 (bg=6.96%) | HepG2 | + | chr11 | | 65267353 | 65267396 |
| 2.79553264789 | uchl5 (bg=18.56%) | K562 | + | chr11 | | 65267361 | 65267370 |
| 3.2252004879 | uchl5 (bg=18.56%) | K562 | + | chr11 | | 65267364 | 65267380 |
| 2.45084252983 | uchl5 (bg=18.56%) | HepG2 | + | chr11 | | 65267367 | 65267376 |
| 3.03943634241 | uchl5 (bg=18.56%) | K562 | + | chr11 | | 65267370 | 65267395 |
| 2.0004648287 | YWHAG (bg=9.14%) | K562 | + | chr11 | | 65267367 | 65267384 |
| 3.30069882235 | zc3h8 (bg=12.78%) | K562 | + | chr11 | | 65267293 | 65267375 |
| 4.22522178211 | znf622 (bg=18.79%) | K562 | + | chr11 | | 65267359 | 65267369 |
| 3.81053729551 | znf622 (bg=18.79%) | K562 | + | chr11 | | 65267359 | 65267371 |
| 4.40530054561 | znf622 (bg=18.79%) | K562 | + | chr11 | | 65267369 | 65267395 |
| 3.71411816167 | znf622 (bg=18.79%) | K562 | + | chr11 | | 65267371 | 65267378 |
| 2.02605380232 | ZNF800 (bg=3.2%) | HepG2 | + | chr11 | | 65267350 | 65267430 |

  
  

| Match 64 in HUMAN | | | | | | | |
| --- | --- | --- | --- | --- | --- | --- | --- |
| Motif | Start in Seq (1 Indexed) | End in Seq (1 Indexed) | Strand | Chrm | Exon | Start in Chrm (0 Indexed) | End in Chrm (1 Indexed) |
| GCTAAGACAAGTATTGGA | 895 | 912 | + | chr11 | 1 | 65267384 | 65267402 |
| eCLIP Fold-Enrichment | Binding Protein | Cell Line | Strand | Chrm | | Start in Chrm (0 Indexed) | End in Chrm (1 Indexed) |
| 2.77676040026 | aggf1 (bg=15.15%) | K562 | + | chr11 | | 65267380 | 65267402 |
| 3.28032945158 | aggf1 (bg=15.15%) | K562 | + | chr11 | | 65267402 | 65267418 |
| 3.59518465363 | bclaf1 (bg=17.67%) | HepG2 | + | chr11 | | 65267363 | 65267396 |
| 4.06206977484 | bclaf1 (bg=17.67%) | HepG2 | + | chr11 | | 65267379 | 65267473 |
| 4.11713735682 | bclaf1 (bg=17.67%) | HepG2 | + | chr11 | | 65267396 | 65267459 |
| 2.33829612357 | bud13 (bg=12.85%) | K562 | + | chr11 | | 65267365 | 65267386 |
| 2.08811123109 | bud13 (bg=12.85%) | HepG2 | + | chr11 | | 65267379 | 65267427 |
| 2.18754999924 | bud13 (bg=12.85%) | K562 | + | chr11 | | 65267380 | 65267391 |
| 2.36562991708 | bud13 (bg=12.85%) | K562 | + | chr11 | | 65267386 | 65267391 |
| 2.34598167936 | bud13 (bg=12.85%) | K562 | + | chr11 | | 65267391 | 65267395 |
| 2.23518251959 | bud13 (bg=12.85%) | K562 | + | chr11 | | 65267391 | 65267403 |
| 2.44892920099 | bud13 (bg=12.85%) | K562 | + | chr11 | | 65267395 | 65267397 |
| 2.59524520429 | bud13 (bg=12.85%) | K562 | + | chr11 | | 65267397 | 65267405 |
| 3.31989387324 | cpsf6 (bg=13.45%) | K562 | + | chr11 | | 65267368 | 65267400 |
| 2.79418062892 | cpsf6 (bg=13.45%) | K562 | + | chr11 | | 65267376 | 65267394 |
| 2.98153837035 | cpsf6 (bg=13.45%) | K562 | + | chr11 | | 65267394 | 65267420 |
| 3.34282194703 | cpsf6 (bg=13.45%) | K562 | + | chr11 | | 65267400 | 65267424 |
| 2.37885701838 | FASTKD2 (bg=4.54%) | K562 | + | chr11 | | 65267370 | 65267395 |
| 2.58145475808 | FASTKD2 (bg=4.54%) | K562 | + | chr11 | | 65267395 | 65267447 |
| 2.07350414972 | FTO (bg=1.11%) | K562 | + | chr11 | | 65267372 | 65267398 |
| 2.23572962633 | FTO (bg=1.11%) | K562 | + | chr11 | | 65267398 | 65267408 |
| 2.65639076089 | fxr2 (bg=10.1%) | HepG2 | + | chr11 | | 65267382 | 65267492 |
| 2.42752002977 | GPKOW (bg=5.66%) | K562 | + | chr11 | | 65267369 | 65267395 |
| 2.3933768929 | GPKOW (bg=5.66%) | K562 | + | chr11 | | 65267395 | 65267443 |
| 2.58481942532 | hltf (bg=24.28%) | HepG2 | + | chr11 | | 65267363 | 65267396 |
| 2.60282753299 | hltf (bg=24.28%) | HepG2 | + | chr11 | | 65267380 | 65267404 |
| 2.3317452171 | hltf (bg=24.28%) | K562 | + | chr11 | | 65267396 | 65267403 |
| 2.52027421551 | hltf (bg=24.28%) | HepG2 | + | chr11 | | 65267396 | 65267406 |
| 3.42942926062 | larp4 (bg=13.51%) | K562 | + | chr11 | | 65267369 | 65267395 |
| 3.4797325896 | larp4 (bg=13.51%) | K562 | + | chr11 | | 65267395 | 65267436 |
| 2.89288198274 | MTPAP (bg=9.55%) | K562 | + | chr11 | | 65267362 | 65267391 |
| 2.08228935078 | MTPAP (bg=9.55%) | K562 | + | chr11 | | 65267379 | 65267388 |
| 3.172488726 | MTPAP (bg=9.55%) | K562 | + | chr11 | | 65267391 | 65267405 |
| 2.44402503497 | MTPAP (bg=9.55%) | K562 | + | chr11 | | 65267394 | 65267414 |
| 2.00393913525 | rbm15 (bg=11.59%) | K562 | + | chr11 | | 65267386 | 65267393 |
| 2.15048649754 | rbm15 (bg=11.59%) | K562 | + | chr11 | | 65267396 | 65267422 |
| 3.47549875898 | rbm22 (bg=12.69%) | HepG2 | + | chr11 | | 65267393 | 65267413 |
| 3.61057254536 | safb2 (bg=26.89%) | K562 | + | chr11 | | 65267371 | 65267387 |
| 4.05602456061 | safb2 (bg=26.89%) | K562 | + | chr11 | | 65267380 | 65267385 |
| 4.02561713675 | safb2 (bg=26.89%) | K562 | + | chr11 | | 65267385 | 65267388 |
| 3.77017519835 | safb2 (bg=26.89%) | K562 | + | chr11 | | 65267387 | 65267391 |
| 4.25692235925 | safb2 (bg=26.89%) | K562 | + | chr11 | | 65267388 | 65267391 |
| 3.71850431226 | safb2 (bg=26.89%) | K562 | + | chr11 | | 65267391 | 65267395 |
| 4.04307795273 | safb2 (bg=26.89%) | K562 | + | chr11 | | 65267391 | 65267407 |
| 3.76121849486 | safb2 (bg=26.89%) | K562 | + | chr11 | | 65267395 | 65267406 |
| 2.91121247028 | srsf1 (bg=30.28%) | K562 | + | chr11 | | 65267376 | 65267396 |
| 2.90077267386 | srsf1 (bg=30.28%) | K562 | + | chr11 | | 65267396 | 65267422 |
| 2.43721328919 | srsf7 (bg=22.53%) | K562 | + | chr11 | | 65267360 | 65267395 |
| 2.53092737624 | srsf7 (bg=22.53%) | K562 | + | chr11 | | 65267369 | 65267391 |
| 2.66715689203 | srsf7 (bg=22.53%) | HepG2 | + | chr11 | | 65267382 | 65267410 |
| 2.26830048768 | srsf7 (bg=22.53%) | K562 | + | chr11 | | 65267391 | 65267395 |
| 2.60452991794 | srsf7 (bg=22.53%) | K562 | + | chr11 | | 65267395 | 65267436 |
| 2.64980437023 | srsf7 (bg=22.53%) | K562 | + | chr11 | | 65267395 | 65267457 |
| 4.4834548098 | SUPV3L1 (bg=9.63%) | K562 | + | chr11 | | 65267386 | 65267417 |
| 3.66803803247 | tra2a (bg=37.02%) | HepG2 | + | chr11 | | 65267366 | 65267392 |
| 5.72894430737 | tra2a (bg=37.02%) | HepG2 | + | chr11 | | 65267366 | 65267472 |
| 6.24504527661 | tra2a (bg=37.02%) | K562 | + | chr11 | | 65267372 | 65267386 |
| 5.970457729 | tra2a (bg=37.02%) | K562 | + | chr11 | | 65267380 | 65267384 |
| 5.99761954528 | tra2a (bg=37.02%) | K562 | + | chr11 | | 65267384 | 65267388 |
| 6.271353129 | tra2a (bg=37.02%) | K562 | + | chr11 | | 65267386 | 65267396 |
| 6.1820227225 | tra2a (bg=37.02%) | K562 | + | chr11 | | 65267388 | 65267391 |
| 6.01112914154 | tra2a (bg=37.02%) | K562 | + | chr11 | | 65267391 | 65267396 |
| 3.71680750425 | tra2a (bg=37.02%) | HepG2 | + | chr11 | | 65267392 | 65267455 |
| 6.34349595348 | tra2a (bg=37.02%) | K562 | + | chr11 | | 65267396 | 65267412 |
| 6.43309235766 | tra2a (bg=37.02%) | K562 | + | chr11 | | 65267396 | 65267412 |
| 2.07516244617 | TROVE2 (bg=6.96%) | HepG2 | + | chr11 | | 65267353 | 65267396 |
| 3.03943634241 | uchl5 (bg=18.56%) | K562 | + | chr11 | | 65267370 | 65267395 |
| 3.28306109775 | uchl5 (bg=18.56%) | K562 | + | chr11 | | 65267380 | 65267386 |
| 3.34656404006 | uchl5 (bg=18.56%) | K562 | + | chr11 | | 65267386 | 65267391 |
| 2.32762966363 | uchl5 (bg=18.56%) | HepG2 | + | chr11 | | 65267388 | 65267413 |
| 3.1904930477 | uchl5 (bg=18.56%) | K562 | + | chr11 | | 65267391 | 65267395 |
| 3.451687139 | uchl5 (bg=18.56%) | K562 | + | chr11 | | 65267395 | 65267421 |
| 3.04607515788 | uchl5 (bg=18.56%) | K562 | + | chr11 | | 65267395 | 65267421 |
| 2.0004648287 | YWHAG (bg=9.14%) | K562 | + | chr11 | | 65267367 | 65267384 |
| 2.22198156258 | YWHAG (bg=9.14%) | K562 | + | chr11 | | 65267384 | 65267432 |
| 3.69418017826 | zc3h8 (bg=12.78%) | K562 | + | chr11 | | 65267392 | 65267407 |
| 4.40530054561 | znf622 (bg=18.79%) | K562 | + | chr11 | | 65267369 | 65267395 |
| 3.39396764017 | znf622 (bg=18.79%) | K562 | + | chr11 | | 65267378 | 65267406 |
| 4.31992870485 | znf622 (bg=18.79%) | K562 | + | chr11 | | 65267395 | 65267407 |
| 2.02605380232 | ZNF800 (bg=3.2%) | HepG2 | + | chr11 | | 65267350 | 65267430 |

  
  

| Match 65 in HUMAN | | | | | | | |
| --- | --- | --- | --- | --- | --- | --- | --- |
| Motif | Start in Seq (1 Indexed) | End in Seq (1 Indexed) | Strand | Chrm | Exon | Start in Chrm (0 Indexed) | End in Chrm (1 Indexed) |
| ATAGAAGATAG | 918 | 928 | + | chr11 | 1 | 65267407 | 65267418 |
| eCLIP Fold-Enrichment | Binding Protein | Cell Line | Strand | Chrm | | Start in Chrm (0 Indexed) | End in Chrm (1 Indexed) |
| 3.28032945158 | aggf1 (bg=15.15%) | K562 | + | chr11 | | 65267402 | 65267418 |
| 3.39735765993 | aggf1 (bg=15.15%) | K562 | + | chr11 | | 65267418 | 65267422 |
| 4.06206977484 | bclaf1 (bg=17.67%) | HepG2 | + | chr11 | | 65267379 | 65267473 |
| 4.11713735682 | bclaf1 (bg=17.67%) | HepG2 | + | chr11 | | 65267396 | 65267459 |
| 2.08811123109 | bud13 (bg=12.85%) | HepG2 | + | chr11 | | 65267379 | 65267427 |
| 2.58096488149 | bud13 (bg=12.85%) | K562 | + | chr11 | | 65267405 | 65267408 |
| 2.56047185595 | bud13 (bg=12.85%) | K562 | + | chr11 | | 65267406 | 65267408 |
| 2.58260548118 | bud13 (bg=12.85%) | K562 | + | chr11 | | 65267408 | 65267412 |
| 2.65055629912 | bud13 (bg=12.85%) | K562 | + | chr11 | | 65267408 | 65267412 |
| 2.73591401546 | bud13 (bg=12.85%) | K562 | + | chr11 | | 65267412 | 65267415 |
| 2.71223390097 | bud13 (bg=12.85%) | K562 | + | chr11 | | 65267412 | 65267421 |
| 2.66103449456 | bud13 (bg=12.85%) | K562 | + | chr11 | | 65267415 | 65267422 |
| 2.98153837035 | cpsf6 (bg=13.45%) | K562 | + | chr11 | | 65267394 | 65267420 |
| 3.34282194703 | cpsf6 (bg=13.45%) | K562 | + | chr11 | | 65267400 | 65267424 |
| 2.58145475808 | FASTKD2 (bg=4.54%) | K562 | + | chr11 | | 65267395 | 65267447 |
| 2.23572962633 | FTO (bg=1.11%) | K562 | + | chr11 | | 65267398 | 65267408 |
| 2.65639076089 | fxr2 (bg=10.1%) | HepG2 | + | chr11 | | 65267382 | 65267492 |
| 2.3933768929 | GPKOW (bg=5.66%) | K562 | + | chr11 | | 65267395 | 65267443 |
| 2.8162311711 | hltf (bg=24.28%) | HepG2 | + | chr11 | | 65267404 | 65267420 |
| 2.36408989572 | hltf (bg=24.28%) | K562 | + | chr11 | | 65267406 | 65267408 |
| 2.49873577591 | hltf (bg=24.28%) | HepG2 | + | chr11 | | 65267406 | 65267421 |
| 2.40751457806 | hltf (bg=24.28%) | K562 | + | chr11 | | 65267408 | 65267412 |
| 2.51963956022 | hltf (bg=24.28%) | K562 | + | chr11 | | 65267412 | 65267415 |
| 2.45358648734 | hltf (bg=24.28%) | K562 | + | chr11 | | 65267415 | 65267422 |
| 3.4797325896 | larp4 (bg=13.51%) | K562 | + | chr11 | | 65267395 | 65267436 |
| 2.44402503497 | MTPAP (bg=9.55%) | K562 | + | chr11 | | 65267394 | 65267414 |
| 3.09018342312 | MTPAP (bg=9.55%) | K562 | + | chr11 | | 65267405 | 65267413 |
| 3.22095977692 | MTPAP (bg=9.55%) | K562 | + | chr11 | | 65267413 | 65267434 |
| 2.15048649754 | rbm15 (bg=11.59%) | K562 | + | chr11 | | 65267396 | 65267422 |
| 3.47549875898 | rbm22 (bg=12.69%) | HepG2 | + | chr11 | | 65267393 | 65267413 |
| 4.04307795273 | safb2 (bg=26.89%) | K562 | + | chr11 | | 65267391 | 65267407 |
| 3.74500314388 | safb2 (bg=26.89%) | K562 | + | chr11 | | 65267406 | 65267412 |
| 4.30370203643 | safb2 (bg=26.89%) | K562 | + | chr11 | | 65267407 | 65267412 |
| 3.76221649612 | safb2 (bg=26.89%) | K562 | + | chr11 | | 65267412 | 65267415 |
| 4.29716162186 | safb2 (bg=26.89%) | K562 | + | chr11 | | 65267412 | 65267420 |
| 3.52876711164 | safb2 (bg=26.89%) | K562 | + | chr11 | | 65267415 | 65267421 |
| 2.90077267386 | srsf1 (bg=30.28%) | K562 | + | chr11 | | 65267396 | 65267422 |
| 2.66715689203 | srsf7 (bg=22.53%) | HepG2 | + | chr11 | | 65267382 | 65267410 |
| 2.60452991794 | srsf7 (bg=22.53%) | K562 | + | chr11 | | 65267395 | 65267436 |
| 2.64980437023 | srsf7 (bg=22.53%) | K562 | + | chr11 | | 65267395 | 65267457 |
| 4.4834548098 | SUPV3L1 (bg=9.63%) | K562 | + | chr11 | | 65267386 | 65267417 |
| 5.72894430737 | tra2a (bg=37.02%) | HepG2 | + | chr11 | | 65267366 | 65267472 |
| 3.71680750425 | tra2a (bg=37.02%) | HepG2 | + | chr11 | | 65267392 | 65267455 |
| 6.34349595348 | tra2a (bg=37.02%) | K562 | + | chr11 | | 65267396 | 65267412 |
| 6.43309235766 | tra2a (bg=37.02%) | K562 | + | chr11 | | 65267396 | 65267412 |
| 6.14952415451 | tra2a (bg=37.02%) | K562 | + | chr11 | | 65267412 | 65267415 |
| 6.23891666916 | tra2a (bg=37.02%) | K562 | + | chr11 | | 65267412 | 65267421 |
| 5.74189163916 | tra2a (bg=37.02%) | K562 | + | chr11 | | 65267415 | 65267427 |
| 2.32762966363 | uchl5 (bg=18.56%) | HepG2 | + | chr11 | | 65267388 | 65267413 |
| 3.451687139 | uchl5 (bg=18.56%) | K562 | + | chr11 | | 65267395 | 65267421 |
| 3.04607515788 | uchl5 (bg=18.56%) | K562 | + | chr11 | | 65267395 | 65267421 |
| 2.22198156258 | YWHAG (bg=9.14%) | K562 | + | chr11 | | 65267384 | 65267432 |
| 3.69418017826 | zc3h8 (bg=12.78%) | K562 | + | chr11 | | 65267392 | 65267407 |
| 4.31992870485 | znf622 (bg=18.79%) | K562 | + | chr11 | | 65267395 | 65267407 |
| 3.53671978739 | znf622 (bg=18.79%) | K562 | + | chr11 | | 65267406 | 65267424 |
| 4.42893551796 | znf622 (bg=18.79%) | K562 | + | chr11 | | 65267407 | 65267421 |
| 2.02605380232 | ZNF800 (bg=3.2%) | HepG2 | + | chr11 | | 65267350 | 65267430 |

  
  

| Match 66 in HUMAN | | | | | | | |
| --- | --- | --- | --- | --- | --- | --- | --- |
| Motif | Start in Seq (1 Indexed) | End in Seq (1 Indexed) | Strand | Chrm | Exon | Start in Chrm (0 Indexed) | End in Chrm (1 Indexed) |
| AAGATAG | 922 | 928 | + | chr11 | 1 | 65267411 | 65267418 |
| eCLIP Fold-Enrichment | Binding Protein | Cell Line | Strand | Chrm | | Start in Chrm (0 Indexed) | End in Chrm (1 Indexed) |
| 3.28032945158 | aggf1 (bg=15.15%) | K562 | + | chr11 | | 65267402 | 65267418 |
| 3.39735765993 | aggf1 (bg=15.15%) | K562 | + | chr11 | | 65267418 | 65267422 |
| 4.06206977484 | bclaf1 (bg=17.67%) | HepG2 | + | chr11 | | 65267379 | 65267473 |
| 4.11713735682 | bclaf1 (bg=17.67%) | HepG2 | + | chr11 | | 65267396 | 65267459 |
| 2.08811123109 | bud13 (bg=12.85%) | HepG2 | + | chr11 | | 65267379 | 65267427 |
| 2.58260548118 | bud13 (bg=12.85%) | K562 | + | chr11 | | 65267408 | 65267412 |
| 2.65055629912 | bud13 (bg=12.85%) | K562 | + | chr11 | | 65267408 | 65267412 |
| 2.73591401546 | bud13 (bg=12.85%) | K562 | + | chr11 | | 65267412 | 65267415 |
| 2.71223390097 | bud13 (bg=12.85%) | K562 | + | chr11 | | 65267412 | 65267421 |
| 2.66103449456 | bud13 (bg=12.85%) | K562 | + | chr11 | | 65267415 | 65267422 |
| 2.98153837035 | cpsf6 (bg=13.45%) | K562 | + | chr11 | | 65267394 | 65267420 |
| 3.34282194703 | cpsf6 (bg=13.45%) | K562 | + | chr11 | | 65267400 | 65267424 |
| 2.58145475808 | FASTKD2 (bg=4.54%) | K562 | + | chr11 | | 65267395 | 65267447 |
| 2.65639076089 | fxr2 (bg=10.1%) | HepG2 | + | chr11 | | 65267382 | 65267492 |
| 2.3933768929 | GPKOW (bg=5.66%) | K562 | + | chr11 | | 65267395 | 65267443 |
| 2.8162311711 | hltf (bg=24.28%) | HepG2 | + | chr11 | | 65267404 | 65267420 |
| 2.49873577591 | hltf (bg=24.28%) | HepG2 | + | chr11 | | 65267406 | 65267421 |
| 2.40751457806 | hltf (bg=24.28%) | K562 | + | chr11 | | 65267408 | 65267412 |
| 2.51963956022 | hltf (bg=24.28%) | K562 | + | chr11 | | 65267412 | 65267415 |
| 2.45358648734 | hltf (bg=24.28%) | K562 | + | chr11 | | 65267415 | 65267422 |
| 3.4797325896 | larp4 (bg=13.51%) | K562 | + | chr11 | | 65267395 | 65267436 |
| 2.44402503497 | MTPAP (bg=9.55%) | K562 | + | chr11 | | 65267394 | 65267414 |
| 3.09018342312 | MTPAP (bg=9.55%) | K562 | + | chr11 | | 65267405 | 65267413 |
| 3.22095977692 | MTPAP (bg=9.55%) | K562 | + | chr11 | | 65267413 | 65267434 |
| 2.15048649754 | rbm15 (bg=11.59%) | K562 | + | chr11 | | 65267396 | 65267422 |
| 3.47549875898 | rbm22 (bg=12.69%) | HepG2 | + | chr11 | | 65267393 | 65267413 |
| 3.74500314388 | safb2 (bg=26.89%) | K562 | + | chr11 | | 65267406 | 65267412 |
| 4.30370203643 | safb2 (bg=26.89%) | K562 | + | chr11 | | 65267407 | 65267412 |
| 3.76221649612 | safb2 (bg=26.89%) | K562 | + | chr11 | | 65267412 | 65267415 |
| 4.29716162186 | safb2 (bg=26.89%) | K562 | + | chr11 | | 65267412 | 65267420 |
| 3.52876711164 | safb2 (bg=26.89%) | K562 | + | chr11 | | 65267415 | 65267421 |
| 2.90077267386 | srsf1 (bg=30.28%) | K562 | + | chr11 | | 65267396 | 65267422 |
| 2.60452991794 | srsf7 (bg=22.53%) | K562 | + | chr11 | | 65267395 | 65267436 |
| 2.64980437023 | srsf7 (bg=22.53%) | K562 | + | chr11 | | 65267395 | 65267457 |
| 4.4834548098 | SUPV3L1 (bg=9.63%) | K562 | + | chr11 | | 65267386 | 65267417 |
| 5.72894430737 | tra2a (bg=37.02%) | HepG2 | + | chr11 | | 65267366 | 65267472 |
| 3.71680750425 | tra2a (bg=37.02%) | HepG2 | + | chr11 | | 65267392 | 65267455 |
| 6.34349595348 | tra2a (bg=37.02%) | K562 | + | chr11 | | 65267396 | 65267412 |
| 6.43309235766 | tra2a (bg=37.02%) | K562 | + | chr11 | | 65267396 | 65267412 |
| 6.14952415451 | tra2a (bg=37.02%) | K562 | + | chr11 | | 65267412 | 65267415 |
| 6.23891666916 | tra2a (bg=37.02%) | K562 | + | chr11 | | 65267412 | 65267421 |
| 5.74189163916 | tra2a (bg=37.02%) | K562 | + | chr11 | | 65267415 | 65267427 |
| 2.32762966363 | uchl5 (bg=18.56%) | HepG2 | + | chr11 | | 65267388 | 65267413 |
| 3.451687139 | uchl5 (bg=18.56%) | K562 | + | chr11 | | 65267395 | 65267421 |
| 3.04607515788 | uchl5 (bg=18.56%) | K562 | + | chr11 | | 65267395 | 65267421 |
| 2.22198156258 | YWHAG (bg=9.14%) | K562 | + | chr11 | | 65267384 | 65267432 |
| 3.53671978739 | znf622 (bg=18.79%) | K562 | + | chr11 | | 65267406 | 65267424 |
| 4.42893551796 | znf622 (bg=18.79%) | K562 | + | chr11 | | 65267407 | 65267421 |
| 2.02605380232 | ZNF800 (bg=3.2%) | HepG2 | + | chr11 | | 65267350 | 65267430 |

  
  

| Match 67 in HUMAN | | | | | | | |
| --- | --- | --- | --- | --- | --- | --- | --- |
| Motif | Start in Seq (1 Indexed) | End in Seq (1 Indexed) | Strand | Chrm | Exon | Start in Chrm (0 Indexed) | End in Chrm (1 Indexed) |
| AAAAATTGGA | 943 | 952 | + | chr11 | 1 | 65267432 | 65267442 |
| eCLIP Fold-Enrichment | Binding Protein | Cell Line | Strand | Chrm | | Start in Chrm (0 Indexed) | End in Chrm (1 Indexed) |
| 3.14150188925 | aggf1 (bg=15.15%) | K562 | + | chr11 | | 65267429 | 65267435 |
| 2.11977325604 | aggf1 (bg=15.15%) | K562 | + | chr11 | | 65267434 | 65267461 |
| 3.12267411758 | aggf1 (bg=15.15%) | K562 | + | chr11 | | 65267435 | 65267441 |
| 3.36077424711 | aggf1 (bg=15.15%) | K562 | + | chr11 | | 65267441 | 65267445 |
| 4.06206977484 | bclaf1 (bg=17.67%) | HepG2 | + | chr11 | | 65267379 | 65267473 |
| 4.11713735682 | bclaf1 (bg=17.67%) | HepG2 | + | chr11 | | 65267396 | 65267459 |
| 2.79794952754 | bud13 (bg=12.85%) | K562 | + | chr11 | | 65267427 | 65267437 |
| 2.67015130269 | bud13 (bg=12.85%) | K562 | + | chr11 | | 65267428 | 65267436 |
| 2.83373580501 | bud13 (bg=12.85%) | K562 | + | chr11 | | 65267436 | 65267442 |
| 2.7496922002 | bud13 (bg=12.85%) | K562 | + | chr11 | | 65267437 | 65267453 |
| 2.72676696169 | bud13 (bg=12.85%) | K562 | + | chr11 | | 65267442 | 65267447 |
| 2.58145475808 | FASTKD2 (bg=4.54%) | K562 | + | chr11 | | 65267395 | 65267447 |
| 2.65639076089 | fxr2 (bg=10.1%) | HepG2 | + | chr11 | | 65267382 | 65267492 |
| 2.3933768929 | GPKOW (bg=5.66%) | K562 | + | chr11 | | 65267395 | 65267443 |
| 2.95355021879 | hltf (bg=24.28%) | HepG2 | + | chr11 | | 65267420 | 65267436 |
| 2.77744079948 | hltf (bg=24.28%) | HepG2 | + | chr11 | | 65267421 | 65267441 |
| 2.08547609306 | hltf (bg=24.28%) | K562 | + | chr11 | | 65267427 | 65267437 |
| 2.47046235478 | hltf (bg=24.28%) | K562 | + | chr11 | | 65267431 | 65267436 |
| 2.64465713745 | hltf (bg=24.28%) | K562 | + | chr11 | | 65267436 | 65267440 |
| 3.5005134377 | hltf (bg=24.28%) | HepG2 | + | chr11 | | 65267436 | 65267467 |
| 2.0957626889 | hltf (bg=24.28%) | K562 | + | chr11 | | 65267437 | 65267440 |
| 2.83734526404 | hltf (bg=24.28%) | K562 | + | chr11 | | 65267440 | 65267442 |
| 2.29329123085 | hltf (bg=24.28%) | K562 | + | chr11 | | 65267440 | 65267442 |
| 3.09902467159 | hltf (bg=24.28%) | HepG2 | + | chr11 | | 65267441 | 65267467 |
| 2.23542980548 | hltf (bg=24.28%) | K562 | + | chr11 | | 65267442 | 65267446 |
| 2.72215845158 | hltf (bg=24.28%) | K562 | + | chr11 | | 65267442 | 65267447 |
| 3.4797325896 | larp4 (bg=13.51%) | K562 | + | chr11 | | 65267395 | 65267436 |
| 3.64756651184 | larp4 (bg=13.51%) | K562 | + | chr11 | | 65267436 | 65267449 |
| 3.22095977692 | MTPAP (bg=9.55%) | K562 | + | chr11 | | 65267413 | 65267434 |
| 2.01955682652 | NIPBL (bg=8.2%) | K562 | + | chr11 | | 65267426 | 65267437 |
| 2.04471212869 | NIPBL (bg=8.2%) | K562 | + | chr11 | | 65267437 | 65267447 |
| 3.25637176171 | npm1 (bg=10.22%) | K562 | + | chr11 | | 65267439 | 65267461 |
| 2.18921421168 | rbm15 (bg=11.59%) | K562 | + | chr11 | | 65267429 | 65267436 |
| 2.18467475606 | rbm15 (bg=11.59%) | K562 | + | chr11 | | 65267436 | 65267440 |
| 2.26563639677 | rbm15 (bg=11.59%) | K562 | + | chr11 | | 65267440 | 65267446 |
| 4.3041826006 | safb2 (bg=26.89%) | K562 | + | chr11 | | 65267428 | 65267437 |
| 3.86118733057 | safb2 (bg=26.89%) | K562 | + | chr11 | | 65267428 | 65267438 |
| 4.3163148117 | safb2 (bg=26.89%) | K562 | + | chr11 | | 65267437 | 65267452 |
| 3.68471510014 | safb2 (bg=26.89%) | K562 | + | chr11 | | 65267438 | 65267451 |
| 2.60452991794 | srsf7 (bg=22.53%) | K562 | + | chr11 | | 65267395 | 65267436 |
| 2.64980437023 | srsf7 (bg=22.53%) | K562 | + | chr11 | | 65267395 | 65267457 |
| 5.72894430737 | tra2a (bg=37.02%) | HepG2 | + | chr11 | | 65267366 | 65267472 |
| 3.71680750425 | tra2a (bg=37.02%) | HepG2 | + | chr11 | | 65267392 | 65267455 |
| 5.49249290927 | tra2a (bg=37.02%) | K562 | + | chr11 | | 65267427 | 65267441 |
| 5.28432009604 | tra2a (bg=37.02%) | K562 | + | chr11 | | 65267431 | 65267438 |
| 4.85435045999 | tra2a (bg=37.02%) | K562 | + | chr11 | | 65267438 | 65267462 |
| 4.84516180341 | tra2a (bg=37.02%) | K562 | + | chr11 | | 65267441 | 65267470 |
| 3.27207657793 | uchl5 (bg=18.56%) | K562 | + | chr11 | | 65267427 | 65267432 |
| 3.59722567671 | uchl5 (bg=18.56%) | K562 | + | chr11 | | 65267427 | 65267436 |
| 3.30703821369 | uchl5 (bg=18.56%) | K562 | + | chr11 | | 65267432 | 65267437 |
| 3.87836032969 | uchl5 (bg=18.56%) | K562 | + | chr11 | | 65267436 | 65267447 |
| 3.36037529974 | uchl5 (bg=18.56%) | K562 | + | chr11 | | 65267437 | 65267446 |
| 2.22198156258 | YWHAG (bg=9.14%) | K562 | + | chr11 | | 65267384 | 65267432 |
| 2.817072064 | YWHAG (bg=9.14%) | K562 | + | chr11 | | 65267432 | 65267470 |
| 4.44025618001 | znf622 (bg=18.79%) | K562 | + | chr11 | | 65267421 | 65267440 |
| 3.44284166372 | znf622 (bg=18.79%) | K562 | + | chr11 | | 65267424 | 65267438 |
| 4.08451337213 | znf622 (bg=18.79%) | K562 | + | chr11 | | 65267440 | 65267448 |

  
  

| Match 68 in HUMAN | | | | | | | |
| --- | --- | --- | --- | --- | --- | --- | --- |
| Motif | Start in Seq (1 Indexed) | End in Seq (1 Indexed) | Strand | Chrm | Exon | Start in Chrm (0 Indexed) | End in Chrm (1 Indexed) |
| AAAAATTGGATAAAATAGCAC | 943 | 963 | + | chr11 | 1 | 65267432 | 65267453 |
| eCLIP Fold-Enrichment | Binding Protein | Cell Line | Strand | Chrm | | Start in Chrm (0 Indexed) | End in Chrm (1 Indexed) |
| 3.14150188925 | aggf1 (bg=15.15%) | K562 | + | chr11 | | 65267429 | 65267435 |
| 2.11977325604 | aggf1 (bg=15.15%) | K562 | + | chr11 | | 65267434 | 65267461 |
| 3.12267411758 | aggf1 (bg=15.15%) | K562 | + | chr11 | | 65267435 | 65267441 |
| 3.36077424711 | aggf1 (bg=15.15%) | K562 | + | chr11 | | 65267441 | 65267445 |
| 3.3593702381 | aggf1 (bg=15.15%) | K562 | + | chr11 | | 65267445 | 65267452 |
| 3.42899211287 | aggf1 (bg=15.15%) | K562 | + | chr11 | | 65267452 | 65267460 |
| 4.06206977484 | bclaf1 (bg=17.67%) | HepG2 | + | chr11 | | 65267379 | 65267473 |
| 4.11713735682 | bclaf1 (bg=17.67%) | HepG2 | + | chr11 | | 65267396 | 65267459 |
| 2.79794952754 | bud13 (bg=12.85%) | K562 | + | chr11 | | 65267427 | 65267437 |
| 2.67015130269 | bud13 (bg=12.85%) | K562 | + | chr11 | | 65267428 | 65267436 |
| 2.83373580501 | bud13 (bg=12.85%) | K562 | + | chr11 | | 65267436 | 65267442 |
| 2.7496922002 | bud13 (bg=12.85%) | K562 | + | chr11 | | 65267437 | 65267453 |
| 2.72676696169 | bud13 (bg=12.85%) | K562 | + | chr11 | | 65267442 | 65267447 |
| 2.85181970353 | bud13 (bg=12.85%) | K562 | + | chr11 | | 65267447 | 65267452 |
| 2.86965184785 | bud13 (bg=12.85%) | K562 | + | chr11 | | 65267452 | 65267460 |
| 2.78328608273 | bud13 (bg=12.85%) | K562 | + | chr11 | | 65267453 | 65267460 |
| 2.58145475808 | FASTKD2 (bg=4.54%) | K562 | + | chr11 | | 65267395 | 65267447 |
| 2.46005079769 | FASTKD2 (bg=4.54%) | K562 | + | chr11 | | 65267447 | 65267483 |
| 2.65639076089 | fxr2 (bg=10.1%) | HepG2 | + | chr11 | | 65267382 | 65267492 |
| 2.3933768929 | GPKOW (bg=5.66%) | K562 | + | chr11 | | 65267395 | 65267443 |
| 2.27011601333 | GPKOW (bg=5.66%) | K562 | + | chr11 | | 65267443 | 65267467 |
| 2.21366204567 | GPKOW (bg=5.66%) | K562 | + | chr11 | | 65267445 | 65267469 |
| 2.95355021879 | hltf (bg=24.28%) | HepG2 | + | chr11 | | 65267420 | 65267436 |
| 2.77744079948 | hltf (bg=24.28%) | HepG2 | + | chr11 | | 65267421 | 65267441 |
| 2.08547609306 | hltf (bg=24.28%) | K562 | + | chr11 | | 65267427 | 65267437 |
| 2.47046235478 | hltf (bg=24.28%) | K562 | + | chr11 | | 65267431 | 65267436 |
| 2.64465713745 | hltf (bg=24.28%) | K562 | + | chr11 | | 65267436 | 65267440 |
| 3.5005134377 | hltf (bg=24.28%) | HepG2 | + | chr11 | | 65267436 | 65267467 |
| 2.0957626889 | hltf (bg=24.28%) | K562 | + | chr11 | | 65267437 | 65267440 |
| 2.83734526404 | hltf (bg=24.28%) | K562 | + | chr11 | | 65267440 | 65267442 |
| 2.29329123085 | hltf (bg=24.28%) | K562 | + | chr11 | | 65267440 | 65267442 |
| 3.09902467159 | hltf (bg=24.28%) | HepG2 | + | chr11 | | 65267441 | 65267467 |
| 2.23542980548 | hltf (bg=24.28%) | K562 | + | chr11 | | 65267442 | 65267446 |
| 2.72215845158 | hltf (bg=24.28%) | K562 | + | chr11 | | 65267442 | 65267447 |
| 2.32536825362 | hltf (bg=24.28%) | K562 | + | chr11 | | 65267446 | 65267451 |
| 2.78564611708 | hltf (bg=24.28%) | K562 | + | chr11 | | 65267447 | 65267452 |
| 2.53187572681 | hltf (bg=24.28%) | K562 | + | chr11 | | 65267451 | 65267472 |
| 2.94880420542 | hltf (bg=24.28%) | K562 | + | chr11 | | 65267452 | 65267473 |
| 3.4797325896 | larp4 (bg=13.51%) | K562 | + | chr11 | | 65267395 | 65267436 |
| 3.64756651184 | larp4 (bg=13.51%) | K562 | + | chr11 | | 65267436 | 65267449 |
| 3.52648201794 | larp4 (bg=13.51%) | K562 | + | chr11 | | 65267449 | 65267472 |
| 3.22095977692 | MTPAP (bg=9.55%) | K562 | + | chr11 | | 65267413 | 65267434 |
| 3.05985234715 | MTPAP (bg=9.55%) | K562 | + | chr11 | | 65267445 | 65267459 |
| 2.01955682652 | NIPBL (bg=8.2%) | K562 | + | chr11 | | 65267426 | 65267437 |
| 2.04471212869 | NIPBL (bg=8.2%) | K562 | + | chr11 | | 65267437 | 65267447 |
| 3.25637176171 | npm1 (bg=10.22%) | K562 | + | chr11 | | 65267439 | 65267461 |
| 2.18921421168 | rbm15 (bg=11.59%) | K562 | + | chr11 | | 65267429 | 65267436 |
| 2.18467475606 | rbm15 (bg=11.59%) | K562 | + | chr11 | | 65267436 | 65267440 |
| 2.26563639677 | rbm15 (bg=11.59%) | K562 | + | chr11 | | 65267440 | 65267446 |
| 2.35138561298 | rbm15 (bg=11.59%) | K562 | + | chr11 | | 65267446 | 65267452 |
| 2.37700924681 | rbm15 (bg=11.59%) | K562 | + | chr11 | | 65267452 | 65267471 |
| 4.3041826006 | safb2 (bg=26.89%) | K562 | + | chr11 | | 65267428 | 65267437 |
| 3.86118733057 | safb2 (bg=26.89%) | K562 | + | chr11 | | 65267428 | 65267438 |
| 4.3163148117 | safb2 (bg=26.89%) | K562 | + | chr11 | | 65267437 | 65267452 |
| 3.68471510014 | safb2 (bg=26.89%) | K562 | + | chr11 | | 65267438 | 65267451 |
| 3.54624903198 | safb2 (bg=26.89%) | K562 | + | chr11 | | 65267451 | 65267458 |
| 4.27380759183 | safb2 (bg=26.89%) | K562 | + | chr11 | | 65267452 | 65267460 |
| 2.60452991794 | srsf7 (bg=22.53%) | K562 | + | chr11 | | 65267395 | 65267436 |
| 2.64980437023 | srsf7 (bg=22.53%) | K562 | + | chr11 | | 65267395 | 65267457 |
| 5.72894430737 | tra2a (bg=37.02%) | HepG2 | + | chr11 | | 65267366 | 65267472 |
| 3.71680750425 | tra2a (bg=37.02%) | HepG2 | + | chr11 | | 65267392 | 65267455 |
| 5.49249290927 | tra2a (bg=37.02%) | K562 | + | chr11 | | 65267427 | 65267441 |
| 5.28432009604 | tra2a (bg=37.02%) | K562 | + | chr11 | | 65267431 | 65267438 |
| 4.85435045999 | tra2a (bg=37.02%) | K562 | + | chr11 | | 65267438 | 65267462 |
| 4.84516180341 | tra2a (bg=37.02%) | K562 | + | chr11 | | 65267441 | 65267470 |
| 3.27207657793 | uchl5 (bg=18.56%) | K562 | + | chr11 | | 65267427 | 65267432 |
| 3.59722567671 | uchl5 (bg=18.56%) | K562 | + | chr11 | | 65267427 | 65267436 |
| 3.30703821369 | uchl5 (bg=18.56%) | K562 | + | chr11 | | 65267432 | 65267437 |
| 3.87836032969 | uchl5 (bg=18.56%) | K562 | + | chr11 | | 65267436 | 65267447 |
| 3.36037529974 | uchl5 (bg=18.56%) | K562 | + | chr11 | | 65267437 | 65267446 |
| 3.40479073585 | uchl5 (bg=18.56%) | K562 | + | chr11 | | 65267446 | 65267452 |
| 3.91761053194 | uchl5 (bg=18.56%) | K562 | + | chr11 | | 65267447 | 65267471 |
| 3.41974305638 | uchl5 (bg=18.56%) | K562 | + | chr11 | | 65267452 | 65267472 |
| 2.22198156258 | YWHAG (bg=9.14%) | K562 | + | chr11 | | 65267384 | 65267432 |
| 2.817072064 | YWHAG (bg=9.14%) | K562 | + | chr11 | | 65267432 | 65267470 |
| 4.44025618001 | znf622 (bg=18.79%) | K562 | + | chr11 | | 65267421 | 65267440 |
| 3.44284166372 | znf622 (bg=18.79%) | K562 | + | chr11 | | 65267424 | 65267438 |
| 4.08451337213 | znf622 (bg=18.79%) | K562 | + | chr11 | | 65267440 | 65267448 |
| 4.07090631877 | znf622 (bg=18.79%) | K562 | + | chr11 | | 65267448 | 65267466 |
| 2.25098841822 | ZNF800 (bg=3.2%) | HepG2 | + | chr11 | | 65267447 | 65267473 |

  
  

| Match 69 in HUMAN | | | | | | | |
| --- | --- | --- | --- | --- | --- | --- | --- |
| Motif | Start in Seq (1 Indexed) | End in Seq (1 Indexed) | Strand | Chrm | Exon | Start in Chrm (0 Indexed) | End in Chrm (1 Indexed) |
| AAAATTGGA | 944 | 952 | + | chr11 | 1 | 65267433 | 65267442 |
| eCLIP Fold-Enrichment | Binding Protein | Cell Line | Strand | Chrm | | Start in Chrm (0 Indexed) | End in Chrm (1 Indexed) |
| 3.14150188925 | aggf1 (bg=15.15%) | K562 | + | chr11 | | 65267429 | 65267435 |
| 2.11977325604 | aggf1 (bg=15.15%) | K562 | + | chr11 | | 65267434 | 65267461 |
| 3.12267411758 | aggf1 (bg=15.15%) | K562 | + | chr11 | | 65267435 | 65267441 |
| 3.36077424711 | aggf1 (bg=15.15%) | K562 | + | chr11 | | 65267441 | 65267445 |
| 4.06206977484 | bclaf1 (bg=17.67%) | HepG2 | + | chr11 | | 65267379 | 65267473 |
| 4.11713735682 | bclaf1 (bg=17.67%) | HepG2 | + | chr11 | | 65267396 | 65267459 |
| 2.79794952754 | bud13 (bg=12.85%) | K562 | + | chr11 | | 65267427 | 65267437 |
| 2.67015130269 | bud13 (bg=12.85%) | K562 | + | chr11 | | 65267428 | 65267436 |
| 2.83373580501 | bud13 (bg=12.85%) | K562 | + | chr11 | | 65267436 | 65267442 |
| 2.7496922002 | bud13 (bg=12.85%) | K562 | + | chr11 | | 65267437 | 65267453 |
| 2.72676696169 | bud13 (bg=12.85%) | K562 | + | chr11 | | 65267442 | 65267447 |
| 2.58145475808 | FASTKD2 (bg=4.54%) | K562 | + | chr11 | | 65267395 | 65267447 |
| 2.65639076089 | fxr2 (bg=10.1%) | HepG2 | + | chr11 | | 65267382 | 65267492 |
| 2.3933768929 | GPKOW (bg=5.66%) | K562 | + | chr11 | | 65267395 | 65267443 |
| 2.95355021879 | hltf (bg=24.28%) | HepG2 | + | chr11 | | 65267420 | 65267436 |
| 2.77744079948 | hltf (bg=24.28%) | HepG2 | + | chr11 | | 65267421 | 65267441 |
| 2.08547609306 | hltf (bg=24.28%) | K562 | + | chr11 | | 65267427 | 65267437 |
| 2.47046235478 | hltf (bg=24.28%) | K562 | + | chr11 | | 65267431 | 65267436 |
| 2.64465713745 | hltf (bg=24.28%) | K562 | + | chr11 | | 65267436 | 65267440 |
| 3.5005134377 | hltf (bg=24.28%) | HepG2 | + | chr11 | | 65267436 | 65267467 |
| 2.0957626889 | hltf (bg=24.28%) | K562 | + | chr11 | | 65267437 | 65267440 |
| 2.83734526404 | hltf (bg=24.28%) | K562 | + | chr11 | | 65267440 | 65267442 |
| 2.29329123085 | hltf (bg=24.28%) | K562 | + | chr11 | | 65267440 | 65267442 |
| 3.09902467159 | hltf (bg=24.28%) | HepG2 | + | chr11 | | 65267441 | 65267467 |
| 2.23542980548 | hltf (bg=24.28%) | K562 | + | chr11 | | 65267442 | 65267446 |
| 2.72215845158 | hltf (bg=24.28%) | K562 | + | chr11 | | 65267442 | 65267447 |
| 3.4797325896 | larp4 (bg=13.51%) | K562 | + | chr11 | | 65267395 | 65267436 |
| 3.64756651184 | larp4 (bg=13.51%) | K562 | + | chr11 | | 65267436 | 65267449 |
| 3.22095977692 | MTPAP (bg=9.55%) | K562 | + | chr11 | | 65267413 | 65267434 |
| 2.01955682652 | NIPBL (bg=8.2%) | K562 | + | chr11 | | 65267426 | 65267437 |
| 2.04471212869 | NIPBL (bg=8.2%) | K562 | + | chr11 | | 65267437 | 65267447 |
| 3.25637176171 | npm1 (bg=10.22%) | K562 | + | chr11 | | 65267439 | 65267461 |
| 2.18921421168 | rbm15 (bg=11.59%) | K562 | + | chr11 | | 65267429 | 65267436 |
| 2.18467475606 | rbm15 (bg=11.59%) | K562 | + | chr11 | | 65267436 | 65267440 |
| 2.26563639677 | rbm15 (bg=11.59%) | K562 | + | chr11 | | 65267440 | 65267446 |
| 4.3041826006 | safb2 (bg=26.89%) | K562 | + | chr11 | | 65267428 | 65267437 |
| 3.86118733057 | safb2 (bg=26.89%) | K562 | + | chr11 | | 65267428 | 65267438 |
| 4.3163148117 | safb2 (bg=26.89%) | K562 | + | chr11 | | 65267437 | 65267452 |
| 3.68471510014 | safb2 (bg=26.89%) | K562 | + | chr11 | | 65267438 | 65267451 |
| 2.60452991794 | srsf7 (bg=22.53%) | K562 | + | chr11 | | 65267395 | 65267436 |
| 2.64980437023 | srsf7 (bg=22.53%) | K562 | + | chr11 | | 65267395 | 65267457 |
| 5.72894430737 | tra2a (bg=37.02%) | HepG2 | + | chr11 | | 65267366 | 65267472 |
| 3.71680750425 | tra2a (bg=37.02%) | HepG2 | + | chr11 | | 65267392 | 65267455 |
| 5.49249290927 | tra2a (bg=37.02%) | K562 | + | chr11 | | 65267427 | 65267441 |
| 5.28432009604 | tra2a (bg=37.02%) | K562 | + | chr11 | | 65267431 | 65267438 |
| 4.85435045999 | tra2a (bg=37.02%) | K562 | + | chr11 | | 65267438 | 65267462 |
| 4.84516180341 | tra2a (bg=37.02%) | K562 | + | chr11 | | 65267441 | 65267470 |
| 3.59722567671 | uchl5 (bg=18.56%) | K562 | + | chr11 | | 65267427 | 65267436 |
| 3.30703821369 | uchl5 (bg=18.56%) | K562 | + | chr11 | | 65267432 | 65267437 |
| 3.87836032969 | uchl5 (bg=18.56%) | K562 | + | chr11 | | 65267436 | 65267447 |
| 3.36037529974 | uchl5 (bg=18.56%) | K562 | + | chr11 | | 65267437 | 65267446 |
| 2.817072064 | YWHAG (bg=9.14%) | K562 | + | chr11 | | 65267432 | 65267470 |
| 4.44025618001 | znf622 (bg=18.79%) | K562 | + | chr11 | | 65267421 | 65267440 |
| 3.44284166372 | znf622 (bg=18.79%) | K562 | + | chr11 | | 65267424 | 65267438 |
| 4.08451337213 | znf622 (bg=18.79%) | K562 | + | chr11 | | 65267440 | 65267448 |

  
  

| Match 70 in HUMAN | | | | | | | |
| --- | --- | --- | --- | --- | --- | --- | --- |
| Motif | Start in Seq (1 Indexed) | End in Seq (1 Indexed) | Strand | Chrm | Exon | Start in Chrm (0 Indexed) | End in Chrm (1 Indexed) |
| GAAAAAATGA | 965 | 974 | + | chr11 | 1 | 65267454 | 65267464 |
| eCLIP Fold-Enrichment | Binding Protein | Cell Line | Strand | Chrm | | Start in Chrm (0 Indexed) | End in Chrm (1 Indexed) |
| 2.11977325604 | aggf1 (bg=15.15%) | K562 | + | chr11 | | 65267434 | 65267461 |
| 3.42899211287 | aggf1 (bg=15.15%) | K562 | + | chr11 | | 65267452 | 65267460 |
| 3.25990808787 | aggf1 (bg=15.15%) | K562 | + | chr11 | | 65267460 | 65267466 |
| 2.09052957568 | aggf1 (bg=15.15%) | K562 | + | chr11 | | 65267461 | 65267484 |
| 4.06206977484 | bclaf1 (bg=17.67%) | HepG2 | + | chr11 | | 65267379 | 65267473 |
| 4.11713735682 | bclaf1 (bg=17.67%) | HepG2 | + | chr11 | | 65267396 | 65267459 |
| 2.86965184785 | bud13 (bg=12.85%) | K562 | + | chr11 | | 65267452 | 65267460 |
| 2.78328608273 | bud13 (bg=12.85%) | K562 | + | chr11 | | 65267453 | 65267460 |
| 2.84817183883 | bud13 (bg=12.85%) | K562 | + | chr11 | | 65267460 | 65267468 |
| 2.67510646481 | bud13 (bg=12.85%) | K562 | + | chr11 | | 65267460 | 65267471 |
| 2.46005079769 | FASTKD2 (bg=4.54%) | K562 | + | chr11 | | 65267447 | 65267483 |
| 2.65639076089 | fxr2 (bg=10.1%) | HepG2 | + | chr11 | | 65267382 | 65267492 |
| 2.27011601333 | GPKOW (bg=5.66%) | K562 | + | chr11 | | 65267443 | 65267467 |
| 2.21366204567 | GPKOW (bg=5.66%) | K562 | + | chr11 | | 65267445 | 65267469 |
| 3.5005134377 | hltf (bg=24.28%) | HepG2 | + | chr11 | | 65267436 | 65267467 |
| 3.09902467159 | hltf (bg=24.28%) | HepG2 | + | chr11 | | 65267441 | 65267467 |
| 2.53187572681 | hltf (bg=24.28%) | K562 | + | chr11 | | 65267451 | 65267472 |
| 2.94880420542 | hltf (bg=24.28%) | K562 | + | chr11 | | 65267452 | 65267473 |
| 3.52648201794 | larp4 (bg=13.51%) | K562 | + | chr11 | | 65267449 | 65267472 |
| 3.05985234715 | MTPAP (bg=9.55%) | K562 | + | chr11 | | 65267445 | 65267459 |
| 3.25637176171 | npm1 (bg=10.22%) | K562 | + | chr11 | | 65267439 | 65267461 |
| 2.37700924681 | rbm15 (bg=11.59%) | K562 | + | chr11 | | 65267452 | 65267471 |
| 3.54624903198 | safb2 (bg=26.89%) | K562 | + | chr11 | | 65267451 | 65267458 |
| 4.27380759183 | safb2 (bg=26.89%) | K562 | + | chr11 | | 65267452 | 65267460 |
| 3.28173419356 | safb2 (bg=26.89%) | K562 | + | chr11 | | 65267458 | 65267471 |
| 4.14383253832 | safb2 (bg=26.89%) | K562 | + | chr11 | | 65267460 | 65267465 |
| 2.64980437023 | srsf7 (bg=22.53%) | K562 | + | chr11 | | 65267395 | 65267457 |
| 5.72894430737 | tra2a (bg=37.02%) | HepG2 | + | chr11 | | 65267366 | 65267472 |
| 3.71680750425 | tra2a (bg=37.02%) | HepG2 | + | chr11 | | 65267392 | 65267455 |
| 4.85435045999 | tra2a (bg=37.02%) | K562 | + | chr11 | | 65267438 | 65267462 |
| 4.84516180341 | tra2a (bg=37.02%) | K562 | + | chr11 | | 65267441 | 65267470 |
| 3.91761053194 | uchl5 (bg=18.56%) | K562 | + | chr11 | | 65267447 | 65267471 |
| 3.41974305638 | uchl5 (bg=18.56%) | K562 | + | chr11 | | 65267452 | 65267472 |
| 2.817072064 | YWHAG (bg=9.14%) | K562 | + | chr11 | | 65267432 | 65267470 |
| 4.07090631877 | znf622 (bg=18.79%) | K562 | + | chr11 | | 65267448 | 65267466 |
| 3.47578198039 | znf622 (bg=18.79%) | K562 | + | chr11 | | 65267458 | 65267461 |
| 2.25098841822 | ZNF800 (bg=3.2%) | HepG2 | + | chr11 | | 65267447 | 65267473 |

  
  

| Match 71 in HUMAN | | | | | | | |
| --- | --- | --- | --- | --- | --- | --- | --- |
| Motif | Start in Seq (1 Indexed) | End in Seq (1 Indexed) | Strand | Chrm | Exon | Start in Chrm (0 Indexed) | End in Chrm (1 Indexed) |
| AATTATTG | 978 | 985 | + | chr11 | 1 | 65267467 | 65267475 |
| eCLIP Fold-Enrichment | Binding Protein | Cell Line | Strand | Chrm | | Start in Chrm (0 Indexed) | End in Chrm (1 Indexed) |
| 2.09052957568 | aggf1 (bg=15.15%) | K562 | + | chr11 | | 65267461 | 65267484 |
| 3.29915939311 | aggf1 (bg=15.15%) | K562 | + | chr11 | | 65267466 | 65267472 |
| 2.69979295693 | aggf1 (bg=15.15%) | HepG2 | + | chr11 | | 65267468 | 65267484 |
| 2.96727317124 | aggf1 (bg=15.15%) | K562 | + | chr11 | | 65267472 | 65267486 |
| 2.81680503972 | AQR (bg=4.89%) | K562 | + | chr11 | | 65267469 | 65267513 |
| 4.06206977484 | bclaf1 (bg=17.67%) | HepG2 | + | chr11 | | 65267379 | 65267473 |
| 2.84817183883 | bud13 (bg=12.85%) | K562 | + | chr11 | | 65267460 | 65267468 |
| 2.67510646481 | bud13 (bg=12.85%) | K562 | + | chr11 | | 65267460 | 65267471 |
| 2.79391302998 | bud13 (bg=12.85%) | K562 | + | chr11 | | 65267468 | 65267472 |
| 2.45656180391 | bud13 (bg=12.85%) | K562 | + | chr11 | | 65267471 | 65267486 |
| 2.57905854152 | bud13 (bg=12.85%) | K562 | + | chr11 | | 65267472 | 65267487 |
| 2.46005079769 | FASTKD2 (bg=4.54%) | K562 | + | chr11 | | 65267447 | 65267483 |
| 2.65639076089 | fxr2 (bg=10.1%) | HepG2 | + | chr11 | | 65267382 | 65267492 |
| 2.27011601333 | GPKOW (bg=5.66%) | K562 | + | chr11 | | 65267443 | 65267467 |
| 2.21366204567 | GPKOW (bg=5.66%) | K562 | + | chr11 | | 65267445 | 65267469 |
| 2.32352207326 | GPKOW (bg=5.66%) | K562 | + | chr11 | | 65267467 | 65267481 |
| 2.15422428228 | GPKOW (bg=5.66%) | K562 | + | chr11 | | 65267469 | 65267479 |
| 3.5005134377 | hltf (bg=24.28%) | HepG2 | + | chr11 | | 65267436 | 65267467 |
| 3.09902467159 | hltf (bg=24.28%) | HepG2 | + | chr11 | | 65267441 | 65267467 |
| 2.53187572681 | hltf (bg=24.28%) | K562 | + | chr11 | | 65267451 | 65267472 |
| 2.94880420542 | hltf (bg=24.28%) | K562 | + | chr11 | | 65267452 | 65267473 |
| 3.52927092602 | hltf (bg=24.28%) | HepG2 | + | chr11 | | 65267467 | 65267471 |
| 3.2887151306 | hltf (bg=24.28%) | HepG2 | + | chr11 | | 65267467 | 65267481 |
| 2.85342041163 | hltf (bg=24.28%) | K562 | + | chr11 | | 65267472 | 65267487 |
| 3.11738337714 | hltf (bg=24.28%) | K562 | + | chr11 | | 65267473 | 65267486 |
| 3.52648201794 | larp4 (bg=13.51%) | K562 | + | chr11 | | 65267449 | 65267472 |
| 3.40566278602 | larp4 (bg=13.51%) | K562 | + | chr11 | | 65267472 | 65267486 |
| 2.94656811516 | MTPAP (bg=9.55%) | K562 | + | chr11 | | 65267472 | 65267475 |
| 2.37700924681 | rbm15 (bg=11.59%) | K562 | + | chr11 | | 65267452 | 65267471 |
| 2.17666646268 | rbm15 (bg=11.59%) | K562 | + | chr11 | | 65267471 | 65267486 |
| 3.28173419356 | safb2 (bg=26.89%) | K562 | + | chr11 | | 65267458 | 65267471 |
| 3.91676047777 | safb2 (bg=26.89%) | K562 | + | chr11 | | 65267465 | 65267468 |
| 3.86609267649 | safb2 (bg=26.89%) | K562 | + | chr11 | | 65267468 | 65267471 |
| 2.85171285032 | safb2 (bg=26.89%) | K562 | + | chr11 | | 65267471 | 65267480 |
| 3.56128368369 | safb2 (bg=26.89%) | K562 | + | chr11 | | 65267471 | 65267482 |
| 5.72894430737 | tra2a (bg=37.02%) | HepG2 | + | chr11 | | 65267366 | 65267472 |
| 4.84516180341 | tra2a (bg=37.02%) | K562 | + | chr11 | | 65267441 | 65267470 |
| 3.92720665799 | tra2a (bg=37.02%) | K562 | + | chr11 | | 65267473 | 65267479 |
| 3.91761053194 | uchl5 (bg=18.56%) | K562 | + | chr11 | | 65267447 | 65267471 |
| 3.41974305638 | uchl5 (bg=18.56%) | K562 | + | chr11 | | 65267452 | 65267472 |
| 3.86761692793 | uchl5 (bg=18.56%) | K562 | + | chr11 | | 65267471 | 65267487 |
| 3.29142125745 | uchl5 (bg=18.56%) | K562 | + | chr11 | | 65267472 | 65267486 |
| 2.817072064 | YWHAG (bg=9.14%) | K562 | + | chr11 | | 65267432 | 65267470 |
| 2.25098841822 | ZNF800 (bg=3.2%) | HepG2 | + | chr11 | | 65267447 | 65267473 |

  
  

| Match 72 in HUMAN | | | | | | | |
| --- | --- | --- | --- | --- | --- | --- | --- |
| Motif | Start in Seq (1 Indexed) | End in Seq (1 Indexed) | Strand | Chrm | Exon | Start in Chrm (0 Indexed) | End in Chrm (1 Indexed) |
| AACCAA | 988 | 993 | + | chr11 | 1 | 65267477 | 65267483 |
| eCLIP Fold-Enrichment | Binding Protein | Cell Line | Strand | Chrm | | Start in Chrm (0 Indexed) | End in Chrm (1 Indexed) |
| 2.09052957568 | aggf1 (bg=15.15%) | K562 | + | chr11 | | 65267461 | 65267484 |
| 2.69979295693 | aggf1 (bg=15.15%) | HepG2 | + | chr11 | | 65267468 | 65267484 |
| 2.96727317124 | aggf1 (bg=15.15%) | K562 | + | chr11 | | 65267472 | 65267486 |
| 2.81680503972 | AQR (bg=4.89%) | K562 | + | chr11 | | 65267469 | 65267513 |
| 2.45656180391 | bud13 (bg=12.85%) | K562 | + | chr11 | | 65267471 | 65267486 |
| 2.57905854152 | bud13 (bg=12.85%) | K562 | + | chr11 | | 65267472 | 65267487 |
| 2.46005079769 | FASTKD2 (bg=4.54%) | K562 | + | chr11 | | 65267447 | 65267483 |
| 2.65639076089 | fxr2 (bg=10.1%) | HepG2 | + | chr11 | | 65267382 | 65267492 |
| 2.32352207326 | GPKOW (bg=5.66%) | K562 | + | chr11 | | 65267467 | 65267481 |
| 2.15422428228 | GPKOW (bg=5.66%) | K562 | + | chr11 | | 65267469 | 65267479 |
| 3.2887151306 | hltf (bg=24.28%) | HepG2 | + | chr11 | | 65267467 | 65267481 |
| 2.85342041163 | hltf (bg=24.28%) | K562 | + | chr11 | | 65267472 | 65267487 |
| 3.11738337714 | hltf (bg=24.28%) | K562 | + | chr11 | | 65267473 | 65267486 |
| 3.61227659651 | hltf (bg=24.28%) | HepG2 | + | chr11 | | 65267476 | 65267479 |
| 3.40566278602 | larp4 (bg=13.51%) | K562 | + | chr11 | | 65267472 | 65267486 |
| 2.17666646268 | rbm15 (bg=11.59%) | K562 | + | chr11 | | 65267471 | 65267486 |
| 2.85171285032 | safb2 (bg=26.89%) | K562 | + | chr11 | | 65267471 | 65267480 |
| 3.56128368369 | safb2 (bg=26.89%) | K562 | + | chr11 | | 65267471 | 65267482 |
| 3.92720665799 | tra2a (bg=37.02%) | K562 | + | chr11 | | 65267473 | 65267479 |
| 3.86761692793 | uchl5 (bg=18.56%) | K562 | + | chr11 | | 65267471 | 65267487 |
| 3.29142125745 | uchl5 (bg=18.56%) | K562 | + | chr11 | | 65267472 | 65267486 |

  
  

| Match 73 in HUMAN | | | | | | | |
| --- | --- | --- | --- | --- | --- | --- | --- |
| Motif | Start in Seq (1 Indexed) | End in Seq (1 Indexed) | Strand | Chrm | Exon | Start in Chrm (0 Indexed) | End in Chrm (1 Indexed) |
| TTTAAAAGCCCATCAATTTAATTTCTG | 999 | 1025 | + | chr11 | 1 | 65267488 | 65267515 |
| eCLIP Fold-Enrichment | Binding Protein | Cell Line | Strand | Chrm | | Start in Chrm (0 Indexed) | End in Chrm (1 Indexed) |
| 2.13552944888 | aggf1 (bg=15.15%) | HepG2 | + | chr11 | | 65267484 | 65267504 |
| 2.4096316699 | aggf1 (bg=15.15%) | K562 | + | chr11 | | 65267486 | 65267504 |
| 2.19234703043 | aggf1 (bg=15.15%) | K562 | + | chr11 | | 65267487 | 65267494 |
| 2.14517732346 | aggf1 (bg=15.15%) | K562 | + | chr11 | | 65267494 | 65267503 |
| 2.25311412488 | aggf1 (bg=15.15%) | K562 | + | chr11 | | 65267503 | 65267518 |
| 2.21338340346 | aggf1 (bg=15.15%) | K562 | + | chr11 | | 65267504 | 65267507 |
| 2.20763295339 | aggf1 (bg=15.15%) | K562 | + | chr11 | | 65267507 | 65267525 |
| 2.81680503972 | AQR (bg=4.89%) | K562 | + | chr11 | | 65267469 | 65267513 |
| 3.20161517423 | bclaf1 (bg=17.67%) | HepG2 | + | chr11 | | 65267489 | 65267501 |
| 2.93221964091 | bclaf1 (bg=17.67%) | HepG2 | + | chr11 | | 65267490 | 65267499 |
| 2.50599076197 | bclaf1 (bg=17.67%) | HepG2 | + | chr11 | | 65267504 | 65267535 |
| 2.39405135166 | bclaf1 (bg=17.67%) | HepG2 | + | chr11 | | 65267505 | 65267531 |
| 2.30377420787 | cpsf6 (bg=13.45%) | K562 | + | chr11 | | 65267510 | 65267521 |
| 4.09215993342 | DDX24 (bg=0.47%) | K562 | + | chr11 | | 65267487 | 65267522 |
| 2.65639076089 | fxr2 (bg=10.1%) | HepG2 | + | chr11 | | 65267382 | 65267492 |
| 2.48633160746 | hltf (bg=24.28%) | K562 | + | chr11 | | 65267487 | 65267501 |
| 2.779020092 | hltf (bg=24.28%) | HepG2 | + | chr11 | | 65267489 | 65267504 |
| 2.54214639598 | hltf (bg=24.28%) | HepG2 | + | chr11 | | 65267490 | 65267502 |
| 2.49777555172 | hltf (bg=24.28%) | HepG2 | + | chr11 | | 65267502 | 65267531 |
| 2.51867192659 | hltf (bg=24.28%) | HepG2 | + | chr11 | | 65267507 | 65267516 |
| 2.45181143229 | larp4 (bg=13.51%) | K562 | + | chr11 | | 65267486 | 65267493 |
| 2.2756117069 | larp4 (bg=13.51%) | K562 | + | chr11 | | 65267493 | 65267504 |
| 2.09305189747 | larp4 (bg=13.51%) | K562 | + | chr11 | | 65267504 | 65267532 |
| 2.1437828125 | MTPAP (bg=9.55%) | K562 | + | chr11 | | 65267488 | 65267503 |
| 2.25007828237 | npm1 (bg=10.22%) | K562 | + | chr11 | | 65267500 | 65267519 |
| 3.40598682518 | ppil4 (bg=43.39%) | K562 | + | chr11 | | 65267489 | 65267494 |
| 3.33033753875 | ppil4 (bg=43.39%) | K562 | + | chr11 | | 65267494 | 65267504 |
| 3.36611944207 | ppil4 (bg=43.39%) | K562 | + | chr11 | | 65267504 | 65267527 |
| 2.85533883799 | ppil4 (bg=43.39%) | K562 | + | chr11 | | 65267509 | 65267529 |
| 3.34579620296 | safb (bg=40.39%) | K562 | + | chr11 | | 65267488 | 65267504 |
| 3.60721181464 | safb (bg=40.39%) | HepG2 | + | chr11 | | 65267489 | 65267496 |
| 3.0038165449 | safb (bg=40.39%) | K562 | + | chr11 | | 65267489 | 65267504 |
| 3.65014561064 | safb (bg=40.39%) | HepG2 | + | chr11 | | 65267496 | 65267504 |
| 3.29306107034 | safb (bg=40.39%) | HepG2 | + | chr11 | | 65267504 | 65267516 |
| 3.0527905642 | safb (bg=40.39%) | K562 | + | chr11 | | 65267504 | 65267517 |
| 3.38623323044 | safb (bg=40.39%) | K562 | + | chr11 | | 65267504 | 65267518 |
| 2.03487675179 | safb (bg=40.39%) | HepG2 | + | chr11 | | 65267508 | 65267520 |
| 3.35138729793 | safb2 (bg=26.89%) | K562 | + | chr11 | | 65267489 | 65267496 |
| 2.81509031729 | safb2 (bg=26.89%) | K562 | + | chr11 | | 65267489 | 65267504 |
| 3.20695911625 | safb2 (bg=26.89%) | K562 | + | chr11 | | 65267496 | 65267505 |
| 2.63393610335 | safb2 (bg=26.89%) | K562 | + | chr11 | | 65267504 | 65267516 |
| 3.20086187242 | safb2 (bg=26.89%) | K562 | + | chr11 | | 65267505 | 65267515 |
| 3.25311618842 | safb2 (bg=26.89%) | K562 | + | chr11 | | 65267515 | 65267531 |
| 2.01197222807 | SND1 (bg=0.87%) | K562 | + | chr11 | | 65267489 | 65267554 |
| 2.73061902869 | srsf1 (bg=30.28%) | K562 | + | chr11 | | 65267491 | 65267499 |
| 2.6232820038 | srsf1 (bg=30.28%) | K562 | + | chr11 | | 65267506 | 65267516 |
| 2.08116492289 | SUPV3L1 (bg=9.63%) | K562 | + | chr11 | | 65267514 | 65267529 |
| 2.35406745661 | TAF15 (bg=9.06%) | HepG2 | + | chr11 | | 65267512 | 65267550 |
| 2.95517699518 | tra2a (bg=37.02%) | K562 | + | chr11 | | 65267489 | 65267495 |
| 2.86359148468 | tra2a (bg=37.02%) | K562 | + | chr11 | | 65267490 | 65267503 |
| 2.80331893672 | tra2a (bg=37.02%) | K562 | + | chr11 | | 65267495 | 65267504 |
| 3.47445788701 | tra2a (bg=37.02%) | K562 | + | chr11 | | 65267503 | 65267515 |
| 3.47378539815 | tra2a (bg=37.02%) | K562 | + | chr11 | | 65267504 | 65267515 |
| 4.16281099872 | tra2a (bg=37.02%) | HepG2 | + | chr11 | | 65267508 | 65267576 |
| 2.86751694933 | tra2a (bg=37.02%) | HepG2 | + | chr11 | | 65267510 | 65267556 |
| 3.5843365823 | tra2a (bg=37.02%) | K562 | + | chr11 | | 65267515 | 65267531 |
| 3.6366550069 | tra2a (bg=37.02%) | K562 | + | chr11 | | 65267515 | 65267533 |
| 2.96437569485 | uchl5 (bg=18.56%) | K562 | + | chr11 | | 65267487 | 65267505 |
| 2.3951142605 | uchl5 (bg=18.56%) | K562 | + | chr11 | | 65267488 | 65267505 |
| 2.61895547621 | uchl5 (bg=18.56%) | K562 | + | chr11 | | 65267505 | 65267517 |
| 2.17871249754 | uchl5 (bg=18.56%) | K562 | + | chr11 | | 65267505 | 65267532 |
| 3.40983206657 | YBX3 (bg=0.84%) | K562 | + | chr11 | | 65267485 | 65267504 |
| 2.73176016146 | YBX3 (bg=0.84%) | K562 | + | chr11 | | 65267504 | 65267536 |
| 2.89607887635 | YBX3 (bg=0.84%) | K562 | + | chr11 | | 65267504 | 65267548 |
| 2.21472307471 | znf622 (bg=18.79%) | K562 | + | chr11 | | 65267489 | 65267495 |
| 2.11711665248 | znf622 (bg=18.79%) | K562 | + | chr11 | | 65267495 | 65267504 |
| 2.54710338519 | znf622 (bg=18.79%) | K562 | + | chr11 | | 65267504 | 65267529 |

  
  

| Match 74 in HUMAN | | | | | | | |
| --- | --- | --- | --- | --- | --- | --- | --- |
| Motif | Start in Seq (1 Indexed) | End in Seq (1 Indexed) | Strand | Chrm | Exon | Start in Chrm (0 Indexed) | End in Chrm (1 Indexed) |
| AAAAGCC | 1002 | 1008 | + | chr11 | 1 | 65267491 | 65267498 |
| eCLIP Fold-Enrichment | Binding Protein | Cell Line | Strand | Chrm | | Start in Chrm (0 Indexed) | End in Chrm (1 Indexed) |
| 2.13552944888 | aggf1 (bg=15.15%) | HepG2 | + | chr11 | | 65267484 | 65267504 |
| 2.4096316699 | aggf1 (bg=15.15%) | K562 | + | chr11 | | 65267486 | 65267504 |
| 2.19234703043 | aggf1 (bg=15.15%) | K562 | + | chr11 | | 65267487 | 65267494 |
| 2.14517732346 | aggf1 (bg=15.15%) | K562 | + | chr11 | | 65267494 | 65267503 |
| 2.81680503972 | AQR (bg=4.89%) | K562 | + | chr11 | | 65267469 | 65267513 |
| 3.20161517423 | bclaf1 (bg=17.67%) | HepG2 | + | chr11 | | 65267489 | 65267501 |
| 2.93221964091 | bclaf1 (bg=17.67%) | HepG2 | + | chr11 | | 65267490 | 65267499 |
| 4.09215993342 | DDX24 (bg=0.47%) | K562 | + | chr11 | | 65267487 | 65267522 |
| 2.65639076089 | fxr2 (bg=10.1%) | HepG2 | + | chr11 | | 65267382 | 65267492 |
| 2.48633160746 | hltf (bg=24.28%) | K562 | + | chr11 | | 65267487 | 65267501 |
| 2.779020092 | hltf (bg=24.28%) | HepG2 | + | chr11 | | 65267489 | 65267504 |
| 2.54214639598 | hltf (bg=24.28%) | HepG2 | + | chr11 | | 65267490 | 65267502 |
| 2.45181143229 | larp4 (bg=13.51%) | K562 | + | chr11 | | 65267486 | 65267493 |
| 2.2756117069 | larp4 (bg=13.51%) | K562 | + | chr11 | | 65267493 | 65267504 |
| 2.1437828125 | MTPAP (bg=9.55%) | K562 | + | chr11 | | 65267488 | 65267503 |
| 3.40598682518 | ppil4 (bg=43.39%) | K562 | + | chr11 | | 65267489 | 65267494 |
| 3.33033753875 | ppil4 (bg=43.39%) | K562 | + | chr11 | | 65267494 | 65267504 |
| 3.34579620296 | safb (bg=40.39%) | K562 | + | chr11 | | 65267488 | 65267504 |
| 3.60721181464 | safb (bg=40.39%) | HepG2 | + | chr11 | | 65267489 | 65267496 |
| 3.0038165449 | safb (bg=40.39%) | K562 | + | chr11 | | 65267489 | 65267504 |
| 3.65014561064 | safb (bg=40.39%) | HepG2 | + | chr11 | | 65267496 | 65267504 |
| 3.35138729793 | safb2 (bg=26.89%) | K562 | + | chr11 | | 65267489 | 65267496 |
| 2.81509031729 | safb2 (bg=26.89%) | K562 | + | chr11 | | 65267489 | 65267504 |
| 3.20695911625 | safb2 (bg=26.89%) | K562 | + | chr11 | | 65267496 | 65267505 |
| 2.01197222807 | SND1 (bg=0.87%) | K562 | + | chr11 | | 65267489 | 65267554 |
| 2.73061902869 | srsf1 (bg=30.28%) | K562 | + | chr11 | | 65267491 | 65267499 |
| 2.95517699518 | tra2a (bg=37.02%) | K562 | + | chr11 | | 65267489 | 65267495 |
| 2.86359148468 | tra2a (bg=37.02%) | K562 | + | chr11 | | 65267490 | 65267503 |
| 2.80331893672 | tra2a (bg=37.02%) | K562 | + | chr11 | | 65267495 | 65267504 |
| 2.96437569485 | uchl5 (bg=18.56%) | K562 | + | chr11 | | 65267487 | 65267505 |
| 2.3951142605 | uchl5 (bg=18.56%) | K562 | + | chr11 | | 65267488 | 65267505 |
| 3.40983206657 | YBX3 (bg=0.84%) | K562 | + | chr11 | | 65267485 | 65267504 |
| 2.21472307471 | znf622 (bg=18.79%) | K562 | + | chr11 | | 65267489 | 65267495 |
| 2.11711665248 | znf622 (bg=18.79%) | K562 | + | chr11 | | 65267495 | 65267504 |

  
  

| Match 75 in HUMAN | | | | | | | |
| --- | --- | --- | --- | --- | --- | --- | --- |
| Motif | Start in Seq (1 Indexed) | End in Seq (1 Indexed) | Strand | Chrm | Exon | Start in Chrm (0 Indexed) | End in Chrm (1 Indexed) |
| AAAAGCCCAT | 1002 | 1011 | + | chr11 | 1 | 65267491 | 65267501 |
| eCLIP Fold-Enrichment | Binding Protein | Cell Line | Strand | Chrm | | Start in Chrm (0 Indexed) | End in Chrm (1 Indexed) |
| 2.13552944888 | aggf1 (bg=15.15%) | HepG2 | + | chr11 | | 65267484 | 65267504 |
| 2.4096316699 | aggf1 (bg=15.15%) | K562 | + | chr11 | | 65267486 | 65267504 |
| 2.19234703043 | aggf1 (bg=15.15%) | K562 | + | chr11 | | 65267487 | 65267494 |
| 2.14517732346 | aggf1 (bg=15.15%) | K562 | + | chr11 | | 65267494 | 65267503 |
| 2.81680503972 | AQR (bg=4.89%) | K562 | + | chr11 | | 65267469 | 65267513 |
| 3.20161517423 | bclaf1 (bg=17.67%) | HepG2 | + | chr11 | | 65267489 | 65267501 |
| 2.93221964091 | bclaf1 (bg=17.67%) | HepG2 | + | chr11 | | 65267490 | 65267499 |
| 4.09215993342 | DDX24 (bg=0.47%) | K562 | + | chr11 | | 65267487 | 65267522 |
| 2.65639076089 | fxr2 (bg=10.1%) | HepG2 | + | chr11 | | 65267382 | 65267492 |
| 2.48633160746 | hltf (bg=24.28%) | K562 | + | chr11 | | 65267487 | 65267501 |
| 2.779020092 | hltf (bg=24.28%) | HepG2 | + | chr11 | | 65267489 | 65267504 |
| 2.54214639598 | hltf (bg=24.28%) | HepG2 | + | chr11 | | 65267490 | 65267502 |
| 2.45181143229 | larp4 (bg=13.51%) | K562 | + | chr11 | | 65267486 | 65267493 |
| 2.2756117069 | larp4 (bg=13.51%) | K562 | + | chr11 | | 65267493 | 65267504 |
| 2.1437828125 | MTPAP (bg=9.55%) | K562 | + | chr11 | | 65267488 | 65267503 |
| 2.25007828237 | npm1 (bg=10.22%) | K562 | + | chr11 | | 65267500 | 65267519 |
| 3.40598682518 | ppil4 (bg=43.39%) | K562 | + | chr11 | | 65267489 | 65267494 |
| 3.33033753875 | ppil4 (bg=43.39%) | K562 | + | chr11 | | 65267494 | 65267504 |
| 3.34579620296 | safb (bg=40.39%) | K562 | + | chr11 | | 65267488 | 65267504 |
| 3.60721181464 | safb (bg=40.39%) | HepG2 | + | chr11 | | 65267489 | 65267496 |
| 3.0038165449 | safb (bg=40.39%) | K562 | + | chr11 | | 65267489 | 65267504 |
| 3.65014561064 | safb (bg=40.39%) | HepG2 | + | chr11 | | 65267496 | 65267504 |
| 3.35138729793 | safb2 (bg=26.89%) | K562 | + | chr11 | | 65267489 | 65267496 |
| 2.81509031729 | safb2 (bg=26.89%) | K562 | + | chr11 | | 65267489 | 65267504 |
| 3.20695911625 | safb2 (bg=26.89%) | K562 | + | chr11 | | 65267496 | 65267505 |
| 2.01197222807 | SND1 (bg=0.87%) | K562 | + | chr11 | | 65267489 | 65267554 |
| 2.73061902869 | srsf1 (bg=30.28%) | K562 | + | chr11 | | 65267491 | 65267499 |
| 2.95517699518 | tra2a (bg=37.02%) | K562 | + | chr11 | | 65267489 | 65267495 |
| 2.86359148468 | tra2a (bg=37.02%) | K562 | + | chr11 | | 65267490 | 65267503 |
| 2.80331893672 | tra2a (bg=37.02%) | K562 | + | chr11 | | 65267495 | 65267504 |
| 2.96437569485 | uchl5 (bg=18.56%) | K562 | + | chr11 | | 65267487 | 65267505 |
| 2.3951142605 | uchl5 (bg=18.56%) | K562 | + | chr11 | | 65267488 | 65267505 |
| 3.40983206657 | YBX3 (bg=0.84%) | K562 | + | chr11 | | 65267485 | 65267504 |
| 2.21472307471 | znf622 (bg=18.79%) | K562 | + | chr11 | | 65267489 | 65267495 |
| 2.11711665248 | znf622 (bg=18.79%) | K562 | + | chr11 | | 65267495 | 65267504 |

  
  

| Match 76 in HUMAN | | | | | | | |
| --- | --- | --- | --- | --- | --- | --- | --- |
| Motif | Start in Seq (1 Indexed) | End in Seq (1 Indexed) | Strand | Chrm | Exon | Start in Chrm (0 Indexed) | End in Chrm (1 Indexed) |
| AATTTAATTTCTG | 1013 | 1025 | + | chr11 | 1 | 65267502 | 65267515 |
| eCLIP Fold-Enrichment | Binding Protein | Cell Line | Strand | Chrm | | Start in Chrm (0 Indexed) | End in Chrm (1 Indexed) |
| 2.13552944888 | aggf1 (bg=15.15%) | HepG2 | + | chr11 | | 65267484 | 65267504 |
| 2.4096316699 | aggf1 (bg=15.15%) | K562 | + | chr11 | | 65267486 | 65267504 |
| 2.14517732346 | aggf1 (bg=15.15%) | K562 | + | chr11 | | 65267494 | 65267503 |
| 2.25311412488 | aggf1 (bg=15.15%) | K562 | + | chr11 | | 65267503 | 65267518 |
| 2.21338340346 | aggf1 (bg=15.15%) | K562 | + | chr11 | | 65267504 | 65267507 |
| 2.20763295339 | aggf1 (bg=15.15%) | K562 | + | chr11 | | 65267507 | 65267525 |
| 2.81680503972 | AQR (bg=4.89%) | K562 | + | chr11 | | 65267469 | 65267513 |
| 2.50599076197 | bclaf1 (bg=17.67%) | HepG2 | + | chr11 | | 65267504 | 65267535 |
| 2.39405135166 | bclaf1 (bg=17.67%) | HepG2 | + | chr11 | | 65267505 | 65267531 |
| 2.30377420787 | cpsf6 (bg=13.45%) | K562 | + | chr11 | | 65267510 | 65267521 |
| 4.09215993342 | DDX24 (bg=0.47%) | K562 | + | chr11 | | 65267487 | 65267522 |
| 2.779020092 | hltf (bg=24.28%) | HepG2 | + | chr11 | | 65267489 | 65267504 |
| 2.54214639598 | hltf (bg=24.28%) | HepG2 | + | chr11 | | 65267490 | 65267502 |
| 2.49777555172 | hltf (bg=24.28%) | HepG2 | + | chr11 | | 65267502 | 65267531 |
| 2.51867192659 | hltf (bg=24.28%) | HepG2 | + | chr11 | | 65267507 | 65267516 |
| 2.2756117069 | larp4 (bg=13.51%) | K562 | + | chr11 | | 65267493 | 65267504 |
| 2.09305189747 | larp4 (bg=13.51%) | K562 | + | chr11 | | 65267504 | 65267532 |
| 2.1437828125 | MTPAP (bg=9.55%) | K562 | + | chr11 | | 65267488 | 65267503 |
| 2.25007828237 | npm1 (bg=10.22%) | K562 | + | chr11 | | 65267500 | 65267519 |
| 3.33033753875 | ppil4 (bg=43.39%) | K562 | + | chr11 | | 65267494 | 65267504 |
| 3.36611944207 | ppil4 (bg=43.39%) | K562 | + | chr11 | | 65267504 | 65267527 |
| 2.85533883799 | ppil4 (bg=43.39%) | K562 | + | chr11 | | 65267509 | 65267529 |
| 3.34579620296 | safb (bg=40.39%) | K562 | + | chr11 | | 65267488 | 65267504 |
| 3.0038165449 | safb (bg=40.39%) | K562 | + | chr11 | | 65267489 | 65267504 |
| 3.65014561064 | safb (bg=40.39%) | HepG2 | + | chr11 | | 65267496 | 65267504 |
| 3.29306107034 | safb (bg=40.39%) | HepG2 | + | chr11 | | 65267504 | 65267516 |
| 3.0527905642 | safb (bg=40.39%) | K562 | + | chr11 | | 65267504 | 65267517 |
| 3.38623323044 | safb (bg=40.39%) | K562 | + | chr11 | | 65267504 | 65267518 |
| 2.03487675179 | safb (bg=40.39%) | HepG2 | + | chr11 | | 65267508 | 65267520 |
| 2.81509031729 | safb2 (bg=26.89%) | K562 | + | chr11 | | 65267489 | 65267504 |
| 3.20695911625 | safb2 (bg=26.89%) | K562 | + | chr11 | | 65267496 | 65267505 |
| 2.63393610335 | safb2 (bg=26.89%) | K562 | + | chr11 | | 65267504 | 65267516 |
| 3.20086187242 | safb2 (bg=26.89%) | K562 | + | chr11 | | 65267505 | 65267515 |
| 3.25311618842 | safb2 (bg=26.89%) | K562 | + | chr11 | | 65267515 | 65267531 |
| 2.01197222807 | SND1 (bg=0.87%) | K562 | + | chr11 | | 65267489 | 65267554 |
| 2.6232820038 | srsf1 (bg=30.28%) | K562 | + | chr11 | | 65267506 | 65267516 |
| 2.08116492289 | SUPV3L1 (bg=9.63%) | K562 | + | chr11 | | 65267514 | 65267529 |
| 2.35406745661 | TAF15 (bg=9.06%) | HepG2 | + | chr11 | | 65267512 | 65267550 |
| 2.86359148468 | tra2a (bg=37.02%) | K562 | + | chr11 | | 65267490 | 65267503 |
| 2.80331893672 | tra2a (bg=37.02%) | K562 | + | chr11 | | 65267495 | 65267504 |
| 3.47445788701 | tra2a (bg=37.02%) | K562 | + | chr11 | | 65267503 | 65267515 |
| 3.47378539815 | tra2a (bg=37.02%) | K562 | + | chr11 | | 65267504 | 65267515 |
| 4.16281099872 | tra2a (bg=37.02%) | HepG2 | + | chr11 | | 65267508 | 65267576 |
| 2.86751694933 | tra2a (bg=37.02%) | HepG2 | + | chr11 | | 65267510 | 65267556 |
| 3.5843365823 | tra2a (bg=37.02%) | K562 | + | chr11 | | 65267515 | 65267531 |
| 3.6366550069 | tra2a (bg=37.02%) | K562 | + | chr11 | | 65267515 | 65267533 |
| 2.96437569485 | uchl5 (bg=18.56%) | K562 | + | chr11 | | 65267487 | 65267505 |
| 2.3951142605 | uchl5 (bg=18.56%) | K562 | + | chr11 | | 65267488 | 65267505 |
| 2.61895547621 | uchl5 (bg=18.56%) | K562 | + | chr11 | | 65267505 | 65267517 |
| 2.17871249754 | uchl5 (bg=18.56%) | K562 | + | chr11 | | 65267505 | 65267532 |
| 3.40983206657 | YBX3 (bg=0.84%) | K562 | + | chr11 | | 65267485 | 65267504 |
| 2.73176016146 | YBX3 (bg=0.84%) | K562 | + | chr11 | | 65267504 | 65267536 |
| 2.89607887635 | YBX3 (bg=0.84%) | K562 | + | chr11 | | 65267504 | 65267548 |
| 2.11711665248 | znf622 (bg=18.79%) | K562 | + | chr11 | | 65267495 | 65267504 |
| 2.54710338519 | znf622 (bg=18.79%) | K562 | + | chr11 | | 65267504 | 65267529 |

  
  

| Match 77 in HUMAN | | | | | | | |
| --- | --- | --- | --- | --- | --- | --- | --- |
| Motif | Start in Seq (1 Indexed) | End in Seq (1 Indexed) | Strand | Chrm | Exon | Start in Chrm (0 Indexed) | End in Chrm (1 Indexed) |
| TTTAATTT | 1015 | 1022 | + | chr11 | 1 | 65267504 | 65267512 |
| eCLIP Fold-Enrichment | Binding Protein | Cell Line | Strand | Chrm | | Start in Chrm (0 Indexed) | End in Chrm (1 Indexed) |
| 2.13552944888 | aggf1 (bg=15.15%) | HepG2 | + | chr11 | | 65267484 | 65267504 |
| 2.4096316699 | aggf1 (bg=15.15%) | K562 | + | chr11 | | 65267486 | 65267504 |
| 2.25311412488 | aggf1 (bg=15.15%) | K562 | + | chr11 | | 65267503 | 65267518 |
| 2.21338340346 | aggf1 (bg=15.15%) | K562 | + | chr11 | | 65267504 | 65267507 |
| 2.20763295339 | aggf1 (bg=15.15%) | K562 | + | chr11 | | 65267507 | 65267525 |
| 2.81680503972 | AQR (bg=4.89%) | K562 | + | chr11 | | 65267469 | 65267513 |
| 2.50599076197 | bclaf1 (bg=17.67%) | HepG2 | + | chr11 | | 65267504 | 65267535 |
| 2.39405135166 | bclaf1 (bg=17.67%) | HepG2 | + | chr11 | | 65267505 | 65267531 |
| 2.30377420787 | cpsf6 (bg=13.45%) | K562 | + | chr11 | | 65267510 | 65267521 |
| 4.09215993342 | DDX24 (bg=0.47%) | K562 | + | chr11 | | 65267487 | 65267522 |
| 2.779020092 | hltf (bg=24.28%) | HepG2 | + | chr11 | | 65267489 | 65267504 |
| 2.49777555172 | hltf (bg=24.28%) | HepG2 | + | chr11 | | 65267502 | 65267531 |
| 2.51867192659 | hltf (bg=24.28%) | HepG2 | + | chr11 | | 65267507 | 65267516 |
| 2.2756117069 | larp4 (bg=13.51%) | K562 | + | chr11 | | 65267493 | 65267504 |
| 2.09305189747 | larp4 (bg=13.51%) | K562 | + | chr11 | | 65267504 | 65267532 |
| 2.25007828237 | npm1 (bg=10.22%) | K562 | + | chr11 | | 65267500 | 65267519 |
| 3.33033753875 | ppil4 (bg=43.39%) | K562 | + | chr11 | | 65267494 | 65267504 |
| 3.36611944207 | ppil4 (bg=43.39%) | K562 | + | chr11 | | 65267504 | 65267527 |
| 2.85533883799 | ppil4 (bg=43.39%) | K562 | + | chr11 | | 65267509 | 65267529 |
| 3.34579620296 | safb (bg=40.39%) | K562 | + | chr11 | | 65267488 | 65267504 |
| 3.0038165449 | safb (bg=40.39%) | K562 | + | chr11 | | 65267489 | 65267504 |
| 3.65014561064 | safb (bg=40.39%) | HepG2 | + | chr11 | | 65267496 | 65267504 |
| 3.29306107034 | safb (bg=40.39%) | HepG2 | + | chr11 | | 65267504 | 65267516 |
| 3.0527905642 | safb (bg=40.39%) | K562 | + | chr11 | | 65267504 | 65267517 |
| 3.38623323044 | safb (bg=40.39%) | K562 | + | chr11 | | 65267504 | 65267518 |
| 2.03487675179 | safb (bg=40.39%) | HepG2 | + | chr11 | | 65267508 | 65267520 |
| 2.81509031729 | safb2 (bg=26.89%) | K562 | + | chr11 | | 65267489 | 65267504 |
| 3.20695911625 | safb2 (bg=26.89%) | K562 | + | chr11 | | 65267496 | 65267505 |
| 2.63393610335 | safb2 (bg=26.89%) | K562 | + | chr11 | | 65267504 | 65267516 |
| 3.20086187242 | safb2 (bg=26.89%) | K562 | + | chr11 | | 65267505 | 65267515 |
| 2.01197222807 | SND1 (bg=0.87%) | K562 | + | chr11 | | 65267489 | 65267554 |
| 2.6232820038 | srsf1 (bg=30.28%) | K562 | + | chr11 | | 65267506 | 65267516 |
| 2.35406745661 | TAF15 (bg=9.06%) | HepG2 | + | chr11 | | 65267512 | 65267550 |
| 2.80331893672 | tra2a (bg=37.02%) | K562 | + | chr11 | | 65267495 | 65267504 |
| 3.47445788701 | tra2a (bg=37.02%) | K562 | + | chr11 | | 65267503 | 65267515 |
| 3.47378539815 | tra2a (bg=37.02%) | K562 | + | chr11 | | 65267504 | 65267515 |
| 4.16281099872 | tra2a (bg=37.02%) | HepG2 | + | chr11 | | 65267508 | 65267576 |
| 2.86751694933 | tra2a (bg=37.02%) | HepG2 | + | chr11 | | 65267510 | 65267556 |
| 2.96437569485 | uchl5 (bg=18.56%) | K562 | + | chr11 | | 65267487 | 65267505 |
| 2.3951142605 | uchl5 (bg=18.56%) | K562 | + | chr11 | | 65267488 | 65267505 |
| 2.61895547621 | uchl5 (bg=18.56%) | K562 | + | chr11 | | 65267505 | 65267517 |
| 2.17871249754 | uchl5 (bg=18.56%) | K562 | + | chr11 | | 65267505 | 65267532 |
| 3.40983206657 | YBX3 (bg=0.84%) | K562 | + | chr11 | | 65267485 | 65267504 |
| 2.73176016146 | YBX3 (bg=0.84%) | K562 | + | chr11 | | 65267504 | 65267536 |
| 2.89607887635 | YBX3 (bg=0.84%) | K562 | + | chr11 | | 65267504 | 65267548 |
| 2.11711665248 | znf622 (bg=18.79%) | K562 | + | chr11 | | 65267495 | 65267504 |
| 2.54710338519 | znf622 (bg=18.79%) | K562 | + | chr11 | | 65267504 | 65267529 |

  
  

| Match 78 in HUMAN | | | | | | | |
| --- | --- | --- | --- | --- | --- | --- | --- |
| Motif | Start in Seq (1 Indexed) | End in Seq (1 Indexed) | Strand | Chrm | Exon | Start in Chrm (0 Indexed) | End in Chrm (1 Indexed) |
| TGGTGCAGAAGTTAGAAGGTAAAG | 1027 | 1050 | + | chr11 | 1 | 65267516 | 65267540 |
| eCLIP Fold-Enrichment | Binding Protein | Cell Line | Strand | Chrm | | Start in Chrm (0 Indexed) | End in Chrm (1 Indexed) |
| 2.25311412488 | aggf1 (bg=15.15%) | K562 | + | chr11 | | 65267503 | 65267518 |
| 2.20763295339 | aggf1 (bg=15.15%) | K562 | + | chr11 | | 65267507 | 65267525 |
| 2.30888527741 | aggf1 (bg=15.15%) | K562 | + | chr11 | | 65267518 | 65267525 |
| 2.09297736712 | aggf1 (bg=15.15%) | K562 | + | chr11 | | 65267532 | 65267563 |
| 2.50599076197 | bclaf1 (bg=17.67%) | HepG2 | + | chr11 | | 65267504 | 65267535 |
| 2.39405135166 | bclaf1 (bg=17.67%) | HepG2 | + | chr11 | | 65267505 | 65267531 |
| 2.20975361643 | bclaf1 (bg=17.67%) | HepG2 | + | chr11 | | 65267531 | 65267552 |
| 2.60872886756 | bclaf1 (bg=17.67%) | HepG2 | + | chr11 | | 65267535 | 65267575 |
| 2.30377420787 | cpsf6 (bg=13.45%) | K562 | + | chr11 | | 65267510 | 65267521 |
| 2.22176205668 | cpsf6 (bg=13.45%) | K562 | + | chr11 | | 65267521 | 65267534 |
| 2.03011485335 | cpsf6 (bg=13.45%) | K562 | + | chr11 | | 65267533 | 65267556 |
| 2.40135797387 | cpsf6 (bg=13.45%) | K562 | + | chr11 | | 65267534 | 65267556 |
| 4.09215993342 | DDX24 (bg=0.47%) | K562 | + | chr11 | | 65267487 | 65267522 |
| 2.49988929144 | fxr2 (bg=10.1%) | HepG2 | + | chr11 | | 65267536 | 65267597 |
| 2.33477314346 | gtf2f1 (bg=10.18%) | HepG2 | + | chr11 | | 65267517 | 65267527 |
| 2.49777555172 | hltf (bg=24.28%) | HepG2 | + | chr11 | | 65267502 | 65267531 |
| 2.51867192659 | hltf (bg=24.28%) | HepG2 | + | chr11 | | 65267507 | 65267516 |
| 2.42178649935 | hltf (bg=24.28%) | HepG2 | + | chr11 | | 65267516 | 65267534 |
| 2.16920349499 | hltf (bg=24.28%) | HepG2 | + | chr11 | | 65267531 | 65267550 |
| 2.36134927052 | hltf (bg=24.28%) | HepG2 | + | chr11 | | 65267534 | 65267553 |
| 2.09305189747 | larp4 (bg=13.51%) | K562 | + | chr11 | | 65267504 | 65267532 |
| 2.08175452135 | larp4 (bg=13.51%) | K562 | + | chr11 | | 65267538 | 65267548 |
| 2.25007828237 | npm1 (bg=10.22%) | K562 | + | chr11 | | 65267500 | 65267519 |
| 2.34264029742 | npm1 (bg=10.22%) | K562 | + | chr11 | | 65267525 | 65267645 |
| 3.36611944207 | ppil4 (bg=43.39%) | K562 | + | chr11 | | 65267504 | 65267527 |
| 2.85533883799 | ppil4 (bg=43.39%) | K562 | + | chr11 | | 65267509 | 65267529 |
| 3.17042196302 | ppil4 (bg=43.39%) | K562 | + | chr11 | | 65267527 | 65267547 |
| 3.11805818376 | ppil4 (bg=43.39%) | K562 | + | chr11 | | 65267529 | 65267547 |
| 3.29306107034 | safb (bg=40.39%) | HepG2 | + | chr11 | | 65267504 | 65267516 |
| 3.0527905642 | safb (bg=40.39%) | K562 | + | chr11 | | 65267504 | 65267517 |
| 3.38623323044 | safb (bg=40.39%) | K562 | + | chr11 | | 65267504 | 65267518 |
| 2.03487675179 | safb (bg=40.39%) | HepG2 | + | chr11 | | 65267508 | 65267520 |
| 3.26312248753 | safb (bg=40.39%) | HepG2 | + | chr11 | | 65267516 | 65267525 |
| 3.00865159087 | safb (bg=40.39%) | K562 | + | chr11 | | 65267517 | 65267532 |
| 3.35434843229 | safb (bg=40.39%) | K562 | + | chr11 | | 65267518 | 65267533 |
| 3.24352035118 | safb (bg=40.39%) | HepG2 | + | chr11 | | 65267525 | 65267532 |
| 2.90050320371 | safb (bg=40.39%) | K562 | + | chr11 | | 65267532 | 65267547 |
| 3.12711085906 | safb (bg=40.39%) | HepG2 | + | chr11 | | 65267532 | 65267548 |
| 3.33740922307 | safb (bg=40.39%) | K562 | + | chr11 | | 65267533 | 65267537 |
| 2.13194416975 | safb (bg=40.39%) | HepG2 | + | chr11 | | 65267536 | 65267555 |
| 3.33398725492 | safb (bg=40.39%) | K562 | + | chr11 | | 65267537 | 65267547 |
| 2.63393610335 | safb2 (bg=26.89%) | K562 | + | chr11 | | 65267504 | 65267516 |
| 3.25311618842 | safb2 (bg=26.89%) | K562 | + | chr11 | | 65267515 | 65267531 |
| 2.66763641885 | safb2 (bg=26.89%) | K562 | + | chr11 | | 65267516 | 65267527 |
| 2.64345911754 | safb2 (bg=26.89%) | K562 | + | chr11 | | 65267527 | 65267533 |
| 3.02084830386 | safb2 (bg=26.89%) | K562 | + | chr11 | | 65267531 | 65267533 |
| 3.1377674407 | safb2 (bg=26.89%) | K562 | + | chr11 | | 65267533 | 65267539 |
| 2.50202359471 | safb2 (bg=26.89%) | K562 | + | chr11 | | 65267533 | 65267539 |
| 2.53527051403 | safb2 (bg=26.89%) | K562 | + | chr11 | | 65267539 | 65267547 |
| 3.19516562824 | safb2 (bg=26.89%) | K562 | + | chr11 | | 65267539 | 65267554 |
| 2.01197222807 | SND1 (bg=0.87%) | K562 | + | chr11 | | 65267489 | 65267554 |
| 2.6232820038 | srsf1 (bg=30.28%) | K562 | + | chr11 | | 65267506 | 65267516 |
| 2.6182983872 | srsf1 (bg=30.28%) | K562 | + | chr11 | | 65267516 | 65267533 |
| 2.59229897163 | srsf1 (bg=30.28%) | K562 | + | chr11 | | 65267533 | 65267549 |
| 2.24334918312 | srsf7 (bg=22.53%) | HepG2 | + | chr11 | | 65267525 | 65267577 |
| 2.08116492289 | SUPV3L1 (bg=9.63%) | K562 | + | chr11 | | 65267514 | 65267529 |
| 2.35406745661 | TAF15 (bg=9.06%) | HepG2 | + | chr11 | | 65267512 | 65267550 |
| 4.16281099872 | tra2a (bg=37.02%) | HepG2 | + | chr11 | | 65267508 | 65267576 |
| 2.86751694933 | tra2a (bg=37.02%) | HepG2 | + | chr11 | | 65267510 | 65267556 |
| 3.5843365823 | tra2a (bg=37.02%) | K562 | + | chr11 | | 65267515 | 65267531 |
| 3.6366550069 | tra2a (bg=37.02%) | K562 | + | chr11 | | 65267515 | 65267533 |
| 3.63481707751 | tra2a (bg=37.02%) | K562 | + | chr11 | | 65267531 | 65267538 |
| 4.14747674477 | tra2a (bg=37.02%) | K562 | + | chr11 | | 65267533 | 65267538 |
| 4.37571872015 | tra2a (bg=37.02%) | K562 | + | chr11 | | 65267538 | 65267548 |
| 4.36111383561 | tra2a (bg=37.02%) | K562 | + | chr11 | | 65267538 | 65267556 |
| 2.61895547621 | uchl5 (bg=18.56%) | K562 | + | chr11 | | 65267505 | 65267517 |
| 2.17871249754 | uchl5 (bg=18.56%) | K562 | + | chr11 | | 65267505 | 65267532 |
| 2.53576779674 | uchl5 (bg=18.56%) | K562 | + | chr11 | | 65267517 | 65267525 |
| 2.46887731269 | uchl5 (bg=18.56%) | K562 | + | chr11 | | 65267525 | 65267537 |
| 2.04498336215 | uchl5 (bg=18.56%) | K562 | + | chr11 | | 65267532 | 65267534 |
| 2.11967680593 | uchl5 (bg=18.56%) | K562 | + | chr11 | | 65267534 | 65267539 |
| 2.54673816523 | uchl5 (bg=18.56%) | K562 | + | chr11 | | 65267537 | 65267547 |
| 2.27300925835 | uchl5 (bg=18.56%) | K562 | + | chr11 | | 65267539 | 65267547 |
| 2.73176016146 | YBX3 (bg=0.84%) | K562 | + | chr11 | | 65267504 | 65267536 |
| 2.89607887635 | YBX3 (bg=0.84%) | K562 | + | chr11 | | 65267504 | 65267548 |
| 2.54710338519 | znf622 (bg=18.79%) | K562 | + | chr11 | | 65267504 | 65267529 |
| 2.37388356297 | znf622 (bg=18.79%) | K562 | + | chr11 | | 65267529 | 65267549 |
| 2.02241493501 | znf622 (bg=18.79%) | K562 | + | chr11 | | 65267533 | 65267552 |

  
  

| Match 79 in HUMAN | | | | | | | |
| --- | --- | --- | --- | --- | --- | --- | --- |
| Motif | Start in Seq (1 Indexed) | End in Seq (1 Indexed) | Strand | Chrm | Exon | Start in Chrm (0 Indexed) | End in Chrm (1 Indexed) |
| GTGCAGAAG | 1029 | 1037 | + | chr11 | 1 | 65267518 | 65267527 |
| eCLIP Fold-Enrichment | Binding Protein | Cell Line | Strand | Chrm | | Start in Chrm (0 Indexed) | End in Chrm (1 Indexed) |
| 2.25311412488 | aggf1 (bg=15.15%) | K562 | + | chr11 | | 65267503 | 65267518 |
| 2.20763295339 | aggf1 (bg=15.15%) | K562 | + | chr11 | | 65267507 | 65267525 |
| 2.30888527741 | aggf1 (bg=15.15%) | K562 | + | chr11 | | 65267518 | 65267525 |
| 2.50599076197 | bclaf1 (bg=17.67%) | HepG2 | + | chr11 | | 65267504 | 65267535 |
| 2.39405135166 | bclaf1 (bg=17.67%) | HepG2 | + | chr11 | | 65267505 | 65267531 |
| 2.30377420787 | cpsf6 (bg=13.45%) | K562 | + | chr11 | | 65267510 | 65267521 |
| 2.22176205668 | cpsf6 (bg=13.45%) | K562 | + | chr11 | | 65267521 | 65267534 |
| 4.09215993342 | DDX24 (bg=0.47%) | K562 | + | chr11 | | 65267487 | 65267522 |
| 2.33477314346 | gtf2f1 (bg=10.18%) | HepG2 | + | chr11 | | 65267517 | 65267527 |
| 2.49777555172 | hltf (bg=24.28%) | HepG2 | + | chr11 | | 65267502 | 65267531 |
| 2.42178649935 | hltf (bg=24.28%) | HepG2 | + | chr11 | | 65267516 | 65267534 |
| 2.09305189747 | larp4 (bg=13.51%) | K562 | + | chr11 | | 65267504 | 65267532 |
| 2.25007828237 | npm1 (bg=10.22%) | K562 | + | chr11 | | 65267500 | 65267519 |
| 2.34264029742 | npm1 (bg=10.22%) | K562 | + | chr11 | | 65267525 | 65267645 |
| 3.36611944207 | ppil4 (bg=43.39%) | K562 | + | chr11 | | 65267504 | 65267527 |
| 2.85533883799 | ppil4 (bg=43.39%) | K562 | + | chr11 | | 65267509 | 65267529 |
| 3.17042196302 | ppil4 (bg=43.39%) | K562 | + | chr11 | | 65267527 | 65267547 |
| 3.38623323044 | safb (bg=40.39%) | K562 | + | chr11 | | 65267504 | 65267518 |
| 2.03487675179 | safb (bg=40.39%) | HepG2 | + | chr11 | | 65267508 | 65267520 |
| 3.26312248753 | safb (bg=40.39%) | HepG2 | + | chr11 | | 65267516 | 65267525 |
| 3.00865159087 | safb (bg=40.39%) | K562 | + | chr11 | | 65267517 | 65267532 |
| 3.35434843229 | safb (bg=40.39%) | K562 | + | chr11 | | 65267518 | 65267533 |
| 3.24352035118 | safb (bg=40.39%) | HepG2 | + | chr11 | | 65267525 | 65267532 |
| 3.25311618842 | safb2 (bg=26.89%) | K562 | + | chr11 | | 65267515 | 65267531 |
| 2.66763641885 | safb2 (bg=26.89%) | K562 | + | chr11 | | 65267516 | 65267527 |
| 2.64345911754 | safb2 (bg=26.89%) | K562 | + | chr11 | | 65267527 | 65267533 |
| 2.01197222807 | SND1 (bg=0.87%) | K562 | + | chr11 | | 65267489 | 65267554 |
| 2.6182983872 | srsf1 (bg=30.28%) | K562 | + | chr11 | | 65267516 | 65267533 |
| 2.24334918312 | srsf7 (bg=22.53%) | HepG2 | + | chr11 | | 65267525 | 65267577 |
| 2.08116492289 | SUPV3L1 (bg=9.63%) | K562 | + | chr11 | | 65267514 | 65267529 |
| 2.35406745661 | TAF15 (bg=9.06%) | HepG2 | + | chr11 | | 65267512 | 65267550 |
| 4.16281099872 | tra2a (bg=37.02%) | HepG2 | + | chr11 | | 65267508 | 65267576 |
| 2.86751694933 | tra2a (bg=37.02%) | HepG2 | + | chr11 | | 65267510 | 65267556 |
| 3.5843365823 | tra2a (bg=37.02%) | K562 | + | chr11 | | 65267515 | 65267531 |
| 3.6366550069 | tra2a (bg=37.02%) | K562 | + | chr11 | | 65267515 | 65267533 |
| 2.17871249754 | uchl5 (bg=18.56%) | K562 | + | chr11 | | 65267505 | 65267532 |
| 2.53576779674 | uchl5 (bg=18.56%) | K562 | + | chr11 | | 65267517 | 65267525 |
| 2.46887731269 | uchl5 (bg=18.56%) | K562 | + | chr11 | | 65267525 | 65267537 |
| 2.73176016146 | YBX3 (bg=0.84%) | K562 | + | chr11 | | 65267504 | 65267536 |
| 2.89607887635 | YBX3 (bg=0.84%) | K562 | + | chr11 | | 65267504 | 65267548 |
| 2.54710338519 | znf622 (bg=18.79%) | K562 | + | chr11 | | 65267504 | 65267529 |

  
  

| Match 80 in HUMAN | | | | | | | |
| --- | --- | --- | --- | --- | --- | --- | --- |
| Motif | Start in Seq (1 Indexed) | End in Seq (1 Indexed) | Strand | Chrm | Exon | Start in Chrm (0 Indexed) | End in Chrm (1 Indexed) |
| AGAAGATGAGGGT | 1055 | 1067 | + | chr11 | 1 | 65267544 | 65267557 |
| eCLIP Fold-Enrichment | Binding Protein | Cell Line | Strand | Chrm | | Start in Chrm (0 Indexed) | End in Chrm (1 Indexed) |
| 2.09297736712 | aggf1 (bg=15.15%) | K562 | + | chr11 | | 65267532 | 65267563 |
| 2.04991391735 | aggf1 (bg=15.15%) | K562 | + | chr11 | | 65267549 | 65267577 |
| 2.20975361643 | bclaf1 (bg=17.67%) | HepG2 | + | chr11 | | 65267531 | 65267552 |
| 2.60872886756 | bclaf1 (bg=17.67%) | HepG2 | + | chr11 | | 65267535 | 65267575 |
| 2.30945510747 | bclaf1 (bg=17.67%) | HepG2 | + | chr11 | | 65267552 | 65267577 |
| 2.03011485335 | cpsf6 (bg=13.45%) | K562 | + | chr11 | | 65267533 | 65267556 |
| 2.40135797387 | cpsf6 (bg=13.45%) | K562 | + | chr11 | | 65267534 | 65267556 |
| 2.72949170768 | cpsf6 (bg=13.45%) | K562 | + | chr11 | | 65267556 | 65267576 |
| 2.32514179035 | cpsf6 (bg=13.45%) | K562 | + | chr11 | | 65267556 | 65267577 |
| 2.49988929144 | fxr2 (bg=10.1%) | HepG2 | + | chr11 | | 65267536 | 65267597 |
| 2.22662433961 | gtf2f1 (bg=10.18%) | HepG2 | + | chr11 | | 65267552 | 65267574 |
| 2.16920349499 | hltf (bg=24.28%) | HepG2 | + | chr11 | | 65267531 | 65267550 |
| 2.36134927052 | hltf (bg=24.28%) | HepG2 | + | chr11 | | 65267534 | 65267553 |
| 2.06498283277 | hltf (bg=24.28%) | HepG2 | + | chr11 | | 65267550 | 65267553 |
| 2.6253314732 | hltf (bg=24.28%) | HepG2 | + | chr11 | | 65267553 | 65267576 |
| 2.69420426748 | hltf (bg=24.28%) | HepG2 | + | chr11 | | 65267553 | 65267577 |
| 2.08175452135 | larp4 (bg=13.51%) | K562 | + | chr11 | | 65267538 | 65267548 |
| 2.00436989587 | larp4 (bg=13.51%) | K562 | + | chr11 | | 65267548 | 65267555 |
| 2.18828874785 | larp4 (bg=13.51%) | K562 | + | chr11 | | 65267555 | 65267563 |
| 2.05640886697 | MTPAP (bg=9.55%) | K562 | + | chr11 | | 65267547 | 65267550 |
| 2.23097278142 | MTPAP (bg=9.55%) | K562 | + | chr11 | | 65267550 | 65267575 |
| 2.34264029742 | npm1 (bg=10.22%) | K562 | + | chr11 | | 65267525 | 65267645 |
| 3.17042196302 | ppil4 (bg=43.39%) | K562 | + | chr11 | | 65267527 | 65267547 |
| 3.11805818376 | ppil4 (bg=43.39%) | K562 | + | chr11 | | 65267529 | 65267547 |
| 3.61161743919 | ppil4 (bg=43.39%) | K562 | + | chr11 | | 65267547 | 65267551 |
| 3.14609910264 | ppil4 (bg=43.39%) | K562 | + | chr11 | | 65267547 | 65267552 |
| 3.88245570227 | ppil4 (bg=43.39%) | K562 | + | chr11 | | 65267551 | 65267565 |
| 3.39759328668 | ppil4 (bg=43.39%) | K562 | + | chr11 | | 65267552 | 65267563 |
| 2.26681258031 | rbm22 (bg=12.69%) | HepG2 | + | chr11 | | 65267551 | 65267577 |
| 2.90050320371 | safb (bg=40.39%) | K562 | + | chr11 | | 65267532 | 65267547 |
| 3.12711085906 | safb (bg=40.39%) | HepG2 | + | chr11 | | 65267532 | 65267548 |
| 2.13194416975 | safb (bg=40.39%) | HepG2 | + | chr11 | | 65267536 | 65267555 |
| 3.33398725492 | safb (bg=40.39%) | K562 | + | chr11 | | 65267537 | 65267547 |
| 2.78163711729 | safb (bg=40.39%) | K562 | + | chr11 | | 65267547 | 65267550 |
| 3.37850104977 | safb (bg=40.39%) | K562 | + | chr11 | | 65267547 | 65267554 |
| 3.22599210074 | safb (bg=40.39%) | HepG2 | + | chr11 | | 65267548 | 65267555 |
| 2.71824023032 | safb (bg=40.39%) | K562 | + | chr11 | | 65267550 | 65267555 |
| 3.40373053228 | safb (bg=40.39%) | K562 | + | chr11 | | 65267554 | 65267556 |
| 2.86592343481 | safb (bg=40.39%) | K562 | + | chr11 | | 65267555 | 65267563 |
| 3.56902987616 | safb (bg=40.39%) | HepG2 | + | chr11 | | 65267555 | 65267565 |
| 2.45429269158 | safb (bg=40.39%) | HepG2 | + | chr11 | | 65267555 | 65267574 |
| 3.43468452787 | safb (bg=40.39%) | K562 | + | chr11 | | 65267556 | 65267565 |
| 2.53527051403 | safb2 (bg=26.89%) | K562 | + | chr11 | | 65267539 | 65267547 |
| 3.19516562824 | safb2 (bg=26.89%) | K562 | + | chr11 | | 65267539 | 65267554 |
| 2.52727075155 | safb2 (bg=26.89%) | K562 | + | chr11 | | 65267547 | 65267549 |
| 2.54324159469 | safb2 (bg=26.89%) | K562 | + | chr11 | | 65267549 | 65267555 |
| 3.24584798733 | safb2 (bg=26.89%) | K562 | + | chr11 | | 65267554 | 65267556 |
| 2.73850167932 | safb2 (bg=26.89%) | K562 | + | chr11 | | 65267555 | 65267564 |
| 3.45846643515 | safb2 (bg=26.89%) | K562 | + | chr11 | | 65267556 | 65267564 |
| 2.01197222807 | SND1 (bg=0.87%) | K562 | + | chr11 | | 65267489 | 65267554 |
| 2.59229897163 | srsf1 (bg=30.28%) | K562 | + | chr11 | | 65267533 | 65267549 |
| 2.53041822638 | srsf1 (bg=30.28%) | K562 | + | chr11 | | 65267549 | 65267555 |
| 2.48372993169 | srsf1 (bg=30.28%) | K562 | + | chr11 | | 65267555 | 65267565 |
| 2.24334918312 | srsf7 (bg=22.53%) | HepG2 | + | chr11 | | 65267525 | 65267577 |
| 2.20453219774 | srsf7 (bg=22.53%) | K562 | + | chr11 | | 65267555 | 65267563 |
| 2.64676885348 | SUPV3L1 (bg=9.63%) | K562 | + | chr11 | | 65267548 | 65267624 |
| 2.35406745661 | TAF15 (bg=9.06%) | HepG2 | + | chr11 | | 65267512 | 65267550 |
| 2.55424426019 | TAF15 (bg=9.06%) | HepG2 | + | chr11 | | 65267550 | 65267575 |
| 2.21387411843 | TAF15 (bg=9.06%) | HepG2 | + | chr11 | | 65267556 | 65267578 |
| 4.16281099872 | tra2a (bg=37.02%) | HepG2 | + | chr11 | | 65267508 | 65267576 |
| 2.86751694933 | tra2a (bg=37.02%) | HepG2 | + | chr11 | | 65267510 | 65267556 |
| 4.37571872015 | tra2a (bg=37.02%) | K562 | + | chr11 | | 65267538 | 65267548 |
| 4.36111383561 | tra2a (bg=37.02%) | K562 | + | chr11 | | 65267538 | 65267556 |
| 4.34303758146 | tra2a (bg=37.02%) | K562 | + | chr11 | | 65267548 | 65267555 |
| 4.56432438323 | tra2a (bg=37.02%) | K562 | + | chr11 | | 65267555 | 65267574 |
| 4.56473880678 | tra2a (bg=37.02%) | K562 | + | chr11 | | 65267556 | 65267569 |
| 2.95421610241 | tra2a (bg=37.02%) | HepG2 | + | chr11 | | 65267556 | 65267576 |
| 2.54673816523 | uchl5 (bg=18.56%) | K562 | + | chr11 | | 65267537 | 65267547 |
| 2.27300925835 | uchl5 (bg=18.56%) | K562 | + | chr11 | | 65267539 | 65267547 |
| 2.23624941686 | uchl5 (bg=18.56%) | K562 | + | chr11 | | 65267547 | 65267550 |
| 2.5809677098 | uchl5 (bg=18.56%) | K562 | + | chr11 | | 65267547 | 65267554 |
| 2.19519282285 | uchl5 (bg=18.56%) | K562 | + | chr11 | | 65267550 | 65267556 |
| 2.57326282218 | uchl5 (bg=18.56%) | K562 | + | chr11 | | 65267554 | 65267556 |
| 2.16932672752 | uchl5 (bg=18.56%) | K562 | + | chr11 | | 65267556 | 65267564 |
| 2.59117891825 | uchl5 (bg=18.56%) | K562 | + | chr11 | | 65267556 | 65267569 |
| 2.89607887635 | YBX3 (bg=0.84%) | K562 | + | chr11 | | 65267504 | 65267548 |
| 2.37388356297 | znf622 (bg=18.79%) | K562 | + | chr11 | | 65267529 | 65267549 |
| 2.02241493501 | znf622 (bg=18.79%) | K562 | + | chr11 | | 65267533 | 65267552 |
| 2.90114942638 | znf622 (bg=18.79%) | K562 | + | chr11 | | 65267549 | 65267569 |
| 2.41856794445 | znf622 (bg=18.79%) | K562 | + | chr11 | | 65267552 | 65267568 |

  
  

| Match 81 in HUMAN | | | | | | | |
| --- | --- | --- | --- | --- | --- | --- | --- |
| Motif | Start in Seq (1 Indexed) | End in Seq (1 Indexed) | Strand | Chrm | Exon | Start in Chrm (0 Indexed) | End in Chrm (1 Indexed) |
| TGAGGGT | 1061 | 1067 | + | chr11 | 1 | 65267550 | 65267557 |
| eCLIP Fold-Enrichment | Binding Protein | Cell Line | Strand | Chrm | | Start in Chrm (0 Indexed) | End in Chrm (1 Indexed) |
| 2.09297736712 | aggf1 (bg=15.15%) | K562 | + | chr11 | | 65267532 | 65267563 |
| 2.04991391735 | aggf1 (bg=15.15%) | K562 | + | chr11 | | 65267549 | 65267577 |
| 2.20975361643 | bclaf1 (bg=17.67%) | HepG2 | + | chr11 | | 65267531 | 65267552 |
| 2.60872886756 | bclaf1 (bg=17.67%) | HepG2 | + | chr11 | | 65267535 | 65267575 |
| 2.30945510747 | bclaf1 (bg=17.67%) | HepG2 | + | chr11 | | 65267552 | 65267577 |
| 2.03011485335 | cpsf6 (bg=13.45%) | K562 | + | chr11 | | 65267533 | 65267556 |
| 2.40135797387 | cpsf6 (bg=13.45%) | K562 | + | chr11 | | 65267534 | 65267556 |
| 2.72949170768 | cpsf6 (bg=13.45%) | K562 | + | chr11 | | 65267556 | 65267576 |
| 2.32514179035 | cpsf6 (bg=13.45%) | K562 | + | chr11 | | 65267556 | 65267577 |
| 2.49988929144 | fxr2 (bg=10.1%) | HepG2 | + | chr11 | | 65267536 | 65267597 |
| 2.22662433961 | gtf2f1 (bg=10.18%) | HepG2 | + | chr11 | | 65267552 | 65267574 |
| 2.16920349499 | hltf (bg=24.28%) | HepG2 | + | chr11 | | 65267531 | 65267550 |
| 2.36134927052 | hltf (bg=24.28%) | HepG2 | + | chr11 | | 65267534 | 65267553 |
| 2.06498283277 | hltf (bg=24.28%) | HepG2 | + | chr11 | | 65267550 | 65267553 |
| 2.6253314732 | hltf (bg=24.28%) | HepG2 | + | chr11 | | 65267553 | 65267576 |
| 2.69420426748 | hltf (bg=24.28%) | HepG2 | + | chr11 | | 65267553 | 65267577 |
| 2.00436989587 | larp4 (bg=13.51%) | K562 | + | chr11 | | 65267548 | 65267555 |
| 2.18828874785 | larp4 (bg=13.51%) | K562 | + | chr11 | | 65267555 | 65267563 |
| 2.05640886697 | MTPAP (bg=9.55%) | K562 | + | chr11 | | 65267547 | 65267550 |
| 2.23097278142 | MTPAP (bg=9.55%) | K562 | + | chr11 | | 65267550 | 65267575 |
| 2.34264029742 | npm1 (bg=10.22%) | K562 | + | chr11 | | 65267525 | 65267645 |
| 3.61161743919 | ppil4 (bg=43.39%) | K562 | + | chr11 | | 65267547 | 65267551 |
| 3.14609910264 | ppil4 (bg=43.39%) | K562 | + | chr11 | | 65267547 | 65267552 |
| 3.88245570227 | ppil4 (bg=43.39%) | K562 | + | chr11 | | 65267551 | 65267565 |
| 3.39759328668 | ppil4 (bg=43.39%) | K562 | + | chr11 | | 65267552 | 65267563 |
| 2.26681258031 | rbm22 (bg=12.69%) | HepG2 | + | chr11 | | 65267551 | 65267577 |
| 2.13194416975 | safb (bg=40.39%) | HepG2 | + | chr11 | | 65267536 | 65267555 |
| 2.78163711729 | safb (bg=40.39%) | K562 | + | chr11 | | 65267547 | 65267550 |
| 3.37850104977 | safb (bg=40.39%) | K562 | + | chr11 | | 65267547 | 65267554 |
| 3.22599210074 | safb (bg=40.39%) | HepG2 | + | chr11 | | 65267548 | 65267555 |
| 2.71824023032 | safb (bg=40.39%) | K562 | + | chr11 | | 65267550 | 65267555 |
| 3.40373053228 | safb (bg=40.39%) | K562 | + | chr11 | | 65267554 | 65267556 |
| 2.86592343481 | safb (bg=40.39%) | K562 | + | chr11 | | 65267555 | 65267563 |
| 3.56902987616 | safb (bg=40.39%) | HepG2 | + | chr11 | | 65267555 | 65267565 |
| 2.45429269158 | safb (bg=40.39%) | HepG2 | + | chr11 | | 65267555 | 65267574 |
| 3.43468452787 | safb (bg=40.39%) | K562 | + | chr11 | | 65267556 | 65267565 |
| 3.19516562824 | safb2 (bg=26.89%) | K562 | + | chr11 | | 65267539 | 65267554 |
| 2.54324159469 | safb2 (bg=26.89%) | K562 | + | chr11 | | 65267549 | 65267555 |
| 3.24584798733 | safb2 (bg=26.89%) | K562 | + | chr11 | | 65267554 | 65267556 |
| 2.73850167932 | safb2 (bg=26.89%) | K562 | + | chr11 | | 65267555 | 65267564 |
| 3.45846643515 | safb2 (bg=26.89%) | K562 | + | chr11 | | 65267556 | 65267564 |
| 2.01197222807 | SND1 (bg=0.87%) | K562 | + | chr11 | | 65267489 | 65267554 |
| 2.53041822638 | srsf1 (bg=30.28%) | K562 | + | chr11 | | 65267549 | 65267555 |
| 2.48372993169 | srsf1 (bg=30.28%) | K562 | + | chr11 | | 65267555 | 65267565 |
| 2.24334918312 | srsf7 (bg=22.53%) | HepG2 | + | chr11 | | 65267525 | 65267577 |
| 2.20453219774 | srsf7 (bg=22.53%) | K562 | + | chr11 | | 65267555 | 65267563 |
| 2.64676885348 | SUPV3L1 (bg=9.63%) | K562 | + | chr11 | | 65267548 | 65267624 |
| 2.35406745661 | TAF15 (bg=9.06%) | HepG2 | + | chr11 | | 65267512 | 65267550 |
| 2.55424426019 | TAF15 (bg=9.06%) | HepG2 | + | chr11 | | 65267550 | 65267575 |
| 2.21387411843 | TAF15 (bg=9.06%) | HepG2 | + | chr11 | | 65267556 | 65267578 |
| 4.16281099872 | tra2a (bg=37.02%) | HepG2 | + | chr11 | | 65267508 | 65267576 |
| 2.86751694933 | tra2a (bg=37.02%) | HepG2 | + | chr11 | | 65267510 | 65267556 |
| 4.36111383561 | tra2a (bg=37.02%) | K562 | + | chr11 | | 65267538 | 65267556 |
| 4.34303758146 | tra2a (bg=37.02%) | K562 | + | chr11 | | 65267548 | 65267555 |
| 4.56432438323 | tra2a (bg=37.02%) | K562 | + | chr11 | | 65267555 | 65267574 |
| 4.56473880678 | tra2a (bg=37.02%) | K562 | + | chr11 | | 65267556 | 65267569 |
| 2.95421610241 | tra2a (bg=37.02%) | HepG2 | + | chr11 | | 65267556 | 65267576 |
| 2.23624941686 | uchl5 (bg=18.56%) | K562 | + | chr11 | | 65267547 | 65267550 |
| 2.5809677098 | uchl5 (bg=18.56%) | K562 | + | chr11 | | 65267547 | 65267554 |
| 2.19519282285 | uchl5 (bg=18.56%) | K562 | + | chr11 | | 65267550 | 65267556 |
| 2.57326282218 | uchl5 (bg=18.56%) | K562 | + | chr11 | | 65267554 | 65267556 |
| 2.16932672752 | uchl5 (bg=18.56%) | K562 | + | chr11 | | 65267556 | 65267564 |
| 2.59117891825 | uchl5 (bg=18.56%) | K562 | + | chr11 | | 65267556 | 65267569 |
| 2.02241493501 | znf622 (bg=18.79%) | K562 | + | chr11 | | 65267533 | 65267552 |
| 2.90114942638 | znf622 (bg=18.79%) | K562 | + | chr11 | | 65267549 | 65267569 |
| 2.41856794445 | znf622 (bg=18.79%) | K562 | + | chr11 | | 65267552 | 65267568 |

  
  

| Match 82 in HUMAN | | | | | | | |
| --- | --- | --- | --- | --- | --- | --- | --- |
| Motif | Start in Seq (1 Indexed) | End in Seq (1 Indexed) | Strand | Chrm | Exon | Start in Chrm (0 Indexed) | End in Chrm (1 Indexed) |
| GAGGGT | 1062 | 1067 | + | chr11 | 1 | 65267551 | 65267557 |
| eCLIP Fold-Enrichment | Binding Protein | Cell Line | Strand | Chrm | | Start in Chrm (0 Indexed) | End in Chrm (1 Indexed) |
| 2.09297736712 | aggf1 (bg=15.15%) | K562 | + | chr11 | | 65267532 | 65267563 |
| 2.04991391735 | aggf1 (bg=15.15%) | K562 | + | chr11 | | 65267549 | 65267577 |
| 2.20975361643 | bclaf1 (bg=17.67%) | HepG2 | + | chr11 | | 65267531 | 65267552 |
| 2.60872886756 | bclaf1 (bg=17.67%) | HepG2 | + | chr11 | | 65267535 | 65267575 |
| 2.30945510747 | bclaf1 (bg=17.67%) | HepG2 | + | chr11 | | 65267552 | 65267577 |
| 2.03011485335 | cpsf6 (bg=13.45%) | K562 | + | chr11 | | 65267533 | 65267556 |
| 2.40135797387 | cpsf6 (bg=13.45%) | K562 | + | chr11 | | 65267534 | 65267556 |
| 2.72949170768 | cpsf6 (bg=13.45%) | K562 | + | chr11 | | 65267556 | 65267576 |
| 2.32514179035 | cpsf6 (bg=13.45%) | K562 | + | chr11 | | 65267556 | 65267577 |
| 2.49988929144 | fxr2 (bg=10.1%) | HepG2 | + | chr11 | | 65267536 | 65267597 |
| 2.22662433961 | gtf2f1 (bg=10.18%) | HepG2 | + | chr11 | | 65267552 | 65267574 |
| 2.36134927052 | hltf (bg=24.28%) | HepG2 | + | chr11 | | 65267534 | 65267553 |
| 2.06498283277 | hltf (bg=24.28%) | HepG2 | + | chr11 | | 65267550 | 65267553 |
| 2.6253314732 | hltf (bg=24.28%) | HepG2 | + | chr11 | | 65267553 | 65267576 |
| 2.69420426748 | hltf (bg=24.28%) | HepG2 | + | chr11 | | 65267553 | 65267577 |
| 2.00436989587 | larp4 (bg=13.51%) | K562 | + | chr11 | | 65267548 | 65267555 |
| 2.18828874785 | larp4 (bg=13.51%) | K562 | + | chr11 | | 65267555 | 65267563 |
| 2.23097278142 | MTPAP (bg=9.55%) | K562 | + | chr11 | | 65267550 | 65267575 |
| 2.34264029742 | npm1 (bg=10.22%) | K562 | + | chr11 | | 65267525 | 65267645 |
| 3.61161743919 | ppil4 (bg=43.39%) | K562 | + | chr11 | | 65267547 | 65267551 |
| 3.14609910264 | ppil4 (bg=43.39%) | K562 | + | chr11 | | 65267547 | 65267552 |
| 3.88245570227 | ppil4 (bg=43.39%) | K562 | + | chr11 | | 65267551 | 65267565 |
| 3.39759328668 | ppil4 (bg=43.39%) | K562 | + | chr11 | | 65267552 | 65267563 |
| 2.26681258031 | rbm22 (bg=12.69%) | HepG2 | + | chr11 | | 65267551 | 65267577 |
| 2.13194416975 | safb (bg=40.39%) | HepG2 | + | chr11 | | 65267536 | 65267555 |
| 3.37850104977 | safb (bg=40.39%) | K562 | + | chr11 | | 65267547 | 65267554 |
| 3.22599210074 | safb (bg=40.39%) | HepG2 | + | chr11 | | 65267548 | 65267555 |
| 2.71824023032 | safb (bg=40.39%) | K562 | + | chr11 | | 65267550 | 65267555 |
| 3.40373053228 | safb (bg=40.39%) | K562 | + | chr11 | | 65267554 | 65267556 |
| 2.86592343481 | safb (bg=40.39%) | K562 | + | chr11 | | 65267555 | 65267563 |
| 3.56902987616 | safb (bg=40.39%) | HepG2 | + | chr11 | | 65267555 | 65267565 |
| 2.45429269158 | safb (bg=40.39%) | HepG2 | + | chr11 | | 65267555 | 65267574 |
| 3.43468452787 | safb (bg=40.39%) | K562 | + | chr11 | | 65267556 | 65267565 |
| 3.19516562824 | safb2 (bg=26.89%) | K562 | + | chr11 | | 65267539 | 65267554 |
| 2.54324159469 | safb2 (bg=26.89%) | K562 | + | chr11 | | 65267549 | 65267555 |
| 3.24584798733 | safb2 (bg=26.89%) | K562 | + | chr11 | | 65267554 | 65267556 |
| 2.73850167932 | safb2 (bg=26.89%) | K562 | + | chr11 | | 65267555 | 65267564 |
| 3.45846643515 | safb2 (bg=26.89%) | K562 | + | chr11 | | 65267556 | 65267564 |
| 2.01197222807 | SND1 (bg=0.87%) | K562 | + | chr11 | | 65267489 | 65267554 |
| 2.53041822638 | srsf1 (bg=30.28%) | K562 | + | chr11 | | 65267549 | 65267555 |
| 2.48372993169 | srsf1 (bg=30.28%) | K562 | + | chr11 | | 65267555 | 65267565 |
| 2.24334918312 | srsf7 (bg=22.53%) | HepG2 | + | chr11 | | 65267525 | 65267577 |
| 2.20453219774 | srsf7 (bg=22.53%) | K562 | + | chr11 | | 65267555 | 65267563 |
| 2.64676885348 | SUPV3L1 (bg=9.63%) | K562 | + | chr11 | | 65267548 | 65267624 |
| 2.55424426019 | TAF15 (bg=9.06%) | HepG2 | + | chr11 | | 65267550 | 65267575 |
| 2.21387411843 | TAF15 (bg=9.06%) | HepG2 | + | chr11 | | 65267556 | 65267578 |
| 4.16281099872 | tra2a (bg=37.02%) | HepG2 | + | chr11 | | 65267508 | 65267576 |
| 2.86751694933 | tra2a (bg=37.02%) | HepG2 | + | chr11 | | 65267510 | 65267556 |
| 4.36111383561 | tra2a (bg=37.02%) | K562 | + | chr11 | | 65267538 | 65267556 |
| 4.34303758146 | tra2a (bg=37.02%) | K562 | + | chr11 | | 65267548 | 65267555 |
| 4.56432438323 | tra2a (bg=37.02%) | K562 | + | chr11 | | 65267555 | 65267574 |
| 4.56473880678 | tra2a (bg=37.02%) | K562 | + | chr11 | | 65267556 | 65267569 |
| 2.95421610241 | tra2a (bg=37.02%) | HepG2 | + | chr11 | | 65267556 | 65267576 |
| 2.5809677098 | uchl5 (bg=18.56%) | K562 | + | chr11 | | 65267547 | 65267554 |
| 2.19519282285 | uchl5 (bg=18.56%) | K562 | + | chr11 | | 65267550 | 65267556 |
| 2.57326282218 | uchl5 (bg=18.56%) | K562 | + | chr11 | | 65267554 | 65267556 |
| 2.16932672752 | uchl5 (bg=18.56%) | K562 | + | chr11 | | 65267556 | 65267564 |
| 2.59117891825 | uchl5 (bg=18.56%) | K562 | + | chr11 | | 65267556 | 65267569 |
| 2.02241493501 | znf622 (bg=18.79%) | K562 | + | chr11 | | 65267533 | 65267552 |
| 2.90114942638 | znf622 (bg=18.79%) | K562 | + | chr11 | | 65267549 | 65267569 |
| 2.41856794445 | znf622 (bg=18.79%) | K562 | + | chr11 | | 65267552 | 65267568 |

  
  

| Match 83 in HUMAN | | | | | | | |
| --- | --- | --- | --- | --- | --- | --- | --- |
| Motif | Start in Seq (1 Indexed) | End in Seq (1 Indexed) | Strand | Chrm | Exon | Start in Chrm (0 Indexed) | End in Chrm (1 Indexed) |
| GTAGACCAGA | 1074 | 1083 | + | chr11 | 1 | 65267563 | 65267573 |
| eCLIP Fold-Enrichment | Binding Protein | Cell Line | Strand | Chrm | | Start in Chrm (0 Indexed) | End in Chrm (1 Indexed) |
| 2.09297736712 | aggf1 (bg=15.15%) | K562 | + | chr11 | | 65267532 | 65267563 |
| 2.04991391735 | aggf1 (bg=15.15%) | K562 | + | chr11 | | 65267549 | 65267577 |
| 2.54569286011 | aggf1 (bg=15.15%) | K562 | + | chr11 | | 65267563 | 65267565 |
| 2.54088576497 | aggf1 (bg=15.15%) | K562 | + | chr11 | | 65267565 | 65267568 |
| 2.52463523645 | aggf1 (bg=15.15%) | K562 | + | chr11 | | 65267568 | 65267570 |
| 2.57616792003 | aggf1 (bg=15.15%) | K562 | + | chr11 | | 65267570 | 65267573 |
| 2.48694473731 | aggf1 (bg=15.15%) | K562 | + | chr11 | | 65267573 | 65267577 |
| 2.60872886756 | bclaf1 (bg=17.67%) | HepG2 | + | chr11 | | 65267535 | 65267575 |
| 2.30945510747 | bclaf1 (bg=17.67%) | HepG2 | + | chr11 | | 65267552 | 65267577 |
| 2.72949170768 | cpsf6 (bg=13.45%) | K562 | + | chr11 | | 65267556 | 65267576 |
| 2.32514179035 | cpsf6 (bg=13.45%) | K562 | + | chr11 | | 65267556 | 65267577 |
| 2.49988929144 | fxr2 (bg=10.1%) | HepG2 | + | chr11 | | 65267536 | 65267597 |
| 2.22662433961 | gtf2f1 (bg=10.18%) | HepG2 | + | chr11 | | 65267552 | 65267574 |
| 2.6253314732 | hltf (bg=24.28%) | HepG2 | + | chr11 | | 65267553 | 65267576 |
| 2.69420426748 | hltf (bg=24.28%) | HepG2 | + | chr11 | | 65267553 | 65267577 |
| 2.18828874785 | larp4 (bg=13.51%) | K562 | + | chr11 | | 65267555 | 65267563 |
| 2.09412126124 | larp4 (bg=13.51%) | K562 | + | chr11 | | 65267563 | 65267576 |
| 2.23097278142 | MTPAP (bg=9.55%) | K562 | + | chr11 | | 65267550 | 65267575 |
| 2.34264029742 | npm1 (bg=10.22%) | K562 | + | chr11 | | 65267525 | 65267645 |
| 3.88245570227 | ppil4 (bg=43.39%) | K562 | + | chr11 | | 65267551 | 65267565 |
| 3.39759328668 | ppil4 (bg=43.39%) | K562 | + | chr11 | | 65267552 | 65267563 |
| 3.36877344595 | ppil4 (bg=43.39%) | K562 | + | chr11 | | 65267563 | 65267567 |
| 3.92048720925 | ppil4 (bg=43.39%) | K562 | + | chr11 | | 65267565 | 65267568 |
| 3.41981213022 | ppil4 (bg=43.39%) | K562 | + | chr11 | | 65267567 | 65267574 |
| 3.84520307775 | ppil4 (bg=43.39%) | K562 | + | chr11 | | 65267568 | 65267578 |
| 2.03117360003 | rbm15 (bg=11.59%) | K562 | + | chr11 | | 65267570 | 65267573 |
| 2.26681258031 | rbm22 (bg=12.69%) | HepG2 | + | chr11 | | 65267551 | 65267577 |
| 2.86592343481 | safb (bg=40.39%) | K562 | + | chr11 | | 65267555 | 65267563 |
| 3.56902987616 | safb (bg=40.39%) | HepG2 | + | chr11 | | 65267555 | 65267565 |
| 2.45429269158 | safb (bg=40.39%) | HepG2 | + | chr11 | | 65267555 | 65267574 |
| 3.43468452787 | safb (bg=40.39%) | K562 | + | chr11 | | 65267556 | 65267565 |
| 2.88033429696 | safb (bg=40.39%) | K562 | + | chr11 | | 65267563 | 65267569 |
| 3.43004593924 | safb (bg=40.39%) | K562 | + | chr11 | | 65267565 | 65267569 |
| 3.56485185967 | safb (bg=40.39%) | HepG2 | + | chr11 | | 65267565 | 65267576 |
| 2.89684600843 | safb (bg=40.39%) | K562 | + | chr11 | | 65267569 | 65267574 |
| 3.28677032249 | safb (bg=40.39%) | K562 | + | chr11 | | 65267569 | 65267577 |
| 2.73850167932 | safb2 (bg=26.89%) | K562 | + | chr11 | | 65267555 | 65267564 |
| 3.45846643515 | safb2 (bg=26.89%) | K562 | + | chr11 | | 65267556 | 65267564 |
| 2.91362574309 | safb2 (bg=26.89%) | K562 | + | chr11 | | 65267564 | 65267567 |
| 3.4876793734 | safb2 (bg=26.89%) | K562 | + | chr11 | | 65267564 | 65267569 |
| 2.88162684404 | safb2 (bg=26.89%) | K562 | + | chr11 | | 65267567 | 65267573 |
| 3.39307996452 | safb2 (bg=26.89%) | K562 | + | chr11 | | 65267569 | 65267579 |
| 2.74977754088 | safb2 (bg=26.89%) | K562 | + | chr11 | | 65267573 | 65267577 |
| 2.48372993169 | srsf1 (bg=30.28%) | K562 | + | chr11 | | 65267555 | 65267565 |
| 2.51647516039 | srsf1 (bg=30.28%) | K562 | + | chr11 | | 65267565 | 65267576 |
| 2.24334918312 | srsf7 (bg=22.53%) | HepG2 | + | chr11 | | 65267525 | 65267577 |
| 2.20453219774 | srsf7 (bg=22.53%) | K562 | + | chr11 | | 65267555 | 65267563 |
| 2.16389549269 | srsf7 (bg=22.53%) | K562 | + | chr11 | | 65267563 | 65267569 |
| 2.04623489792 | srsf7 (bg=22.53%) | K562 | + | chr11 | | 65267569 | 65267577 |
| 2.64676885348 | SUPV3L1 (bg=9.63%) | K562 | + | chr11 | | 65267548 | 65267624 |
| 2.55424426019 | TAF15 (bg=9.06%) | HepG2 | + | chr11 | | 65267550 | 65267575 |
| 2.21387411843 | TAF15 (bg=9.06%) | HepG2 | + | chr11 | | 65267556 | 65267578 |
| 4.16281099872 | tra2a (bg=37.02%) | HepG2 | + | chr11 | | 65267508 | 65267576 |
| 4.56432438323 | tra2a (bg=37.02%) | K562 | + | chr11 | | 65267555 | 65267574 |
| 4.56473880678 | tra2a (bg=37.02%) | K562 | + | chr11 | | 65267556 | 65267569 |
| 2.95421610241 | tra2a (bg=37.02%) | HepG2 | + | chr11 | | 65267556 | 65267576 |
| 4.56601475342 | tra2a (bg=37.02%) | K562 | + | chr11 | | 65267569 | 65267579 |
| 2.16932672752 | uchl5 (bg=18.56%) | K562 | + | chr11 | | 65267556 | 65267564 |
| 2.59117891825 | uchl5 (bg=18.56%) | K562 | + | chr11 | | 65267556 | 65267569 |
| 2.15656876111 | uchl5 (bg=18.56%) | K562 | + | chr11 | | 65267564 | 65267577 |
| 2.57886472672 | uchl5 (bg=18.56%) | K562 | + | chr11 | | 65267569 | 65267578 |
| 2.90114942638 | znf622 (bg=18.79%) | K562 | + | chr11 | | 65267549 | 65267569 |
| 2.41856794445 | znf622 (bg=18.79%) | K562 | + | chr11 | | 65267552 | 65267568 |
| 2.55460171955 | znf622 (bg=18.79%) | K562 | + | chr11 | | 65267568 | 65267578 |
| 3.19416449573 | znf622 (bg=18.79%) | K562 | + | chr11 | | 65267569 | 65267573 |
| 3.29117047828 | znf622 (bg=18.79%) | K562 | + | chr11 | | 65267573 | 65267577 |

  
  

| Match 84 in HUMAN | | | | | | | |
| --- | --- | --- | --- | --- | --- | --- | --- |
| Motif | Start in Seq (1 Indexed) | End in Seq (1 Indexed) | Strand | Chrm | Exon | Start in Chrm (0 Indexed) | End in Chrm (1 Indexed) |
| GTAGACC | 1074 | 1080 | + | chr11 | 1 | 65267563 | 65267570 |
| eCLIP Fold-Enrichment | Binding Protein | Cell Line | Strand | Chrm | | Start in Chrm (0 Indexed) | End in Chrm (1 Indexed) |
| 2.09297736712 | aggf1 (bg=15.15%) | K562 | + | chr11 | | 65267532 | 65267563 |
| 2.04991391735 | aggf1 (bg=15.15%) | K562 | + | chr11 | | 65267549 | 65267577 |
| 2.54569286011 | aggf1 (bg=15.15%) | K562 | + | chr11 | | 65267563 | 65267565 |
| 2.54088576497 | aggf1 (bg=15.15%) | K562 | + | chr11 | | 65267565 | 65267568 |
| 2.52463523645 | aggf1 (bg=15.15%) | K562 | + | chr11 | | 65267568 | 65267570 |
| 2.57616792003 | aggf1 (bg=15.15%) | K562 | + | chr11 | | 65267570 | 65267573 |
| 2.60872886756 | bclaf1 (bg=17.67%) | HepG2 | + | chr11 | | 65267535 | 65267575 |
| 2.30945510747 | bclaf1 (bg=17.67%) | HepG2 | + | chr11 | | 65267552 | 65267577 |
| 2.72949170768 | cpsf6 (bg=13.45%) | K562 | + | chr11 | | 65267556 | 65267576 |
| 2.32514179035 | cpsf6 (bg=13.45%) | K562 | + | chr11 | | 65267556 | 65267577 |
| 2.49988929144 | fxr2 (bg=10.1%) | HepG2 | + | chr11 | | 65267536 | 65267597 |
| 2.22662433961 | gtf2f1 (bg=10.18%) | HepG2 | + | chr11 | | 65267552 | 65267574 |
| 2.6253314732 | hltf (bg=24.28%) | HepG2 | + | chr11 | | 65267553 | 65267576 |
| 2.69420426748 | hltf (bg=24.28%) | HepG2 | + | chr11 | | 65267553 | 65267577 |
| 2.18828874785 | larp4 (bg=13.51%) | K562 | + | chr11 | | 65267555 | 65267563 |
| 2.09412126124 | larp4 (bg=13.51%) | K562 | + | chr11 | | 65267563 | 65267576 |
| 2.23097278142 | MTPAP (bg=9.55%) | K562 | + | chr11 | | 65267550 | 65267575 |
| 2.34264029742 | npm1 (bg=10.22%) | K562 | + | chr11 | | 65267525 | 65267645 |
| 3.88245570227 | ppil4 (bg=43.39%) | K562 | + | chr11 | | 65267551 | 65267565 |
| 3.39759328668 | ppil4 (bg=43.39%) | K562 | + | chr11 | | 65267552 | 65267563 |
| 3.36877344595 | ppil4 (bg=43.39%) | K562 | + | chr11 | | 65267563 | 65267567 |
| 3.92048720925 | ppil4 (bg=43.39%) | K562 | + | chr11 | | 65267565 | 65267568 |
| 3.41981213022 | ppil4 (bg=43.39%) | K562 | + | chr11 | | 65267567 | 65267574 |
| 3.84520307775 | ppil4 (bg=43.39%) | K562 | + | chr11 | | 65267568 | 65267578 |
| 2.03117360003 | rbm15 (bg=11.59%) | K562 | + | chr11 | | 65267570 | 65267573 |
| 2.26681258031 | rbm22 (bg=12.69%) | HepG2 | + | chr11 | | 65267551 | 65267577 |
| 2.86592343481 | safb (bg=40.39%) | K562 | + | chr11 | | 65267555 | 65267563 |
| 3.56902987616 | safb (bg=40.39%) | HepG2 | + | chr11 | | 65267555 | 65267565 |
| 2.45429269158 | safb (bg=40.39%) | HepG2 | + | chr11 | | 65267555 | 65267574 |
| 3.43468452787 | safb (bg=40.39%) | K562 | + | chr11 | | 65267556 | 65267565 |
| 2.88033429696 | safb (bg=40.39%) | K562 | + | chr11 | | 65267563 | 65267569 |
| 3.43004593924 | safb (bg=40.39%) | K562 | + | chr11 | | 65267565 | 65267569 |
| 3.56485185967 | safb (bg=40.39%) | HepG2 | + | chr11 | | 65267565 | 65267576 |
| 2.89684600843 | safb (bg=40.39%) | K562 | + | chr11 | | 65267569 | 65267574 |
| 3.28677032249 | safb (bg=40.39%) | K562 | + | chr11 | | 65267569 | 65267577 |
| 2.73850167932 | safb2 (bg=26.89%) | K562 | + | chr11 | | 65267555 | 65267564 |
| 3.45846643515 | safb2 (bg=26.89%) | K562 | + | chr11 | | 65267556 | 65267564 |
| 2.91362574309 | safb2 (bg=26.89%) | K562 | + | chr11 | | 65267564 | 65267567 |
| 3.4876793734 | safb2 (bg=26.89%) | K562 | + | chr11 | | 65267564 | 65267569 |
| 2.88162684404 | safb2 (bg=26.89%) | K562 | + | chr11 | | 65267567 | 65267573 |
| 3.39307996452 | safb2 (bg=26.89%) | K562 | + | chr11 | | 65267569 | 65267579 |
| 2.48372993169 | srsf1 (bg=30.28%) | K562 | + | chr11 | | 65267555 | 65267565 |
| 2.51647516039 | srsf1 (bg=30.28%) | K562 | + | chr11 | | 65267565 | 65267576 |
| 2.24334918312 | srsf7 (bg=22.53%) | HepG2 | + | chr11 | | 65267525 | 65267577 |
| 2.20453219774 | srsf7 (bg=22.53%) | K562 | + | chr11 | | 65267555 | 65267563 |
| 2.16389549269 | srsf7 (bg=22.53%) | K562 | + | chr11 | | 65267563 | 65267569 |
| 2.04623489792 | srsf7 (bg=22.53%) | K562 | + | chr11 | | 65267569 | 65267577 |
| 2.64676885348 | SUPV3L1 (bg=9.63%) | K562 | + | chr11 | | 65267548 | 65267624 |
| 2.55424426019 | TAF15 (bg=9.06%) | HepG2 | + | chr11 | | 65267550 | 65267575 |
| 2.21387411843 | TAF15 (bg=9.06%) | HepG2 | + | chr11 | | 65267556 | 65267578 |
| 4.16281099872 | tra2a (bg=37.02%) | HepG2 | + | chr11 | | 65267508 | 65267576 |
| 4.56432438323 | tra2a (bg=37.02%) | K562 | + | chr11 | | 65267555 | 65267574 |
| 4.56473880678 | tra2a (bg=37.02%) | K562 | + | chr11 | | 65267556 | 65267569 |
| 2.95421610241 | tra2a (bg=37.02%) | HepG2 | + | chr11 | | 65267556 | 65267576 |
| 4.56601475342 | tra2a (bg=37.02%) | K562 | + | chr11 | | 65267569 | 65267579 |
| 2.16932672752 | uchl5 (bg=18.56%) | K562 | + | chr11 | | 65267556 | 65267564 |
| 2.59117891825 | uchl5 (bg=18.56%) | K562 | + | chr11 | | 65267556 | 65267569 |
| 2.15656876111 | uchl5 (bg=18.56%) | K562 | + | chr11 | | 65267564 | 65267577 |
| 2.57886472672 | uchl5 (bg=18.56%) | K562 | + | chr11 | | 65267569 | 65267578 |
| 2.90114942638 | znf622 (bg=18.79%) | K562 | + | chr11 | | 65267549 | 65267569 |
| 2.41856794445 | znf622 (bg=18.79%) | K562 | + | chr11 | | 65267552 | 65267568 |
| 2.55460171955 | znf622 (bg=18.79%) | K562 | + | chr11 | | 65267568 | 65267578 |
| 3.19416449573 | znf622 (bg=18.79%) | K562 | + | chr11 | | 65267569 | 65267573 |

  
  

| Match 85 in HUMAN | | | | | | | |
| --- | --- | --- | --- | --- | --- | --- | --- |
| Motif | Start in Seq (1 Indexed) | End in Seq (1 Indexed) | Strand | Chrm | Exon | Start in Chrm (0 Indexed) | End in Chrm (1 Indexed) |
| CCAATTTAGAAGAATA | 1085 | 1100 | + | chr11 | 1 | 65267574 | 65267590 |
| eCLIP Fold-Enrichment | Binding Protein | Cell Line | Strand | Chrm | | Start in Chrm (0 Indexed) | End in Chrm (1 Indexed) |
| 2.04991391735 | aggf1 (bg=15.15%) | K562 | + | chr11 | | 65267549 | 65267577 |
| 2.48694473731 | aggf1 (bg=15.15%) | K562 | + | chr11 | | 65267573 | 65267577 |
| 2.57918825284 | aggf1 (bg=15.15%) | K562 | + | chr11 | | 65267577 | 65267586 |
| 2.64205984103 | aggf1 (bg=15.15%) | K562 | + | chr11 | | 65267586 | 65267589 |
| 2.02049744969 | aggf1 (bg=15.15%) | K562 | + | chr11 | | 65267587 | 65267601 |
| 2.36271889345 | aggf1 (bg=15.15%) | K562 | + | chr11 | | 65267589 | 65267603 |
| 2.60872886756 | bclaf1 (bg=17.67%) | HepG2 | + | chr11 | | 65267535 | 65267575 |
| 2.30945510747 | bclaf1 (bg=17.67%) | HepG2 | + | chr11 | | 65267552 | 65267577 |
| 3.36681170889 | bclaf1 (bg=17.67%) | HepG2 | + | chr11 | | 65267575 | 65267590 |
| 2.95285097368 | bclaf1 (bg=17.67%) | HepG2 | + | chr11 | | 65267577 | 65267587 |
| 3.12358516662 | bclaf1 (bg=17.67%) | HepG2 | + | chr11 | | 65267587 | 65267604 |
| 3.11268678464 | bclaf1 (bg=17.67%) | HepG2 | + | chr11 | | 65267590 | 65267598 |
| 2.72949170768 | cpsf6 (bg=13.45%) | K562 | + | chr11 | | 65267556 | 65267576 |
| 2.32514179035 | cpsf6 (bg=13.45%) | K562 | + | chr11 | | 65267556 | 65267577 |
| 2.9060077473 | cpsf6 (bg=13.45%) | K562 | + | chr11 | | 65267576 | 65267587 |
| 2.48113862281 | cpsf6 (bg=13.45%) | K562 | + | chr11 | | 65267577 | 65267607 |
| 2.82100195184 | cpsf6 (bg=13.45%) | K562 | + | chr11 | | 65267587 | 65267608 |
| 2.08815924402 | EIF3H (bg=0.83%) | HepG2 | + | chr11 | | 65267576 | 65267638 |
| 2.49988929144 | fxr2 (bg=10.1%) | HepG2 | + | chr11 | | 65267536 | 65267597 |
| 2.22662433961 | gtf2f1 (bg=10.18%) | HepG2 | + | chr11 | | 65267552 | 65267574 |
| 2.45762989125 | gtf2f1 (bg=10.18%) | HepG2 | + | chr11 | | 65267574 | 65267590 |
| 2.4841507675 | gtf2f1 (bg=10.18%) | HepG2 | + | chr11 | | 65267590 | 65267644 |
| 2.6253314732 | hltf (bg=24.28%) | HepG2 | + | chr11 | | 65267553 | 65267576 |
| 2.69420426748 | hltf (bg=24.28%) | HepG2 | + | chr11 | | 65267553 | 65267577 |
| 2.97537794852 | hltf (bg=24.28%) | HepG2 | + | chr11 | | 65267576 | 65267603 |
| 2.94396796629 | hltf (bg=24.28%) | HepG2 | + | chr11 | | 65267577 | 65267590 |
| 2.01999342638 | hltf (bg=24.28%) | K562 | + | chr11 | | 65267587 | 65267598 |
| 3.19477346748 | hltf (bg=24.28%) | HepG2 | + | chr11 | | 65267590 | 65267629 |
| 2.09412126124 | larp4 (bg=13.51%) | K562 | + | chr11 | | 65267563 | 65267576 |
| 2.18845269115 | larp4 (bg=13.51%) | K562 | + | chr11 | | 65267576 | 65267587 |
| 2.51226467138 | larp4 (bg=13.51%) | K562 | + | chr11 | | 65267587 | 65267603 |
| 2.23097278142 | MTPAP (bg=9.55%) | K562 | + | chr11 | | 65267550 | 65267575 |
| 2.43988482223 | MTPAP (bg=9.55%) | K562 | + | chr11 | | 65267575 | 65267589 |
| 2.37029134231 | MTPAP (bg=9.55%) | K562 | + | chr11 | | 65267589 | 65267598 |
| 2.34264029742 | npm1 (bg=10.22%) | K562 | + | chr11 | | 65267525 | 65267645 |
| 3.41981213022 | ppil4 (bg=43.39%) | K562 | + | chr11 | | 65267567 | 65267574 |
| 3.84520307775 | ppil4 (bg=43.39%) | K562 | + | chr11 | | 65267568 | 65267578 |
| 3.54884392684 | ppil4 (bg=43.39%) | K562 | + | chr11 | | 65267574 | 65267577 |
| 3.95381272048 | ppil4 (bg=43.39%) | K562 | + | chr11 | | 65267577 | 65267589 |
| 4.38143925534 | ppil4 (bg=43.39%) | K562 | + | chr11 | | 65267578 | 65267596 |
| 4.11704306935 | ppil4 (bg=43.39%) | K562 | + | chr11 | | 65267589 | 65267603 |
| 2.18031694968 | rbm15 (bg=11.59%) | K562 | + | chr11 | | 65267577 | 65267595 |
| 2.26681258031 | rbm22 (bg=12.69%) | HepG2 | + | chr11 | | 65267551 | 65267577 |
| 3.01022755801 | rbm22 (bg=12.69%) | HepG2 | + | chr11 | | 65267577 | 65267589 |
| 2.49670567809 | rbm22 (bg=12.69%) | HepG2 | + | chr11 | | 65267589 | 65267597 |
| 2.45429269158 | safb (bg=40.39%) | HepG2 | + | chr11 | | 65267555 | 65267574 |
| 3.56485185967 | safb (bg=40.39%) | HepG2 | + | chr11 | | 65267565 | 65267576 |
| 2.89684600843 | safb (bg=40.39%) | K562 | + | chr11 | | 65267569 | 65267574 |
| 3.28677032249 | safb (bg=40.39%) | K562 | + | chr11 | | 65267569 | 65267577 |
| 2.84398553974 | safb (bg=40.39%) | K562 | + | chr11 | | 65267574 | 65267577 |
| 2.7560173788 | safb (bg=40.39%) | HepG2 | + | chr11 | | 65267574 | 65267608 |
| 3.64753675814 | safb (bg=40.39%) | HepG2 | + | chr11 | | 65267576 | 65267588 |
| 3.38957583632 | safb (bg=40.39%) | K562 | + | chr11 | | 65267577 | 65267588 |
| 2.87341522093 | safb (bg=40.39%) | K562 | + | chr11 | | 65267577 | 65267589 |
| 3.39991261082 | safb (bg=40.39%) | K562 | + | chr11 | | 65267588 | 65267595 |
| 3.63342544785 | safb (bg=40.39%) | HepG2 | + | chr11 | | 65267588 | 65267607 |
| 2.7622405587 | safb (bg=40.39%) | K562 | + | chr11 | | 65267589 | 65267595 |
| 3.39307996452 | safb2 (bg=26.89%) | K562 | + | chr11 | | 65267569 | 65267579 |
| 2.74977754088 | safb2 (bg=26.89%) | K562 | + | chr11 | | 65267573 | 65267577 |
| 3.0431734121 | safb2 (bg=26.89%) | K562 | + | chr11 | | 65267577 | 65267586 |
| 3.42354563682 | safb2 (bg=26.89%) | K562 | + | chr11 | | 65267579 | 65267595 |
| 2.96329848577 | safb2 (bg=26.89%) | K562 | + | chr11 | | 65267586 | 65267598 |
| 2.31705570547 | SLTM (bg=7.5%) | HepG2 | + | chr11 | | 65267582 | 65267655 |
| 2.51647516039 | srsf1 (bg=30.28%) | K562 | + | chr11 | | 65267565 | 65267576 |
| 2.6378082581 | srsf1 (bg=30.28%) | K562 | + | chr11 | | 65267576 | 65267588 |
| 2.85214840714 | srsf1 (bg=30.28%) | K562 | + | chr11 | | 65267588 | 65267606 |
| 2.24334918312 | srsf7 (bg=22.53%) | HepG2 | + | chr11 | | 65267525 | 65267577 |
| 2.04623489792 | srsf7 (bg=22.53%) | K562 | + | chr11 | | 65267569 | 65267577 |
| 2.16548667636 | srsf7 (bg=22.53%) | K562 | + | chr11 | | 65267576 | 65267589 |
| 2.33978131434 | srsf7 (bg=22.53%) | K562 | + | chr11 | | 65267577 | 65267585 |
| 2.53330049418 | srsf7 (bg=22.53%) | K562 | + | chr11 | | 65267585 | 65267607 |
| 2.31347407901 | srsf7 (bg=22.53%) | K562 | + | chr11 | | 65267589 | 65267606 |
| 2.62818845739 | SRSF9 (bg=9.67%) | HepG2 | + | chr11 | | 65267590 | 65267620 |
| 2.64676885348 | SUPV3L1 (bg=9.63%) | K562 | + | chr11 | | 65267548 | 65267624 |
| 2.55424426019 | TAF15 (bg=9.06%) | HepG2 | + | chr11 | | 65267550 | 65267575 |
| 2.21387411843 | TAF15 (bg=9.06%) | HepG2 | + | chr11 | | 65267556 | 65267578 |
| 2.26818752069 | TAF15 (bg=9.06%) | HepG2 | + | chr11 | | 65267575 | 65267601 |
| 4.16281099872 | tra2a (bg=37.02%) | HepG2 | + | chr11 | | 65267508 | 65267576 |
| 4.56432438323 | tra2a (bg=37.02%) | K562 | + | chr11 | | 65267555 | 65267574 |
| 2.95421610241 | tra2a (bg=37.02%) | HepG2 | + | chr11 | | 65267556 | 65267576 |
| 4.56601475342 | tra2a (bg=37.02%) | K562 | + | chr11 | | 65267569 | 65267579 |
| 4.66682416027 | tra2a (bg=37.02%) | K562 | + | chr11 | | 65267574 | 65267579 |
| 3.79945079622 | tra2a (bg=37.02%) | HepG2 | + | chr11 | | 65267576 | 65267636 |
| 3.23813804326 | tra2a (bg=37.02%) | HepG2 | + | chr11 | | 65267576 | 65267648 |
| 4.97314365153 | tra2a (bg=37.02%) | K562 | + | chr11 | | 65267579 | 65267583 |
| 4.76597171423 | tra2a (bg=37.02%) | K562 | + | chr11 | | 65267579 | 65267587 |
| 5.03100035317 | tra2a (bg=37.02%) | K562 | + | chr11 | | 65267583 | 65267587 |
| 5.06804589431 | tra2a (bg=37.02%) | K562 | + | chr11 | | 65267587 | 65267595 |
| 5.13890539895 | tra2a (bg=37.02%) | K562 | + | chr11 | | 65267587 | 65267606 |
| 2.15656876111 | uchl5 (bg=18.56%) | K562 | + | chr11 | | 65267564 | 65267577 |
| 2.57886472672 | uchl5 (bg=18.56%) | K562 | + | chr11 | | 65267569 | 65267578 |
| 2.24534029701 | uchl5 (bg=18.56%) | K562 | + | chr11 | | 65267577 | 65267589 |
| 2.74730259503 | uchl5 (bg=18.56%) | K562 | + | chr11 | | 65267578 | 65267588 |
| 2.96831104045 | uchl5 (bg=18.56%) | K562 | + | chr11 | | 65267588 | 65267598 |
| 2.41064167964 | uchl5 (bg=18.56%) | K562 | + | chr11 | | 65267589 | 65267598 |
| 2.33390505957 | YWHAG (bg=9.14%) | K562 | + | chr11 | | 65267578 | 65267586 |
| 2.47231223323 | YWHAG (bg=9.14%) | K562 | + | chr11 | | 65267578 | 65267602 |
| 2.40308938685 | YWHAG (bg=9.14%) | K562 | + | chr11 | | 65267586 | 65267590 |
| 2.55460171955 | znf622 (bg=18.79%) | K562 | + | chr11 | | 65267568 | 65267578 |
| 3.29117047828 | znf622 (bg=18.79%) | K562 | + | chr11 | | 65267573 | 65267577 |
| 3.56855486137 | znf622 (bg=18.79%) | K562 | + | chr11 | | 65267577 | 65267596 |
| 2.65680717374 | znf622 (bg=18.79%) | K562 | + | chr11 | | 65267578 | 65267581 |
| 2.66388249703 | znf622 (bg=18.79%) | K562 | + | chr11 | | 65267581 | 65267585 |
| 2.78872932817 | znf622 (bg=18.79%) | K562 | + | chr11 | | 65267585 | 65267590 |
| 2.39957255062 | znf622 (bg=18.79%) | K562 | + | chr11 | | 65267590 | 65267597 |

  
  

| Match 86 in HUMAN | | | | | | | |
| --- | --- | --- | --- | --- | --- | --- | --- |
| Motif | Start in Seq (1 Indexed) | End in Seq (1 Indexed) | Strand | Chrm | Exon | Start in Chrm (0 Indexed) | End in Chrm (1 Indexed) |
| TTGAAGCTAGAAGGG | 1102 | 1116 | + | chr11 | 1 | 65267591 | 65267606 |
| eCLIP Fold-Enrichment | Binding Protein | Cell Line | Strand | Chrm | | Start in Chrm (0 Indexed) | End in Chrm (1 Indexed) |
| 2.02049744969 | aggf1 (bg=15.15%) | K562 | + | chr11 | | 65267587 | 65267601 |
| 2.36271889345 | aggf1 (bg=15.15%) | K562 | + | chr11 | | 65267589 | 65267603 |
| 2.82484073992 | aggf1 (bg=15.15%) | K562 | + | chr11 | | 65267603 | 65267609 |
| 3.12358516662 | bclaf1 (bg=17.67%) | HepG2 | + | chr11 | | 65267587 | 65267604 |
| 3.11268678464 | bclaf1 (bg=17.67%) | HepG2 | + | chr11 | | 65267590 | 65267598 |
| 3.47017247096 | bclaf1 (bg=17.67%) | HepG2 | + | chr11 | | 65267598 | 65267656 |
| 3.40112401216 | bclaf1 (bg=17.67%) | HepG2 | + | chr11 | | 65267604 | 65267655 |
| 2.30955231469 | bud13 (bg=12.85%) | K562 | + | chr11 | | 65267604 | 65267615 |
| 2.10165235238 | bud13 (bg=12.85%) | K562 | + | chr11 | | 65267604 | 65267617 |
| 2.48113862281 | cpsf6 (bg=13.45%) | K562 | + | chr11 | | 65267577 | 65267607 |
| 2.82100195184 | cpsf6 (bg=13.45%) | K562 | + | chr11 | | 65267587 | 65267608 |
| 2.08815924402 | EIF3H (bg=0.83%) | HepG2 | + | chr11 | | 65267576 | 65267638 |
| 2.49988929144 | fxr2 (bg=10.1%) | HepG2 | + | chr11 | | 65267536 | 65267597 |
| 2.35149745155 | fxr2 (bg=10.1%) | HepG2 | + | chr11 | | 65267597 | 65267659 |
| 2.4841507675 | gtf2f1 (bg=10.18%) | HepG2 | + | chr11 | | 65267590 | 65267644 |
| 2.97537794852 | hltf (bg=24.28%) | HepG2 | + | chr11 | | 65267576 | 65267603 |
| 2.01999342638 | hltf (bg=24.28%) | K562 | + | chr11 | | 65267587 | 65267598 |
| 3.19477346748 | hltf (bg=24.28%) | HepG2 | + | chr11 | | 65267590 | 65267629 |
| 2.97688423684 | hltf (bg=24.28%) | HepG2 | + | chr11 | | 65267603 | 65267608 |
| 2.27951050572 | hltf (bg=24.28%) | K562 | + | chr11 | | 65267605 | 65267609 |
| 2.51226467138 | larp4 (bg=13.51%) | K562 | + | chr11 | | 65267587 | 65267603 |
| 2.7252312195 | larp4 (bg=13.51%) | K562 | + | chr11 | | 65267603 | 65267615 |
| 2.37029134231 | MTPAP (bg=9.55%) | K562 | + | chr11 | | 65267589 | 65267598 |
| 2.39983561792 | MTPAP (bg=9.55%) | K562 | + | chr11 | | 65267598 | 65267602 |
| 2.69278508905 | MTPAP (bg=9.55%) | K562 | + | chr11 | | 65267602 | 65267621 |
| 2.15597137445 | MTPAP (bg=9.55%) | K562 | + | chr11 | | 65267604 | 65267622 |
| 2.34264029742 | npm1 (bg=10.22%) | K562 | + | chr11 | | 65267525 | 65267645 |
| 4.38143925534 | ppil4 (bg=43.39%) | K562 | + | chr11 | | 65267578 | 65267596 |
| 4.11704306935 | ppil4 (bg=43.39%) | K562 | + | chr11 | | 65267589 | 65267603 |
| 4.47549762161 | ppil4 (bg=43.39%) | K562 | + | chr11 | | 65267596 | 65267598 |
| 4.52701645608 | ppil4 (bg=43.39%) | K562 | + | chr11 | | 65267598 | 65267602 |
| 4.92843680564 | ppil4 (bg=43.39%) | K562 | + | chr11 | | 65267602 | 65267624 |
| 4.67817442895 | ppil4 (bg=43.39%) | K562 | + | chr11 | | 65267603 | 65267637 |
| 2.18031694968 | rbm15 (bg=11.59%) | K562 | + | chr11 | | 65267577 | 65267595 |
| 2.13744128839 | rbm15 (bg=11.59%) | K562 | + | chr11 | | 65267595 | 65267603 |
| 2.19927042638 | rbm15 (bg=11.59%) | K562 | + | chr11 | | 65267603 | 65267609 |
| 2.49670567809 | rbm22 (bg=12.69%) | HepG2 | + | chr11 | | 65267589 | 65267597 |
| 2.82905196501 | rbm22 (bg=12.69%) | HepG2 | + | chr11 | | 65267597 | 65267651 |
| 2.7560173788 | safb (bg=40.39%) | HepG2 | + | chr11 | | 65267574 | 65267608 |
| 3.39991261082 | safb (bg=40.39%) | K562 | + | chr11 | | 65267588 | 65267595 |
| 3.63342544785 | safb (bg=40.39%) | HepG2 | + | chr11 | | 65267588 | 65267607 |
| 2.7622405587 | safb (bg=40.39%) | K562 | + | chr11 | | 65267589 | 65267595 |
| 3.33165218486 | safb (bg=40.39%) | K562 | + | chr11 | | 65267595 | 65267598 |
| 2.79699585094 | safb (bg=40.39%) | K562 | + | chr11 | | 65267595 | 65267598 |
| 3.1653513968 | safb (bg=40.39%) | K562 | + | chr11 | | 65267598 | 65267605 |
| 2.61874682425 | safb (bg=40.39%) | K562 | + | chr11 | | 65267598 | 65267605 |
| 3.22310389901 | safb (bg=40.39%) | K562 | + | chr11 | | 65267605 | 65267608 |
| 2.75185238521 | safb (bg=40.39%) | K562 | + | chr11 | | 65267605 | 65267626 |
| 3.42354563682 | safb2 (bg=26.89%) | K562 | + | chr11 | | 65267579 | 65267595 |
| 2.96329848577 | safb2 (bg=26.89%) | K562 | + | chr11 | | 65267586 | 65267598 |
| 3.59826107011 | safb2 (bg=26.89%) | K562 | + | chr11 | | 65267595 | 65267598 |
| 3.54795765739 | safb2 (bg=26.89%) | K562 | + | chr11 | | 65267598 | 65267605 |
| 2.93347833723 | safb2 (bg=26.89%) | K562 | + | chr11 | | 65267598 | 65267605 |
| 3.04630897349 | safb2 (bg=26.89%) | K562 | + | chr11 | | 65267605 | 65267609 |
| 3.67603851756 | safb2 (bg=26.89%) | K562 | + | chr11 | | 65267605 | 65267615 |
| 2.31705570547 | SLTM (bg=7.5%) | HepG2 | + | chr11 | | 65267582 | 65267655 |
| 2.85214840714 | srsf1 (bg=30.28%) | K562 | + | chr11 | | 65267588 | 65267606 |
| 2.2253345695 | srsf1 (bg=30.28%) | HepG2 | + | chr11 | | 65267593 | 65267611 |
| 3.0458761831 | srsf1 (bg=30.28%) | K562 | + | chr11 | | 65267606 | 65267640 |
| 2.53330049418 | srsf7 (bg=22.53%) | K562 | + | chr11 | | 65267585 | 65267607 |
| 2.31347407901 | srsf7 (bg=22.53%) | K562 | + | chr11 | | 65267589 | 65267606 |
| 2.48742176052 | srsf7 (bg=22.53%) | K562 | + | chr11 | | 65267606 | 65267647 |
| 2.62818845739 | SRSF9 (bg=9.67%) | HepG2 | + | chr11 | | 65267590 | 65267620 |
| 2.64676885348 | SUPV3L1 (bg=9.63%) | K562 | + | chr11 | | 65267548 | 65267624 |
| 2.26818752069 | TAF15 (bg=9.06%) | HepG2 | + | chr11 | | 65267575 | 65267601 |
| 2.34250721804 | TAF15 (bg=9.06%) | HepG2 | + | chr11 | | 65267601 | 65267633 |
| 3.79945079622 | tra2a (bg=37.02%) | HepG2 | + | chr11 | | 65267576 | 65267636 |
| 3.23813804326 | tra2a (bg=37.02%) | HepG2 | + | chr11 | | 65267576 | 65267648 |
| 5.06804589431 | tra2a (bg=37.02%) | K562 | + | chr11 | | 65267587 | 65267595 |
| 5.13890539895 | tra2a (bg=37.02%) | K562 | + | chr11 | | 65267587 | 65267606 |
| 5.12395042257 | tra2a (bg=37.02%) | K562 | + | chr11 | | 65267595 | 65267608 |
| 5.09088657143 | tra2a (bg=37.02%) | K562 | + | chr11 | | 65267606 | 65267636 |
| 2.96831104045 | uchl5 (bg=18.56%) | K562 | + | chr11 | | 65267588 | 65267598 |
| 2.41064167964 | uchl5 (bg=18.56%) | K562 | + | chr11 | | 65267589 | 65267598 |
| 2.96485280806 | uchl5 (bg=18.56%) | K562 | + | chr11 | | 65267598 | 65267602 |
| 2.47100185636 | uchl5 (bg=18.56%) | K562 | + | chr11 | | 65267598 | 65267602 |
| 3.11625989451 | uchl5 (bg=18.56%) | K562 | + | chr11 | | 65267602 | 65267605 |
| 2.56136906453 | uchl5 (bg=18.56%) | K562 | + | chr11 | | 65267602 | 65267605 |
| 2.73038263891 | uchl5 (bg=18.56%) | K562 | + | chr11 | | 65267605 | 65267609 |
| 3.22500206018 | uchl5 (bg=18.56%) | K562 | + | chr11 | | 65267605 | 65267618 |
| 2.17566847702 | XRCC6 (bg=3.81%) | HepG2 | + | chr11 | | 65267602 | 65267653 |
| 2.47231223323 | YWHAG (bg=9.14%) | K562 | + | chr11 | | 65267578 | 65267602 |
| 2.41088775741 | YWHAG (bg=9.14%) | K562 | + | chr11 | | 65267598 | 65267653 |
| 2.91481349648 | YWHAG (bg=9.14%) | K562 | + | chr11 | | 65267602 | 65267616 |
| 3.56855486137 | znf622 (bg=18.79%) | K562 | + | chr11 | | 65267577 | 65267596 |
| 2.39957255062 | znf622 (bg=18.79%) | K562 | + | chr11 | | 65267590 | 65267597 |
| 3.72461742704 | znf622 (bg=18.79%) | K562 | + | chr11 | | 65267596 | 65267602 |
| 2.29799079869 | znf622 (bg=18.79%) | K562 | + | chr11 | | 65267597 | 65267600 |
| 2.55164856794 | znf622 (bg=18.79%) | K562 | + | chr11 | | 65267600 | 65267613 |
| 3.71634464773 | znf622 (bg=18.79%) | K562 | + | chr11 | | 65267602 | 65267608 |

  
  

| Match 87 in HUMAN | | | | | | | |
| --- | --- | --- | --- | --- | --- | --- | --- |
| Motif | Start in Seq (1 Indexed) | End in Seq (1 Indexed) | Strand | Chrm | Exon | Start in Chrm (0 Indexed) | End in Chrm (1 Indexed) |
| AGCTAGAAGGG | 1106 | 1116 | + | chr11 | 1 | 65267595 | 65267606 |
| eCLIP Fold-Enrichment | Binding Protein | Cell Line | Strand | Chrm | | Start in Chrm (0 Indexed) | End in Chrm (1 Indexed) |
| 2.02049744969 | aggf1 (bg=15.15%) | K562 | + | chr11 | | 65267587 | 65267601 |
| 2.36271889345 | aggf1 (bg=15.15%) | K562 | + | chr11 | | 65267589 | 65267603 |
| 2.82484073992 | aggf1 (bg=15.15%) | K562 | + | chr11 | | 65267603 | 65267609 |
| 3.12358516662 | bclaf1 (bg=17.67%) | HepG2 | + | chr11 | | 65267587 | 65267604 |
| 3.11268678464 | bclaf1 (bg=17.67%) | HepG2 | + | chr11 | | 65267590 | 65267598 |
| 3.47017247096 | bclaf1 (bg=17.67%) | HepG2 | + | chr11 | | 65267598 | 65267656 |
| 3.40112401216 | bclaf1 (bg=17.67%) | HepG2 | + | chr11 | | 65267604 | 65267655 |
| 2.30955231469 | bud13 (bg=12.85%) | K562 | + | chr11 | | 65267604 | 65267615 |
| 2.10165235238 | bud13 (bg=12.85%) | K562 | + | chr11 | | 65267604 | 65267617 |
| 2.48113862281 | cpsf6 (bg=13.45%) | K562 | + | chr11 | | 65267577 | 65267607 |
| 2.82100195184 | cpsf6 (bg=13.45%) | K562 | + | chr11 | | 65267587 | 65267608 |
| 2.08815924402 | EIF3H (bg=0.83%) | HepG2 | + | chr11 | | 65267576 | 65267638 |
| 2.49988929144 | fxr2 (bg=10.1%) | HepG2 | + | chr11 | | 65267536 | 65267597 |
| 2.35149745155 | fxr2 (bg=10.1%) | HepG2 | + | chr11 | | 65267597 | 65267659 |
| 2.4841507675 | gtf2f1 (bg=10.18%) | HepG2 | + | chr11 | | 65267590 | 65267644 |
| 2.97537794852 | hltf (bg=24.28%) | HepG2 | + | chr11 | | 65267576 | 65267603 |
| 2.01999342638 | hltf (bg=24.28%) | K562 | + | chr11 | | 65267587 | 65267598 |
| 3.19477346748 | hltf (bg=24.28%) | HepG2 | + | chr11 | | 65267590 | 65267629 |
| 2.97688423684 | hltf (bg=24.28%) | HepG2 | + | chr11 | | 65267603 | 65267608 |
| 2.27951050572 | hltf (bg=24.28%) | K562 | + | chr11 | | 65267605 | 65267609 |
| 2.51226467138 | larp4 (bg=13.51%) | K562 | + | chr11 | | 65267587 | 65267603 |
| 2.7252312195 | larp4 (bg=13.51%) | K562 | + | chr11 | | 65267603 | 65267615 |
| 2.37029134231 | MTPAP (bg=9.55%) | K562 | + | chr11 | | 65267589 | 65267598 |
| 2.39983561792 | MTPAP (bg=9.55%) | K562 | + | chr11 | | 65267598 | 65267602 |
| 2.69278508905 | MTPAP (bg=9.55%) | K562 | + | chr11 | | 65267602 | 65267621 |
| 2.15597137445 | MTPAP (bg=9.55%) | K562 | + | chr11 | | 65267604 | 65267622 |
| 2.34264029742 | npm1 (bg=10.22%) | K562 | + | chr11 | | 65267525 | 65267645 |
| 4.38143925534 | ppil4 (bg=43.39%) | K562 | + | chr11 | | 65267578 | 65267596 |
| 4.11704306935 | ppil4 (bg=43.39%) | K562 | + | chr11 | | 65267589 | 65267603 |
| 4.47549762161 | ppil4 (bg=43.39%) | K562 | + | chr11 | | 65267596 | 65267598 |
| 4.52701645608 | ppil4 (bg=43.39%) | K562 | + | chr11 | | 65267598 | 65267602 |
| 4.92843680564 | ppil4 (bg=43.39%) | K562 | + | chr11 | | 65267602 | 65267624 |
| 4.67817442895 | ppil4 (bg=43.39%) | K562 | + | chr11 | | 65267603 | 65267637 |
| 2.18031694968 | rbm15 (bg=11.59%) | K562 | + | chr11 | | 65267577 | 65267595 |
| 2.13744128839 | rbm15 (bg=11.59%) | K562 | + | chr11 | | 65267595 | 65267603 |
| 2.19927042638 | rbm15 (bg=11.59%) | K562 | + | chr11 | | 65267603 | 65267609 |
| 2.49670567809 | rbm22 (bg=12.69%) | HepG2 | + | chr11 | | 65267589 | 65267597 |
| 2.82905196501 | rbm22 (bg=12.69%) | HepG2 | + | chr11 | | 65267597 | 65267651 |
| 2.7560173788 | safb (bg=40.39%) | HepG2 | + | chr11 | | 65267574 | 65267608 |
| 3.39991261082 | safb (bg=40.39%) | K562 | + | chr11 | | 65267588 | 65267595 |
| 3.63342544785 | safb (bg=40.39%) | HepG2 | + | chr11 | | 65267588 | 65267607 |
| 2.7622405587 | safb (bg=40.39%) | K562 | + | chr11 | | 65267589 | 65267595 |
| 3.33165218486 | safb (bg=40.39%) | K562 | + | chr11 | | 65267595 | 65267598 |
| 2.79699585094 | safb (bg=40.39%) | K562 | + | chr11 | | 65267595 | 65267598 |
| 3.1653513968 | safb (bg=40.39%) | K562 | + | chr11 | | 65267598 | 65267605 |
| 2.61874682425 | safb (bg=40.39%) | K562 | + | chr11 | | 65267598 | 65267605 |
| 3.22310389901 | safb (bg=40.39%) | K562 | + | chr11 | | 65267605 | 65267608 |
| 2.75185238521 | safb (bg=40.39%) | K562 | + | chr11 | | 65267605 | 65267626 |
| 3.42354563682 | safb2 (bg=26.89%) | K562 | + | chr11 | | 65267579 | 65267595 |
| 2.96329848577 | safb2 (bg=26.89%) | K562 | + | chr11 | | 65267586 | 65267598 |
| 3.59826107011 | safb2 (bg=26.89%) | K562 | + | chr11 | | 65267595 | 65267598 |
| 3.54795765739 | safb2 (bg=26.89%) | K562 | + | chr11 | | 65267598 | 65267605 |
| 2.93347833723 | safb2 (bg=26.89%) | K562 | + | chr11 | | 65267598 | 65267605 |
| 3.04630897349 | safb2 (bg=26.89%) | K562 | + | chr11 | | 65267605 | 65267609 |
| 3.67603851756 | safb2 (bg=26.89%) | K562 | + | chr11 | | 65267605 | 65267615 |
| 2.31705570547 | SLTM (bg=7.5%) | HepG2 | + | chr11 | | 65267582 | 65267655 |
| 2.85214840714 | srsf1 (bg=30.28%) | K562 | + | chr11 | | 65267588 | 65267606 |
| 2.2253345695 | srsf1 (bg=30.28%) | HepG2 | + | chr11 | | 65267593 | 65267611 |
| 3.0458761831 | srsf1 (bg=30.28%) | K562 | + | chr11 | | 65267606 | 65267640 |
| 2.53330049418 | srsf7 (bg=22.53%) | K562 | + | chr11 | | 65267585 | 65267607 |
| 2.31347407901 | srsf7 (bg=22.53%) | K562 | + | chr11 | | 65267589 | 65267606 |
| 2.48742176052 | srsf7 (bg=22.53%) | K562 | + | chr11 | | 65267606 | 65267647 |
| 2.62818845739 | SRSF9 (bg=9.67%) | HepG2 | + | chr11 | | 65267590 | 65267620 |
| 2.64676885348 | SUPV3L1 (bg=9.63%) | K562 | + | chr11 | | 65267548 | 65267624 |
| 2.26818752069 | TAF15 (bg=9.06%) | HepG2 | + | chr11 | | 65267575 | 65267601 |
| 2.34250721804 | TAF15 (bg=9.06%) | HepG2 | + | chr11 | | 65267601 | 65267633 |
| 3.79945079622 | tra2a (bg=37.02%) | HepG2 | + | chr11 | | 65267576 | 65267636 |
| 3.23813804326 | tra2a (bg=37.02%) | HepG2 | + | chr11 | | 65267576 | 65267648 |
| 5.06804589431 | tra2a (bg=37.02%) | K562 | + | chr11 | | 65267587 | 65267595 |
| 5.13890539895 | tra2a (bg=37.02%) | K562 | + | chr11 | | 65267587 | 65267606 |
| 5.12395042257 | tra2a (bg=37.02%) | K562 | + | chr11 | | 65267595 | 65267608 |
| 5.09088657143 | tra2a (bg=37.02%) | K562 | + | chr11 | | 65267606 | 65267636 |
| 2.96831104045 | uchl5 (bg=18.56%) | K562 | + | chr11 | | 65267588 | 65267598 |
| 2.41064167964 | uchl5 (bg=18.56%) | K562 | + | chr11 | | 65267589 | 65267598 |
| 2.96485280806 | uchl5 (bg=18.56%) | K562 | + | chr11 | | 65267598 | 65267602 |
| 2.47100185636 | uchl5 (bg=18.56%) | K562 | + | chr11 | | 65267598 | 65267602 |
| 3.11625989451 | uchl5 (bg=18.56%) | K562 | + | chr11 | | 65267602 | 65267605 |
| 2.56136906453 | uchl5 (bg=18.56%) | K562 | + | chr11 | | 65267602 | 65267605 |
| 2.73038263891 | uchl5 (bg=18.56%) | K562 | + | chr11 | | 65267605 | 65267609 |
| 3.22500206018 | uchl5 (bg=18.56%) | K562 | + | chr11 | | 65267605 | 65267618 |
| 2.17566847702 | XRCC6 (bg=3.81%) | HepG2 | + | chr11 | | 65267602 | 65267653 |
| 2.47231223323 | YWHAG (bg=9.14%) | K562 | + | chr11 | | 65267578 | 65267602 |
| 2.41088775741 | YWHAG (bg=9.14%) | K562 | + | chr11 | | 65267598 | 65267653 |
| 2.91481349648 | YWHAG (bg=9.14%) | K562 | + | chr11 | | 65267602 | 65267616 |
| 3.56855486137 | znf622 (bg=18.79%) | K562 | + | chr11 | | 65267577 | 65267596 |
| 2.39957255062 | znf622 (bg=18.79%) | K562 | + | chr11 | | 65267590 | 65267597 |
| 3.72461742704 | znf622 (bg=18.79%) | K562 | + | chr11 | | 65267596 | 65267602 |
| 2.29799079869 | znf622 (bg=18.79%) | K562 | + | chr11 | | 65267597 | 65267600 |
| 2.55164856794 | znf622 (bg=18.79%) | K562 | + | chr11 | | 65267600 | 65267613 |
| 3.71634464773 | znf622 (bg=18.79%) | K562 | + | chr11 | | 65267602 | 65267608 |

  
  

| Match 88 in HUMAN | | | | | | | |
| --- | --- | --- | --- | --- | --- | --- | --- |
| Motif | Start in Seq (1 Indexed) | End in Seq (1 Indexed) | Strand | Chrm | Exon | Start in Chrm (0 Indexed) | End in Chrm (1 Indexed) |
| GGTTAA | 1123 | 1128 | + | chr11 | 1 | 65267612 | 65267618 |
| eCLIP Fold-Enrichment | Binding Protein | Cell Line | Strand | Chrm | | Start in Chrm (0 Indexed) | End in Chrm (1 Indexed) |
| 2.93808581543 | aggf1 (bg=15.15%) | K562 | + | chr11 | | 65267609 | 65267615 |
| 2.81583689718 | aggf1 (bg=15.15%) | K562 | + | chr11 | | 65267615 | 65267618 |
| 2.74333958887 | aggf1 (bg=15.15%) | K562 | + | chr11 | | 65267618 | 65267620 |
| 3.47017247096 | bclaf1 (bg=17.67%) | HepG2 | + | chr11 | | 65267598 | 65267656 |
| 3.40112401216 | bclaf1 (bg=17.67%) | HepG2 | + | chr11 | | 65267604 | 65267655 |
| 2.30955231469 | bud13 (bg=12.85%) | K562 | + | chr11 | | 65267604 | 65267615 |
| 2.10165235238 | bud13 (bg=12.85%) | K562 | + | chr11 | | 65267604 | 65267617 |
| 2.4293277022 | bud13 (bg=12.85%) | K562 | + | chr11 | | 65267615 | 65267620 |
| 2.14297651084 | bud13 (bg=12.85%) | K562 | + | chr11 | | 65267617 | 65267632 |
| 2.59404009629 | cpsf6 (bg=13.45%) | K562 | + | chr11 | | 65267607 | 65267640 |
| 2.79933777456 | cpsf6 (bg=13.45%) | K562 | + | chr11 | | 65267608 | 65267644 |
| 2.08815924402 | EIF3H (bg=0.83%) | HepG2 | + | chr11 | | 65267576 | 65267638 |
| 2.35149745155 | fxr2 (bg=10.1%) | HepG2 | + | chr11 | | 65267597 | 65267659 |
| 2.4841507675 | gtf2f1 (bg=10.18%) | HepG2 | + | chr11 | | 65267590 | 65267644 |
| 3.19477346748 | hltf (bg=24.28%) | HepG2 | + | chr11 | | 65267590 | 65267629 |
| 3.1667446722 | hltf (bg=24.28%) | HepG2 | + | chr11 | | 65267608 | 65267613 |
| 2.41438087104 | hltf (bg=24.28%) | K562 | + | chr11 | | 65267609 | 65267615 |
| 3.25020556749 | hltf (bg=24.28%) | HepG2 | + | chr11 | | 65267613 | 65267619 |
| 2.56014398953 | hltf (bg=24.28%) | K562 | + | chr11 | | 65267615 | 65267620 |
| 2.7252312195 | larp4 (bg=13.51%) | K562 | + | chr11 | | 65267603 | 65267615 |
| 2.87421410329 | larp4 (bg=13.51%) | K562 | + | chr11 | | 65267615 | 65267658 |
| 2.69278508905 | MTPAP (bg=9.55%) | K562 | + | chr11 | | 65267602 | 65267621 |
| 2.15597137445 | MTPAP (bg=9.55%) | K562 | + | chr11 | | 65267604 | 65267622 |
| 2.00111287804 | NIPBL (bg=8.2%) | K562 | + | chr11 | | 65267608 | 65267619 |
| 2.34264029742 | npm1 (bg=10.22%) | K562 | + | chr11 | | 65267525 | 65267645 |
| 4.92843680564 | ppil4 (bg=43.39%) | K562 | + | chr11 | | 65267602 | 65267624 |
| 4.67817442895 | ppil4 (bg=43.39%) | K562 | + | chr11 | | 65267603 | 65267637 |
| 2.0067546483 | rbm15 (bg=11.59%) | K562 | + | chr11 | | 65267608 | 65267629 |
| 2.21717145988 | rbm15 (bg=11.59%) | K562 | + | chr11 | | 65267609 | 65267616 |
| 2.24080270087 | rbm15 (bg=11.59%) | K562 | + | chr11 | | 65267616 | 65267619 |
| 2.82905196501 | rbm22 (bg=12.69%) | HepG2 | + | chr11 | | 65267597 | 65267651 |
| 2.75185238521 | safb (bg=40.39%) | K562 | + | chr11 | | 65267605 | 65267626 |
| 3.51884190323 | safb (bg=40.39%) | HepG2 | + | chr11 | | 65267607 | 65267645 |
| 3.32480699699 | safb (bg=40.39%) | K562 | + | chr11 | | 65267608 | 65267620 |
| 2.78694923835 | safb (bg=40.39%) | HepG2 | + | chr11 | | 65267608 | 65267631 |
| 3.67603851756 | safb2 (bg=26.89%) | K562 | + | chr11 | | 65267605 | 65267615 |
| 3.07328697203 | safb2 (bg=26.89%) | K562 | + | chr11 | | 65267609 | 65267617 |
| 3.79633334413 | safb2 (bg=26.89%) | K562 | + | chr11 | | 65267615 | 65267625 |
| 3.12352569327 | safb2 (bg=26.89%) | K562 | + | chr11 | | 65267617 | 65267632 |
| 2.31705570547 | SLTM (bg=7.5%) | HepG2 | + | chr11 | | 65267582 | 65267655 |
| 2.15259022169 | SLTM (bg=7.5%) | K562 | + | chr11 | | 65267609 | 65267627 |
| 3.0458761831 | srsf1 (bg=30.28%) | K562 | + | chr11 | | 65267606 | 65267640 |
| 2.57749486581 | srsf1 (bg=30.28%) | HepG2 | + | chr11 | | 65267611 | 65267621 |
| 2.48742176052 | srsf7 (bg=22.53%) | K562 | + | chr11 | | 65267606 | 65267647 |
| 2.61924536128 | srsf7 (bg=22.53%) | K562 | + | chr11 | | 65267607 | 65267643 |
| 2.62818845739 | SRSF9 (bg=9.67%) | HepG2 | + | chr11 | | 65267590 | 65267620 |
| 2.64676885348 | SUPV3L1 (bg=9.63%) | K562 | + | chr11 | | 65267548 | 65267624 |
| 3.11626460393 | SUPV3L1 (bg=9.63%) | K562 | + | chr11 | | 65267611 | 65267635 |
| 2.34250721804 | TAF15 (bg=9.06%) | HepG2 | + | chr11 | | 65267601 | 65267633 |
| 3.79945079622 | tra2a (bg=37.02%) | HepG2 | + | chr11 | | 65267576 | 65267636 |
| 3.23813804326 | tra2a (bg=37.02%) | HepG2 | + | chr11 | | 65267576 | 65267648 |
| 5.09088657143 | tra2a (bg=37.02%) | K562 | + | chr11 | | 65267606 | 65267636 |
| 5.063182712 | tra2a (bg=37.02%) | K562 | + | chr11 | | 65267608 | 65267637 |
| 3.22500206018 | uchl5 (bg=18.56%) | K562 | + | chr11 | | 65267605 | 65267618 |
| 2.69630844322 | uchl5 (bg=18.56%) | K562 | + | chr11 | | 65267609 | 65267617 |
| 2.72167293399 | uchl5 (bg=18.56%) | K562 | + | chr11 | | 65267617 | 65267643 |
| 3.20367199731 | uchl5 (bg=18.56%) | K562 | + | chr11 | | 65267618 | 65267656 |
| 2.17566847702 | XRCC6 (bg=3.81%) | HepG2 | + | chr11 | | 65267602 | 65267653 |
| 2.41088775741 | YWHAG (bg=9.14%) | K562 | + | chr11 | | 65267598 | 65267653 |
| 2.91481349648 | YWHAG (bg=9.14%) | K562 | + | chr11 | | 65267602 | 65267616 |
| 3.03265309331 | YWHAG (bg=9.14%) | K562 | + | chr11 | | 65267616 | 65267653 |
| 2.55164856794 | znf622 (bg=18.79%) | K562 | + | chr11 | | 65267600 | 65267613 |
| 3.8669520469 | znf622 (bg=18.79%) | K562 | + | chr11 | | 65267608 | 65267615 |
| 2.95933661148 | znf622 (bg=18.79%) | K562 | + | chr11 | | 65267613 | 65267628 |
| 3.86095839183 | znf622 (bg=18.79%) | K562 | + | chr11 | | 65267615 | 65267619 |

  
  

| Match 89 in HUMAN | | | | | | | |
| --- | --- | --- | --- | --- | --- | --- | --- |
| Motif | Start in Seq (1 Indexed) | End in Seq (1 Indexed) | Strand | Chrm | Exon | Start in Chrm (0 Indexed) | End in Chrm (1 Indexed) |
| CATCAAAAAGCT | 1135 | 1146 | + | chr11 | 1 | 65267624 | 65267636 |
| eCLIP Fold-Enrichment | Binding Protein | Cell Line | Strand | Chrm | | Start in Chrm (0 Indexed) | End in Chrm (1 Indexed) |
| 3.00525764843 | aggf1 (bg=15.15%) | K562 | + | chr11 | | 65267620 | 65267629 |
| 2.58394809533 | aggf1 (bg=15.15%) | K562 | + | chr11 | | 65267629 | 65267635 |
| 2.73223402194 | aggf1 (bg=15.15%) | K562 | + | chr11 | | 65267635 | 65267647 |
| 3.47017247096 | bclaf1 (bg=17.67%) | HepG2 | + | chr11 | | 65267598 | 65267656 |
| 3.40112401216 | bclaf1 (bg=17.67%) | HepG2 | + | chr11 | | 65267604 | 65267655 |
| 2.14297651084 | bud13 (bg=12.85%) | K562 | + | chr11 | | 65267617 | 65267632 |
| 2.40024347525 | bud13 (bg=12.85%) | K562 | + | chr11 | | 65267620 | 65267626 |
| 2.47890643645 | bud13 (bg=12.85%) | K562 | + | chr11 | | 65267626 | 65267643 |
| 2.20926946342 | bud13 (bg=12.85%) | K562 | + | chr11 | | 65267632 | 65267657 |
| 2.59404009629 | cpsf6 (bg=13.45%) | K562 | + | chr11 | | 65267607 | 65267640 |
| 2.79933777456 | cpsf6 (bg=13.45%) | K562 | + | chr11 | | 65267608 | 65267644 |
| 2.08815924402 | EIF3H (bg=0.83%) | HepG2 | + | chr11 | | 65267576 | 65267638 |
| 2.35149745155 | fxr2 (bg=10.1%) | HepG2 | + | chr11 | | 65267597 | 65267659 |
| 2.4841507675 | gtf2f1 (bg=10.18%) | HepG2 | + | chr11 | | 65267590 | 65267644 |
| 3.19477346748 | hltf (bg=24.28%) | HepG2 | + | chr11 | | 65267590 | 65267629 |
| 3.13444885293 | hltf (bg=24.28%) | HepG2 | + | chr11 | | 65267619 | 65267625 |
| 2.58829248238 | hltf (bg=24.28%) | K562 | + | chr11 | | 65267620 | 65267625 |
| 2.15029770655 | hltf (bg=24.28%) | K562 | + | chr11 | | 65267625 | 65267654 |
| 2.76615639752 | hltf (bg=24.28%) | K562 | + | chr11 | | 65267625 | 65267655 |
| 3.23281833788 | hltf (bg=24.28%) | HepG2 | + | chr11 | | 65267625 | 65267657 |
| 3.46501688949 | hltf (bg=24.28%) | HepG2 | + | chr11 | | 65267629 | 65267636 |
| 3.55258790472 | hltf (bg=24.28%) | HepG2 | + | chr11 | | 65267636 | 65267657 |
| 2.0955986381 | khdrbs1 (bg=10.41%) | K562 | + | chr11 | | 65267623 | 65267634 |
| 2.21422405145 | khdrbs1 (bg=10.41%) | K562 | + | chr11 | | 65267634 | 65267646 |
| 2.87421410329 | larp4 (bg=13.51%) | K562 | + | chr11 | | 65267615 | 65267658 |
| 2.73809649475 | MTPAP (bg=9.55%) | K562 | + | chr11 | | 65267621 | 65267644 |
| 2.20007765763 | MTPAP (bg=9.55%) | K562 | + | chr11 | | 65267622 | 65267634 |
| 2.10171106286 | MTPAP (bg=9.55%) | K562 | + | chr11 | | 65267634 | 65267652 |
| 2.28180184717 | NIPBL (bg=8.2%) | K562 | + | chr11 | | 65267621 | 65267648 |
| 2.0285333905 | NIPBL (bg=8.2%) | K562 | + | chr11 | | 65267625 | 65267633 |
| 2.34264029742 | npm1 (bg=10.22%) | K562 | + | chr11 | | 65267525 | 65267645 |
| 4.92843680564 | ppil4 (bg=43.39%) | K562 | + | chr11 | | 65267602 | 65267624 |
| 4.67817442895 | ppil4 (bg=43.39%) | K562 | + | chr11 | | 65267603 | 65267637 |
| 5.00574691839 | ppil4 (bg=43.39%) | K562 | + | chr11 | | 65267624 | 65267632 |
| 4.76547970104 | ppil4 (bg=43.39%) | K562 | + | chr11 | | 65267632 | 65267646 |
| 2.0067546483 | rbm15 (bg=11.59%) | K562 | + | chr11 | | 65267608 | 65267629 |
| 2.08340566276 | rbm15 (bg=11.59%) | K562 | + | chr11 | | 65267619 | 65267625 |
| 2.40145309885 | rbm15 (bg=11.59%) | K562 | + | chr11 | | 65267625 | 65267632 |
| 2.30386922296 | rbm15 (bg=11.59%) | K562 | + | chr11 | | 65267632 | 65267636 |
| 2.25765093782 | rbm15 (bg=11.59%) | K562 | + | chr11 | | 65267636 | 65267647 |
| 2.82905196501 | rbm22 (bg=12.69%) | HepG2 | + | chr11 | | 65267597 | 65267651 |
| 2.75185238521 | safb (bg=40.39%) | K562 | + | chr11 | | 65267605 | 65267626 |
| 3.51884190323 | safb (bg=40.39%) | HepG2 | + | chr11 | | 65267607 | 65267645 |
| 2.78694923835 | safb (bg=40.39%) | HepG2 | + | chr11 | | 65267608 | 65267631 |
| 3.31344048871 | safb (bg=40.39%) | K562 | + | chr11 | | 65267623 | 65267626 |
| 3.39044630415 | safb (bg=40.39%) | K562 | + | chr11 | | 65267626 | 65267635 |
| 2.74050397926 | safb (bg=40.39%) | K562 | + | chr11 | | 65267626 | 65267636 |
| 3.39694256543 | safb (bg=40.39%) | K562 | + | chr11 | | 65267635 | 65267642 |
| 2.7219389762 | safb (bg=40.39%) | K562 | + | chr11 | | 65267636 | 65267644 |
| 3.79633334413 | safb2 (bg=26.89%) | K562 | + | chr11 | | 65267615 | 65267625 |
| 3.12352569327 | safb2 (bg=26.89%) | K562 | + | chr11 | | 65267617 | 65267632 |
| 3.67458290031 | safb2 (bg=26.89%) | K562 | + | chr11 | | 65267625 | 65267633 |
| 3.27199918242 | safb2 (bg=26.89%) | K562 | + | chr11 | | 65267632 | 65267636 |
| 3.70155773856 | safb2 (bg=26.89%) | K562 | + | chr11 | | 65267633 | 65267636 |
| 4.06531521625 | safb2 (bg=26.89%) | K562 | + | chr11 | | 65267636 | 65267643 |
| 3.4199853084 | safb2 (bg=26.89%) | K562 | + | chr11 | | 65267636 | 65267644 |
| 2.31705570547 | SLTM (bg=7.5%) | HepG2 | + | chr11 | | 65267582 | 65267655 |
| 2.15259022169 | SLTM (bg=7.5%) | K562 | + | chr11 | | 65267609 | 65267627 |
| 2.06971083977 | SLTM (bg=7.5%) | K562 | + | chr11 | | 65267627 | 65267639 |
| 3.0458761831 | srsf1 (bg=30.28%) | K562 | + | chr11 | | 65267606 | 65267640 |
| 2.48742176052 | srsf7 (bg=22.53%) | K562 | + | chr11 | | 65267606 | 65267647 |
| 2.61924536128 | srsf7 (bg=22.53%) | K562 | + | chr11 | | 65267607 | 65267643 |
| 2.64676885348 | SUPV3L1 (bg=9.63%) | K562 | + | chr11 | | 65267548 | 65267624 |
| 3.11626460393 | SUPV3L1 (bg=9.63%) | K562 | + | chr11 | | 65267611 | 65267635 |
| 2.34250721804 | TAF15 (bg=9.06%) | HepG2 | + | chr11 | | 65267601 | 65267633 |
| 3.79945079622 | tra2a (bg=37.02%) | HepG2 | + | chr11 | | 65267576 | 65267636 |
| 3.23813804326 | tra2a (bg=37.02%) | HepG2 | + | chr11 | | 65267576 | 65267648 |
| 5.09088657143 | tra2a (bg=37.02%) | K562 | + | chr11 | | 65267606 | 65267636 |
| 5.063182712 | tra2a (bg=37.02%) | K562 | + | chr11 | | 65267608 | 65267637 |
| 4.20358642024 | tra2a (bg=37.02%) | K562 | + | chr11 | | 65267636 | 65267642 |
| 2.72167293399 | uchl5 (bg=18.56%) | K562 | + | chr11 | | 65267617 | 65267643 |
| 3.20367199731 | uchl5 (bg=18.56%) | K562 | + | chr11 | | 65267618 | 65267656 |
| 2.17566847702 | XRCC6 (bg=3.81%) | HepG2 | + | chr11 | | 65267602 | 65267653 |
| 2.41088775741 | YWHAG (bg=9.14%) | K562 | + | chr11 | | 65267598 | 65267653 |
| 3.03265309331 | YWHAG (bg=9.14%) | K562 | + | chr11 | | 65267616 | 65267653 |
| 2.95933661148 | znf622 (bg=18.79%) | K562 | + | chr11 | | 65267613 | 65267628 |
| 3.44379653771 | znf622 (bg=18.79%) | K562 | + | chr11 | | 65267619 | 65267624 |
| 3.48293031635 | znf622 (bg=18.79%) | K562 | + | chr11 | | 65267624 | 65267632 |
| 2.26845070316 | znf622 (bg=18.79%) | K562 | + | chr11 | | 65267628 | 65267637 |
| 3.1374729661 | znf622 (bg=18.79%) | K562 | + | chr11 | | 65267632 | 65267655 |

  
  

| Match 90 in HUMAN | | | | | | | |
| --- | --- | --- | --- | --- | --- | --- | --- |
| Motif | Start in Seq (1 Indexed) | End in Seq (1 Indexed) | Strand | Chrm | Exon | Start in Chrm (0 Indexed) | End in Chrm (1 Indexed) |
| CAAAAAG | 1138 | 1144 | + | chr11 | 1 | 65267627 | 65267634 |
| eCLIP Fold-Enrichment | Binding Protein | Cell Line | Strand | Chrm | | Start in Chrm (0 Indexed) | End in Chrm (1 Indexed) |
| 3.00525764843 | aggf1 (bg=15.15%) | K562 | + | chr11 | | 65267620 | 65267629 |
| 2.58394809533 | aggf1 (bg=15.15%) | K562 | + | chr11 | | 65267629 | 65267635 |
| 3.47017247096 | bclaf1 (bg=17.67%) | HepG2 | + | chr11 | | 65267598 | 65267656 |
| 3.40112401216 | bclaf1 (bg=17.67%) | HepG2 | + | chr11 | | 65267604 | 65267655 |
| 2.14297651084 | bud13 (bg=12.85%) | K562 | + | chr11 | | 65267617 | 65267632 |
| 2.47890643645 | bud13 (bg=12.85%) | K562 | + | chr11 | | 65267626 | 65267643 |
| 2.20926946342 | bud13 (bg=12.85%) | K562 | + | chr11 | | 65267632 | 65267657 |
| 2.59404009629 | cpsf6 (bg=13.45%) | K562 | + | chr11 | | 65267607 | 65267640 |
| 2.79933777456 | cpsf6 (bg=13.45%) | K562 | + | chr11 | | 65267608 | 65267644 |
| 2.08815924402 | EIF3H (bg=0.83%) | HepG2 | + | chr11 | | 65267576 | 65267638 |
| 2.35149745155 | fxr2 (bg=10.1%) | HepG2 | + | chr11 | | 65267597 | 65267659 |
| 2.4841507675 | gtf2f1 (bg=10.18%) | HepG2 | + | chr11 | | 65267590 | 65267644 |
| 3.19477346748 | hltf (bg=24.28%) | HepG2 | + | chr11 | | 65267590 | 65267629 |
| 2.15029770655 | hltf (bg=24.28%) | K562 | + | chr11 | | 65267625 | 65267654 |
| 2.76615639752 | hltf (bg=24.28%) | K562 | + | chr11 | | 65267625 | 65267655 |
| 3.23281833788 | hltf (bg=24.28%) | HepG2 | + | chr11 | | 65267625 | 65267657 |
| 3.46501688949 | hltf (bg=24.28%) | HepG2 | + | chr11 | | 65267629 | 65267636 |
| 2.0955986381 | khdrbs1 (bg=10.41%) | K562 | + | chr11 | | 65267623 | 65267634 |
| 2.21422405145 | khdrbs1 (bg=10.41%) | K562 | + | chr11 | | 65267634 | 65267646 |
| 2.87421410329 | larp4 (bg=13.51%) | K562 | + | chr11 | | 65267615 | 65267658 |
| 2.73809649475 | MTPAP (bg=9.55%) | K562 | + | chr11 | | 65267621 | 65267644 |
| 2.20007765763 | MTPAP (bg=9.55%) | K562 | + | chr11 | | 65267622 | 65267634 |
| 2.10171106286 | MTPAP (bg=9.55%) | K562 | + | chr11 | | 65267634 | 65267652 |
| 2.28180184717 | NIPBL (bg=8.2%) | K562 | + | chr11 | | 65267621 | 65267648 |
| 2.0285333905 | NIPBL (bg=8.2%) | K562 | + | chr11 | | 65267625 | 65267633 |
| 2.34264029742 | npm1 (bg=10.22%) | K562 | + | chr11 | | 65267525 | 65267645 |
| 4.67817442895 | ppil4 (bg=43.39%) | K562 | + | chr11 | | 65267603 | 65267637 |
| 5.00574691839 | ppil4 (bg=43.39%) | K562 | + | chr11 | | 65267624 | 65267632 |
| 4.76547970104 | ppil4 (bg=43.39%) | K562 | + | chr11 | | 65267632 | 65267646 |
| 2.0067546483 | rbm15 (bg=11.59%) | K562 | + | chr11 | | 65267608 | 65267629 |
| 2.40145309885 | rbm15 (bg=11.59%) | K562 | + | chr11 | | 65267625 | 65267632 |
| 2.30386922296 | rbm15 (bg=11.59%) | K562 | + | chr11 | | 65267632 | 65267636 |
| 2.82905196501 | rbm22 (bg=12.69%) | HepG2 | + | chr11 | | 65267597 | 65267651 |
| 3.51884190323 | safb (bg=40.39%) | HepG2 | + | chr11 | | 65267607 | 65267645 |
| 2.78694923835 | safb (bg=40.39%) | HepG2 | + | chr11 | | 65267608 | 65267631 |
| 3.39044630415 | safb (bg=40.39%) | K562 | + | chr11 | | 65267626 | 65267635 |
| 2.74050397926 | safb (bg=40.39%) | K562 | + | chr11 | | 65267626 | 65267636 |
| 3.12352569327 | safb2 (bg=26.89%) | K562 | + | chr11 | | 65267617 | 65267632 |
| 3.67458290031 | safb2 (bg=26.89%) | K562 | + | chr11 | | 65267625 | 65267633 |
| 3.27199918242 | safb2 (bg=26.89%) | K562 | + | chr11 | | 65267632 | 65267636 |
| 3.70155773856 | safb2 (bg=26.89%) | K562 | + | chr11 | | 65267633 | 65267636 |
| 2.31705570547 | SLTM (bg=7.5%) | HepG2 | + | chr11 | | 65267582 | 65267655 |
| 2.15259022169 | SLTM (bg=7.5%) | K562 | + | chr11 | | 65267609 | 65267627 |
| 2.06971083977 | SLTM (bg=7.5%) | K562 | + | chr11 | | 65267627 | 65267639 |
| 3.0458761831 | srsf1 (bg=30.28%) | K562 | + | chr11 | | 65267606 | 65267640 |
| 2.48742176052 | srsf7 (bg=22.53%) | K562 | + | chr11 | | 65267606 | 65267647 |
| 2.61924536128 | srsf7 (bg=22.53%) | K562 | + | chr11 | | 65267607 | 65267643 |
| 3.11626460393 | SUPV3L1 (bg=9.63%) | K562 | + | chr11 | | 65267611 | 65267635 |
| 2.34250721804 | TAF15 (bg=9.06%) | HepG2 | + | chr11 | | 65267601 | 65267633 |
| 3.79945079622 | tra2a (bg=37.02%) | HepG2 | + | chr11 | | 65267576 | 65267636 |
| 3.23813804326 | tra2a (bg=37.02%) | HepG2 | + | chr11 | | 65267576 | 65267648 |
| 5.09088657143 | tra2a (bg=37.02%) | K562 | + | chr11 | | 65267606 | 65267636 |
| 5.063182712 | tra2a (bg=37.02%) | K562 | + | chr11 | | 65267608 | 65267637 |
| 2.72167293399 | uchl5 (bg=18.56%) | K562 | + | chr11 | | 65267617 | 65267643 |
| 3.20367199731 | uchl5 (bg=18.56%) | K562 | + | chr11 | | 65267618 | 65267656 |
| 2.17566847702 | XRCC6 (bg=3.81%) | HepG2 | + | chr11 | | 65267602 | 65267653 |
| 2.41088775741 | YWHAG (bg=9.14%) | K562 | + | chr11 | | 65267598 | 65267653 |
| 3.03265309331 | YWHAG (bg=9.14%) | K562 | + | chr11 | | 65267616 | 65267653 |
| 2.95933661148 | znf622 (bg=18.79%) | K562 | + | chr11 | | 65267613 | 65267628 |
| 3.48293031635 | znf622 (bg=18.79%) | K562 | + | chr11 | | 65267624 | 65267632 |
| 2.26845070316 | znf622 (bg=18.79%) | K562 | + | chr11 | | 65267628 | 65267637 |
| 3.1374729661 | znf622 (bg=18.79%) | K562 | + | chr11 | | 65267632 | 65267655 |

  
  

| Match 91 in HUMAN | | | | | | | |
| --- | --- | --- | --- | --- | --- | --- | --- |
| Motif | Start in Seq (1 Indexed) | End in Seq (1 Indexed) | Strand | Chrm | Exon | Start in Chrm (0 Indexed) | End in Chrm (1 Indexed) |
| AAAAACTAAGGCAGAAGGCTTTTGGAAGAGTTAGAAGAATTTGGAAGGCCTTAAA | 1172 | 1226 | + | chr11 | 1 | 65267661 | 65267716 |
| eCLIP Fold-Enrichment | Binding Protein | Cell Line | Strand | Chrm | | Start in Chrm (0 Indexed) | End in Chrm (1 Indexed) |
| 2.39206461581 | aggf1 (bg=15.15%) | K562 | + | chr11 | | 65267673 | 65267693 |
| 2.26728761824 | aggf1 (bg=15.15%) | K562 | + | chr11 | | 65267693 | 65267699 |
| 2.63164621557 | aggf1 (bg=15.15%) | K562 | + | chr11 | | 65267699 | 65267705 |
| 2.80855881553 | aggf1 (bg=15.15%) | K562 | + | chr11 | | 65267705 | 65267716 |
| 2.34386890885 | aggf1 (bg=15.15%) | K562 | + | chr11 | | 65267716 | 65267720 |
| 3.53721181885 | AQR (bg=4.89%) | HepG2 | + | chr11 | | 65267639 | 65267714 |
| 3.50972676373 | bclaf1 (bg=17.67%) | HepG2 | + | chr11 | | 65267657 | 65267691 |
| 3.16757048805 | bclaf1 (bg=17.67%) | HepG2 | + | chr11 | | 65267658 | 65267712 |
| 3.552958825 | bclaf1 (bg=17.67%) | HepG2 | + | chr11 | | 65267691 | 65267700 |
| 3.57035329757 | bclaf1 (bg=17.67%) | HepG2 | + | chr11 | | 65267700 | 65267728 |
| 3.18855939417 | bclaf1 (bg=17.67%) | HepG2 | + | chr11 | | 65267712 | 65267727 |
| 2.00508090901 | bud13 (bg=12.85%) | K562 | + | chr11 | | 65267679 | 65267704 |
| 2.13107236919 | bud13 (bg=12.85%) | K562 | + | chr11 | | 65267700 | 65267704 |
| 2.32070259946 | bud13 (bg=12.85%) | K562 | + | chr11 | | 65267704 | 65267716 |
| 2.26887578952 | bud13 (bg=12.85%) | K562 | + | chr11 | | 65267704 | 65267716 |
| 2.37561418817 | bud13 (bg=12.85%) | K562 | + | chr11 | | 65267716 | 65267724 |
| 2.21516353751 | bud13 (bg=12.85%) | K562 | + | chr11 | | 65267716 | 65267729 |
| 2.35921987815 | cpsf6 (bg=13.45%) | K562 | + | chr11 | | 65267680 | 65267689 |
| 2.22787345402 | cpsf6 (bg=13.45%) | K562 | + | chr11 | | 65267682 | 65267688 |
| 2.35812937408 | cpsf6 (bg=13.45%) | K562 | + | chr11 | | 65267688 | 65267727 |
| 2.45284549895 | cpsf6 (bg=13.45%) | K562 | + | chr11 | | 65267689 | 65267719 |
| 2.13516780009 | FUBP3 (bg=1.53%) | HepG2 | + | chr11 | | 65267686 | 65267715 |
| 2.04259239197 | gtf2f1 (bg=10.18%) | HepG2 | + | chr11 | | 65267680 | 65267698 |
| 3.11768190552 | hltf (bg=24.28%) | HepG2 | + | chr11 | | 65267659 | 65267679 |
| 2.57241328154 | hltf (bg=24.28%) | HepG2 | + | chr11 | | 65267660 | 65267665 |
| 2.48290556527 | hltf (bg=24.28%) | K562 | + | chr11 | | 65267664 | 65267701 |
| 2.68250620683 | hltf (bg=24.28%) | HepG2 | + | chr11 | | 65267665 | 65267677 |
| 3.01191708454 | hltf (bg=24.28%) | HepG2 | + | chr11 | | 65267677 | 65267715 |
| 2.15250878361 | hltf (bg=24.28%) | K562 | + | chr11 | | 65267678 | 65267687 |
| 3.24602156721 | hltf (bg=24.28%) | HepG2 | + | chr11 | | 65267679 | 65267690 |
| 2.32059470481 | hltf (bg=24.28%) | K562 | + | chr11 | | 65267687 | 65267704 |
| 3.29033972907 | hltf (bg=24.28%) | HepG2 | + | chr11 | | 65267690 | 65267701 |
| 2.57851952129 | hltf (bg=24.28%) | K562 | + | chr11 | | 65267701 | 65267704 |
| 3.10360033354 | hltf (bg=24.28%) | HepG2 | + | chr11 | | 65267701 | 65267716 |
| 2.55982008968 | hltf (bg=24.28%) | K562 | + | chr11 | | 65267704 | 65267717 |
| 2.2525083015 | hltf (bg=24.28%) | K562 | + | chr11 | | 65267704 | 65267720 |
| 3.02454294356 | hltf (bg=24.28%) | HepG2 | + | chr11 | | 65267715 | 65267728 |
| 3.40733586751 | hltf (bg=24.28%) | HepG2 | + | chr11 | | 65267716 | 65267729 |
| 2.84333479041 | larp4 (bg=13.51%) | K562 | + | chr11 | | 65267658 | 65267677 |
| 2.97032660674 | larp4 (bg=13.51%) | K562 | + | chr11 | | 65267677 | 65267719 |
| 2.05334665493 | MTPAP (bg=9.55%) | K562 | + | chr11 | | 65267678 | 65267686 |
| 2.10035044927 | MTPAP (bg=9.55%) | K562 | + | chr11 | | 65267686 | 65267699 |
| 2.273598175 | MTPAP (bg=9.55%) | K562 | + | chr11 | | 65267699 | 65267709 |
| 2.32744679803 | MTPAP (bg=9.55%) | K562 | + | chr11 | | 65267709 | 65267721 |
| 2.18060772905 | NIPBL (bg=8.2%) | K562 | + | chr11 | | 65267658 | 65267679 |
| 2.16153453282 | NIPBL (bg=8.2%) | K562 | + | chr11 | | 65267679 | 65267690 |
| 2.57289277341 | NIPBL (bg=8.2%) | K562 | + | chr11 | | 65267679 | 65267690 |
| 2.31525271045 | NIPBL (bg=8.2%) | K562 | + | chr11 | | 65267690 | 65267699 |
| 2.78883140717 | NIPBL (bg=8.2%) | K562 | + | chr11 | | 65267690 | 65267701 |
| 2.42059621013 | NIPBL (bg=8.2%) | K562 | + | chr11 | | 65267701 | 65267713 |
| 2.31705890409 | NIPBL (bg=8.2%) | K562 | + | chr11 | | 65267713 | 65267728 |
| 3.02788444105 | NIPBL (bg=8.2%) | K562 | + | chr11 | | 65267713 | 65267729 |
| 3.73105963275 | ppil4 (bg=43.39%) | K562 | + | chr11 | | 65267662 | 65267666 |
| 3.67474426105 | ppil4 (bg=43.39%) | K562 | + | chr11 | | 65267666 | 65267677 |
| 3.97898846988 | ppil4 (bg=43.39%) | K562 | + | chr11 | | 65267677 | 65267691 |
| 3.43447880293 | ppil4 (bg=43.39%) | K562 | + | chr11 | | 65267682 | 65267688 |
| 3.51009536414 | ppil4 (bg=43.39%) | K562 | + | chr11 | | 65267688 | 65267696 |
| 4.01480522059 | ppil4 (bg=43.39%) | K562 | + | chr11 | | 65267691 | 65267699 |
| 3.44758022344 | ppil4 (bg=43.39%) | K562 | + | chr11 | | 65267696 | 65267700 |
| 4.17636016742 | ppil4 (bg=43.39%) | K562 | + | chr11 | | 65267699 | 65267712 |
| 3.68144101701 | ppil4 (bg=43.39%) | K562 | + | chr11 | | 65267700 | 65267714 |
| 4.37565356457 | ppil4 (bg=43.39%) | K562 | + | chr11 | | 65267712 | 65267719 |
| 3.86010481549 | ppil4 (bg=43.39%) | K562 | + | chr11 | | 65267714 | 65267721 |
| 2.94920578364 | rbm15 (bg=11.59%) | K562 | + | chr11 | | 65267677 | 65267688 |
| 3.66404704718 | rbm15 (bg=11.59%) | K562 | + | chr11 | | 65267678 | 65267689 |
| 2.59968931361 | rbm15 (bg=11.59%) | HepG2 | + | chr11 | | 65267681 | 65267688 |
| 3.13706913022 | rbm15 (bg=11.59%) | K562 | + | chr11 | | 65267688 | 65267699 |
| 2.80754004338 | rbm15 (bg=11.59%) | HepG2 | + | chr11 | | 65267688 | 65267710 |
| 3.66054732229 | rbm15 (bg=11.59%) | K562 | + | chr11 | | 65267689 | 65267719 |
| 3.04939652618 | rbm15 (bg=11.59%) | K562 | + | chr11 | | 65267699 | 65267719 |
| 3.18456581421 | rbm15 (bg=11.59%) | HepG2 | + | chr11 | | 65267710 | 65267716 |
| 2.97003551065 | rbm15 (bg=11.59%) | HepG2 | + | chr11 | | 65267716 | 65267720 |
| 2.71585900943 | rbm22 (bg=12.69%) | HepG2 | + | chr11 | | 65267663 | 65267690 |
| 3.20967653578 | rbm22 (bg=12.69%) | HepG2 | + | chr11 | | 65267690 | 65267702 |
| 3.12396666563 | rbm22 (bg=12.69%) | HepG2 | + | chr11 | | 65267702 | 65267718 |
| 3.49949465066 | safb (bg=40.39%) | K562 | + | chr11 | | 65267656 | 65267666 |
| 2.91927013498 | safb (bg=40.39%) | K562 | + | chr11 | | 65267659 | 65267680 |
| 3.10786950902 | safb (bg=40.39%) | HepG2 | + | chr11 | | 65267660 | 65267669 |
| 3.35425207597 | safb (bg=40.39%) | K562 | + | chr11 | | 65267666 | 65267673 |
| 3.15183749217 | safb (bg=40.39%) | HepG2 | + | chr11 | | 65267669 | 65267680 |
| 2.11742098822 | safb (bg=40.39%) | HepG2 | + | chr11 | | 65267672 | 65267713 |
| 3.4009533966 | safb (bg=40.39%) | K562 | + | chr11 | | 65267673 | 65267679 |
| 3.28540423823 | safb (bg=40.39%) | K562 | + | chr11 | | 65267679 | 65267685 |
| 2.85842820297 | safb (bg=40.39%) | K562 | + | chr11 | | 65267680 | 65267685 |
| 3.08001815108 | safb (bg=40.39%) | HepG2 | + | chr11 | | 65267680 | 65267687 |
| 3.28485956945 | safb (bg=40.39%) | K562 | + | chr11 | | 65267685 | 65267701 |
| 2.87992864189 | safb (bg=40.39%) | K562 | + | chr11 | | 65267685 | 65267701 |
| 2.97207370553 | safb (bg=40.39%) | HepG2 | + | chr11 | | 65267687 | 65267705 |
| 3.09845020774 | safb (bg=40.39%) | K562 | + | chr11 | | 65267701 | 65267704 |
| 2.65506431131 | safb (bg=40.39%) | K562 | + | chr11 | | 65267701 | 65267704 |
| 3.11558455452 | safb (bg=40.39%) | K562 | + | chr11 | | 65267704 | 65267707 |
| 2.73631168497 | safb (bg=40.39%) | K562 | + | chr11 | | 65267704 | 65267713 |
| 3.09015563815 | safb (bg=40.39%) | HepG2 | + | chr11 | | 65267705 | 65267712 |
| 3.15503962407 | safb (bg=40.39%) | K562 | + | chr11 | | 65267707 | 65267712 |
| 3.13040106264 | safb (bg=40.39%) | K562 | + | chr11 | | 65267712 | 65267717 |
| 3.12465958833 | safb (bg=40.39%) | HepG2 | + | chr11 | | 65267712 | 65267718 |
| 2.37952492318 | safb (bg=40.39%) | HepG2 | + | chr11 | | 65267713 | 65267723 |
| 2.70129273303 | safb (bg=40.39%) | K562 | + | chr11 | | 65267713 | 65267727 |
| 3.36622446325 | safb2 (bg=26.89%) | K562 | + | chr11 | | 65267658 | 65267667 |
| 2.67215736529 | safb2 (bg=26.89%) | K562 | + | chr11 | | 65267658 | 65267667 |
| 3.18750481913 | safb2 (bg=26.89%) | K562 | + | chr11 | | 65267667 | 65267679 |
| 2.47043505187 | safb2 (bg=26.89%) | K562 | + | chr11 | | 65267667 | 65267680 |
| 3.77029859122 | safb2 (bg=26.89%) | K562 | + | chr11 | | 65267679 | 65267704 |
| 2.96958196121 | safb2 (bg=26.89%) | K562 | + | chr11 | | 65267680 | 65267687 |
| 3.11943226617 | safb2 (bg=26.89%) | K562 | + | chr11 | | 65267687 | 65267704 |
| 4.00636486817 | safb2 (bg=26.89%) | K562 | + | chr11 | | 65267704 | 65267707 |
| 3.36589686773 | safb2 (bg=26.89%) | K562 | + | chr11 | | 65267704 | 65267716 |
| 4.01444313473 | safb2 (bg=26.89%) | K562 | + | chr11 | | 65267707 | 65267715 |
| 4.23608343113 | safb2 (bg=26.89%) | K562 | + | chr11 | | 65267715 | 65267718 |
| 3.48750161851 | safb2 (bg=26.89%) | K562 | + | chr11 | | 65267716 | 65267720 |
| 2.1618117191 | SLTM (bg=7.5%) | K562 | + | chr11 | | 65267715 | 65267723 |
| 3.13591174384 | srsf1 (bg=30.28%) | K562 | + | chr11 | | 65267680 | 65267685 |
| 3.12882315 | srsf1 (bg=30.28%) | K562 | + | chr11 | | 65267685 | 65267718 |
| 2.01624812679 | srsf7 (bg=22.53%) | K562 | + | chr11 | | 65267687 | 65267717 |
| 3.39842090079 | SRSF9 (bg=9.67%) | HepG2 | + | chr11 | | 65267671 | 65267721 |
| 2.1225325833 | SUPV3L1 (bg=9.63%) | K562 | + | chr11 | | 65267691 | 65267722 |
| 3.92643080692 | tra2a (bg=37.02%) | K562 | + | chr11 | | 65267661 | 65267679 |
| 3.74032109875 | tra2a (bg=37.02%) | HepG2 | + | chr11 | | 65267662 | 65267727 |
| 4.24915216475 | tra2a (bg=37.02%) | K562 | + | chr11 | | 65267667 | 65267688 |
| 4.3269063174 | tra2a (bg=37.02%) | HepG2 | + | chr11 | | 65267674 | 65267734 |
| 4.37485249289 | tra2a (bg=37.02%) | K562 | + | chr11 | | 65267679 | 65267689 |
| 4.2750341392 | tra2a (bg=37.02%) | K562 | + | chr11 | | 65267688 | 65267716 |
| 4.28992698738 | tra2a (bg=37.02%) | K562 | + | chr11 | | 65267689 | 65267716 |
| 3.79393257828 | tra2a (bg=37.02%) | K562 | + | chr11 | | 65267716 | 65267724 |
| 3.72285844699 | tra2a (bg=37.02%) | K562 | + | chr11 | | 65267716 | 65267728 |
| 2.29592388209 | uchl5 (bg=18.56%) | K562 | + | chr11 | | 65267660 | 65267704 |
| 2.08920472514 | uchl5 (bg=18.56%) | HepG2 | + | chr11 | | 65267688 | 65267699 |
| 2.31503394643 | uchl5 (bg=18.56%) | K562 | + | chr11 | | 65267704 | 65267715 |
| 2.3140147262 | uchl5 (bg=18.56%) | K562 | + | chr11 | | 65267715 | 65267720 |
| 2.07381317049 | XRCC6 (bg=3.81%) | K562 | + | chr11 | | 65267678 | 65267704 |
| 2.09413607166 | XRCC6 (bg=3.81%) | K562 | + | chr11 | | 65267704 | 65267713 |
| 2.10807182265 | XRCC6 (bg=3.81%) | K562 | + | chr11 | | 65267713 | 65267724 |
| 2.27320063573 | YWHAG (bg=9.14%) | K562 | + | chr11 | | 65267675 | 65267695 |
| 2.53050409297 | YWHAG (bg=9.14%) | K562 | + | chr11 | | 65267676 | 65267697 |
| 2.15504928401 | YWHAG (bg=9.14%) | K562 | + | chr11 | | 65267695 | 65267729 |
| 2.39623106134 | YWHAG (bg=9.14%) | K562 | + | chr11 | | 65267697 | 65267710 |
| 2.6394130453 | YWHAG (bg=9.14%) | K562 | + | chr11 | | 65267710 | 65267716 |
| 2.48721779749 | zc3h8 (bg=12.78%) | K562 | + | chr11 | | 65267673 | 65267708 |
| 2.63510665207 | znf622 (bg=18.79%) | K562 | + | chr11 | | 65267660 | 65267664 |
| 2.36470304566 | znf622 (bg=18.79%) | K562 | + | chr11 | | 65267661 | 65267678 |
| 2.9011803277 | znf622 (bg=18.79%) | K562 | + | chr11 | | 65267664 | 65267679 |
| 2.5768062671 | znf622 (bg=18.79%) | K562 | + | chr11 | | 65267678 | 65267690 |
| 2.96968696131 | znf622 (bg=18.79%) | K562 | + | chr11 | | 65267679 | 65267699 |
| 2.60061167411 | znf622 (bg=18.79%) | K562 | + | chr11 | | 65267690 | 65267701 |
| 2.83634964725 | znf622 (bg=18.79%) | K562 | + | chr11 | | 65267699 | 65267701 |
| 2.25014888786 | znf622 (bg=18.79%) | K562 | + | chr11 | | 65267701 | 65267707 |
| 2.71335431416 | znf622 (bg=18.79%) | K562 | + | chr11 | | 65267701 | 65267713 |
| 2.67905805002 | znf622 (bg=18.79%) | K562 | + | chr11 | | 65267707 | 65267710 |
| 2.53112571557 | znf622 (bg=18.79%) | K562 | + | chr11 | | 65267710 | 65267718 |
| 2.98973446828 | znf622 (bg=18.79%) | K562 | + | chr11 | | 65267713 | 65267728 |

  
  

| Match 92 in HUMAN | | | | | | | |
| --- | --- | --- | --- | --- | --- | --- | --- |
| Motif | Start in Seq (1 Indexed) | End in Seq (1 Indexed) | Strand | Chrm | Exon | Start in Chrm (0 Indexed) | End in Chrm (1 Indexed) |
| GAGTTA | 1199 | 1204 | + | chr11 | 1 | 65267688 | 65267694 |
| eCLIP Fold-Enrichment | Binding Protein | Cell Line | Strand | Chrm | | Start in Chrm (0 Indexed) | End in Chrm (1 Indexed) |
| 2.39206461581 | aggf1 (bg=15.15%) | K562 | + | chr11 | | 65267673 | 65267693 |
| 2.26728761824 | aggf1 (bg=15.15%) | K562 | + | chr11 | | 65267693 | 65267699 |
| 3.53721181885 | AQR (bg=4.89%) | HepG2 | + | chr11 | | 65267639 | 65267714 |
| 3.50972676373 | bclaf1 (bg=17.67%) | HepG2 | + | chr11 | | 65267657 | 65267691 |
| 3.16757048805 | bclaf1 (bg=17.67%) | HepG2 | + | chr11 | | 65267658 | 65267712 |
| 3.552958825 | bclaf1 (bg=17.67%) | HepG2 | + | chr11 | | 65267691 | 65267700 |
| 2.00508090901 | bud13 (bg=12.85%) | K562 | + | chr11 | | 65267679 | 65267704 |
| 2.35921987815 | cpsf6 (bg=13.45%) | K562 | + | chr11 | | 65267680 | 65267689 |
| 2.22787345402 | cpsf6 (bg=13.45%) | K562 | + | chr11 | | 65267682 | 65267688 |
| 2.35812937408 | cpsf6 (bg=13.45%) | K562 | + | chr11 | | 65267688 | 65267727 |
| 2.45284549895 | cpsf6 (bg=13.45%) | K562 | + | chr11 | | 65267689 | 65267719 |
| 2.13516780009 | FUBP3 (bg=1.53%) | HepG2 | + | chr11 | | 65267686 | 65267715 |
| 2.04259239197 | gtf2f1 (bg=10.18%) | HepG2 | + | chr11 | | 65267680 | 65267698 |
| 2.48290556527 | hltf (bg=24.28%) | K562 | + | chr11 | | 65267664 | 65267701 |
| 3.01191708454 | hltf (bg=24.28%) | HepG2 | + | chr11 | | 65267677 | 65267715 |
| 3.24602156721 | hltf (bg=24.28%) | HepG2 | + | chr11 | | 65267679 | 65267690 |
| 2.32059470481 | hltf (bg=24.28%) | K562 | + | chr11 | | 65267687 | 65267704 |
| 3.29033972907 | hltf (bg=24.28%) | HepG2 | + | chr11 | | 65267690 | 65267701 |
| 2.97032660674 | larp4 (bg=13.51%) | K562 | + | chr11 | | 65267677 | 65267719 |
| 2.10035044927 | MTPAP (bg=9.55%) | K562 | + | chr11 | | 65267686 | 65267699 |
| 2.16153453282 | NIPBL (bg=8.2%) | K562 | + | chr11 | | 65267679 | 65267690 |
| 2.57289277341 | NIPBL (bg=8.2%) | K562 | + | chr11 | | 65267679 | 65267690 |
| 2.31525271045 | NIPBL (bg=8.2%) | K562 | + | chr11 | | 65267690 | 65267699 |
| 2.78883140717 | NIPBL (bg=8.2%) | K562 | + | chr11 | | 65267690 | 65267701 |
| 3.97898846988 | ppil4 (bg=43.39%) | K562 | + | chr11 | | 65267677 | 65267691 |
| 3.43447880293 | ppil4 (bg=43.39%) | K562 | + | chr11 | | 65267682 | 65267688 |
| 3.51009536414 | ppil4 (bg=43.39%) | K562 | + | chr11 | | 65267688 | 65267696 |
| 4.01480522059 | ppil4 (bg=43.39%) | K562 | + | chr11 | | 65267691 | 65267699 |
| 2.94920578364 | rbm15 (bg=11.59%) | K562 | + | chr11 | | 65267677 | 65267688 |
| 3.66404704718 | rbm15 (bg=11.59%) | K562 | + | chr11 | | 65267678 | 65267689 |
| 2.59968931361 | rbm15 (bg=11.59%) | HepG2 | + | chr11 | | 65267681 | 65267688 |
| 3.13706913022 | rbm15 (bg=11.59%) | K562 | + | chr11 | | 65267688 | 65267699 |
| 2.80754004338 | rbm15 (bg=11.59%) | HepG2 | + | chr11 | | 65267688 | 65267710 |
| 3.66054732229 | rbm15 (bg=11.59%) | K562 | + | chr11 | | 65267689 | 65267719 |
| 2.71585900943 | rbm22 (bg=12.69%) | HepG2 | + | chr11 | | 65267663 | 65267690 |
| 3.20967653578 | rbm22 (bg=12.69%) | HepG2 | + | chr11 | | 65267690 | 65267702 |
| 2.11742098822 | safb (bg=40.39%) | HepG2 | + | chr11 | | 65267672 | 65267713 |
| 3.28485956945 | safb (bg=40.39%) | K562 | + | chr11 | | 65267685 | 65267701 |
| 2.87992864189 | safb (bg=40.39%) | K562 | + | chr11 | | 65267685 | 65267701 |
| 2.97207370553 | safb (bg=40.39%) | HepG2 | + | chr11 | | 65267687 | 65267705 |
| 3.77029859122 | safb2 (bg=26.89%) | K562 | + | chr11 | | 65267679 | 65267704 |
| 3.11943226617 | safb2 (bg=26.89%) | K562 | + | chr11 | | 65267687 | 65267704 |
| 3.12882315 | srsf1 (bg=30.28%) | K562 | + | chr11 | | 65267685 | 65267718 |
| 2.01624812679 | srsf7 (bg=22.53%) | K562 | + | chr11 | | 65267687 | 65267717 |
| 3.39842090079 | SRSF9 (bg=9.67%) | HepG2 | + | chr11 | | 65267671 | 65267721 |
| 2.1225325833 | SUPV3L1 (bg=9.63%) | K562 | + | chr11 | | 65267691 | 65267722 |
| 3.74032109875 | tra2a (bg=37.02%) | HepG2 | + | chr11 | | 65267662 | 65267727 |
| 4.24915216475 | tra2a (bg=37.02%) | K562 | + | chr11 | | 65267667 | 65267688 |
| 4.3269063174 | tra2a (bg=37.02%) | HepG2 | + | chr11 | | 65267674 | 65267734 |
| 4.37485249289 | tra2a (bg=37.02%) | K562 | + | chr11 | | 65267679 | 65267689 |
| 4.2750341392 | tra2a (bg=37.02%) | K562 | + | chr11 | | 65267688 | 65267716 |
| 4.28992698738 | tra2a (bg=37.02%) | K562 | + | chr11 | | 65267689 | 65267716 |
| 2.29592388209 | uchl5 (bg=18.56%) | K562 | + | chr11 | | 65267660 | 65267704 |
| 2.08920472514 | uchl5 (bg=18.56%) | HepG2 | + | chr11 | | 65267688 | 65267699 |
| 2.07381317049 | XRCC6 (bg=3.81%) | K562 | + | chr11 | | 65267678 | 65267704 |
| 2.27320063573 | YWHAG (bg=9.14%) | K562 | + | chr11 | | 65267675 | 65267695 |
| 2.53050409297 | YWHAG (bg=9.14%) | K562 | + | chr11 | | 65267676 | 65267697 |
| 2.48721779749 | zc3h8 (bg=12.78%) | K562 | + | chr11 | | 65267673 | 65267708 |
| 2.5768062671 | znf622 (bg=18.79%) | K562 | + | chr11 | | 65267678 | 65267690 |
| 2.96968696131 | znf622 (bg=18.79%) | K562 | + | chr11 | | 65267679 | 65267699 |
| 2.60061167411 | znf622 (bg=18.79%) | K562 | + | chr11 | | 65267690 | 65267701 |

  
  

| Match 93 in HUMAN | | | | | | | |
| --- | --- | --- | --- | --- | --- | --- | --- |
| Motif | Start in Seq (1 Indexed) | End in Seq (1 Indexed) | Strand | Chrm | Exon | Start in Chrm (0 Indexed) | End in Chrm (1 Indexed) |
| GTAGCTT | 1231 | 1237 | + | chr11 | 1 | 65267720 | 65267727 |
| eCLIP Fold-Enrichment | Binding Protein | Cell Line | Strand | Chrm | | Start in Chrm (0 Indexed) | End in Chrm (1 Indexed) |
| 2.34386890885 | aggf1 (bg=15.15%) | K562 | + | chr11 | | 65267716 | 65267720 |
| 2.24133228574 | aggf1 (bg=15.15%) | K562 | + | chr11 | | 65267720 | 65267724 |
| 2.7709433939 | aggf1 (bg=15.15%) | K562 | + | chr11 | | 65267724 | 65267729 |
| 3.57035329757 | bclaf1 (bg=17.67%) | HepG2 | + | chr11 | | 65267700 | 65267728 |
| 3.18855939417 | bclaf1 (bg=17.67%) | HepG2 | + | chr11 | | 65267712 | 65267727 |
| 3.52559438144 | bclaf1 (bg=17.67%) | HepG2 | + | chr11 | | 65267727 | 65267743 |
| 2.37561418817 | bud13 (bg=12.85%) | K562 | + | chr11 | | 65267716 | 65267724 |
| 2.21516353751 | bud13 (bg=12.85%) | K562 | + | chr11 | | 65267716 | 65267729 |
| 2.35812937408 | cpsf6 (bg=13.45%) | K562 | + | chr11 | | 65267688 | 65267727 |
| 2.7841618731 | cpsf6 (bg=13.45%) | K562 | + | chr11 | | 65267719 | 65267723 |
| 2.2525083015 | hltf (bg=24.28%) | K562 | + | chr11 | | 65267704 | 65267720 |
| 3.02454294356 | hltf (bg=24.28%) | HepG2 | + | chr11 | | 65267715 | 65267728 |
| 3.40733586751 | hltf (bg=24.28%) | HepG2 | + | chr11 | | 65267716 | 65267729 |
| 2.13200919457 | hltf (bg=24.28%) | K562 | + | chr11 | | 65267717 | 65267720 |
| 2.10814077797 | hltf (bg=24.28%) | K562 | + | chr11 | | 65267720 | 65267723 |
| 2.00654351738 | hltf (bg=24.28%) | K562 | + | chr11 | | 65267720 | 65267727 |
| 2.80615242536 | larp4 (bg=13.51%) | K562 | + | chr11 | | 65267719 | 65267729 |
| 2.32744679803 | MTPAP (bg=9.55%) | K562 | + | chr11 | | 65267709 | 65267721 |
| 2.08764926641 | MTPAP (bg=9.55%) | K562 | + | chr11 | | 65267721 | 65267744 |
| 2.31705890409 | NIPBL (bg=8.2%) | K562 | + | chr11 | | 65267713 | 65267728 |
| 3.02788444105 | NIPBL (bg=8.2%) | K562 | + | chr11 | | 65267713 | 65267729 |
| 3.86010481549 | ppil4 (bg=43.39%) | K562 | + | chr11 | | 65267714 | 65267721 |
| 4.35152724645 | ppil4 (bg=43.39%) | K562 | + | chr11 | | 65267719 | 65267726 |
| 3.66336650453 | ppil4 (bg=43.39%) | K562 | + | chr11 | | 65267721 | 65267729 |
| 4.23133455108 | ppil4 (bg=43.39%) | K562 | + | chr11 | | 65267726 | 65267730 |
| 2.97003551065 | rbm15 (bg=11.59%) | HepG2 | + | chr11 | | 65267716 | 65267720 |
| 3.07565763726 | rbm15 (bg=11.59%) | K562 | + | chr11 | | 65267719 | 65267730 |
| 3.5498931996 | rbm15 (bg=11.59%) | K562 | + | chr11 | | 65267719 | 65267730 |
| 2.55313826887 | rbm15 (bg=11.59%) | HepG2 | + | chr11 | | 65267720 | 65267743 |
| 3.35739527114 | rbm22 (bg=12.69%) | HepG2 | + | chr11 | | 65267718 | 65267746 |
| 2.37952492318 | safb (bg=40.39%) | HepG2 | + | chr11 | | 65267713 | 65267723 |
| 2.70129273303 | safb (bg=40.39%) | K562 | + | chr11 | | 65267713 | 65267727 |
| 3.15162495966 | safb (bg=40.39%) | K562 | + | chr11 | | 65267717 | 65267724 |
| 2.97563152494 | safb (bg=40.39%) | HepG2 | + | chr11 | | 65267718 | 65267726 |
| 2.51688276689 | safb (bg=40.39%) | K562 | + | chr11 | | 65267724 | 65267728 |
| 3.48750161851 | safb2 (bg=26.89%) | K562 | + | chr11 | | 65267716 | 65267720 |
| 4.13748687622 | safb2 (bg=26.89%) | K562 | + | chr11 | | 65267718 | 65267724 |
| 3.40558535927 | safb2 (bg=26.89%) | K562 | + | chr11 | | 65267720 | 65267729 |
| 3.52059035881 | safb2 (bg=26.89%) | K562 | + | chr11 | | 65267724 | 65267728 |
| 2.1618117191 | SLTM (bg=7.5%) | K562 | + | chr11 | | 65267715 | 65267723 |
| 3.26365574047 | srsf1 (bg=30.28%) | K562 | + | chr11 | | 65267718 | 65267728 |
| 2.48762321446 | srsf7 (bg=22.53%) | K562 | + | chr11 | | 65267717 | 65267724 |
| 2.1610278099 | srsf7 (bg=22.53%) | K562 | + | chr11 | | 65267719 | 65267727 |
| 3.39842090079 | SRSF9 (bg=9.67%) | HepG2 | + | chr11 | | 65267671 | 65267721 |
| 2.1225325833 | SUPV3L1 (bg=9.63%) | K562 | + | chr11 | | 65267691 | 65267722 |
| 3.74032109875 | tra2a (bg=37.02%) | HepG2 | + | chr11 | | 65267662 | 65267727 |
| 4.3269063174 | tra2a (bg=37.02%) | HepG2 | + | chr11 | | 65267674 | 65267734 |
| 3.79393257828 | tra2a (bg=37.02%) | K562 | + | chr11 | | 65267716 | 65267724 |
| 3.72285844699 | tra2a (bg=37.02%) | K562 | + | chr11 | | 65267716 | 65267728 |
| 3.2396136773 | tra2a (bg=37.02%) | K562 | + | chr11 | | 65267724 | 65267728 |
| 3.40394125911 | tra2a (bg=37.02%) | HepG2 | + | chr11 | | 65267727 | 65267742 |
| 2.3140147262 | uchl5 (bg=18.56%) | K562 | + | chr11 | | 65267715 | 65267720 |
| 2.31524788262 | uchl5 (bg=18.56%) | K562 | + | chr11 | | 65267720 | 65267724 |
| 2.10807182265 | XRCC6 (bg=3.81%) | K562 | + | chr11 | | 65267713 | 65267724 |
| 2.15504928401 | YWHAG (bg=9.14%) | K562 | + | chr11 | | 65267695 | 65267729 |
| 2.98973446828 | znf622 (bg=18.79%) | K562 | + | chr11 | | 65267713 | 65267728 |
| 2.36377009273 | znf622 (bg=18.79%) | K562 | + | chr11 | | 65267718 | 65267729 |

  
  

| Match 94 in HUMAN | | | | | | | |
| --- | --- | --- | --- | --- | --- | --- | --- |
| Motif | Start in Seq (1 Indexed) | End in Seq (1 Indexed) | Strand | Chrm | Exon | Start in Chrm (0 Indexed) | End in Chrm (1 Indexed) |
| GTAGCTTAGTTTGAAAAAT | 1231 | 1249 | + | chr11 | 1 | 65267720 | 65267739 |
| eCLIP Fold-Enrichment | Binding Protein | Cell Line | Strand | Chrm | | Start in Chrm (0 Indexed) | End in Chrm (1 Indexed) |
| 2.34386890885 | aggf1 (bg=15.15%) | K562 | + | chr11 | | 65267716 | 65267720 |
| 2.24133228574 | aggf1 (bg=15.15%) | K562 | + | chr11 | | 65267720 | 65267724 |
| 2.7709433939 | aggf1 (bg=15.15%) | K562 | + | chr11 | | 65267724 | 65267729 |
| 2.49068342089 | aggf1 (bg=15.15%) | K562 | + | chr11 | | 65267729 | 65267738 |
| 2.09401296346 | aggf1 (bg=15.15%) | K562 | + | chr11 | | 65267738 | 65267748 |
| 3.57035329757 | bclaf1 (bg=17.67%) | HepG2 | + | chr11 | | 65267700 | 65267728 |
| 3.18855939417 | bclaf1 (bg=17.67%) | HepG2 | + | chr11 | | 65267712 | 65267727 |
| 3.52559438144 | bclaf1 (bg=17.67%) | HepG2 | + | chr11 | | 65267727 | 65267743 |
| 3.71470123622 | bclaf1 (bg=17.67%) | HepG2 | + | chr11 | | 65267728 | 65267738 |
| 3.82842145051 | bclaf1 (bg=17.67%) | HepG2 | + | chr11 | | 65267738 | 65267746 |
| 2.37561418817 | bud13 (bg=12.85%) | K562 | + | chr11 | | 65267716 | 65267724 |
| 2.21516353751 | bud13 (bg=12.85%) | K562 | + | chr11 | | 65267716 | 65267729 |
| 2.25537452853 | bud13 (bg=12.85%) | K562 | + | chr11 | | 65267728 | 65267738 |
| 2.04050465908 | bud13 (bg=12.85%) | K562 | + | chr11 | | 65267738 | 65267746 |
| 2.35812937408 | cpsf6 (bg=13.45%) | K562 | + | chr11 | | 65267688 | 65267727 |
| 2.7841618731 | cpsf6 (bg=13.45%) | K562 | + | chr11 | | 65267719 | 65267723 |
| 2.2525083015 | hltf (bg=24.28%) | K562 | + | chr11 | | 65267704 | 65267720 |
| 3.02454294356 | hltf (bg=24.28%) | HepG2 | + | chr11 | | 65267715 | 65267728 |
| 3.40733586751 | hltf (bg=24.28%) | HepG2 | + | chr11 | | 65267716 | 65267729 |
| 2.13200919457 | hltf (bg=24.28%) | K562 | + | chr11 | | 65267717 | 65267720 |
| 2.10814077797 | hltf (bg=24.28%) | K562 | + | chr11 | | 65267720 | 65267723 |
| 2.00654351738 | hltf (bg=24.28%) | K562 | + | chr11 | | 65267720 | 65267727 |
| 2.96573222816 | hltf (bg=24.28%) | HepG2 | + | chr11 | | 65267728 | 65267738 |
| 3.54390825312 | hltf (bg=24.28%) | HepG2 | + | chr11 | | 65267729 | 65267738 |
| 3.44624406879 | hltf (bg=24.28%) | HepG2 | + | chr11 | | 65267738 | 65267746 |
| 3.09702593706 | hltf (bg=24.28%) | HepG2 | + | chr11 | | 65267738 | 65267746 |
| 2.31709049342 | hnrnpa1 (bg=18.32%) | K562 | + | chr11 | | 65267732 | 65267735 |
| 2.80615242536 | larp4 (bg=13.51%) | K562 | + | chr11 | | 65267719 | 65267729 |
| 2.79979675664 | larp4 (bg=13.51%) | K562 | + | chr11 | | 65267729 | 65267737 |
| 2.72411715146 | larp4 (bg=13.51%) | K562 | + | chr11 | | 65267737 | 65267746 |
| 2.32744679803 | MTPAP (bg=9.55%) | K562 | + | chr11 | | 65267709 | 65267721 |
| 2.08764926641 | MTPAP (bg=9.55%) | K562 | + | chr11 | | 65267721 | 65267744 |
| 2.31705890409 | NIPBL (bg=8.2%) | K562 | + | chr11 | | 65267713 | 65267728 |
| 3.02788444105 | NIPBL (bg=8.2%) | K562 | + | chr11 | | 65267713 | 65267729 |
| 2.25007903714 | NIPBL (bg=8.2%) | K562 | + | chr11 | | 65267728 | 65267744 |
| 3.18072253389 | NIPBL (bg=8.2%) | K562 | + | chr11 | | 65267729 | 65267736 |
| 2.99146009534 | NIPBL (bg=8.2%) | K562 | + | chr11 | | 65267736 | 65267746 |
| 3.86010481549 | ppil4 (bg=43.39%) | K562 | + | chr11 | | 65267714 | 65267721 |
| 4.35152724645 | ppil4 (bg=43.39%) | K562 | + | chr11 | | 65267719 | 65267726 |
| 3.66336650453 | ppil4 (bg=43.39%) | K562 | + | chr11 | | 65267721 | 65267729 |
| 4.23133455108 | ppil4 (bg=43.39%) | K562 | + | chr11 | | 65267726 | 65267730 |
| 3.8637126615 | ppil4 (bg=43.39%) | K562 | + | chr11 | | 65267729 | 65267738 |
| 4.34747014115 | ppil4 (bg=43.39%) | K562 | + | chr11 | | 65267730 | 65267737 |
| 4.237277399 | ppil4 (bg=43.39%) | K562 | + | chr11 | | 65267737 | 65267742 |
| 2.97003551065 | rbm15 (bg=11.59%) | HepG2 | + | chr11 | | 65267716 | 65267720 |
| 3.07565763726 | rbm15 (bg=11.59%) | K562 | + | chr11 | | 65267719 | 65267730 |
| 3.5498931996 | rbm15 (bg=11.59%) | K562 | + | chr11 | | 65267719 | 65267730 |
| 2.55313826887 | rbm15 (bg=11.59%) | HepG2 | + | chr11 | | 65267720 | 65267743 |
| 2.44650174321 | rbm15 (bg=11.59%) | K562 | + | chr11 | | 65267730 | 65267738 |
| 2.69204491911 | rbm15 (bg=11.59%) | K562 | + | chr11 | | 65267730 | 65267738 |
| 2.31584349525 | rbm15 (bg=11.59%) | K562 | + | chr11 | | 65267738 | 65267746 |
| 3.35739527114 | rbm22 (bg=12.69%) | HepG2 | + | chr11 | | 65267718 | 65267746 |
| 2.37952492318 | safb (bg=40.39%) | HepG2 | + | chr11 | | 65267713 | 65267723 |
| 2.70129273303 | safb (bg=40.39%) | K562 | + | chr11 | | 65267713 | 65267727 |
| 3.15162495966 | safb (bg=40.39%) | K562 | + | chr11 | | 65267717 | 65267724 |
| 2.97563152494 | safb (bg=40.39%) | HepG2 | + | chr11 | | 65267718 | 65267726 |
| 2.51688276689 | safb (bg=40.39%) | K562 | + | chr11 | | 65267724 | 65267728 |
| 3.10559499854 | safb (bg=40.39%) | K562 | + | chr11 | | 65267728 | 65267738 |
| 3.00445687938 | safb (bg=40.39%) | HepG2 | + | chr11 | | 65267730 | 65267734 |
| 2.88138335789 | safb (bg=40.39%) | K562 | + | chr11 | | 65267730 | 65267737 |
| 2.27998924963 | safb (bg=40.39%) | HepG2 | + | chr11 | | 65267734 | 65267739 |
| 3.17707598999 | safb (bg=40.39%) | HepG2 | + | chr11 | | 65267734 | 65267743 |
| 2.85296008496 | safb (bg=40.39%) | K562 | + | chr11 | | 65267737 | 65267743 |
| 3.08577083607 | safb (bg=40.39%) | K562 | + | chr11 | | 65267738 | 65267747 |
| 3.48750161851 | safb2 (bg=26.89%) | K562 | + | chr11 | | 65267716 | 65267720 |
| 4.13748687622 | safb2 (bg=26.89%) | K562 | + | chr11 | | 65267718 | 65267724 |
| 3.40558535927 | safb2 (bg=26.89%) | K562 | + | chr11 | | 65267720 | 65267729 |
| 3.52059035881 | safb2 (bg=26.89%) | K562 | + | chr11 | | 65267724 | 65267728 |
| 3.88097055512 | safb2 (bg=26.89%) | K562 | + | chr11 | | 65267728 | 65267737 |
| 3.10708811472 | safb2 (bg=26.89%) | K562 | + | chr11 | | 65267729 | 65267738 |
| 3.75950510395 | safb2 (bg=26.89%) | K562 | + | chr11 | | 65267737 | 65267741 |
| 2.8421283133 | safb2 (bg=26.89%) | K562 | + | chr11 | | 65267738 | 65267744 |
| 2.1618117191 | SLTM (bg=7.5%) | K562 | + | chr11 | | 65267715 | 65267723 |
| 3.26365574047 | srsf1 (bg=30.28%) | K562 | + | chr11 | | 65267718 | 65267728 |
| 2.92809726037 | srsf1 (bg=30.28%) | K562 | + | chr11 | | 65267728 | 65267737 |
| 2.89024660836 | srsf1 (bg=30.28%) | K562 | + | chr11 | | 65267737 | 65267743 |
| 2.48762321446 | srsf7 (bg=22.53%) | K562 | + | chr11 | | 65267717 | 65267724 |
| 2.1610278099 | srsf7 (bg=22.53%) | K562 | + | chr11 | | 65267719 | 65267727 |
| 2.05515248792 | srsf7 (bg=22.53%) | K562 | + | chr11 | | 65267729 | 65267742 |
| 3.39842090079 | SRSF9 (bg=9.67%) | HepG2 | + | chr11 | | 65267671 | 65267721 |
| 2.58112899934 | SRSF9 (bg=9.67%) | HepG2 | + | chr11 | | 65267739 | 65267774 |
| 2.1225325833 | SUPV3L1 (bg=9.63%) | K562 | + | chr11 | | 65267691 | 65267722 |
| 3.74032109875 | tra2a (bg=37.02%) | HepG2 | + | chr11 | | 65267662 | 65267727 |
| 4.3269063174 | tra2a (bg=37.02%) | HepG2 | + | chr11 | | 65267674 | 65267734 |
| 3.79393257828 | tra2a (bg=37.02%) | K562 | + | chr11 | | 65267716 | 65267724 |
| 3.72285844699 | tra2a (bg=37.02%) | K562 | + | chr11 | | 65267716 | 65267728 |
| 3.2396136773 | tra2a (bg=37.02%) | K562 | + | chr11 | | 65267724 | 65267728 |
| 3.40394125911 | tra2a (bg=37.02%) | HepG2 | + | chr11 | | 65267727 | 65267742 |
| 3.27429788682 | tra2a (bg=37.02%) | K562 | + | chr11 | | 65267728 | 65267736 |
| 3.31216281803 | tra2a (bg=37.02%) | K562 | + | chr11 | | 65267728 | 65267737 |
| 4.337725168 | tra2a (bg=37.02%) | HepG2 | + | chr11 | | 65267734 | 65267774 |
| 2.99058037098 | tra2a (bg=37.02%) | K562 | + | chr11 | | 65267736 | 65267745 |
| 3.12451957698 | tra2a (bg=37.02%) | K562 | + | chr11 | | 65267737 | 65267746 |
| 2.3140147262 | uchl5 (bg=18.56%) | K562 | + | chr11 | | 65267715 | 65267720 |
| 2.31524788262 | uchl5 (bg=18.56%) | K562 | + | chr11 | | 65267720 | 65267724 |
| 2.10132311033 | uchl5 (bg=18.56%) | K562 | + | chr11 | | 65267728 | 65267738 |
| 2.04912545093 | uchl5 (bg=18.56%) | K562 | + | chr11 | | 65267729 | 65267738 |
| 2.07394862811 | uchl5 (bg=18.56%) | K562 | + | chr11 | | 65267738 | 65267744 |
| 2.08927973636 | uchl5 (bg=18.56%) | K562 | + | chr11 | | 65267738 | 65267747 |
| 2.10807182265 | XRCC6 (bg=3.81%) | K562 | + | chr11 | | 65267713 | 65267724 |
| 2.15504928401 | YWHAG (bg=9.14%) | K562 | + | chr11 | | 65267695 | 65267729 |
| 2.11077017083 | YWHAG (bg=9.14%) | K562 | + | chr11 | | 65267729 | 65267741 |
| 2.00270692935 | YWHAG (bg=9.14%) | K562 | + | chr11 | | 65267738 | 65267757 |
| 2.98973446828 | znf622 (bg=18.79%) | K562 | + | chr11 | | 65267713 | 65267728 |
| 2.36377009273 | znf622 (bg=18.79%) | K562 | + | chr11 | | 65267718 | 65267729 |
| 2.99355243431 | znf622 (bg=18.79%) | K562 | + | chr11 | | 65267728 | 65267738 |
| 2.34742505423 | znf622 (bg=18.79%) | K562 | + | chr11 | | 65267729 | 65267737 |
| 2.14585873865 | znf622 (bg=18.79%) | K562 | + | chr11 | | 65267737 | 65267745 |
| 2.6648147076 | znf622 (bg=18.79%) | K562 | + | chr11 | | 65267738 | 65267744 |

  
  

| Match 95 in HUMAN | | | | | | | |
| --- | --- | --- | --- | --- | --- | --- | --- |
| Motif | Start in Seq (1 Indexed) | End in Seq (1 Indexed) | Strand | Chrm | Exon | Start in Chrm (0 Indexed) | End in Chrm (1 Indexed) |
| AAGGACTTT | 1253 | 1261 | + | chr11 | 1 | 65267742 | 65267751 |
| eCLIP Fold-Enrichment | Binding Protein | Cell Line | Strand | Chrm | | Start in Chrm (0 Indexed) | End in Chrm (1 Indexed) |
| 2.09401296346 | aggf1 (bg=15.15%) | K562 | + | chr11 | | 65267738 | 65267748 |
| 2.46147905701 | aggf1 (bg=15.15%) | K562 | + | chr11 | | 65267748 | 65267756 |
| 3.52559438144 | bclaf1 (bg=17.67%) | HepG2 | + | chr11 | | 65267727 | 65267743 |
| 3.82842145051 | bclaf1 (bg=17.67%) | HepG2 | + | chr11 | | 65267738 | 65267746 |
| 3.81560348363 | bclaf1 (bg=17.67%) | HepG2 | + | chr11 | | 65267743 | 65267758 |
| 4.05045848447 | bclaf1 (bg=17.67%) | HepG2 | + | chr11 | | 65267746 | 65267759 |
| 2.04050465908 | bud13 (bg=12.85%) | K562 | + | chr11 | | 65267738 | 65267746 |
| 2.15418876191 | bud13 (bg=12.85%) | K562 | + | chr11 | | 65267746 | 65267757 |
| 3.44624406879 | hltf (bg=24.28%) | HepG2 | + | chr11 | | 65267738 | 65267746 |
| 3.09702593706 | hltf (bg=24.28%) | HepG2 | + | chr11 | | 65267738 | 65267746 |
| 3.65295101242 | hltf (bg=24.28%) | HepG2 | + | chr11 | | 65267746 | 65267756 |
| 3.40923227241 | hltf (bg=24.28%) | HepG2 | + | chr11 | | 65267746 | 65267762 |
| 2.01151789922 | hnrnpa1 (bg=18.32%) | K562 | + | chr11 | | 65267740 | 65267759 |
| 2.72411715146 | larp4 (bg=13.51%) | K562 | + | chr11 | | 65267737 | 65267746 |
| 2.91325261346 | larp4 (bg=13.51%) | K562 | + | chr11 | | 65267746 | 65267759 |
| 2.93710973687 | LARP7 (bg=2.17%) | HepG2 | + | chr11 | | 65267748 | 65267760 |
| 2.08764926641 | MTPAP (bg=9.55%) | K562 | + | chr11 | | 65267721 | 65267744 |
| 2.05472788533 | MTPAP (bg=9.55%) | K562 | + | chr11 | | 65267744 | 65267759 |
| 2.25007903714 | NIPBL (bg=8.2%) | K562 | + | chr11 | | 65267728 | 65267744 |
| 2.99146009534 | NIPBL (bg=8.2%) | K562 | + | chr11 | | 65267736 | 65267746 |
| 2.08974673349 | NIPBL (bg=8.2%) | K562 | + | chr11 | | 65267744 | 65267763 |
| 3.05848632467 | NIPBL (bg=8.2%) | K562 | + | chr11 | | 65267746 | 65267755 |
| 4.237277399 | ppil4 (bg=43.39%) | K562 | + | chr11 | | 65267737 | 65267742 |
| 4.38370643834 | ppil4 (bg=43.39%) | K562 | + | chr11 | | 65267742 | 65267746 |
| 4.45195353023 | ppil4 (bg=43.39%) | K562 | + | chr11 | | 65267746 | 65267758 |
| 3.86346731643 | ppil4 (bg=43.39%) | K562 | + | chr11 | | 65267749 | 65267758 |
| 2.55313826887 | rbm15 (bg=11.59%) | HepG2 | + | chr11 | | 65267720 | 65267743 |
| 2.31584349525 | rbm15 (bg=11.59%) | K562 | + | chr11 | | 65267738 | 65267746 |
| 2.68678788392 | rbm15 (bg=11.59%) | HepG2 | + | chr11 | | 65267743 | 65267758 |
| 2.06041828388 | rbm15 (bg=11.59%) | K562 | + | chr11 | | 65267745 | 65267757 |
| 2.61970010341 | rbm15 (bg=11.59%) | K562 | + | chr11 | | 65267746 | 65267759 |
| 3.35739527114 | rbm22 (bg=12.69%) | HepG2 | + | chr11 | | 65267718 | 65267746 |
| 2.8420861989 | rbm22 (bg=12.69%) | HepG2 | + | chr11 | | 65267746 | 65267758 |
| 3.17707598999 | safb (bg=40.39%) | HepG2 | + | chr11 | | 65267734 | 65267743 |
| 2.85296008496 | safb (bg=40.39%) | K562 | + | chr11 | | 65267737 | 65267743 |
| 3.08577083607 | safb (bg=40.39%) | K562 | + | chr11 | | 65267738 | 65267747 |
| 3.28986370785 | safb (bg=40.39%) | HepG2 | + | chr11 | | 65267747 | 65267759 |
| 3.17782879351 | safb (bg=40.39%) | K562 | + | chr11 | | 65267748 | 65267757 |
| 2.9926084451 | safb (bg=40.39%) | K562 | + | chr11 | | 65267748 | 65267757 |
| 2.43802057249 | safb (bg=40.39%) | HepG2 | + | chr11 | | 65267751 | 65267759 |
| 2.8421283133 | safb2 (bg=26.89%) | K562 | + | chr11 | | 65267738 | 65267744 |
| 3.71709478847 | safb2 (bg=26.89%) | K562 | + | chr11 | | 65267741 | 65267747 |
| 2.78188275678 | safb2 (bg=26.89%) | K562 | + | chr11 | | 65267744 | 65267758 |
| 3.7438719994 | safb2 (bg=26.89%) | K562 | + | chr11 | | 65267747 | 65267756 |
| 2.89024660836 | srsf1 (bg=30.28%) | K562 | + | chr11 | | 65267737 | 65267743 |
| 3.01864901808 | srsf1 (bg=30.28%) | K562 | + | chr11 | | 65267747 | 65267757 |
| 2.05515248792 | srsf7 (bg=22.53%) | K562 | + | chr11 | | 65267729 | 65267742 |
| 2.34037104202 | srsf7 (bg=22.53%) | K562 | + | chr11 | | 65267749 | 65267762 |
| 2.58112899934 | SRSF9 (bg=9.67%) | HepG2 | + | chr11 | | 65267739 | 65267774 |
| 3.40394125911 | tra2a (bg=37.02%) | HepG2 | + | chr11 | | 65267727 | 65267742 |
| 4.337725168 | tra2a (bg=37.02%) | HepG2 | + | chr11 | | 65267734 | 65267774 |
| 2.99058037098 | tra2a (bg=37.02%) | K562 | + | chr11 | | 65267736 | 65267745 |
| 3.12451957698 | tra2a (bg=37.02%) | K562 | + | chr11 | | 65267737 | 65267746 |
| 3.22397272735 | tra2a (bg=37.02%) | HepG2 | + | chr11 | | 65267742 | 65267780 |
| 3.31556352559 | tra2a (bg=37.02%) | K562 | + | chr11 | | 65267745 | 65267758 |
| 3.33411365891 | tra2a (bg=37.02%) | K562 | + | chr11 | | 65267746 | 65267757 |
| 2.07394862811 | uchl5 (bg=18.56%) | K562 | + | chr11 | | 65267738 | 65267744 |
| 2.08927973636 | uchl5 (bg=18.56%) | K562 | + | chr11 | | 65267738 | 65267747 |
[truncated: 1,667,551 more chars]
