# Supplementary material for: Uncovering deeply conserved motif combinations in rapidly evolving noncoding sequences
Source: Genome Biol. 2021 Jan 11;22:29. doi: 10.1186/s13059-020-02247-1 (PMC7798263; doi:10.1186/s13059-020-02247-1)
Supplement: Supplementary file 6 — Additional file 6: Table S1. Order of sequences analyzed by LncLOOM. Table S3. Oligonucleotide sequences used for RNA pulldown. Mutated bases are underlined. Table S4. Oligonucleotide sequences of ASOs and LNA GapmeRs. Table S5. Primer sequences. [file 13059_2020_2247_MOESM6_ESM.docx]

**Table S1. Order of sequences analysed by LncLOOM.**

| ***Layer*** | ***Cyrano*** | ***libra*** | ***Chaserr*** | ***DICER1*** | ***PUM1*** | ***PUM2*** |
| --- | --- | --- | --- | --- | --- | --- |
| 1 | Human | Human | Human | Human | Human | Human |
| 2 | Rhesus | Dog | Dog | Cow | Dog | Dog |
| 3 | Cow | Mouse | Ferret | Dog | Cow | Cow |
| 4 | Dog | Opossum | Pig | Opossum | Opossum | Mouse |
| 5 | Rabbit | Chicken | Rabbit | Xenopus | Chicken | Chicken |
| 6 | Rat | Xenopus | Armadillo | Zebrafish | Lizzard | Lizzard |
| 7 | Mouse | Spotted Gar | Mouse | Medaka | Mouse | Shark |
| 8 | Opossum | Zebrafish | Opossum | Mouse | Zebrafish | Opossum |
| 9 | Chicken |  | Platypus | Lancelet | Tetraodon | Xenopus |
| 10 | Xenopus |  | Lizard | Sea Urchin | Stickleback | Tetraodon |
| 11 | Spotted Gar |  | Chicken | Fly (*DICER1*) | Xenopus | Stickleback |
| 12 | Nile Tilapia |  | Nile Tilapia | Fly (*DICER2*) | Shark | Zebrafish |
| 13 | Fugu |  | Stickleback |  | Lamprey | Lamprey |
| 14 | Medaka |  | Medaka |  | Lancelet | Lancelet |
| 15 | Stickleback |  | Zebrafish |  | Ciona | Ciona |
| 16 | Atlantic Cod |  | Xenopus |  | Fly | Fly |
| 17 | Zebrafish |  |  |  |  |  |
| 18 | Elephant Shark |  |  |  |  |  |

**Table S3**. **Oligonucleotide sequences used for RNA pulldown.** Mutated bases are underlined

| **Oligo name** | **Description** | **Sequence** |
| --- | --- | --- |
| Exon5-WT | WT sequence of Mouse *Chaserr* Exon 5 | caccccgcttgaagagtttgaaatggactttaccactgagaaatcaagatggcagcccattatggggaattgaggaaaatggattaatgcaagaatgctgtaatattatacaaccaacacaggattcttttaatgtggattccatgaaatgaatgattcttacccaacacaaatggacagtggaatttacttcctaaagacttgttacatgtcatgtacatttttgacatctggagaagactctacaattctacaaatggtagtttgtattcctggaatttcttgcagtttgatctgaagtgaccttatggaatgttaactttaataaaat |
| Exon5-MC | Mouse *Chaserr* Exon 5 with four ATGG->TACC mutations. All four are located within conserved motif identified by LncLOOM | caccccgcttgaagagtttgaaatggactttaccactgagaaatcaagTACCcagcccattTACCggaattgaggaaaTACCattaatgcaagaatgctgtaatattatacaaccaacacaggattcttttaatgtggattccatgaaatgaatgattcttacccaacacaaTACCacagtggaatttacttcctaaagacttgttacatgtcatgtacatttttgacatctggagaagactctacaattctacaaatggtagtttgtattcctggaatttcttgcagtttgatctgaagtgaccttatggaatgttaactttaataaaat |
| Exon5-MA | Mouse *Chaserr* Exon 5 with all ATGG sites mutated to TACC. In total 7 ATGG->TACC mutations. | caccccgcttgaagagtttgaaTACCactttaccactgagaaatcaagTACCcagcccattTACCggaattgaggaaaTACCattaatgcaagaatgctgtaatattatacaaccaacacaggattcttttaatgtggattccatgaaatgaatgattcttacccaacacaaTACCacagtggaatttacttcctaaagacttgttacatgtcatgtacatttttgacatctggagaagactctacaattctacaaTACCtagtttgtattcctggaatttcttgcagtttgatctgaagtgaccttTACCaatgttaactttaataaaat |

**Table S4**. **Oligonucleotide sequences of ASOs and LNA GapmeRs**

| **Name** | **Sequence** |
| --- | --- |
| ASO NTC (Control ASO) | CTCTCTCTCTTTCTATCCCTTC |
| ASO1 | CCATAATGGGCTGCCATCTT |
| ASO2 | GCATTAATCCATTTTCCT |
| ASO3 | TTCCACTGTCCATTTGTG |
| LNA NTC (Control GapmeR) | AACACGTCTATACGC (Cat#: LG00000002) |
| LNA1 | ATAGCGTGCATAAATT |
| LNA2 | GCAGAATGAAGACAAA |
| LNA3 | ATCAATGAATTCACAT |
| LNA4 | CAACGACTGATCCTAA |

**Table S5**. Primer sequences

| Gene | Forward primer | Reverse primer |
| --- | --- | --- |
| *Chaserr* (Primer 1) | GCCATTTTGAAGACTGAGACCA | TCTATGGTGCAGGCCTTTCA |
| *Chaserr* (Primer 2) | TGACATCTGGAGAAGACTCTACAA | AGGTCACTTCAGATCAAACTGC |
| *Chd2* | GGAGATCATAGAACGGGCCA | AAAAGGGTTTGAGTTGGATCTTC |
| *Actin* | TTGGGTATGGAATCCTGTGG | CTTCTGCATCCTGTCAGCAA |
| *Gapdh* | GTCGGTGTGAACGGATTTG | GAATTTGCCGTGAGTGGAGT |
| *Malat1* | GTTACCAGCCCAAACCTCAA | CACTTGTGGGGAGACCTTGT |
| For amplification of Exon5_WT and Exon5_MC for T7 in vitro transcription | TAATACGACTCACTATAGGGCACCCCGCTTGAAGAG | AAGTTAACATTCCATAAGGTCACTTCAG |
| For amplification of Exon5_WT and Exon5_MC Antisense for T7 in vitro transcription | TAATACGACTCACTATAGGGAAGTTAACATTCCATAAGGTCACTTCAG | CACCCCGCTTGAAGAG |
| For amplification of Exon5_MA for T7 in vitro transcription | TAATACGACTCACTATAGGGCACCCCGCTTGAAGAG | AAGTTAACATTGGTAAAGGTCACTTCAG |
| For amplification of Exon5_MA Antisense for T7 in vitro transcription | TAATACGACTCACTATAGGGAAGTTAACATTGGTAAAGGTCACTTCAG | CACCCCGCTTGAAGAG |
