# Supplementary material for: Uncovering deeply conserved motif combinations in rapidly evolving noncoding sequences
Source: Genome Biol. 2021 Jan 11;22:29. doi: 10.1186/s13059-020-02247-1 (PMC7798263; doi:10.1186/s13059-020-02247-1)
Supplement: Supplementary file 7 — Additional file 7: Review history. [file 13059_2020_2247_MOESM7_ESM.docx]

Review History

**First round of review**

**Reviewer 1**

**Are you able to assess all statistics in the manuscript, including the appropriateness of statistical tests used?**

No, I do not feel adequately qualified to assess the statistics.

**Comments to author:**

The manuscript by Ross et al., describes lncLOOM, a novel graph-based algorithm, enabling identification of short conserved RNA sequences (so-called k-mers of 6-15 nt) within rapidly evolving noncoding RNA sequences. As a proof-of-concept, the authors used two well- characterized and deeply conserved long noncoding RNAs (lncRNAs) Cyrano and libra to demonstrate that lncLOOM can reliably identify their regulatory motifs. More specifically, both, libra and Cyrano, trigger degradation of miR-29 and miR-7, respectively, by their near-perfect miRNA binding sites. As demonstrated in the manuscript, lncLOOM identifies the high complementarity miRNA sites in both lncRNA transcripts. Moreover, lncLOOM detects additional conserved sequence elements within these two lncRNAs. While the functional importance of these new motifs is yet unclear, it demonstrates the potential of lncLOOM to find both, already known functionally characterized elements and novel sequence motifs with yet unknown function. This challenging task which is key for dissection of lncRNA mechanism of action has not been achieved with other algorithms that usually require longer stretches of sequence conservation. Importantly, the authors show that lncLOOM enables identification of novel functional motifs within the lncRNA CHASERR that has been recently shown by the Ulitsky team to be essential for embryonic viability in mice. In a series of experiments, the authors demonstrated functional requirement of these CHASERR sequence elements to control CHD2 gene expression and identified a set of proteins binding to these elements. Moreover, general applicability of lncLOOM was additionally demonstrated by identifying sequence motifs withing 3'UTRs.

While I am not in a position to comment on the lncLOOM pipeline per se (i.e. if any improvements or modifications to the pipeline are required), I believe there is sufficient evidence presented in the manuscript that lncLOOM enables identification of conserved short RNA sequence motifs facilitating discovery of the mode of action of lncRNAs and other noncoding transcripts or transcript parts such as 3'UTRs. The study is very timely and will be appreciated not only by the lncRNA field but will be of interest to the whole RNA community. I recommend the manuscript for publication at Genome Biology; please find below my specific comments to clarify some minor points.

-	The authors should introduce the SEEKR k-mer identifying algorithm earlier in the manuscript as the difference between these two algorithms and what they can do is mentioned only in the discussion.

-	Is there any particular reason to use 6-15 mers for lncLOOM? Can the sequence be extended? Would lncLOOM enable identification of bipartite motifs, which many RBPs are binding to (Dominguez et al., 2018, Mol Cell)?

-	I am curios if lncLOOM can be used to some other well-characterized lncRNA examples that do not show such a deep evolutionary conservation such as NORAD, Firre, Xist etc but have sets of well-characterized functional sequence elements. The authors should elaborate on how deeply conserved in terms of putative orothologs (i.e. syntenic sequences with no sequence conservation) test lncRNA should be for lncLOOM to identify motif elements.

-	The authors should discuss in more detail why do they believe that linear sequence conservation is important (i.e. that the order of short sequence motifs is important). For instance, the SEEKR paper believes that a combination of k-mers within a lncRNA transcript but not their order is not essential.

-	I would suggest to use "canonical miRNA sites" instead of "regular" miRNA sites (page 6).

-	The authors should specify how long are the fragments of the Chaserr transcript used for their in vitro transcription and RNA pull-down experiments.

**Reviewer 2**

**Are you able to assess all statistics in the manuscript, including the appropriateness of statistical tests used?**

Yes, and I have assessed the statistics in my report.

**Comments to author:**

The study entitled "Uncovering deeply conserved motif combinations in rapidly evolving noncoding sequences" develop a resource, termed LncLOOM, to identify elusive conserved RNA sequence motifs in otherwise poorly conserved (on a primary sequence level) lncRNA genes and 3' UTR regions. LncLOOM organizes "homologous" sequences from multiple species, these sequences are surveyed for kmers(6-15), indexing unique Khmers as nodes and linear path analysis across the network. Interestingly, the authors then iterate (iterative Kmeans) of the graph to iterate until optimal network is derived. The authors further use multiple sequence alignments, with permutation analysis to derive an experimental null and significance of a given motif. Overall, to my knowledge, this a very sound way to analyze any primary sequence -- if not quite clever, with a html suite to boot, for user friendly dissemination and exploration!

The authors test LncLOOM predictions across many well known motifs in multiple different data contexts (e.g. UTRs, lncRNA motifs, RNA binding motifs) -- all with compelling validation from the unbiased predictions of LncLOOM. The authors further identify an element in the lncRNA CHASSER that the authors have previously studied. Overall, the authors clearly demonstrate the utility of LncLOOM. The authors give very clear explanations of the methodology, their GitHub has LncLOOM Py code well organized and accessible and the applications are seemingly boundless.

I don't have any additional suggestions or concerns -- rather congratulate the authors. I look forward to using this resource soon!

**Authors Response**

**Point-by-point responses to the reviewers’ comments:**

Point-by-point response to references for “Uncovering deeply conserved motif combinations in rapidly evolving noncoding sequences” by Ross et al.

Reviewer #1: The manuscript by Ross et al., describes lncLOOM, a novel graph-based algorithm, enabling identification of short conserved RNA sequences (so-called k-mers of 6-15 nt) within rapidly evolving noncoding RNA sequences. As a proof-of-concept, the authors used two well- characterized and deeply conserved long noncoding RNAs (lncRNAs) Cyrano and libra to demonstrate that lncLOOM can reliably identify their regulatory motifs. More specifically, both, libra and Cyrano, trigger degradation of miR-29 and miR-7, respectively, by their near-perfect miRNA binding sites. As demonstrated in the manuscript, lncLOOM identifies the high complementarity miRNA sites in both lncRNA transcripts. Moreover, lncLOOM detects additional conserved sequence elements within these two lncRNAs. While the functional importance of these new motifs is yet unclear, it demonstrates the potential of lncLOOM to find both, already known functionally characterized elements and novel sequence motifs with yet unknown function. This challenging task which is key for dissection of lncRNA mechanism of action has not been achieved with other algorithms that usually require longer stretches of sequence conservation. Importantly, the authors show that lncLOOM enables identification of novel functional motifs within the lncRNA CHASERR that has been recently shown by the Ulitsky team to be essential for embryonic viability in mice. In a series of experiments, the authors demonstrated functional requirement of these CHASERR sequence elements to control CHD2 gene expression and identified a set of proteins binding to these elements. Moreover, general applicability of lncLOOM was additionally demonstrated by identifying sequence motifs withing 3'UTRs.

While I am not in a position to comment on the lncLOOM pipeline per se (i.e. if any improvements or modifications to the pipeline are required), I believe there is sufficient evidence presented in the manuscript that lncLOOM enables identification of conserved short RNA sequence motifs facilitating discovery of the mode of action of lncRNAs and other noncoding transcripts or transcript parts such as 3'UTRs. The study is very timely and will be appreciated not only by the lncRNA field but will be of interest to the whole RNA community. I recommend the manuscript for publication at Genome Biology; please find below my specific comments to clarify some minor points.

-	The authors should introduce the SEEKR k-mer identifying algorithm earlier in the manuscript as the difference between these two algorithms and what they can do is mentioned only in the discussion.

*We now mention SEEKR in the Introduction (Page 2):*

*“One alternative to conventional sequence analysis is the SEEKR algorithm [6]. Developed primarily for the analysis of lncRNAs, SEEKR evaluates the functional similarity of sequences based on the abundance profiles of short k-mers. Such k-mers may correspond to specific protein binding sites, and SEEKR was shown to be effective for grouping of different lncRNAs with putatively related functions within the same species [6]. However, more sensitive approaches are needed to confidently detect specific functional elements that have been evolutionarily conserved in orthologous lncRNAs in distantly related species.”*

-	Is there any particular reason to use 6-15 mers for lncLOOM? Can the sequence be extended? Would lncLOOM enable identification of bipartite motifs, which many RBPs are binding to (Dominguez et al., 2018, Mol Cell)?

*The range of motif lengths we consider, 5–15, is constrained on the one hand by the infeasibility of solving the large graphs imposed by the 5mers, and the fact that stretches longer than 15 bases are rare. We note that LncLOOM is capable of finding conserved sequences longer than 15, as those will be composed of multiple overlapping conserved 15mers. There is also no upper limit imposed on the starting motif length, which allows motif discovery to be initiated with much longer k-mers if the user would like to analyze longer and more similar sequences, as this can reduce the computational complexity. Bipartite motifs can also be found if the length of each part is at least 6 bases. Finding bipartite motifs with a shorter side is challenging due to the very large possible numbers of combinations to consider. We discuss this in the text (Page 3):*

*“For the analysis presented here, we used k between 6 and 15, as k-mers shorter than 5 will typically result in graphs that are too large for efficient computation, and conserved k-mers longer than 15 are rare enough to be mostly found only once in each sequence (longer conserved k-mers will be recovered by merging of shorter k-mers (see below). Although 6 is the minimum k-mer length allowed by LncLOOM, it is possible to initiate motif discovery with k-mers of any length to reduce graph complexity for the analysis of longer and/or more similar sequences. ”*

-	I am curios if lncLOOM can be used to some other well-characterized lncRNA examples that do not show such a deep evolutionary conservation such as NORAD, Firre, Xist etc but have sets of well-characterized functional sequence elements. The authors should elaborate on how deeply conserved in terms of putative orothologs (i.e. syntenic sequences with no sequence conservation) test lncRNA should be for lncLOOM to identify motif elements.

*This is an excellent suggestion. We now mention in the discussion and show in Additional Files examples of LncLOOM outputs on additional lncRNAs – NORAD, MALAT1, and XIST.*

*Pages 11-12: “Once a set of putatively homologous sequences is available, in our experience so far, we find that LncLOOM is most effective in cases where the sequences span a wide phylogenetic range, ideally one where for the the most distant species there is limited or no homology detectable by BLAST (as we show for Cyrano and CHASERR). In these cases LncLOOM can detect a few highly specific elements that can serve as a basis for functional experiments. When applied to more shallowly conserved functional lncRNAs, the large number of detected elements makes LncLOOM output more challenging to use. For example, when applied to NORAD [33,34] sequences from nine mammals (4917–5458 nt in length), LncLOOM detects 46 significantly conserved elements shared in all species, including some as long as 34 nt (Additional File 4). Reassuringly, these elements included five binding sites for the Pumilio proteins, known functional binders of NORAD [33,34]. Similarly, when applied to the longer XIST sequences from six mammals (10,466–25,215 nt in length), LncLOOM identifies 23 significantly conserved elements (Additional File 5).*

*Analysis of 19 sequences of MALAT1 (4992–12676 nt in length, Additional File 6), a deeply conserved and extremely abundant lncRNA [13,35,36] illustrates a more complex scenario of high conservation only up to a certain evolutionary depth. Only six motifs are conserved in all sequences, including three that form part of the triple-helical motif at the 3' end of MALAT1 [37,38], and another motif in that region is conserved in 18 of the species. Nine additional short motifs are significantly conserved outside of amniotes (i.e., in at least 11 of the input species). In contrast to this relative scarcity of very deeply conserved motifs (given the length of the RNA), 122 k-mers are significantly conserved in at least one amniote species (turtle, alligator, or lizard).”*

-	The authors should discuss in more detail why do they believe that linear sequence conservation is important (i.e. that the order of short sequence motifs is important). For instance, the SEEKR paper believes that a combination of k-mers within a lncRNA transcript but not their order is not essential.

*We now address this point in the discussion and in the new conclusions part:*

*Page 10: “Contrary to SEEKR, LncLOOM requires that the linear order of conserved k-mers is maintained. Although the order of k-mers may not always be important for function, this constraint increases the precision of motif discovery as an ordered set of conserved kmers is less likely to appear by chance and allows us to consider sets of conserved elements within the context of their rapidly evolving sequences. As we show here, in many cases, those k-mers correspond to functionally relevant elements.”*

*Page 11: “LncLOOM is a powerful new framework for identification of conserved combinations of short sequence motifs in noncoding elements, capable of recovering both known and novel functional elements in lncRNAs and 3'UTRs, that can also be applied to other biological sequences. The assumption of linear order conservation is restrictive, but it is shared with alignment-based methods, and it is the basis for the statistical power to identify particularly deeply conserved motifs, shared between all vertebrates in several lncRNAs and conserved in 3'UTRs between vertebrates and insects. As we further demonstrate, once short functional motifs are identified they can serve as a starting point for experimental determination of specific RNA binding partners and for design of effective antisense reagents for perturbation of lncRNA function.”*

-	I would suggest to use "canonical miRNA sites" instead of "regular" miRNA sites (page 6).

*Fixed.*

-	The authors should specify how long are the fragments of the Chaserr transcript used for their in vitro transcription and RNA pull-down experiments.

*This has now been mentioned on page 7:*

*“In order to identify proteins potentially binding the conserved regions, we used in vitro transcription to generate biotinylated RNAs containing the first 322 nt of the WT sequence of the last exon of mouse Chaserr (bases 559–882 of the RefSeq NR_037569.1 isoform) , the same sequence with AUGG→UACC mutations in four conserved motifs, and a second mutant in which all seven of the AUGG sites in the last exon were mutated to UACC”*

Reviewer #2: The study entitled "Uncovering deeply conserved motif combinations in rapidly evolving noncoding sequences" develop a resource, termed LncLOOM, to identify elusive conserved RNA sequence motifs in otherwise poorly conserved (on a primary sequence level) lncRNA genes and 3' UTR regions. LncLOOM organizes "homologous" sequences from multiple species, these sequences are surveyed for kmers(6-15), indexing unique Khmers as nodes and linear path analysis across the network. Interestingly, the authors then iterate (iterative Kmeans) of the graph to iterate until optimal network is derived. The authors further use multiple sequence alignments, with permutation analysis to derive an experimental null and significance of a given motif. Overall, to my knowledge, this a very sound way to analyze any primary sequence -- if not quite clever, with a html suite to boot, for user friendly dissemination and exploration!

The authors test LncLOOM predictions across many well known motifs in multiple different data contexts (e.g. UTRs, lncRNA motifs, RNA binding motifs) -- all with compelling validation from the unbiased predictions of LncLOOM. The authors further identify an element in the lncRNA CHASSER that the authors have previously studied. Overall, the authors clearly demonstrate the utility of LncLOOM. The authors give very clear explanations of the methodology, their GitHub has LncLOOM Py code well organized and accessible and the applications are seemingly boundless.

I don't have any additional suggestions or concerns -- rather congratulate the authors. I look forward to using this resource soon!

*We thank the reviewer for the very positive evaluation of our manuscript.*
